# Immediate Adaptation Analysis Implicates BCL6 as an EGFR-TKI Combination Therapy Target in NSCLC*[Fn FN2]

**DOI:** 10.1074/mcp.RA120.002036

**Published:** 2020-03-30

**Authors:** Yan Zhou Tran, Rezan Minozada, Xiaofang Cao, Henrik J. Johansson, Rui M. Branca, Brinton Seashore-Ludlow, Lukas M. Orre

**Affiliations:** Department of Oncology-Pathology, Science for Life Laboratory, Karolinska Institutet, Stockholm, Sweden

**Keywords:** Mass spectrometry, RNA SEQ, cancer therapeutics, targeted therapies, personalized medicine, receptor tyrosine kinases, lung cancer, bioinformatics, cancer biomarker(s), drug targets

## Abstract

Drug resistance is a major obstacle to curative cancer therapies, and increased understanding of the molecular events contributing to resistance would enable better prediction of therapy response, as well as contribute to new targets for combination therapy. Here we have analyzed the early molecular response to epidermal growth factor receptor (EGFR) inhibition using RNA sequencing data covering 13,486 genes and mass spectrometry data covering 10,138 proteins. This analysis revealed a massive response to EGFR inhibition already within the first 24 h, including significant regulation of hundreds of genes known to control downstream signaling, such as transcription factors, kinases, phosphatases and ubiquitin E3-ligases. Importantly, this response included upregulation of key genes in multiple oncogenic signaling pathways that promote proliferation and survival, such as ERBB3, FGFR2, JAK3, and BCL6, indicating an early adaptive response to EGFR inhibition. Using a library of more than 500 approved and experimental compounds in a combination therapy screen, we could show that several kinase inhibitors with targets including JAK3 and FGFR2 increased the response to EGFR inhibitors. Further, we investigated the functional impact of BCL6 upregulation in response to EGFR inhibition using siRNA-based silencing of BCL6. Proteomics profiling revealed that BCL6 inhibited transcription of multiple target genes including p53, resulting in reduced apoptosis which implicates BCL6 upregulation as a new EGFR inhibitor treatment escape mechanism. Finally, we demonstrate that combined treatment targeting both EGFR and BCL6 act synergistically in killing lung cancer cells. In conclusion, or data indicates that multiple different adaptive mechanisms may act in concert to blunt the cellular impact of EGFR inhibition, and we suggest BCL6 as a potential target for EGFR inhibitor-based combination therapy.

## Abstract (precis)

Drug resistance is a major obstacle to targeted cancer therapies. Here we have used in-depth molecular response profiling and drug screening to investigate early adaptive responses after EGFR-inhibition and to identify new combination therapy targets. Response profiling at both mRNA and protein level revealed increased signaling in multiple pathways with the potential to blunt efficacy and cause drug resistance. Inhibition of several of these pathways resulted in synergistic effects together with EGFR-inhibitors, suggesting potential new combination therapy strategies.

## Graphical Abstract

**Figure:**
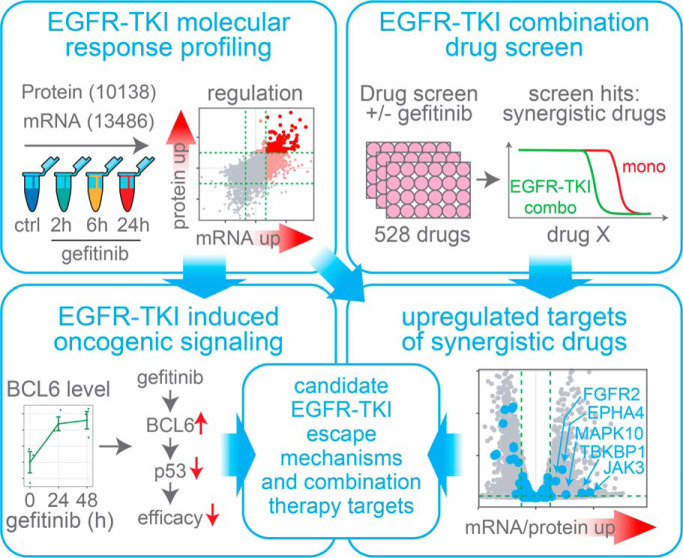

**Highlights**

- EGFR-TKI molecular response profiling covering 10138 proteins and 13486 mRNAs.
- EGFR-TKI combination therapy screen using a library of 528 compounds.
- Several new candidate EGFR-TKI escape mechanisms and combination therapy targets.
- Combined targeting of the oncogene BCL6 and EGFR results in synergy in NSCLC cells.

Epidermal growth factor receptor (EGFR)^1^ targeting therapy has been the prototype example of successful precision medicine ever since it revolutionized the treatment of non-small cell lung cancer (NSCLC) 15 years ago. Even though EGFR tyrosine kinase inhibitors (TKIs) continue to be a cornerstone in the therapy, the efficacy of the treatment constantly needs to be re-evaluated for individual patients as resistance inevitably develops. Today, drug resistance is considered the principal limiting factor to curing cancer patients (1). Lung cancer (85% NSCLC) is the leading cause of cancer-related death worldwide and is responsible for 18.4% of the total deaths to cancer (2, 3). In a majority of all cases, NSCLC is diagnosed at an advanced stage when the tumor has metastasized, and curative surgery or radiotherapy is no longer an option (4). Consequently, for patients with spread disease, drug treatment is the only alternative. Even though targeted therapy (*e.g.* EGFR-TKIs), immune checkpoint inhibitors or combination chemotherapy can delay disease progression for these patients, low initial response rates, as well as resistance development results in a 5-year survival rate of 5% (4). To improve the survival for these patients, a deeper understanding of the complex biology behind drug resistance is needed.

Oncogenic activation of receptor tyrosine kinases (RTKs), such as EGFR, is common in cancer and results in abnormal signaling through downstream pathways (5). Typically, the activation of RTKs leads to signaling through the Mitogen-activated protein kinase (MAPK) pathway resulting in increased cell proliferation, as well as through the phosphoinositide 3-kinase (PI3K)-AKT-mammalian target of rapamycin (mTOR) pathway leading to increased survival (6, 7). Increasing molecular knowledge about cancer spurred the development of drugs that could inhibit oncogenic signaling and kill the cancer cells, resulting in the first-generation EGFR-TKIs gefitinib (8) and erlotinib (9). Response to monotherapy with EGFR-TKIs is dependent on the presence of activating EGFR mutations, such as exon 19 deletions or L858R mutations, present in 16.6% of lung adenocarcinoma patients (10). Since the first approval of EGFR-TKIs, second-generation TKIs such as afatinib (11) (targeting EGFR and ERBB2) and the third-generation TKI osimertinib (12) (targeting EGFR carrying the T790M resistance mutation) have been developed and approved for use in NSCLC. Nevertheless, resistance (13–16) to all these therapies is observed clinically, underscoring an urgent need for improved treatment strategies.

In addition to “intrinsic resistance”, where the cells are resistant already before treatment, resistance can be divided into “early adaptive responses” or “acquired resistance” that occurs after longer drug exposure (1). These can be further classified as “on-target” resistance where the actual target of the drug is altered, and “off-target” resistance where downstream or parallel pathways are modified (17). A prototype example of acquired on-target resistance toward EGFR-TKIs is the occurrence of the T790M gatekeeper mutation in the ATP binding pocket of EGFR that has been found in 50% of patients with acquired resistance to first generation EGFR-TKIs. When understood, such resistance can be combatted through the development of new drugs that can inhibit the altered target as exemplified by the development of osimertinib (12). Early adaptive off-target responses that limit or completely abolish the effect of EGFR-TKIs are commonly driven by complex feedback processes in pathways that controls the oncogenic growth and survival. This type of adaptation can result in lack of, or only short-term, clinical response because it occurs so rapidly that initial effects on the tumor may not even be clinically quantifiable (1). If detected however, rationally designed combinations of different targeted therapies could inhibit the escape of tumor cells from monotherapy treatment and provide patient benefit. EGFR-TKI based combination therapy in NSCLC is currently not applied in the clinic, however a large number of clinical studies have been performed or are currently ongoing and showing promising results (17).

The aim of this study was to explore the immediate adaptive response to EGFR-TKIs and to suggest novel relevant targets for EGFR-TKI based combination therapy for improved treatment of NSCLC patients. Using in-depth transcriptomics and proteomics data from gefitinib treated cells we could show dramatic changes in mRNA and protein levels over treatment duration, with engagement of multiple signaling pathways already within the first 24 h. Importantly, this molecular response profiling experiment revealed that key components in several pathways with growth/survival promoting capacity was increased including ERBB3, FGFR2, JAK3 and BCL6. Next, combination therapy drug screening was used to identify synergistic effects between gefitinib and a library of 528 different compounds, resulting in the identification of multiple candidates for combination therapy including the kinase inhibitors, nintedanib and momelotinib with targets including FGFR2 and JAK3 respectively. Further, we investigated the molecular effects of BCL6 in response to EGFR inhibition using BCL6 silencing coupled to in-depth proteomics profiling. Through this data we could identify many BCL6-regulated candidate proteins including the tumor supressor p53. Finally, we used clonogenic assays to demonstrate the synergy in combined targeting of EGFR and BCL6- mediated adaptive response in multiple cell lines.

## MATERIALS AND METHODS

### 

#### 

##### Experimental Design and Statistical Rationale

Overall, the experimental design for analysis included here is according to standard practice. For each experiment biological triplicates were used is indicated in respective figure and/or materials and methods section. The only is the MS profiling after gefitinib treatment, in which the 2 h and 6 h gefitinib treatment samples were biological duplicates. By replicates we refer to biological replicates *i.e.* replicate cell culture dishes. DESeq2 (18) was used to perform differential expression analysis (DEA) of RNA sequencing data. DEqMS (19) was used to perform differential expression analysis of proteome data. Both methods use the Benjamini-Hochberg (BH) adjustment (20) to calculate for each gene an adjusted *p* value. Cut-offs used for significantly regulated mRNAs were the following: absolute value of log2 fold change > 1 and adjusted *p* value < 0.01. Cut-offs used for significantly regulated proteins were the following: absolute value of log2 fold change > 0.5 and adjusted *p* value < 0.01. All Western blotting quantifications (based on densitometric analysis) and clonogenic assay quantifications (based on colony area analysis) were analyzed from biological triplicates and the *p* values were calculated by student's *t* test. *p* values < 0.05 were considered as statistically significant. Pearson correlation method was used to calculate the distance matrix in hierarchical clustering.

##### Omics Based Molecular Response Profiling After Gefitinib Treatment

A431 gefitinib molecular response profiling data generated using RNA sequencing for mRNA level analysis and HiRIEF LC-MS for protein level analysis as described in our previous publications (21, 22) was downloaded from ArrayExpress (mRNA data, identifier E-MTAB-5285) and ProteomeXchange (protein level, identifier PXD006291). Briefly, for omics based molecular response profiling after gefitinib treatment, raw count table for mRNA and gene centric protein table were used for analysis. The protein quantities in gene centric protein table were calculated using median Sweeping method (23) from PSM raw intensity table. Biological triplicates of RNA sequencing data of all conditions (control (untreated), gefitinib treated 2 h, 6 h or 24 h) were used for mRNA differential expression analysis. Biological triplicates of proteomics data for control (untreated) and gefitinib treated (24 h) were used in protein differential expression analysis, and these two triplicates together with biological duplicates of proteomics data for gefitinib treated (2 h and 6 h) were used for heatmap visualization.

##### Cell Lines and Treatment

A431 (DSMZ, Braunschweig, Germany, ACC-91), HCC827 (ATCC, CRL-2868), NCI-H1869 (ATCC, Teddington, Middlesex, United Kingdom, CRL-5900), NCI-H1666 (ATCC, CRL-5885) cells were cultured in RPMI 1640 medium (Sigma-Aldrich, St. Louis, Missouri, R2405) with 10% FBS (Sigma-Aldrich, F7524) and 1% penicillin-streptomycin (Sigma-Aldrich, P4333) at 37 °C, 5% CO_2_. All cell lines were tested and found free of Mycoplasma using MycoAlert Mycoplasma detection kit (Lonza, Basel, Switzerland, S1025). For Western blotting to select BCL6 siRNA, A431 cells were transfected with control siRNA or BCL6 siRNA (Dharmarcon, Lafayette, Colorado, MQ-011591-01-0020, contains 4 of BCL6 siRNAs (#1-#4) ranging from d-0011591-02-0020 to d-0011591-05-0020), for 24 h. Cells were then untreated or treated with 2.5 μm gefitinib (Selleckchem, Munich, Germany, S1025) for 48 h. For BCL6 dependent gefitinib response proteomics experiment, A431 cells were transfected with non-targeting control siRNA (Dharmarcon, d-001210–05-20) or BCL6 siRNA (Dharmarcon, d-011591-02-0020), for 24 h. After transfection, old medium was replaced by fresh medium with or without 2.5 μm gefitinib. Cells were harvested after 24 or 48 h, according to the setting in Fig. 4*A*. For Western blotting to validate BCL6 and apoptosis relations, A431 cells were transfected with non-targeting control siRNA (Dharmarcon, d-001210-05-20) or BCL6 siRNA (Dharmarcon, d-011591-02-0020), for 24 h. After transfection, cells were incubated in fresh medium with or without 2.5 μm gefitinib for 48 h. For Western blotting to detect BCL6 level in NSCLC cells, A431, HCC827, and H1869 were treated with or without gefitinib for 24, 48 and 72 h. The concentration of gefitinib correspond to an estimated cell line specific EC_50_ value, for A431 was 1.4 μm, HCC827 was 8.1 nm, and NCI-H1869 was 1.1 μm. Non-targeting control siRNA (Dharmarcon, d-001210-05-20) was used as control siRNA in all siRNA related experiments. Cells transfected with AllStars cell death control siRNA (Qiagen, Hilden, Germany, 1027298) were used as positive control in all siRNA related experiments. All cells were harvested with accutase (Sigma-Aldrich, A6964).

##### Drug Sensitivity and Resistance Testing (DSRT)

DSRT^27^, ^61^ assay was used in this study. Briefly, A431 cell were exposed to a small molecule library consisting of 528 drugs from the Institute for Molecular Medicine Finland (FIMM) oncology set with or without 1.5 μm gefitinib. Compounds and viability controls (DMSO, 100 μm benzethonium chloride) were predispensed on tissue culture treated 384 well plates (Corning, Hickory, North Carolina, 3764). Each compound was plated in 5 concentrations spanning a 10,000-fold concentration range (10-fold dilution). Assay ready plates were stored in pressurized StoragePods (Roylan Developments) under inert atmosphere until used. Using a MultiDrop Combi (Thermo Scientific, Waltham, Massachusetts) 5 μl media with or without 1.5 μm gefitinib was first dispensed into assay ready plates and centrifuged briefly. Twenty microliters of a single-cell suspension was then seeded using a peristaltic pump to the plates at a density of 1500 cells/per well. As a surrogate for cell viability, cellular ATP levels were assessed 72 h after plating using CellTiterGlo (Promega, Madison, Wisconsin) with detection on an EnSight plate reader (PerkinElmer, Waltham, Massachusetts). Drug response curves were fitted after per plate normalization from the viability of cells and the concentration of drugs. Drug Sensitivity Score (DSS) for drug combination or control was calculated based on the model described in 62 using Breeze (https://www.fimm.fi/en/software-tools). DSS values with standard error of estimate of the curve greater than 19 were not considered in further analysis.

##### Western Blot Analysis

Cells were lysed with lysis buffer (4% (w/v) sodium dodecyl sulfate (SDS), 25 mm HEPES (pH 7.6), 1 mm dithiothreitol (DTT)). Extracted proteins were separated in SDS-PAGE gels and transferred to nitrocellulose membranes (Invitrogen, Waltham, Massachusetts). After blotting, membranes were blocked in 5% non-fat skim milk in Tris-Buffered Saline (TBS) containing 0.1% Tween20. Membranes were incubated with the following primary antibodies as specified in figures: rabbit BCL6 (1:1000. Cell Signaling, Leiden, The Netherlands, 14895), rabbit p53 (1:1000. Cell Signaling, 2527), rabbit cleaved PARP1 (1:10,000. Abcam, Cambridge, United Kingdom, ab32064), mouse GAPDH (1:10,000. Sigma Aldrich, G8795), followed with incubation with relevant HRP-conjugated secondary antibody. Membranes were visualized with Clarity western ECL substrate (BIO-RAD, Hercules, California, 1705061) and the final image was taken by iBright CL1000 imaging system (Thermo Fisher). The quantification of the Western blotting band intensity was performed by following Davarinejad H. 's protocol (24). All Western blots were performed in biological triplicates except the samples for the Western blotting to select BCL6 siRNA.

##### Clonogenic Assay

In the clonogenic assay to quantify the synergy of gefitinib and FX1 (Selleckchem, S8591), A431, HCC827, and H1869 were incubated 10 days either untreated as control, or treated with gefitinib, FX1 or a combination of the two drugs. Drug concentrations used were based on the estimated cell line specific EC_50_ values for gefitinib/FX1: 1.4uM/6 μm for A431 cells, 8.1 nm/3 μm for HCC827 and 1.1uM/3 μm for H1869. Medium was replaced every 3 days. Cells were stained with staining solution (0.5% crystal violet, 6% glutaraldehyde in distilled water) for 30 min, air dried and the photos of the plates were taken with iBright CL1000 imaging systems (Thermo Fisher). Colony area was calculated using ImageJ plug ColonyArea (25) according to the user manual.

##### Sample Preparation for MS

Cell pellets were lysed by addition of lysis buffer (4% SDS, 25 mm HEPES pH 7.6, 1 mm DTT), followed by heating to 95 °C for 5 min and sonicating for 1 min. After centrifuging at 14,000 × *g* (11,000 rpm) for 15 min, supernatants were collected to new vials and protein concentration was measured by Bio-Rad DCC protein assay. For each sample, 200 μg protein lysate was diluted with fresh lysis buffer to reach 200 μl of sample volume and protein concentration of 1 μg/μl. Protein cleanup was performed following a slightly modified standard SP3 protocol as previously described (26, 27) and followed by lysyl endopeptidase (Lys-C, Wako, Neuss, Germany, 129–02541, enzyme/protein = 1:50, diluted in 50 mm HEPES pH 7.6, 4 m Urea, diluted in 50 mm HEPES, pH 7.6) digestion for 4 h and trypsin (enzyme/protein = 1:50, Thermo Fisher Scientific) digestion for 14 h, both in 37 °C. Digested peptides′ solution was collected to new vials, peptide concentration was determined with Bio-Rad DCC assay. 100 μg of peptides from each sample was labeled with TMT-10plex (Thermo Scientific, 90110) isobaric label reagent following the manufacturer's instruction. Labeled samples were pooled, cleaned by strata-X-C-cartridges (Phenomenex, Torrance, California) and dried with speed-vac.

##### Peptide Level Sample Fractionation Through HiRIEF

Four hundred micrograms of the TMT labeled peptides were separated by immobilized pH gradient - isoelectric focusing (IPG-IEF) on pH 3–10 strips using HiRIEF method as described previously (21). Peptides were extracted from the strips by a liquid handling robot (Etan digester from GE Healthcare Bio-Sciences AB, which is a modified Gilson liquid handler 215), supplied by GE Healthcare Bio-Sciences AB, Uppsala, Sweden. A polypropylene well former with 72 wells was put onto each strip and 50 μl of MQ was added to each well. After 30 min incubation, the liquid was transferred to a 96-well plate and the extraction was repeated 2 more times using 35% acetonitrile in the second round, and 35% acetonitrile, 0.1% formic acid in the third round. The extracted peptides were dried in speed-vac for storage.

##### LC-MS-based Quantitative Proteomics

For each LC-MS run of a HiRIEF fraction, the auto sampler (Ultimate 3000 RSLC system, Thermo Scientific Dionex) dispensed 20 μl of mobile phase A (95% water, 5% dimethyl sulfoxide (DMSO), 0.1% formic acid) into the corresponding well of the microtiter plate, mixed by aspirating/dispensing 10 μl ten times, and finally injected 10 μl into a C18 trap desalting column (Acclaim pepmap, C18, 3 μm bead size, 100Å, 75 μm × 20 mm, nanoViper, Thermo). After 5min of flow at 5 μl/min with the loading pump, the 10-port valve switched to analysis mode in which the NC pump provided a flow of 250 nL/min through the trap column. The curved gradient (curve 6 in the Chromeleon software) then proceeded from 3% mobile phase B (90% acetonitrile, 5% DMSO, 5% water, 0.1% formic acid) to 45% B in 50–110min (depending on IPG-IEF fraction complexity) followed by wash at 99%B and re-equilibration. Total LC-MS run time is 24 min longer than the gradient time. We used a nano EASY-Spray column (pepmap RSLC, C18, 2 μm bead size, 100Å, 75 μm × 50 cm, Thermo) on the nano electrospray ionization (NSI) EASY-Spray source (Thermo) at 60 °C. Online LC-MS was performed using a hybrid Q-Exactive HF mass spectrometer (Thermo Scientific). FTMS master scans with 60,000 resolution (and mass range 300–1500 *m*/*z*) were followed by data-dependent MS/MS (30,000 resolution) on the top 5 ions using higher energy collision dissociation (HCD) at 30% normalized collision energy. Precursors were isolated with a 2 *m*/*z* window and an isolation offset of 0.5 *m*/*z*. Automatic gain control (AGC) targets were 1e6 for MS1 and 1e5 for MS2. Maximum injection times were 100ms for MS1 and MS2. The entire duty cycle lasted ∼1s. Dynamic exclusion was used with 30s duration. Precursors with charge states 2–7 were included. An underfill ratio of 1% was used.

##### Peptide and Protein Identification

Orbitrap raw MS/MS files were converted to mzML format using msConvert (v3.0) from the ProteoWizard (v3.0) tool suite used code as: ProteoWizard 3.0.18250.994311be0 > msconvert *.raw - numpressLinear [=arg(= 2e-09)]. For all subsequent steps we used the ddamsproteomics pipeline (v1.1) in nextflow (v.19.04.0) (28). Spectra were searched using MSGF+(v2017.07.21) and Percolator (v3.01), where search results from all fractions were grouped for Percolator target/decoy analysis. All searches were done against the human protein subset of Ensembl 92 (107844 protein entries) using target/decoy concatenation allowing for one tryptic missed cleavage. MSGF+ settings included precursor mass tolerance of 10 ppm, fragment mass tolerance was according to MSGF+ option 3 (Q-Exactive HCD spectra), fully tryptic peptides, isotope error −1, 2, peptide length 7–50 amino acids and precursor charge states 2–6. Fixed modifications were TMT-10plex on lysines and peptide N termini, and carbamidomethylation on cysteine residues, a variable modification was used for oxidation on methionine residues. Quantification of TMT-10plex reporter ions was done using OpenMS (v2.4.0) project's IsobaricAnalyzer. Median Sweeping method (23) was used to calculate protein relative abundance. Briefly, relative quantification for each protein is calculated by taking the median TMT ratio from the set of PSMs unique to that protein. Protein TMT ratios are then normalized by column median centering. FDR (false discovery rate) was estimated by target/decoy competition. PSMs and peptides found at 1% FDR were used to infer gene identities. Protein false discovery rates were calculated using the picked-FDR method (29) using gene symbols as protein groups and limited to 1% FDR. Hardklor (v2.3.0) was used for peptide feature identification and isotope deconvolution. Peptide areas were then estimated by Kronik (v2.20) and integrated to the data tables using msstitch (v2.15). Protein areas are calculated by averaging the areas of the Top 3 peptides mapping to the protein.

##### Bioinformatics Analysis

Kinase maps were generated using KinoViewer (30) with light modifications. Input for each map was significantly regulated mRNAs or proteins from the comparison pairs such as gefitinib treated 2h/6h/24h *versus* control.

Genes annotated in different regulatory categories were retrieved from various sources; transcription factors (TFs, 1569 unique gene symbols retrieved from animalTFDB (31)); transcription co-factors (413 genes, animalTFDB); chromatin remodeling factors (129 genes, animalTFDB); protein kinases (514 genes, 2007 update of (32)); Ubiquitin E3 ligases (614 genes (33)) and protein phosphatases (189 genes (34)).

Target annotation for the drug library used in the DSRT experiments were based on drug target information from the original FIMM oncology set, complemented by target information from DrugBank (v5.0) (35), high confident targets from a recent publication describing the target landscape of clinical kinase drugs (36), and selleckchem.com.

WebGestalt (37) was used for KEGG and Reactome pathway enrichment analysis. In gefitinib profiling experiments, KEGG pathway enrichment analysis was performed as follows. As input for the enrichment analysis we used regulated mRNAs (log2FC > 1 or < −1 and adjust *p* value < 0.01) or proteins (log2FC > 0.5 or < −0.5 and adjust *p* value < 0.01). Gene symbol of 99 significantly regulated mRNAs (for gefitinib treated 2 h *versus* control), 474 significantly regulated mRNAs for gefitinib treated 6 h *versus* control), 3160 significantly regulated mRNAs (for the gefitinib treated 24 h *versus* control) or 628 proteins (for the gefitinib treated 24 h *versus* control) were used for the analysis. The reference gene lists were 13489 profiled mRNAs or 10138 profiled proteins, respectively. In the BCL6 siRNA experiment KEGG pathway enrichment did not generate any significant results. Instead we used Reactome pathway enrichment analysis. Enrichment analysis was based on the 447 significantly regulated proteins from untreated siBCL6 *versus* siCtrl cells, 448 significantly regulated proteins from 24 h gefitinib treated siBCL6 *versus* siCtrl cells or 698 significantly regulated proteins from 48 h gefitinib treated siBCL6 *versus* siCtrl cells. The reference gene lists were 9944 profiled proteins from MS profiling of BCL6 silenced EGFR TKI induced response experiment. For the comparison of untreated cells, no significant pathways were identified. The method used for enrichment was Over-Representation Analysis (ORA). The enriched pathways with FDR < 0.05 were selected for visualization as bubble plot and the bubbles were ranked based on -log10 of FDR values for the pathway enrichment.

## RESULTS

### 

#### 

##### Molecular Profiling of EGFR-TKI Response Indicates Rapid Upregulation of Genes Potentially Involved in Treatment Escape Mechanisms

**Fig. 1.**
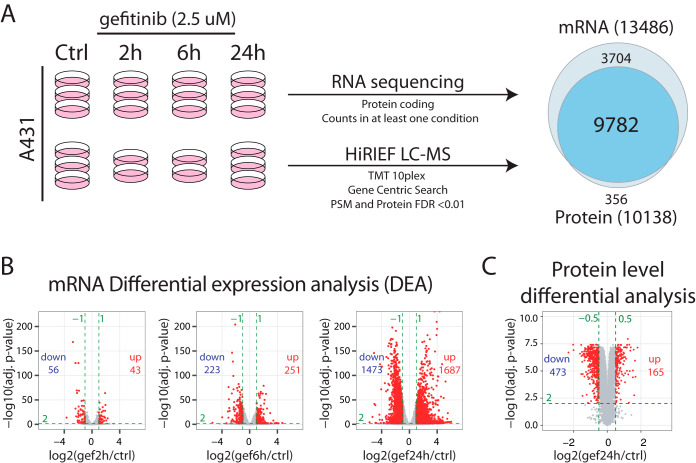
**EGFR-TKI molecular response profiling.** *A*, A431 cells in replicate cultures were untreated or treated with 2.5 μm gefitinib for 2/6/24 h. Transcriptomics profiling was performed using RNA sequencing of triplicate samples for each condition. Proteomics profiling was performed using HiRIEF LC-MS of triplicate control and 24 h gefitinib samples and duplicate 2 h and 6 h samples. The profiling experiments resulted in quantification of 13 486 genes at mRNA level and 10,138 genes at protein level, with an overlap of 9782 genes. For additional details see materials and methods. *B*, Volcano plots indicating results from mRNA-level differential expression analysis performed at different timepoints after gefitinib treatment. Green dotted lines indicate the cutoffs used to define regulated mRNAs (log2 FC>±1, adjusted *p* value<0.01). Indicated in each plot is also the number of upregulated and downregulated mRNAs. *C*, Volcano plot indicating results from protein-level differential analysis comparing 24 h gefitinib treated samples to control samples. Green dotted lines indicate the cutoffs used to define regulated mRNAs (log2 FC>±0.5, adjusted *p* value<0.01). Indicated in the plot is also the number of upregulated and downregulated proteins.

To identify potential early EGFR-inhibitor escape mechanisms, we used a previously published data set (21, 22) where we performed mRNA and protein level molecular profiling at several timepoints after gefitinib treatment in A431 cells as illustrated in Fig. 1*A*. The epidermoid carcinoma cell line A431 is amplified for *wild-type* EGFR and is often used as a model system to study EGFR signaling. Briefly, transcriptome analysis was performed by RNA sequencing where triplicate samples were analyzed as untreated and at 2 h, 6 h and 24 h after gefitinib treatment. Filtering the data based on protein coding genes with counts in all replicates in at least one experimental condition resulted in the identification and quantification of mRNAs mapping to 13,486 genes (supplemental Table S1). For protein level profiling we used our in-house developed method for in-depth MS-based proteomics, HiRIEF (High-Resolution Iso-Electric Focusing) LC-MS^1^, and TMT (tandem mass tag) 10-plex isobaric labeling for relative quantification between samples. Gene-centric search of the MS-data resulted in the identification and quantification of 10 138 proteins (PSM, peptide and protein FDR<1%, supplemental Table S2). The gene-level identification overlap between mRNA and protein level analysis was 9782 genes (Fig. 1*A*), with high correlation in overall abundance between mRNAs (average number of reads) and proteins (average number of PSMs, supplemental Fig. S1).

For mRNA-level differential expression analysis we used the DESeq2 (39) method, resulting in the identification of 43, 251, and 1687 upregulated; and 56, 223, and 1473 downregulated genes at the 2 h, 6 h and 24 h time points respectively (abs. log2 FC>1, adjusted *p* value<0.01, Fig. 1*B*). These results indicate a dramatic alteration in the cellular signaling already during the first 24 h after EGFR inhibition. For the protein level differential analysis, we used the DEqMS (19) method to compare gefitinib-24 h samples with untreated control samples as triplicate samples were available for these two conditions. This analysis resulted in the identification of 165 upregulated proteins and 473 downregulated proteins (abs. log2 FC>0.5, adjusted *p* value<0.01, Fig. 1*C*). For the protein level analysis of 2 h and 6 h timepoints after gefitinib treatment only duplicate samples were available, limiting the possibility to perform statistical analysis of altered protein levels. Still, a heatmap visualization of the protein level quantification at all timepoints indicates a gradual increase/decrease of protein levels with clearly visible patterns already 2 h after EGFR-TKI treatment (supplemental Fig. S2*A*). In total, 3219 and 638 genes were significantly regulated in response to EGFR inhibition at mRNA and protein levels respectively, with an overlap of 404 genes (supplemental Fig. S2*B* and supplemental Table S3). Importantly, these genes included many transcriptional regulators (302 transcription factors, 95 co-factors and 29 chromatin remodeling factors) as well as many ubiquitin E3-ligases (95), kinases (86) and phosphatases (42, supplemental Fig. S2*C*). Further, an evaluation of the subcellular localization of proteins encoded by regulated genes using the SubCellBarCode resource (40) showed that all major compartments of the cell was engaged (supplemental Fig. S2*D*). Our deep molecular profiling thus indicates a vastly complex cellular response to EGFR inhibition, already 24 h after treatment. For a general overview of the molecular response to EGFR inhibition, pathway enrichment analysis was then performed at both mRNA (2 h, 6 h and 24 h treatment) and protein (24 h treatment) levels (supplemental Fig. S3). This analysis showed as expected that gefitinib resulted in a rapid impact on the MAPK pathway, detectable at mRNA level already 2 h after treatment. At later timepoints broader terms were enriched such as “Cell Cycle,” “DNA replication” and “Senescence,” as well as specific pathways such as the p53 pathway and the JAK-STAT pathway. No overlap was seen in the mRNA level pathway enrichment analysis at 6h and 24h. These findings are in line with wave-like transcriptional response to EGFR activation, with immediate and delayed early genes, as well as secondary response genes, which are regulated through multiple levels of feedback (41). Interestingly, the protein-level analysis of samples treated with gefitinib for 24 h included terms enriched at mRNA level at all three timepoints, indicating that the proteome-level analysis generated a phenotype-level summary of all transcriptional events.

**Fig. 2.**
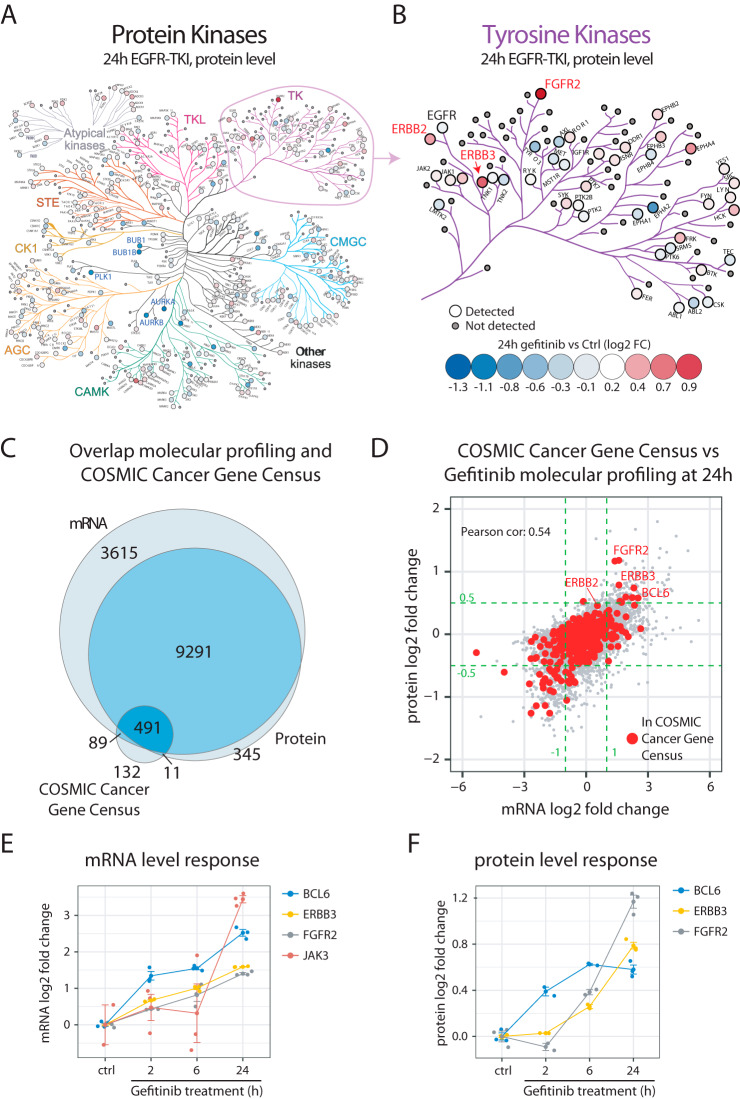
**EGFR-TKI treatment results in upregulation of proteins potentially involved in treatment escape.** *A*, Kinase regulation in response to EGFR-TKI treatment (gefitinib 2.5 μm, 24h) of A431 cells as measured at protein level. The map shows all kinases as visualized by the KinoViewer tool (30). Indicated in the map is also the fold regulation (blue-red scale). *B*, Zoom in on tyrosine kinases. Indicated in the map is EGFR (unchanged) as well as a few upregulated receptor tyrosine kinases with potential impact on therapy response (ERBB2, ERBB3 and FGFR2). *C*, Venn diagram indicating the overlap between the molecular response profiling and the COSMIC cancer gene census catalogue of genes that are causally implicated in cancer (43). *D*, Scatterplot showing the mRNA and protein level regulation at 24 h post treatment with gefitinib. Indicated in red are the COSMIC cancer gene census genes. Green dotted lines indicate the cutoffs used to define regulated mRNAs (log2 FC>±1), and proteins (log2 FC>±0.5). Indicated in the plot are a few upregulated cancer-associated genes potentially involved in EGFR-TKI treatment escape. *E*, mRNA level response to EGFR-inhibition by gefitinib shown for BCL6, ERBB3, FGFR2 and JAK3. *F*, Protein level response to EGFR-inhibition by gefitinib shown for BCL6, ERBB3 and FGFR2.

One of the previously shown non-genetic mechanisms of acquired resistance includes upregulation of alternative tyrosine kinases that can reactivate the downstream signaling after inhibition of a primary tyrosine kinase drug target such as EGFR (42). Evaluation of protein kinases in our data indicated that the protein levels of many kinases were altered already 24 h after EGFR inhibition (Fig. 2*A*). In some cases, the altered protein levels were a consequence of the cell cycle arrest accompanying EGFR inhibition, such as for example decreased level of the mitotic kinases PLK1, AURKA, and BUB1. Importantly however, the analysis also indicated increased protein levels of multiple receptor tyrosine kinases such as ERBB2, ERBB3, and FGFR2 that could potentially contribute to EGFR-TKI treatment escape (Fig. 2*B*). Overall, the transcriptomics analysis of kinase regulation supported the findings from the protein-level analysis but indicated upregulation of additional tyrosine kinases not covered by the protein-level analysis, such as JAK3 and ALK (supplemental Fig. S4).

The cellular response to EGFR-TKIs includes cell cycle inhibition, as well as induction of apoptosis. The regulation of cell cycle progression and apoptosis takes place at multiple different levels and includes large and complex networks of proteins and, importantly, deregulation of these processes is closely linked to cancer. To identify additional potential mechanisms of treatment escape in response to EGFR-TKIs, we therefore investigated regulation of genes causally linked to cancer according to the COSMIC Cancer Gene Census effort (43). Out of the 723 genes included in this list, 491 were identified and quantified at both mRNA and protein levels in our data, whereas 89 and 11 genes were present only at mRNA or protein levels respectively (Fig. 2*C* and supplemental Table S4). Out of these, 52 and 10 genes were here found significantly increased at mRNA level and protein level respectively 24 h after gefitinib treatment, with an overlap of 9 genes (Fig. 2*D*). Twenty-one of the genes that were found significantly increased in our analysis were also annotated as oncogenes in COSMIC, including FGFR2, ERBB3 and JAK3 as discussed above. Interestingly, this analysis also implicated upregulation of the BCL6 oncogene at both mRNA and protein level in response to gefitinib (Fig. 2*E*–2*F*). In conclusion, our profiling data clearly describes the direct and anticipated effects of EGFR inhibition, but in addition our more directed investigations suggest that some of the regulated genes may help cells escape the effects of therapy.

##### HTS Combination Drug Testing Indicate Synergistic Effects Between EGFR and FGFR2 or JAK3 Inhibition

The aim of this study was to identify potential targets for EGFR-TKI based combination therapy by analyzing the molecular response to EGFR-TKIs to better understand cellular treatment escape mechanisms. To examine the regulation of drug targets in general in response to EGFR-TKIs we used a recently published list of 667 proteins that are targets of FDA approved drugs (44). Three hundred eighty-one of these targets were identified and quantified at mRNA and/or protein level in our data, with 38 targets significantly upregulated at mRNA level only, and 4 targets (ERBB3, FGFR2, EPHA4, and HDAC5) at both protein and mRNA level (supplemental Fig. S5 and supplemental Table S5).

**Fig. 3.**
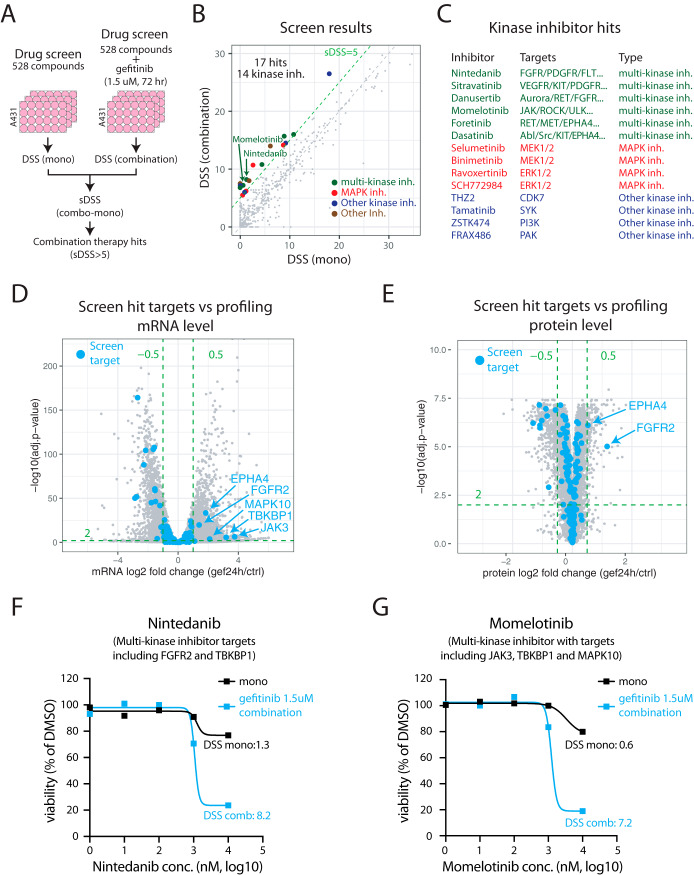
**EGFR-TKI combination therapy screen indicates synergistic effects between EGFR and FGFR2 or JAK3 inhibition.** *A*, Experimental setup for gefitinib combination therapy screen. *B*, Scatterplot showing drug sensitivity by drug sensitivity score (DSS) for monotherapy and combination therapy screens. Each point in the plot represent one compound in screen library. Indicated in the plot are the 17 hits from the combination therapy screen (sDSS>5), color coded by type. *C*, Kinase inhibitor screen hits color coded by type with targets indicated. *D*, Volcano plot indicating results from mRNA-level differential analysis comparing 24 h gefitinib treated samples to control samples. Highlighted in blue are targets of hits from the combination therapy screen with significantly upregulated mRNAs indicated. *E*, Volcano plot indicating results from protein-level differential analysis comparing 24 h gefitinib treated samples to control samples. Highlighted in blue are targets of hits from the combination therapy screen with significantly upregulated proteins indicated. *F*, Drug response data for the multi-kinase inhibitor nintedanib from the combination therapy screen. *G*, Drug response data for the multi-kinase inhibitor momelotinib from the combination therapy screen.

To evaluate potential combination therapies experimentally we performed an EGFR-TKI based combination therapy drug screen (Fig. 3*A*). Briefly, A431 cells were treated using a drug library including 528 different compounds, each in five different concentrations (the FIMM drug sensitivity and resistance test (DSRT) library (45, 46)). To generate a comprehensive compound target annotation, the original FIMM target annotation of the 528 library compounds was complemented in three ways. First, the FIMM annotation was complemented with target data available in the Selleckchem website (www.selleckchem.com) resulting in 985 targets for the 528 compounds in the library. Second, targets identified as high confident in a recent study investigating the target landscape of kinase targeting drugs was included (47), adding 2597 targets for 122 of the library compounds. Third, annotated targets from the DrugBank database were included (48) resulting in 973 targets for 215 library compounds (supplemental Fig. S6 and supplemental Table S6). The screen was performed both using the library compounds alone (mono), or in a combination with gefitinib at a fixed concentration of 1.5 μm (combination). As a readout of treatment effect on the cells we calculated the drug sensitivity score (DSS) as previously described (49), and for each compound a synergy score (sDSS) was also calculated (sDSS = DSS_combo_ − DSS_mono_). The analysis of the drug screen data indicated 17 compounds as combination therapy hits (cutoff of sDSS>5, Fig. 3*B*, supplemental Table S7). Interestingly, 14/17 screen hits (82%, Fig. 3*C*, supplemental Fig. S7) were kinase inhibitors indicating an enrichment of this compound class compared with others based on the library composition (255 kinase inhibitors in total, 48%). Many of the kinase inhibitor hits were multi-targeting kinase inhibitors that could potentially block cellular escape mechanisms driven by upregulation of alternative upstream kinases. Other hits were targeting the MAPK pathway downstream of EGFR, that could also inhibit escape mechanisms where switching to other upstream kinases is used.

Next, we investigated all reported targets of the drug screen hits in relation to mRNA and protein level regulation 24 h after gefitinib treatment. In total, 190 targets were annotated for these 17 compounds, and out of these targets 153 and 141 were identified and quantified at mRNA and protein levels respectively in our gefitinib profiling data (supplemental Table S8). Only five of these targets were significantly upregulated according to the defined thresholds in response to EGFR inhibition at either mRNA level (Fig. 3*D*) or protein level (Fig. 3*E*), FGFR2 and EPHA4 (both mRNA and protein level) and JAK3, TBKBP1, and MAPK10 (only covered by the mRNA level analysis). Out of these five targets, FGFR2 (targeted by nintedanib, Fig. 3*A*) and JAK3 (targeted by momelotinib, Fig. 3*G*) have a well described role in cancer and they are both annotated as hallmark oncogenes in COSMIC. In summary, our drug screen identified several kinase inhibitors that should be further evaluated in an EGFR-TKI based combination therapy setting. In addition, our profiling data revealed that several targets of these kinase inhibitors were upregulated in response to EGFR-TKI treatment, indicating potential involvement of these genes in EGFR-TKI treatment escape mechanisms.

##### BCL6 Silencing in EGFR-TKI Treated Cells Results in Increased Protein Levels of Multiple BCL6 Target Genes Including TP53

**Fig. 4.**
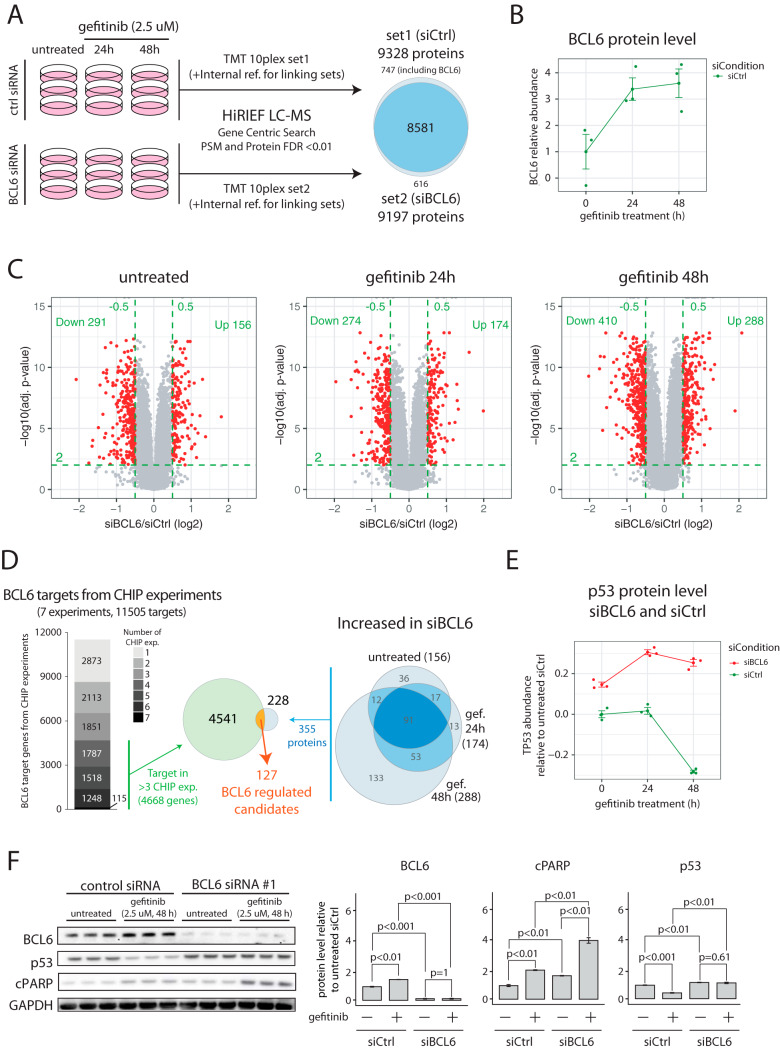
**MS profiling on BCL6 silenced EGFR TKI induced response.** *A*, Experimental setup for the proteomics profiling of BCL6-silencing effects in EGFR-TKI treated A431 cells. Cells were first transfected with non-targeting control siRNA or BCL6 siRNA for 24 h and were then treated with gefitinib for 24 h or 48 h or left untreated. Cells were collected and proteins were extracted for proteomics analysis. The right part of the figure shows the overlap in identified and quantified proteins between the two MS-experiments. *B*, Plot showing BCL6 protein levels in response to gefitinib treatment at 24 h and 48 h in cells treated with non-targeting control siRNA. BCL6 was not identified in cells treated with BCL6 siRNA. *C*, Volcano plots indicating results from proteomics differential analysis performed between cells treated with BCL6 siRNA and non-targeting control siRNA at different conditions (untreated, 24 h gefitinib and 48 h gefitinib). Green dotted lines indicate the cutoffs used to define regulated mRNAs (log2 FC>±0.5, adjusted *p* value<0.01). Indicated in each plot is also the number of regulated proteins. *D*, The left part shows public domain data from 7 different BCL6 CHIP experiments retrieved through the ChIPBase database (51). 4668 genes identified as BCL6 targets in at least 4 different experiments were considered for further investigation. The right Venn diagram indicates the 355 significantly upregulated proteins identified in the proteomics profiling experiment. The Venn diagram in the center shows the overlap at gene level, indicating 127 genes as BCL6 regulated candidates. *E*, Plot showing TP53 protein levels in response to gefitinib treatment at 24 h and 48 h in cells treated with non-targeting control siRNA or BCL6 siRNA. TP53 was identified as a BCL6 target gene in 5/7 CHIP experiments. *F*, Western blots showing BCL6, p53 and cleaved Caspase protein expression for A431 after siRNA silencing of BCL6 in untreated or gefitinib (48 h) treated cells. The bar plots indicate relative protein levels normalized to GAPDH as determined by densitometry analysis of Western blotting results. All quantifications were based on three independent experiments with barplot error bars indicating S.D. and *p* values calculated by Student's *t* test.

Neither the list of targets of FDA approved drugs nor the targets of the compounds tested in our combination therapy screen includes all potential or experimental drug targets. As an example, BCL6, one of the oncogenes that were found significantly increased at both mRNA and protein level after gefitinib treatment, was not present. To further investigate the potential of BCL6 as an EGFR-TKI based combination therapy target, we evaluated the impact of siRNA based BCL6 silencing on gefitinib response. Four different siRNAs targeting BCL6 were evaluated against non-targeting control siRNA in A431 cells for selection of an siRNA causing efficient BCL6 silencing (supplemental Fig. S8). Next we performed proteomics-based profiling of gefitinib response in A431 cells that were pretreated with either non-targeting control siRNA or BCL6 siRNA as illustrated in Fig. 4*A*. After harvesting cells in triplicates for each of the six conditions, proteins were extracted and digested into peptides followed by TMT 10plex labeling for relative quantification using two TMT-sets (TMTset1: siCtrl untreated/24 h gef/48 h gef and TMTset2: siBCL6 untreated/24 h gef/48 h gef). For comparison between TMT experiments, the 10th label in each TMTset was used for labeling an internal reference sample consisting of a pool of all the other 18 samples. HiRIEF LC-MS based profiling resulted in the identification and quantification of 9328 and 9197 proteins in set1 and set2 respectively with an overlap of 8581 proteins (gene centric search, protein and peptide FDR<1%, supplemental Table S9). BCL6 itself was only identified in TMT set1 (ctrl siRNA), where it was found increased in response to gefitinib treatment as expected (Fig. 4*B*). In total, the levels of 447, 448, and 698 proteins were altered in BCL6 silenced cells as compared with control cells in untreated, 24 h gefitinib and 48 h gefitinib treated cells respectively (Fig. 4*C*). For a general overview of the effects of BCL6 silencing, pathway enrichment analysis was performed based on the altered proteins compared with siCtrl treated cells in each of the three conditions. This analysis did not result in any significant pathway enrichment of in untreated cells. In cells treated with gefitinib for 24 h multiple pathway terms were enriched including several terms related to cell cycle regulation (supplemental Fig. S9*A*). Interestingly, in cells treated with gefitinib for 48h, the pathway enrichment analysis indicated altered p53 regulated transcription of genes involved in cell cycle arrest as well as apoptosis (supplemental Fig. S9*B*).

The described molecular function of BCL6 has primarily been that it acts as a transcriptional repressor (50), and hence we focused our attention on proteins with increased protein levels in BCL6 silenced cells compared with control cells. Interestingly, we could see an increasing number of proteins with higher level in BCL6 silenced cells over the treatment time course with 156, 174, and 288 significantly higher proteins in the untreated, gefitinib 24 h and gefitinib 48 h conditions respectively (Fig. 4*C*). This finding is in line with the increasing BCL6 levels detected after gefitinib treatment in control siRNA cells. The regulation in response to BCL6 silencing can be a direct effect of BCL6 binding to DNA resulting in transcriptional repression, or secondary effects. To investigate this further we downloaded data from seven different BCL6 ChIP (Chromatin Immuno-Precipitation) experiments available through the ChIPBase resource (51). ChIP analysis is used to identify target genes of DNA binding transcriptional regulators, and in total the seven experiments here analyzed identified 11,505 potential BCL6 target genes (Fig. 4*D*). To remove low confident targets, we focused our continued analysis on targets that were identified in at least four different ChIP experiments. Out of these 4668 genes, 127 showed significantly higher protein levels in BCL6-silenced A431 cells in at least one of the tested conditions (supplemental Table S10). BCL6 has been shown to target both tumor suppressors and oncogenes, thereby regulating the balance between proliferation and growth arrest, as well as between survival and apoptosis (52). Only six of the 127 candidate BCL6 regulated targets were linked to cancer according to COSMIC, including MYC and p53, both previously described as BCL6 target genes (52). We directed our further attention to p53 because it was implicated in the pathway enrichment analysis, and because it is a tumor suppressor, deeply involved in regulation of the cell cycle as well as apoptosis. p53 was decreased at the protein level 48h after gefitinib treatment in control siRNA cells, in agreement with p53 being a target for BCL6-dependent transcriptional repression (Fig. 4*E*). In BCL6 silenced cells however, this decrease in p53 level was not detected. The impact of BCL6 silencing on p53 levels after gefitinib treatment was validated using Western blotting (Fig. 4*F*). Further, gefitinib treatment in BCL6-silenced cells was associated with a stronger induction of apoptosis as assayed by cleaved PARP (Fig. 4*F*). These results are fully supported by previous reports where BCL6 was shown to suppress the expression of p53 (53). In summary, this proteomics analysis indicates that BCL6 upregulation in response to EGFR-TKIs results in inhibition of p53 transcription and apoptosis.

##### Inhibition of BCL6 Sensitizes NSCLC Cells to EGFR-TKI Treatment

**Fig. 5.**
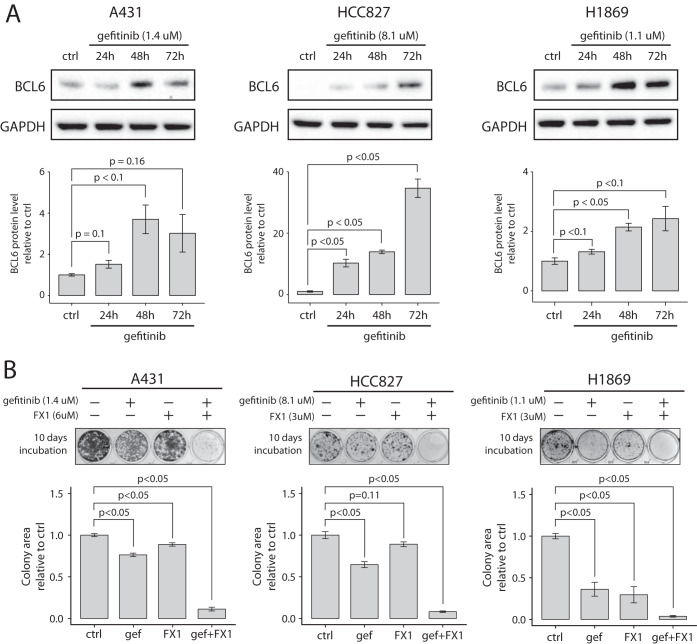
**Inhibition of BCL6 sensitizes NSCLC cells EGFR-TKI treatment** *A*, Western blots showing BCL6 protein expression for A431, HCC827 and H1869 cells untreated or treated with gefitinib for 24, 48, or 72 h. The barplots indicate relative protein levels normalized to GAPDH as determined by densitometry analysis of Western blotting results. *B*, Clonogenic assay results for A431, HCC827 and H1869 cells treated with gefitinib and the BCL6 inhibitor FX1, either alone or in combination for 10 days. Barplots indicate the results from colony area-based quantification as a measurement of cell survival. All quantifications were based on three independent experiments with barplot error bars indicating S.D. and *p* values calculated by Student's *t* test.

The drug response molecular profiling and the EGFR-TKI combination target discovery described above was performed using A431 cells (epidermoid carcinoma) as a model of EGFR signaling and EGFR inhibition. Further, BCL6 has been implicated as an oncogene primarily in B-cell malignancies such as diffuse large B-cell lymphoma (DLBCL) (53). To evaluate if BCL6 is expressed also in NSCLC we investigated BCL6 mRNA levels in 31 different cancer types using the cancer genome atlas (TCGA) PanCancer data set (54). As expected, this analysis indicated that the highest BCL6 expression was found in DLBCL samples, but also that relatively high BCL6 levels were seen in both lung adenocarcinoma and squamous cell carcinoma (supplemental Fig. S10). Inspired by this we investigated if BCL6 was regulated in response to EGFR inhibition also in NSCLC cells using three NSCLC cell lines in addition to A431 cells. HCC827 is a lung adenocarcinoma cell line harboring an activating mutation in EGFR (exon 19 deletion (55)), H1869 is a squamous cell carcinoma cell line with wild-type EGFR previously reported to be sensitive to EGFR-TKIs (56), and H1666 is a lung adenocarcinoma cell line also with wild-type EGFR and reported to be sensitive to EGFR-TKIs (57). Cells were either left untreated or treated for 24 h, 48 h, or 72 h with gefitinib at a concentration corresponding to an estimated cell line specific EC_50_ value. In three of the cell lines (A431, HCC827, and H1869), the BCL6 protein level gradually increased after gefitinib treatment indicating that the findings in A431 cells were true also in some NSCLC cells (Fig. 5*A* and supplemental Fig. S11*A*). However, no increase in BCL6 level was identified in H1666 cells indicating that the results are not generalizable to all NSCLC cells (supplemental Fig. S11*B*).

Our initial molecular response profiling, as well as our BCL6 silencing experiments suggested that BCL6 was a potential EGFR-TKI escape mechanism. Consequently, co-targeting of EGFR and BCL6 should result in synergy and increased drug-induced cell killing. For BCL6 inhibition, only pre-clinical drugs are available including two small molecular inhibitors 79–6 (58) and FX1 (59), and a peptidomimetic inhibitor RI-BPI (60). To investigate if combination therapy targeting EGFR and BCL6 would be more effective in killing cancer cells than monotherapy we treated all three cell lines with gefitinib and FX1, alone or in combination, and evaluated survival using clonogenic assay. Gefitinib and FX1 concentrations for each cell line were estimated to cell line specific EC_50_ values based on pilot experiments (data not shown). Importantly, significant drug synergy was observed in all three cell lines, and in addition, the combination treatment resulted in a near complete ablation of cells in all three cell lines tested (Fig. 5*B* and supplemental Fig. S12).

## DISCUSSION

Here we have used in-depth transcriptomics and proteomics data to investigate the immediate molecular effects of EGFR-TKIs and demonstrate that multiple distinct pathways are activated through upregulation of key pathway components including ERBB2/ERBB3, FGFR2, JAK3 and BCL6 already within 24 h after treatment initiation. Each of these pathways has the potential to attenuate the cytostatic/cytotoxic effects of EGFR-TKIs resulting in reduced efficacy of the treatment. Further, using drug screening we could show synergistic effects when treating cells with gefitinib in combination with kinase inhibitors with targets including FGFR2 and JAK3, supporting this approach in a combination therapy setting. Finally, we investigated the role of BCL6 in response to EGFR inhibition, and showed that gefitinib-induced BCL6 upregulation results in transcriptional repression of multiple target genes including p53, ultimately leading to increased survival. Importantly, combined targeting of EGFR using gefitinib and BCL6 using FX1 produced strong synergy and effective killing of NSCLC cells.

BCL6 has previously been demonstrated as a drug resistance mechanism through protective feedback signaling in response to the BCR-ABL1 targeting TKI imatinib in leukemia cells (61). It was shown that imatinib treatment caused upregulation of BCL6, resulting in reduced transcription of the BCL6 target genes including TP53 and CDKN1A. Combined treatment using BCR-ABL inhibitor and the peptidomimetic BCL6 inhibitor RI-BPI resulted in synergistic effects both *in vitro* and in xenograft models. Also supporting our current findings, it has been shown that BCL6 depletion in glioblastoma cells sensitized these cells to the EGFR-TKI erlotinib (62). Further, BCL6 was suggested as a drug target in combination with STAT3 in NSCLC cells (63), as well as in breast cancer cells (64). Intriguingly, our data suggests that both BCL6 and STAT3 signaling (through JAK) contribute to escape from EGFR-TKI response.

The molecular function of BCL6 and its role in cancer has mainly been described in B-cell lymphomas, but the importance of BCL6 as an oncogene also in other malignancies is currently gaining more attention (50). In normal physiology BCL6 is upregulated during the humoral immune response to allow for massive expansion and maturation of B-cells in germinal centers of secondary lymphoid organs. BCL6 mediates its effect through binding to and repressing the transcription of hundreds of different target genes, resulting in increased proliferation and reduced DNA damage sensing, which is needed to allow for hypermutation of the B cell antibody genes. This function of BCL6 also explains why uncontrolled expression of BCL6 is oncogenic (52). It has been shown that the BCL6 target genes are different in B-cell lymphoma and breast cancer cells, indicating cell-type specific activity of BCL6 (64). Our analysis here contributes a list of 127 candidate BCL6 targets based on our own silencing experiment, as well as previously performed ChIP experiments. More specifically, we show that BCL6 is upregulated in response to EGFR inhibition, resulting in reduced p53 transcription and inhibition of apoptosis. We cannot rule out that other BCL6 target genes of the many here suggested are contributing to the cellular effects of BCL6 upregulation in response to EGFR inhibition, and the complete investigation of BCL6 in this setting warrants further investigation. The data presented here indicates for the first time that BCL6 should be evaluated as a combination therapy target together with EGFR-TKIs in NSCLC.

Even though transcriptional regulators in general are poor drug targets, structural characterization of BCL6-corepressor complexes indicated that BCL6 could be druggable (50). Indeed, several different BCL6 inhibitors have been developed including peptidomimetics such as RI-BPI (60) and small-molecule inhibitors including 79–6 (58) and the more potent FX1 (59). Importantly, all of these inhibitors act by blocking the interaction between BCL6 and its co-repressors, resulting in re-expression of BCL6 target genes, proliferation arrest and apoptosis in B-cell lymphoma xenografts. In addition, it was shown using *ex vivo* screening that BCL6 inhibitors were active in killing primary human B-cell lymphoma cells (58–60).

Increased signaling through alternative EGFR family members ERBB2 and ERBB3 has previously been recognized as resistance mechanisms in different types of cancer including NSCLC (65–67). Our data supports this, as increased levels of ERBB2 and ERBB3 at both mRNA and protein level are observed already 24h after EGFR inhibition, indicating a rapid transcriptional feedback mechanism. In line with this, simultaneous inhibition of EGFR/ERBB2 by the dual targeting inhibitor afatinib is more effective than gefitinib in patients with EGFR mutant NSCLC (68), and afatinib is now the standard first-line therapy in patients with activating EGFR mutations.

FGFR signaling has also been proposed previously as an EGFR-TKI treatment escape mechanism and therefore suggested as a combination therapy target (69) (70). Our data supports also these findings and indicate that combined targeting of EGFR by gefitinib and FGFR by the multi-kinase targeting drug nintedanib increases the treatment effect. In NSCLC, nintedanib was shown effective in a second line setting in combination with docetaxel in patients after progression on platinum-based chemotherapy (71), and is now approved by the European medical agency (EMA). Selective FGFR2 inhibitors have so far not reached the clinic, but an allosteric inhibitor specific for FGFR2, alofanib, has been developed, showing antitumor activity in preclinical models (72). Our data support a clinical evaluation of combined targeting of EGFR and FGFR2, which would be feasible now with nintedanib approved for use in NSCLC.

The JAK-STAT pathway has also been implicated previously as an escape mechanism for EGFR targeting therapy. The JAK family of kinases are implicated in cancer through phosphorylation and activation of the STAT family of transcription factors, most notably STAT3 or STAT5, resulting in increased tumor cell proliferation and survival (73, 74). Importantly, Lee and coworkers showed that inhibition of oncogenic RTK/MAPK signaling activated both FGFR-PI3K and IL6-JAK pathways resulting in STAT3 activation and treatment escape (70). Previous research around the involvement of JAK-STAT in resistance to EGFR-TKIs has focused on JAK1 and JAK2, and the role of JAK3 in solid malignancies is less well described. JAK3 has been shown expressed primarily in hematopoietic cells and is frequently mutated in T-lineage acute lymphoblastic leukemia (75). The data here presented suggests that transcriptional upregulation of JAK3 could be part of a JAK-STAT dependent EGFR-TKI resistance mechanism.

Immediate adaptation to EGFR inhibition as demonstrated here would likely result in complete lack of efficacy of EGFR-TKIs as monotherapy in a clinical setting. Importantly, EGFR-TKIs could still contribute benefit to the patient in a combination therapy setting, given that the correct combination therapy target is identified. Further, our analysis show that multiple survival mechanisms act in concert to reduce the effect of EGFR inhibition, which poses the question of how many drugs we need to combine in order to kill the cancer cells. Incomplete killing of the cancer cells produces a selective pressure that will contribute to the clonal expansion of resistant cells already present in the tumor, or to generation of *de novo* resistant clones through genetic alterations (76). To cure cancer, such opportunities need to be blocked, and finding the correct combinations of targeted therapies is crucial to achieve this goal.

## DATA AVAILABILITY

The mass spectrometry proteomics data have been deposited to ProteomeXchange Consortium via the PRIDE (38) partner repository with the data set identifier PXD016605. All other data supporting the finding of the study are available from the corresponding authors on request.

## Supplementary Material

Supplementary Figures

Supplementary Tables

Annotated spectra

## Footnotes

^1^ The abbreviations used are:

EGFR: epidermal growth factor receptor
NSCLC: non-small cell lung cancer
TKIs: tyrosine kinase inhibitors
RTKs: receptor tyrosine kinases
MAPK: Mitogen-activated protein kinase
mTOR: mammalian target of rapamycin
DSRT: drug sensitivity and resistance testing.
