# Supplementary material for: Immediate Adaptation Analysis Implicates BCL6 as an EGFR-TKI Combination Therapy Target in NSCLC
Source: Mol Cell Proteomics. 2020 Mar 30;19(6):928–43. doi: 10.1074/mcp.RA120.002036 (PMC7261823; doi:10.1074/mcp.RA120.002036)
Supplement: Supplementary Figures [file 159477_0_supp_492406_q74q99.pdf]

Supplementary Figures related to:

Immediate adaptation analysis implicates BCL6 as an EGFR-TKI combination therapy target in NSCLC

Yan Zhou Tran[1], Rezan Minozada[1], Xiaofang Cao[1], Henrik J. Johansson[1], Rui M. Mamede Branca[1], Brinton Seashore-Ludlow[1], Lukas M. Orre[1$]

[1]Department of Oncology-Pathology, Science for Life Laboratory, Karolinska Institutet, Stockholm, Sweden.

[$]Correspondence should be addressed to lukas.orre@ki.se

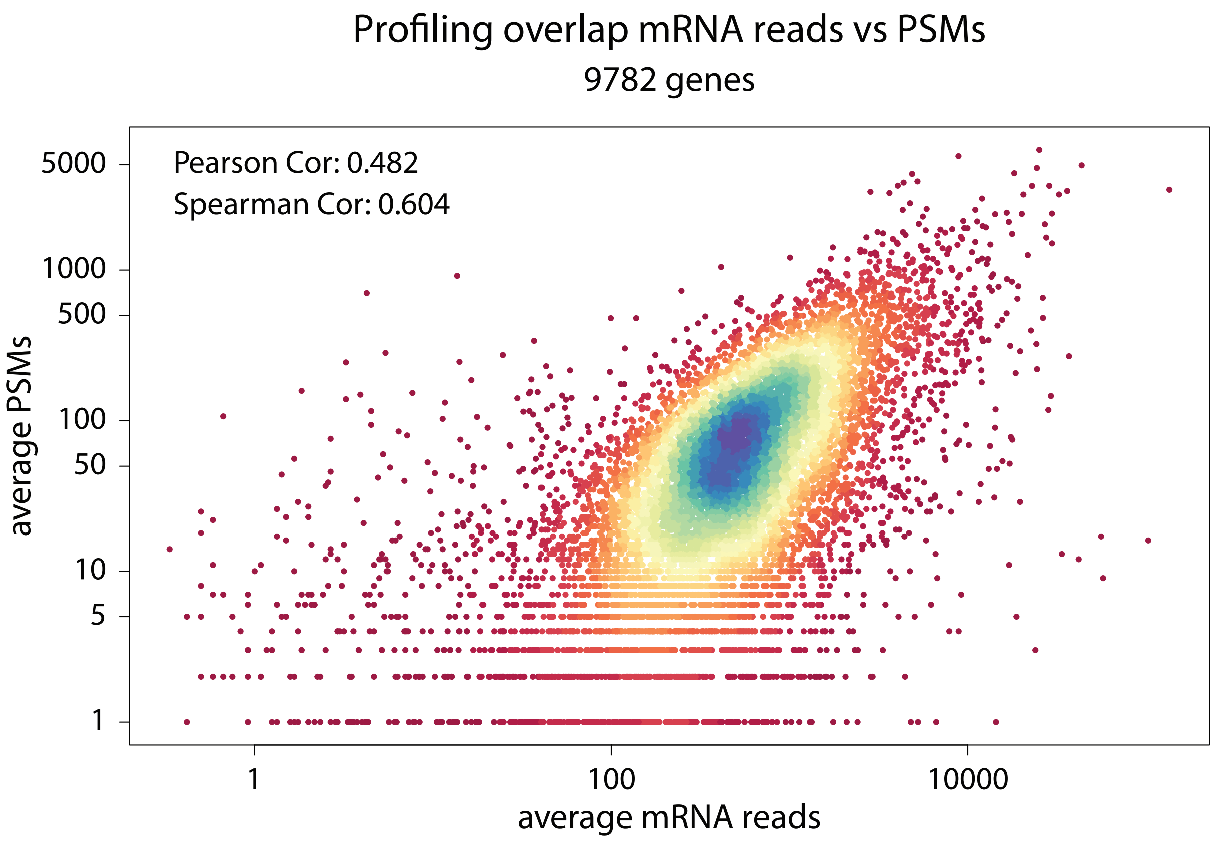

Supplementary Figure 1. mRNA and protein abundance estimation for profiling overlap. Scatterplot showing for each gene the average number of mRNA reads across the 12 samples analyzed by RNA sequencing and the average number of PSMs used for quantification in MS-based proteomics. Indicated in the plot is also the Pearson and Spearman correlation between the two measures across the 9782 genes.

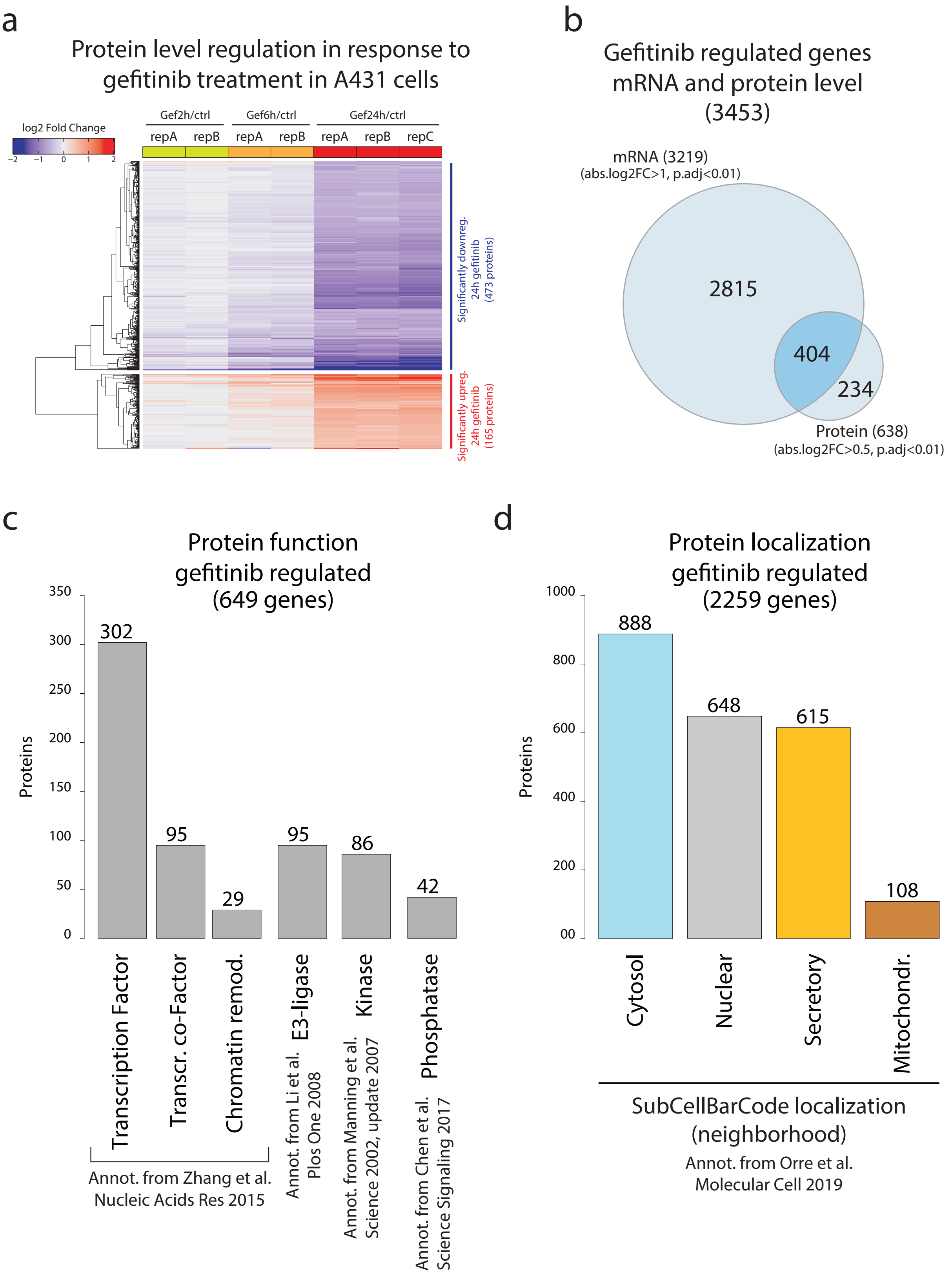

Supplementary Figure 2. Evaluation of significant regulation after EGFR inhibition. **a.** Heatmap showing hierarchical clustering of proteins in A431 cells treated with gefitinib at different timepoints (2h, 6h and 24h). Proteins significantly regulated (abs. log2 FC > 0.5 and p. adj. < 0.01) 24h after gefitinib treatment were selected

and visualized in the figure. All the treated samples are shown as a ratio to the mean of the 3 untreated samples (ctrl). The distance matrix for the clustering was calculated using Pearson correlation coefficient. **b.** Gene-centric overlap of significant mRNA and protein level regulation in response to gefitinib treatment. **c.** Barplot indicating functional annotation for significantly regulated mRNAs and proteins. Annotations were taken from resources indicated in the figure. **d.** Barplot indicating subcellular localization of proteins encoded by genes significantly regulated after gefitinib treatment. Subcellular localization information was retrieved from the SubCellBarCode resource.

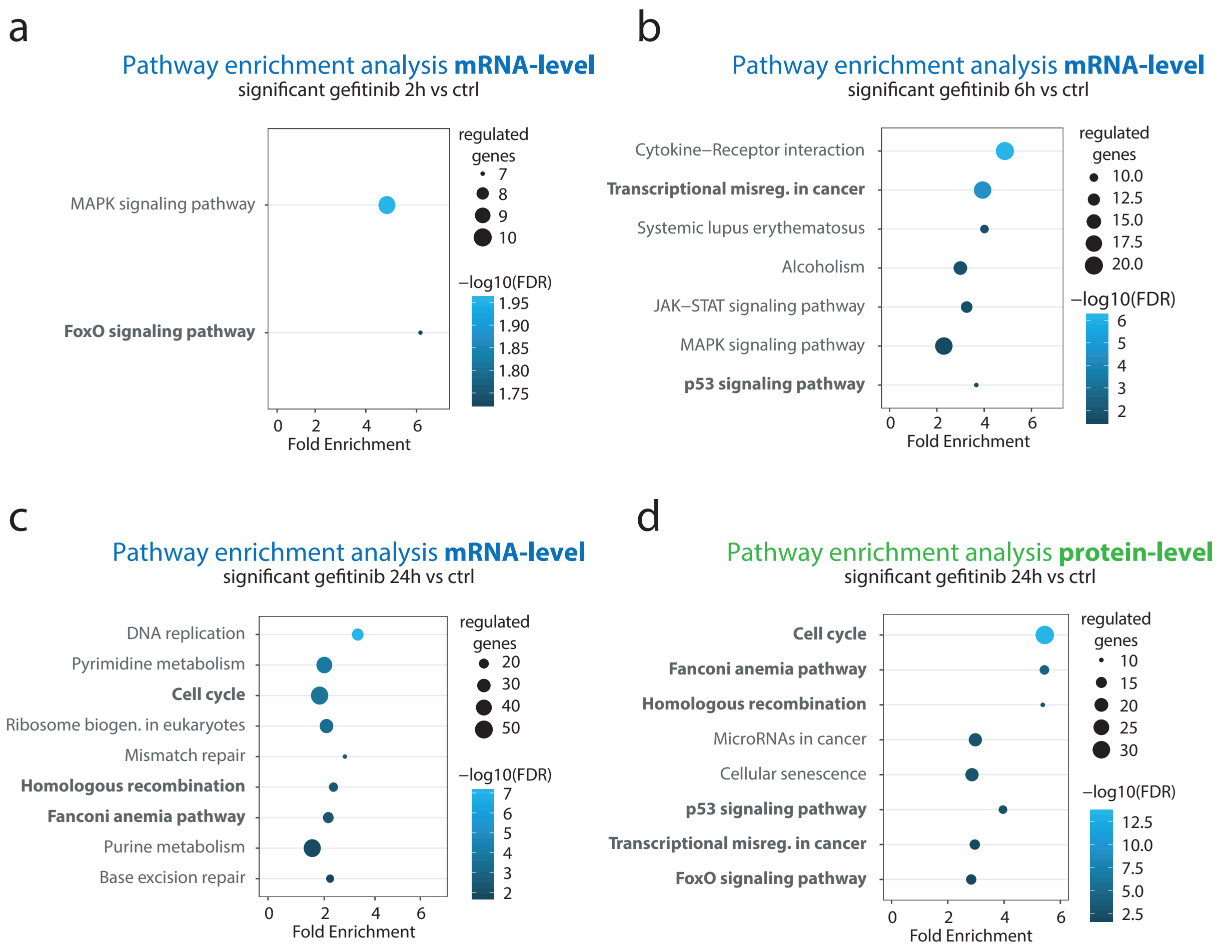

Supplementary Figure 3. Pathway enrichment analysis of significant gefitinib induced effects at mRNA and protein levels. a. mRNA-level KEGG pathway enrichment analysis of significant regulated genes 2h after gefitinib treatment. **b.** mRNA-level pathway analysis of significant regulated genes 6h after gefitinib treatment. **c.** mRNA-level pathway analysis of significant regulated genes 24h after gefitinib treatment. **d.** protein-level pathway analysis of significant regulated genes 24h after gefitinib treatment. For each plot the size of bubbles represents the number of regulated genes in the enriched pathway, the color represents the FDR of the enrichment calculated using Benjamini & Hochberg method. Indicated in the x-axis is the fold of the enrichment.

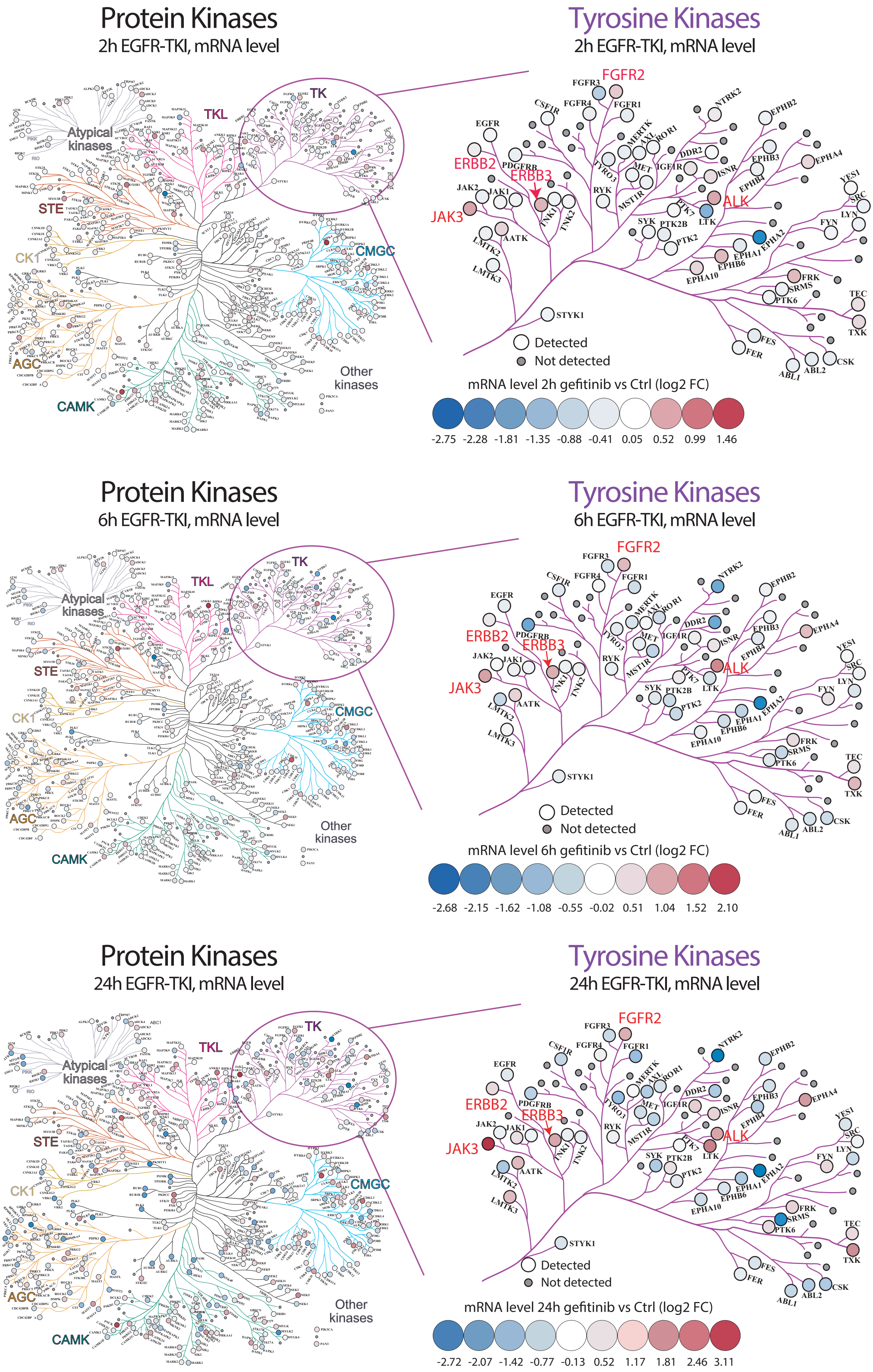
Protein Kinases
2h EGFR-TKI, mRNA level
Tyrosine Kinases
2h EGFR-TKI, mRNA level
Atypical kinases
TKL
TK
STE
CK1
CMGC
AGC
CAMK
Other kinases
FGFR2
ERBB2
ERBB3
JAK3
ALK
Detected
Not detected
mRNA level 2h gefitinib vs Ctrl (log2 FC)
-2.75 -2.28 -1.81 -1.35 -0.88 -0.41 0.05 0.52 0.99 1.46
Protein Kinases
6h EGFR-TKI, mRNA level
Tyrosine Kinases
6h EGFR-TKI, mRNA level
Detected
Not detected
mRNA level 6h gefitinib vs Ctrl (log2 FC)
-2.68 -2.15 -1.62 -1.08 -0.55 -0.02 0.51 1.04 1.52 2.10
Protein Kinases
24h EGFR-TKI, mRNA level
Tyrosine Kinases
24h EGFR-TKI, mRNA level
Detected
Not detected
mRNA level 24h gefitinib vs Ctrl (log2 FC)
-2.72 -2.07 -1.42 -0.77 -0.13 0.52 1.17 1.81 2.46 3.11

Supplementary Figure 4. mRNA-level kinase mapping after gefitinib treatment. Kinase regulation in response to EGFR-TKI treatment (2h, 6h, or 24h) of A431 cells as measured at mRNA level. Left maps show all kinases as visualized by the KinoViewer tool, and right maps show a zoom in on tyrosine kinases. Indicated in the maps are also the fold regulation (blue-red scale), as well as a few upregulated receptor tyrosine kinases with potential impact on therapy response (ERBB2, ERBB3 and FGFR2) and two additional tyrosine kinases (ALK, JAK3) that were not covered in the protein level analysis (**Figure 2a-b**).

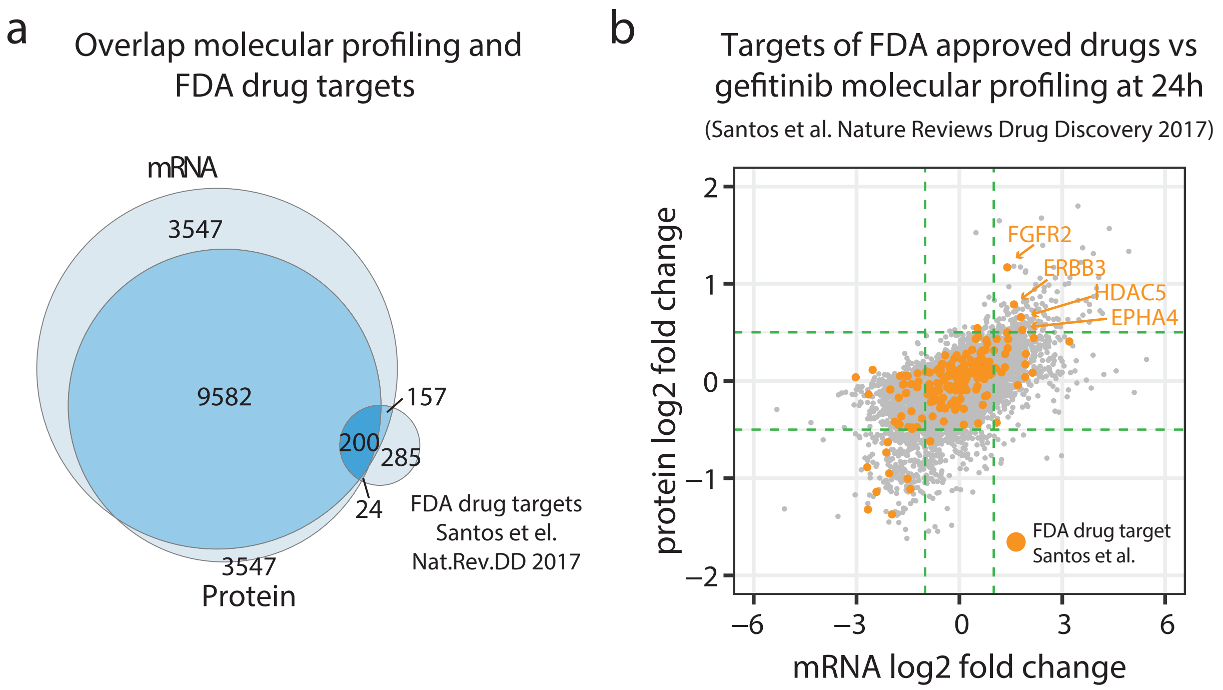

Supplementary Figure 5. Overlap between molecular response profiling and FDA drug targets reveals potential EGFR-TKI combination targets. a. Venn diagram indicating the overlap between the molecular response profiling and FDA drug targets. **b.** Scatterplot showing the mRNA and protein level regulation at 24h post treatment with gefitinib. Indicated in blue are the previously reported FDA drug targets. Red dotted lines indicate the cutoffs used to define regulated mRNAs (abs. log2 FC>1), and proteins (abs. log2 FC>0.5). Indicated in the plot are a few upregulated genes with a potential as EGFR-TKI combination therapy targets.

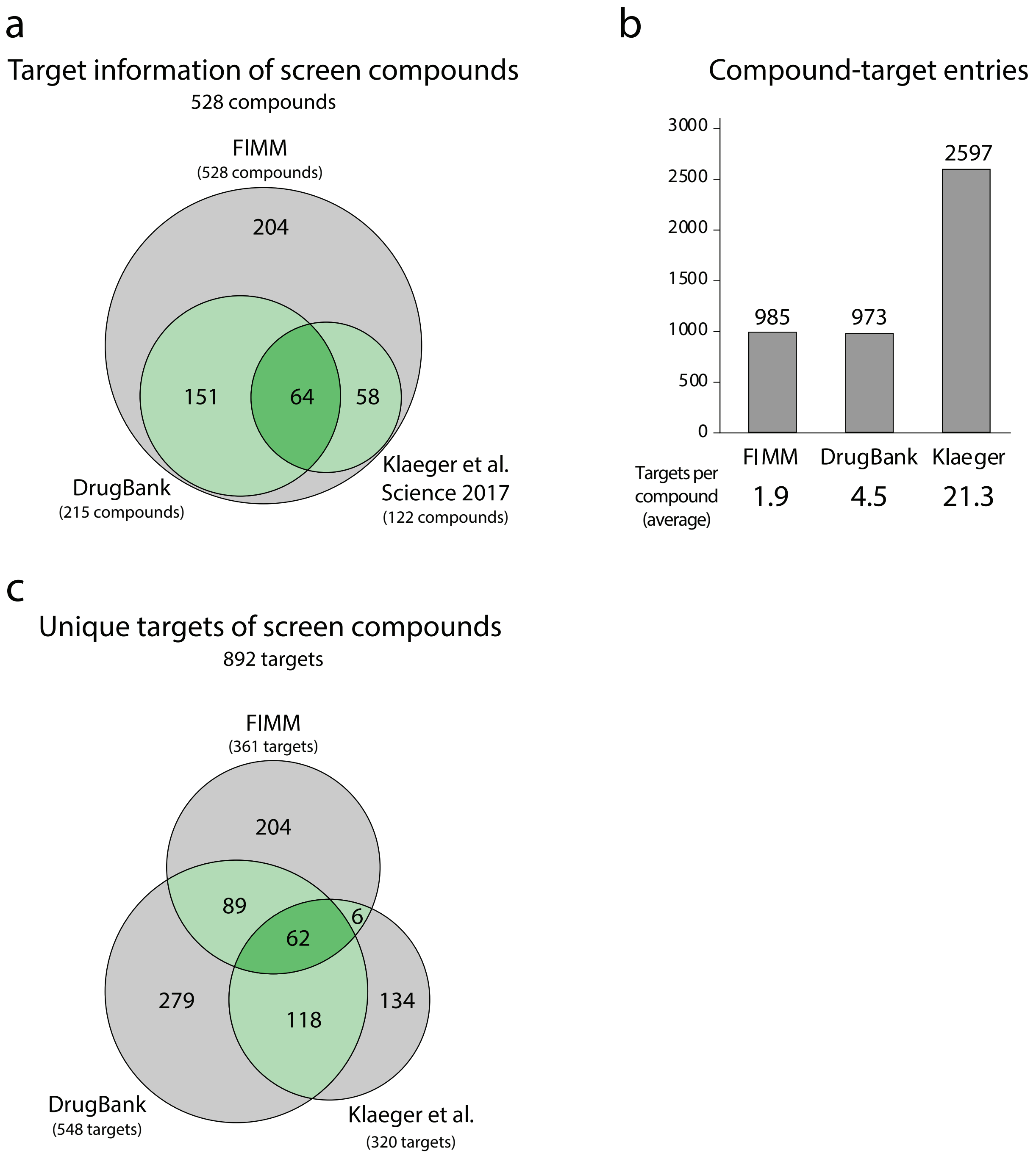

Supplementary Figure 6. Screening library target annotation. a. Venn diagram showing target informatin sources for the 528 compounds in the drug library used for EGFR-TKI combination therapy screens. **b.** Barplot showing the total drug-target entries for the three resources. **c.** Venn diagram showing the number of unique drug targets for the compounds in the screen and the overlap between sources.

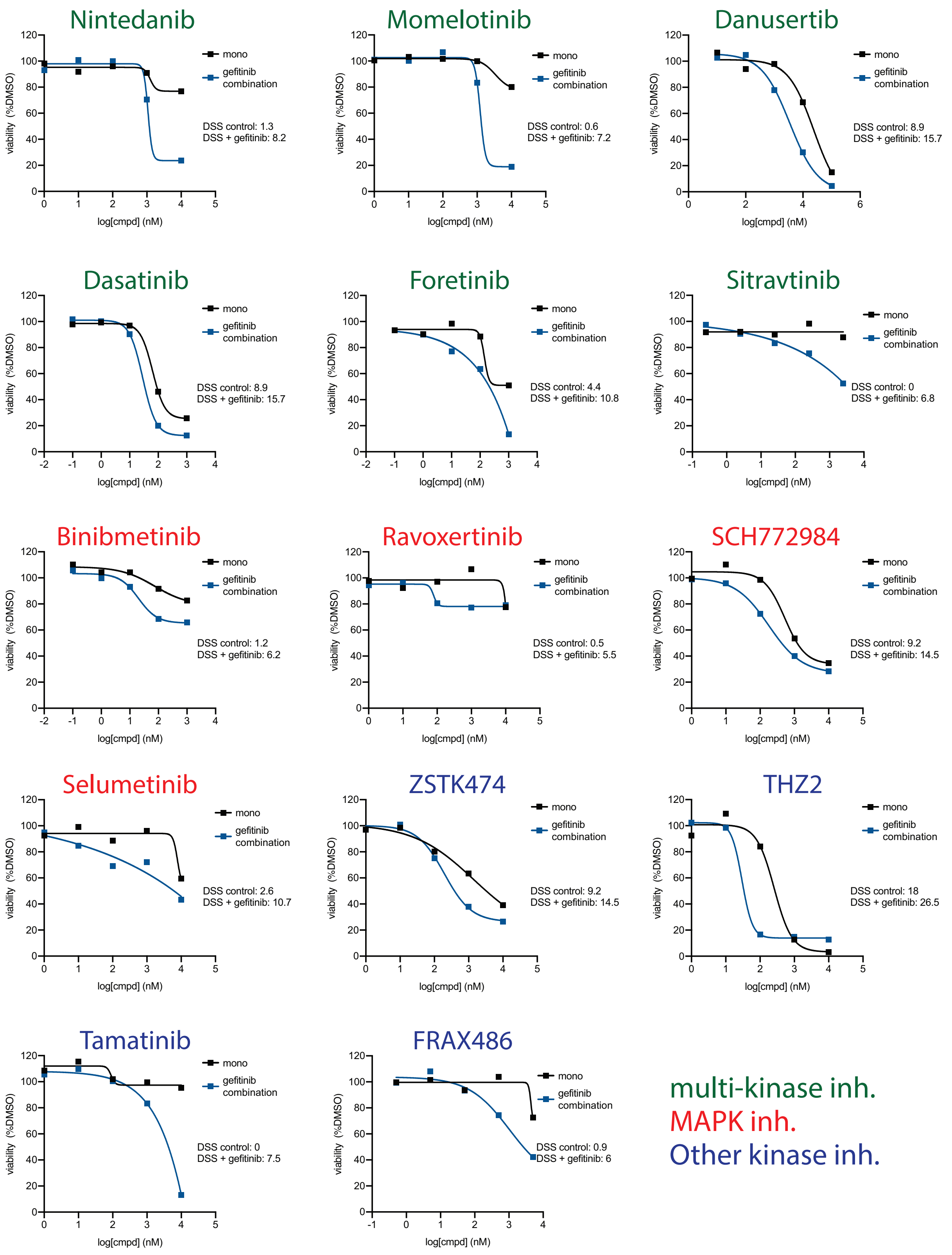

Supplementary Figure 7. Kinase inhibitor hits from the EGFR-TKI combination therapy screen. Drug response data from the combination therapy screen. Indicated in the plots are the drug sensitivity scores (DSS) for the monotherapy as well as for the combination with gefitinib.

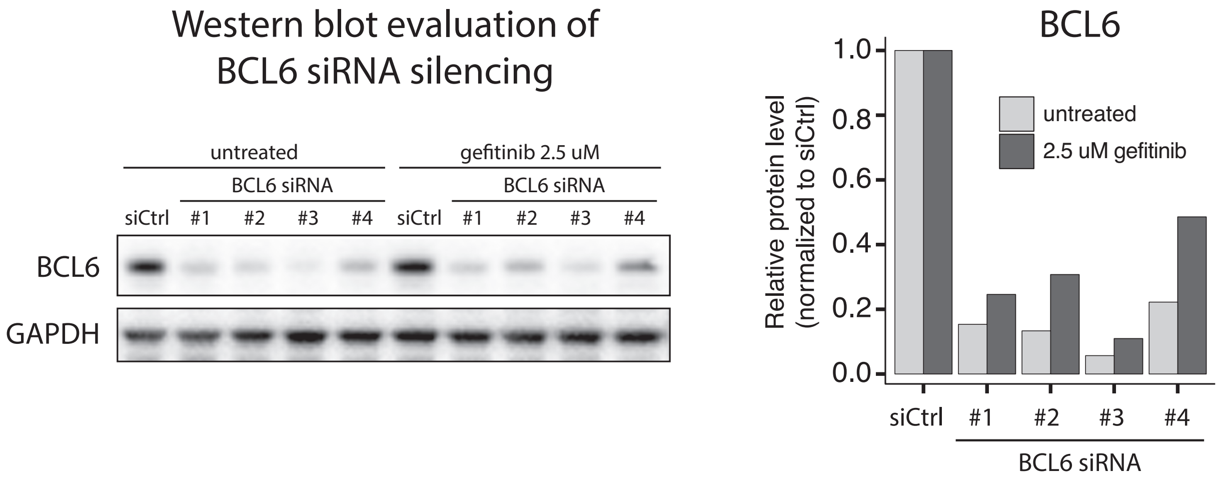

Supplementary Figure 8. Evaluation of silencing effect for different BCL6 targeting siRNAs. The left figure shows a western blot analysis of BCL6 protein expression in A431 cells after transfection with non-targeting control siRNA or one of four different BCL6 targeting siRNAs either in untreated cells or combined with 48 hours of gefitinib treatment. The right part show a bar plot indicating the relative BCL6 protein levels normalized to GAPDH as determined by densitometry analysis of western blot results.

a

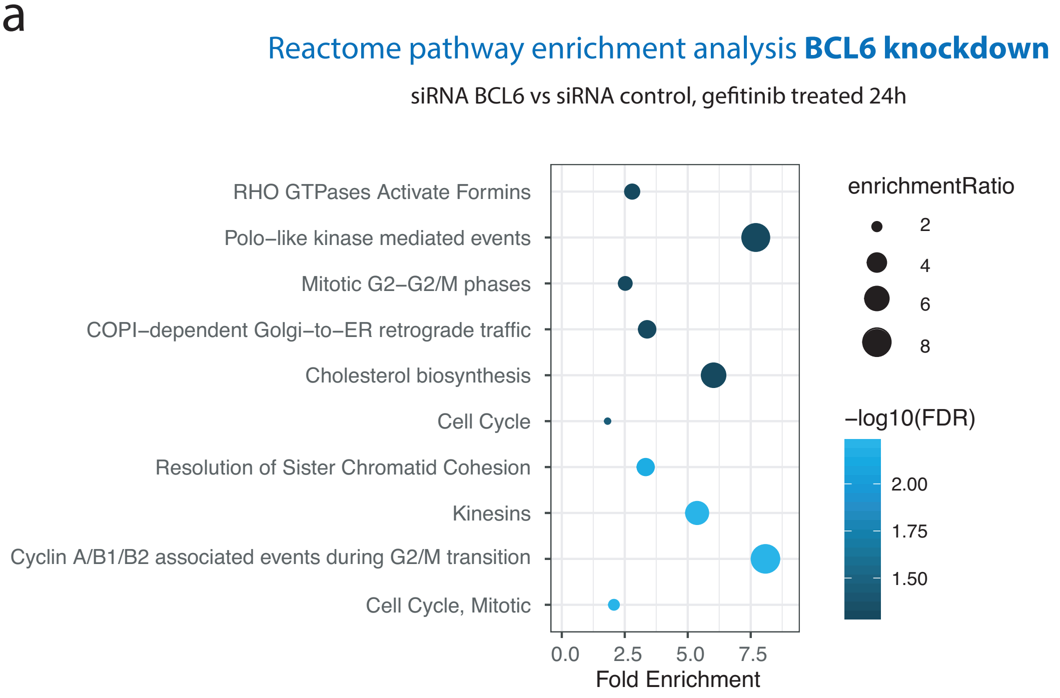

b

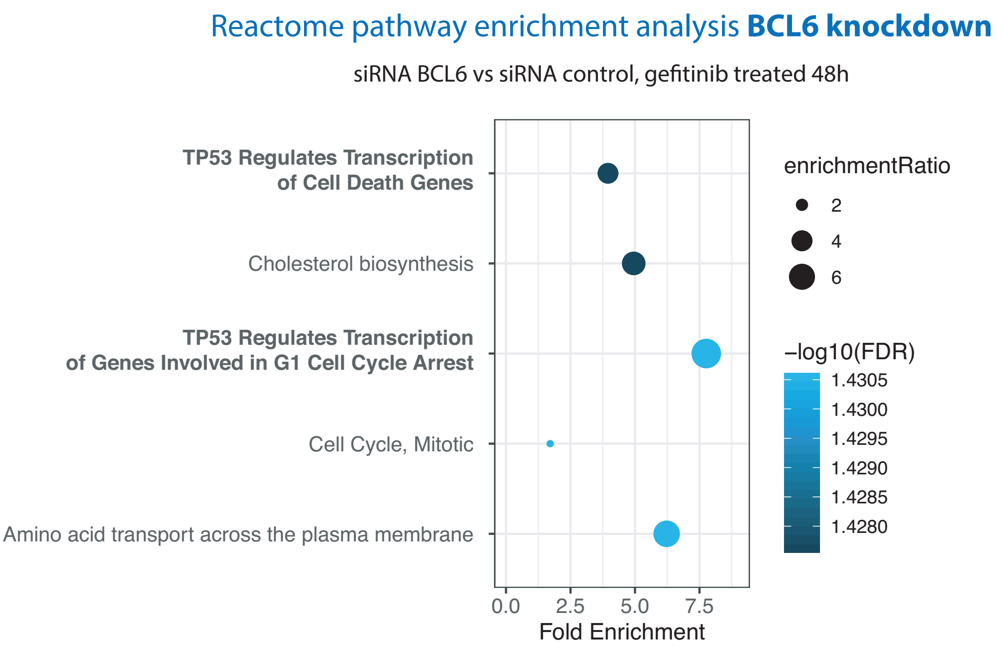

Supplementary Figure 9. Pathway enrichment analysis for evaluation of BCL6 silencing impact. a. Pathway analysis of significantly regulated proteins in BCL6 siRNA cells compared to siCtrl cells 24h after gefitinib treatment. **b.** Pathway analysis of significant regulated proteins in BCL6 siRNA cells compared to siCtrl cells 48h after gefitinib treatment. For each plot the size of bubbles represents the number of regulated genes in the

enriched pathway, the color represents the FDR of the enrichment calculated using Benjamini & Hochberg method. Indicated in the x-axis is the fold of the enrichment.

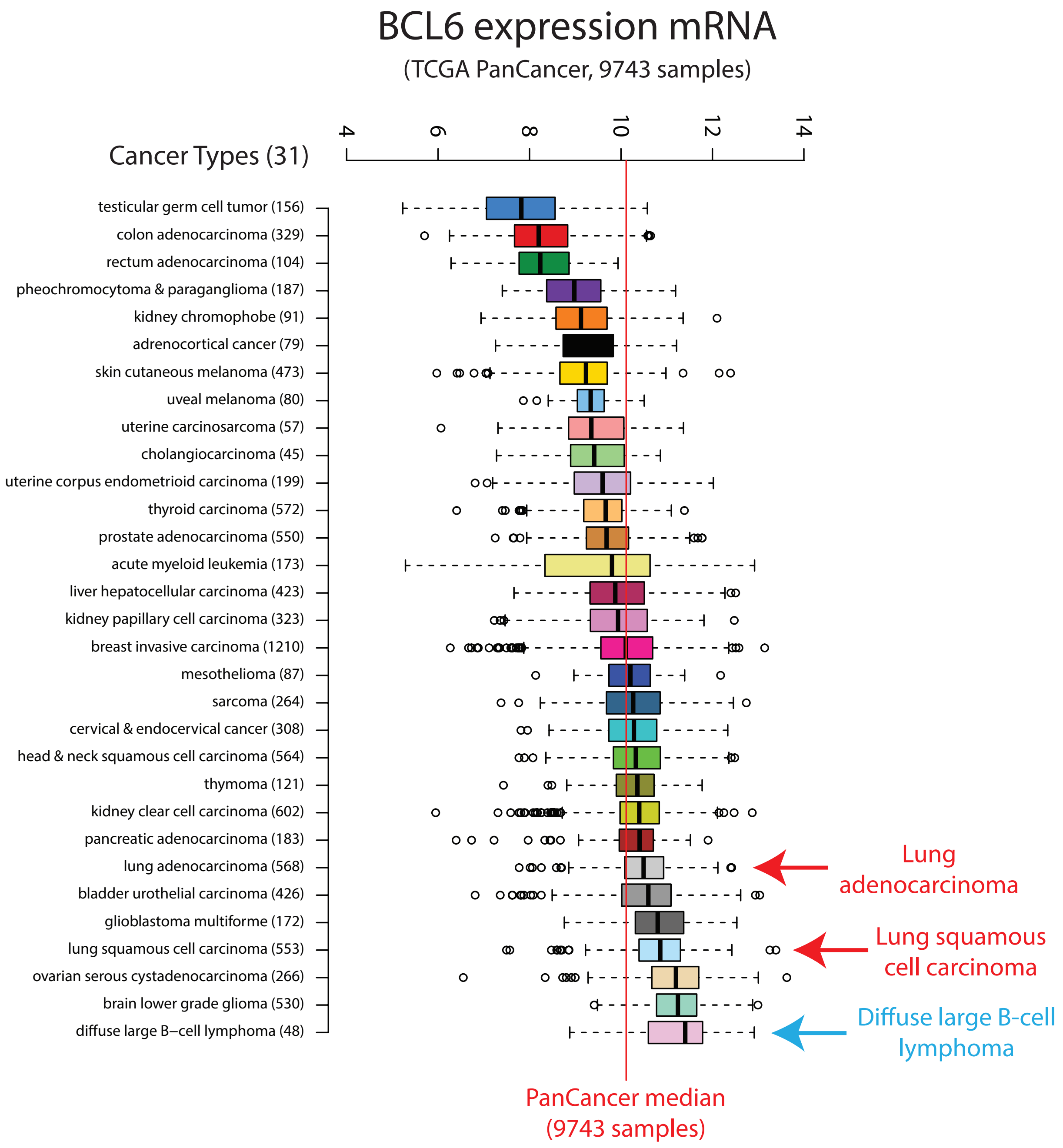

Supplementary Figure 10. Evaluation of BCL6 expression across different cancer types. Boxplot indicating the mRNA expression level of BCL6 across 31 different cancer types according to The Cancer Genome Atlas (TCGA) PanCancer dataset. Indicated in the plot is Diffuse large B-cell lymphoma (DLBCL), the cancer type with the highest BCL6 expression, as well as the NSCLC histological types adenocarcinoma and squamous cell carcinoma. Indicated is also the median BCL6 expression across all 9743 samples included in the dataset.

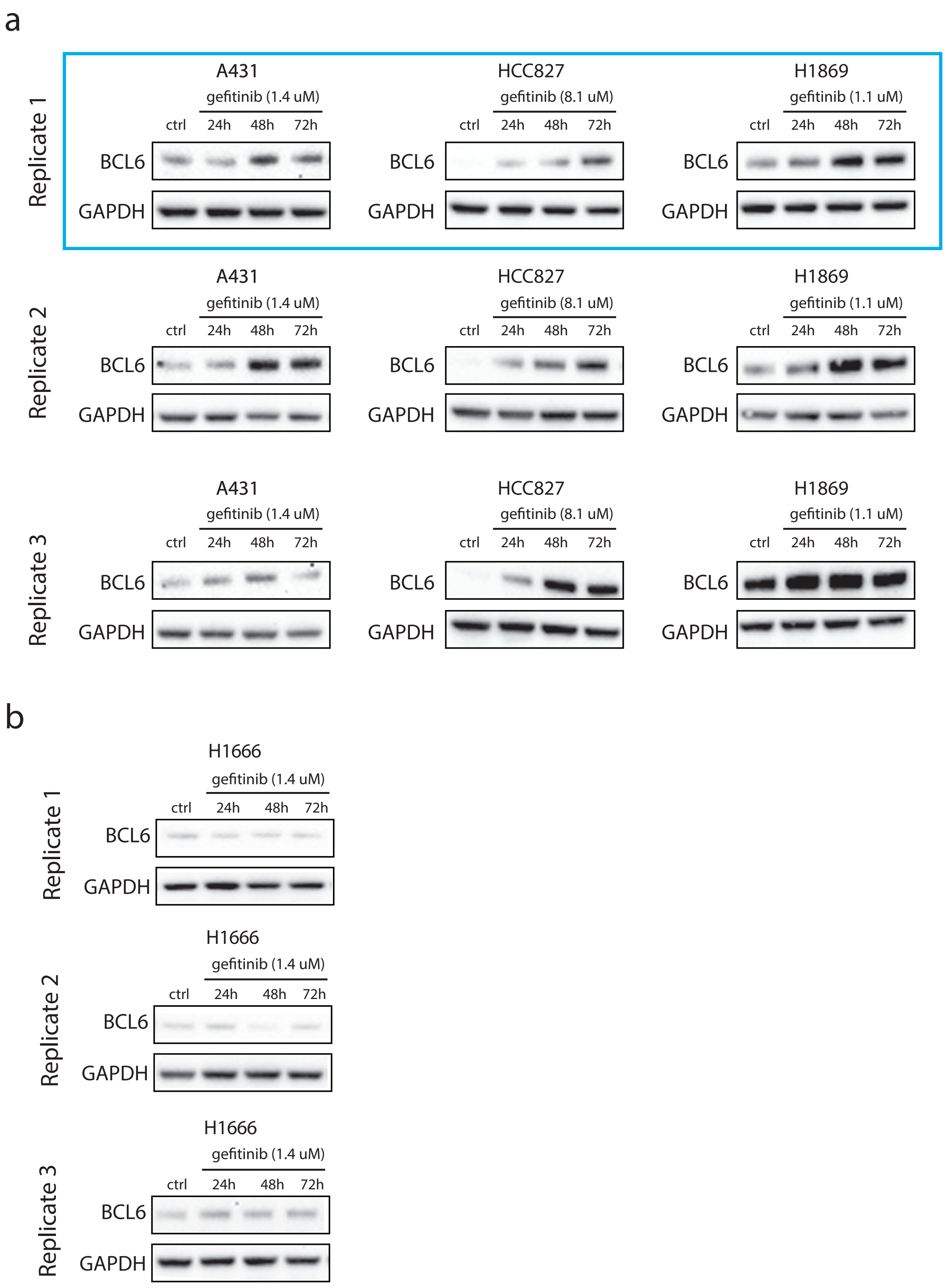

Supplementary Figure 11. Validation of BCL6 upregulation after EGFR-TKI treatment in NSCLC cells. a. Western blots showing BCL6 protein expression for A431, HCC827 and H1869 cells untreated or treated with gefitinib for

24, 48, or 72 hours. Replicate 1 (selected by blue box) from all 3 cell lines were used in **Figure 5a** as representative result of each western blot. **b.** Western blots showing BCL6 protein expression for H1666 cells untreated or treated with gefitinib for 24, 48, or 72 hours.

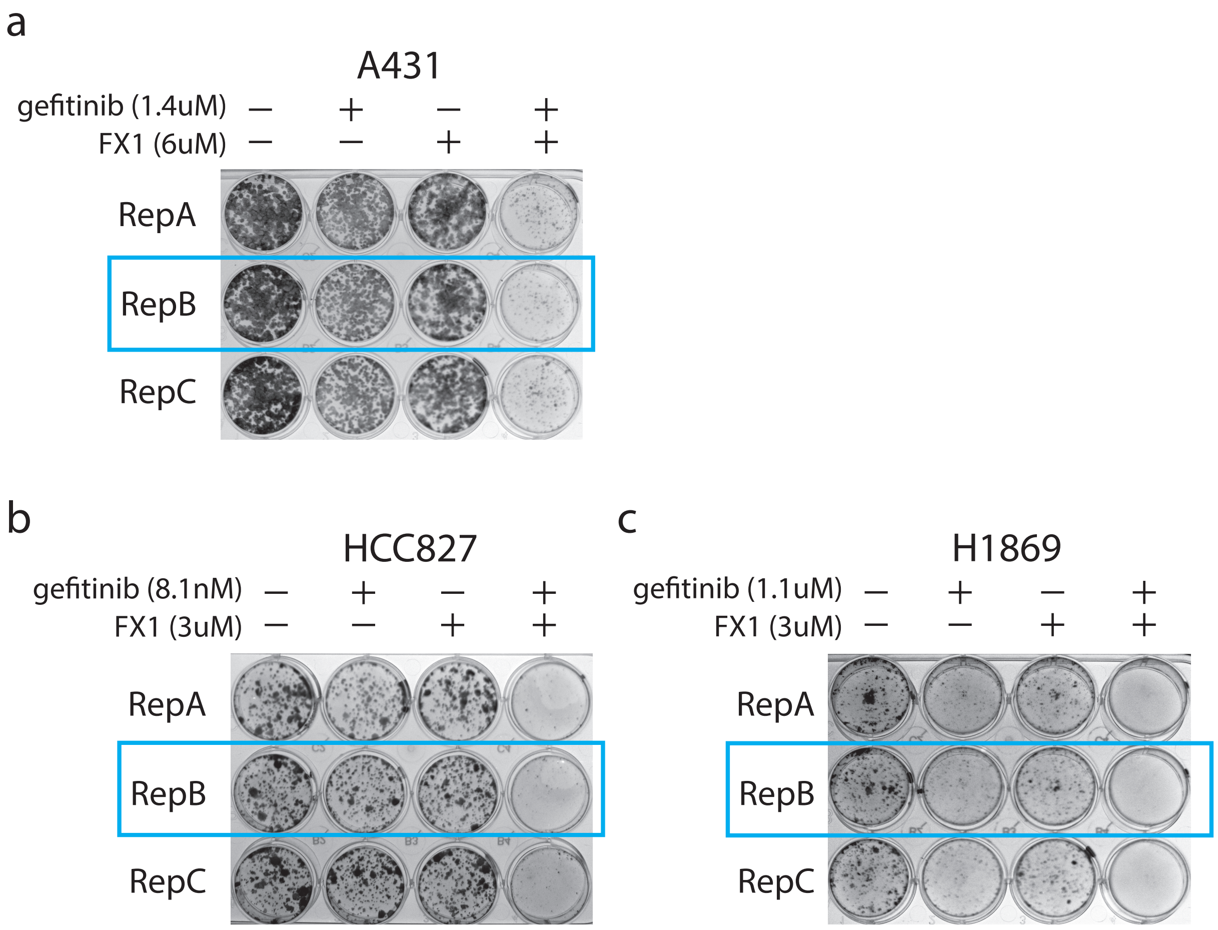

Supplementary Figure 12. Inhibition of BCL6 sensitizes NSCLC cells to EGFR-TKI treatment. a. Clonogenic assay results for A431 cells treated with gefitinib and the BCL6 inhibitor FX1, either alone or in combination for 10 days. **b.** Clonogenic assay results for HCC827 cells treated with gefitinib and the BCL6 inhibitor FX1, either alone or in combination for 10 days. **c.** Clonogenic assay results for H1869 cells treated with gefitinib and the BCL6 inhibitor FX1, either alone or in combination for 10 days. Replicate B (selected by blue box) from all 3 cell lines were used in **Figure 5b** as representative result of each clonogenic assay.
