# Supplementary material for: Immediate Adaptation Analysis Implicates BCL6 as an EGFR-TKI Combination Therapy Target in NSCLC
Source: Mol Cell Proteomics. 2020 Mar 30;19(6):928–43. doi: 10.1074/mcp.RA120.002036 (PMC7261823; doi:10.1074/mcp.RA120.002036)
Supplement: Annotated spectra [file 159477_0_supp_492414_q74qpj.zip › PSM.annotated.spectra_HIRIEFII.pdf]

ABCD2 | +229.163LWYIM+15.995IEQFLM+15.995K+229.163

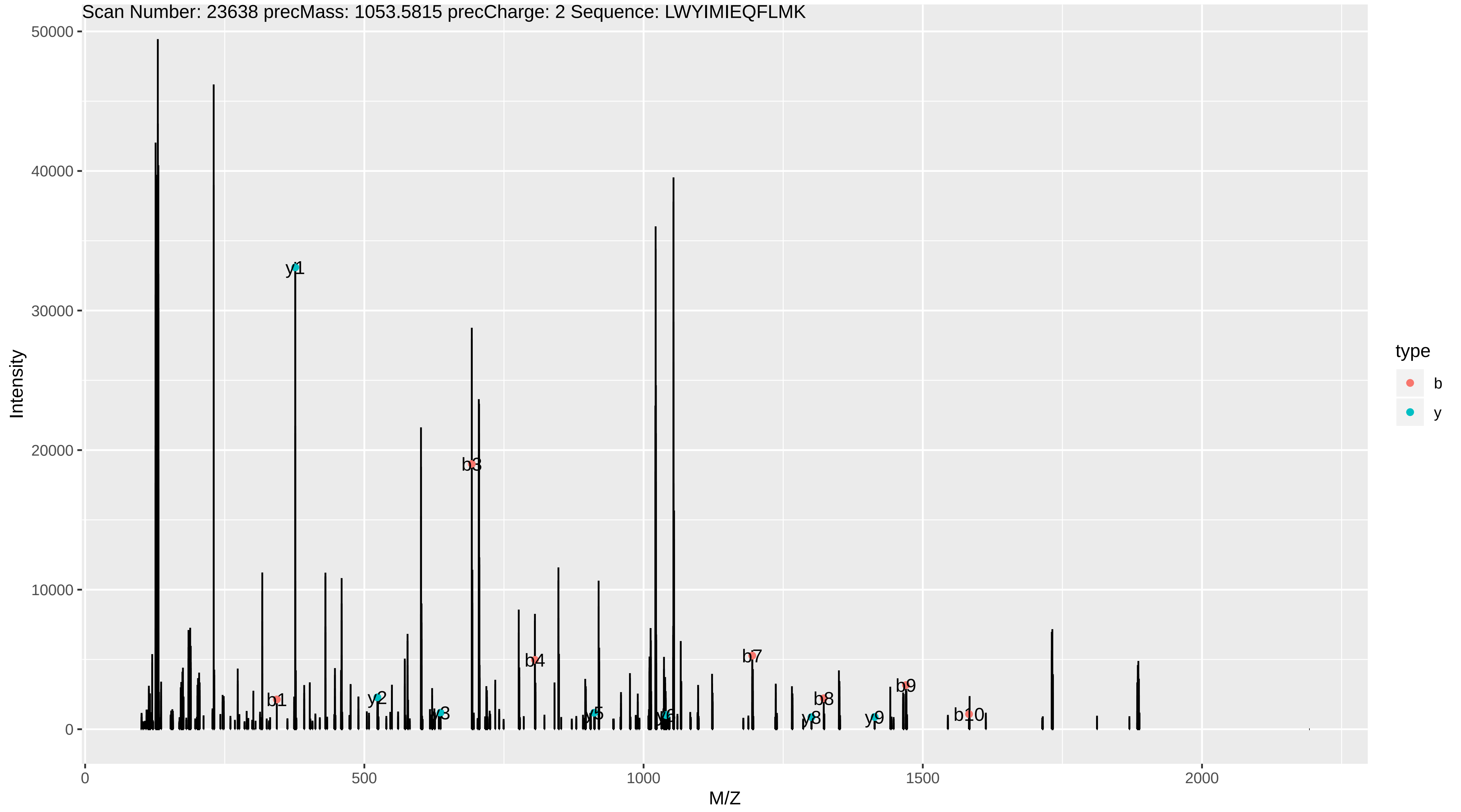

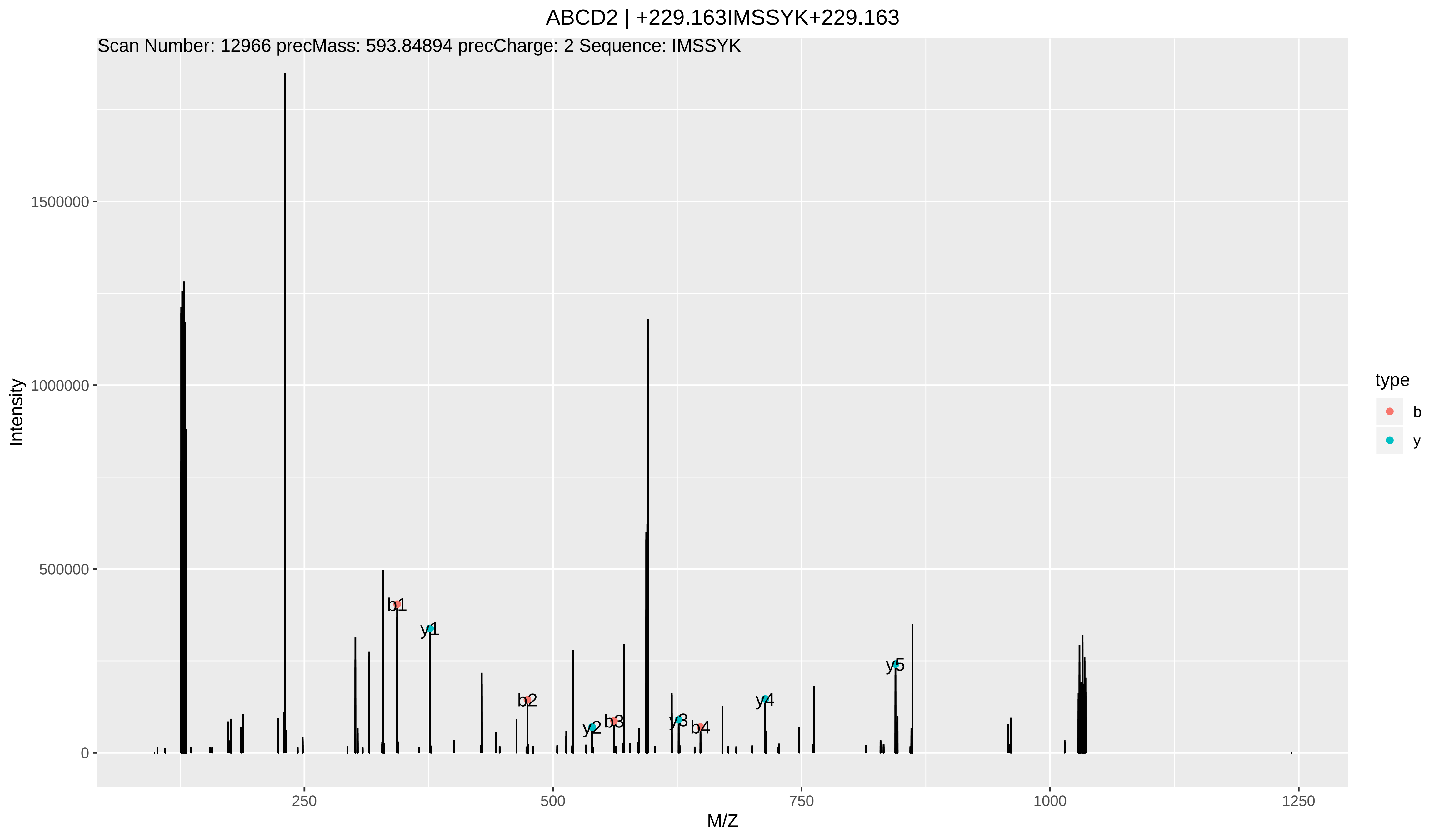
ABCD2 | +229.163IMSSYK+229.163
Scan Number: 12966 precMass: 593.84894 precCharge: 2 Sequence: IMSSYK
Intensity
1500000
1000000
500000
0
M/Z
250
500
750
1000
1250
b1
y1
b2
y2
b3
y3
b4
y4
y5
type
b
y

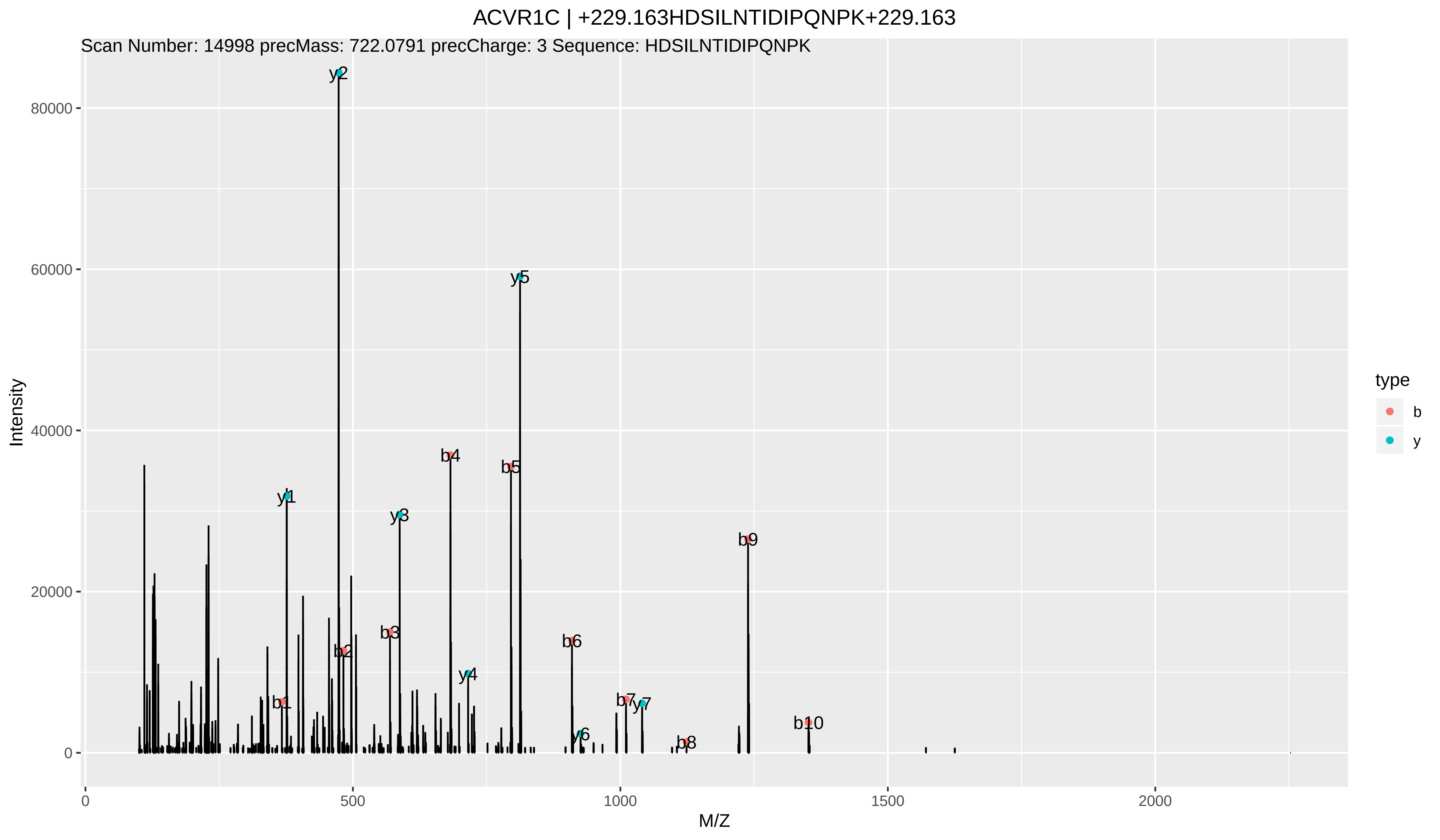

ACVR1C | +229.163HDSILNTIDIPQNPK+229.163
Scan Number: 14998 precMass: 722.0791 precCharge: 3 Sequence: HDSILNTIDIPQNPK
Intensity
M/Z
0
20000
40000
60000
80000
500
1000
1500
2000
y2
y5
b4
b5
y1
y3
b9
b3
b6
b2
y4
b1
b7
y7
b10
y6
b8
type
b
y

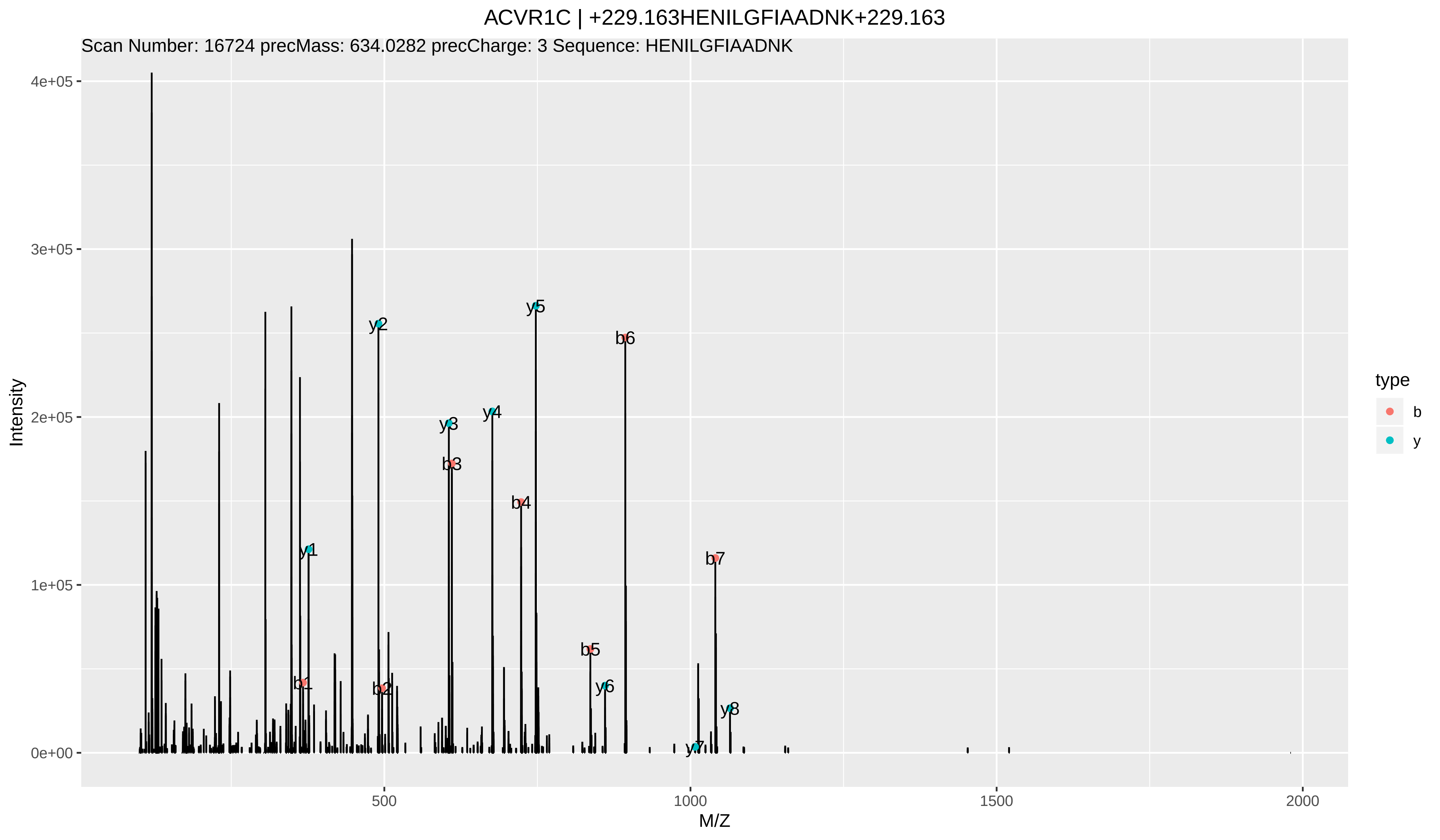

ACVR1C | +229.163HENILGFIAADNK+229.163
Scan Number: 16724 precMass: 634.0282 precCharge: 3 Sequence: HENILGFIAADNK
Intensity
4e+05
3e+05
2e+05
1e+05
0e+00
M/Z
500
1000
1500
2000
y1
y2
y3
y4
y5
y6
y7
y8
b1
b2
b3
b4
b5
b6
b7
type
b
y

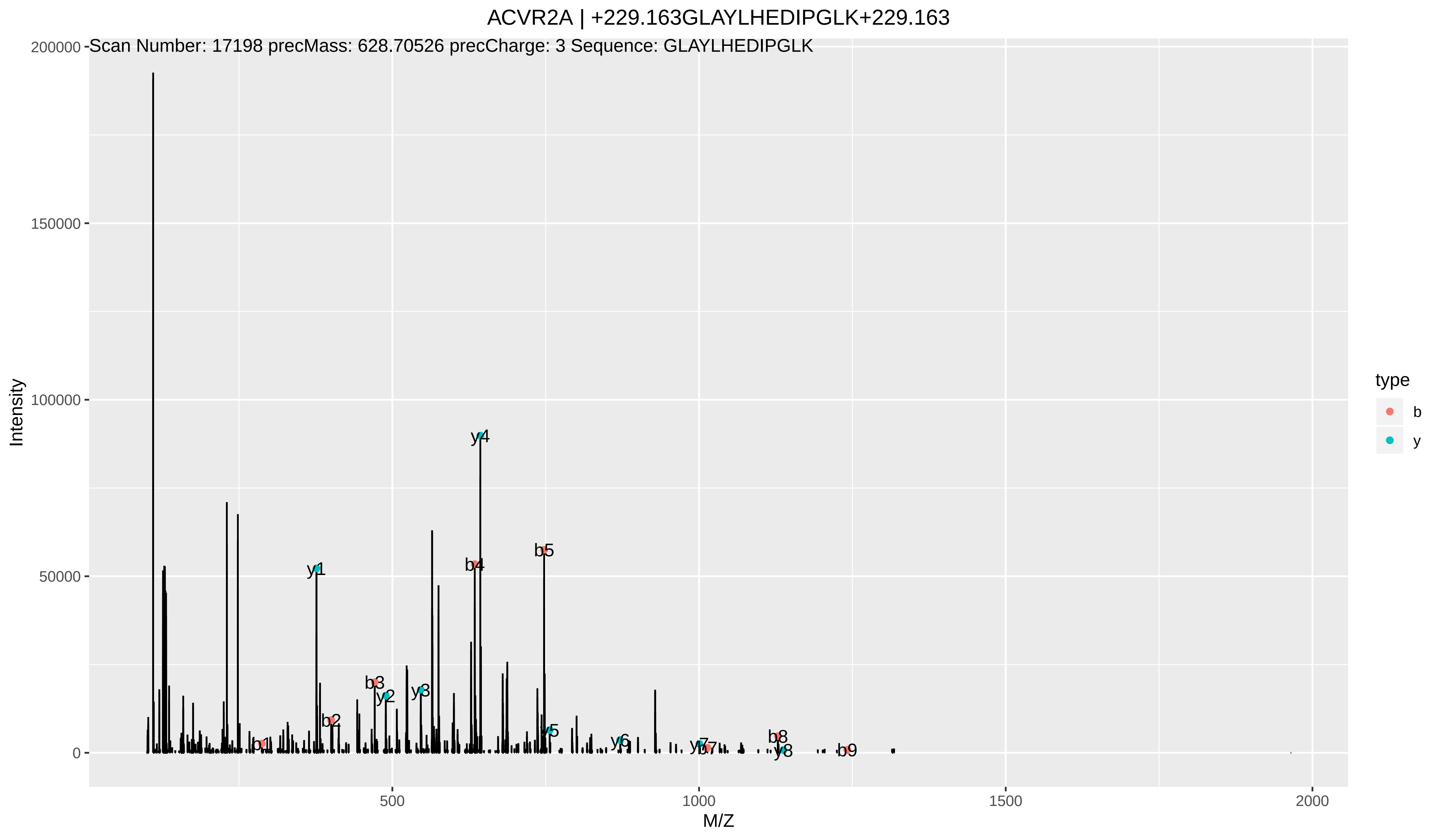

ACVR2A | +229.163GLAYLHEDIPGLK+229.163
Scan Number: 17198 precMass: 628.70526 precCharge: 3 Sequence: GLAYLHEDIPGLK
Intensity
M/Z
200000
150000
100000
50000
0
500
1000
1500
2000
y4
b5
b4
y1
b3
y3
y2
b2
b1
y5
y6
y7
b7
b8
y8
b9
type
b
y

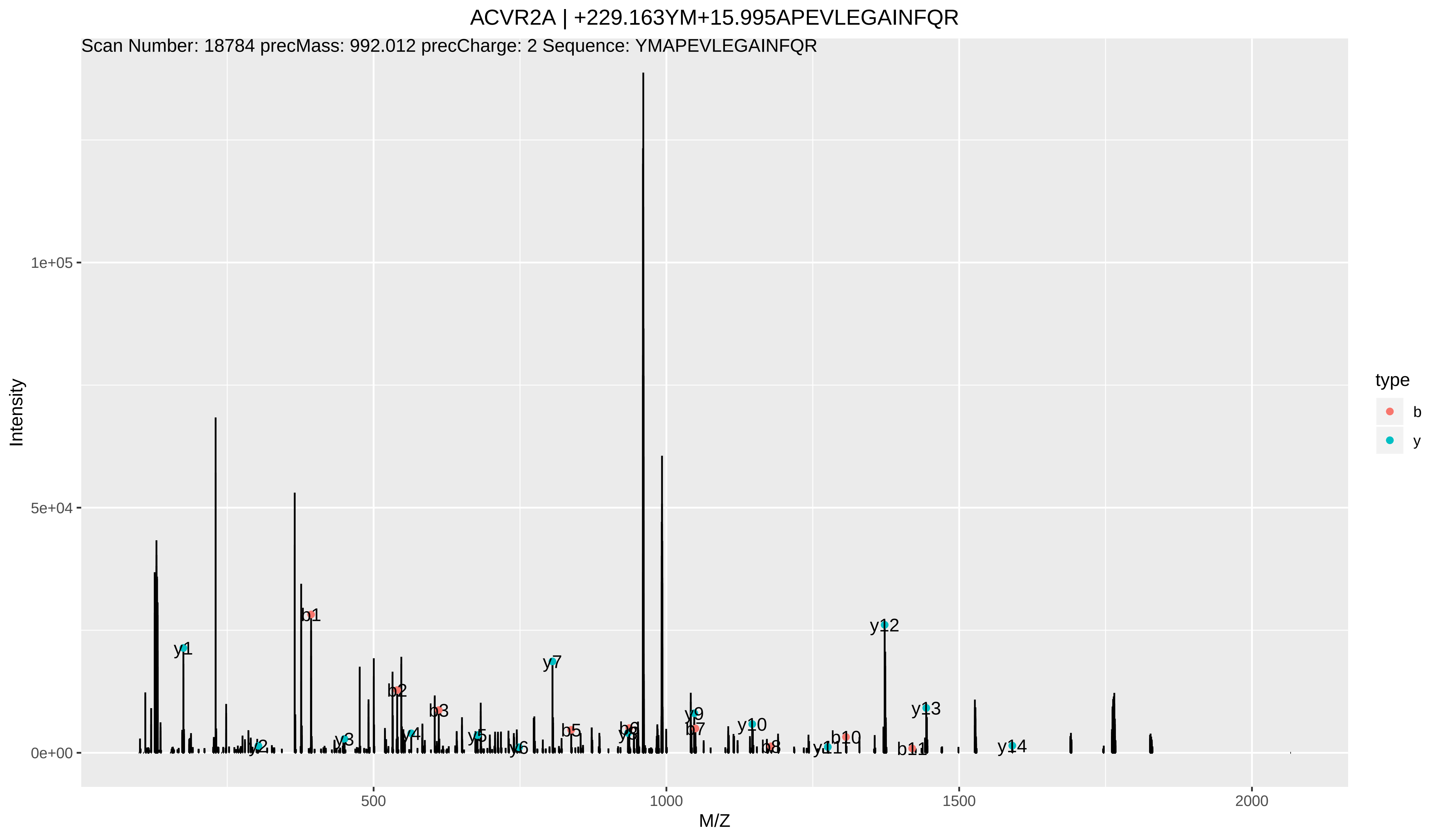
ACVR2A | +229.163YM+15.995APEVLEGAINFQR
Scan Number: 18784 precMass: 992.012 precCharge: 2 Sequence: YMAPEVLEGAINFQR
Intensity
M/Z
1e+05
5e+04
0e+00
500
1000
1500
2000
type
b
y
y1
y2
y3
y4
y5
y6
y7
y9
y10
y11
y12
y13
y14
b1
b2
b3
b5
b7
b8
b10
b11

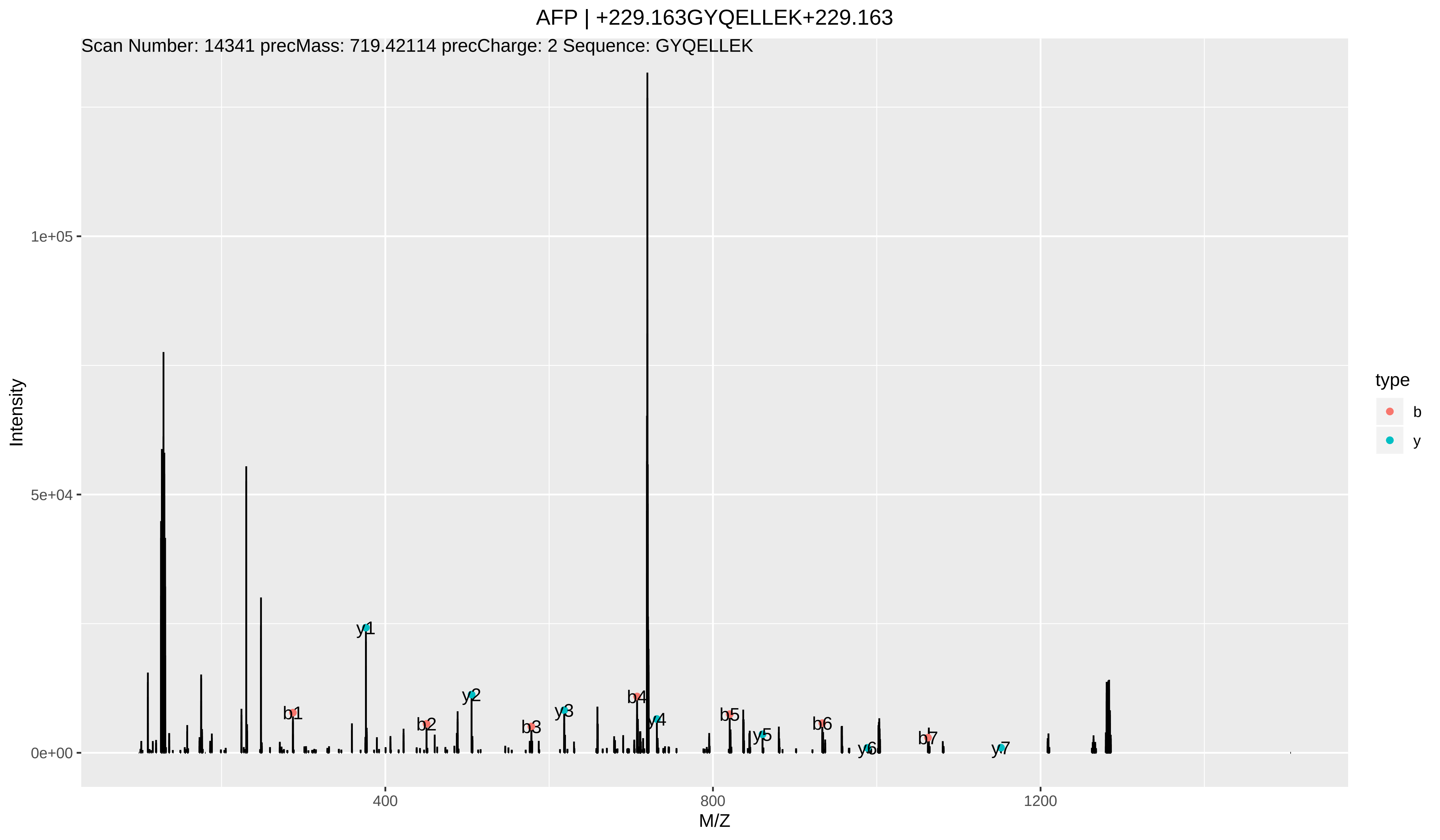

AFP | +229.163GYQELLEK+229.163
Scan Number: 14341 precMass: 719.42114 precCharge: 2 Sequence: GYQELLEK
Intensity
1e+05
5e+04
0e+00
400
800
1200
M/Z
y1
b1
b2
y2
b3
y3
b4
y4
b5
y5
b6
y6
b7
y7
type
b
y

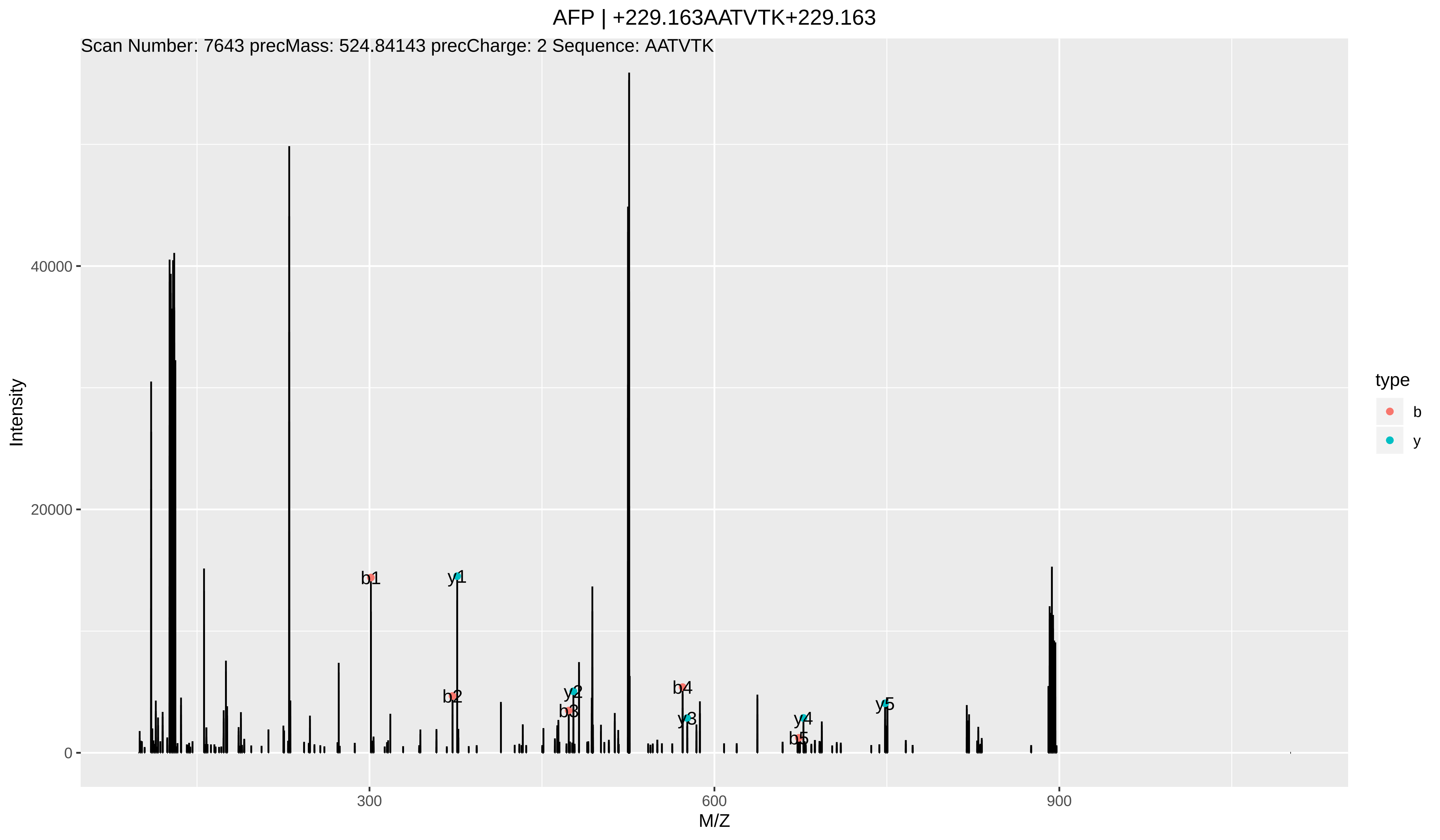
AFP | +229.163AATVTK+229.163
Scan Number: 7643 precMass: 524.84143 precCharge: 2 Sequence: AATVTK
Intensity
40000
20000
0
300
600
900
M/Z
b1
y1
b2
y2
b3
y3
b4
y4
b5
y5
type
b
y

AIF1L | +229.163YMEFDLNNEGEIDLMSLK+229.163

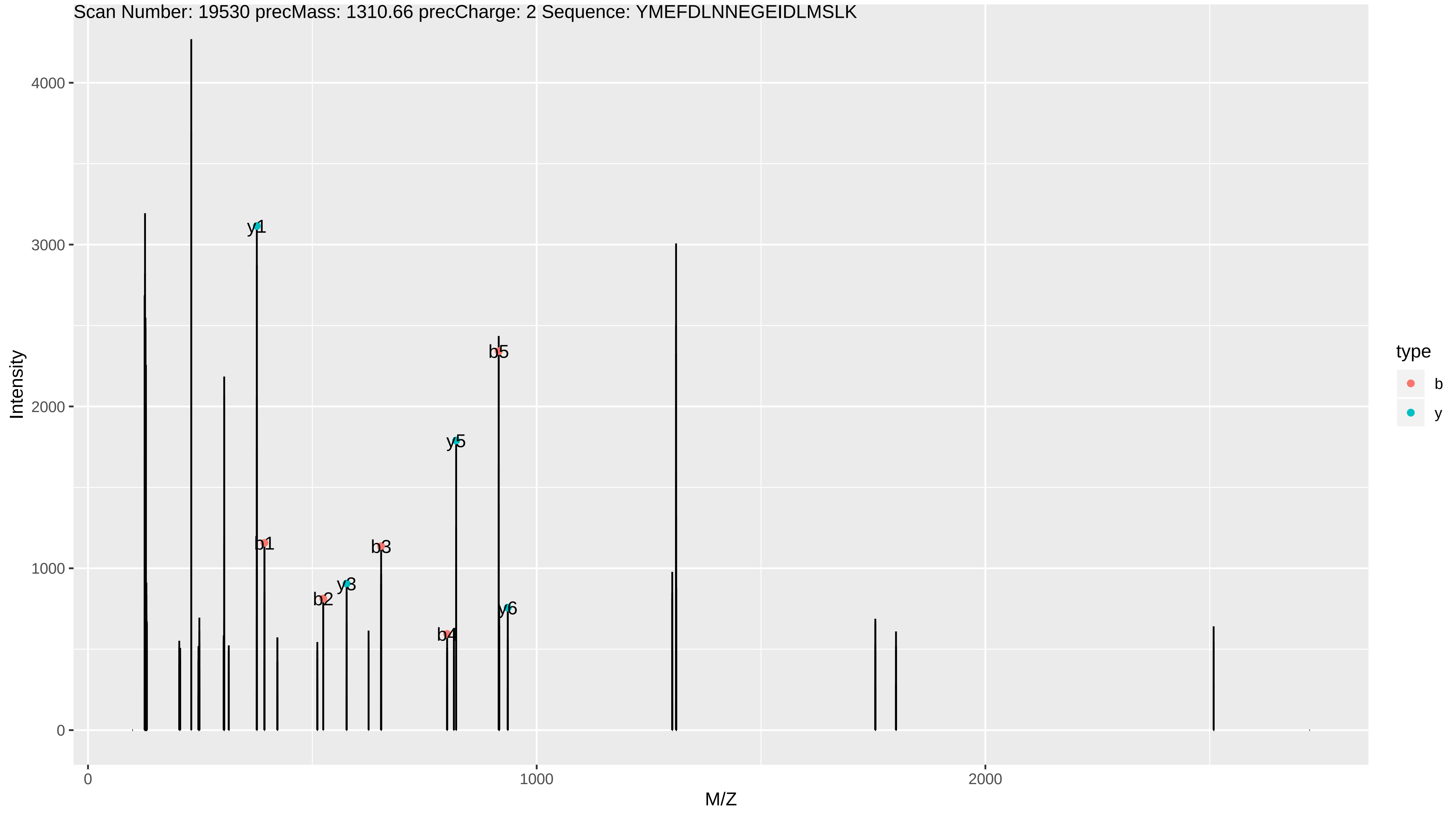

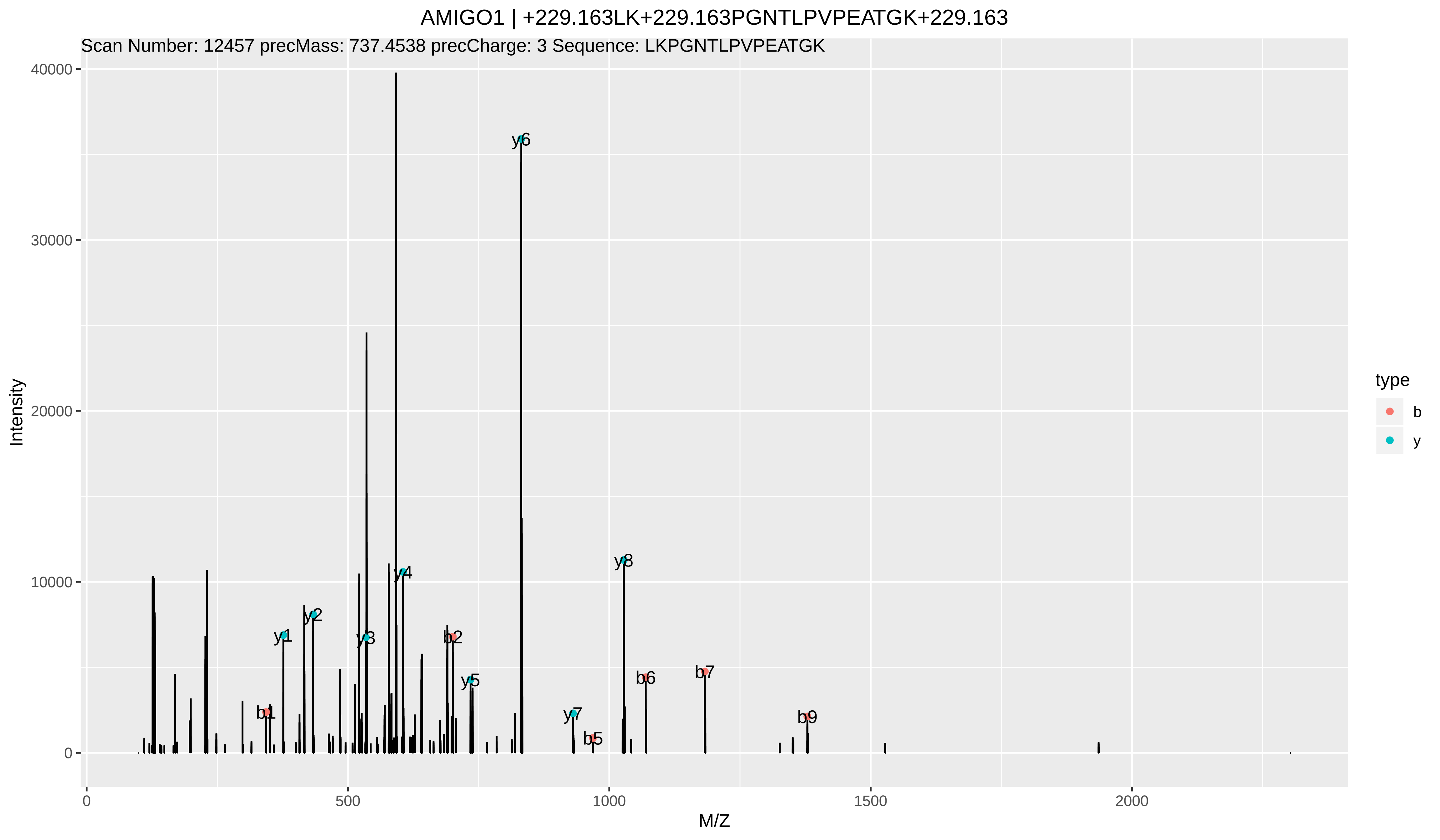

AMIGO1 | +229.163LK+229.163PGNTLPVPEATGK+229.163
Scan Number: 12457 precMass: 737.4538 precCharge: 3 Sequence: LKPGNTLPVPEATGK
Intensity
M/Z
0
10000
20000
30000
40000
500
1000
1500
2000
y6
y8
y4
y2
y1
y3
b2
y5
b6
b7
b1
y7
b5
b9
type
b
y

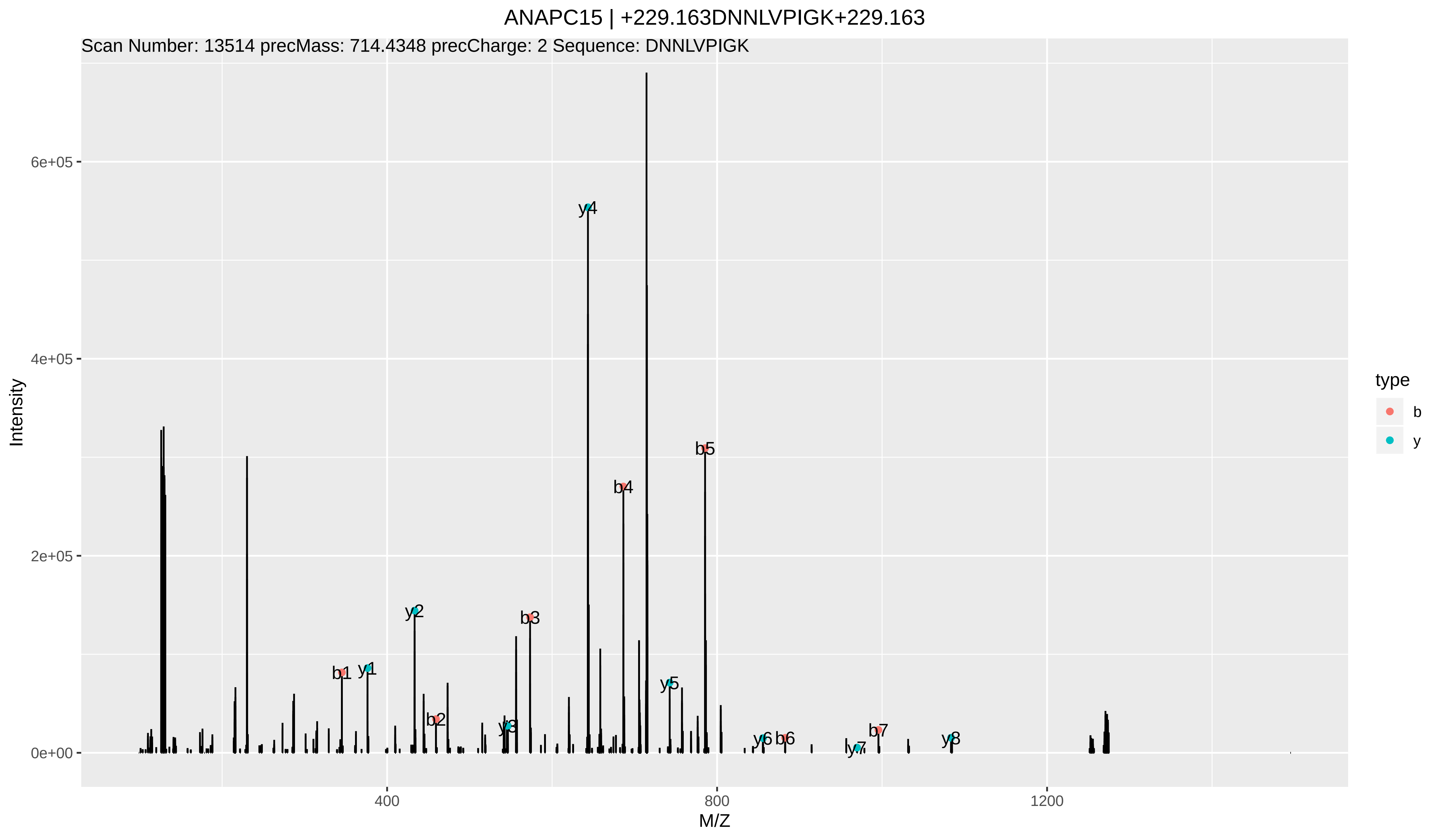
ANAPC15 | +229.163DNNLVPIGK+229.163
Scan Number: 13514 precMass: 714.4348 precCharge: 2 Sequence: DNNLVPIGK
Intensity
6e+05
4e+05
2e+05
0e+00
M/Z
400
800
1200
y4
b5
b4
y2
b3
b1
y1
y5
b2
y3
y6
b6
b7
y7
y8
type
b
y

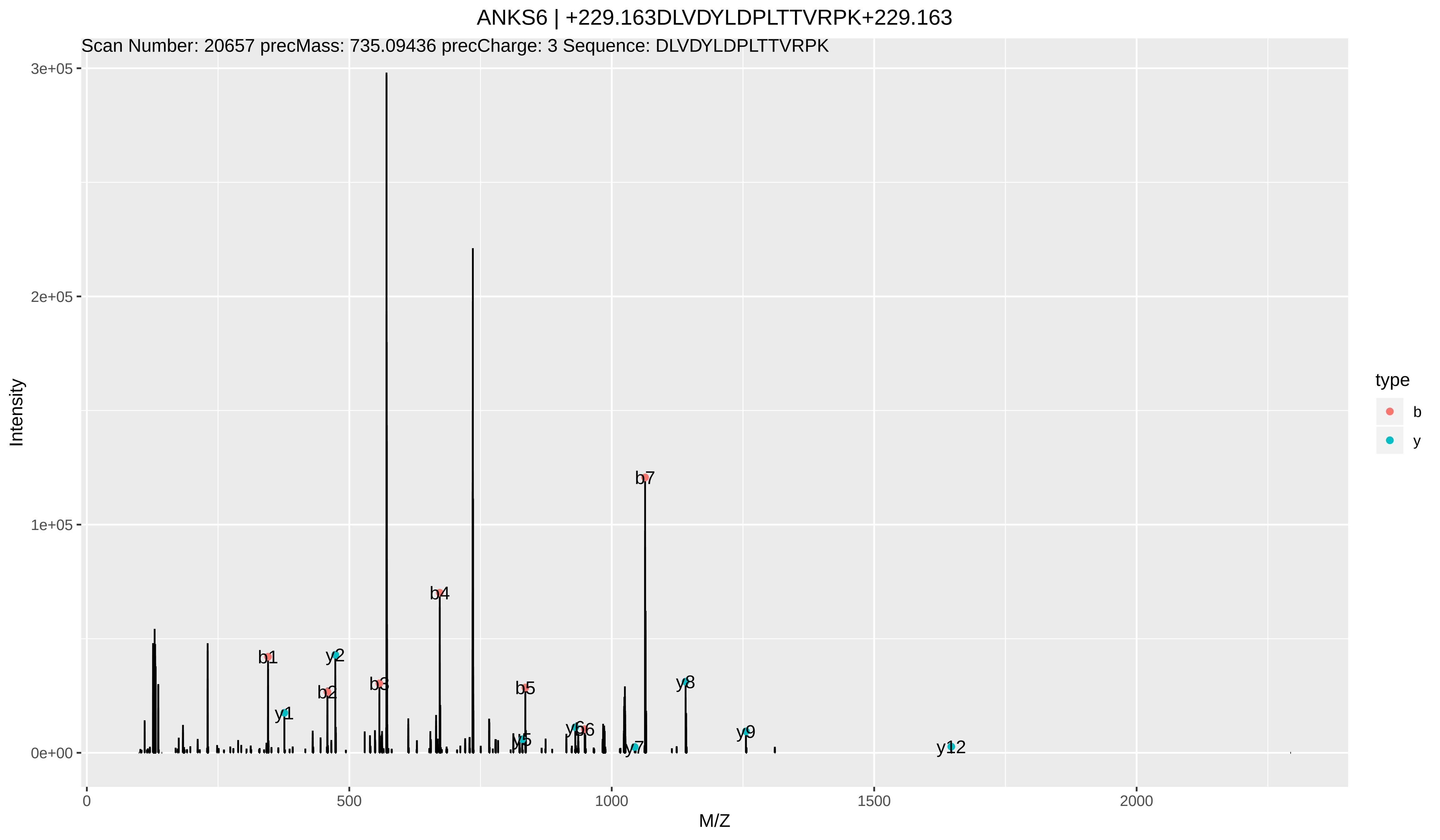

ANKS6 | +229.163DLVDYLDPLTTVRPK+229.163
Scan Number: 20657 precMass: 735.09436 precCharge: 3 Sequence: DLVDYLDPLTTVRPK
Intensity
M/Z
3e+05
2e+05
1e+05
0e+00
0
500
1000
1500
2000
type
b
y
b1
b2
b3
b4
b5
b6
b7
y1
y2
y5
y6
y7
y8
y9
y12

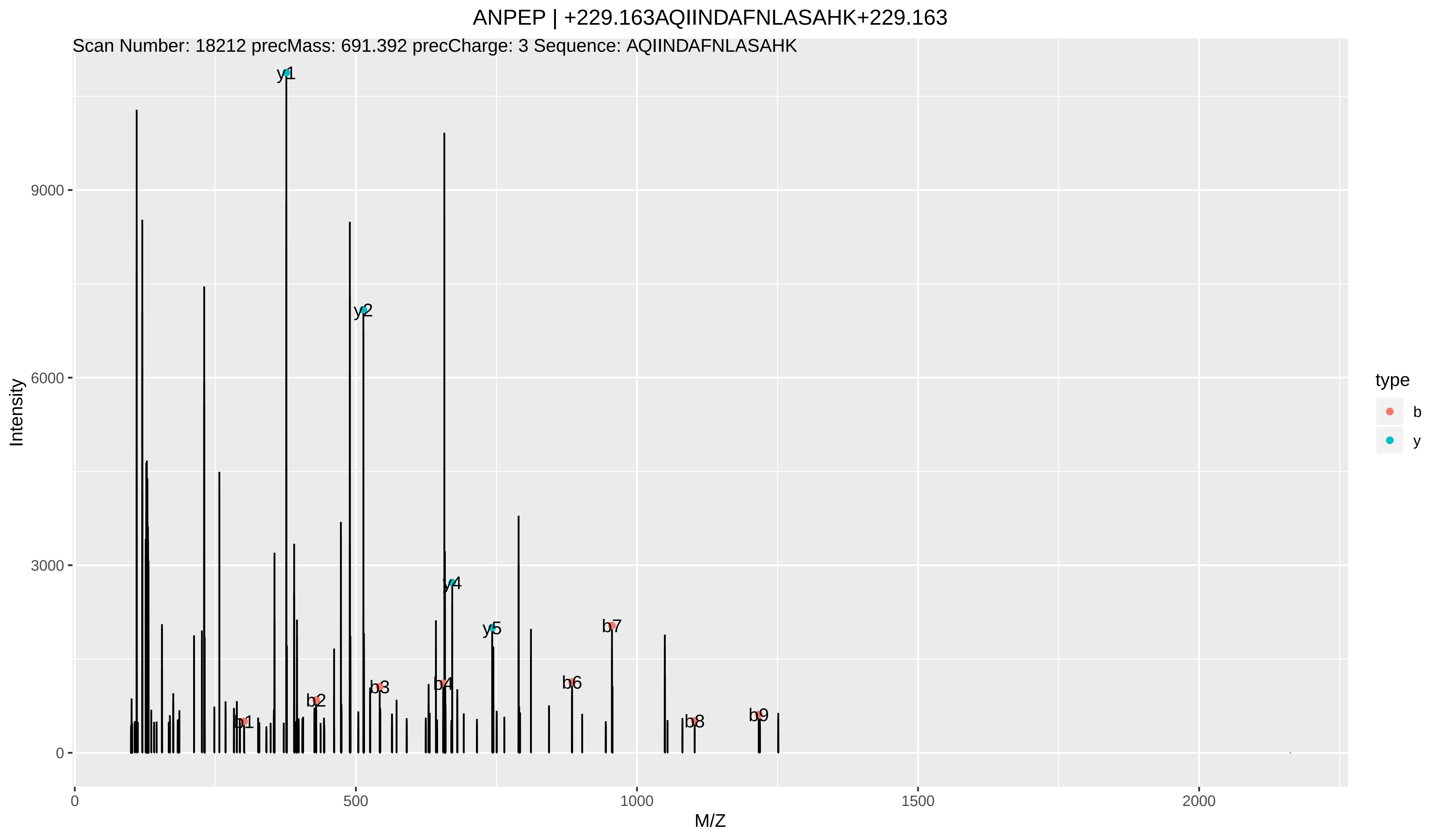

ANPEP | +229.163AQIINDAFNLASAHK+229.163
Scan Number: 18212 precMass: 691.392 precCharge: 3 Sequence: AQIINDAFNLASAHK
y1
y2
y4
y5
b1
b2
b3
b4
b6
b7
b8
b9
Intensity
9000
6000
3000
0
0
500
1000
1500
2000
M/Z
type
b
y

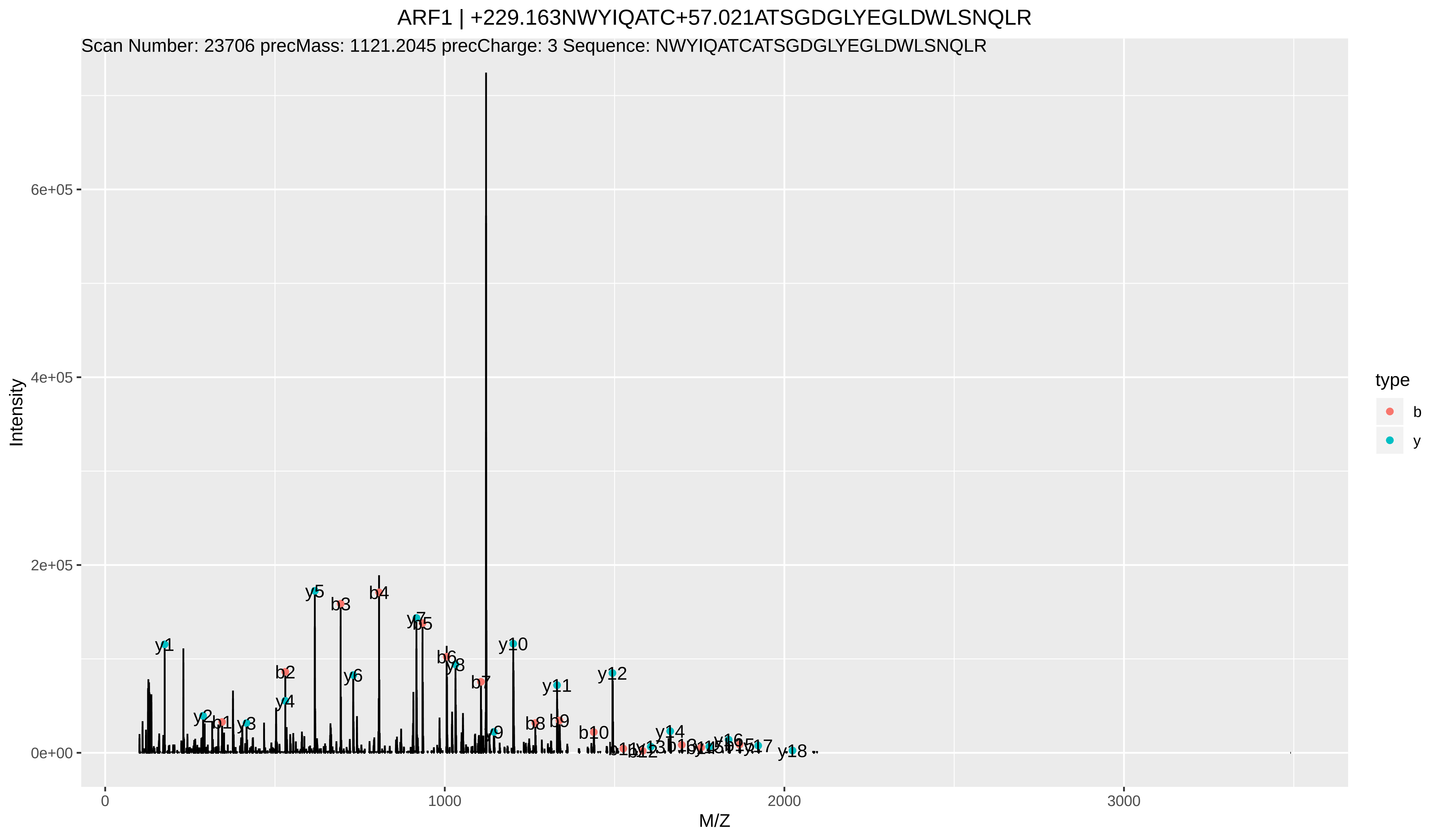
ARF1 | +229.163NWYIQATC+57.021ATSGDGLYEGLDWLSNQLR
Scan Number: 23706 precMass: 1121.2045 precCharge: 3 Sequence: NWYIQATCATSGDGLYEGLDWLSNQLR
Intensity
M/Z
type
b
y

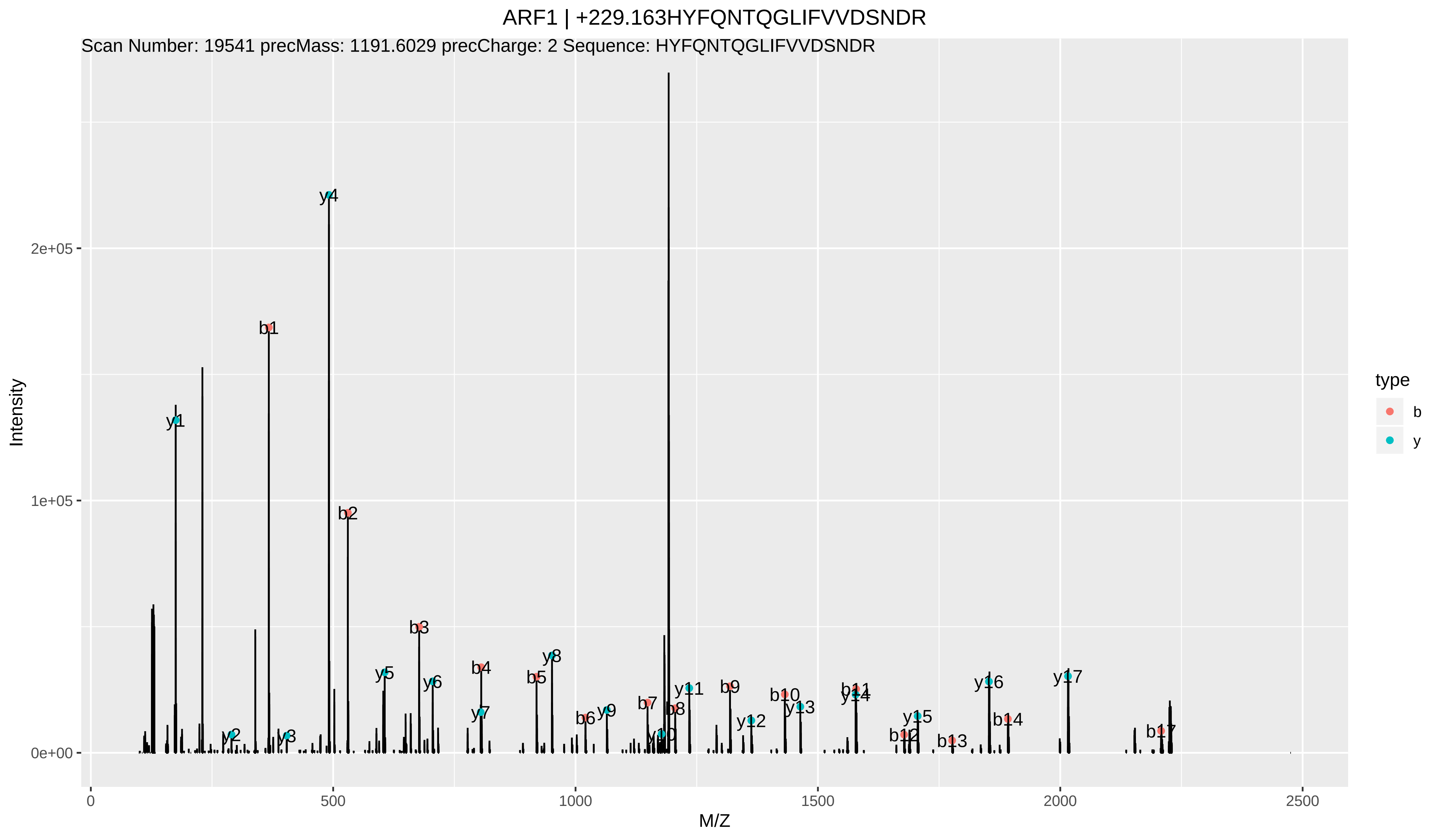

ARF1 | +229.163HYFQNTQGLIFVVDSNDR
Scan Number: 19541 precMass: 1191.6029 precCharge: 2 Sequence: HYFQNTQGLIFVVDSNDR
Intensity
2e+05
1e+05
0e+00
M/Z
0
500
1000
1500
2000
2500
y1
y2
y3
y4
y5
y6
y7
y8
y9
y10
y11
y12
y13
y14
y15
y16
y17
b1
b2
b3
b4
b5
b6
b7
b8
b9
b10
b11
b12
b13
b14
b17
type
b
y

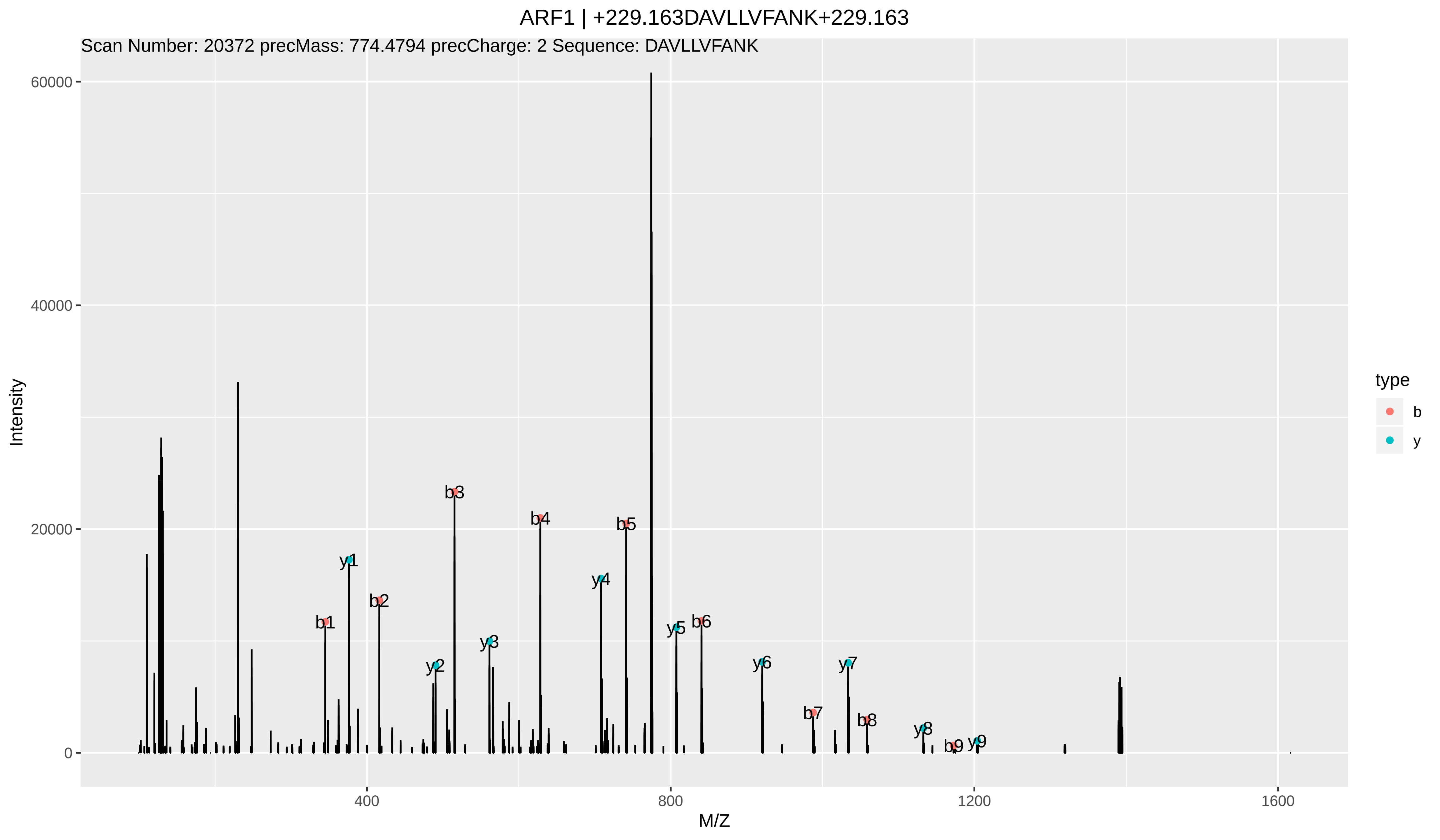
ARF1 | +229.163DAVLLVFANK+229.163
Scan Number: 20372 precMass: 774.4794 precCharge: 2 Sequence: DAVLLVFANK
Intensity
60000
40000
20000
0
M/Z
400
800
1200
1600
b1
y1
b2
y2
b3
y3
b4
y4
b5
y5
b6
y6
b7
y7
b8
y8
b9
y9
type
b
y

ARF1 | +229.163QDLPNAM+15.995NAAEITDK+229.163

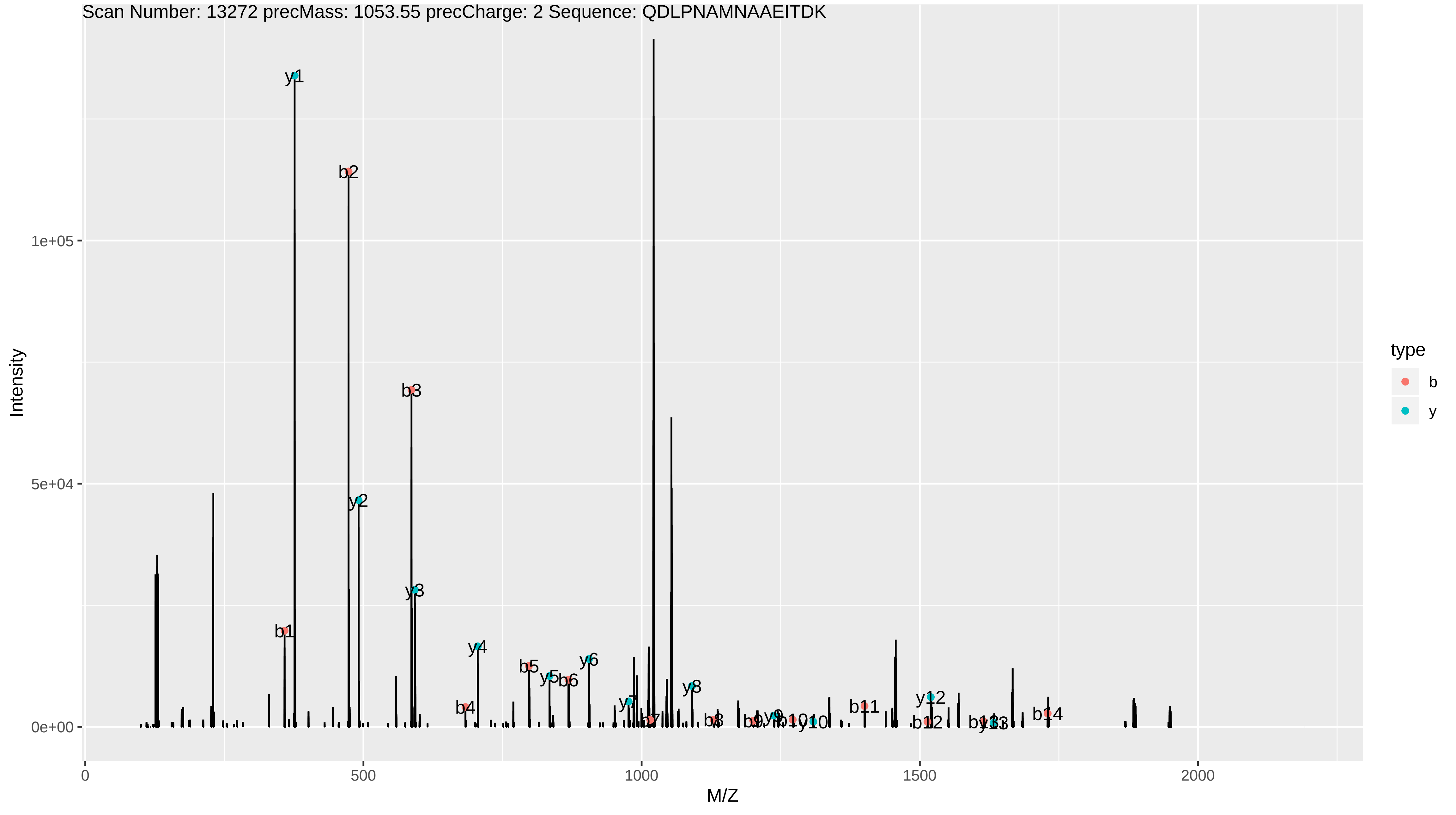

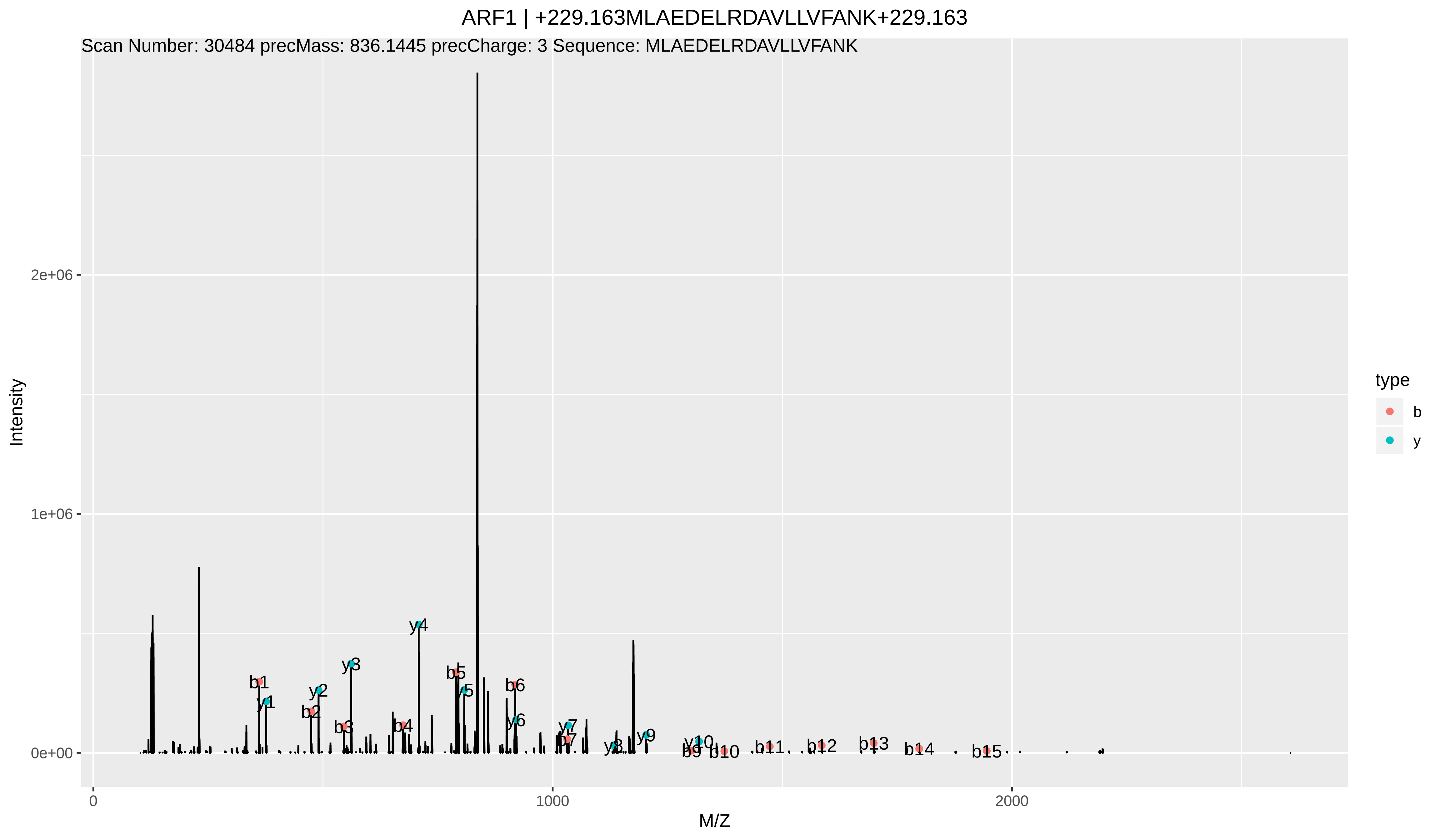

ARF1 | +229.163MLAEDELRDAVLLVFANK+229.163
Scan Number: 30484 precMass: 836.1445 precCharge: 3 Sequence: MLAEDELRDAVLLVFANK
Intensity
2e+06
1e+06
0e+00
M/Z
0
1000
2000
type
b
y
b1
y1
b2
y2
b3
y3
b4
y4
b5
y5
b6
y6
b7
y7
y8
y9
b9
y10
b10
b11
b12
b13
b14
b15

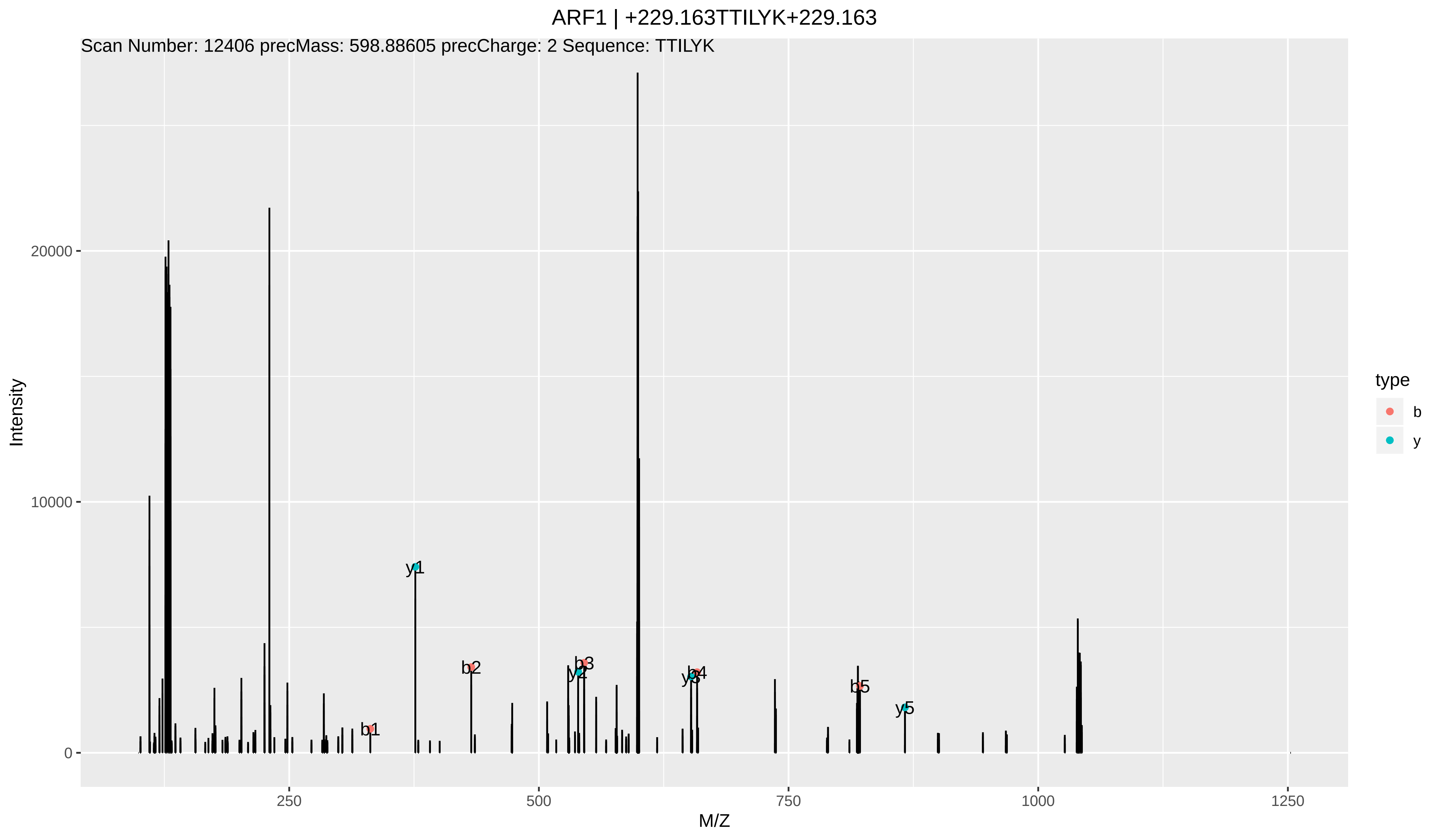

ARF1 | +229.163TTILYK+229.163
Scan Number: 12406 precMass: 598.88605 precCharge: 2 Sequence: TTILYK
Intensity
20000
10000
0
250
500
750
1000
1250
M/Z
y1
b1
b2
b3
y2
b4
y3
b5
y5
type
b
y

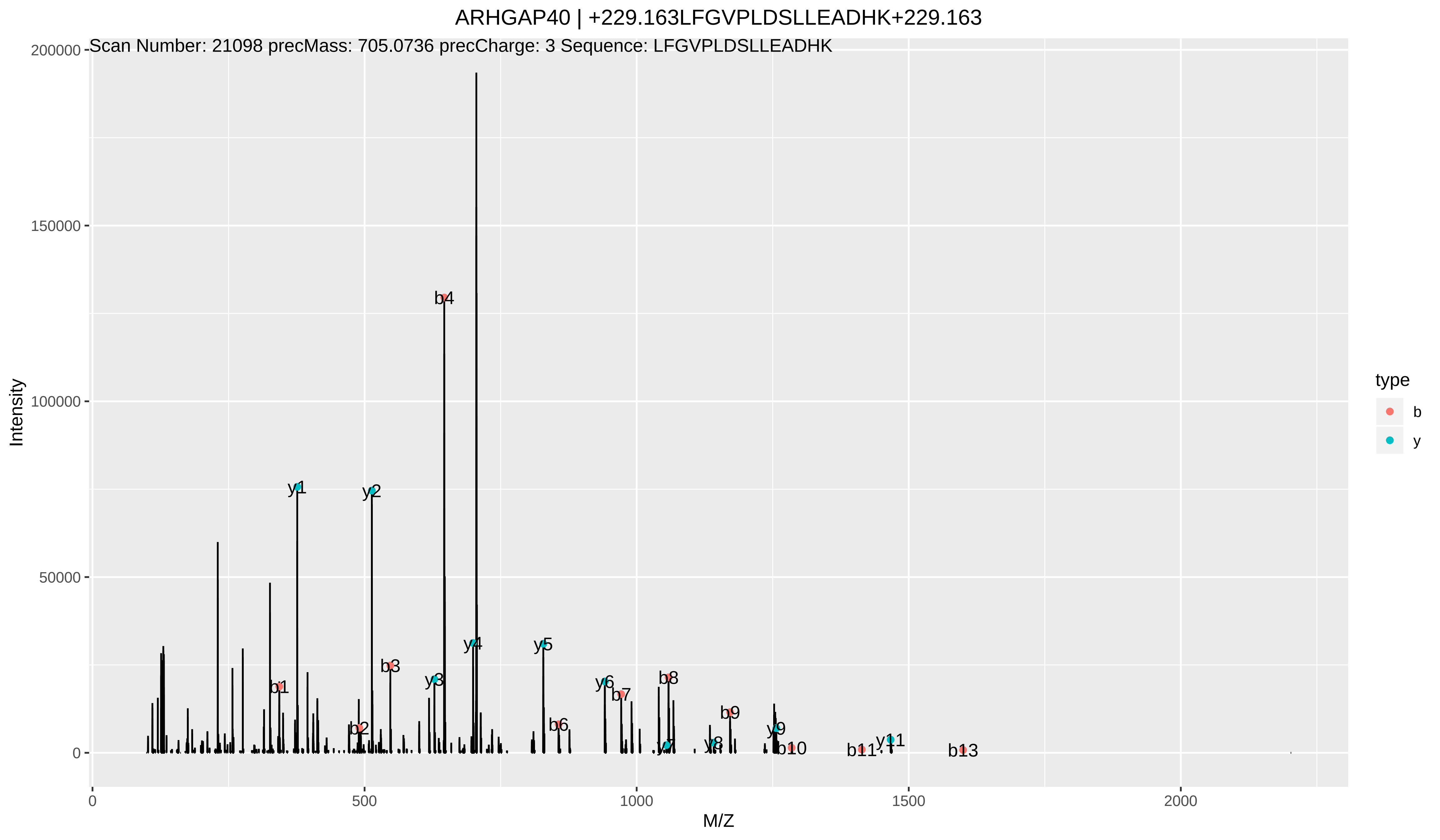

ARHGAP40 | +229.163LFGVPLDSLLEADHK+229.163
Scan Number: 21098 precMass: 705.0736 precCharge: 3 Sequence: LFGVPLDSLLEADHK
Intensity
M/Z
type
b
y
b1
b2
b3
b4
b6
b7
b8
b9
b10
b11
b13
y1
y2
y3
y4
y5
y6
y7
y8
y9
y11
0
50000
100000
150000
200000
500
1000
1500
2000

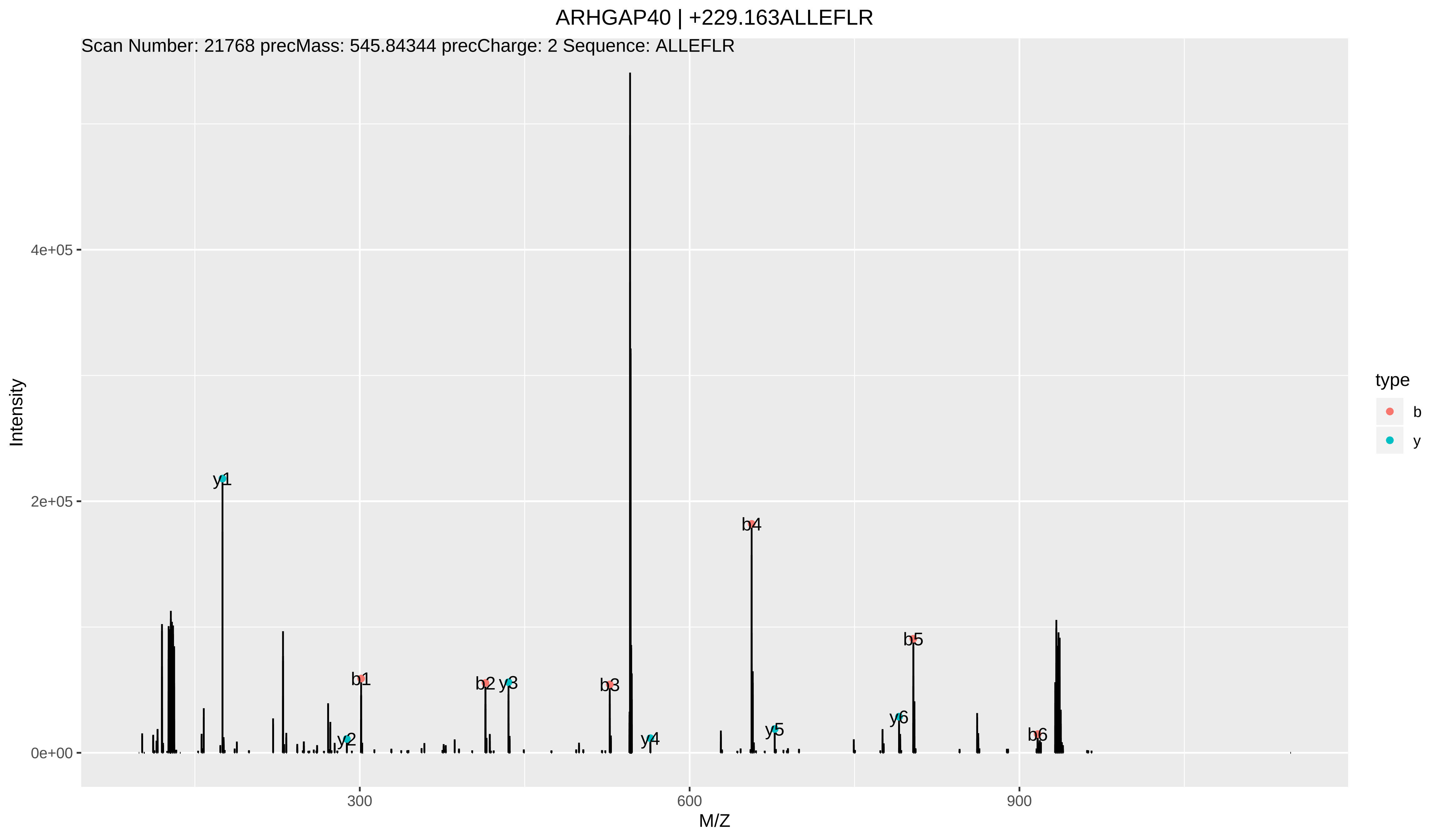

ARHGAP40 | +229.163ALLEFLR
Scan Number: 21768 precMass: 545.84344 precCharge: 2 Sequence: ALLEFLR
Intensity
4e+05
2e+05
0e+00
M/Z
300
600
900
y1
y2
y3
y4
y5
y6
b1
b2
b3
b4
b5
b6
type
b
y

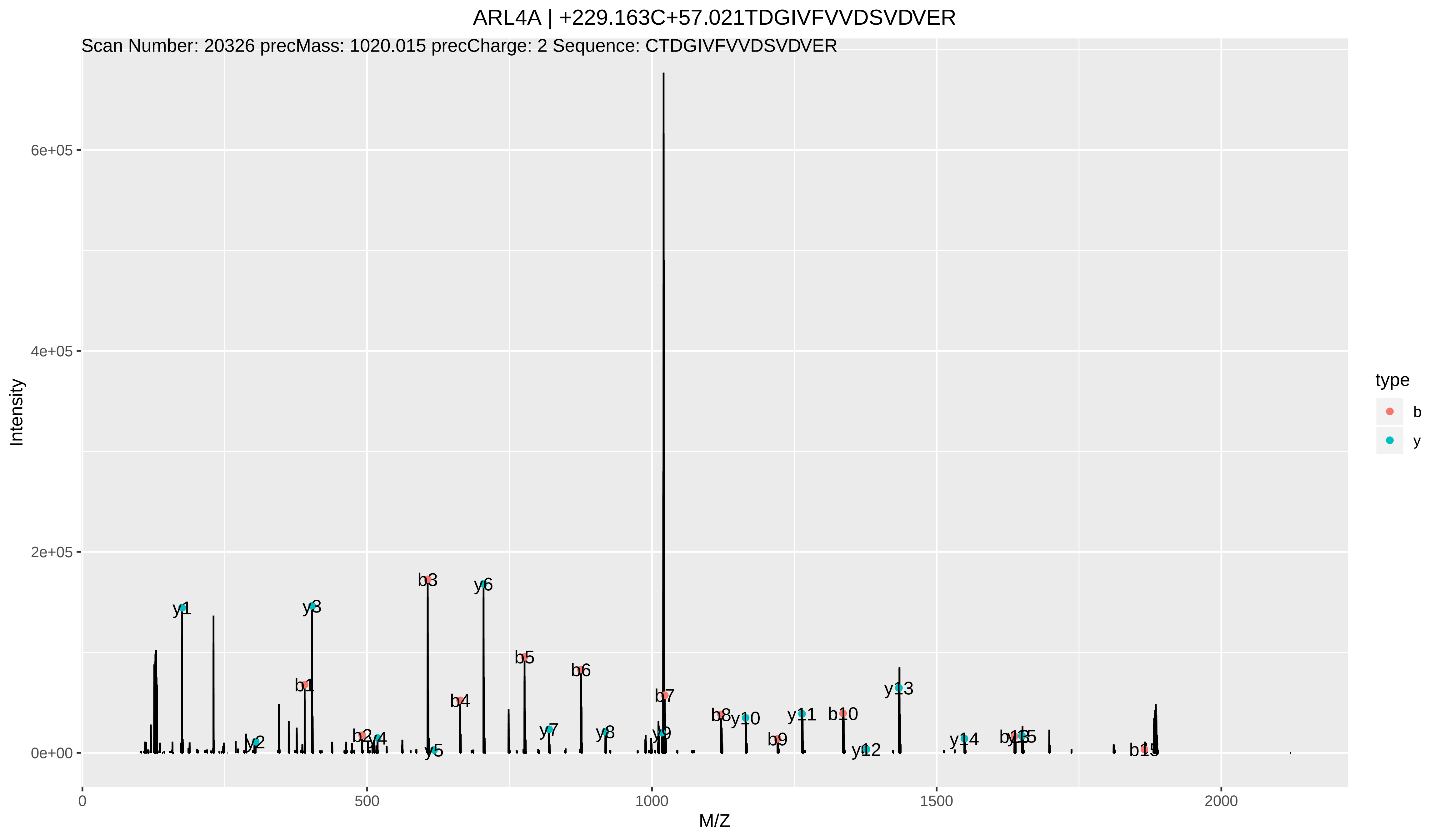

ARL4A | +229.163C+57.021TDGIVFVVDSVDVER
Scan Number: 20326 precMass: 1020.015 precCharge: 2 Sequence: CTDGIVFVVDSVDVER
Intensity
M/Z
6e+05
4e+05
2e+05
0e+00
0
500
1000
1500
2000
type
b
y
y1
y2
y3
b1
b2
y4
y5
b3
b4
y6
b5
y7
b6
y8
b7
y9
b8
y10
b9
y11
b10
y12
y13
y14
b13
y15
b15

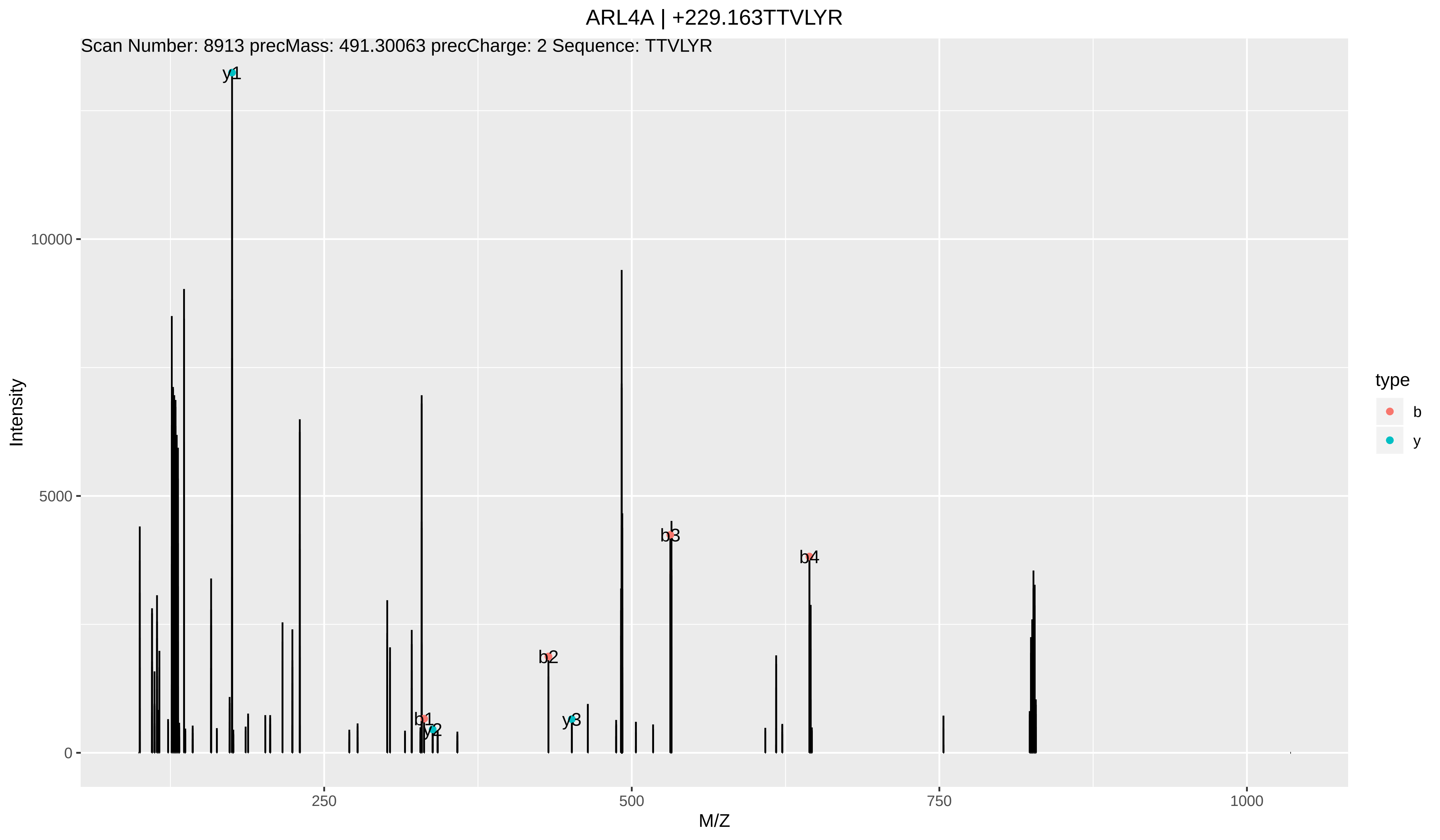

ARL4A | +229.163TTVLYR
Scan Number: 8913 precMass: 491.30063 precCharge: 2 Sequence: TTVLYR
y1
b1
y2
b2
y3
b3
b4
10000
5000
0
250
500
750
1000
Intensity
M/Z
type
b
y

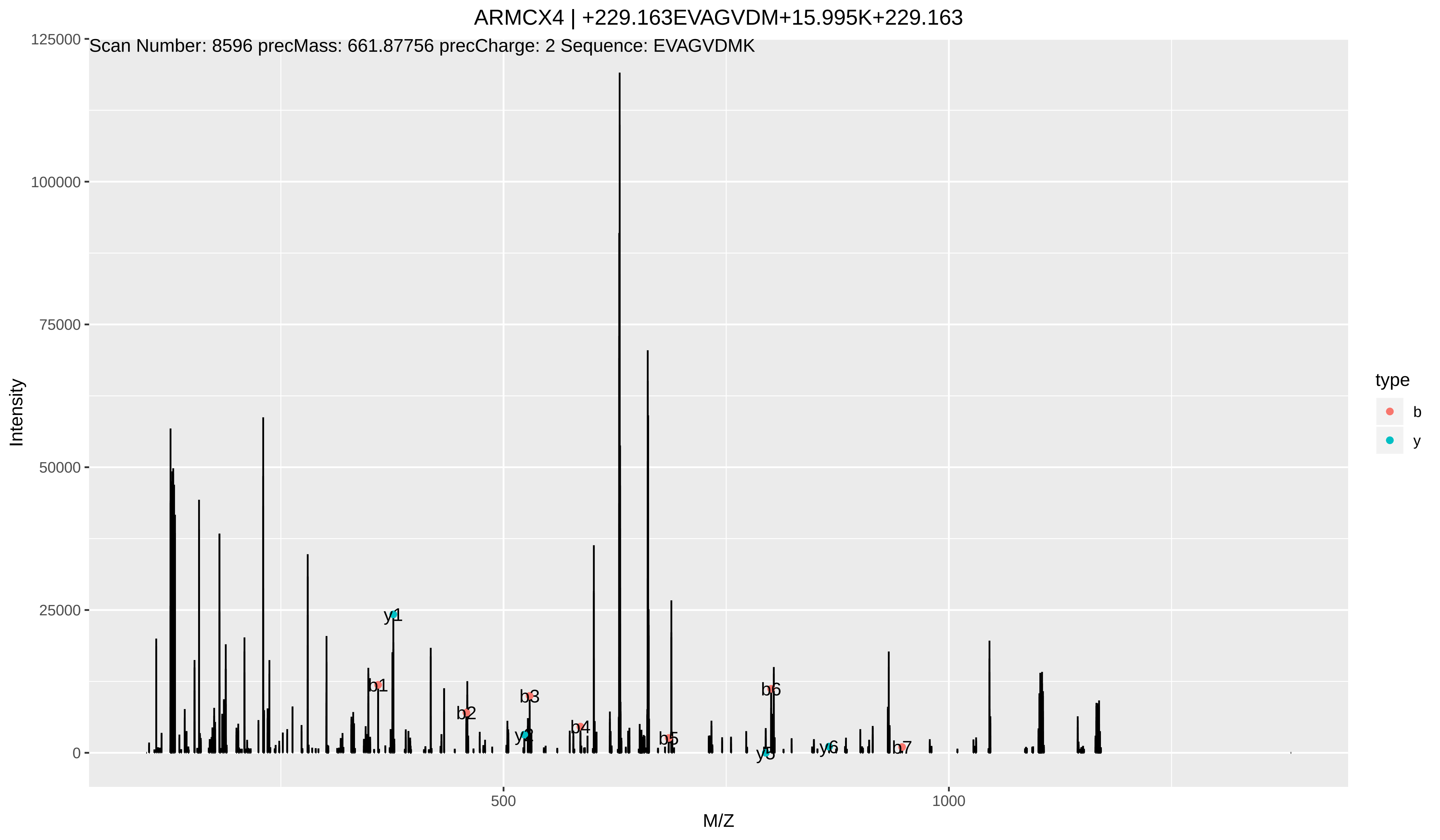

ARMCX4 | +229.163EVAGVDM+15.995K+229.163
Scan Number: 8596 precMass: 661.87756 precCharge: 2 Sequence: EVAGVDMK
Intensity
125000
100000
75000
50000
25000
0
500
1000
M/Z
y1
b1
b2
b3
y2
b4
b5
y5
b6
y6
b7
type
b
y

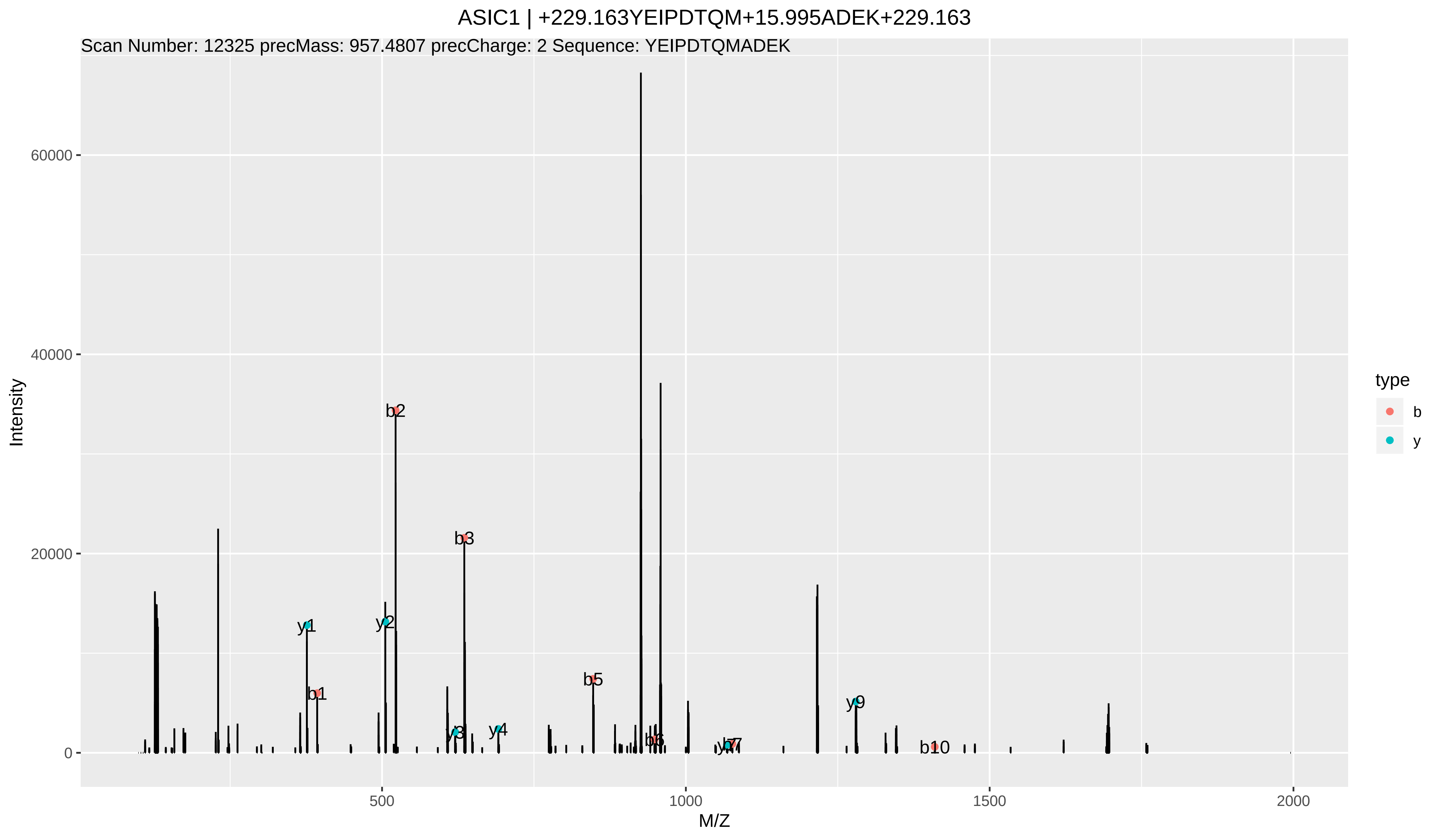

ASIC1 | +229.163YEIPDTQM+15.995ADEK+229.163
Scan Number: 12325 precMass: 957.4807 precCharge: 2 Sequence: YEIPDTQMADEK
Intensity
60000
40000
20000
0
M/Z
500
1000
1500
2000
b2
b3
y1
y2
b1
b5
y9
y4
y3
b6
y7
b7
b10
type
b
y

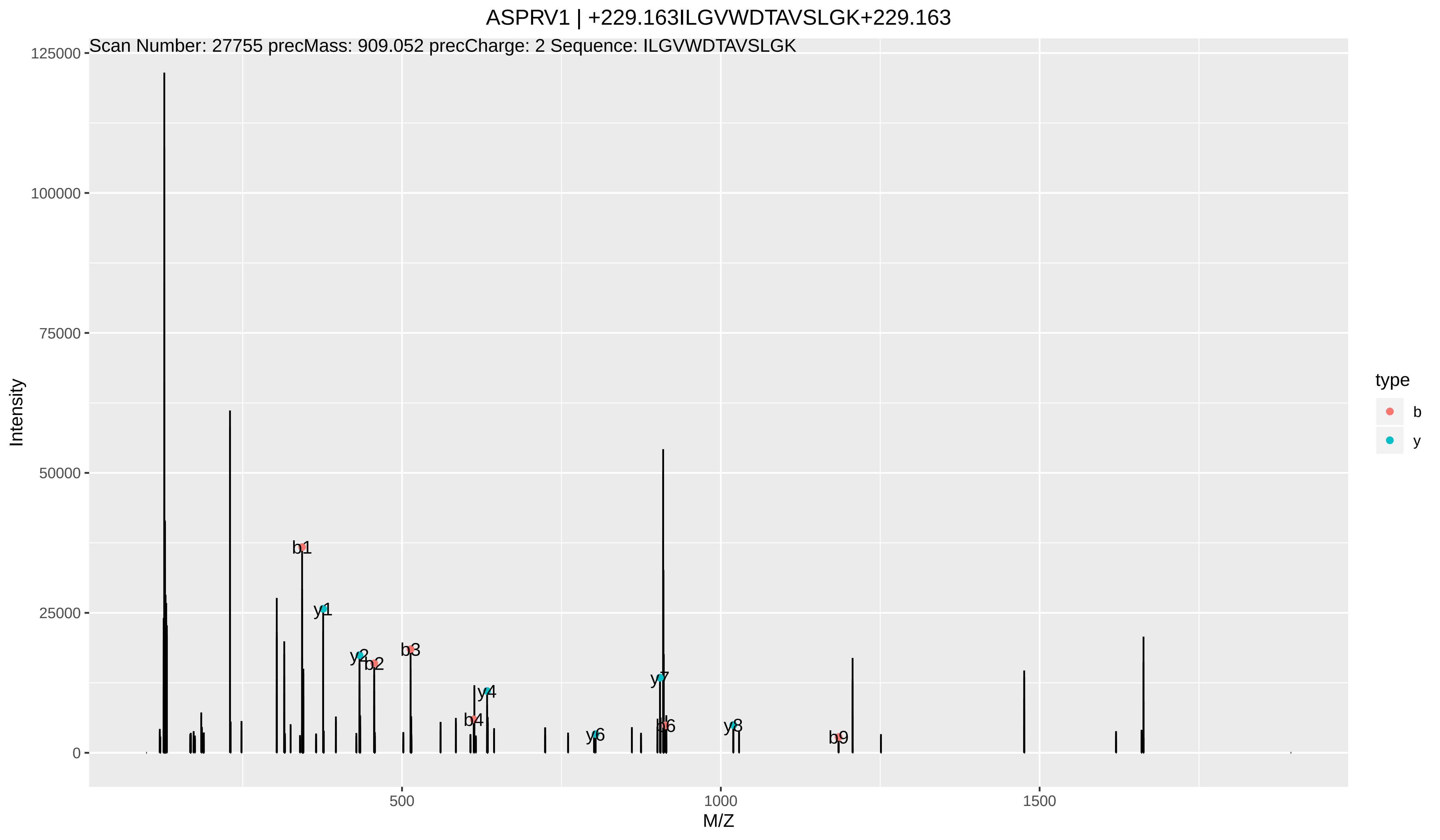

ASPRV1 | +229.163ILGVWDTAVSLGK+229.163
Scan Number: 27755 precMass: 909.052 precCharge: 2 Sequence: ILGVWDTAVSLGK
Intensity
M/Z
125000
100000
75000
50000
25000
0
500
1000
1500
b1
y1
y2
b2
b3
y4
b4
y6
y7
b6
y8
b9
type
b
y

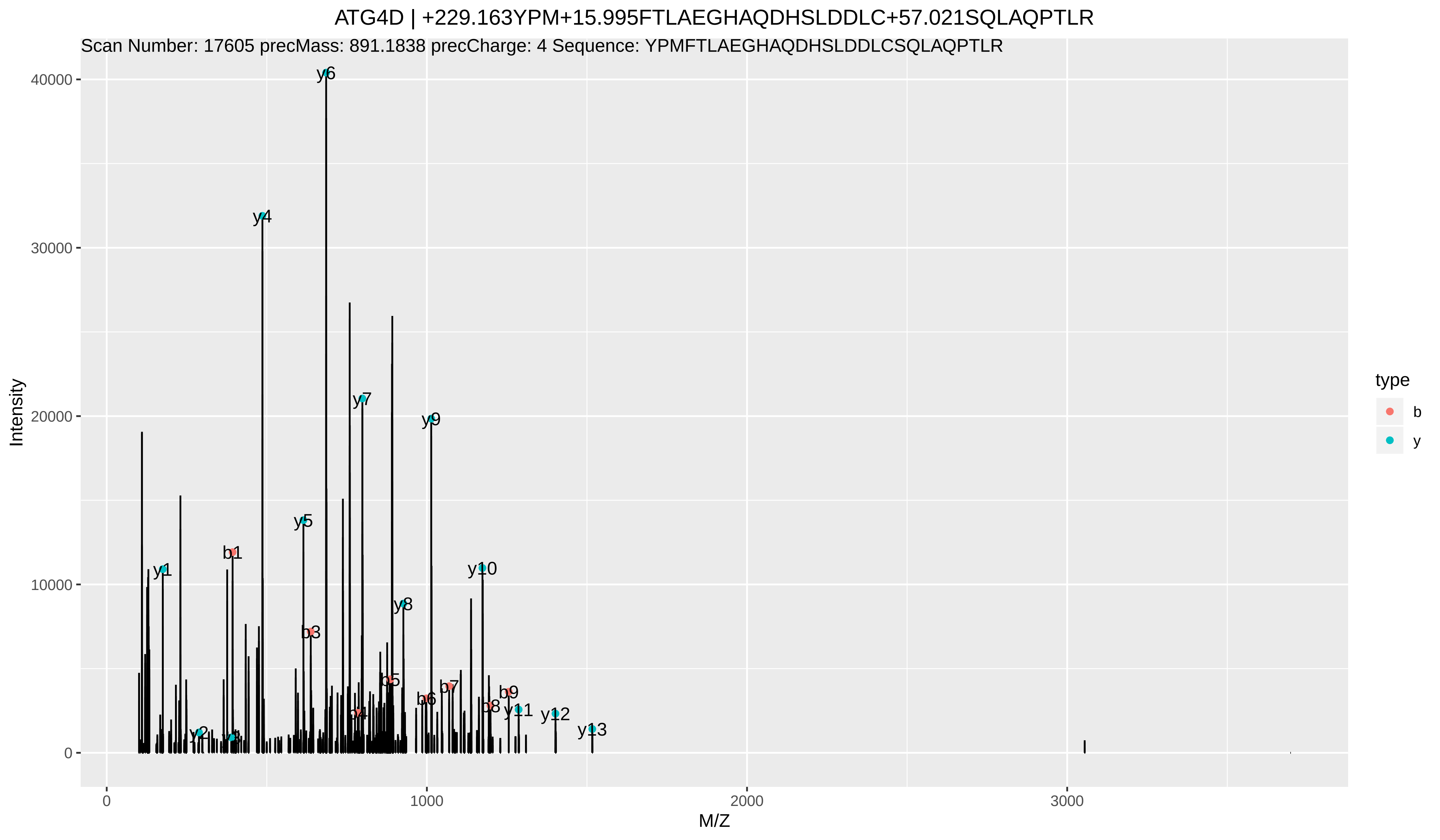

ATG4D | +229.163YPM+15.995FTLAEGHAQDHSLDDLC+57.021SQLAQPTLR
Scan Number: 17605 precMass: 891.1838 precCharge: 4 Sequence: YPMFTLAEGHAQDHSLDDLCSQLAQPTLR
Intensity
M/Z
type
b
y
y6
y4
y7
y9
y5
b1
y1
y10
y8
b3
b5
b7
b6
b9
b8
y11
y12
y13
y2
40000
30000
20000
10000
0
1000
2000
3000

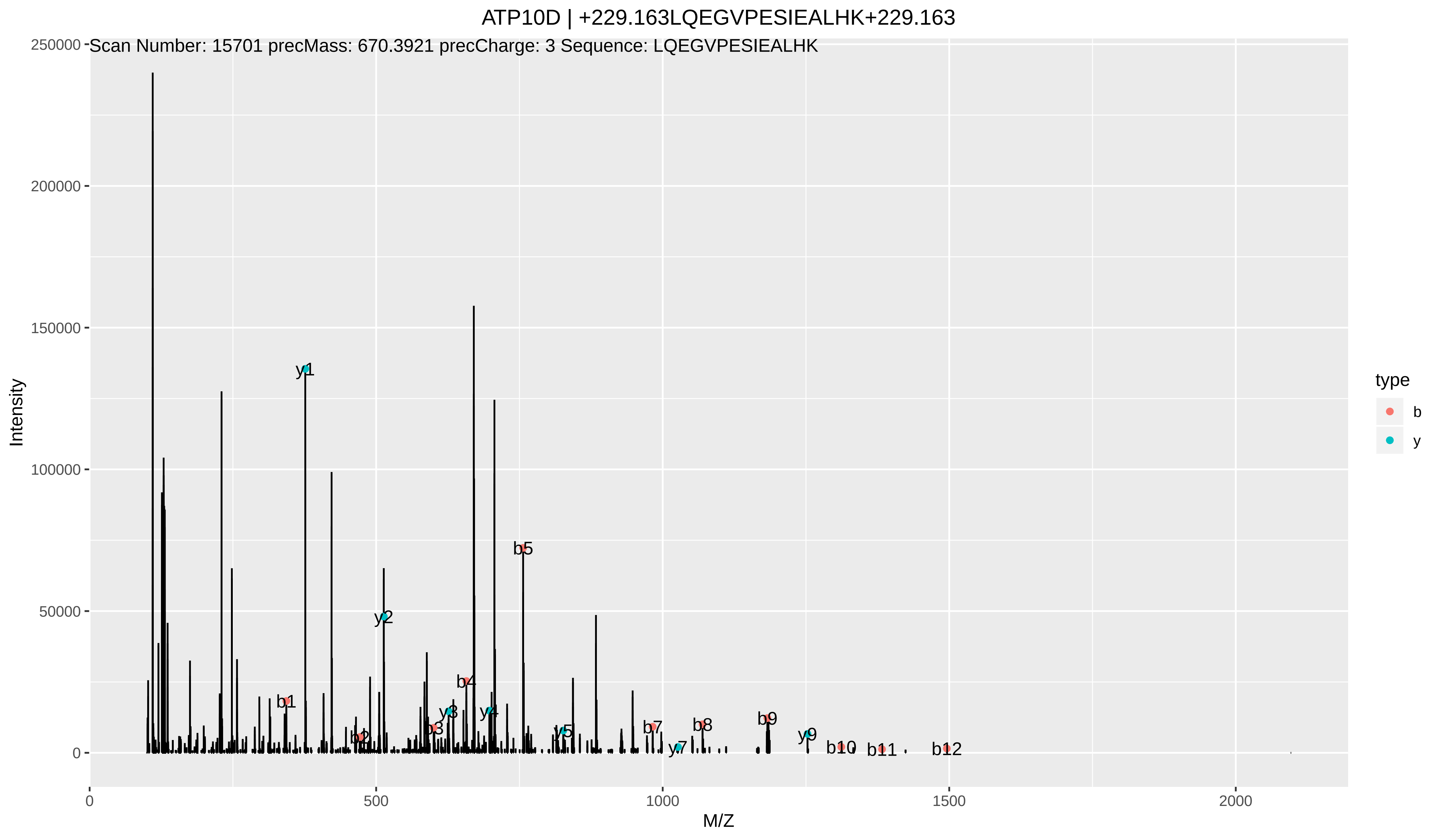
ATP10D | +229.163LQEGVPESIEALHK+229.163
Scan Number: 15701 precMass: 670.3921 precCharge: 3 Sequence: LQEGVPESIEALHK
Intensity
M/Z
0
50000
100000
150000
200000
250000
500
1000
1500
2000
y1
y2
y3
y4
y5
y7
y9
b1
b2
b3
b4
b5
b7
b8
b9
b10
b11
b12
type
b
y

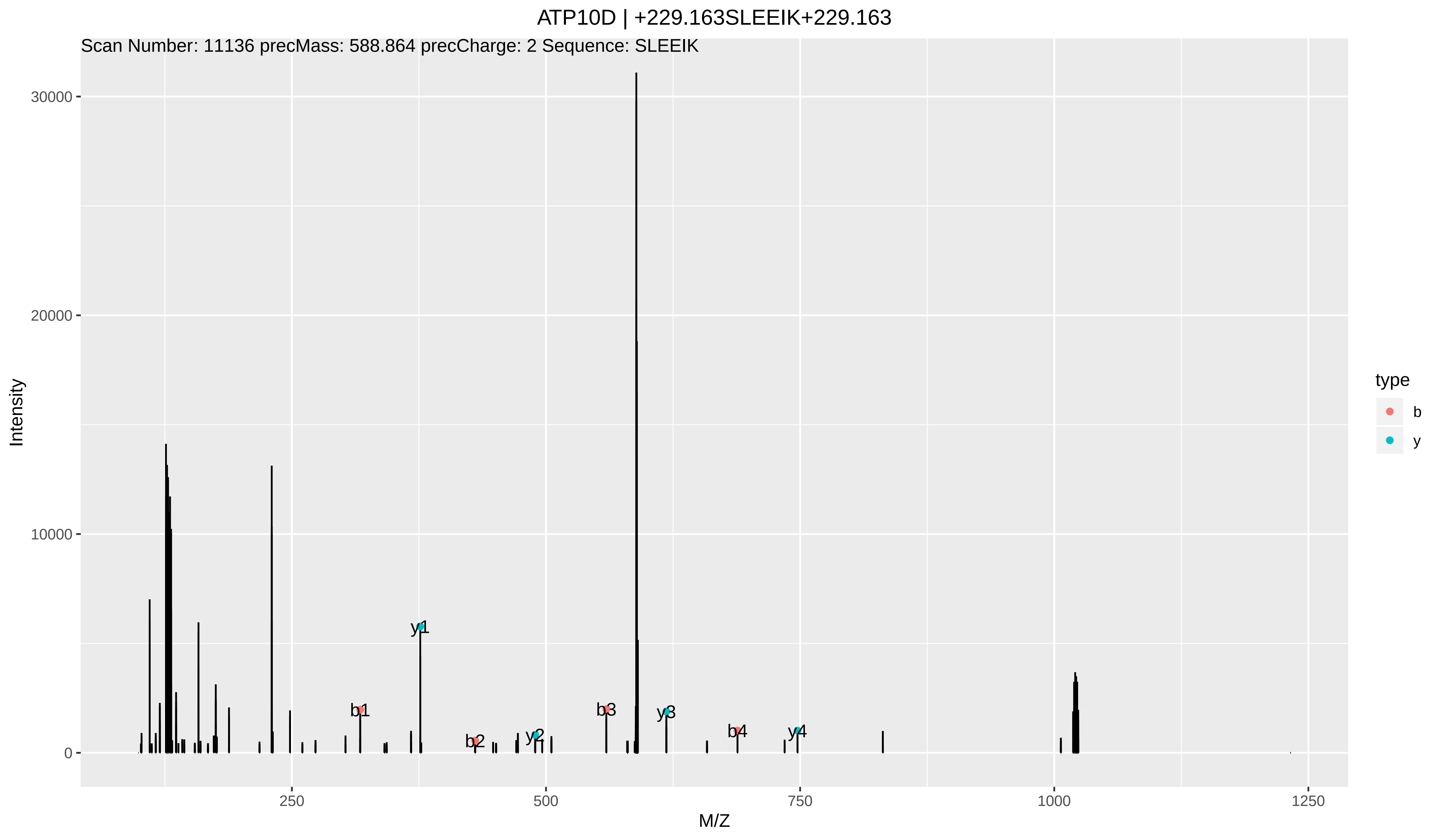
ATP10D | +229.163SLEEIK+229.163
Scan Number: 11136 precMass: 588.864 precCharge: 2 Sequence: SLEEIK
Intensity
30000
20000
10000
0
M/Z
250
500
750
1000
1250
y1
b1
b2
y2
b3
y3
b4
y4
type
b
y

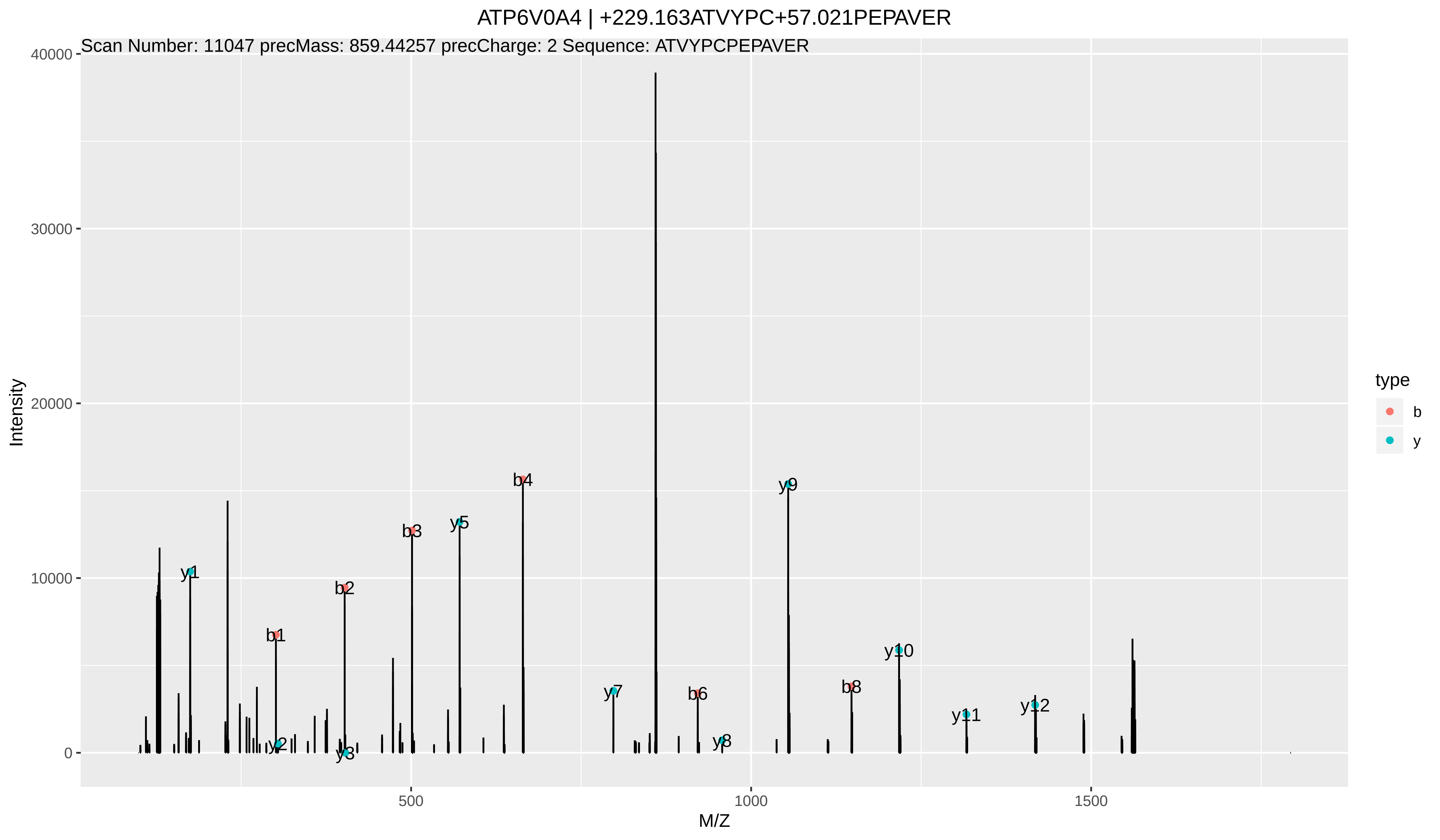

ATP6V0A4 | +229.163ATVYPC+57.021PEPAVER
Scan Number: 11047 precMass: 859.44257 precCharge: 2 Sequence: ATVYPCPEPAVER
Intensity
40000
30000
20000
10000
0
M/Z
500
1000
1500
y1
b1
y2
y3
b2
b3
y5
b4
y7
b6
y8
y9
b8
y10
y11
y12
type
b
y

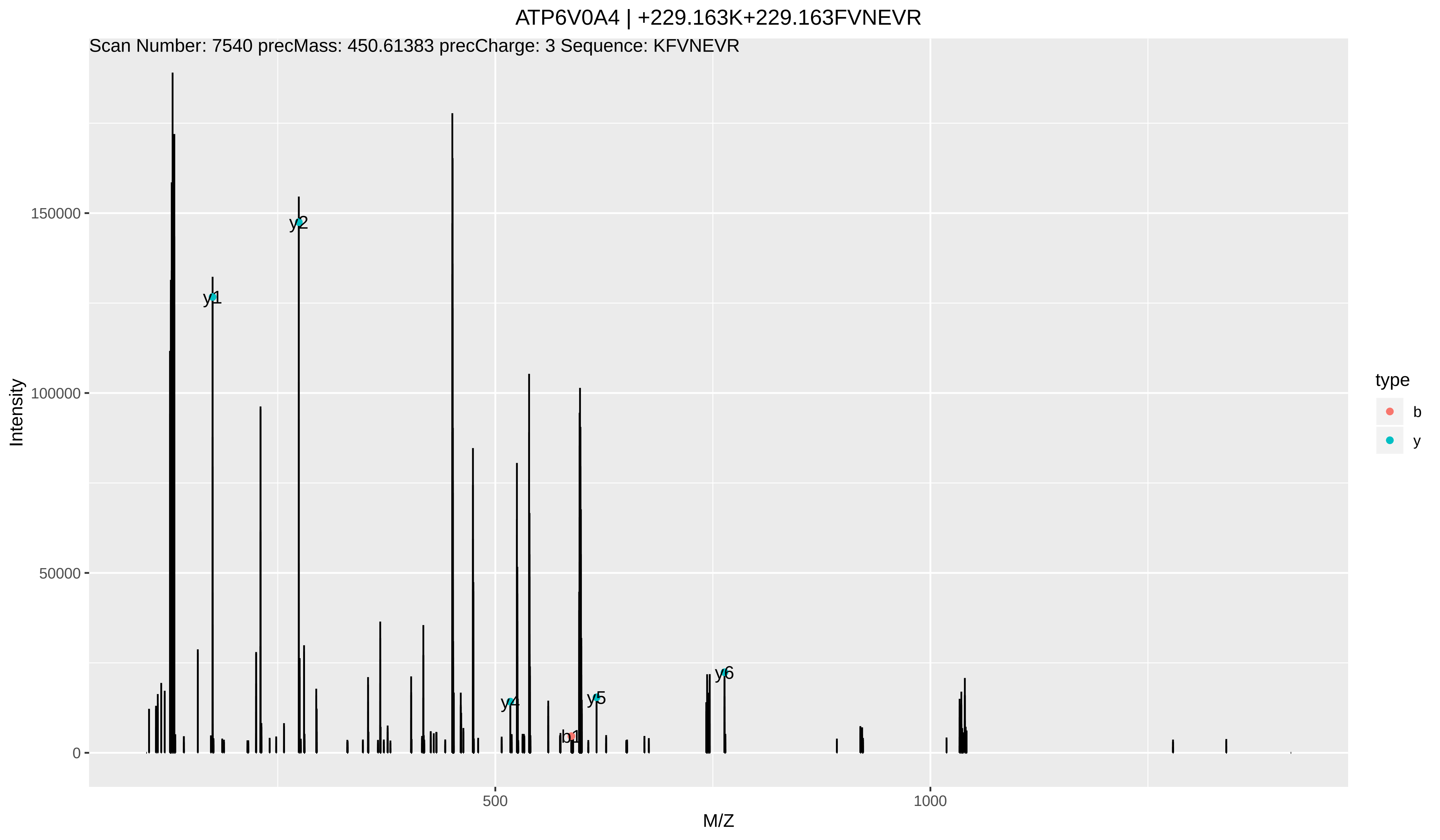

ATP6V0A4 | +229.163K+229.163FVNEVR
Scan Number: 7540 precMass: 450.61383 precCharge: 3 Sequence: KFVNEVR
Intensity
M/Z
150000
100000
50000
0
500
1000
y1
y2
y4
b1
y5
y6
type
b
y

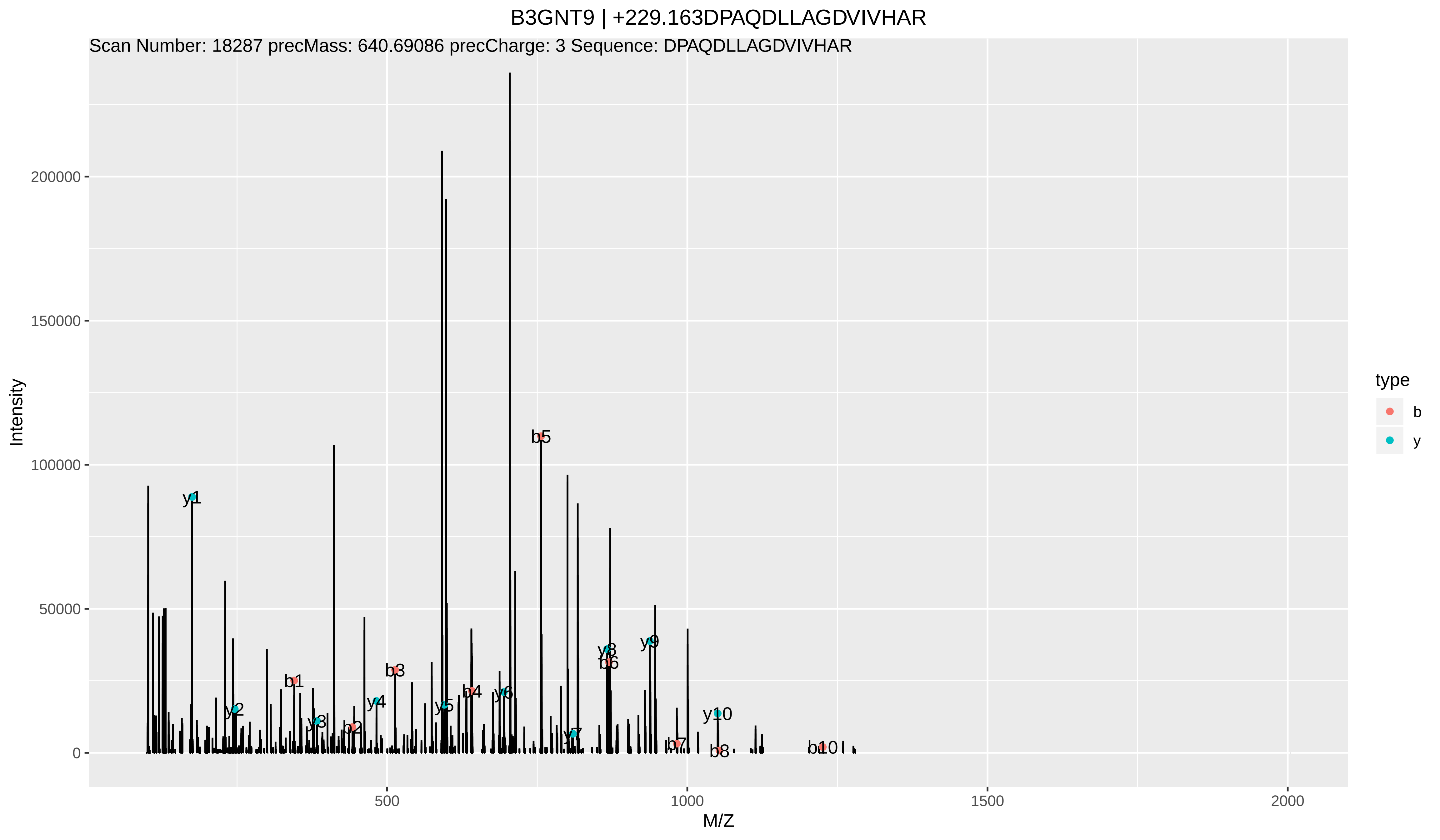

B3GNT9 | +229.163DPAQDLLAGDVIVHAR
Scan Number: 18287 precMass: 640.69086 precCharge: 3 Sequence: DPAQDLLAGDVIVHAR
Intensity
M/Z
type
b
y
0
50000
100000
150000
200000
500
1000
1500
2000
y1
y2
y3
y4
y5
y6
y7
y8
y9
y10
b1
b2
b3
b4
b5
b6
b7
b8
b10

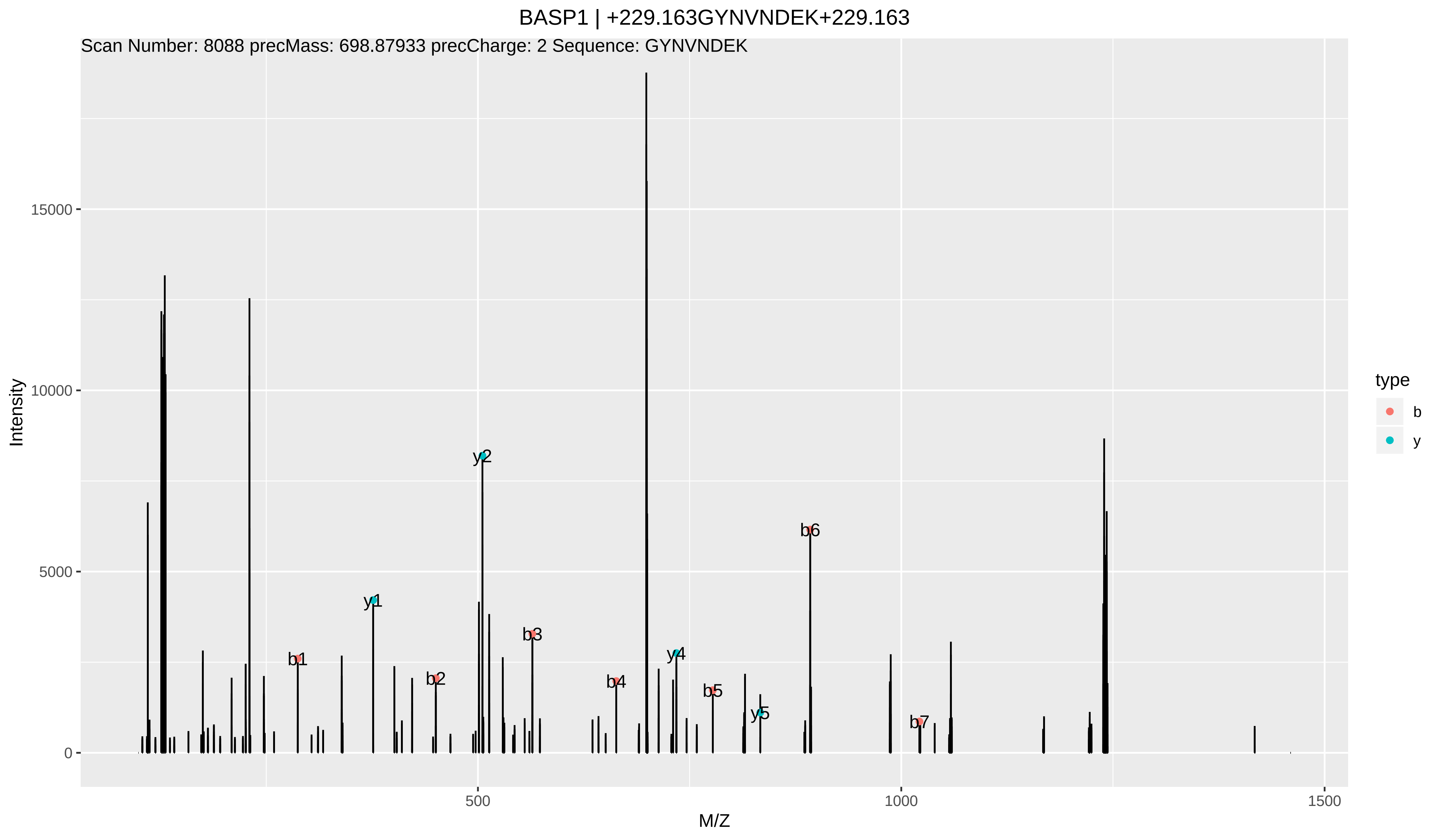

BASP1 | +229.163GYNVNDEK+229.163
Scan Number: 8088 precMass: 698.87933 precCharge: 2 Sequence: GYNVNDEK
Intensity
M/Z
15000
10000
5000
0
500
1000
1500
y2
y1
b1
b2
b3
b4
y4
b5
y5
b6
b7
type
b
y

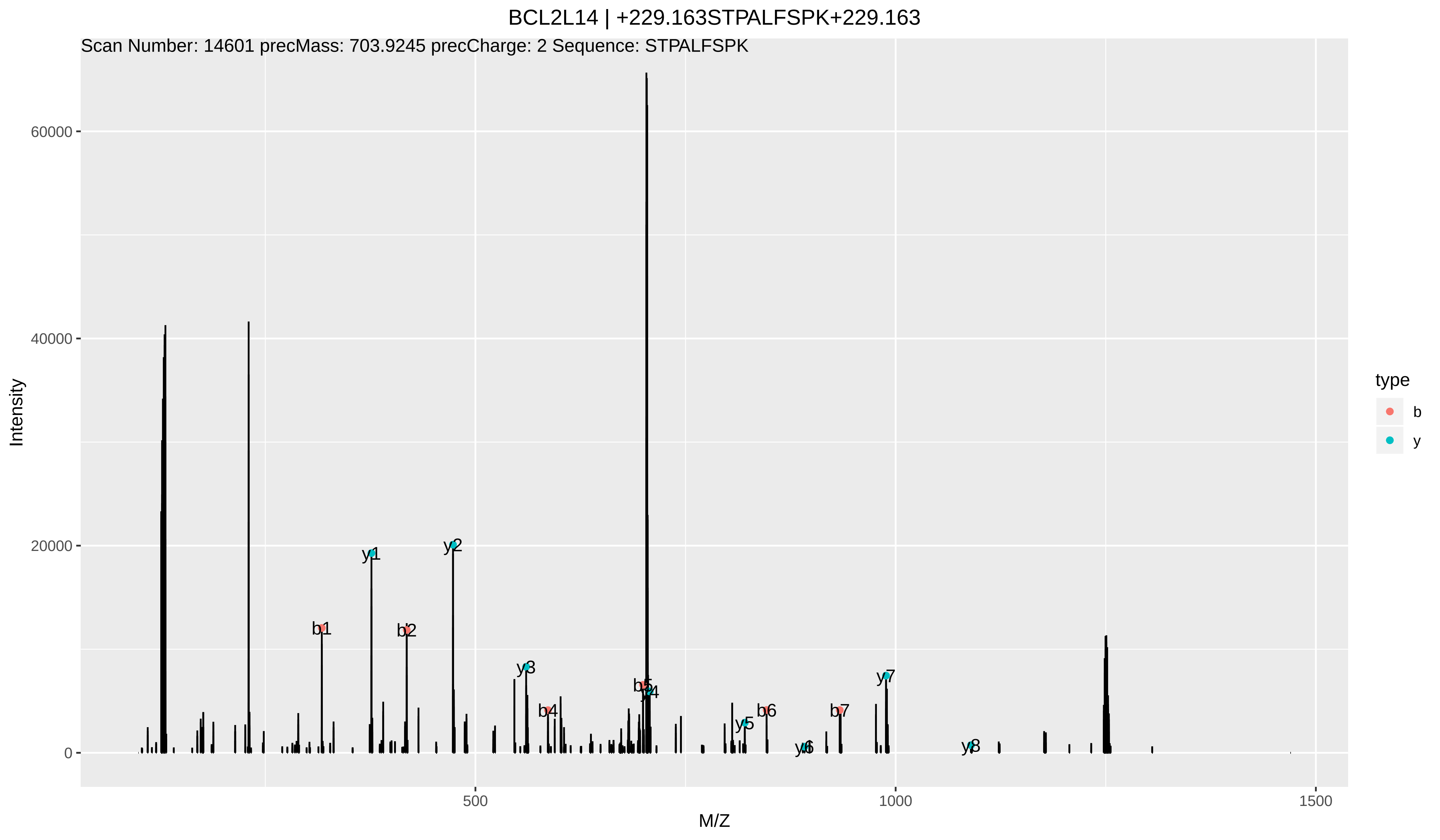

BCL2L14 | +229.163STPALFSPK+229.163
Scan Number: 14601 precMass: 703.9245 precCharge: 2 Sequence: STPALFSPK
Intensity
M/Z
60000
40000
20000
0
500
1000
1500
b1
b2
b4
b5
b6
b7
y1
y2
y3
y4
y5
y6
y7
y8
type
b
y

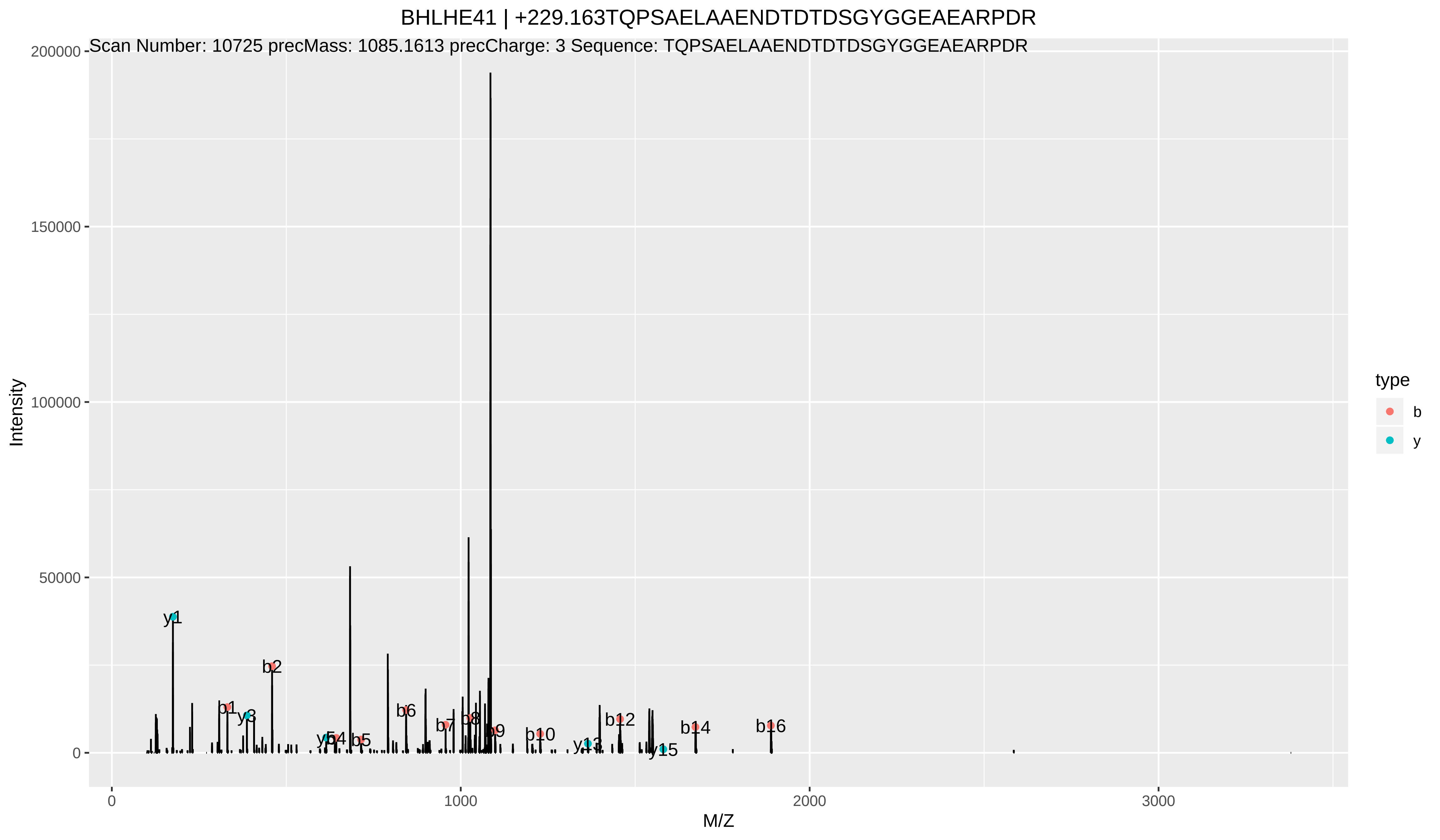

BHLHE41 | +229.163TQPSAELAAENDTDTDSGYGGEAEARPDR
Scan Number: 10725 precMass: 1085.1613 precCharge: 3 Sequence: TQPSAELAAENDTDTDSGYGGEAEARPDR
Intensity
200000
150000
100000
50000
0
M/Z
0
1000
2000
3000
y1
b2
b1
y3
y5
b4
b5
b6
b7
b8
b9
b10
y13
b12
y15
b14
b16
type
b
y

BHLHE41 | +229.163DLLPEHLK+229.163

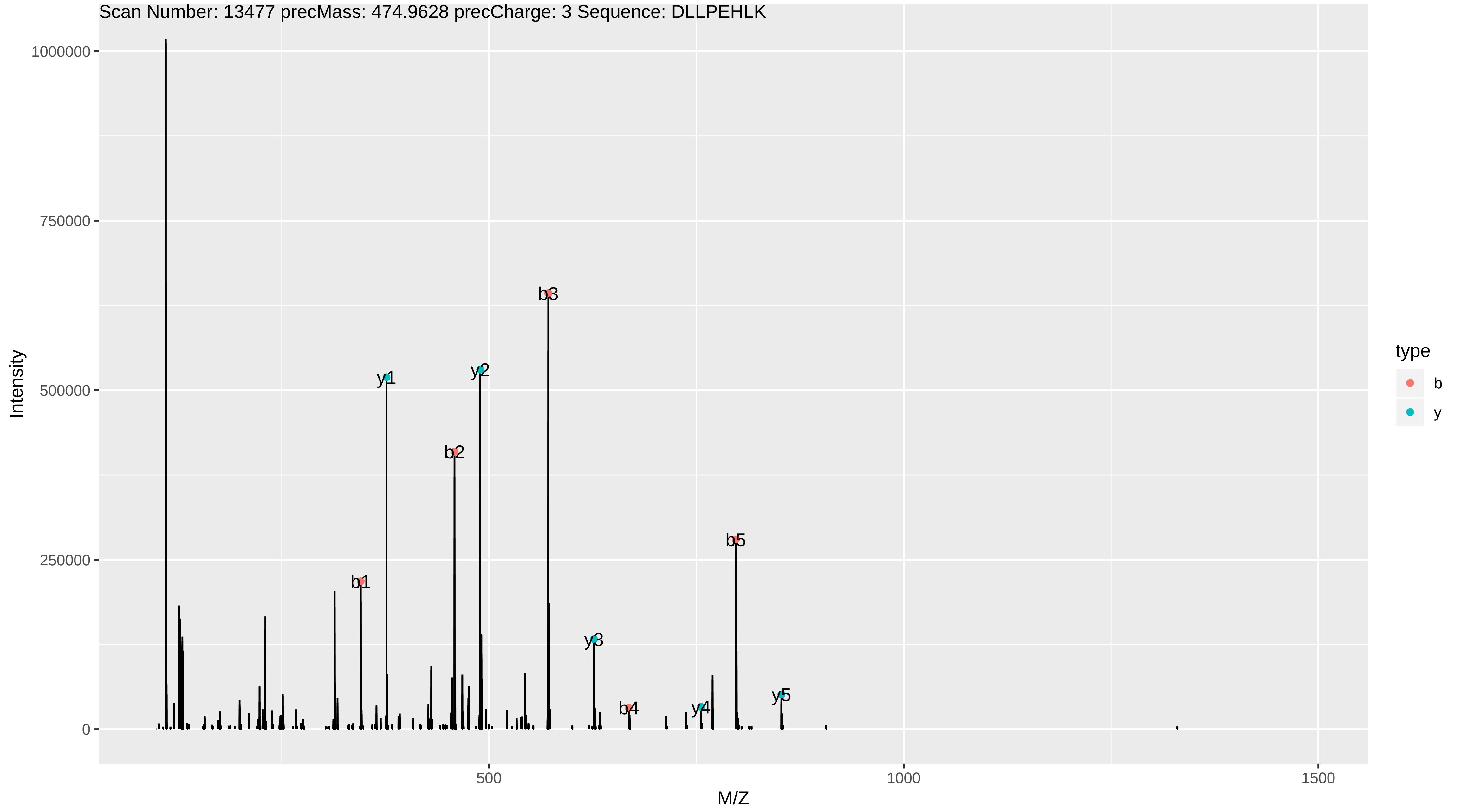

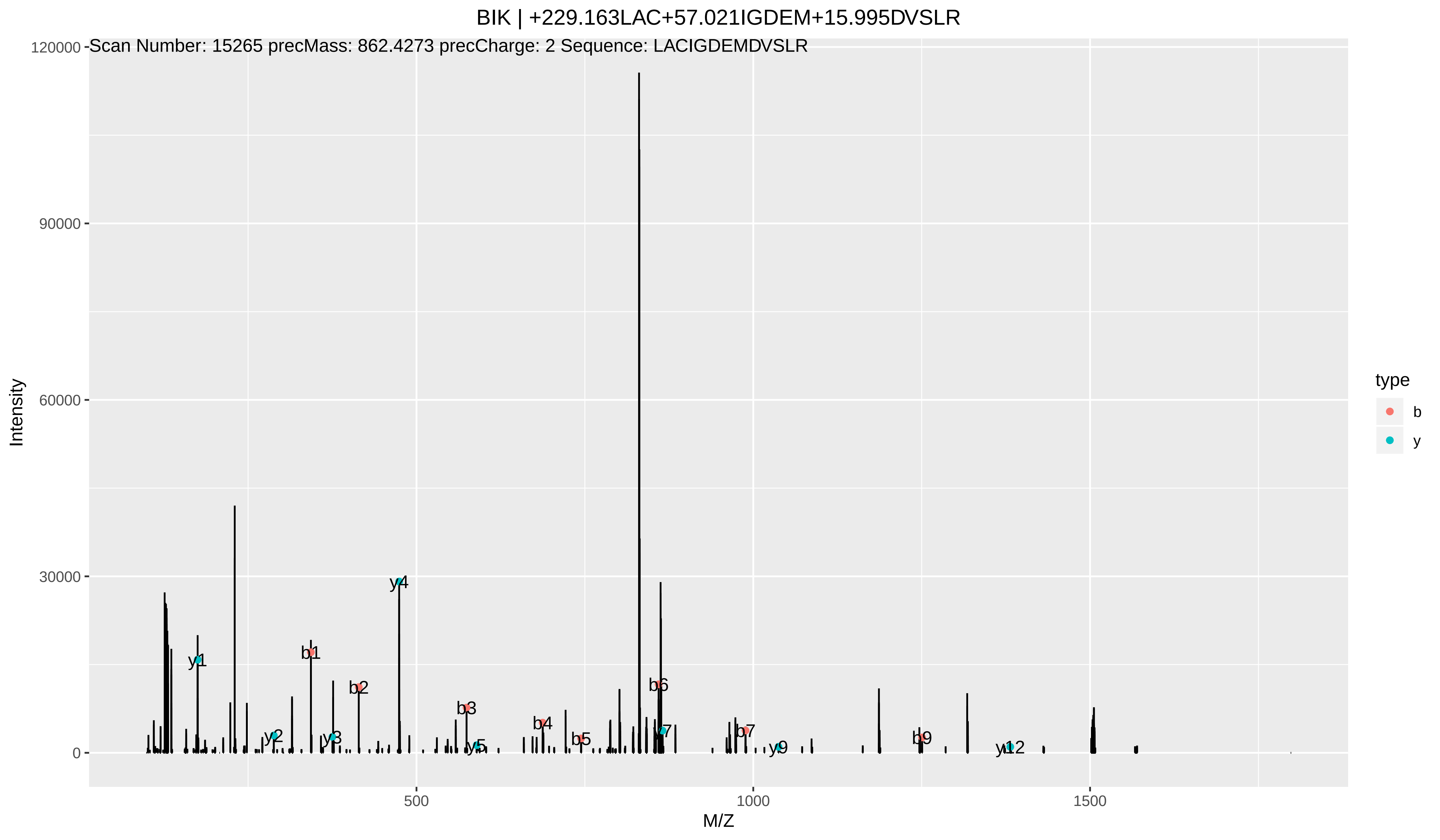

BIK | +229.163LAC+57.021IGDEM+15.995DVSLR
Scan Number: 15265 precMass: 862.4273 precCharge: 2 Sequence: LACIGDEMDVSLR
Intensity
M/Z
type
b
y
0
30000
60000
90000
120000
500
1000
1500
y1
y2
y3
y4
y5
y7
y9
y12
b1
b2
b3
b4
b5
b6
b7
b9

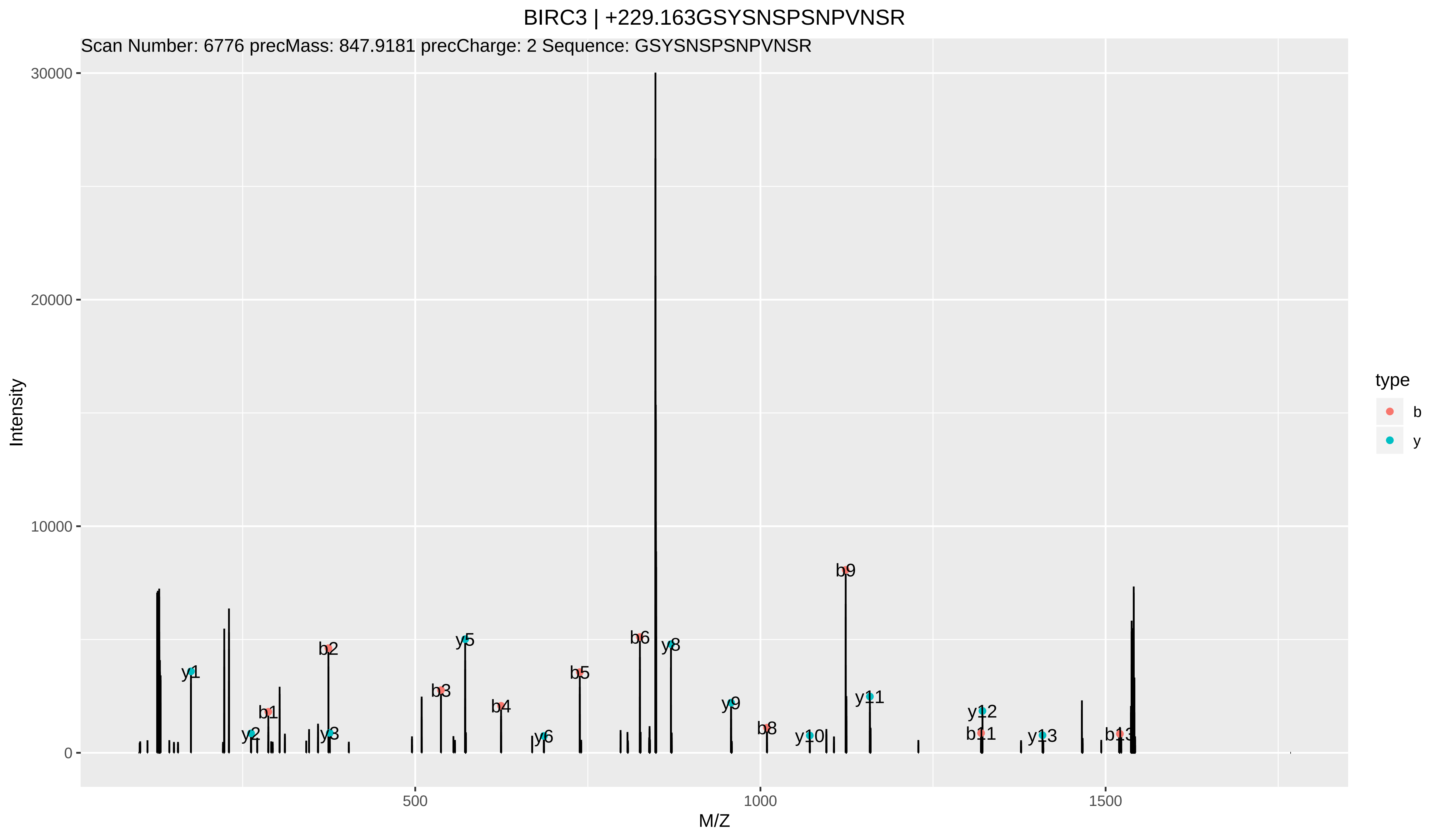
BIRC3 | +229.163GSYSNSPSNPVNSR
Scan Number: 6776 precMass: 847.9181 precCharge: 2 Sequence: GSYSNSPSNPVNSR
Intensity
M/Z
type
b
y
30000
20000
10000
0
500
1000
1500
y1
b1
y2
b2
y3
b3
y5
b4
y6
b5
b6
y8
y9
b8
y10
b9
y11
y12
b11
y13
b13

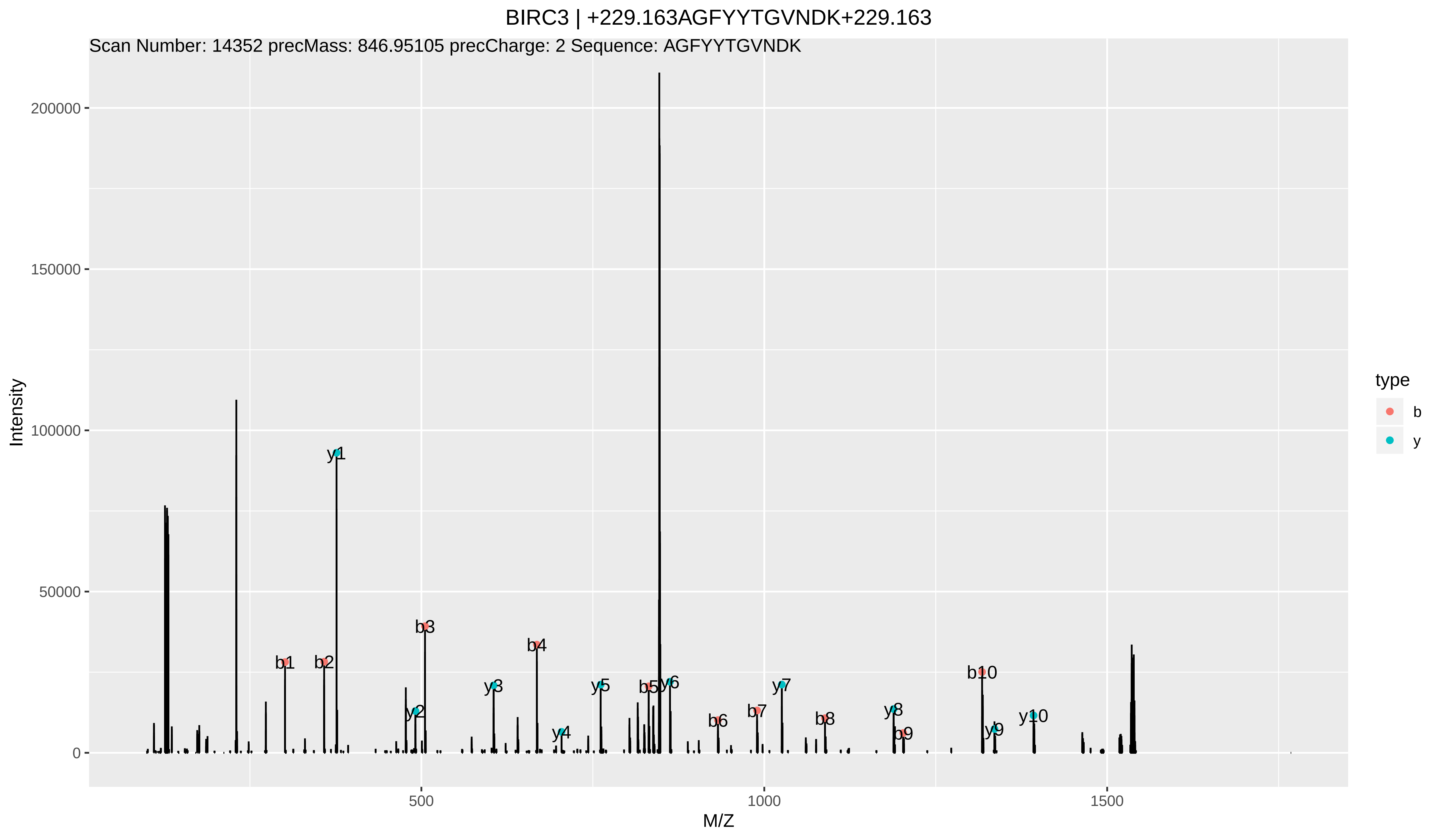

BIRC3 | +229.163AGFYYTGVNDK+229.163
Scan Number: 14352 precMass: 846.95105 precCharge: 2 Sequence: AGFYYTGVNDK
Intensity
200000
150000
100000
50000
0
500
1000
1500
M/Z
type
b
y
y1
b1
b2
b3
b4
y2
y3
y4
y5
b5
y6
b6
b7
y7
b8
y8
b9
b10
y9
y10

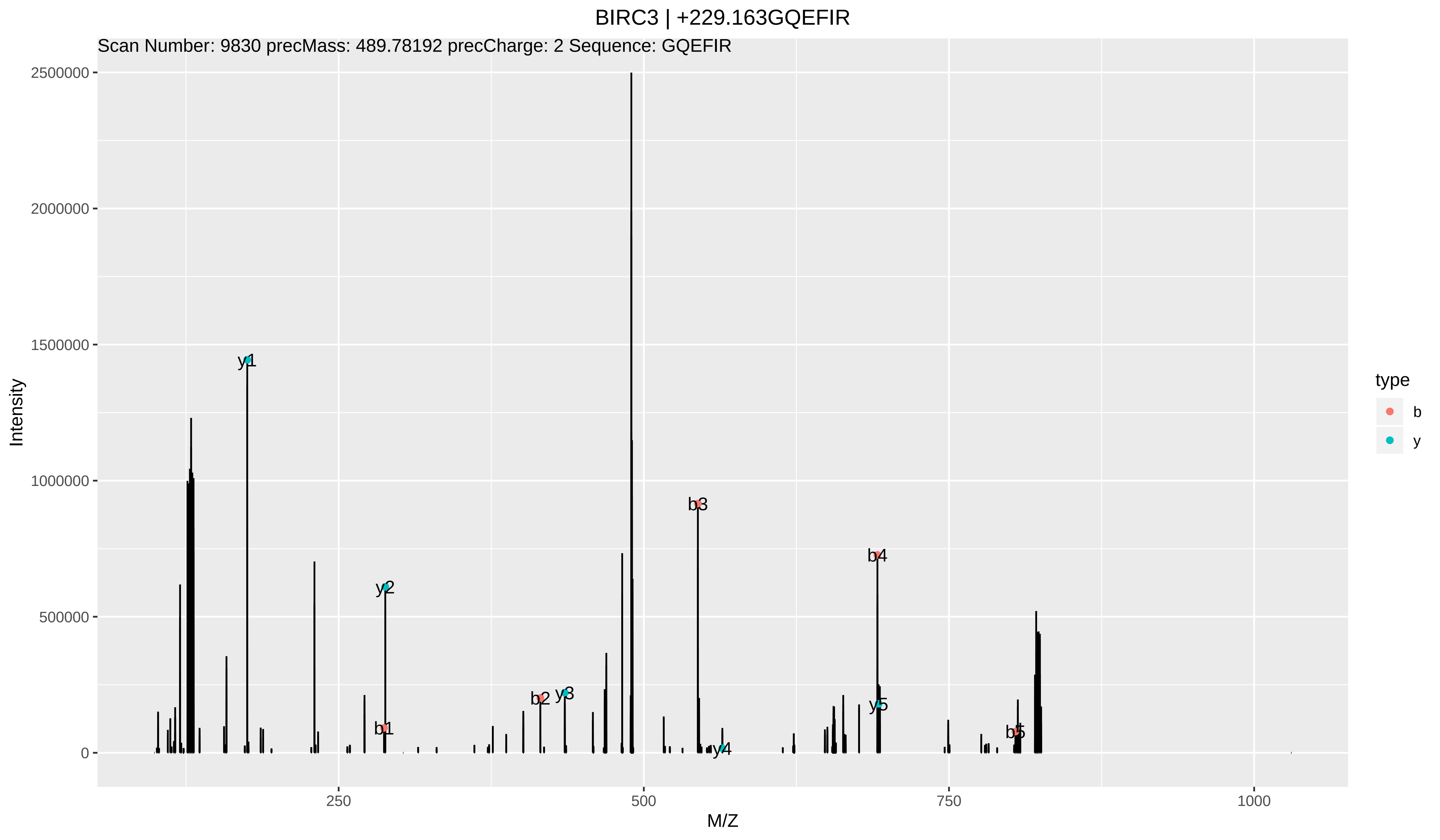

BIRC3 | +229.163GQEFIR
Scan Number: 9830 precMass: 489.78192 precCharge: 2 Sequence: GQEFIR
Intensity
M/Z
0
500000
1000000
1500000
2000000
2500000
250
500
750
1000
y1
y2
b1
b2
y3
b3
y4
b4
y5
b5
type
b
y

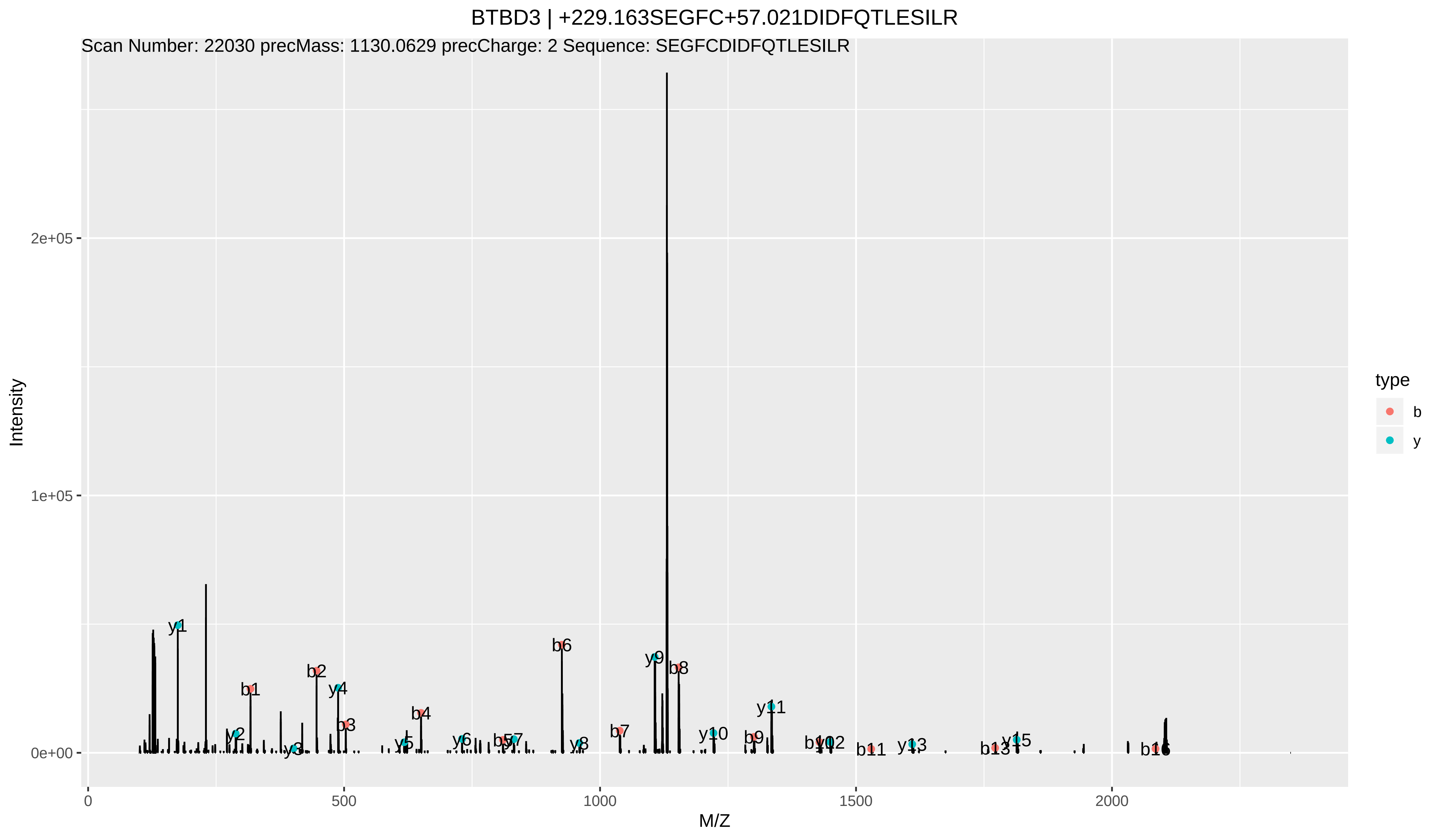
BTBD3 | +229.163SEGFC+57.021DIDFQTLESILR
Scan Number: 22030 precMass: 1130.0629 precCharge: 2 Sequence: SEGFCDIDFQTLESILR
Intensity
2e+05
1e+05
0e+00
M/Z
0
500
1000
1500
2000
type
b
y
y1
b1
y2
y3
b2
y4
b3
y5
b4
y6
b5
y7
b6
y8
b7
y9
b8
y10
b9
y11
b10
y12
b11
y13
b13
y15
b16

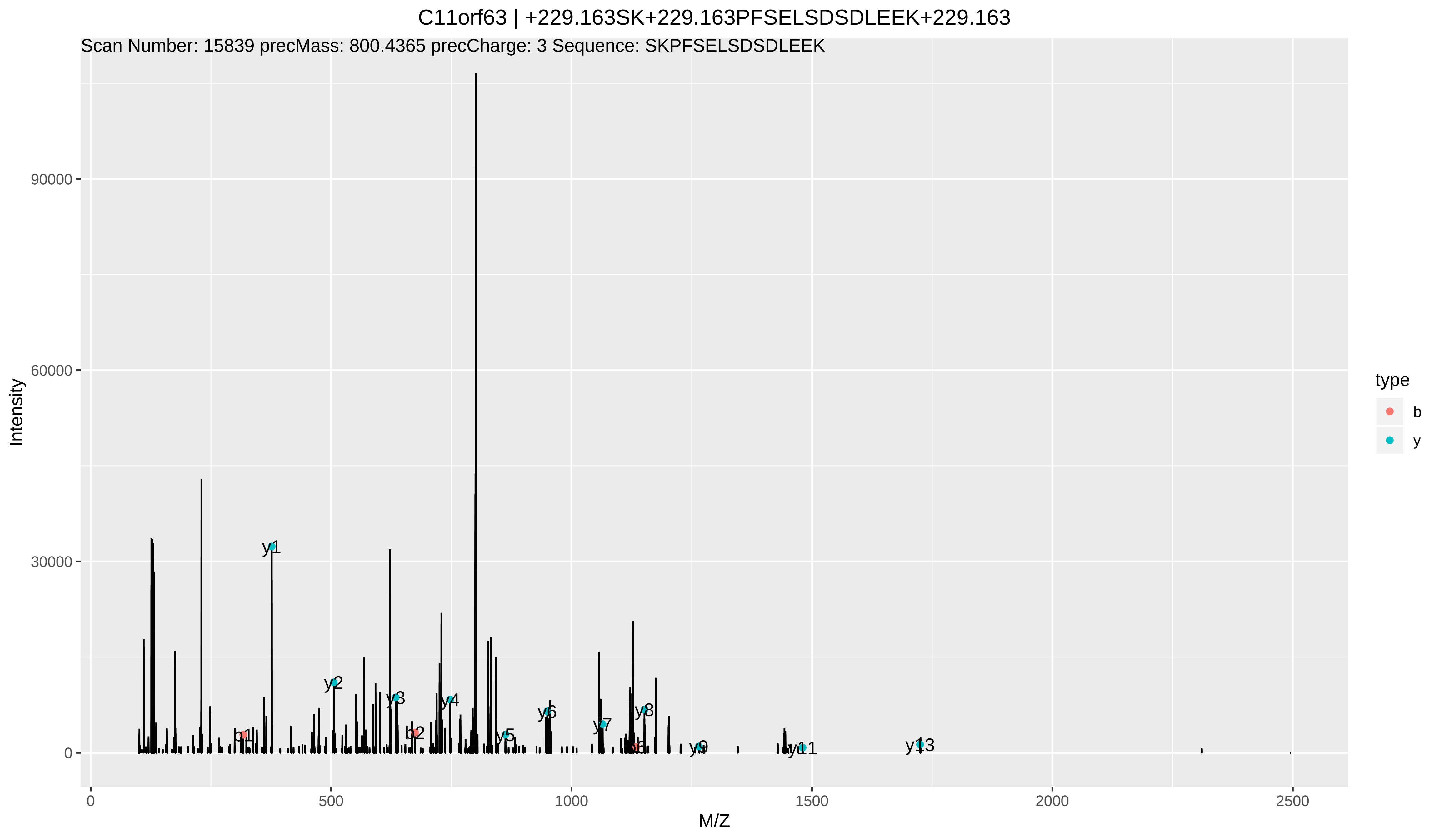
C11orf63 | +229.163SK+229.163PFSELSDSDLEEK+229.163
Scan Number: 15839 precMass: 800.4365 precCharge: 3 Sequence: SKPFSELSDSDLEEK
Intensity
M/Z
0
30000
60000
90000
500
1000
1500
2000
2500
y1
y2
y3
y4
y5
y6
y7
y8
y9
y11
y13
b1
b2
type
b
y

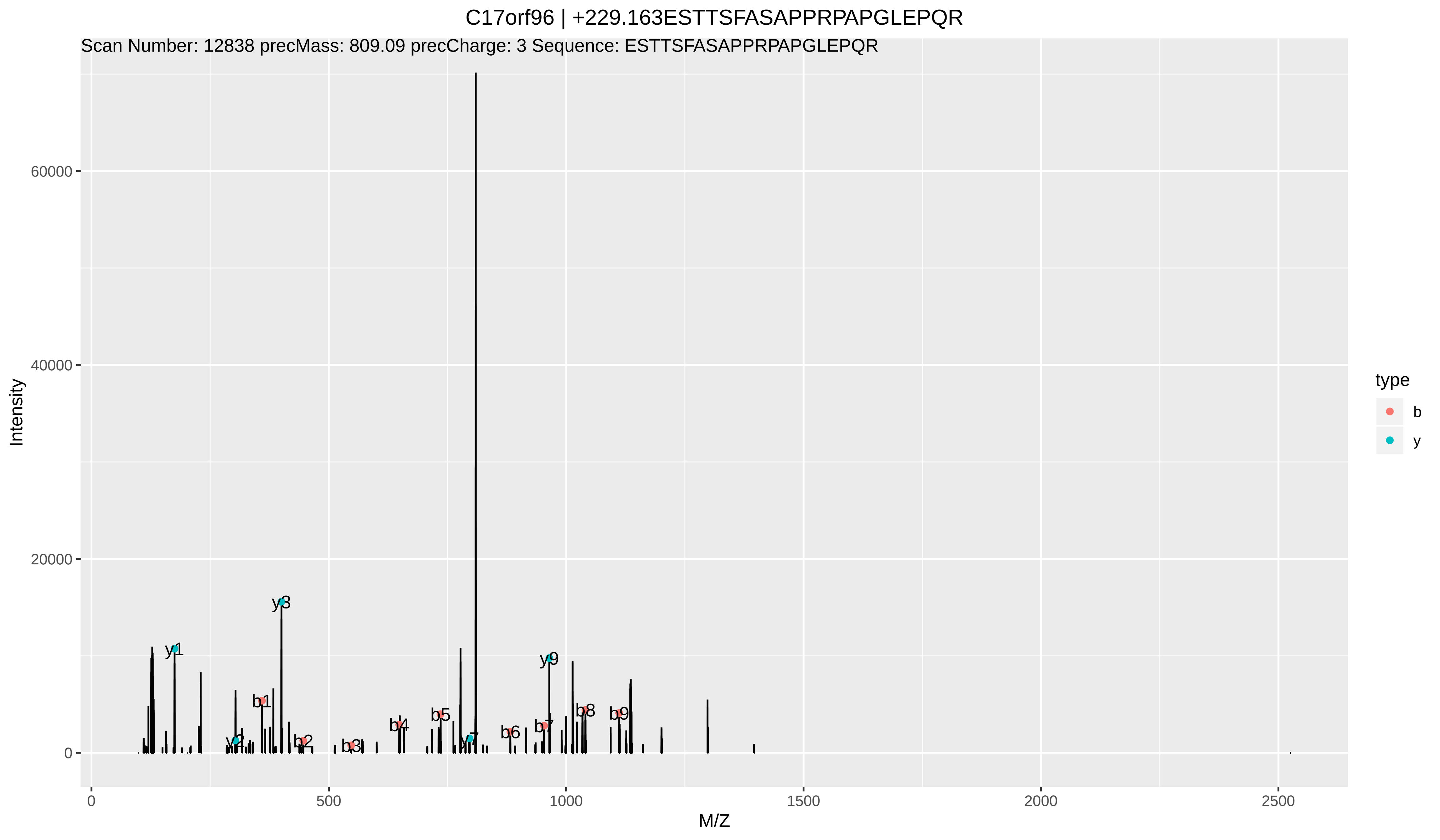

C17orf96 | +229.163ESTTSFASAPPRPAPGLEPQR
Scan Number: 12838 precMass: 809.09 precCharge: 3 Sequence: ESTTSFASAPPRPAPGLEPQR
Intensity
M/Z
0
20000
40000
60000
0
500
1000
1500
2000
2500
y1
y2
y3
y7
y9
b1
b2
b3
b4
b5
b6
b7
b8
b9
type
b
y

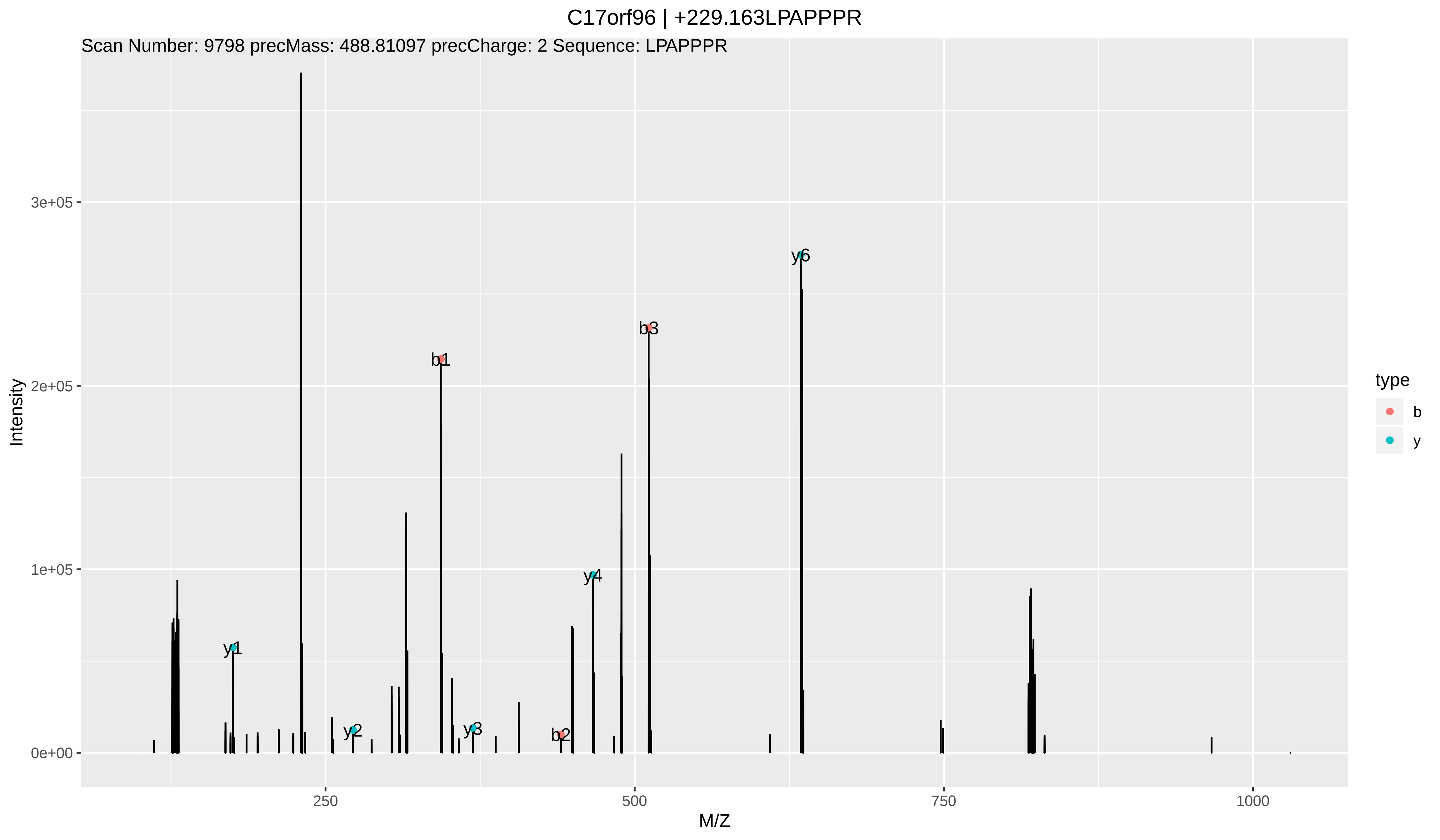
C17orf96 | +229.163LPAPPPR
Scan Number: 9798 precMass: 488.81097 precCharge: 2 Sequence: LPAPPPR
Intensity
M/Z
3e+05
2e+05
1e+05
0e+00
250
500
750
1000
y1
y2
y3
y4
y6
b1
b2
b3
type
b
y

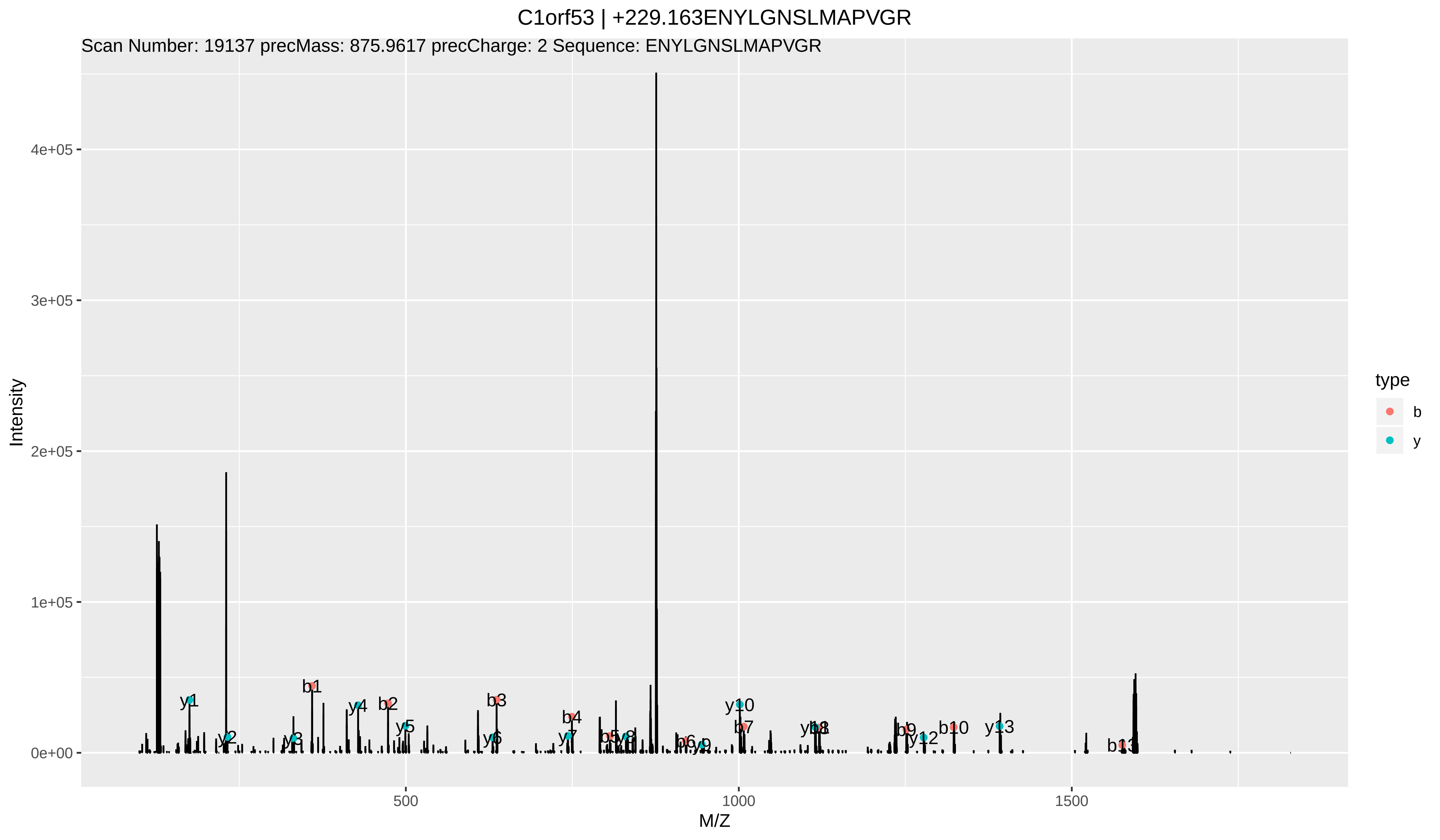

C1orf53 | +229.163ENYLGNSLMAPVGR
Scan Number: 19137 precMass: 875.9617 precCharge: 2 Sequence: ENYLGNSLMAPVGR
Intensity
4e+05
3e+05
2e+05
1e+05
0e+00
M/Z
500
1000
1500
y1
y2
y3
b1
y4
b2
y5
y6
b3
y7
b4
b5
y8
b6
y9
y10
b7
y11
b8
b9
y12
b10
y13
b13
type
b
y

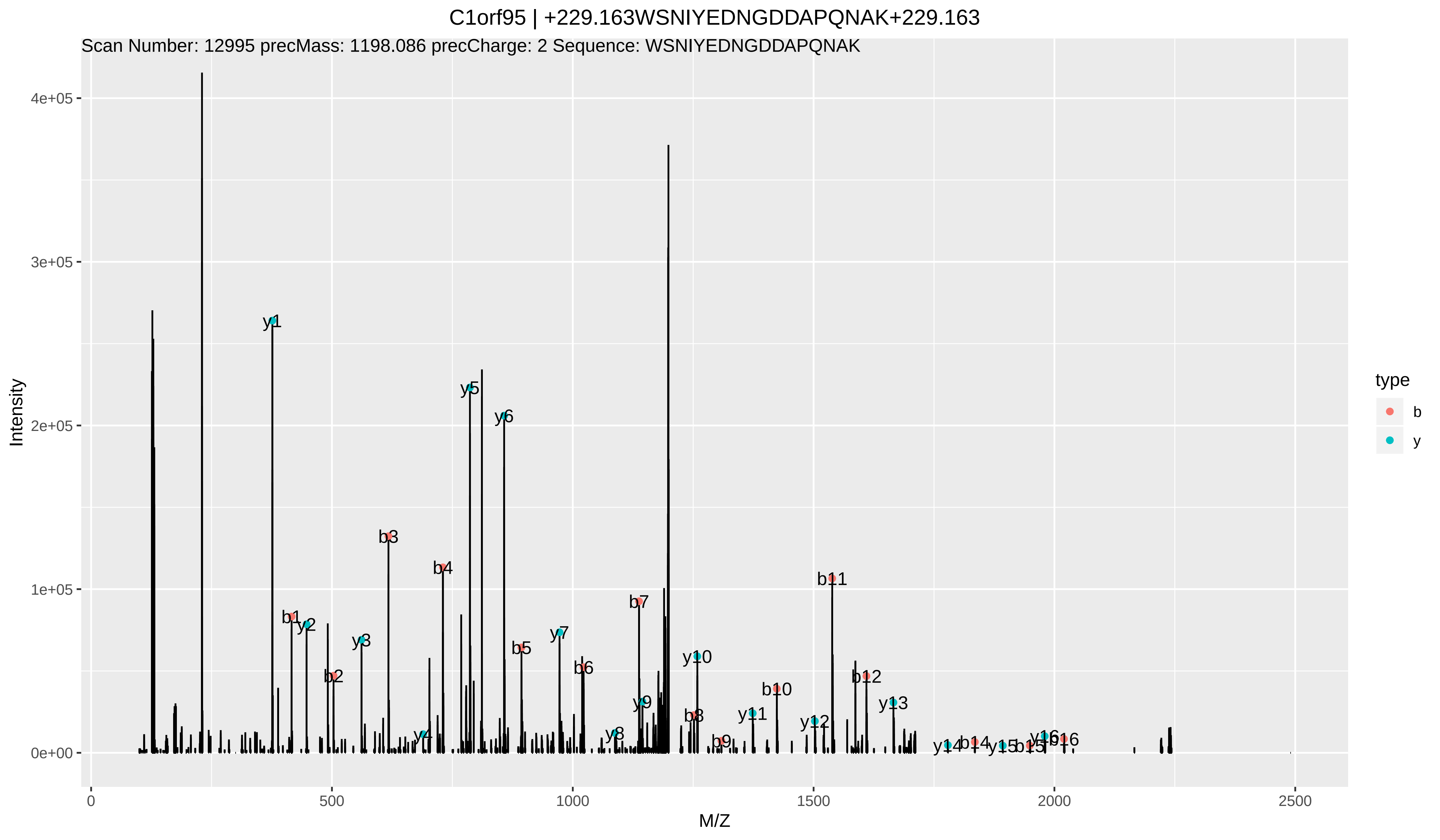
C1orf95 | +229.163WSNIYEDNGDDAPQNAK+229.163
Scan Number: 12995 precMass: 1198.086 precCharge: 2 Sequence: WSNIYEDNGDDAPQNAK
Intensity
M/Z
4e+05
3e+05
2e+05
1e+05
0e+00
0
500
1000
1500
2000
2500
type
b
y
y1
b1
y2
b2
y3
b3
y4
b4
y5
y6
b5
y7
b6
y8
b7
y9
y10
b8
b9
y11
b10
y12
b11
b12
y13
y14
b14
y15
b15
y16
b16

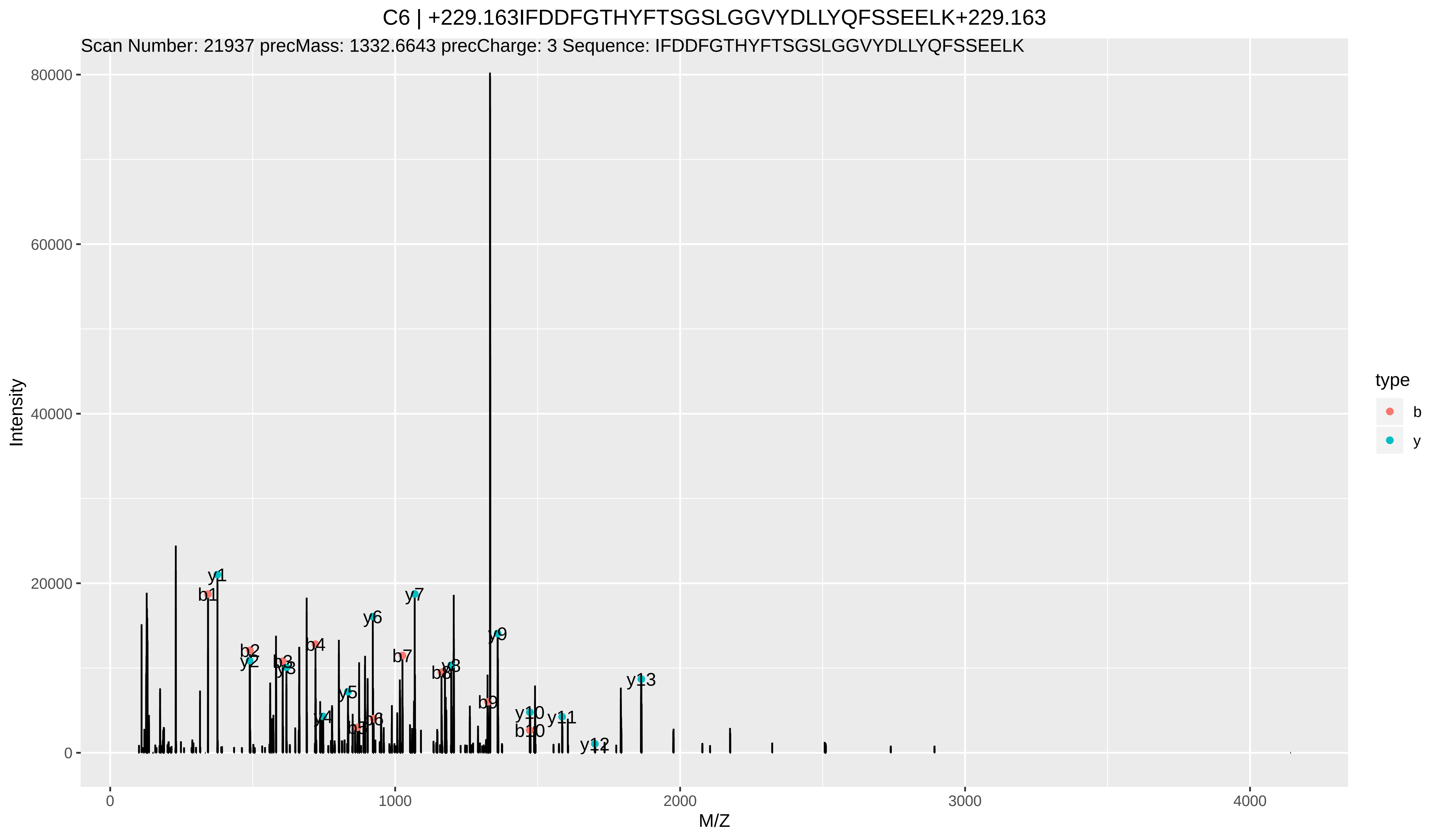
C6 | +229.163IFDDFGTHYFTSGSLGGVYDLLYQFSSEELK+229.163
Scan Number: 21937 precMass: 1332.6643 precCharge: 3 Sequence: IFDDFGTHYFTSGSLGGVYDLLYQFSSEELK
Intensity
80000
60000
40000
20000
0
M/Z
0
1000
2000
3000
4000
type
b
y
y1
b1
b2
y2
b3
y3
b4
y4
y5
b5
b6
y6
y7
b7
b8
y8
y9
b9
y10
b10
y11
y12
y13

CACFD1 | +229.163K+229.163GDAISYAR

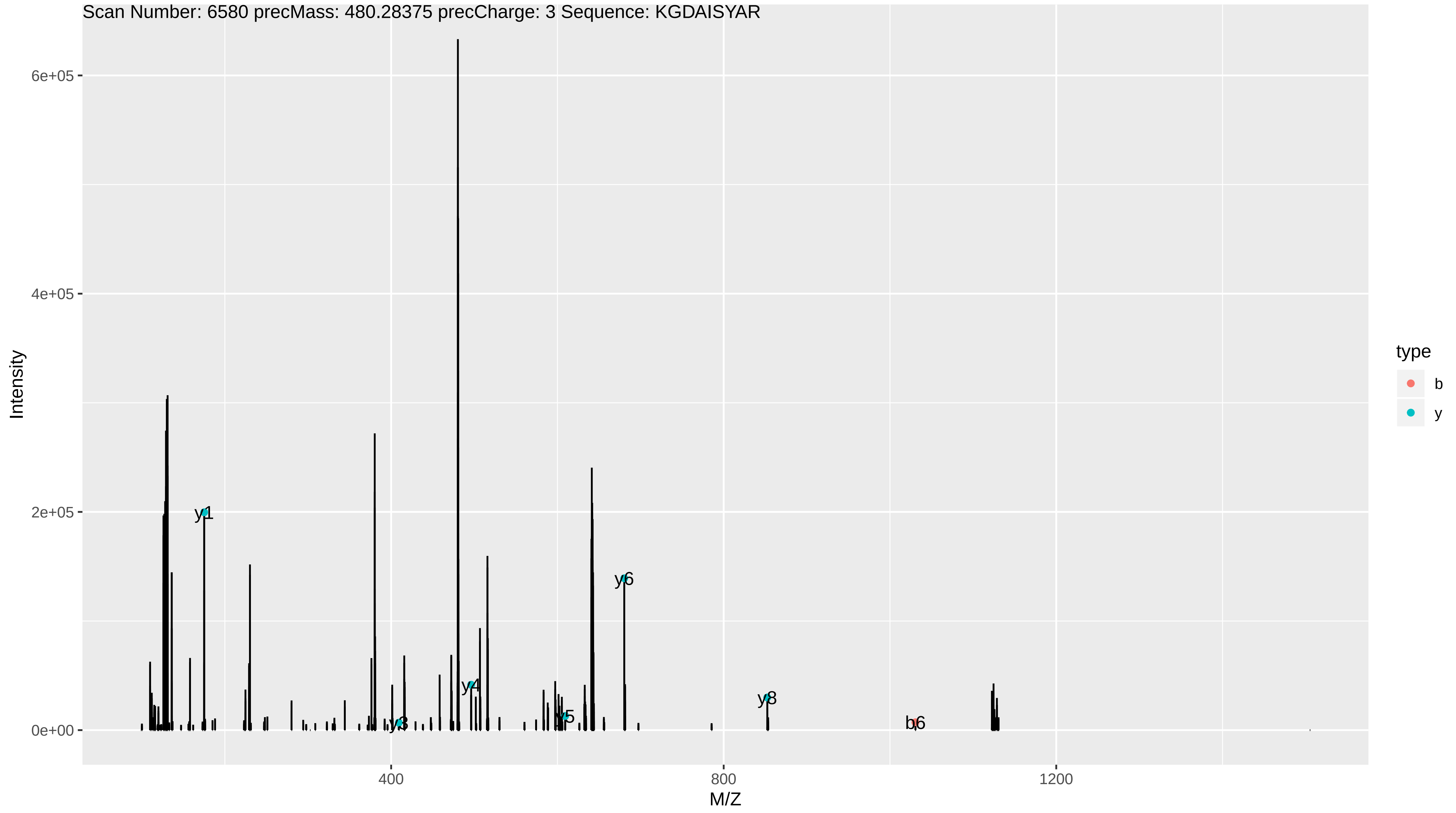

CACNB1 | +229.163HLNVQIAASEK+229.163

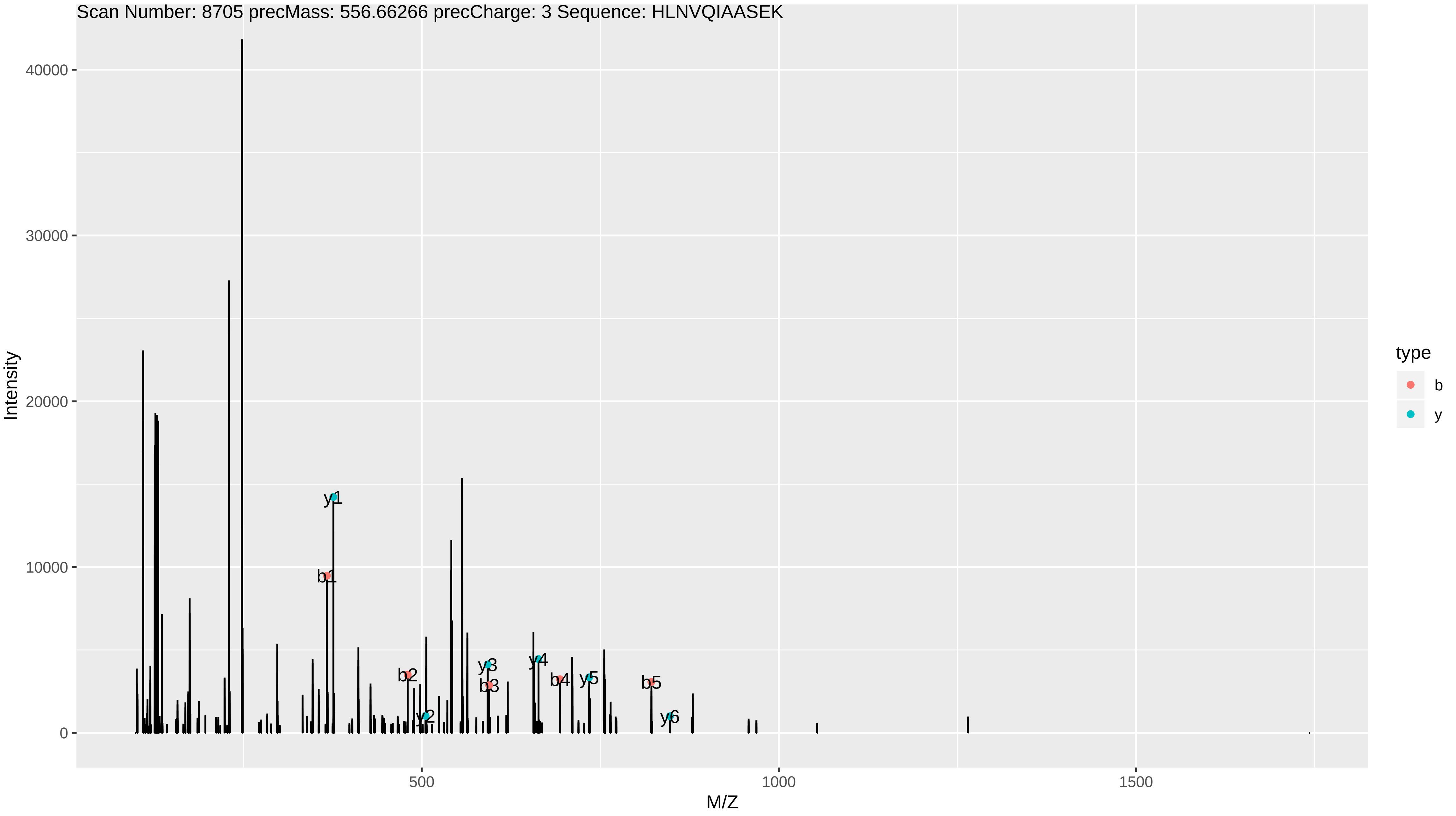

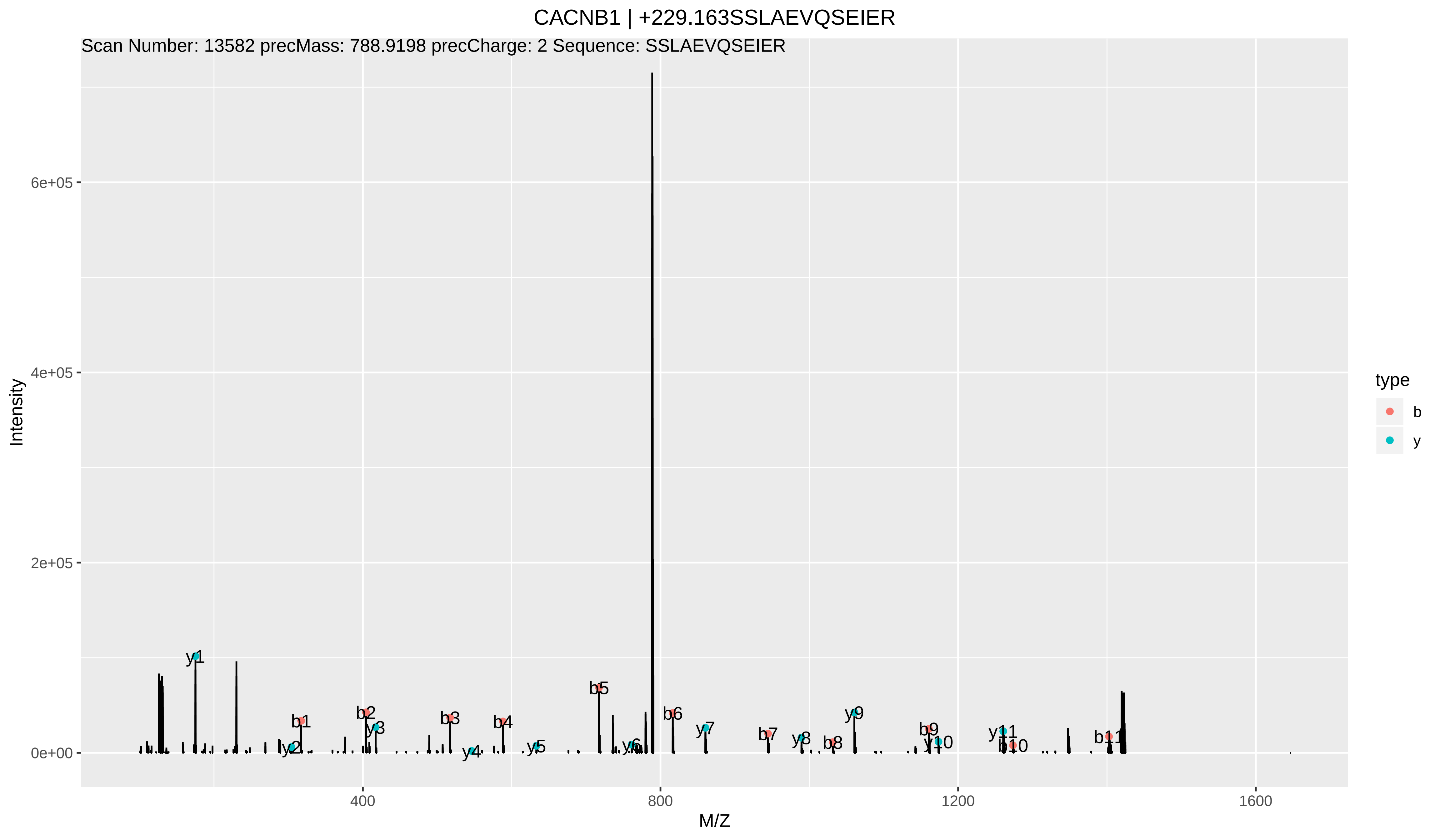

CACNB1 | +229.163SSLAEVQSEIER
Scan Number: 13582 precMass: 788.9198 precCharge: 2 Sequence: SSLAEVQSEIER
Intensity
6e+05
4e+05
2e+05
0e+00
M/Z
400
800
1200
1600
y1
y2
b1
b2
y3
b3
y4
b4
y5
b5
y6
b6
y7
b7
y8
b8
y9
b9
y10
b10
y11
b11
type
b
y

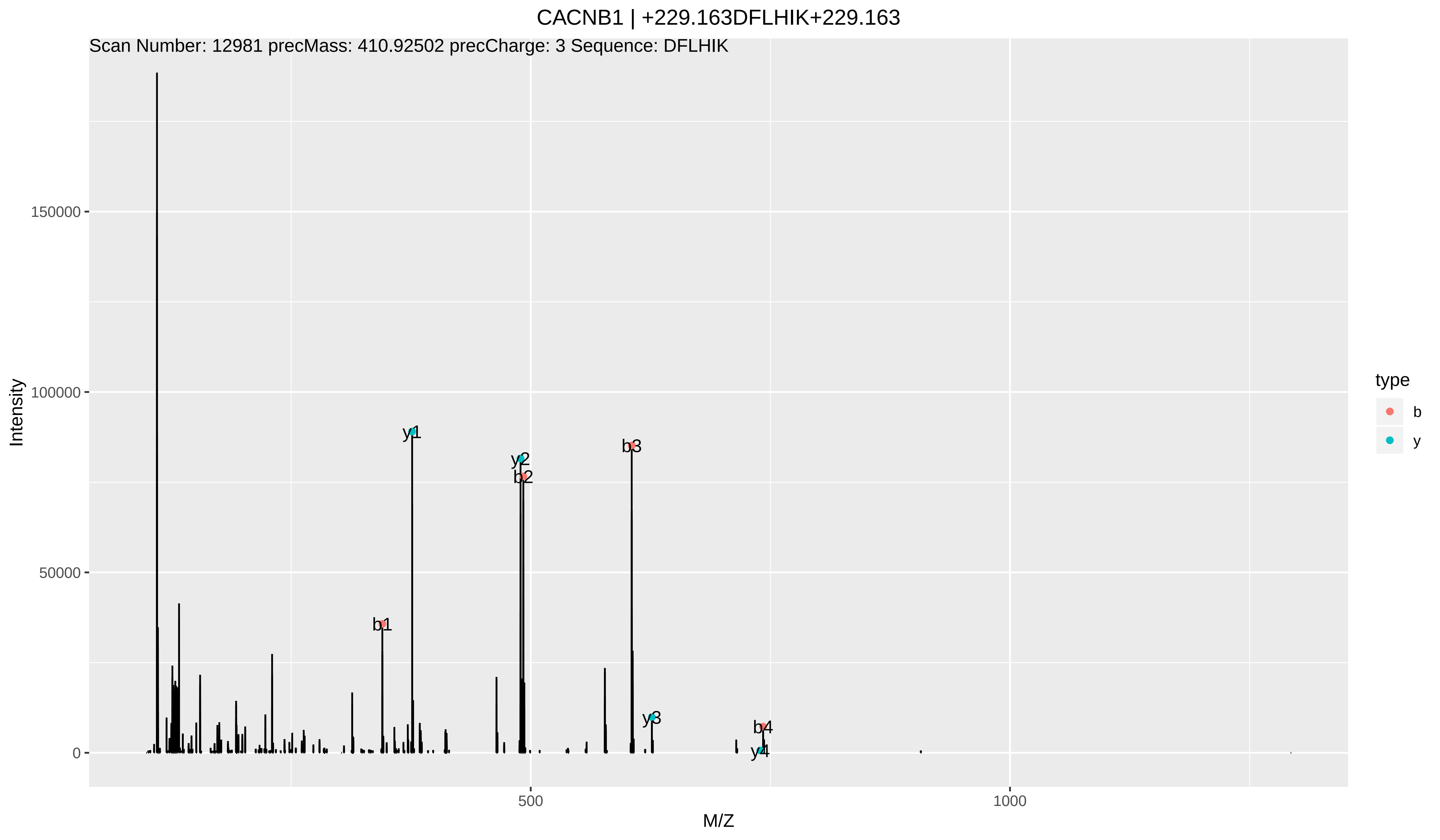
CACNB1 | +229.163DFLHIK+229.163
Scan Number: 12981 precMass: 410.92502 precCharge: 3 Sequence: DFLHIK
Intensity
150000
100000
50000
0
M/Z
500
1000
y1
y2
b2
b3
b1
y3
b4
y4
type
b
y

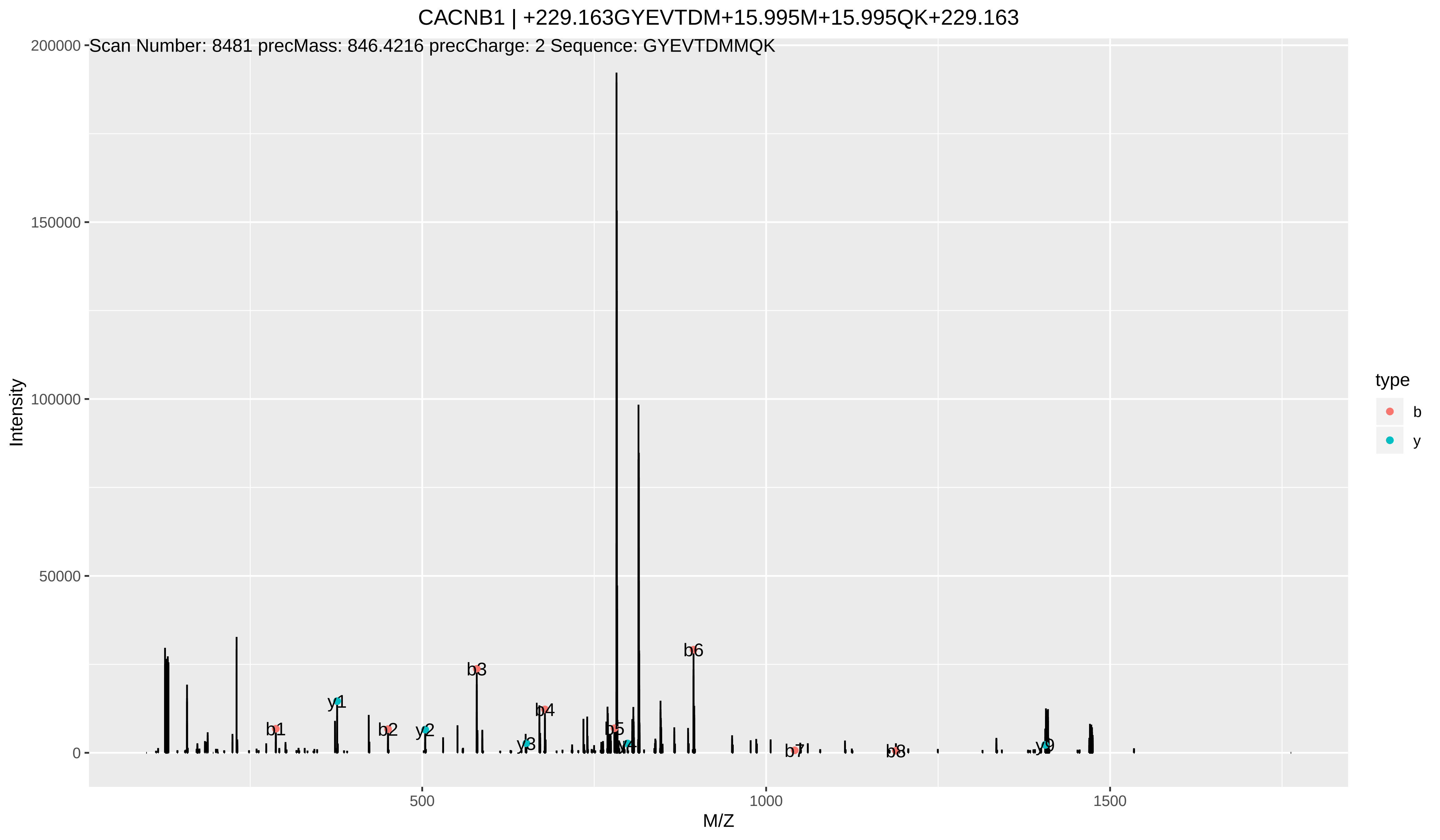

CACNB1 | +229.163GYEVTDM+15.995M+15.995QK+229.163
Scan Number: 8481 precMass: 846.4216 precCharge: 2 Sequence: GYEVTDMMQK
Intensity
200000
150000
100000
50000
0
M/Z
500
1000
1500
b1
b2
b3
b4
b5
b6
b7
b8
y1
y2
y3
y9
type
b
y

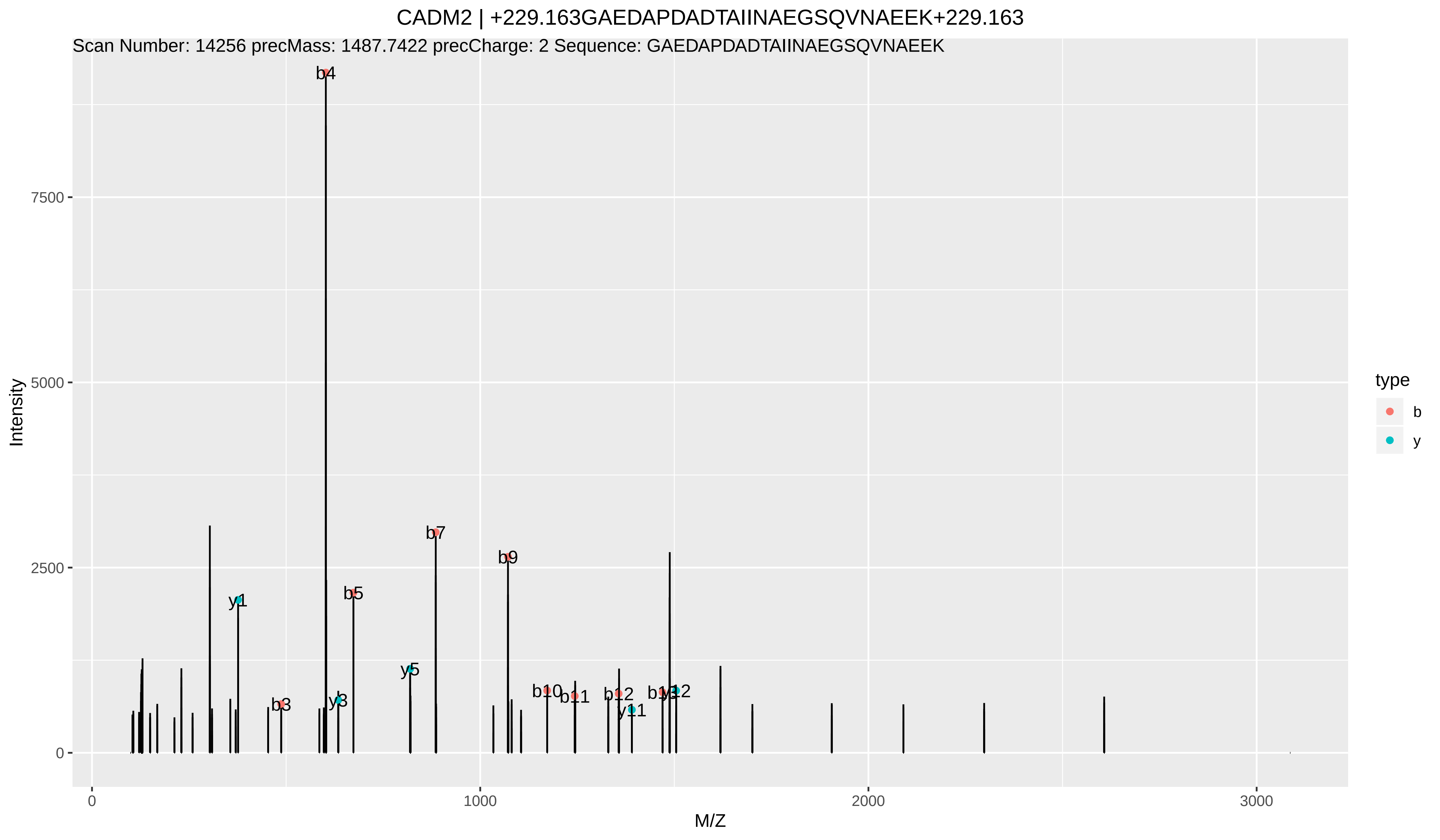
CADM2 | +229.163GAEDAPDADTAIINAEGSQVNAEEK+229.163
Scan Number: 14256 precMass: 1487.7422 precCharge: 2 Sequence: GAEDAPDADTAIINAEGSQVNAEEK
Intensity
M/Z
type
b
y
b4
b7
b9
b5
y1
y5
b3
y3
b10
b11
b12
y11
y12
0
2500
5000
7500
0
1000
2000
3000

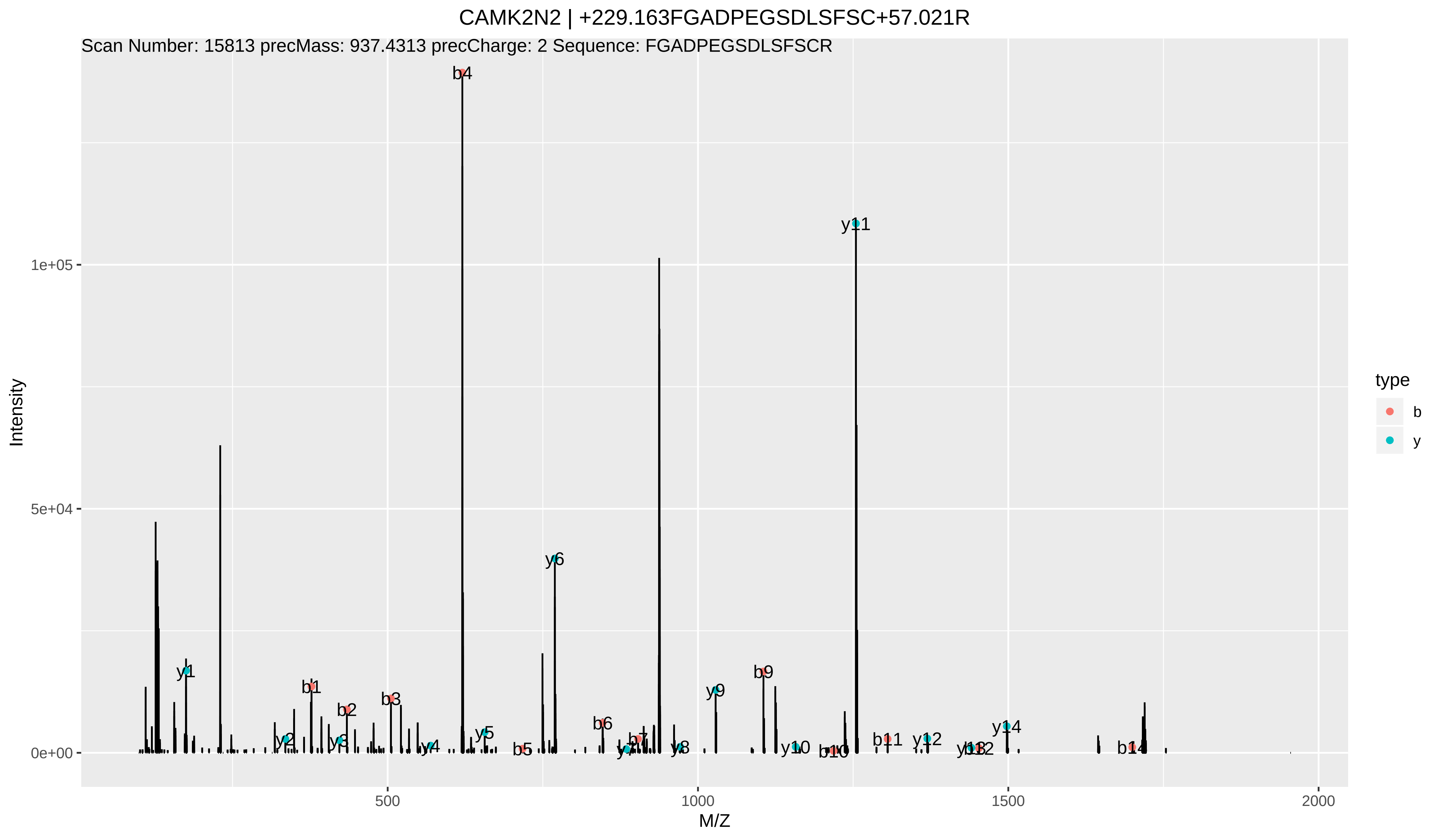

CAMK2N2 | +229.163FGADPEGSDLSFSC+57.021R
Scan Number: 15813 precMass: 937.4313 precCharge: 2 Sequence: FGADPEGSDLSFSCR
Intensity
1e+05
5e+04
0e+00
M/Z
500
1000
1500
2000
type
b
y
b4
y11
y6
y1
b1
b2
b3
b6
b9
y9
y14
y12
b11
b14
y5
b5
y4
y3
y2
y8
y10
b10
b7

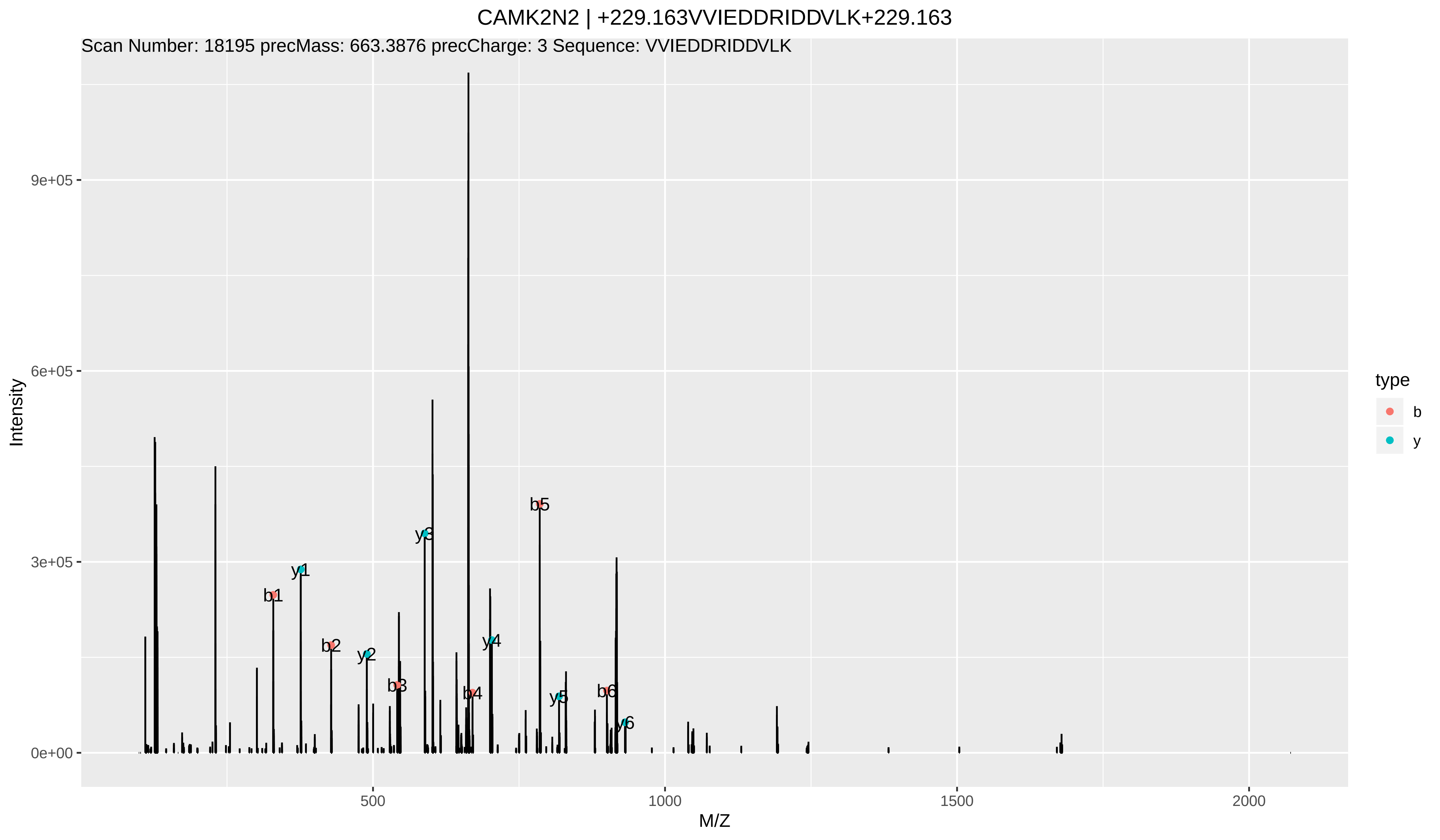

CAMK2N2 | +229.163VVIEDDRIDDVLK+229.163
Scan Number: 18195 precMass: 663.3876 precCharge: 3 Sequence: VVIEDDRIDDVLK
Intensity
9e+05
6e+05
3e+05
0e+00
M/Z
500
1000
1500
2000
b1
b2
b3
b4
b5
b6
y1
y2
y3
y4
y5
y6
type
b
y

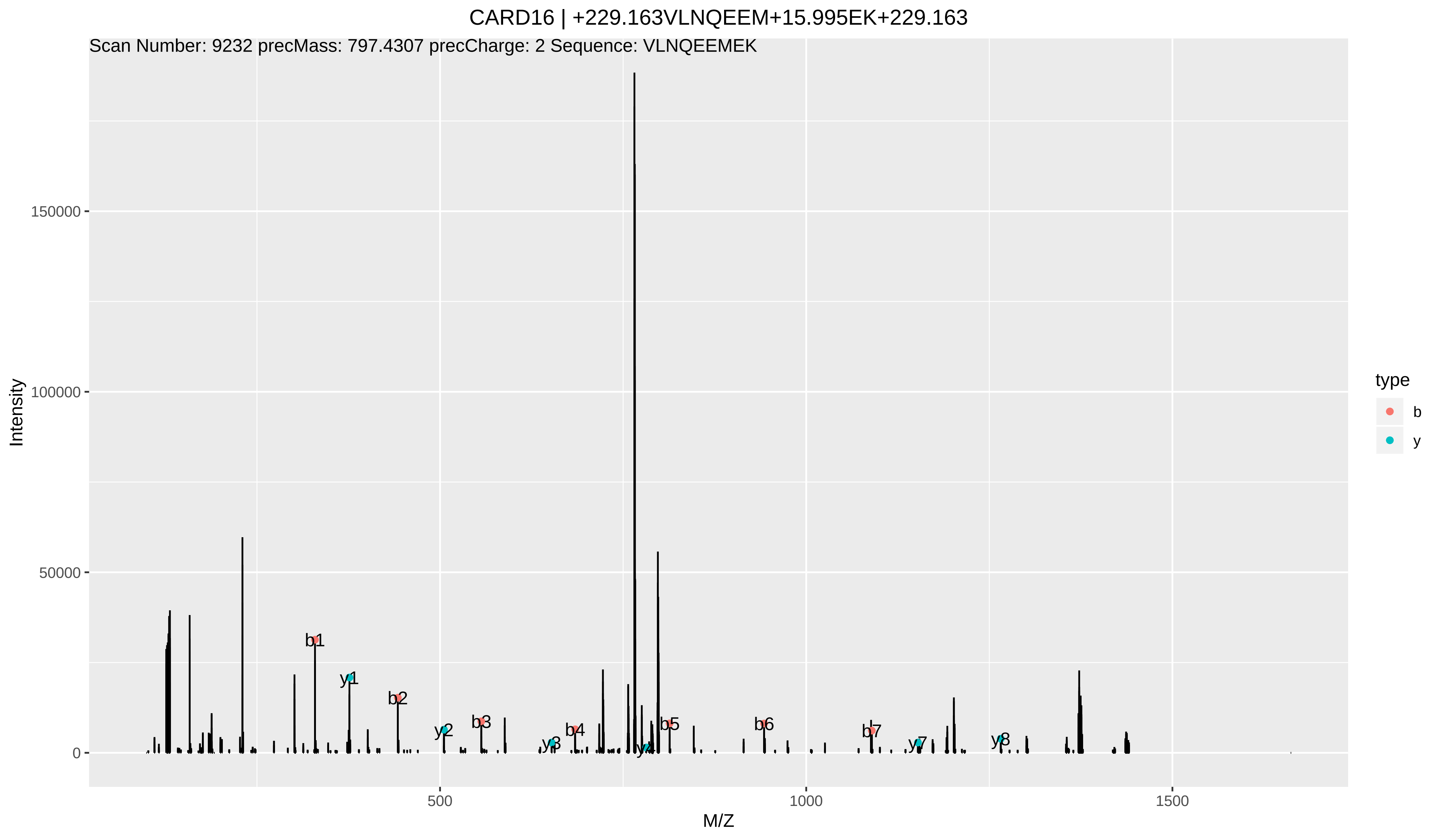
CARD16 | +229.163VLNQEEM+15.995EK+229.163
Scan Number: 9232 precMass: 797.4307 precCharge: 2 Sequence: VLNQEEMEK
Intensity
150000
100000
50000
0
500
1000
1500
M/Z
b1
y1
b2
y2
b3
y3
b4
b5
b6
b7
y7
y8
type
b
y

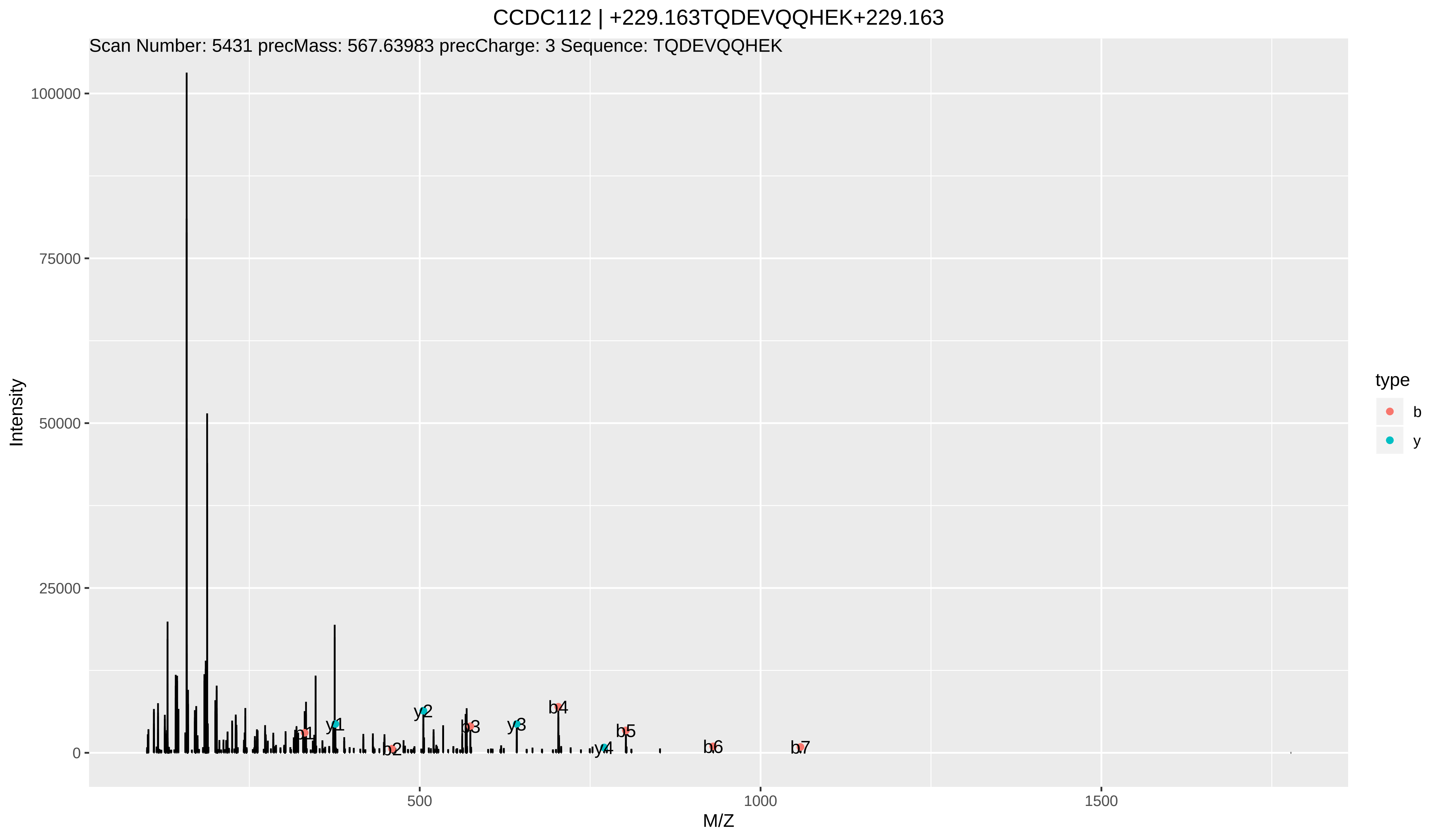

CCDC112 | +229.163TQDEVQQHEK+229.163
Scan Number: 5431 precMass: 567.63983 precCharge: 3 Sequence: TQDEVQQHEK
Intensity
100000
75000
50000
25000
0
M/Z
500
1000
1500
b1
y1
b2
y2
b3
y3
b4
y4
b5
b6
b7
type
b
y

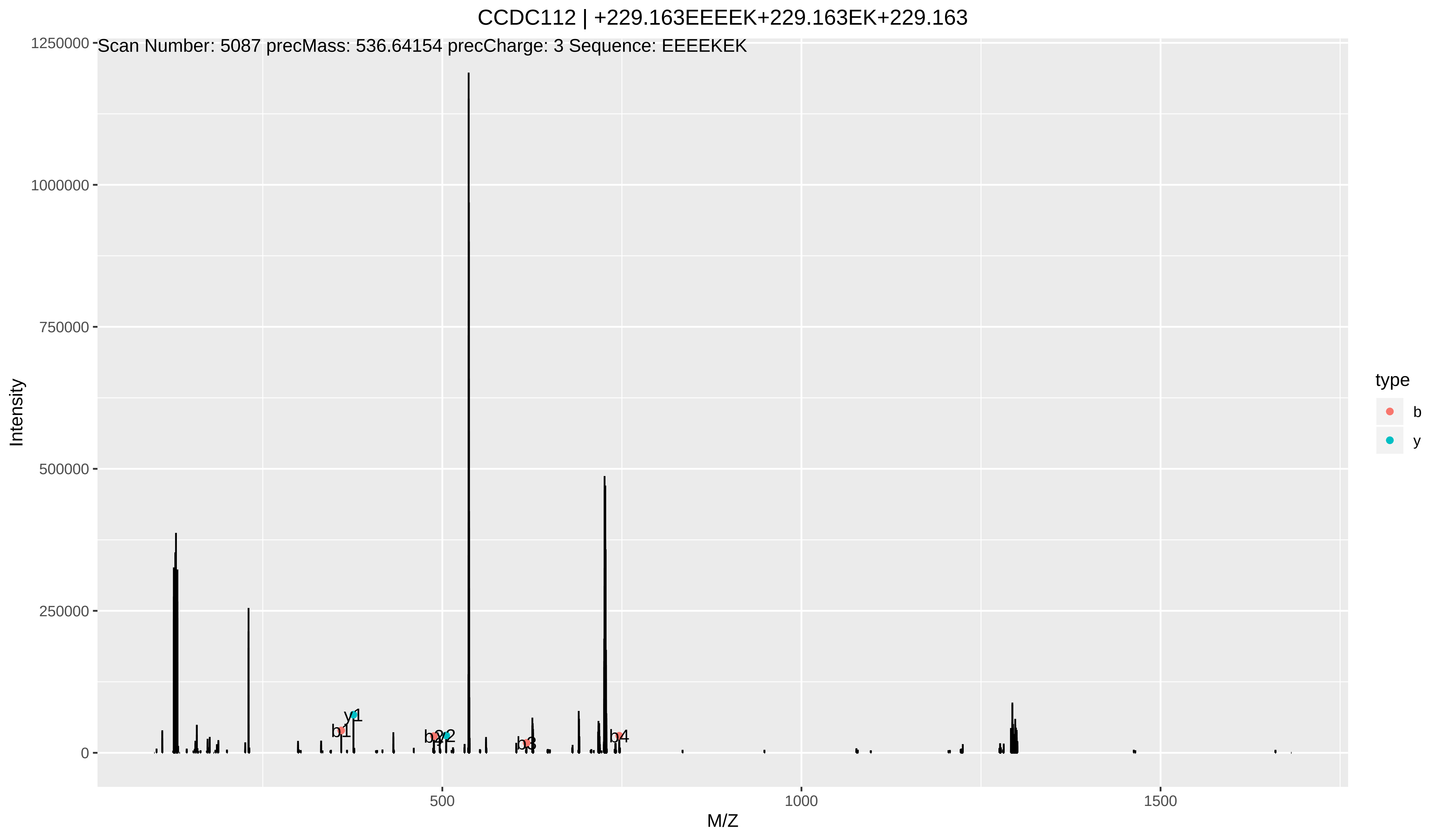

CCDC112 | +229.163EEEEK+229.163EK+229.163
Scan Number: 5087 precMass: 536.64154 precCharge: 3 Sequence: EEEEKEK
Intensity
1250000
1000000
750000
500000
250000
0
M/Z
500
1000
1500
y1
b1
b2
y2
b3
b4
type
b
y

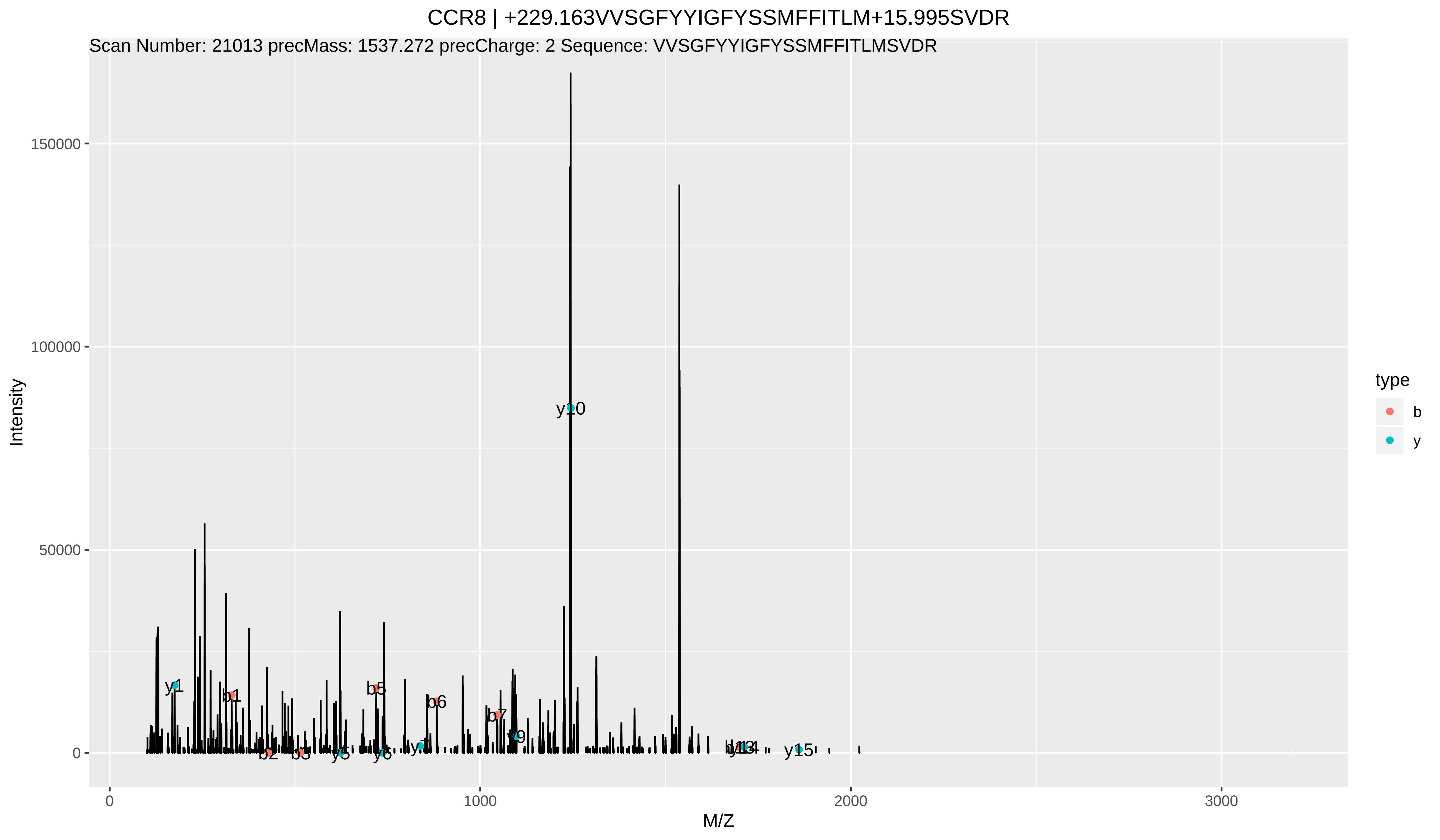

CCR8 | +229.163VVSGFYYIGFYSSMFFITLM+15.995SVDR
Scan Number: 21013 precMass: 1537.272 precCharge: 2 Sequence: VVSGFYYIGFYSSMFFITLMSVDR
Intensity
M/Z
150000
100000
50000
0
1000
2000
3000
y10
y1
b1
b2
b3
y5
y6
b5
b6
y7
b7
y9
y12
y14
y15
type
b
y

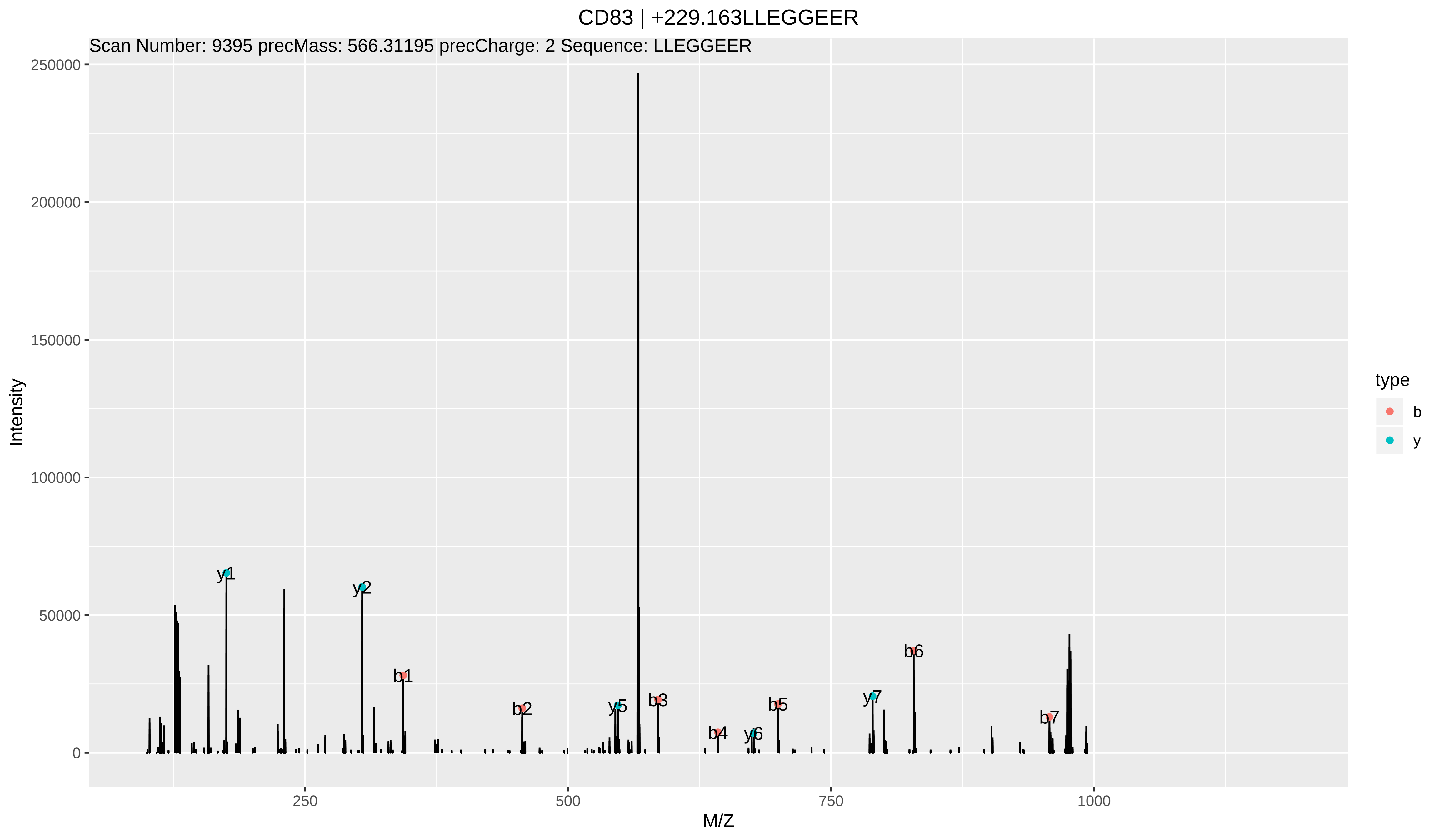

CD83 | +229.163LLEGGEER
Scan Number: 9395 precMass: 566.31195 precCharge: 2 Sequence: LLEGGEER
Intensity
M/Z
250000
200000
150000
100000
50000
0
250
500
750
1000
y1
y2
b1
b2
y5
b3
b4
y6
b5
y7
b6
b7
type
b
y

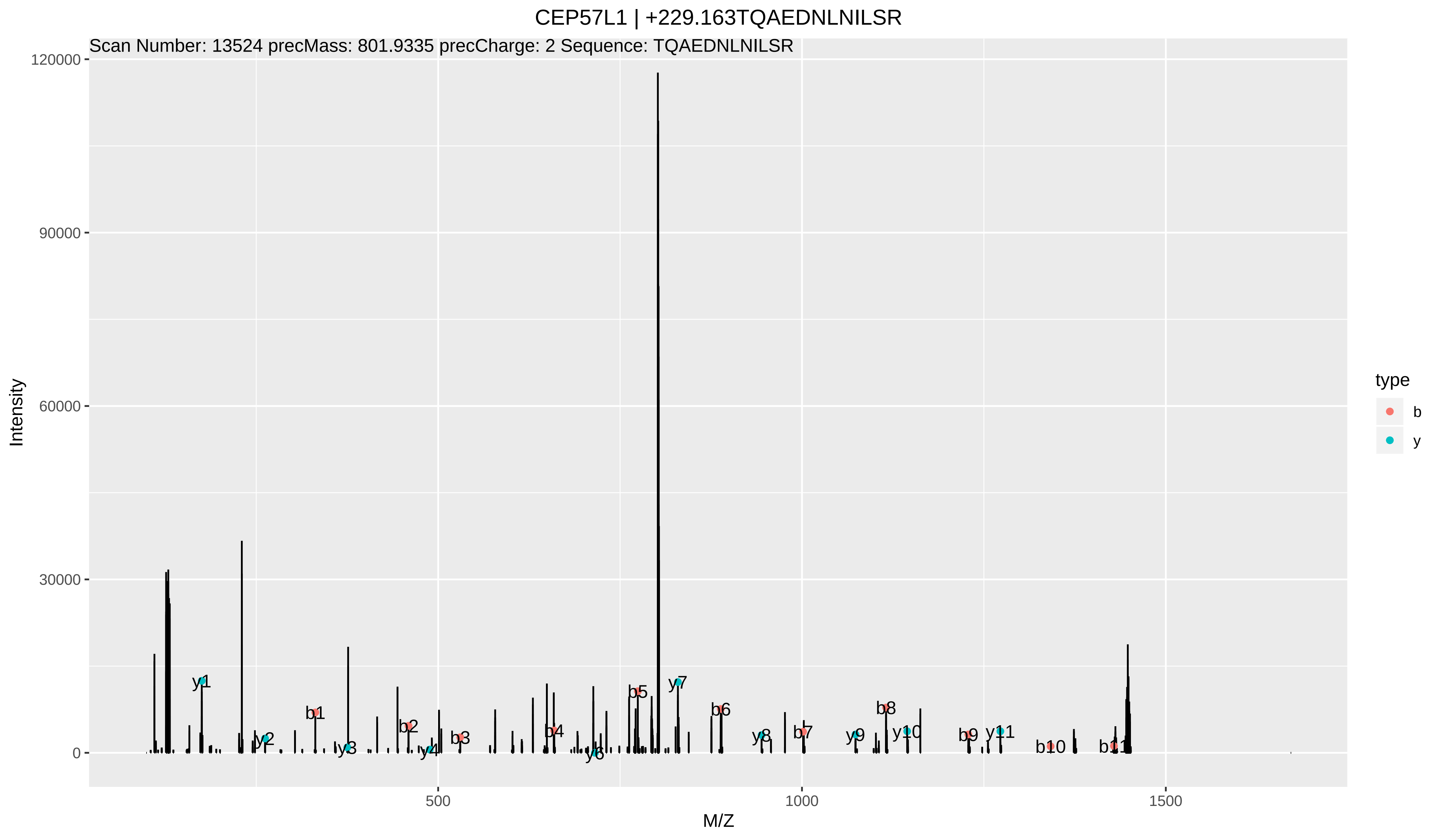
CEP57L1 | +229.163TQAEDNLNILSR
Scan Number: 13524 precMass: 801.9335 precCharge: 2 Sequence: TQAEDNLNILSR
Intensity
M/Z
0
30000
60000
90000
120000
500
1000
1500
type
b
y
y1
y2
y3
y4
y6
y7
y8
y9
y10
y11
b1
b2
b3
b4
b5
b6
b7
b8
b9
b10
b11

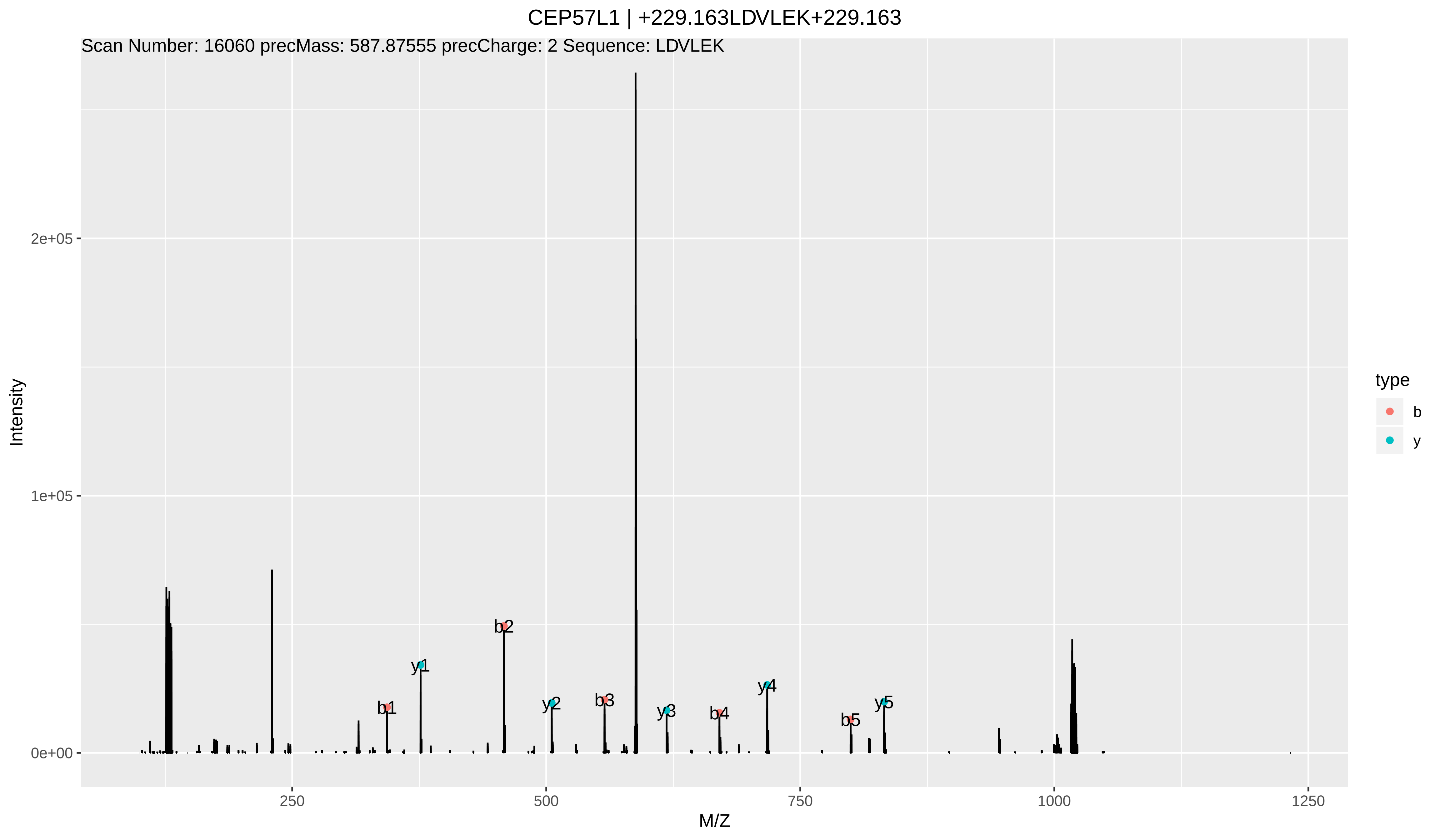

CEP57L1 | +229.163LDVLEK+229.163
Scan Number: 16060 precMass: 587.87555 precCharge: 2 Sequence: LDVLEK
Intensity
2e+05
1e+05
0e+00
M/Z
250
500
750
1000
1250
b1
y1
b2
y2
b3
y3
b4
y4
b5
y5
type
b
y

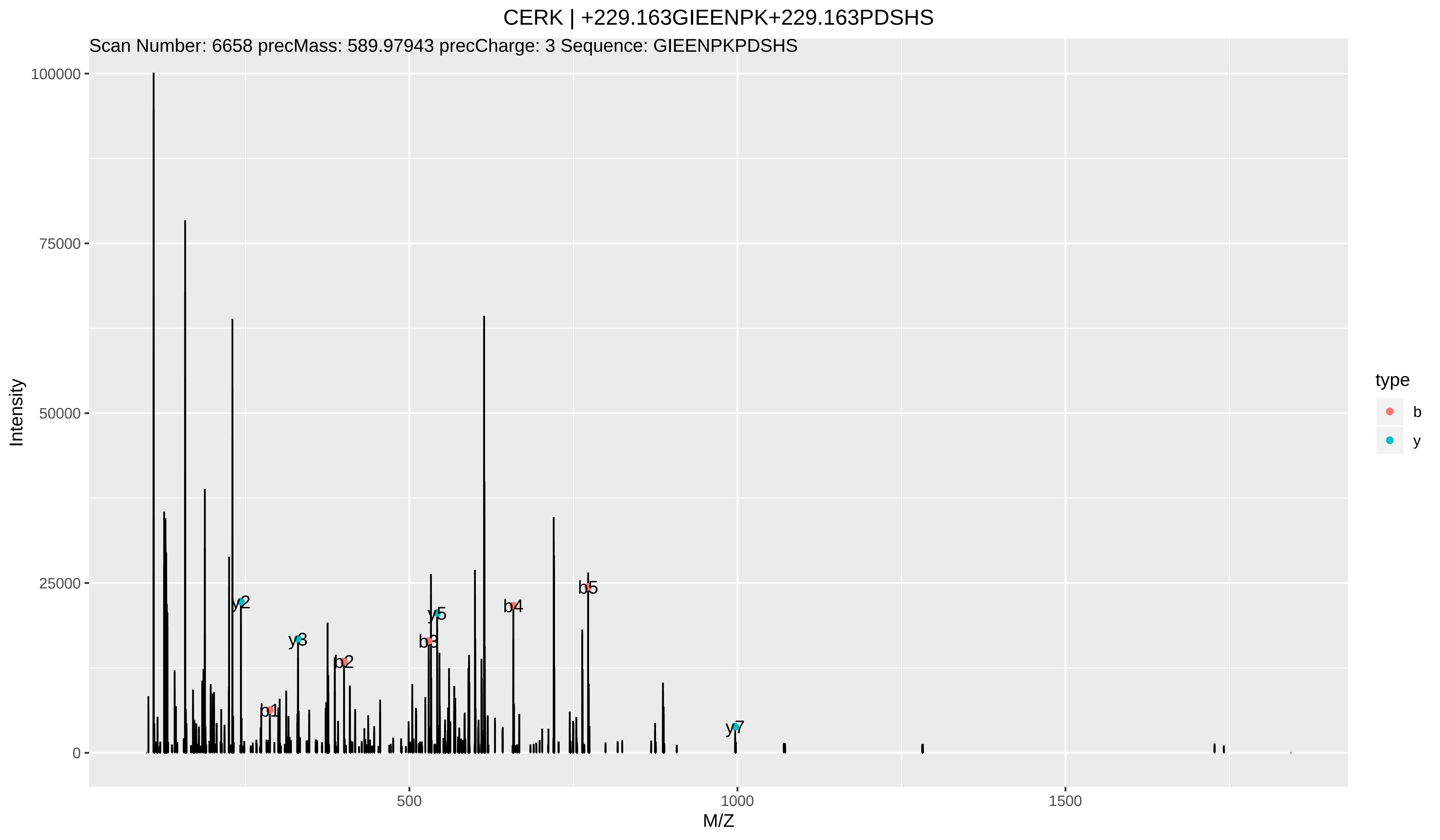

CERK | +229.163GIEENPK+229.163PDSHS
Scan Number: 6658 precMass: 589.97943 precCharge: 3 Sequence: GIEENPKPDSHS
Intensity
M/Z
100000
75000
50000
25000
0
500
1000
1500
y2
y3
b1
b2
b3
y5
b4
b5
y7
type
b
y

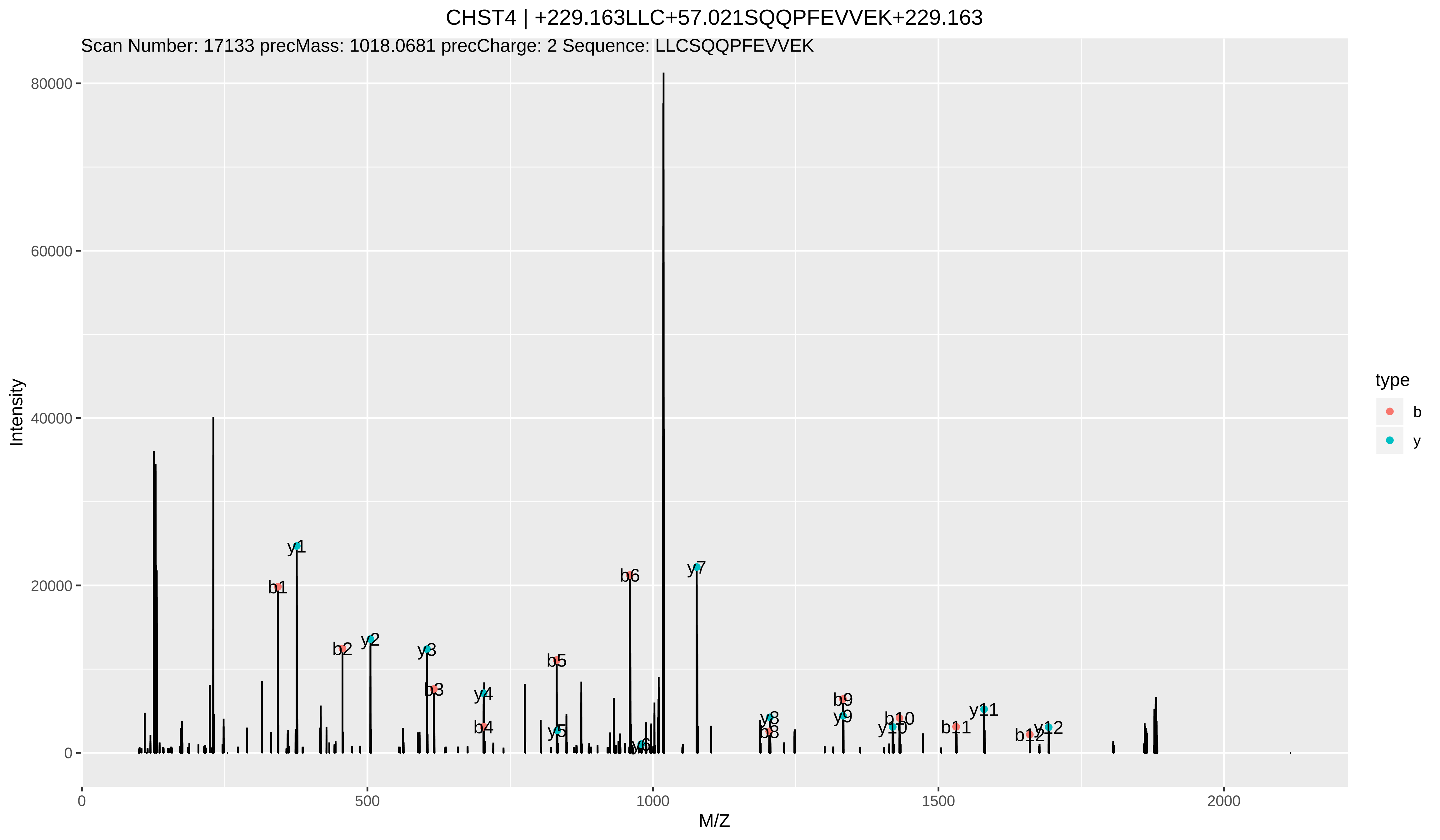
CHST4 | +229.163LLC+57.021SQQPFEVVEK+229.163
Scan Number: 17133 precMass: 1018.0681 precCharge: 2 Sequence: LLCSQQPFEVVEK
Intensity
M/Z
type
b
y
y1
b1
b2
y2
y3
b3
y4
b4
b5
y5
b6
y6
y7
y8
b8
b9
y9
b10
y10
b11
y11
b12
y12
0
20000
40000
60000
80000
500
1000
1500
2000

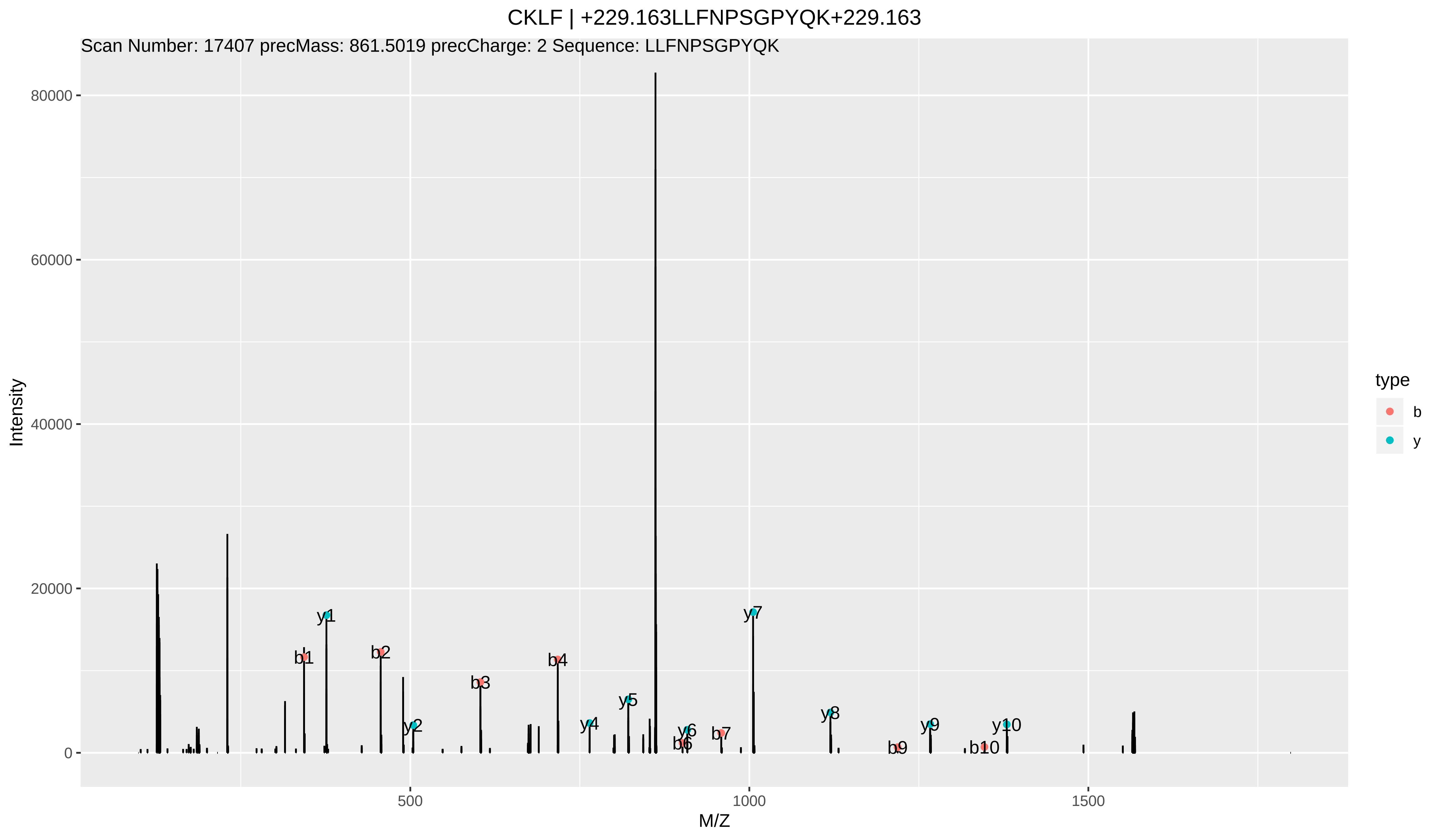
CKLF | +229.163LLFNPSGPYQK+229.163
Scan Number: 17407 precMass: 861.5019 precCharge: 2 Sequence: LLFNPSGPYQK
Intensity
80000
60000
40000
20000
0
500
1000
1500
M/Z
type
b
y
y1
b1
b2
y2
b3
b4
y4
y5
y6
b6
b7
y7
y8
b9
y9
b10
y10

CNKSR3 | +229.163APNEFLTSVVELIGAAK+229.163

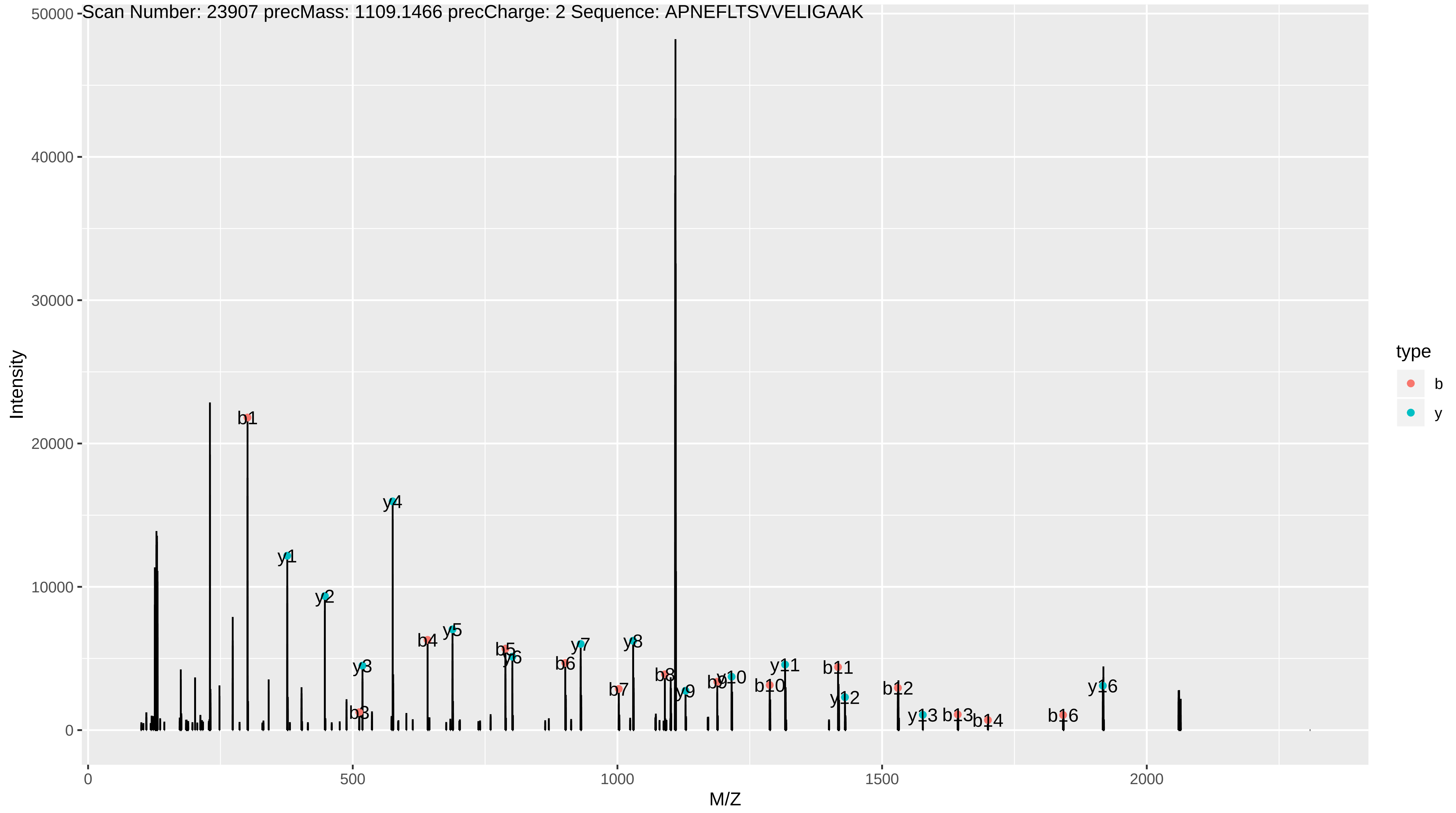

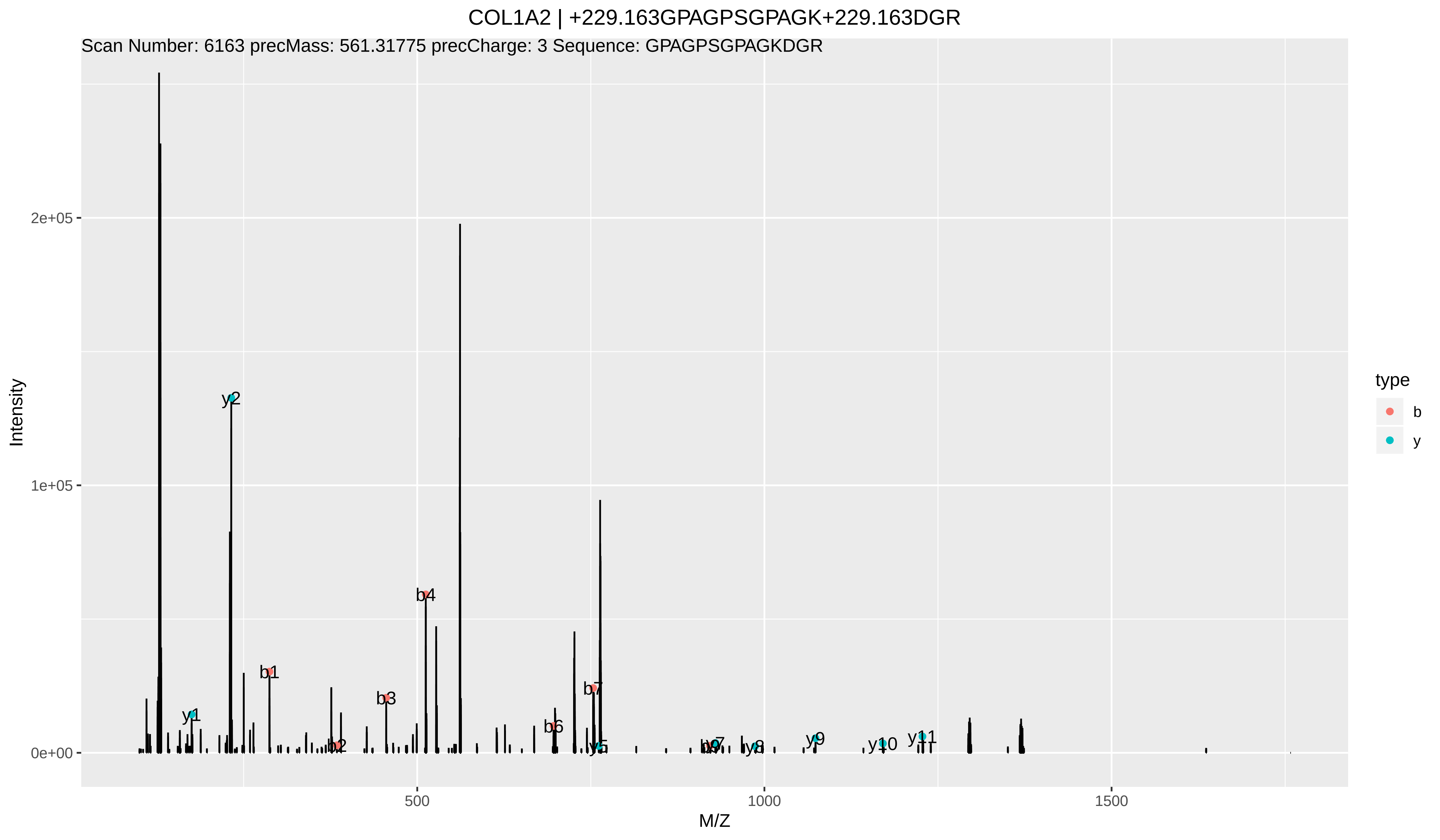
COL1A2 | +229.163GPAGPSGPAGK+229.163DGR
Scan Number: 6163 precMass: 561.31775 precCharge: 3 Sequence: GPAGPSGPAGKDGR
Intensity
2e+05
1e+05
0e+00
M/Z
500
1000
1500
y1
y2
b1
b2
b3
b4
b6
b7
y5
y7
y8
y9
y10
y11
type
b
y

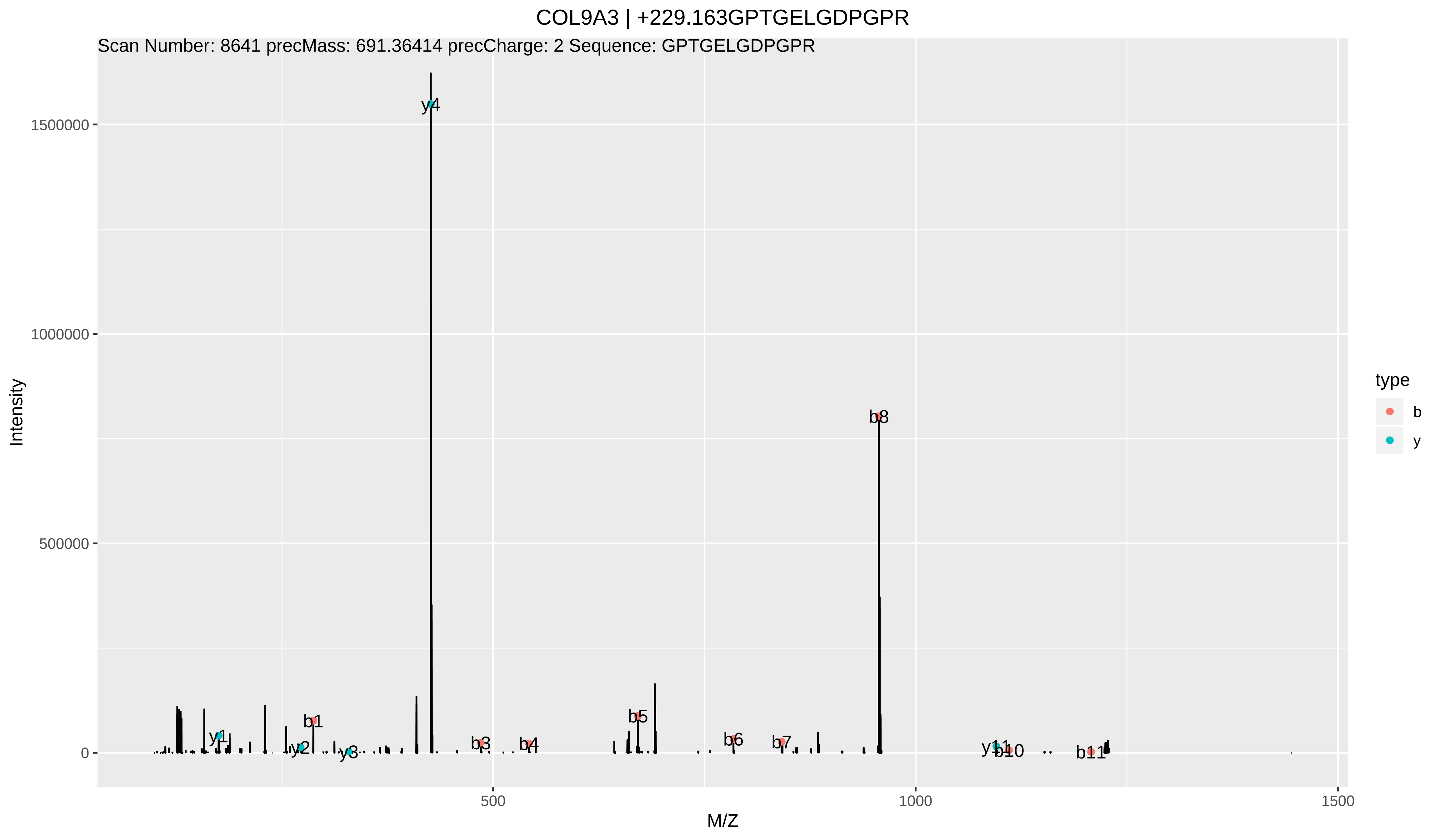
COL9A3 | +229.163GPTGELGDPGPR
Scan Number: 8641 precMass: 691.36414 precCharge: 2 Sequence: GPTGELGDPGPR
Intensity
M/Z
type
b
y
y4
b8
b1
y1
y2
y3
b3
b4
b5
b6
b7
y11
b10
b11
1500000
1000000
500000
0
500
1000
1500

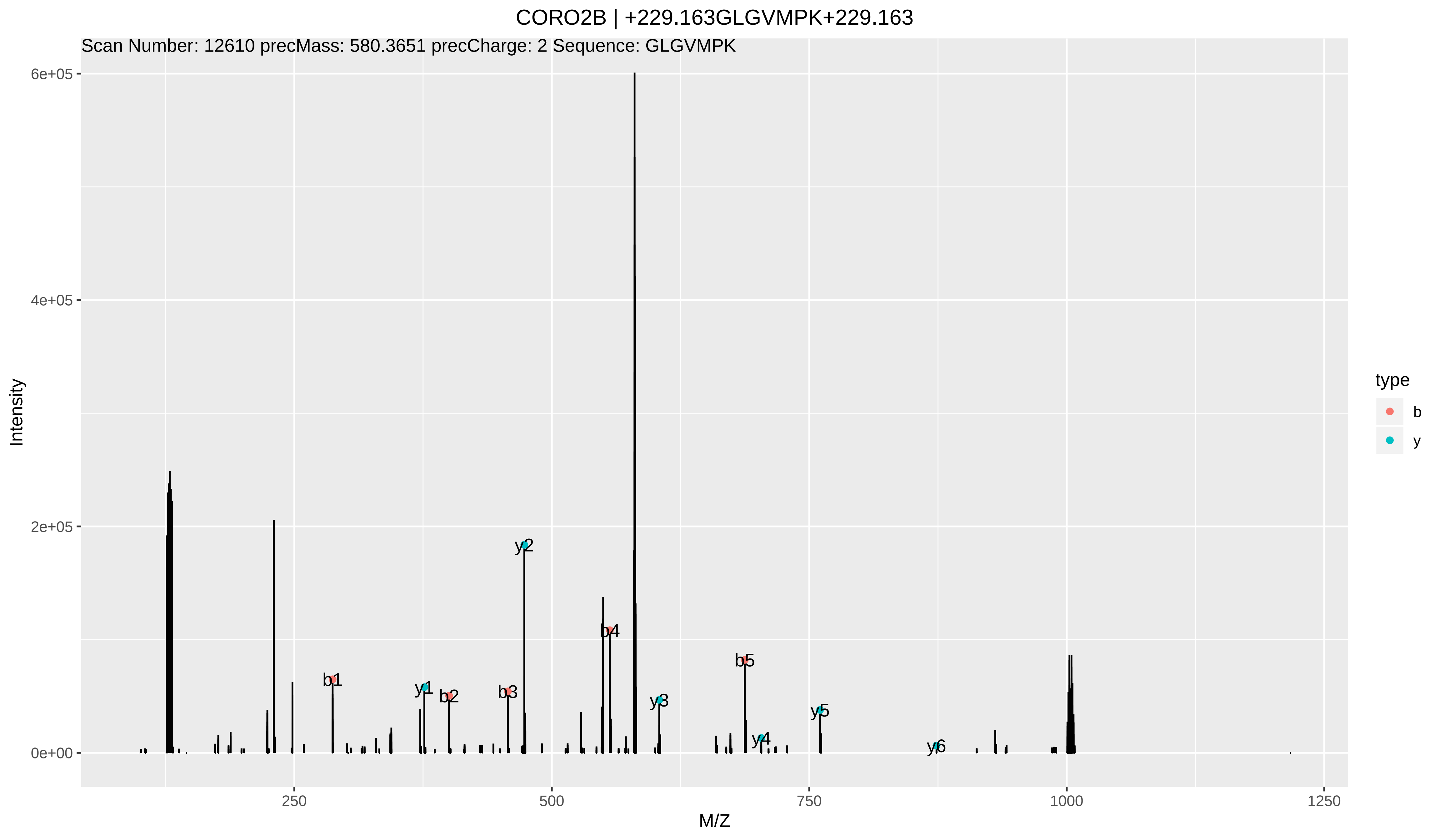

CORO2B | +229.163GLGVMPK+229.163
Scan Number: 12610 precMass: 580.3651 precCharge: 2 Sequence: GLGVMPK
Intensity
M/Z
6e+05
4e+05
2e+05
0e+00
250
500
750
1000
1250
b1
y1
b2
b3
y2
b4
y3
b5
y4
y5
y6
type
b
y

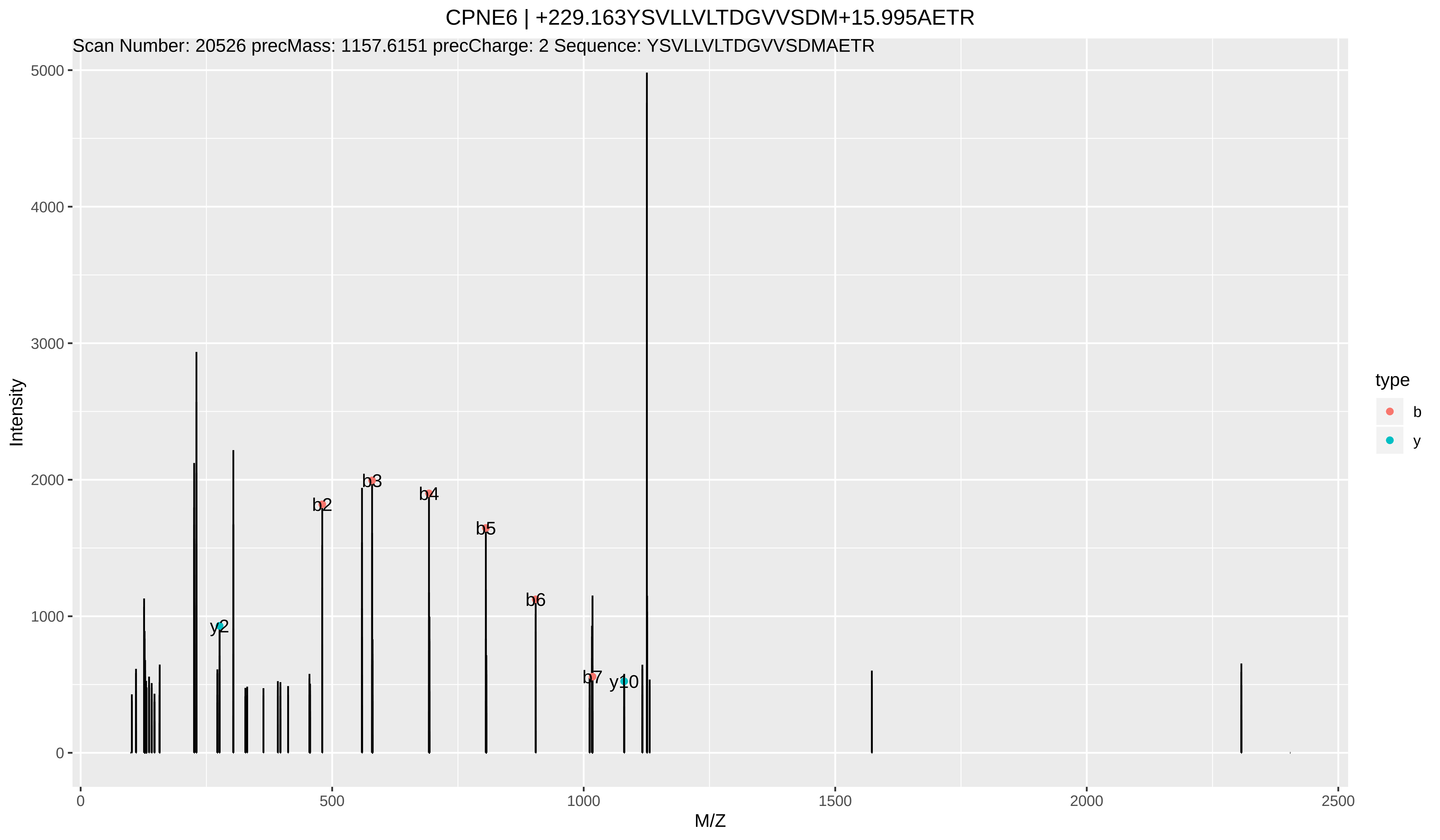

CPNE6 | +229.163YSVLLVLTDGVVSDM+15.995AETR
Scan Number: 20526 precMass: 1157.6151 precCharge: 2 Sequence: YSVLLVLTDGVVSDMAETR
Intensity
5000
4000
3000
2000
1000
0
M/Z
0
500
1000
1500
2000
2500
y2
b2
b3
b4
b5
b6
b7
y10
type
b
y

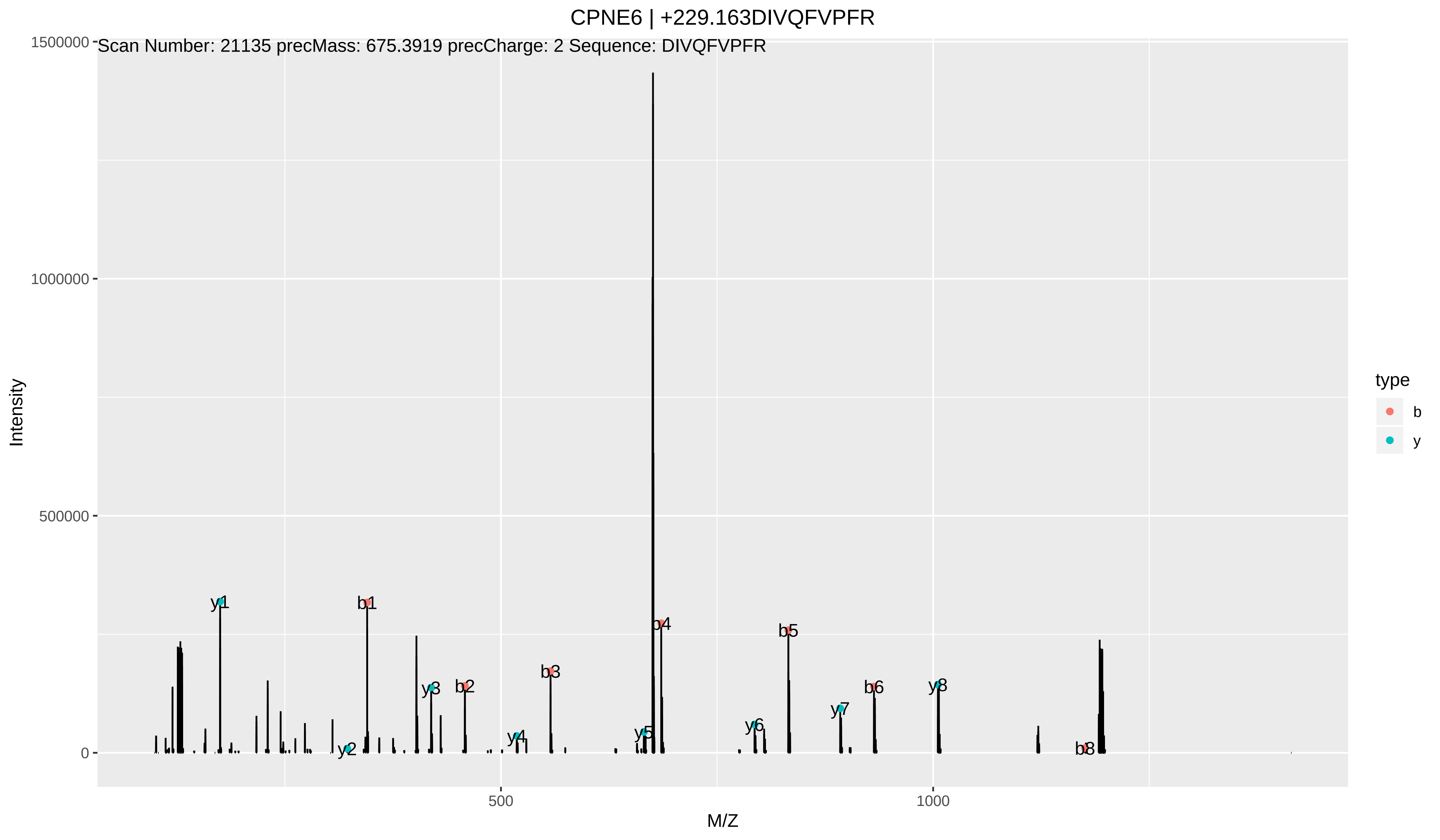
CPNE6 | +229.163DIVQFVPFR
Scan Number: 21135 precMass: 675.3919 precCharge: 2 Sequence: DIVQFVPFR
Intensity
M/Z
1500000
1000000
500000
0
500
1000
y1
b1
b4
b5
b3
y3
b2
b6
y8
y7
y6
y4
y5
y2
b8
type
b
y

CREB3 | +229.163EGLILPETLPLTK+229.163

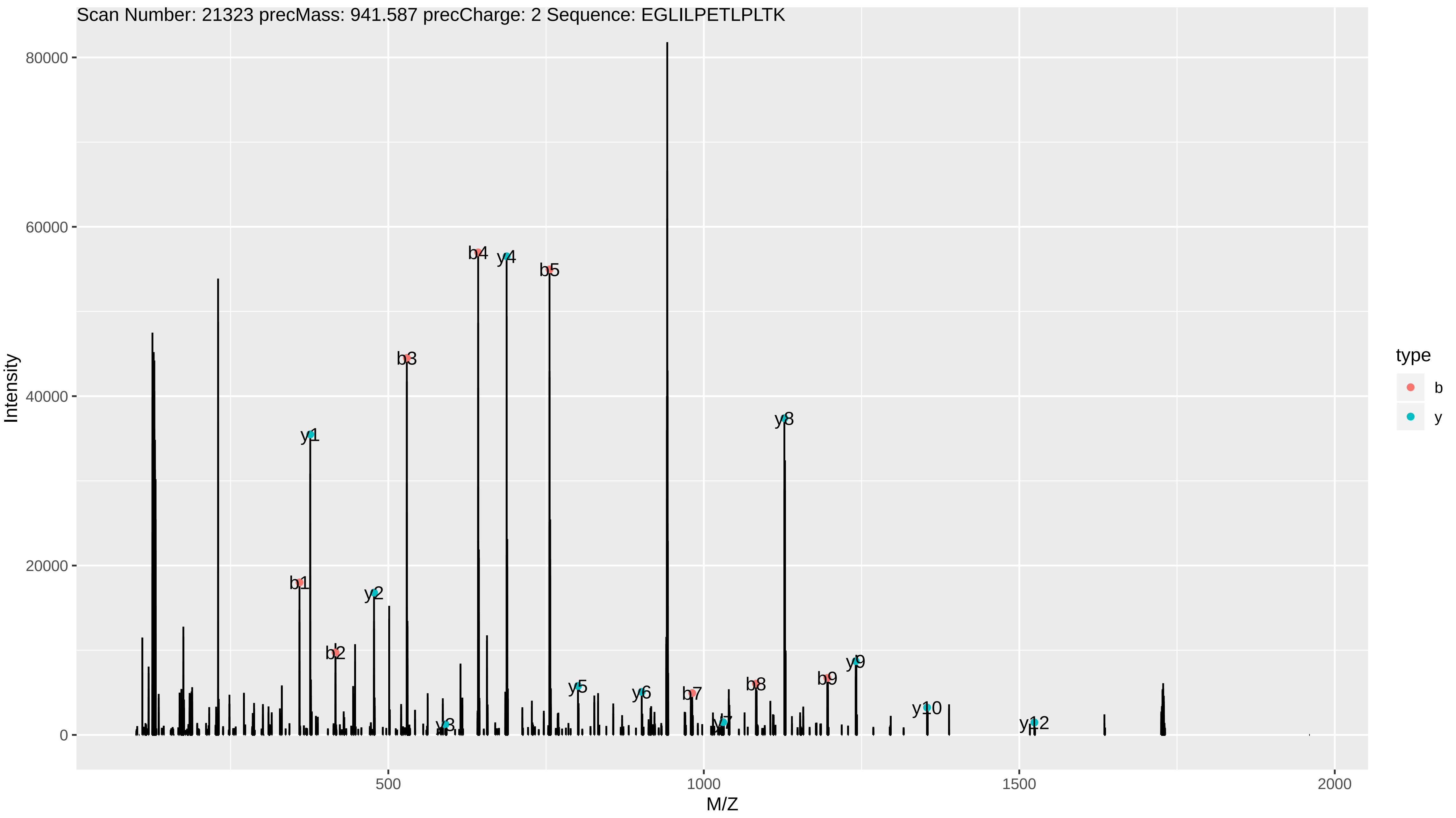

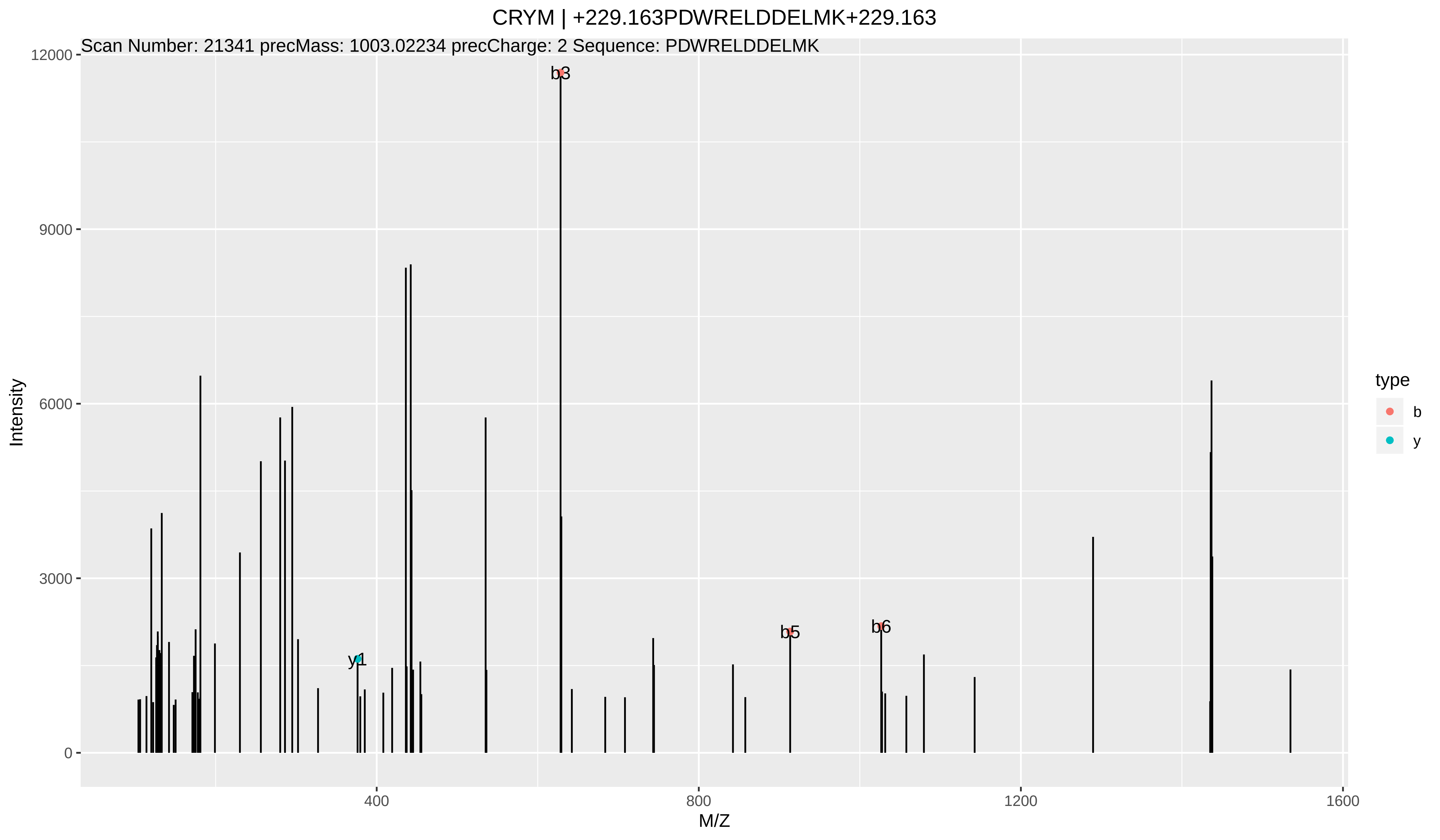
CRYM | +229.163PDWRELDDELMK+229.163
Scan Number: 21341 precMass: 1003.02234 precCharge: 2 Sequence: PDWRELDDELMK
Intensity
M/Z
b3
y1
b5
b6
type
b
y
0
3000
6000
9000
12000
400
800
1200
1600

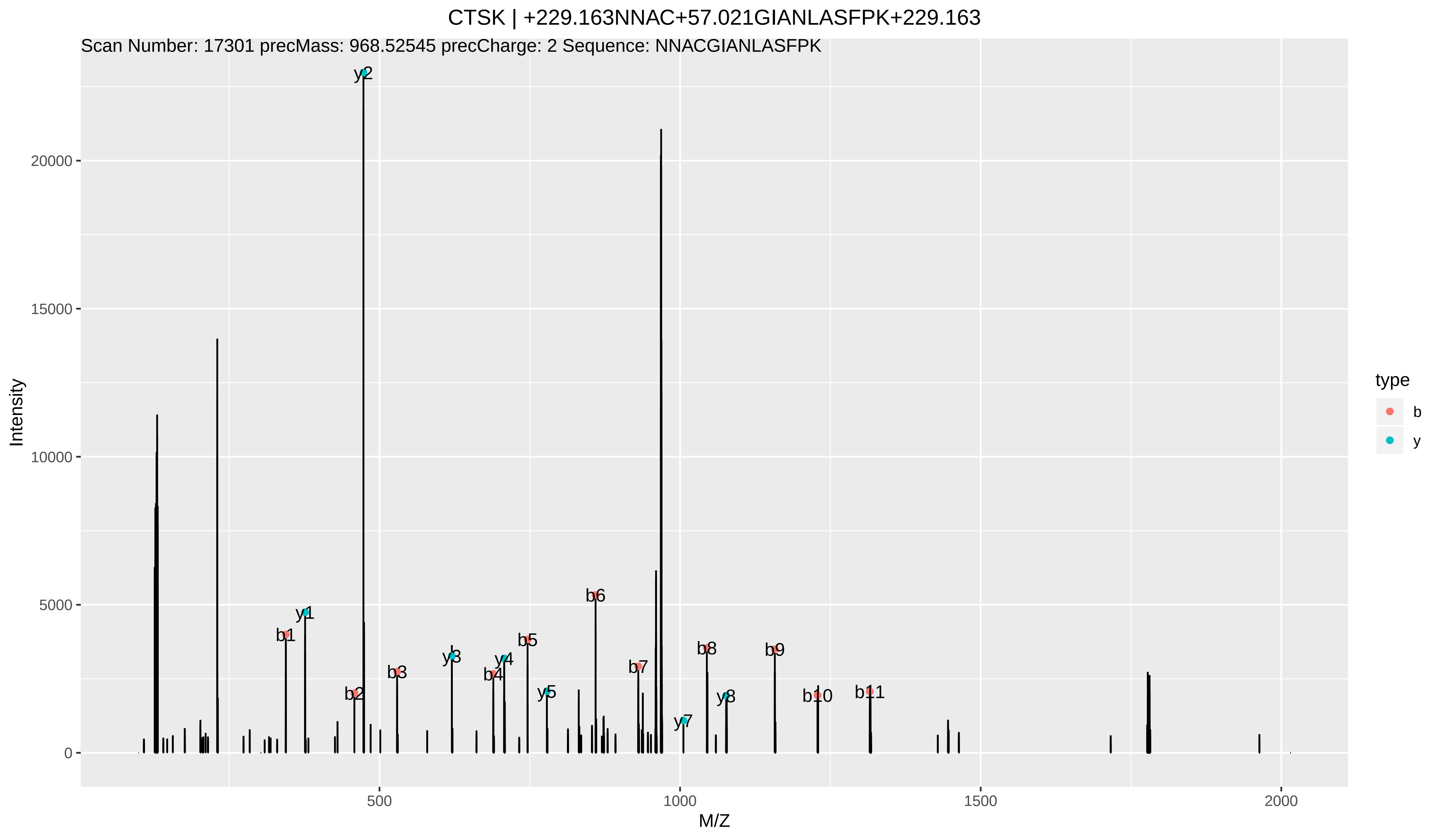
CTSK | +229.163NNAC+57.021GIANLASFPK+229.163
Scan Number: 17301 precMass: 968.52545 precCharge: 2 Sequence: NNACGIANLASFPK
Intensity
M/Z
type
b
y
y2
y1
b1
b2
b3
y3
b4
y4
b5
y5
b6
b7
y7
b8
y8
b9
b10
b11
0
5000
10000
15000
20000
500
1000
1500
2000

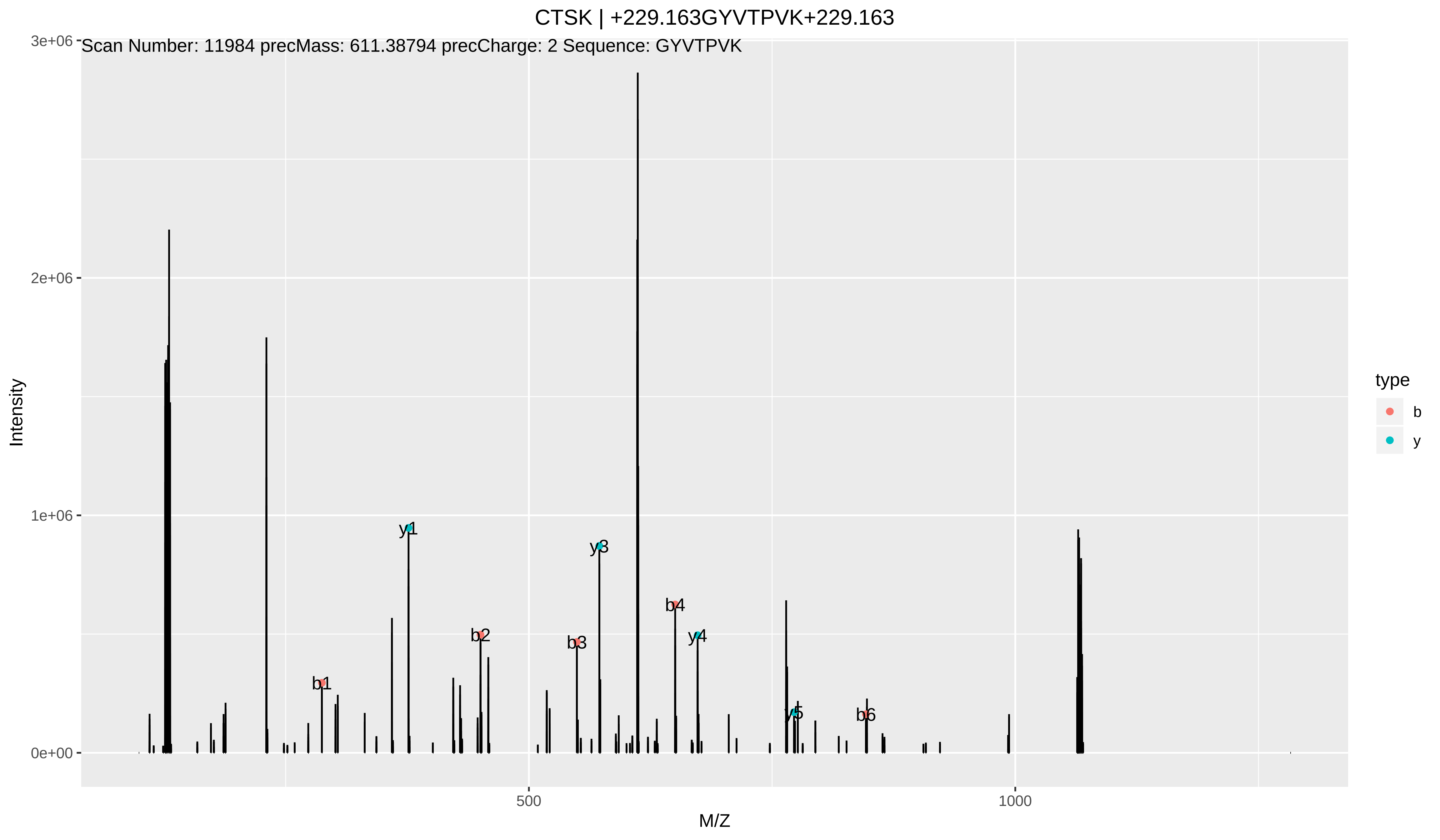

CTSK | +229.163GYVTPVK+229.163
Scan Number: 11984 precMass: 611.38794 precCharge: 2 Sequence: GYVTPVK
Intensity
3e+06
2e+06
1e+06
0e+00
M/Z
500
1000
y1
y3
b4
b2
b3
y4
b1
y5
b6
type
b
y

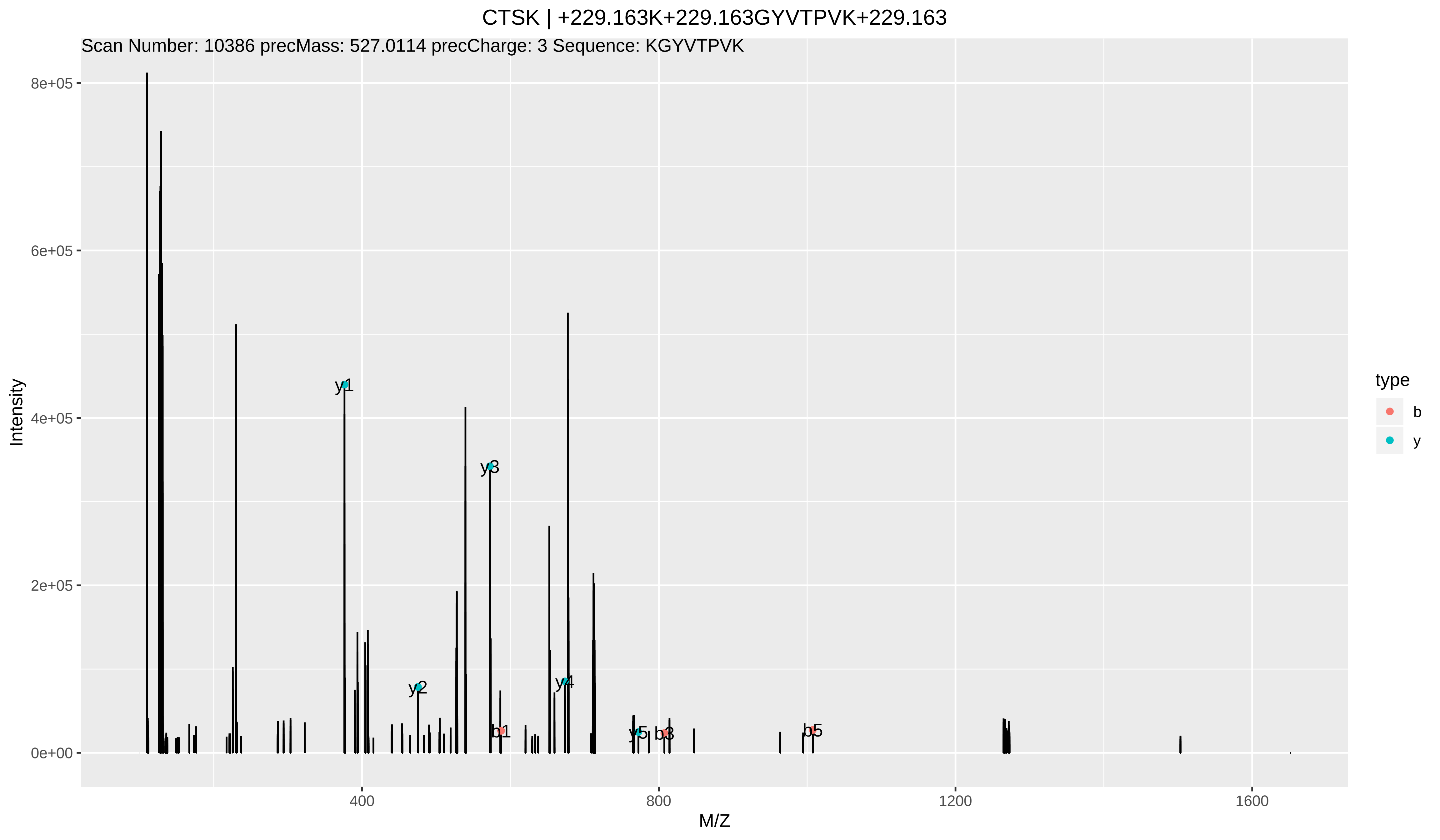

CTSK | +229.163K+229.163GYVTPVK+229.163
Scan Number: 10386 precMass: 527.0114 precCharge: 3 Sequence: KGYVTPVK
Intensity
8e+05
6e+05
4e+05
2e+05
0e+00
M/Z
400
800
1200
1600
y1
y2
y3
y4
y5
b1
b3
b5
type
b
y

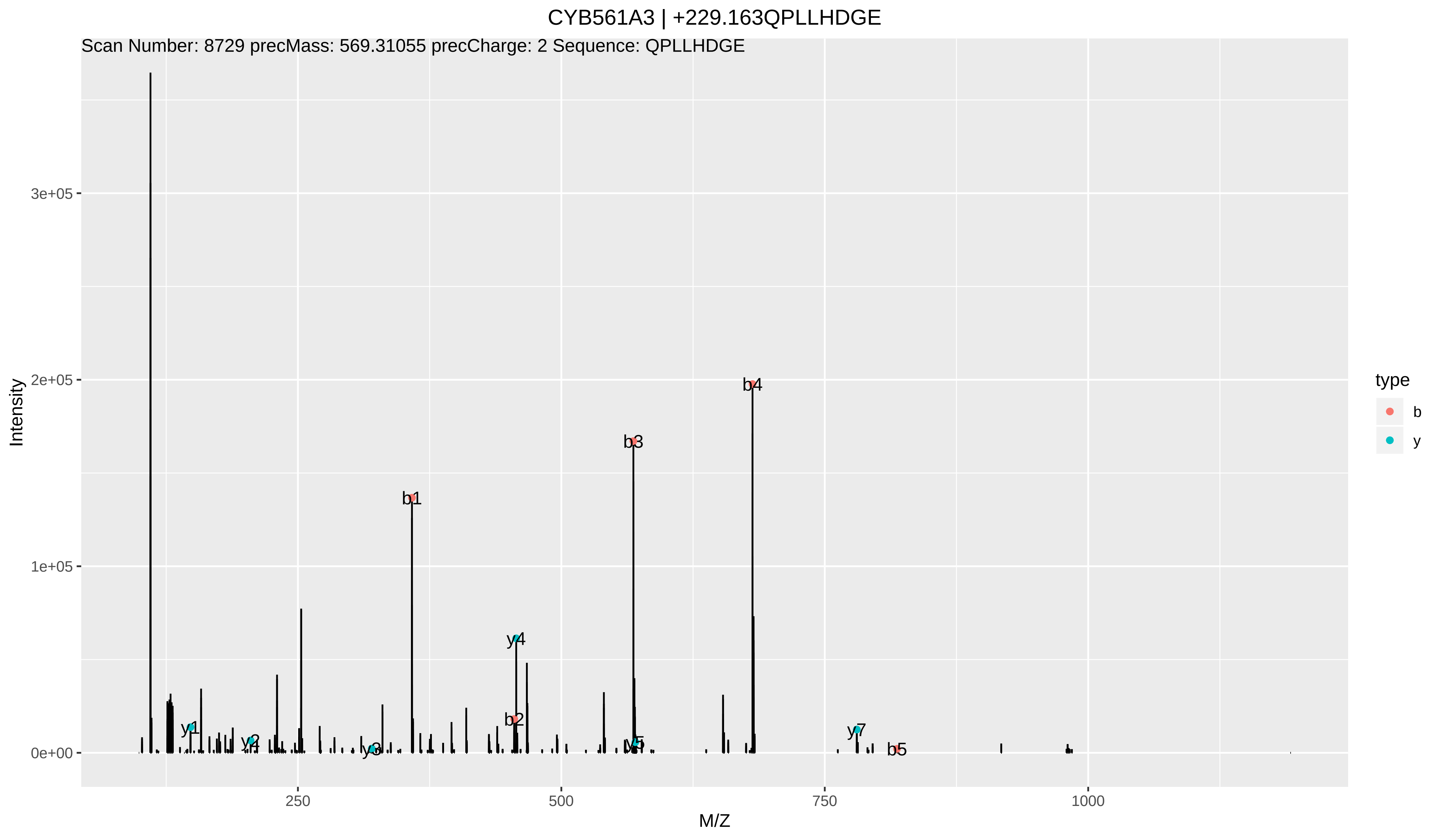
CYB561A3 | +229.163QPLLHDGE
Scan Number: 8729 precMass: 569.31055 precCharge: 2 Sequence: QPLLHDGE
Intensity
3e+05
2e+05
1e+05
0e+00
M/Z
250
500
750
1000
type
b
y
b1
b2
b3
b4
b5
y1
y3
y4
y7

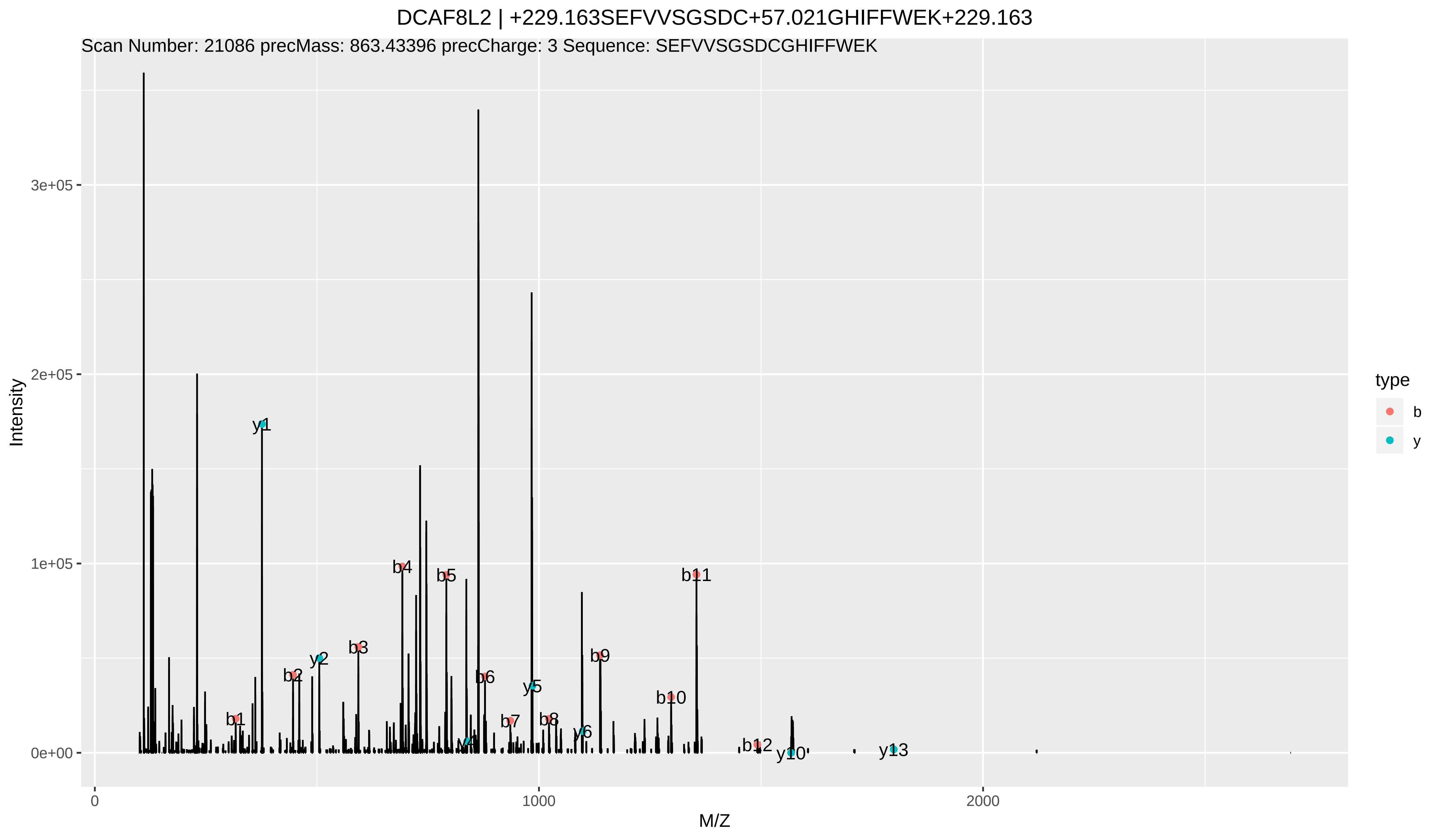
DCAF8L2 | +229.163SEFVVSGSDC+57.021GHIFFWEK+229.163
Scan Number: 21086 precMass: 863.43396 precCharge: 3 Sequence: SEFVVSGSDCGHIFFWEK
Intensity
0e+00
1e+05
2e+05
3e+05
M/Z
0
1000
2000
type
b
y
y1
y2
b1
b2
b3
b4
b5
b6
b7
b8
b9
b10
b11
b12
y5
y6
y10
y13

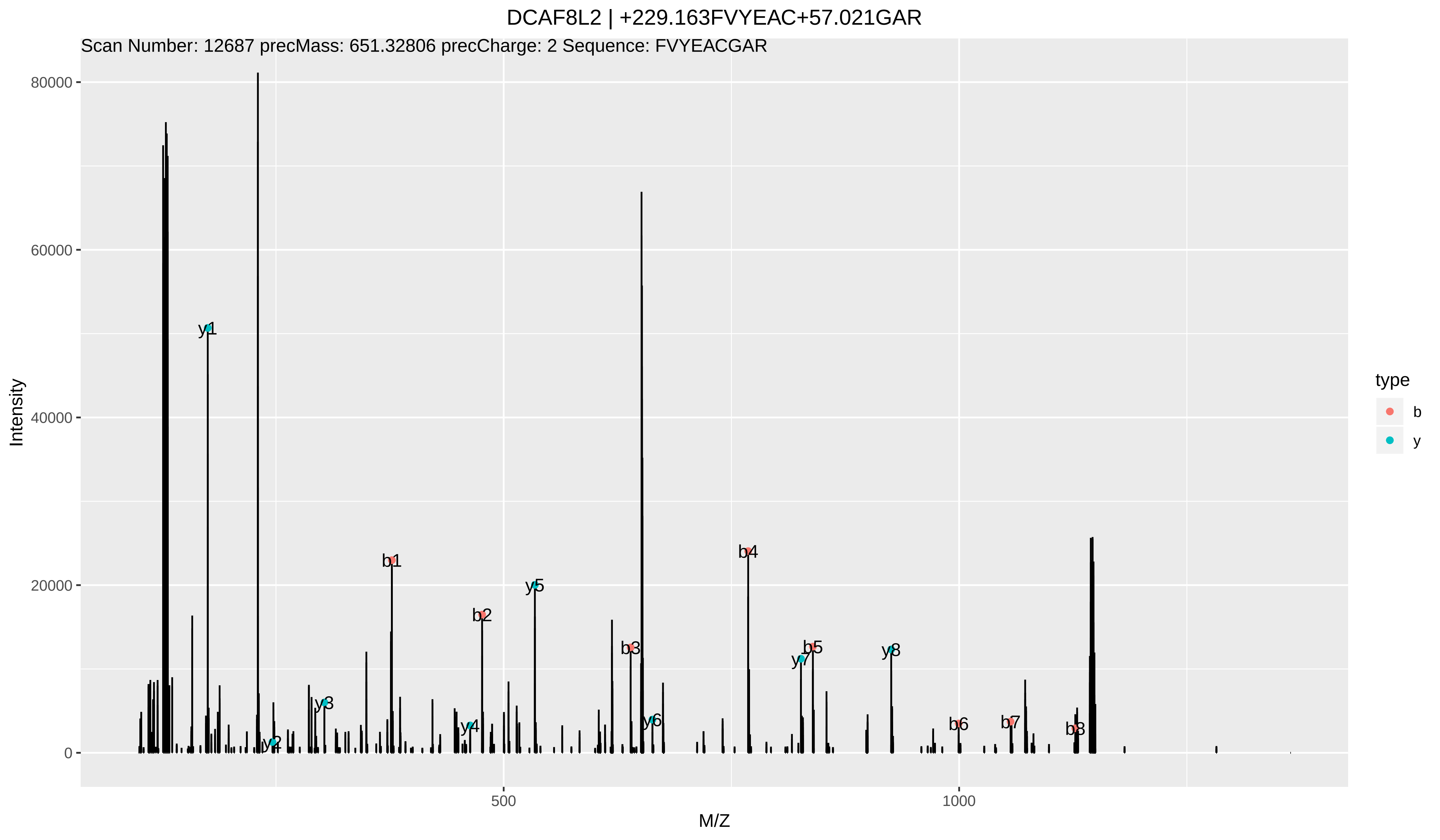
DCAF8L2 | +229.163FVYEAC+57.021GAR
Scan Number: 12687 precMass: 651.32806 precCharge: 2 Sequence: FVYEACGAR
Intensity
M/Z
80000
60000
40000
20000
0
500
1000
y1
y2
y3
y4
y5
y6
y7
y8
b1
b2
b3
b4
b5
b6
b7
b8
type
b
y

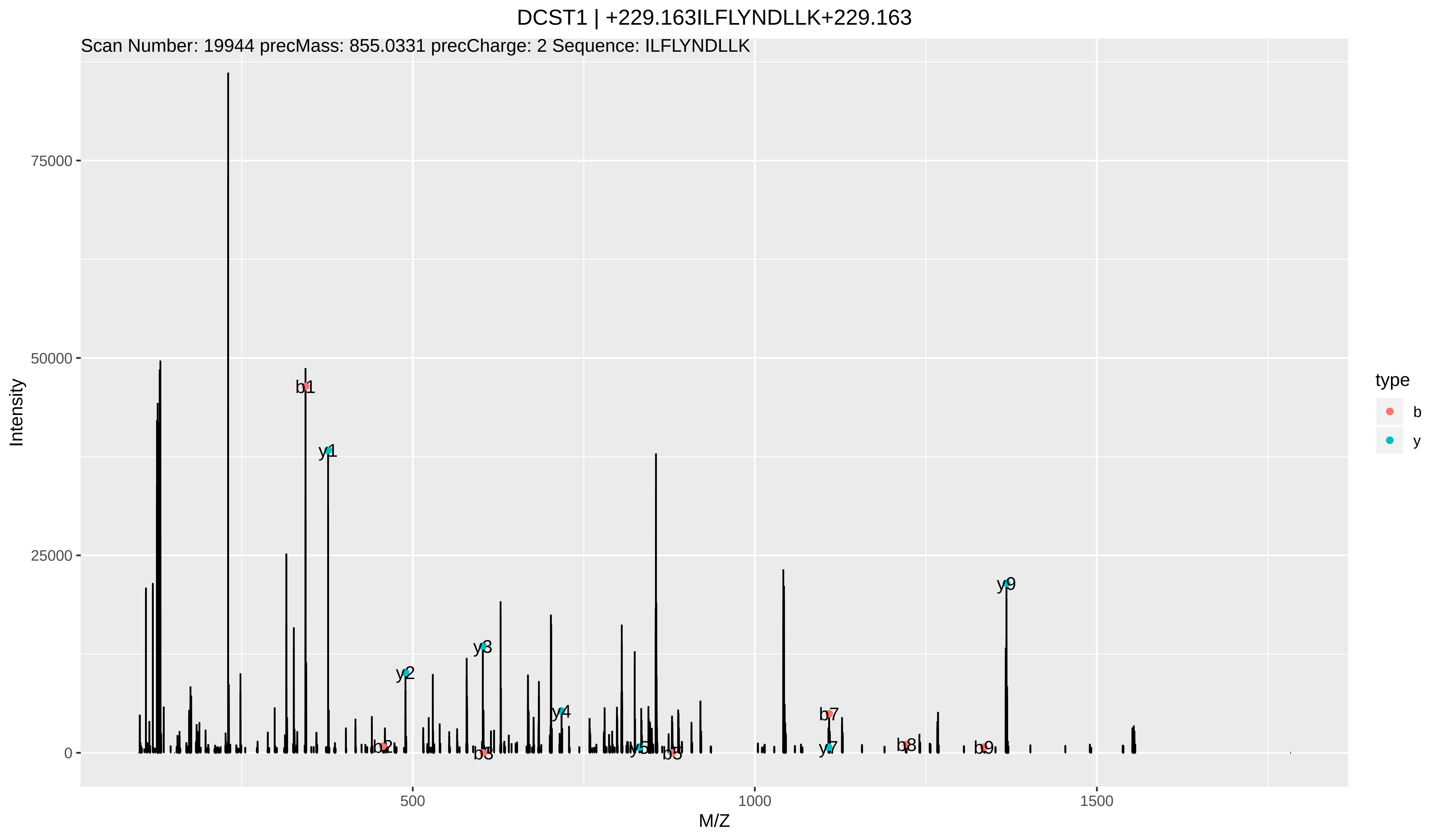

DCST1 | +229.163ILFLYNDLLK+229.163
Scan Number: 19944 precMass: 855.0331 precCharge: 2 Sequence: ILFLYNDLLK
Intensity
M/Z
75000
50000
25000
0
500
1000
1500
b1
y1
y2
b2
y3
b3
y4
y5
b5
b7
y7
b8
b9
y9
type
b
y

DDIT4 | +229.163FSSSSTSSSPSSLPR

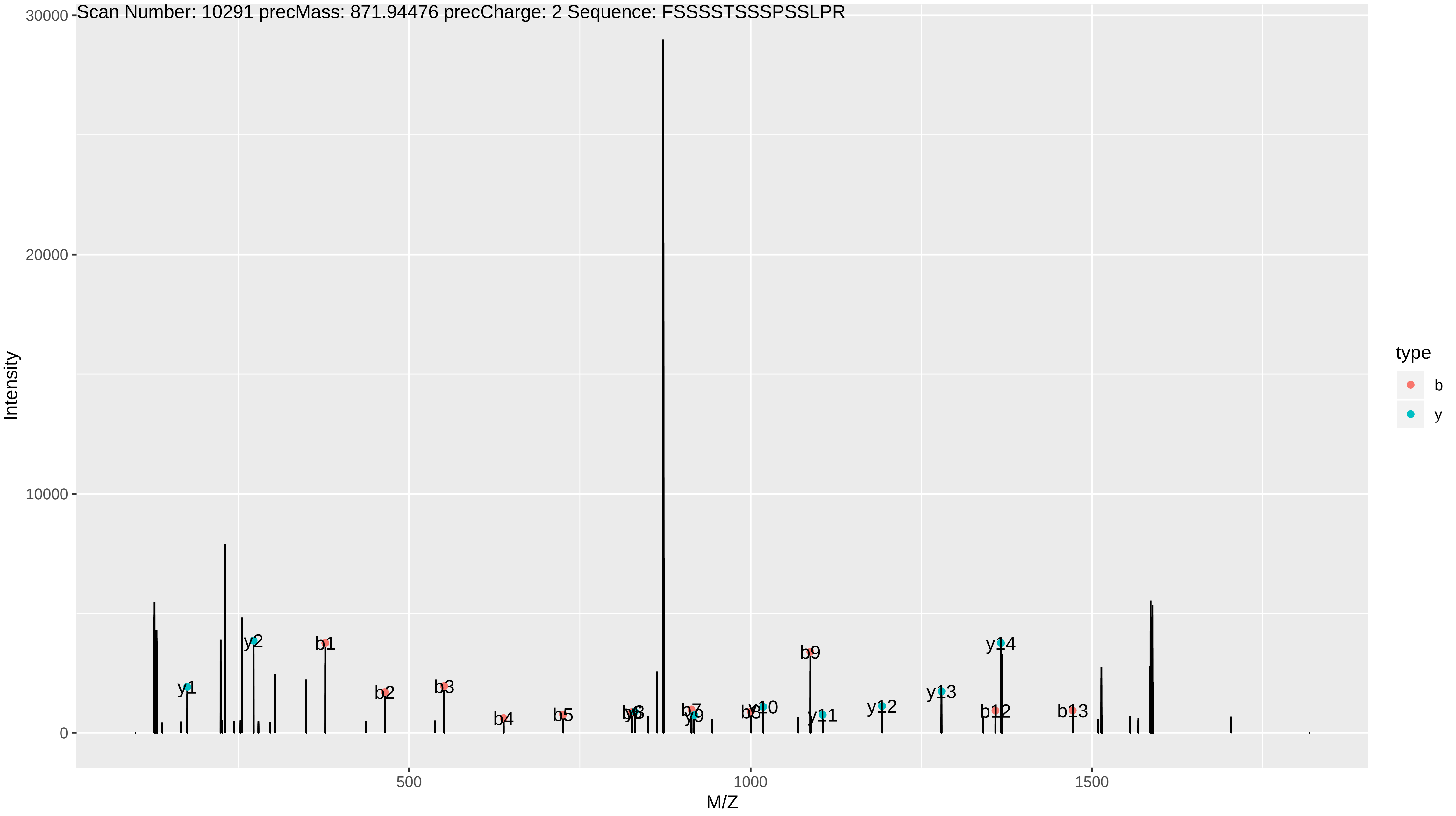

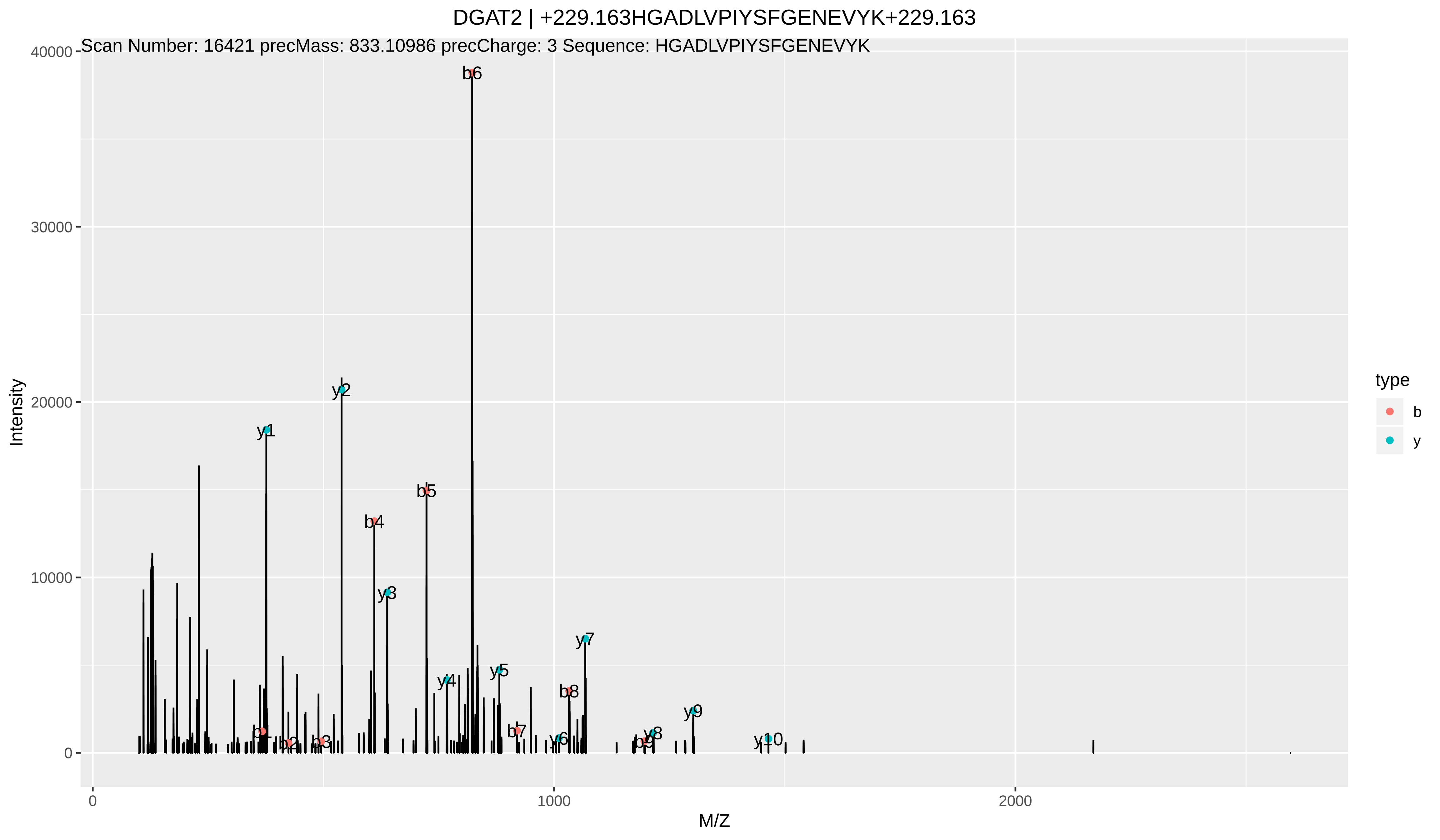
DGAT2 | +229.163HGADLVPIYSFGENEVYK+229.163
Scan Number: 16421 precMass: 833.10986 precCharge: 3 Sequence: HGADLVPIYSFGENEVYK
Intensity
M/Z
type
b
y
b6
y2
y1
b5
b4
y3
y7
y5
y4
b8
y9
b7
b1
b2
b3
y6
y8
b9
y10
0
10000
20000
30000
40000
1000
2000

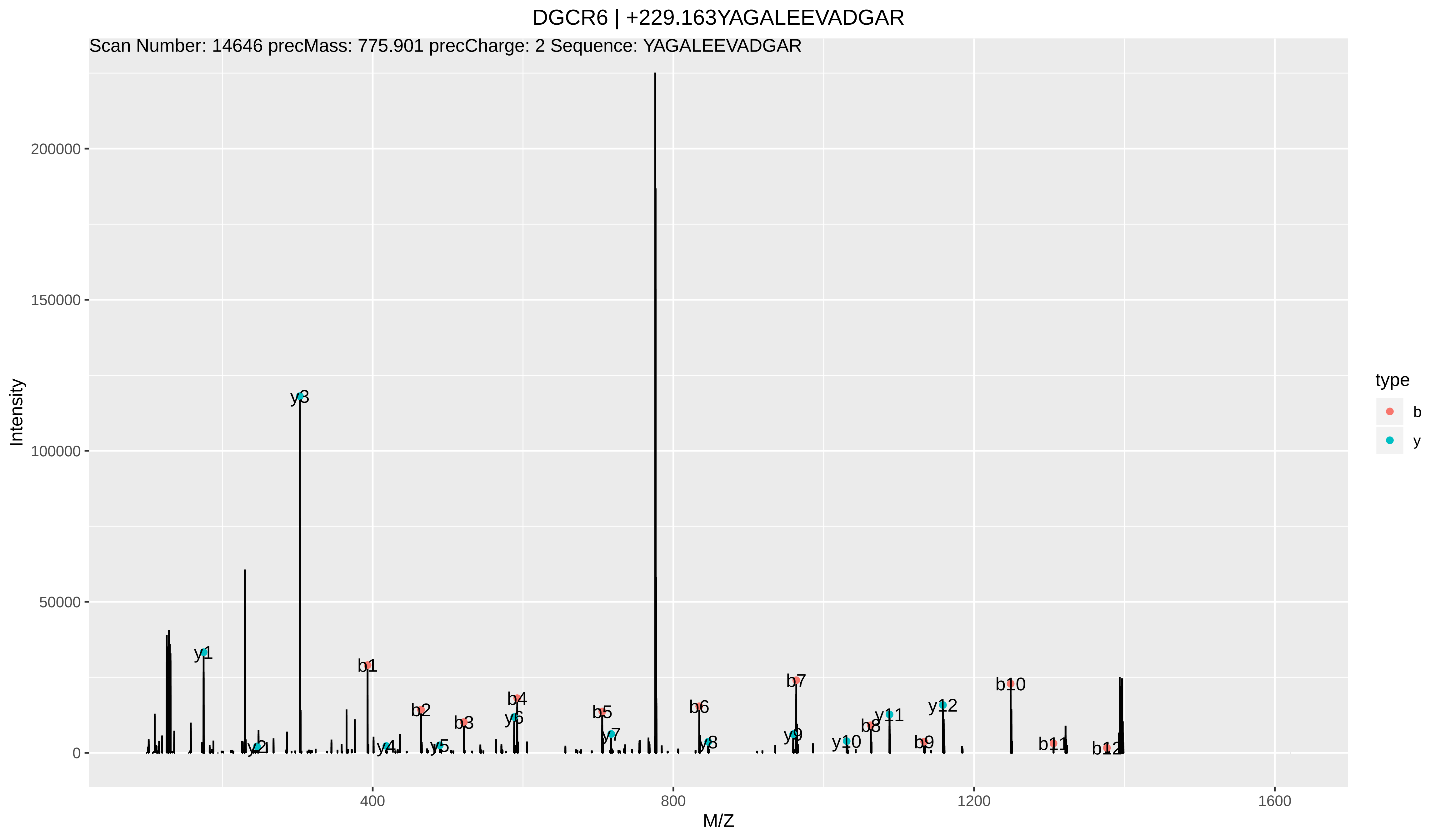
DGCR6 | +229.163YAGALEEVADGAR
Scan Number: 14646 precMass: 775.901 precCharge: 2 Sequence: YAGALEEVADGAR
Intensity
M/Z
type
b
y
y1
y2
y3
y4
y5
y6
y7
y8
y9
y10
y11
y12
b1
b2
b3
b4
b5
b6
b7
b8
b9
b10
b11
b12
0
50000
100000
150000
200000
400
800
1200
1600

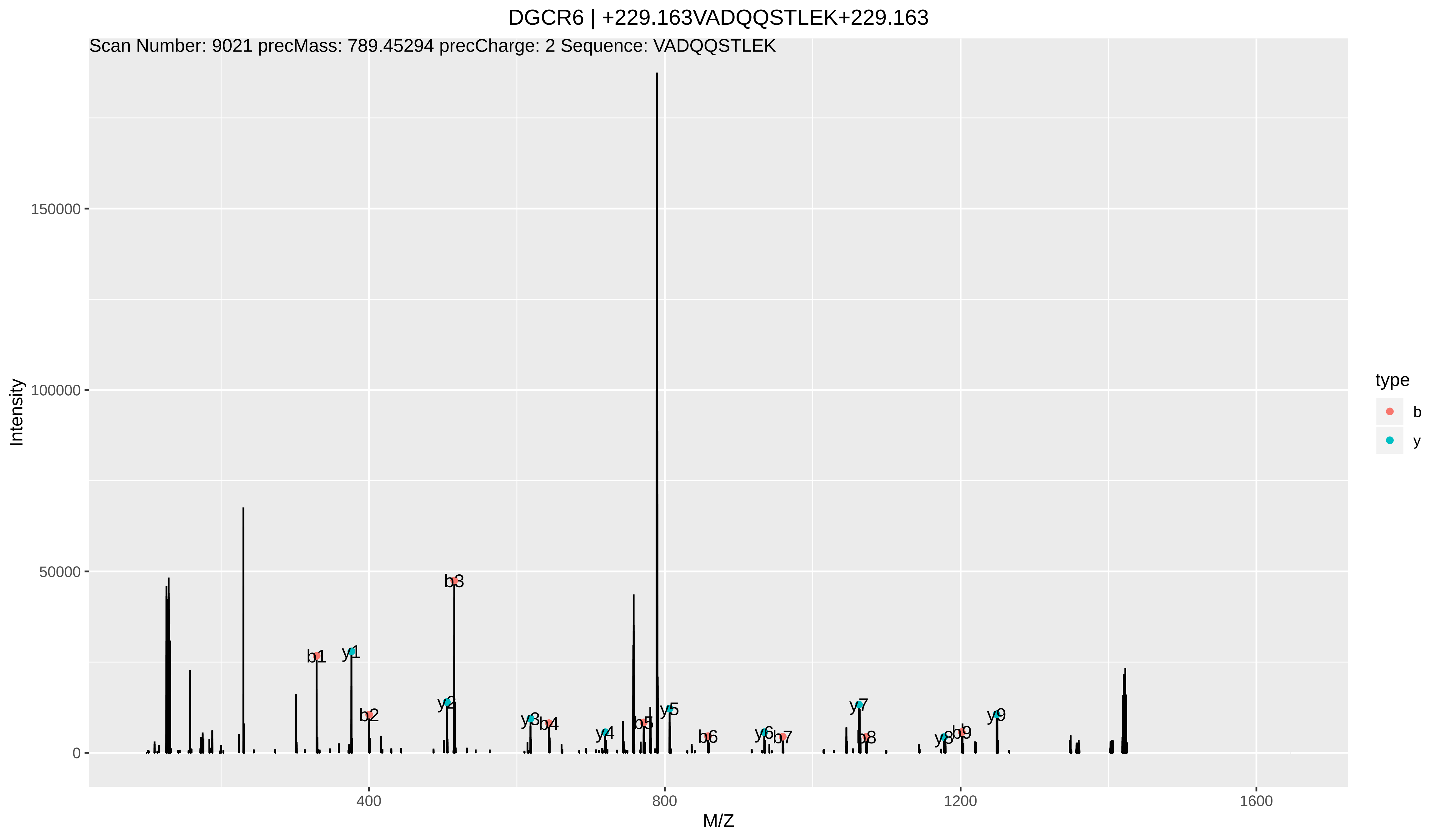
DGCR6 | +229.163VADQQSTLEK+229.163
Scan Number: 9021 precMass: 789.45294 precCharge: 2 Sequence: VADQQSTLEK
Intensity
150000
100000
50000
0
M/Z
400
800
1200
1600
b1
y1
b2
y2
b3
y3
b4
y4
b5
y5
b6
y6
b7
y7
b8
y8
b9
y9
type
b
y

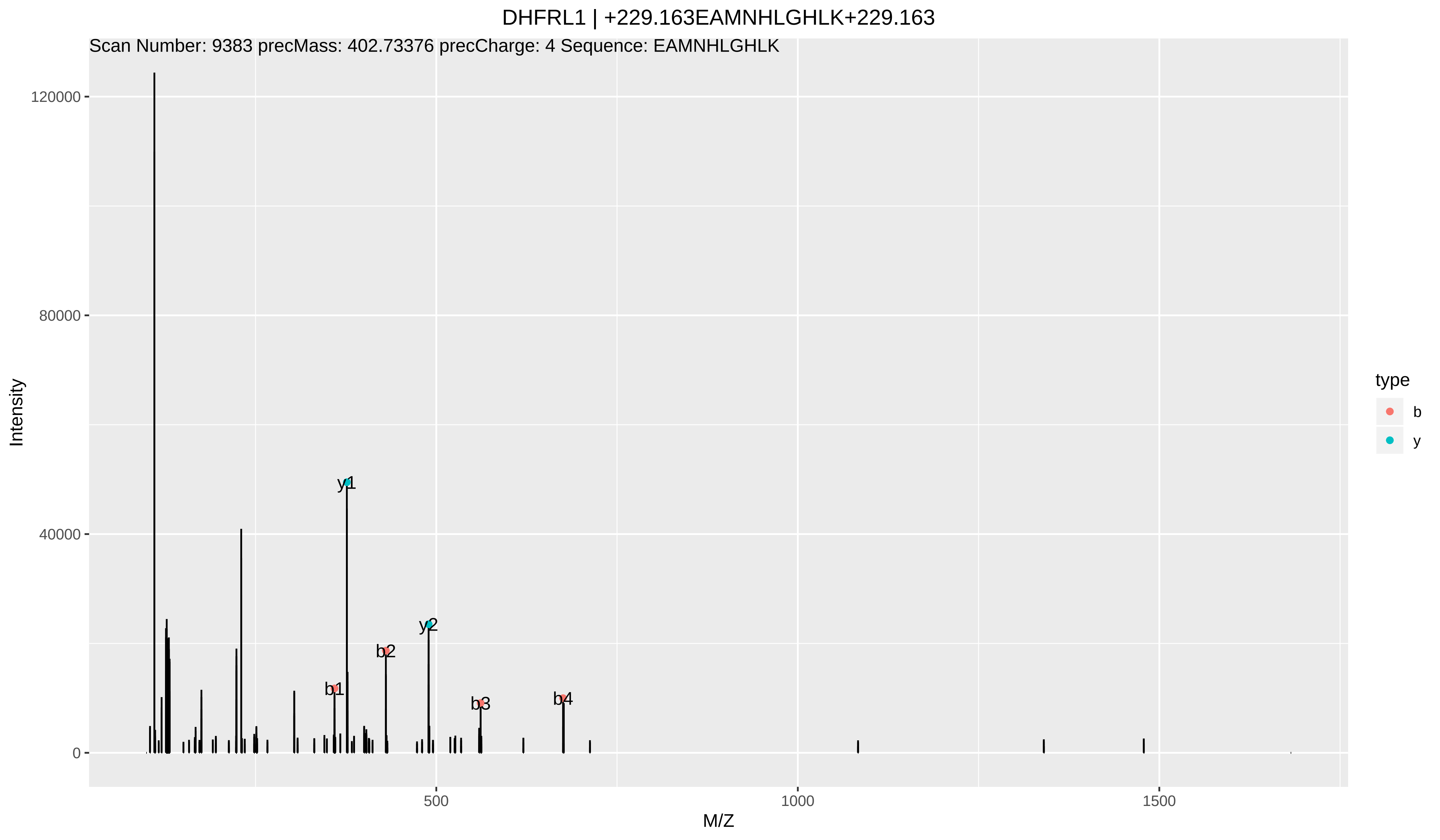
DHFRL1 | +229.163EAMNHLGHLK+229.163
Scan Number: 9383 precMass: 402.73376 precCharge: 4 Sequence: EAMNHLGHLK
Intensity
M/Z
120000
80000
40000
0
500
1000
1500
y1
y2
b1
b2
b3
b4
type
b
y

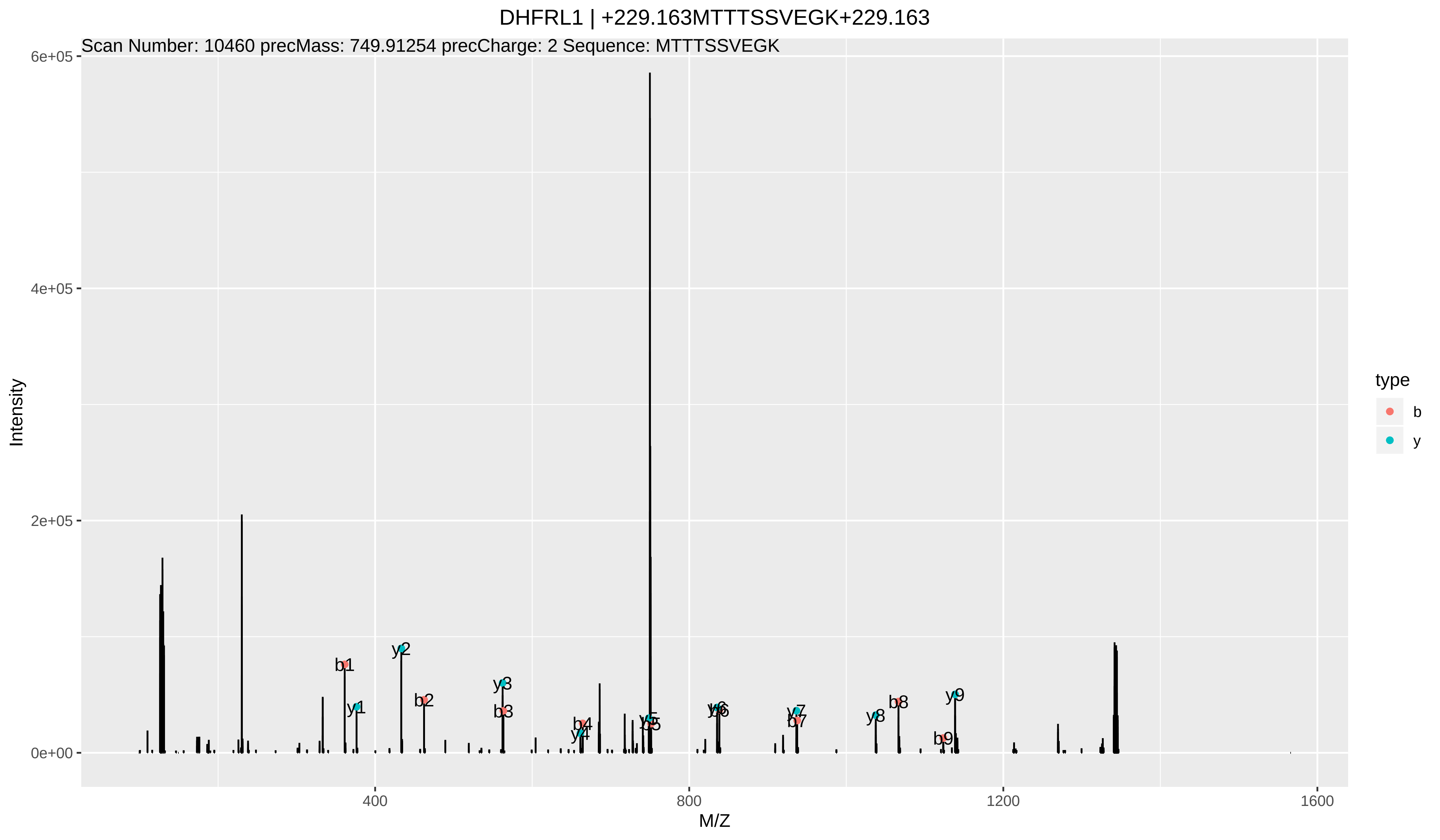

DHFRL1 | +229.163MTTTSSVEGK+229.163
Scan Number: 10460 precMass: 749.91254 precCharge: 2 Sequence: MTTTSSVEGK
Intensity
M/Z
6e+05
4e+05
2e+05
0e+00
400
800
1200
1600
type
b
y
b1
y1
y2
b2
y3
b3
b4
y4
b5
y5
y6
b6
y7
b7
y8
b8
y9
b9

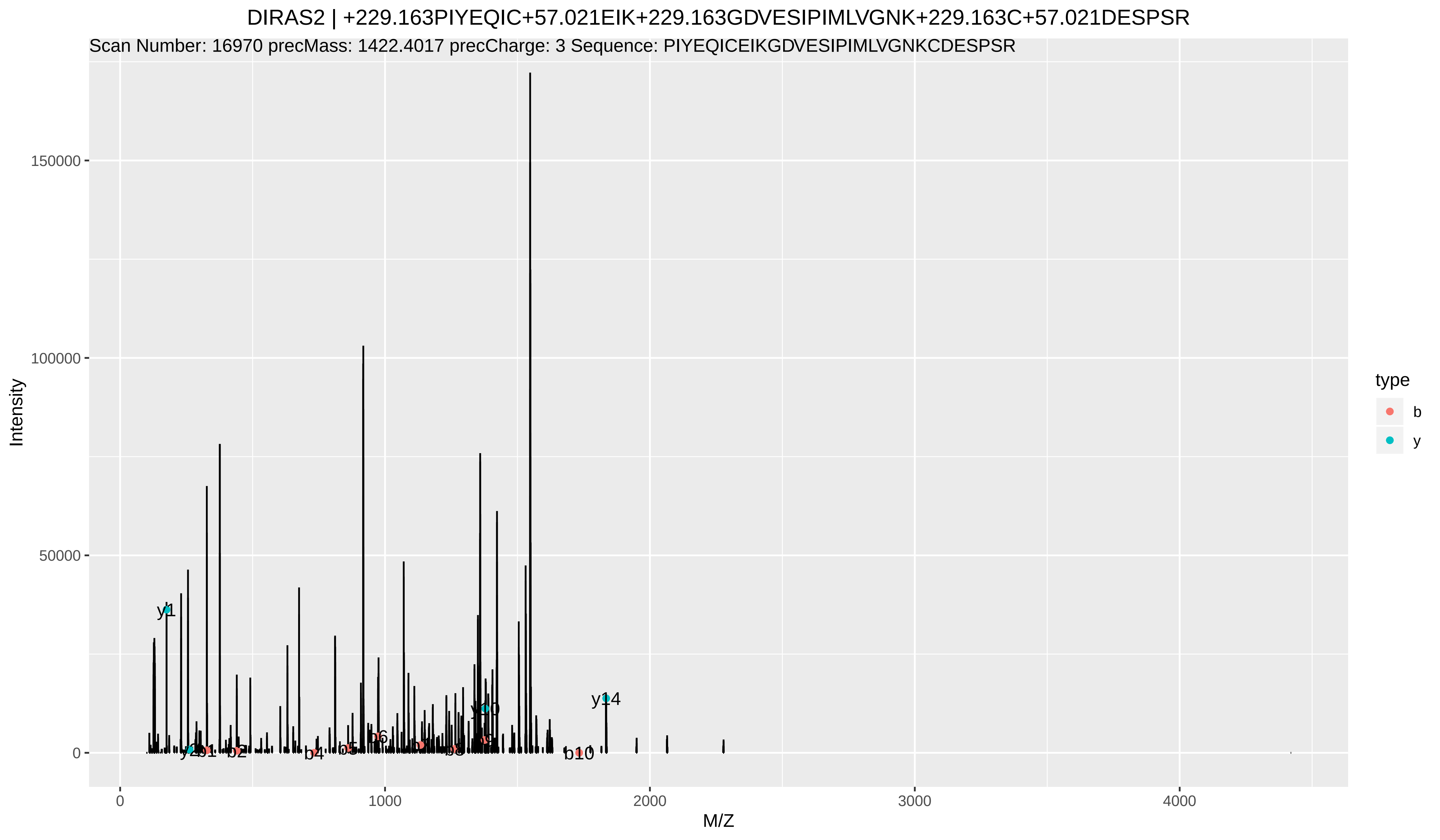
DIRAS2 | +229.163PIYEQIC+57.021EIK+229.163GDVESIPIMLVGNK+229.163C+57.021DESPSR
Scan Number: 16970 precMass: 1422.4017 precCharge: 3 Sequence: PIYEQICEIKGDVESIPIMLVGNKCDESPSR
Intensity
150000
100000
50000
0
M/Z
0
1000
2000
3000
4000
y1
y2
b1
b2
b4
b5
b6
b8
y10
y14
b10
type
b
y

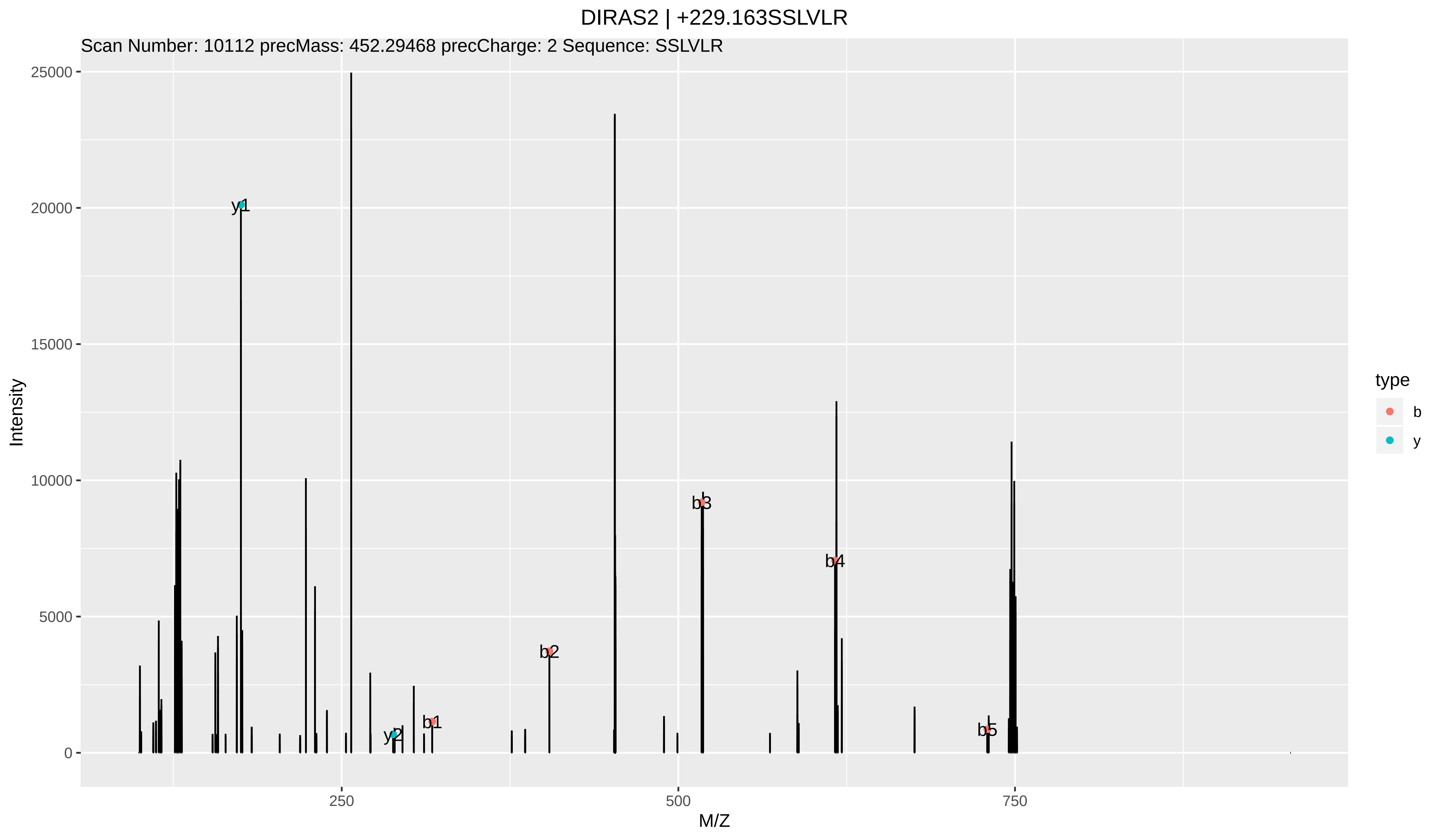

DIRAS2 | +229.163SSLVLR
Scan Number: 10112 precMass: 452.29468 precCharge: 2 Sequence: SSLVLR
Intensity
M/Z
type
b
y
y1
y2
b1
b2
b3
b4
b5
0
5000
10000
15000
20000
25000
250
500
750

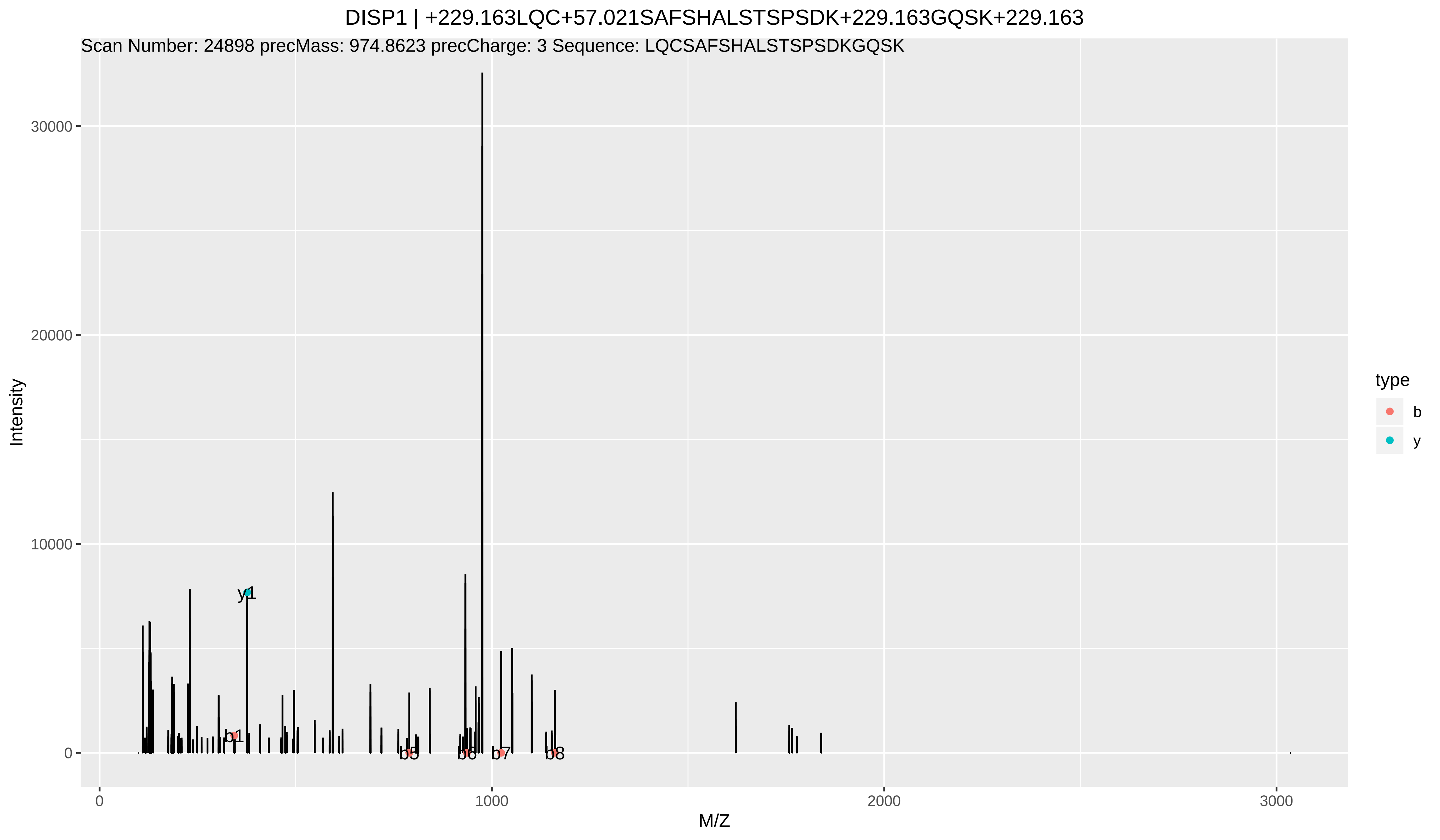

DISP1 | +229.163LQC+57.021SAFSHALSTSPSDK+229.163GQSK+229.163
Scan Number: 24898 precMass: 974.8623 precCharge: 3 Sequence: LQCSAFSHALSTSPSDKGQSK
Intensity
M/Z
type
b
y
y1
b1
b5
b6
b7
b8
0
10000
20000
30000
1000
2000
3000

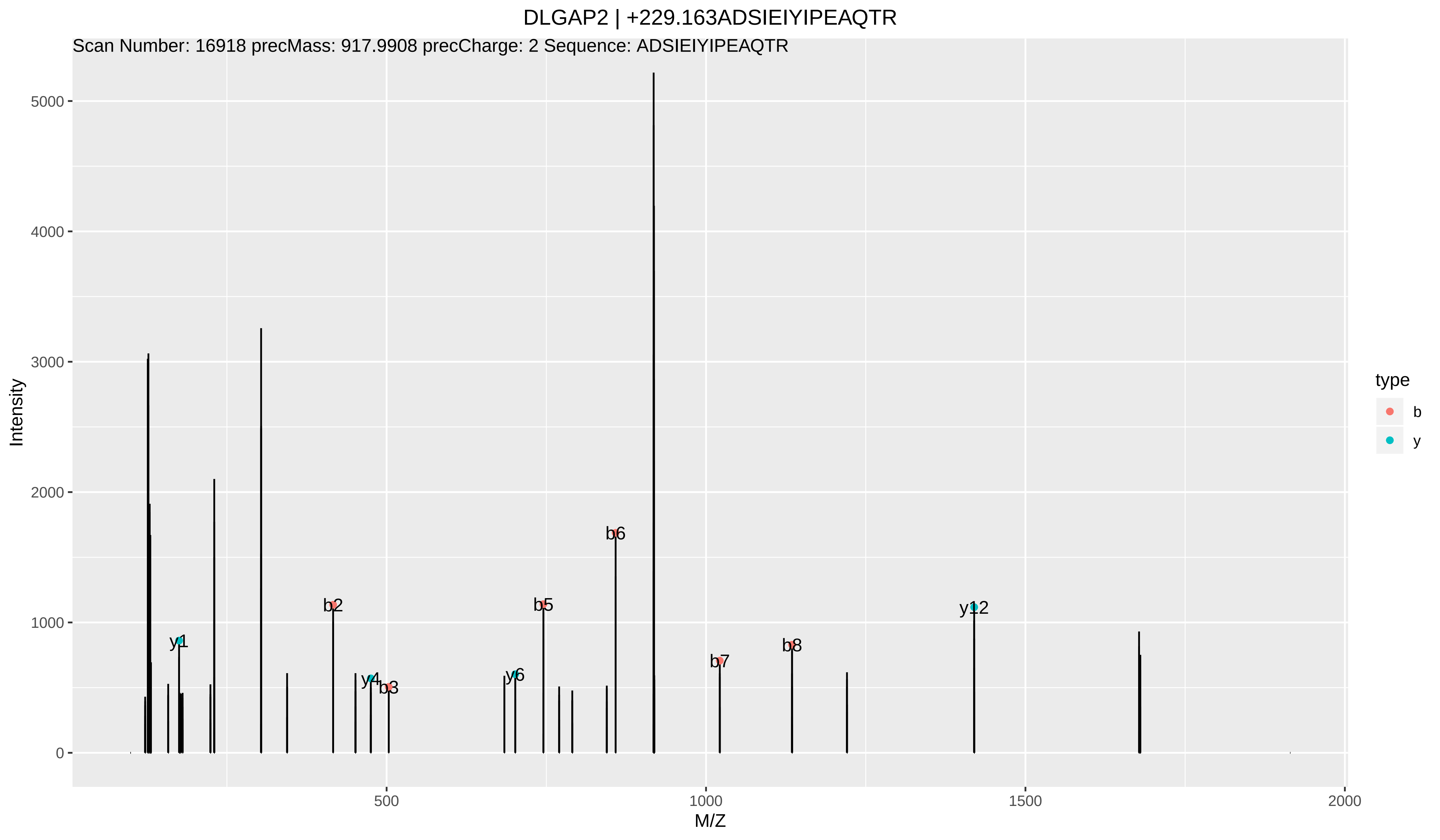

DLGAP2 | +229.163ADSIEIYIPEAQTR
Scan Number: 16918 precMass: 917.9908 precCharge: 2 Sequence: ADSIEIYIPEAQTR
Intensity
M/Z
0
1000
2000
3000
4000
5000
500
1000
1500
2000
y1
b2
y4
b3
y6
b5
b6
b7
b8
y12
type
b
y

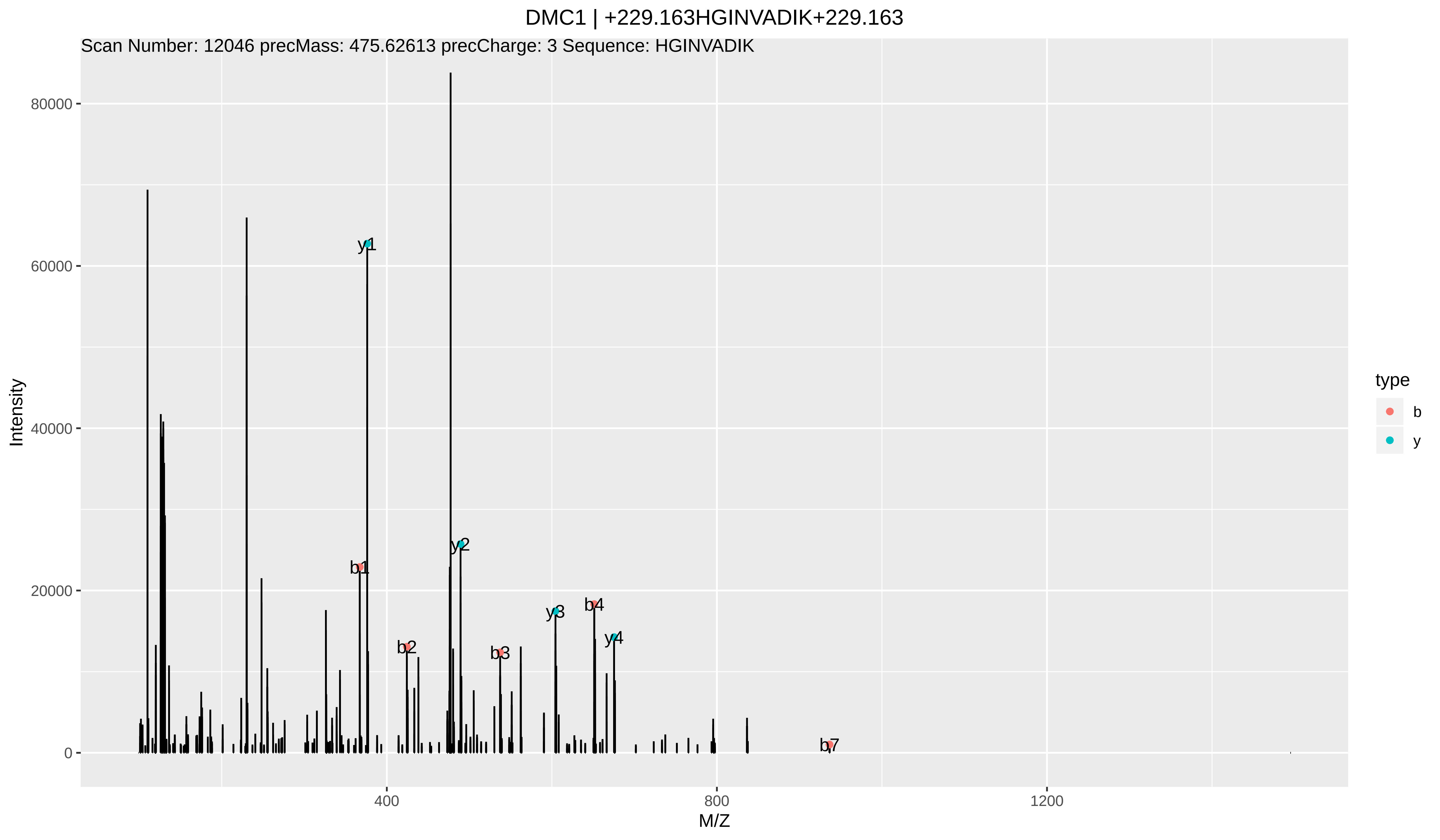
DMC1 | +229.163HGINVADIK+229.163
Scan Number: 12046 precMass: 475.62613 precCharge: 3 Sequence: HGINVADIK
Intensity
M/Z
type
b
y
y1
y2
y3
y4
b1
b2
b3
b4
b7
0
20000
40000
60000
80000
400
800
1200

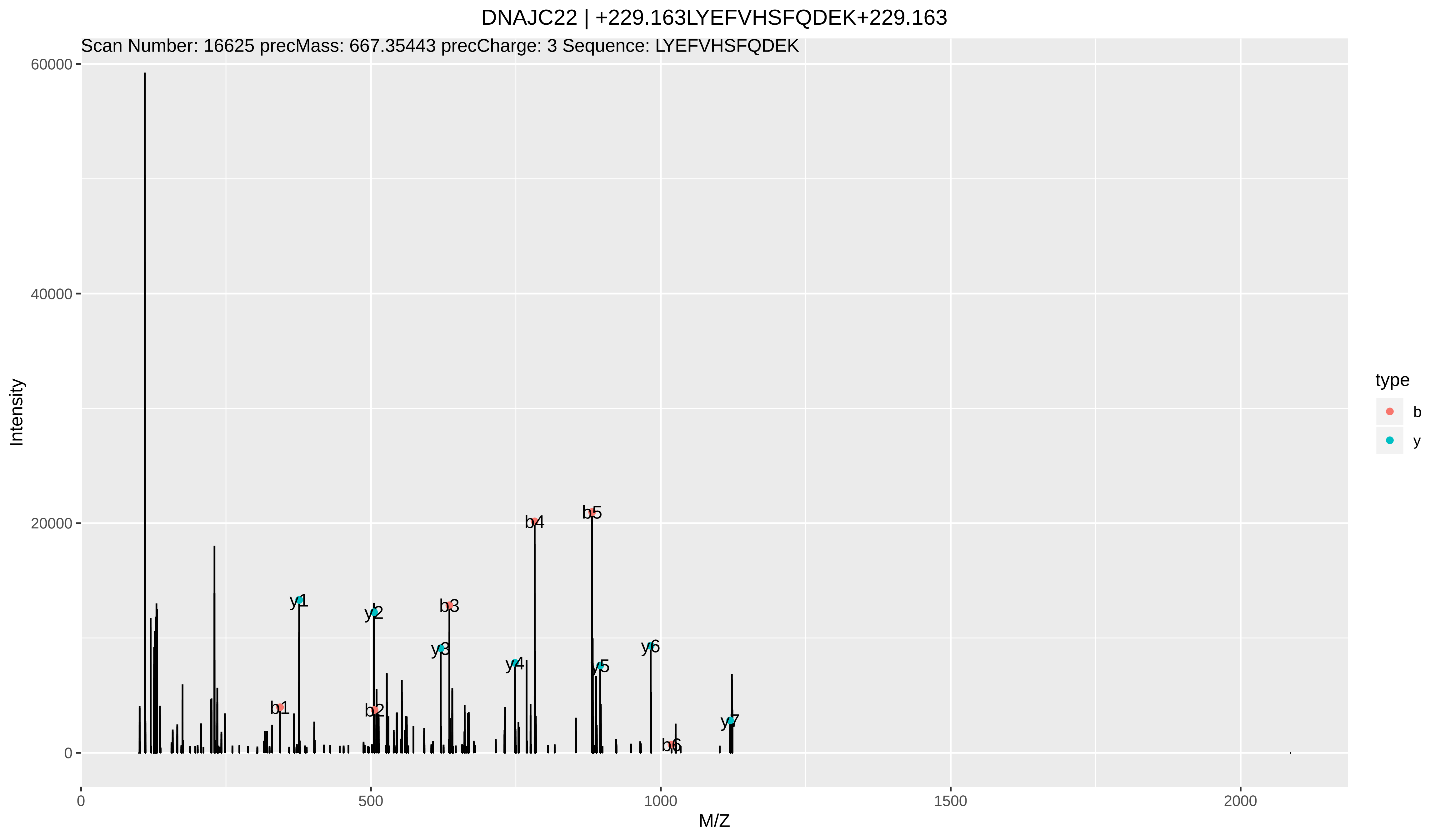

DNAJC22 | +229.163LYEFVHSFQDEK+229.163
Scan Number: 16625 precMass: 667.35443 precCharge: 3 Sequence: LYEFVHSFQDEK
Intensity
M/Z
60000
40000
20000
0
0
500
1000
1500
2000
b1
b2
b3
b4
b5
b6
y1
y2
y3
y4
y5
y6
y7
type
b
y

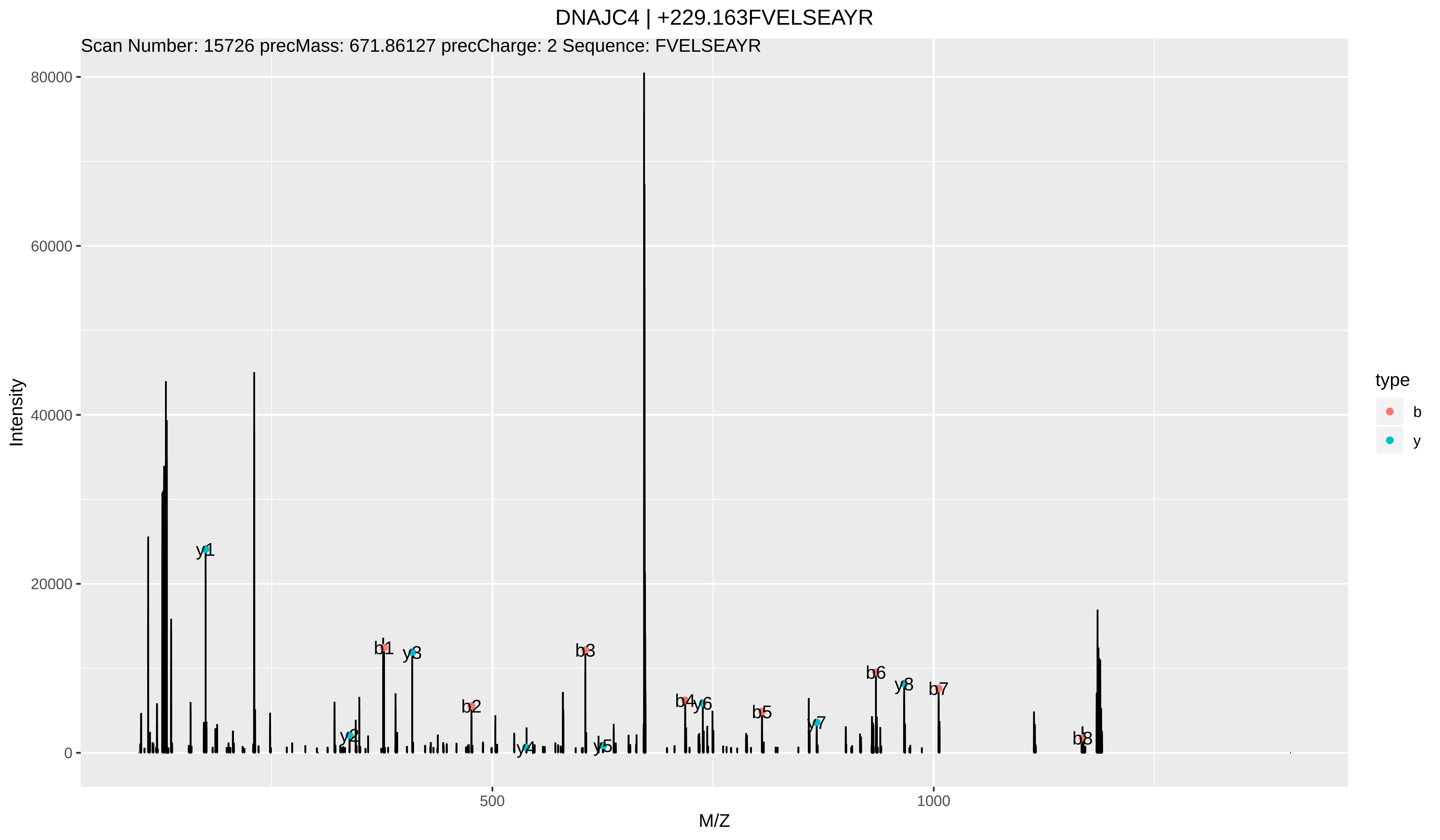

DNAJC4 | +229.163FVELSEAYR
Scan Number: 15726 precMass: 671.86127 precCharge: 2 Sequence: FVELSEAYR
Intensity
M/Z
80000
60000
40000
20000
0
500
1000
y1
y2
b1
y3
b2
y4
b3
y5
b4
y6
b5
y7
b6
y8
b7
b8
type
b
y

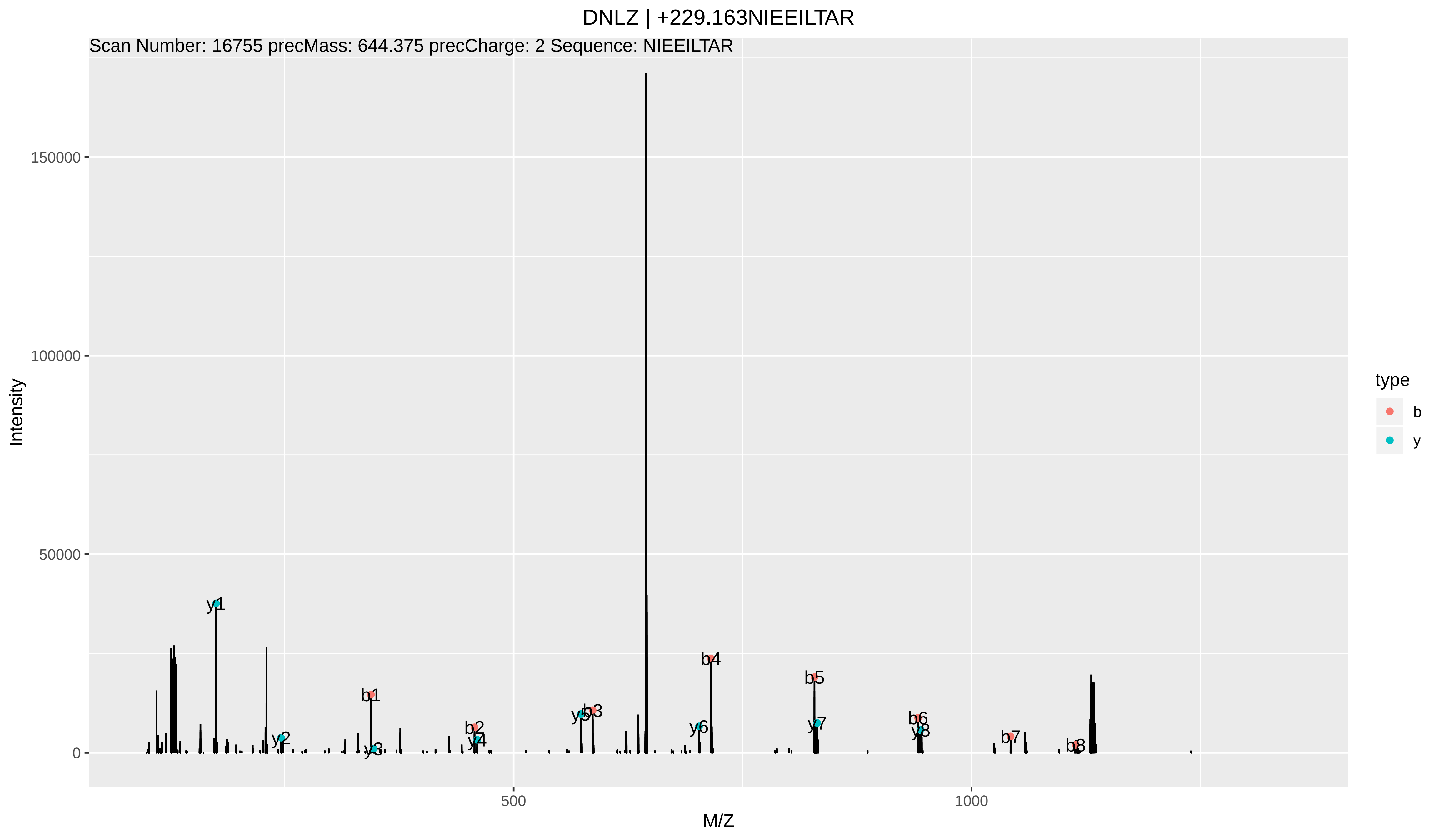

DNLZ | +229.163NIEEILTAR
Scan Number: 16755 precMass: 644.375 precCharge: 2 Sequence: NIEEILTAR
Intensity
M/Z
150000
100000
50000
0
500
1000
y1
y2
y3
y4
y5
y6
y7
y8
b1
b2
b3
b4
b5
b6
b7
b8
type
b
y

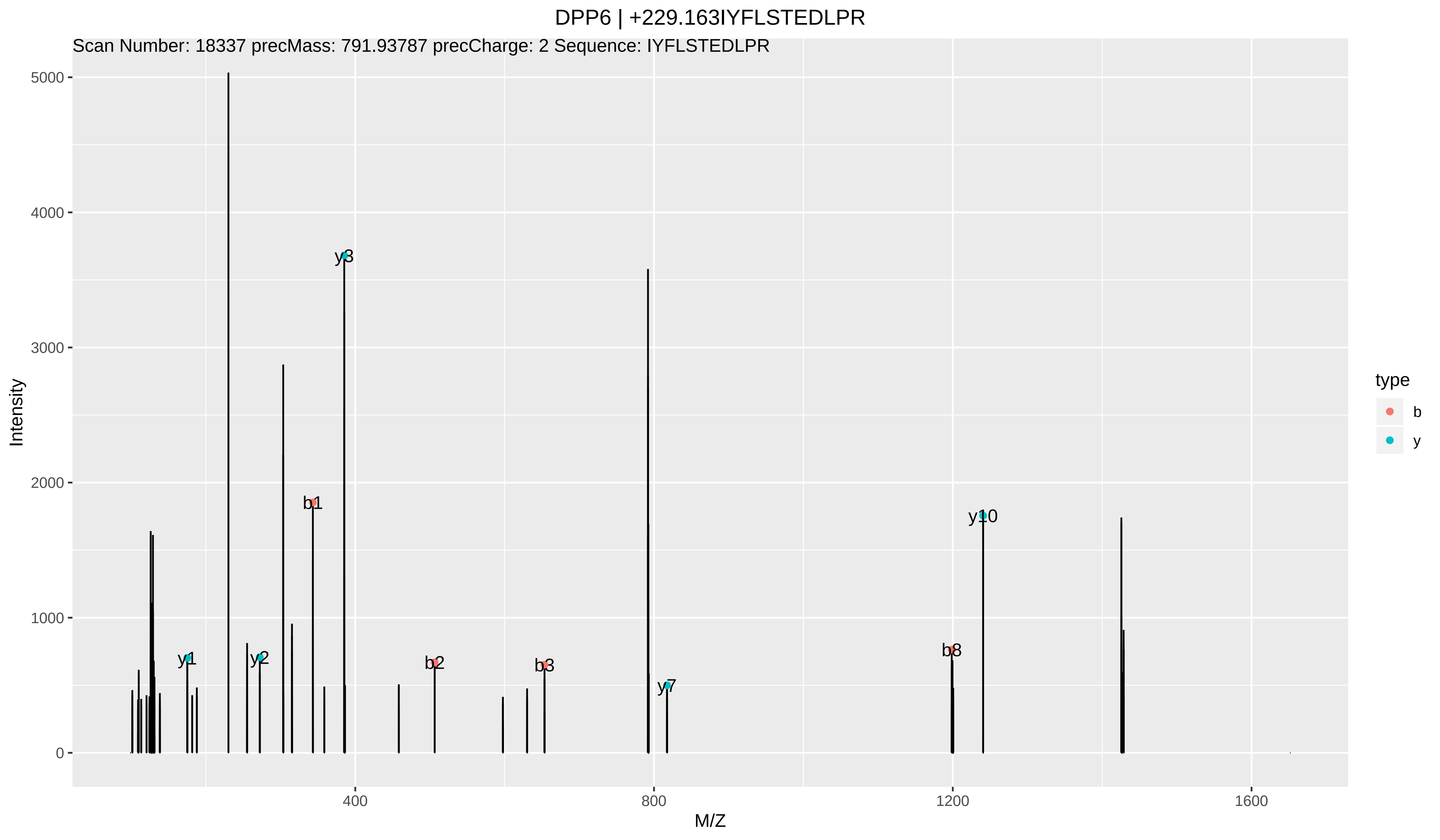

DPP6 | +229.163IYFLSTEDLPR
Scan Number: 18337 precMass: 791.93787 precCharge: 2 Sequence: IYFLSTEDLPR
Intensity
0
1000
2000
3000
4000
5000
M/Z
400
800
1200
1600
y1
y2
b1
y3
b2
b3
y7
b8
y10
type
b
y

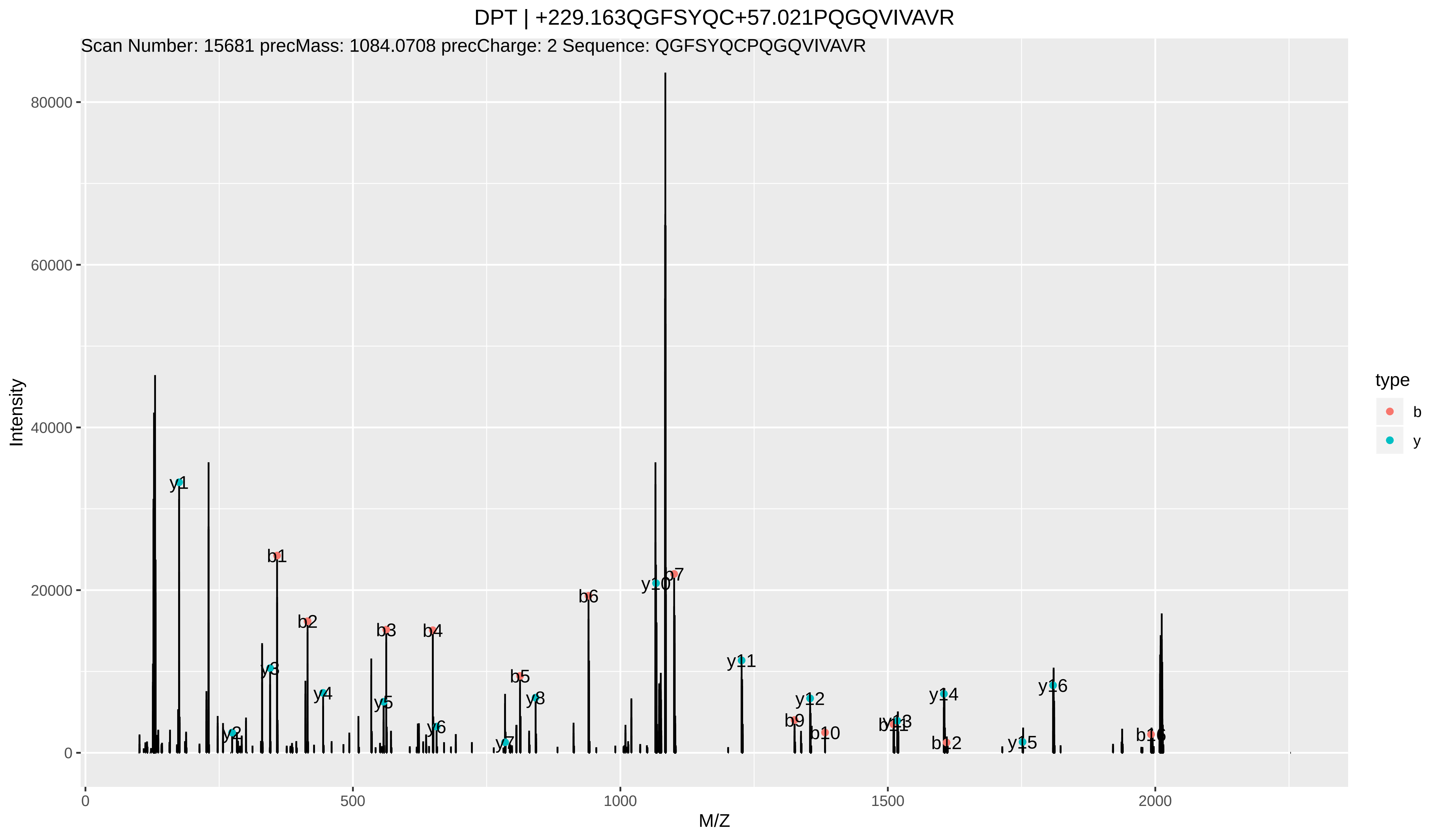

DPT | +229.163QGFSYQC+57.021PQGQVIVAVR
Scan Number: 15681 precMass: 1084.0708 precCharge: 2 Sequence: QGFSYQCPQGQVIVAVR
Intensity
M/Z
0
20000
40000
60000
80000
0
500
1000
1500
2000
type
b
y
y1
b1
b2
b3
b4
b5
b6
b7
y3
y4
y5
y6
y7
y8
y10
y11
y12
y14
y15
y16
b9
b10
b12

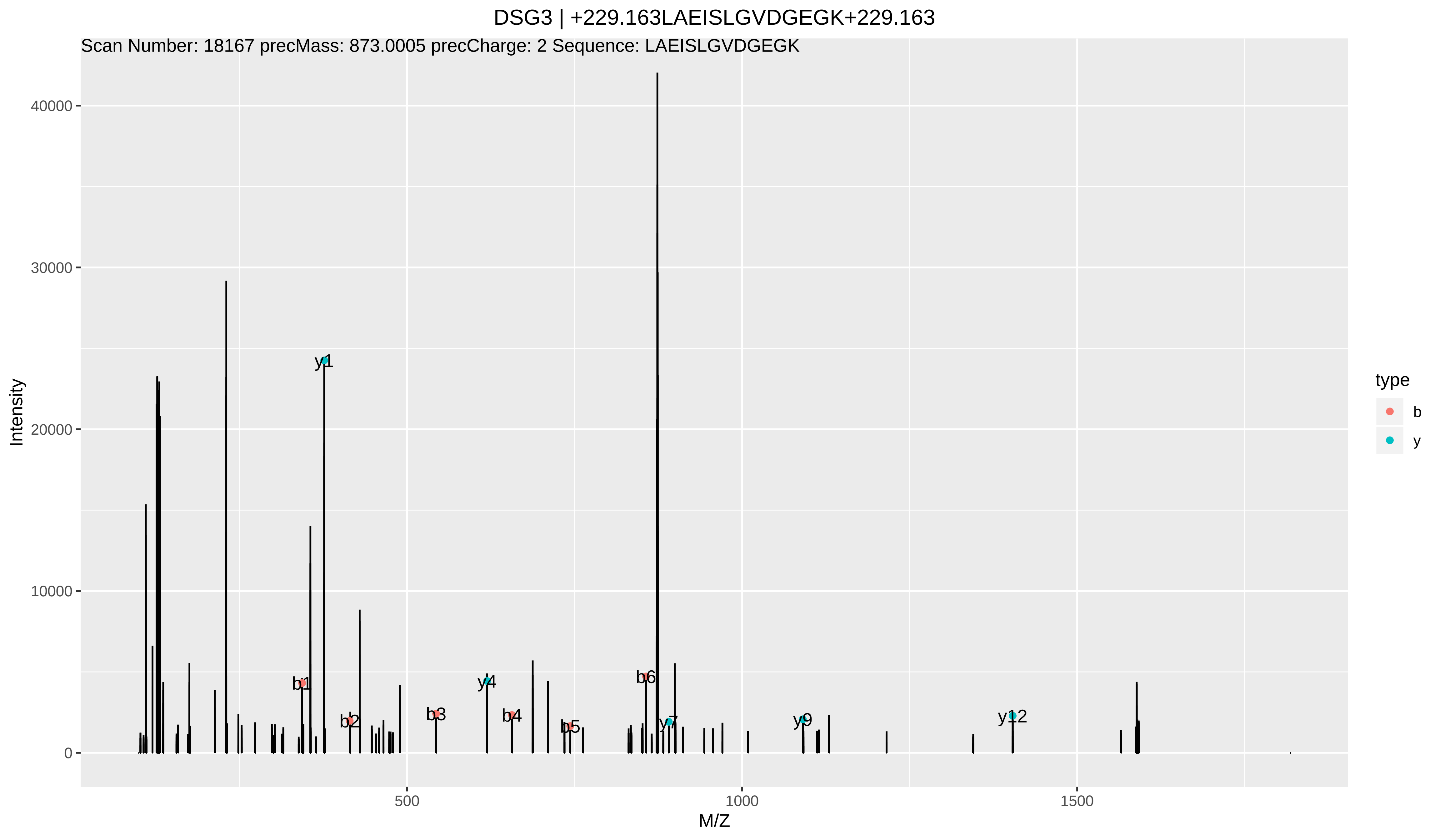

DSG3 | +229.163LAEISLGVDGEGK+229.163
Scan Number: 18167 precMass: 873.0005 precCharge: 2 Sequence: LAEISLGVDGEGK
Intensity
40000
30000
20000
10000
0
500
1000
1500
M/Z
y1
b1
b2
b3
y4
b4
b5
b6
y7
y9
y12
type
b
y

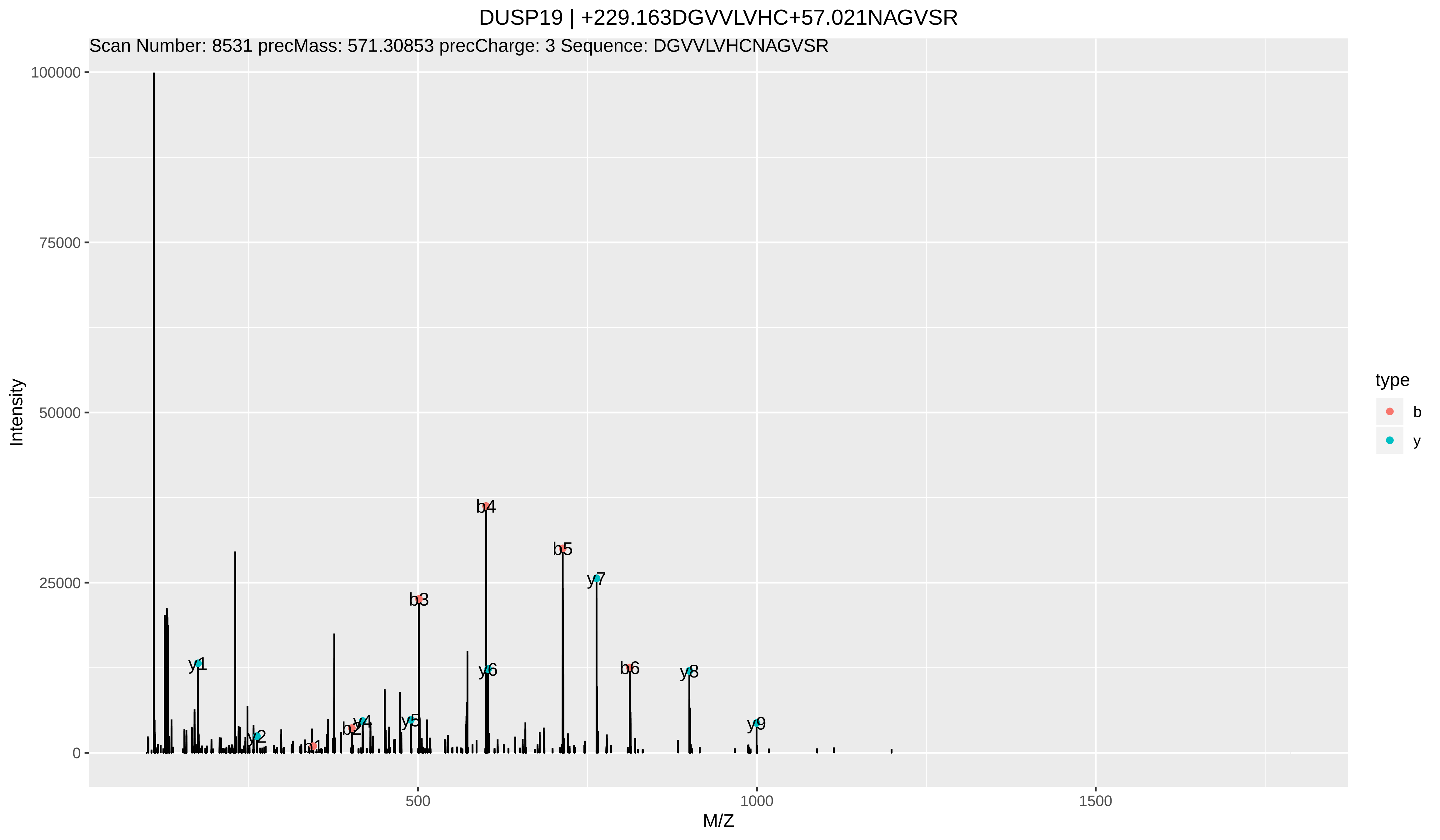

DUSP19 | +229.163DGVVLVHC+57.021NAGVSR
Scan Number: 8531 precMass: 571.30853 precCharge: 3 Sequence: DGVVLVHCNAGVSR
Intensity
100000
75000
50000
25000
0
M/Z
500
1000
1500
type
b
y
y1
y2
b1
b2
y4
y5
b3
b4
y6
b5
y7
b6
y8
y9

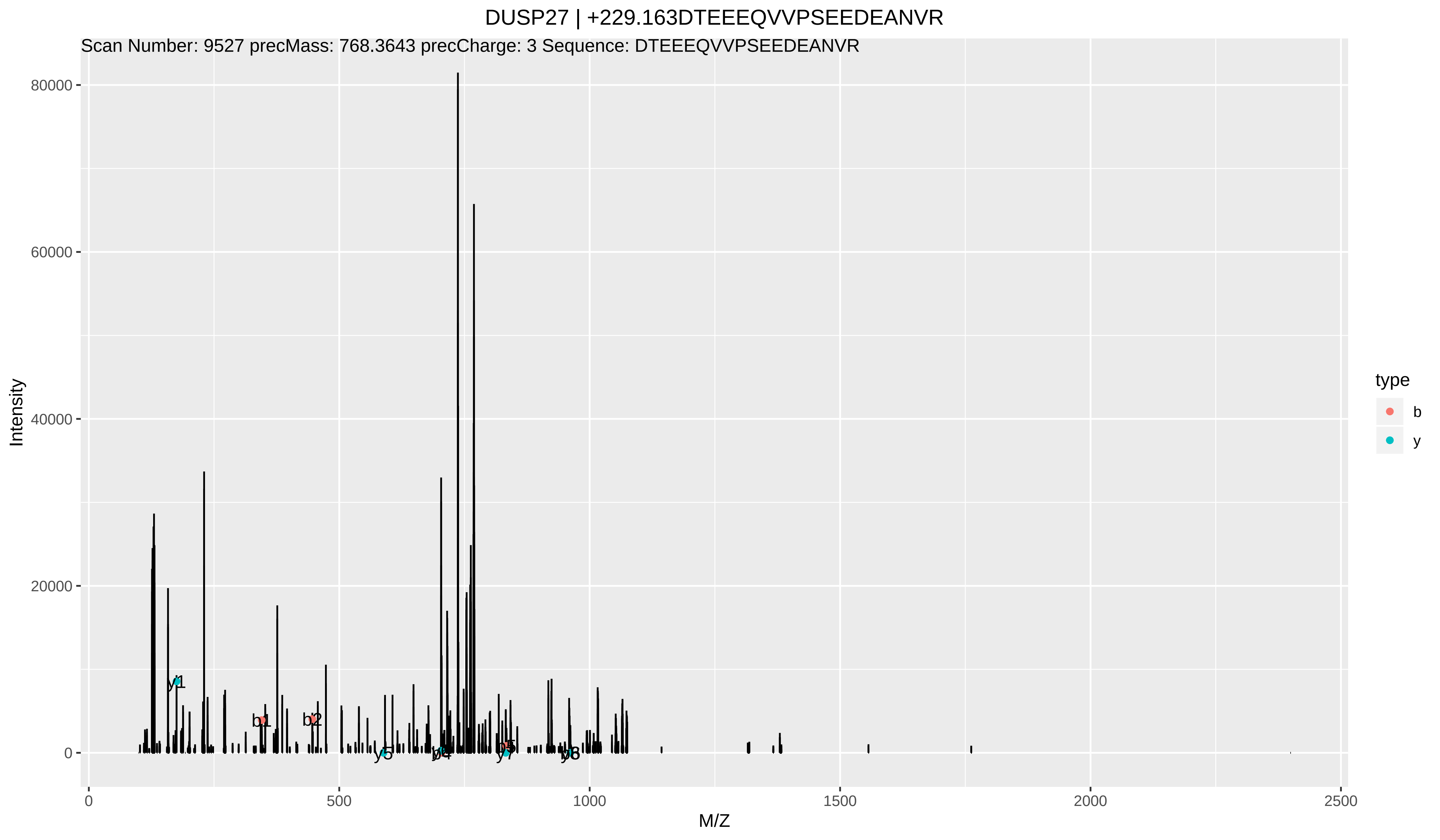

DUSP27 | +229.163DTEEEQVVPSEEDEANVR
Scan Number: 9527 precMass: 768.3643 precCharge: 3 Sequence: DTEEEQVVPSEEDEANVR
Intensity
M/Z
type
b
y

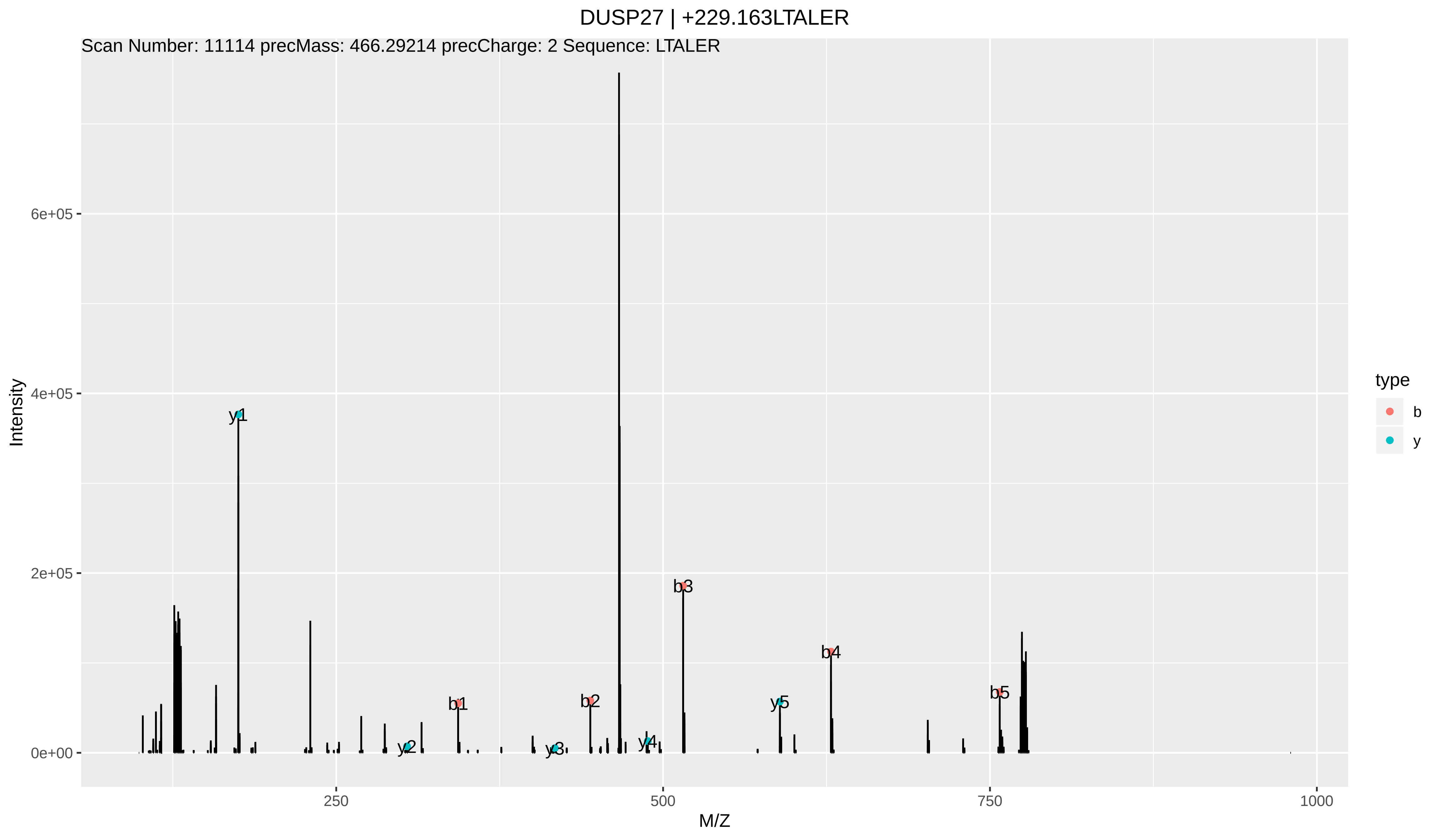
DUSP27 | +229.163LTALER
Scan Number: 11114 precMass: 466.29214 precCharge: 2 Sequence: LTALER
Intensity
6e+05
4e+05
2e+05
0e+00
y1
b1
y2
y3
b2
y4
b3
y5
b4
b5
250
500
750
1000
M/Z
type
b
y

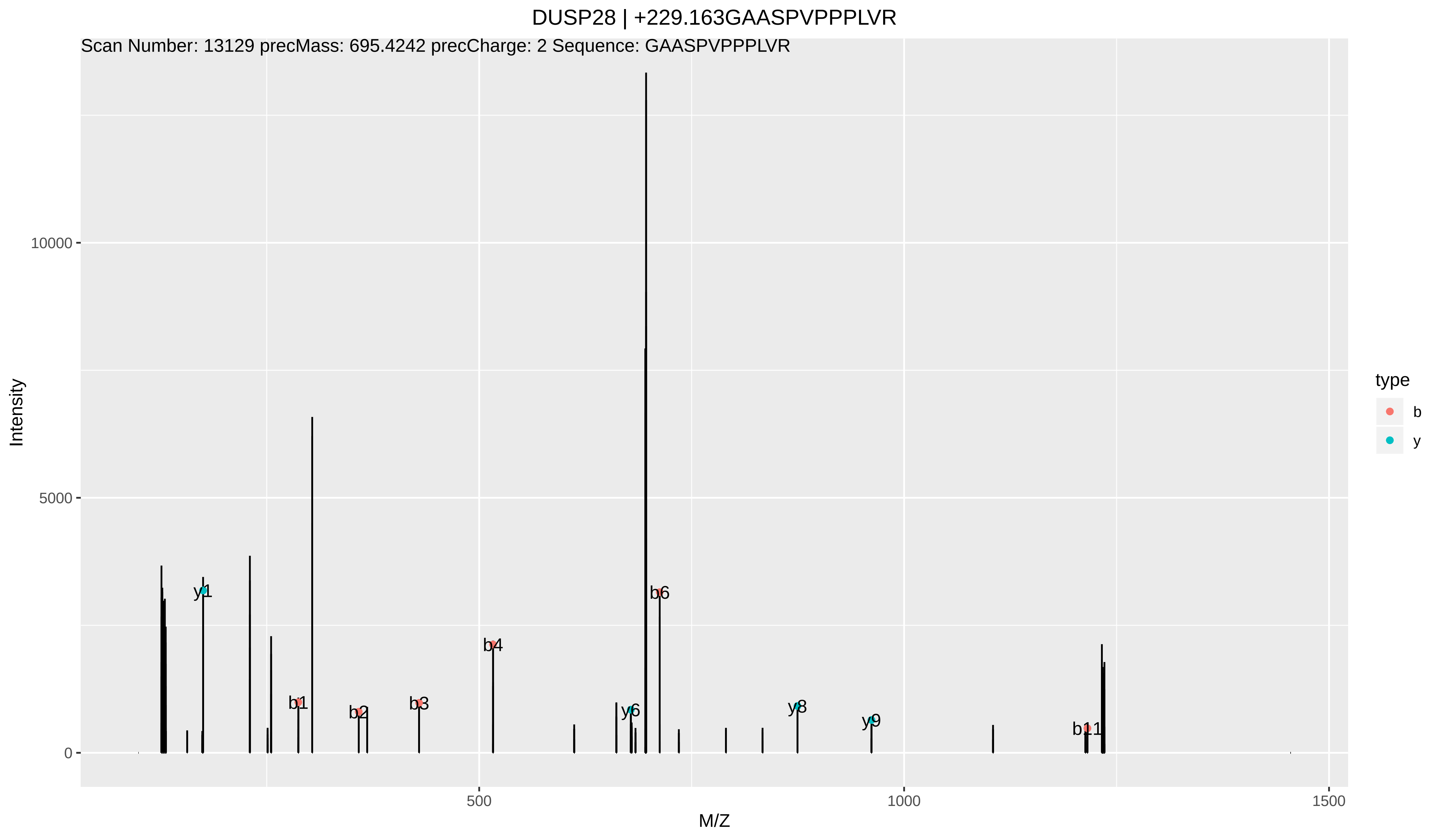

DUSP28 | +229.163GAASPVPPPLVR
Scan Number: 13129 precMass: 695.4242 precCharge: 2 Sequence: GAASPVPPPLVR
Intensity
10000
5000
0
500
1000
1500
M/Z
y1
b1
b2
b3
b4
y6
b6
y8
y9
b11
type
b
y

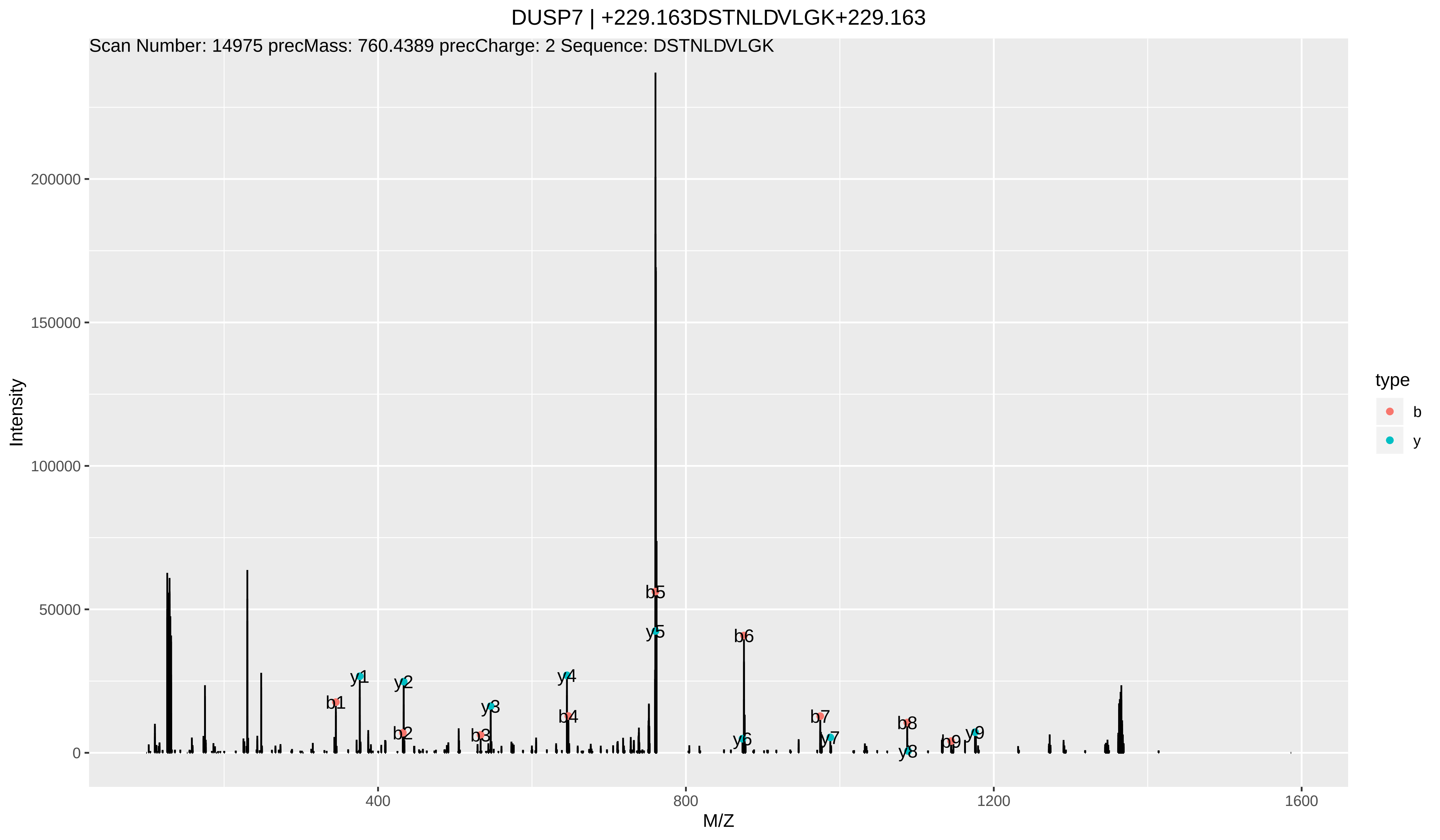
DUSP7 | +229.163DSTNLDVLGK+229.163
Scan Number: 14975 precMass: 760.4389 precCharge: 2 Sequence: DSTNLDVLGK
Intensity
200000
150000
100000
50000
0
M/Z
400
800
1200
1600
type
b
y
b1
b2
b3
b4
b5
b6
b7
b8
b9
y1
y2
y3
y4
y5
y6
y7
y8
y9

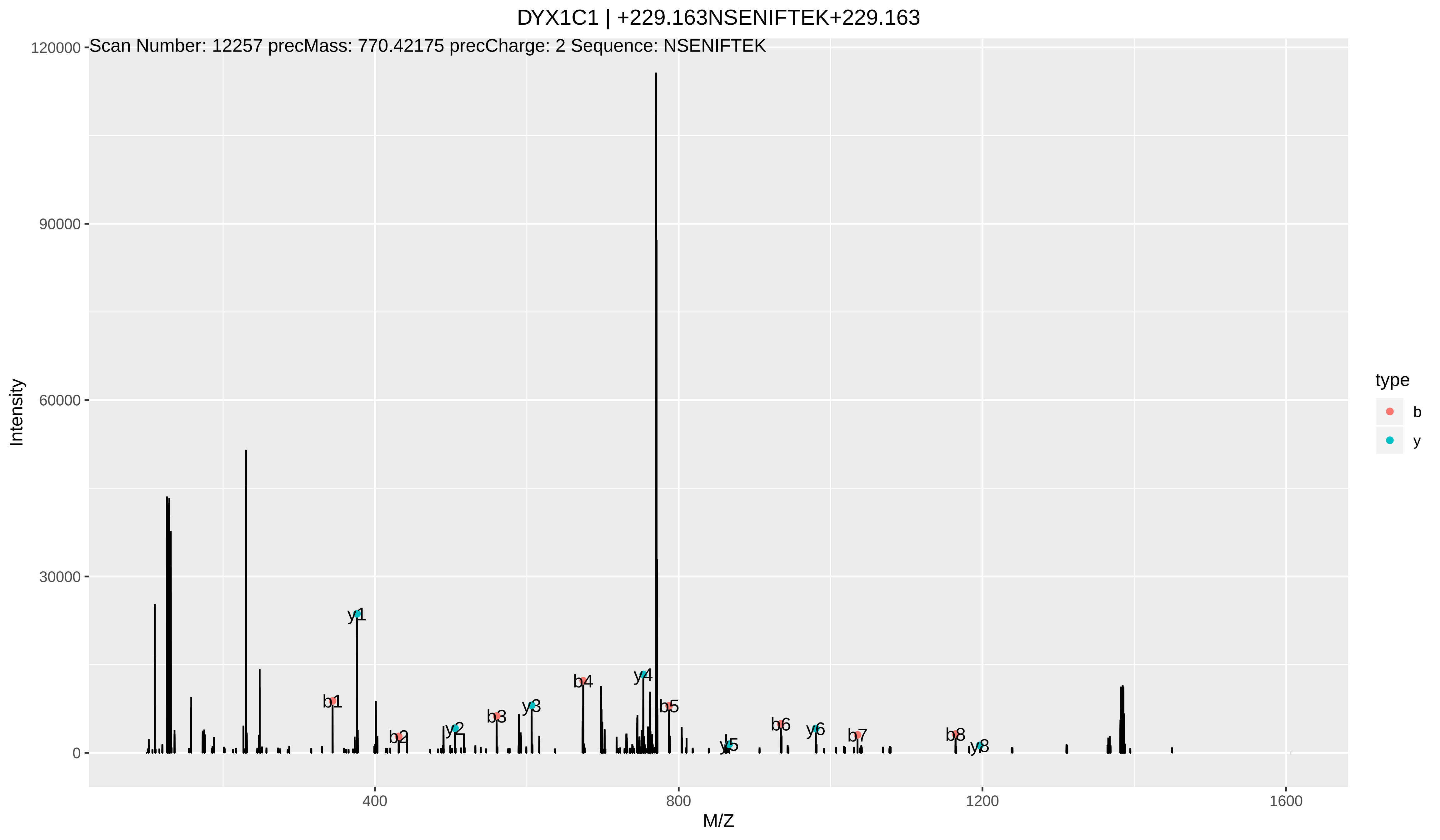

DYX1C1 | +229.163NSENIFTEK+229.163
Scan Number: 12257 precMass: 770.42175 precCharge: 2 Sequence: NSENIFTEK
Intensity
M/Z
0
30000
60000
90000
120000
400
800
1200
1600
y1
b1
b2
y2
b3
y3
b4
y4
b5
y5
b6
y6
b7
b8
y8
type
b
y

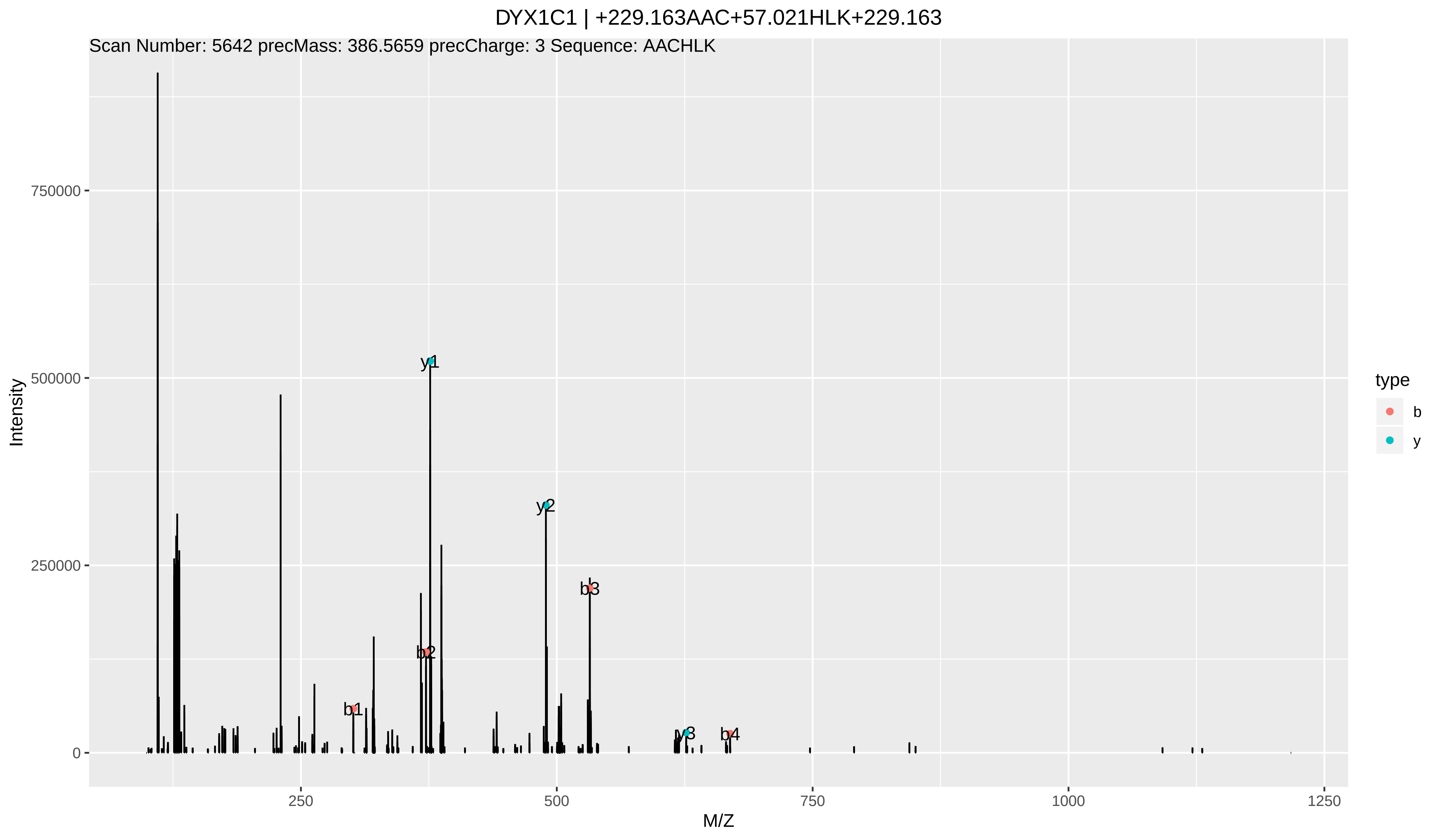
DYX1C1 | +229.163AAC+57.021HLK+229.163
Scan Number: 5642 precMass: 386.5659 precCharge: 3 Sequence: AACHLK
Intensity
750000
500000
250000
0
M/Z
250
500
750
1000
1250
y1
y2
b3
b2
b1
y3
b4
type
b
y

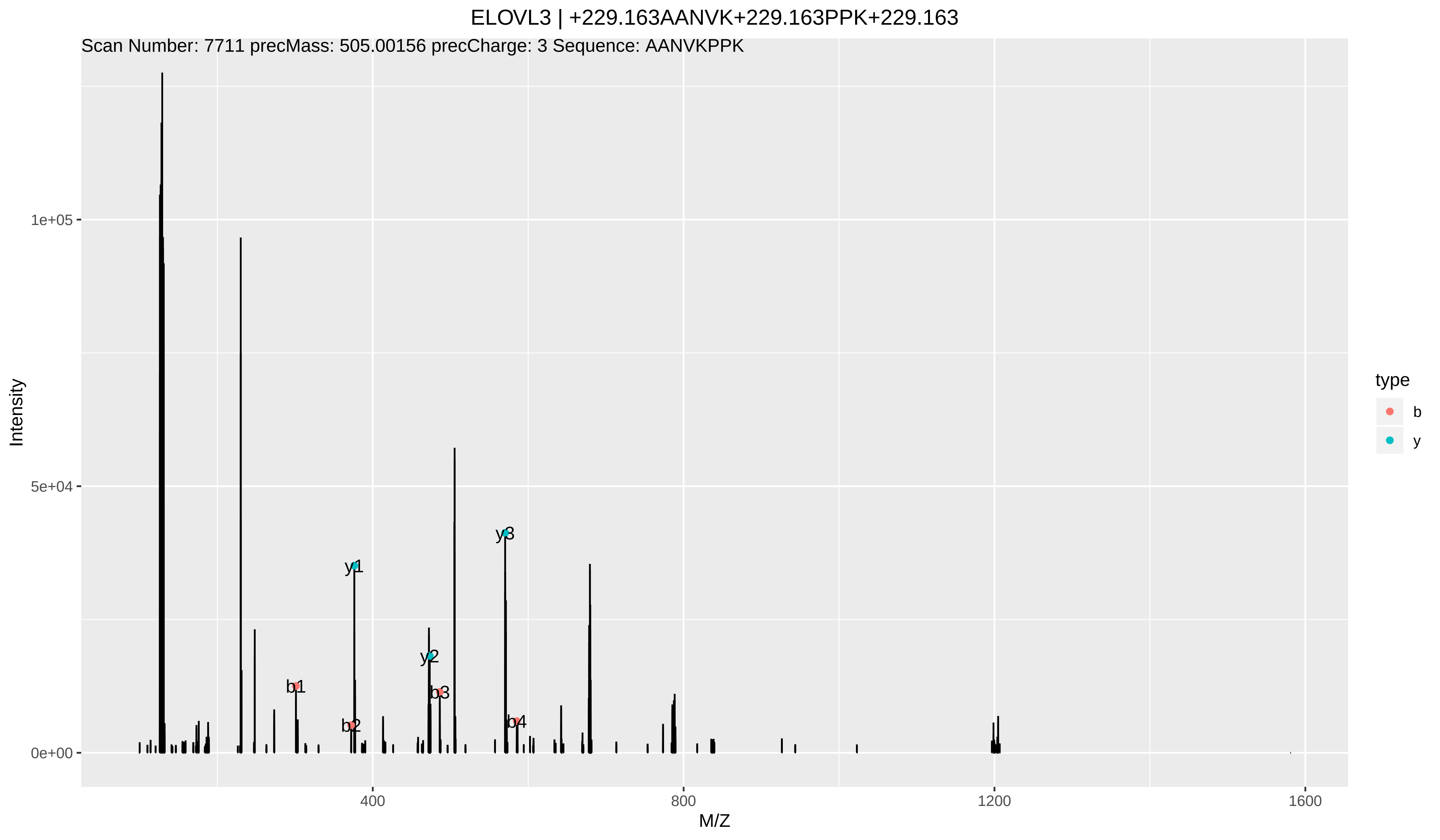
ELOVL3 | +229.163AANVK+229.163PPK+229.163
Scan Number: 7711 precMass: 505.00156 precCharge: 3 Sequence: AANVKPPK
Intensity
1e+05
5e+04
0e+00
M/Z
400
800
1200
1600
y3
y1
y2
b1
b2
b3
b4
type
b
y

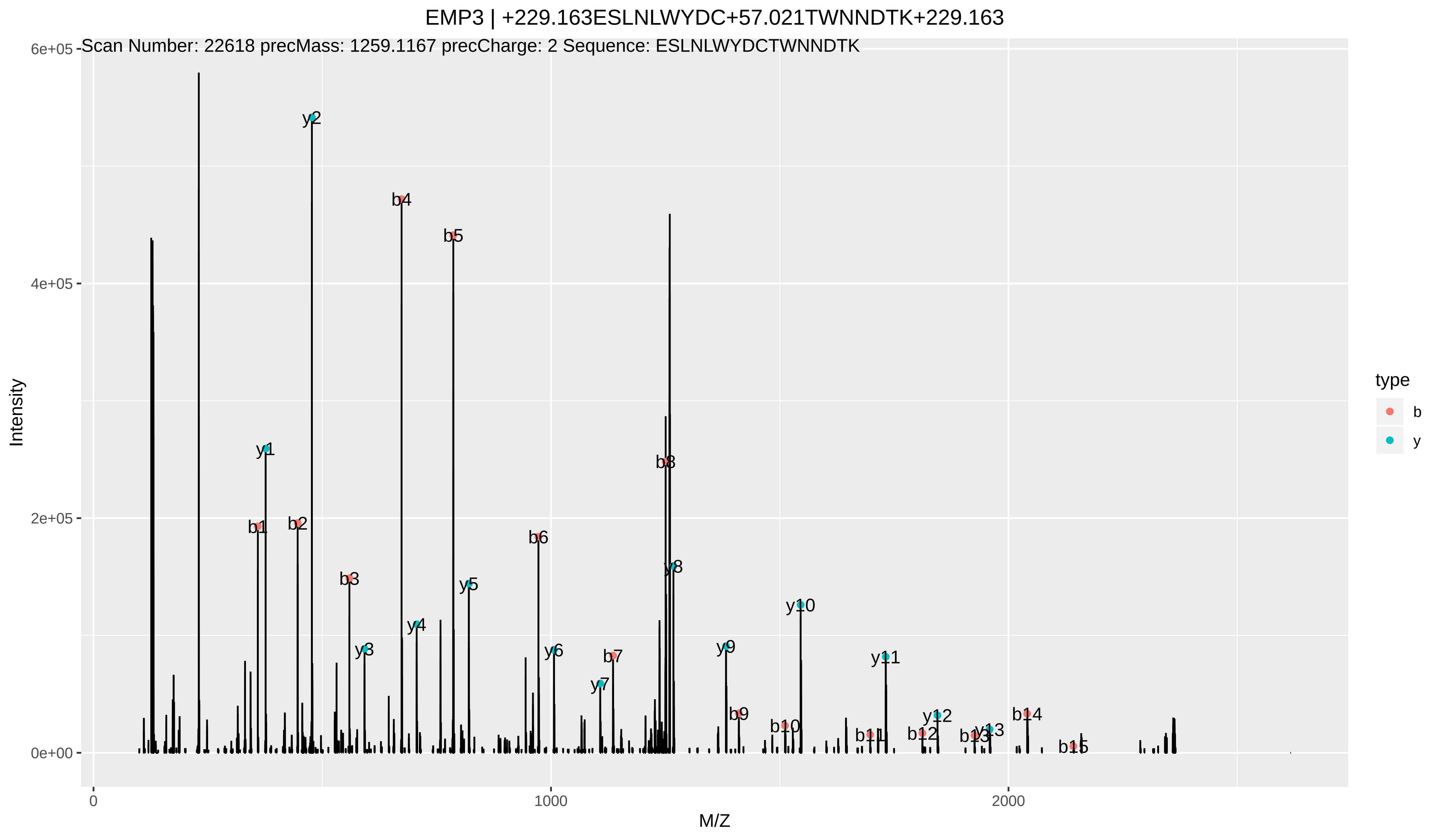

EMP3 | +229.163ESLNLWYDC+57.021TWNNDTK+229.163
Scan Number: 22618 precMass: 1259.1167 precCharge: 2 Sequence: ESLNLWYDCTWNNDTK
Intensity
M/Z
0e+00
2e+05
4e+05
6e+05
0
1000
2000
type
b
y
y1
y2
y3
y4
y5
y6
y7
y8
y9
y10
y11
y12
y13
b1
b2
b3
b4
b5
b6
b7
b8
b9
b10
b11
b12
b13
b14
b15

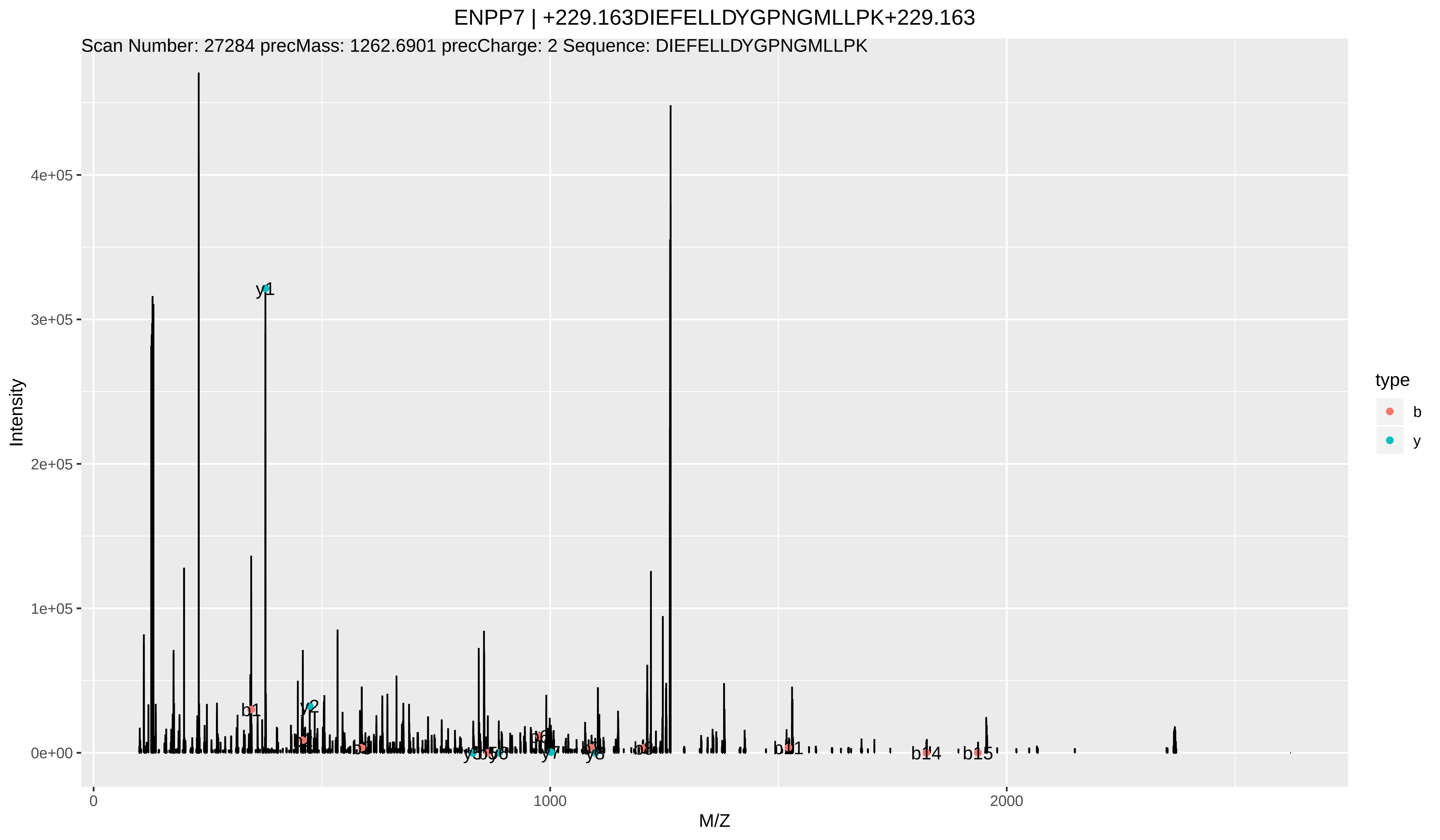
ENPP7 | +229.163DIEFELLDYGPNGMLLPK+229.163
Scan Number: 27284 precMass: 1262.6901 precCharge: 2 Sequence: DIEFELLDYGPNGMLLPK
Intensity
M/Z
4e+05
3e+05
2e+05
1e+05
0e+00
0
1000
2000
y1
y2
b1
y7
y8
b11
b14
b15
type
b
y

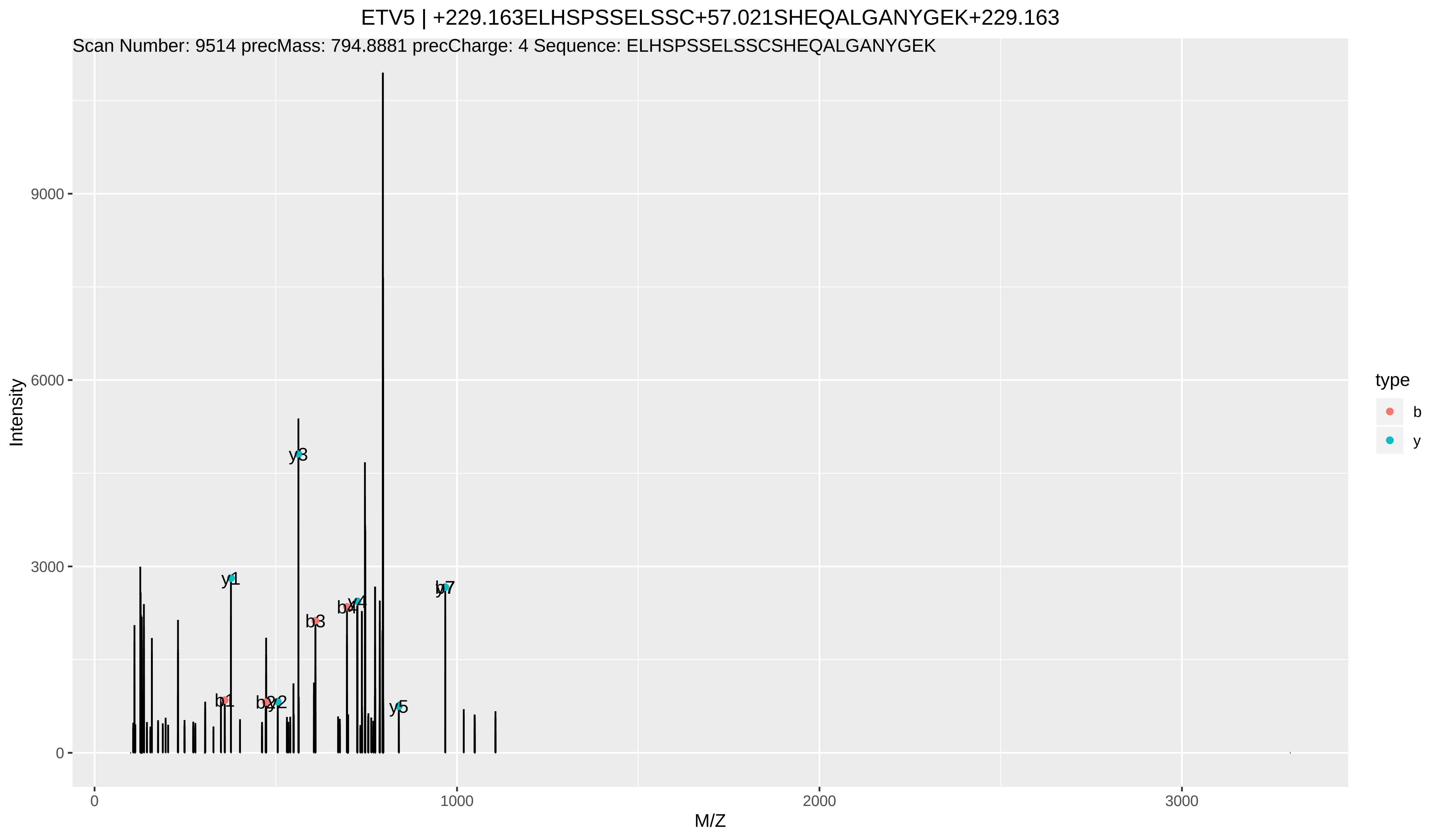
ETV5 | +229.163ELHSPSSELSSC+57.021SHEQALGANYGEK+229.163
Scan Number: 9514 precMass: 794.8881 precCharge: 4 Sequence: ELHSPSSELSSCSHEQALGANYGEK
Intensity
M/Z
type
b
y
y1
y3
b1
b2
y2
b3
b4
y4
y5
b7
y7
9000
6000
3000
0
0
1000
2000
3000

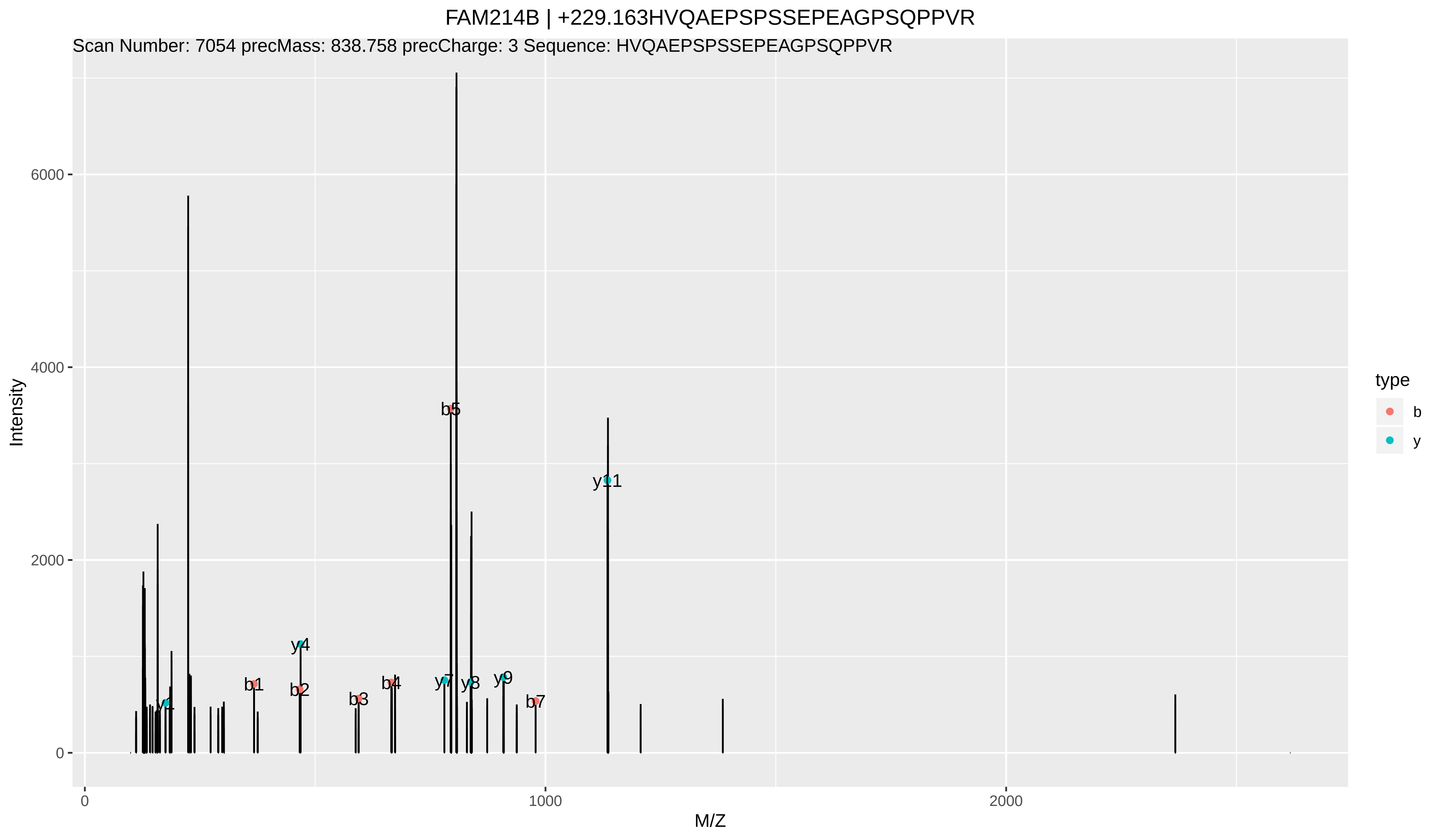

FAM214B | +229.163HVQAEPSPSSEPEAGPSQPPVR
Scan Number: 7054 precMass: 838.758 precCharge: 3 Sequence: HVQAEPSPSSEPEAGPSQPPVR
Intensity
M/Z
0
2000
4000
6000
0
1000
2000
b5
y11
y4
b1
b2
b3
b4
y7
y8
y9
b7
y1
type
b
y

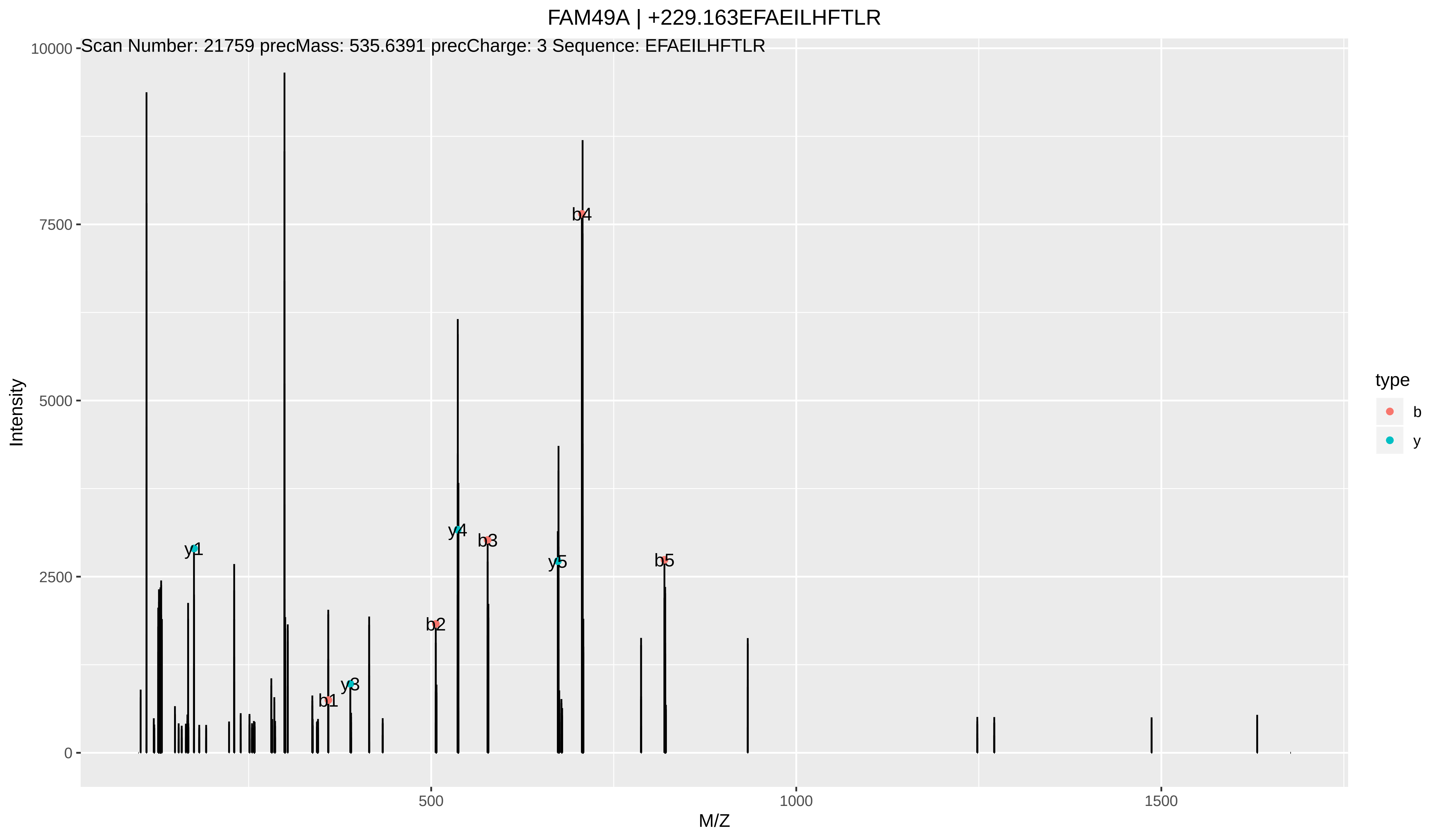
FAM49A | +229.163EFAEILHFTLR
Scan Number: 21759 precMass: 535.6391 precCharge: 3 Sequence: EFAEILHFTLR
Intensity
10000
7500
5000
2500
0
M/Z
500
1000
1500
y1
y3
y4
y5
b1
b2
b3
b4
b5
type
b
y

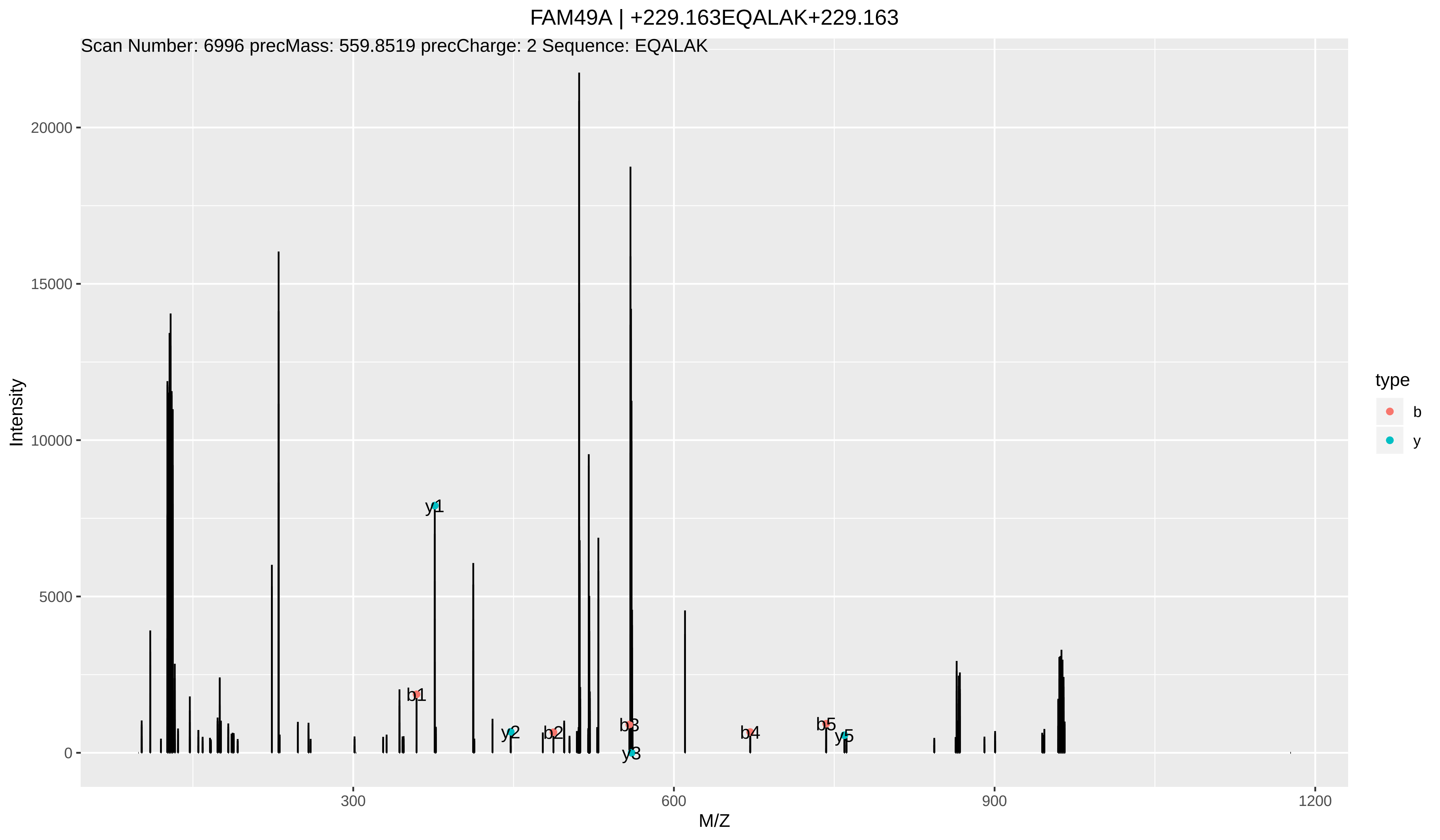

FAM49A | +229.163EQALAK+229.163
Scan Number: 6996 precMass: 559.8519 precCharge: 2 Sequence: EQALAK
Intensity
20000
15000
10000
5000
0
M/Z
300
600
900
1200
y1
b1
y2
b2
b3
y3
b4
b5
y5
type
b
y

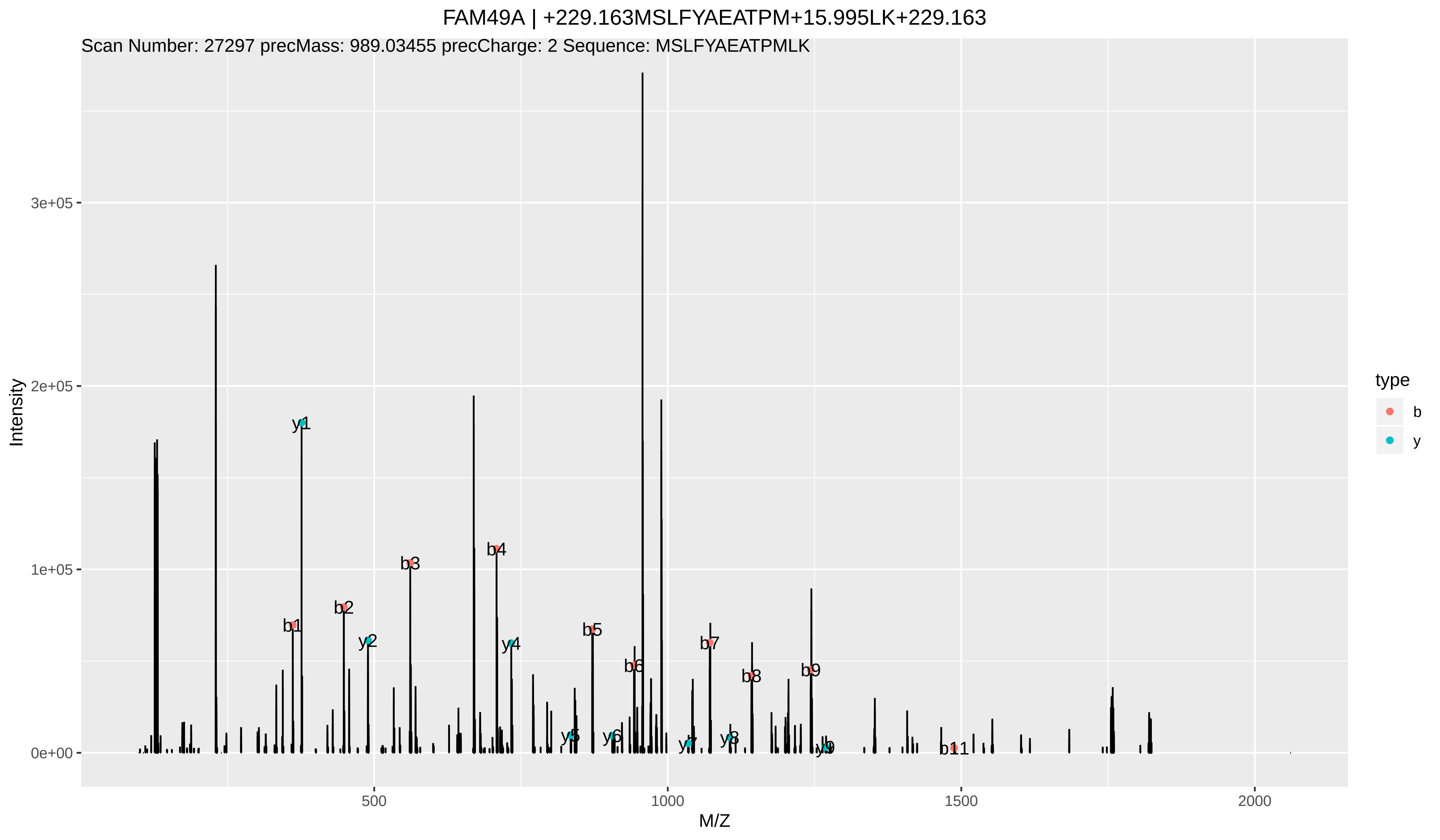
FAM49A | +229.163MSLFYAEATPM+15.995LK+229.163
Scan Number: 27297 precMass: 989.03455 precCharge: 2 Sequence: MSLFYAEATPMLK
Intensity
M/Z
type
b
y
0e+00
1e+05
2e+05
3e+05
500
1000
1500
2000
y1
y2
y4
y5
y6
y7
y8
y9
b1
b2
b3
b4
b5
b6
b7
b8
b9
b11

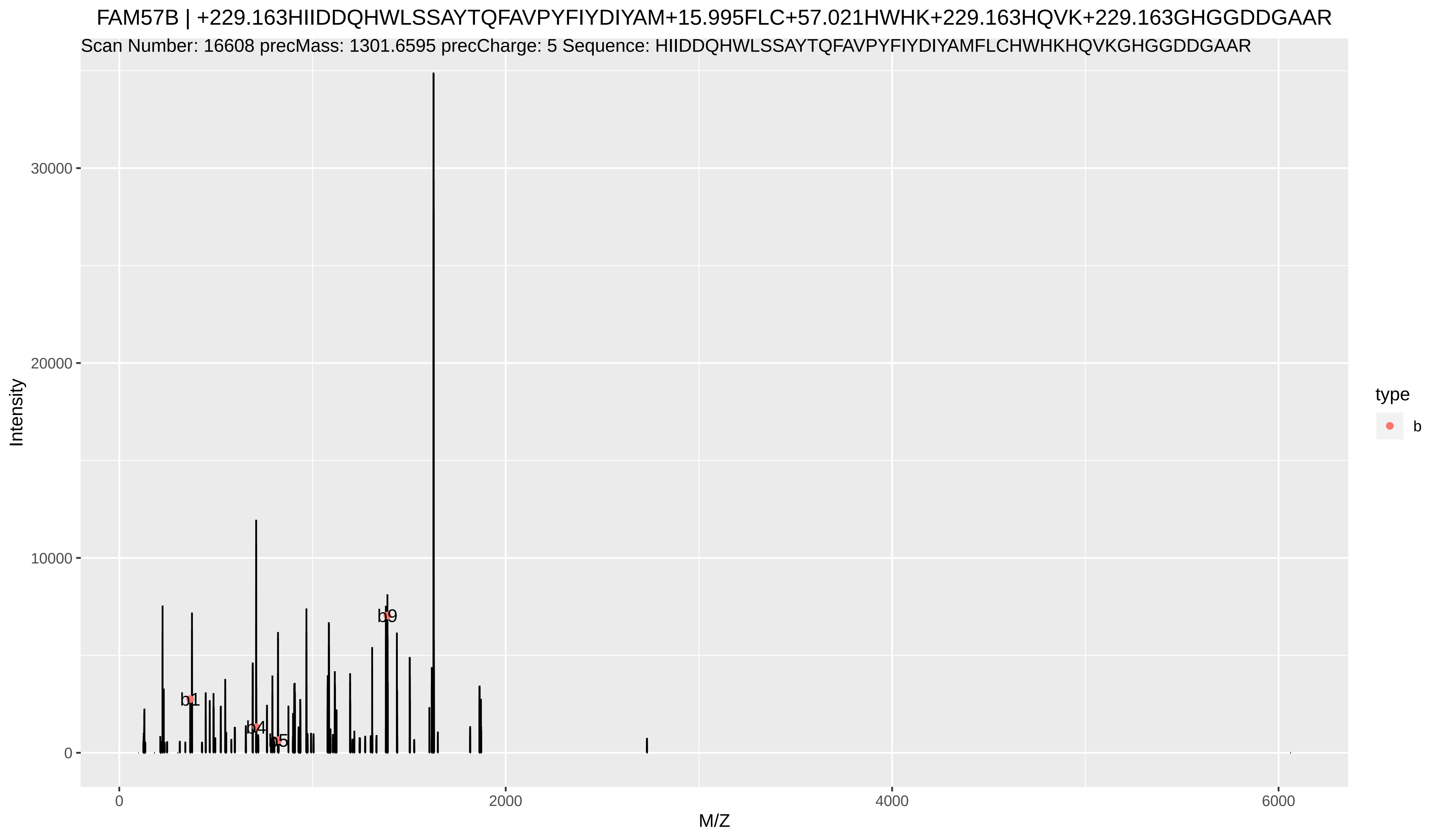
FAM57B | +229.163HIIDDQHWLSSAYTQFAVPYFIYDIYAM+15.995FLC+57.021HWHK+229.163HQVK+229.163GHGGDDGAAR
Scan Number: 16608 precMass: 1301.6595 precCharge: 5 Sequence: HIIDDQHWLSSAYTQFAVPYFIYDIYAMFLCHWHKHQVKGHGGDDGAAR
Intensity
M/Z
type
b
b1
b4
b5
b9
0
10000
20000
30000
2000
4000
6000

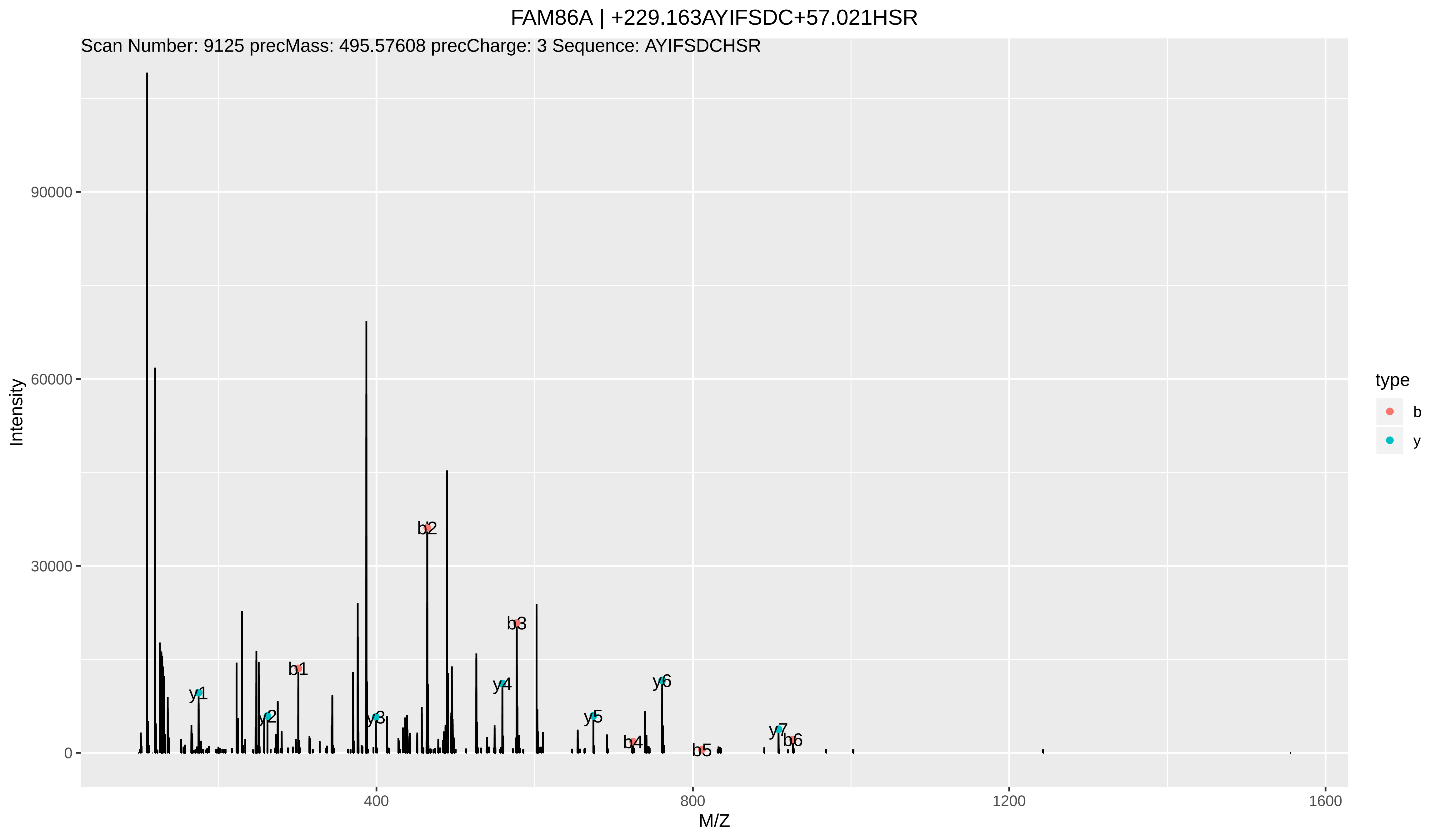

FAM86A | +229.163AYIFSDC+57.021HSR
Scan Number: 9125 precMass: 495.57608 precCharge: 3 Sequence: AYIFSDCHSR
Intensity
90000
60000
30000
0
M/Z
400
800
1200
1600
y1
y2
b1
y3
b2
y4
b3
y5
b4
y6
b5
y7
b6
type
b
y

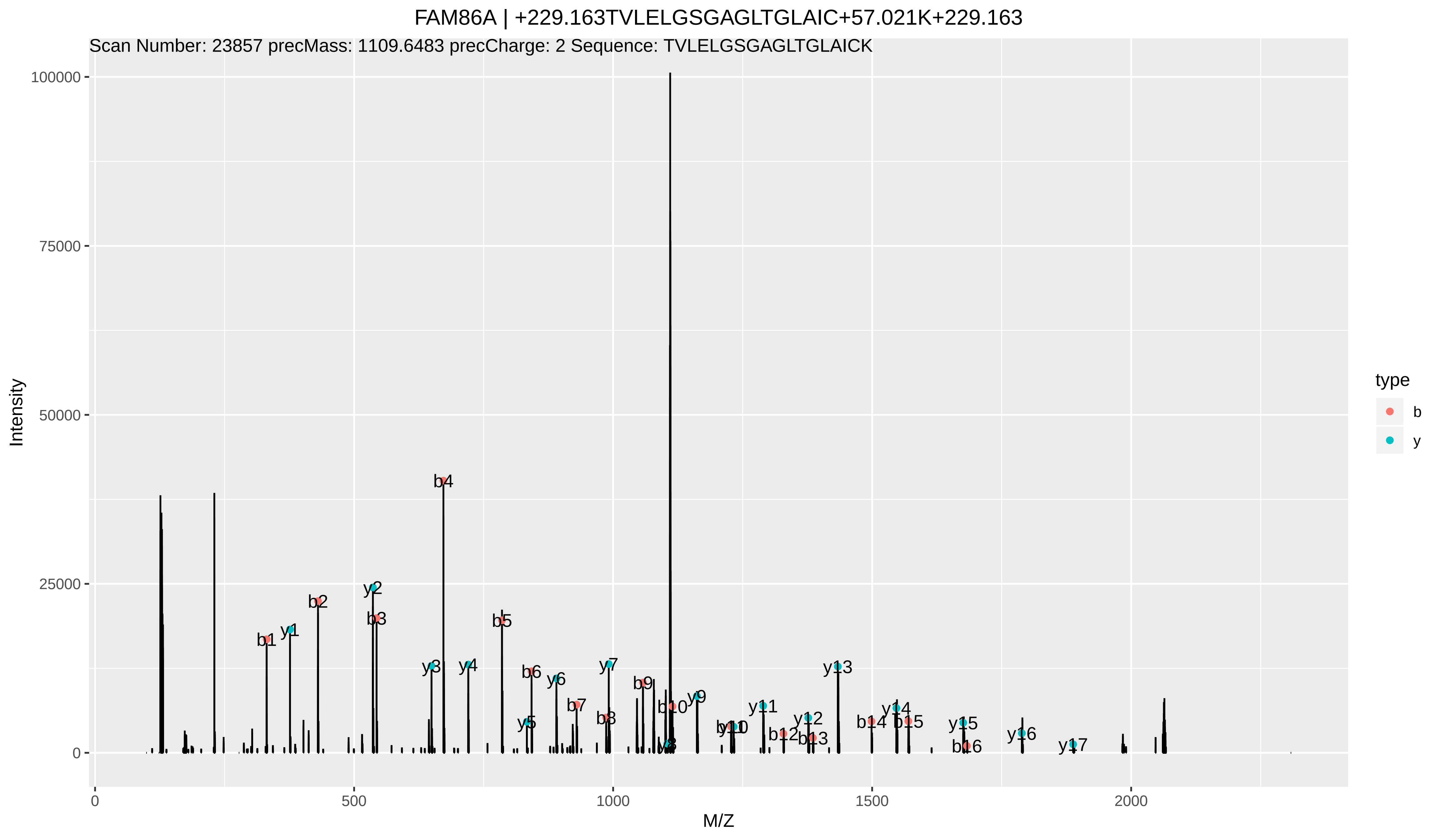
FAM86A | +229.163TVLELGSGAGLTGLAIC+57.021K+229.163
Scan Number: 23857 precMass: 1109.6483 precCharge: 2 Sequence: TVLELGSGAGLTGLAICK
Intensity
100000
75000
50000
25000
0
M/Z
0
500
1000
1500
2000
type
b
y
b1
y1
b2
y2
b3
y3
b4
y4
b5
y5
b6
y6
b7
y7
b8
b9
y9
b10
y11
y12
b12
b13
y13
b14
y14
b15
y15
b16
y16
y17

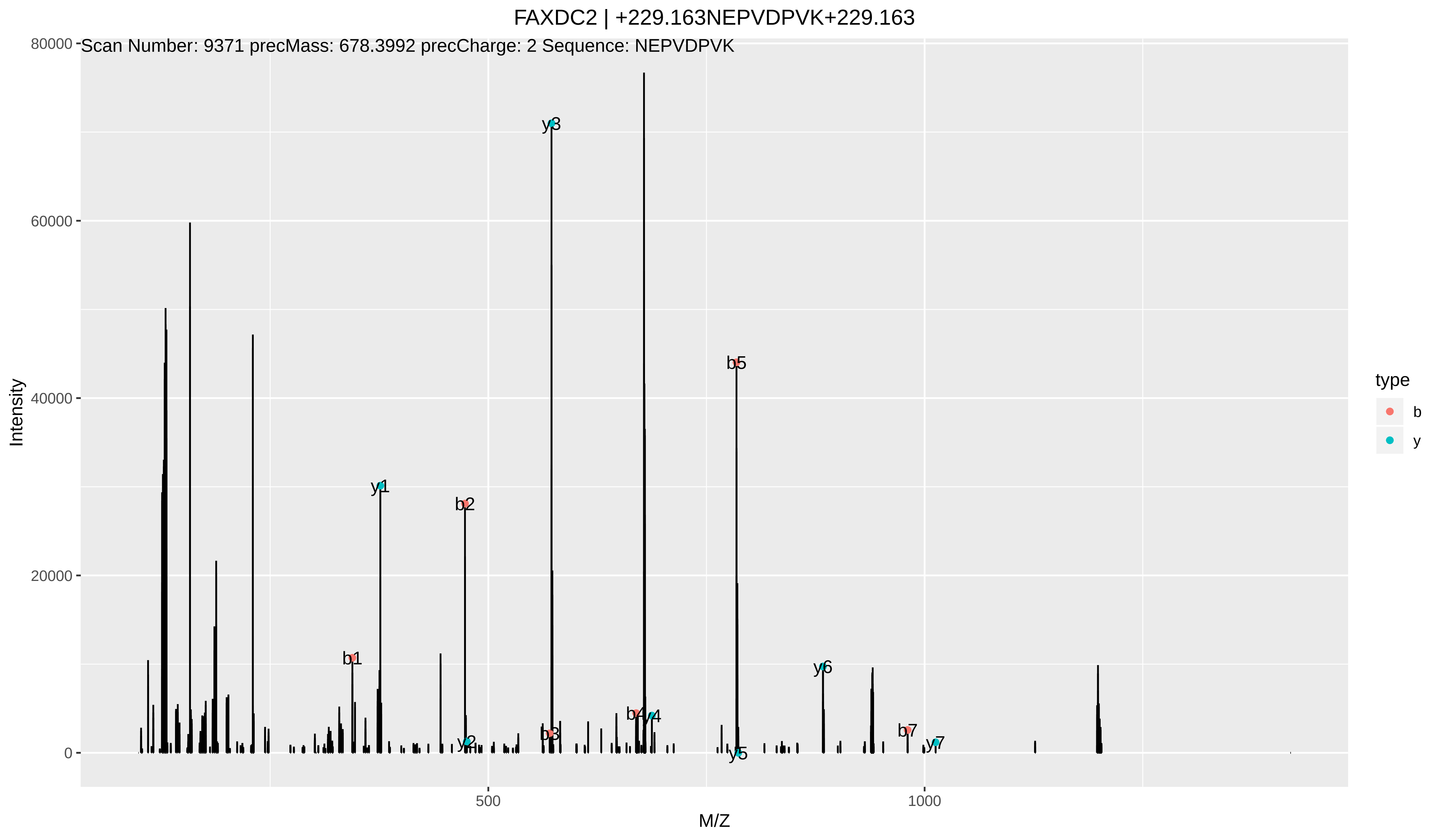

FAXDC2 | +229.163NEPVDPVK+229.163
Scan Number: 9371 precMass: 678.3992 precCharge: 2 Sequence: NEPVDPVK
Intensity
M/Z
type
b
y
y3
b5
y1
b2
b1
y6
b4
y4
b3
y2
b7
y7
y5

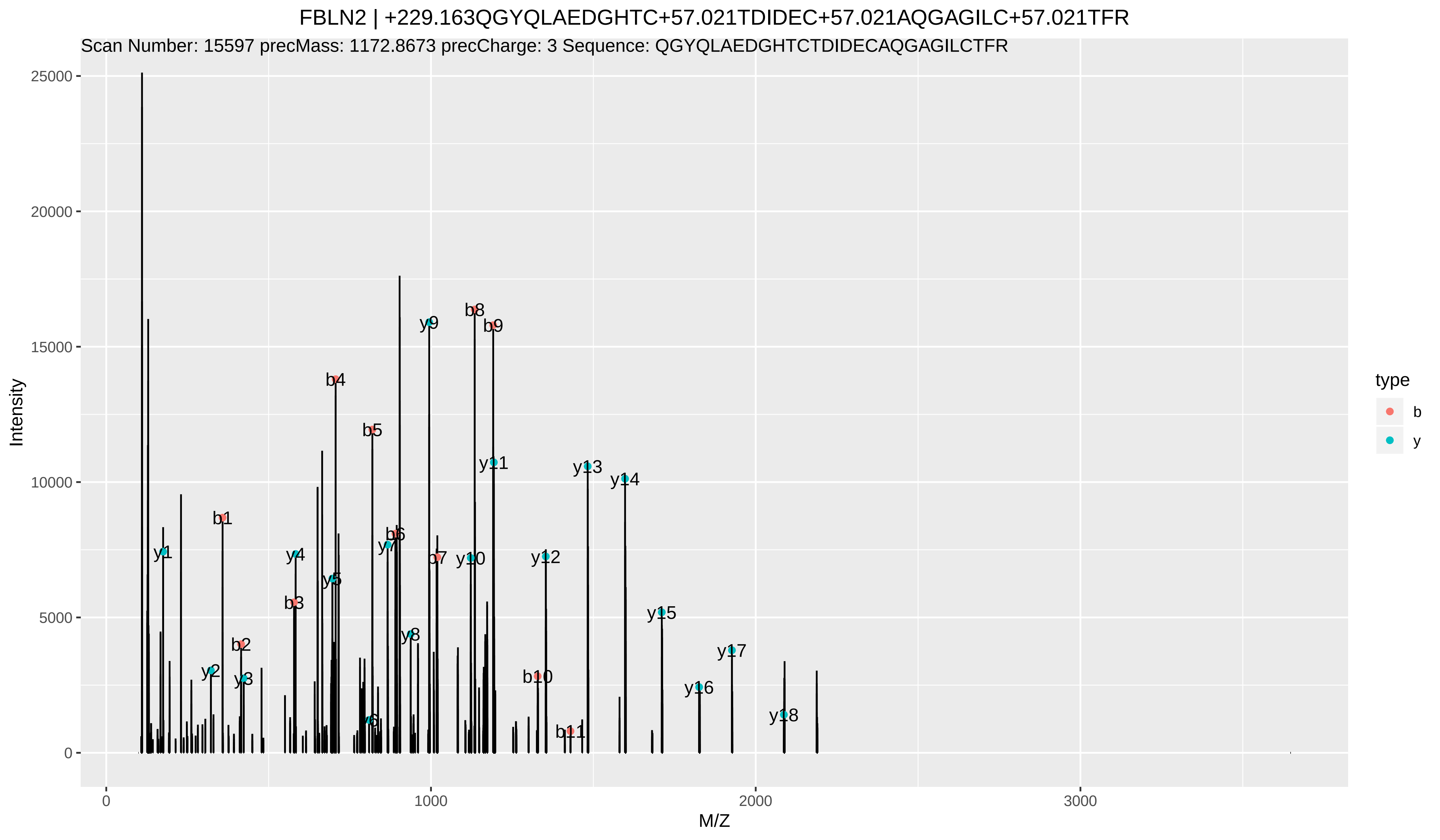
FBLN2 | +229.163QGYQLAEDGHTC+57.021TDIDEC+57.021AQGAGILC+57.021TFR
Scan Number: 15597 precMass: 1172.8673 precCharge: 3 Sequence: QGYQLAEDGHTCTDIDECAQGAGILCTFR
Intensity
M/Z
type
b
y
b1
b2
b3
b4
b5
b6
b7
b8
b9
b10
b11
y1
y2
y3
y4
y5
y6
y7
y8
y9
y10
y11
y12
y13
y14
y15
y16
y17
y18
25000
20000
15000
10000
5000
0
0
1000
2000
3000

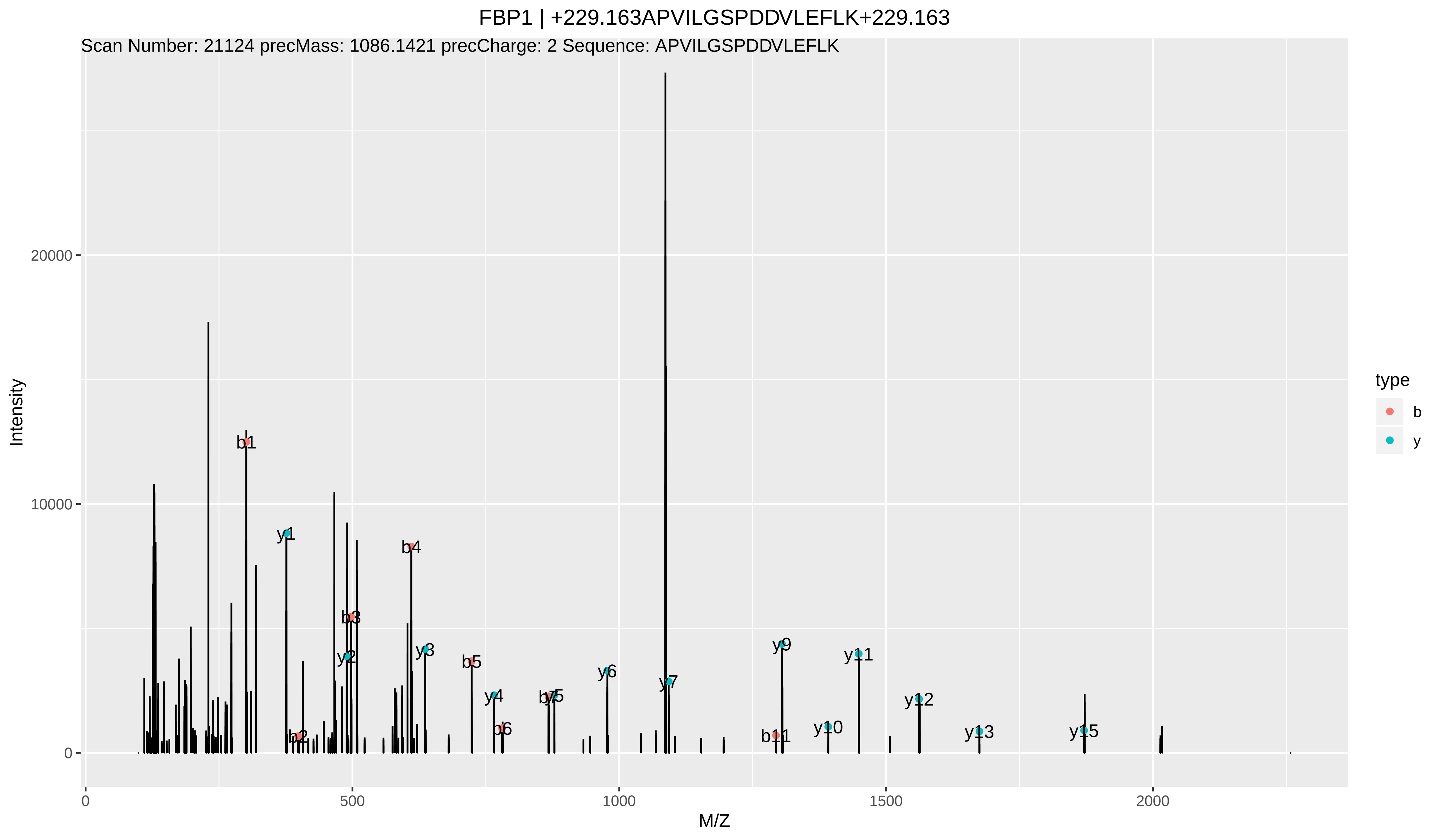

FBP1 | +229.163APVILGSPDDVLEFLK+229.163
Scan Number: 21124 precMass: 1086.1421 precCharge: 2 Sequence: APVILGSPDDVLEFLK
Intensity
20000
10000
0
M/Z
0
500
1000
1500
2000
b1
y1
b2
b3
y2
b4
y3
b5
y4
b6
b7
y5
y6
y7
b11
y9
y10
y11
y12
y13
y15
type
b
y

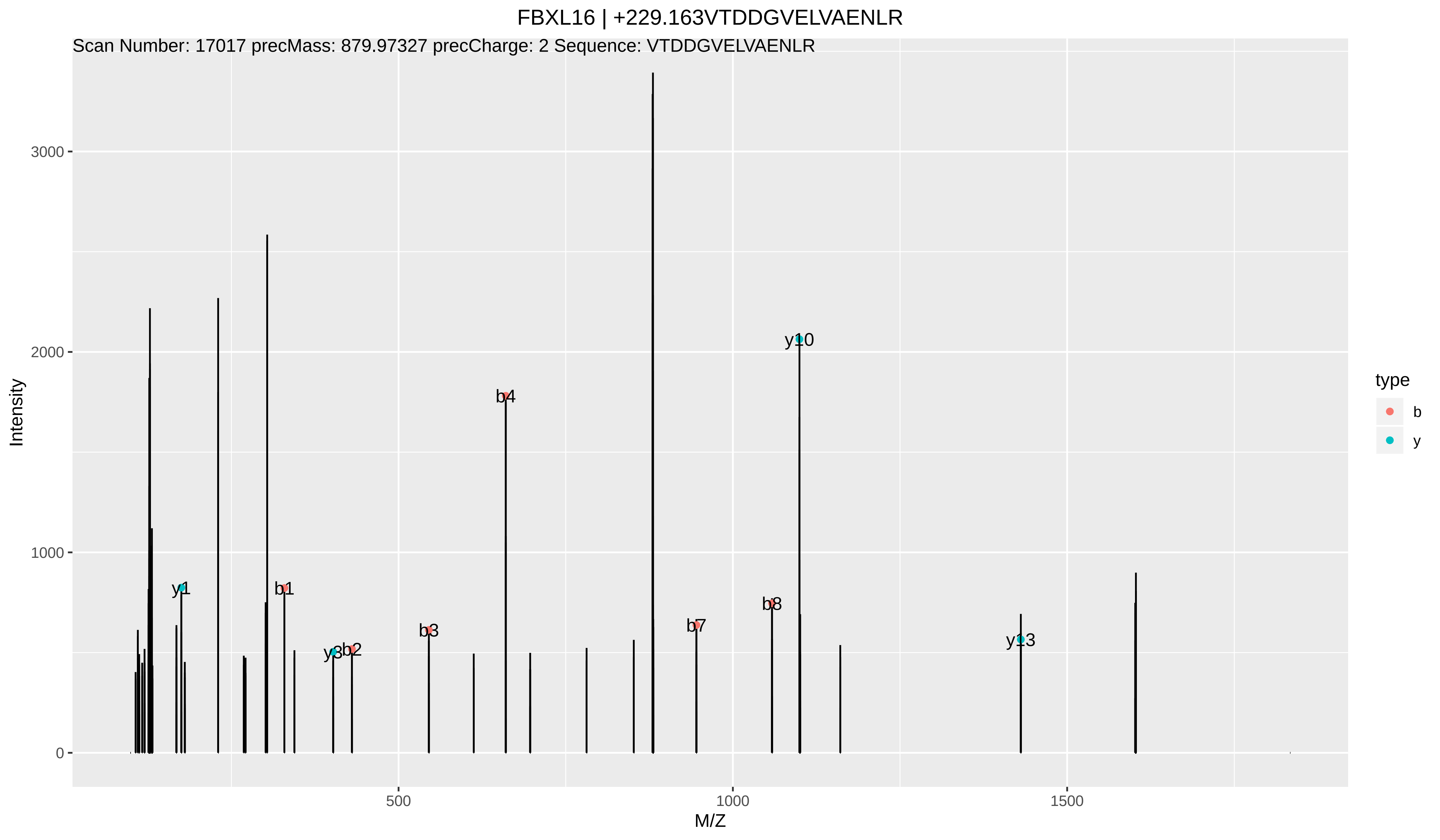
FBXL16 | +229.163VTDDGVELVAENLR
Scan Number: 17017 precMass: 879.97327 precCharge: 2 Sequence: VTDDGVELVAENLR
Intensity
M/Z
0
1000
2000
3000
500
1000
1500
y1
b1
y3
b2
b3
b4
b7
b8
y10
y13
type
b
y

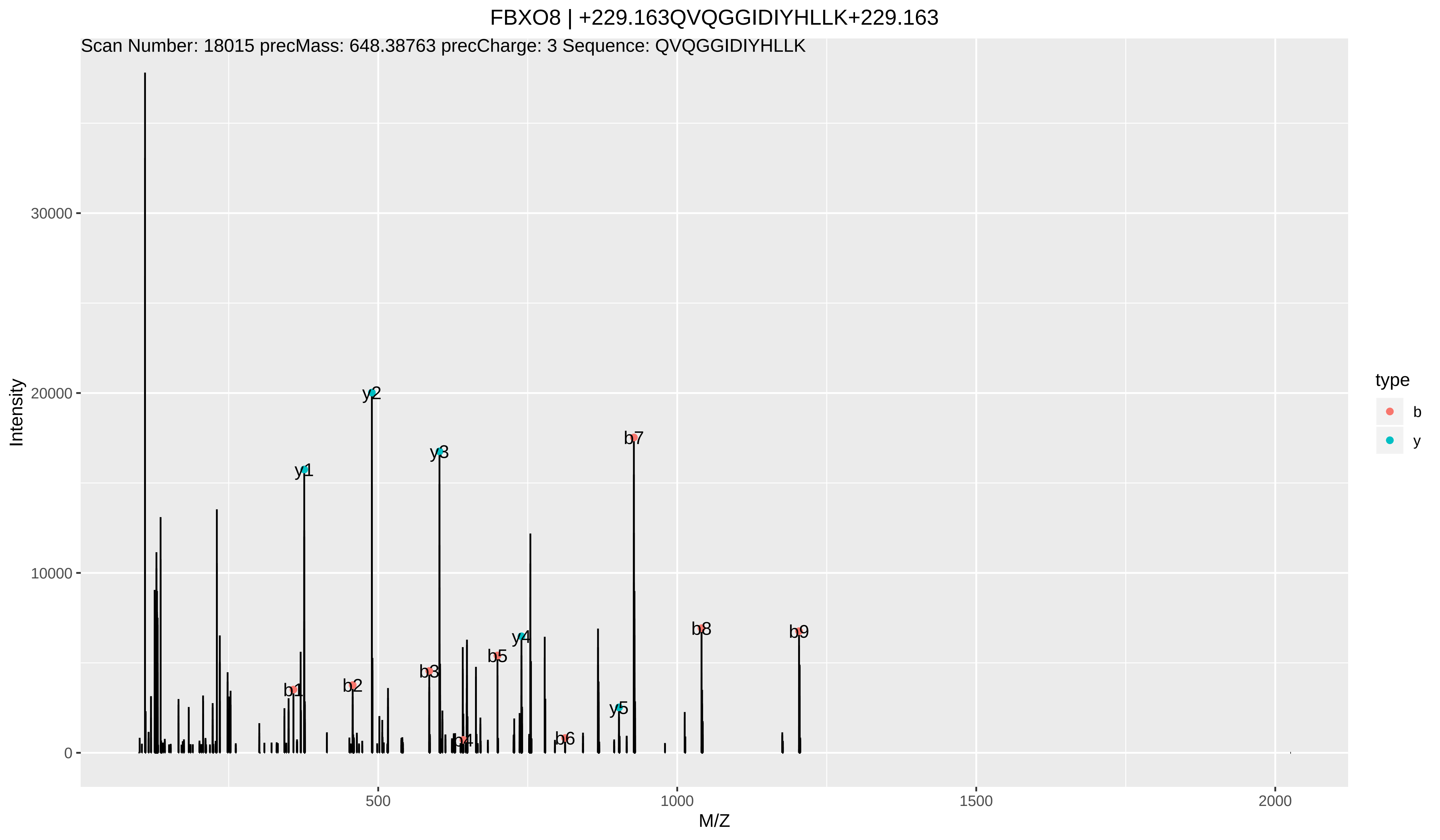
FBXO8 | +229.163QVQGGIDIYHLLK+229.163
Scan Number: 18015 precMass: 648.38763 precCharge: 3 Sequence: QVQGGIDIYHLLK
Intensity
30000
20000
10000
0
M/Z
500
1000
1500
2000
y1
y2
y3
y4
y5
b1
b2
b3
b4
b5
b6
b7
b8
b9
type
b
y

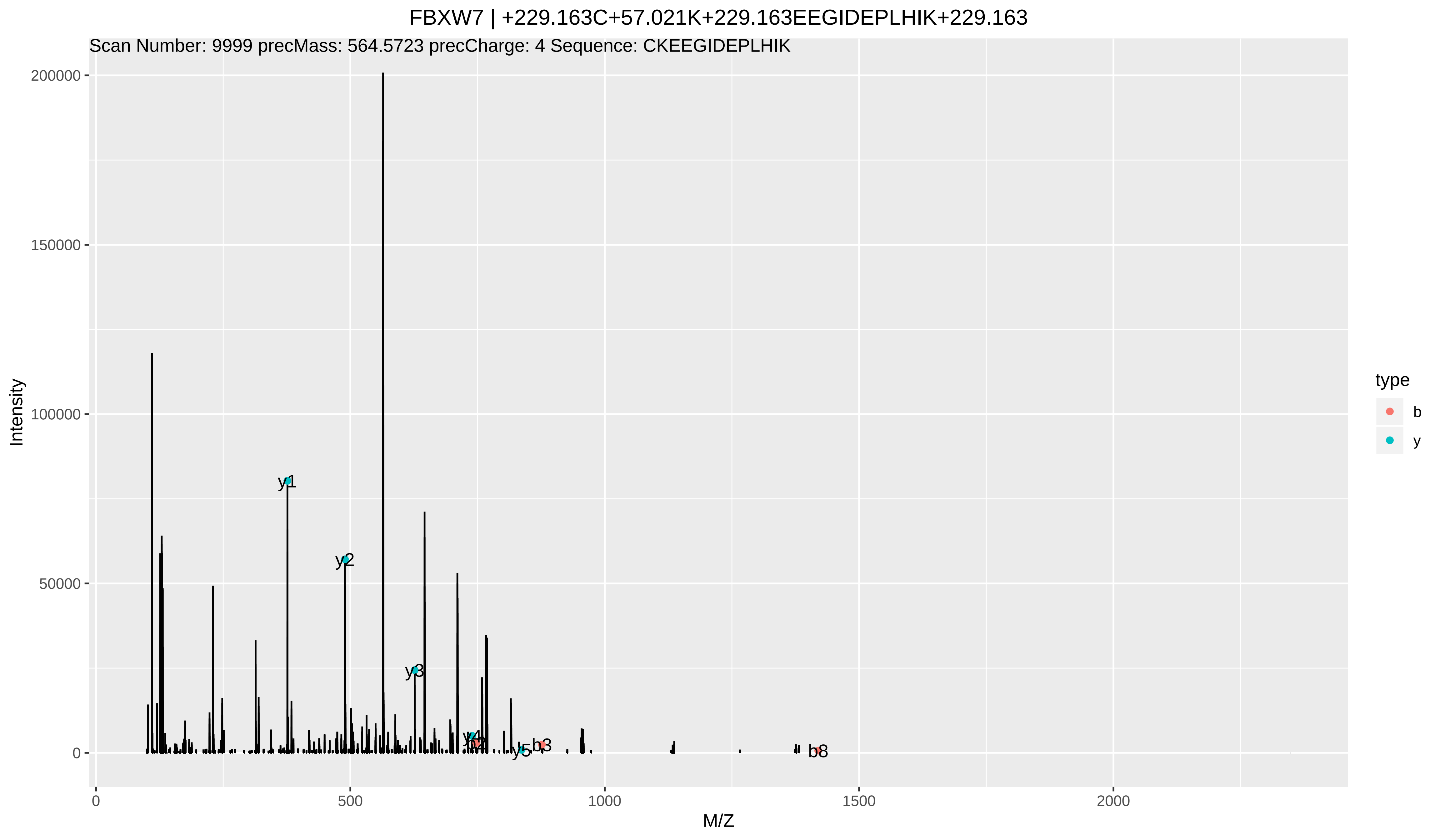

FBXW7 | +229.163C+57.021K+229.163EEGIDEPLHIK+229.163
Scan Number: 9999 precMass: 564.5723 precCharge: 4 Sequence: CKEEGIDEPLHIK
Intensity
200000
150000
100000
50000
0
M/Z
0
500
1000
1500
2000
y1
y2
y3
y4
y5
b2
b3
b8
type
b
y

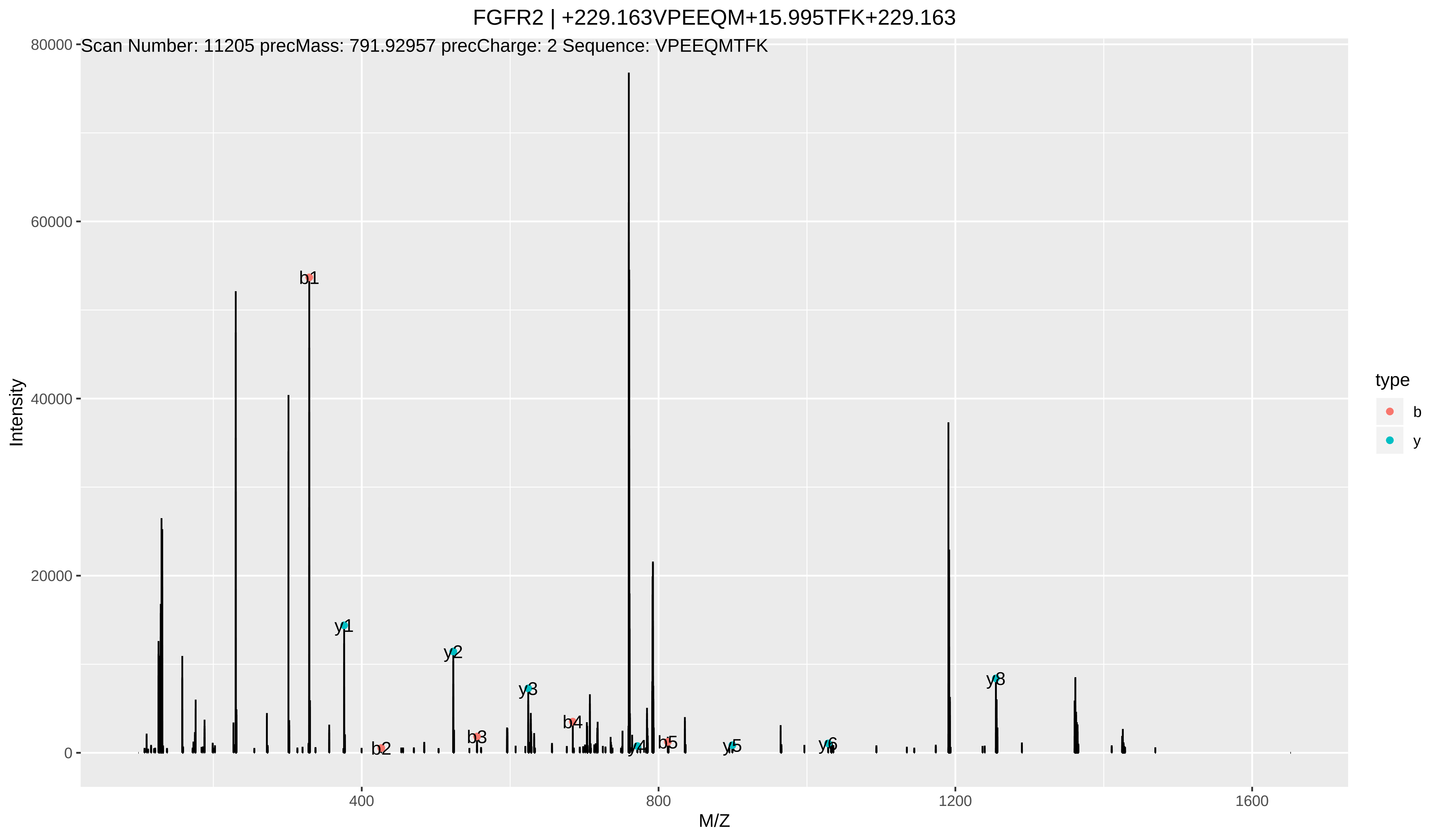
FGFR2 | +229.163VPEEQM+15.995TFK+229.163
Scan Number: 11205 precMass: 791.92957 precCharge: 2 Sequence: VPEEQMTFK
Intensity
M/Z
0
20000
40000
60000
80000
400
800
1200
1600
b1
b2
b3
b4
b5
y1
y2
y3
y4
y5
y6
y8
type
b
y

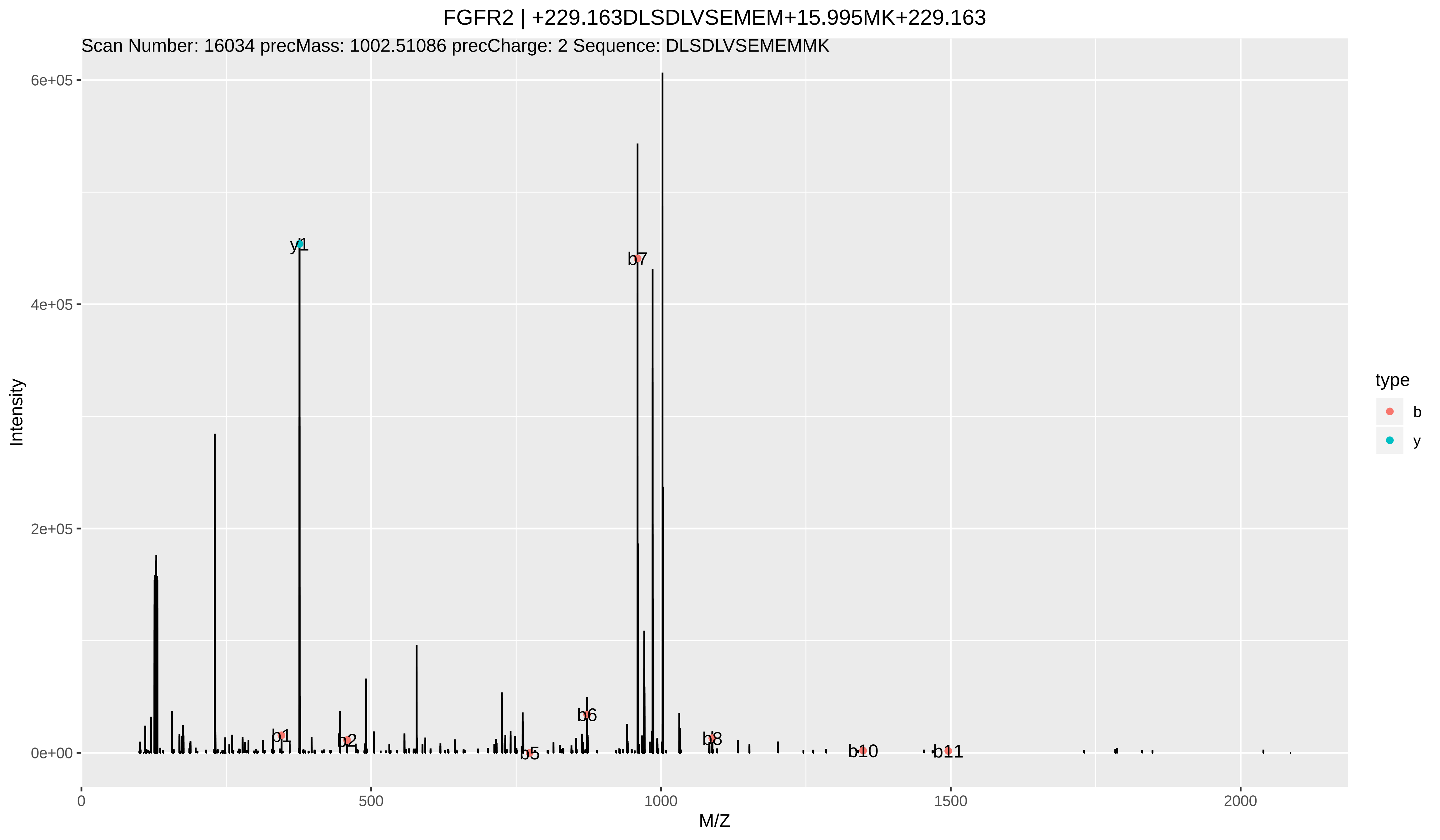
FGFR2 | +229.163DLSDLVSEMEM+15.995MK+229.163
Scan Number: 16034 precMass: 1002.51086 precCharge: 2 Sequence: DLSDLVSEMEMMK
Intensity
M/Z
type
b
y
y1
b7
b1
b2
b5
b6
b8
b10
b11
6e+05
4e+05
2e+05
0e+00
0
500
1000
1500
2000

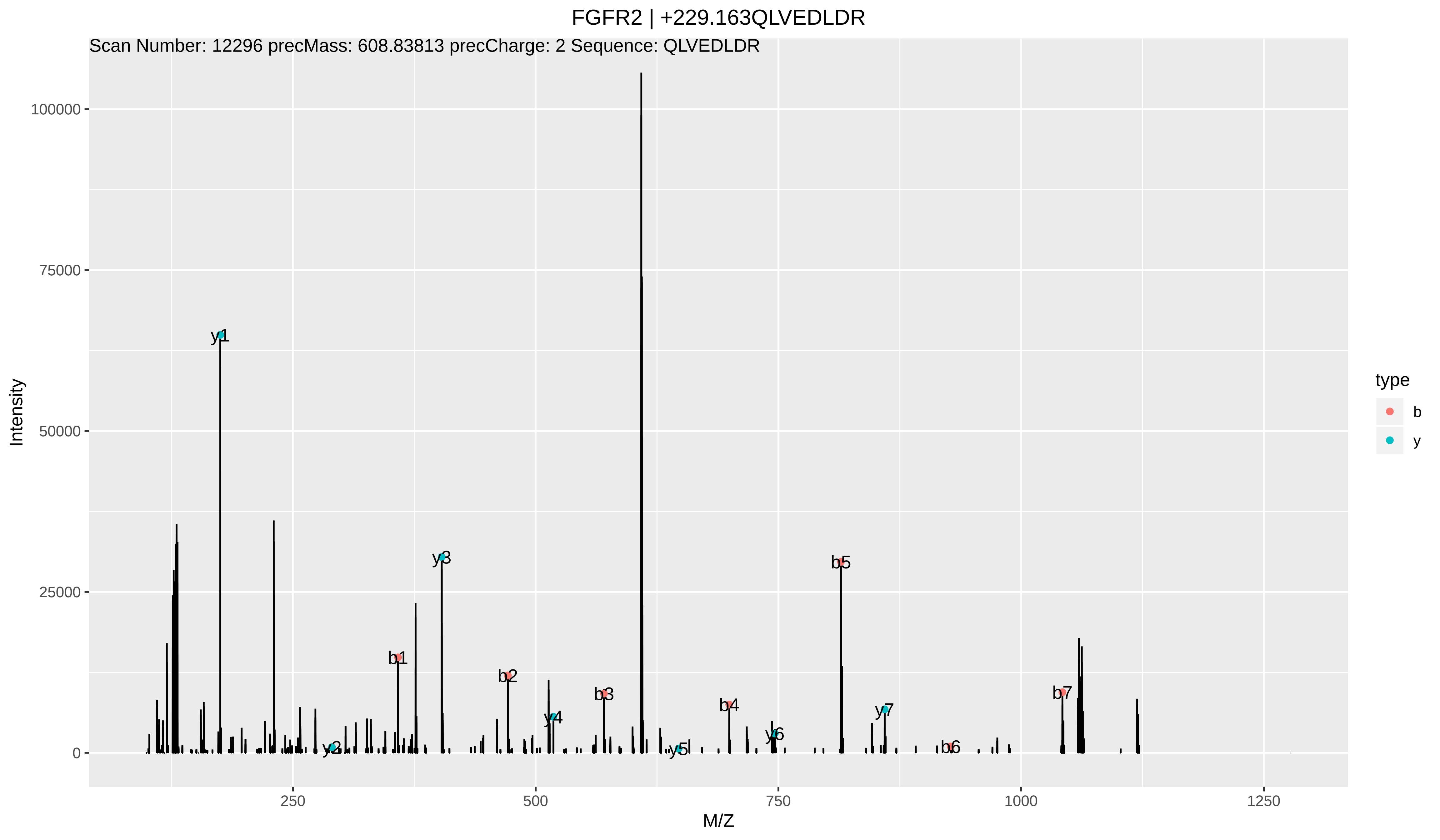

FGFR2 | +229.163QLVEDLDR
Scan Number: 12296 precMass: 608.83813 precCharge: 2 Sequence: QLVEDLDR
Intensity
M/Z
100000
75000
50000
25000
0
250
500
750
1000
1250
y1
y2
y3
y4
y5
y6
y7
b1
b2
b3
b4
b5
b6
b7
type
b
y

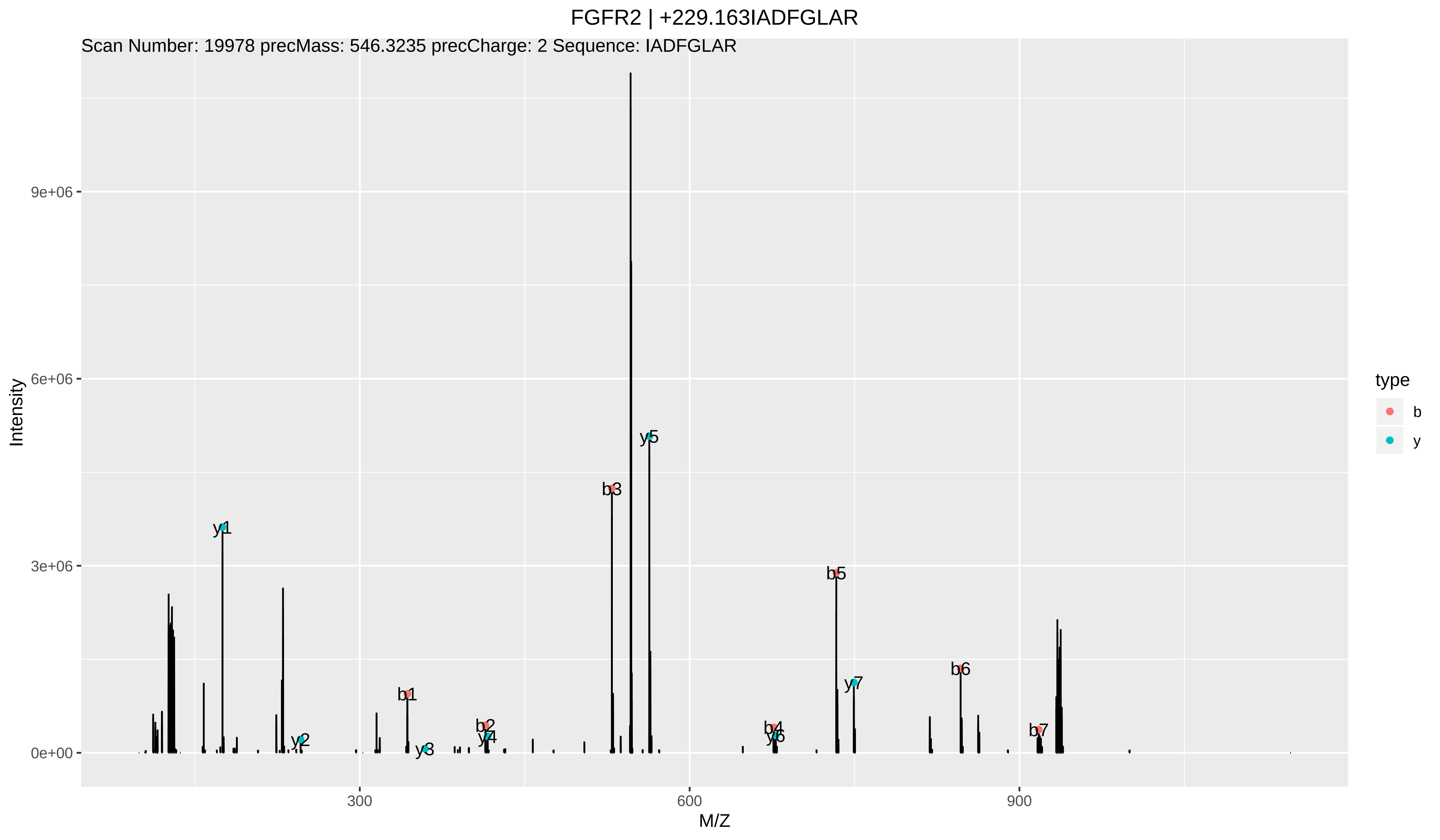

FGFR2 | +229.163IADFGLAR
Scan Number: 19978 precMass: 546.3235 precCharge: 2 Sequence: IADFGLAR
Intensity
9e+06
6e+06
3e+06
0e+00
M/Z
300
600
900
y1
y2
y3
b1
b2
y4
b3
y5
b4
y6
b5
y7
b6
b7
type
b
y

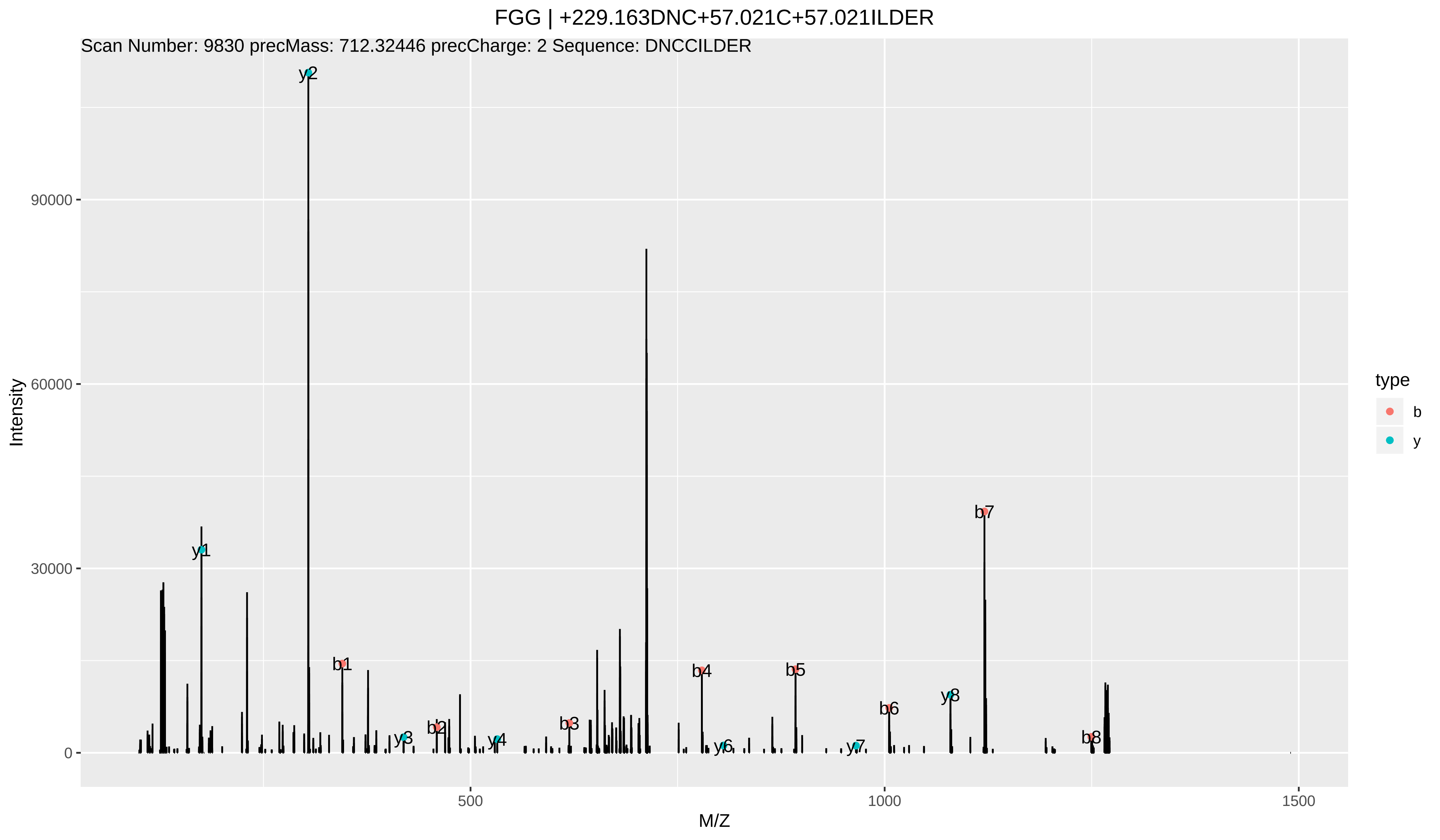
FGG | +229.163DNC+57.021C+57.021ILDER
Scan Number: 9830 precMass: 712.32446 precCharge: 2 Sequence: DNCCILDER
Intensity
M/Z
0
30000
60000
90000
500
1000
1500
y1
y2
b1
y3
b2
y4
b3
b4
y6
b5
y7
b6
y8
b7
b8
type
b
y

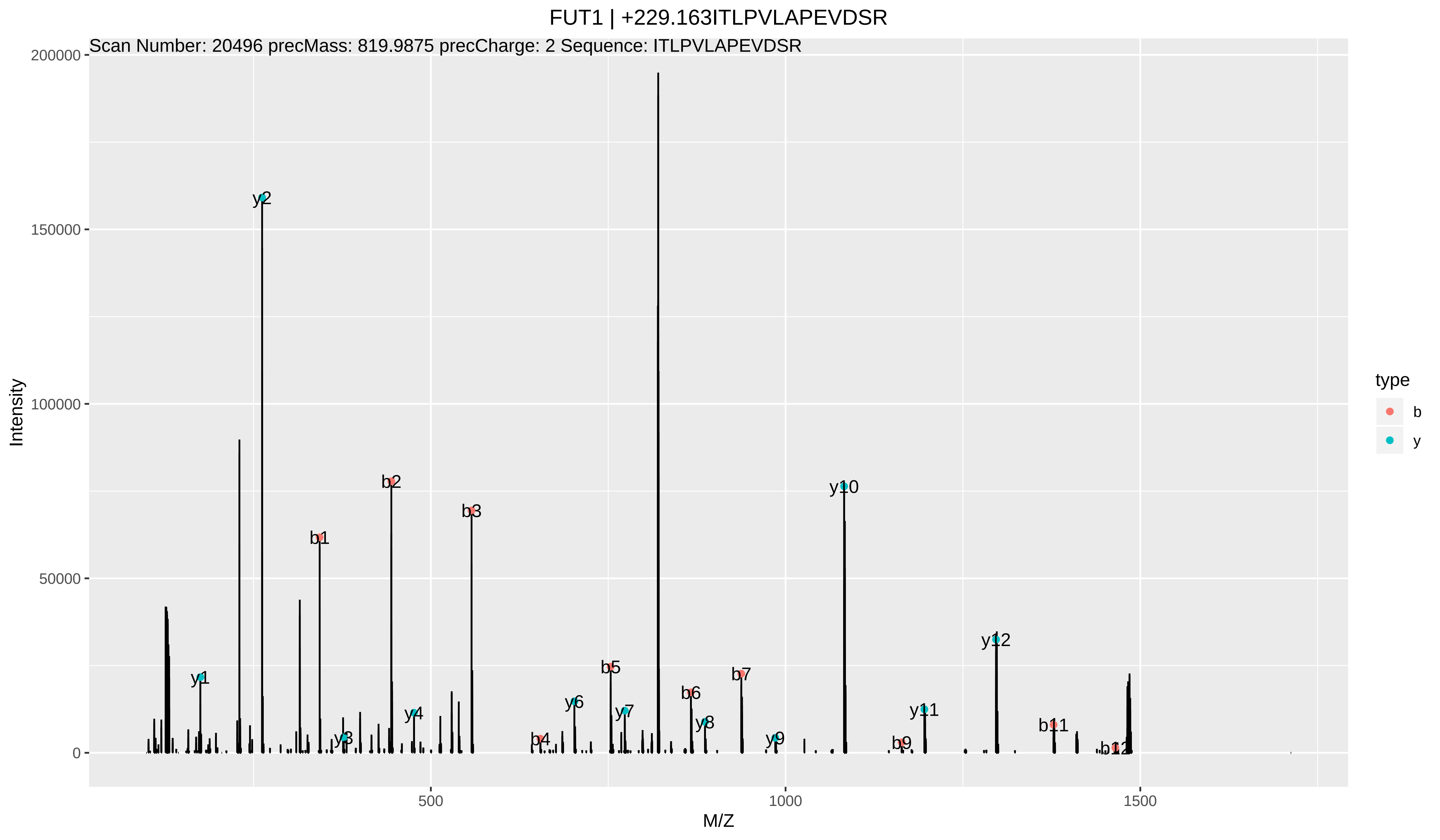

FUT1 | +229.163ITLPVLAPEVDSR
Scan Number: 20496 precMass: 819.9875 precCharge: 2 Sequence: ITLPVLAPEVDSR
Intensity
200000
150000
100000
50000
0
M/Z
500
1000
1500
y1
y2
y3
y4
y6
y7
y8
y9
y10
y11
y12
b1
b2
b3
b4
b5
b6
b7
b9
b11
b12
type
b
y

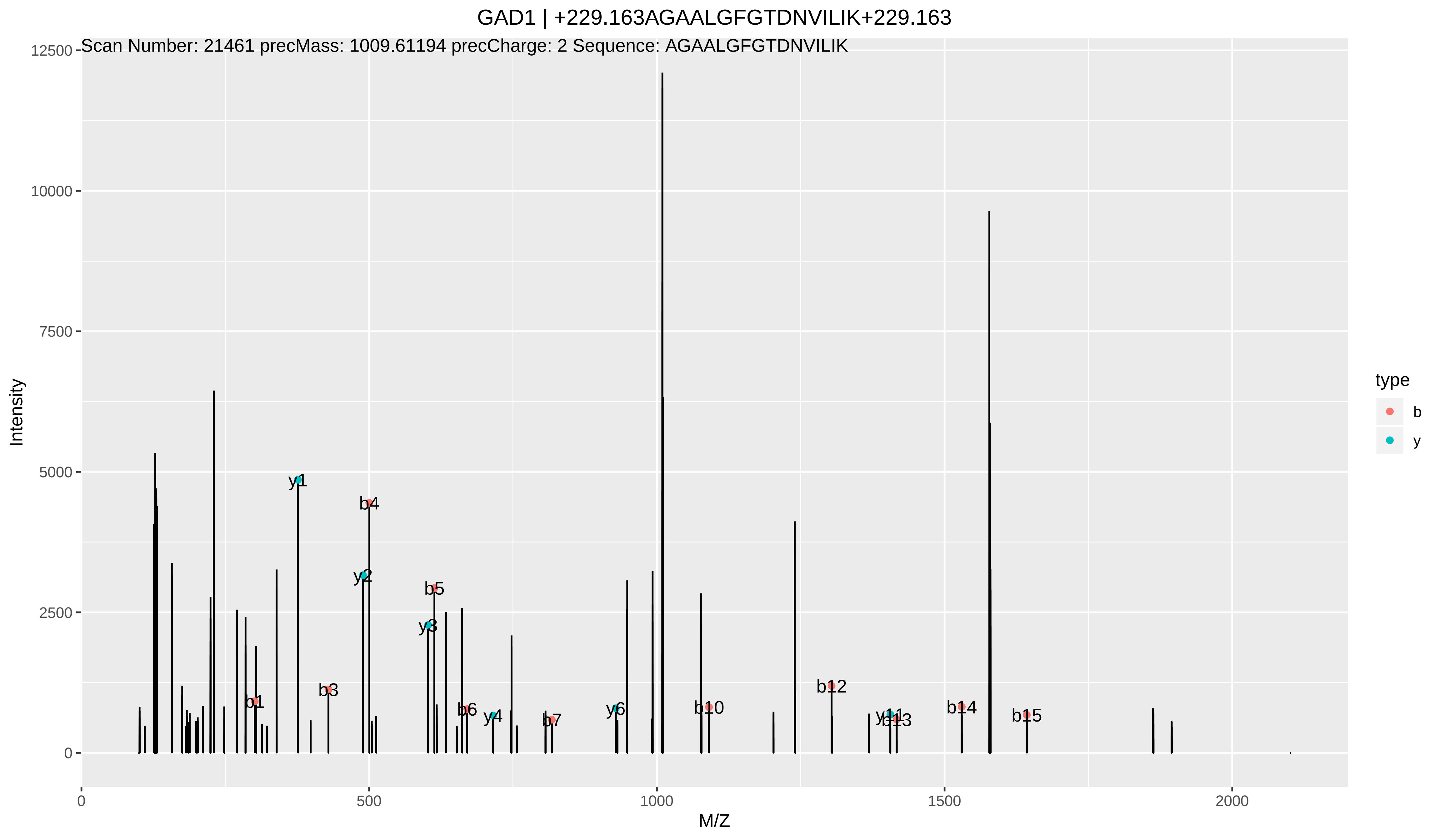
GAD1 | +229.163AGAALGFGTDNVILIK+229.163
Scan Number: 21461 precMass: 1009.61194 precCharge: 2 Sequence: AGAALGFGTDNVILIK
Intensity
M/Z
type
b
y
y1
b4
y2
b5
y3
b3
b1
b6
y4
b7
y6
b10
b12
y11
b13
b14
b15
0
2500
5000
7500
10000
12500
500
1000
1500
2000

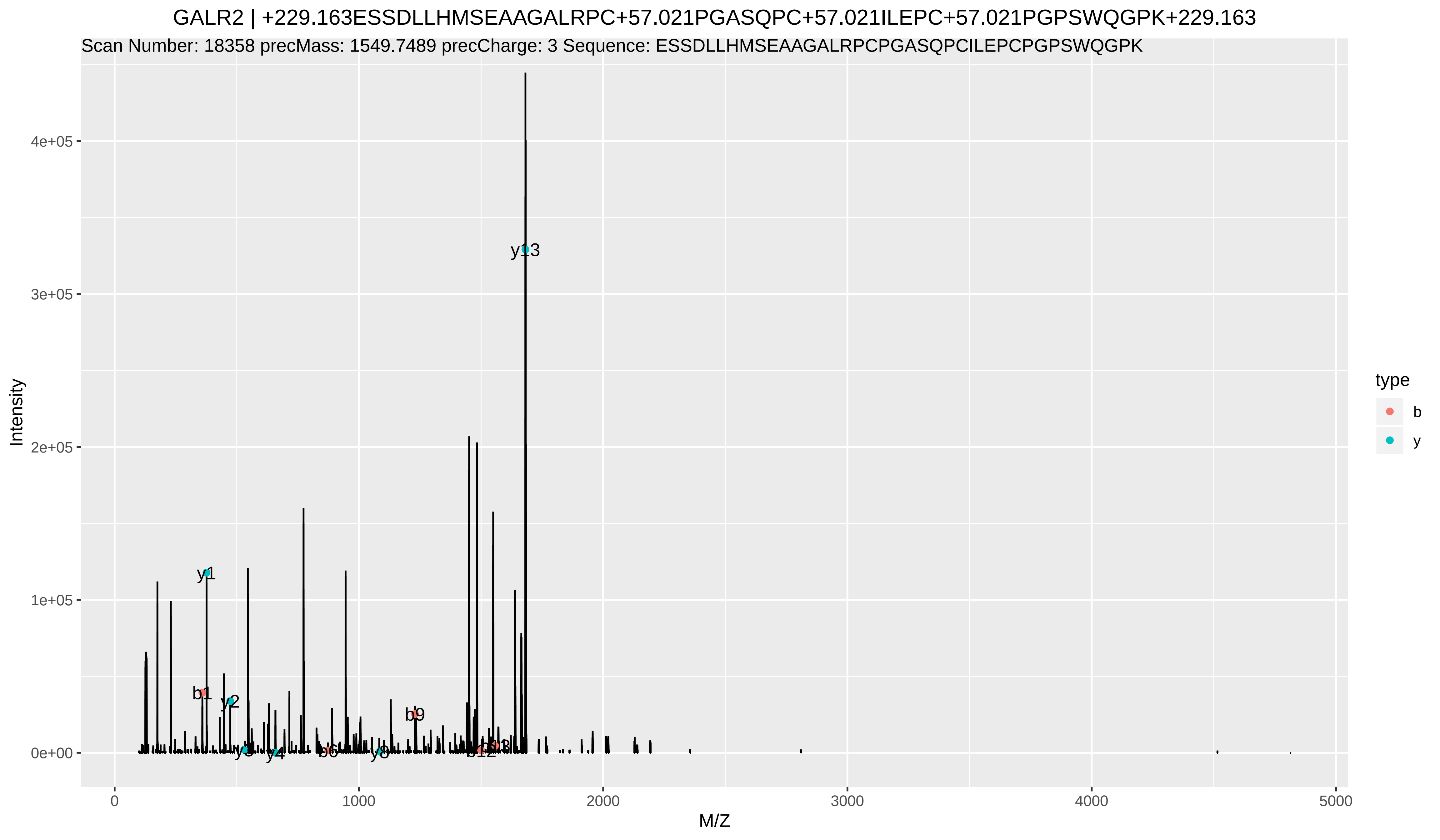

GALR2 | +229.163ESSDLLHMSEAAGALRPC+57.021PGASQPC+57.021ILEPC+57.021PGPSWQGPK+229.163
Scan Number: 18358 precMass: 1549.7489 precCharge: 3 Sequence: ESSDLLHMSEAAGALRPCPGASQPCILEPCPGPSWQGPK
Intensity
M/Z
0e+00
1e+05
2e+05
3e+05
4e+05
0
1000
2000
3000
4000
5000
y13
y1
b1
y2
b9
y3
y4
b6
y8
b12
type
b
y

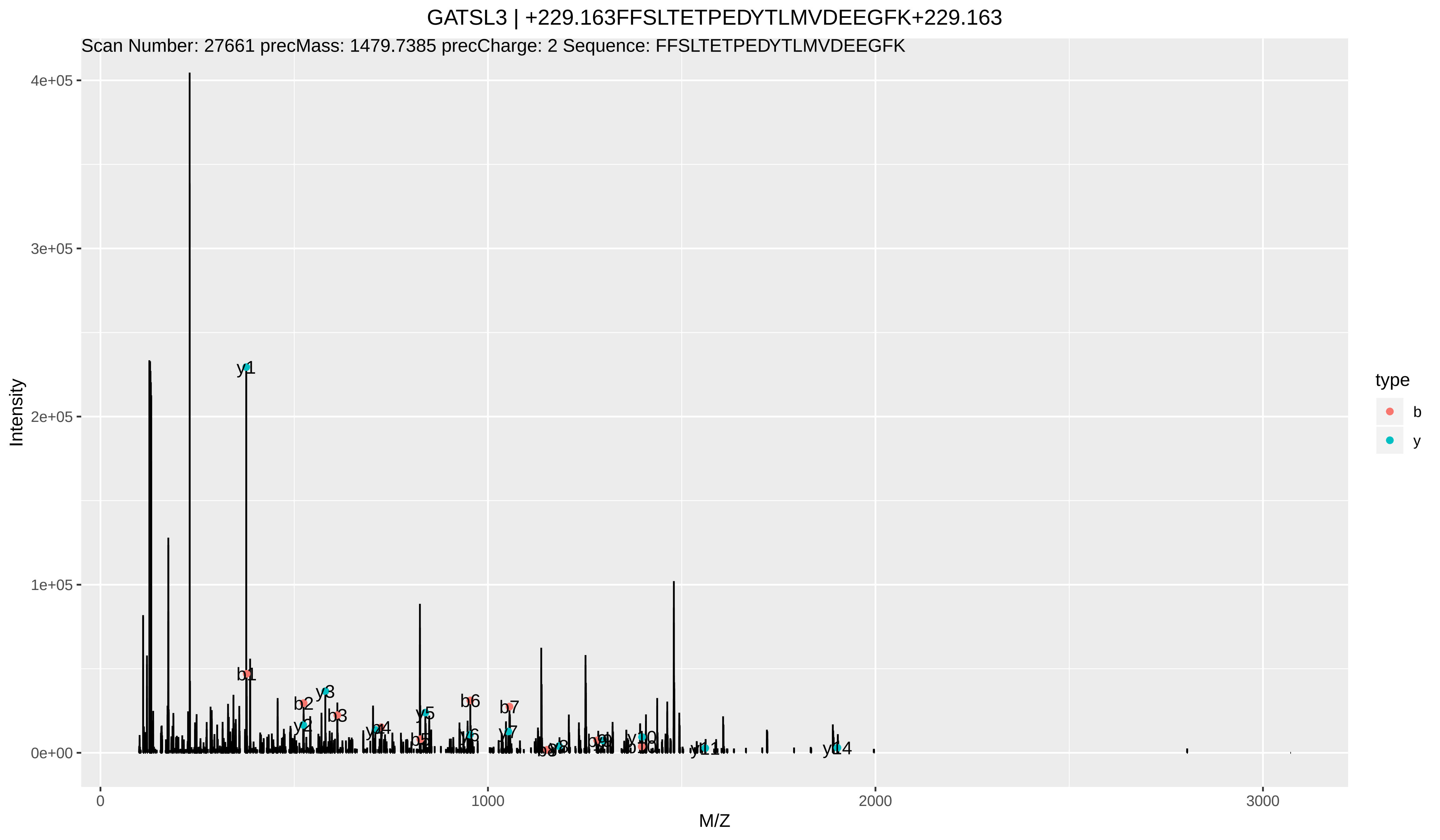
GATSL3 | +229.163FFSLTETPEDYTLMVDEEGFK+229.163
Scan Number: 27661 precMass: 1479.7385 precCharge: 2 Sequence: FFSLTETPEDYTLMVDEEGFK
Intensity
4e+05
3e+05
2e+05
1e+05
0e+00
M/Z
0
1000
2000
3000
type
b
y
y1
b1
b2
y2
y3
b3
y5
b6
b7
y7
y6
y10
y11
y14

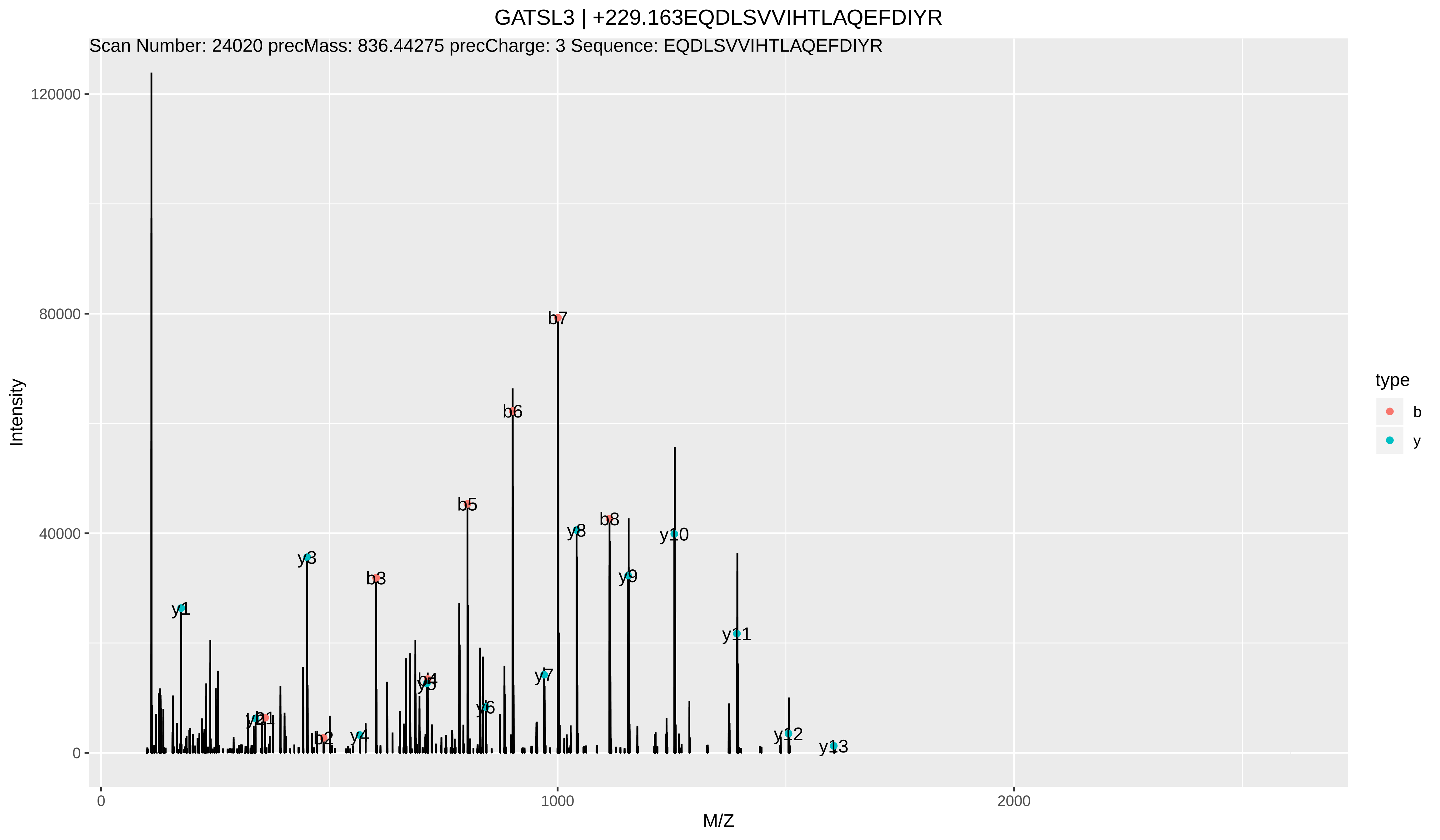

GATSL3 | +229.163EQDLSVVIHTLAQEFDIYR
Scan Number: 24020 precMass: 836.44275 precCharge: 3 Sequence: EQDLSVVIHTLAQEFDIYR
Intensity
120000
80000
40000
0
M/Z
0
1000
2000
b7
b6
b5
b8
y8
y10
y3
b3
y9
y1
y11
b4
y5
y7
y6
y2
b1
b2
y4
y12
y13
type
b
y

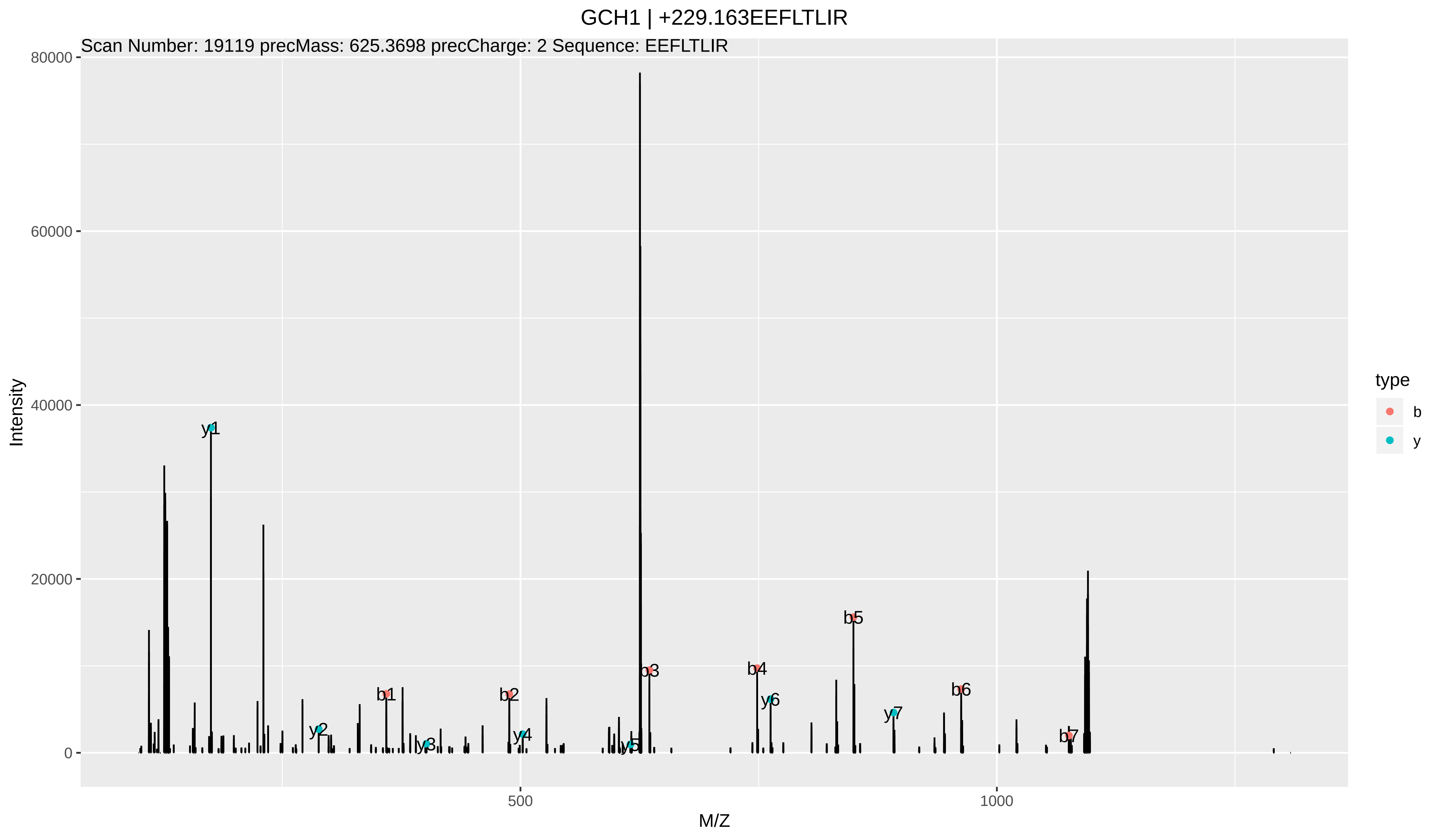
GCH1 | +229.163EEFLTLIR
Scan Number: 19119 precMass: 625.3698 precCharge: 2 Sequence: EEFLTLIR
80000
60000
40000
20000
0
Intensity
500
1000
M/Z
y1
y2
y3
y4
y5
y6
y7
b1
b2
b3
b4
b5
b6
b7
type
b
y

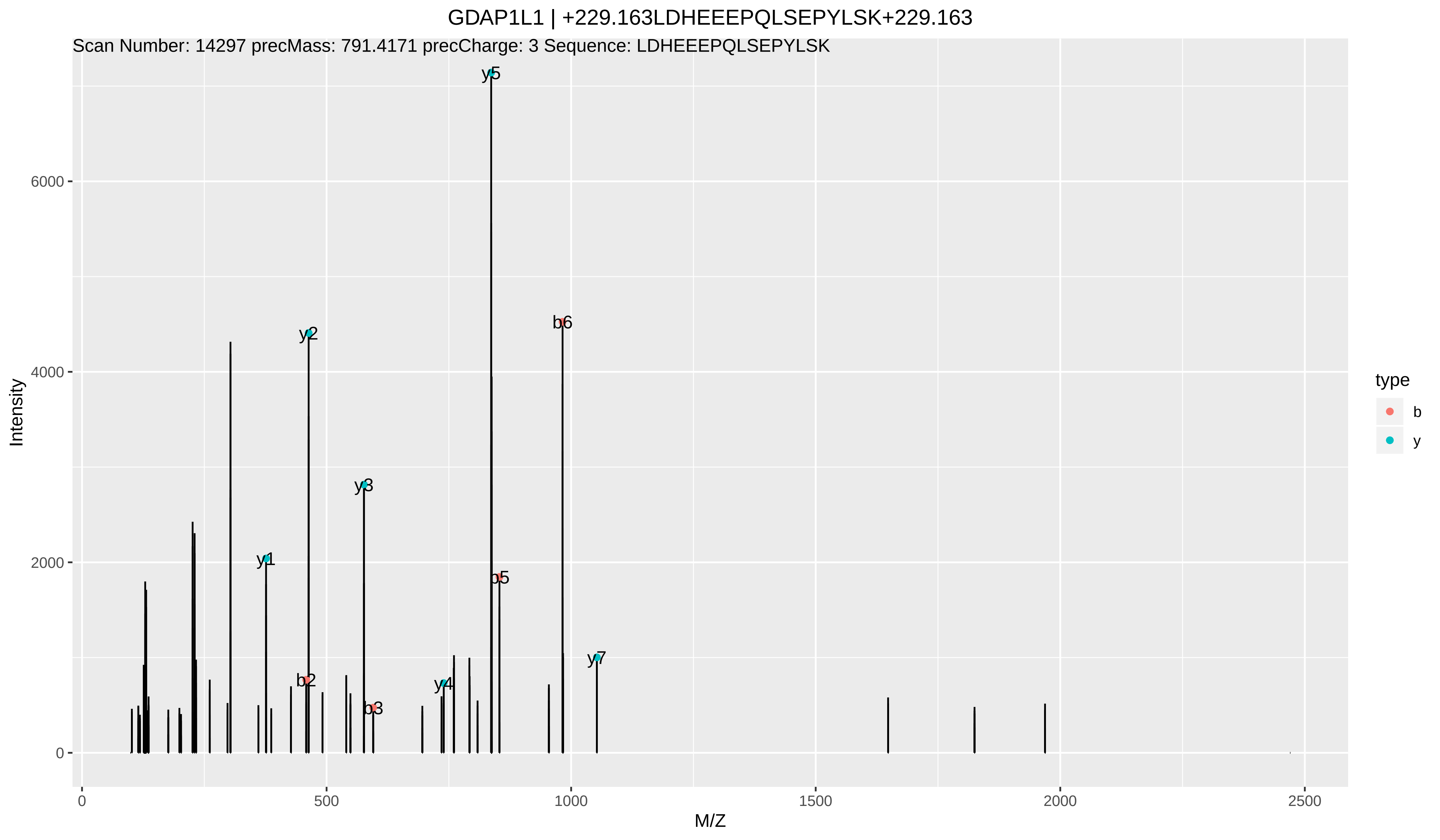

GDAP1L1 | +229.163LDHEEEPQLSEPYLSK+229.163
Scan Number: 14297 precMass: 791.4171 precCharge: 3 Sequence: LDHEEEPQLSEPYLSK
Intensity
M/Z
y5
b6
y2
y3
y1
b5
y7
b2
y4
b3
type
b
y
0
2000
4000
6000
0
500
1000
1500
2000
2500

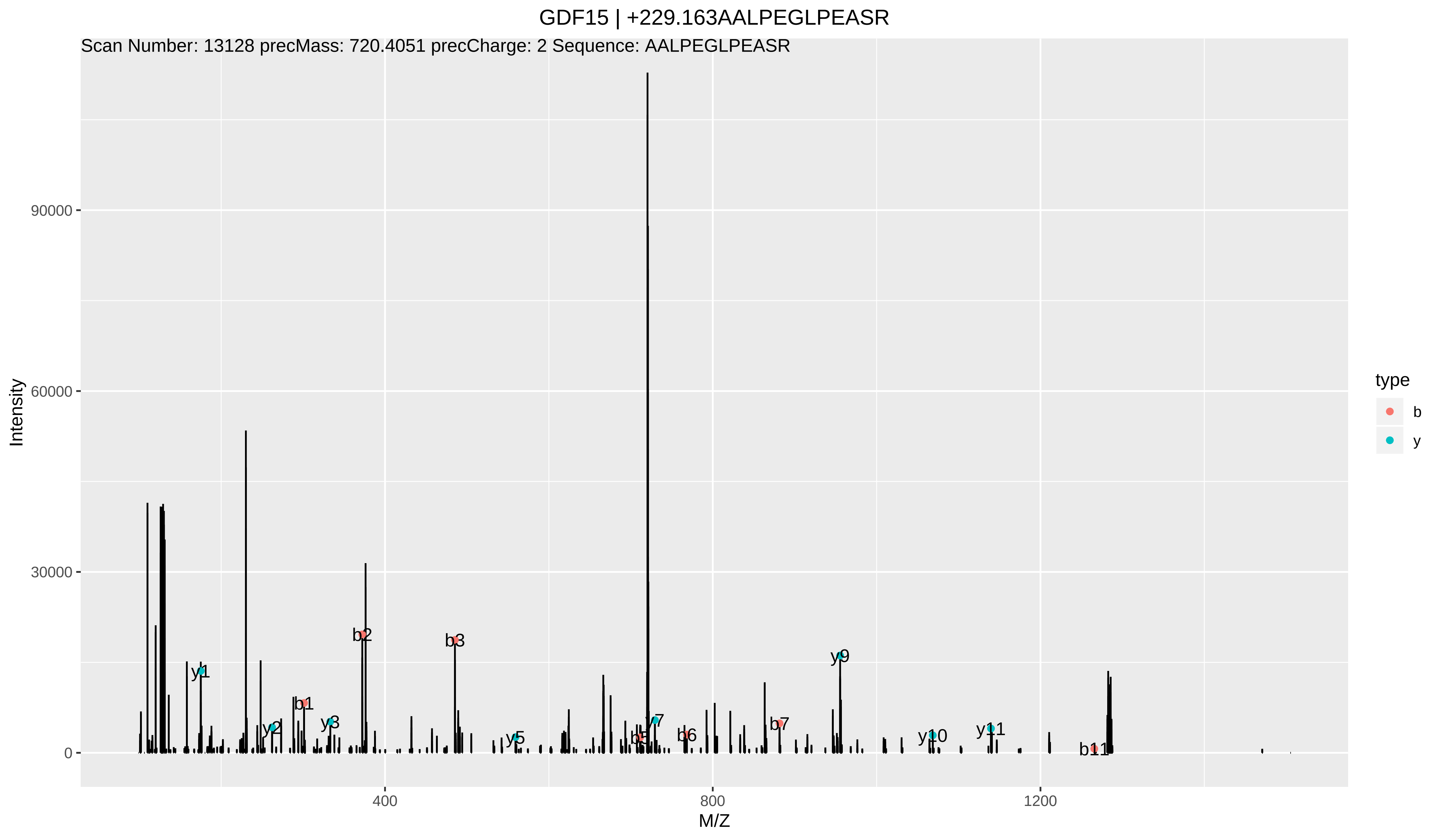

GDF15 | +229.163AALPEGLPEASR
Scan Number: 13128 precMass: 720.4051 precCharge: 2 Sequence: AALPEGLPEASR
Intensity
90000
60000
30000
0
M/Z
400
800
1200
type
b
y
y1
y2
y3
y5
y9
y10
y11
b1
b2
b3
b6
b7
b11

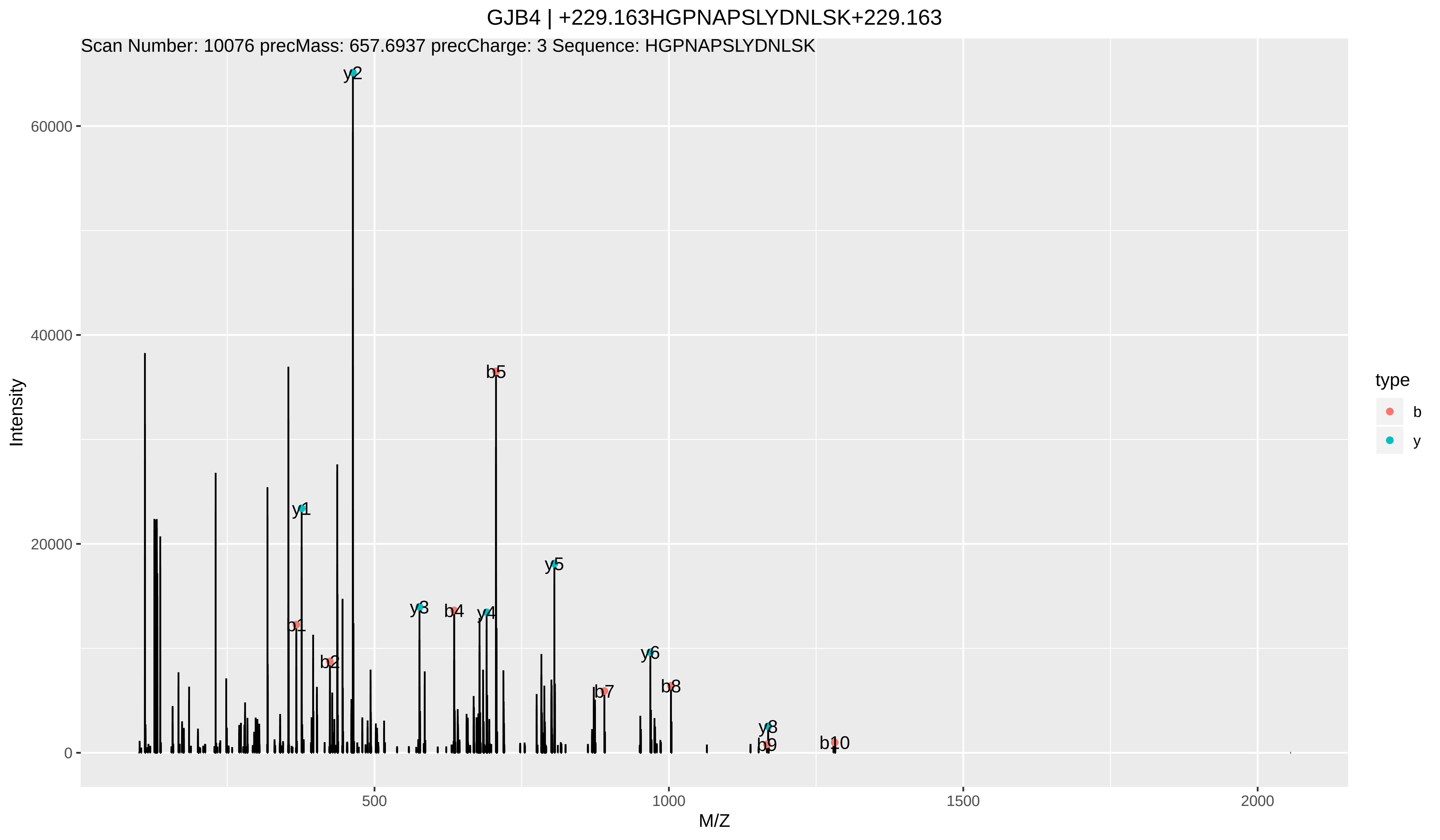

GJB4 | +229.163HGPNAPSLYDNLSK+229.163
Scan Number: 10076 precMass: 657.6937 precCharge: 3 Sequence: HGPNAPSLYDNLSK
Intensity
M/Z
0
20000
40000
60000
500
1000
1500
2000
y2
b5
y1
y5
y3
b4
y4
b1
b2
y6
b8
b7
y8
b9
b10
type
b
y

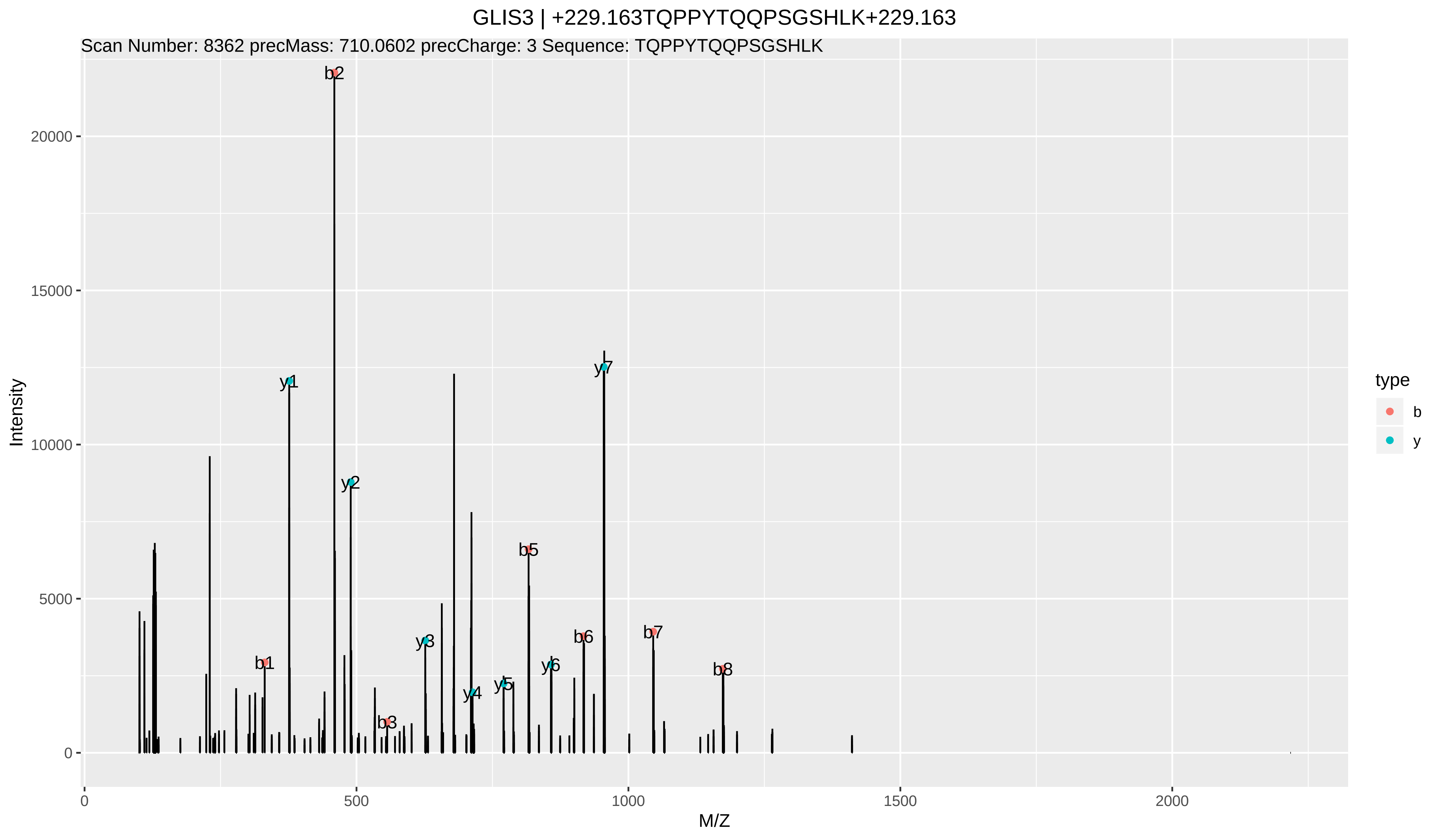
GLIS3 | +229.163TQPPYTQQPSGSHLK+229.163
Scan Number: 8362 precMass: 710.0602 precCharge: 3 Sequence: TQPPYTQQPSGSHLK
Intensity
M/Z
0
5000
10000
15000
20000
500
1000
1500
2000
b2
y1
y2
y7
b5
y3
b1
b3
y4
y5
y6
b6
b7
b8
type
b
y

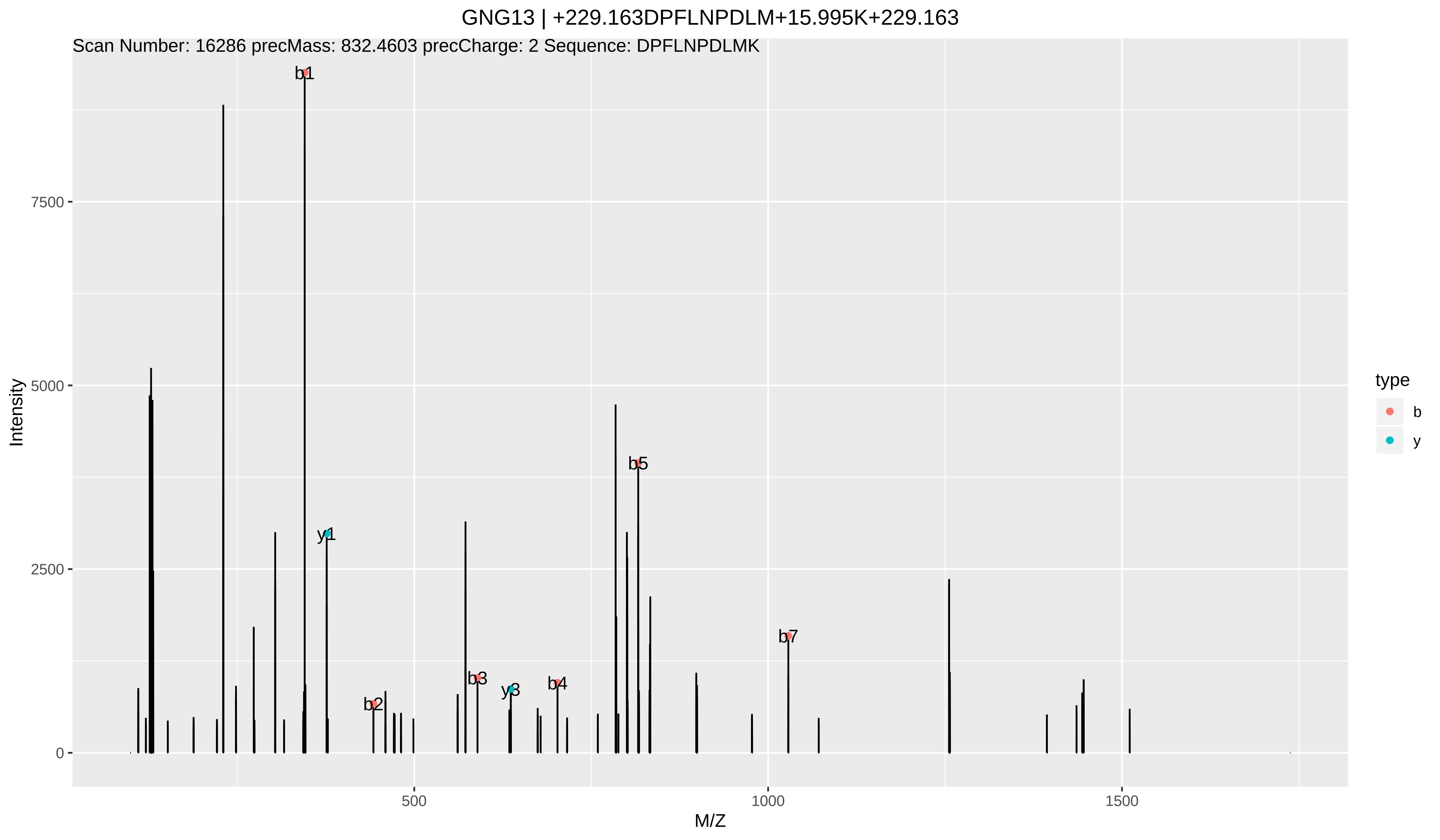

GNG13 | +229.163DPFLNPDLM+15.995K+229.163
Scan Number: 16286 precMass: 832.4603 precCharge: 2 Sequence: DPFLNPDLMK
Intensity
M/Z
type
b
y
b1
y1
b2
b3
y3
b4
b5
b7
0
2500
5000
7500
500
1000
1500

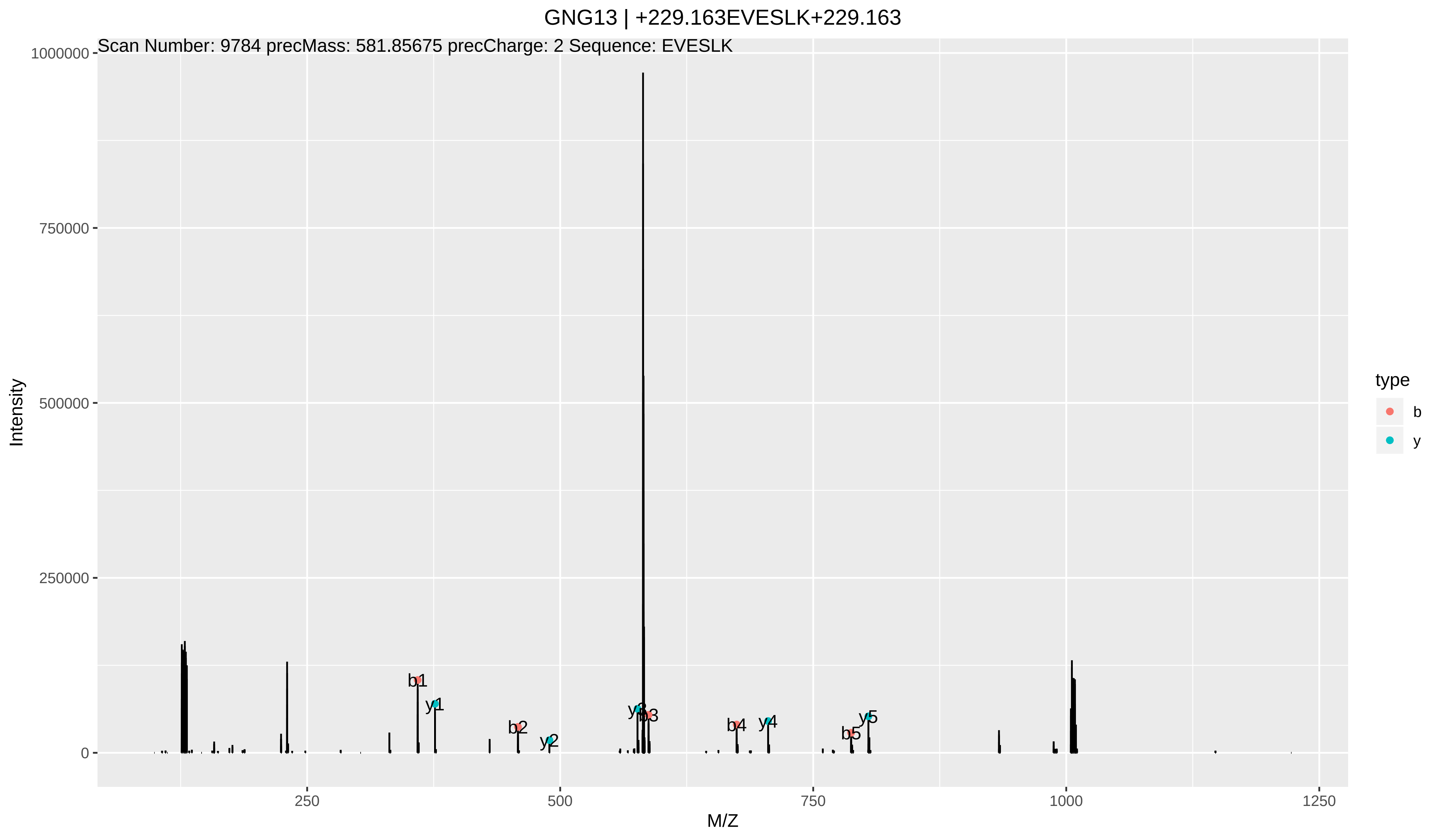
GNG13 | +229.163EVESLK+229.163
Scan Number: 9784 precMass: 581.85675 precCharge: 2 Sequence: EVESLK
Intensity
M/Z
1000000
750000
500000
250000
0
250
500
750
1000
1250
b1
y1
b2
y2
y3
b3
b4
y4
b5
y5
type
b
y

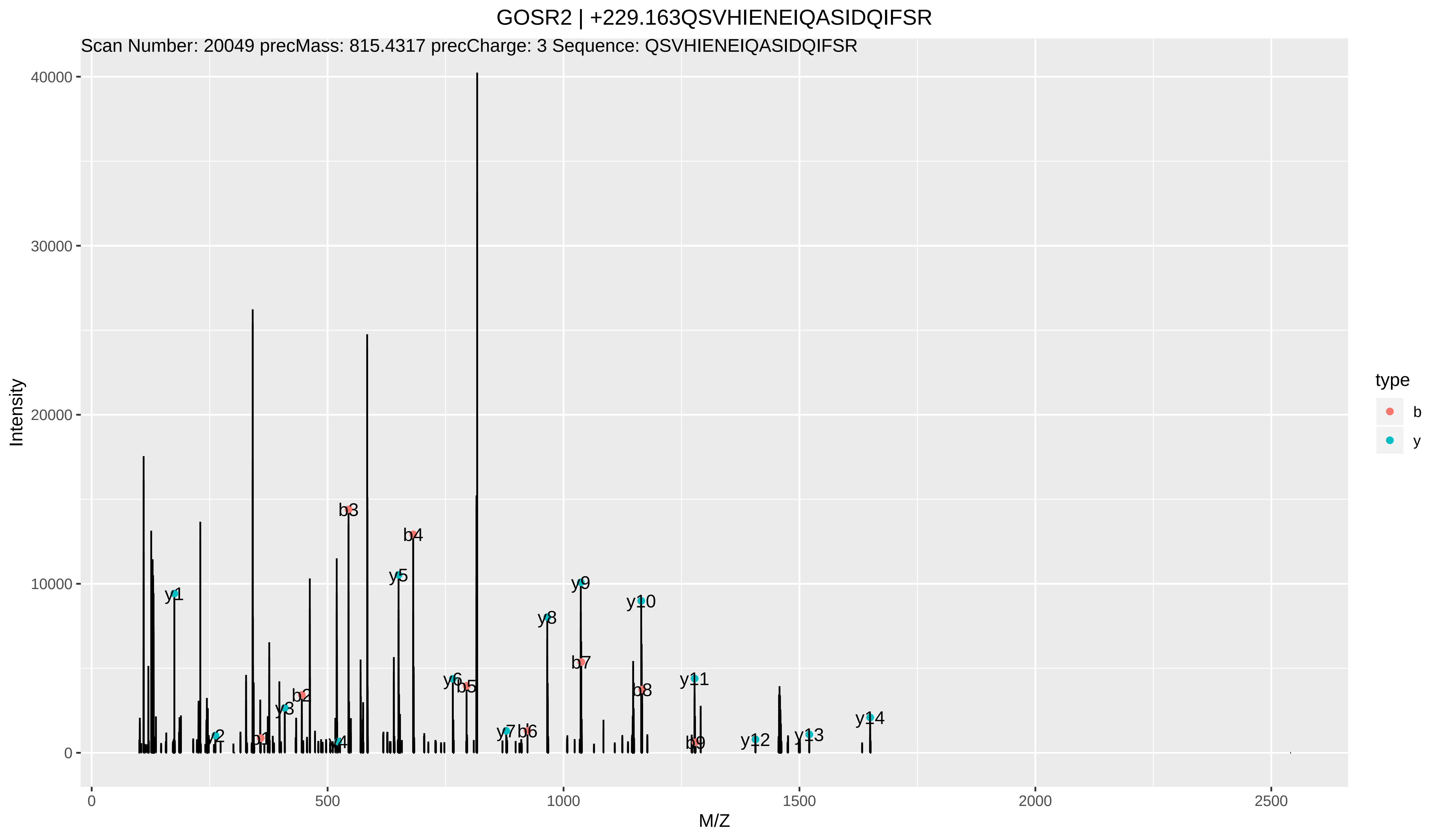

GOSR2 | +229.163QSVHIENEIQASIDQIFSR
Scan Number: 20049 precMass: 815.4317 precCharge: 3 Sequence: QSVHIENEIQASIDQIFSR
Intensity
M/Z
type
b
y

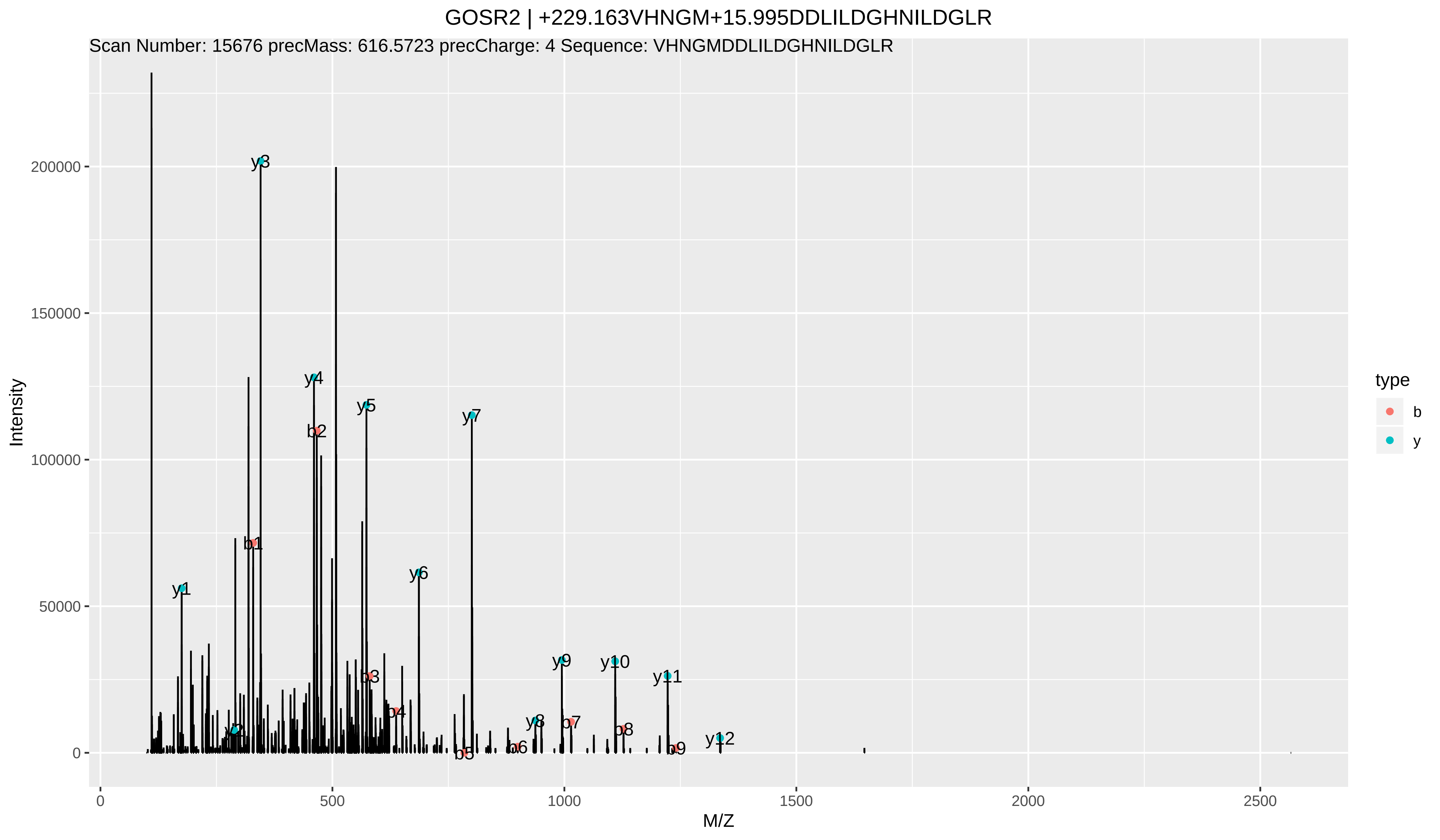

GOSR2 | +229.163VHNGM+15.995DDLILDGHNILDGLR
Scan Number: 15676 precMass: 616.5723 precCharge: 4 Sequence: VHNGMDDLILDGHNILDGLR
Intensity
M/Z
type
b
y
y1
y2
y3
y4
y5
y6
y7
y8
y9
y10
y11
y12
b1
b2
b3
b4
b5
b6
b7
b8
b9
0
50000
100000
150000
200000
500
1000
1500
2000
2500

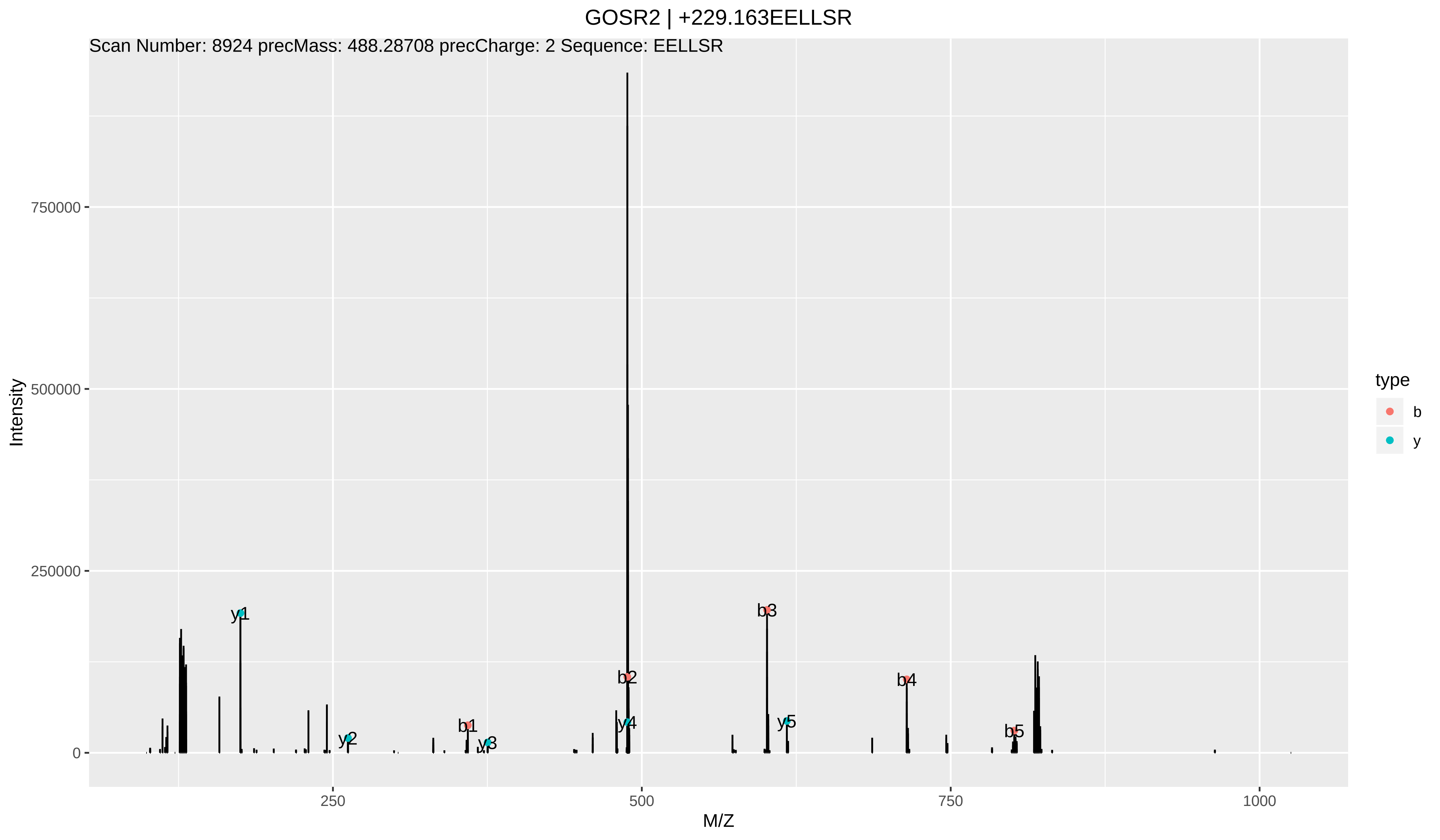

GOSR2 | +229.163EELLSR
Scan Number: 8924 precMass: 488.28708 precCharge: 2 Sequence: EELLSR
Intensity
M/Z
750000
500000
250000
0
250
500
750
1000
y1
y2
b1
y3
b2
y4
b3
y5
b4
b5
type
b
y

GPD1 | +229.163ITVVQEVDTVEIC+57.021GALK+229.163

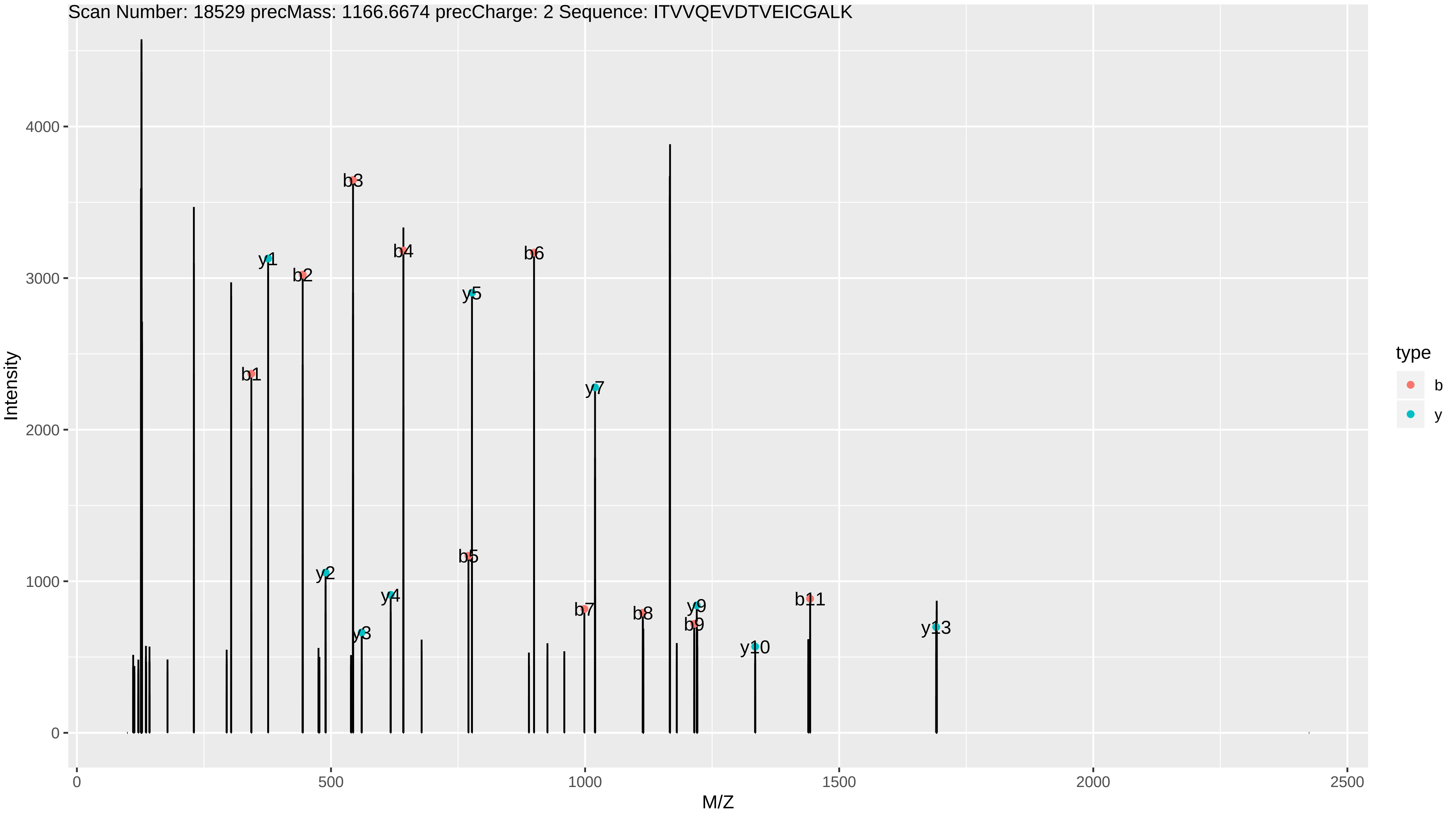

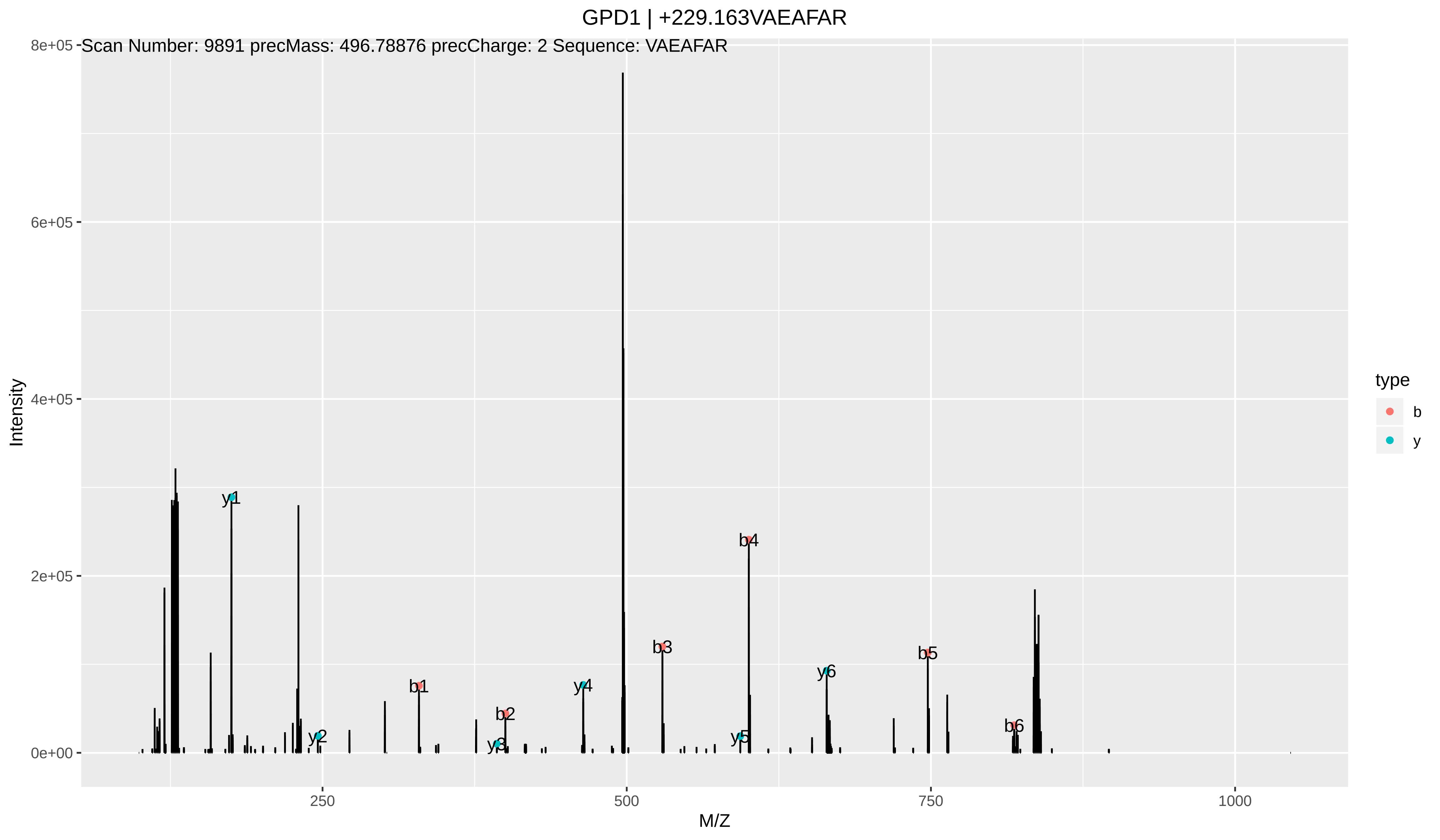
GPD1 | +229.163VAEAFAR
Scan Number: 9891 precMass: 496.78876 precCharge: 2 Sequence: VAEAFAR
Intensity
M/Z
0e+00
2e+05
4e+05
6e+05
8e+05
250
500
750
1000
y1
y2
y3
y4
y5
y6
b1
b2
b3
b4
b5
b6
type
b
y

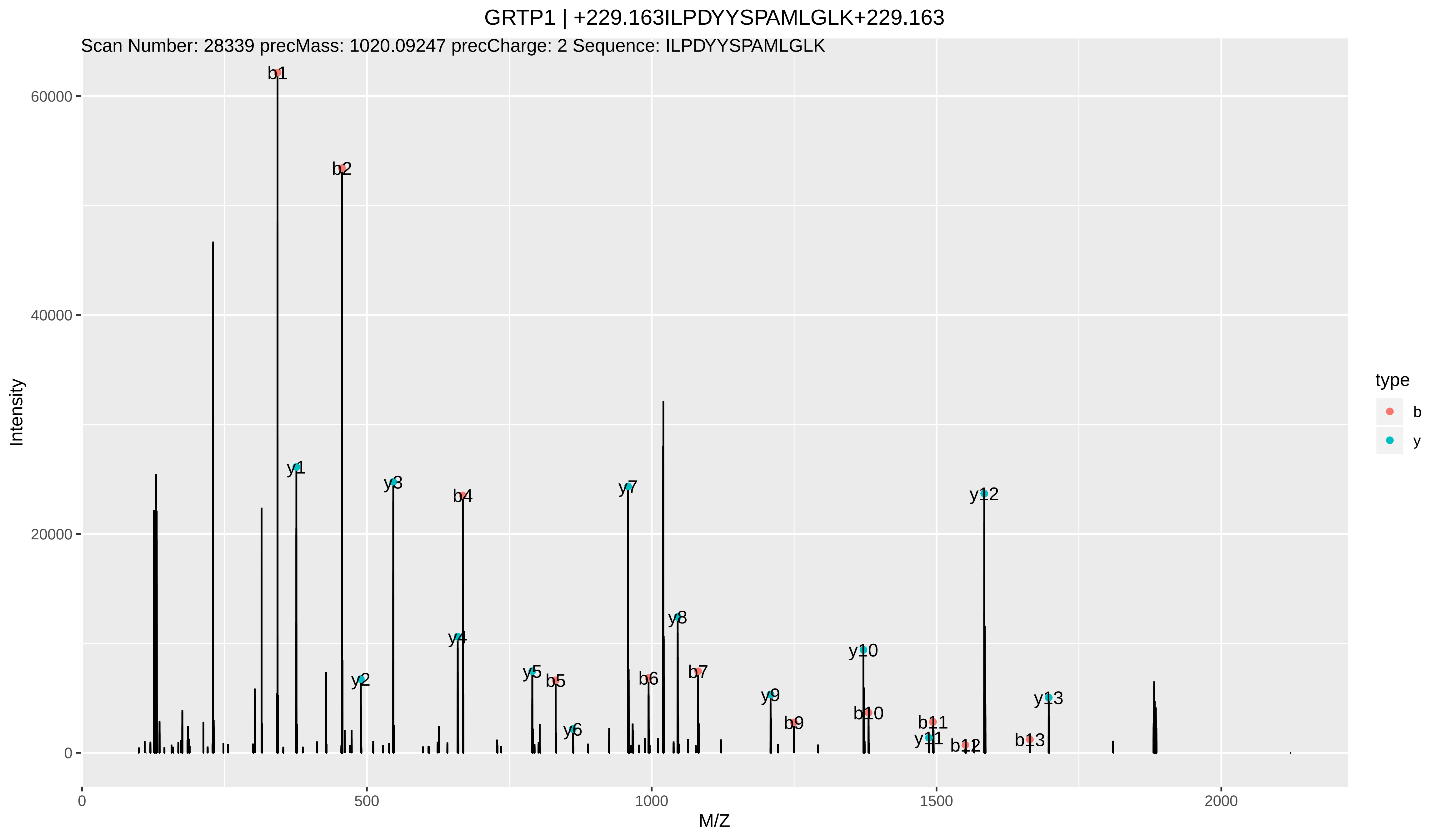

GRTP1 | +229.163ILPDYYSPAMLGLK+229.163
Scan Number: 28339 precMass: 1020.09247 precCharge: 2 Sequence: ILPDYYSPAMLGLK
Intensity
M/Z
0
20000
40000
60000
0
500
1000
1500
2000
b1
b2
y1
y3
b4
y7
y12
y8
y4
y10
b7
y5
b5
b6
y2
y9
y13
b10
b9
b11
y6
y11
b12
b13
type
b
y

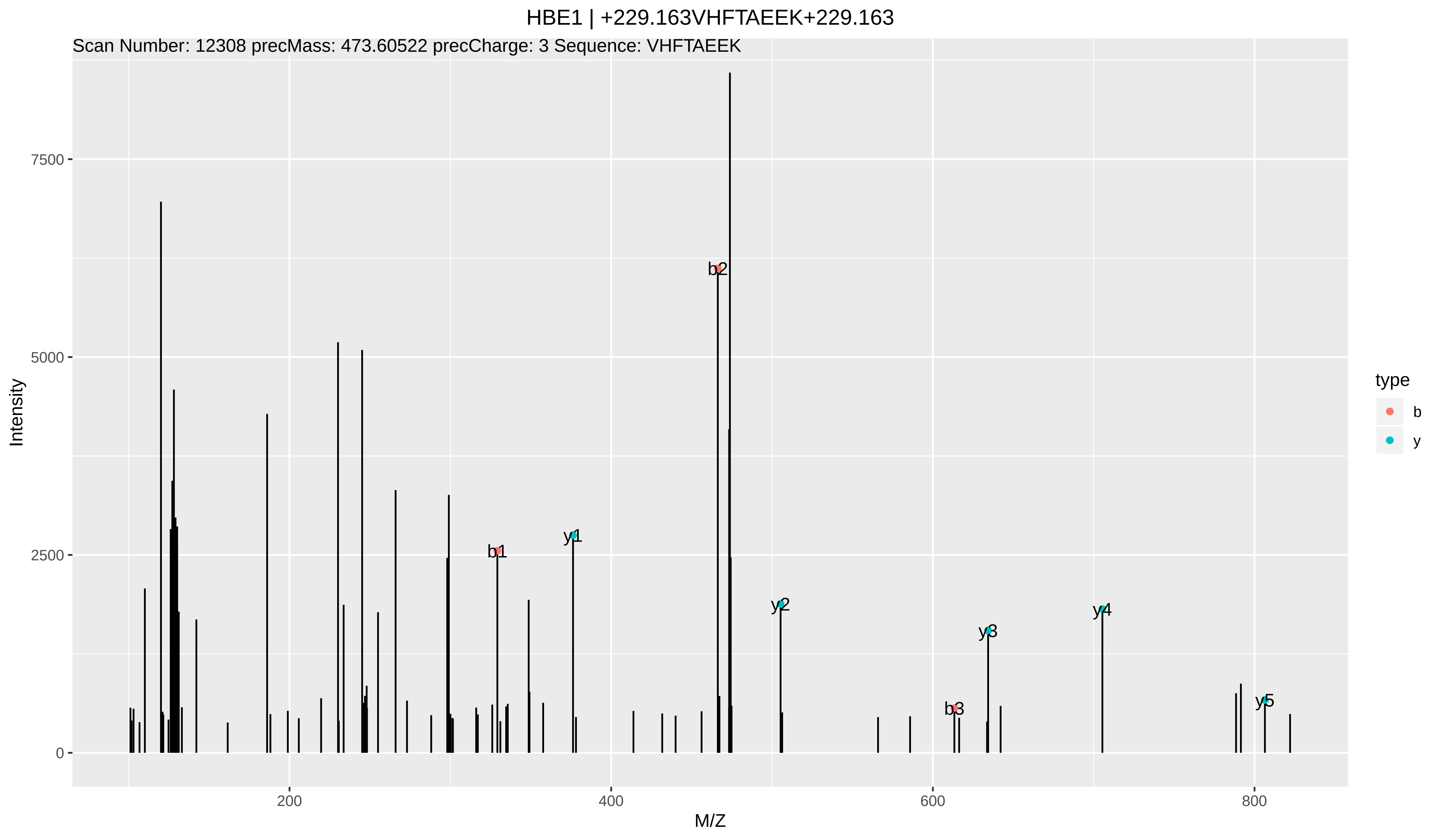

HBE1 | +229.163VHFTAEEK+229.163
Scan Number: 12308 precMass: 473.60522 precCharge: 3 Sequence: VHFTAEEK
Intensity
M/Z
0
2500
5000
7500
200
400
600
800
b1
b2
b3
y1
y2
y3
y4
y5
type
b
y

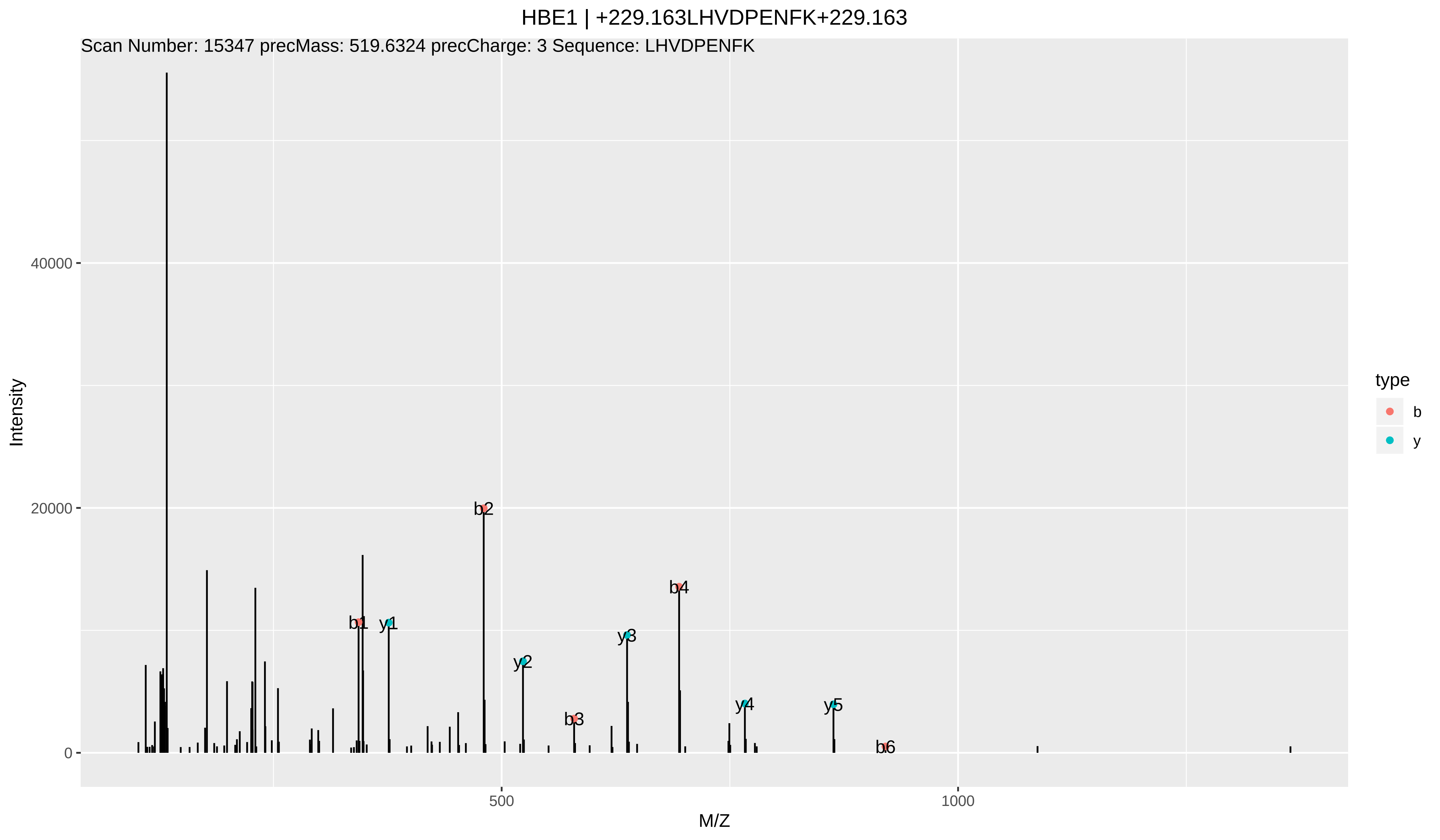

HBE1 | +229.163LHVDPENFK+229.163
Scan Number: 15347 precMass: 519.6324 precCharge: 3 Sequence: LHVDPENFK
Intensity
40000
20000
0
M/Z
500
1000
b1
y1
b2
y2
b3
y3
b4
y4
y5
b6
type
b
y

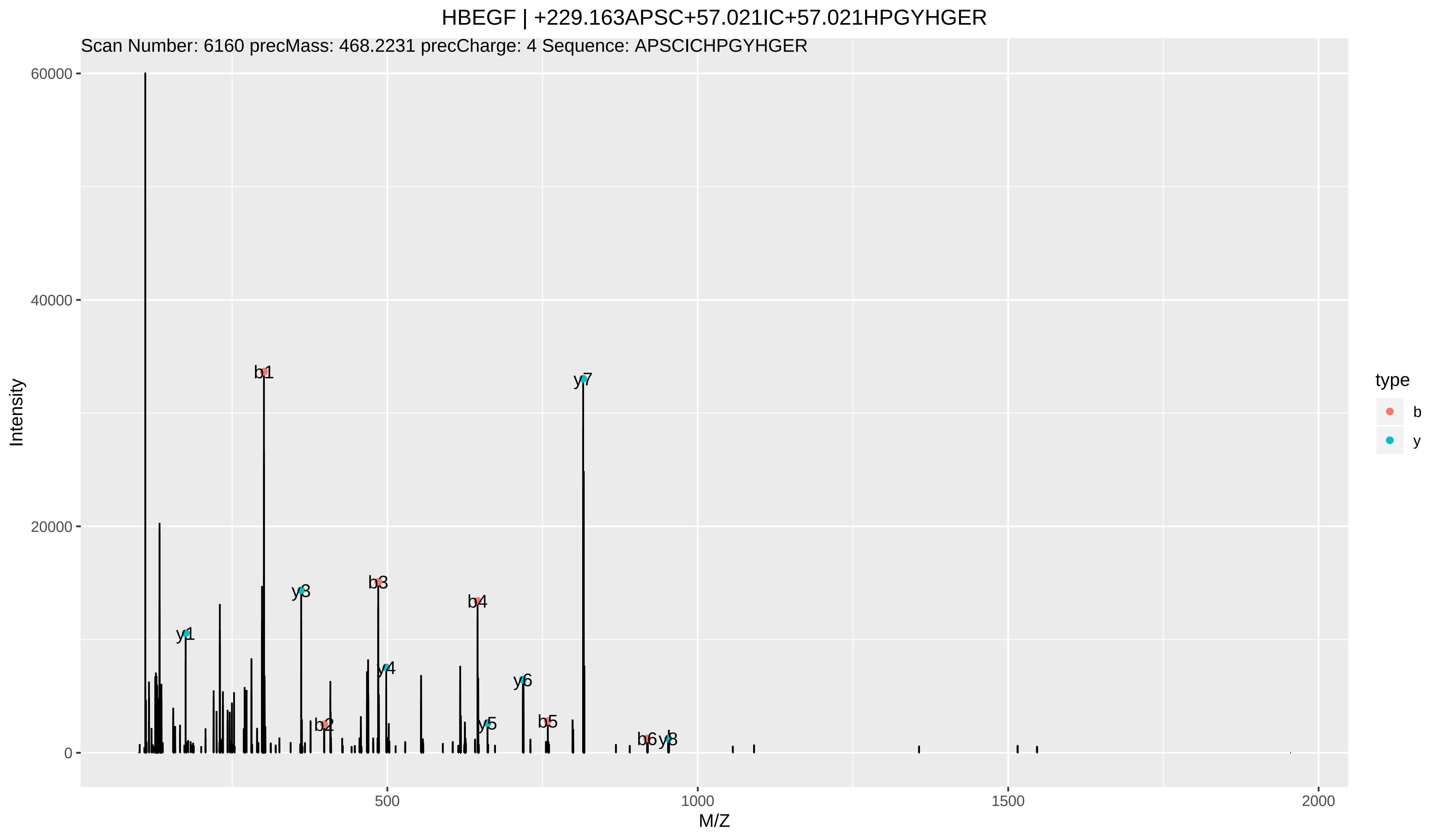

HBEGF | +229.163APSC+57.021IC+57.021HPGYHGER
Scan Number: 6160 precMass: 468.2231 precCharge: 4 Sequence: APSCICHPGYHGER
Intensity
60000
40000
20000
0
M/Z
500
1000
1500
2000
b1
y7
y3
b3
b4
y1
y4
y6
b2
y5
b5
b6
y8
type
b
y

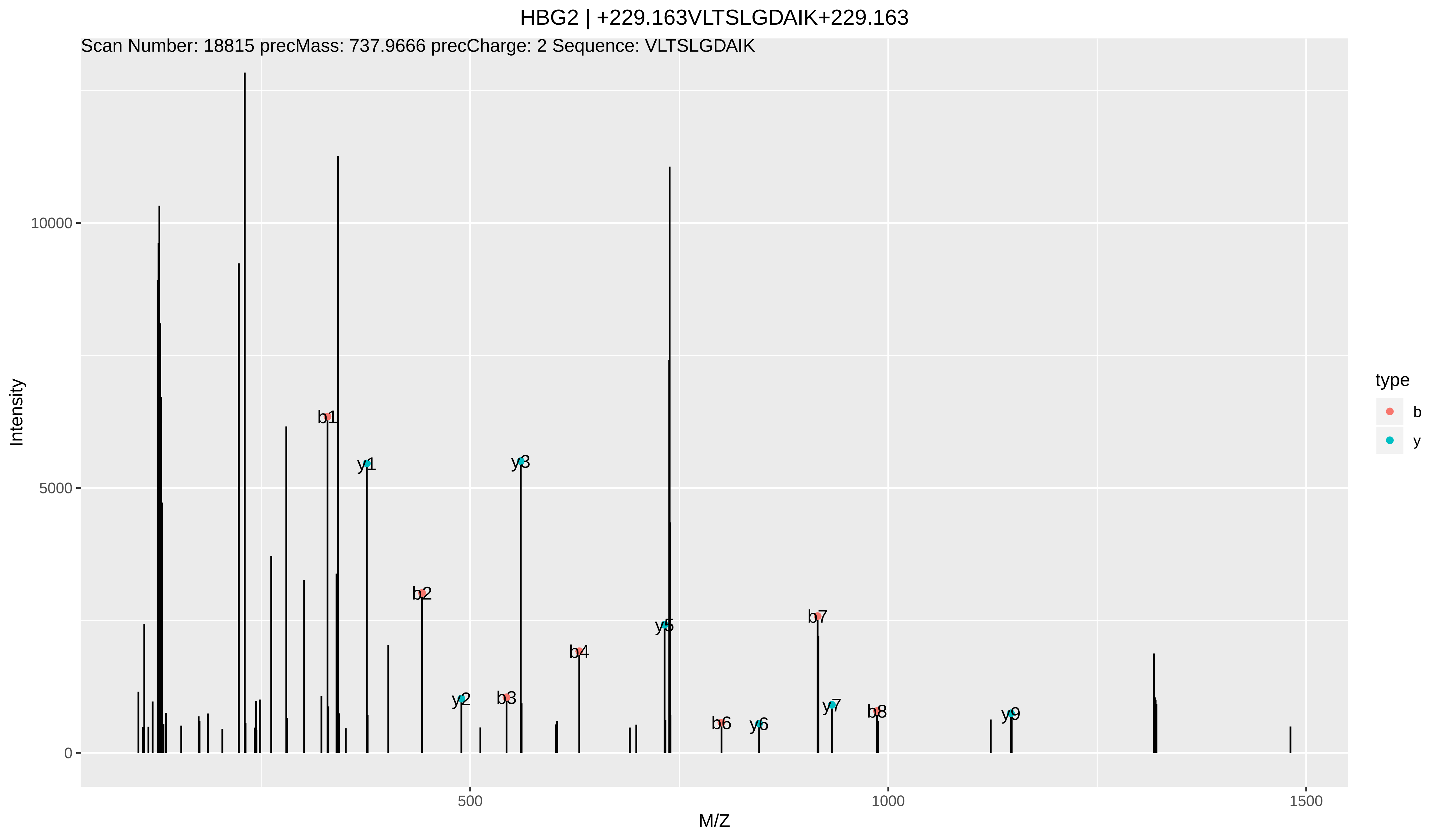

HBG2 | +229.163VLTSLGDAIK+229.163
Scan Number: 18815 precMass: 737.9666 precCharge: 2 Sequence: VLTSLGDAIK
Intensity
10000
5000
0
500
1000
1500
M/Z
b1
y1
y3
b2
y5
b7
b4
y2
b3
b6
y6
y7
b8
y9
type
b
y

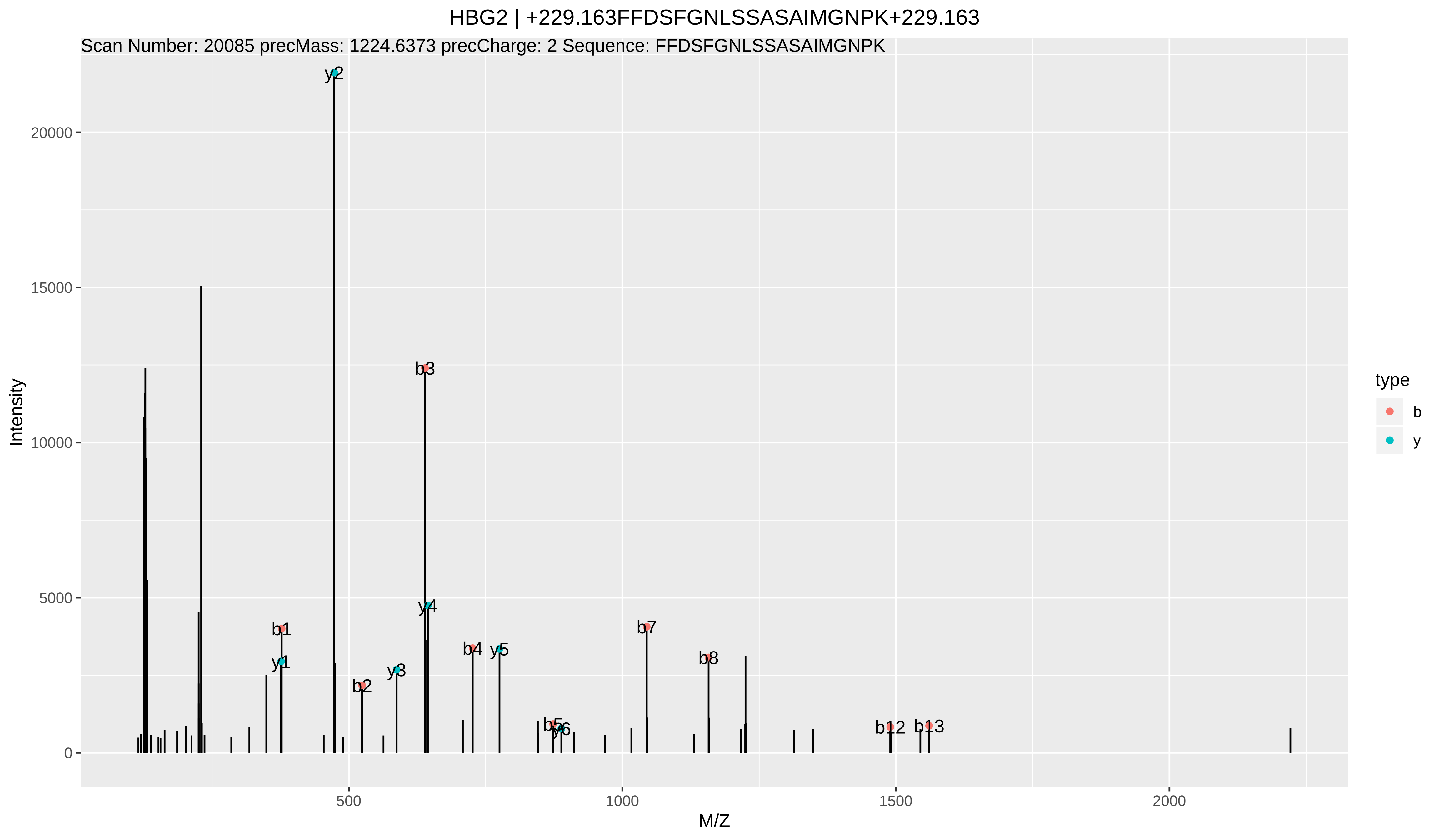
HBG2 | +229.163FFDSFGNLSSASAIMGNPK+229.163
Scan Number: 20085 precMass: 1224.6373 precCharge: 2 Sequence: FFDSFGNLSSASAIMGNPK
Intensity
M/Z
0
5000
10000
15000
20000
500
1000
1500
2000
y2
b3
y4
b1
y1
b2
y3
b4
y5
b5
y6
b7
b8
b12
b13
type
b
y

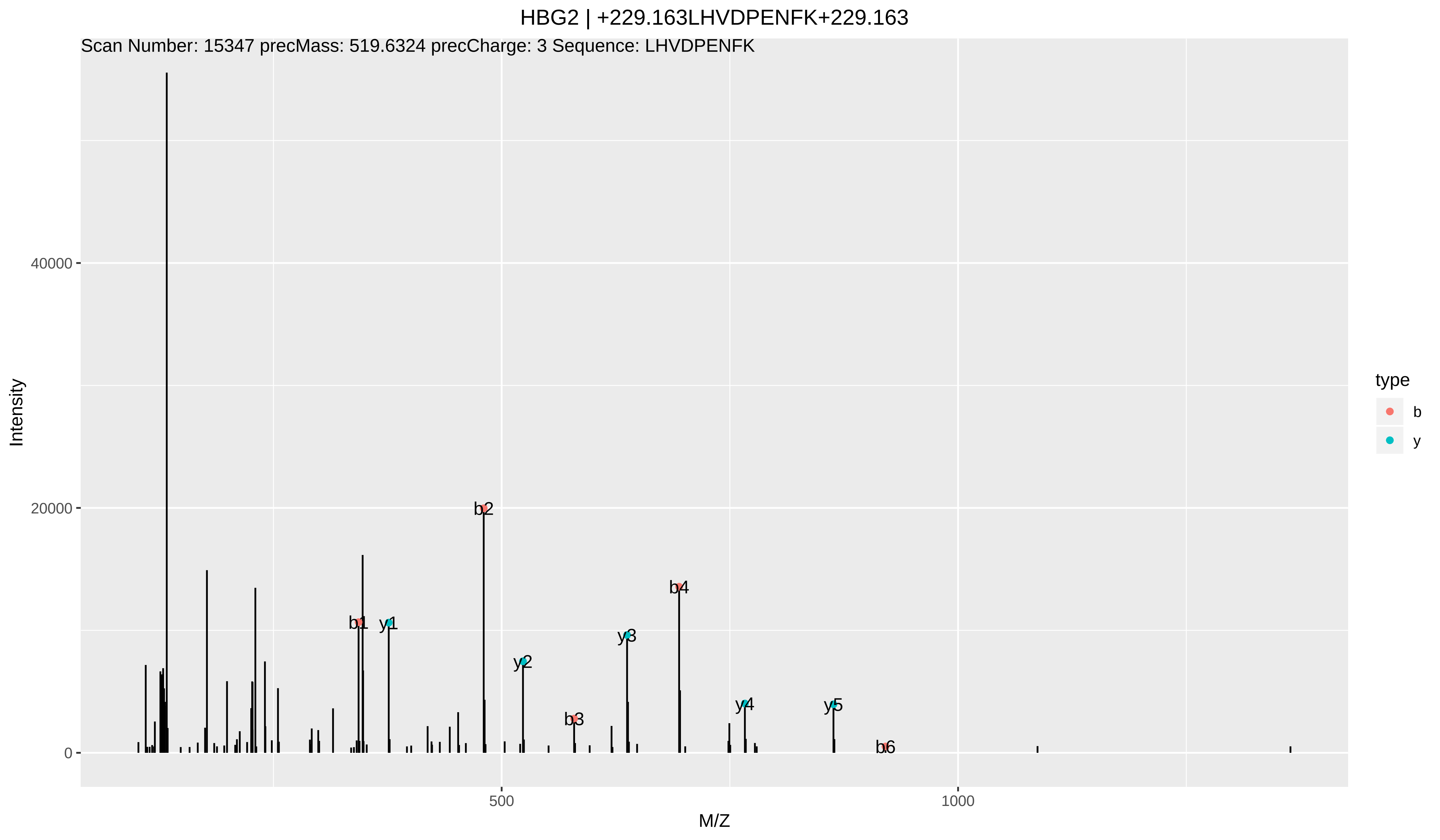

HBG2 | +229.163LHVDPENFK+229.163
Scan Number: 15347 precMass: 519.6324 precCharge: 3 Sequence: LHVDPENFK
Intensity
40000
20000
0
500
1000
M/Z
b1
y1
b2
y2
b3
y3
b4
y4
y5
b6
type
b
y

HCK | +229.163LIDFSAQIAEGM+15.995AFIEQR

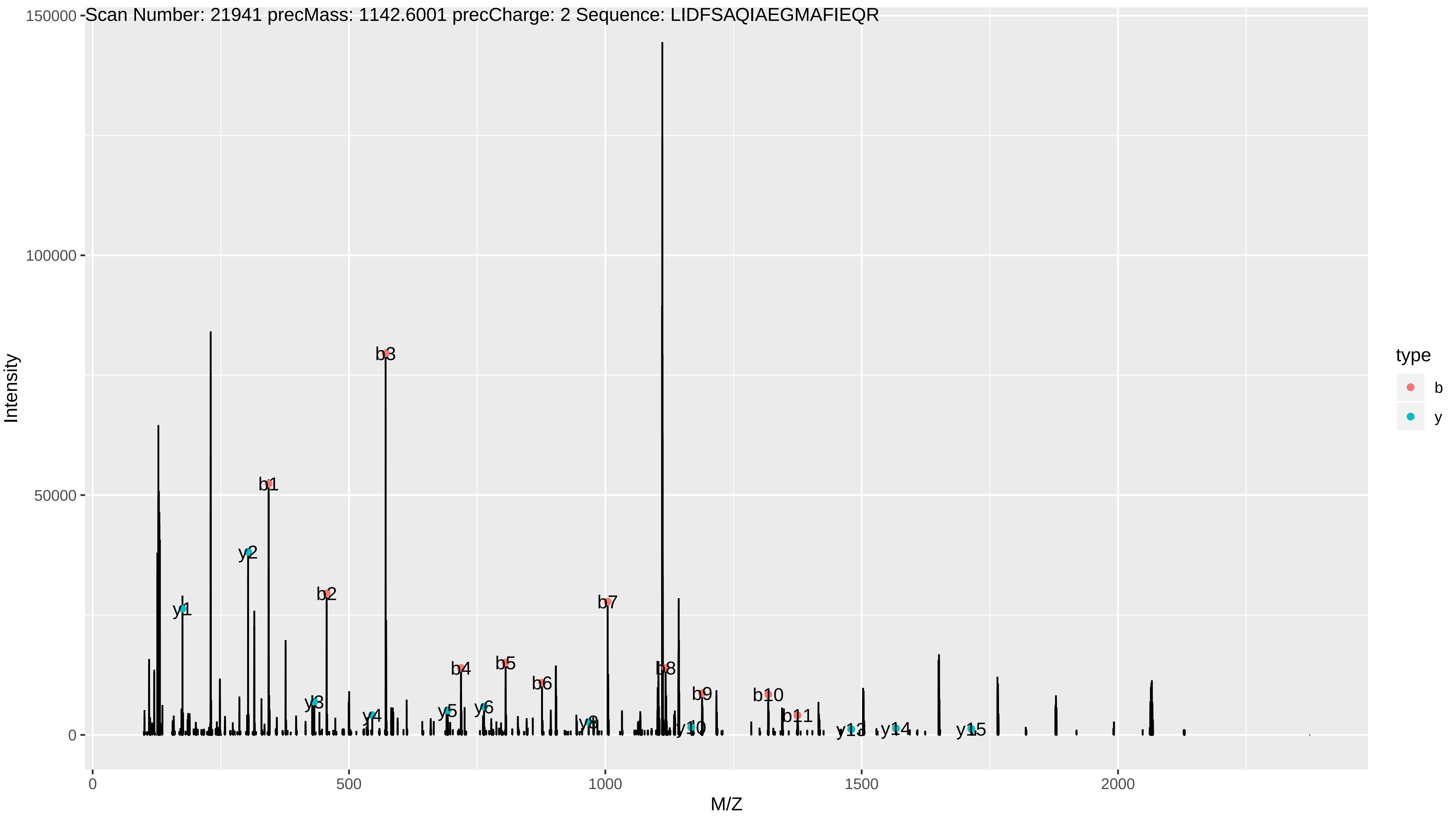

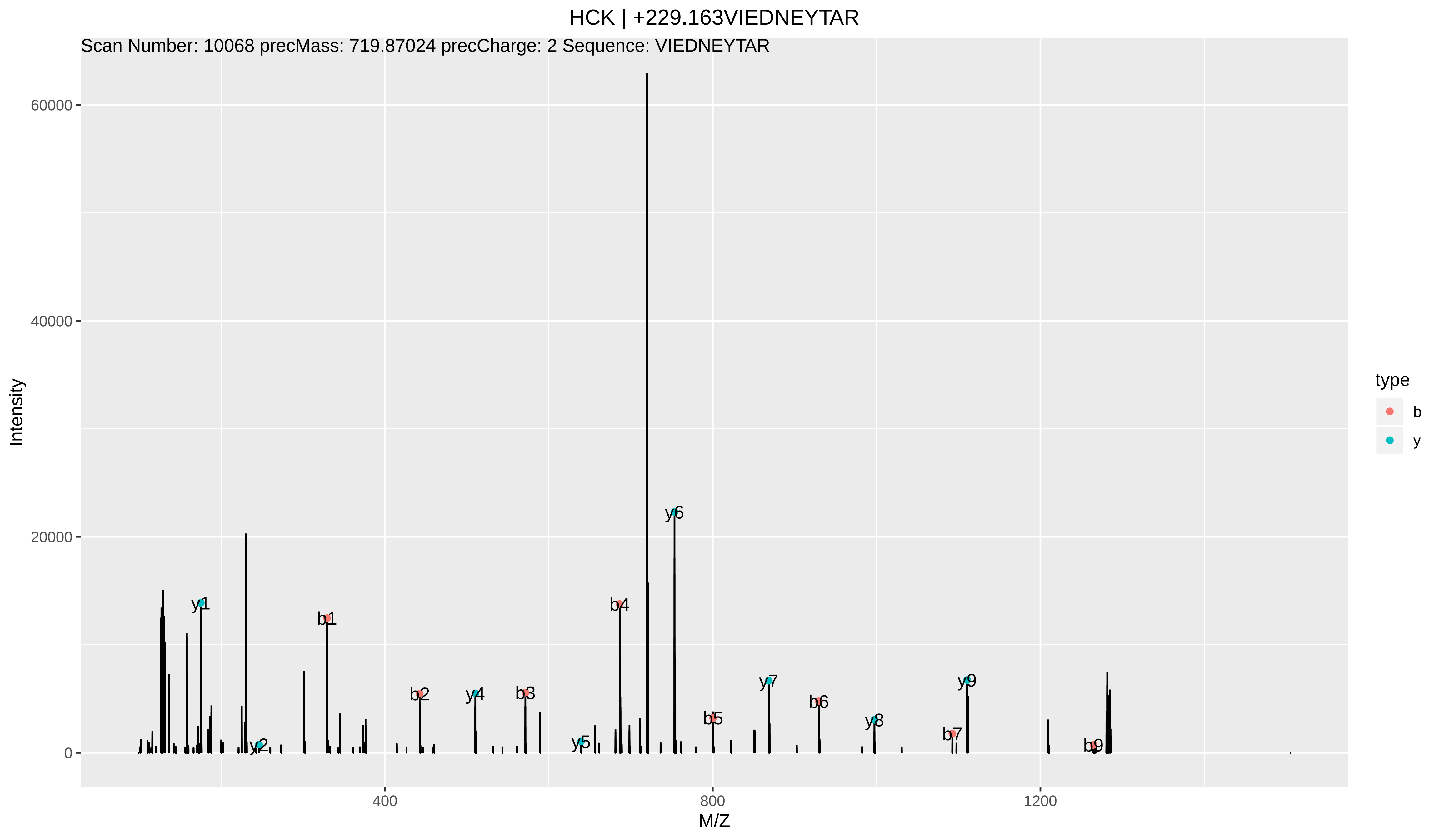

HCK | +229.163VIEDNEYTAR
Scan Number: 10068 precMass: 719.87024 precCharge: 2 Sequence: VIEDNEYTAR
Intensity
60000
40000
20000
0
M/Z
400
800
1200
y1
y2
b1
b2
y4
b3
y5
b4
y6
b5
y7
b6
y8
b7
y9
b9
type
b
y

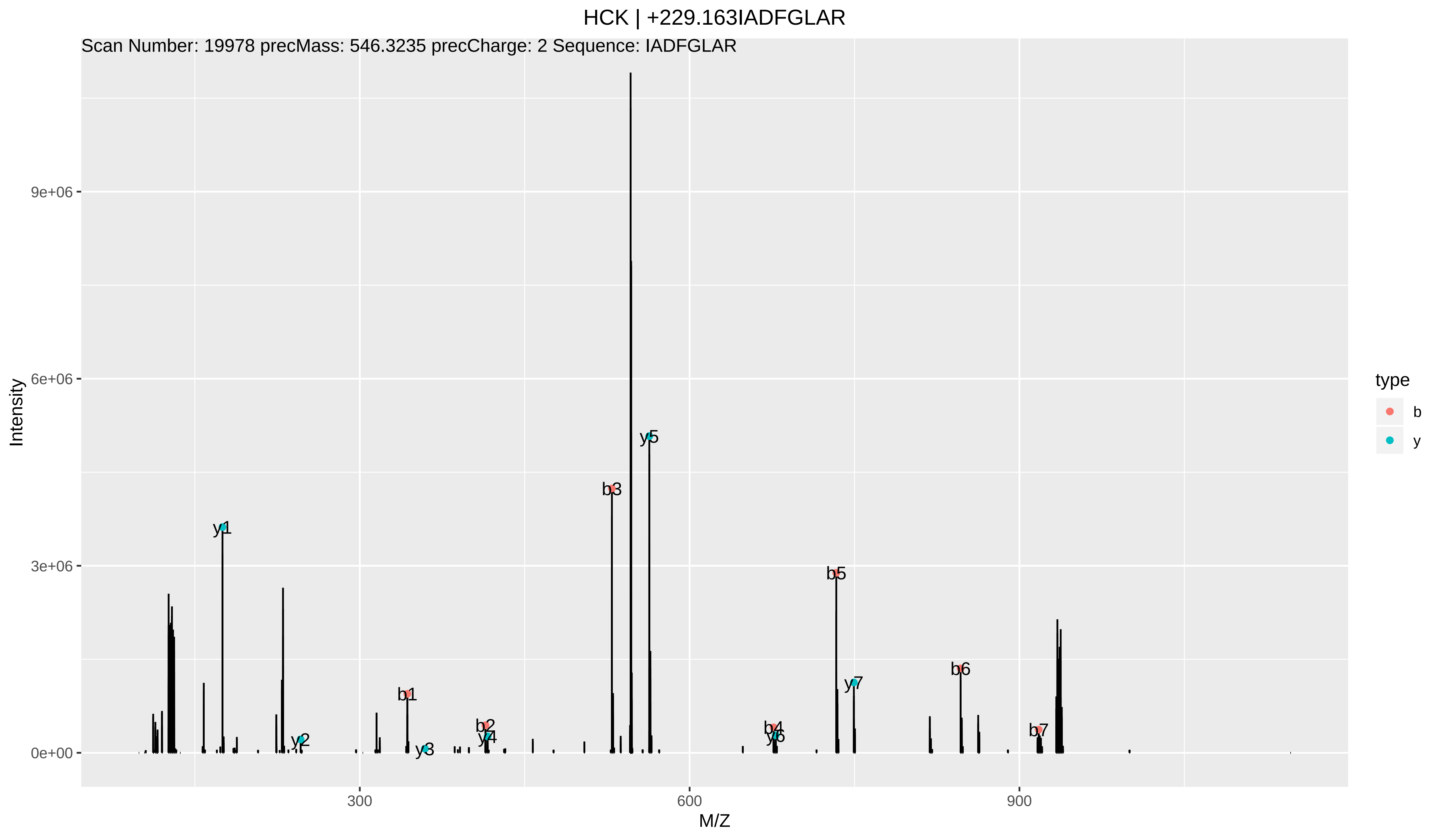
HCK | +229.163IADFGLAR
Scan Number: 19978 precMass: 546.3235 precCharge: 2 Sequence: IADFGLAR
Intensity
9e+06
6e+06
3e+06
0e+00
300
600
900
M/Z
y1
y2
b1
y3
b2
y4
b3
y5
b4
y6
b5
y7
b6
b7
type
b
y

HCK | +229.163GSLLDFLK+229.163

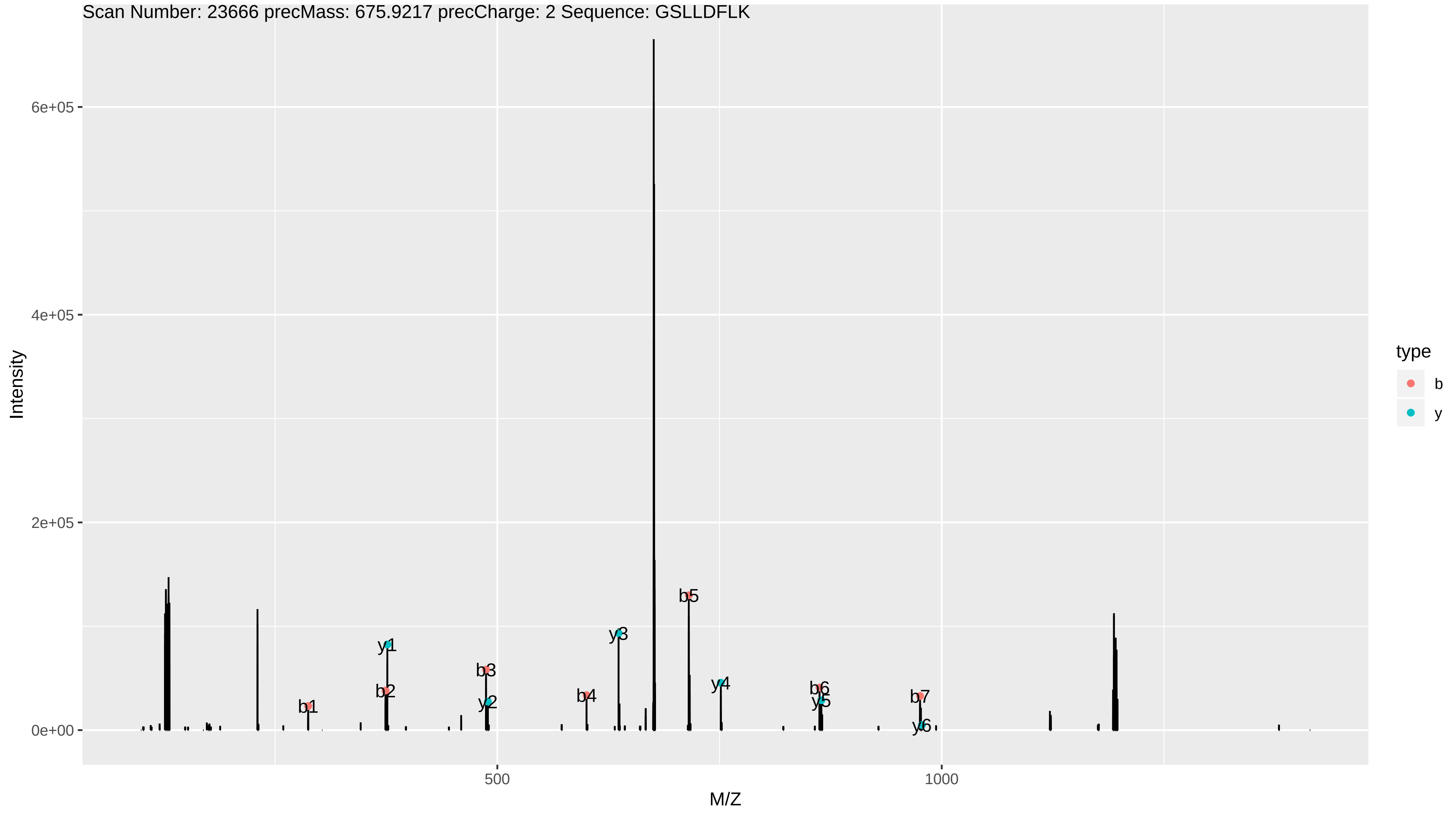

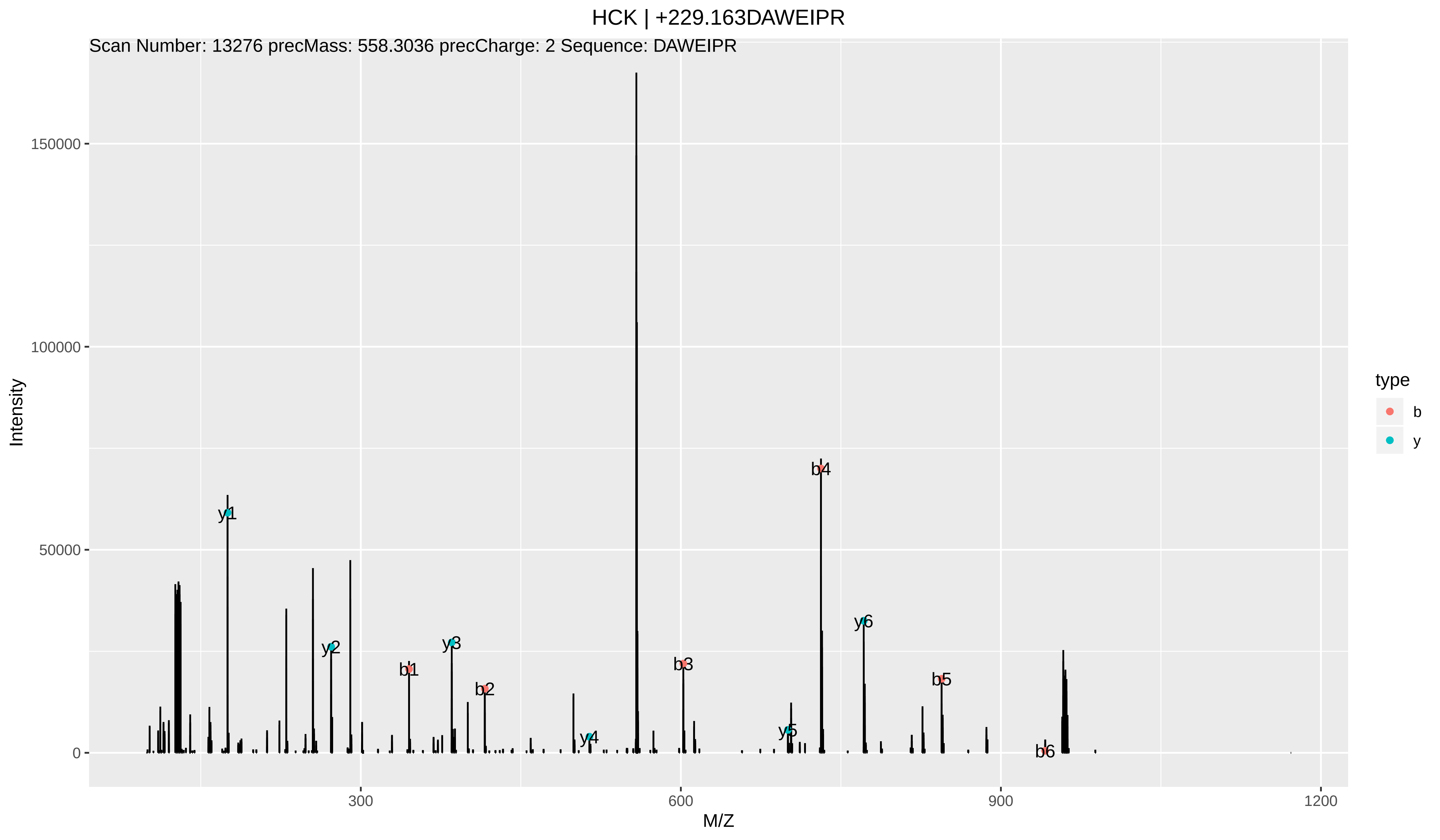

HCK | +229.163DAWEIPR
Scan Number: 13276 precMass: 558.3036 precCharge: 2 Sequence: DAWEIPR
Intensity
150000
100000
50000
0
M/Z
300
600
900
1200
y1
y2
b1
y3
b2
y4
b3
y5
b4
y6
b5
b6
type
b
y

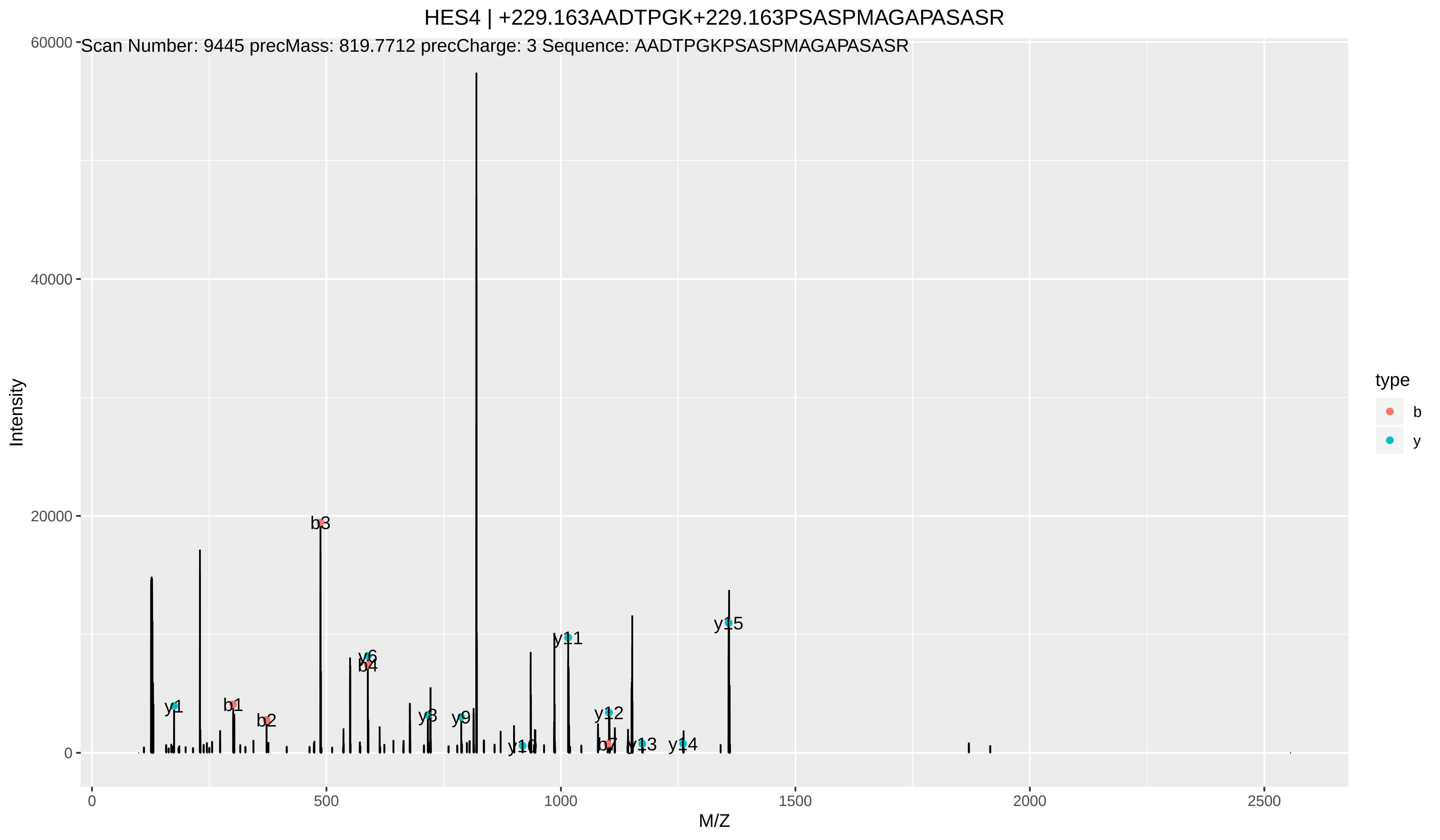

HES4 | +229.163AADTPGK+229.163PSASPMAGAPASASR
Scan Number: 9445 precMass: 819.7712 precCharge: 3 Sequence: AADTPGKPSASPMAGAPASASR
Intensity
M/Z
type
b
y

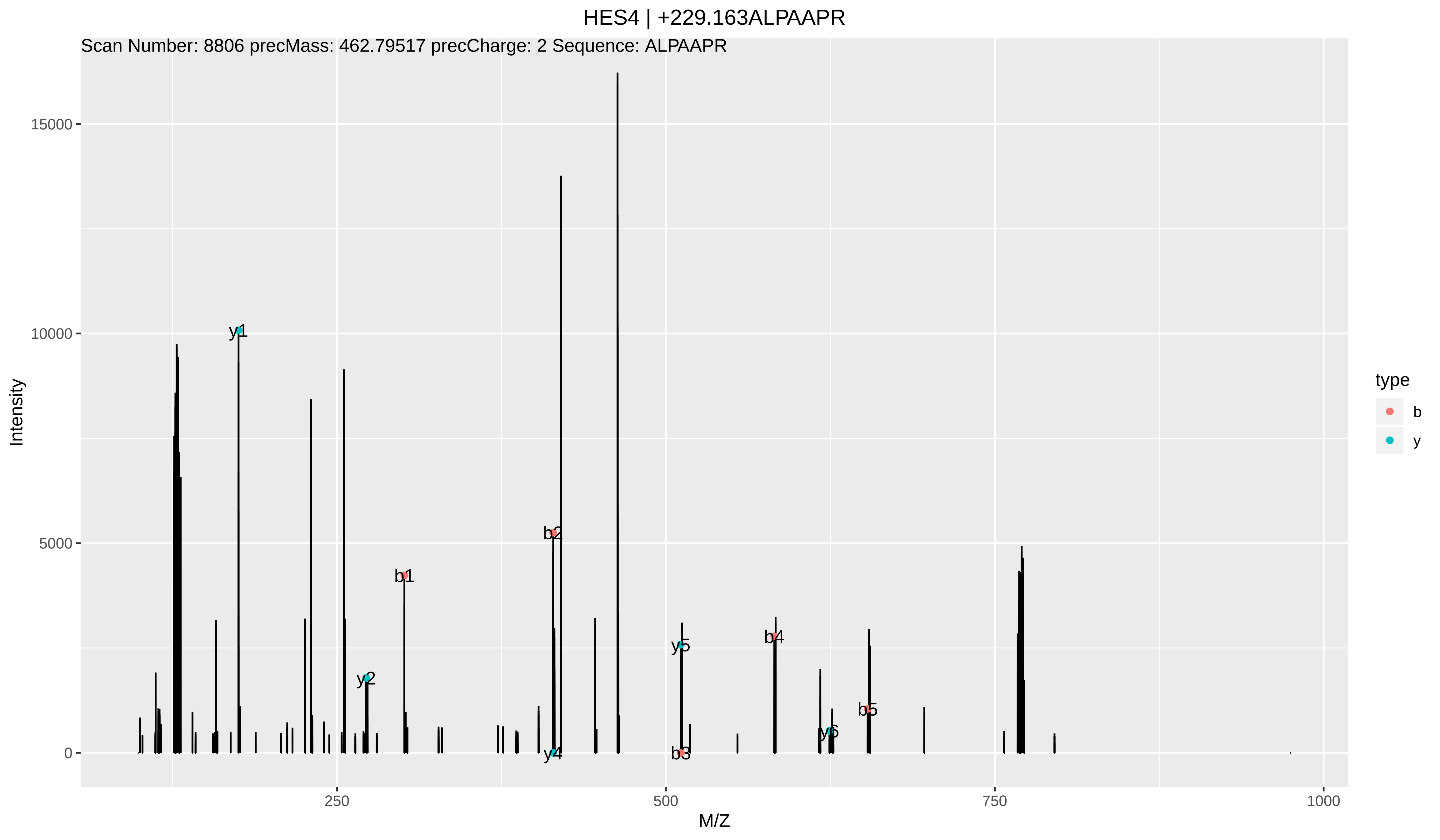
HES4 | +229.163ALPAAPR
Scan Number: 8806 precMass: 462.79517 precCharge: 2 Sequence: ALPAAPR
Intensity
15000
10000
5000
0
250
500
750
1000
M/Z
y1
y2
b1
b2
y4
y5
b3
b4
y6
b5
type
b
y

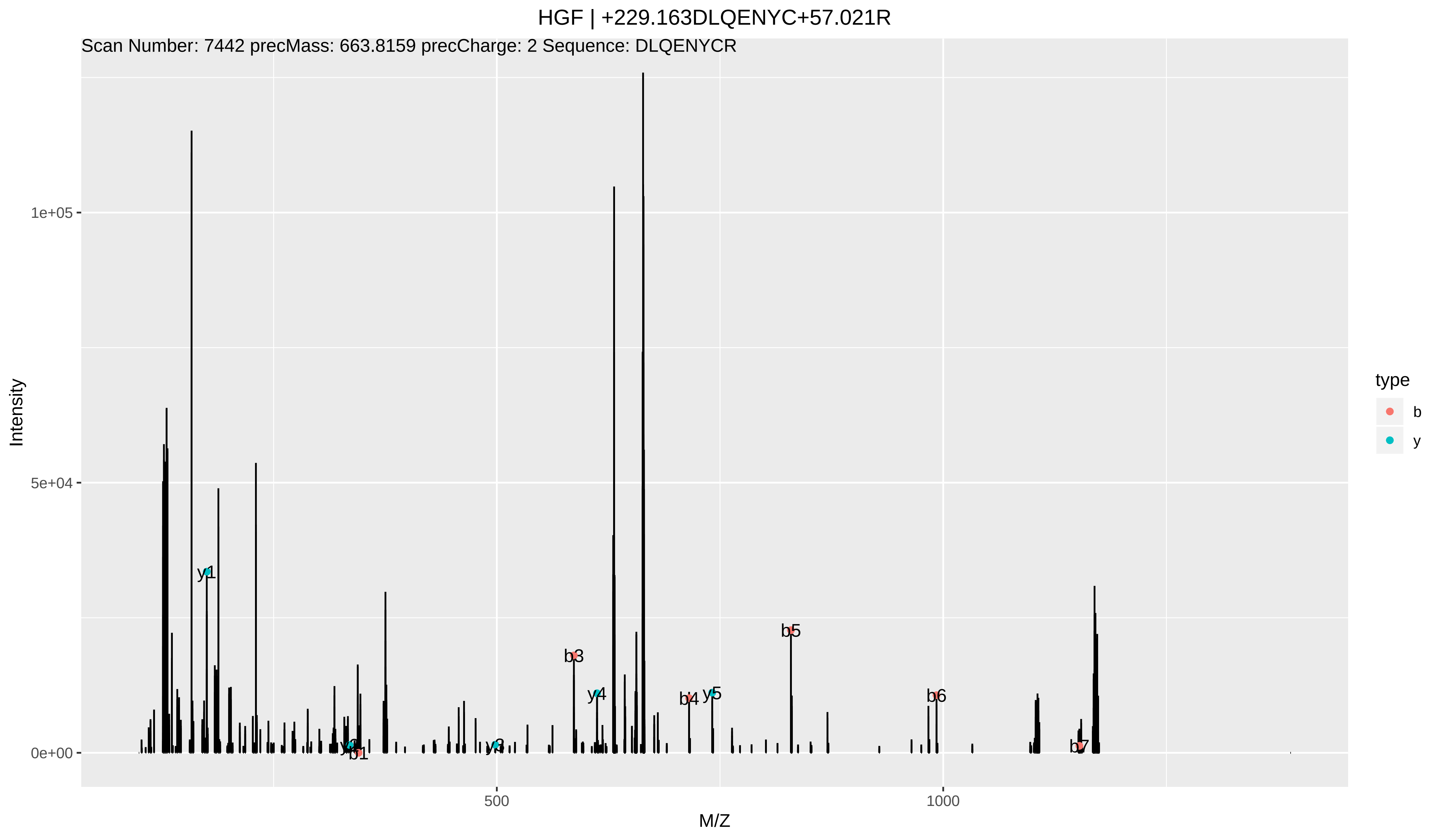
HGF | +229.163DLQENYC+57.021R
Scan Number: 7442 precMass: 663.8159 precCharge: 2 Sequence: DLQENYCR
Intensity
1e+05
5e+04
0e+00
M/Z
500
1000
y1
y2
b1
y3
b3
y4
b4
y5
b5
b6
b7
type
b
y

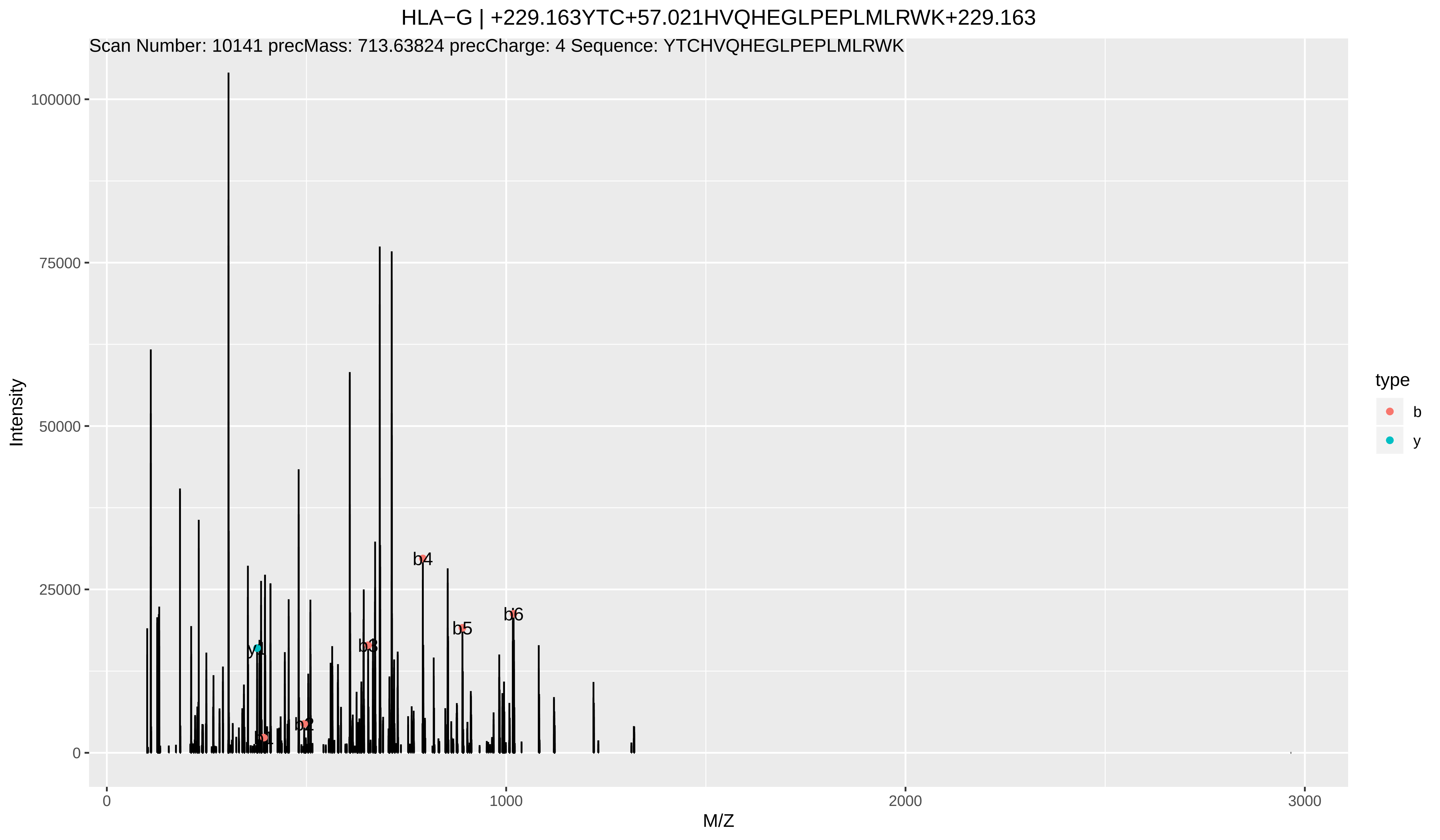

HLA−G | +229.163YTC+57.021HVQHEGLPEPLMLRWK+229.163
Scan Number: 10141 precMass: 713.63824 precCharge: 4 Sequence: YTCHVQHEGLPEPLMLRWK
Intensity
M/Z
type
b
y
b4
b5
b6

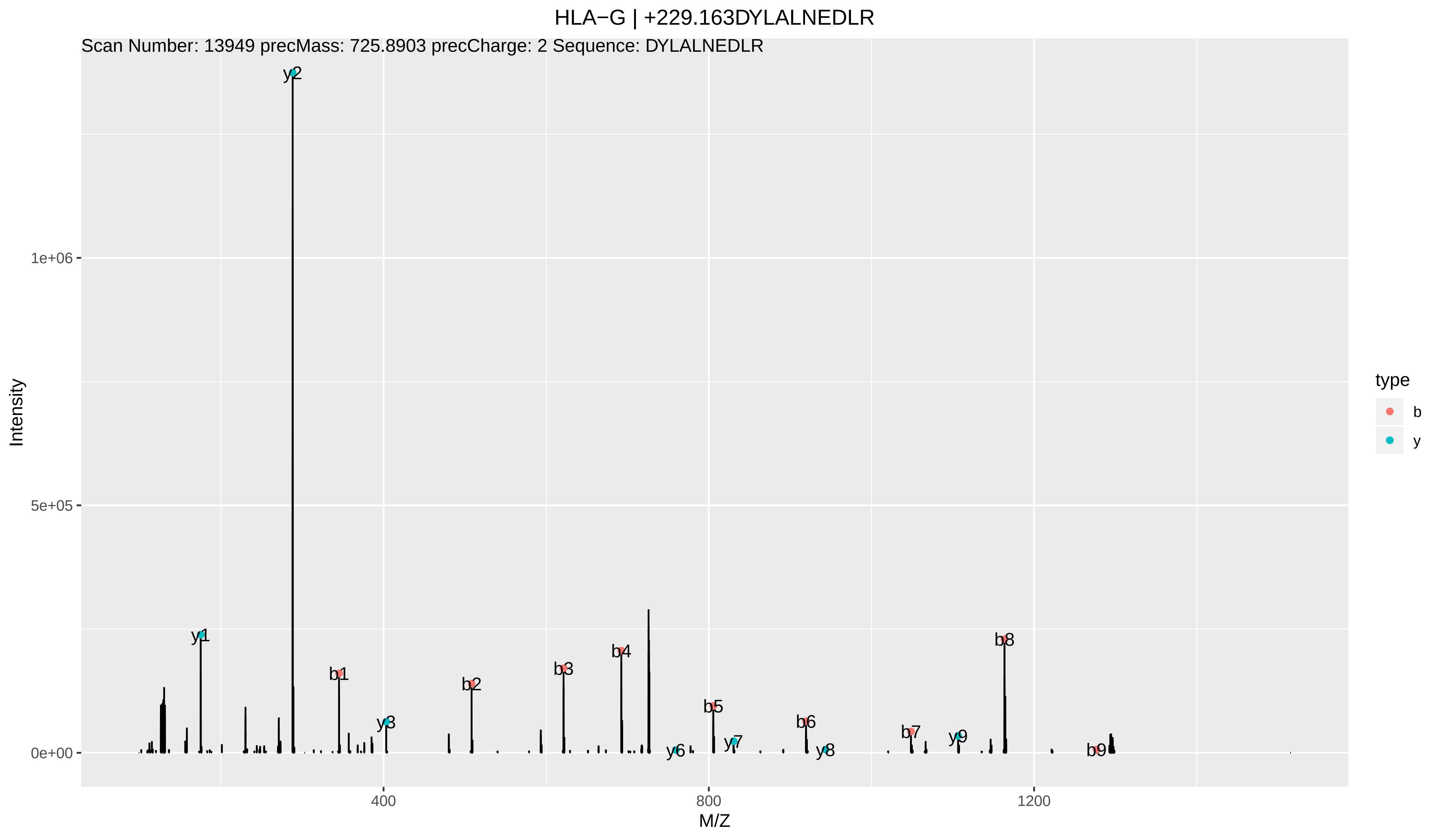
HLA−G | +229.163DYLALNEDLR
Scan Number: 13949 precMass: 725.8903 precCharge: 2 Sequence: DYLALNEDLR
Intensity
M/Z
0e+00
5e+05
1e+06
400
800
1200
y1
y2
y3
y6
y7
y8
y9
b1
b2
b3
b4
b5
b6
b7
b8
b9
type
b
y

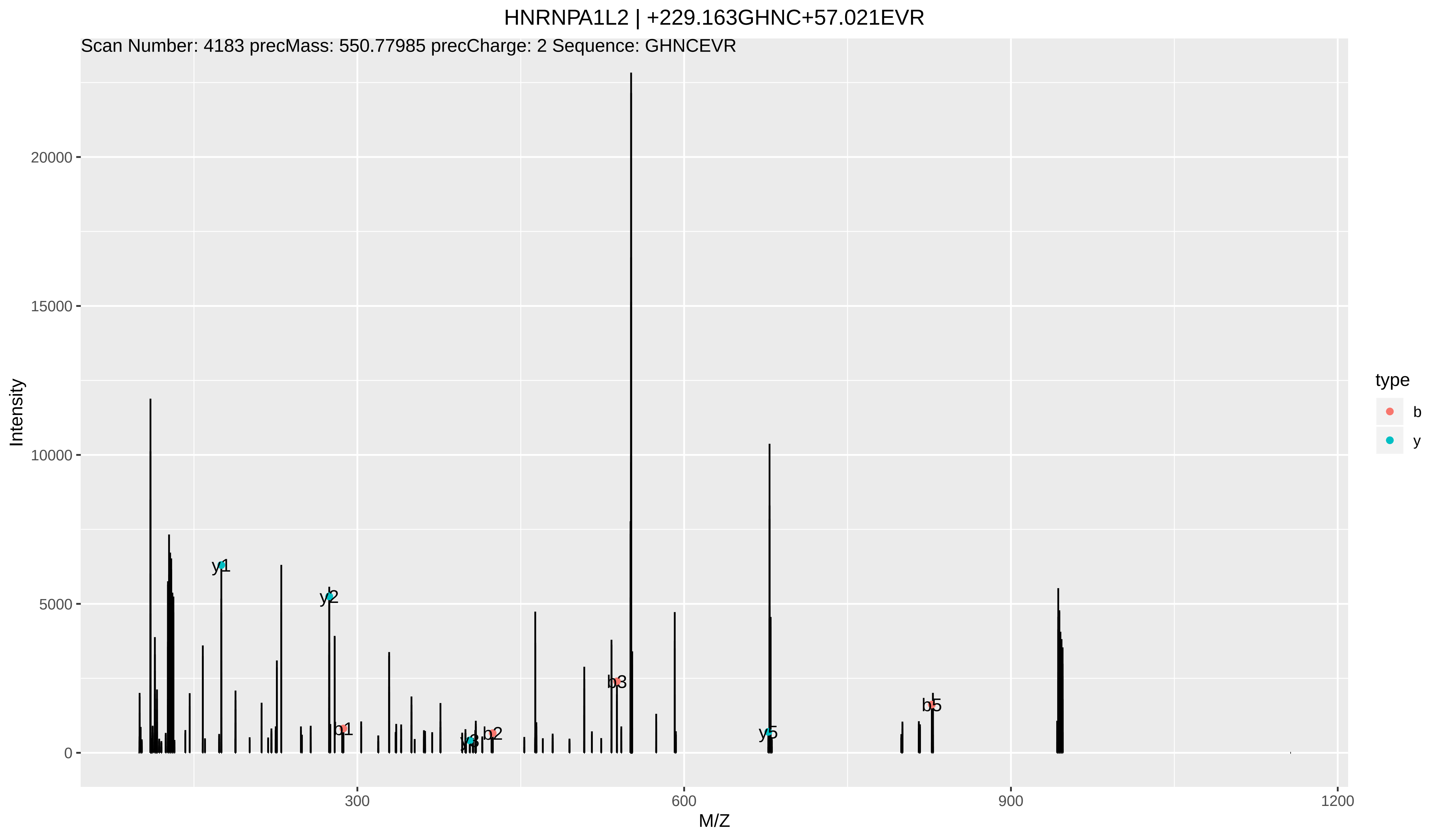

HNRNPA1L2 | +229.163GHNC+57.021EVR
Scan Number: 4183 precMass: 550.77985 precCharge: 2 Sequence: GHNCEVR
Intensity
M/Z
type
b
y
y1
y2
b1
y3
b2
b3
y5
b5
0
5000
10000
15000
20000
300
600
900
1200

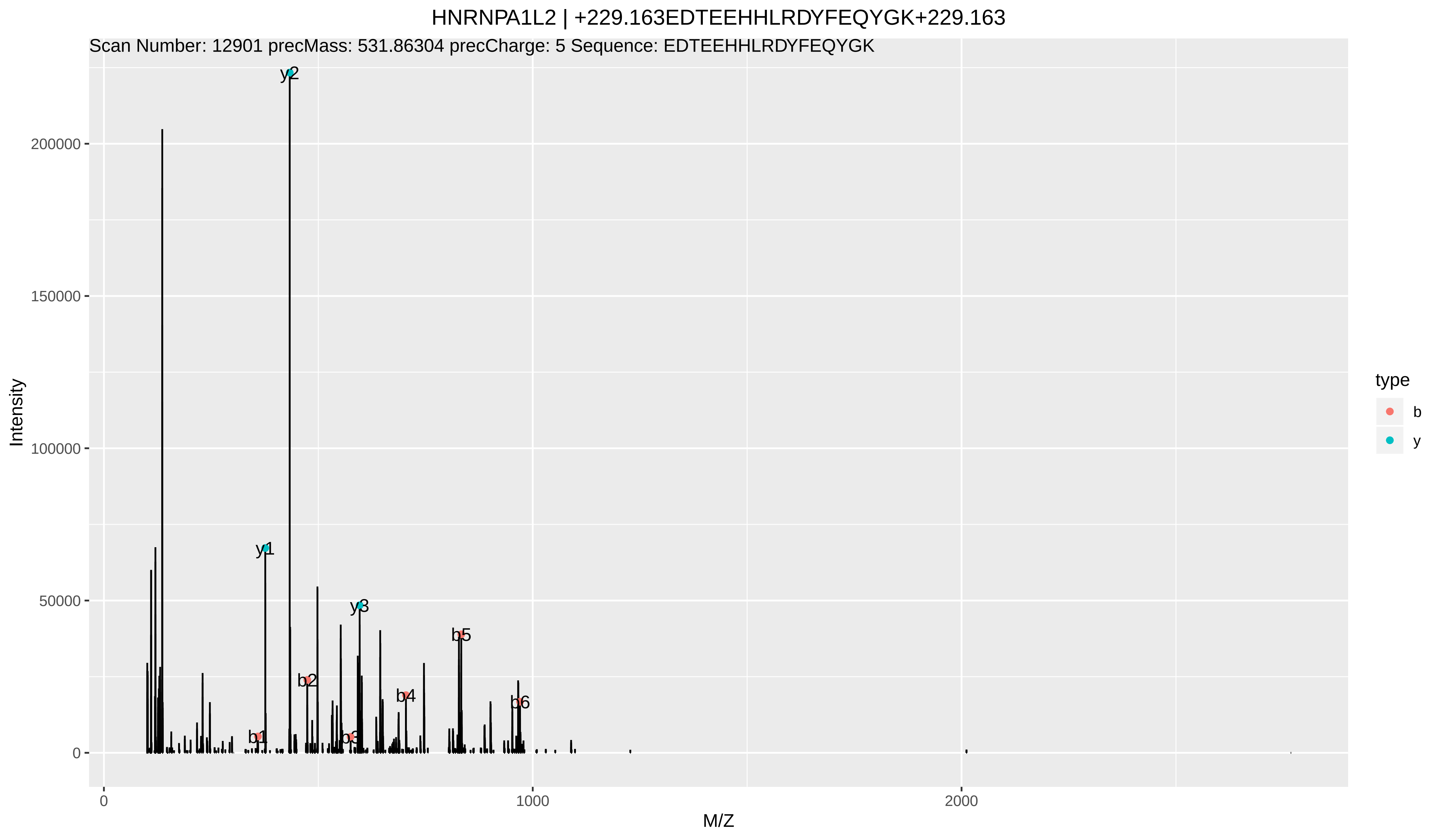

HNRNPA1L2 | +229.163EDTEEHHLRDYFEQYGK+229.163
Scan Number: 12901 precMass: 531.86304 precCharge: 5 Sequence: EDTEEHHLRDYFEQYGK
y2
y1
y3
b1
b2
b3
b4
b5
b6
200000
150000
100000
50000
0
0
1000
2000
Intensity
M/Z
type
b
y

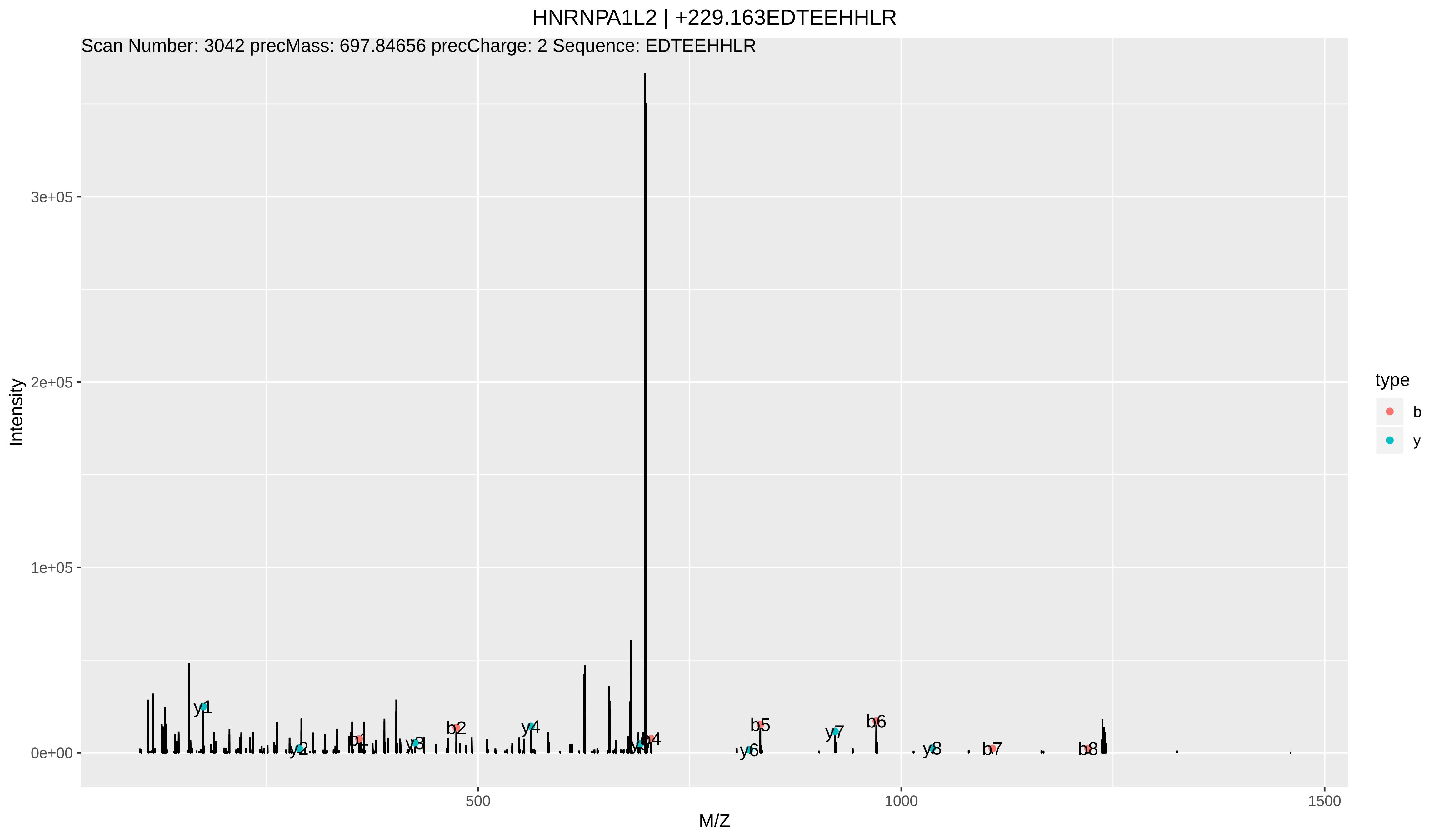

HNRNPA1L2 | +229.163EDTEEHHLR
Scan Number: 3042 precMass: 697.84656 precCharge: 2 Sequence: EDTEEHHLR
Intensity
3e+05
2e+05
1e+05
0e+00
M/Z
500
1000
1500
y1
y2
b1
y3
b2
y4
b4
b5
y6
y7
b6
y8
b7
b8
type
b
y

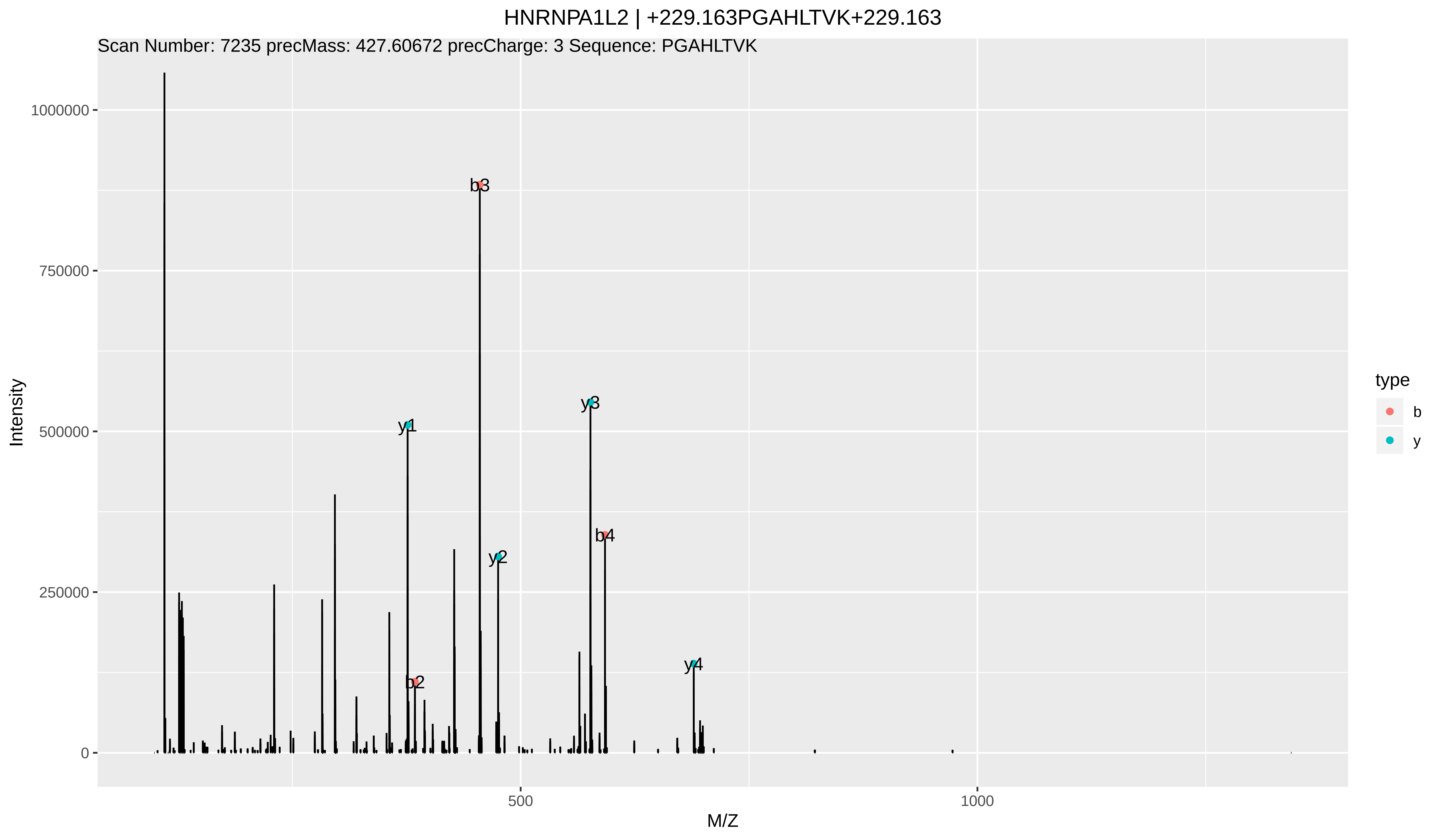

HNRNPA1L2 | +229.163PGAHLTVK+229.163
Scan Number: 7235 precMass: 427.60672 precCharge: 3 Sequence: PGAHLTVK
Intensity
1000000
750000
500000
250000
0
500
1000
M/Z
b3
y1
b2
y3
b4
y2
y4
type
b
y

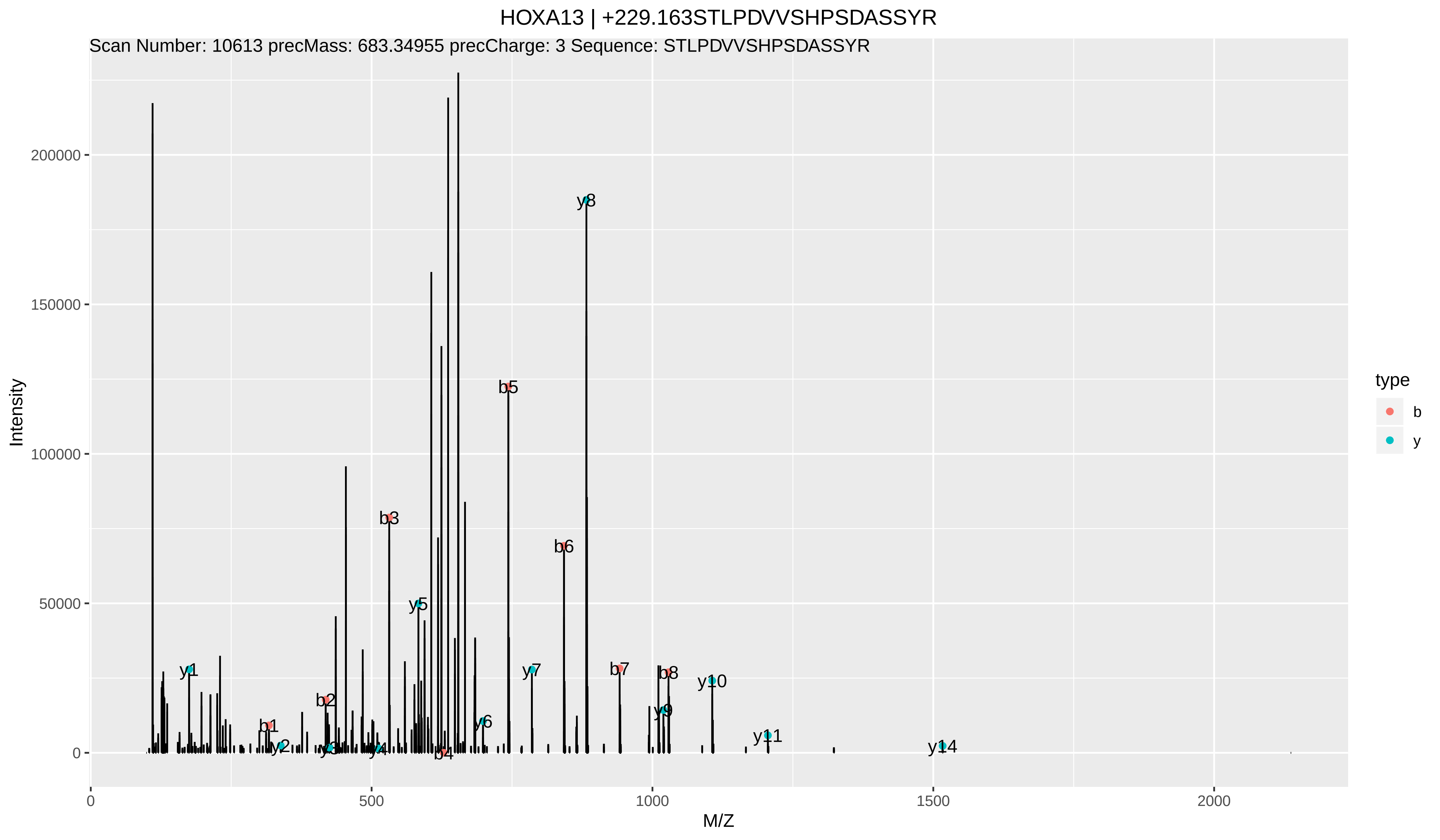

HOXA13 | +229.163STLPDVVSHPSDASSYR
Scan Number: 10613 precMass: 683.34955 precCharge: 3 Sequence: STLPDVVSHPSDASSYR
Intensity
M/Z
0
50000
100000
150000
200000
0
500
1000
1500
2000
y1
b1
y2
b2
y3
y4
b3
y5
b4
y6
b5
y7
b6
y8
b7
y9
b8
y10
y11
y14
type
b
y

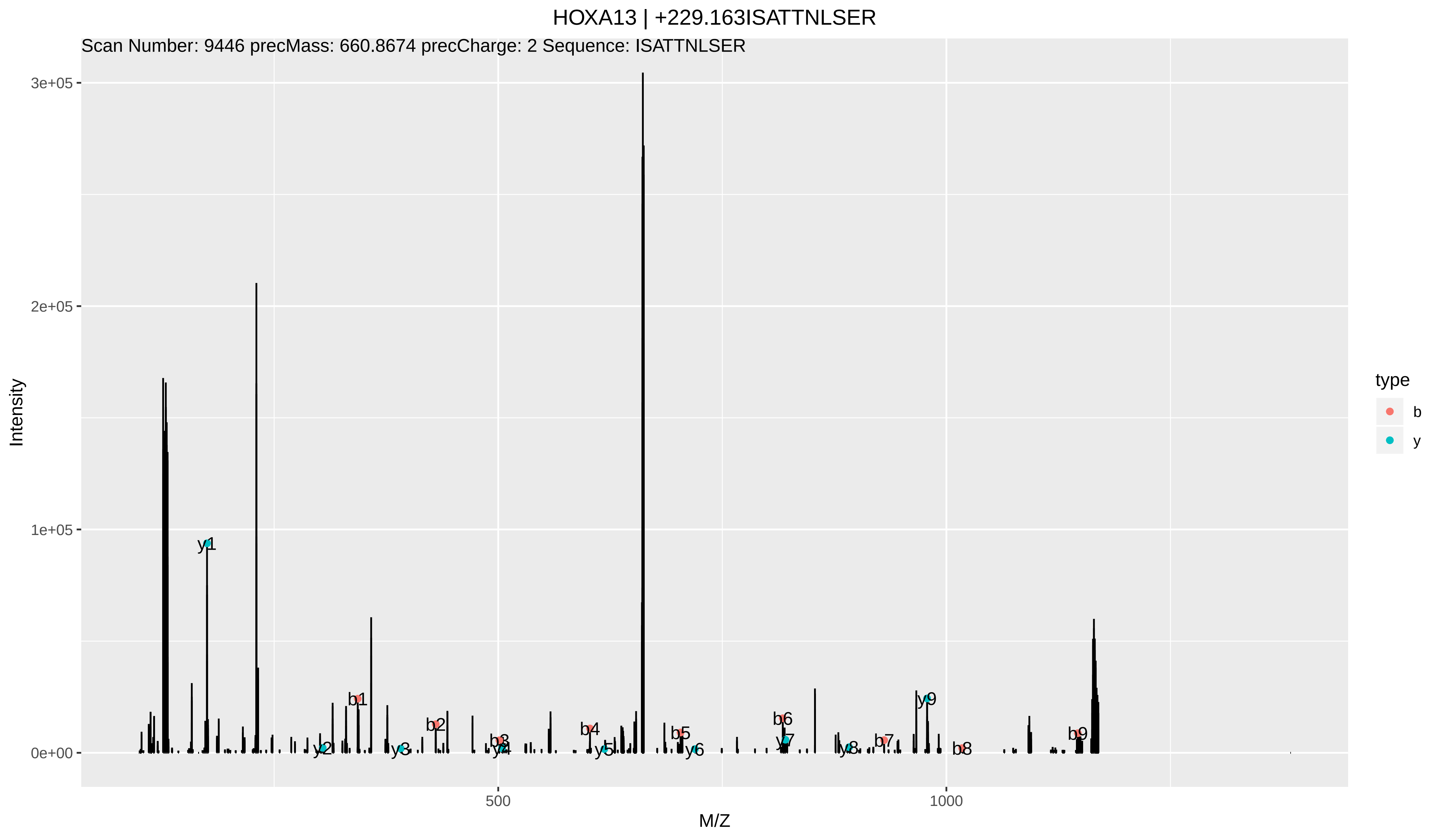

HOXA13 | +229.163ISATTNLSER
Scan Number: 9446 precMass: 660.8674 precCharge: 2 Sequence: ISATTNLSER
Intensity
3e+05
2e+05
1e+05
0e+00
M/Z
500
1000
y1
y2
y3
y4
y5
y6
y7
y8
y9
b1
b2
b3
b4
b5
b6
b7
b8
b9
type
b
y

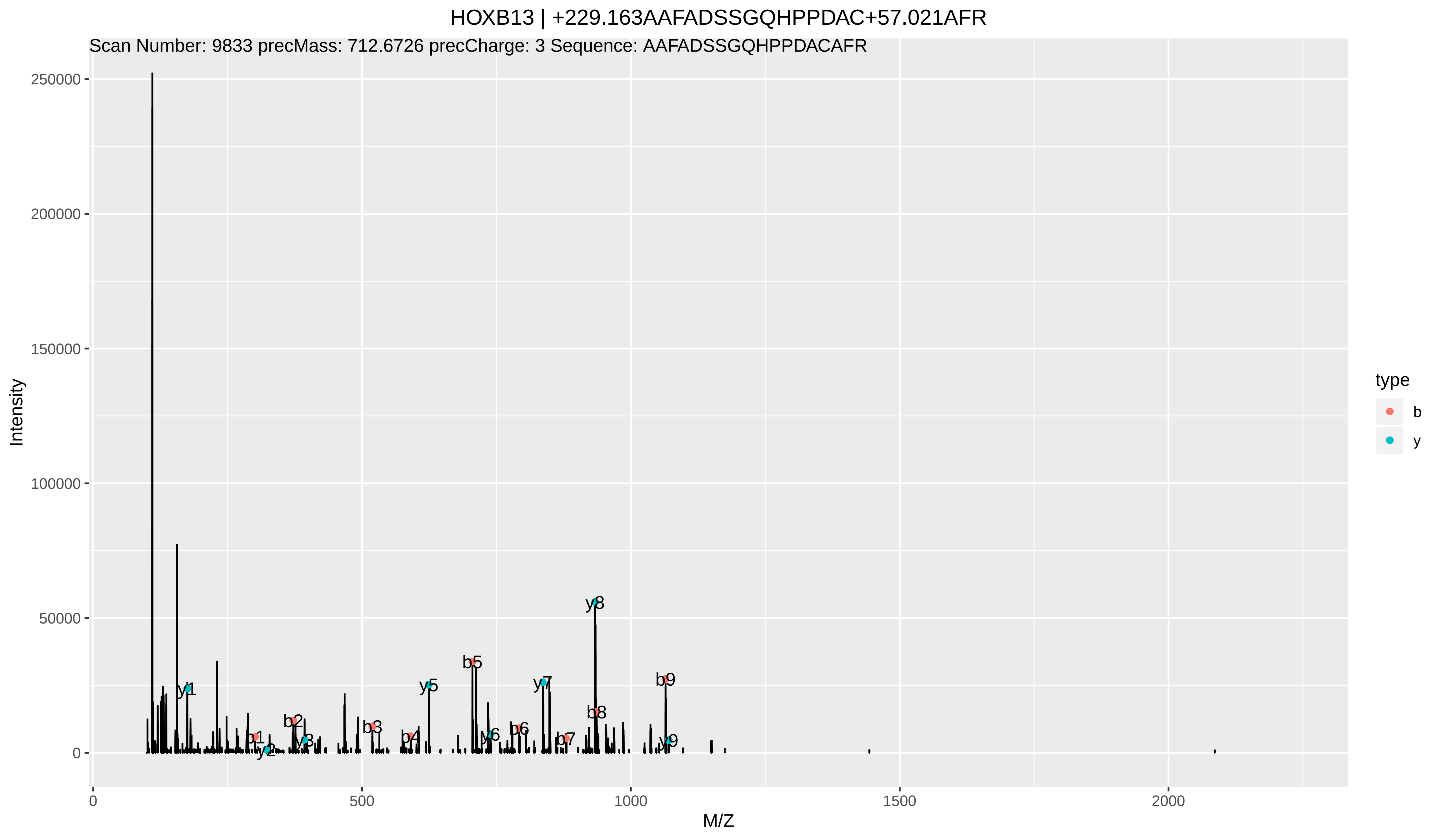

HOXB13 | +229.163AAFADSSGQHPPDAC+57.021AFR
Scan Number: 9833 precMass: 712.6726 precCharge: 3 Sequence: AAFADSSGQHPPDACAFR
Intensity
M/Z
0
50000
100000
150000
200000
250000
0
500
1000
1500
2000
y1
y2
y3
y5
y6
y7
y8
y9
b1
b2
b3
b4
b5
b6
b7
b8
b9
type
b
y

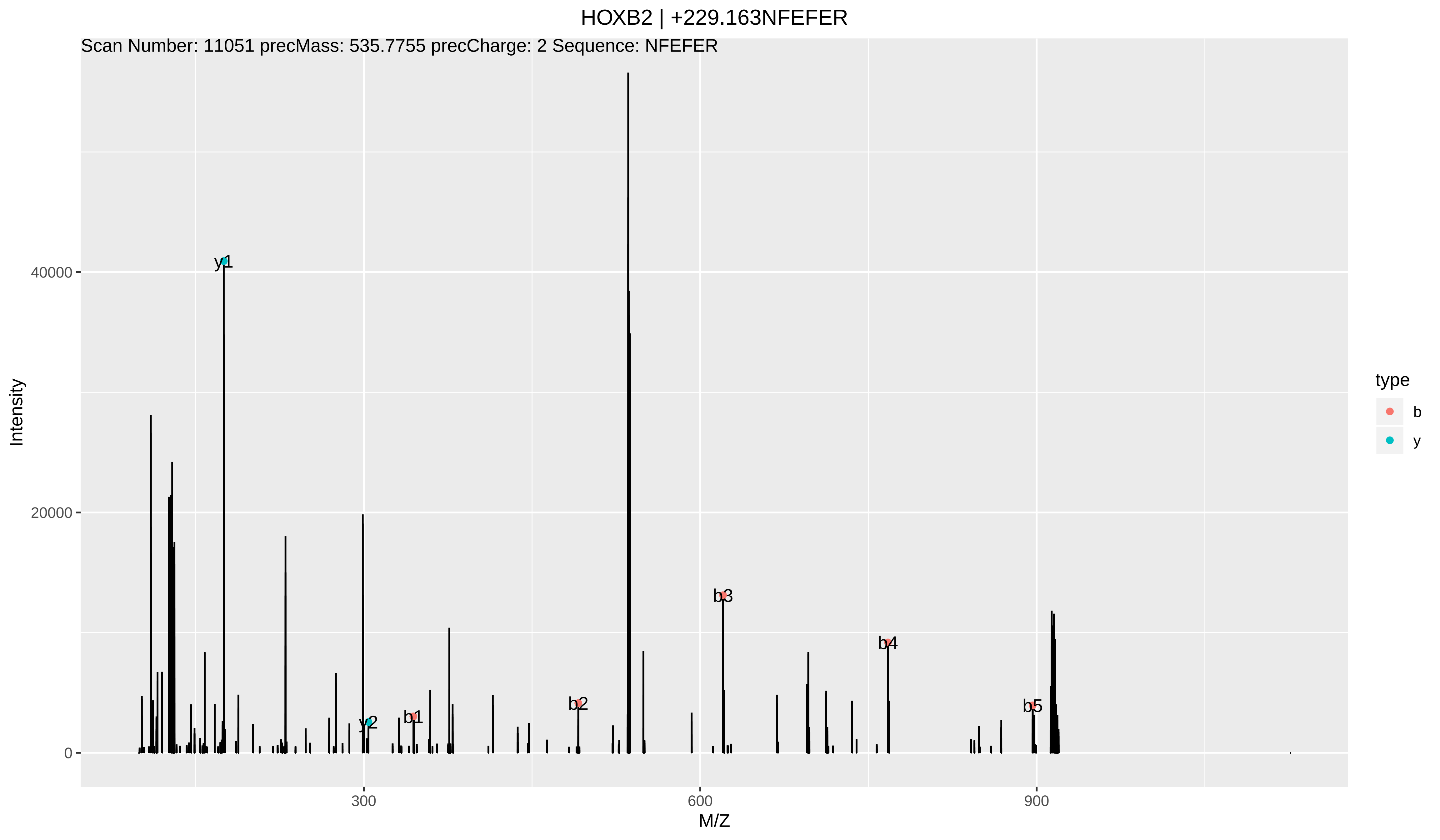
HOXB2 | +229.163NFEFER
Scan Number: 11051 precMass: 535.7755 precCharge: 2 Sequence: NFEFER
Intensity
M/Z
0
20000
40000
300
600
900
y1
y2
b1
b2
b3
b4
b5
type
b
y

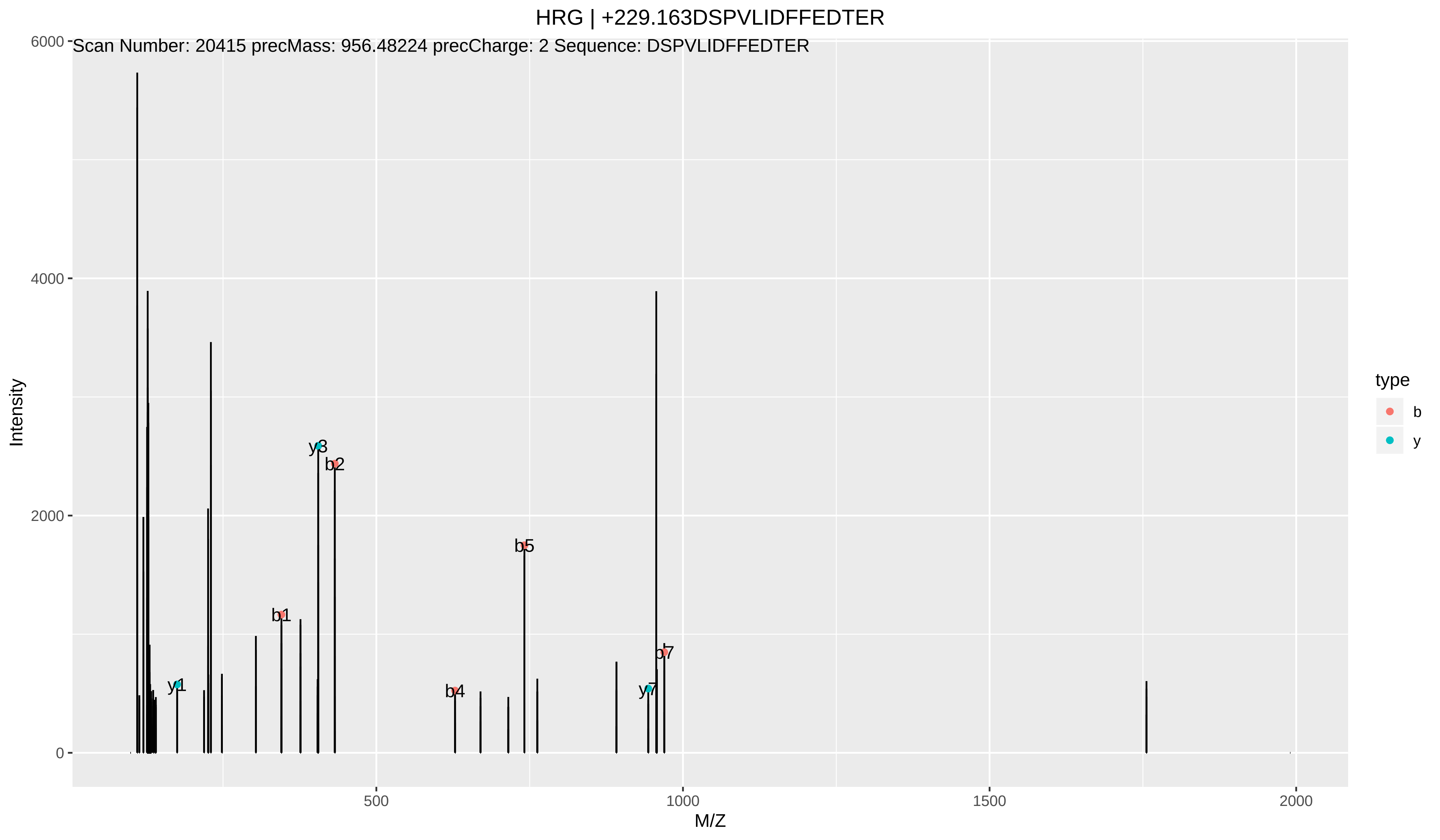

HRG | +229.163DSPVLIDFFEDTER
Scan Number: 20415 precMass: 956.48224 precCharge: 2 Sequence: DSPVLIDFFEDTER
Intensity
M/Z
6000
4000
2000
0
500
1000
1500
2000
y1
b1
y3
b2
b4
b5
y7
b7
type
b
y

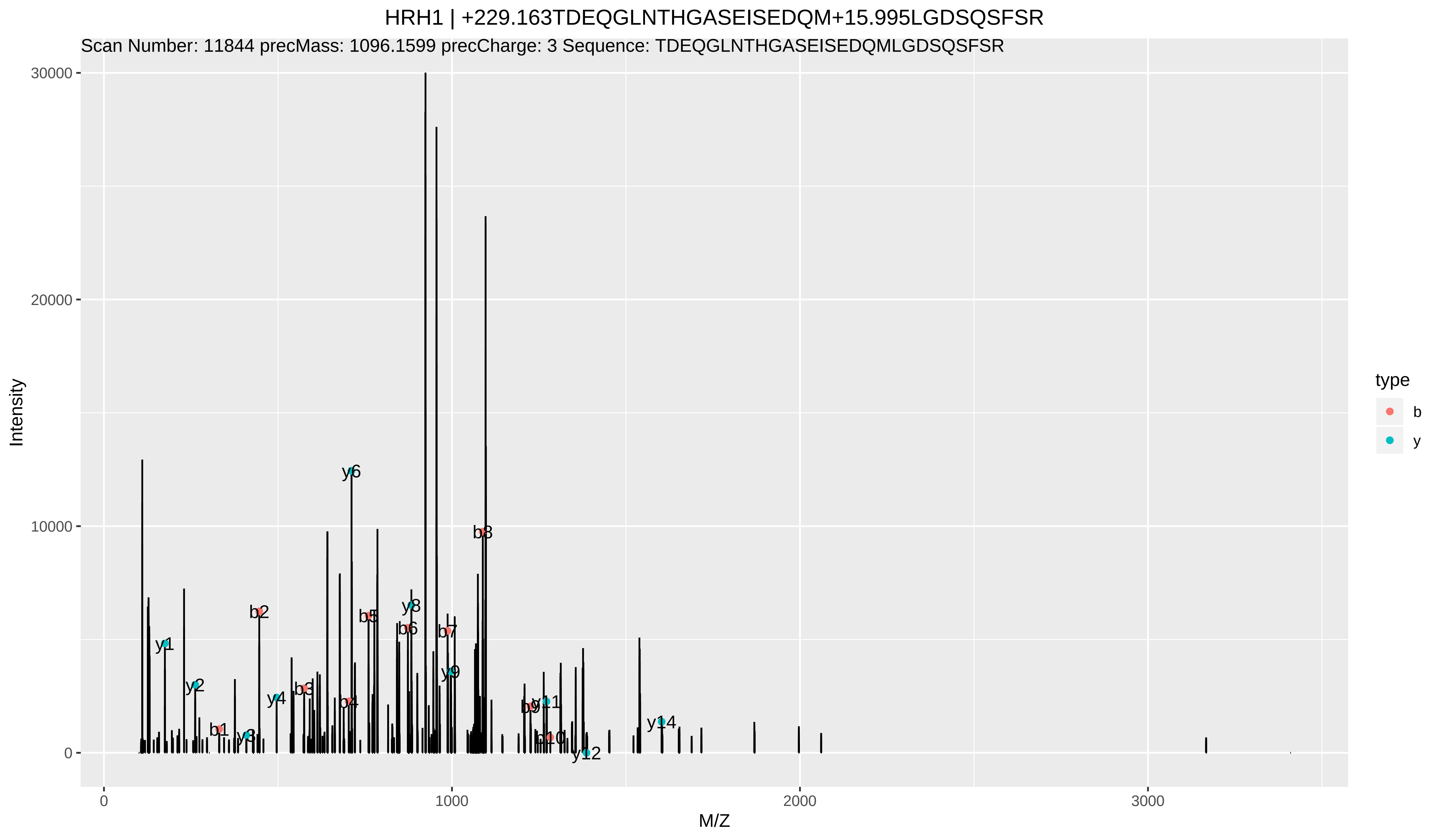

HRH1 | +229.163TDEQGLNTHGASEISEDQM+15.995LGDSQSFSR
Scan Number: 11844 precMass: 1096.1599 precCharge: 3 Sequence: TDEQGLNTHGASEISEDQMLGDSQSFSR
Intensity
M/Z
type
b
y

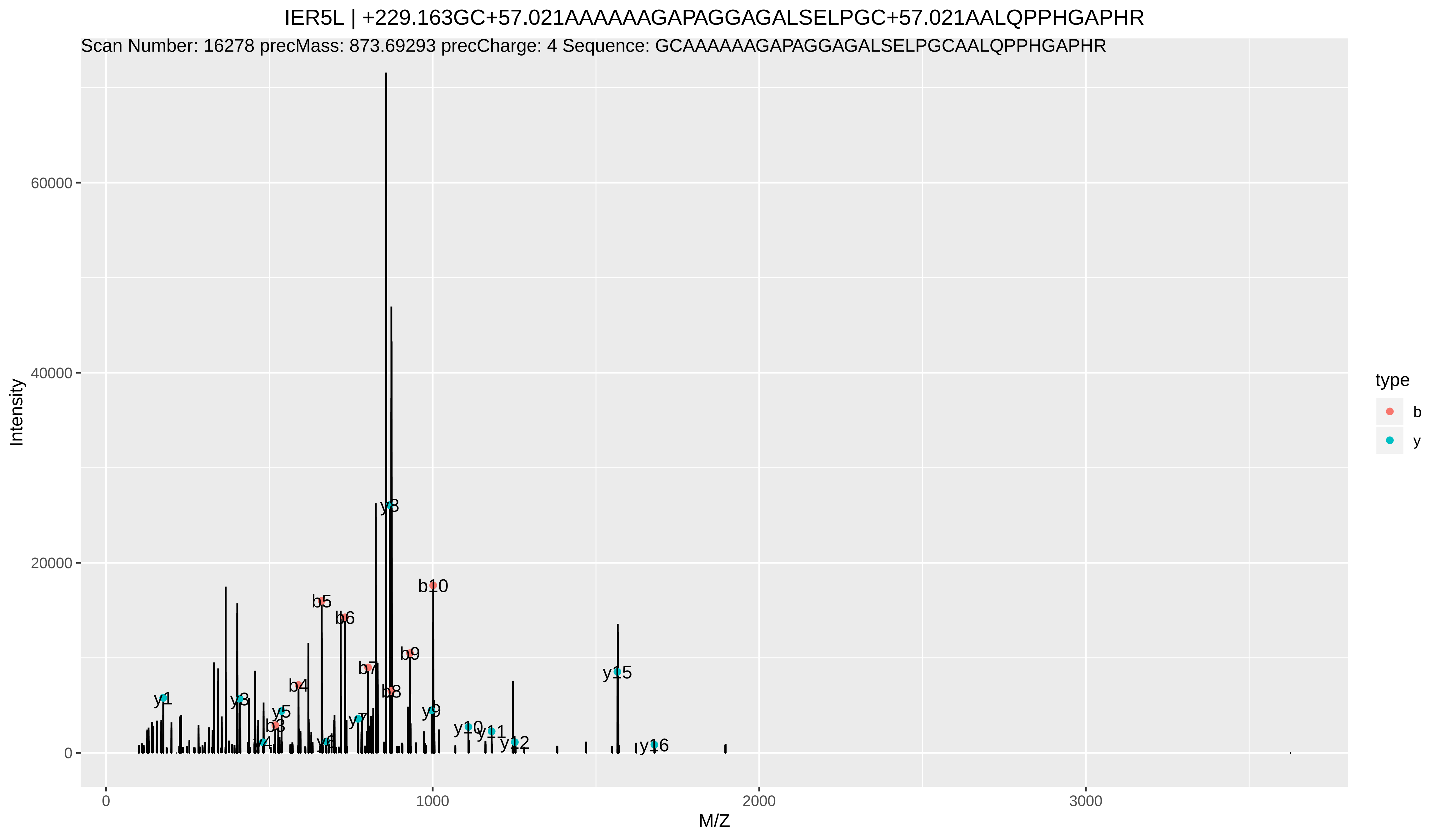

IER5L | +229.163GC+57.021AAAAAAGAPAGGAGALSELPGC+57.021AALQPPHGAPHR
Scan Number: 16278 precMass: 873.69293 precCharge: 4 Sequence: GCAAAAAAGAPAGGAGALSELPGCAALQPPHGAPHR
Intensity
60000
40000
20000
0
M/Z
0
1000
2000
3000
type
b
y
y1
y3
b3
y4
y5
b4
b5
y6
b6
y7
b7
y8
b8
b9
y9
b10
y10
y11
y12
y15
y16

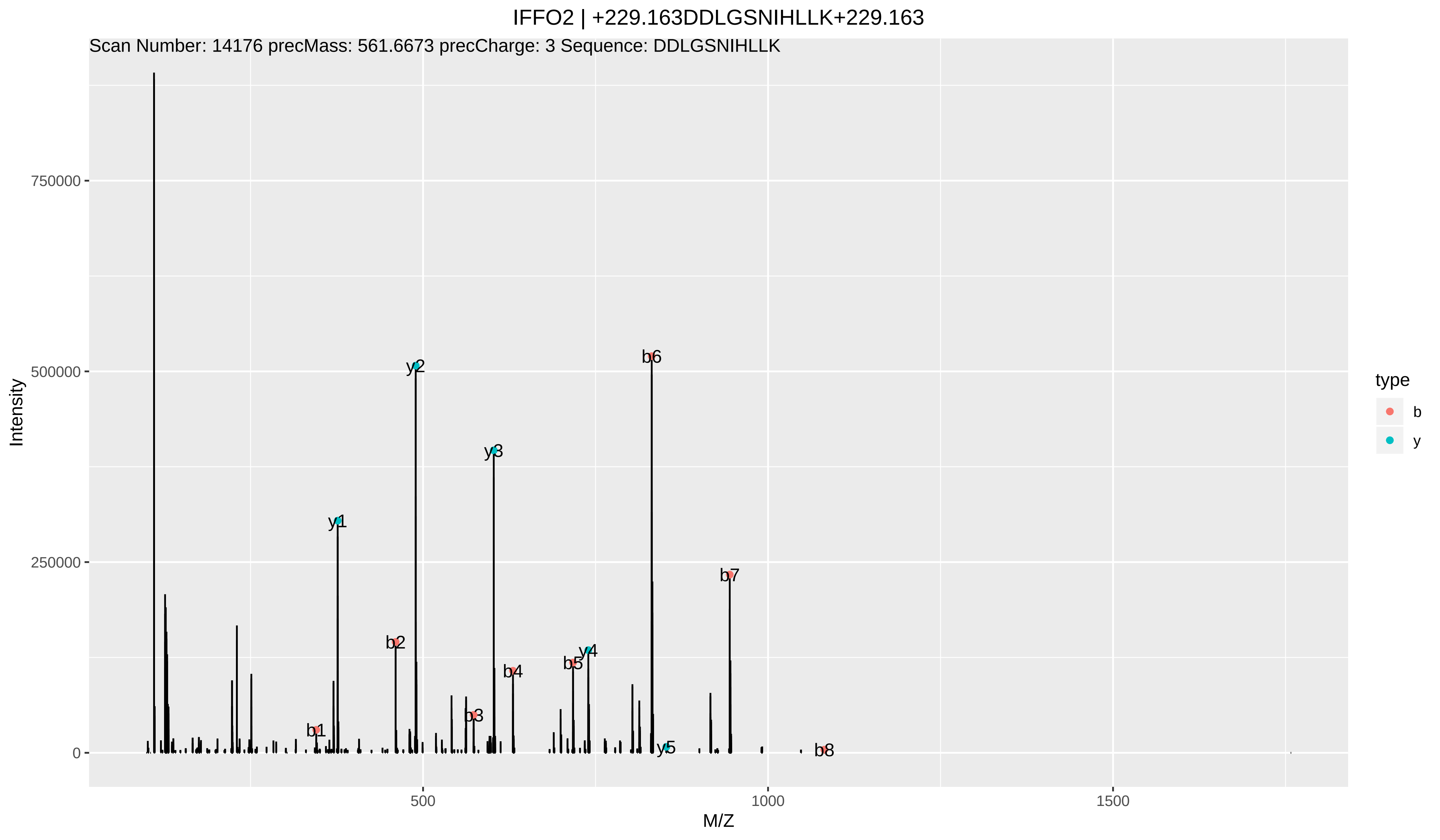

IFFO2 | +229.163DDLGSNIHLLK+229.163
Scan Number: 14176 precMass: 561.6673 precCharge: 3 Sequence: DDLGSNIHLLK
Intensity
750000
500000
250000
0
M/Z
500
1000
1500
y1
y2
y3
y4
y5
b1
b2
b3
b4
b5
b6
b7
b8
type
b
y

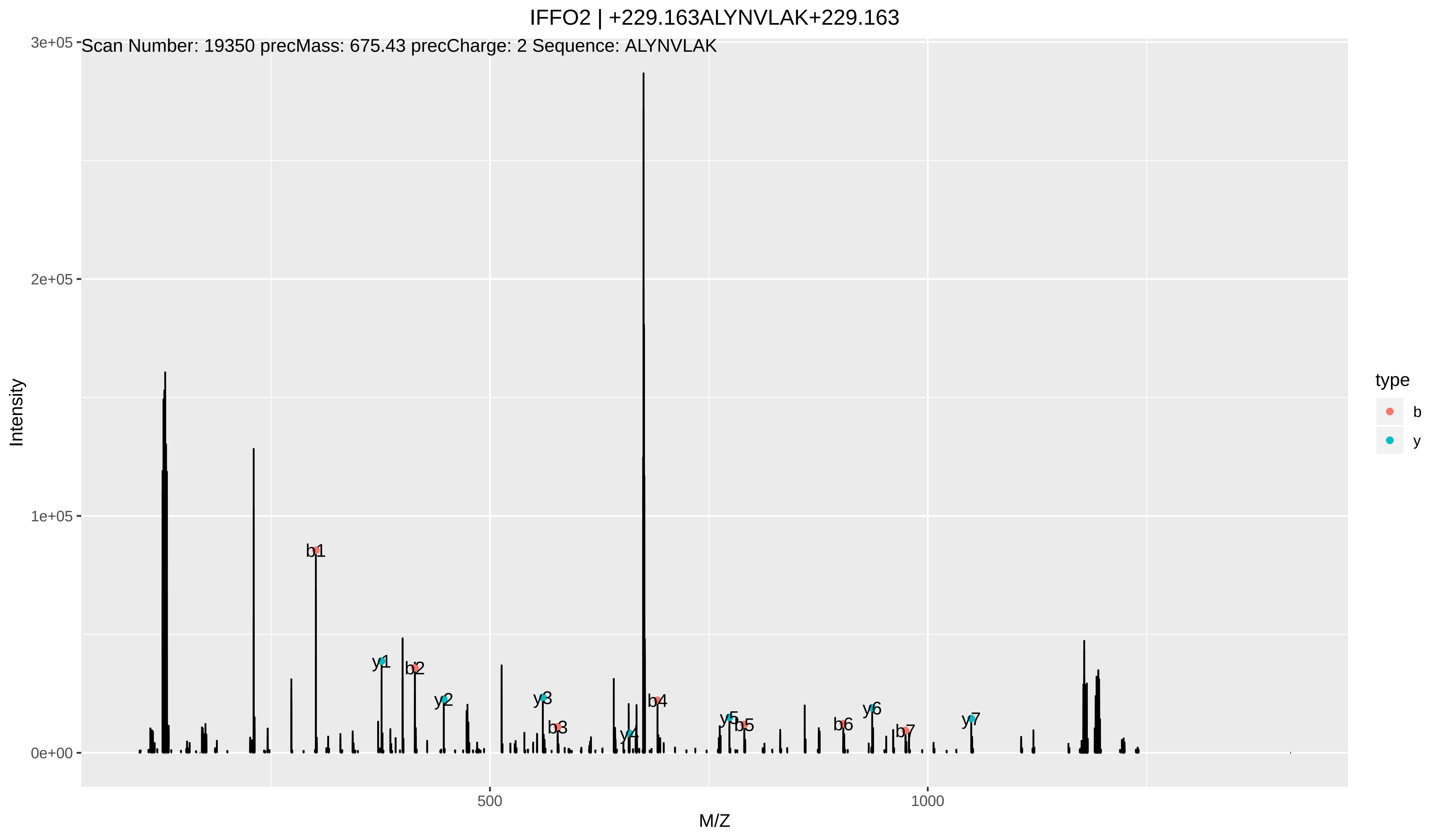
IFFO2 | +229.163ALYNVLAK+229.163
Scan Number: 19350 precMass: 675.43 precCharge: 2 Sequence: ALYNVLAK
Intensity
3e+05
2e+05
1e+05
0e+00
M/Z
500
1000
b1
y1
b2
y2
y3
b3
y4
b4
y5
b5
b6
y6
b7
y7
type
b
y

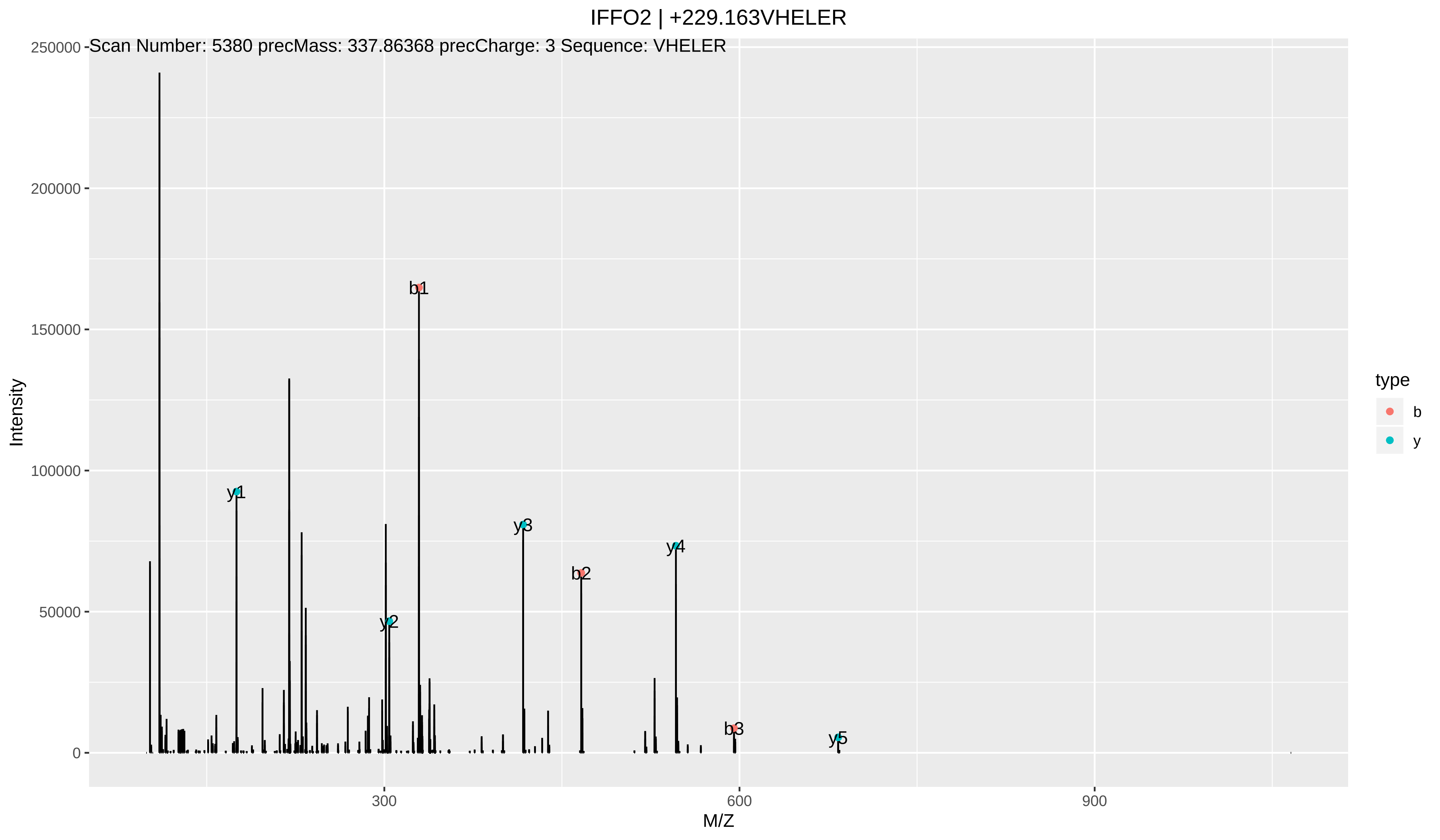
IFFO2 | +229.163VHELER
Scan Number: 5380 precMass: 337.86368 precCharge: 3 Sequence: VHELER
Intensity
M/Z
0
50000
100000
150000
200000
250000
300
600
900
b1
b2
b3
y1
y2
y3
y4
y5
type
b
y

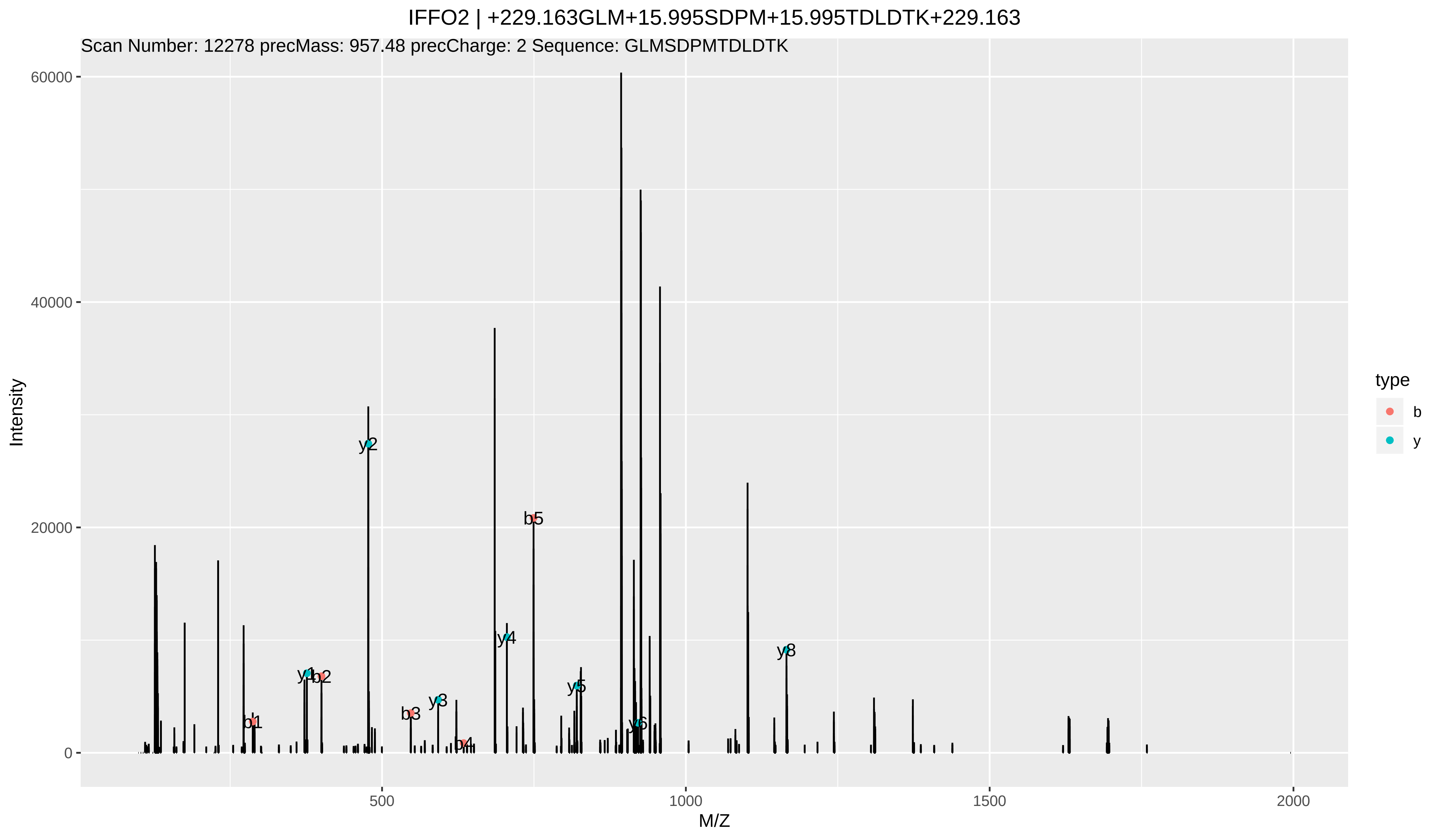

IFFO2 | +229.163GLM+15.995SDPM+15.995TDLDTK+229.163
Scan Number: 12278 precMass: 957.48 precCharge: 2 Sequence: GLMSDPMTDLDTK
Intensity
M/Z
60000
40000
20000
0
500
1000
1500
2000
type
b
y
b1
y1
b2
y2
b3
y3
b4
y4
b5
y5
y6
y8

IFFO2 | +229.163GLDVQM+15.995ETC+57.021R

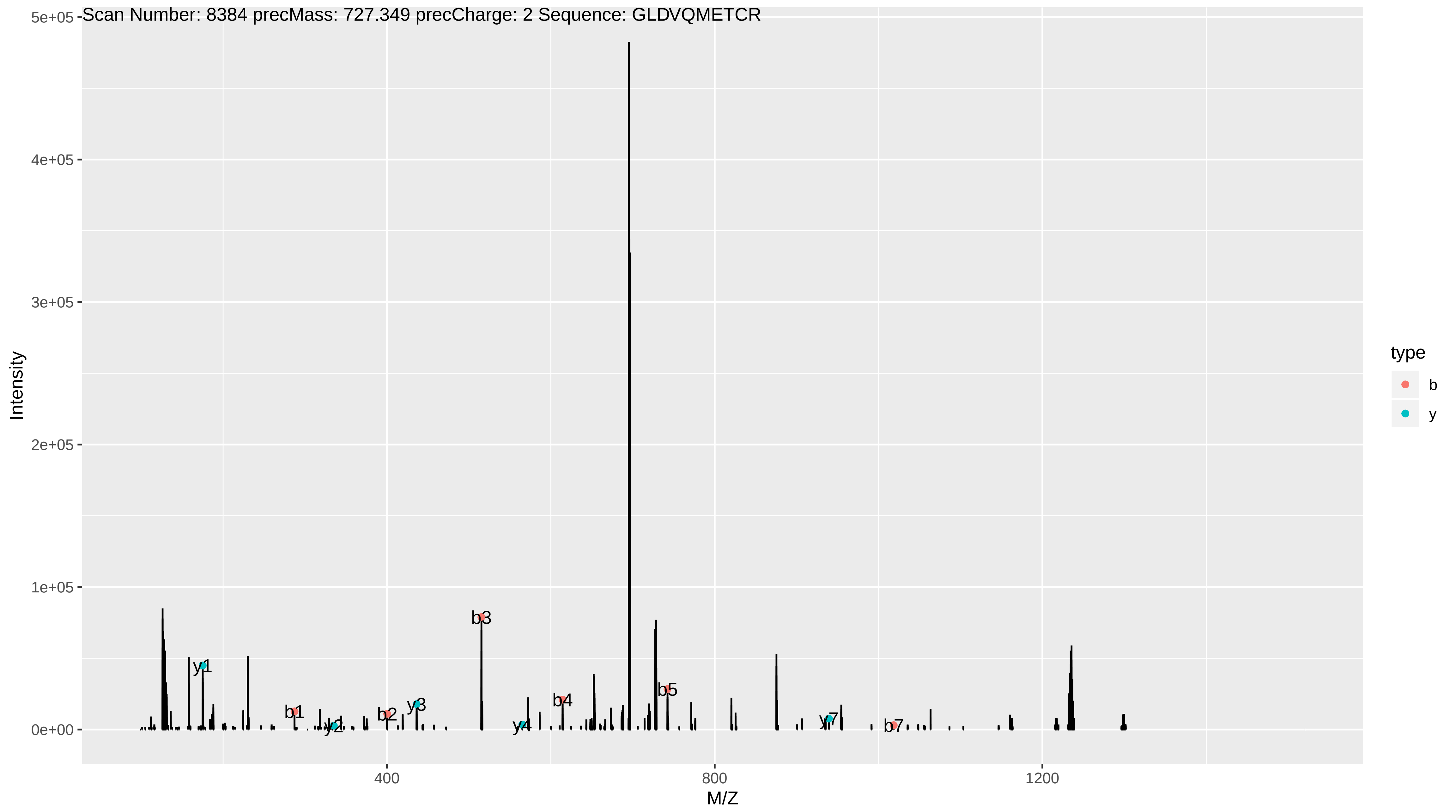

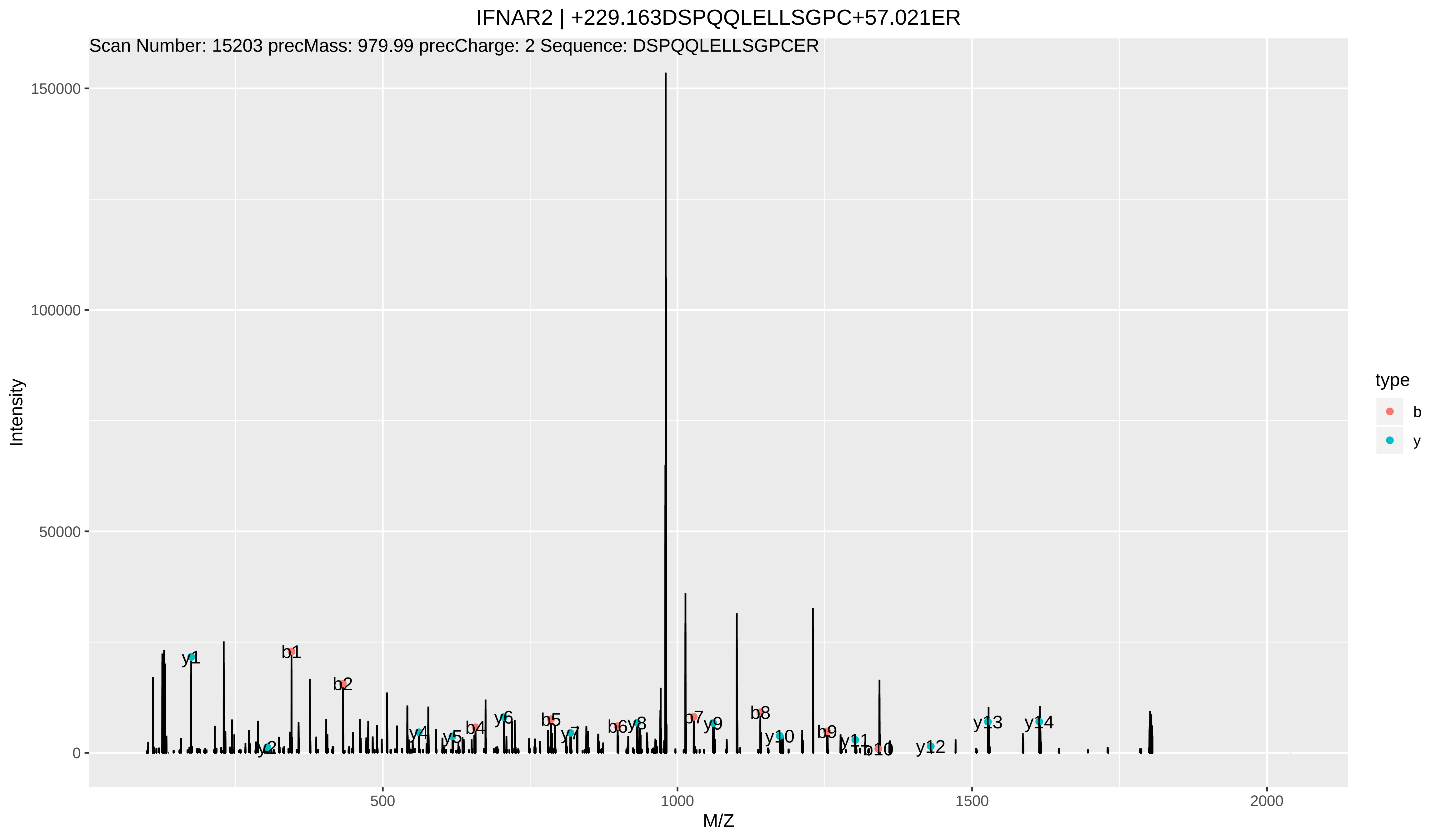

IFNAR2 | +229.163DSPQQLELLSGPC+57.021ER
Scan Number: 15203 precMass: 979.99 precCharge: 2 Sequence: DSPQQLELLSGPCER
Intensity
M/Z
type
b
y

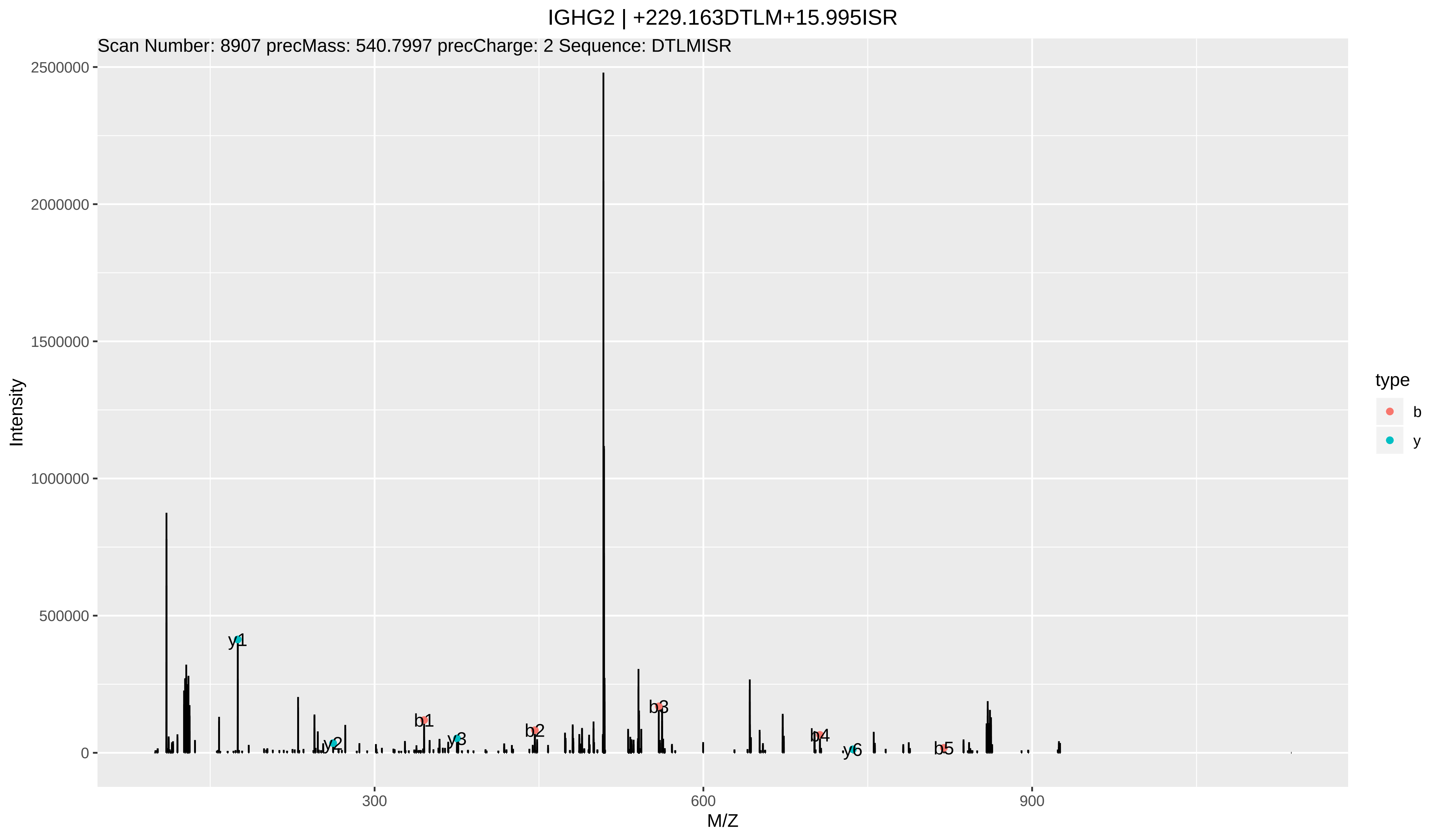
IGHG2 | +229.163DTLM+15.995ISR
Scan Number: 8907 precMass: 540.7997 precCharge: 2 Sequence: DTLMISR
Intensity
2500000
2000000
1500000
1000000
500000
0
M/Z
300
600
900
y1
y2
b1
y3
b2
b3
b4
y6
b5
type
b
y

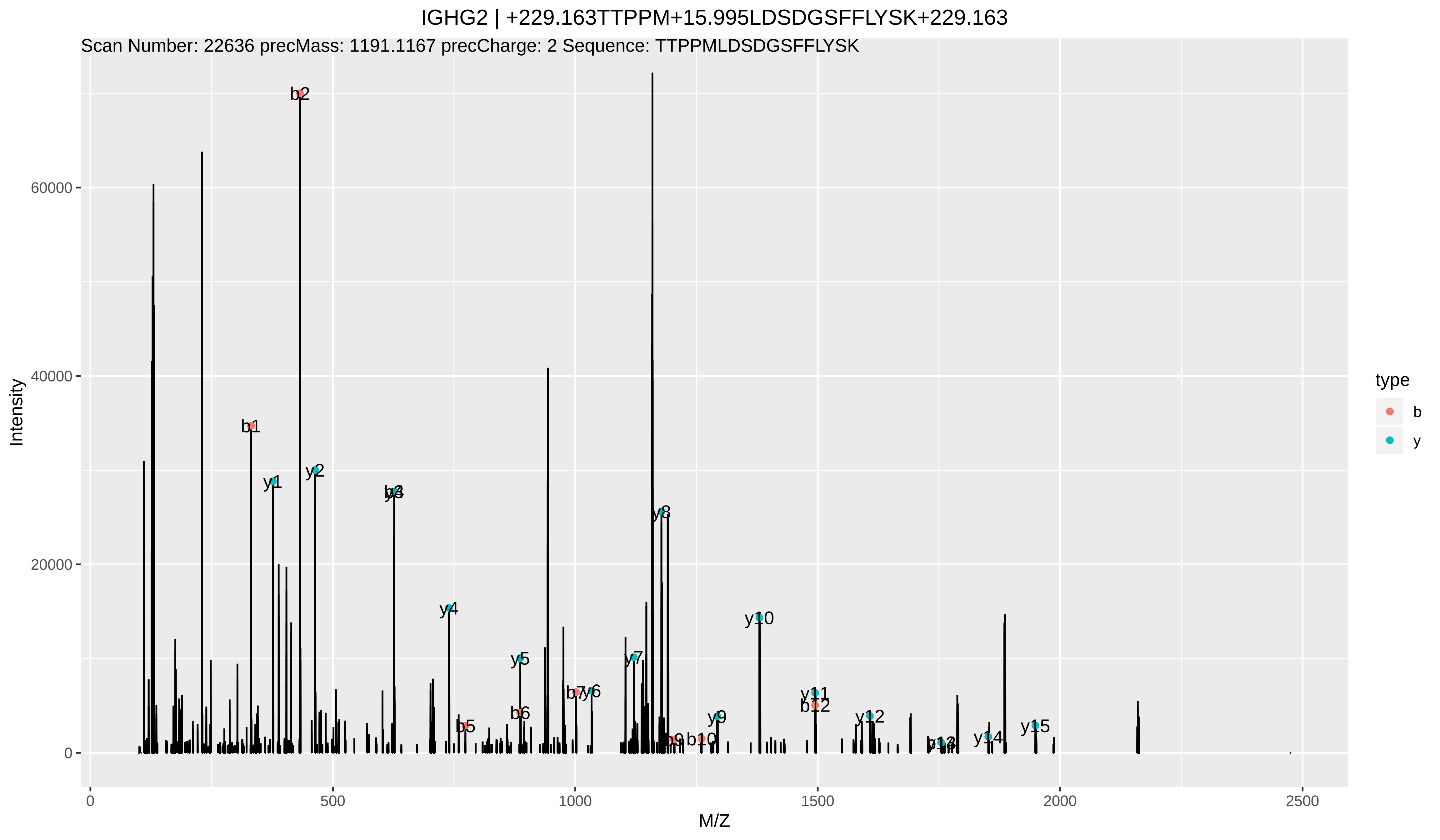
IGHG2 | +229.163TTPPM+15.995LDSDGSFFLYSK+229.163
Scan Number: 22636 precMass: 1191.1167 precCharge: 2 Sequence: TTPPMLDSDGSFFLYSK
Intensity
M/Z
0
20000
40000
60000
500
1000
1500
2000
2500
type
b
y
b1
b2
b5
b6
b10
b12
y1
y2
y4
y5
y6
y7
y8
y9
y10
y11
y12
y14
y15

IGHM | +229.163YAATSQVLLPSK+229.163

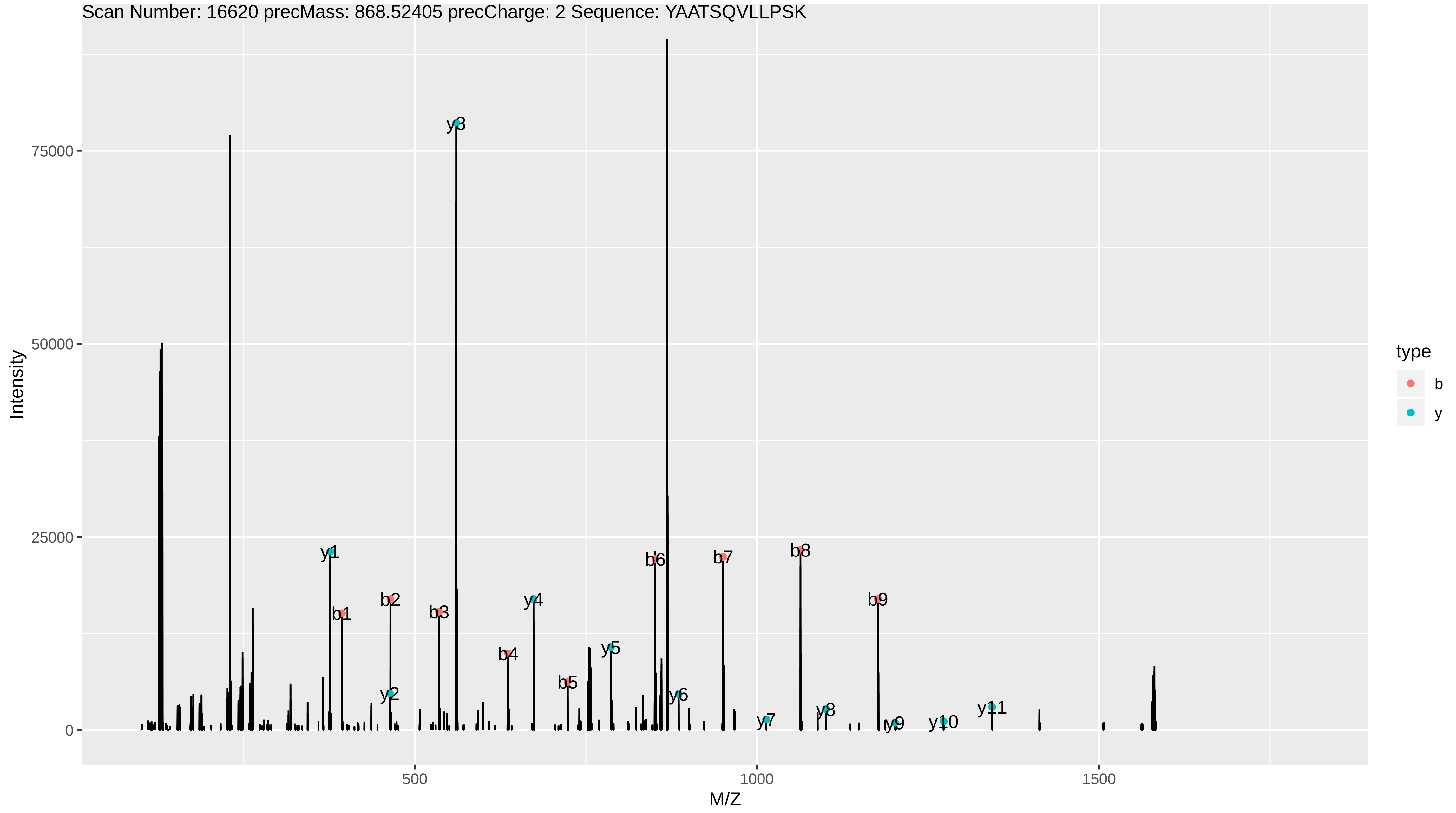

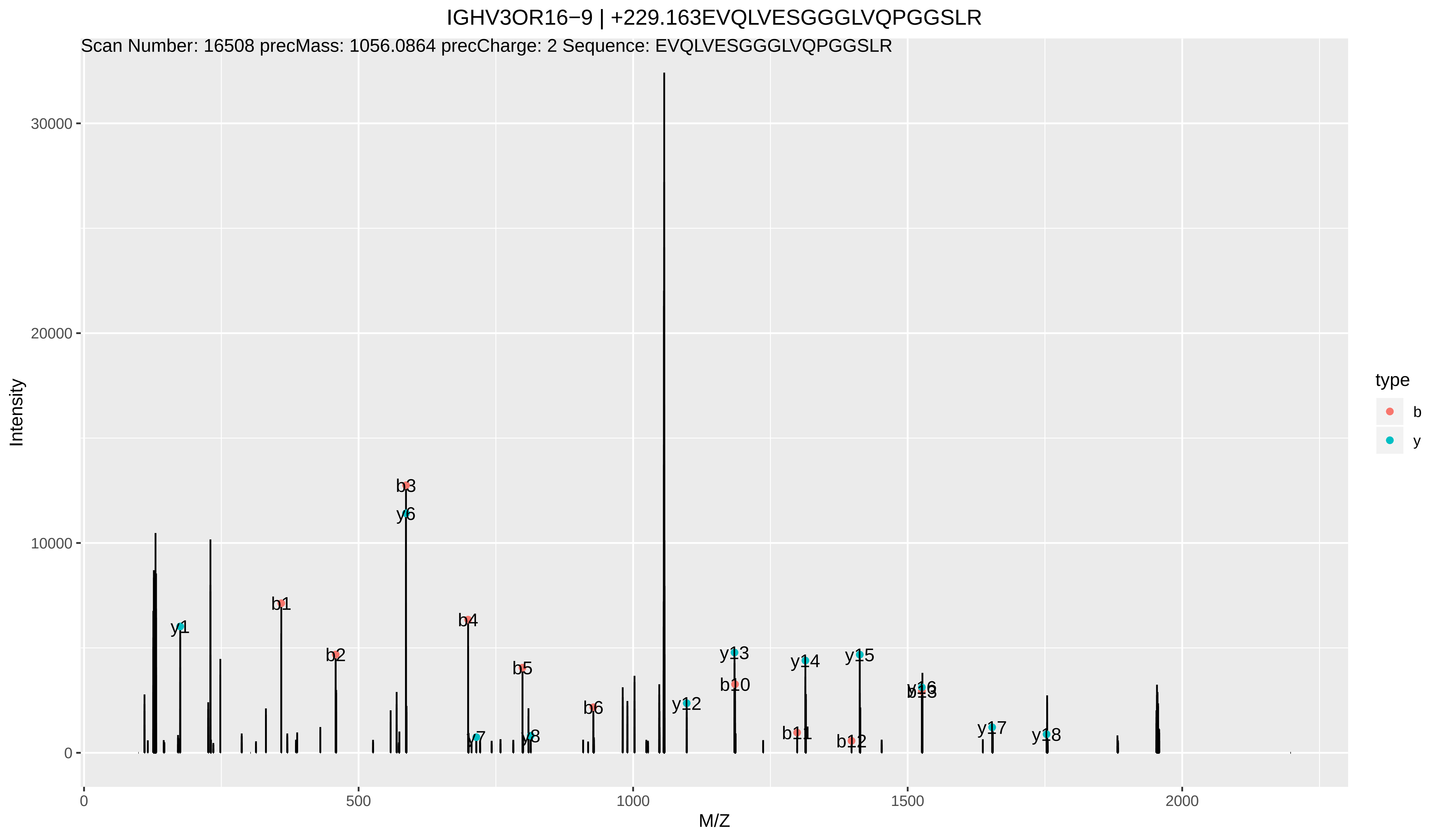
IGHV3OR16−9 | +229.163EVQLVESGGGLVQPGGSLR
Scan Number: 16508 precMass: 1056.0864 precCharge: 2 Sequence: EVQLVESGGGLVQPGGSLR
Intensity
M/Z
type
b
y
y1
b1
b2
b3
y6
b4
y7
b5
y8
b6
y12
y13
b10
b11
y14
y15
b12
y16
b13
y17
y18
0
10000
20000
30000
0
500
1000
1500
2000

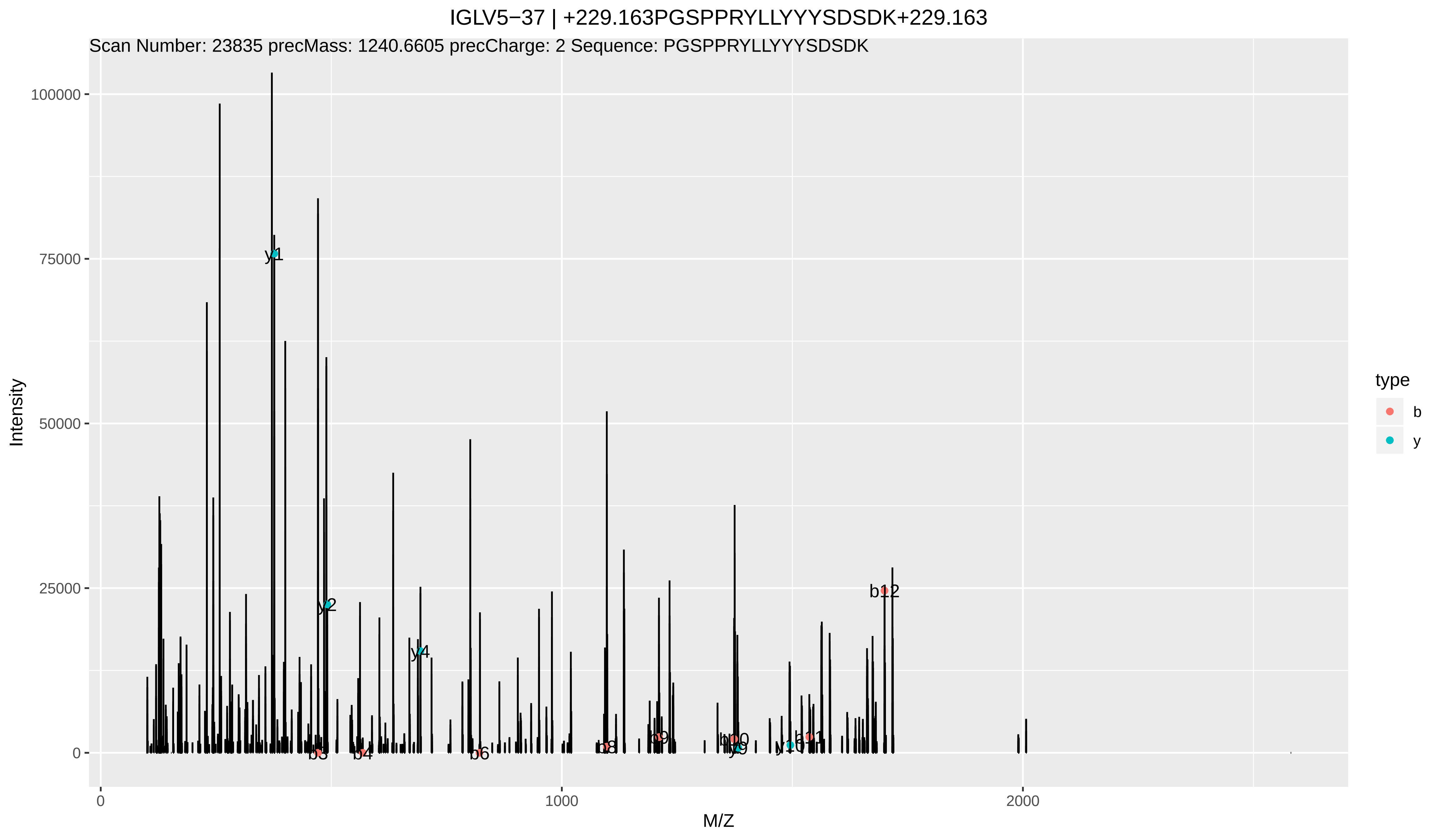
IGLV5−37 | +229.163PGSPPRYLLYYYSDSDK+229.163
Scan Number: 23835 precMass: 1240.6605 precCharge: 2 Sequence: PGSPPRYLLYYYSDSDK
Intensity
M/Z
type
b
y

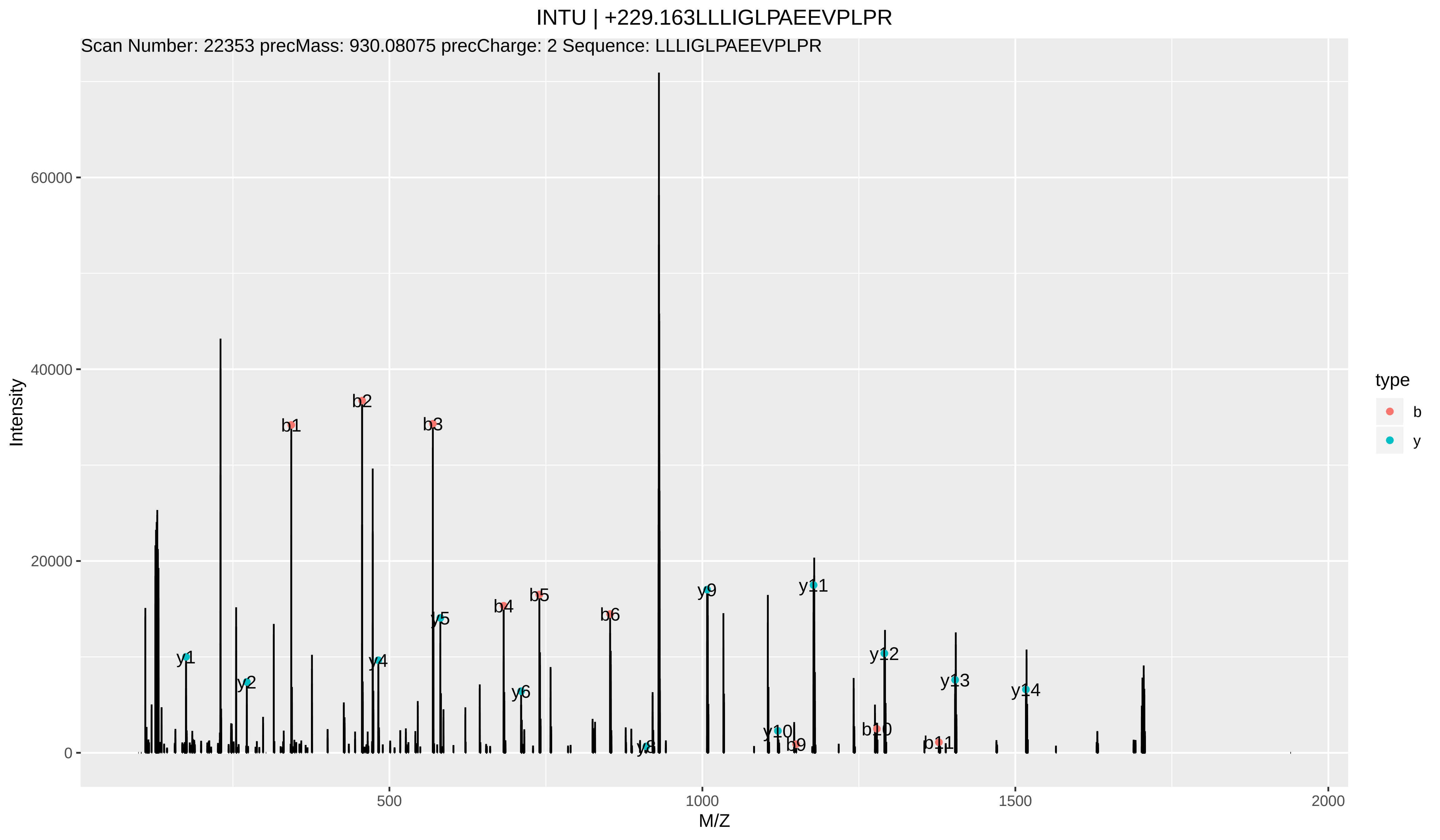
INTU | +229.163LLLIGLPAEEVPLPR
Scan Number: 22353 precMass: 930.08075 precCharge: 2 Sequence: LLLIGLPAEEVPLPR
Intensity
M/Z
type
b
y
b1
b2
b3
b4
b5
b6
b9
b10
b11
y1
y2
y4
y5
y6
y8
y9
y10
y11
y12
y13
y14
0
20000
40000
60000
500
1000
1500
2000

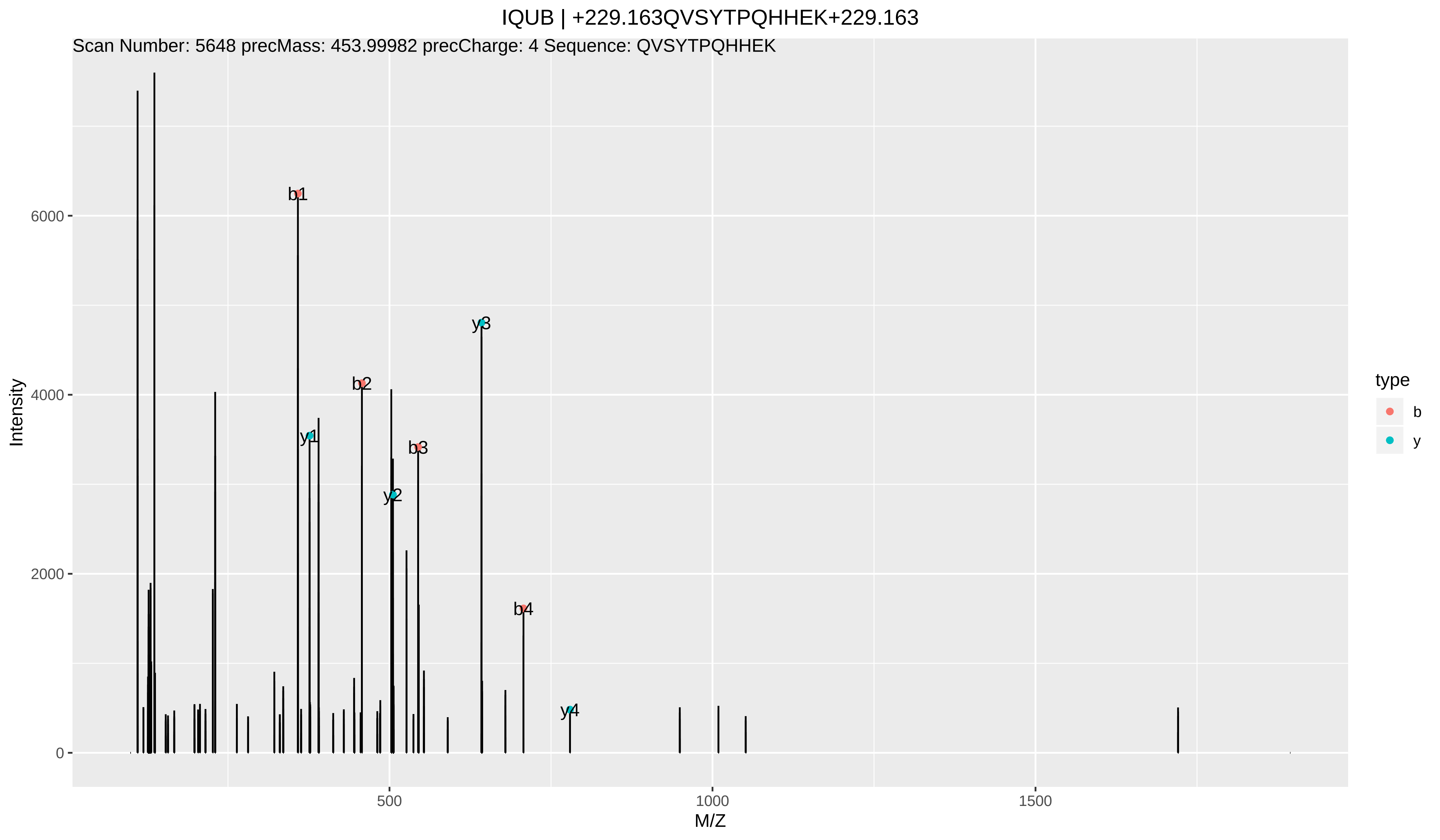
IQUB | +229.163QVSYTPQHHEK+229.163
Scan Number: 5648 precMass: 453.99982 precCharge: 4 Sequence: QVSYTPQHHEK
Intensity
M/Z
0
2000
4000
6000
500
1000
1500
b1
b2
b3
b4
y1
y2
y3
y4
type
b
y

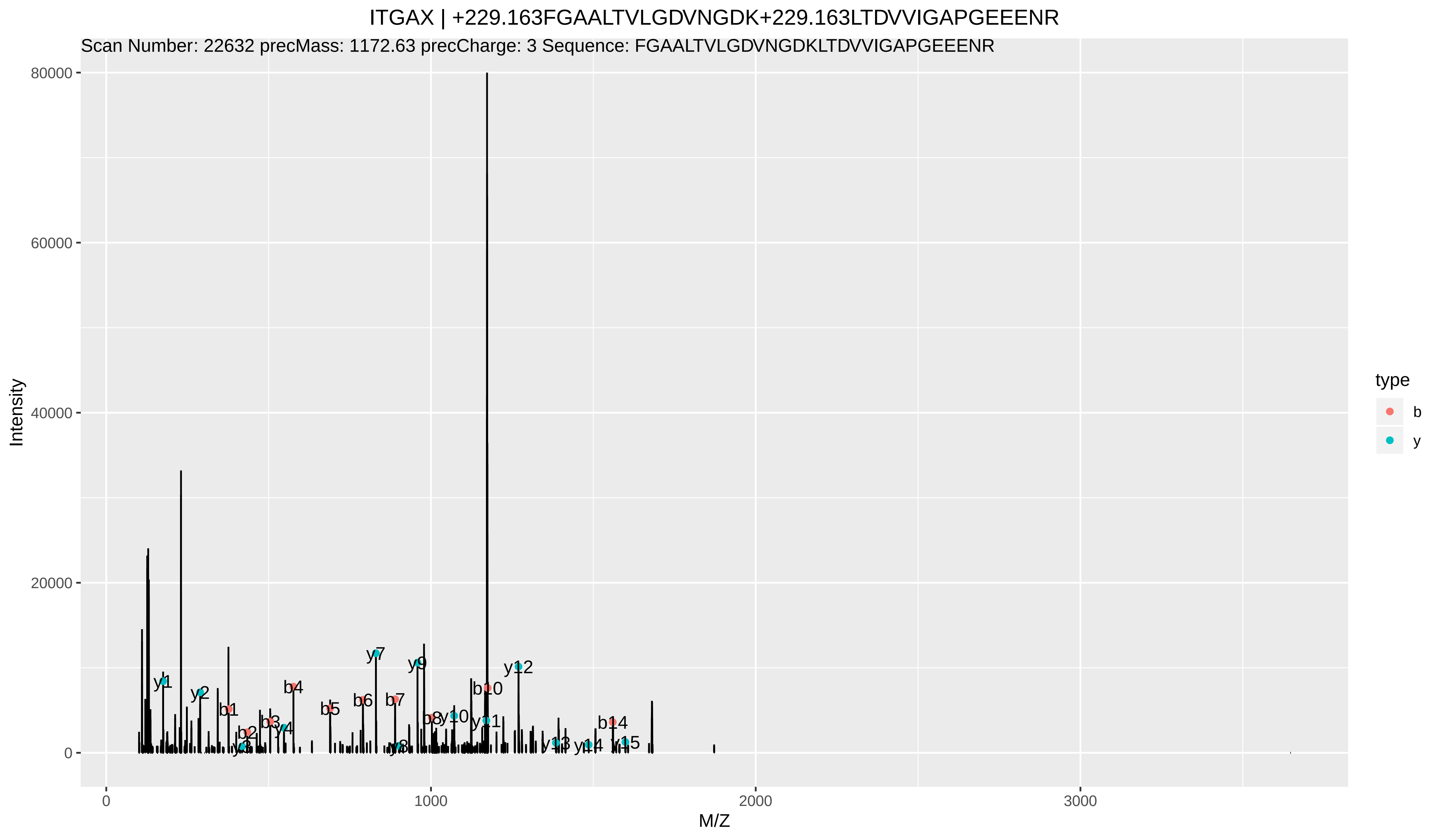

ITGAX | +229.163FGAALTVLGDVNGDK+229.163LTDVVIGAPGEEENR
Scan Number: 22632 precMass: 1172.63 precCharge: 3 Sequence: FGAALTVLGDVNGDKLTDVVIGAPGEEENR
Intensity
M/Z
type
b
y
80000
60000
40000
20000
0
0
1000
2000
3000
y1
y2
b1
b2
b3
y3
y4
b4
b5
b6
y7
b7
y8
y9
b8
y10
b10
y11
y12
y13
y14
b14
y15

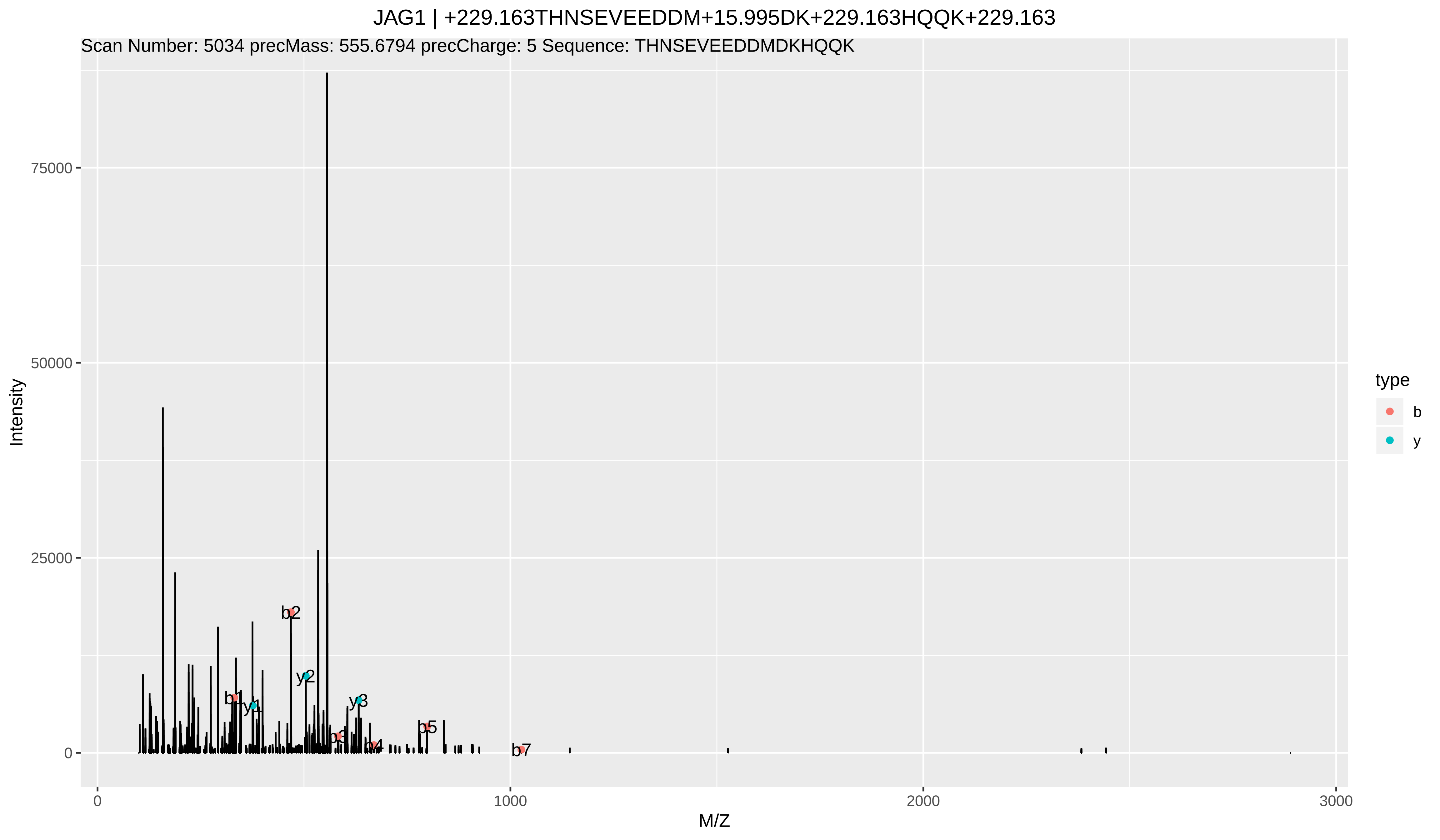
JAG1 | +229.163THNSEVEEDDM+15.995DK+229.163HQQK+229.163
Scan Number: 5034 precMass: 555.6794 precCharge: 5 Sequence: THNSEVEEDDMDKHQQK
Intensity
M/Z
type
b
y
b1
b2
b5
b7
y1
y2
y3
0
25000
50000
75000
1000
2000
3000

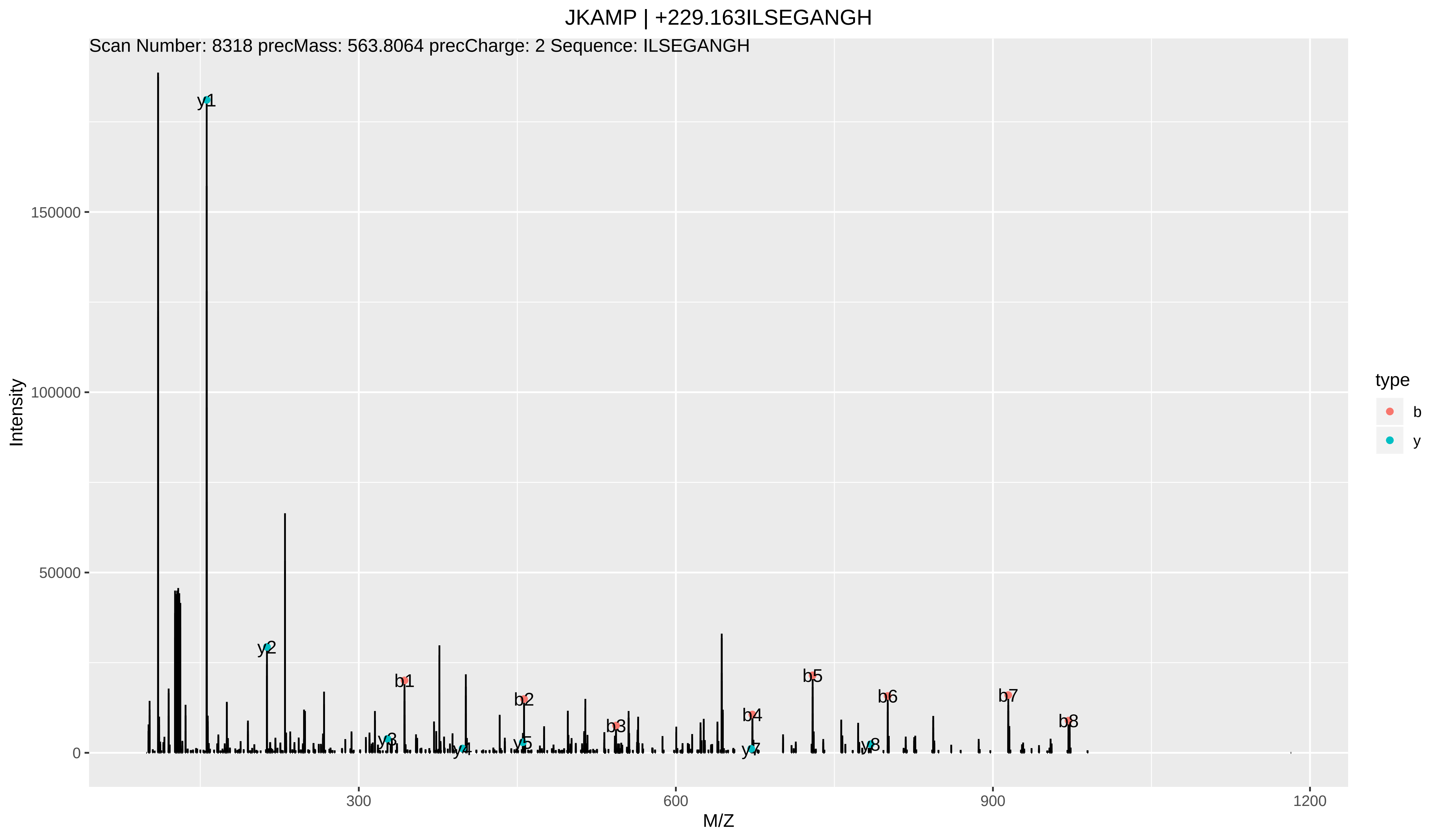

JKAMP | +229.163ILSEGANGH
Scan Number: 8318 precMass: 563.8064 precCharge: 2 Sequence: ILSEGANGH
Intensity
M/Z
type
b
y
150000
100000
50000
0
300
600
900
1200
y1
y2
y3
y4
y5
y7
y8
b1
b2
b3
b4
b5
b6
b7
b8

JMJD7 | +229.163VPWIPLDPLAPDLAR

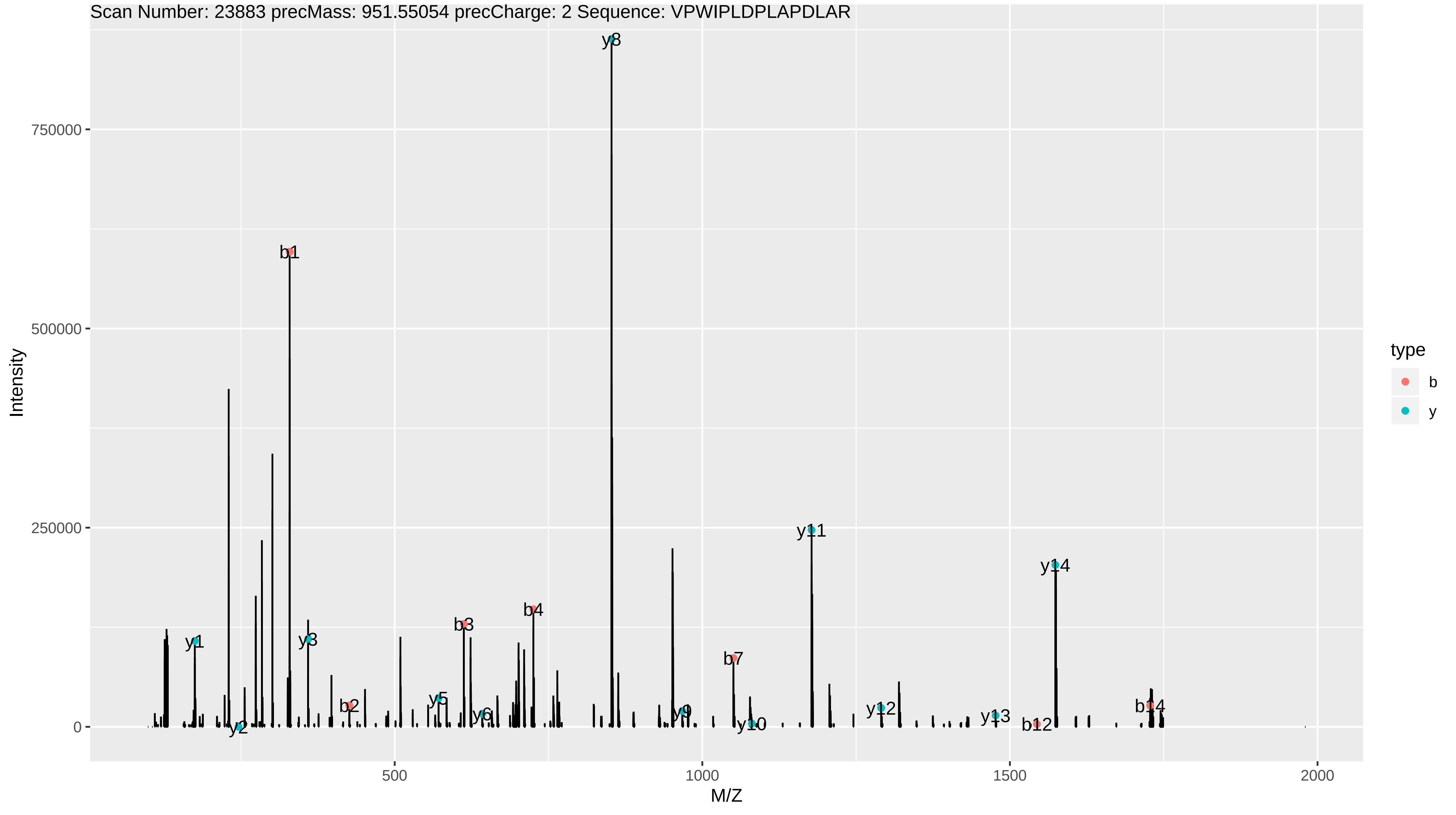

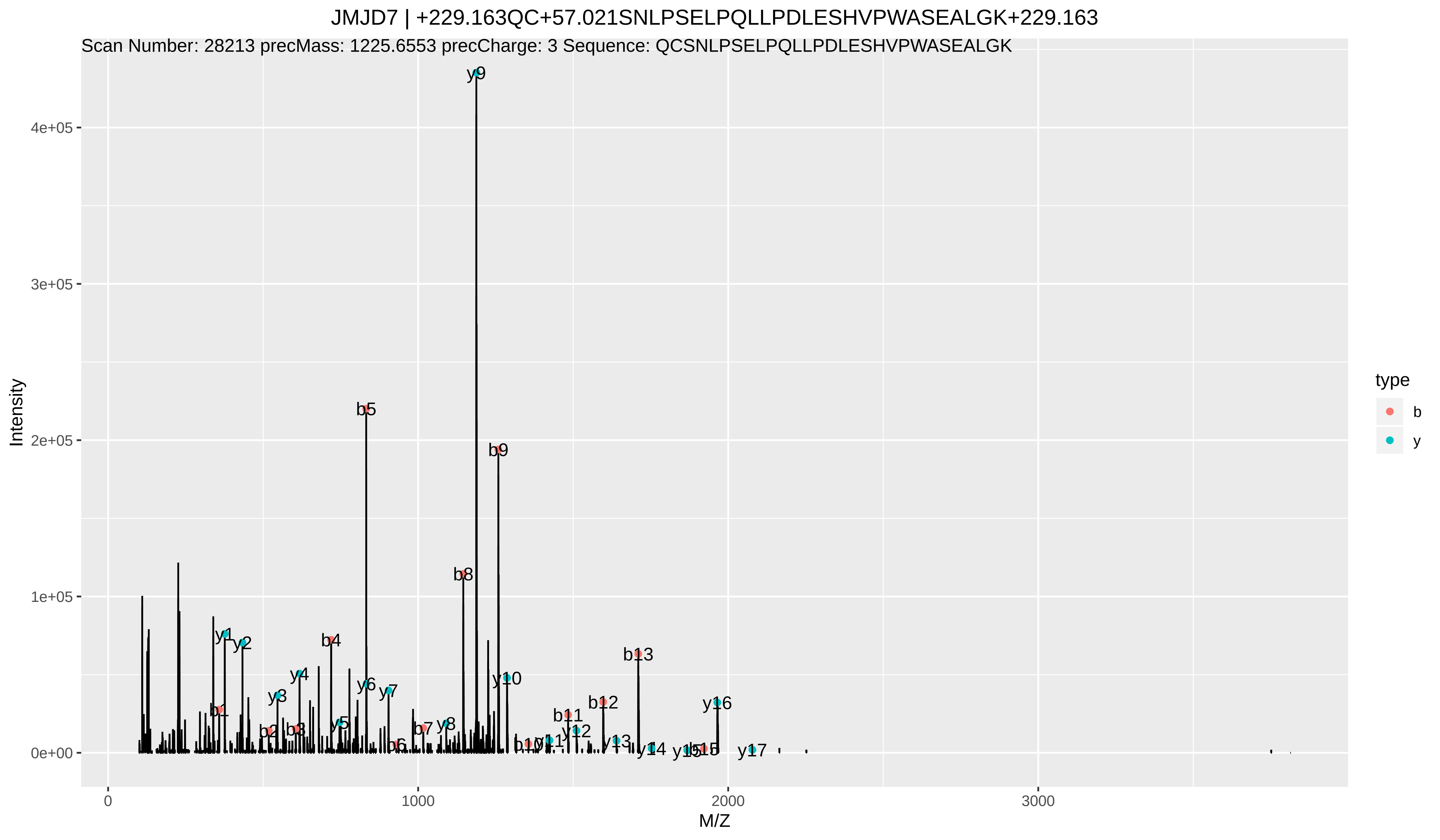

JMJD7 | +229.163QC+57.021SNLPSELPQLLPDLESHVPWASEALGK+229.163
Scan Number: 28213 precMass: 1225.6553 precCharge: 3 Sequence: QCSNLPSELPQLLPDLESHVPWASEALGK
Intensity
M/Z
type
b
y
y9
b5
b9
b8
y1
y2
b4
b13
y4
y10
y6
y7
y3
b12
y16
b1
b11
y12
y5
b2
b3
b7
y8
b6
b10
y11
y13
y14
y15
b15
y17
0e+00
1e+05
2e+05
3e+05
4e+05
0
1000
2000
3000

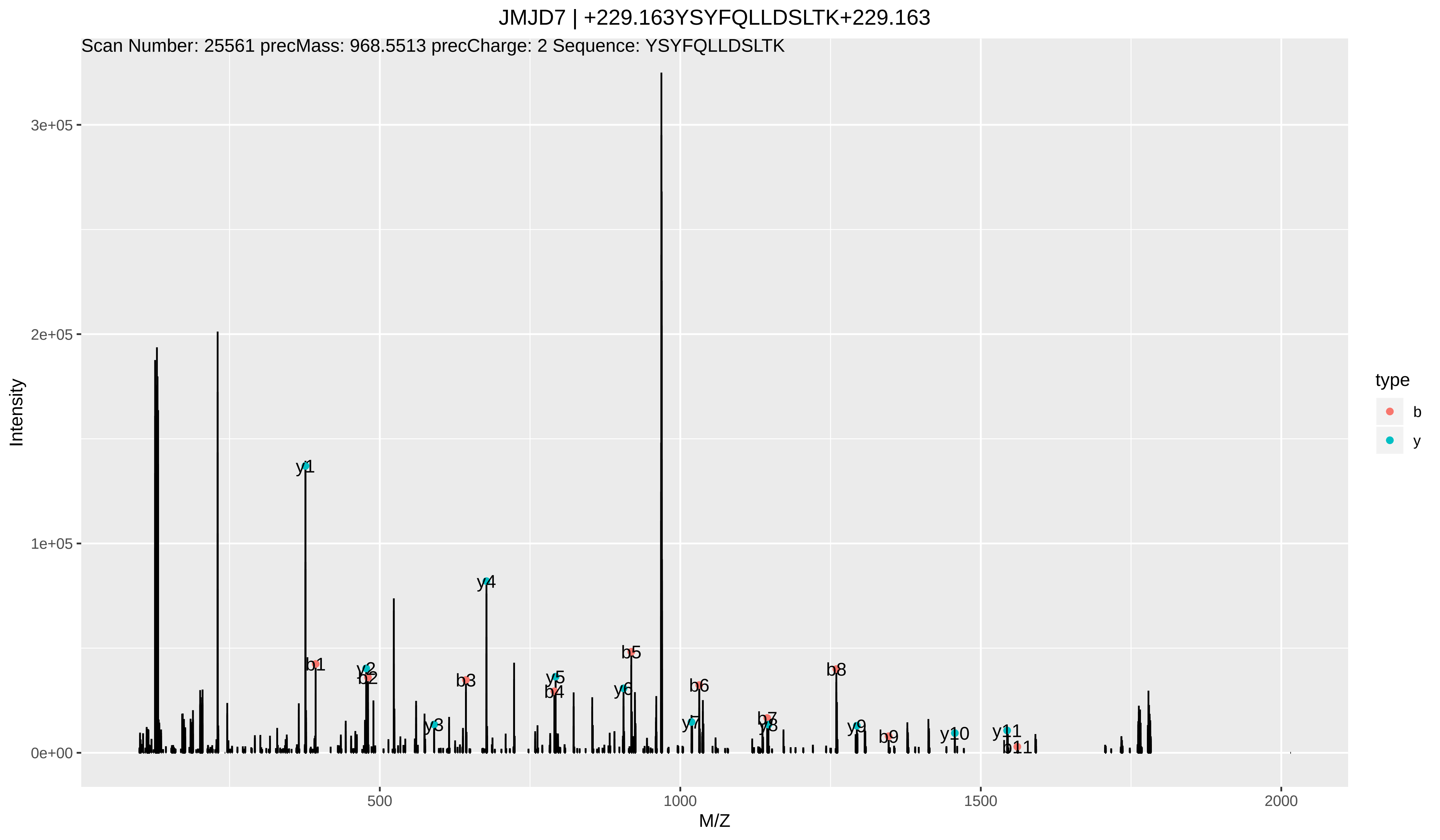
JMJD7 | +229.163YSYFQLLDSLTK+229.163
Scan Number: 25561 precMass: 968.5513 precCharge: 2 Sequence: YSYFQLLDSLTK
Intensity
3e+05
2e+05
1e+05
0e+00
M/Z
500
1000
1500
2000
y1
b1
y2
b2
y3
b3
y4
b4
y5
y6
b5
y7
b6
b7
y8
b8
y9
b9
y10
y11
b11
type
b
y

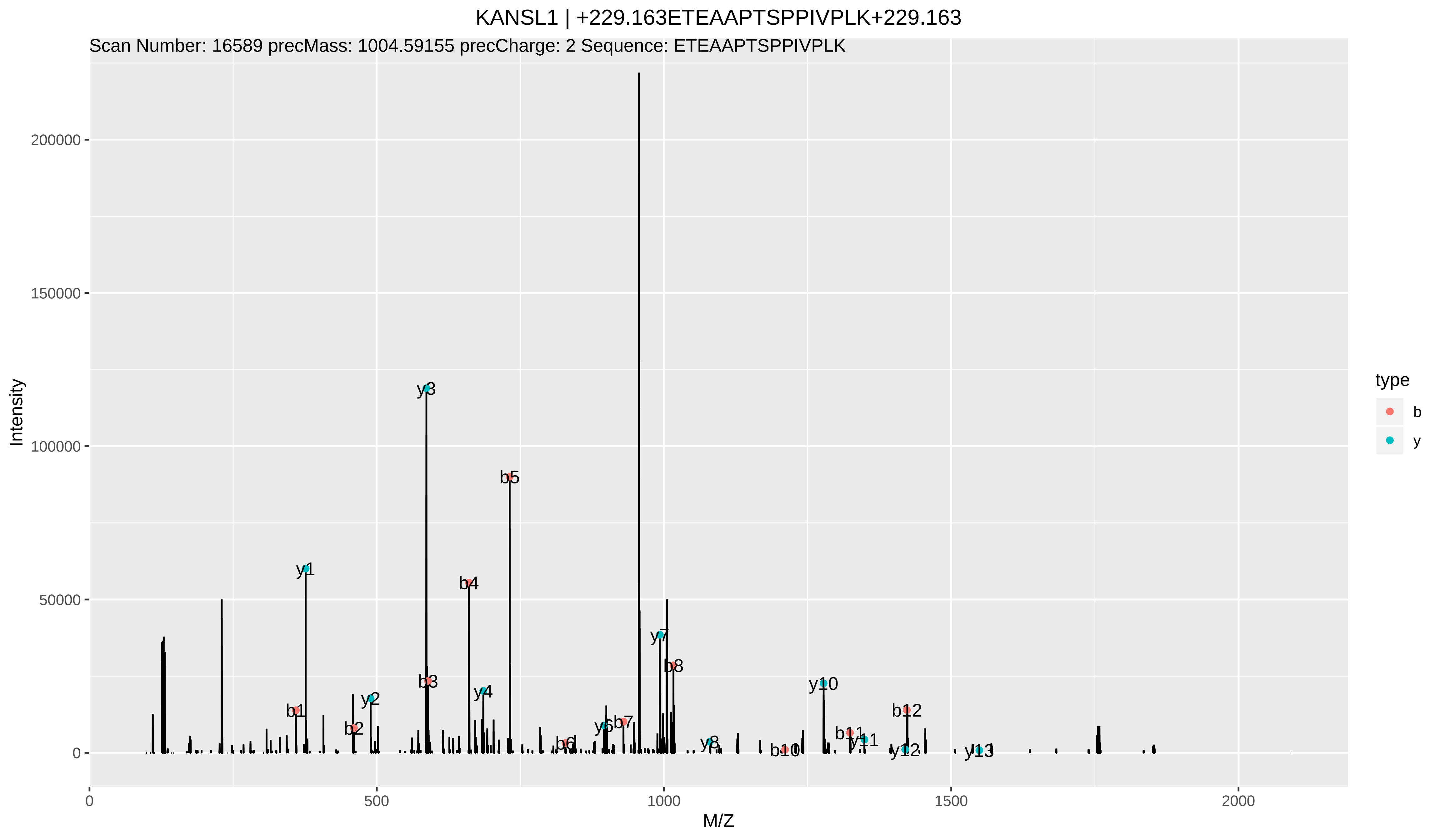

KANSL1 | +229.163ETEAAPTSPPIVPLK+229.163
Scan Number: 16589 precMass: 1004.59155 precCharge: 2 Sequence: ETEAAPTSPPIVPLK
Intensity
M/Z
type
b
y
y1
y2
y3
y4
y6
y7
y8
y10
y11
y12
y13
b1
b2
b3
b4
b5
b6
b7
b8
b10
b11
b12

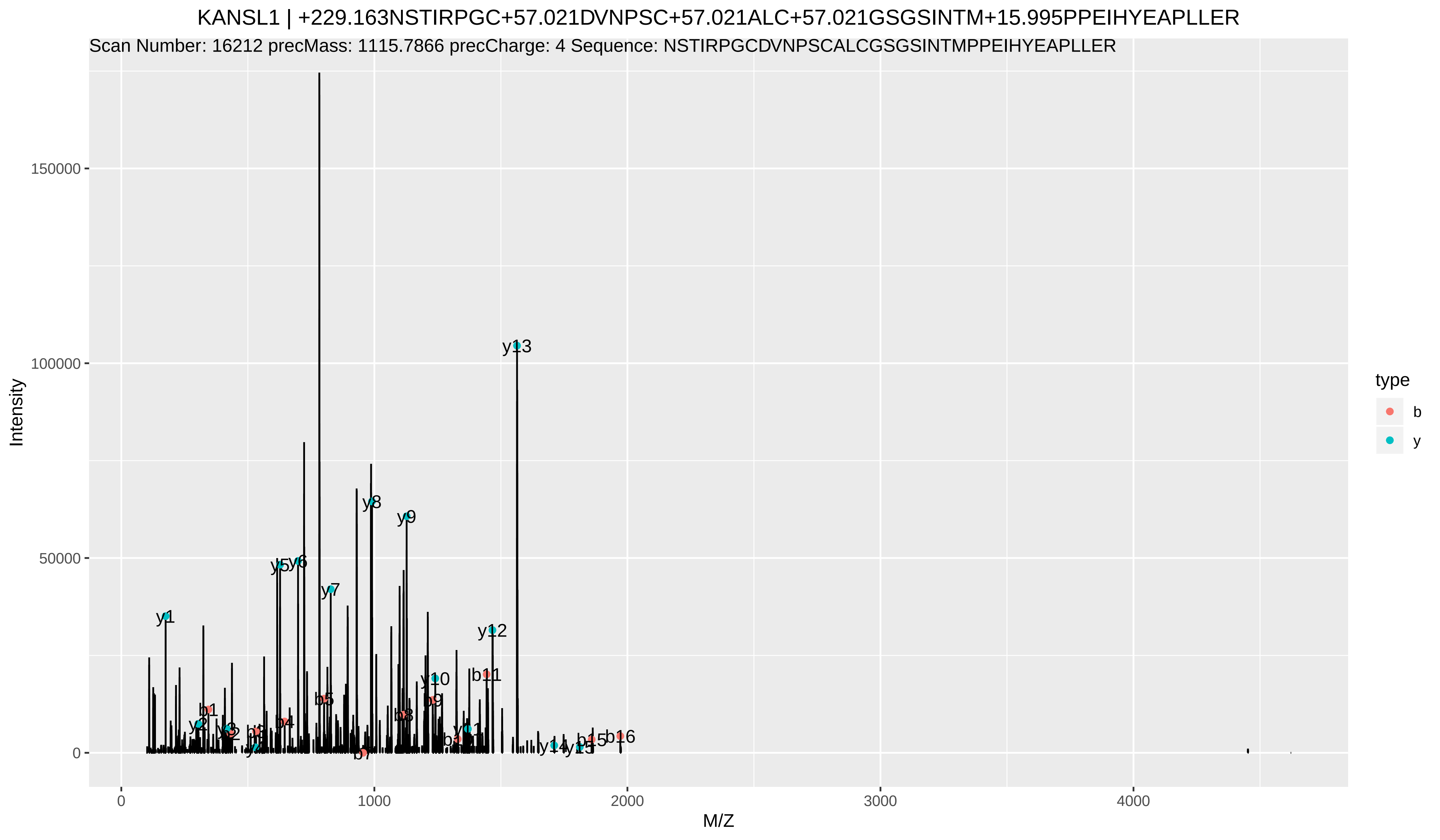
KANSL1 | +229.163NSTIRPGC+57.021DVNPSC+57.021ALC+57.021GSGSINTM+15.995PPEIHYEAPLLER
Scan Number: 16212 precMass: 1115.7866 precCharge: 4 Sequence: NSTIRPGCDVNPSCALCGSGSINTMPPEIHYEAPLLER
Intensity
M/Z
type
b
y
y13
y8
y9
y5
y6
y7
y1
y12
y10
b11
b5
b9
b1
b4
b8
y2
b16
0
50000
100000
150000
1000
2000
3000
4000

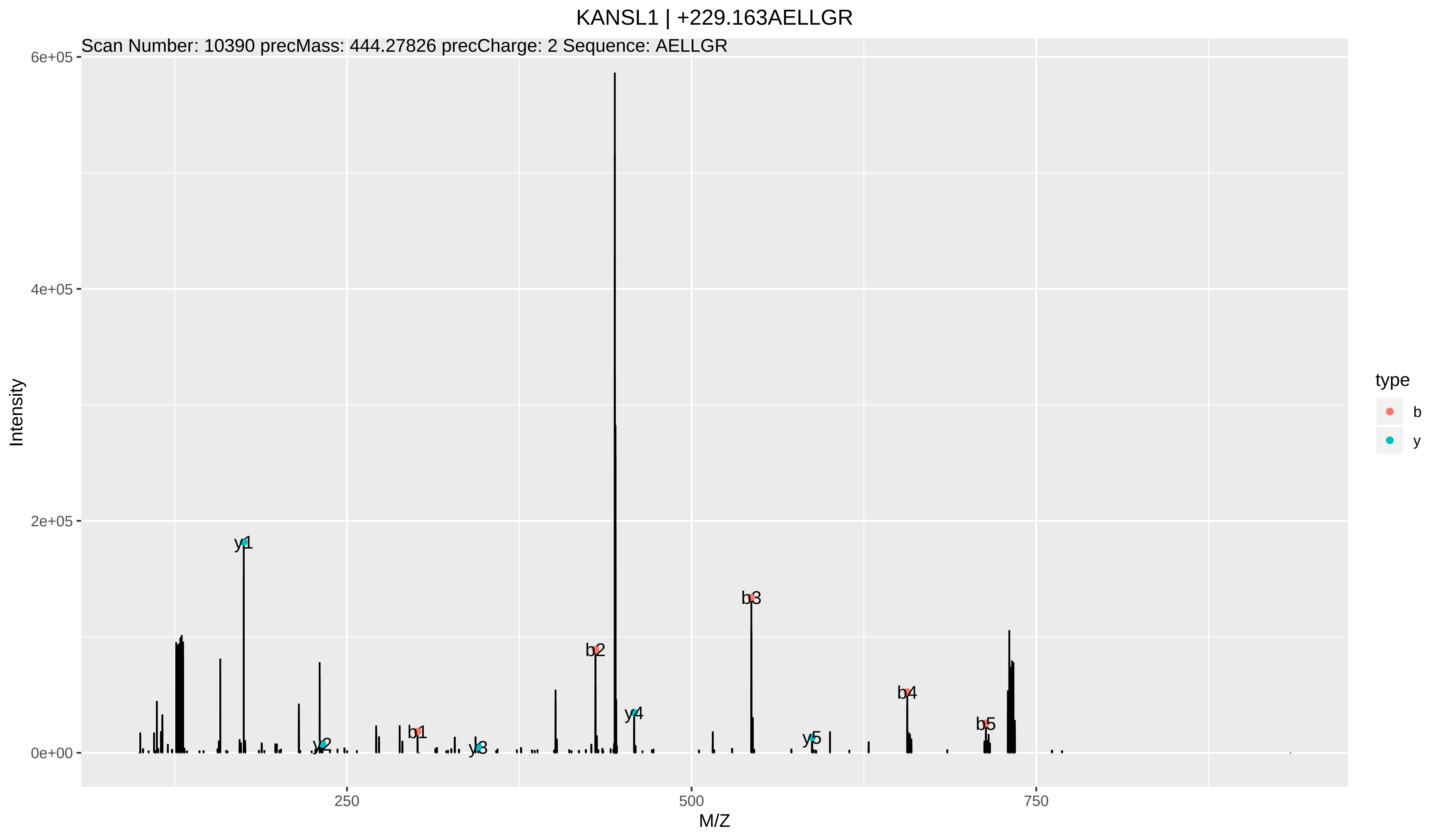

KANSL1 | +229.163AELLGR
Scan Number: 10390 precMass: 444.27826 precCharge: 2 Sequence: AELLGR
Intensity
6e+05
4e+05
2e+05
0e+00
y1
y2
b1
y3
b2
y4
b3
y5
b4
b5
250
500
750
M/Z
type
b
y

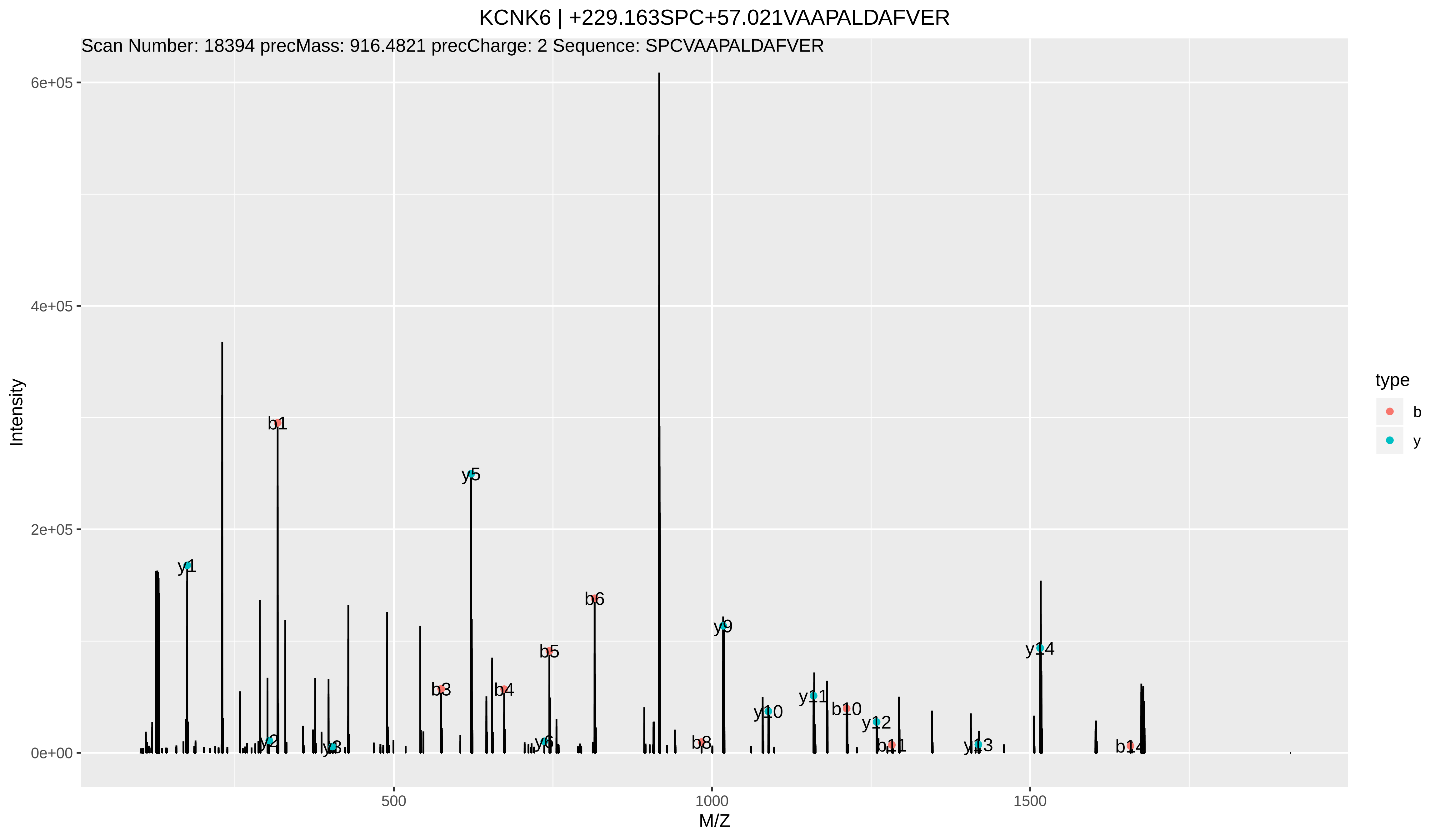

KCNK6 | +229.163SPC+57.021VAAPALDAFVER
Scan Number: 18394 precMass: 916.4821 precCharge: 2 Sequence: SPCVAAPALDAFVER
Intensity
M/Z
6e+05
4e+05
2e+05
0e+00
500
1000
1500
type
b
y
y1
b1
y2
y3
b3
y5
b4
y6
b5
b6
b8
y9
y10
y11
b10
y12
b11
y13
y14
b14

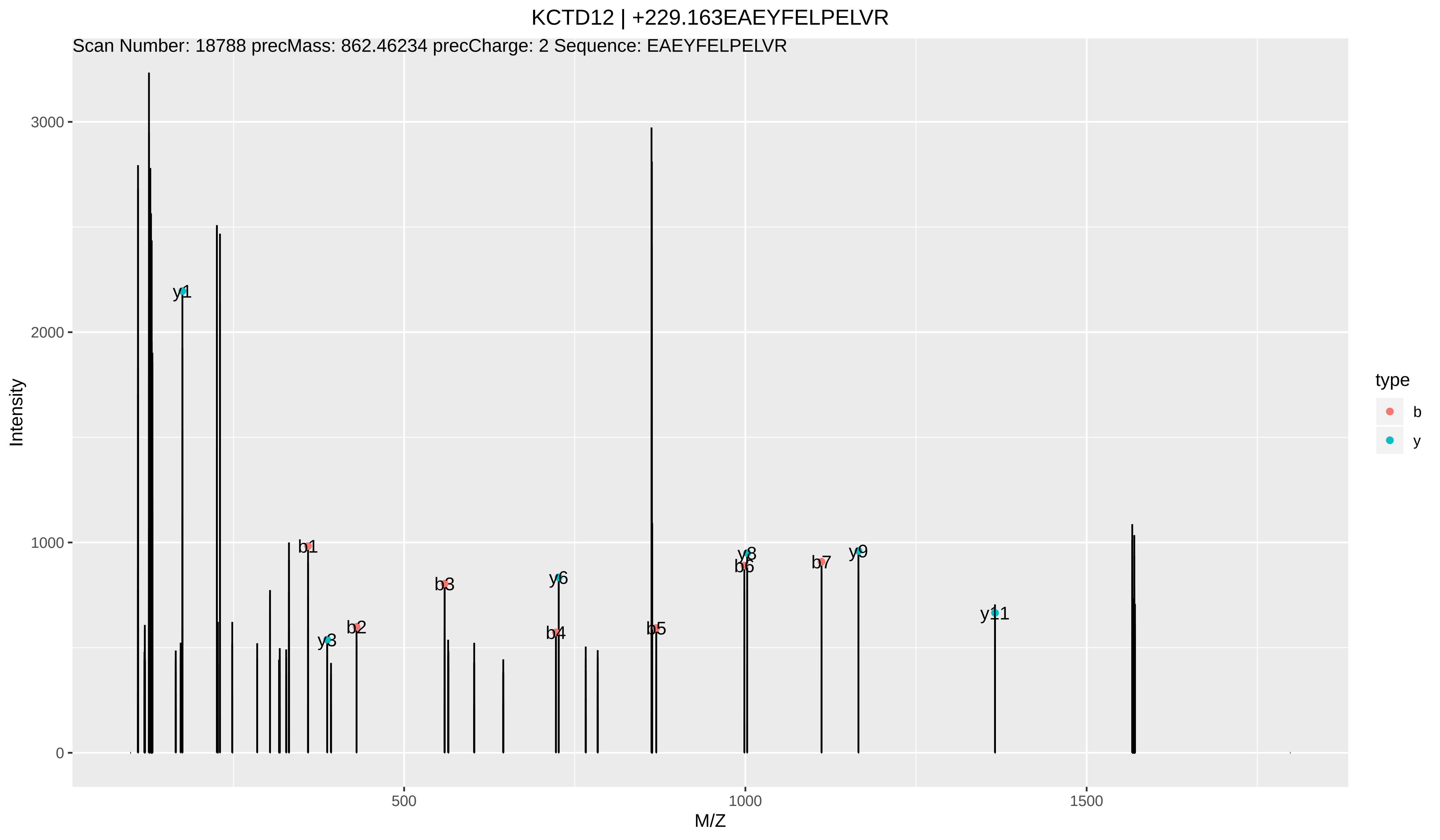
KCTD12 | +229.163EAEYFELPELVR
Scan Number: 18788 precMass: 862.46234 precCharge: 2 Sequence: EAEYFELPELVR
Intensity
M/Z
0
1000
2000
3000
500
1000
1500
y1
b1
y3
b2
b3
y6
b4
b5
y8
b6
b7
y9
y11
type
b
y

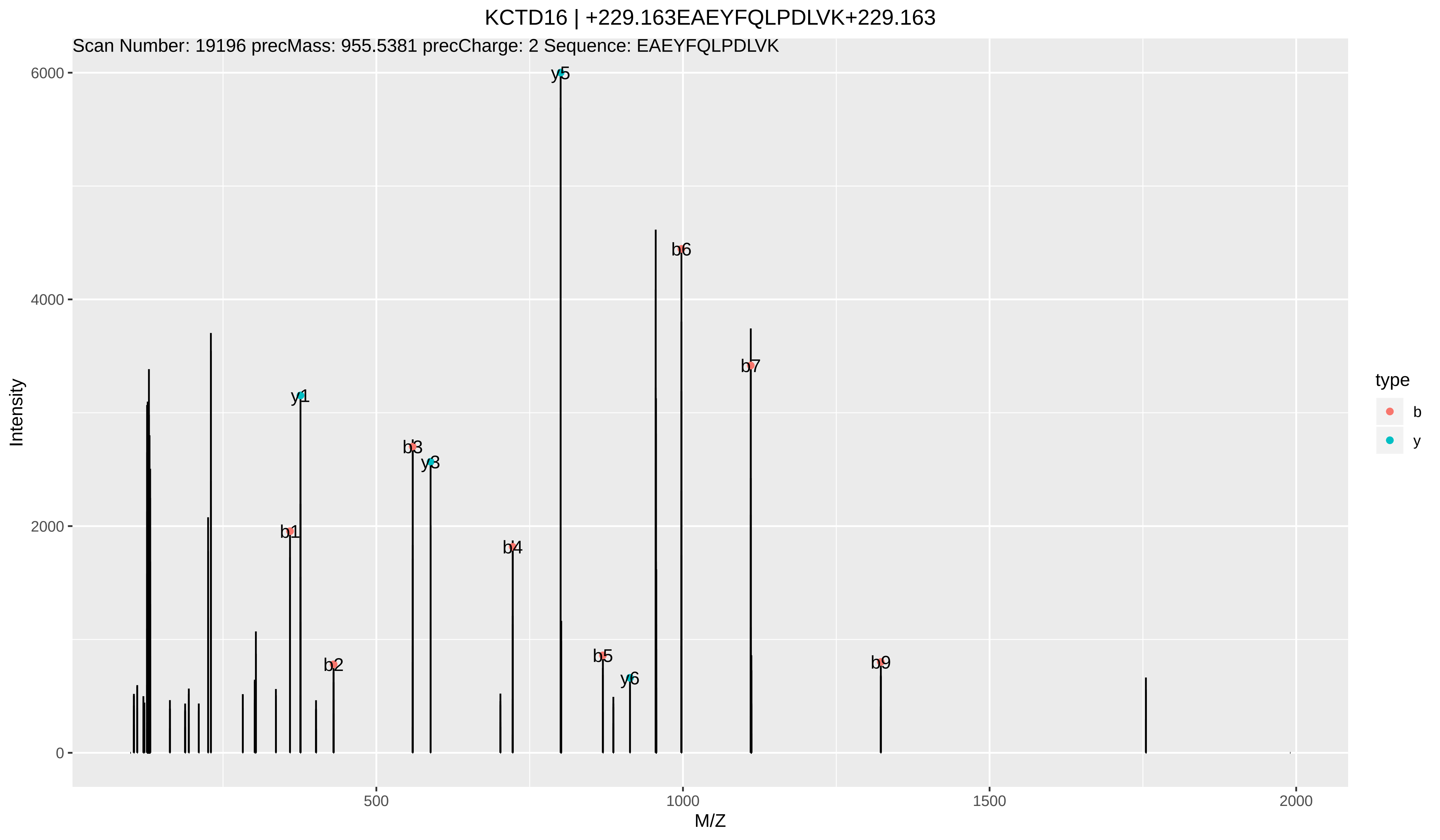
KCTD16 | +229.163EAEYFQLPDLVK+229.163
Scan Number: 19196 precMass: 955.5381 precCharge: 2 Sequence: EAEYFQLPDLVK
Intensity
M/Z
6000
4000
2000
0
500
1000
1500
2000
y5
b6
b7
y1
b3
y3
b1
b4
b5
y6
b2
b9
type
b
y

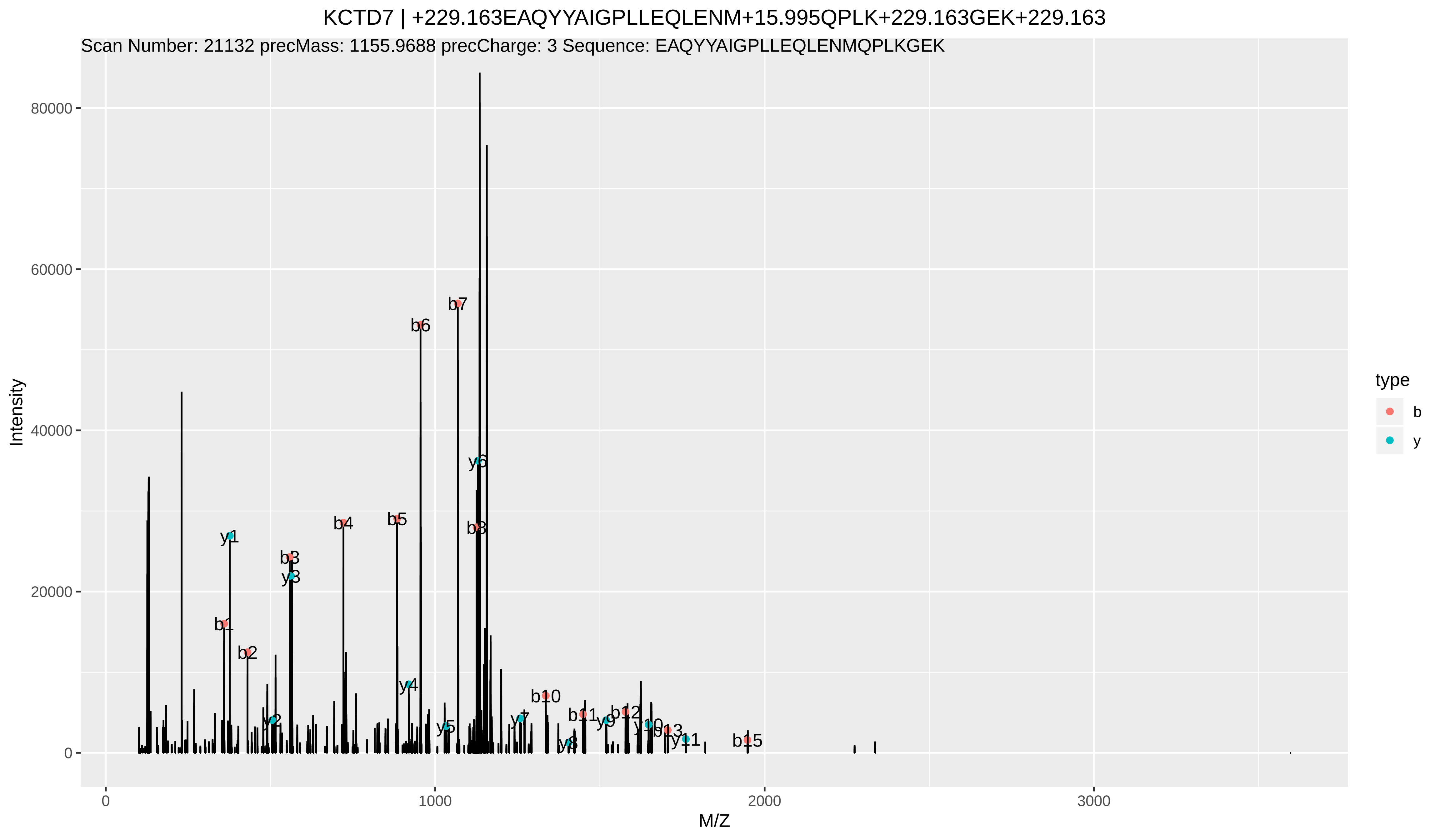

KCTD7 | +229.163EAQYYAIGPLLEQLENM+15.995QPLK+229.163GEK+229.163
Scan Number: 21132 precMass: 1155.9688 precCharge: 3 Sequence: EAQYYAIGPLLEQLENMQPLKGEK
Intensity
M/Z
type
b
y
80000
60000
40000
20000
0
0
1000
2000
3000
b1
b2
b3
b4
b5
b6
b7
b8
b10
b11
b12
b13
b15
y1
y2
y3
y4
y5
y6
y7
y8
y9
y10
y11

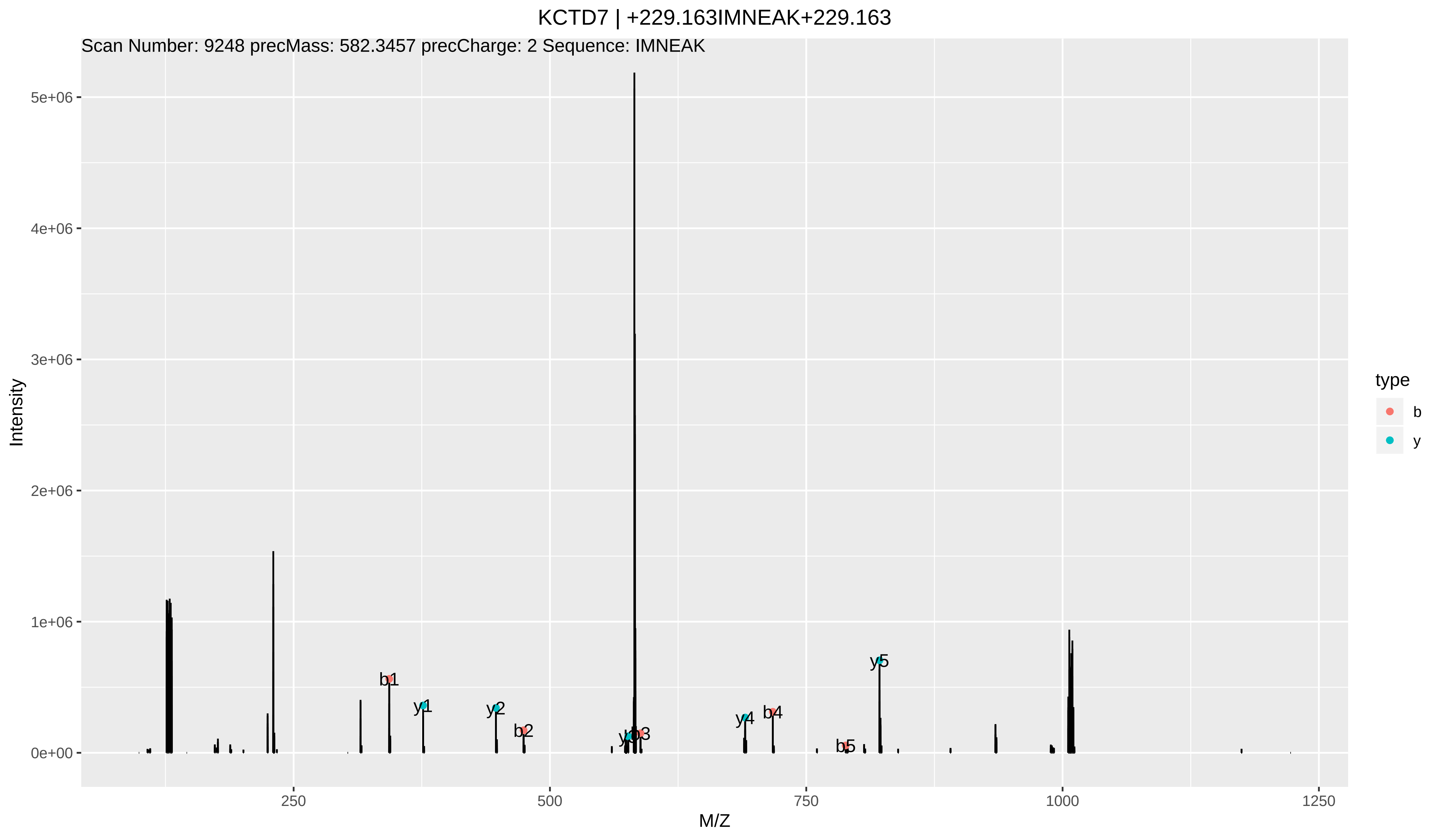

KCTD7 | +229.163IMNEAK+229.163
Scan Number: 9248 precMass: 582.3457 precCharge: 2 Sequence: IMNEAK
Intensity
5e+06
4e+06
3e+06
2e+06
1e+06
0e+00
b1
y1
y2
b2
y3
b3
y4
b4
b5
y5
250
500
750
1000
1250
M/Z
type
b
y

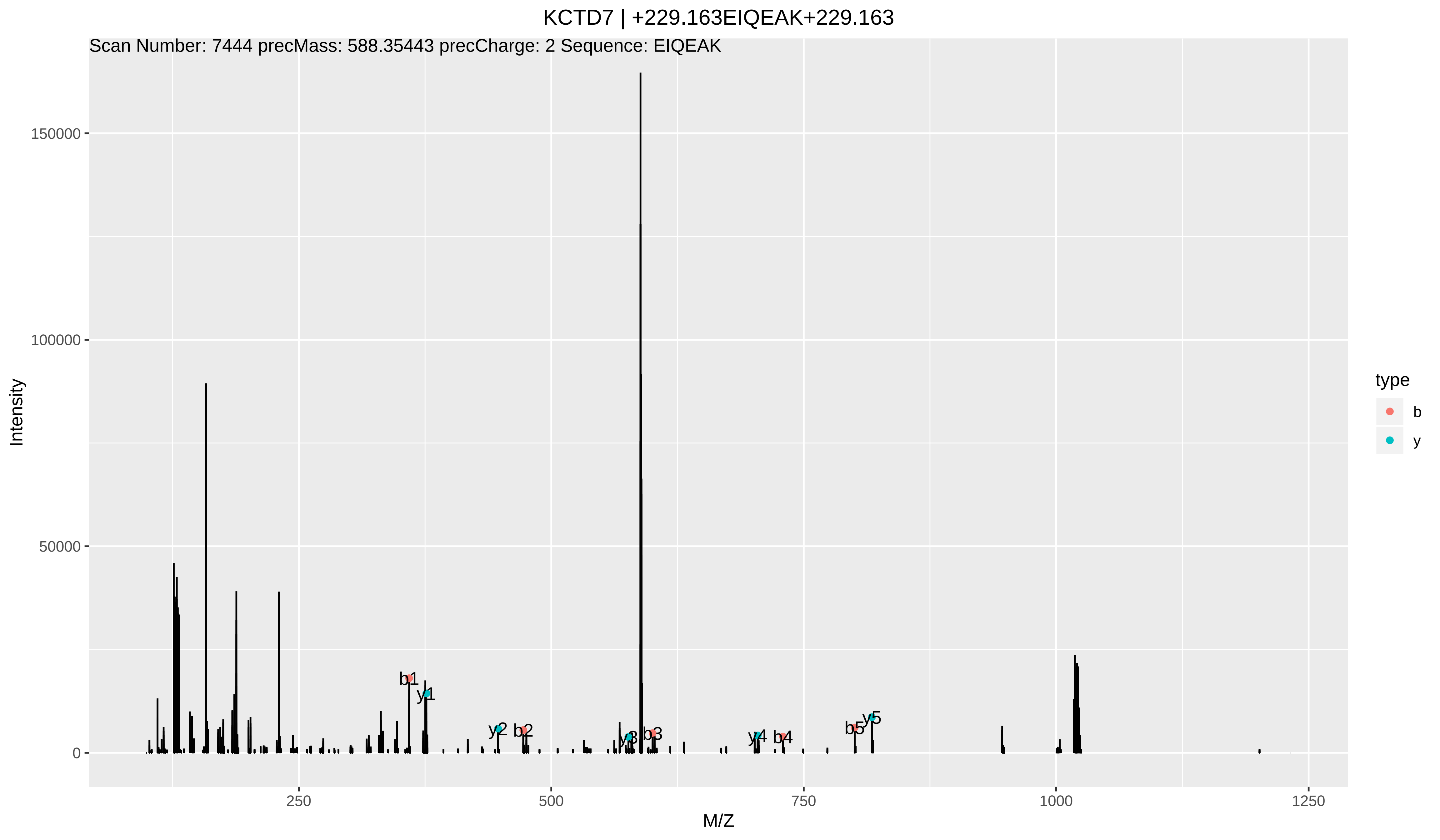

KCTD7 | +229.163EIQEAK+229.163
Scan Number: 7444 precMass: 588.35443 precCharge: 2 Sequence: EIQEAK
Intensity
150000
100000
50000
0
M/Z
250
500
750
1000
1250
b1
y1
y2
b2
y3
b3
y4
b4
b5
y5
type
b
y

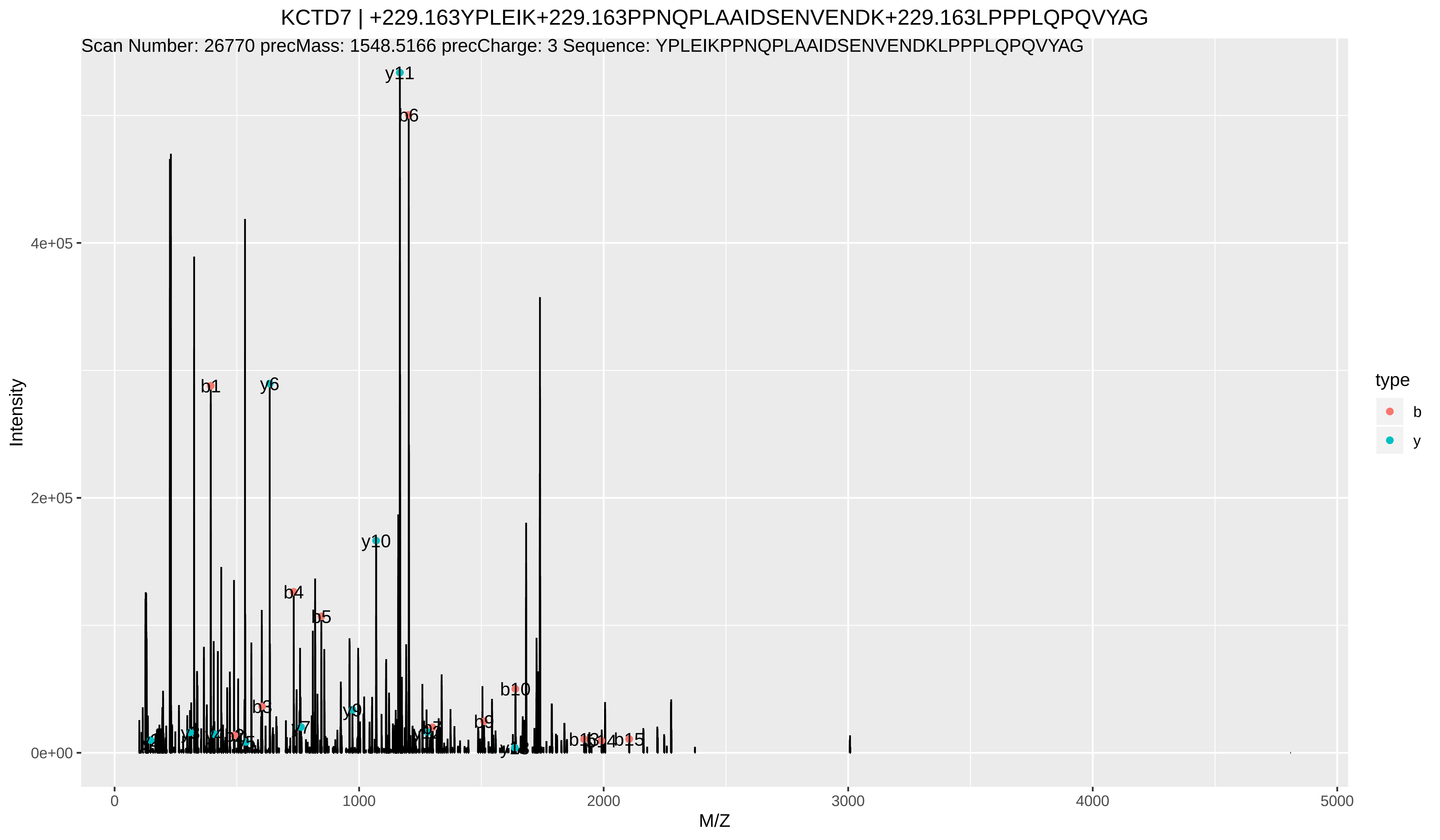

KCTD7 | +229.163YPLEIK+229.163PPNQPLAAIDSENVENDK+229.163LPPPLQPQVYAG
Scan Number: 26770 precMass: 1548.5166 precCharge: 3 Sequence: YPLEIKPPNQPLAAIDSENVENDKLPPPLQPQVYAG
y11
b6
b1
y6
y10
b4
b5
b10
b3
y9
b9
y7
b15
Intensity
4e+05
2e+05
0e+00
0
1000
2000
3000
4000
5000
M/Z
type
b
y

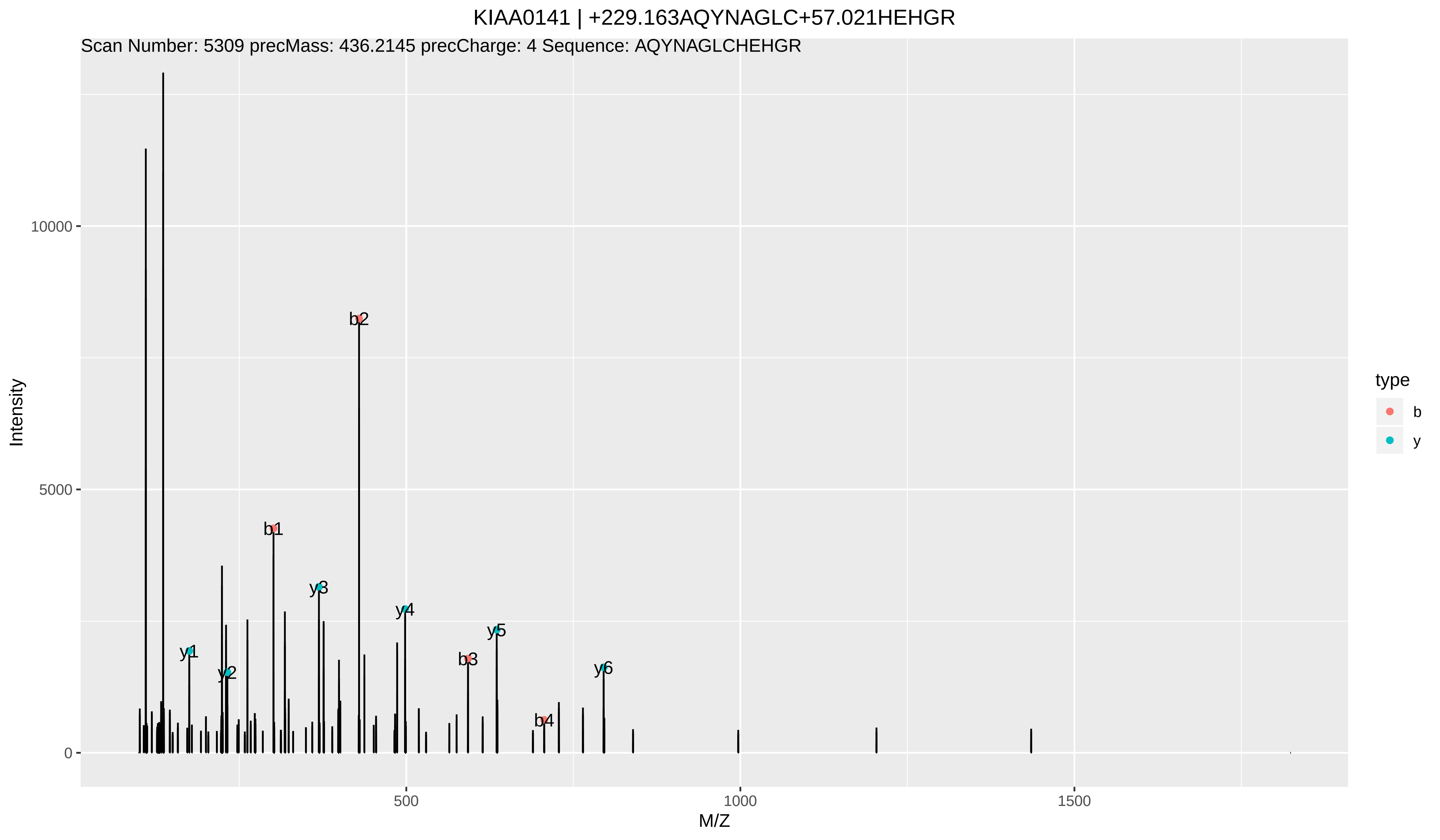

KIAA0141 | +229.163AQYNAGLC+57.021HEHGR
Scan Number: 5309 precMass: 436.2145 precCharge: 4 Sequence: AQYNAGLCHEHGR
Intensity
10000
5000
0
500
1000
1500
M/Z
b1
b2
b3
b4
y1
y2
y3
y4
y5
y6
type
b
y

KIAA1324 | +229.163LIGVTTDM+15.995TLDGITSPAELFHLESLGIPDVIFFYR

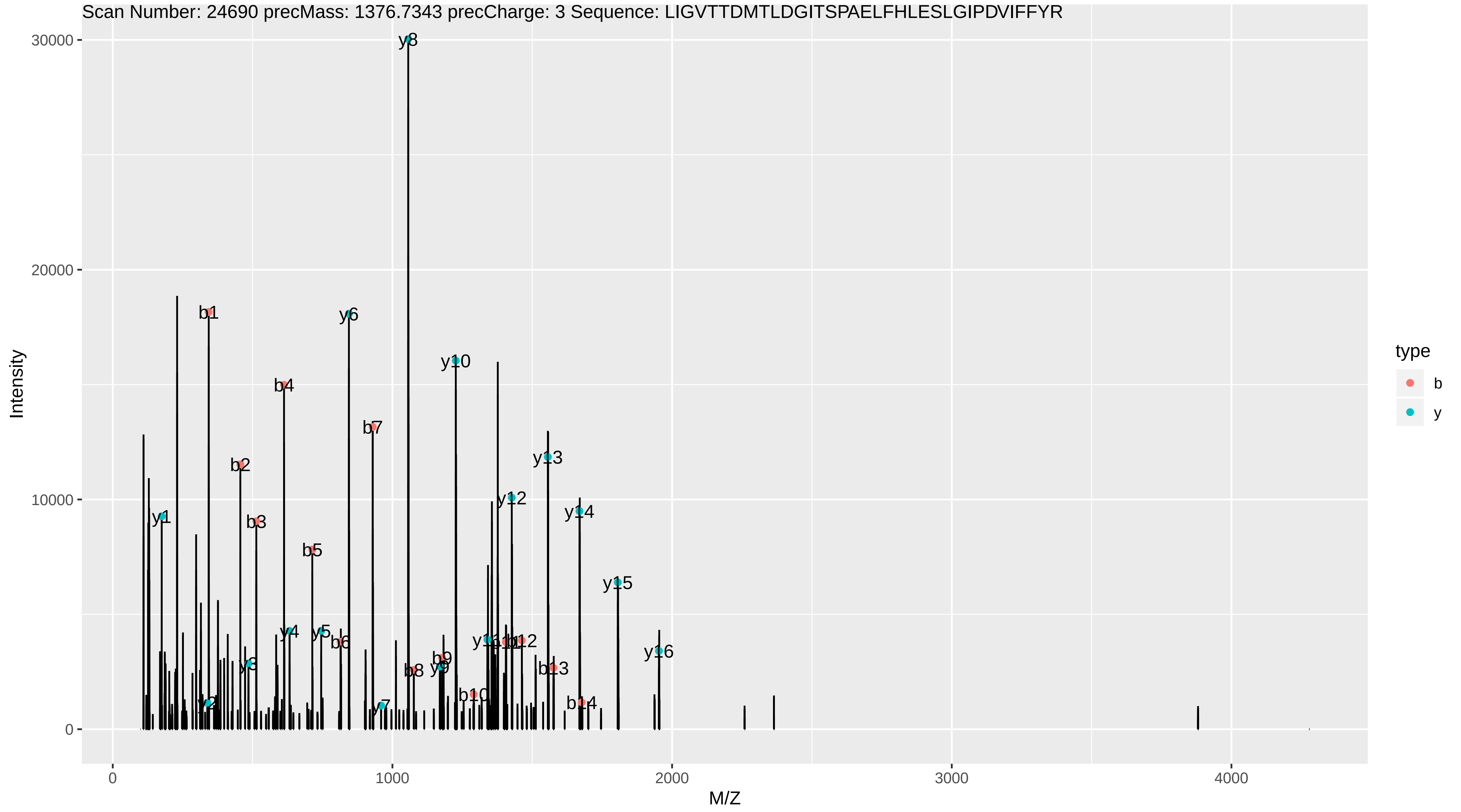

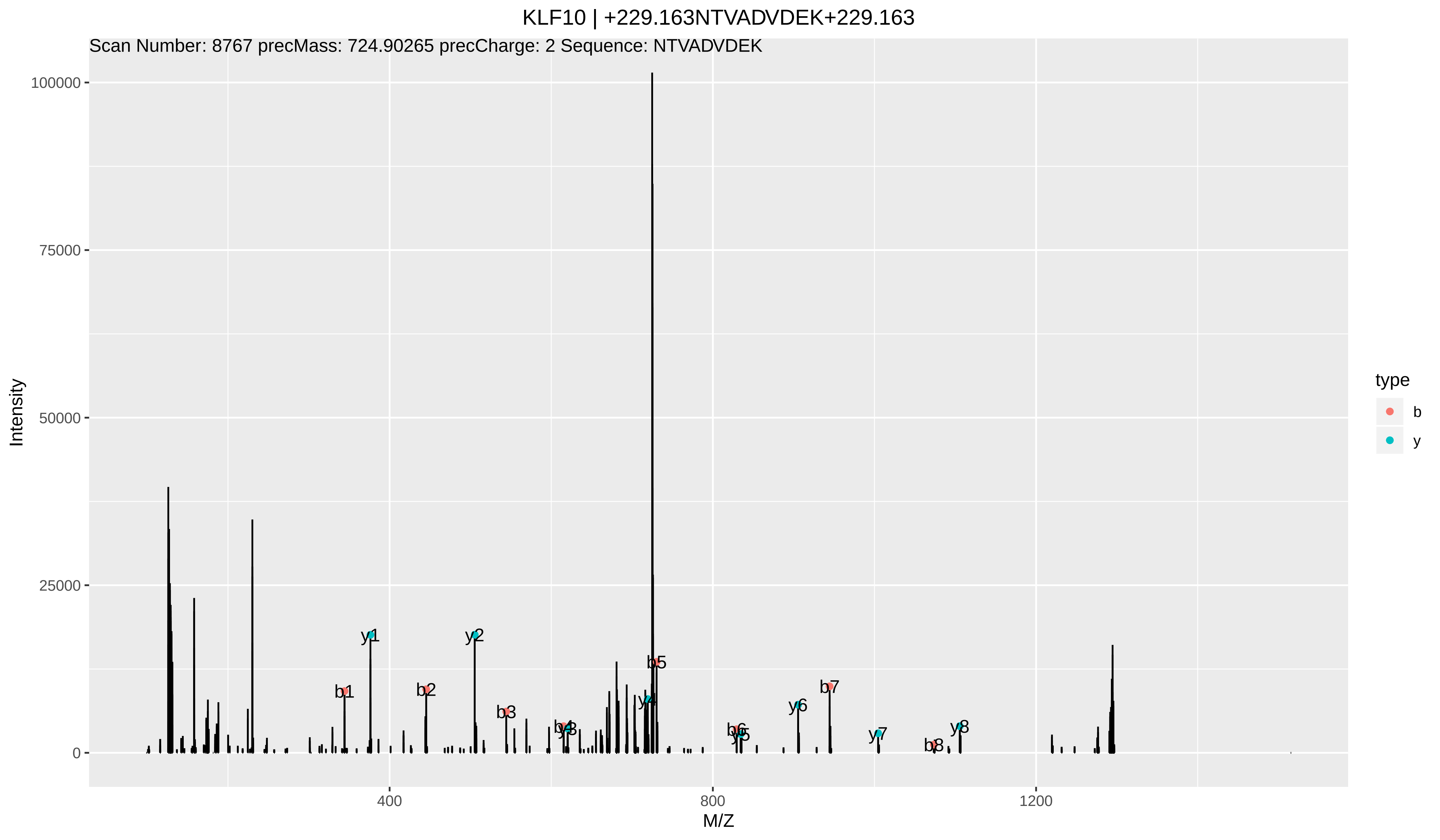

KLF10 | +229.163NTVADVDEK+229.163
Scan Number: 8767 precMass: 724.90265 precCharge: 2 Sequence: NTVADVDEK
Intensity
100000
75000
50000
25000
0
400
800
1200
M/Z
type
b
y
y1
y2
b1
b2
b3
b4
y3
y4
b5
b6
y5
y6
b7
y7
b8
y8

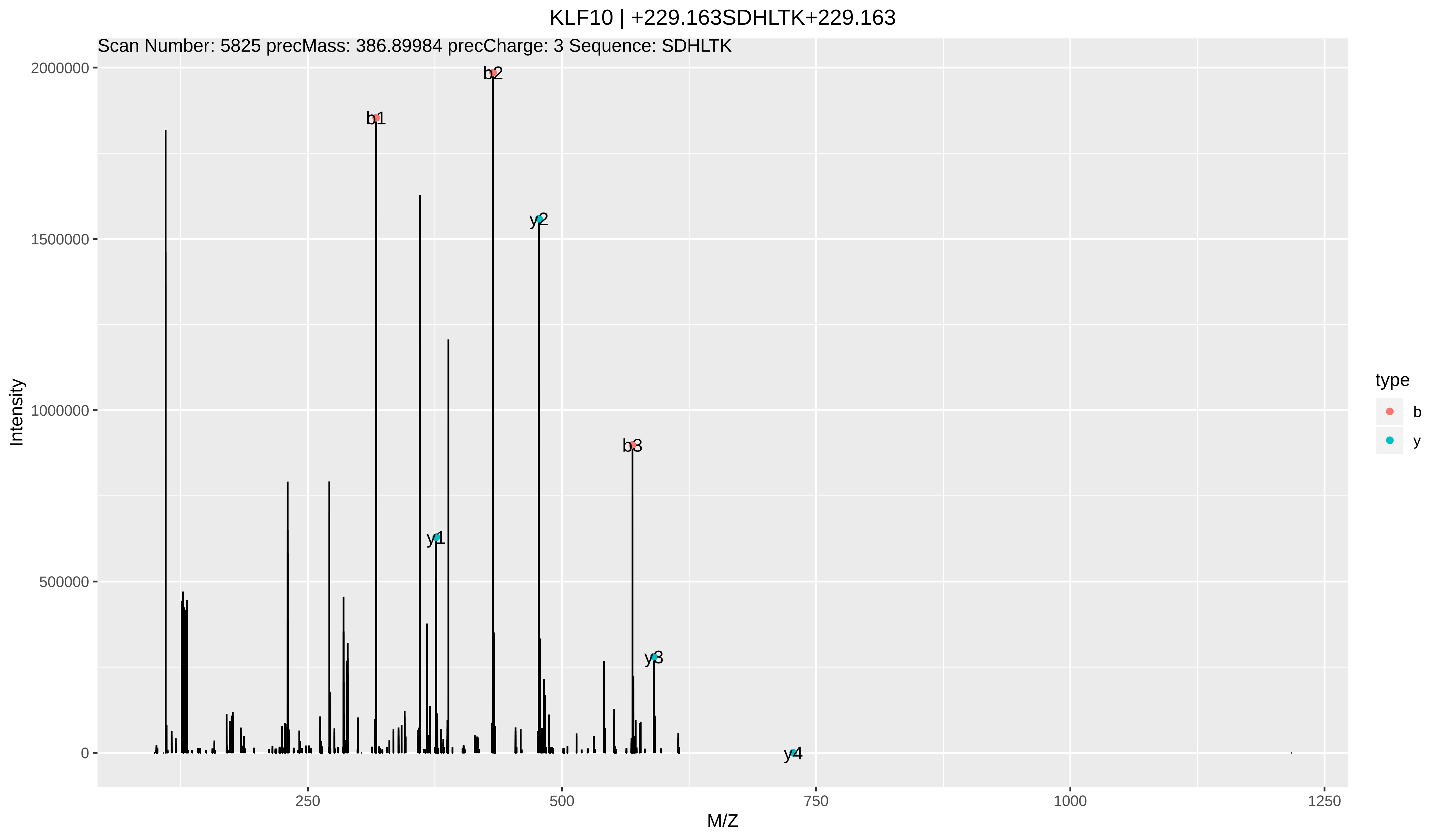

KLF10 | +229.163SDHLTK+229.163
Scan Number: 5825 precMass: 386.89984 precCharge: 3 Sequence: SDHLTK
Intensity
2000000
1500000
1000000
500000
0
M/Z
250
500
750
1000
1250
b1
b2
b3
y1
y2
y3
y4
type
b
y

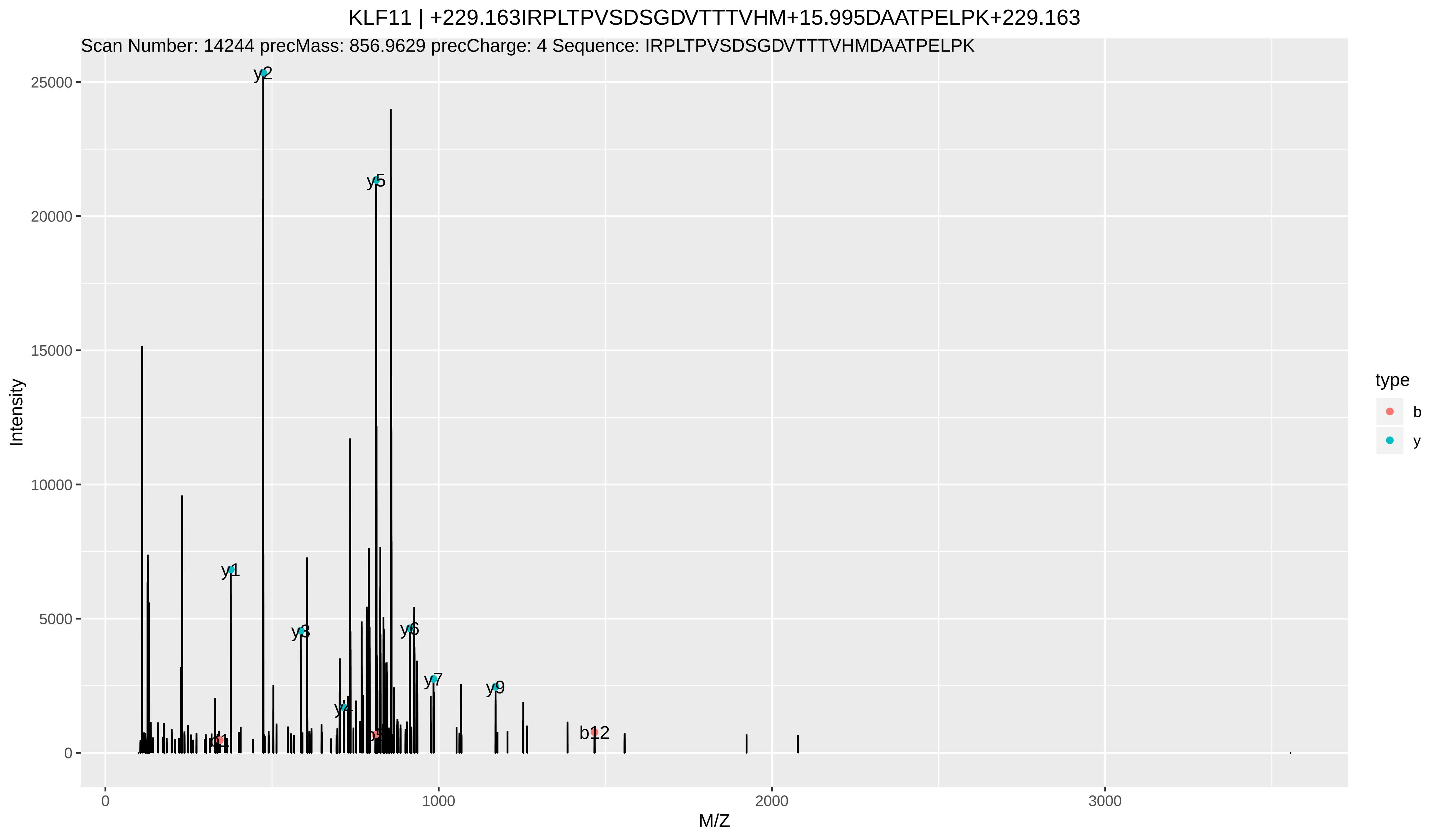

KLF11 | +229.163IRPLTPVSDSGDVTTTVHM+15.995DAATPELPK+229.163
Scan Number: 14244 precMass: 856.9629 precCharge: 4 Sequence: IRPLTPVSDSGDVTTTVHMDAATPELPK
Intensity
M/Z
0
5000
10000
15000
20000
25000
1000
2000
3000
y1
y2
y3
y4
y5
y6
y7
y9
b12
type
b
y

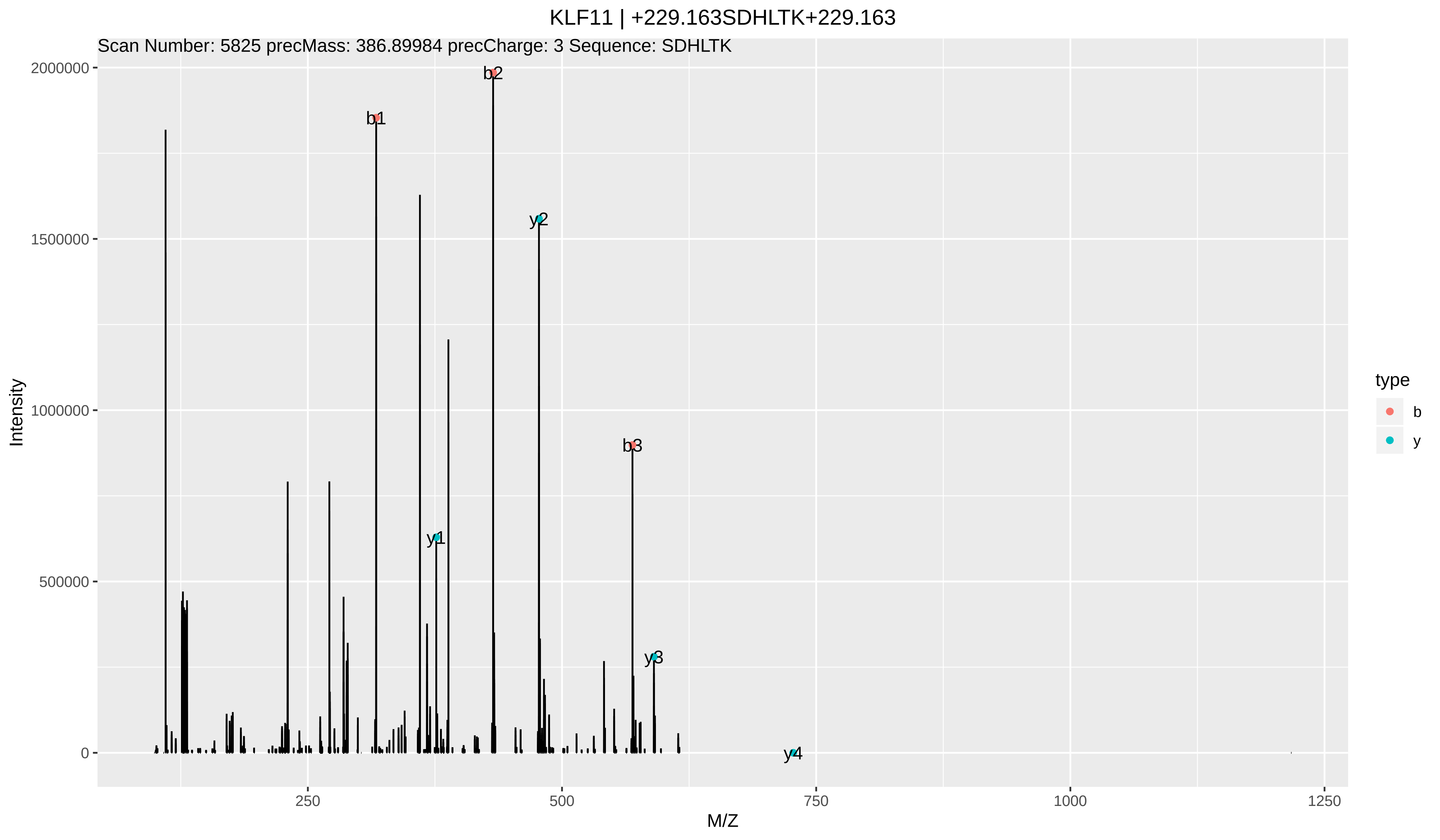

KLF11 | +229.163SDHLTK+229.163
Scan Number: 5825 precMass: 386.89984 precCharge: 3 Sequence: SDHLTK
Intensity
M/Z
0
500000
1000000
1500000
2000000
250
500
750
1000
1250
b1
b2
b3
y1
y2
y3
y4
type
b
y

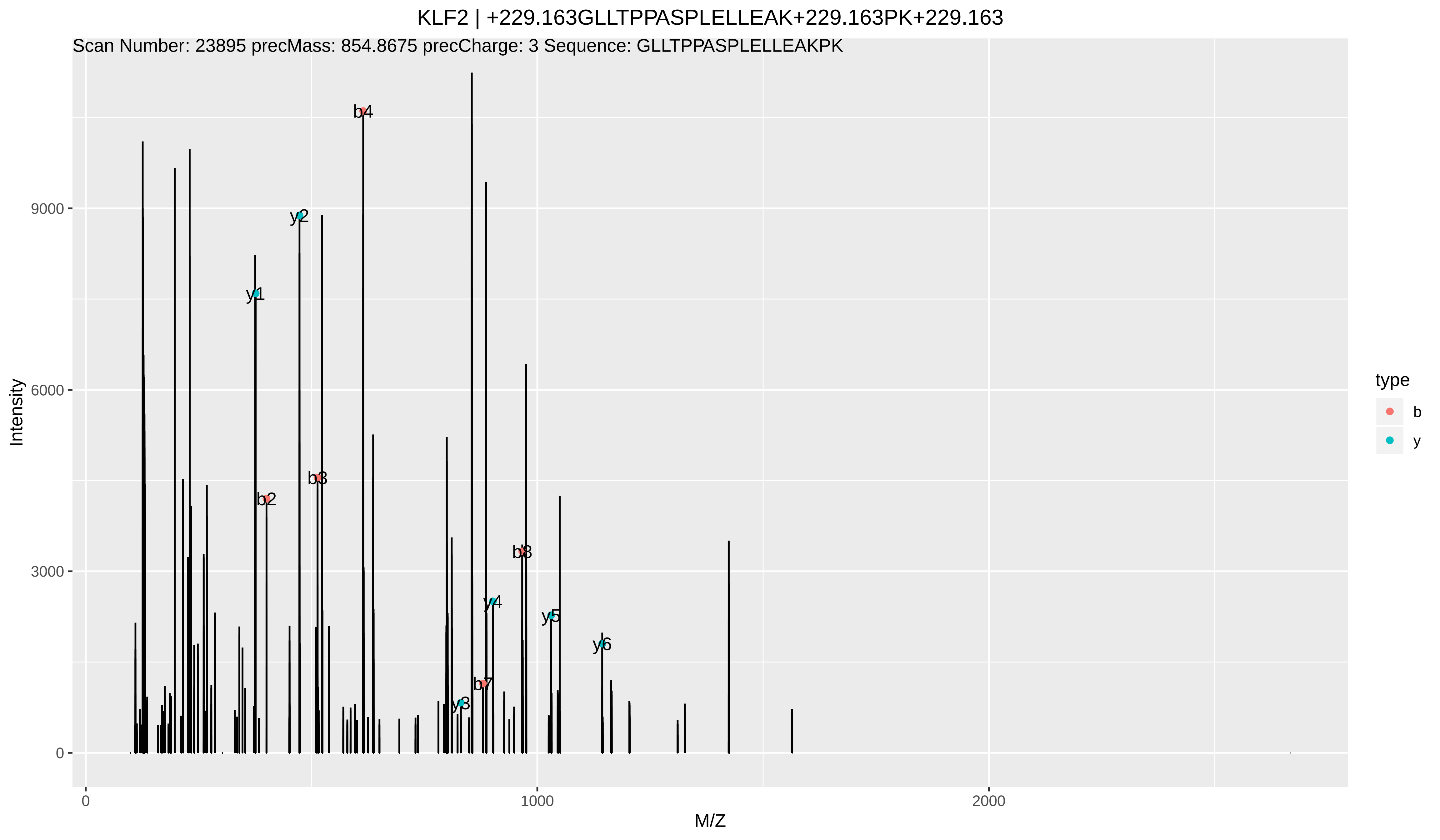

KLF2 | +229.163GLLTPPASPLELLEAK+229.163PK+229.163
Scan Number: 23895 precMass: 854.8675 precCharge: 3 Sequence: GLLTPPASPLELLEAKPK
Intensity
0
3000
6000
9000
M/Z
0
1000
2000
b4
y2
y1
b3
b2
b8
y4
y5
y6
b7
y3
type
b
y

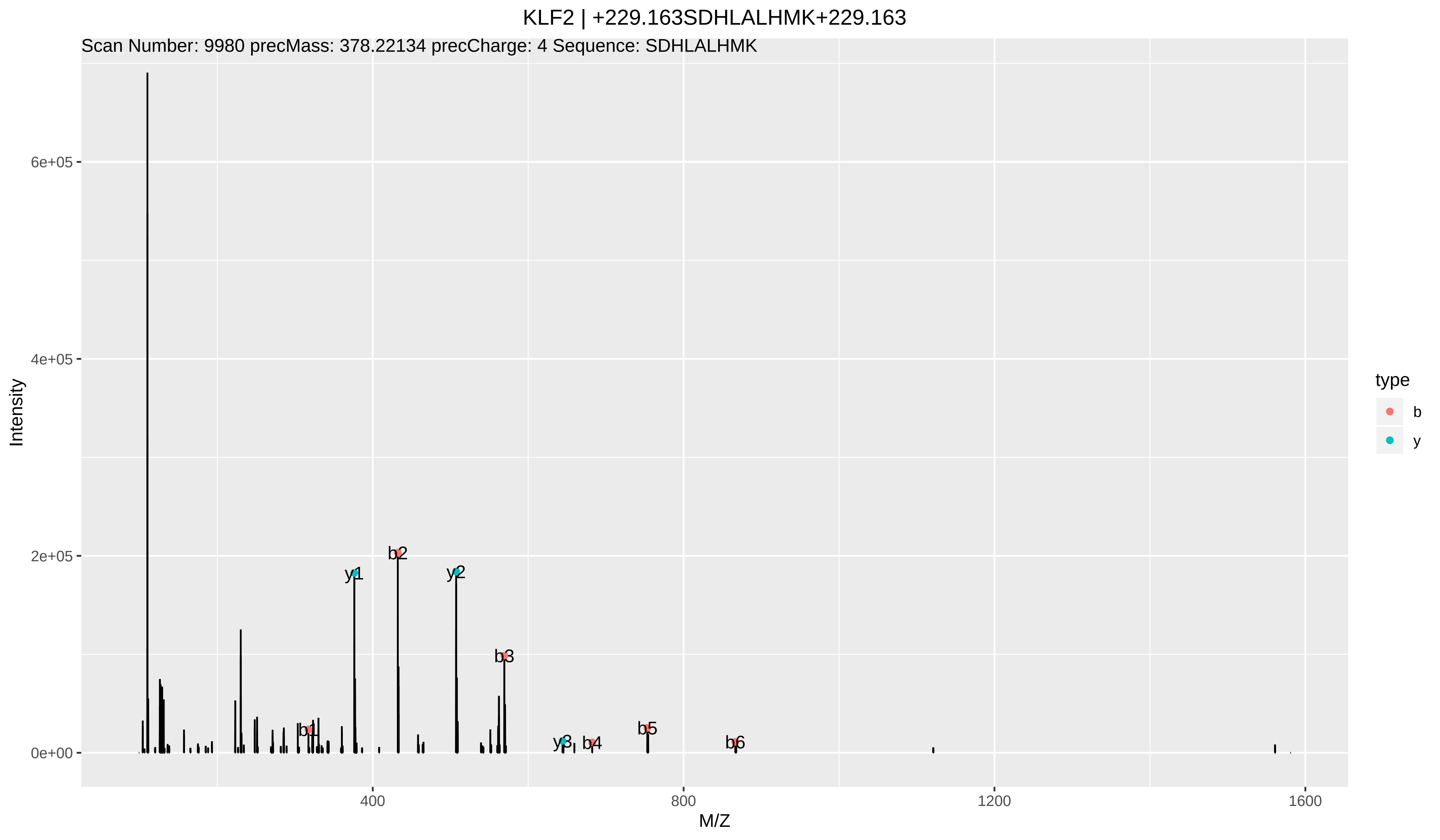
KLF2 | +229.163SDHLALHMK+229.163
Scan Number: 9980 precMass: 378.22134 precCharge: 4 Sequence: SDHLALHMK
Intensity
M/Z
6e+05
4e+05
2e+05
0e+00
400
800
1200
1600
b2
y1
y2
b3
b1
y3
b4
b5
b6
type
b
y

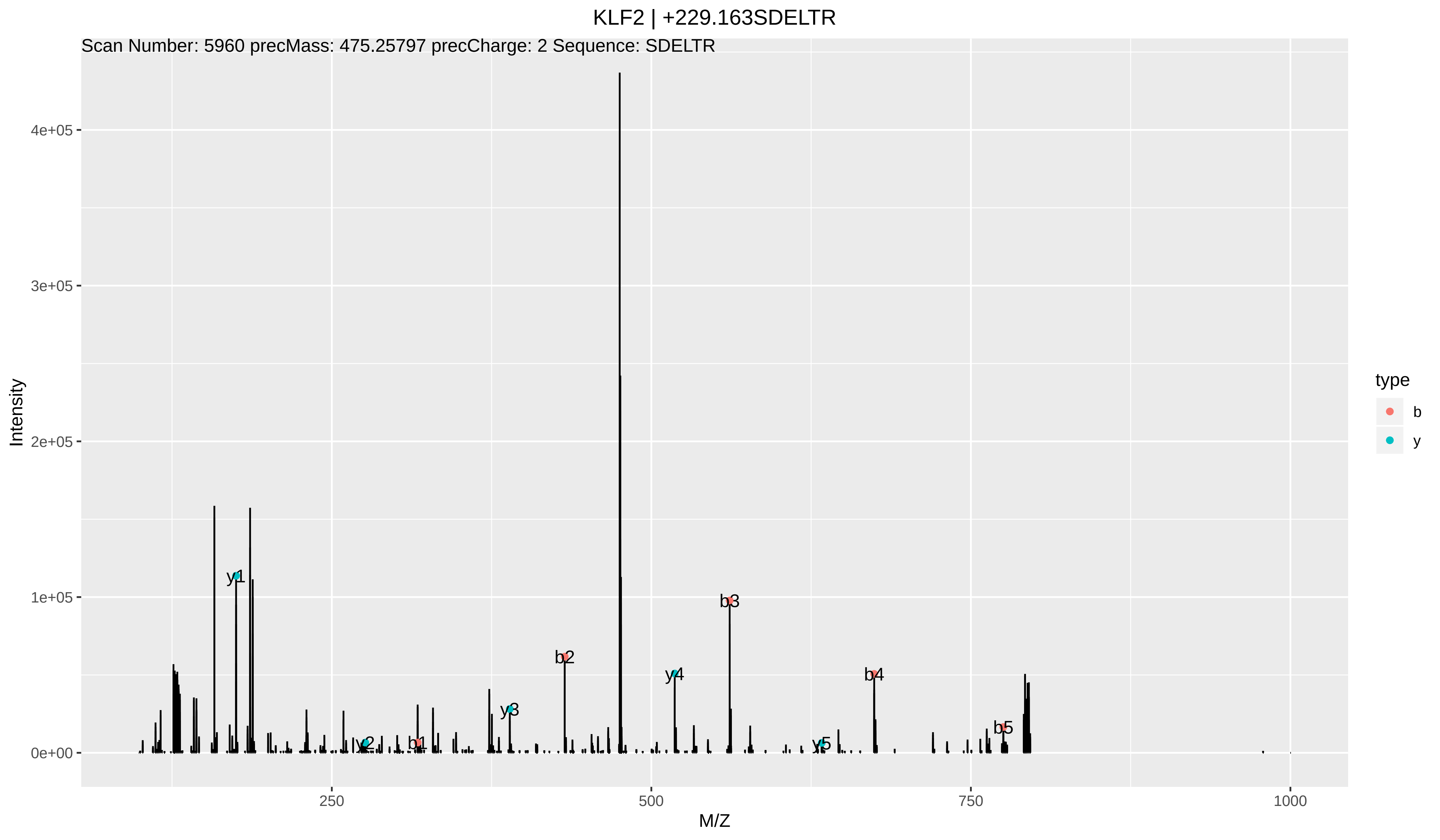
KLF2 | +229.163SDELTR
Scan Number: 5960 precMass: 475.25797 precCharge: 2 Sequence: SDELTR
Intensity
M/Z
type
b
y
y1
y2
b1
y3
b2
y4
b3
y5
b4
b5
0e+00
1e+05
2e+05
3e+05
4e+05
250
500
750
1000

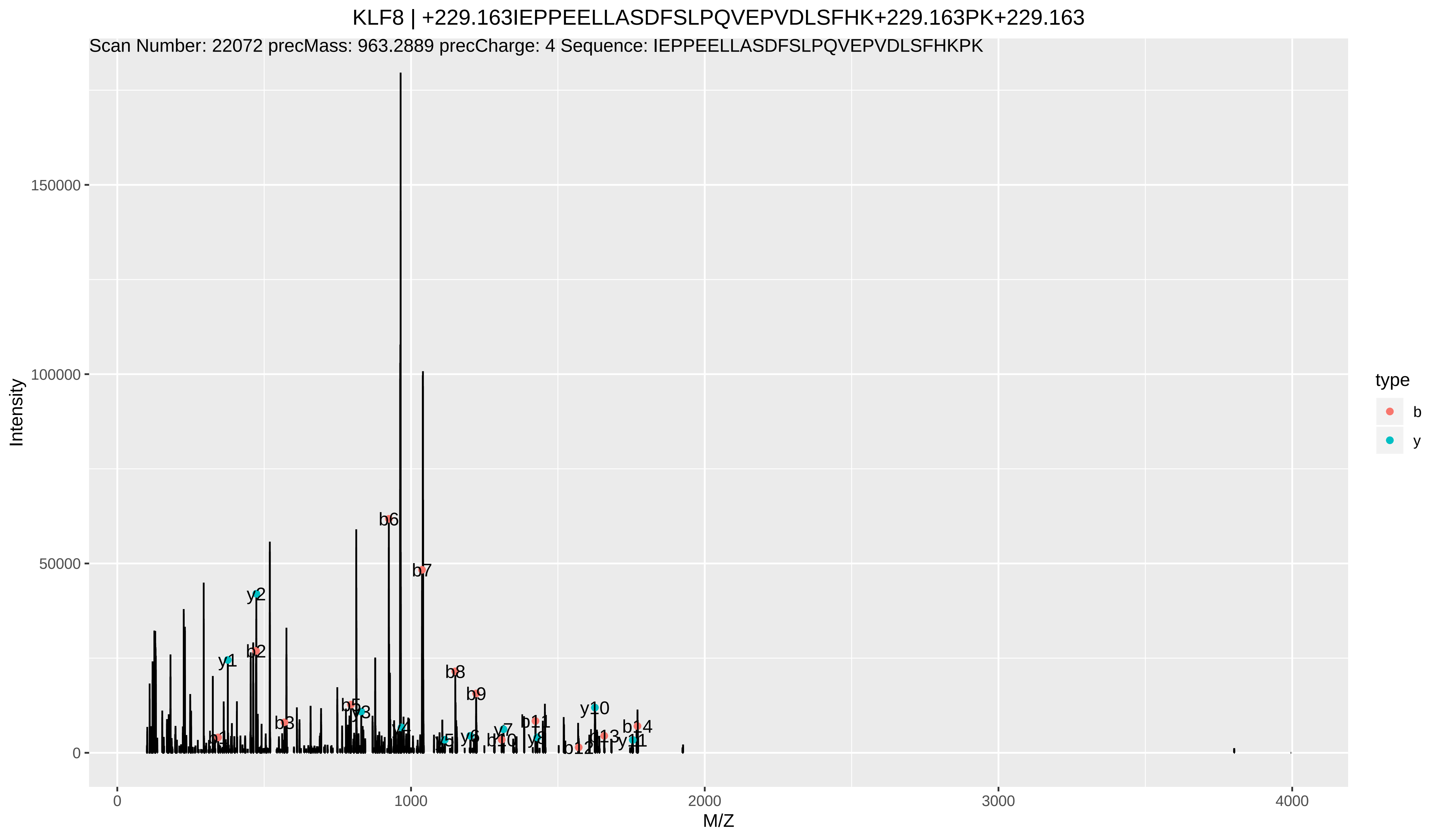

KLF8 | +229.163IEPPEELLASDFSLPQVEPVDLSFHK+229.163PK+229.163
Scan Number: 22072 precMass: 963.2889 precCharge: 4 Sequence: IEPPEELLASDFSLPQVEPVDLSFHKPK
Intensity
M/Z
150000
100000
50000
0
1000
2000
3000
4000
type
b
y
b6
b7
y2
b2
y1
b8
b9
y10
b3
b5
y3
b11
b14
b13
y11
b12
y7
y8
y6
b10

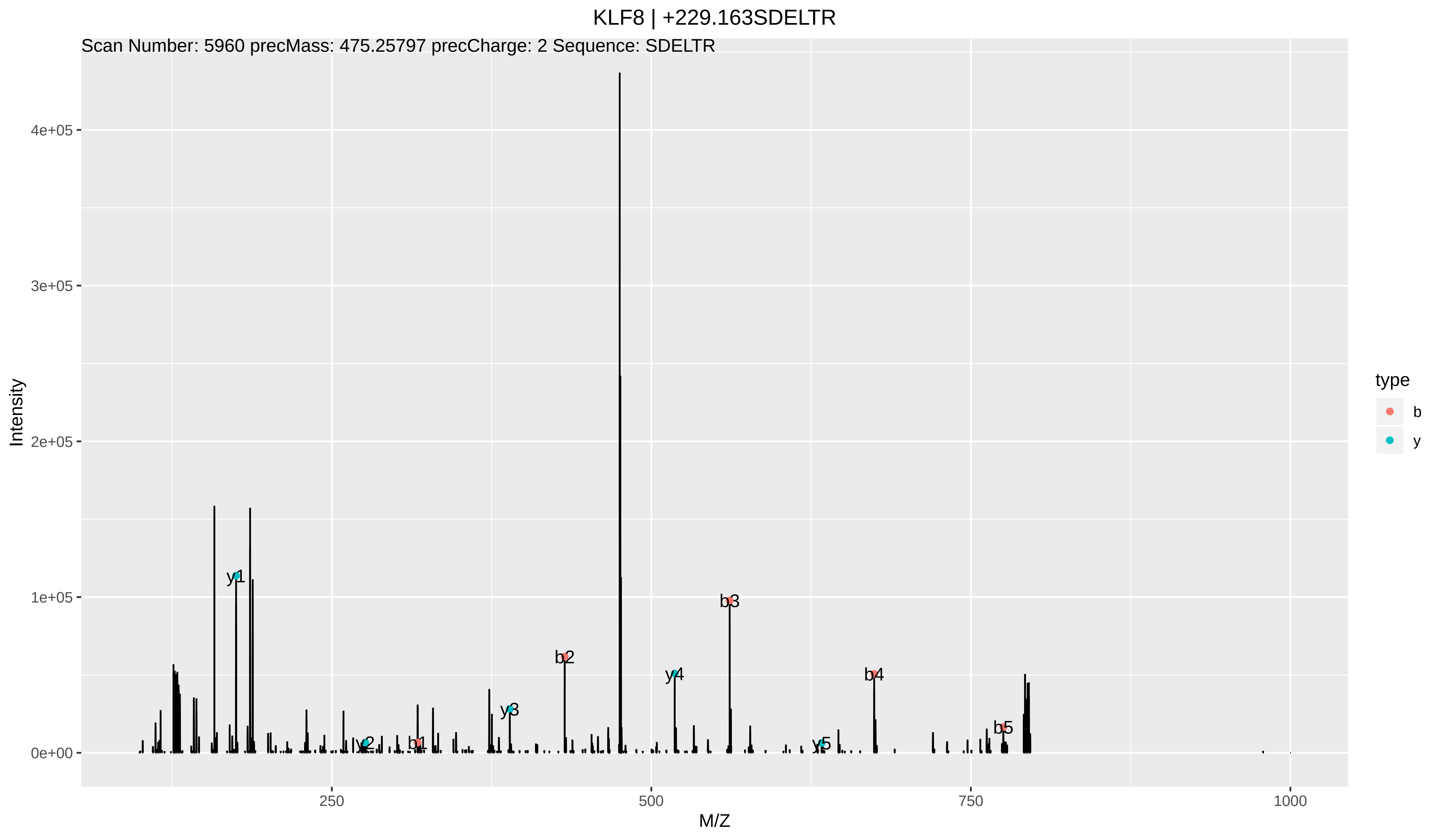

KLF8 | +229.163SDELTR
Scan Number: 5960 precMass: 475.25797 precCharge: 2 Sequence: SDELTR
Intensity
4e+05
3e+05
2e+05
1e+05
0e+00
y1
y2
b1
y3
b2
y4
b3
y5
b4
b5
250
500
750
1000
M/Z
type
b
y

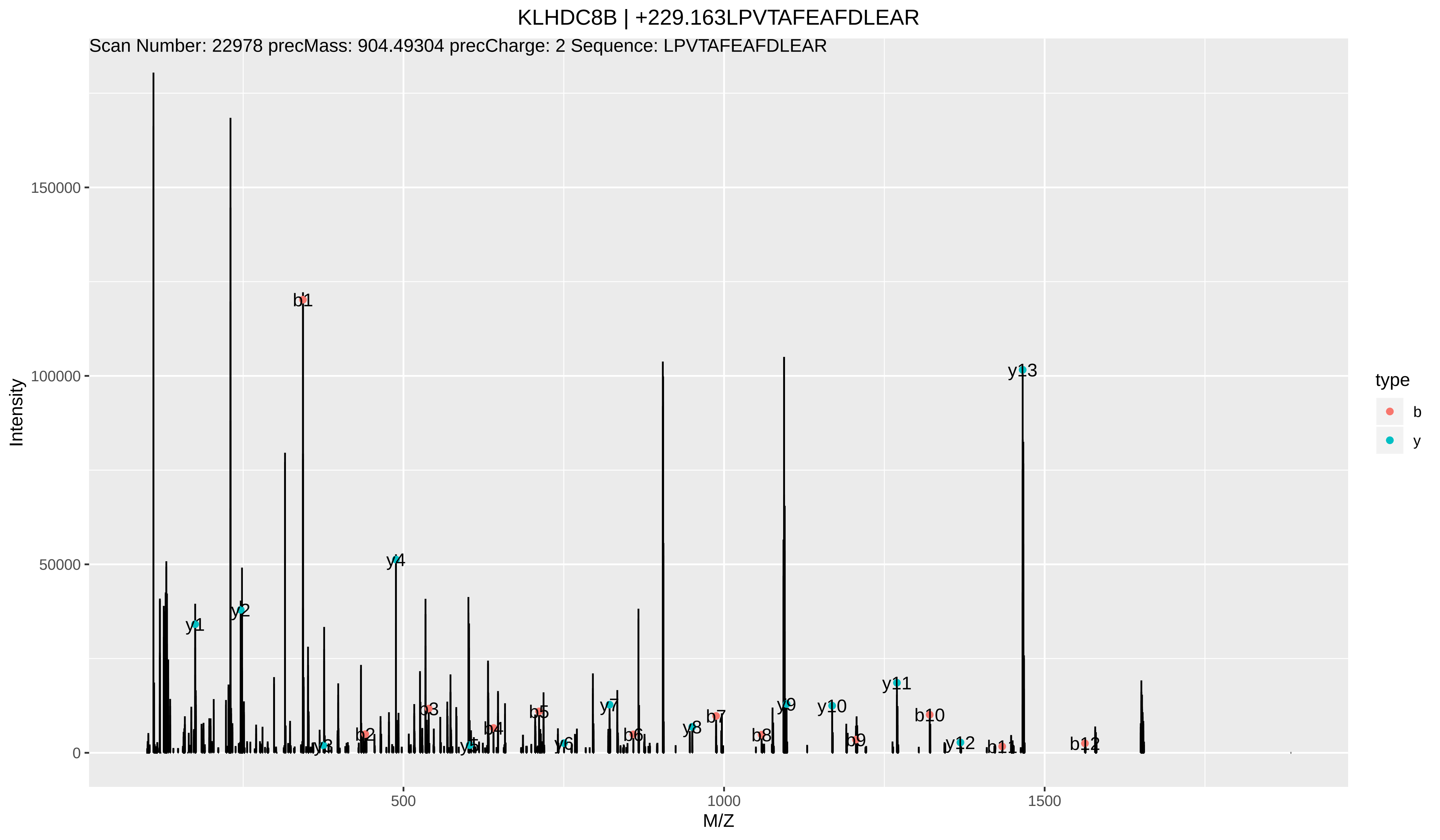

KLHDC8B | +229.163LPVTAFEAFDLEAR
Scan Number: 22978 precMass: 904.49304 precCharge: 2 Sequence: LPVTAFEAFDLEAR
Intensity
M/Z
150000
100000
50000
0
500
1000
1500
type
b
y
b1
b2
b3
b4
b5
b6
b7
b8
b9
b10
b11
b12
y1
y2
y3
y4
y5
y6
y7
y8
y9
y10
y11
y12
y13

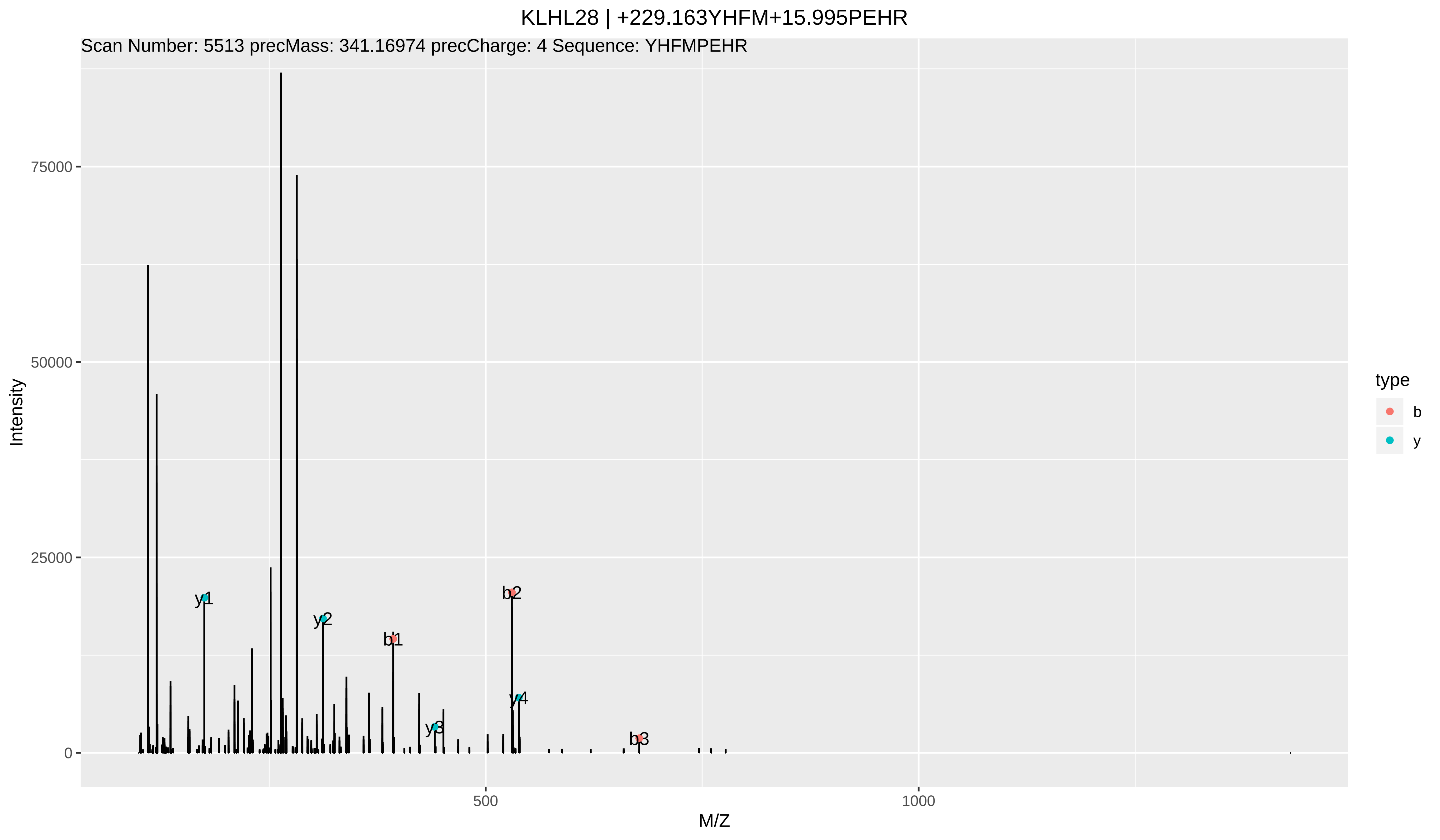
KLHL28 | +229.163YHFM+15.995PEHR
Scan Number: 5513 precMass: 341.16974 precCharge: 4 Sequence: YHFMPEHR
Intensity
75000
50000
25000
0
M/Z
500
1000
y1
y2
b1
b2
y3
y4
b3
type
b
y

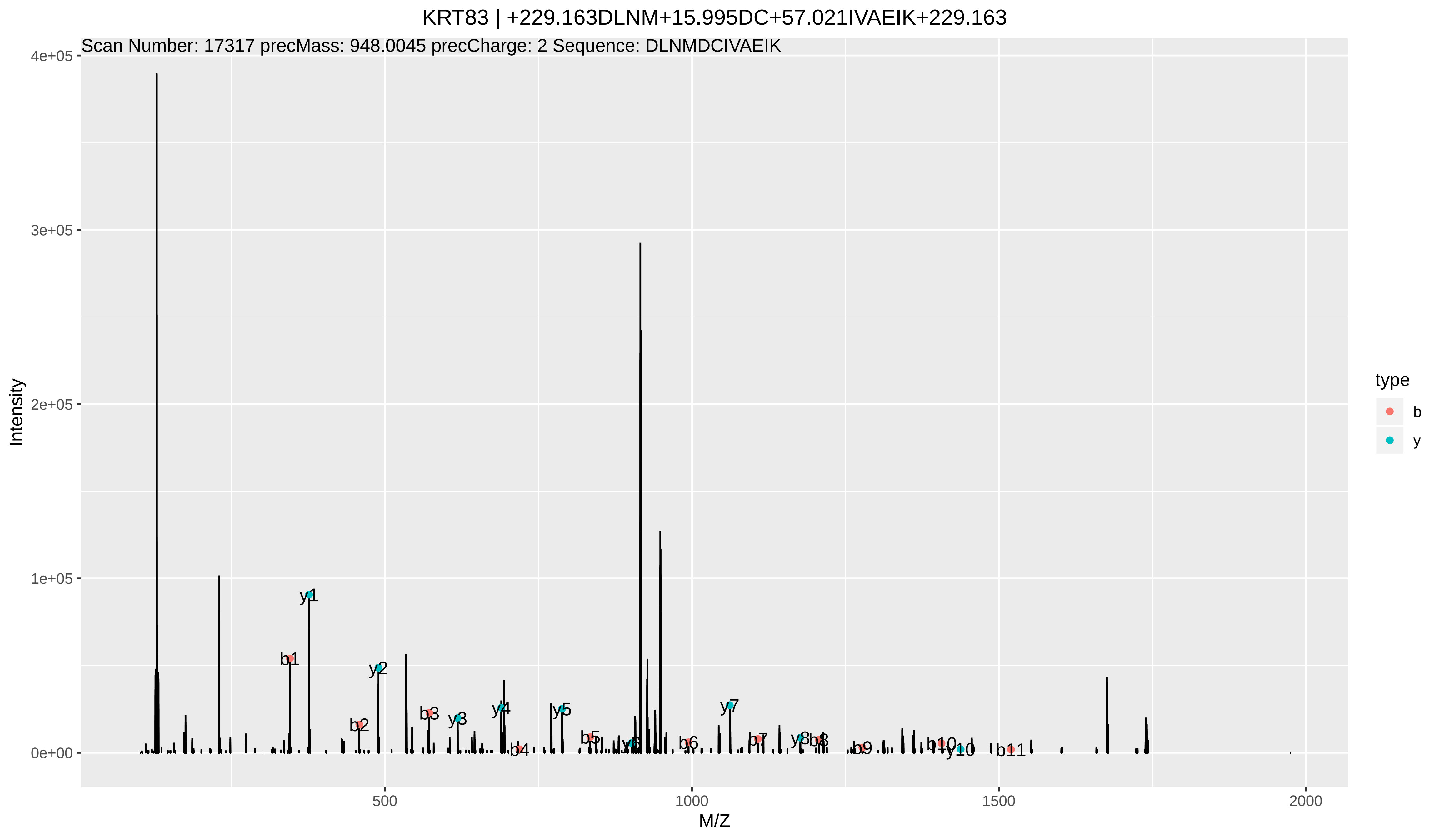

KRT83 | +229.163DLNM+15.995DC+57.021IVAEIK+229.163
Scan Number: 17317 precMass: 948.0045 precCharge: 2 Sequence: DLNMDCIVAEIK
Intensity
4e+05
3e+05
2e+05
1e+05
0e+00
M/Z
500
1000
1500
2000
type
b
y
y1
b1
y2
b2
b3
y3
y4
b4
y5
b5
y6
b6
y7
b7
y8
b8
b9
b10
y10
b11

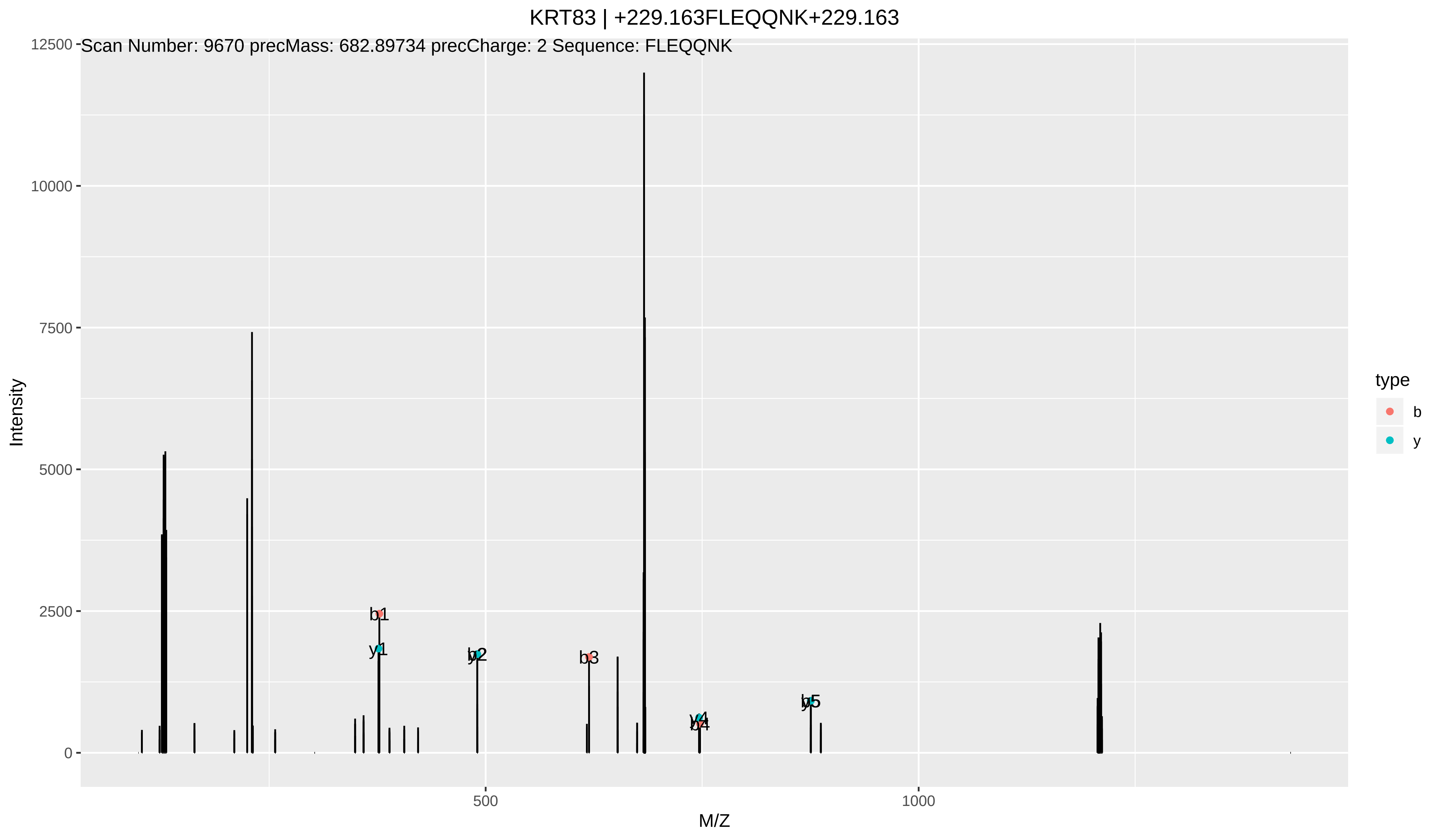

KRT83 | +229.163FLEQQNK+229.163
Scan Number: 9670 precMass: 682.89734 precCharge: 2 Sequence: FLEQQNK
Intensity
M/Z
12500
10000
7500
5000
2500
0
500
1000
b1
y1
b2
y2
b3
y4
b4
b5
y5
type
b
y

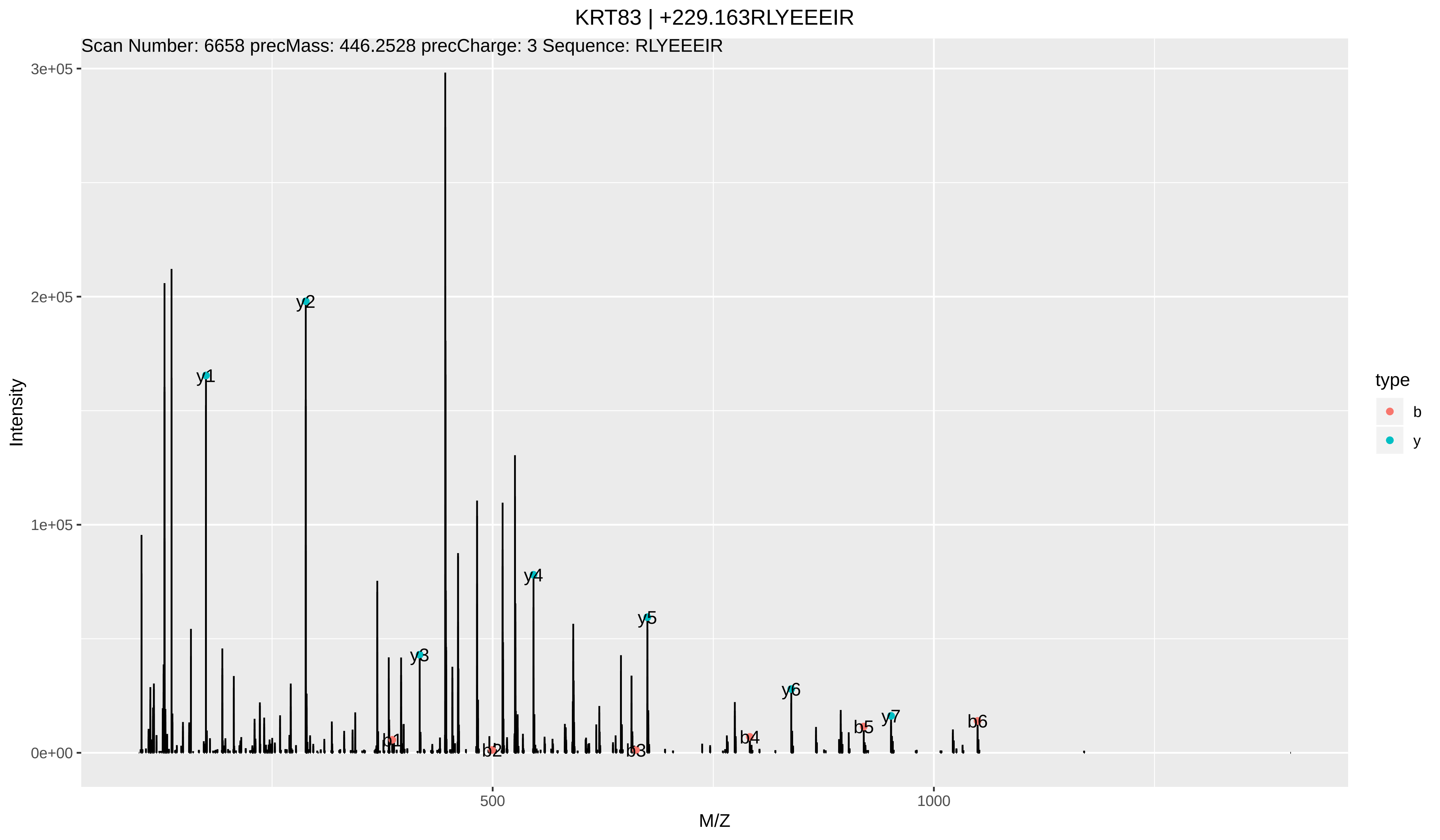

KRT83 | +229.163RLYEEEIR
Scan Number: 6658 precMass: 446.2528 precCharge: 3 Sequence: RLYEEEIR
Intensity
M/Z
3e+05
2e+05
1e+05
0e+00
500
1000
y1
y2
y3
y4
y5
y6
y7
b1
b2
b3
b4
b5
b6
type
b
y

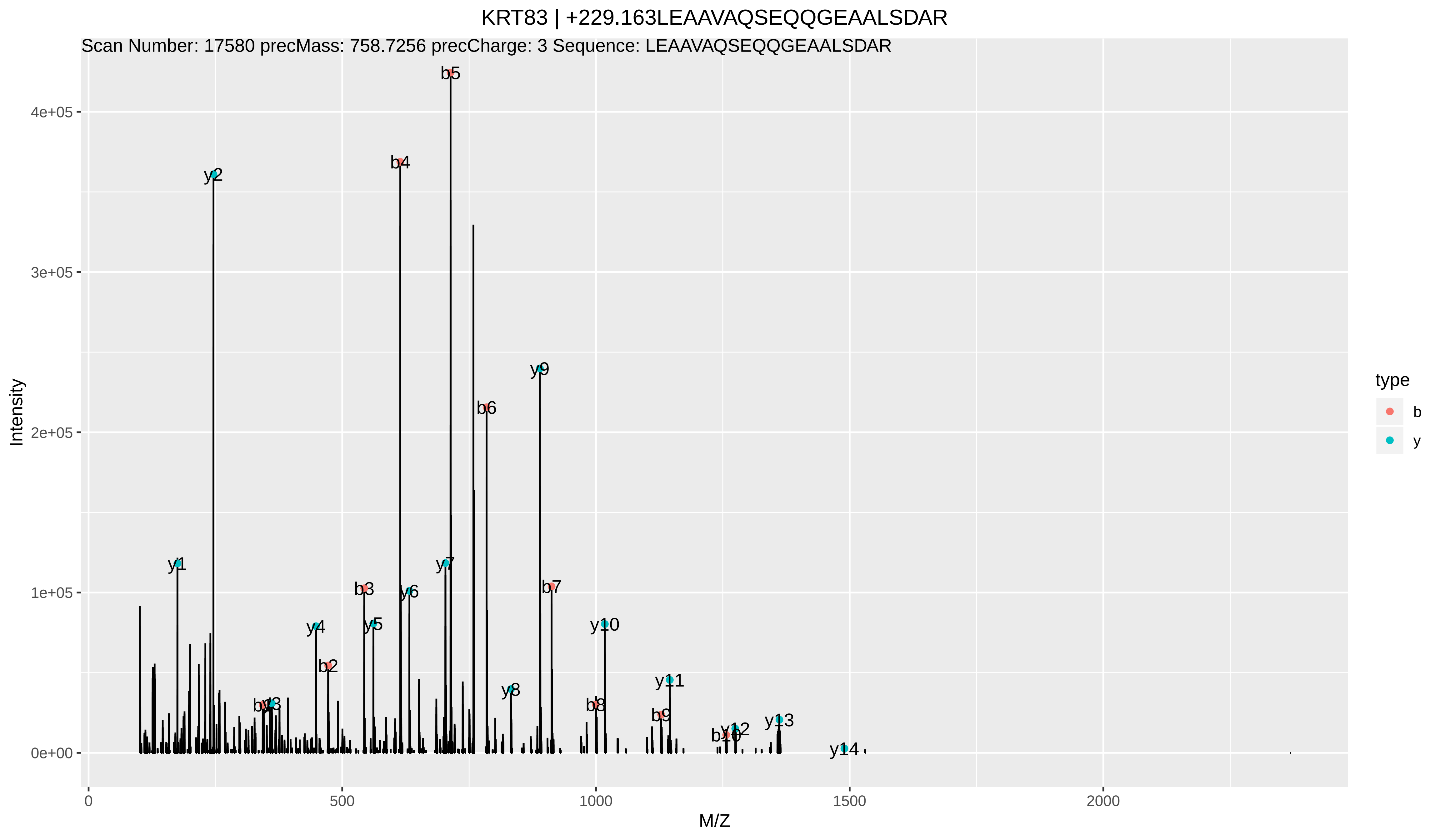

KRT83 | +229.163LEAAVAQSEQQGEAALSDAR
Scan Number: 17580 precMass: 758.7256 precCharge: 3 Sequence: LEAAVAQSEQQGEAALSDAR
b5
b4
y2
y9
b6
y1
y7
b3
y6
b7
y4
y5
y10
b2
y11
y8
b1
y3
b8
b9
y12
b10
y13
y14
4e+05
3e+05
2e+05
1e+05
0e+00
0
500
1000
1500
2000
Intensity
M/Z
type
b
y

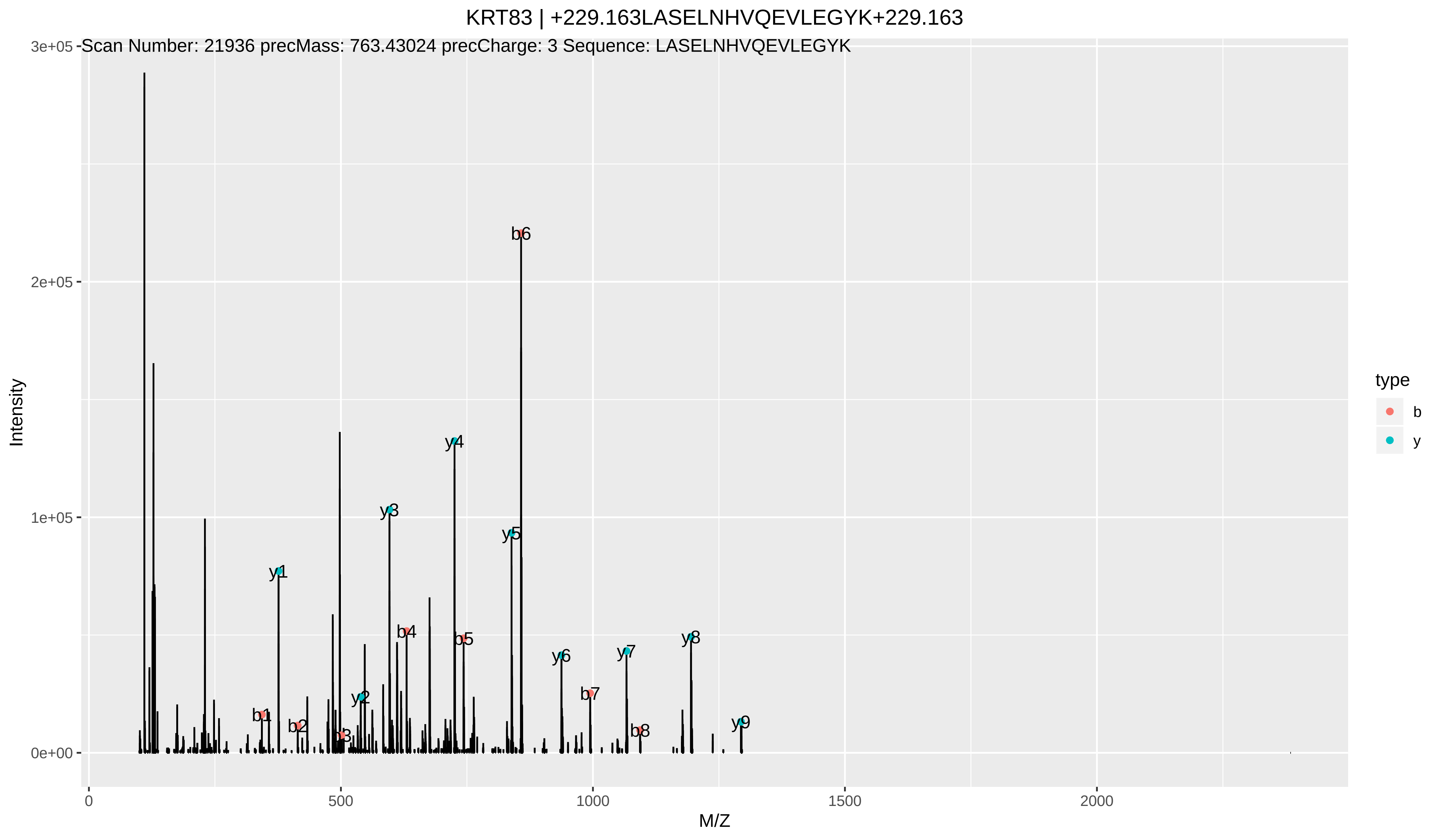
KRT83 | +229.163LASELNHVQEVLEGYK+229.163
Scan Number: 21936 precMass: 763.43024 precCharge: 3 Sequence: LASELNHVQEVLEGYK
Intensity
M/Z
3e+05
2e+05
1e+05
0e+00
0
500
1000
1500
2000
b6
y4
y3
y5
y1
b4
b5
y6
y7
y8
b7
y2
b1
b2
b3
b8
y9
type
b
y

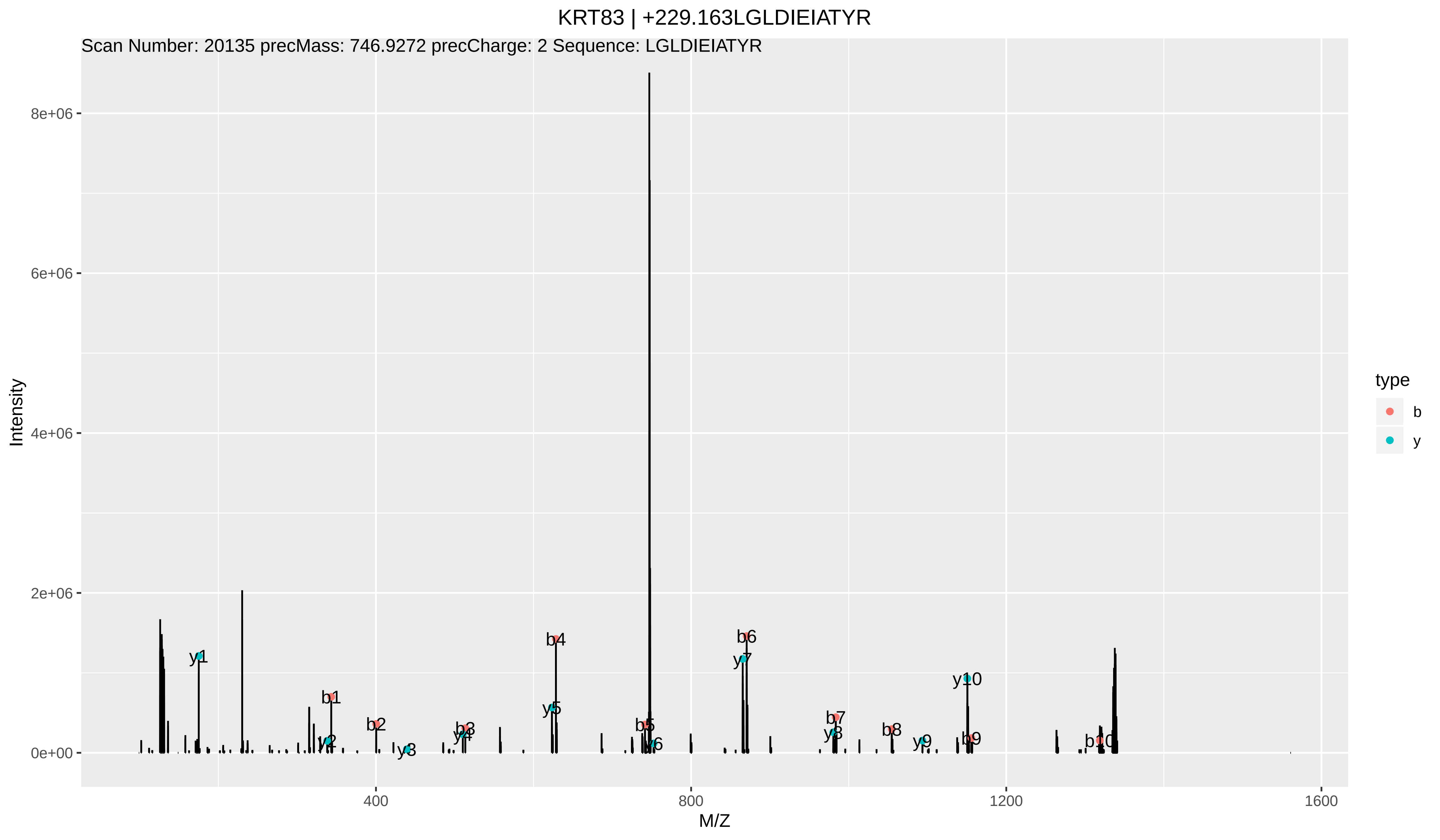

KRT83 | +229.163LGLDIEIATYR
Scan Number: 20135 precMass: 746.9272 precCharge: 2 Sequence: LGLDIEIATYR
Intensity
M/Z
8e+06
6e+06
4e+06
2e+06
0e+00
400
800
1200
1600
type
b
y
y1
b1
y2
b2
y3
b3
y4
b4
y5
b5
y6
b6
y7
b7
y8
b8
y9
b9
y10
b10

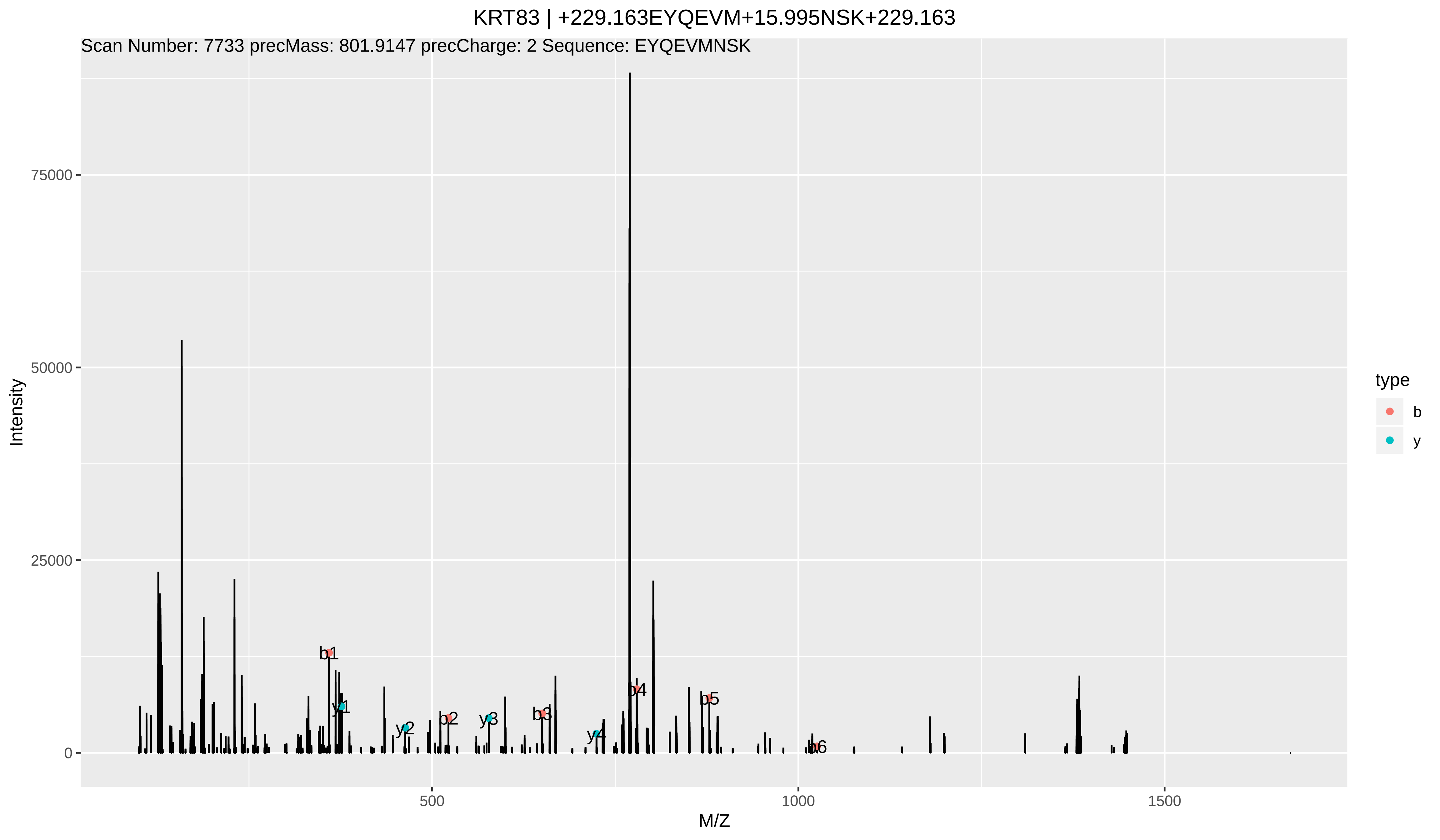
KRT83 | +229.163EYQEVM+15.995NSK+229.163
Scan Number: 7733 precMass: 801.9147 precCharge: 2 Sequence: EYQEVMNSK
Intensity
75000
50000
25000
0
M/Z
500
1000
1500
b1
y1
y2
b2
y3
b3
y4
b4
b5
b6
type
b
y

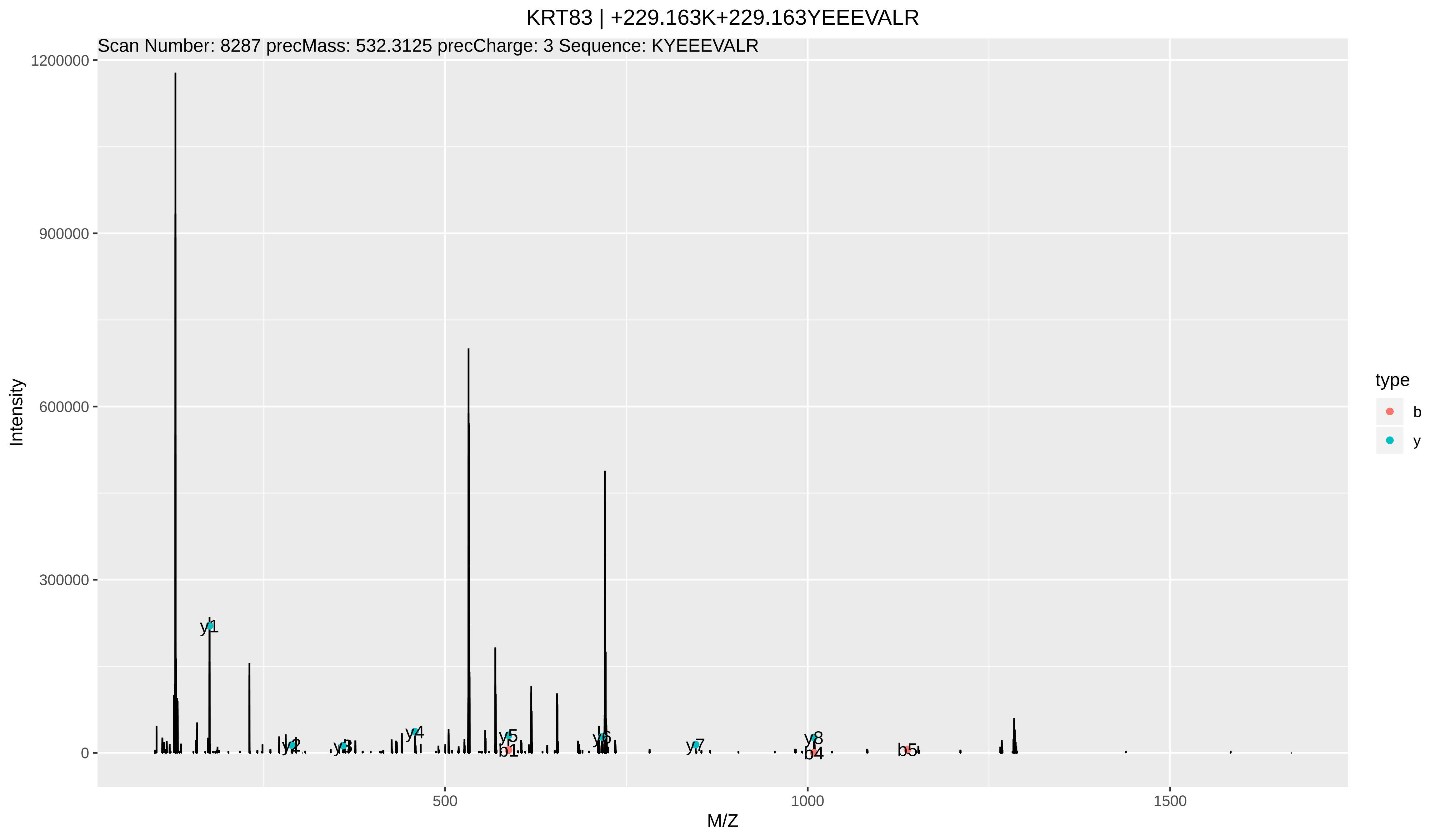

KRT83 | +229.163K+229.163YEEEVALR
Scan Number: 8287 precMass: 532.3125 precCharge: 3 Sequence: KYEEEVALR
Intensity
1200000
900000
600000
300000
0
M/Z
500
1000
1500
y1
y2
y3
y4
y5
b1
y6
y7
y8
b4
b5
type
b
y

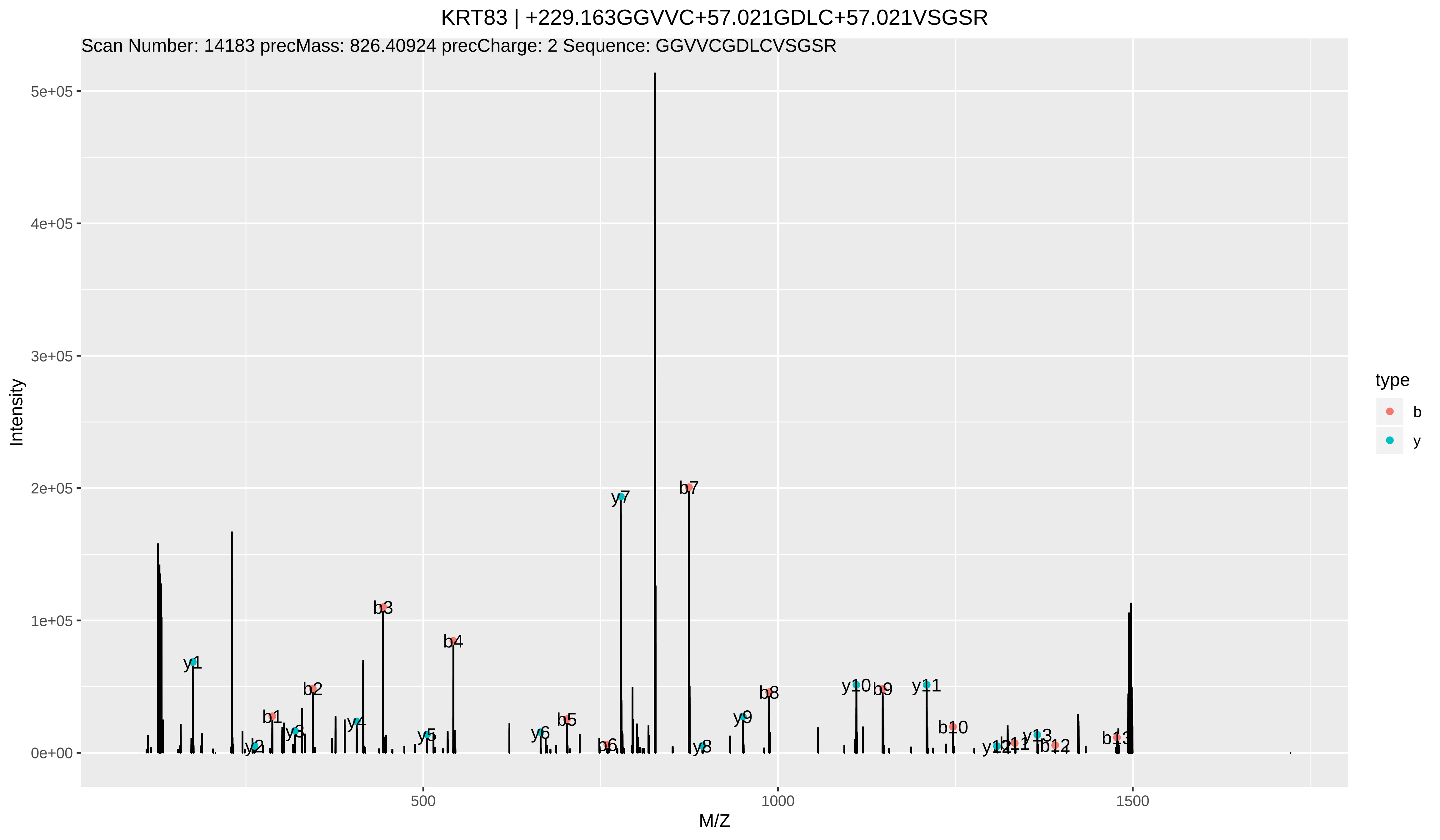
KRT83 | +229.163GGVVC+57.021GDLC+57.021VSGSR
Scan Number: 14183 precMass: 826.40924 precCharge: 2 Sequence: GGVVCGDLCVSGSR
Intensity
M/Z
type
b
y

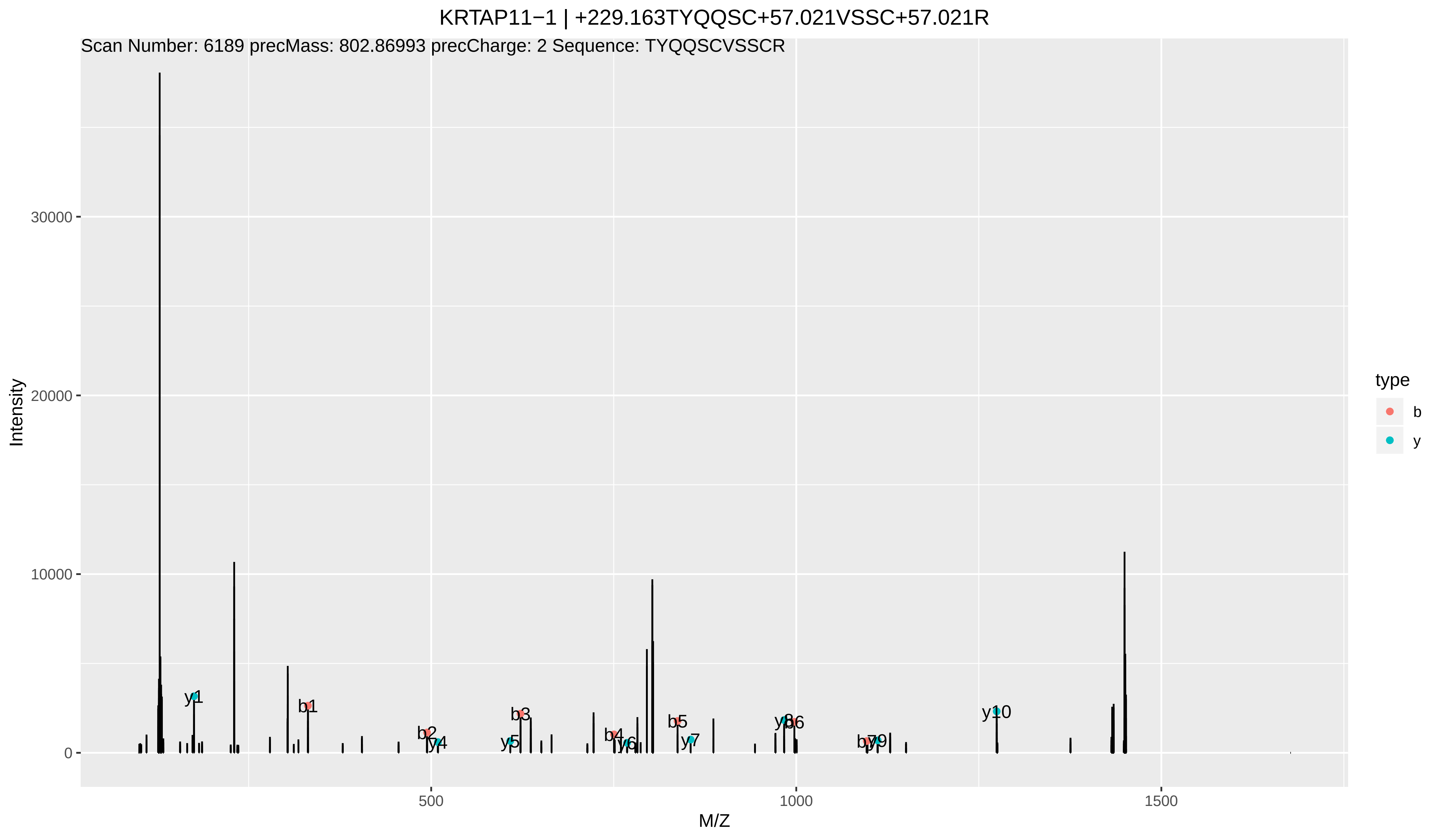

KRTAP11−1 | +229.163TYQQSC+57.021VSSC+57.021R
Scan Number: 6189 precMass: 802.86993 precCharge: 2 Sequence: TYQQSCVSSCR
Intensity
30000
20000
10000
0
M/Z
500
1000
1500
y1
b1
b2
y4
b3
y5
b4
y6
b5
y7
y8
b6
b7
y9
y10
type
b
y

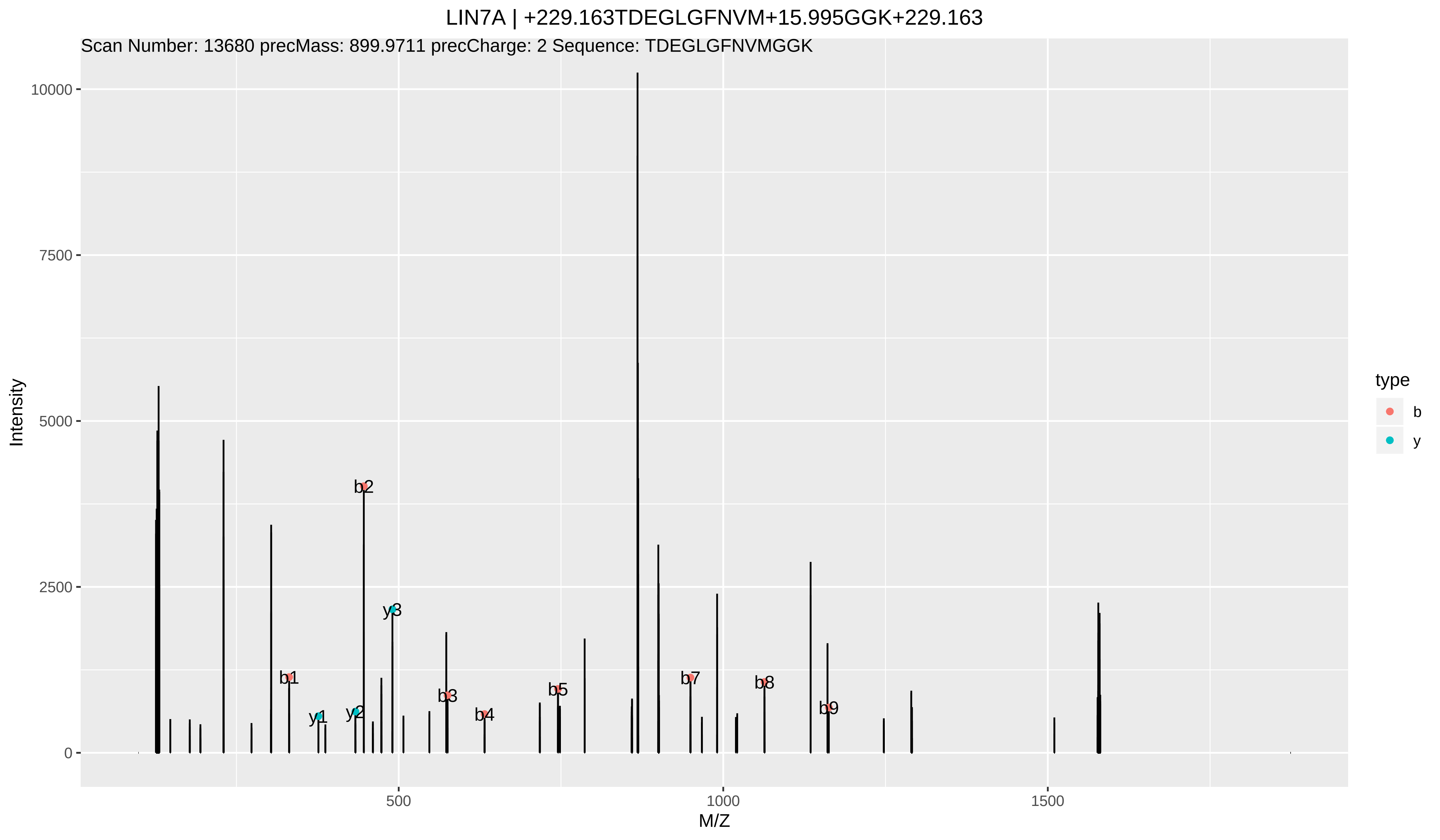

LIN7A | +229.163TDEGLGFNVM+15.995GGK+229.163
Scan Number: 13680 precMass: 899.9711 precCharge: 2 Sequence: TDEGLGFNVMGGK
Intensity
10000
7500
5000
2500
0
M/Z
500
1000
1500
b1
b2
b3
b4
b5
b7
b8
b9
y1
y2
y3
type
b
y

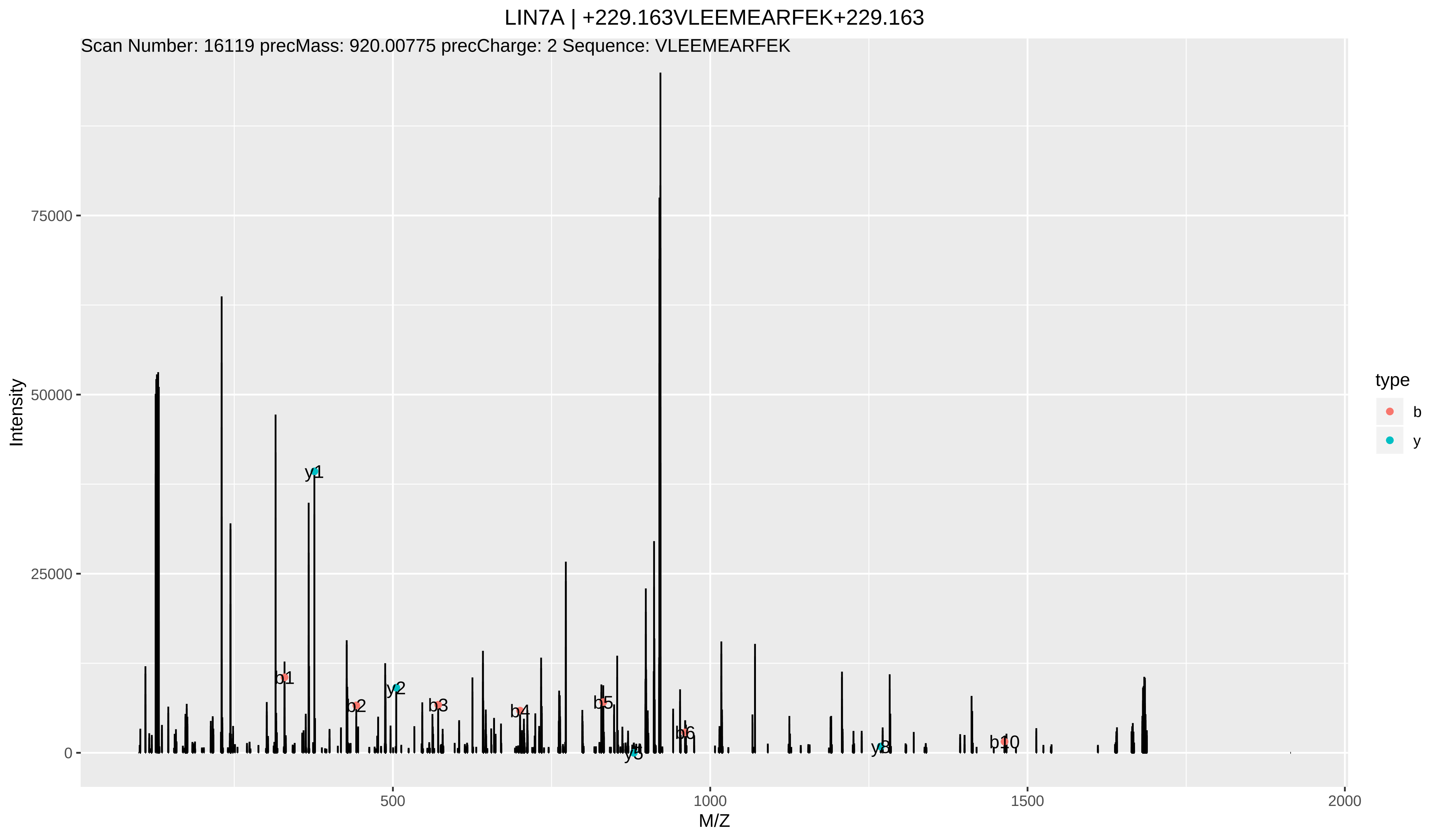

LIN7A | +229.163VLEEMEARFEK+229.163
Scan Number: 16119 precMass: 920.00775 precCharge: 2 Sequence: VLEEMEARFEK
Intensity
M/Z
75000
50000
25000
0
500
1000
1500
2000
y1
y2
y3
y8
b1
b2
b3
b4
b5
b6
b10
type
b
y

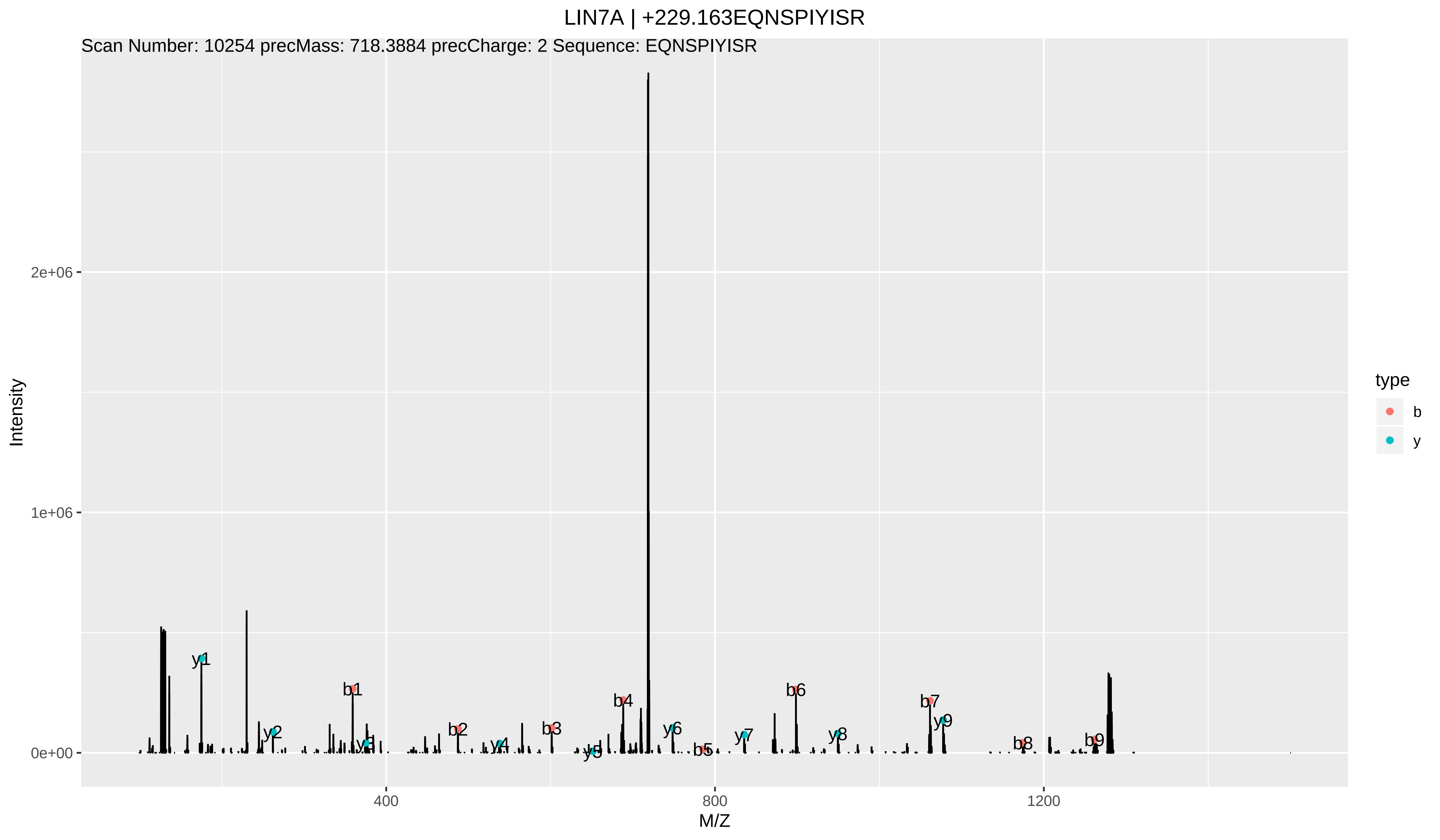

LIN7A | +229.163EQNSPIYISR
Scan Number: 10254 precMass: 718.3884 precCharge: 2 Sequence: EQNSPIYISR
Intensity
2e+06
1e+06
0e+00
400
800
1200
M/Z
y1
b1
y2
y3
b2
y4
b3
y5
b4
y6
b5
y7
b6
y8
b7
y9
b8
b9
type
b
y

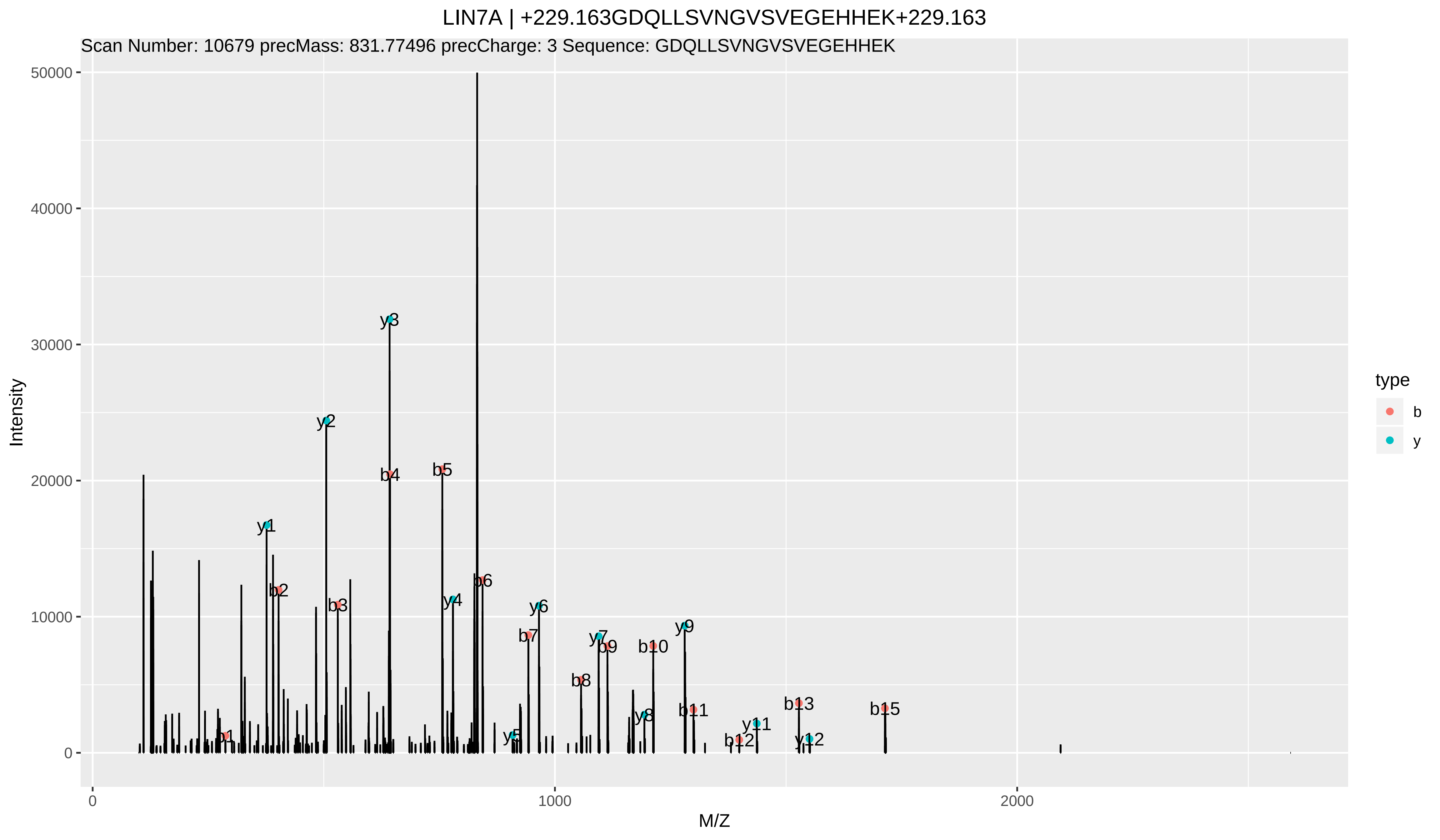

LIN7A | +229.163GDQLLSVNGVSVEGEHHEK+229.163
Scan Number: 10679 precMass: 831.77496 precCharge: 3 Sequence: GDQLLSVNGVSVEGEHHEK
Intensity
M/Z
type
b
y
y1
y2
y3
y4
y5
y6
y7
y8
y9
y11
y12
b1
b2
b3
b4
b5
b6
b7
b8
b9
b10
b11
b12
b13
b15
0
10000
20000
30000
40000
50000
1000
2000

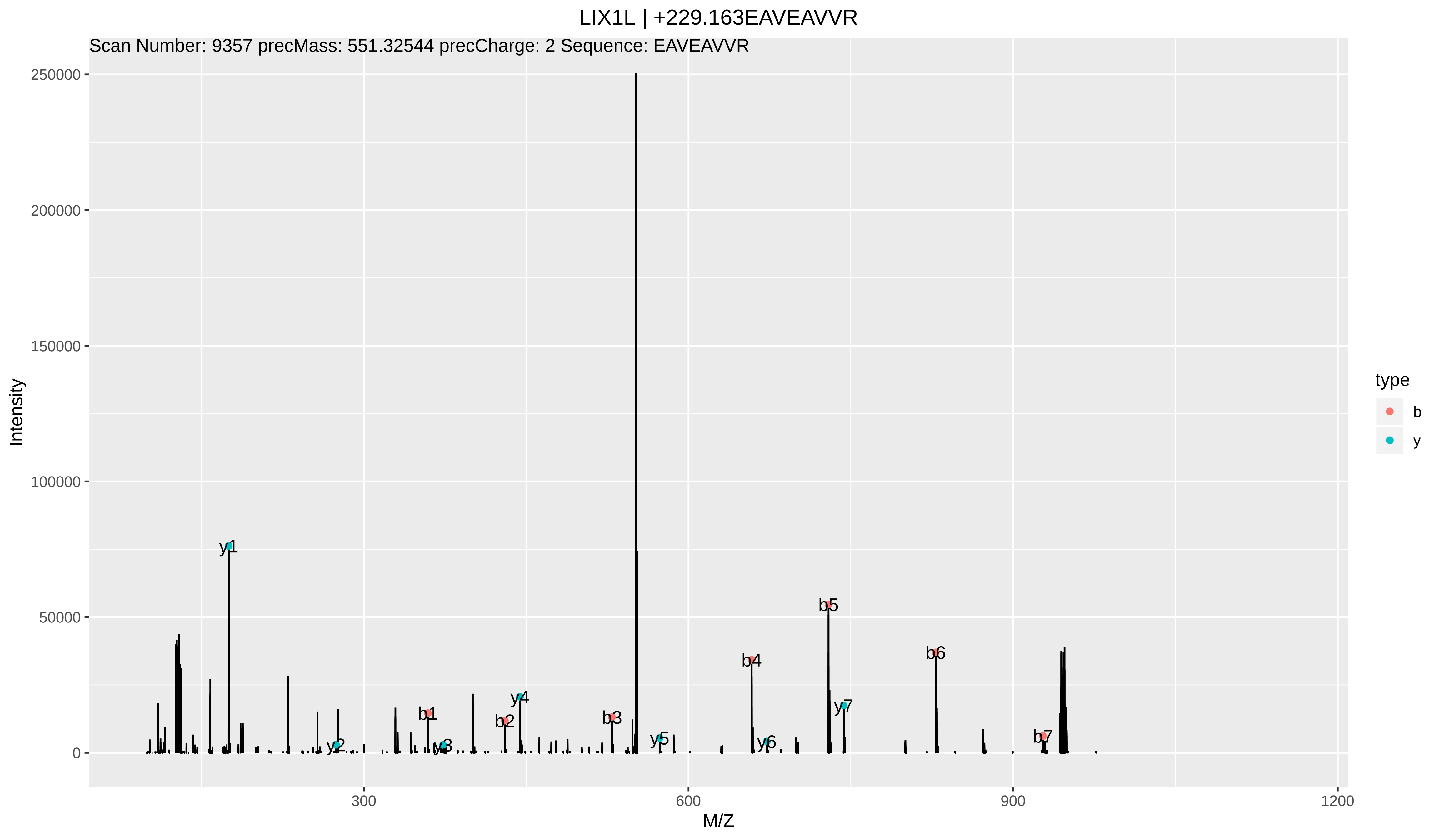

LIX1L | +229.163EAVEAVVR
Scan Number: 9357 precMass: 551.32544 precCharge: 2 Sequence: EAVEAVVR
Intensity
250000
200000
150000
100000
50000
0
M/Z
300
600
900
1200
y1
y2
b1
y3
y4
b2
b3
y5
b4
y6
b5
y7
b6
b7
type
b
y

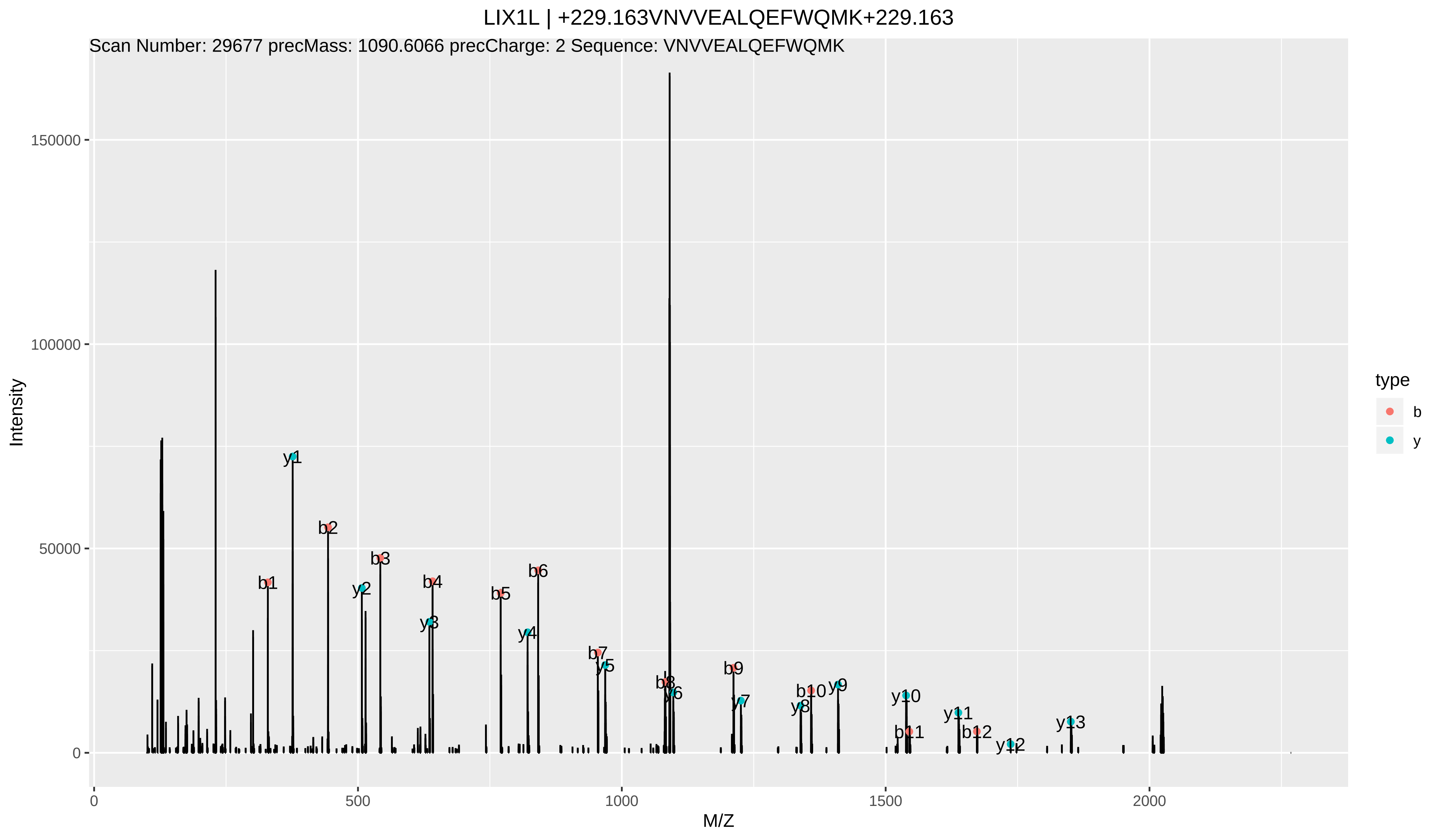

LIX1L | +229.163VNVVEALQEFWQMK+229.163
Scan Number: 29677 precMass: 1090.6066 precCharge: 2 Sequence: VNVVEALQEFWQMK
Intensity
M/Z
type
b
y
b1
b2
b3
b4
b5
b6
b7
b8
b9
b10
b11
b12
y1
y2
y3
y4
y5
y6
y7
y8
y9
y10
y11
y12
y13
0
50000
100000
150000
500
1000
1500
2000

LMF1 | +229.163TGYSDPEPESPPAPGR

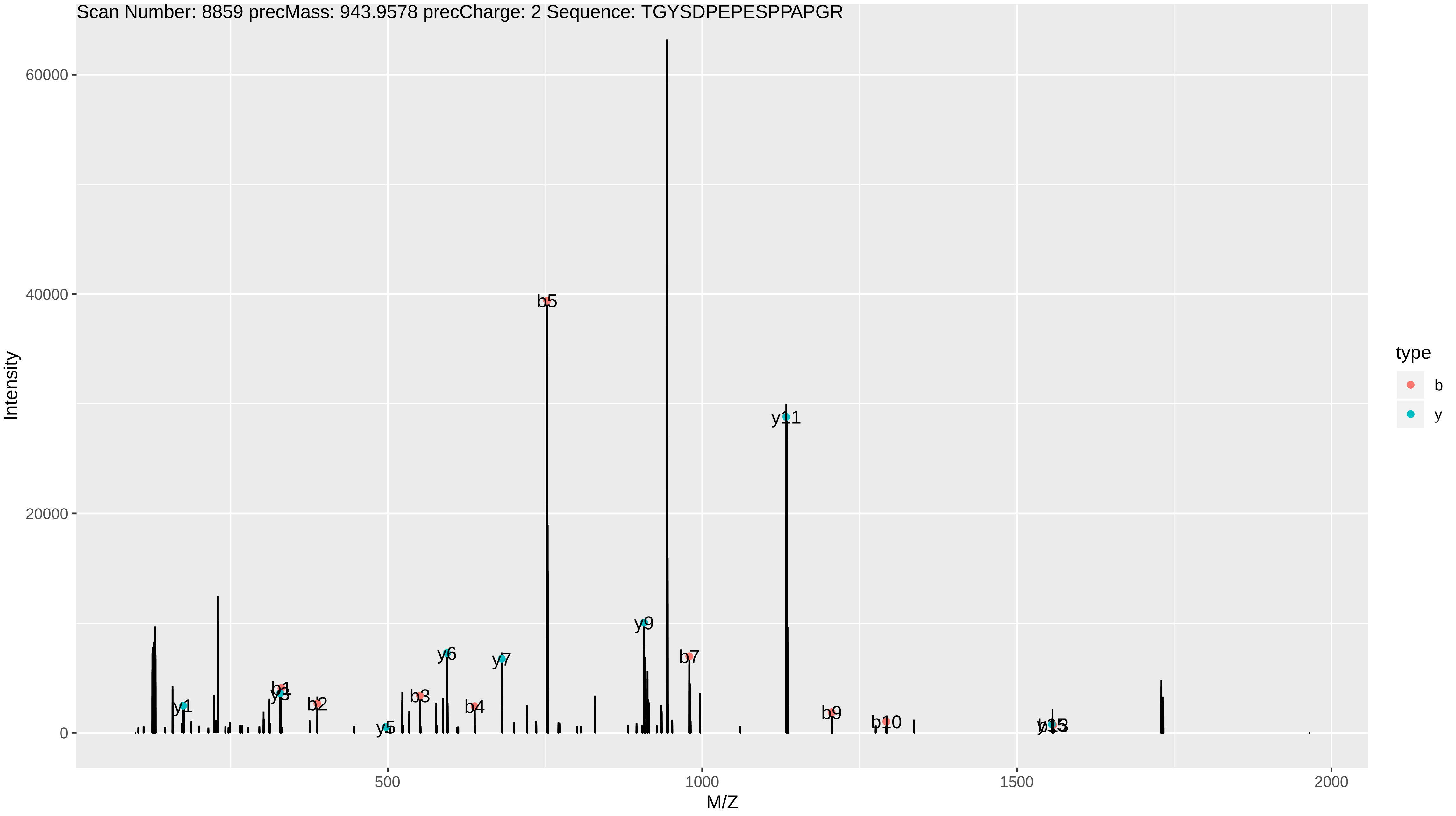

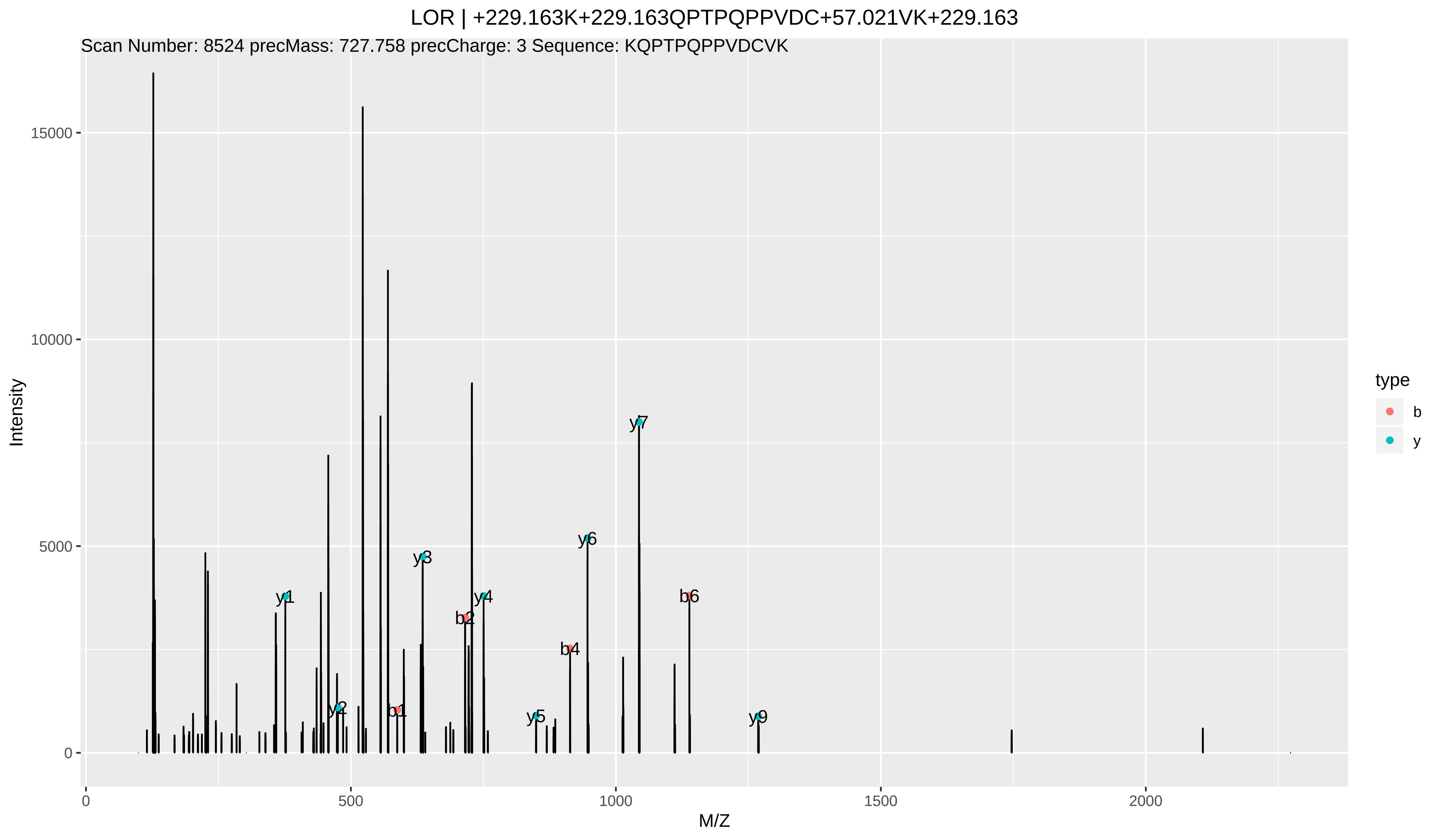

LOR | +229.163K+229.163QPTPQPPVDC+57.021VK+229.163
Scan Number: 8524 precMass: 727.758 precCharge: 3 Sequence: KQPTPQPPVDCVK
Intensity
M/Z
0
5000
10000
15000
0
500
1000
1500
2000
y1
y2
b1
y3
b2
y4
y5
b4
y6
y7
b6
y9
type
b
y

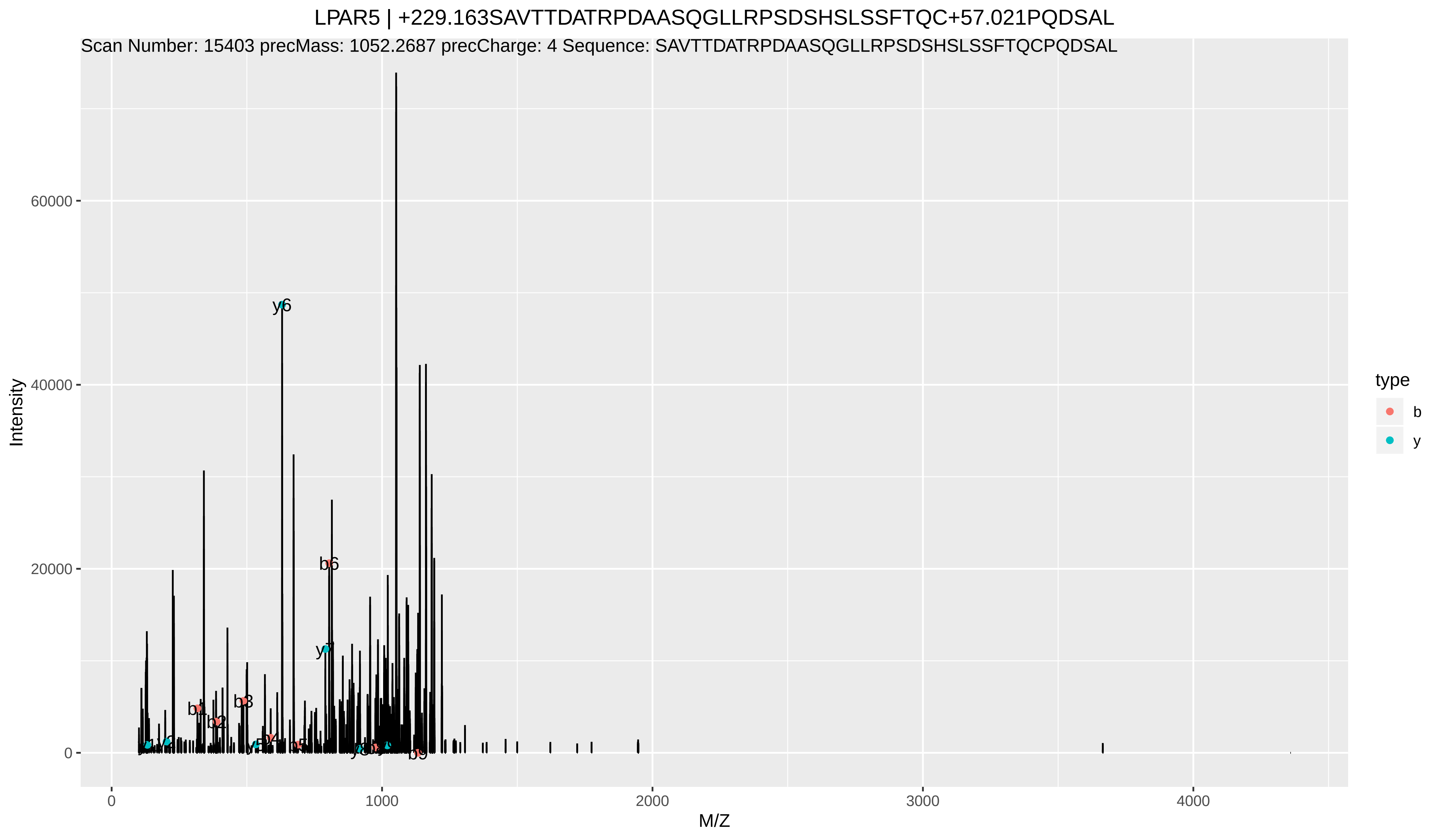

LPAR5 | +229.163SAVTTDATRPDAASQGLLRPSDSHSLSSFTQC+57.021PQDSAL
Scan Number: 15403 precMass: 1052.2687 precCharge: 4 Sequence: SAVTTDATRPDAASQGLLRPSDSHSLSSFTQCPQDSAL
Intensity
M/Z
type
b
y
y6
b6
y7
b1
b2
b3
b9

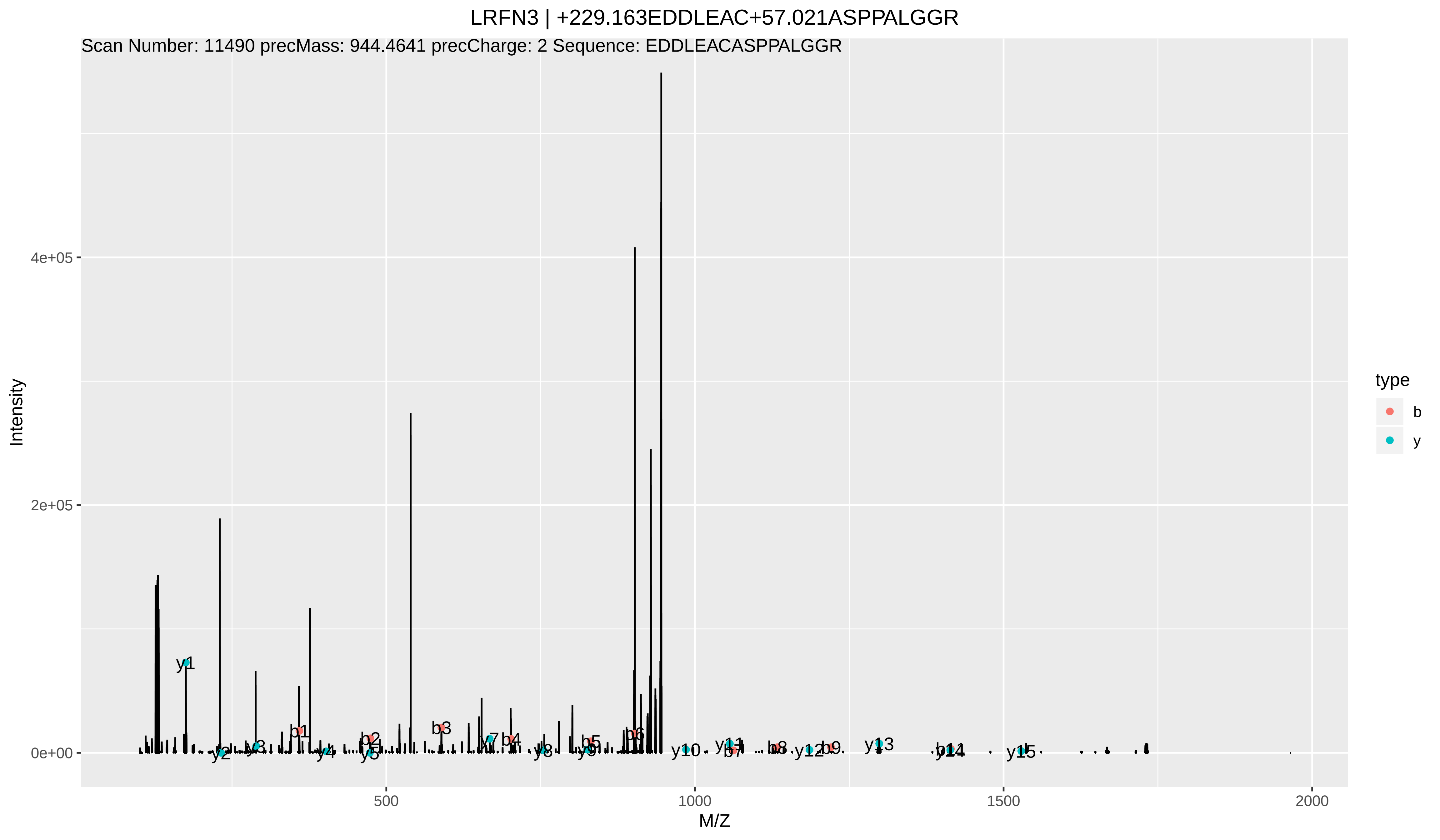

LRFN3 | +229.163EDDLEAC+57.021ASPPALGGR
Scan Number: 11490 precMass: 944.4641 precCharge: 2 Sequence: EDDLEACASPPALGGR
Intensity
M/Z
4e+05
2e+05
0e+00
500
1000
1500
2000
type
b
y

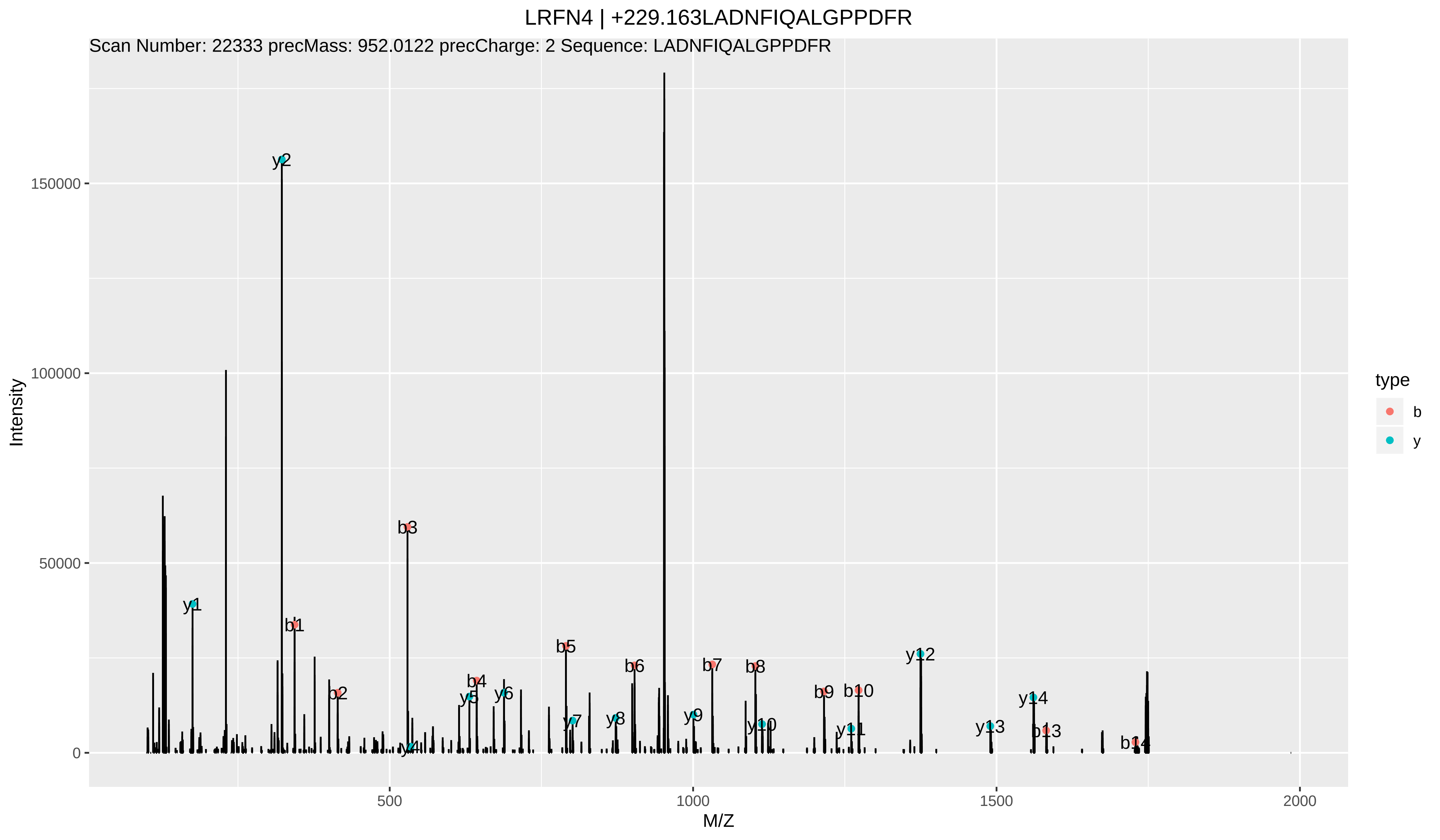

LRFN4 | +229.163LADNFIQALGPPDFR
Scan Number: 22333 precMass: 952.0122 precCharge: 2 Sequence: LADNFIQALGPPDFR
Intensity
M/Z
type
b
y
y1
y2
b1
b2
b3
y4
b4
y5
y6
b5
y7
y8
b6
y9
b7
b8
y10
b9
b10
y11
y12
y13
y14
b13
b14
150000
100000
50000
0
500
1000
1500
2000

LRRC24 | +229.163EALQPLASLQVLR

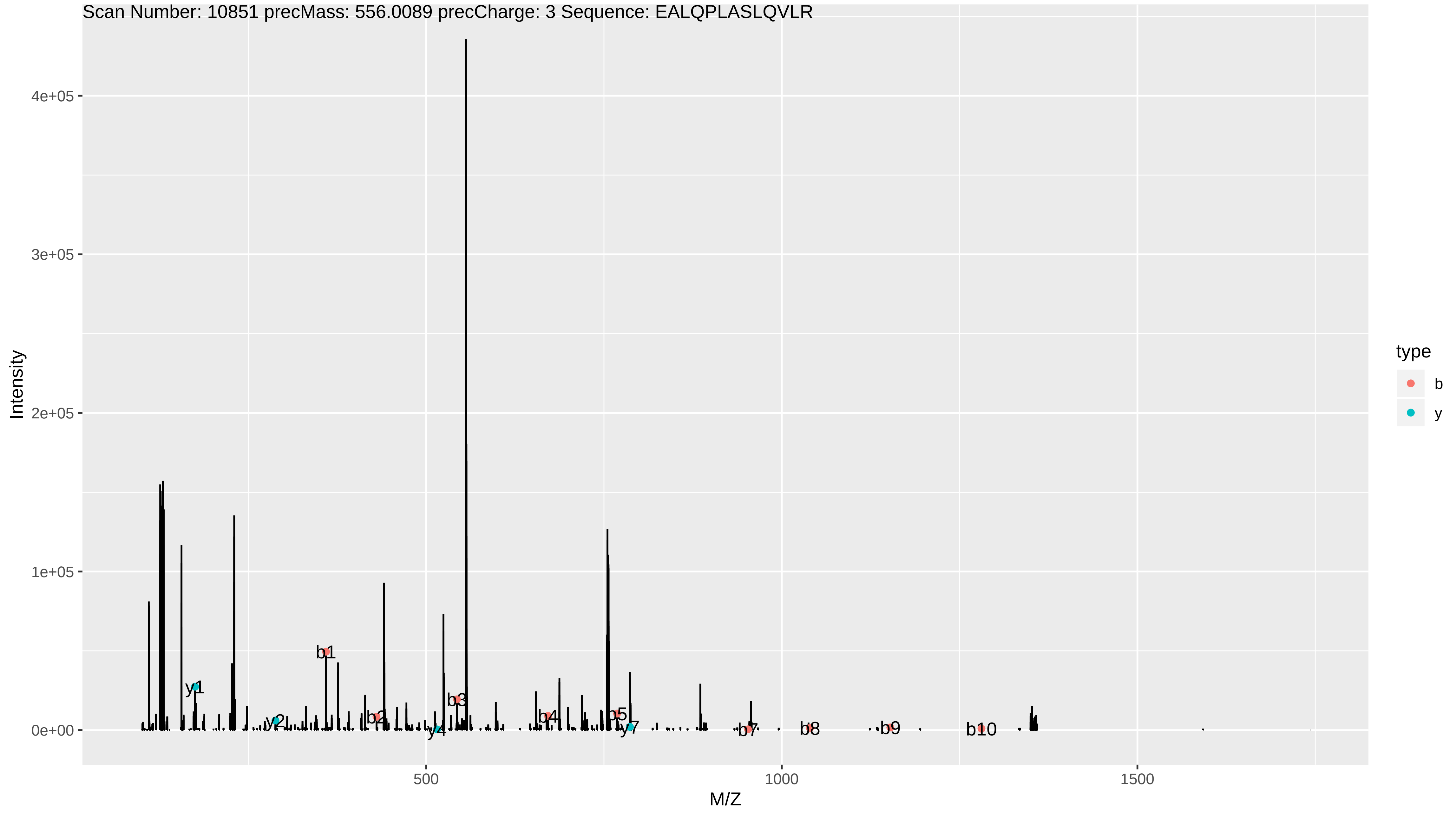

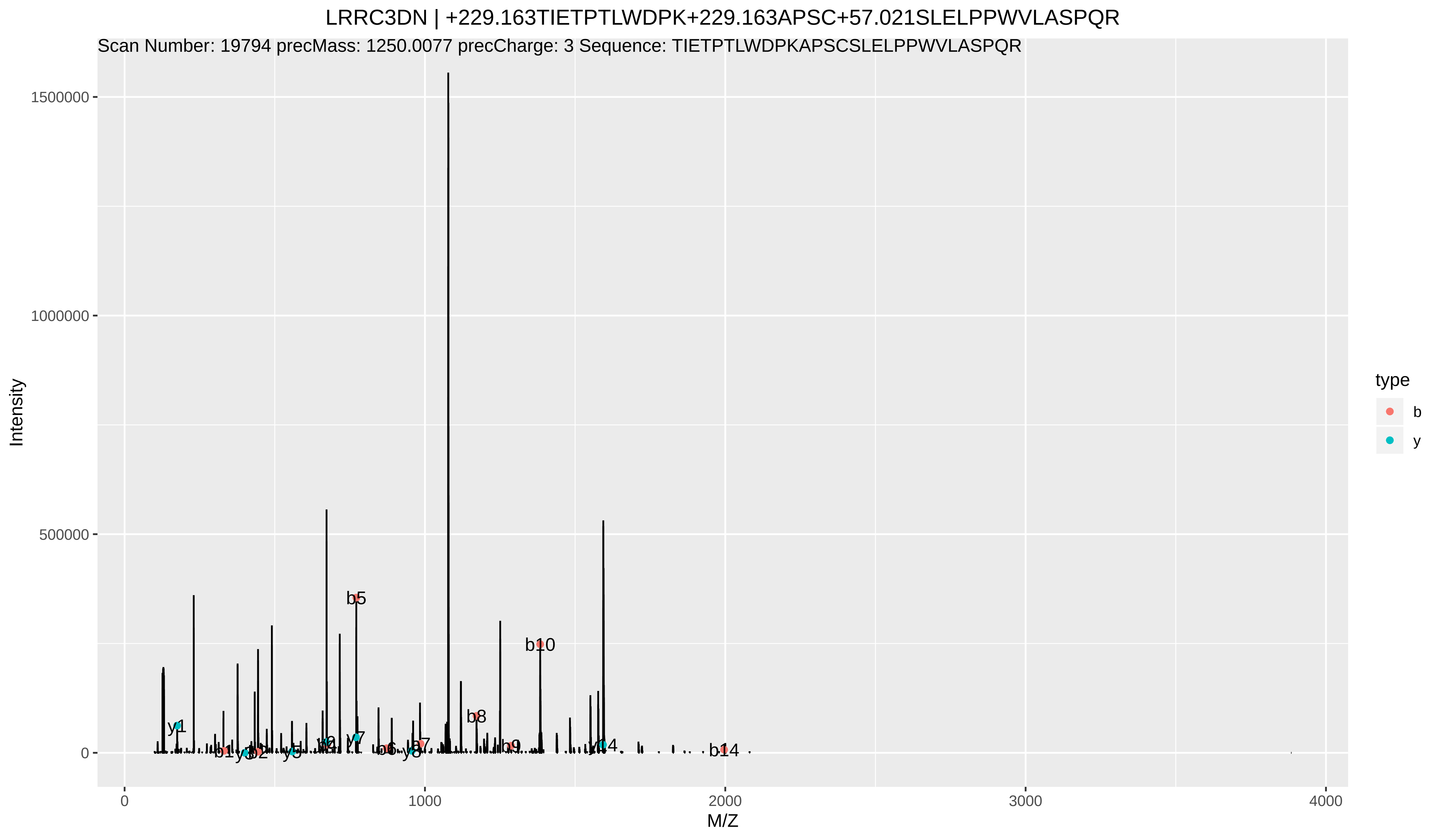
LRRC3DN | +229.163TIETPTLWDPK+229.163APSC+57.021SLELPPWVLASPQR
Scan Number: 19794 precMass: 1250.0077 precCharge: 3 Sequence: TIETPTLWDPKAPSCSLELPPWVLASPQR
Intensity
1500000
1000000
500000
0
M/Z
0
1000
2000
3000
4000
b5
b10
b8
y1
y7
b14
type
b
y

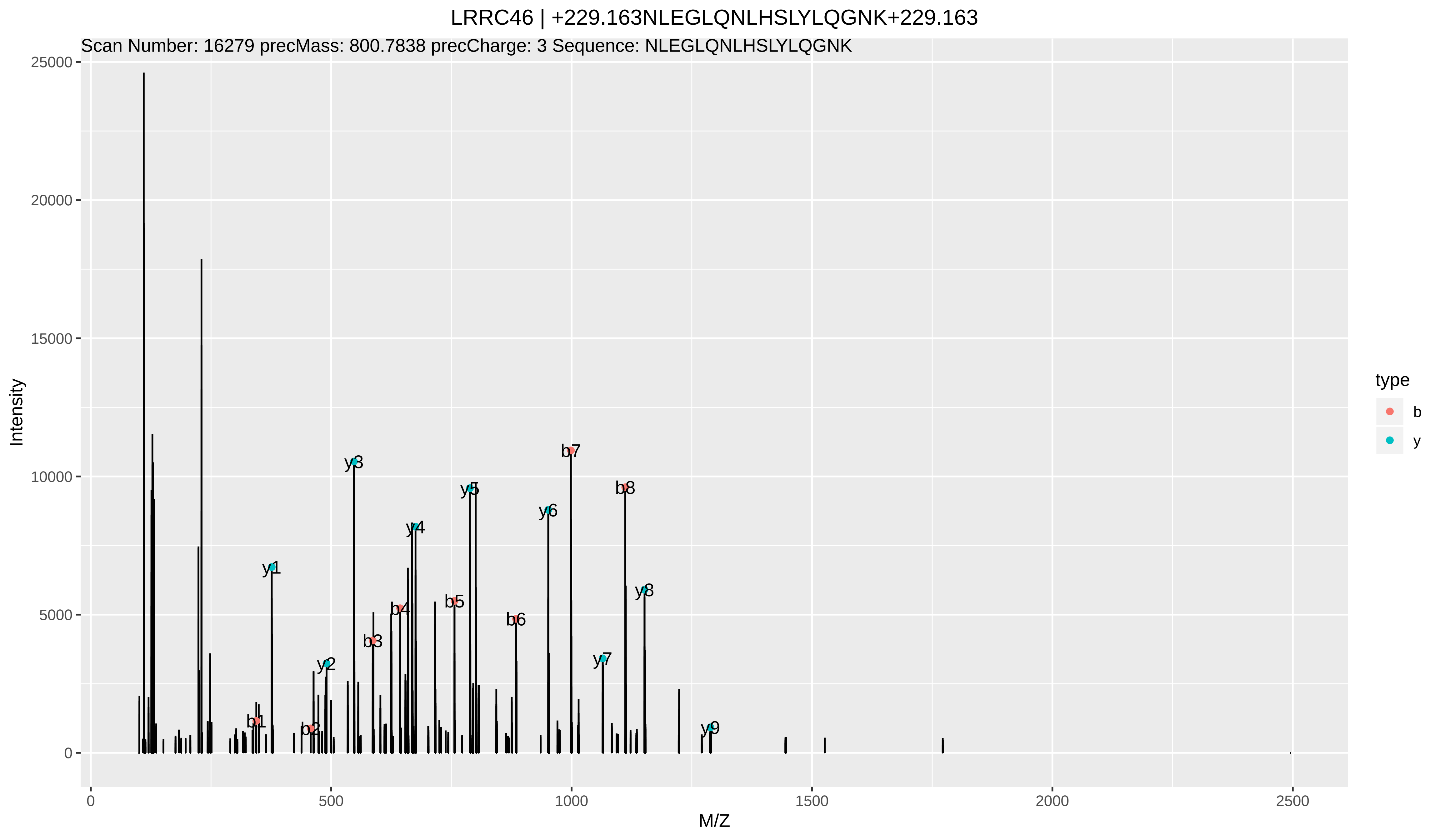

LRRC46 | +229.163NLEGLQNLHSLYLQGNK+229.163
Scan Number: 16279 precMass: 800.7838 precCharge: 3 Sequence: NLEGLQNLHSLYLQGNK
Intensity
M/Z
0
5000
10000
15000
20000
25000
500
1000
1500
2000
2500
y1
y2
y3
y4
y5
y6
y7
y8
y9
b1
b2
b3
b4
b5
b6
b7
b8
type
b
y

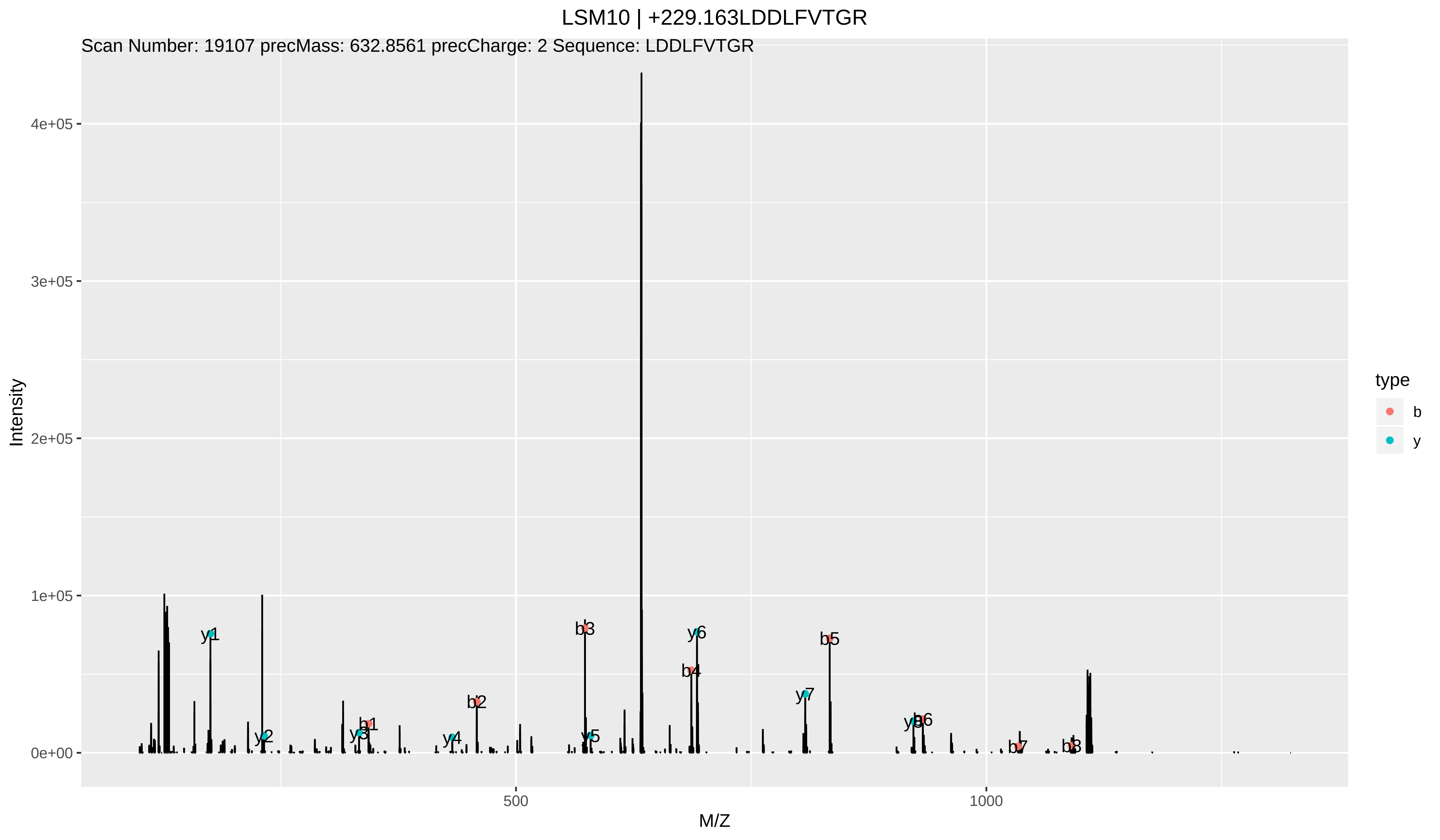

LSM10 | +229.163LDDLFVTGR
Scan Number: 19107 precMass: 632.8561 precCharge: 2 Sequence: LDDLFVTGR
Intensity
4e+05
3e+05
2e+05
1e+05
0e+00
M/Z
500
1000
type
b
y
y1
y2
y3
b1
y4
b2
b3
y5
y6
b4
y7
b5
y8
b6
b7
b8

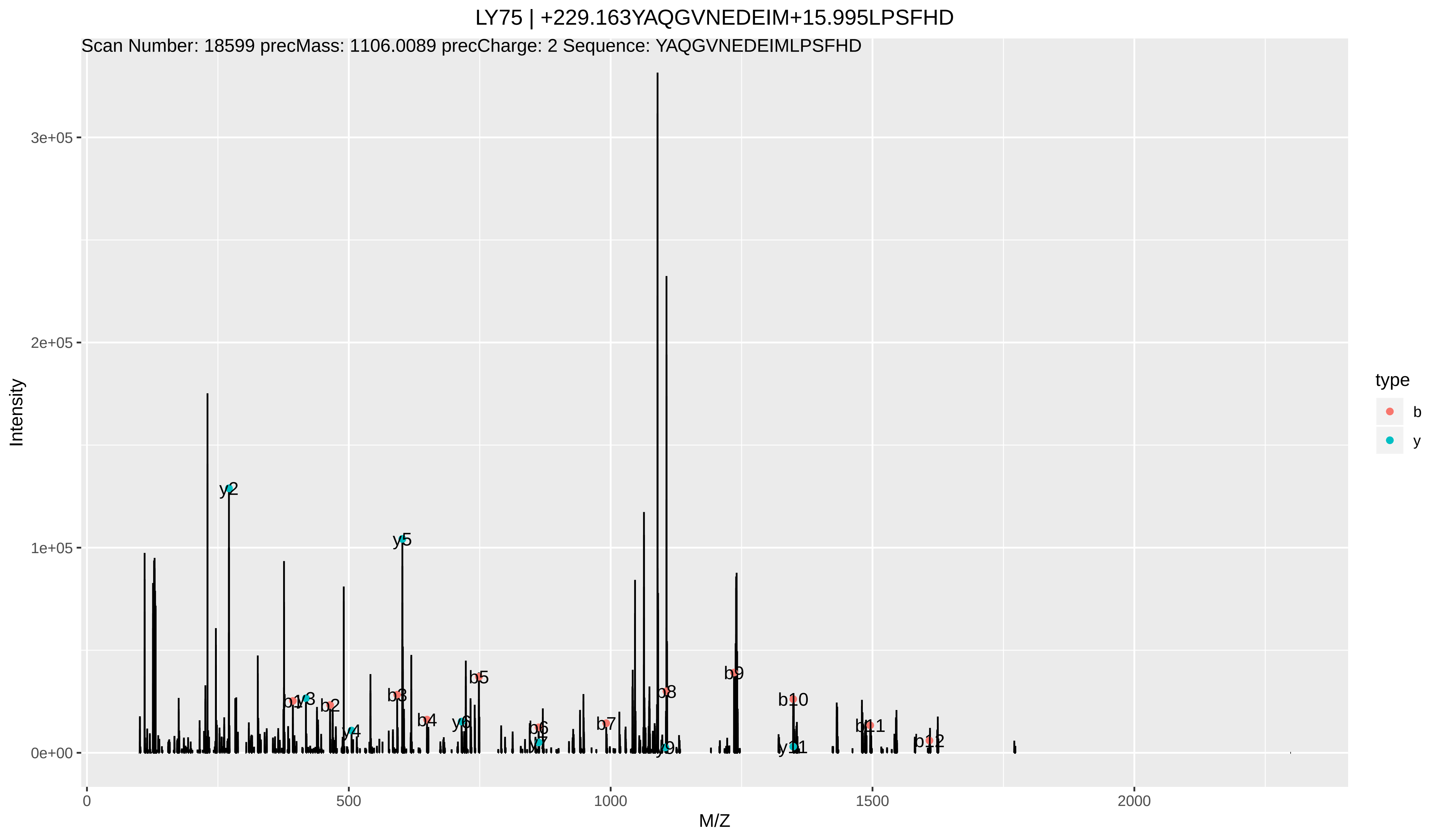
LY75 | +229.163YAQGVNEDEIM+15.995LPSFHD
Scan Number: 18599 precMass: 1106.0089 precCharge: 2 Sequence: YAQGVNEDEIMLPSFHD
Intensity
3e+05
2e+05
1e+05
0e+00
0
500
1000
1500
2000
M/Z
y2
y5
b1
y3
b2
y4
b3
b4
y6
b5
b6
y7
b7
y9
b8
b9
b10
y11
b11
b12
type
b
y

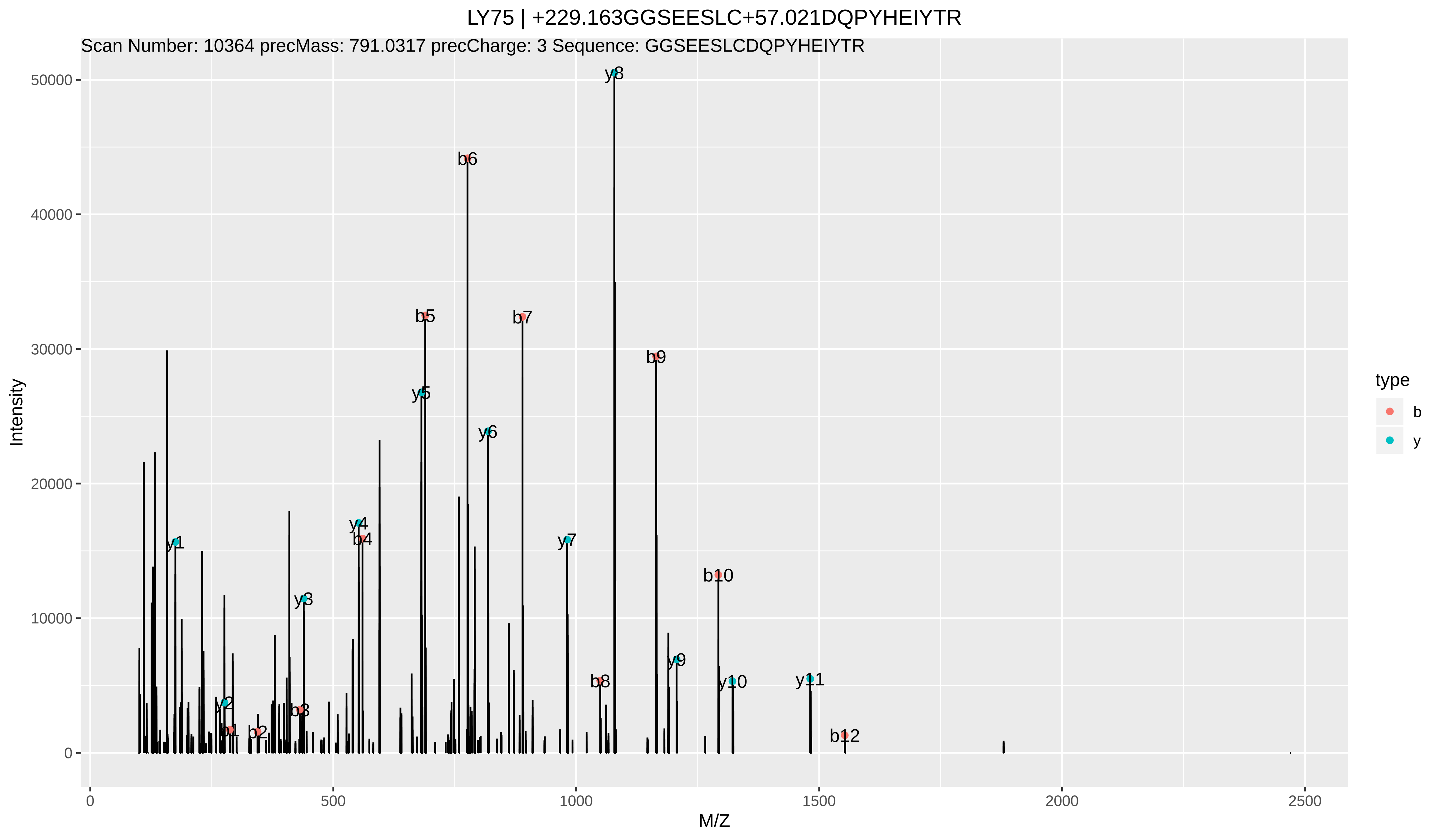
LY75 | +229.163GGSEESLC+57.021DQPYHEIYTR
Scan Number: 10364 precMass: 791.0317 precCharge: 3 Sequence: GGSEESLCDQPYHEIYTR
Intensity
M/Z
0
10000
20000
30000
40000
50000
500
1000
1500
2000
2500
y8
b6
b5
b7
b9
y5
y6
y4
b4
y1
y7
b10
y3
y9
b8
y10
y11
y2
b3
b1
b2
b12
type
b
y

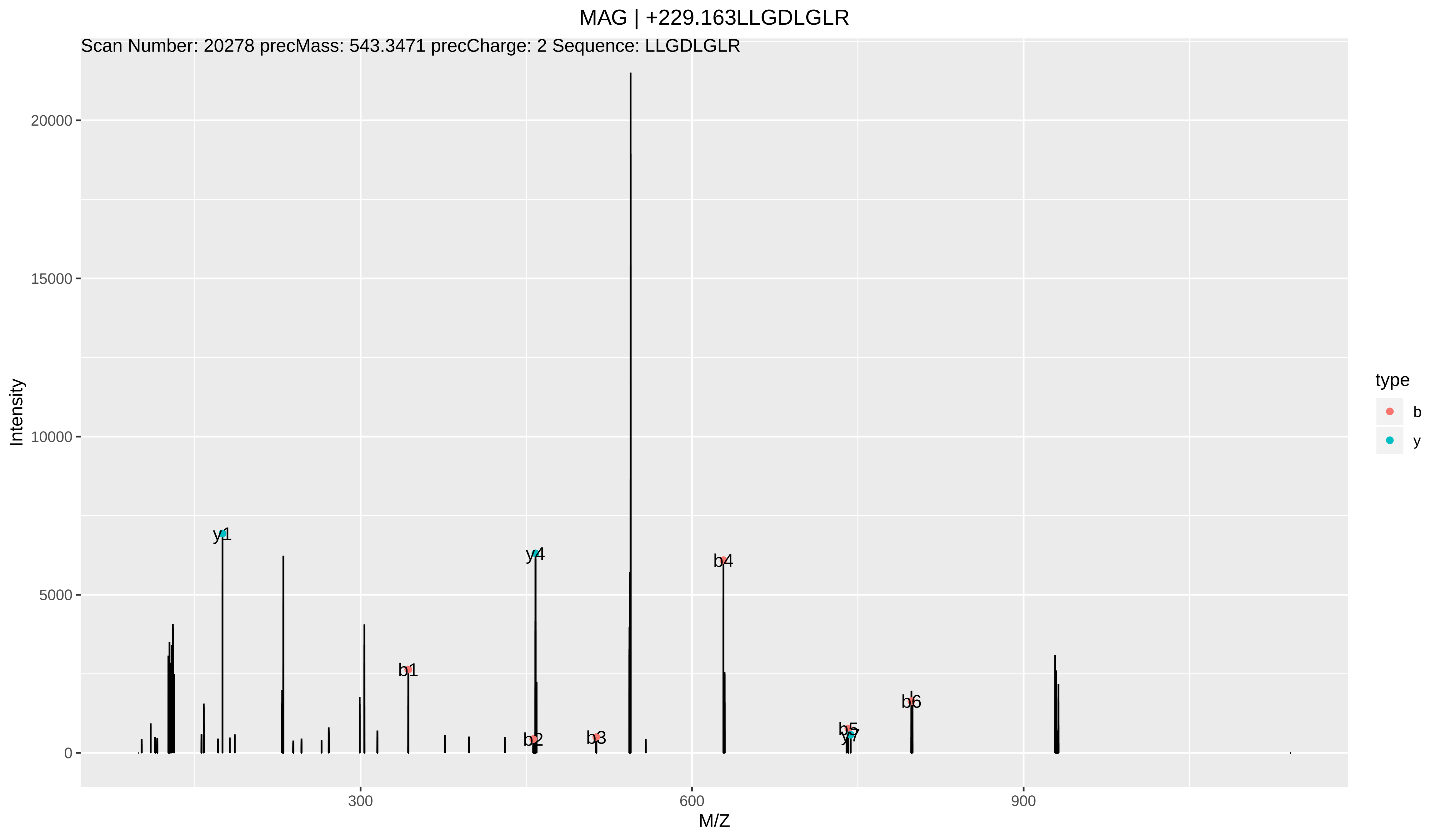

MAG | +229.163LLGDLGLR
Scan Number: 20278 precMass: 543.3471 precCharge: 2 Sequence: LLGDLGLR
Intensity
20000
15000
10000
5000
0
300
600
900
M/Z
y1
y4
b4
b1
b2
b3
b5
y7
b6
type
b
y

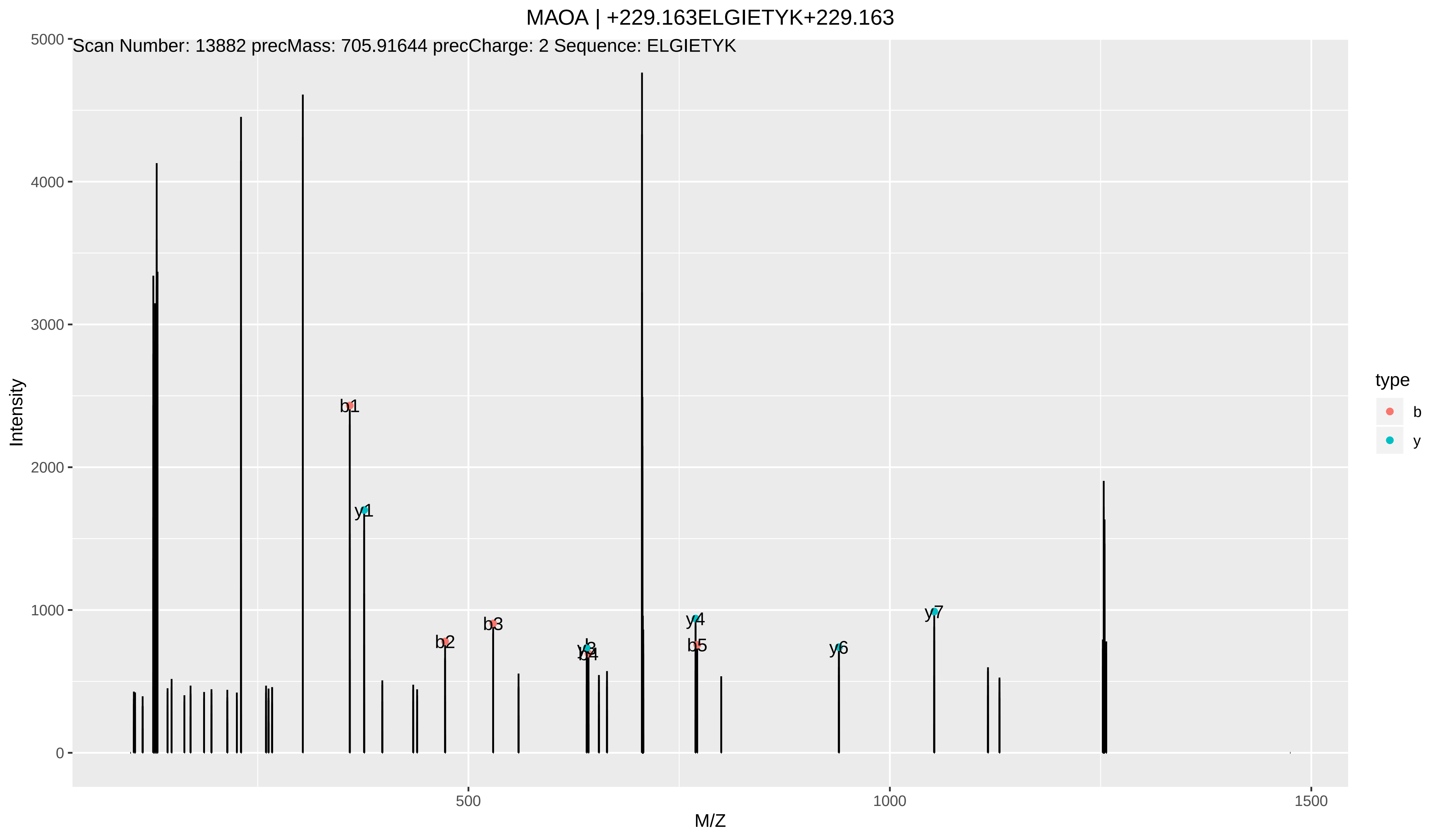

MAOA | +229.163ELGIETYK+229.163
Scan Number: 13882 precMass: 705.91644 precCharge: 2 Sequence: ELGIETYK
Intensity
M/Z
type
b
y
b1
y1
b2
b3
y3
b4
y4
b5
y6
y7
0
1000
2000
3000
4000
5000
500
1000
1500

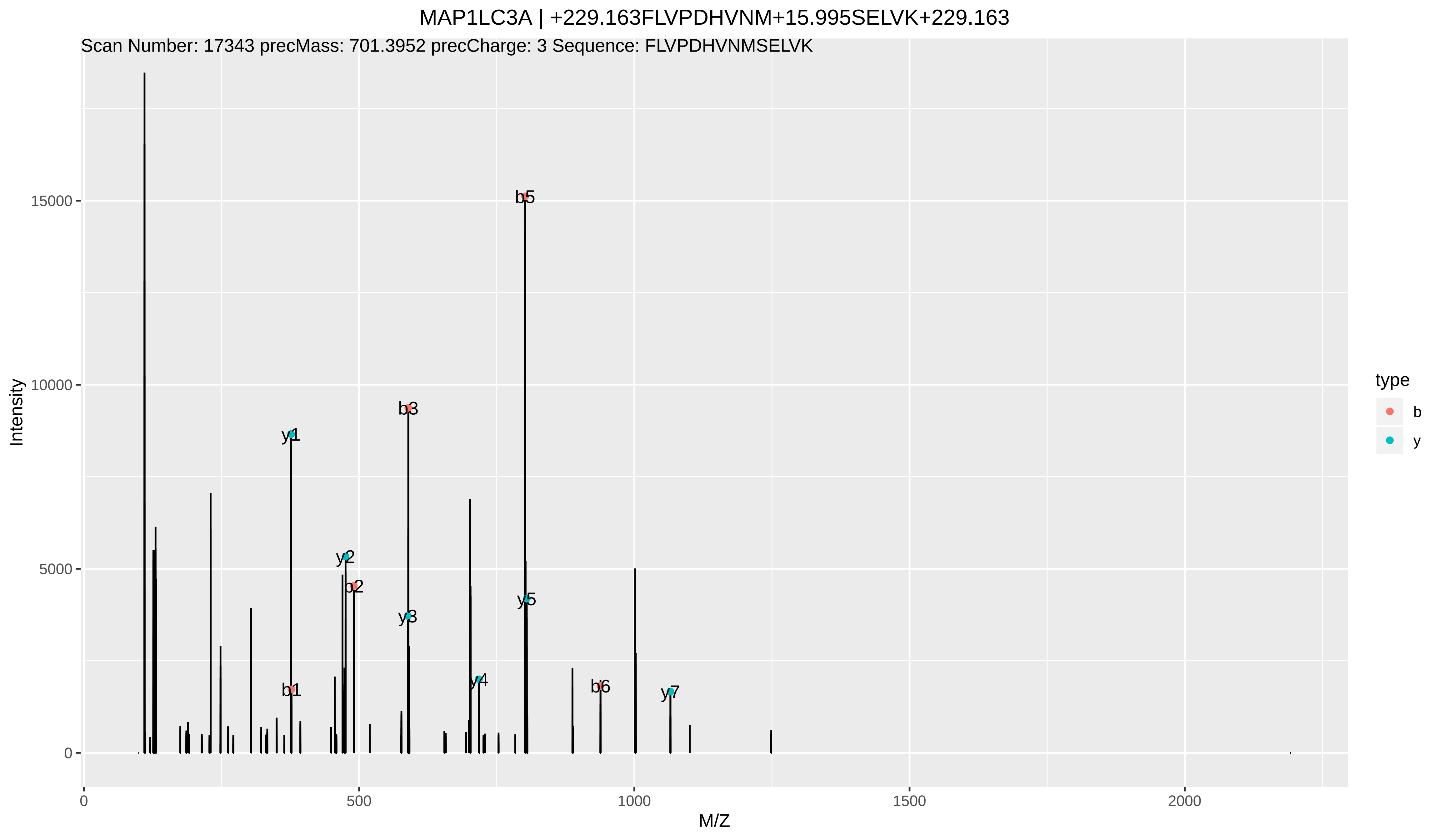

MAP1LC3A | +229.163FLVPDHVNM+15.995SELVK+229.163
Scan Number: 17343 precMass: 701.3952 precCharge: 3 Sequence: FLVPDHVNMSELVK
Intensity
M/Z
type
b
y
b5
b3
y1
y2
b2
y3
y5
y4
b1
b6
y7

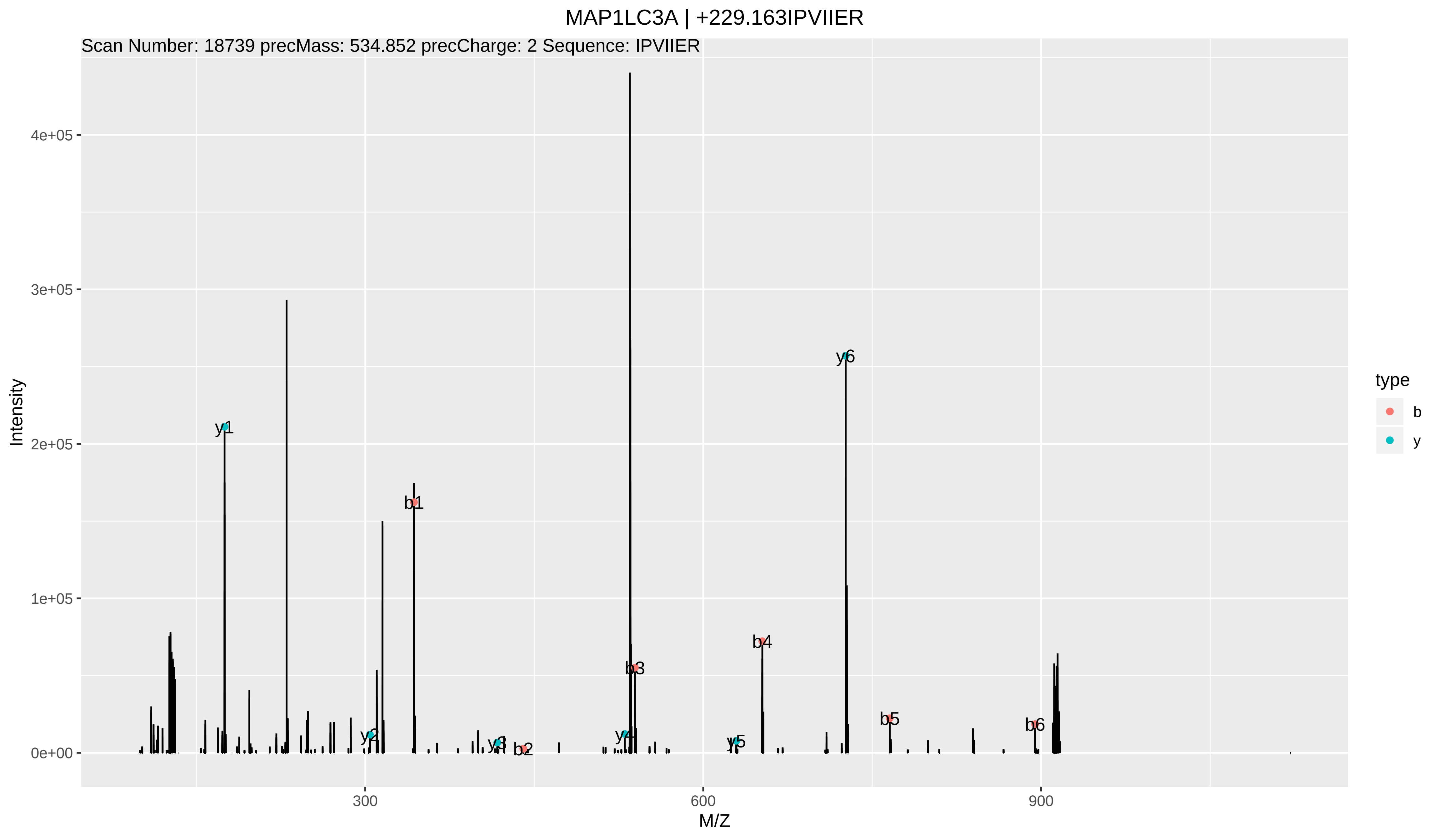

MAP1LC3A | +229.163IPVIIER
Scan Number: 18739 precMass: 534.852 precCharge: 2 Sequence: IPVIIER
Intensity
4e+05
3e+05
2e+05
1e+05
0e+00
300
600
900
M/Z
y1
y2
y3
y4
y5
y6
b1
b2
b3
b4
b5
b6
type
b
y

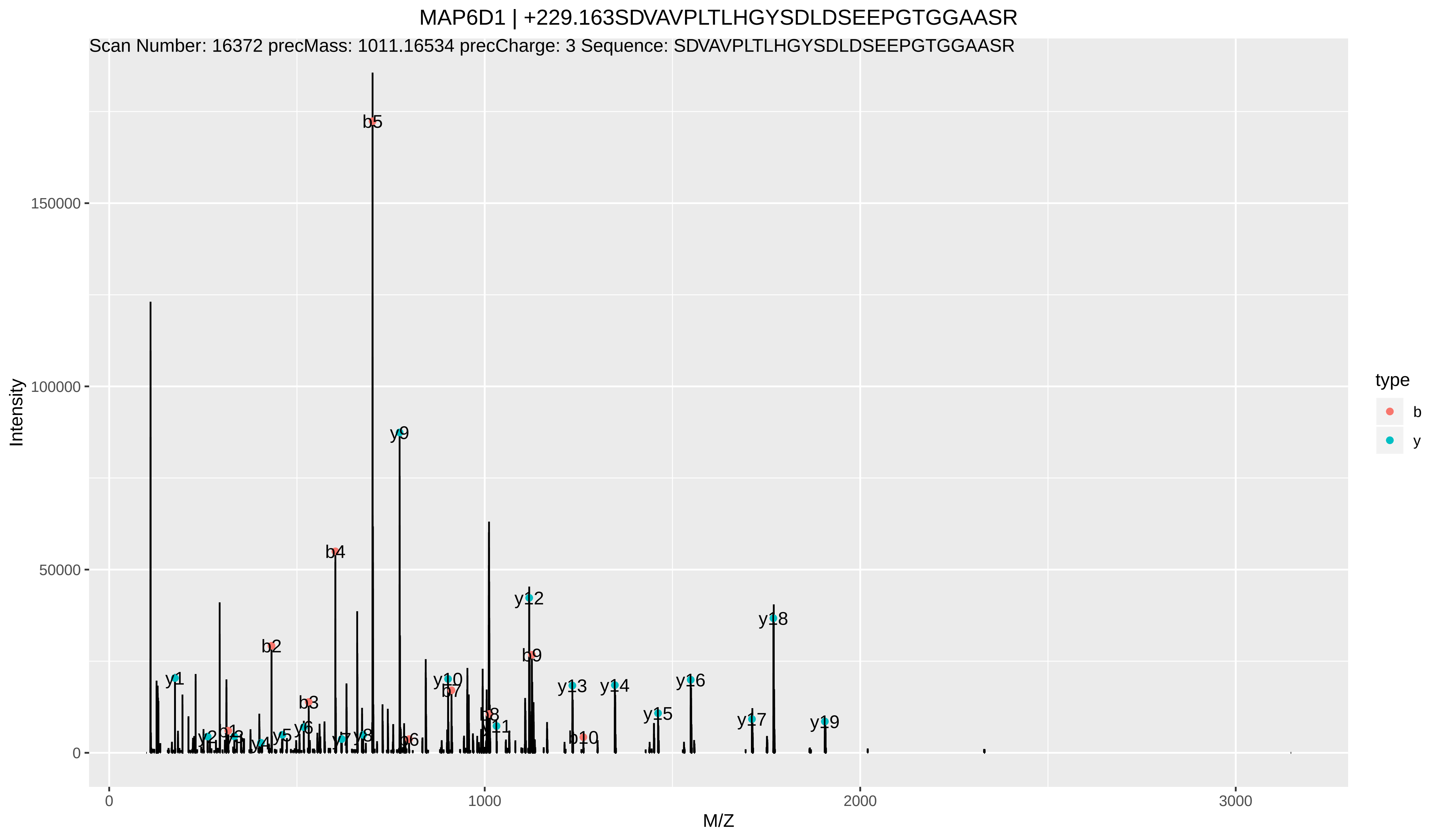

MAP6D1 | +229.163SDVAVPLTLHGYSDLDSEEPGTGGAASR
Scan Number: 16372 precMass: 1011.16534 precCharge: 3 Sequence: SDVAVPLTLHGYSDLDSEEPGTGGAASR
Intensity
M/Z
type
b
y
b5
y9
b4
y12
y18
b2
b9
y1
y10
b7
y13
y14
y16
b3
y15
b8
y17
y19
b10
y5
y6
y8
b6

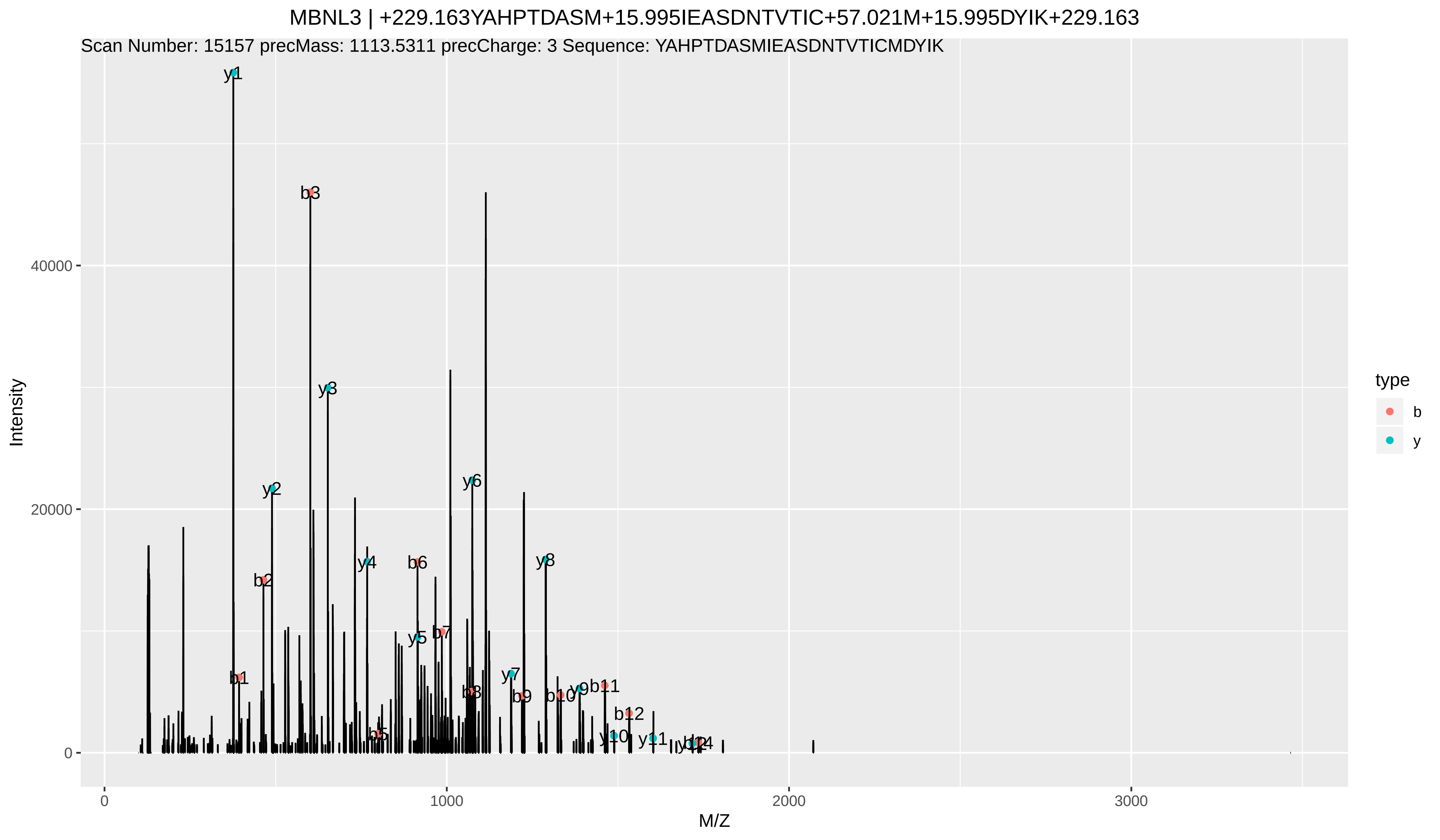
MBNL3 | +229.163YAHPTDASM+15.995IEASDNTVTIC+57.021M+15.995DYIK+229.163
Scan Number: 15157 precMass: 1113.5311 precCharge: 3 Sequence: YAHPTDASMIEASDNTVTICMDYIK
Intensity
M/Z
type
b
y
y1
b3
y3
y2
y6
y4
b6
y8
b2
y5
b7
b1
y7
b8
b9
b10
y9
b11
b12
y10
y11
b5
0
20000
40000
1000
2000
3000

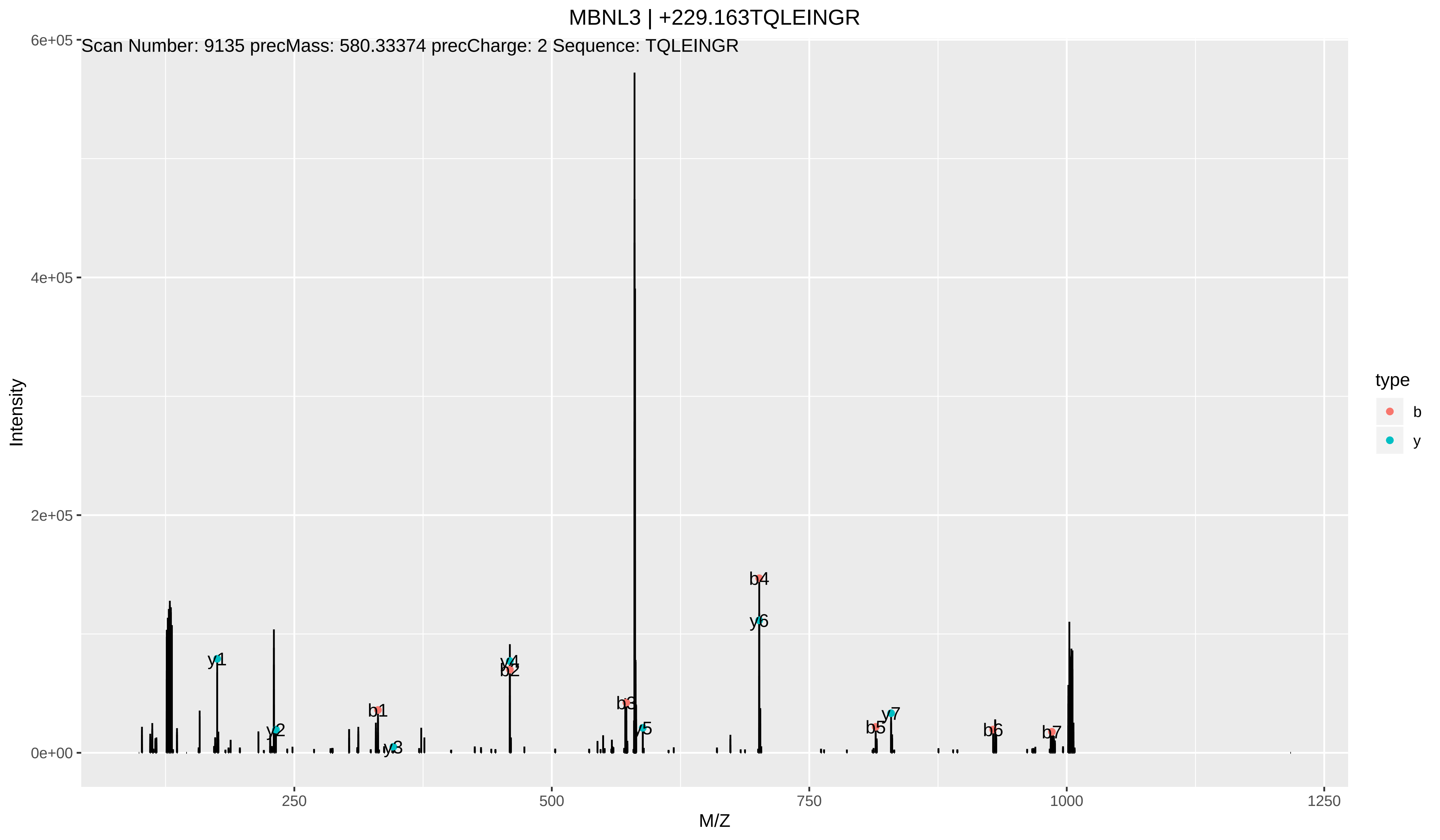

MBNL3 | +229.163TQLEINGR
Scan Number: 9135 precMass: 580.33374 precCharge: 2 Sequence: TQLEINGR
Intensity
6e+05
4e+05
2e+05
0e+00
M/Z
250
500
750
1000
1250
type
b
y
y1
y2
y3
y4
y5
y6
y7
b1
b2
b3
b4
b5
b6
b7

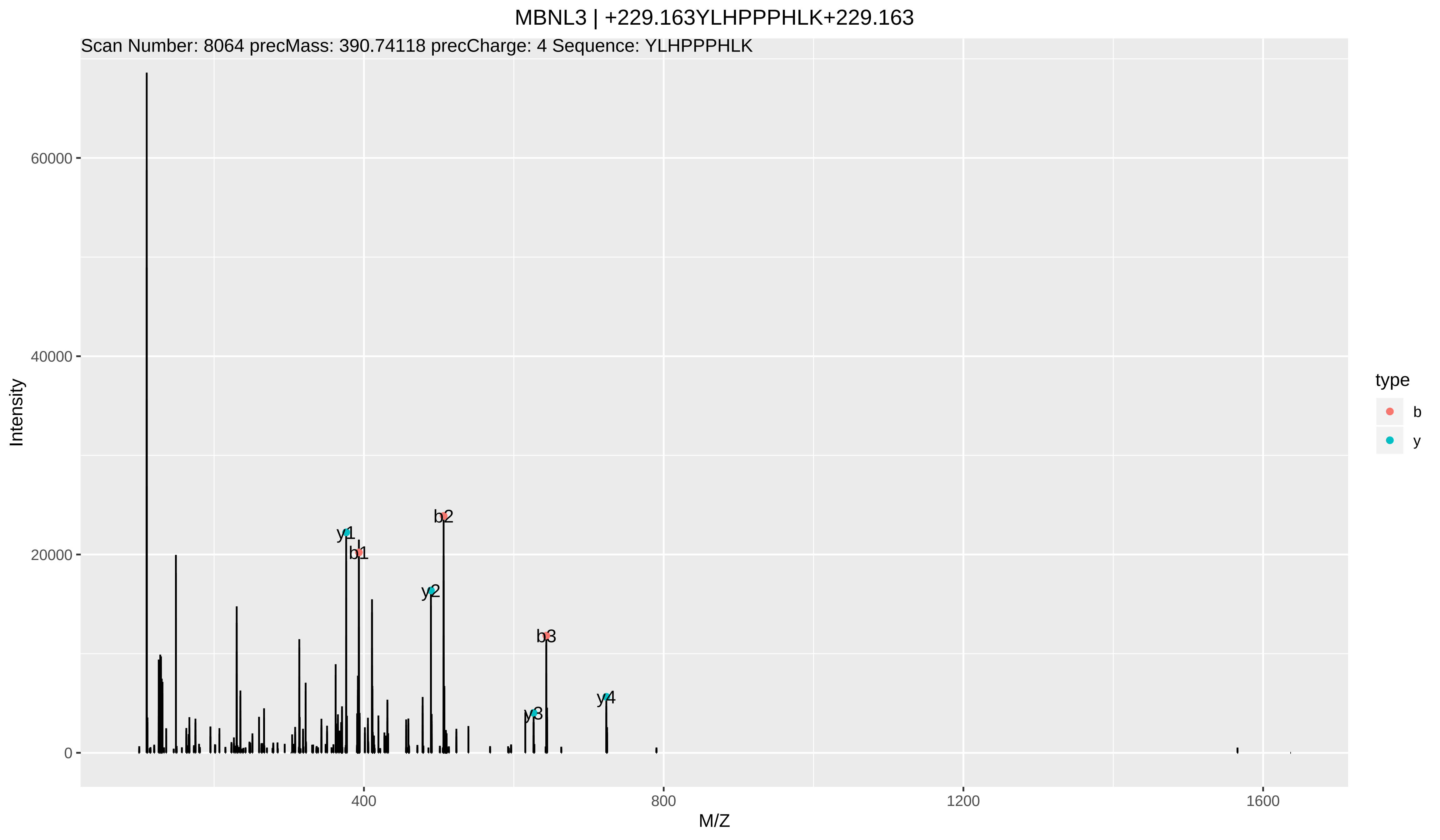

MBNL3 | +229.163YLHPPPHLK+229.163
Scan Number: 8064 precMass: 390.74118 precCharge: 4 Sequence: YLHPPPHLK
Intensity
60000
40000
20000
0
400
800
1200
1600
M/Z
y1
b1
b2
y2
b3
y3
y4
type
b
y

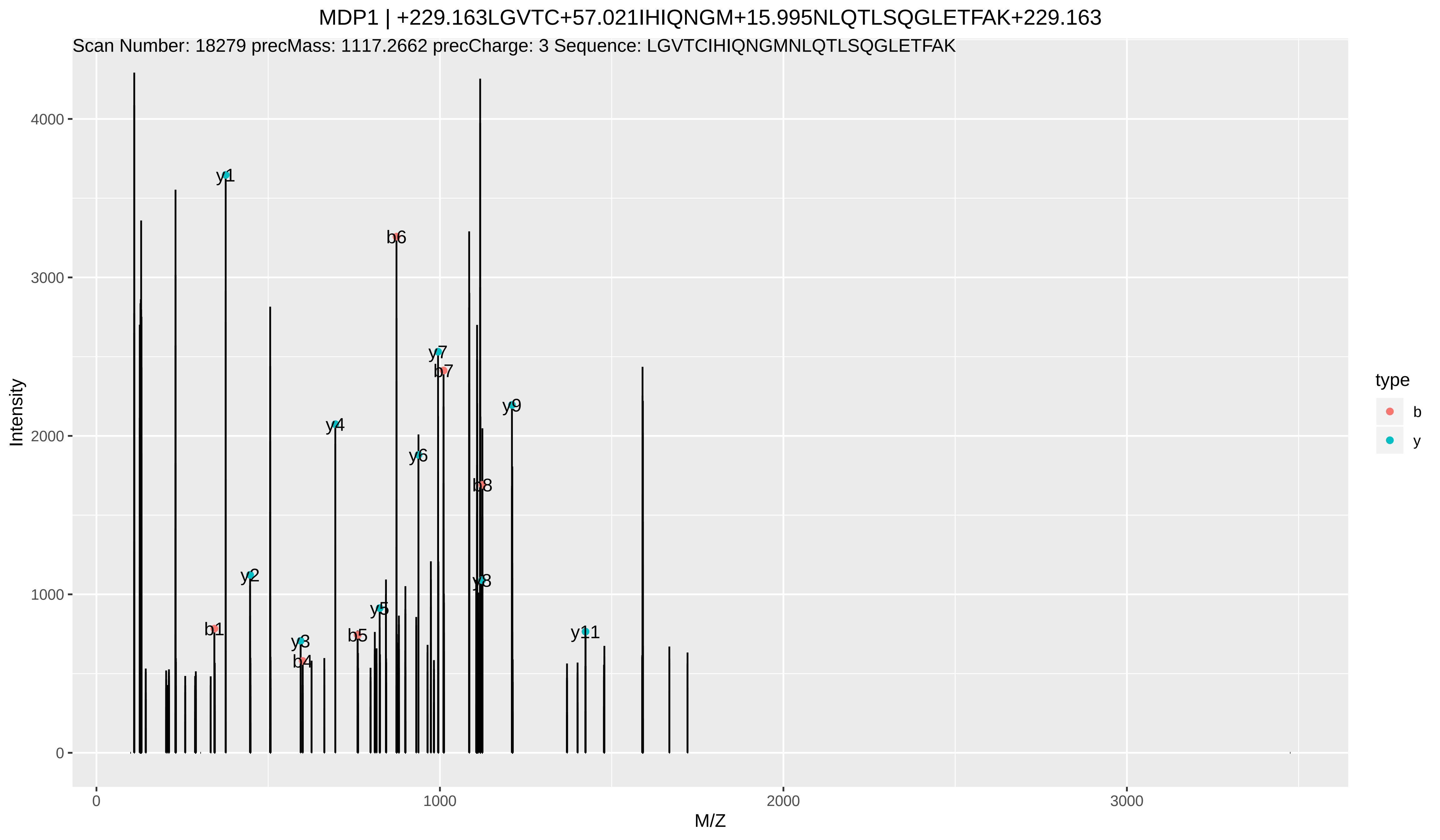

MDP1 | +229.163LGVTC+57.021IHIQNGM+15.995NLQTLSQGLETFAK+229.163
Scan Number: 18279 precMass: 1117.2662 precCharge: 3 Sequence: LGVTCIHIQNGMNLQTLSQGLETFAK
Intensity
M/Z
type
b
y
y1
y2
y3
y4
y5
y6
y7
y8
y9
y11
b1
b4
b5
b6
b7
b8
0
1000
2000
3000
4000
0
1000
2000
3000

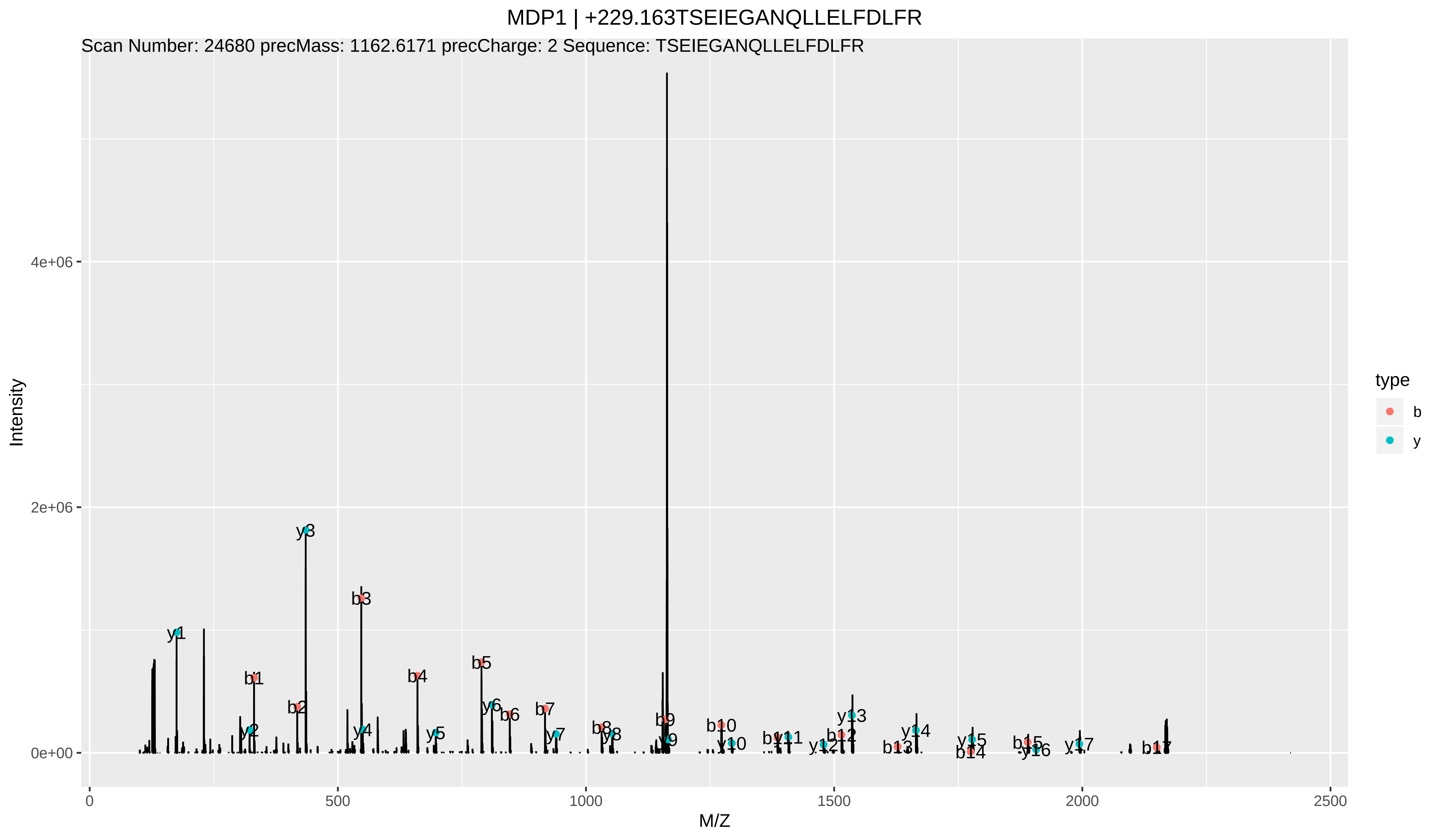

MDP1 | +229.163TSEIEGANQLLELFDLFR
Scan Number: 24680 precMass: 1162.6171 precCharge: 2 Sequence: TSEIEGANQLLELFDLFR
Intensity
M/Z
type
b
y

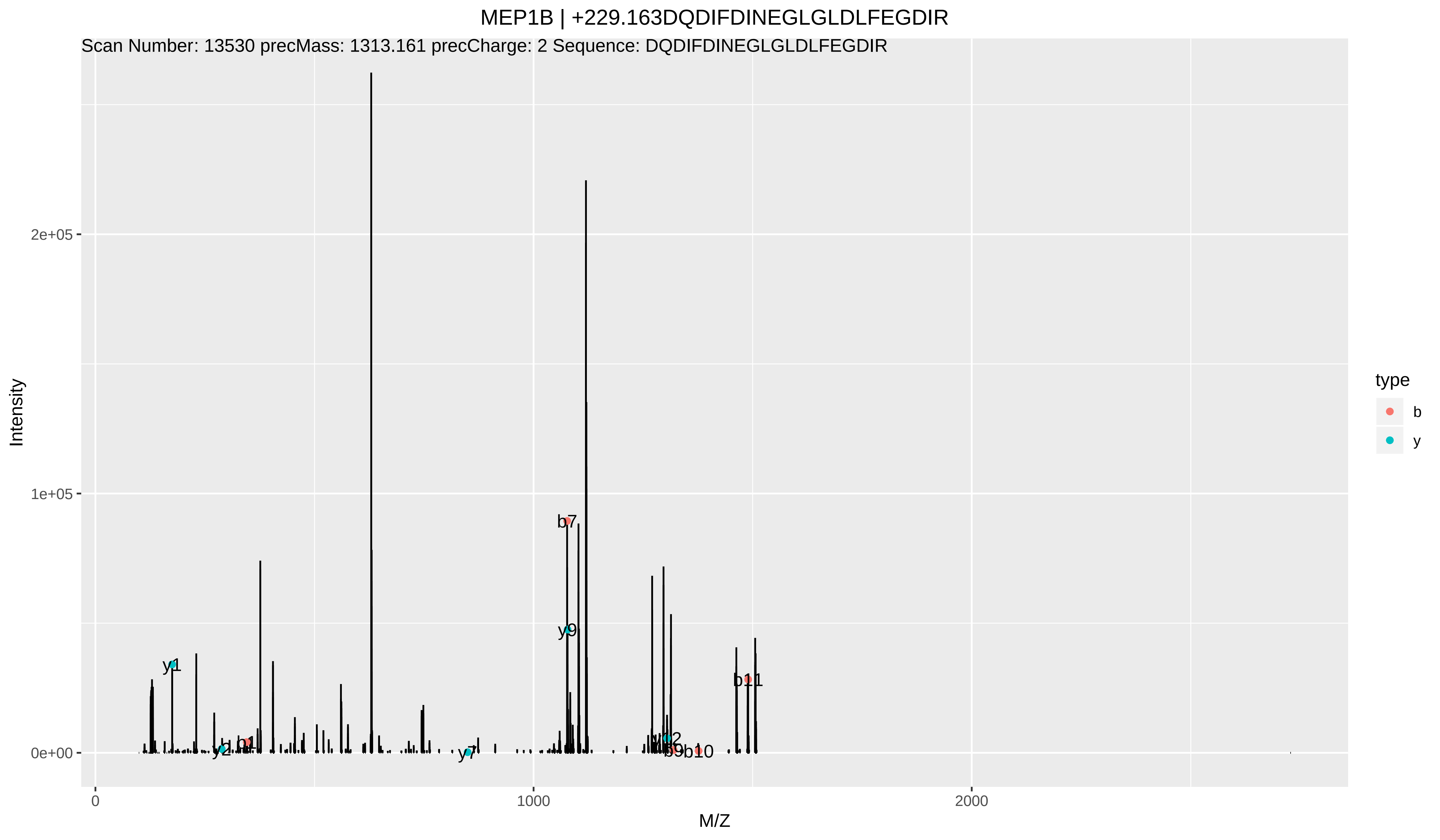

MEP1B | +229.163DQDIFDINEGLGLDLFEGDIR
Scan Number: 13530 precMass: 1313.161 precCharge: 2 Sequence: DQDIFDINEGLGLDLFEGDIR
Intensity
2e+05
1e+05
0e+00
M/Z
0
1000
2000
b7
y9
y1
b11
y2
b1
y7
y12
b9
b10
type
b
y

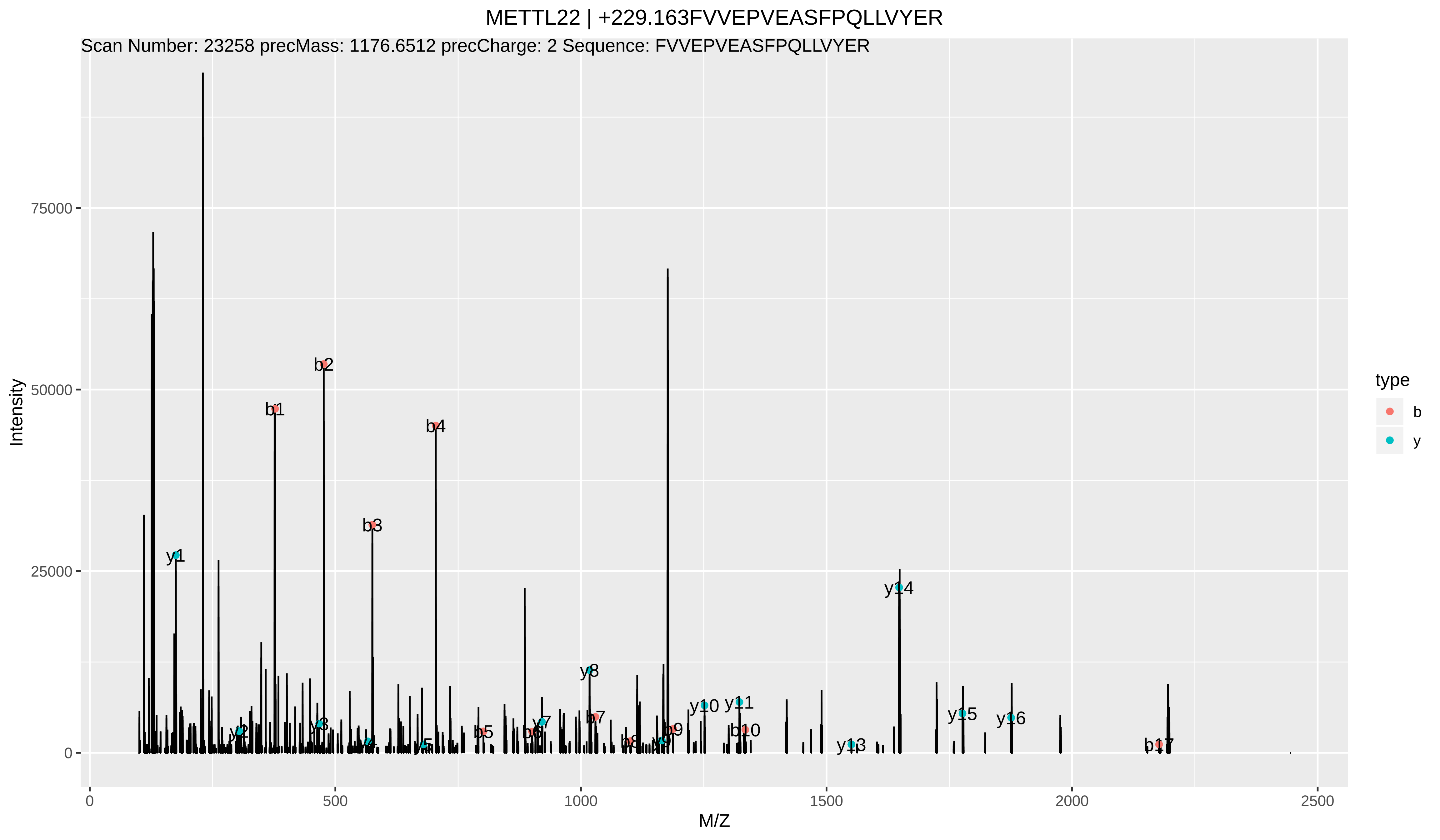

METTL22 | +229.163FVVEPVEASFPQLLVYER
Scan Number: 23258 precMass: 1176.6512 precCharge: 2 Sequence: FVVEPVEASFPQLLVYER
Intensity
M/Z
type
b
y
b1
b2
b3
b4
b5
b6
b7
b8
b9
b10
b17
y1
y2
y3
y4
y5
y7
y8
y9
y10
y11
y13
y14
y15
y16
0
25000
50000
75000
0
500
1000
1500
2000
2500

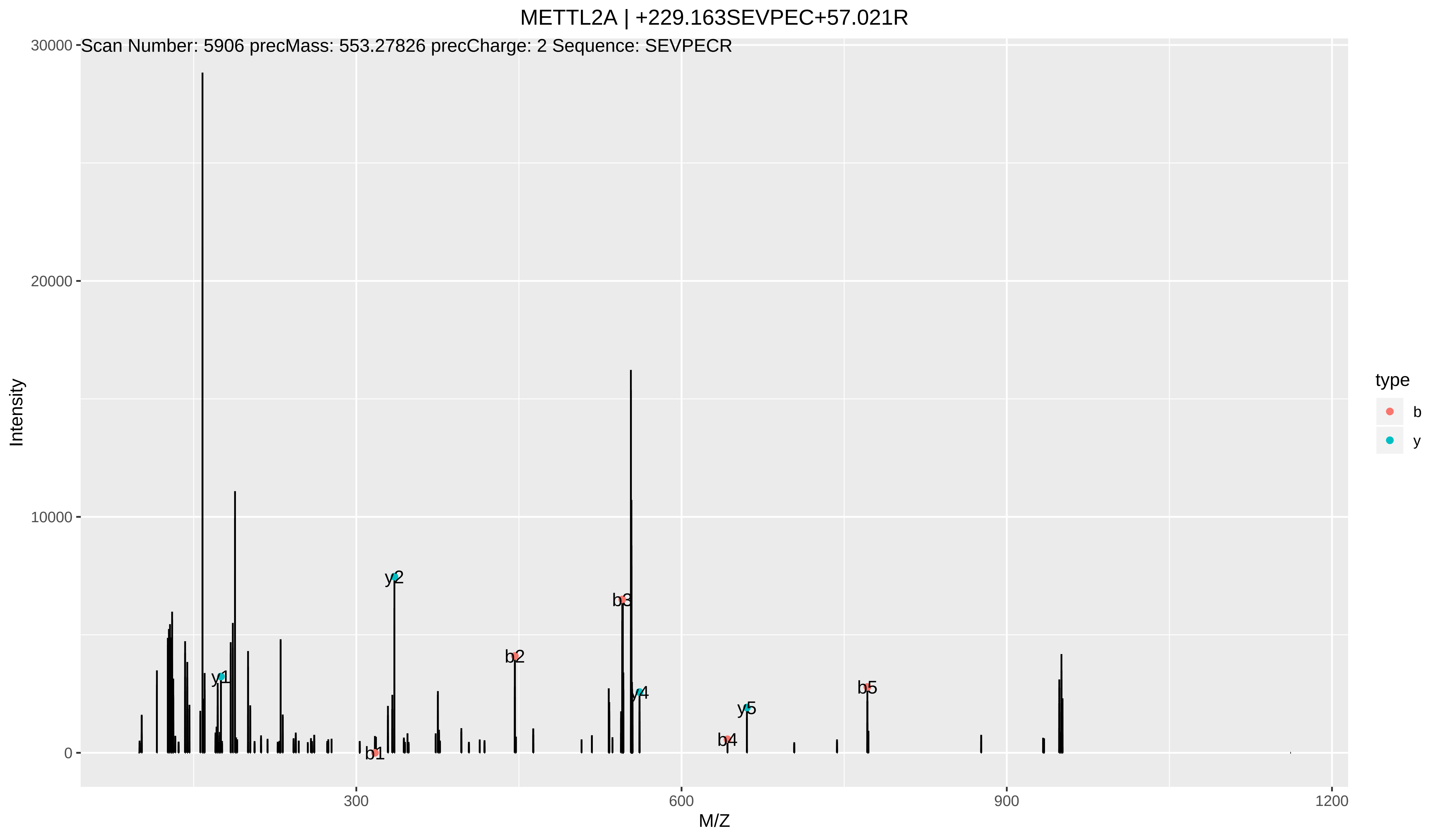

METTL2A | +229.163SEVPEC+57.021R
Scan Number: 5906 precMass: 553.27826 precCharge: 2 Sequence: SEVPECR
Intensity
30000
20000
10000
0
M/Z
300
600
900
1200
y1
y2
b1
b2
b3
y4
b4
y5
b5
type
b
y

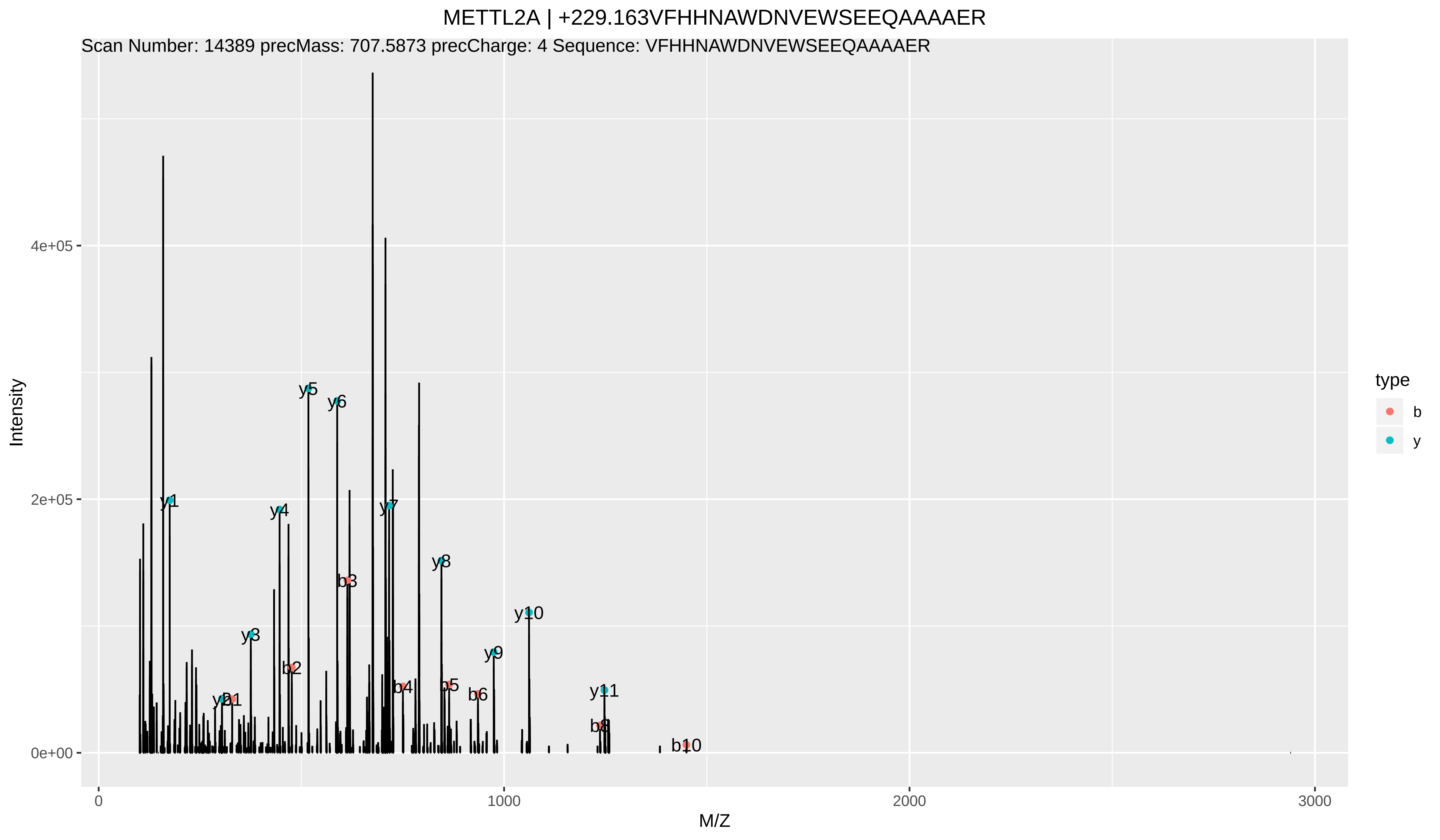

METTL2A | +229.163VFHHNAWDNVEWSEEQAAAAER
Scan Number: 14389 precMass: 707.5873 precCharge: 4 Sequence: VFHHNAWDNVEWSEEQAAAAER
Intensity
4e+05
2e+05
0e+00
M/Z
0
1000
2000
3000
y1
y2
b1
y3
y4
b2
y5
y6
b3
y7
b4
y8
b5
b6
y9
y10
y11
b8
b10
type
b
y

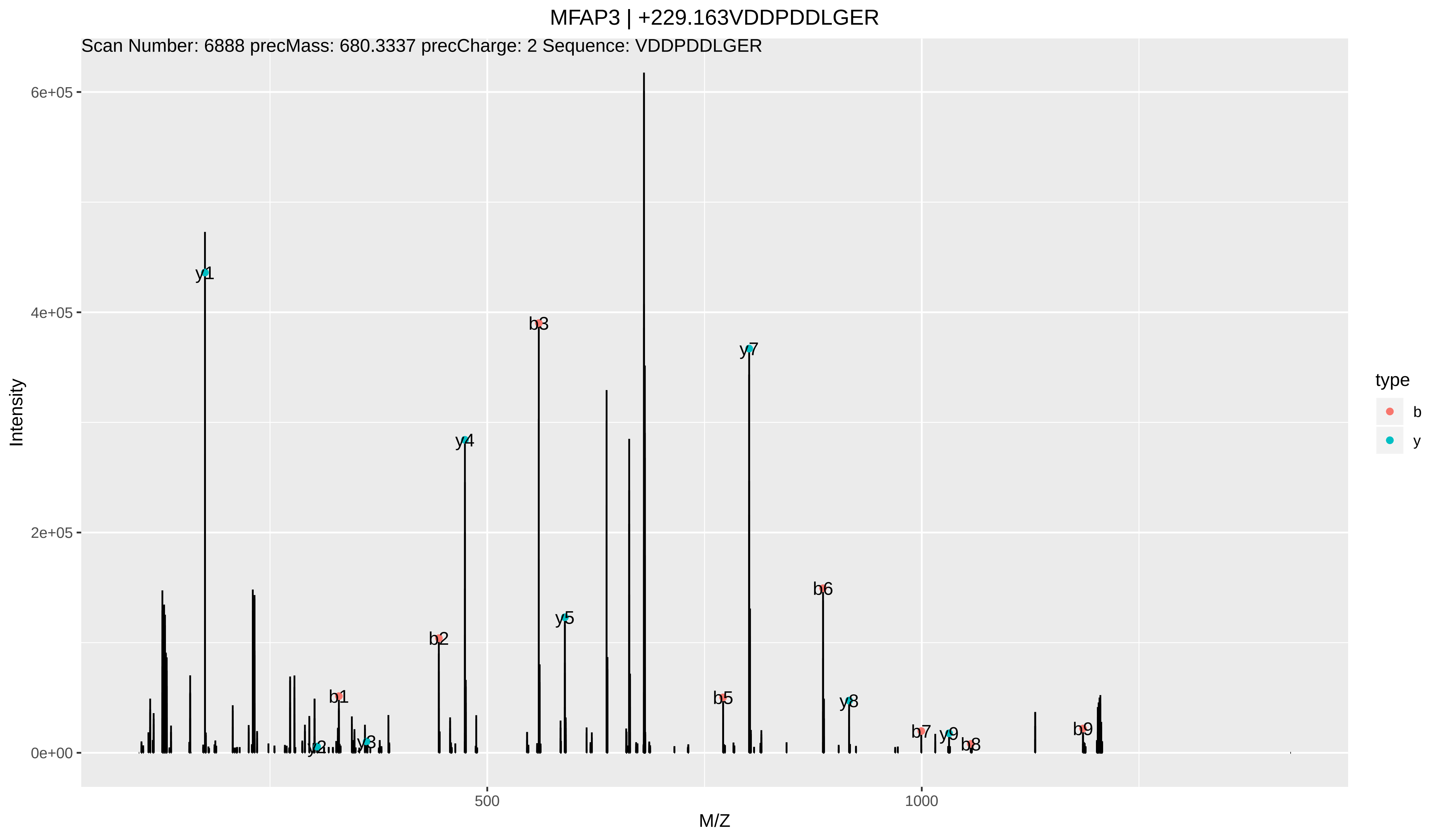

MFAP3 | +229.163VDDPDDLGER
Scan Number: 6888 precMass: 680.3337 precCharge: 2 Sequence: VDDPDDLGER
Intensity
6e+05
4e+05
2e+05
0e+00
y1
y2
y3
b1
b2
y4
b3
y5
y7
b5
b6
y8
b7
y9
b8
b9
500
1000
M/Z
type
b
y

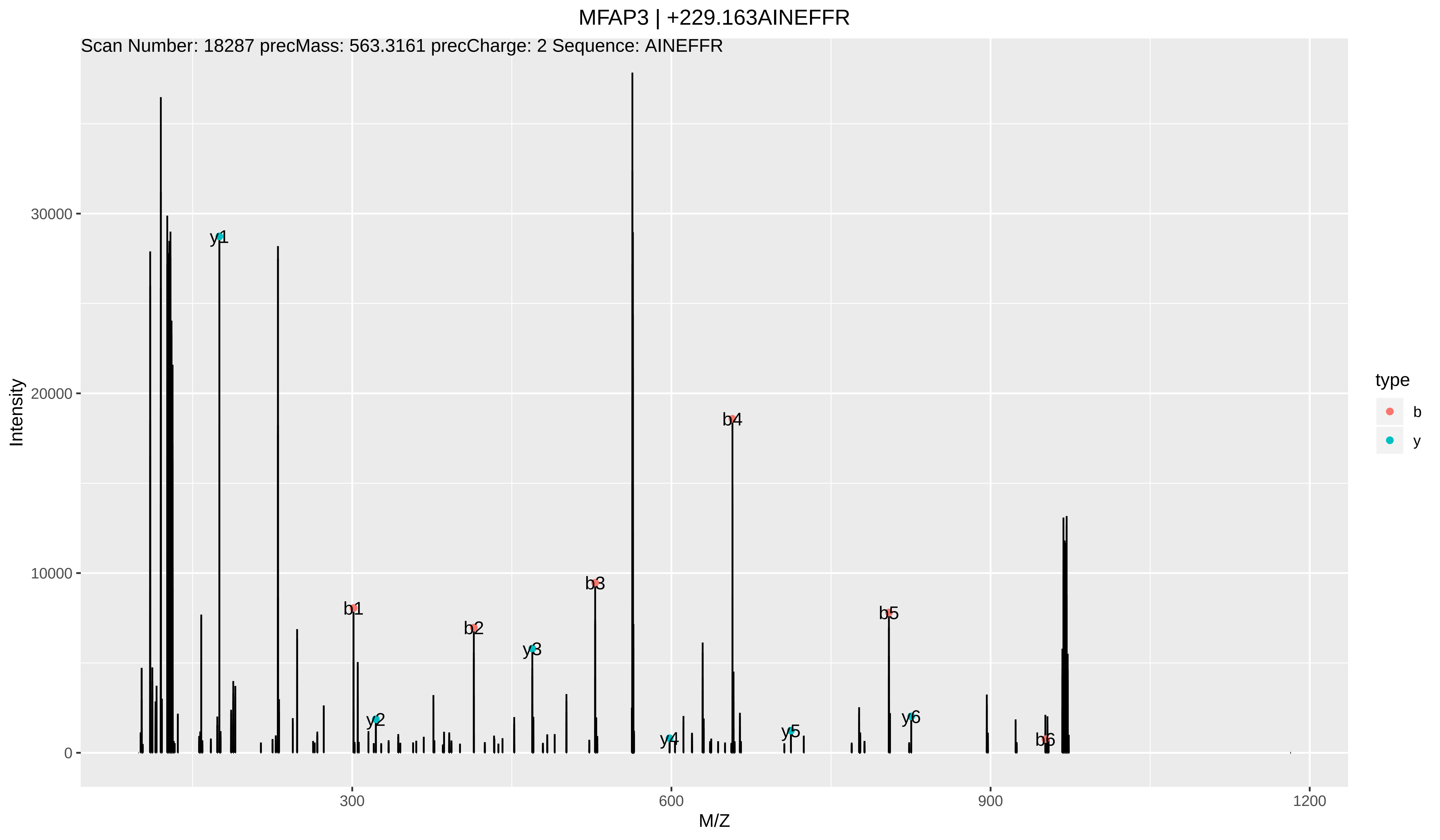
MFAP3 | +229.163AINEFFR
Scan Number: 18287 precMass: 563.3161 precCharge: 2 Sequence: AINEFFR
Intensity
M/Z
0
10000
20000
30000
300
600
900
1200
y1
y2
y3
y4
y5
y6
b1
b2
b3
b4
b5
b6
type
b
y

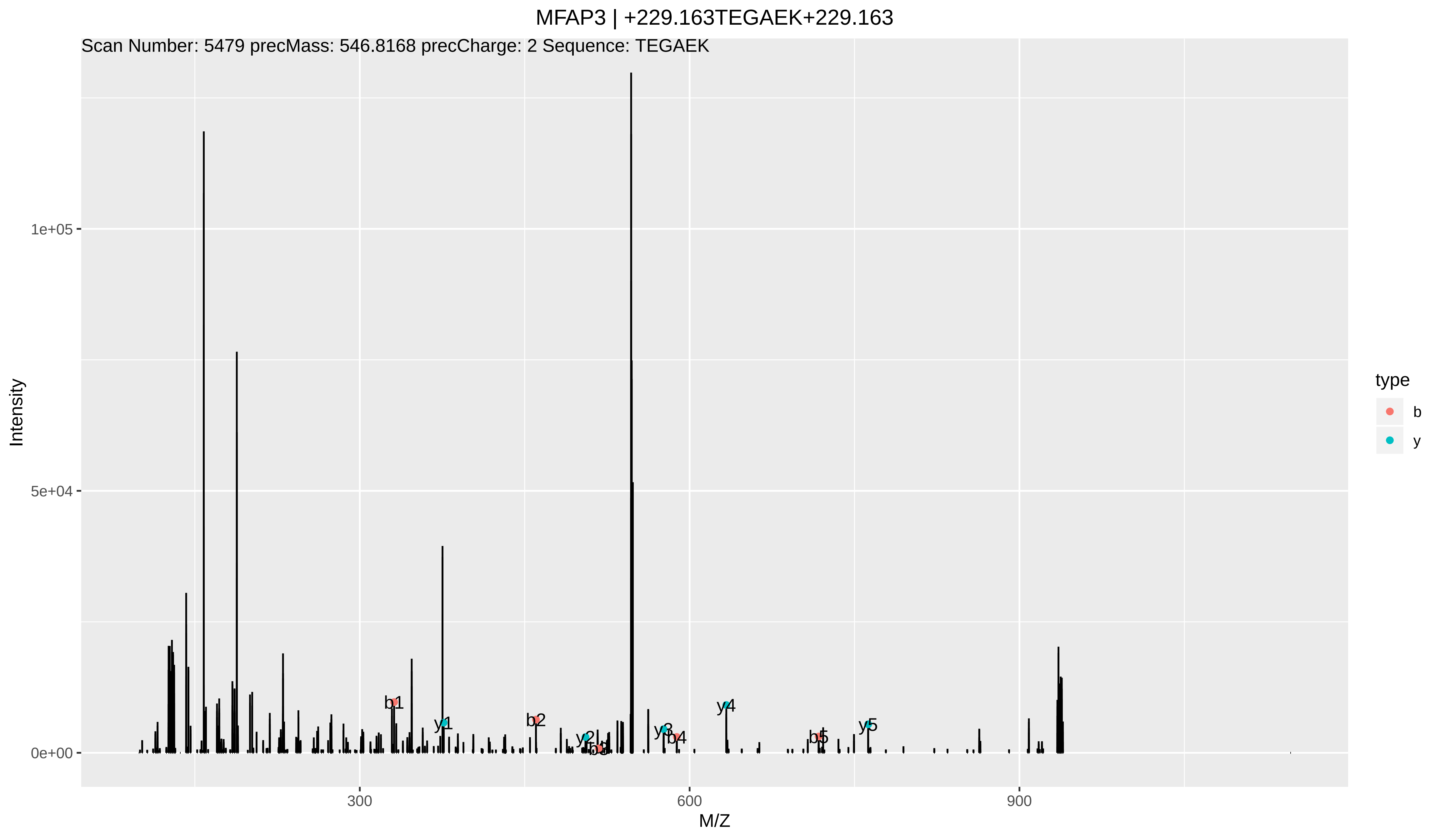

MFAP3 | +229.163TEGAEK+229.163
Scan Number: 5479 precMass: 546.8168 precCharge: 2 Sequence: TEGAEK
Intensity
1e+05
5e+04
0e+00
M/Z
300
600
900
b1
y1
b2
y2
b3
y3
b4
y4
b5
y5
type
b
y

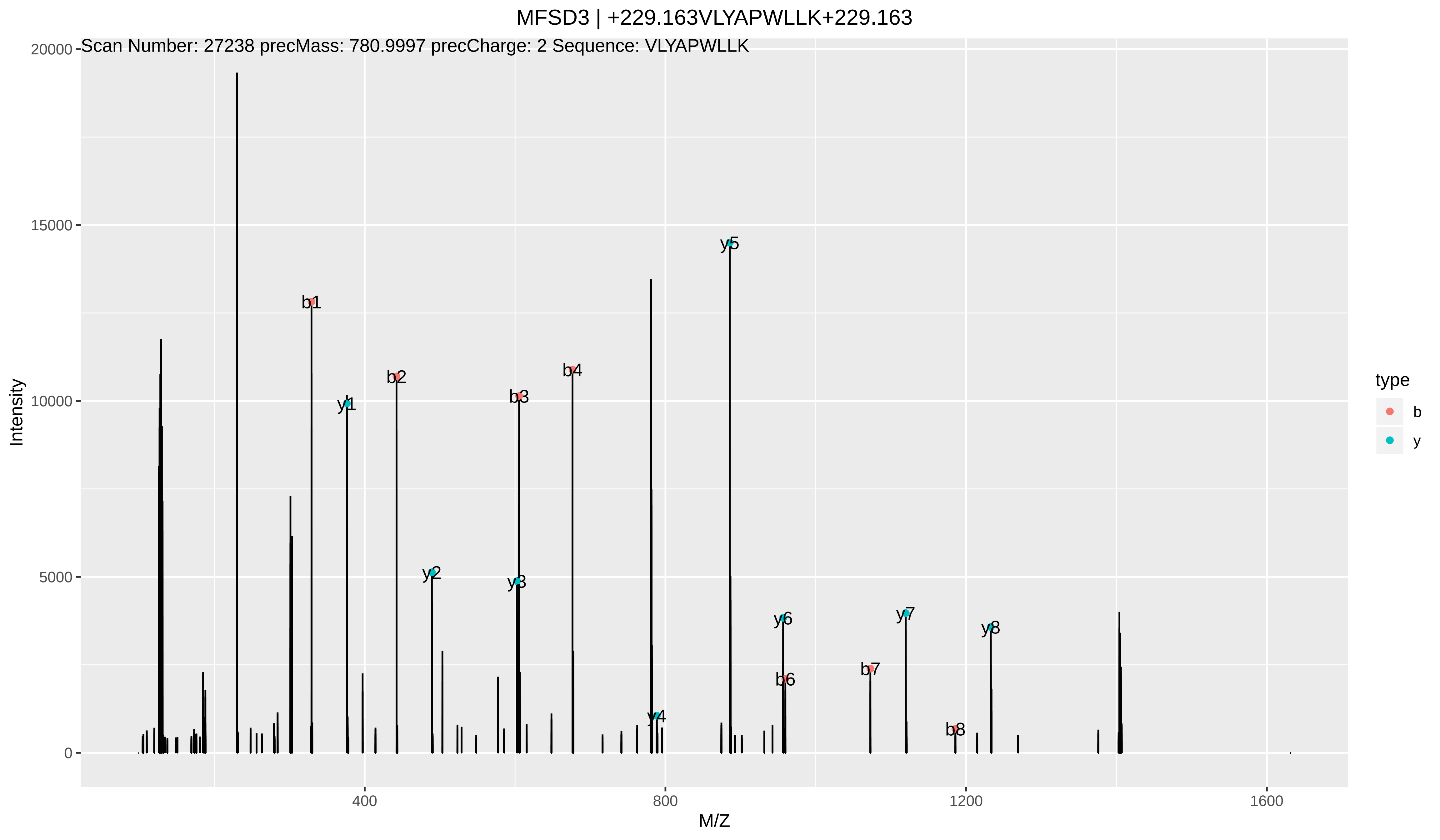

MFSD3 | +229.163VLYAPWLLK+229.163
Scan Number: 27238 precMass: 780.9997 precCharge: 2 Sequence: VLYAPWLLK
Intensity
M/Z
type
b
y
b1
b2
b3
b4
b6
b7
b8
y1
y2
y3
y4
y5
y6
y7
y8
0
5000
10000
15000
20000
400
800
1200
1600

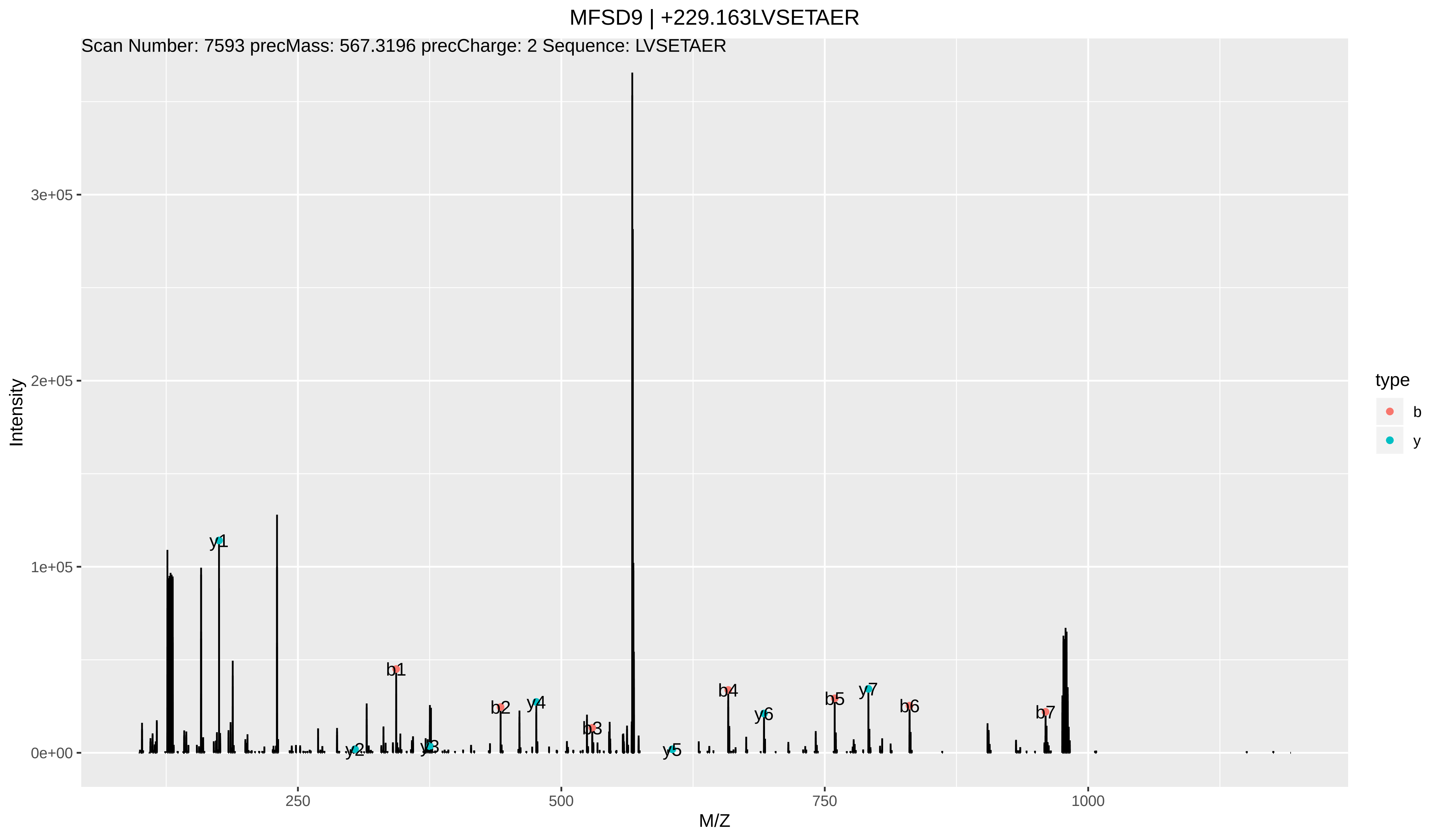

MFSD9 | +229.163LVSETAER
Scan Number: 7593 precMass: 567.3196 precCharge: 2 Sequence: LVSETAER
Intensity
3e+05
2e+05
1e+05
0e+00
M/Z
250
500
750
1000
y1
y2
y3
y4
y5
y6
y7
b1
b2
b3
b4
b5
b6
b7
type
b
y

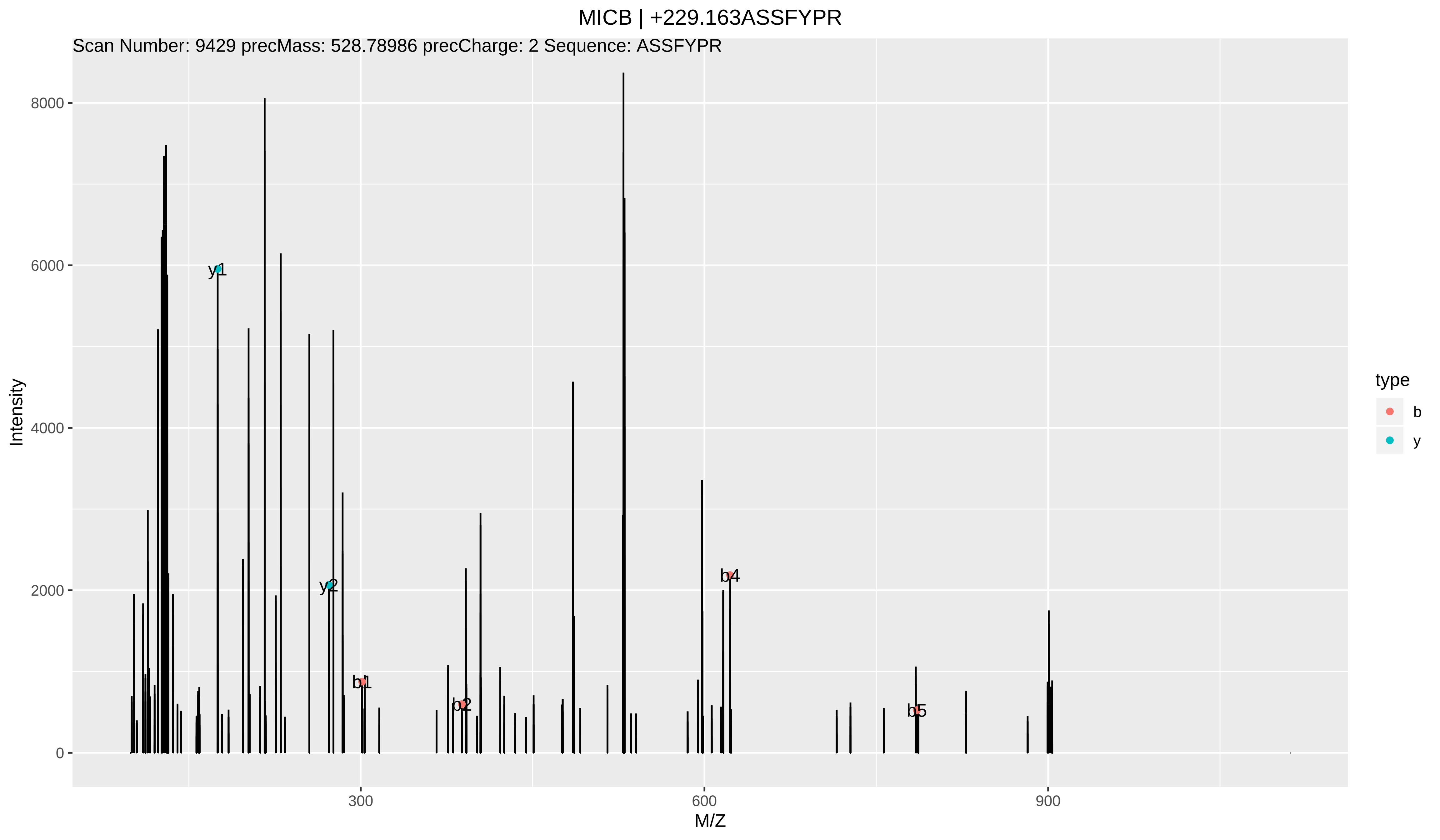

MICB | +229.163ASSFYPR
Scan Number: 9429 precMass: 528.78986 precCharge: 2 Sequence: ASSFYPR
Intensity
8000
6000
4000
2000
0
300
600
900
M/Z
y1
y2
b1
b2
b4
b5
type
b
y

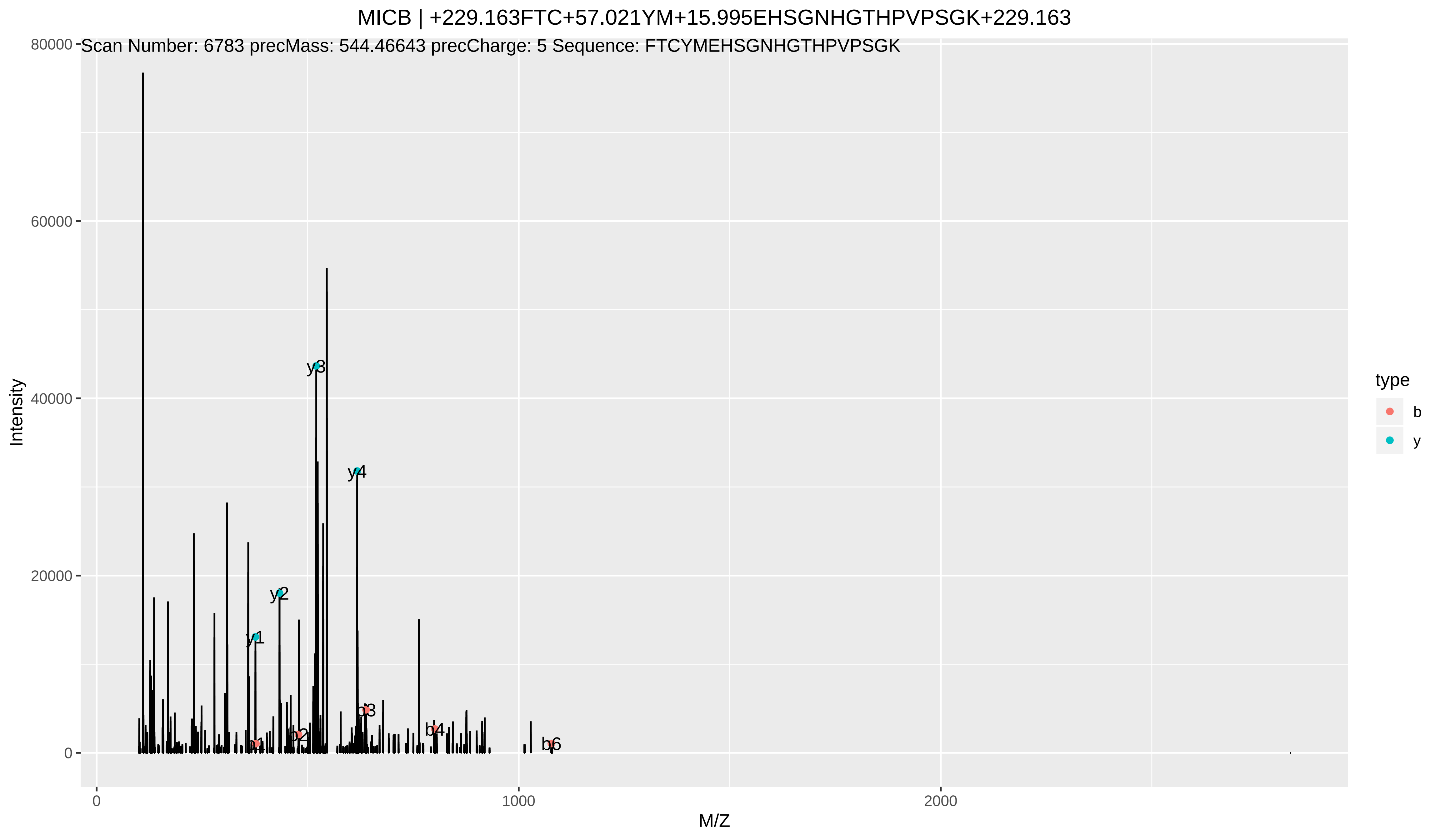
MICB | +229.163FTC+57.021YM+15.995EHSGNHGTHPVPSGK+229.163
Scan Number: 6783 precMass: 544.46643 precCharge: 5 Sequence: FTCYMEHSGNHGTHPVPSGK
Intensity
80000
60000
40000
20000
0
M/Z
0
1000
2000
y1
y2
y3
y4
b1
b2
b3
b4
b6
type
b
y

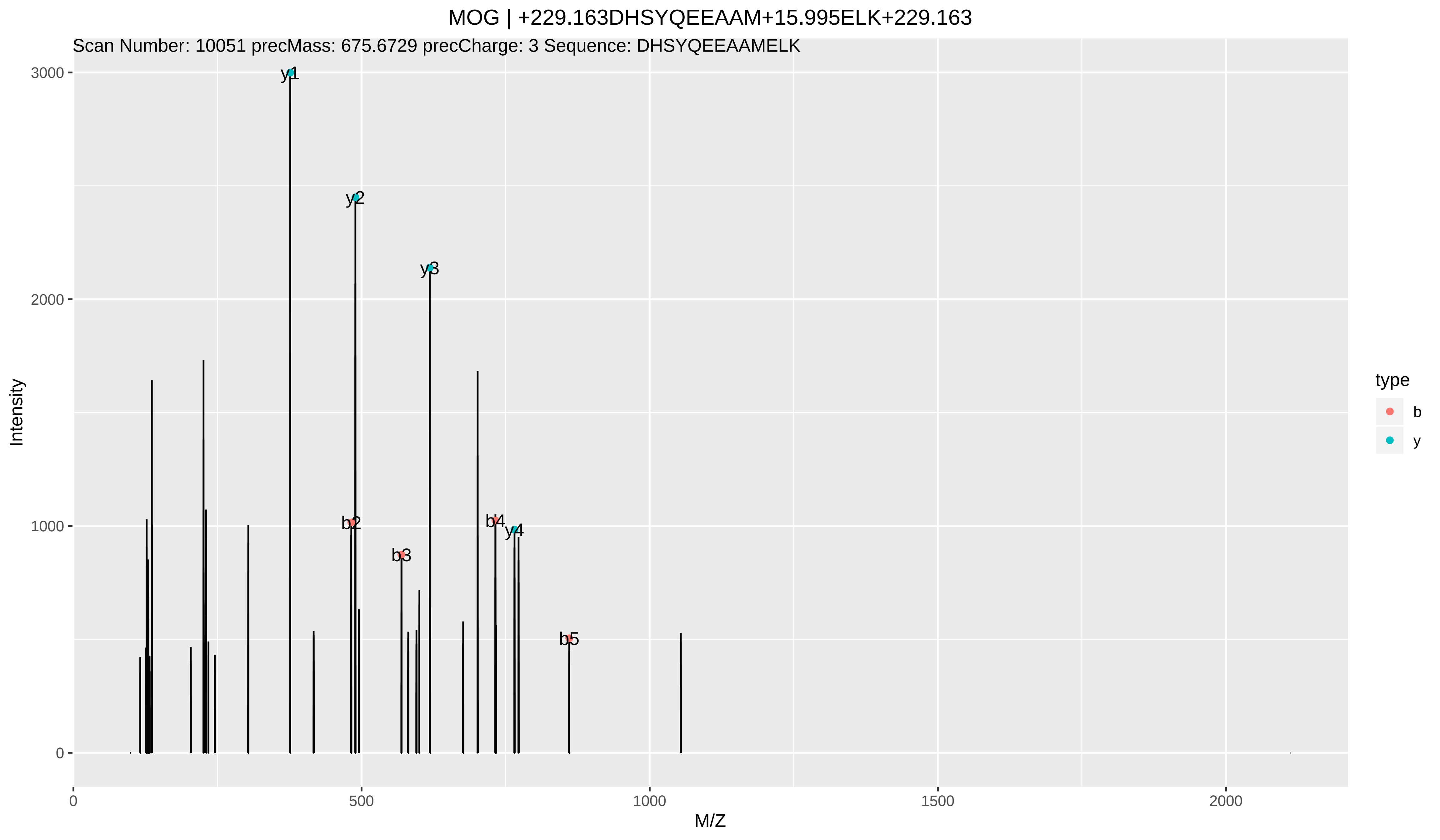
MOG | +229.163DHSYQEEAAM+15.995ELK+229.163
Scan Number: 10051 precMass: 675.6729 precCharge: 3 Sequence: DHSYQEEAAMELK
Intensity
M/Z
y1
y2
y3
b2
b3
b4
y4
b5
type
b
y

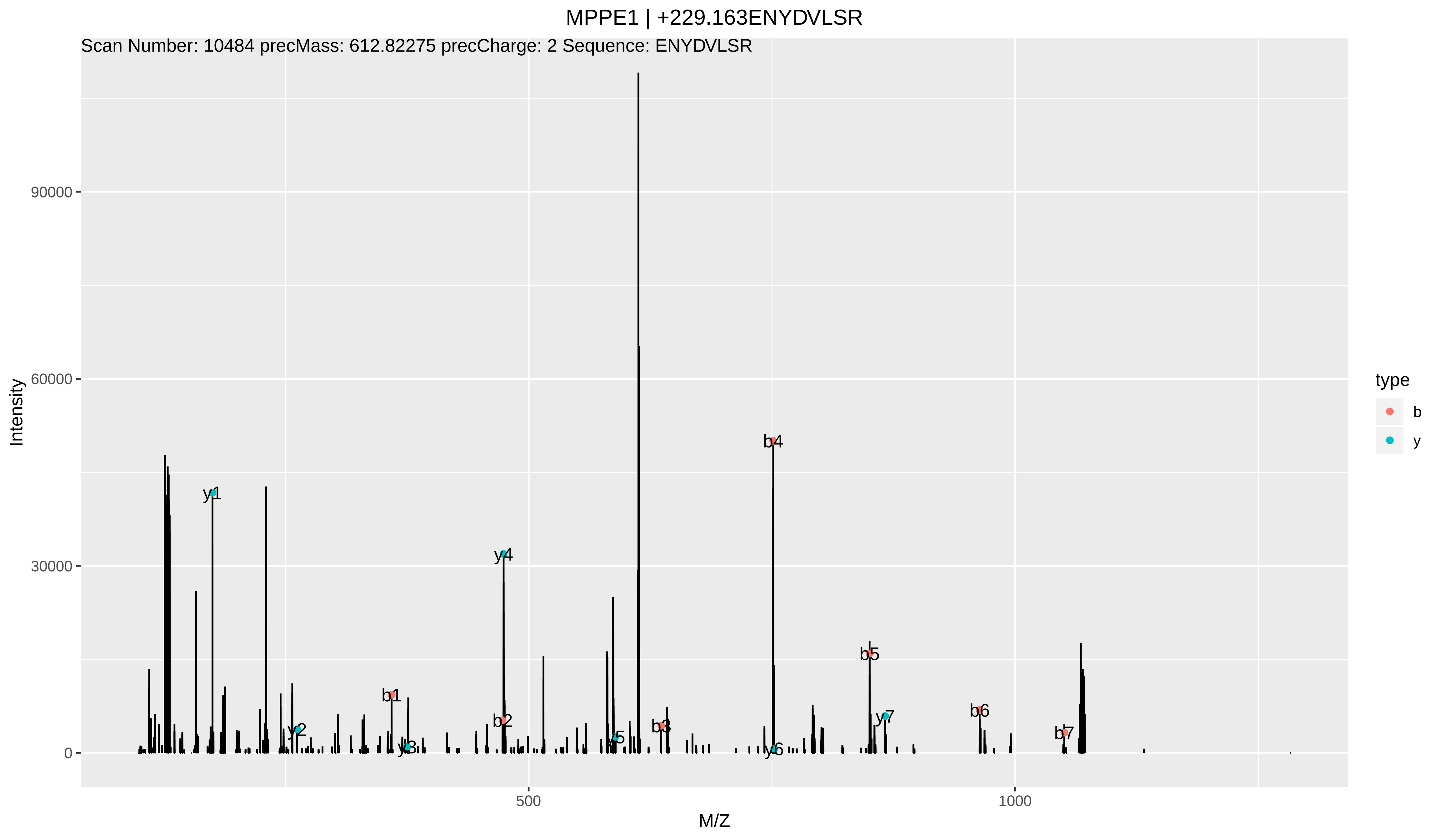
MPPE1 | +229.163ENYDVLSR
Scan Number: 10484 precMass: 612.82275 precCharge: 2 Sequence: ENYDVLSR
Intensity
90000
60000
30000
0
M/Z
500
1000
type
b
y
y1
y2
y3
y4
y5
y6
y7
b1
b2
b3
b4
b5
b6
b7

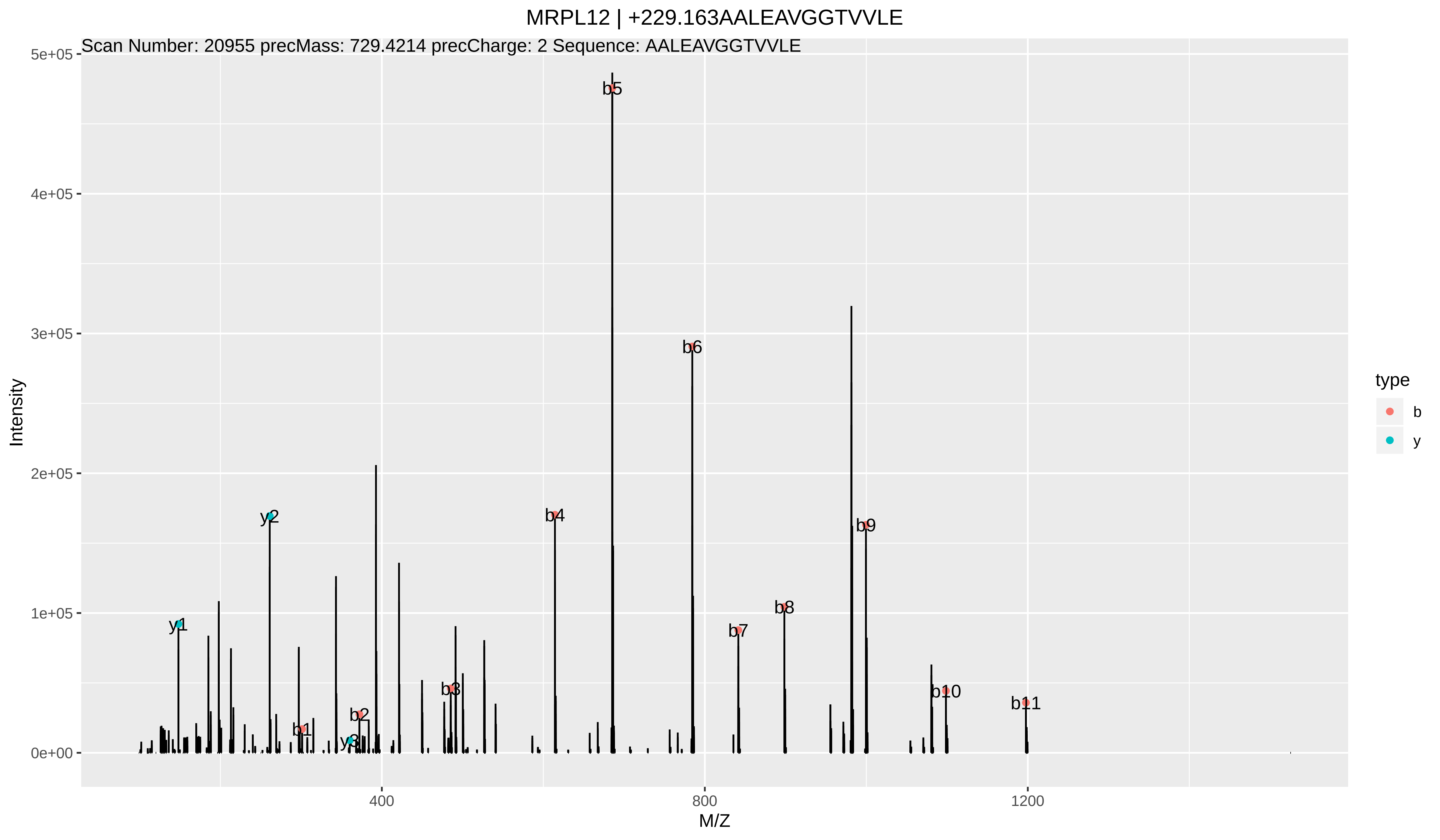
MRPL12 | +229.163AALEAVGGTVVLE
Scan Number: 20955 precMass: 729.4214 precCharge: 2 Sequence: AALEAVGGTVVLE
Intensity
M/Z
5e+05
4e+05
3e+05
2e+05
1e+05
0e+00
400
800
1200
y1
y2
y3
b1
b2
b3
b4
b5
b6
b7
b8
b9
b10
b11
type
b
y

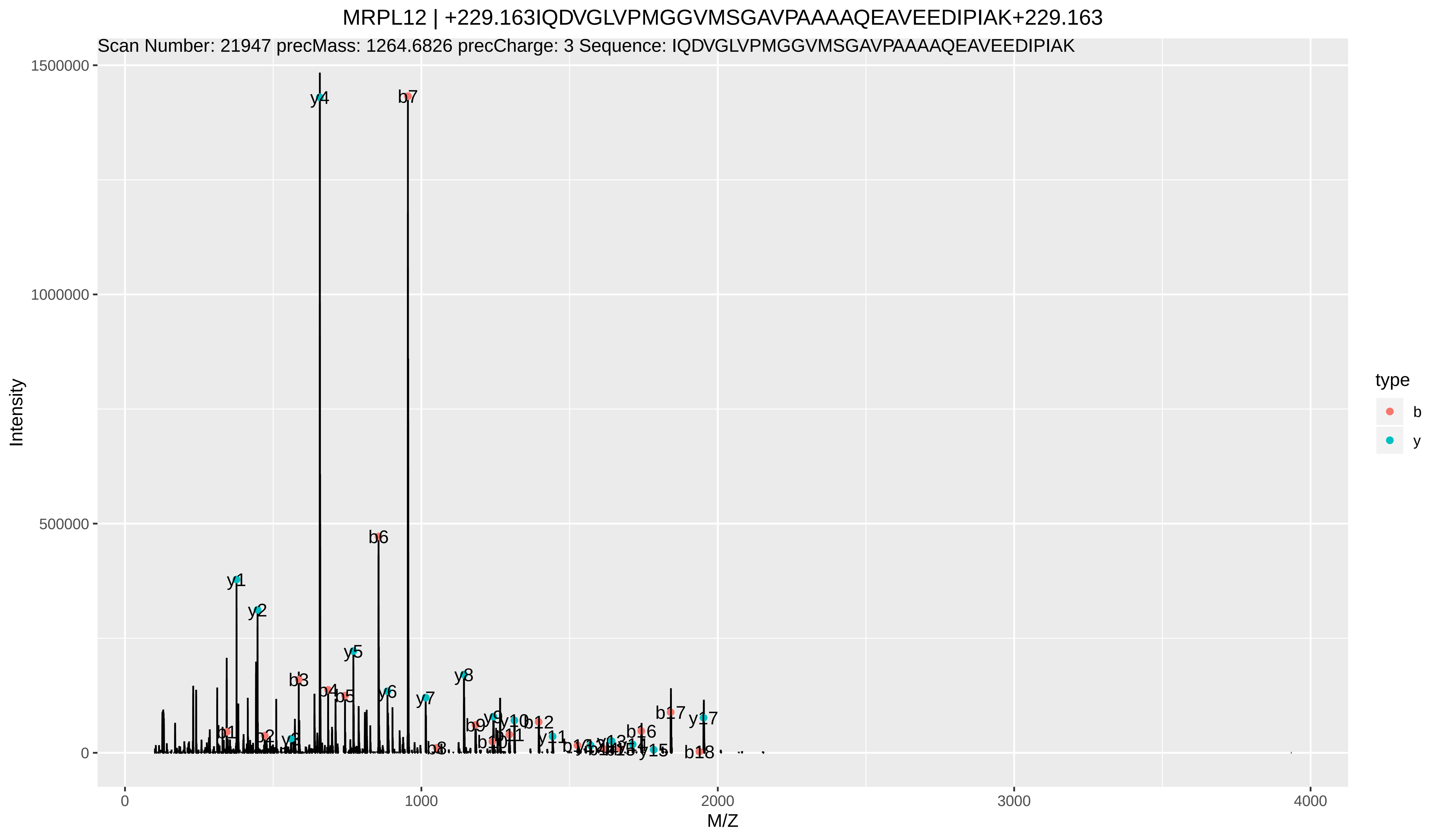
MRPL12 | +229.163IQDVGLVPMGGVMSGAVPAAAAQEAVEEDIPIAK+229.163
Scan Number: 21947 precMass: 1264.6826 precCharge: 3 Sequence: IQDVGLVPMGGVMSGAVPAAAAQEAVEEDIPIAK
Intensity
M/Z
type
b
y

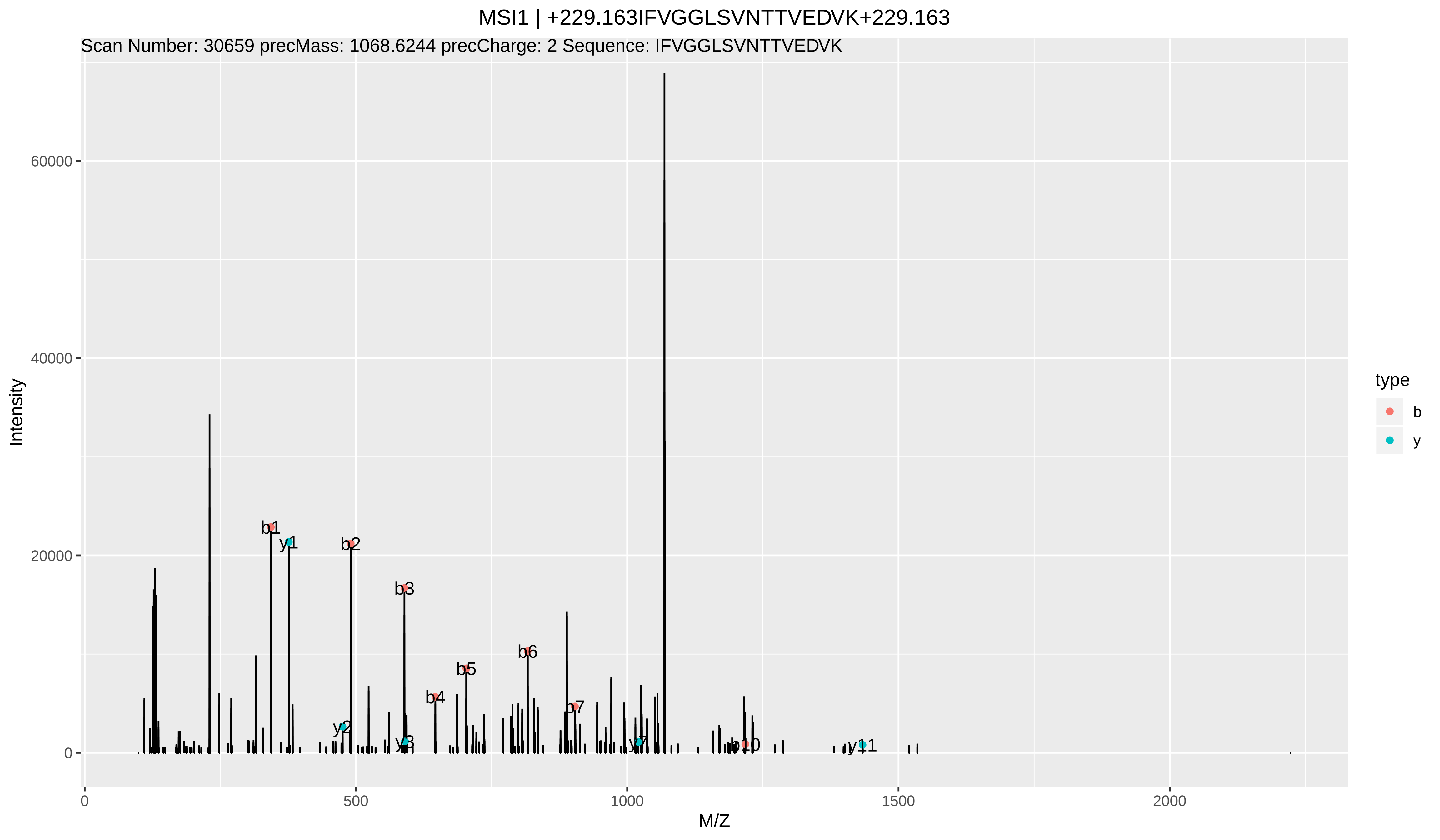
MSI1 | +229.163IFVGGLSVNTTVEDVK+229.163
Scan Number: 30659 precMass: 1068.6244 precCharge: 2 Sequence: IFVGGLSVNTTVEDVK
Intensity
M/Z
type
b
y
b1
y1
b2
b3
b4
b5
b6
b7
y2
y3
y7
b10
y11
0
20000
40000
60000
0
500
1000
1500
2000

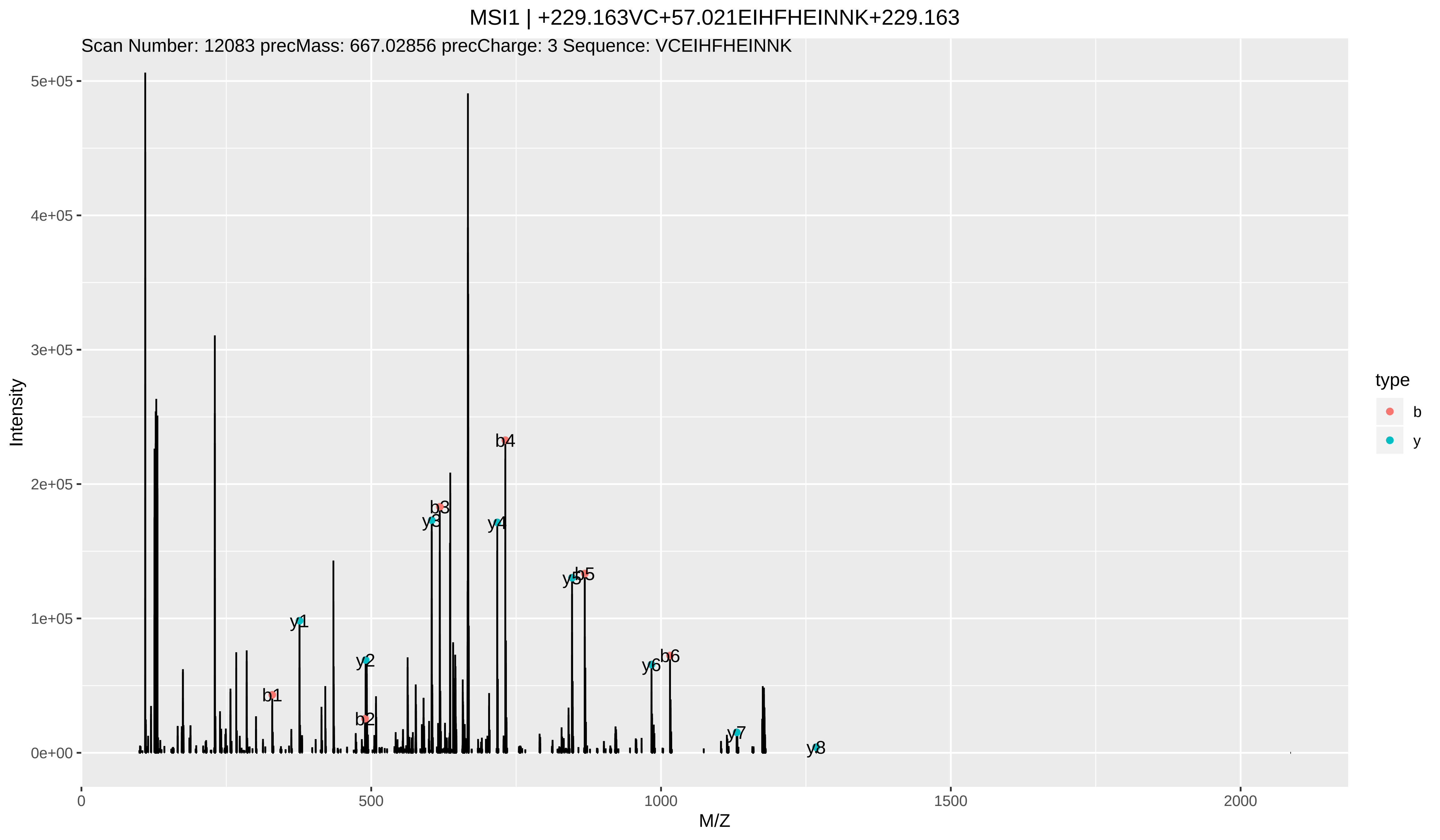

MSI1 | +229.163VC+57.021EIHFHEINNK+229.163
Scan Number: 12083 precMass: 667.02856 precCharge: 3 Sequence: VCEIHFHEINNK
Intensity
M/Z
type
b
y
5e+05
4e+05
3e+05
2e+05
1e+05
0e+00
0
500
1000
1500
2000
b1
b2
b3
b4
b5
b6
y1
y2
y3
y4
y5
y6
y7
y8

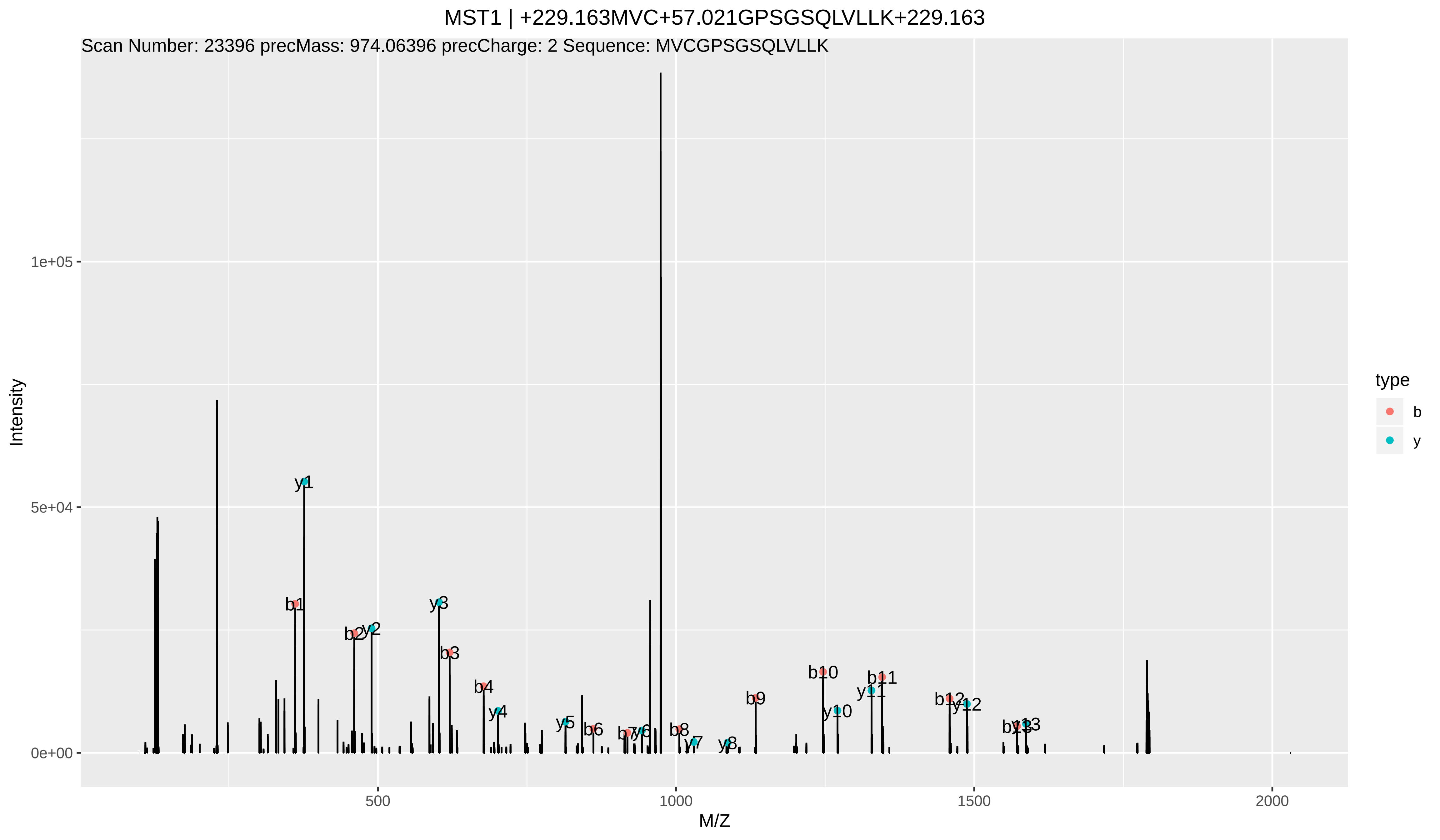

MST1 | +229.163MVC+57.021GPSGSQLVLLK+229.163
Scan Number: 23396 precMass: 974.06396 precCharge: 2 Sequence: MVCGPSGSQLVLLK
Intensity
M/Z
1e+05
5e+04
0e+00
500
1000
1500
2000
type
b
y
y1
b1
b2
y2
y3
b3
b4
y4
y5
b6
y6
b8
y8
b9
b10
b11
y10
y11
b12
y12

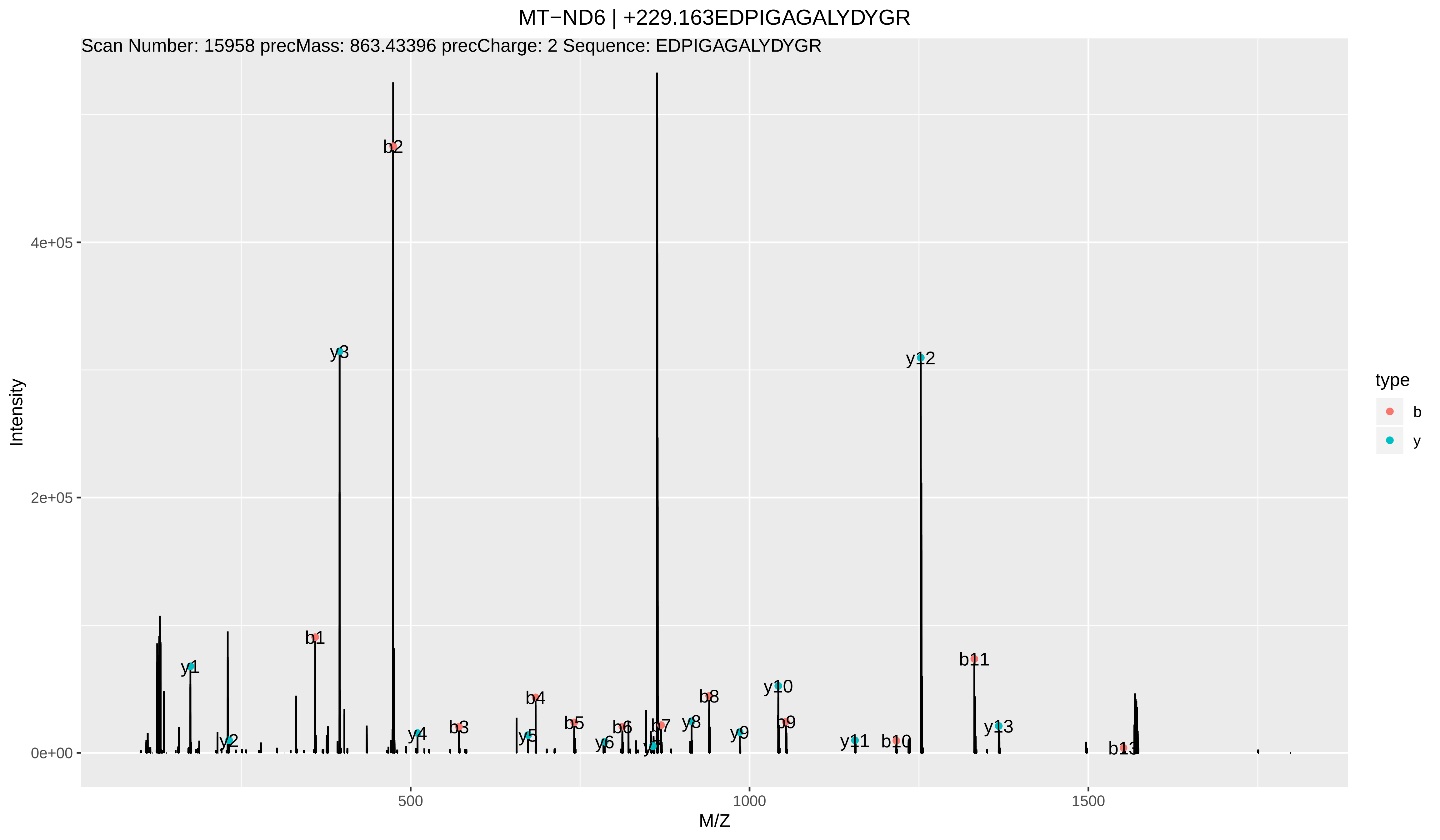
MT−ND6 | +229.163EDPIGAGALYDYGR
Scan Number: 15958 precMass: 863.43396 precCharge: 2 Sequence: EDPIGAGALYDYGR
Intensity
4e+05
2e+05
0e+00
M/Z
500
1000
1500
type
b
y
b1
b2
b3
b4
b5
b6
b7
b8
b9
b10
b11
b13
y1
y2
y3
y4
y5
y6
y8
y9
y10
y11
y12
y13

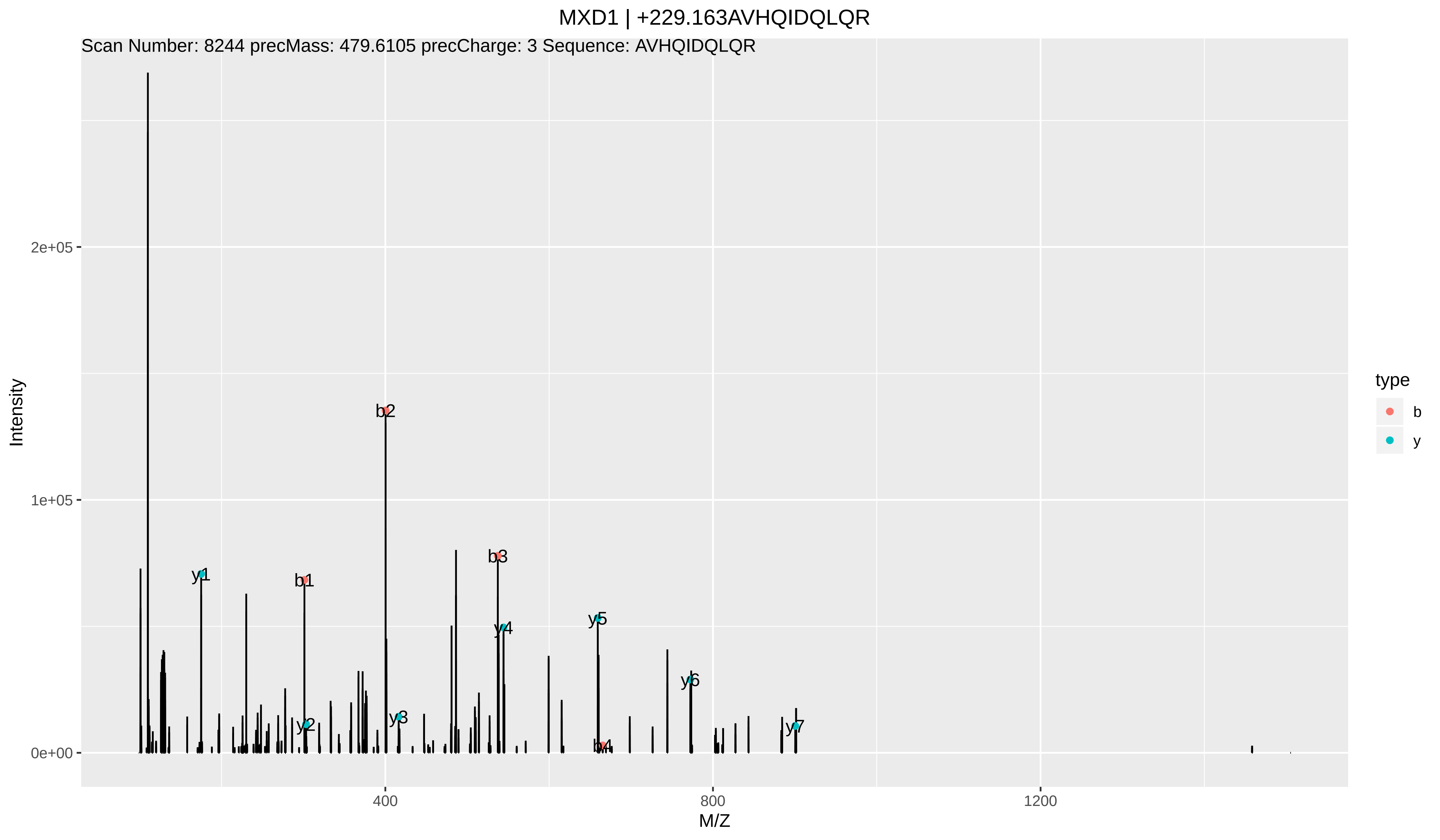
MXD1 | +229.163AVHQIDQLQR
Scan Number: 8244 precMass: 479.6105 precCharge: 3 Sequence: AVHQIDQLQR
Intensity
2e+05
1e+05
0e+00
M/Z
400
800
1200
y1
b1
b2
b3
y2
y3
y4
y5
b4
y6
y7
type
b
y

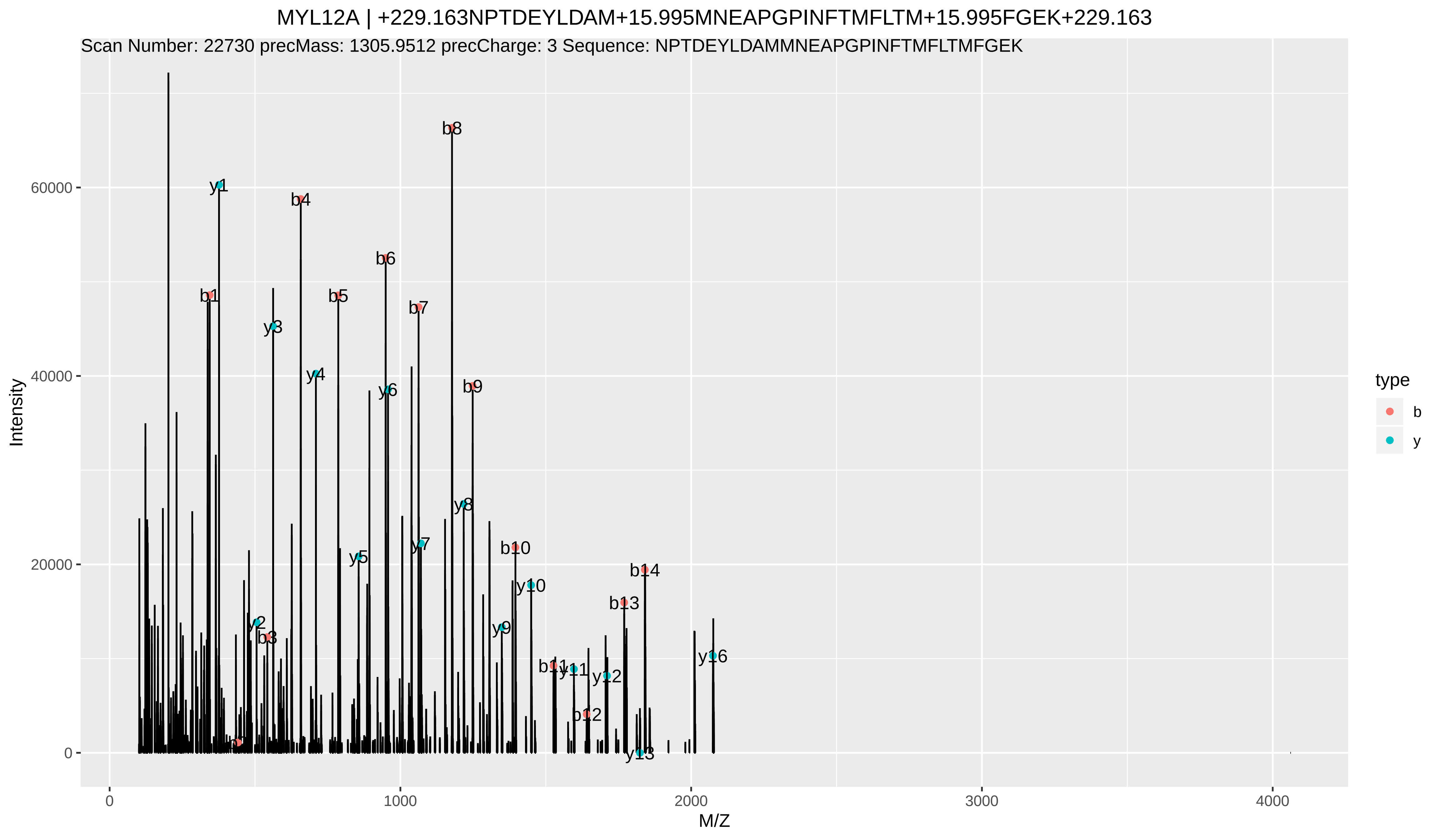

MYL12A | +229.163NPTDEYLDAM+15.995MNEAPGPINFTMFLTM+15.995FGEK+229.163
Scan Number: 22730 precMass: 1305.9512 precCharge: 3 Sequence: NPTDEYLDAMMNEAPGPINFTMFLTMFGEK
Intensity
M/Z
type
b
y
b8
y1
b4
b6
b1
b5
b7
y3
y4
y6
b9
y8
b10
y7
y5
b14
y10
b13
y9
y2
b3
y16
b11
y11
y12
b12
y13
0
20000
40000
60000
1000
2000
3000
4000

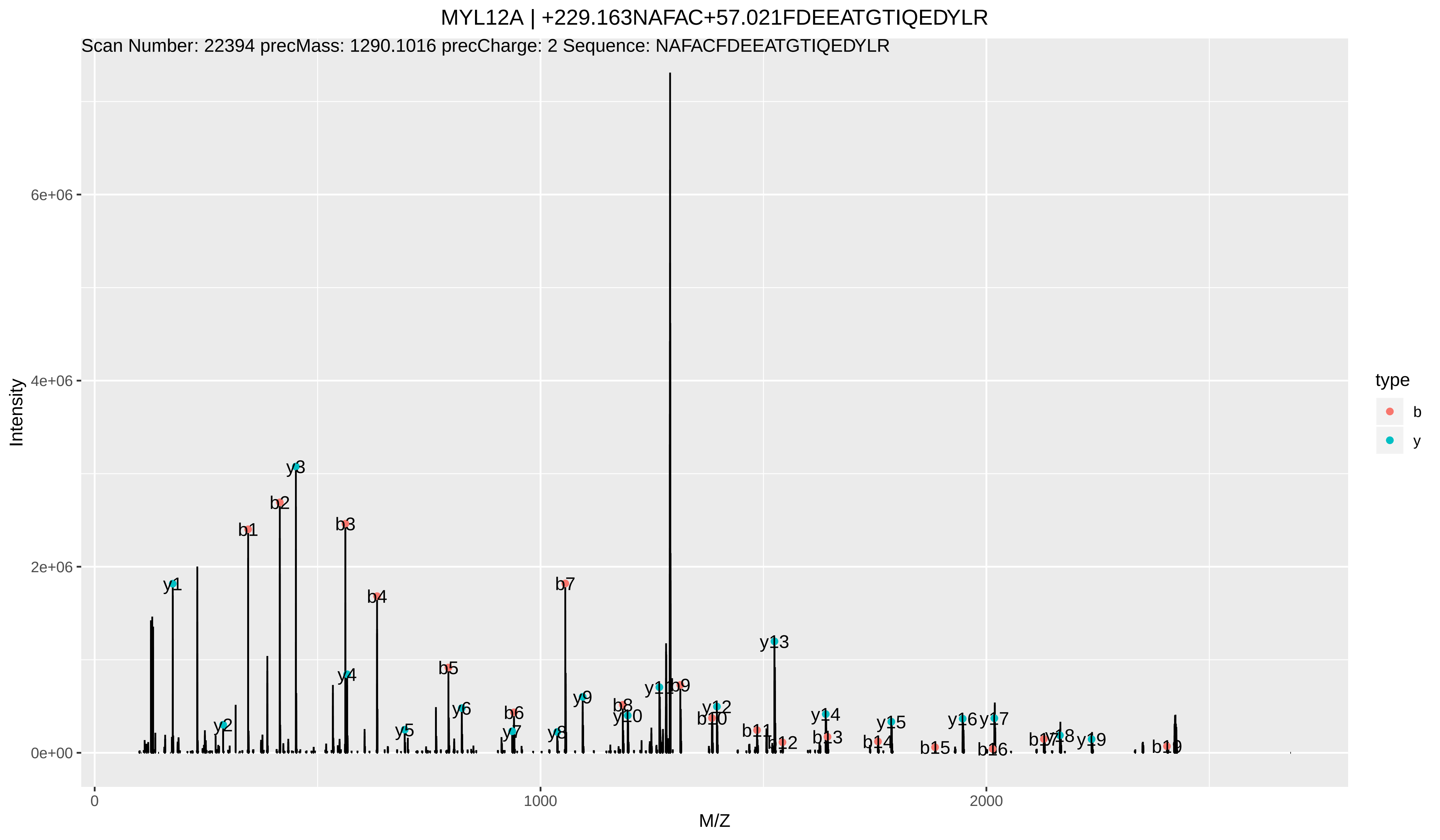
MYL12A | +229.163NAFAC+57.021FDEEATGTIQEDYLR
Scan Number: 22394 precMass: 1290.1016 precCharge: 2 Sequence: NAFACFDEEATGTIQEDYLR
Intensity
6e+06
4e+06
2e+06
0e+00
M/Z
0
1000
2000
type
b
y
y1
y2
y3
y4
y5
y6
y7
y8
y9
y10
y12
y13
y14
y15
y16
y17
y18
y19
b1
b2
b3
b4
b5
b6
b7
b8
b9
b10
b11
b13
b14
b15
b16

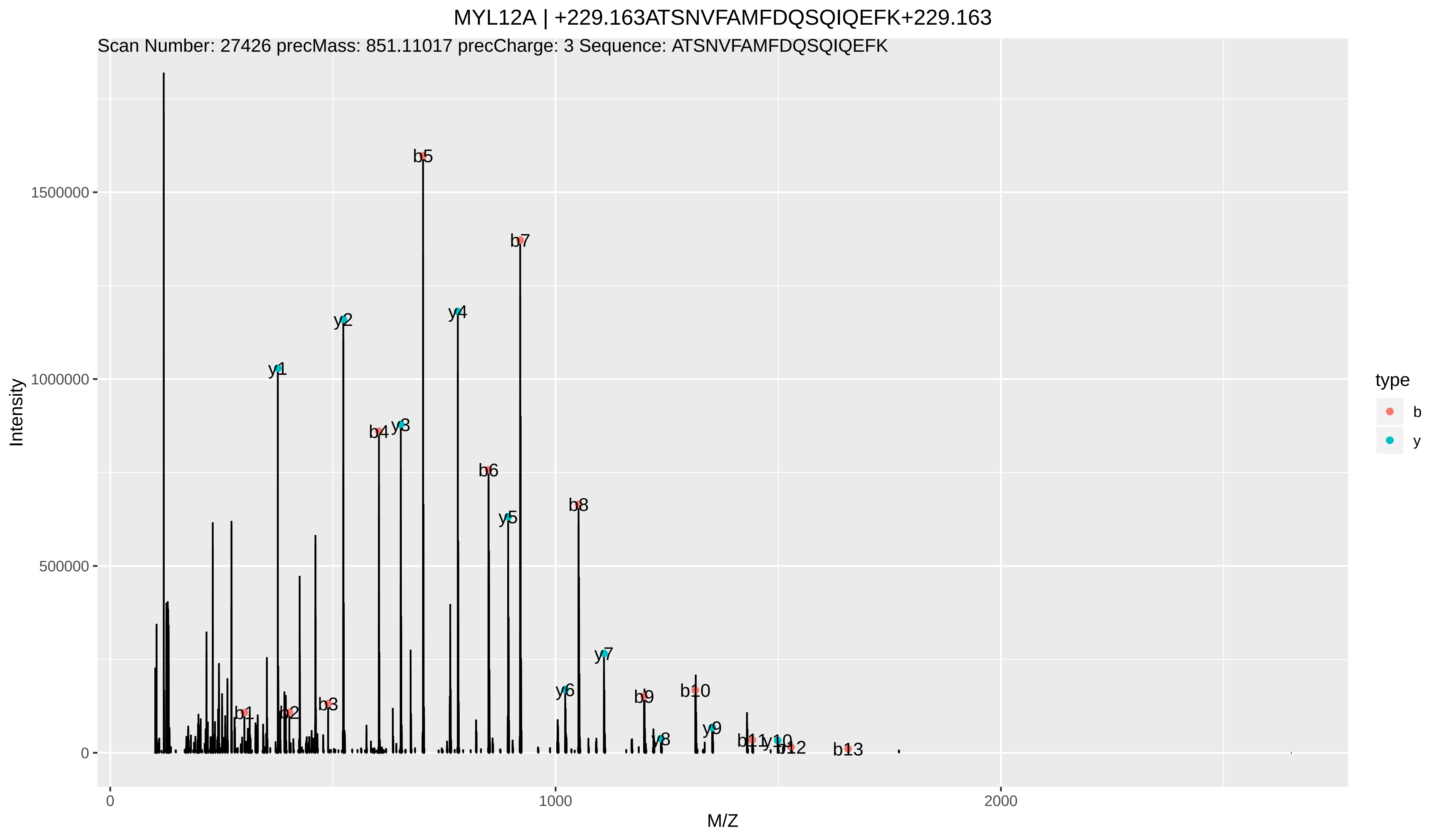

MYL12A | +229.163ATSNVFAMFDQSQIQEFK+229.163
Scan Number: 27426 precMass: 851.11017 precCharge: 3 Sequence: ATSNVFAMFDQSQIQEFK
Intensity
M/Z
type
b
y
b1
b2
b3
b4
b5
b6
b7
b8
b9
b10
b11
b12
b13
y1
y2
y3
y4
y5
y6
y7
y8
y9
y10
0
500000
1000000
1500000
1000
2000

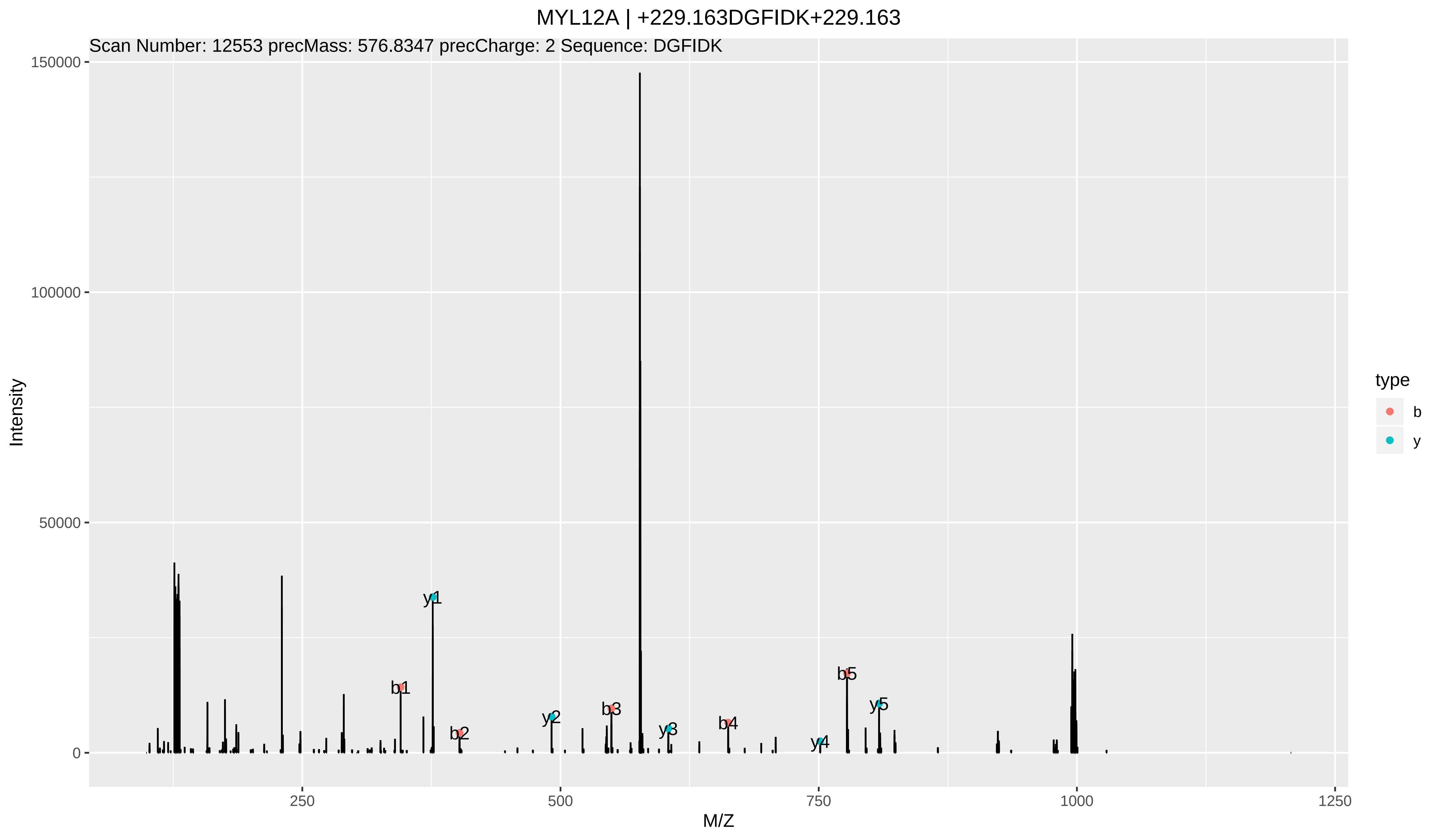

MYL12A | +229.163DGFIDK+229.163
Scan Number: 12553 precMass: 576.8347 precCharge: 2 Sequence: DGFIDK
Intensity
M/Z
150000
100000
50000
0
250
500
750
1000
1250
y1
b1
b2
y2
b3
y3
b4
y4
b5
y5
type
b
y

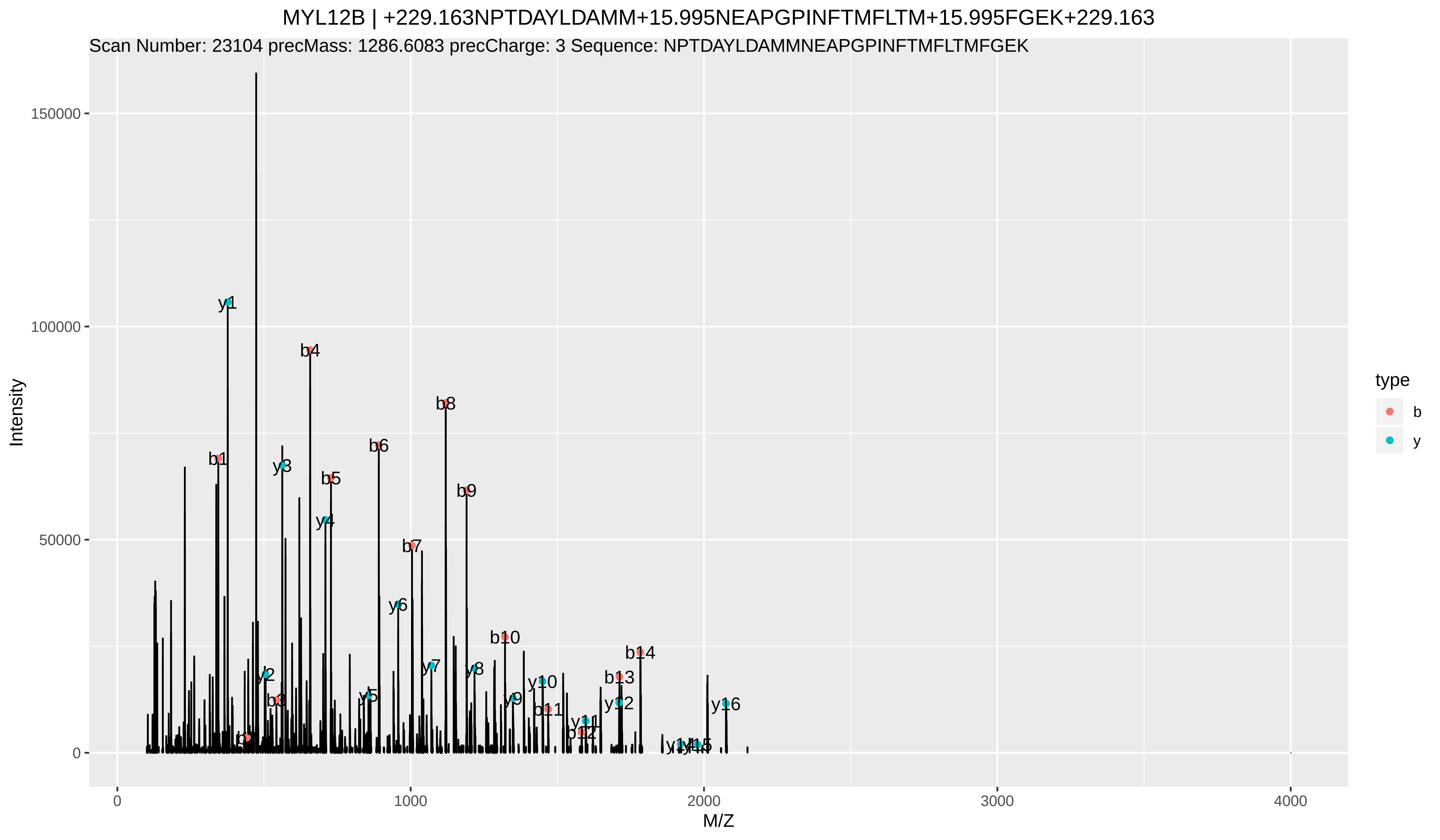
MYL12B | +229.163NPTDAYLDAMM+15.995NEAPGPINFTMFLTM+15.995FGEK+229.163
Scan Number: 23104 precMass: 1286.6083 precCharge: 3 Sequence: NPTDAYLDAMMNEAPGPINFTMFLTMFGEK
Intensity
M/Z
type
b
y
0
50000
100000
150000
0
1000
2000
3000
4000
y1
b4
b8
b6
b1
y3
b5
b9
y4
b7
y6
b10
b14
y7
y8
y2
b13
y10
b3
y5
y9
y12
y16
b11
y11
b12
y14
y15

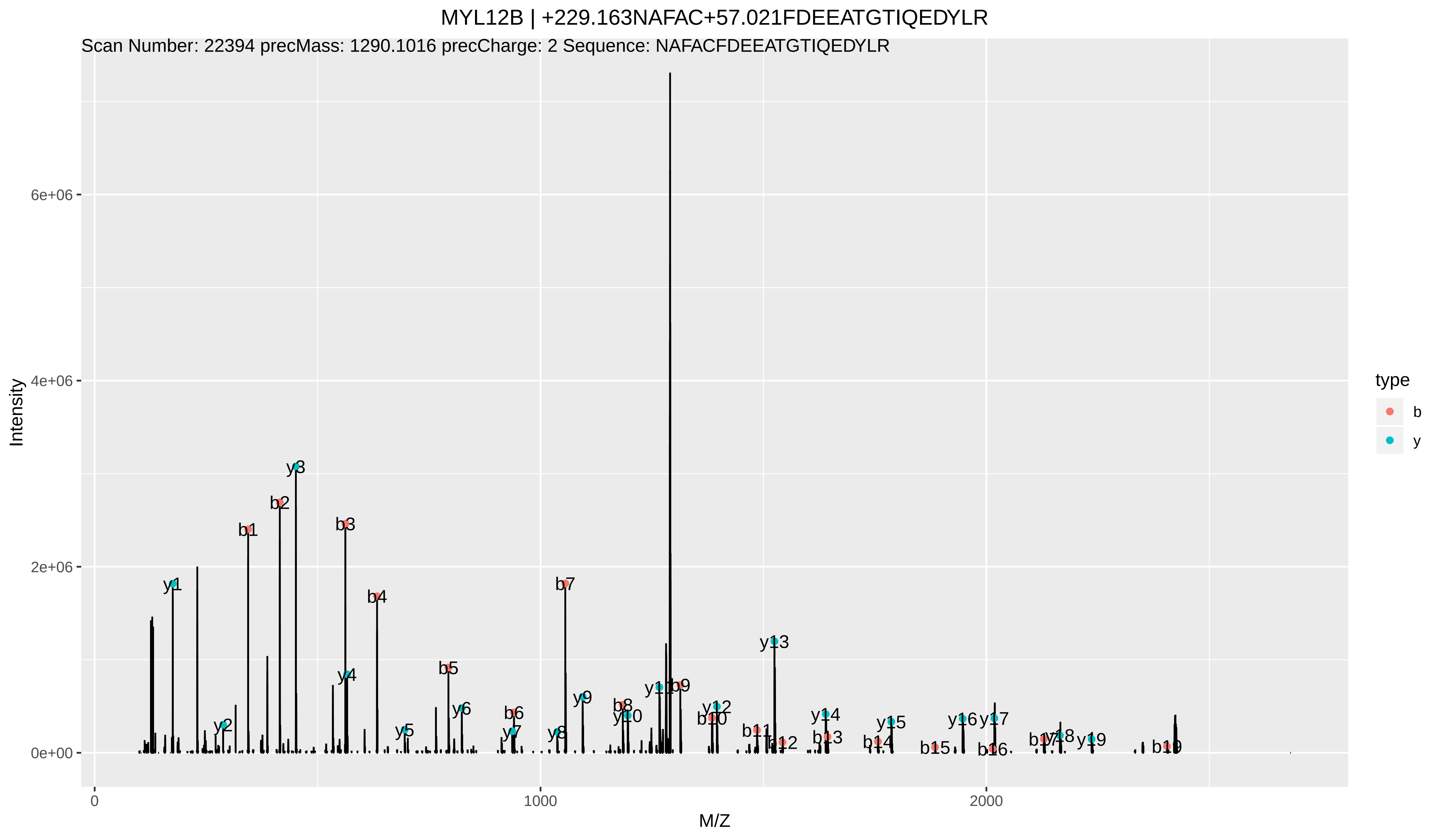

MYL12B | +229.163NAFAC+57.021FDEEATGTIQEDYLR
Scan Number: 22394 precMass: 1290.1016 precCharge: 2 Sequence: NAFACFDEEATGTIQEDYLR
Intensity
M/Z
type
b
y

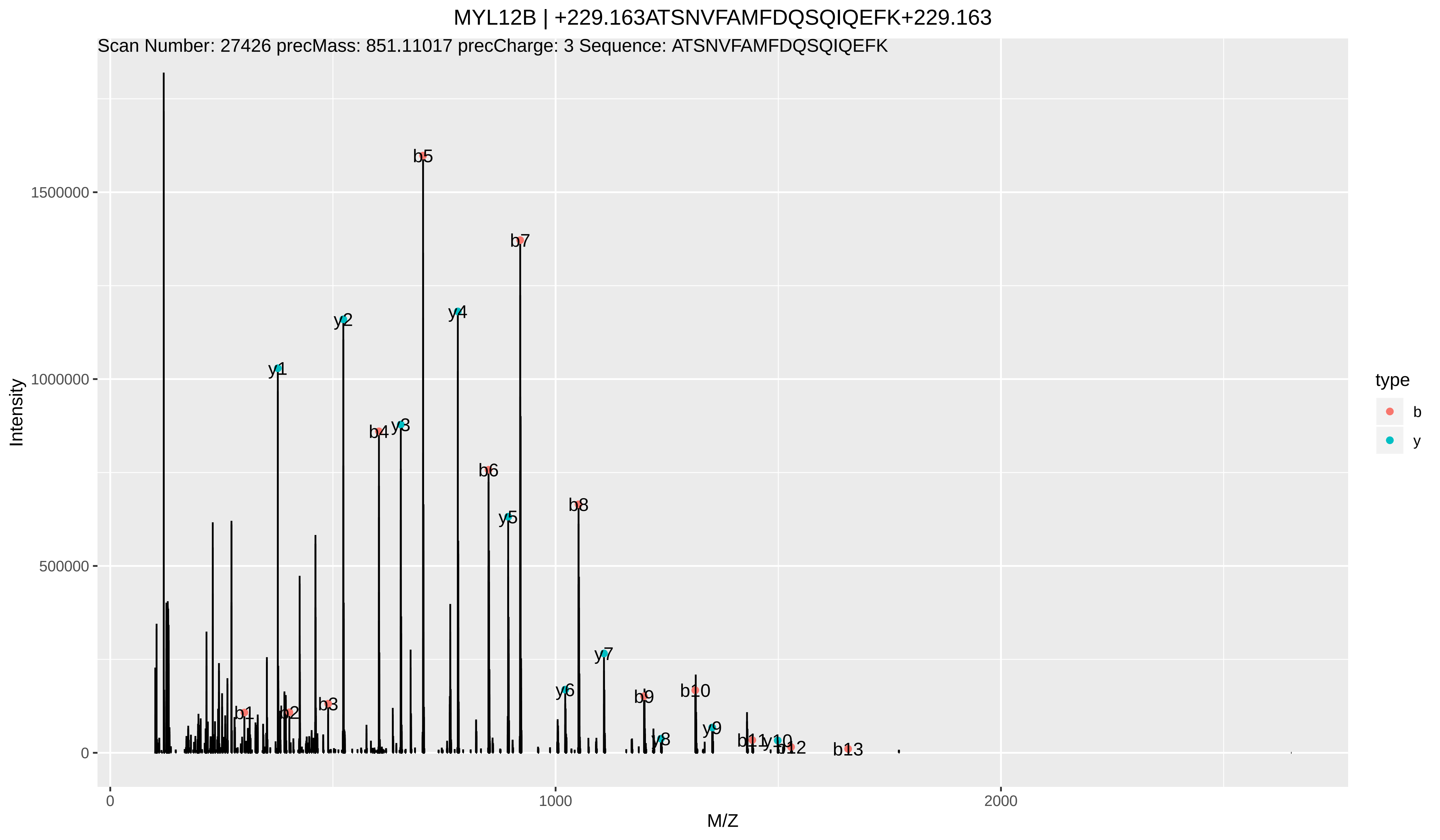
MYL12B | +229.163ATSNVFAMFDQSQIQEFK+229.163
Scan Number: 27426 precMass: 851.11017 precCharge: 3 Sequence: ATSNVFAMFDQSQIQEFK
Intensity
1500000
1000000
500000
0
M/Z
0
1000
2000
b5
b7
y2
y4
y1
b4
y3
b6
y5
b8
y7
y6
b10
b9
b1
b2
b3
y8
y9
b11
y10
b12
b13
type
b
y

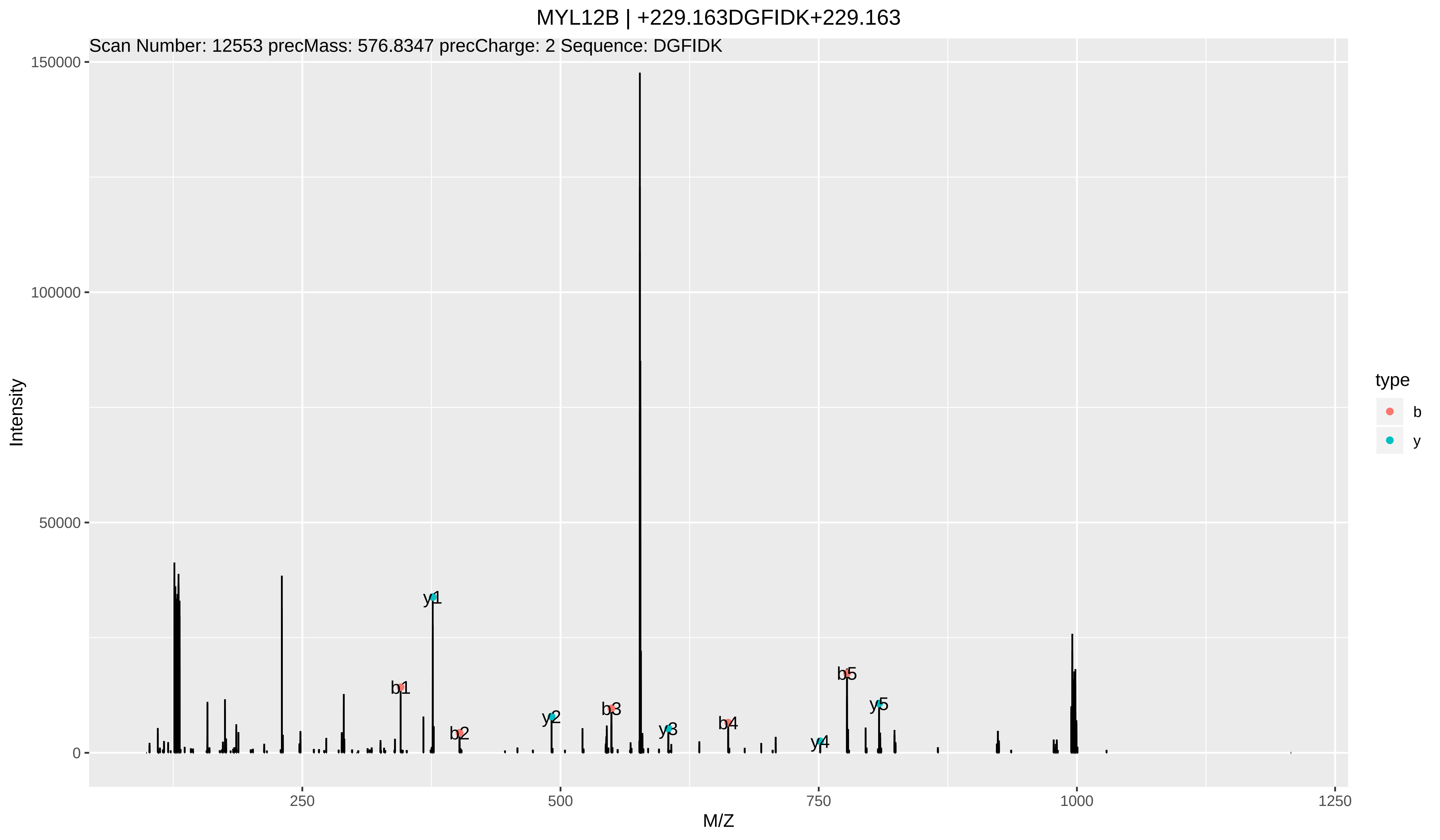

MYL12B | +229.163DGFIDK+229.163
Scan Number: 12553 precMass: 576.8347 precCharge: 2 Sequence: DGFIDK
Intensity
150000
100000
50000
0
M/Z
250
500
750
1000
1250
y1
b1
b2
y2
b3
y3
b4
y4
b5
y5
type
b
y

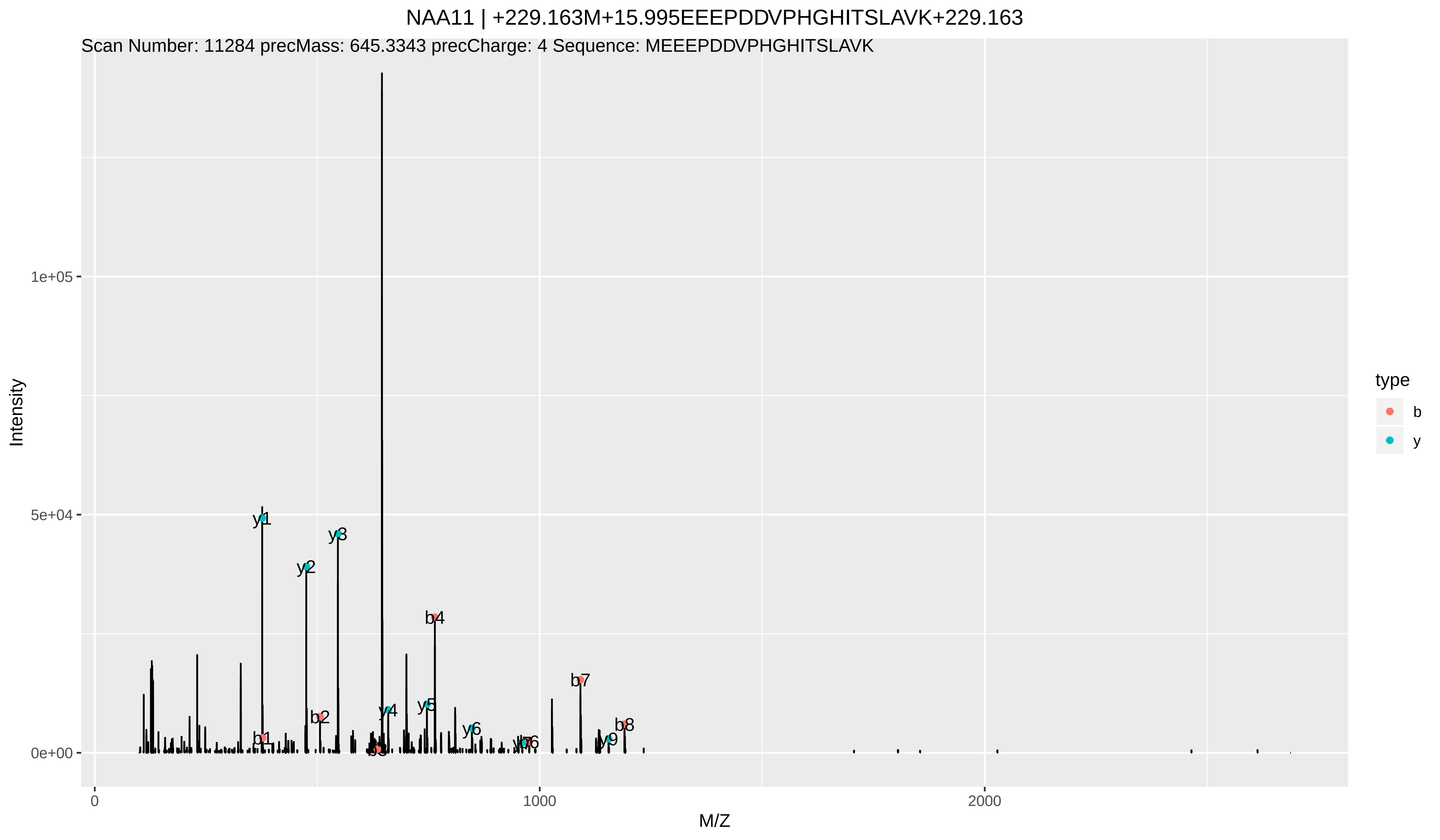

NAA11 | +229.163M+15.995EEEPDDVPHGHITSLAVK+229.163
Scan Number: 11284 precMass: 645.3343 precCharge: 4 Sequence: MEEEPDDVPHGHITSLAVK
Intensity
1e+05
5e+04
0e+00
0
1000
2000
M/Z
y1
y2
y3
y4
y5
y6
y9
b1
b2
b4
b7
b8
type
b
y

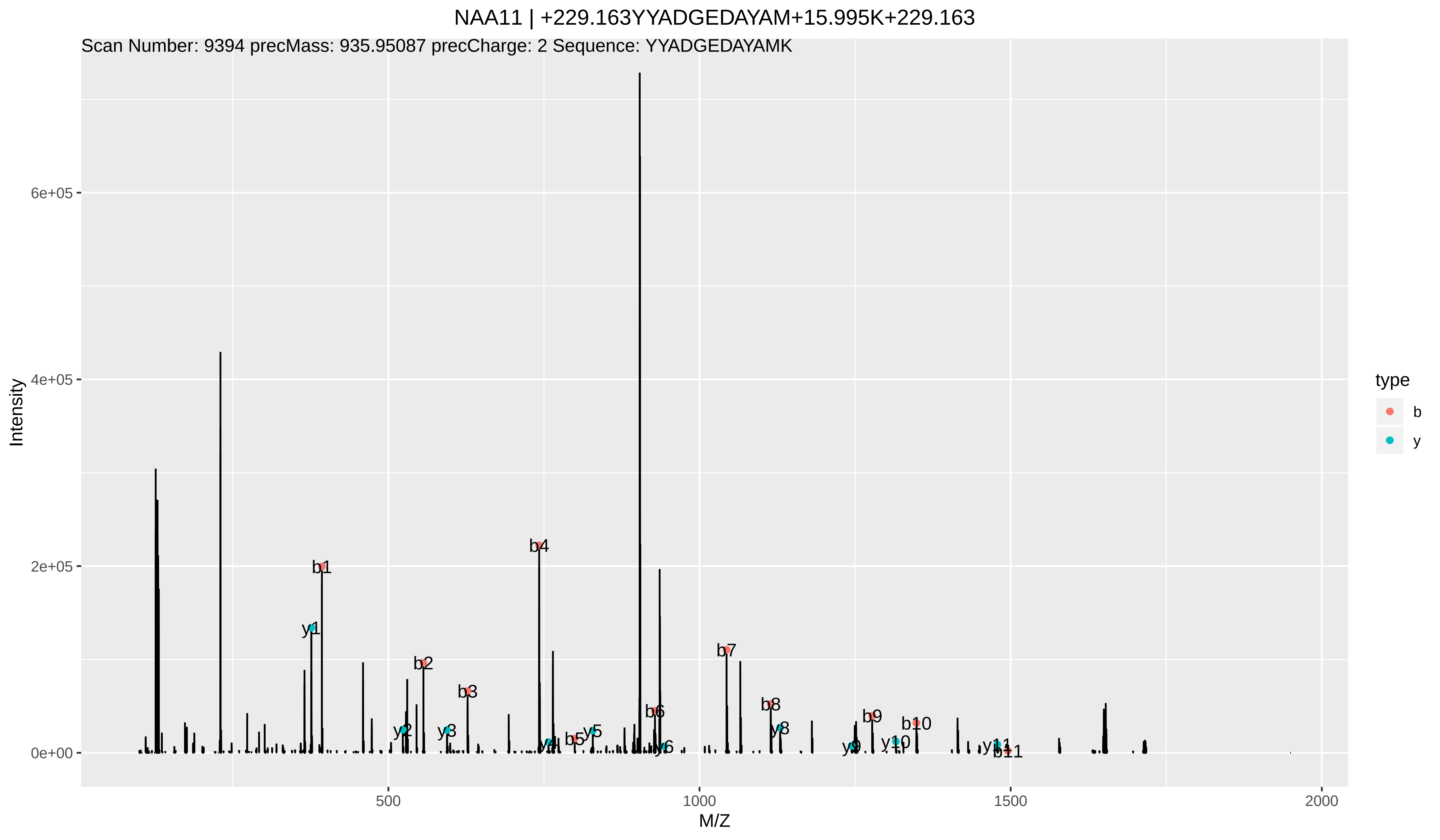

NAA11 | +229.163YYADGEDAYAM+15.995K+229.163
Scan Number: 9394 precMass: 935.95087 precCharge: 2 Sequence: YYADGEDAYAMK
Intensity
6e+05
4e+05
2e+05
0e+00
M/Z
500
1000
1500
2000
b1
b2
b3
b4
b5
b6
b7
b8
b9
b10
b11
y1
y2
y3
y4
y5
y6
y8
y9
y10
y11
type
b
y

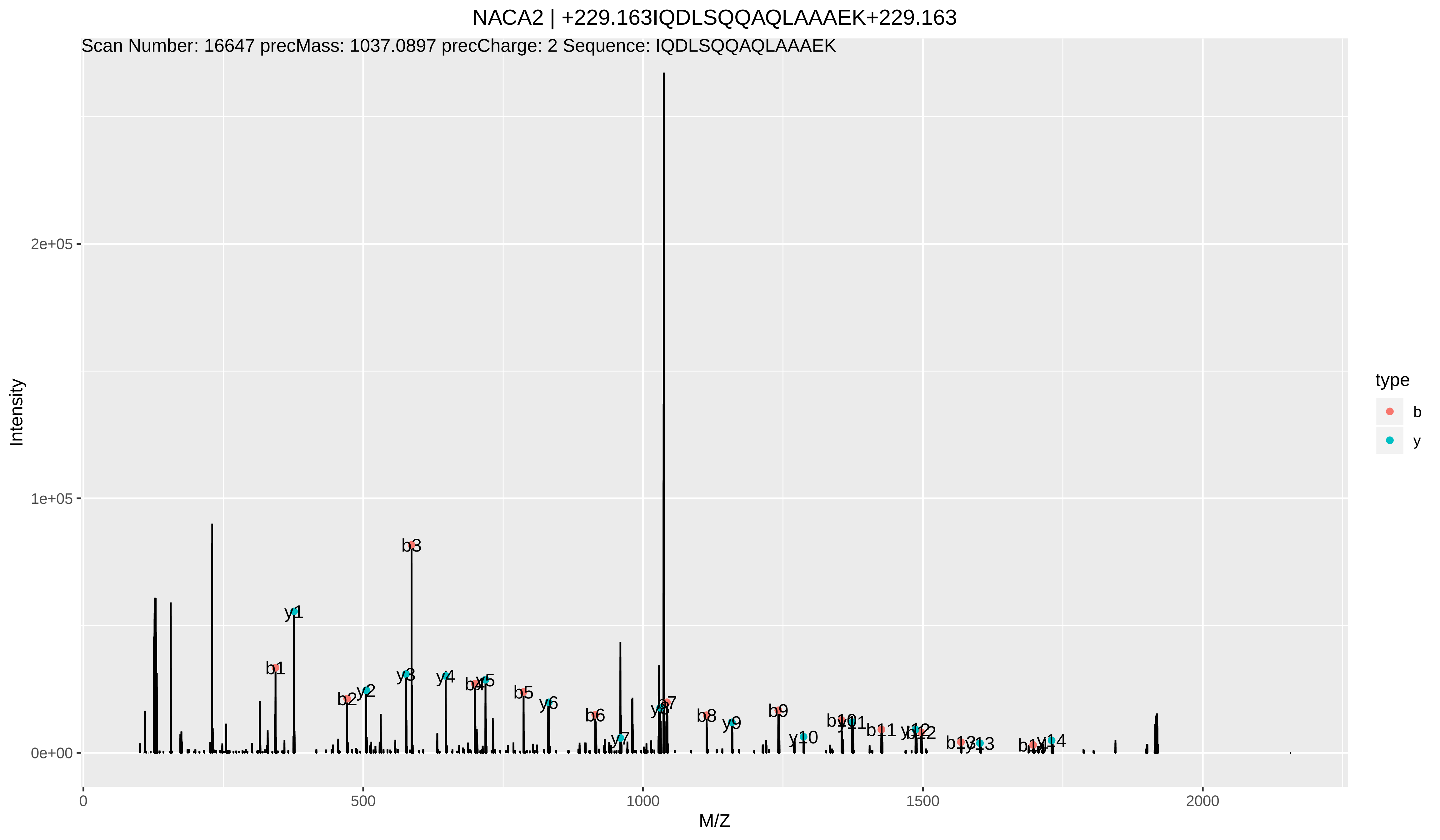

NACA2 | +229.163IQDLSQQAQLAAAEK+229.163
Scan Number: 16647 precMass: 1037.0897 precCharge: 2 Sequence: IQDLSQQAQLAAAEK
Intensity
M/Z
type
b
y
0e+00
1e+05
2e+05
0
500
1000
1500
2000
b1
b2
b3
b4
b5
b6
b7
b8
b9
b10
b11
b12
b13
b14
y1
y2
y3
y4
y5
y6
y7
y8
y9
y10
y11
y12
y13
y14

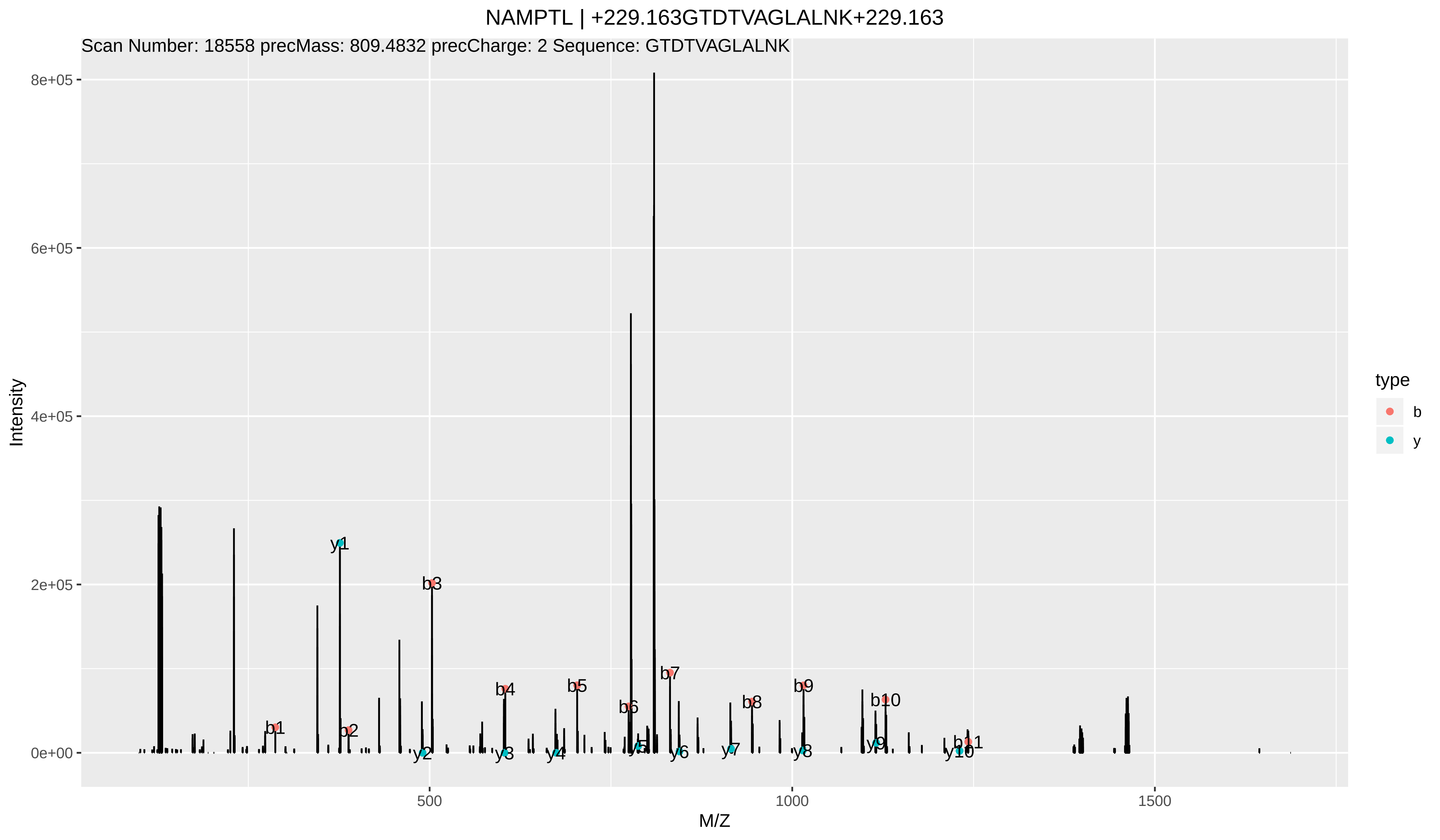

NAMPTL | +229.163GTDTVAGLALNK+229.163
Scan Number: 18558 precMass: 809.4832 precCharge: 2 Sequence: GTDTVAGLALNK
Intensity
8e+05
6e+05
4e+05
2e+05
0e+00
M/Z
500
1000
1500
y1
b1
b2
b3
b4
b5
b6
b7
b8
b9
b10
b11
y2
y3
y4
y5
y6
y7
y8
y9
y10
type
b
y

NAMPTL | +229.163TPAGNFVTLEEGK+229.163GDLEEYGQDLLHTVFK+229.163

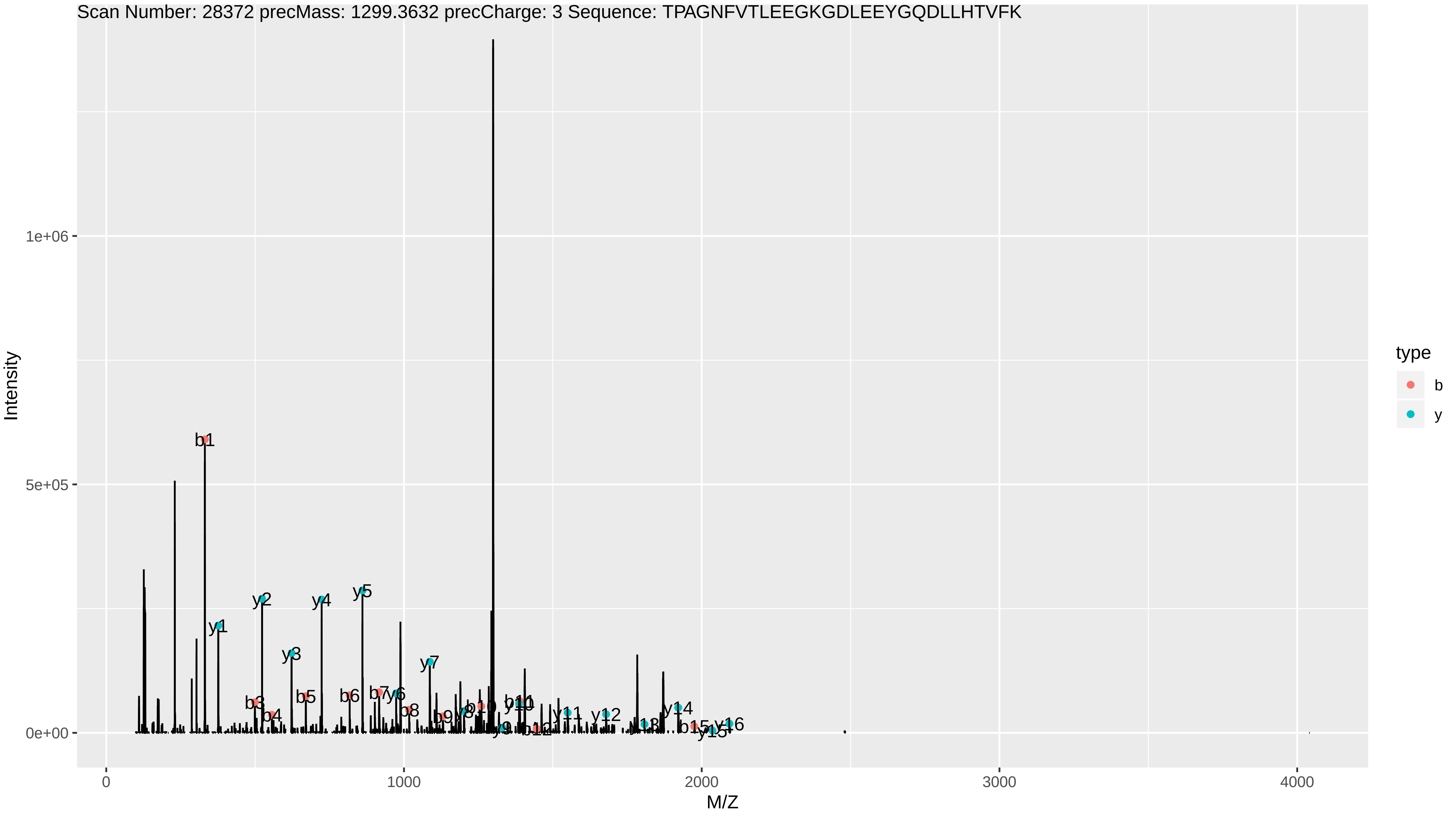

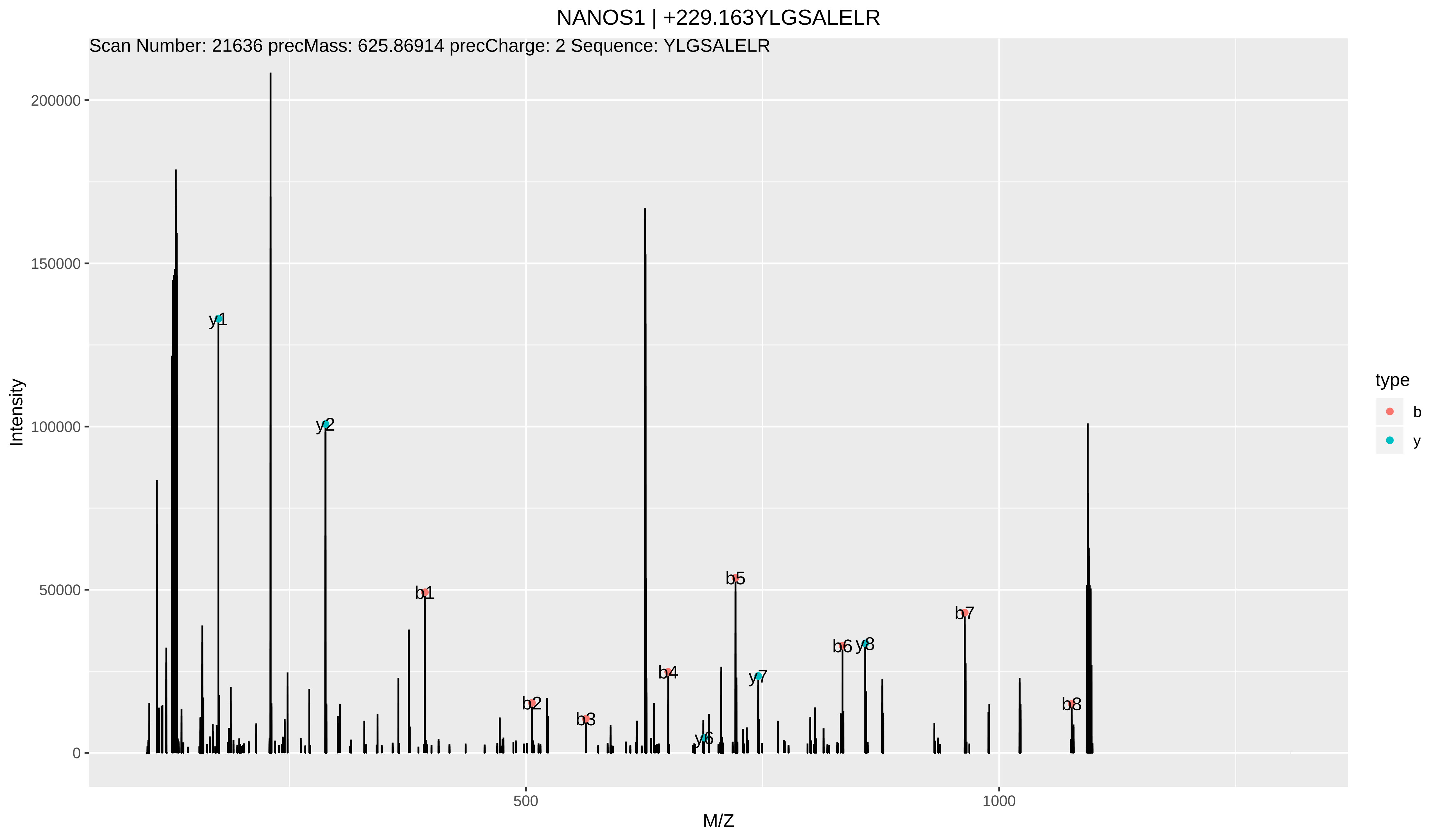

NANOS1 | +229.163YLGSALELR
Scan Number: 21636 precMass: 625.86914 precCharge: 2 Sequence: YLGSALELR
Intensity
200000
150000
100000
50000
0
M/Z
500
1000
y1
y2
b1
b2
b3
b4
y6
b5
y7
b6
y8
b7
b8
type
b
y

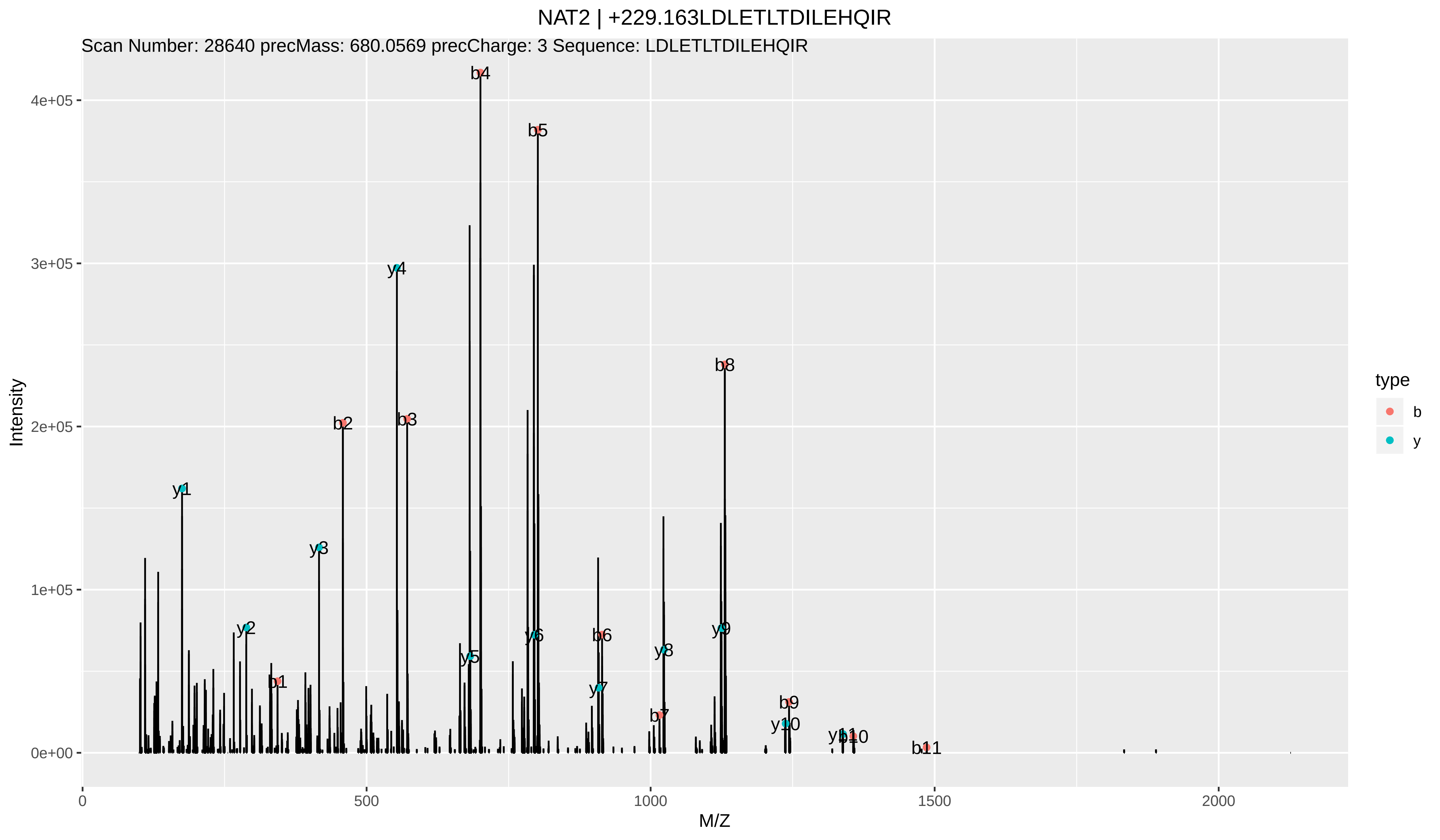
NAT2 | +229.163LDLETLTDILEHQIR
Scan Number: 28640 precMass: 680.0569 precCharge: 3 Sequence: LDLETLTDILEHQIR
Intensity
M/Z
type
b
y
b4
b5
y4
b8
b2
b3
y1
y3
y2
b1
y5
y6
b6
y8
y7
b7
y9
b9
y10
y11
b10
b11
0e+00
1e+05
2e+05
3e+05
4e+05
0
500
1000
1500
2000

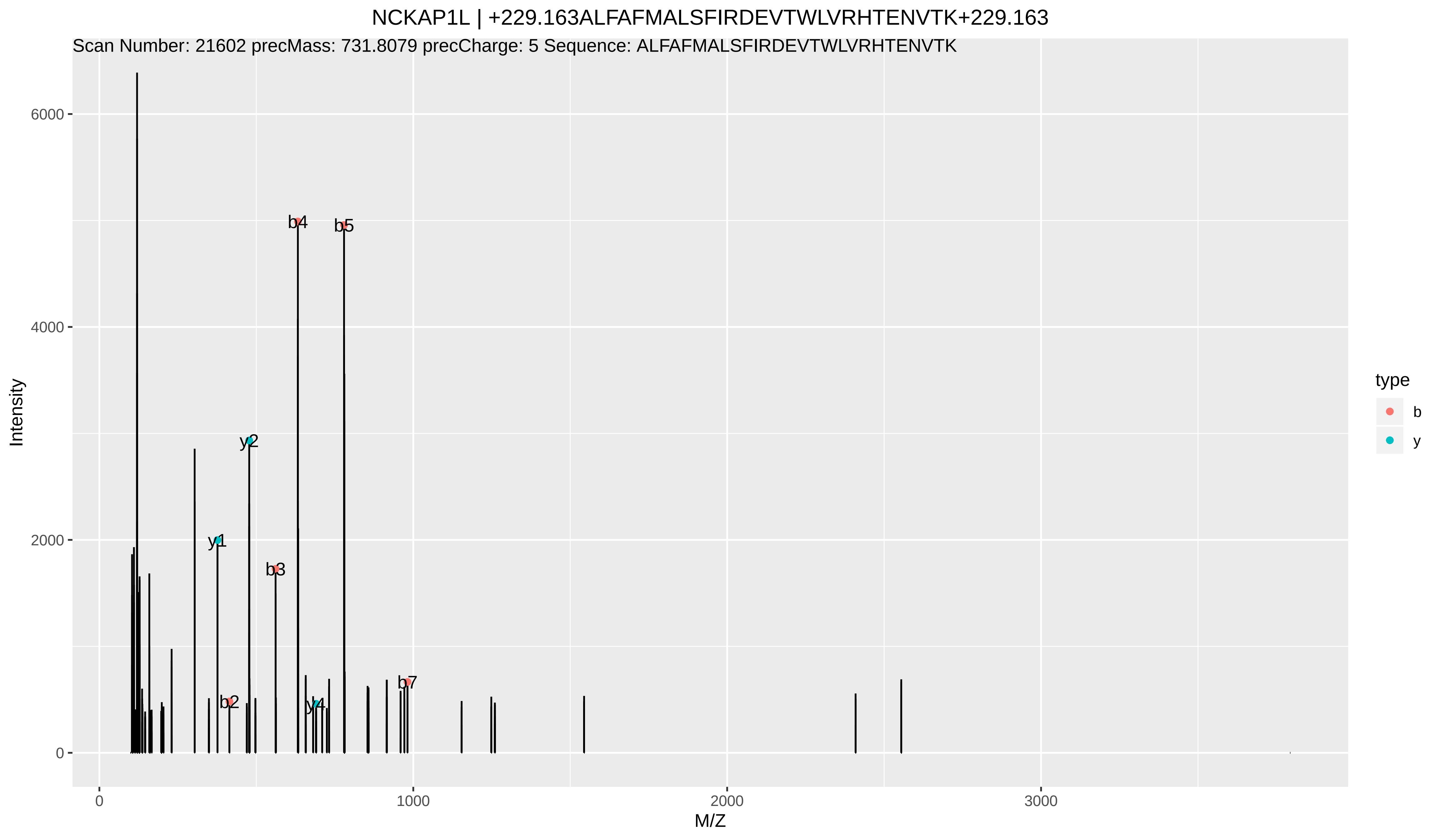

NCKAP1L | +229.163ALFAFMALSFIRDEVTWLVRHTENVTK+229.163
Scan Number: 21602 precMass: 731.8079 precCharge: 5 Sequence: ALFAFMALSFIRDEVTWLVRHTENVTK
Intensity
6000
4000
2000
0
M/Z
0
1000
2000
3000
b4
b5
y2
y1
b3
b7
b2
y4
type
b
y

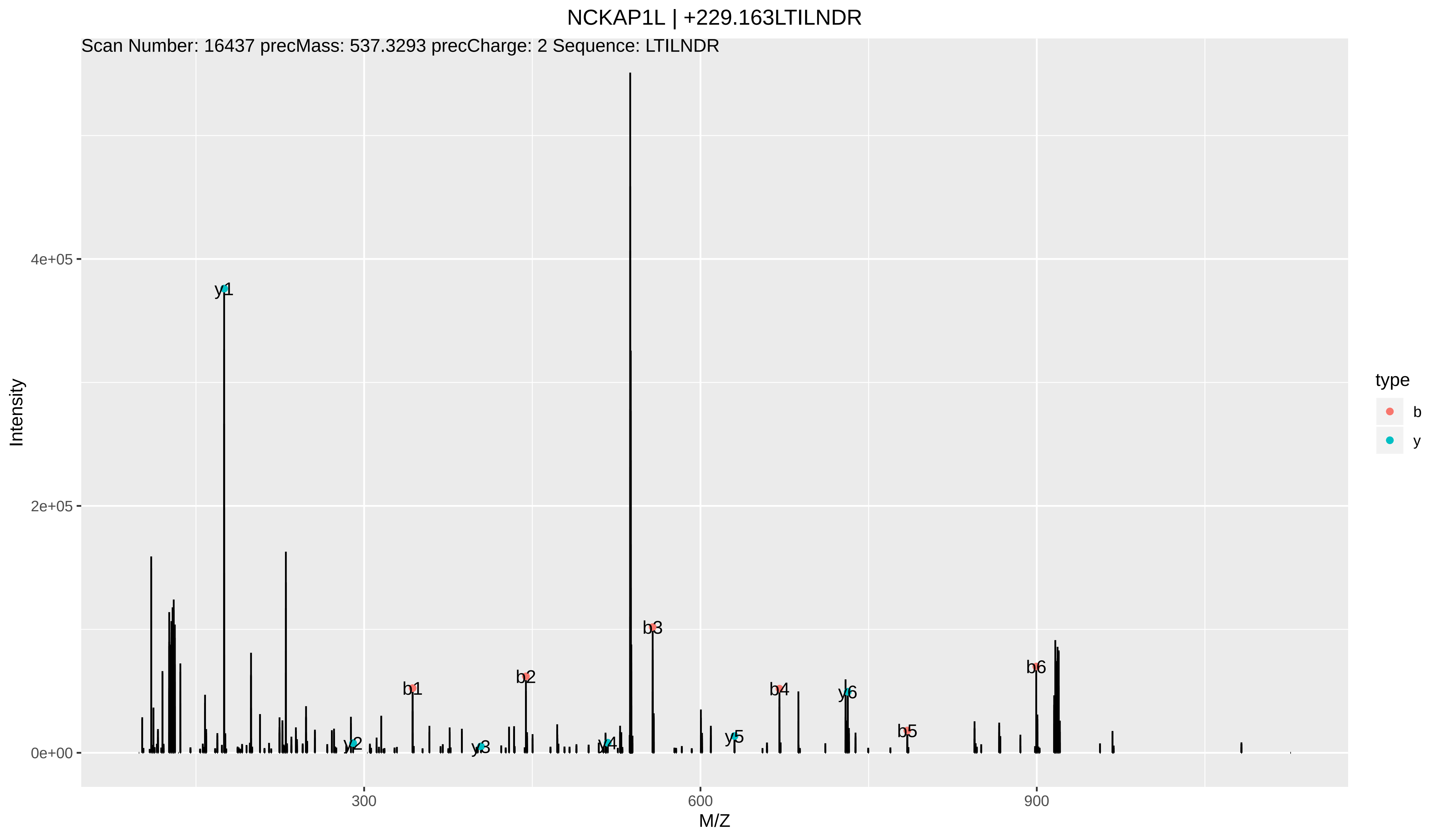
NCKAP1L | +229.163LTILNDR
Scan Number: 16437 precMass: 537.3293 precCharge: 2 Sequence: LTILNDR
Intensity
4e+05
2e+05
0e+00
M/Z
300
600
900
y1
y2
y3
y4
y5
y6
b1
b2
b3
b4
b5
b6
type
b
y

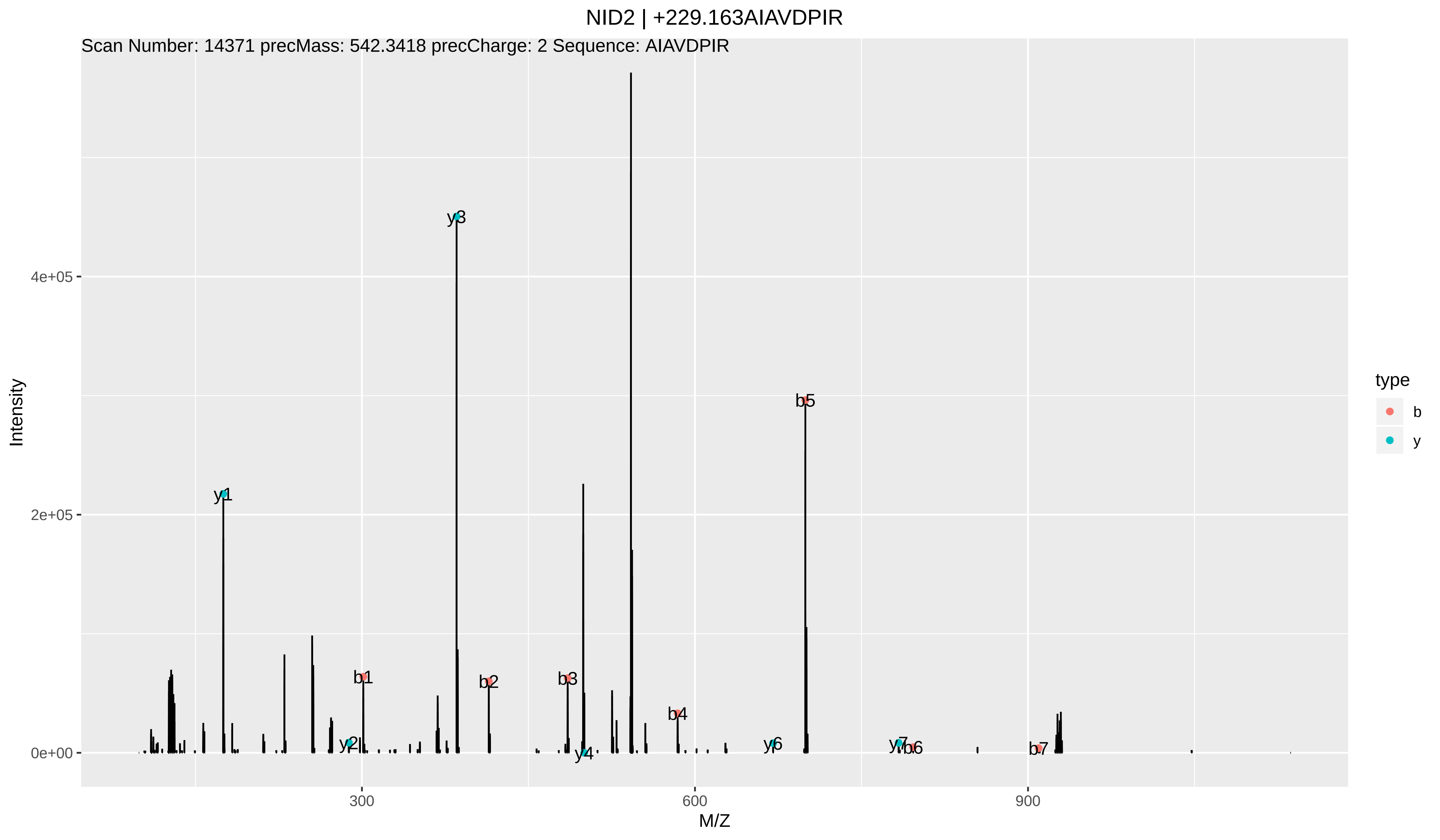

NID2 | +229.163AIAVDPIR
Scan Number: 14371 precMass: 542.3418 precCharge: 2 Sequence: AIAVDPIR
Intensity
M/Z
0e+00
2e+05
4e+05
300
600
900
y1
y2
y3
y4
y6
y7
b1
b2
b3
b4
b5
b6
b7
type
b
y

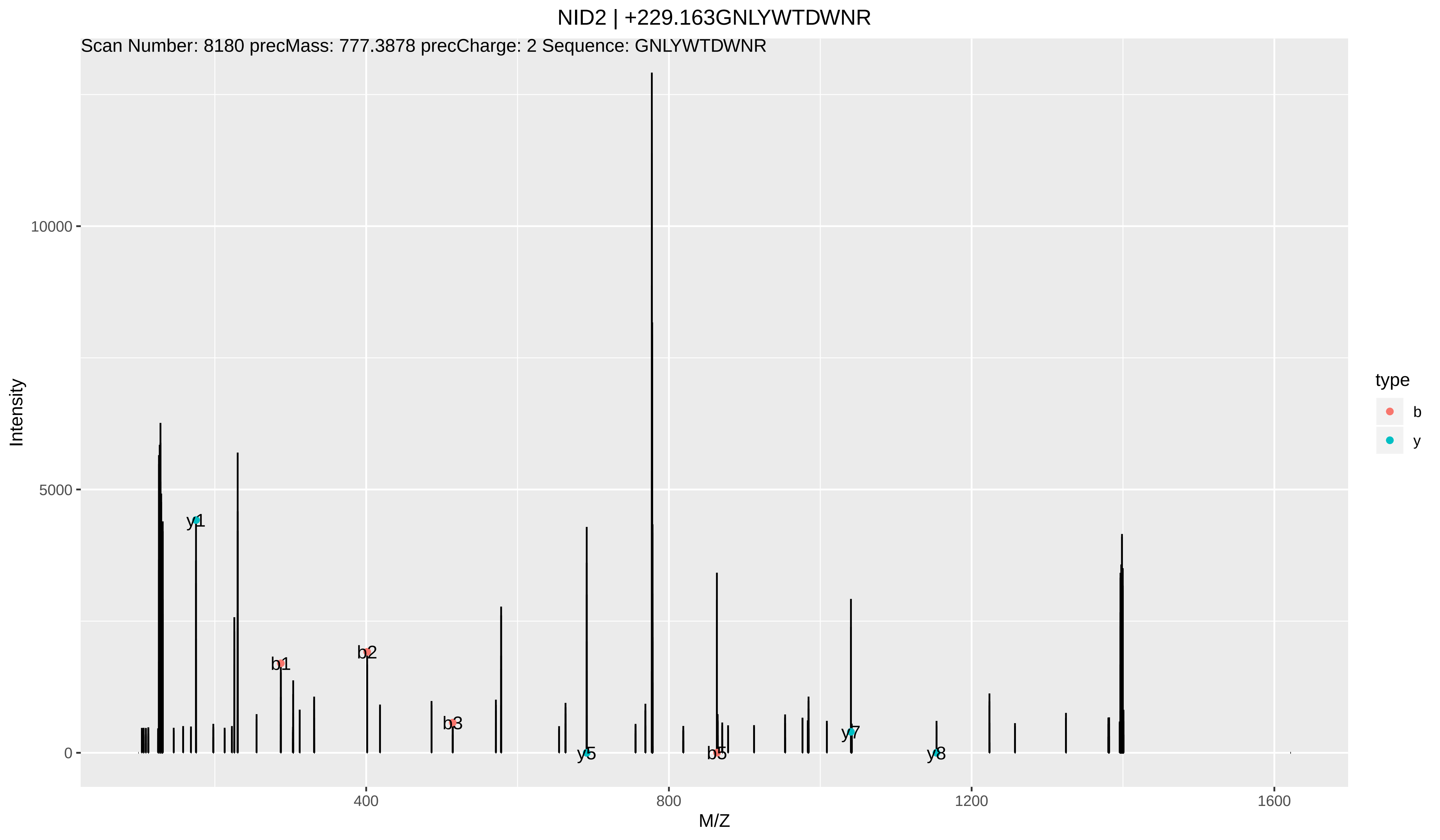

NID2 | +229.163GNLYWTDWNR
Scan Number: 8180 precMass: 777.3878 precCharge: 2 Sequence: GNLYWTDWNR
Intensity
M/Z
type
b
y
y1
b1
b2
b3
y5
b5
y7
y8
10000
5000
0
400
800
1200
1600

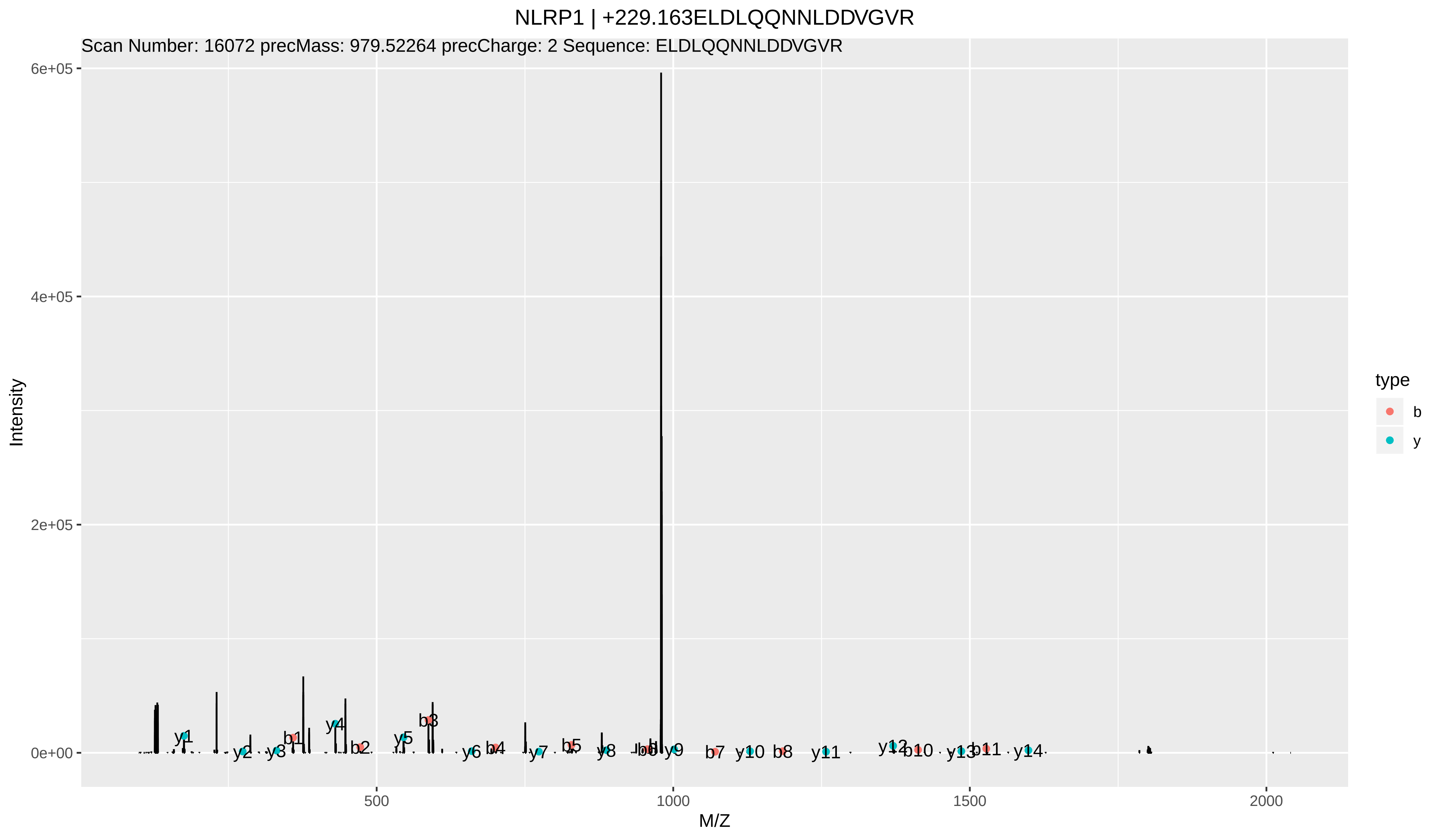

NLRP1 | +229.163ELDLQQNNLDDVGVR
Scan Number: 16072 precMass: 979.52264 precCharge: 2 Sequence: ELDLQQNNLDDVGVR
Intensity
6e+05
4e+05
2e+05
0e+00
M/Z
500
1000
1500
2000
y1
y2
y3
b1
y4
b2
y5
b3
y6
b4
y7
b5
y8
b6
y9
b7
y10
b8
y11
y12
b10
y13
b11
y14
type
b
y

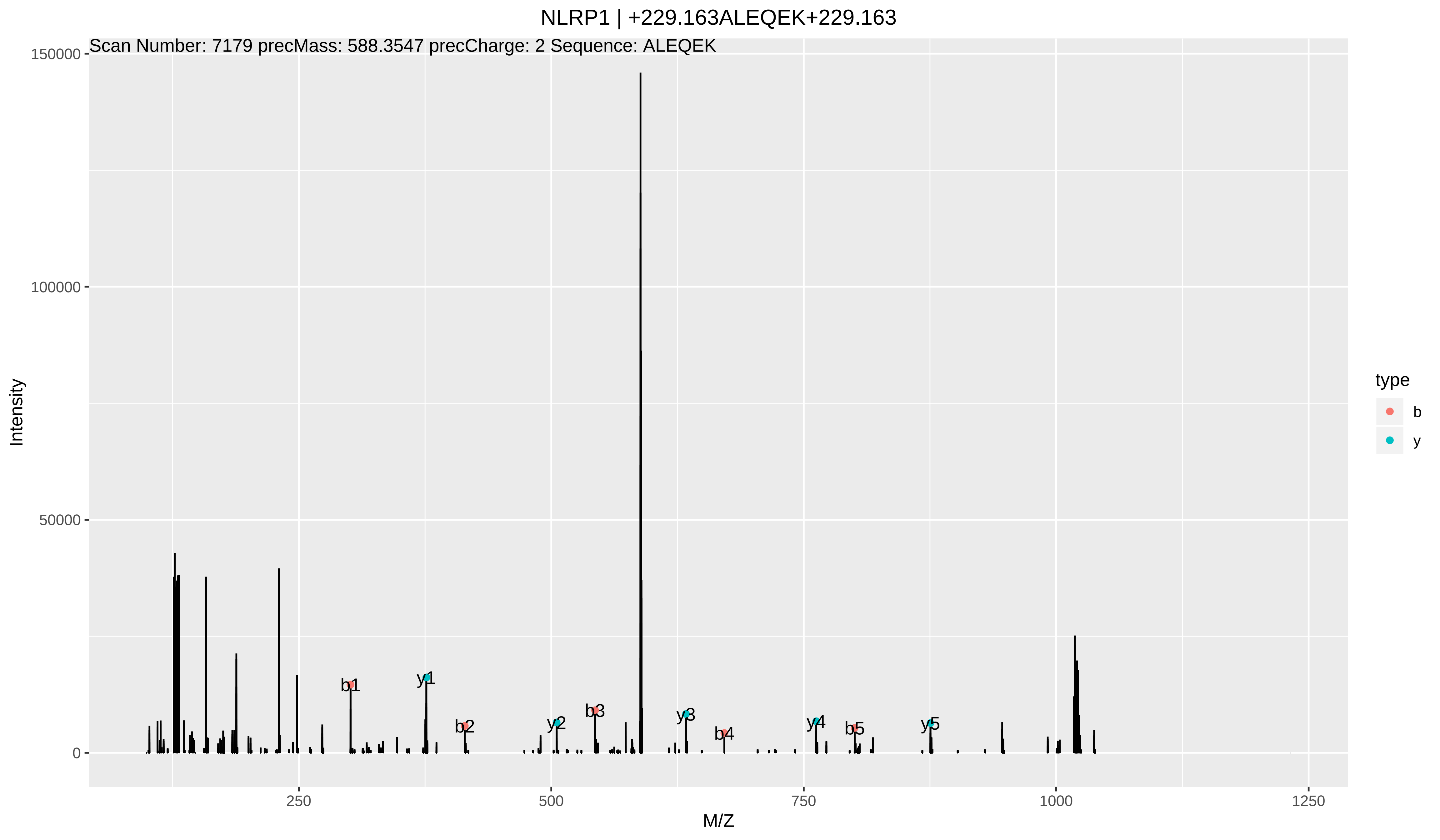
NLRP1 | +229.163ALEQEK+229.163
Scan Number: 7179 precMass: 588.3547 precCharge: 2 Sequence: ALEQEK
Intensity
150000
100000
50000
0
M/Z
250
500
750
1000
1250
b1
y1
b2
y2
b3
y3
b4
y4
b5
y5
type
b
y

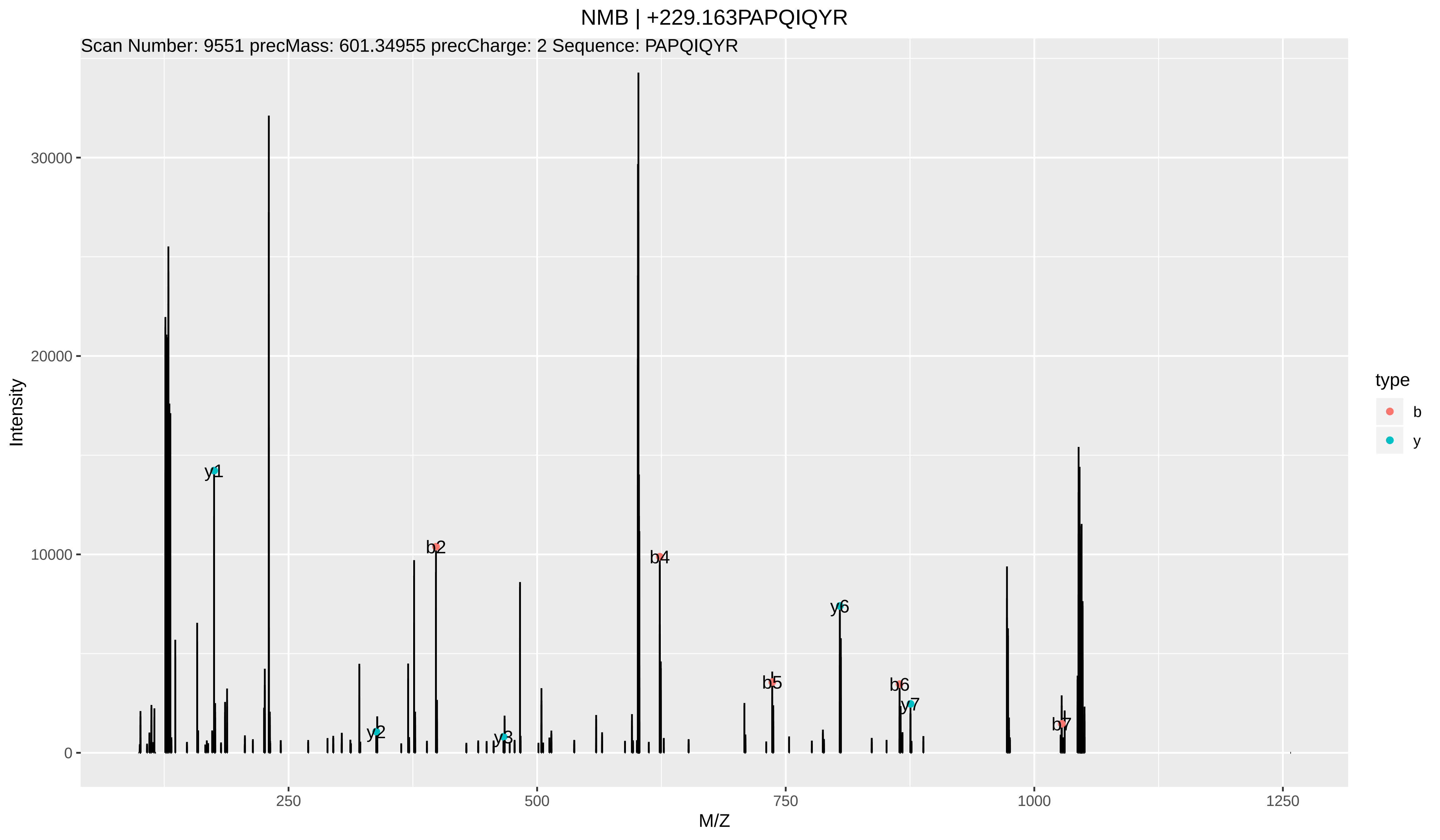

NMB | +229.163PAPQIQYR
Scan Number: 9551 precMass: 601.34955 precCharge: 2 Sequence: PAPQIQYR
Intensity
30000
20000
10000
0
M/Z
250
500
750
1000
1250
y1
b2
y2
y3
b4
b5
y6
b6
y7
b7
type
b
y

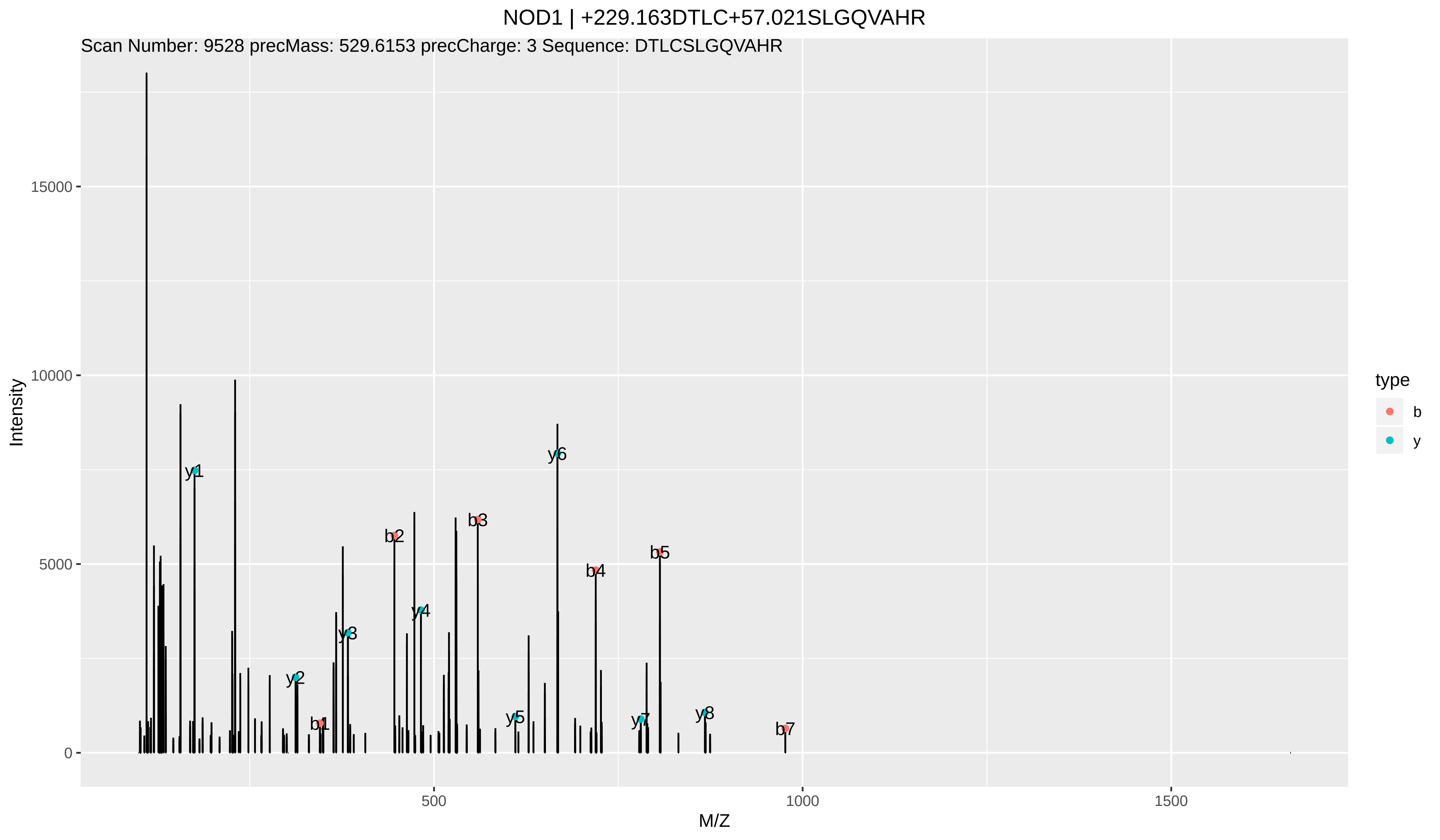

NOD1 | +229.163DTLC+57.021SLGQVAHR
Scan Number: 9528 precMass: 529.6153 precCharge: 3 Sequence: DTLCSLGQVAHR
Intensity
15000
10000
5000
0
500
1000
1500
M/Z
y1
y2
y3
y4
y5
y6
y7
y8
b1
b2
b3
b4
b5
b7
type
b
y

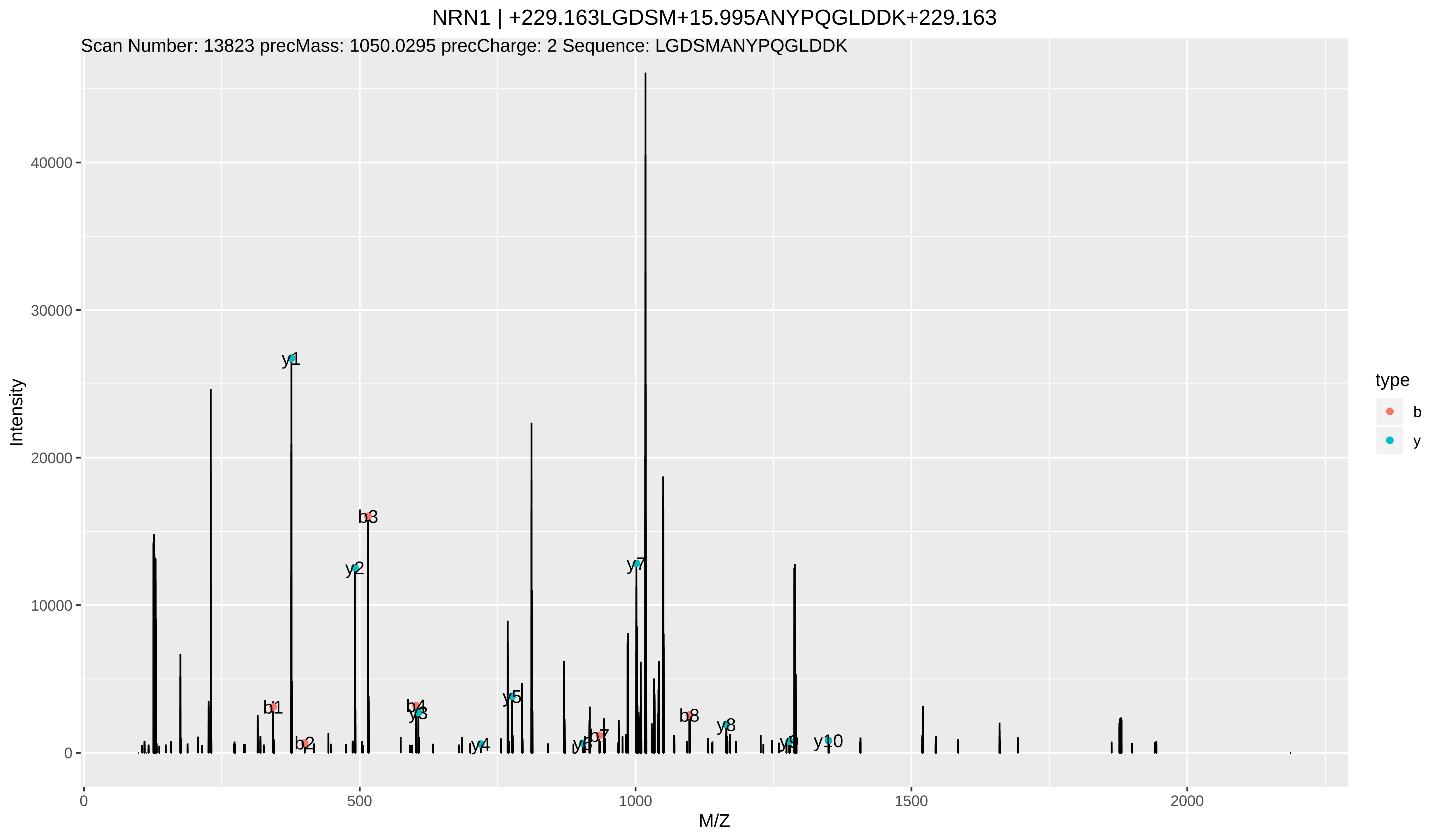

NRN1 | +229.163LGDSM+15.995ANYPQGLDDK+229.163
Scan Number: 13823 precMass: 1050.0295 precCharge: 2 Sequence: LGDSMANYPQGLDDK
Intensity
M/Z
0
10000
20000
30000
40000
0
500
1000
1500
2000
y1
b3
y2
y7
b1
b2
b4
y3
y4
y5
y6
b7
b8
y8
y9
y10
type
b
y

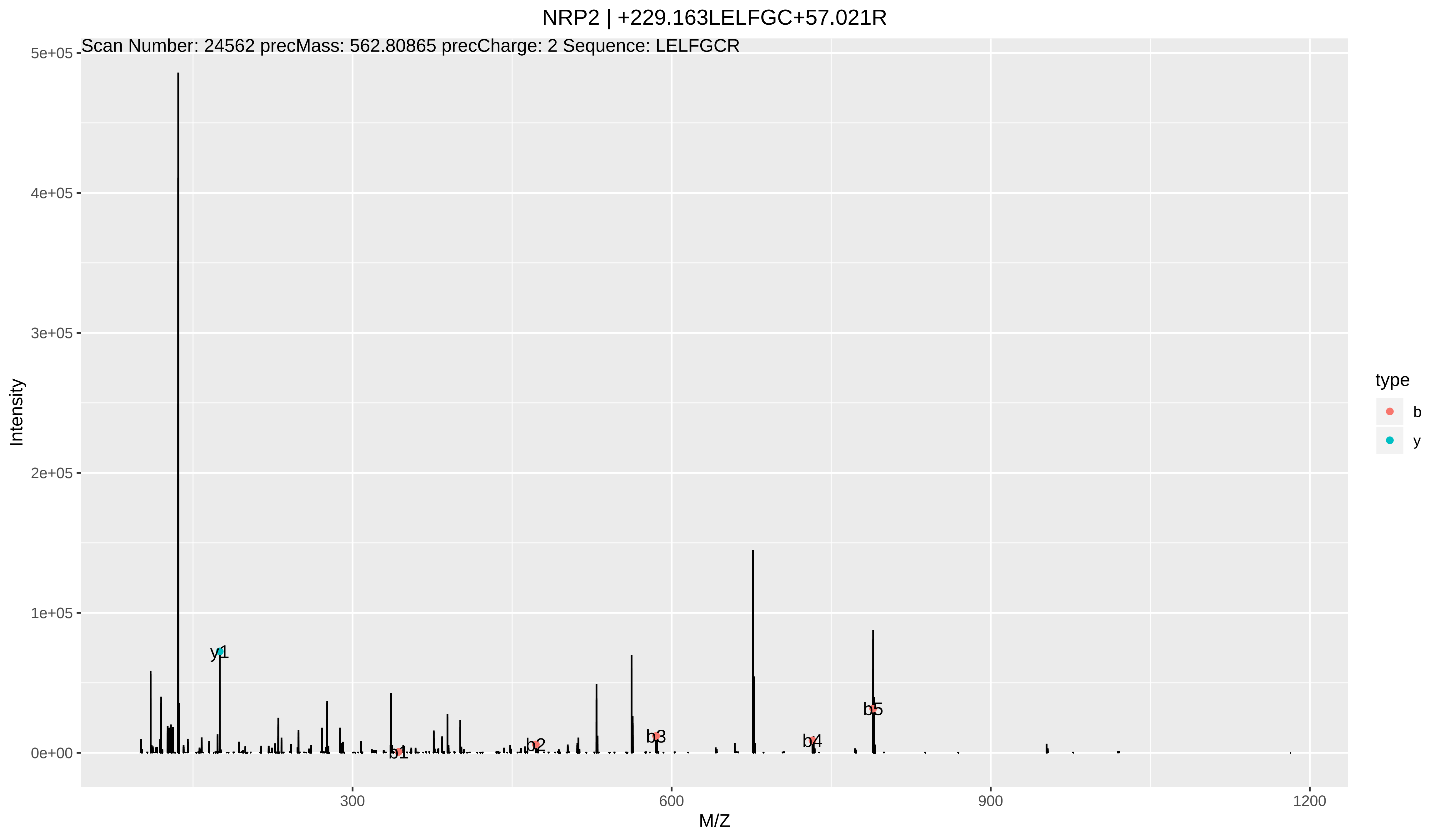
NRP2 | +229.163LELFGC+57.021R
Scan Number: 24562 precMass: 562.80865 precCharge: 2 Sequence: LELFGCR
Intensity
M/Z
0e+00
1e+05
2e+05
3e+05
4e+05
5e+05
300
600
900
1200
y1
b1
b2
b3
b4
b5
type
b
y

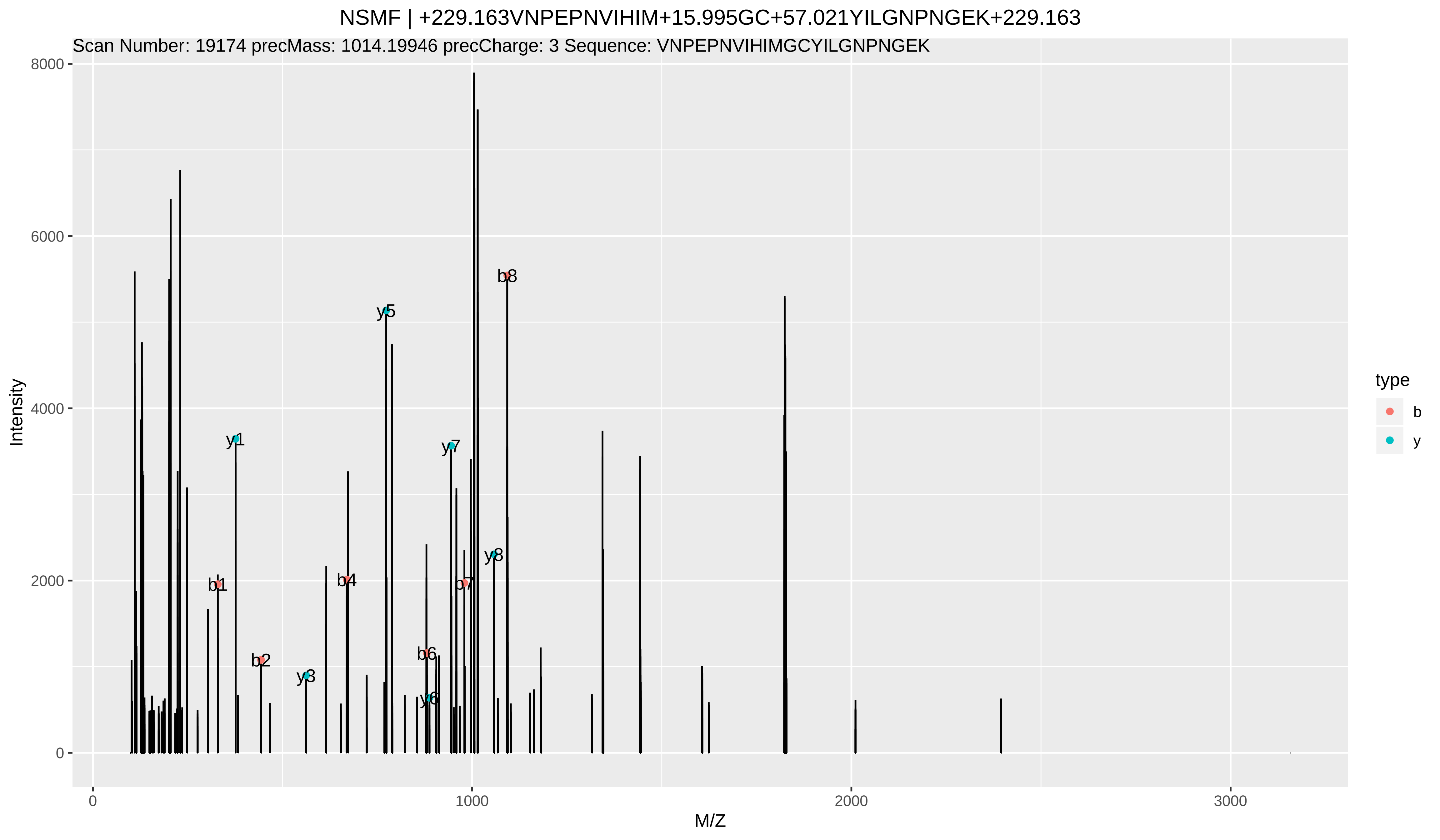

NSMF | +229.163VNPEPNVIHIM+15.995GC+57.021YILGNPNGEK+229.163
Scan Number: 19174 precMass: 1014.19946 precCharge: 3 Sequence: VNPEPNVIHIMGCYILGNPNGEK
Intensity
M/Z
0
2000
4000
6000
8000
1000
3000
b1
b2
b4
b6
b7
b8
y1
y3
y5
y6
y7
y8
type
b
y

NUDT13 | +229.163HSLLELER

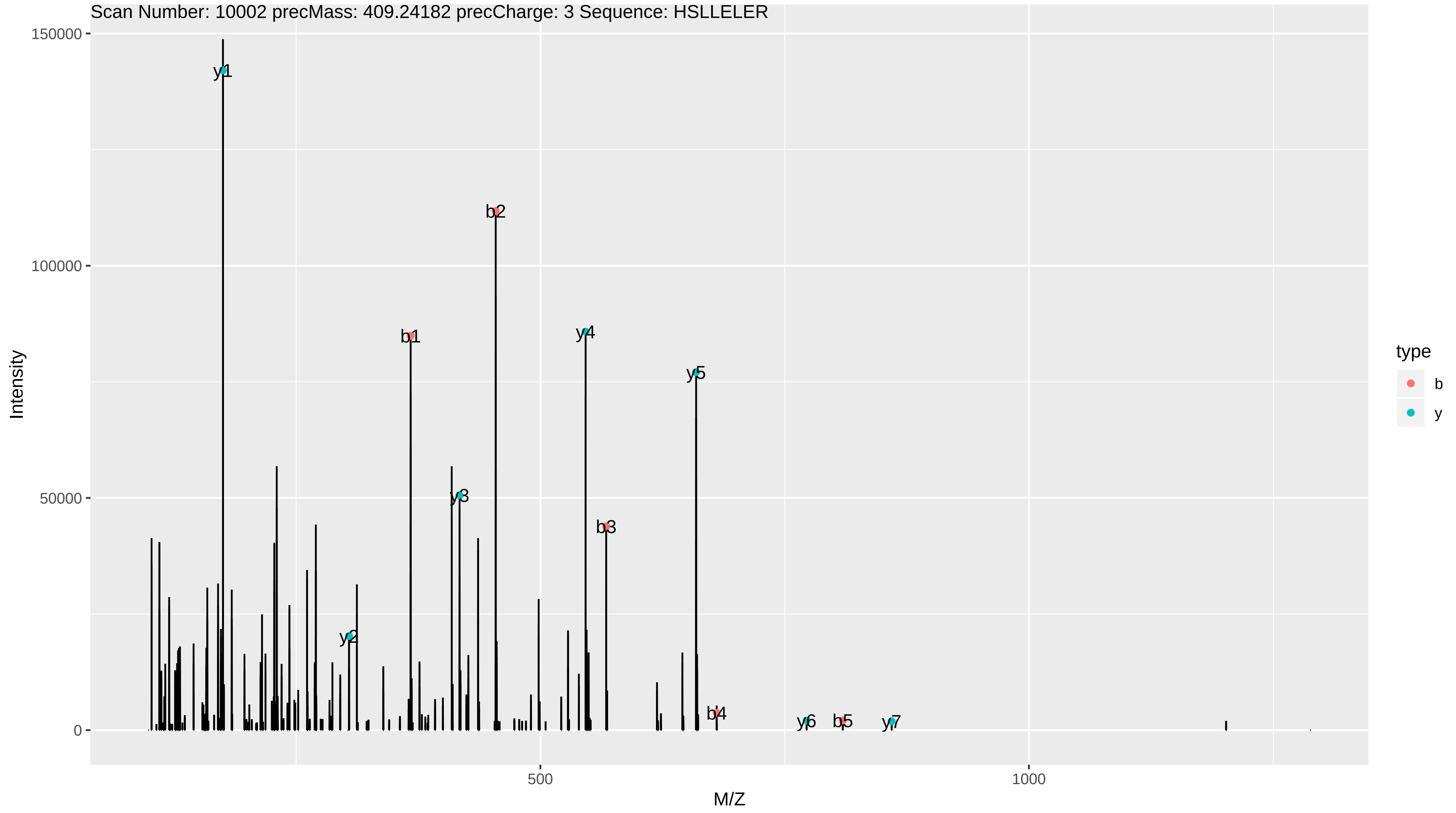

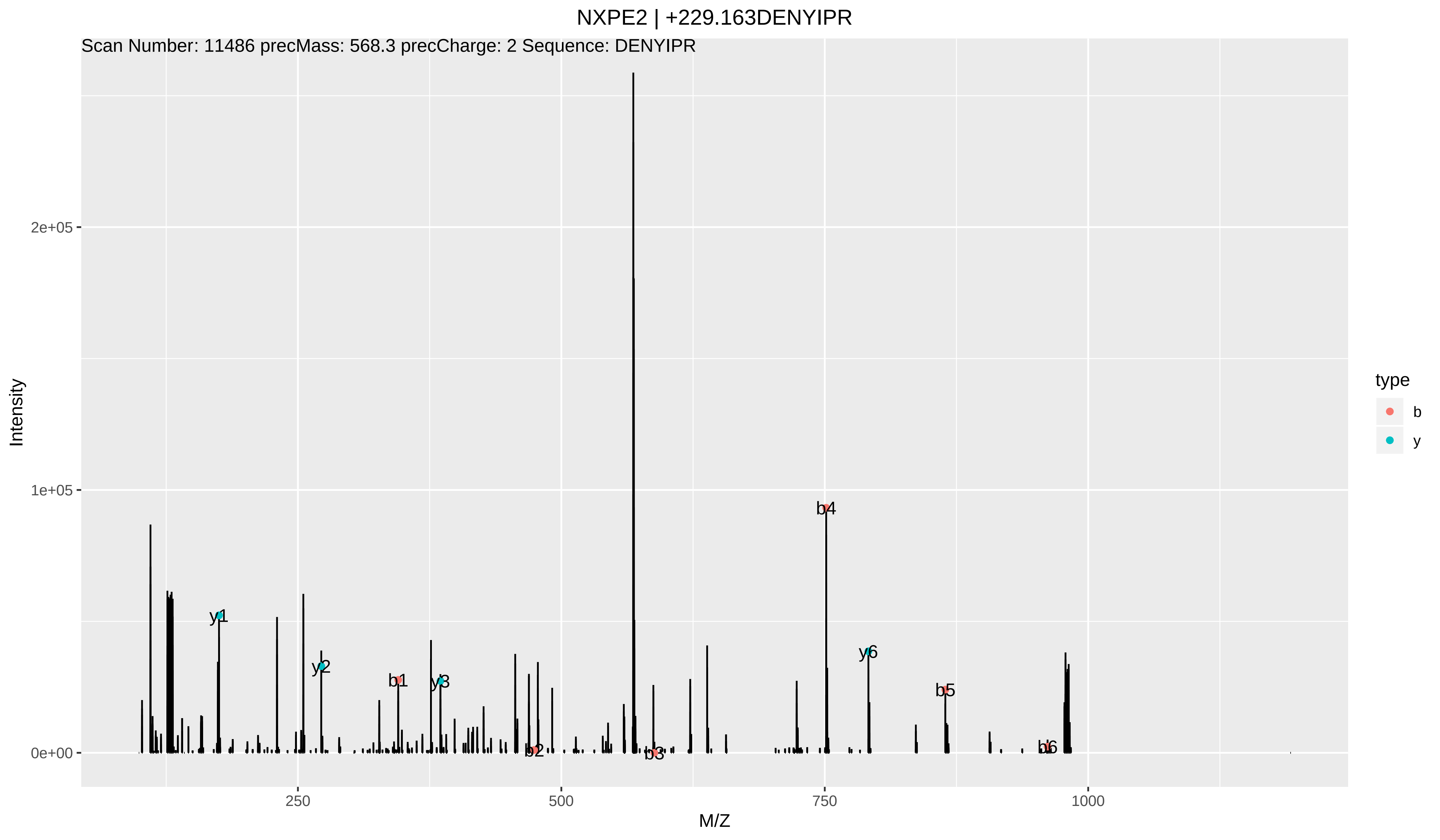
NXPE2 | +229.163DENYIPR
Scan Number: 11486 precMass: 568.3 precCharge: 2 Sequence: DENYIPR
Intensity
M/Z
0e+00
1e+05
2e+05
250
500
750
1000
y1
y2
b1
y3
b2
b3
b4
y6
b5
b6
type
b
y

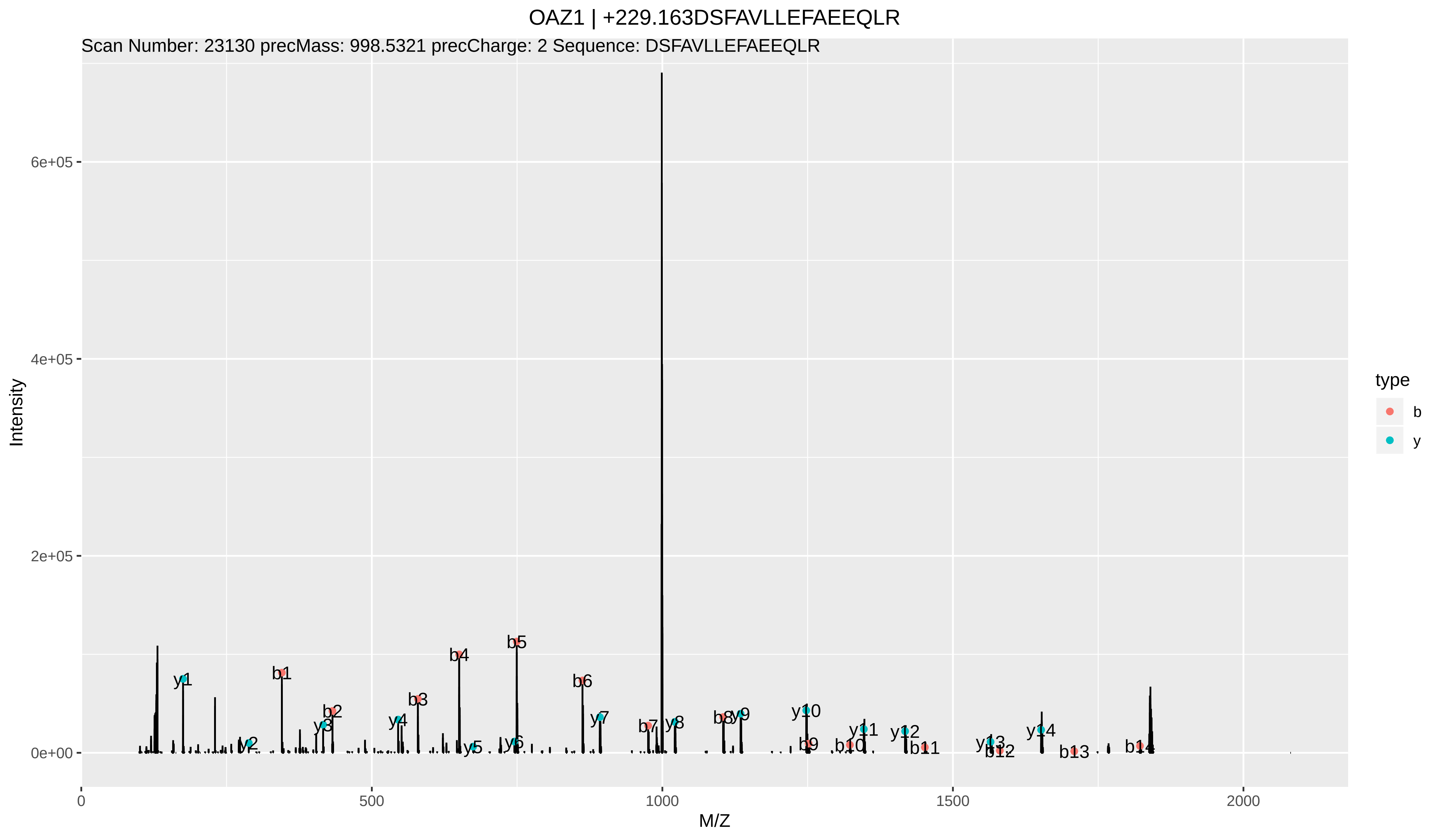

OAZ1 | +229.163DSFAVLLEFAEEQLR
Scan Number: 23130 precMass: 998.5321 precCharge: 2 Sequence: DSFAVLLEFAEEQLR
Intensity
6e+05
4e+05
2e+05
0e+00
0
500
1000
1500
2000
M/Z
type
b
y
y1
b1
b2
y2
y3
b3
y4
b4
y5
b5
y6
b6
y7
b7
y8
b8
y9
y10
b9
b10
y11
y12
b11
y13
b12
y14
b13
b14

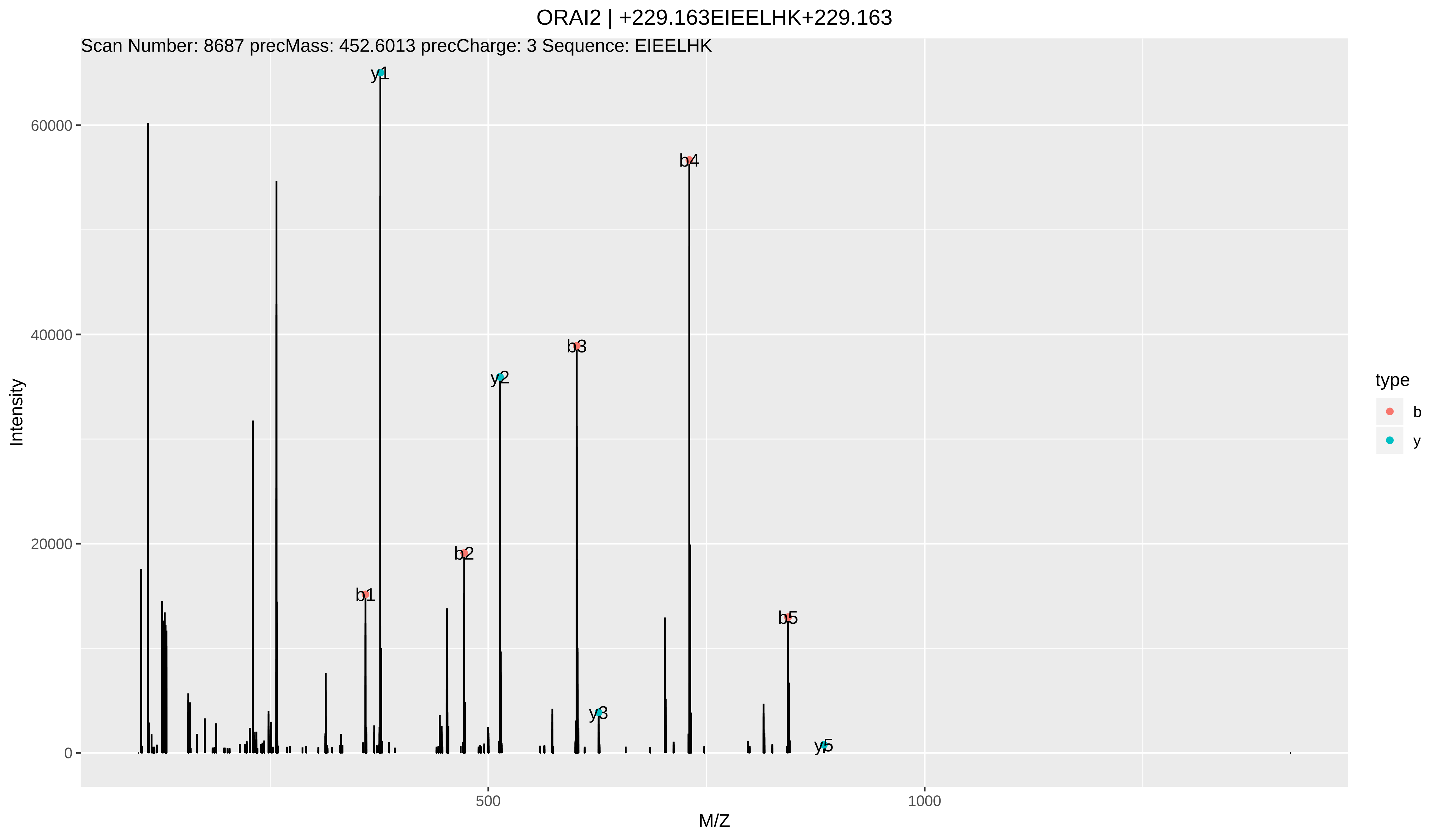
ORAI2 | +229.163EIEELHK+229.163
Scan Number: 8687 precMass: 452.6013 precCharge: 3 Sequence: EIEELHK
Intensity
M/Z
0
20000
40000
60000
500
1000
y1
b4
b3
y2
b2
b1
b5
y3
y5
type
b
y

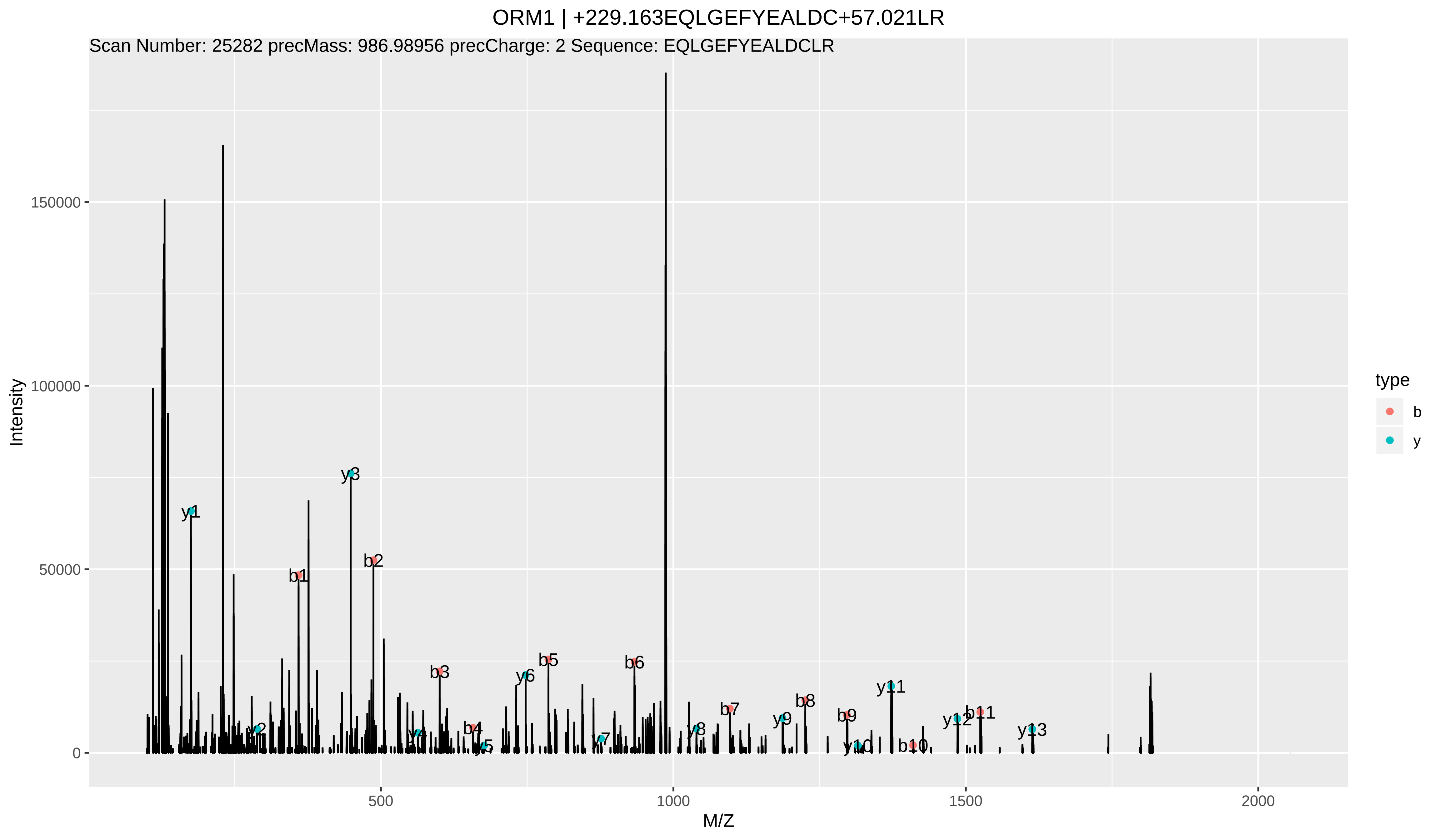

ORM1 | +229.163EQLGEFYEALDC+57.021LR
Scan Number: 25282 precMass: 986.98956 precCharge: 2 Sequence: EQLGEFYEALDCLR
Intensity
150000
100000
50000
0
500
1000
1500
2000
M/Z
type
b
y
y1
y2
y3
y4
y5
y6
y7
y8
y9
y10
y11
y12
y13
b1
b2
b3
b4
b5
b6
b7
b8
b9
b10
b11

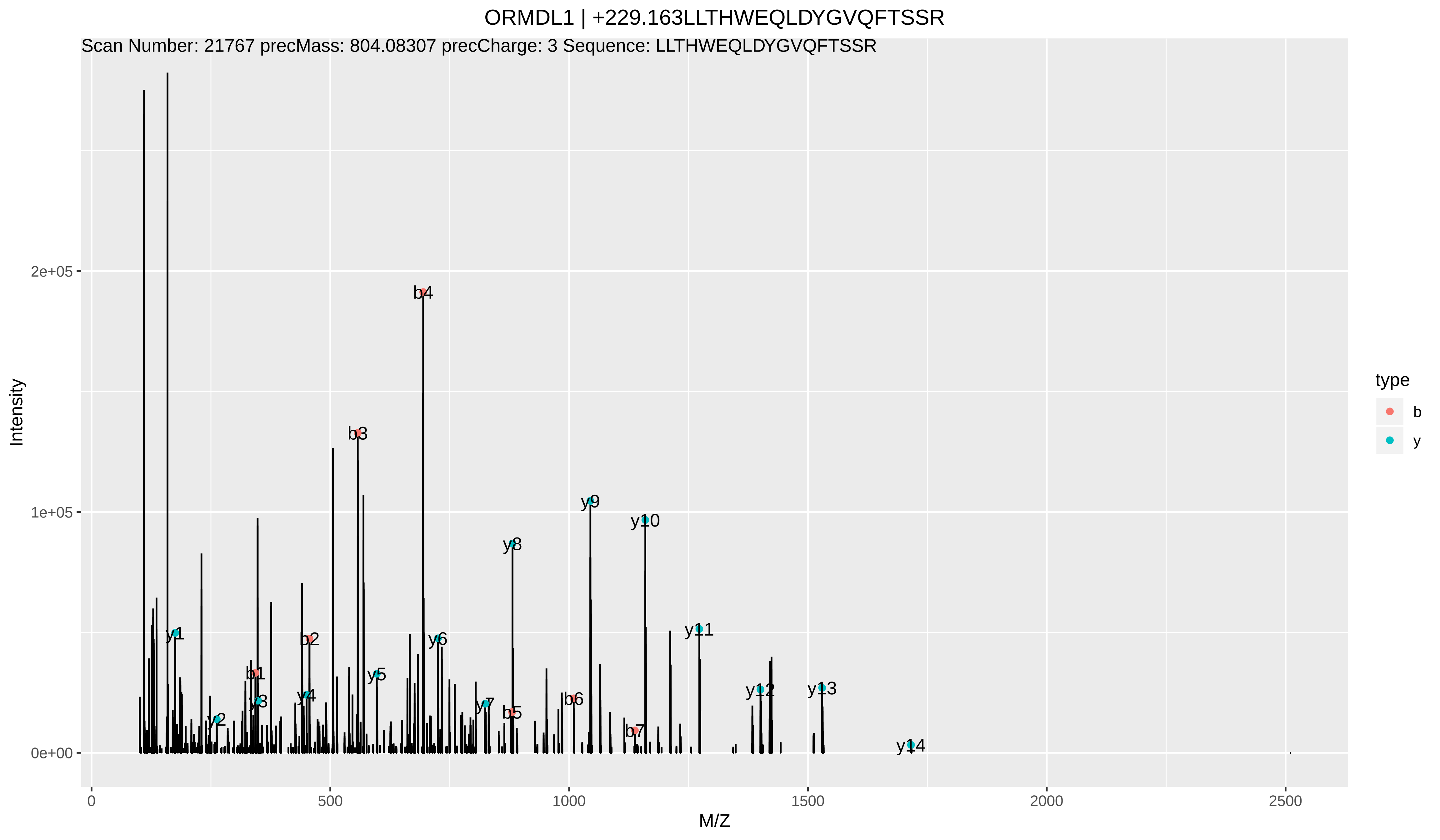

ORMDL1 | +229.163LLTHWEQLDYGVQFTSSR
Scan Number: 21767 precMass: 804.08307 precCharge: 3 Sequence: LLTHWEQLDYGVQFTSSR
Intensity
M/Z
0e+00
1e+05
2e+05
0
500
1000
1500
2000
2500
b4
b3
y9
y10
y8
y1
b2
y6
y11
b1
y5
y12
y13
y3
y4
b6
y7
b5
y2
b7
y14
type
b
y

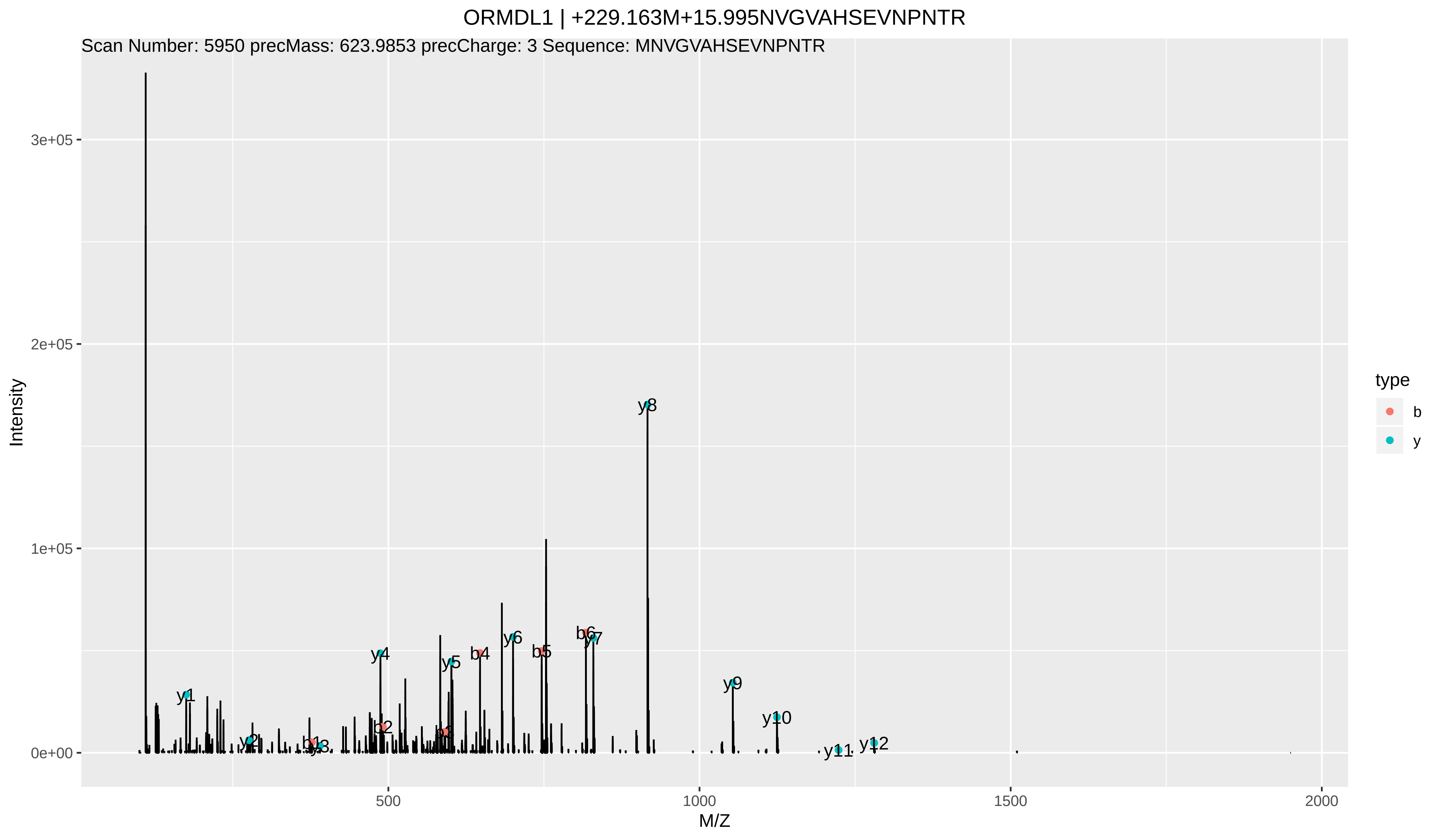
ORMDL1 | +229.163M+15.995NVGVAHSEVNPNTR
Scan Number: 5950 precMass: 623.9853 precCharge: 3 Sequence: MNVGVAHSEVNPNTR
Intensity
M/Z
3e+05
2e+05
1e+05
0e+00
500
1000
1500
2000
type
b
y
y1
y2
b1
y3
y4
b2
b3
y5
b4
y6
b5
b6
y7
y8
y9
y10
y11
y12

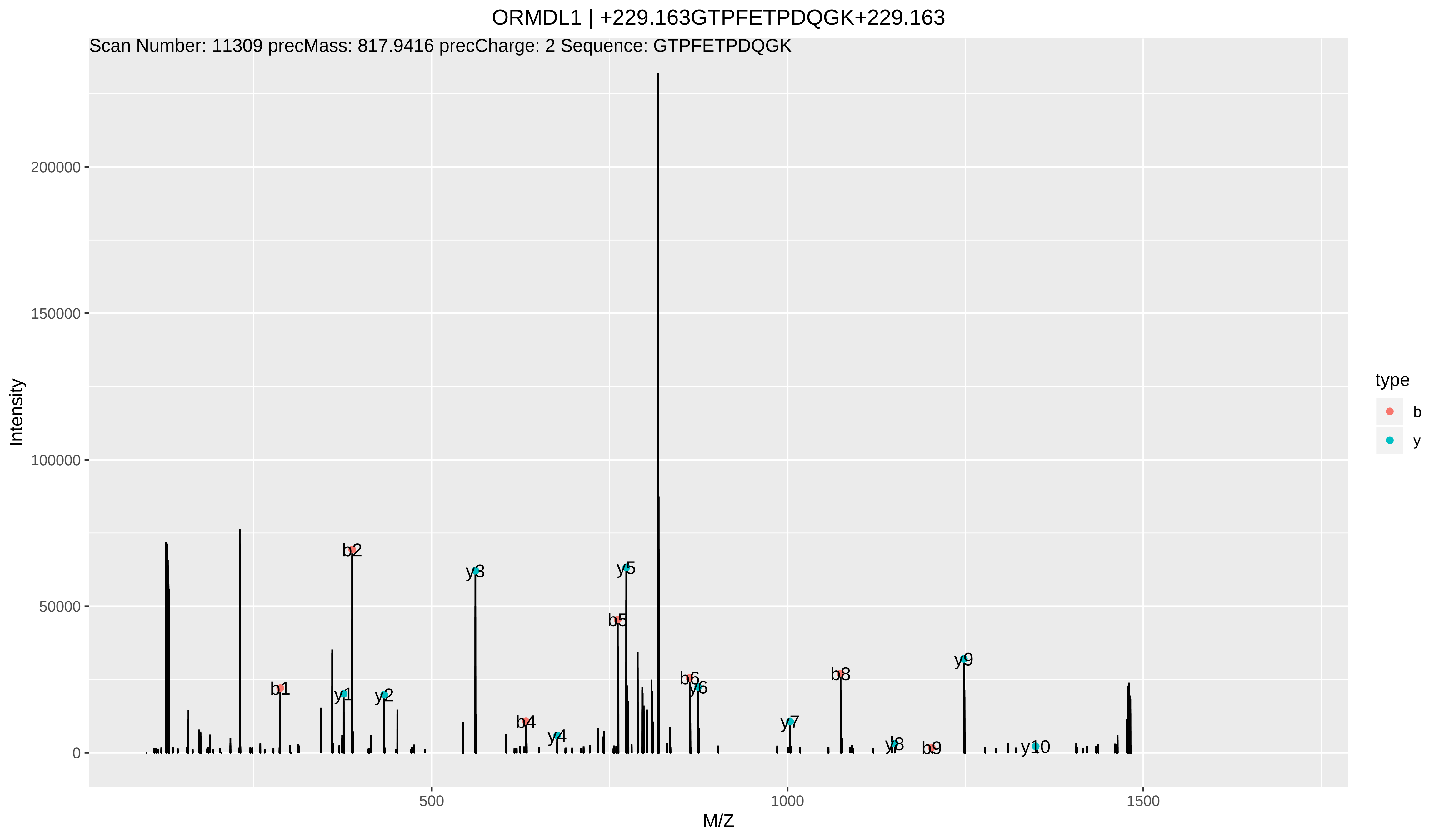
ORMDL1 | +229.163GTPFETPDQGK+229.163
Scan Number: 11309 precMass: 817.9416 precCharge: 2 Sequence: GTPFETPDQGK
Intensity
0
50000
100000
150000
200000
M/Z
500
1000
1500
b1
b2
b4
b5
b6
b8
b9
y1
y2
y3
y4
y5
y6
y7
y8
y9
y10
type
b
y

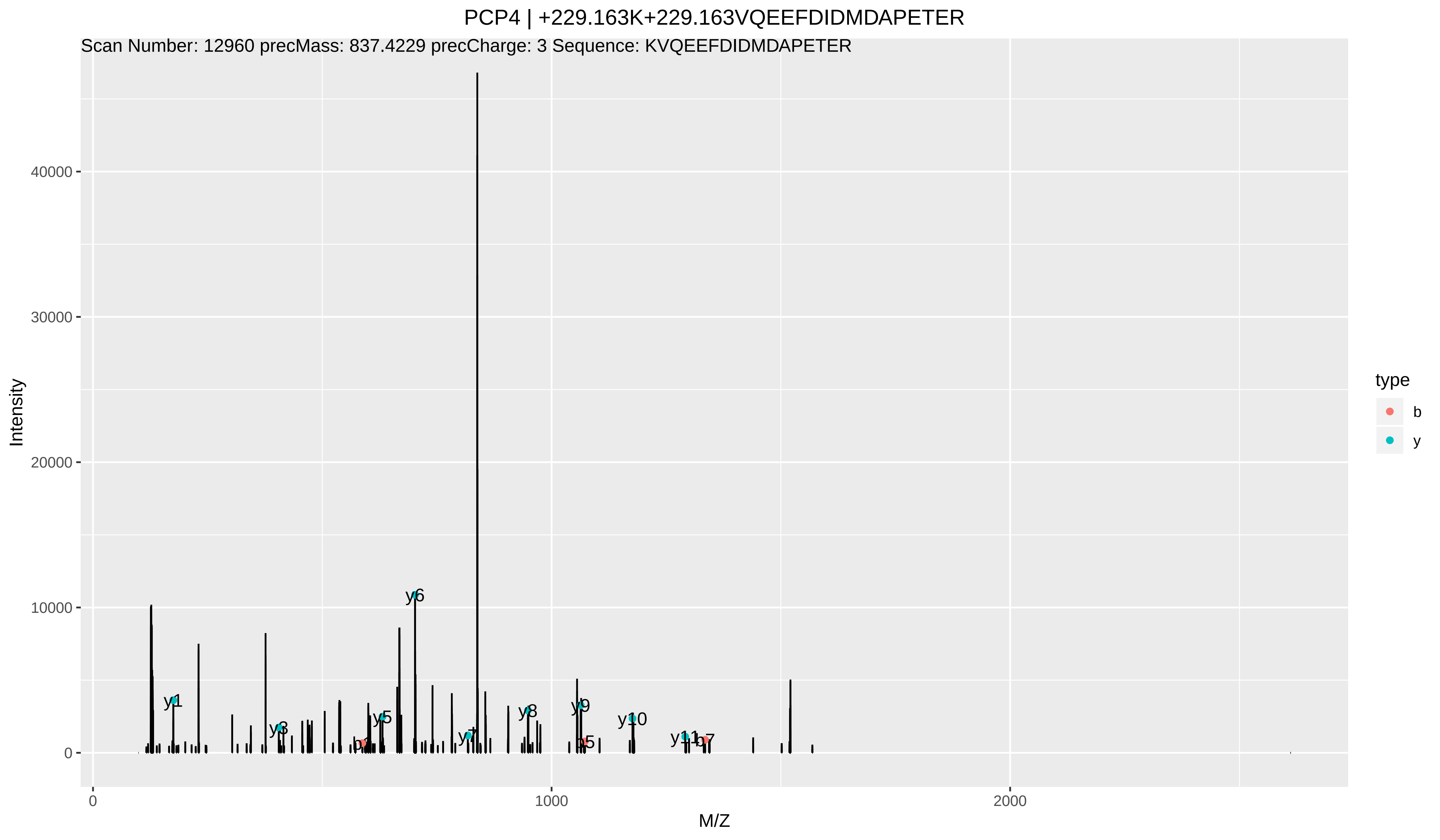

PCP4 | +229.163K+229.163VQEEFDIDMDAPETER
Scan Number: 12960 precMass: 837.4229 precCharge: 3 Sequence: KVQEEFDIDMDAPETER
Intensity
0
10000
20000
30000
40000
M/Z
0
1000
2000
y1
y3
y5
y6
y7
y8
y9
y10
y11
b7
type
b
y

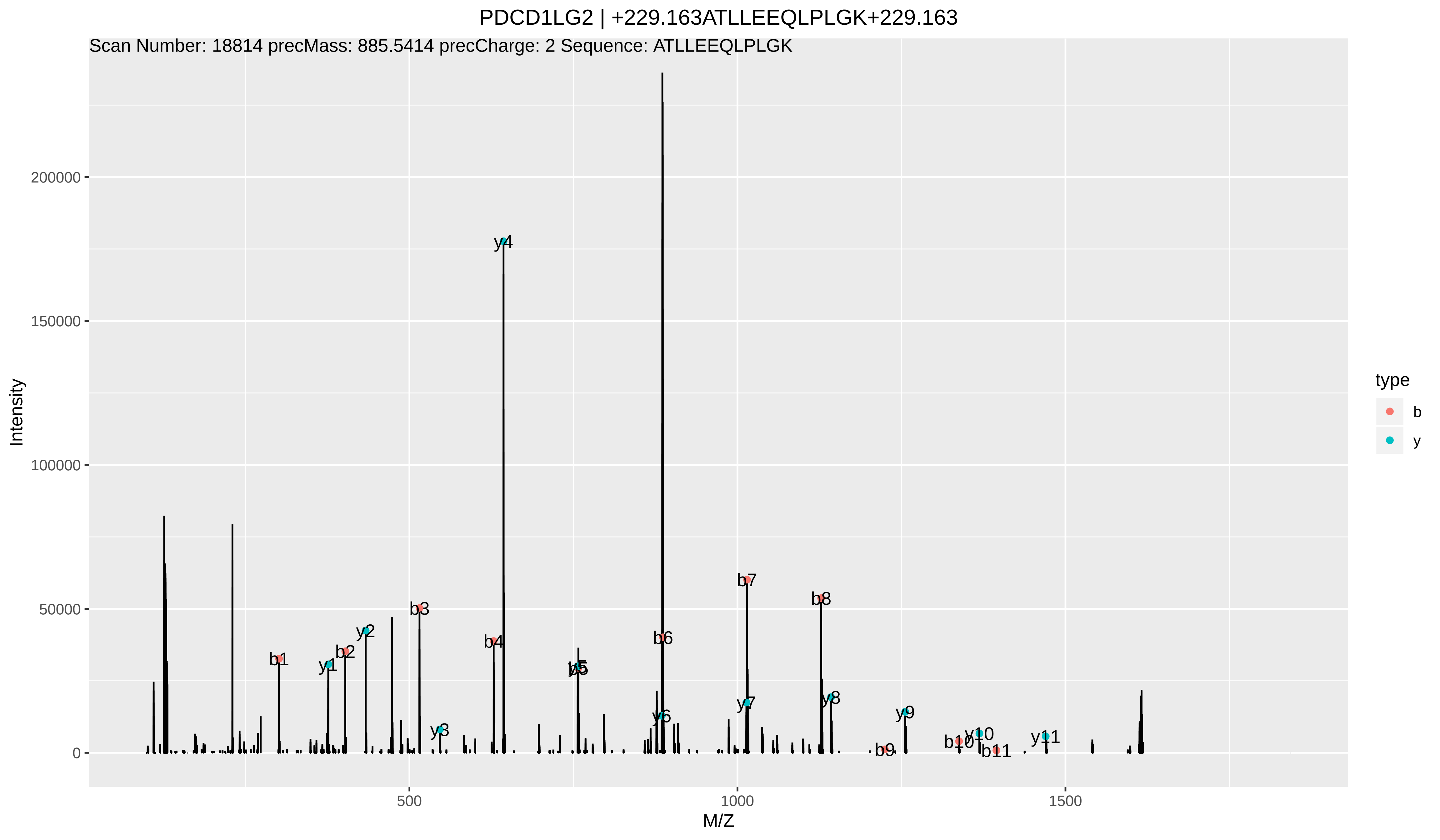

PDCD1LG2 | +229.163ATLLEEQLPLGK+229.163
Scan Number: 18814 precMass: 885.5414 precCharge: 2 Sequence: ATLLEEQLPLGK
Intensity
M/Z
0
50000
100000
150000
200000
500
1000
1500
y4
b7
b8
b3
y2
b6
b4
b2
b1
y1
y5
b5
y7
y8
y6
y9
y3
y10
y11
b10
b11
b9
type
b
y

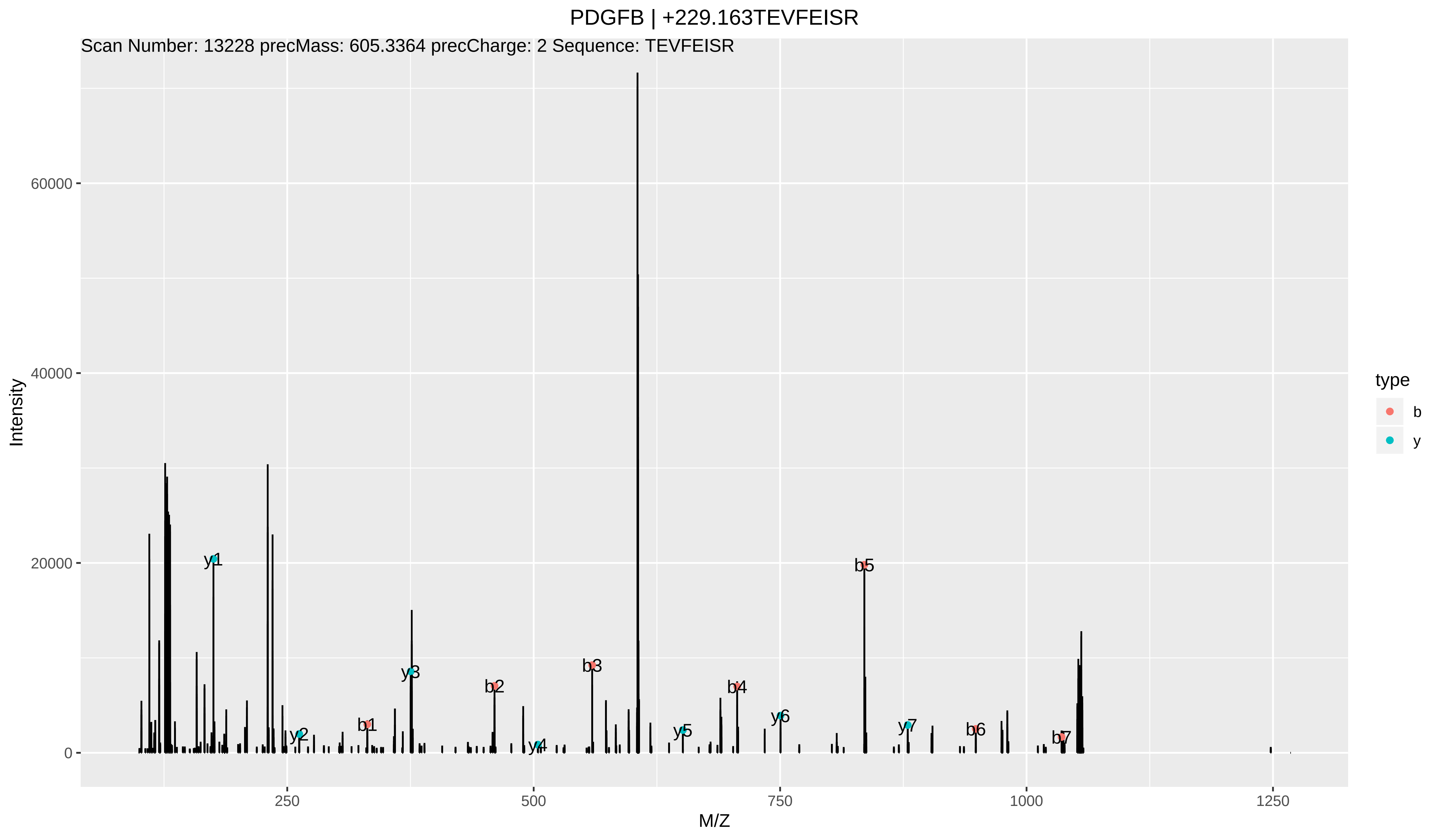

PDGFB | +229.163TEVFEISR
Scan Number: 13228 precMass: 605.3364 precCharge: 2 Sequence: TEVFEISR
Intensity
60000
40000
20000
0
M/Z
250
500
750
1000
1250
y1
y2
b1
y3
b2
y4
b3
y5
b4
y6
b5
y7
b6
b7
type
b
y

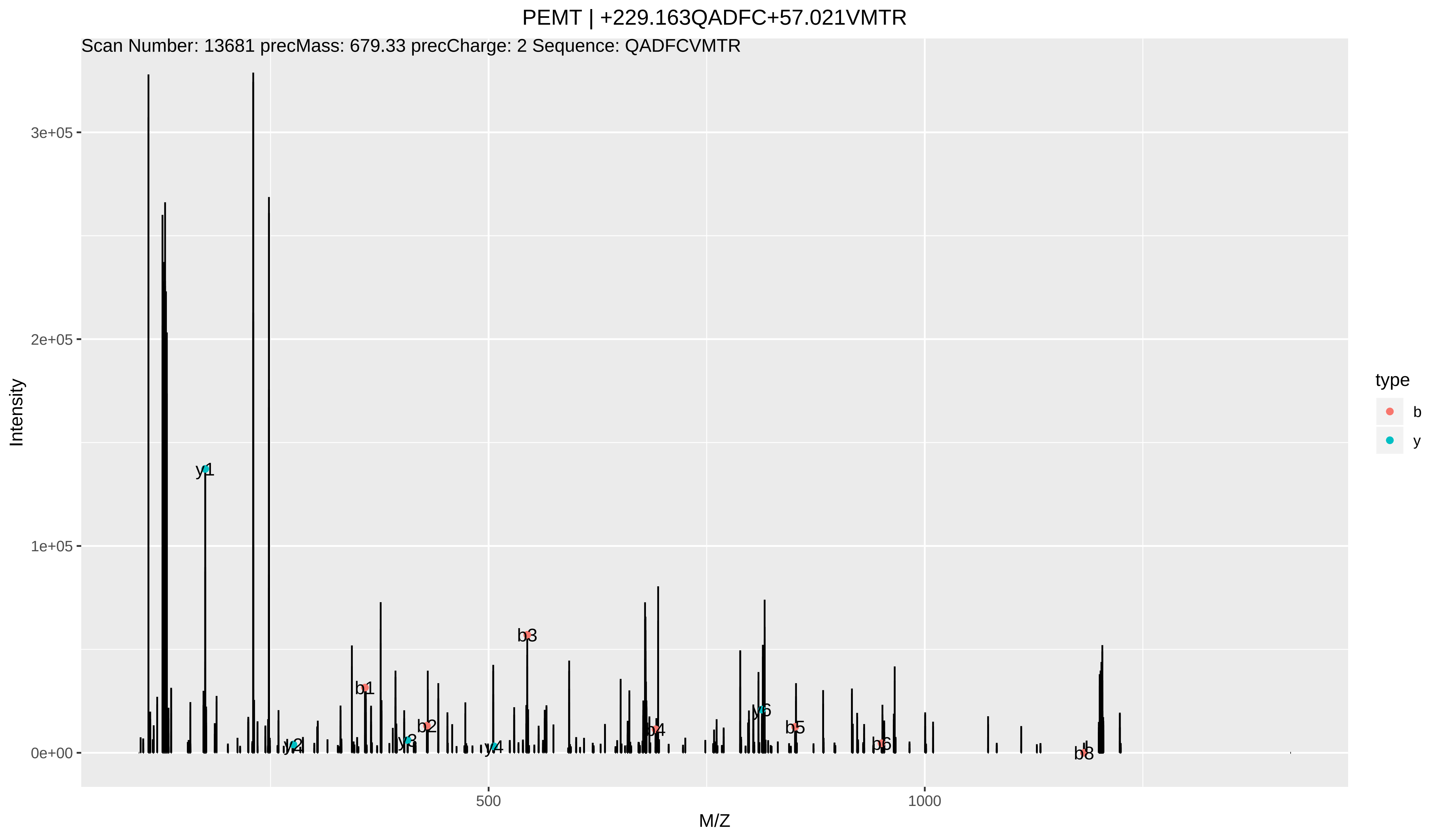

PEMT | +229.163QADFC+57.021VMTR
Scan Number: 13681 precMass: 679.33 precCharge: 2 Sequence: QADFCVMTR
Intensity
3e+05
2e+05
1e+05
0e+00
M/Z
500
1000
y1
y2
y3
y4
y6
b1
b2
b3
b4
b5
b6
b8
type
b
y

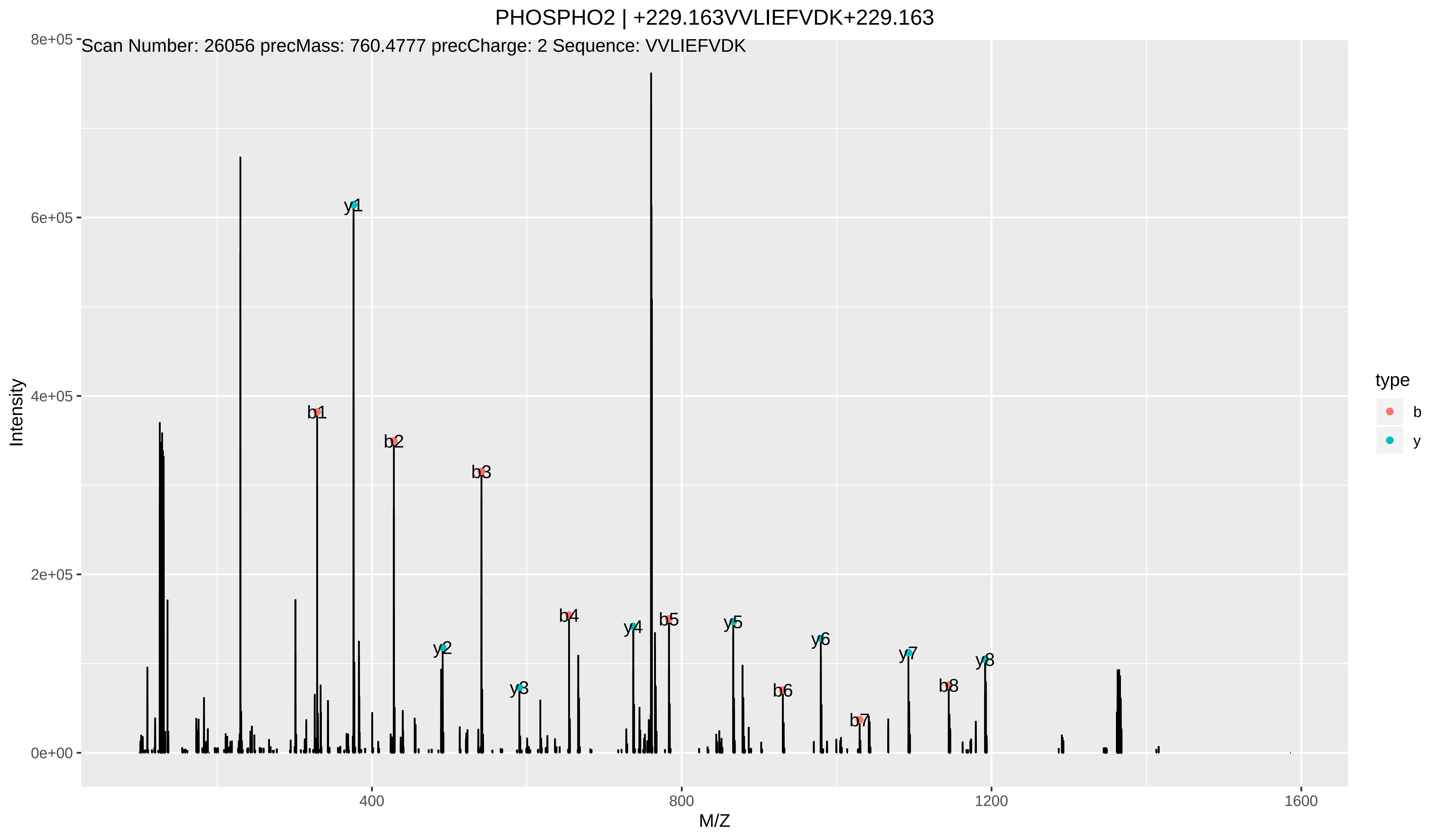
PHOSPHO2 | +229.163VVLIEFVDK+229.163
Scan Number: 26056 precMass: 760.4777 precCharge: 2 Sequence: VVLIEFVDK
Intensity
M/Z
0e+00
2e+05
4e+05
6e+05
8e+05
400
800
1200
1600
y1
b1
b2
b3
y2
y3
b4
y4
b5
y5
b6
y6
b7
y7
b8
y8
type
b
y

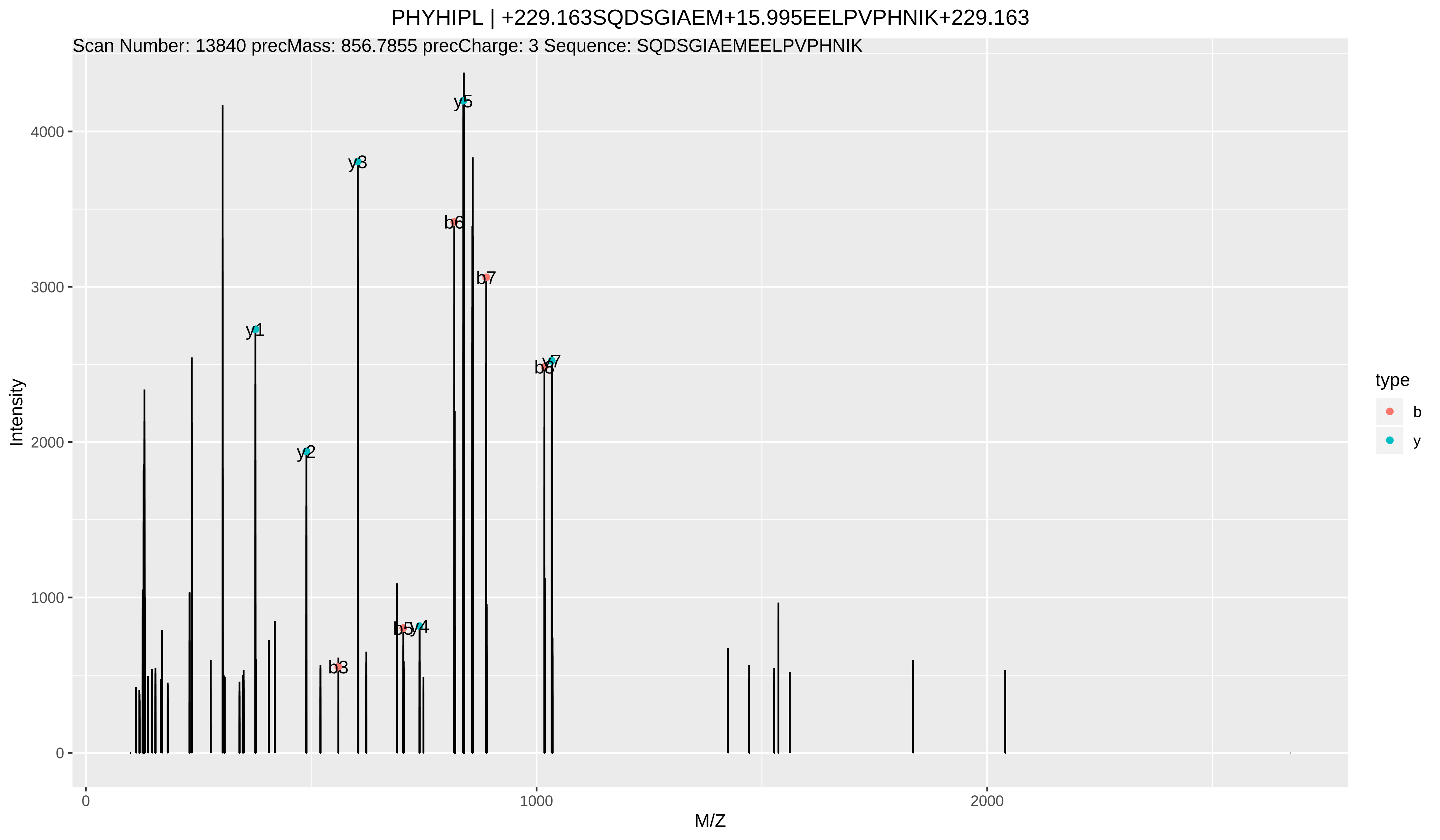

PHYHIPL | +229.163SQDSGIAEM+15.995EELPVPHNIK+229.163
Scan Number: 13840 precMass: 856.7855 precCharge: 3 Sequence: SQDSGIAEMEELPVPHNIK
Intensity
M/Z
0
1000
2000
3000
4000
y1
y2
y3
y4
y5
y7
b3
b5
b6
b7
b8
type
b
y

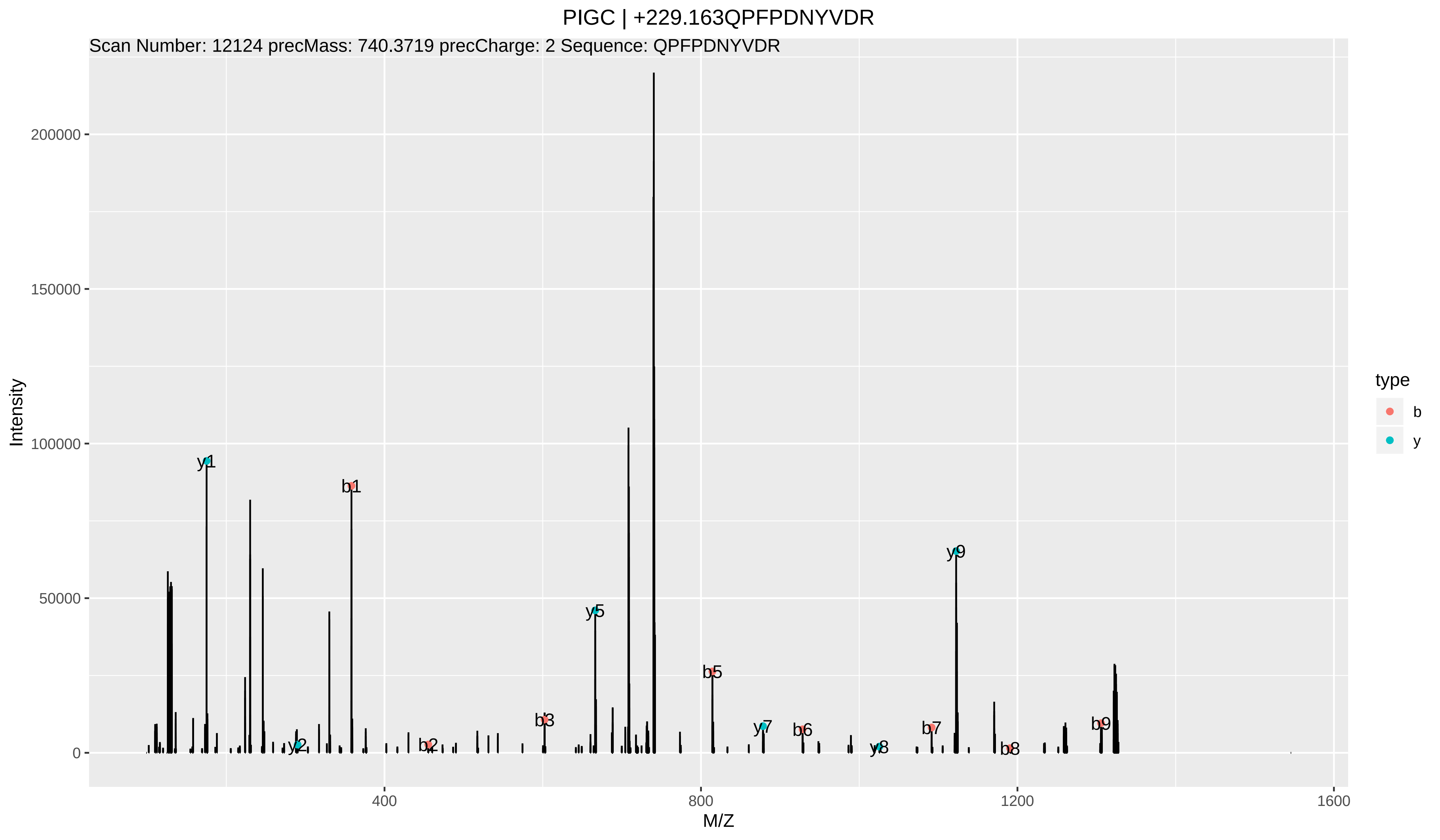
PIGC | +229.163QPFPDNYVDR
Scan Number: 12124 precMass: 740.3719 precCharge: 2 Sequence: QPFPDNYVDR
Intensity
200000
150000
100000
50000
0
400
800
1200
1600
M/Z
y1
b1
y2
b2
b3
y5
b5
y7
b6
y8
b7
b8
y9
b9
type
b
y

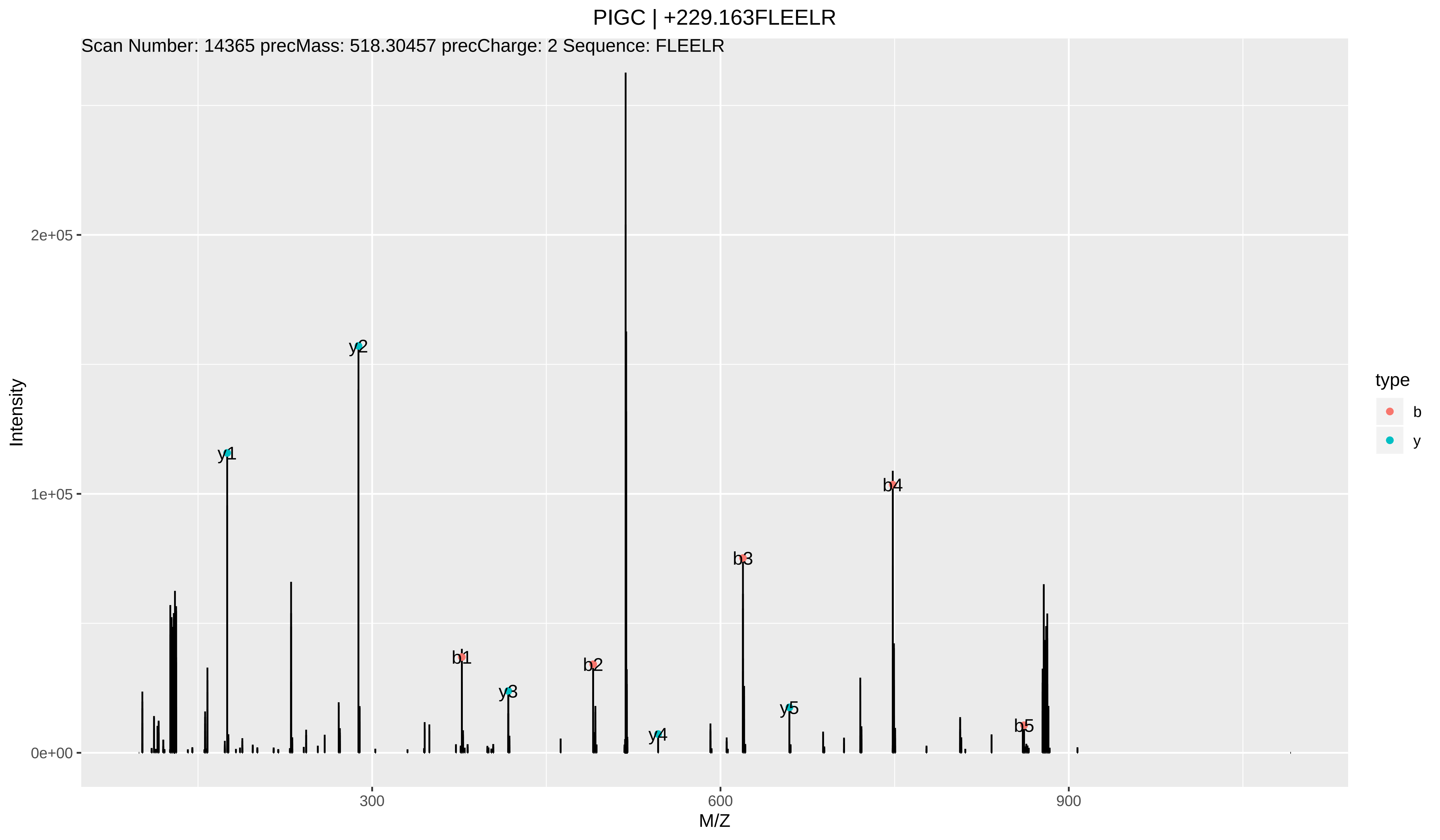

PIGC | +229.163FLEELR
Scan Number: 14365 precMass: 518.30457 precCharge: 2 Sequence: FLEELR
Intensity
M/Z
2e+05
1e+05
0e+00
300
600
900
y1
y2
y3
y4
y5
b1
b2
b3
b4
b5
type
b
y

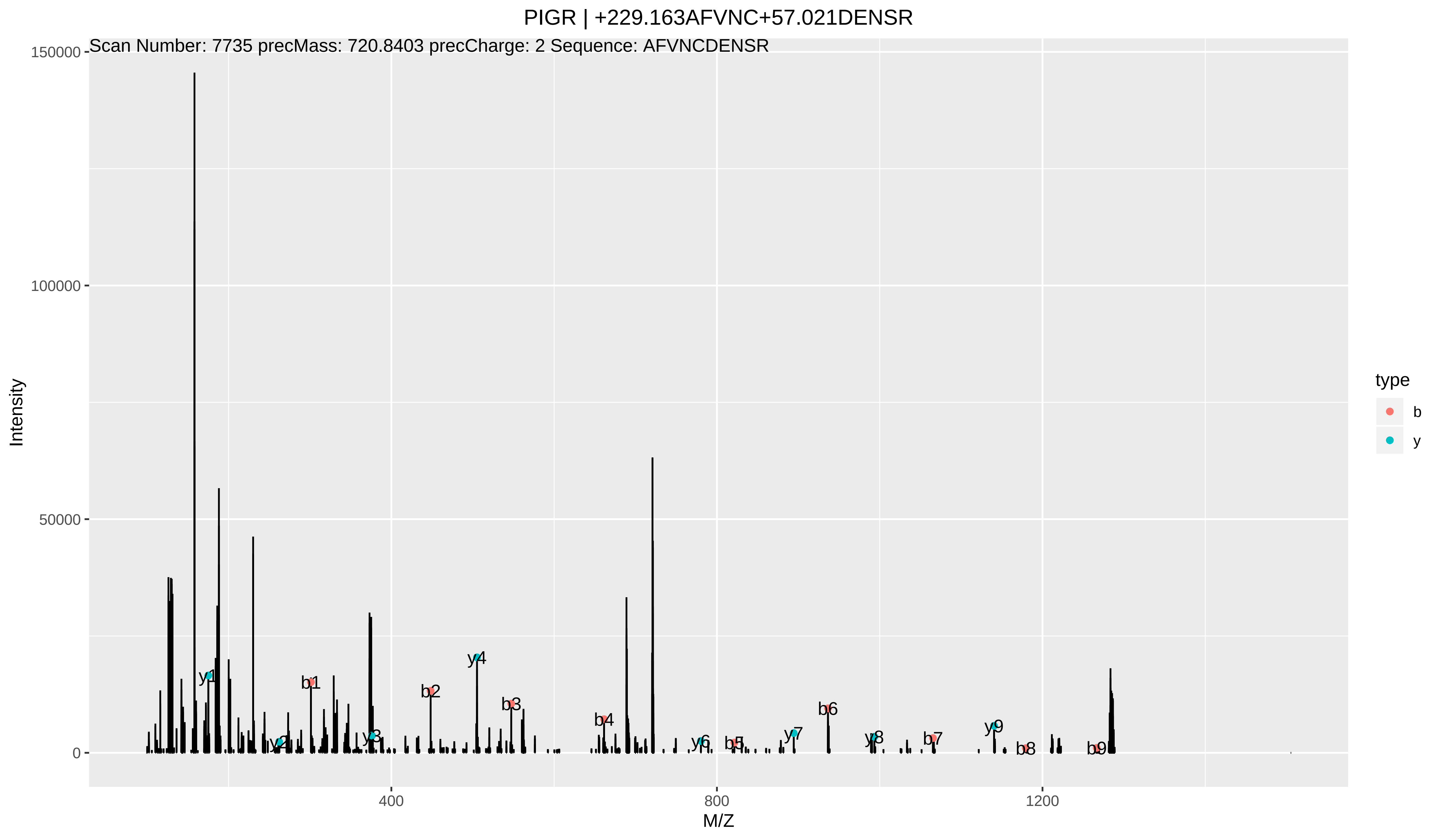

PIGR | +229.163AFVNC+57.021DENSR
Scan Number: 7735 precMass: 720.8403 precCharge: 2 Sequence: AFVNCDENSR
Intensity
150000
100000
50000
0
M/Z
400
800
1200
y1
y2
y3
y4
y6
y7
y8
y9
b1
b2
b3
b4
b5
b6
b7
b8
b9
type
b
y

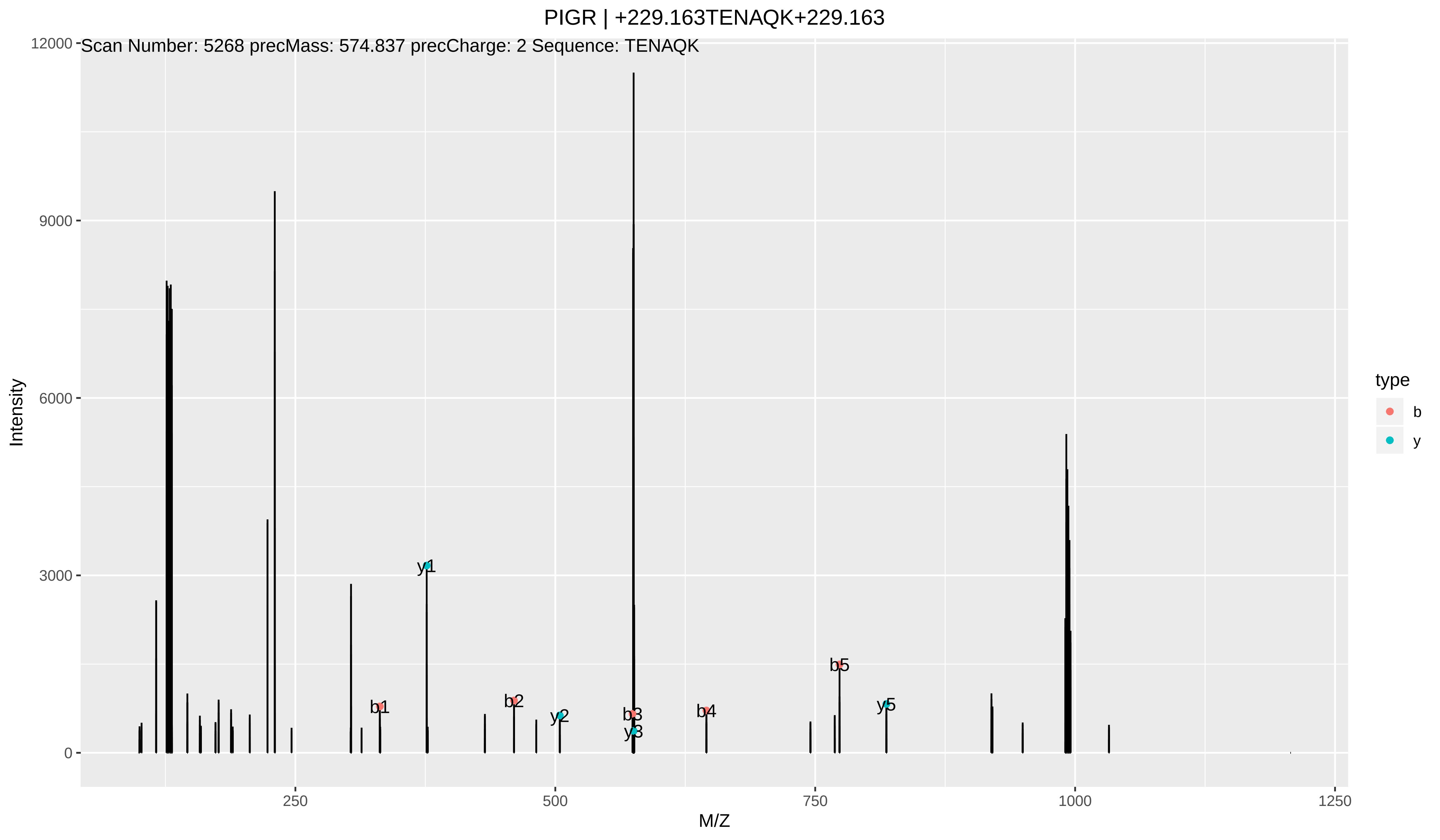

PIGR | +229.163TENAQK+229.163
Scan Number: 5268 precMass: 574.837 precCharge: 2 Sequence: TENAQK
Intensity
12000
9000
6000
3000
0
M/Z
250
500
750
1000
1250
y1
b1
b2
y2
b3
y3
b4
b5
y5
type
b
y

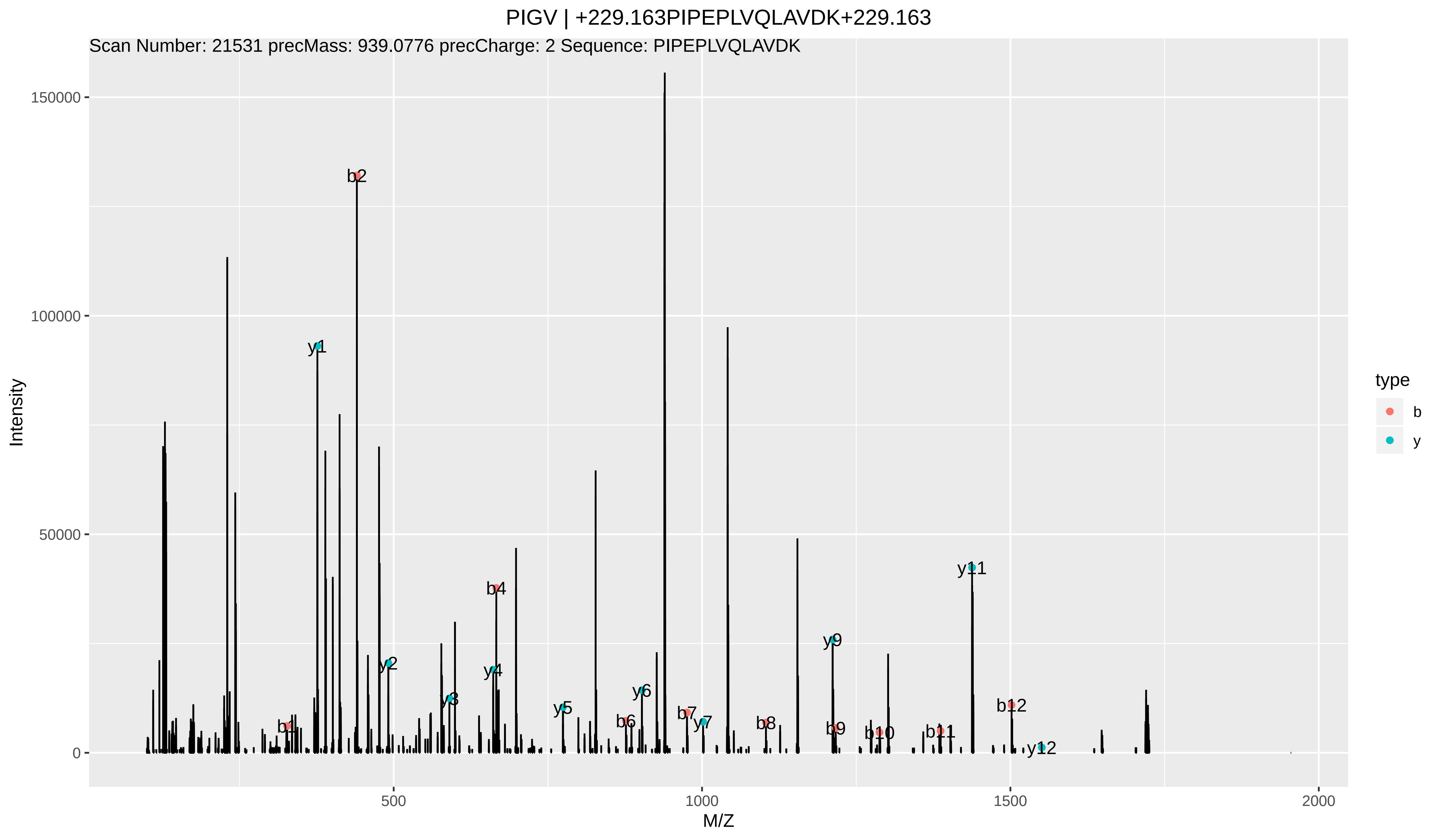

PIGV | +229.163PIPEPLVQLAVDK+229.163
Scan Number: 21531 precMass: 939.0776 precCharge: 2 Sequence: PIPEPLVQLAVDK
Intensity
150000
100000
50000
0
M/Z
500
1000
1500
2000
b2
y1
b4
y2
y4
y3
b1
y5
b6
y6
b7
y7
b8
y9
b9
b10
b11
y11
b12
y12
type
b
y

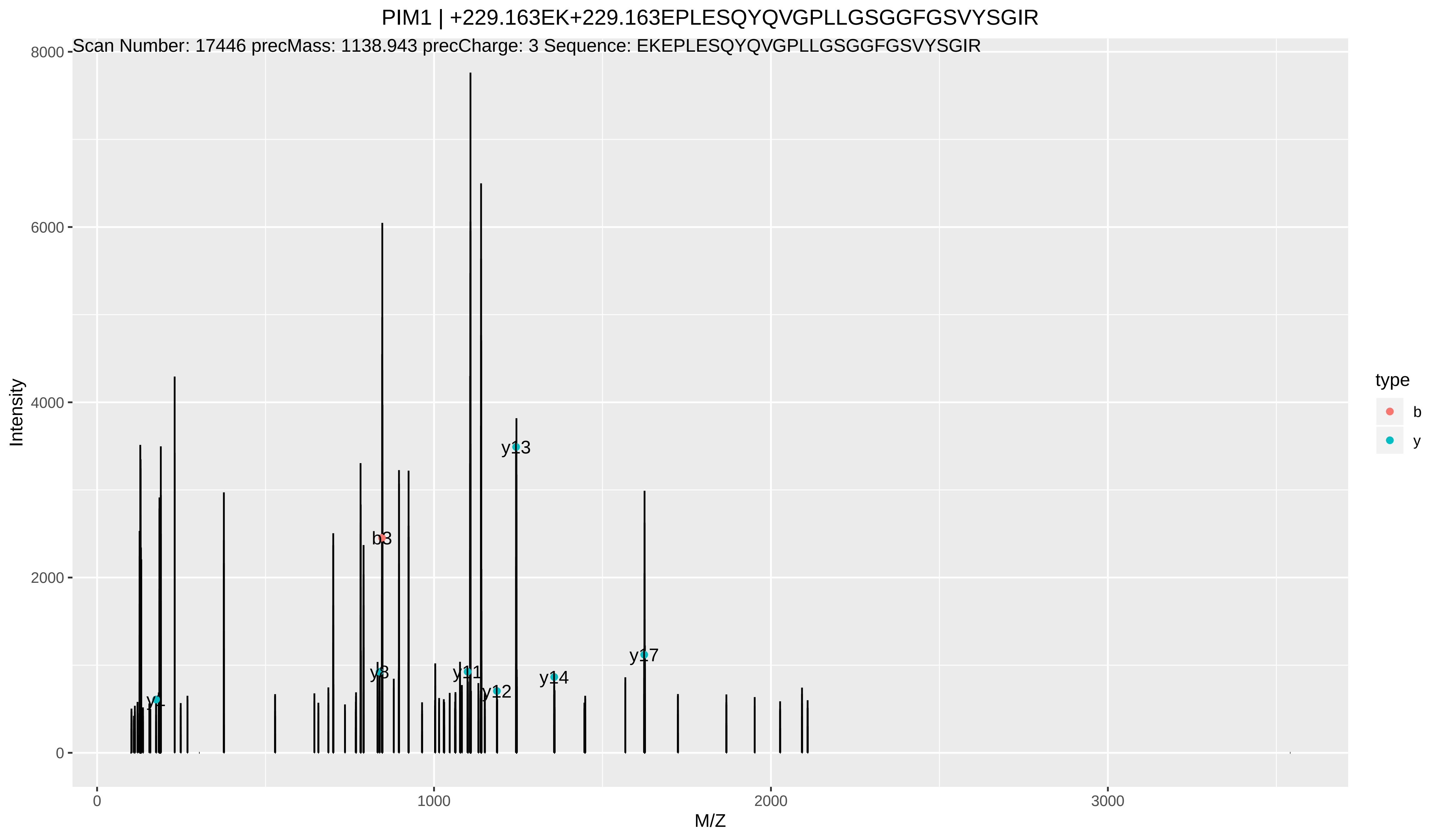
PIM1 | +229.163EK+229.163EPLESQYQVGPLLGSGGFGSVYSGIR
Scan Number: 17446 precMass: 1138.943 precCharge: 3 Sequence: EKEPLESQYQVGPLLGSGGFGSVYSGIR
Intensity
8000
6000
4000
2000
0
M/Z
0
1000
2000
3000
b3
y8
y11
y12
y13
y14
y17
type
b
y

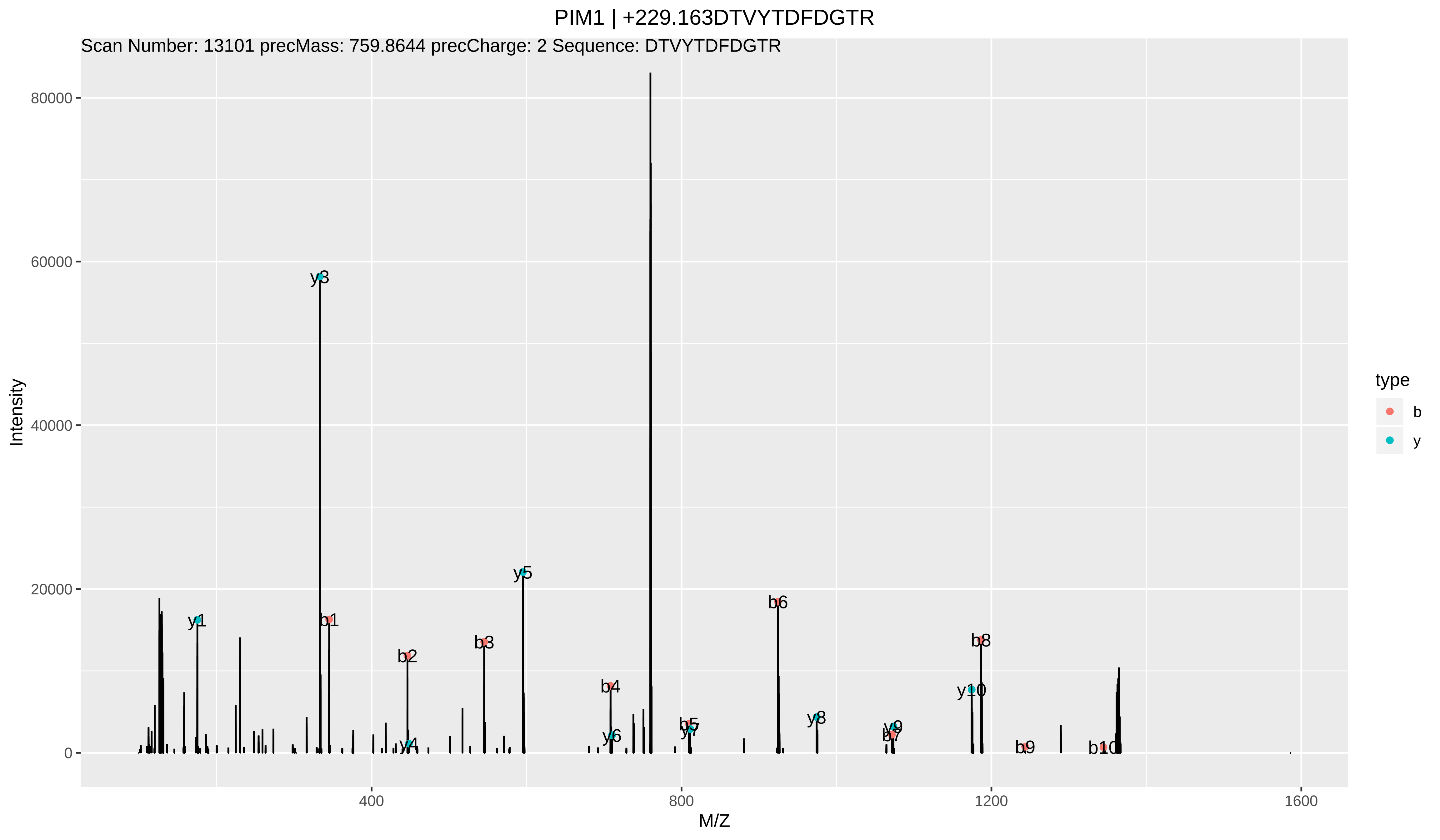
PIM1 | +229.163DTVYTDFDGTR
Scan Number: 13101 precMass: 759.8644 precCharge: 2 Sequence: DTVYTDFDGTR
Intensity
M/Z
0
20000
40000
60000
80000
400
800
1200
1600
y1
y3
b1
b2
b3
y4
y5
b4
y6
b5
y7
b6
y8
y9
b7
y10
b8
b9
b10
type
b
y

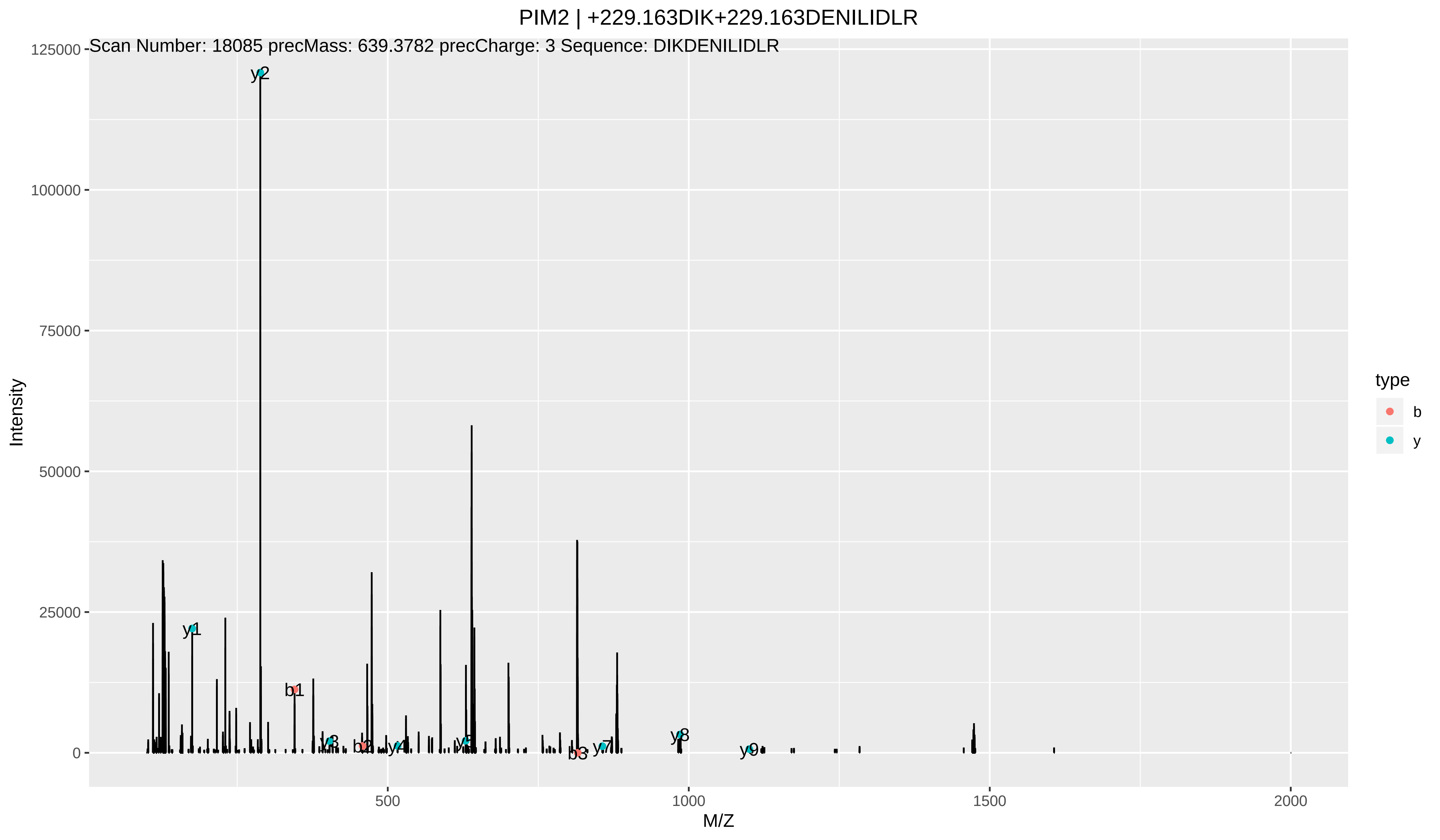
PIM2 | +229.163DIK+229.163DENILIDLR
Scan Number: 18085 precMass: 639.3782 precCharge: 3 Sequence: DIKDENILIDLR
Intensity
M/Z
type
b
y
y1
y2
b1
y3
b2
y4
y5
b3
y7
y8
y9
0
25000
50000
75000
100000
125000
500
1000
1500
2000

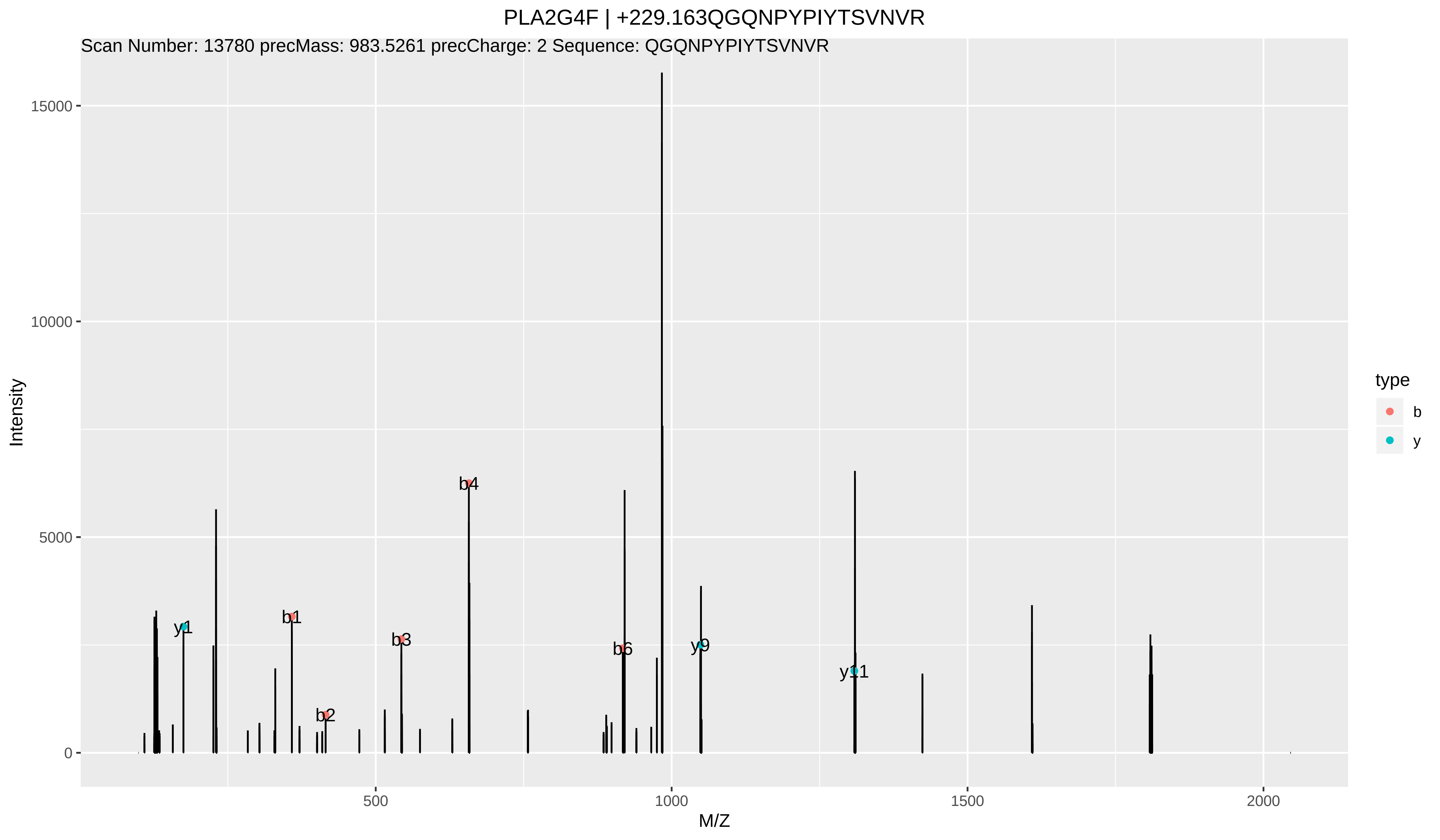

PLA2G4F | +229.163QGQNPYPIYTSVNVR
Scan Number: 13780 precMass: 983.5261 precCharge: 2 Sequence: QGQNPYPIYTSVNVR
Intensity
M/Z
0
5000
10000
15000
500
1000
1500
2000
y1
b1
b2
b3
b4
b6
y9
y11
type
b
y

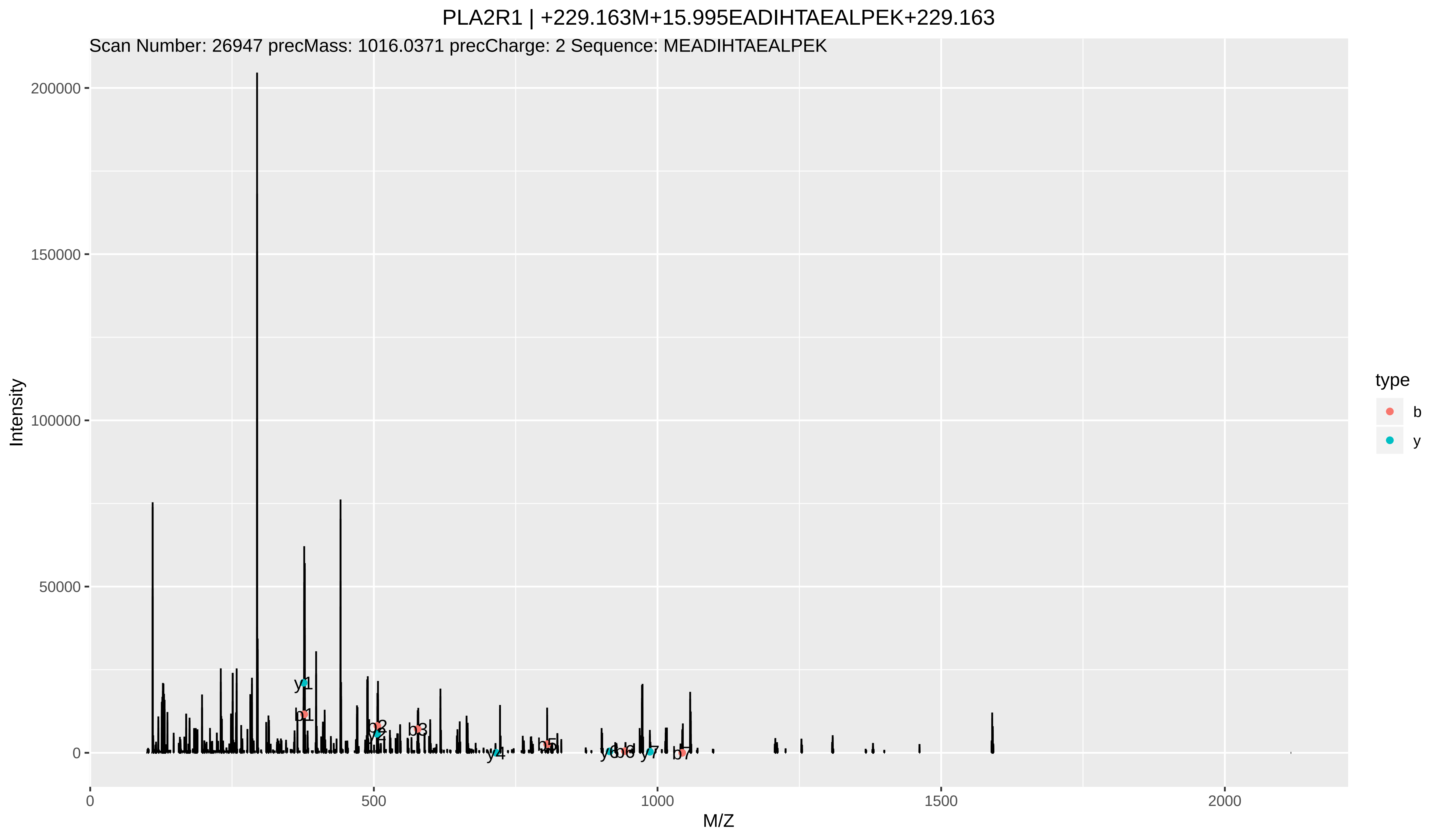
PLA2R1 | +229.163M+15.995EADIHTAEALPEK+229.163
Scan Number: 26947 precMass: 1016.0371 precCharge: 2 Sequence: MEADIHTAEALPEK
Intensity
M/Z
type
b
y
y1
b1
b2
y2
b3
y4
b5
y6
b6
y7
b7
200000
150000
100000
50000
0
0
500
1000
1500
2000

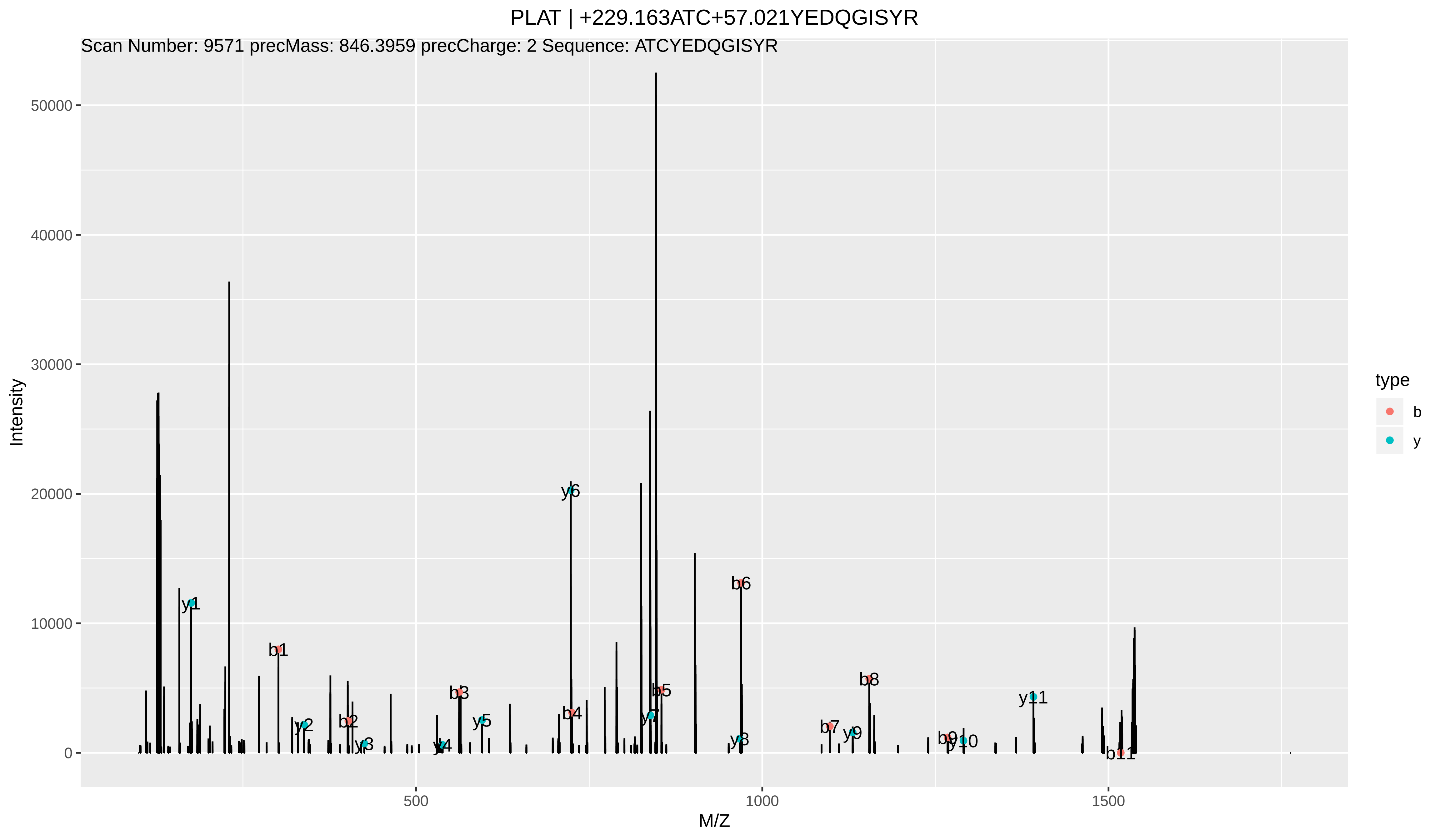
PLAT | +229.163ATC+57.021YEDQGISYR
Scan Number: 9571 precMass: 846.3959 precCharge: 2 Sequence: ATCYEDQGISYR
Intensity
M/Z
type
b
y
y1
b1
y2
b2
y3
b3
y4
y5
y6
b4
y7
b5
y8
b6
b7
y9
b8
b9
y10
y11
b11
0
10000
20000
30000
40000
50000
500
1000
1500

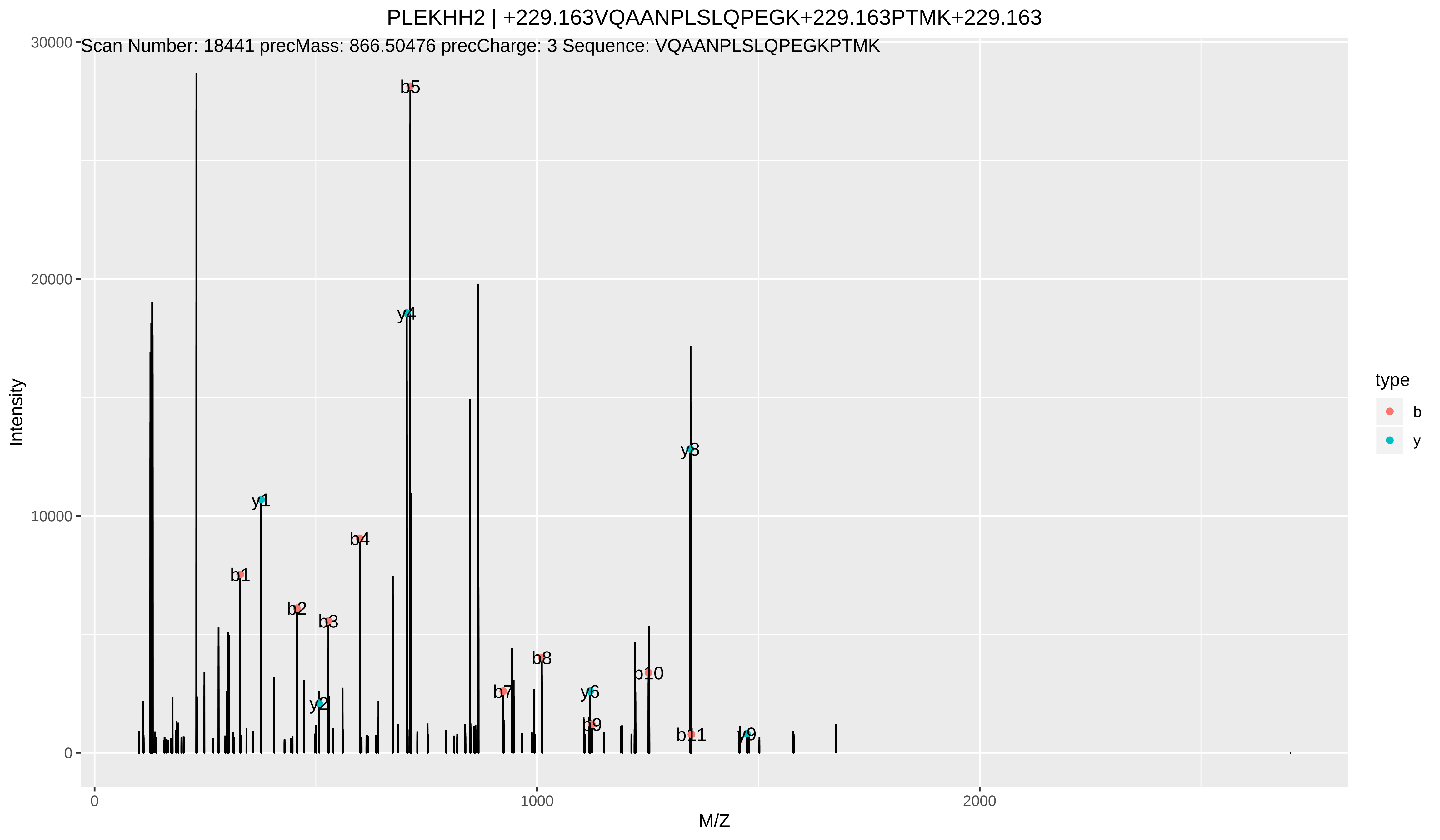
PLEKHH2 | +229.163VQAANPLSLQPEGK+229.163PTMK+229.163
Scan Number: 18441 precMass: 866.50476 precCharge: 3 Sequence: VQAANPLSLQPEGKPTMK
Intensity
M/Z
0
10000
20000
30000
0
1000
2000
b5
y4
y1
b4
b1
b2
b3
y2
b7
b8
y6
b9
b10
y8
b11
y9
type
b
y

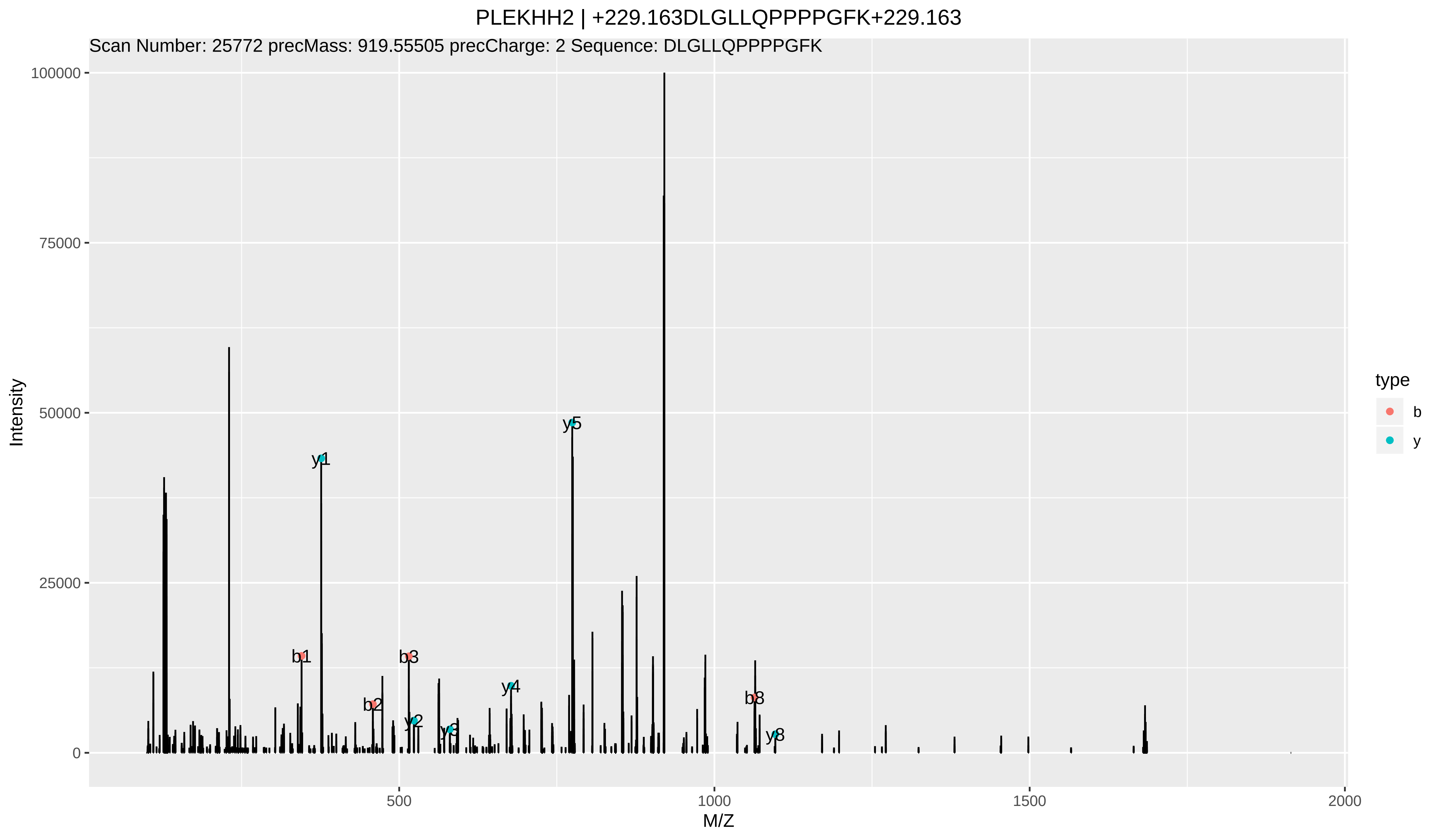

PLEKHH2 | +229.163DLGLLQPPPPGFK+229.163
Scan Number: 25772 precMass: 919.55505 precCharge: 2 Sequence: DLGLLQPPPPGFK
Intensity
100000
75000
50000
25000
0
M/Z
500
1000
1500
2000
y1
y2
y3
y4
y5
y8
b1
b2
b3
b8
type
b
y

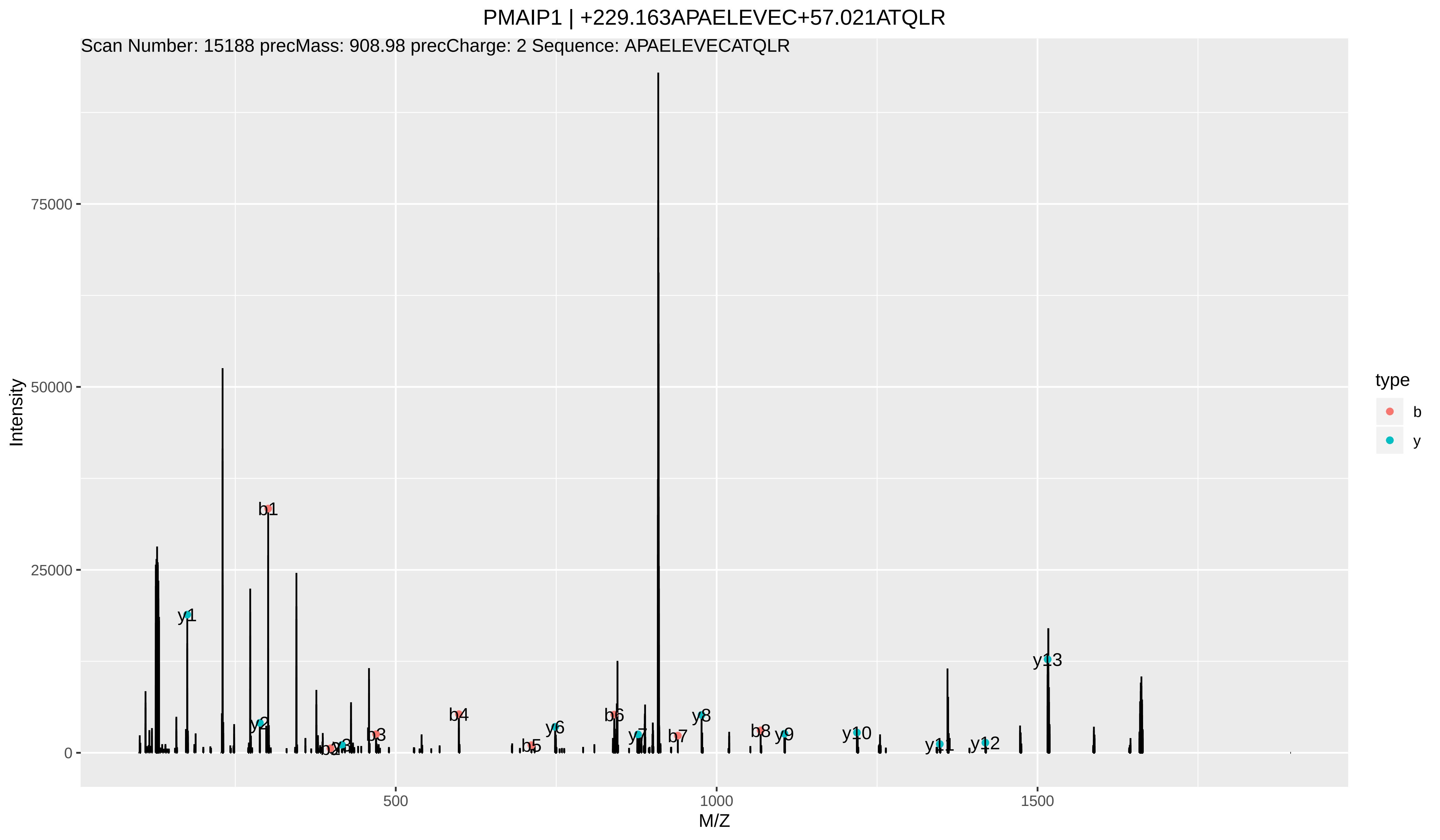
PMAIP1 | +229.163APAELEVEC+57.021ATQLR
Scan Number: 15188 precMass: 908.98 precCharge: 2 Sequence: APAELEVECATQLR
Intensity
M/Z
type
b
y
y1
y2
b1
b2
y3
b3
b4
b5
y6
b6
y7
b7
y8
b8
y9
y10
y11
y12
y13
75000
50000
25000
0
500
1000
1500

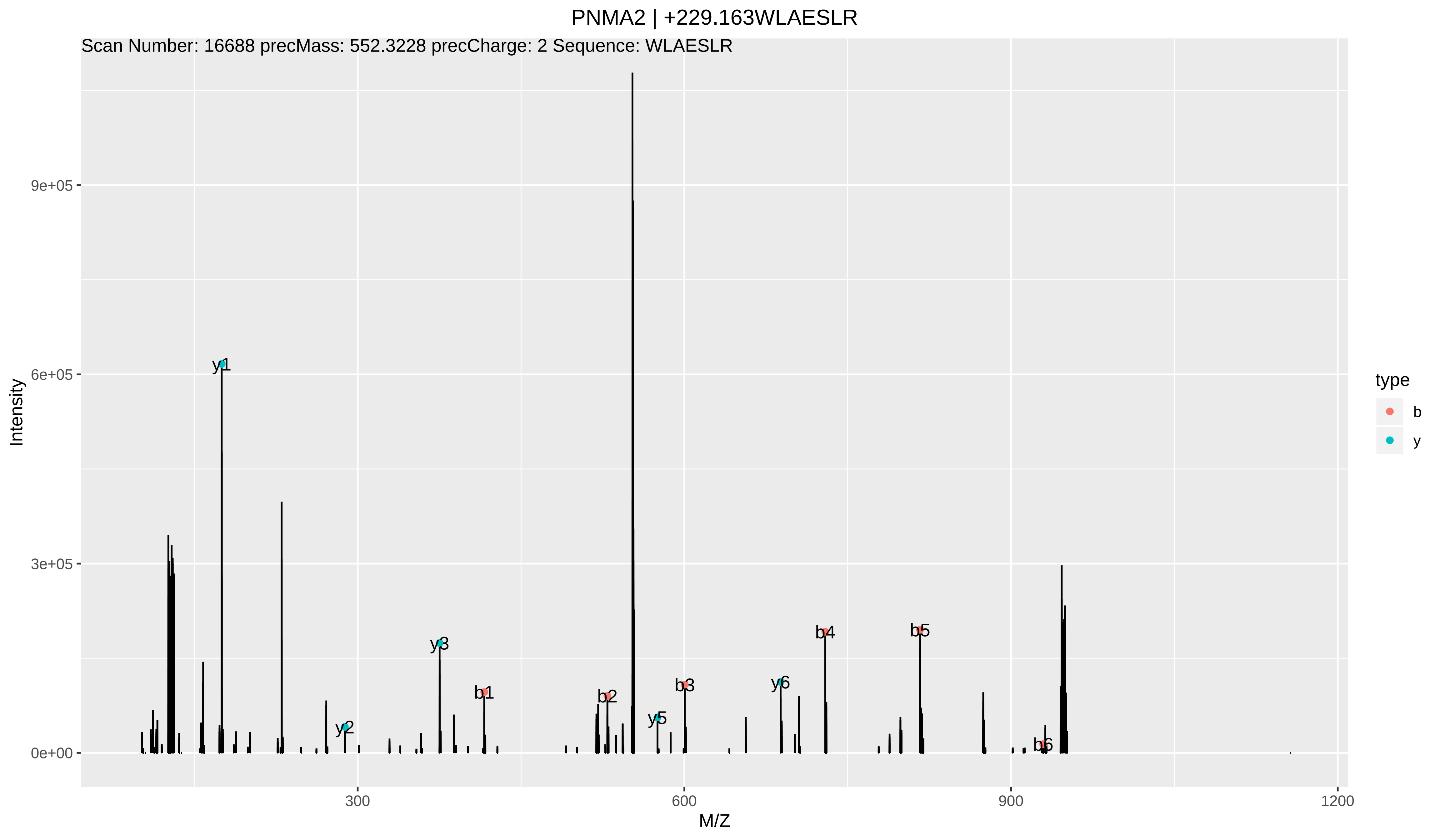
PNMA2 | +229.163WLAESLR
Scan Number: 16688 precMass: 552.3228 precCharge: 2 Sequence: WLAESLR
Intensity
9e+05
6e+05
3e+05
0e+00
M/Z
300
600
900
1200
y1
y2
y3
b1
b2
y5
b3
y6
b4
b5
b6
type
b
y

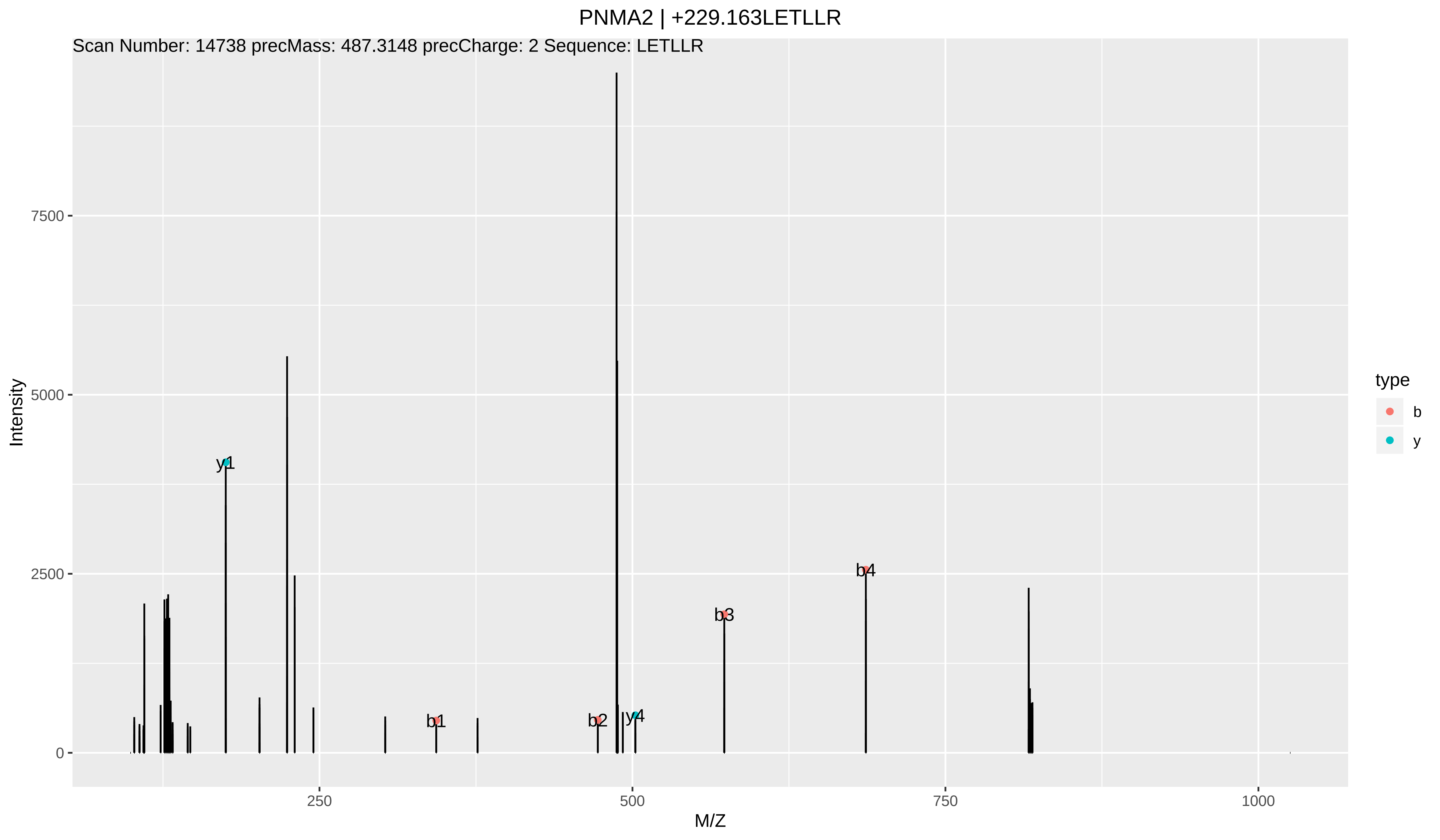

PNMA2 | +229.163LETLLR
Scan Number: 14738 precMass: 487.3148 precCharge: 2 Sequence: LETLLR
Intensity
7500
5000
2500
0
250
500
750
1000
M/Z
y1
b1
b2
y4
b3
b4
type
b
y

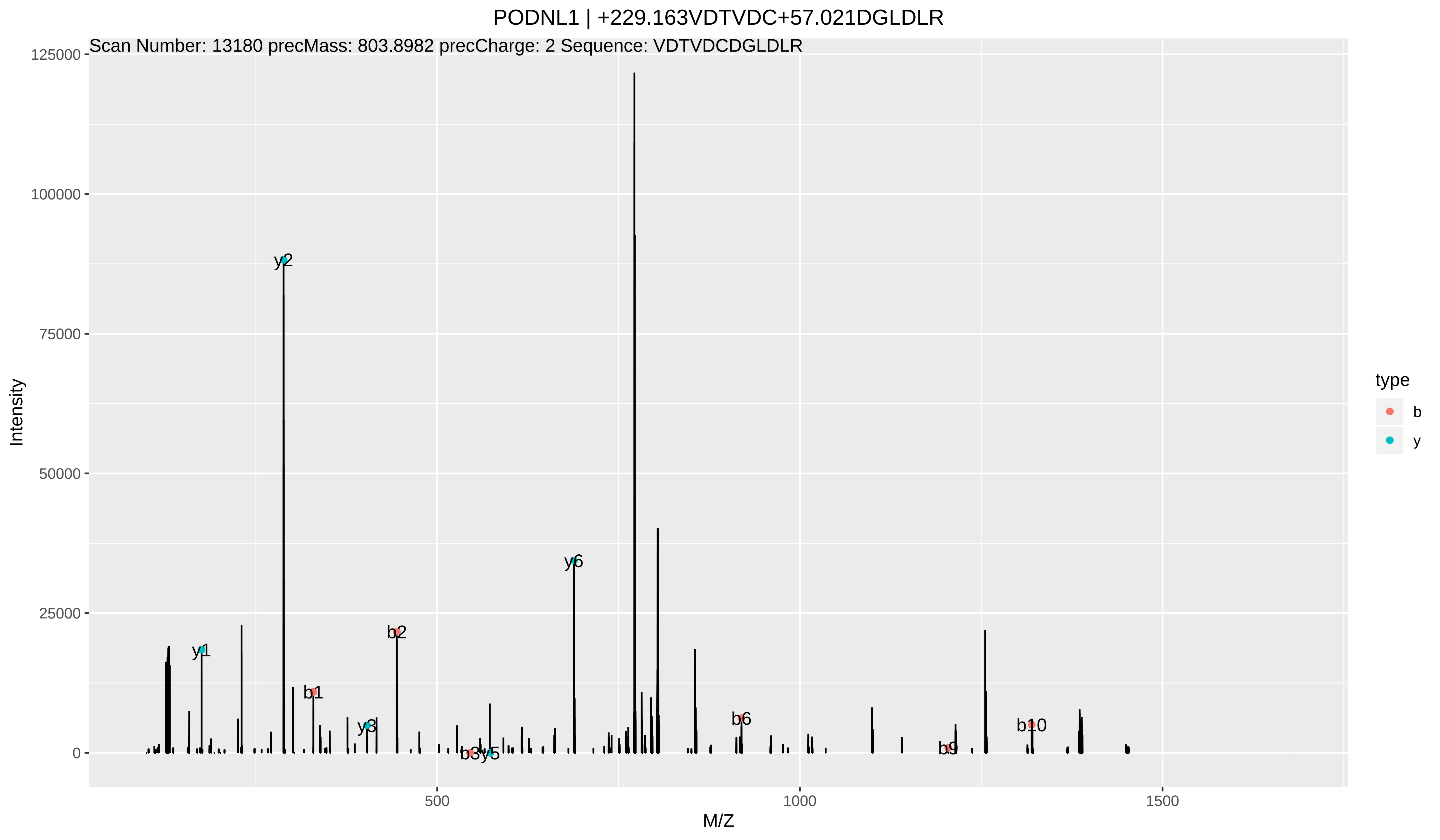

PODNL1 | +229.163VDTVDC+57.021DGLDLR
Scan Number: 13180 precMass: 803.8982 precCharge: 2 Sequence: VDTVDCDGLDLR
Intensity
M/Z
y2
y6
b2
y1
b1
y3
b3
y5
b6
b9
b10
type
b
y

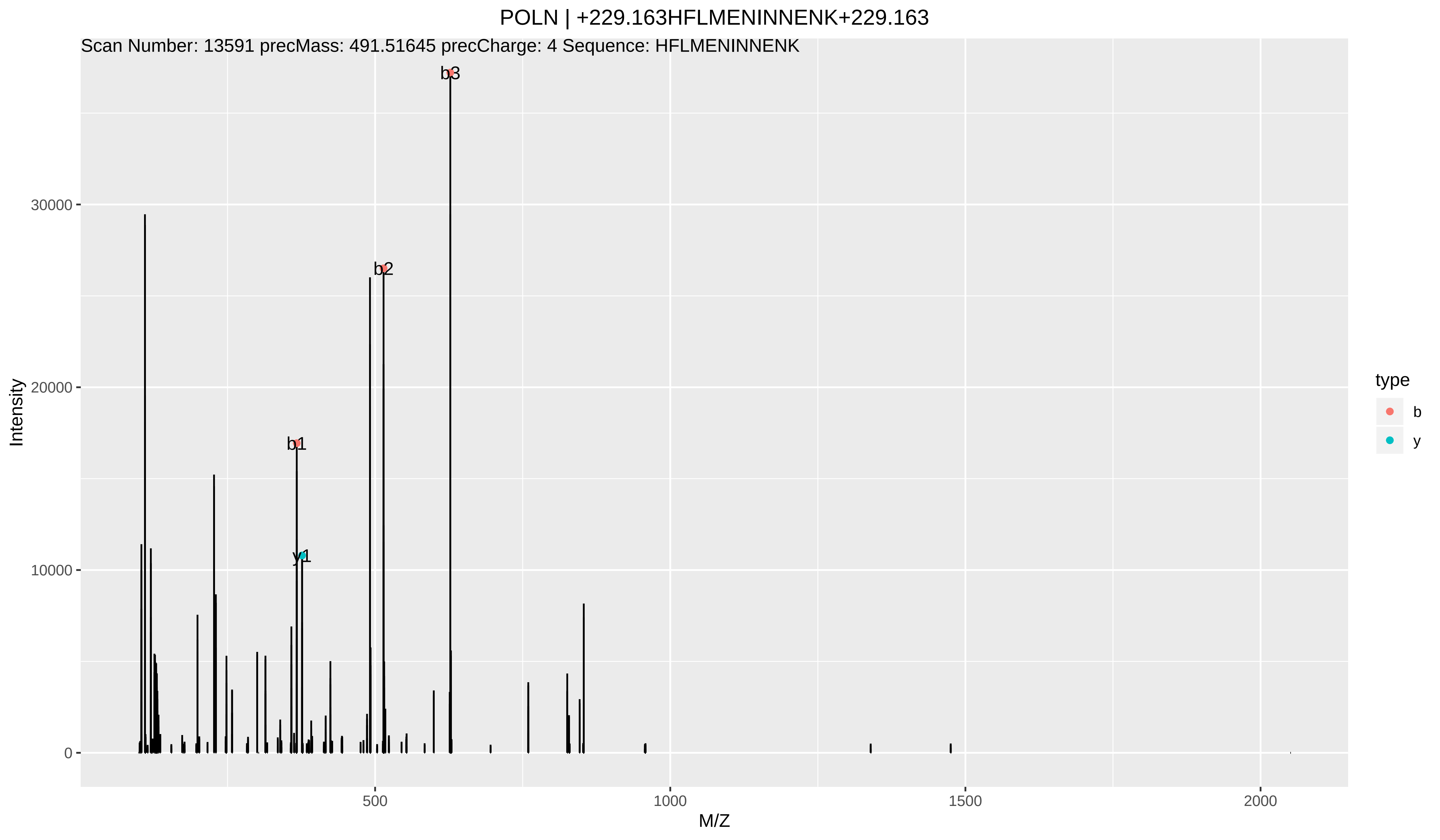

POLN | +229.163HFLMENINNENK+229.163
Scan Number: 13591 precMass: 491.51645 precCharge: 4 Sequence: HFLMENINNENK
b3
b2
b1
y1
Intensity
30000
20000
10000
0
500
1000
1500
2000
M/Z
type
b
y

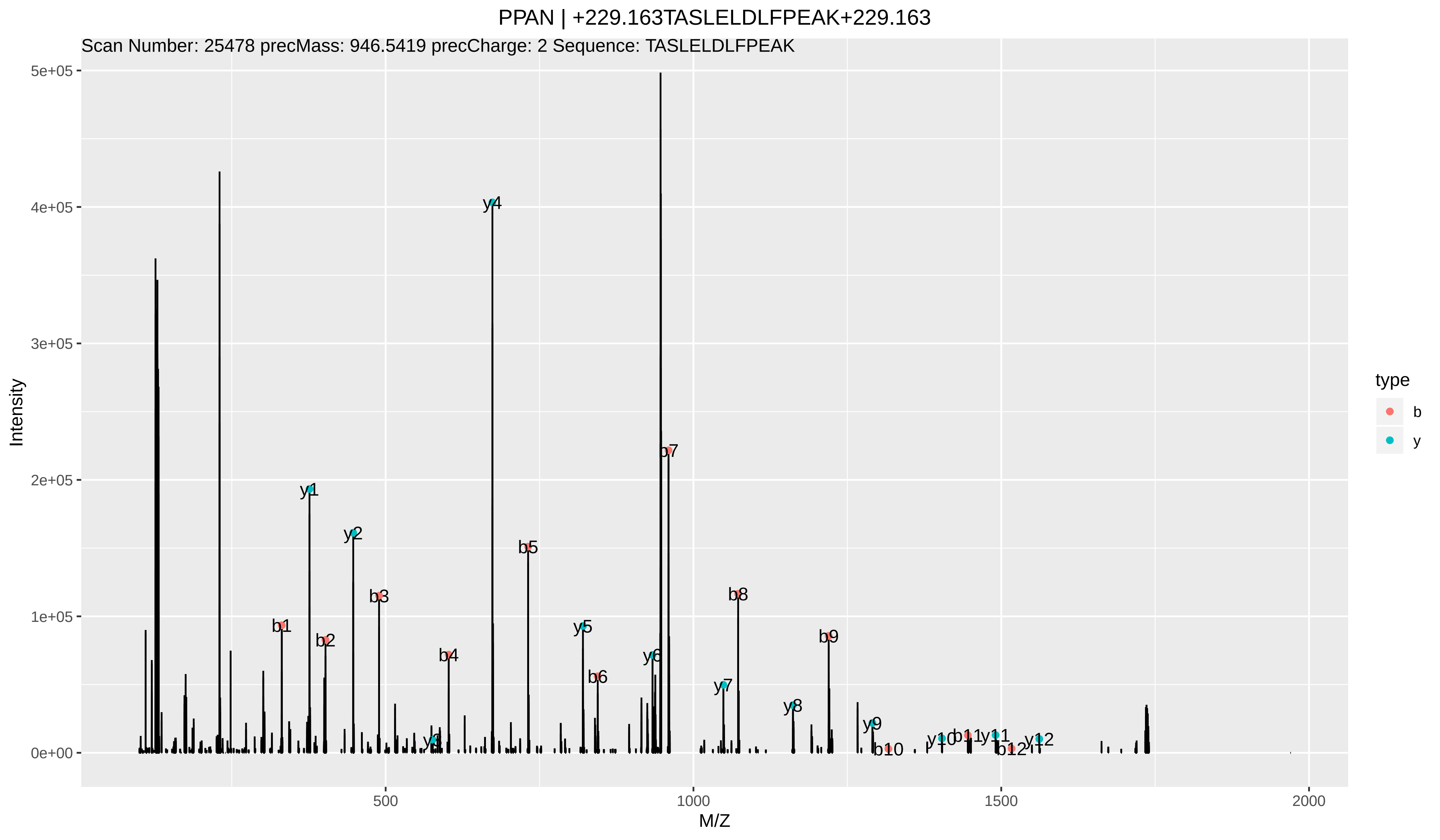

PPAN | +229.163TASLELDLFPEAK+229.163
Scan Number: 25478 precMass: 946.5419 precCharge: 2 Sequence: TASLELDLFPEAK
Intensity
M/Z
type
b
y

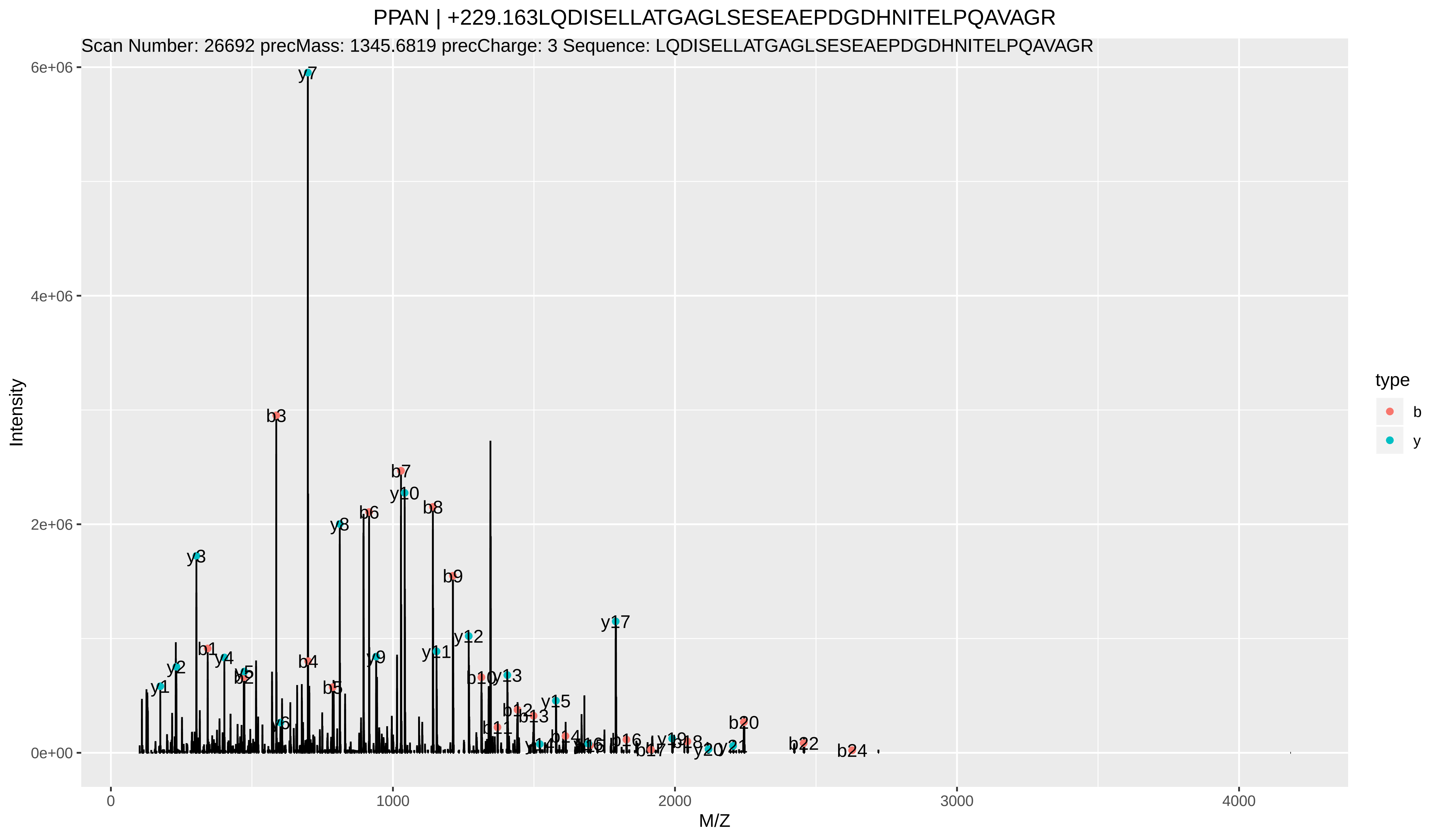

PPAN | +229.163LQDISELLATGAGLSESEAEPDGDHNITELPQAVAGR
Scan Number: 26692 precMass: 1345.6819 precCharge: 3 Sequence: LQDISELLATGAGLSESEAEPDGDHNITELPQAVAGR
Intensity
M/Z
6e+06
4e+06
2e+06
0e+00
0
1000
2000
3000
4000
type
b
y
y7
b3
b7
y10
b8
b6
y8
b9
y3
y17
y12
y11
b1
y4
y9
b4
y2
y5
b2
y1
b5
b10
y13
y15
b12
b13
y6
b11
b20
b14
b16
y19
b18
b22
y21
y20
b17
b24

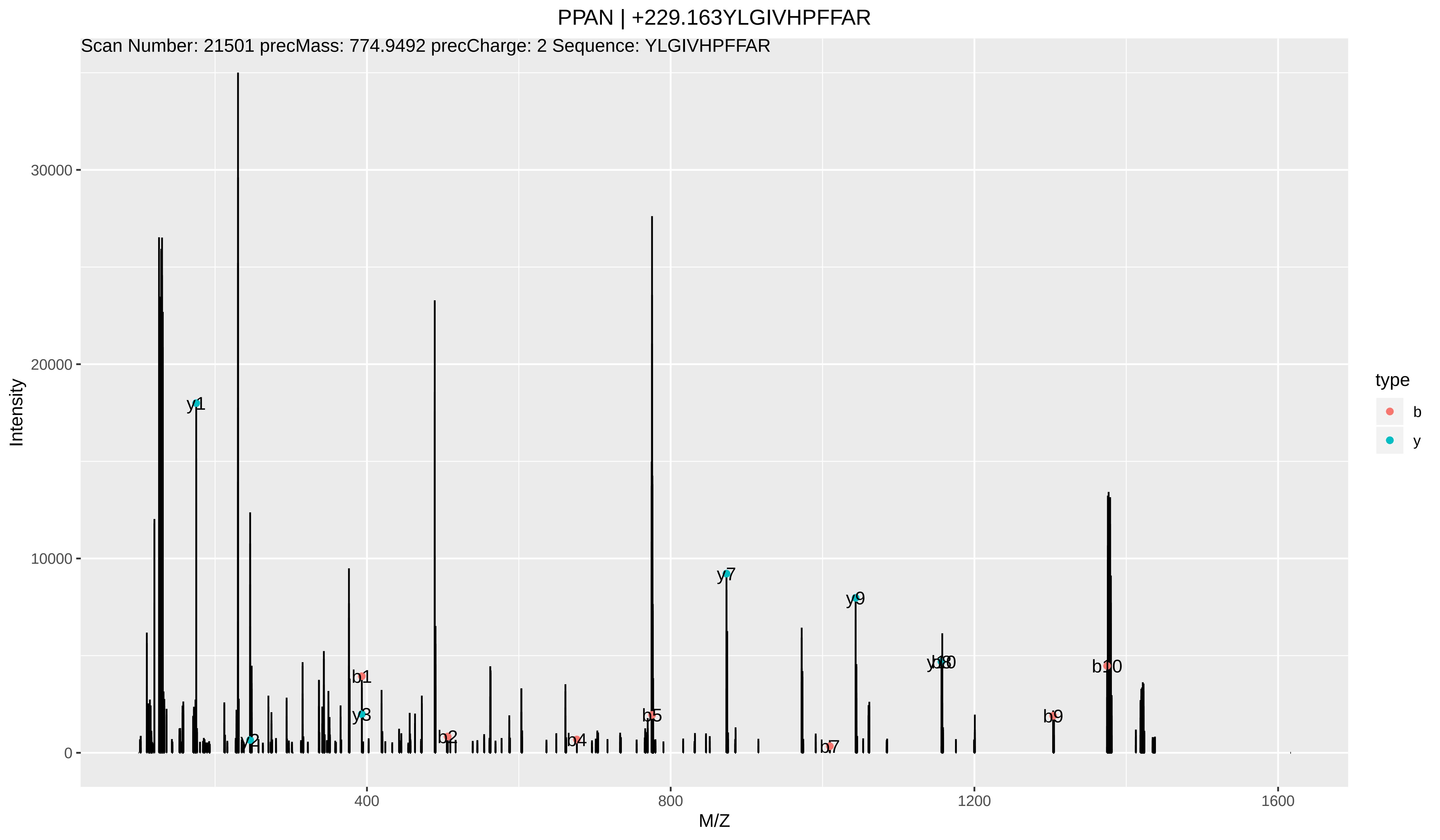

PPAN | +229.163YLGIVHPFFAR
Scan Number: 21501 precMass: 774.9492 precCharge: 2 Sequence: YLGIVHPFFAR
Intensity
30000
20000
10000
0
M/Z
400
800
1200
1600
y1
y2
y3
b1
b2
b4
b5
y7
b7
y9
y8
b8
b9
b10
type
b
y

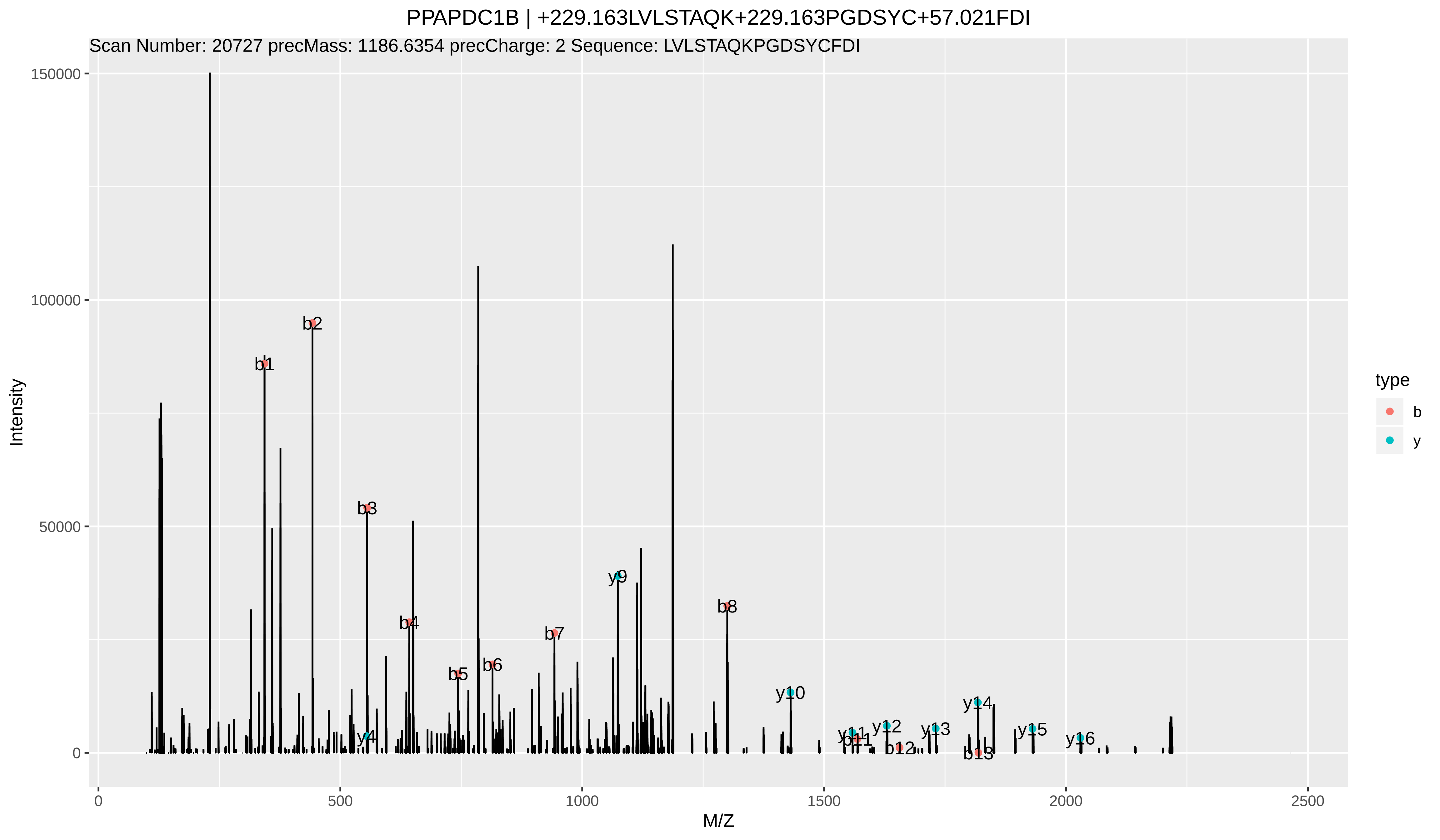

PPAPDC1B | +229.163LVLSTAQK+229.163PGDSYC+57.021FDI
Scan Number: 20727 precMass: 1186.6354 precCharge: 2 Sequence: LVLSTAQKPGDSYCFDI
Intensity
M/Z
0
50000
100000
150000
0
500
1000
1500
2000
2500
b1
b2
b3
b4
b5
b6
b7
b8
b11
b12
b13
y4
y9
y10
y11
y12
y13
y14
y15
y16
type
b
y

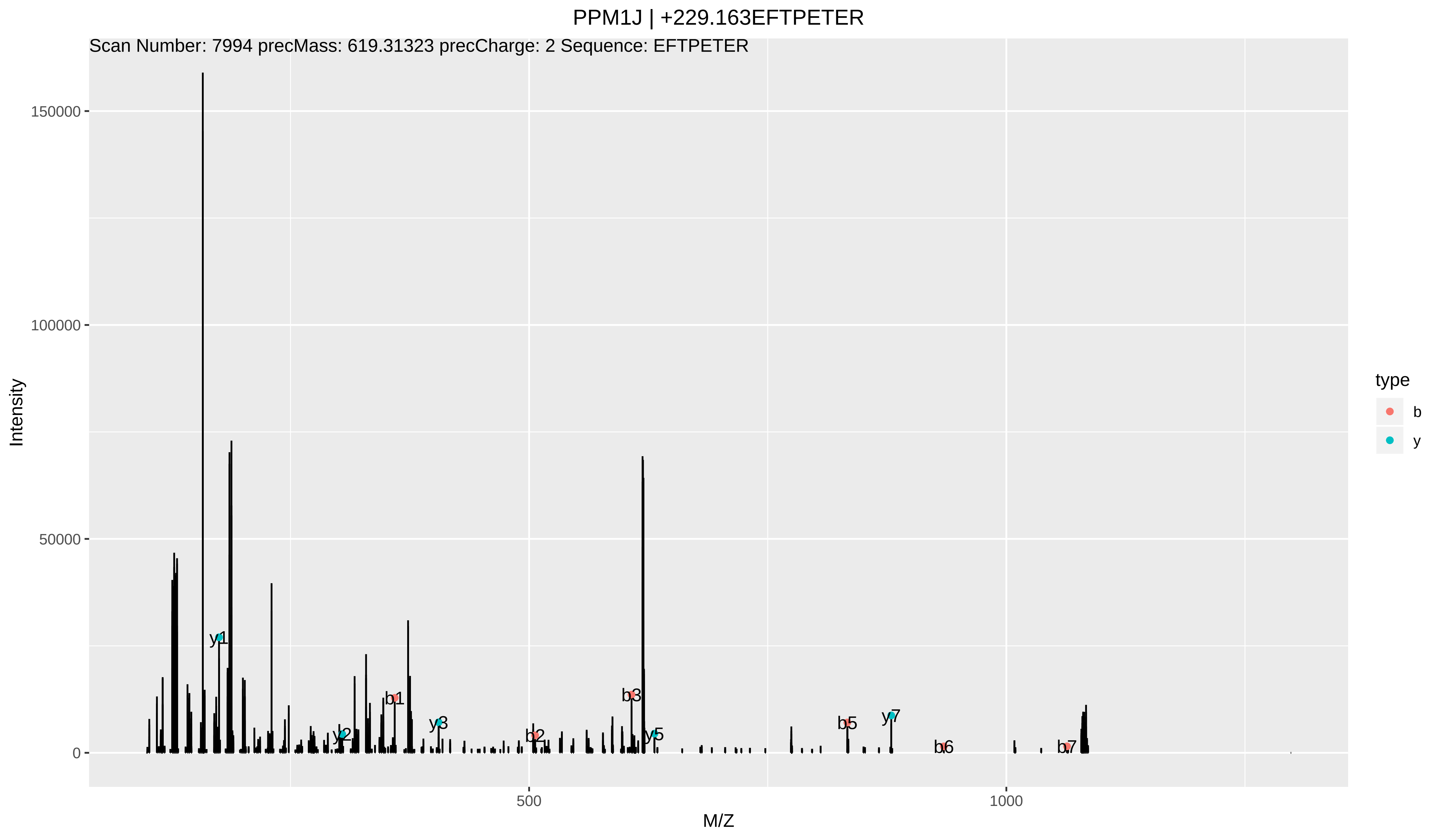
PPM1J | +229.163EFTPETER
Scan Number: 7994 precMass: 619.31323 precCharge: 2 Sequence: EFTPETER
Intensity
150000
100000
50000
0
M/Z
500
1000
y1
y2
b1
y3
b2
b3
y5
b5
y7
b6
b7
type
b
y

PRR7 | +229.163ALELEPLELEGSLAGSPPGLAPPQPPPHR

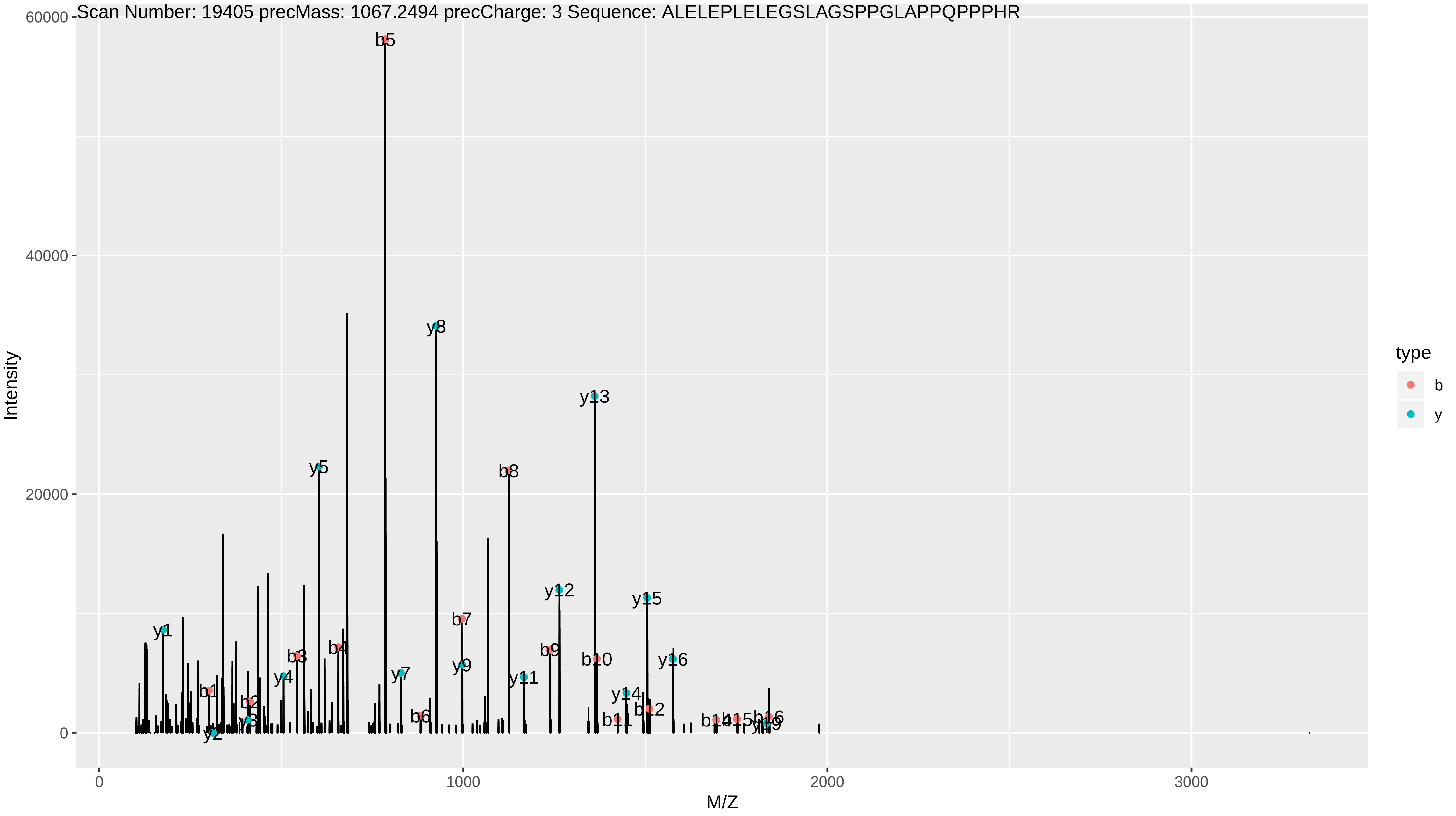

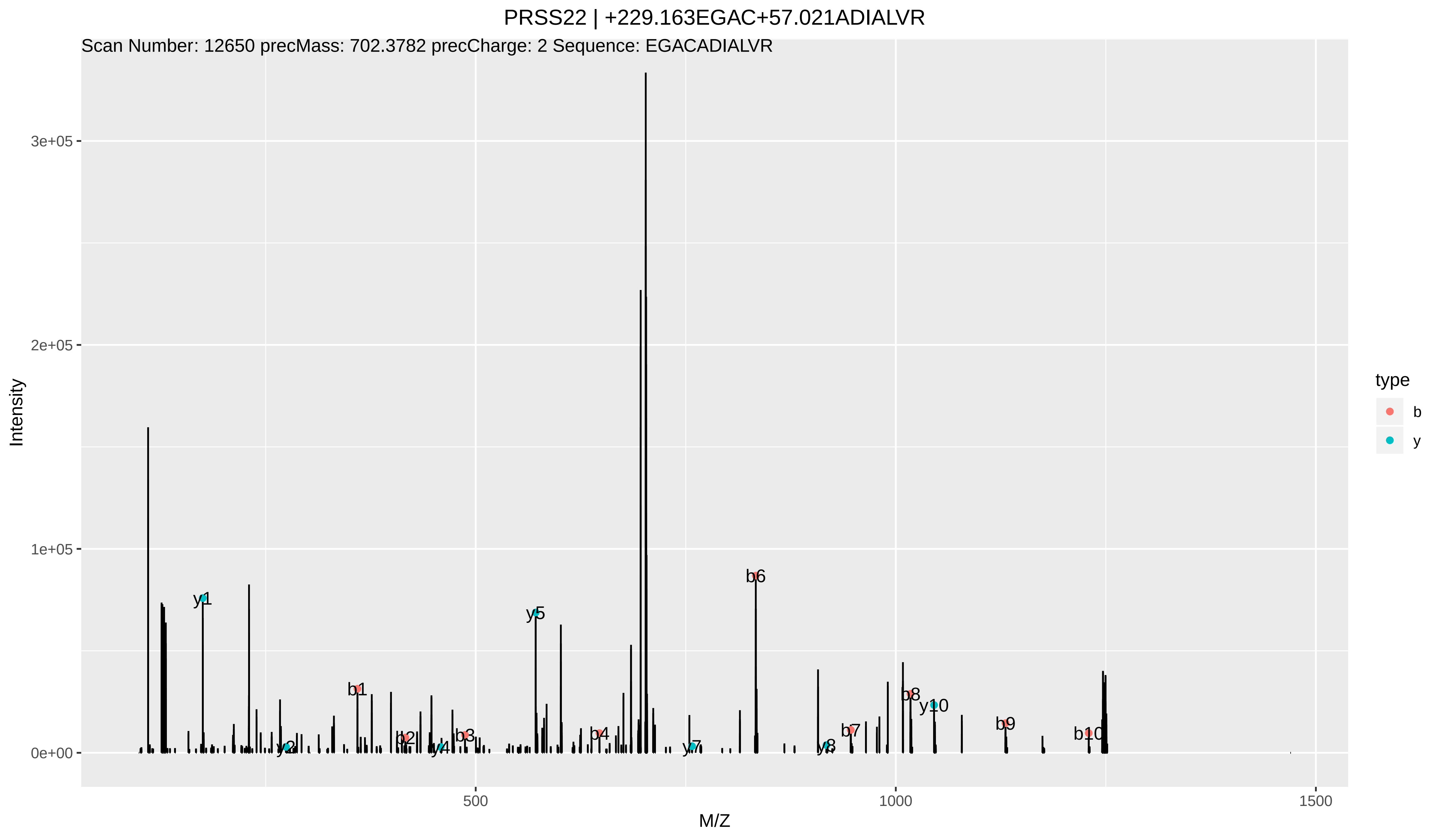
PRSS22 | +229.163EGAC+57.021ADIALVR
Scan Number: 12650 precMass: 702.3782 precCharge: 2 Sequence: EGACADIALVR
Intensity
3e+05
2e+05
1e+05
0e+00
M/Z
500
1000
1500
y1
y2
y4
y5
y7
y8
y10
b1
b2
b3
b4
b6
b7
b8
b9
b10
type
b
y

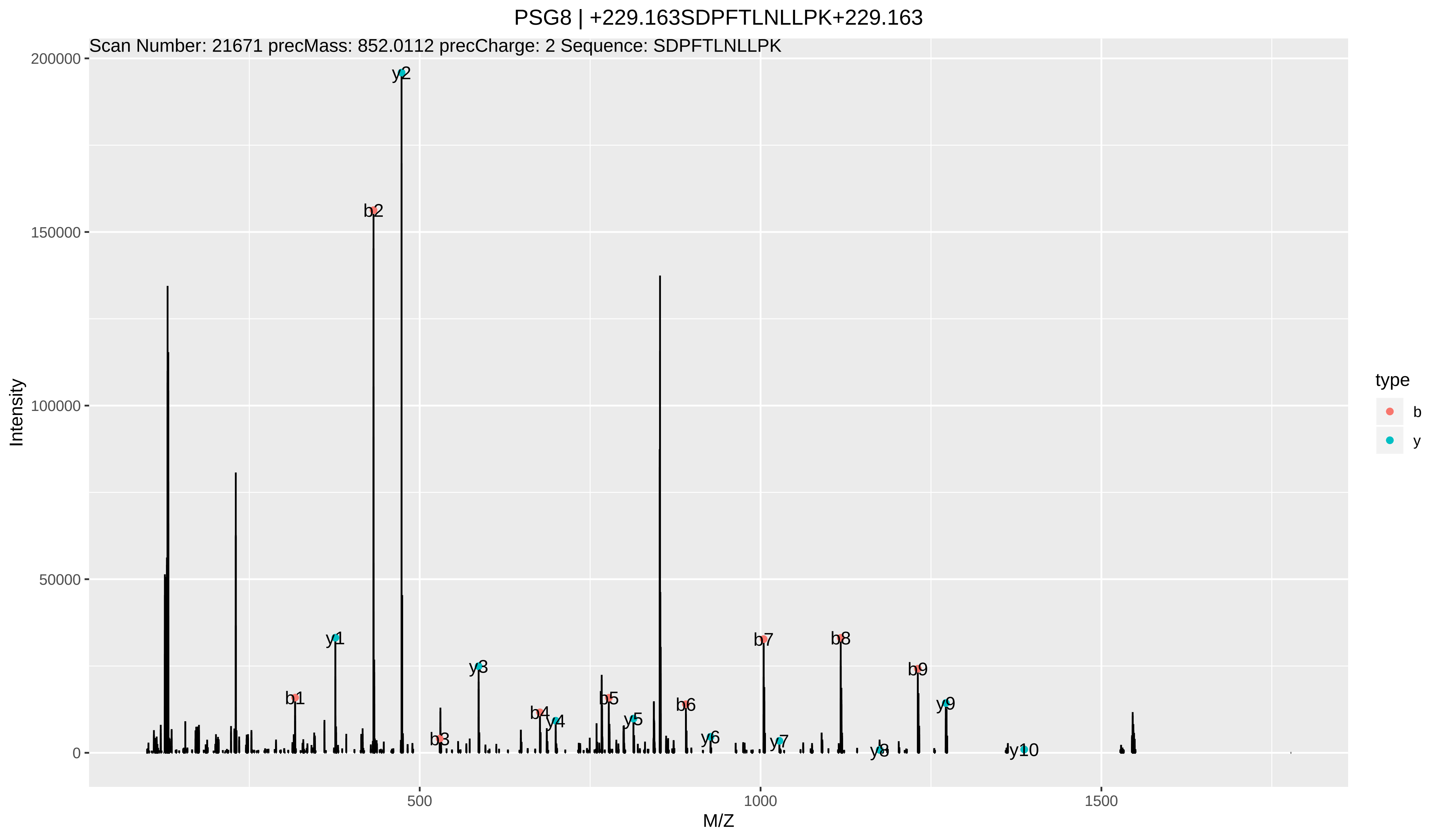
PSG8 | +229.163SDPFTLNLLPK+229.163
Scan Number: 21671 precMass: 852.0112 precCharge: 2 Sequence: SDPFTLNLLPK
Intensity
M/Z
type
b
y
y2
b2
y1
b1
y3
b3
b4
y4
b5
y5
b6
y6
b7
y7
b8
y8
b9
y9
y10
200000
150000
100000
50000
0
500
1000
1500

PSG8 | +229.163IYPSFTYYR

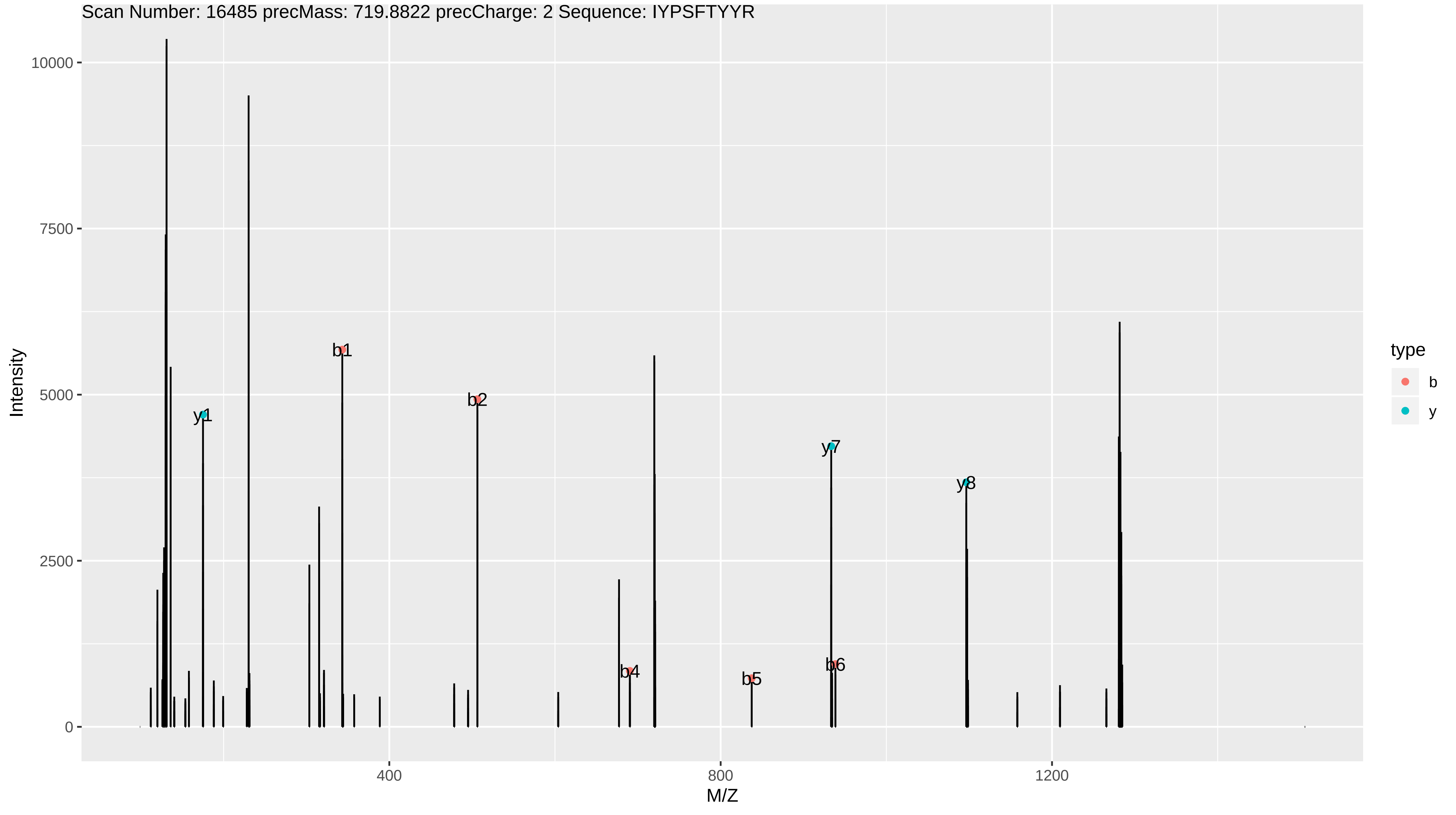

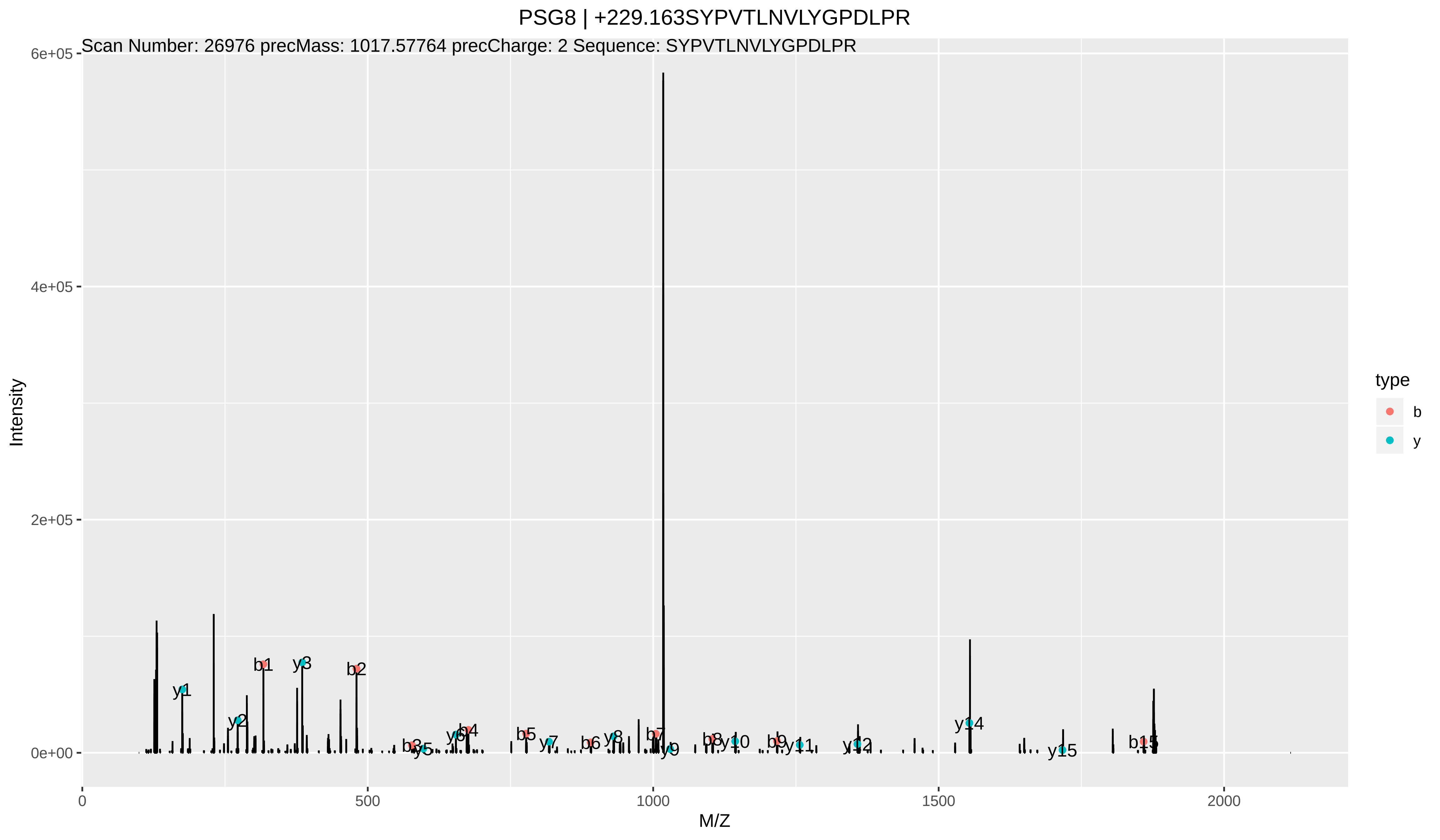

PSG8 | +229.163SYPVTLNVLYGPDLPR
Scan Number: 26976 precMass: 1017.57764 precCharge: 2 Sequence: SYPVTLNVLYGPDLPR
Intensity
M/Z
6e+05
4e+05
2e+05
0e+00
0
500
1000
1500
2000
type
b
y
b1
b2
b3
b4
b5
b6
b7
b8
b9
b15
y1
y2
y3
y5
y6
y7
y8
y9
y10
y11
y12
y14
y15

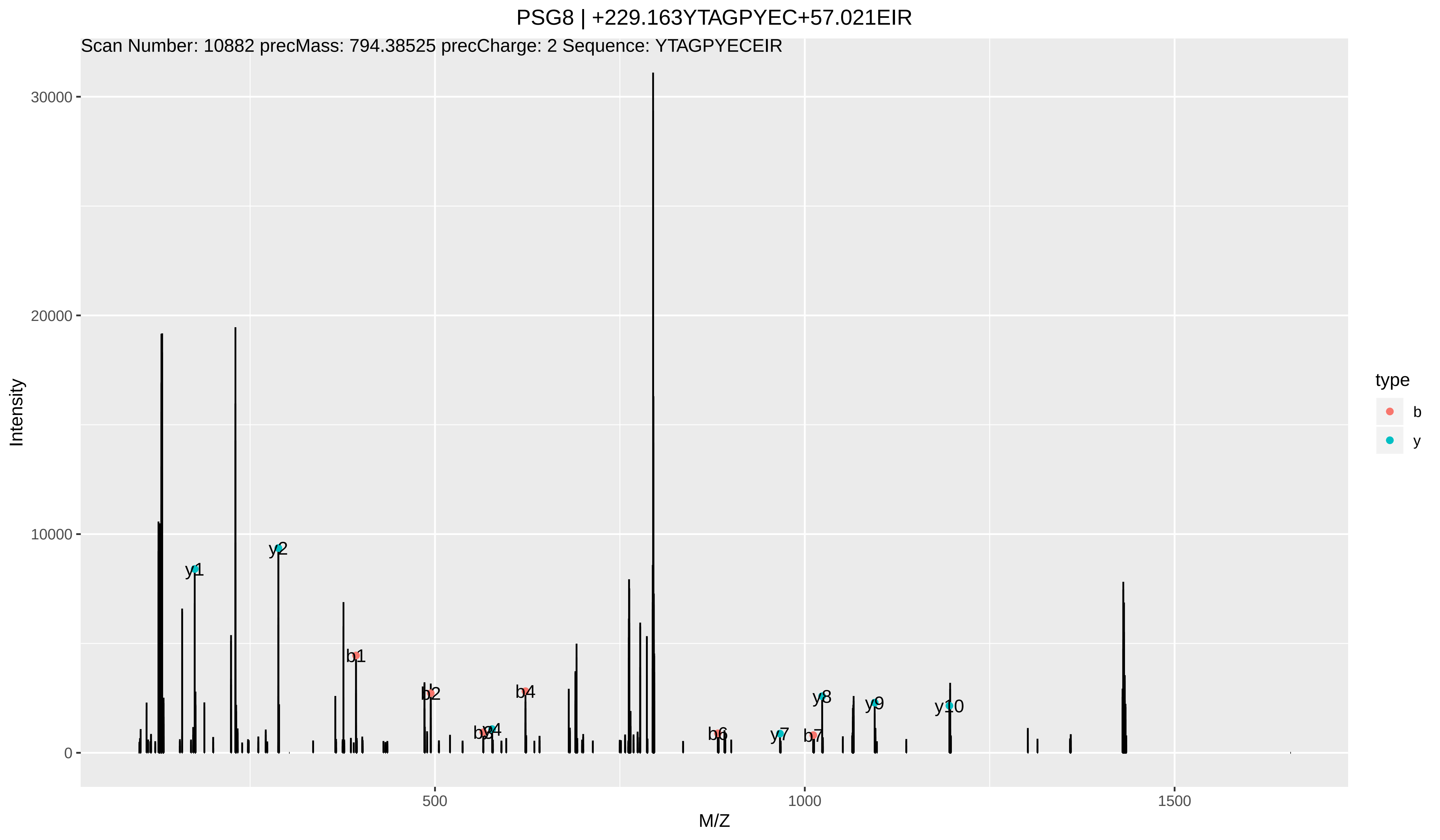
PSG8 | +229.163YTAGPYEC+57.021EIR
Scan Number: 10882 precMass: 794.38525 precCharge: 2 Sequence: YTAGPYECEIR
Intensity
M/Z
30000
20000
10000
0
500
1000
1500
y1
y2
b1
b2
b3
y4
b4
b6
y7
b7
y8
y9
y10
type
b
y

PTPRU | +229.163SFTDQSTLQEDER

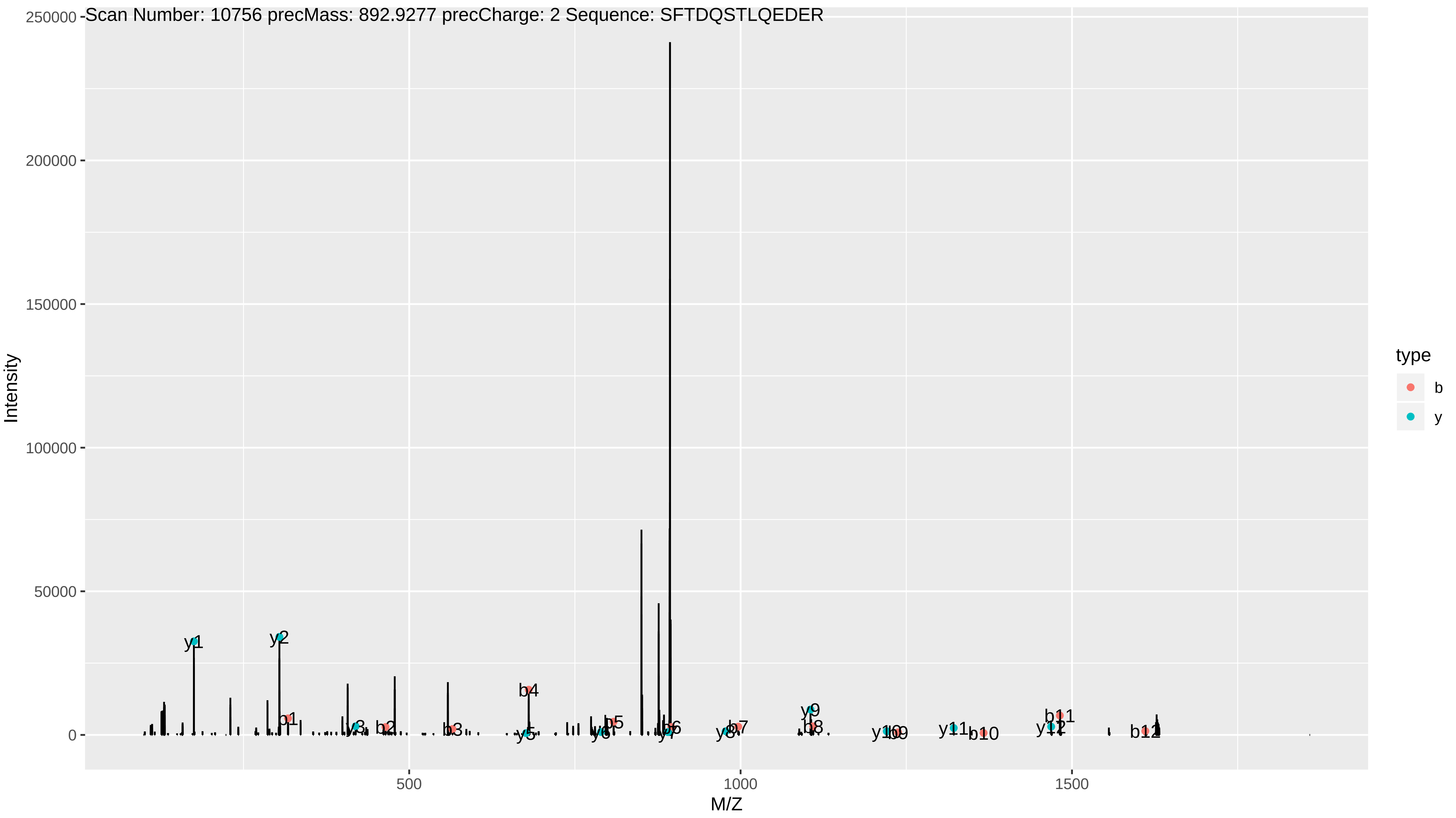

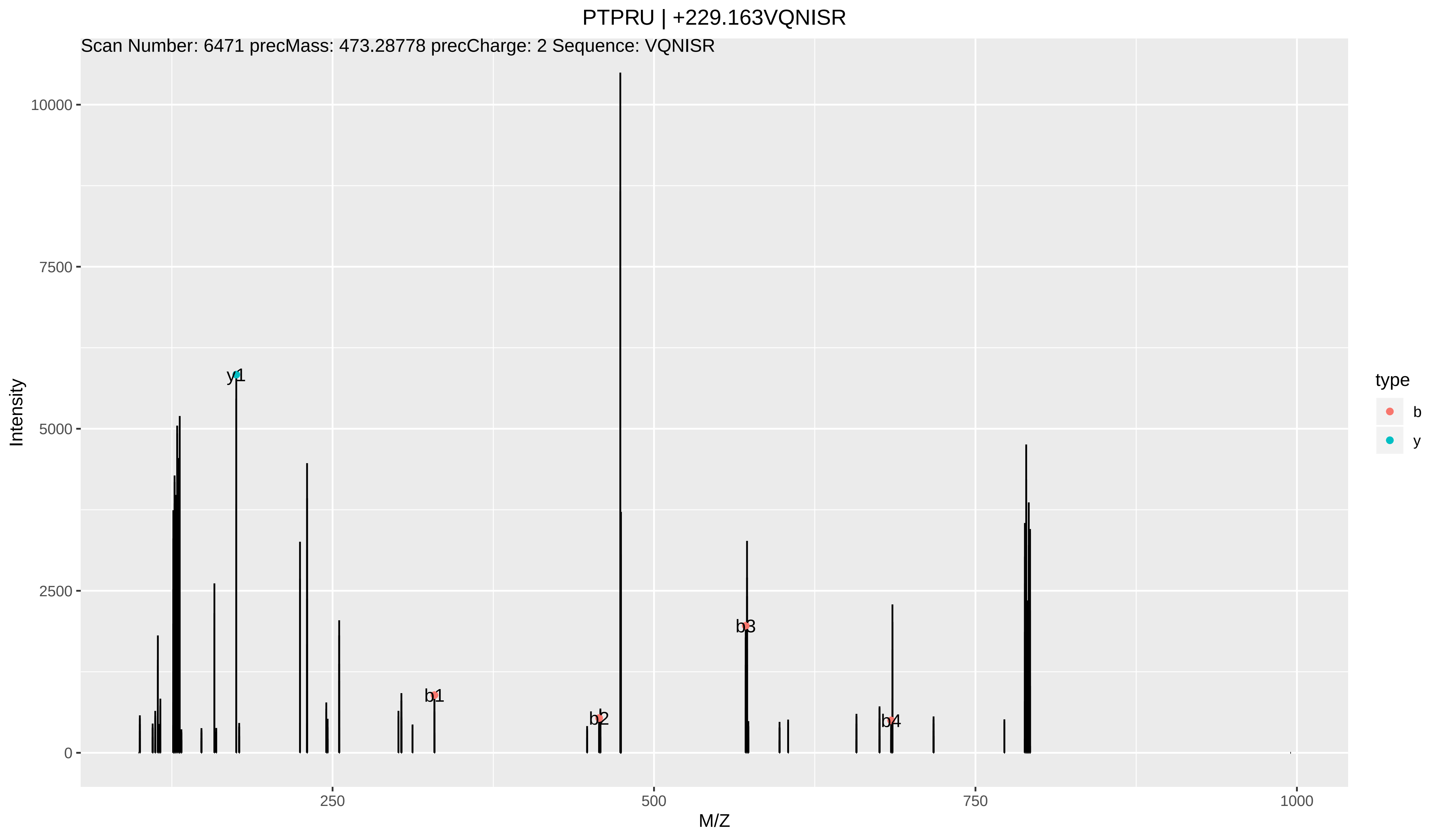
PTPRU | +229.163VQNISR
Scan Number: 6471 precMass: 473.28778 precCharge: 2 Sequence: VQNISR
Intensity
10000
7500
5000
2500
0
250
500
750
1000
M/Z
y1
b1
b2
b3
b4
type
b
y

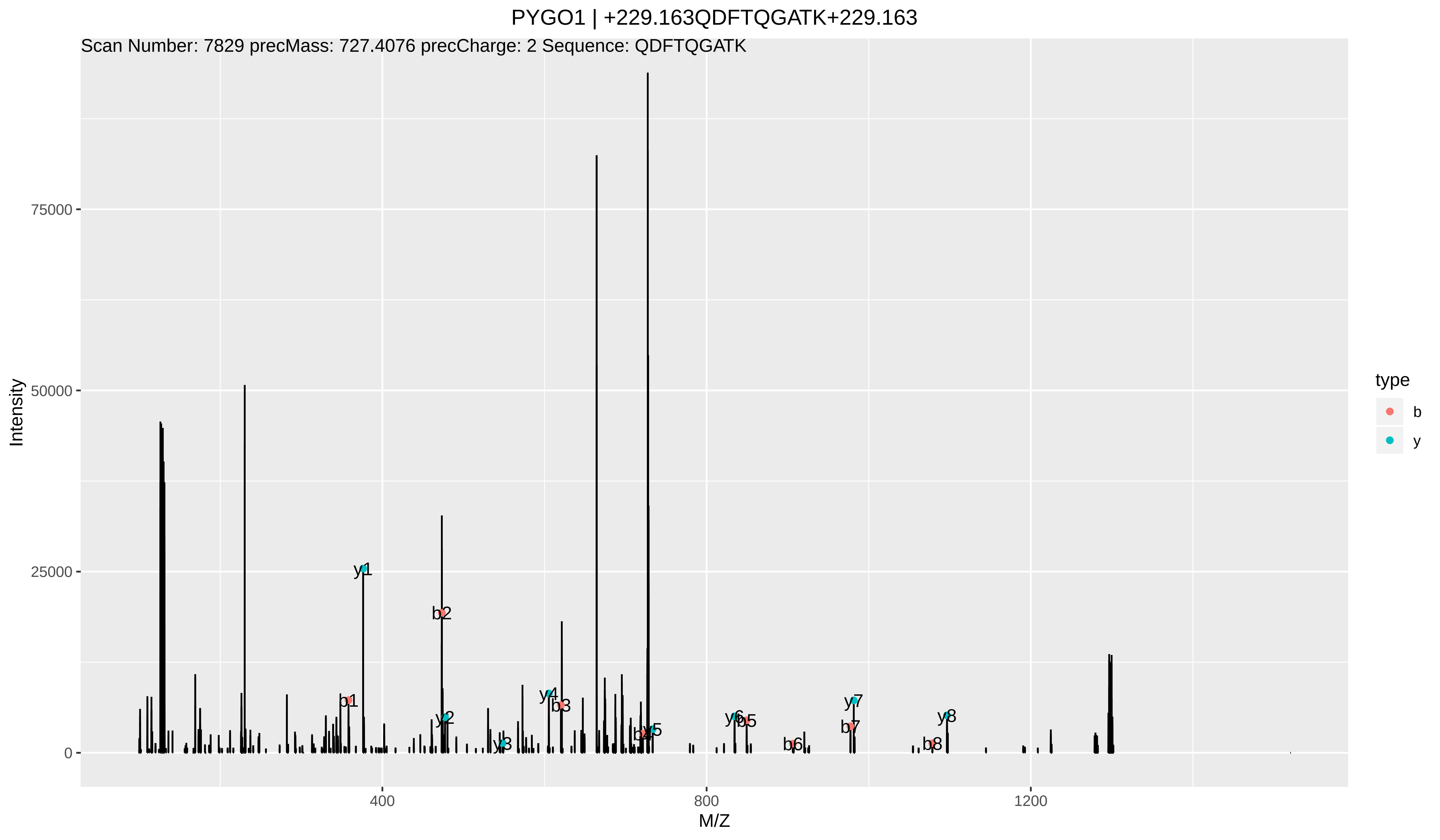

PYGO1 | +229.163QDFTQGATK+229.163
Scan Number: 7829 precMass: 727.4076 precCharge: 2 Sequence: QDFTQGATK
Intensity
75000
50000
25000
0
400
800
1200
M/Z
y1
b2
b1
y2
y3
y4
b3
b4
y5
y6
b5
b6
y7
b7
b8
y8
type
b
y

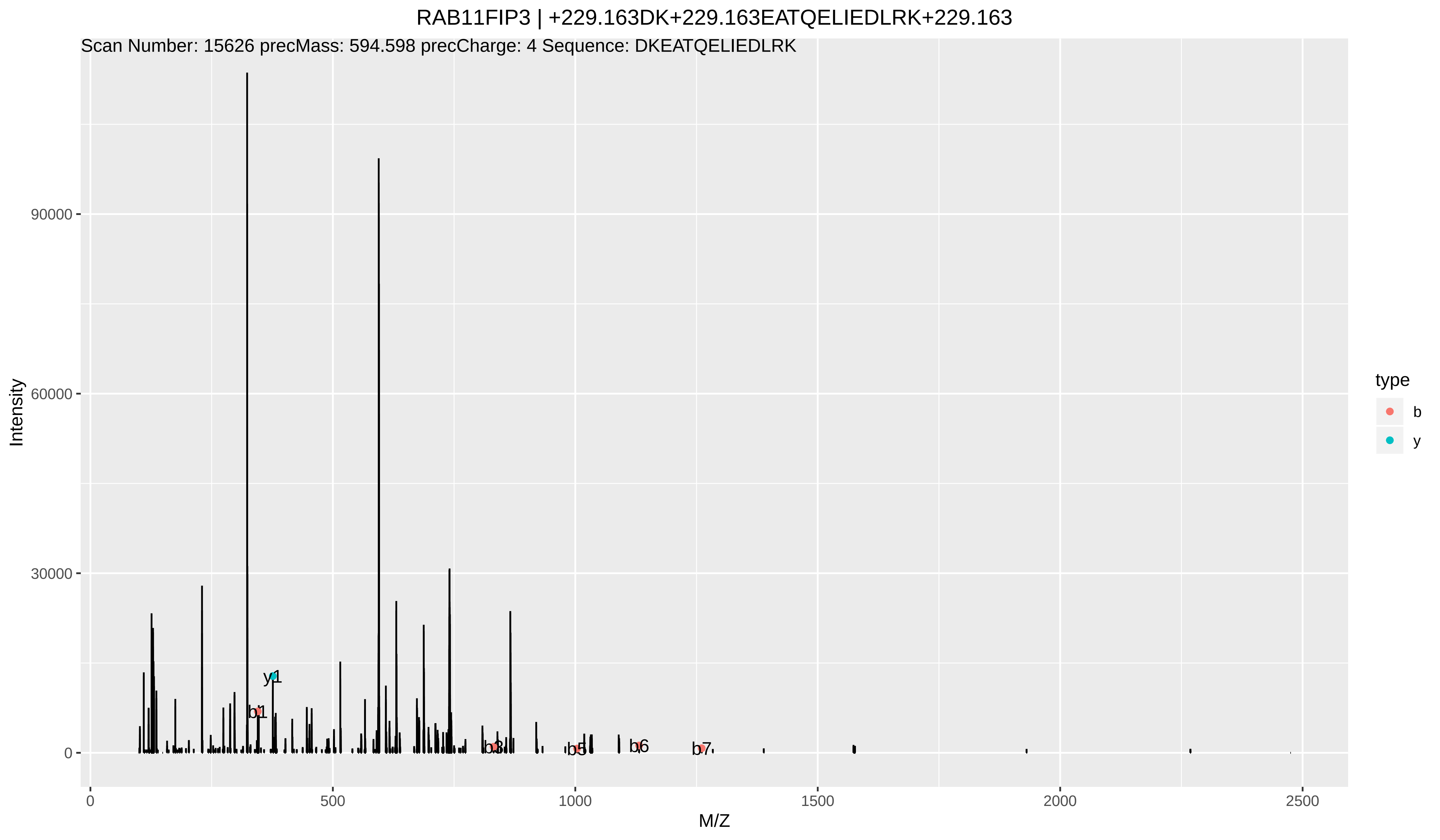

RAB11FIP3 | +229.163DK+229.163EATQELIEDLRK+229.163
Scan Number: 15626 precMass: 594.598 precCharge: 4 Sequence: DKEATQELIEDLRK
Intensity
90000
60000
30000
0
M/Z
0
500
1000
1500
2000
2500
y1
b1
b3
b5
b6
b7
type
b
y

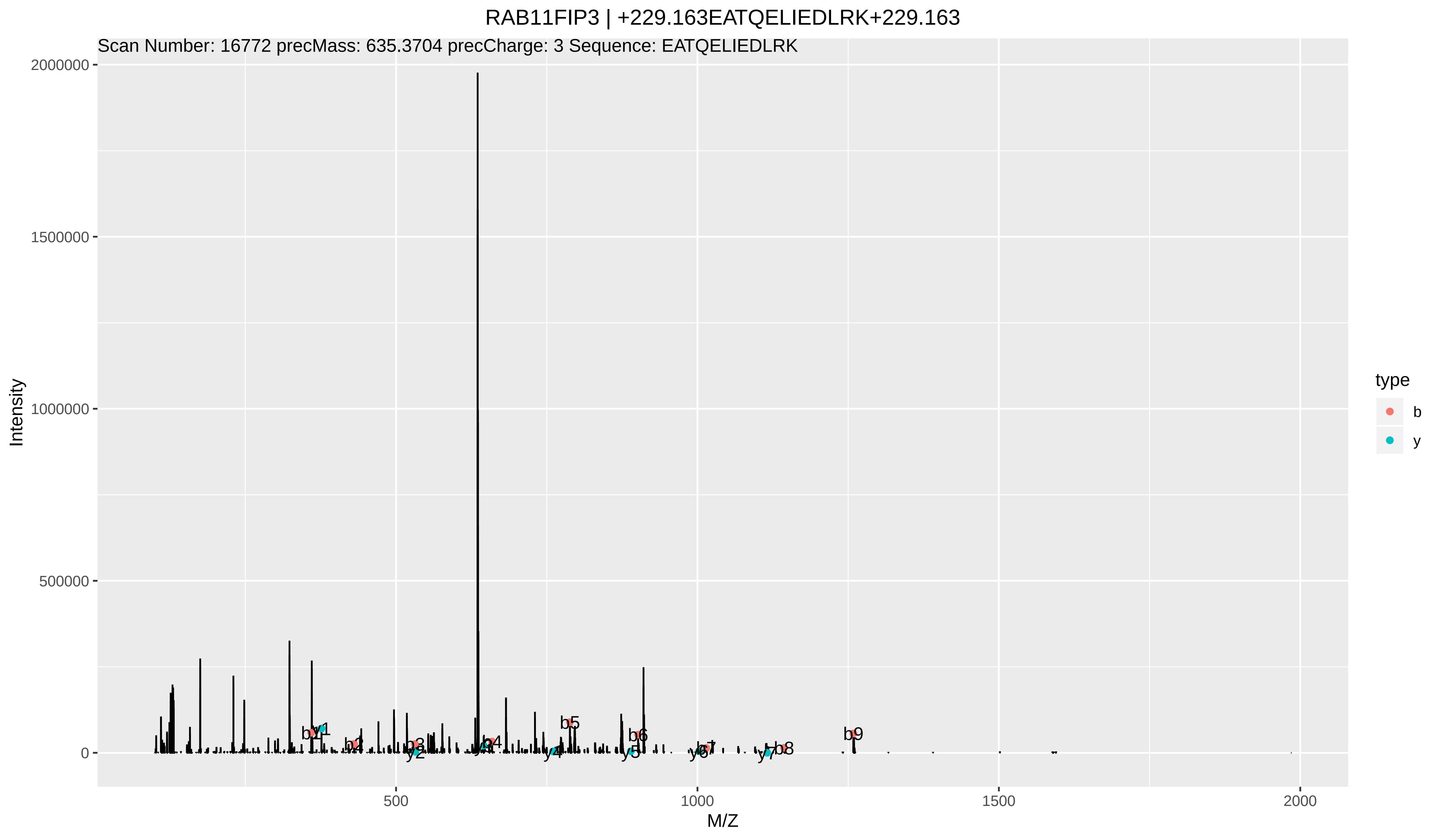

RAB11FIP3 | +229.163EATQELIEDLRK+229.163
Scan Number: 16772 precMass: 635.3704 precCharge: 3 Sequence: EATQELIEDLRK
Intensity
2000000
1500000
1000000
500000
0
500
1000
1500
2000
M/Z
type
b
y
b5
b6
b9

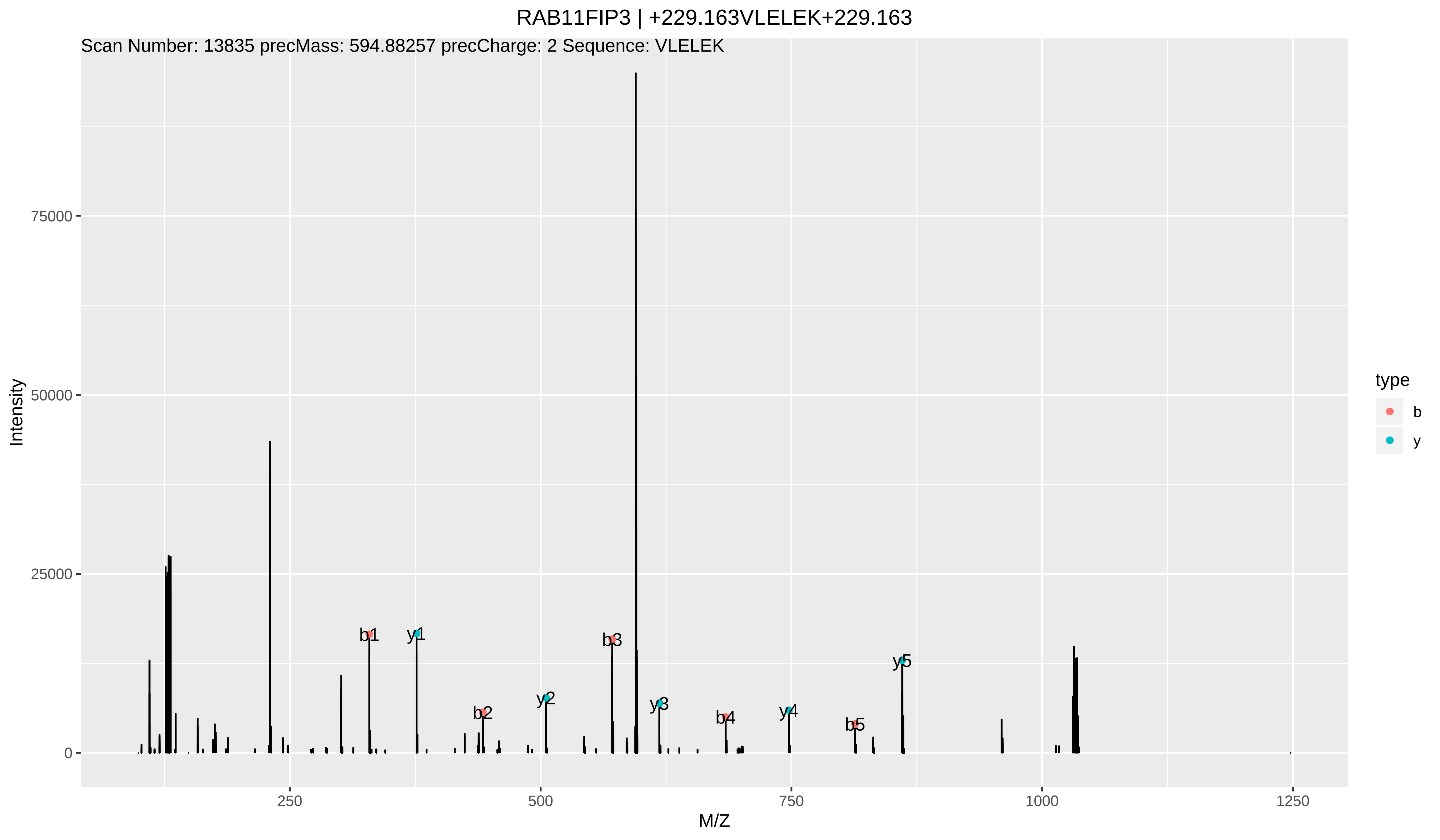

RAB11FIP3 | +229.163VLELEK+229.163
Scan Number: 13835 precMass: 594.88257 precCharge: 2 Sequence: VLELEK
Intensity
75000
50000
25000
0
M/Z
250
500
750
1000
1250
b1
y1
b2
y2
b3
y3
b4
y4
b5
y5
type
b
y

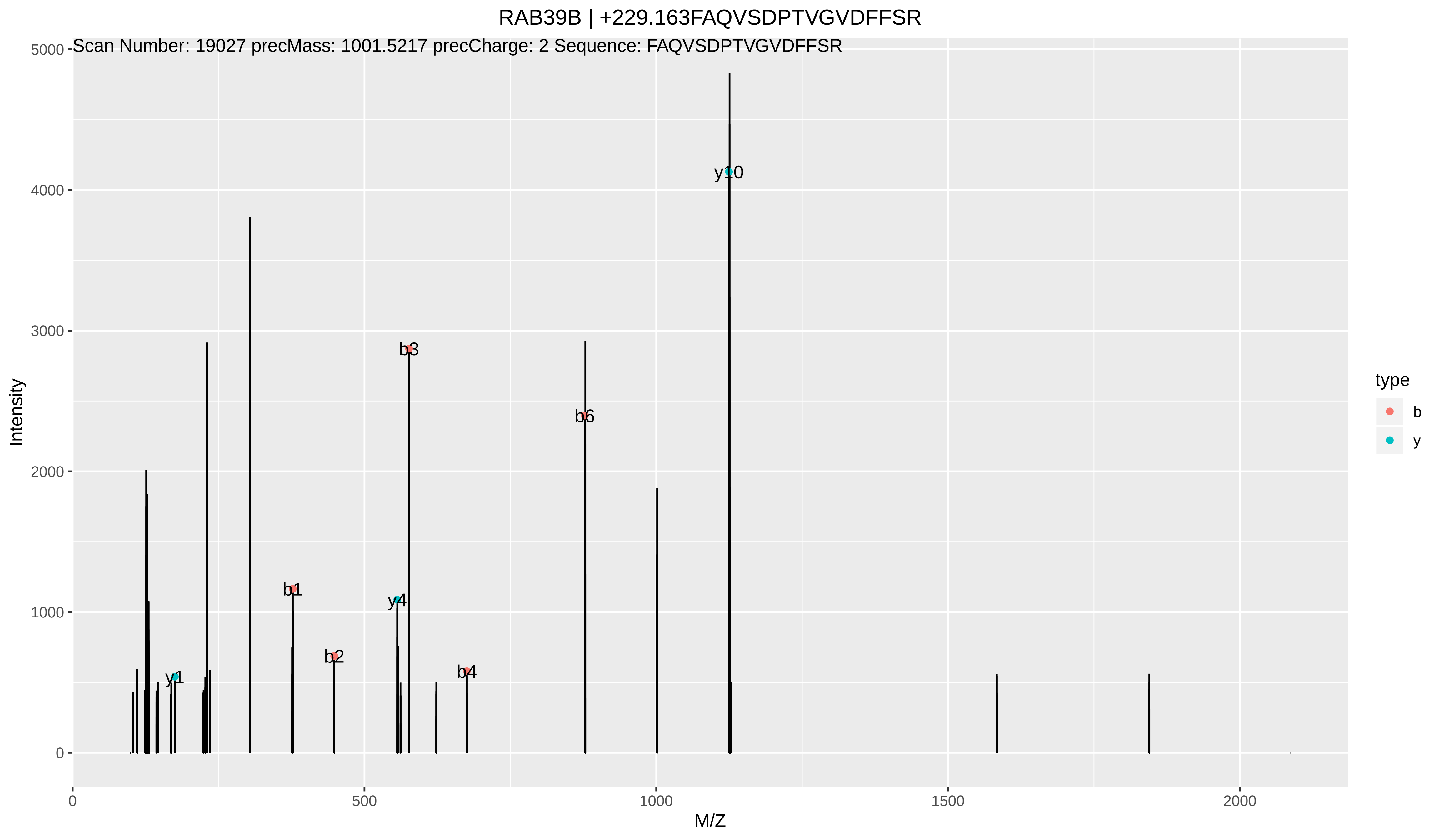

RAB39B | +229.163FAQVSDPTVGVDFFSR
Scan Number: 19027 precMass: 1001.5217 precCharge: 2 Sequence: FAQVSDPTVGVDFFSR
Intensity
0
1000
2000
3000
4000
5000
M/Z
0
500
1000
1500
2000
y10
b3
b6
b1
y4
b2
b4
y1
type
b
y

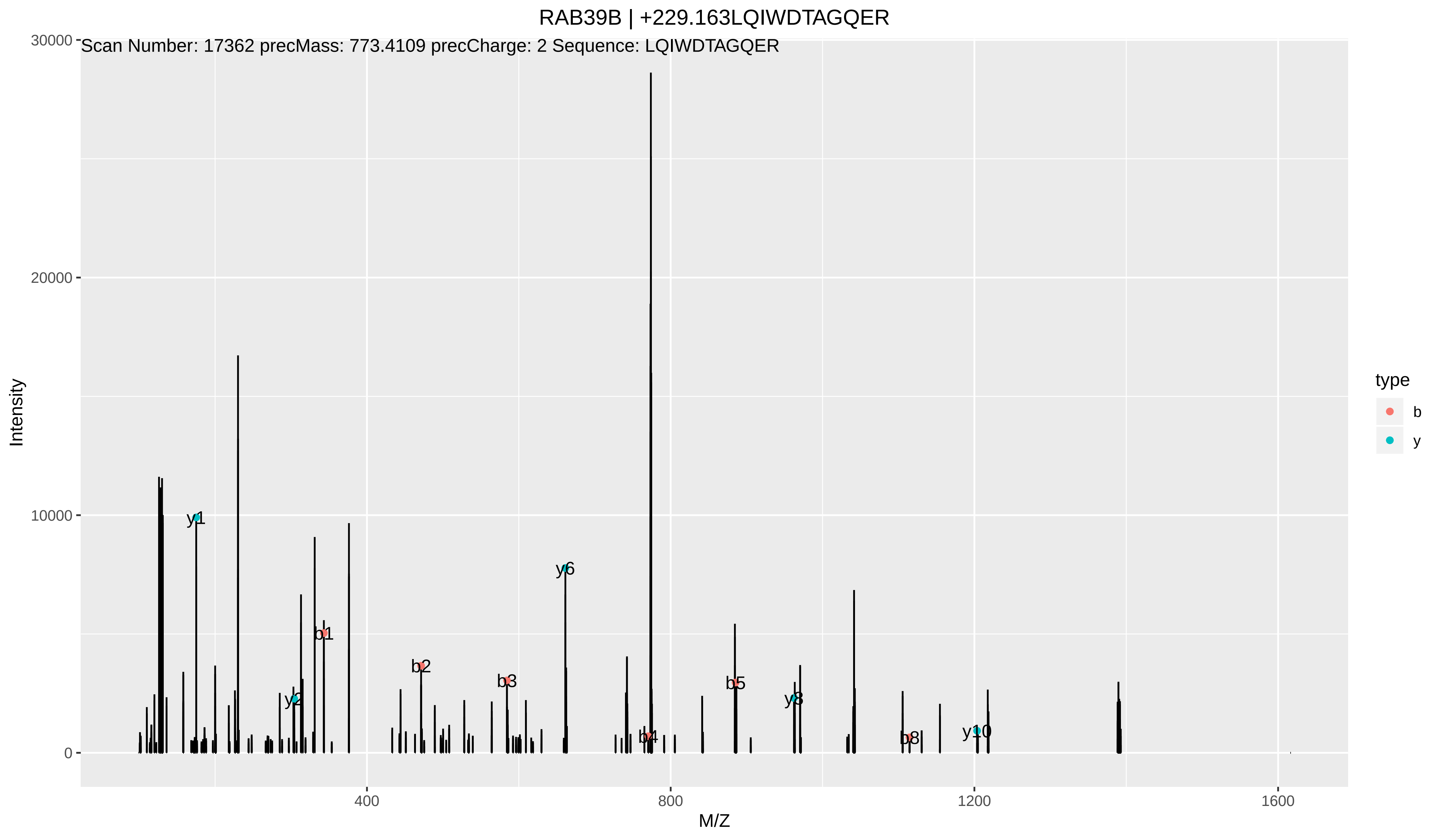

RAB39B | +229.163LQIWDTAGQER
Scan Number: 17362 precMass: 773.4109 precCharge: 2 Sequence: LQIWDTAGQER
Intensity
M/Z
30000
20000
10000
0
400
800
1200
1600
y1
y2
b1
b2
b3
y6
b4
b5
y8
b8
y10
type
b
y

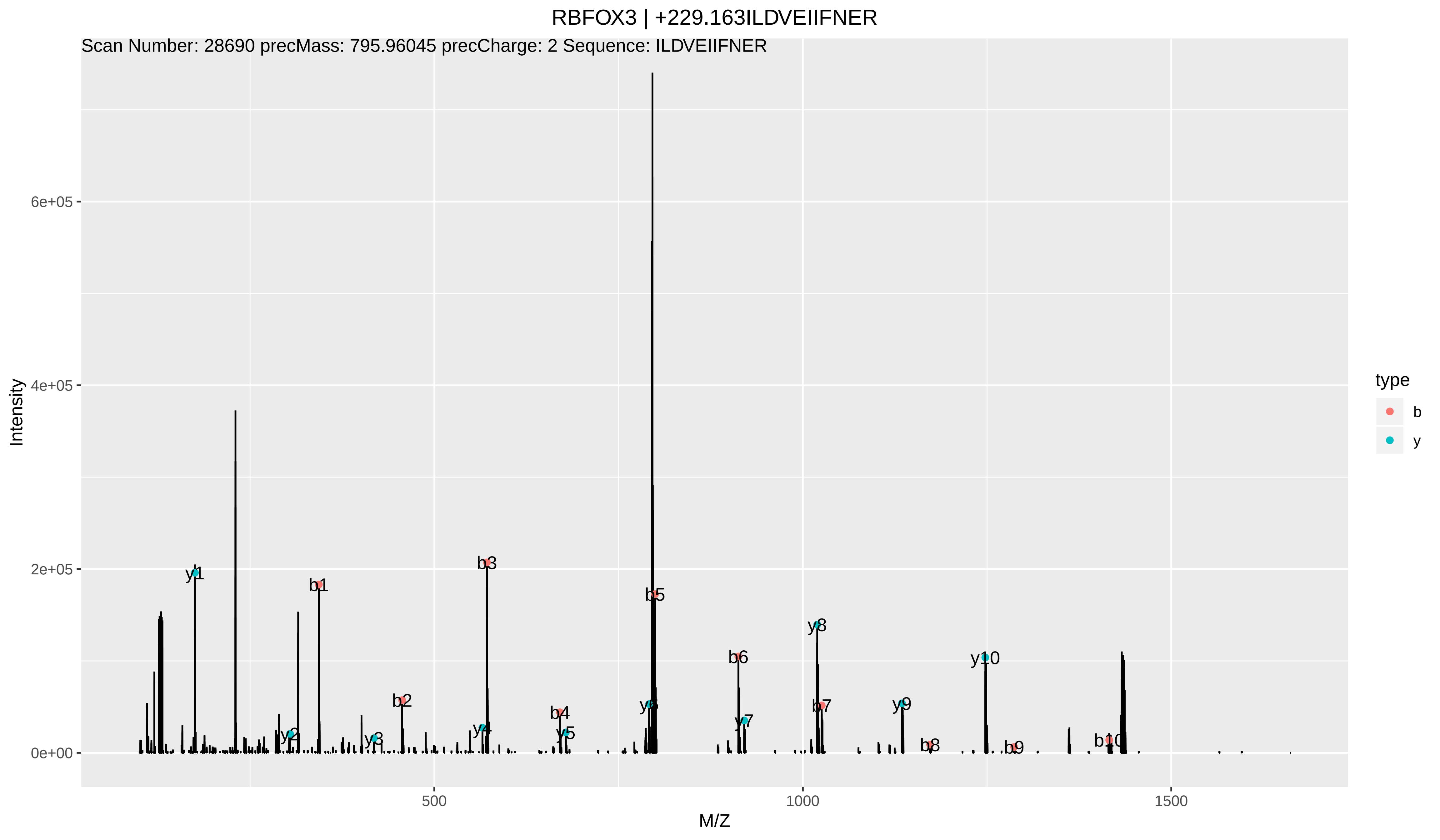
RBFOX3 | +229.163ILDVEIIFNER
Scan Number: 28690 precMass: 795.96045 precCharge: 2 Sequence: ILDVEIIFNER
Intensity
M/Z
6e+05
4e+05
2e+05
0e+00
500
1000
1500
y1
b1
b3
b5
y8
b6
y10
b2
b4
y6
b7
y9
y4
y5
y7
y2
y3
b8
b9
b10
type
b
y

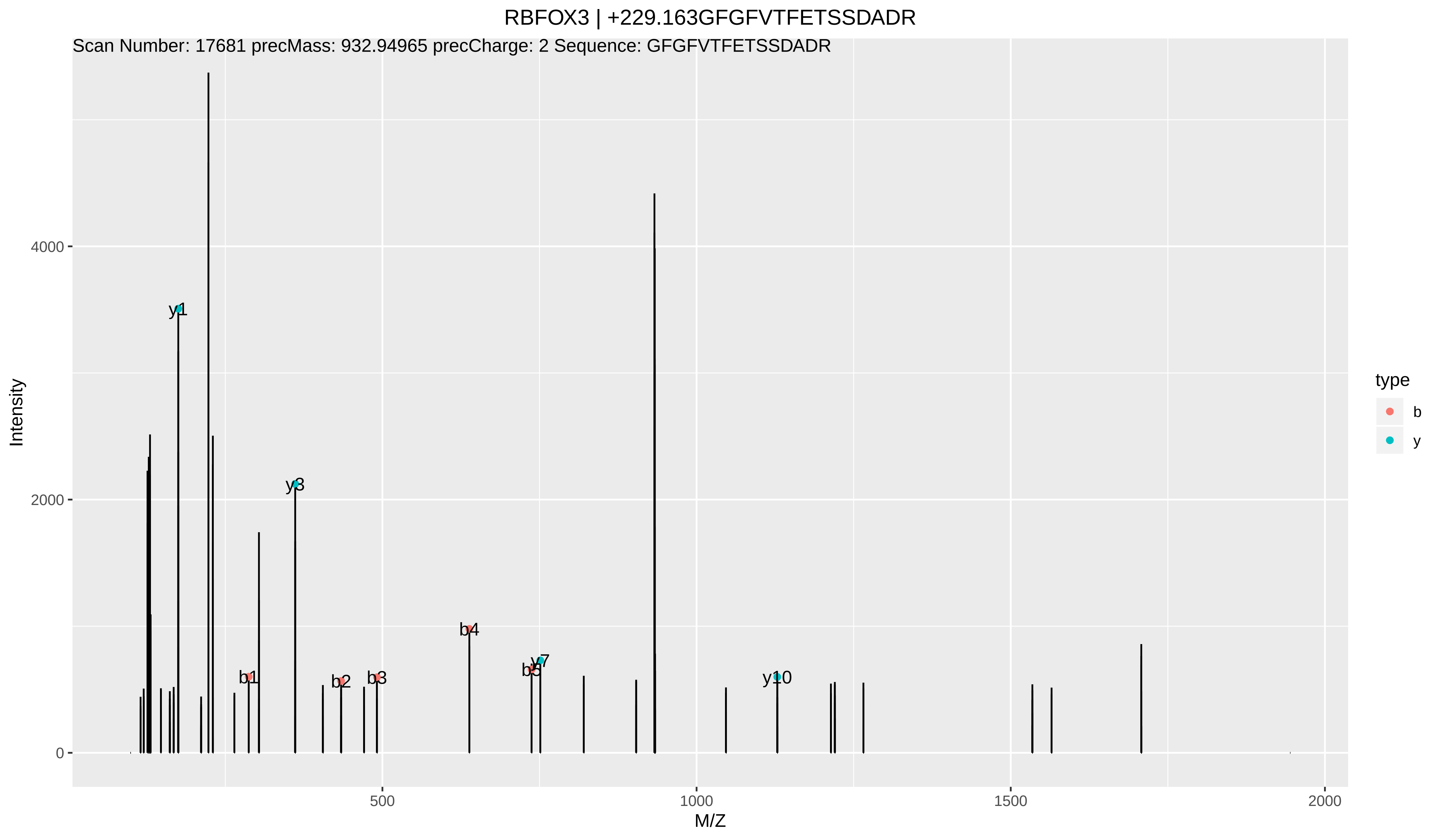

RBFOX3 | +229.163GFGFVTFETSSDADR
Scan Number: 17681 precMass: 932.94965 precCharge: 2 Sequence: GFGFVTFETSSDADR
Intensity
M/Z
0
2000
4000
500
1000
1500
2000
y1
y3
b1
b2
b3
b4
b5
y7
y10
type
b
y

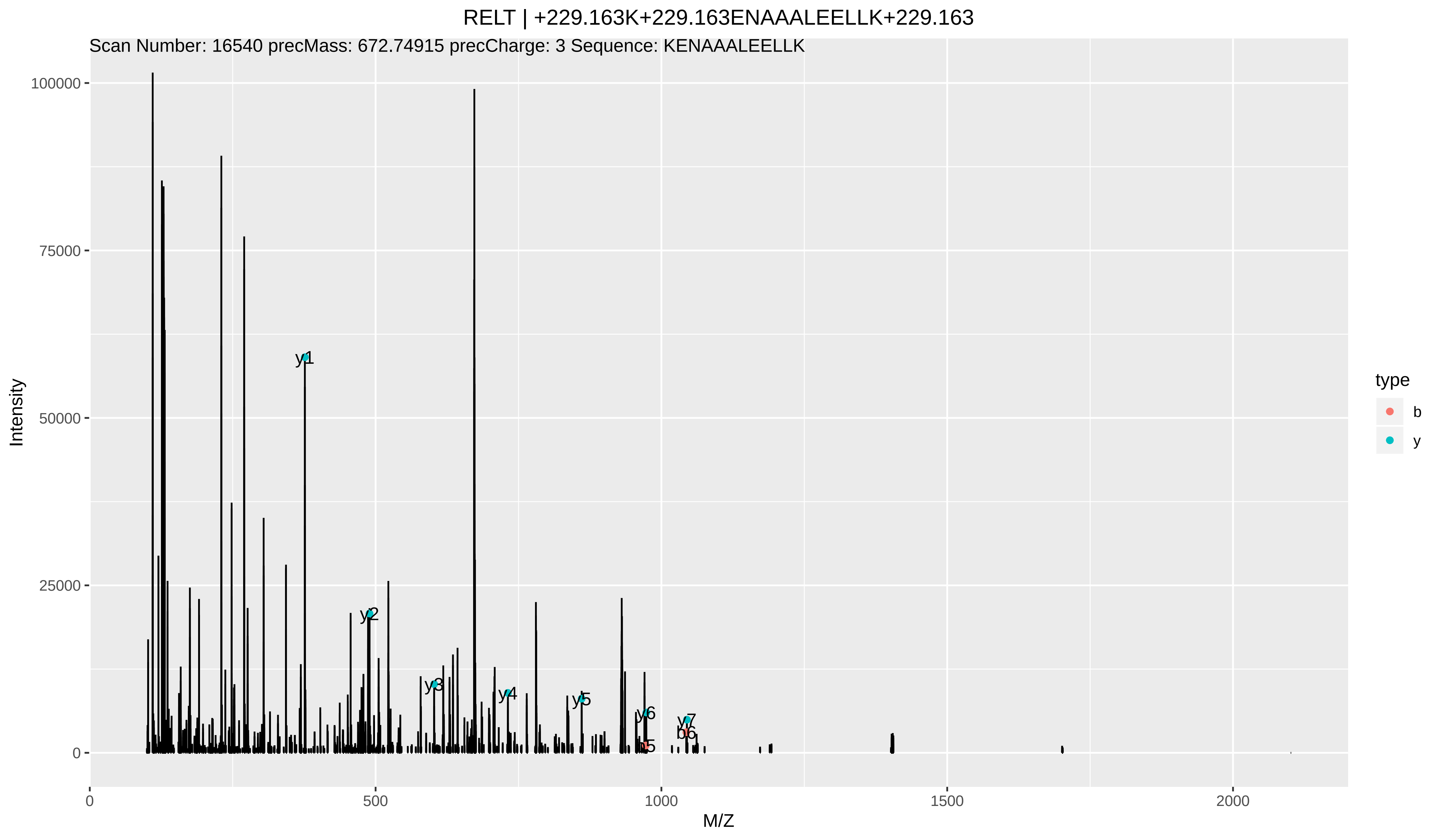

RELT | +229.163K+229.163ENAAALEELLK+229.163
Scan Number: 16540 precMass: 672.74915 precCharge: 3 Sequence: KENAAALEELLK
Intensity
100000
75000
50000
25000
0
0
500
1000
1500
2000
M/Z
y1
y2
y3
y4
y5
y6
y7
b6
b5
type
b
y

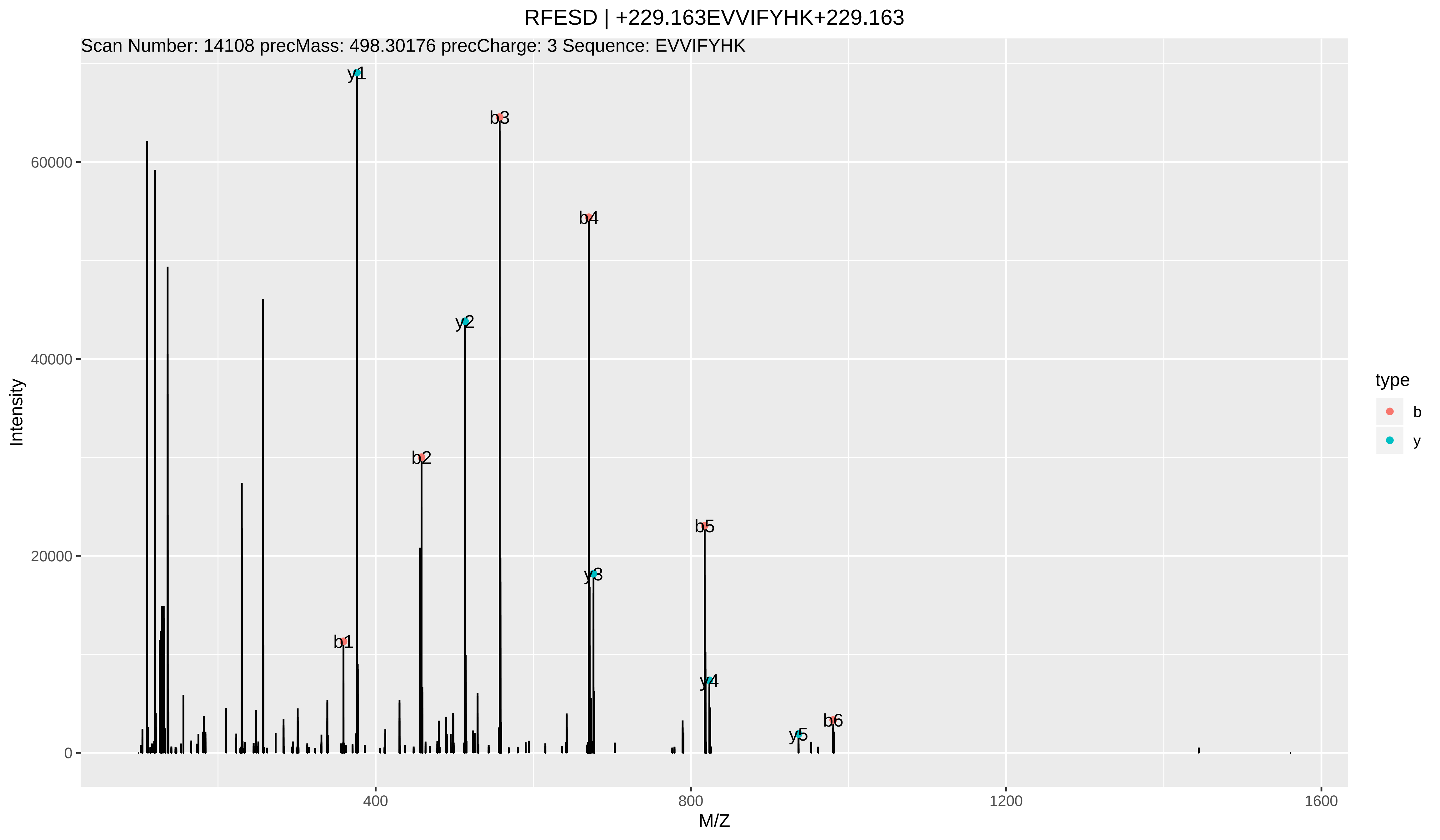

RFESD | +229.163EVVIFYHK+229.163
Scan Number: 14108 precMass: 498.30176 precCharge: 3 Sequence: EVVIFYHK
Intensity
M/Z
0
20000
40000
60000
400
800
1200
1600
y1
b3
b4
y2
b2
b5
y3
b1
y4
b6
y5
type
b
y

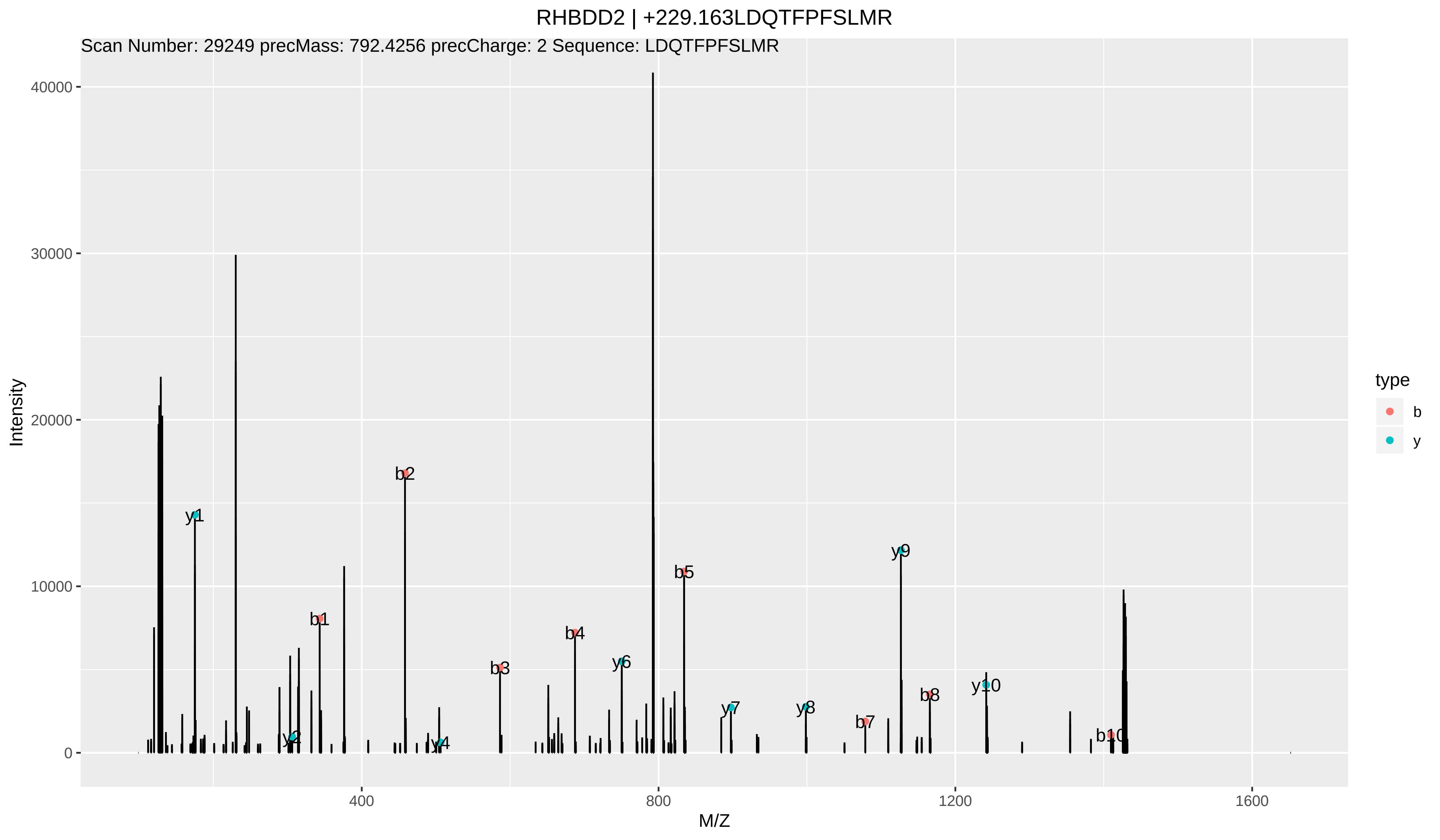

RHBDD2 | +229.163LDQTFPFSLMR
Scan Number: 29249 precMass: 792.4256 precCharge: 2 Sequence: LDQTFPFSLMR
Intensity
M/Z
0
10000
20000
30000
40000
400
800
1200
1600
y1
y2
b1
b2
y4
b3
b4
y6
b5
y7
y8
b7
y9
b8
y10
b10
type
b
y

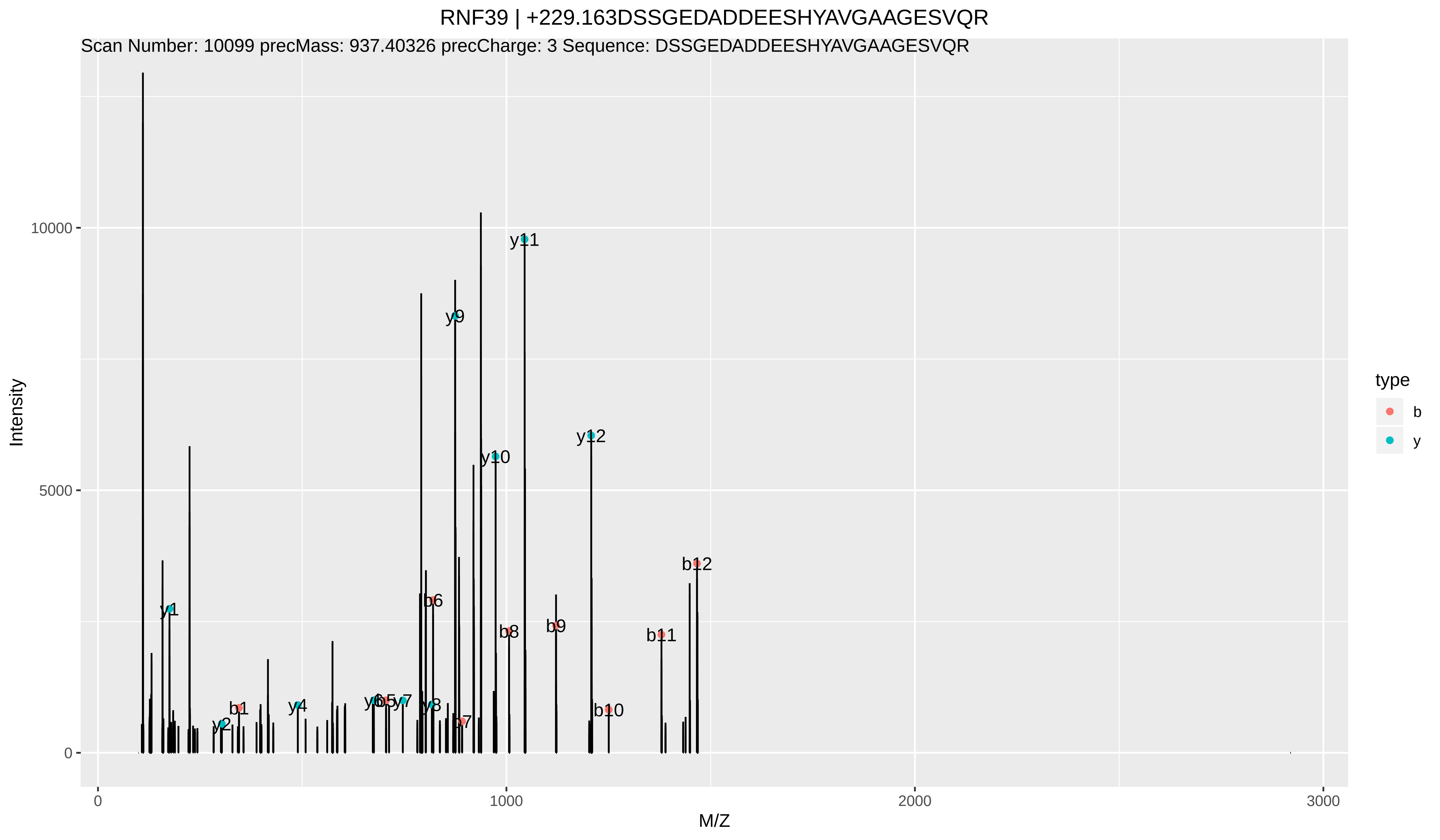

RNF39 | +229.163DSSGEDADDEESHYAVGAAGESVQR
Scan Number: 10099 precMass: 937.40326 precCharge: 3 Sequence: DSSGEDADDEESHYAVGAAGESVQR
Intensity
M/Z
type
b
y
y1
y2
b1
y4
y6
b5
y7
y8
b6
b7
y9
y10
y11
b8
b9
y12
b10
b11
b12

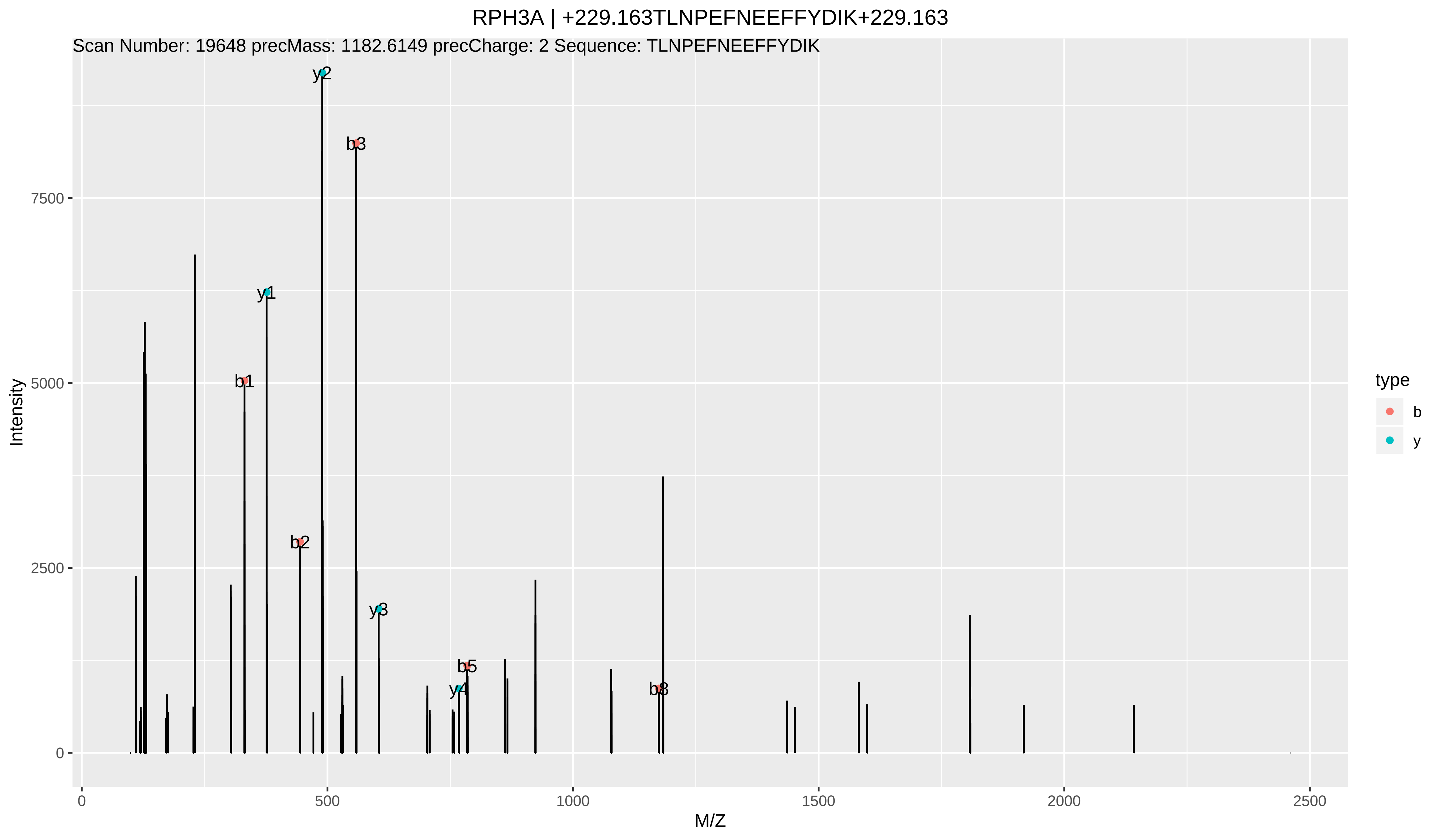

RPH3A | +229.163TLNPEFNEEFFYDIK+229.163
Scan Number: 19648 precMass: 1182.6149 precCharge: 2 Sequence: TLNPEFNEEFFYDIK
y2
b3
y1
b1
b2
y3
b5
y4
b8
Intensity
7500
5000
2500
0
0
500
1000
1500
2000
2500
M/Z
type
b
y

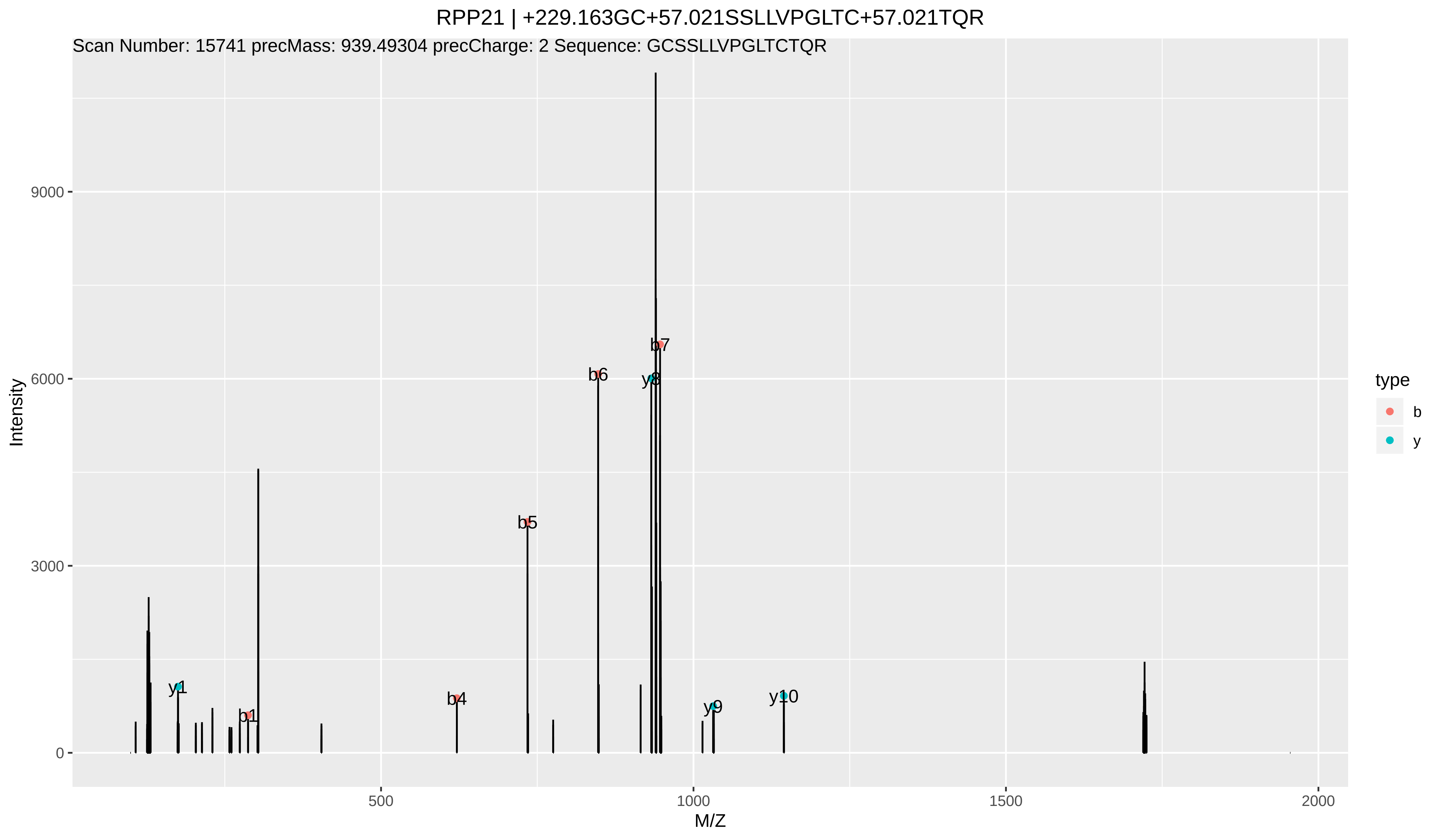

RPP21 | +229.163GC+57.021SSLLVPGLTC+57.021TQR
Scan Number: 15741 precMass: 939.49304 precCharge: 2 Sequence: GCSSLLVPGLTCTQR
Intensity
9000
6000
3000
0
500
1000
1500
2000
M/Z
y1
b1
b4
b5
b6
y8
b7
y9
y10
type
b
y

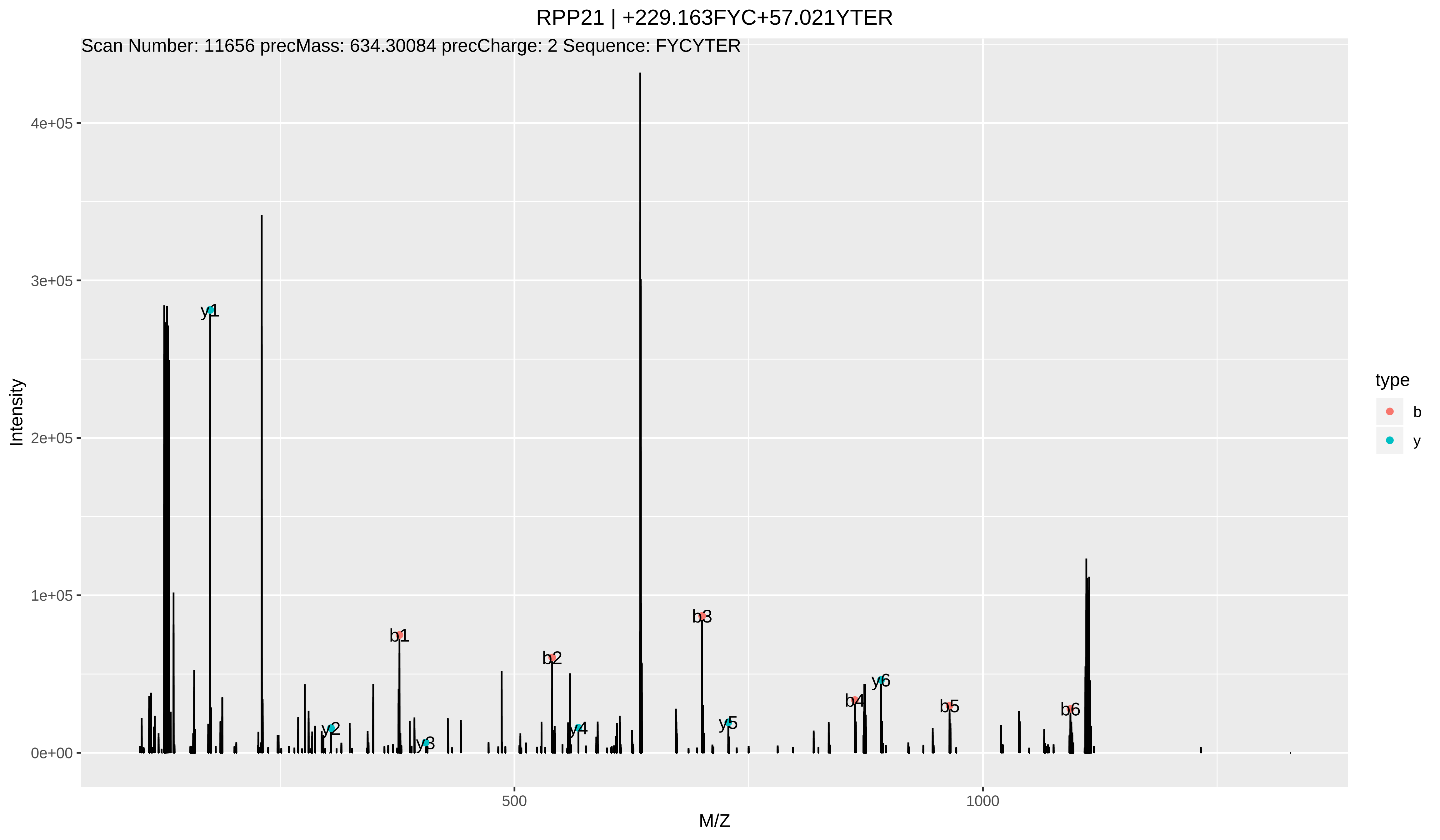

RPP21 | +229.163FYC+57.021YTER
Scan Number: 11656 precMass: 634.30084 precCharge: 2 Sequence: FYCYTER
Intensity
4e+05
3e+05
2e+05
1e+05
0e+00
500
1000
M/Z
y1
y2
y3
y4
y5
y6
b1
b2
b3
b4
b5
b6
type
b
y

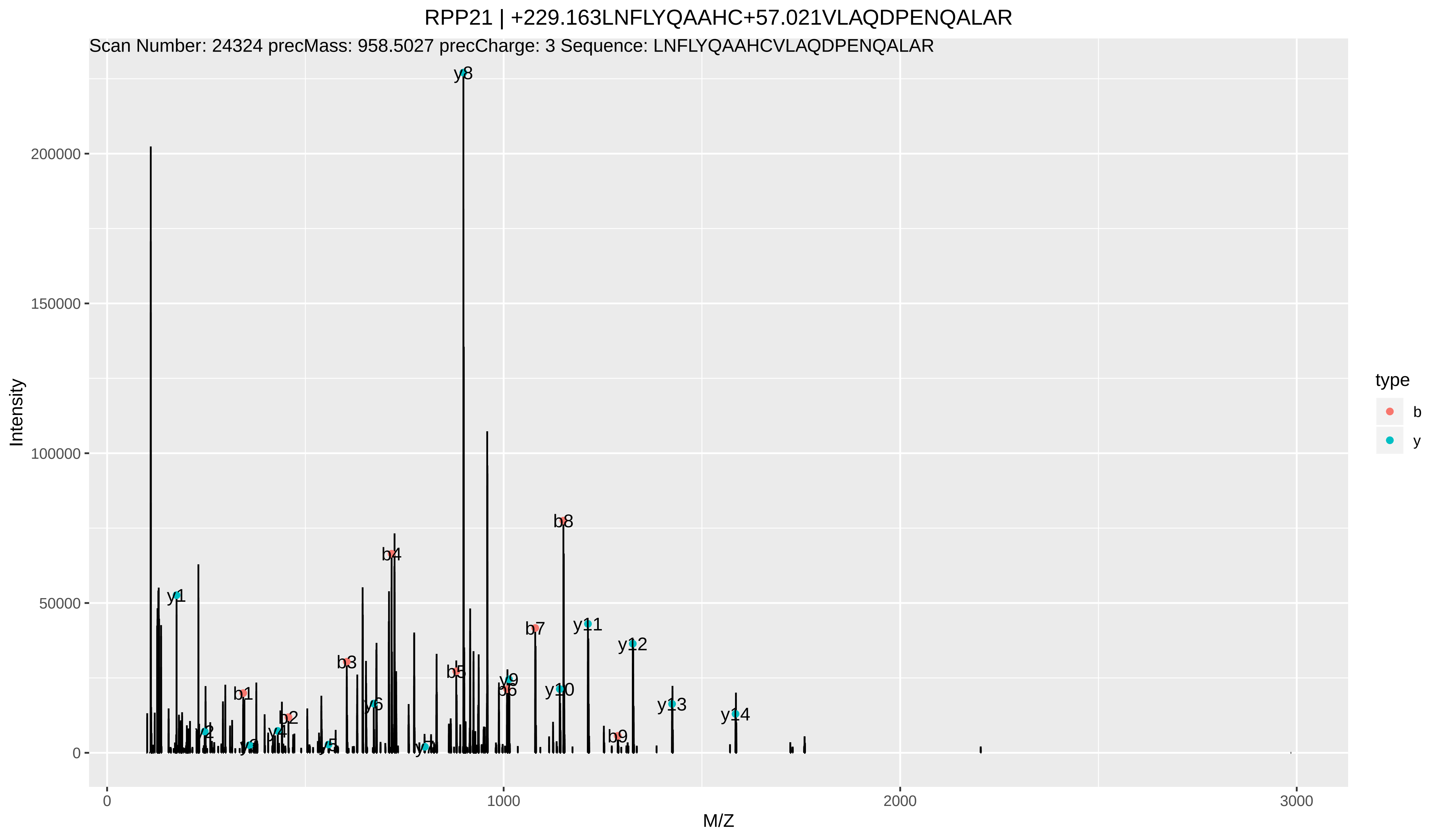

RPP21 | +229.163LNFLYQAAHC+57.021VLAQDPENQALAR
Scan Number: 24324 precMass: 958.5027 precCharge: 3 Sequence: LNFLYQAAHCVLAQDPENQALAR
Intensity
M/Z
type
b
y

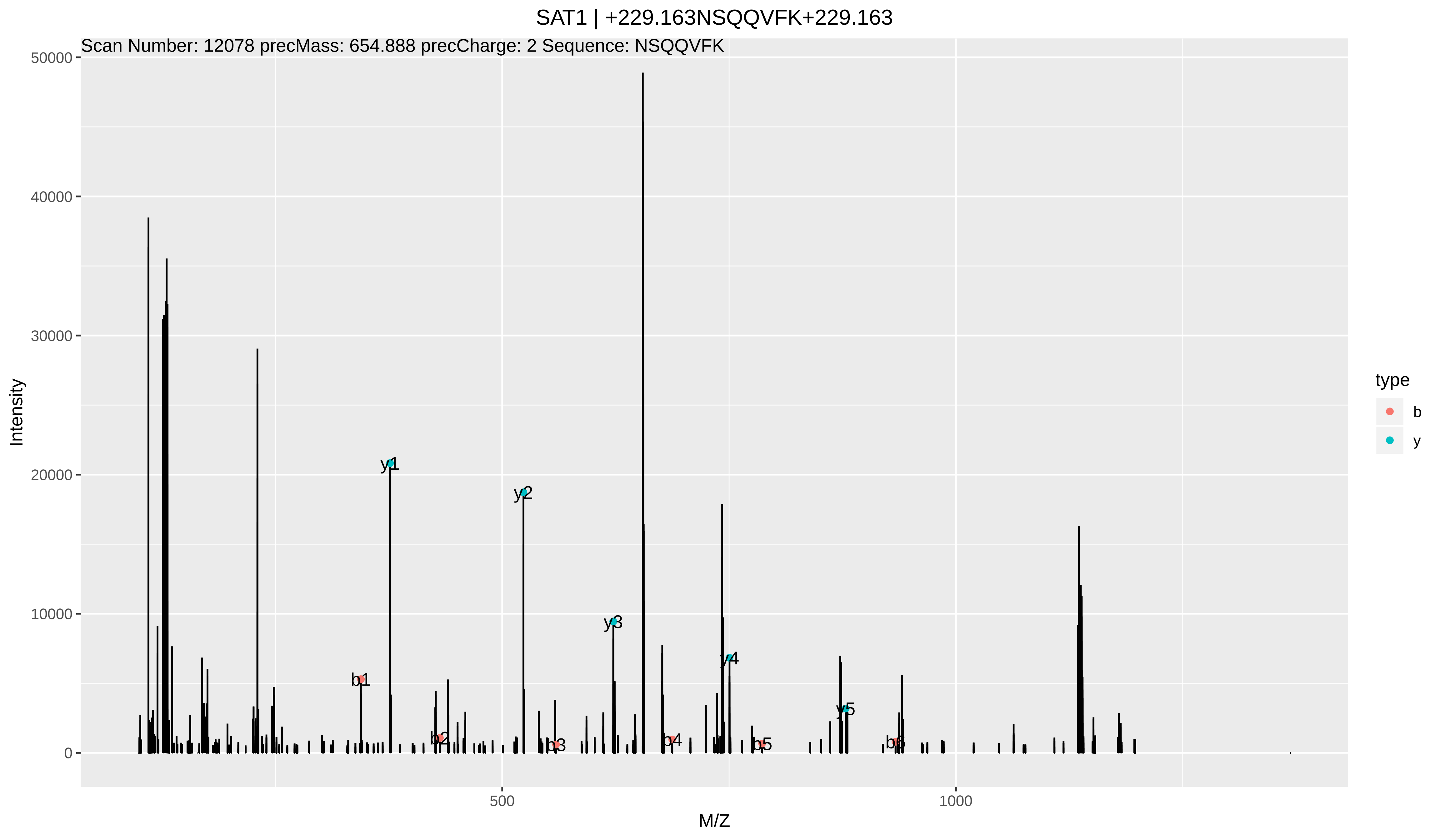

SAT1 | +229.163NSQQVFK+229.163
Scan Number: 12078 precMass: 654.888 precCharge: 2 Sequence: NSQQVFK
Intensity
50000
40000
30000
20000
10000
0
M/Z
500
1000
y1
y2
y3
y4
y5
b1
b2
b3
b4
b5
b6
type
b
y

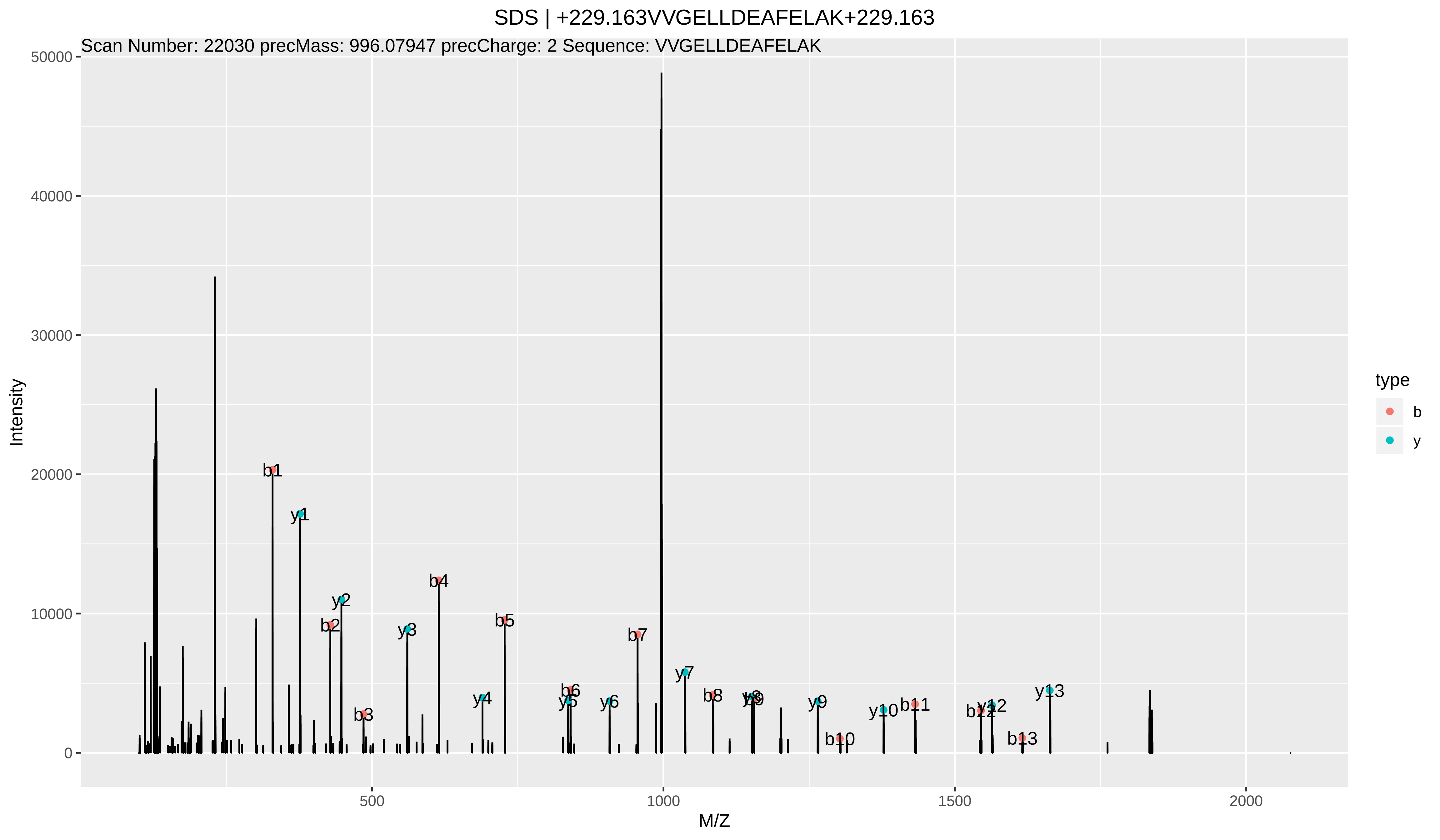

SDS | +229.163VVGELLDEAFELAK+229.163
Scan Number: 22030 precMass: 996.07947 precCharge: 2 Sequence: VVGELLDEAFELAK
Intensity
M/Z
type
b
y
b1
y1
b2
y2
b3
y3
b4
y4
b5
y5
b6
y6
b7
y7
b8
y9
b10
y10
b11
b12
y12
b13
y13
0
10000
20000
30000
40000
50000
500
1000
1500
2000

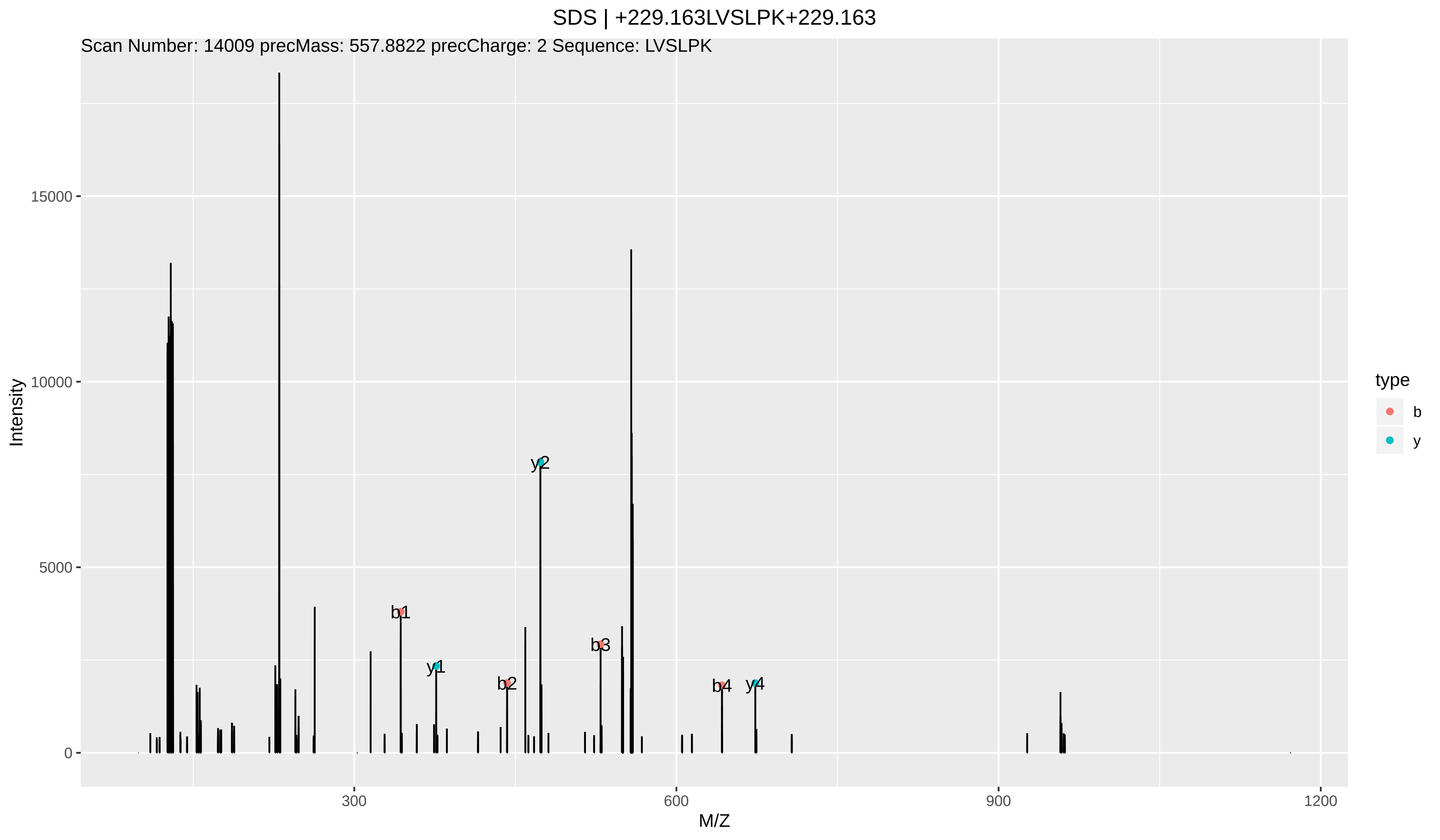

SDS | +229.163LVSLPK+229.163
Scan Number: 14009 precMass: 557.8822 precCharge: 2 Sequence: LVSLPK
Intensity
15000
10000
5000
0
M/Z
300
600
900
1200
b1
y1
b2
y2
b3
b4
y4
type
b
y

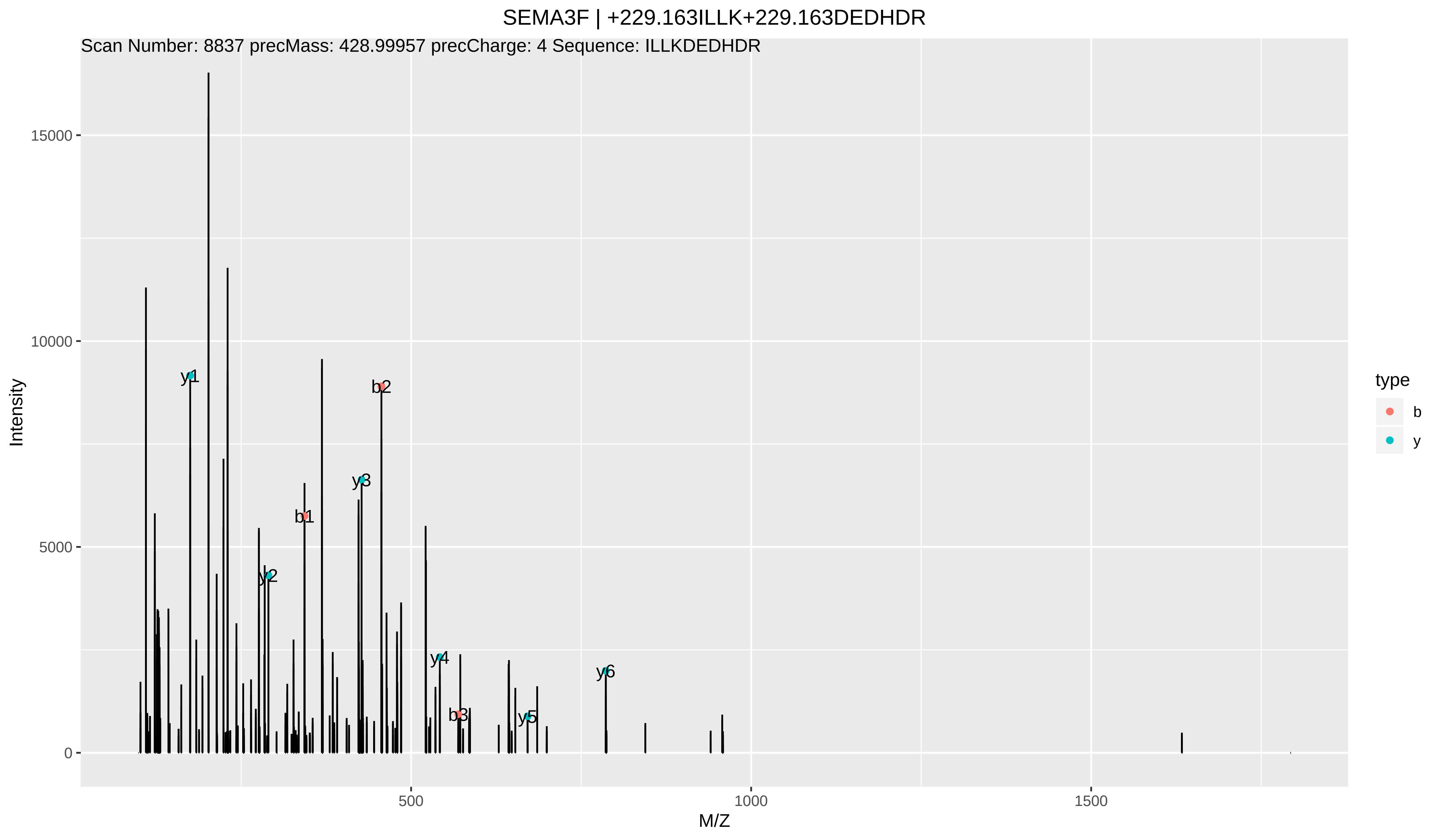
SEMA3F | +229.163ILLK+229.163DEDHDR
Scan Number: 8837 precMass: 428.99957 precCharge: 4 Sequence: ILLKDEDHDR
Intensity
M/Z
type
b
y
y1
b2
y3
b1
y2
y4
y6
b3
y5
15000
10000
5000
0
500
1000
1500

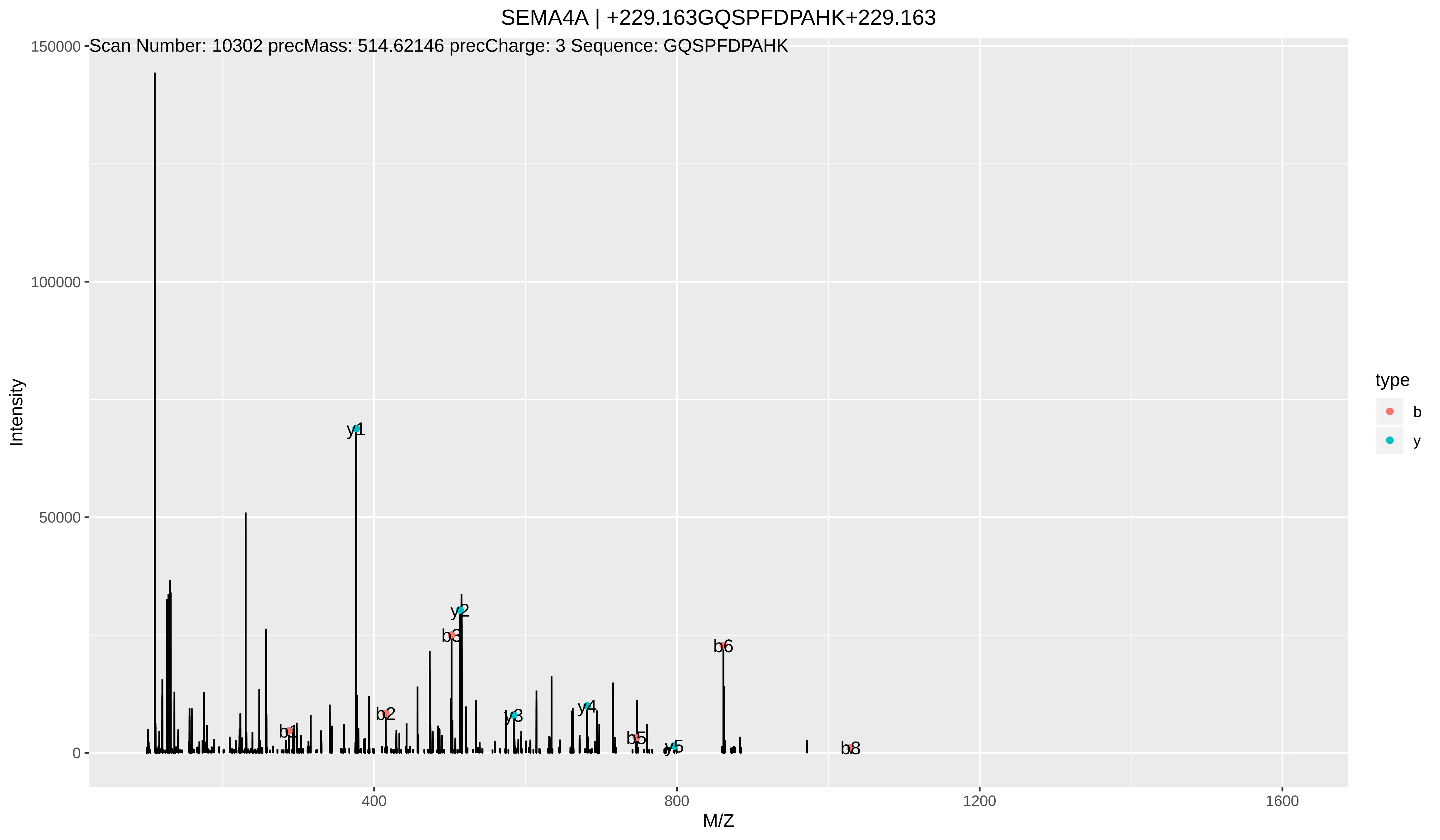
SEMA4A | +229.163GQSPFDPAHK+229.163
Scan Number: 10302 precMass: 514.62146 precCharge: 3 Sequence: GQSPFDPAHK
Intensity
150000
100000
50000
0
M/Z
400
800
1200
1600
y1
y2
b3
b6
b2
y3
y4
b1
b5
y5
b8
type
b
y

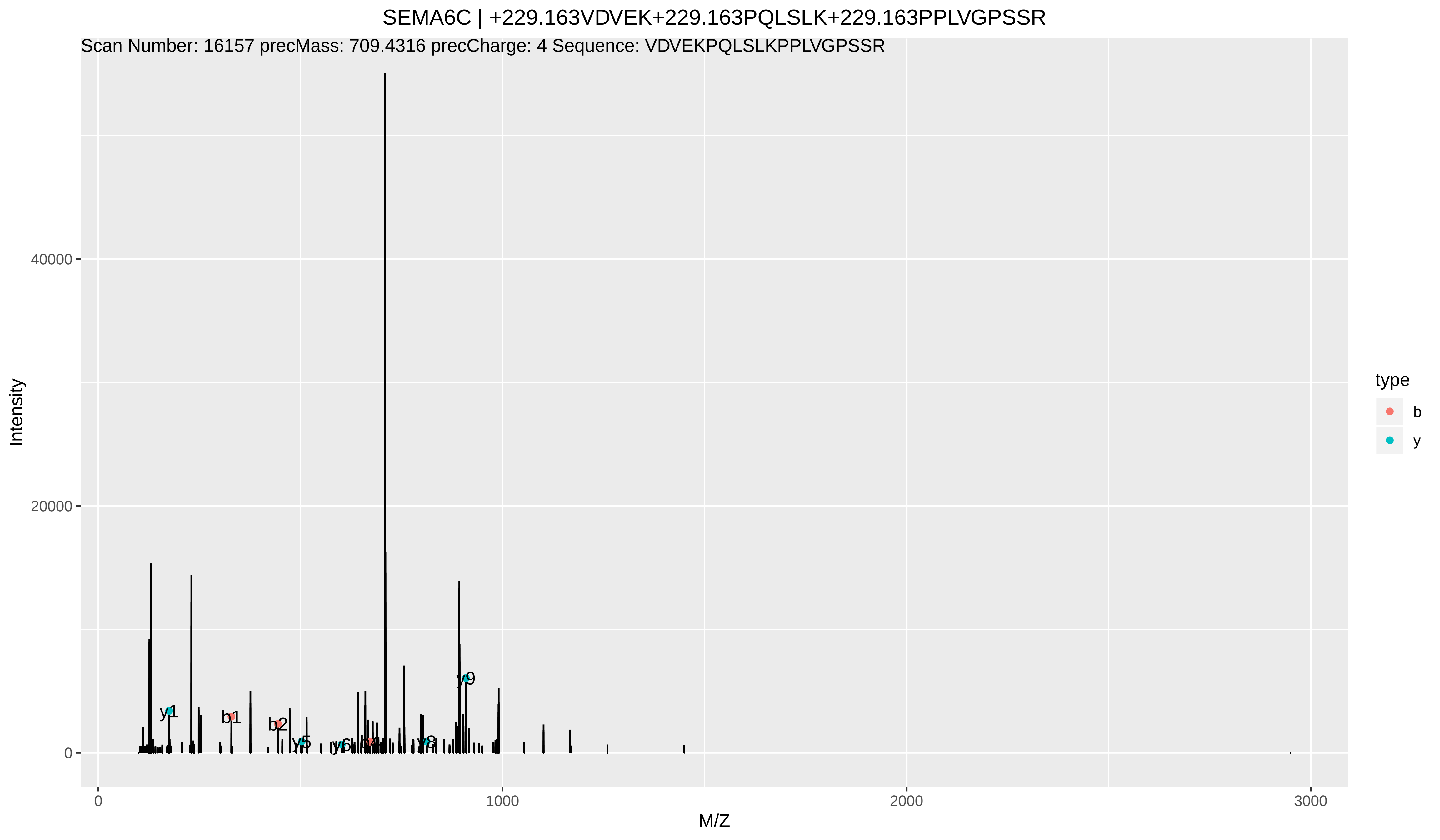

SEMA6C | +229.163VDVEK+229.163PQLSLK+229.163PPLVGPSSR
Scan Number: 16157 precMass: 709.4316 precCharge: 4 Sequence: VDVEKPQLSLKPPLVGPSSR
Intensity
M/Z
type
b
y
y1
b1
b2
y5
y9

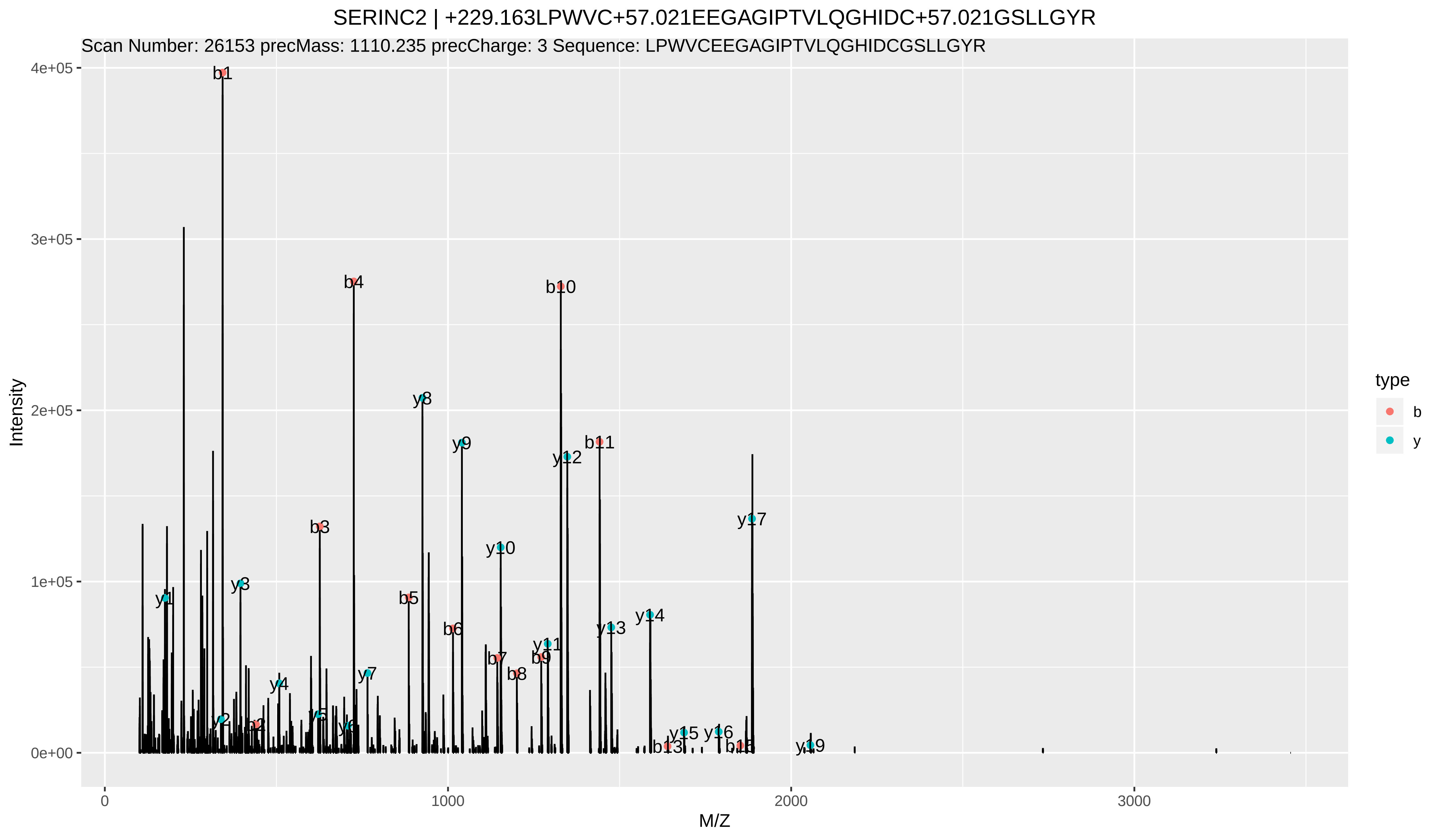
SERINC2 | +229.163LPWVC+57.021EEGAGIPTVLQGHIDC+57.021GSLLGYR
Scan Number: 26153 precMass: 1110.235 precCharge: 3 Sequence: LPWVCEEGAGIPTVLQGHIDCGSLLGYR
Intensity
M/Z
4e+05
3e+05
2e+05
1e+05
0e+00
0
1000
2000
3000
type
b
y
b1
b4
b10
y8
y9
b11
y12
y17
b3
y10
y1
y3
b5
y14
b6
y13
y11
b7
b9
b8
y4
y7
y2
b2
y5
y6
y15
y16
b13
b15
y19

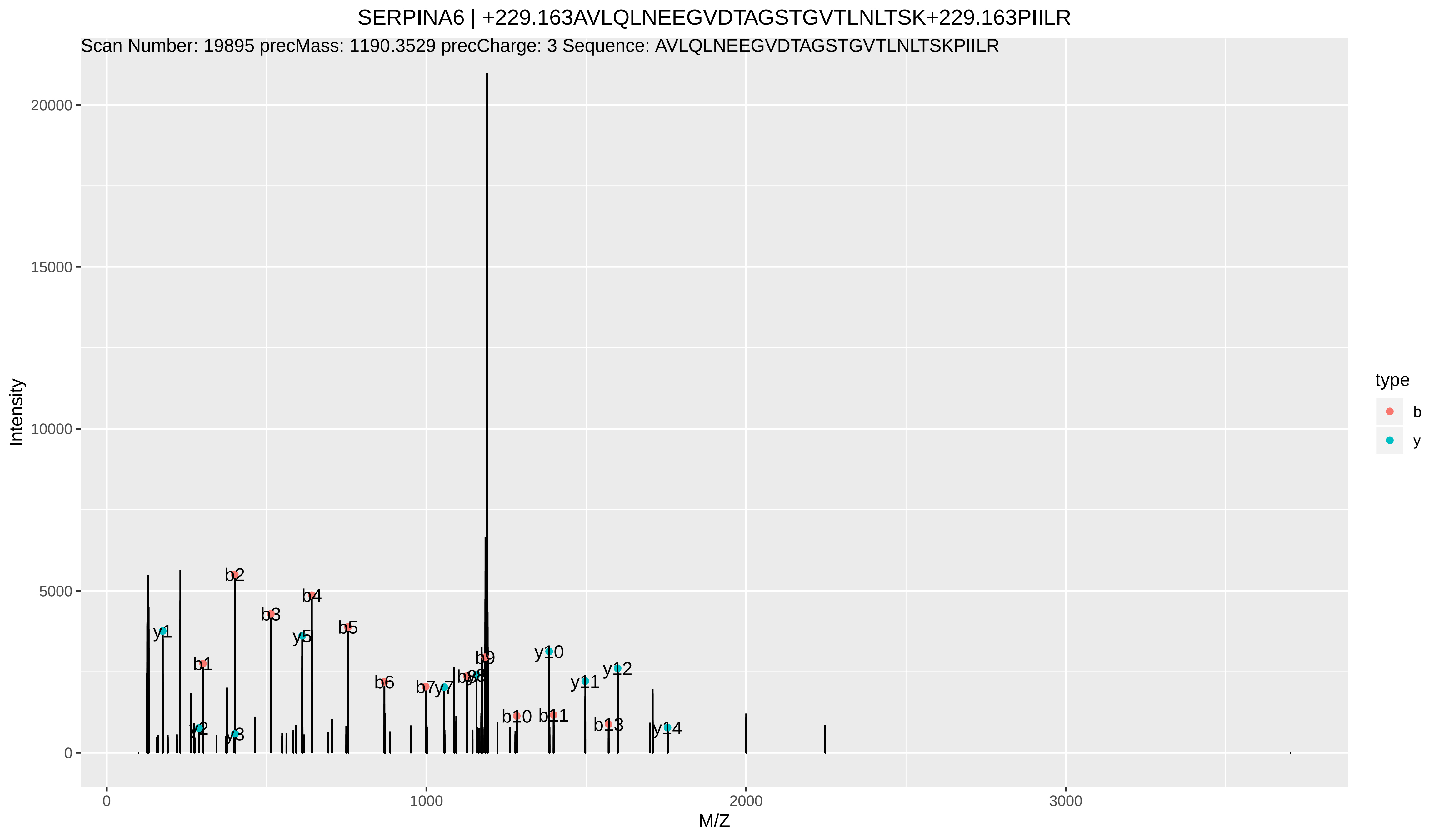

SERPINA6 | +229.163AVLQLNEEGVDTAGSTGVTLNLTSK+229.163PIILR
Scan Number: 19895 precMass: 1190.3529 precCharge: 3 Sequence: AVLQLNEEGVDTAGSTGVTLNLTSKPIILR
Intensity
M/Z
0
5000
10000
15000
20000
1000
2000
3000
type
b
y
b1
b2
b3
b4
b5
b6
b7
b9
b10
b11
b13
y1
y2
y3
y5
y7
y10
y11
y12
y14

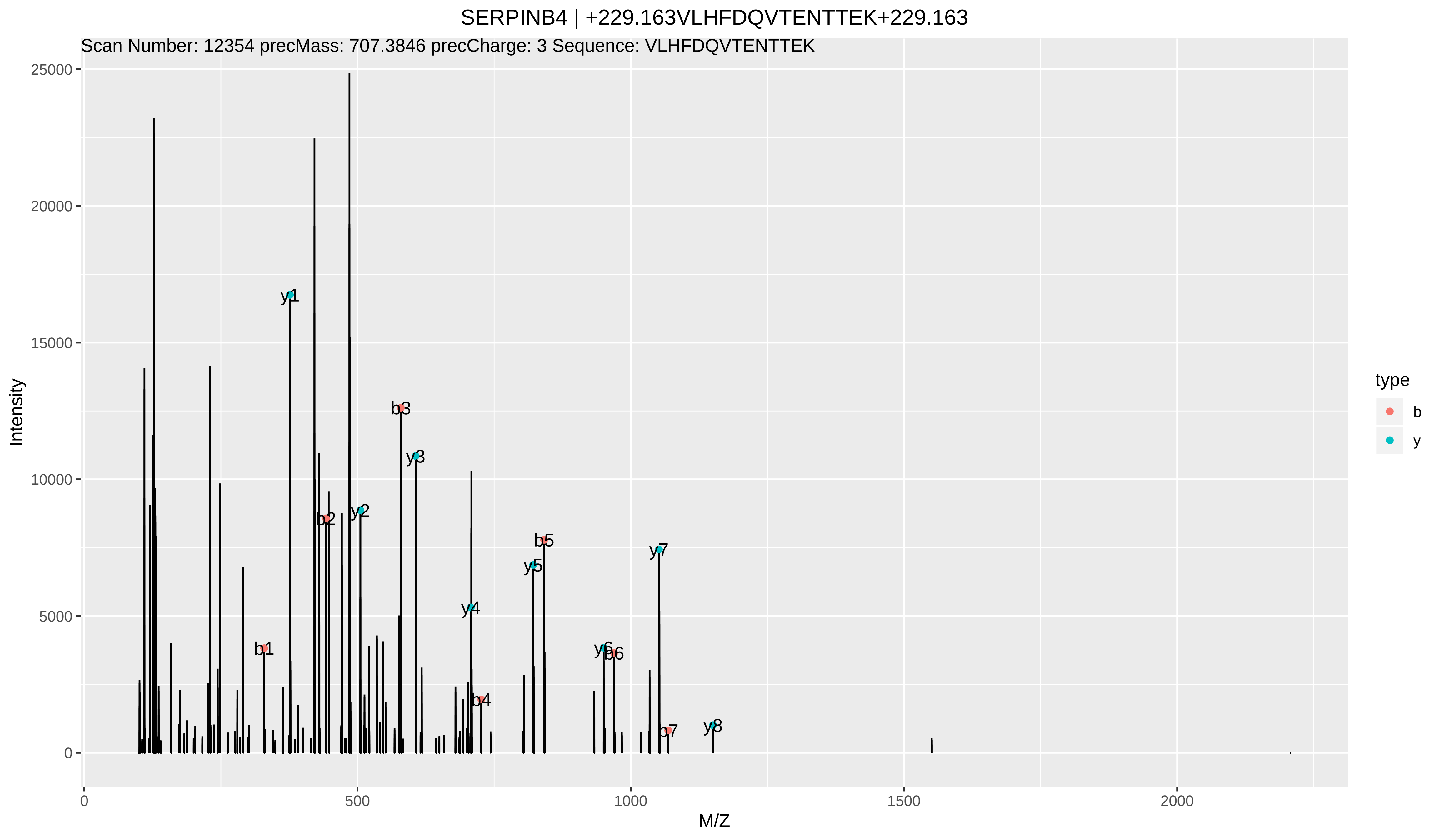
SERPINB4 | +229.163VLHFDQVTENTTEK+229.163
Scan Number: 12354 precMass: 707.3846 precCharge: 3 Sequence: VLHFDQVTENTTEK
Intensity
M/Z
type
b
y
y1
b1
b2
y2
b3
y3
y4
b4
y5
b5
y6
b6
y7
b7
y8
0
5000
10000
15000
20000
25000
0
500
1000
1500
2000

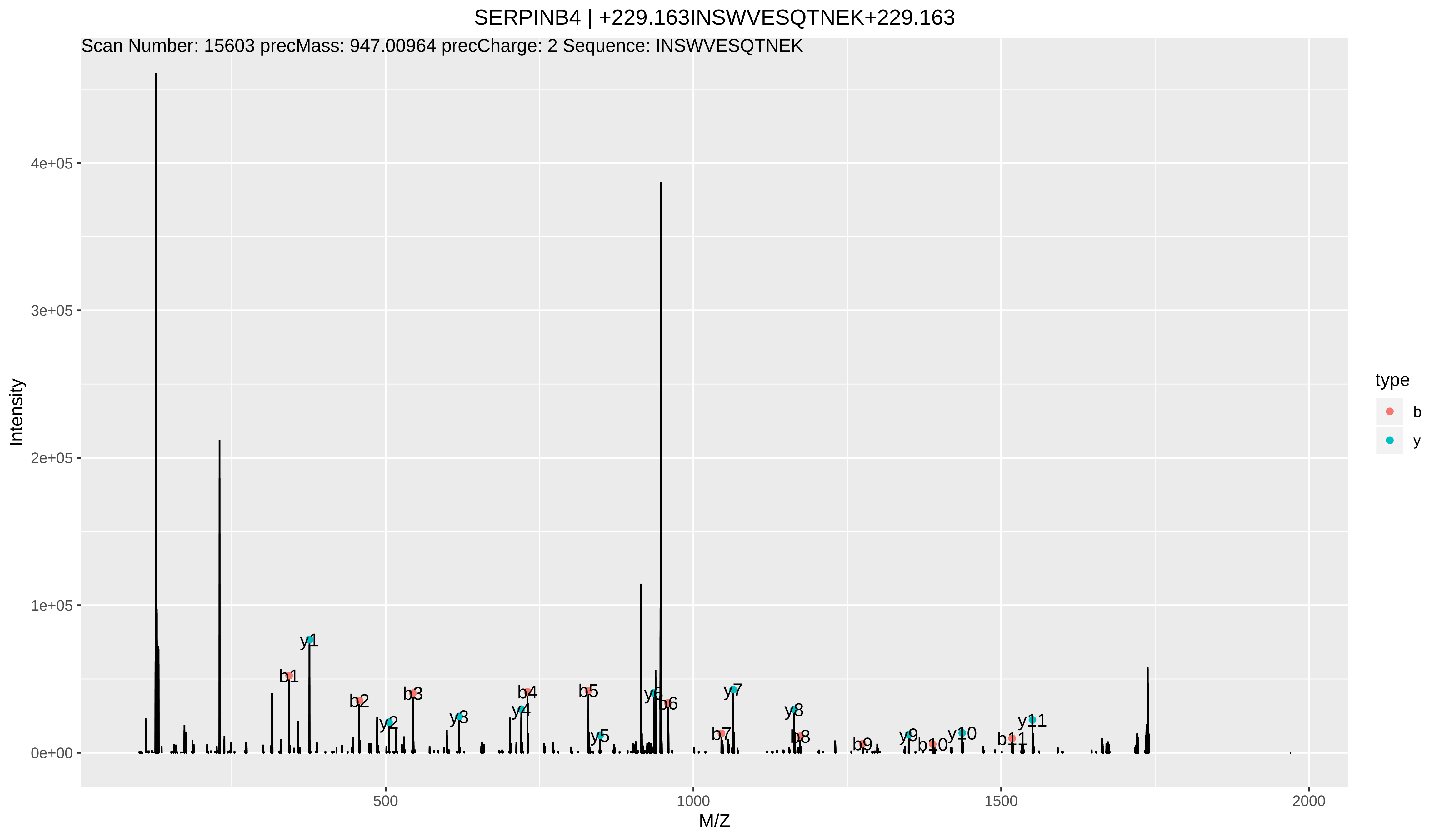
SERPINB4 | +229.163INSWVESQTNEK+229.163
Scan Number: 15603 precMass: 947.00964 precCharge: 2 Sequence: INSWVESQTNEK
Intensity
4e+05
3e+05
2e+05
1e+05
0e+00
M/Z
500
1000
1500
2000
type
b
y
y1
b1
b2
y2
b3
y3
b4
y4
b5
y5
y6
b6
b7
y7
y8
b8
b9
y9
b10
y10
b11
y11

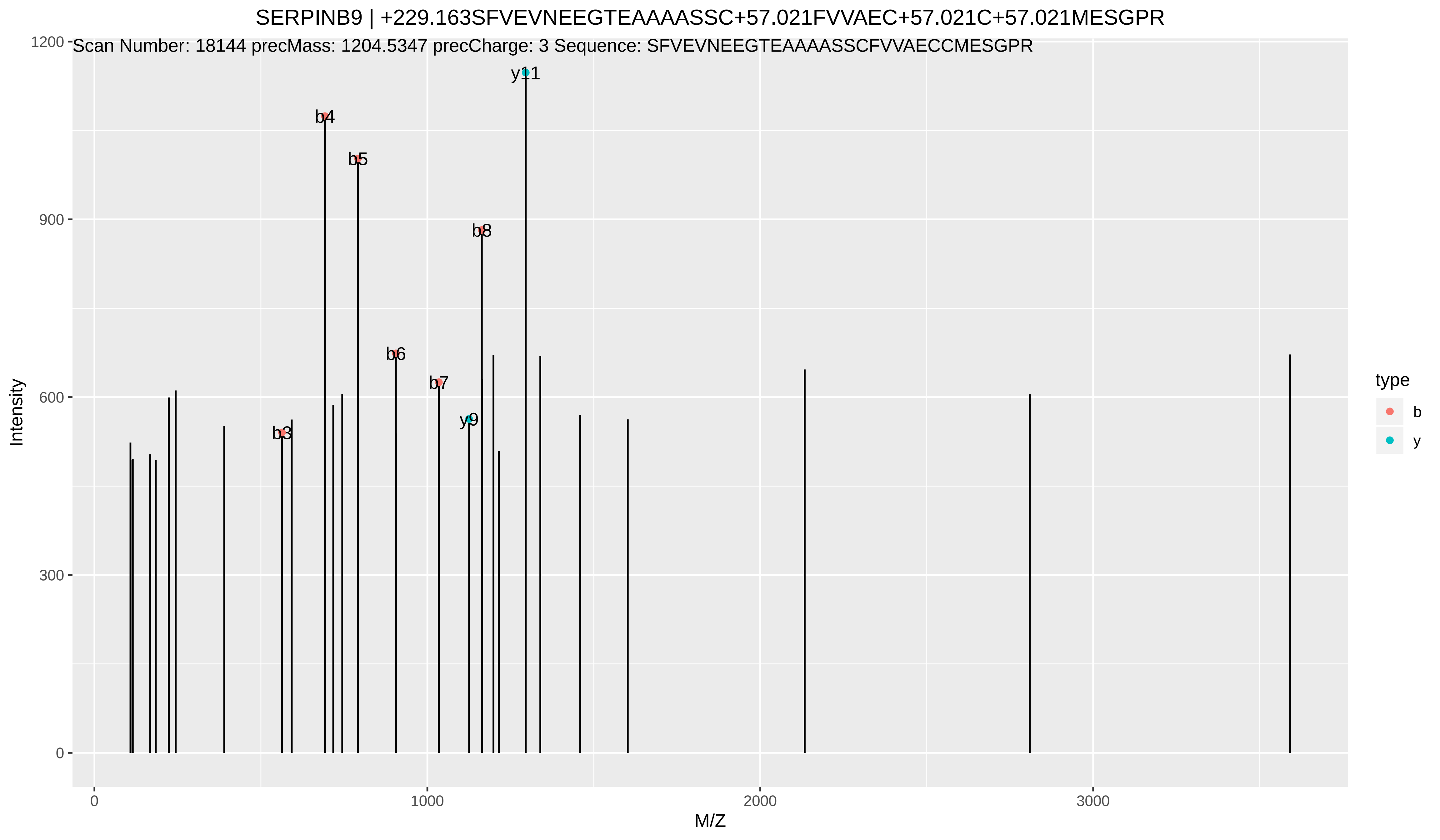

SERPINB9 | +229.163SFVEVNEEGTEAAAASSC+57.021FVVAEC+57.021C+57.021MESGPR
Scan Number: 18144 precMass: 1204.5347 precCharge: 3 Sequence: SFVEVNEEGTEAAAASSCFVVAECCMESGPR
y11
b4
b5
b8
b6
b7
y9
b3
Intensity
M/Z
type
b
y
0
300
600
900
1200
0
1000
2000
3000

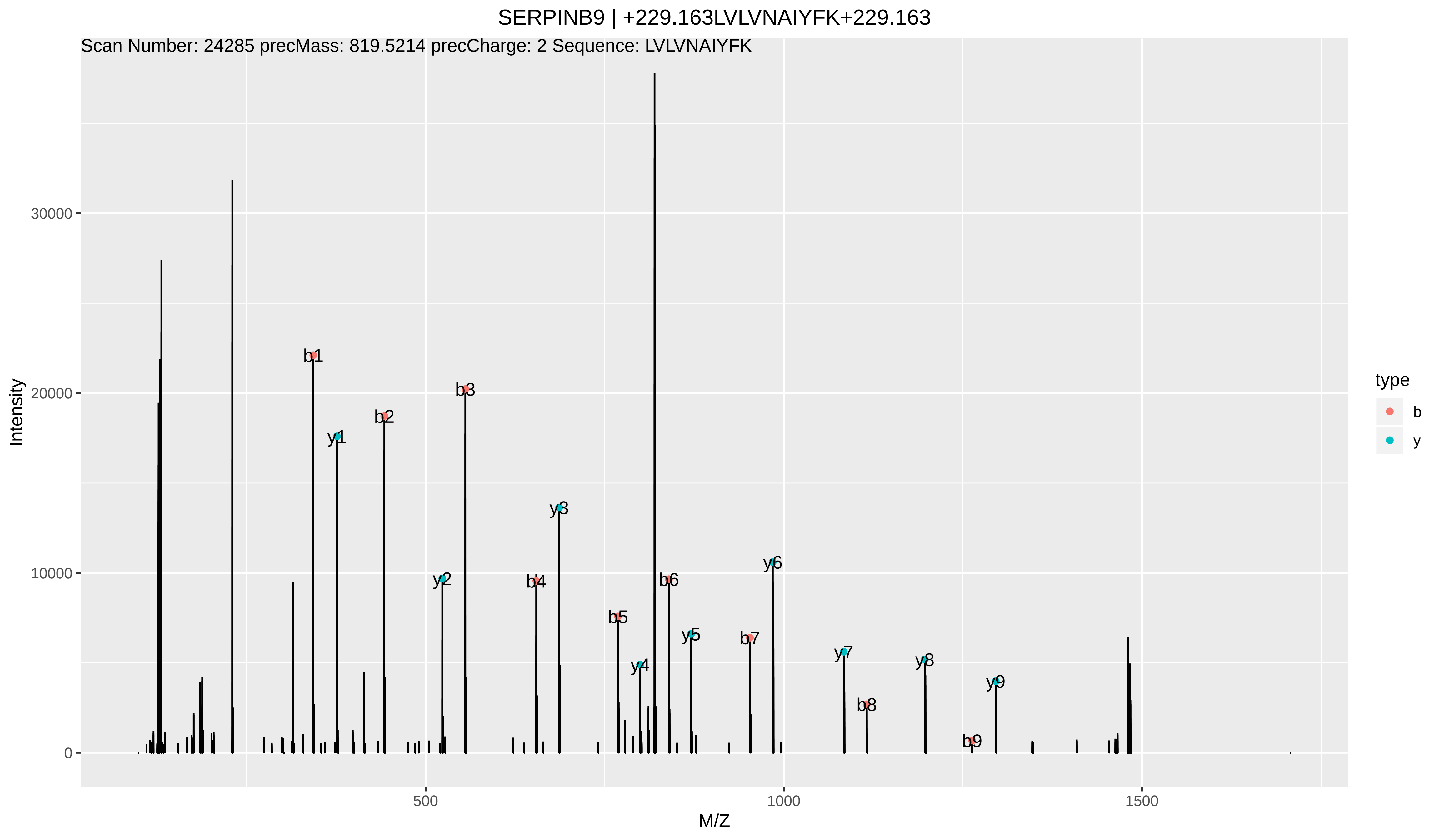
SERPINB9 | +229.163LVLVNAIYFK+229.163
Scan Number: 24285 precMass: 819.5214 precCharge: 2 Sequence: LVLVNAIYFK
Intensity
M/Z
type
b
y
b1
b2
b3
b4
b5
b6
b7
b8
b9
y1
y2
y3
y4
y5
y6
y7
y8
y9
0
10000
20000
30000
500
1000
1500

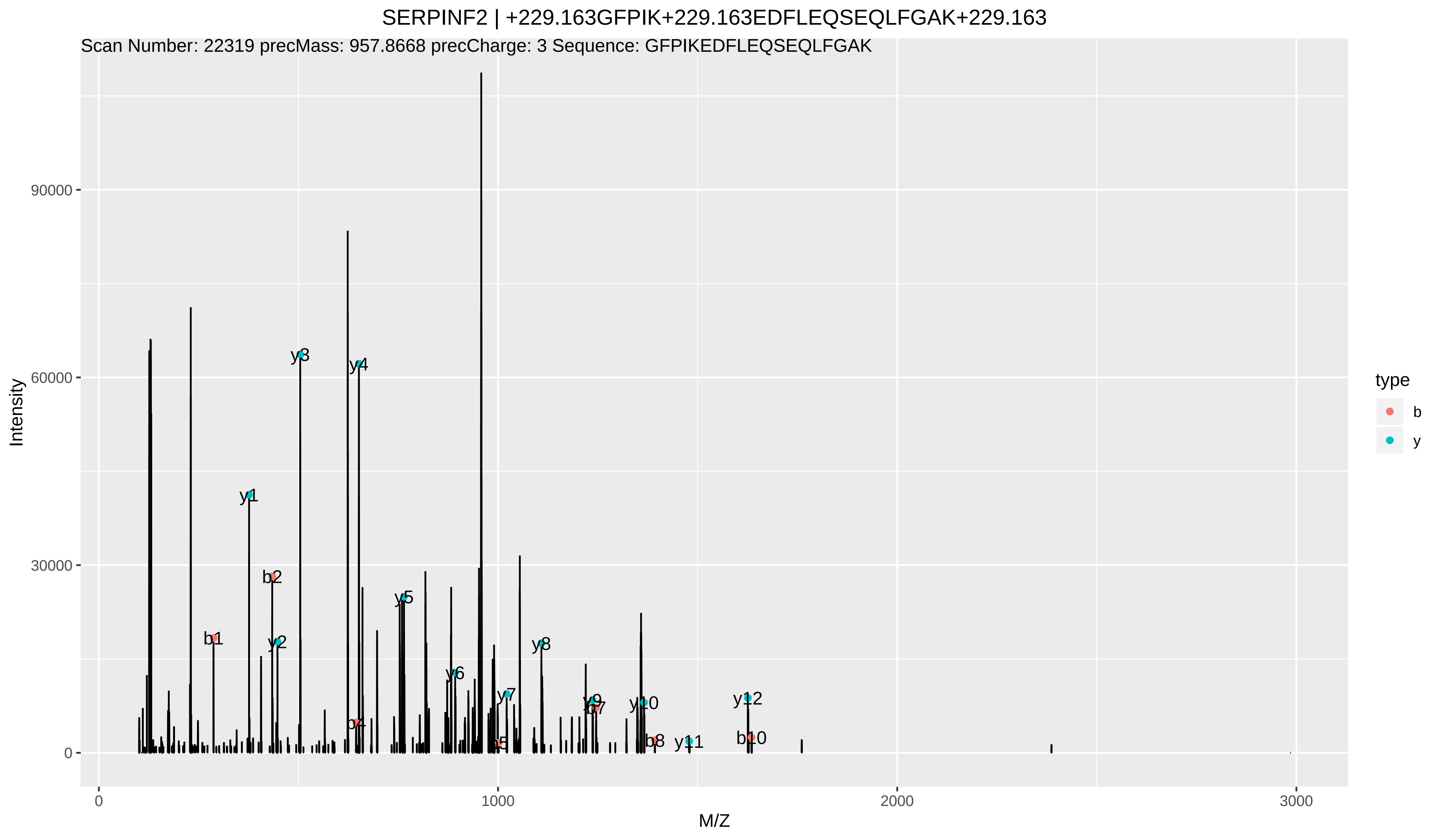
SERPINF2 | +229.163GFPIK+229.163EDFLEQSEQLFGAK+229.163
Scan Number: 22319 precMass: 957.8668 precCharge: 3 Sequence: GFPIKEDFLEQSEQLFGAK
Intensity
M/Z
0
30000
60000
90000
0
1000
2000
3000
y3
y4
y1
b2
y5
b1
y2
y8
y6
y7
y9
b7
y10
y12
b4
b5
b8
y11
b10
type
b
y

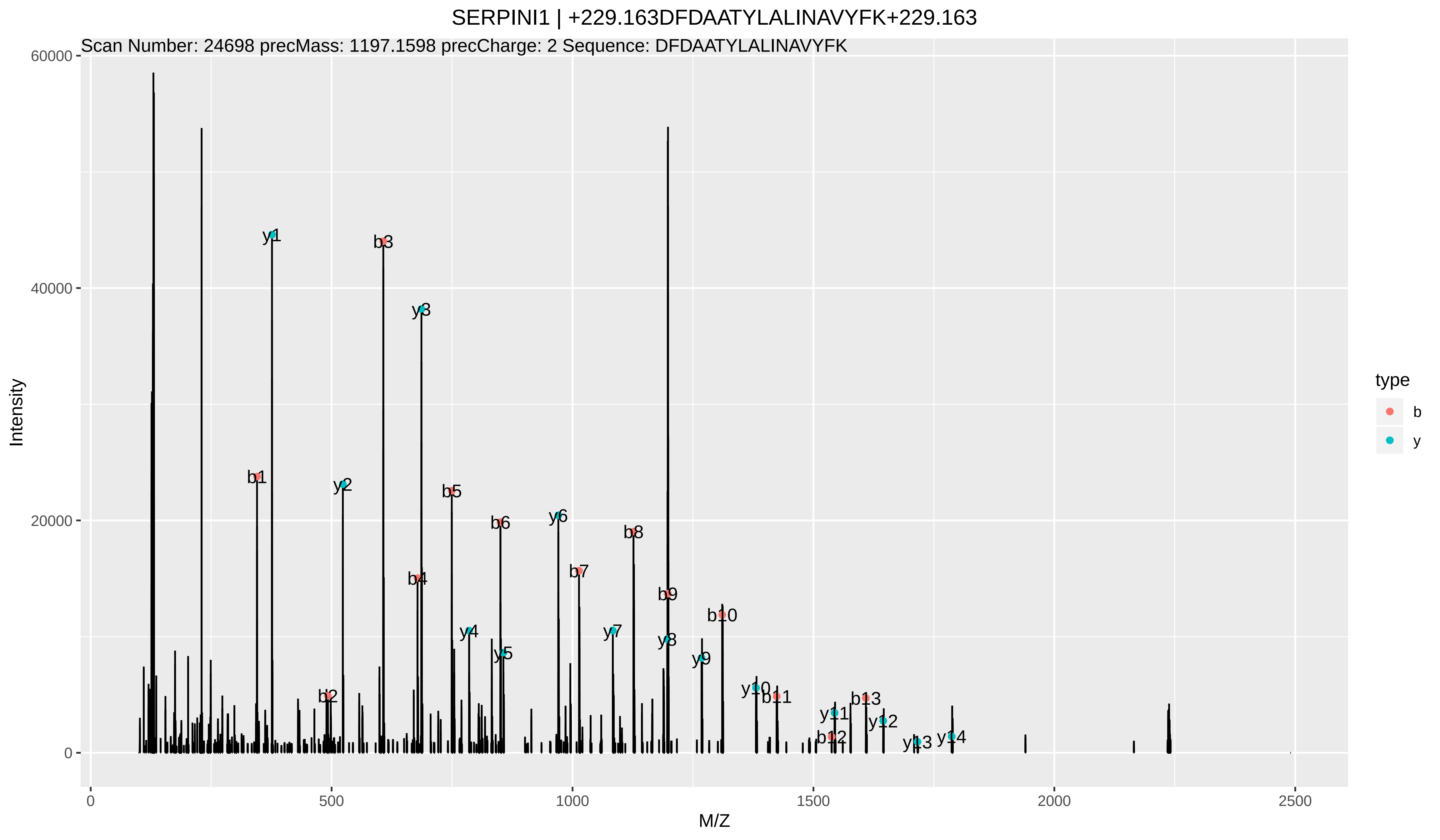
SERPINI1 | +229.163DFDAATYLALINAVYFK+229.163
Scan Number: 24698 precMass: 1197.1598 precCharge: 2 Sequence: DFDAATYLALINAVYFK
Intensity
M/Z
0
20000
40000
60000
0
500
1000
1500
2000
2500
y1
b3
y3
b1
y2
b5
b6
y6
b8
b7
b4
b9
b10
y4
y7
y8
y5
y9
b2
y10
b11
b13
y11
y12
b12
y13
y14
type
b
y

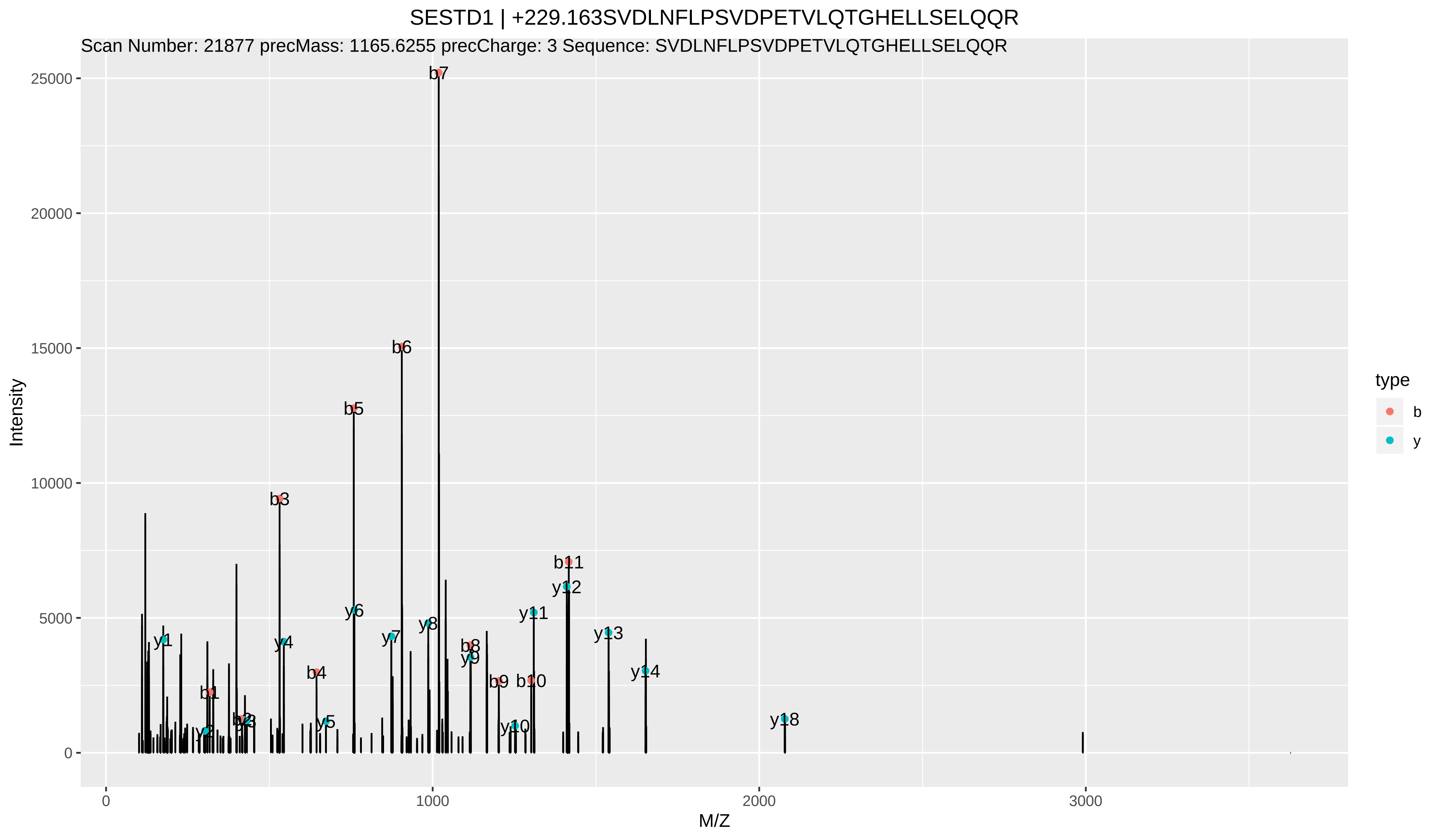

SESTD1 | +229.163SVDLNFLPSVDPETVLQTGHELLSELQQR
Scan Number: 21877 precMass: 1165.6255 precCharge: 3 Sequence: SVDLNFLPSVDPETVLQTGHELLSELQQR
Intensity
M/Z
type
b
y
b7
b6
b5
b3
b11
y12
y6
y11
y8
y13
y7
y4
y1
b8
y9
b4
y14
b9
b10
b1
y18
y5
y10
y2
0
5000
10000
15000
20000
25000
0
1000
2000
3000

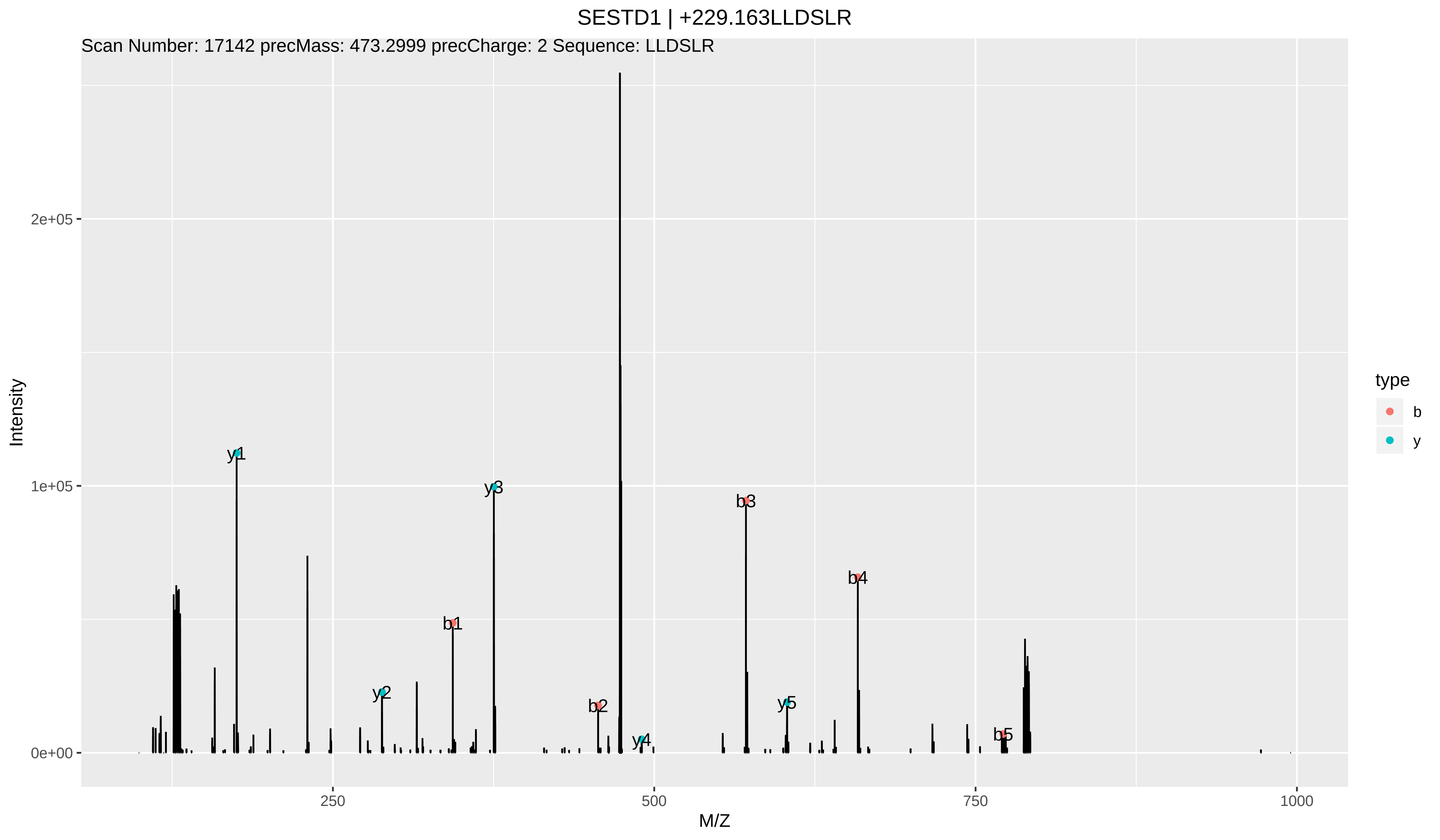

SESTD1 | +229.163LLDSLR
Scan Number: 17142 precMass: 473.2999 precCharge: 2 Sequence: LLDSLR
Intensity
M/Z
0e+00
1e+05
2e+05
250
500
750
1000
y1
y2
y3
y4
y5
b1
b2
b3
b4
b5
type
b
y

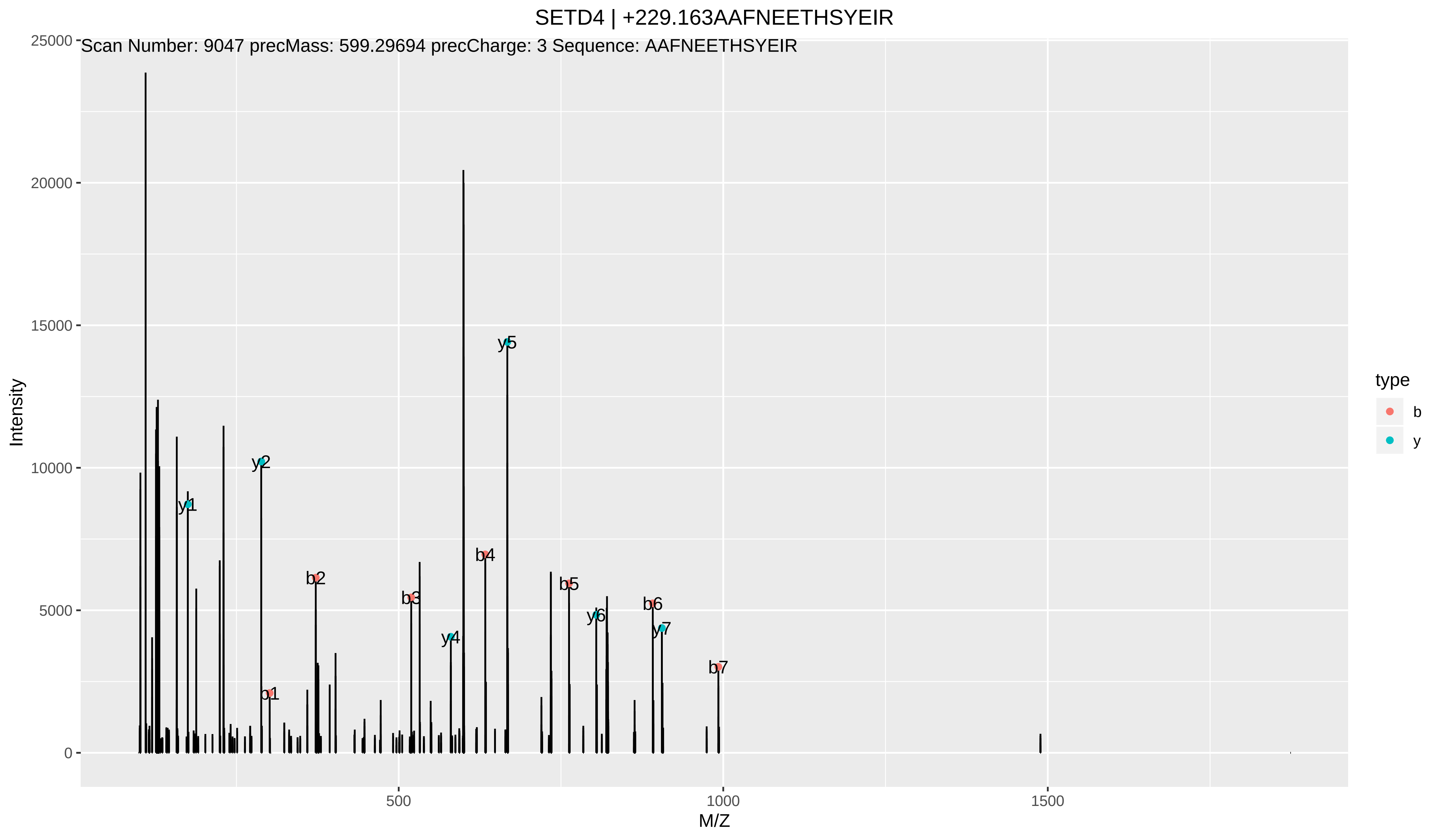

SETD4 | +229.163AAFNEETHSYEIR
Scan Number: 9047 precMass: 599.29694 precCharge: 3 Sequence: AAFNEETHSYEIR
Intensity
M/Z
0
5000
10000
15000
20000
25000
500
1000
1500
y1
y2
b1
b2
b3
y4
b4
y5
b5
y6
b6
y7
b7
type
b
y

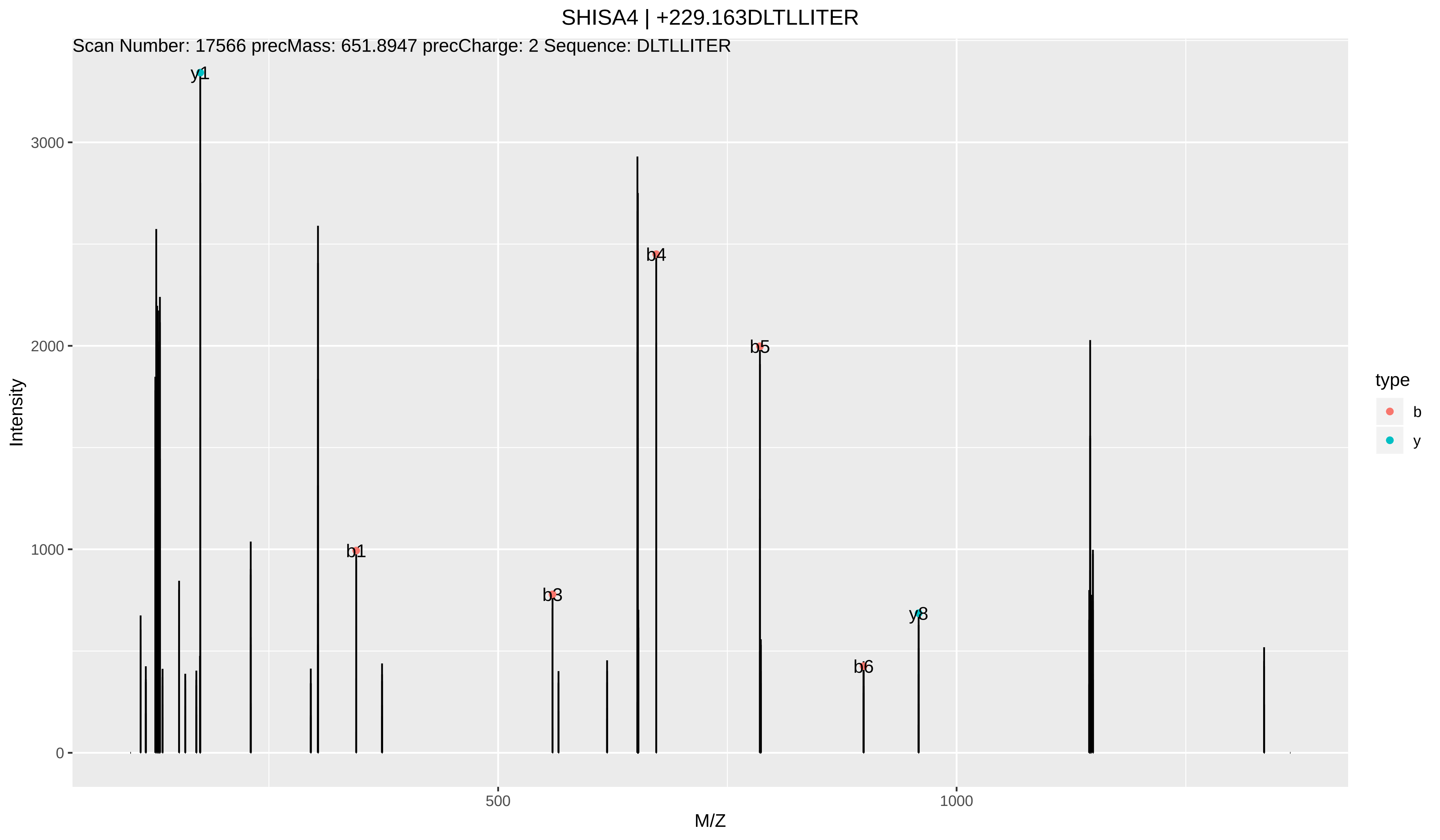

SHISA4 | +229.163DLTLLITER
Scan Number: 17566 precMass: 651.8947 precCharge: 2 Sequence: DLTLLITER
y1
b1
b3
b4
b5
b6
y8
Intensity
M/Z
0
1000
2000
3000
500
1000
type
b
y

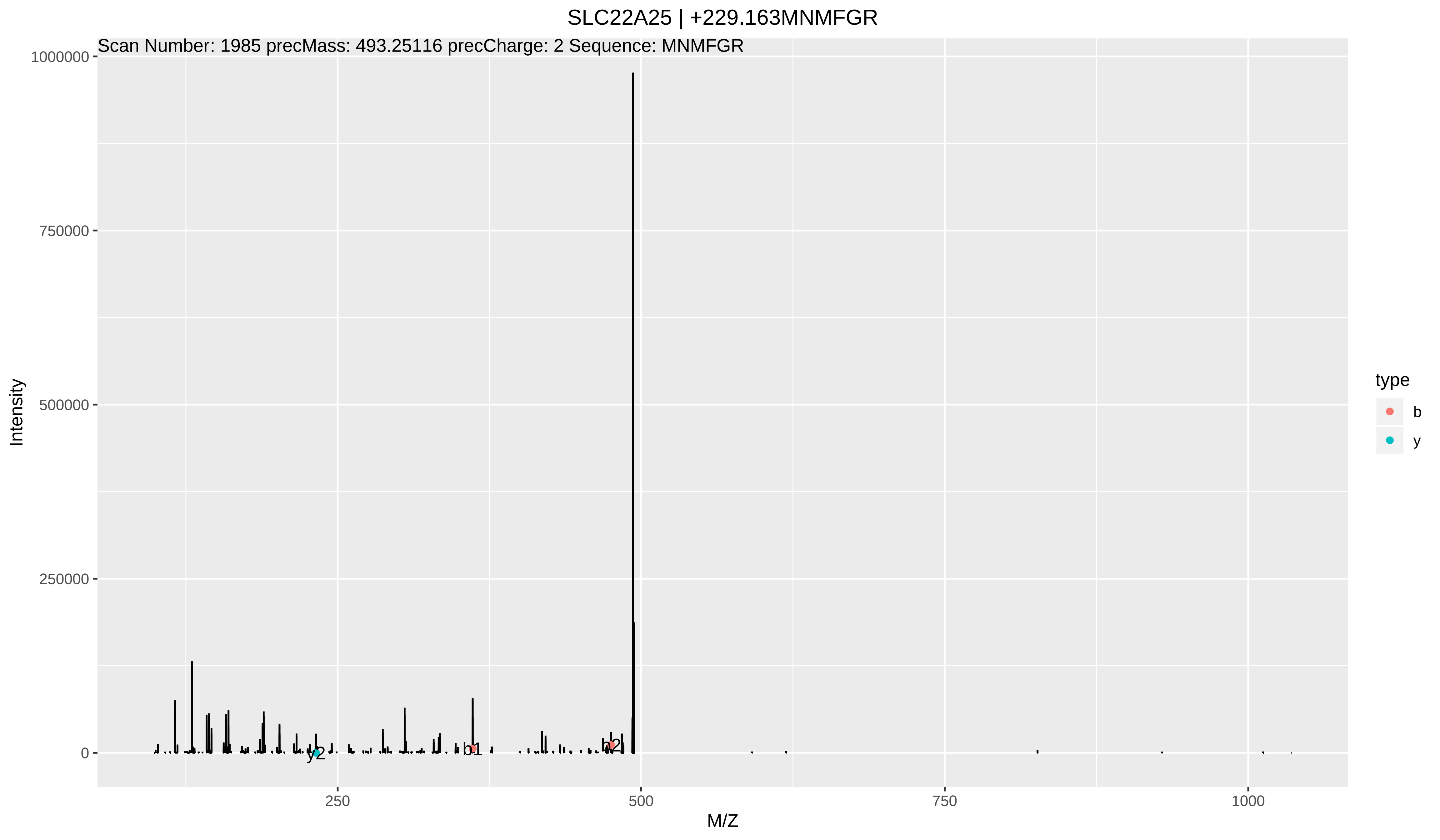

SLC22A25 | +229.163MNMFGR
Scan Number: 1985 precMass: 493.25116 precCharge: 2 Sequence: MNMFGR
Intensity
1000000
750000
500000
250000
0
M/Z
250
500
750
1000
y2
b1
b2
type
b
y

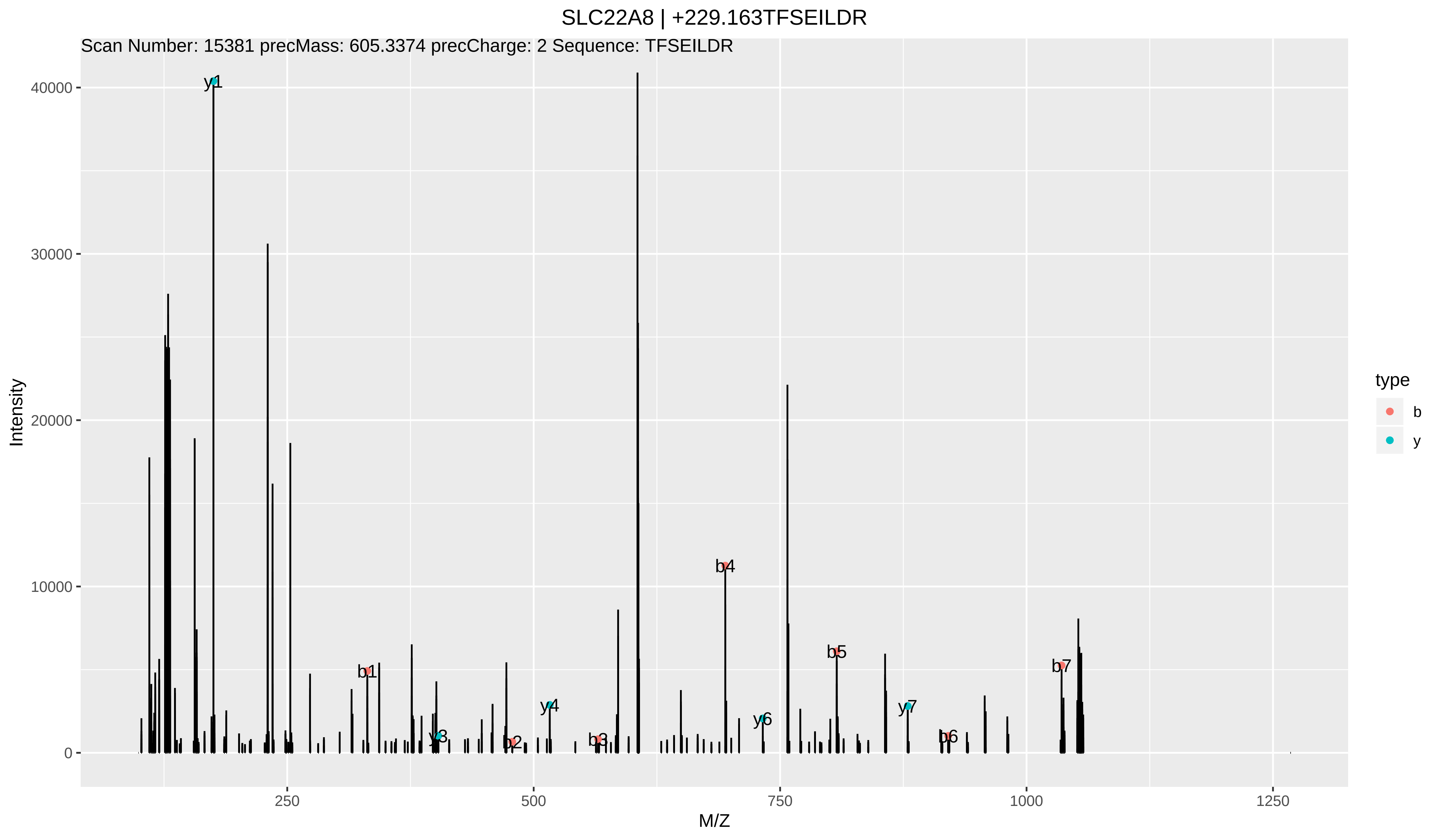
SLC22A8 | +229.163TFSEILDR
Scan Number: 15381 precMass: 605.3374 precCharge: 2 Sequence: TFSEILDR
Intensity
M/Z
0
10000
20000
30000
40000
250
500
750
1000
1250
y1
b1
y3
b2
y4
b3
b4
y6
b5
y7
b6
b7
type
b
y

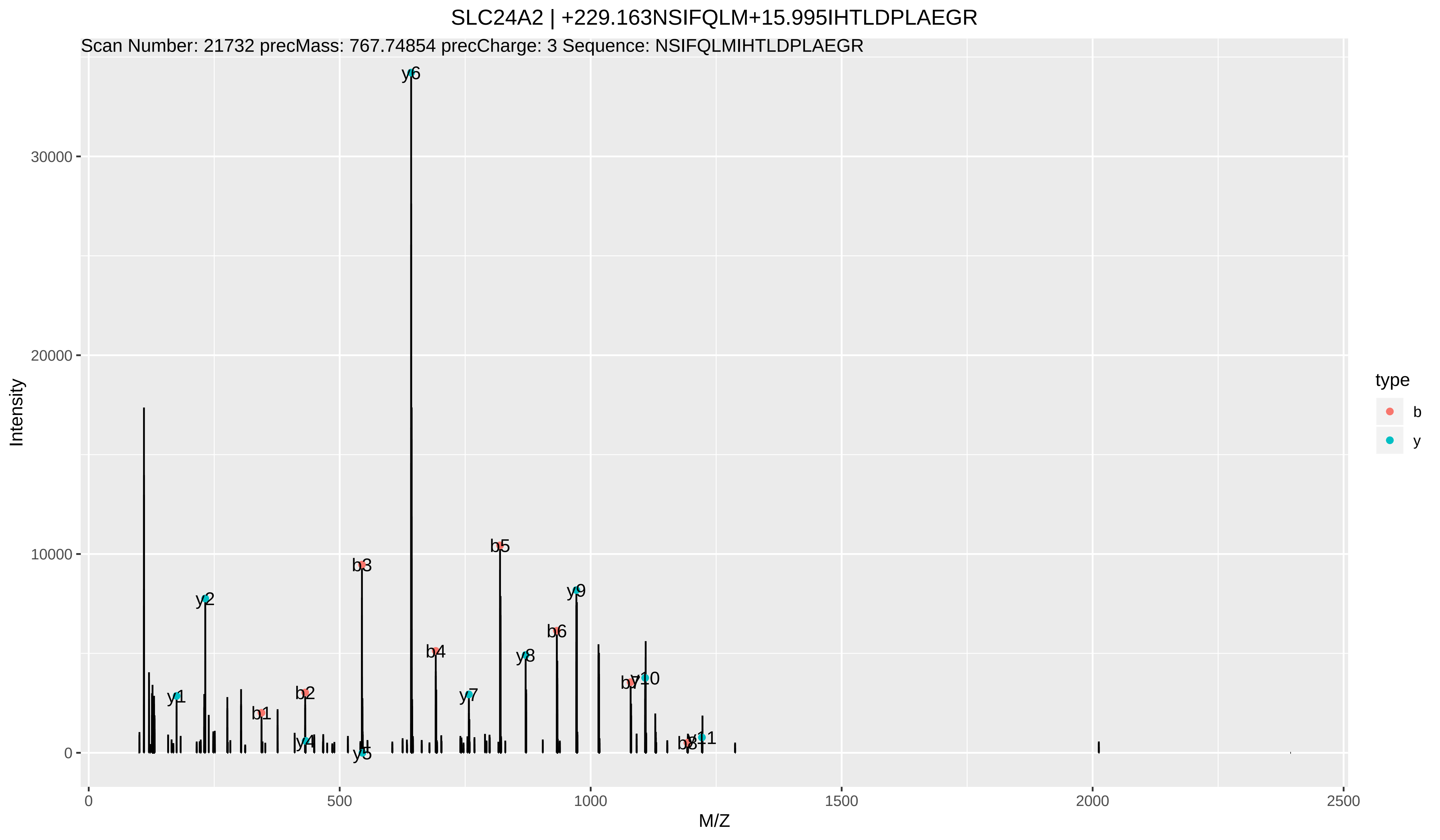

SLC24A2 | +229.163NSIFQLM+15.995IHTLDPLAEGR
Scan Number: 21732 precMass: 767.74854 precCharge: 3 Sequence: NSIFQLMIHTLDPLAEGR
Intensity
M/Z
0
10000
20000
30000
0
500
1000
1500
2000
2500
y6
b5
b3
y9
y2
b6
b4
y8
b7
y10
y1
b2
y7
b1
y4
y5
b8
y11
type
b
y

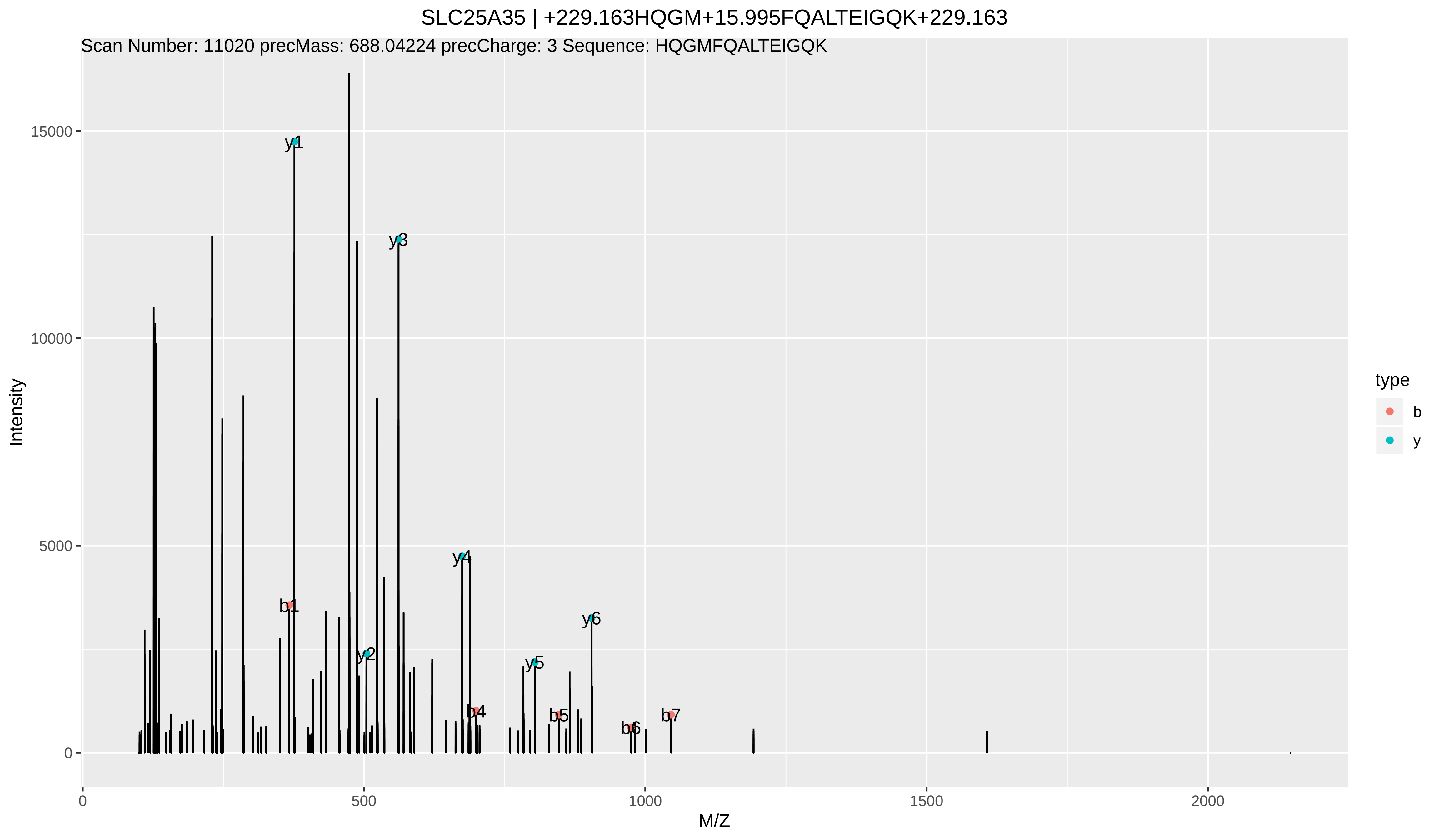

SLC25A35 | +229.163HQGM+15.995FQALTEIGQK+229.163
Scan Number: 11020 precMass: 688.04224 precCharge: 3 Sequence: HQGMFQALTEIGQK
Intensity
M/Z
0
5000
10000
15000
0
500
1000
1500
2000
y1
y2
y3
y4
y5
y6
b1
b4
b5
b6
b7
type
b
y

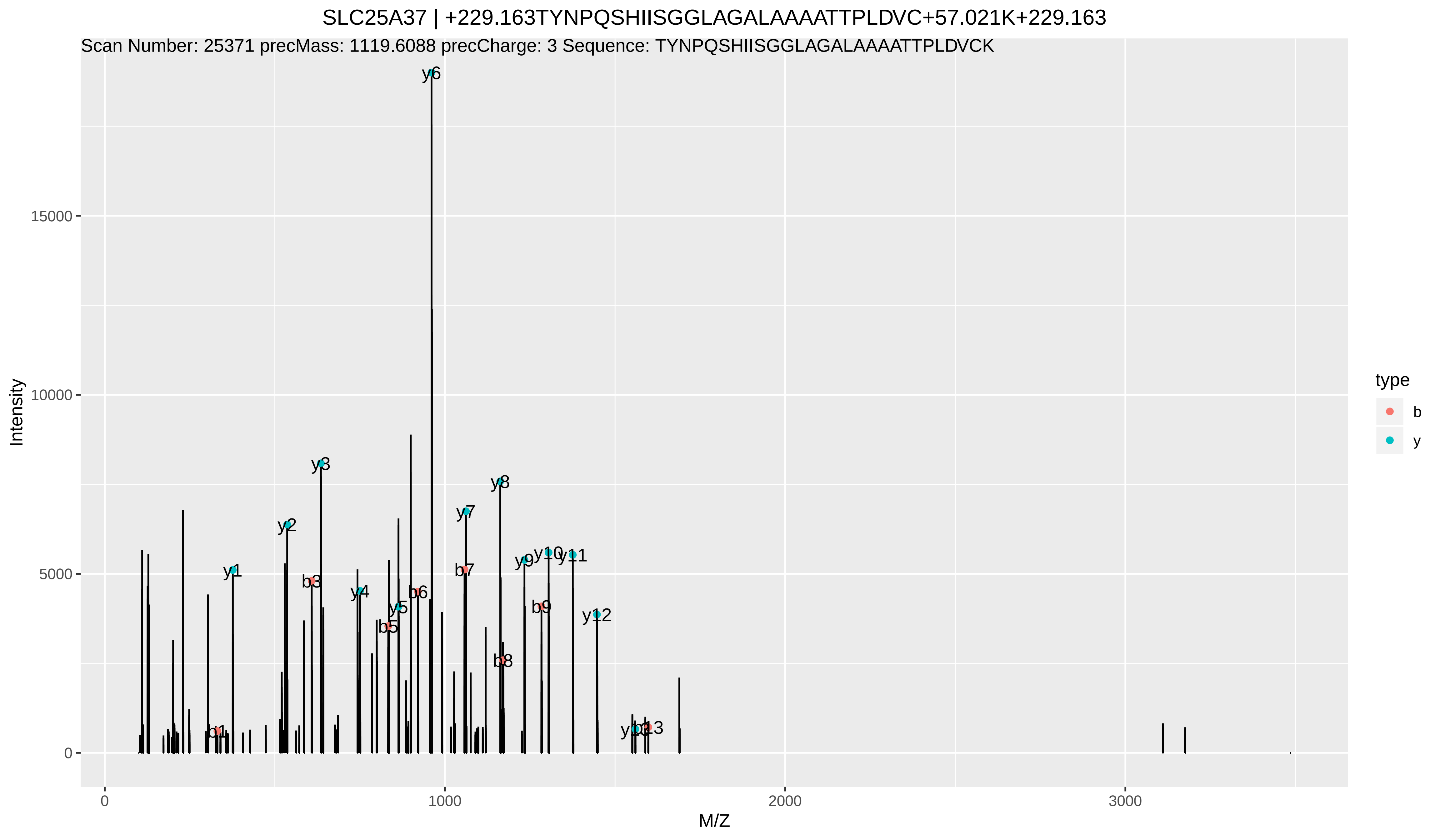

SLC25A37 | +229.163TYNPQSHIISGGLAGALAAAATTPLDVC+57.021K+229.163
Scan Number: 25371 precMass: 1119.6088 precCharge: 3 Sequence: TYNPQSHIISGGLAGALAAAATTPLDVCK
Intensity
M/Z
type
b
y
y6
y3
y8
y7
y2
y10
y11
y9
y1
b7
b3
y4
b6
y5
b9
y12
b5
b8
b1
y13
b13
0
5000
10000
15000
1000
2000
3000

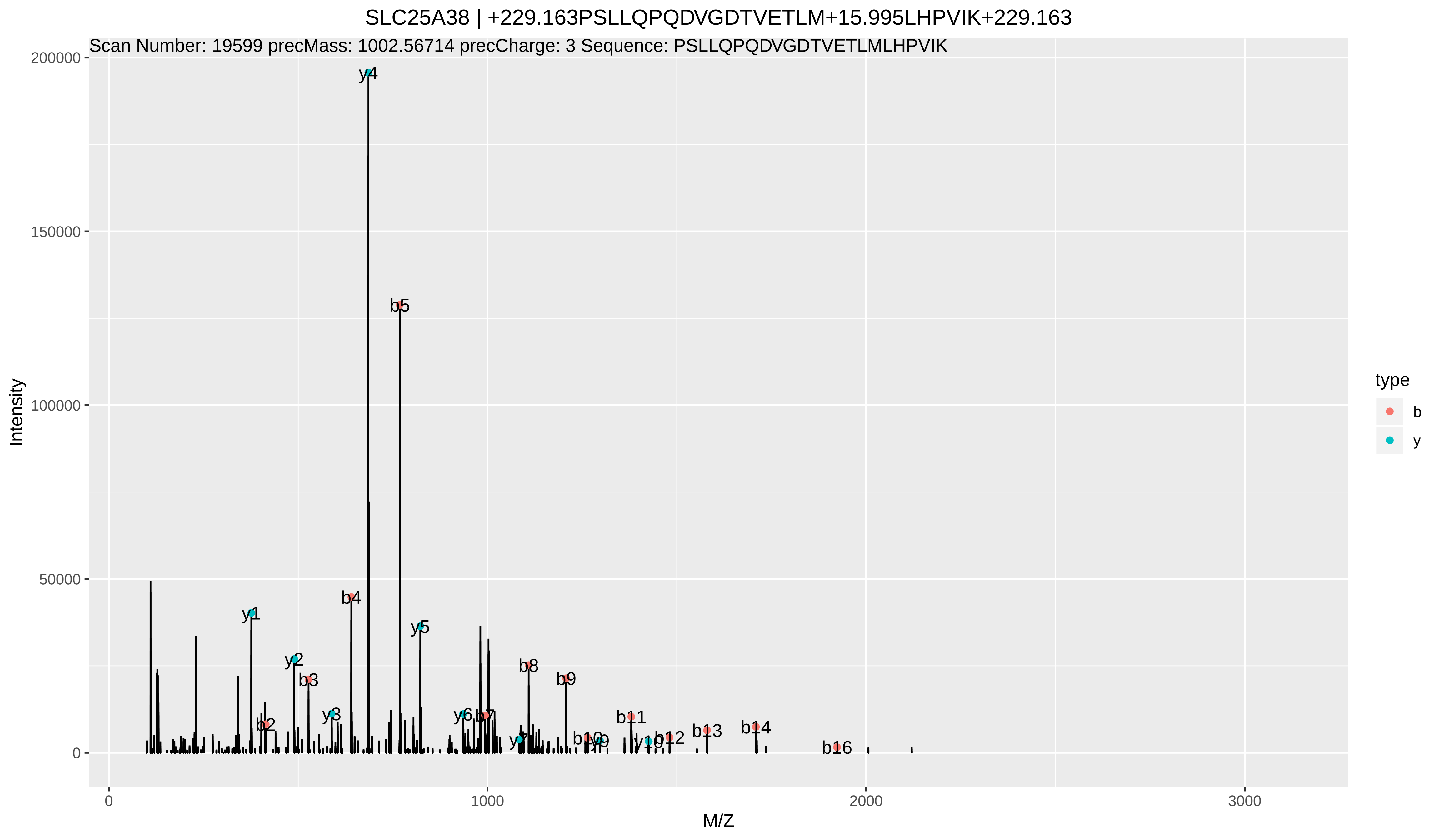

SLC25A38 | +229.163PSLLQPQDVGDTVETLM+15.995LHPVIK+229.163
Scan Number: 19599 precMass: 1002.56714 precCharge: 3 Sequence: PSLLQPQDVGDTVETLMLHPVIK
Intensity
M/Z
200000
150000
100000
50000
0
1000
2000
3000
y4
b5
b4
y1
y5
y2
b3
b8
b9
y3
b2
y6
b7
y7
b10
y9
b11
y10
b12
b13
b14
b16
type
b
y

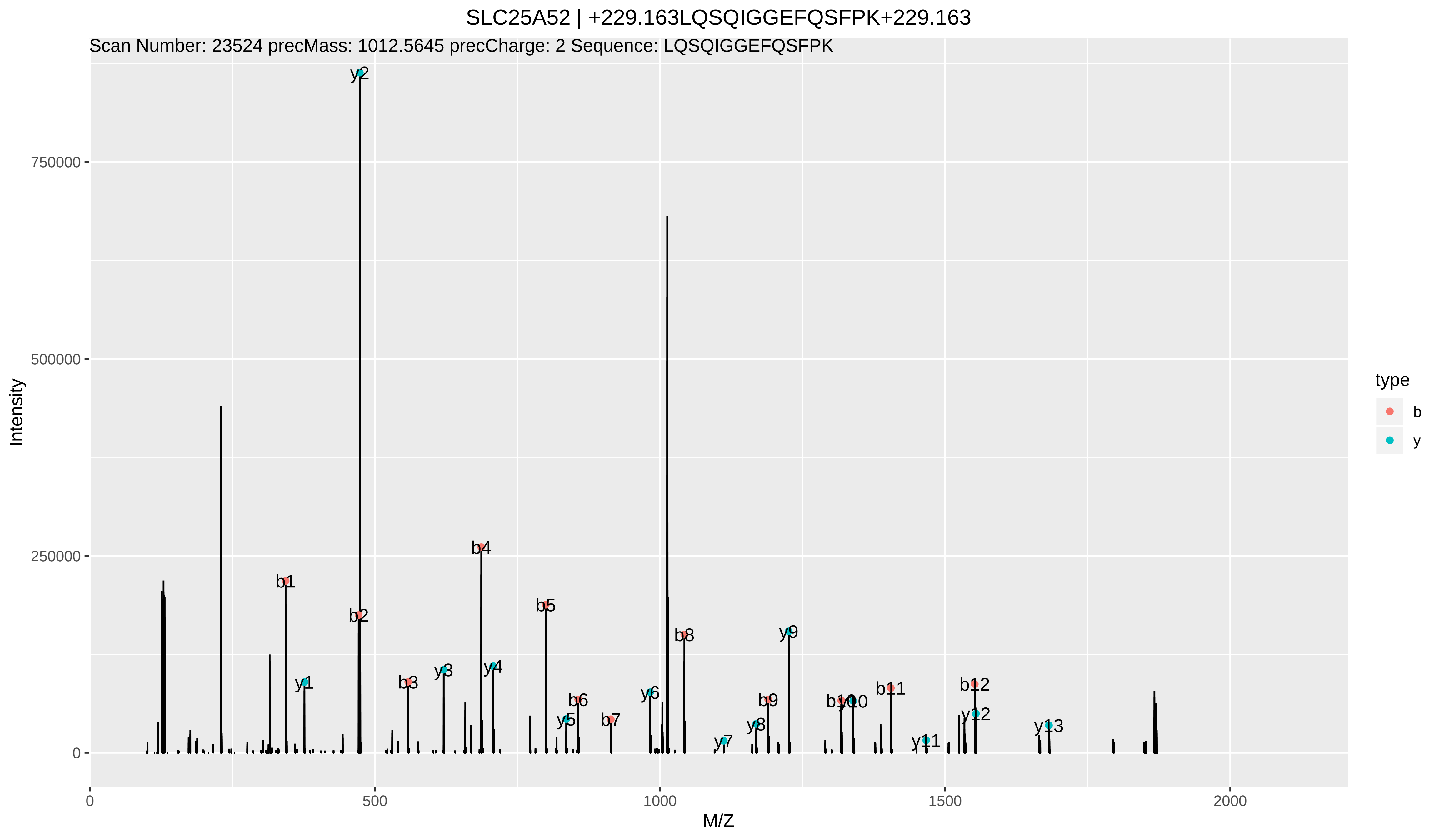
SLC25A52 | +229.163LQSQIGGEFQSFPK+229.163
Scan Number: 23524 precMass: 1012.5645 precCharge: 2 Sequence: LQSQIGGEFQSFPK
Intensity
750000
500000
250000
0
M/Z
0
500
1000
1500
2000
type
b
y
y2
b1
b2
y1
b3
y3
b4
y4
b5
y5
b6
b7
y6
b8
y7
y8
b9
y9
b10
y10
b11
y11
b12
y12
y13

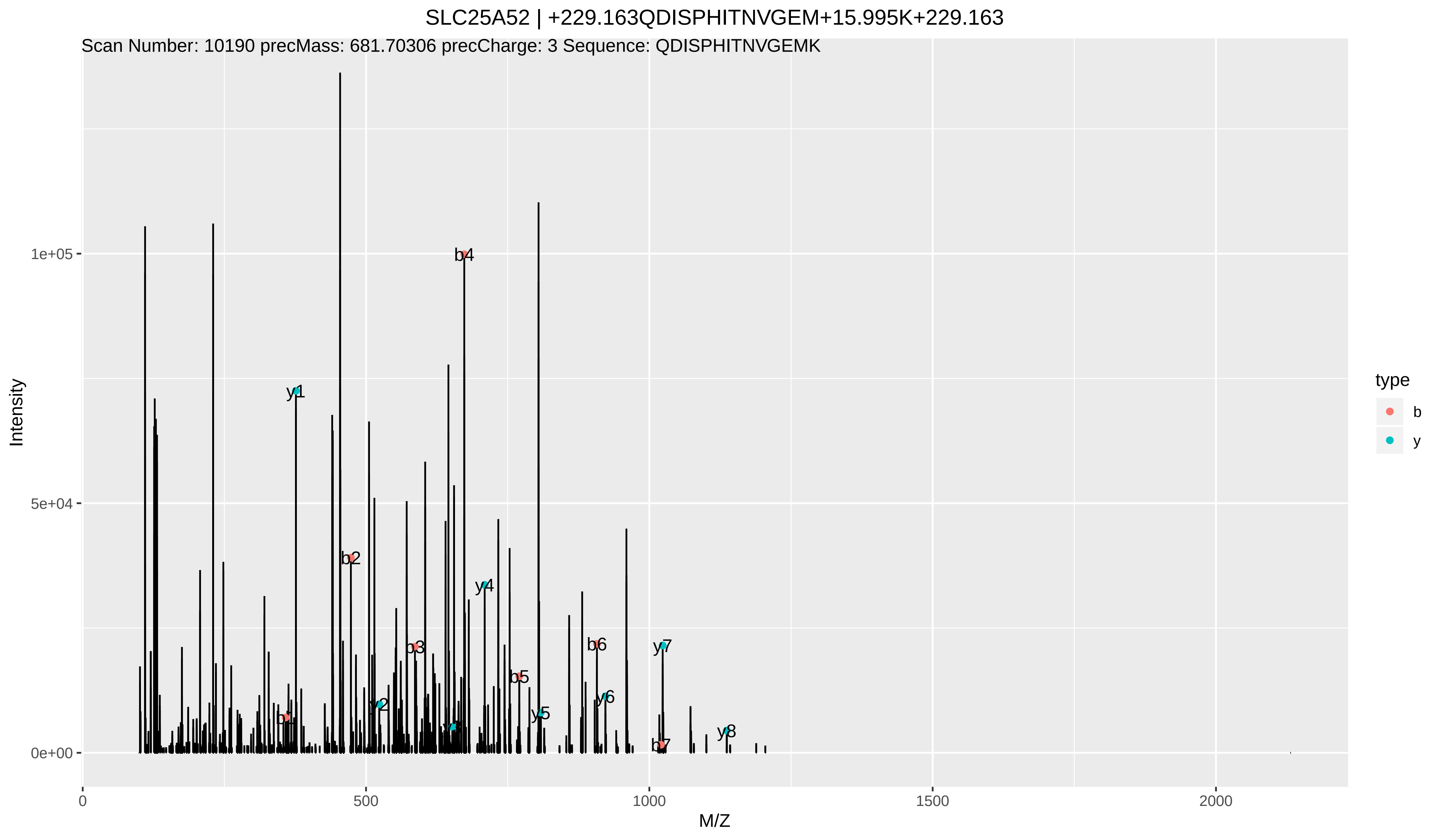

SLC25A52 | +229.163QDISPHITNVGEM+15.995K+229.163
Scan Number: 10190 precMass: 681.70306 precCharge: 3 Sequence: QDISPHITNVGEMK
Intensity
M/Z
0e+00
5e+04
1e+05
0
500
1000
1500
2000
b4
y1
b2
y4
b3
b6
y7
b5
y6
y2
y5
b1
y8
b7
type
b
y

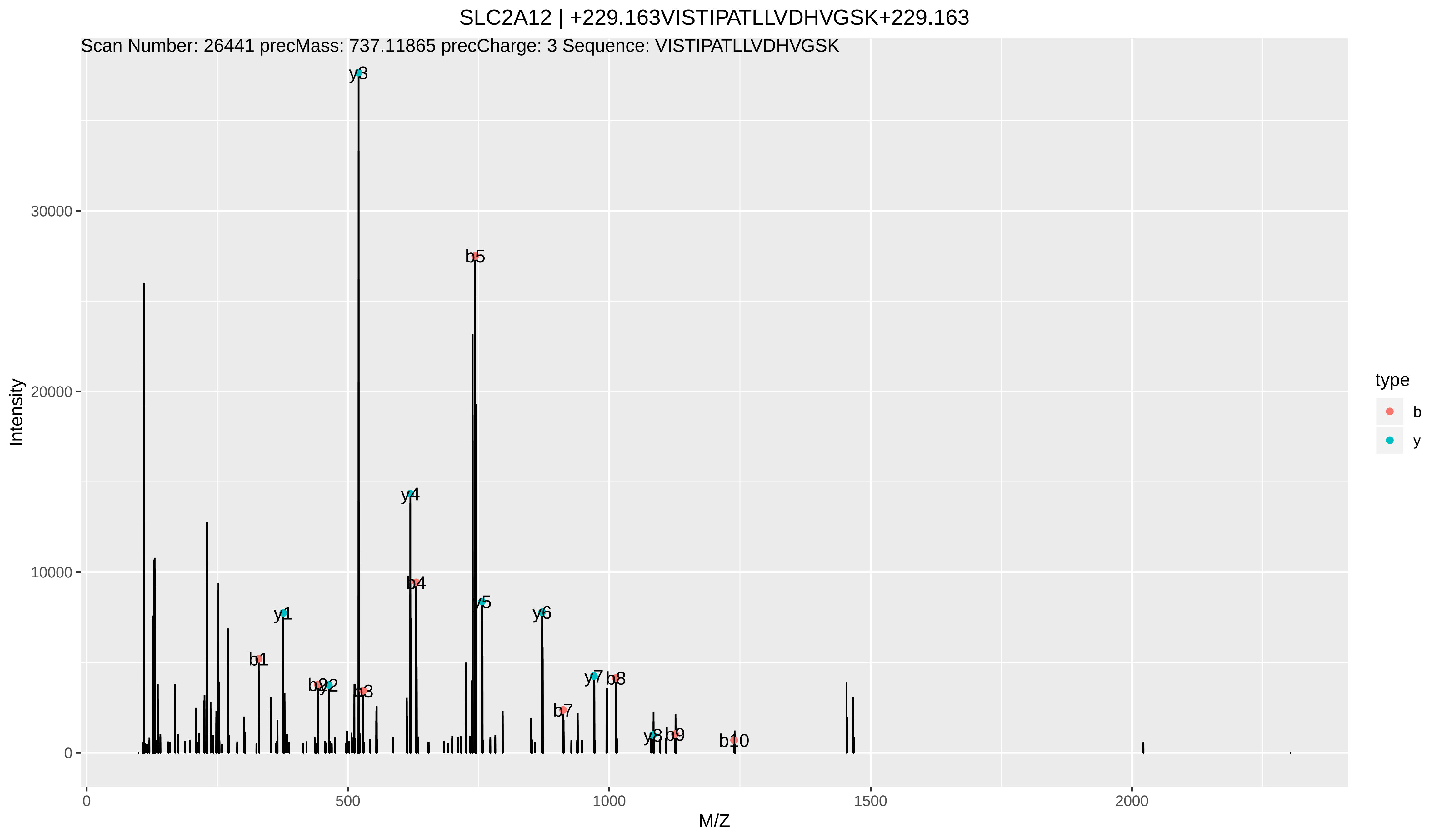

SLC2A12 | +229.163VISTIPATLLVDHVGSK+229.163
Scan Number: 26441 precMass: 737.11865 precCharge: 3 Sequence: VISTIPATLLVDHVGSK
Intensity
M/Z
0
10000
20000
30000
500
1000
1500
2000
type
b
y
y3
b5
y4
b4
y5
y6
y1
b1
b2
y2
b3
b7
y7
b8
y8
b9
b10

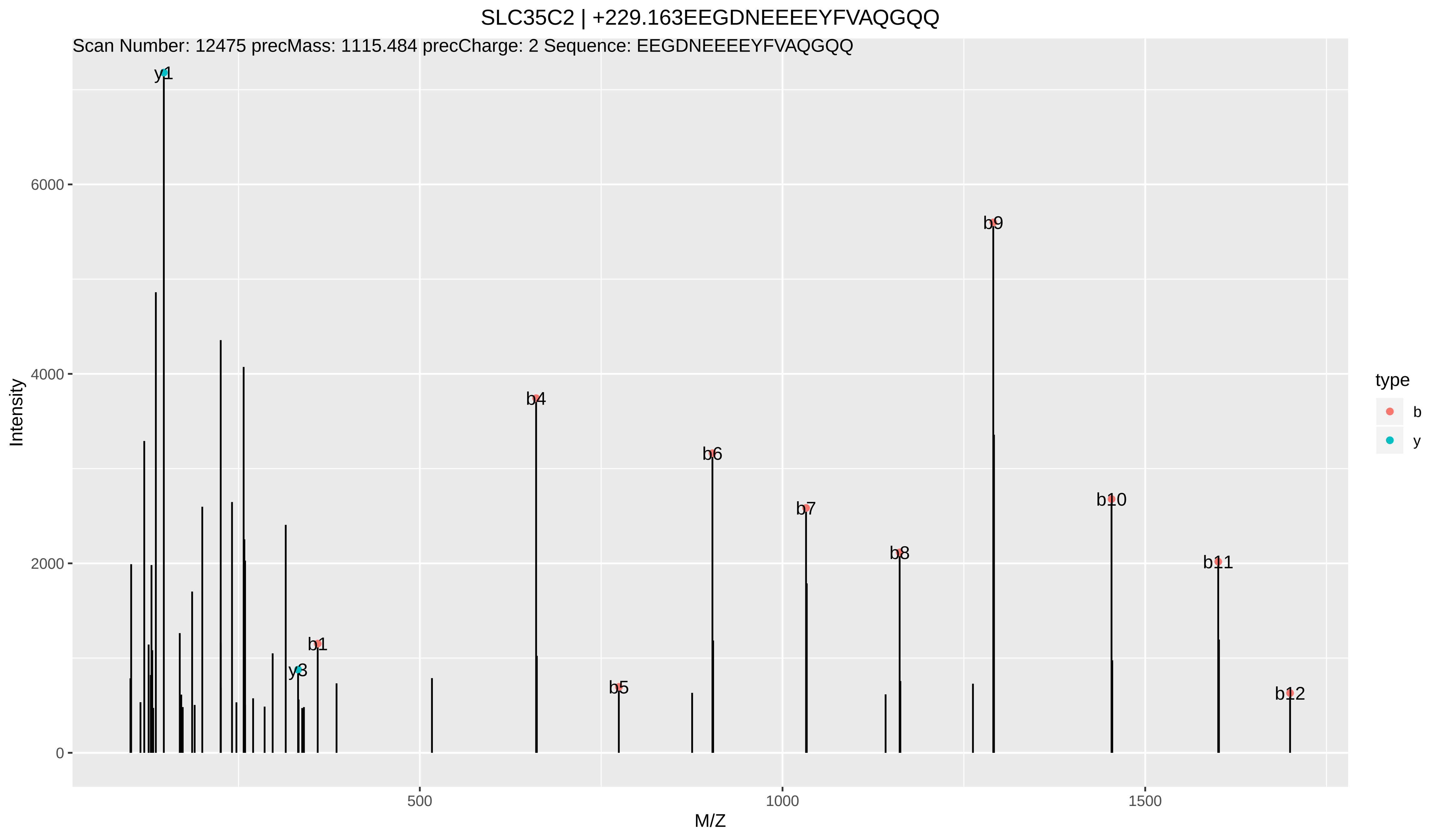
SLC35C2 | +229.163EEGDNEEEEYFVAQGQQ
Scan Number: 12475 precMass: 1115.484 precCharge: 2 Sequence: EEGDNEEEEYFVAQGQQ
y1
b9
b4
b6
b7
b10
b8
b11
b1
y3
b5
b12
Intensity
6000
4000
2000
0
500
1000
1500
M/Z
type
b
y

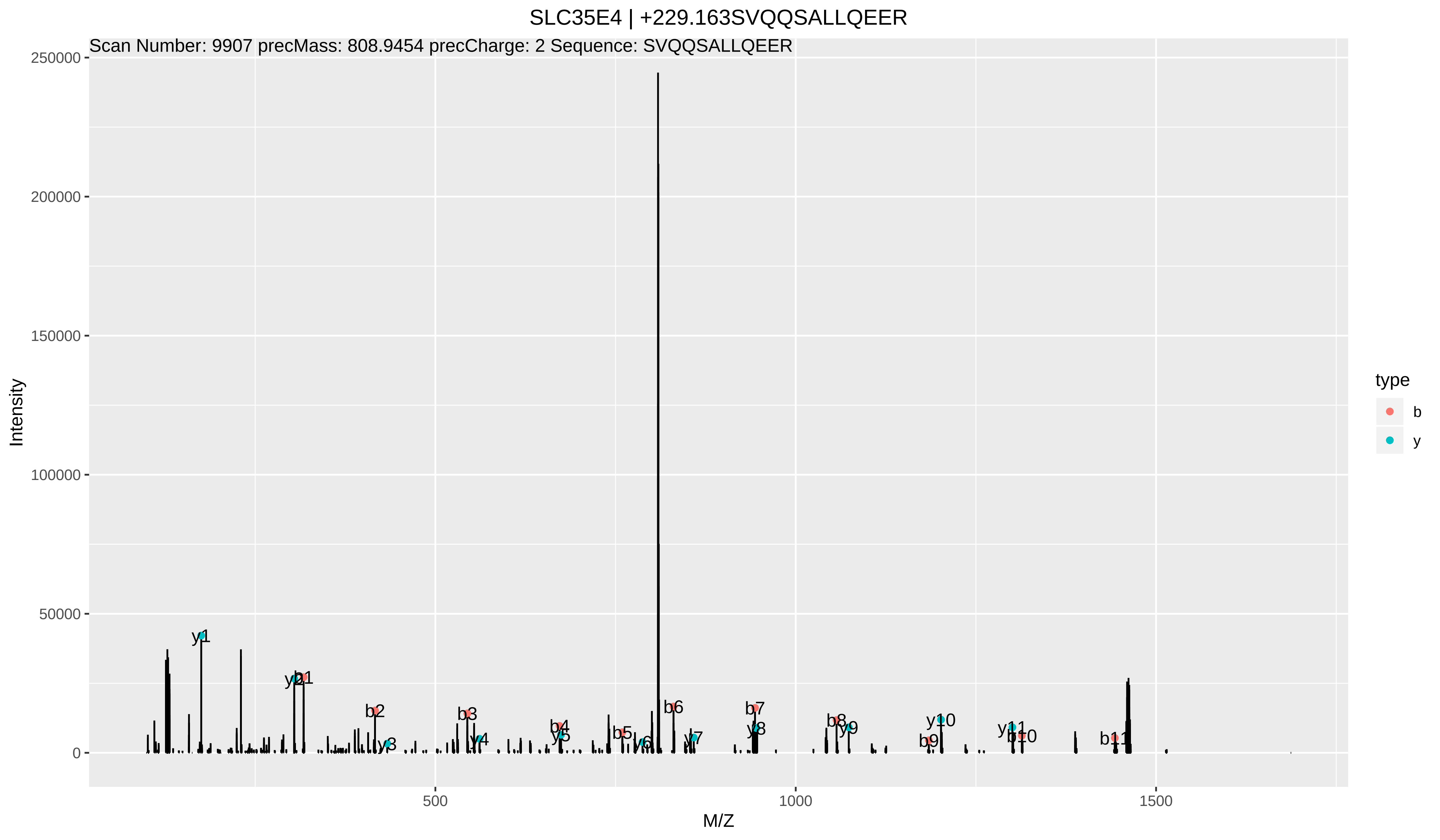

SLC35E4 | +229.163SVQQSALLQEER
Scan Number: 9907 precMass: 808.9454 precCharge: 2 Sequence: SVQQSALLQEER
Intensity
M/Z
type
b
y
0
50000
100000
150000
200000
250000
500
1000
1500
y1
y2
b1
b2
y3
b3
y4
b4
y5
b5
y6
b6
y7
b7
y8
b8
y9
b9
y10
y11
b10
b11

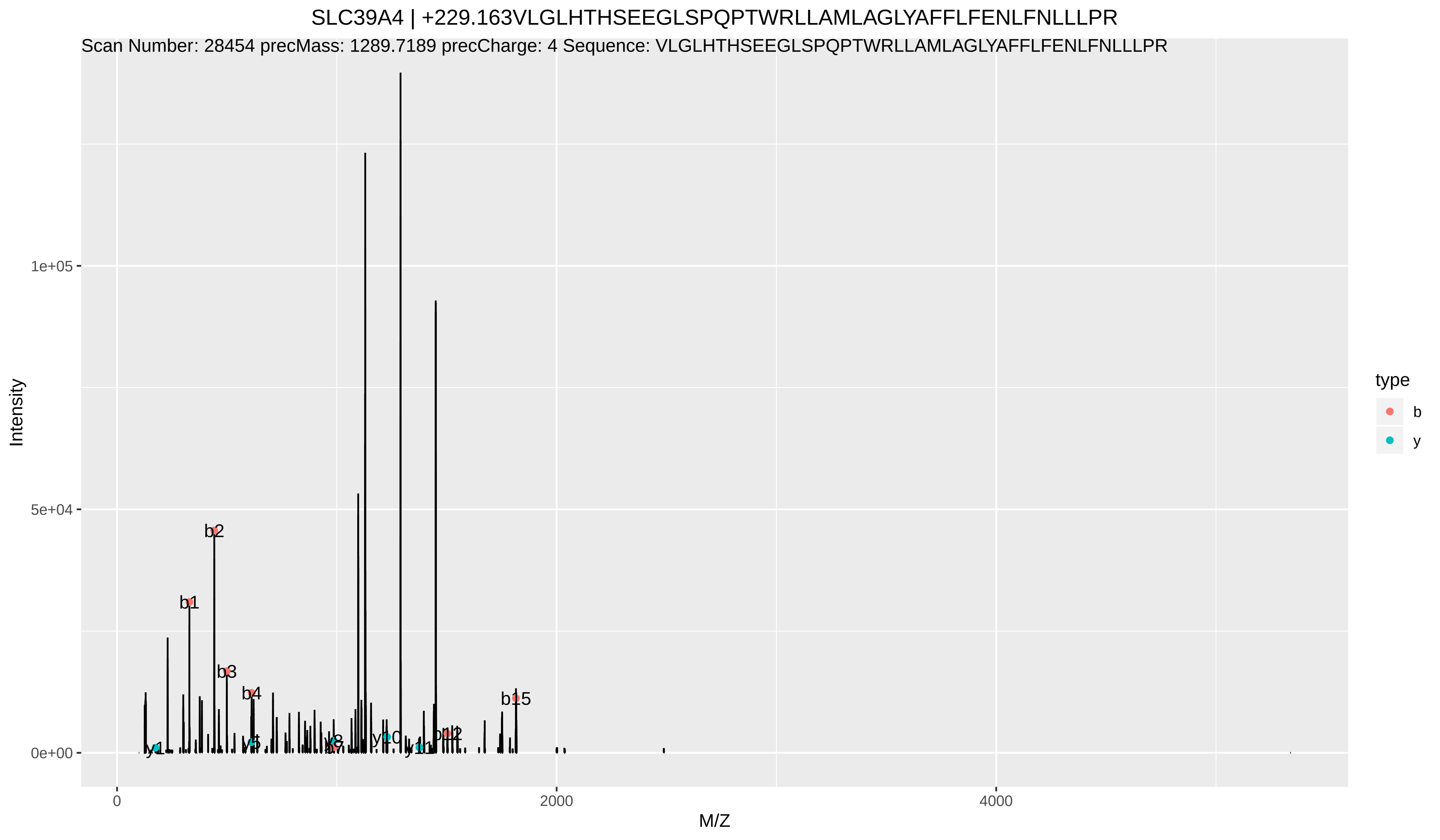

SLC39A4 | +229.163VLGLHTHSEEGLSPQPTWRLLAMLAGLYAFFLFENLFNLLLPR
Scan Number: 28454 precMass: 1289.7189 precCharge: 4 Sequence: VLGLHTHSEEGLSPQPTWRLLAMLAGLYAFFLFENLFNLLLPR
Intensity
1e+05
5e+04
0e+00
M/Z
0
2000
4000
b1
b2
b3
b4
b15
y1
y10
type
b
y

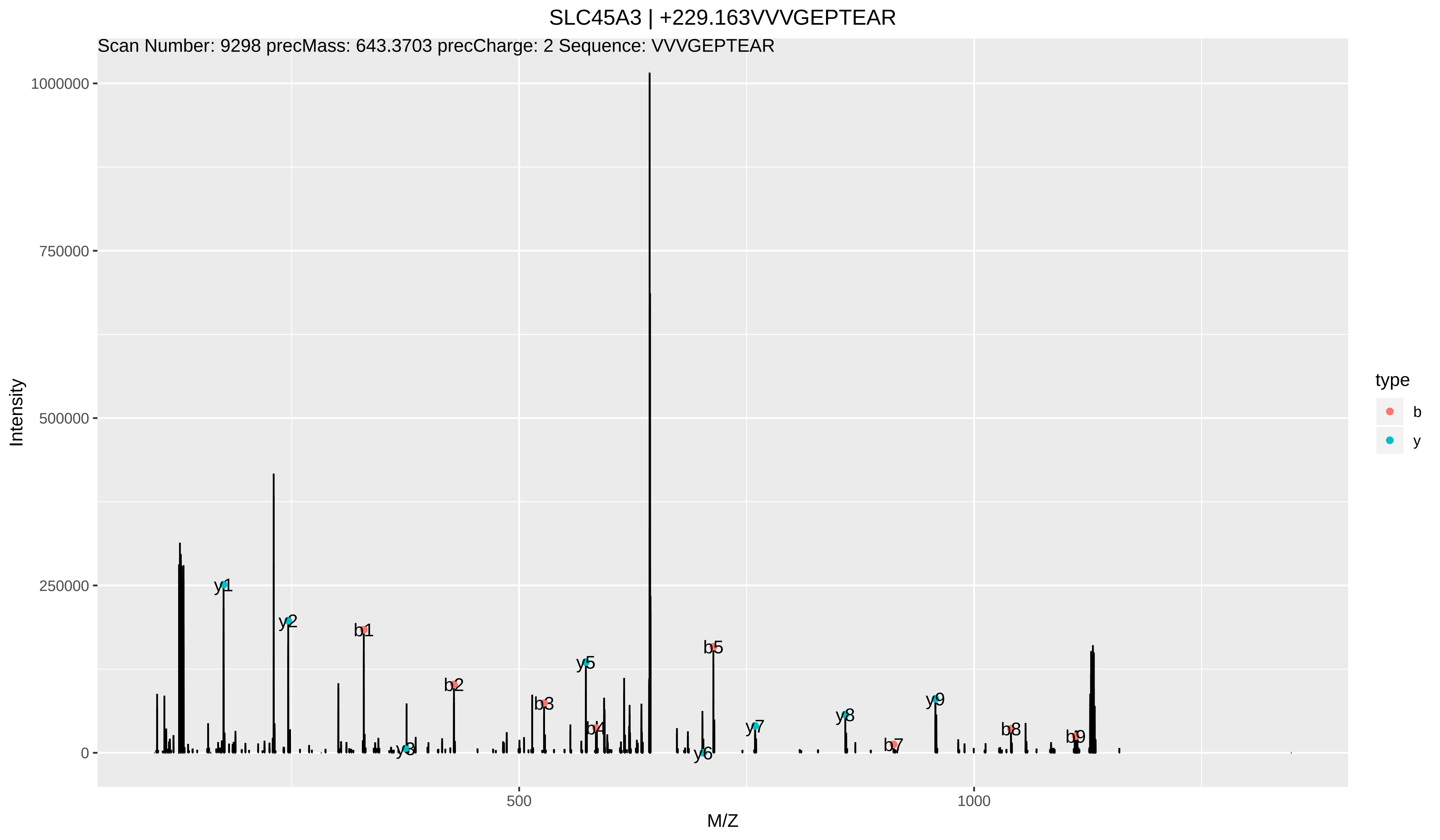
SLC45A3 | +229.163VVVGEPTEAR
Scan Number: 9298 precMass: 643.3703 precCharge: 2 Sequence: VVVGEPTEAR
Intensity
1000000
750000
500000
250000
0
M/Z
500
1000
y1
y2
b1
y3
b2
b3
y5
b4
y6
b5
y7
y8
b7
y9
b8
b9
type
b
y

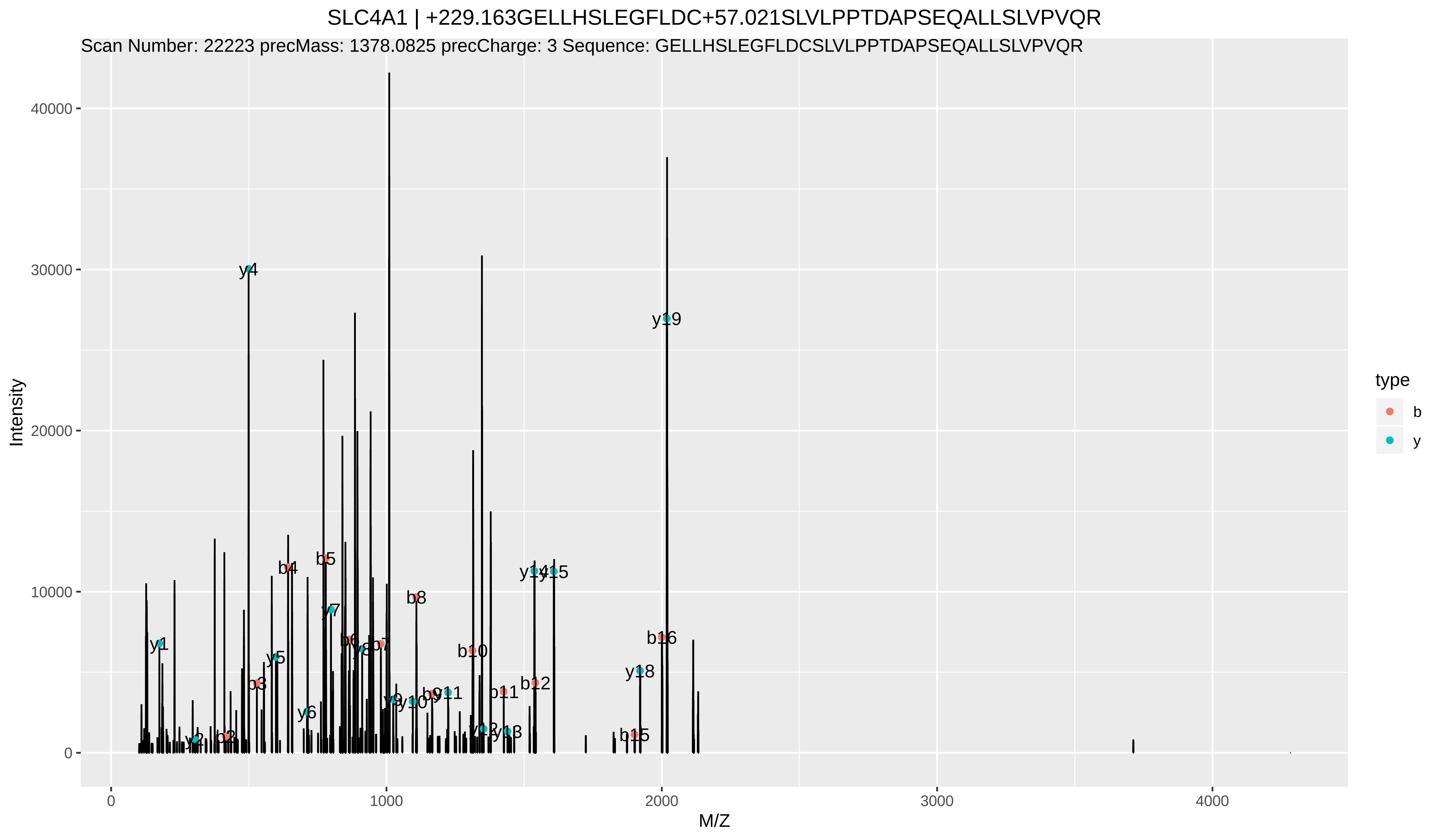
SLC4A1 | +229.163GELLHSLEGFLDC+57.021SLVLPPTDAPSEQALLSLVPVQR
Scan Number: 22223 precMass: 1378.0825 precCharge: 3 Sequence: GELLHSLEGFLDCSLVLPPTDAPSEQALLSLVPVQR
Intensity
M/Z
type
b
y
0
10000
20000
30000
40000
1000
2000
3000
4000
y1
y2
b2
b3
y4
b4
y5
b5
y6
y7
b6
y8
b7
b8
y9
y10
b9
y11
b10
b11
y12
y13
y14
y15
b12
b15
b16
y18
y19

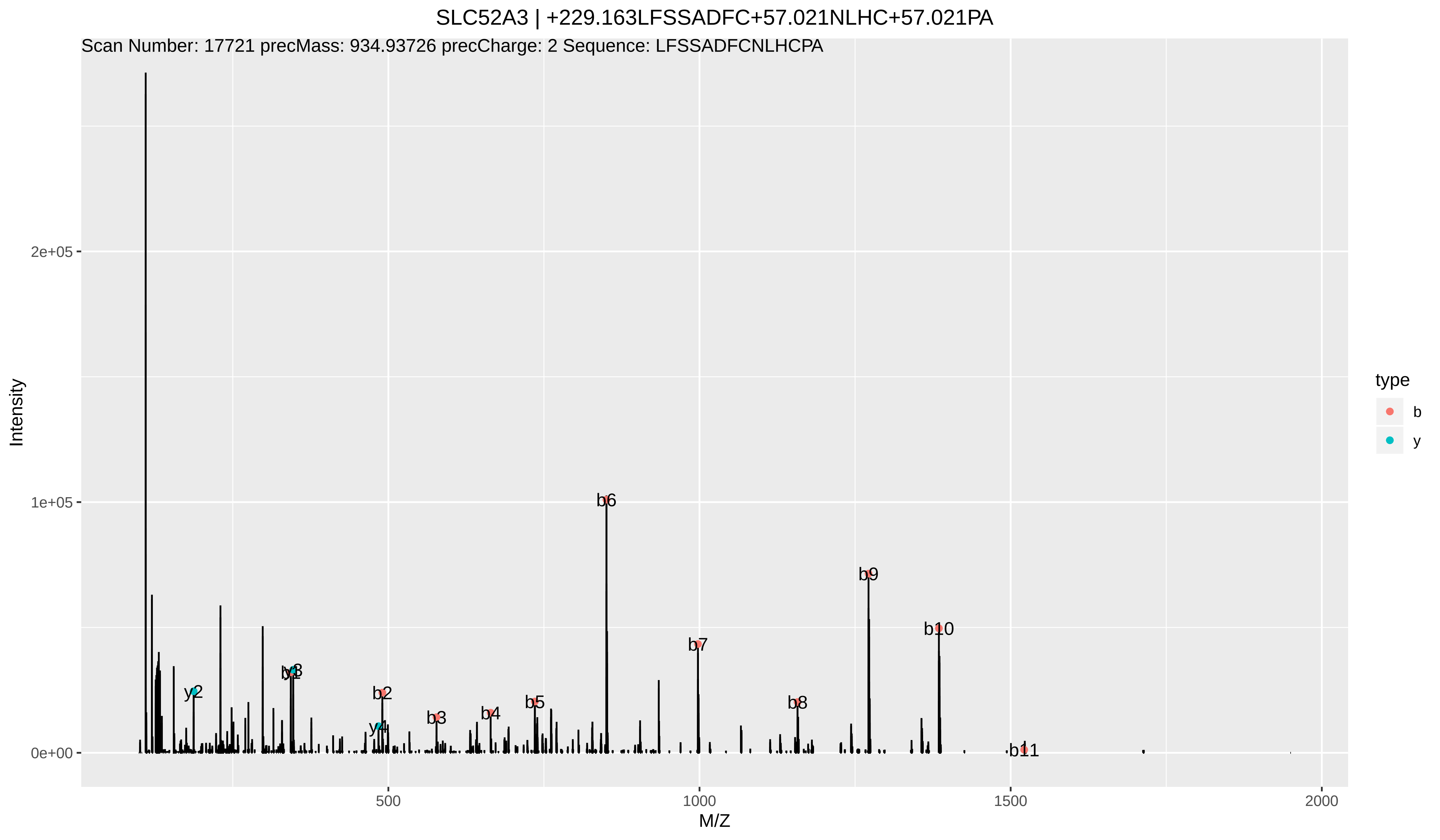

SLC52A3 | +229.163LFSSADFC+57.021NLHC+57.021PA
Scan Number: 17721 precMass: 934.93726 precCharge: 2 Sequence: LFSSADFCNLHCPA
Intensity
2e+05
1e+05
0e+00
M/Z
500
1000
1500
2000
b6
b9
b10
b7
y2
y3
b1
b2
b3
b4
b5
y4
b8
b11
type
b
y

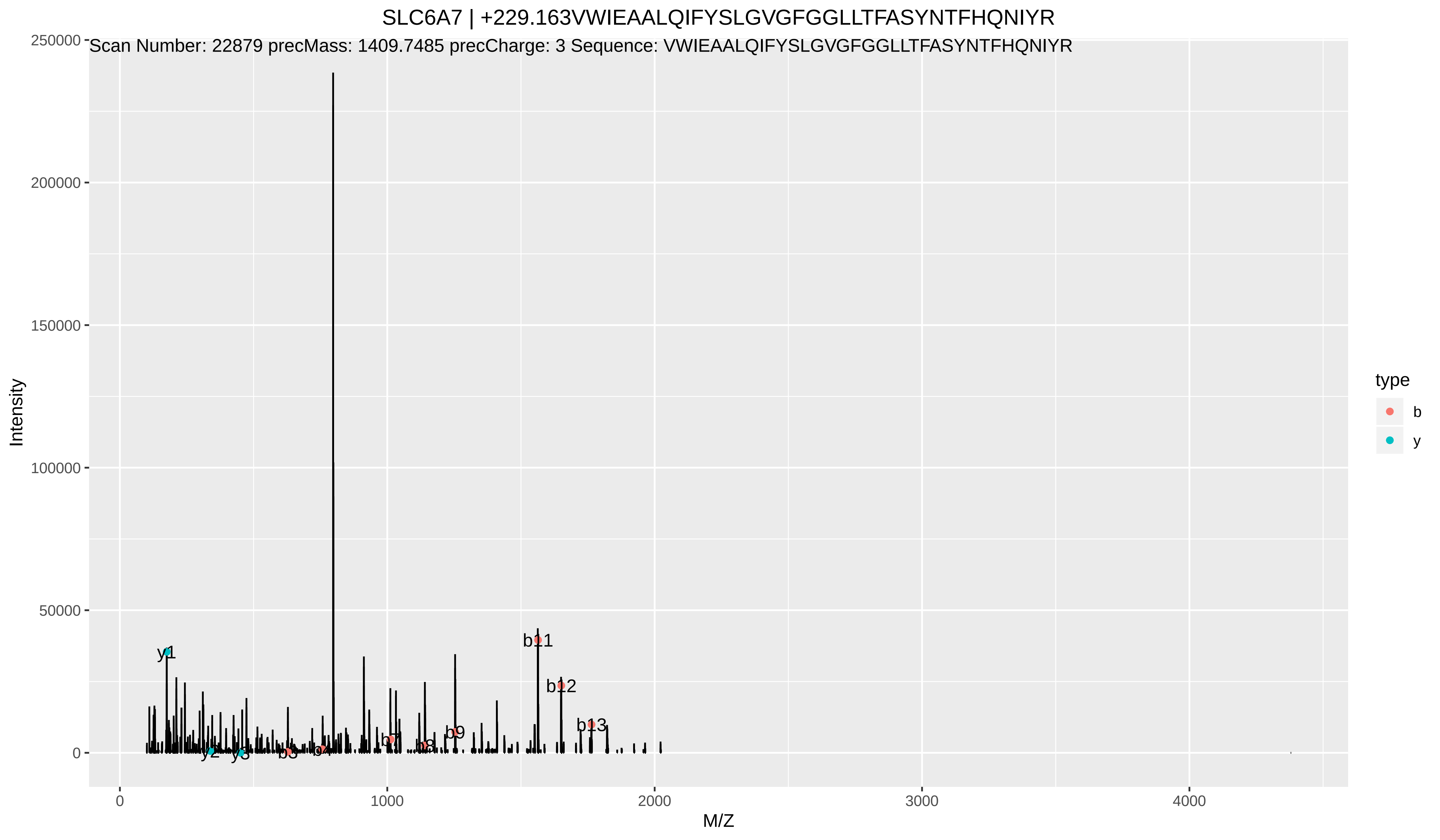
SLC6A7 | +229.163VWIEAALQIFYSLGVGFGGLLTFASYNTFHQNIYR
Scan Number: 22879 precMass: 1409.7485 precCharge: 3 Sequence: VWIEAALQIFYSLGVGFGGLLTFASYNTFHQNIYR
Intensity
M/Z
250000
200000
150000
100000
50000
0
1000
2000
3000
4000
y1
y2
y3
b3
b4
b7
b8
b9
b11
b12
b13
type
b
y

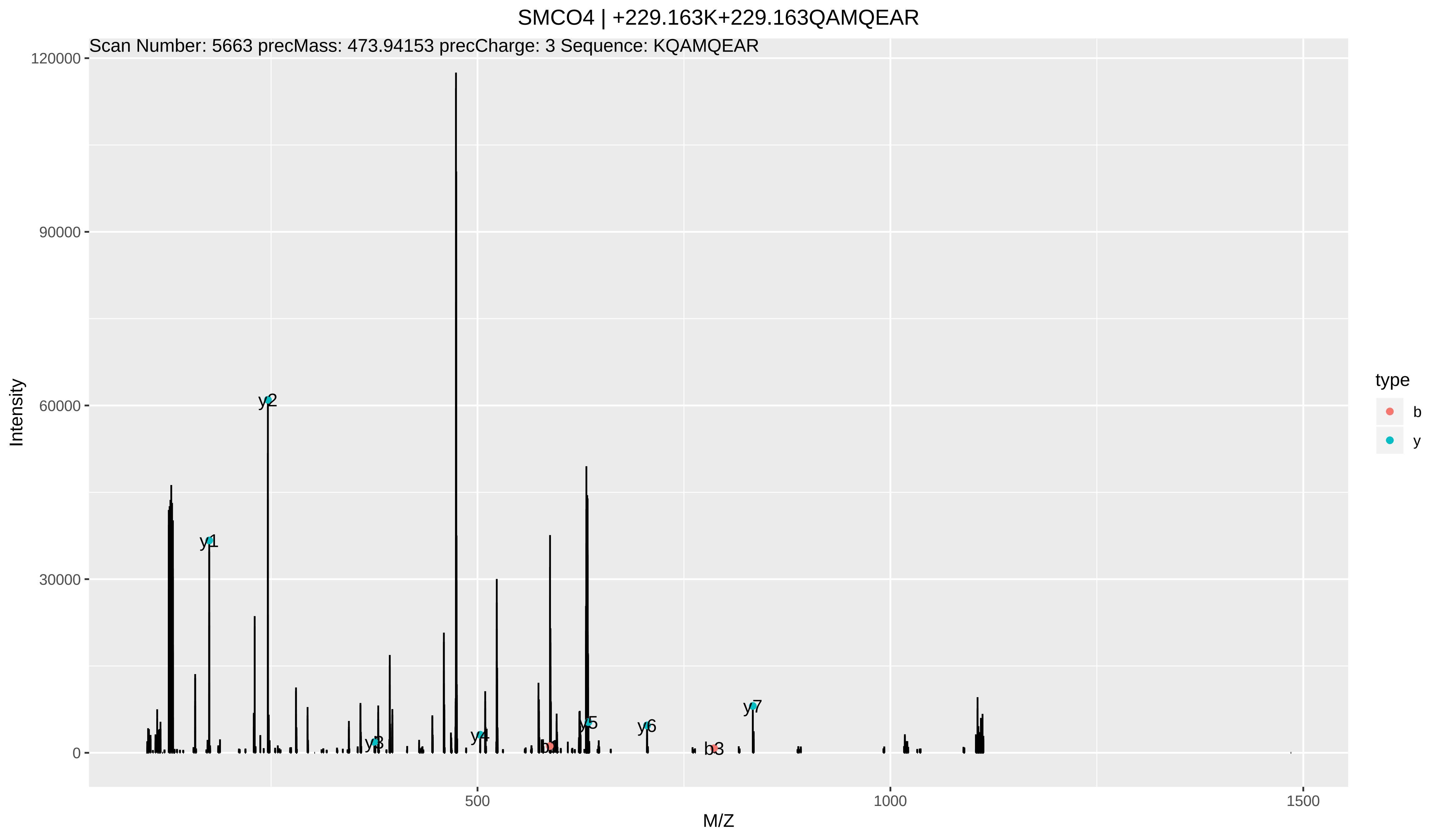
SMCO4 | +229.163K+229.163QAMQEAR
Scan Number: 5663 precMass: 473.94153 precCharge: 3 Sequence: KQAMQEAR
Intensity
120000
90000
60000
30000
0
M/Z
500
1000
1500
y1
y2
y3
y4
y5
y6
y7
b3
type
b
y

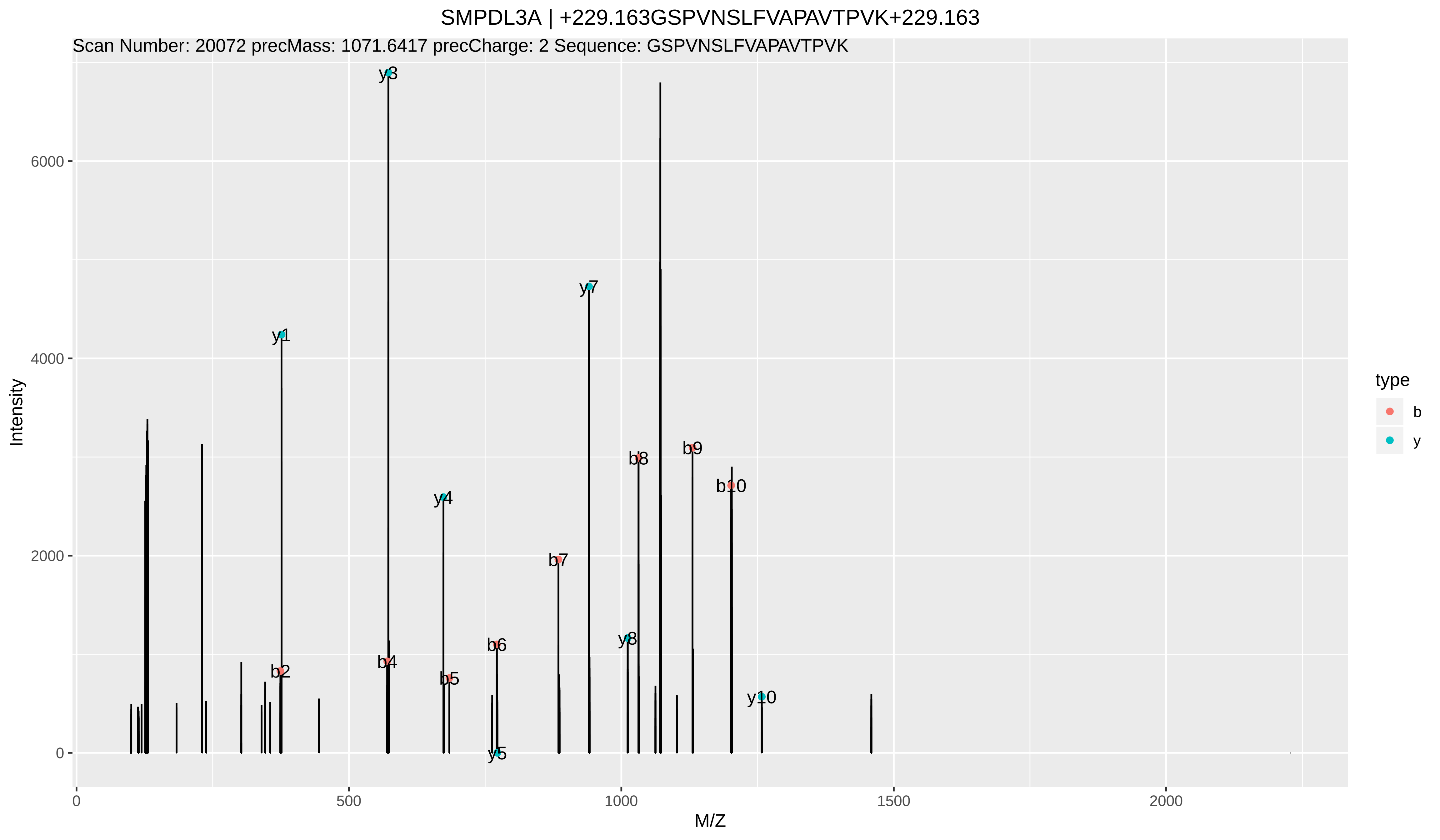
SMPDL3A | +229.163GSPVNSLFVAPAVTPVK+229.163
Scan Number: 20072 precMass: 1071.6417 precCharge: 2 Sequence: GSPVNSLFVAPAVTPVK
Intensity
M/Z
type
b
y
y3
y7
y1
y4
b8
b9
b10
b7
b6
y8
b2
b4
b5
y10
y5
0
2000
4000
6000
0
500
1000
1500
2000

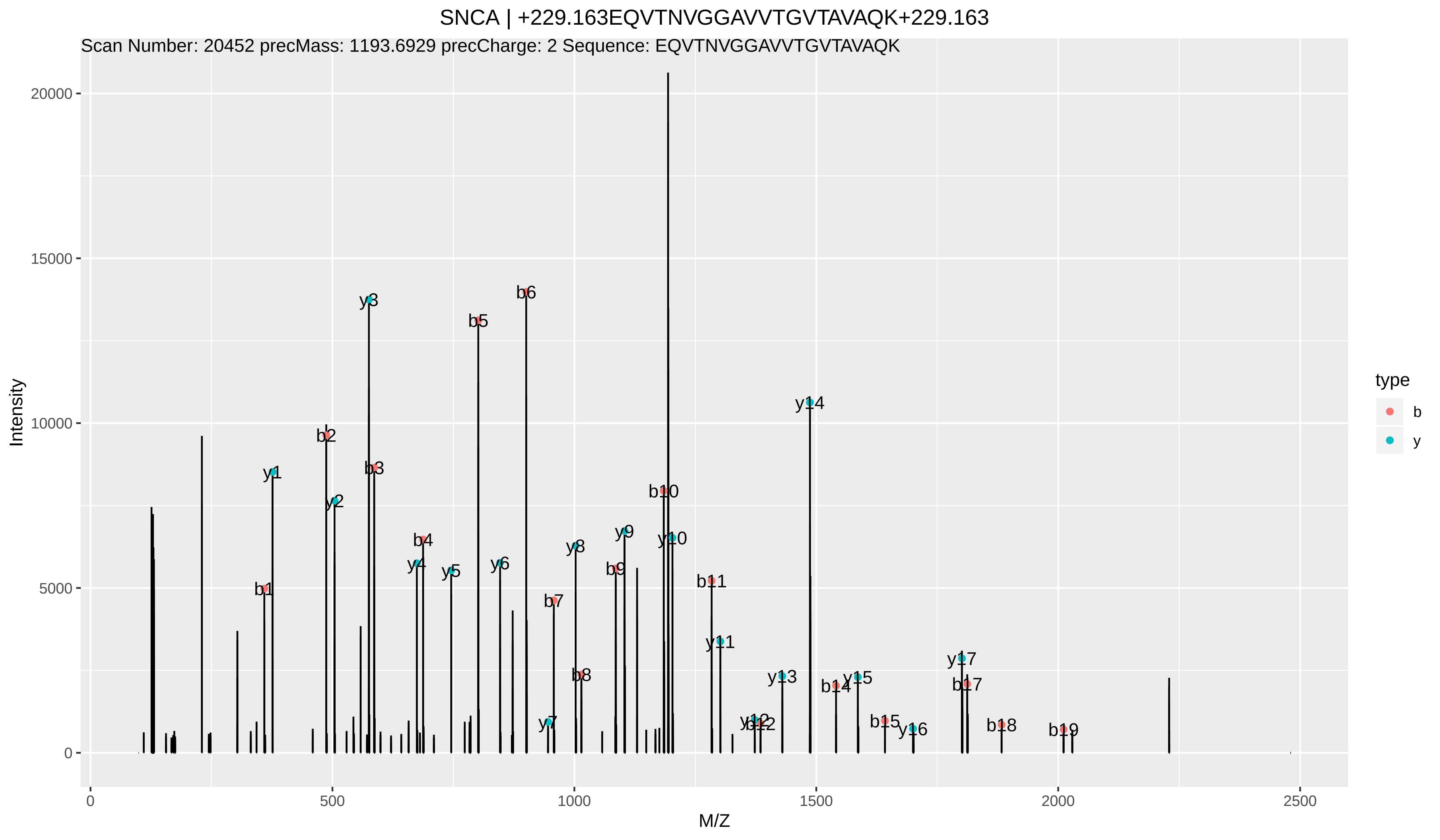

SNCA | +229.163EQVTNVGGAVVTGVTAVAQK+229.163
Scan Number: 20452 precMass: 1193.6929 precCharge: 2 Sequence: EQVTNVGGAVVTGVTAVAQK
Intensity
M/Z
0
5000
10000
15000
20000
500
1000
1500
2000
2500
type
b
y
y1
y2
y3
y4
y5
y6
y7
y8
y9
y10
y11
y12
y13
y14
y15
y16
y17
b1
b2
b3
b4
b5
b6
b7
b8
b9
b10
b11
b12
b14
b15
b17
b18
b19

SNCA | +229.163EGVLYVGSK+229.163

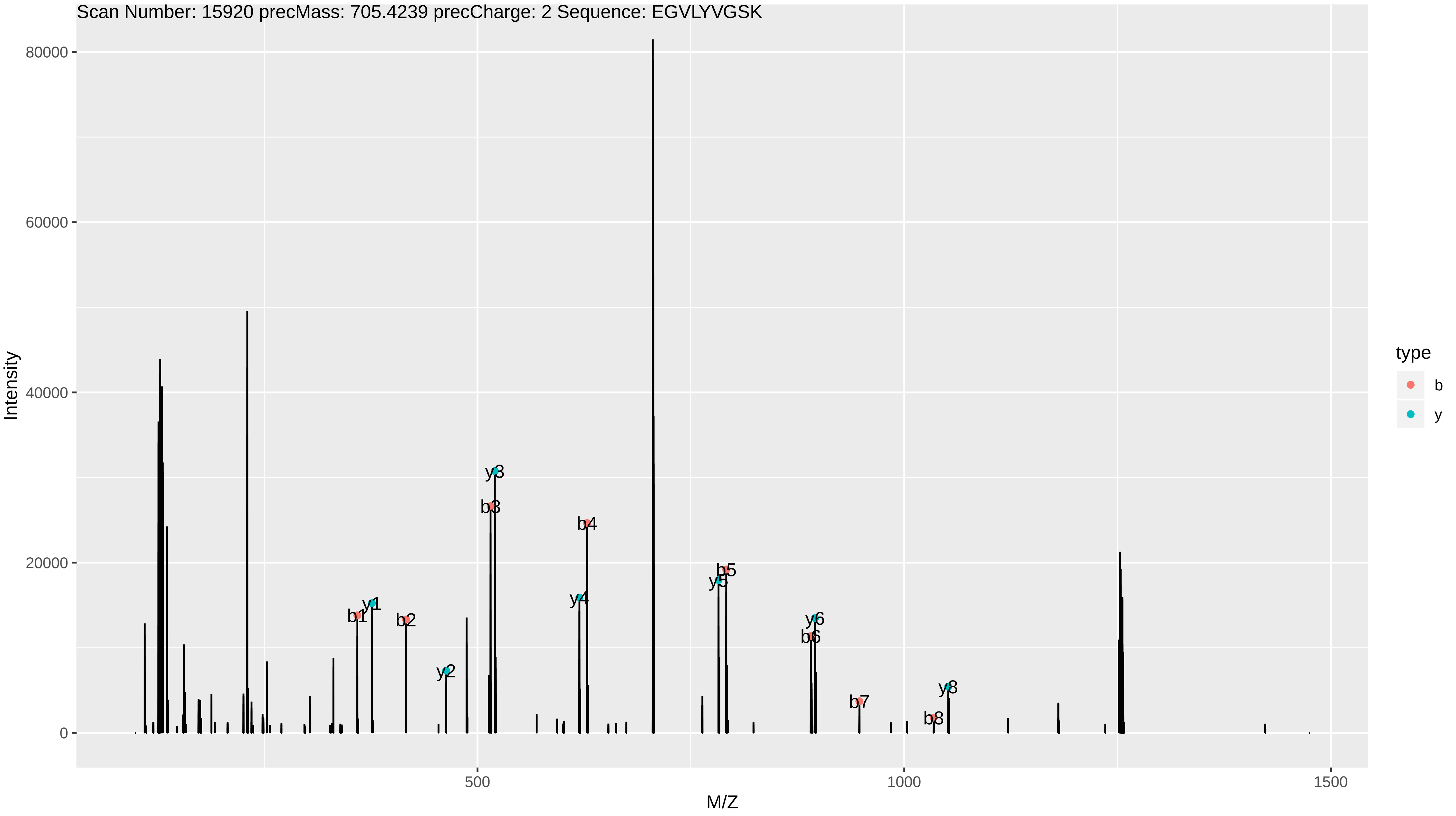

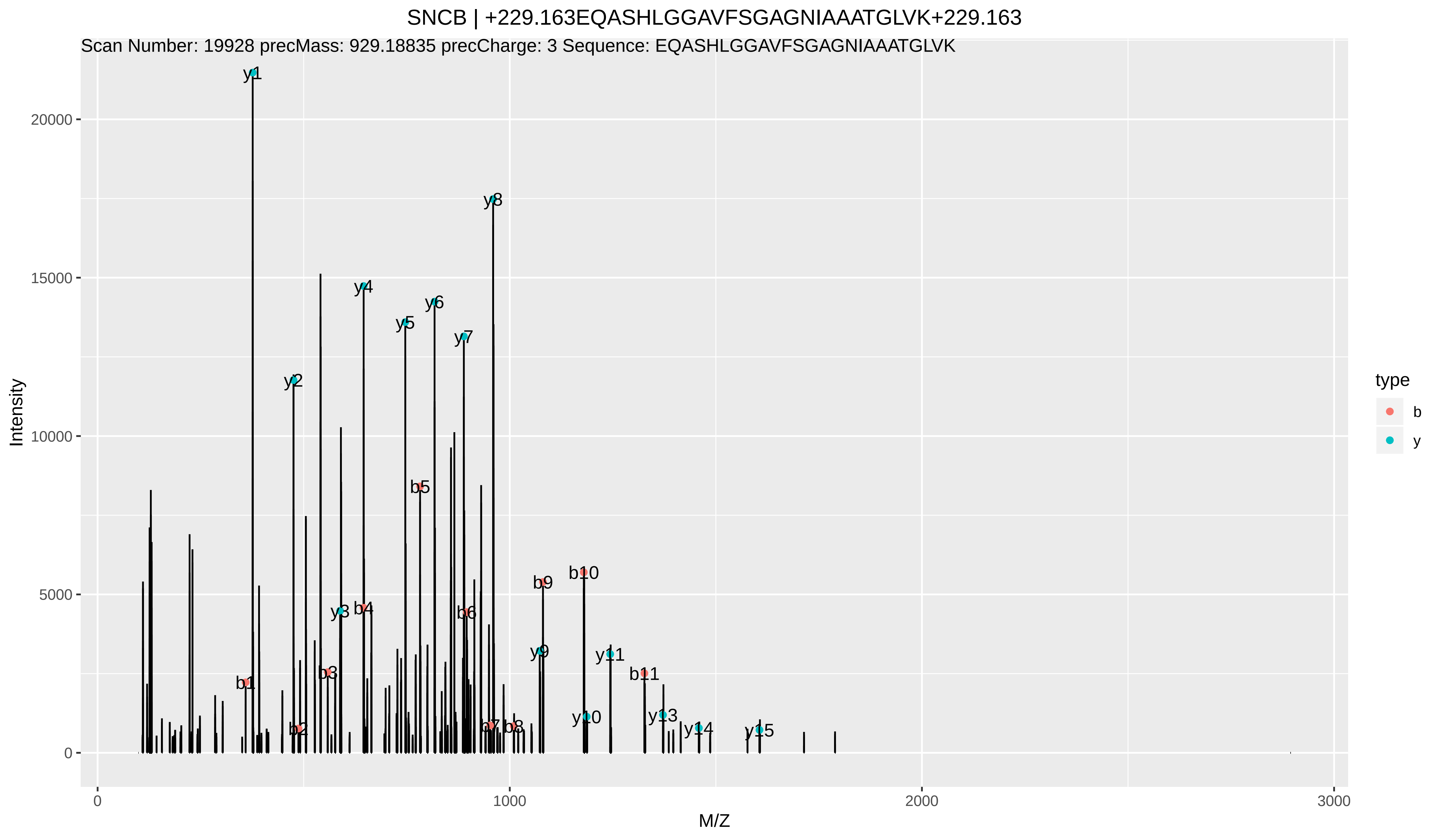

SNCB | +229.163EQASHLGGAVFSGAGNIAAATGLVK+229.163
Scan Number: 19928 precMass: 929.18835 precCharge: 3 Sequence: EQASHLGGAVFSGAGNIAAATGLVK
Intensity
M/Z
0
5000
10000
15000
20000
0
1000
2000
3000
type
b
y
y1
y2
y3
y4
y5
y6
y7
y8
y9
y10
y11
y13
y14
y15
b1
b2
b3
b4
b5
b6
b7
b8
b9
b10
b11

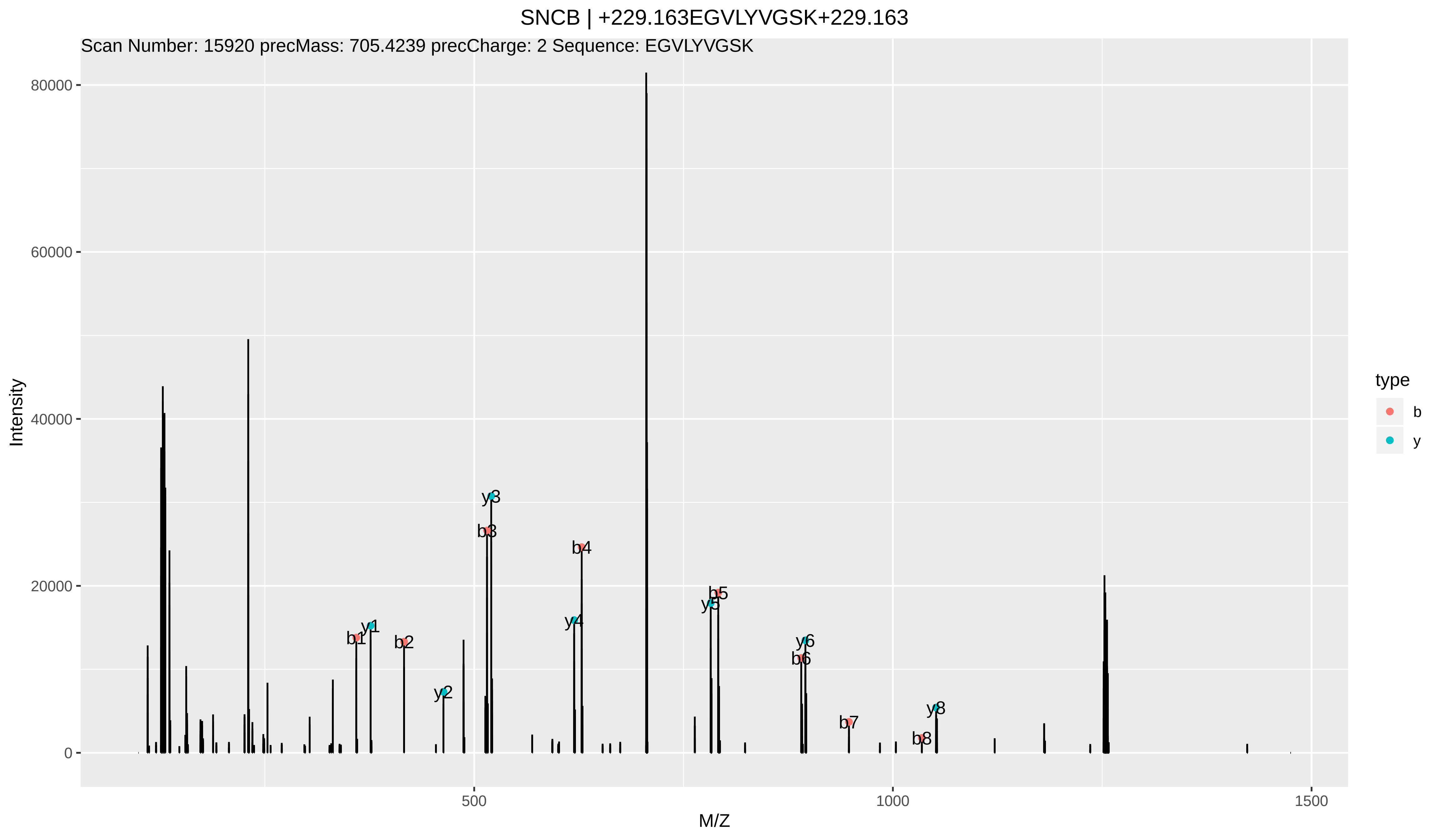

SNCB | +229.163EGVLYVGSK+229.163
Scan Number: 15920 precMass: 705.4239 precCharge: 2 Sequence: EGVLYVGSK
Intensity
80000
60000
40000
20000
0
500
1000
1500
M/Z
y3
b3
b4
y4
b5
y5
y6
b6
b1
y1
b2
y2
b7
b8
y8
type
b
y

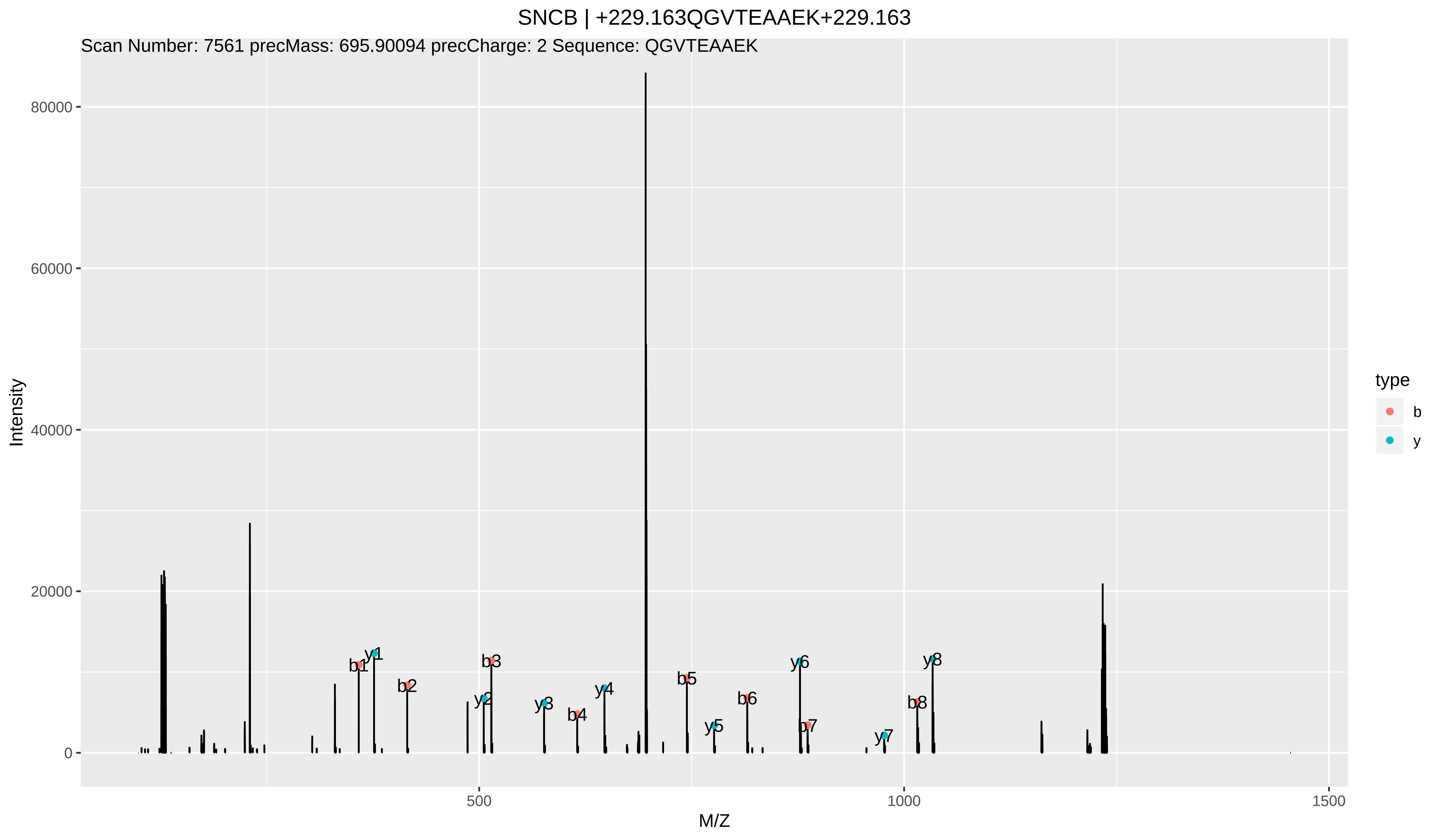

SNCB | +229.163QGVTEAAEK+229.163
Scan Number: 7561 precMass: 695.90094 precCharge: 2 Sequence: QGVTEAAEK
Intensity
80000
60000
40000
20000
0
M/Z
500
1000
1500
b1
y1
b2
y2
b3
y3
b4
y4
b5
y5
b6
y6
b7
y7
b8
y8
type
b
y

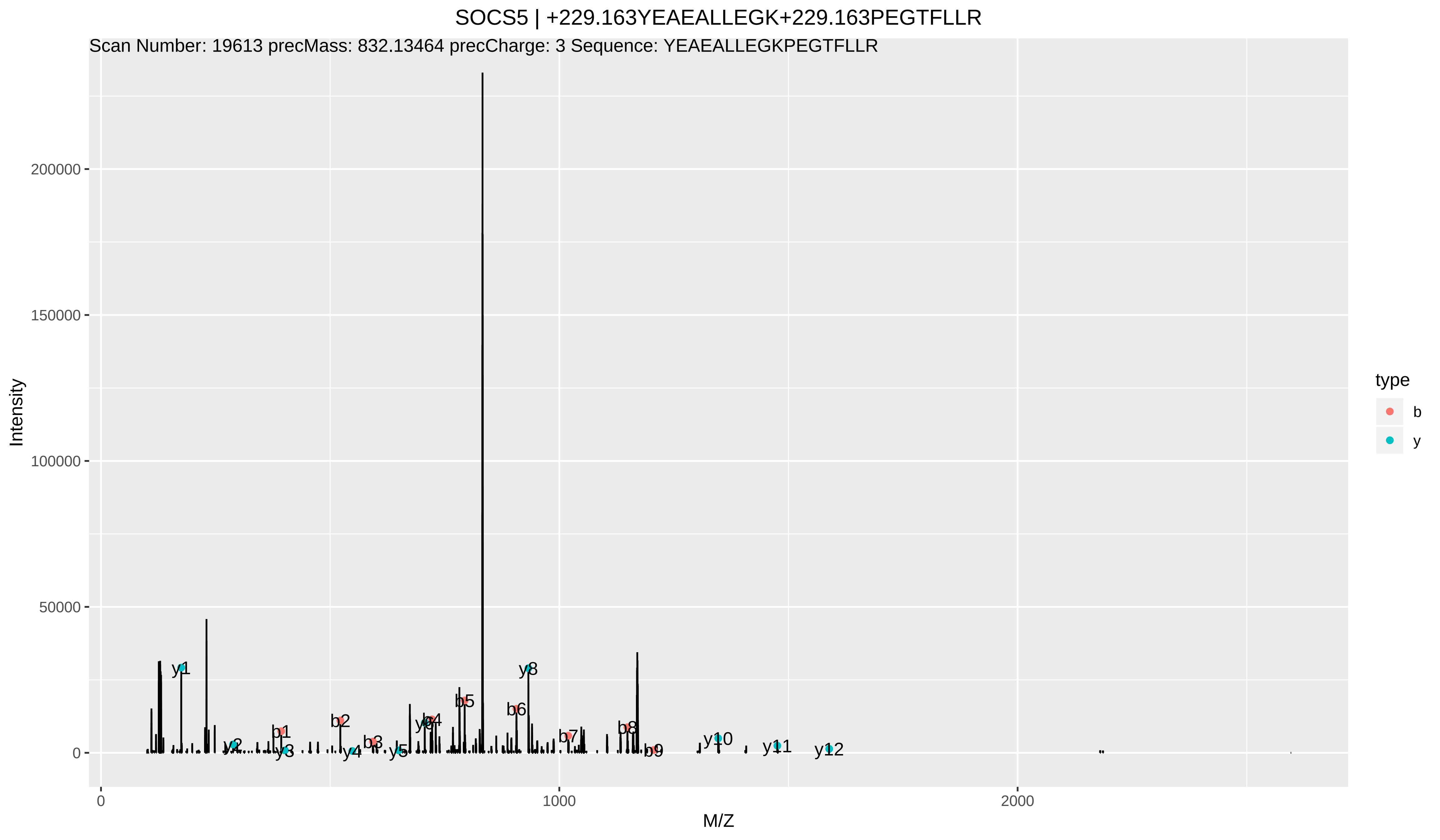
SOCS5 | +229.163YEAEALLEGK+229.163PEGTFLLR
Scan Number: 19613 precMass: 832.13464 precCharge: 3 Sequence: YEAEALLEGKPEGTFLLR
Intensity
0
50000
100000
150000
200000
M/Z
0
1000
2000
type
b
y
y1
y2
y3
y4
y5
y6
y8
y10
y11
y12
b1
b2
b3
b4
b5
b6
b7
b8
b9

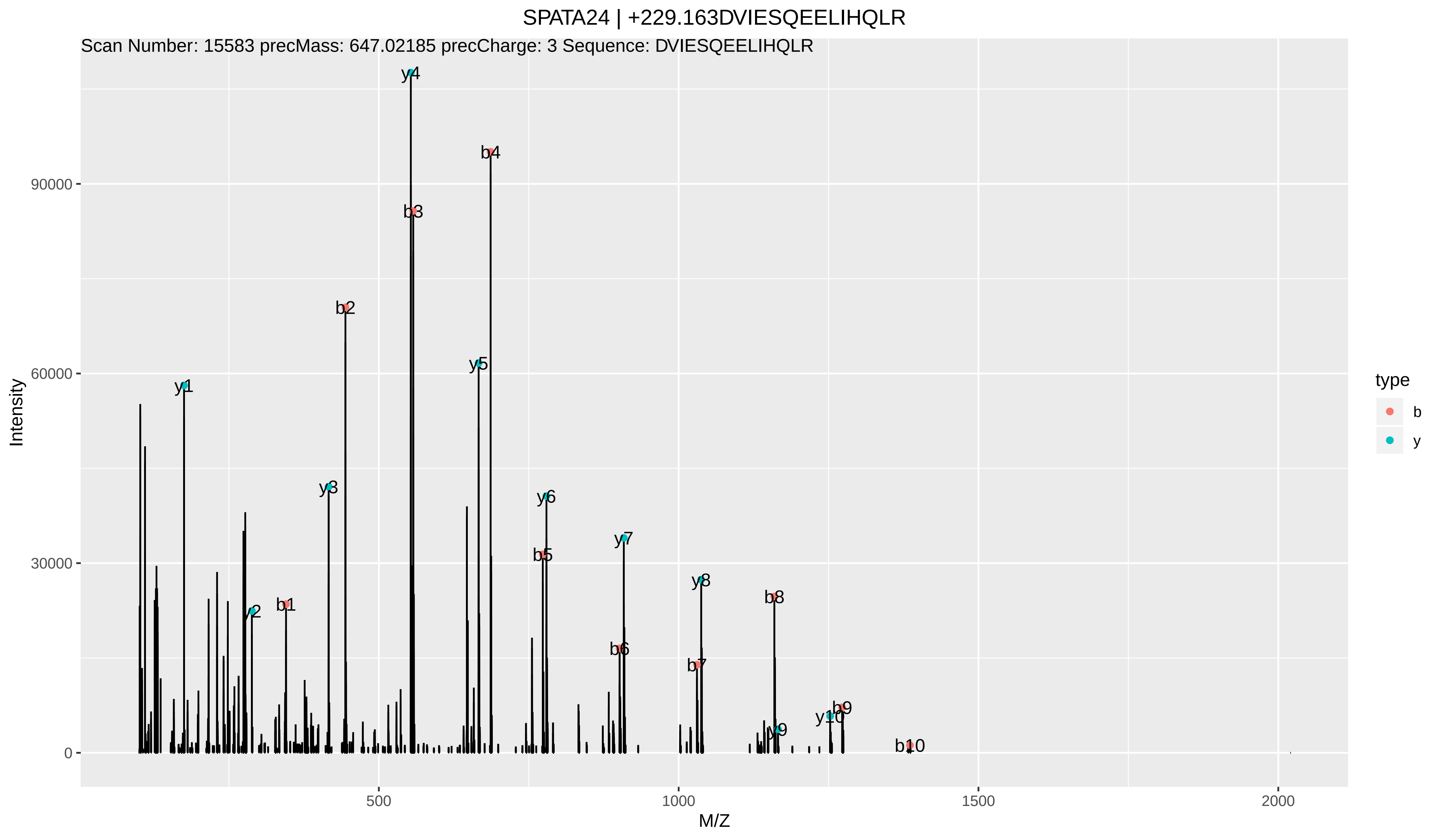

SPATA24 | +229.163DVIESQEELIHQLR
Scan Number: 15583 precMass: 647.02185 precCharge: 3 Sequence: DVIESQEELIHQLR
Intensity
M/Z
0
30000
60000
90000
500
1000
1500
2000
y4
b4
b3
b2
y5
y1
y3
y6
y7
b5
y8
b8
y2
b1
b6
b7
b9
y10
y9
b10
type
b
y

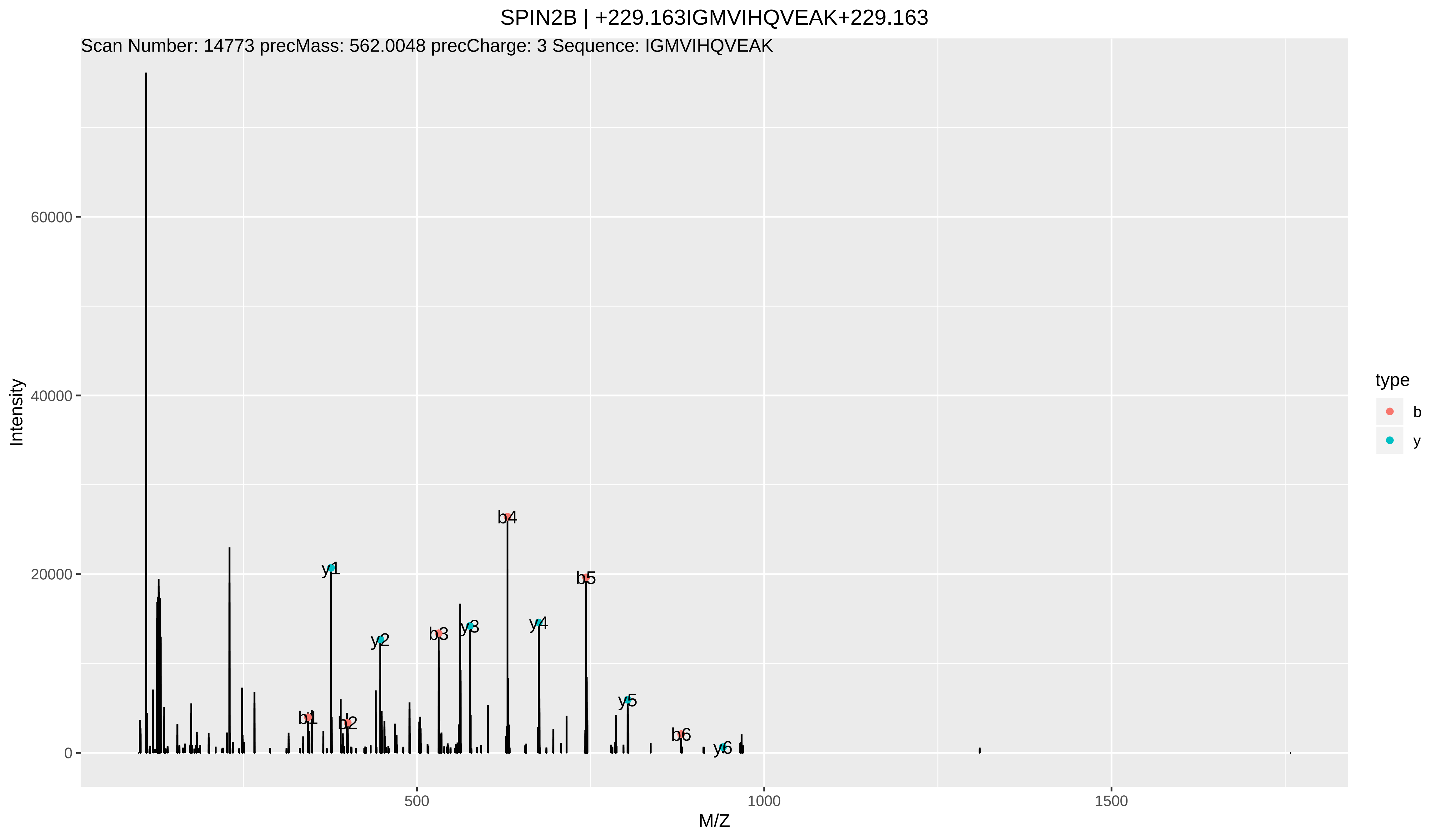
SPIN2B | +229.163IGMVIHQVEAK+229.163
Scan Number: 14773 precMass: 562.0048 precCharge: 3 Sequence: IGMVIHQVEAK
Intensity
60000
40000
20000
0
500
1000
1500
M/Z
b4
y1
b5
y4
y3
y2
b3
y5
b1
b2
b6
y6
type
b
y

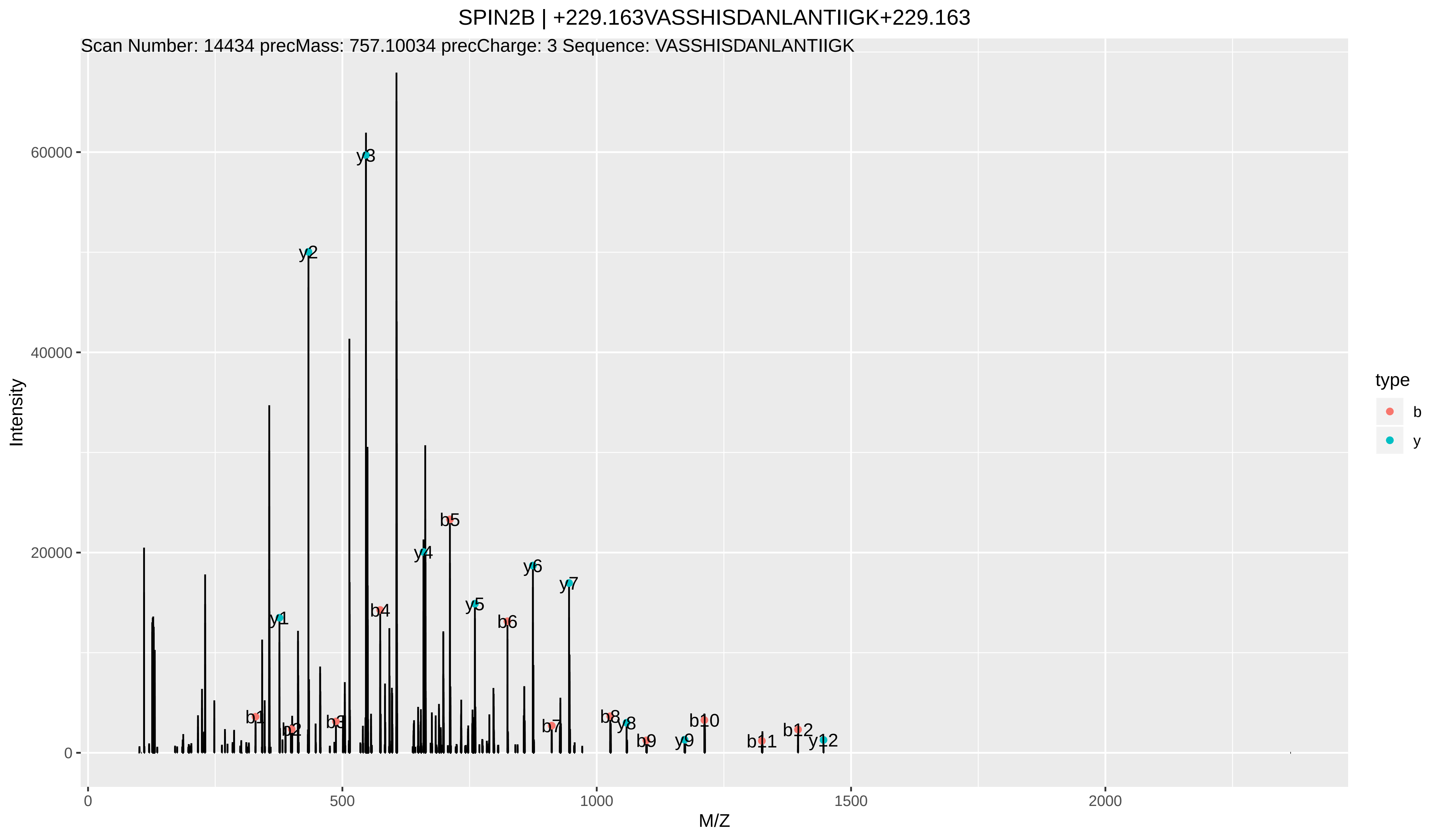

SPIN2B | +229.163VASSHISDANLANTIIGK+229.163
Scan Number: 14434 precMass: 757.10034 precCharge: 3 Sequence: VASSHISDANLANTIIGK
Intensity
M/Z
0
20000
40000
60000
500
1000
1500
2000
y1
y2
y3
y4
y5
y6
y7
y8
y9
y12
b1
b2
b3
b4
b5
b6
b7
b8
b9
b10
b11
b12
type
b
y

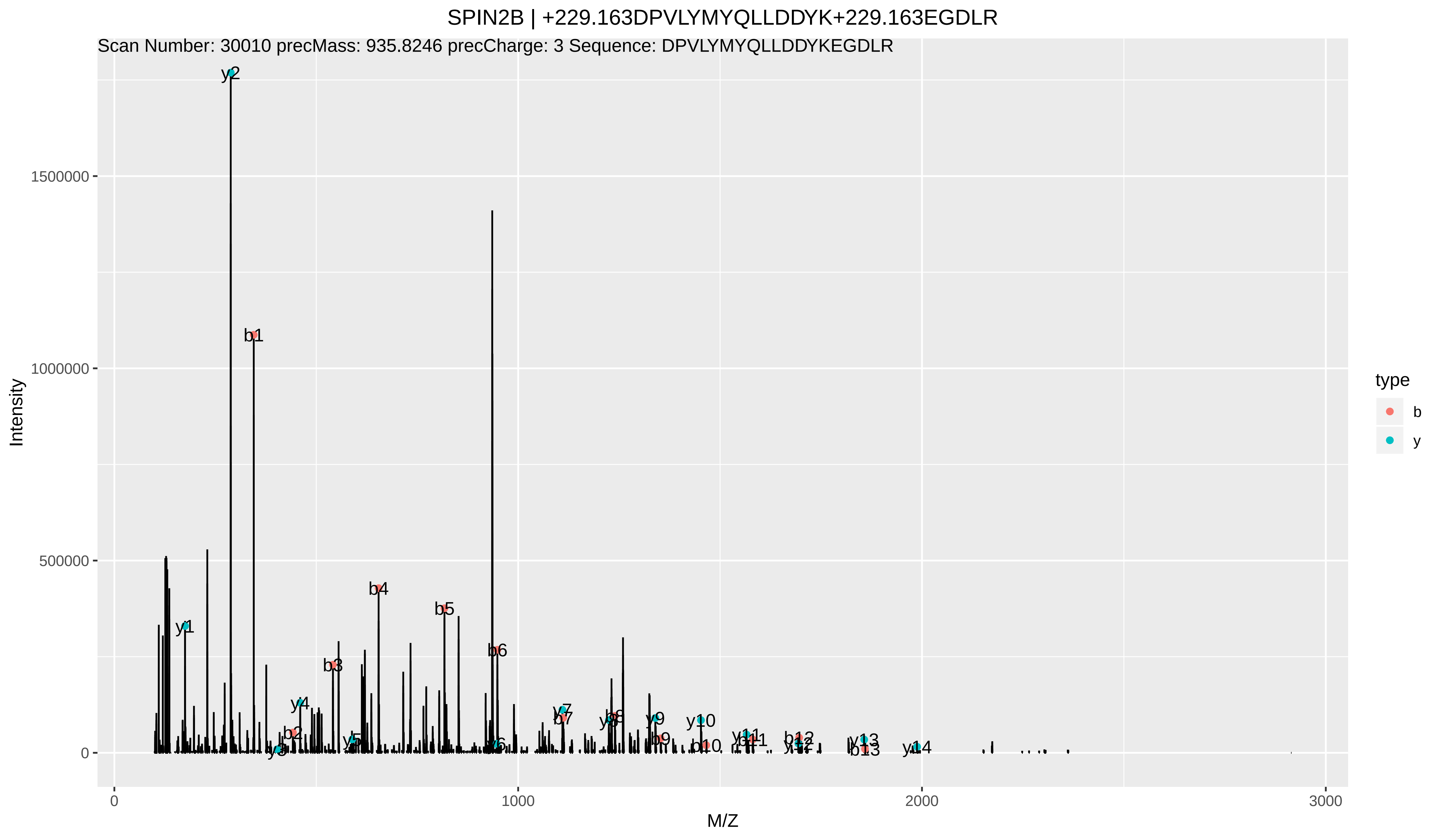

SPIN2B | +229.163DPVLYMYQLLDDYK+229.163EGDLR
Scan Number: 30010 precMass: 935.8246 precCharge: 3 Sequence: DPVLYMYQLLDDYKEGDLR
Intensity
M/Z
0
500000
1000000
1500000
1000
2000
3000
type
b
y
y1
y2
b1
b2
b3
b4
b5
b6
b7
b9
b10
b12
b13
y4
y7
y9
y10
y13
y14

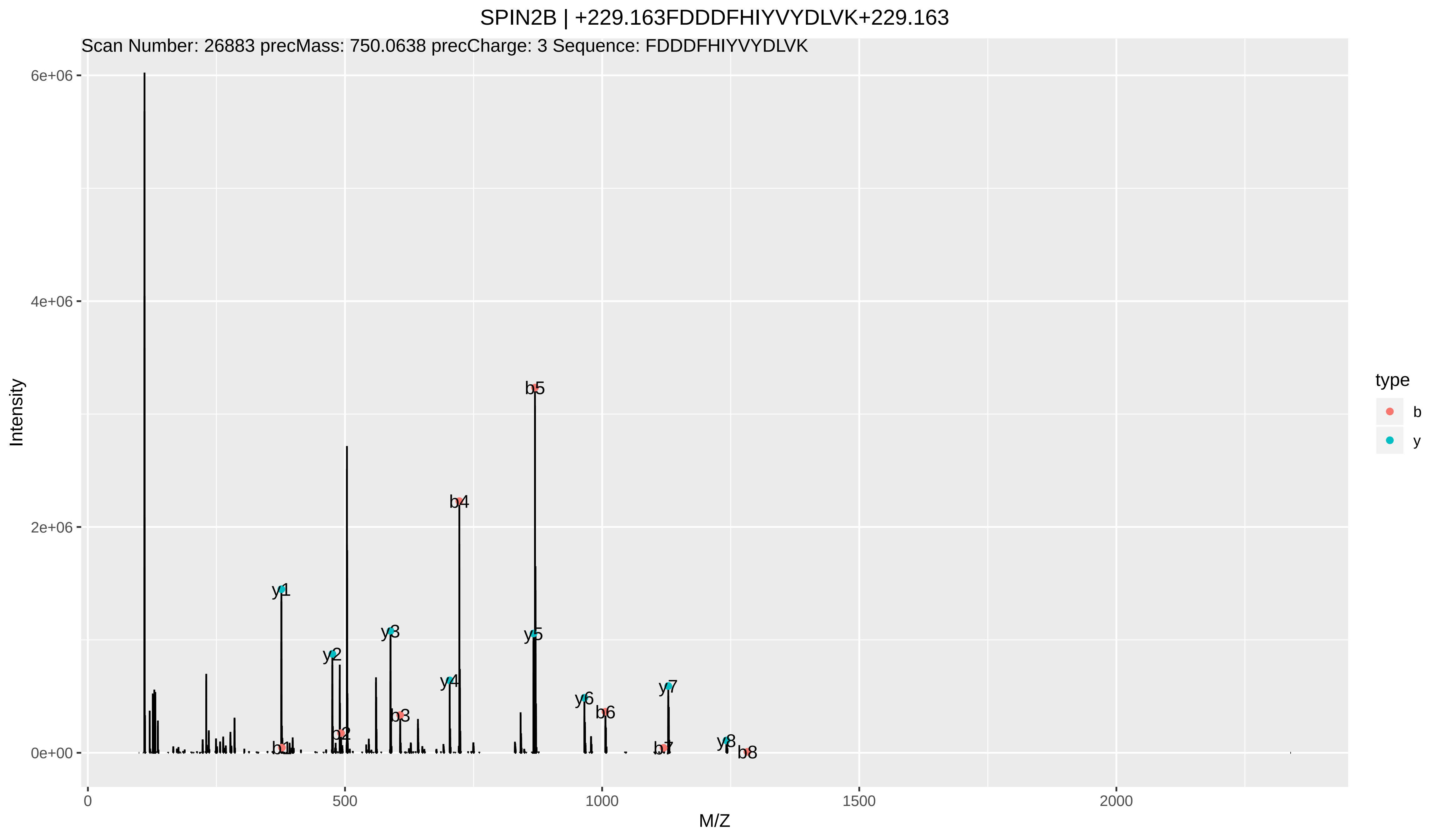

SPIN2B | +229.163FDDDFHIYVYDLVK+229.163
Scan Number: 26883 precMass: 750.0638 precCharge: 3 Sequence: FDDDFHIYVYDLVK
Intensity
6e+06
4e+06
2e+06
0e+00
M/Z
0
500
1000
1500
2000
type
b
y
b1
b2
b3
b4
b5
b6
b7
b8
y1
y2
y3
y4
y5
y6
y7
y8

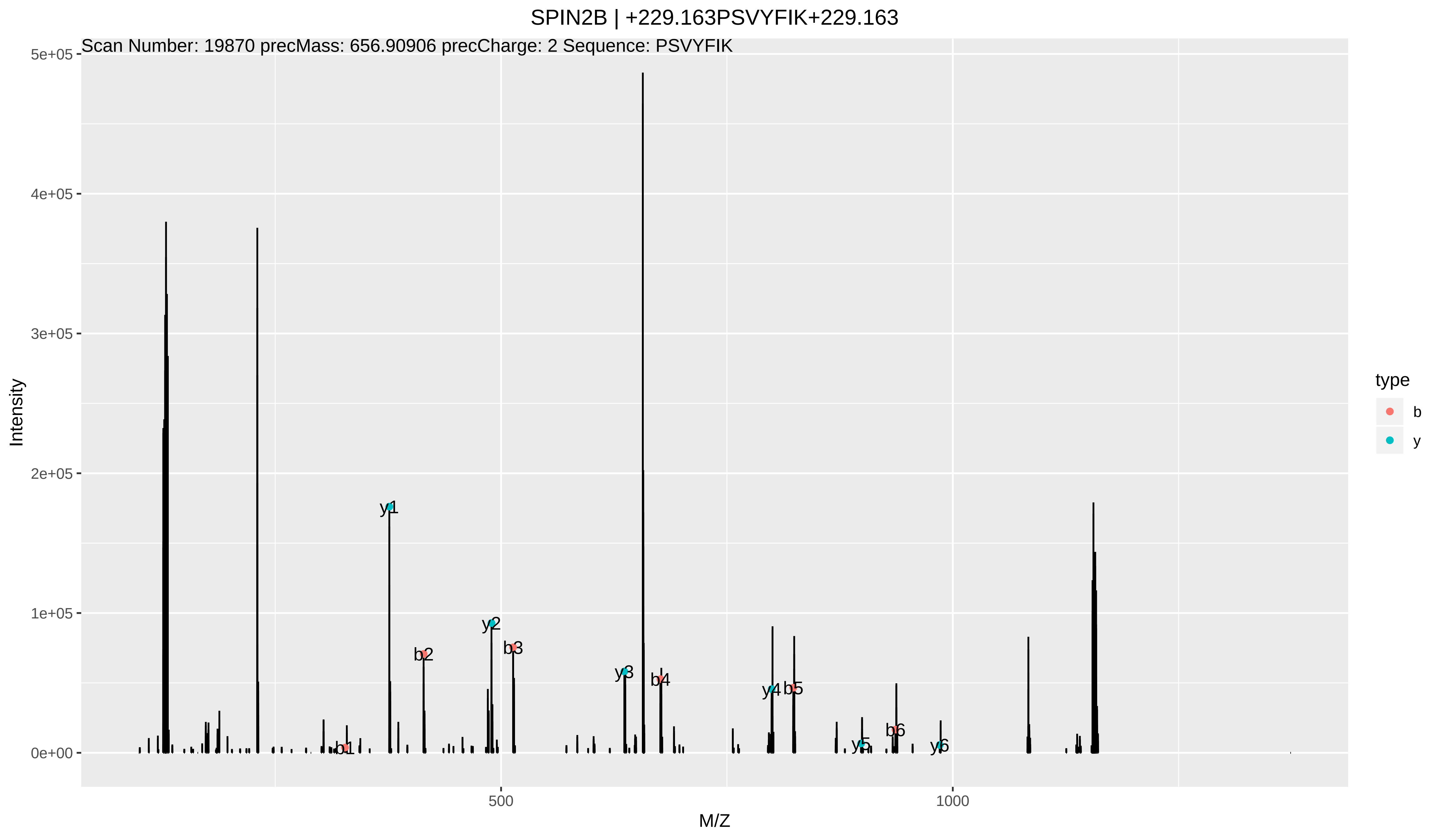
SPIN2B | +229.163PSVYFIK+229.163
Scan Number: 19870 precMass: 656.90906 precCharge: 2 Sequence: PSVYFIK
Intensity
M/Z
0e+00
1e+05
2e+05
3e+05
4e+05
5e+05
500
1000
y1
y2
y3
y4
y5
y6
b1
b2
b3
b4
b5
b6
type
b
y

SRGAP2D | +229.163ANQQETEQFYFTVR

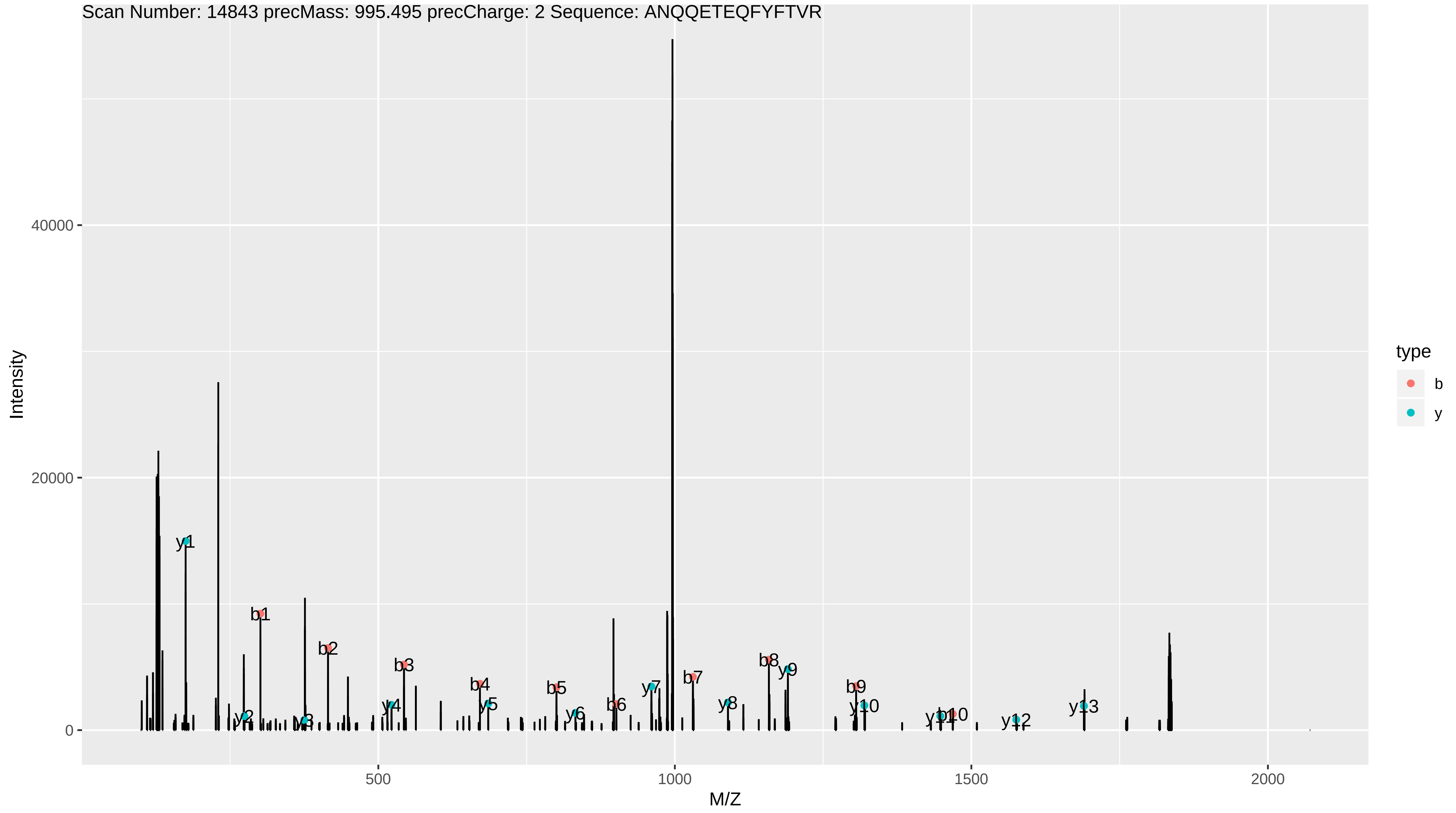

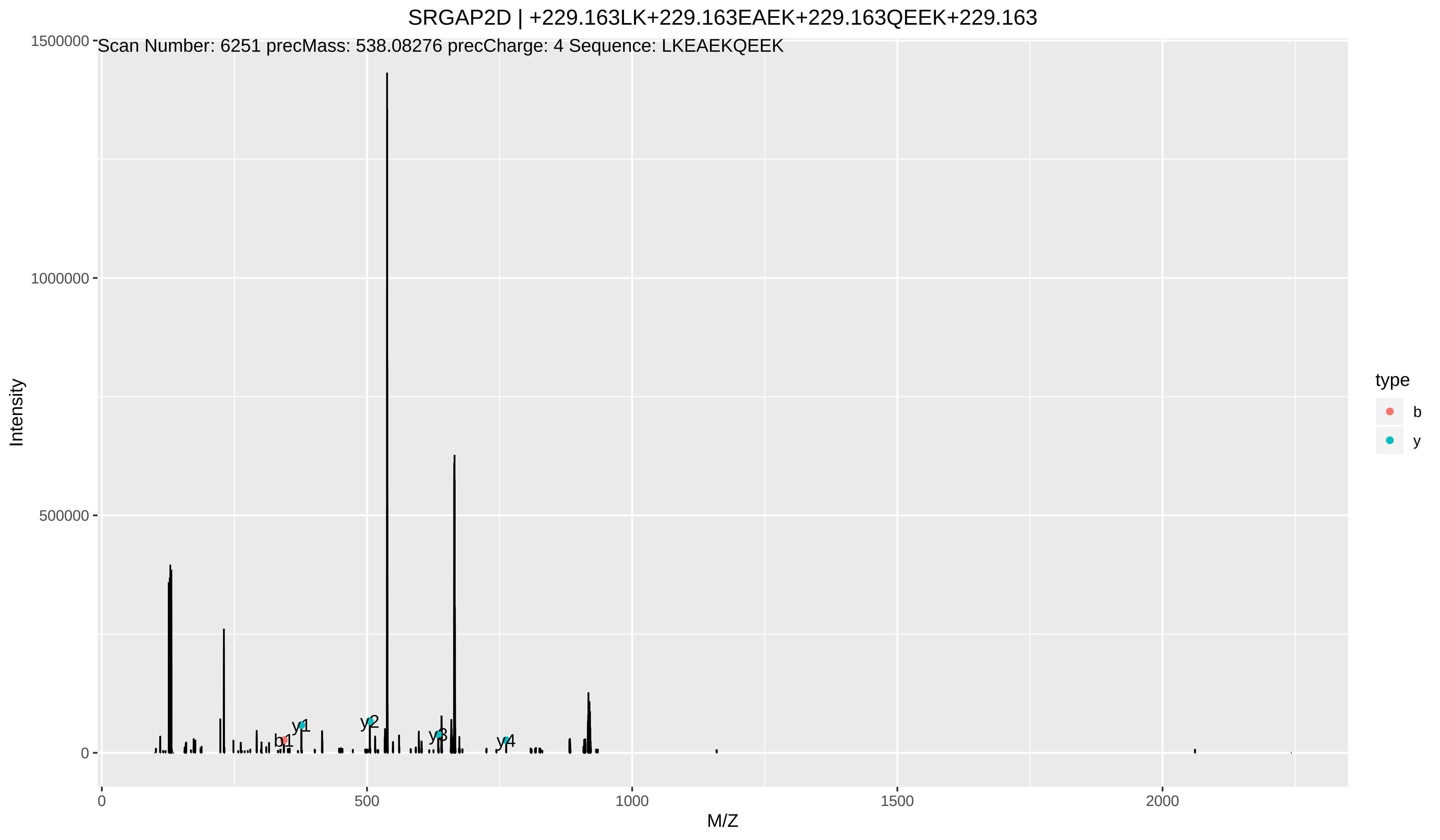

SRGAP2D | +229.163LK+229.163EAEK+229.163QEEK+229.163
Scan Number: 6251 precMass: 538.08276 precCharge: 4 Sequence: LKEAEKQEEK
1500000
1000000
500000
0
Intensity
b1
y1
y2
y3
y4
0
500
1000
1500
2000
M/Z
type
b
y

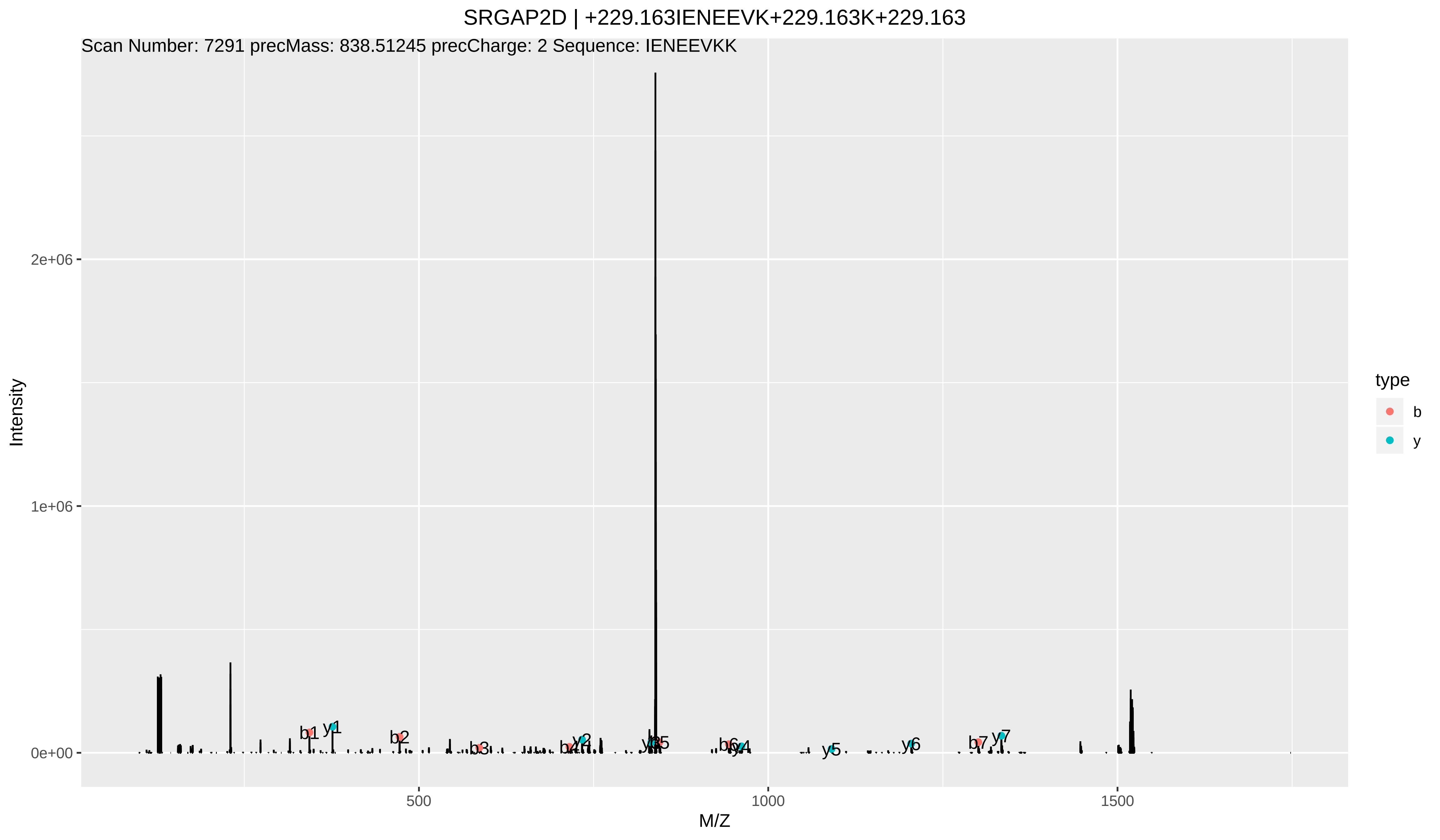

SRGAP2D | +229.163IENEEVK+229.163K+229.163
Scan Number: 7291 precMass: 838.51245 precCharge: 2 Sequence: IENEEVKK
Intensity
2e+06
1e+06
0e+00
M/Z
500
1000
1500
type
b
y
b1
y1
b2
b3
b4
y2
y3
b5
b6
y4
y5
y6
b7
y7

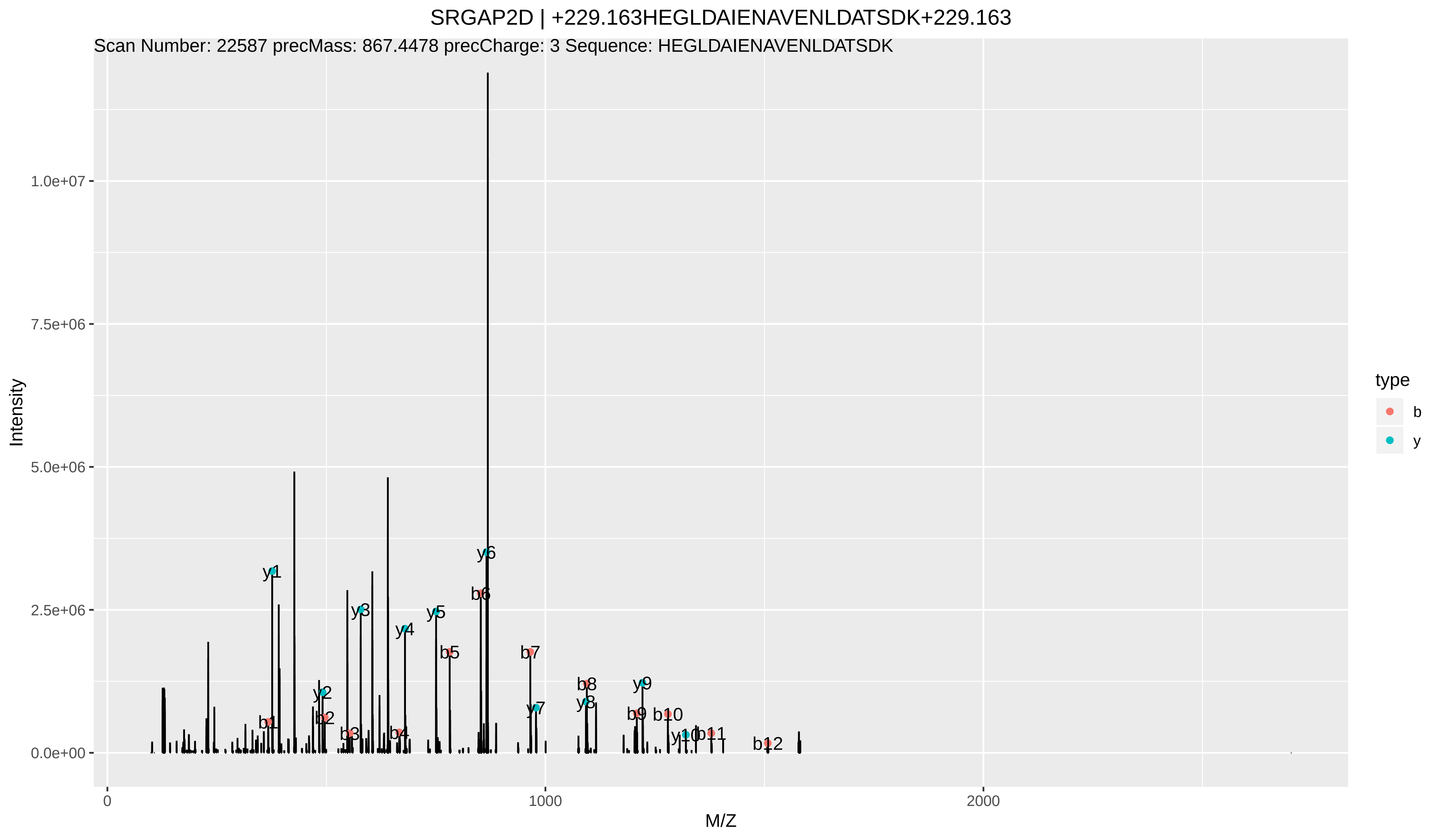
SRGAP2D | +229.163HEGLDAIENAVENLDATSDK+229.163
Scan Number: 22587 precMass: 867.4478 precCharge: 3 Sequence: HEGLDAIENAVENLDATSDK
Intensity
1.0e+07
7.5e+06
5.0e+06
2.5e+06
0.0e+00
0
1000
2000
M/Z
type
b
y
y1
b1
y2
b2
y3
b3
y4
b4
y5
b5
y6
b6
y7
b7
y8
b8
y9
b9
b10
y10
b11
b12

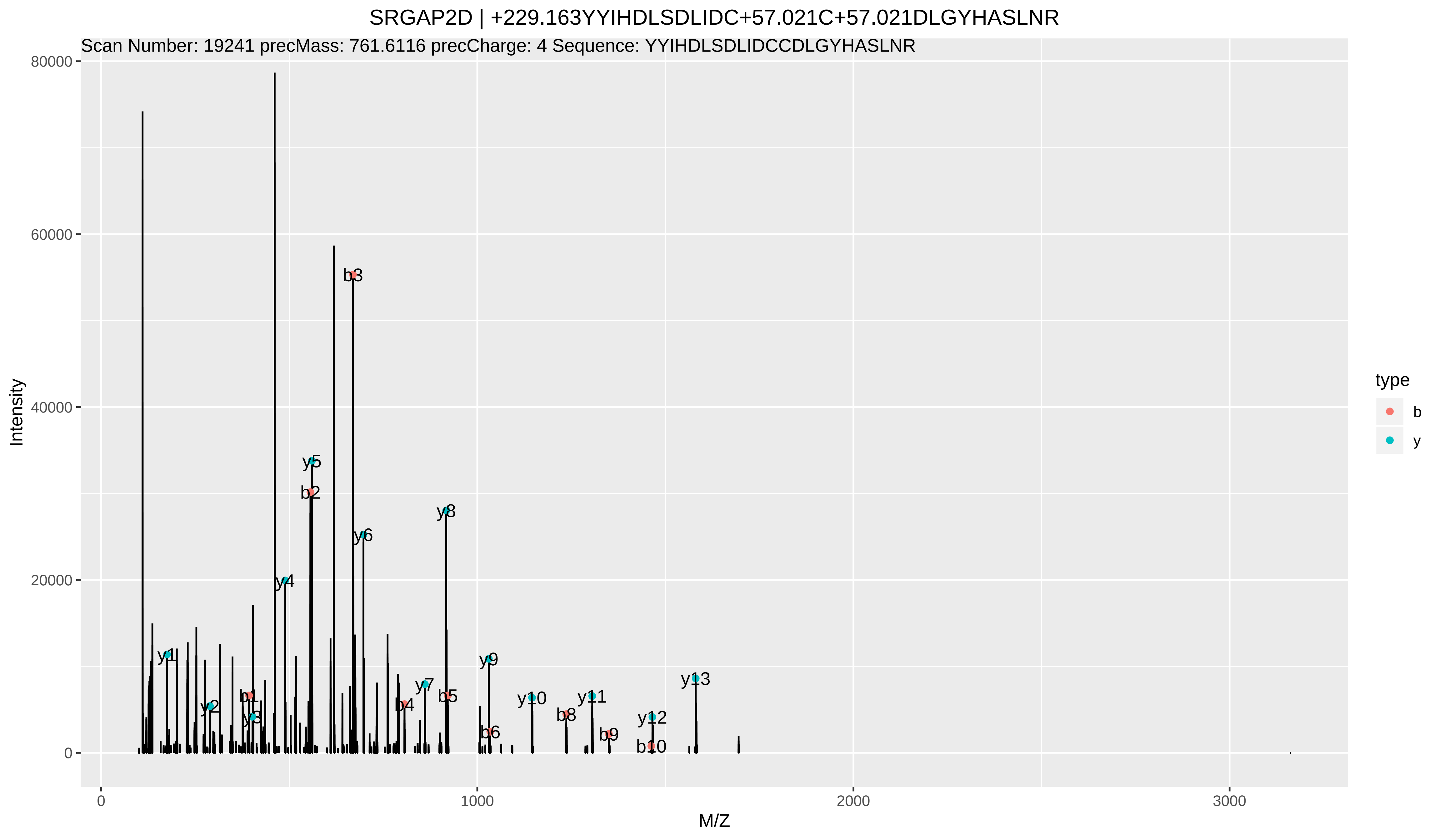

SRGAP2D | +229.163YYIHDLSDLIDC+57.021C+57.021DLGYHASLNR
Scan Number: 19241 precMass: 761.6116 precCharge: 4 Sequence: YYIHDLSDLIDCCDLGYHASLNR
Intensity
M/Z
type
b
y
b3
y5
b2
y8
y6
y4
y1
y9
y13
y7
b1
b5
y10
y11
y2
b4
b8
y3
y12
b6
b9
b10
0
20000
40000
60000
80000
0
1000
2000
3000

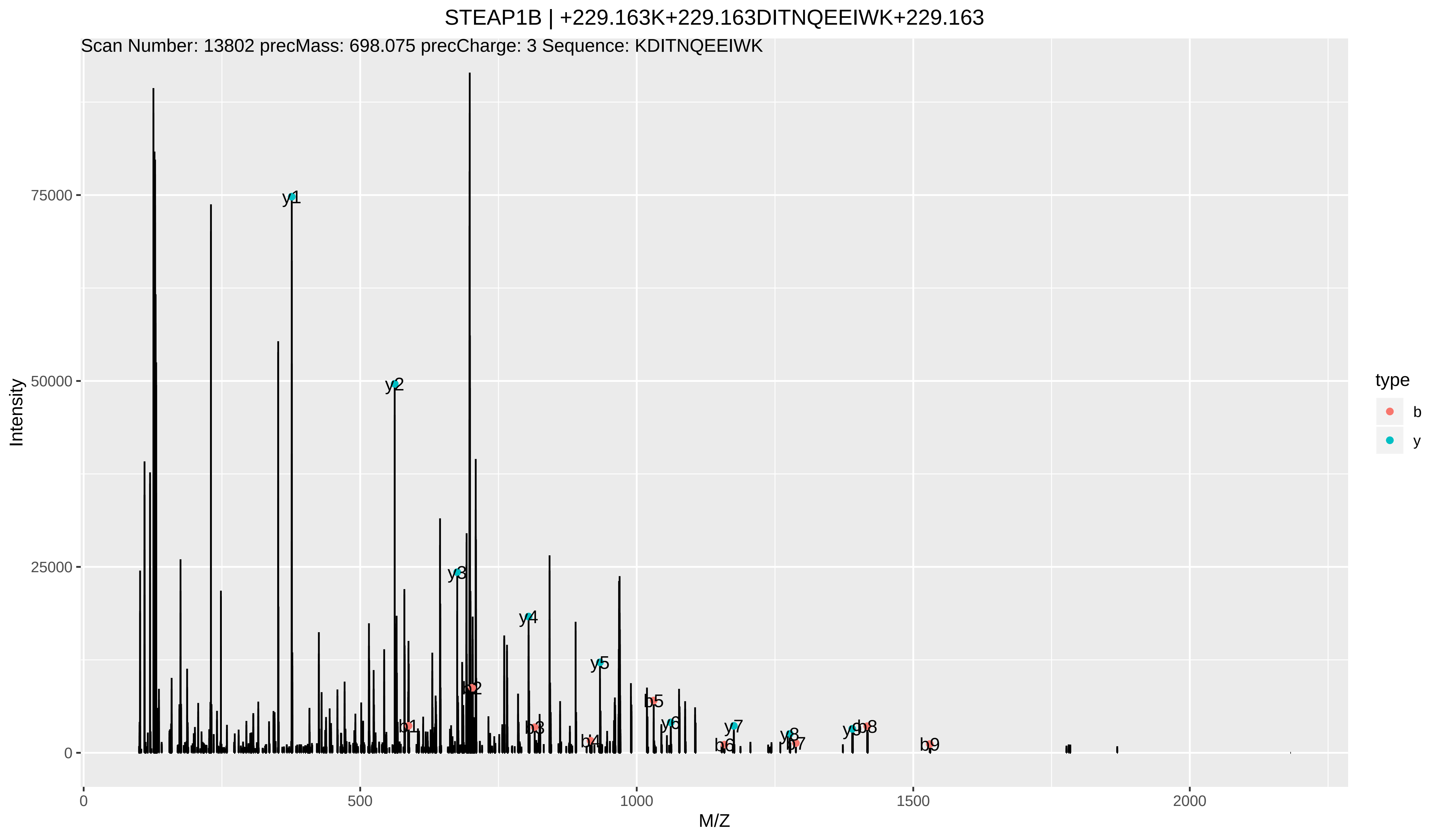

STEAP1B | +229.163K+229.163DITNQEEIWK+229.163
Scan Number: 13802 precMass: 698.075 precCharge: 3 Sequence: KDITNQEEIWK
Intensity
M/Z
type
b
y
y1
y2
y3
y4
y5
y6
y7
y8
y9
b1
b2
b3
b4
b5
b6
b7
b8
b9
0
25000
50000
75000
500
1000
1500
2000

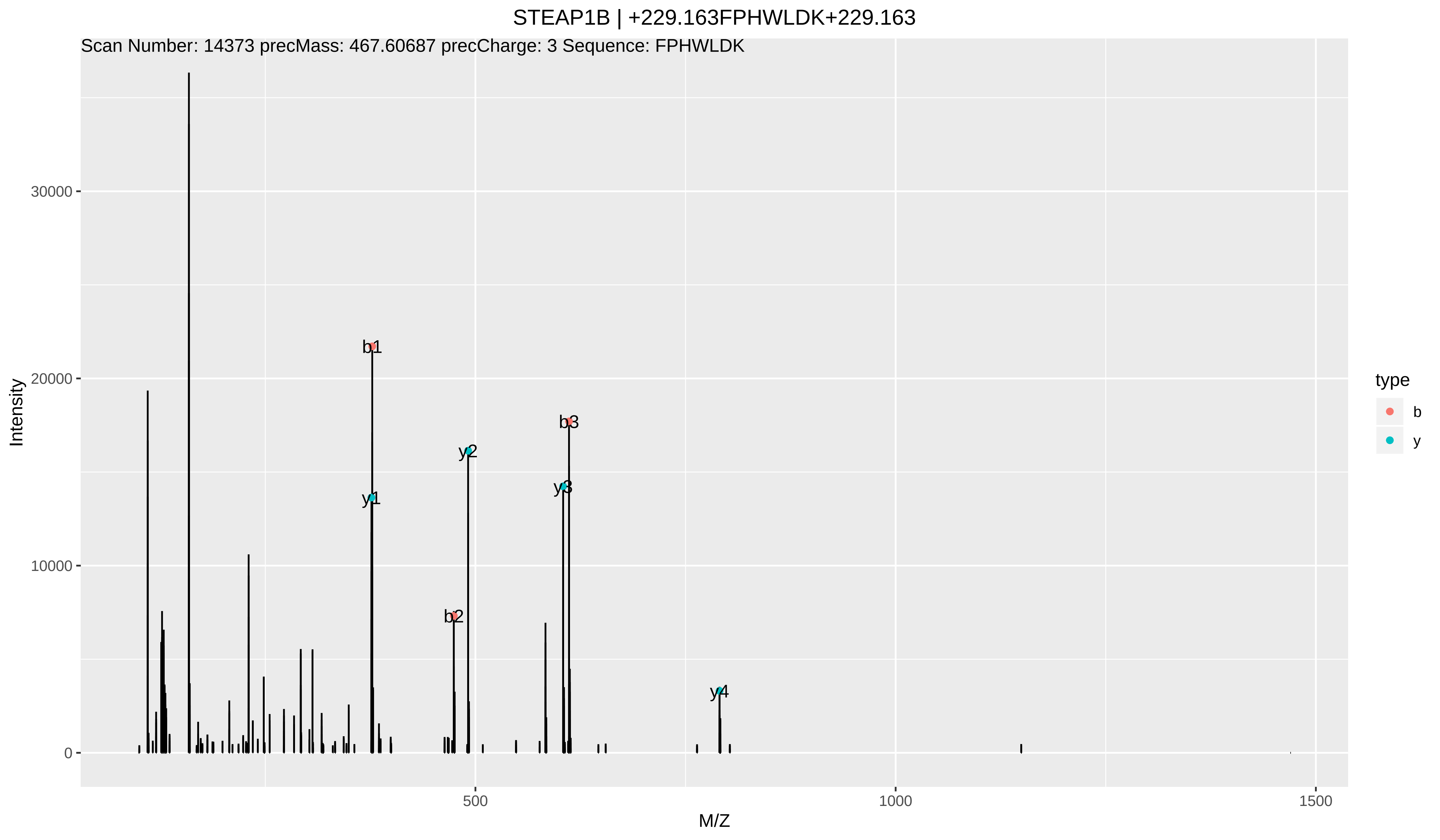

STEAP1B | +229.163FPHWLDK+229.163
Scan Number: 14373 precMass: 467.60687 precCharge: 3 Sequence: FPHWLDK
Intensity
M/Z
30000
20000
10000
0
500
1000
1500
b1
y1
b2
y2
y3
b3
y4
type
b
y

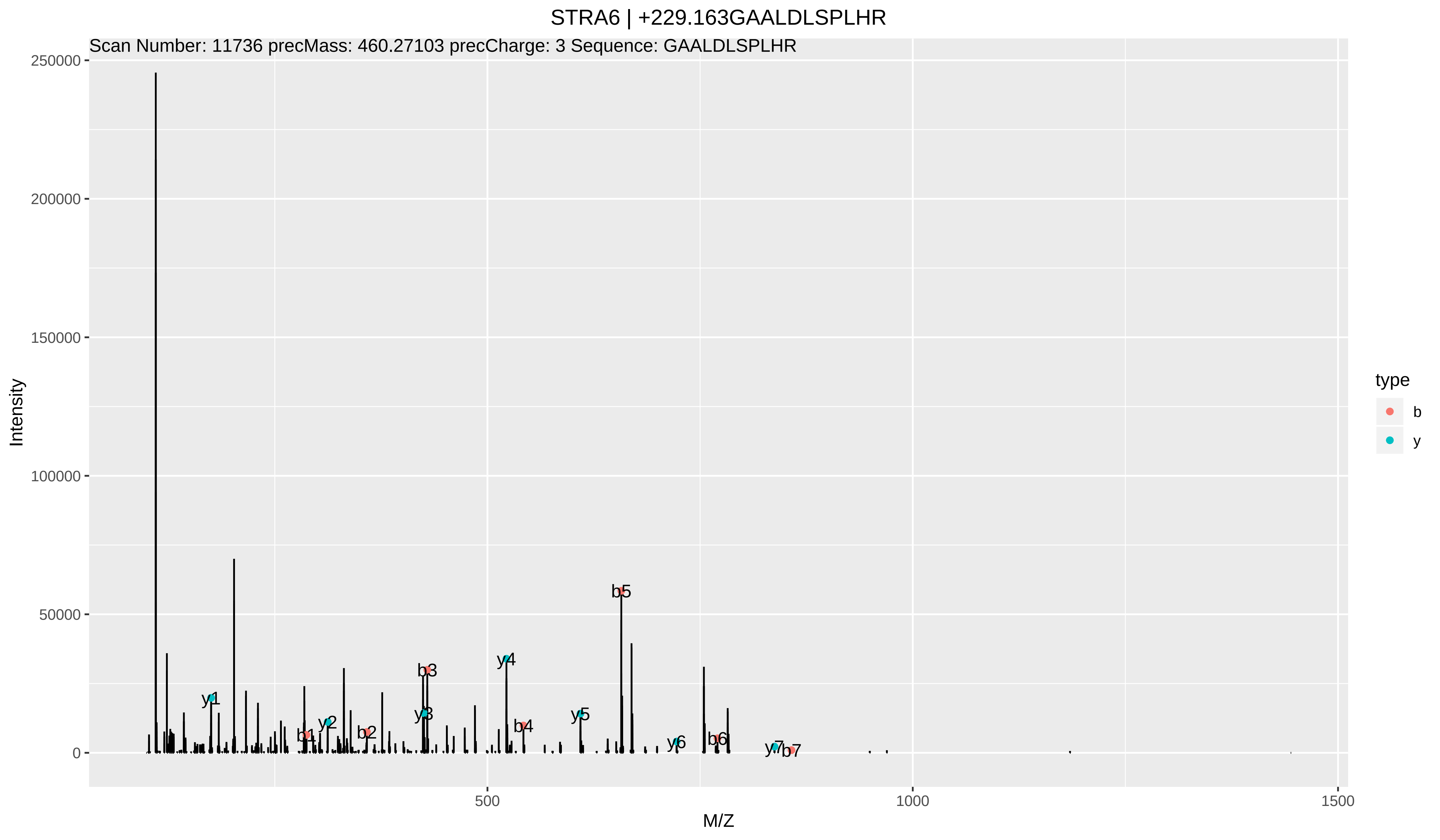
STRA6 | +229.163GAALDLSPLHR
Scan Number: 11736 precMass: 460.27103 precCharge: 3 Sequence: GAALDLSPLHR
Intensity
250000
200000
150000
100000
50000
0
500
1000
1500
M/Z
type
b
y
y1
y2
b1
b2
y3
b3
y4
b4
y5
b5
y6
b6
y7
b7

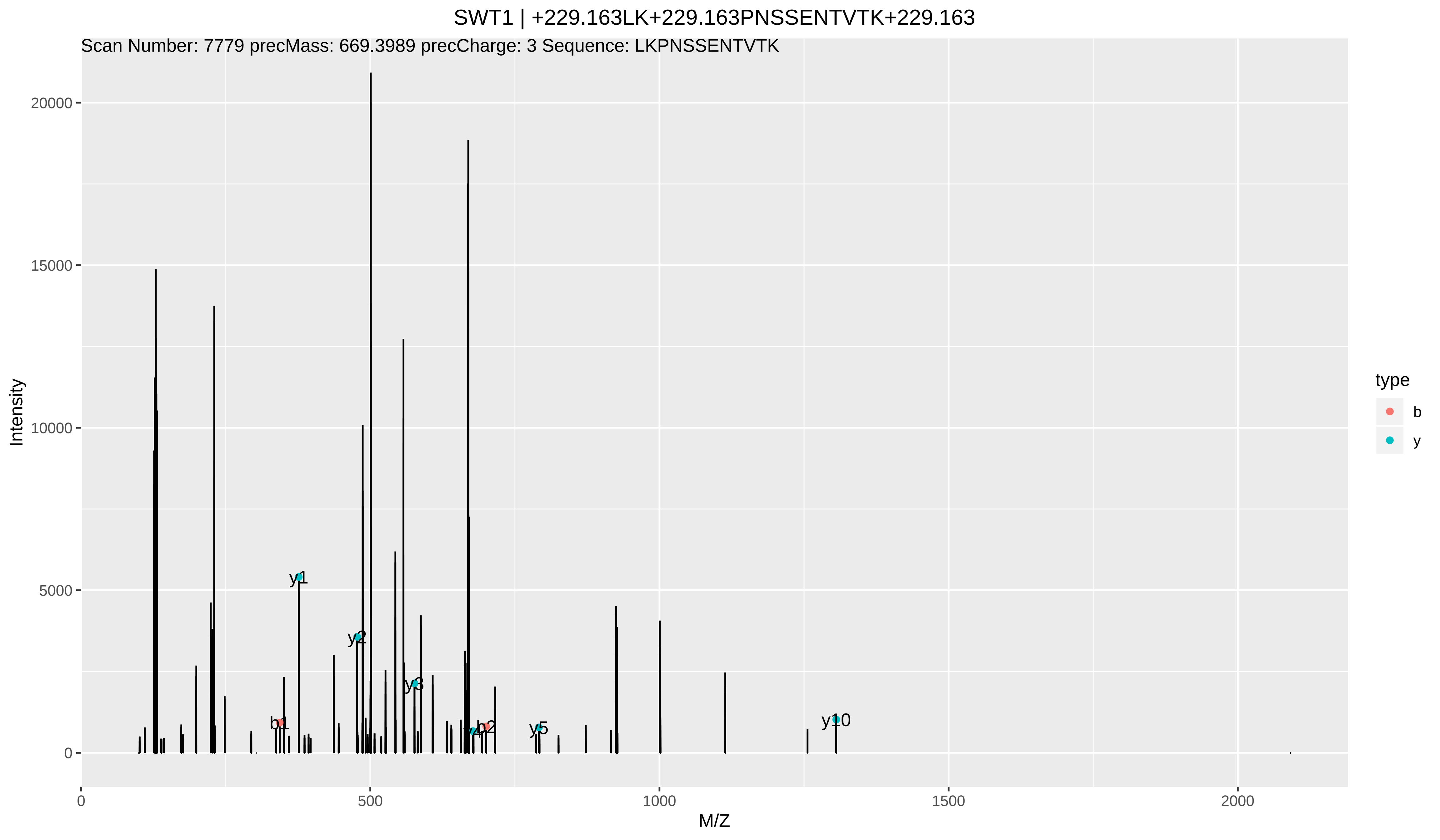
SWT1 | +229.163LK+229.163PNSSENTVTK+229.163
Scan Number: 7779 precMass: 669.3989 precCharge: 3 Sequence: LKPNSSENTVTK
Intensity
M/Z
type
b
y
y1
y2
y3
y5
y10
b1
b2
0
5000
10000
15000
20000
500
1000
1500
2000

SYT14 | +229.163C+57.021EFSHC+57.021SNSPR

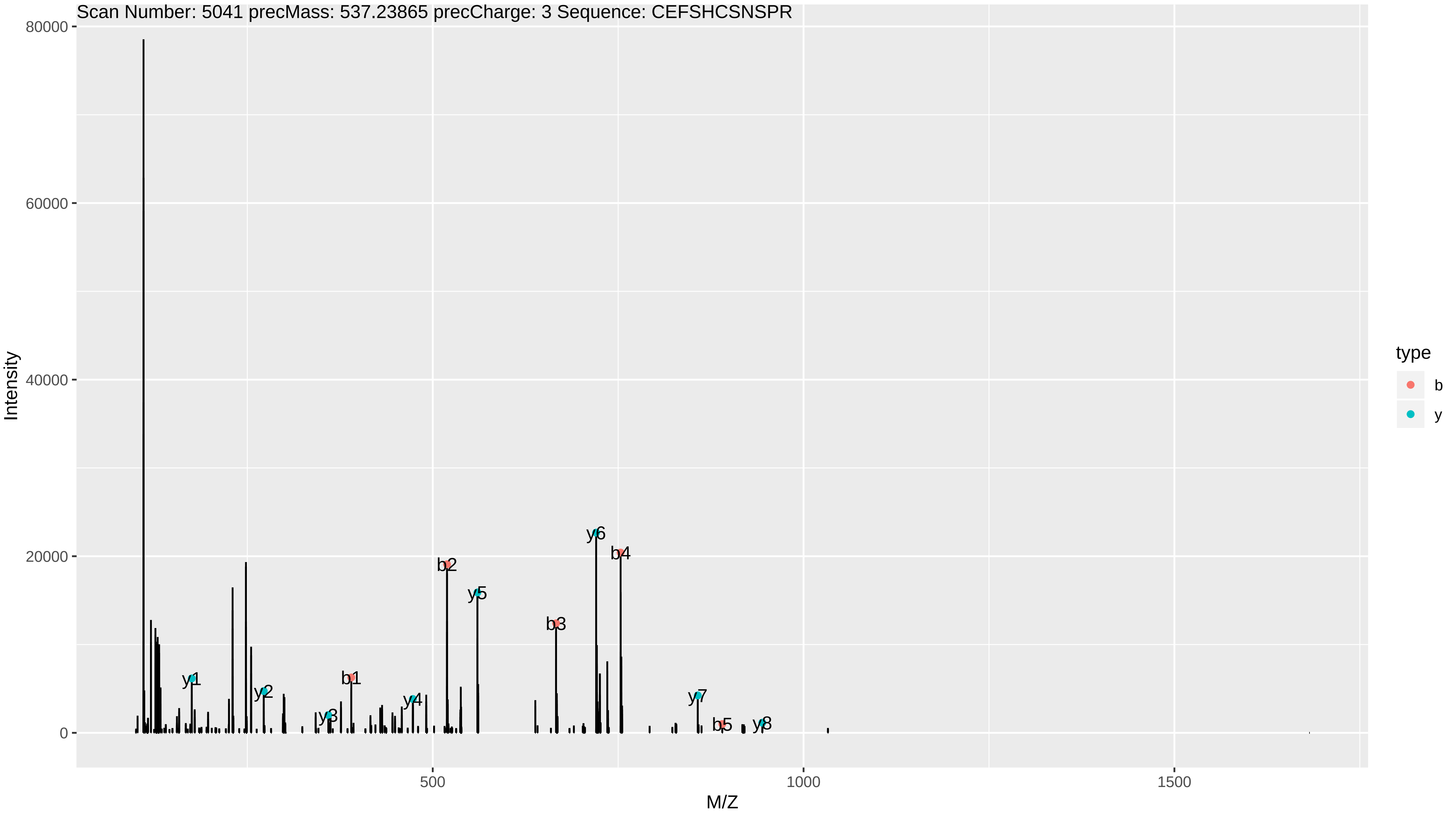

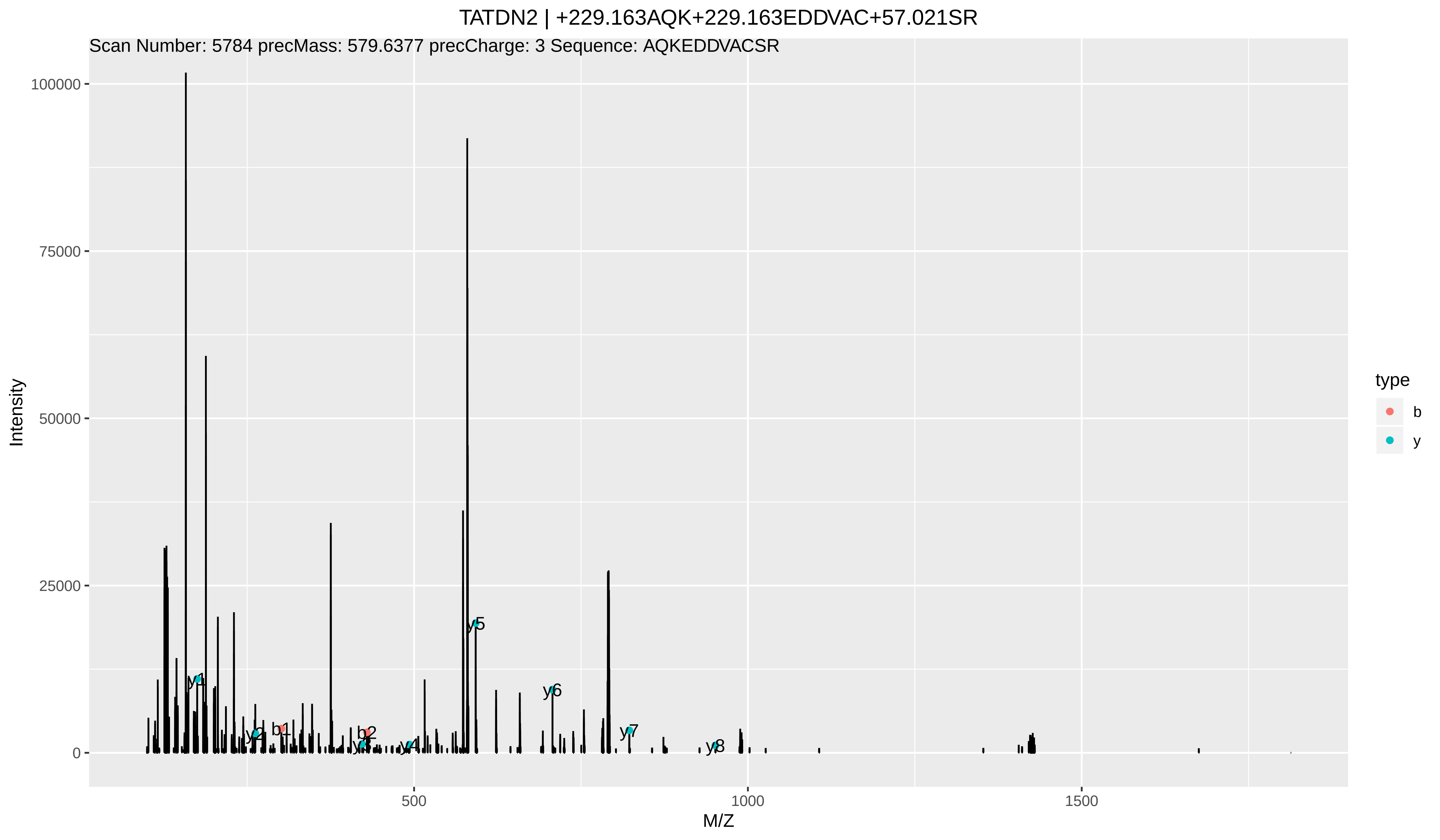

TATDN2 | +229.163AQK+229.163EDDVAC+57.021SR
Scan Number: 5784 precMass: 579.6377 precCharge: 3 Sequence: AQKEDDVACSR
Intensity
100000
75000
50000
25000
0
M/Z
500
1000
1500
type
b
y
y1
y2
b1
b2
y3
y4
y5
y6
y7
y8

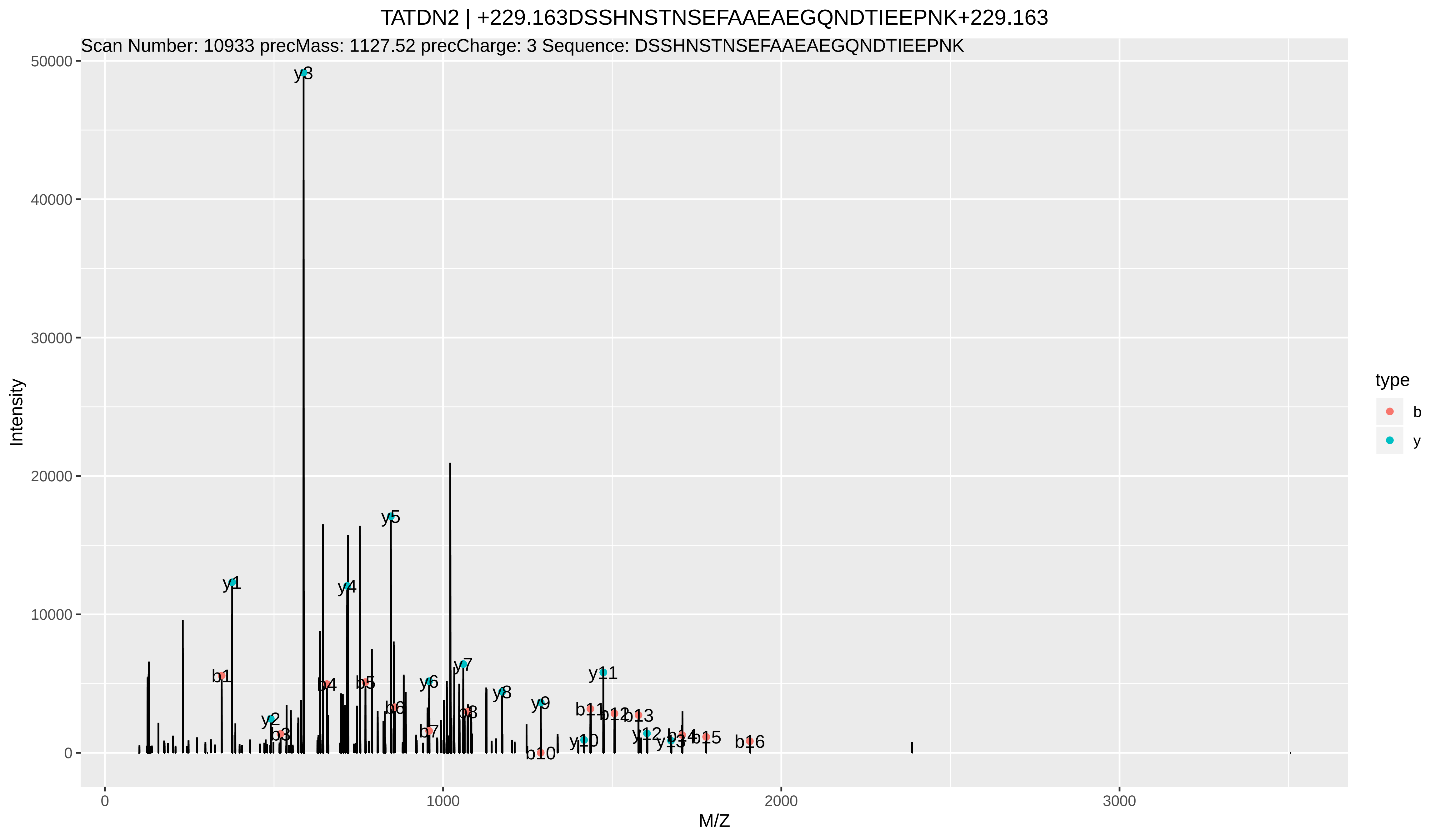

TATDN2 | +229.163DSSHNSTNSEFAAEAEGQNDTIEEPNK+229.163
Scan Number: 10933 precMass: 1127.52 precCharge: 3 Sequence: DSSHNSTNSEFAAEAEGQNDTIEEPNK
Intensity
M/Z
type
b
y
y3
y5
y1
y4
b1
b4
b5
y6
y7
y8
y9
y11
b6
b8
b7
y2
b3
b10
b11
b12
b13
y10
y12
y13
b14
b15
b16
0
10000
20000
30000
40000
50000
1000
2000
3000

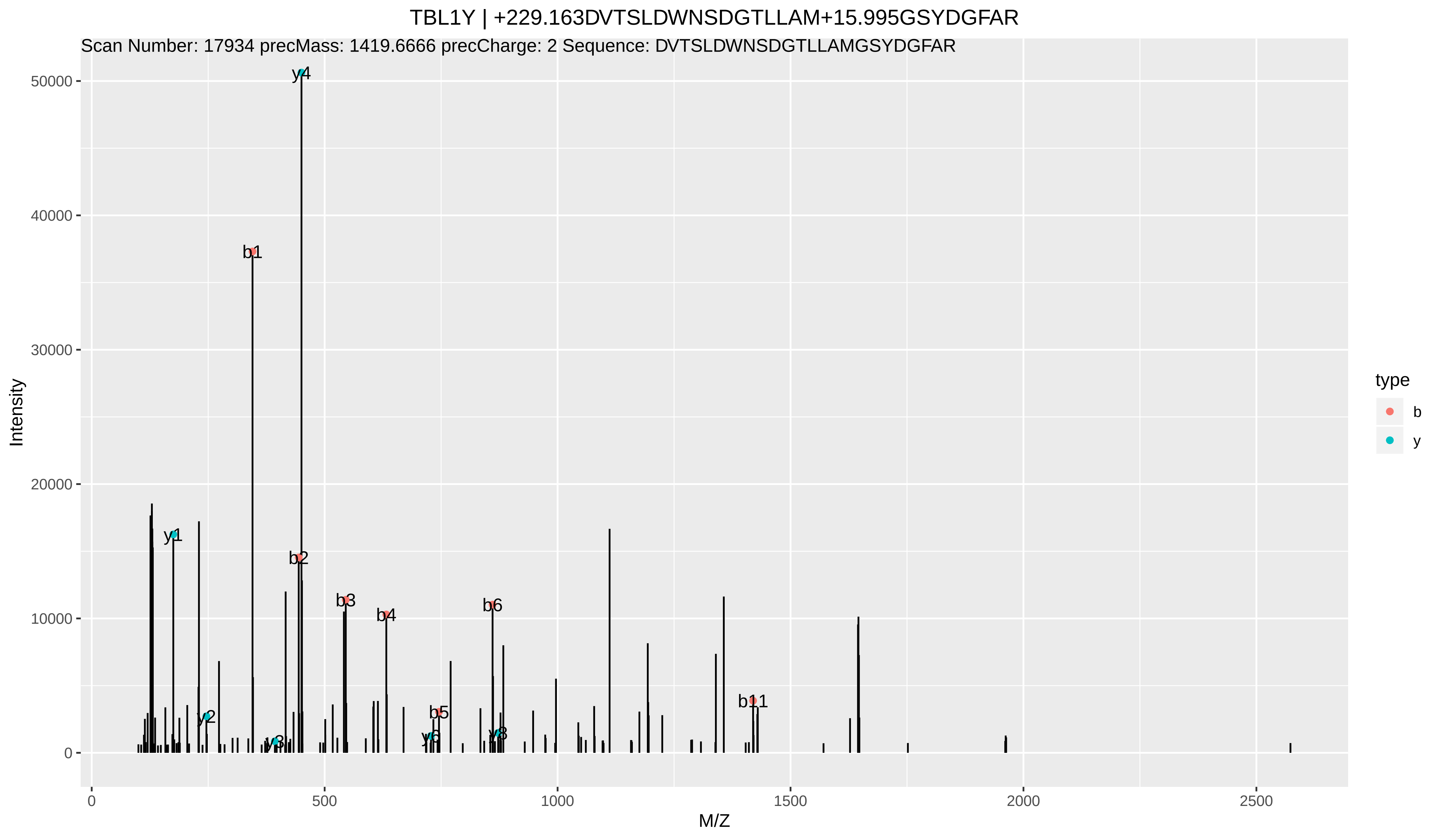

TBL1Y | +229.163DVTSLDWNSDGTLLAM+15.995GSYDGFAR
Scan Number: 17934 precMass: 1419.6666 precCharge: 2 Sequence: DVTSLDWNSDGTLLAMGSYDGFAR
Intensity
M/Z
type
b
y
y4
b1
y1
b2
b3
b4
b6
b11
b5
y2
y3
y6
y8
0
10000
20000
30000
40000
50000
0
500
1000
1500
2000
2500

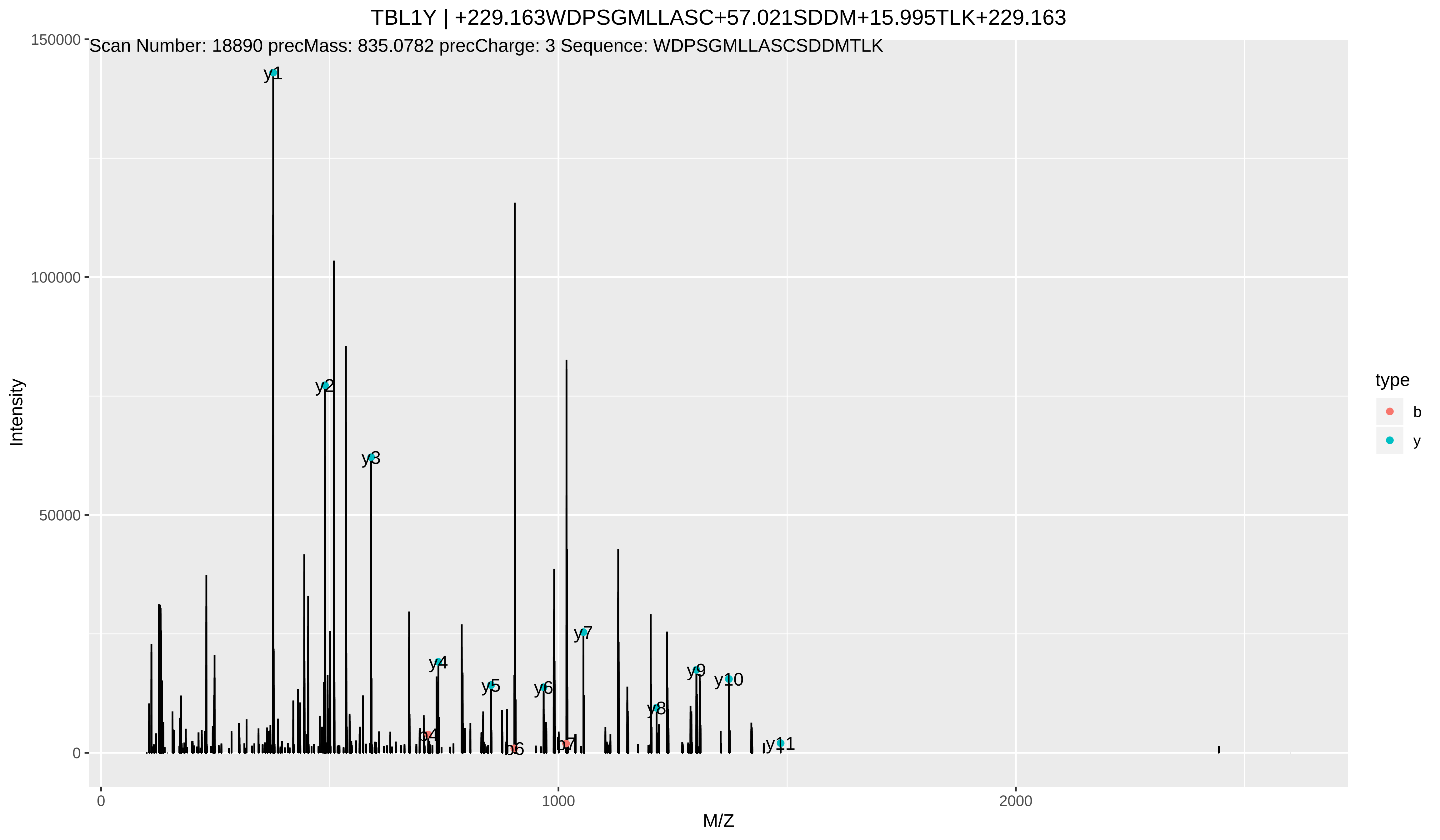

TBL1Y | +229.163WDPSGMLLASC+57.021SDDM+15.995TLK+229.163
Scan Number: 18890 precMass: 835.0782 precCharge: 3 Sequence: WDPSGMLLASCSDDMTLK
150000
100000
50000
0
Intensity
0
1000
2000
M/Z
y1
y2
y3
y4
y5
y6
y7
y8
y9
y10
y11
b4
b6
b7
type
b
y

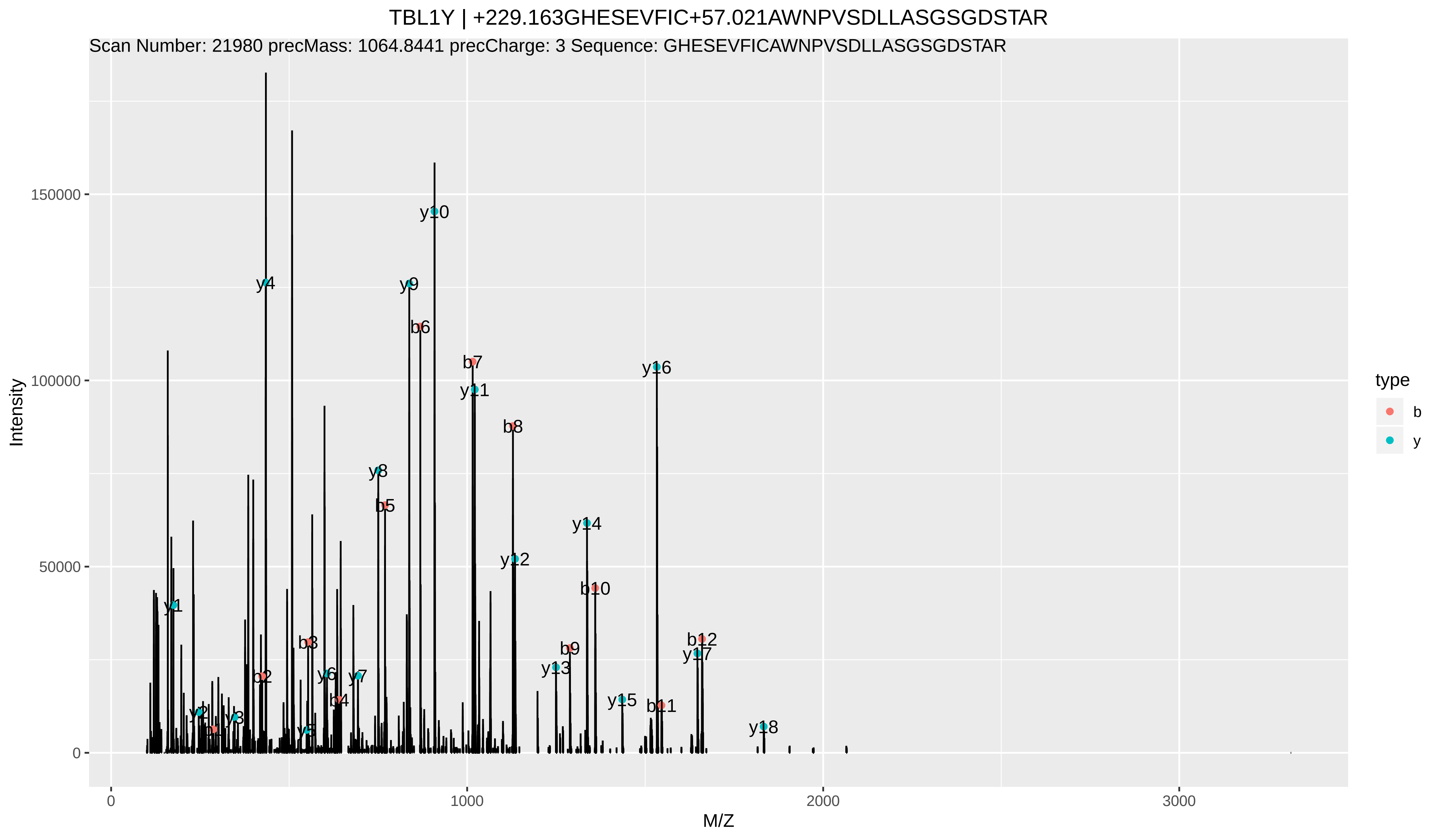
TBL1Y | +229.163GHESEVFIC+57.021AWNPVSDLLASGSGDSTAR
Scan Number: 21980 precMass: 1064.8441 precCharge: 3 Sequence: GHESEVFICAWNPVSDLLASGSGDSTAR
Intensity
M/Z
type
b
y
y1
y2
y3
y4
y5
y6
y7
y8
y9
y10
y11
y12
y13
y14
y15
y16
y17
y18
b1
b2
b3
b4
b5
b6
b7
b8
b9
b10
b11
b12
0
50000
100000
150000
1000
2000
3000

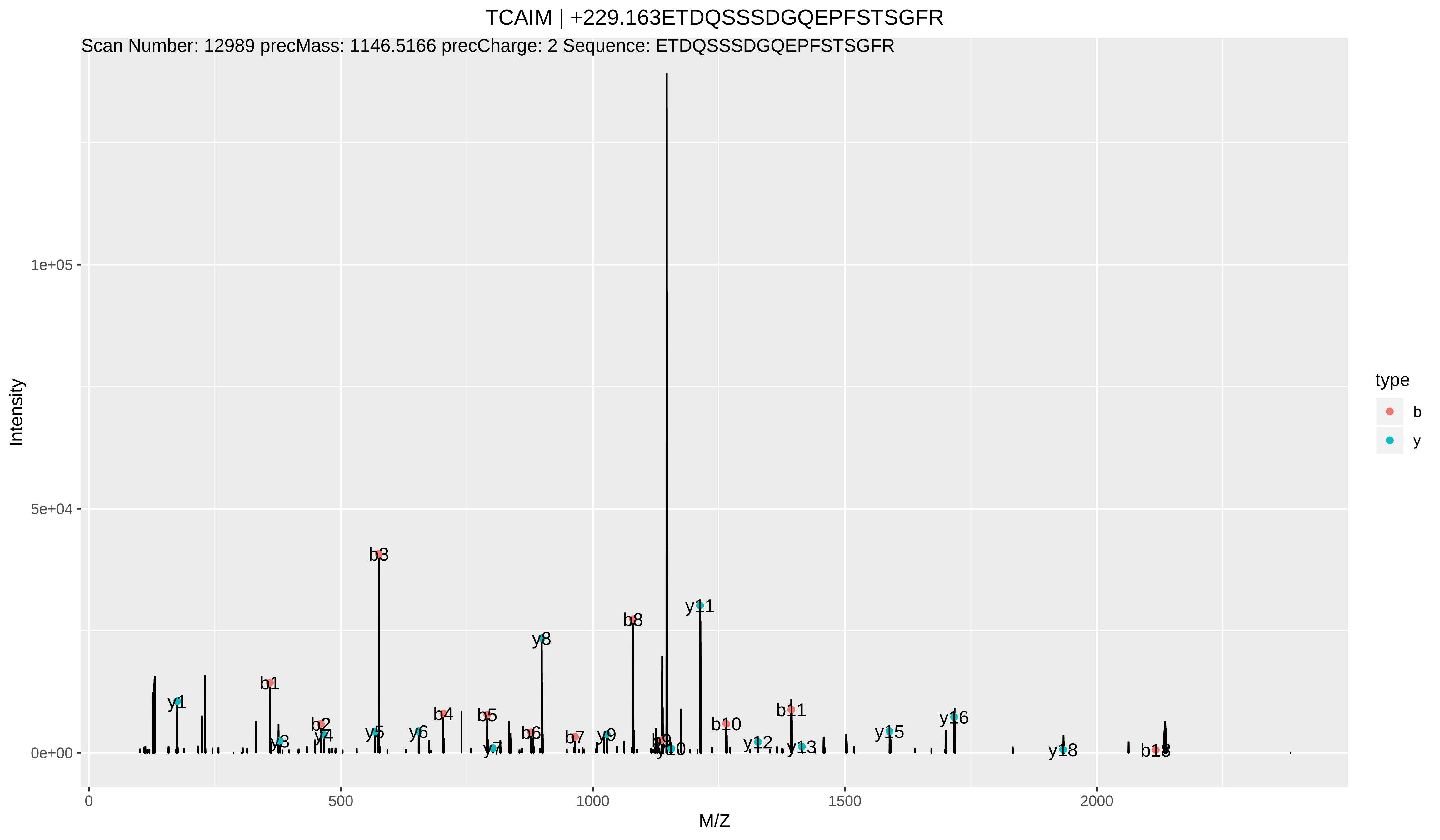

TCAIM | +229.163ETDQSSSDGQEPFSTSGFR
Scan Number: 12989 precMass: 1146.5166 precCharge: 2 Sequence: ETDQSSSDGQEPFSTSGFR
Intensity
1e+05
5e+04
0e+00
M/Z
0
500
1000
1500
2000
type
b
y
b1
b2
b3
b4
b5
b6
b7
b8
b9
b10
b11
b18
y1
y3
y4
y5
y6
y7
y8
y9
y10
y11
y12
y13
y15
y16
y18

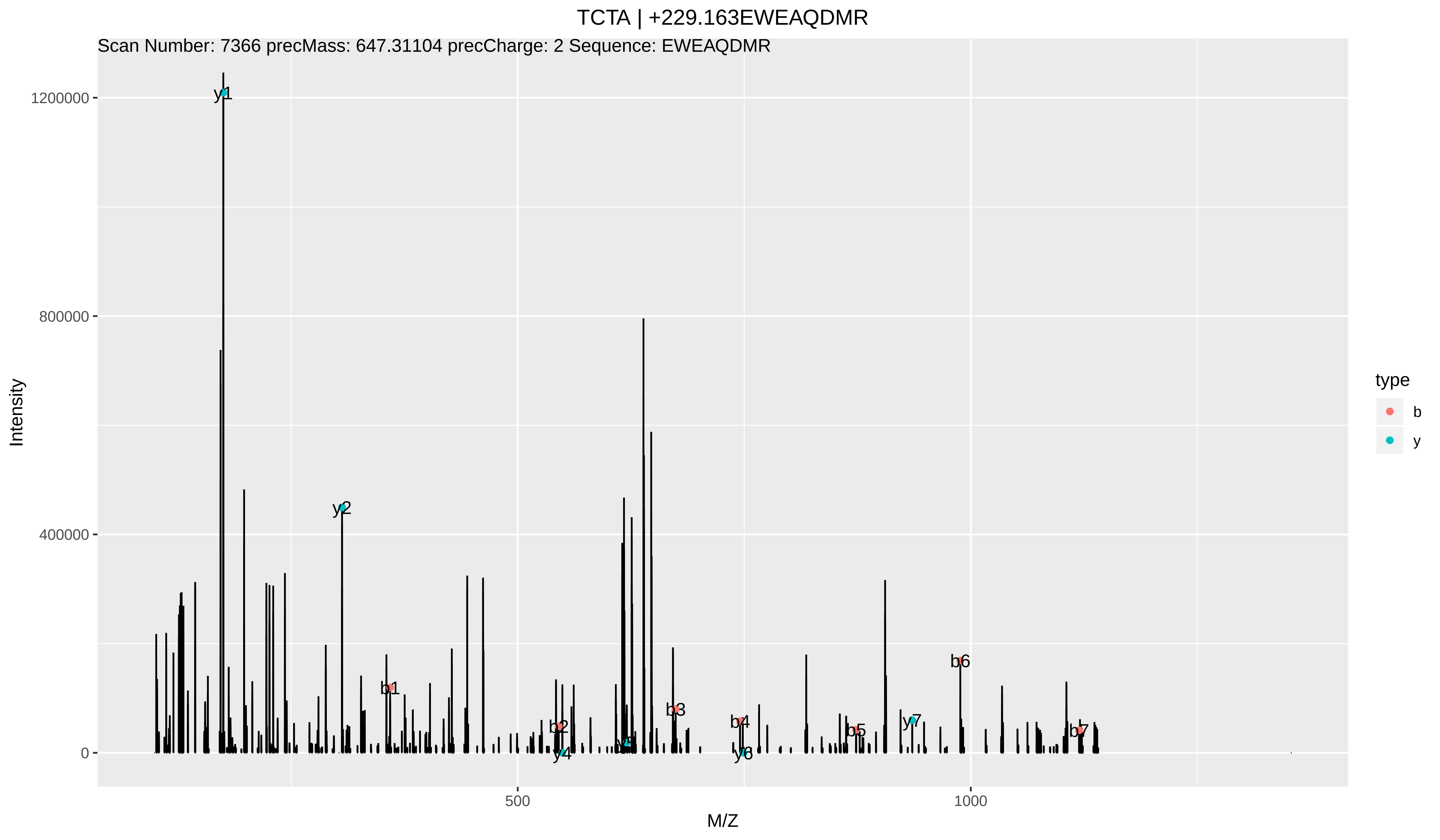

TCTA | +229.163EWEAQDMR
Scan Number: 7366 precMass: 647.31104 precCharge: 2 Sequence: EWEAQDMR
Intensity
1200000
800000
400000
0
500
1000
M/Z
y1
y2
b1
b2
y4
b3
b4
y6
b5
y7
b6
b7
type
b
y

TCTEX1D2 | +229.163GEGVFMASR

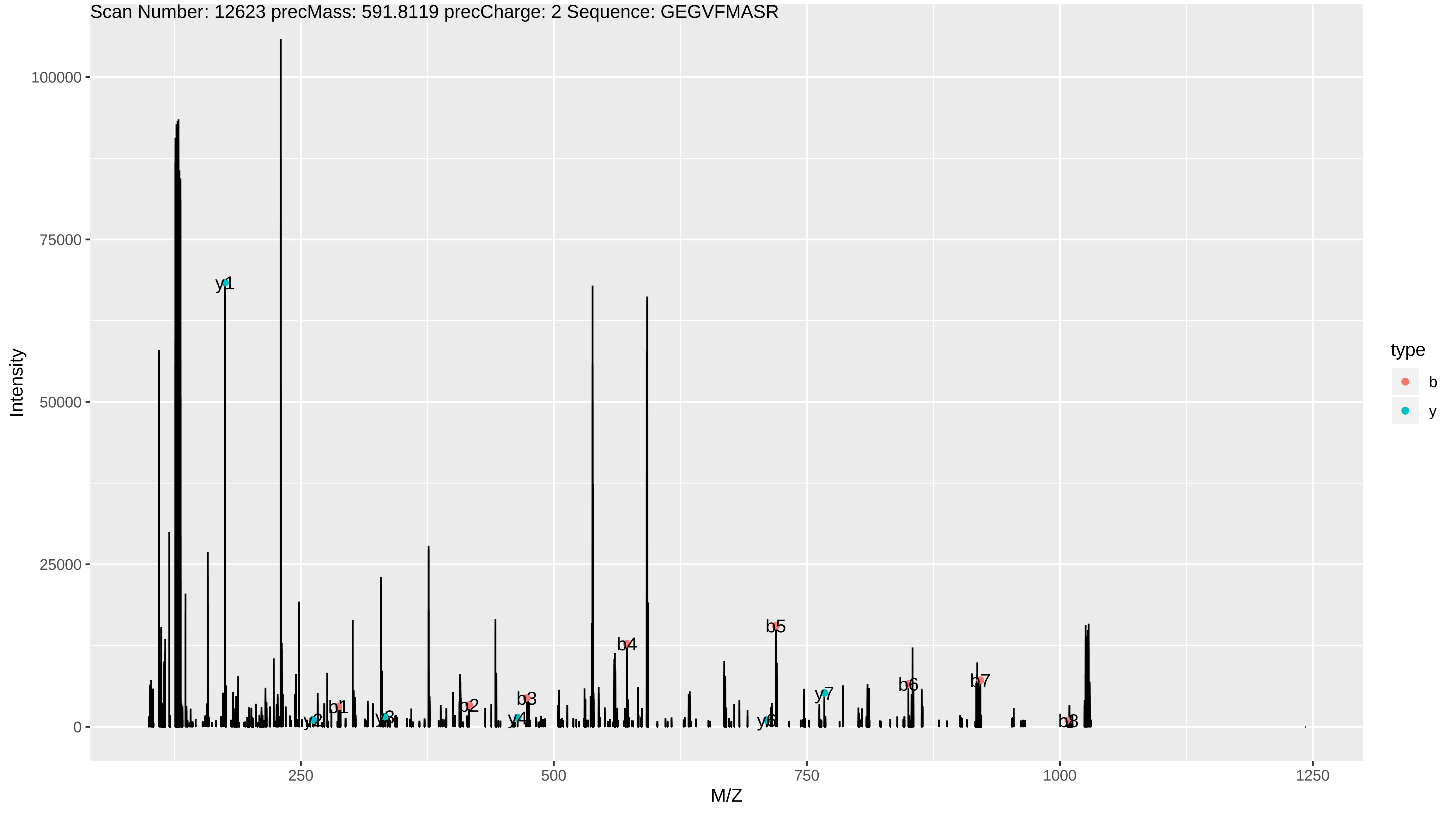

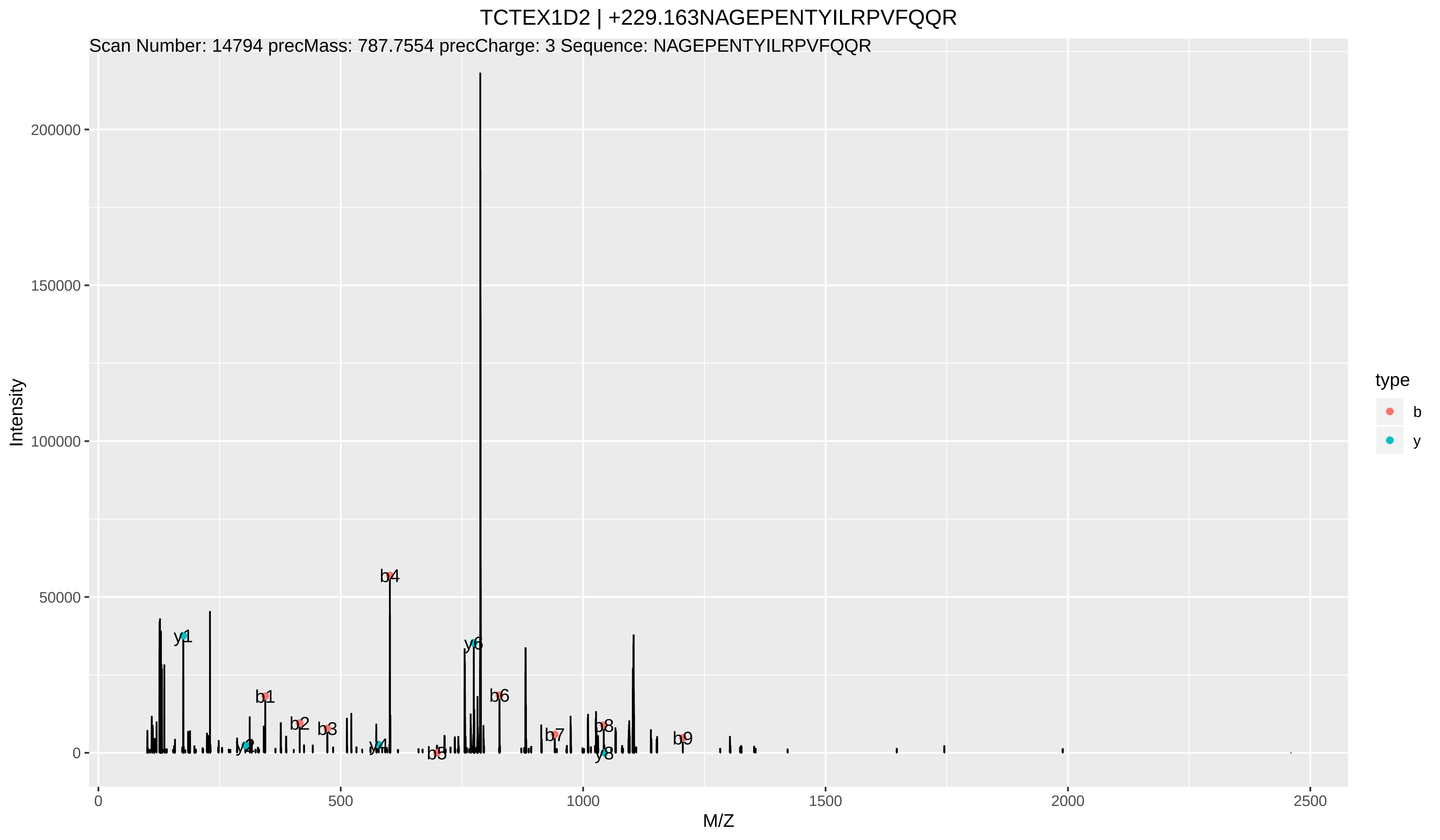
TCTEX1D2 | +229.163NAGEPENTYILRPVFQQR
Scan Number: 14794 precMass: 787.7554 precCharge: 3 Sequence: NAGEPENTYILRPVFQQR
Intensity
M/Z
type
b
y
y1
b1
b2
b3
y4
b4
b5
y6
b6
b7
b8
y8
b9
0
50000
100000
150000
200000
500
1000
1500
2000
2500

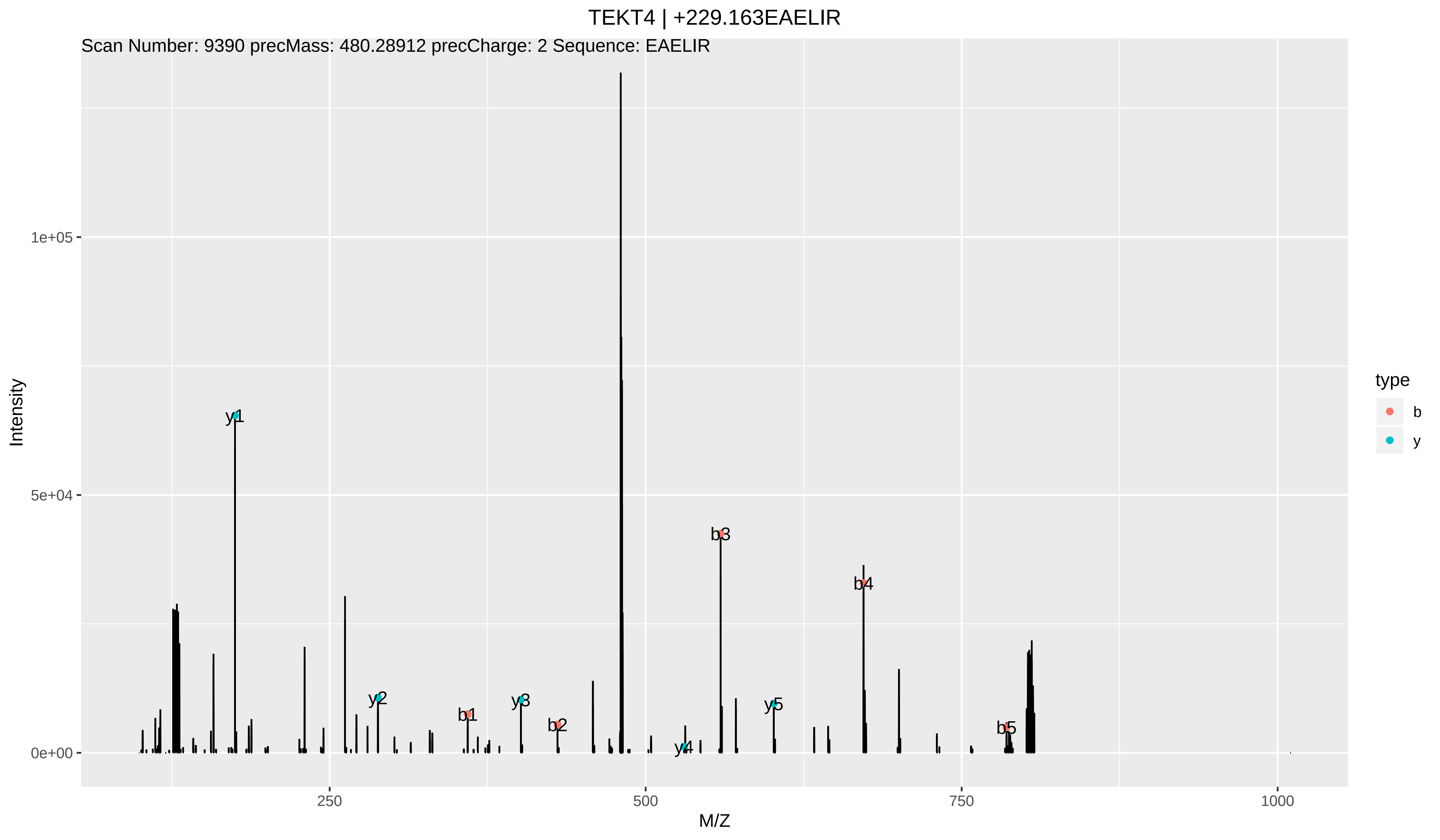

TEKT4 | +229.163EAELIR
Scan Number: 9390 precMass: 480.28912 precCharge: 2 Sequence: EAELIR
Intensity
1e+05
5e+04
0e+00
M/Z
250
500
750
1000
y1
y2
y3
y4
y5
b1
b2
b3
b4
b5
type
b
y

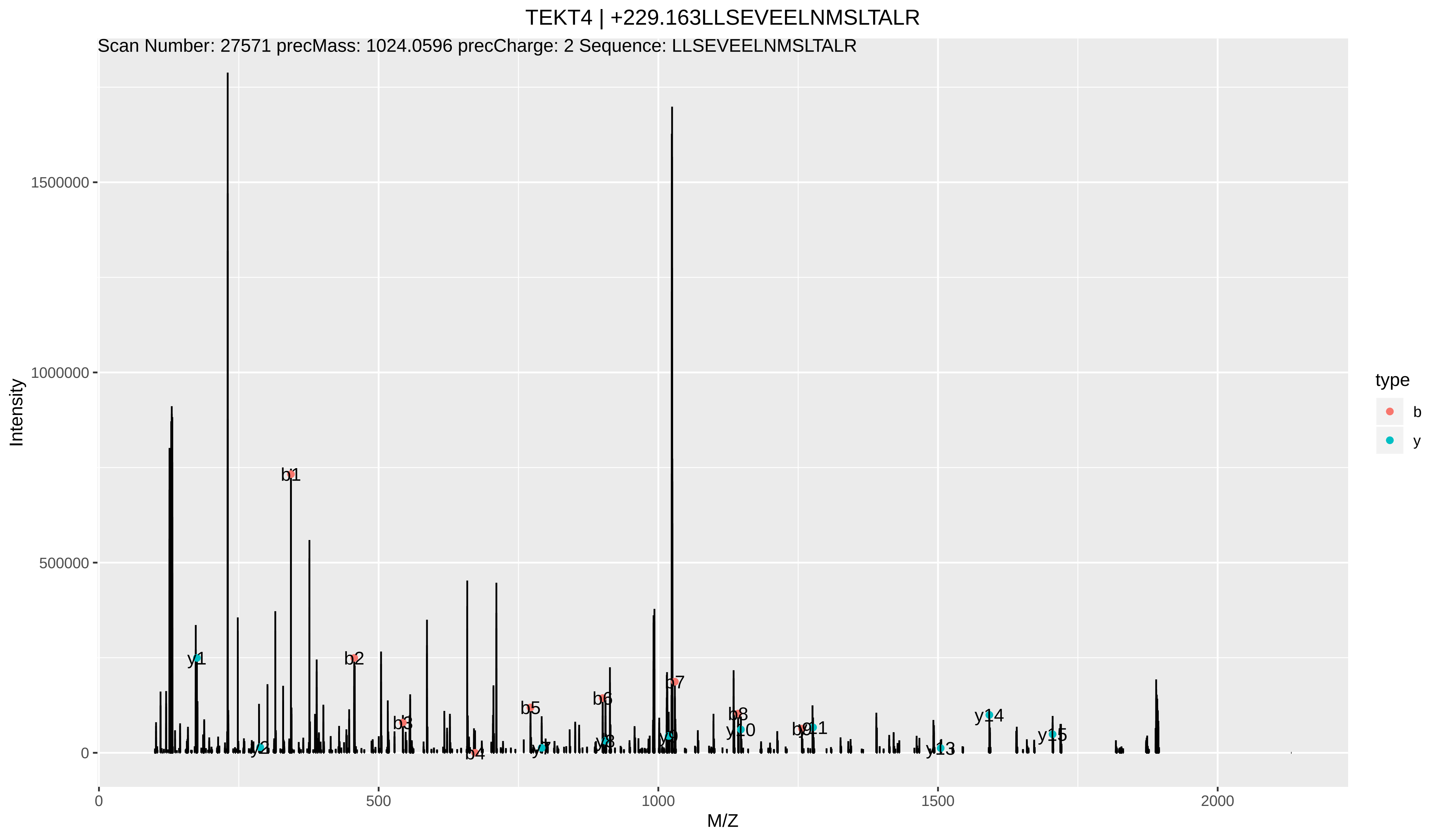

TEKT4 | +229.163LLSEVEELNMSLTALR
Scan Number: 27571 precMass: 1024.0596 precCharge: 2 Sequence: LLSEVEELNMSLTALR
Intensity
1500000
1000000
500000
0
M/Z
0
500
1000
1500
2000
type
b
y
b1
b2
b3
b4
b5
b6
b7
b8
b9
y1
y2
y7
y8
y9
y10
y11
y13
y14
y15

TFAP2E | +229.163DSLVGGITNPGEVFC+57.021SVPGRLSLLSSTSK+229.163

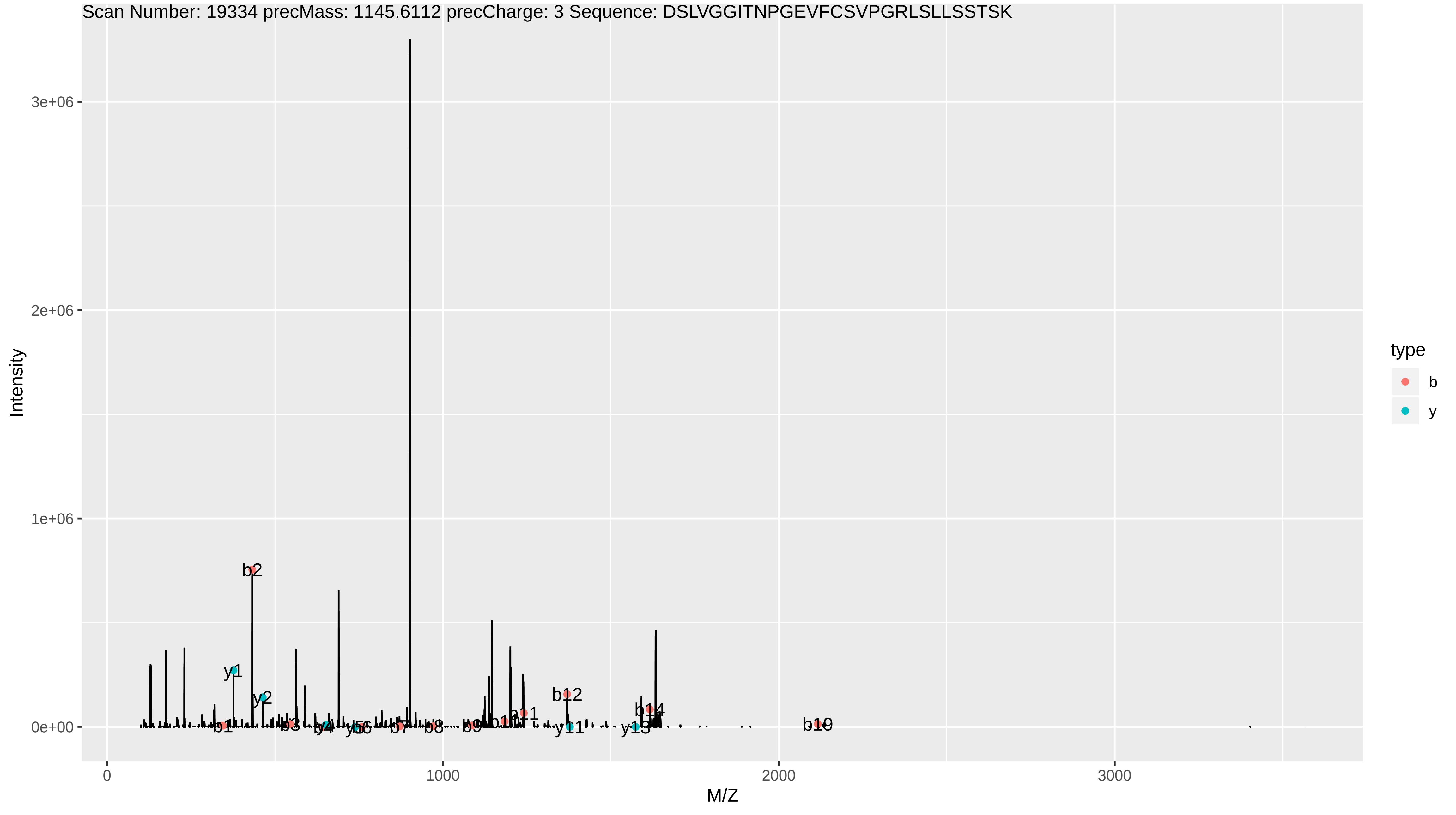

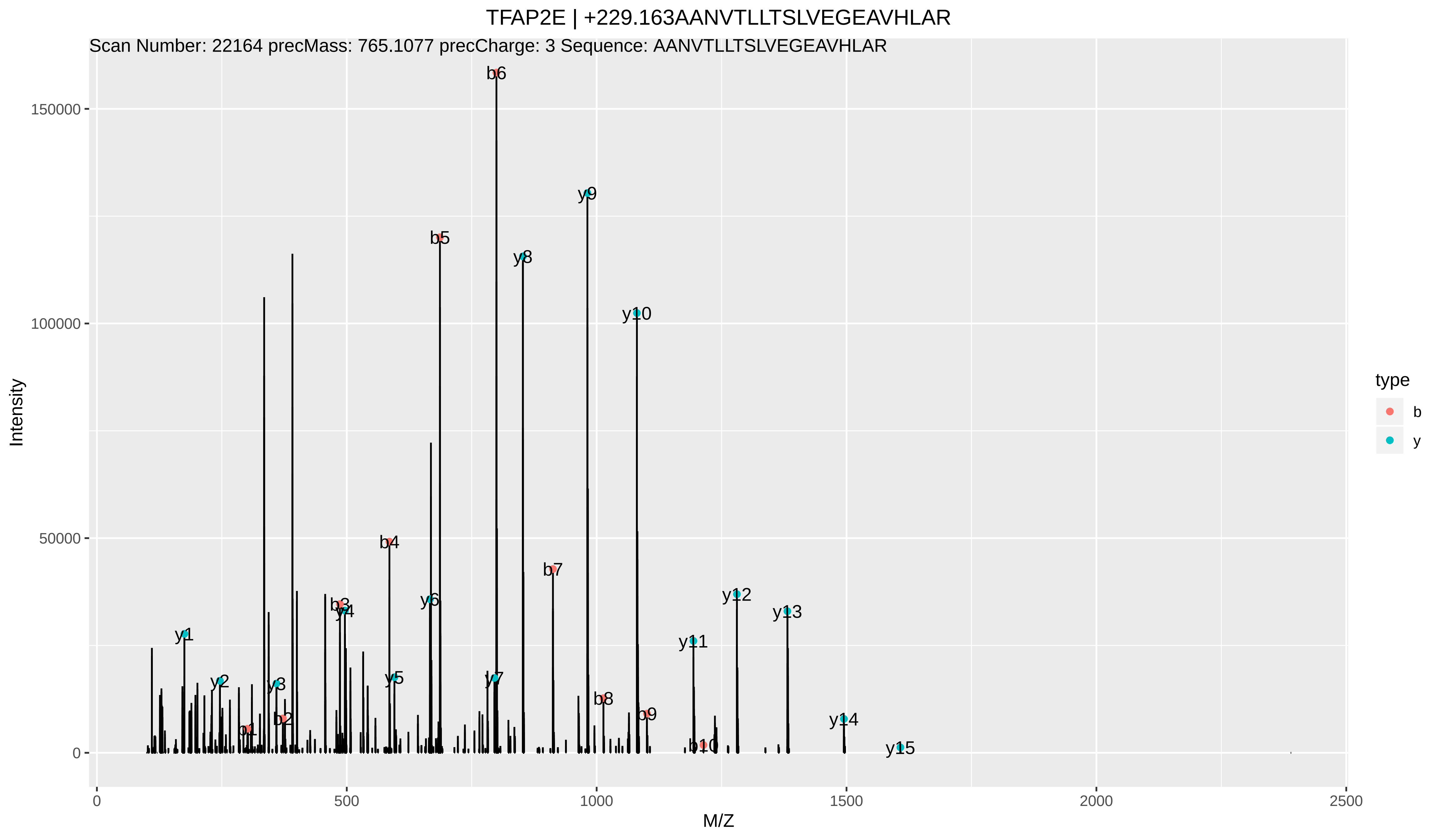

TFAP2E | +229.163AANVTLLTSLVEGEAVHLAR
Scan Number: 22164 precMass: 765.1077 precCharge: 3 Sequence: AANVTLLTSLVEGEAVHLAR
Intensity
M/Z
type
b
y
b6
y9
b5
y8
y10
b4
b7
y6
b3
y4
y12
y13
y1
y11
y2
y3
y5
y7
b8
b2
b1
b9
y14
b10
y15
150000
100000
50000
0
0
500
1000
1500
2000
2500

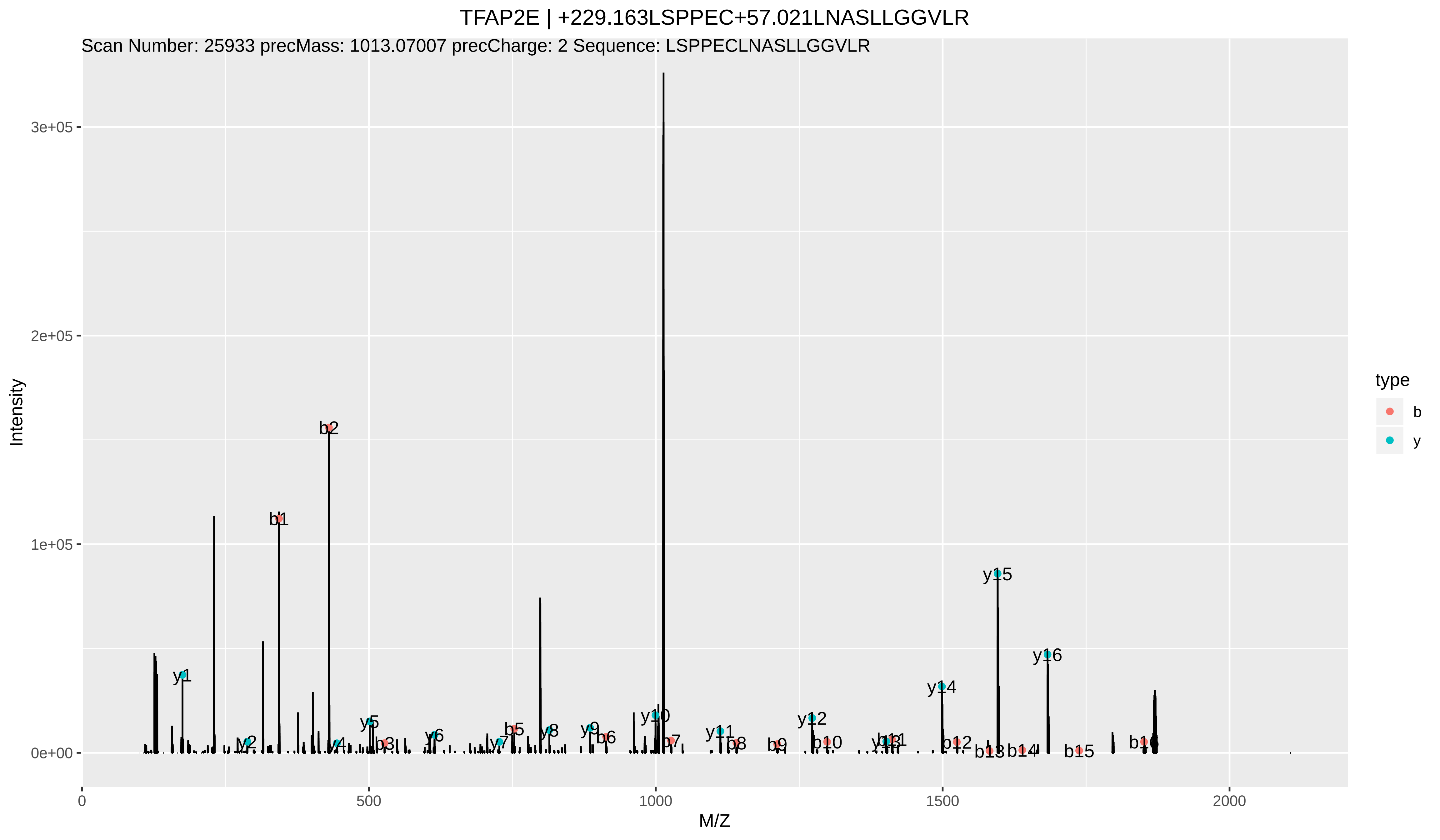

TFAP2E | +229.163LSPPEC+57.021LNASLLGGVLR
Scan Number: 25933 precMass: 1013.07007 precCharge: 2 Sequence: LSPPECLNASLLGGVLR
Intensity
M/Z
type
b
y

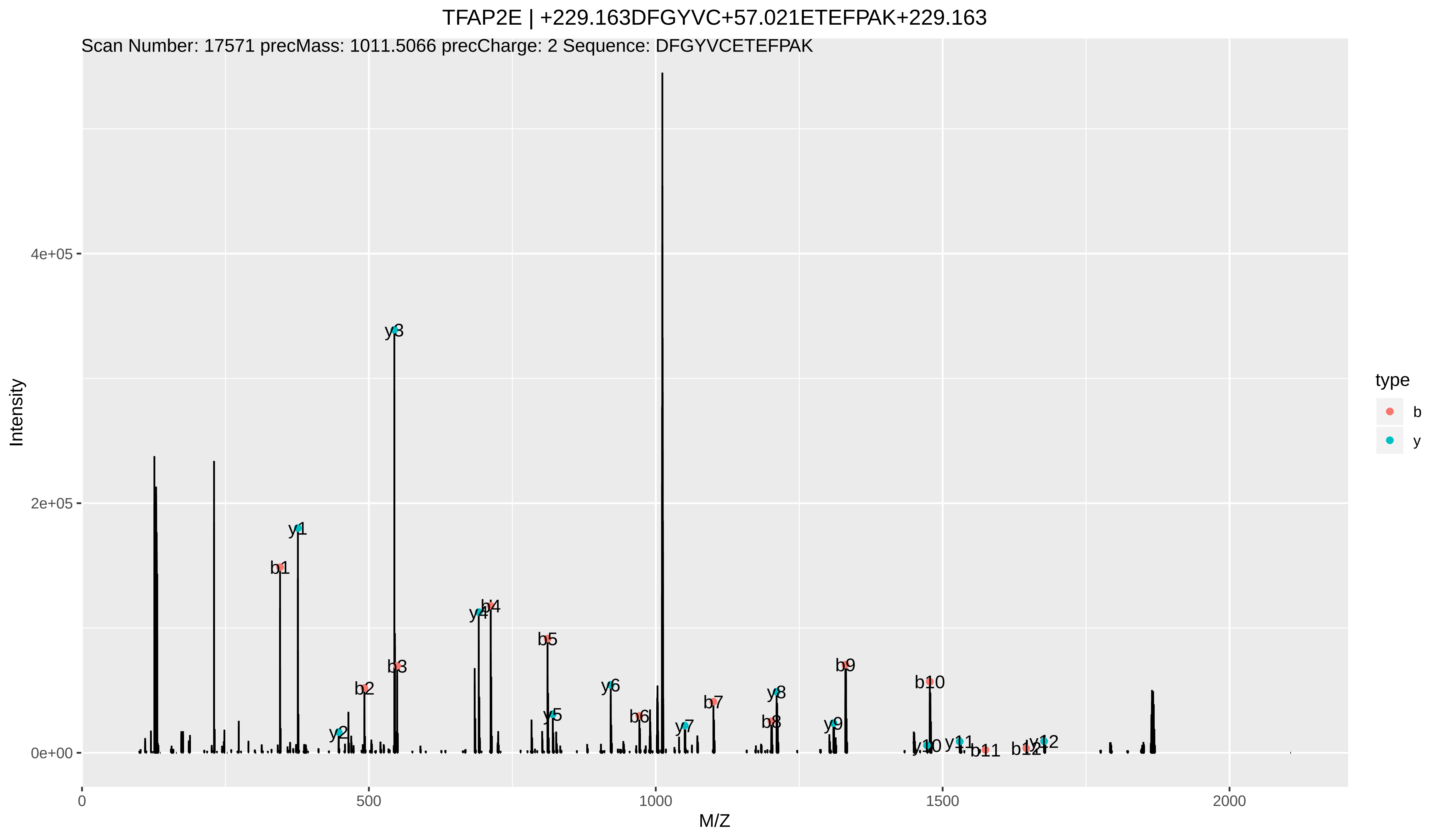
TFAP2E | +229.163DFGYVC+57.021ETEFPAK+229.163
Scan Number: 17571 precMass: 1011.5066 precCharge: 2 Sequence: DFGYVCETEFPAK
Intensity
4e+05
2e+05
0e+00
M/Z
0
500
1000
1500
2000
type
b
y
y3
y1
b1
y4
b4
b5
b3
b2
y6
y5
b6
y7
b7
y8
b8
y9
b9
b10
y10
y11
b11
b12
y12

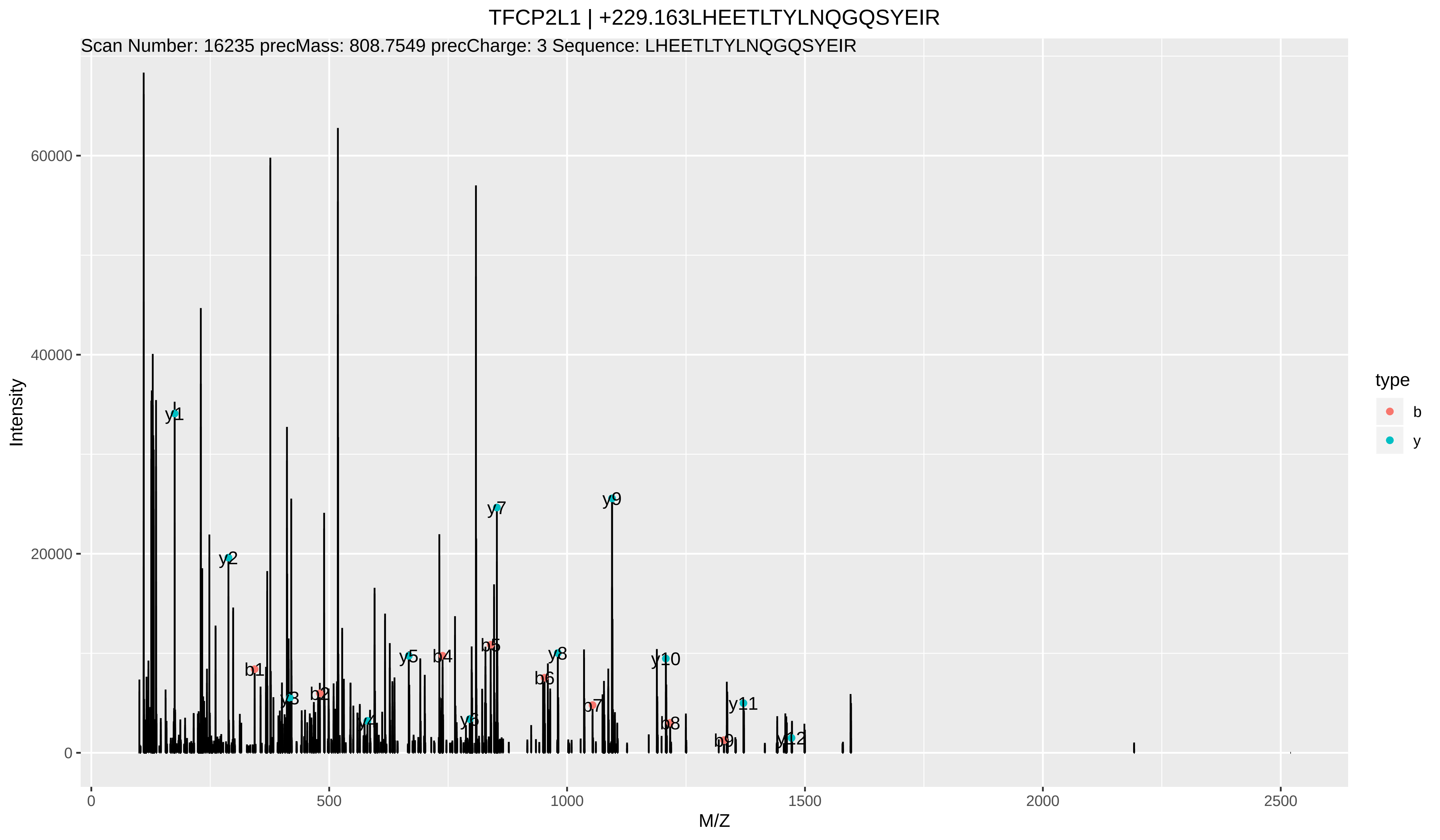
TFCP2L1 | +229.163LHEETLTYLNQGQSYEIR
Scan Number: 16235 precMass: 808.7549 precCharge: 3 Sequence: LHEETLTYLNQGQSYEIR
Intensity
M/Z
type
b
y
y1
y2
y3
y4
y5
y6
y7
y8
y9
y10
y11
y12
b1
b2
b4
b5
b6
b7
b8
b9
0
20000
40000
60000
500
1000
1500
2000
2500

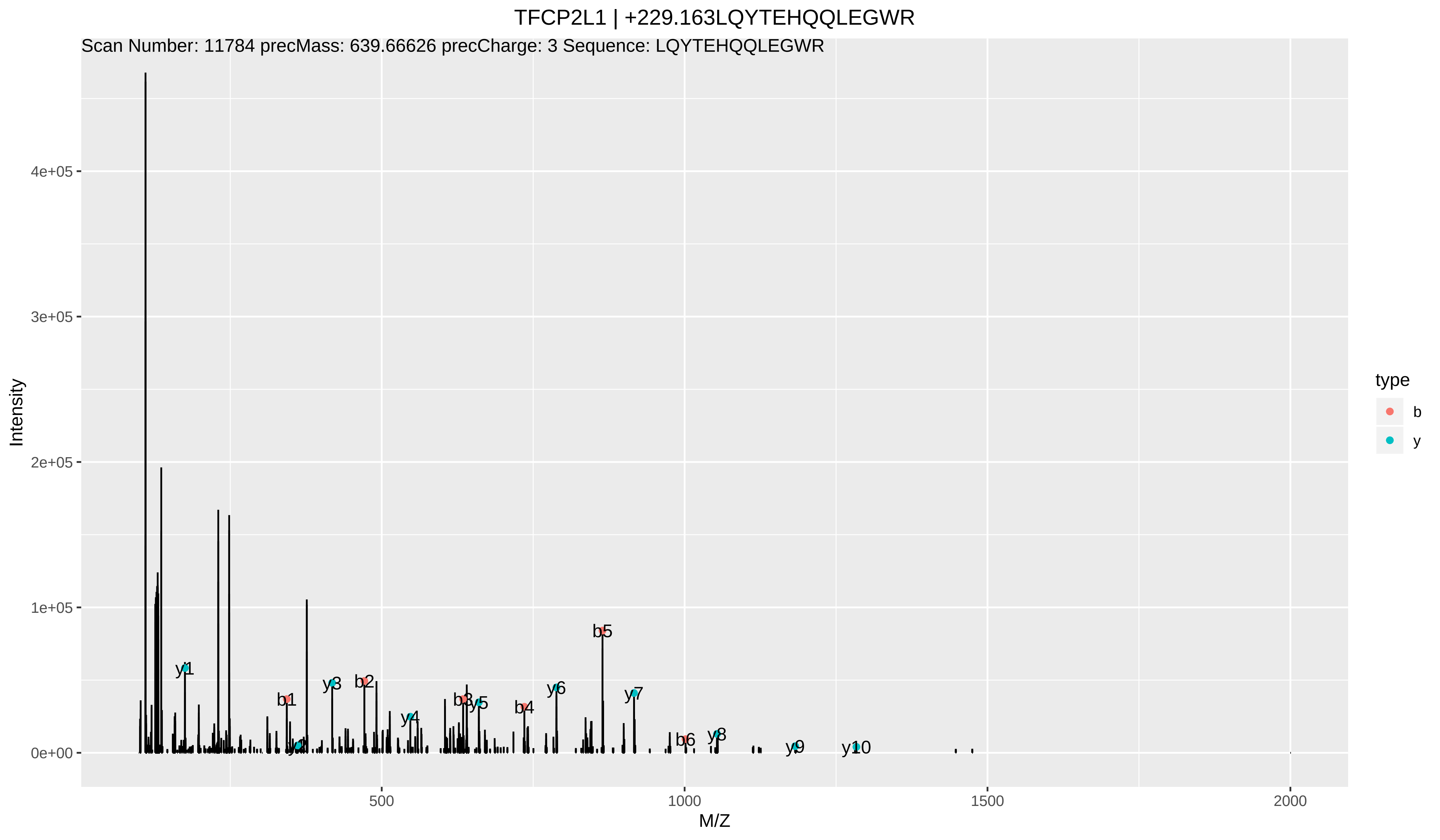

TFCP2L1 | +229.163LQYTEHQQLEGWR
Scan Number: 11784 precMass: 639.66626 precCharge: 3 Sequence: LQYTEHQQLEGWR
Intensity
4e+05
3e+05
2e+05
1e+05
0e+00
M/Z
500
1000
1500
2000
y1
b1
y3
b2
y4
b3
y5
b4
y6
b5
y7
b6
y8
y9
y10
type
b
y

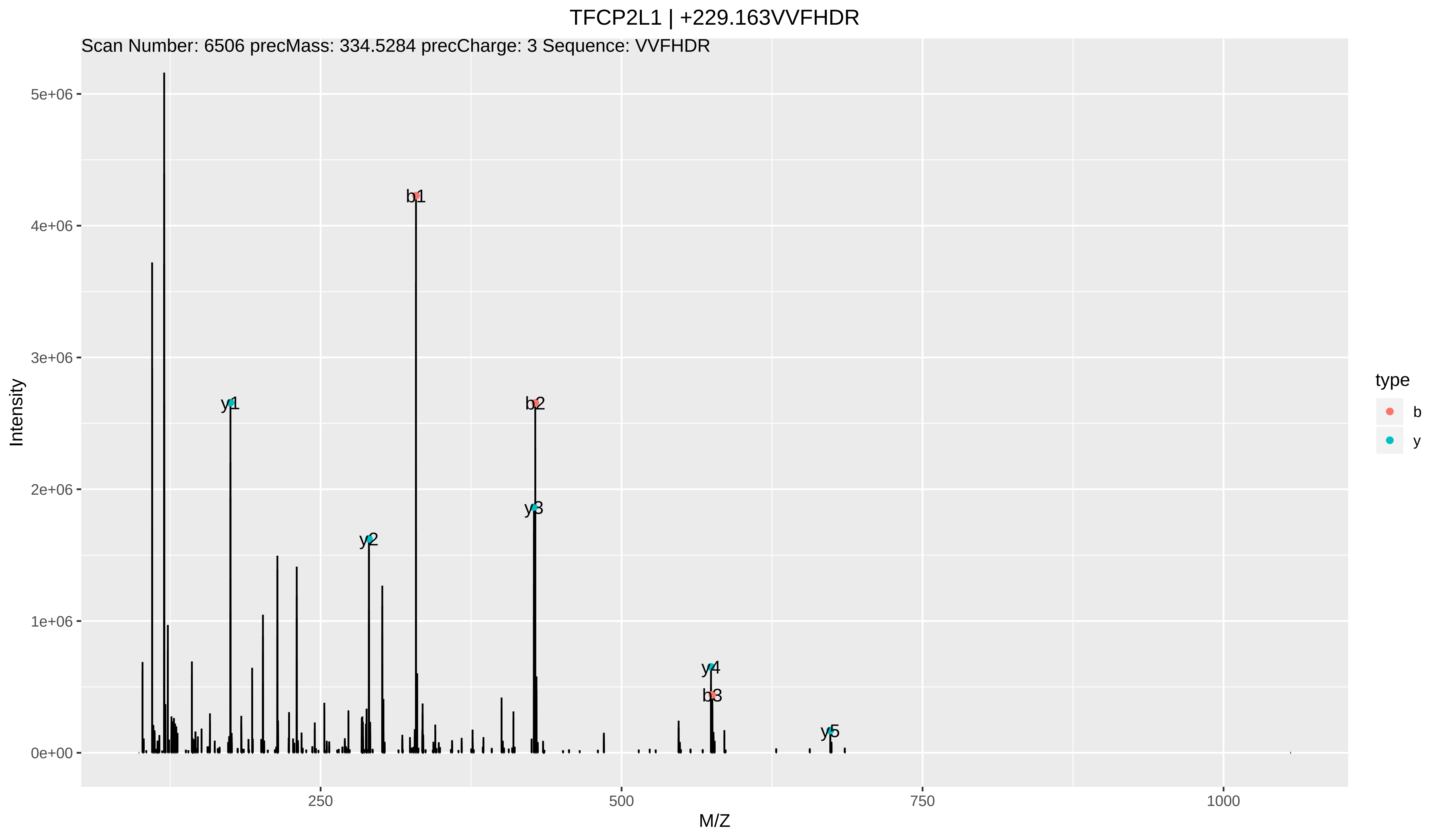

TFCP2L1 | +229.163VVFHDR
Scan Number: 6506 precMass: 334.5284 precCharge: 3 Sequence: VVFHDR
Intensity
5e+06
4e+06
3e+06
2e+06
1e+06
0e+00
M/Z
250
500
750
1000
b1
b2
b3
y1
y2
y3
y4
y5
type
b
y

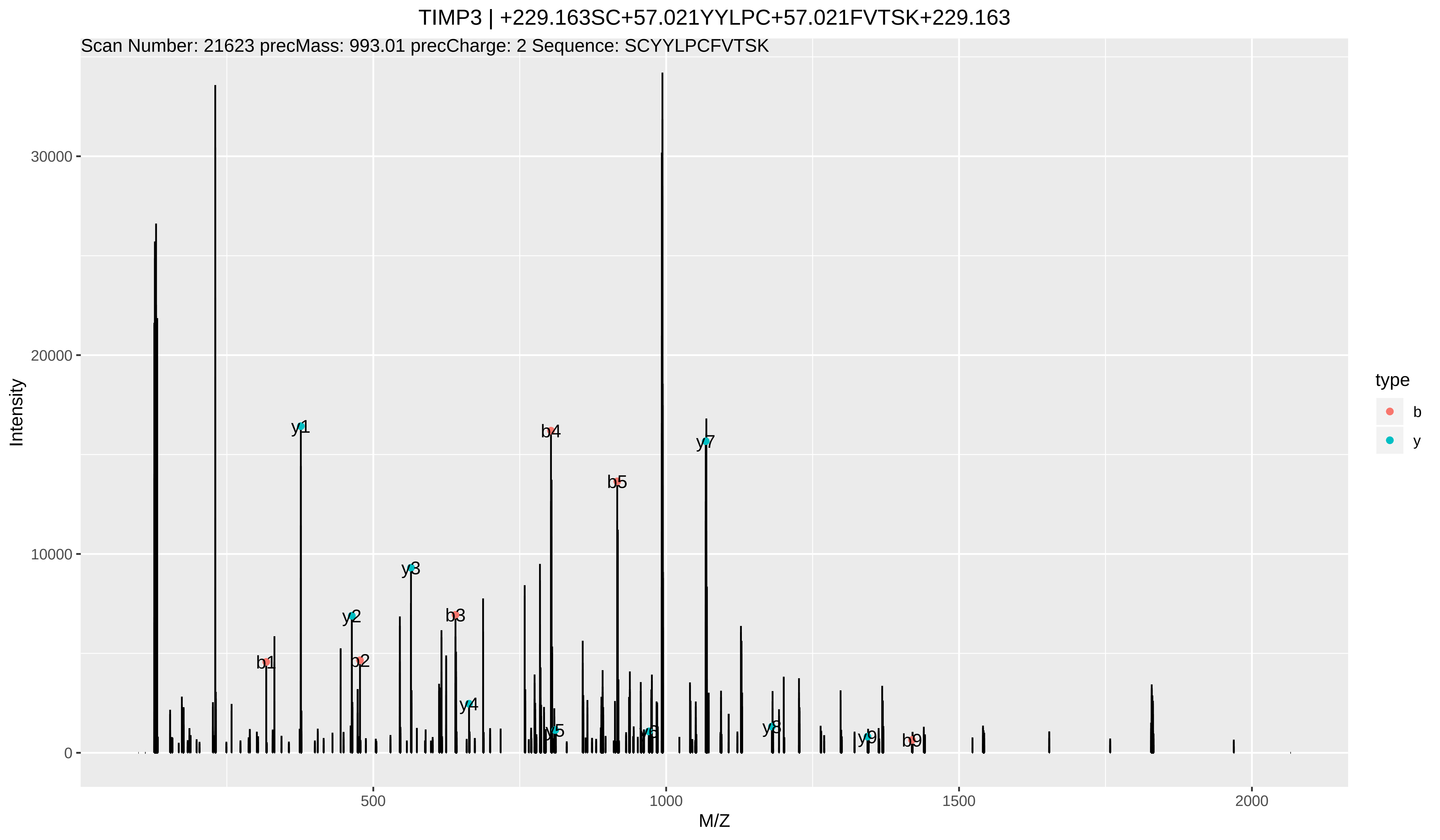

TIMP3 | +229.163SC+57.021YYLPC+57.021FVTSK+229.163
Scan Number: 21623 precMass: 993.01 precCharge: 2 Sequence: SCYYLPCFVTSK
Intensity
M/Z
type
b
y
y1
y2
y3
y4
y5
y6
y7
y8
y9
b1
b2
b3
b4
b5
b9

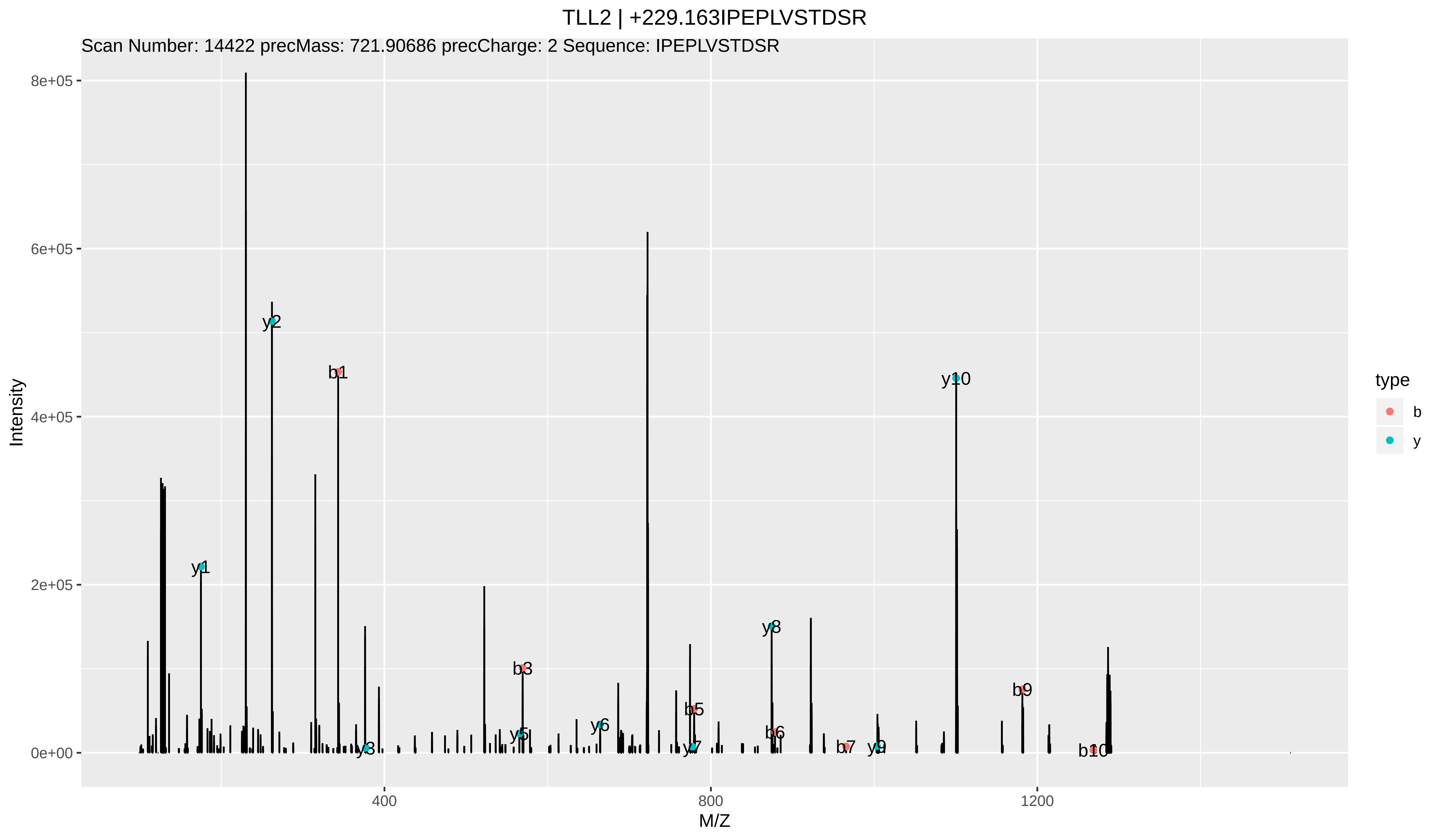
TLL2 | +229.163IPEPLVSTDSR
Scan Number: 14422 precMass: 721.90686 precCharge: 2 Sequence: IPEPLVSTDSR
Intensity
M/Z
8e+05
6e+05
4e+05
2e+05
0e+00
400
800
1200
y1
y2
b1
y3
b3
y5
y6
b5
y7
y8
b6
b7
y9
y10
b9
b10
type
b
y

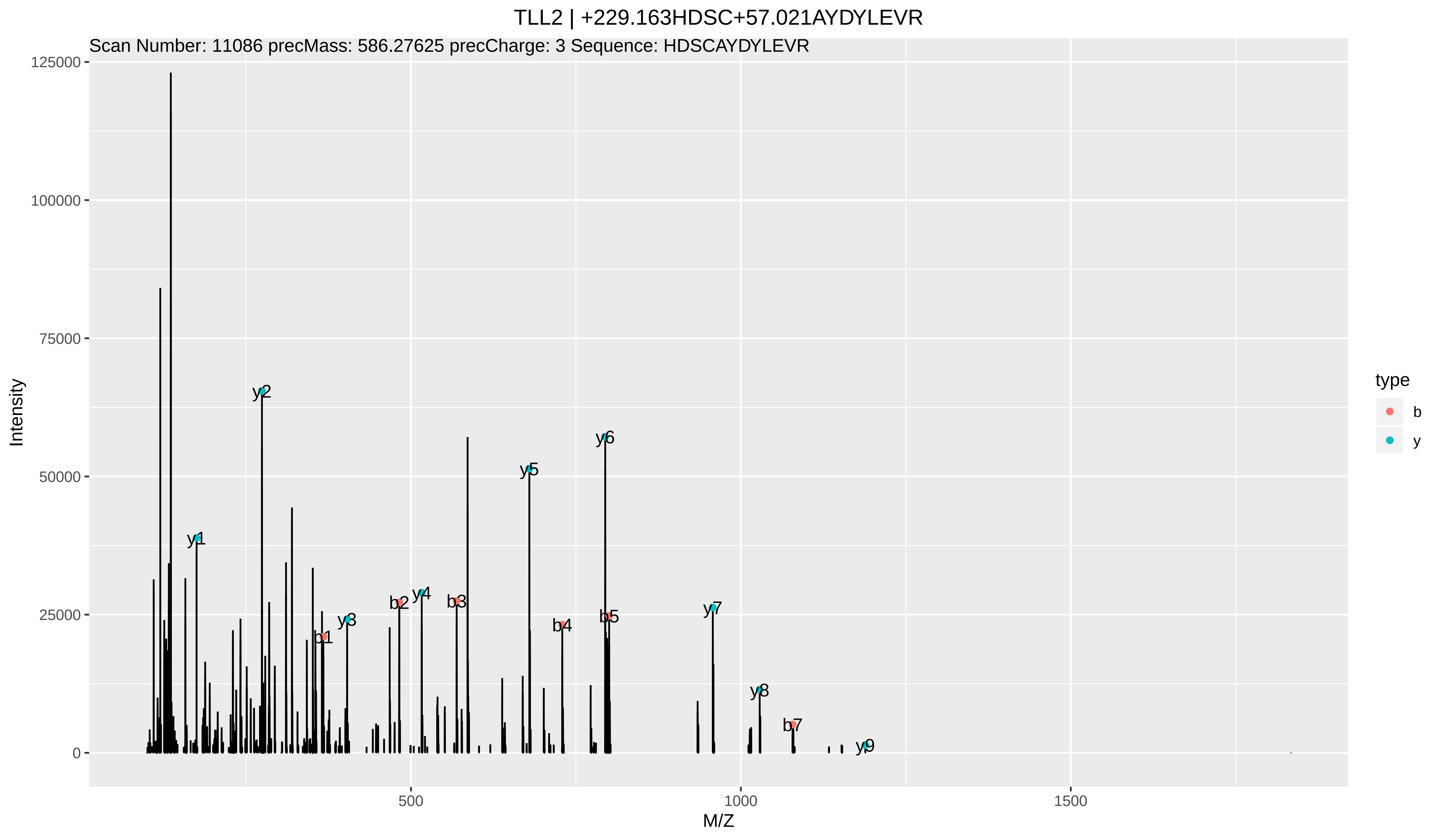

TLL2 | +229.163HDSC+57.021AYDYLEVR
Scan Number: 11086 precMass: 586.27625 precCharge: 3 Sequence: HDSCAYDYLEVR
Intensity
M/Z
125000
100000
75000
50000
25000
0
500
1000
1500
y1
y2
y3
y4
y5
y6
y7
y8
y9
b1
b2
b3
b4
b5
b7
type
b
y

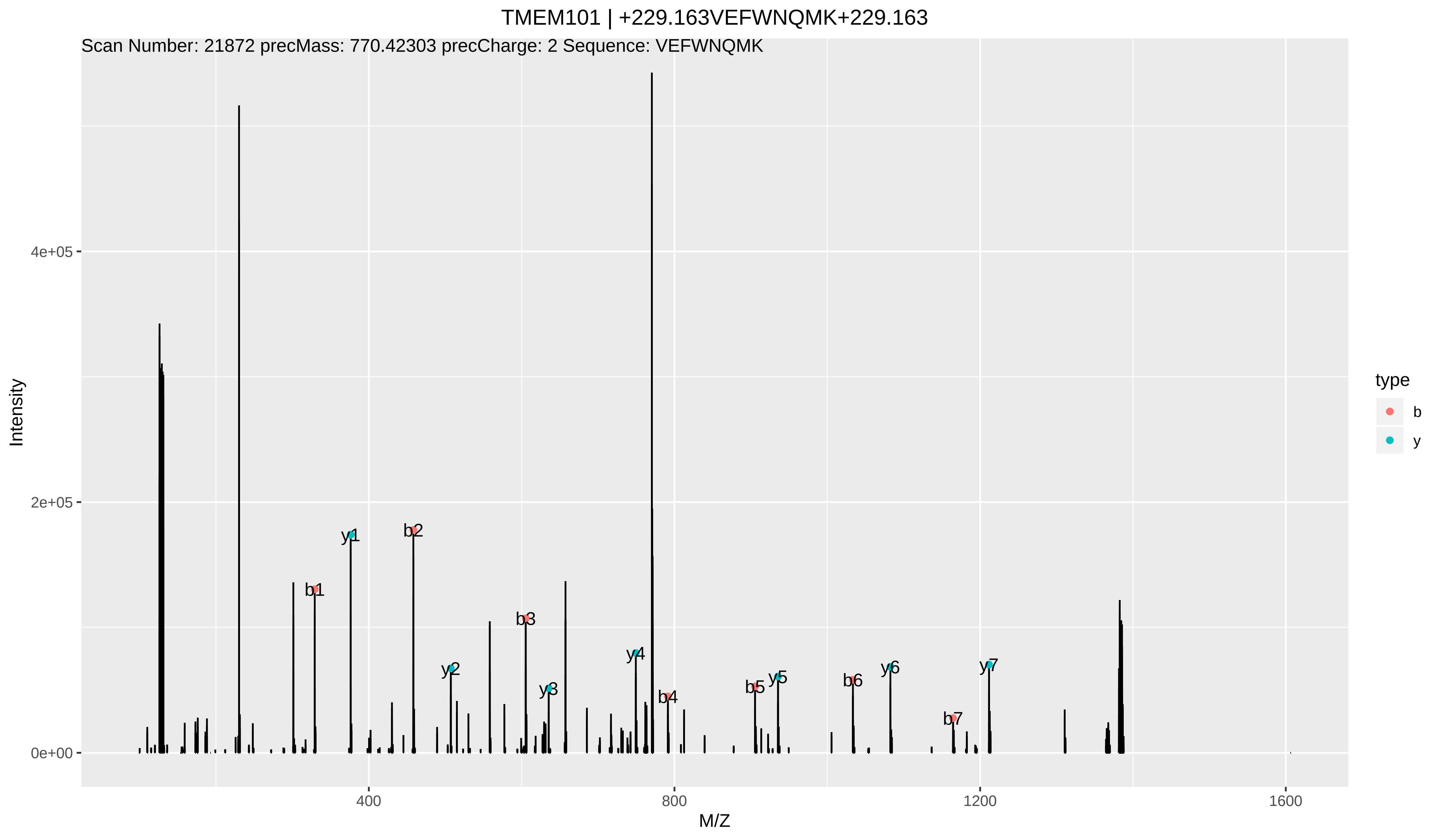
TMEM101 | +229.163VEFWNQMK+229.163
Scan Number: 21872 precMass: 770.42303 precCharge: 2 Sequence: VEFWNQMK
Intensity
4e+05
2e+05
0e+00
M/Z
400
800
1200
1600
y1
b2
b1
b3
y2
y3
y4
b4
b5
y5
b6
y6
b7
y7
type
b
y

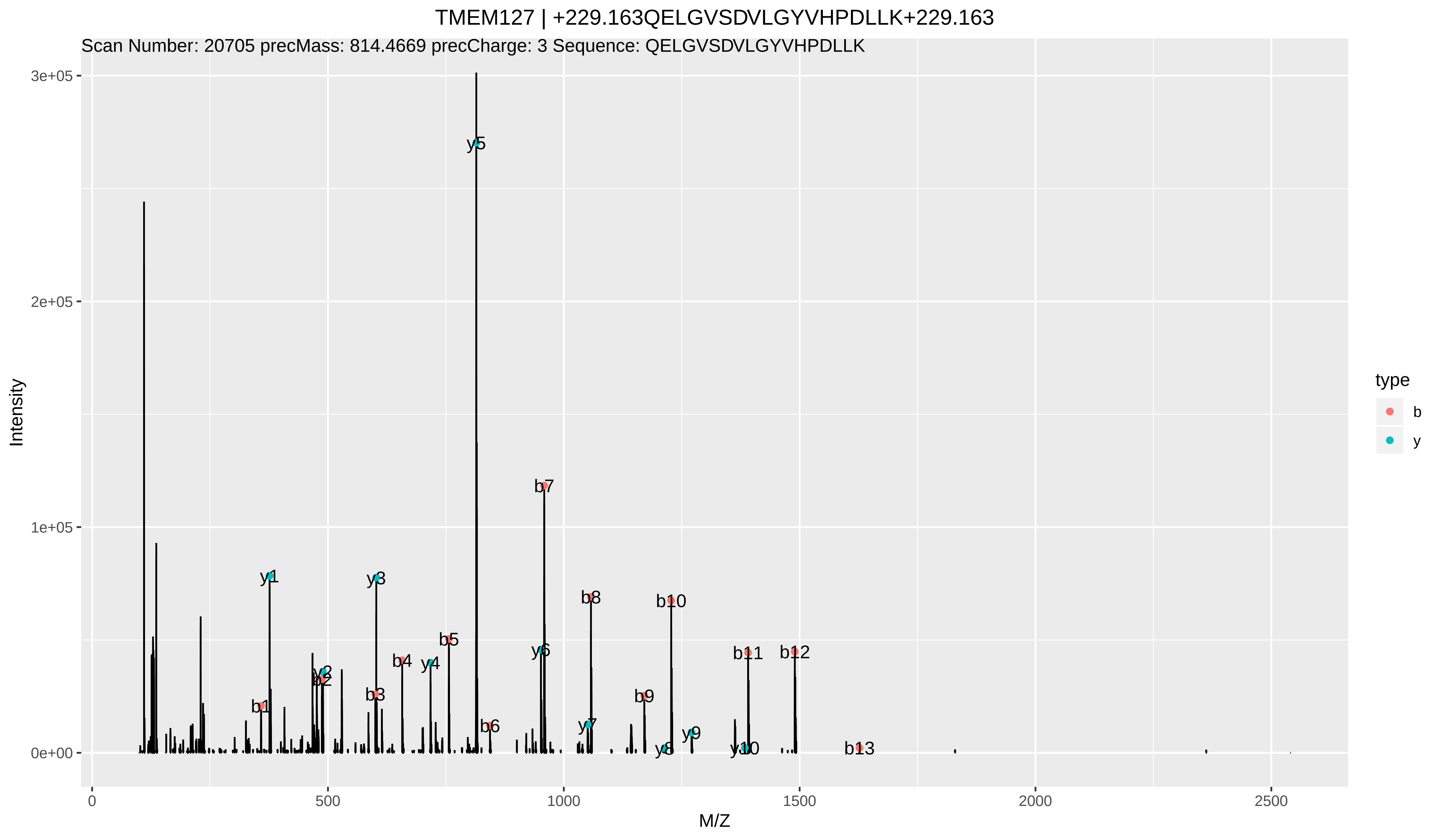

TMEM127 | +229.163QELGVSDVLGYVHPDLLK+229.163
Scan Number: 20705 precMass: 814.4669 precCharge: 3 Sequence: QELGVSDVLGYVHPDLLK
Intensity
M/Z
type
b
y
y5
b7
y1
y3
b8
b10
b5
y6
b11
b12
b4
y4
y2
b2
b1
b3
b9
b6
y7
y9
y8
y10
b13
3e+05
2e+05
1e+05
0e+00
0
500
1000
1500
2000
2500

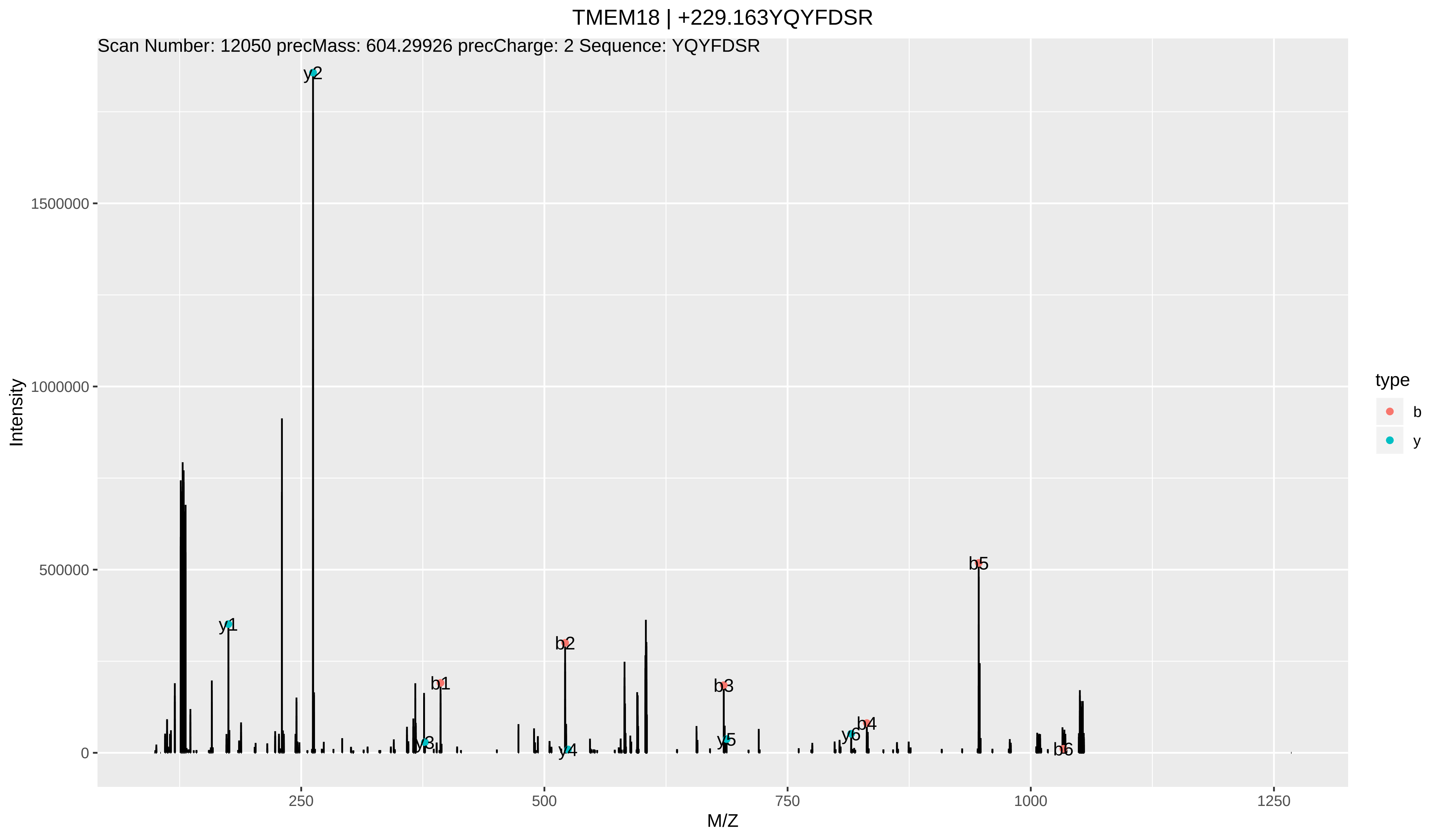
TMEM18 | +229.163YQYFDSR
Scan Number: 12050 precMass: 604.29926 precCharge: 2 Sequence: YQYFDSR
y2
y1
b1
y3
b2
y4
b3
y5
y6
b4
b5
b6
Intensity
1500000
1000000
500000
0
250
500
750
1000
1250
M/Z
type
b
y

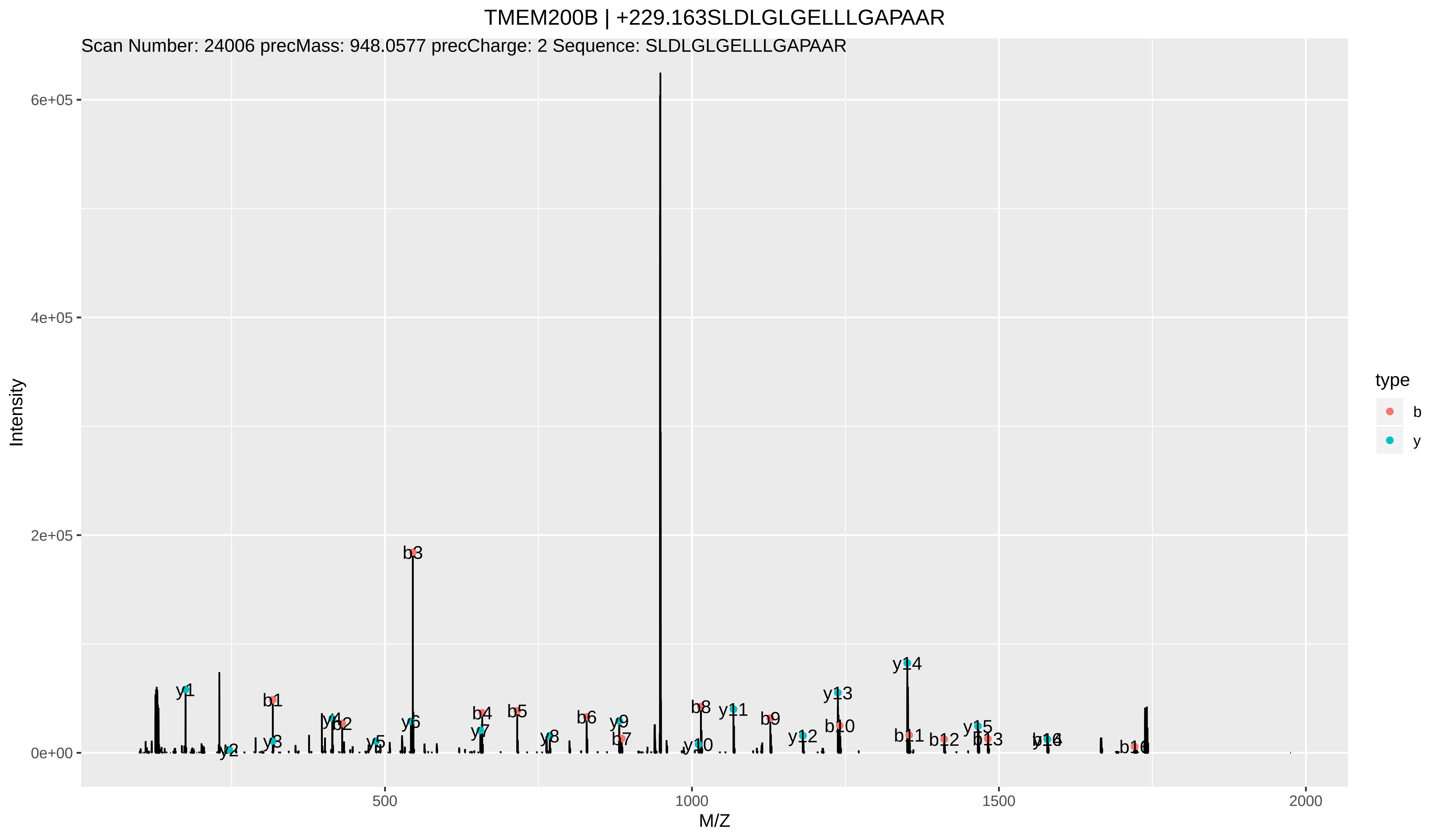

TMEM200B | +229.163SLDLGLGELLLGAPAAR
Scan Number: 24006 precMass: 948.0577 precCharge: 2 Sequence: SLDLGLGELLLGAPAAR
Intensity
M/Z
type
b
y

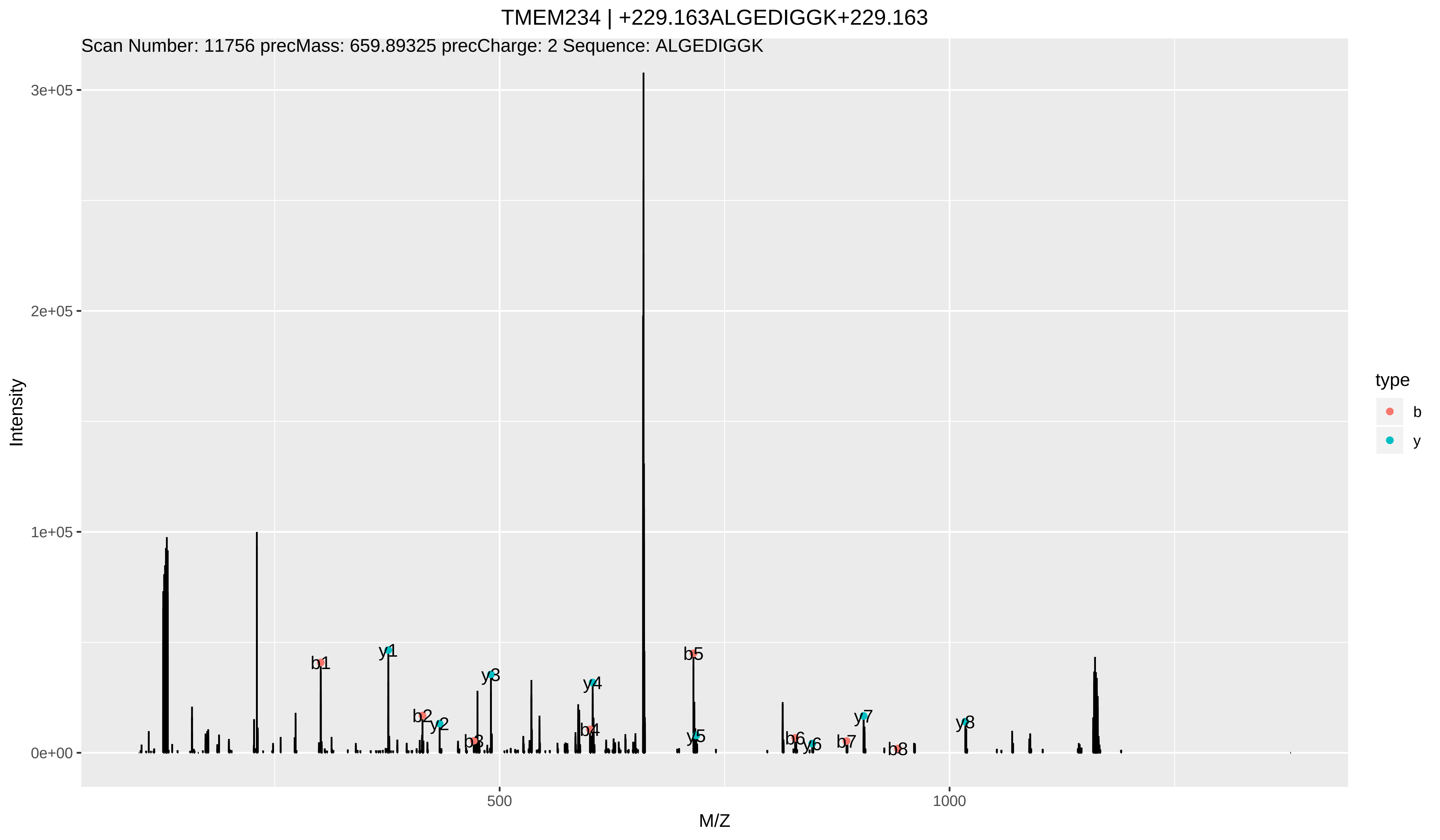

TMEM234 | +229.163ALGEDIGGK+229.163
Scan Number: 11756 precMass: 659.89325 precCharge: 2 Sequence: ALGEDIGGK
Intensity
3e+05
2e+05
1e+05
0e+00
M/Z
500
1000
b1
y1
b2
y2
b3
y3
b4
y4
b5
y5
b6
y6
b7
y7
b8
y8
type
b
y

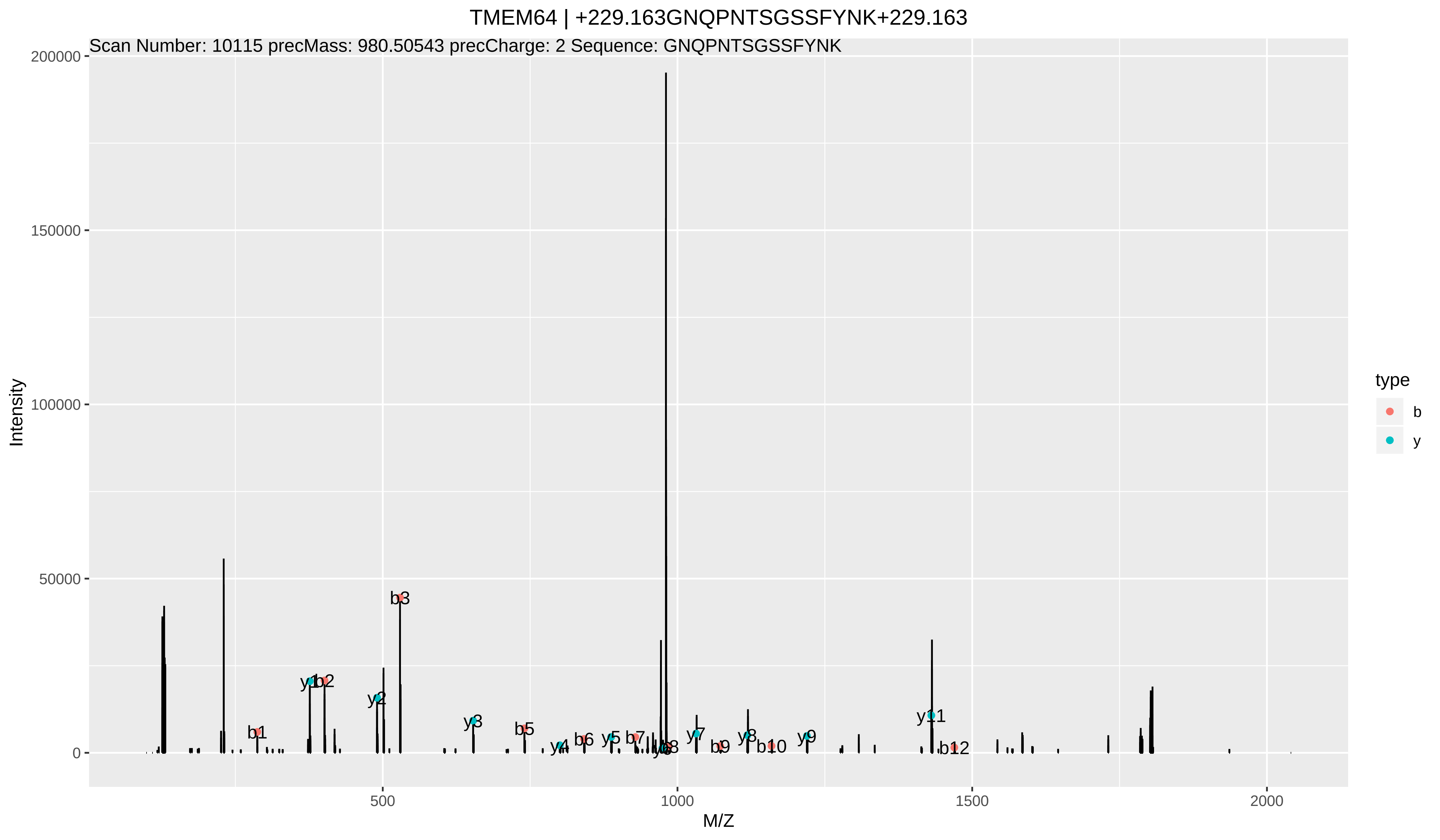

TMEM64 | +229.163GNQPNTSGSSFYNK+229.163
Scan Number: 10115 precMass: 980.50543 precCharge: 2 Sequence: GNQPNTSGSSFYNK
Intensity
M/Z
type
b
y

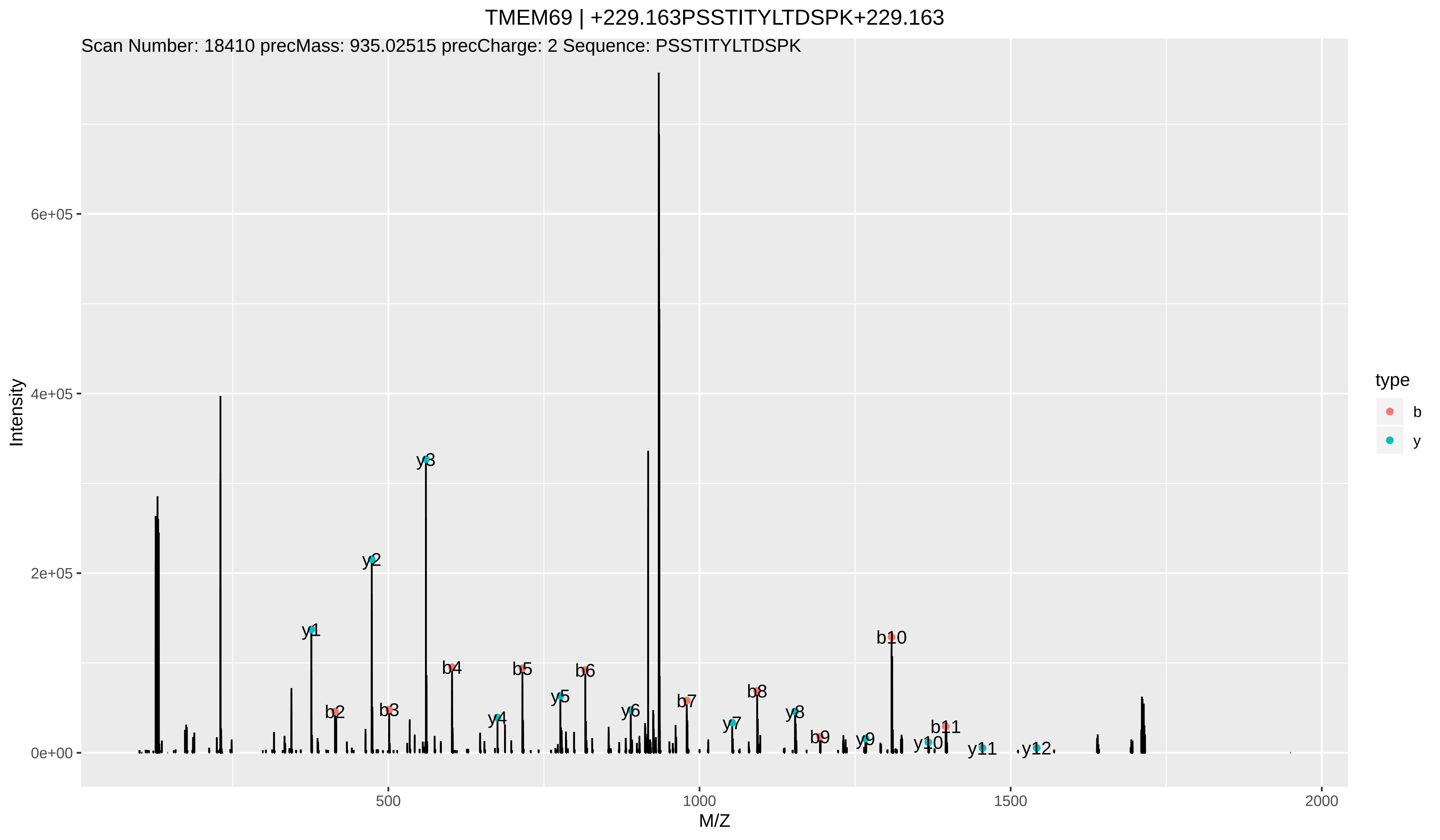
TMEM69 | +229.163PSSTITYLTDSPK+229.163
Scan Number: 18410 precMass: 935.02515 precCharge: 2 Sequence: PSSTITYLTDSPK
Intensity
M/Z
0e+00
2e+05
4e+05
6e+05
500
1000
1500
2000
y1
y2
y3
y4
y5
y6
y7
y8
y9
y10
y11
y12
b2
b3
b4
b5
b6
b7
b8
b9
b10
b11
type
b
y

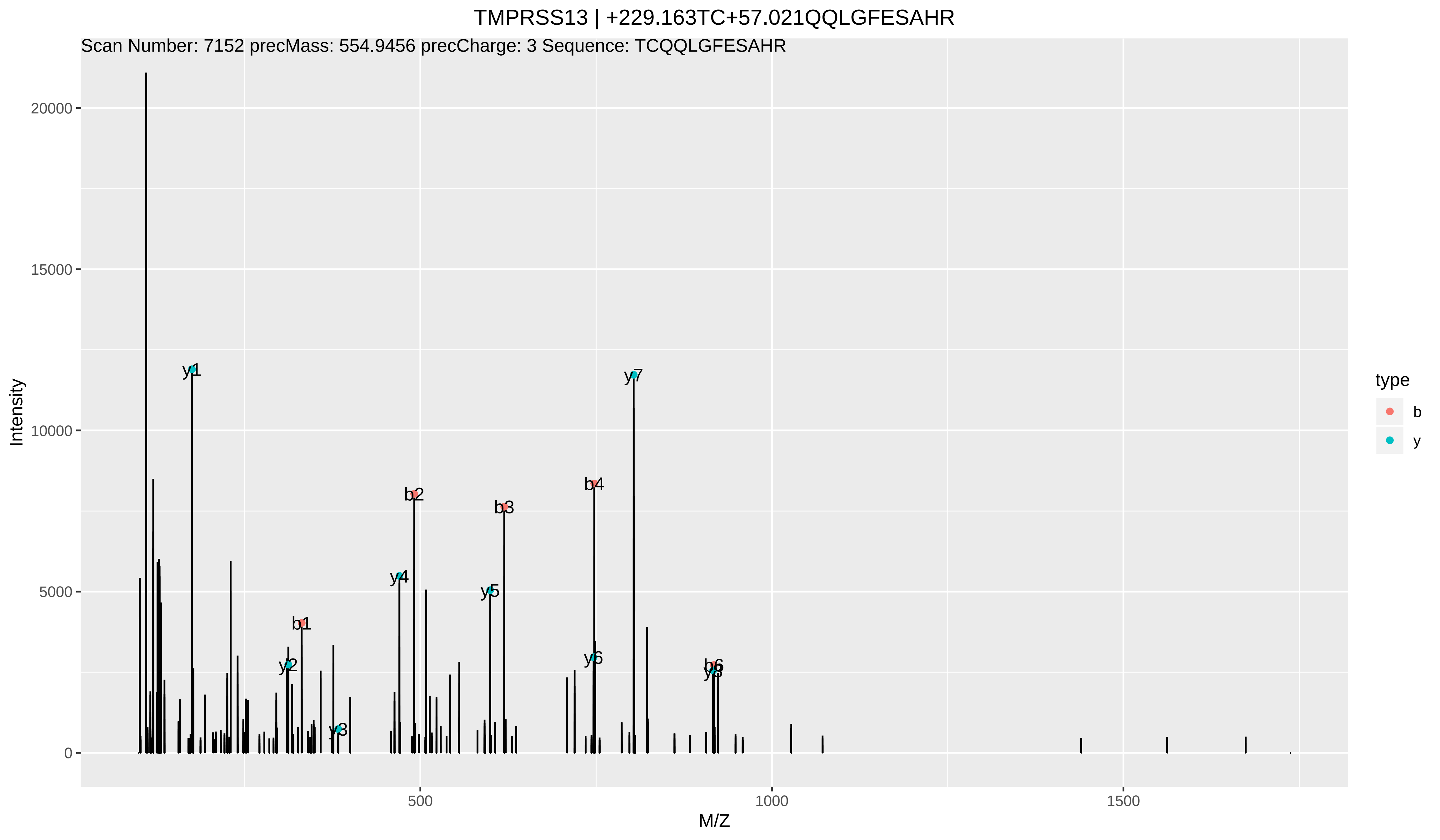
TMPRSS13 | +229.163TC+57.021QQLGFESAHR
Scan Number: 7152 precMass: 554.9456 precCharge: 3 Sequence: TCQQLGFESAHR
Intensity
M/Z
type
b
y
y1
y2
y3
y4
y5
y6
y7
b1
b2
b3
b4
b6
0
5000
10000
15000
20000
500
1000
1500

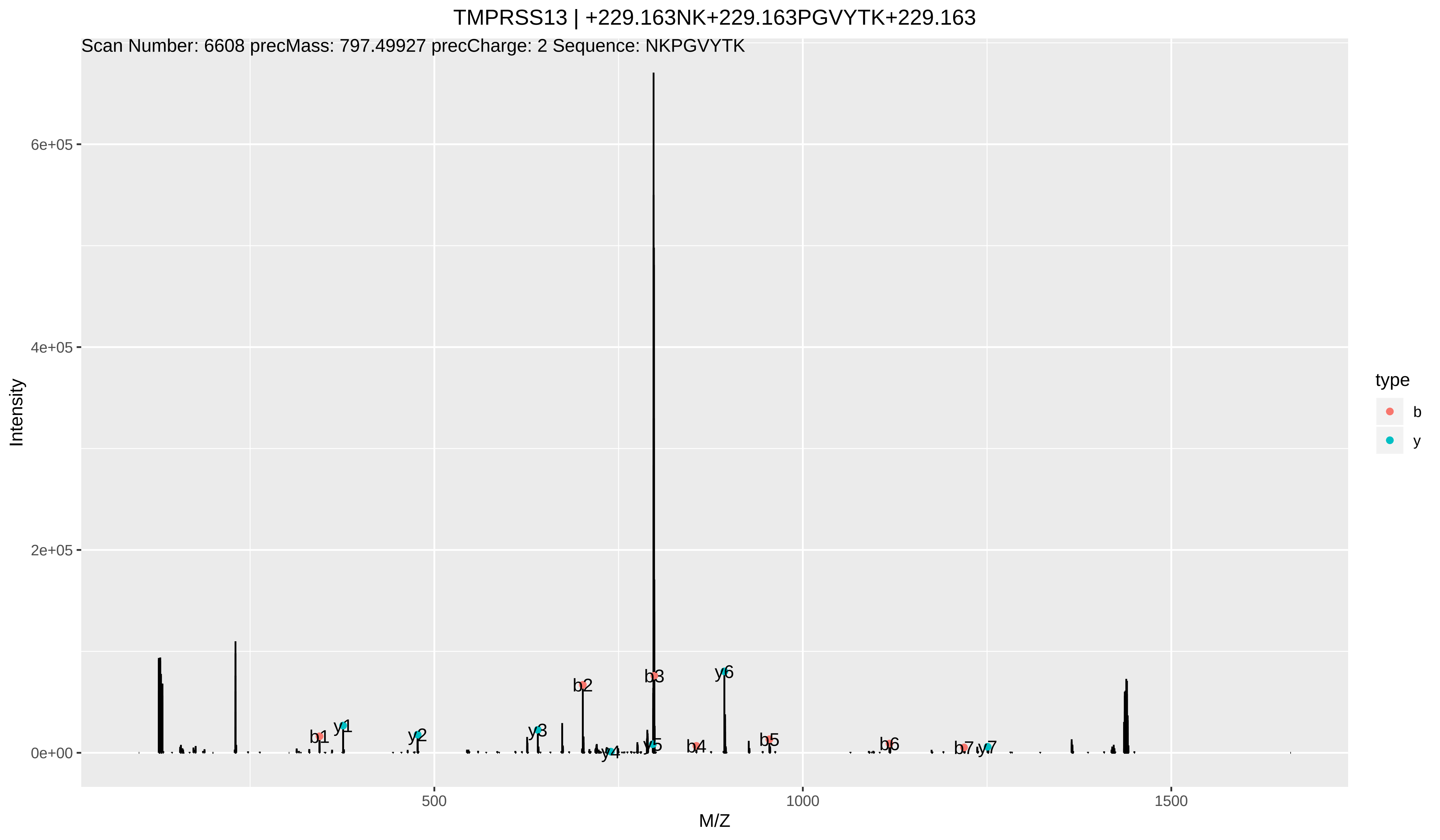

TMPRSS13 | +229.163NK+229.163PGVYTK+229.163
Scan Number: 6608 precMass: 797.49927 precCharge: 2 Sequence: NKPGVYTK
Intensity
6e+05
4e+05
2e+05
0e+00
M/Z
500
1000
1500
b1
y1
y2
y3
b2
y4
b3
y5
b4
y6
b5
b6
b7
y7
type
b
y

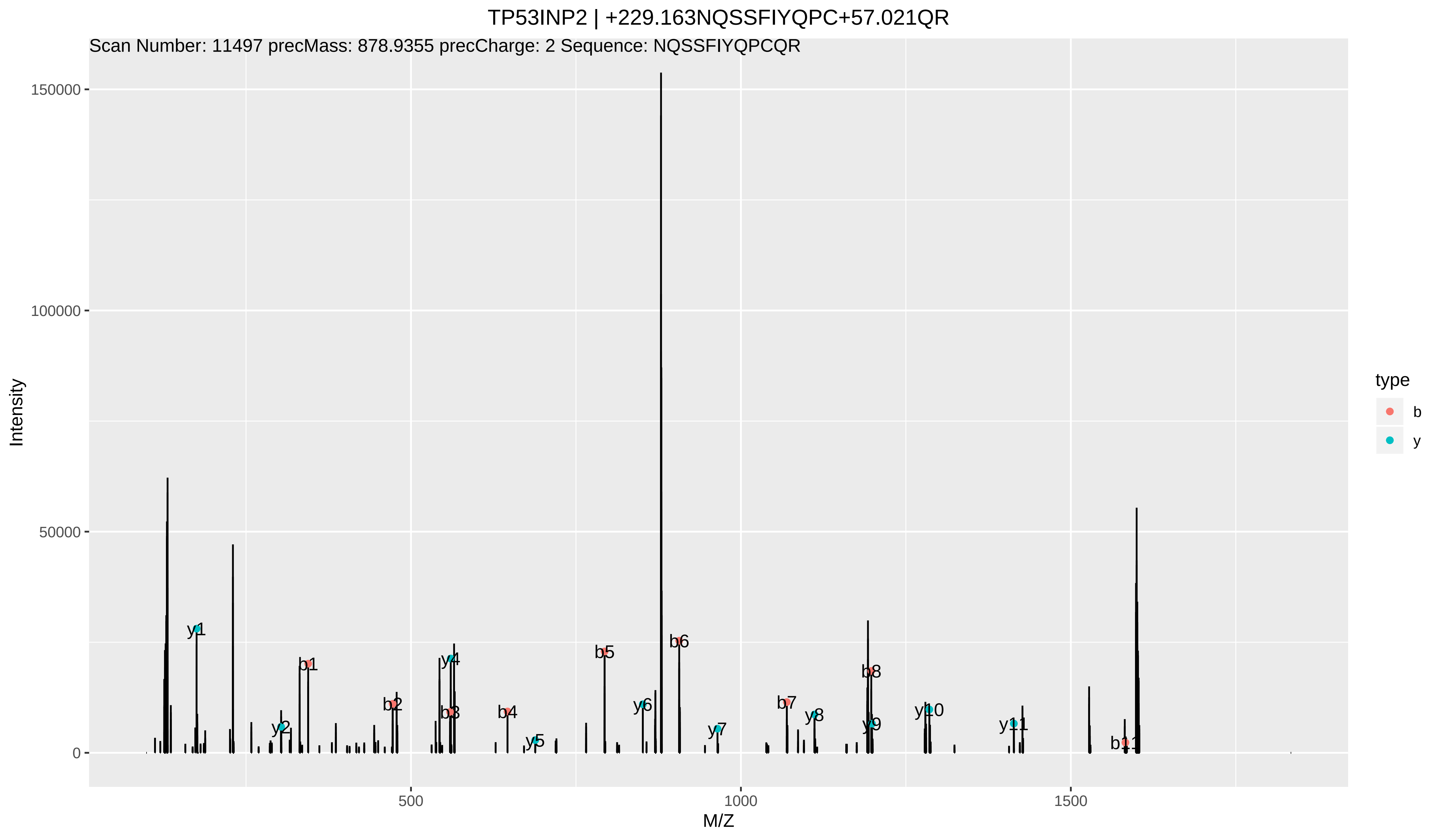

TP53INP2 | +229.163NQSSFIYQPC+57.021QR
Scan Number: 11497 precMass: 878.9355 precCharge: 2 Sequence: NQSSFIYQPCQR
Intensity
150000
100000
50000
0
500
1000
1500
M/Z
y1
y2
b1
b2
y4
b3
b4
y5
b5
y6
b6
y7
b7
y8
b8
y9
y10
y11
b11
type
b
y

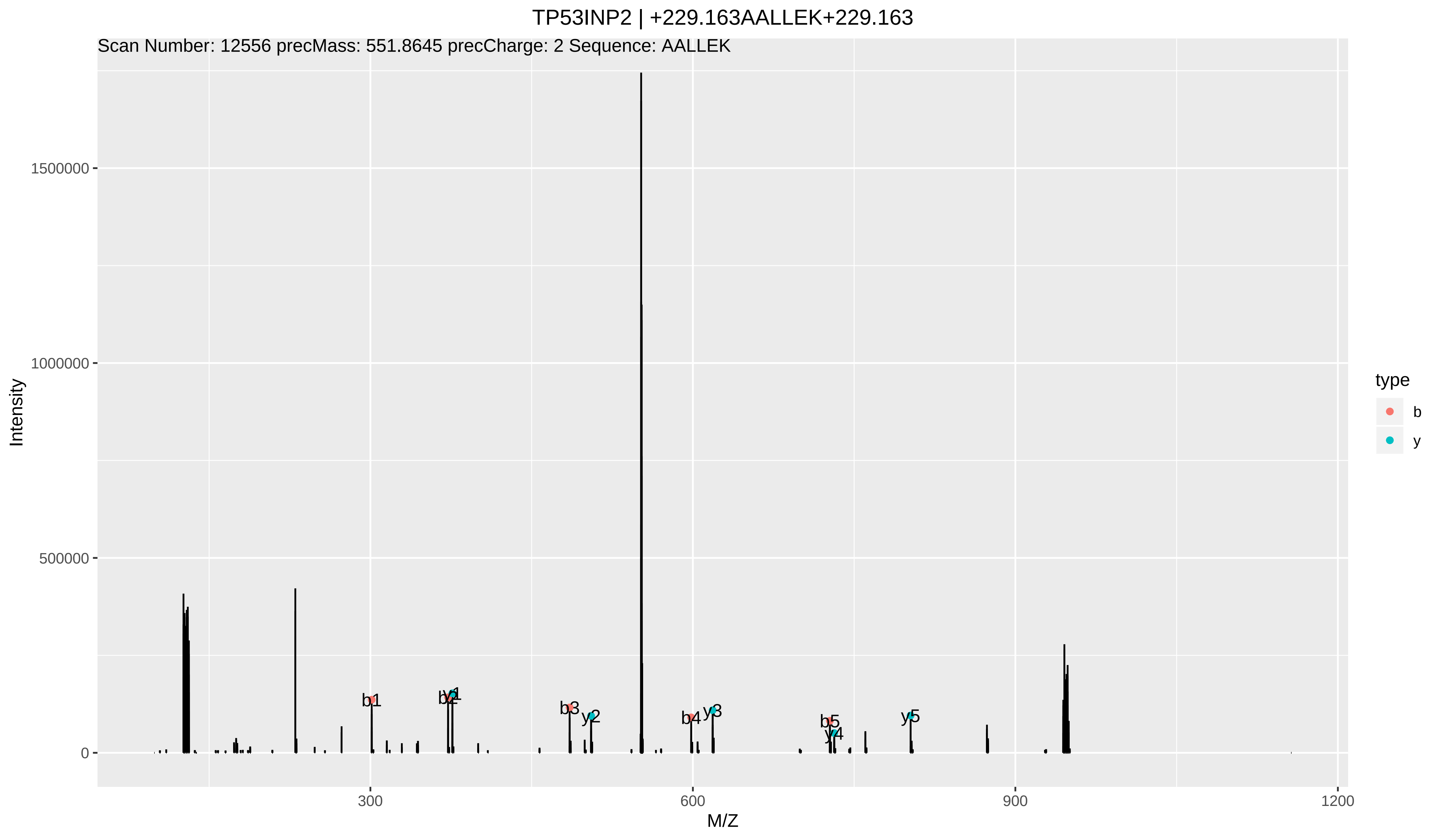

TP53INP2 | +229.163AALLEK+229.163
Scan Number: 12556 precMass: 551.8645 precCharge: 2 Sequence: AALLEK
Intensity
1500000
1000000
500000
0
300
600
900
1200
M/Z
b1
b2
y1
b3
y2
b4
y3
b5
y4
y5
type
b
y

TRIML2 | +229.163HHM+15.995VC+57.021GIQEAAENYR

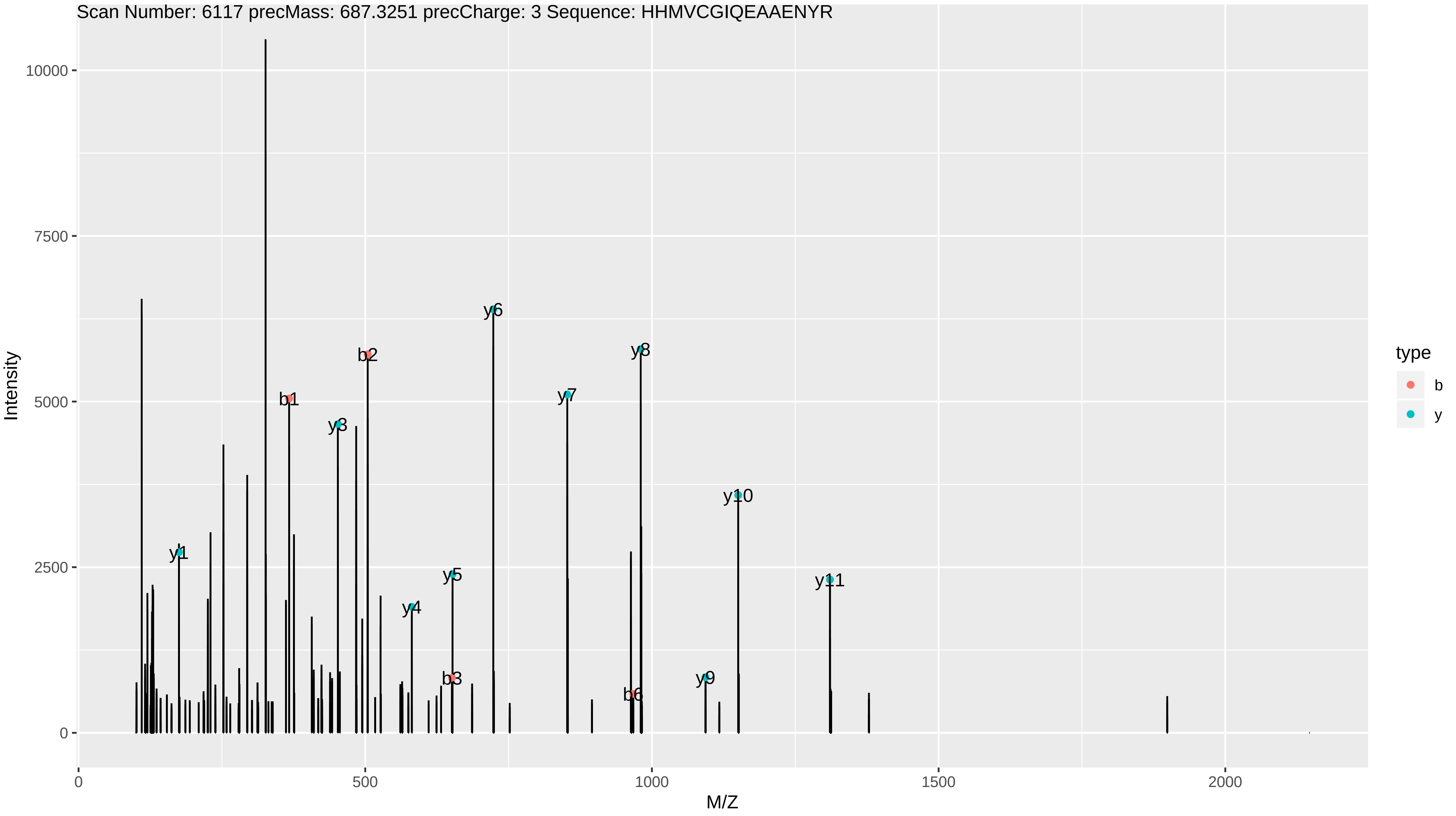

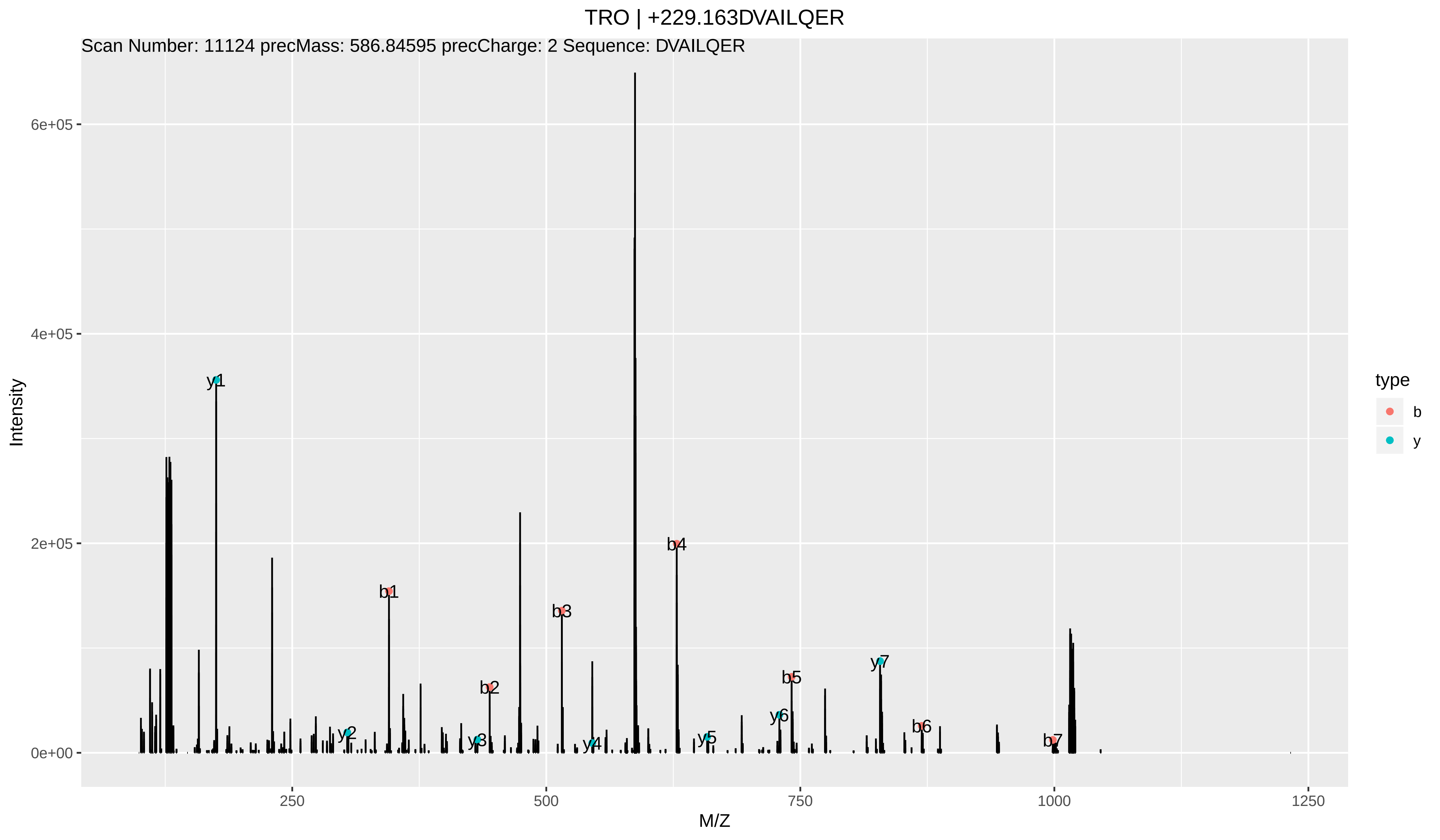
TRO | +229.163DVAILQER
Scan Number: 11124 precMass: 586.84595 precCharge: 2 Sequence: DVAILQER
Intensity
M/Z
6e+05
4e+05
2e+05
0e+00
250
500
750
1000
1250
y1
b1
b2
b3
b4
b5
b6
b7
y2
y3
y4
y5
y6
y7
type
b
y

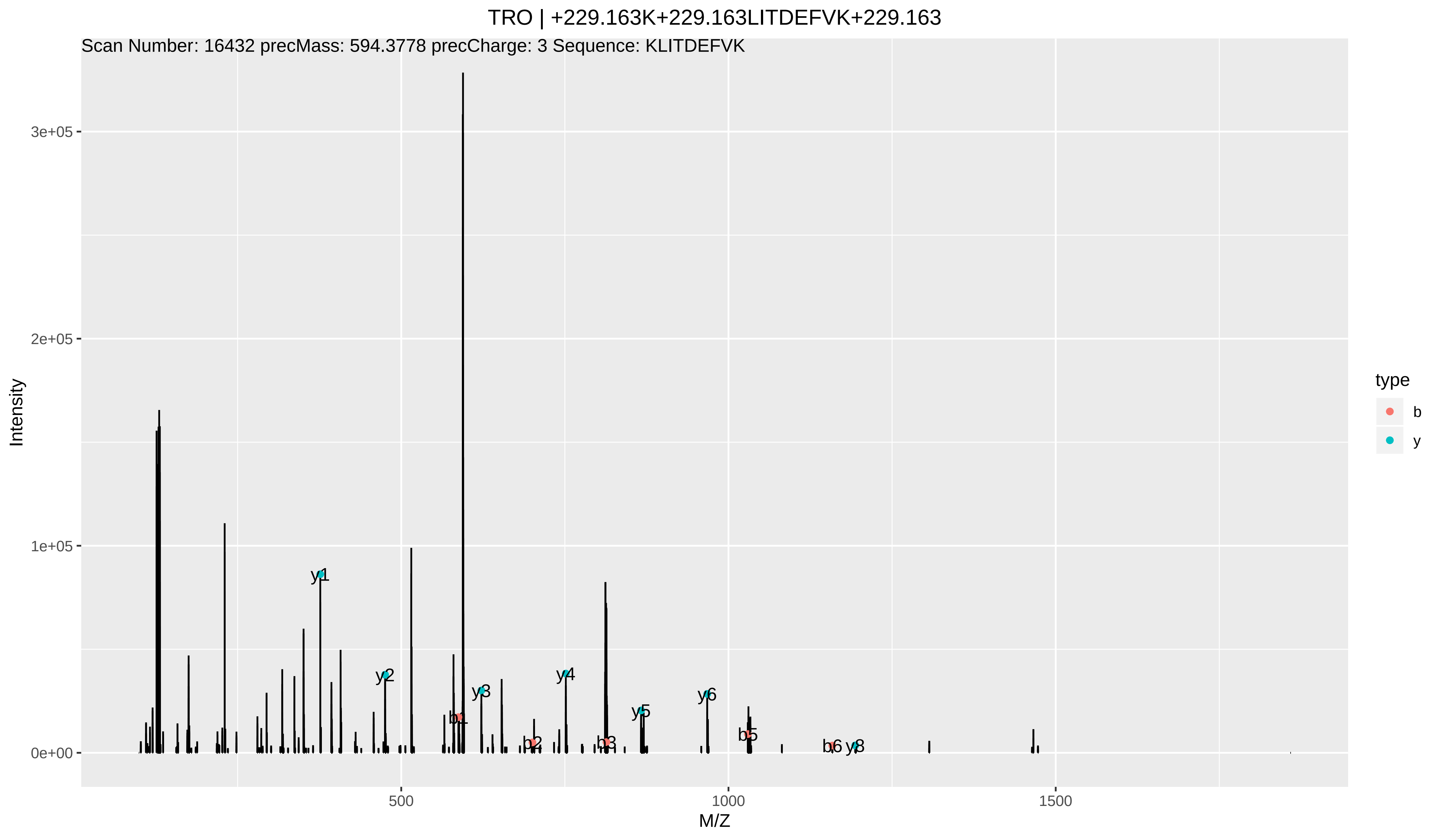

TRO | +229.163K+229.163LITDEFVK+229.163
Scan Number: 16432 precMass: 594.3778 precCharge: 3 Sequence: KLITDEFVK
Intensity
3e+05
2e+05
1e+05
0e+00
M/Z
500
1000
1500
y1
y2
y3
y4
y5
y6
y8
b1
b2
b3
b5
b6
type
b
y

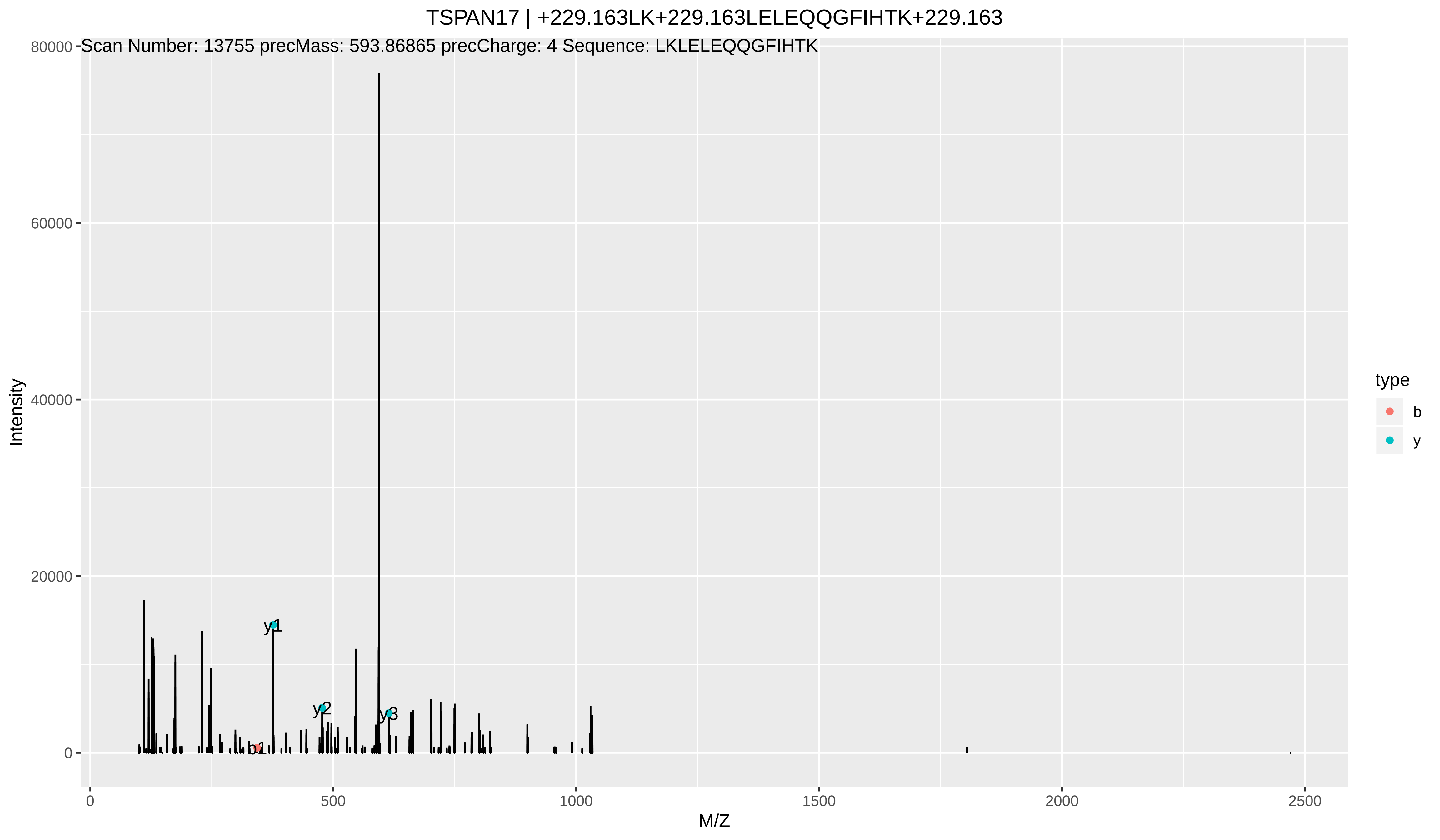

TSPAN17 | +229.163LK+229.163LELEQQGFIHTK+229.163
Scan Number: 13755 precMass: 593.86865 precCharge: 4 Sequence: LKLELEQQGFIHTK
Intensity
M/Z
80000
60000
40000
20000
0
0
500
1000
1500
2000
2500
y1
y2
y3
b1
type
b
y

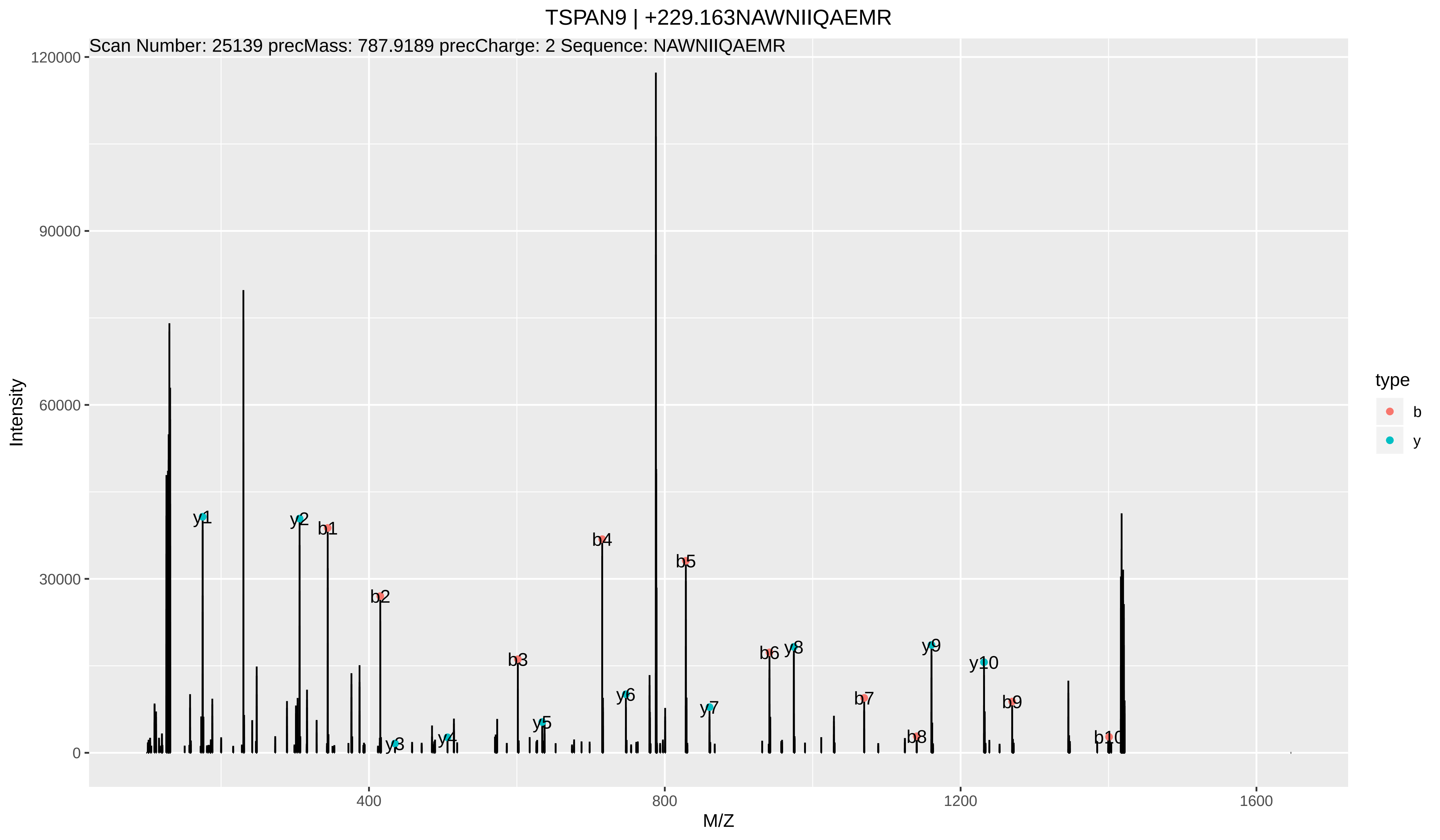

TSPAN9 | +229.163NAWNIIQAEMR
Scan Number: 25139 precMass: 787.9189 precCharge: 2 Sequence: NAWNIIQAEMR
Intensity
M/Z
0
30000
60000
90000
120000
400
800
1200
1600
y1
y2
b1
b2
y3
y4
b3
y5
b4
y6
b5
y7
b6
y8
b7
b8
y9
y10
b9
b10
type
b
y

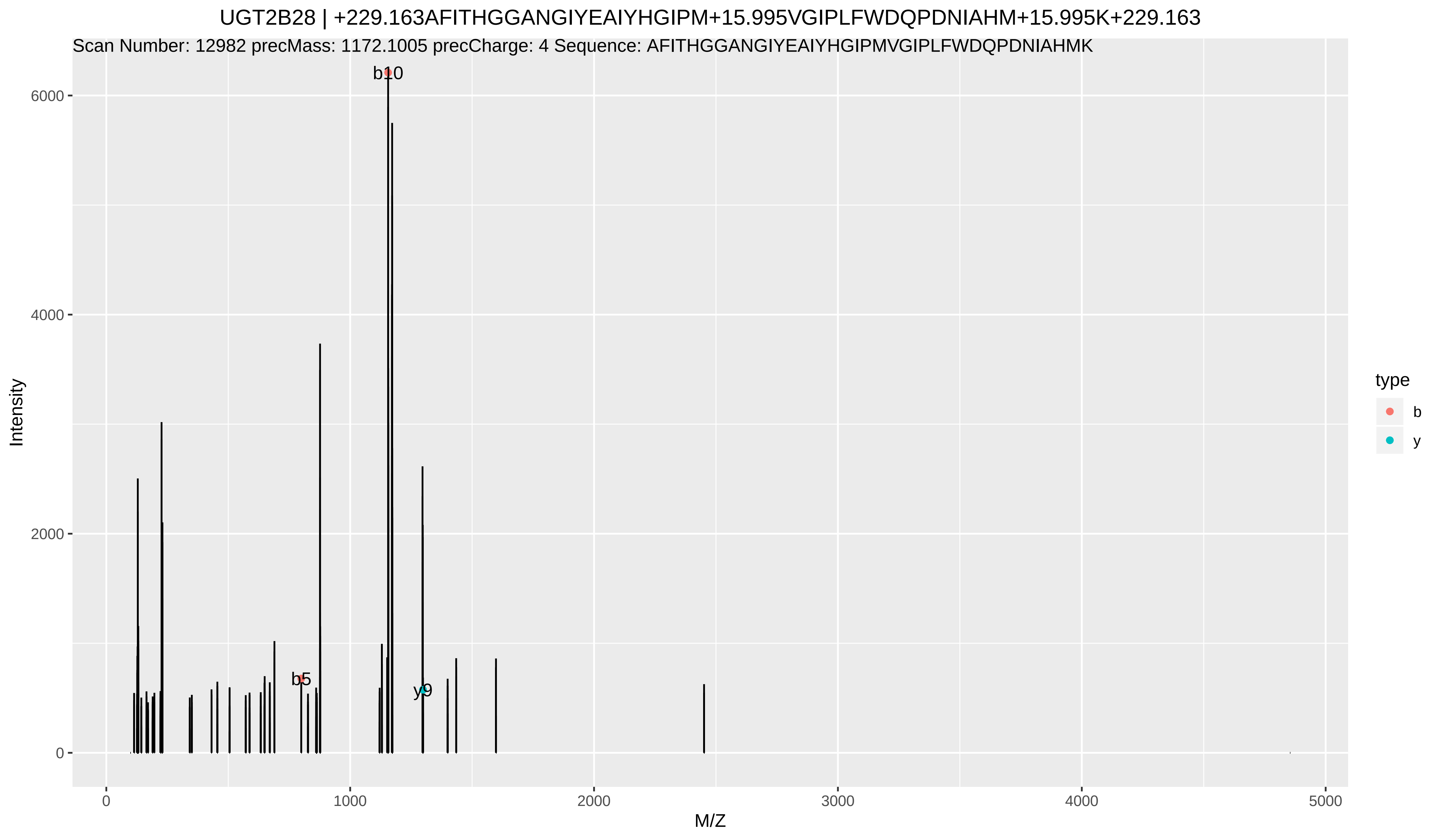
UGT2B28 | +229.163AFITHGGANGIYEAIYHGIPM+15.995VGIPLFWDQPDNIAHM+15.995K+229.163
Scan Number: 12982 precMass: 1172.1005 precCharge: 4 Sequence: AFITHGGANGIYEAIYHGIPMVGIPLFWDQPDNIAHMK
b10
b5
y9
Intensity
M/Z
0
2000
4000
6000
0
1000
2000
3000
4000
5000
type
b
y

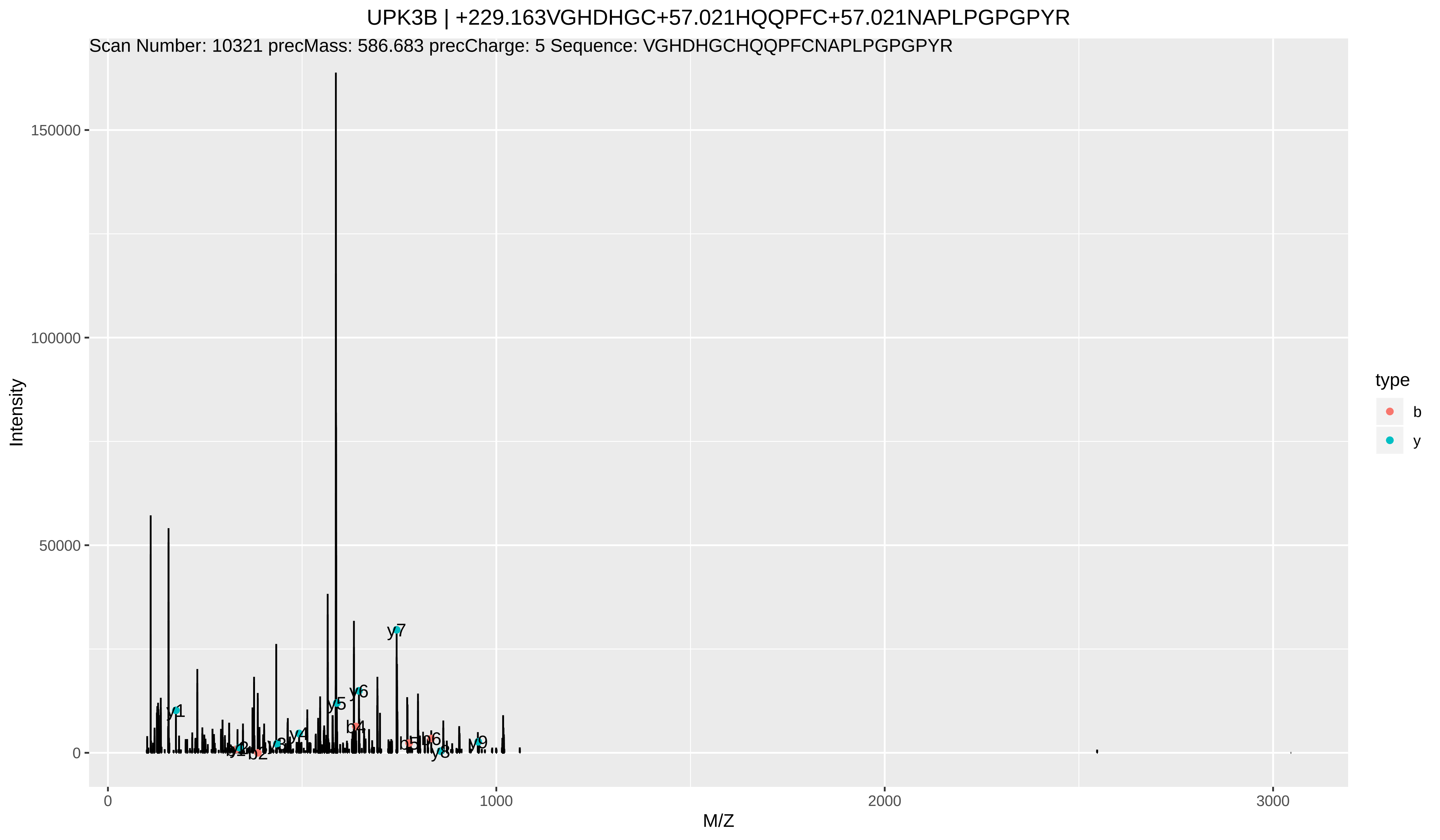
UPK3B | +229.163VGHDHGC+57.021HQQPFC+57.021NAPLPGPGPYR
Scan Number: 10321 precMass: 586.683 precCharge: 5 Sequence: VGHDHGCHQQPFCNAPLPGPGPYR
Intensity
150000
100000
50000
0
0
1000
2000
3000
M/Z
type
b
y
y1
y3
y4
y5
y6
y7
y8
y9
b2
b4
b6

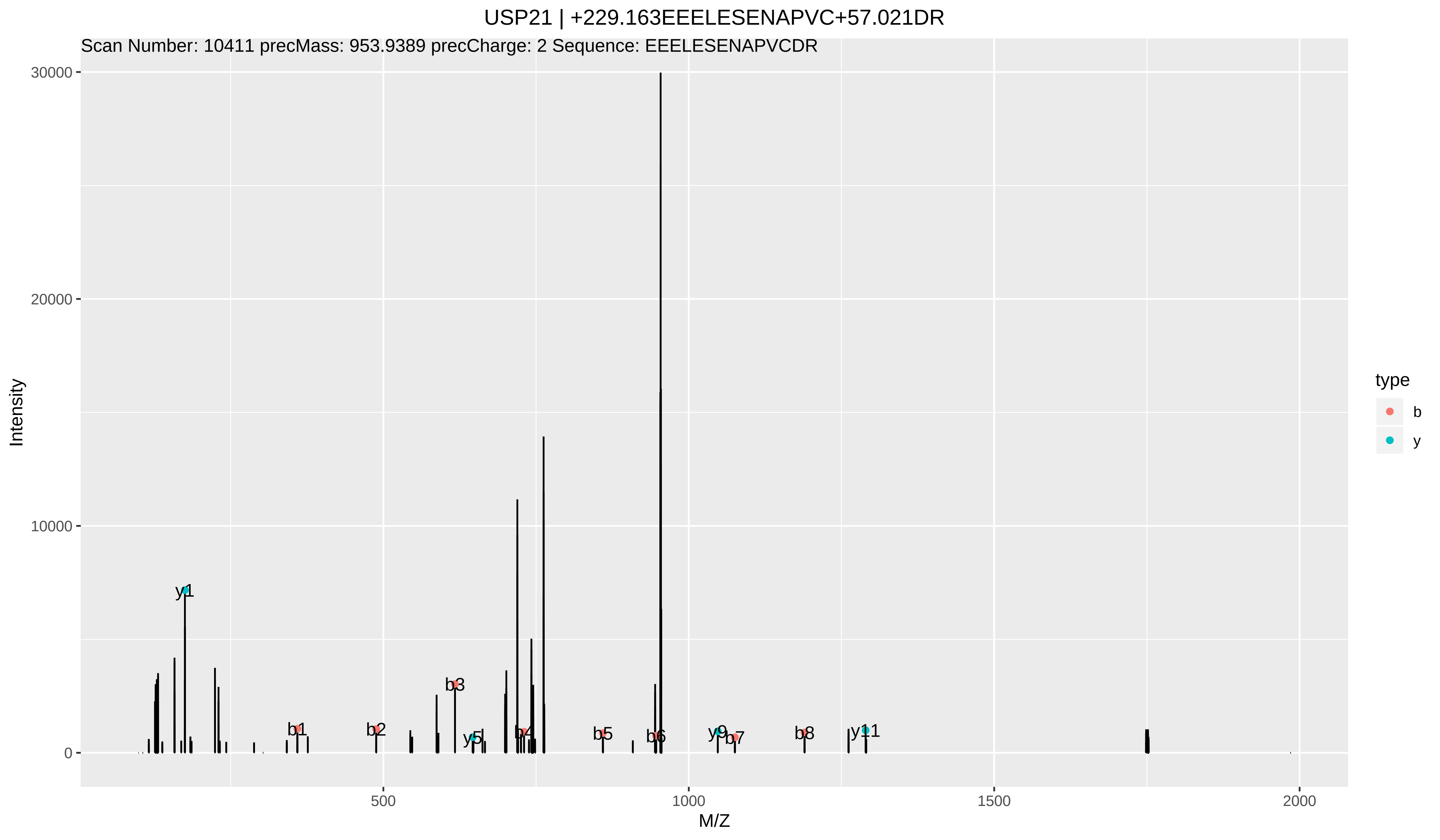

USP21 | +229.163EEELESENAPVC+57.021DR
Scan Number: 10411 precMass: 953.9389 precCharge: 2 Sequence: EEELESENAPVCDR
Intensity
M/Z
30000
20000
10000
0
500
1000
1500
2000
y1
b1
b2
b3
y5
b4
b5
b6
y9
b7
b8
y11
type
b
y

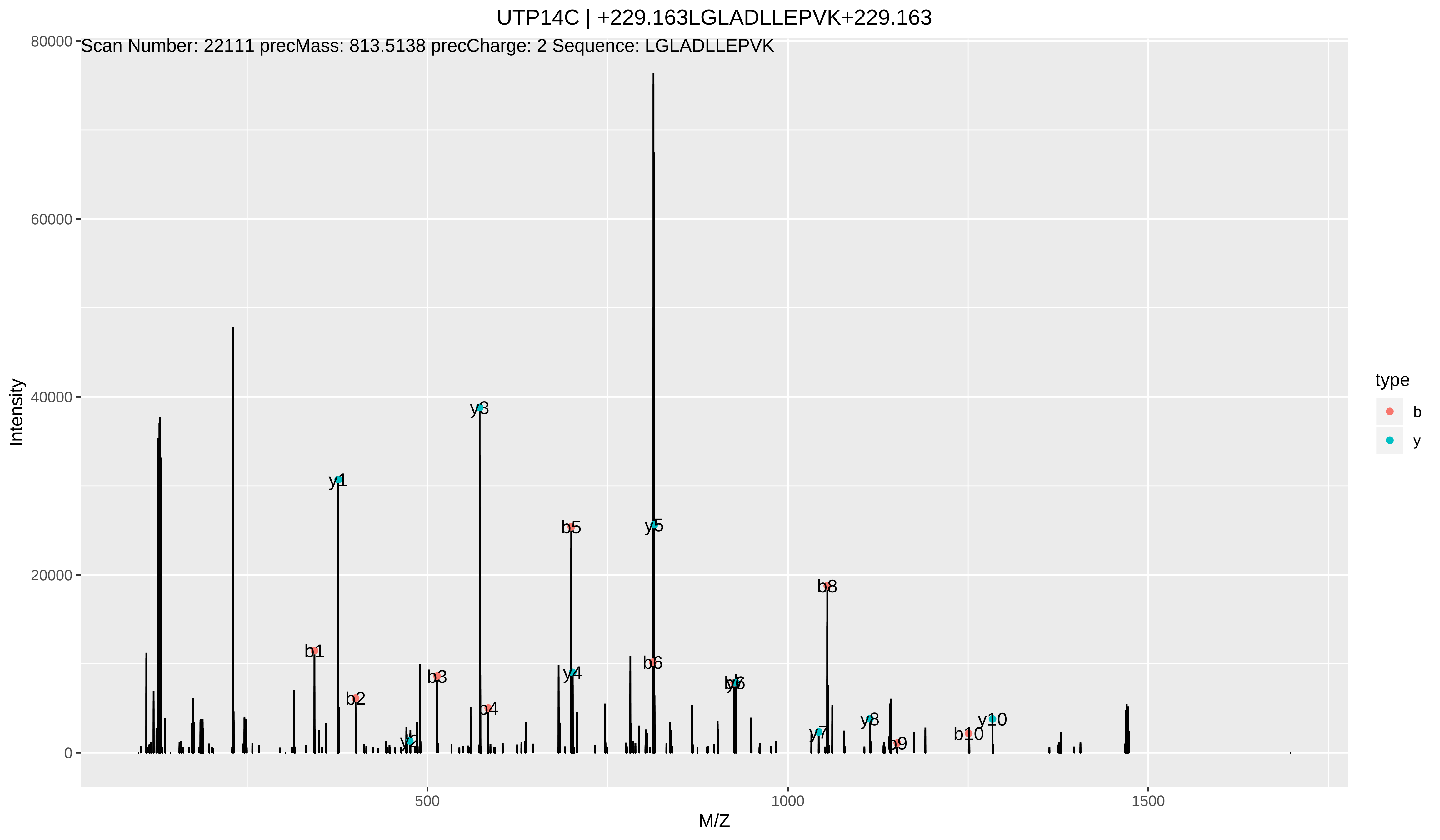

UTP14C | +229.163LGLADLLEPVK+229.163
Scan Number: 22111 precMass: 813.5138 precCharge: 2 Sequence: LGLADLLEPVK
Intensity
M/Z
type
b
y
y1
y2
y3
y4
y5
y6
y7
y8
y10
b1
b2
b3
b4
b5
b6
b7
b8
b9
b10
0
20000
40000
60000
80000
500
1000
1500

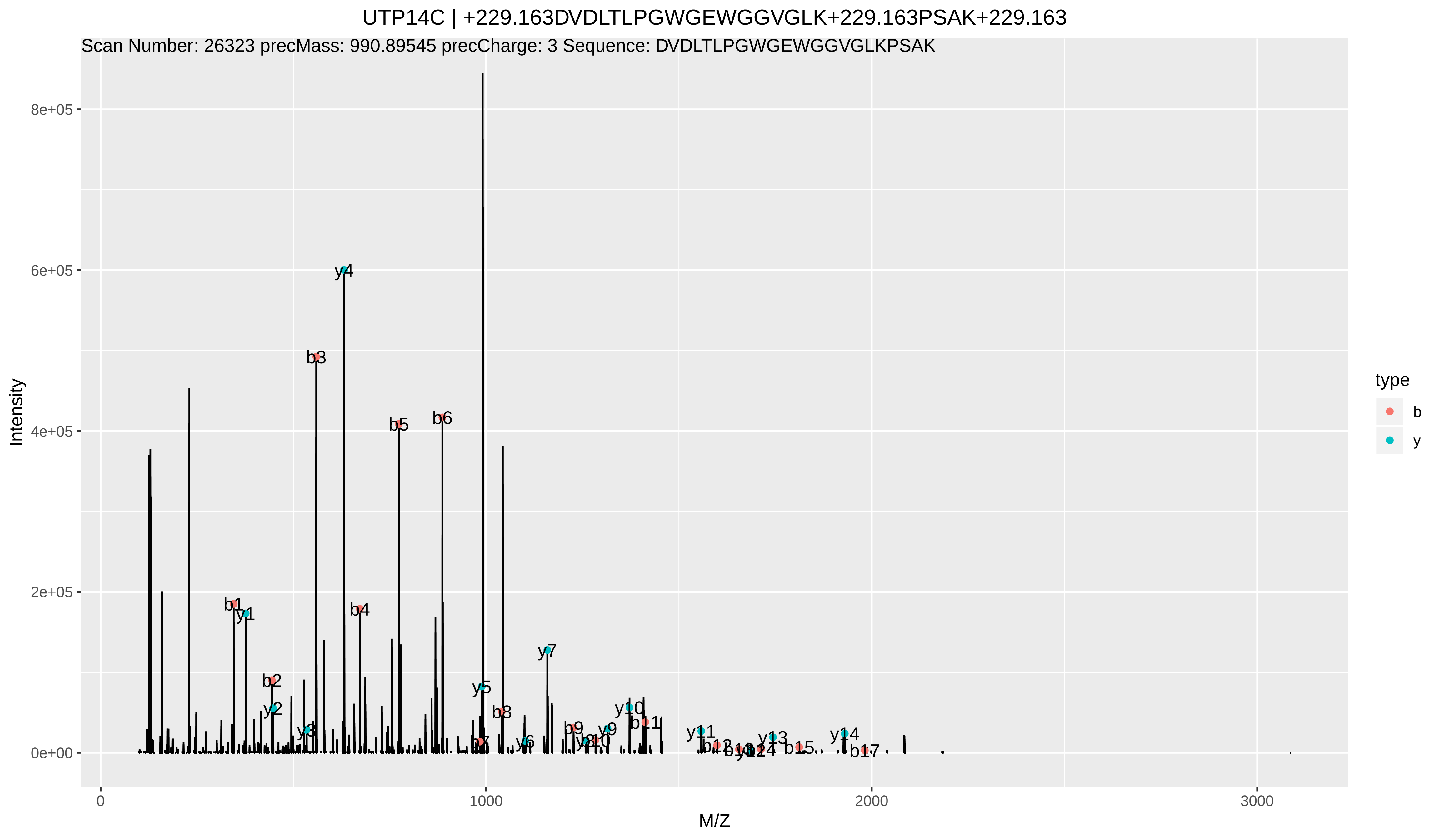
UTP14C | +229.163DVDLTLPGWGEWGGVGLK+229.163PSAK+229.163
Scan Number: 26323 precMass: 990.89545 precCharge: 3 Sequence: DVDLTLPGWGEWGGVGLKPSAK
Intensity
M/Z
type
b
y
8e+05
6e+05
4e+05
2e+05
0e+00
0
1000
2000
3000
y4
b3
b5
b6
b1
y1
b4
y7
b2
y5
y2
b8
y3
y10
b11
b9
y9
y6
y11
y13
y14
b15
b17

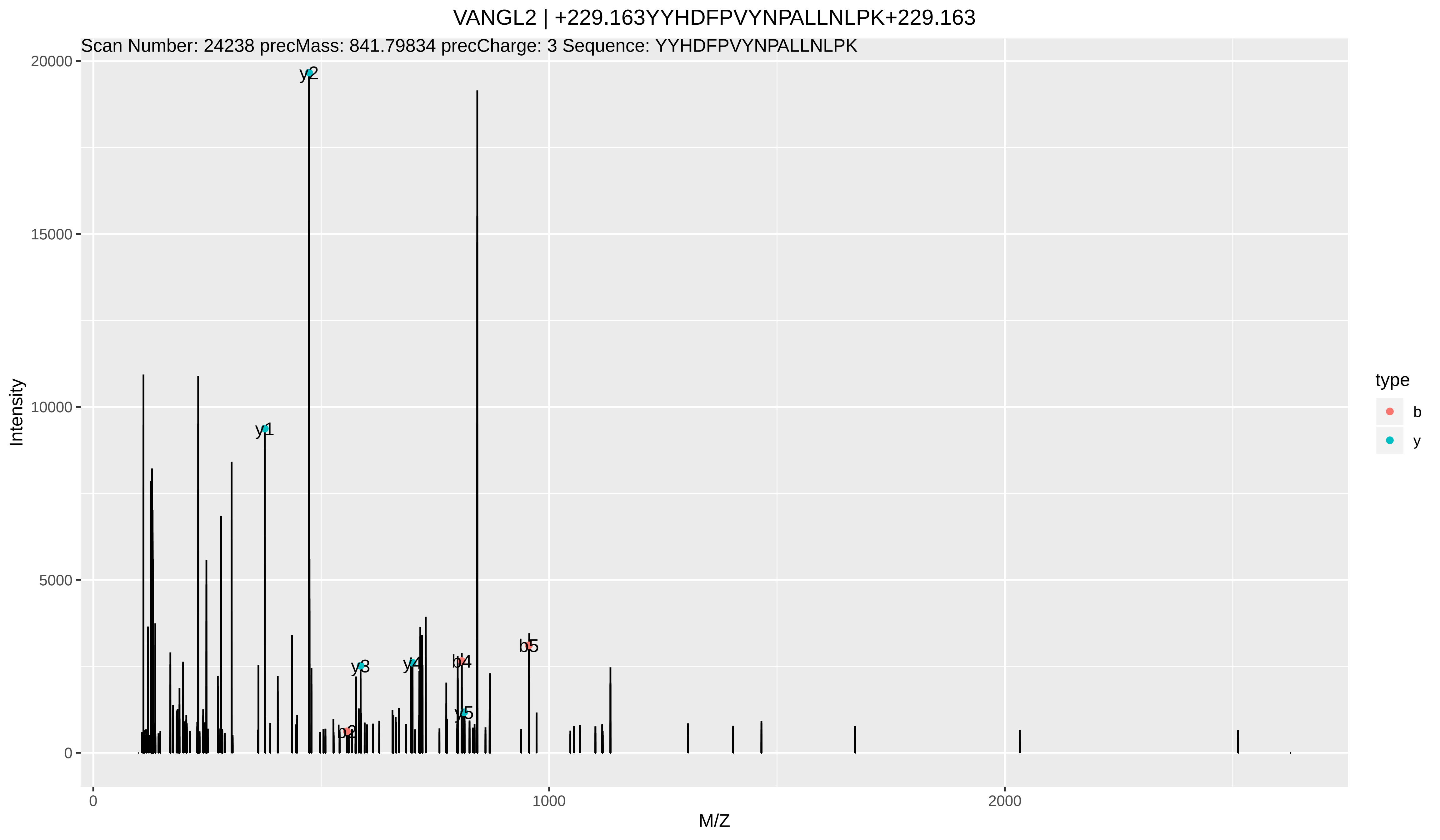

VANGL2 | +229.163YYHDFPVYNPALLNLPK+229.163
Scan Number: 24238 precMass: 841.79834 precCharge: 3 Sequence: YYHDFPVYNPALLNLPK
Intensity
M/Z
0
5000
10000
15000
20000
1000
2000
y1
y2
y3
y4
y5
b2
b4
b5
type
b
y

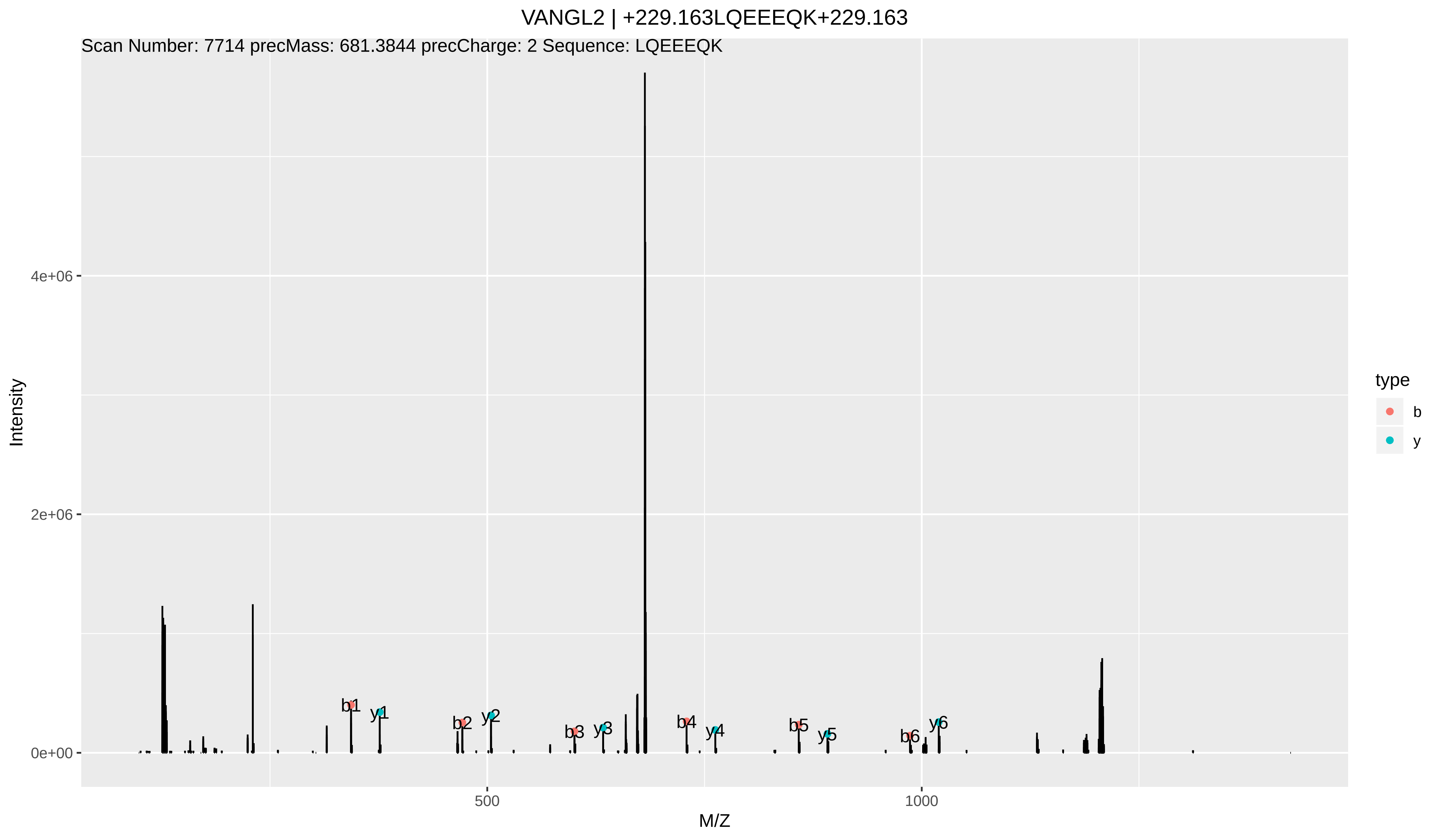

VANGL2 | +229.163LQEEEQK+229.163
Scan Number: 7714 precMass: 681.3844 precCharge: 2 Sequence: LQEEEQK
Intensity
4e+06
2e+06
0e+00
M/Z
500
1000
b1
y1
b2
y2
b3
y3
b4
y4
b5
y5
b6
y6
type
b
y

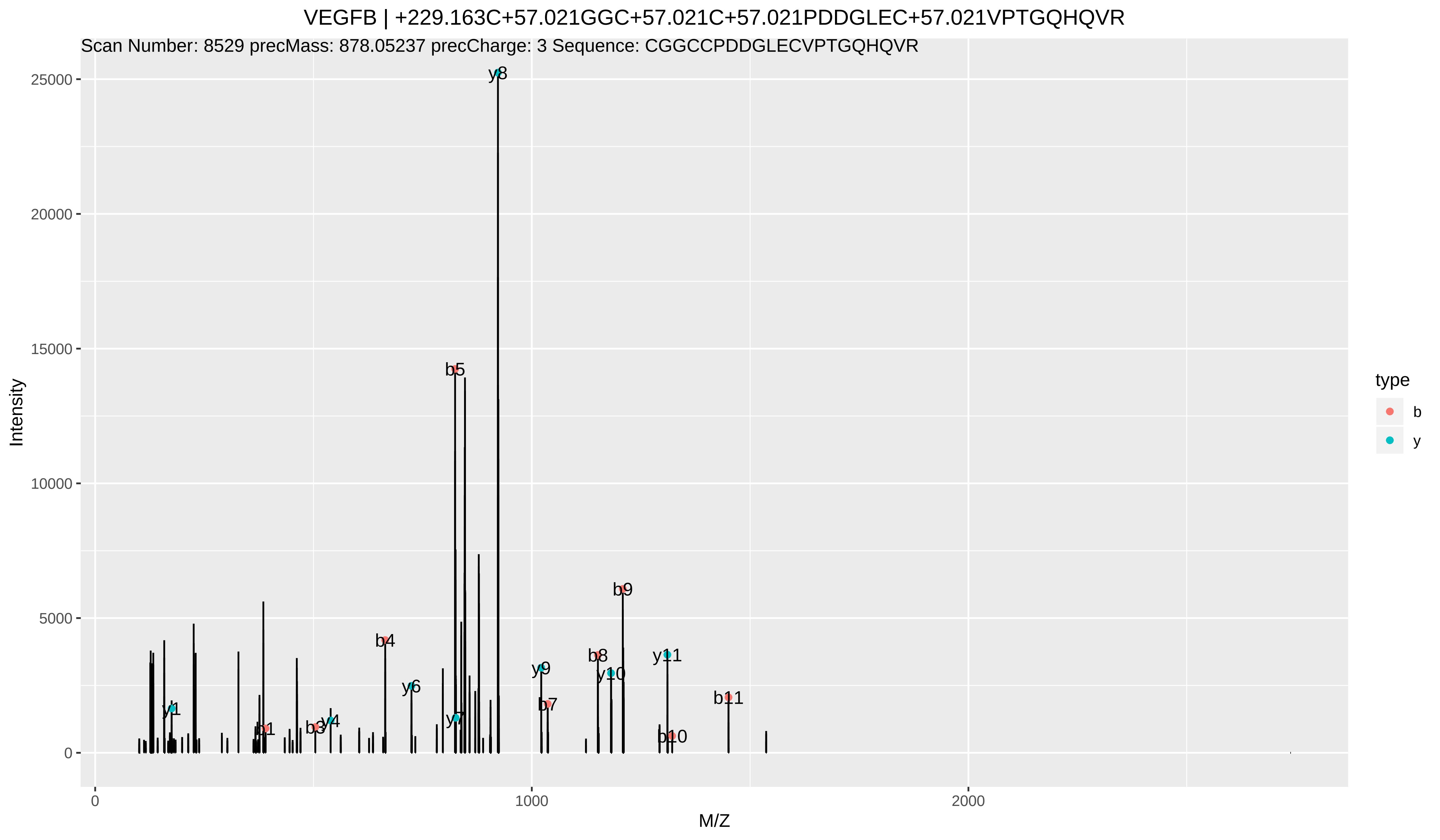

VEGFB | +229.163C+57.021GGC+57.021C+57.021PDDGLEC+57.021VPTGQHQVR
Scan Number: 8529 precMass: 878.05237 precCharge: 3 Sequence: CGGCCPDDGLECVPTGQHQVR
Intensity
M/Z
0
5000
10000
15000
20000
25000
0
1000
2000
y8
b5
b9
b4
b8
y11
y9
y10
y6
b7
b11
y1
b1
b3
y4
y7
b10
type
b
y

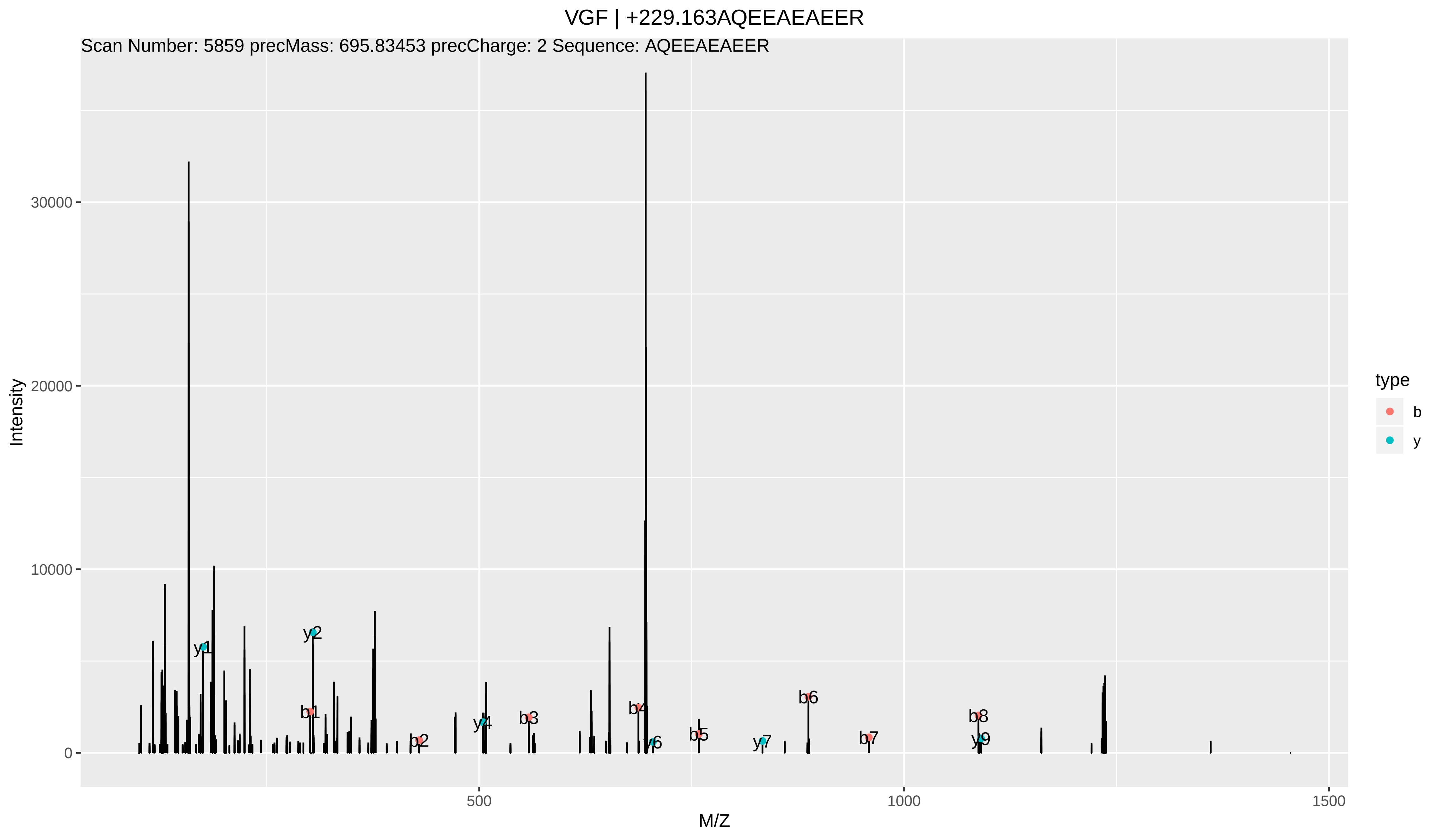

VGF | +229.163AQEEAEAEER
Scan Number: 5859 precMass: 695.83453 precCharge: 2 Sequence: AQEEAEAEER
Intensity
M/Z
type
b
y
y1
y2
b1
b2
y4
b3
b4
y6
b5
y7
b6
b7
b8
y9

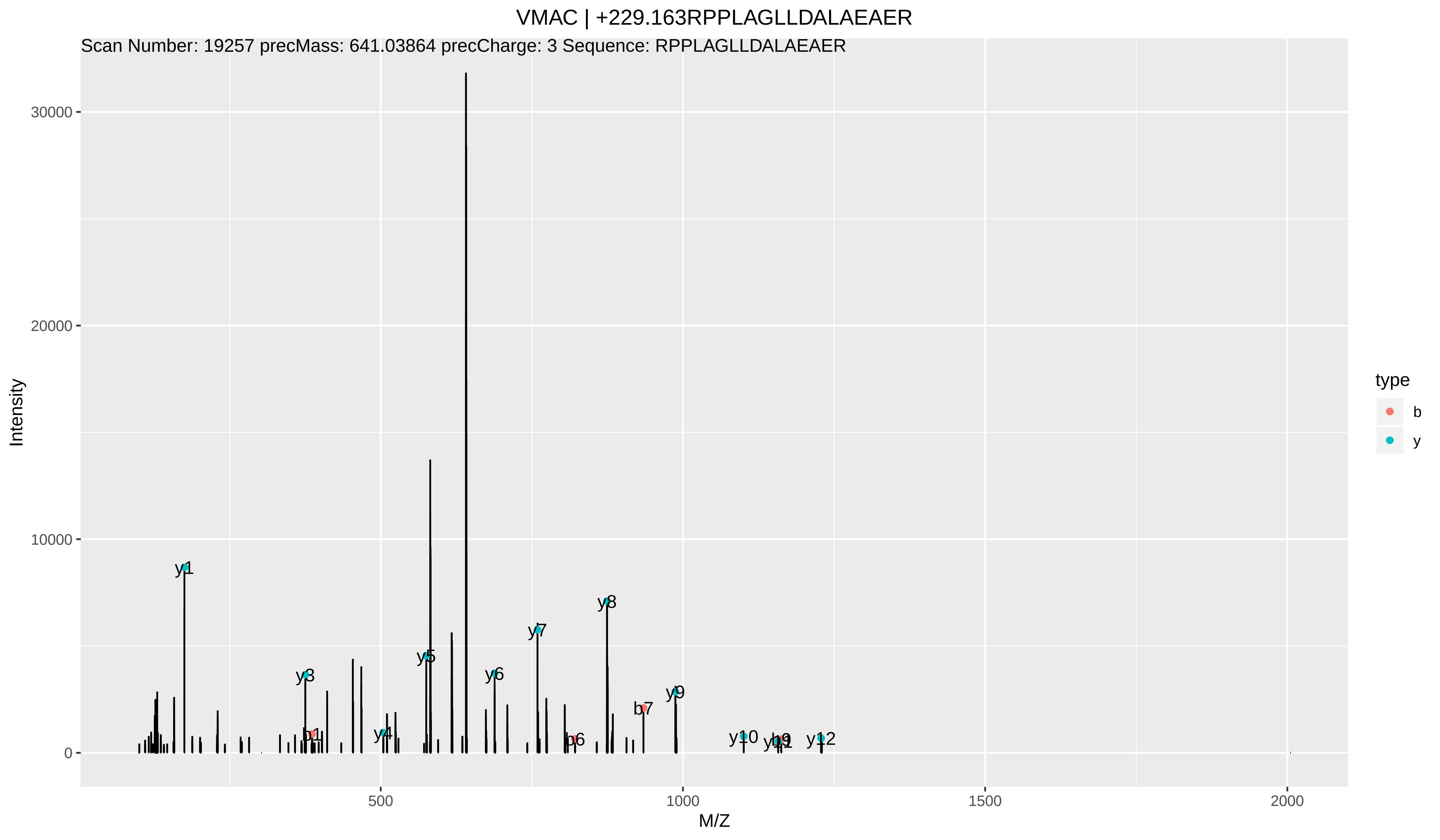

VMAC | +229.163RPPLAGLLDALAEAER
Scan Number: 19257 precMass: 641.03864 precCharge: 3 Sequence: RPPLAGLLDALAEAER
Intensity
M/Z
type
b
y
y1
y3
b1
y4
y5
y6
y7
b6
y8
b7
y9
y10
y12

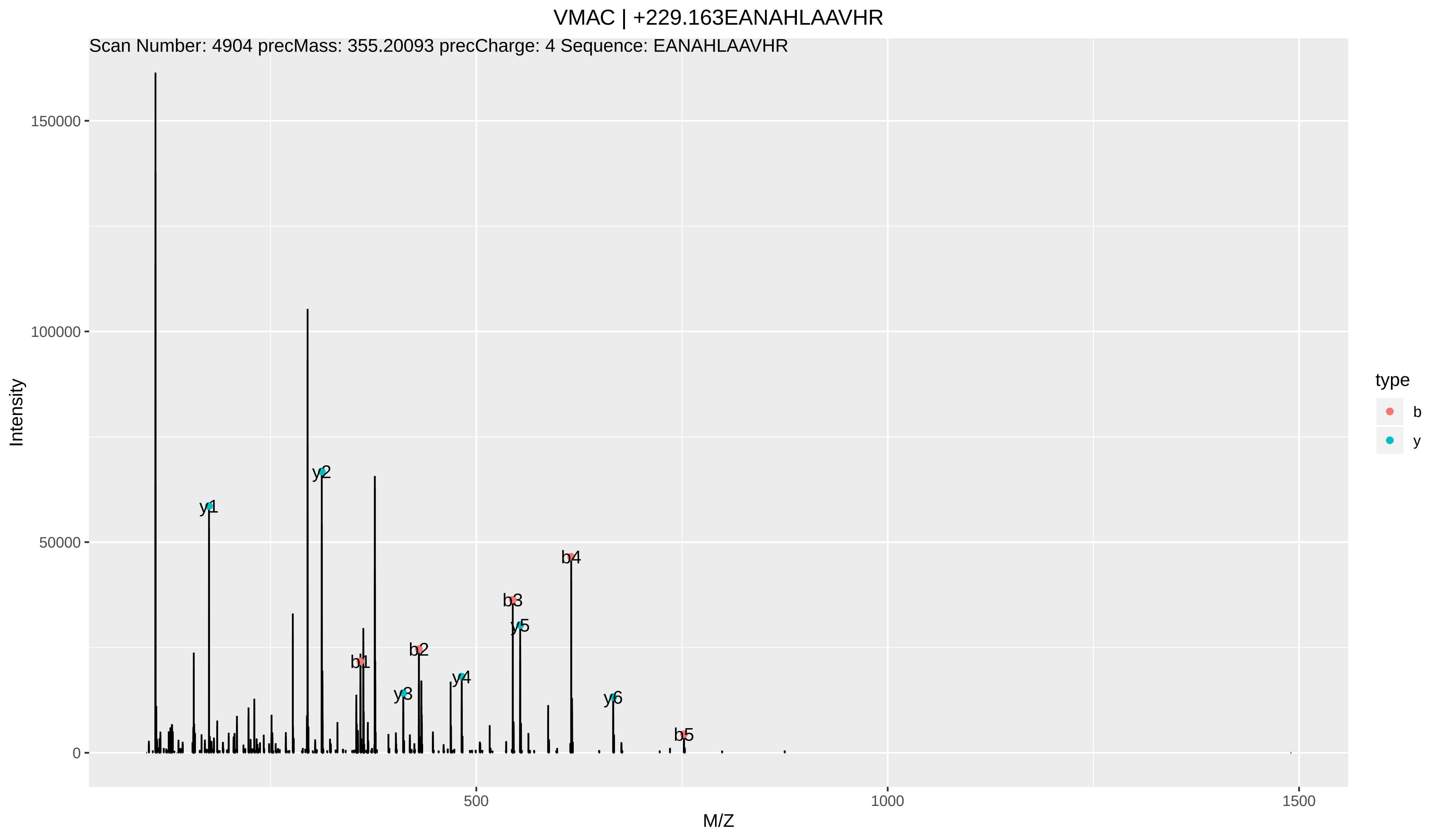

VMAC | +229.163EANAHLAAVHR
Scan Number: 4904 precMass: 355.20093 precCharge: 4 Sequence: EANAHLAAVHR
Intensity
150000
100000
50000
0
M/Z
500
1000
1500
y1
y2
b1
y3
b2
y4
b3
y5
b4
y6
b5
type
b
y

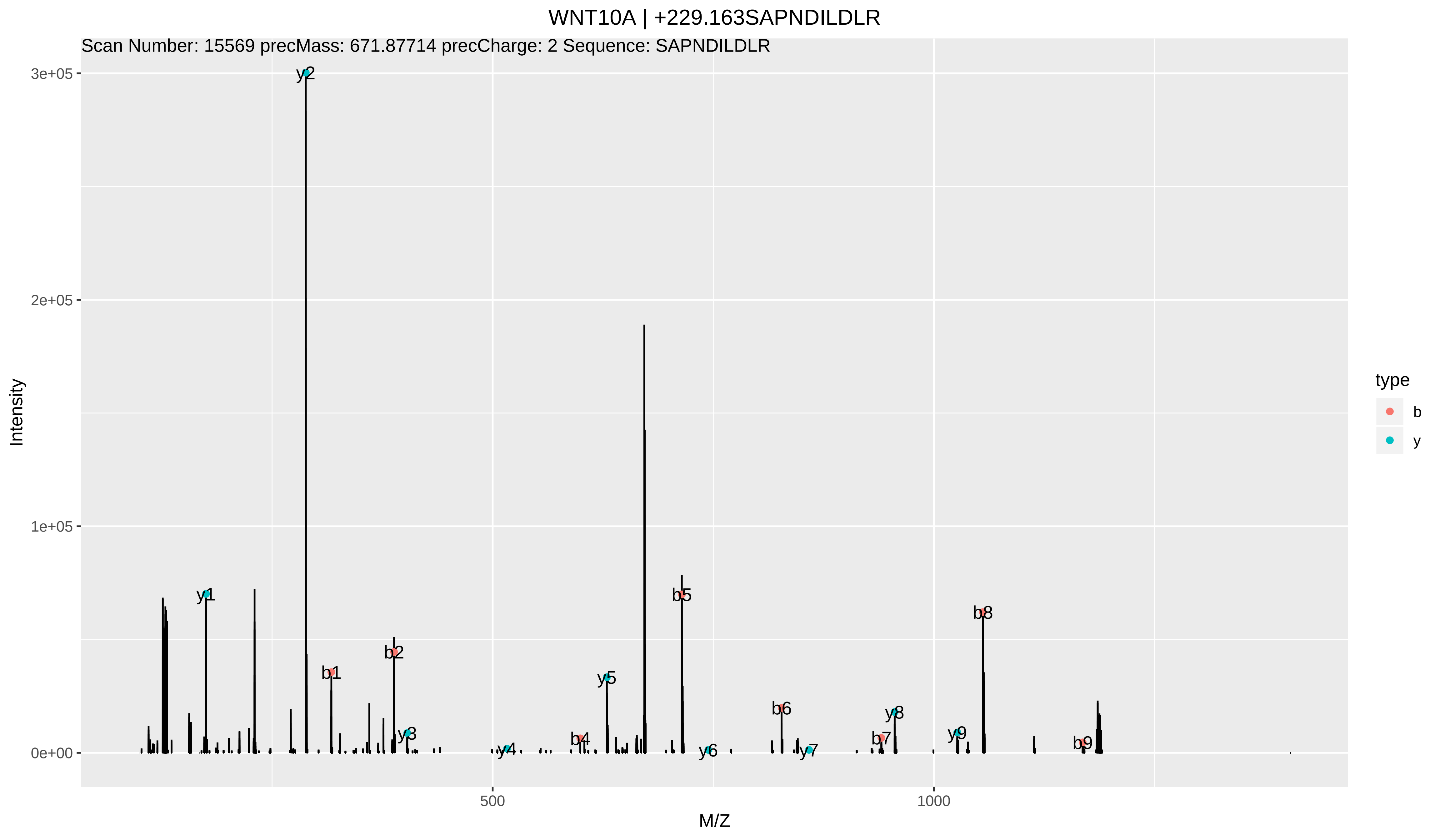
WNT10A | +229.163SAPNDILDLR
Scan Number: 15569 precMass: 671.87714 precCharge: 2 Sequence: SAPNDILDLR
Intensity
M/Z
type
b
y
3e+05
2e+05
1e+05
0e+00
500
1000
y1
y2
b1
b2
y3
y4
b4
y5
b5
y6
b6
y7
b7
y8
y9
b8
b9

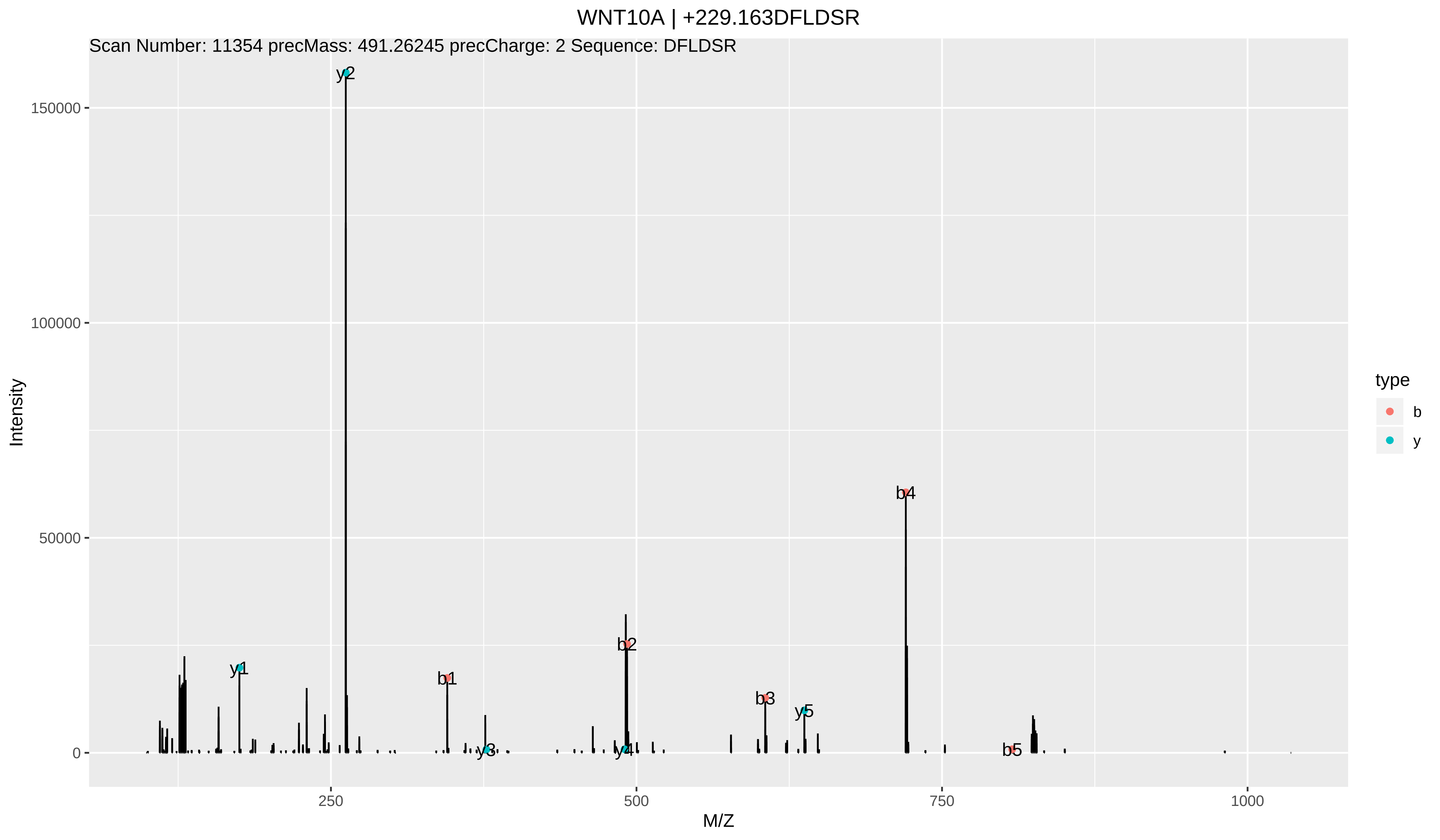

WNT10A | +229.163DFLDSR
Scan Number: 11354 precMass: 491.26245 precCharge: 2 Sequence: DFLDSR
Intensity
150000
100000
50000
0
250
500
750
1000
M/Z
y2
b4
b2
y1
b1
b3
y5
y3
y4
b5
type
b
y

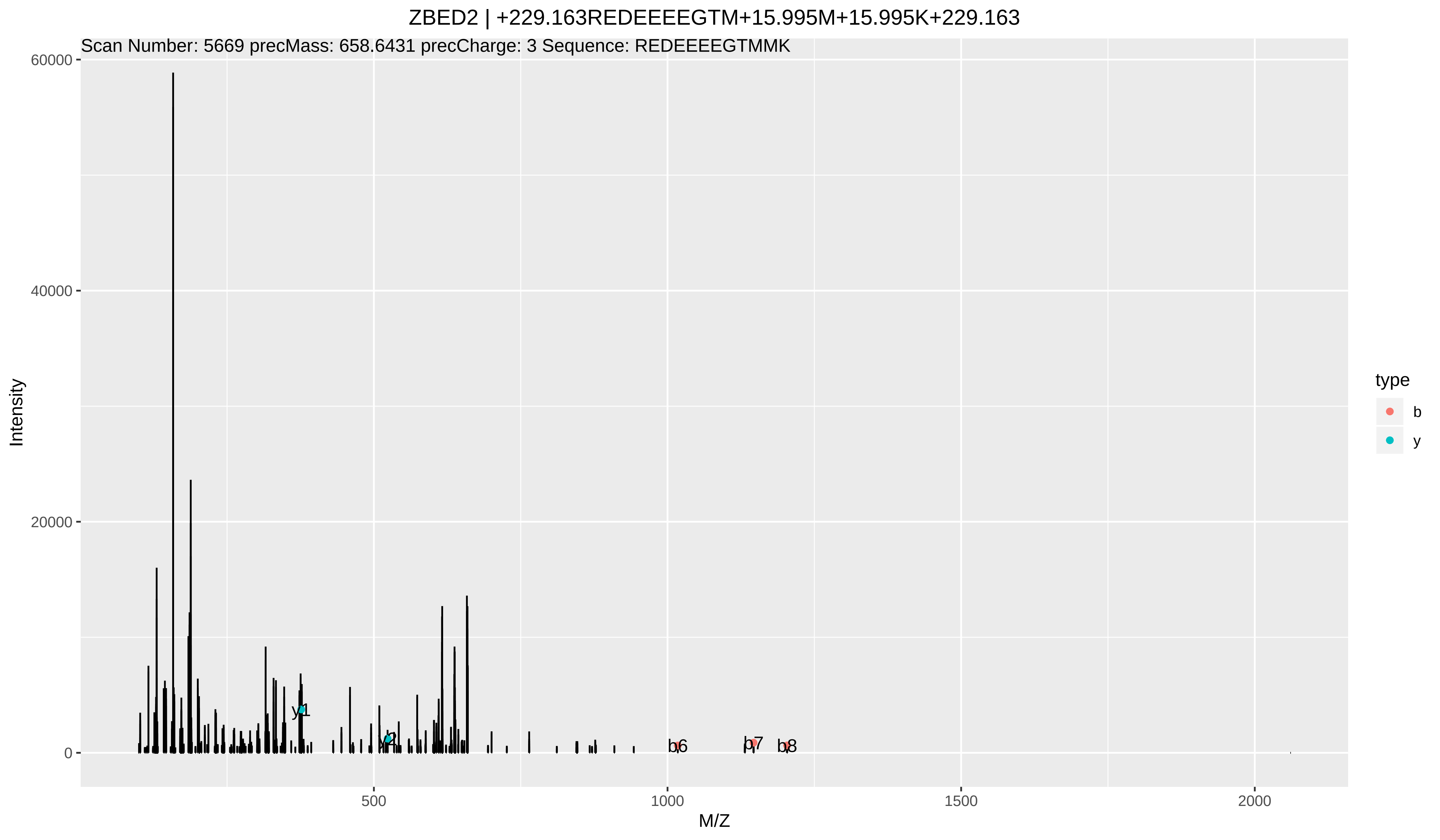

ZBED2 | +229.163REDEEEEGTM+15.995M+15.995K+229.163
Scan Number: 5669 precMass: 658.6431 precCharge: 3 Sequence: REDEEEEGTMMK
Intensity
M/Z
0
20000
40000
60000
500
1000
1500
2000
y1
y2
b6
b7
b8
type
b
y

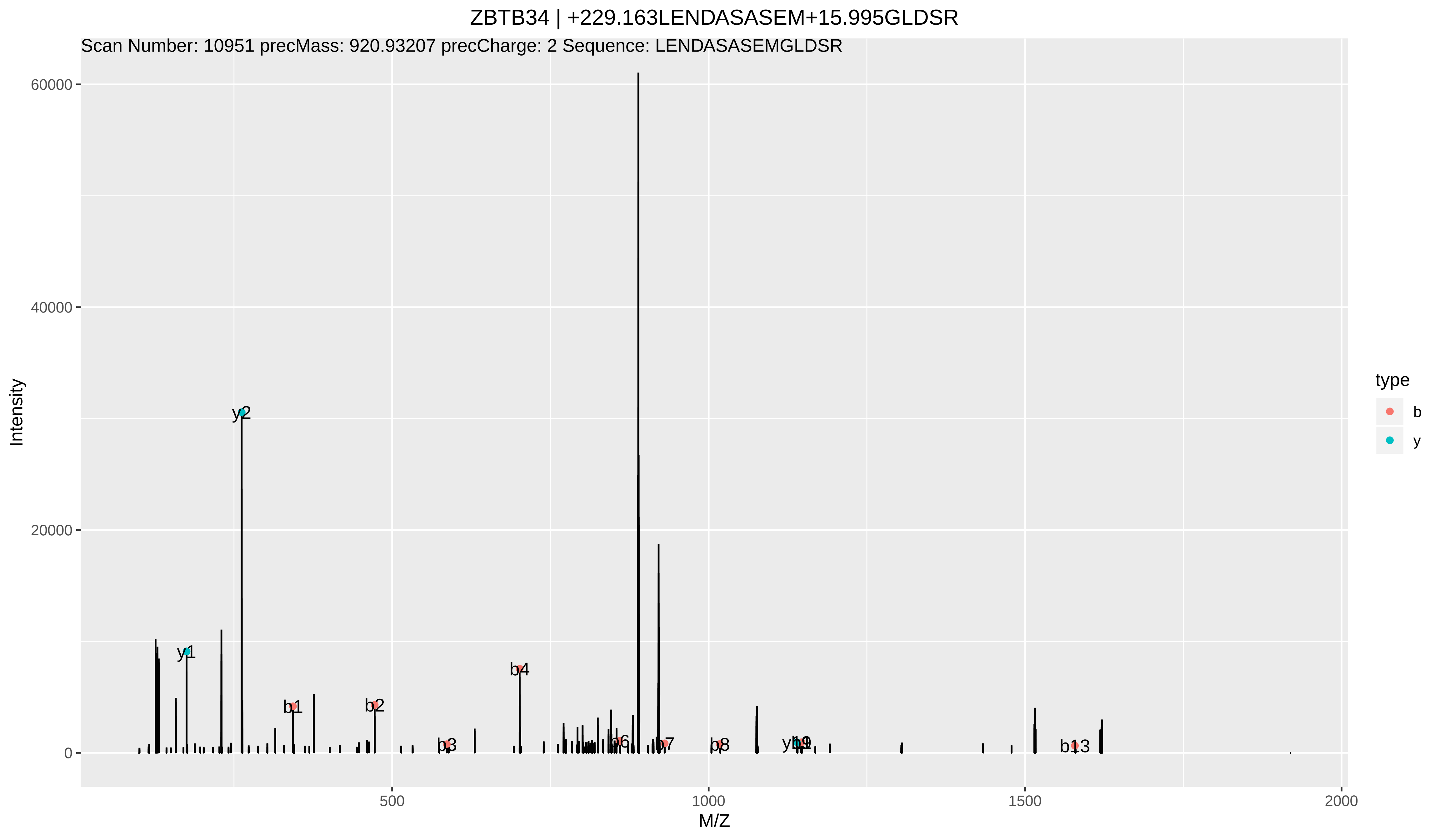
ZBTB34 | +229.163LENDASASEM+15.995GLDSR
Scan Number: 10951 precMass: 920.93207 precCharge: 2 Sequence: LENDASASEMGLDSR
Intensity
M/Z
type
b
y
y1
y2
b1
b2
b3
b4
b6
b7
b8
b13
0
20000
40000
60000
500
1000
1500
2000

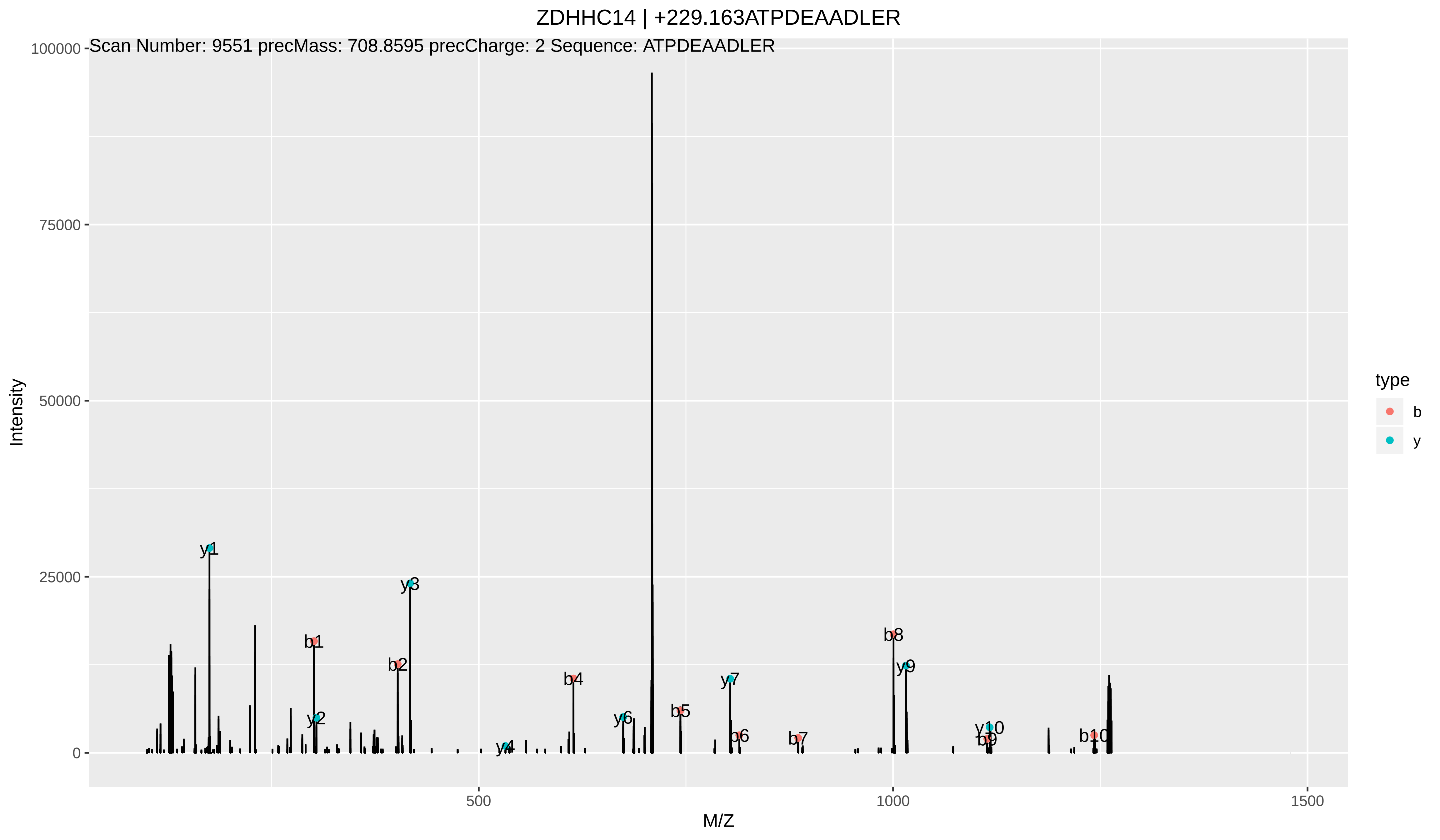

ZDHHC14 | +229.163ATPDEAADLER
Scan Number: 9551 precMass: 708.8595 precCharge: 2 Sequence: ATPDEAADLER
Intensity
100000
75000
50000
25000
0
500
1000
1500
M/Z
y1
y2
y3
y4
y6
y7
y9
y10
b1
b2
b4
b5
b6
b7
b8
b9
b10
type
b
y

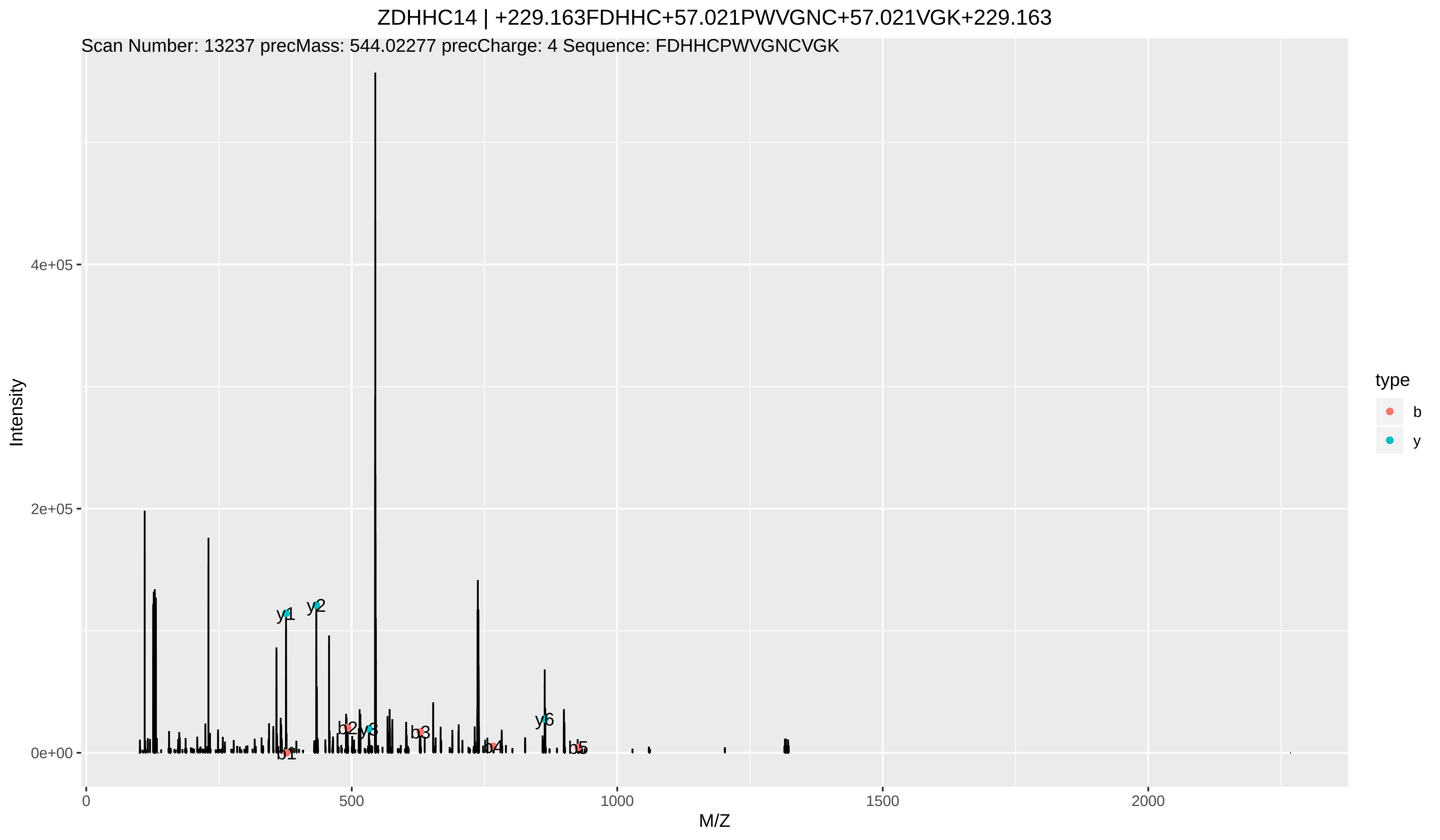

ZDHHC14 | +229.163FDHHC+57.021PWVGNC+57.021VGK+229.163
Scan Number: 13237 precMass: 544.02277 precCharge: 4 Sequence: FDHHCPWVGNCVGK
Intensity
4e+05
2e+05
0e+00
0
500
1000
1500
2000
M/Z
y1
y2
b1
b2
y3
b3
b4
y6
b5
type
b
y

ZDHHC16 | +229.163EAYAAIEK+229.163

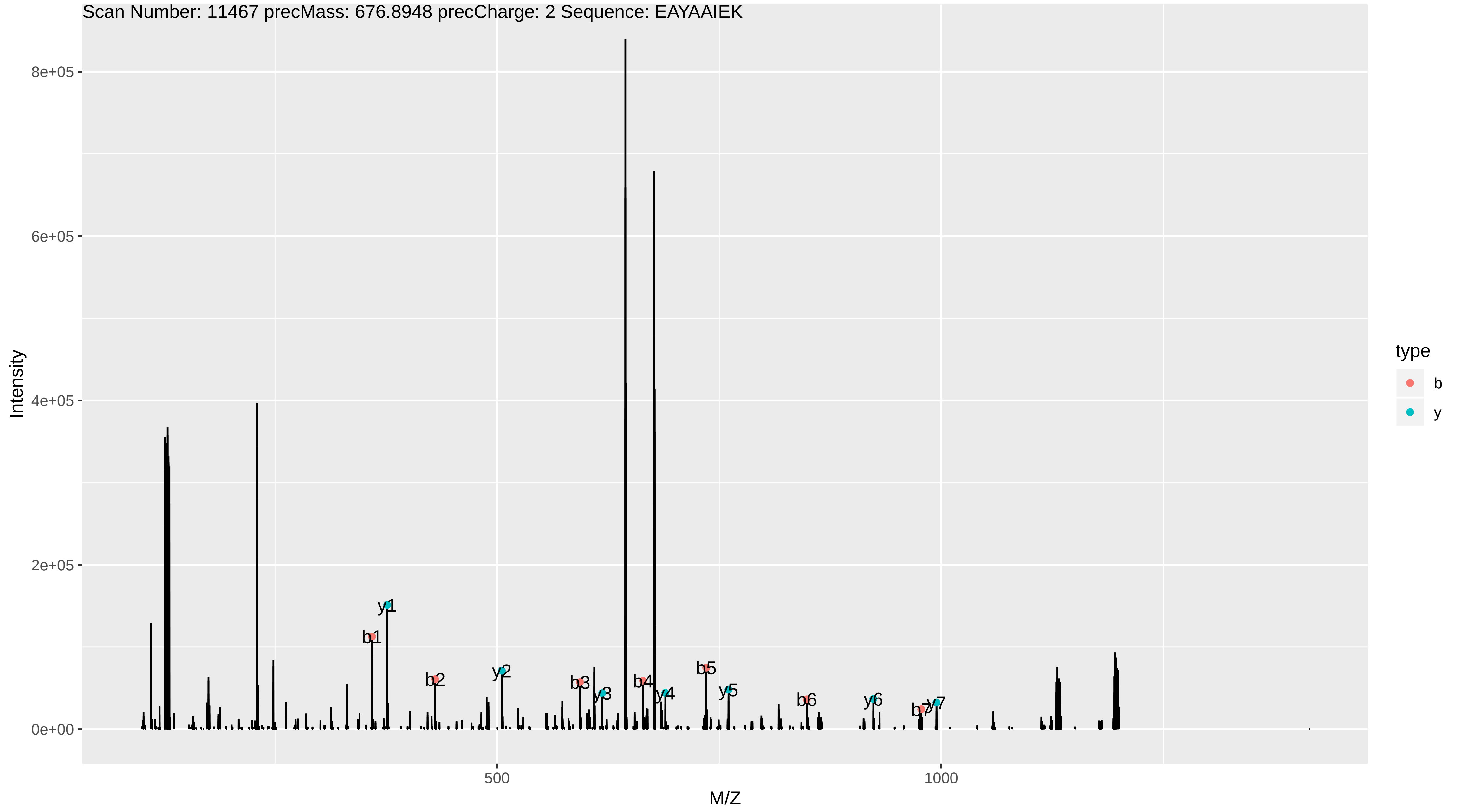

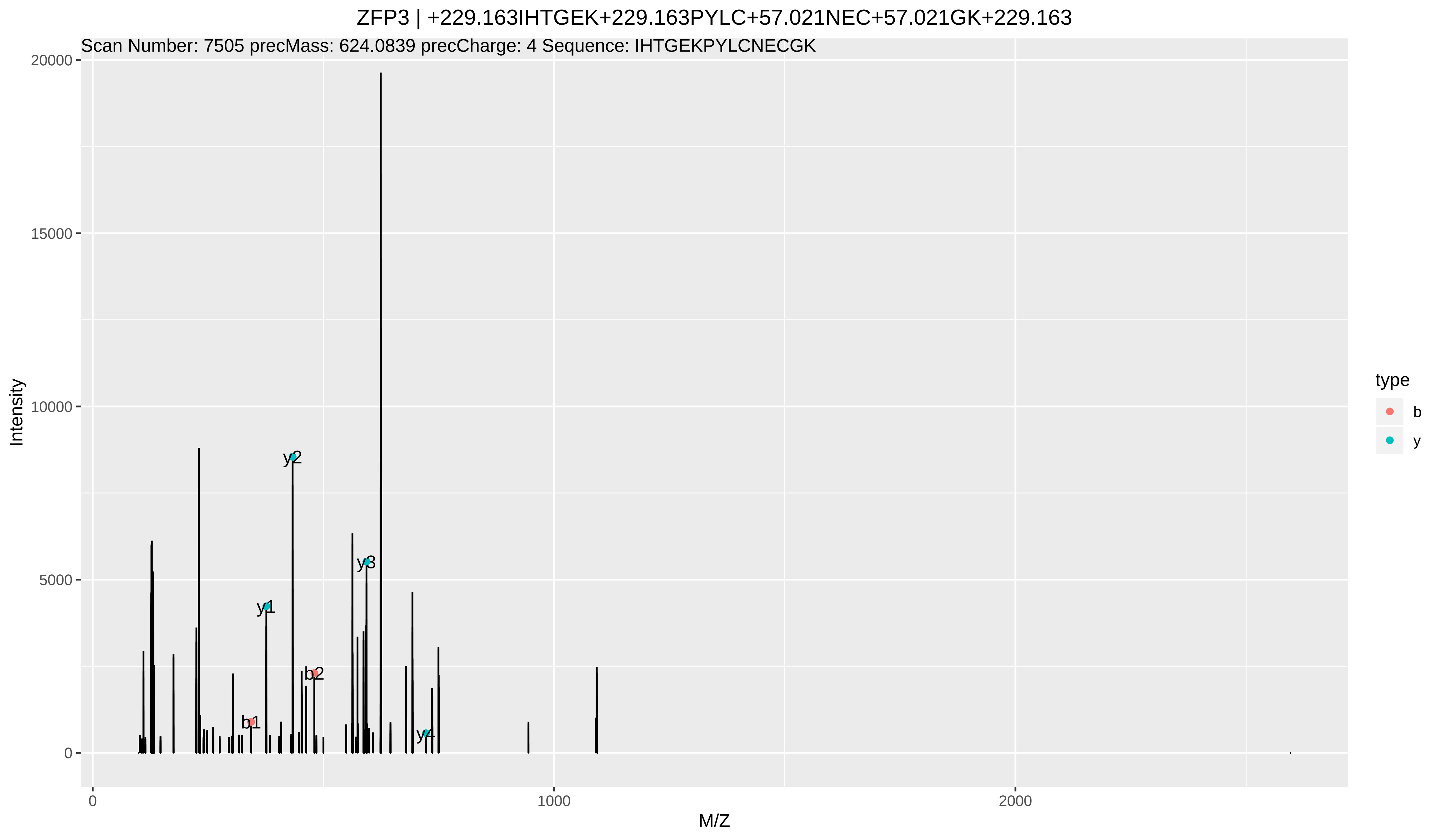

ZFP3 | +229.163IHTGEK+229.163PYLC+57.021NEC+57.021GK+229.163
Scan Number: 7505 precMass: 624.0839 precCharge: 4 Sequence: IHTGEKPYLCNECGK
Intensity
M/Z
0
5000
10000
15000
20000
1000
2000
y1
y2
y3
y4
b1
b2
type
b
y

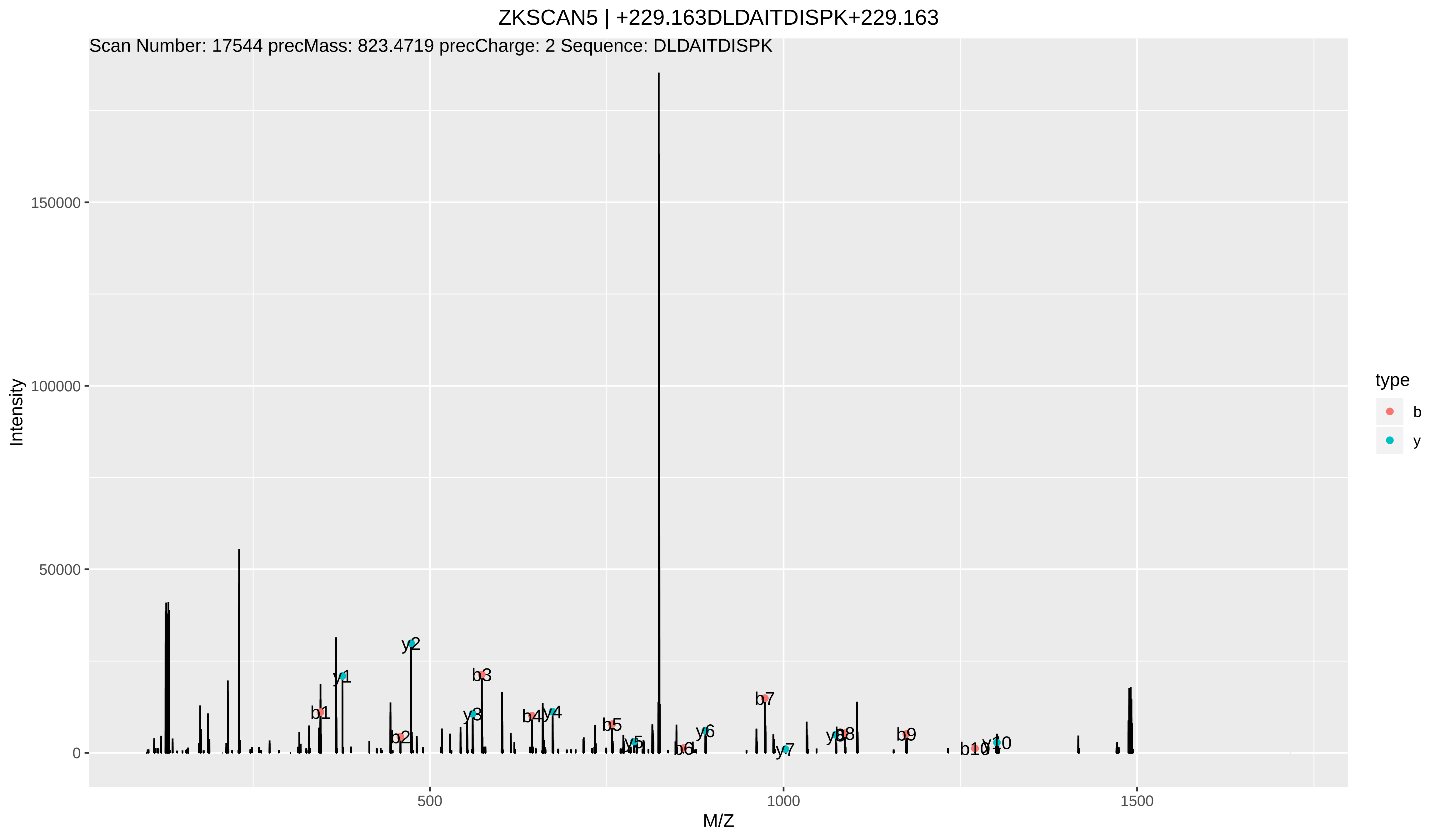

ZKSCAN5 | +229.163DLDAITDISPK+229.163
Scan Number: 17544 precMass: 823.4719 precCharge: 2 Sequence: DLDAITDISPK
Intensity
150000
100000
50000
0
M/Z
500
1000
1500
type
b
y
b1
b2
b3
b4
b5
b6
b7
b8
b9
b10
y1
y2
y3
y4
y5
y6
y7
y8
y10

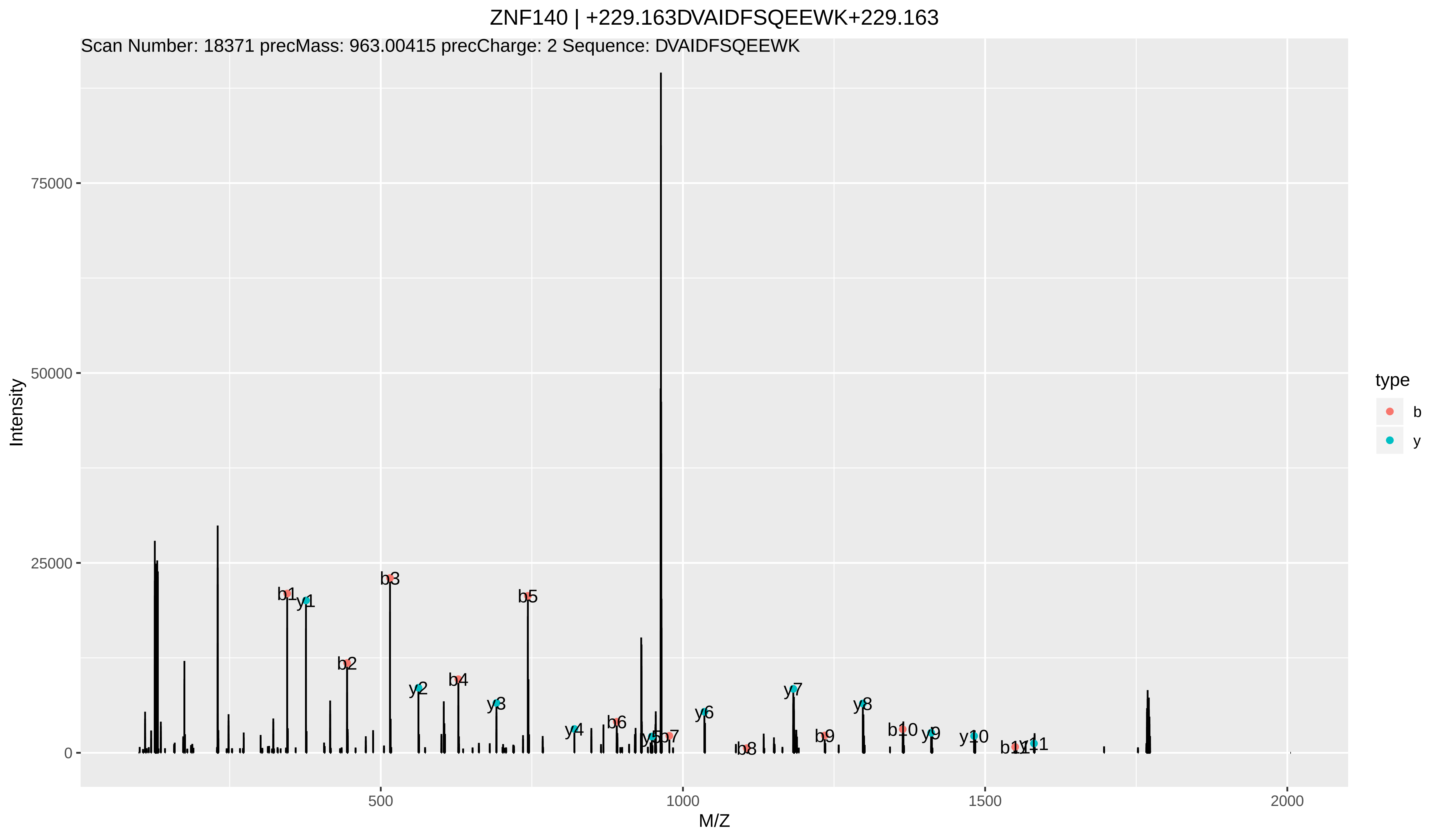
ZNF140 | +229.163DVAIDFSQEEWK+229.163
Scan Number: 18371 precMass: 963.00415 precCharge: 2 Sequence: DVAIDFSQEEWK
Intensity
M/Z
type
b
y
0
25000
50000
75000
500
1000
1500
2000
b1
y1
b2
b3
y2
b4
y3
b5
y4
b6
y5
b7
y6
b8
y7
b9
y8
b10
y9
y10
b11
y11

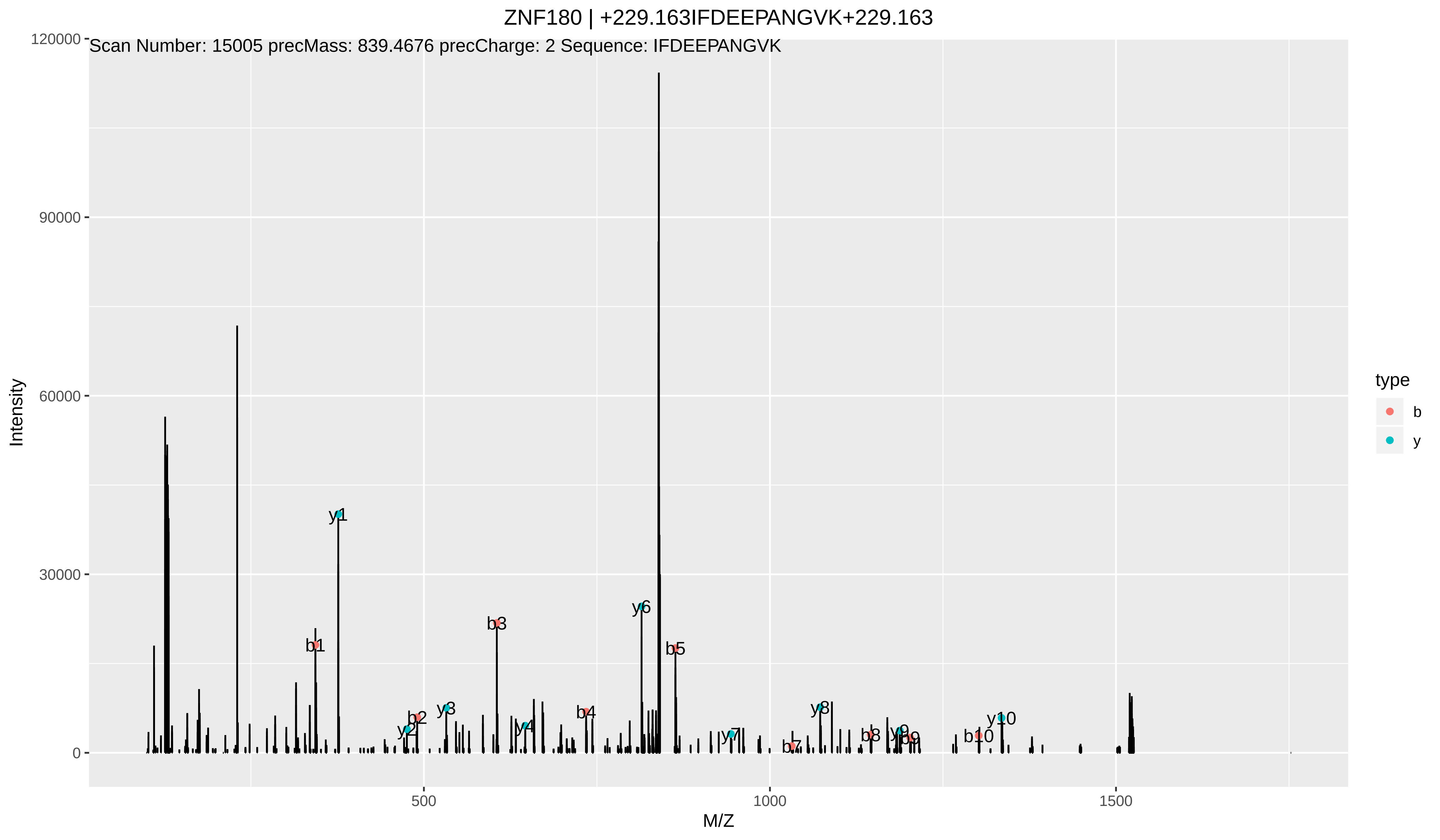
ZNF180 | +229.163IFDEEPANGVK+229.163
Scan Number: 15005 precMass: 839.4676 precCharge: 2 Sequence: IFDEEPANGVK
Intensity
120000
90000
60000
30000
0
500
1000
1500
M/Z
type
b
y
y1
b1
b3
y6
b5
y2
b2
y3
y4
b4
y7
b7
y8
b8
y9
b9
b10
y10

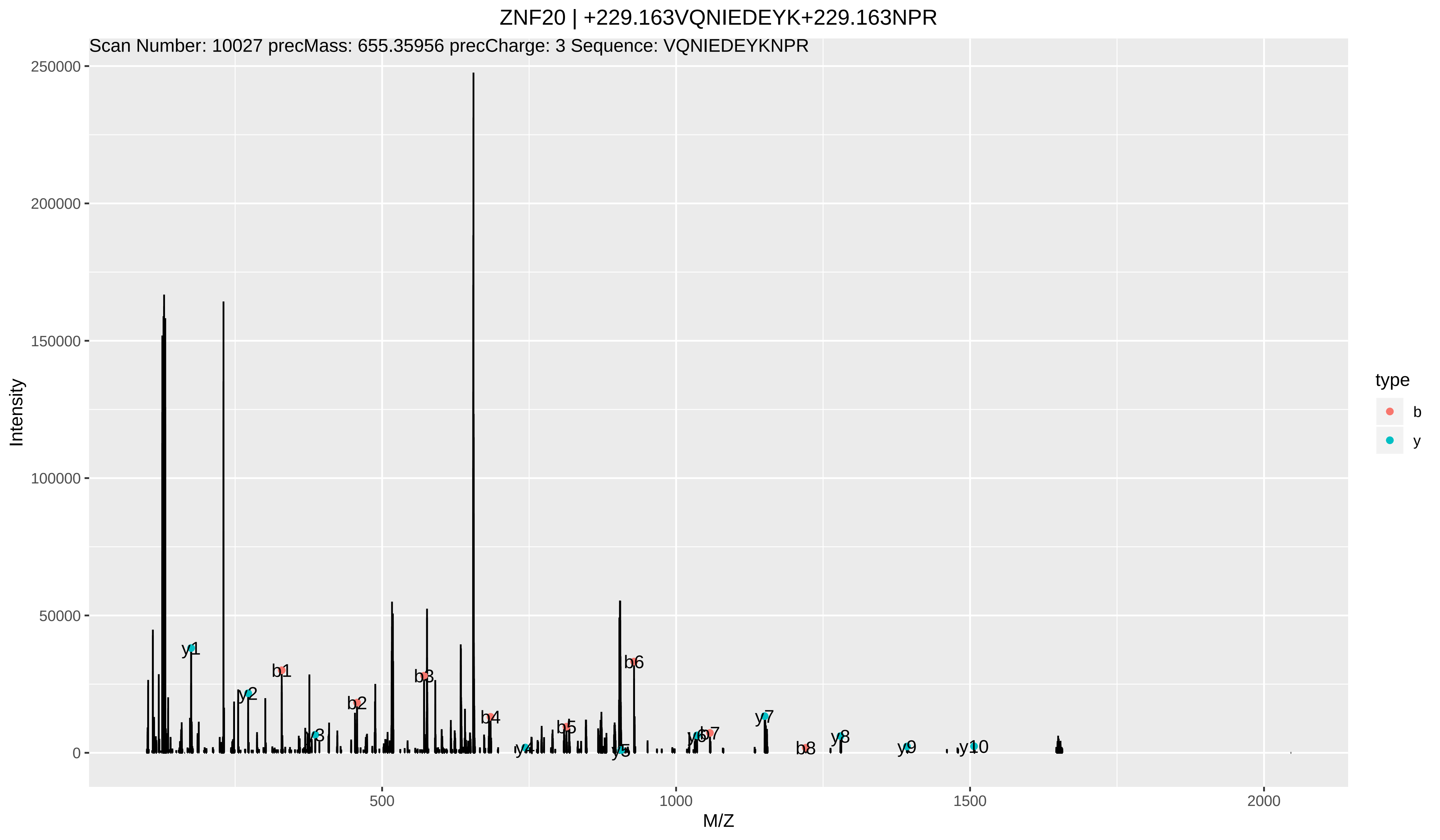
ZNF20 | +229.163VQNIEDEYK+229.163NPR
Scan Number: 10027 precMass: 655.35956 precCharge: 3 Sequence: VQNIEDEYKNPR
Intensity
M/Z
type
b
y
0
50000
100000
150000
200000
250000
500
1000
1500
2000
y1
y2
b1
b2
b3
b4
b5
b6
b7
b8
y3
y4
y5
y6
y7
y8
y9
y10

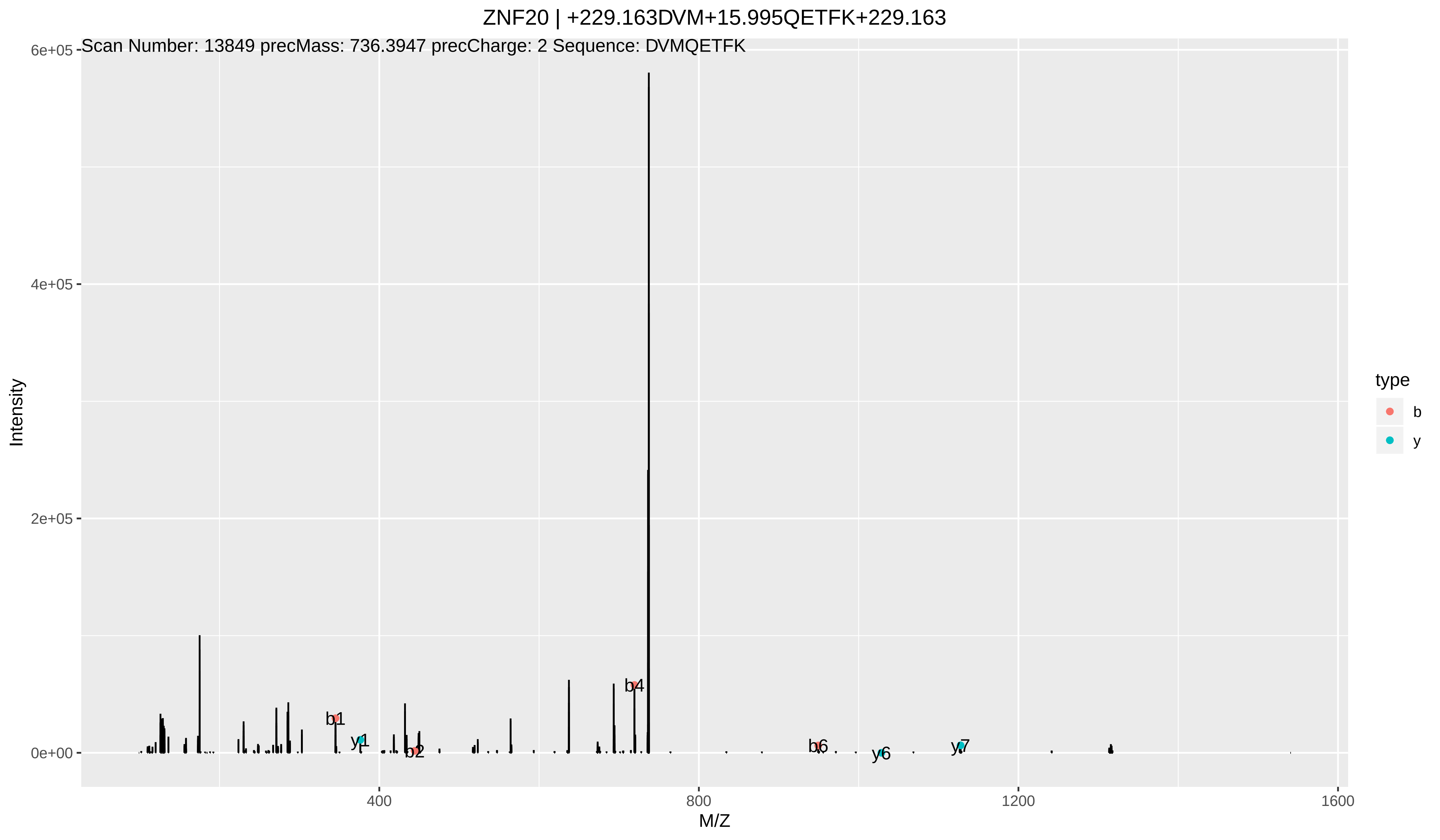
ZNF20 | +229.163DVM+15.995QETFK+229.163
Scan Number: 13849 precMass: 736.3947 precCharge: 2 Sequence: DVMQETFK
Intensity
M/Z
6e+05
4e+05
2e+05
0e+00
400
800
1200
1600
b1
y1
b2
b4
b6
y6
y7
type
b
y

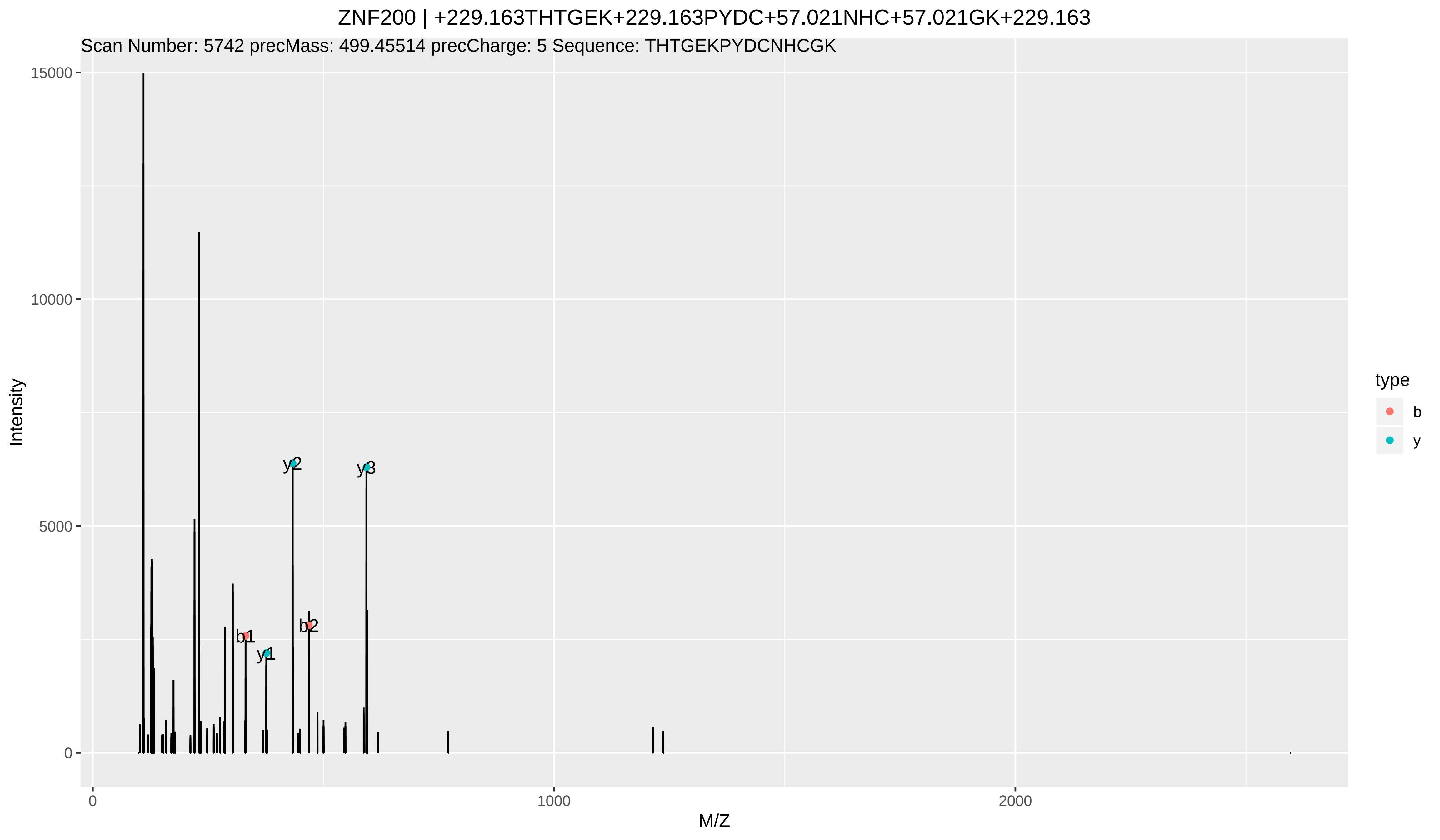
ZNF200 | +229.163THTGEK+229.163PYDC+57.021NHC+57.021GK+229.163
Scan Number: 5742 precMass: 499.45514 precCharge: 5 Sequence: THTGEKPYDCNHCGK
Intensity
15000
10000
5000
0
0
1000
2000
M/Z
y2
y3
b1
b2
y1
type
b
y

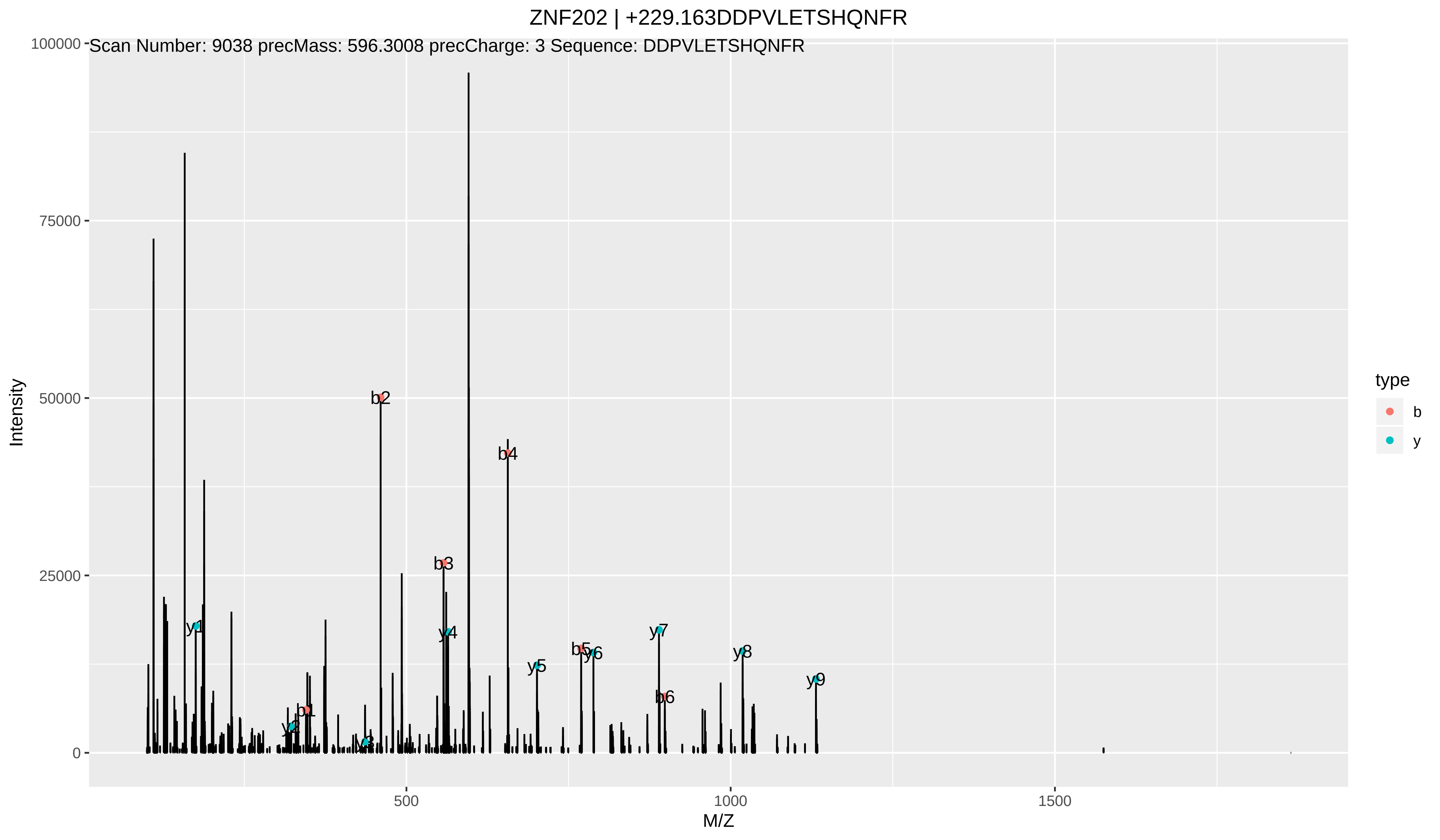

ZNF202 | +229.163DDPVLETSHQNFR
Scan Number: 9038 precMass: 596.3008 precCharge: 3 Sequence: DDPVLETSHQNFR
Intensity
M/Z
0
25000
50000
75000
100000
500
1000
1500
b2
b4
b3
y1
y4
b5
y6
y7
y8
y5
y9
b6
b1
y2
y3
type
b
y

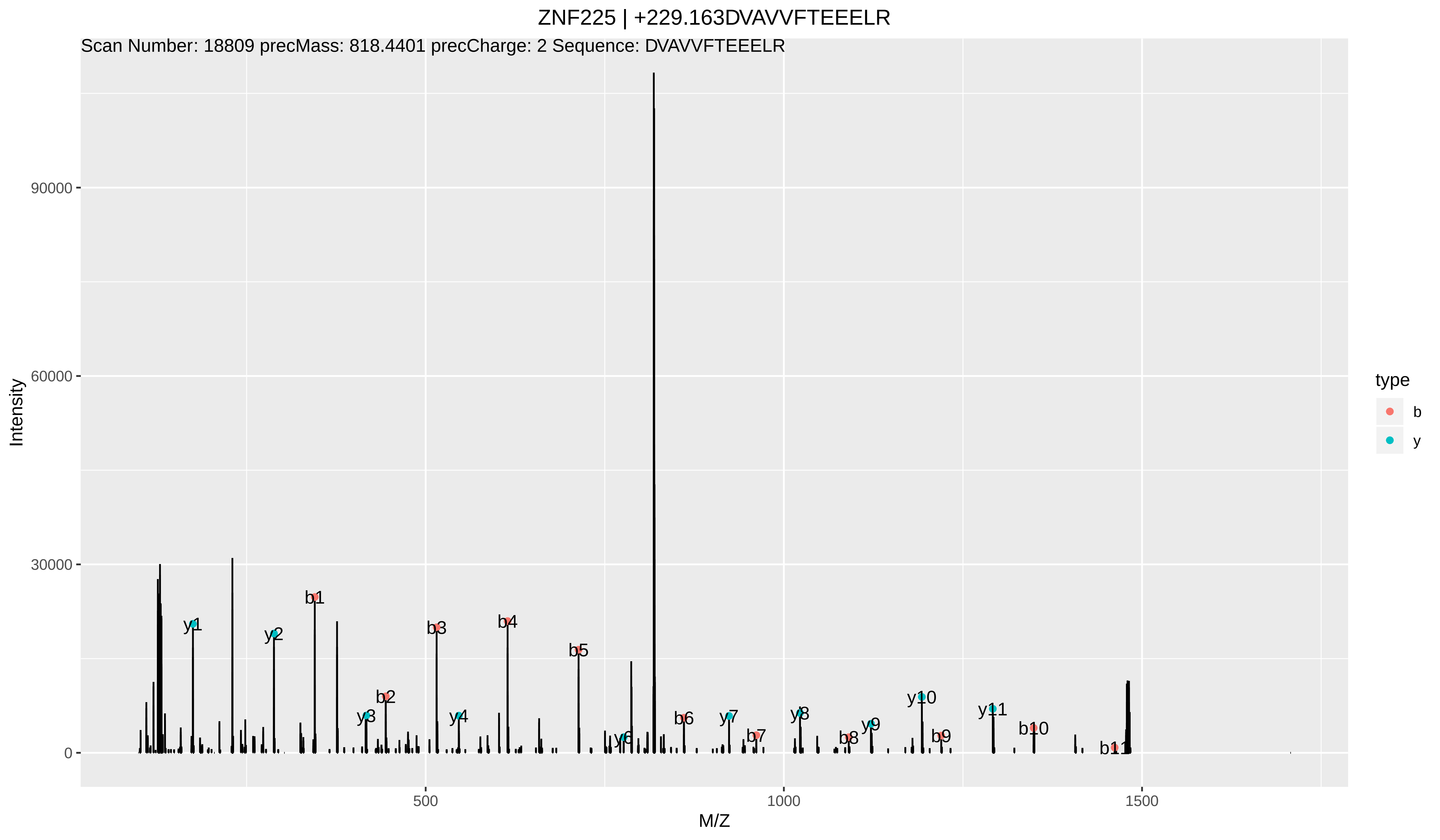

ZNF225 | +229.163DVAVVFTEEELR
Scan Number: 18809 precMass: 818.4401 precCharge: 2 Sequence: DVAVVFTEEELR
Intensity
90000
60000
30000
0
500
1000
1500
M/Z
y1
y2
b1
b2
y3
b3
y4
b4
b5
y6
b6
y7
b7
y8
b8
y9
y10
b9
y11
b10
b11
type
b
y

ZNF232 | +229.163EEEQSC+57.021EYETR

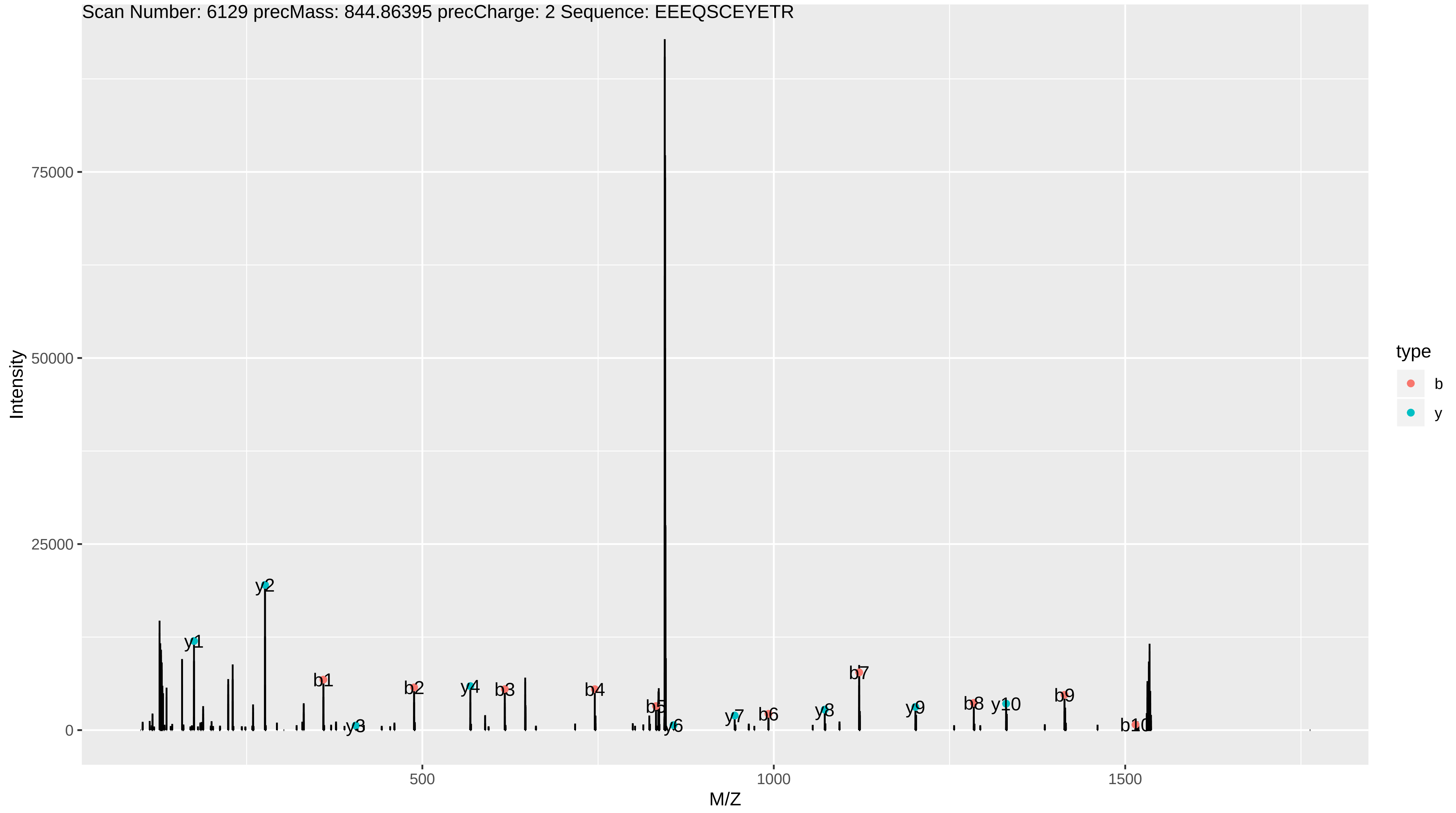

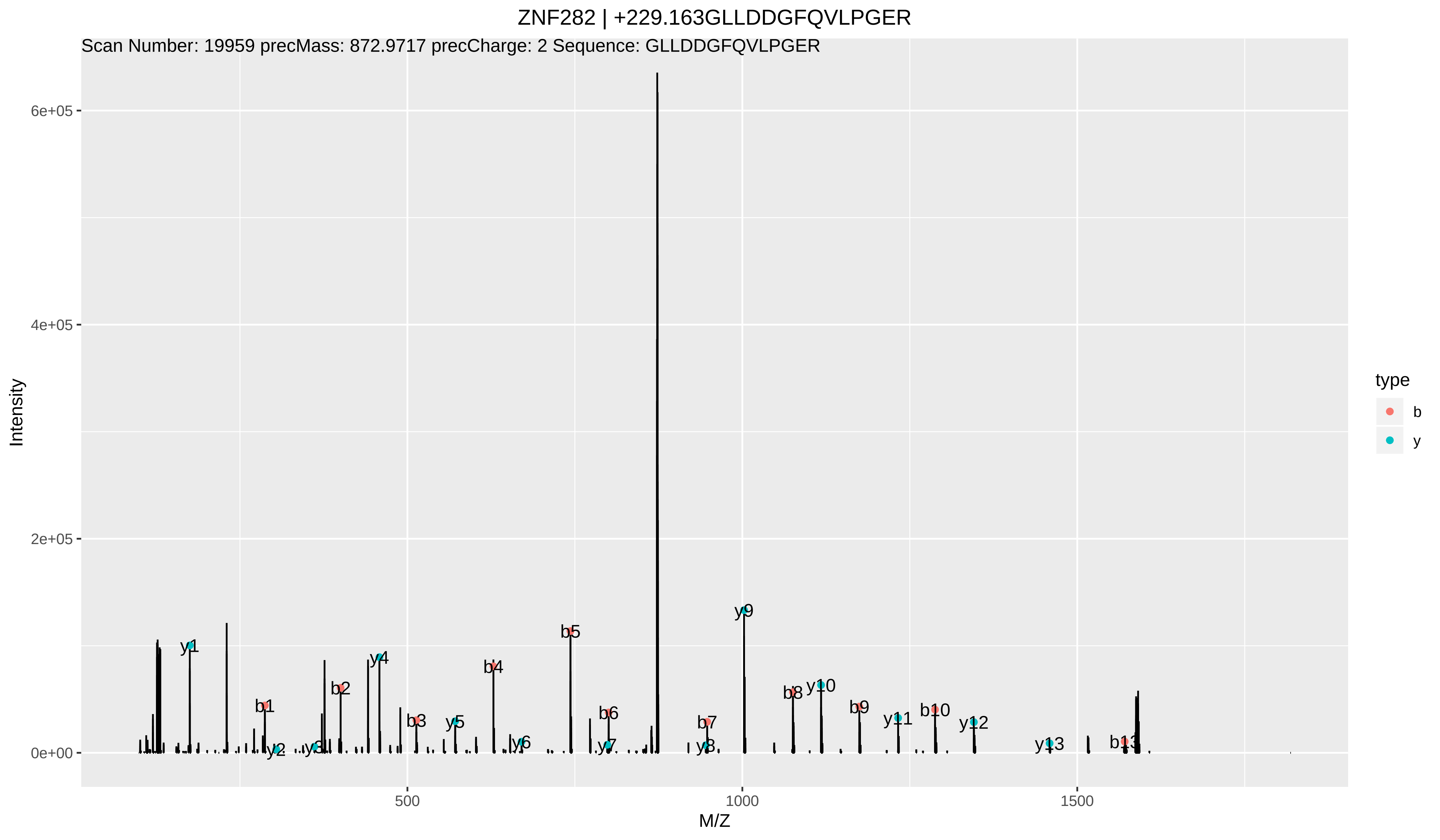

ZNF282 | +229.163GLLDDGFQVLPGER
Scan Number: 19959 precMass: 872.9717 precCharge: 2 Sequence: GLLDDGFQVLPGER
Intensity
M/Z
6e+05
4e+05
2e+05
0e+00
500
1000
1500
y1
b1
y2
y3
b2
y4
b3
y5
b4
y6
b5
b6
y7
b7
y8
y9
b8
y10
b9
y11
b10
y12
y13
b13
type
b
y

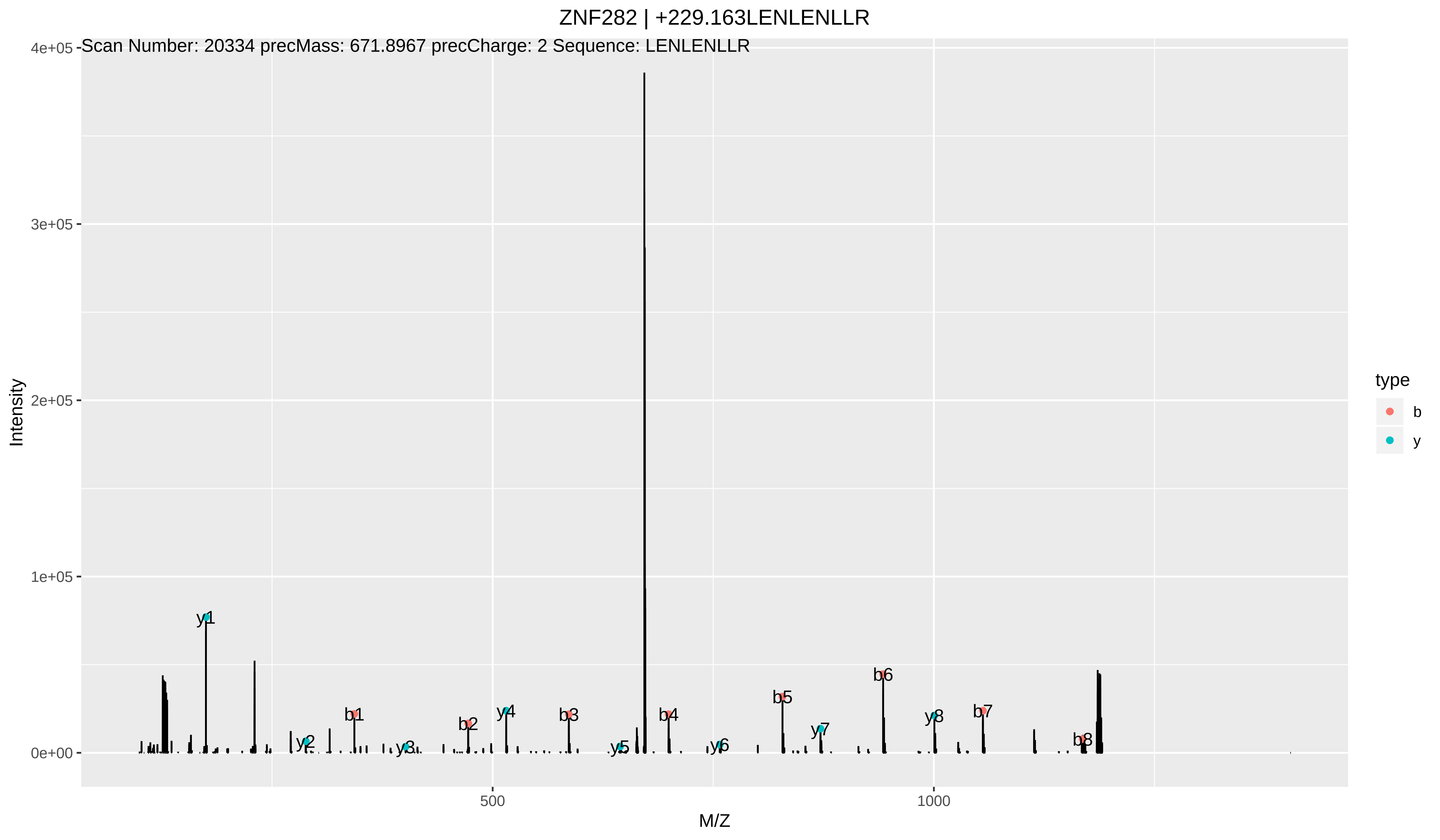

ZNF282 | +229.163LENLENLLR
Scan Number: 20334 precMass: 671.8967 precCharge: 2 Sequence: LENLENLLR
Intensity
4e+05
3e+05
2e+05
1e+05
0e+00
M/Z
500
1000
y1
y2
b1
y3
b2
y4
b3
y5
b4
y6
b5
y7
b6
y8
b7
b8
type
b
y

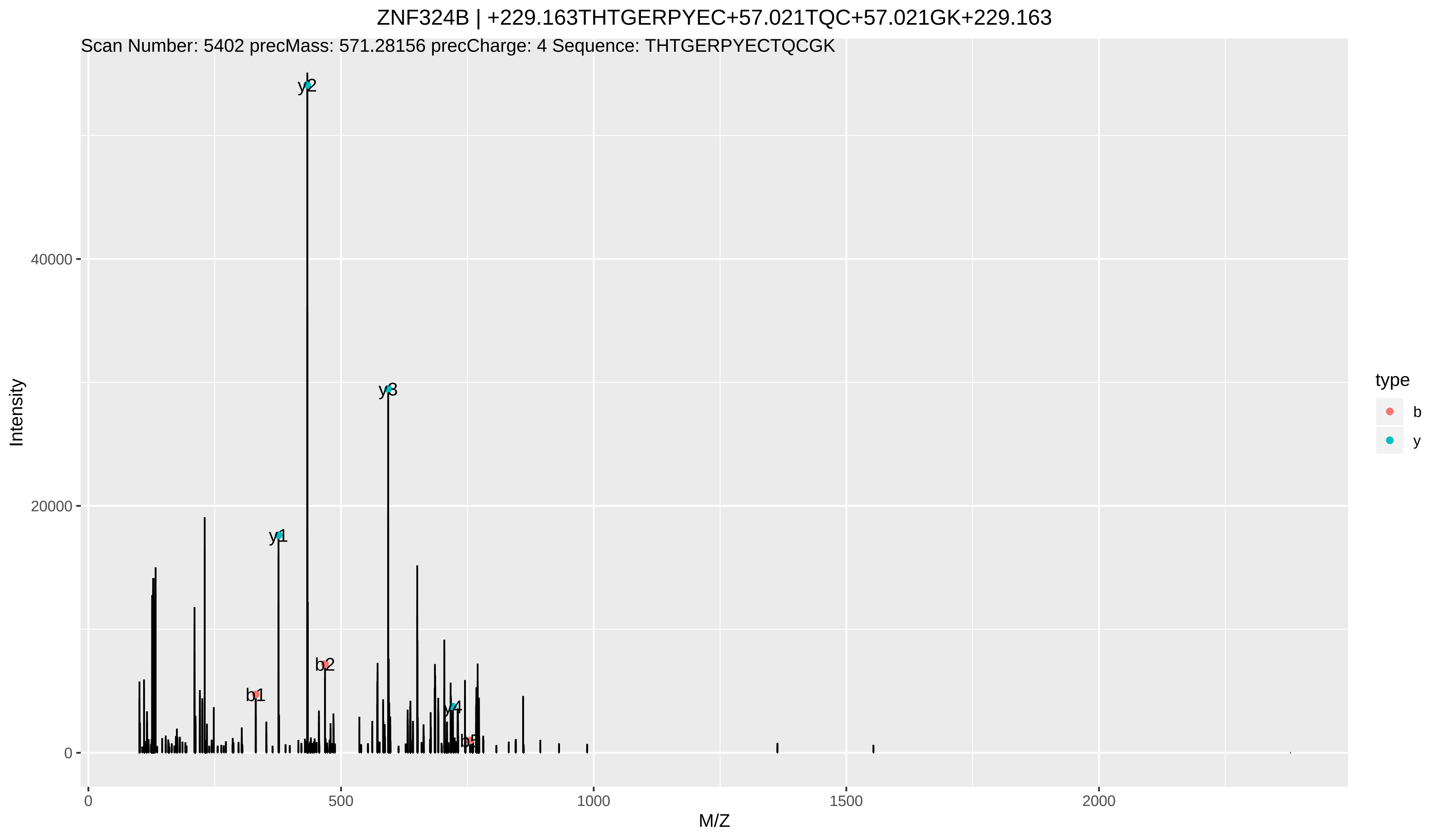

ZNF324B | +229.163THTGERPYEC+57.021TQC+57.021GK+229.163
Scan Number: 5402 precMass: 571.28156 precCharge: 4 Sequence: THTGERPYECTQCGK
y2
y3
y1
b2
b1
y4
b5
Intensity
40000
20000
0
0
500
1000
1500
2000
M/Z
type
b
y

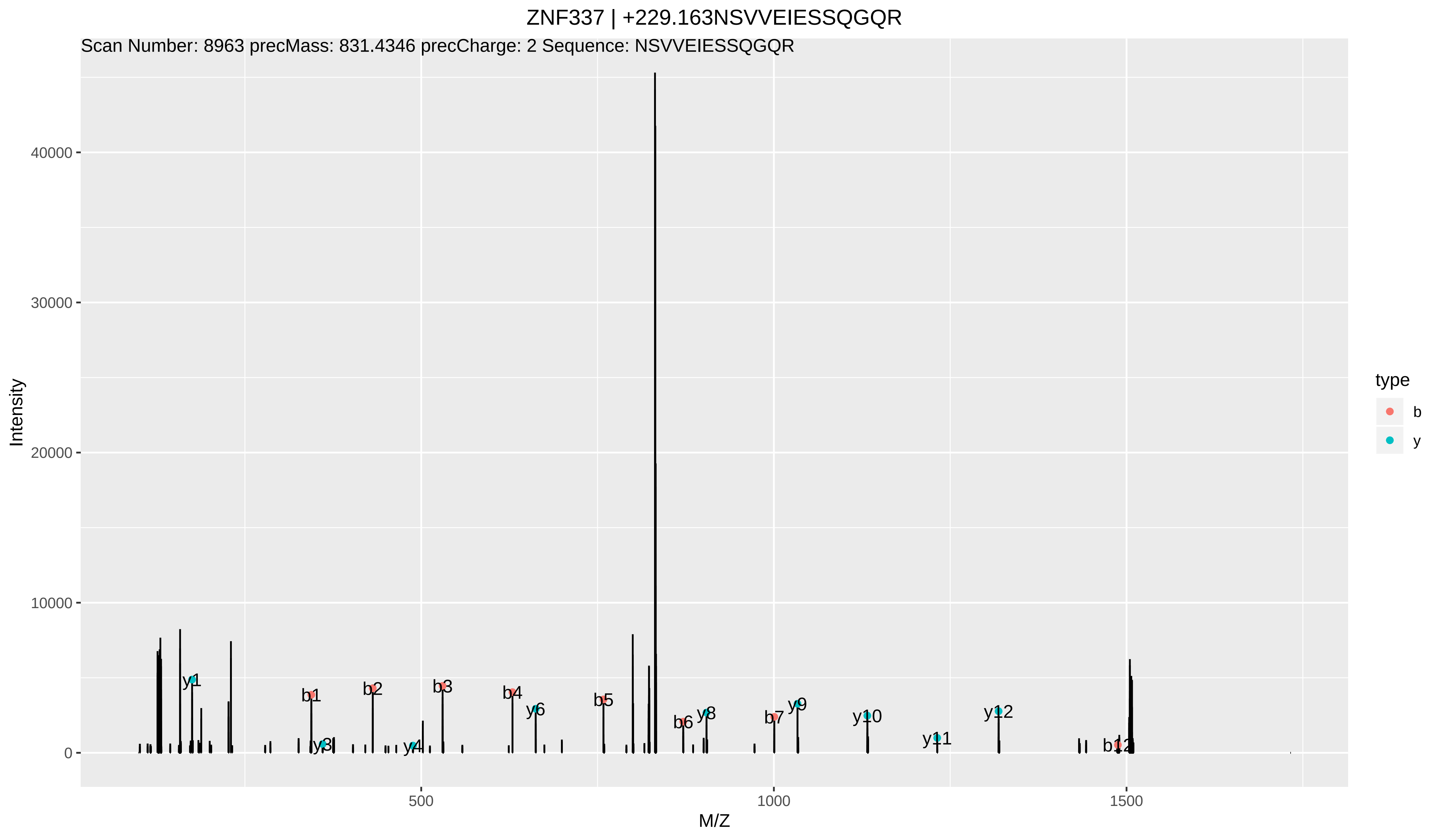
ZNF337 | +229.163NSVVEIESSQGQR
Scan Number: 8963 precMass: 831.4346 precCharge: 2 Sequence: NSVVEIESSQGQR
Intensity
M/Z
0
10000
20000
30000
40000
500
1000
1500
type
b
y
y1
b1
y3
b2
y4
b3
b4
y6
b5
b6
y8
b7
y9
y10
y11
y12
b12

ZNF343 | +229.163EYEPDHNLESNFITNPR

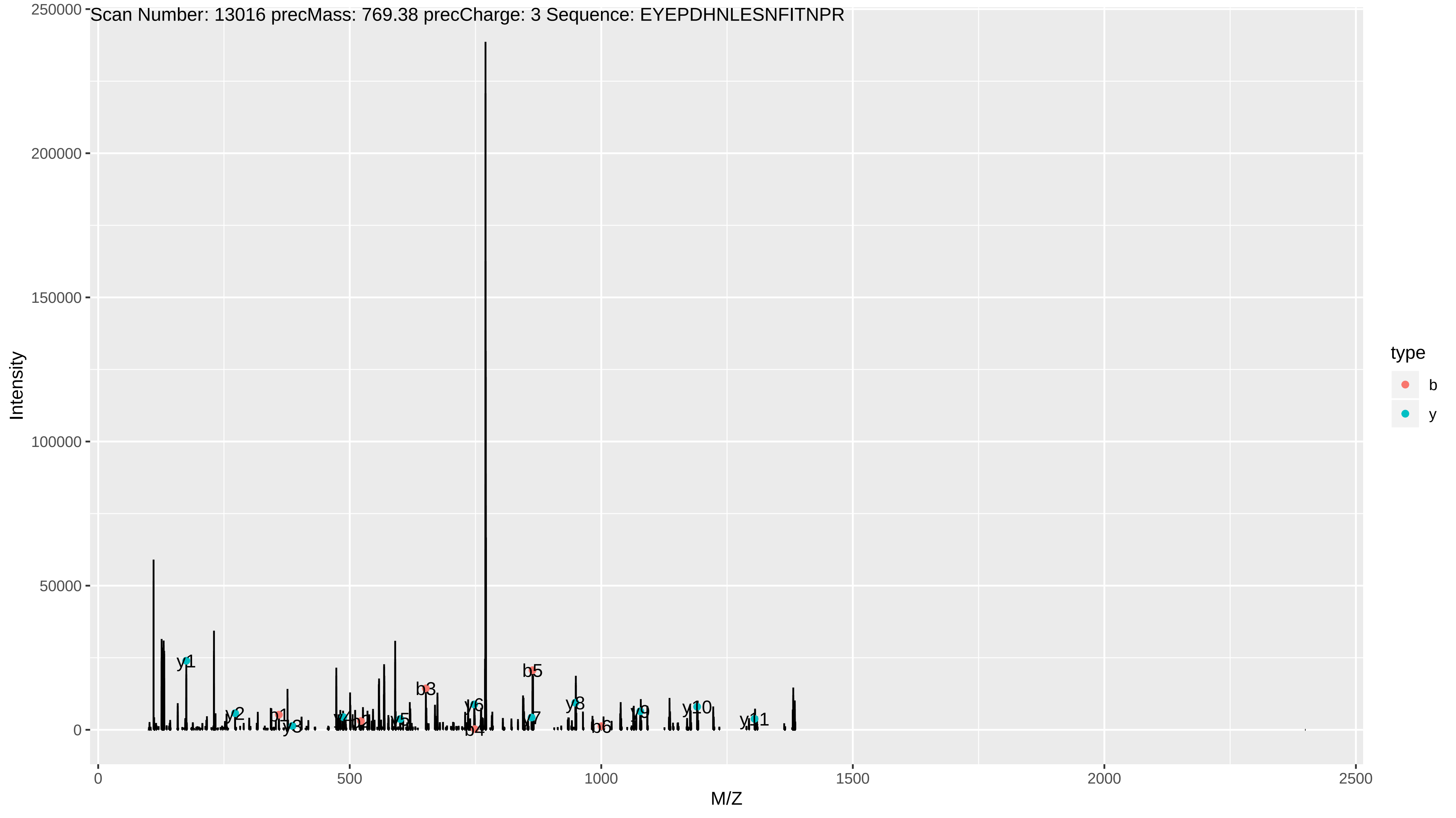

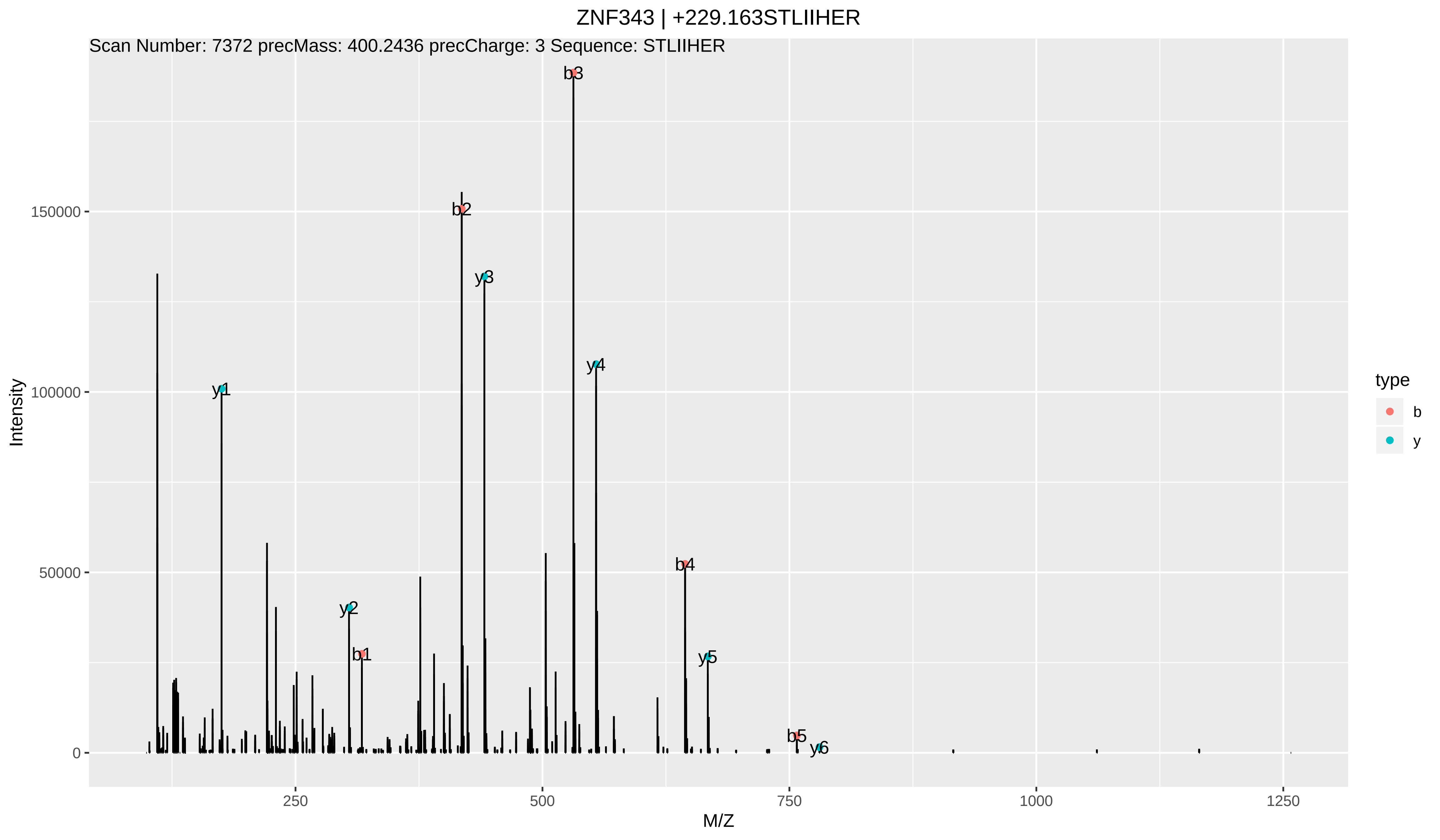

ZNF343 | +229.163STLIIHER
Scan Number: 7372 precMass: 400.2436 precCharge: 3 Sequence: STLIIHER
b3
b2
y3
y4
y1
b4
y2
b1
y5
b5
y6
Intensity
150000
100000
50000
0
250
500
750
1000
1250
M/Z
type
b
y

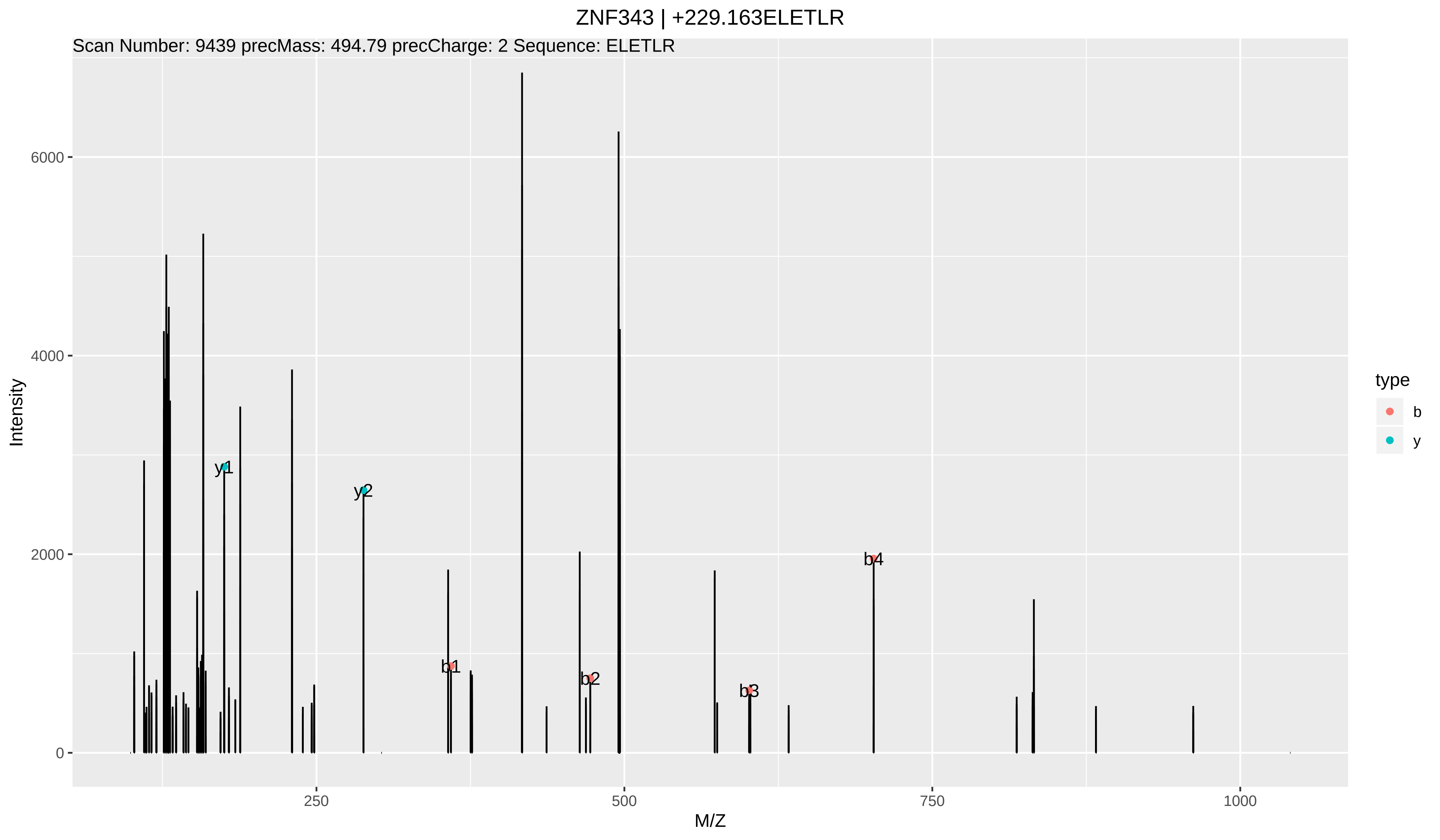

ZNF343 | +229.163ELETLR
Scan Number: 9439 precMass: 494.79 precCharge: 2 Sequence: ELETLR
Intensity
6000
4000
2000
0
M/Z
250
500
750
1000
y1
y2
b1
b2
b3
b4
type
b
y

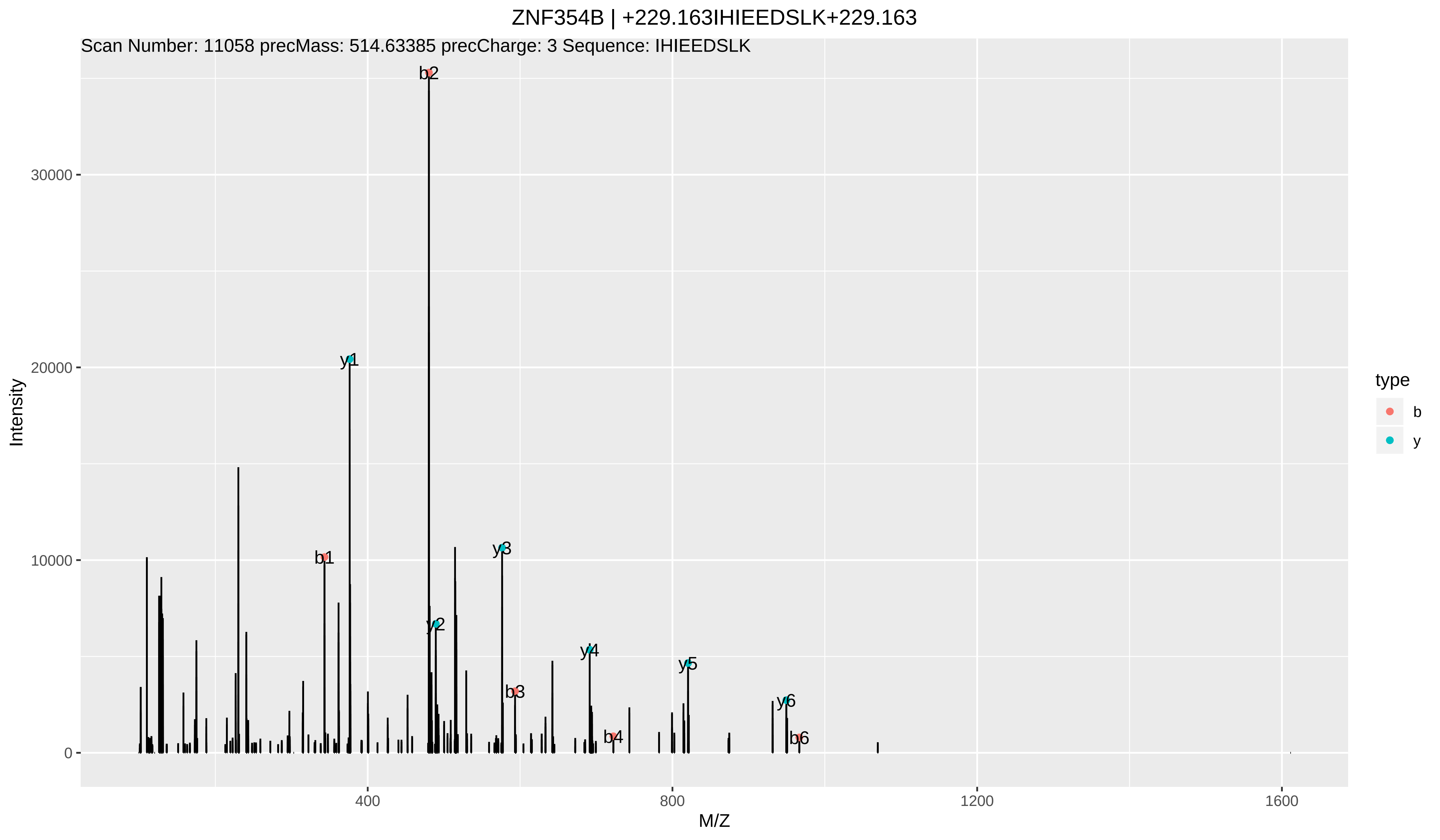

ZNF354B | +229.163IHIEEDSLK+229.163
Scan Number: 11058 precMass: 514.63385 precCharge: 3 Sequence: IHIEEDSLK
b2
y1
b1
y3
y2
y4
y5
b3
y6
b4
b6
Intensity
30000
20000
10000
0
400
800
1200
1600
M/Z
type
b
y

ZNF358 | +229.163GFGQGSALLK+229.163

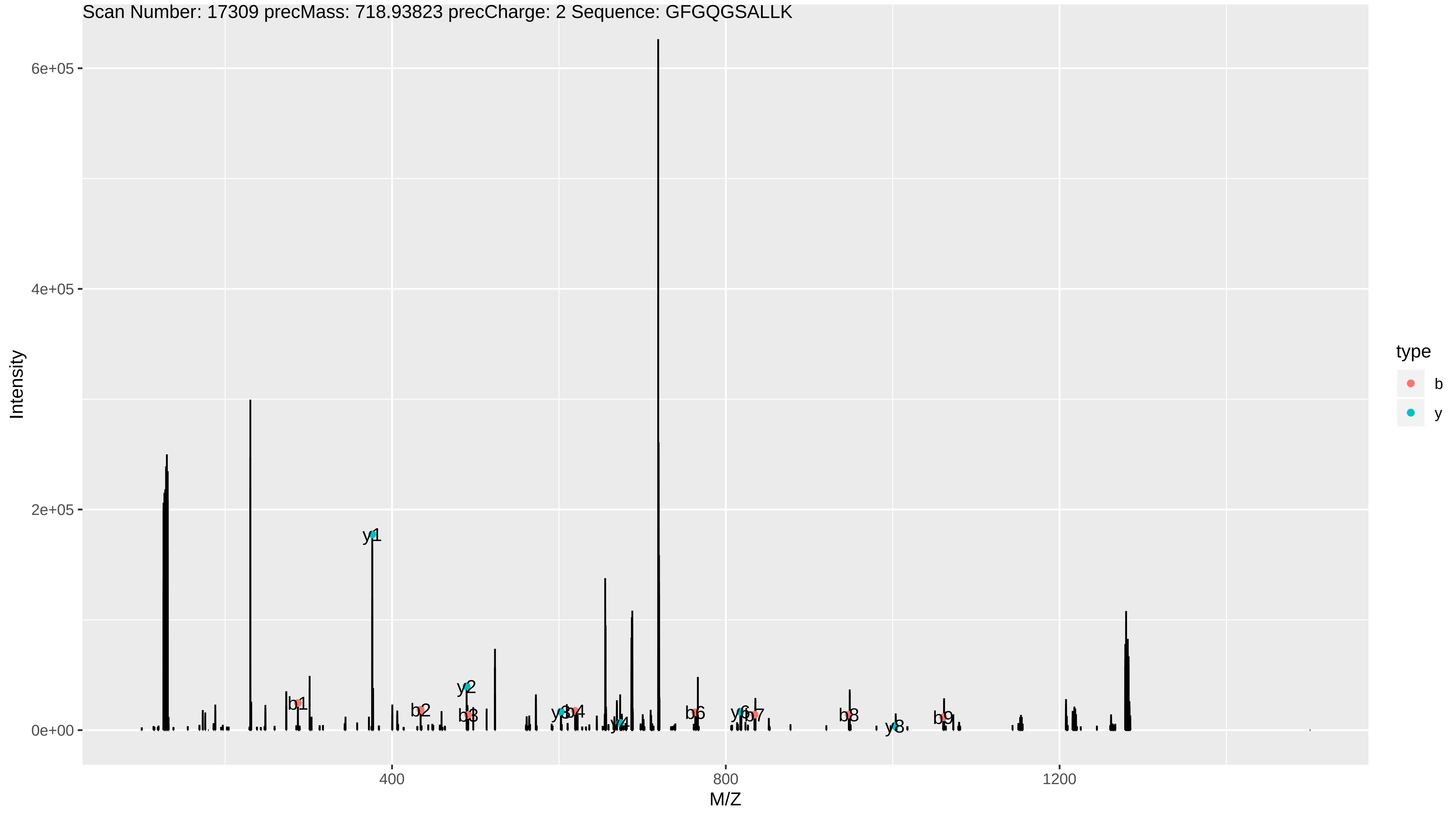

ZNF37A | +229.163AFPENSLFLVHK+229.163

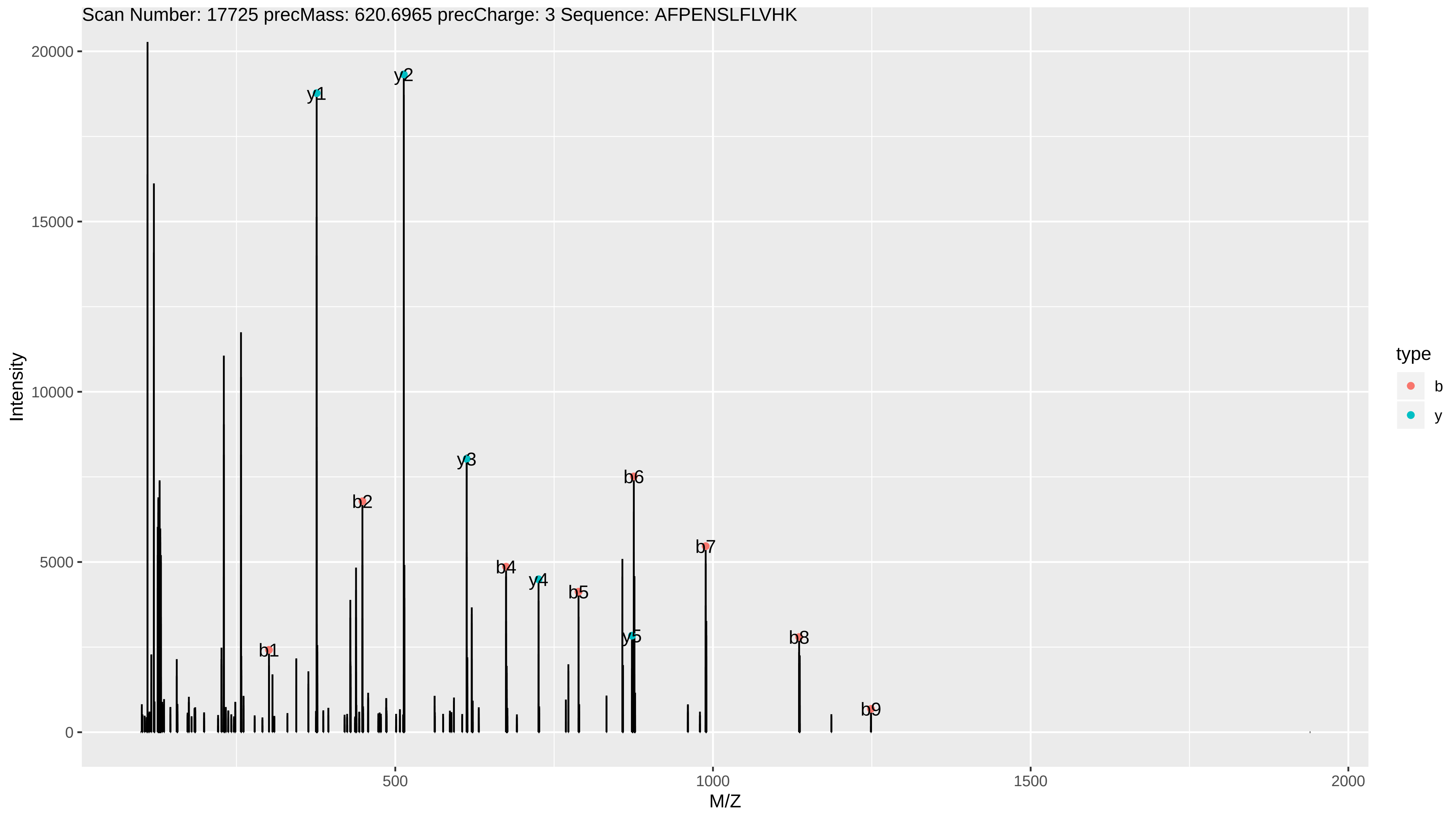

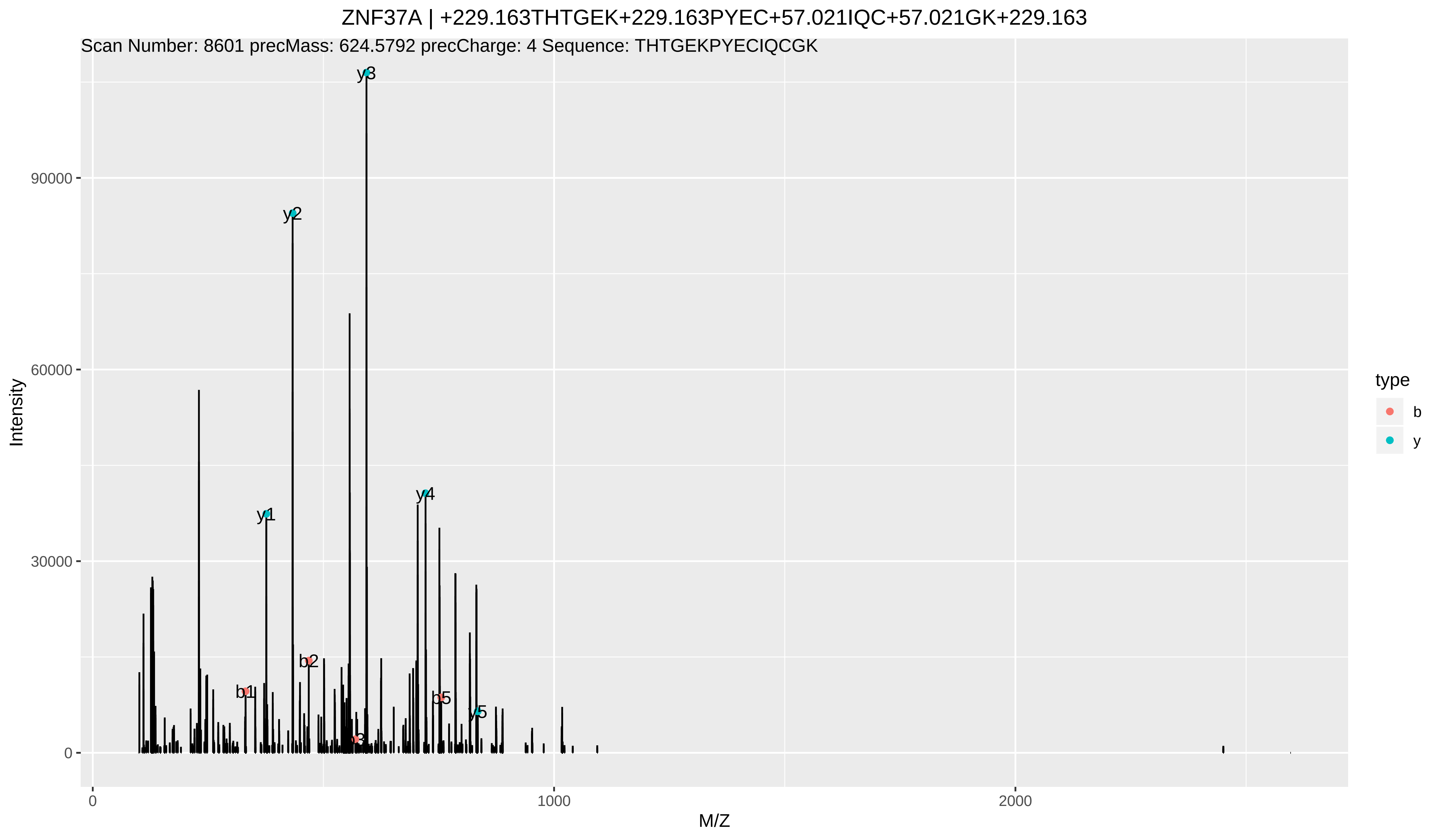

ZNF37A | +229.163THTGEK+229.163PYEC+57.021IQC+57.021GK+229.163
Scan Number: 8601 precMass: 624.5792 precCharge: 4 Sequence: THTGEKPYECIQCGK
Intensity
M/Z
type
b
y
y1
y2
y3
y4
y5
b1
b2
b5
0
30000
60000
90000
1000
2000

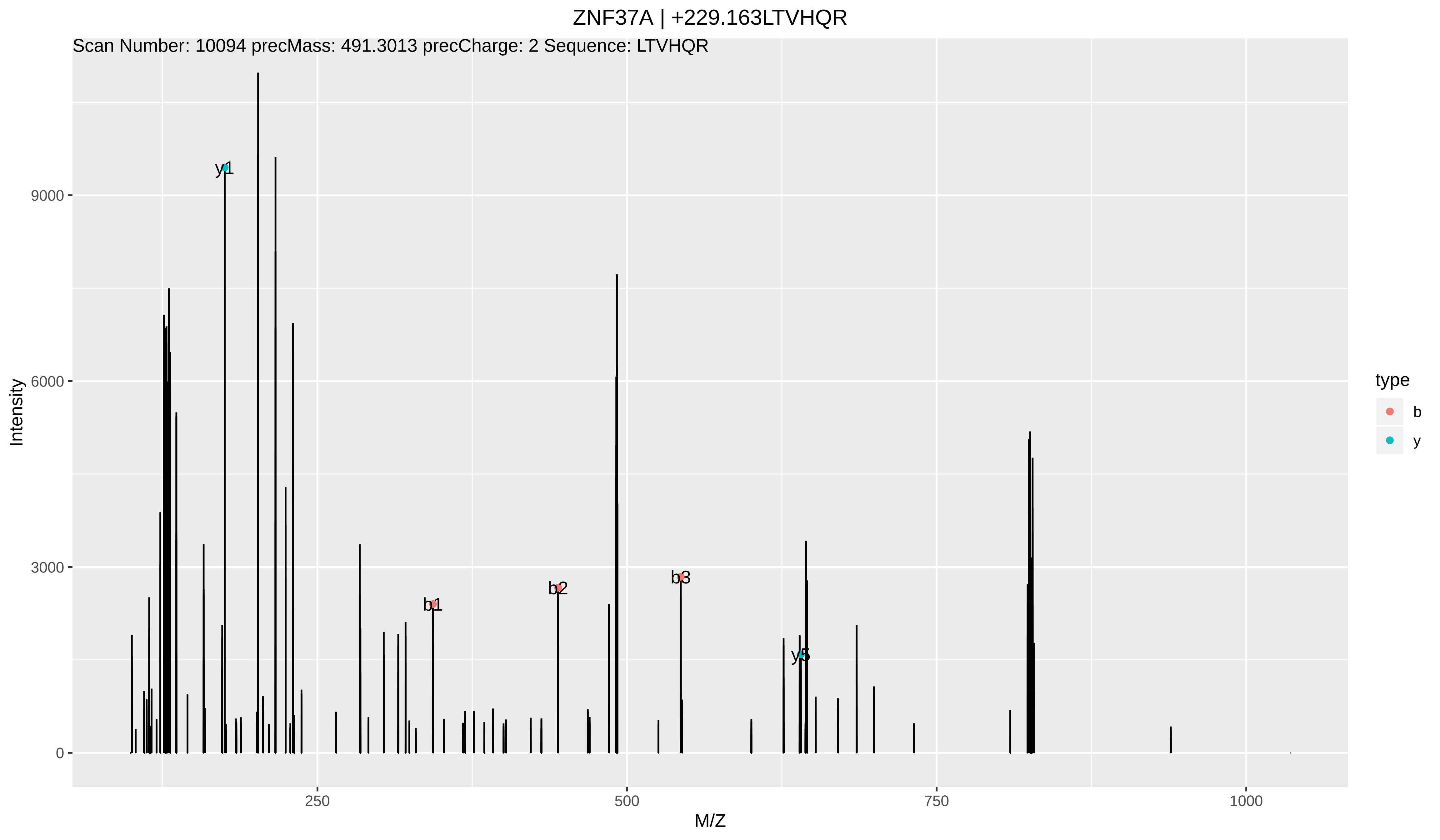

ZNF37A | +229.163LTVHQR
Scan Number: 10094 precMass: 491.3013 precCharge: 2 Sequence: LTVHQR
y1
b1
b2
b3
y5
Intensity
M/Z
type
b
y

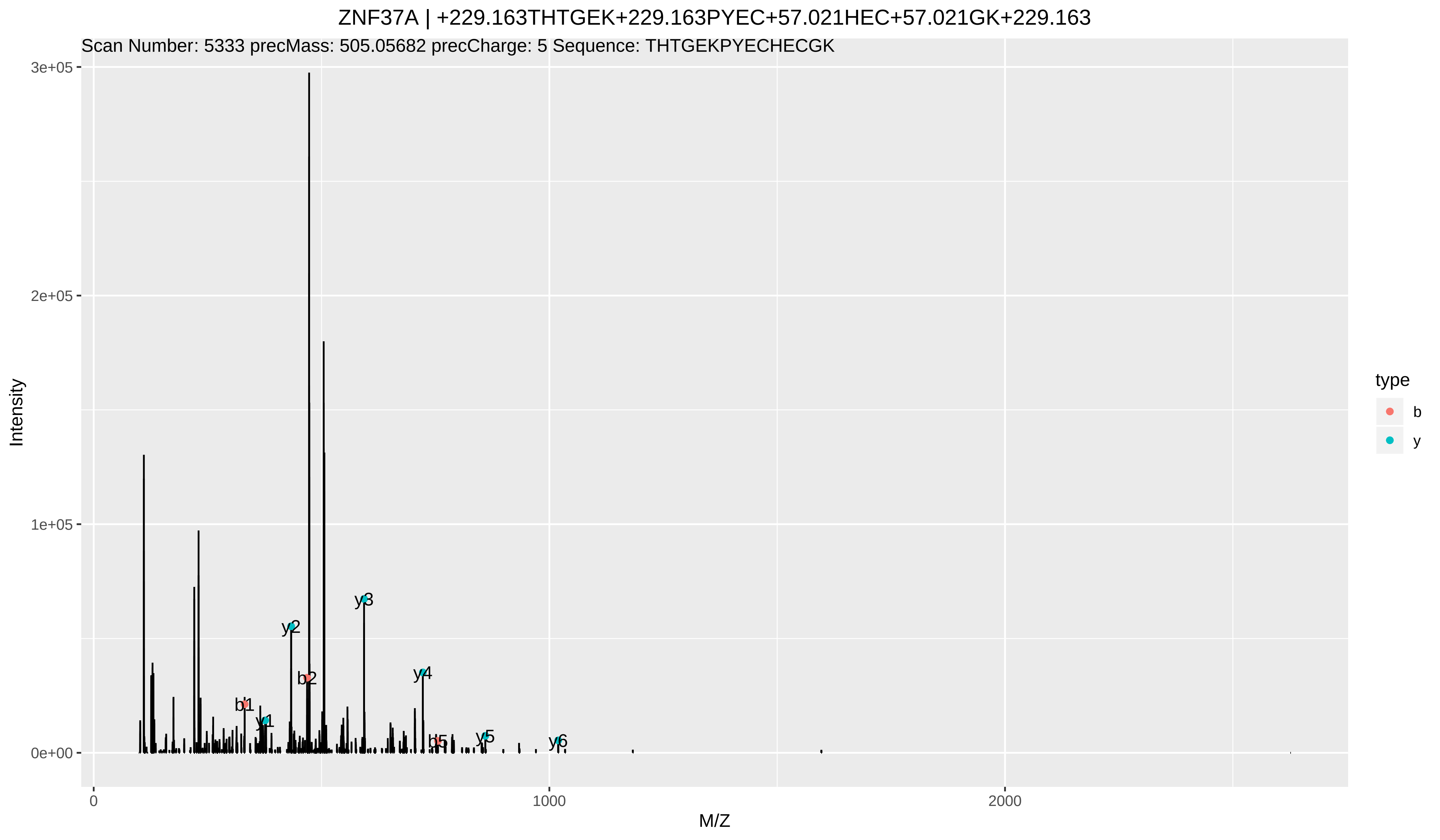
ZNF37A | +229.163THTGEK+229.163PYEC+57.021HEC+57.021GK+229.163
Scan Number: 5333 precMass: 505.05682 precCharge: 5 Sequence: THTGEKPYECHECGK
Intensity
3e+05
2e+05
1e+05
0e+00
M/Z
0
1000
2000
y3
y2
y4
b2
b1
y1
b5
y5
y6
type
b
y

ZNF425 | +229.163LEIPTGPR

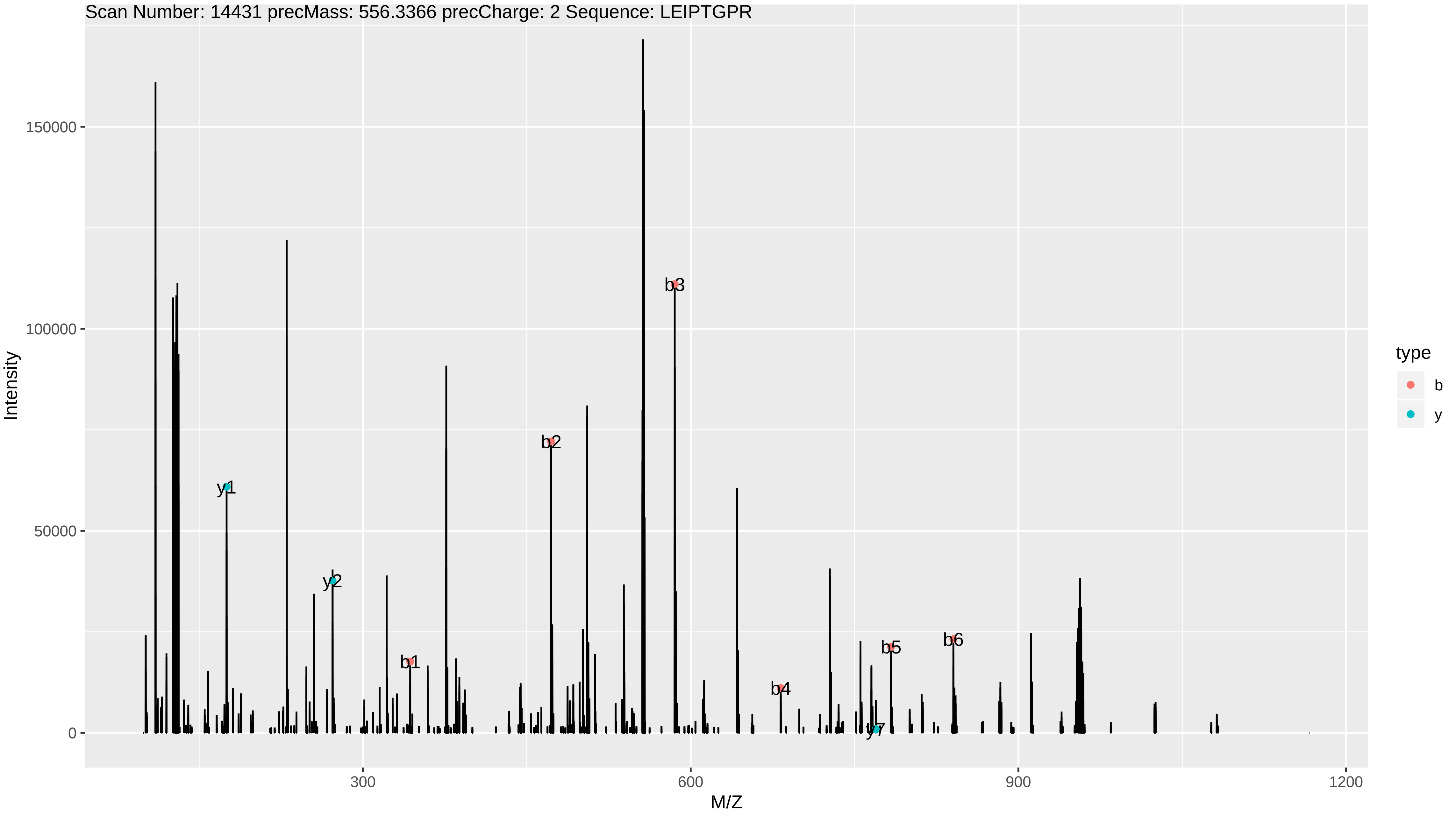

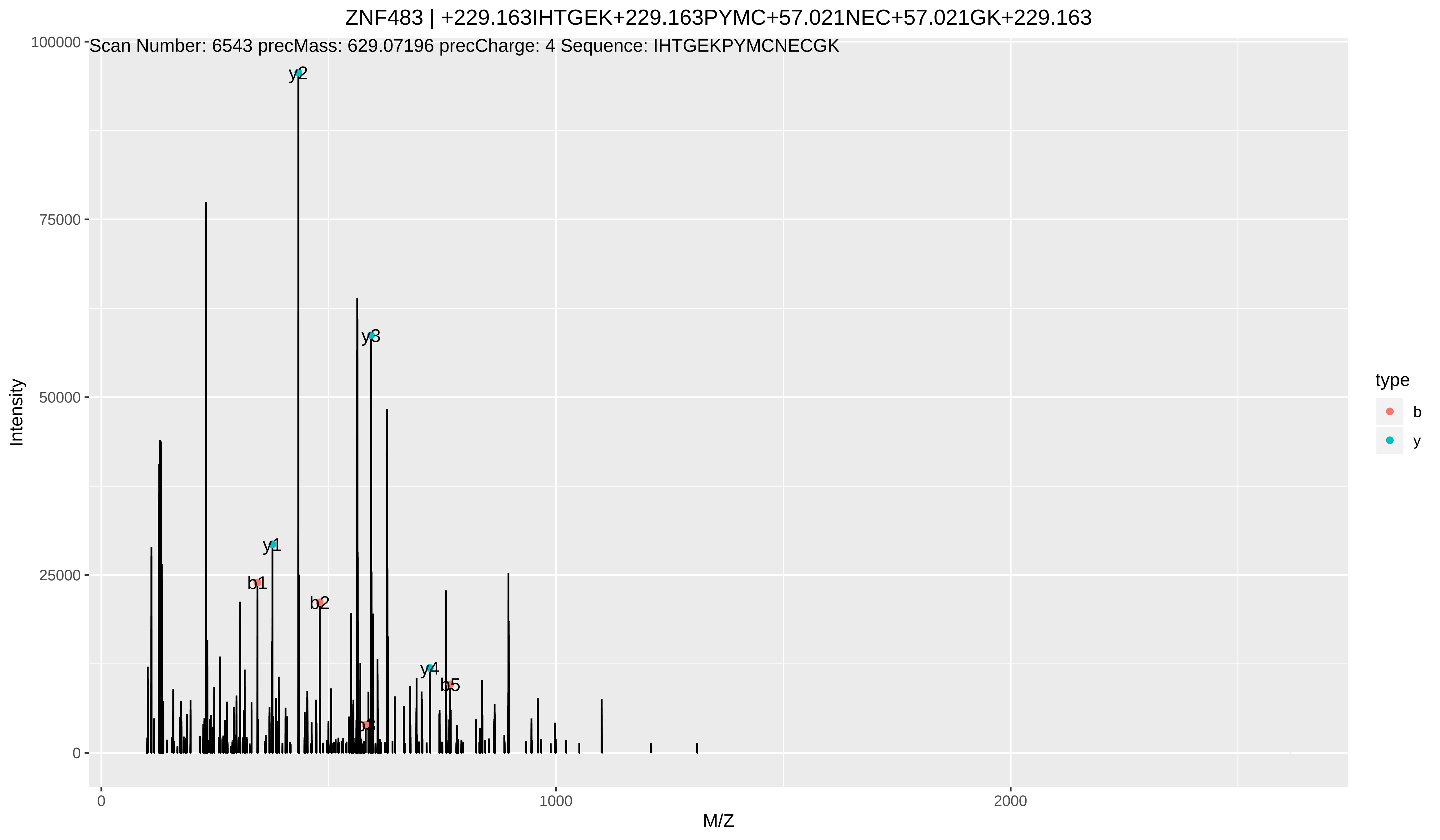

ZNF483 | +229.163IHTGEK+229.163PYMC+57.021NEC+57.021GK+229.163
Scan Number: 6543 precMass: 629.07196 precCharge: 4 Sequence: IHTGEKPYMCNECGK
y2
y3
y1
b1
b2
y4
b5
Intensity
M/Z
100000
75000
50000
25000
0
1000
2000
type
b
y

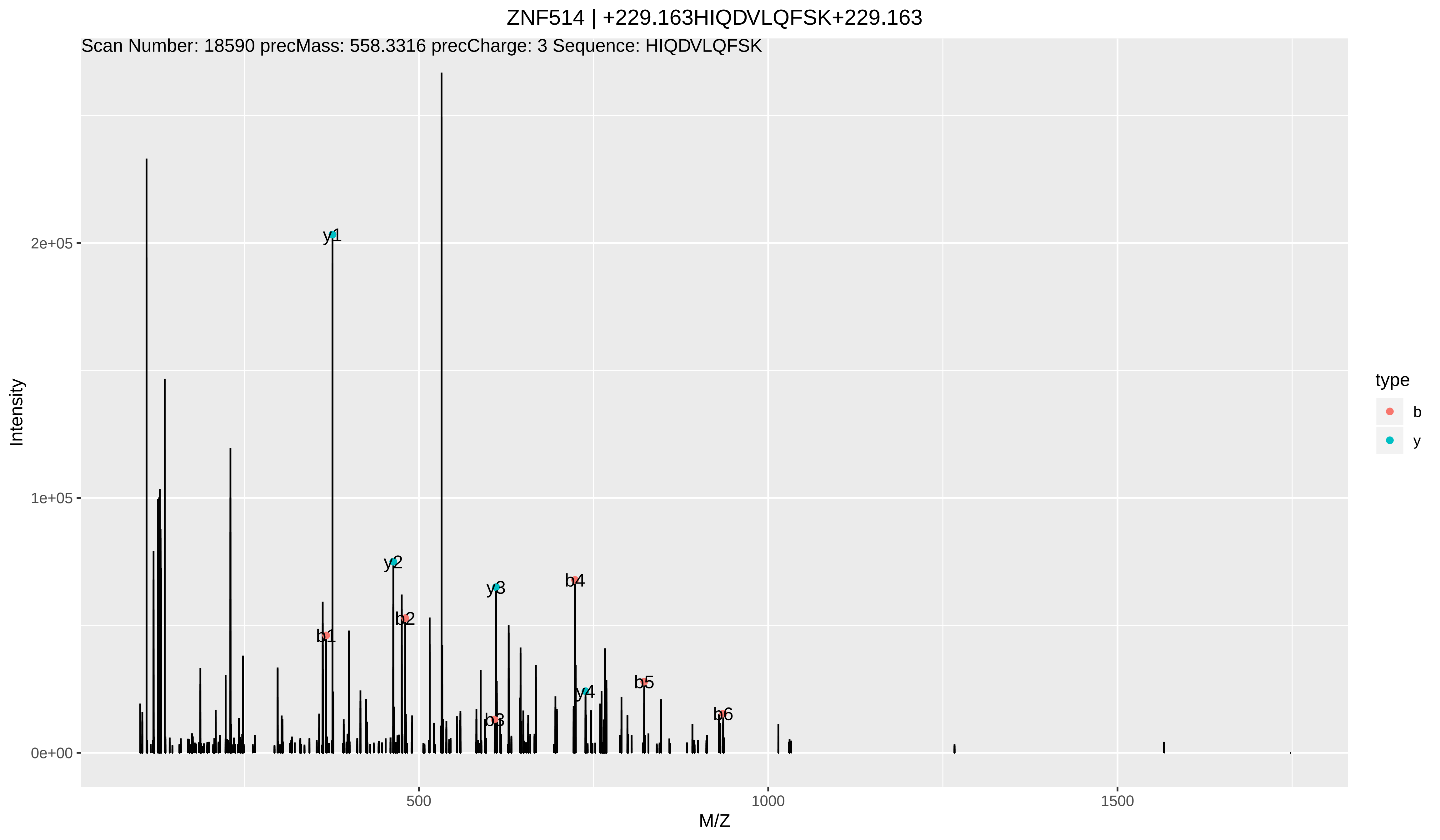
ZNF514 | +229.163HIQDVLQFSK+229.163
Scan Number: 18590 precMass: 558.3316 precCharge: 3 Sequence: HIQDVLQFSK
Intensity
2e+05
1e+05
0e+00
M/Z
500
1000
1500
y1
y2
y3
y4
b1
b2
b3
b4
b5
b6
type
b
y

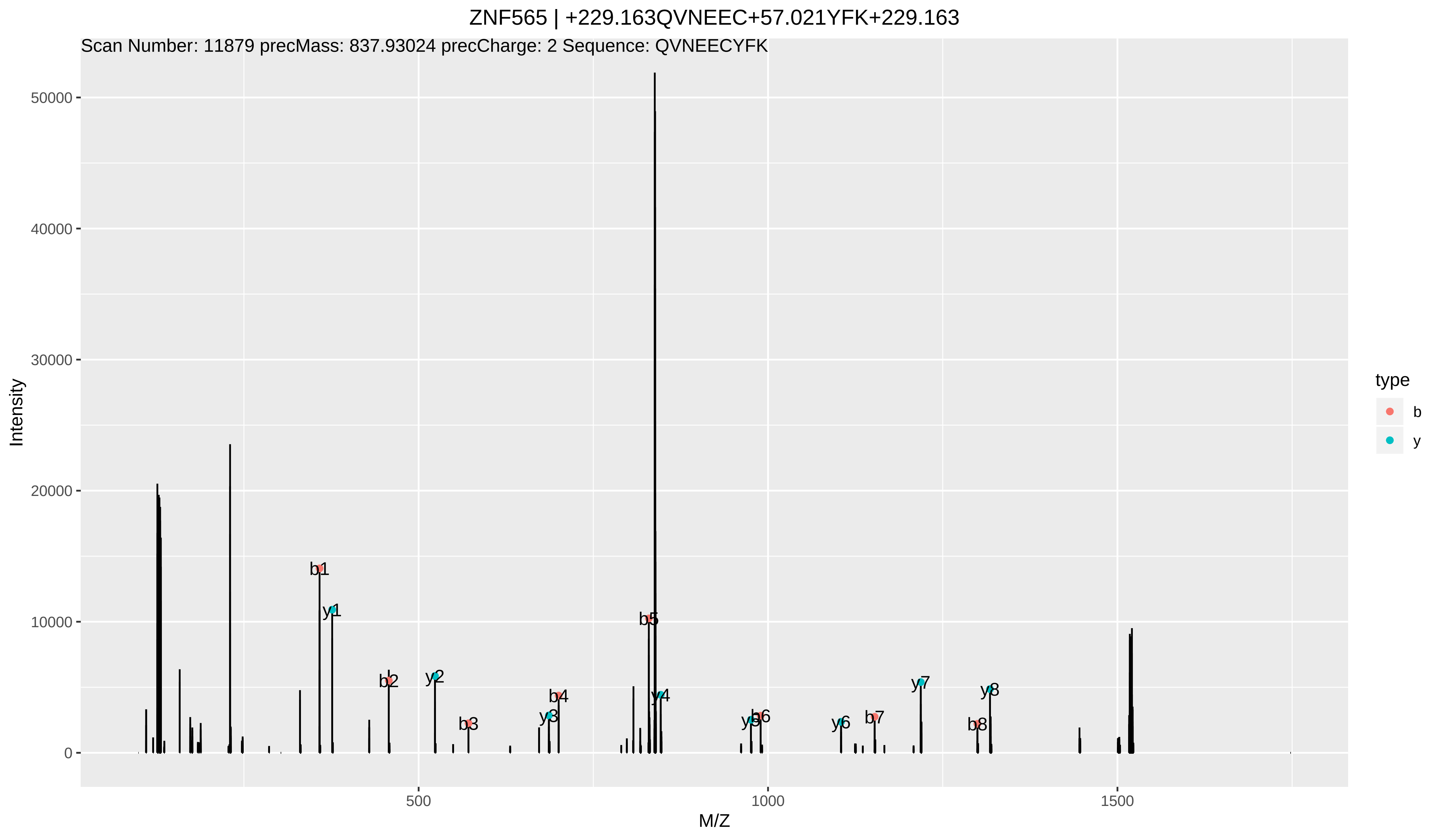
ZNF565 | +229.163QVNEEC+57.021YFK+229.163
Scan Number: 11879 precMass: 837.93024 precCharge: 2 Sequence: QVNEECYFK
Intensity
M/Z
0
10000
20000
30000
40000
50000
500
1000
1500
b1
y1
b2
y2
b3
y3
b4
b5
y4
y5
b6
y6
b7
y7
b8
y8
type
b
y

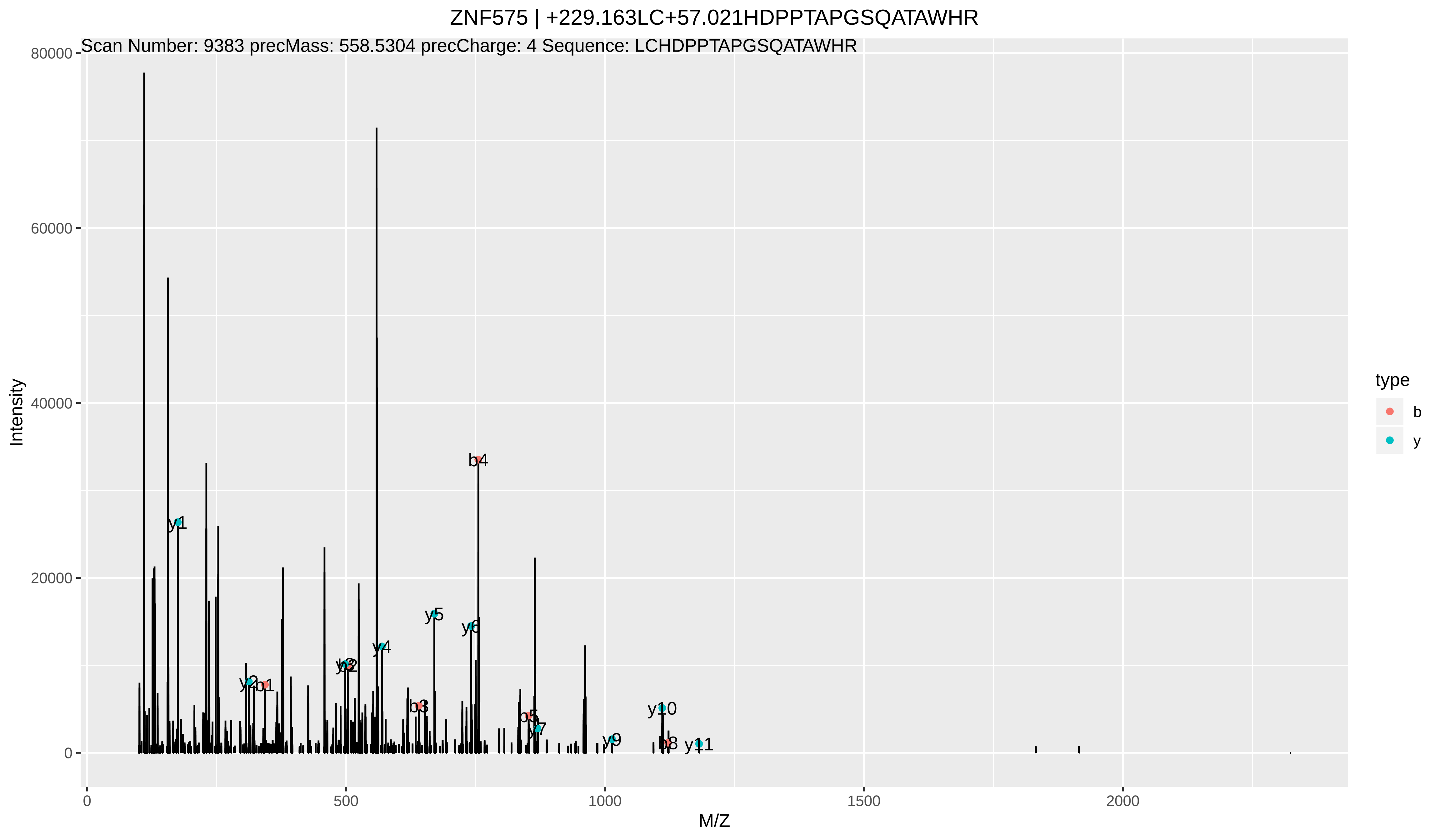

ZNF575 | +229.163LC+57.021HDPPTAPGSQATAWHR
Scan Number: 9383 precMass: 558.5304 precCharge: 4 Sequence: LCHDPPTAPGSQATAWHR
Intensity
M/Z
80000
60000
40000
20000
0
0
500
1000
1500
2000
b4
y1
y5
y6
y4
y2
b1
b2
b3
y10
b5
y7
y9
b8
y11
type
b
y

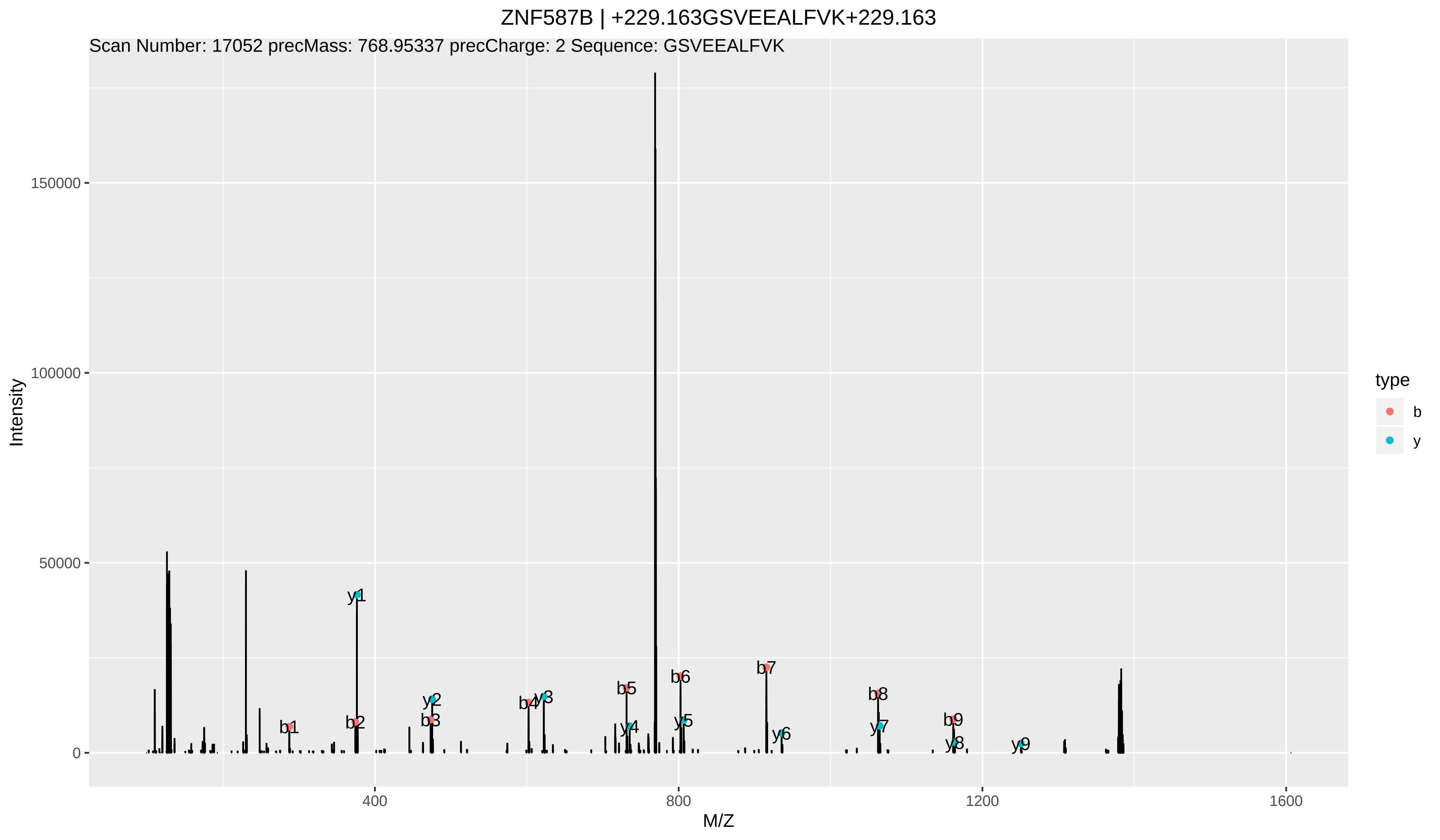

ZNF587B | +229.163GSVEEALFVK+229.163
Scan Number: 17052 precMass: 768.95337 precCharge: 2 Sequence: GSVEEALFVK
Intensity
150000
100000
50000
0
400
800
1200
1600
M/Z
y1
b1
b2
y2
b3
b4
y3
b5
y4
b6
y5
b7
y6
b8
y7
b9
y8
y9
type
b
y

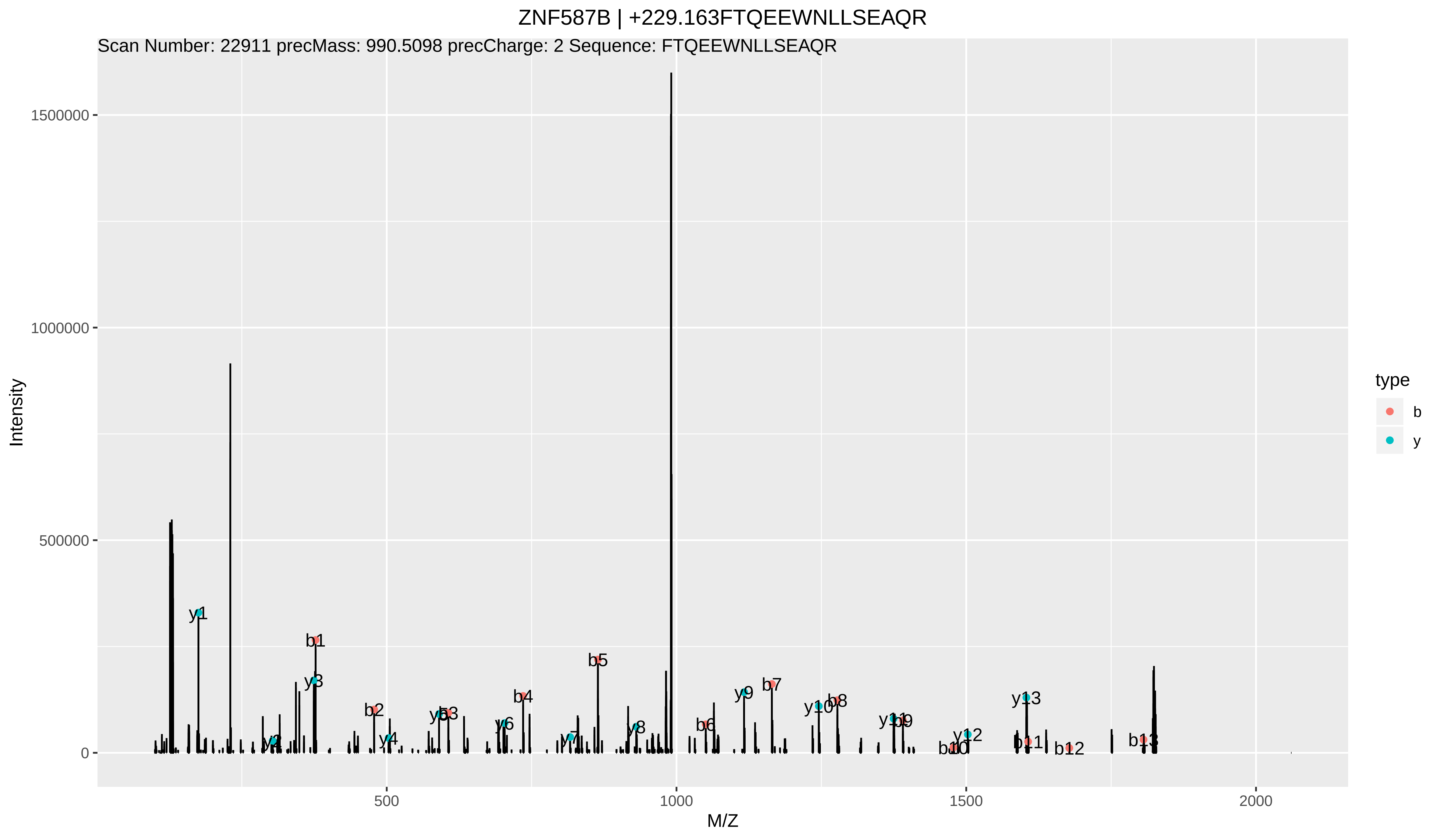

ZNF587B | +229.163FTQEEWNLLSEAQR
Scan Number: 22911 precMass: 990.5098 precCharge: 2 Sequence: FTQEEWNLLSEAQR
Intensity
M/Z
type
b
y

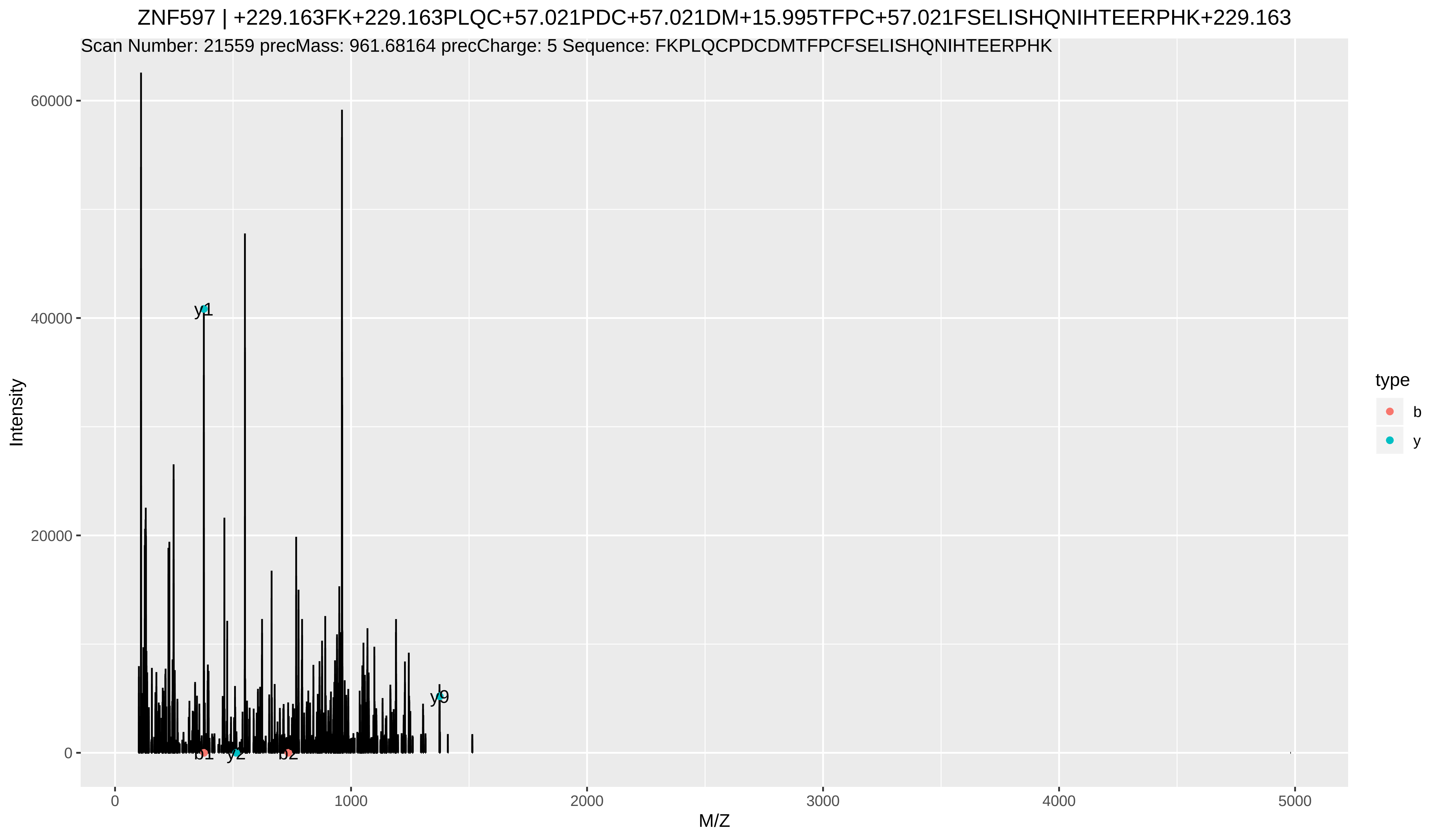

ZNF597 | +229.163FK+229.163PLQC+57.021PDC+57.021DM+15.995TFPC+57.021FSELISHQNIHTEERPHK+229.163
Scan Number: 21559 precMass: 961.68164 precCharge: 5 Sequence: FKPLQCPDCDMTFPCFSELISHQNIHTEERPHK
Intensity
M/Z
type
b
y
y1
y2
y9
b1
b2
0
20000
40000
60000
1000
2000
3000
4000
5000

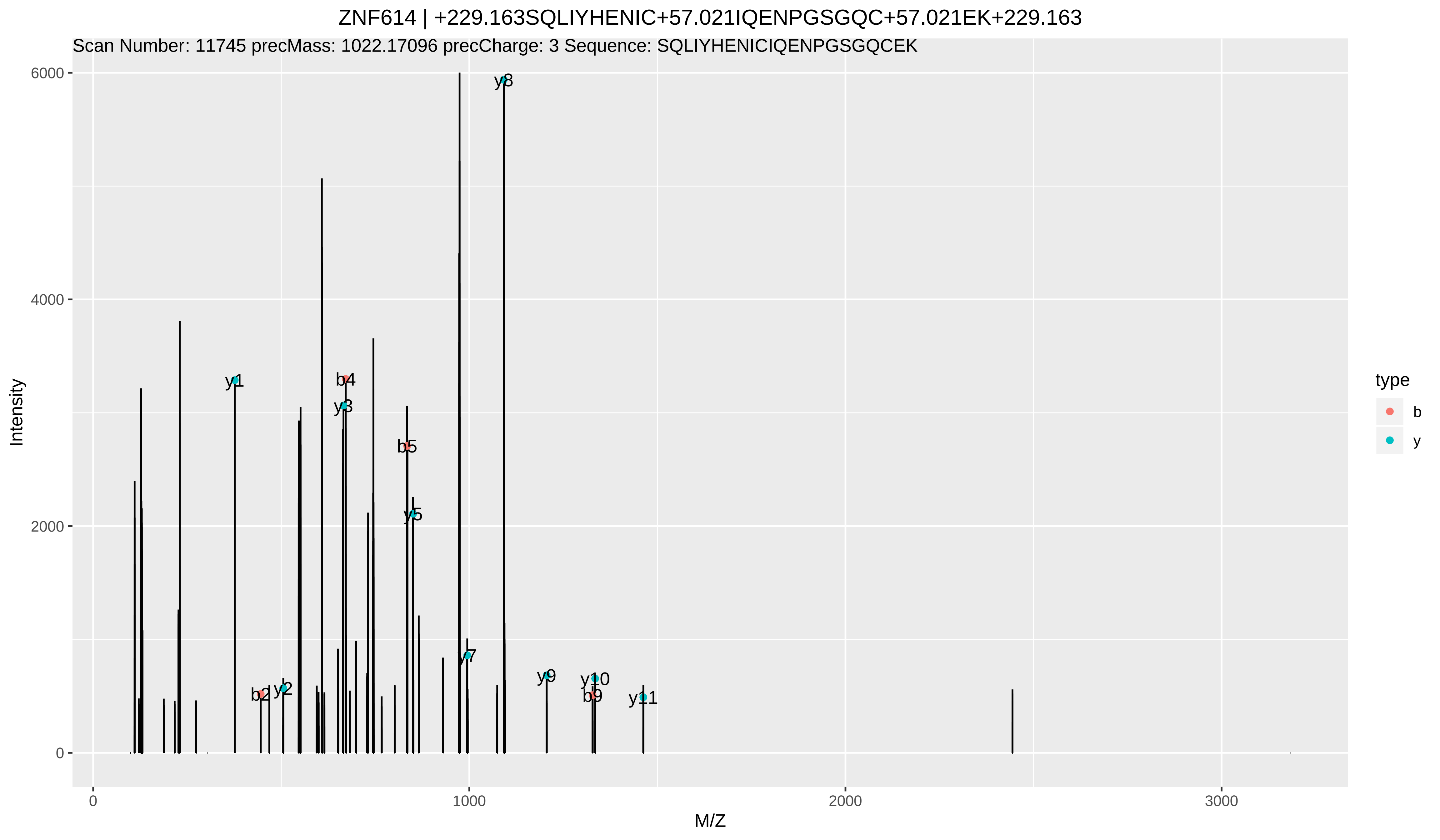

ZNF614 | +229.163SQLIYHENIC+57.021IQENPGSGQC+57.021EK+229.163
Scan Number: 11745 precMass: 1022.17096 precCharge: 3 Sequence: SQLIYHENICIQENPGSGQCEK
Intensity
M/Z
0
2000
4000
6000
0
1000
2000
3000
y1
b2
y2
y3
b4
b5
y5
y7
y8
y9
y10
b9
y11
type
b
y

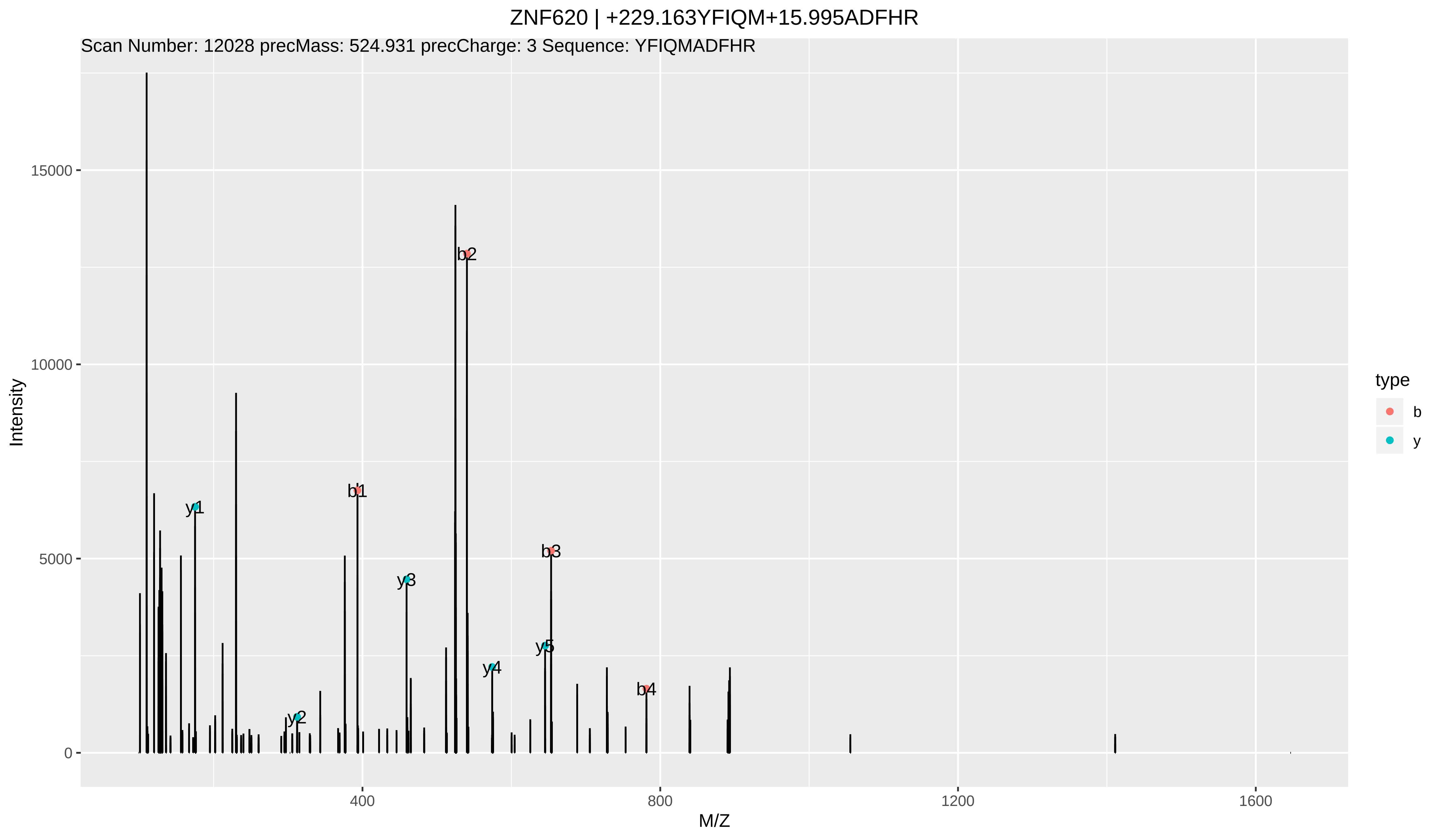

ZNF620 | +229.163YFIQM+15.995ADFHR
Scan Number: 12028 precMass: 524.931 precCharge: 3 Sequence: YFIQMADFHR
Intensity
15000
10000
5000
0
M/Z
400
800
1200
1600
y1
y2
b1
y3
b2
y4
y5
b3
b4
type
b
y

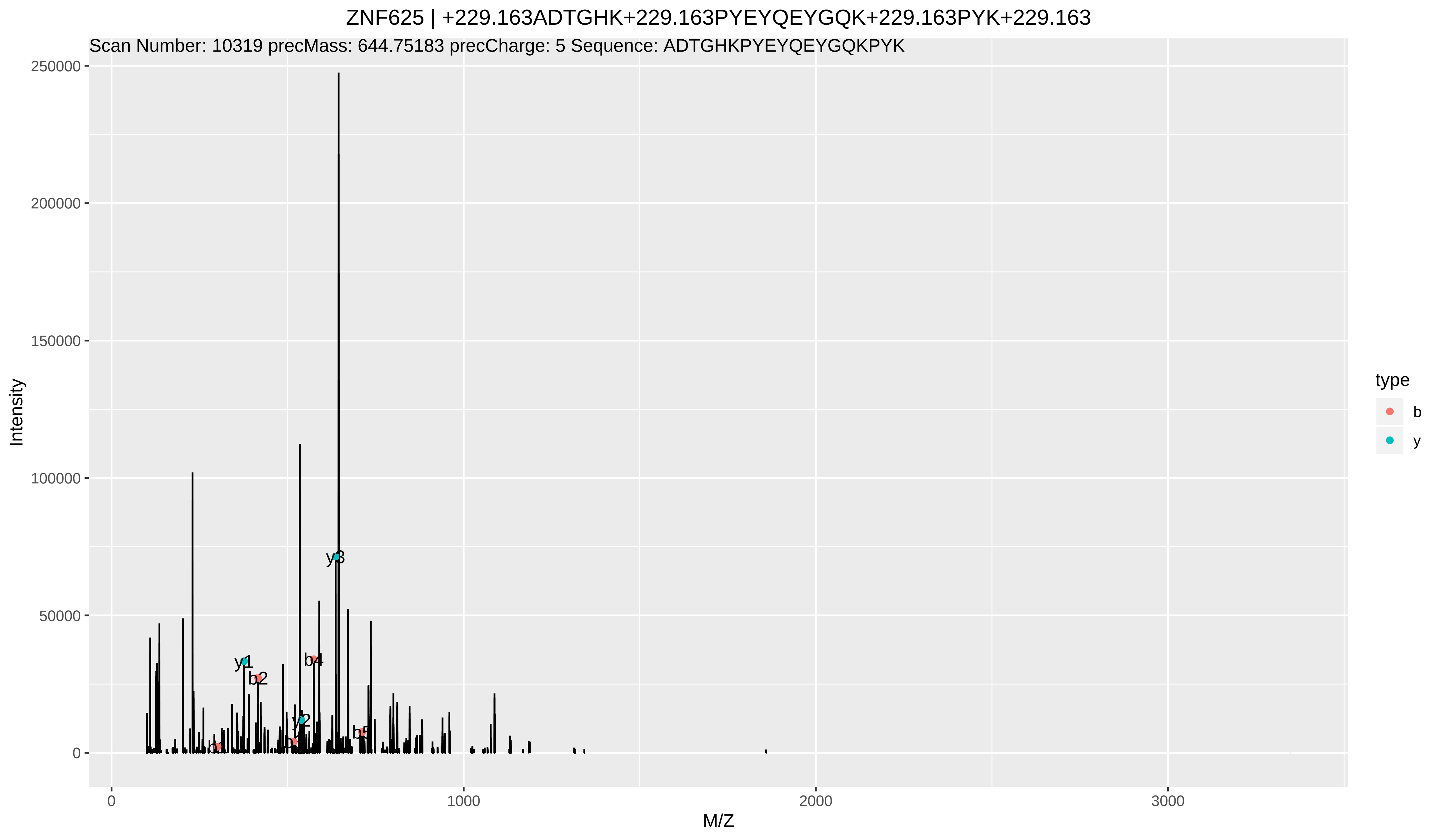
ZNF625 | +229.163ADTGHK+229.163PYEYQEYGQK+229.163PYK+229.163
Scan Number: 10319 precMass: 644.75183 precCharge: 5 Sequence: ADTGHKPYEYQEYGQKPYK
Intensity
250000
200000
150000
100000
50000
0
M/Z
0
1000
2000
3000
y1
b2
b4
y3
y2
b5
type
b
y

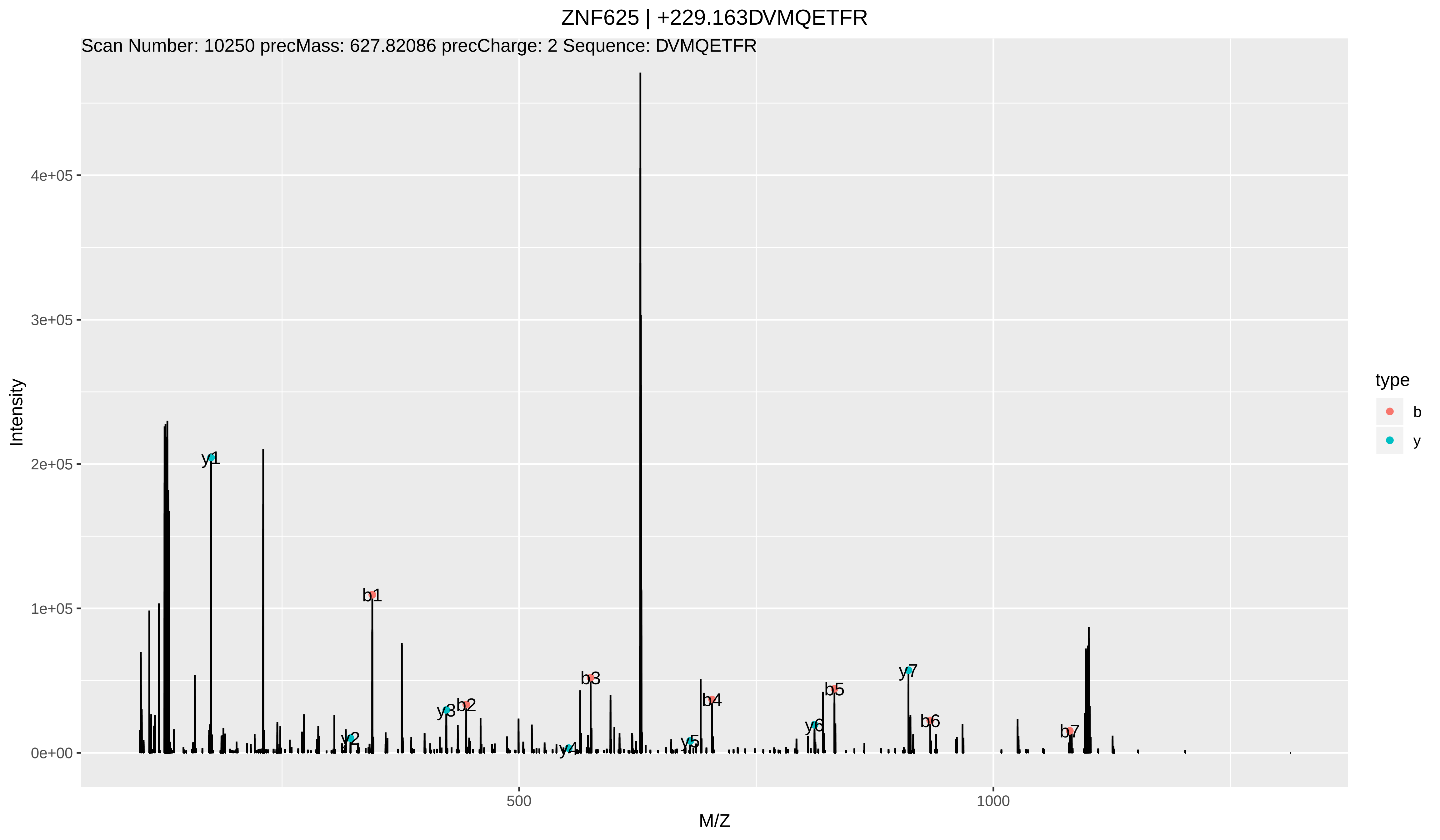
ZNF625 | +229.163DVMQETFR
Scan Number: 10250 precMass: 627.82086 precCharge: 2 Sequence: DVMQETFR
Intensity
4e+05
3e+05
2e+05
1e+05
0e+00
500
1000
M/Z
y1
y2
y3
y4
y5
y6
y7
b1
b2
b3
b4
b5
b6
b7
type
b
y

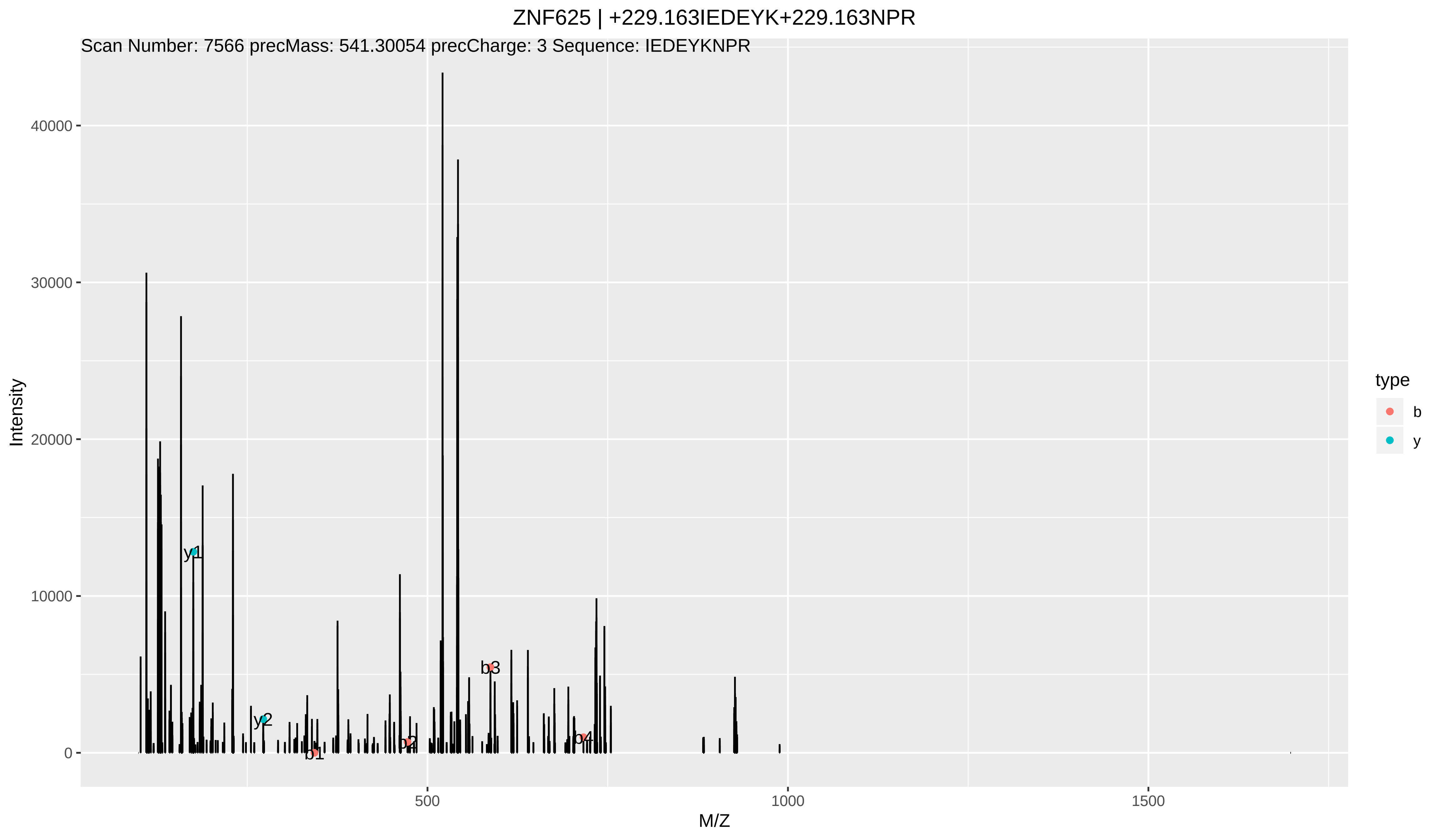
ZNF625 | +229.163IEDEYK+229.163NPR
Scan Number: 7566 precMass: 541.30054 precCharge: 3 Sequence: IEDEYKNPR
Intensity
40000
30000
20000
10000
0
500
1000
1500
M/Z
y1
y2
b1
b2
b3
b4
type
b
y

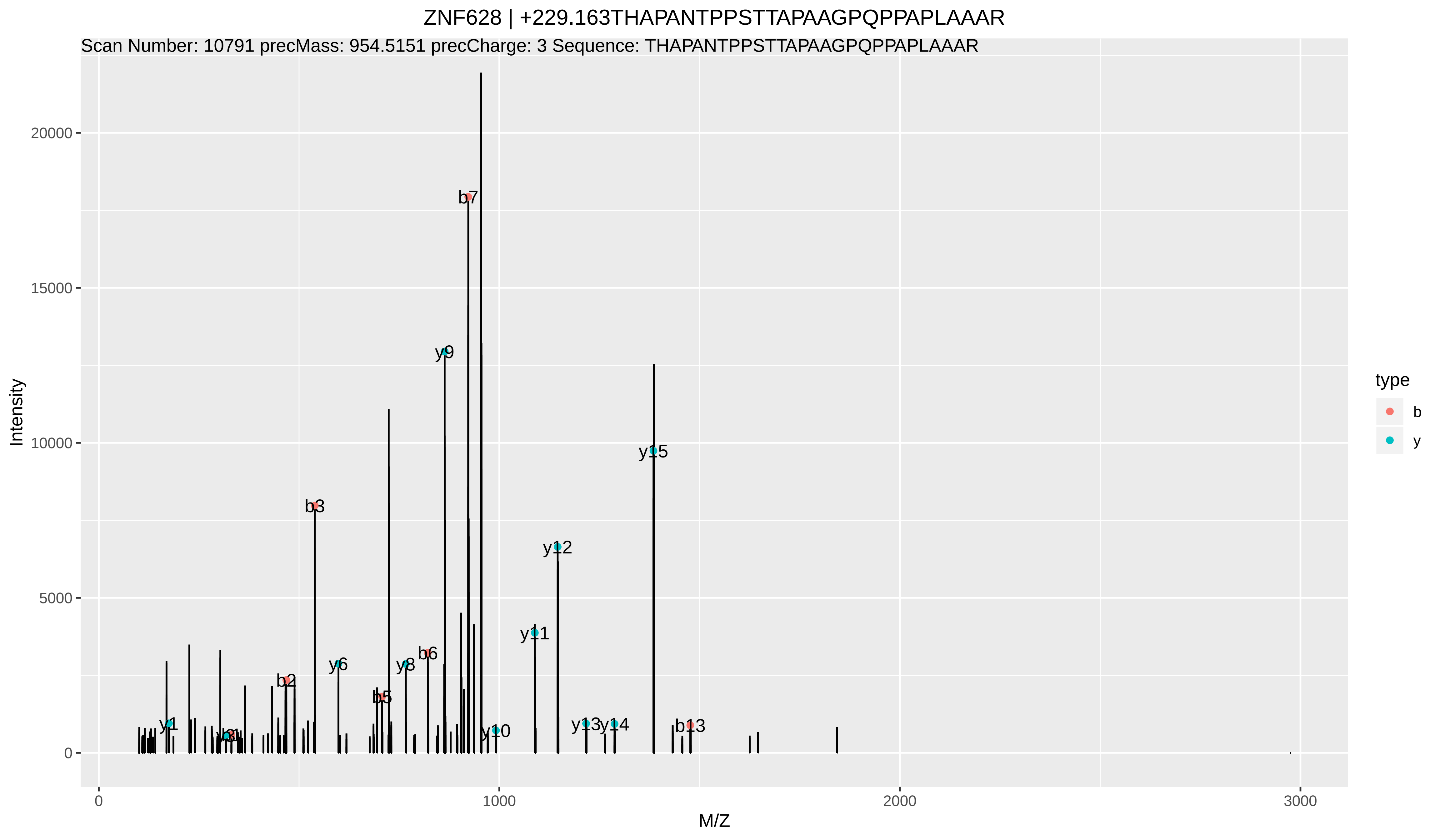

ZNF628 | +229.163THAPANTPPSTTAPAAGPQPPAPLAAAR
Scan Number: 10791 precMass: 954.5151 precCharge: 3 Sequence: THAPANTPPSTTAPAAGPQPPAPLAAAR
Intensity
M/Z
0
5000
10000
15000
20000
0
1000
2000
3000
b7
y9
y15
b3
y12
y11
b6
y6
y8
b2
b5
y1
y13
y14
b13
y10
type
b
y

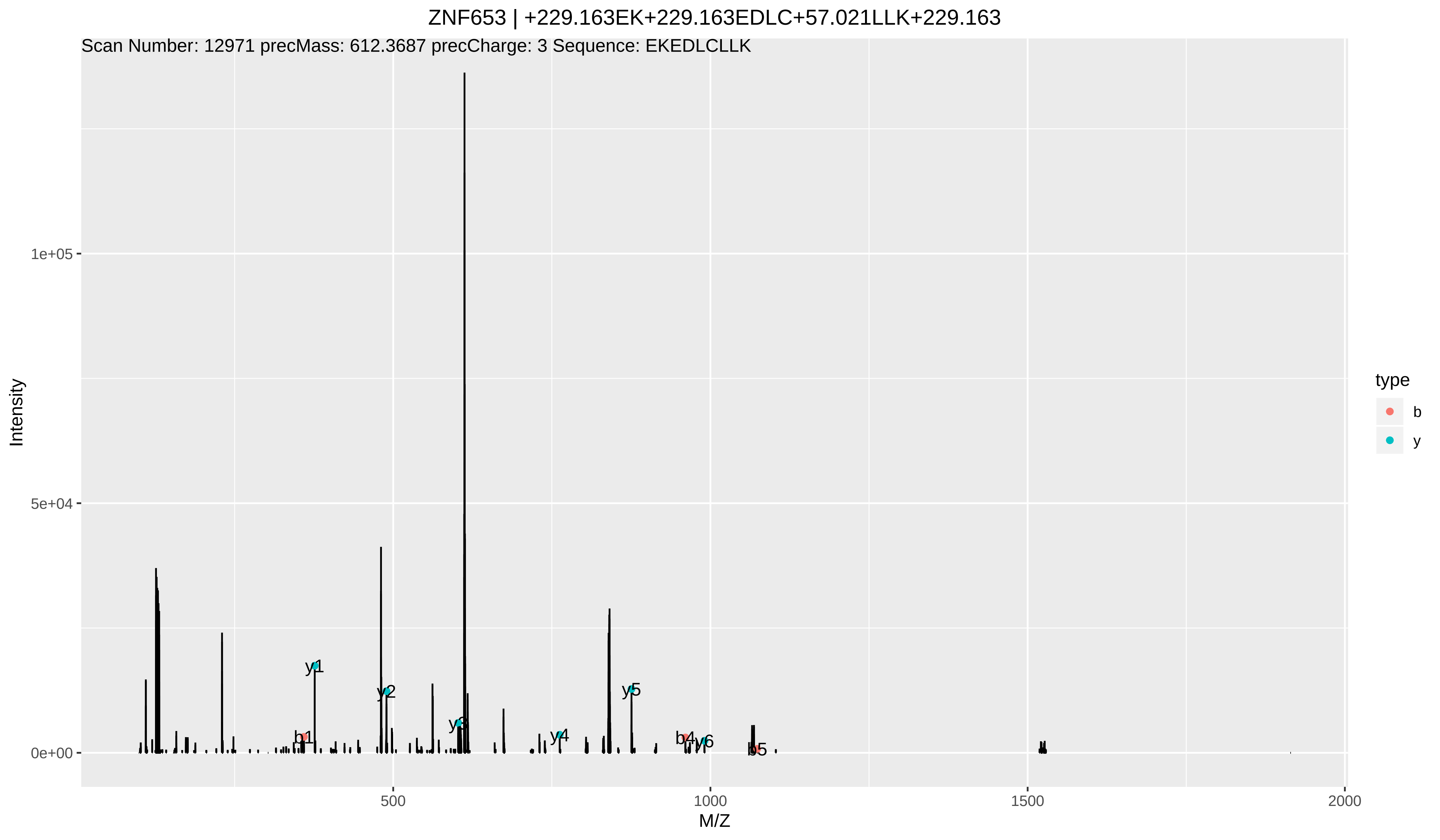

ZNF653 | +229.163EK+229.163EDLC+57.021LLK+229.163
Scan Number: 12971 precMass: 612.3687 precCharge: 3 Sequence: EKEDLCLLK
Intensity
1e+05
5e+04
0e+00
M/Z
500
1000
1500
2000
y1
y2
y3
y4
y5
y6
b1
b4
b5
type
b
y

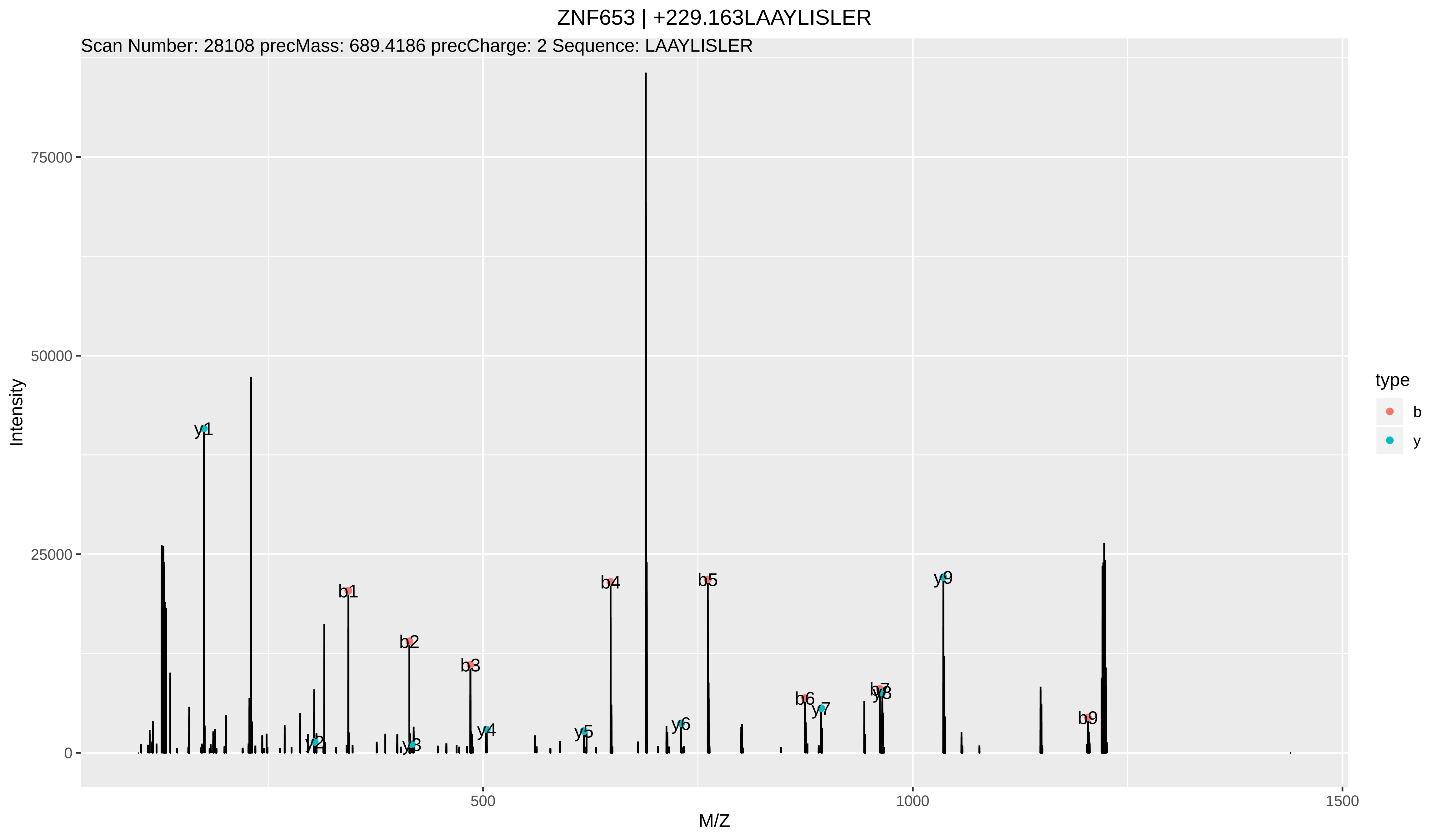

ZNF653 | +229.163LAAYLISLER
Scan Number: 28108 precMass: 689.4186 precCharge: 2 Sequence: LAAYLISLER
Intensity
75000
50000
25000
0
500
1000
1500
M/Z
type
b
y
y1
y2
y3
y4
y5
y6
y7
y8
y9
b1
b2
b3
b4
b5
b6
b7
b9

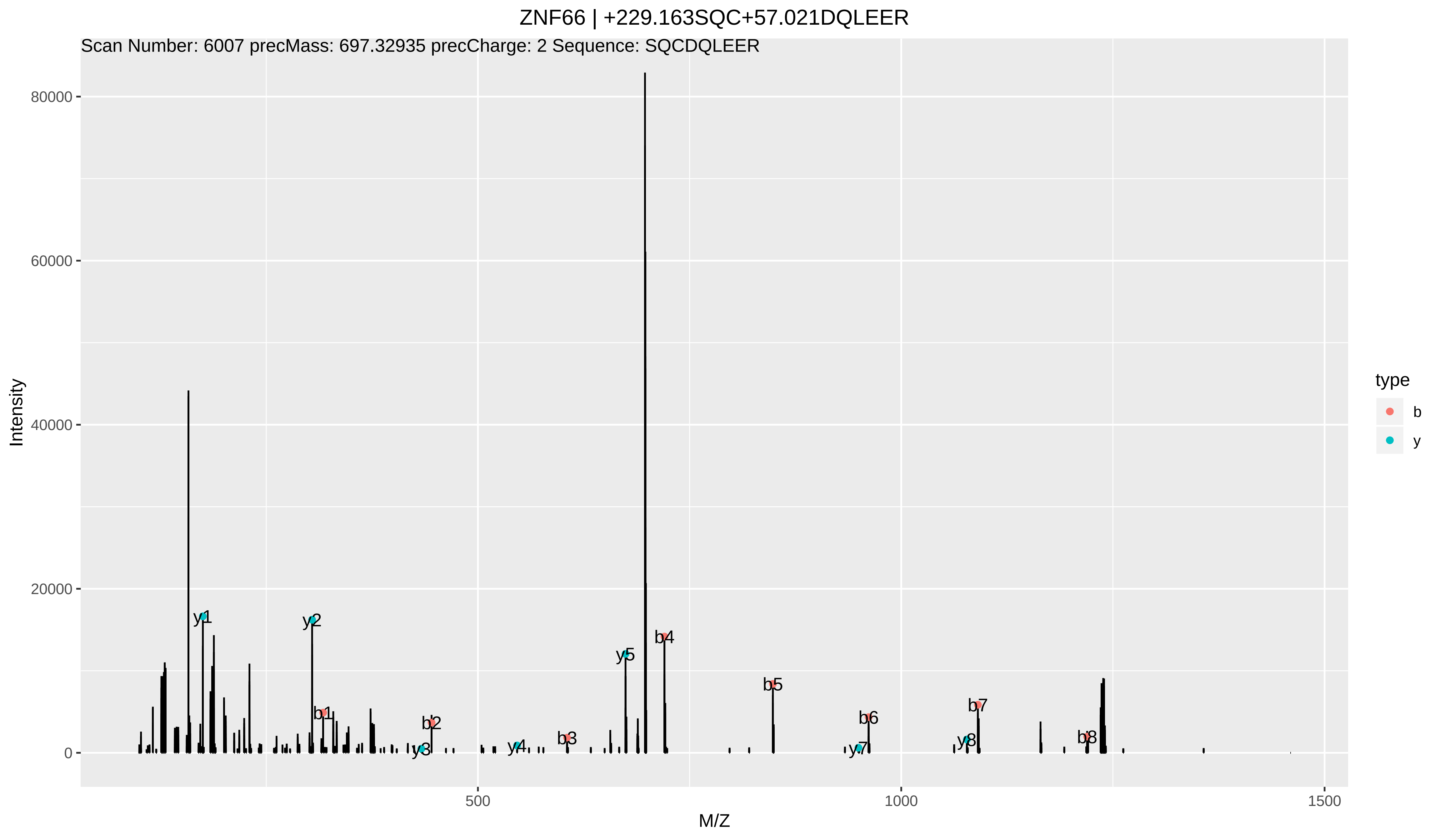

ZNF66 | +229.163SQC+57.021DQLEER
Scan Number: 6007 precMass: 697.32935 precCharge: 2 Sequence: SQCDQLEER
Intensity
80000
60000
40000
20000
0
500
1000
1500
M/Z
y1
y2
b1
b2
y3
y4
b3
y5
b4
b5
y7
b6
b7
y8
b8
type
b
y

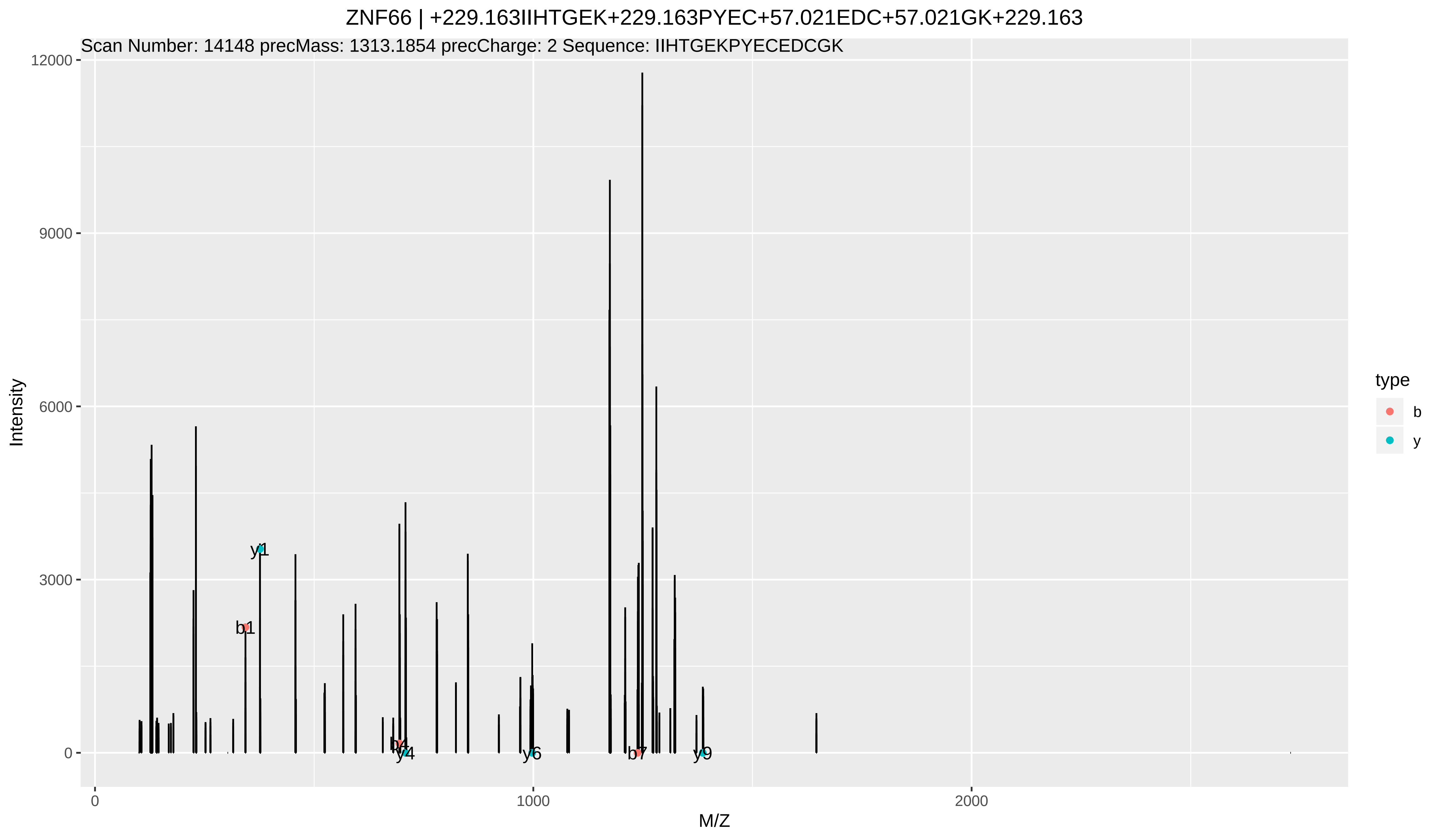
ZNF66 | +229.163IIHTGEK+229.163PYEC+57.021EDC+57.021GK+229.163
Scan Number: 14148 precMass: 1313.1854 precCharge: 2 Sequence: IIHTGEKPYECEDCGK
Intensity
M/Z
type
b
y
y1
b1
b4
y4
y6
b7
y9
0
3000
6000
9000
12000
1000
2000

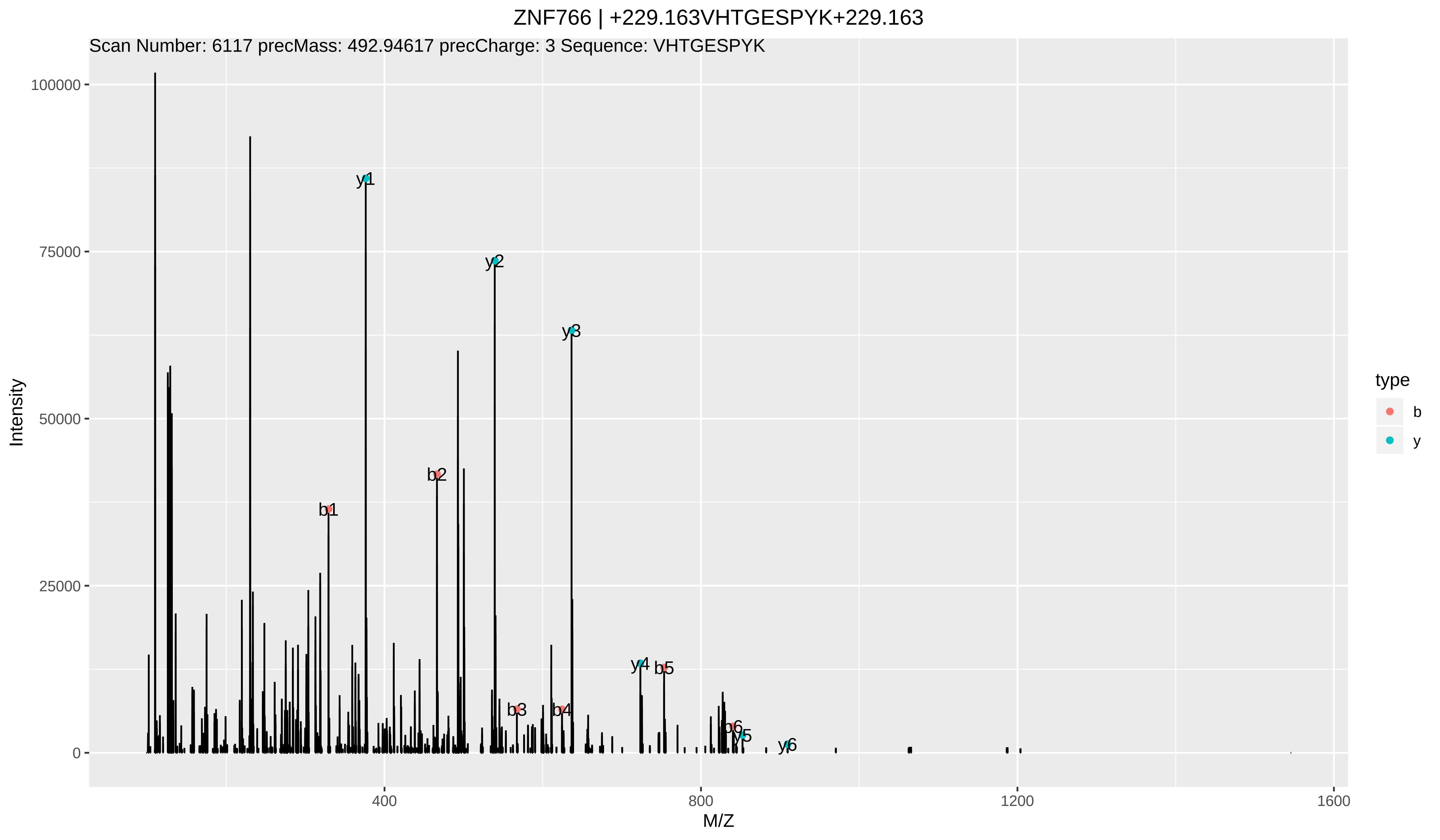

ZNF766 | +229.163VHTGESPYK+229.163
Scan Number: 6117 precMass: 492.94617 precCharge: 3 Sequence: VHTGESPYK
Intensity
100000
75000
50000
25000
0
400
800
1200
1600
M/Z
y1
y2
y3
b2
b1
y4
b5
b3
b4
b6
y5
y6
type
b
y

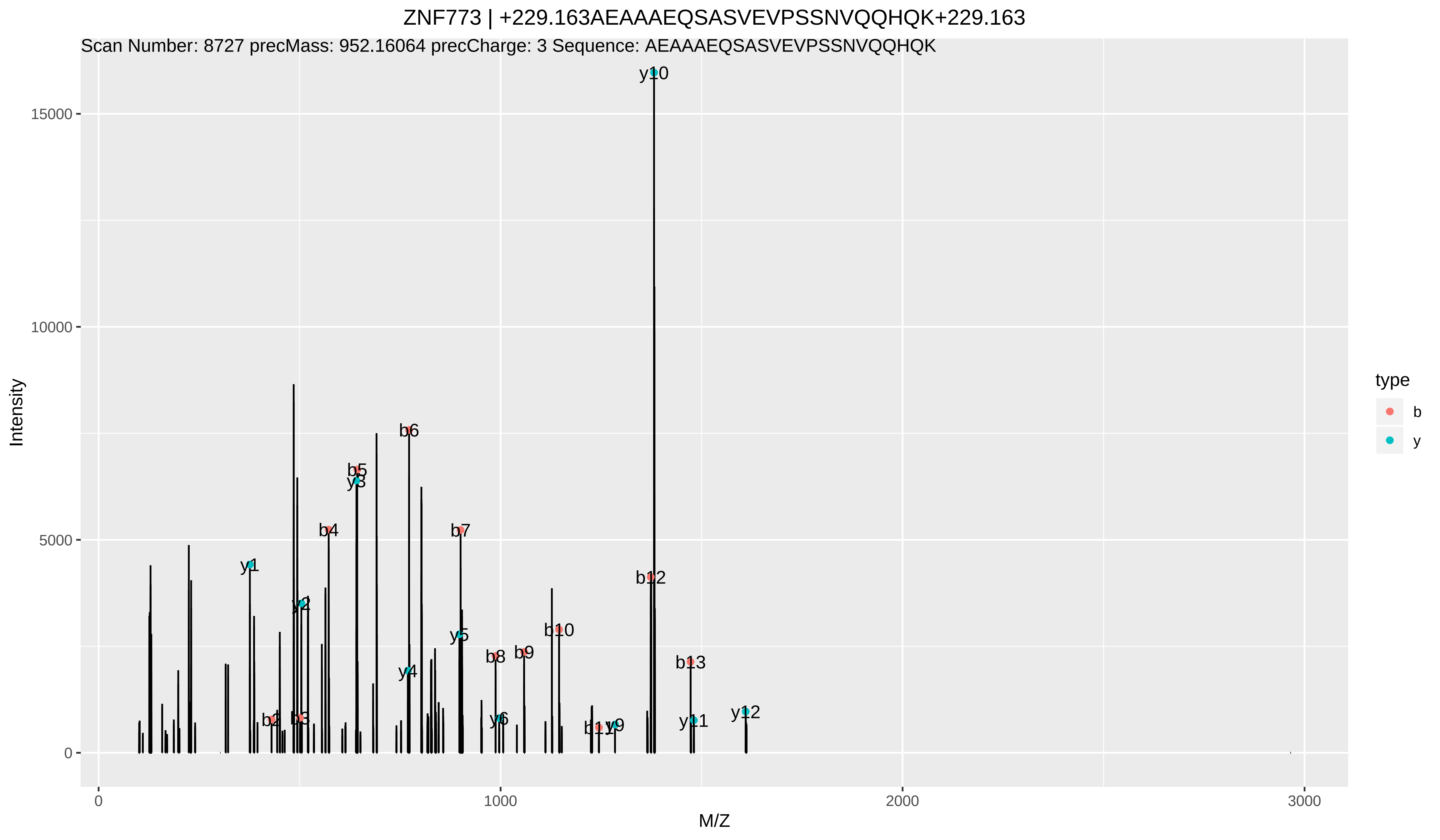
ZNF773 | +229.163AEAAAEQSASVEVPSSNVQQHQK+229.163
Scan Number: 8727 precMass: 952.16064 precCharge: 3 Sequence: AEAAAEQSASVEVPSSNVQQHQK
Intensity
M/Z
type
b
y
y10
b6
b5
y3
b4
b7
y1
b12
y2
y5
b10
b8
b9
b13
y4
y12
b2
b3
y6
b11
y9
y11
0
5000
10000
15000
0
1000
2000
3000

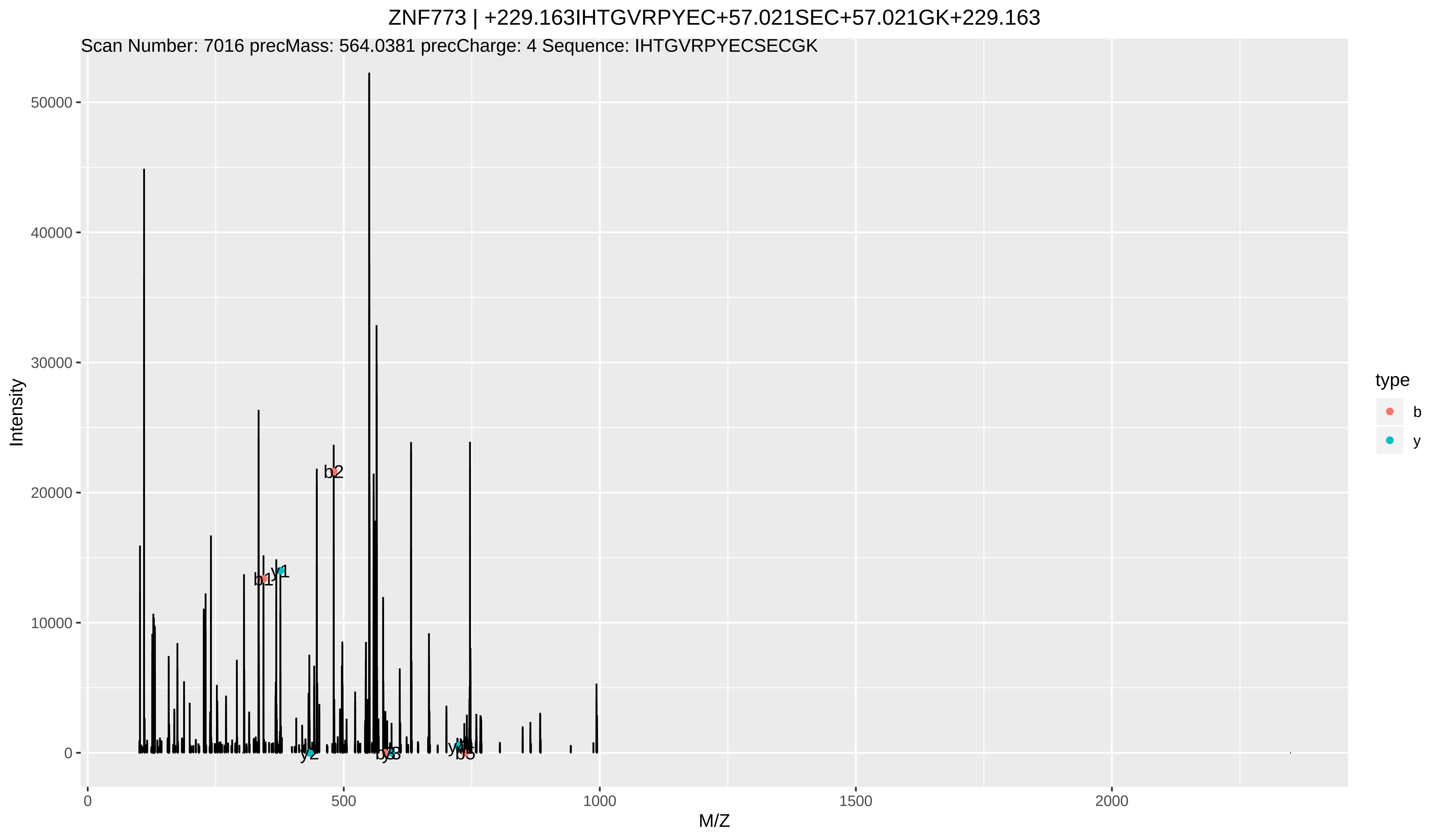
ZNF773 | +229.163IHTGVRPYEC+57.021SEC+57.021GK+229.163
Scan Number: 7016 precMass: 564.0381 precCharge: 4 Sequence: IHTGVRPYECSECGK
Intensity
M/Z
type
b
y
b1
y1
b2
y2
y3
b3
y4
b5
0
10000
20000
30000
40000
50000
0
500
1000
1500
2000

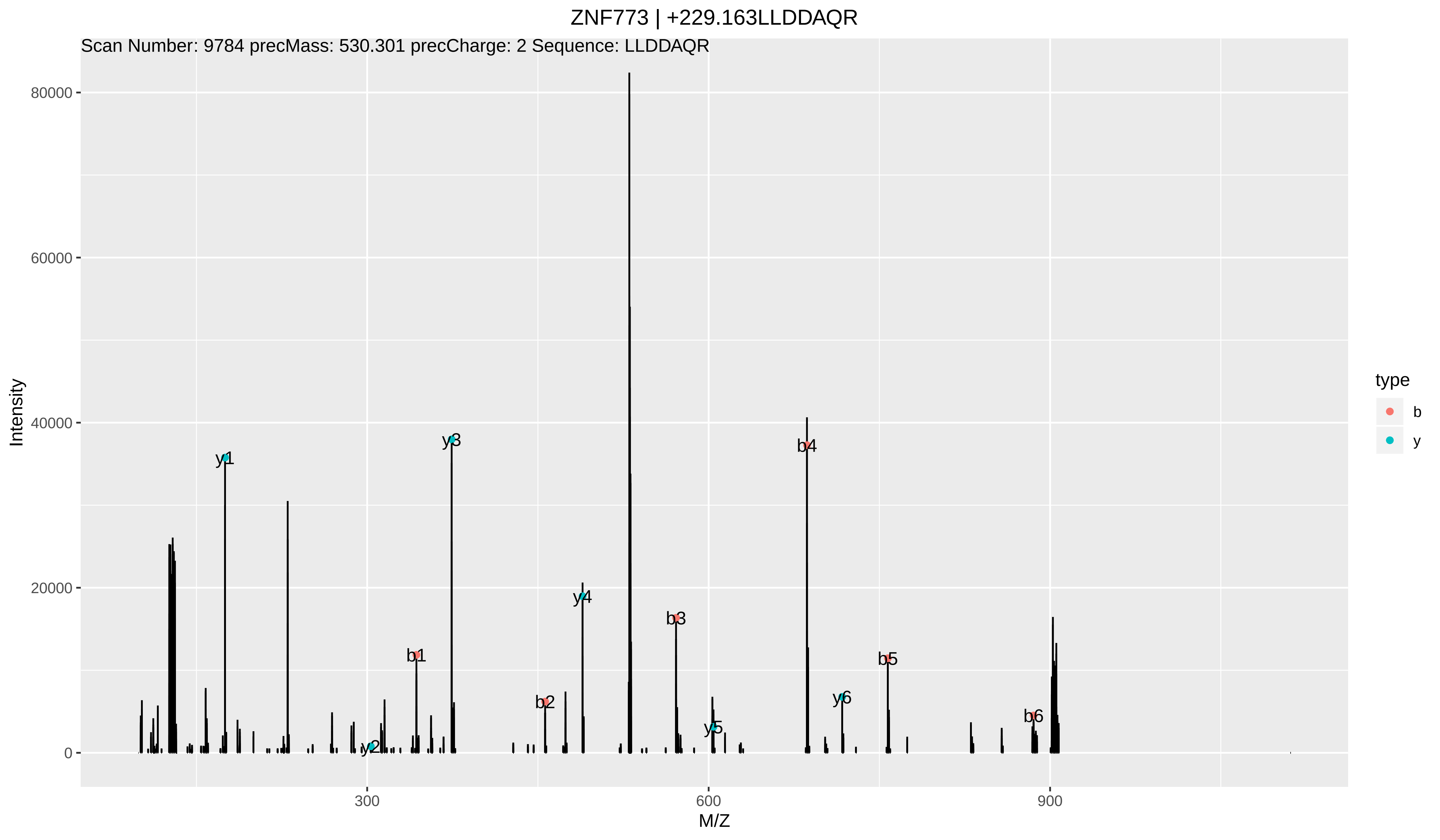
ZNF773 | +229.163LLDDAQR
Scan Number: 9784 precMass: 530.301 precCharge: 2 Sequence: LLDDAQR
Intensity
80000
60000
40000
20000
0
M/Z
300
600
900
y1
y2
y3
y4
y5
y6
b1
b2
b3
b4
b5
b6
type
b
y

ZNF773 | +229.163VHTGERPYEC+57.021SEC+57.021GK+229.163

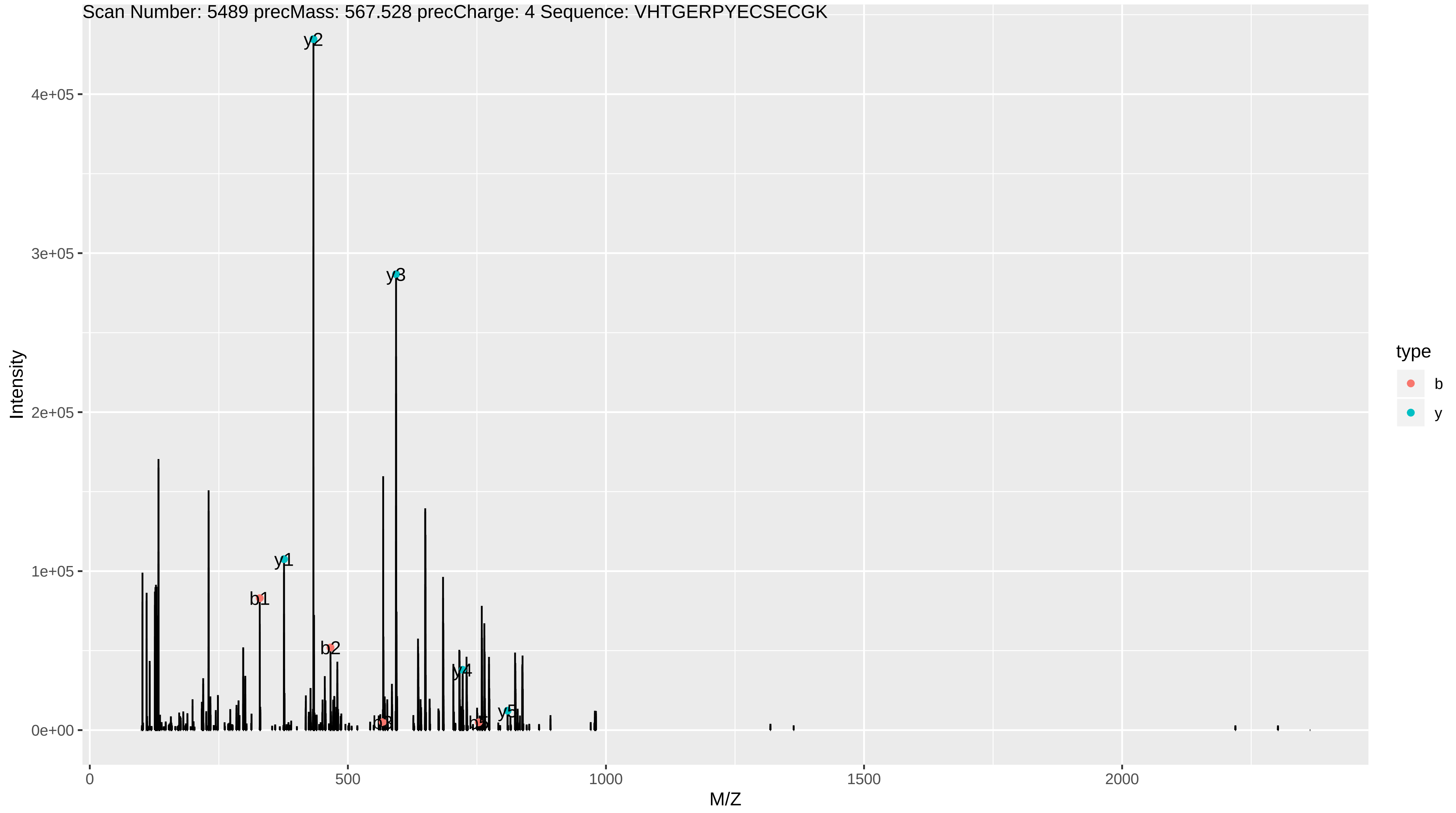

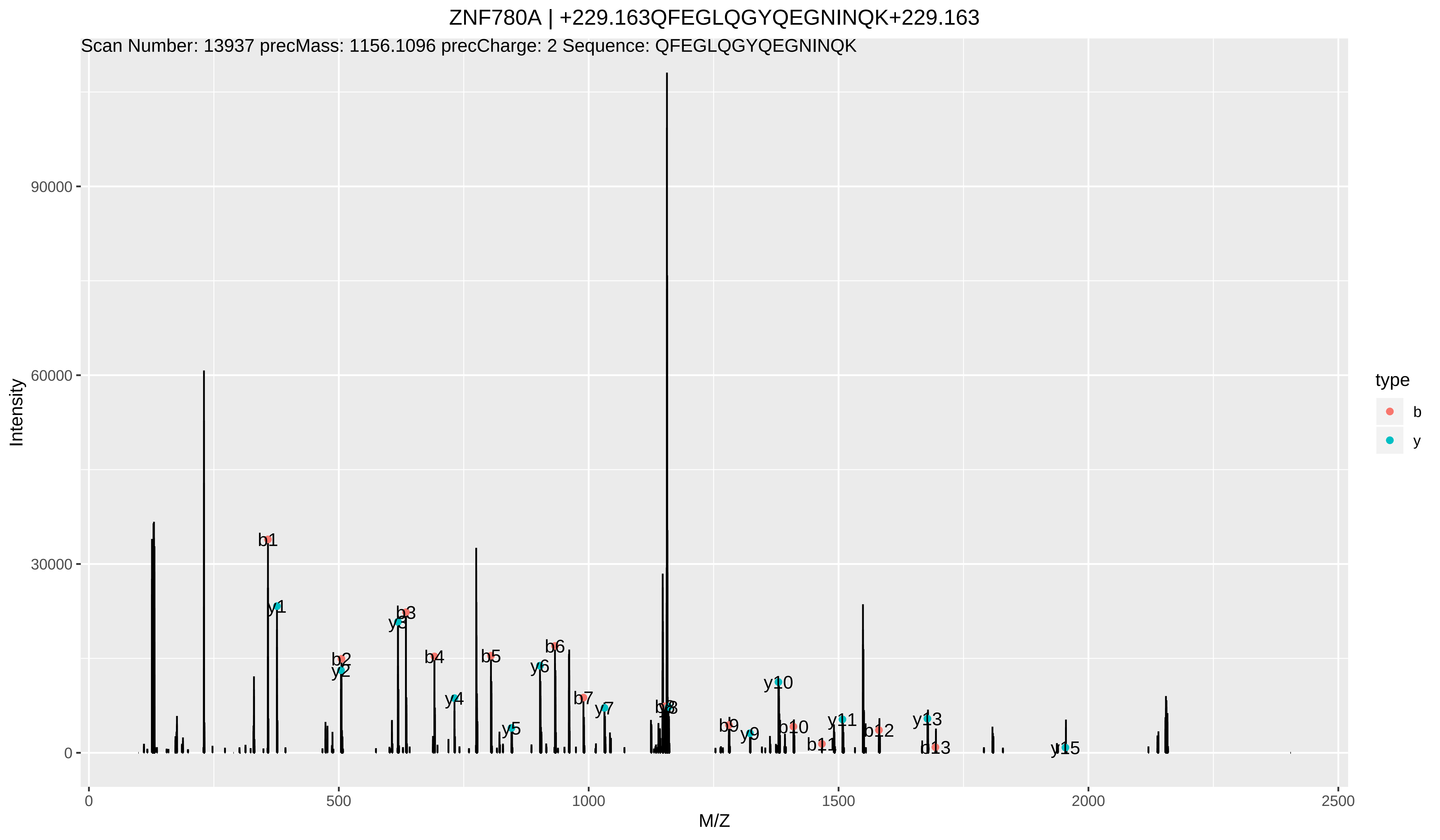
ZNF780A | +229.163QFEGLQGYQEGNINQK+229.163
Scan Number: 13937 precMass: 1156.1096 precCharge: 2 Sequence: QFEGLQGYQEGNINQK
Intensity
M/Z
type
b
y
b1
y1
b2
y2
y3
b3
b4
y4
b5
y5
y6
b6
b7
y7
y8
b8
b9
y9
y10
b10
b11
y11
b12
y13
b13
y15
0
30000
60000
90000
0
500
1000
1500
2000
2500

ZNF862 | +229.163YFEC+57.021SLPTGYSEEALLEEWLGLK+229.163

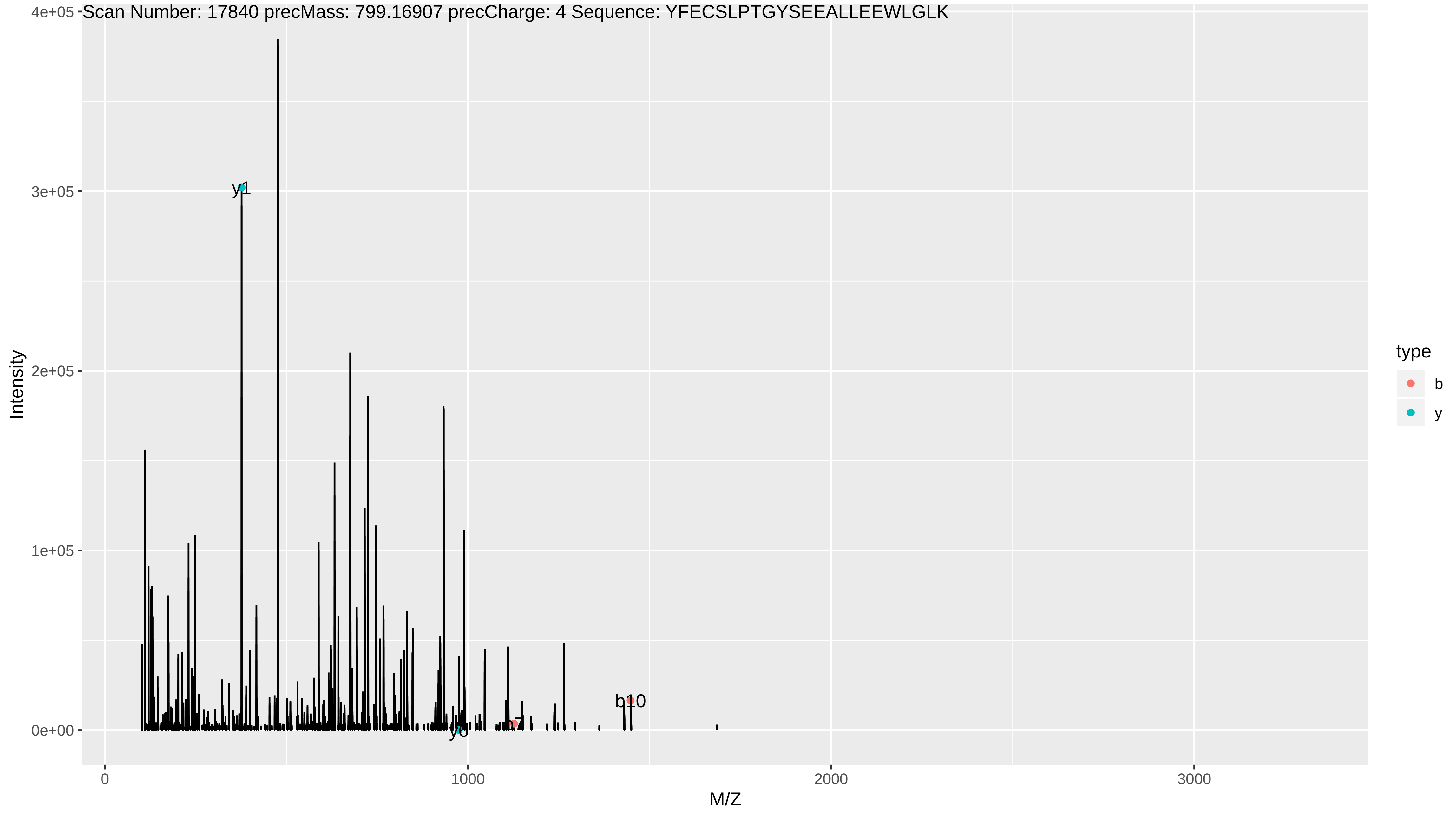

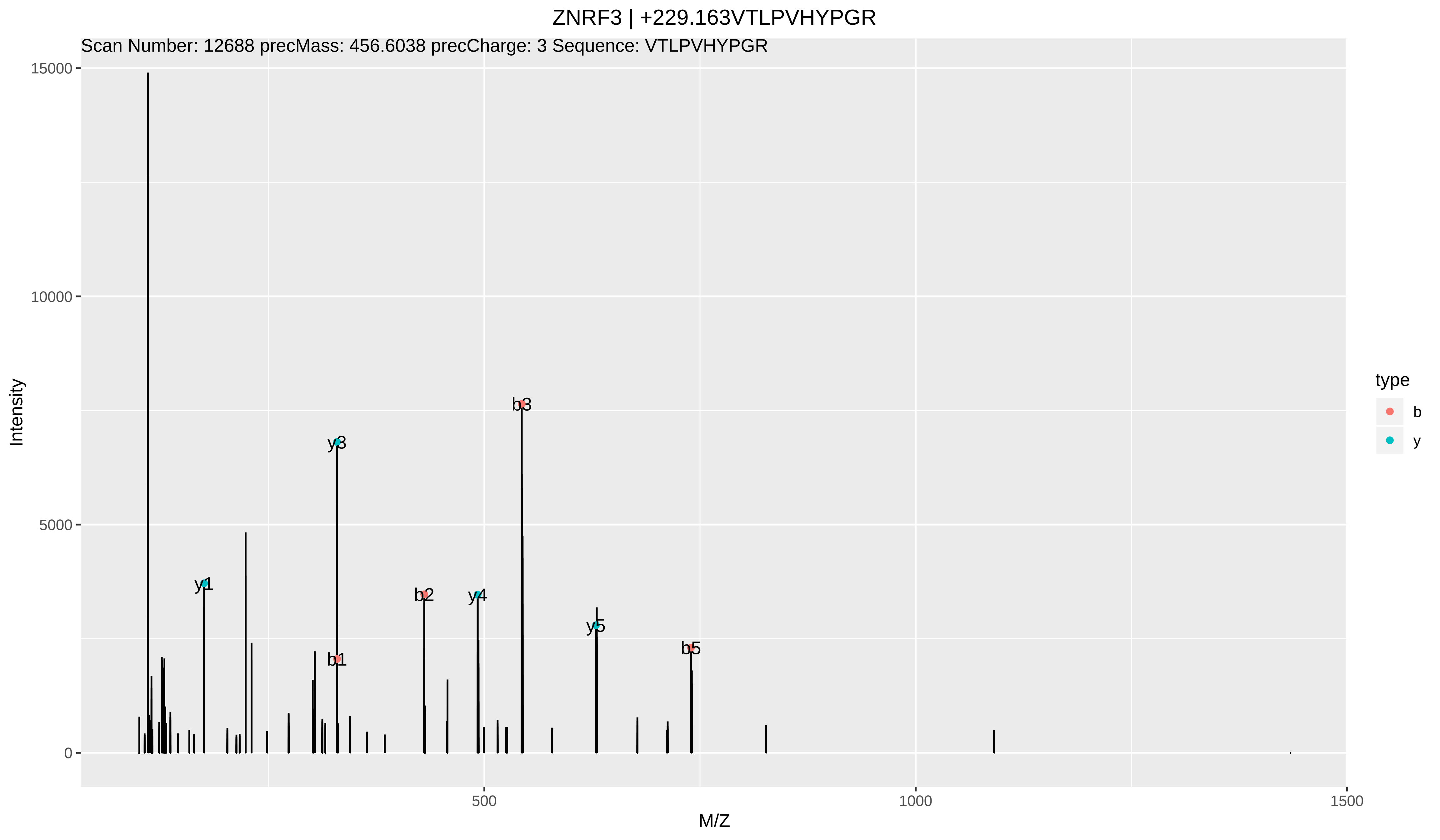
ZNRF3 | +229.163VTLPVHYPGR
Scan Number: 12688 precMass: 456.6038 precCharge: 3 Sequence: VTLPVHYPGR
Intensity
15000
10000
5000
0
M/Z
500
1000
1500
y1
y3
b1
b2
y4
b3
y5
b5
type
b
y

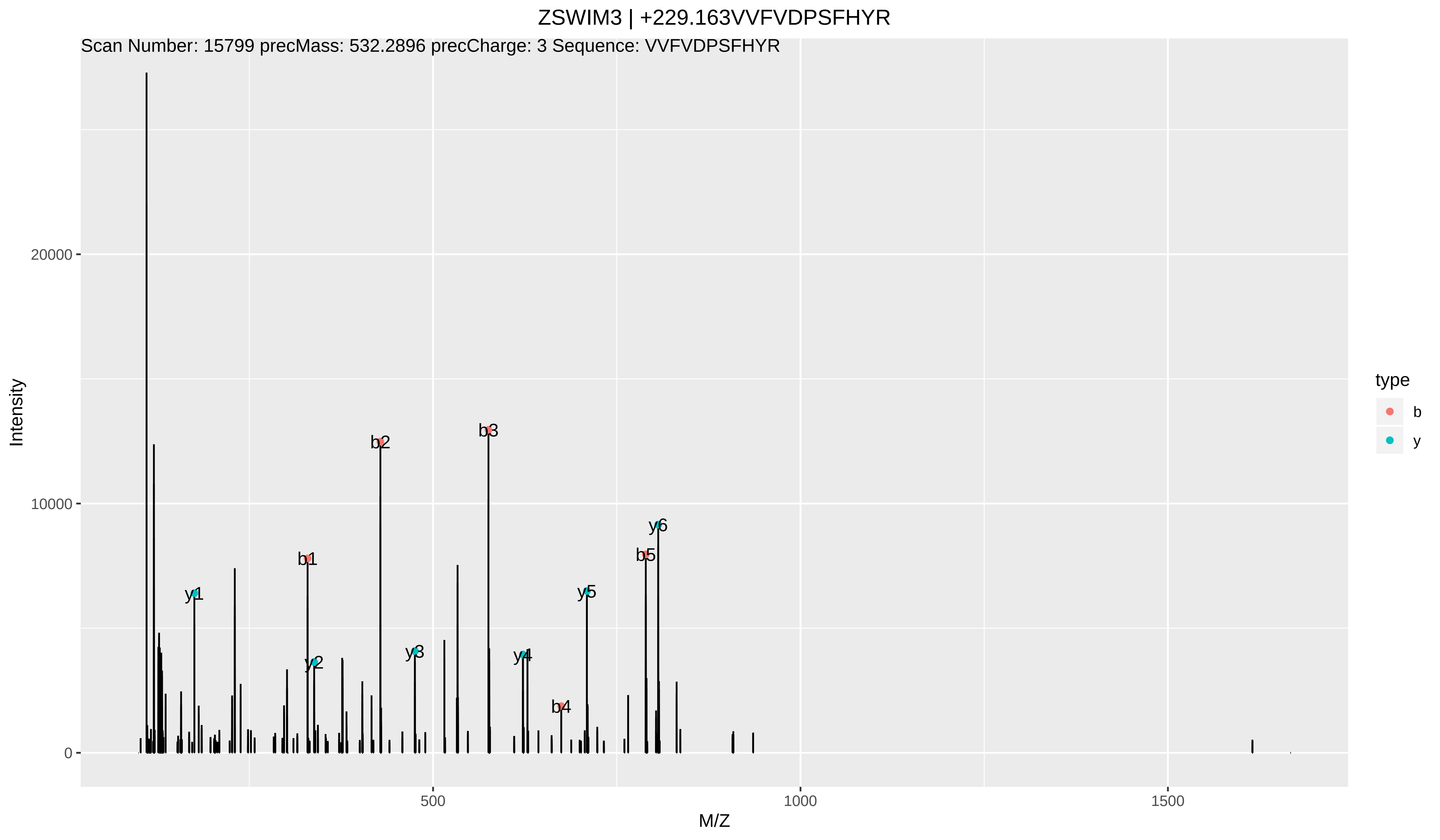

ZSWIM3 | +229.163VVFVDPSFHYR
Scan Number: 15799 precMass: 532.2896 precCharge: 3 Sequence: VVFVDPSFHYR
Intensity
20000
10000
0
M/Z
500
1000
1500
y1
b1
y2
b2
y3
b3
y4
b4
y5
b5
y6
type
b
y
