# Supplementary material for: Immediate Adaptation Analysis Implicates BCL6 as an EGFR-TKI Combination Therapy Target in NSCLC
Source: Mol Cell Proteomics. 2020 Mar 30;19(6):928–43. doi: 10.1074/mcp.RA120.002036 (PMC7261823; doi:10.1074/mcp.RA120.002036)
Supplement: Annotated spectra [file PSM.annotated.spectra_siRNA_BCL6_set2.pdf › PSM.annotated.spectra_siRNA_BCL6_set2.pdf]

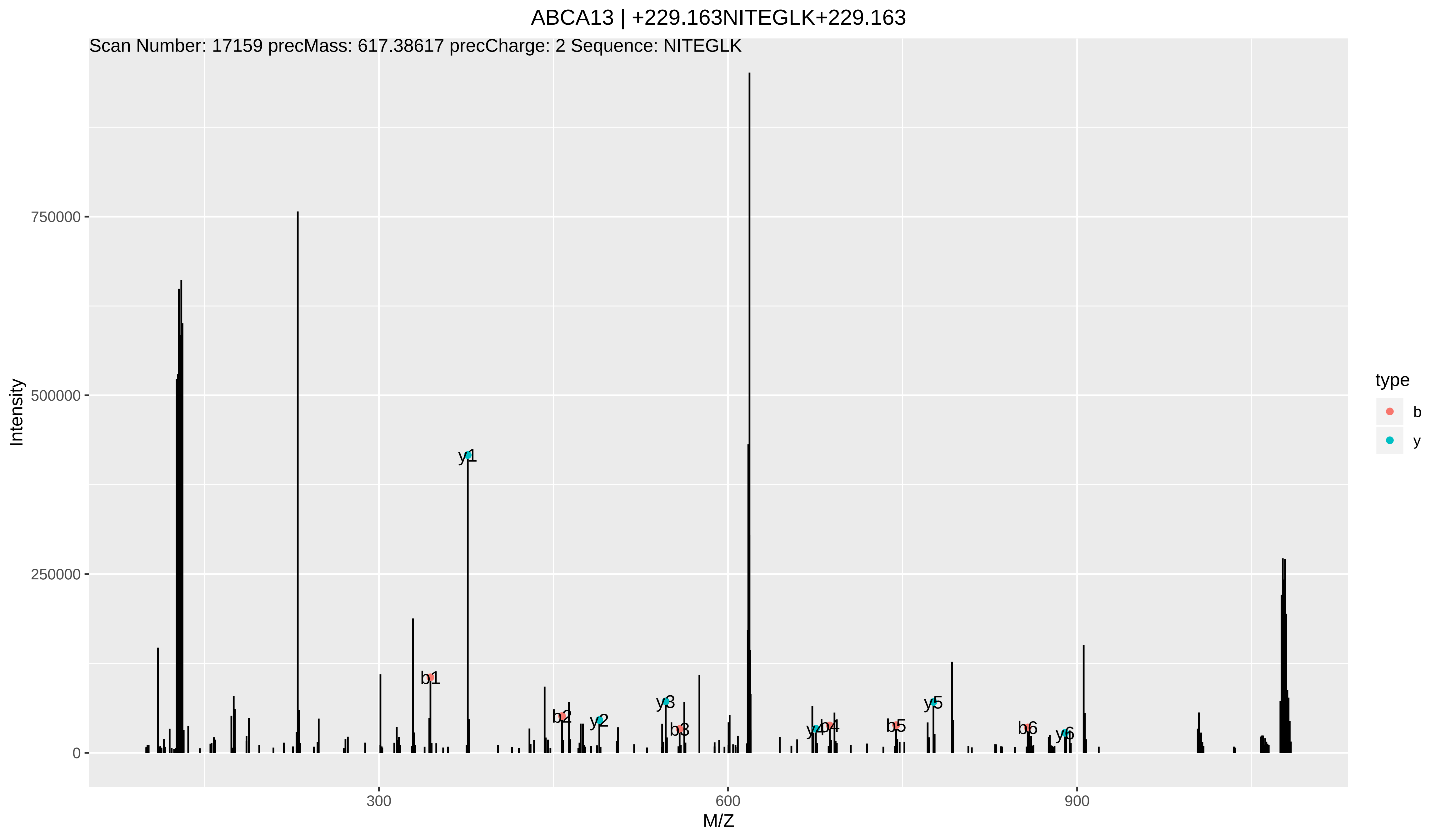
ABCA13 | +229.163NITEGLK+229.163
Scan Number: 17159 precMass: 617.38617 precCharge: 2 Sequence: NITEGLK
Intensity
750000
500000
250000
0
M/Z
300
600
900
y1
b1
b2
y2
y3
b3
y4
b4
b5
y5
b6
y6
type
b
y

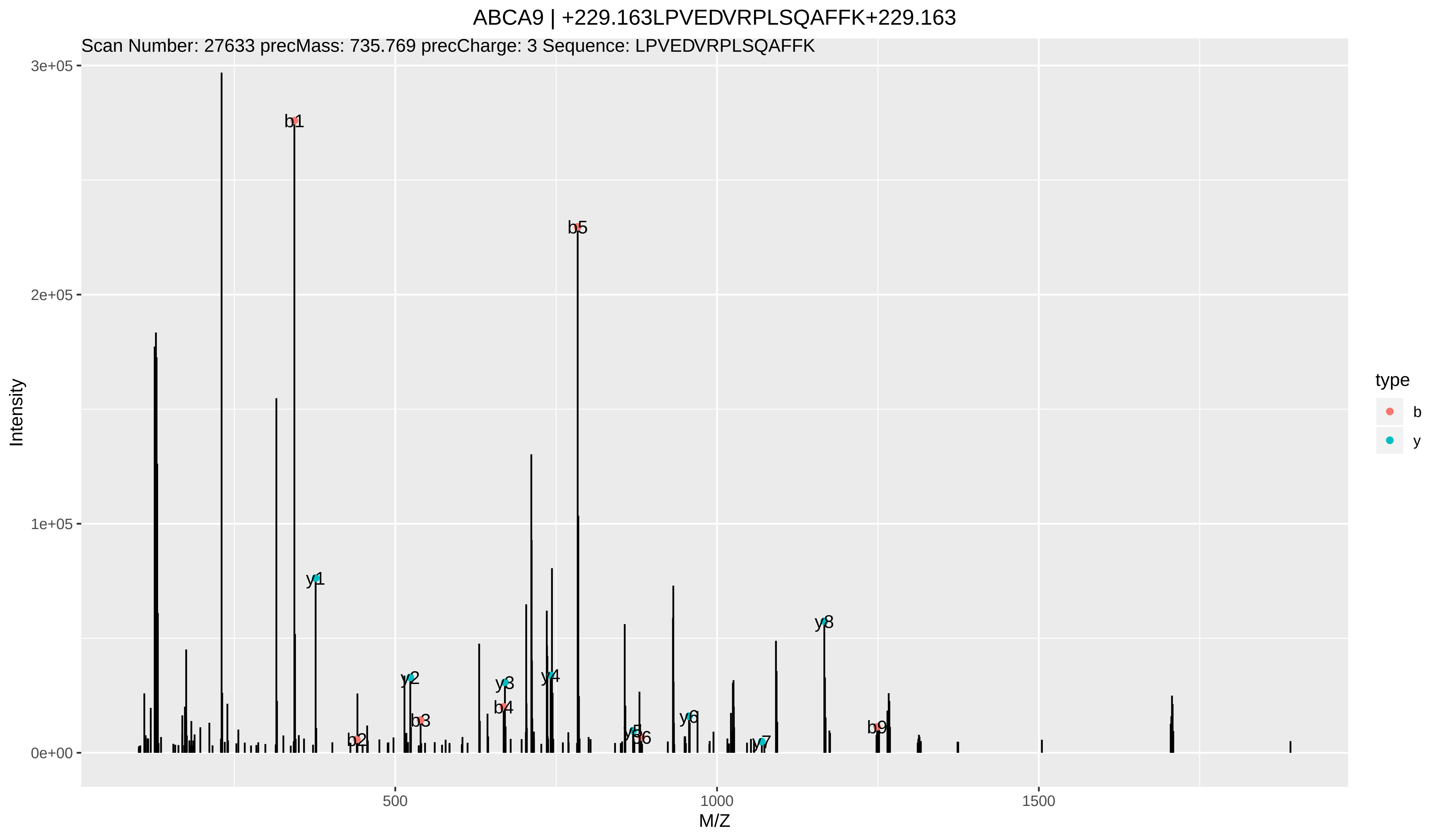

ABCA9 | +229.163LPVEDVRPLSQAFFK+229.163
Scan Number: 27633 precMass: 735.769 precCharge: 3 Sequence: LPVEDVRPLSQAFFK
Intensity
M/Z
3e+05
2e+05
1e+05
0e+00
500
1000
1500
b1
b5
y1
y8
y2
y3
y4
b3
b4
y6
b9
y5
b6
b2
y7
type
b
y

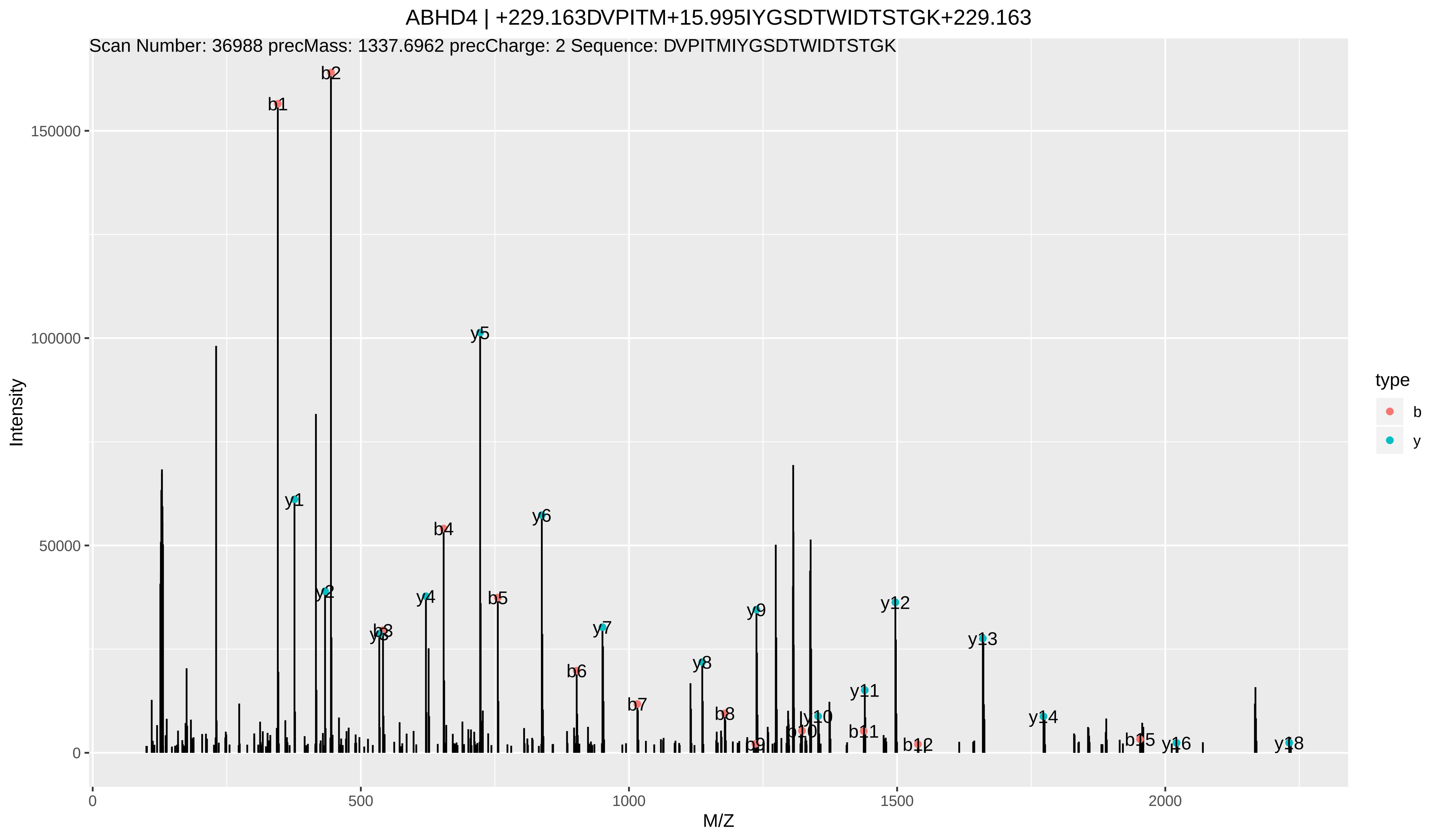

ABHD4 | +229.163DVPITM+15.995IYGSDTWIDTSTGK+229.163
Scan Number: 36988 precMass: 1337.6962 precCharge: 2 Sequence: DVPITMIYGSDTWIDTSTGK
Intensity
M/Z
type
b
y
b1
b2
y1
y2
b3
y3
y4
b4
y5
b5
y6
b6
y7
b7
y8
b8
y9
b9
b10
y10
y11
b11
y12
b12
y13
y14
b15
y16
y18
0
50000
100000
150000
0
500
1000
1500
2000

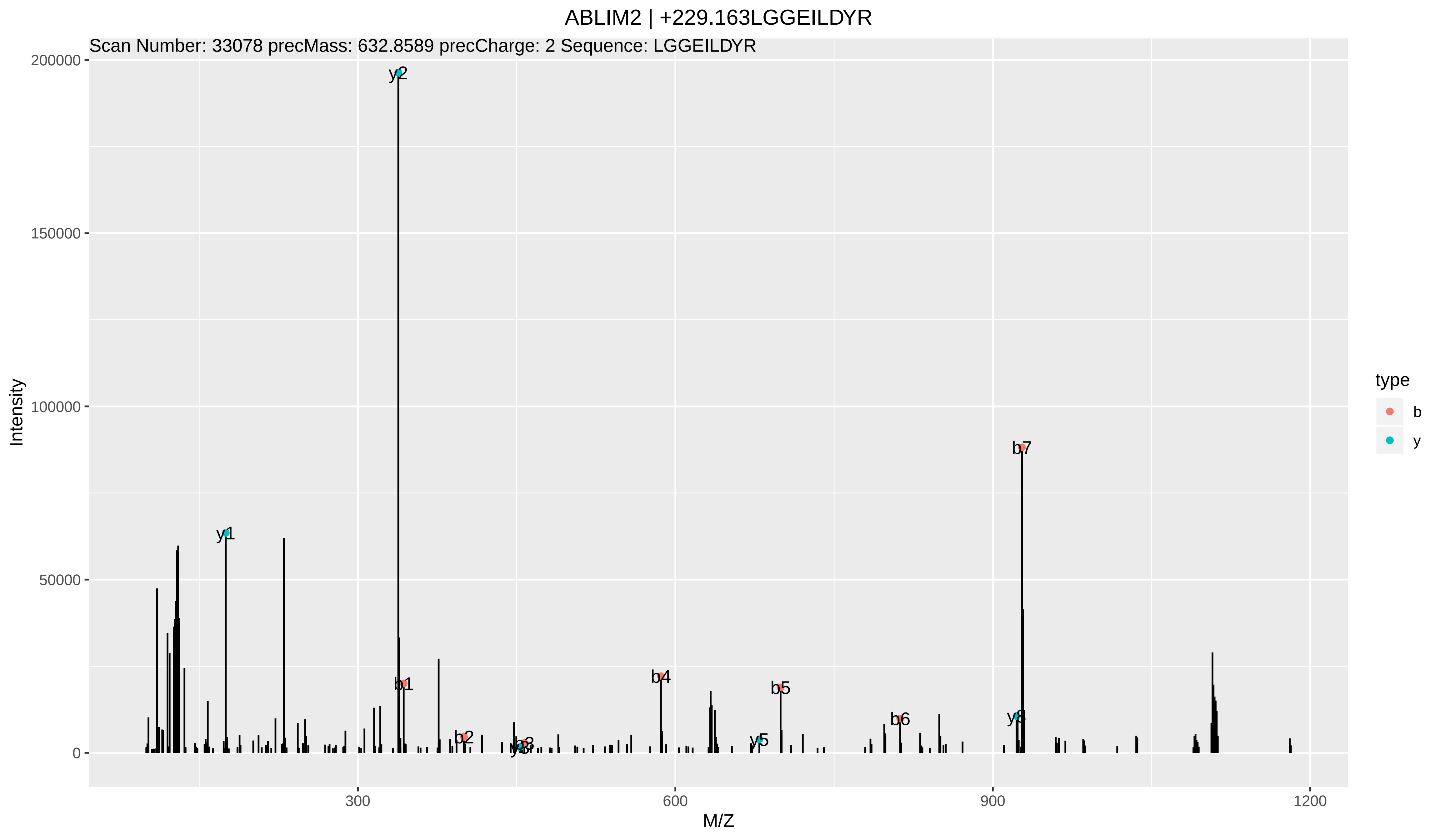

ABLIM2 | +229.163LGGEILDYR
Scan Number: 33078 precMass: 632.8589 precCharge: 2 Sequence: LGGEILDYR
Intensity
200000
150000
100000
50000
0
M/Z
300
600
900
1200
y2
y1
b1
b2
b3
y3
b4
y5
b5
b6
y8
b7
type
b
y

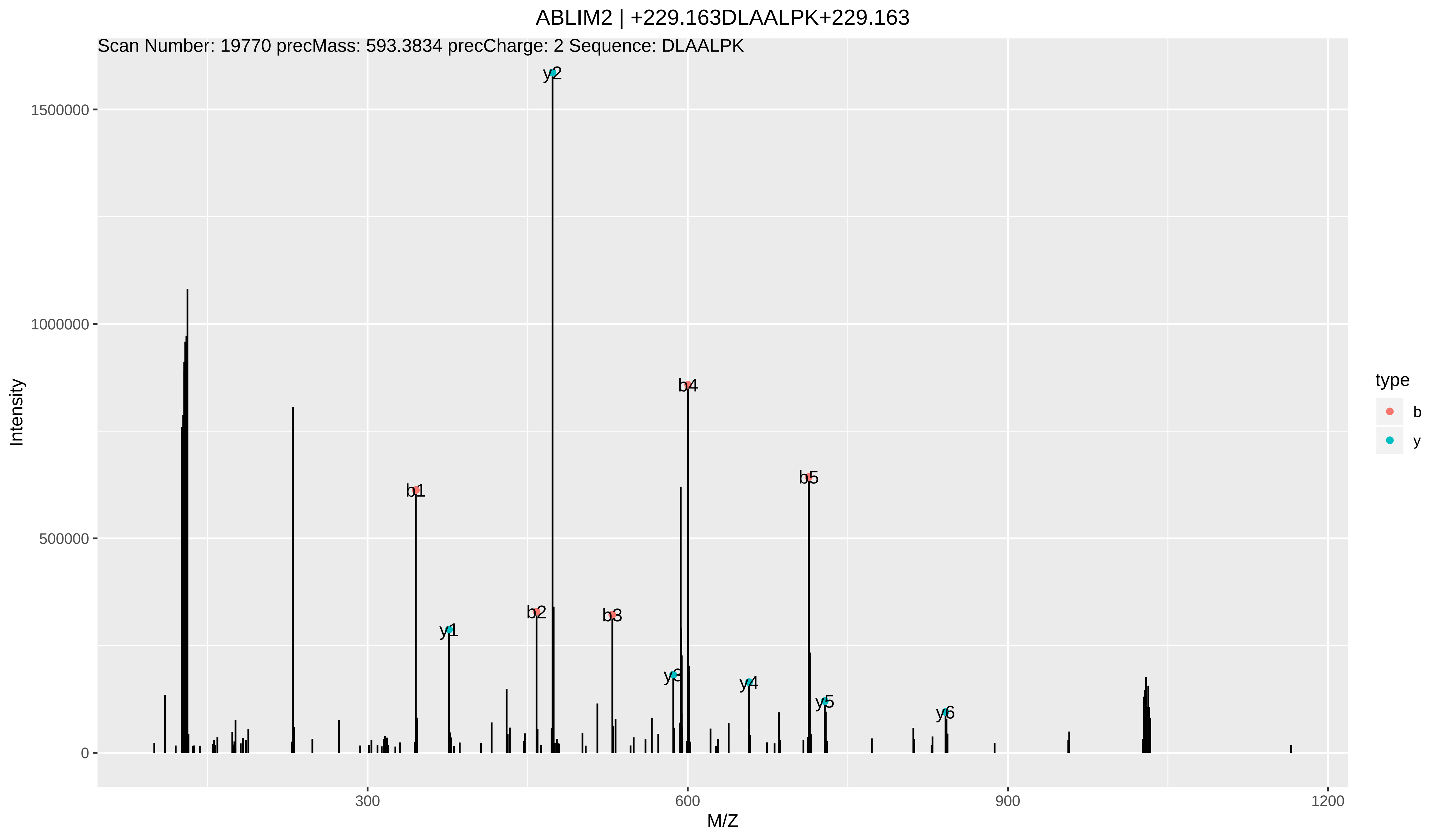

ABLIM2 | +229.163DLAALPK+229.163
Scan Number: 19770 precMass: 593.3834 precCharge: 2 Sequence: DLAALPK
y2
b4
b5
b1
b2
b3
y1
y3
y4
y5
y6
1500000
1000000
500000
0
300
600
900
1200
M/Z
Intensity
type
b
y

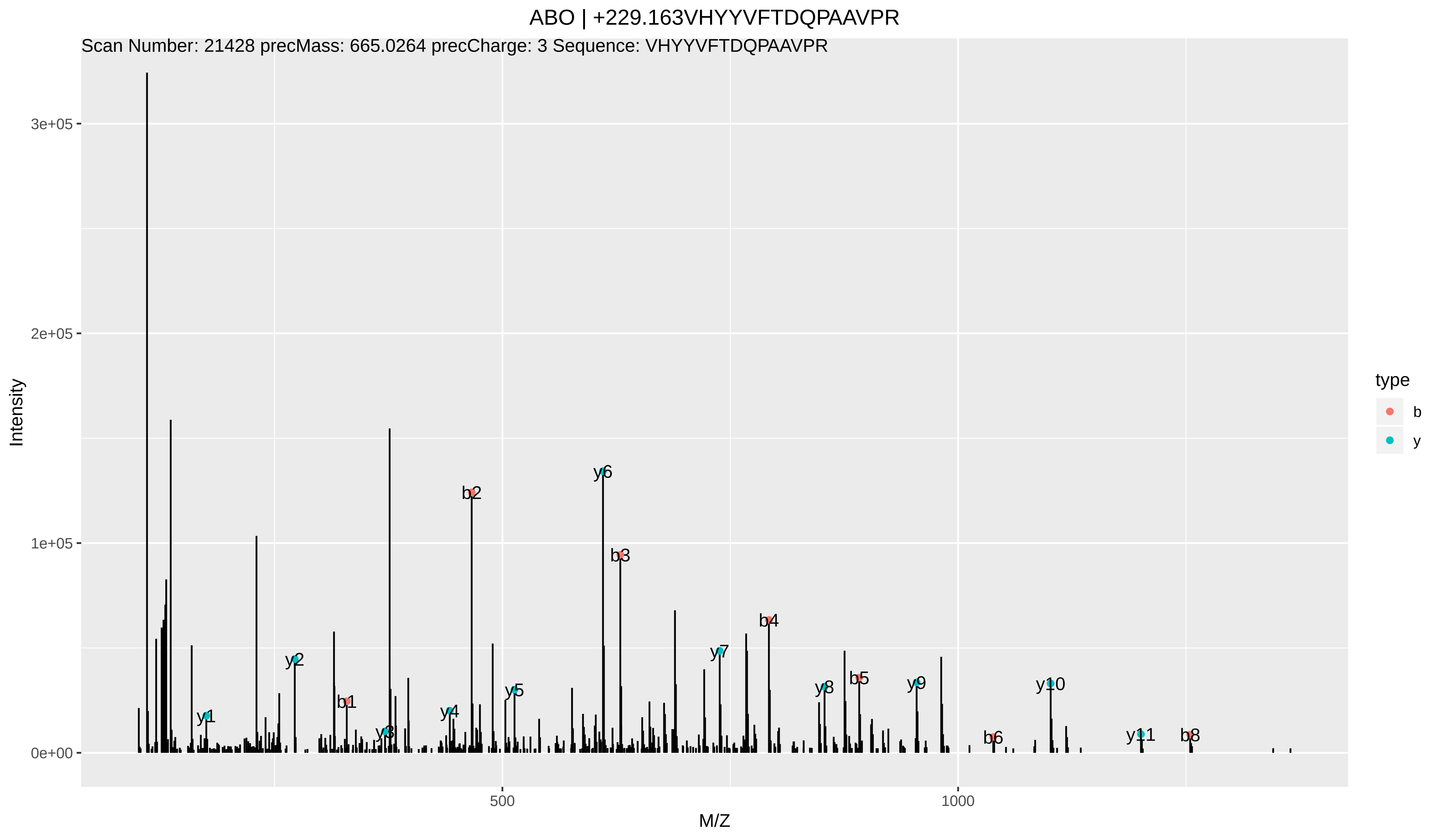

ABO | +229.163VHYYVFTDQPAAVPR
Scan Number: 21428 precMass: 665.0264 precCharge: 3 Sequence: VHYYVFTDQPAAVPR
Intensity
3e+05
2e+05
1e+05
0e+00
M/Z
500
1000
y1
y2
b1
y3
y4
b2
y5
y6
b3
y7
b4
y8
b5
y9
b6
y10
y11
b8
type
b
y

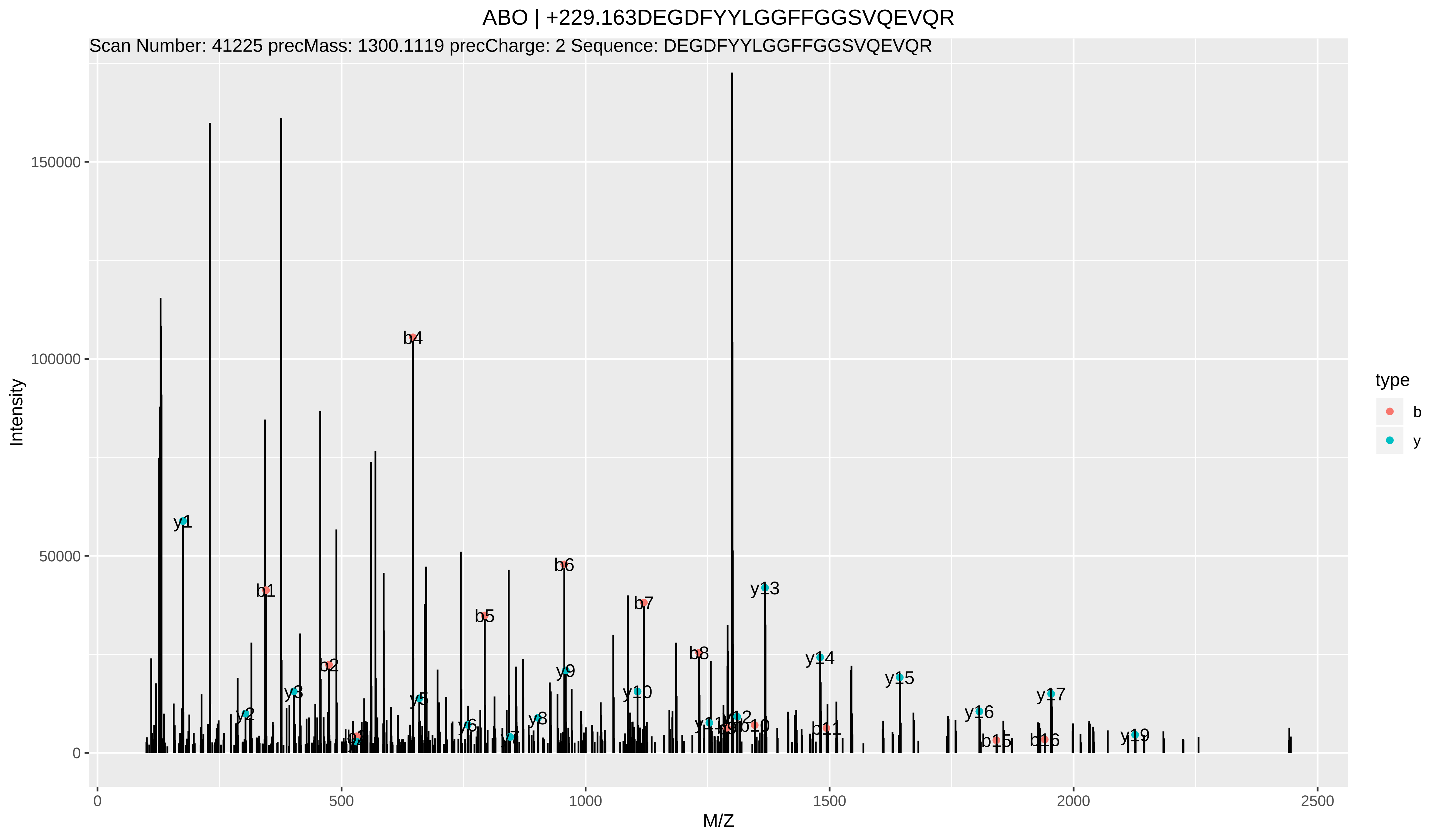
ABO | +229.163DEGDFYYLGGFFGGSVQEVQR
Scan Number: 41225 precMass: 1300.1119 precCharge: 2 Sequence: DEGDFYYLGGFFGGSVQEVQR
Intensity
M/Z
type
b
y
150000
100000
50000
0
0
500
1000
1500
2000
2500
y1
b1
y2
y3
b2
b4
y5
y6
b5
y8
b6
y9
y10
b7
b8
y13
y14
y15
y16
y17
y19
b15
b16
b11
b10

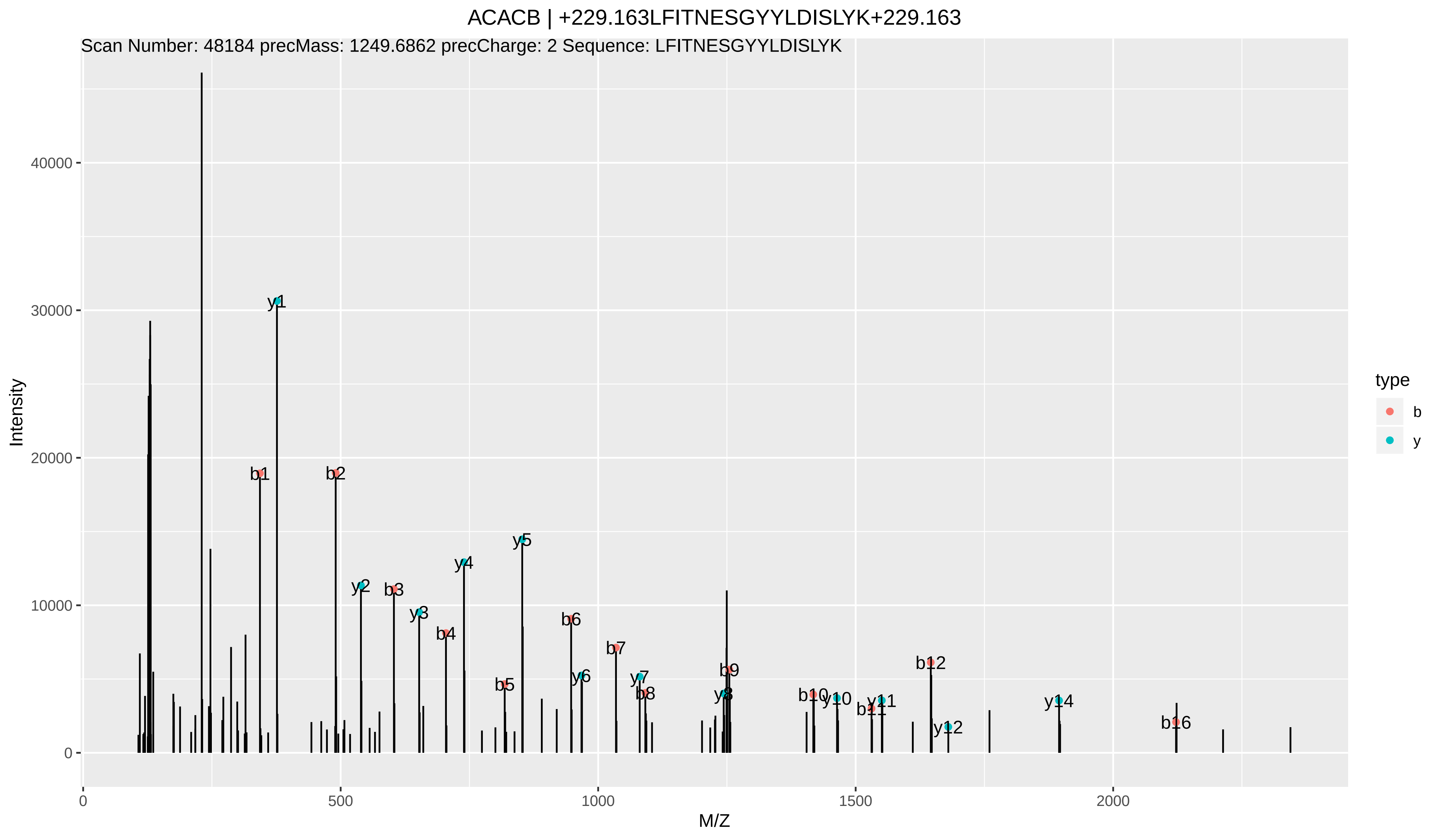

ACACB | +229.163LFITNESGYYLDISLYK+229.163
Scan Number: 48184 precMass: 1249.6862 precCharge: 2 Sequence: LFITNESGYYLDISLYK
Intensity
0
10000
20000
30000
40000
M/Z
0
500
1000
1500
2000
y1
b1
b2
y2
b3
y3
b4
y4
y5
b5
b6
y6
b7
y7
b8
y8
b9
b10
y10
b11
y11
b12
y12
y14
b16
type
b
y

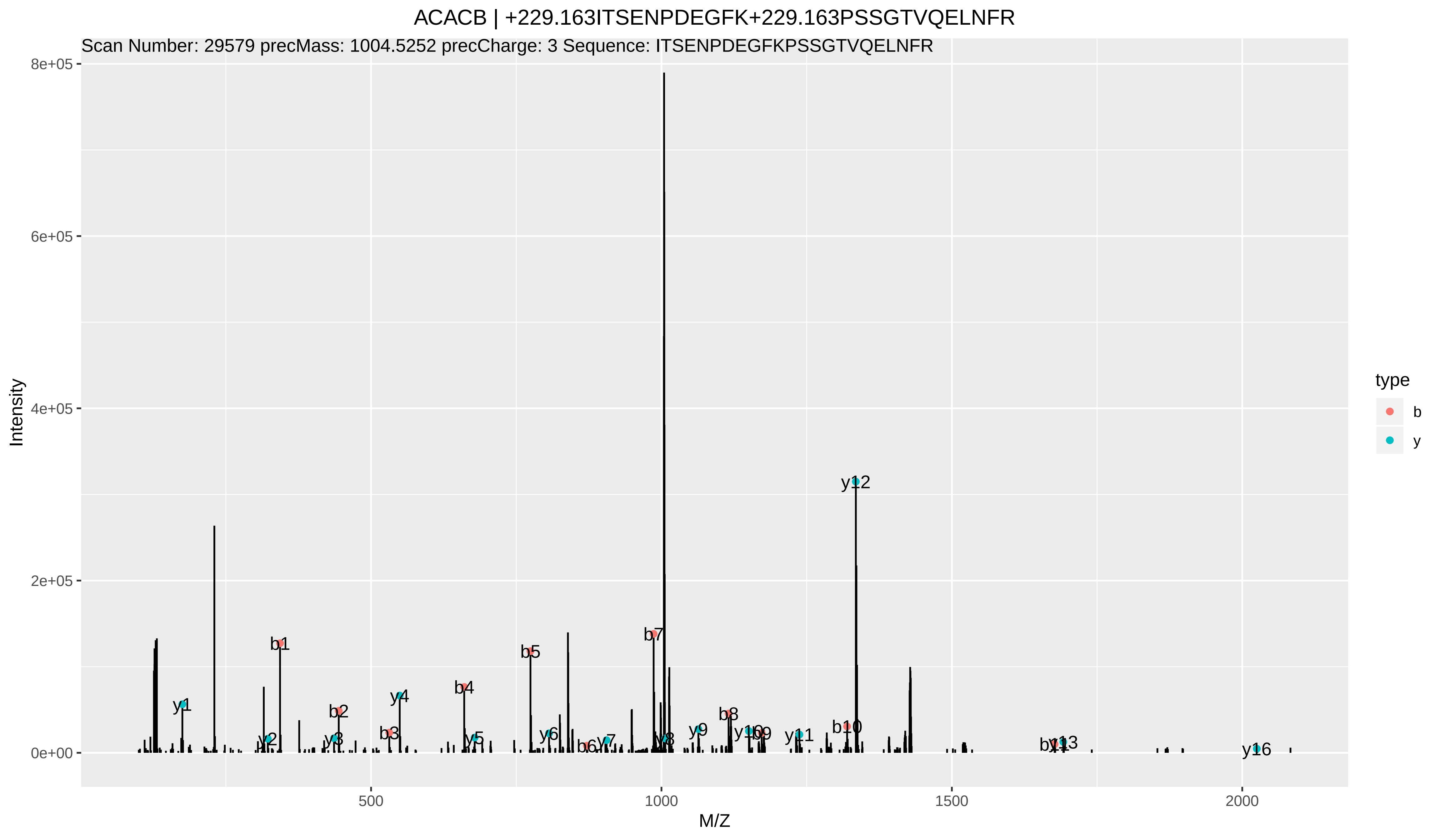
ACACB | +229.163ITSENPDEGFK+229.163PSSGTVQELNFR
Scan Number: 29579 precMass: 1004.5252 precCharge: 3 Sequence: ITSENPDEGFKPSSGTVQELNFR
Intensity
0e+00
2e+05
4e+05
6e+05
8e+05
M/Z
500
1000
1500
2000
type
b
y
y1
y2
y3
y4
y5
y6
y7
y8
y9
y10
y11
y12
y13
y16
b1
b2
b3
b4
b5
b6
b7
b8
b9
b10
b11

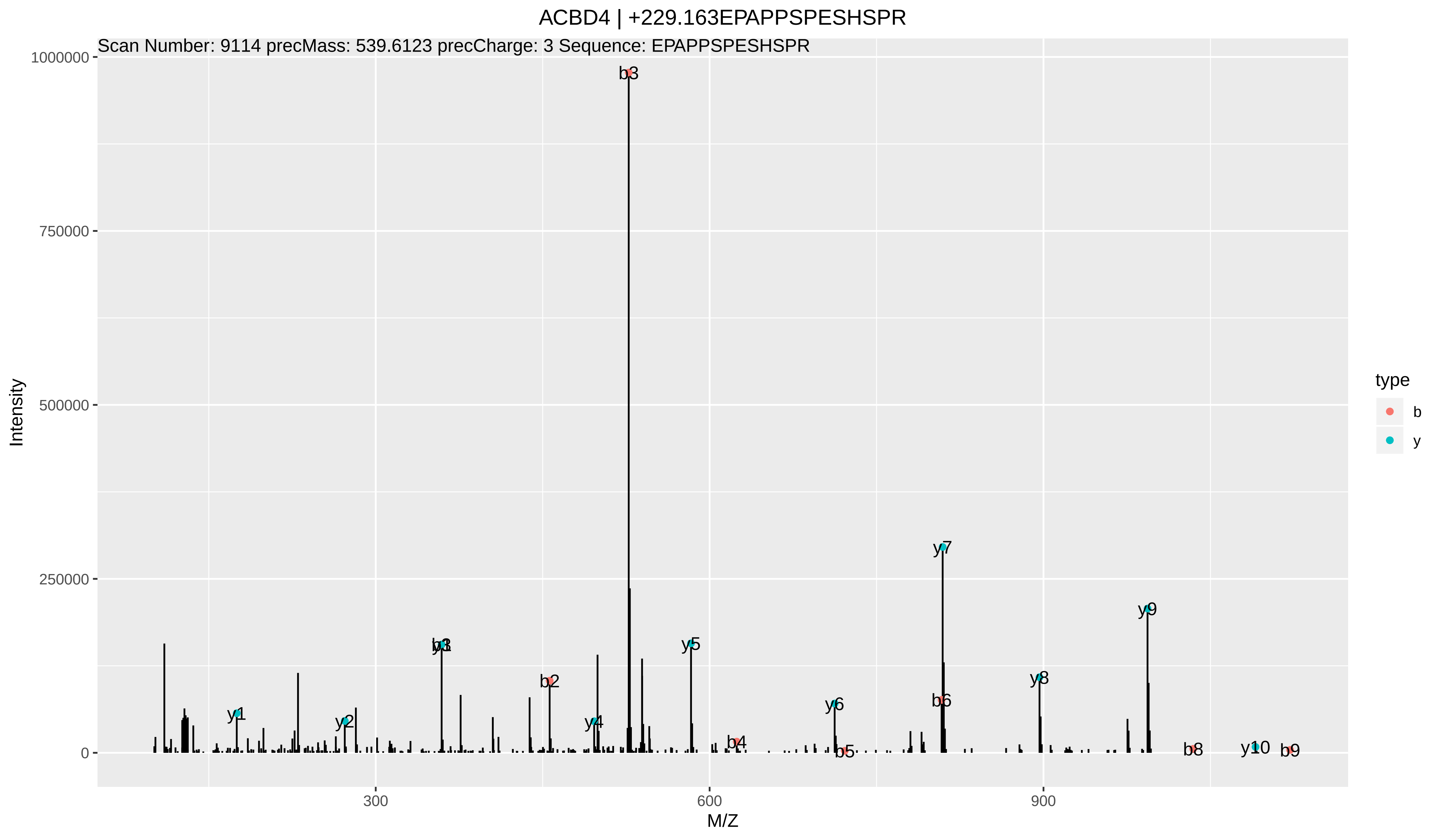

ACBD4 | +229.163EPAPPSPESHSPR
Scan Number: 9114 precMass: 539.6123 precCharge: 3 Sequence: EPAPPSPESHSPR
Intensity
M/Z
1000000
750000
500000
250000
0
300
600
900
b3
y7
y9
y3
b1
y5
b2
y8
b6
y6
y1
y2
y4
b4
b5
b8
y10
b9
type
b
y

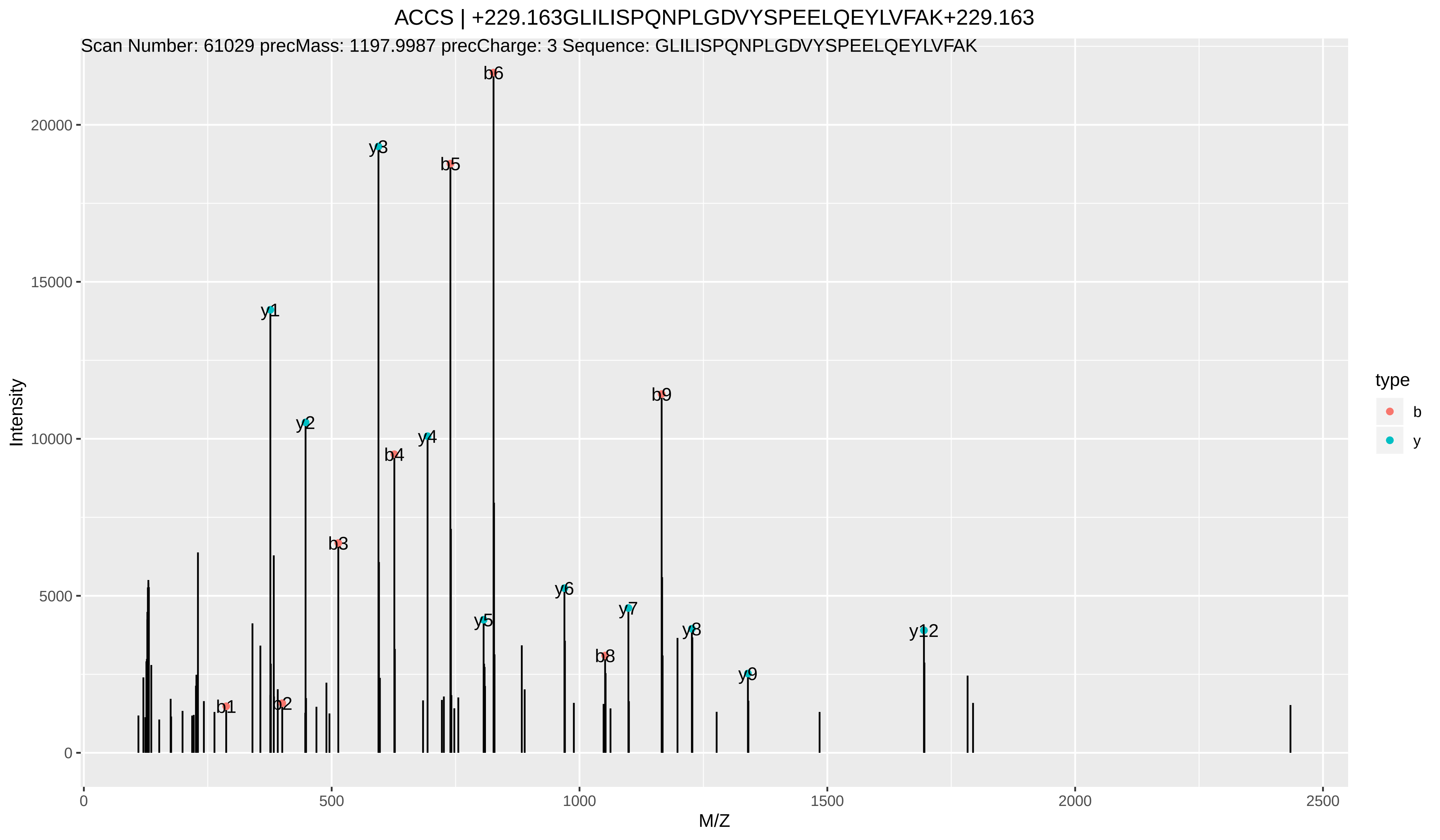

ACCS | +229.163GLILISPQNPLGDVYSPEELQEYLVFAK+229.163
Scan Number: 61029 precMass: 1197.9987 precCharge: 3 Sequence: GLILISPQNPLGDVYSPEELQEYLVFAK
Intensity
M/Z
type
b
y
b1
b2
b3
b4
b5
b6
b8
b9
y1
y2
y3
y4
y5
y6
y7
y8
y9
y12
0
5000
10000
15000
20000
500
1000
1500
2000
2500

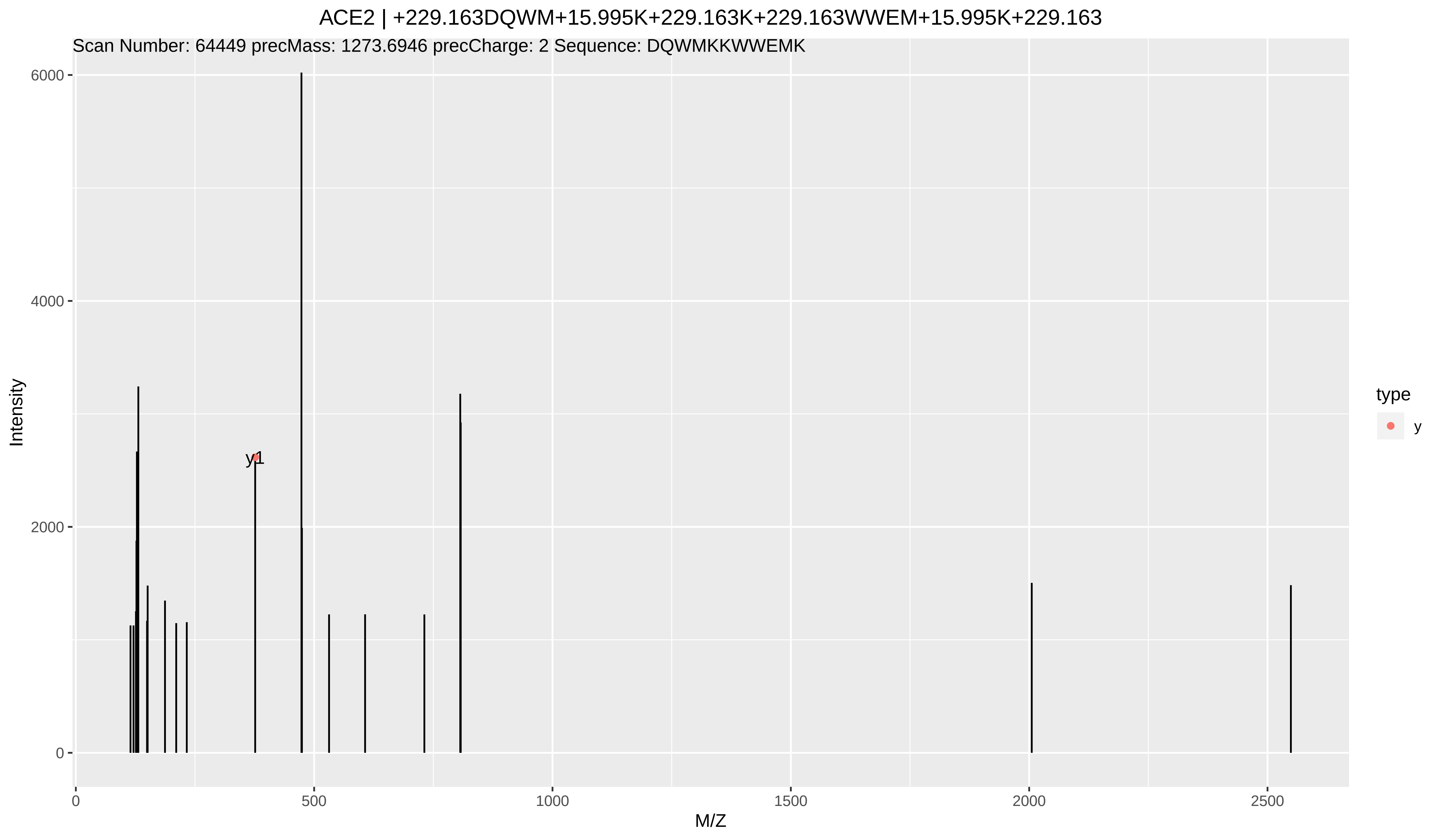

ACE2 | +229.163DQWM+15.995K+229.163K+229.163WWEM+15.995K+229.163
Scan Number: 64449 precMass: 1273.6946 precCharge: 2 Sequence: DQWMKKWWEMK
Intensity
6000
4000
2000
0
M/Z
0
500
1000
1500
2000
2500
y1
type
y

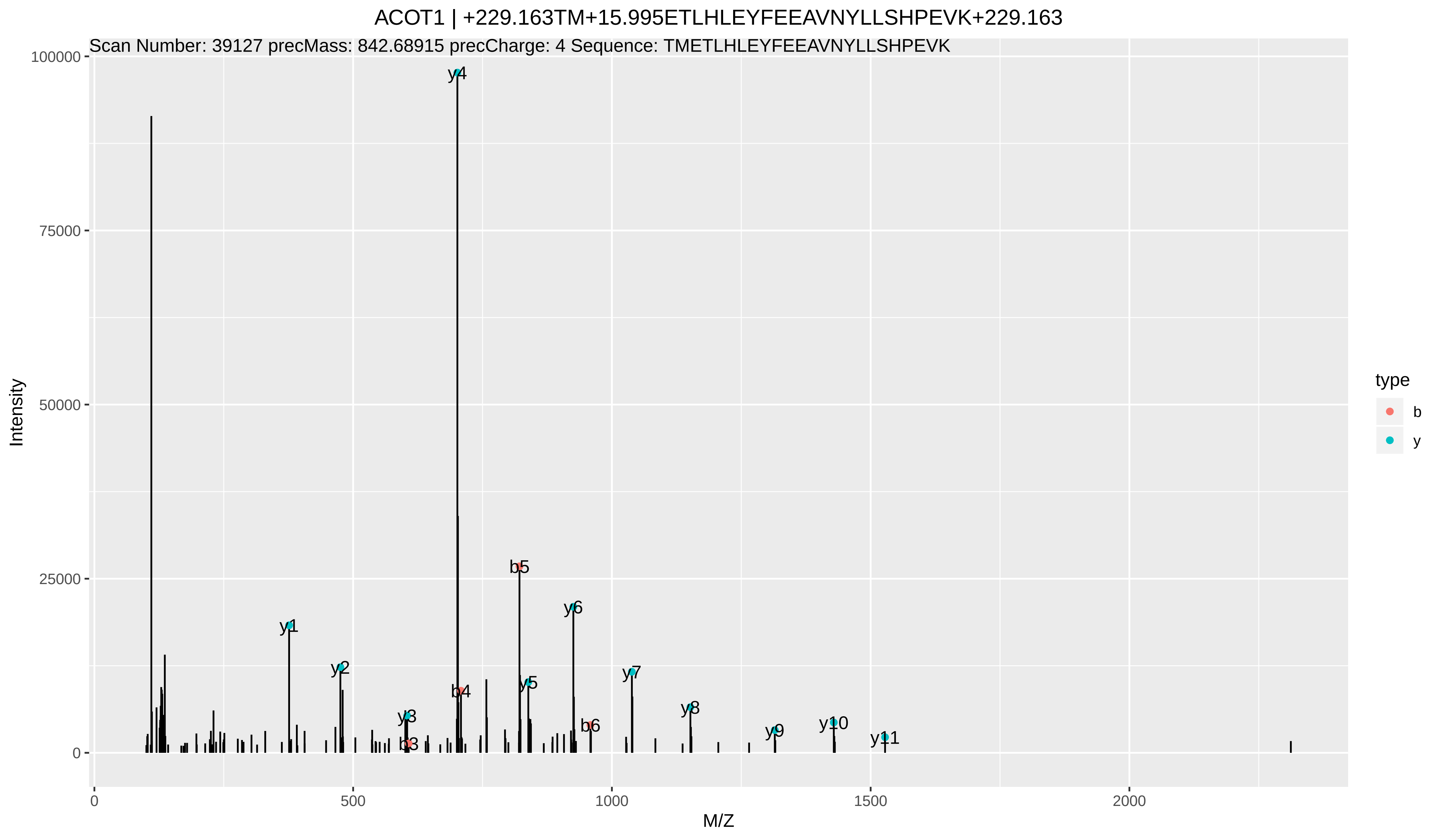

ACOT1 | +229.163TM+15.995ETLHLEYFEEAVNYLLSHPEVK+229.163
Scan Number: 39127 precMass: 842.68915 precCharge: 4 Sequence: TMETLHLEYFEEAVNYLLSHPEVK
Intensity
100000
75000
50000
25000
0
M/Z
0
500
1000
1500
2000
y4
b5
y6
y1
y2
y7
y5
b4
y8
y3
b6
y10
y9
y11
b3
type
b
y

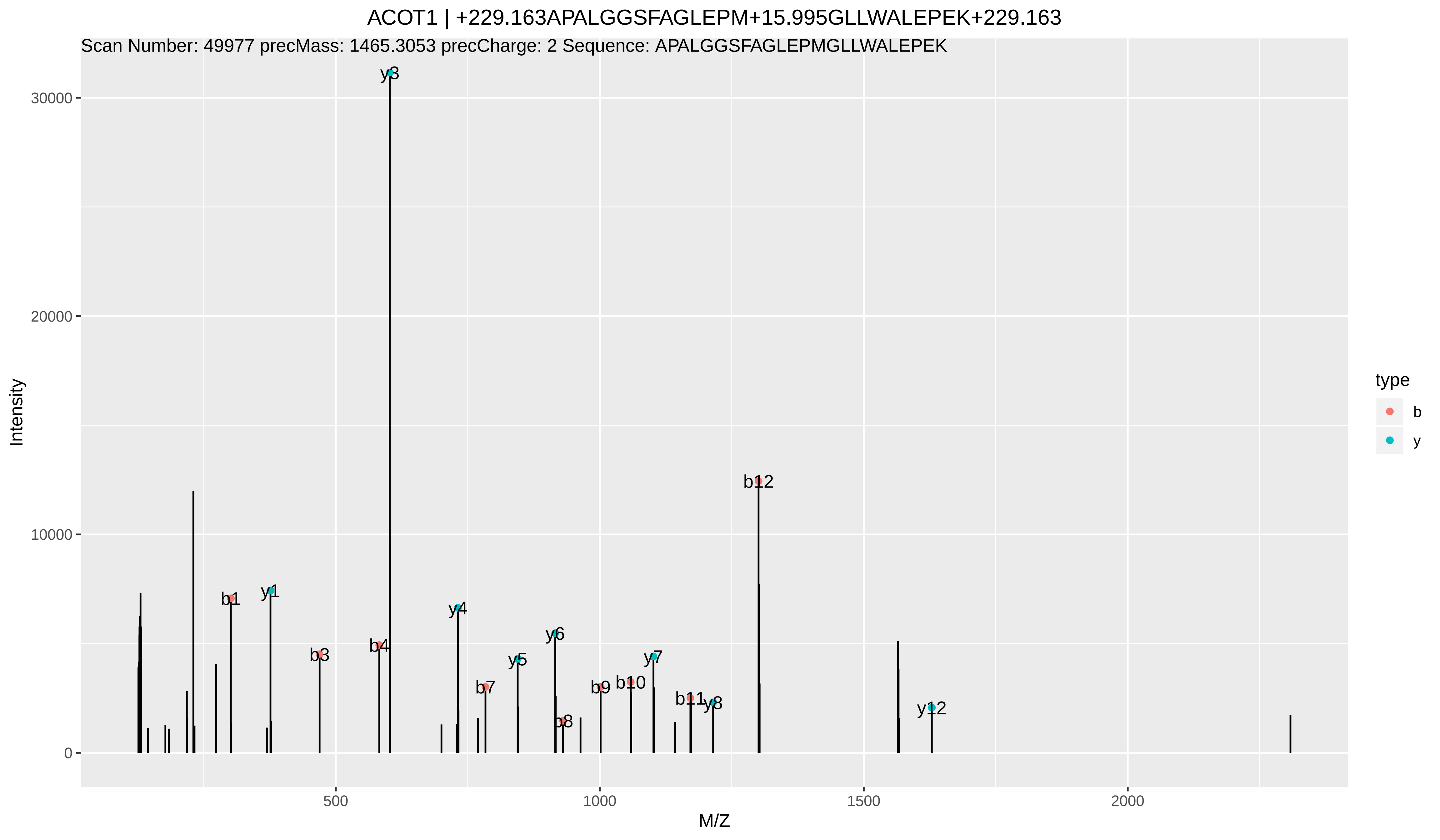
ACOT1 | +229.163APALGGSFAGLEPM+15.995GLLWALEPEK+229.163
Scan Number: 49977 precMass: 1465.3053 precCharge: 2 Sequence: APALGGSFAGLEPMGLLWALEPEK
Intensity
M/Z
type
b
y
y3
b12
b1
y1
y4
b3
b4
y6
y5
y7
b7
b9
b10
b11
y8
b8
y12
0
10000
20000
30000
500
1000
1500
2000

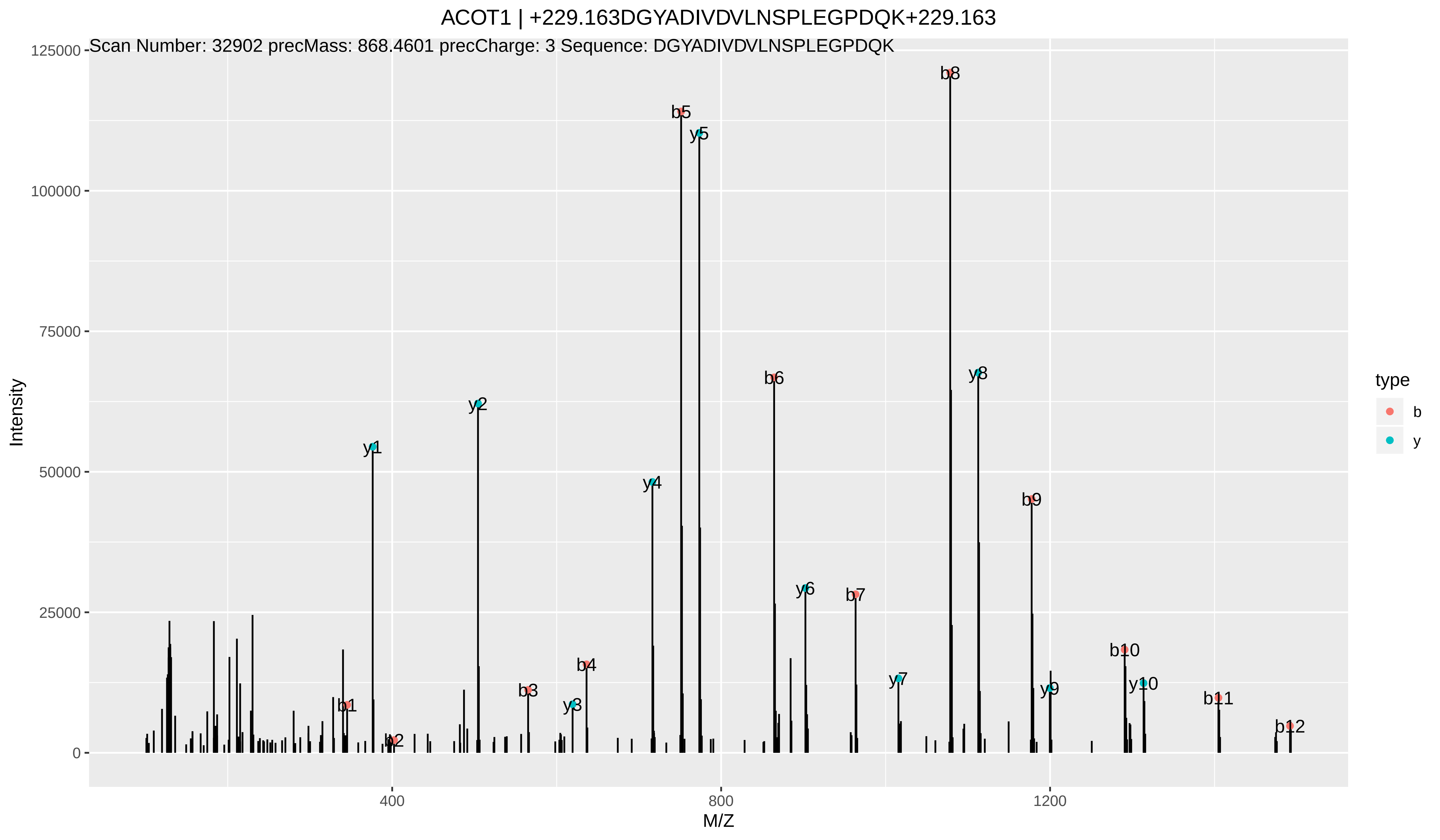

ACOT1 | +229.163DGYADIVDVLNSPLEGPDQK+229.163
Scan Number: 32902 precMass: 868.4601 precCharge: 3 Sequence: DGYADIVDVLNSPLEGPDQK
Intensity
M/Z
125000
100000
75000
50000
25000
0
400
800
1200
b8
b5
y5
b6
y8
y2
y1
y4
b9
y6
b7
b10
y7
y10
b4
b3
y9
b11
y3
b1
b12
b2
type
b
y

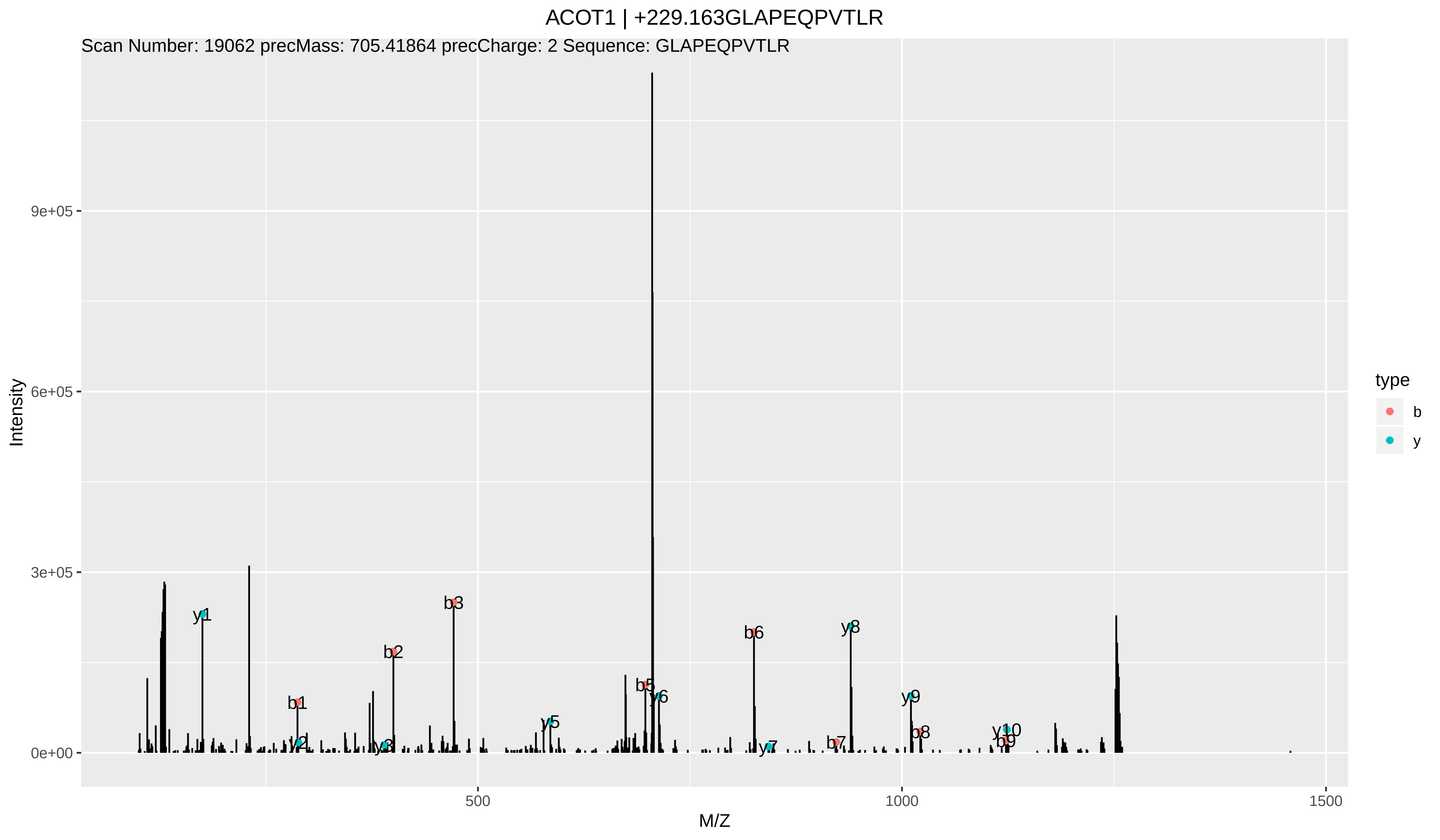

ACOT1 | +229.163GLAPEQPVTLR
Scan Number: 19062 precMass: 705.41864 precCharge: 2 Sequence: GLAPEQPVTLR
Intensity
M/Z
0e+00
3e+05
6e+05
9e+05
500
1000
1500
y1
b1
y2
y3
b2
b3
y5
b5
y6
b6
y7
b7
y8
y9
b8
y10
b9
type
b
y

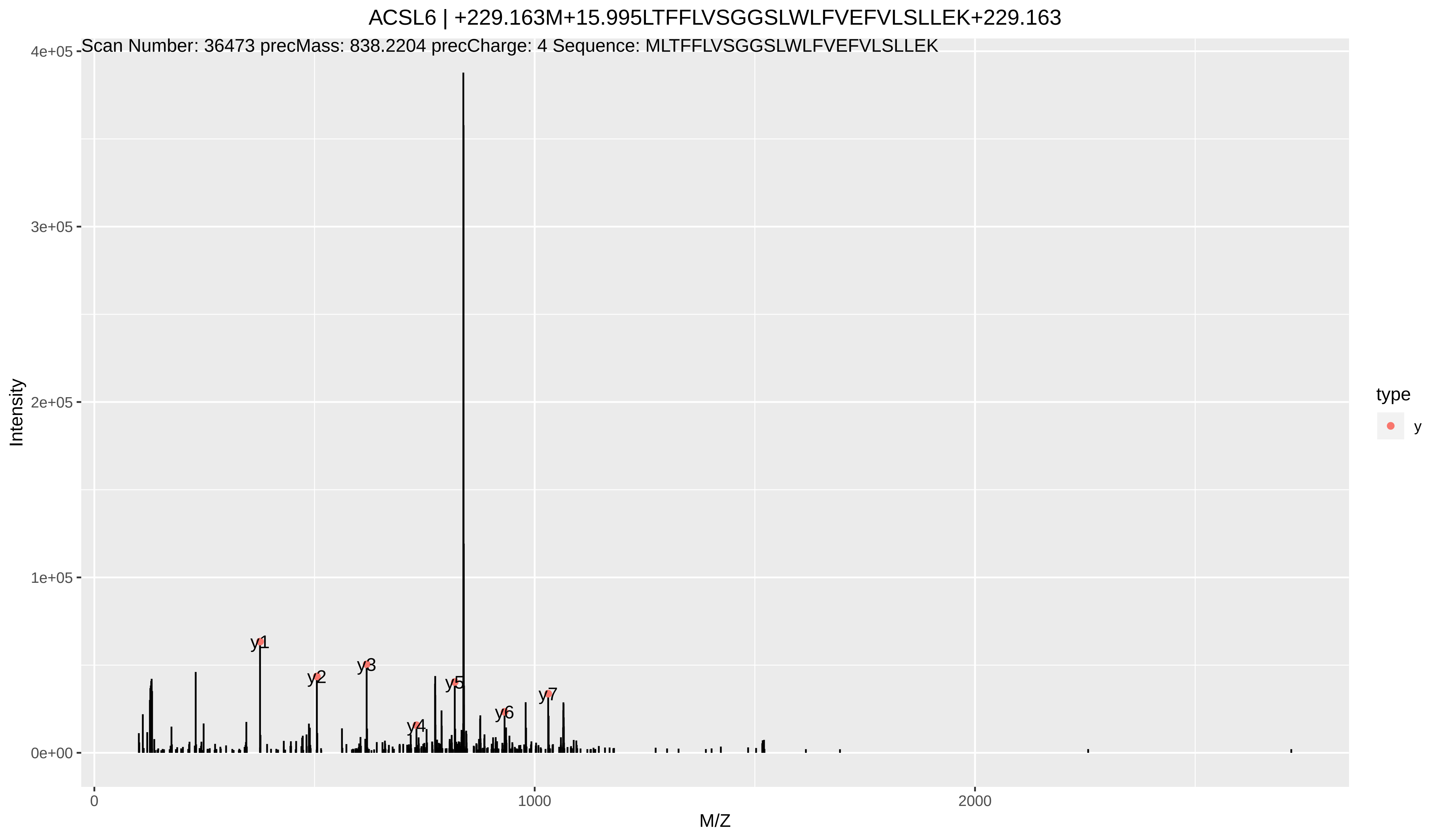

ACSL6 | +229.163M+15.995LTFFLVSGGSLWLFVEFVLSLLEK+229.163
Scan Number: 36473 precMass: 838.2204 precCharge: 4 Sequence: MLTFFLVSGGSLWLFVEFVLSLLEK
Intensity
4e+05
3e+05
2e+05
1e+05
0e+00
y1
y2
y3
y4
y5
y6
y7
0
1000
2000
M/Z
type
y

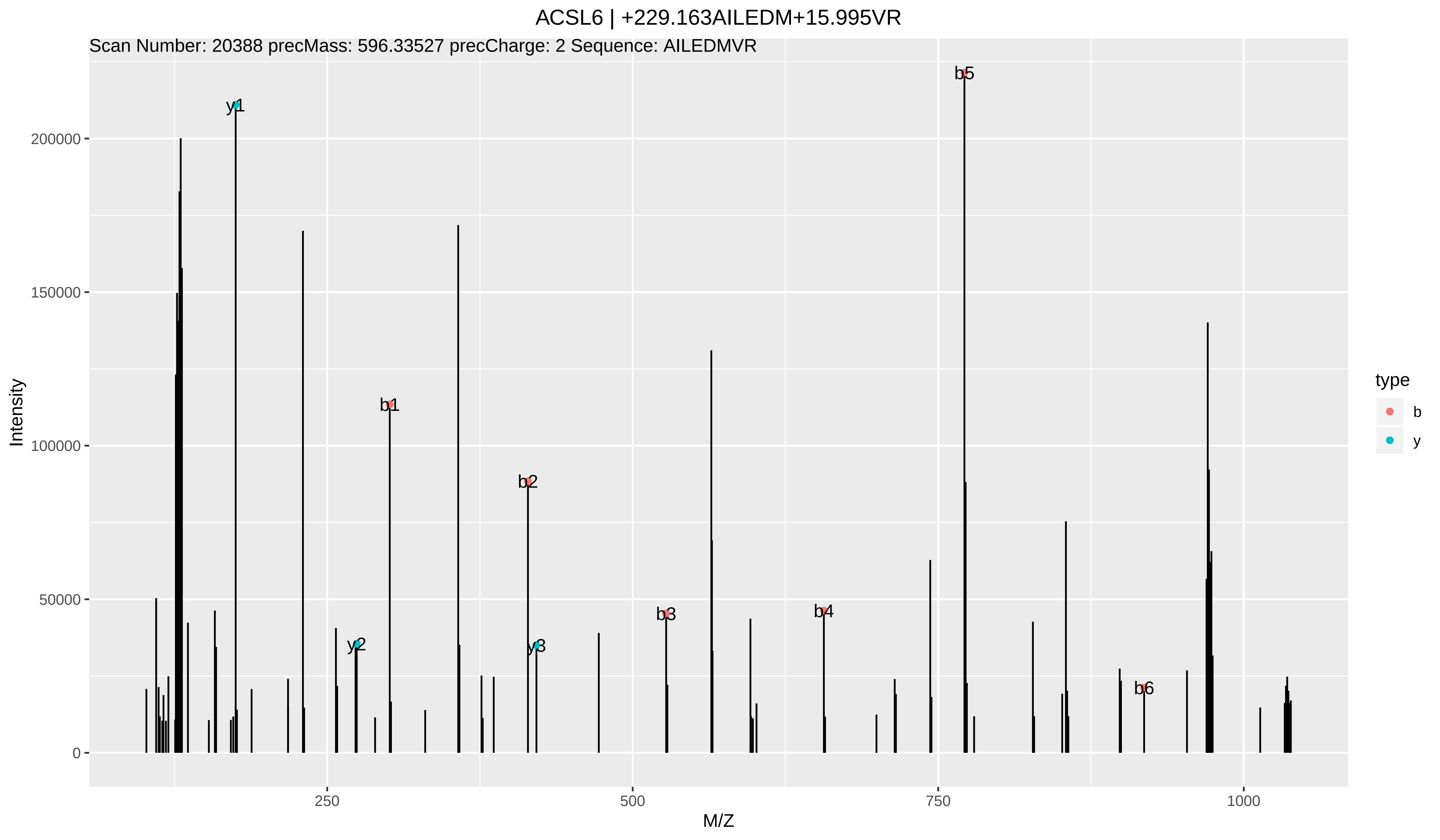

ACSL6 | +229.163AILEDM+15.995VR
Scan Number: 20388 precMass: 596.33527 precCharge: 2 Sequence: AILEDMVR
Intensity
M/Z
type
b
y
y1
b1
b2
b3
b4
b5
b6
y2
y3
0
50000
100000
150000
200000
250
500
750
1000

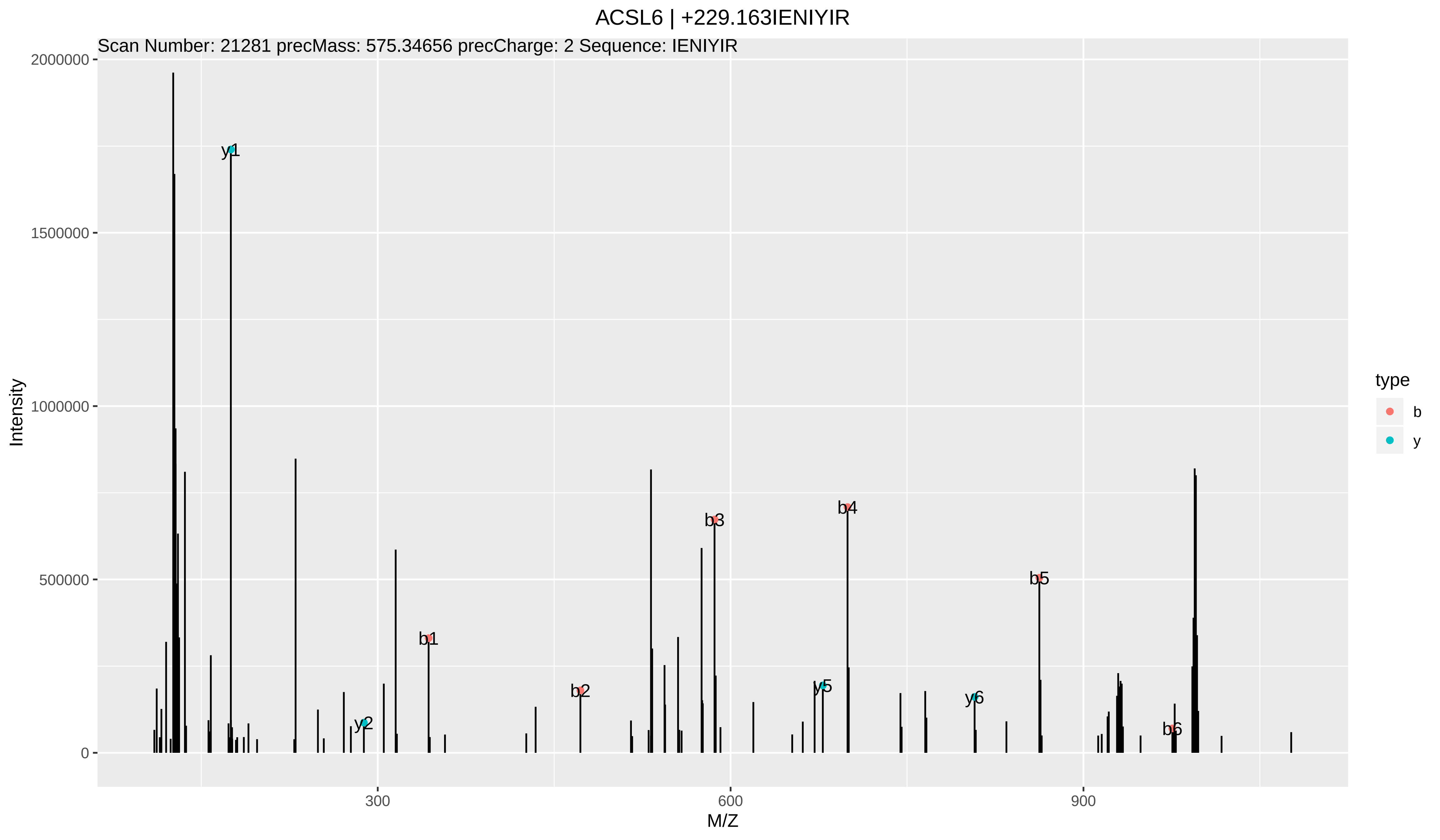

ACSL6 | +229.163IENIYIR
Scan Number: 21281 precMass: 575.34656 precCharge: 2 Sequence: IENIYIR
Intensity
M/Z
2000000
1500000
1000000
500000
0
300
600
900
y1
y2
b1
b2
b3
b4
y5
y6
b5
b6
type
b
y

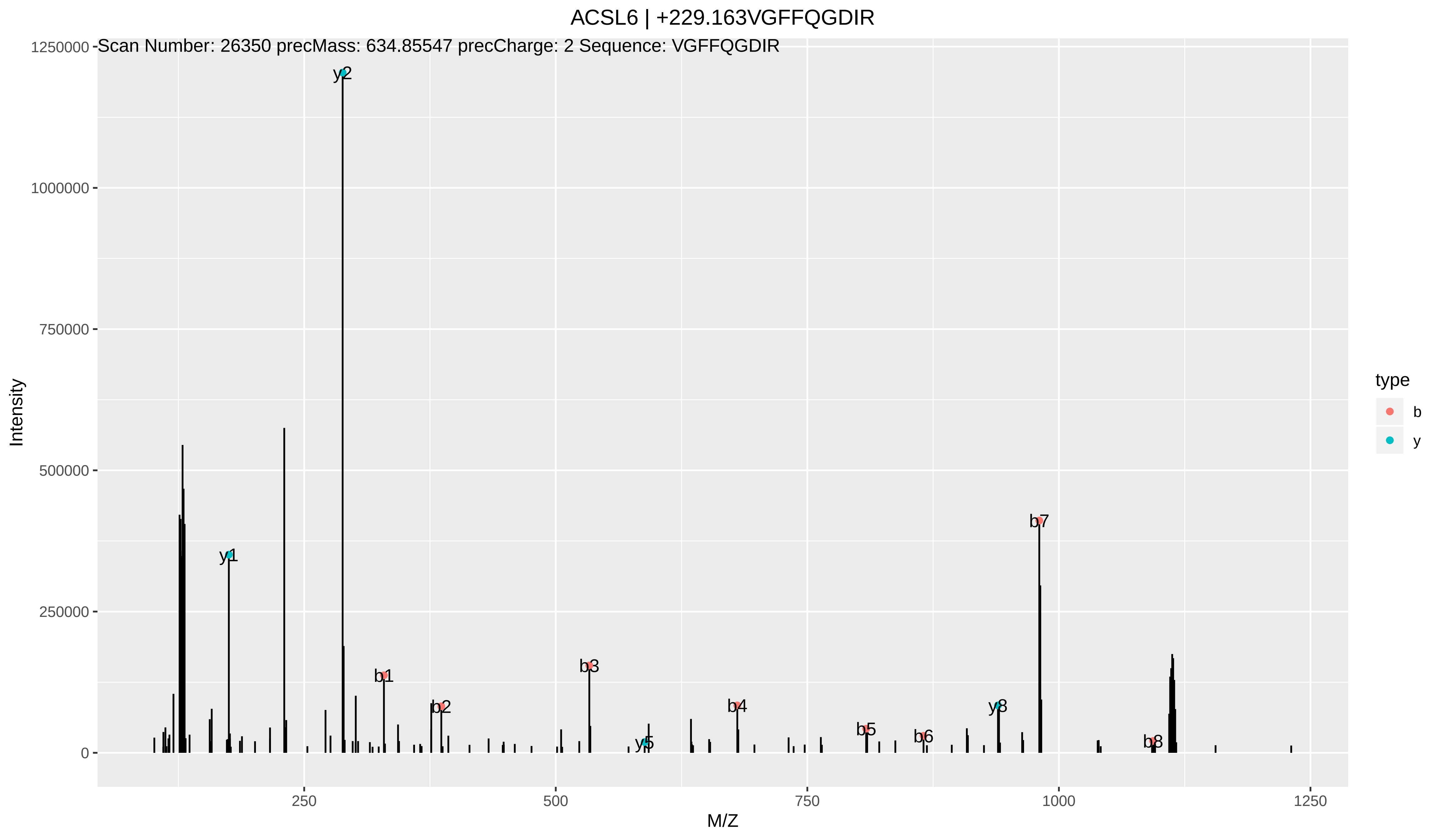

ACSL6 | +229.163VGFFQGDIR
Scan Number: 26350 precMass: 634.85547 precCharge: 2 Sequence: VGFFQGDIR
Intensity
M/Z
0
250000
500000
750000
1000000
1250000
250
500
750
1000
1250
y2
y1
b1
b2
b3
y5
b4
b5
b6
y8
b7
b8
type
b
y

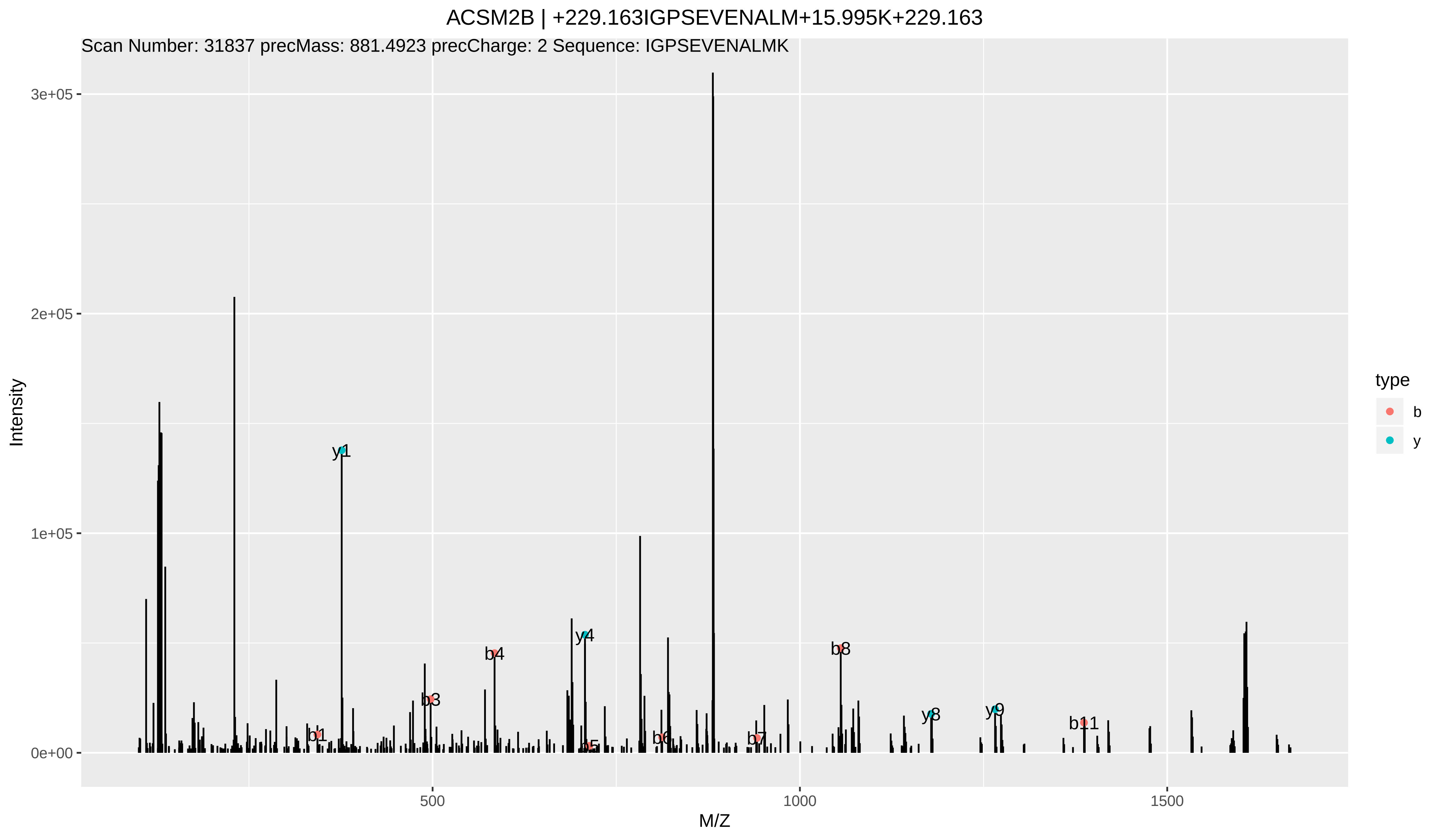

ACSM2B | +229.163IGPSEVENALM+15.995K+229.163
Scan Number: 31837 precMass: 881.4923 precCharge: 2 Sequence: IGPSEVENALMK
Intensity
3e+05
2e+05
1e+05
0e+00
M/Z
500
1000
1500
y1
b1
b3
b4
y4
b5
b6
b7
b8
y8
y9
b11
type
b
y

ACTG1 | +229.163EEEIAALVIDNGSGM+15.995C+57.021K+229.163

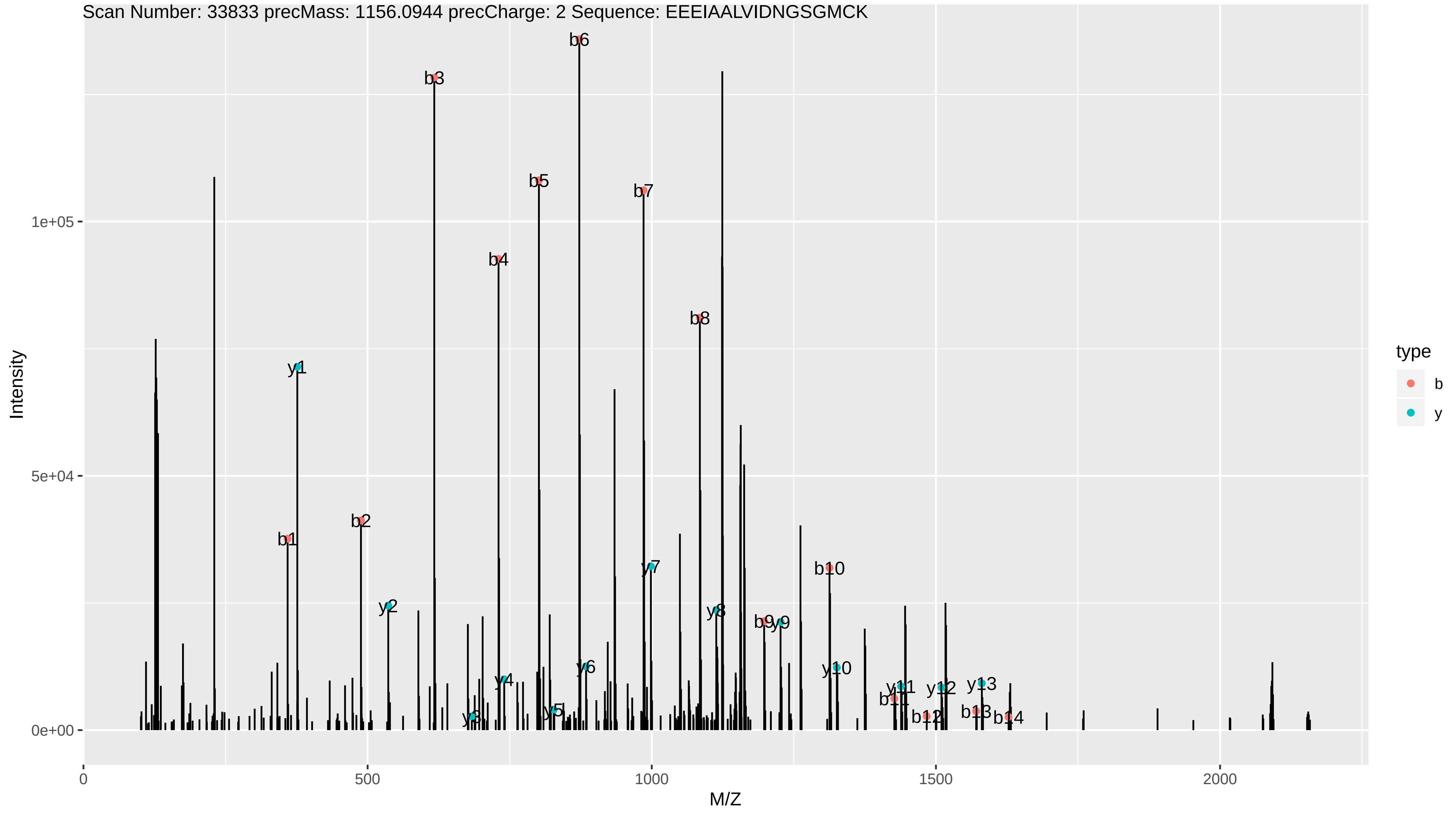

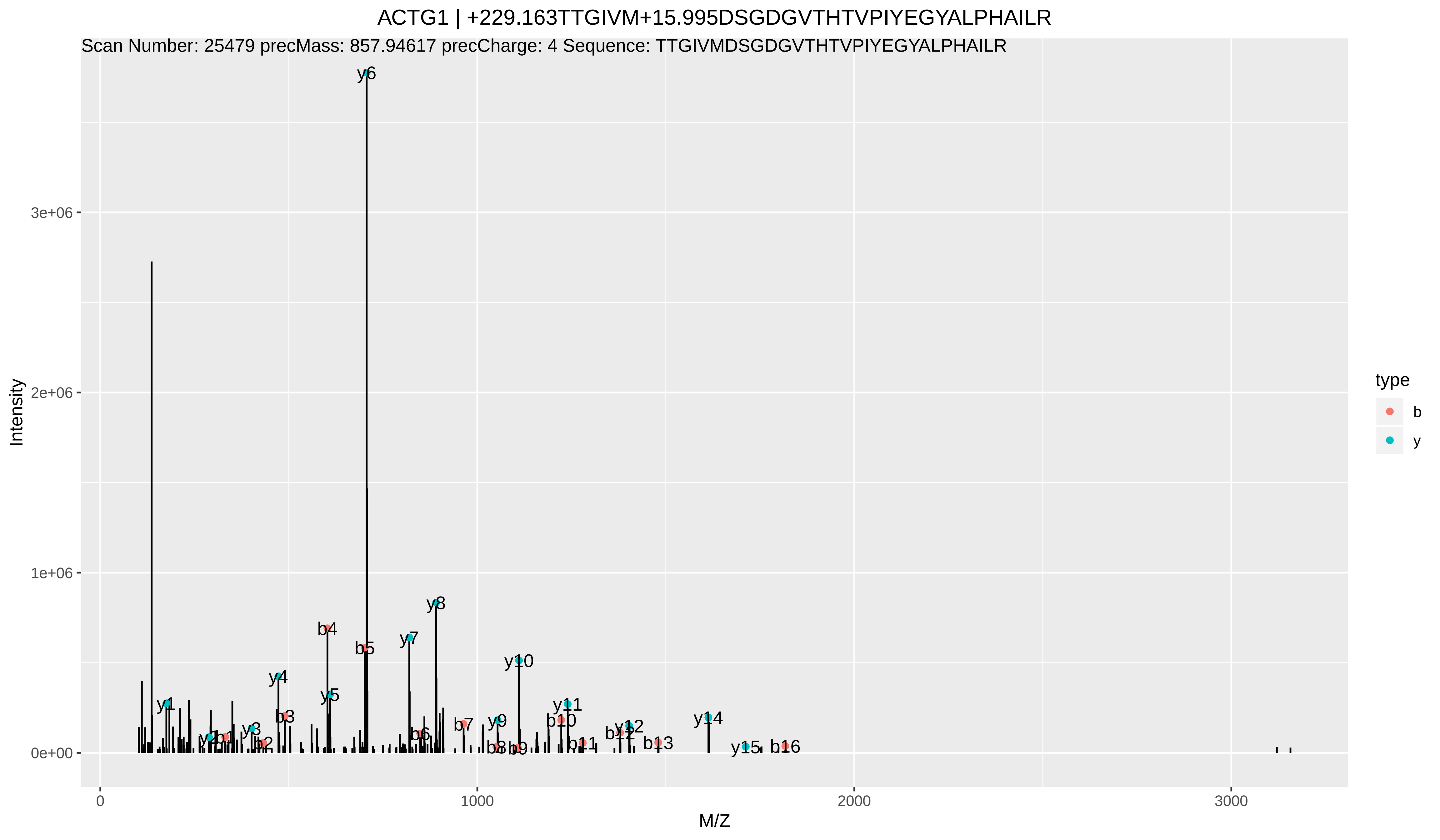

ACTG1 | +229.163TTGIVM+15.995DSGDGVTHTVPIYEGYALPHAILR
Scan Number: 25479 precMass: 857.94617 precCharge: 4 Sequence: TTGIVMDSGDGVTHTVPIYEGYALPHAILR
Intensity
M/Z
0e+00
1e+06
2e+06
3e+06
0
1000
2000
3000
type
b
y
y6
y8
b4
y7
b5
y10
y4
y5
y1
y11
b3
y14
b10
b7
y9
y12
y3
b12
b1
b13
b6
b11
b2
y15
b16
b8
b9

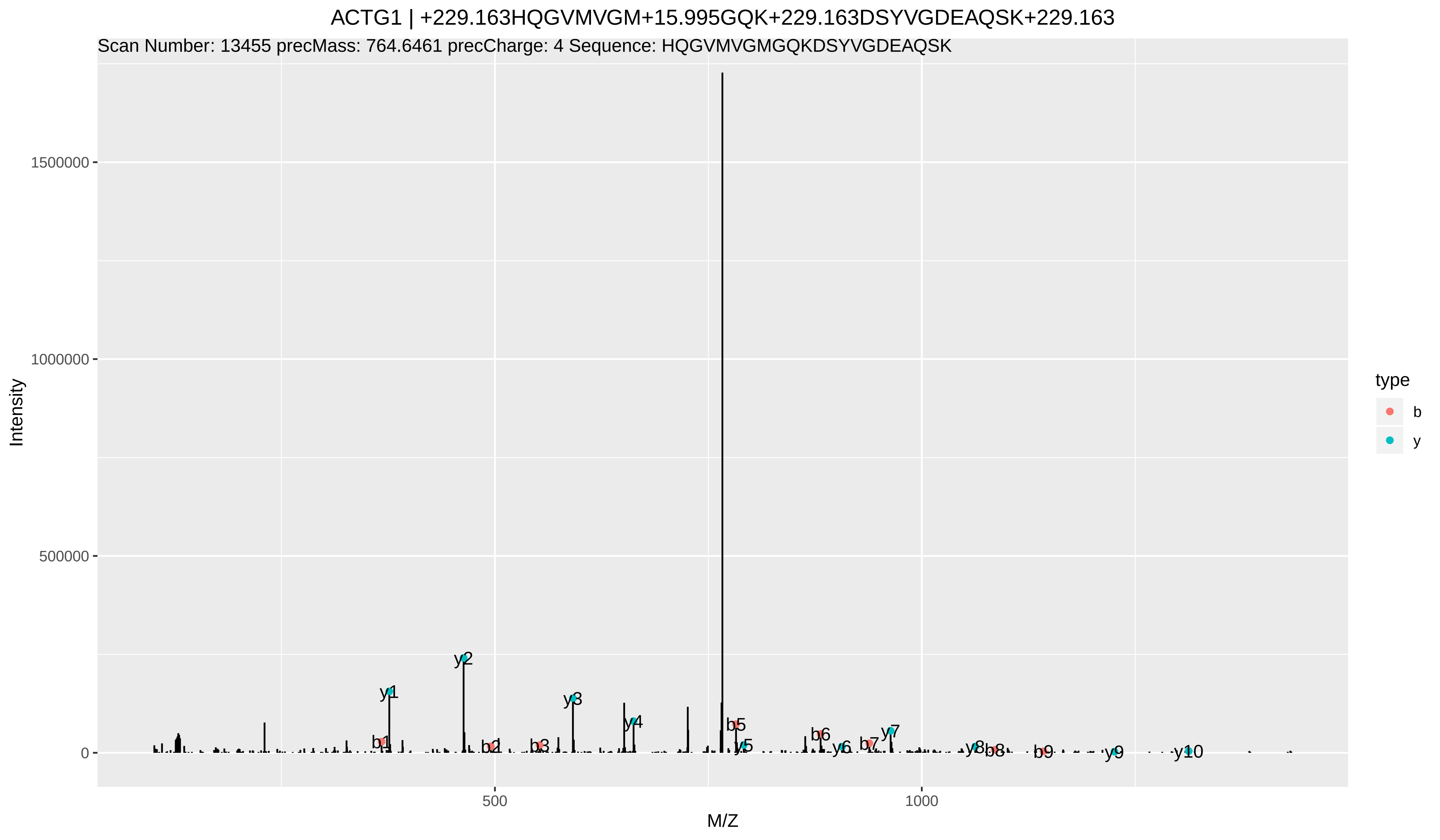
ACTG1 | +229.163HQGVMVGM+15.995GQK+229.163DSYVGDEAQSK+229.163
Scan Number: 13455 precMass: 764.6461 precCharge: 4 Sequence: HQGVMVGMGQKDSYVGDEAQSK
Intensity
1500000
1000000
500000
0
M/Z
500
1000
y1
y2
y3
y4
y5
y6
y7
y8
y9
y10
b1
b2
b3
b5
b6
b7
b8
b9
type
b
y

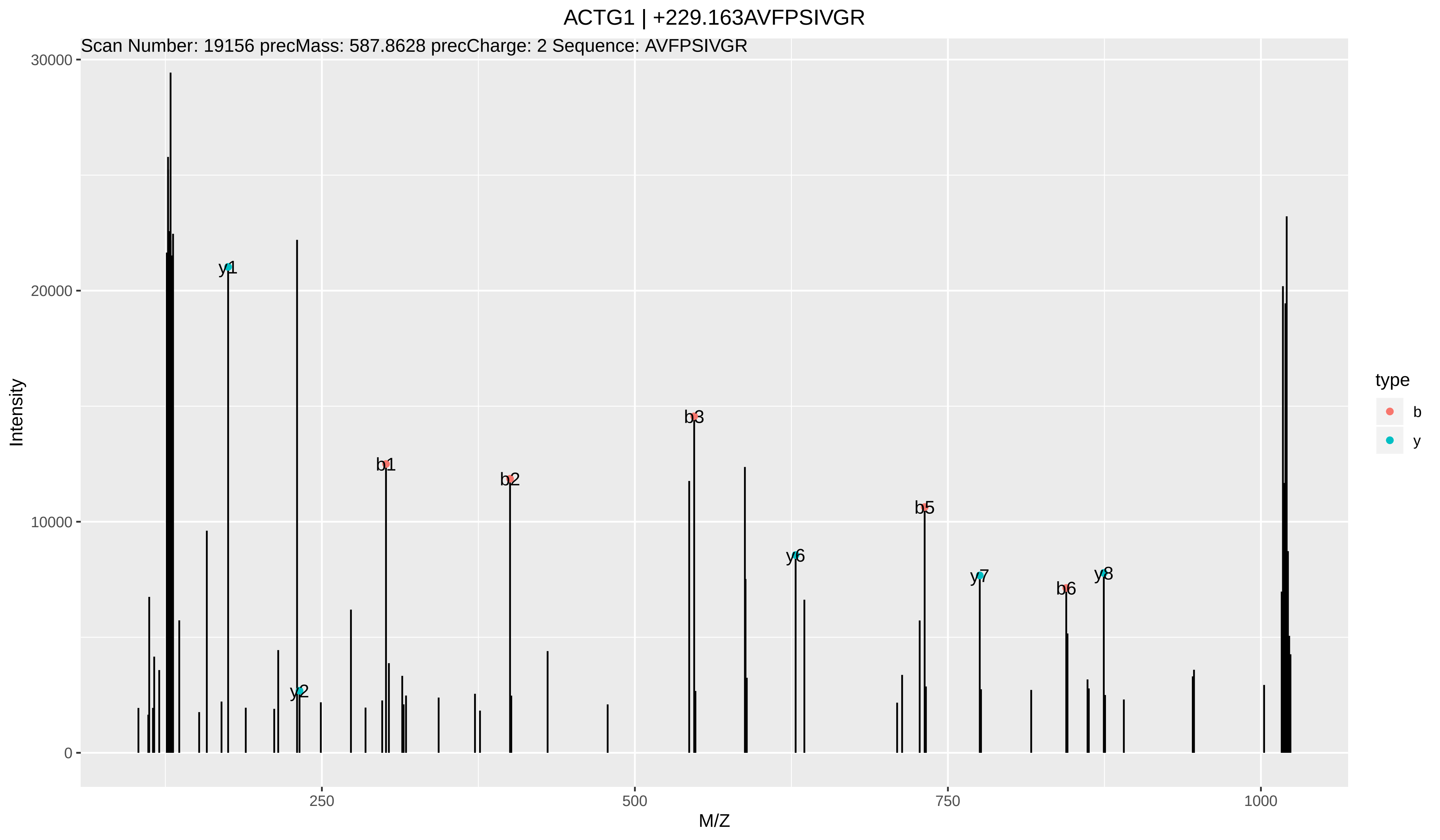
ACTG1 | +229.163AVFPSIVGR
Scan Number: 19156 precMass: 587.8628 precCharge: 2 Sequence: AVFPSIVGR
Intensity
30000
20000
10000
0
M/Z
250
500
750
1000
y1
y2
b1
b2
b3
y6
b5
y7
b6
y8
type
b
y

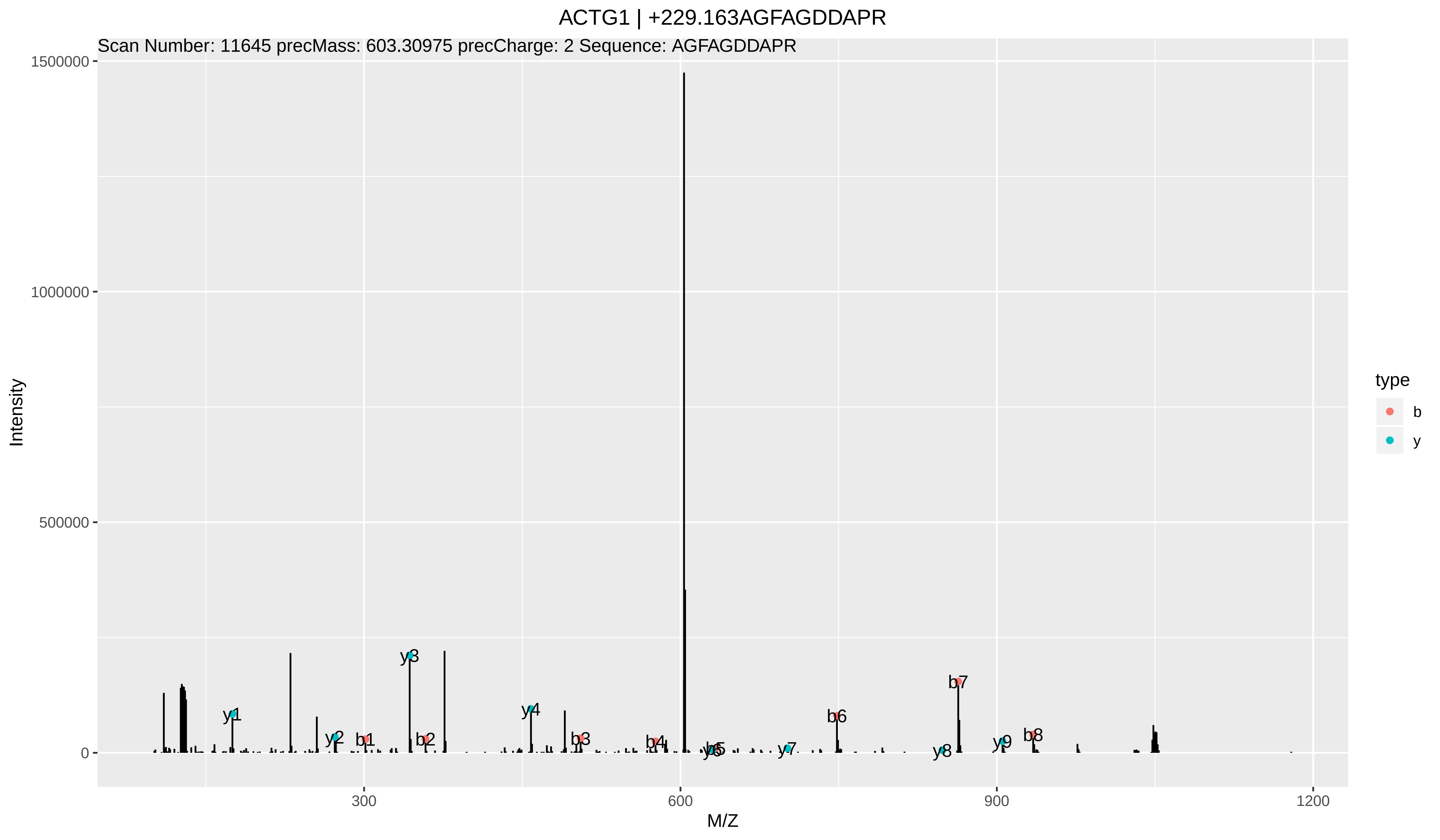

ACTG1 | +229.163AGFAGDDAPR
Scan Number: 11645 precMass: 603.30975 precCharge: 2 Sequence: AGFAGDDAPR
Intensity
M/Z
1500000
1000000
500000
0
300
600
900
1200
y1
y2
b1
y3
b2
y4
b3
b4
y5
b5
y7
b6
b7
y8
y9
b8
type
b
y

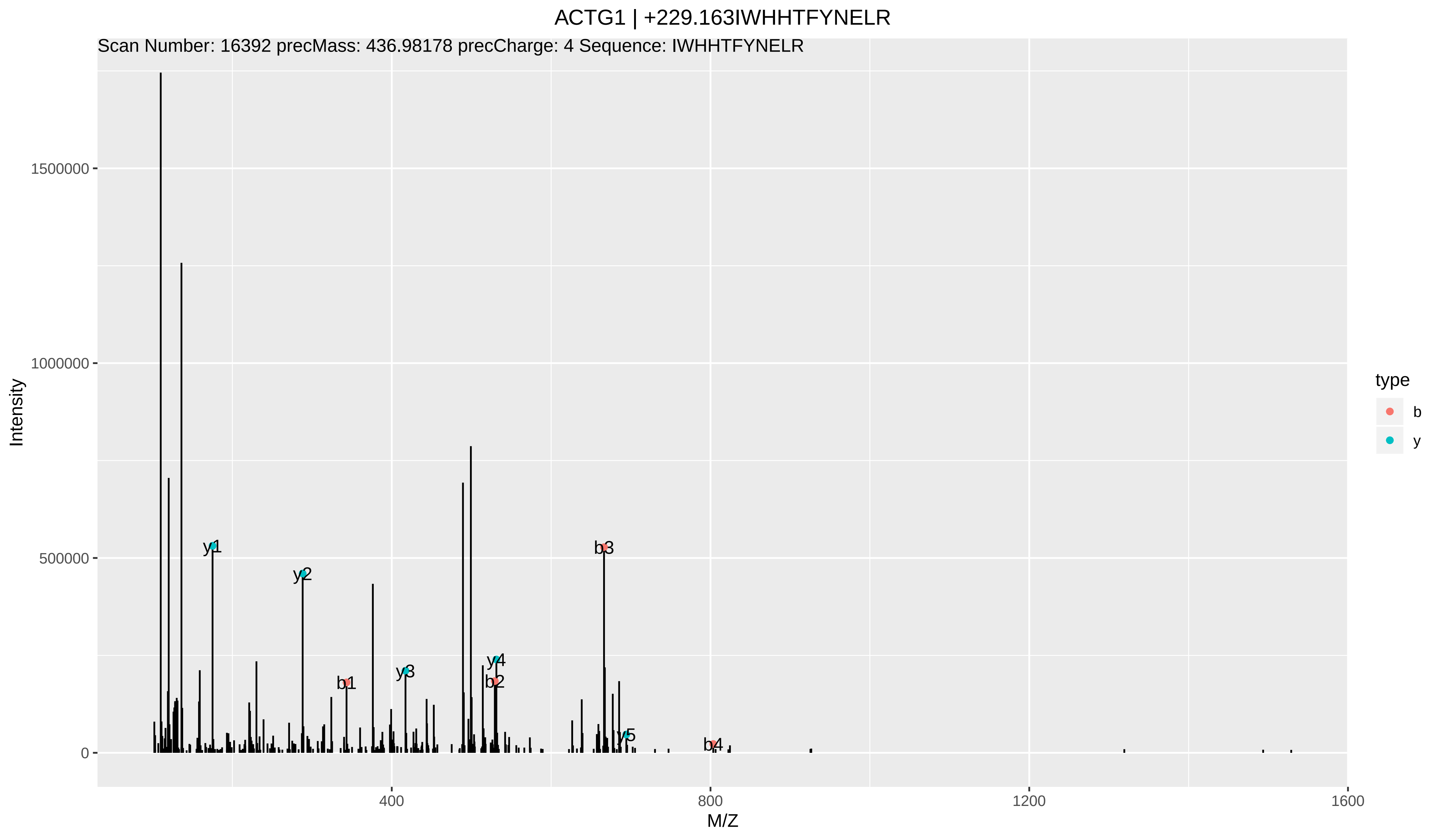

ACTG1 | +229.163IWHHTFYNELR
Scan Number: 16392 precMass: 436.98178 precCharge: 4 Sequence: IWHHTFYNELR
Intensity
1500000
1000000
500000
0
400
800
1200
1600
M/Z
y1
y2
b1
y3
y4
b2
b3
y5
b4
type
b
y

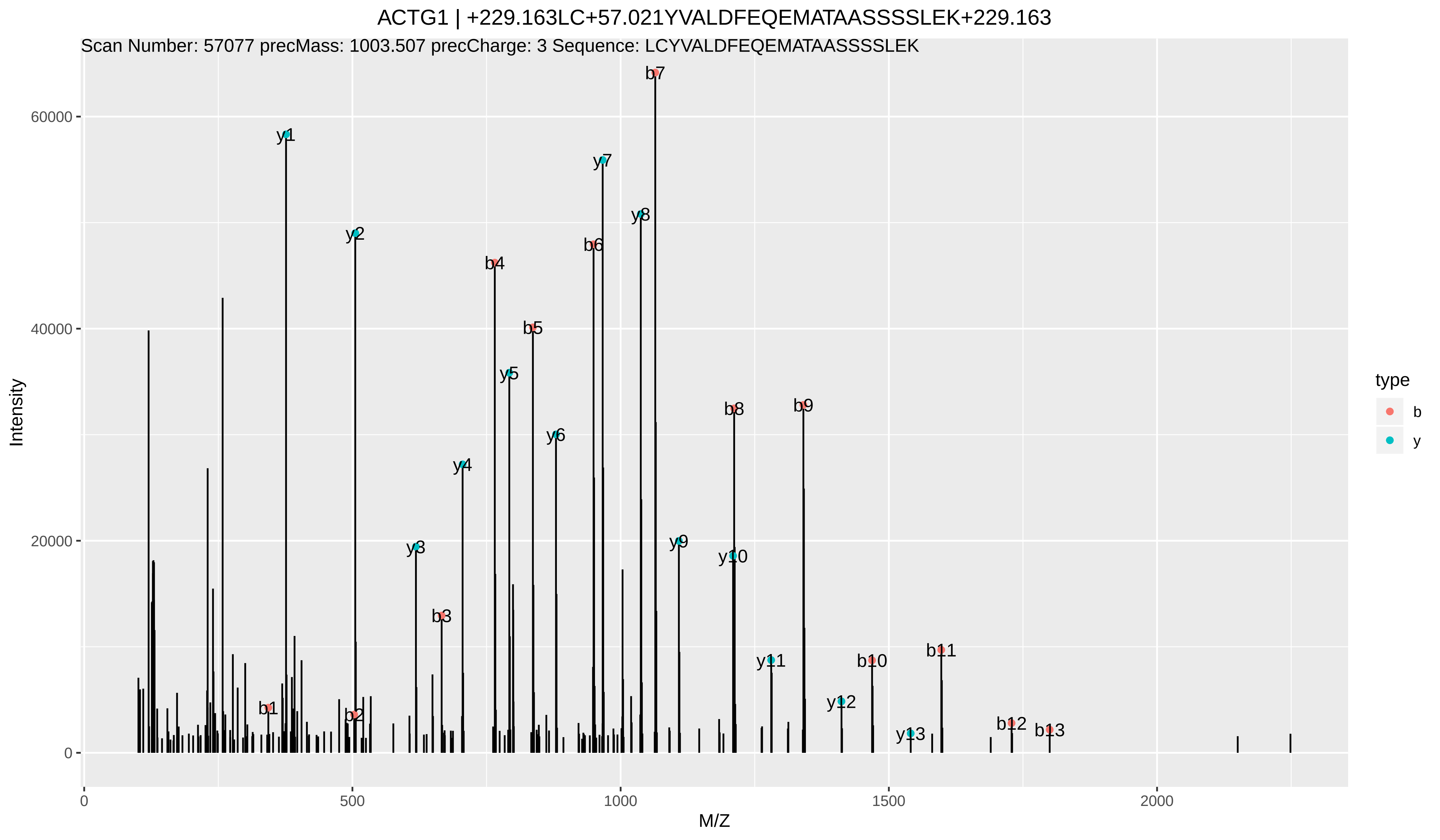
ACTG1 | +229.163LC+57.021YVALDFEQEMATAASSSSLEK+229.163
Scan Number: 57077 precMass: 1003.507 precCharge: 3 Sequence: LCYVALDFEQEMATAASSSSLEK
Intensity
M/Z
type
b
y
b1
b2
b3
b4
b5
b6
b7
b8
b9
b10
b11
b12
b13
y1
y2
y3
y4
y5
y6
y7
y8
y9
y10
y11
y12
y13
0
20000
40000
60000
500
1000
1500
2000

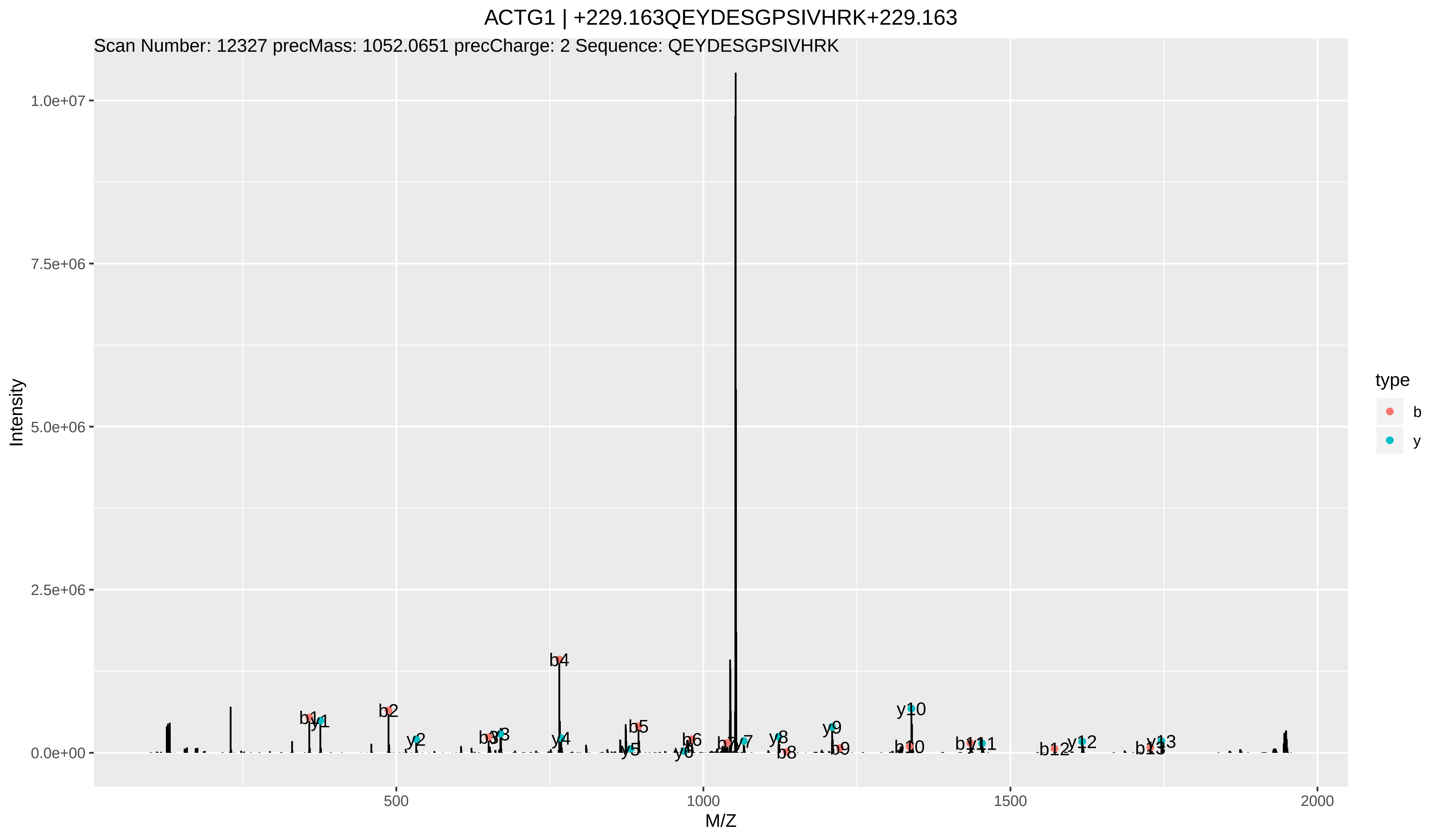

ACTG1 | +229.163QEYDESGPSIVHRK+229.163
Scan Number: 12327 precMass: 1052.0651 precCharge: 2 Sequence: QEYDESGPSIVHRK
Intensity
1.0e+07
7.5e+06
5.0e+06
2.5e+06
0.0e+00
M/Z
500
1000
1500
2000
type
b
y
b1
y1
b2
y2
b3
y3
b4
y4
b5
y5
b6
y6
b7
y7
b8
y8
b9
y9
b10
y10
b11
y11
b12
y12
b13
y13

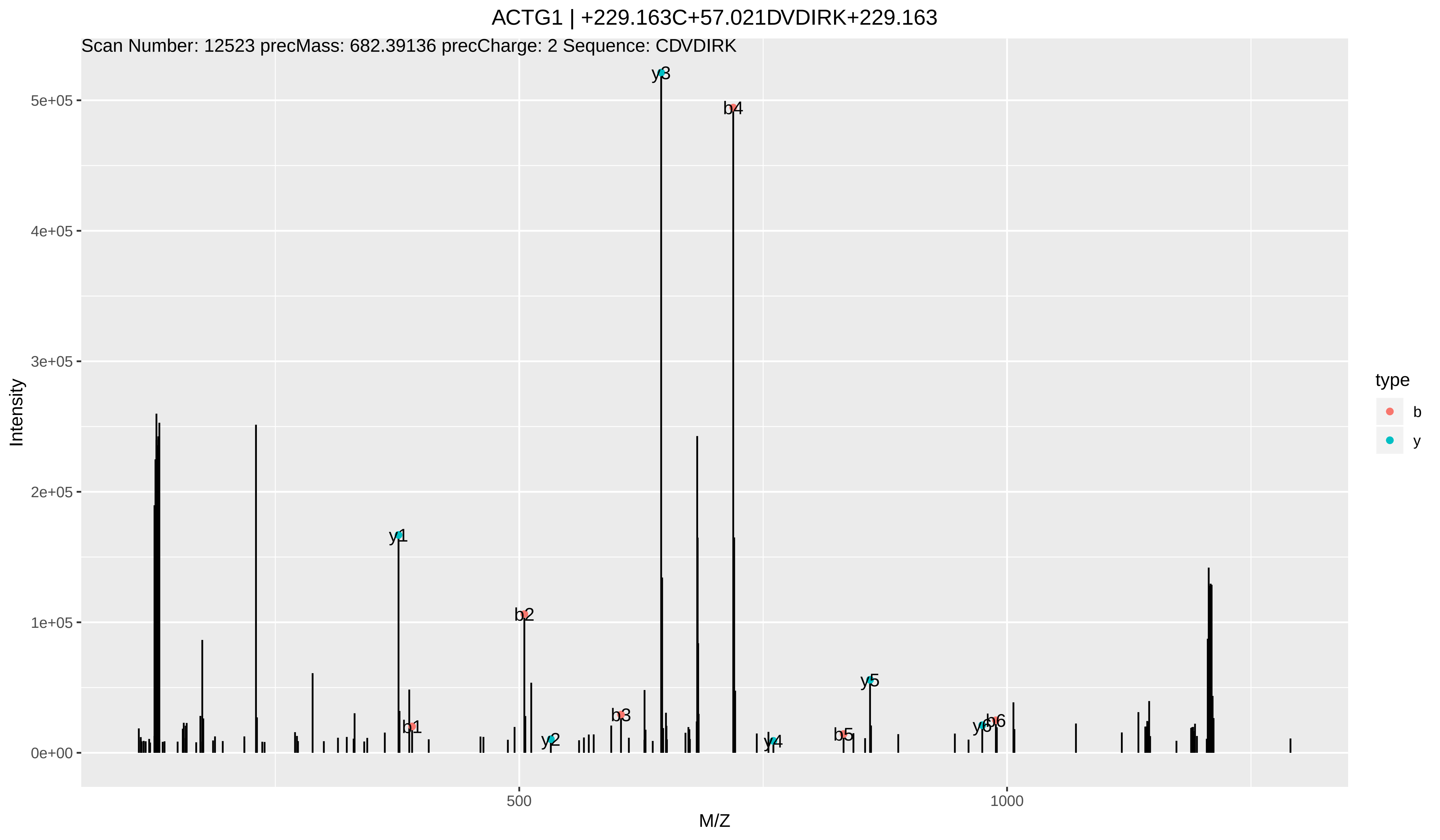
ACTG1 | +229.163C+57.021DVDIRK+229.163
Scan Number: 12523 precMass: 682.39136 precCharge: 2 Sequence: CDVDIRK
y3
b4
y1
b2
y5
b3
b1
y2
y4
b5
y6
b6
Intensity
0e+00
1e+05
2e+05
3e+05
4e+05
5e+05
M/Z
500
1000
type
b
y

ACTG1 | +229.163LDLAGRDLTDYLM+15.995K+229.163

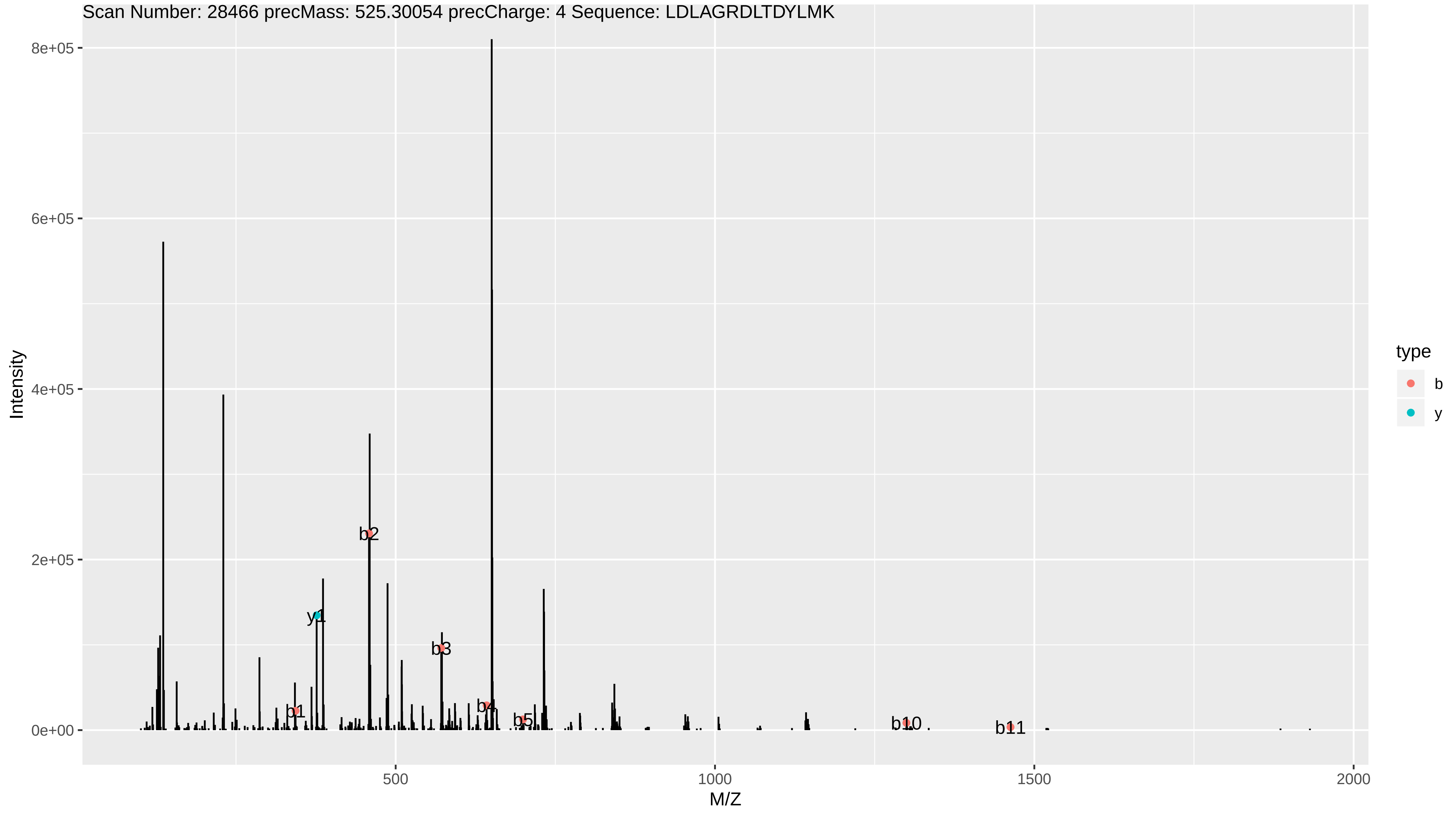

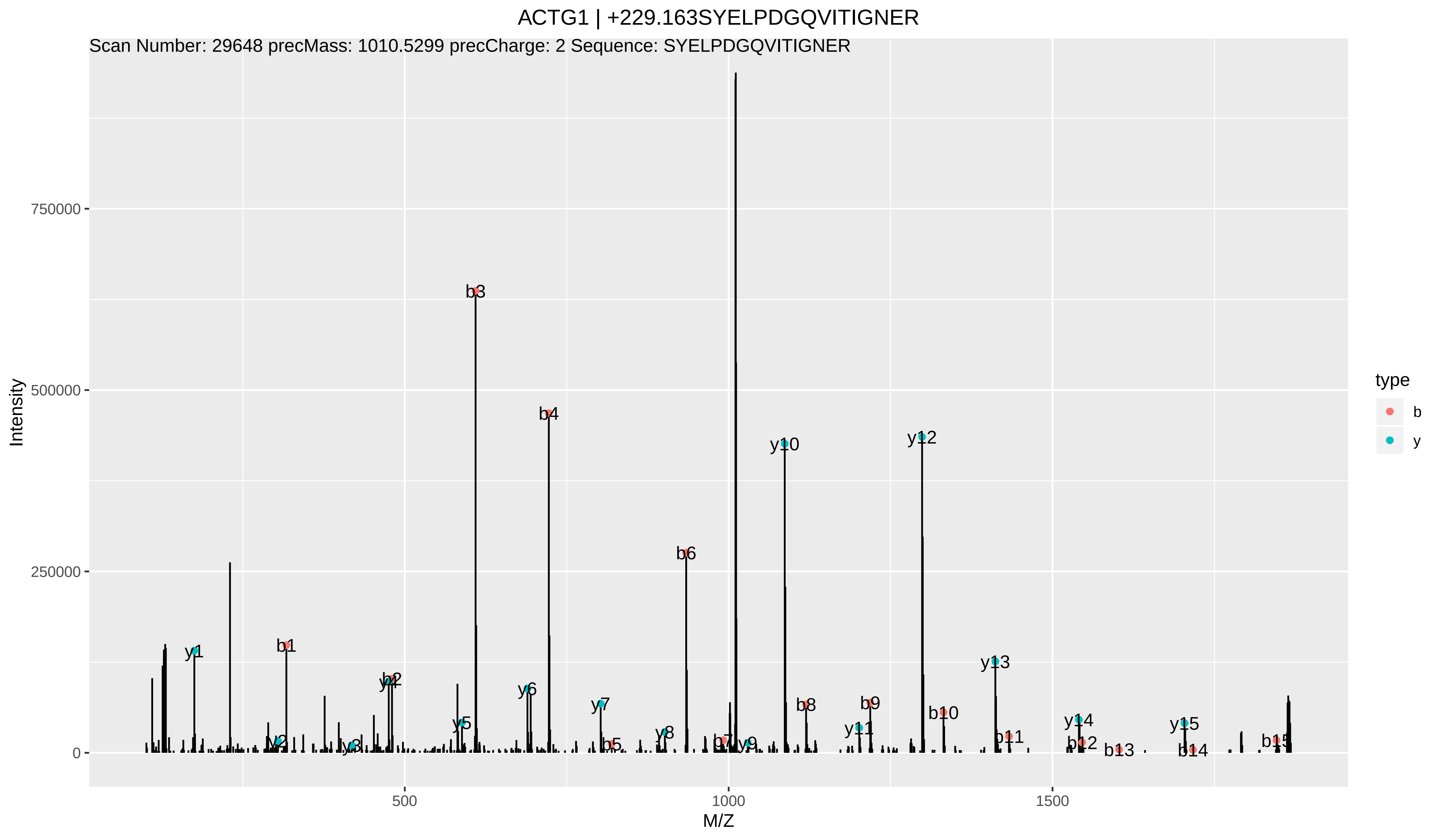

ACTG1 | +229.163SYELPDGQVITIGNER
Scan Number: 29648 precMass: 1010.5299 precCharge: 2 Sequence: SYELPDGQVITIGNER
Intensity
750000
500000
250000
0
M/Z
500
1000
1500
type
b
y
y1
b1
y2
y3
y4
b2
y5
b3
y6
b4
y7
b5
y8
b6
b7
y9
y10
b8
y11
b9
y12
b10
y13
b11
y14
b12
b13
y15
b14
b15

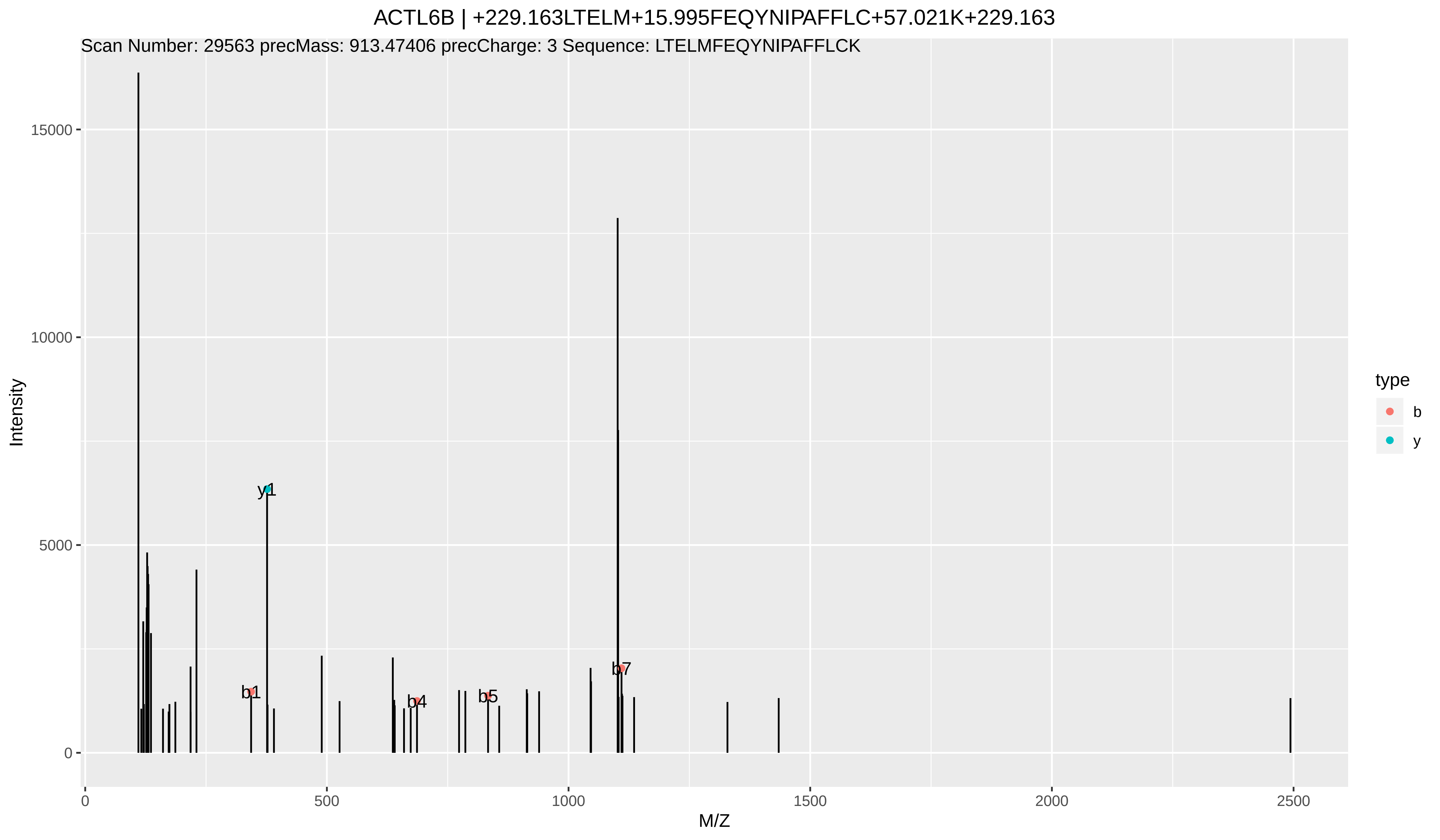

ACTL6B | +229.163LTELM+15.995FEQYNIPAFFLC+57.021K+229.163
Scan Number: 29563 precMass: 913.47406 precCharge: 3 Sequence: LTELMFEQYNIPAFFLCK
Intensity
15000
10000
5000
0
M/Z
0
500
1000
1500
2000
2500
y1
b1
b4
b5
b7
type
b
y

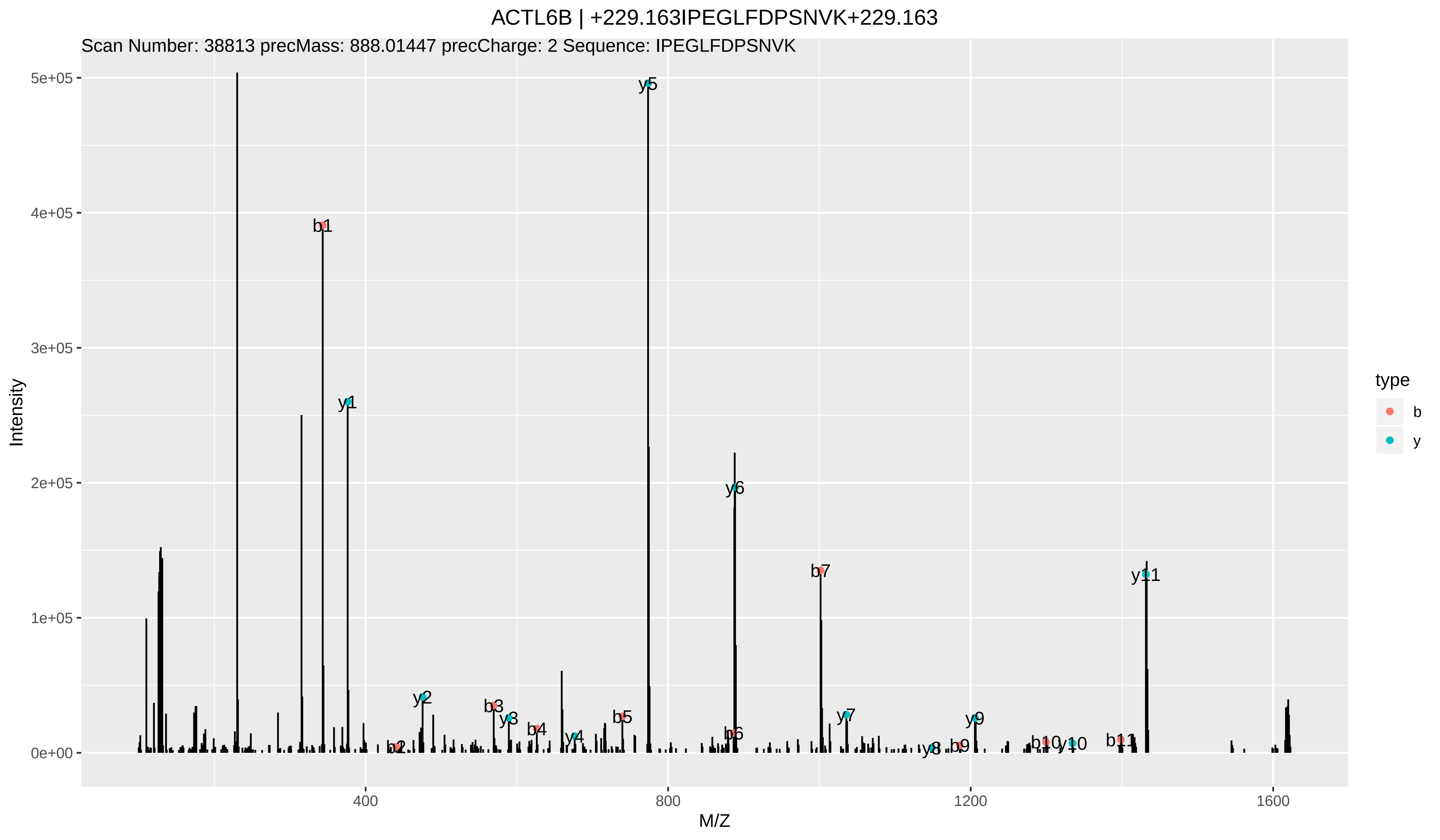
ACTL6B | +229.163IPEGLFDPSNVK+229.163
Scan Number: 38813 precMass: 888.01447 precCharge: 2 Sequence: IPEGLFDPSNVK
Intensity
M/Z
0e+00
1e+05
2e+05
3e+05
4e+05
5e+05
400
800
1200
1600
b1
y1
y2
b2
b3
y3
b4
y4
b5
y5
b6
y6
b7
y7
y8
b9
y9
b10
y10
b11
y11
type
b
y

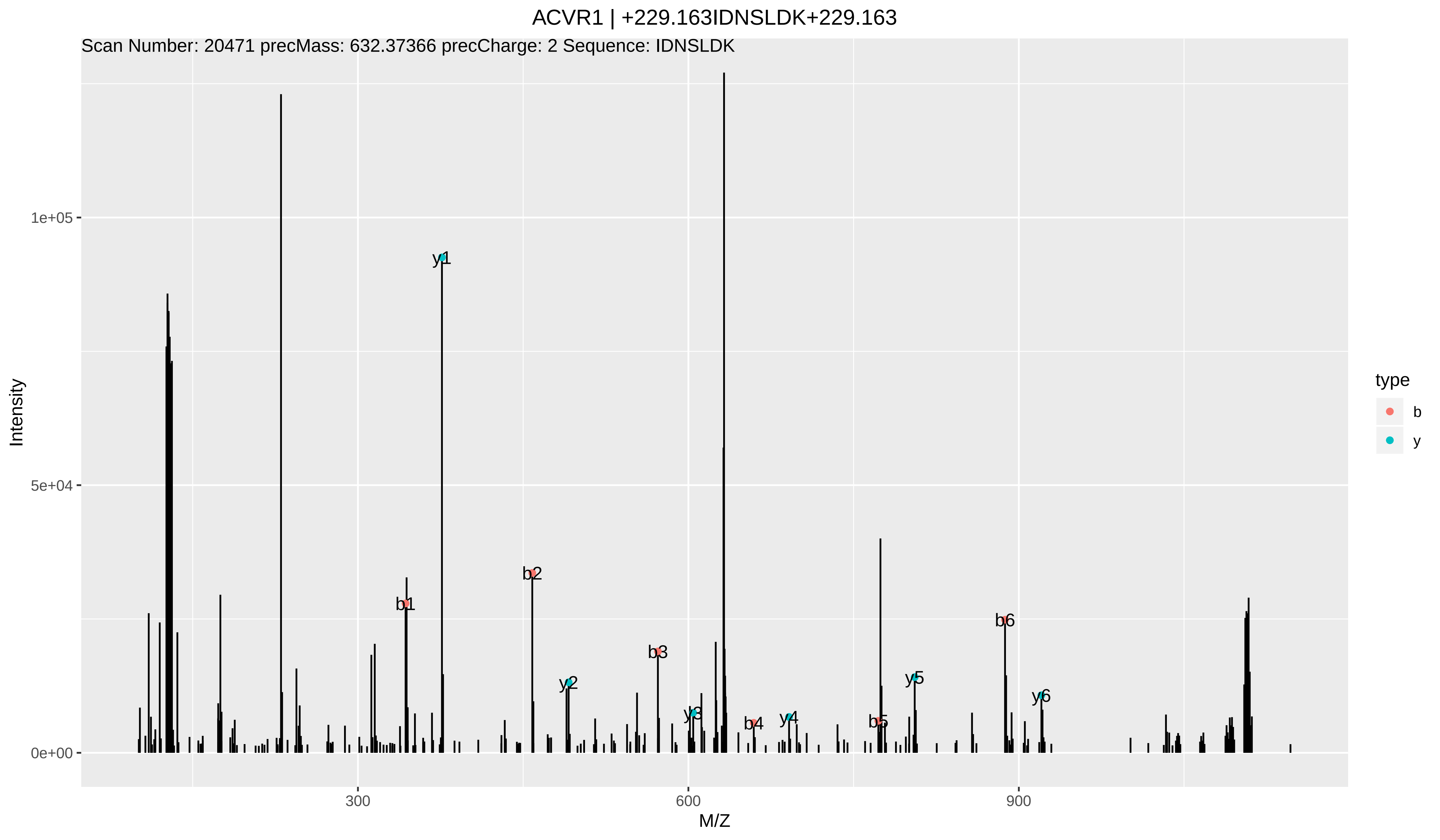

ACVR1 | +229.163IDNSLDK+229.163
Scan Number: 20471 precMass: 632.37366 precCharge: 2 Sequence: IDNSLDK
Intensity
1e+05
5e+04
0e+00
M/Z
300
600
900
y1
b1
b2
y2
b3
y3
b4
y4
b5
y5
b6
y6
type
b
y

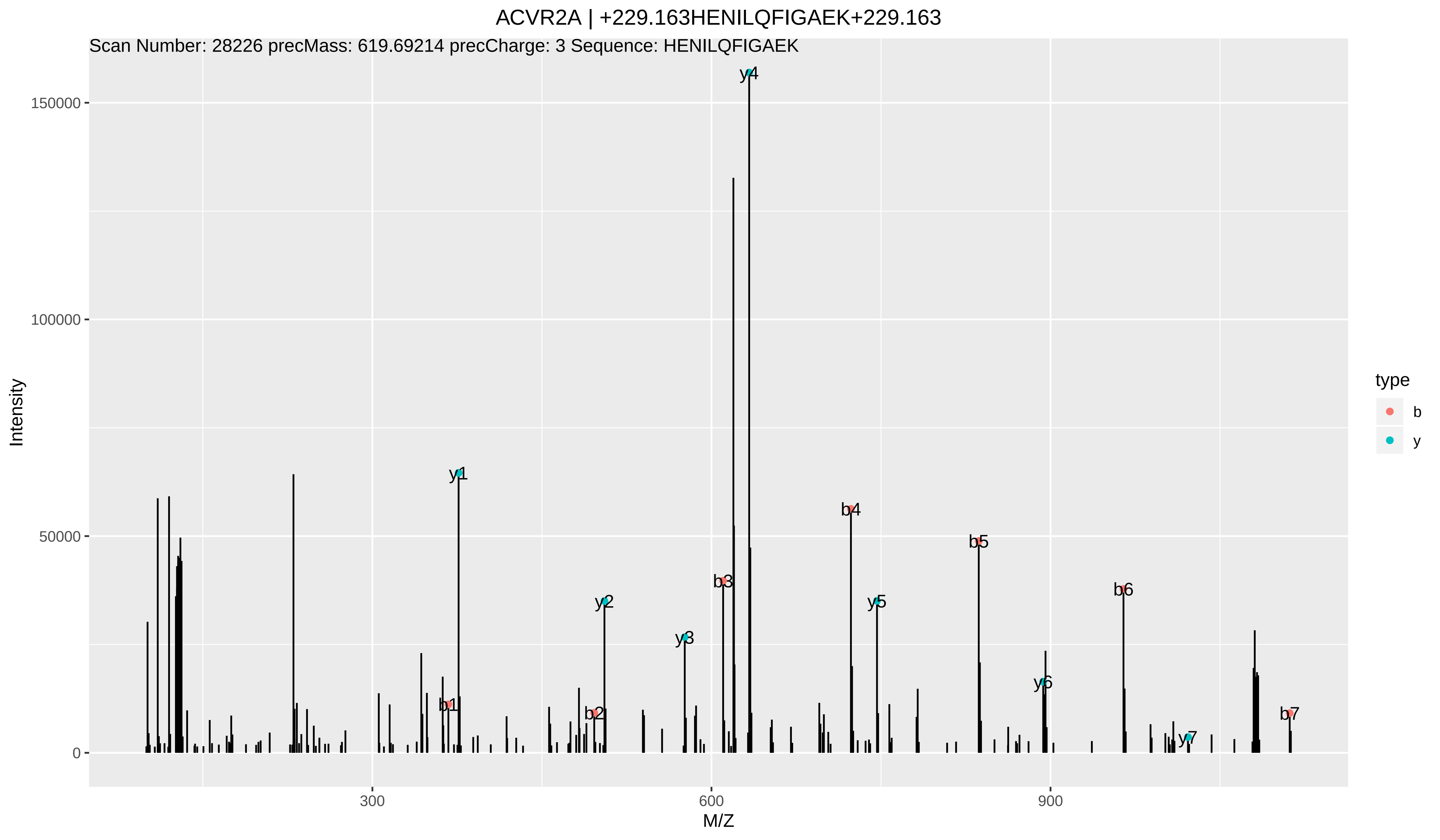

ACVR2A | +229.163HENILQFIGAEK+229.163
Scan Number: 28226 precMass: 619.69214 precCharge: 3 Sequence: HENILQFIGAEK
Intensity
150000
100000
50000
0
300
600
900
M/Z
y4
y1
b4
b5
b3
y2
b6
y5
y3
y6
b1
b2
b7
y7
type
b
y

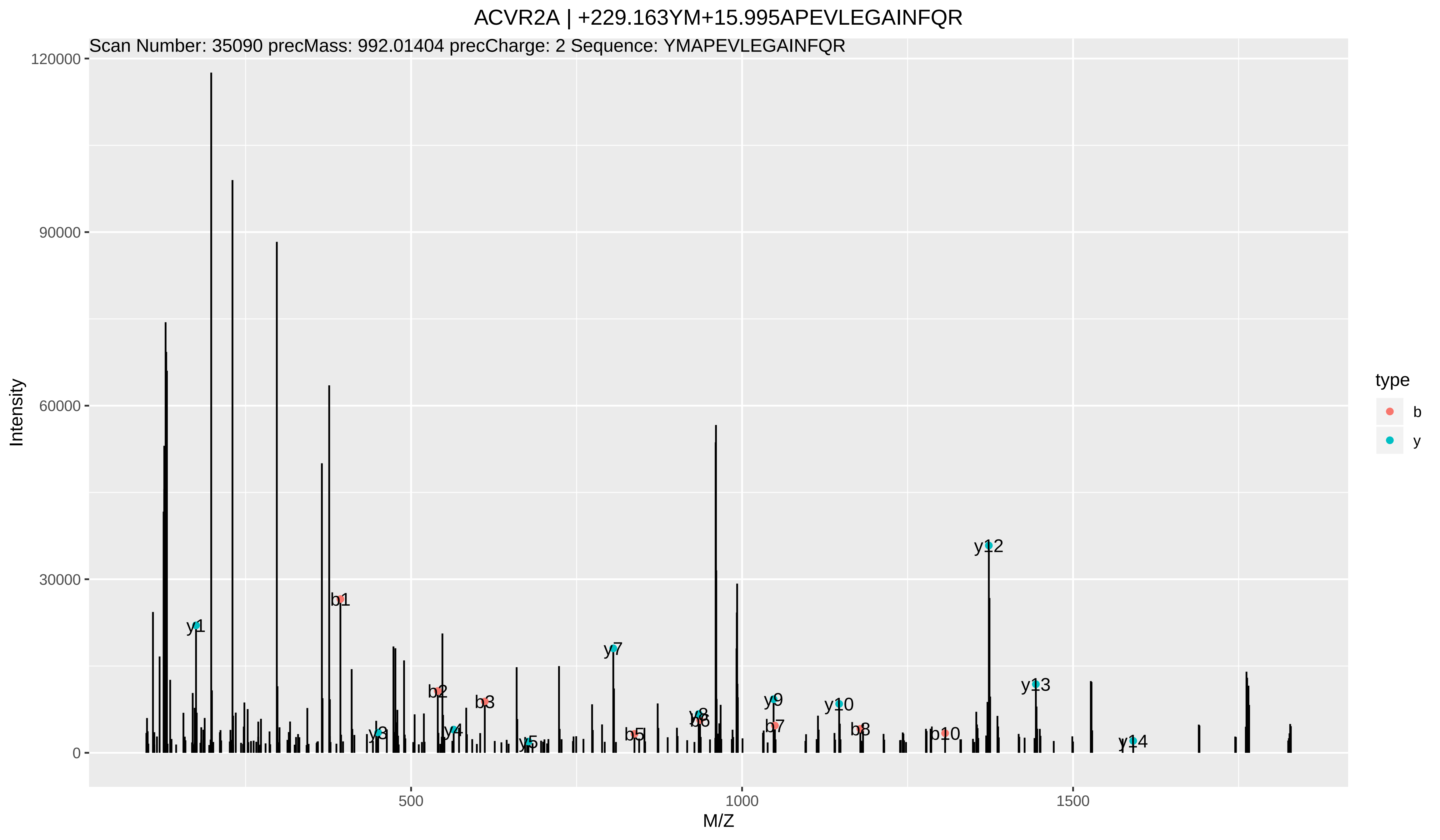
ACVR2A | +229.163YM+15.995APEVLEGAINFQR
Scan Number: 35090 precMass: 992.01404 precCharge: 2 Sequence: YMAPEVLEGAINFQR
Intensity
M/Z
120000
90000
60000
30000
0
500
1000
1500
y1
b1
b2
b3
y3
y4
y5
b5
y7
y8
b6
y9
b7
y10
b8
b10
y12
y13
y14
type
b
y

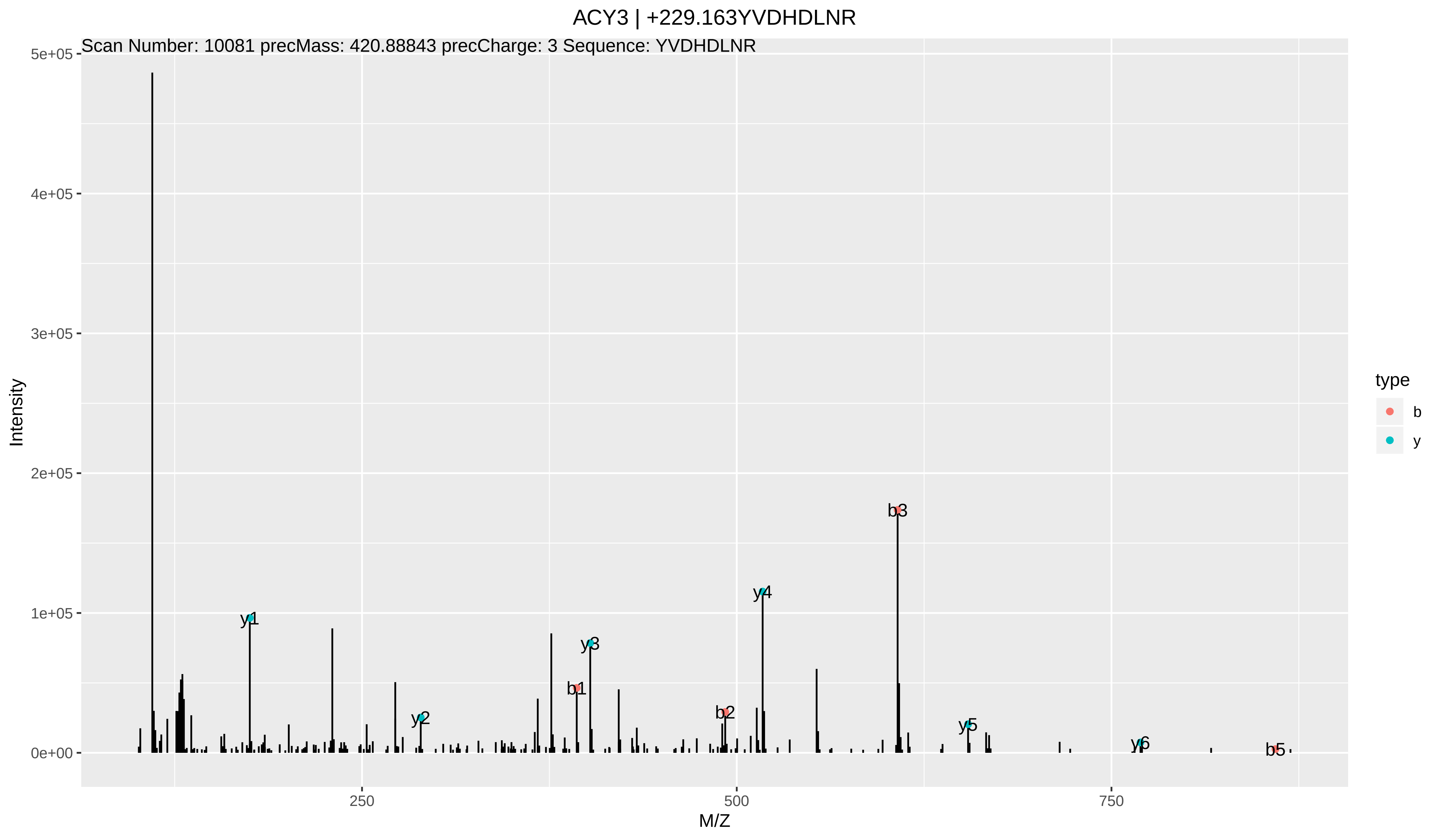
ACY3 | +229.163YVDHDLNR
Scan Number: 10081 precMass: 420.88843 precCharge: 3 Sequence: YVDHDLNR
Intensity
5e+05
4e+05
3e+05
2e+05
1e+05
0e+00
M/Z
250
500
750
y1
y2
y3
y4
y5
y6
b1
b2
b3
b5
type
b
y

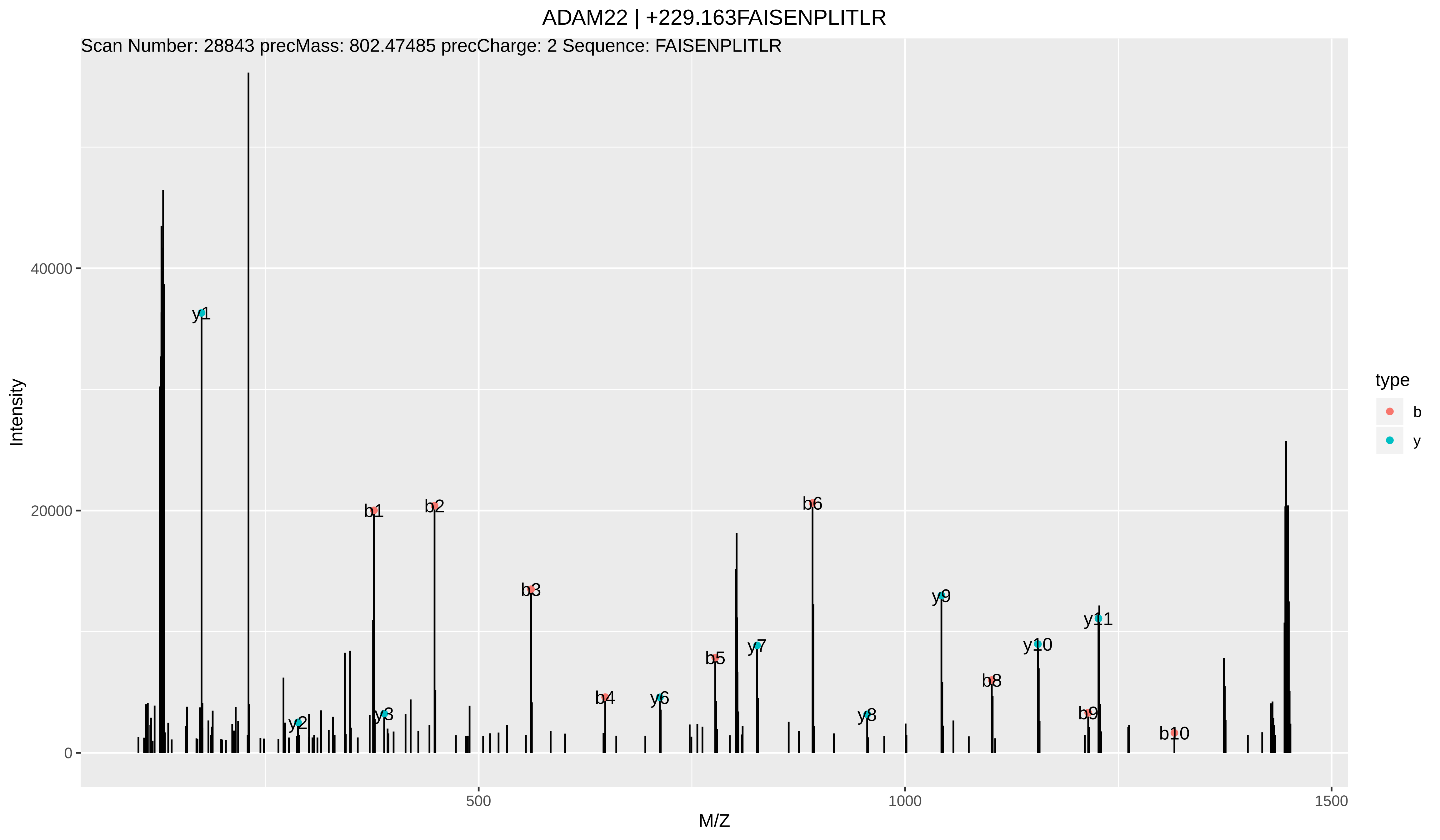
ADAM22 | +229.163FAISENPLITLR
Scan Number: 28843 precMass: 802.47485 precCharge: 2 Sequence: FAISENPLITLR
Intensity
M/Z
0
20000
40000
500
1000
1500
y1
y2
y3
y6
y7
y8
y9
y10
y11
b1
b2
b3
b4
b5
b6
b8
b9
b10
type
b
y

ADAMTS16 | +229.163FYGK+229.163QC+57.021C+57.021K+229.163TC+57.021SK+229.163

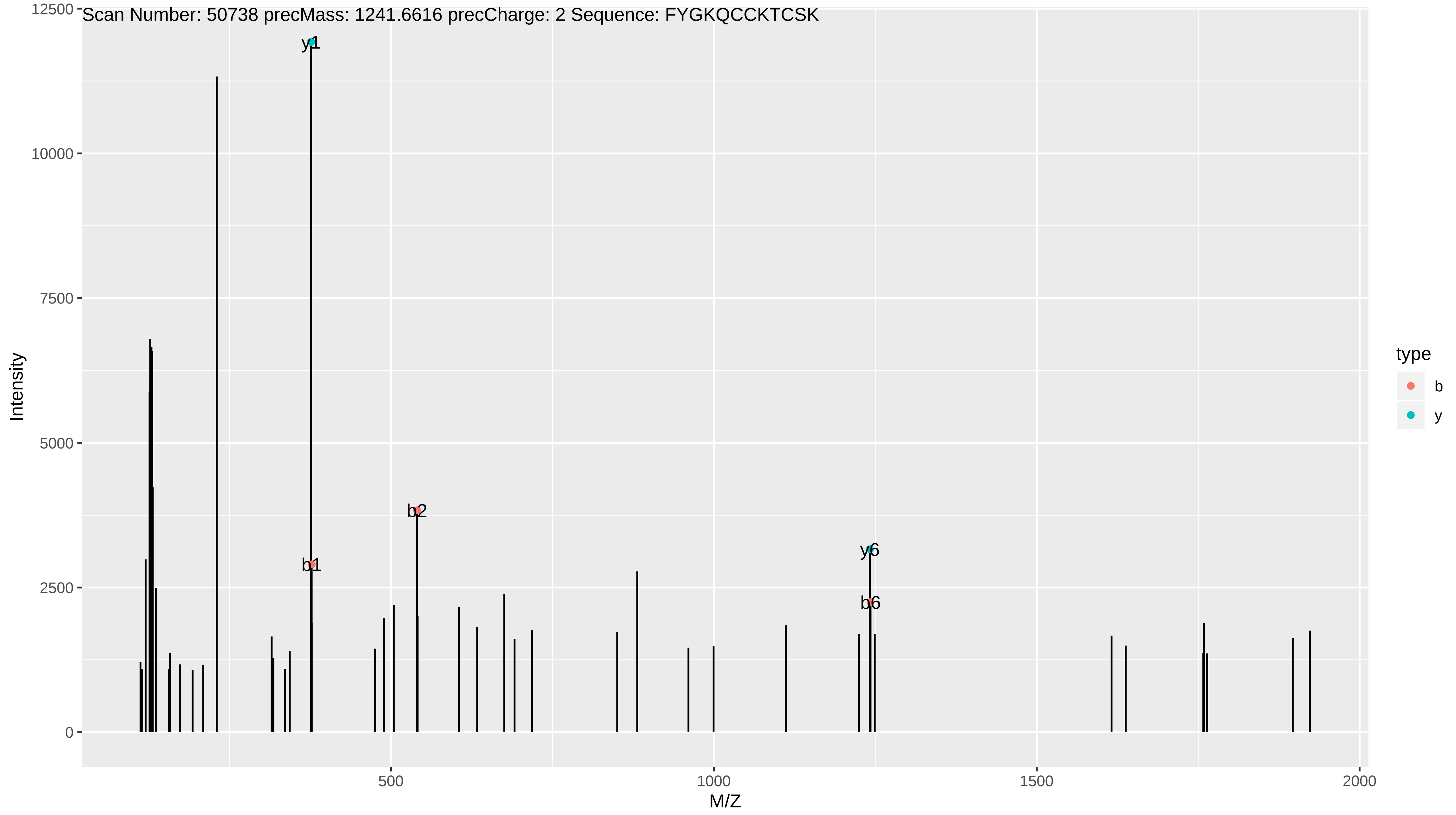

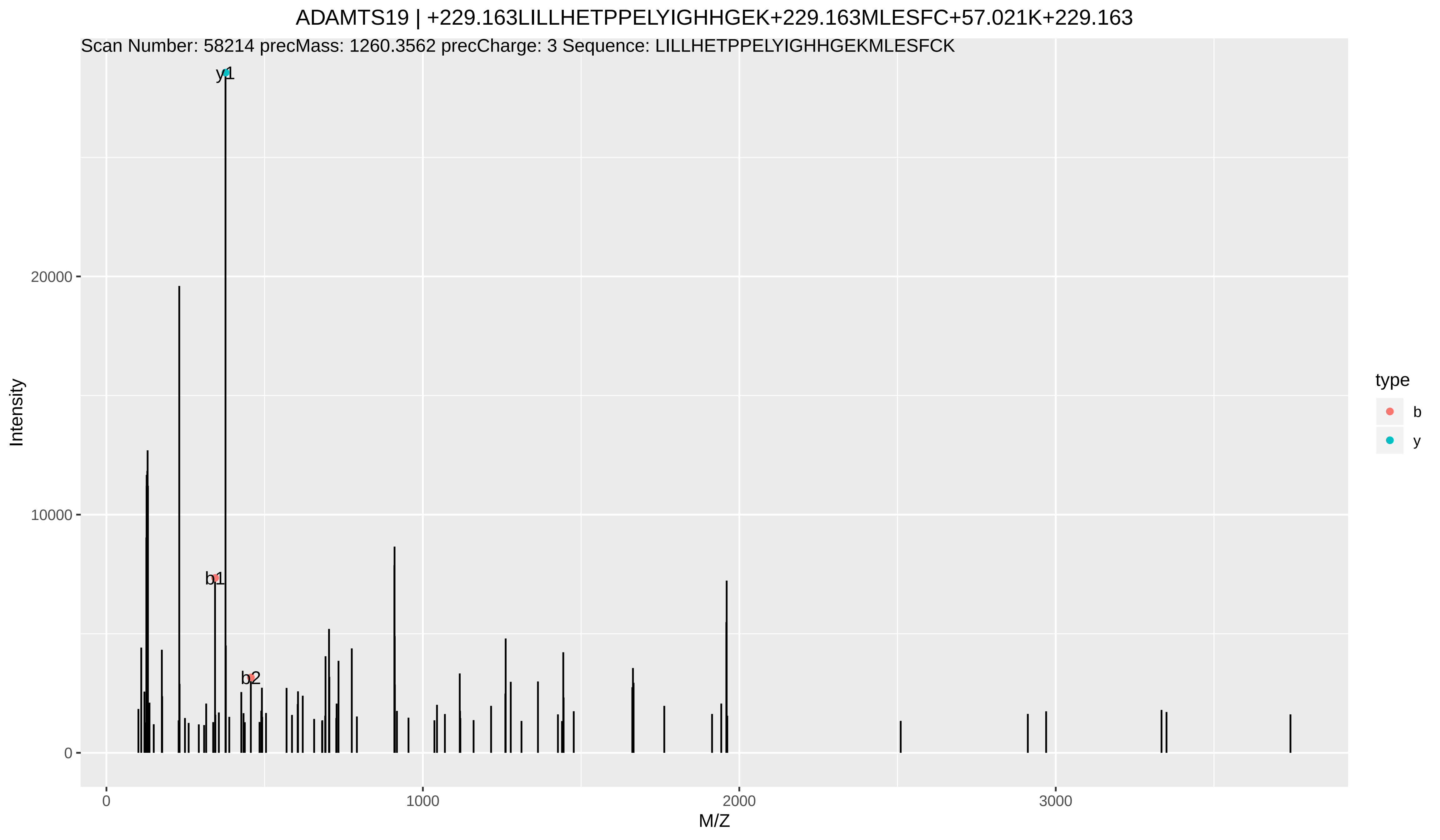

ADAMTS19 | +229.163LILLHETPPELYIGHHGEK+229.163MLESFC+57.021K+229.163
Scan Number: 58214 precMass: 1260.3562 precCharge: 3 Sequence: LILLHETPPELYIGHHGEKMLESFCK
y1
b1
b2
Intensity
20000
10000
0
0
1000
2000
3000
M/Z
type
b
y

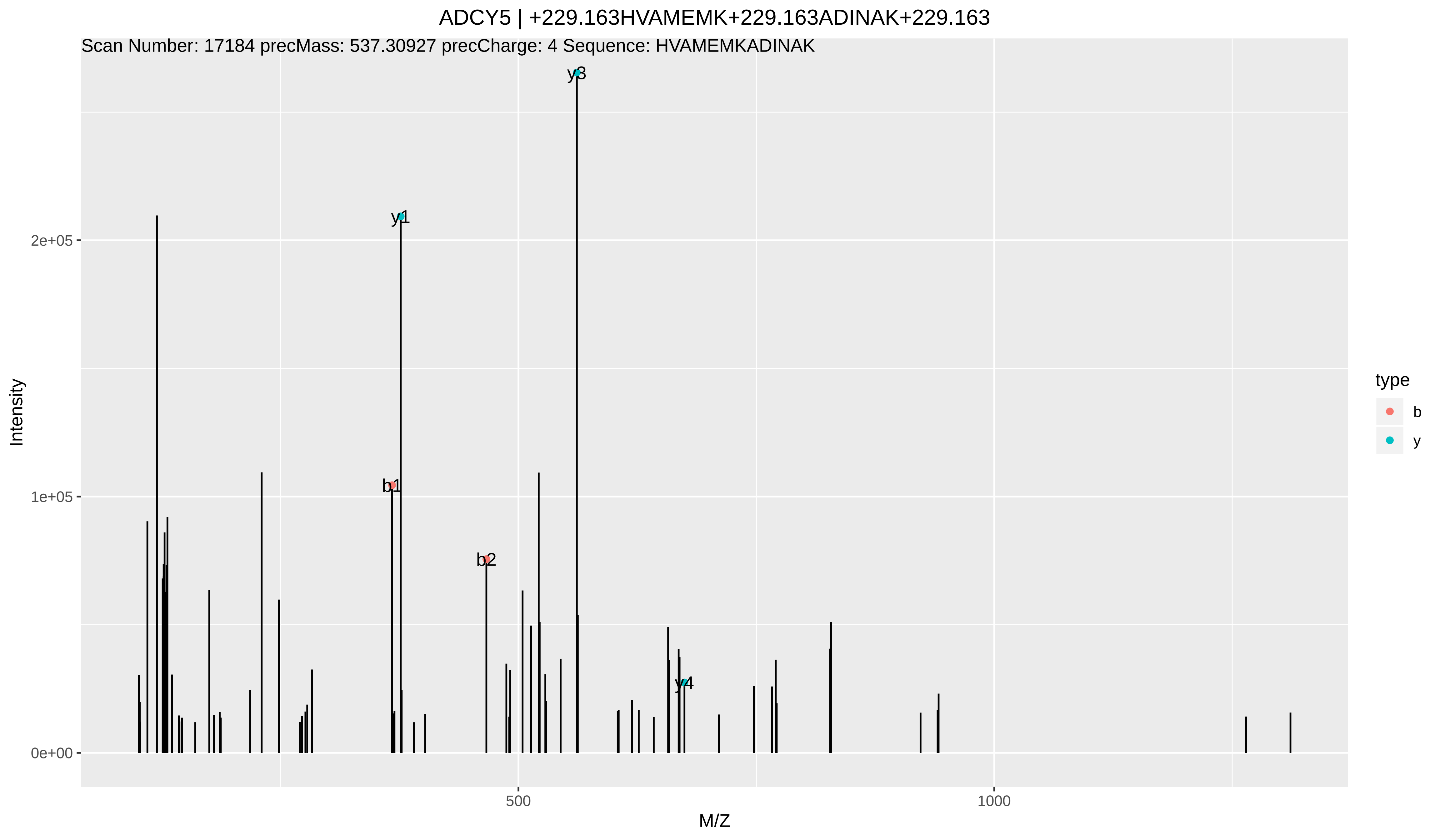

ADCY5 | +229.163HVAMEMK+229.163ADINAK+229.163
Scan Number: 17184 precMass: 537.30927 precCharge: 4 Sequence: HVAMEMKADINAK
y3
y1
b1
b2
y4
2e+05
1e+05
0e+00
Intensity
500
1000
M/Z
type
b
y

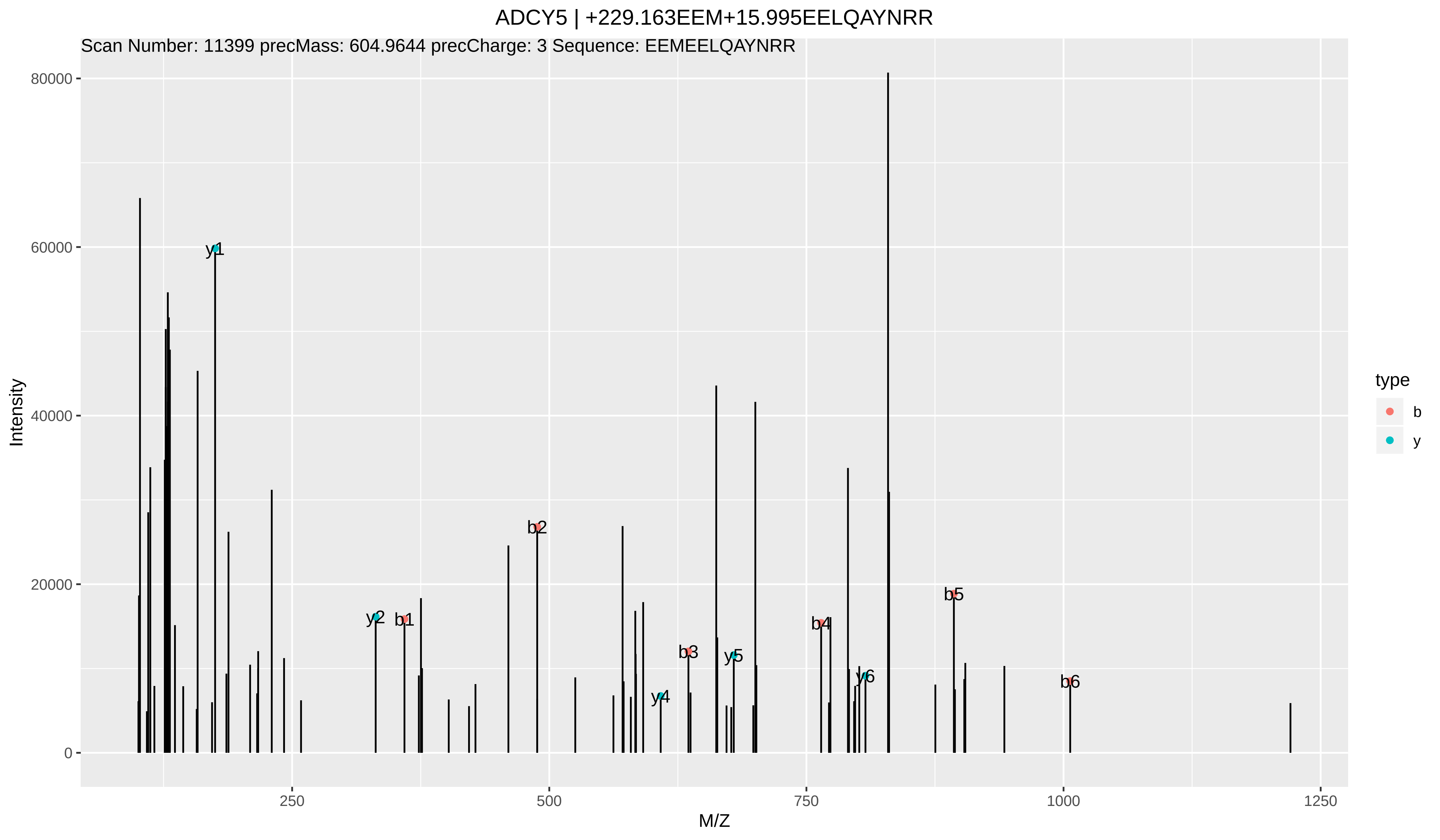
ADCY5 | +229.163EEM+15.995EELQAYNRR
Scan Number: 11399 precMass: 604.9644 precCharge: 3 Sequence: EEMEELQAYNRR
Intensity
80000
60000
40000
20000
0
M/Z
250
500
750
1000
1250
y1
y2
b1
b2
y4
b3
y5
b4
y6
b5
b6
type
b
y

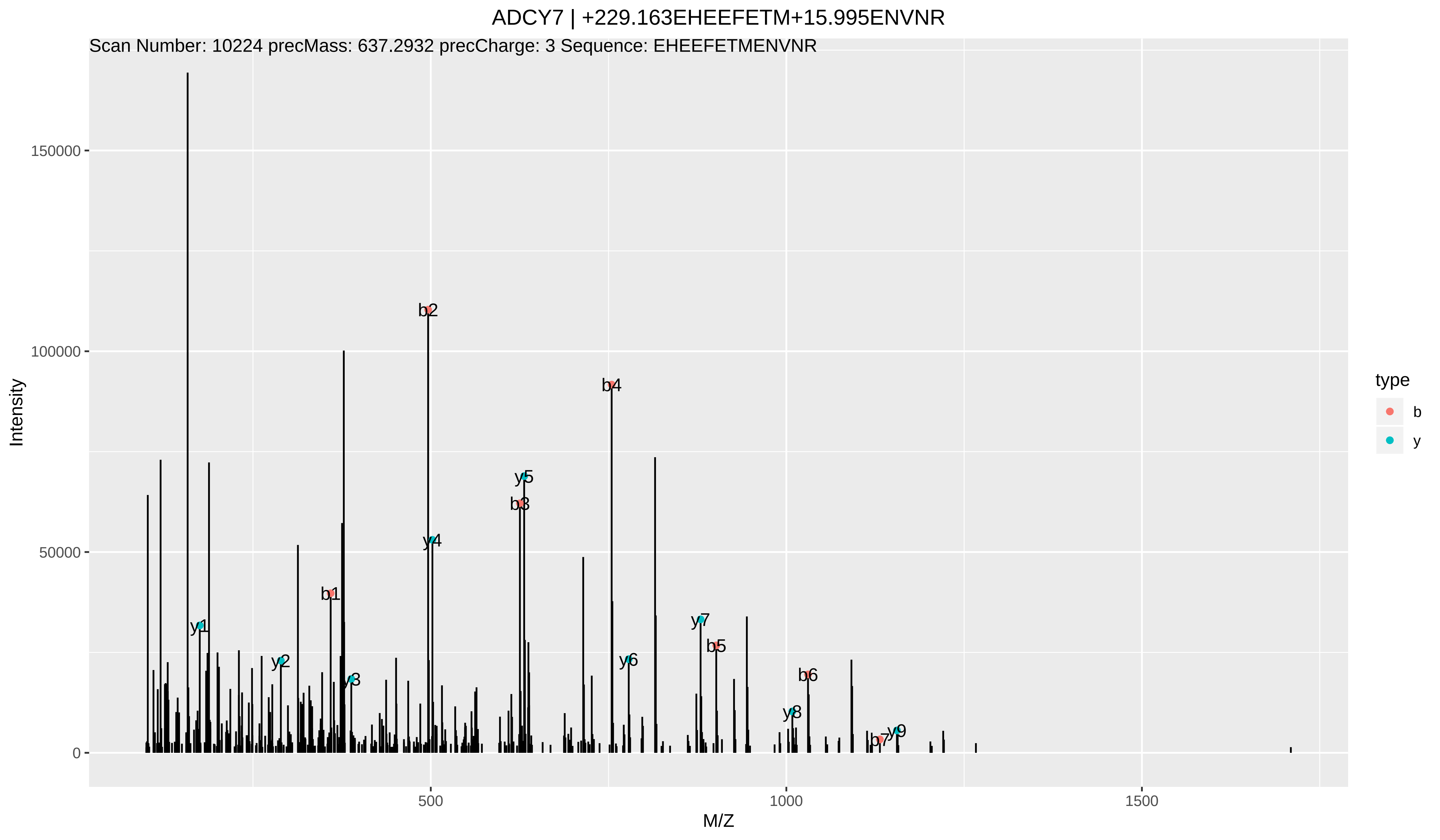

ADCY7 | +229.163EHEEFETM+15.995ENVNR
Scan Number: 10224 precMass: 637.2932 precCharge: 3 Sequence: EHEEFETMENVNR
Intensity
M/Z
0
50000
100000
150000
500
1000
1500
b1
b2
b3
b4
b5
b6
b7
y1
y2
y3
y4
y5
y6
y7
y8
y9
type
b
y

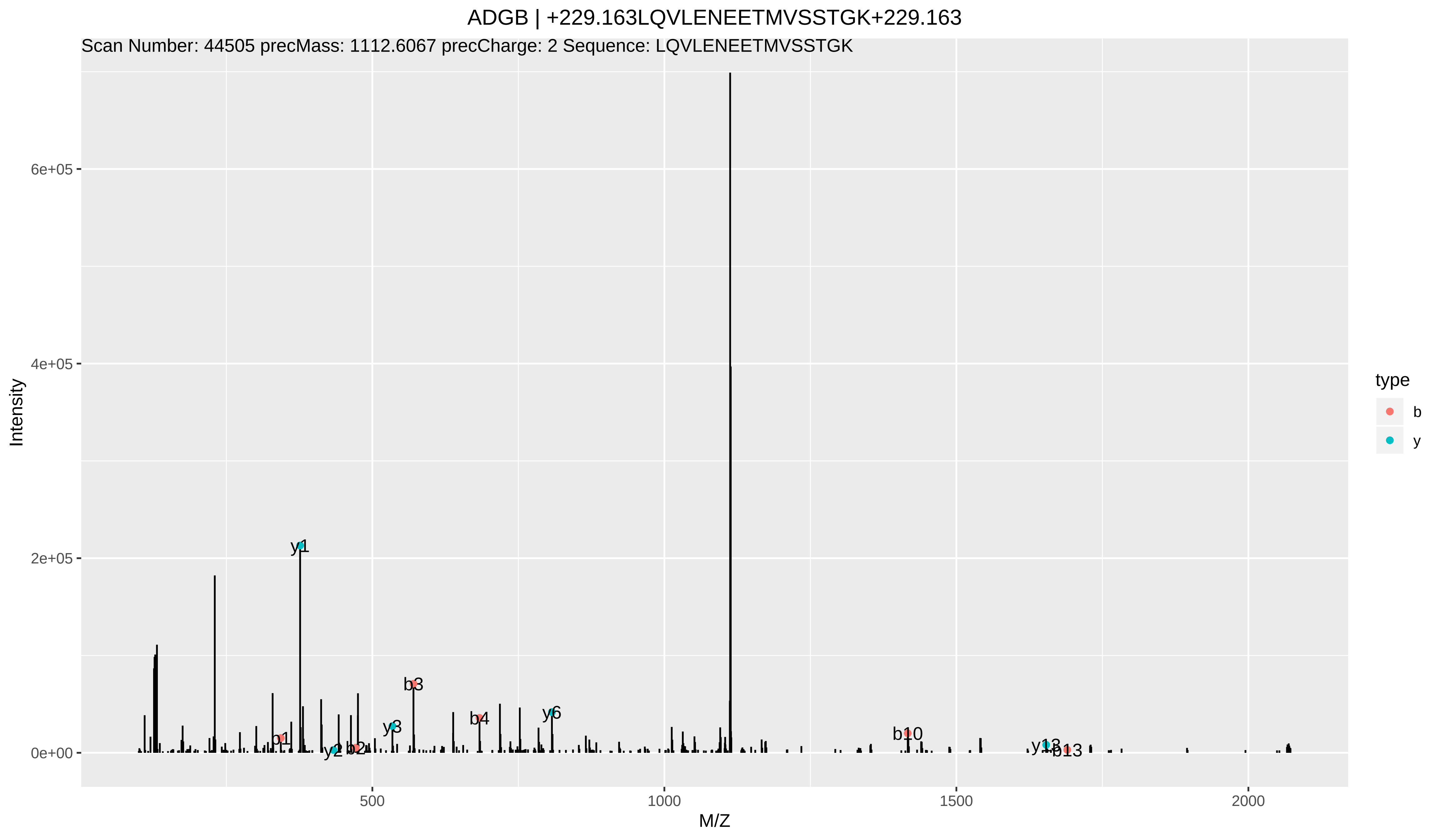

ADGB | +229.163LQVLENEETMVSSTGK+229.163
Scan Number: 44505 precMass: 1112.6067 precCharge: 2 Sequence: LQVLENEETMVSSTGK
Intensity
M/Z
6e+05
4e+05
2e+05
0e+00
500
1000
1500
2000
y1
b1
y2
b2
b3
y3
b4
y6
b10
y13
b13
type
b
y

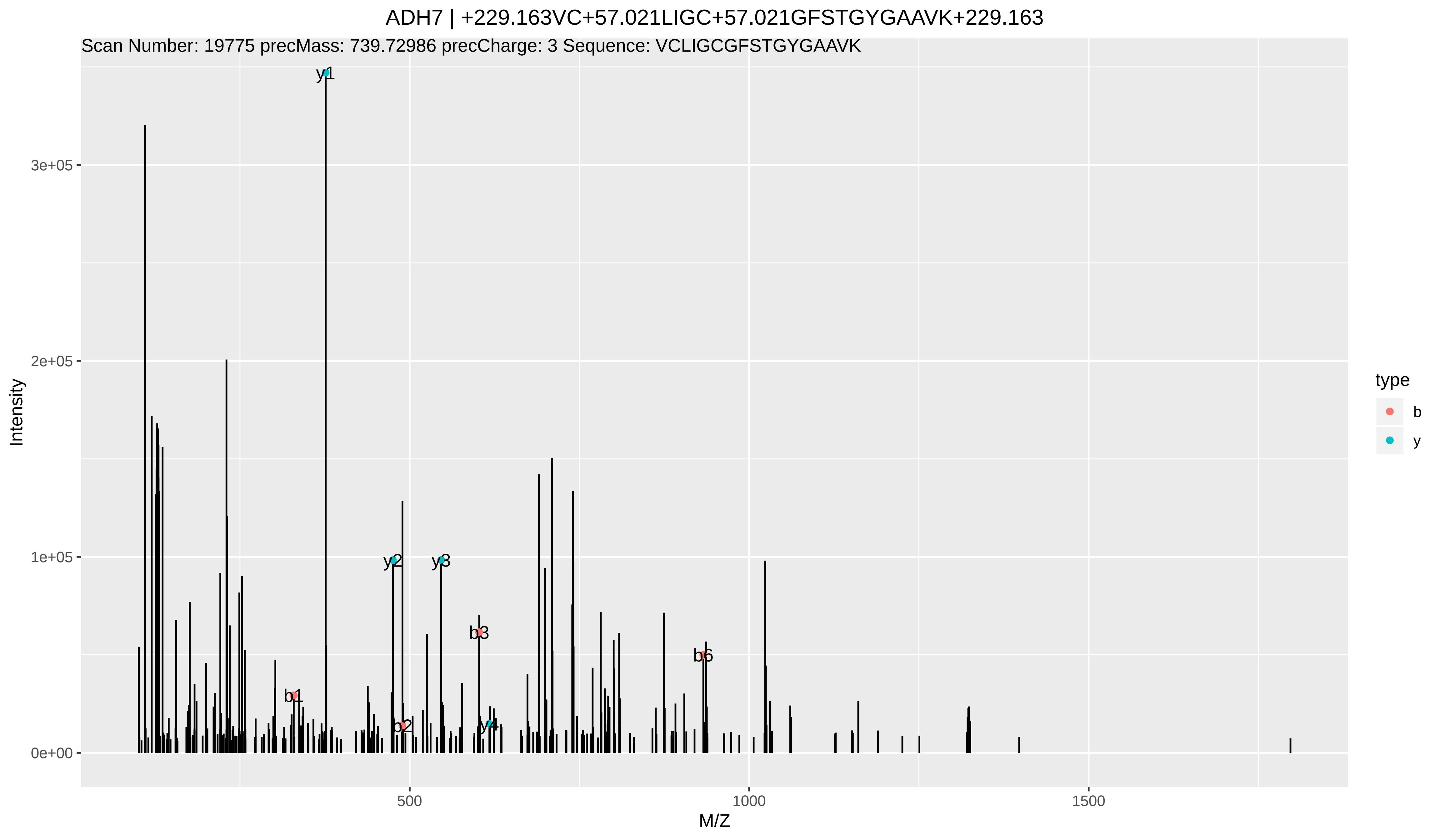

ADH7 | +229.163VC+57.021LIGC+57.021GFSTGYGAAVK+229.163
Scan Number: 19775 precMass: 739.72986 precCharge: 3 Sequence: VCLIGCGFSTGYGAAVK
Intensity
3e+05
2e+05
1e+05
0e+00
M/Z
500
1000
1500
type
b
y
y1
y2
y3
y4
b1
b2
b3
b6

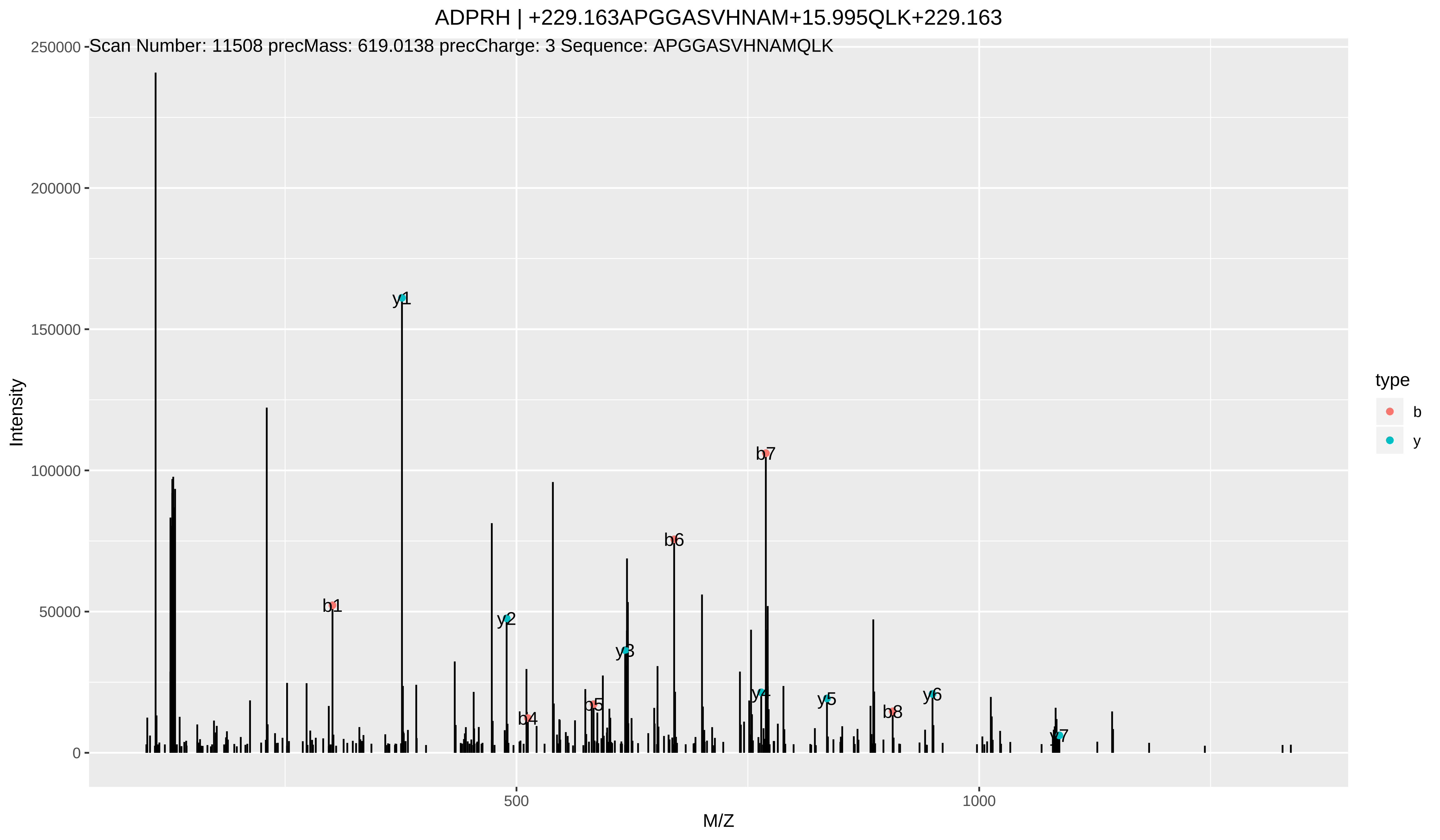

ADPRH | +229.163APGGASVHNAM+15.995QLK+229.163
Scan Number: 11508 precMass: 619.0138 precCharge: 3 Sequence: APGGASVHNAMQLK
Intensity
250000
200000
150000
100000
50000
0
M/Z
500
1000
y1
b1
y2
b4
b5
y3
b6
y4
b7
y5
b8
y6
y7
type
b
y

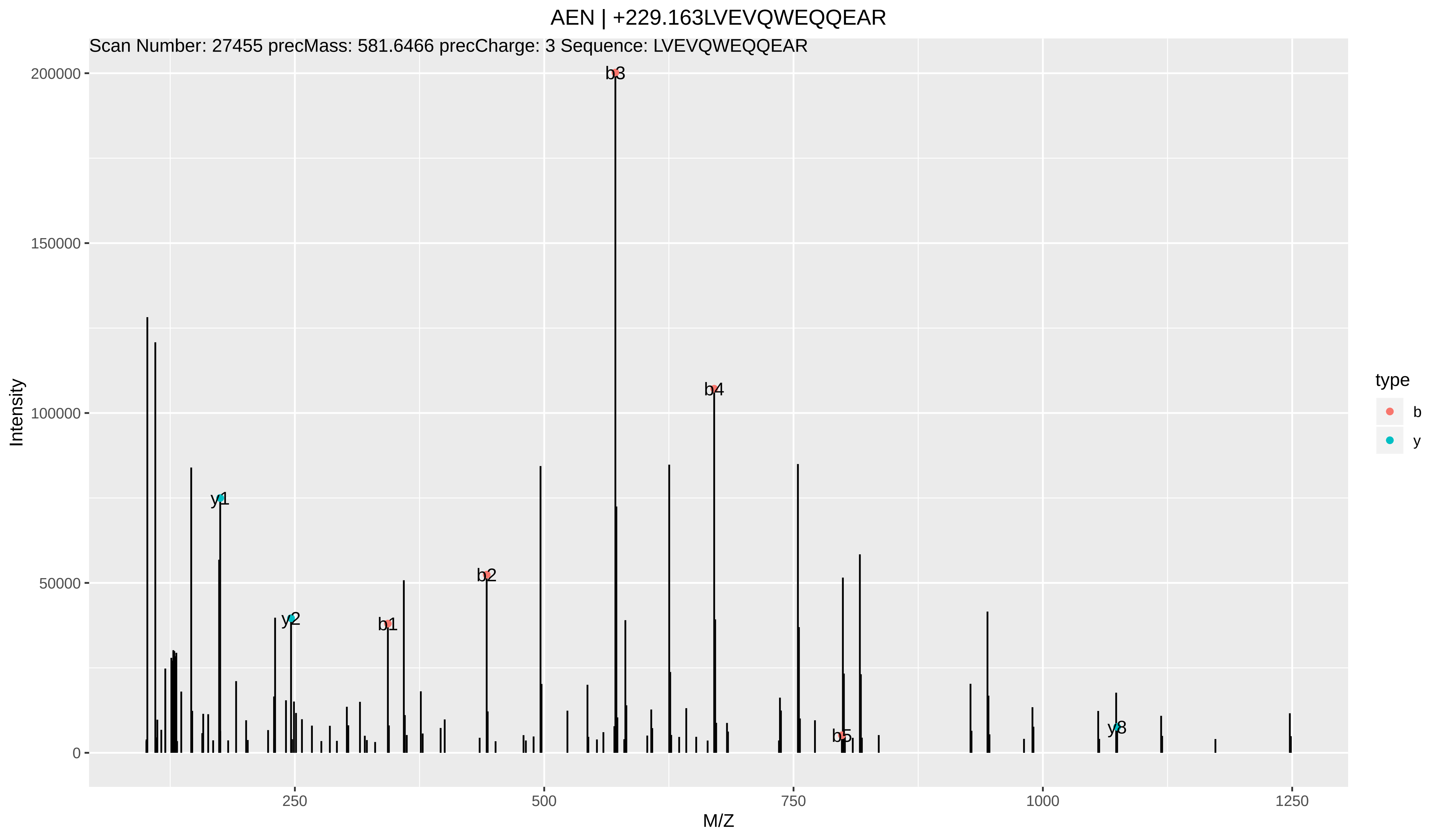

AEN | +229.163LVEVQWEQQEAR
Scan Number: 27455 precMass: 581.6466 precCharge: 3 Sequence: LVEVQWEQQEAR
Intensity
M/Z
type
b
y
b3
b4
y1
b2
y2
b1
b5
y8
200000
150000
100000
50000
0
250
500
750
1000
1250

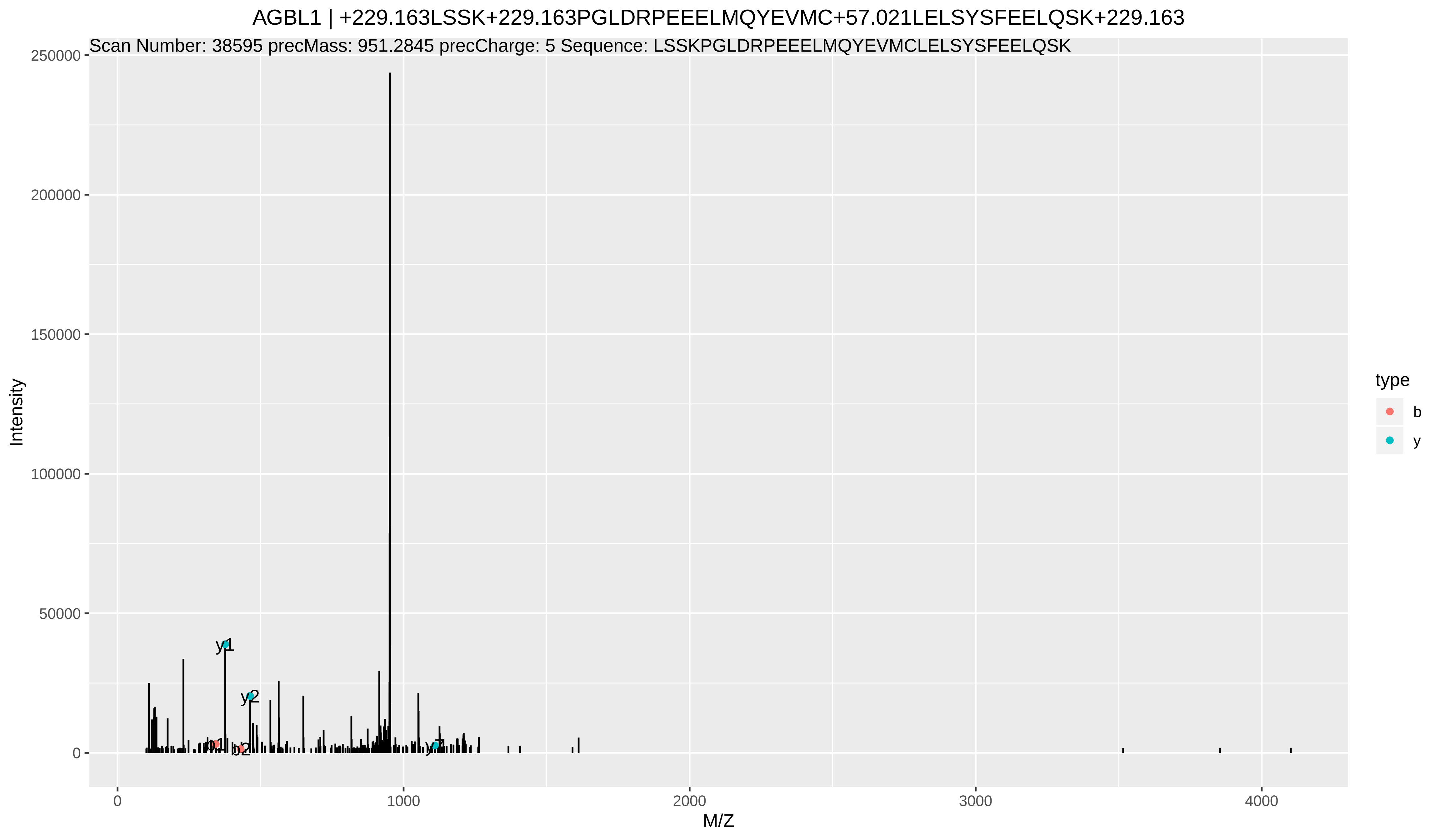
AGBL1 | +229.163LSSK+229.163PGLDRPEEELMQYEVMC+57.021LELSYSFEELQSK+229.163
Scan Number: 38595 precMass: 951.2845 precCharge: 5 Sequence: LSSKPGLDRPEEELMQYEVMCLELSYSFEELQSK
Intensity
250000
200000
150000
100000
50000
0
M/Z
0
1000
2000
3000
4000
y1
y2
b1
b2
y7
type
b
y

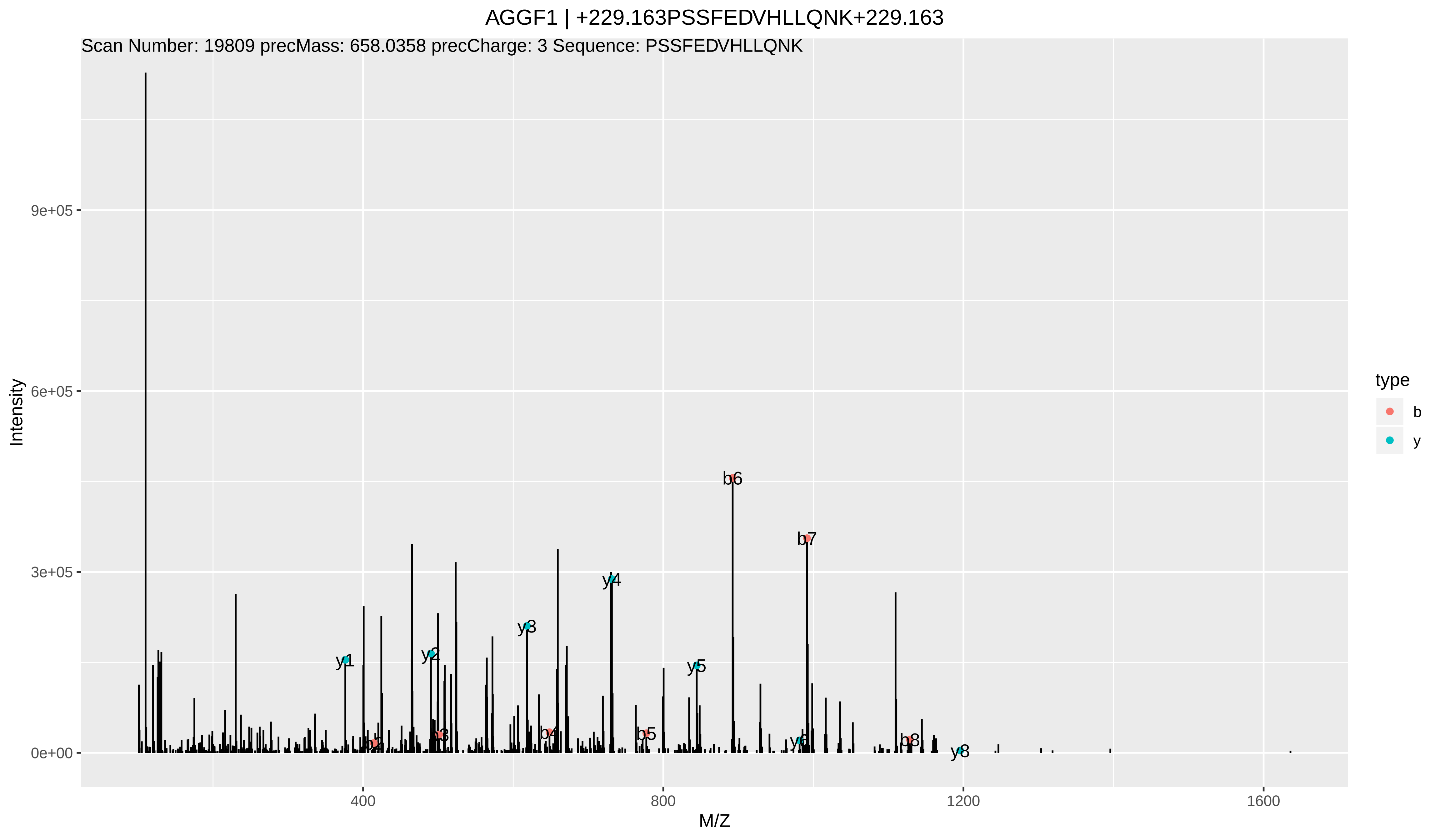
AGGF1 | +229.163PSSFEDVHLLQNK+229.163
Scan Number: 19809 precMass: 658.0358 precCharge: 3 Sequence: PSSFEDVHLLQNK
Intensity
9e+05
6e+05
3e+05
0e+00
M/Z
400
800
1200
1600
y1
y2
y3
y4
y5
y6
y8
b2
b3
b4
b5
b6
b7
b8
type
b
y

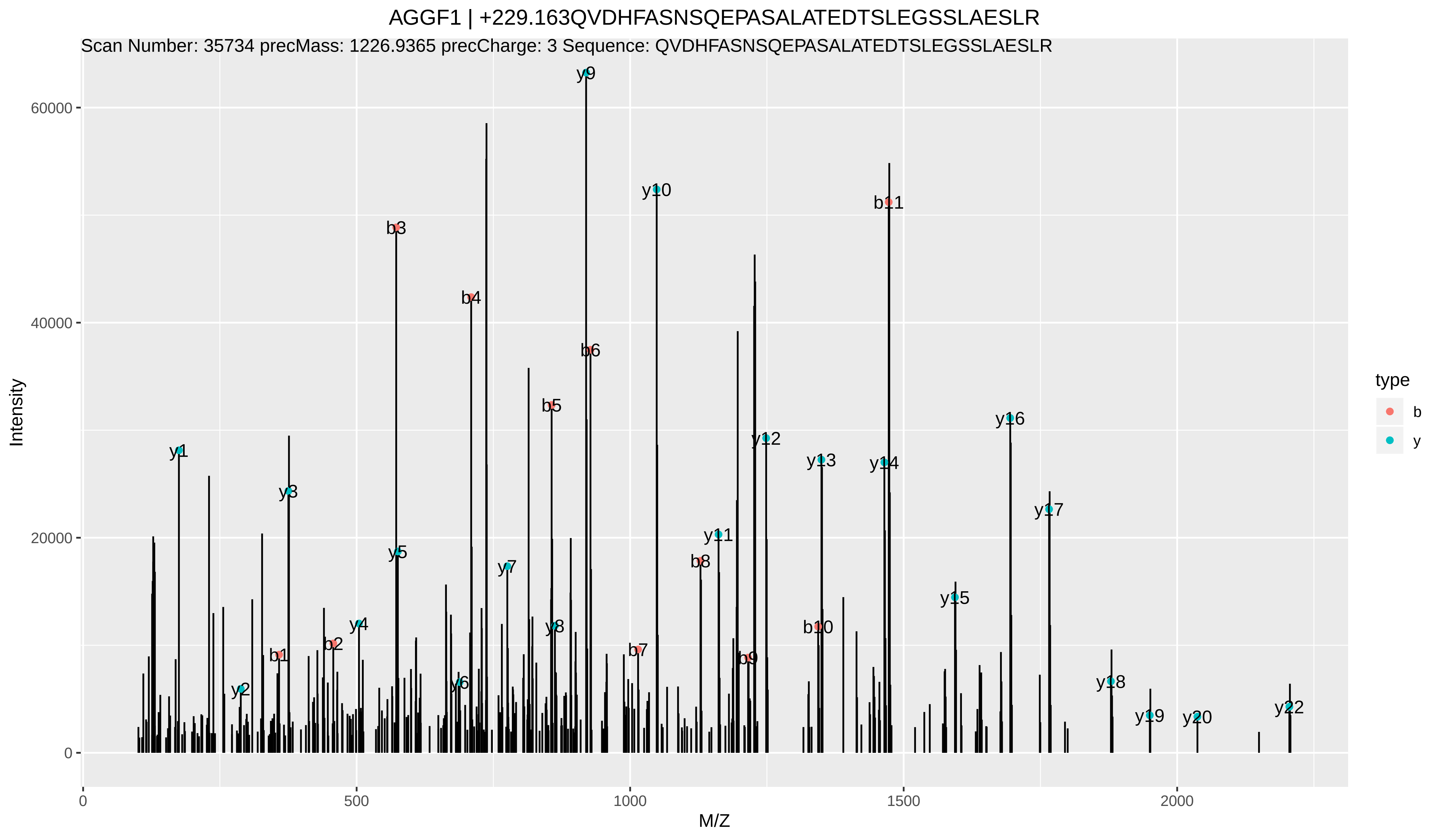

AGGF1 | +229.163QVDHFASNSQEPASALATEDTSLEGSSLAESLR
Scan Number: 35734 precMass: 1226.9365 precCharge: 3 Sequence: QVDHFASNSQEPASALATEDTSLEGSSLAESLR
Intensity
M/Z
type
b
y
y1
y2
y3
y4
y5
y6
y7
y8
y9
y10
y11
y12
y13
y14
y15
y16
y17
y18
y19
y20
y22
b1
b2
b3
b4
b5
b6
b7
b8
b9
b10
b11
0
20000
40000
60000
500
1000
1500
2000

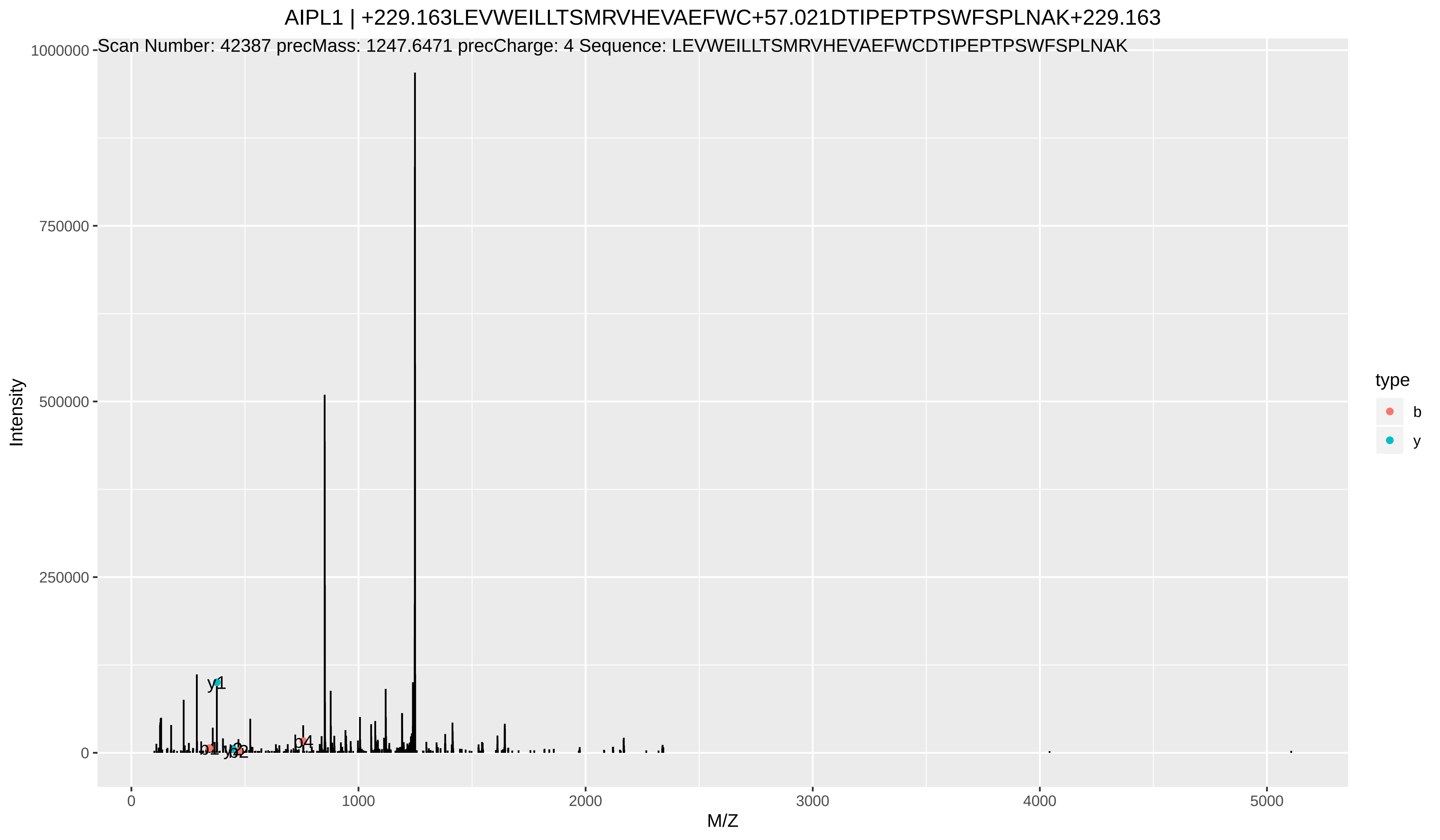

AIPL1 | +229.163LEVWEILLTSMRVHEVAEFWC+57.021DTIPEPTPSWFSPLNAK+229.163
Scan Number: 42387 precMass: 1247.6471 precCharge: 4 Sequence: LEVWEILLTSMRVHEVAEFWCDTIPEPTPSWFSPLNAK
Intensity
M/Z
0
250000
500000
750000
1000000
0
1000
2000
3000
4000
5000
y1
b1
y2
b2
b4
type
b
y

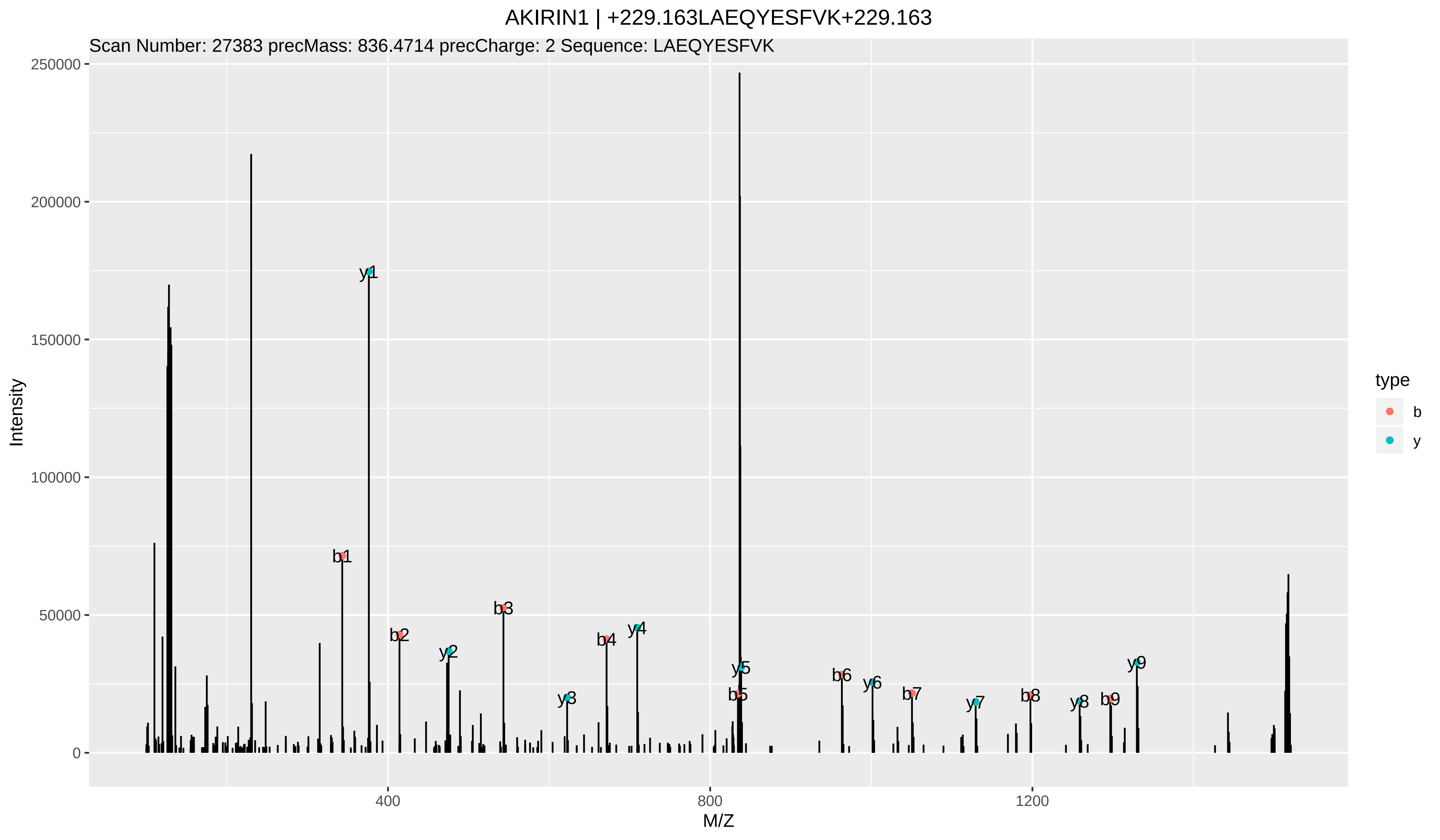

AKIRIN1 | +229.163LAEQYESFVK+229.163
Scan Number: 27383 precMass: 836.4714 precCharge: 2 Sequence: LAEQYESFVK
Intensity
250000
200000
150000
100000
50000
0
M/Z
400
800
1200
y1
b1
b2
y2
b3
y3
b4
y4
y5
b5
b6
y6
b7
y7
b8
y8
b9
y9
type
b
y

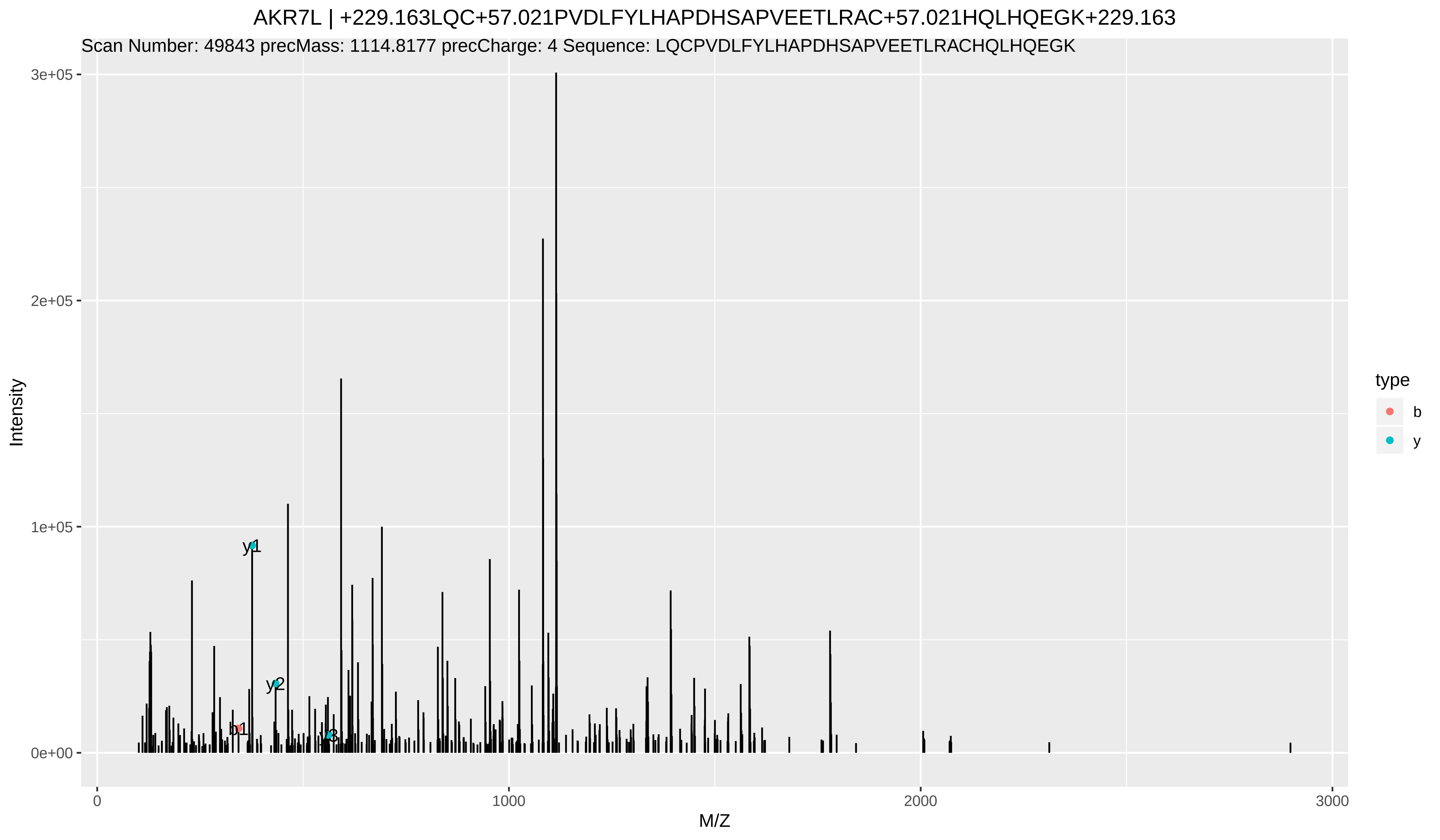
AKR7L | +229.163LQC+57.021PVDLFYLHAPDHSAPVEETLRAC+57.021HQLHQEGK+229.163
Scan Number: 49843 precMass: 1114.8177 precCharge: 4 Sequence: LQCPVDLFYLHAPDHSAPVEETLRACHQLHQEGK
Intensity
3e+05
2e+05
1e+05
0e+00
M/Z
0
1000
2000
3000
y1
y2
b1
y3
type
b
y

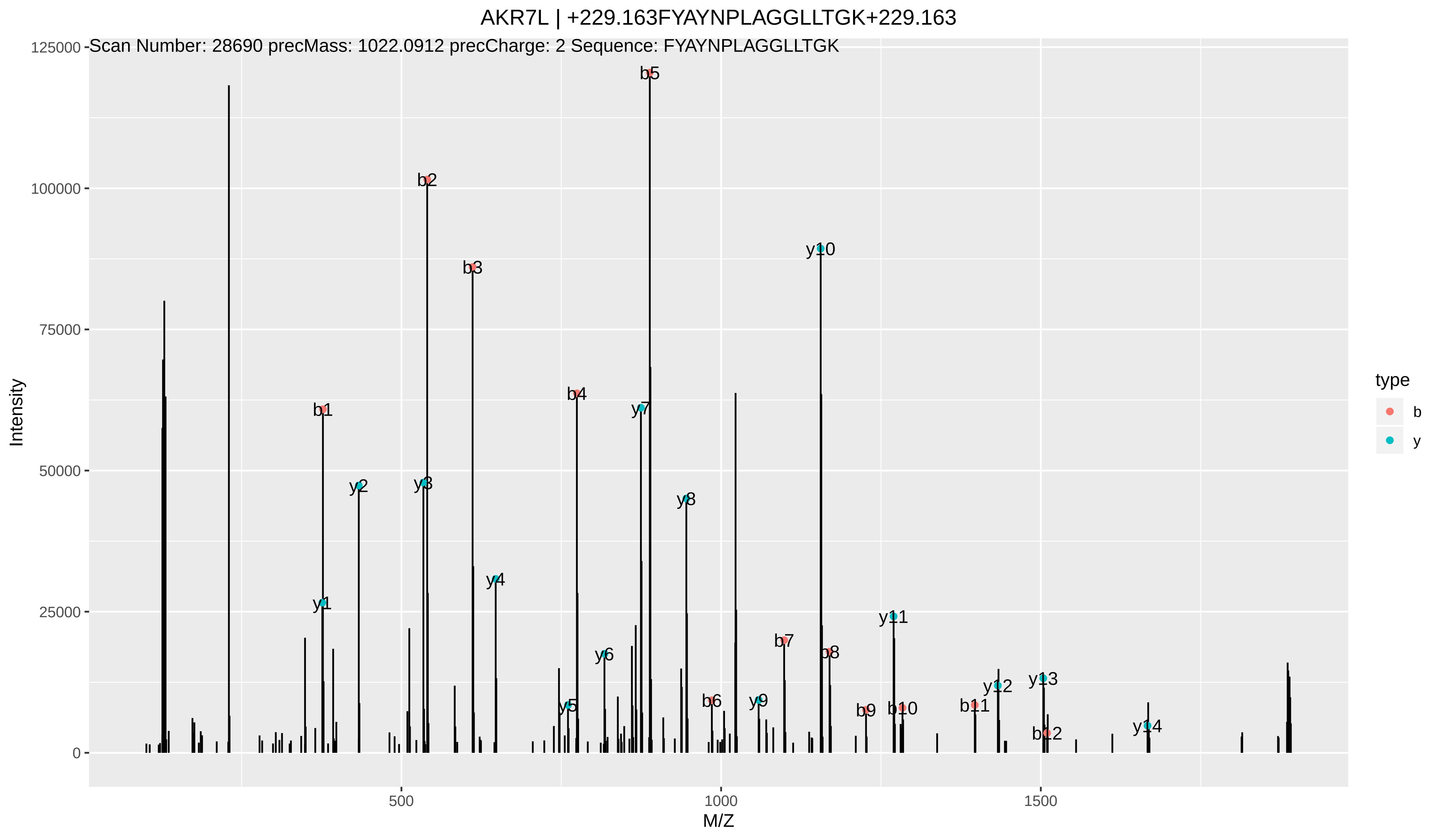

AKR7L | +229.163FYAYNPLAGGLLTGK+229.163
Scan Number: 28690 precMass: 1022.0912 precCharge: 2 Sequence: FYAYNPLAGGLLTGK
Intensity
M/Z
0
25000
50000
75000
100000
125000
500
1000
1500
b1
b2
b3
b4
b5
b6
b7
b8
b9
b10
b11
b12
y1
y2
y3
y4
y5
y6
y7
y8
y9
y10
y11
y12
y13
y14
type
b
y

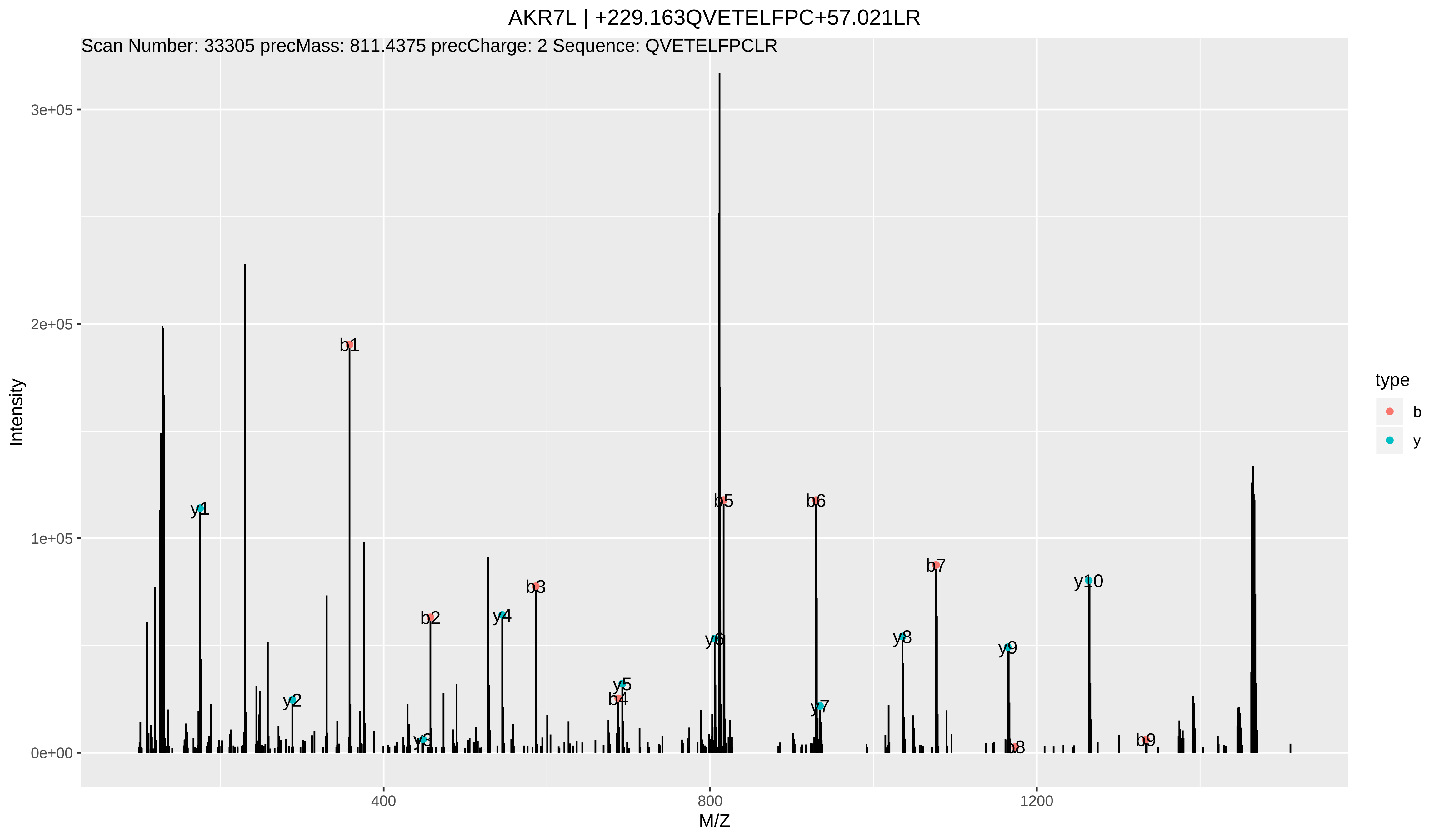

AKR7L | +229.163QVETELFPC+57.021LR
Scan Number: 33305 precMass: 811.4375 precCharge: 2 Sequence: QVETELFPCLR
Intensity
3e+05
2e+05
1e+05
0e+00
M/Z
400
800
1200
b1
b2
b3
b4
b5
b6
b7
b8
b9
y1
y2
y3
y4
y5
y6
y7
y8
y9
y10
type
b
y

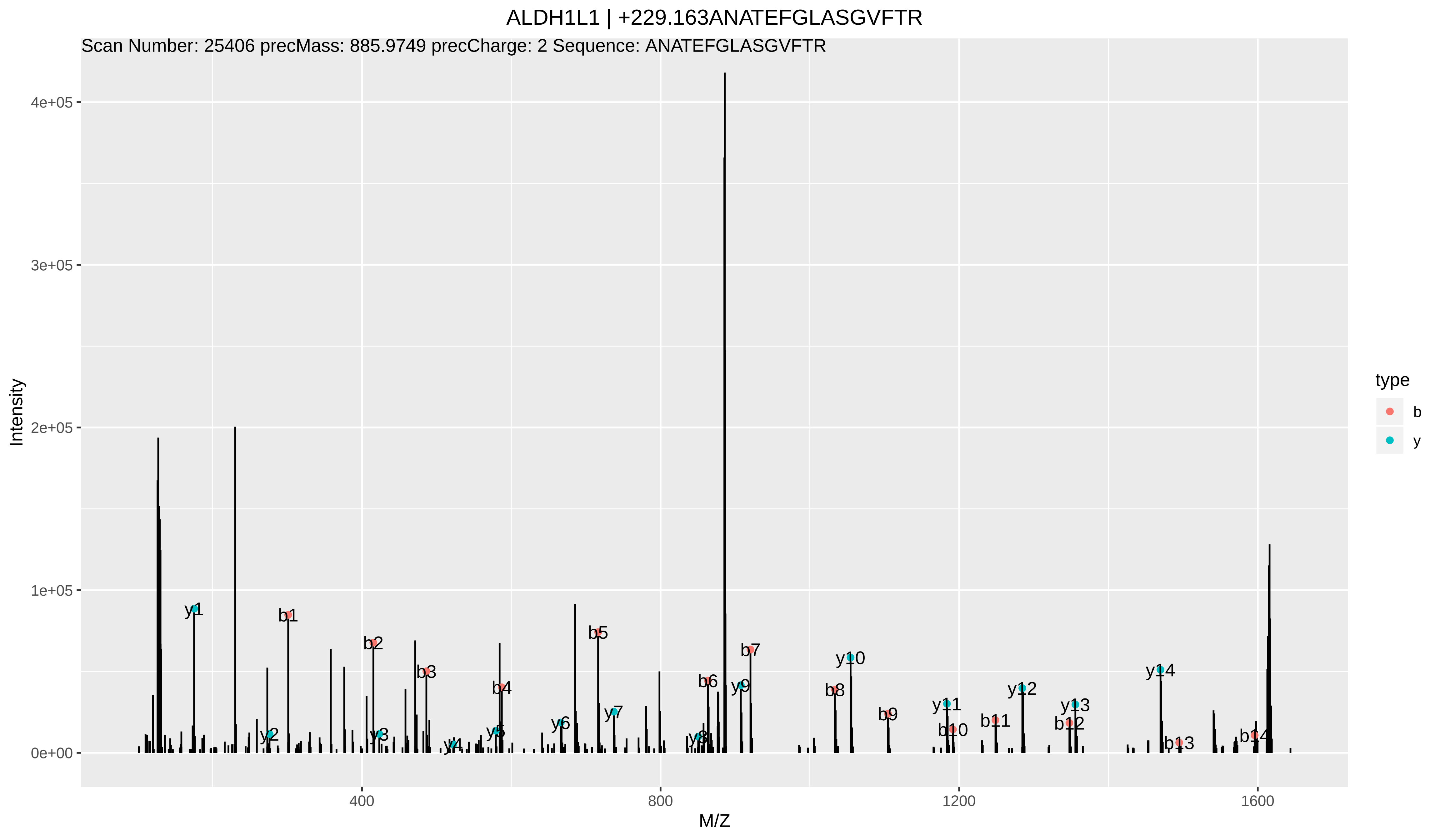

ALDH1L1 | +229.163ANATEFGLASGVFTR
Scan Number: 25406 precMass: 885.9749 precCharge: 2 Sequence: ANATEFGLASGVFTR
Intensity
4e+05
3e+05
2e+05
1e+05
0e+00
M/Z
400
800
1200
1600
y1
b1
b2
b3
b4
y2
y3
y4
y5
y6
b5
y7
y8
b6
y9
b7
b8
y10
b9
y11
b10
b11
y12
b12
y13
y14
b13
b14
type
b
y

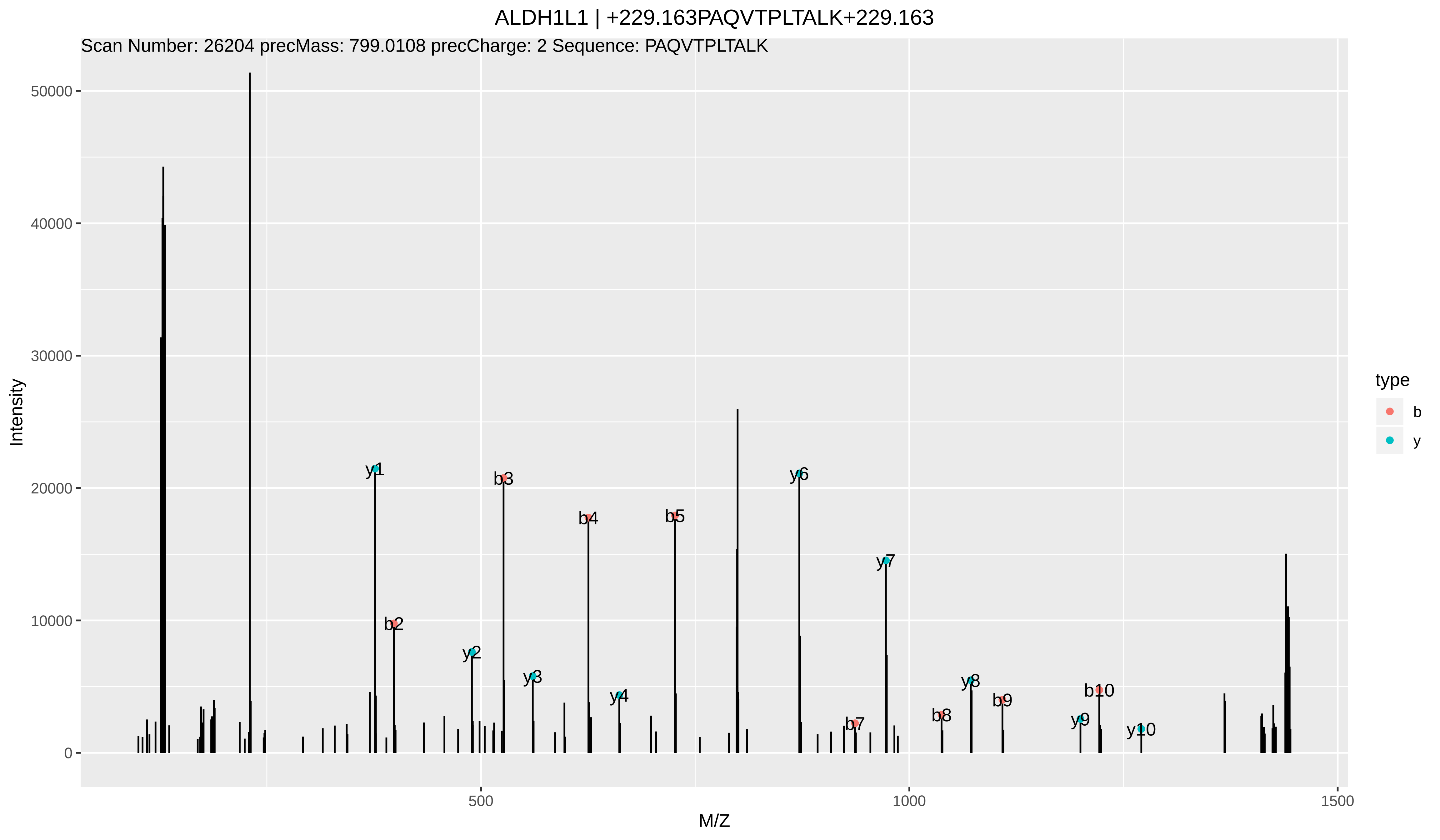

ALDH1L1 | +229.163PAQVTPLTALK+229.163
Scan Number: 26204 precMass: 799.0108 precCharge: 2 Sequence: PAQVTPLTALK
Intensity
M/Z
y1
b2
y2
b3
y3
b4
y4
b5
y6
y7
b7
b8
y8
b9
y9
b10
y10
type
b
y

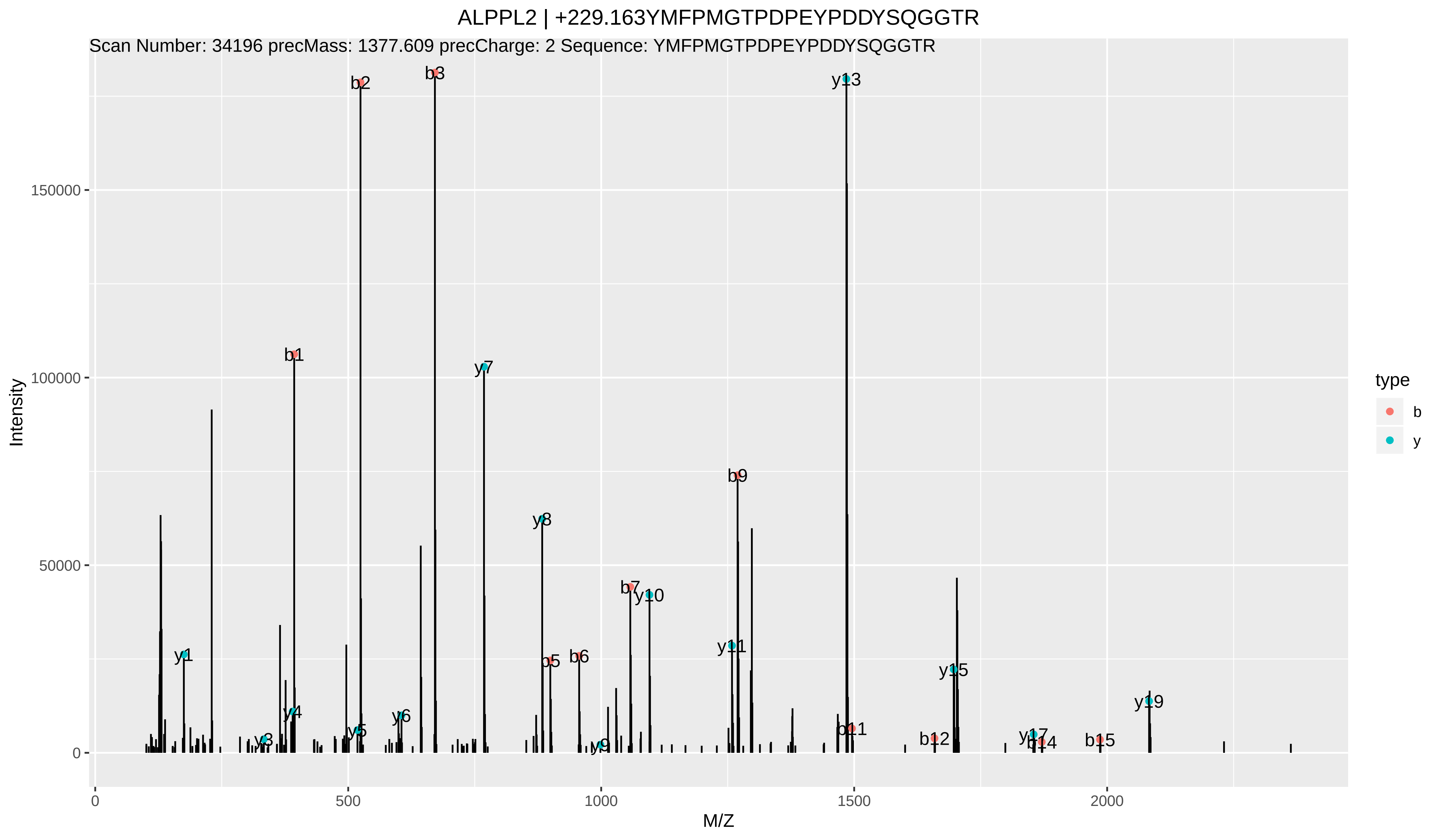

ALPPL2 | +229.163YMFPMGTPDPEYPDDYSQGGTR
Scan Number: 34196 precMass: 1377.609 precCharge: 2 Sequence: YMFPMGTPDPEYPDDYSQGGTR
Intensity
M/Z
type
b
y

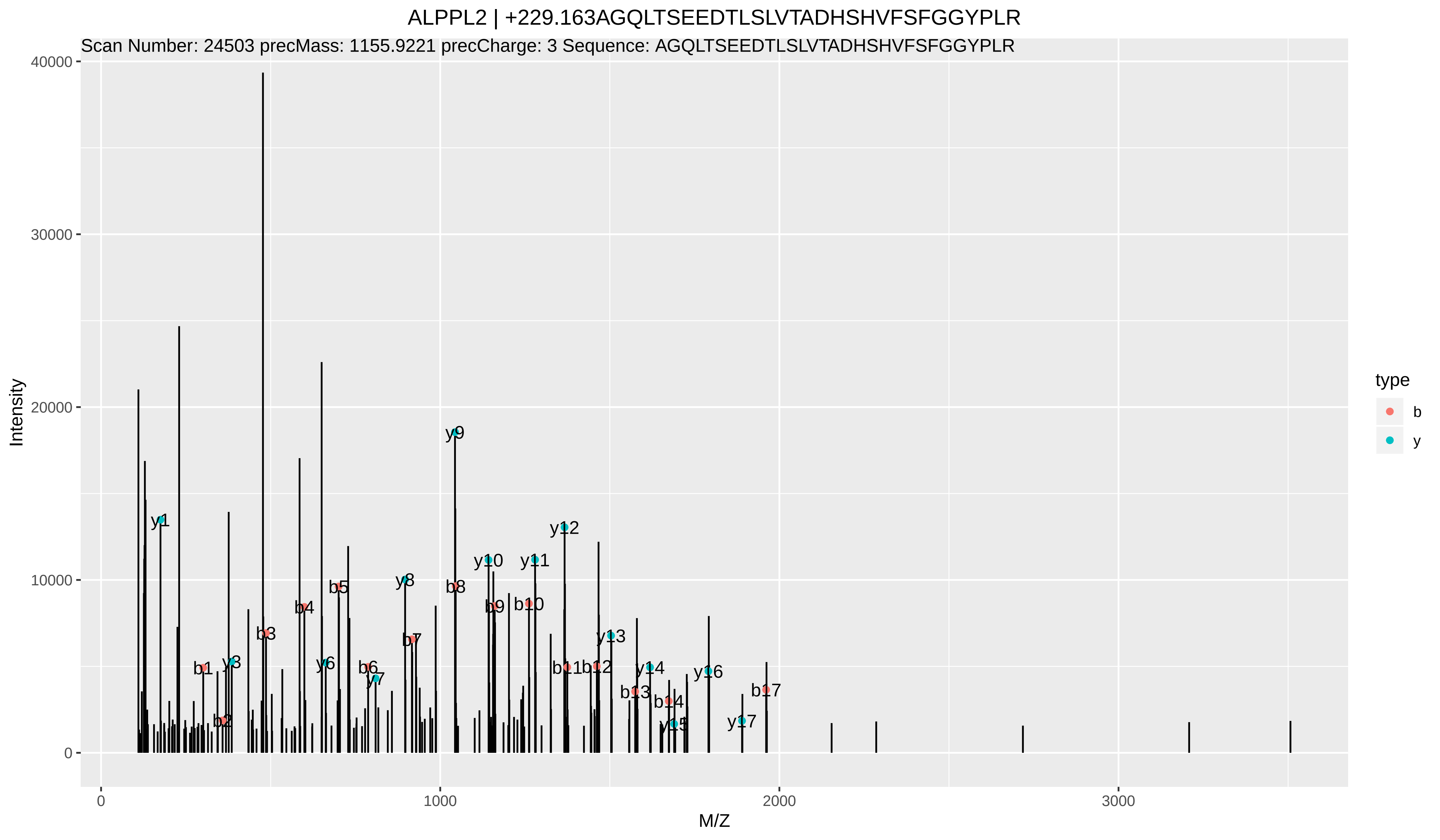

ALPPL2 | +229.163AGQLTSEEDTLSLVTADHSHVFSFGGYPLR
Scan Number: 24503 precMass: 1155.9221 precCharge: 3 Sequence: AGQLTSEEDTLSLVTADHSHVFSFGGYPLR
Intensity
M/Z
0
10000
20000
30000
40000
0
1000
2000
3000
type
b
y
y1
b1
b2
y3
b3
b4
b5
y6
b6
y7
y8
b7
y9
b8
b9
y10
y11
b10
y12
b11
b12
y13
b13
y14
b14
y15
y16
y17
b17

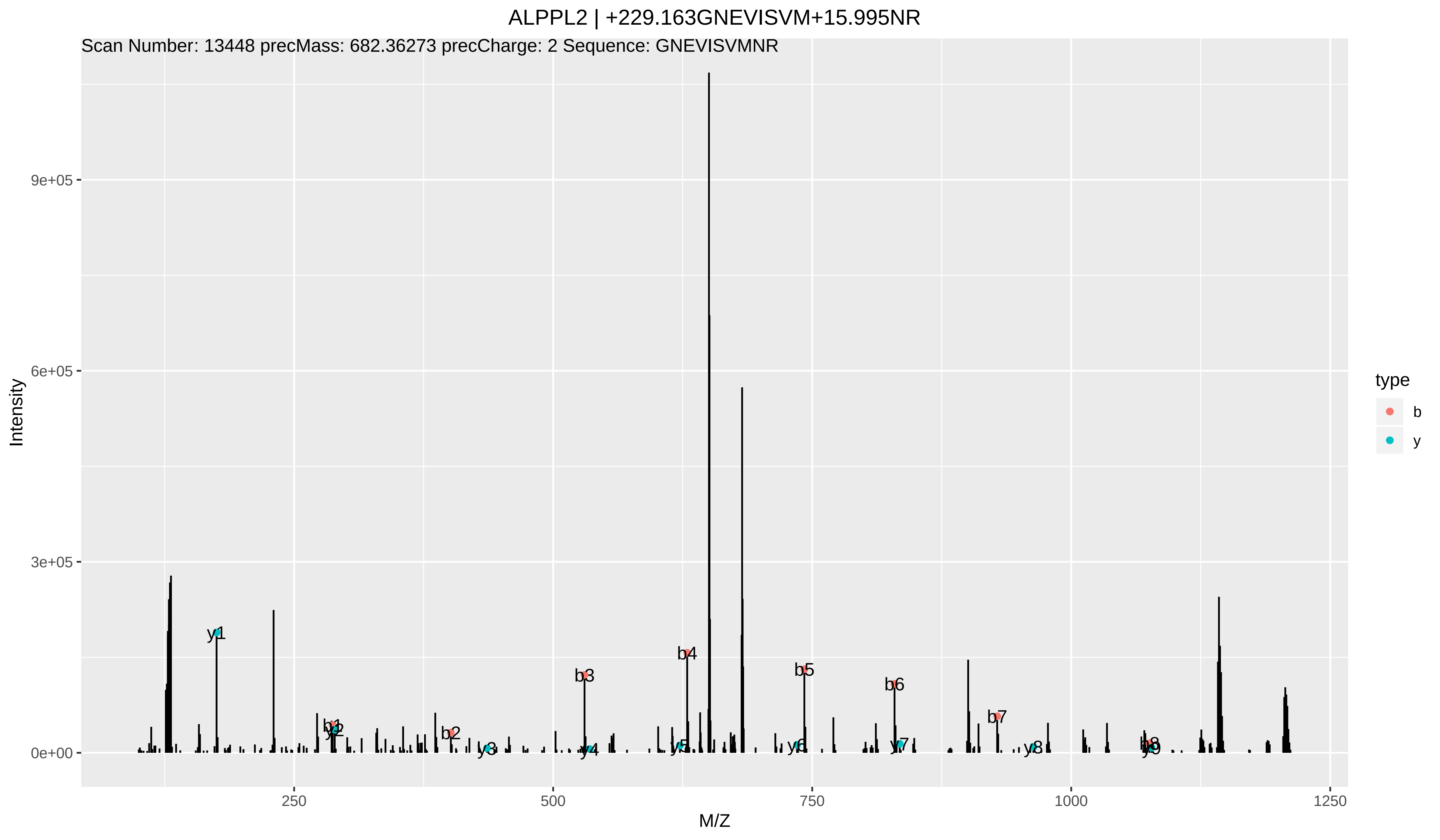
ALPPL2 | +229.163GNEVISVM+15.995NR
Scan Number: 13448 precMass: 682.36273 precCharge: 2 Sequence: GNEVISVMNR
Intensity
9e+05
6e+05
3e+05
0e+00
M/Z
250
500
750
1000
1250
y1
b1
y2
b2
y3
b3
y4
b4
y5
b5
y6
b6
y7
b7
y8
b8
y9
type
b
y

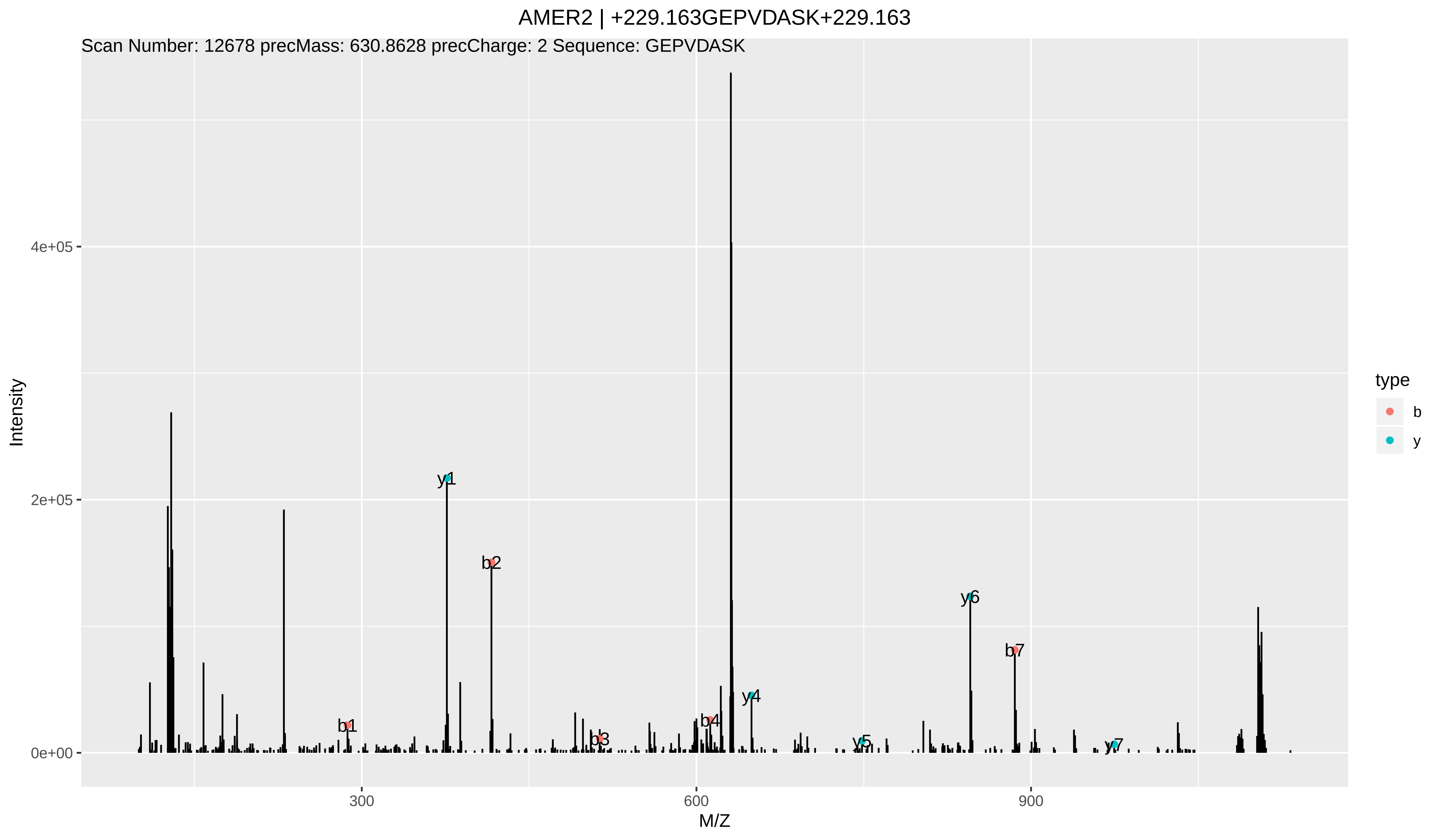
AMER2 | +229.163GEPVDASK+229.163
Scan Number: 12678 precMass: 630.8628 precCharge: 2 Sequence: GEPVDASK
Intensity
M/Z
4e+05
2e+05
0e+00
300
600
900
y1
b2
y6
b7
y4
b4
b1
b3
y5
y7
type
b
y

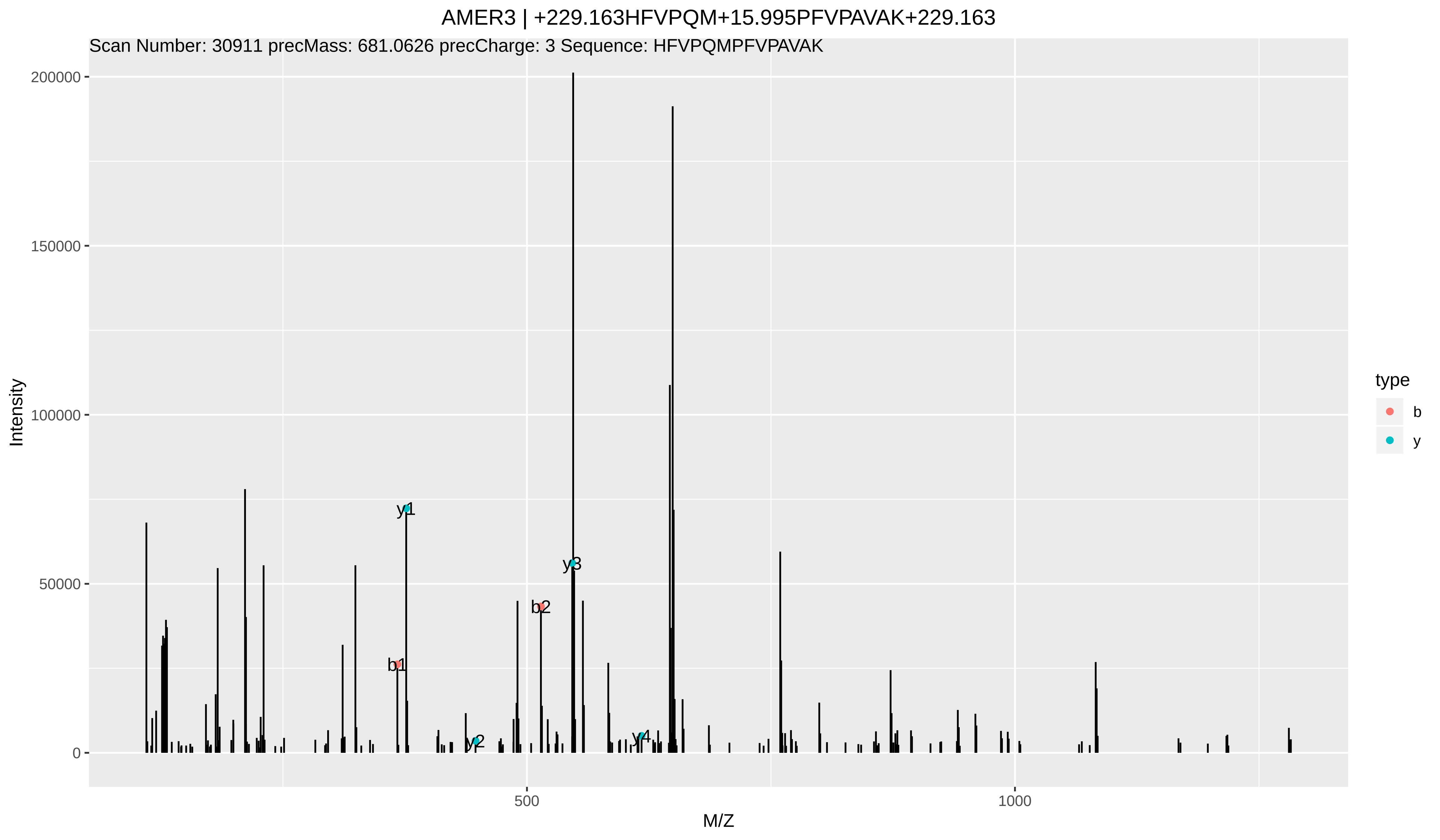

AMER3 | +229.163HFVPQM+15.995PFVPAVAK+229.163
Scan Number: 30911 precMass: 681.0626 precCharge: 3 Sequence: HFVPQMPFVPAVAK
Intensity
200000
150000
100000
50000
0
500
1000
M/Z
y1
y3
b2
b1
y2
y4
type
b
y

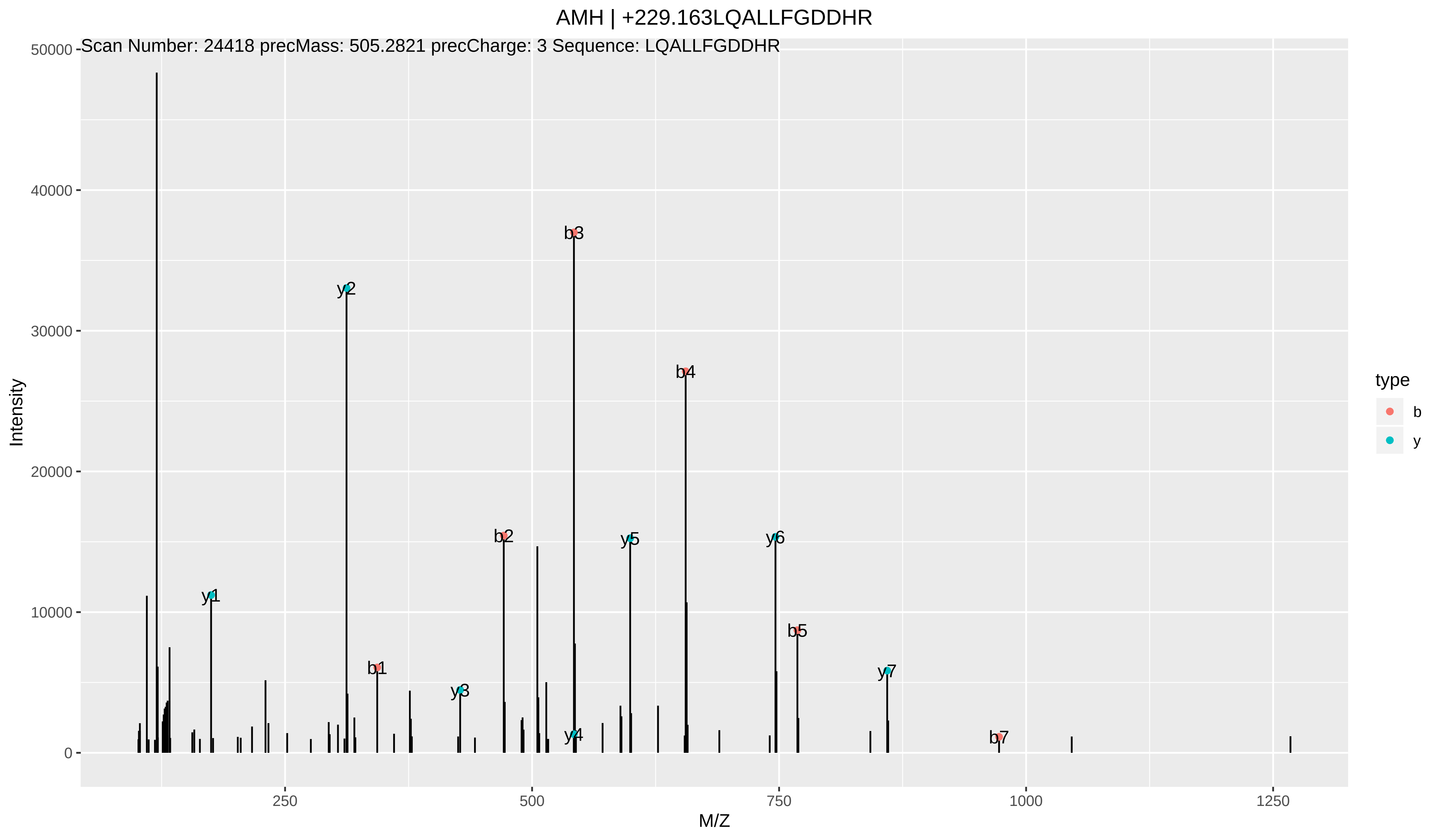
AMH | +229.163LQALLFGDDHR
Scan Number: 24418 precMass: 505.2821 precCharge: 3 Sequence: LQALLFGDDHR
Intensity
M/Z
0
10000
20000
30000
40000
50000
250
500
750
1000
1250
y1
y2
y3
y4
y5
y6
y7
b1
b2
b3
b4
b5
b7
type
b
y

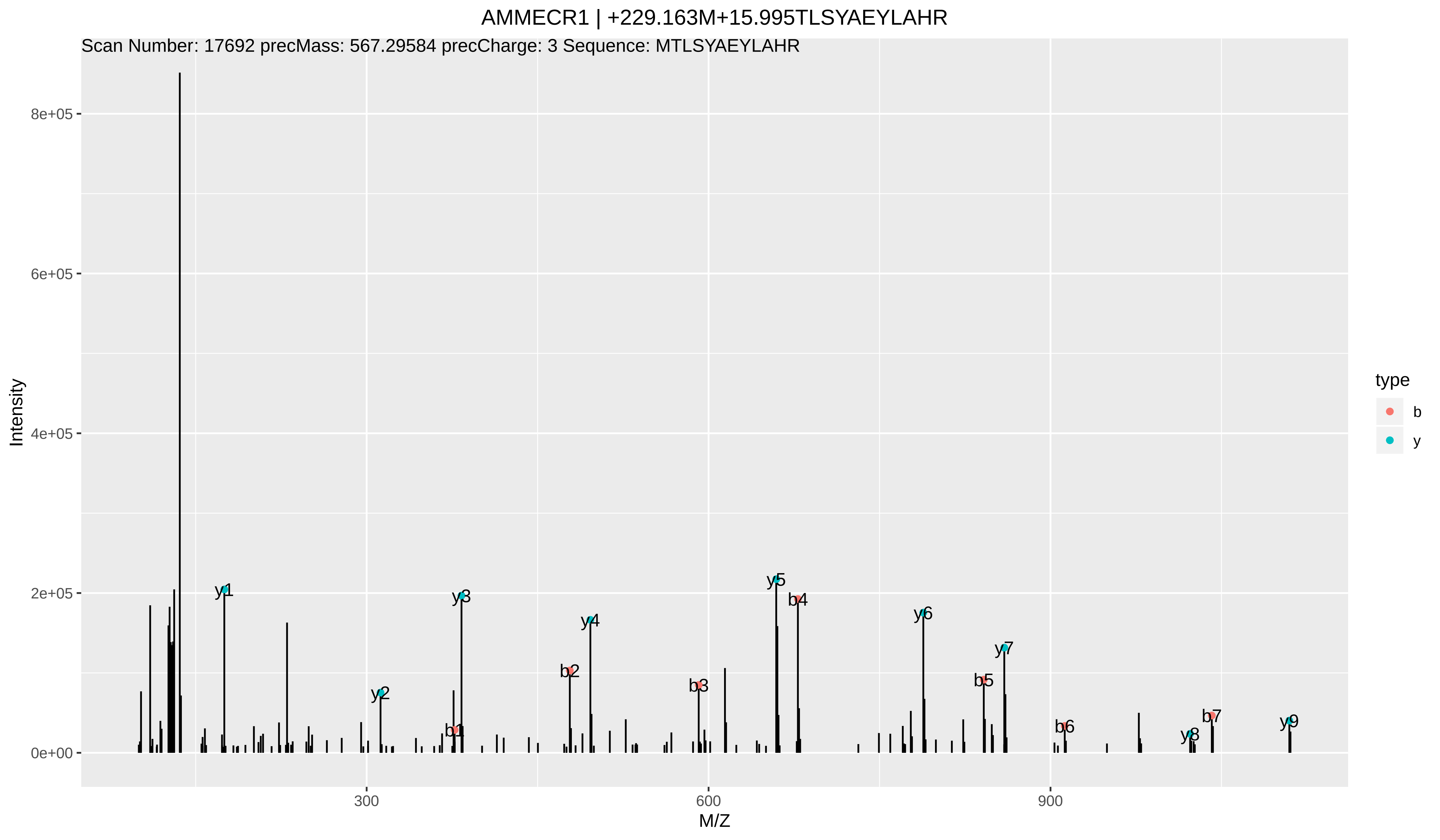

AMMECR1 | +229.163M+15.995TLSYAEYLAHR
Scan Number: 17692 precMass: 567.29584 precCharge: 3 Sequence: MTLSYAEYLAHR
Intensity
M/Z
type
b
y
y1
y2
y3
y4
y5
y6
y7
y8
y9
b1
b2
b3
b4
b5
b6
b7
0e+00
2e+05
4e+05
6e+05
8e+05
300
600
900

AMMECR1 | +229.163TATYLPEVAK+229.163

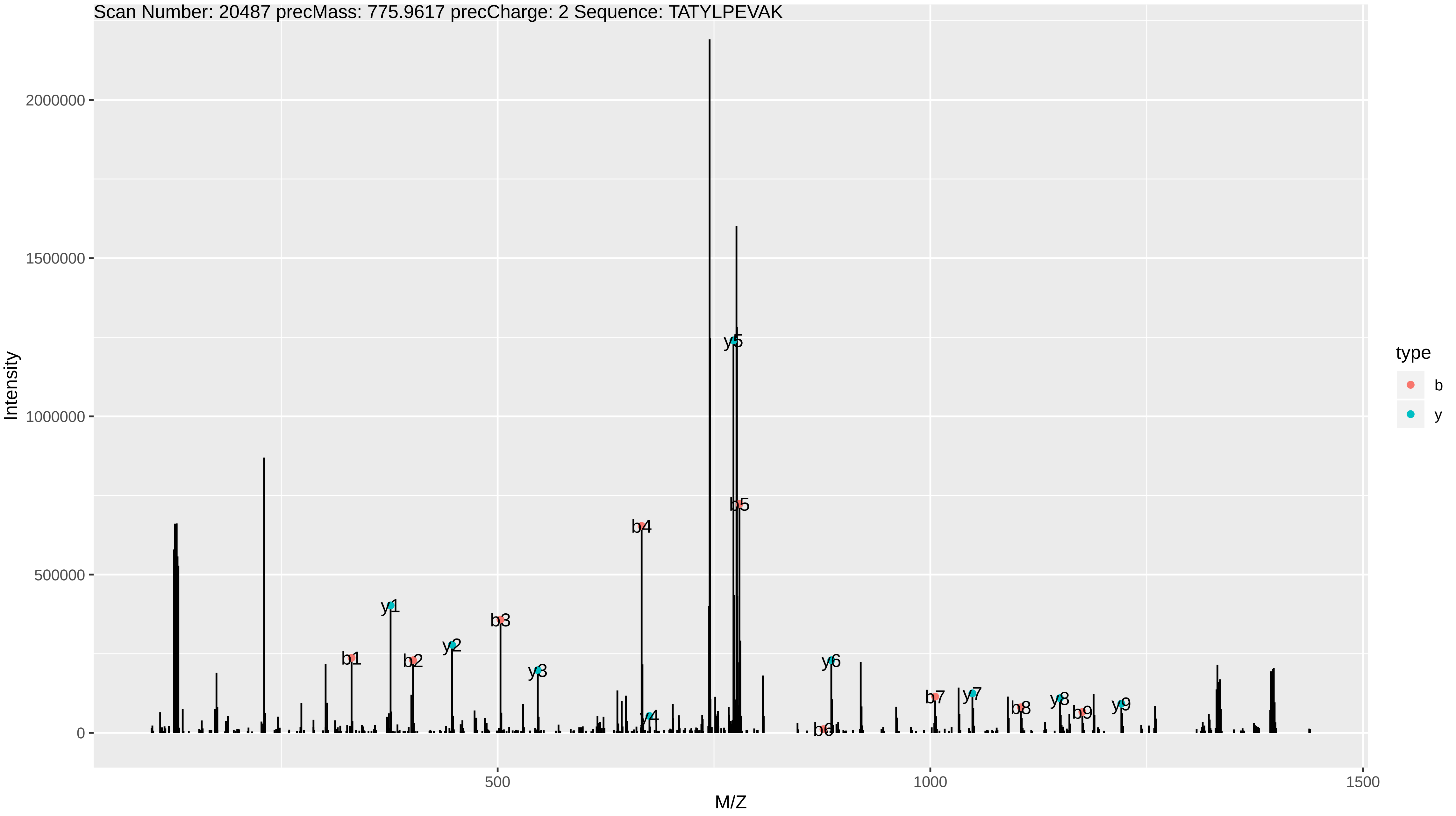

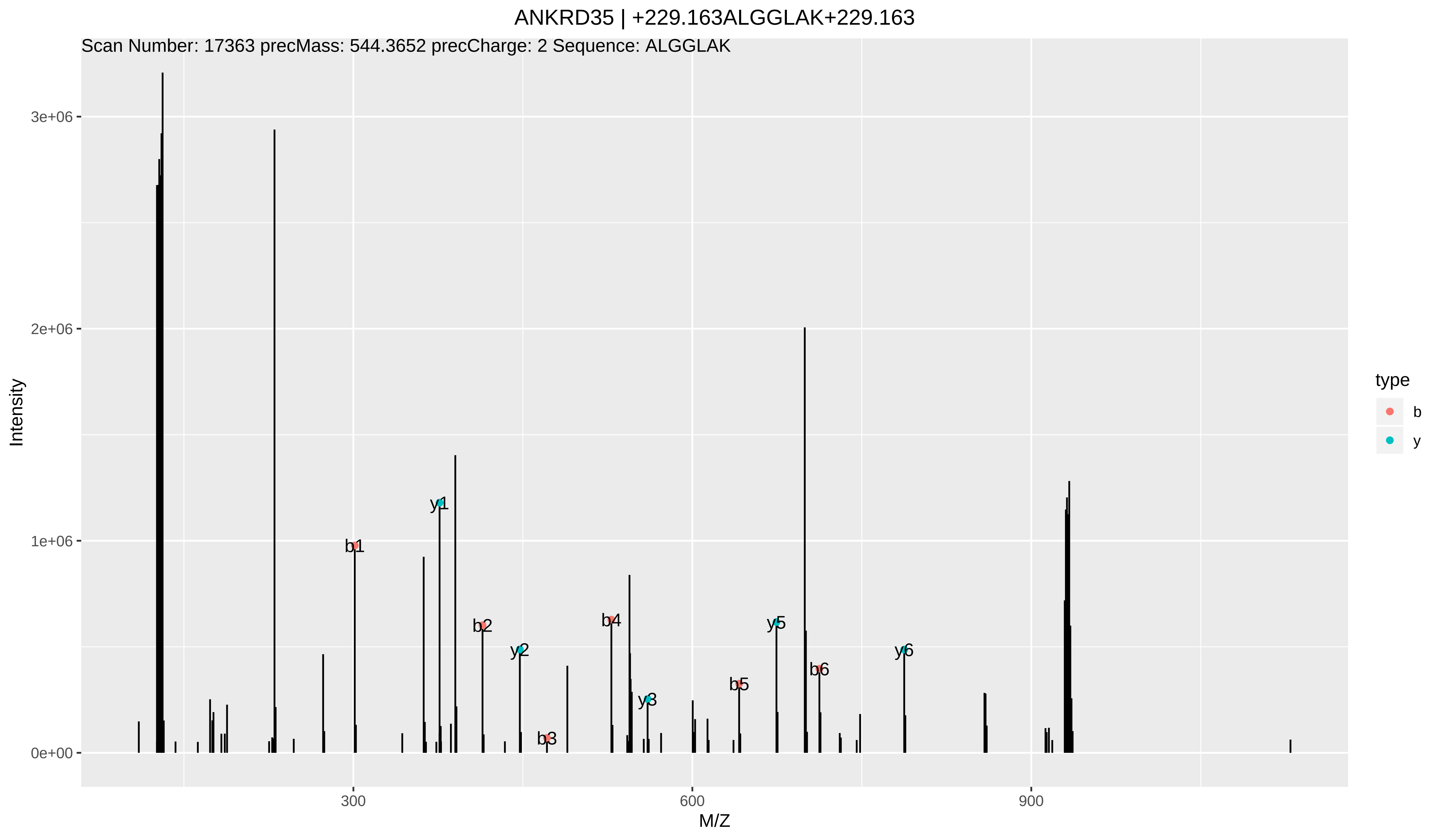
ANKRD35 | +229.163ALGGLAK+229.163
Scan Number: 17363 precMass: 544.3652 precCharge: 2 Sequence: ALGGLAK
Intensity
3e+06
2e+06
1e+06
0e+00
M/Z
300
600
900
y1
b1
b2
y2
b3
b4
y3
b5
y5
b6
y6
type
b
y

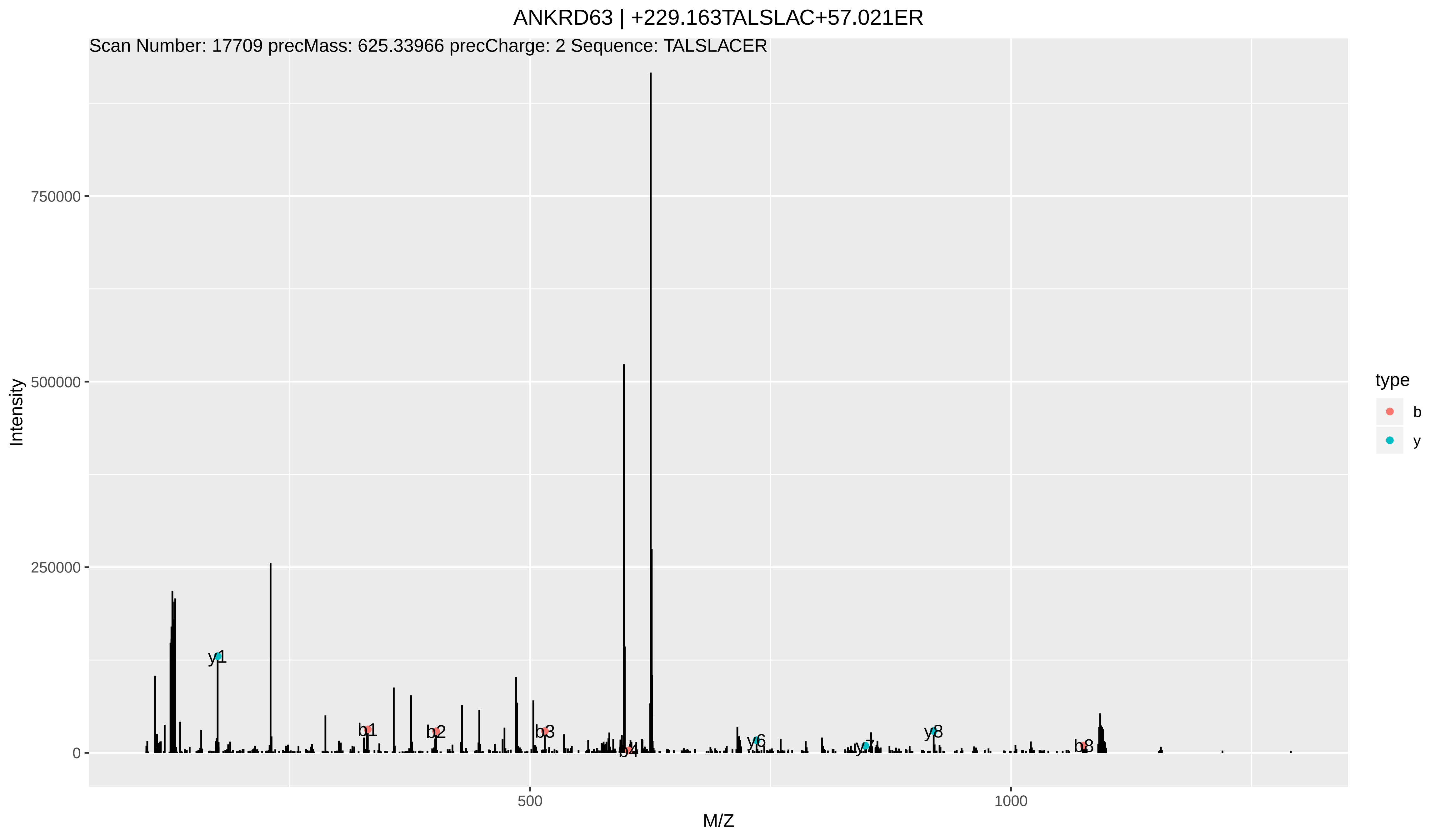

ANKRD63 | +229.163TALSLAC+57.021ER
Scan Number: 17709 precMass: 625.33966 precCharge: 2 Sequence: TALSLACER
Intensity
750000
500000
250000
0
M/Z
500
1000
y1
b1
b2
b3
b4
y6
y7
y8
b8
type
b
y

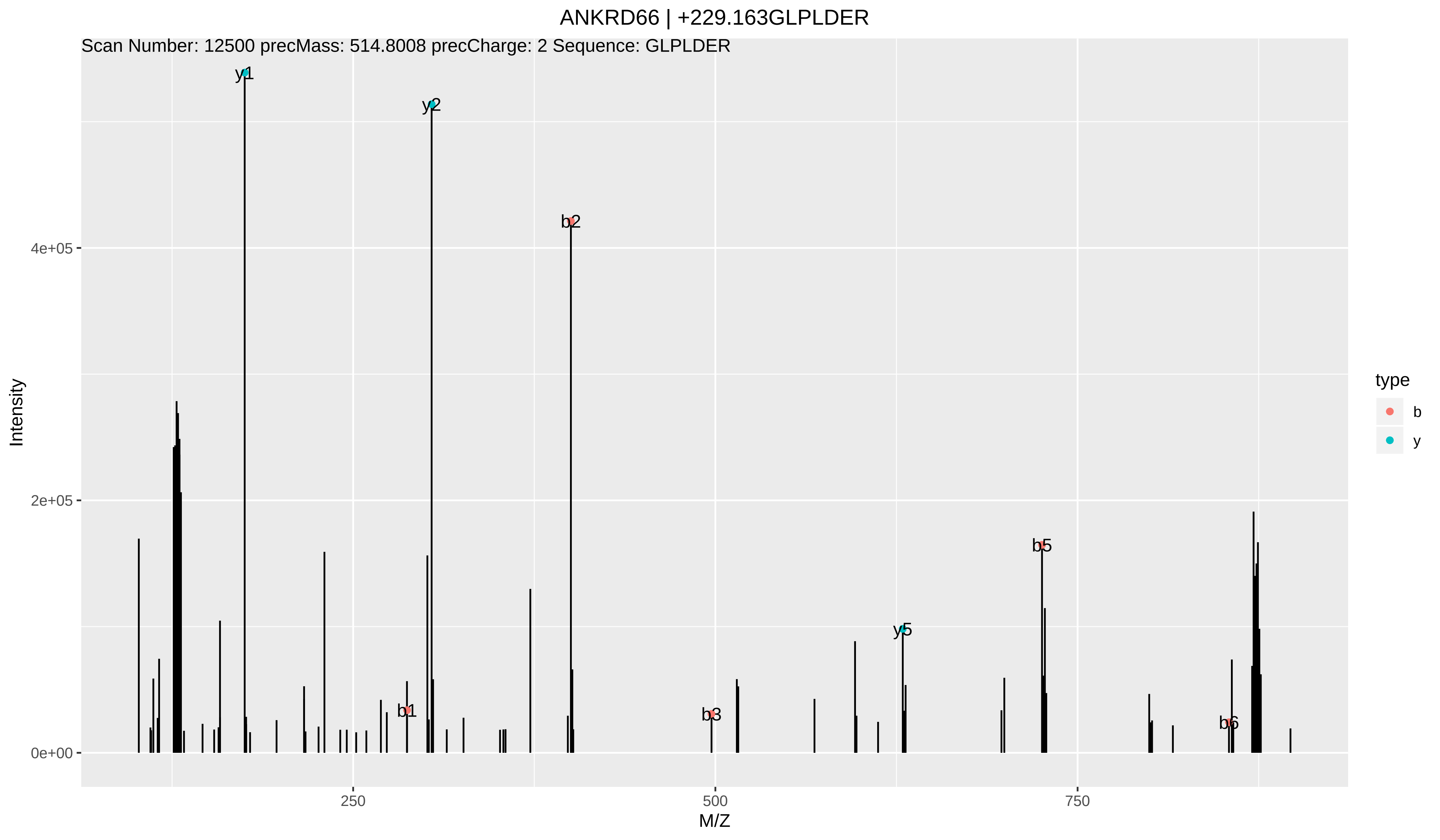

ANKRD66 | +229.163GLPLDER
Scan Number: 12500 precMass: 514.8008 precCharge: 2 Sequence: GLPLDER
y1
y2
b2
b5
y5
b1
b3
b6
Intensity
4e+05
2e+05
0e+00
250
500
750
M/Z
type
b
y

ANKS1B | +229.163FIDATNK+229.163

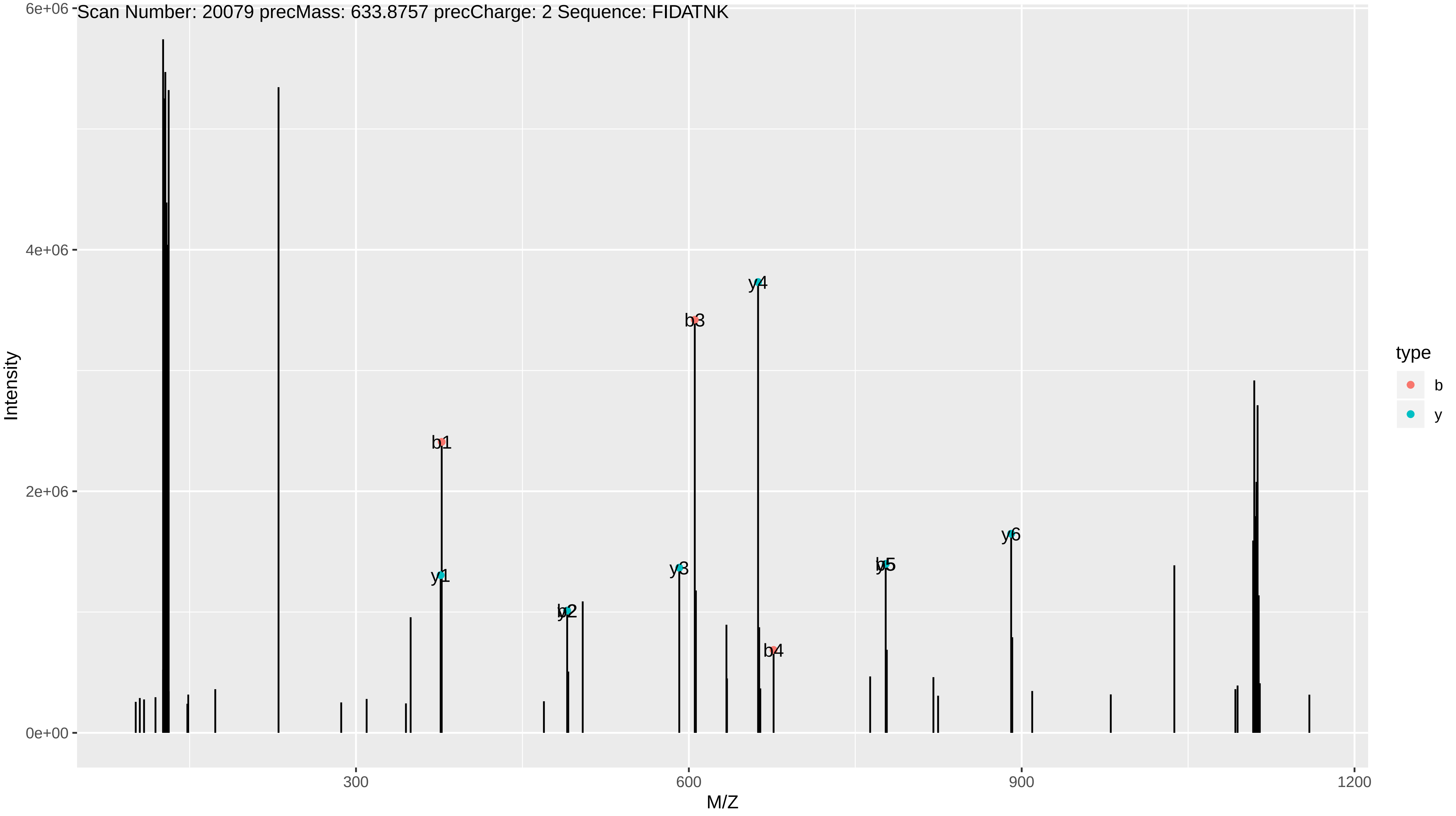

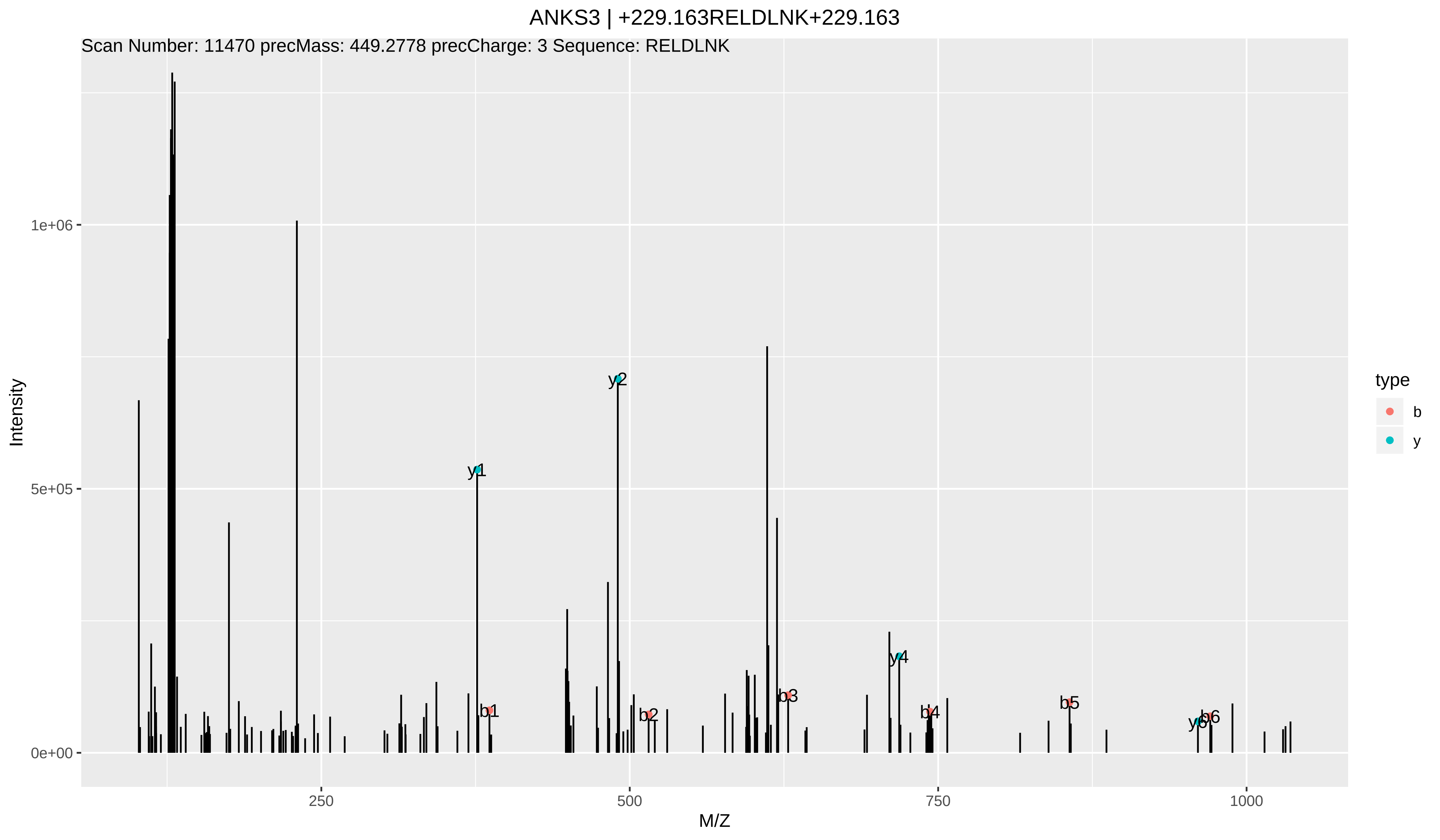
ANKS3 | +229.163RELDLNK+229.163
Scan Number: 11470 precMass: 449.2778 precCharge: 3 Sequence: RELDLNK
Intensity
1e+06
5e+05
0e+00
250
500
750
1000
M/Z
y1
y2
y4
y6
b1
b2
b3
b4
b5
b6
type
b
y

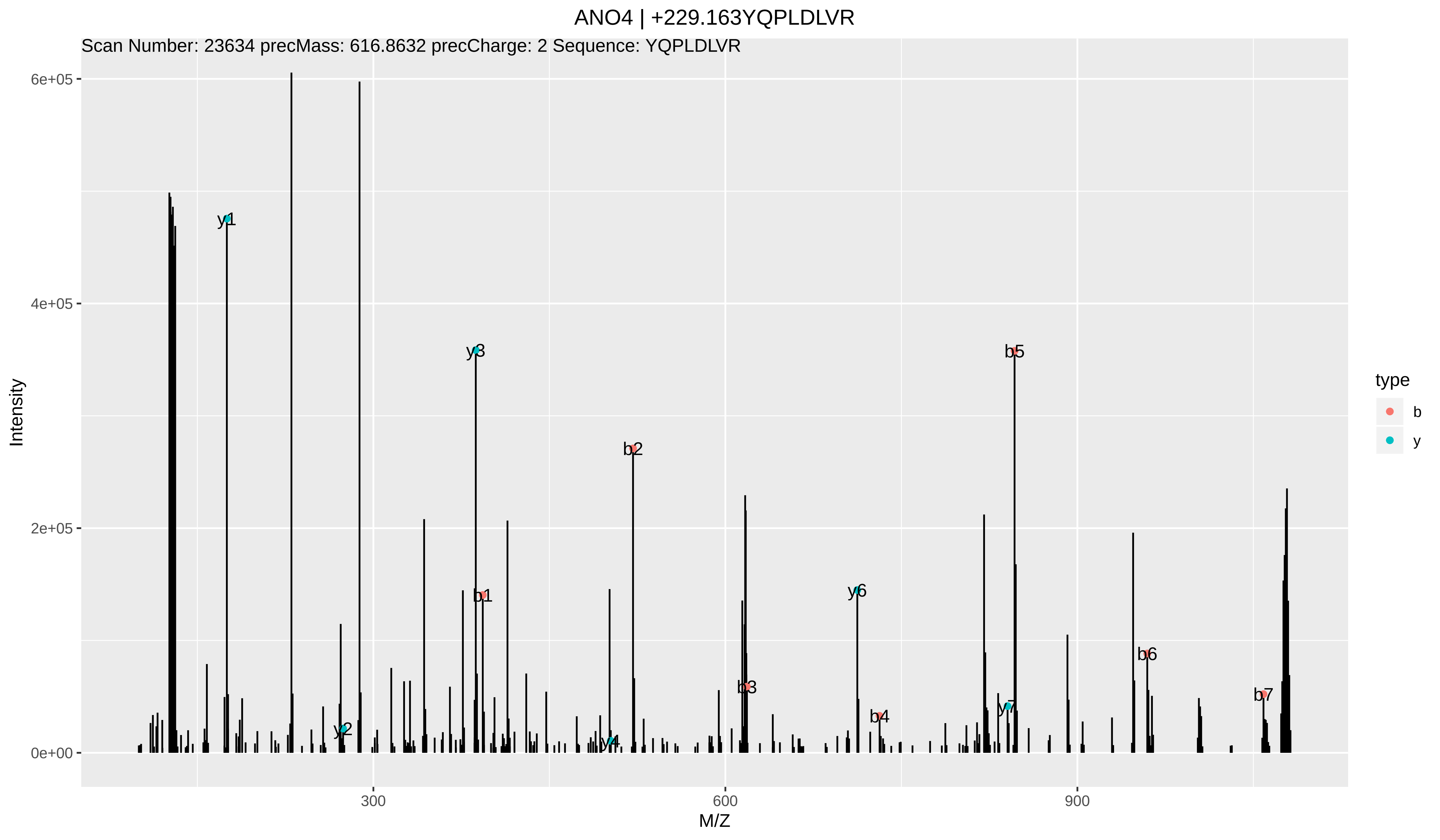

ANO4 | +229.163YQPLDLVR
Scan Number: 23634 precMass: 616.8632 precCharge: 2 Sequence: YQPLDLVR
Intensity
6e+05
4e+05
2e+05
0e+00
M/Z
300
600
900
y1
y2
y3
y4
y6
y7
b1
b2
b3
b4
b5
b6
b7
type
b
y

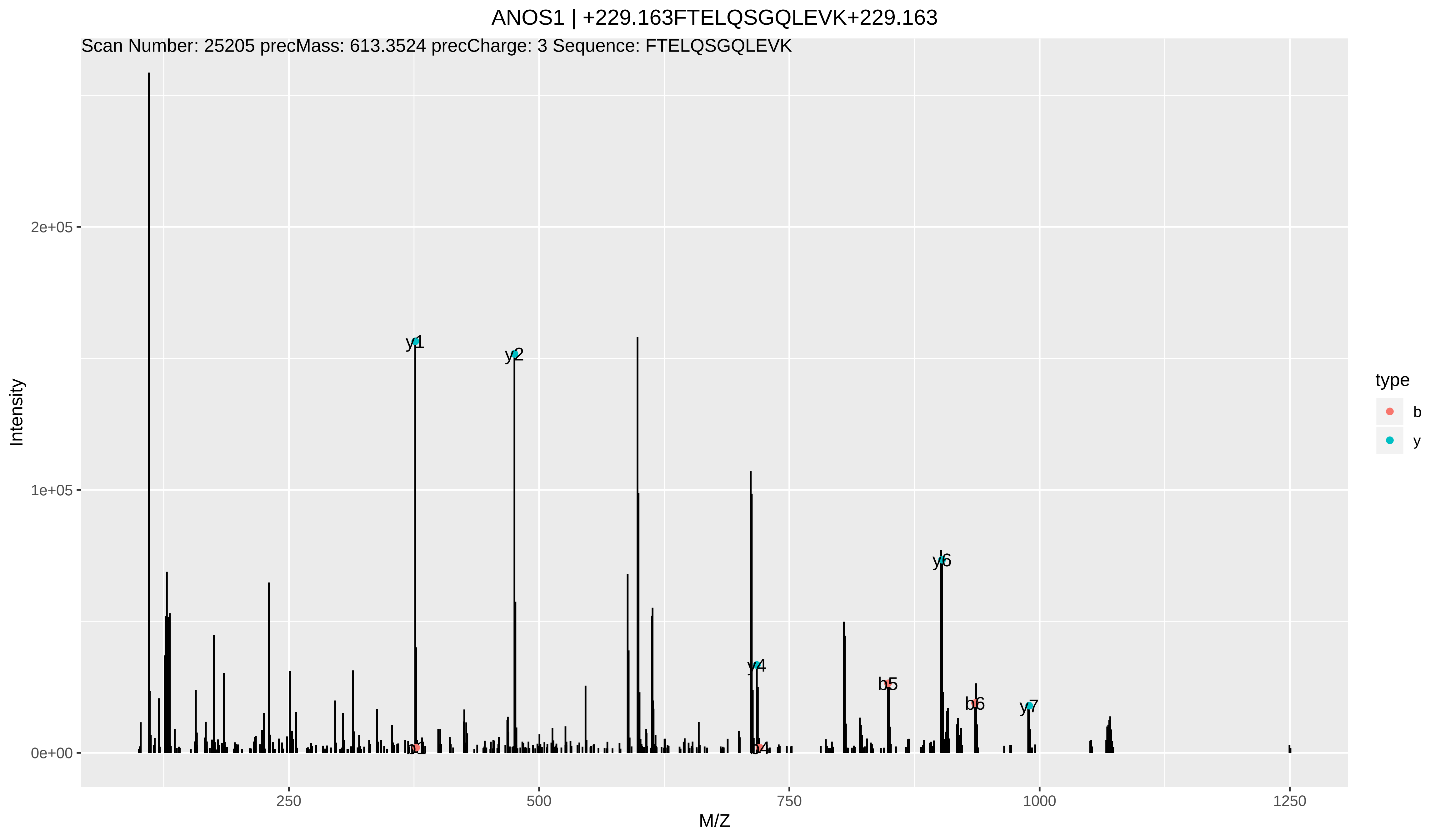

ANOS1 | +229.163FTELQSGQLEVK+229.163
Scan Number: 25205 precMass: 613.3524 precCharge: 3 Sequence: FTELQSGQLEVK
Intensity
2e+05
1e+05
0e+00
M/Z
250
500
750
1000
1250
y1
y2
y4
y6
y7
b1
b4
b5
b6
type
b
y

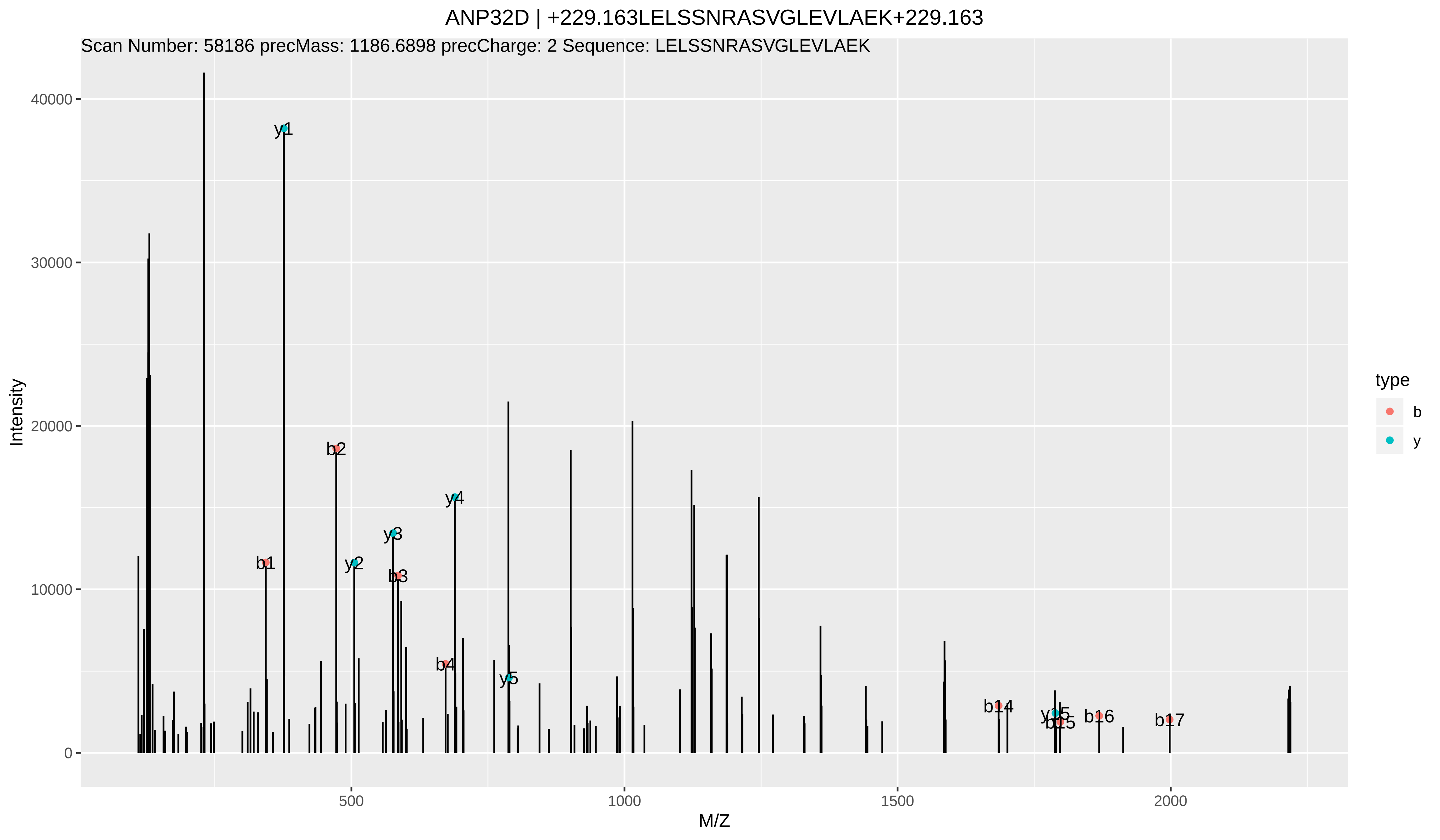

ANP32D | +229.163LELSSNRASVGLEVLAEK+229.163
Scan Number: 58186 precMass: 1186.6898 precCharge: 2 Sequence: LELSSNRASVGLEVLAEK
Intensity
M/Z
type
b
y
y1
b1
b2
y2
y3
b3
y4
b4
y5
b14
y15
b15
b16
b17
0
10000
20000
30000
40000
500
1000
1500
2000

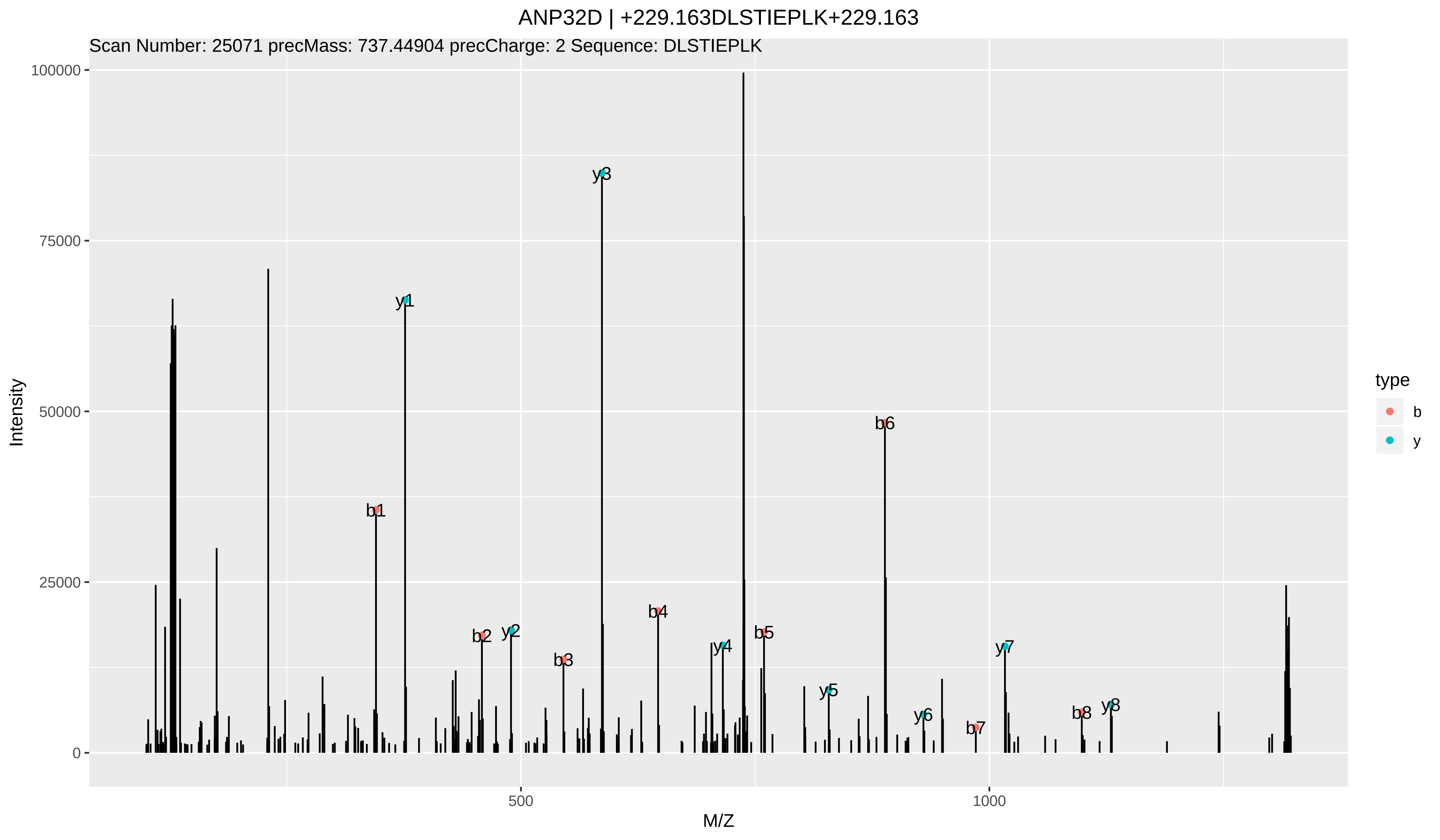

ANP32D | +229.163DLSTIEPLK+229.163
Scan Number: 25071 precMass: 737.44904 precCharge: 2 Sequence: DLSTIEPLK
Intensity
100000
75000
50000
25000
0
M/Z
500
1000
y3
y1
b1
b6
b4
b2
y2
b3
y4
b5
y5
y6
b7
y7
b8
y8
type
b
y

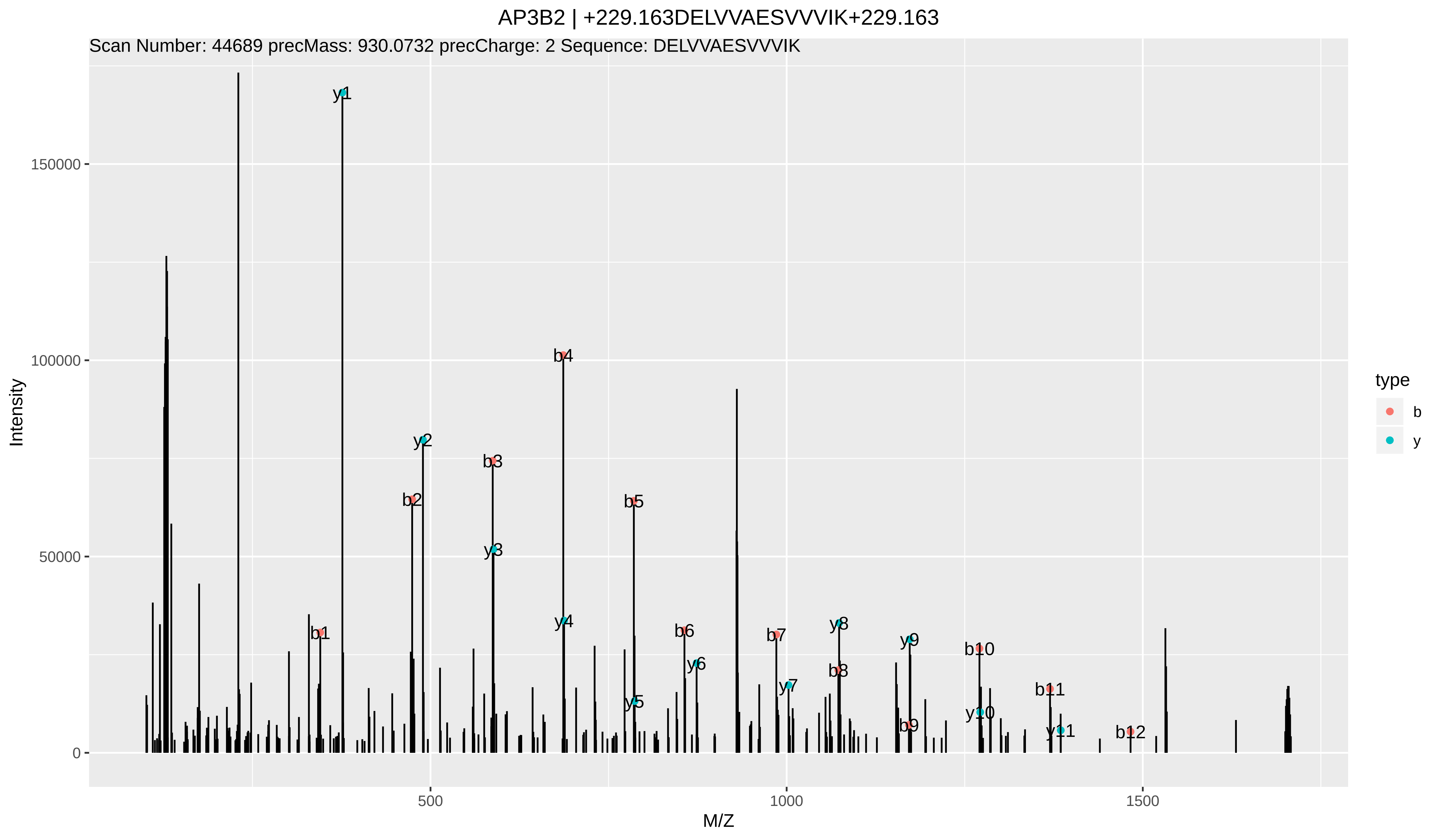

AP3B2 | +229.163DELVVAESVVVIK+229.163
Scan Number: 44689 precMass: 930.0732 precCharge: 2 Sequence: DELVVAESVVVIK
Intensity
M/Z
0
50000
100000
150000
500
1000
1500
y1
y2
b2
b3
y3
b4
y4
b5
y5
b1
b6
y6
b7
y7
y8
b8
y9
b9
b10
y10
b11
y11
b12
type
b
y

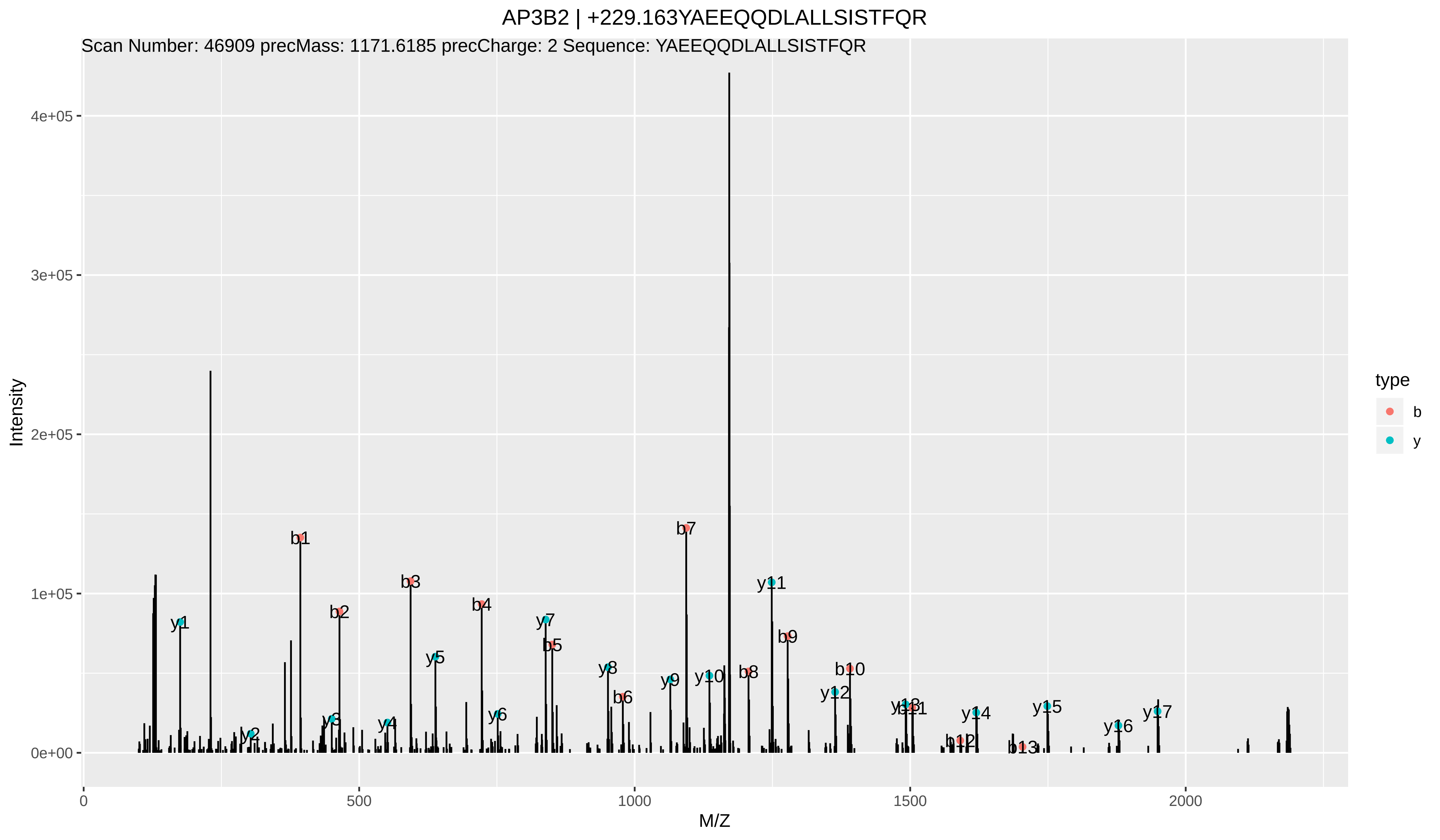

AP3B2 | +229.163YAEEQQDLALLSISTFQR
Scan Number: 46909 precMass: 1171.6185 precCharge: 2 Sequence: YAEEQQDLALLSISTFQR
Intensity
M/Z
type
b
y
0e+00
1e+05
2e+05
3e+05
4e+05
0
500
1000
1500
2000
b1
b2
b3
b4
b5
b6
b7
b8
b9
b10
b11
b12
b13
y1
y2
y3
y4
y5
y6
y7
y8
y9
y10
y11
y12
y13
y14
y15
y16
y17

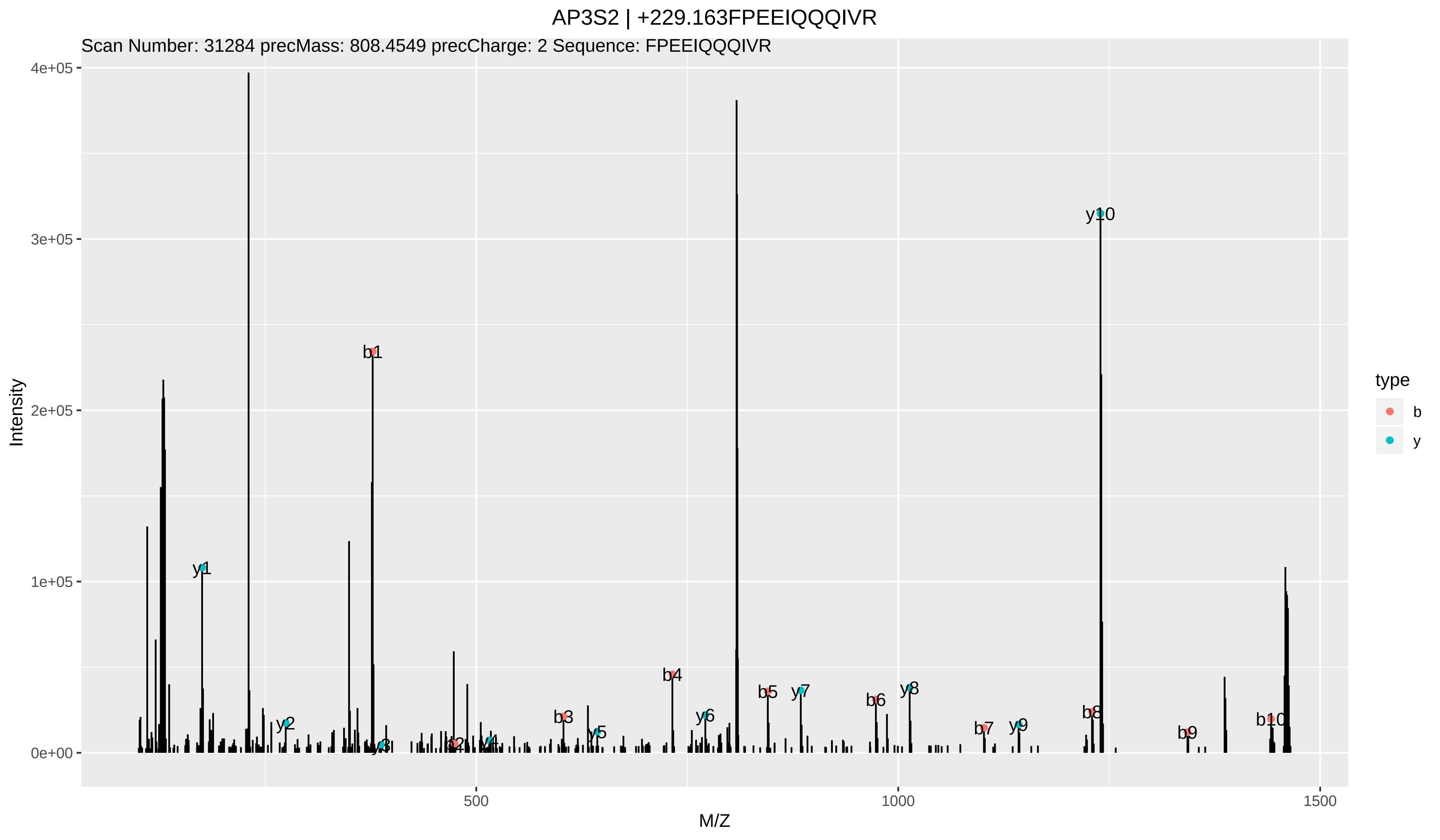

AP3S2 | +229.163FPEEIQQQIVR
Scan Number: 31284 precMass: 808.4549 precCharge: 2 Sequence: FPEEIQQQIVR
Intensity
4e+05
3e+05
2e+05
1e+05
0e+00
M/Z
500
1000
1500
y1
y2
b1
y3
b2
y4
b3
y5
b4
y6
b5
y7
b6
y8
b7
y9
b8
y10
b9
b10
type
b
y

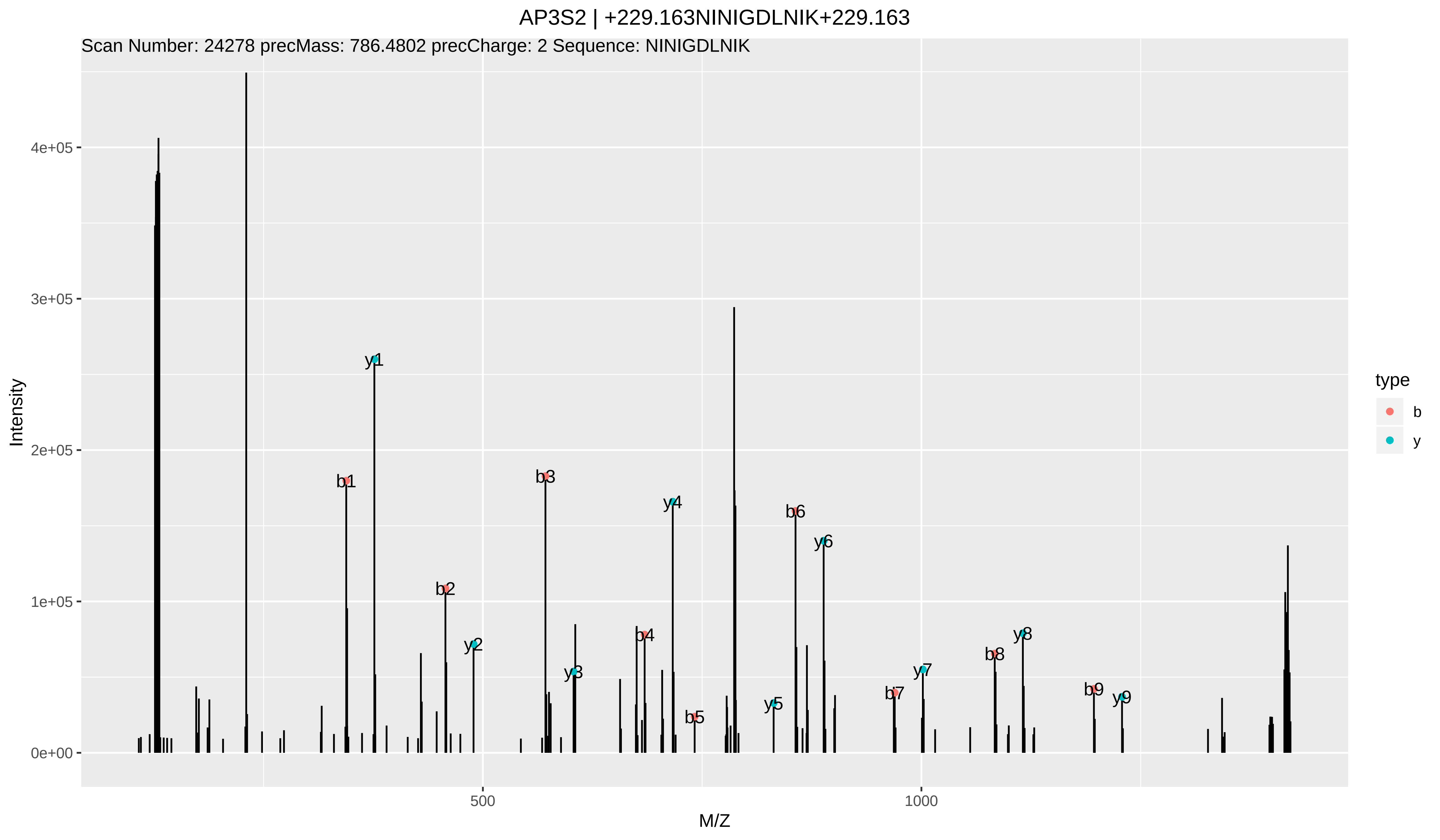

AP3S2 | +229.163NINIGDLNIK+229.163
Scan Number: 24278 precMass: 786.4802 precCharge: 2 Sequence: NINIGDLNIK
Intensity
4e+05
3e+05
2e+05
1e+05
0e+00
M/Z
500
1000
y1
b1
b2
y2
b3
y3
b4
y4
b5
y5
b6
y6
b7
y7
b8
y8
b9
y9
type
b
y

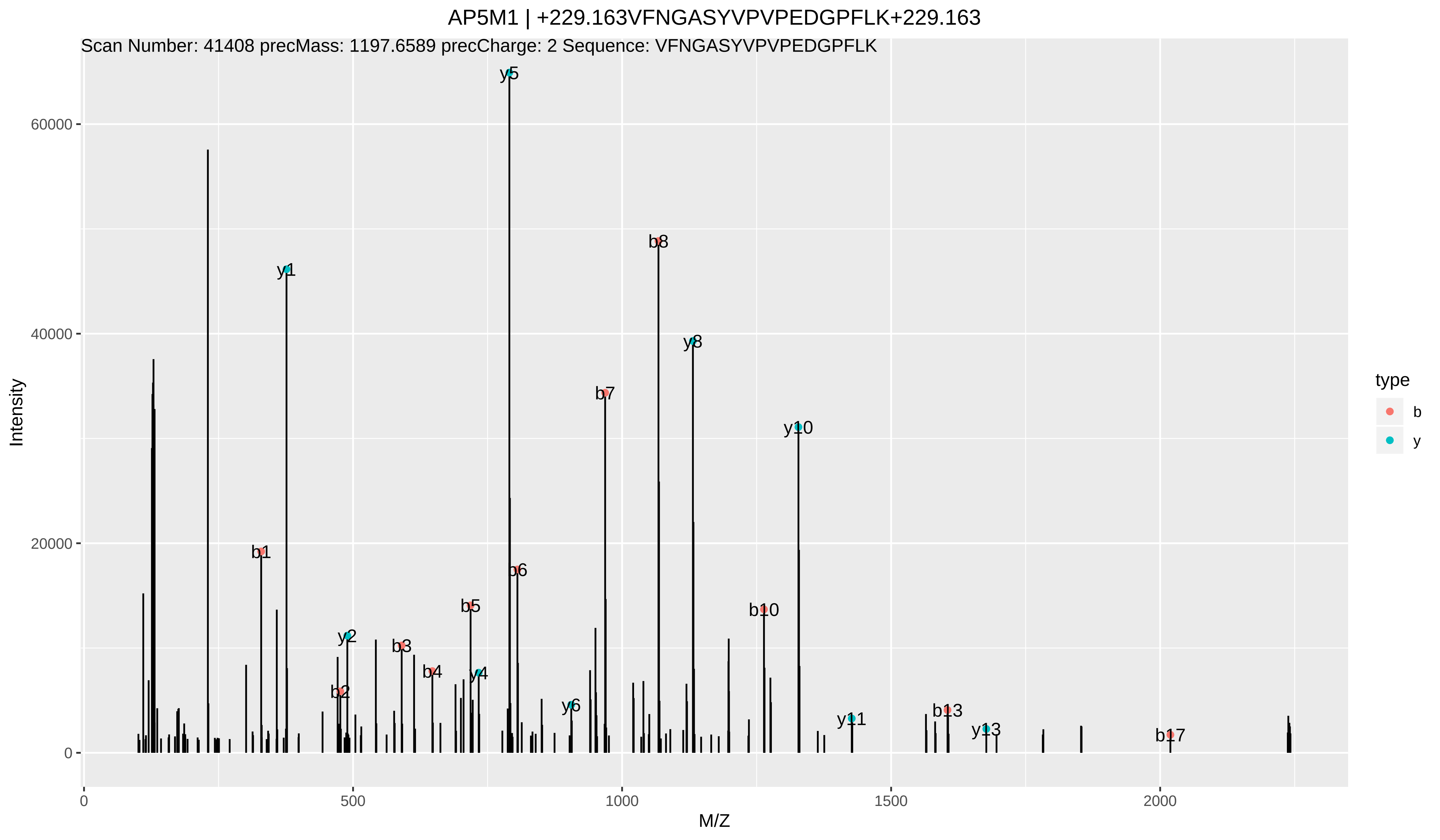

AP5M1 | +229.163VFNGASYVPVPEDGPFLK+229.163
Scan Number: 41408 precMass: 1197.6589 precCharge: 2 Sequence: VFNGASYVPVPEDGPFLK
Intensity
M/Z
0
20000
40000
60000
0
500
1000
1500
2000
y5
y1
b8
y8
b7
y10
b1
b6
b5
b10
y2
b3
b4
y4
b2
y6
y11
b13
y13
b17
type
b
y

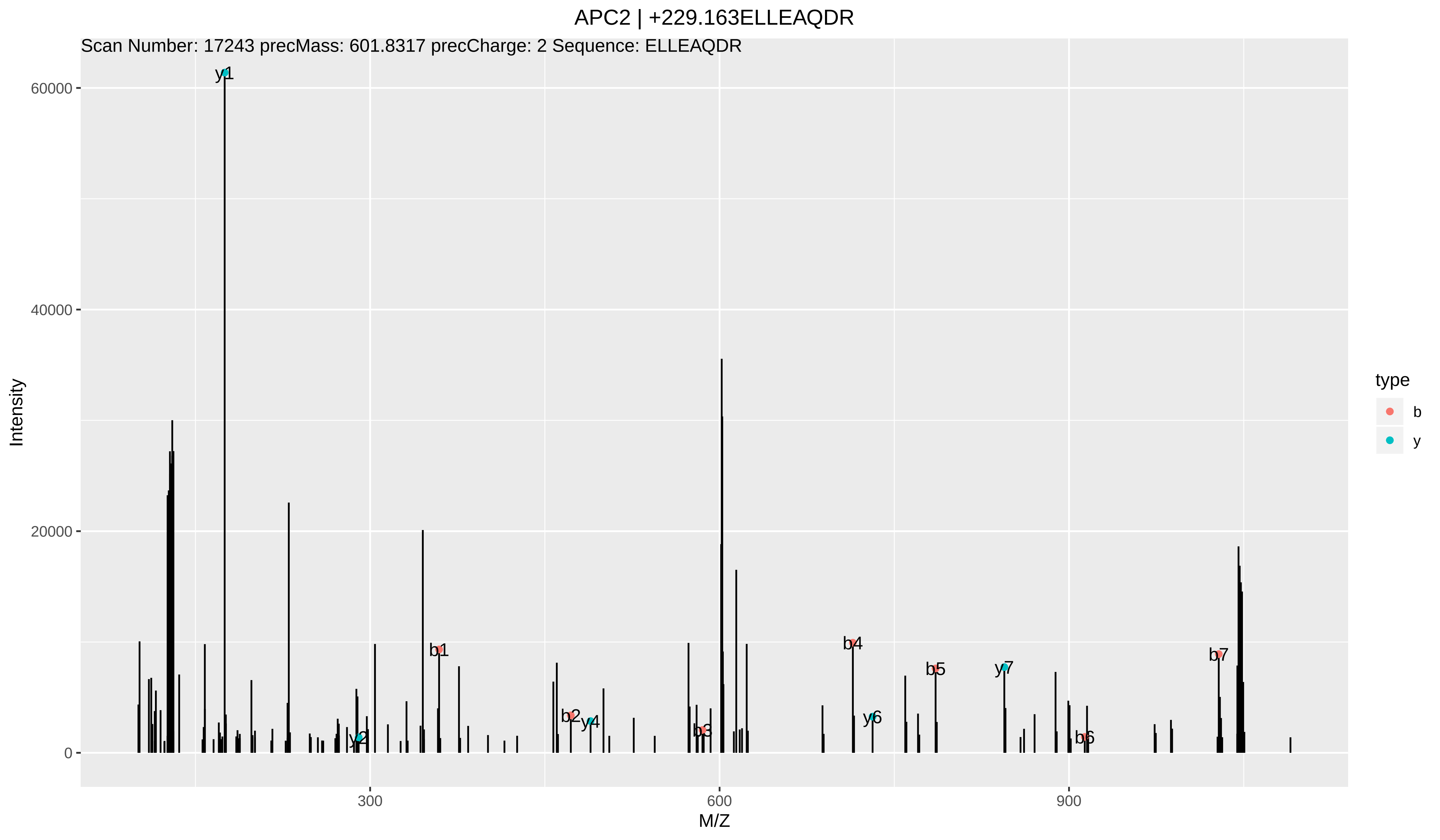

APC2 | +229.163ELLEAQDR
Scan Number: 17243 precMass: 601.8317 precCharge: 2 Sequence: ELLEAQDR
Intensity
60000
40000
20000
0
300
600
900
M/Z
y1
y2
b1
b2
y4
b3
b4
y6
b5
y7
b6
b7
type
b
y

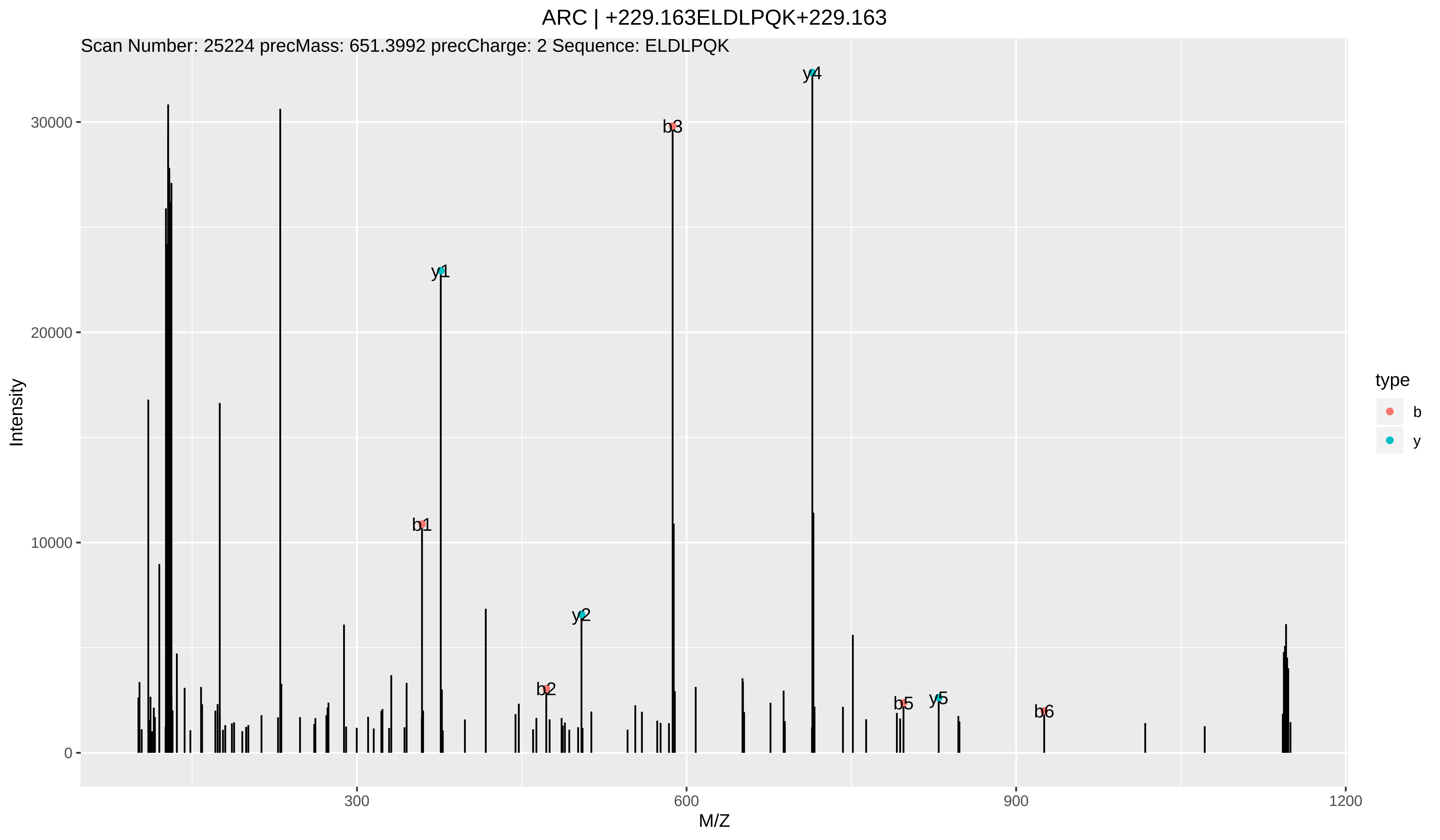

ARC | +229.163ELDLPQK+229.163
Scan Number: 25224 precMass: 651.3992 precCharge: 2 Sequence: ELDLPQK
y4
b3
y1
b1
y2
b2
b5
y5
b6
30000
20000
10000
0
300
600
900
1200
M/Z
Intensity
type
b
y

ARHGAP31 | +229.163ELPNPLLTYELYEK+229.163

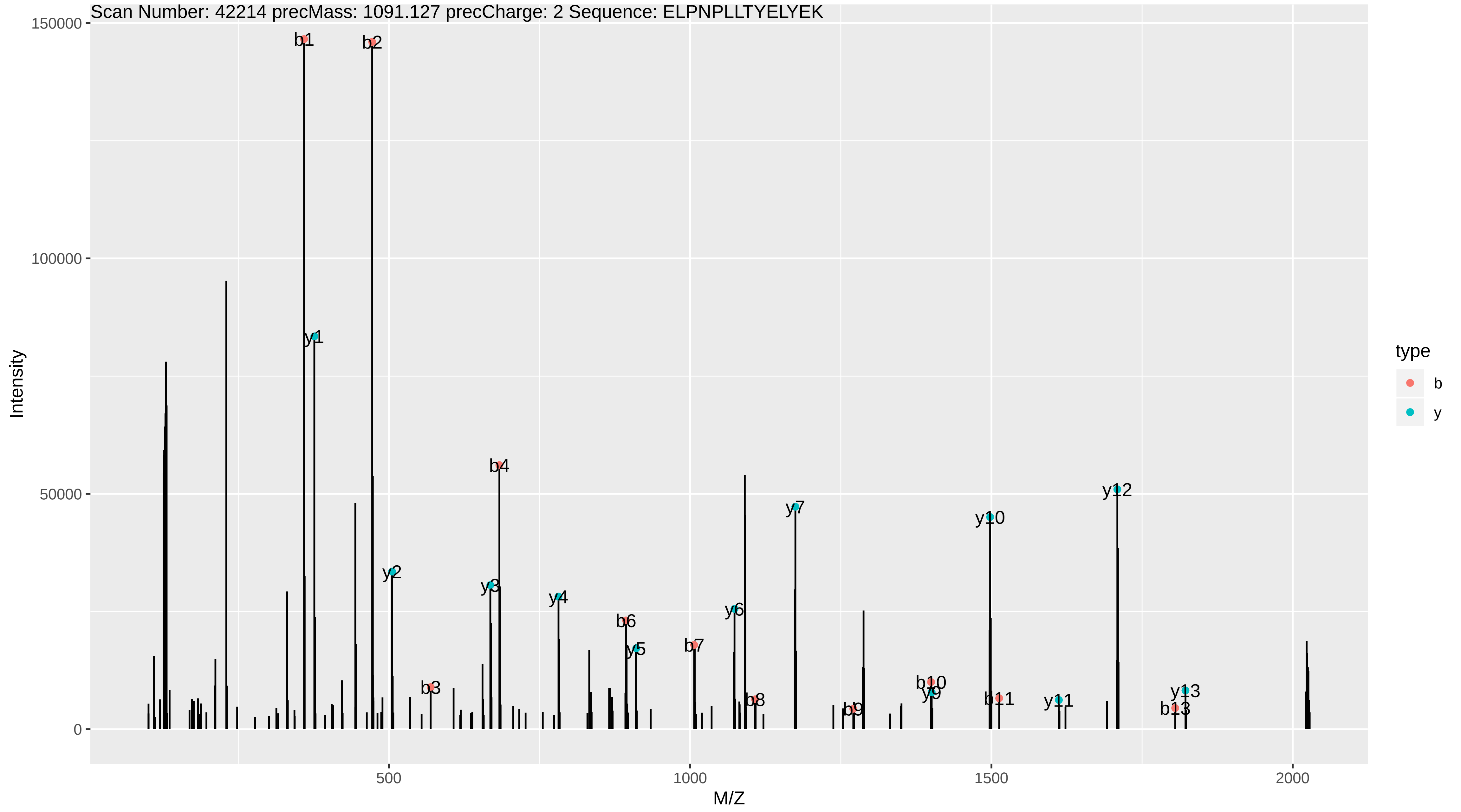

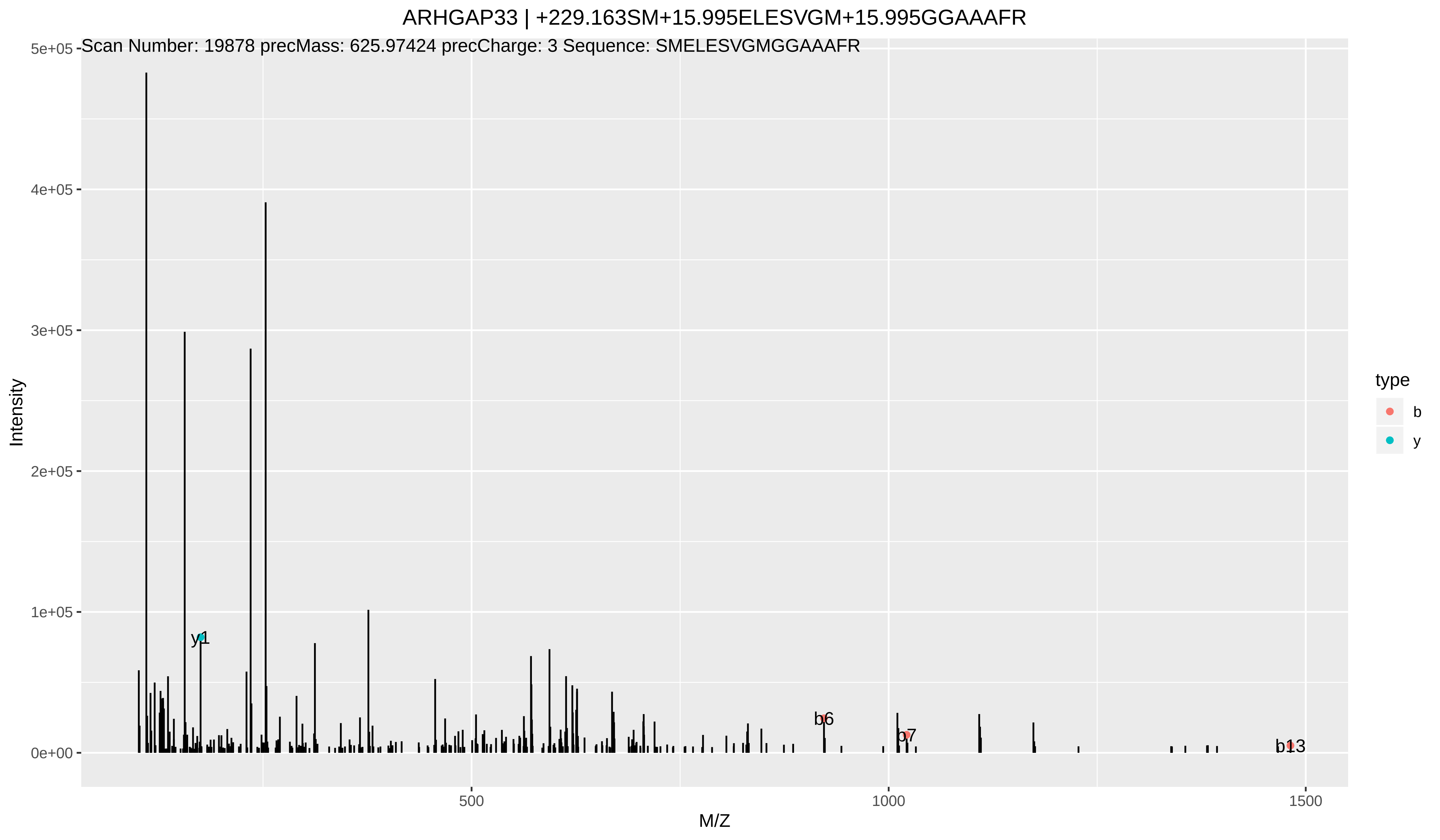
ARHGAP33 | +229.163SM+15.995ELESVGM+15.995GGAAAFR
Scan Number: 19878 precMass: 625.97424 precCharge: 3 Sequence: SMELESVGMGGAAAFR
Intensity
M/Z
type
b
y
y1
b6
b7
b13
5e+05
4e+05
3e+05
2e+05
1e+05
0e+00
500
1000
1500

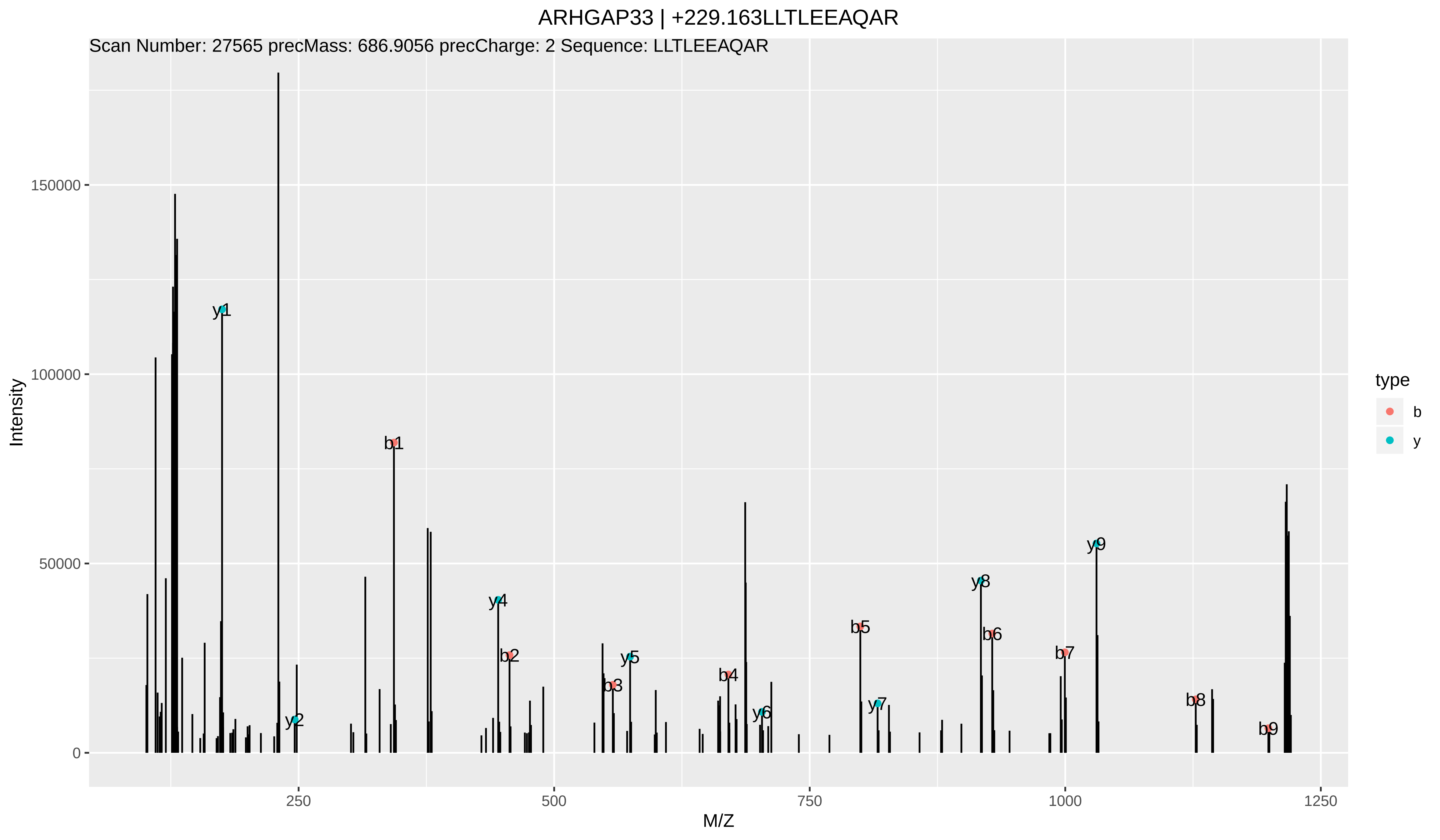

ARHGAP33 | +229.163LLTLEEAQAR
Scan Number: 27565 precMass: 686.9056 precCharge: 2 Sequence: LLTLEEAQAR
Intensity
M/Z
150000
100000
50000
0
250
500
750
1000
1250
y1
y2
y4
y5
y6
y7
y8
y9
b1
b2
b3
b4
b5
b6
b7
b8
b9
type
b
y

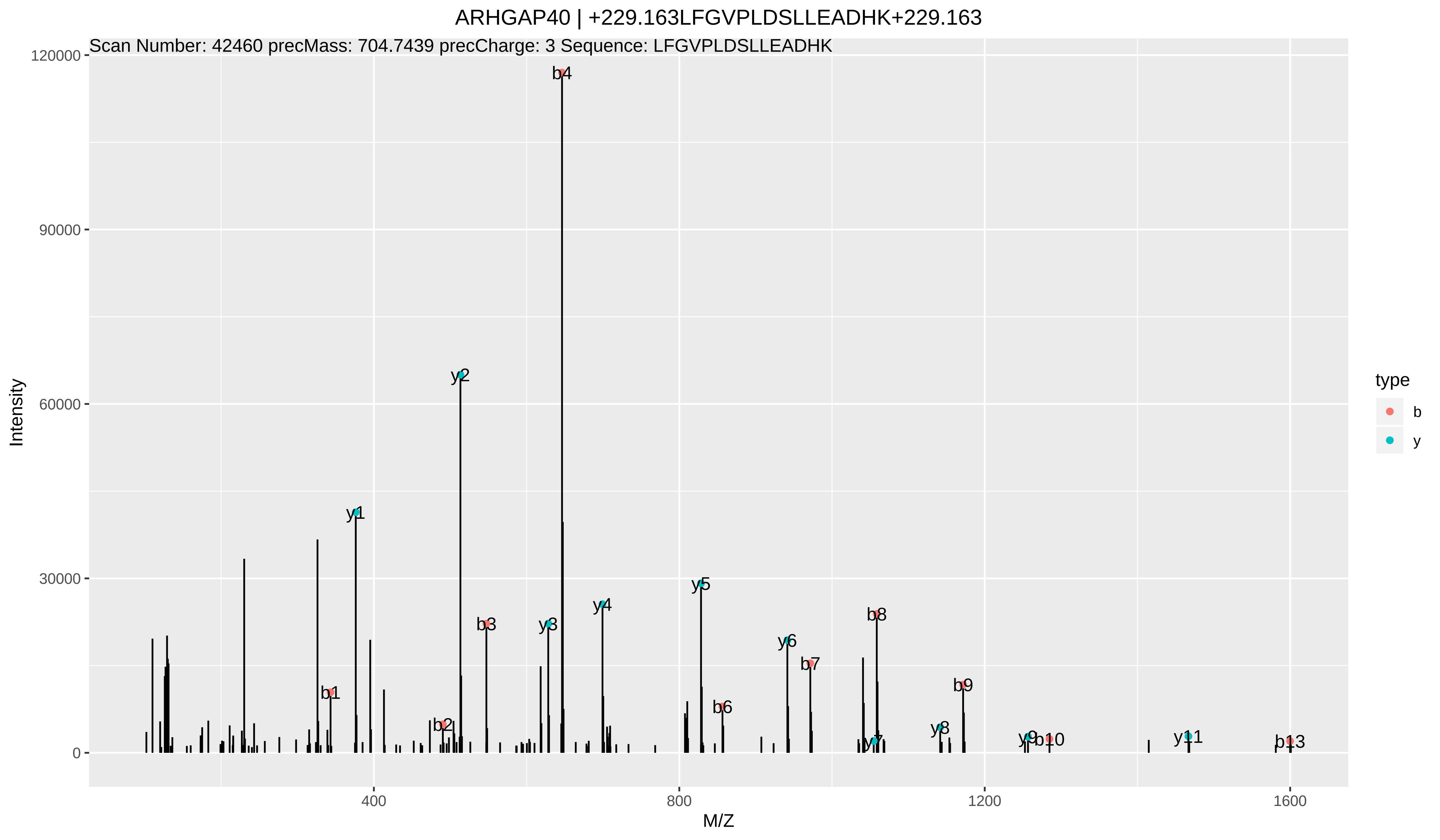
ARHGAP40 | +229.163LFGVPLDSLLEADHK+229.163
Scan Number: 42460 precMass: 704.7439 precCharge: 3 Sequence: LFGVPLDSLLEADHK
Intensity
M/Z
type
b
y
b1
b2
b3
b4
b6
b7
b8
b9
b10
b13
y1
y2
y3
y4
y5
y6
y7
y8
y9
y11
0
30000
60000
90000
120000
400
800
1200
1600

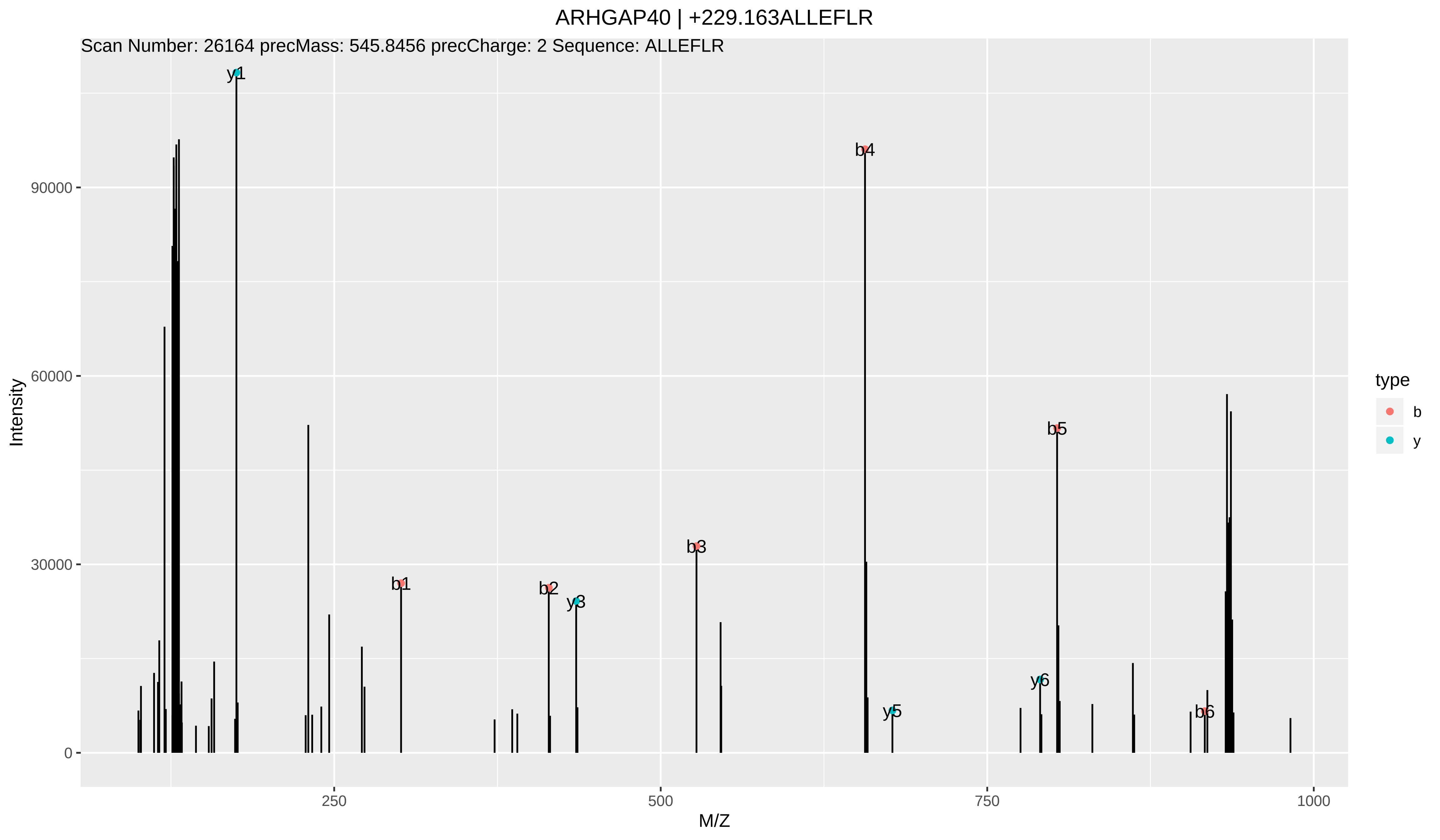
ARHGAP40 | +229.163ALLEFLR
Scan Number: 26164 precMass: 545.8456 precCharge: 2 Sequence: ALLEFLR
y1
b4
b5
b3
b1
b2
y3
y6
y5
b6
90000
60000
30000
0
Intensity
250
500
750
1000
M/Z
type
b
y

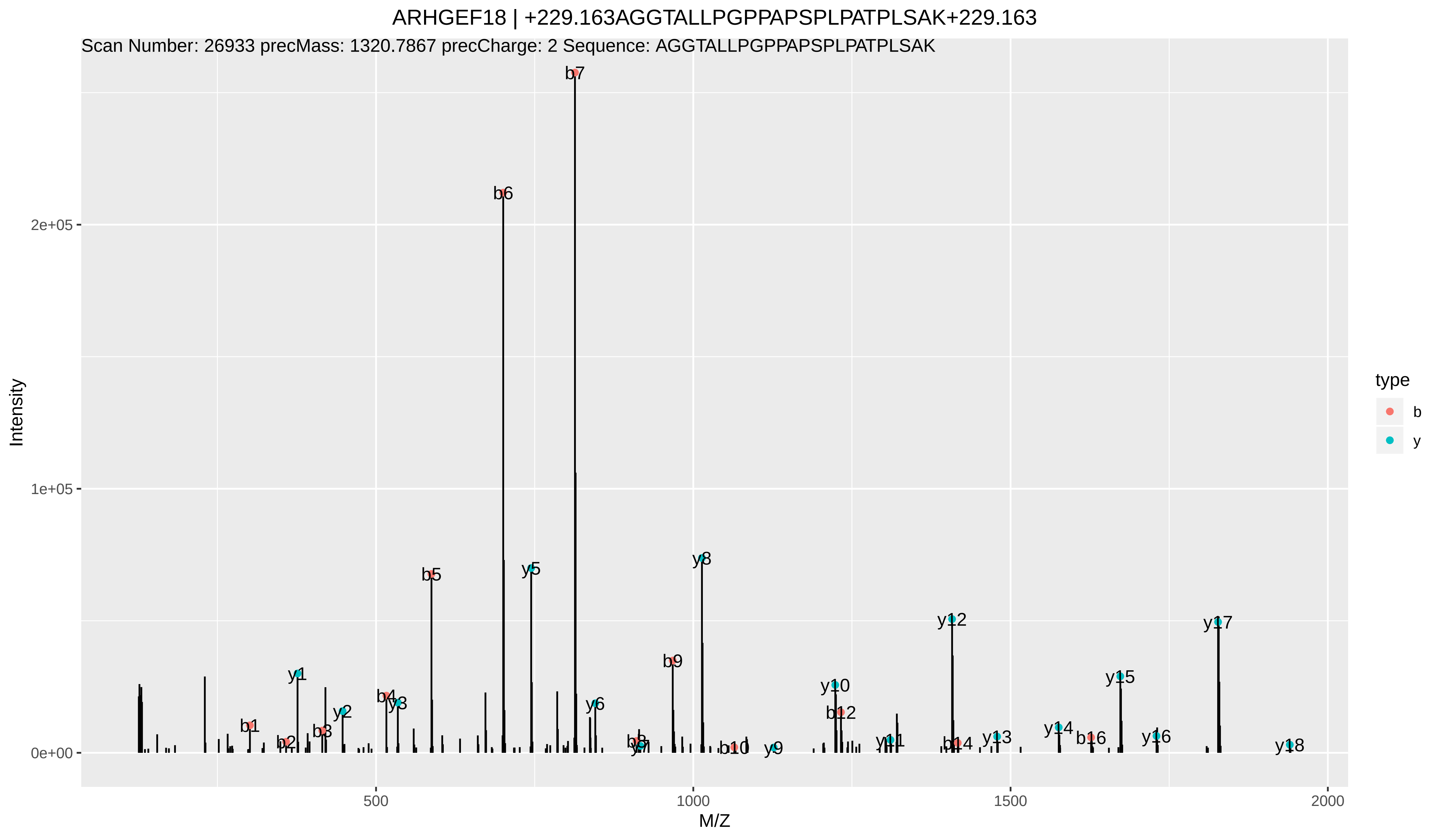

ARHGEF18 | +229.163AGGTALLPGPPAPSPLPATPLSAK+229.163
Scan Number: 26933 precMass: 1320.7867 precCharge: 2 Sequence: AGGTALLPGPPAPSPLPATPLSAK
Intensity
M/Z
2e+05
1e+05
0e+00
500
1000
1500
2000
b7
b6
b5
y5
y8
y12
y17
b9
y15
y10
y1
b4
y3
y2
y6
b12
b1
b3
b2
y14
b16
y16
y13
y11
b14
b8
y7
b10
y9
y18
type
b
y

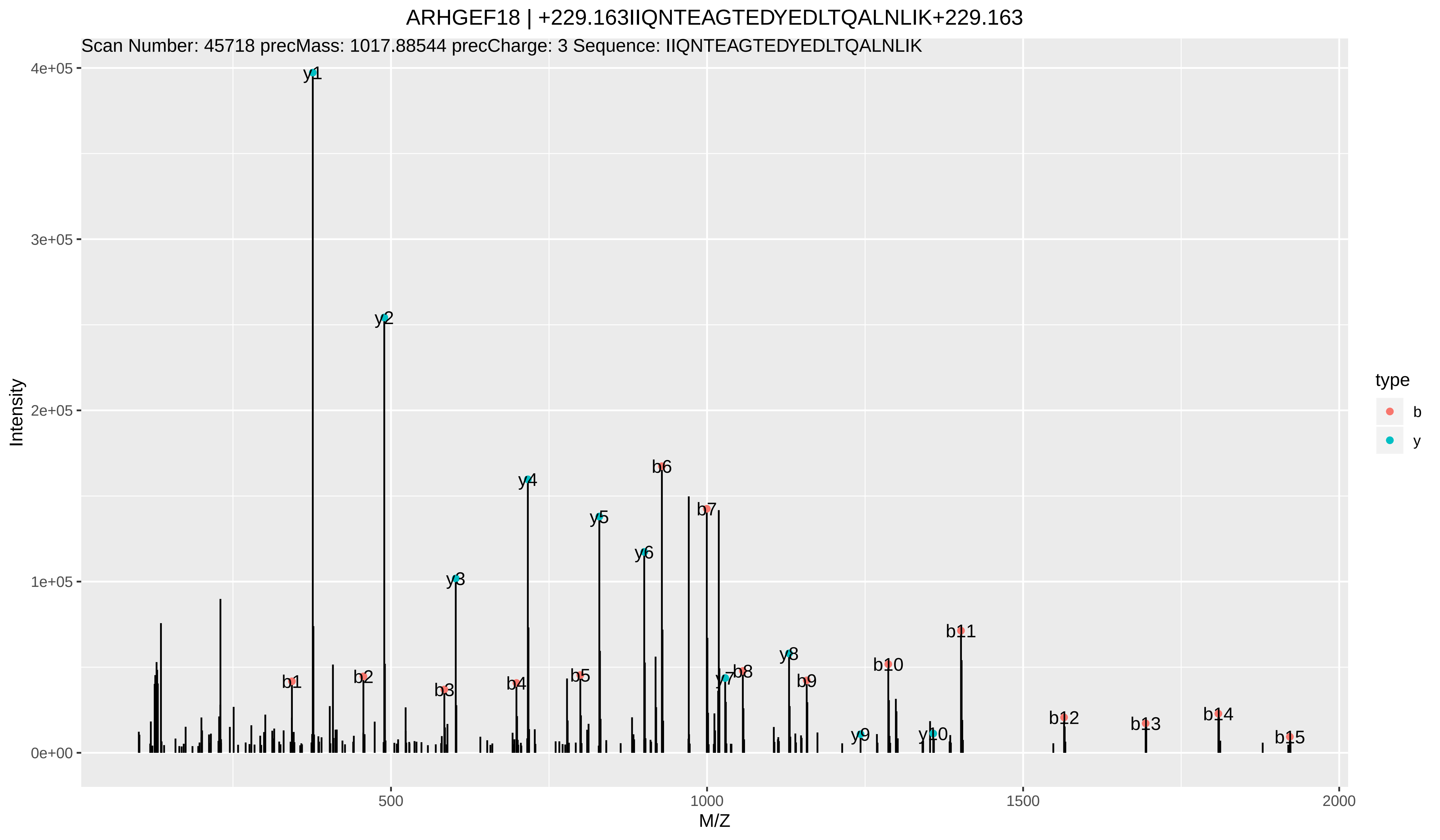

ARHGEF18 | +229.163IIQNTEAGTEDYEDLTQALNLIK+229.163
Scan Number: 45718 precMass: 1017.88544 precCharge: 3 Sequence: IIQNTEAGTEDYEDLTQALNLIK
Intensity
M/Z
type
b
y
y1
y2
y3
y4
y5
y6
y7
y8
y9
y10
b1
b2
b3
b4
b5
b6
b7
b8
b9
b10
b11
b12
b13
b14
b15
0e+00
1e+05
2e+05
3e+05
4e+05
500
1000
1500
2000

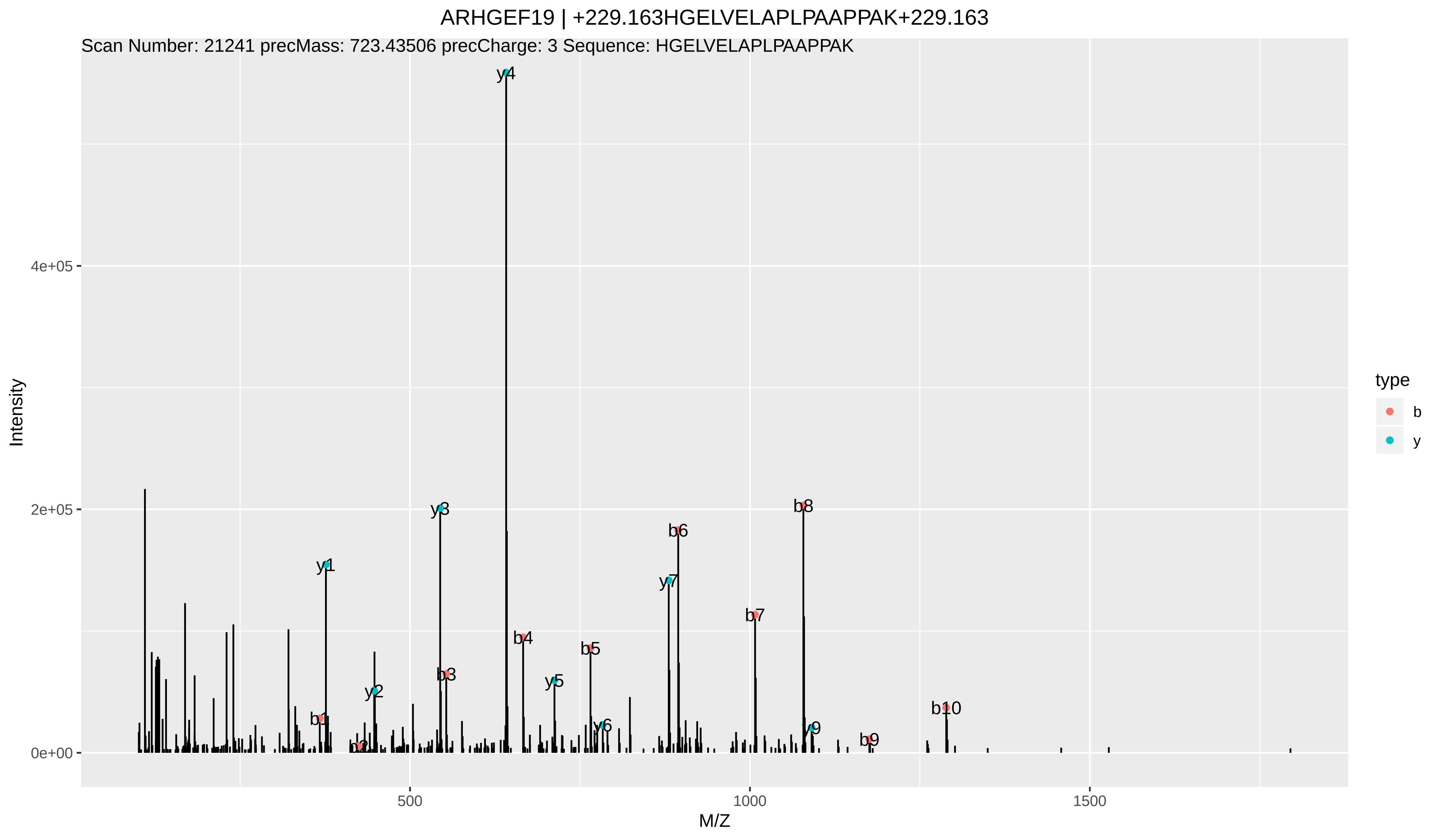
ARHGEF19 | +229.163HGELVELAPLPAAPPAK+229.163
Scan Number: 21241 precMass: 723.43506 precCharge: 3 Sequence: HGELVELAPLPAAPPAK
Intensity
M/Z
type
b
y
y4
y3
y1
y7
y2
y5
y6
y9
b8
b6
b7
b4
b5
b3
b1
b10
b9
4e+05
2e+05
0e+00
500
1000
1500

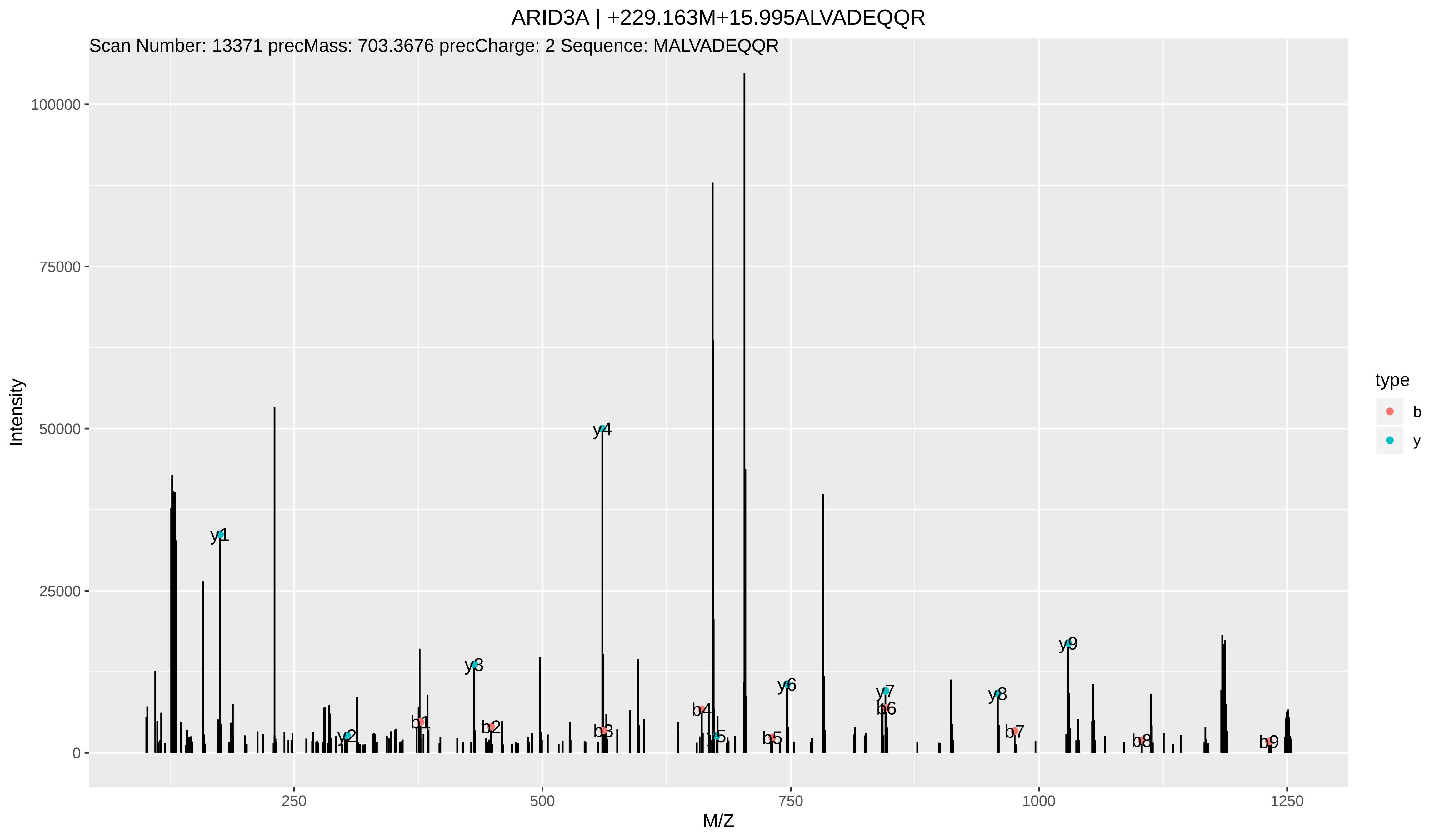
ARID3A | +229.163M+15.995ALVADEQQR
Scan Number: 13371 precMass: 703.3676 precCharge: 2 Sequence: MALVADEQQR
Intensity
M/Z
type
b
y
y1
y2
y3
y4
y6
y7
y8
y9
b1
b2
b3
b4
b5
b6
b7
b8
b9
100000
75000
50000
25000
0
250
500
750
1000
1250

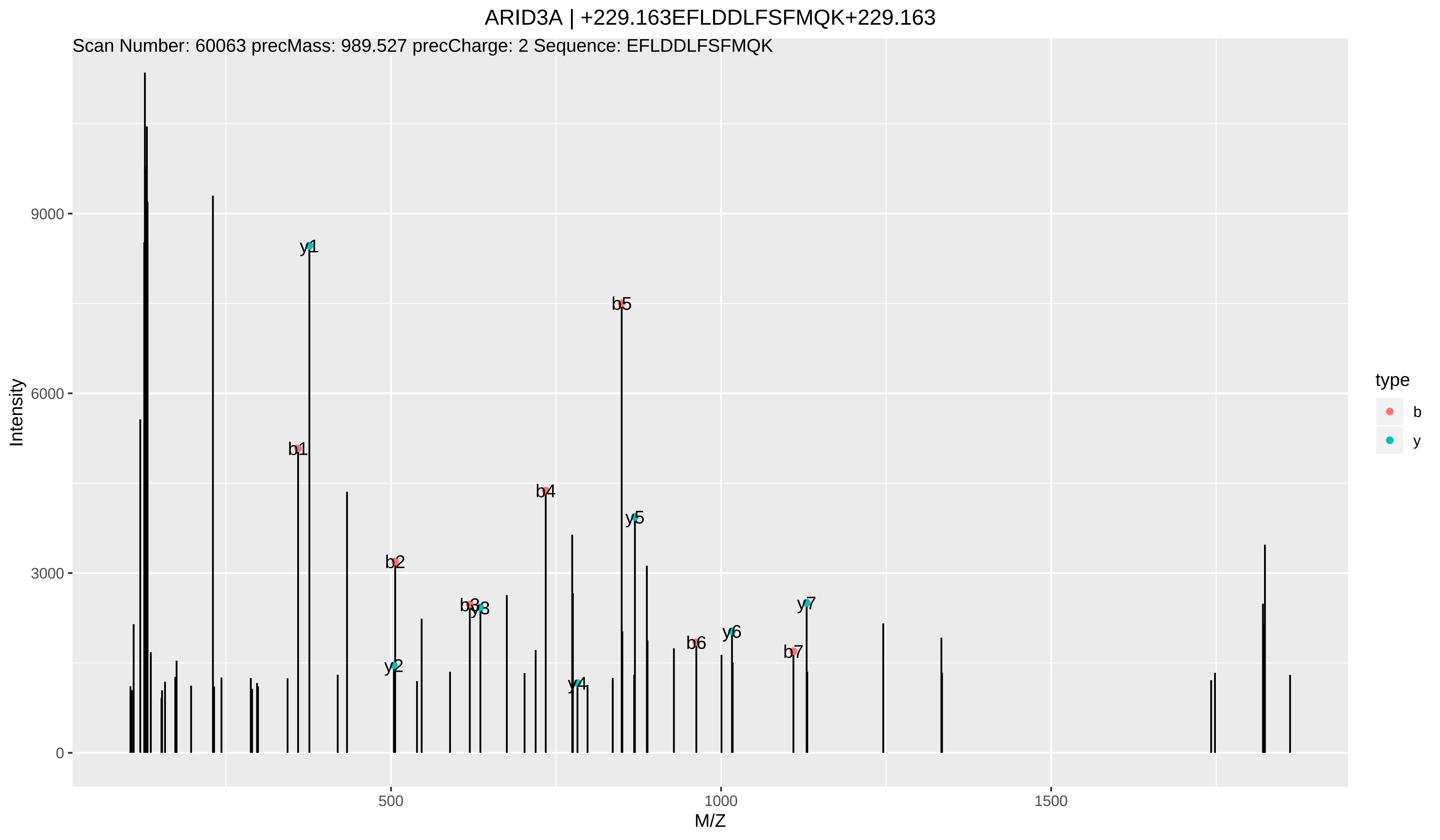
ARID3A | +229.163EFLDDLFSFMQK+229.163
Scan Number: 60063 precMass: 989.527 precCharge: 2 Sequence: EFLDDLFSFMQK
Intensity
9000
6000
3000
0
500
1000
1500
M/Z
y1
b5
b1
b4
y5
b2
b3
y3
y7
y6
b6
b7
y2
y4
type
b
y

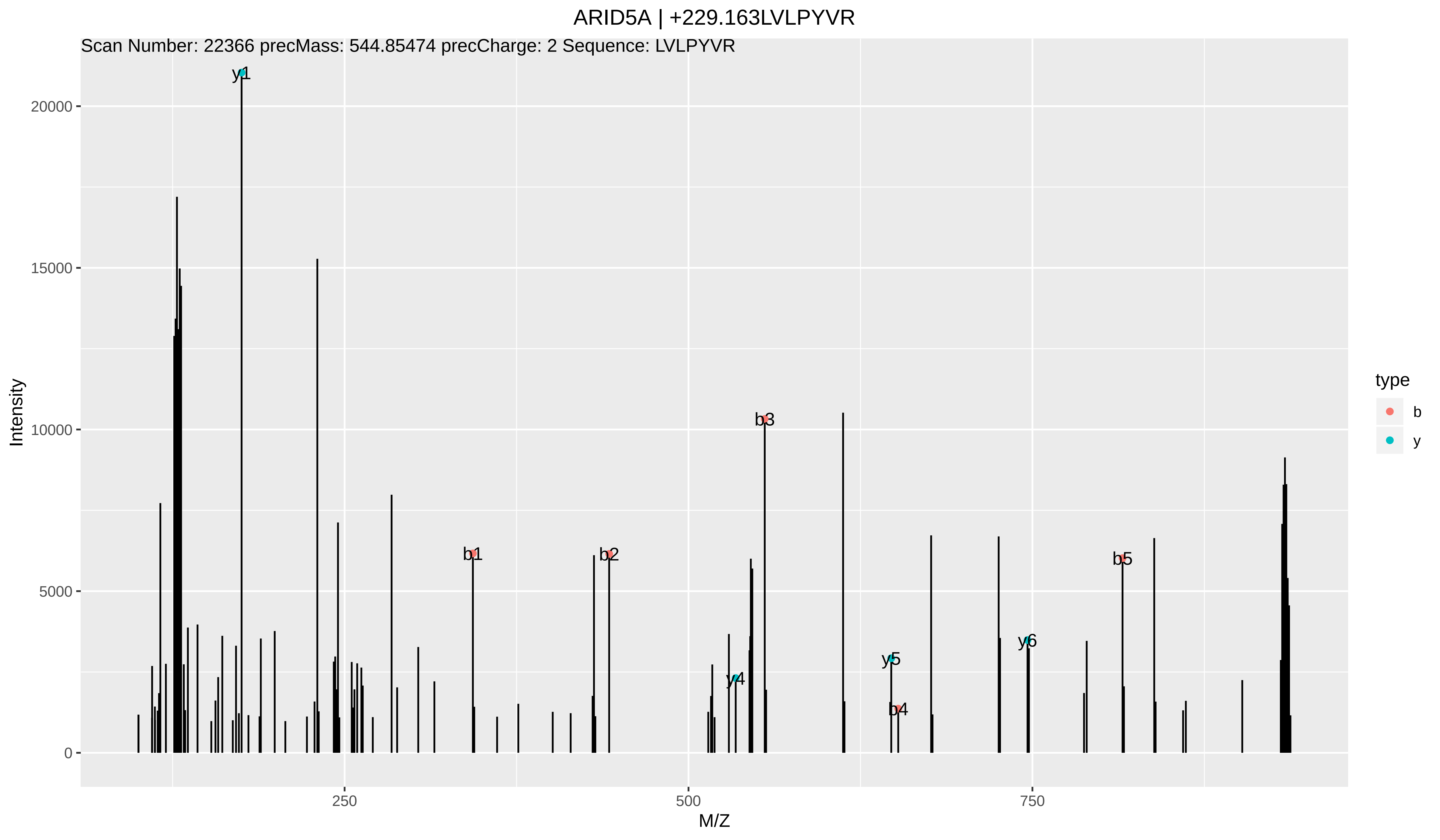

ARID5A | +229.163LVLPYVR
Scan Number: 22366 precMass: 544.85474 precCharge: 2 Sequence: LVLPYVR
Intensity
M/Z
0
5000
10000
15000
20000
250
500
750
y1
b1
b2
b3
y4
y5
b4
y6
b5
type
b
y

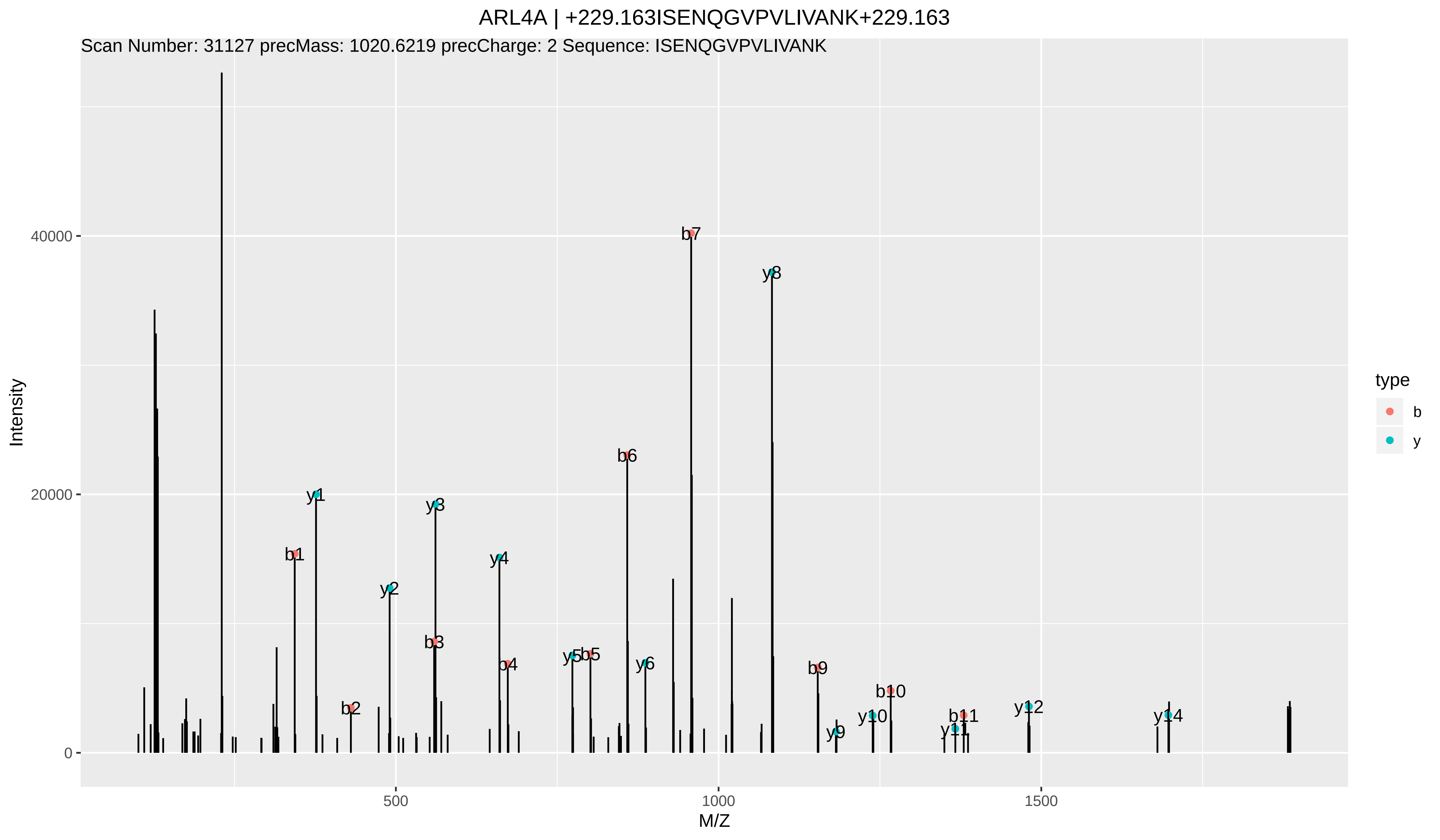

ARL4A | +229.163ISENQGVPVLIVANK+229.163
Scan Number: 31127 precMass: 1020.6219 precCharge: 2 Sequence: ISENQGVPVLIVANK
Intensity
40000
20000
0
500
1000
1500
M/Z
b7
y8
b6
y1
y3
b1
y4
y2
b3
y5
b5
b4
y6
b9
b2
b10
y10
y9
b11
y11
y12
y14
type
b
y

ARMCX5 | +229.163IPPYHGPYYQTLAEIK+229.163

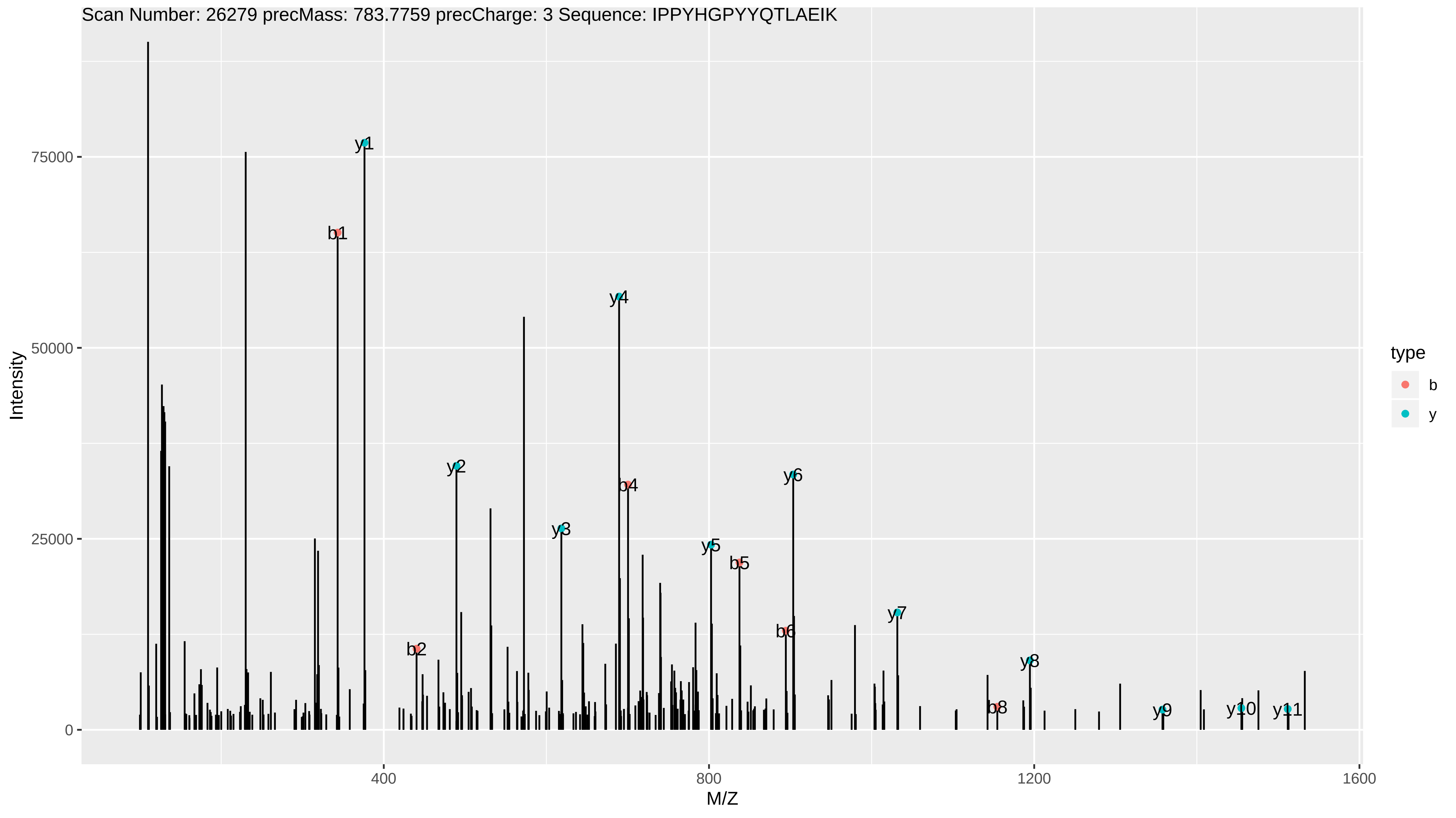

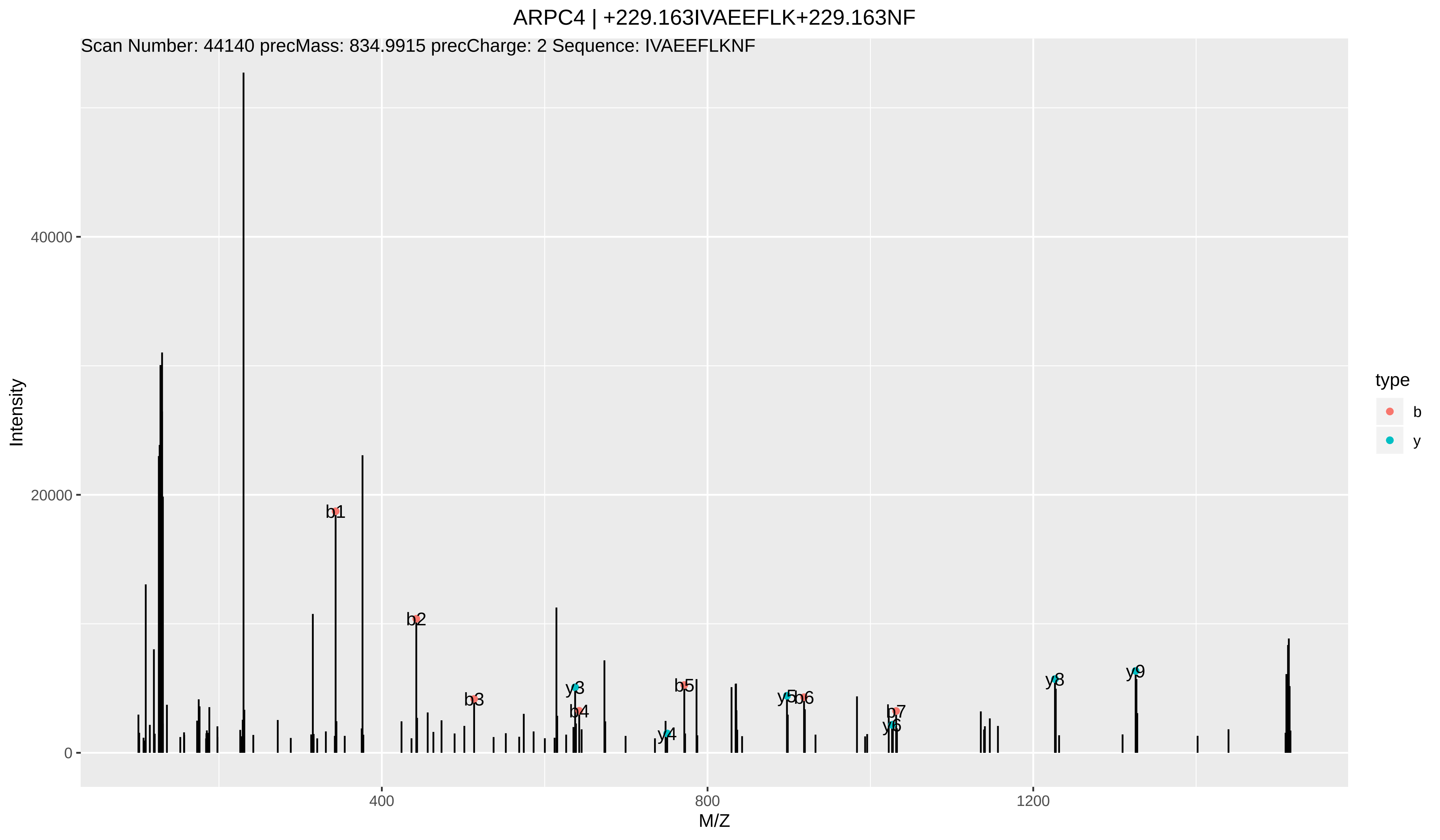

ARPC4 | +229.163IVAEEFLK+229.163NF
Scan Number: 44140 precMass: 834.9915 precCharge: 2 Sequence: IVAEEFLKNF
Intensity
40000
20000
0
400
800
1200
M/Z
b1
b2
b3
b4
b5
b6
b7
y3
y4
y5
y6
y8
y9
type
b
y

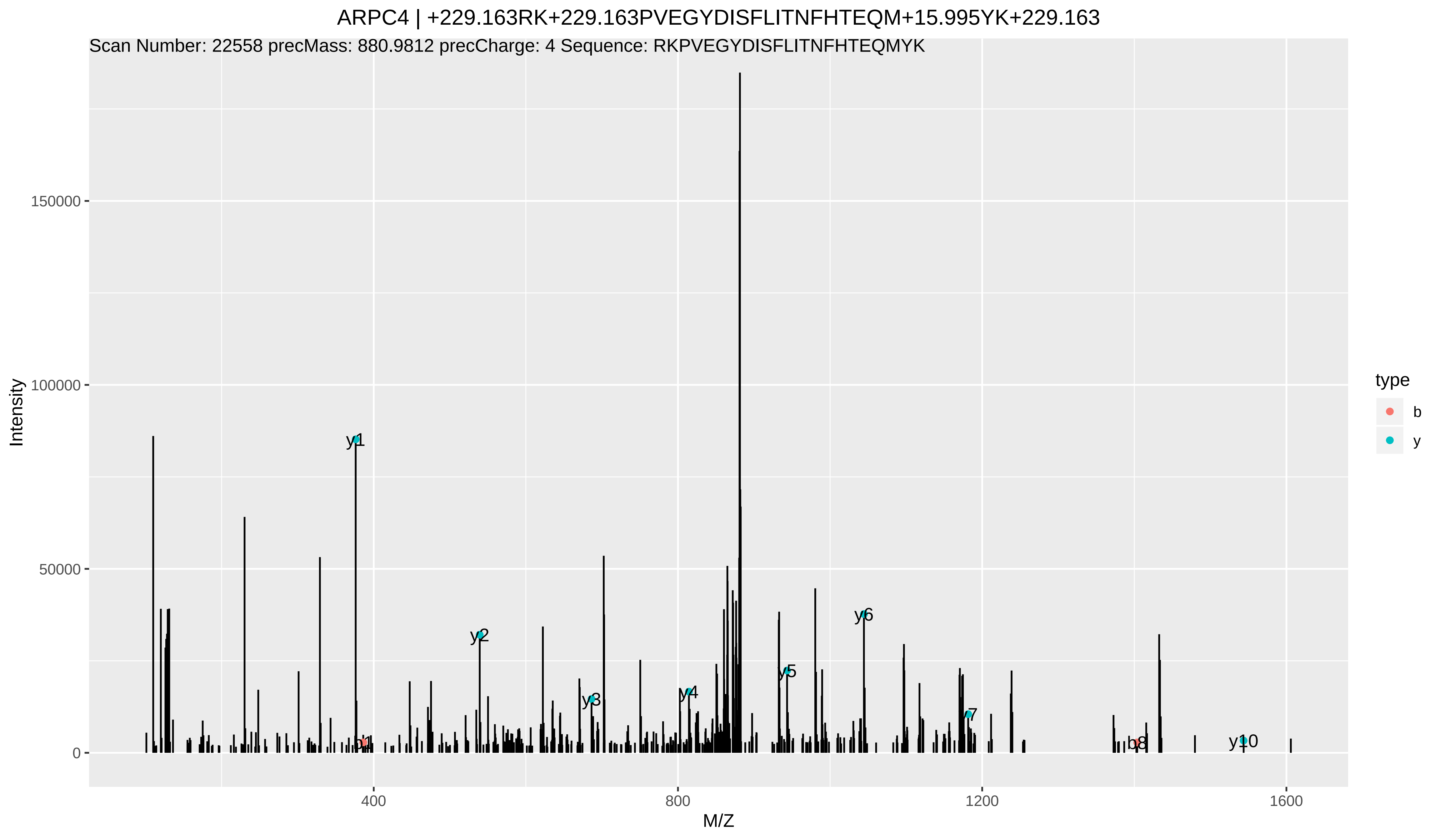
ARPC4 | +229.163RK+229.163PVEGYDISFLITNFHTEQM+15.995YK+229.163
Scan Number: 22558 precMass: 880.9812 precCharge: 4 Sequence: RKPVEGYDISFLITNFHTEQMYK
Intensity
150000
100000
50000
0
M/Z
400
800
1200
1600
y1
y2
y3
y4
y5
y6
y10
b8
type
b
y

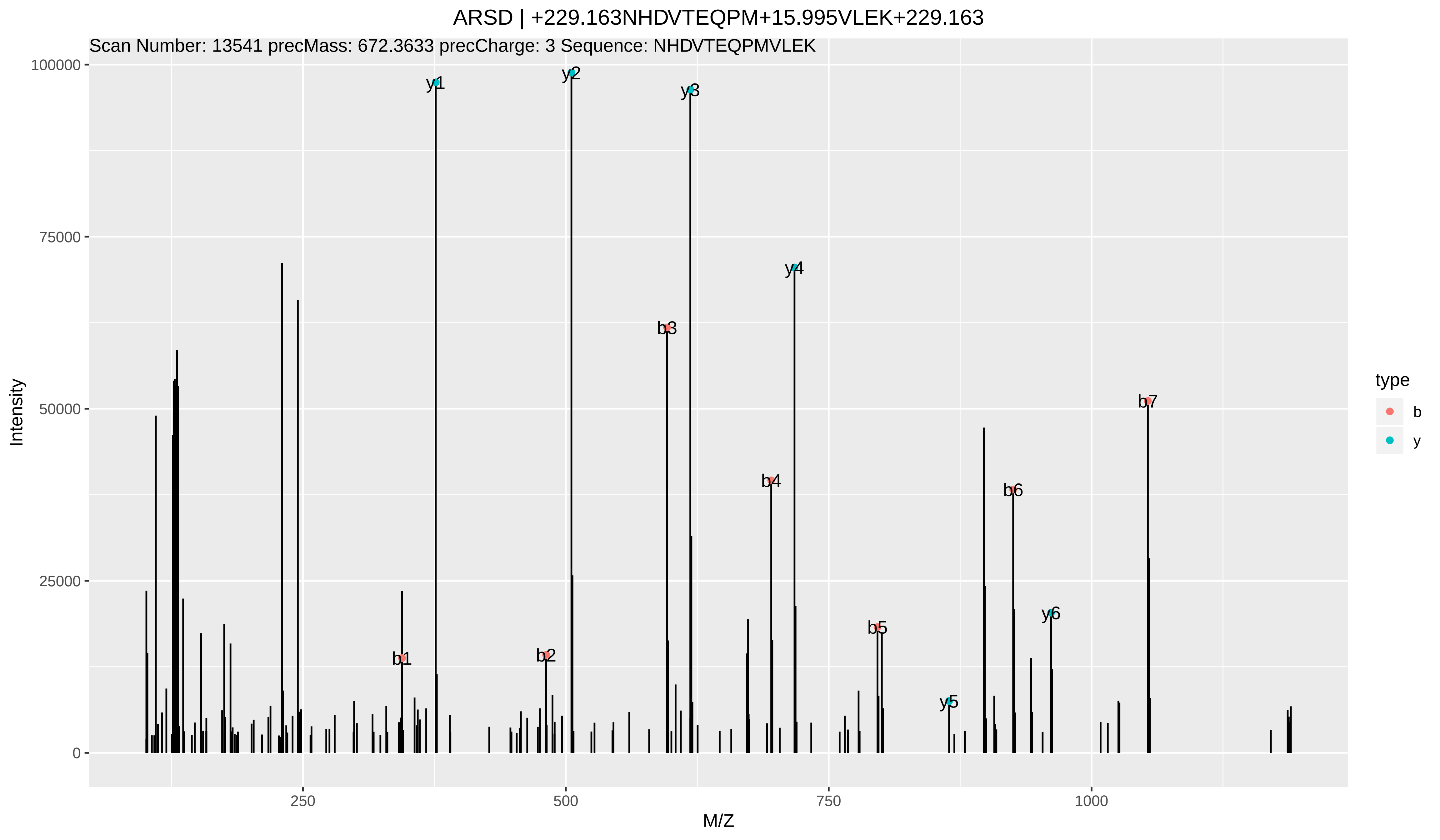
ARSD | +229.163NHDVTEQPM+15.995VLEK+229.163
Scan Number: 13541 precMass: 672.3633 precCharge: 3 Sequence: NHDVTEQPMVLEK
Intensity
100000
75000
50000
25000
0
250
500
750
1000
M/Z
y1
y2
y3
y4
y5
y6
b1
b2
b3
b4
b5
b6
b7
type
b
y

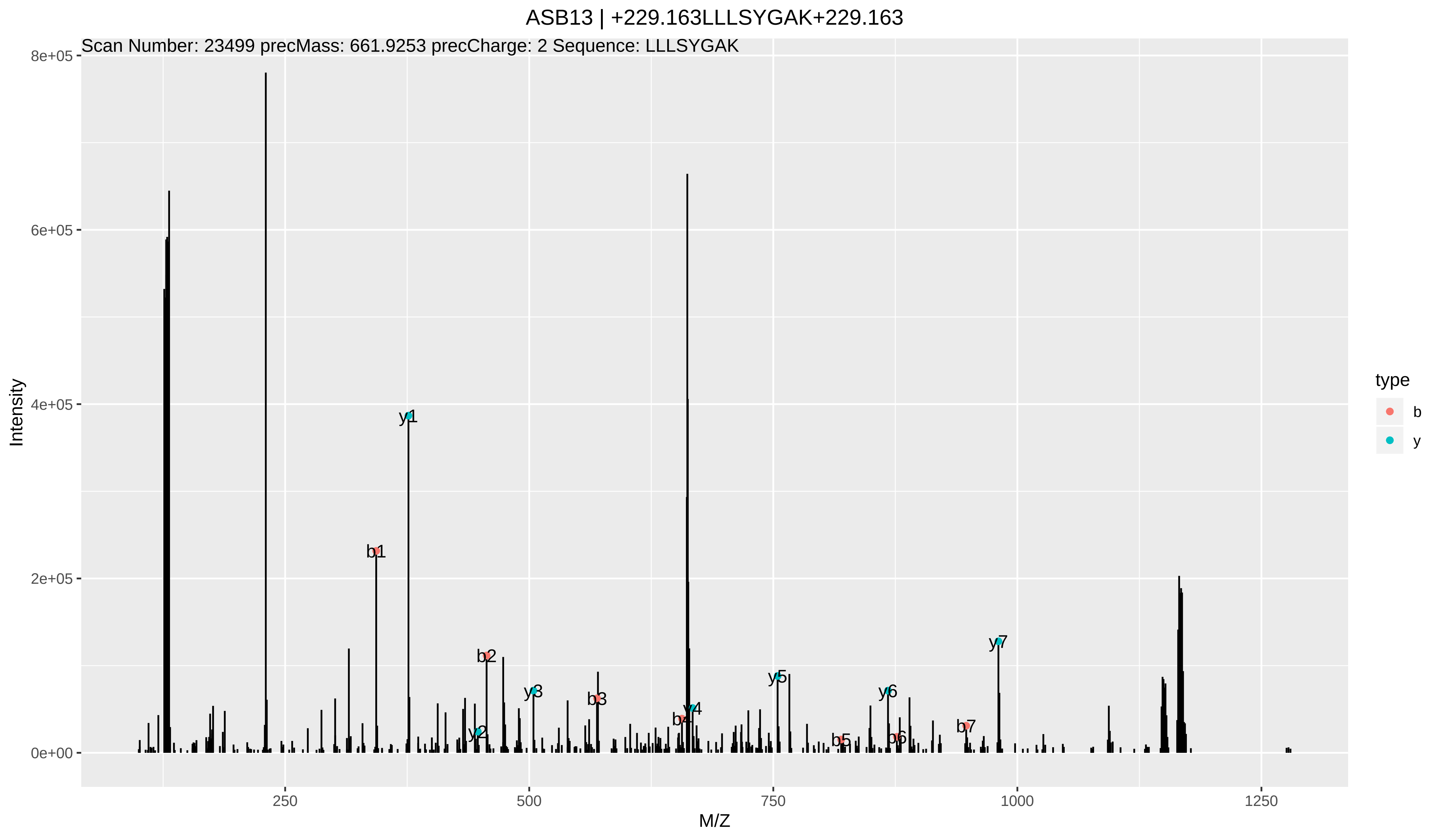
ASB13 | +229.163LLLSYGAK+229.163
Scan Number: 23499 precMass: 661.9253 precCharge: 2 Sequence: LLLSYGAK
Intensity
M/Z
8e+05
6e+05
4e+05
2e+05
0e+00
250
500
750
1000
1250
y1
b1
b2
y2
y3
b3
b4
y4
y5
b5
y6
b6
b7
y7
type
b
y

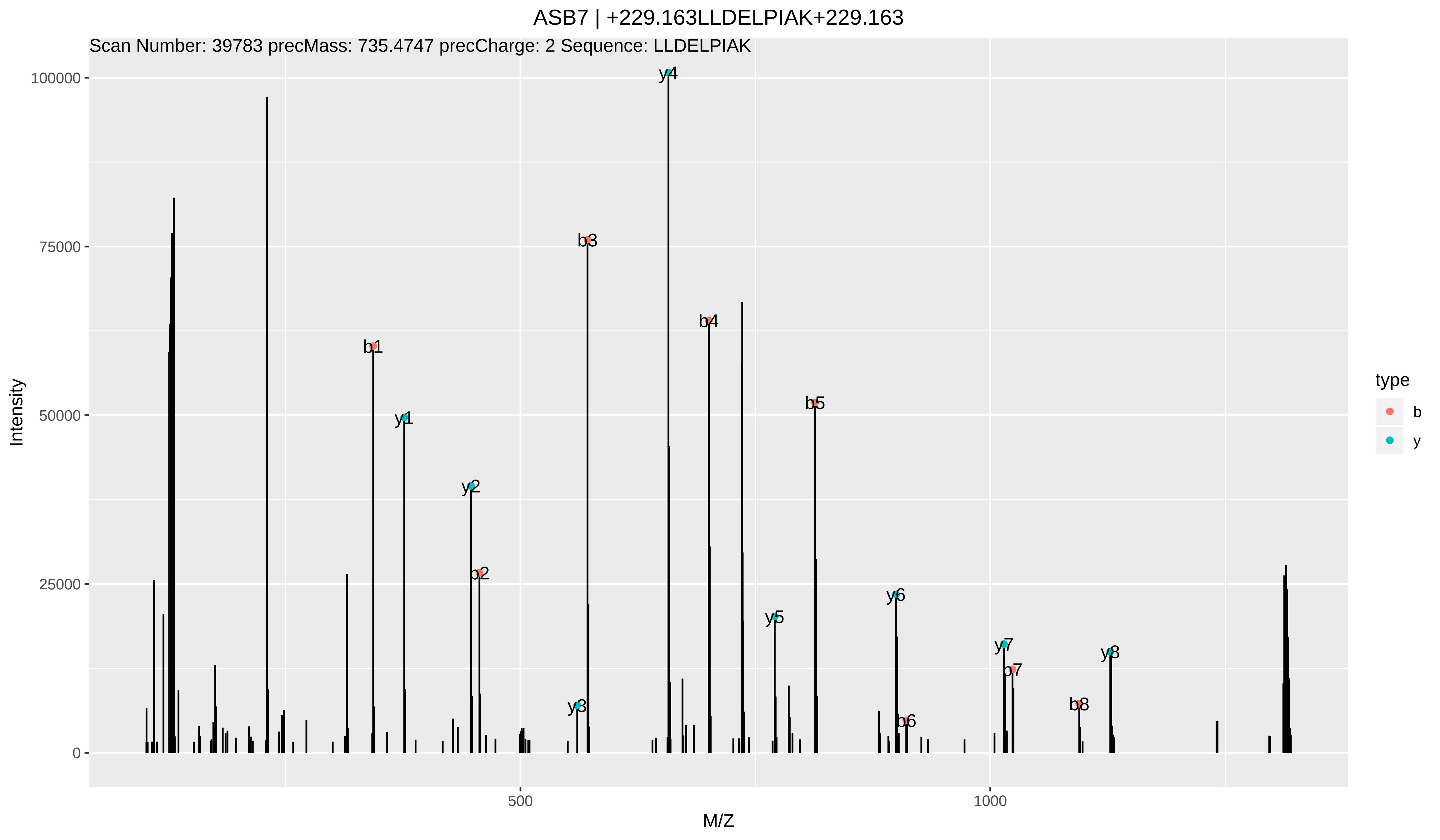
ASB7 | +229.163LLDELPIAK+229.163
Scan Number: 39783 precMass: 735.4747 precCharge: 2 Sequence: LLDELPIAK
Intensity
100000
75000
50000
25000
0
500
1000
M/Z
y4
b3
b4
b1
b5
y1
y2
b2
y6
y5
y7
b7
y8
b8
y3
b6
type
b
y

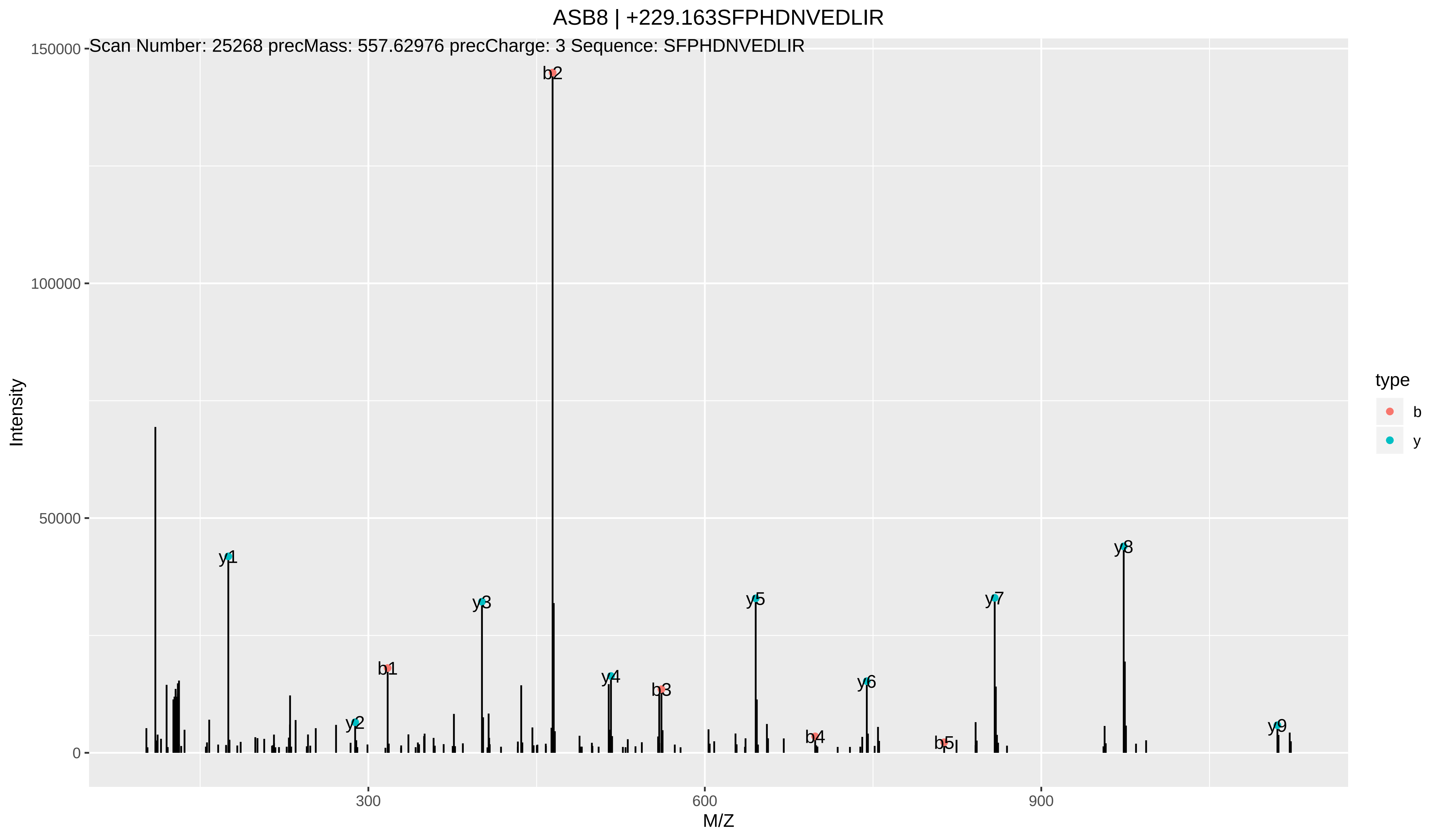
ASB8 | +229.163SFPHDNVEDLIR
Scan Number: 25268 precMass: 557.62976 precCharge: 3 Sequence: SFPHDNVEDLIR
Intensity
M/Z
0
50000
100000
150000
300
600
900
b2
y1
y2
b1
y3
y4
b3
y5
b4
y6
b5
y7
y8
y9
type
b
y

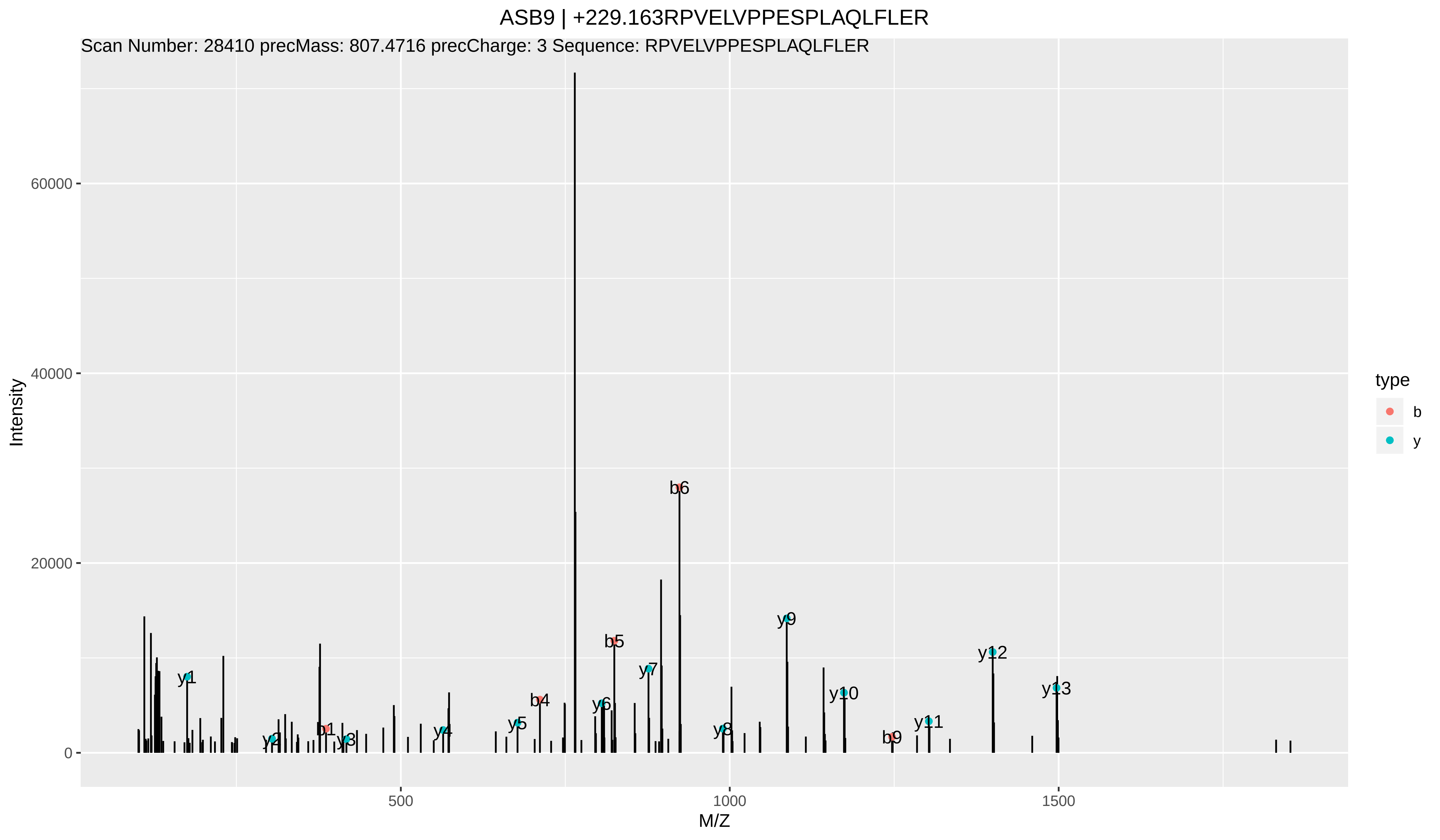

ASB9 | +229.163RPVELVPPESPLAQLFLER
Scan Number: 28410 precMass: 807.4716 precCharge: 3 Sequence: RPVELVPPESPLAQLFLER
Intensity
M/Z
0
20000
40000
60000
500
1000
1500
type
b
y
y1
y2
b1
y3
y4
y5
b4
y6
b5
y7
b6
y8
y9
y10
b9
y11
y12
y13

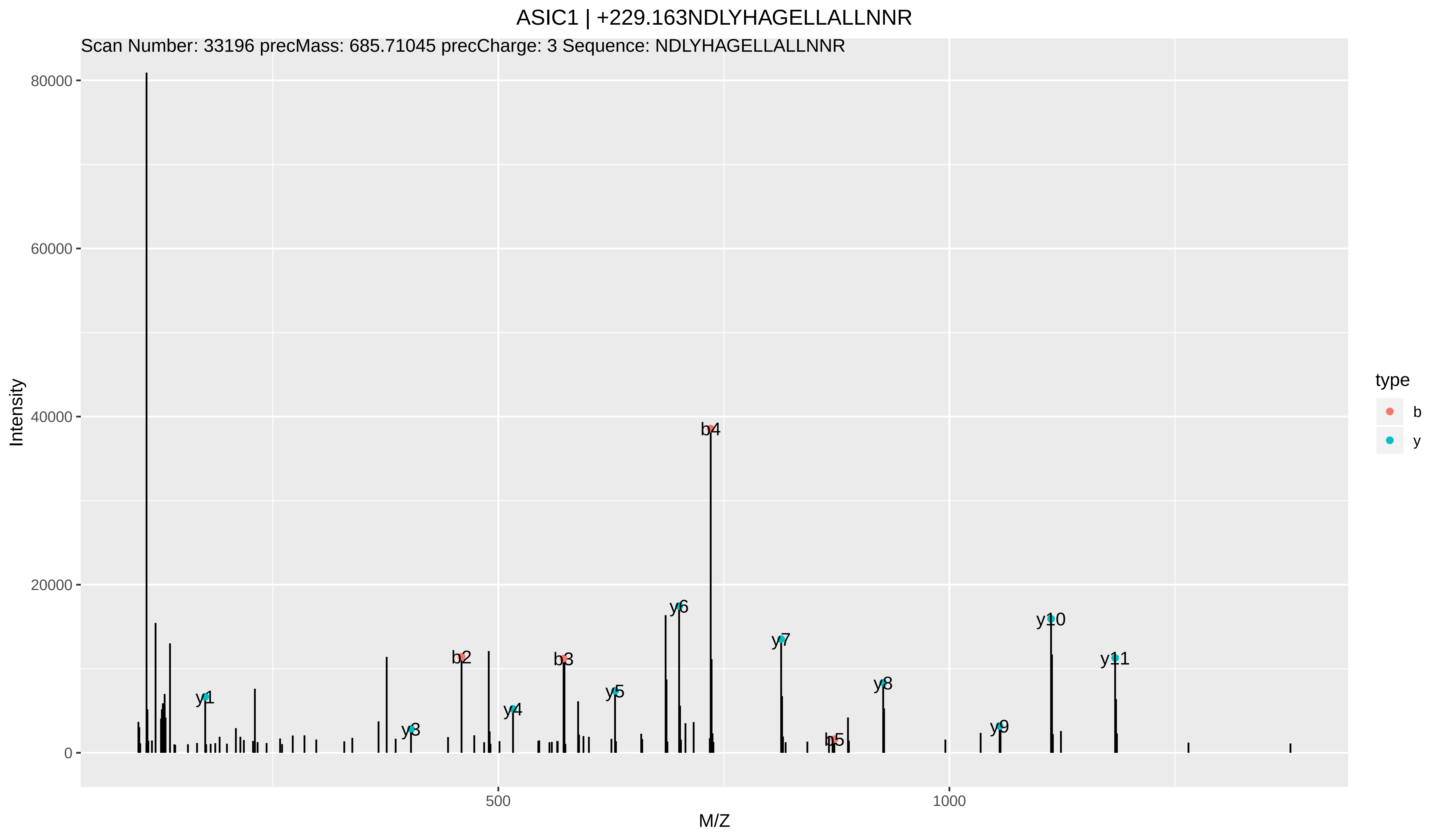

ASIC1 | +229.163NDLYHAGELLALLNNR
Scan Number: 33196 precMass: 685.71045 precCharge: 3 Sequence: NDLYHAGELLALLNNR
Intensity
M/Z
80000
60000
40000
20000
0
500
1000
y1
y3
b2
y4
b3
y5
y6
b4
y7
b5
y8
y9
y10
y11
type
b
y

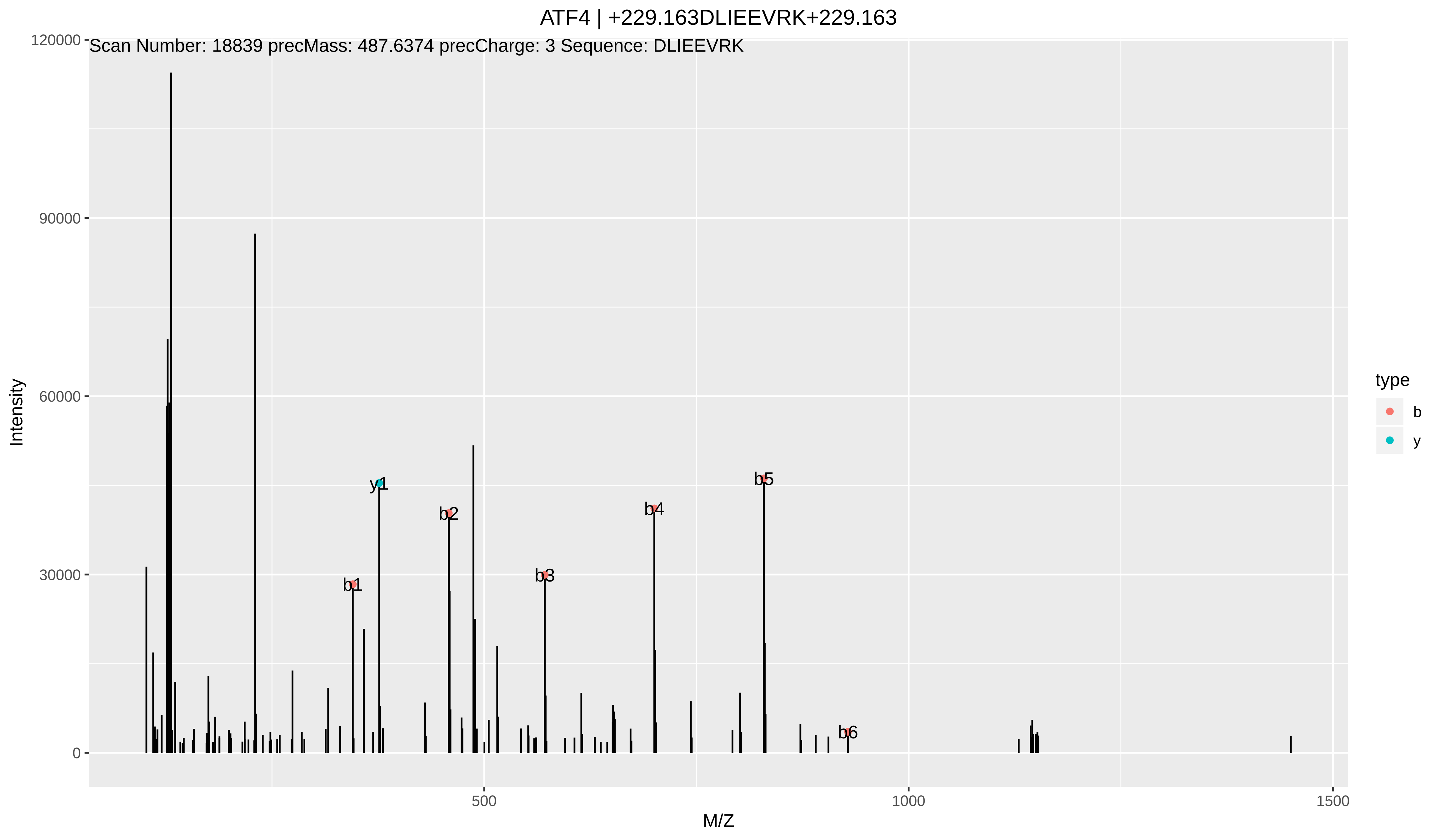
ATF4 | +229.163DLIEEVRK+229.163
Scan Number: 18839 precMass: 487.6374 precCharge: 3 Sequence: DLIEEVRK
Intensity
120000
90000
60000
30000
0
M/Z
500
1000
1500
y1
b1
b2
b3
b4
b5
b6
type
b
y

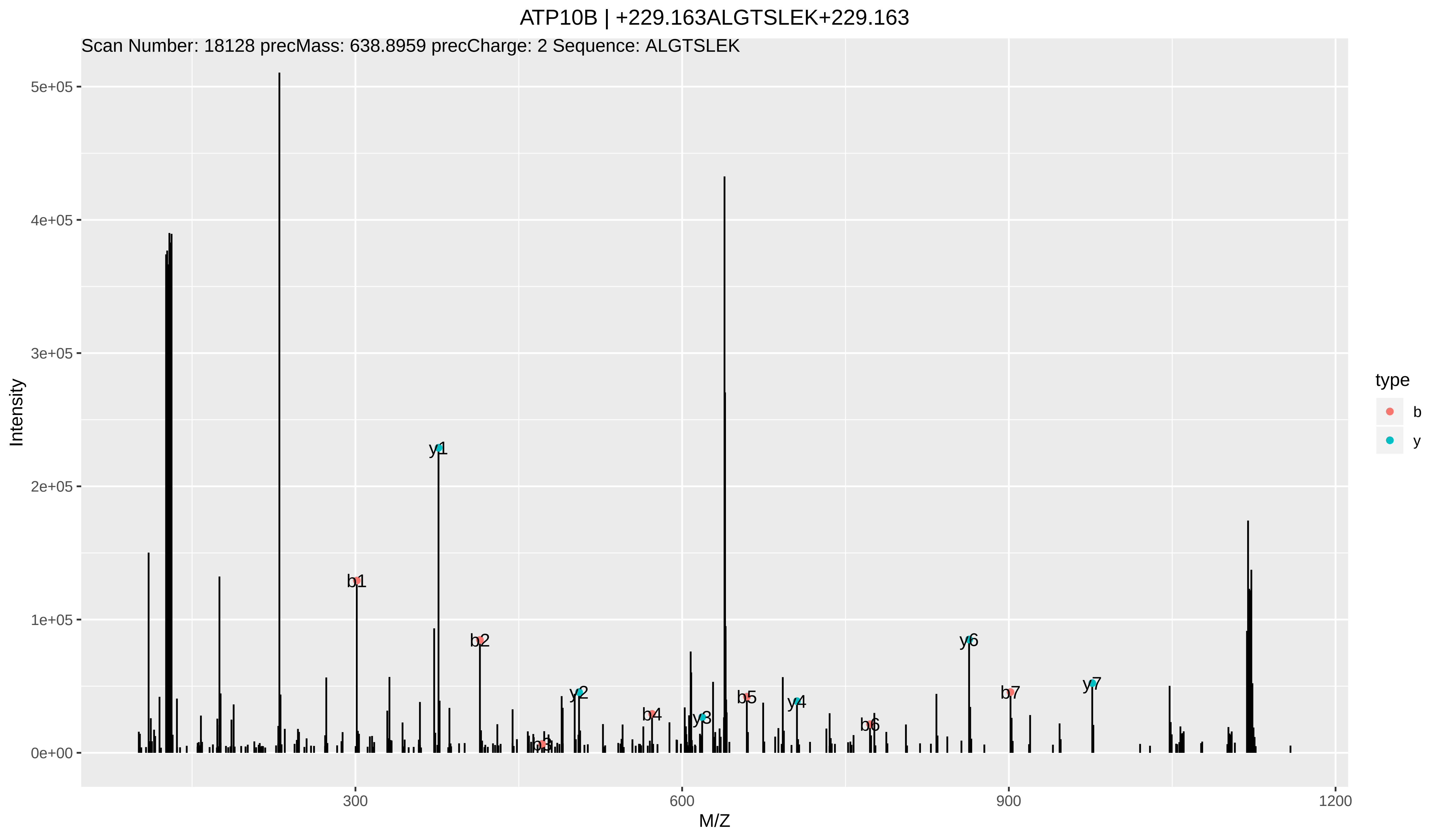

ATP10B | +229.163ALGTSLEK+229.163
Scan Number: 18128 precMass: 638.8959 precCharge: 2 Sequence: ALGTSLEK
Intensity
M/Z
5e+05
4e+05
3e+05
2e+05
1e+05
0e+00
300
600
900
1200
y1
b1
b2
b3
y2
b4
y3
b5
y4
b6
y6
b7
y7
type
b
y

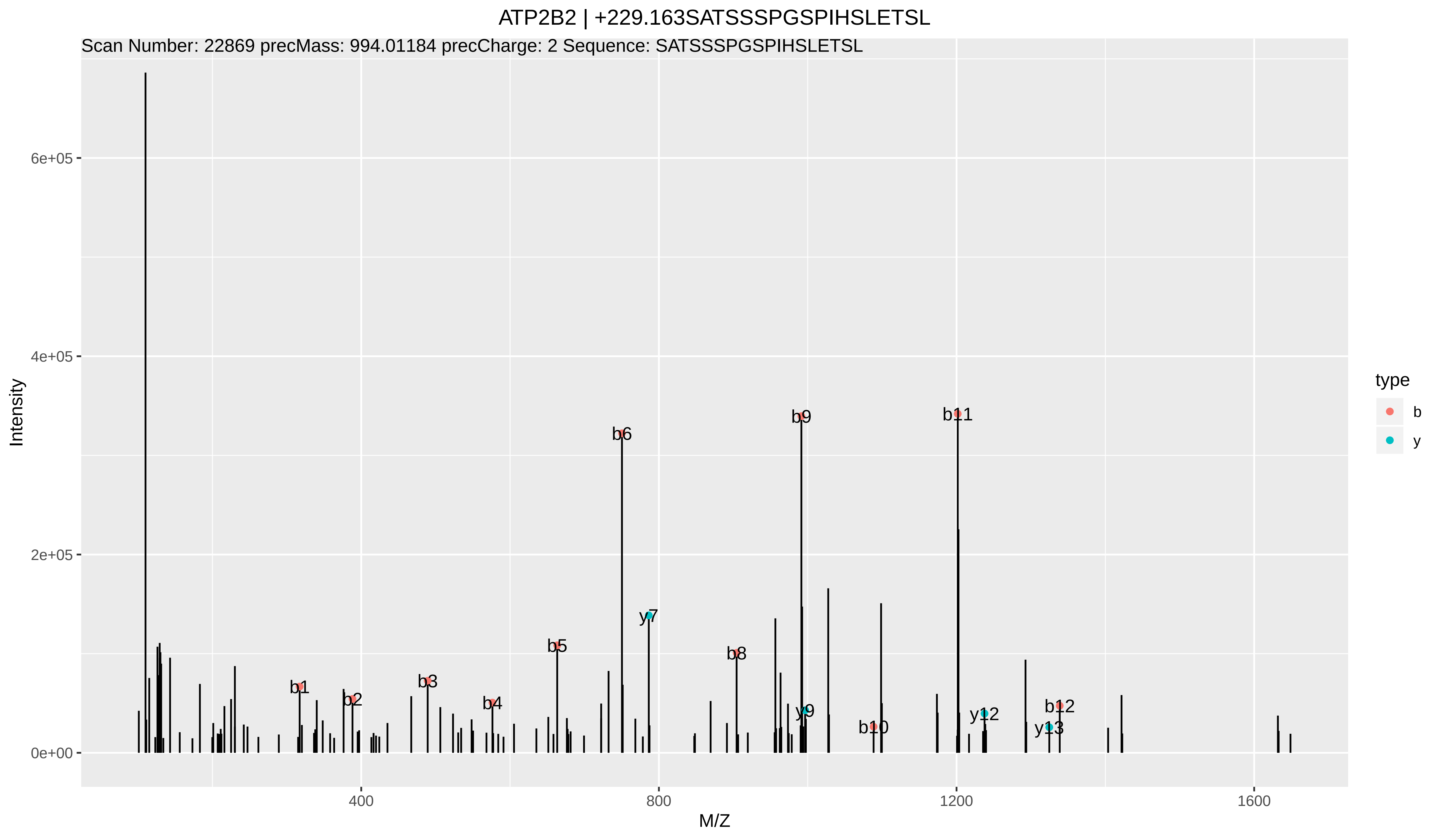

ATP2B2 | +229.163SATSSSPGSPIHSLETSL
Scan Number: 22869 precMass: 994.01184 precCharge: 2 Sequence: SATSSSPGSPIHSLETSL
Intensity
6e+05
4e+05
2e+05
0e+00
M/Z
400
800
1200
1600
b1
b2
b3
b4
b5
b6
y7
b8
b9
y9
b10
b11
y12
b12
y13
type
b
y

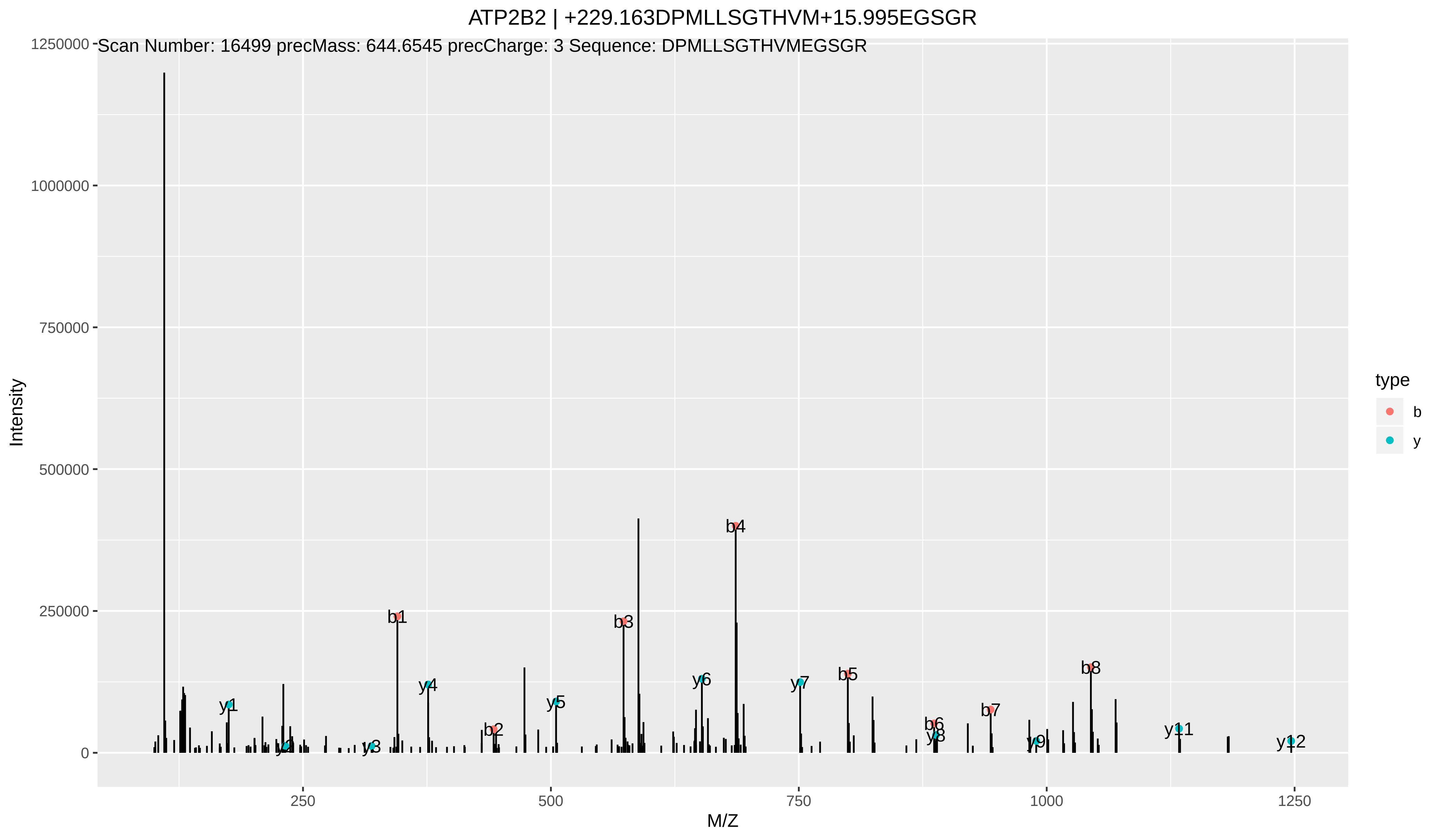

ATP2B2 | +229.163DPMLLSGTHVM+15.995EGSGR
Scan Number: 16499 precMass: 644.6545 precCharge: 3 Sequence: DPMLLSGTHVMEGSGR
Intensity
M/Z
type
b
y
b1
b2
b3
b4
b5
b6
b7
b8
y1
y2
y3
y4
y5
y6
y7
y8
y9
y11
y12

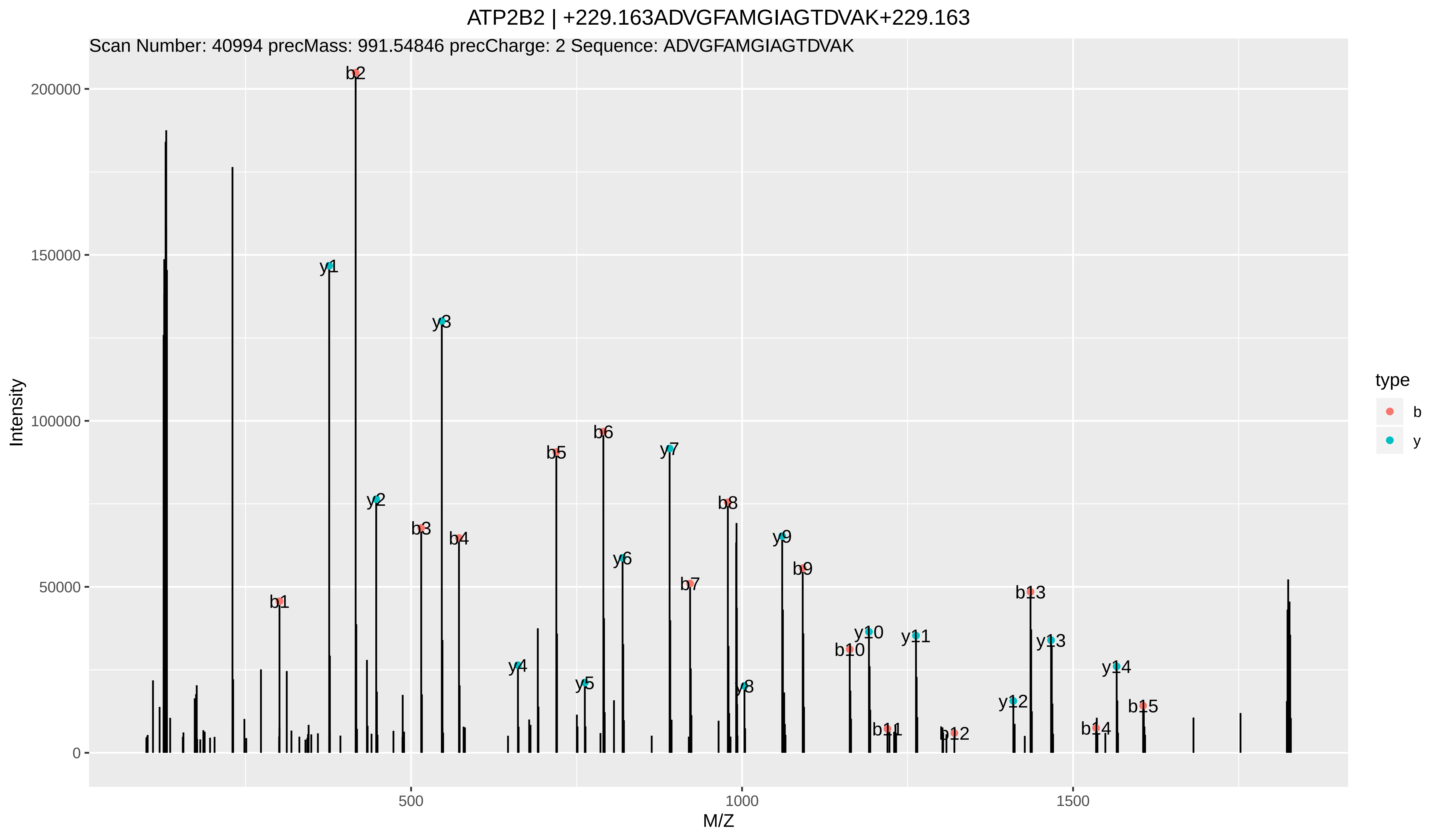

ATP2B2 | +229.163ADVGFAMGIAGTDVAK+229.163
Scan Number: 40994 precMass: 991.54846 precCharge: 2 Sequence: ADVGFAMGIAGTDVAK
b2
y1
y3
y2
b3
b4
b5
b6
y7
b8
y9
y6
b9
b7
b1
b13
y10
y11
b10
y13
y4
y14
y5
y8
b15
y12
b11
b14
b12
Intensity
0
50000
100000
150000
200000
M/Z
500
1000
1500
type
b
y

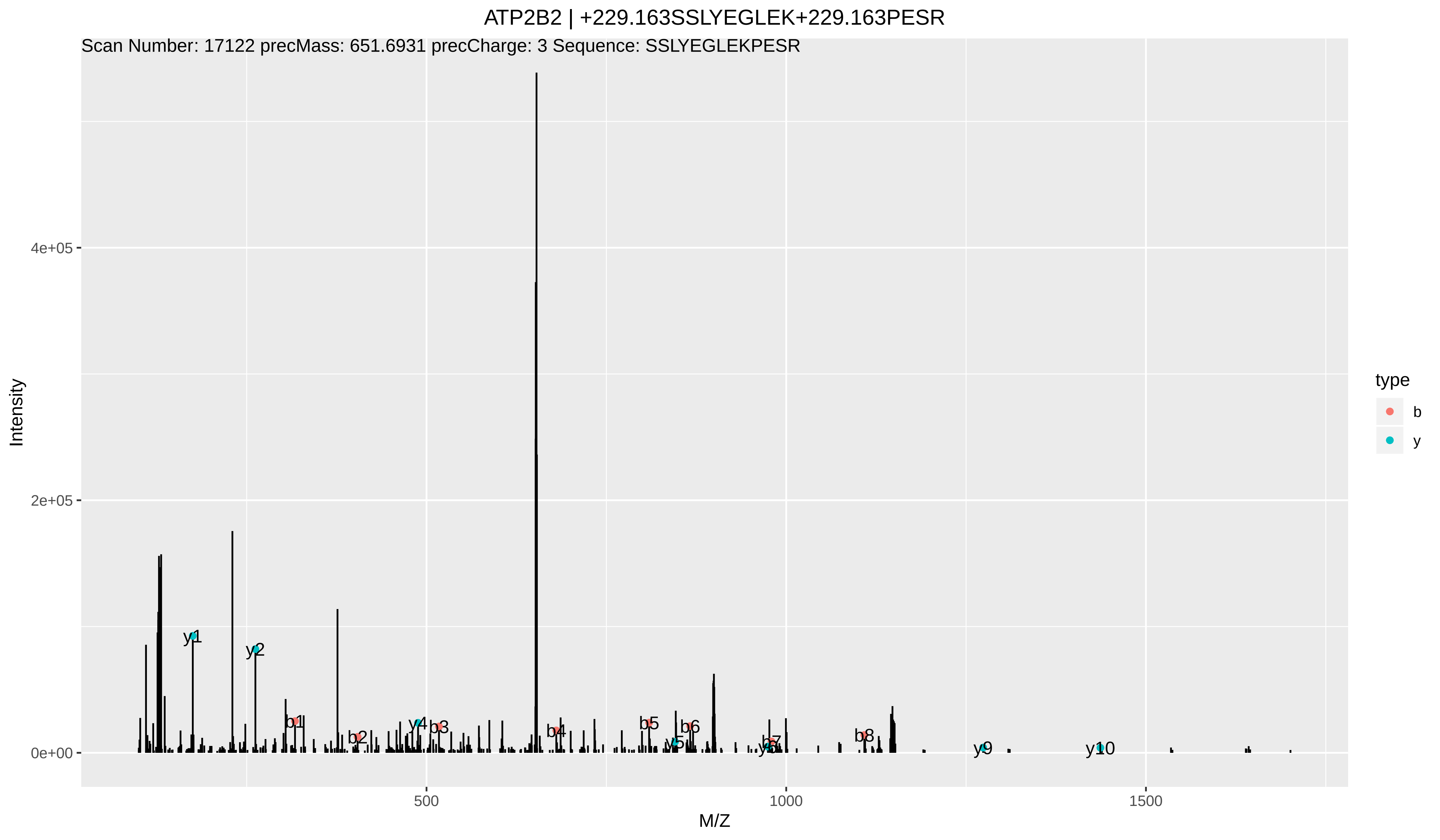

ATP2B2 | +229.163SSLYEGLEK+229.163PESR
Scan Number: 17122 precMass: 651.6931 precCharge: 3 Sequence: SSLYEGLEKPESR
Intensity
4e+05
2e+05
0e+00
M/Z
500
1000
1500
y1
y2
b1
b2
y4
b3
b4
b5
y5
b6
b7
y6
b8
y9
y10
type
b
y

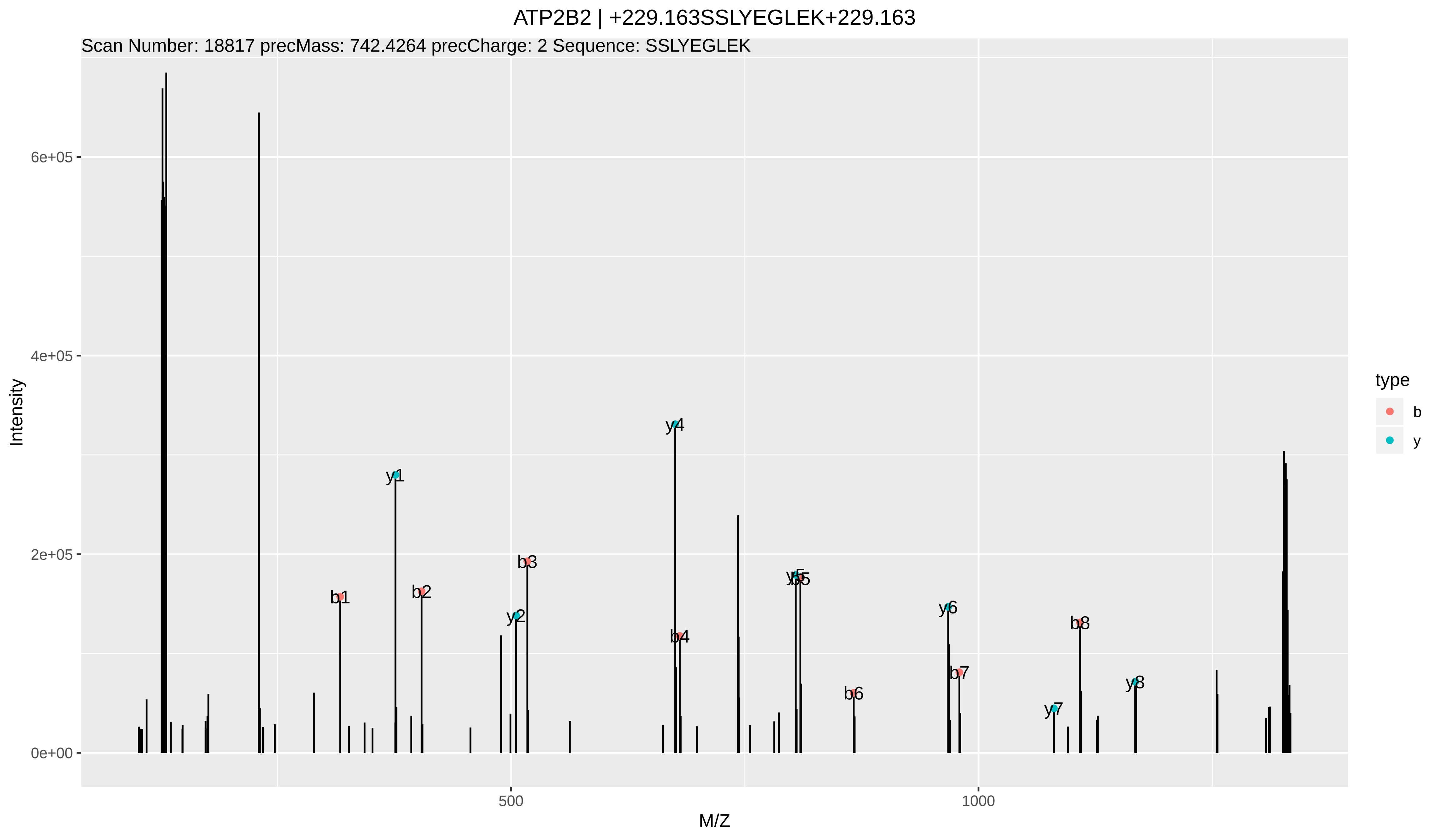

ATP2B2 | +229.163SSLYEGLEK+229.163
Scan Number: 18817 precMass: 742.4264 precCharge: 2 Sequence: SSLYEGLEK
Intensity
6e+05
4e+05
2e+05
0e+00
M/Z
500
1000
y4
y1
b3
y5
b5
b2
b1
y2
b4
y6
b8
b7
y8
b6
y7
type
b
y

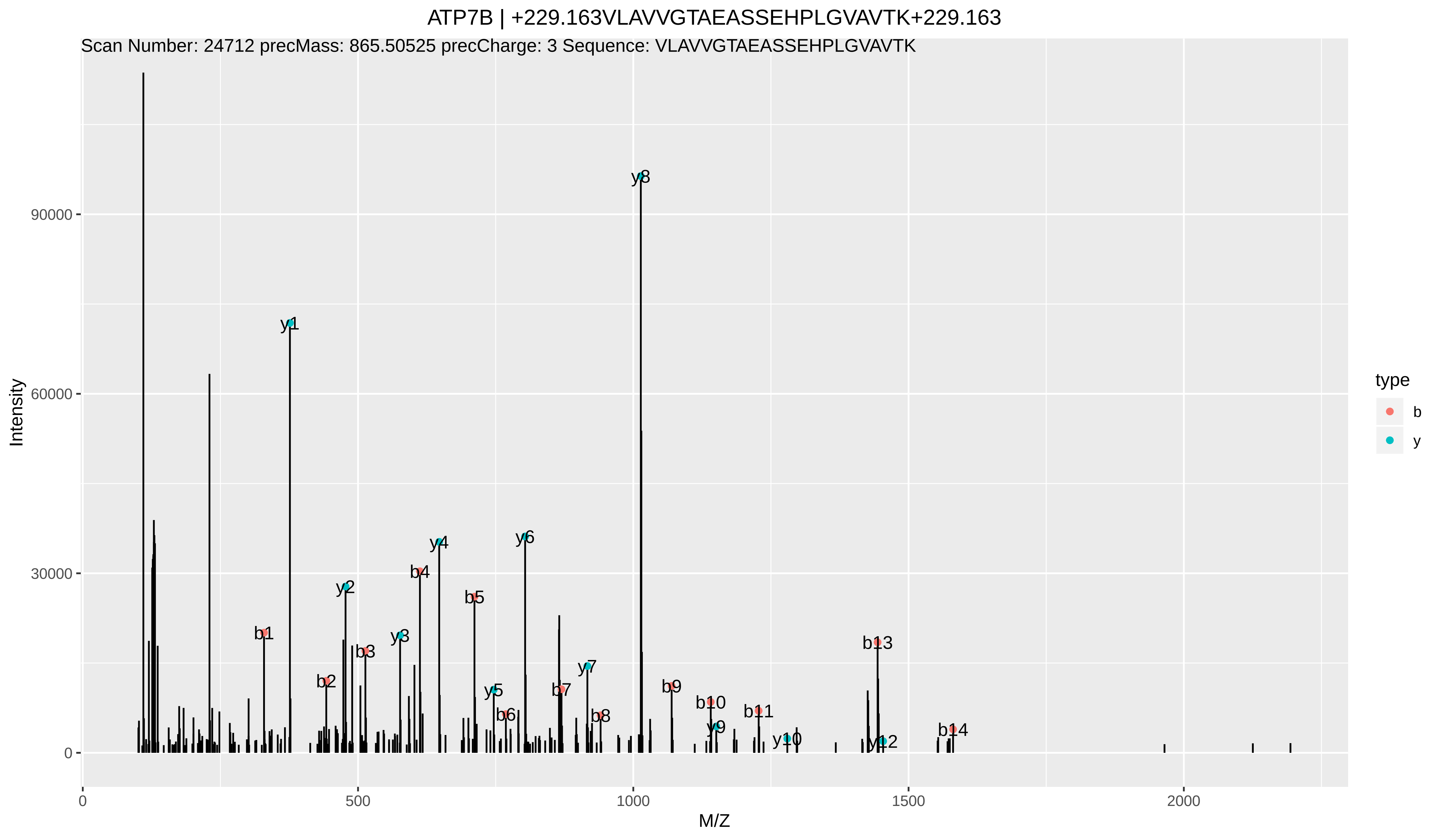
ATP7B | +229.163VLAVVGTAEASSEHPLGVAVTK+229.163
Scan Number: 24712 precMass: 865.50525 precCharge: 3 Sequence: VLAVVGTAEASSEHPLGVAVTK
Intensity
M/Z
type
b
y
y1
y2
y3
y4
y5
y6
y7
y8
y9
y10
y12
b1
b2
b3
b4
b5
b6
b7
b8
b9
b10
b11
b13
b14
0
30000
60000
90000
500
1000
1500
2000

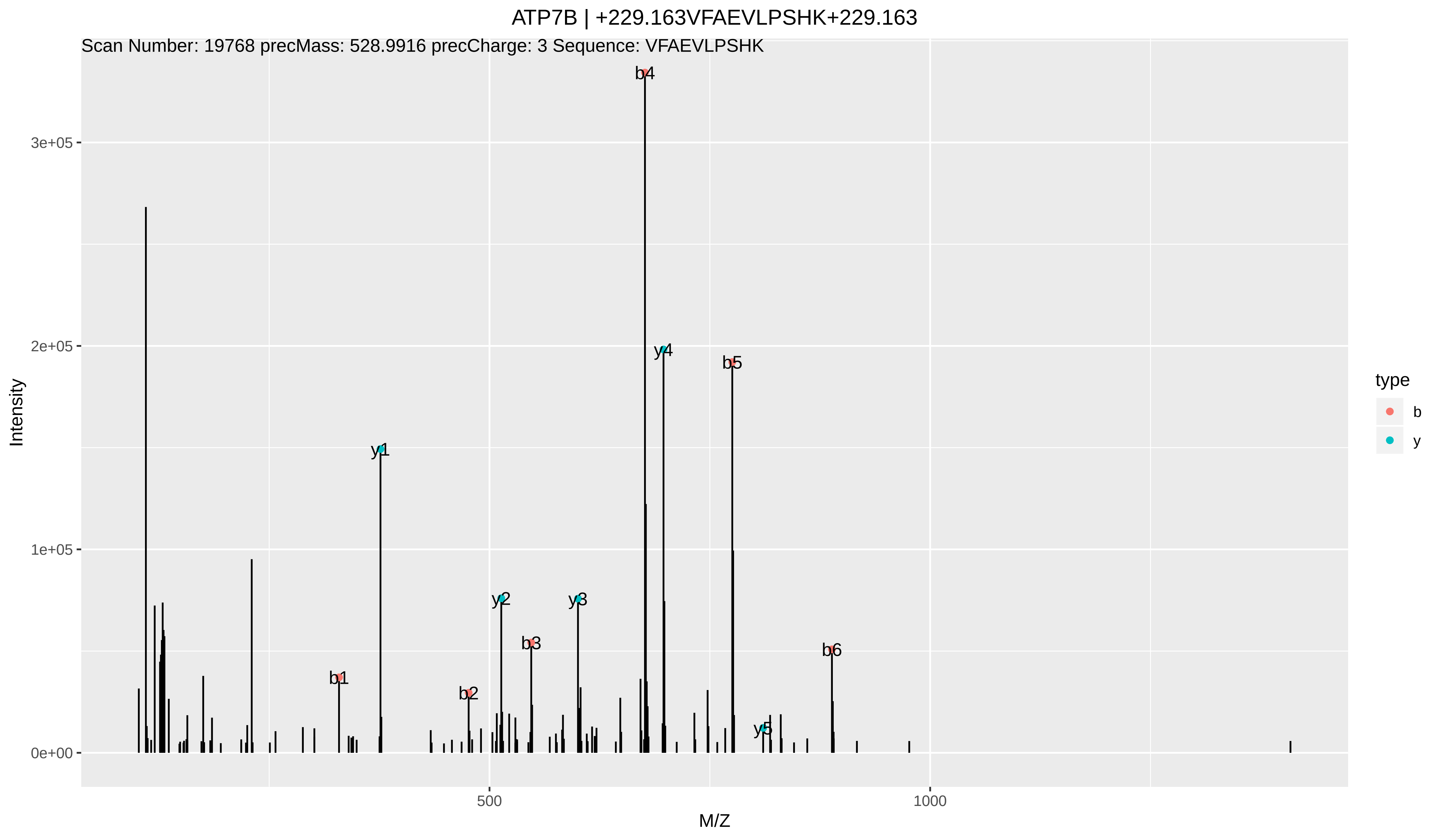
ATP7B | +229.163VFAEVLPSHK+229.163
Scan Number: 19768 precMass: 528.9916 precCharge: 3 Sequence: VFAEVLPSHK
b4
y4
b5
y1
y2
y3
b3
b6
b1
b2
y5
3e+05
2e+05
1e+05
0e+00
Intensity
500
1000
M/Z
type
b
y

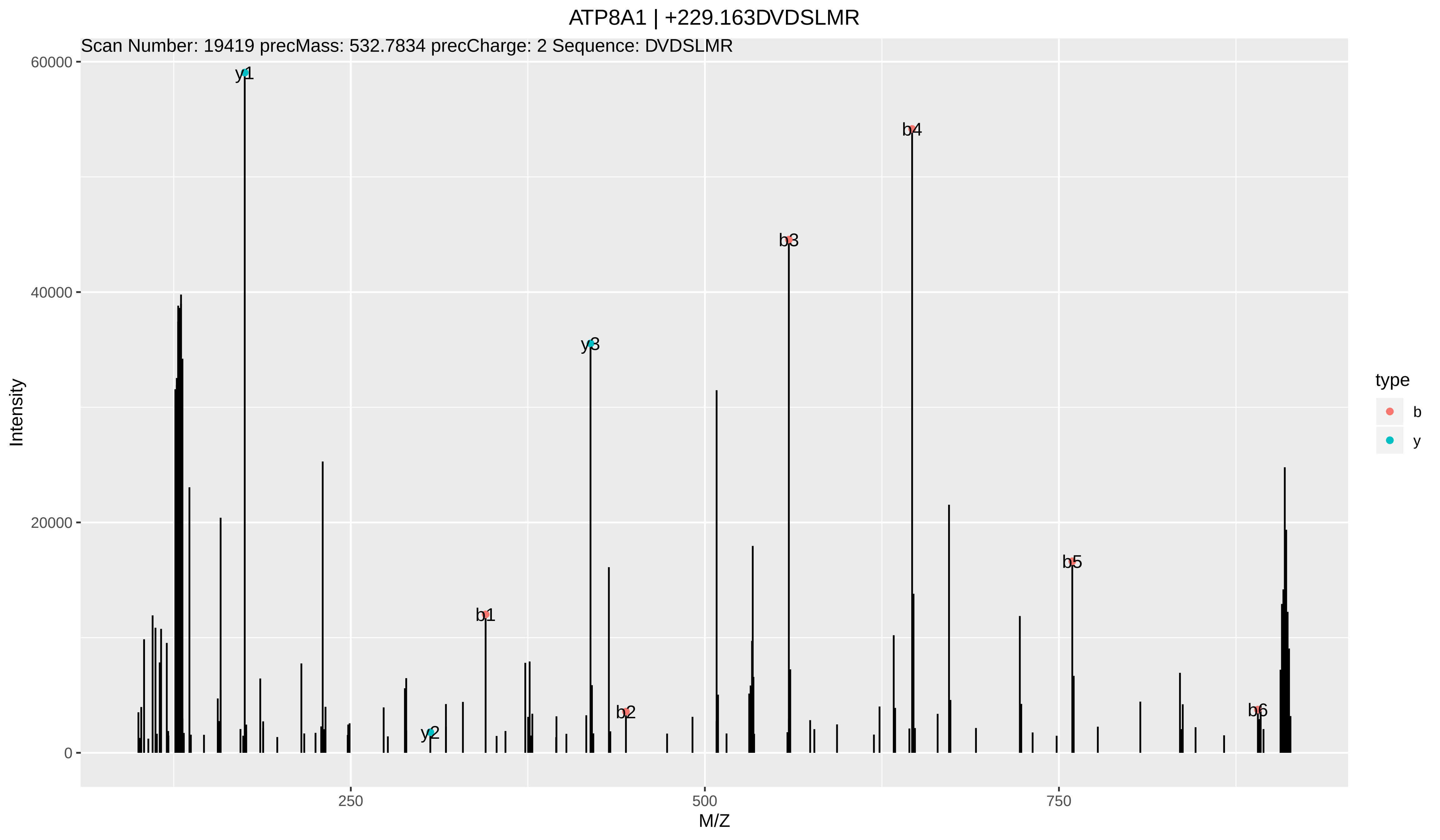
ATP8A1 | +229.163DVDSLMR
Scan Number: 19419 precMass: 532.7834 precCharge: 2 Sequence: DVDSLMR
Intensity
M/Z
60000
40000
20000
0
250
500
750
y1
b4
b3
y3
b5
b1
b2
b6
y2
type
b
y

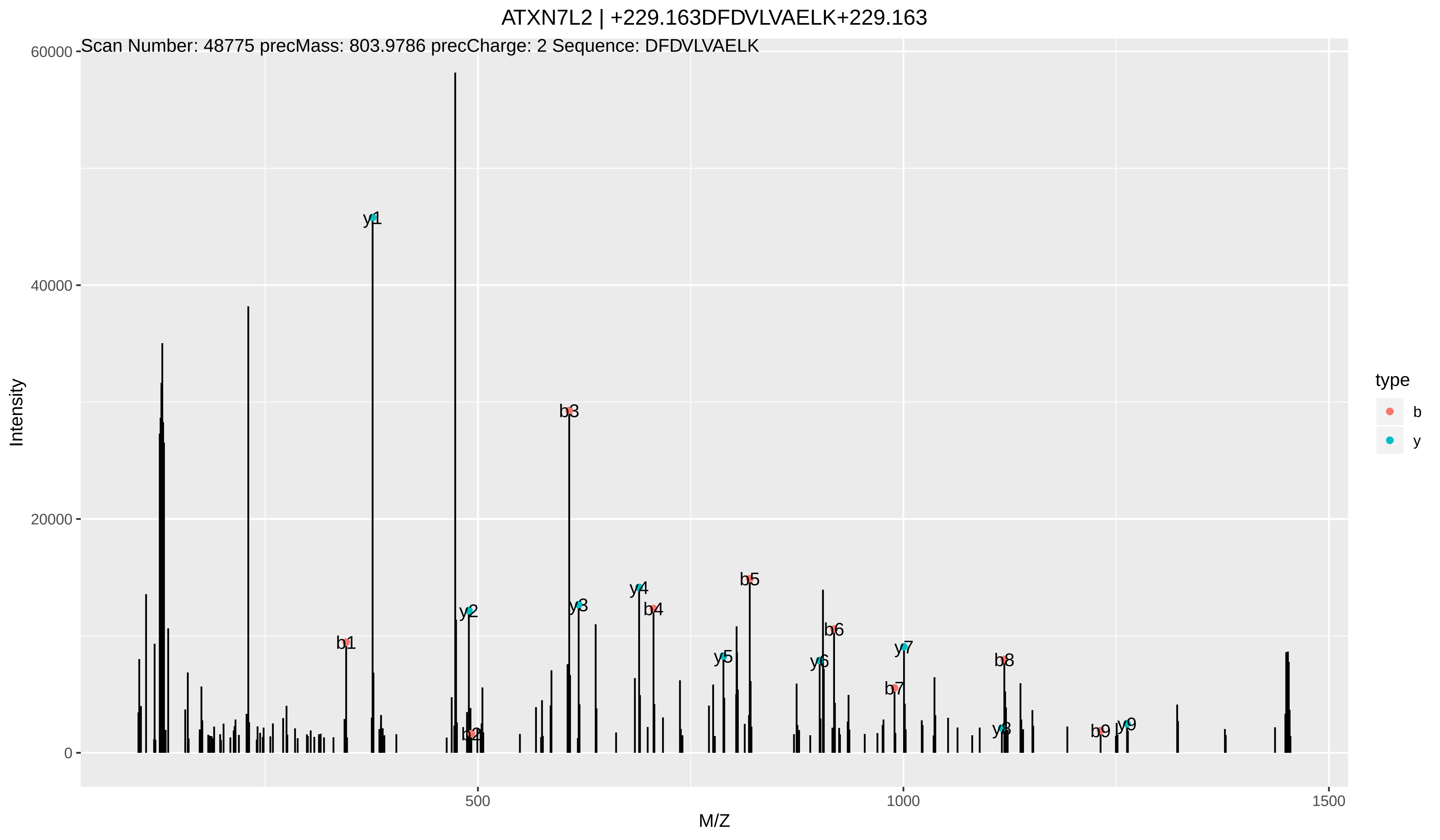

ATXN7L2 | +229.163DFDVLVAELK+229.163
Scan Number: 48775 precMass: 803.9786 precCharge: 2 Sequence: DFDVLVAELK
Intensity
M/Z
60000
40000
20000
0
500
1000
1500
y1
b3
y2
b1
b2
y3
y4
b4
b5
y5
y6
b6
y7
b7
b8
y8
b9
y9
type
b
y

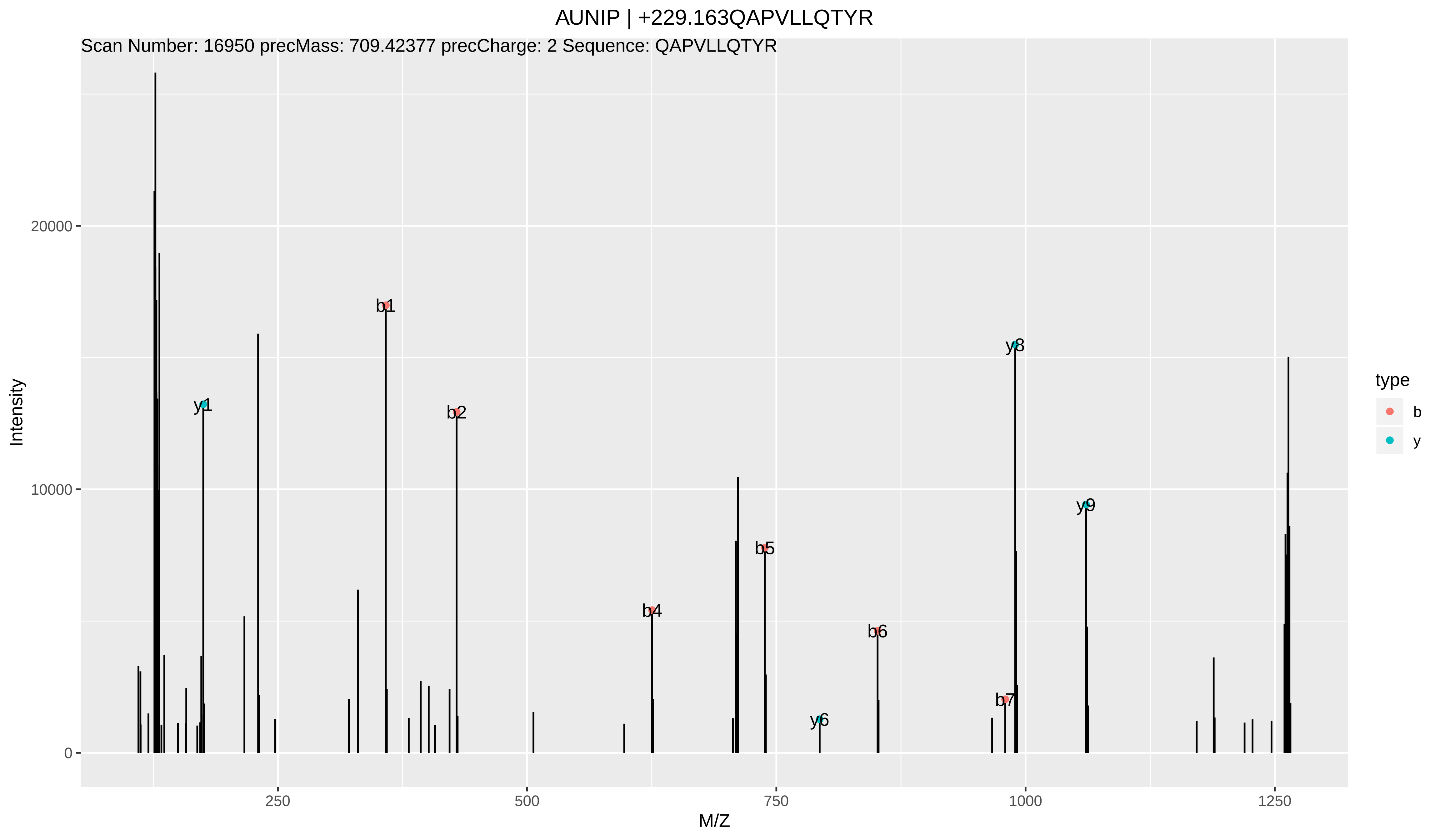
AUNIP | +229.163QAPVLLQTYR
Scan Number: 16950 precMass: 709.42377 precCharge: 2 Sequence: QAPVLLQTYR
Intensity
M/Z
20000
10000
0
250
500
750
1000
1250
y1
b1
b2
b4
b5
y6
b6
b7
y8
y9
type
b
y

AURKAIP1 | +229.163EAPEGWQTPK+229.163

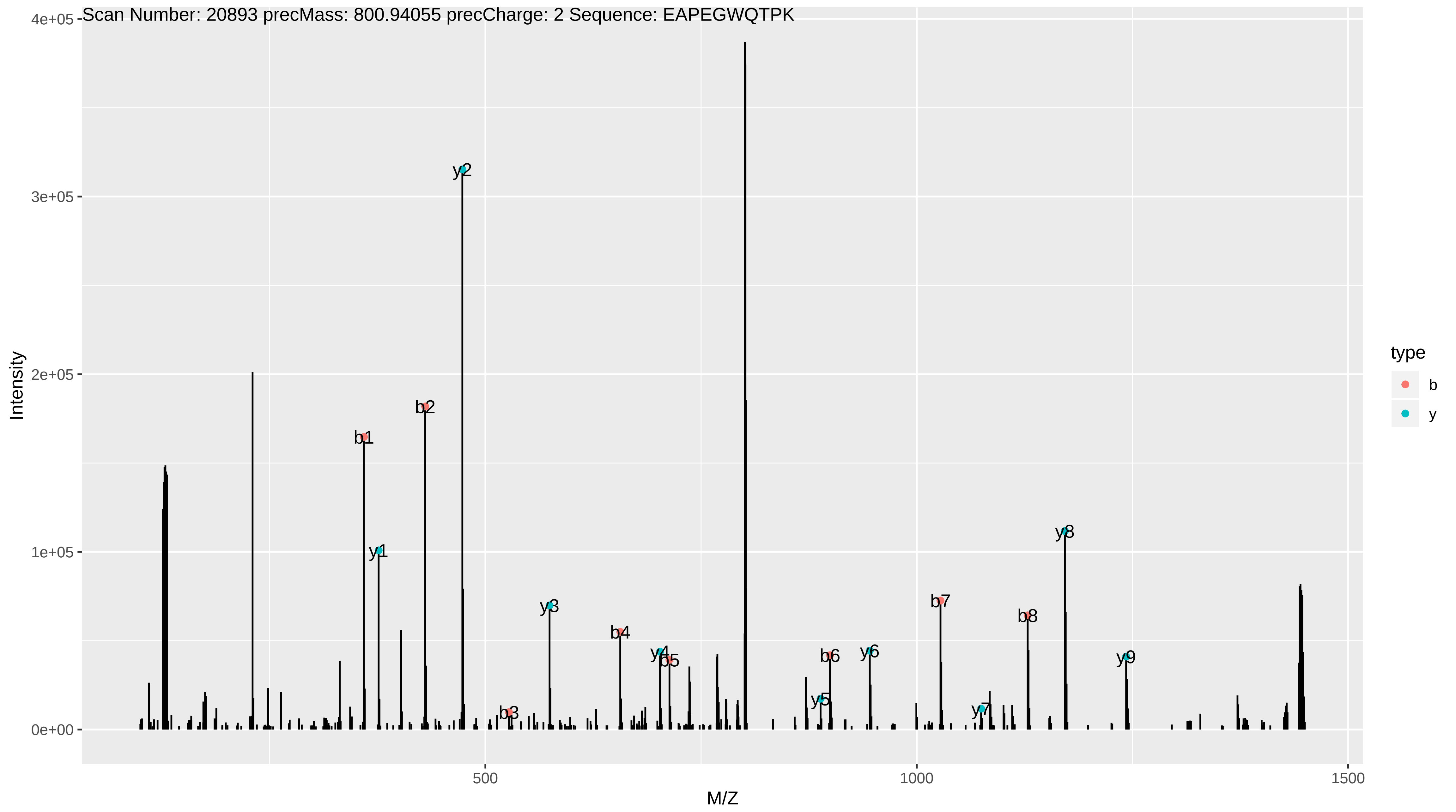

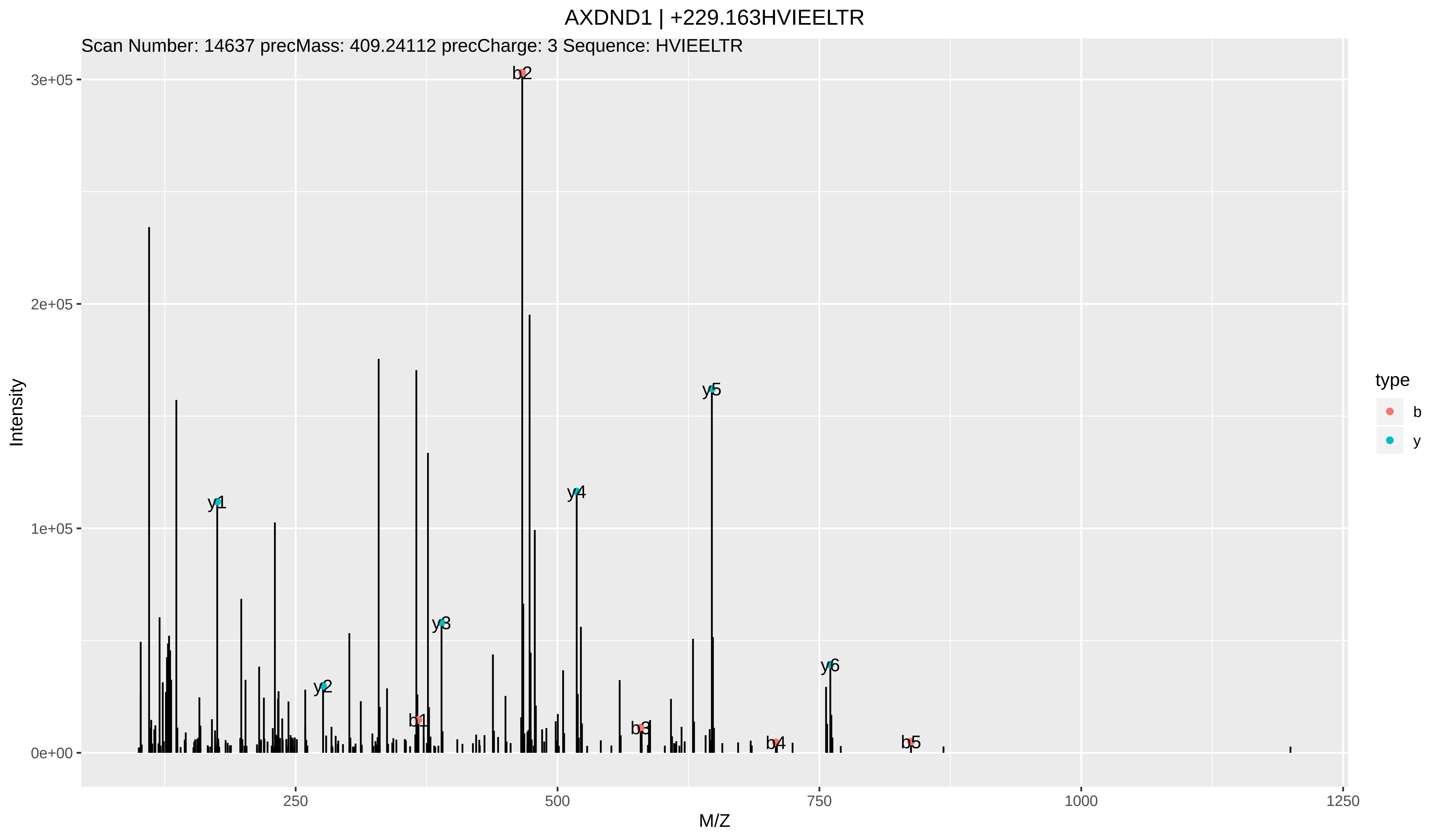

AXDND1 | +229.163HVIEELTR
Scan Number: 14637 precMass: 409.24112 precCharge: 3 Sequence: HVIEELTR
Intensity
M/Z
3e+05
2e+05
1e+05
0e+00
250
500
750
1000
1250
b2
y1
y2
y3
b1
y4
b3
y5
b4
y6
b5
type
b
y

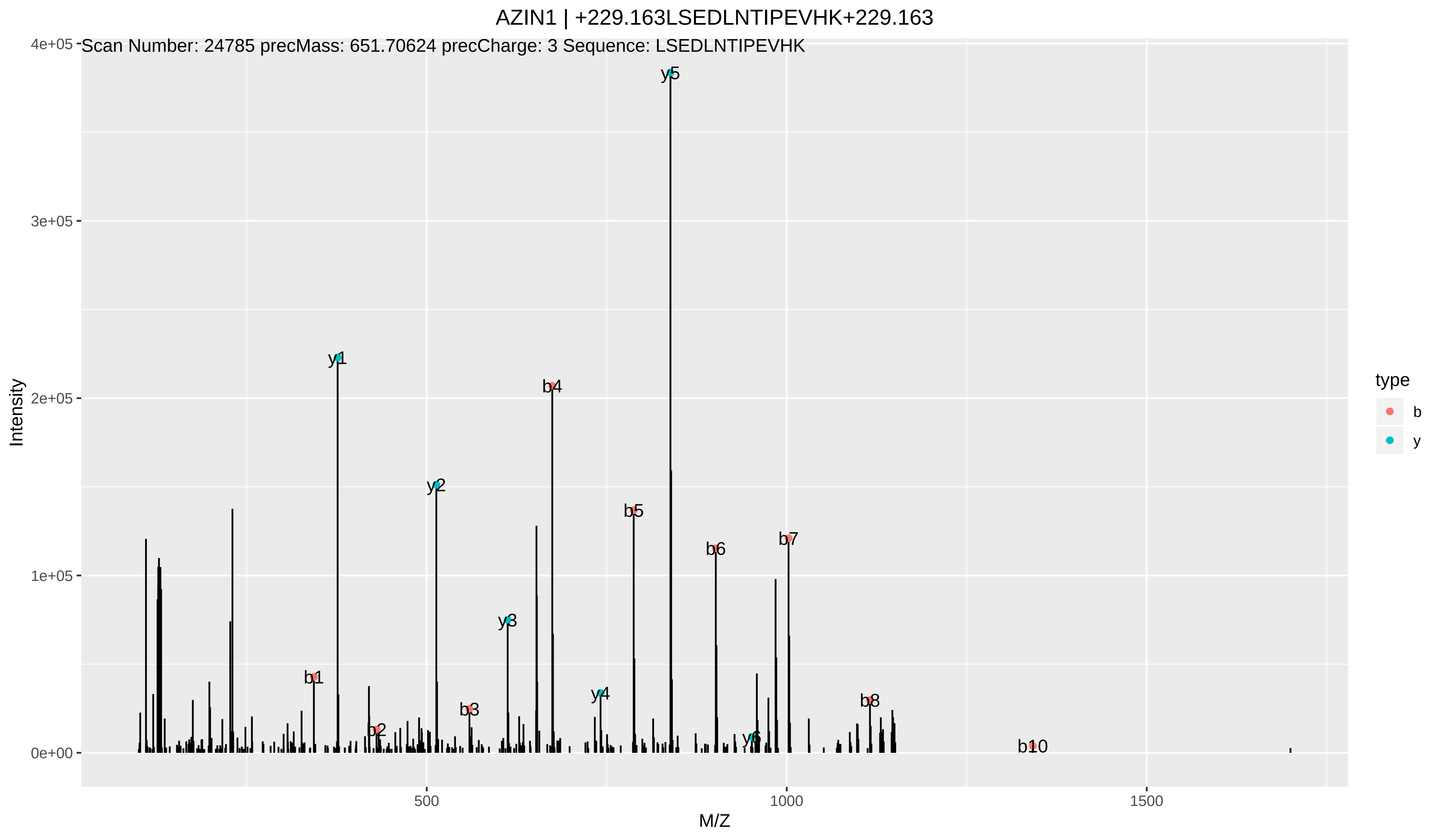

AZIN1 | +229.163LSEDLNTIPEVHK+229.163
Scan Number: 24785 precMass: 651.70624 precCharge: 3 Sequence: LSEDLNTIPEVHK
Intensity
M/Z
4e+05
3e+05
2e+05
1e+05
0e+00
500
1000
1500
y5
y1
b4
y2
b5
b6
b7
y3
b1
y4
b3
b2
y6
b8
b10
type
b
y

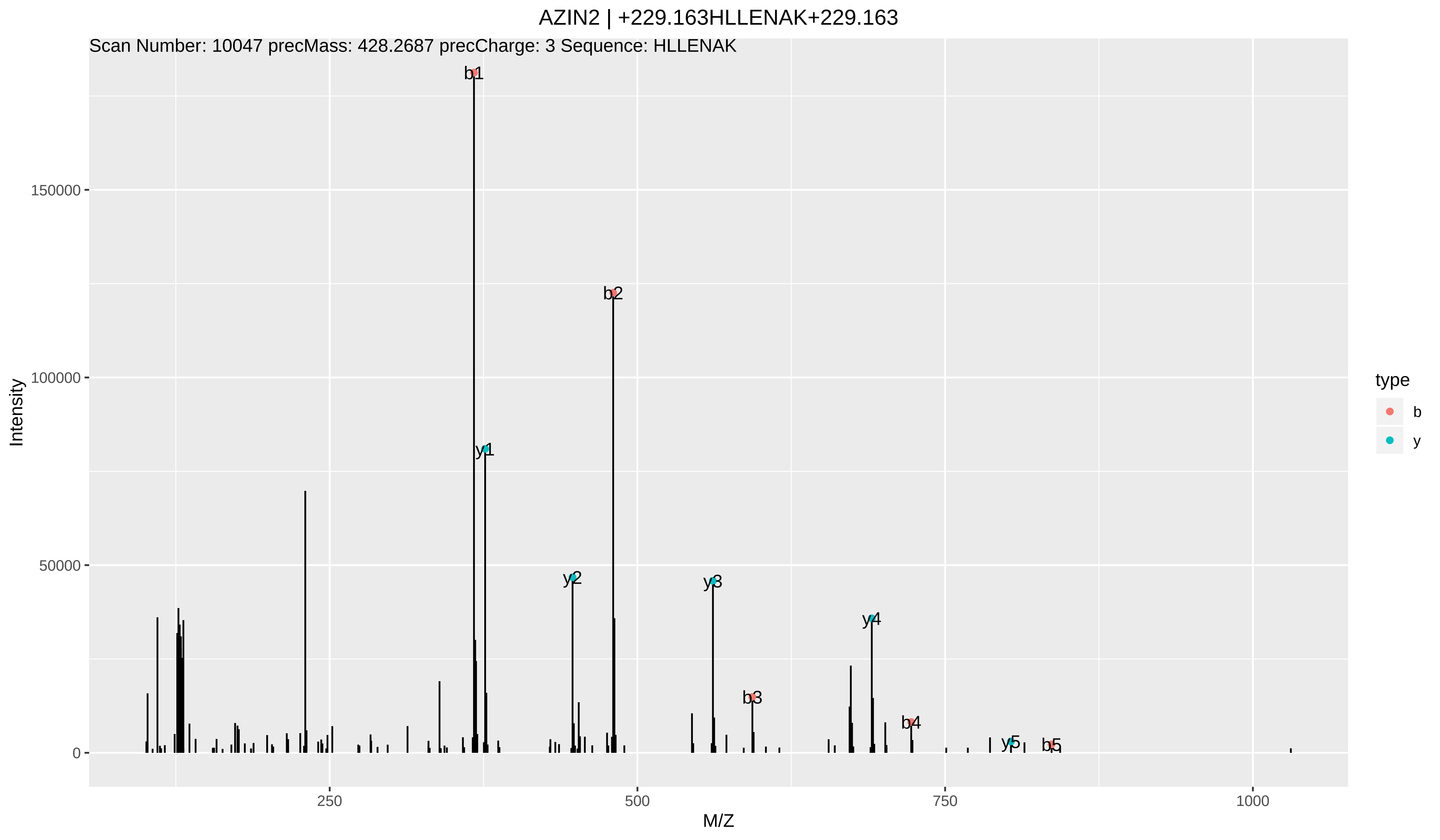
AZIN2 | +229.163HLLENAK+229.163
Scan Number: 10047 precMass: 428.2687 precCharge: 3 Sequence: HLLENAK
b1
b2
y1
y2
y3
b3
y4
b4
y5
b5
Intensity
150000
100000
50000
0
250
500
750
1000
M/Z
type
b
y

B9D2 | +229.163AFVGGGPQLLHGDTIYSGADR

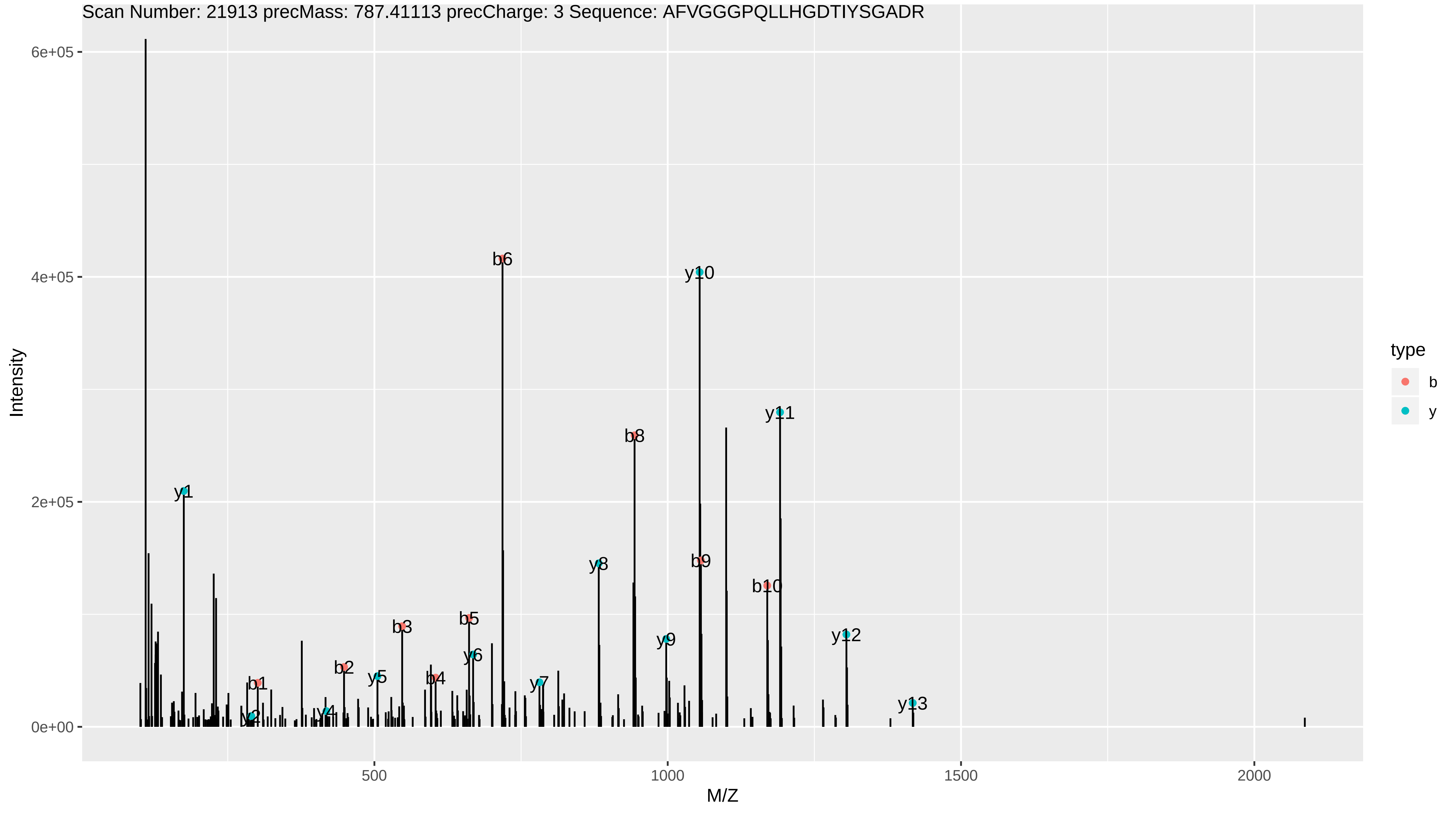

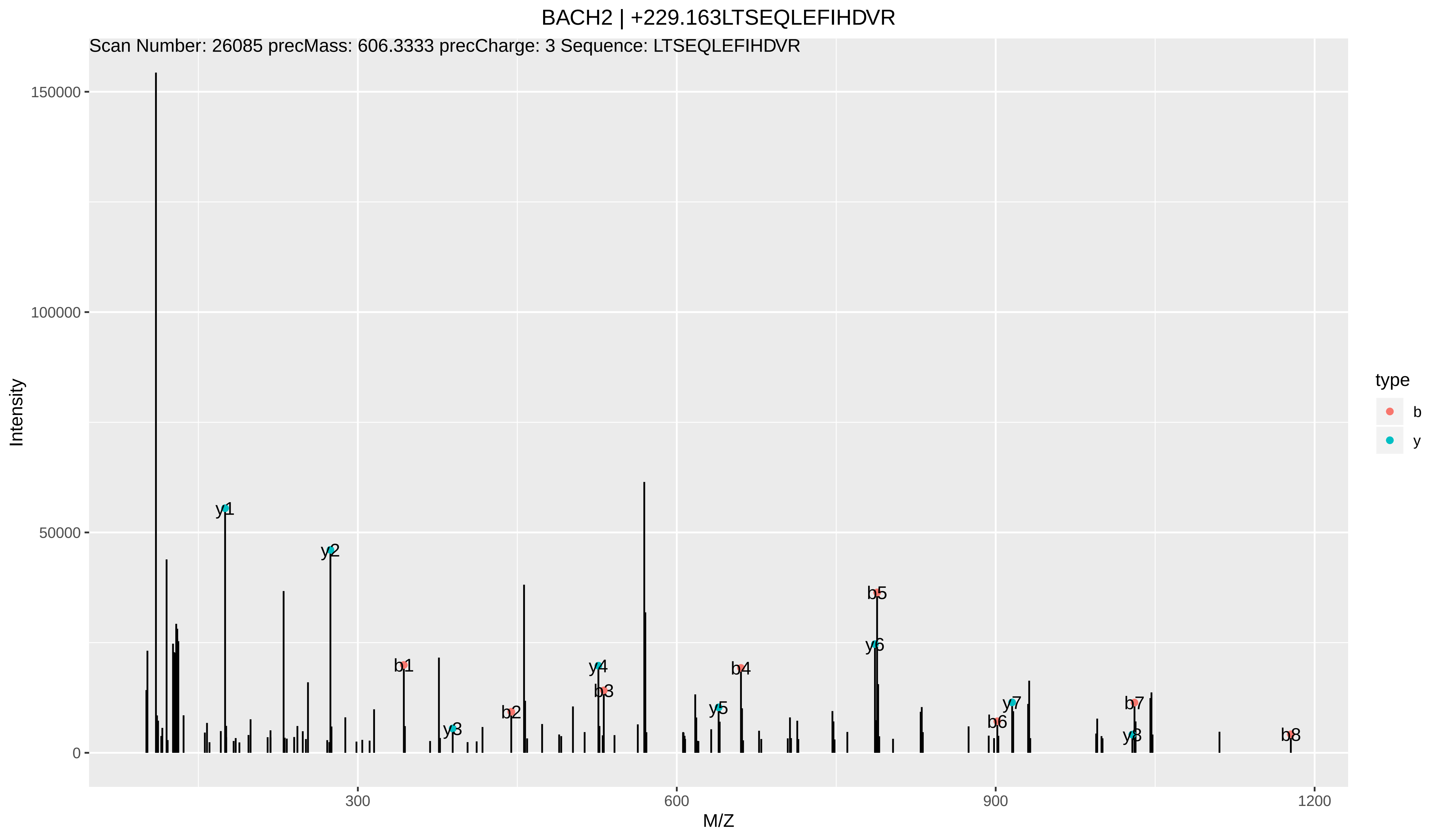
BACH2 | +229.163LTSEQLEFIHDVR
Scan Number: 26085 precMass: 606.3333 precCharge: 3 Sequence: LTSEQLEFIHDVR
Intensity
M/Z
type
b
y
y1
y2
b1
y3
b2
y4
b3
y5
b4
b5
y6
b6
y7
b7
y8
b8
150000
100000
50000
0
300
600
900
1200

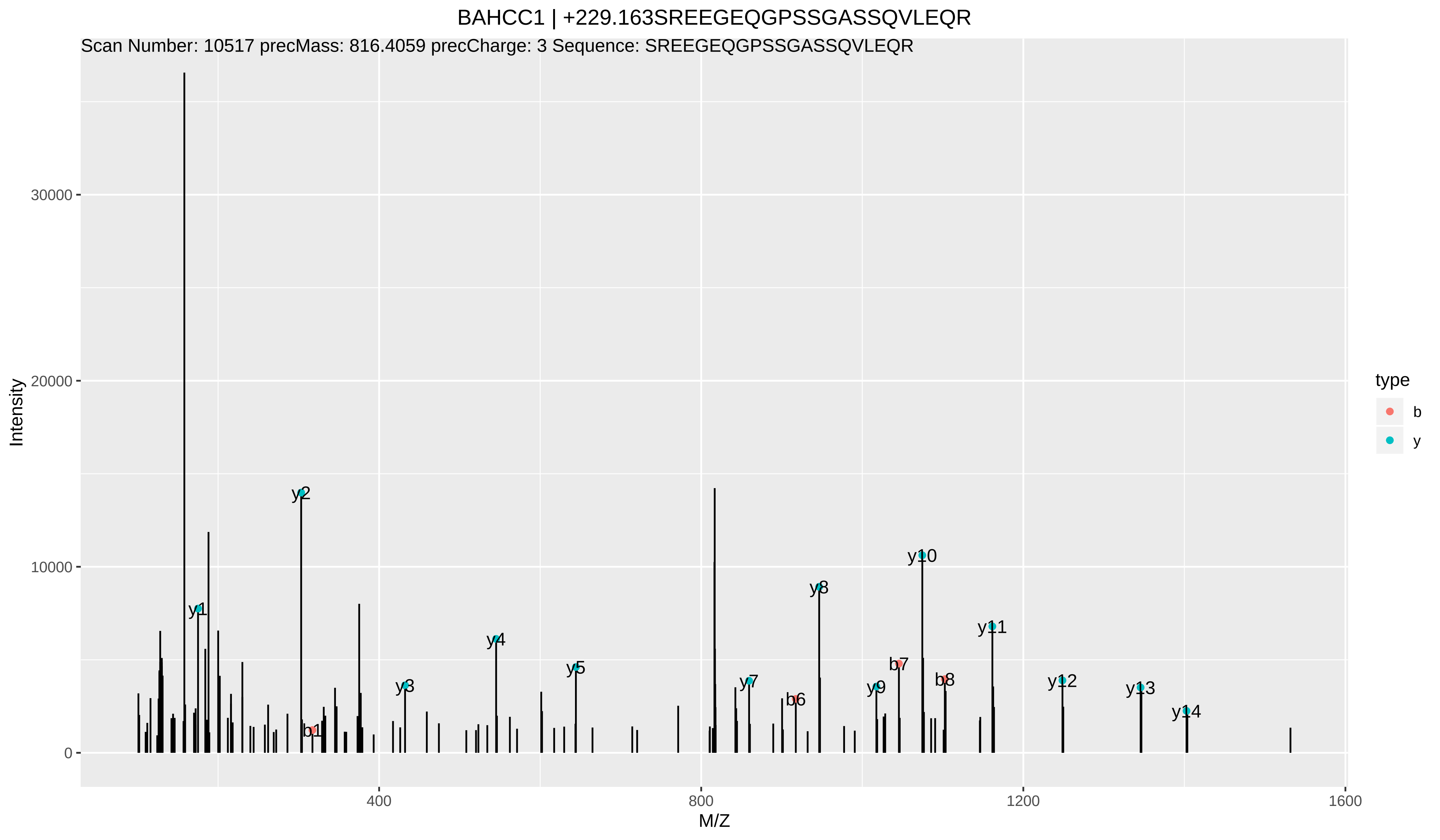
BAHCC1 | +229.163SREEGEQGPSSGASSQVLEQR
Scan Number: 10517 precMass: 816.4059 precCharge: 3 Sequence: SREEGEQGPSSGASSQVLEQR
Intensity
30000
20000
10000
0
M/Z
400
800
1200
1600
y1
y2
b1
y3
y4
y5
y7
b6
y8
y9
b7
y10
b8
y11
y12
y13
y14
type
b
y

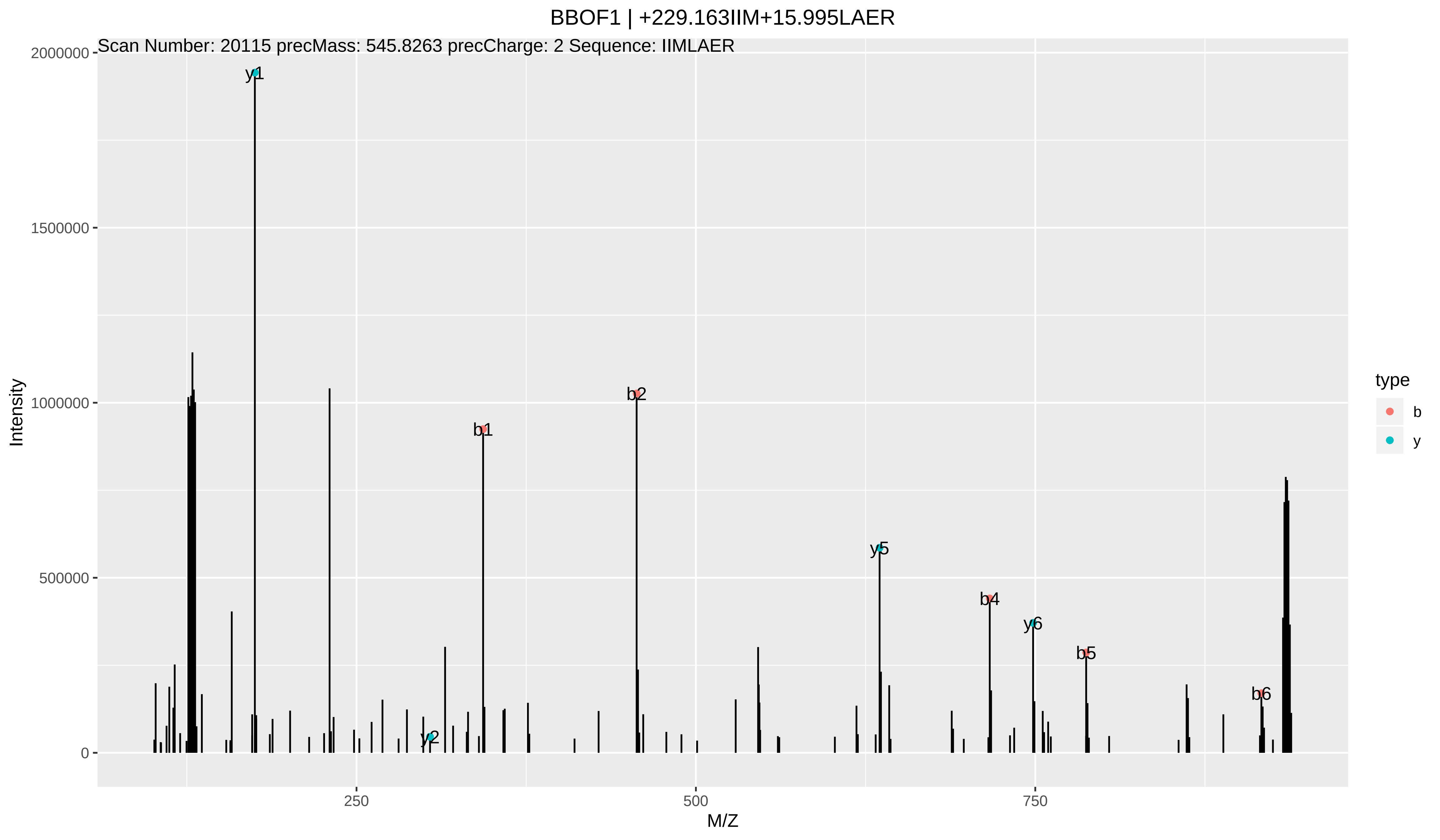

BBOF1 | +229.163IIM+15.995LAER
Scan Number: 20115 precMass: 545.8263 precCharge: 2 Sequence: IIMLAER
Intensity
M/Z
2000000
1500000
1000000
500000
0
250
500
750
y1
y2
b1
b2
y5
b4
y6
b5
b6
type
b
y

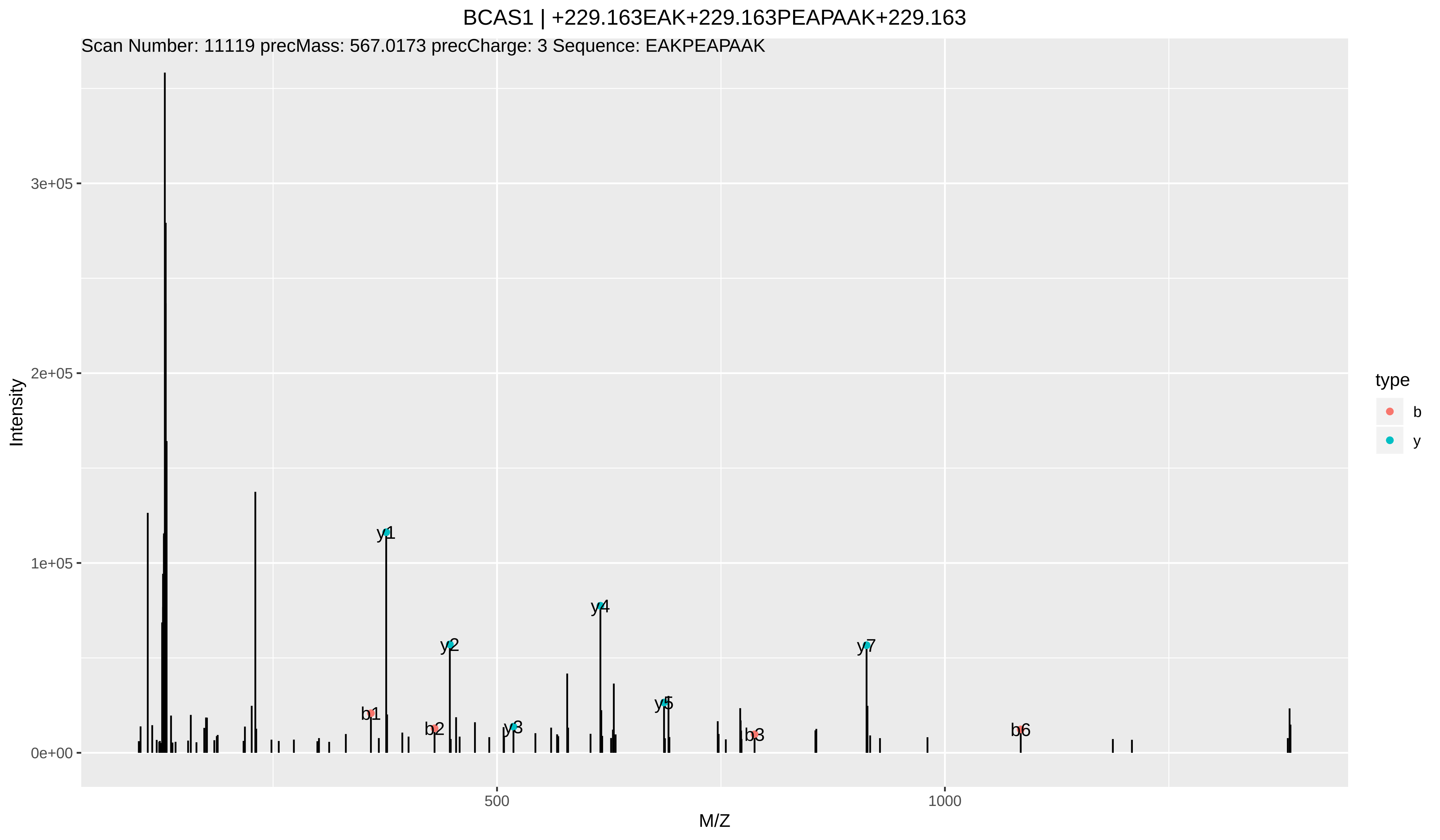
BCAS1 | +229.163EAK+229.163PEAPAAK+229.163
Scan Number: 11119 precMass: 567.0173 precCharge: 3 Sequence: EAKPEAPAAK
Intensity
3e+05
2e+05
1e+05
0e+00
M/Z
500
1000
y1
y2
y3
y4
y5
y7
b1
b2
b3
b6
type
b
y

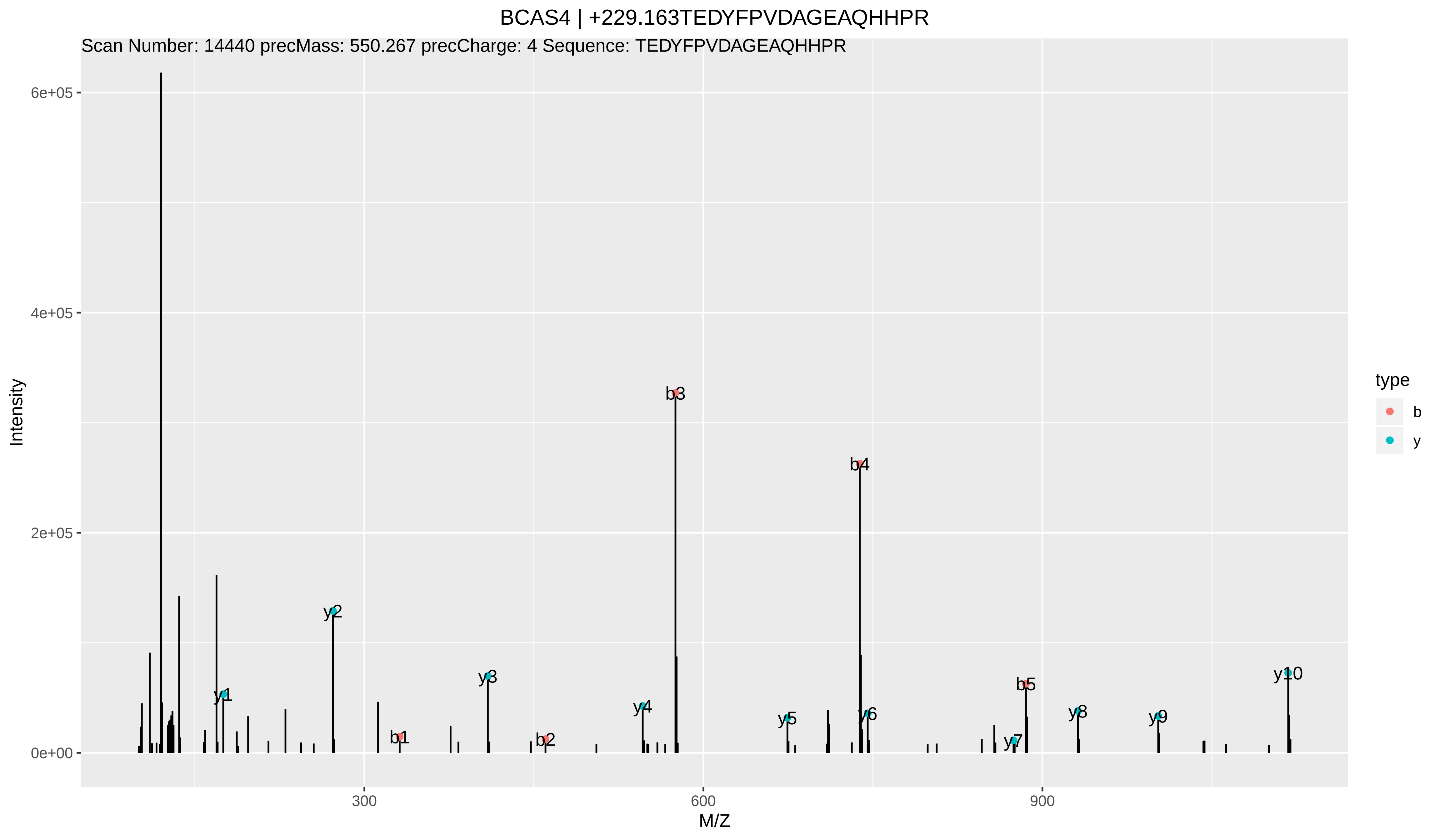
BCAS4 | +229.163TEDYFPVDAGEAQHHPR
Scan Number: 14440 precMass: 550.267 precCharge: 4 Sequence: TEDYFPVDAGEAQHHPR
Intensity
M/Z
6e+05
4e+05
2e+05
0e+00
300
600
900
type
b
y
b1
b2
b3
b4
b5
y1
y2
y3
y4
y5
y6
y7
y8
y9
y10

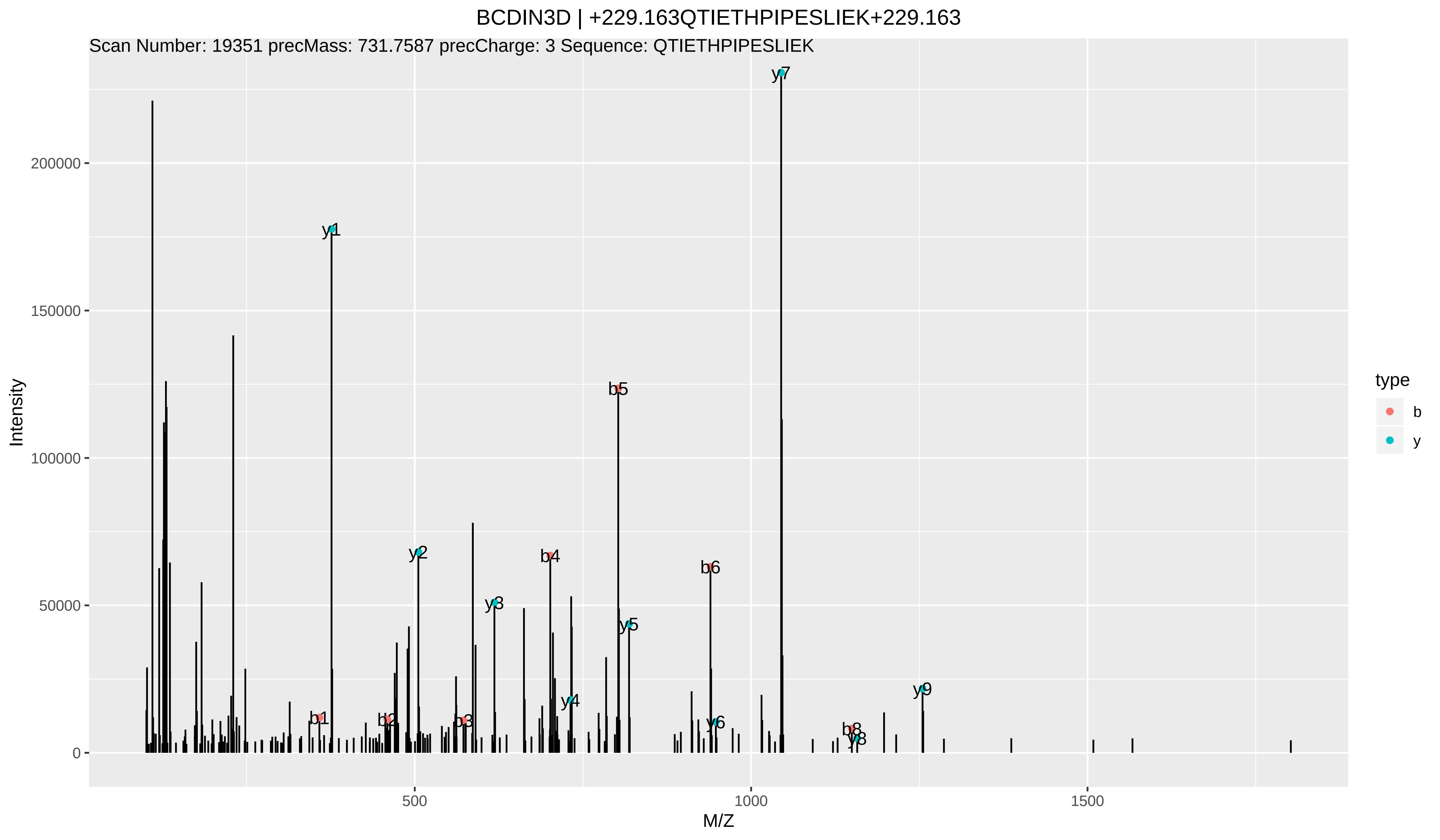

BCDIN3D | +229.163QTIETHPIPESLIEK+229.163
Scan Number: 19351 precMass: 731.7587 precCharge: 3 Sequence: QTIETHPIPESLIEK
Intensity
M/Z
0
50000
100000
150000
200000
500
1000
1500
y7
y1
b5
y2
b4
b6
y3
y5
y9
y4
b1
b2
b3
y6
b8
y8
type
b
y

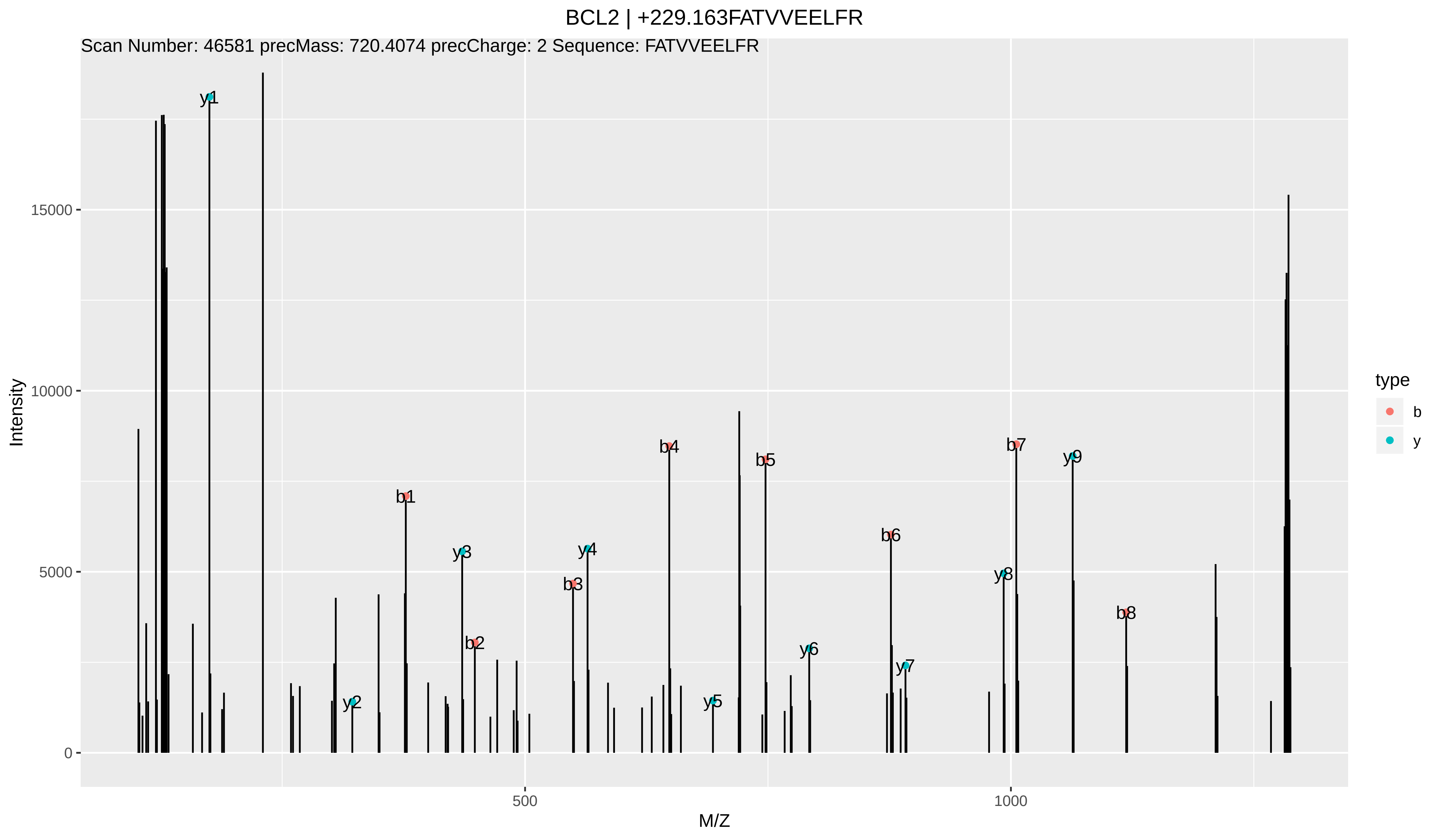
BCL2 | +229.163FATVVEELFR
Scan Number: 46581 precMass: 720.4074 precCharge: 2 Sequence: FATVVEELFR
Intensity
0
5000
10000
15000
M/Z
500
1000
y1
b1
y2
y3
b2
b3
y4
b4
y5
b5
y6
b6
y7
y8
b7
y9
b8
type
b
y

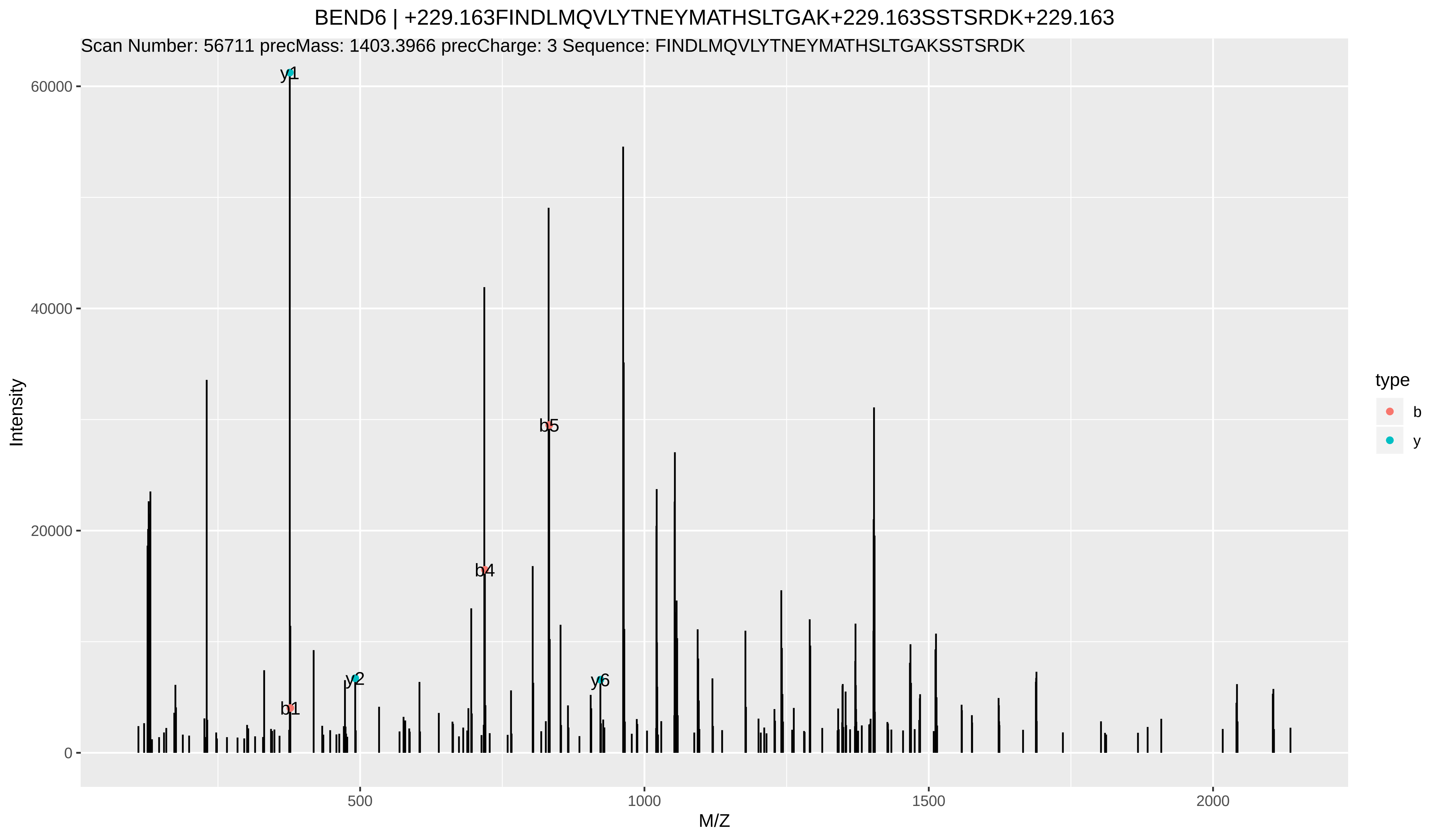

BEND6 | +229.163FINDLMQVLYTNEYMATHSLTGAK+229.163SSTSRDK+229.163
Scan Number: 56711 precMass: 1403.3966 precCharge: 3 Sequence: FINDLMQVLYTNEYMATHSLTGAKSSTSRDK
y1
b5
b4
y2
y6
b1
Intensity
60000
40000
20000
0
500
1000
1500
2000
M/Z
type
b
y

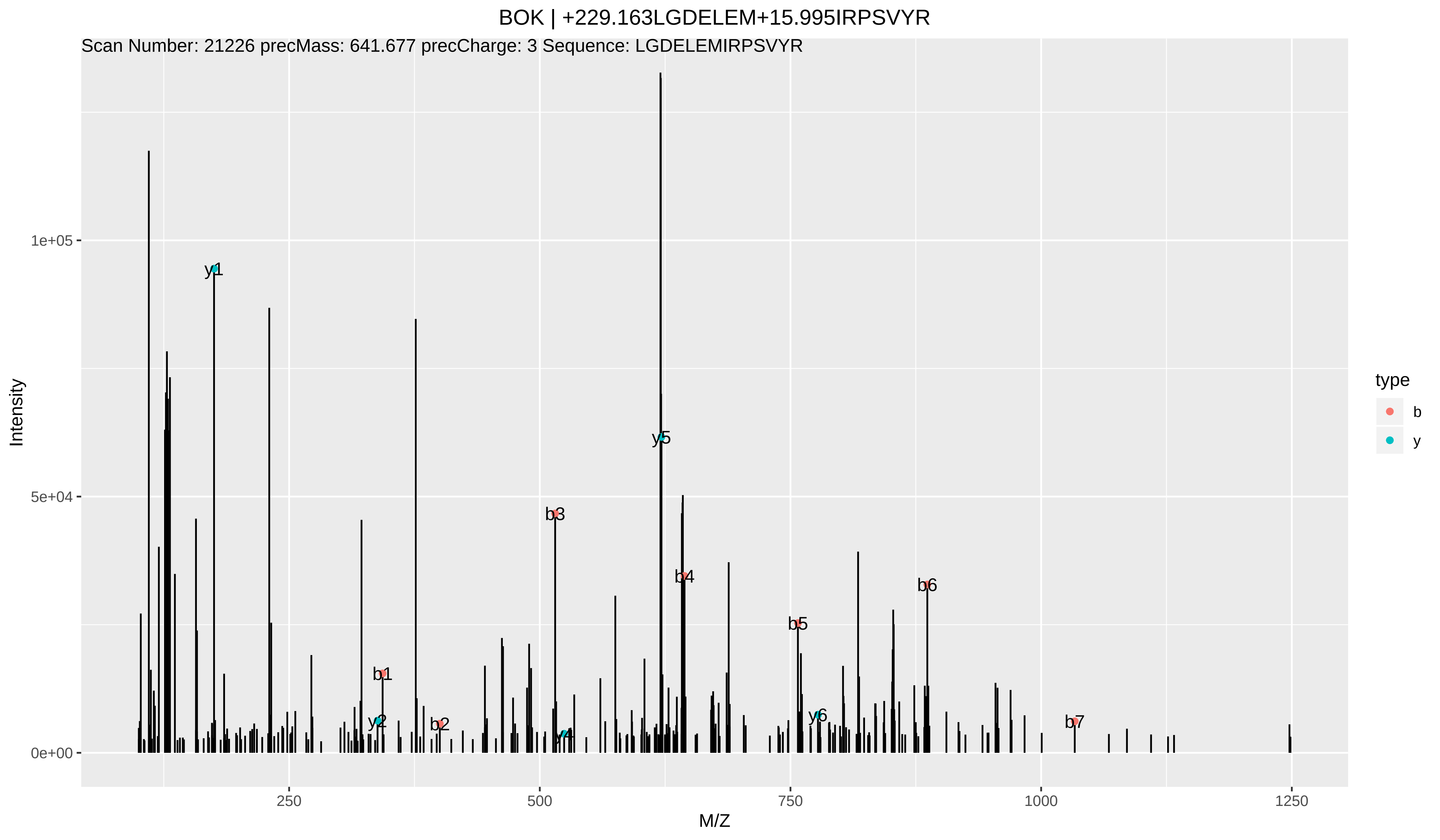

BOK | +229.163LGDELEM+15.995IRPSVYR
Scan Number: 21226 precMass: 641.677 precCharge: 3 Sequence: LGDELEMIRPSVYR
Intensity
1e+05
5e+04
0e+00
M/Z
250
500
750
1000
1250
y1
y2
y4
y5
y6
b1
b2
b3
b4
b5
b6
b7
type
b
y

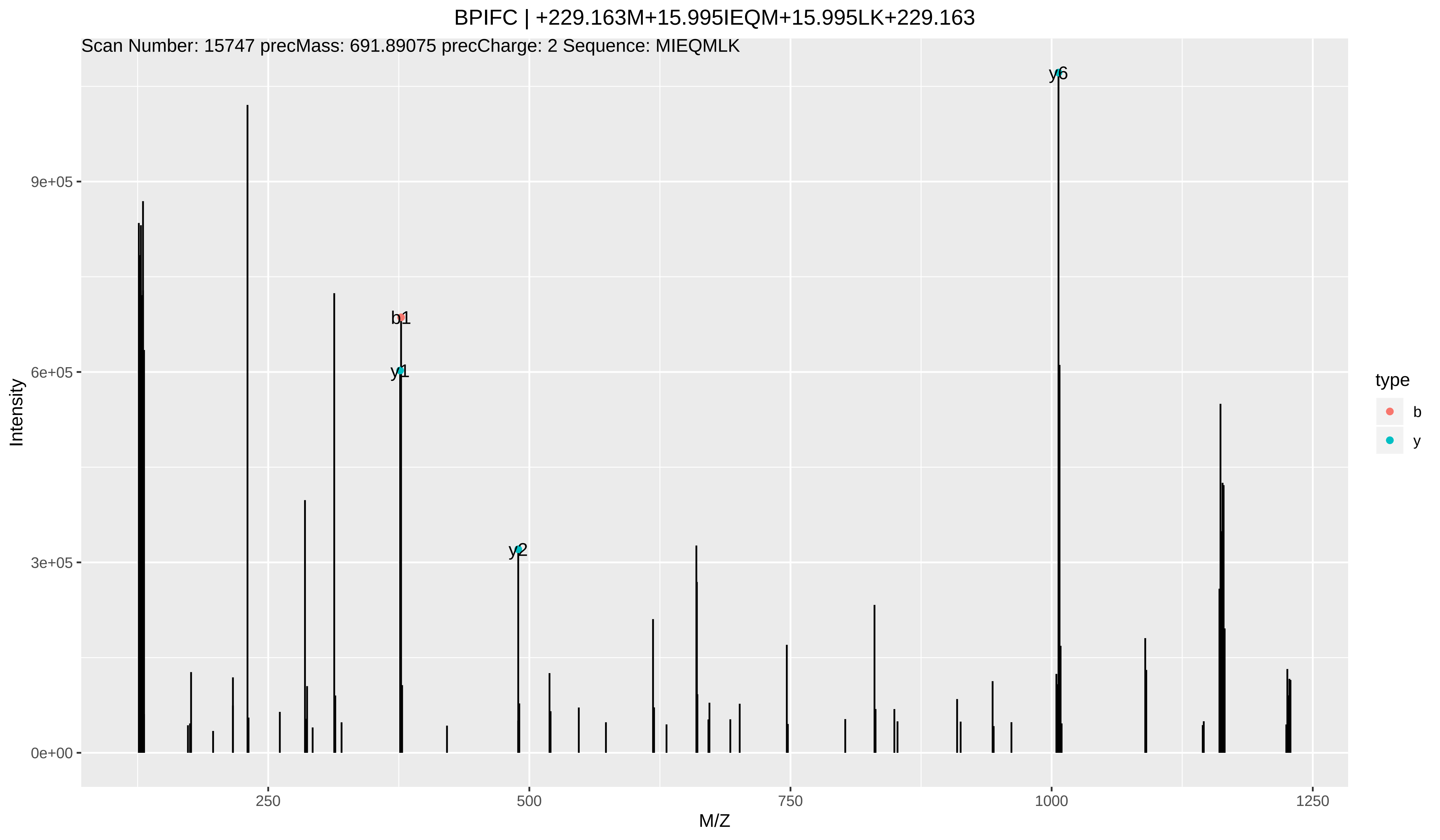

BPIFC | +229.163M+15.995IEQM+15.995LK+229.163
Scan Number: 15747 precMass: 691.89075 precCharge: 2 Sequence: MIEQMLK
y6
b1
y1
y2
Intensity
9e+05
6e+05
3e+05
0e+00
250
500
750
1000
1250
M/Z
type
b
y

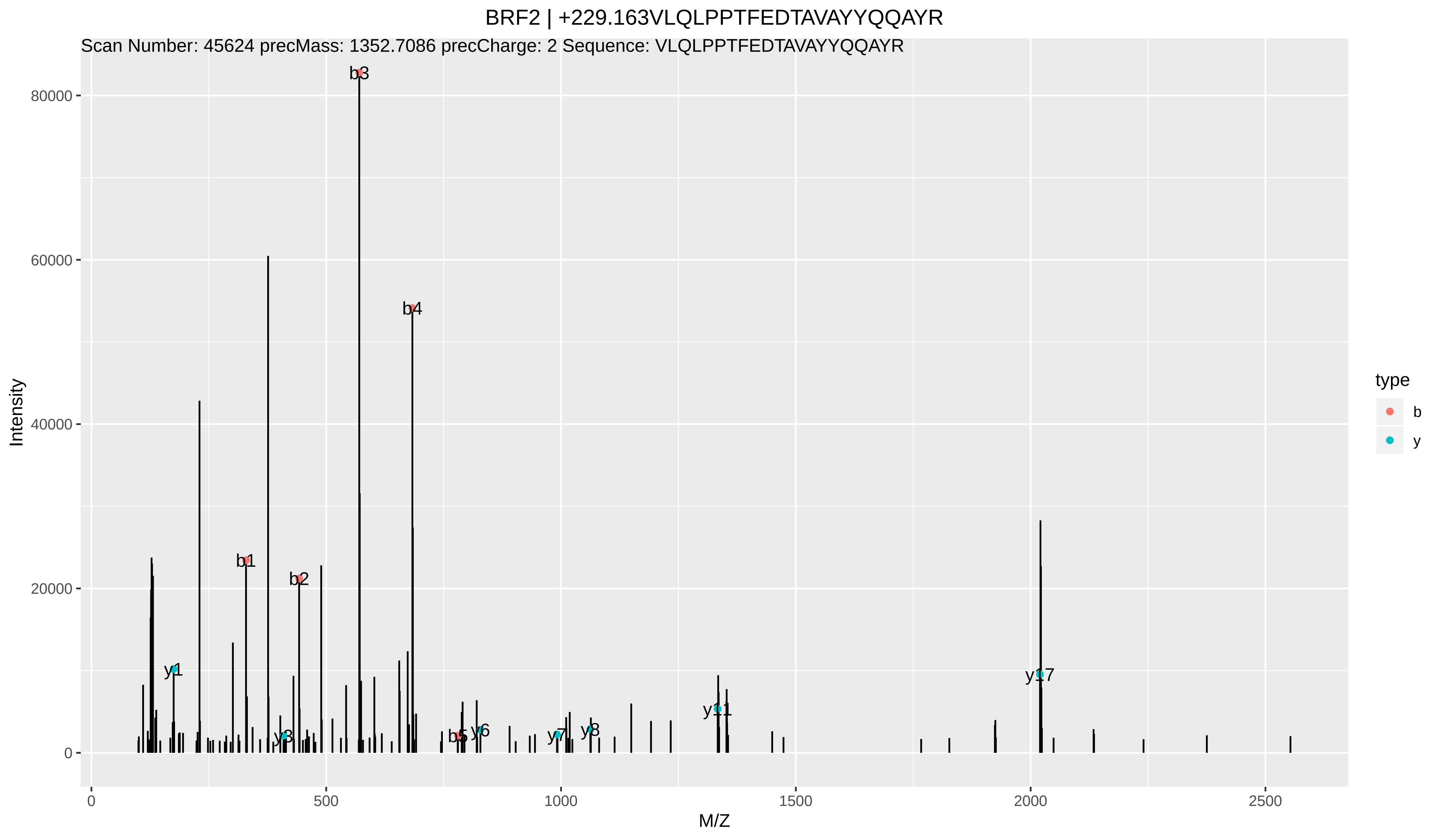

BRF2 | +229.163VLQLPPTFEDTAVAYYQQAYR
Scan Number: 45624 precMass: 1352.7086 precCharge: 2 Sequence: VLQLPPTFEDTAVAYYQQAYR
b3
b4
b1
b2
y1
y3
b5
y6
y7
y8
y11
y17
Intensity
0
20000
40000
60000
80000
M/Z
0
500
1000
1500
2000
2500
type
b
y

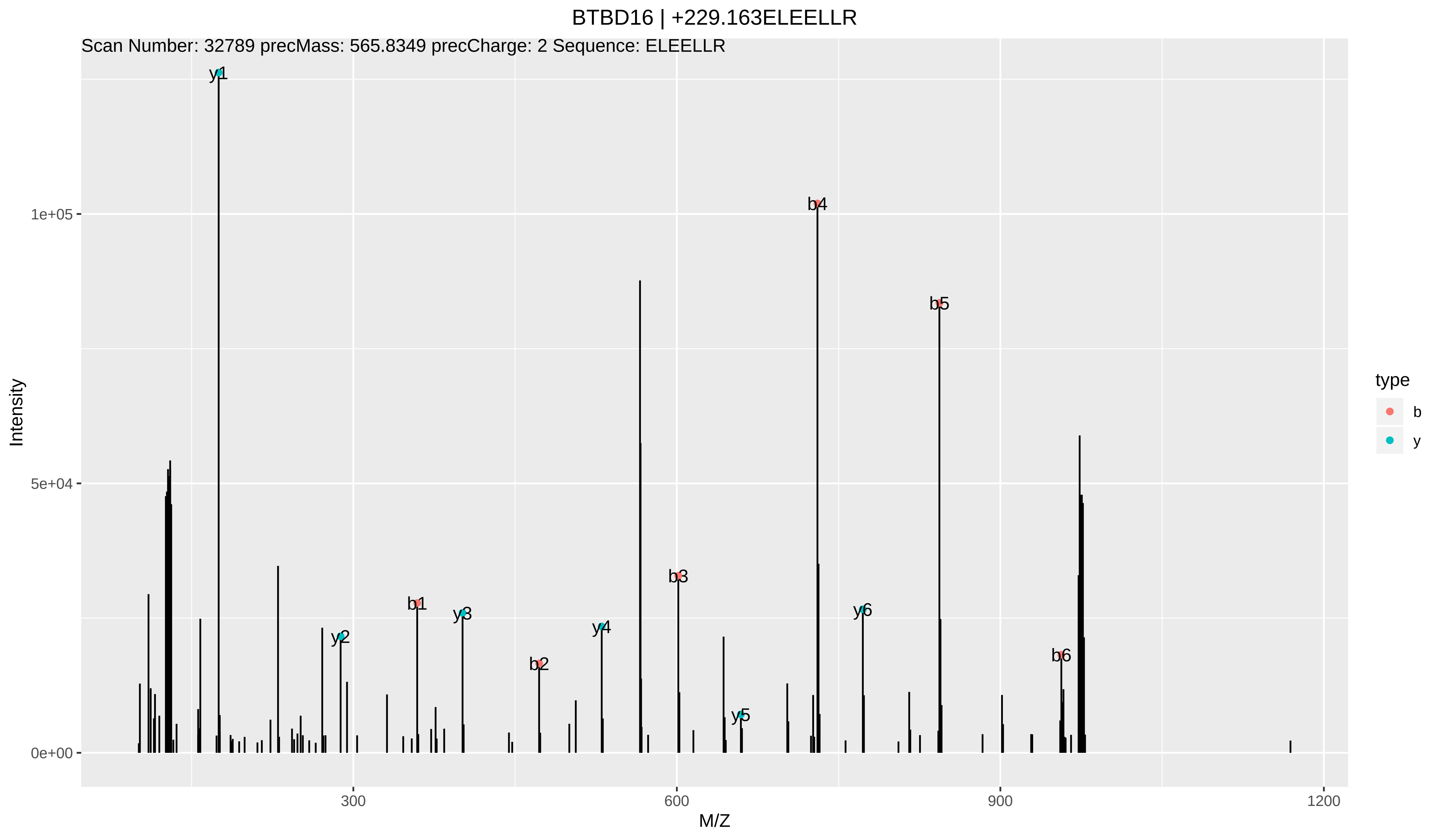
BTBD16 | +229.163ELEELLR
Scan Number: 32789 precMass: 565.8349 precCharge: 2 Sequence: ELEELLR
Intensity
M/Z
1e+05
5e+04
0e+00
300
600
900
1200
y1
y2
y3
y4
y5
y6
b1
b2
b3
b4
b5
b6
type
b
y

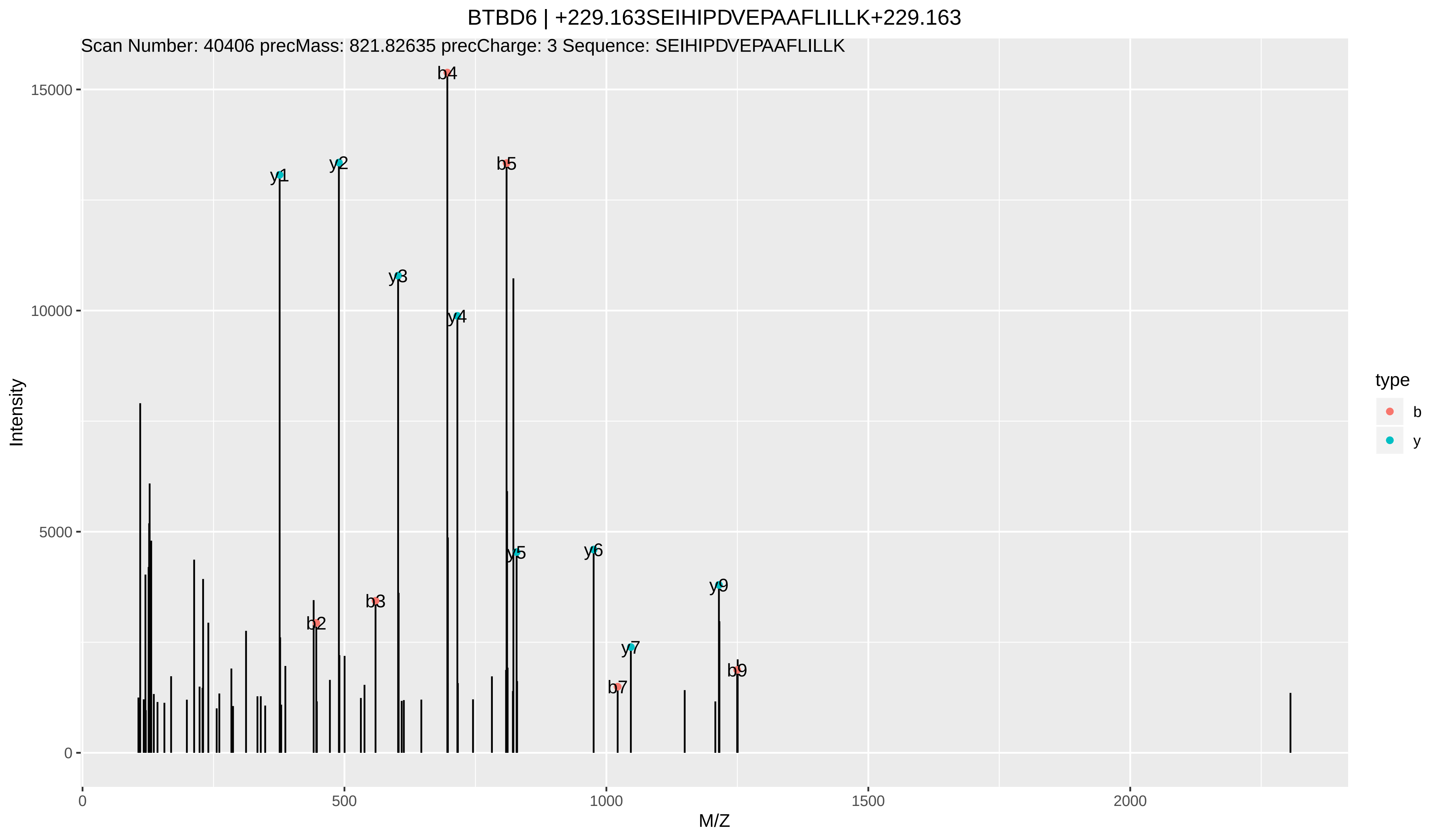

BTBD6 | +229.163SEIHIPDVEPAAFLILLK+229.163
Scan Number: 40406 precMass: 821.82635 precCharge: 3 Sequence: SEIHIPDVEPAAFLILLK
Intensity
M/Z
0
5000
10000
15000
0
500
1000
1500
2000
b4
y1
y2
b5
y3
y4
y5
y6
y9
b3
b2
y7
b9
b7
type
b
y

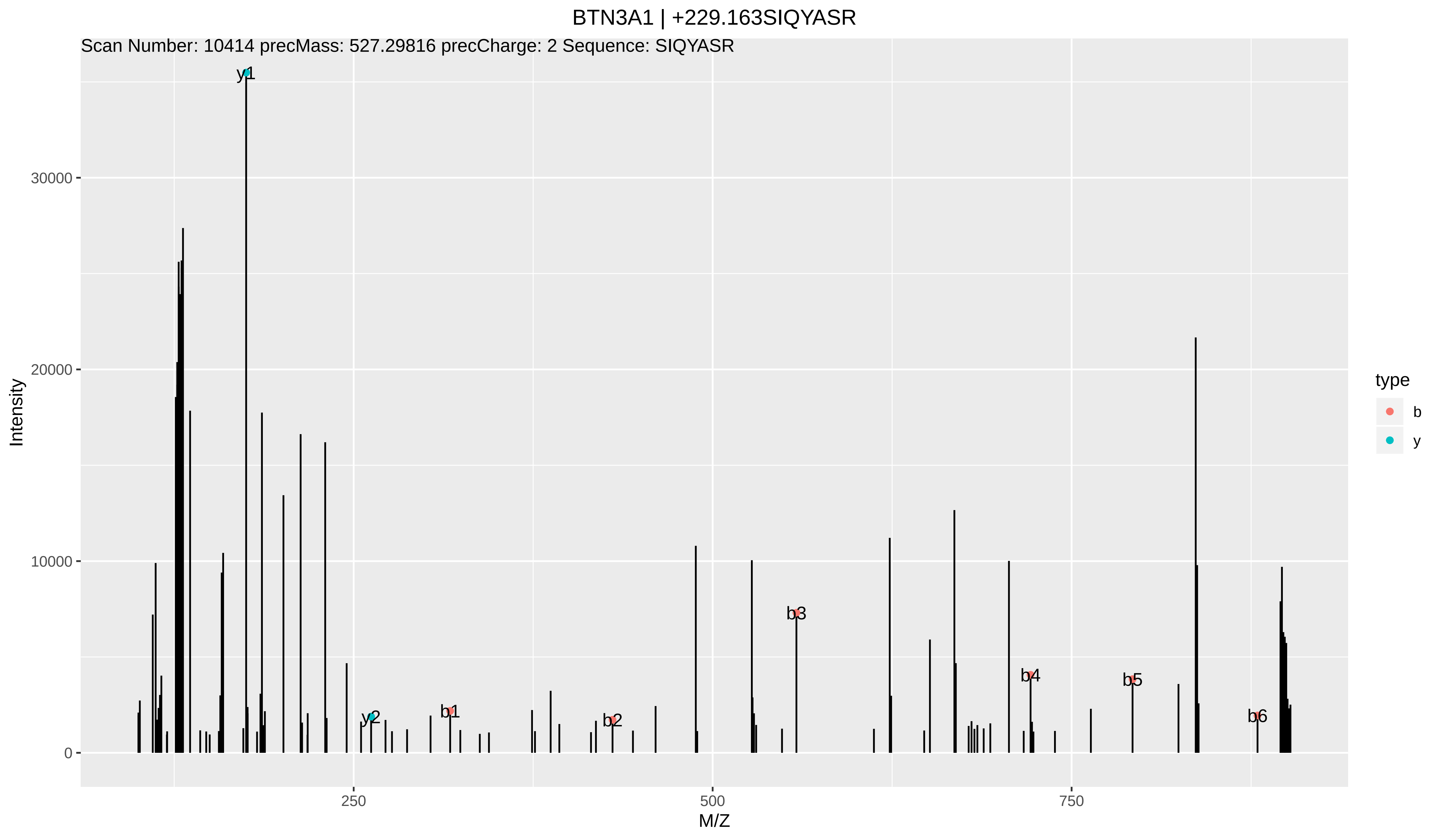
BTN3A1 | +229.163SIQYASR
Scan Number: 10414 precMass: 527.29816 precCharge: 2 Sequence: SIQYASR
y1
y2
b1
b2
b3
b4
b5
b6
Intensity
30000
20000
10000
0
250
500
750
M/Z
type
b
y

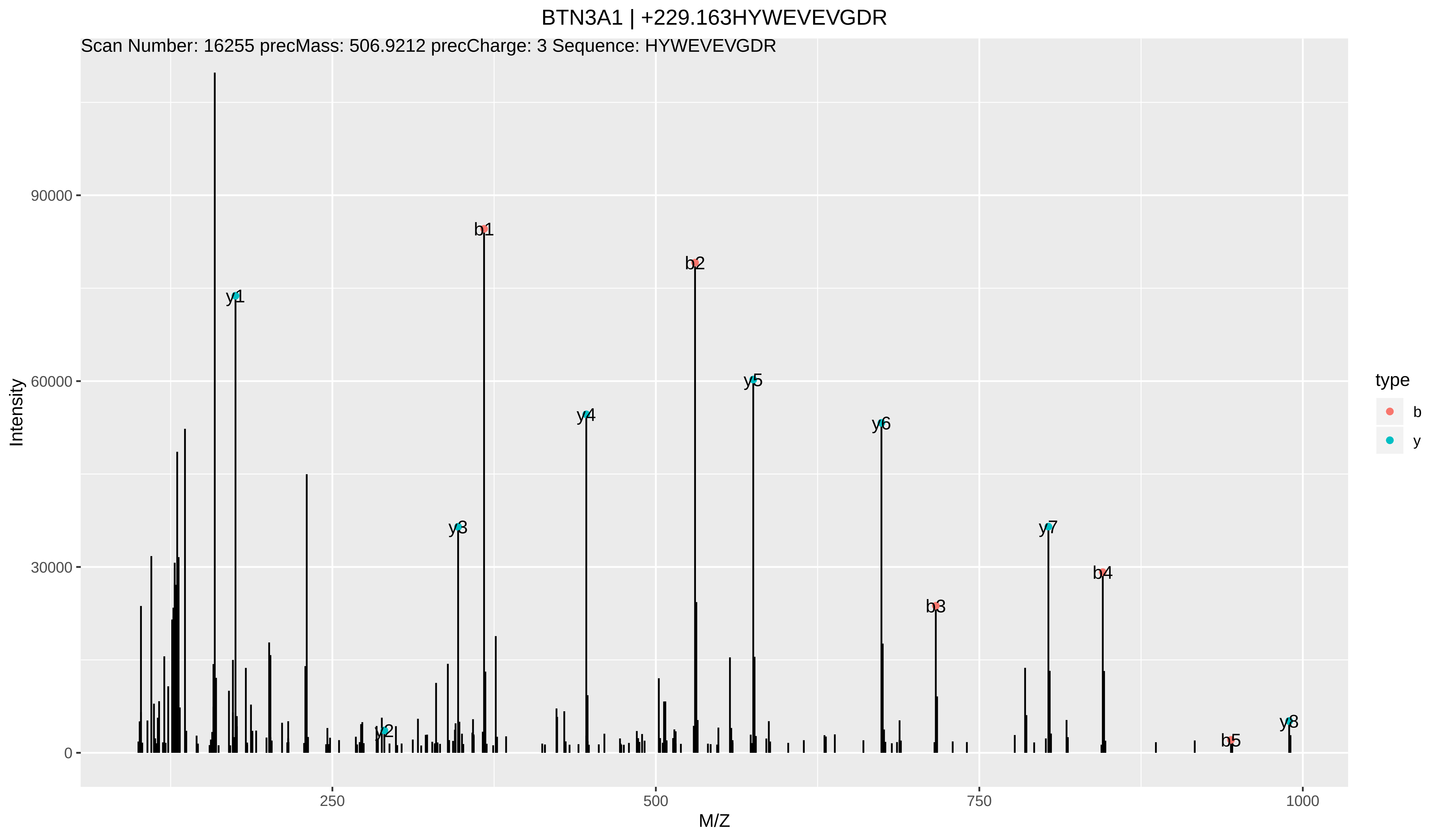
BTN3A1 | +229.163HYWEVEVGDR
Scan Number: 16255 precMass: 506.9212 precCharge: 3 Sequence: HYWEVEVGDR
Intensity
90000
60000
30000
0
M/Z
250
500
750
1000
b1
b2
y1
y5
y4
y6
y3
y7
b4
b3
y2
y8
b5
type
b
y

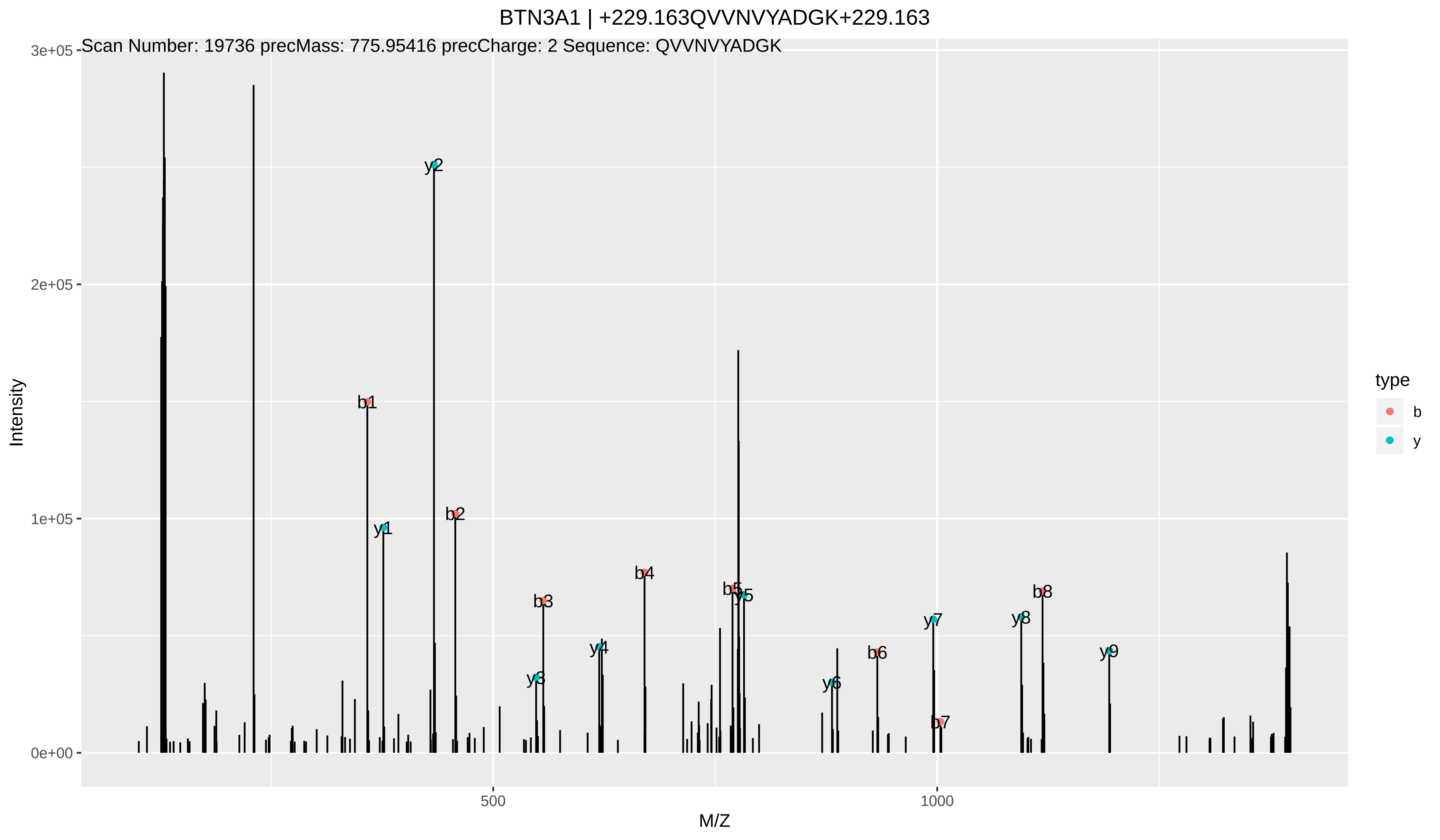

BTN3A1 | +229.163QVVNVYADGK+229.163
Scan Number: 19736 precMass: 775.95416 precCharge: 2 Sequence: QVVNVYADGK
Intensity
M/Z
3e+05
2e+05
1e+05
0e+00
500
1000
y2
b1
y1
b2
b3
y3
y4
b4
b5
y5
y6
b6
y7
b7
y8
b8
y9
type
b
y

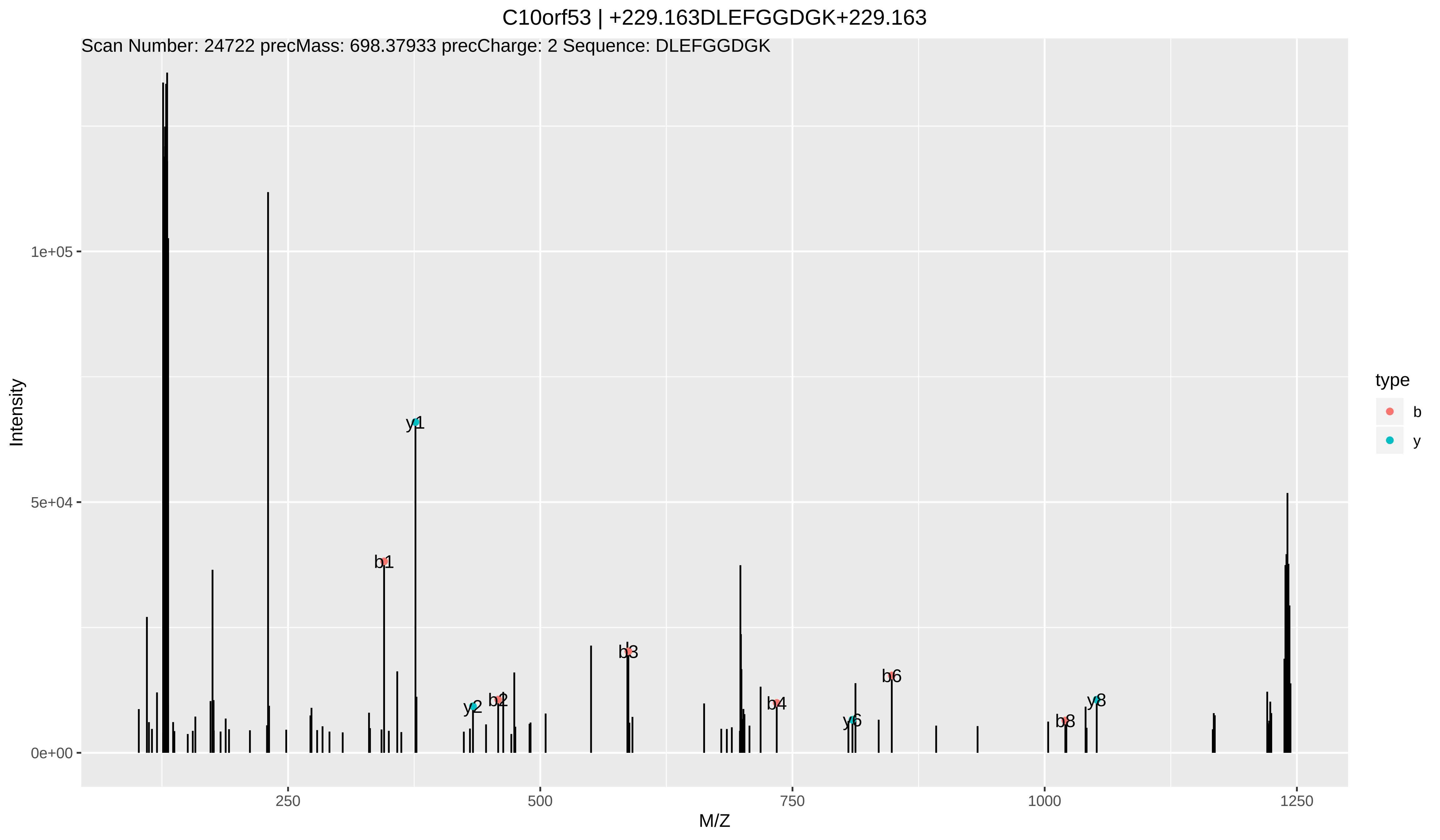

C10orf53 | +229.163DLEFGGDGK+229.163
Scan Number: 24722 precMass: 698.37933 precCharge: 2 Sequence: DLEFGGDGK
Intensity
1e+05
5e+04
0e+00
M/Z
250
500
750
1000
1250
y1
b1
b2
y2
b3
b4
y6
b6
b8
y8
type
b
y

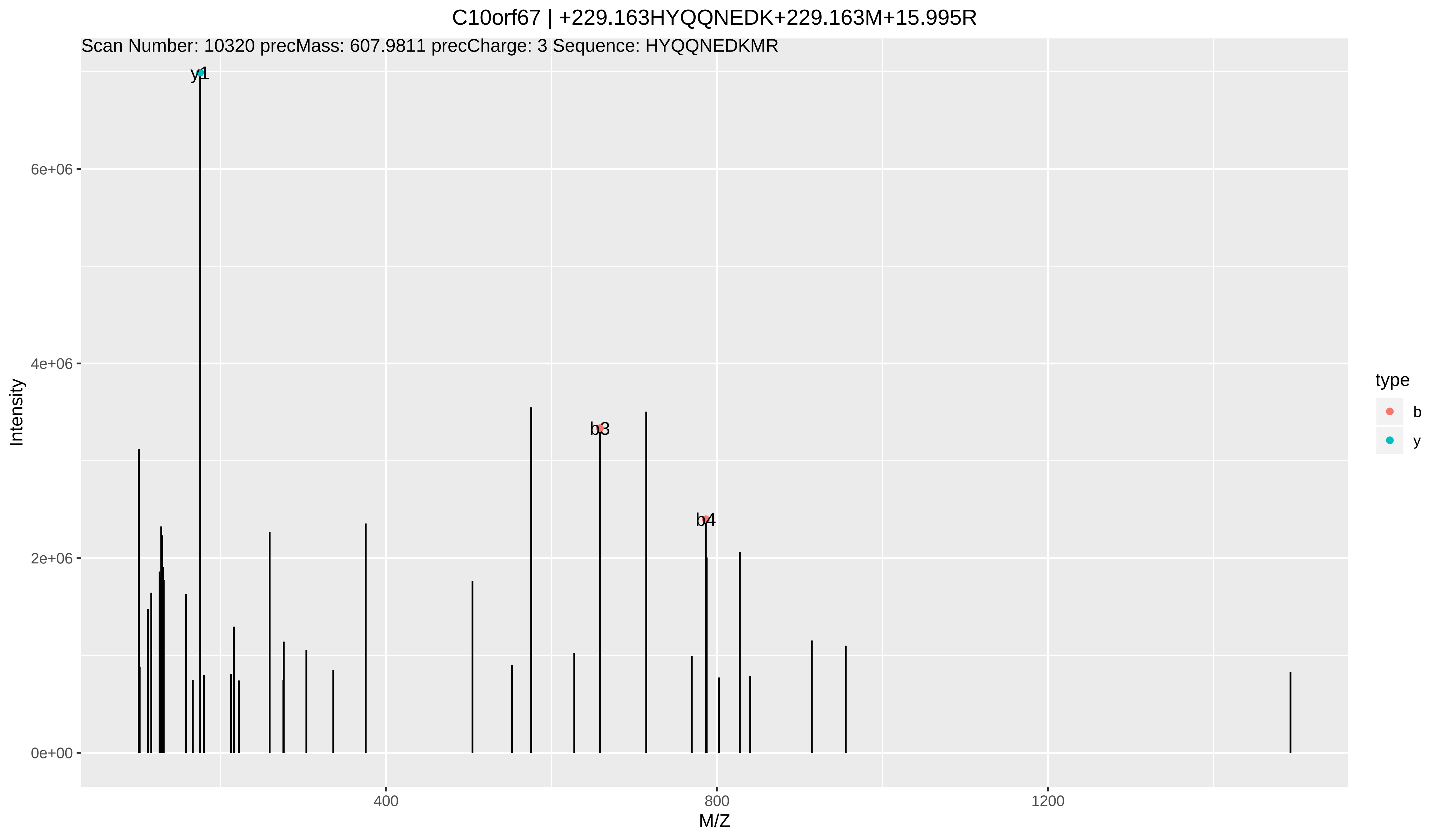

C10orf67 | +229.163HYQQNEDK+229.163M+15.995R
Scan Number: 10320 precMass: 607.9811 precCharge: 3 Sequence: HYQQNEDKMR
y1
b3
b4
Intensity
6e+06
4e+06
2e+06
0e+00
M/Z
400
800
1200
type
b
y

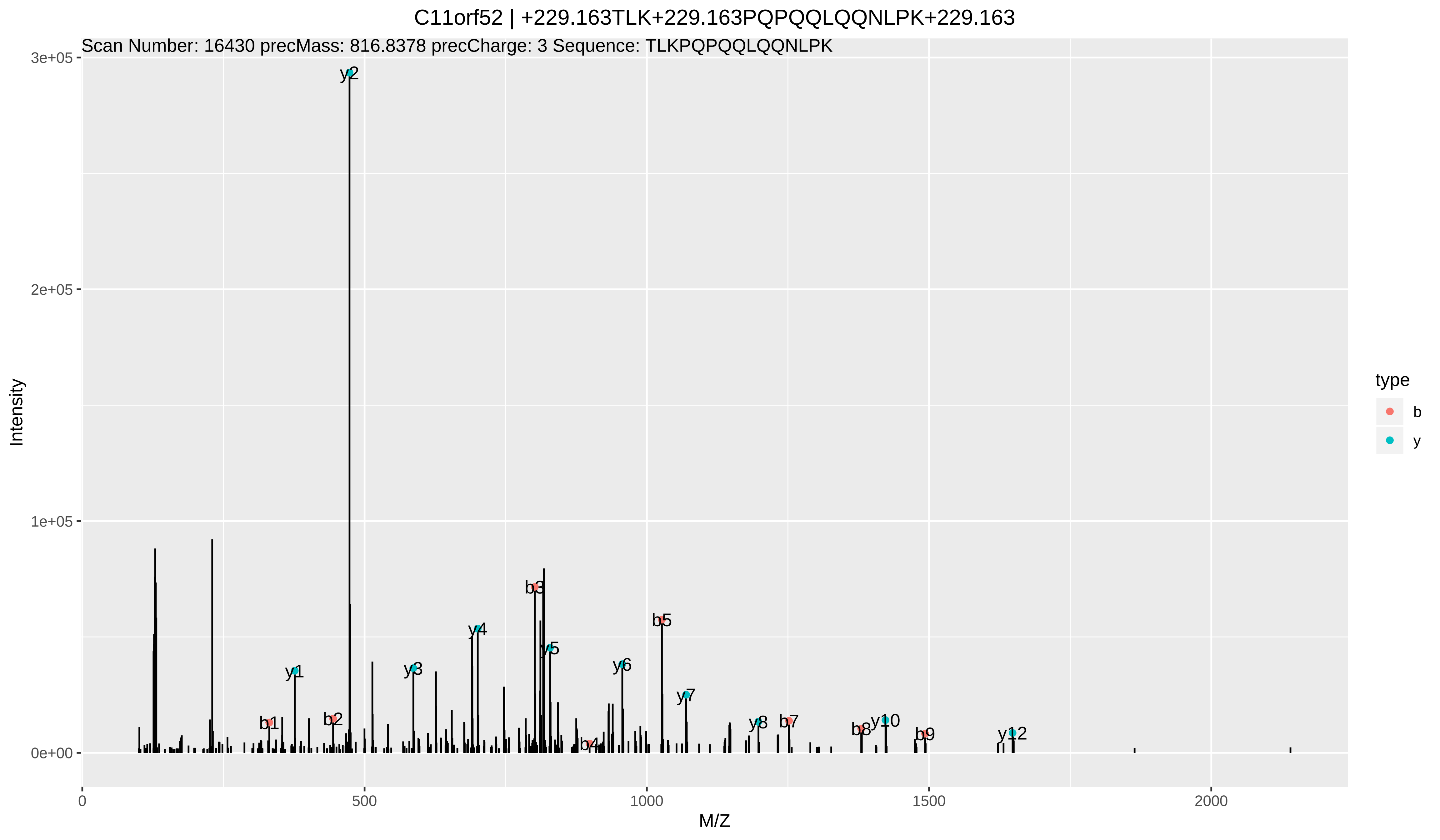

C11orf52 | +229.163TLK+229.163PQPQQLQQNLPK+229.163
Scan Number: 16430 precMass: 816.8378 precCharge: 3 Sequence: TLKPQPQQLQQNLPK
Intensity
M/Z
3e+05
2e+05
1e+05
0e+00
0
500
1000
1500
2000
y2
b3
b5
y4
y5
y6
y1
y3
y7
b1
b2
b4
y8
b7
b8
y10
b9
y12
type
b
y

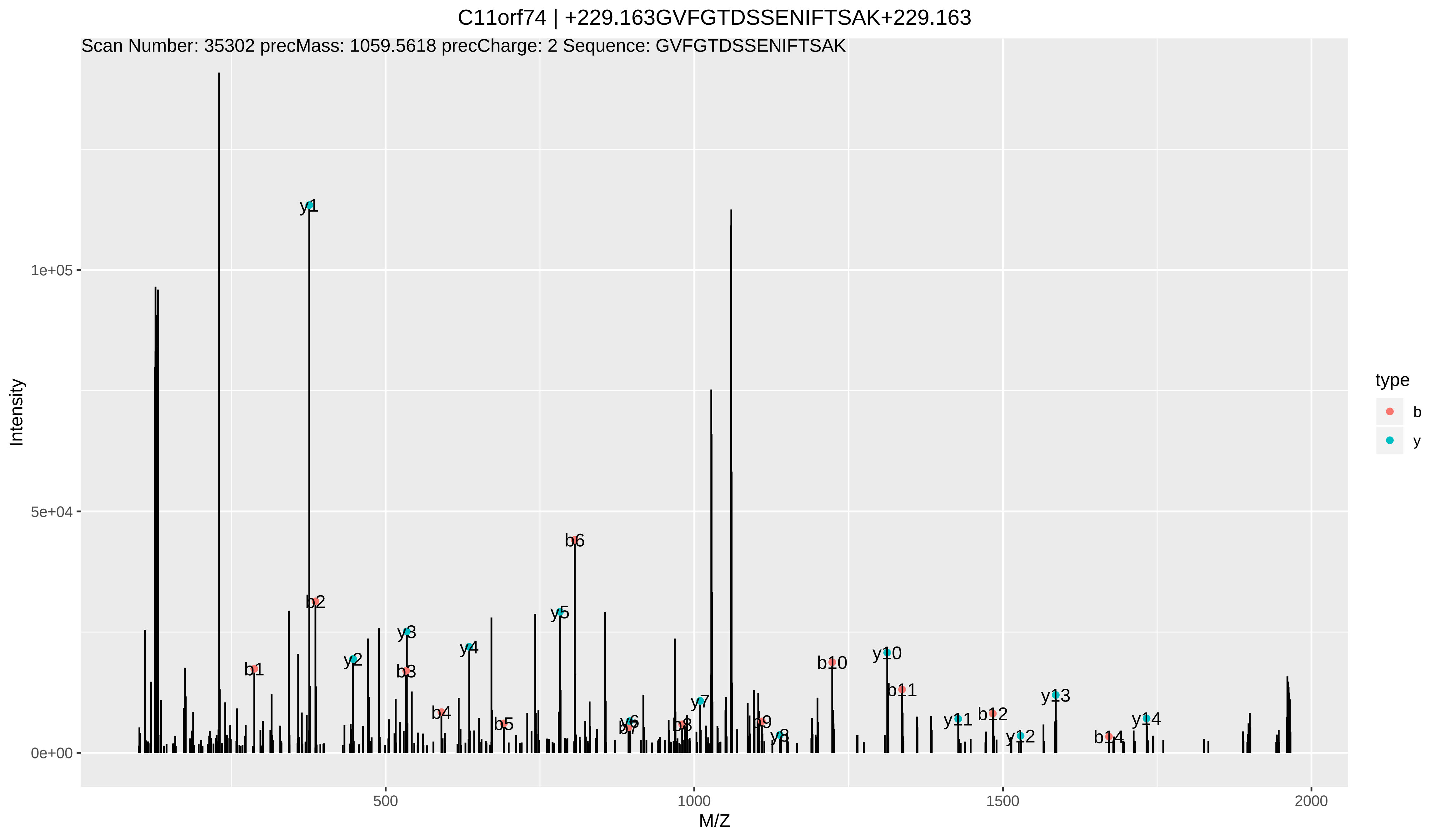

C11orf74 | +229.163GVFGTDSSENIFTSAK+229.163
Scan Number: 35302 precMass: 1059.5618 precCharge: 2 Sequence: GVFGTDSSENIFTSAK
Intensity
M/Z
type
b
y
1e+05
5e+04
0e+00
500
1000
1500
2000
y1
b1
b2
y2
y3
b3
y4
b4
b5
y5
b6
y6
b7
b8
y7
b9
y8
b10
y10
b11
y11
b12
y12
y13
b14
y14

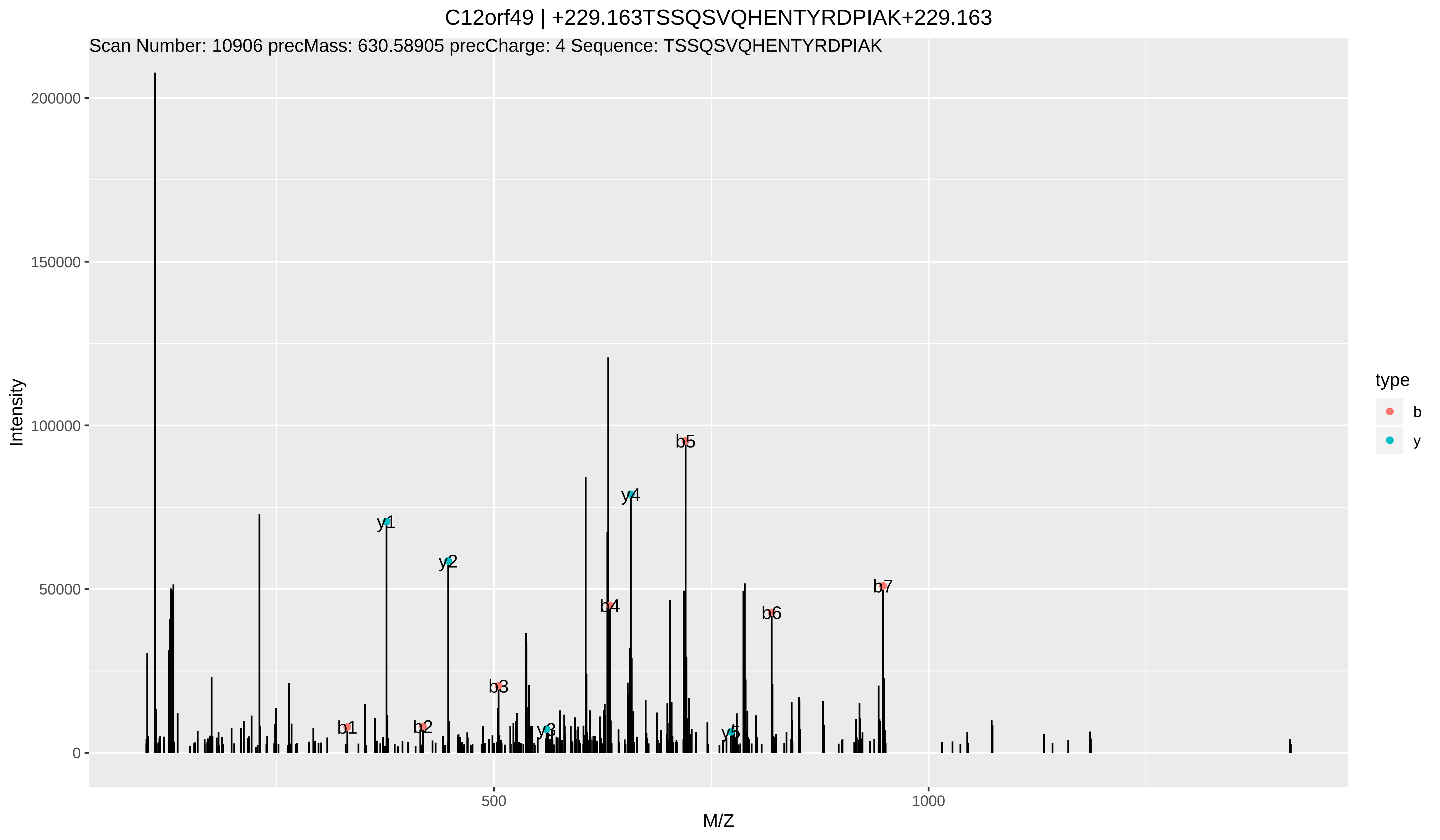

C12orf49 | +229.163TSSQSVQHENTYRDPIAK+229.163
Scan Number: 10906 precMass: 630.58905 precCharge: 4 Sequence: TSSQSVQHENTYRDPIAK
Intensity
200000
150000
100000
50000
0
M/Z
500
1000
y1
y2
y3
y4
y5
b1
b2
b3
b4
b5
b6
b7
type
b
y

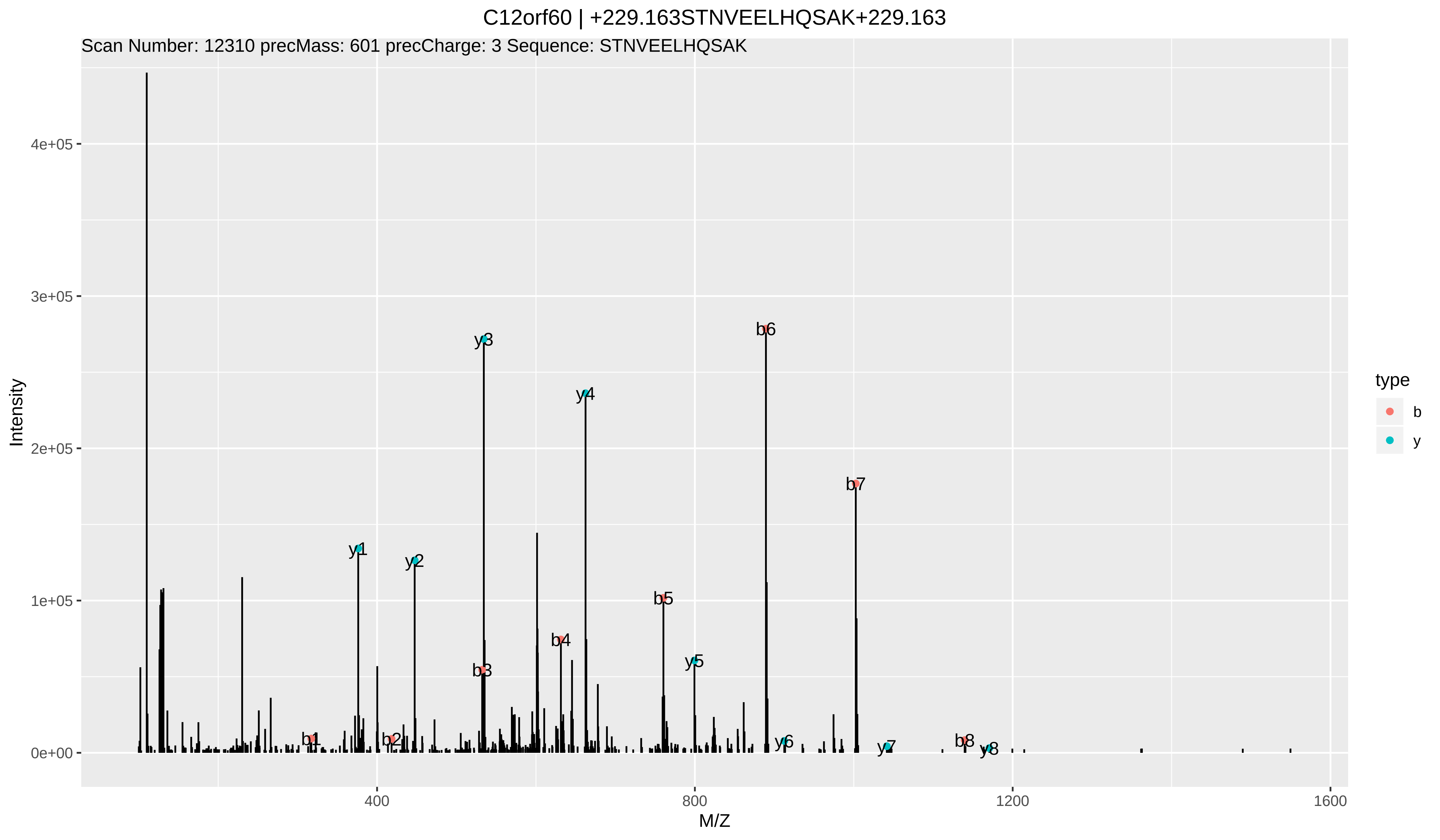

C12orf60 | +229.163STNVEELHQSAK+229.163
Scan Number: 12310 precMass: 601 precCharge: 3 Sequence: STNVEELHQSAK
Intensity
M/Z
0e+00
1e+05
2e+05
3e+05
4e+05
400
800
1200
1600
type
b
y
y1
y2
y3
y4
y5
y6
y7
y8
b1
b2
b3
b4
b5
b6
b7
b8

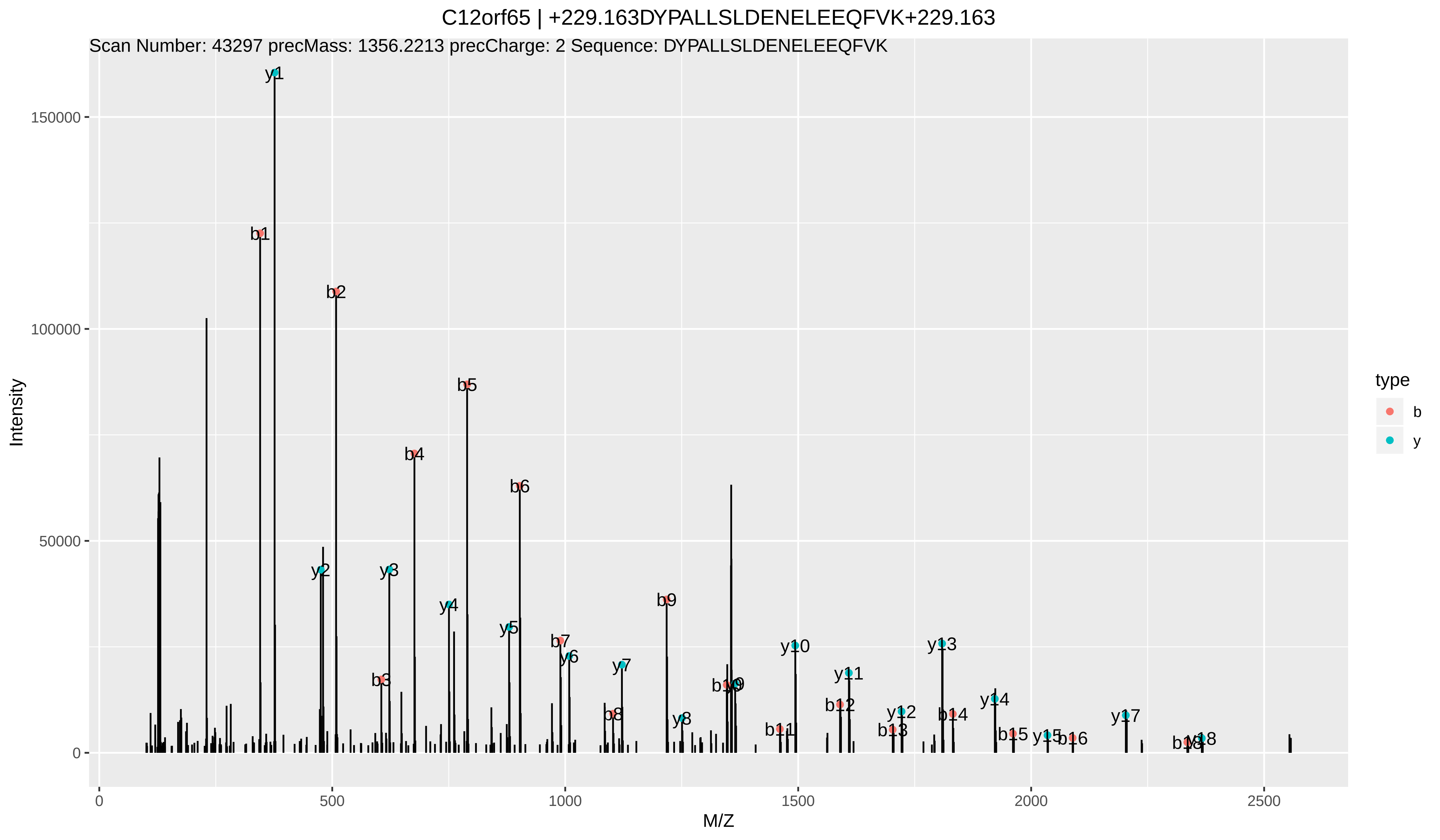

C12orf65 | +229.163DYPALLSLDENELEEQFVK+229.163
Scan Number: 43297 precMass: 1356.2213 precCharge: 2 Sequence: DYPALLSLDENELEEQFVK
Intensity
M/Z
type
b
y

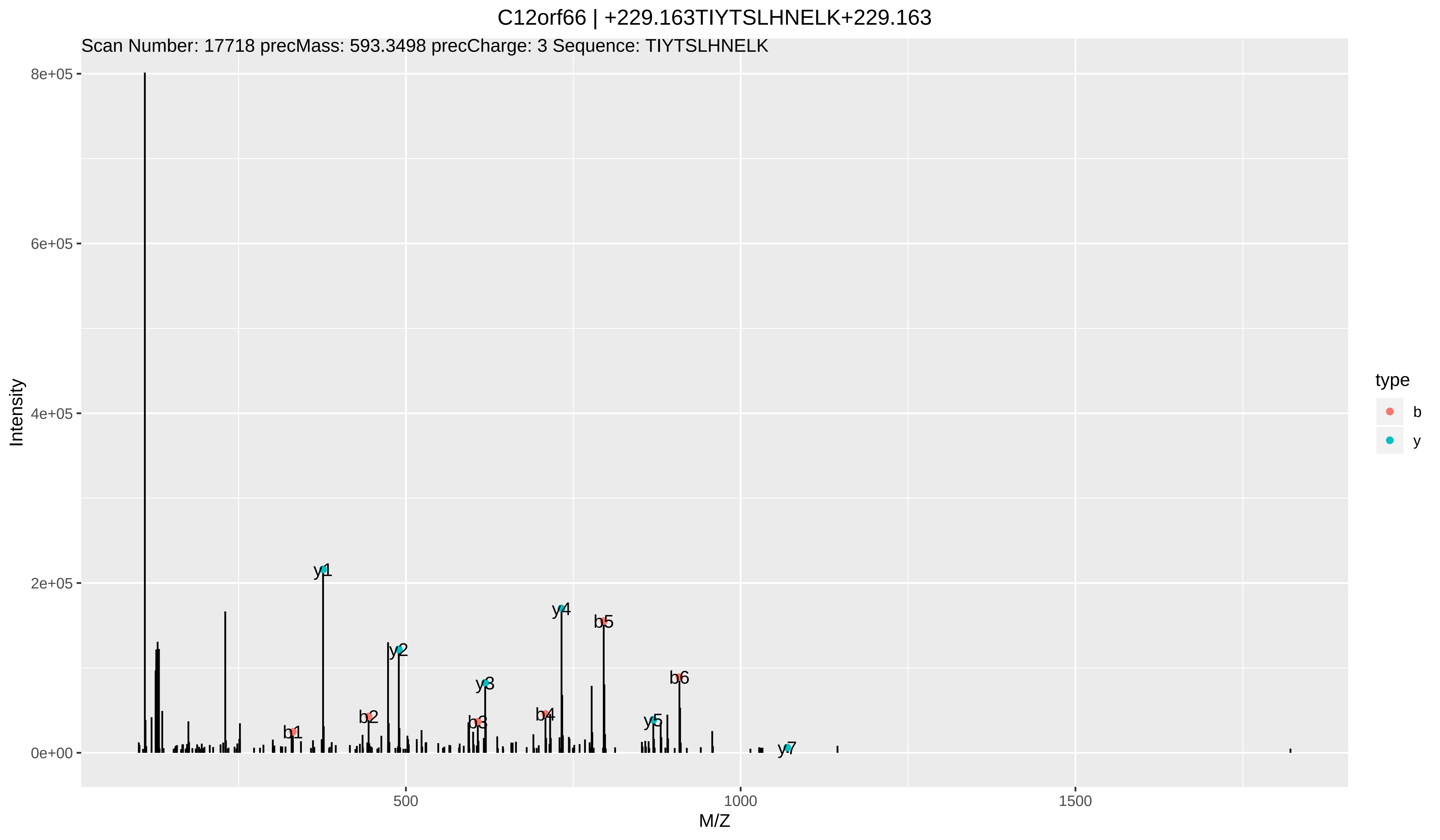

C12orf66 | +229.163TIYTSLHNELK+229.163
Scan Number: 17718 precMass: 593.3498 precCharge: 3 Sequence: TIYTSLHNELK
Intensity
M/Z
8e+05
6e+05
4e+05
2e+05
0e+00
500
1000
1500
y1
y2
y3
y4
y5
y7
b1
b2
b3
b4
b5
b6
type
b
y

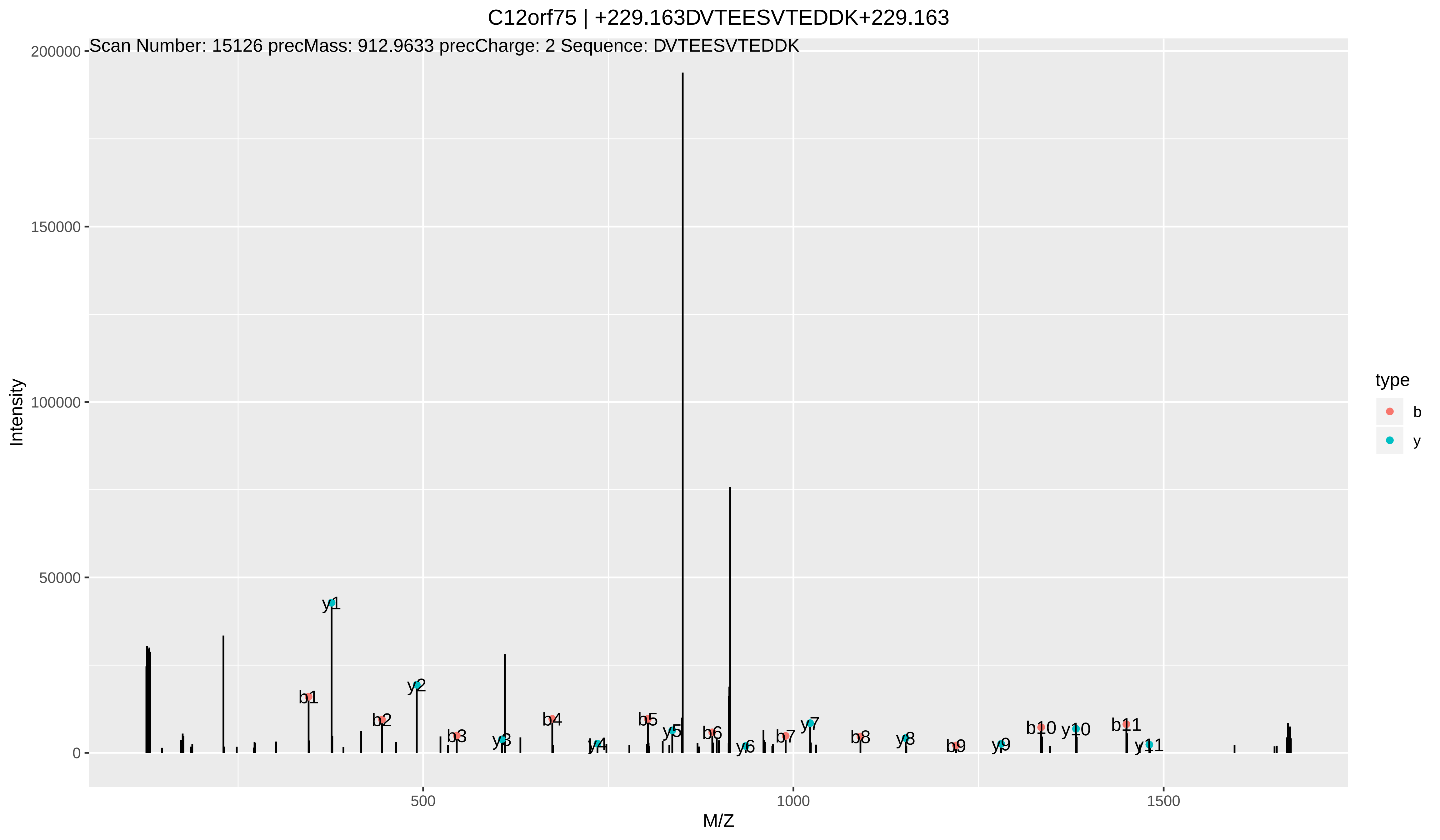

C12orf75 | +229.163DVTEESVTEDDK+229.163
Scan Number: 15126 precMass: 912.9633 precCharge: 2 Sequence: DVTEESVTEDDK
Intensity
M/Z
200000
150000
100000
50000
0
500
1000
1500
type
b
y
y1
y2
y3
y4
y5
y6
y7
y8
y9
y10
y11
b1
b2
b3
b4
b5
b6
b7
b8
b9
b10
b11

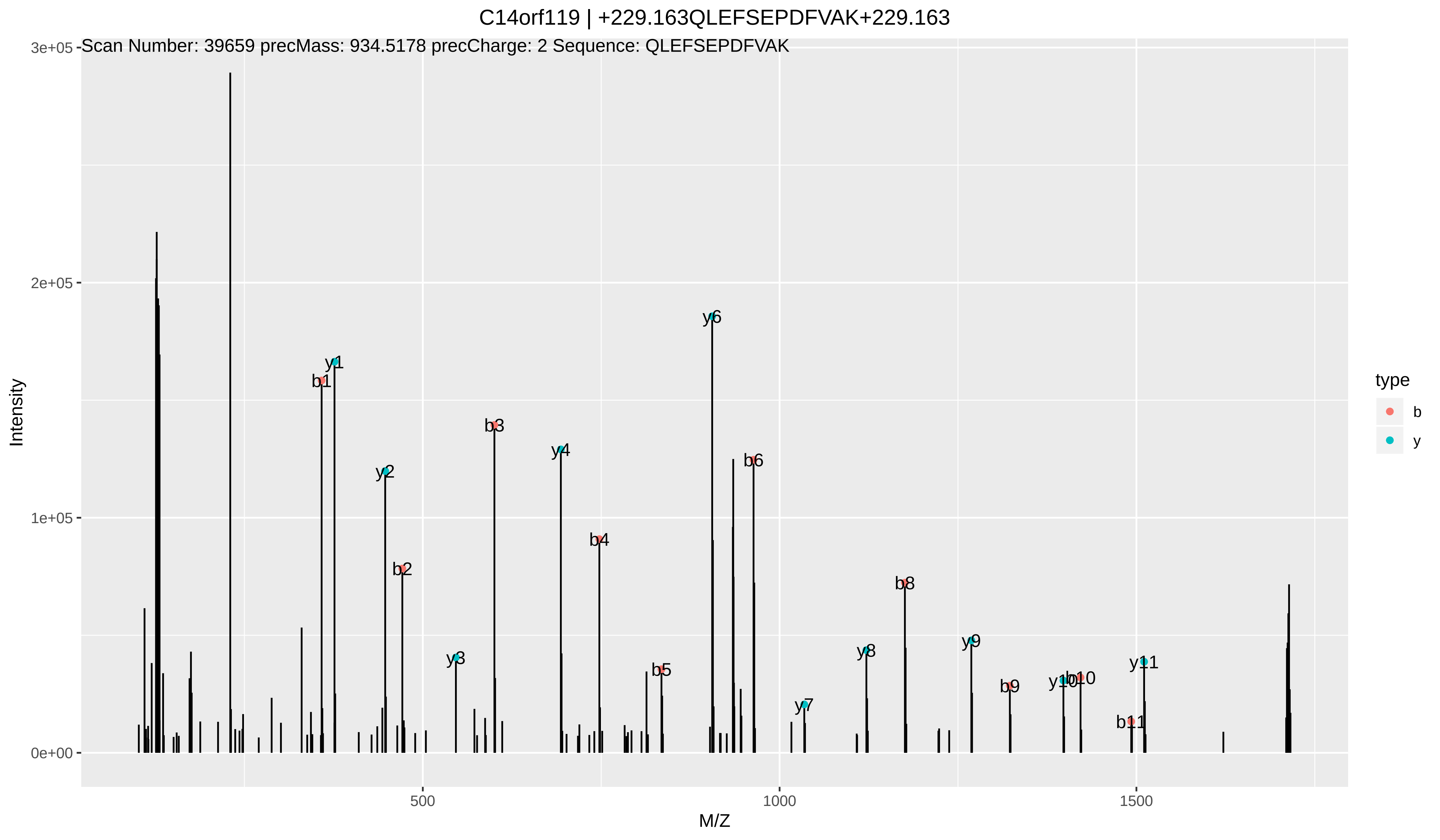
C14orf119 | +229.163QLEFSEPDFVAK+229.163
Scan Number: 39659 precMass: 934.5178 precCharge: 2 Sequence: QLEFSEPDFVAK
Intensity
3e+05
2e+05
1e+05
0e+00
M/Z
500
1000
1500
y1
b1
y2
b2
y3
b3
y4
b4
b5
y6
b6
y7
y8
b8
y9
b9
y10
b10
b11
y11
type
b
y

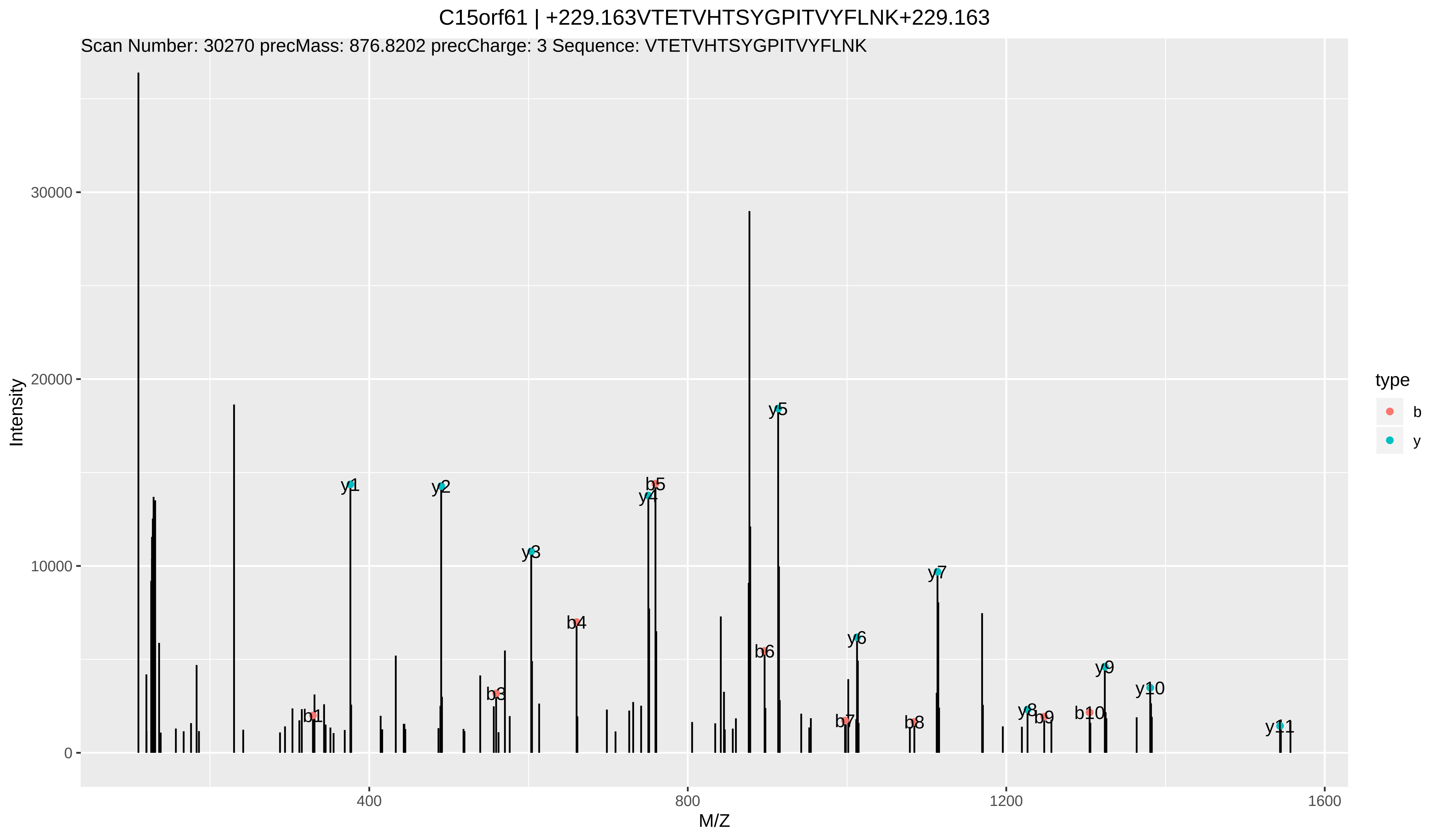

C15orf61 | +229.163VTETVHTSYGPITVYFLNK+229.163
Scan Number: 30270 precMass: 876.8202 precCharge: 3 Sequence: VTETVHTSYGPITVYFLNK
Intensity
30000
20000
10000
0
400
800
1200
1600
M/Z
type
b
y
y1
y2
y3
y4
y5
y6
y7
y8
y9
y10
y11
b1
b3
b4
b5
b6
b7
b8
b9
b10

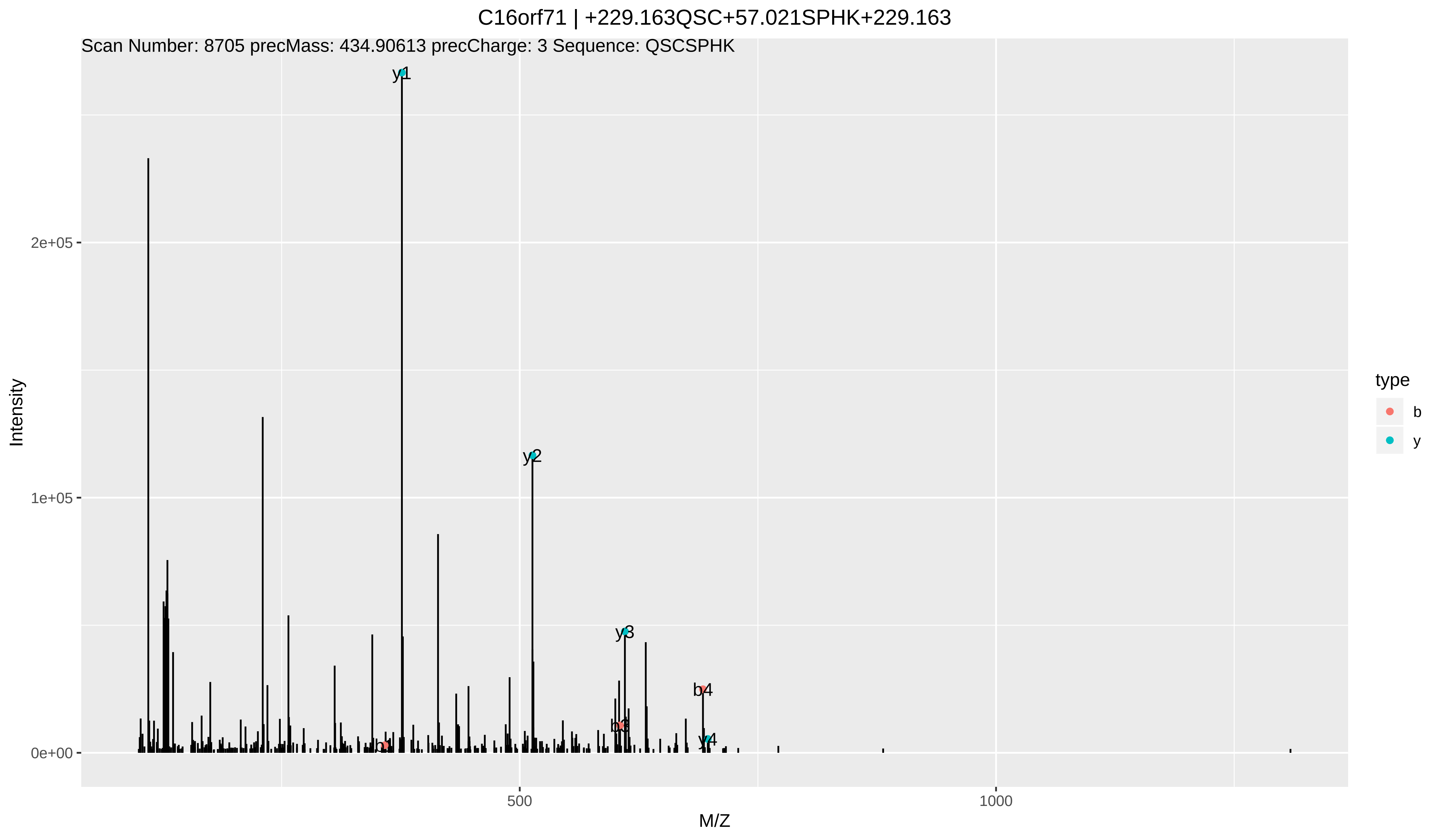

C16orf71 | +229.163QSC+57.021SPHK+229.163
Scan Number: 8705 precMass: 434.90613 precCharge: 3 Sequence: QSCSPHK
y1
y2
y3
b4
y4
2e+05
1e+05
0e+00
Intensity
500
1000
M/Z
type
b
y

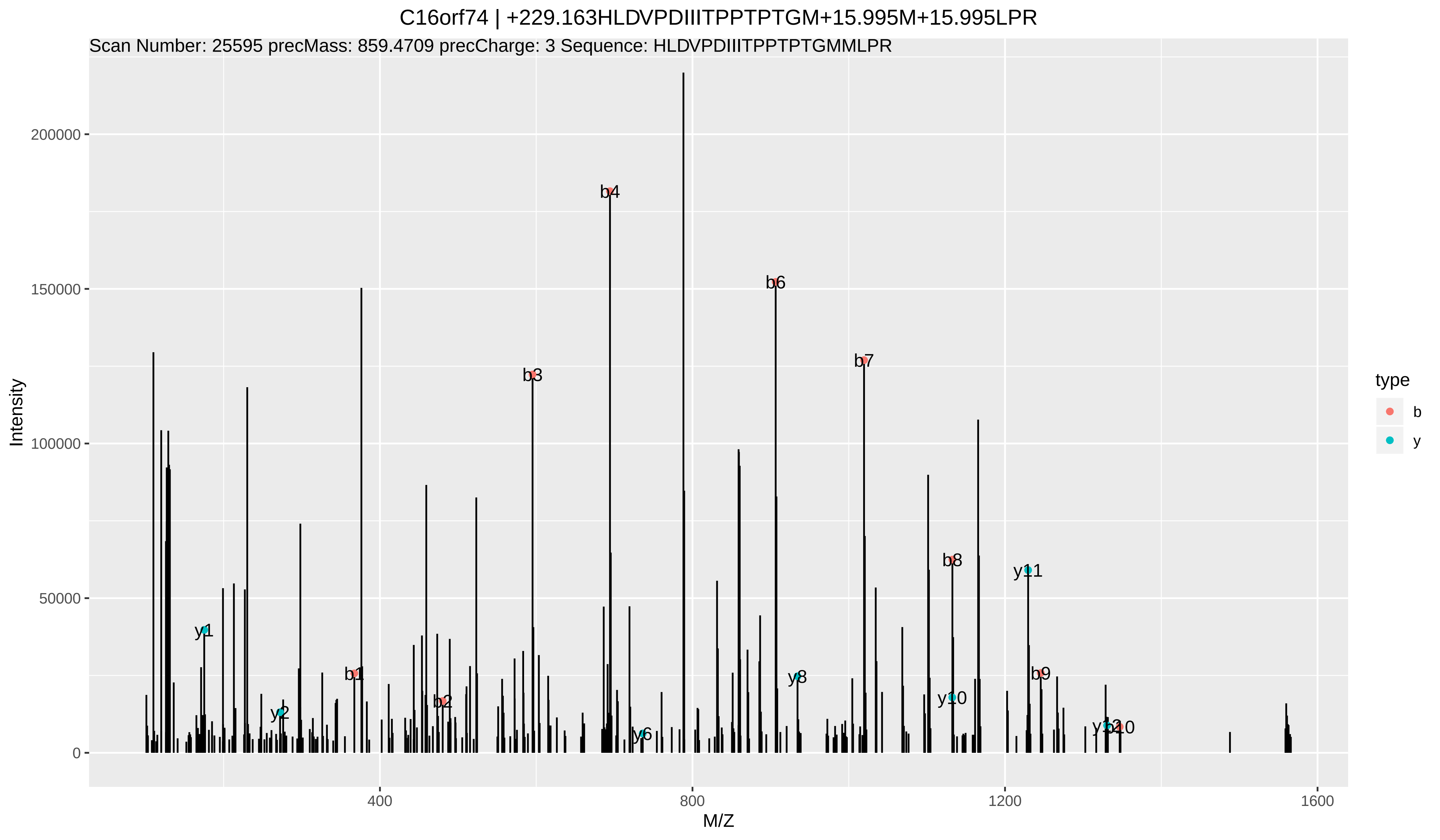
C16orf74 | +229.163HLDVPDIIITPPTPTGM+15.995M+15.995LPR
Scan Number: 25595 precMass: 859.4709 precCharge: 3 Sequence: HLDVPDIIITPPTPTGMMLPR
Intensity
M/Z
type
b
y
b1
b2
b3
b4
b6
b7
b8
b9
b10
y1
y2
y6
y8
y10
y11
y12
0
50000
100000
150000
200000
400
800
1200
1600

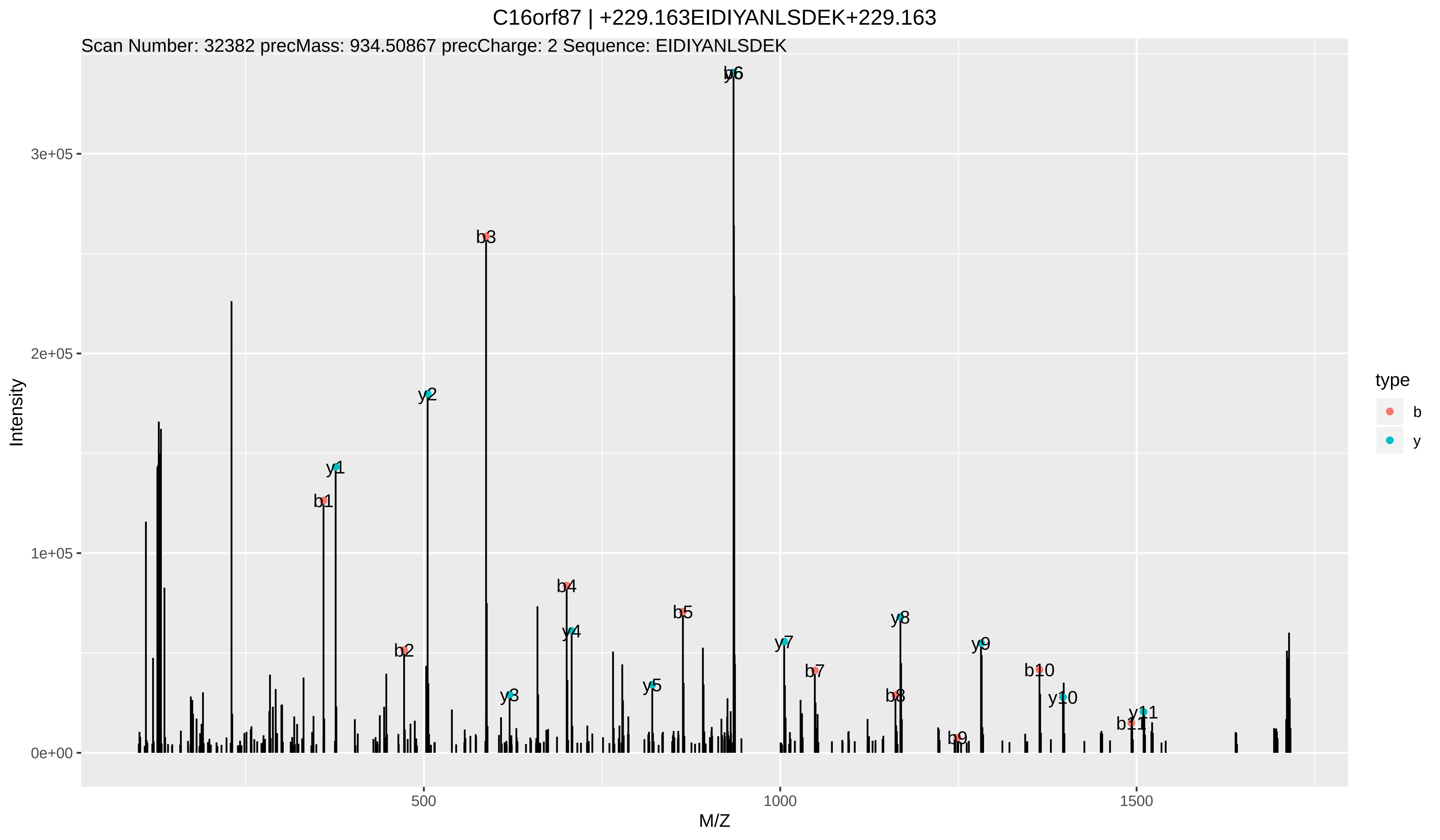
C16orf87 | +229.163EIDIYANLSDEK+229.163
Scan Number: 32382 precMass: 934.50867 precCharge: 2 Sequence: EIDIYANLSDEK
Intensity
M/Z
type
b
y
3e+05
2e+05
1e+05
0e+00
500
1000
1500
b1
b2
b3
b4
b5
b6
b7
b8
b9
b10
b11
y1
y2
y3
y4
y5
y6
y7
y8
y9
y10
y11

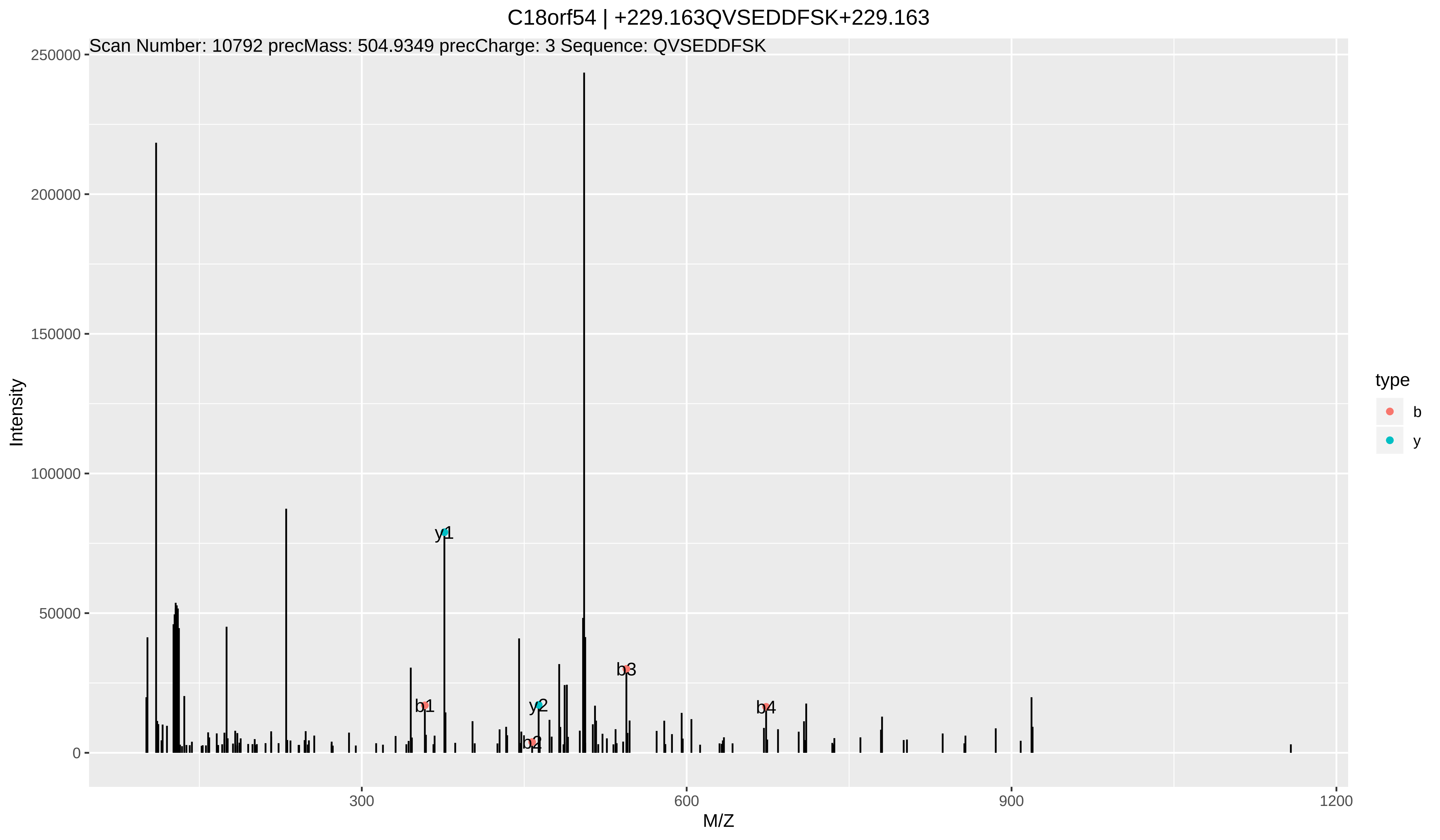

C18orf54 | +229.163QVSEDDFSK+229.163
Scan Number: 10792 precMass: 504.9349 precCharge: 3 Sequence: QVSEDDFSK
Intensity
M/Z
y1
y2
b1
b2
b3
b4
type
b
y
0
50000
100000
150000
200000
250000
300
600
900
1200

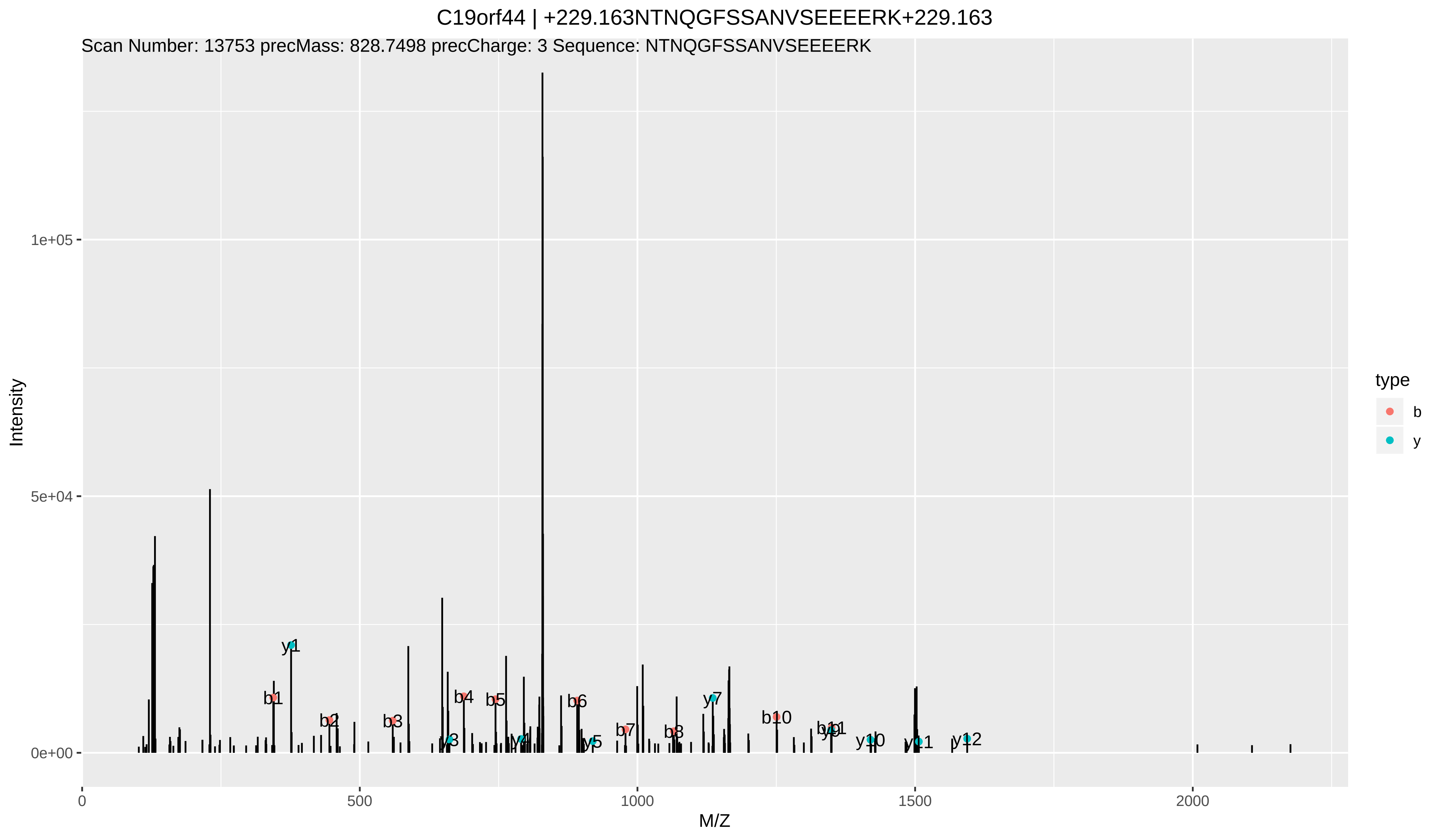
C19orf44 | +229.163NTNQGFSSANVSEEEERK+229.163
Scan Number: 13753 precMass: 828.7498 precCharge: 3 Sequence: NTNQGFSSANVSEEEERK
Intensity
1e+05
5e+04
0e+00
M/Z
0
500
1000
1500
2000
type
b
y
y1
b1
b2
b3
b4
b5
b6
b7
b8
b10
y7
y10
y12

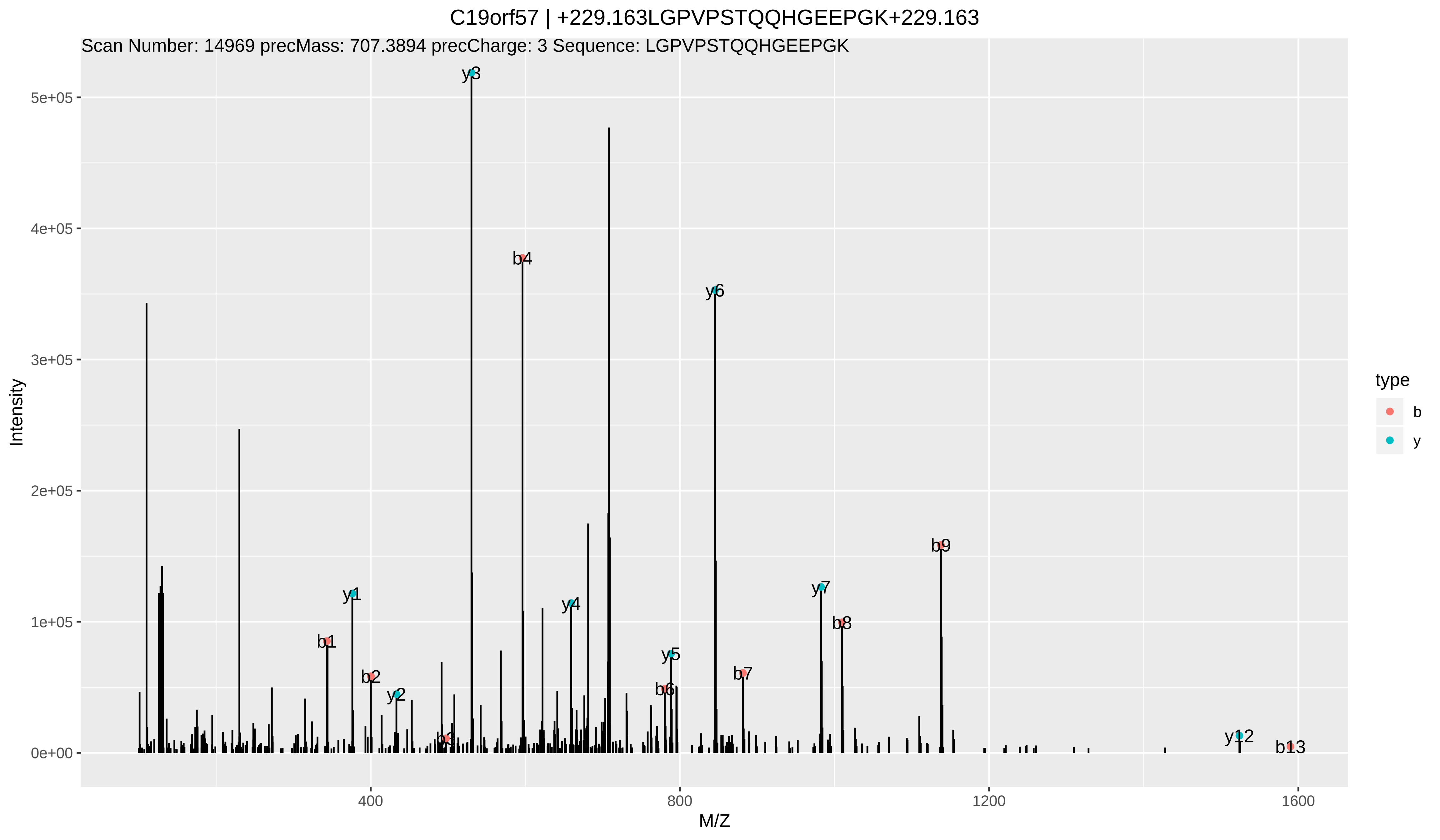

C19orf57 | +229.163LGPVPSTQQHGEEPGK+229.163
Scan Number: 14969 precMass: 707.3894 precCharge: 3 Sequence: LGPVPSTQQHGEEPGK
y3
b4
y6
b9
y7
y1
y4
b8
b1
y5
b2
b7
b6
y2
b3
y12
b13
5e+05
4e+05
3e+05
2e+05
1e+05
0e+00
Intensity
400
800
1200
1600
M/Z
type
b
y

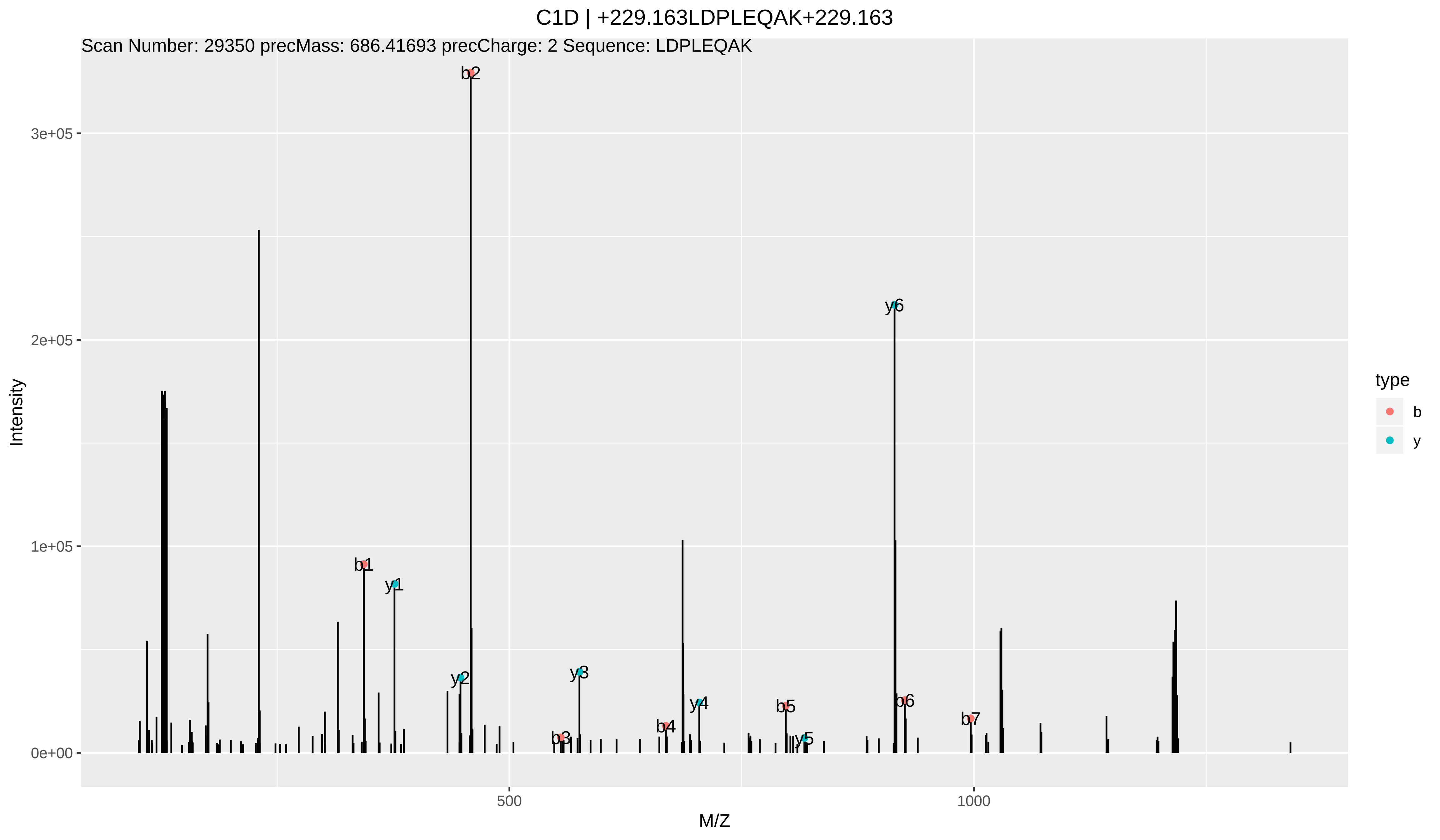
C1D | +229.163LDPLEQAK+229.163
Scan Number: 29350 precMass: 686.41693 precCharge: 2 Sequence: LDPLEQAK
Intensity
M/Z
3e+05
2e+05
1e+05
0e+00
500
1000
b2
y6
b1
y1
y2
y3
b3
b4
y4
b5
y5
b6
b7
type
b
y

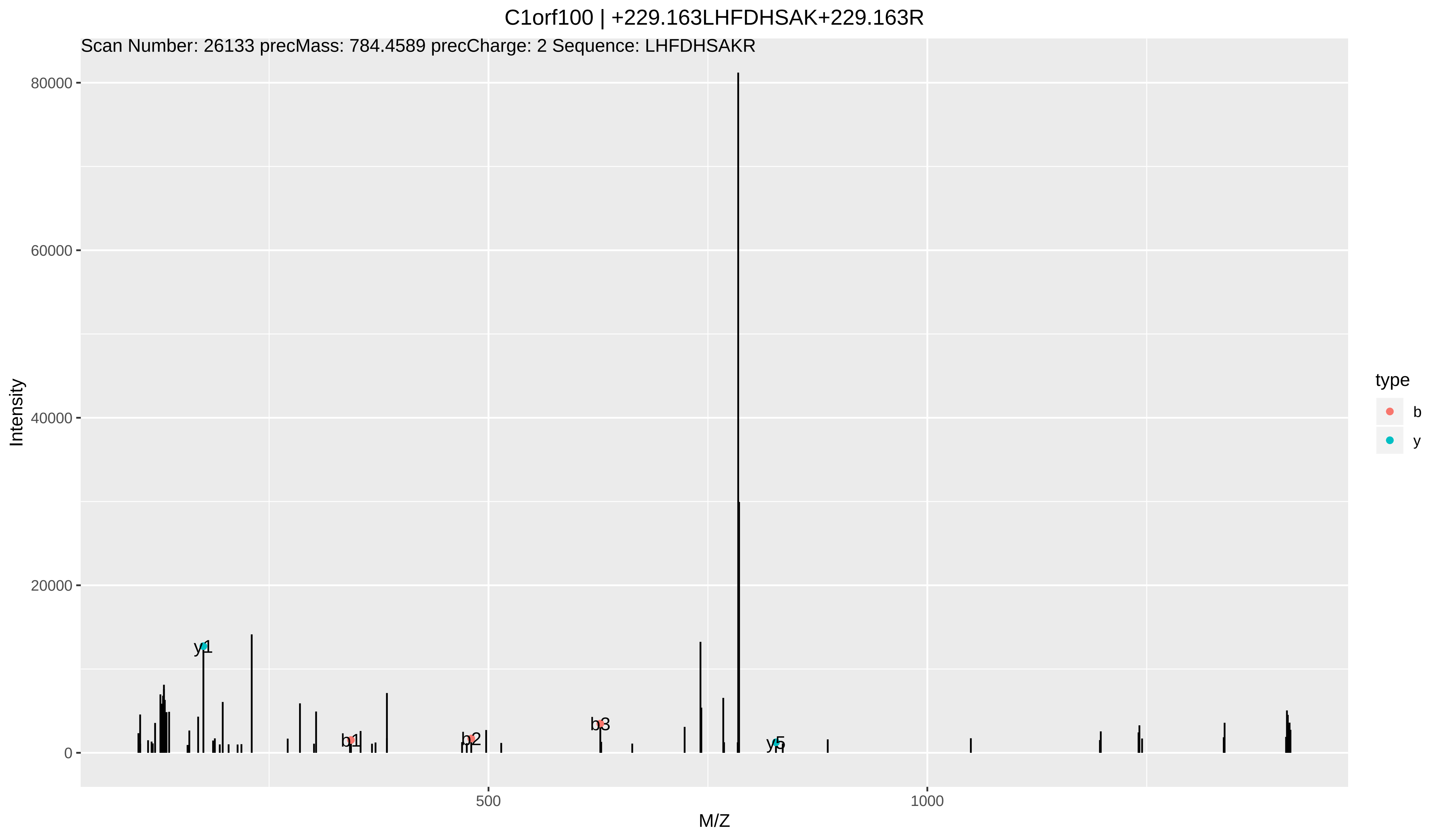
C1orf100 | +229.163LHFDHSAK+229.163R
Scan Number: 26133 precMass: 784.4589 precCharge: 2 Sequence: LHFDHSAKR
Intensity
M/Z
type
b
y
y1
b1
b2
b3
y5
0
20000
40000
60000
80000
500
1000

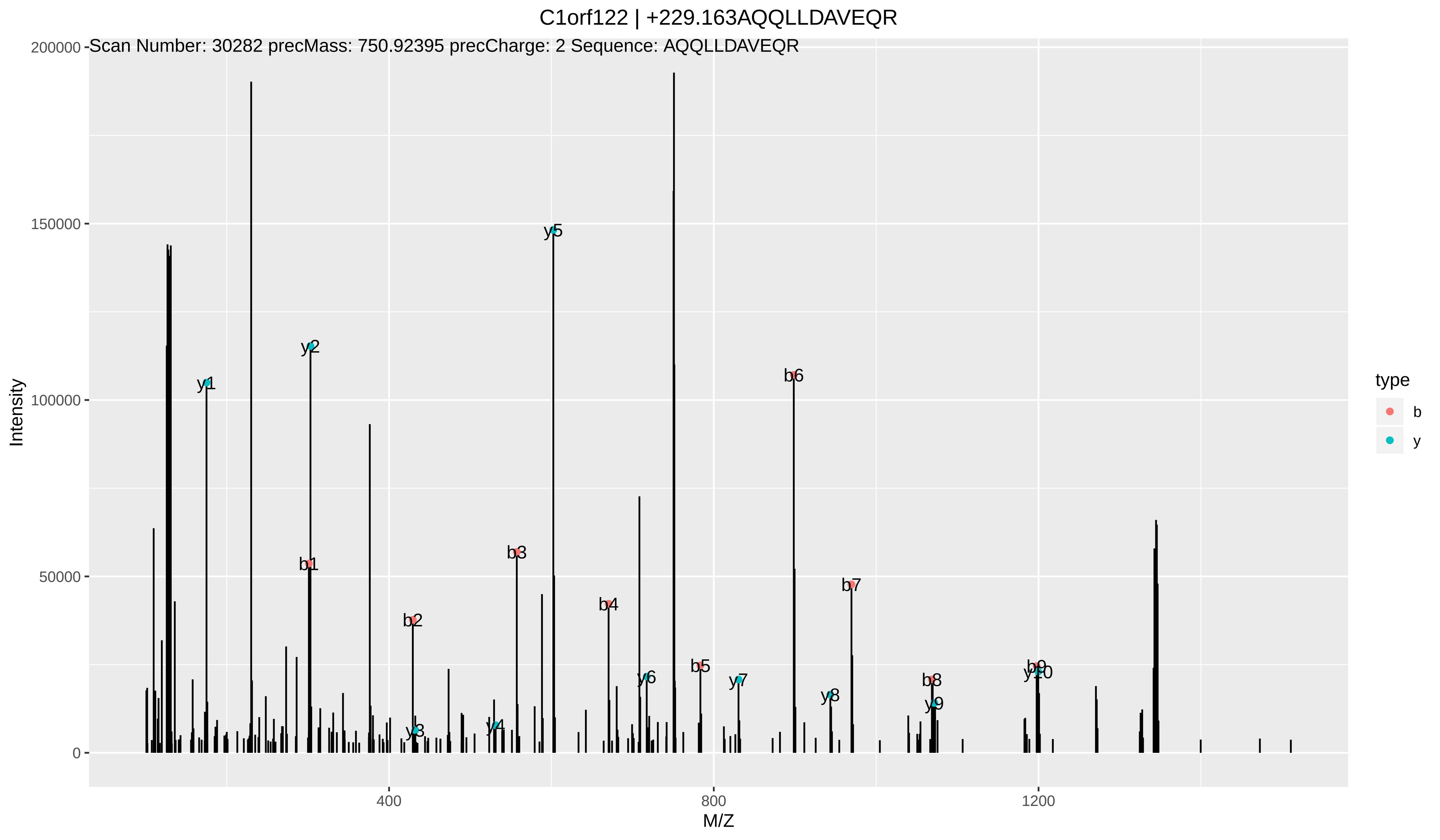

C1orf122 | +229.163AQQLLDAVEQR
Scan Number: 30282 precMass: 750.92395 precCharge: 2 Sequence: AQQLLDAVEQR
Intensity
M/Z
type
b
y
y1
y2
y3
y4
y5
y6
y7
y8
y9
y10
b1
b2
b3
b4
b5
b6
b7
b8
b9

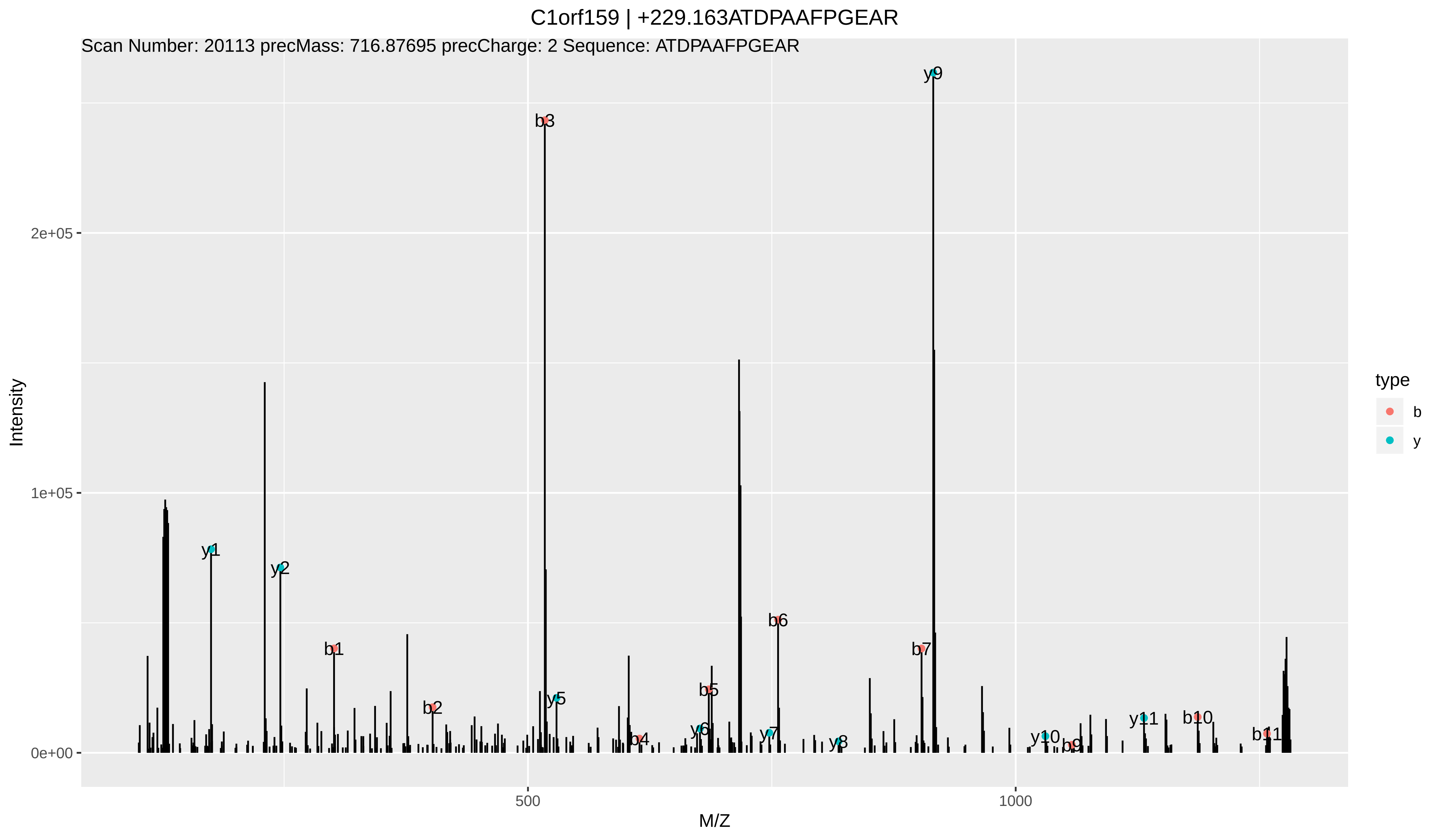

C1orf159 | +229.163ATDPAAFPGEAR
Scan Number: 20113 precMass: 716.87695 precCharge: 2 Sequence: ATDPAAFPGEAR
Intensity
0e+00
1e+05
2e+05
M/Z
500
1000
y9
b3
y1
y2
b1
b2
y5
b4
y6
b5
y7
b6
y8
b7
y10
b9
y11
b10
b11
type
b
y

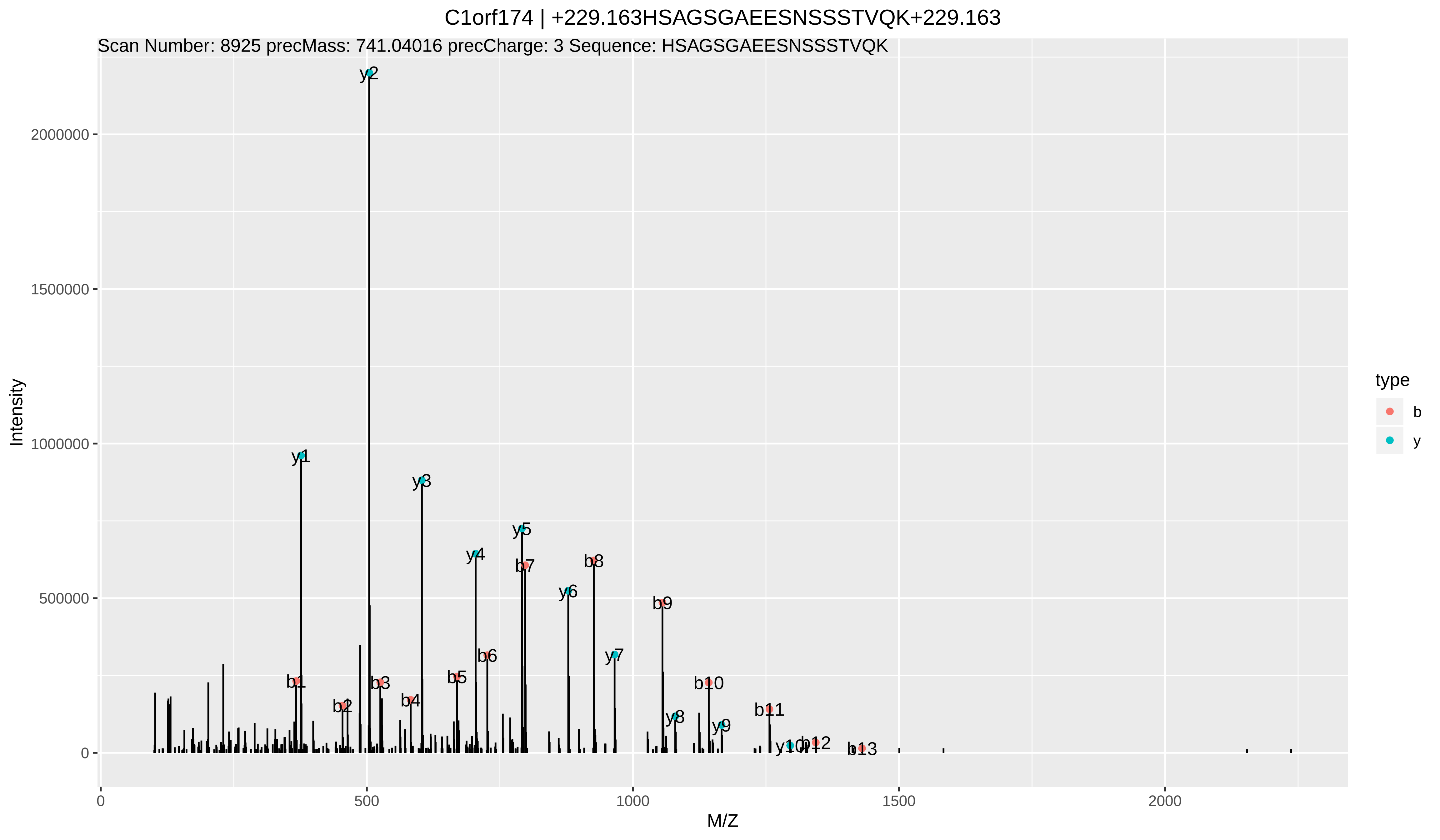

C1orf174 | +229.163HSAGSGAEESNSSSTVQK+229.163
Scan Number: 8925 precMass: 741.04016 precCharge: 3 Sequence: HSAGSGAEESNSSSTVQK
Intensity
M/Z
type
b
y
0
500000
1000000
1500000
2000000
500
1000
1500
2000
y1
y2
y3
y4
y5
y6
y7
y8
y9
y10
b1
b2
b3
b4
b5
b6
b7
b8
b9
b10
b11
b12
b13

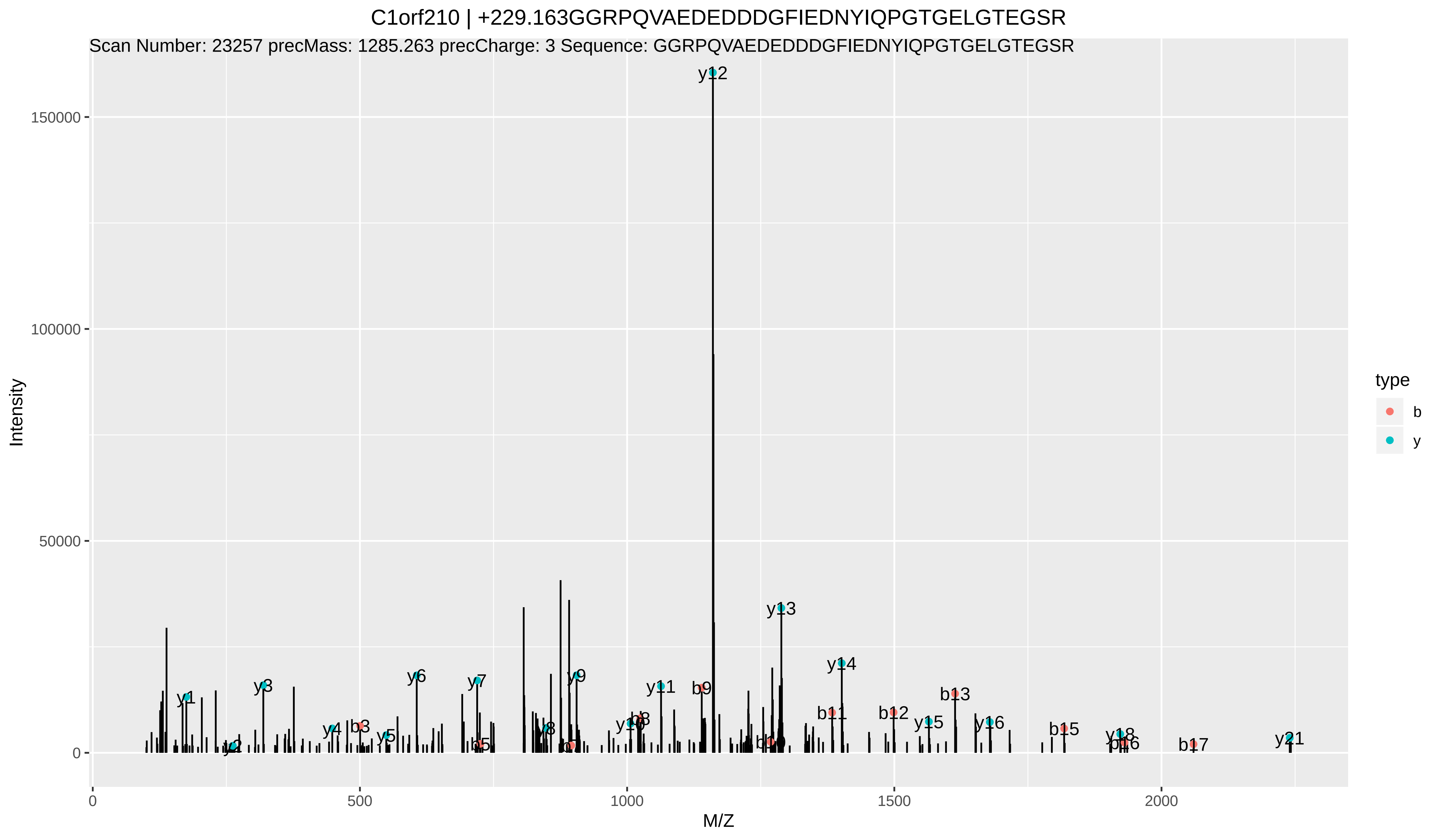

C1orf210 | +229.163GGRPQVAEDEDDDGFIEDNYIQPGTGELGTEGSR
Scan Number: 23257 precMass: 1285.263 precCharge: 3 Sequence: GGRPQVAEDEDDDGFIEDNYIQPGTGELGTEGSR
Intensity
M/Z
type
b
y
y1
y2
y3
y4
b3
y5
y6
y7
b5
y8
y9
y10
b8
y11
b9
y12
b10
y13
b11
y14
b12
y15
b13
y16
b15
y18
b16
b17
y21
0
50000
100000
150000
0
500
1000
1500
2000

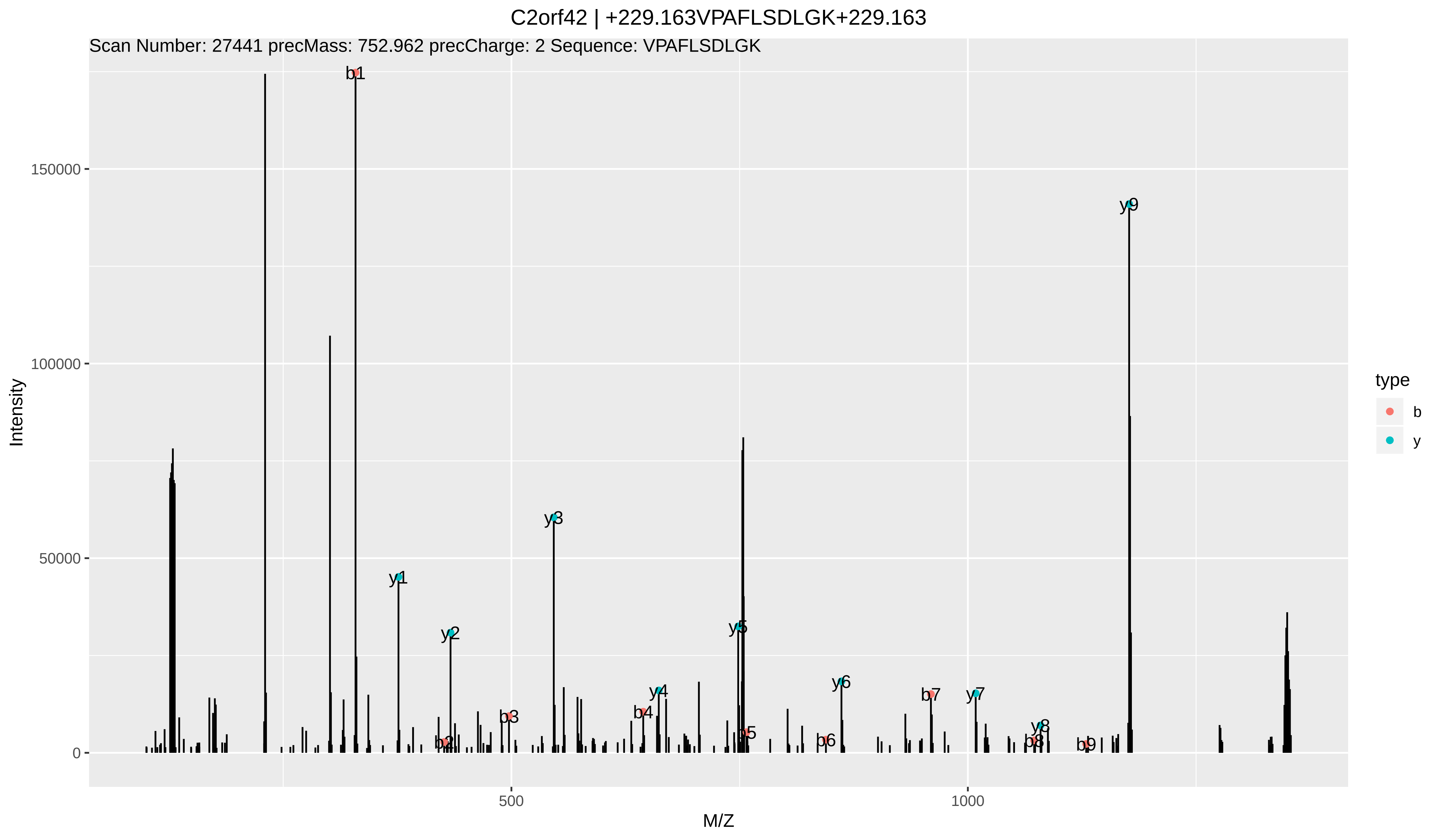

C2orf42 | +229.163VPAFLSDLGK+229.163
Scan Number: 27441 precMass: 752.962 precCharge: 2 Sequence: VPAFLSDLGK
Intensity
M/Z
150000
100000
50000
0
500
1000
b1
y1
y2
b2
b3
y3
b4
y4
y5
b5
b6
y6
b7
y7
y8
b8
b9
y9
type
b
y

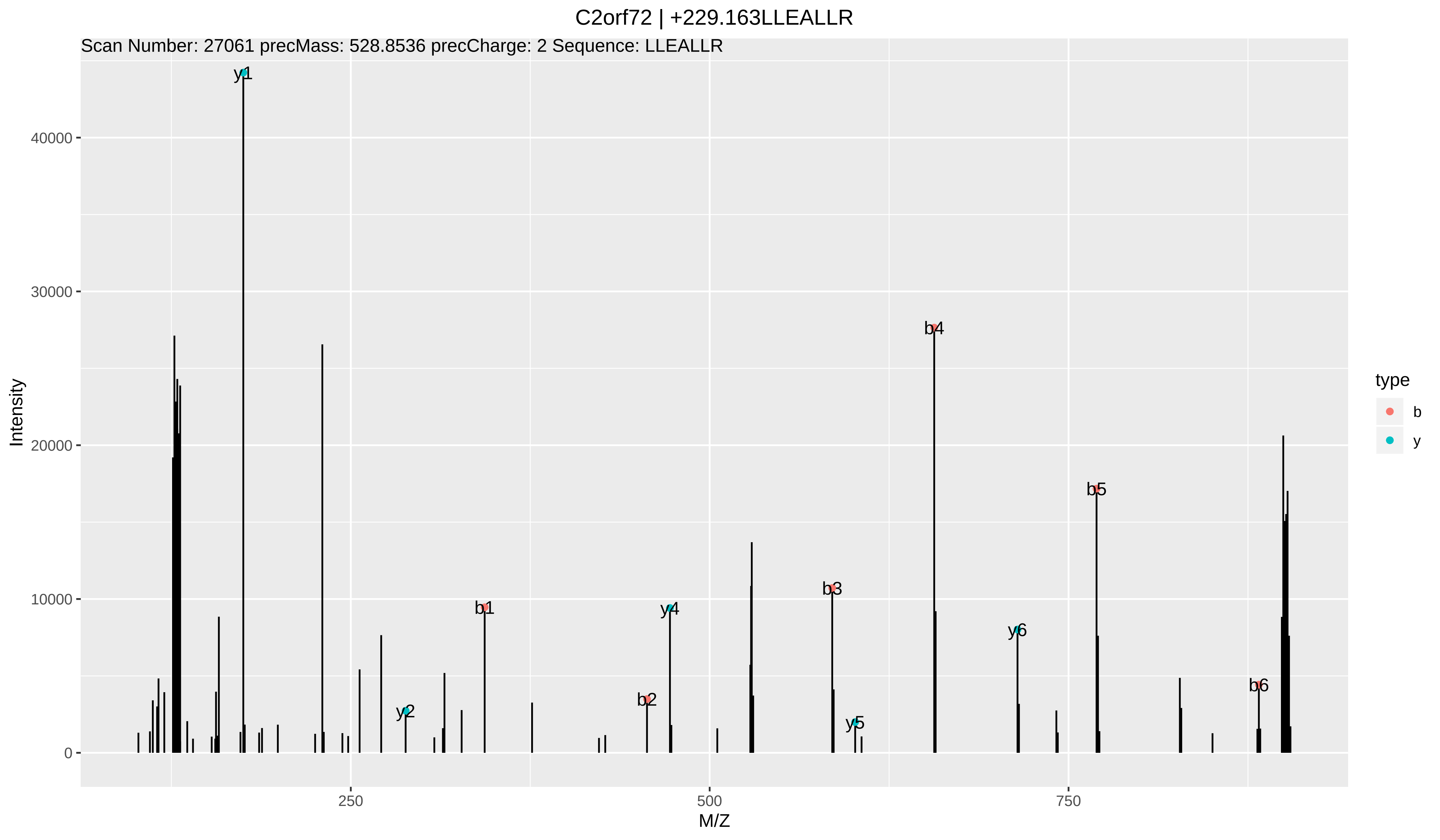
C2orf72 | +229.163LLEALLR
Scan Number: 27061 precMass: 528.8536 precCharge: 2 Sequence: LLEALLR
Intensity
0
10000
20000
30000
40000
M/Z
250
500
750
y1
y2
y4
y5
y6
b1
b2
b3
b4
b5
b6
type
b
y

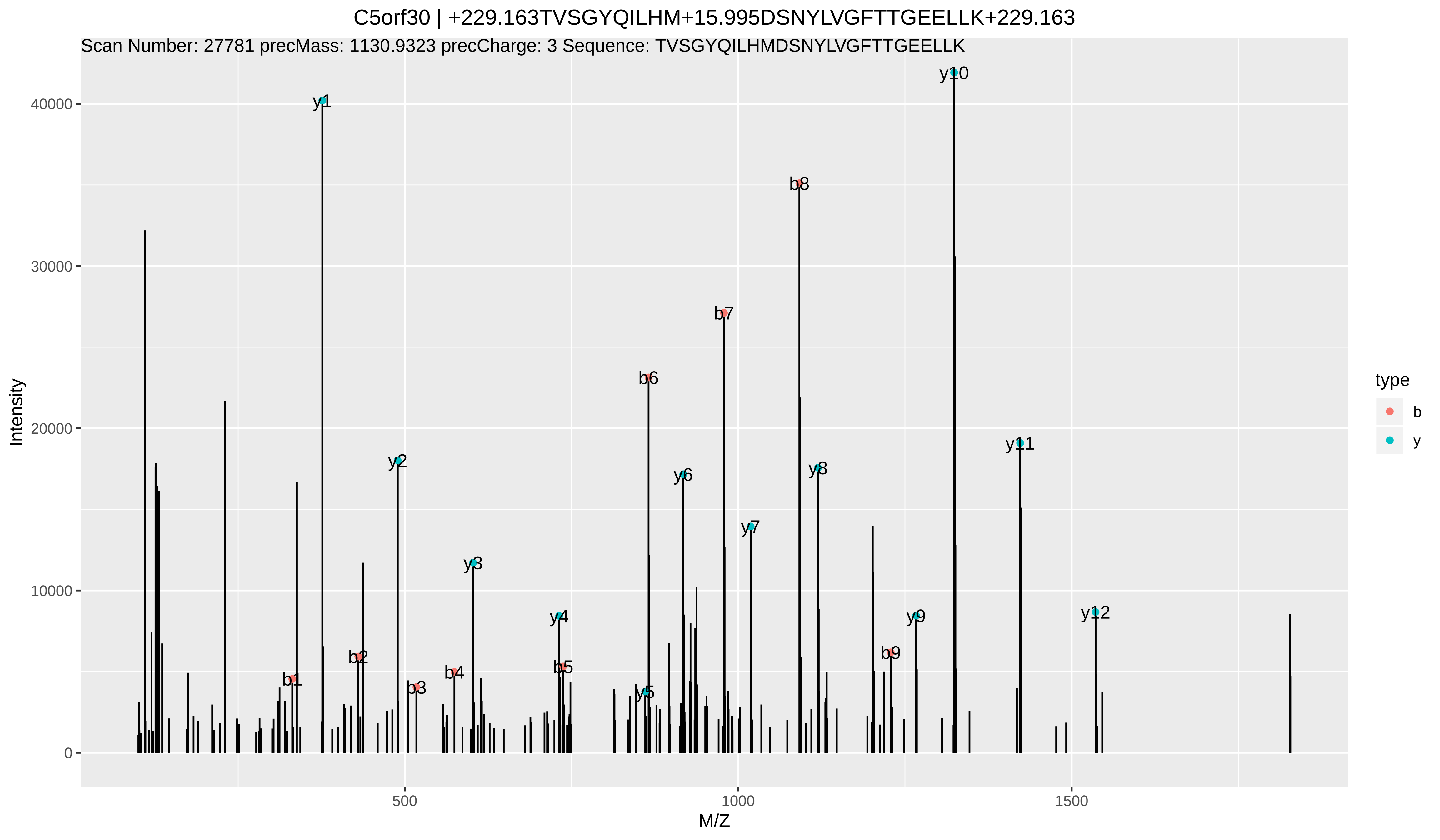
C5orf30 | +229.163TVSGYQILHM+15.995DSNYLVGFTTGEELLK+229.163
Scan Number: 27781 precMass: 1130.9323 precCharge: 3 Sequence: TVSGYQILHMDSNYLVGFTTGEELLK
Intensity
M/Z
0
10000
20000
30000
40000
500
1000
1500
y1
y2
y3
y4
y5
y6
y7
y8
y9
y10
y11
y12
b1
b2
b3
b4
b5
b6
b7
b8
b9
type
b
y

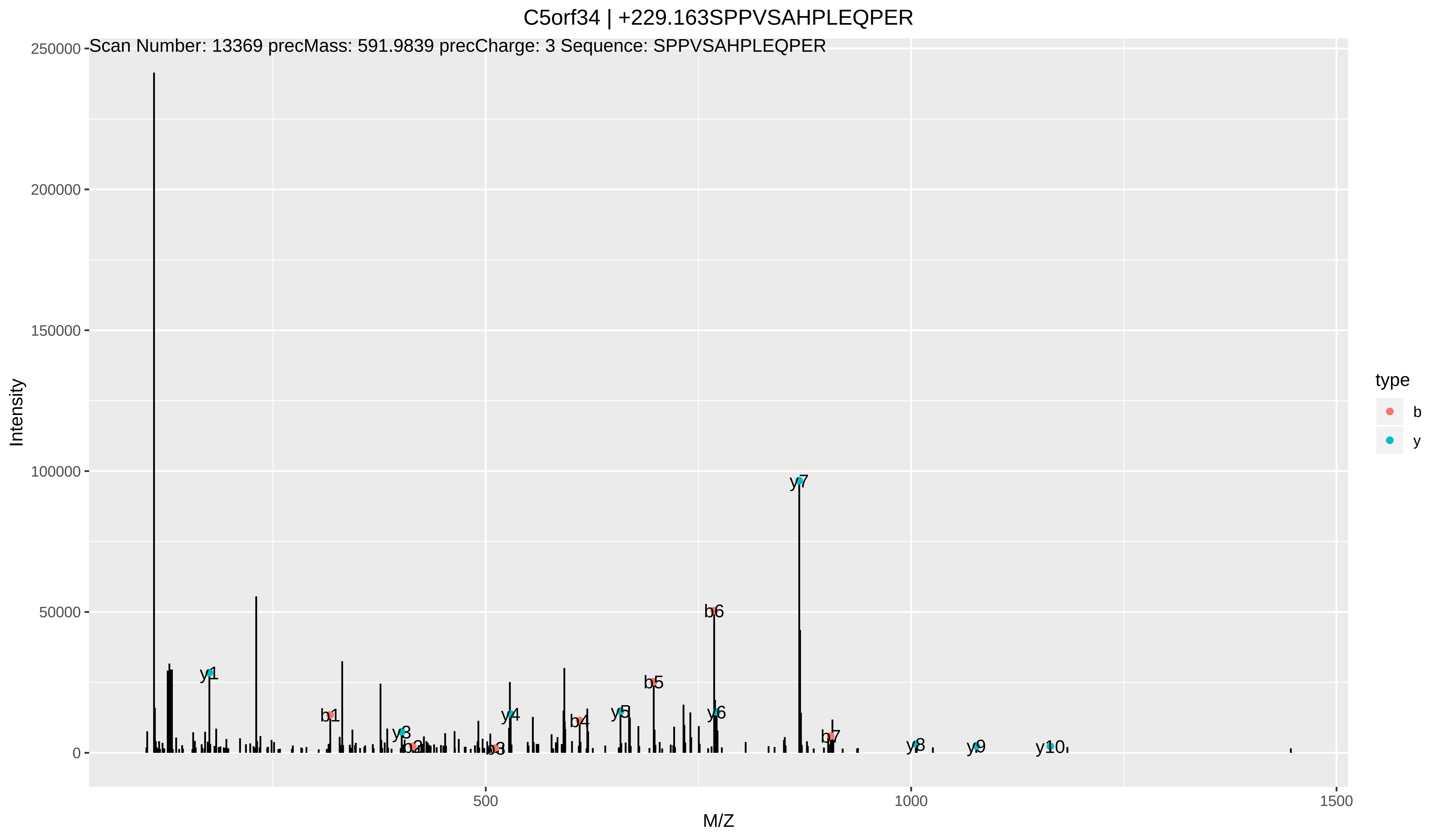

C5orf34 | +229.163SPPVSAHPLEQPER
Scan Number: 13369 precMass: 591.9839 precCharge: 3 Sequence: SPPVSAHPLEQPER
Intensity
M/Z
0
50000
100000
150000
200000
250000
500
1000
1500
type
b
y
y1
b1
y3
b2
b3
y4
b4
y5
b5
b6
y6
y7
b7
y8
y9
y10

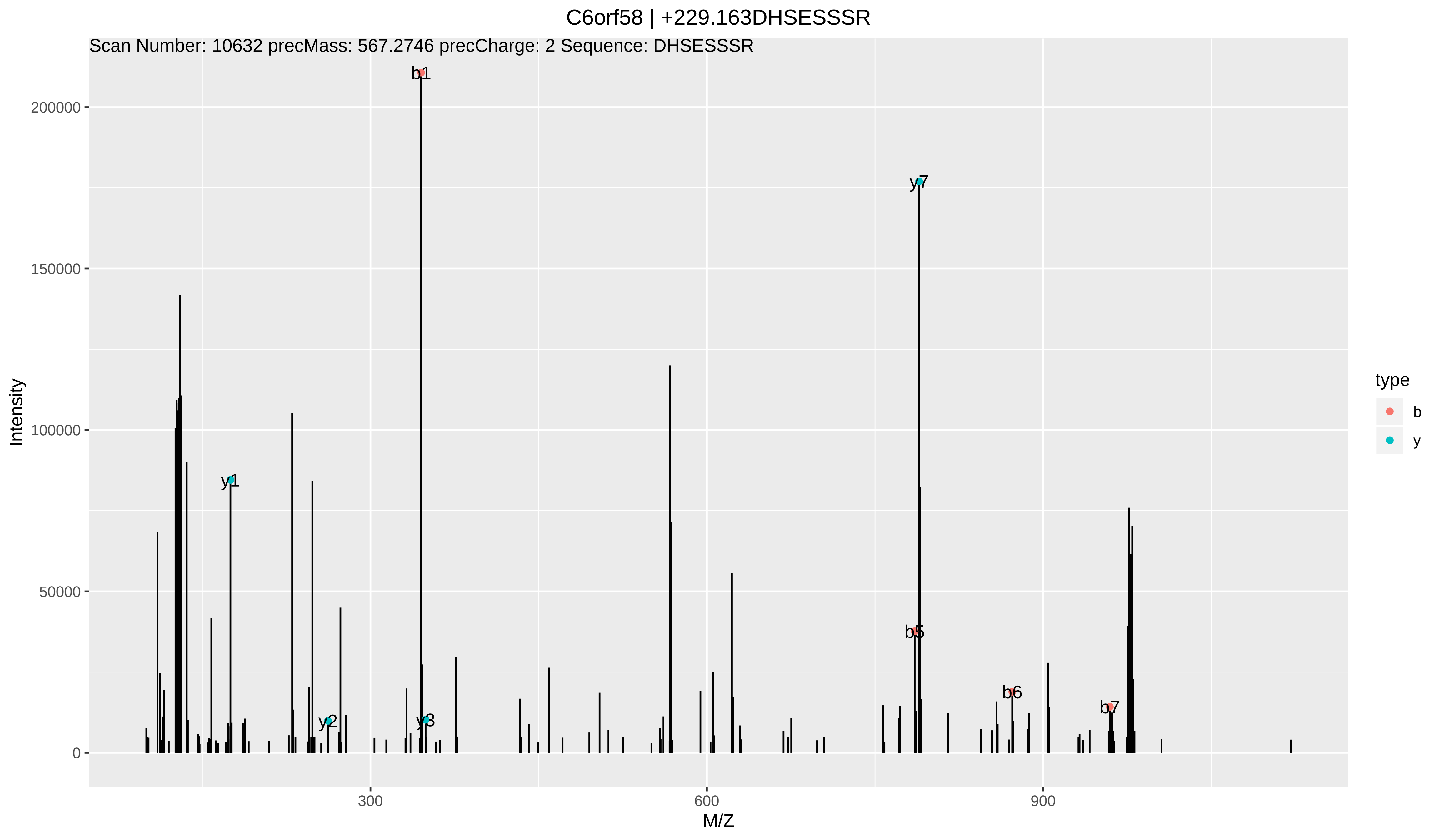
C6orf58 | +229.163DHSESSSR
Scan Number: 10632 precMass: 567.2746 precCharge: 2 Sequence: DHSESSSR
Intensity
M/Z
type
b
y
b1
y7
y1
b5
b6
b7
y2
y3
0
50000
100000
150000
200000
300
600
900

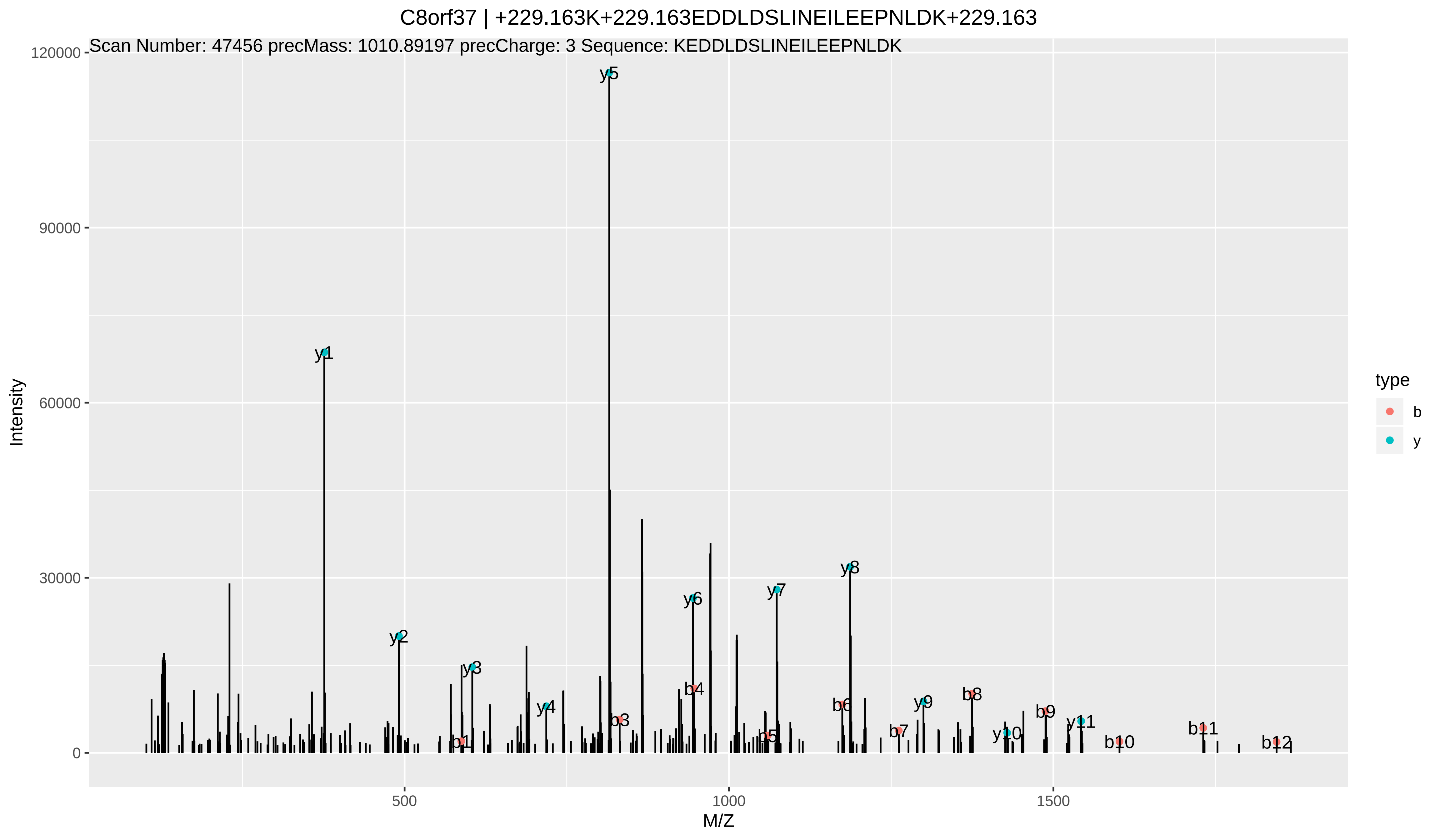
C8orf37 | +229.163K+229.163EDDLDSLINEILEEPNLDK+229.163
Scan Number: 47456 precMass: 1010.89197 precCharge: 3 Sequence: KEDDLDSLINEILEEPNLDK
Intensity
M/Z
type
b
y
y5
y1
y2
y3
y4
y6
y7
y8
y9
y10
y11
b1
b3
b4
b5
b6
b7
b8
b9
b10
b11
b12
0
30000
60000
90000
120000
500
1000
1500

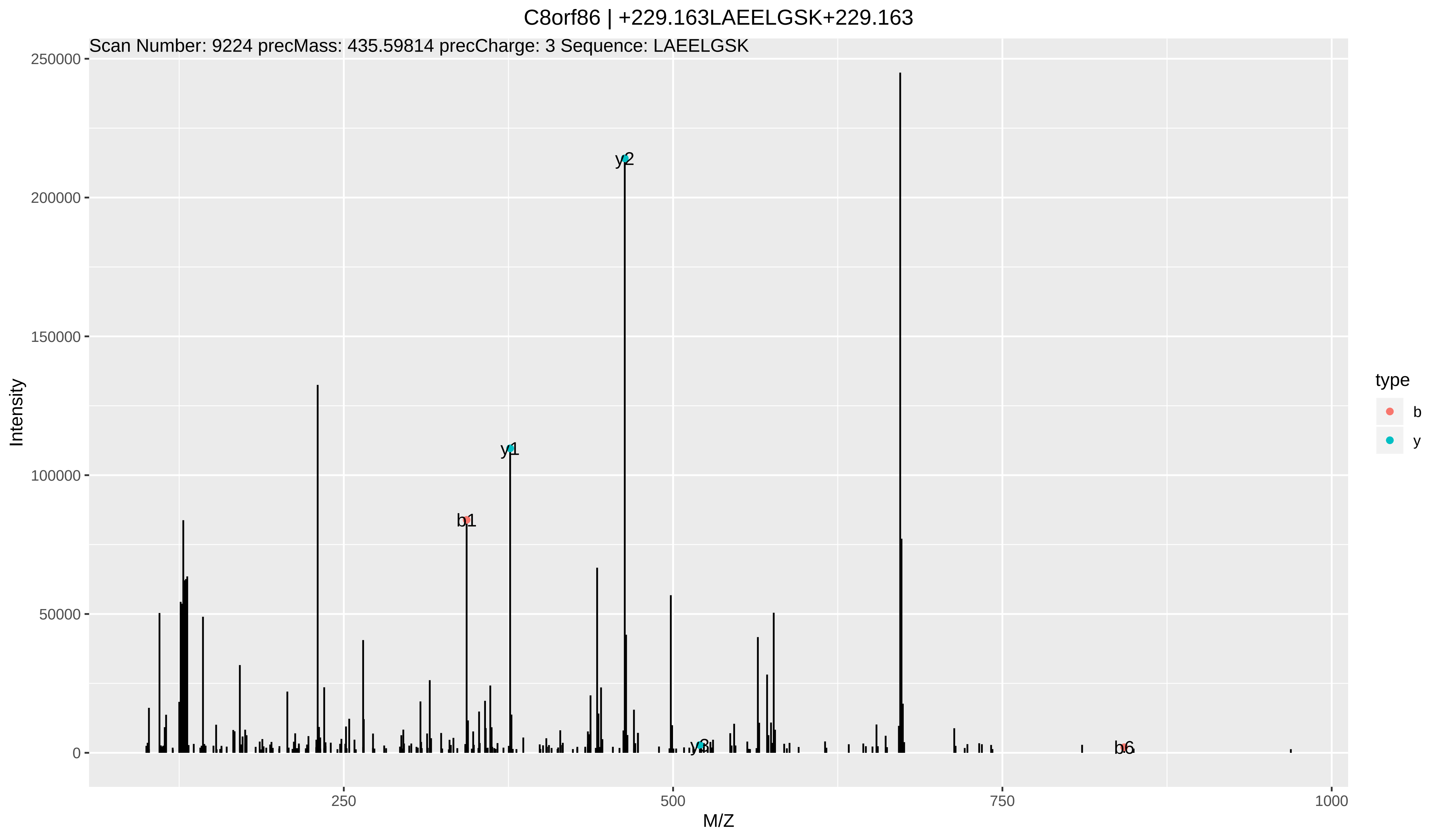

C8orf86 | +229.163LAEELGSK+229.163
Scan Number: 9224 precMass: 435.59814 precCharge: 3 Sequence: LAEELGSK
Intensity
M/Z
type
b
y
y2
y1
b1
y3
b6
250000
200000
150000
100000
50000
0
250
500
750
1000

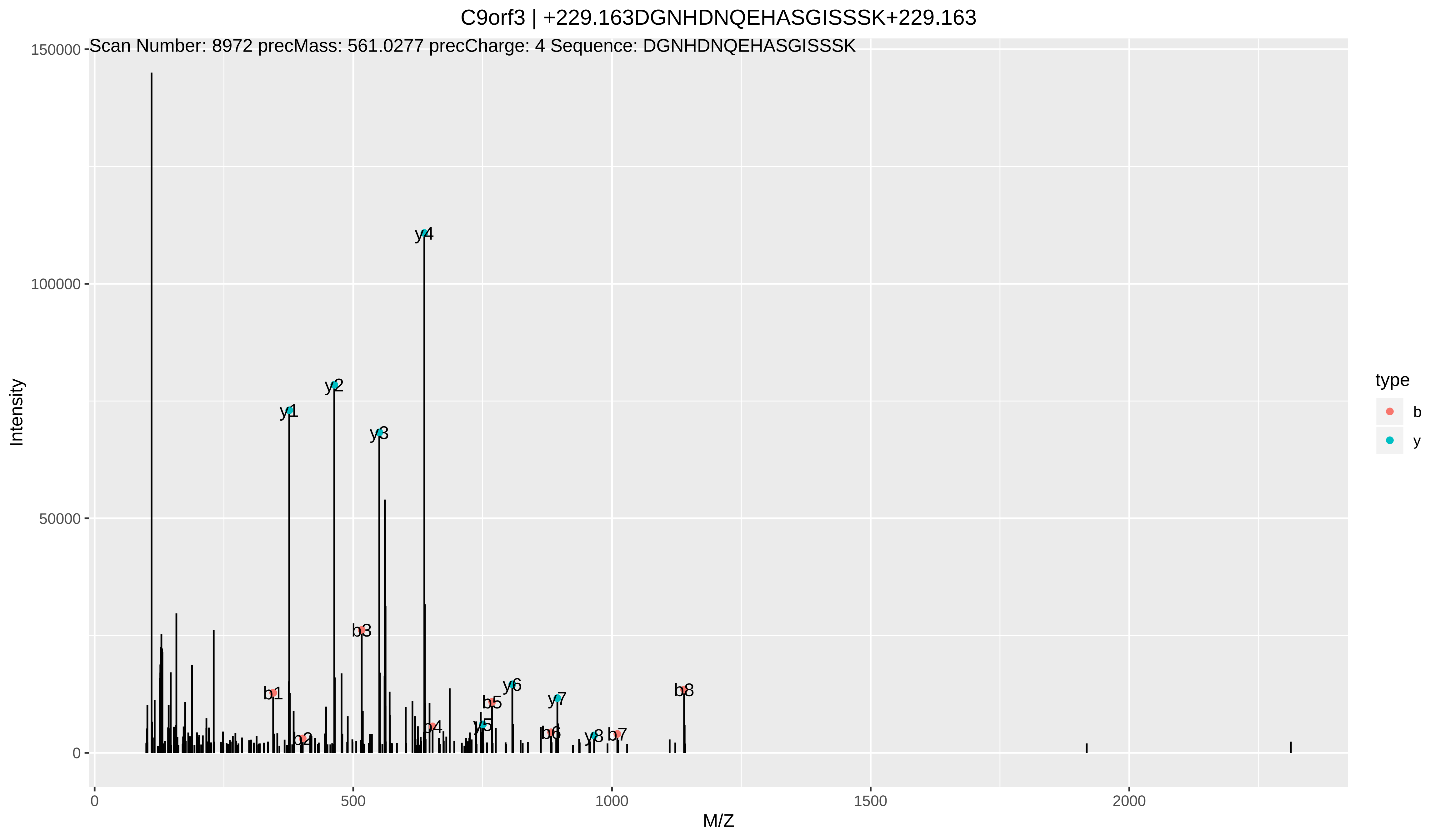

C9orf3 | +229.163DGNHDNQEHASGISSSK+229.163
Scan Number: 8972 precMass: 561.0277 precCharge: 4 Sequence: DGNHDNQEHASGISSSK
Intensity
M/Z
type
b
y
y1
y2
y3
y4
y5
y6
y7
y8
b1
b2
b3
b4
b5
b6
b7
b8

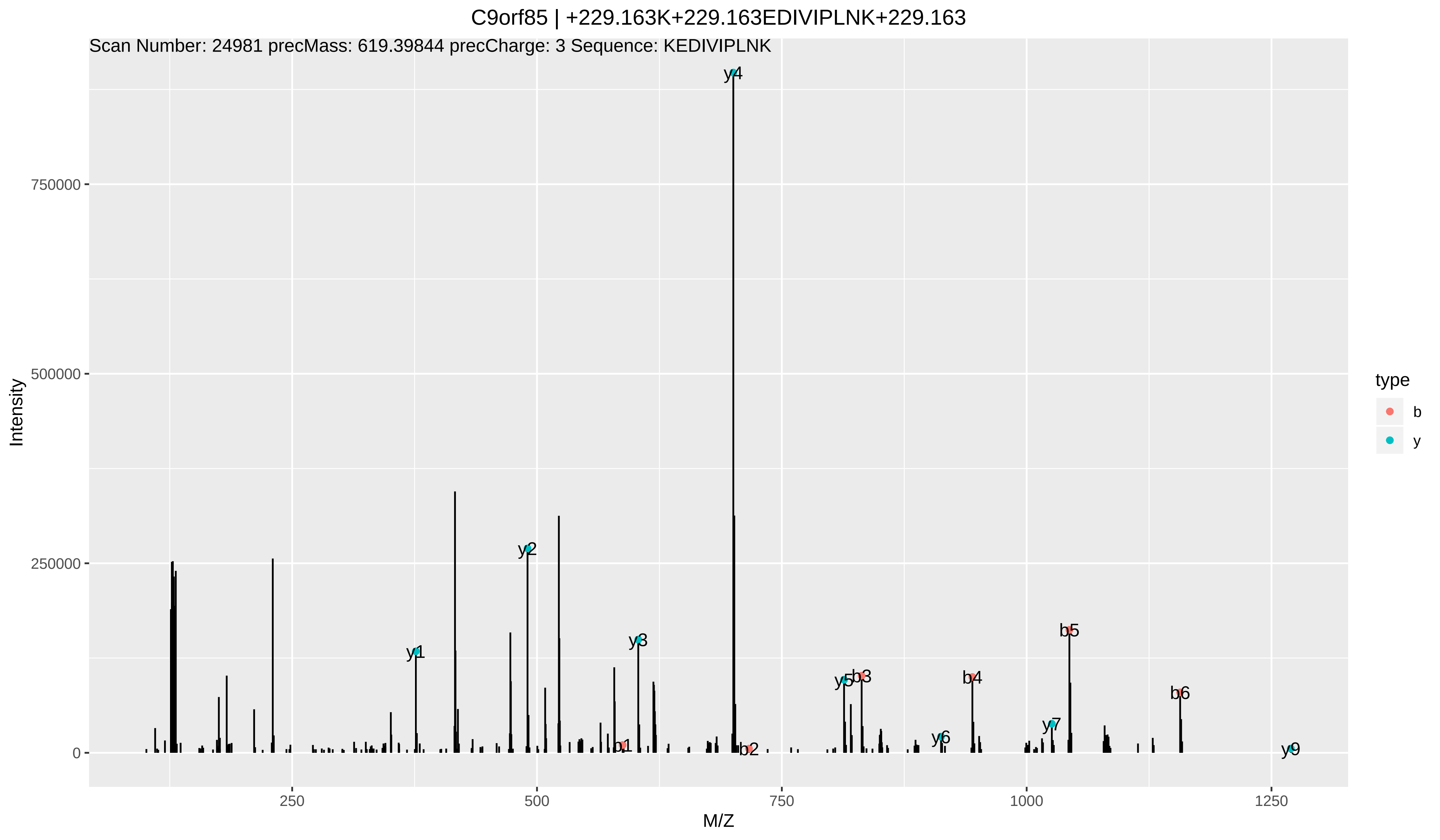

C9orf85 | +229.163K+229.163EDIVIPLNK+229.163
Scan Number: 24981 precMass: 619.39844 precCharge: 3 Sequence: KEDIVIPLNK
Intensity
750000
500000
250000
0
M/Z
250
500
750
1000
1250
y4
y2
y1
y3
b1
b2
y5
b3
b4
y6
y7
b5
b6
y9
type
b
y

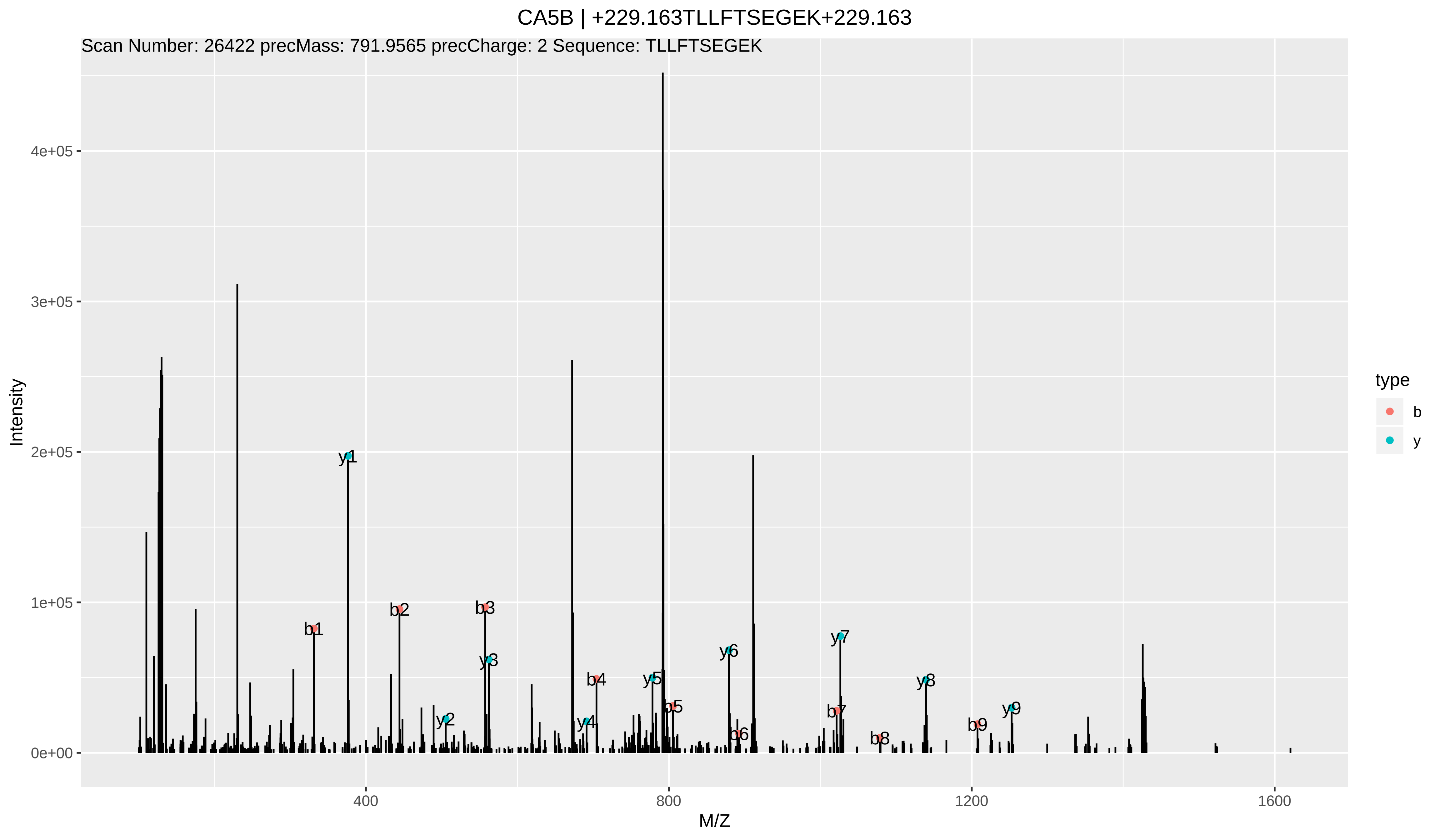

CA5B | +229.163TLLFTSEGEK+229.163
Scan Number: 26422 precMass: 791.9565 precCharge: 2 Sequence: TLLFTSEGEK
Intensity
0e+00
1e+05
2e+05
3e+05
4e+05
M/Z
400
800
1200
1600
y1
b1
b2
b3
y3
y2
y4
b4
y5
b5
y6
b6
y7
b7
b8
y8
b9
y9
type
b
y

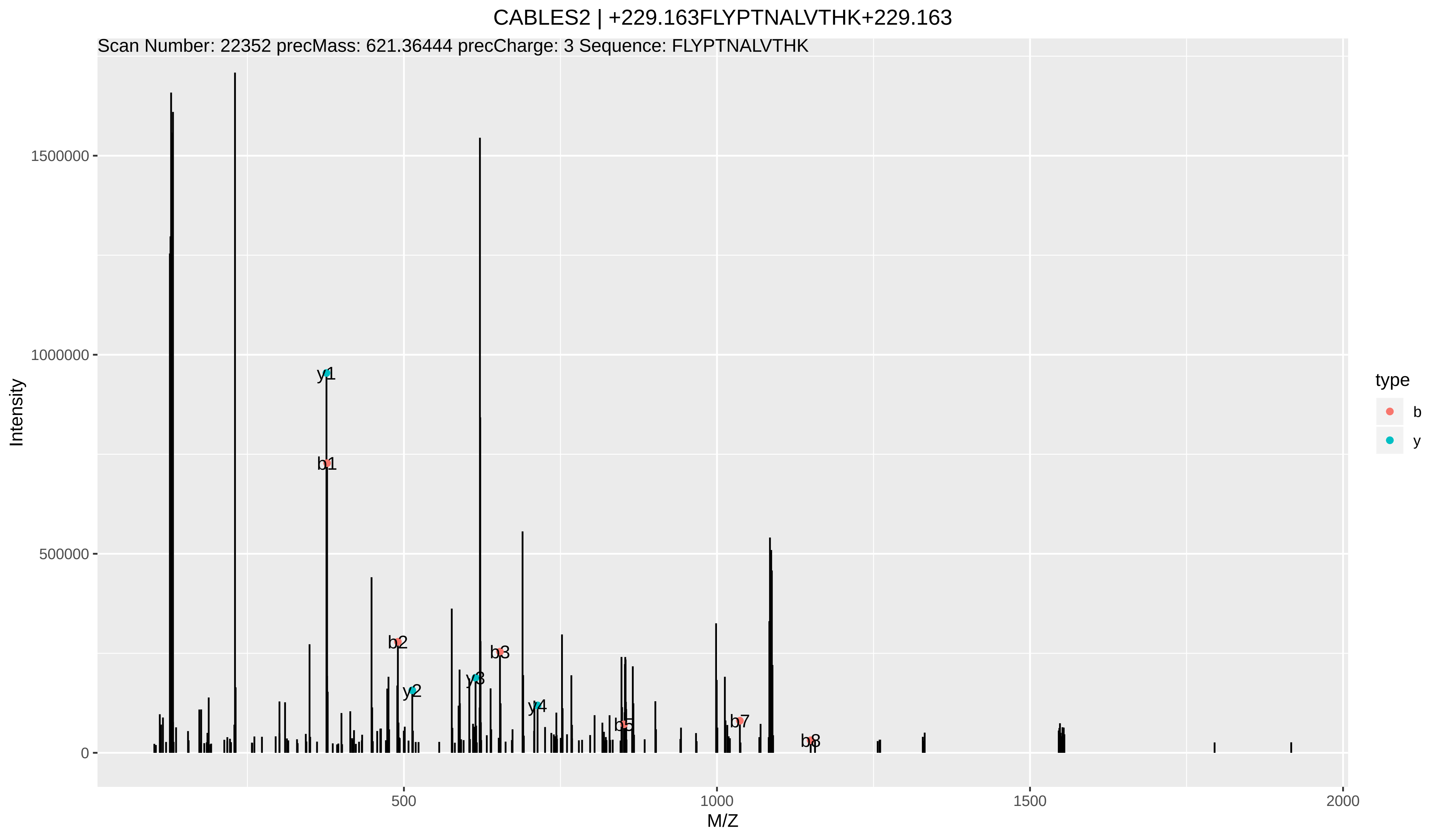

CABLES2 | +229.163FLYPTNALVTHK+229.163
Scan Number: 22352 precMass: 621.36444 precCharge: 3 Sequence: FLYPTNALVTHK
Intensity
M/Z
type
b
y
y1
b1
b2
y2
y3
b3
y4
b5
b7
b8
0
500000
1000000
1500000
500
1000
1500
2000

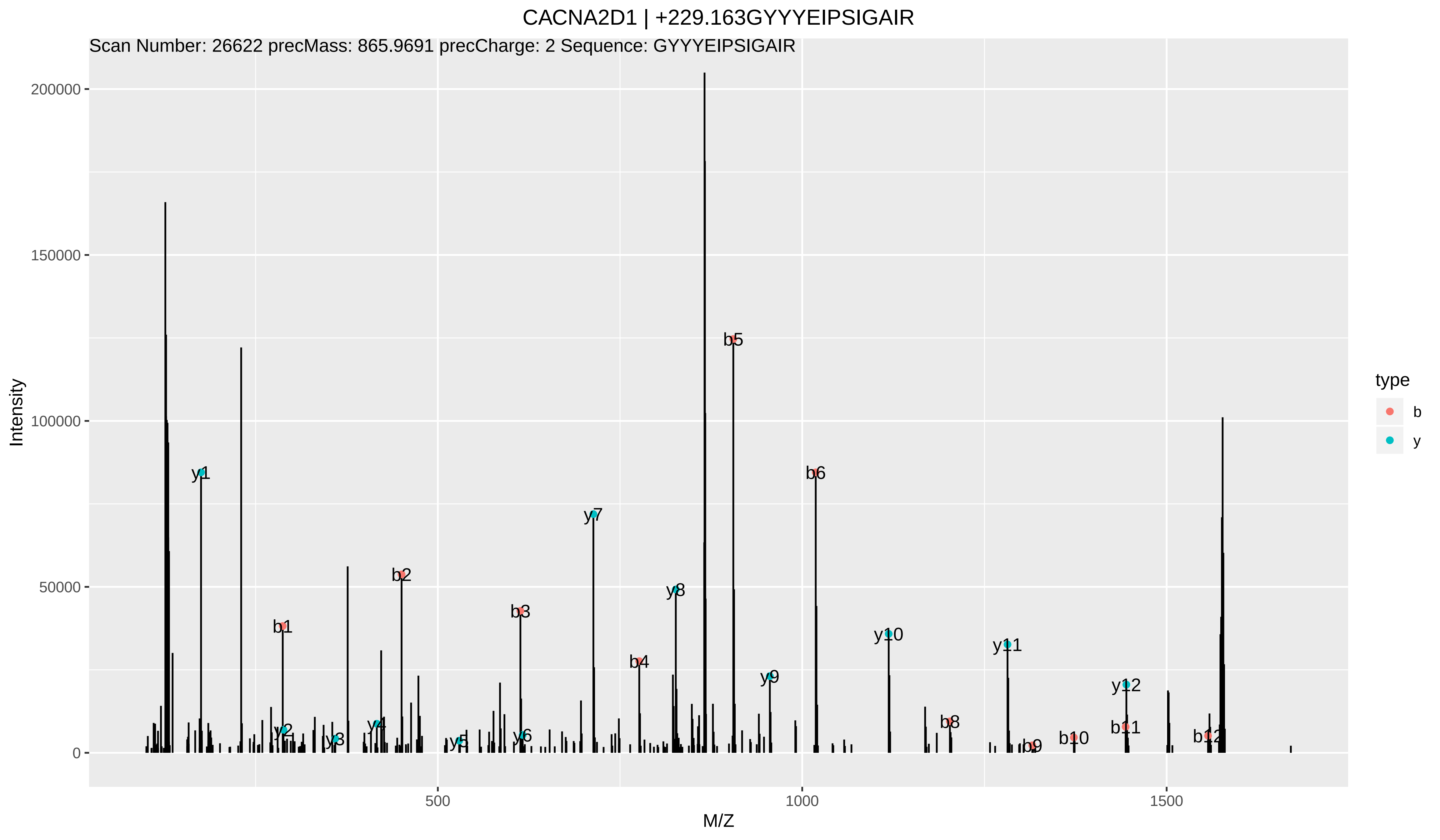

CACNA2D1 | +229.163GYYYEIPSIGAIR
Scan Number: 26622 precMass: 865.9691 precCharge: 2 Sequence: GYYYEIPSIGAIR
Intensity
M/Z
0
50000
100000
150000
200000
500
1000
1500
type
b
y
y1
y2
y3
y4
y5
y6
y7
y8
y9
y10
y11
y12
b1
b2
b3
b4
b5
b6
b8
b9
b10
b11
b12

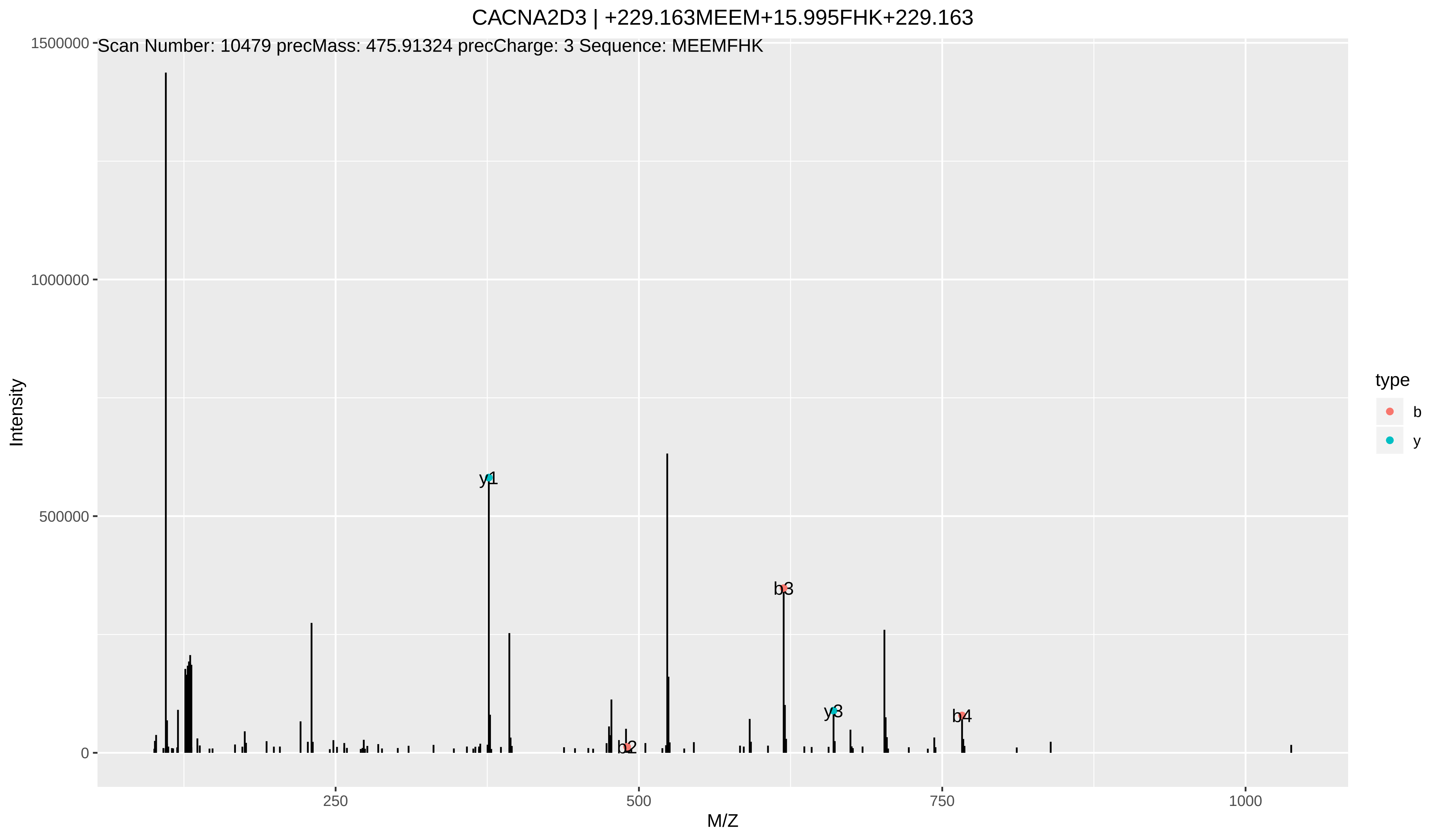

CACNA2D3 | +229.163MEEM+15.995FHK+229.163
Scan Number: 10479 precMass: 475.91324 precCharge: 3 Sequence: MEEMFHK
1500000
1000000
500000
0
Intensity
250
500
750
1000
M/Z
y1
b2
b3
y3
b4
type
b
y

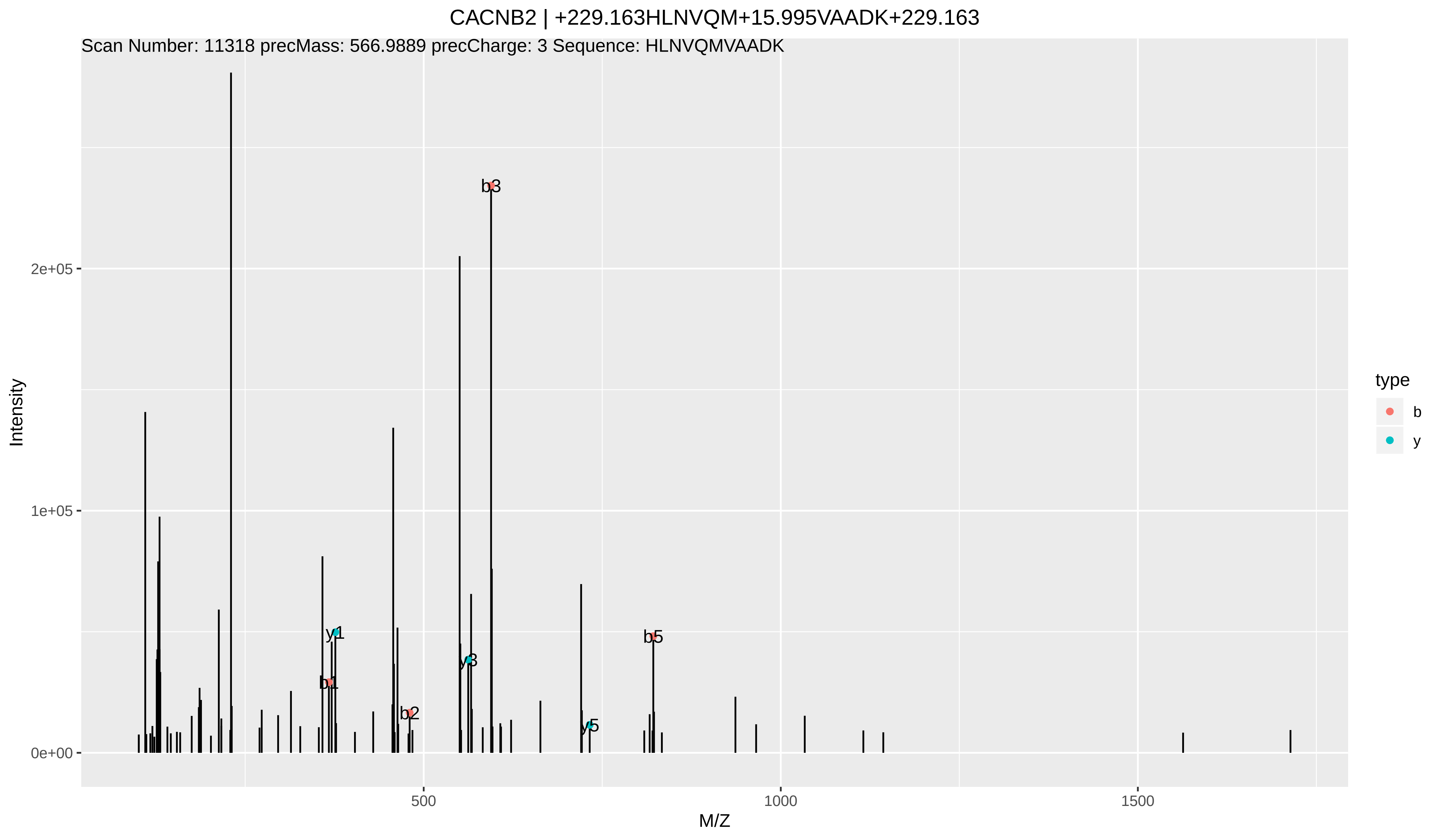

CACNB2 | +229.163HLNVQM+15.995VAADK+229.163
Scan Number: 11318 precMass: 566.9889 precCharge: 3 Sequence: HLNVQMVAADK
Intensity
2e+05
1e+05
0e+00
M/Z
500
1000
1500
b3
y1
b1
b2
y3
y5
b5
type
b
y

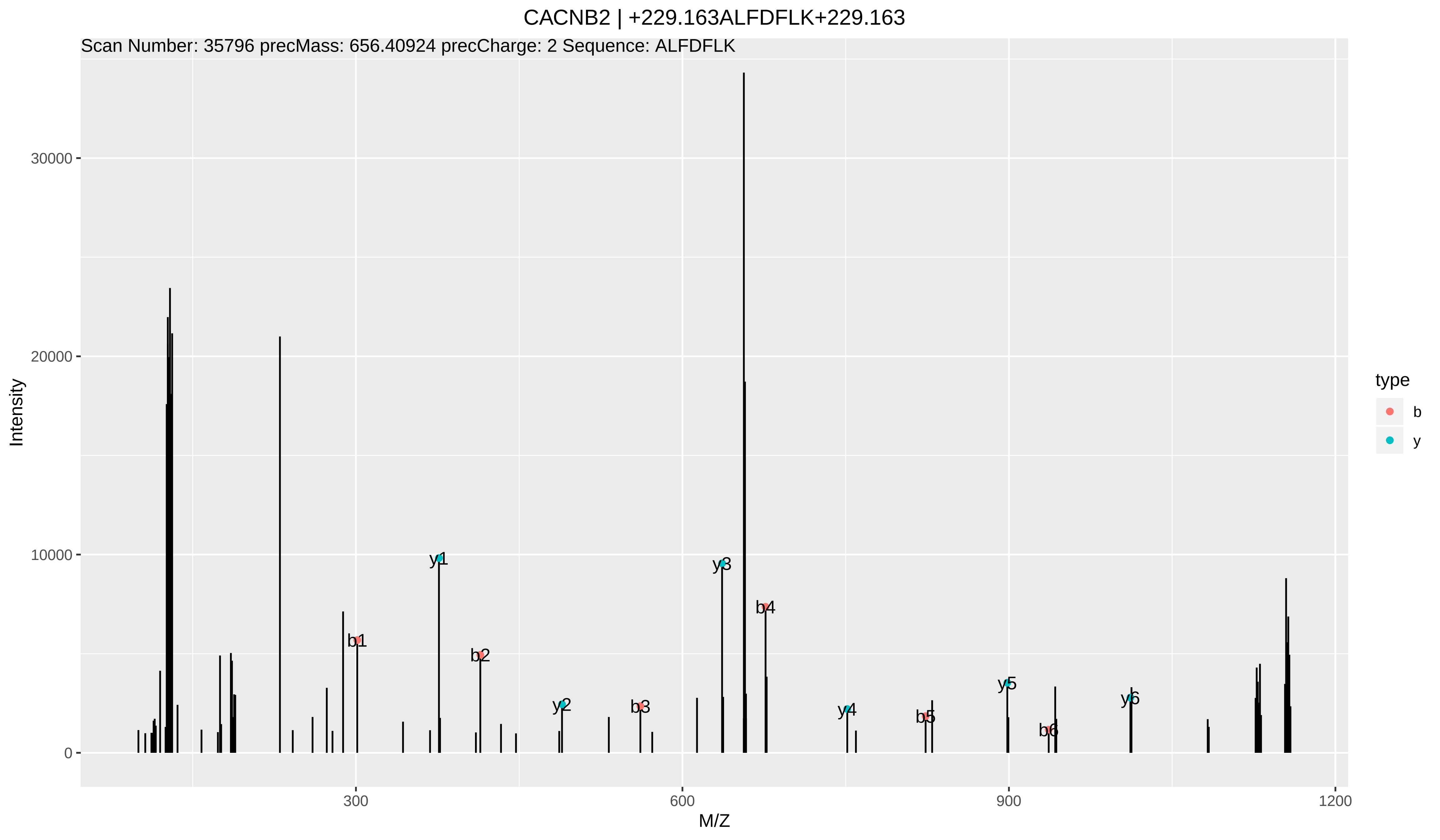

CACNB2 | +229.163ALFDFLK+229.163
Scan Number: 35796 precMass: 656.40924 precCharge: 2 Sequence: ALFDFLK
Intensity
30000
20000
10000
0
M/Z
300
600
900
1200
y1
b1
b2
y2
b3
y3
b4
y4
b5
y5
b6
y6
type
b
y

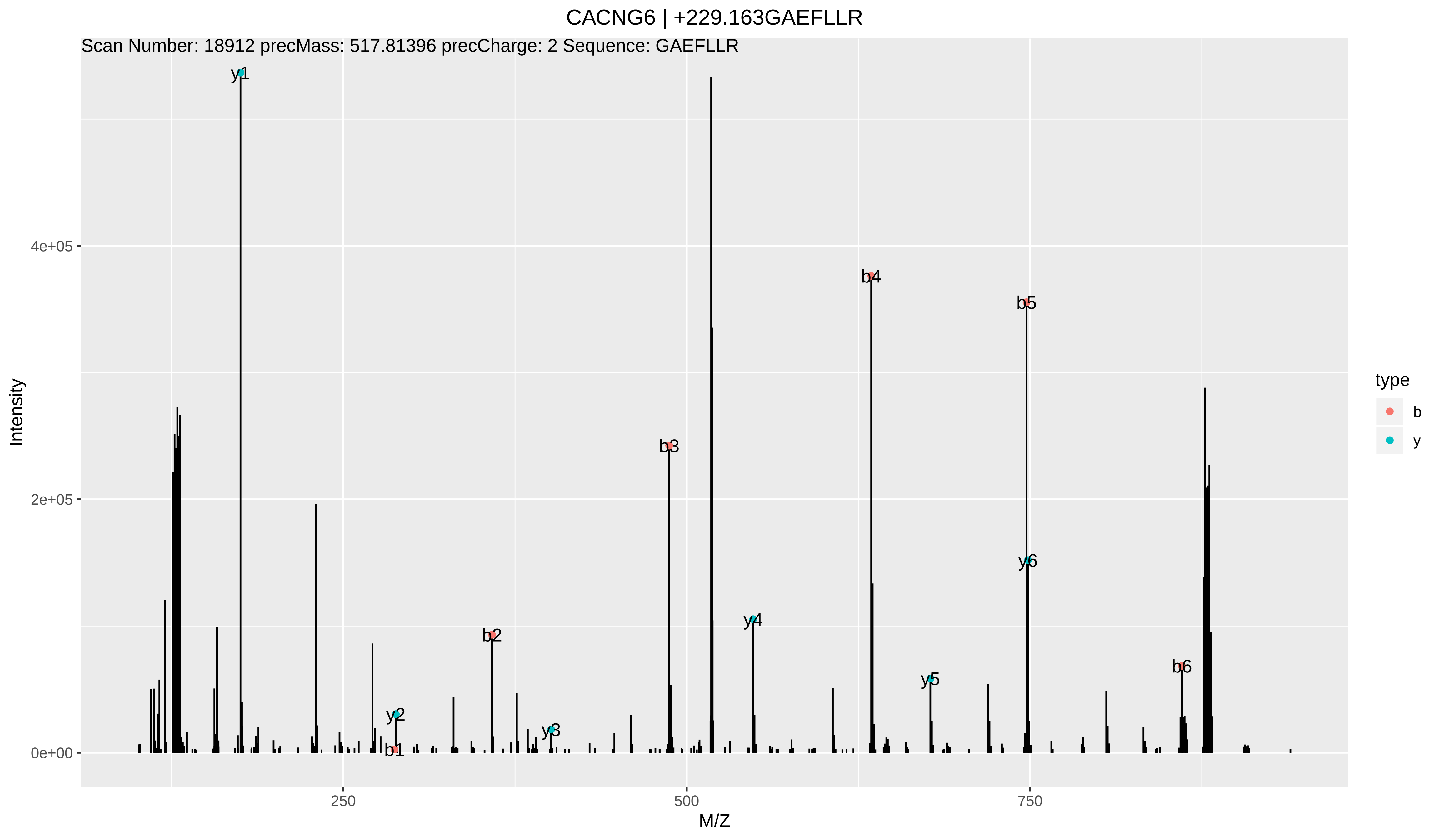
CACNG6 | +229.163GAEFLLR
Scan Number: 18912 precMass: 517.81396 precCharge: 2 Sequence: GAEFLLR
Intensity
M/Z
0e+00
2e+05
4e+05
250
500
750
y1
b1
y2
b2
y3
b3
y4
b4
y5
b5
y6
b6
type
b
y

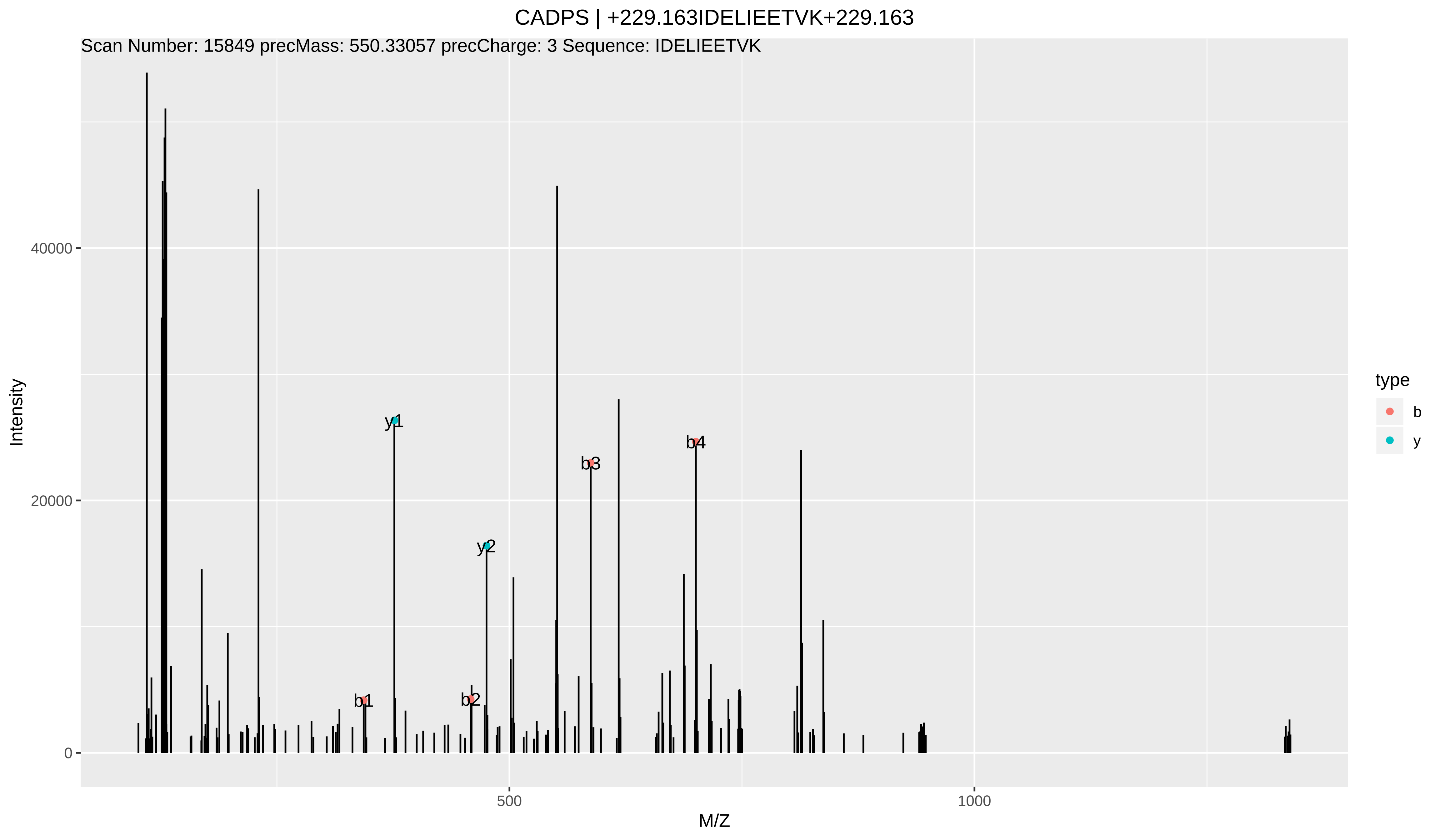

CADPS | +229.163IDELIEETVK+229.163
Scan Number: 15849 precMass: 550.33057 precCharge: 3 Sequence: IDELIEETVK
Intensity
40000
20000
0
500
1000
M/Z
y1
y2
b1
b2
b3
b4
type
b
y

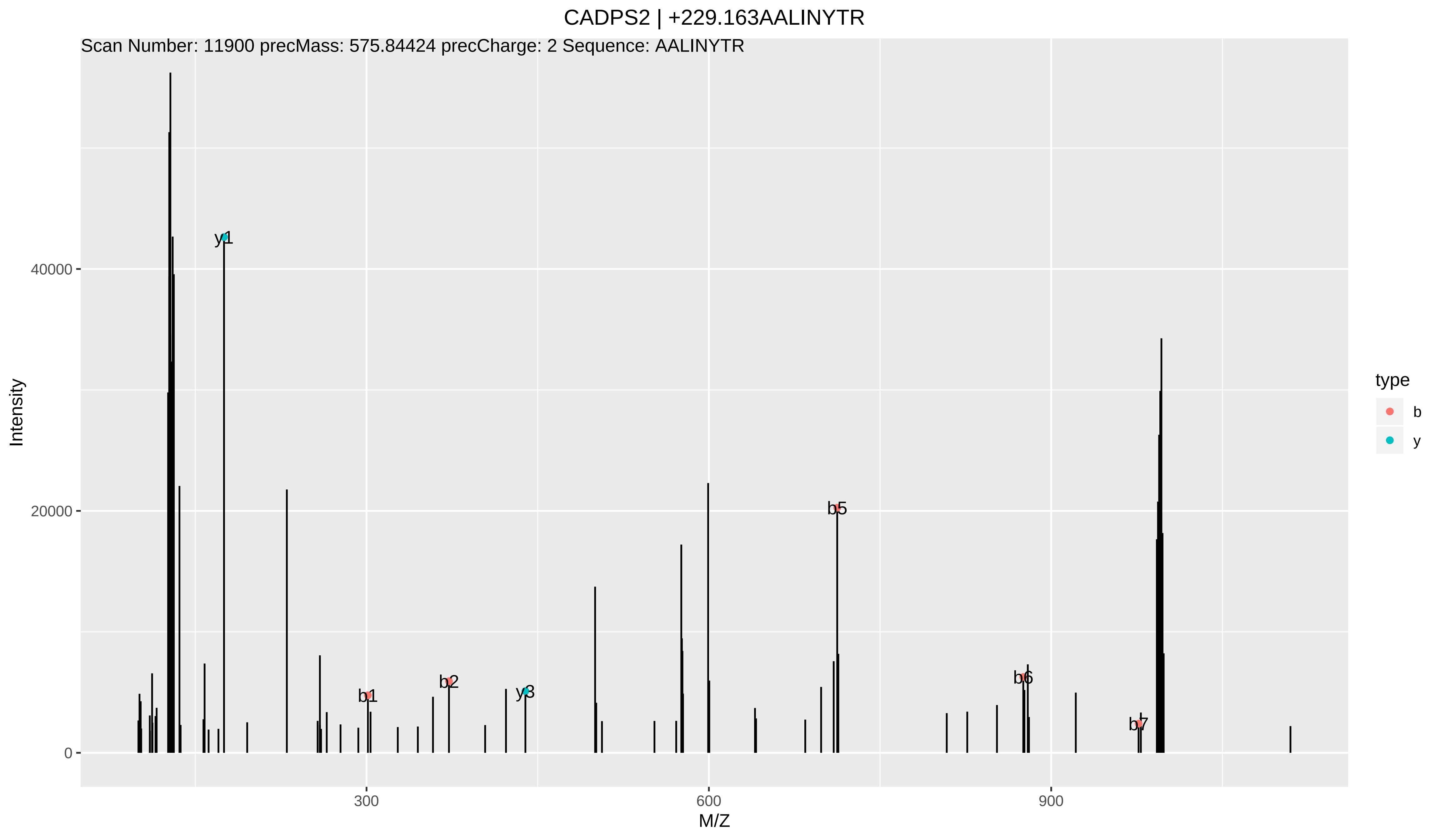

CADPS2 | +229.163AALINYTR
Scan Number: 11900 precMass: 575.84424 precCharge: 2 Sequence: AALINYTR
Intensity
M/Z
0
20000
40000
300
600
900
y1
b1
b2
y3
b5
b6
b7
type
b
y

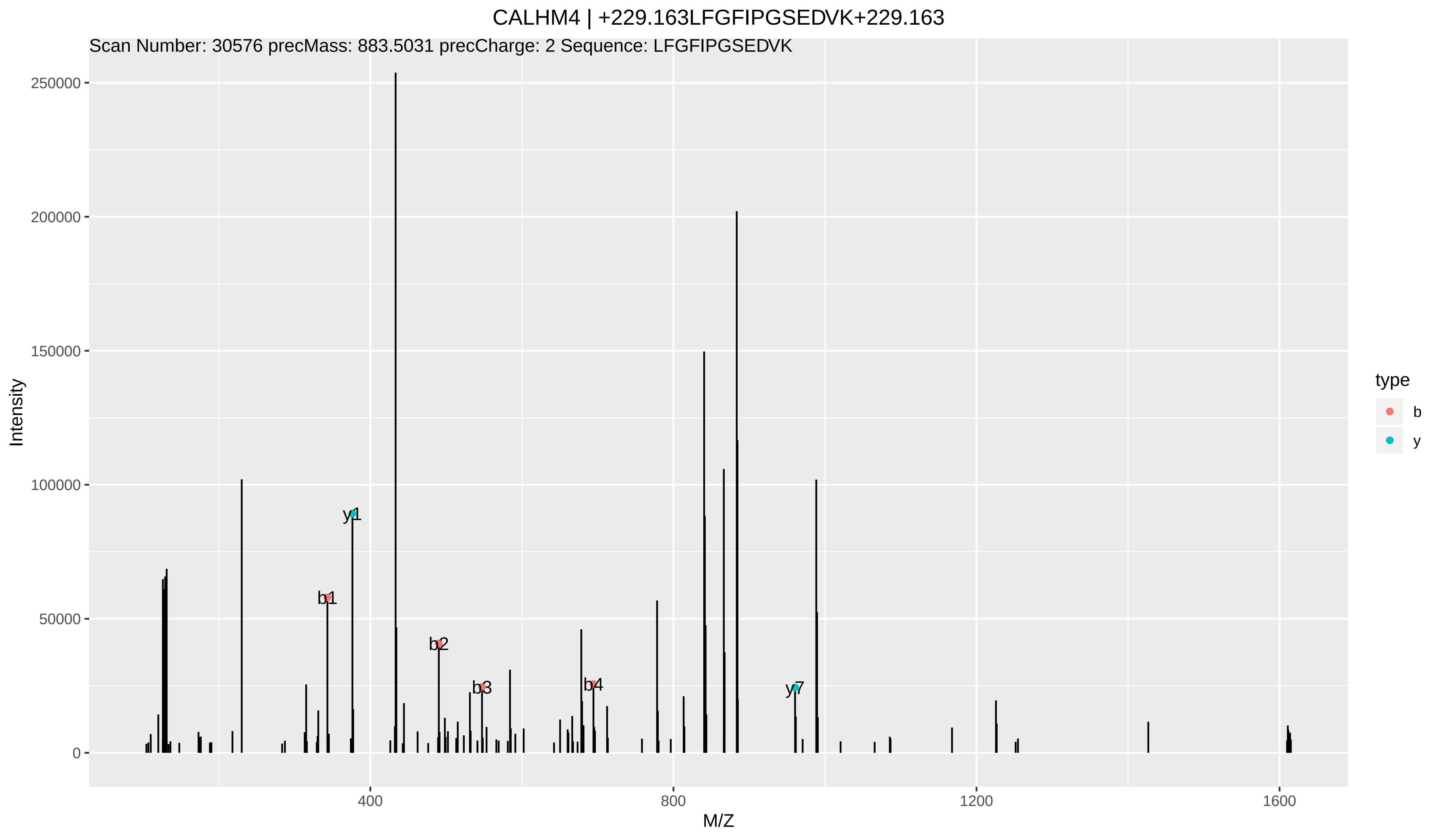

CALHM4 | +229.163LFGFIPGSEDVK+229.163
Scan Number: 30576 precMass: 883.5031 precCharge: 2 Sequence: LFGFIPGSEDVK
Intensity
250000
200000
150000
100000
50000
0
M/Z
400
800
1200
1600
y1
b1
b2
b3
b4
y7
type
b
y

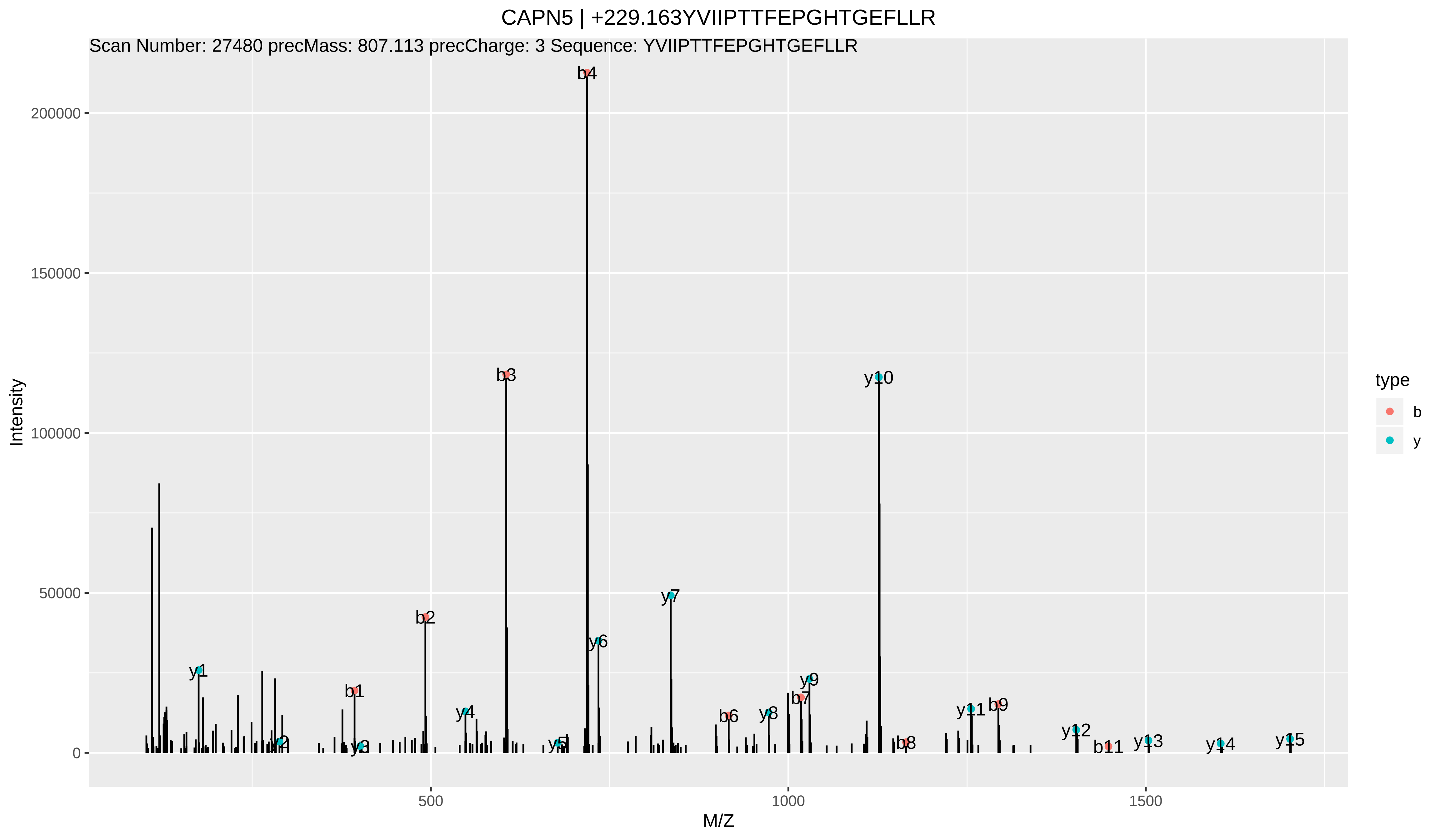

CAPN5 | +229.163YVIIPTTFEPGHTGEFLLR
Scan Number: 27480 precMass: 807.113 precCharge: 3 Sequence: YVIIPTTFEPGHTGEFLLR
Intensity
M/Z
0
50000
100000
150000
200000
500
1000
1500
type
b
y
b1
b2
b3
b4
b6
b7
b8
b9
b11
y1
y2
y3
y4
y5
y6
y7
y8
y9
y10
y11
y12
y13
y14
y15

CARD14 | +229.163AVLEDTTLEEAVGLLR

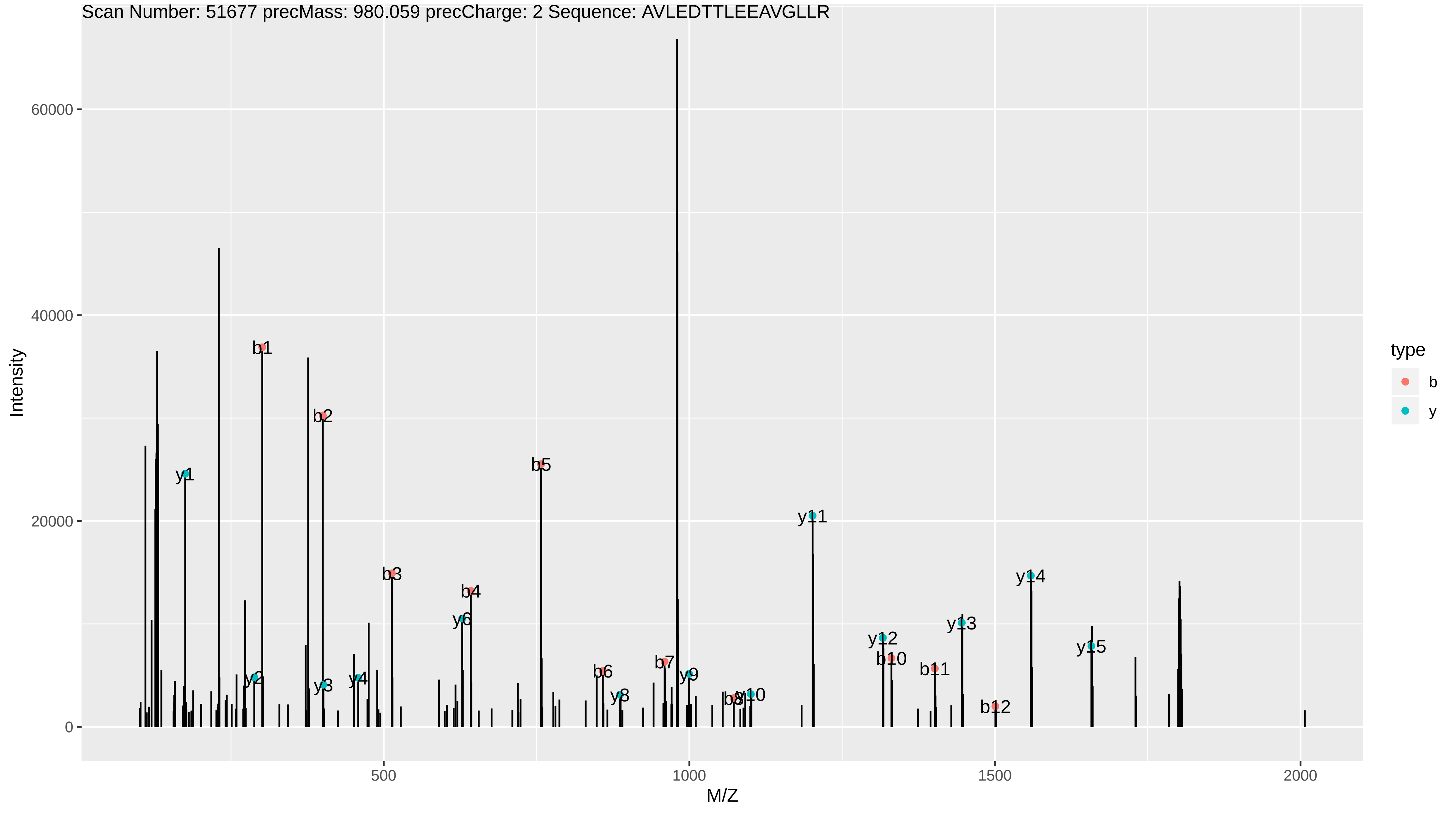

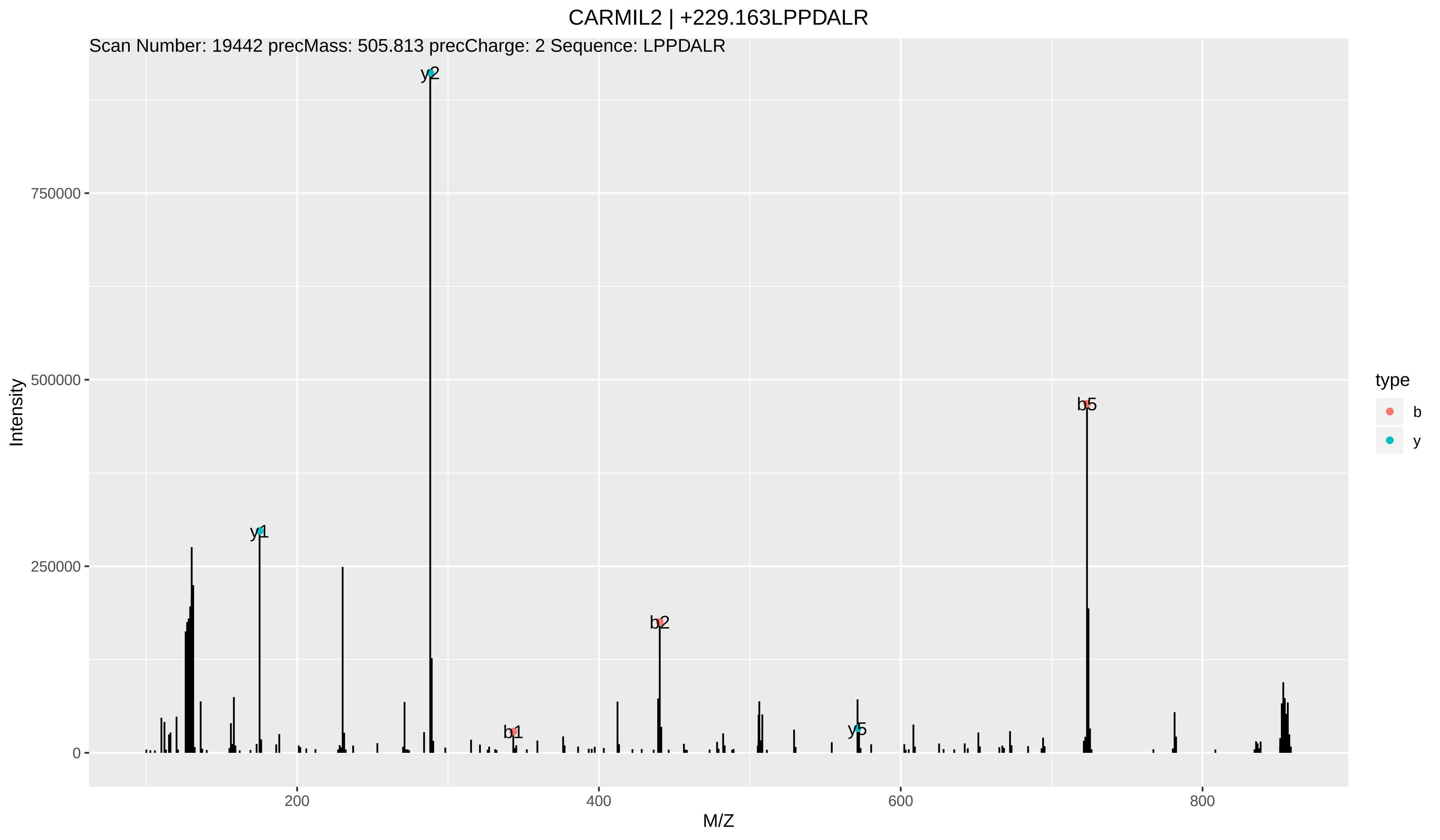

CARMIL2 | +229.163LPPDALR
Scan Number: 19442 precMass: 505.813 precCharge: 2 Sequence: LPPDALR
y2
b5
y1
b2
b1
y5
Intensity
750000
500000
250000
0
200
400
600
800
M/Z
type
b
y

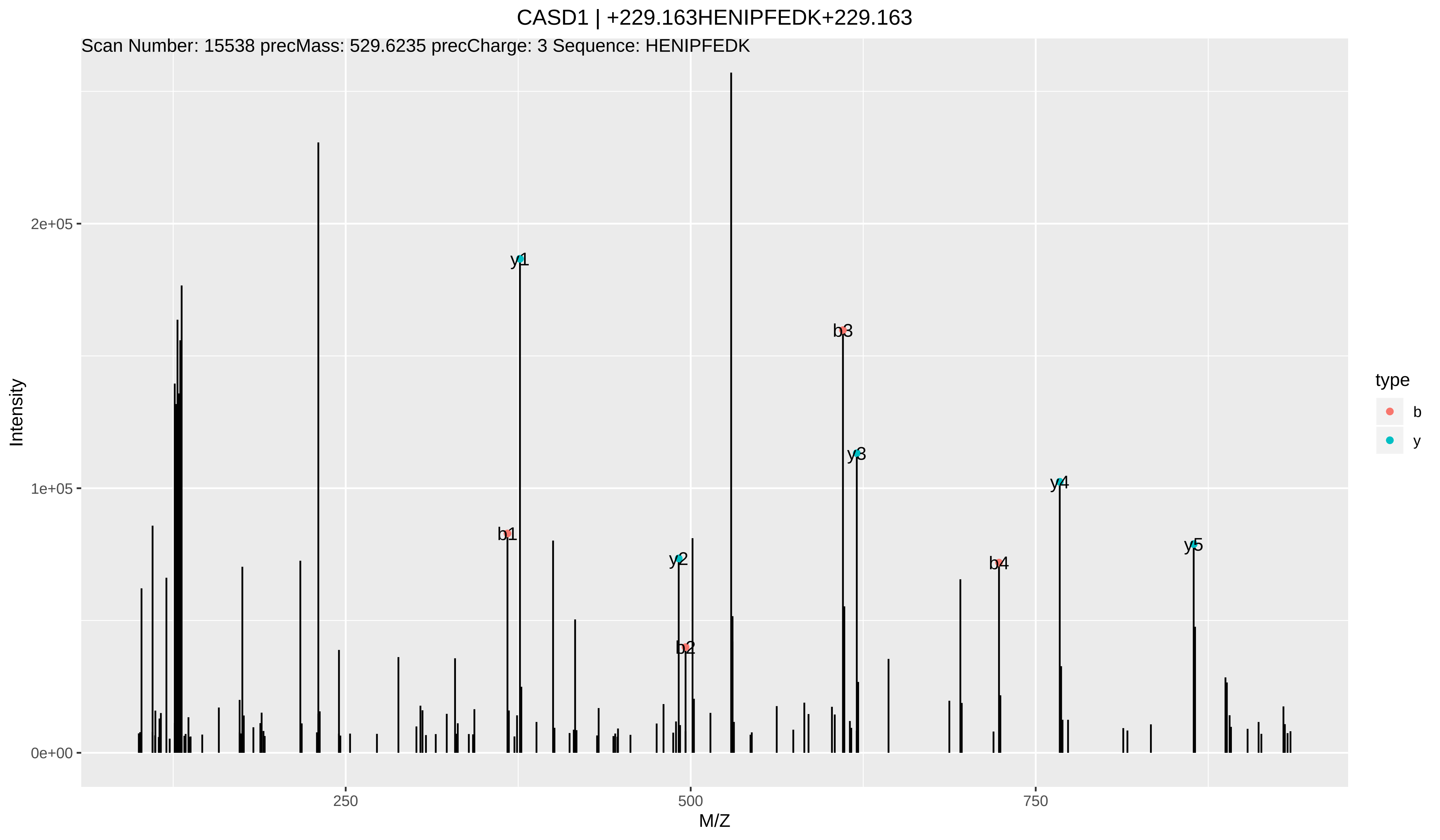

CASD1 | +229.163HENIPFEDK+229.163
Scan Number: 15538 precMass: 529.6235 precCharge: 3 Sequence: HENIPFEDK
Intensity
M/Z
2e+05
1e+05
0e+00
250
500
750
y1
b1
y2
b2
b3
y3
b4
y4
y5
type
b
y

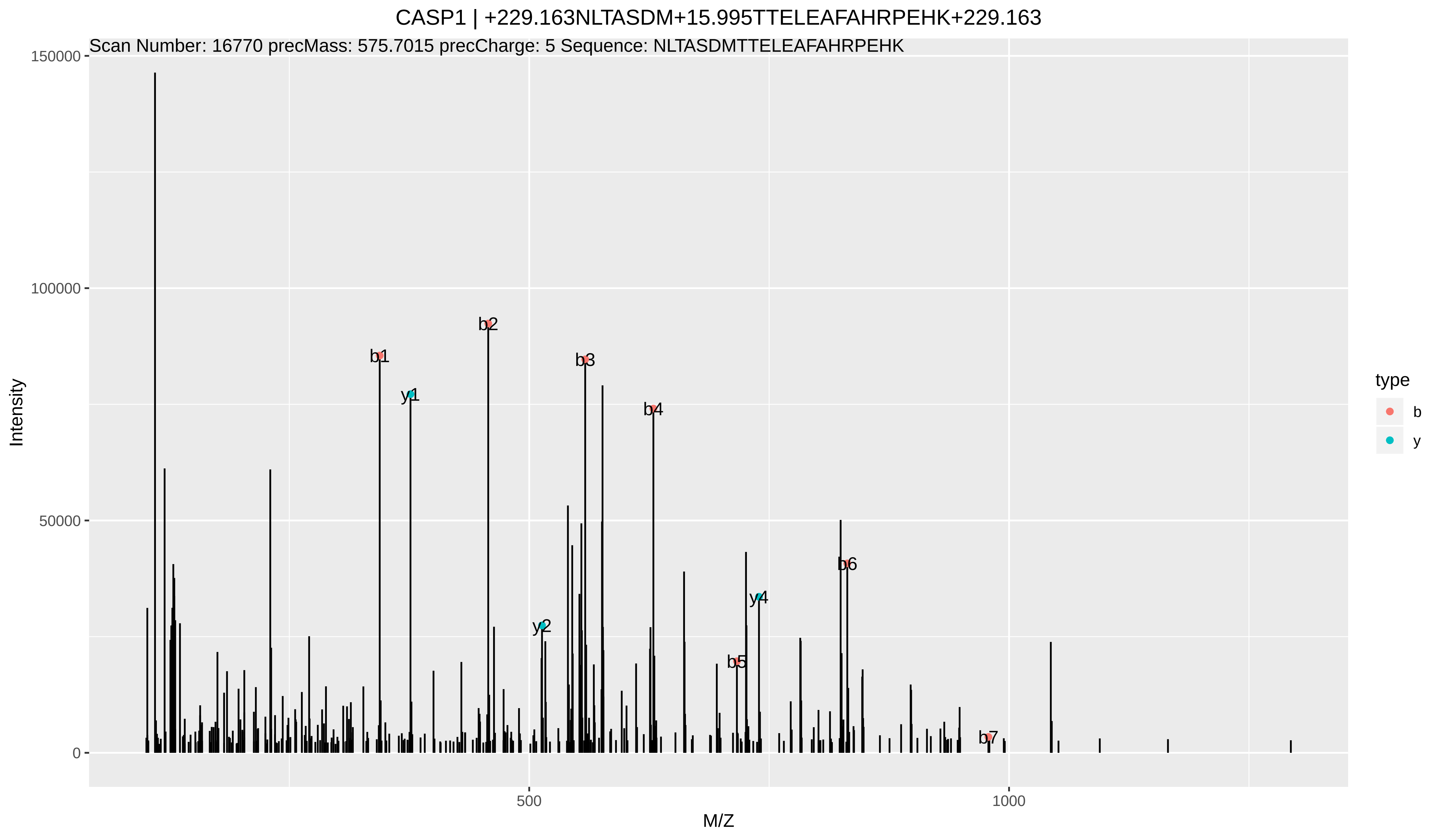
CASP1 | +229.163NLTASDM+15.995TTELEAFAHRPEHK+229.163
Scan Number: 16770 precMass: 575.7015 precCharge: 5 Sequence: NLTASDMTTELEAFAHRPEHK
Intensity
150000
100000
50000
0
500
1000
M/Z
b1
y1
b2
b3
b4
y2
b5
y4
b6
b7
type
b
y

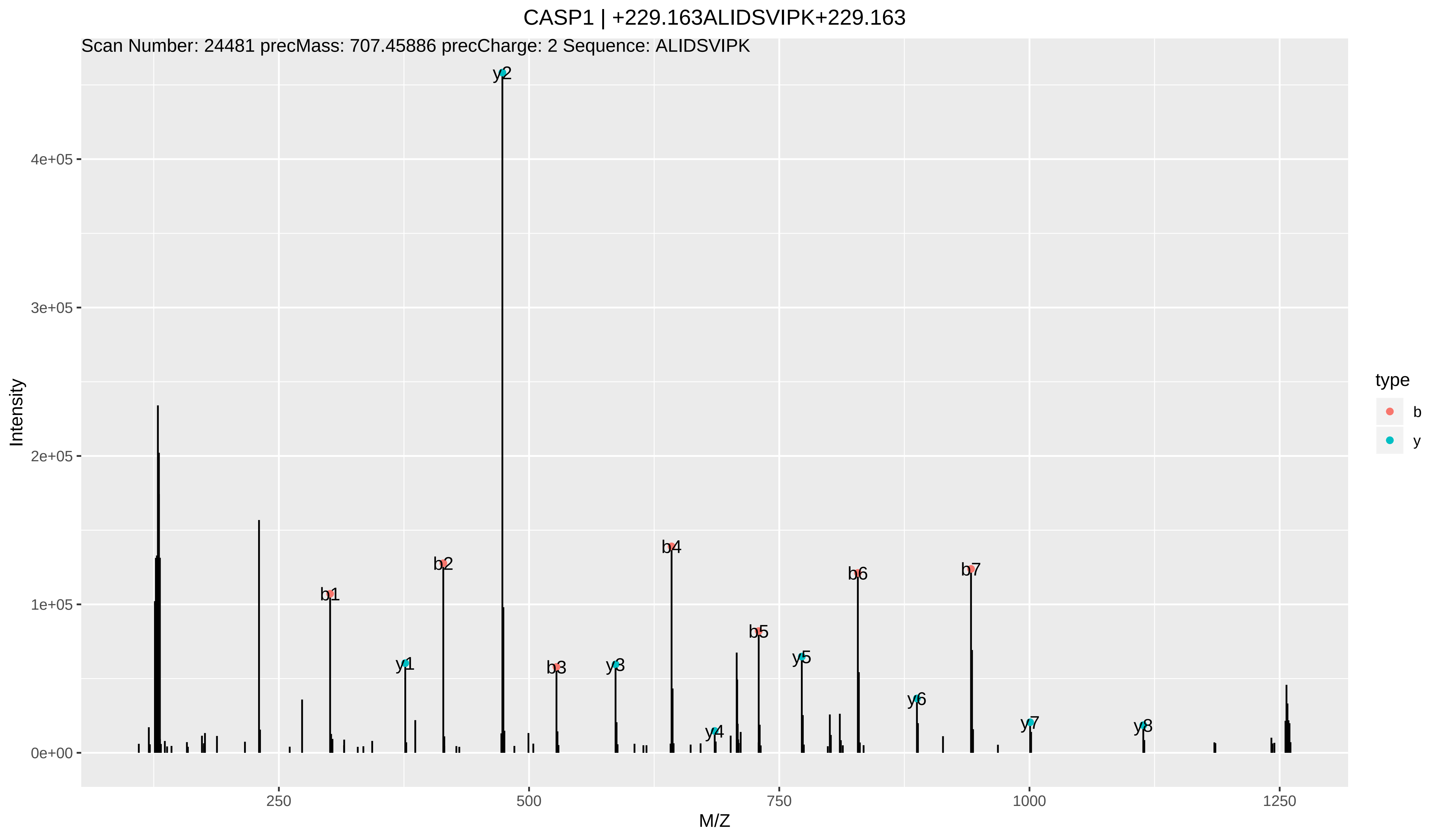

CASP1 | +229.163ALIDSVIPK+229.163
Scan Number: 24481 precMass: 707.45886 precCharge: 2 Sequence: ALIDSVIPK
Intensity
4e+05
3e+05
2e+05
1e+05
0e+00
M/Z
250
500
750
1000
1250
y2
b1
b2
b3
b4
b5
b6
b7
y1
y3
y4
y5
y6
y7
y8
type
b
y

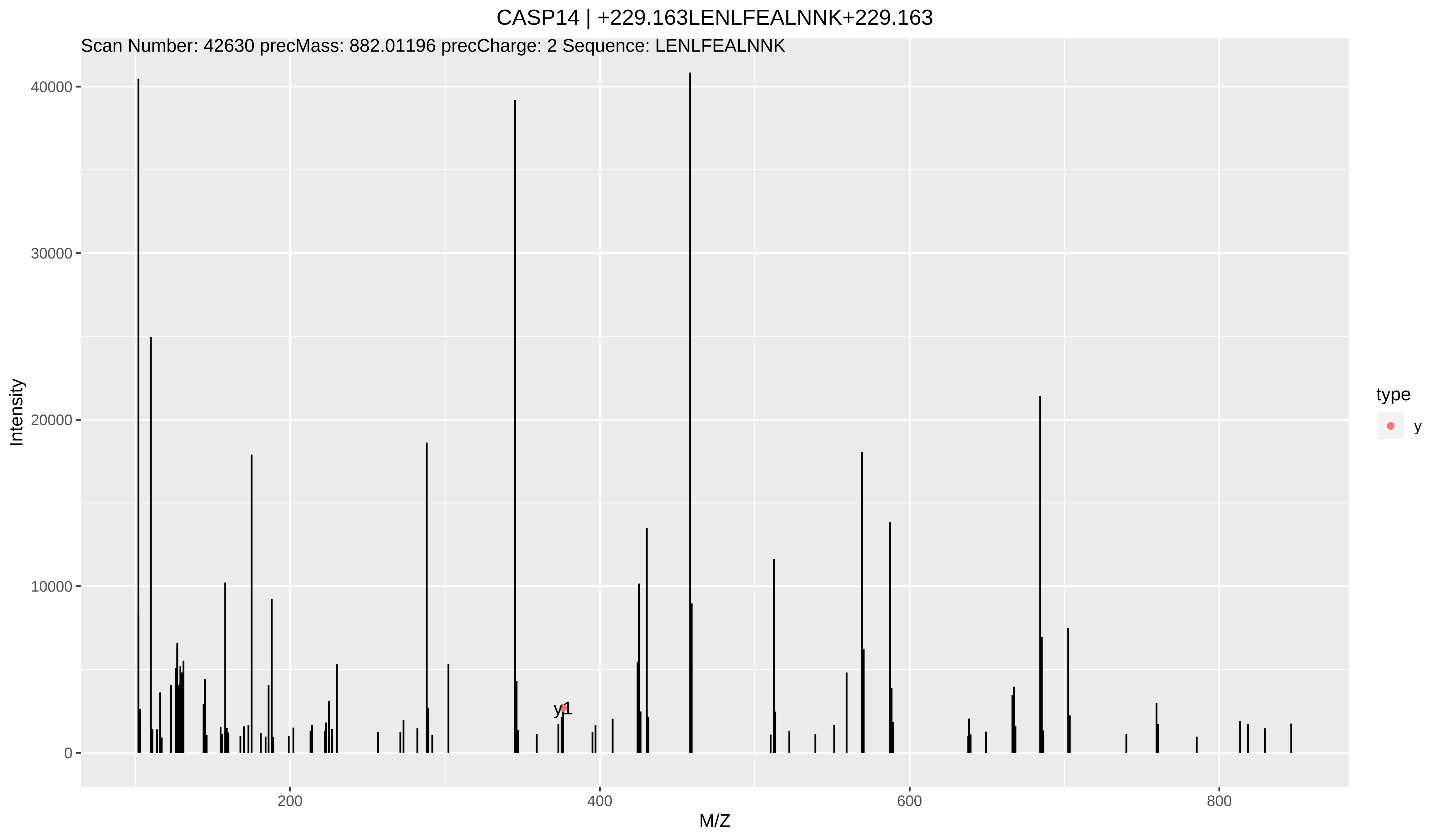

CASP14 | +229.163LENLFEALNNK+229.163
Scan Number: 42630 precMass: 882.01196 precCharge: 2 Sequence: LENLFEALNNK
Intensity
40000
30000
20000
10000
0
M/Z
200
400
600
800
y1
type
y

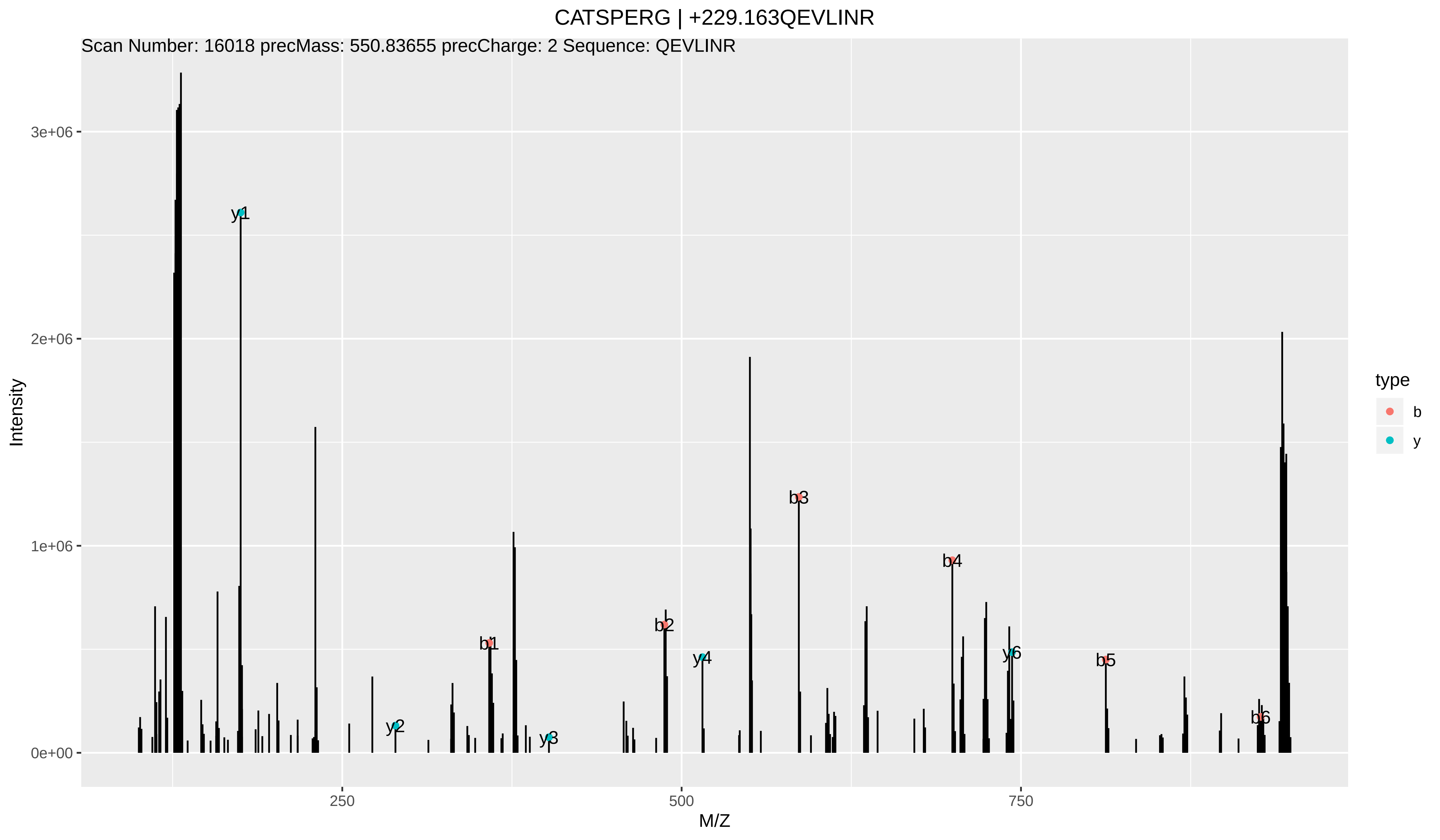
CATSPERG | +229.163QEVLINR
Scan Number: 16018 precMass: 550.83655 precCharge: 2 Sequence: QEVLINR
Intensity
3e+06
2e+06
1e+06
0e+00
M/Z
250
500
750
y1
y2
y3
y4
y6
b1
b2
b3
b4
b5
b6
type
b
y

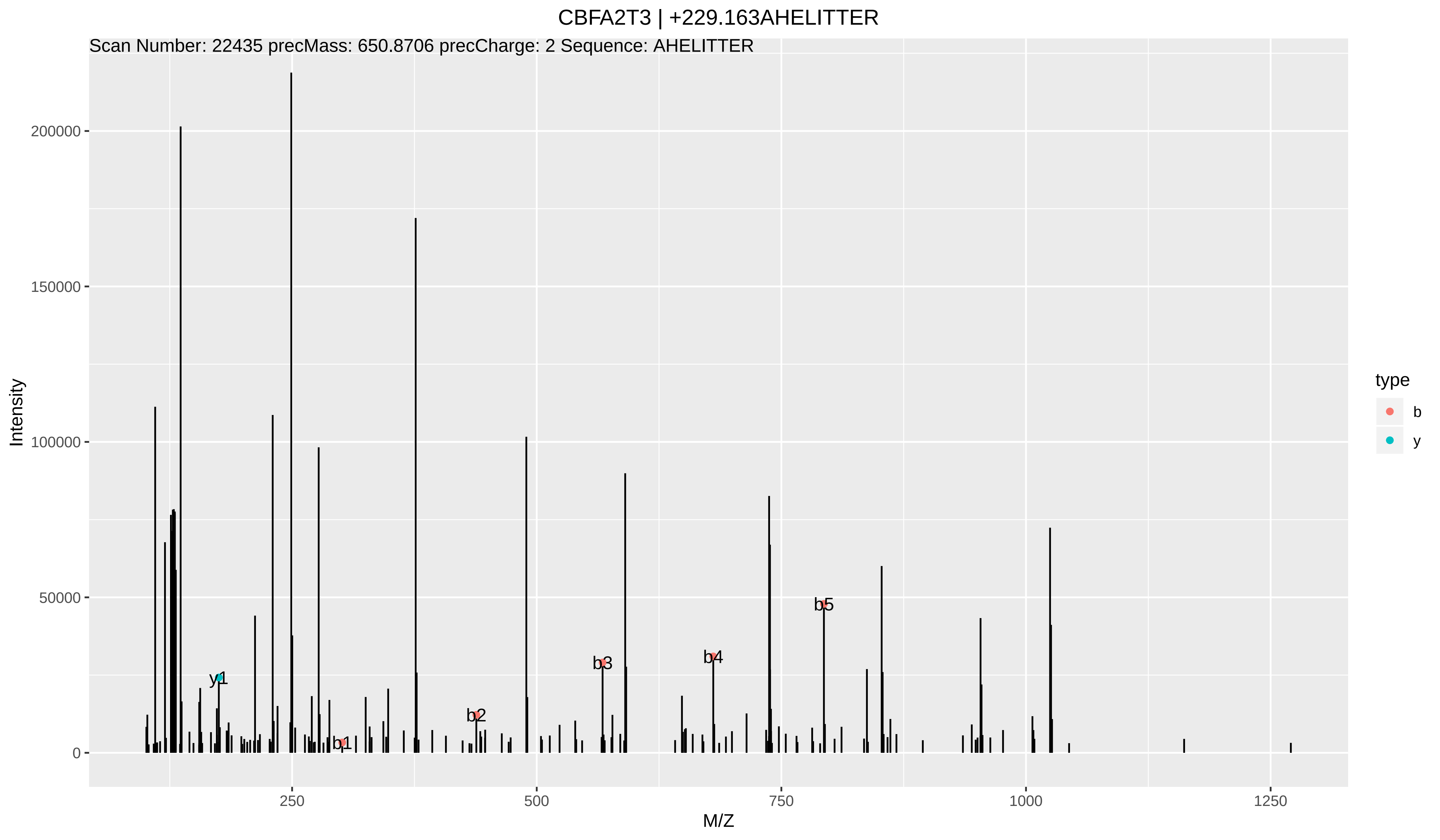
CBFA2T3 | +229.163AHELITTER
Scan Number: 22435 precMass: 650.8706 precCharge: 2 Sequence: AHELITTER
Intensity
200000
150000
100000
50000
0
250
500
750
1000
1250
M/Z
y1
b1
b2
b3
b4
b5
type
b
y

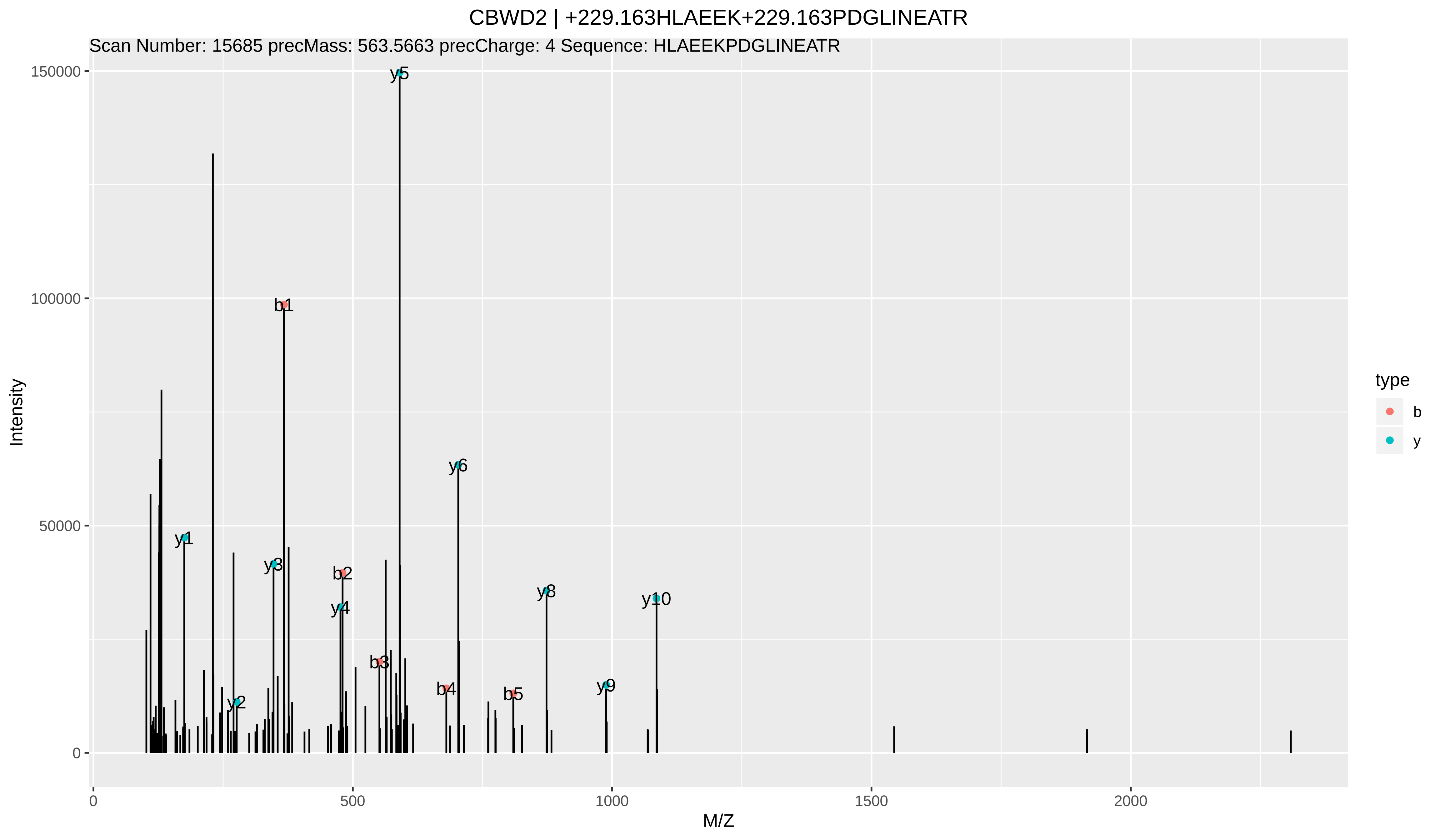

CBWD2 | +229.163HLAEEK+229.163PDGLINEATR
Scan Number: 15685 precMass: 563.5663 precCharge: 4 Sequence: HLAEEKPDGLINEATR
Intensity
M/Z
150000
100000
50000
0
500
1000
1500
2000
y5
b1
y6
y1
y3
b2
y4
y8
y10
b3
b4
b5
y9
y2
type
b
y

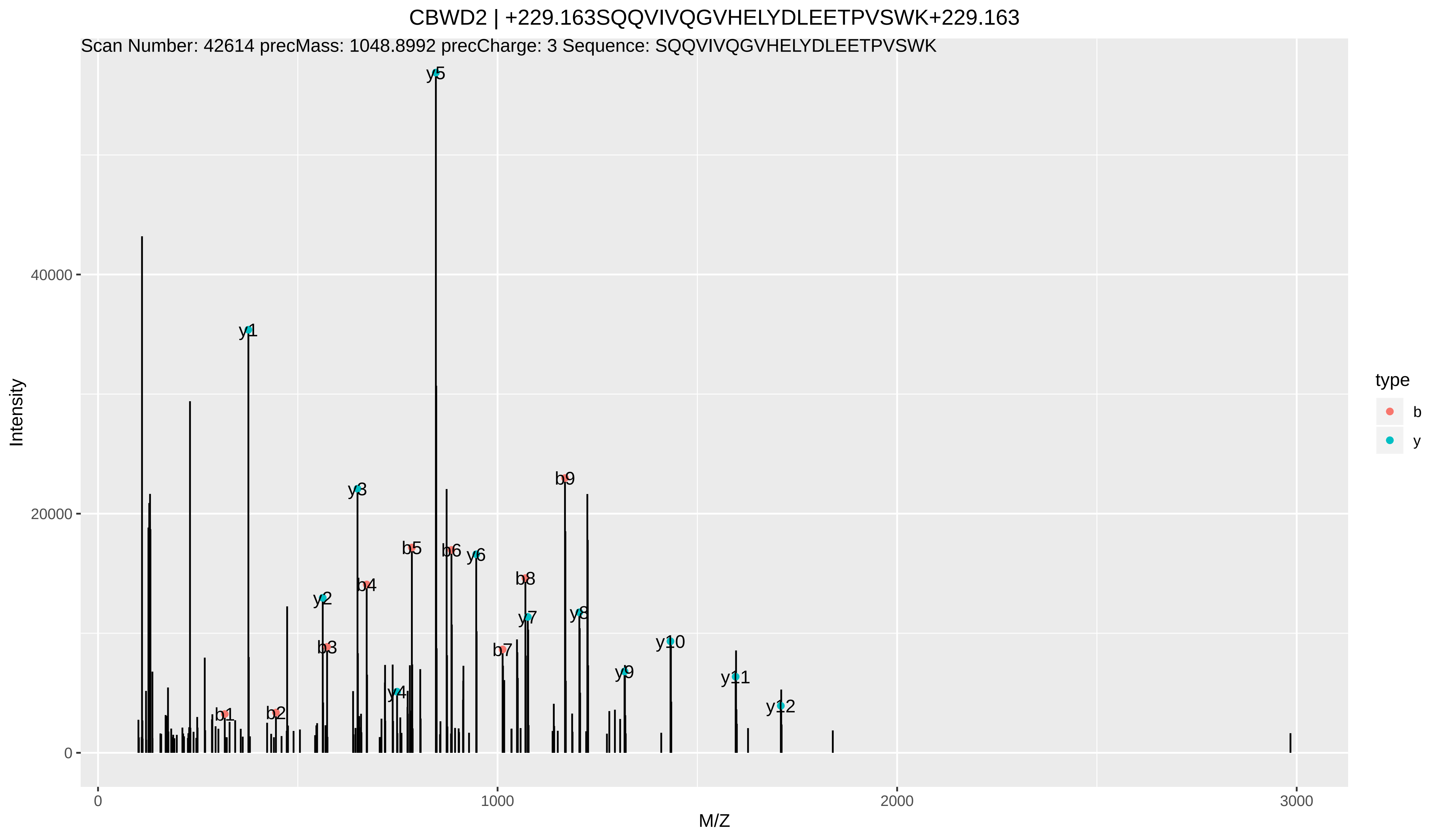
CBWD2 | +229.163SQQVIVQGVHELYDLEETPVSWK+229.163
Scan Number: 42614 precMass: 1048.8992 precCharge: 3 Sequence: SQQVIVQGVHELYDLEETPVSWK
Intensity
M/Z
type
b
y
y5
y1
y3
b9
b5
b6
y6
b4
b8
y2
y7
y8
b3
b7
y10
y9
y11
y4
y12
b1
b2
0
20000
40000
0
1000
2000
3000

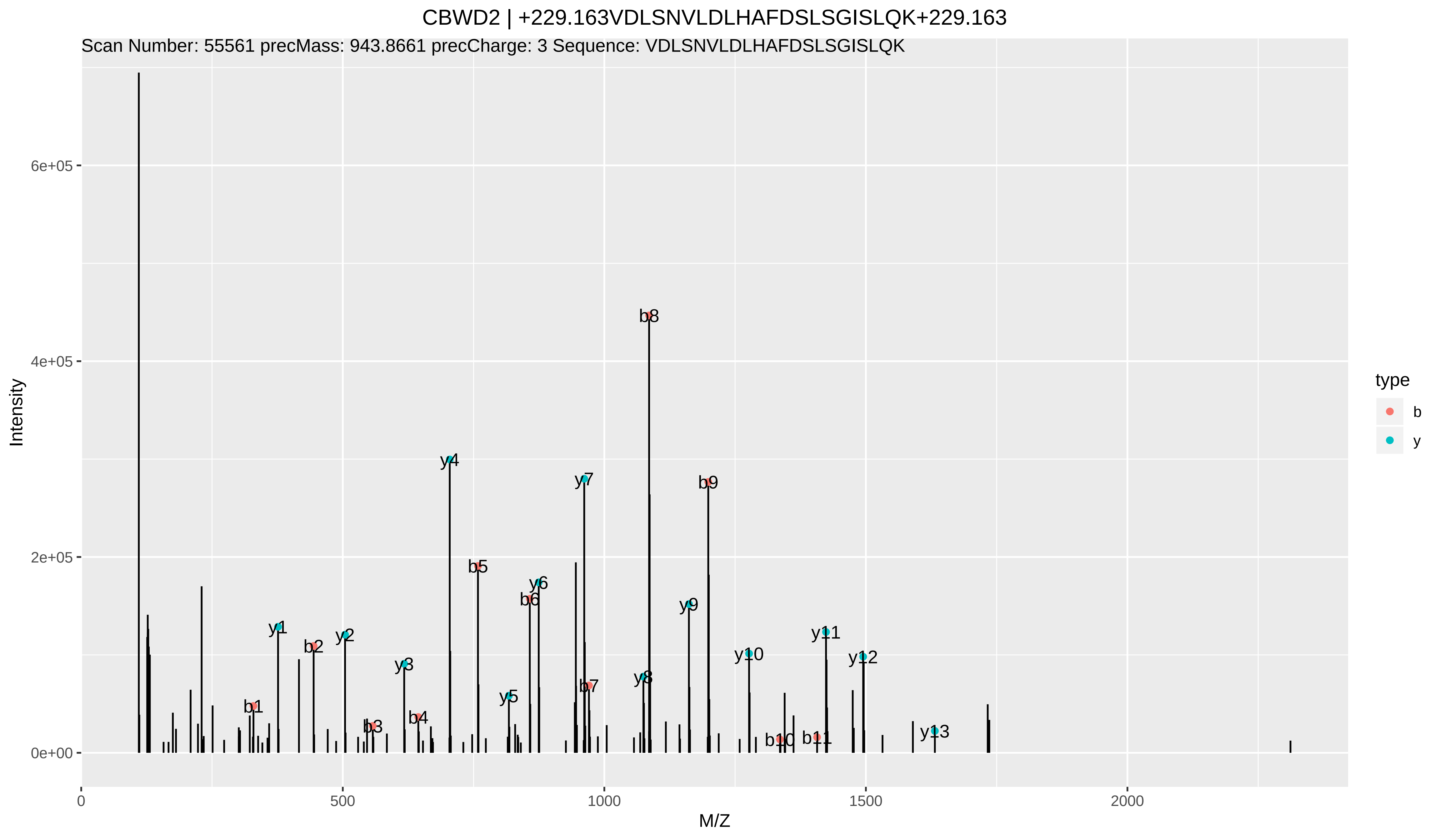

CBWD2 | +229.163VDLSNVLDLHAFDSLSGISLQK+229.163
Scan Number: 55561 precMass: 943.8661 precCharge: 3 Sequence: VDLSNVLDLHAFDSLSGISLQK
Intensity
6e+05
4e+05
2e+05
0e+00
M/Z
0
500
1000
1500
2000
type
b
y
b1
b2
b3
b4
b5
b6
b7
b8
b9
b10
b11
y1
y2
y3
y4
y5
y6
y7
y8
y9
y10
y11
y12
y13

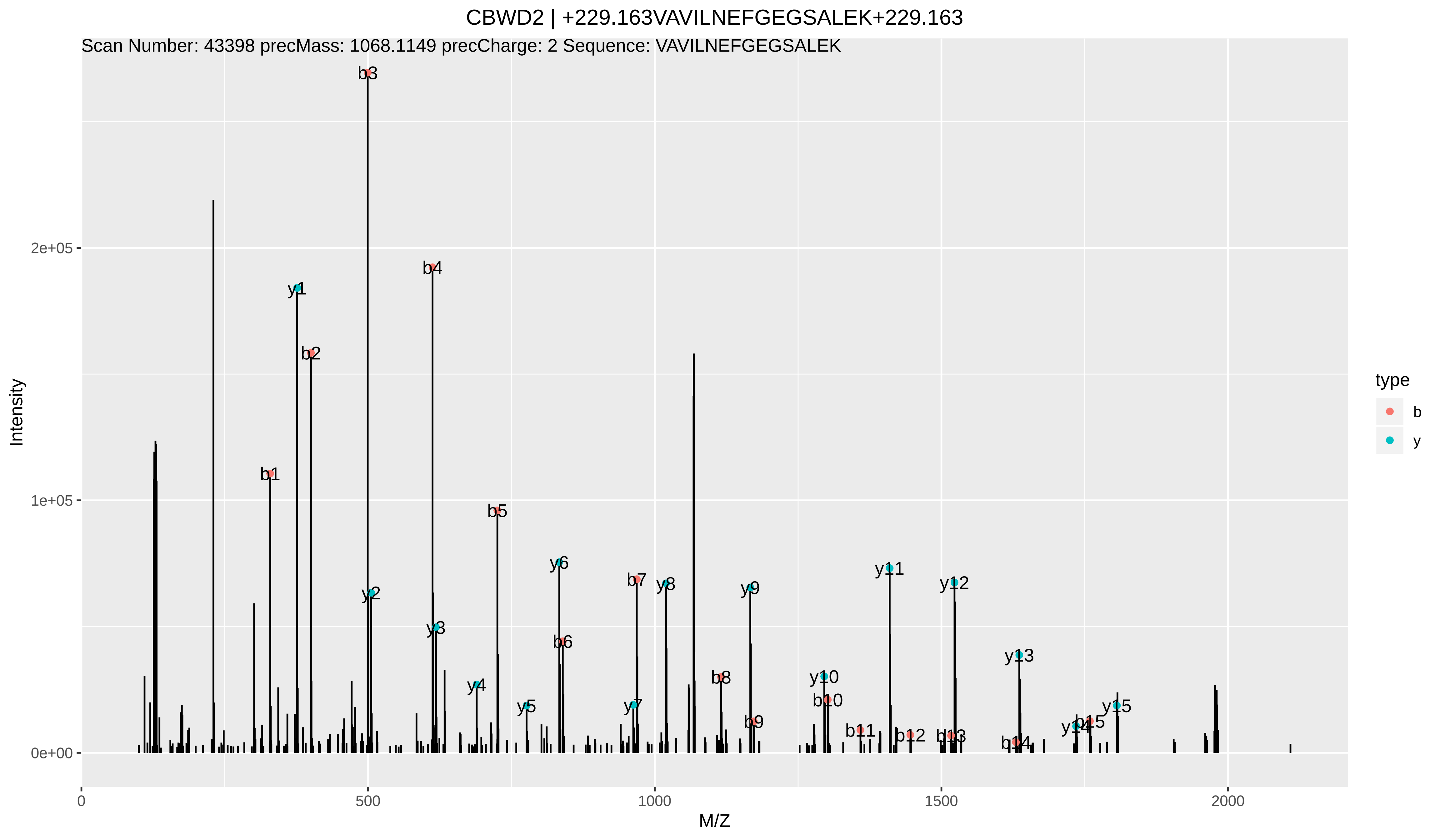

CBWD2 | +229.163VAVILNEFGEGSALEK+229.163
Scan Number: 43398 precMass: 1068.1149 precCharge: 2 Sequence: VAVILNEFGEGSALEK
Intensity
2e+05
1e+05
0e+00
M/Z
0
500
1000
1500
2000
b3
b4
y1
b2
b1
b5
y6
b7
y8
y9
y11
y12
y2
y3
b6
y13
y4
b8
y10
b10
y5
y7
b9
y15
b11
b12
b13
b14
y14
b15
type
b
y

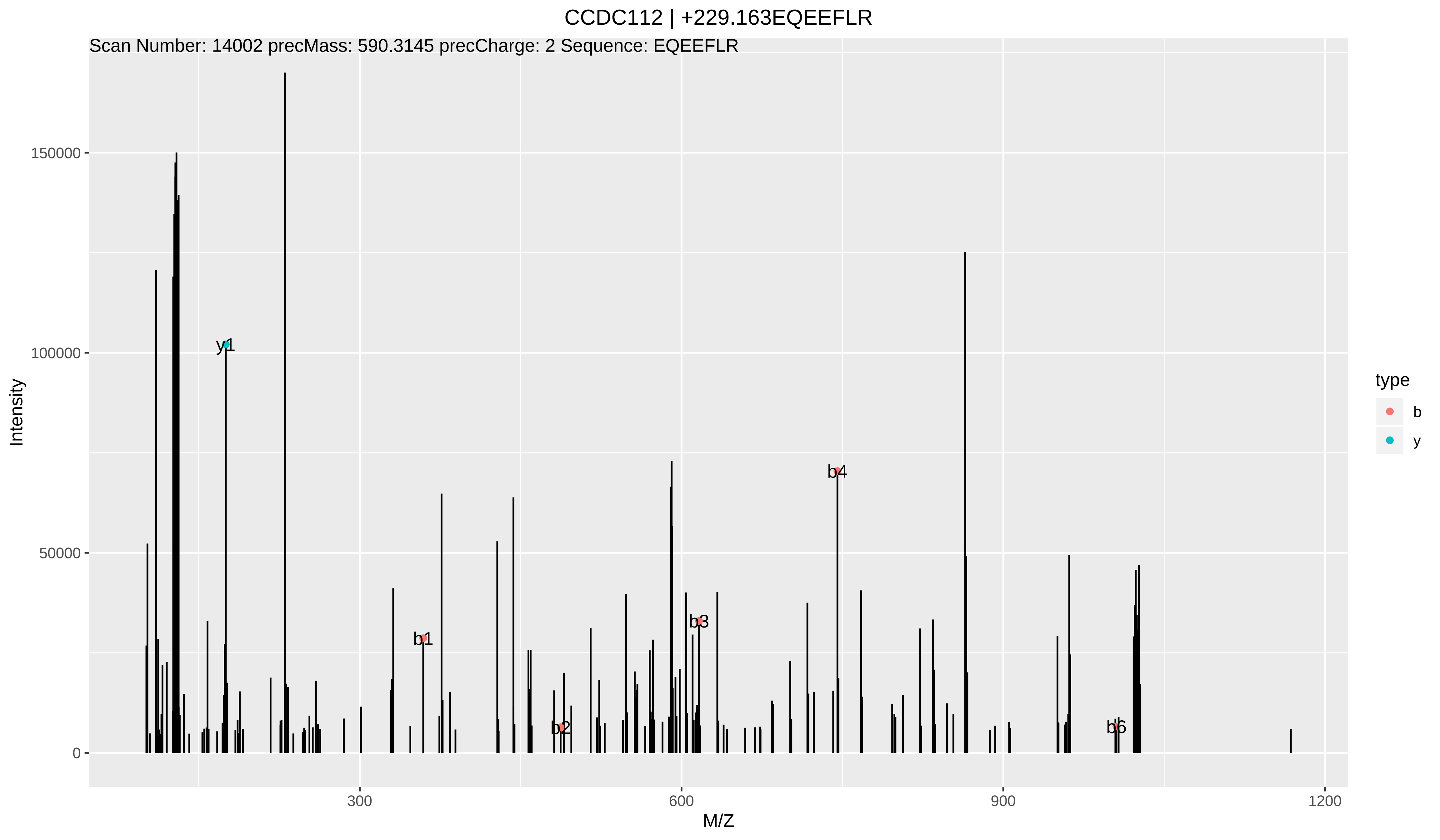

CCDC112 | +229.163EQEEFLR
Scan Number: 14002 precMass: 590.3145 precCharge: 2 Sequence: EQEEFLR
Intensity
M/Z
y1
b1
b2
b3
b4
b6
type
b
y

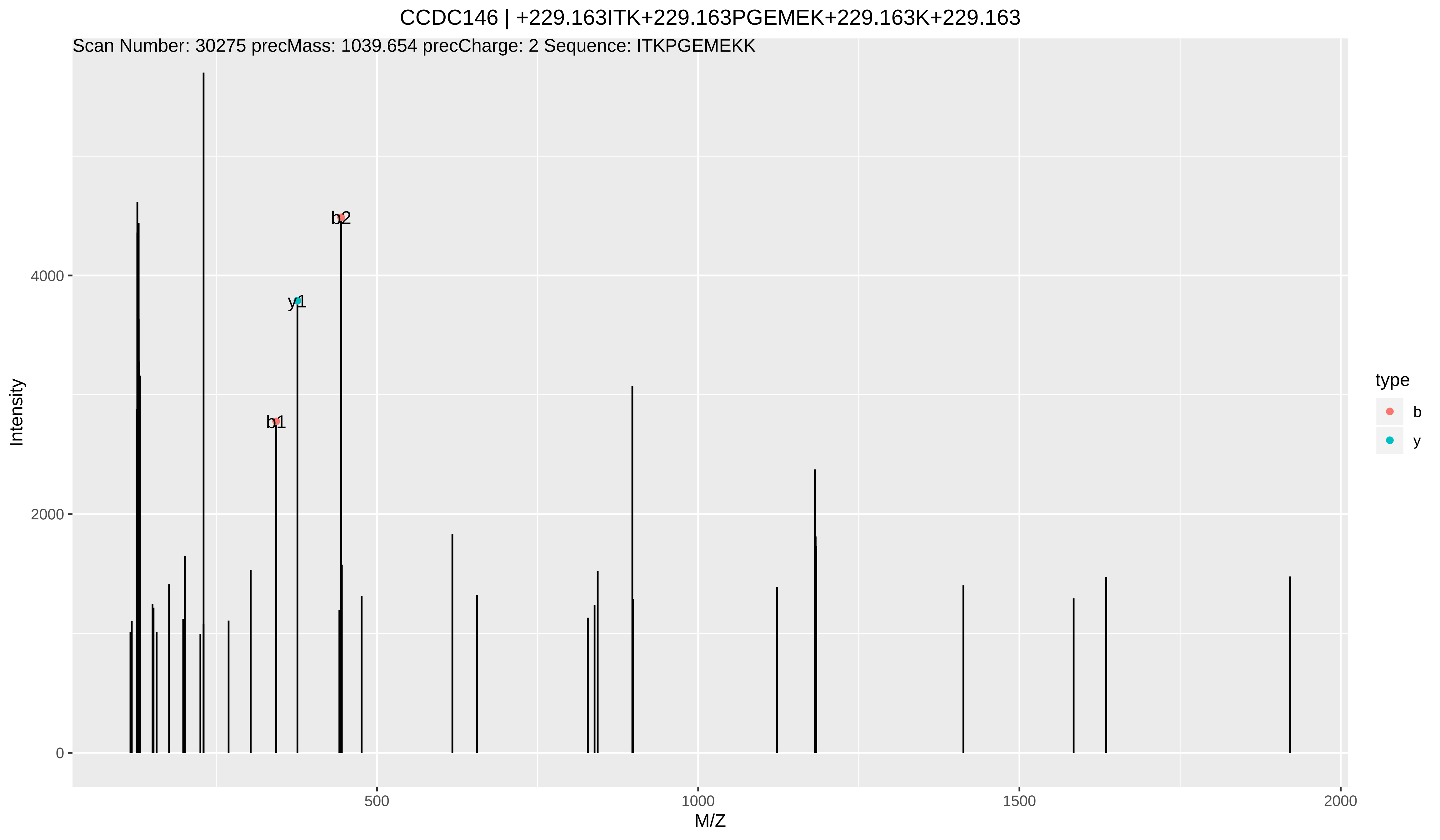
CCDC146 | +229.163ITK+229.163PGEMEK+229.163K+229.163
Scan Number: 30275 precMass: 1039.654 precCharge: 2 Sequence: ITKPGEMEKK
Intensity
M/Z
b2
y1
b1
type
b
y
0
2000
4000
500
1000
1500
2000

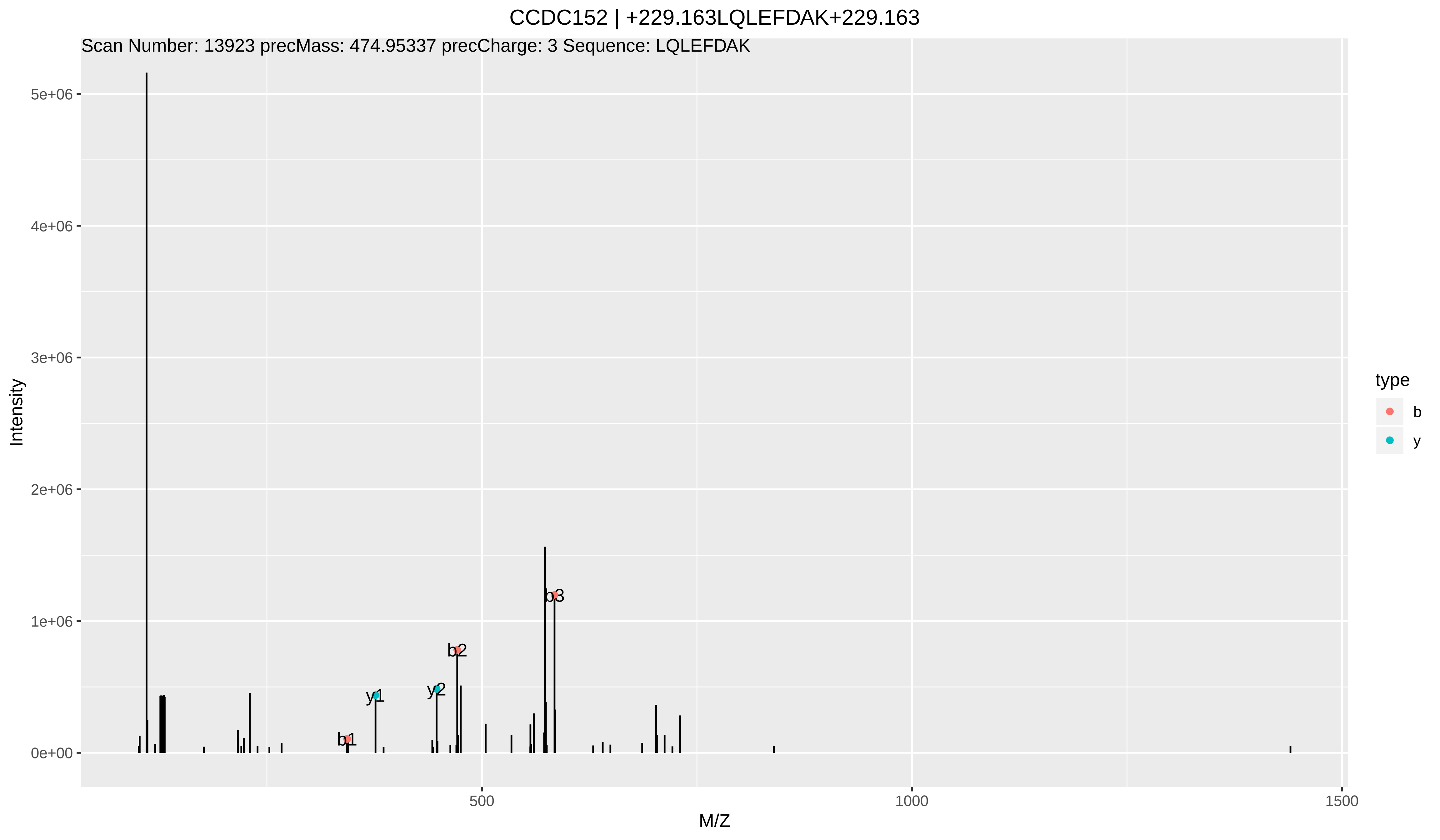
CCDC152 | +229.163LQLEFDAK+229.163
Scan Number: 13923 precMass: 474.95337 precCharge: 3 Sequence: LQLEFDAK
Intensity
M/Z
5e+06
4e+06
3e+06
2e+06
1e+06
0e+00
500
1000
1500
b1
y1
y2
b2
b3
type
b
y

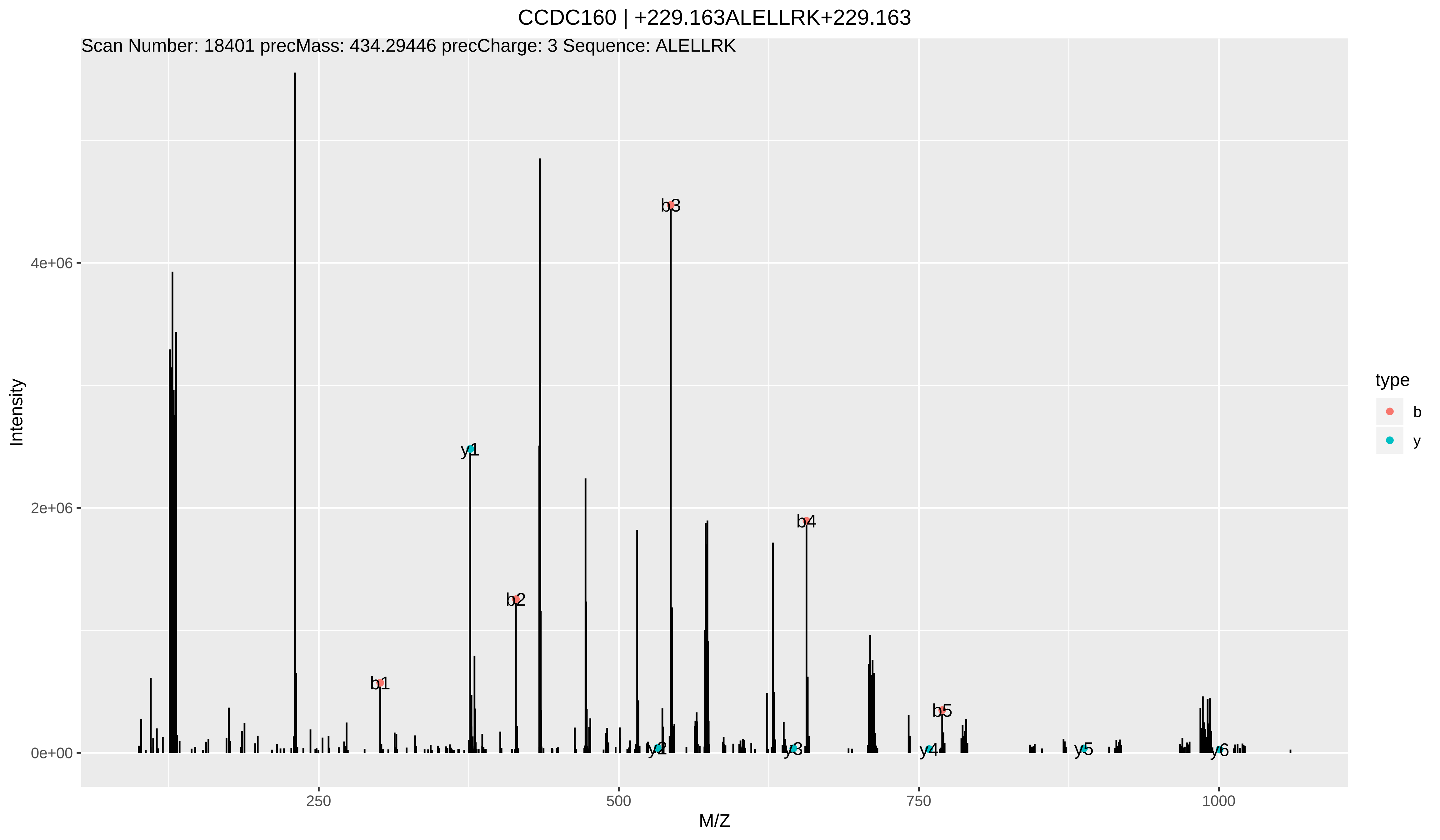

CCDC160 | +229.163ALELLRK+229.163
Scan Number: 18401 precMass: 434.29446 precCharge: 3 Sequence: ALELLRK
Intensity
4e+06
2e+06
0e+00
M/Z
250
500
750
1000
b1
b2
b3
b4
b5
y1
y2
y3
y4
y5
y6
type
b
y

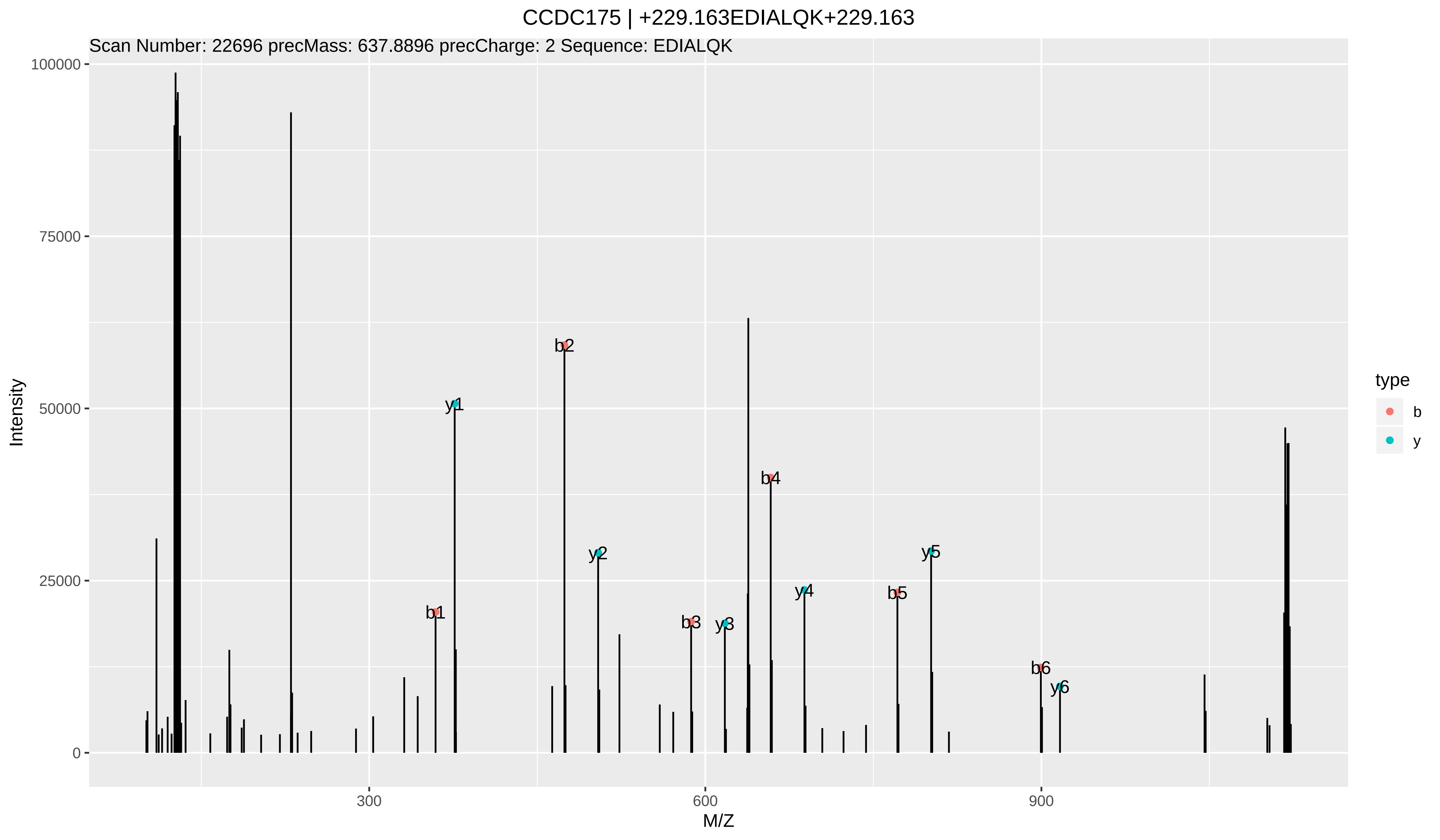

CCDC175 | +229.163EDIALQK+229.163
Scan Number: 22696 precMass: 637.8896 precCharge: 2 Sequence: EDIALQK
Intensity
100000
75000
50000
25000
0
300
600
900
M/Z
b1
y1
b2
y2
b3
y3
b4
y4
b5
y5
b6
y6
type
b
y

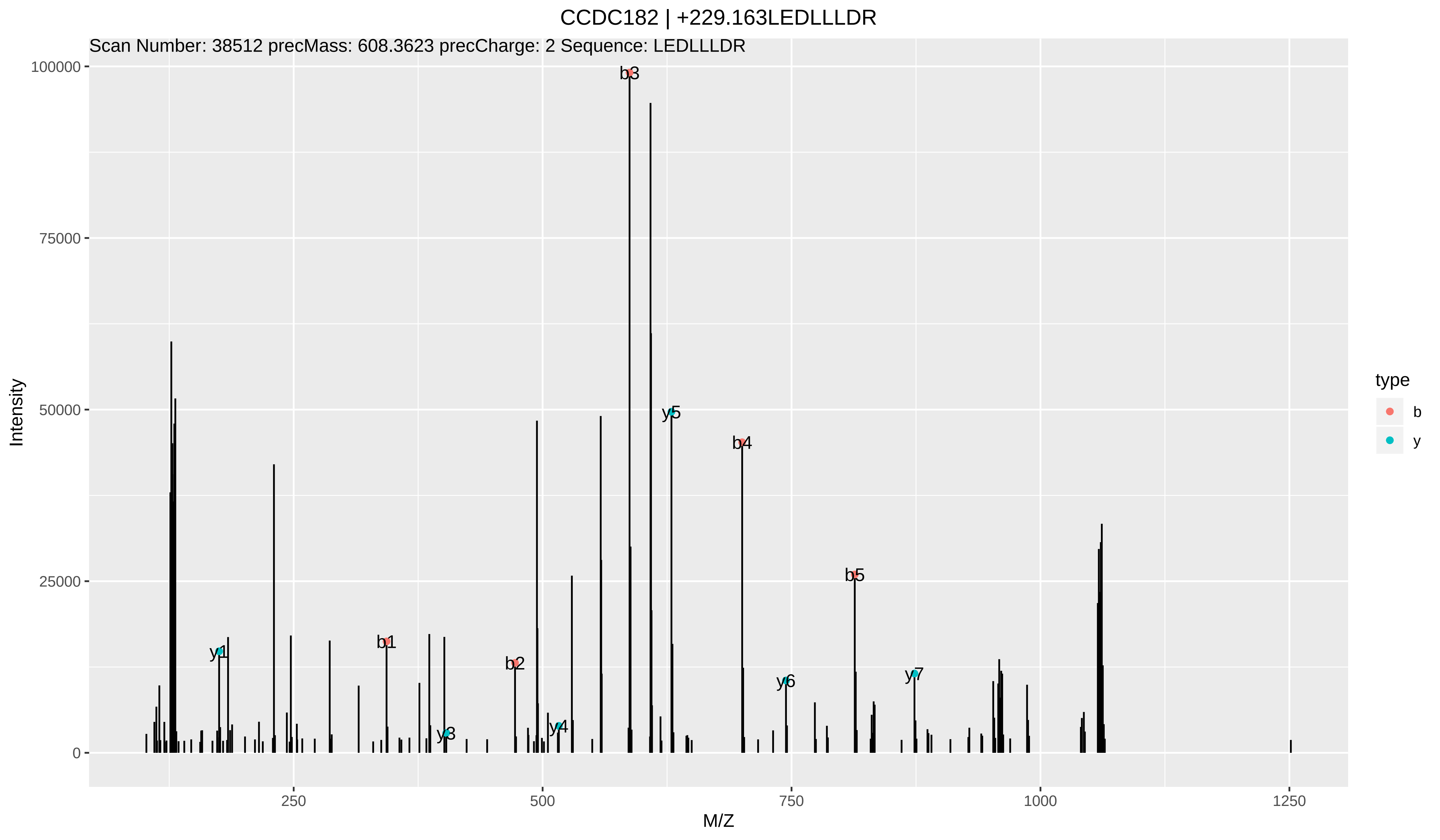

CCDC182 | +229.163LEDLLLDR
Scan Number: 38512 precMass: 608.3623 precCharge: 2 Sequence: LEDLLLDR
Intensity
M/Z
100000
75000
50000
25000
0
250
500
750
1000
1250
b3
y5
b4
b5
y1
b1
b2
y3
y4
y6
y7
type
b
y

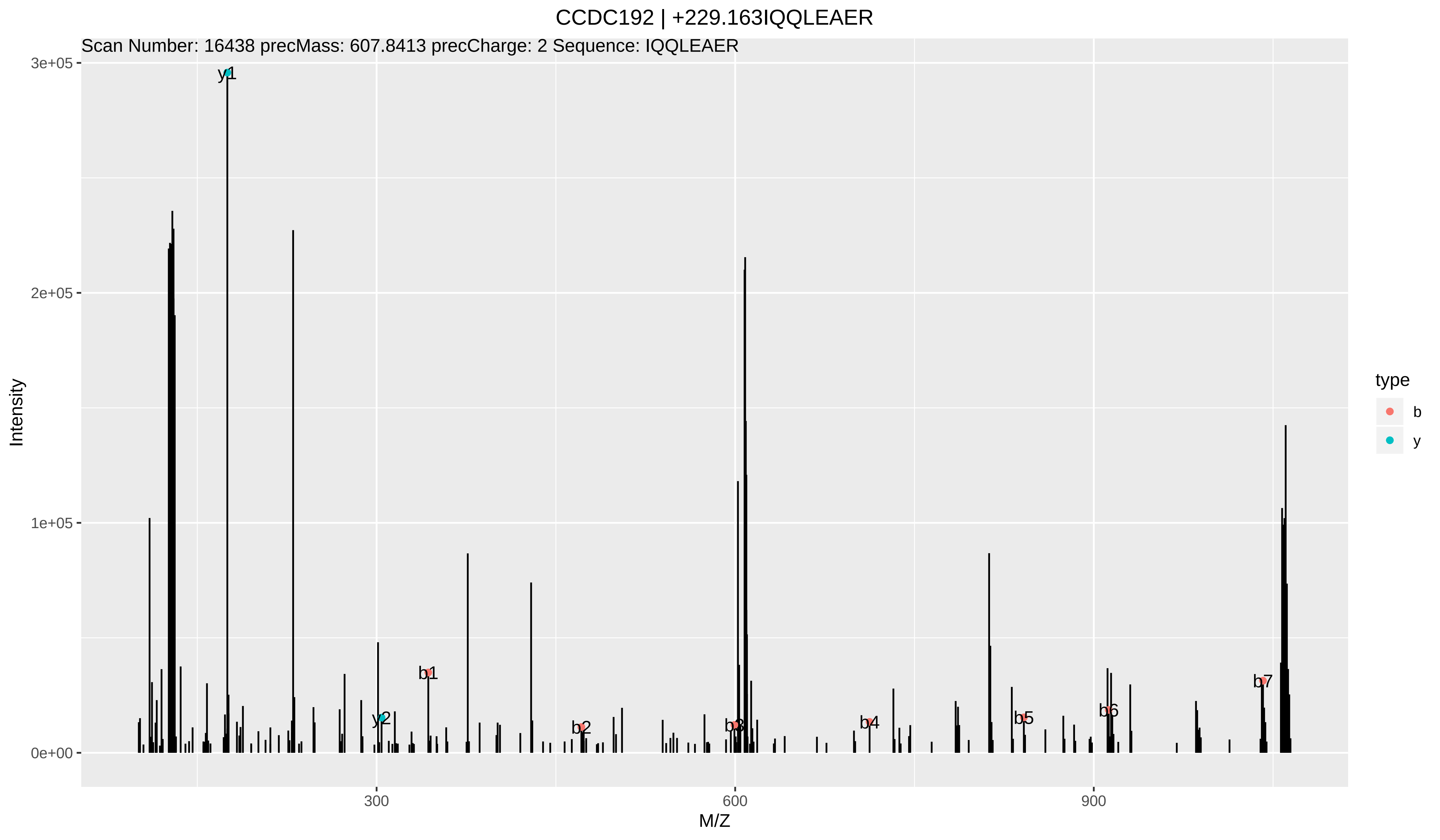

CCDC192 | +229.163IQQLEAER
Scan Number: 16438 precMass: 607.8413 precCharge: 2 Sequence: IQQLEAER
Intensity
M/Z
3e+05
2e+05
1e+05
0e+00
300
600
900
y1
y2
b1
b2
b3
b4
b5
b6
b7
type
b
y

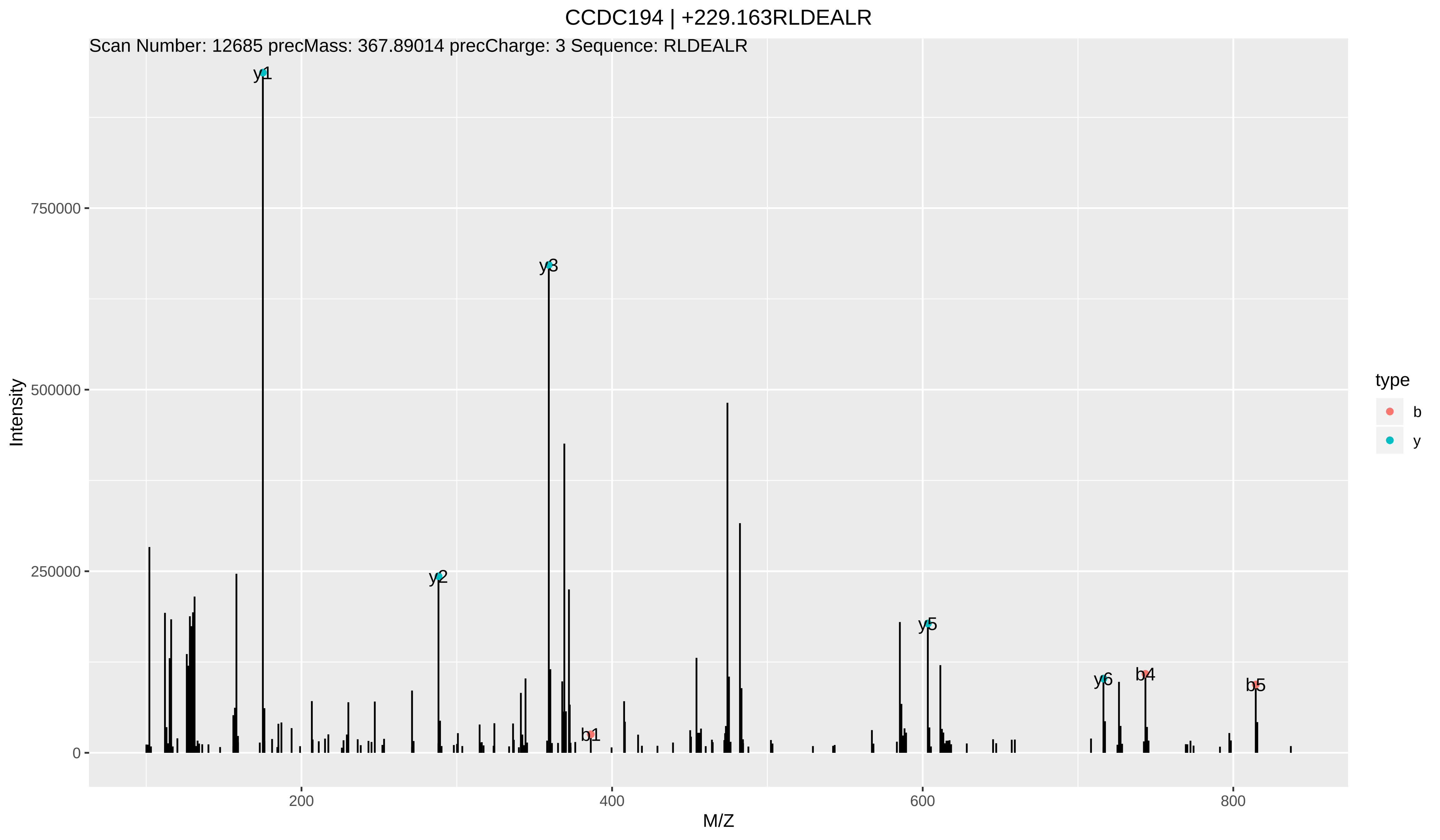

CCDC194 | +229.163RLDEALR
Scan Number: 12685 precMass: 367.89014 precCharge: 3 Sequence: RLDEALR
y1
y3
y2
b1
y5
y6
b4
b5
Intensity
750000
500000
250000
0
200
400
600
800
M/Z
type
b
y

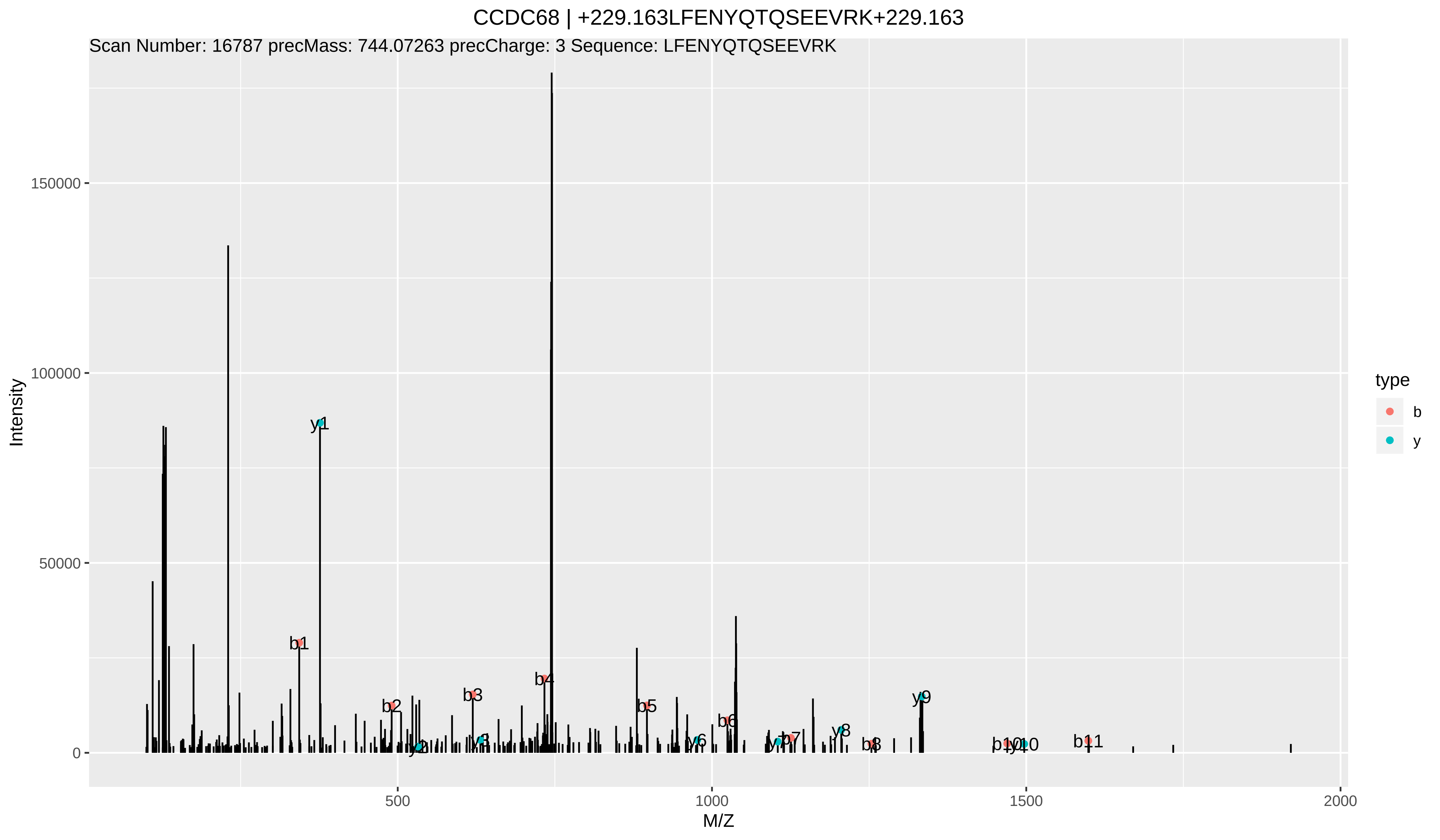

CCDC68 | +229.163LFENYQTQSEEVRK+229.163
Scan Number: 16787 precMass: 744.07263 precCharge: 3 Sequence: LFENYQTQSEEVRK
Intensity
M/Z
type
b
y
y1
b1
b2
y2
b3
y3
b4
b5
y6
b6
y7
b7
y8
b8
y9
b10
y10
b11

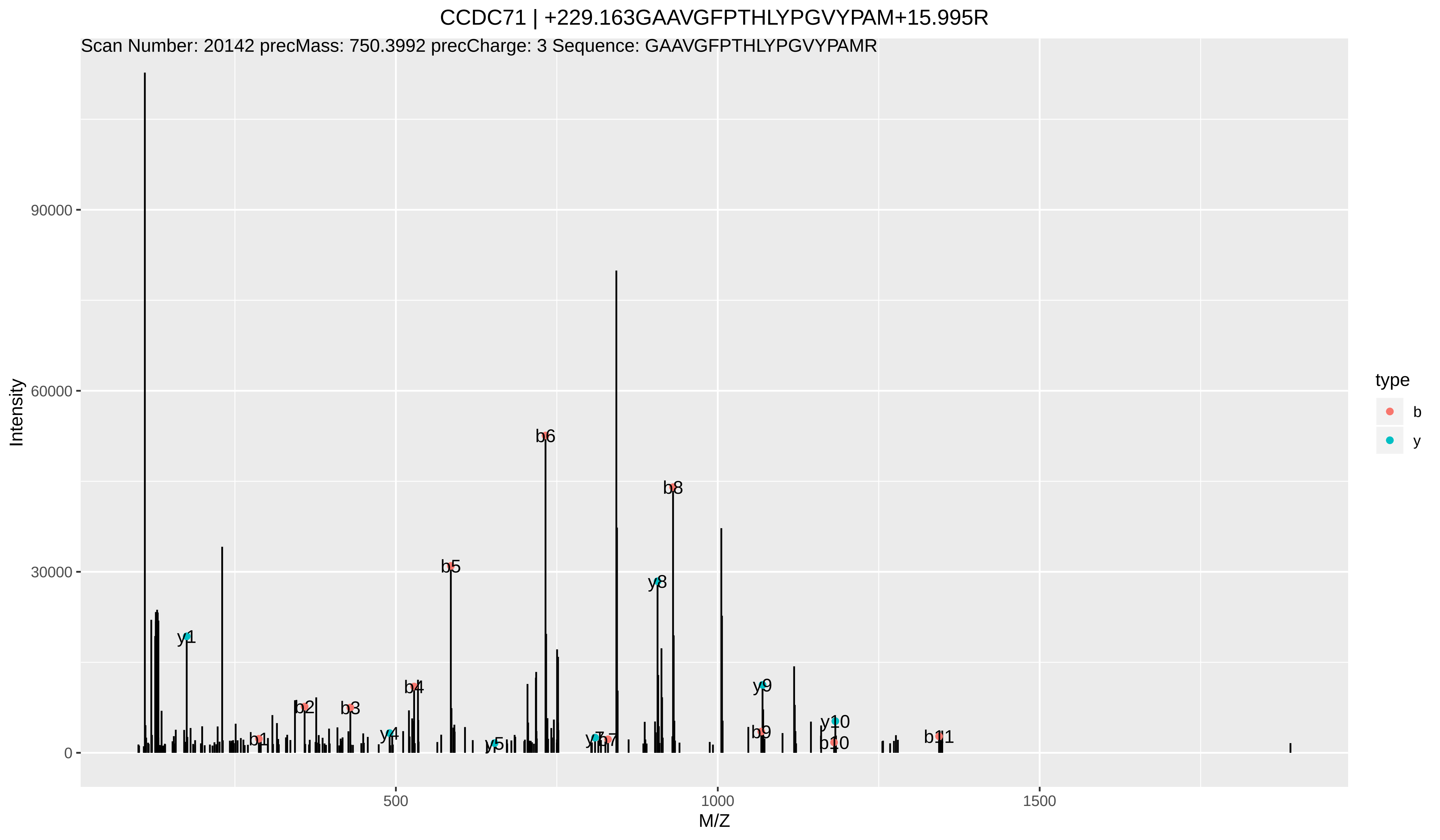
CCDC71 | +229.163GAAVGFPTHLYPGVYPAM+15.995R
Scan Number: 20142 precMass: 750.3992 precCharge: 3 Sequence: GAAVGFPTHLYPGVYPAMR
Intensity
M/Z
type
b
y
90000
60000
30000
0
500
1000
1500
y1
b1
b2
b3
y4
b4
b5
y5
b6
y7
b7
y8
b8
y9
b9
y10
b10
b11

CCDC83 | +229.163M+15.995QISNAEK+229.163

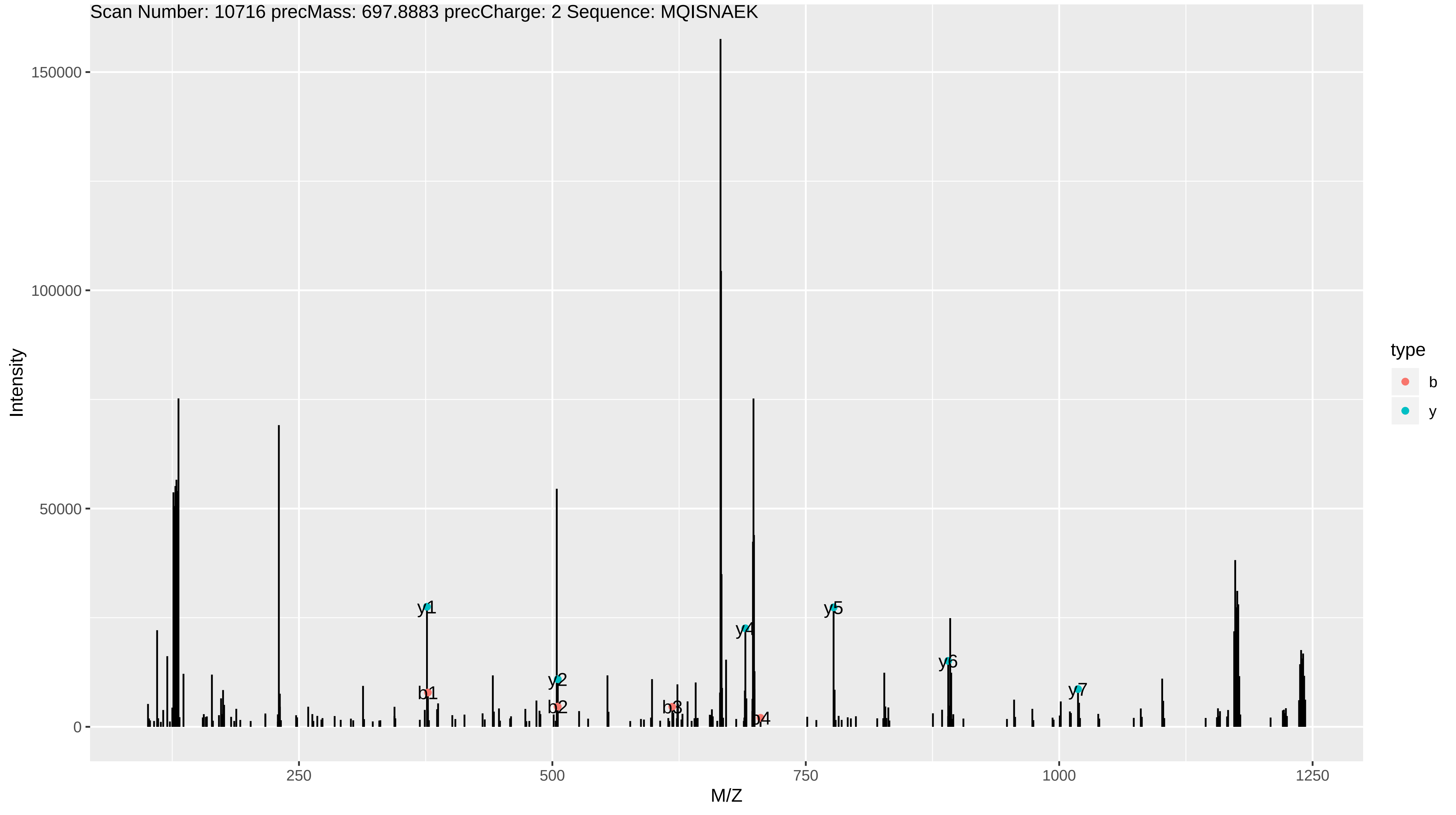

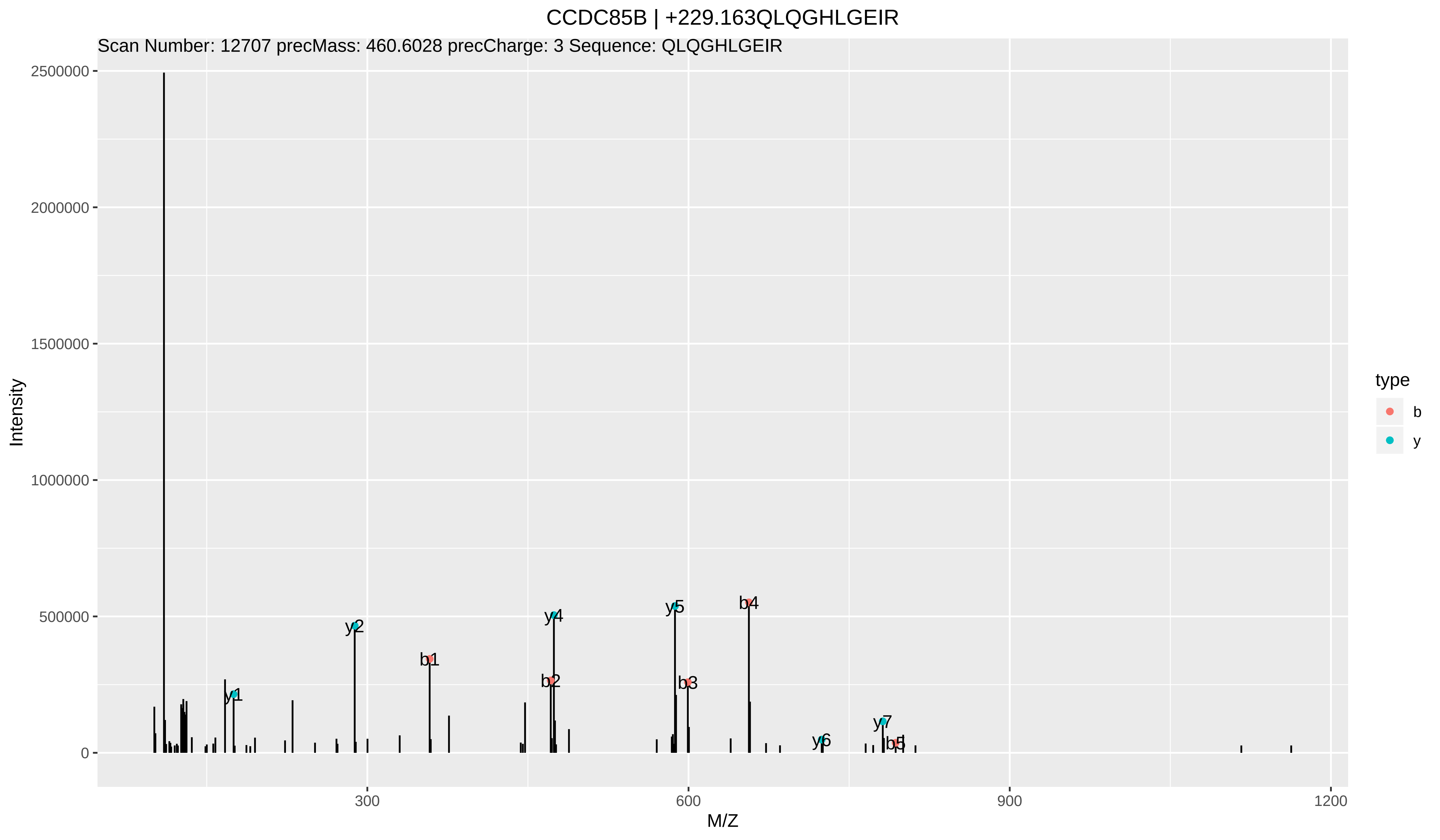

CCDC85B | +229.163QLQGHLGEIR
Scan Number: 12707 precMass: 460.6028 precCharge: 3 Sequence: QLQGHLGEIR
Intensity
2500000
2000000
1500000
1000000
500000
0
M/Z
300
600
900
1200
y1
y2
b1
b2
y4
y5
b3
b4
y6
y7
b5
type
b
y

CCDC85B | +229.163LAELEGR

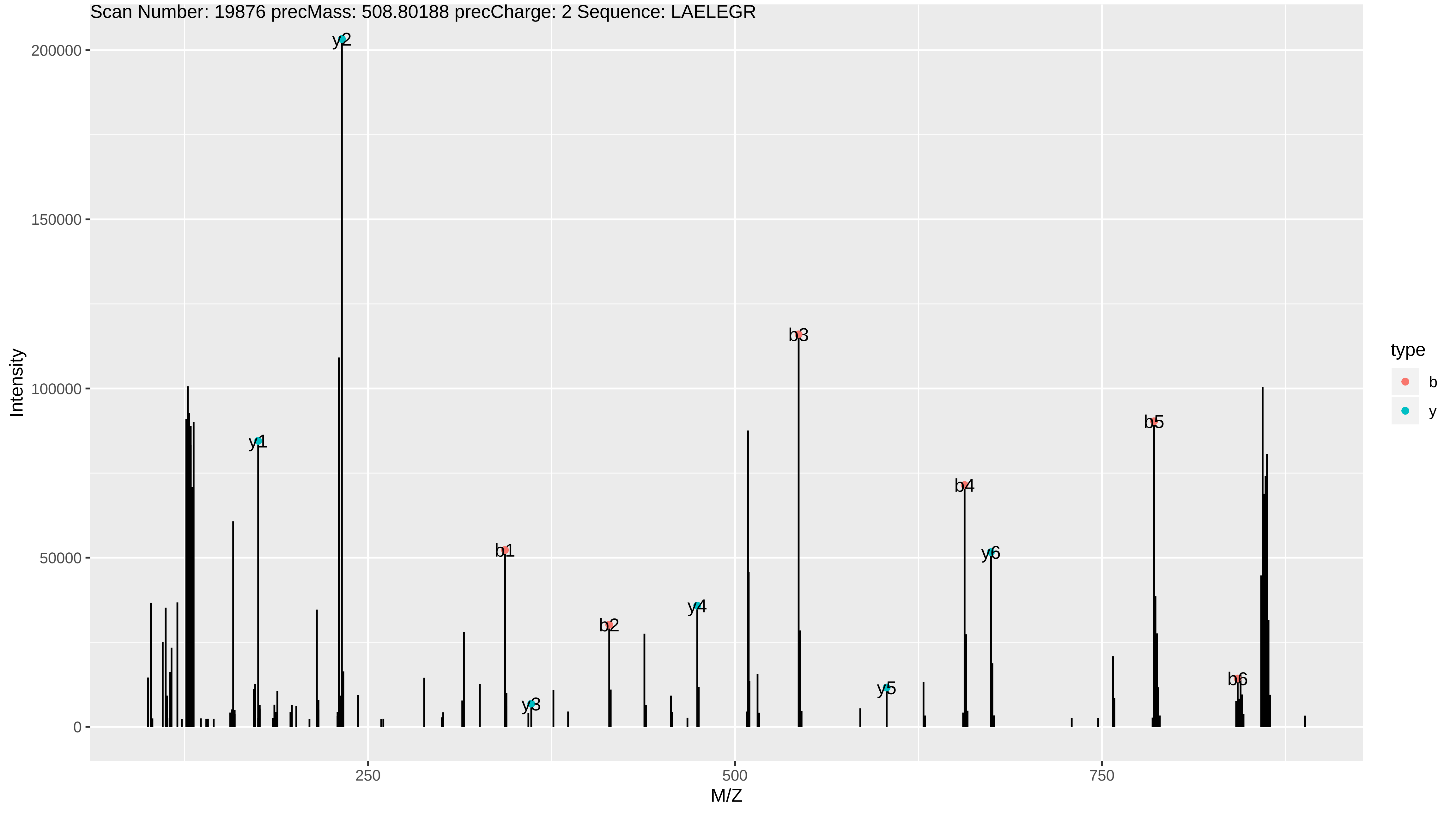

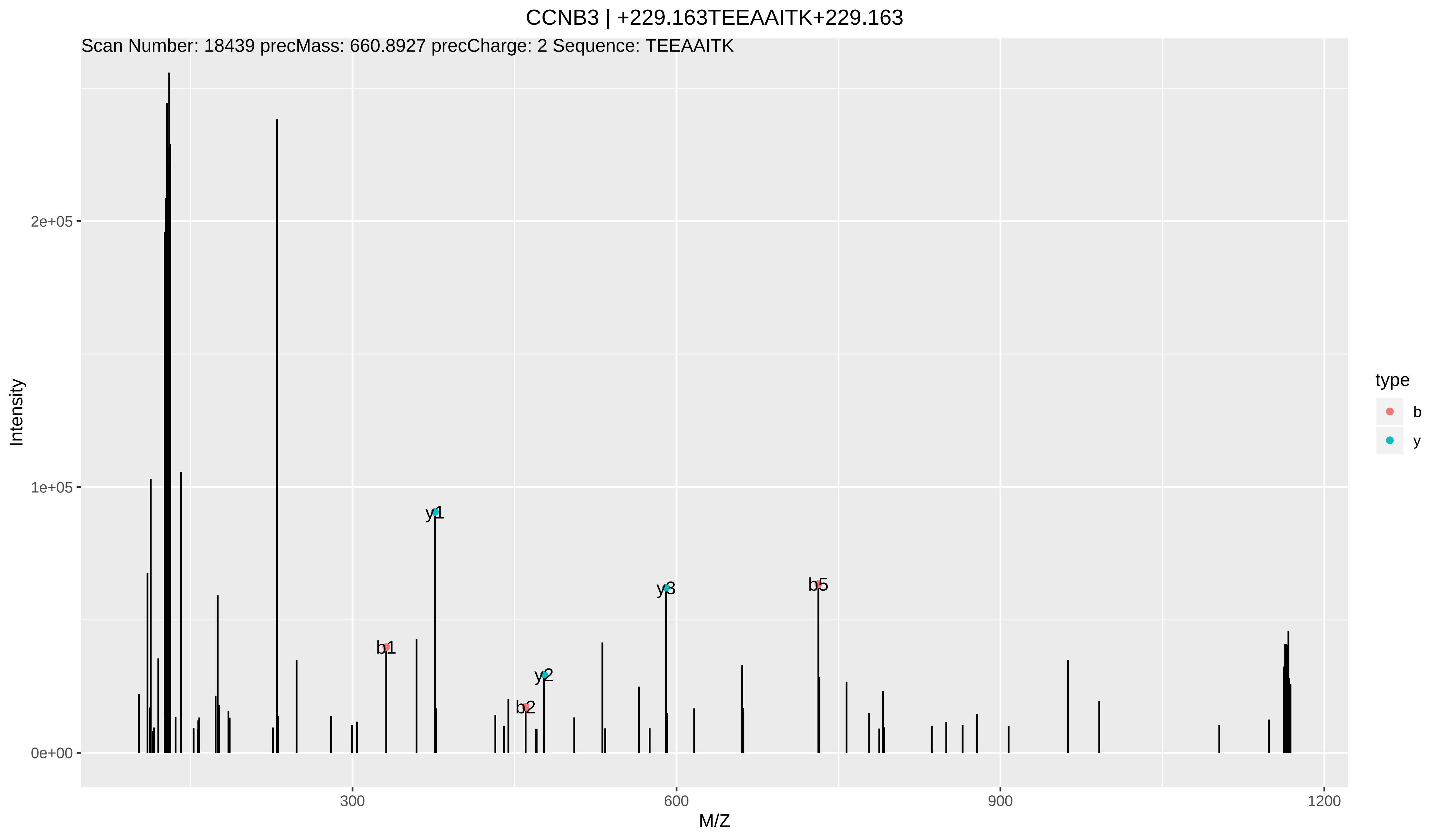

CCNB3 | +229.163TEEAAITK+229.163
Scan Number: 18439 precMass: 660.8927 precCharge: 2 Sequence: TEEAAITK
Intensity
2e+05
1e+05
0e+00
M/Z
300
600
900
1200
y1
b1
y2
b2
y3
b5
type
b
y

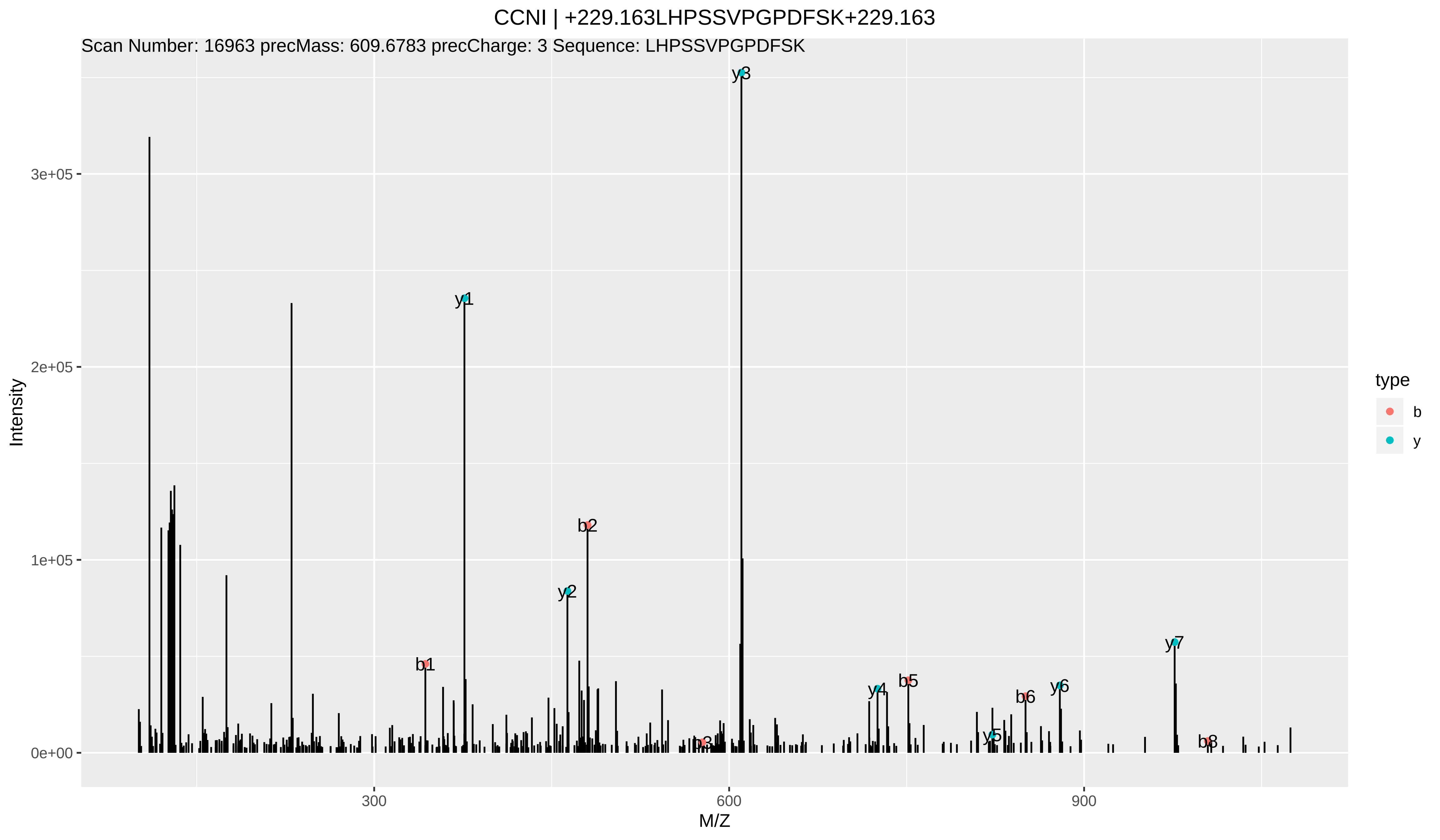

CCNI | +229.163LHPSSVPGPDFSK+229.163
Scan Number: 16963 precMass: 609.6783 precCharge: 3 Sequence: LHPSSVPGPDFSK
Intensity
M/Z
3e+05
2e+05
1e+05
0e+00
300
600
900
y3
y1
b2
y2
b1
b3
y4
b5
y5
b6
y6
y7
b8
type
b
y

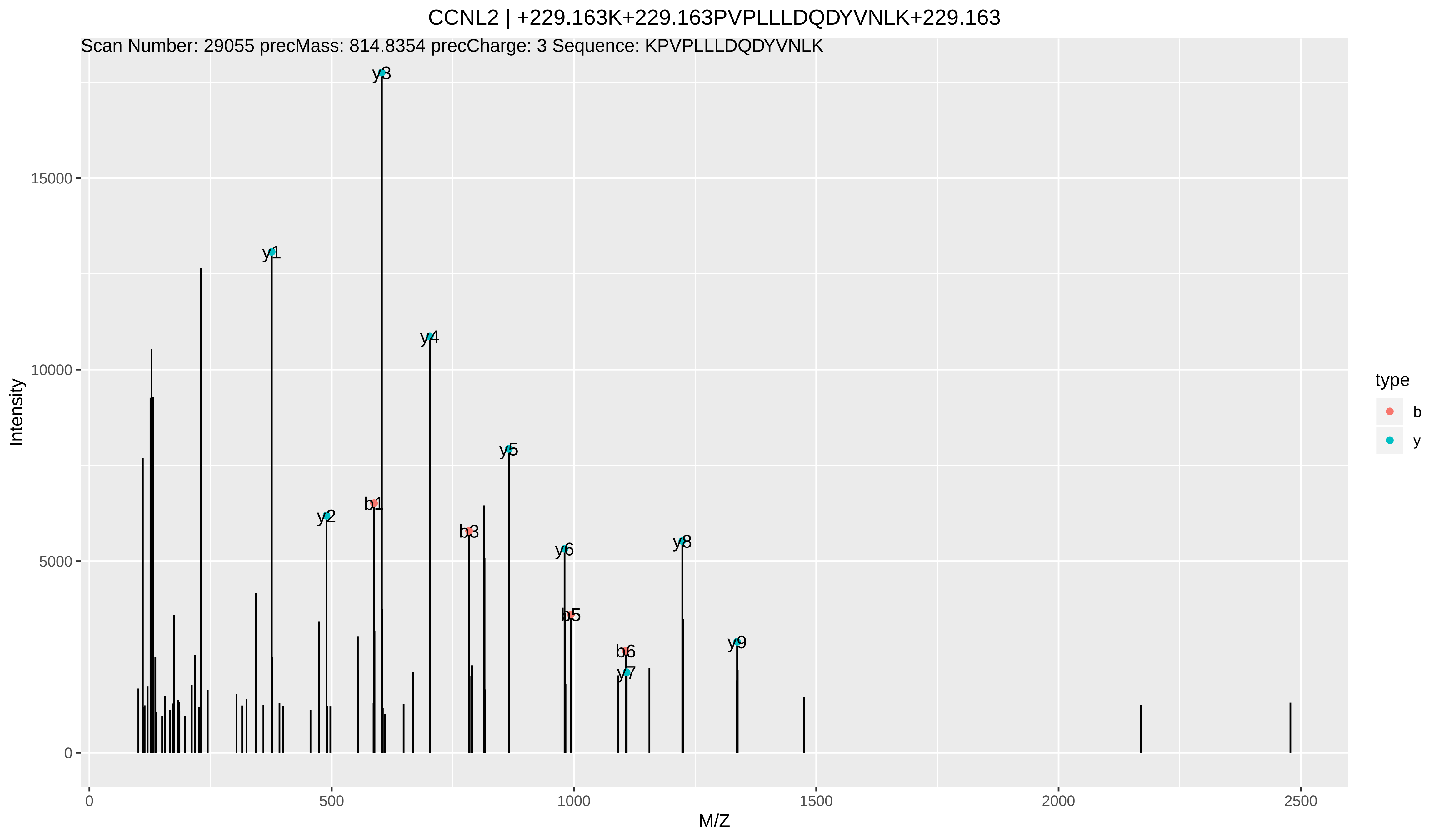
CCNL2 | +229.163K+229.163PVPLLLDQDYVNLK+229.163
Scan Number: 29055 precMass: 814.8354 precCharge: 3 Sequence: KPVPLLLDQDYVNLK
y3
y1
y4
y5
b1
y2
b3
y6
y8
b5
b6
y9
y7
Intensity
15000
10000
5000
0
0
500
1000
1500
2000
2500
M/Z
type
b
y

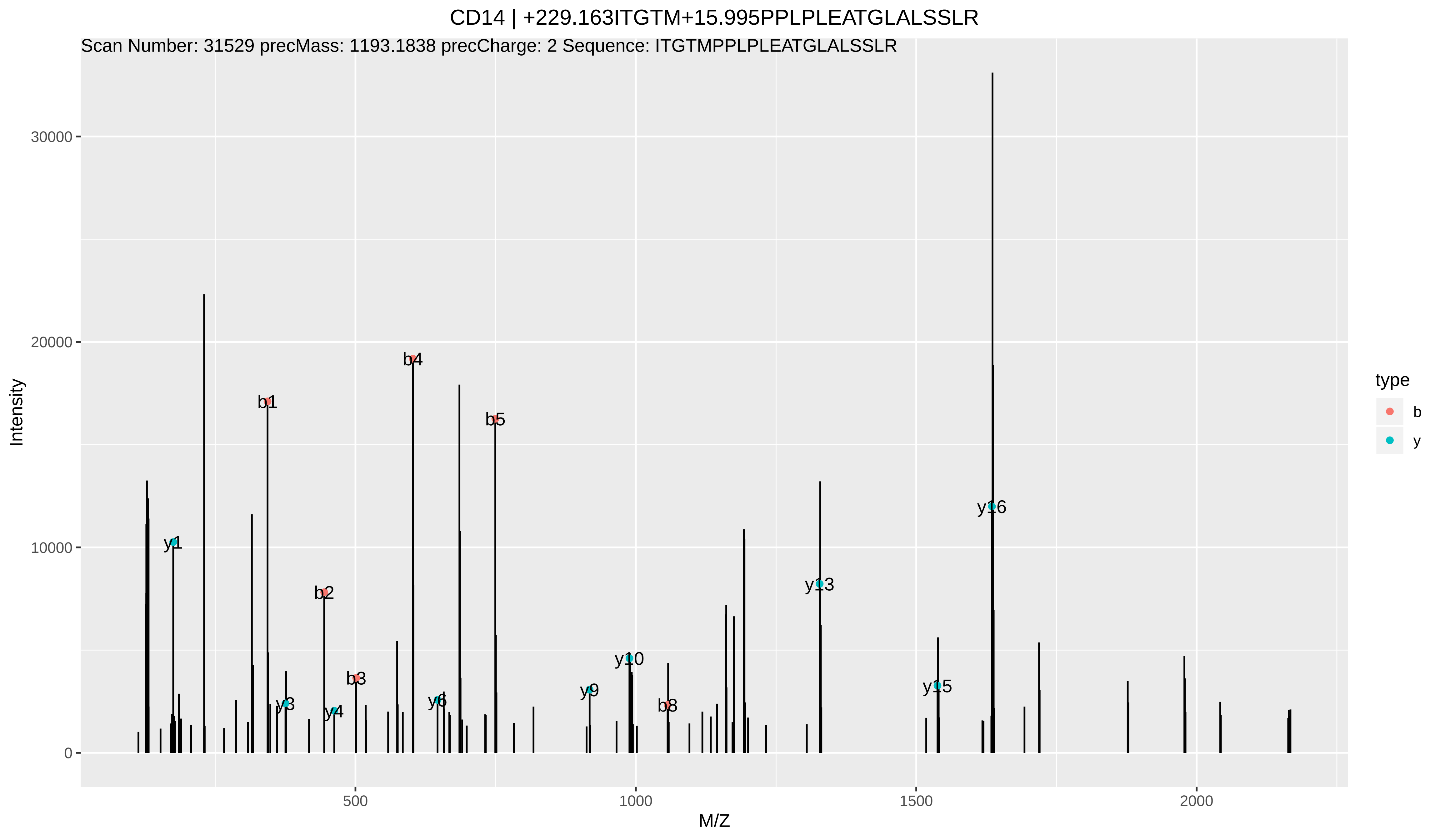
CD14 | +229.163ITGTM+15.995PPLPLEATGLALSSLR
Scan Number: 31529 precMass: 1193.1838 precCharge: 2 Sequence: ITGTMPPLPLEATGLALSSLR
Intensity
M/Z
type
b
y
y1
b1
b2
b3
b4
b5
b8
y3
y4
y6
y9
y10
y13
y15
y16
0
10000
20000
30000
500
1000
1500
2000

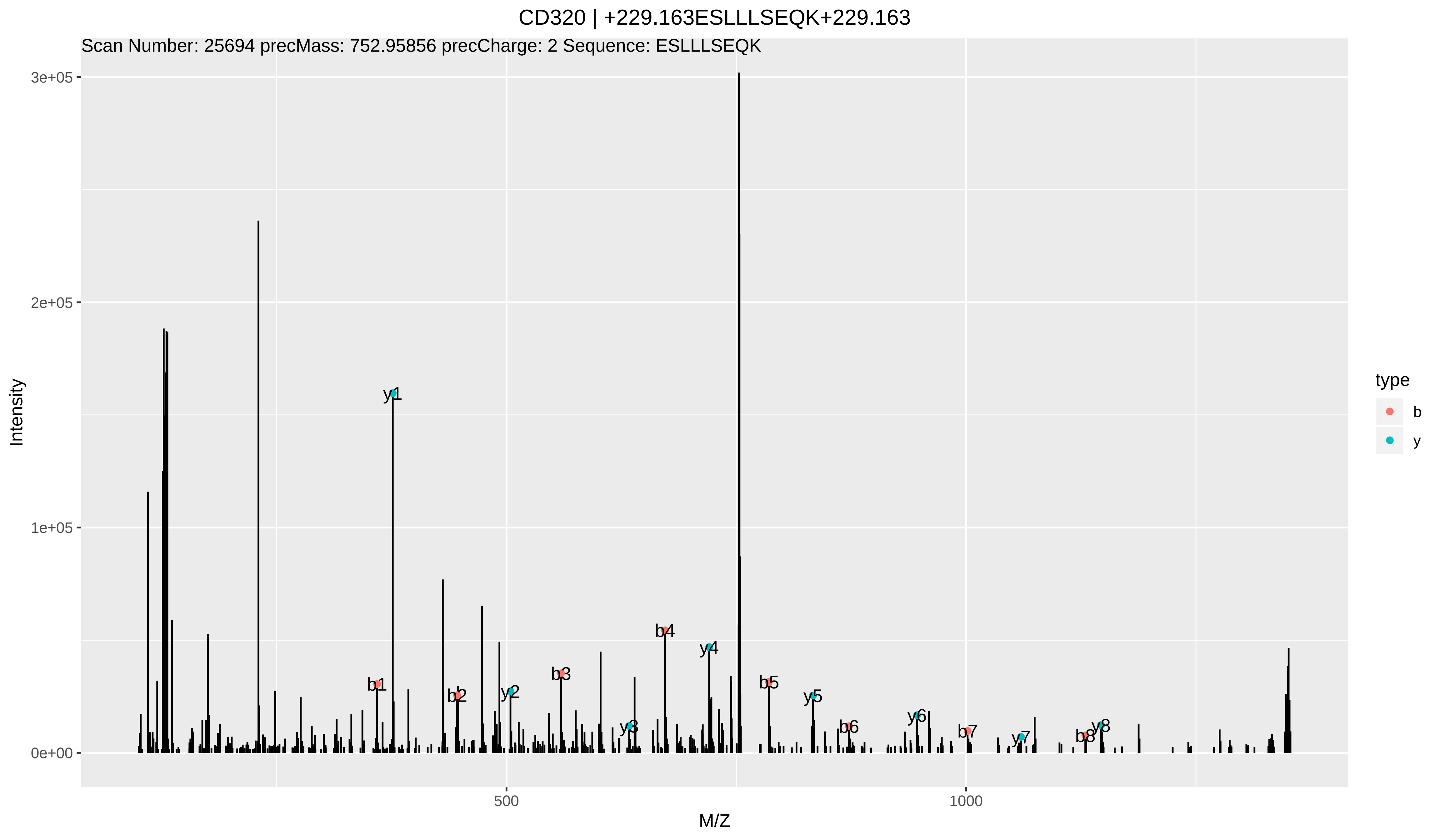

CD320 | +229.163ESLLLSEQK+229.163
Scan Number: 25694 precMass: 752.95856 precCharge: 2 Sequence: ESLLLSEQK
Intensity
3e+05
2e+05
1e+05
0e+00
M/Z
500
1000
y1
b1
b2
y2
b3
y3
b4
y4
b5
y5
b6
y6
b7
y7
b8
y8
type
b
y

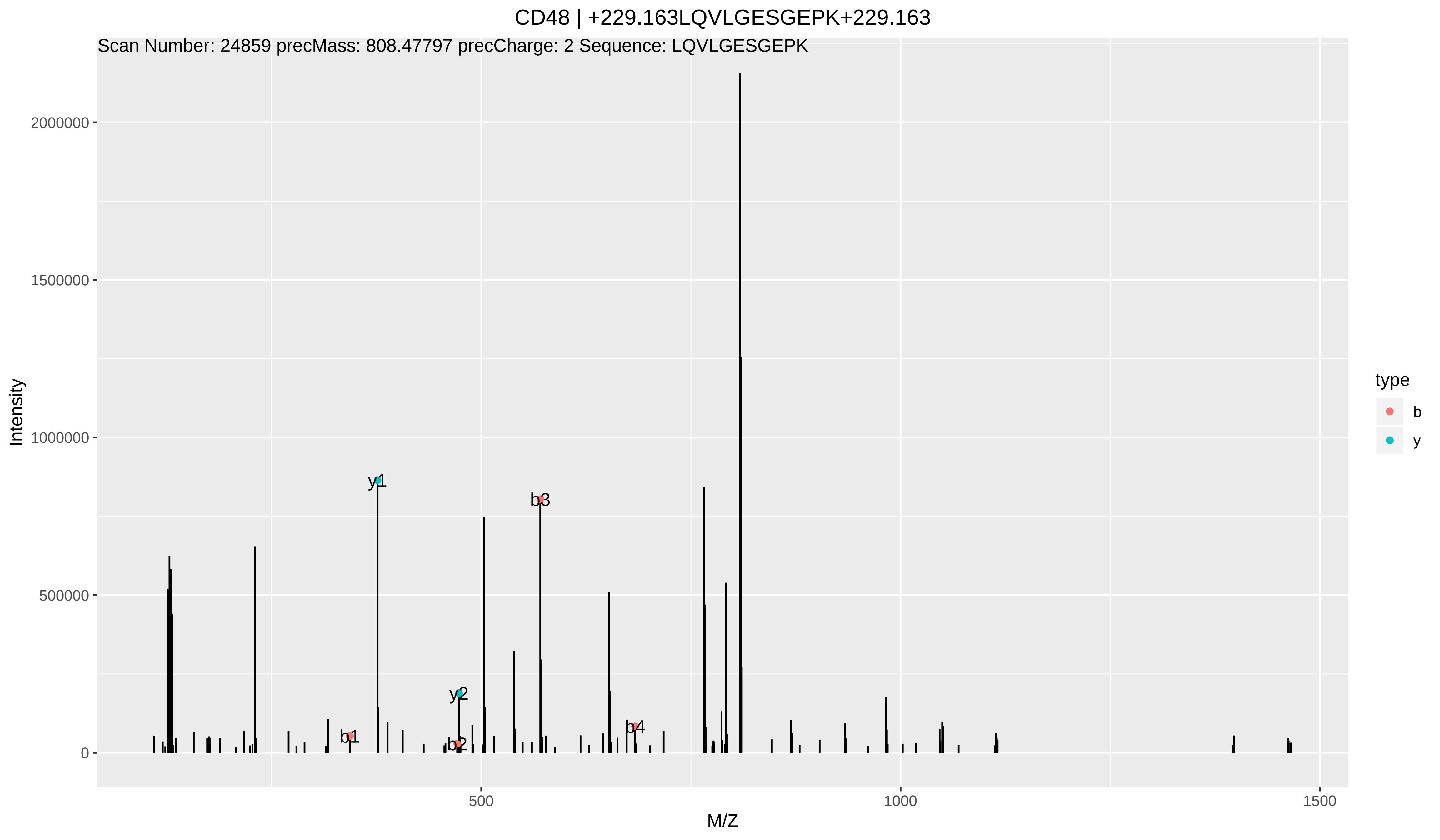
CD48 | +229.163LQVLGESGEPK+229.163
Scan Number: 24859 precMass: 808.47797 precCharge: 2 Sequence: LQVLGESGEPK
Intensity
2000000
1500000
1000000
500000
0
M/Z
500
1000
1500
y1
b3
y2
b1
b2
b4
type
b
y

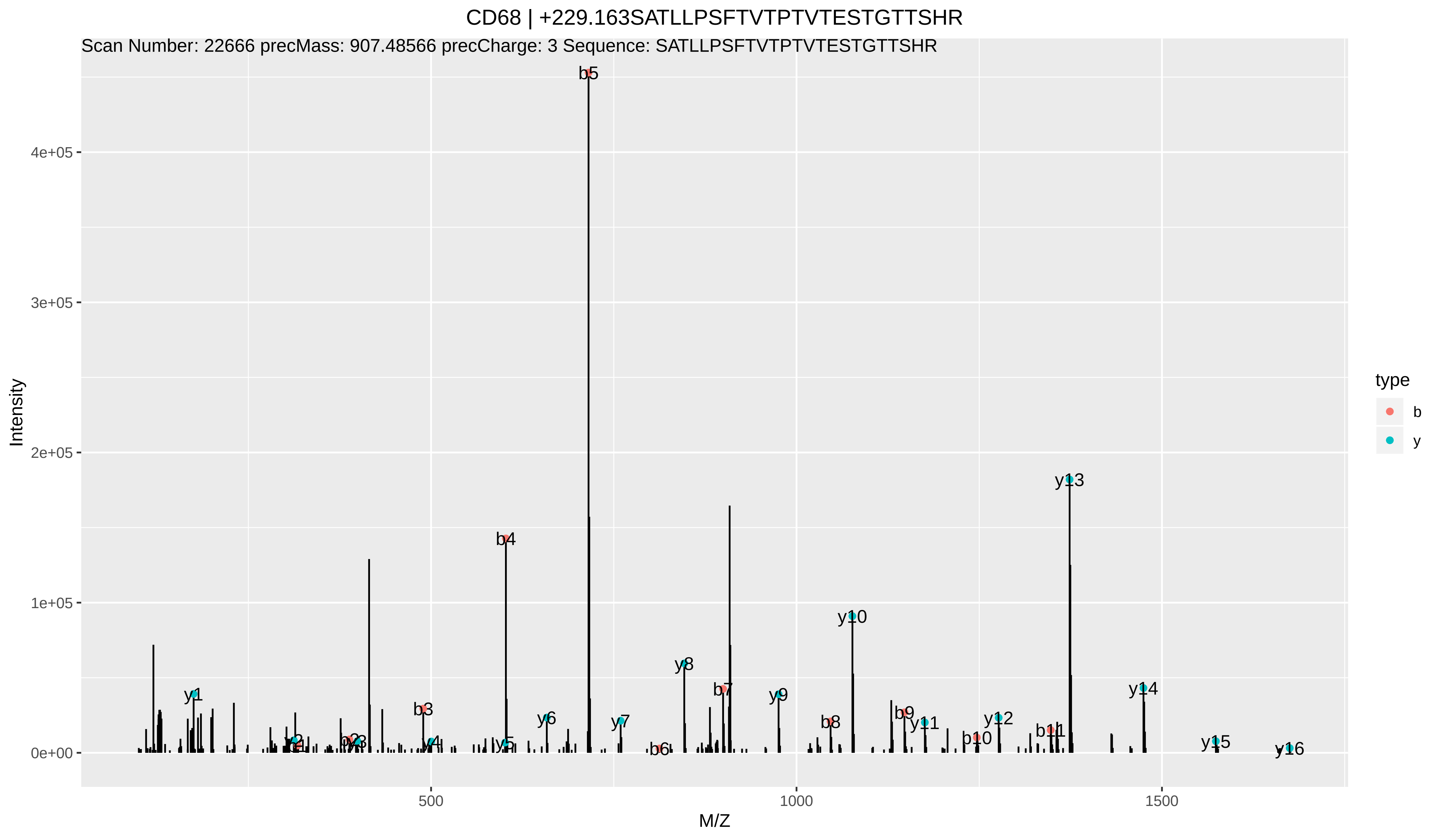

CD68 | +229.163SATLLPSFTVTPTVTESTGTTSHR
Scan Number: 22666 precMass: 907.48566 precCharge: 3 Sequence: SATLLPSFTVTPTVTESTGTTSHR
Intensity
M/Z
0e+00
1e+05
2e+05
3e+05
4e+05
500
1000
1500
b5
b4
b3
b6
b7
b8
b9
b10
b11
y1
y2
y4
y5
y6
y7
y8
y9
y10
y11
y12
y13
y14
y15
y16
type
b
y

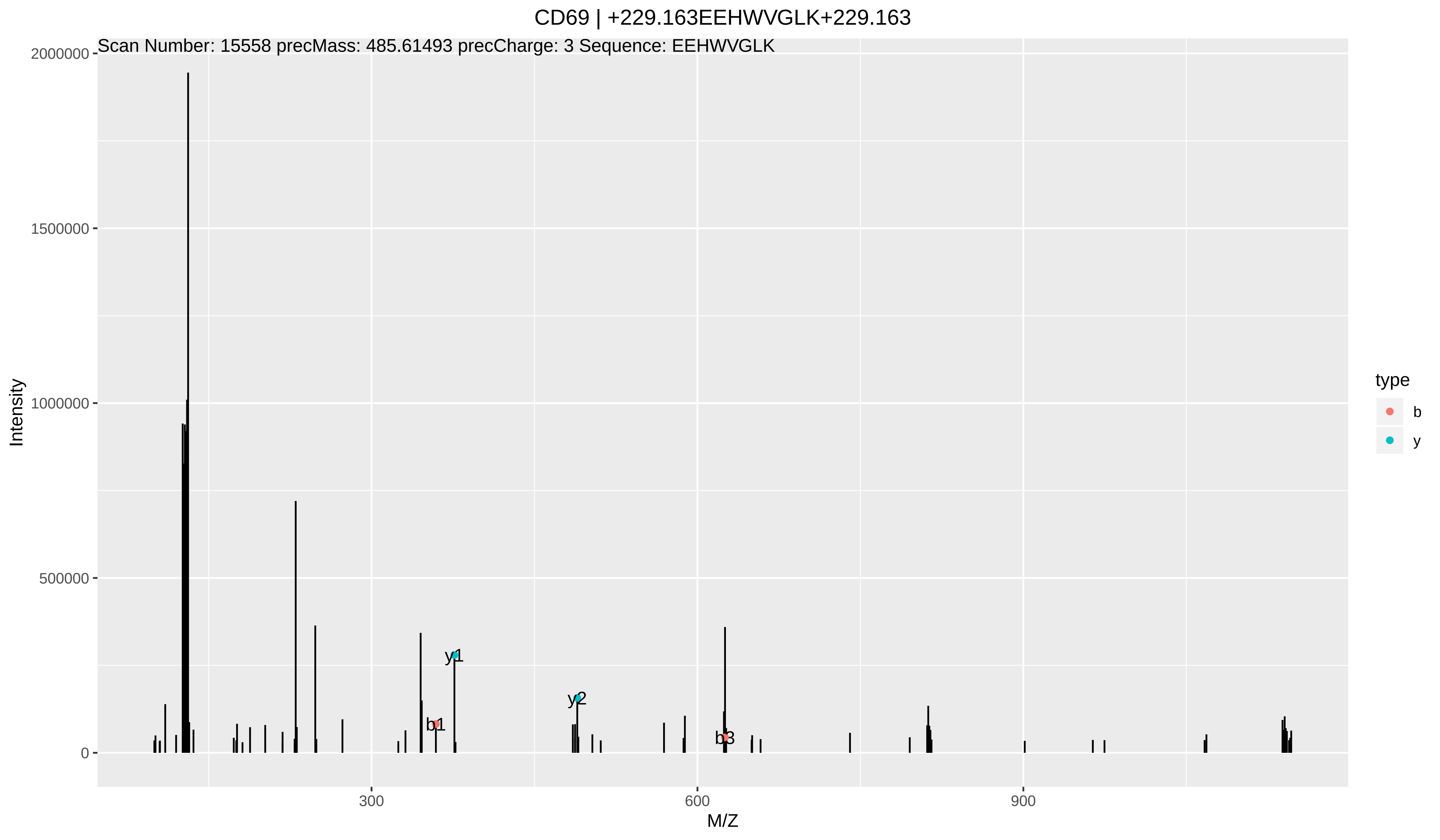

CD69 | +229.163EEHWVGLK+229.163
Scan Number: 15558 precMass: 485.61493 precCharge: 3 Sequence: EEHWVGLK
Intensity
2000000
1500000
1000000
500000
0
M/Z
300
600
900
y1
y2
b1
b3
type
b
y

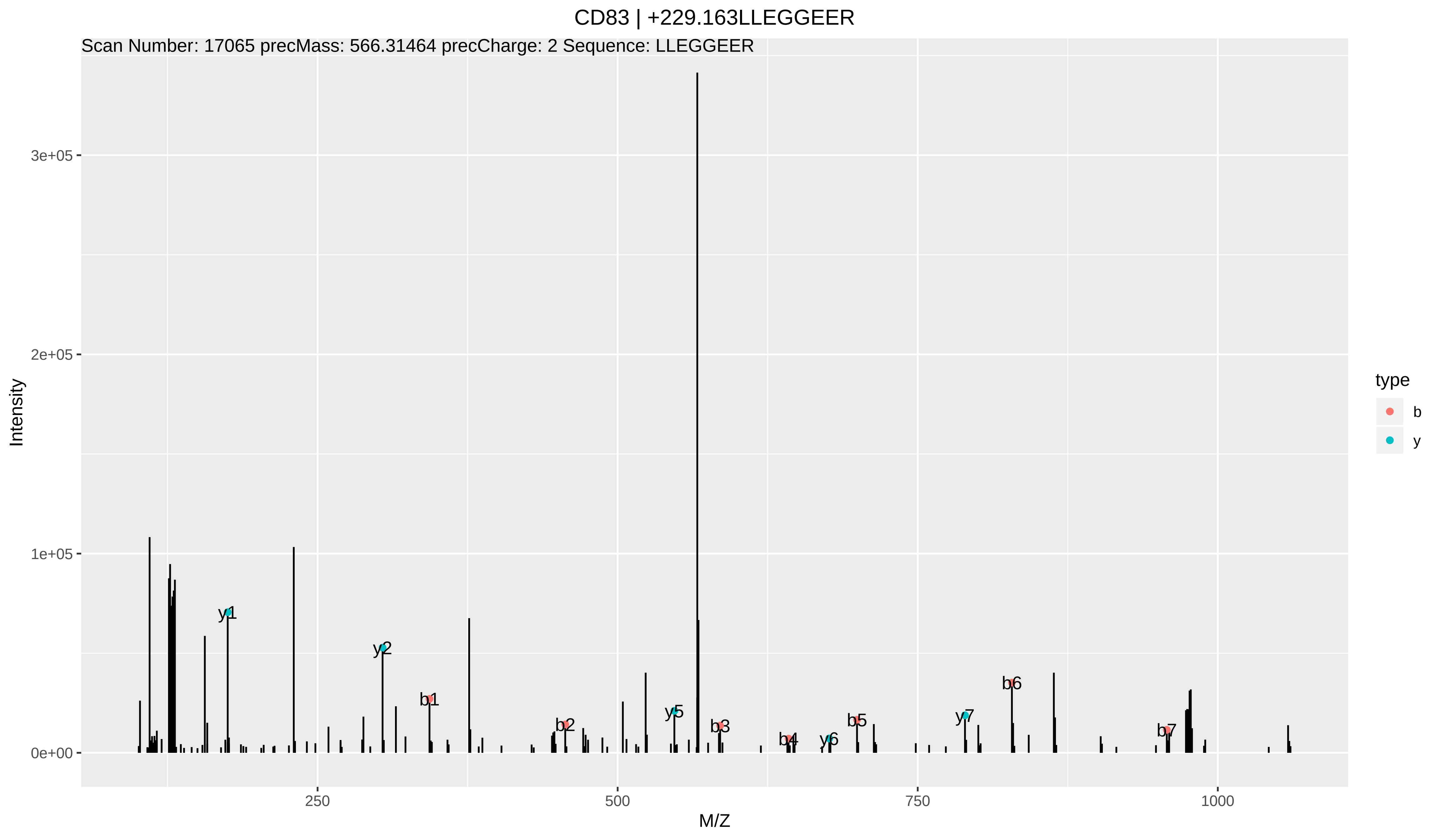
CD83 | +229.163LLEGGEER
Scan Number: 17065 precMass: 566.31464 precCharge: 2 Sequence: LLEGGEER
Intensity
3e+05
2e+05
1e+05
0e+00
M/Z
250
500
750
1000
y1
y2
b1
b2
y5
b3
b4
y6
b5
y7
b6
b7
type
b
y

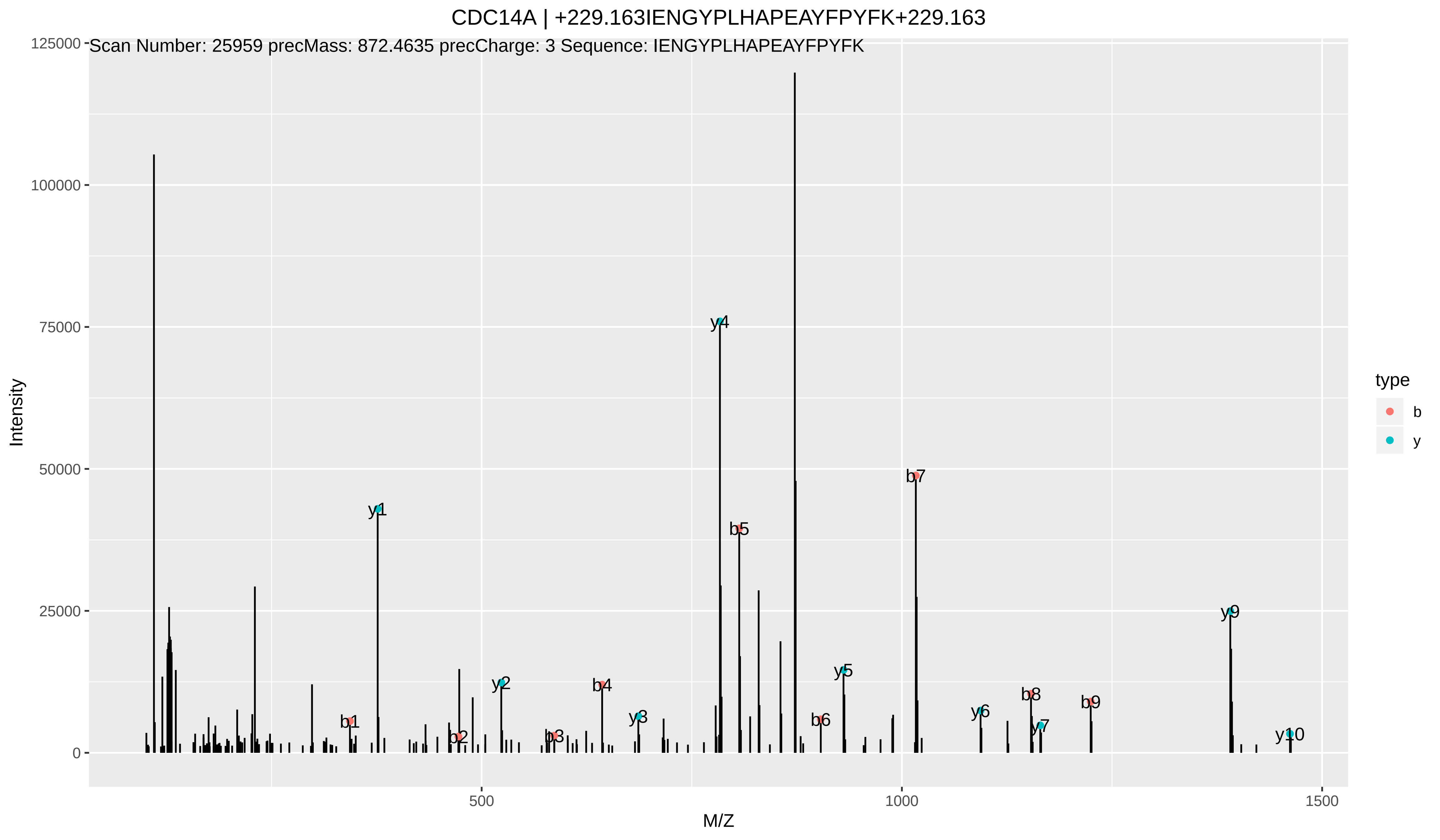
CDC14A | +229.163IENGYPLHAPEAYFPYFK+229.163
Scan Number: 25959 precMass: 872.4635 precCharge: 3 Sequence: IENGYPLHAPEAYFPYFK
Intensity
125000
100000
75000
50000
25000
0
M/Z
500
1000
1500
y1
y2
y3
y4
y5
y6
y7
y9
y10
b1
b2
b3
b4
b5
b6
b7
b8
b9
type
b
y

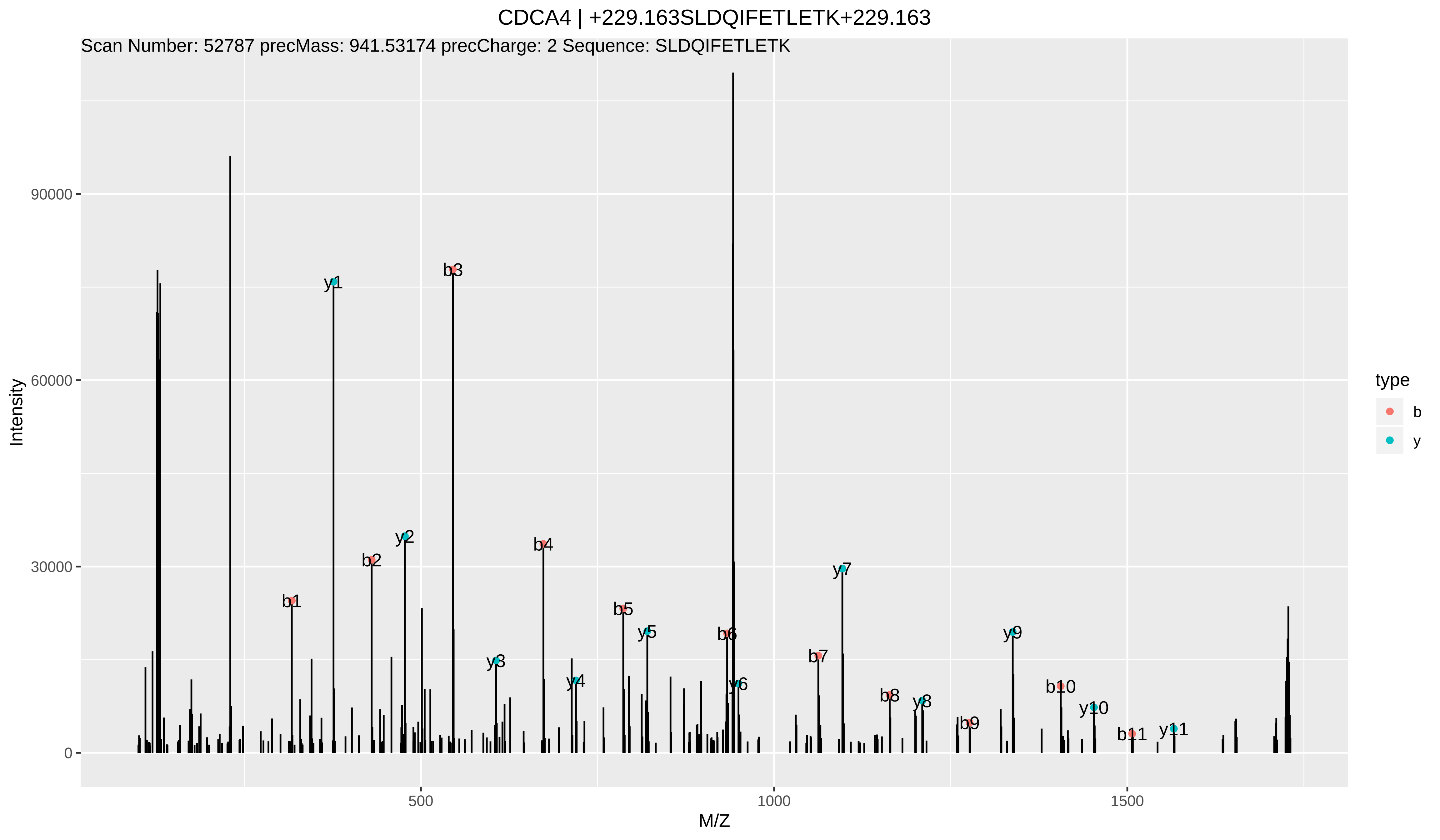
CDCA4 | +229.163SLDQIFETLETK+229.163
Scan Number: 52787 precMass: 941.53174 precCharge: 2 Sequence: SLDQIFETLETK
Intensity
M/Z
0
30000
60000
90000
500
1000
1500
b1
y1
b2
y2
b3
y3
b4
y4
b5
y5
b6
y6
b7
y7
b8
y8
b9
y9
b10
y10
b11
y11
type
b
y

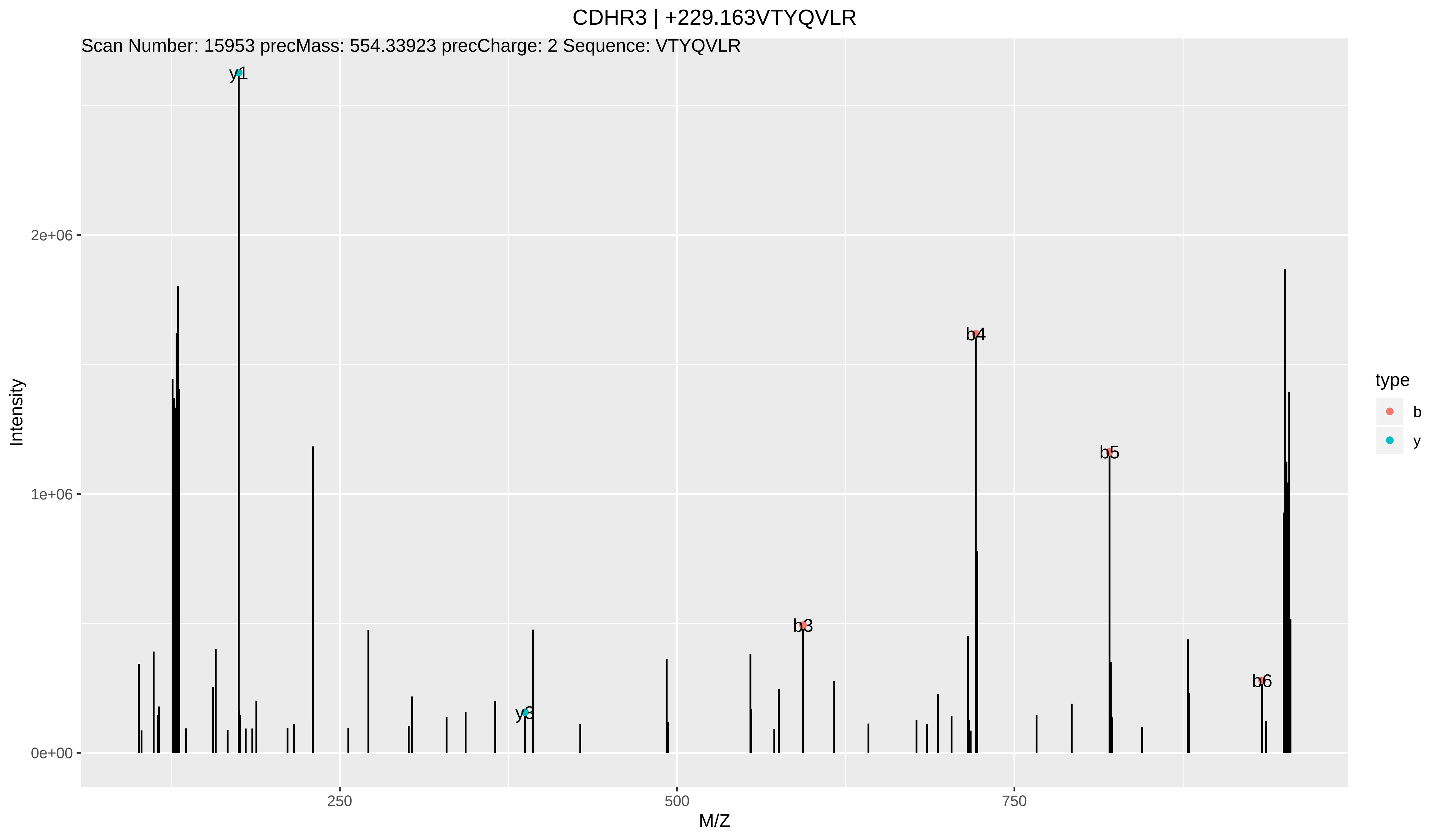

CDHR3 | +229.163VTYQVLR
Scan Number: 15953 precMass: 554.33923 precCharge: 2 Sequence: VTYQVLR
y1
y3
b3
b4
b5
b6
2e+06
1e+06
0e+00
Intensity
250
500
750
M/Z
type
b
y

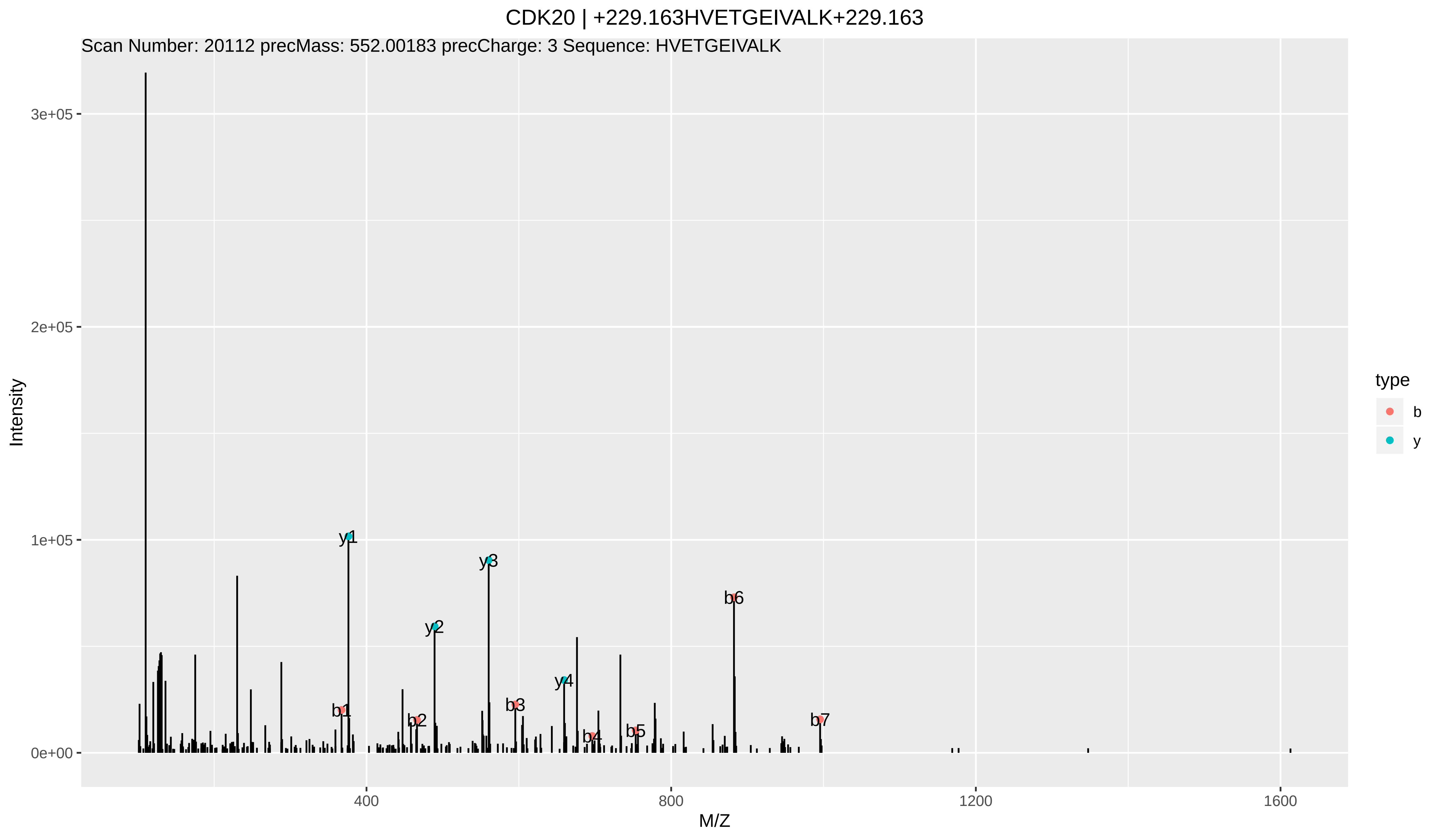
CDK20 | +229.163HVETGEIVALK+229.163
Scan Number: 20112 precMass: 552.00183 precCharge: 3 Sequence: HVETGEIVALK
Intensity
M/Z
0e+00
1e+05
2e+05
3e+05
400
800
1200
1600
y1
y2
y3
y4
b1
b2
b3
b4
b5
b6
b7
type
b
y

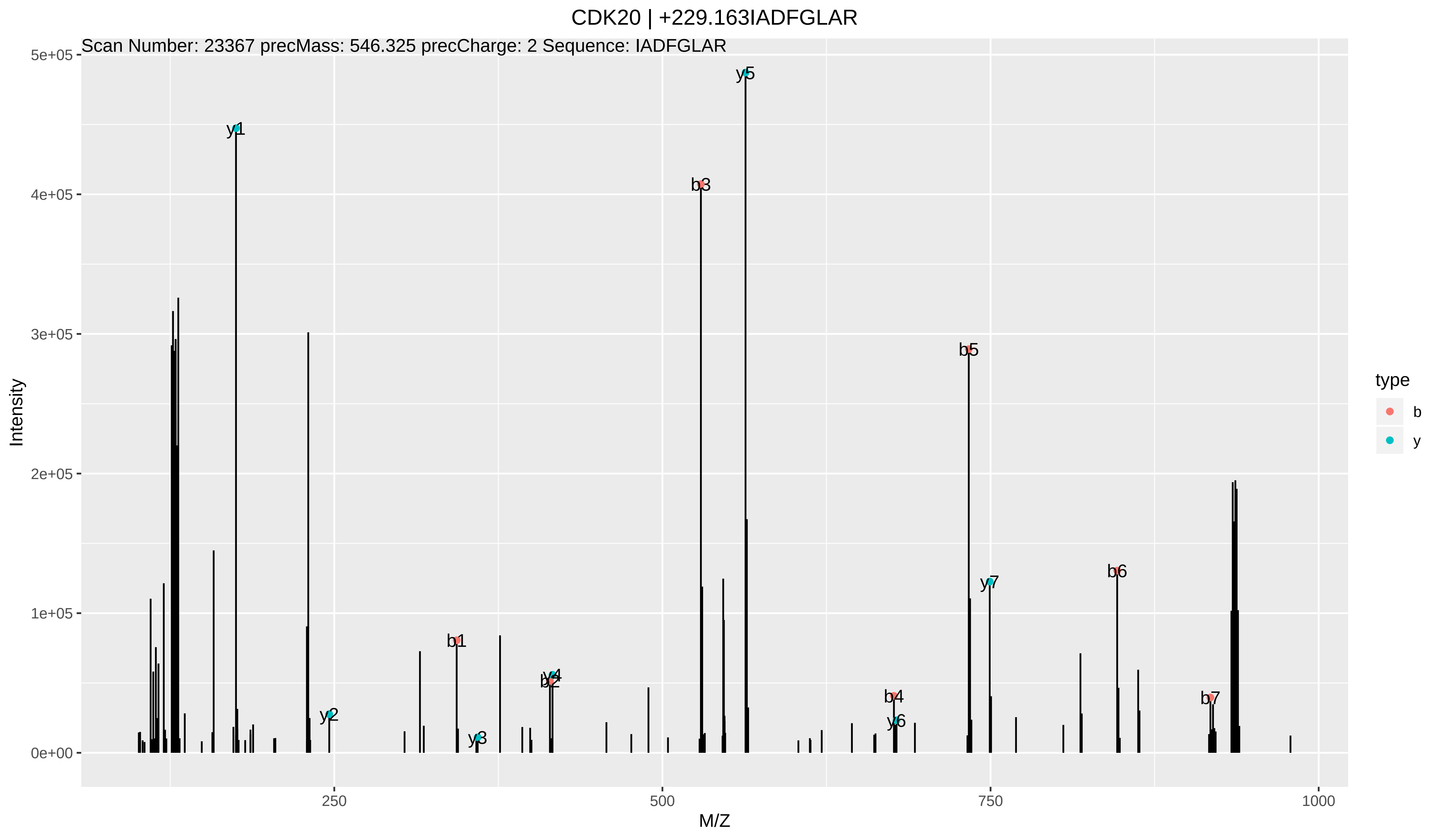

CDK20 | +229.163IADFGLAR
Scan Number: 23367 precMass: 546.325 precCharge: 2 Sequence: IADFGLAR
Intensity
M/Z
0e+00
1e+05
2e+05
3e+05
4e+05
5e+05
250
500
750
1000
y1
y2
y3
y4
y5
y6
y7
b1
b2
b3
b4
b5
b6
b7
type
b
y

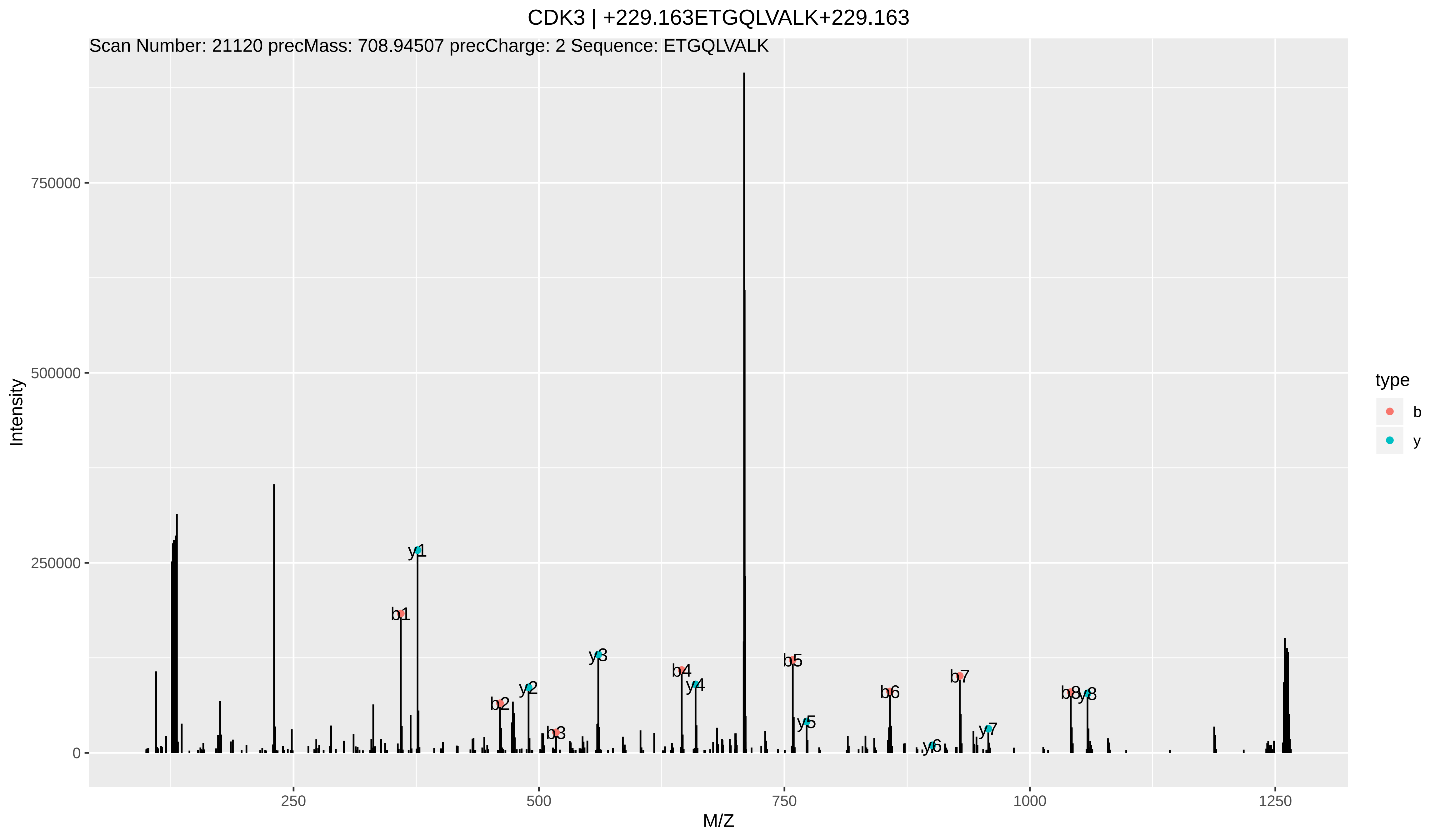
CDK3 | +229.163ETGQLVALK+229.163
Scan Number: 21120 precMass: 708.94507 precCharge: 2 Sequence: ETGQLVALK
Intensity
750000
500000
250000
0
250
500
750
1000
1250
M/Z
y1
b1
b2
y2
b3
y3
b4
y4
b5
y5
b6
y6
b7
y7
b8
y8
type
b
y

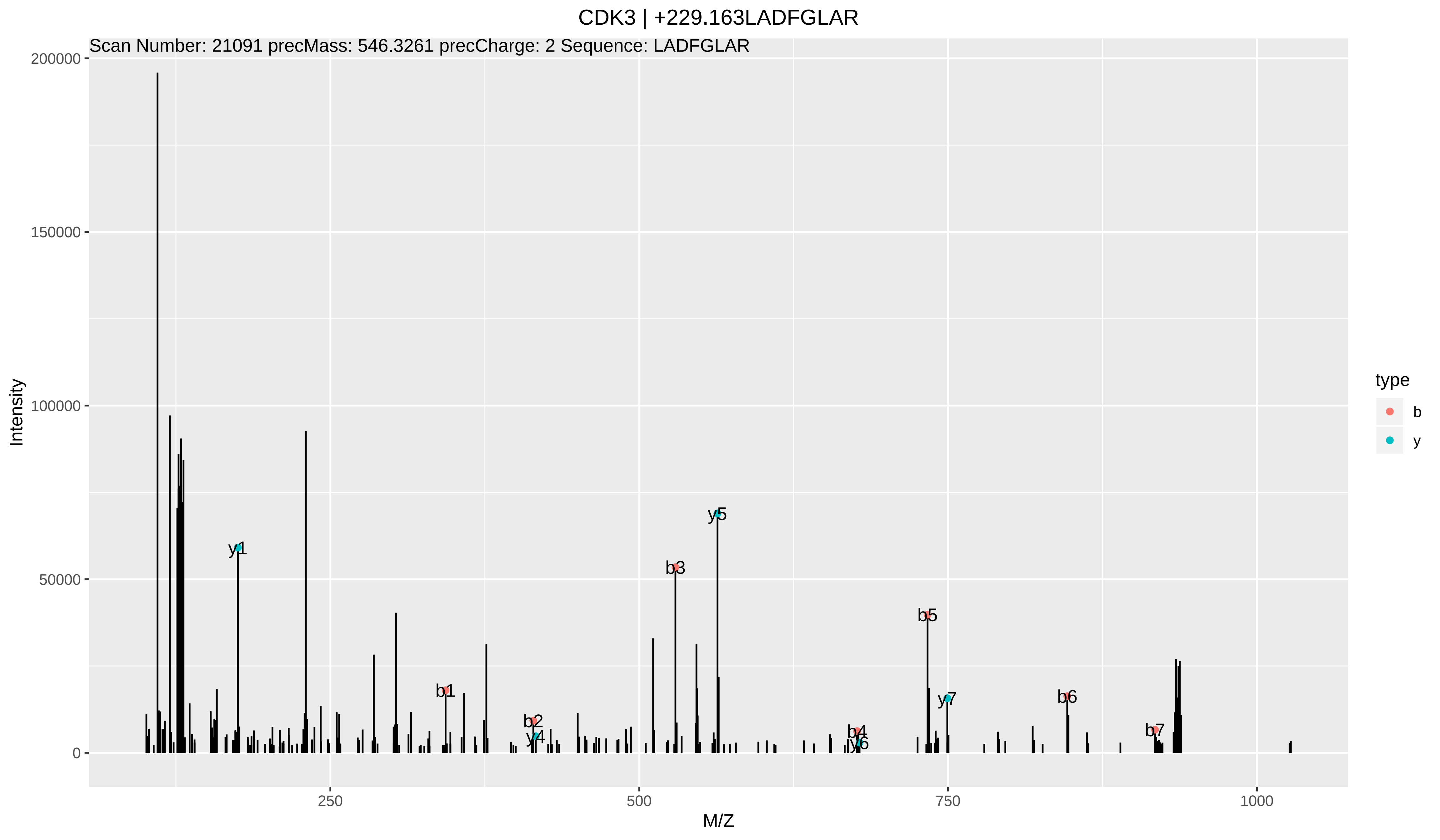

CDK3 | +229.163LADFGLAR
Scan Number: 21091 precMass: 546.3261 precCharge: 2 Sequence: LADFGLAR
Intensity
M/Z
200000
150000
100000
50000
0
250
500
750
1000
y1
b1
b2
y4
b3
y5
b4
y6
b5
y7
b6
b7
type
b
y

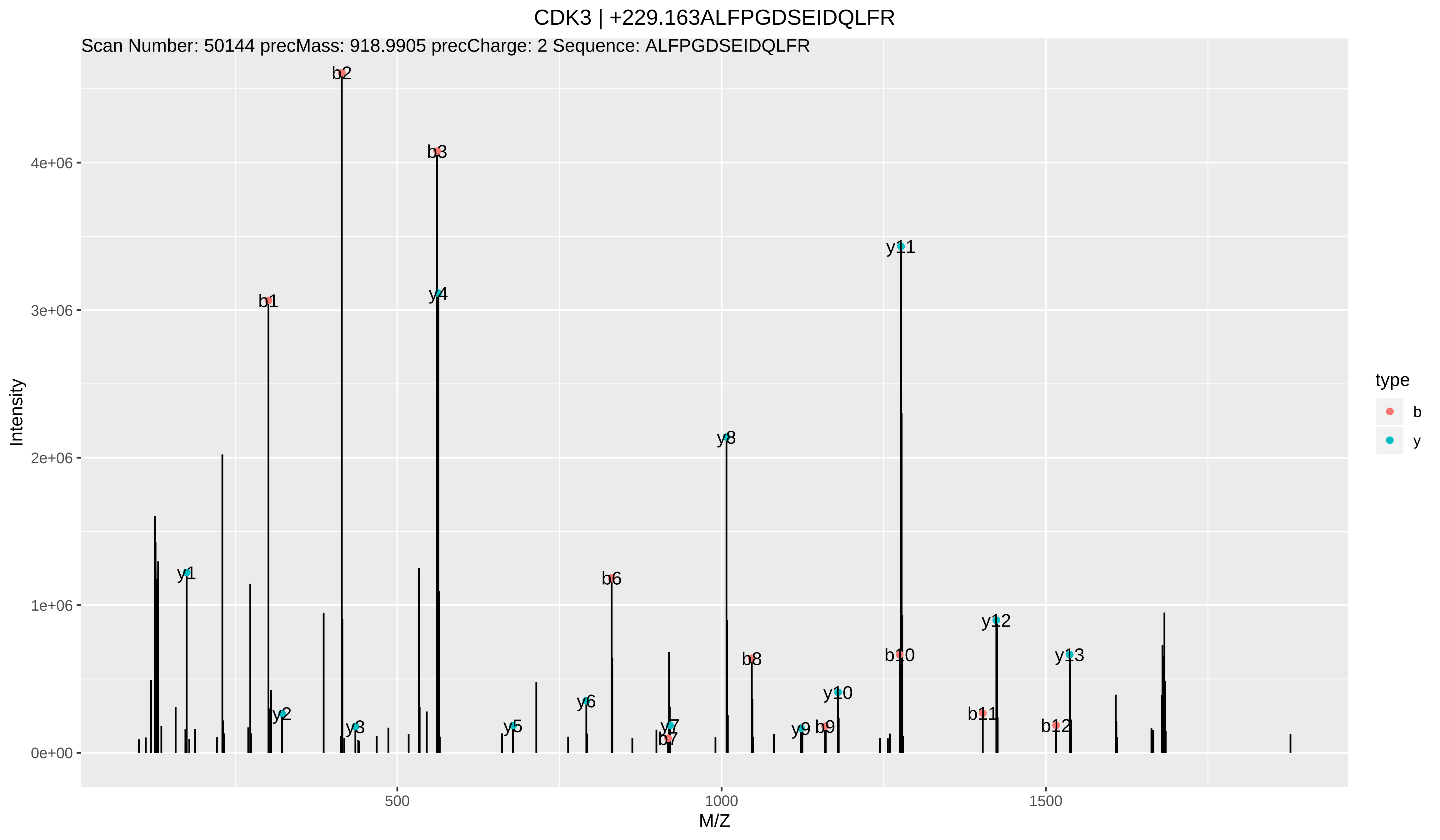

CDK3 | +229.163ALFPGDSEIDQLFR
Scan Number: 50144 precMass: 918.9905 precCharge: 2 Sequence: ALFPGDSEIDQLFR
Intensity
0e+00
1e+06
2e+06
3e+06
4e+06
M/Z
500
1000
1500
b1
b2
b3
b6
b7
b8
b9
b10
b11
b12
y1
y2
y3
y4
y5
y6
y7
y8
y9
y10
y11
y12
y13
type
b
y

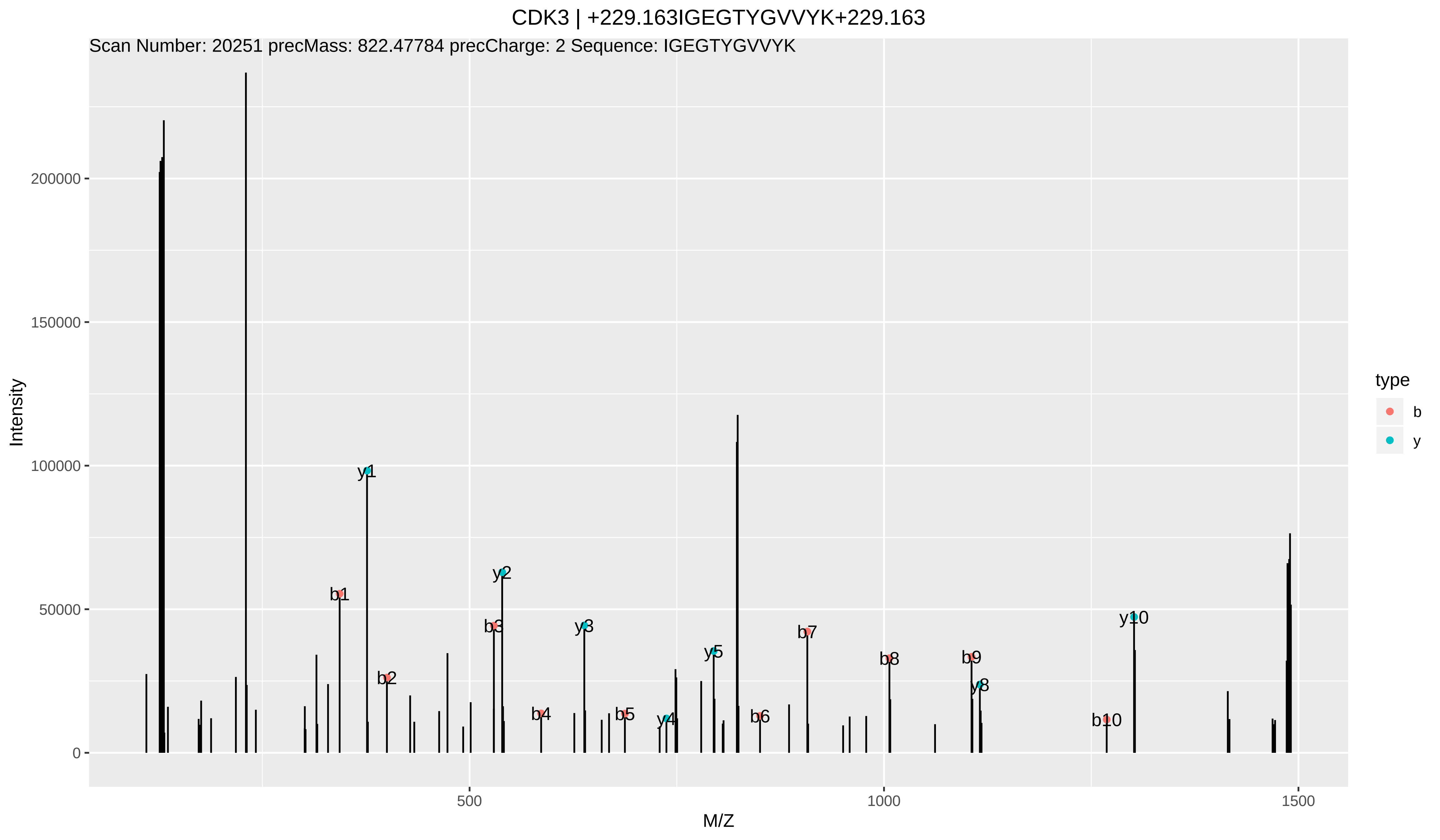

CDK3 | +229.163IGEGTYGVVYK+229.163
Scan Number: 20251 precMass: 822.47784 precCharge: 2 Sequence: IGEGTYGVVYK
Intensity
200000
150000
100000
50000
0
500
1000
1500
M/Z
y1
y2
b1
b3
y3
y10
b7
y5
b8
b9
y8
b2
b4
b5
y4
b6
b10
type
b
y

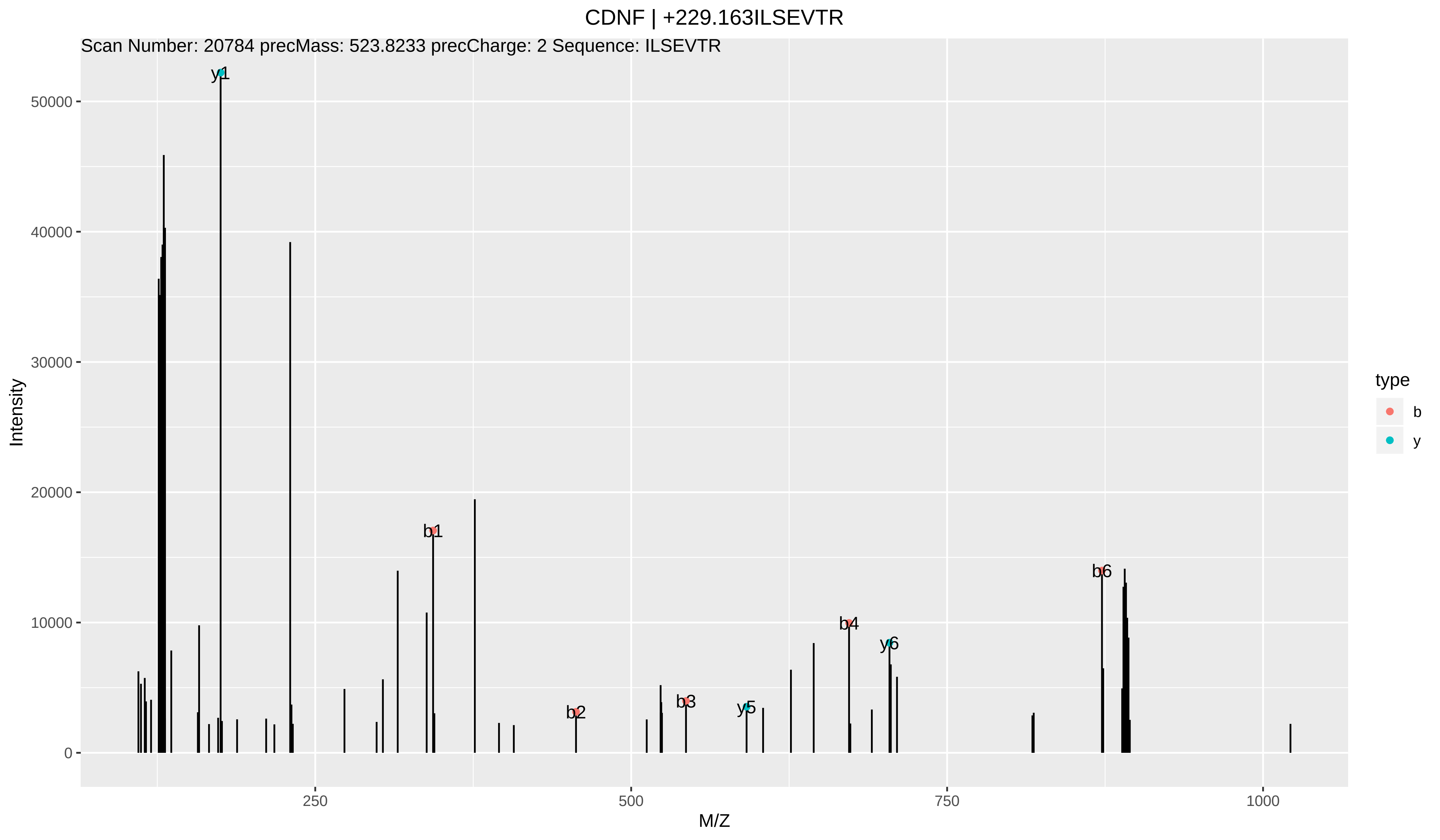

CDNF | +229.163ILSEVTR
Scan Number: 20784 precMass: 523.8233 precCharge: 2 Sequence: ILSEVTR
y1
b1
b2
b3
y5
b4
y6
b6
Intensity
50000
40000
30000
20000
10000
0
250
500
750
1000
M/Z
type
b
y

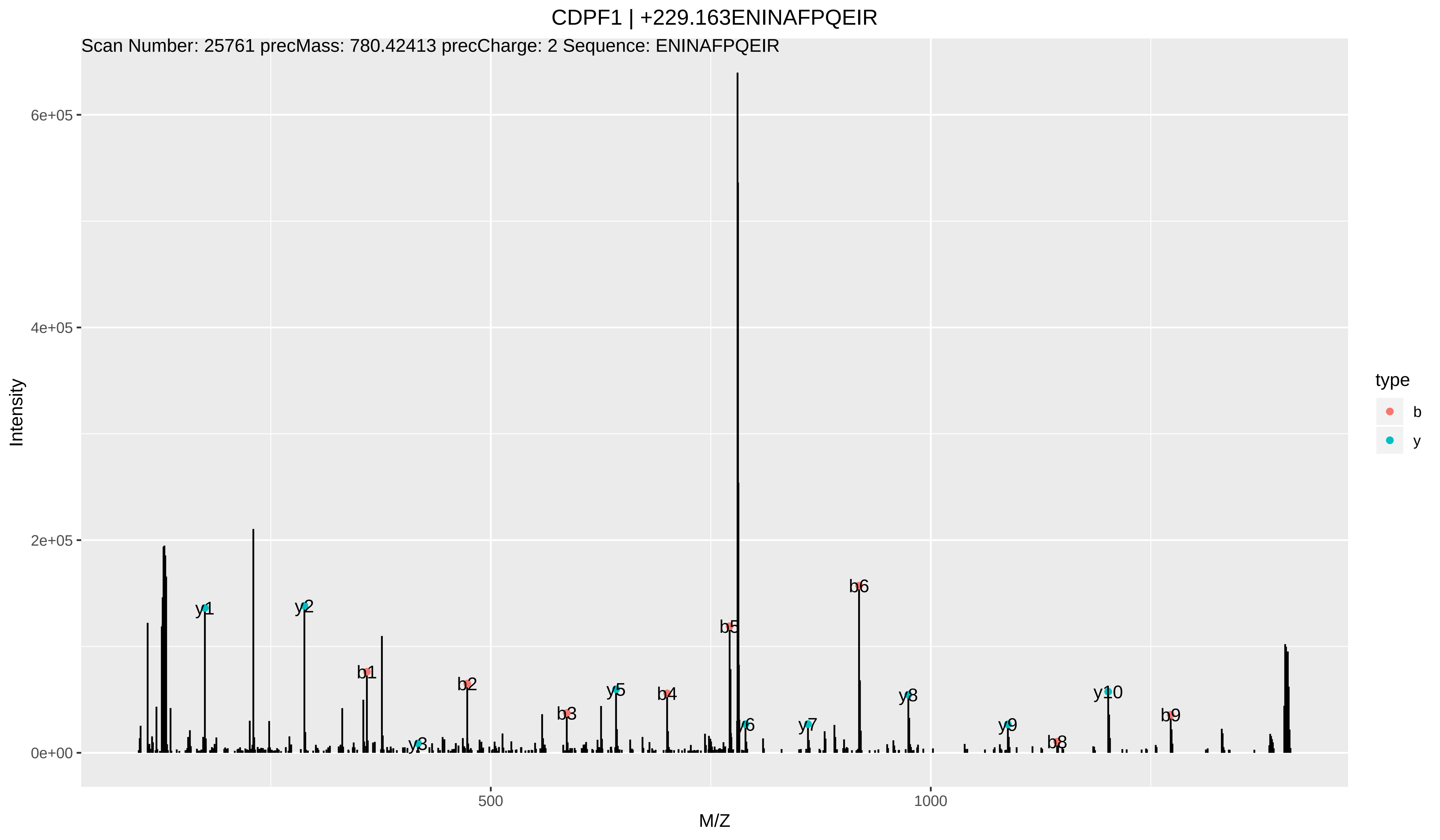

CDPF1 | +229.163ENINAFPQEIR
Scan Number: 25761 precMass: 780.42413 precCharge: 2 Sequence: ENINAFPQEIR
Intensity
M/Z
6e+05
4e+05
2e+05
0e+00
500
1000
y1
y2
b1
b2
y3
b3
y5
b4
b5
y6
y7
b6
y8
y9
b8
y10
b9
type
b
y

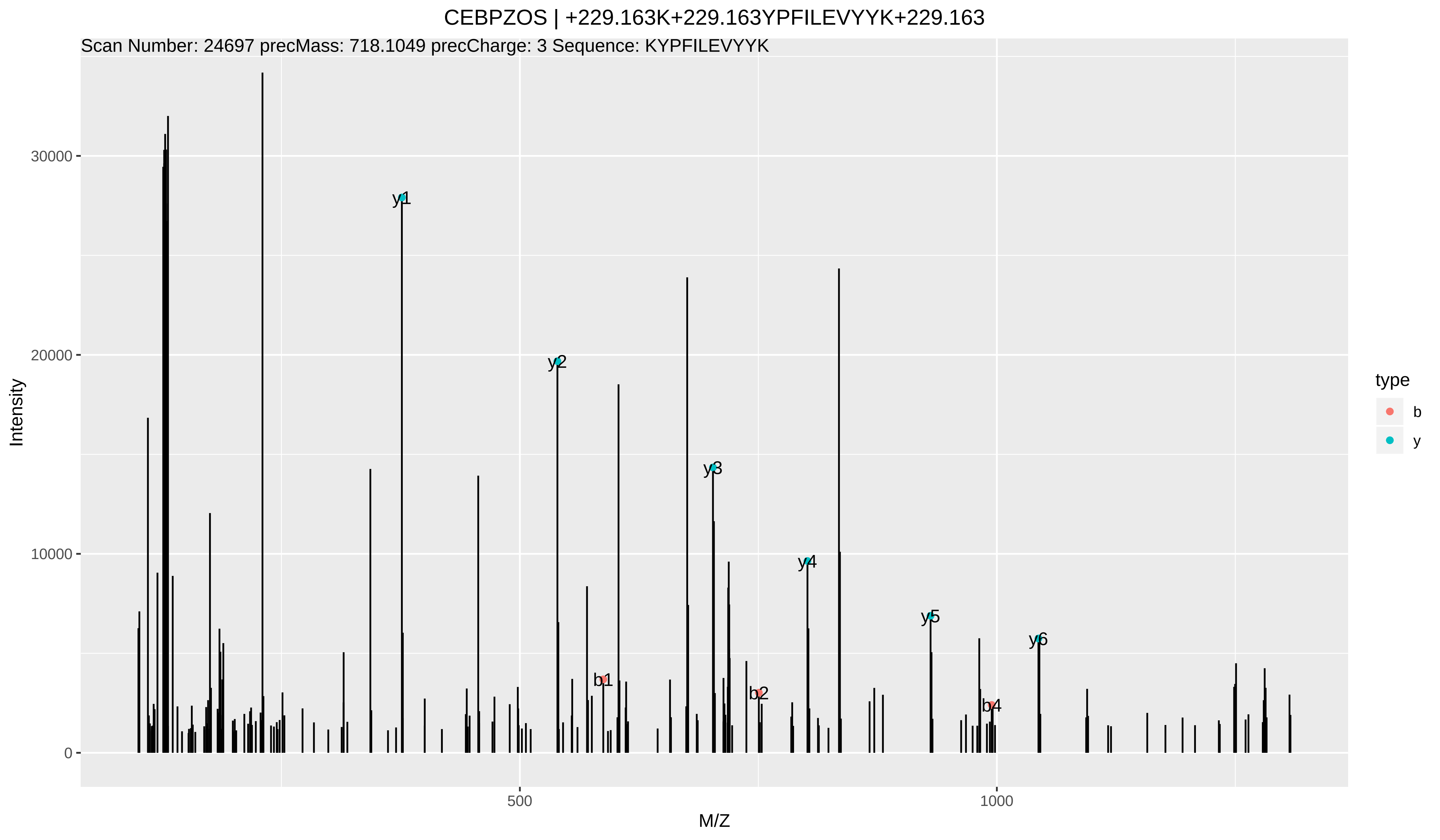

CEBPZOS | +229.163K+229.163YPFILEVYYK+229.163
Scan Number: 24697 precMass: 718.1049 precCharge: 3 Sequence: KYPFILEVYYK
Intensity
30000
20000
10000
0
500
1000
M/Z
y1
y2
y3
y4
y5
y6
b1
b2
b4
type
b
y

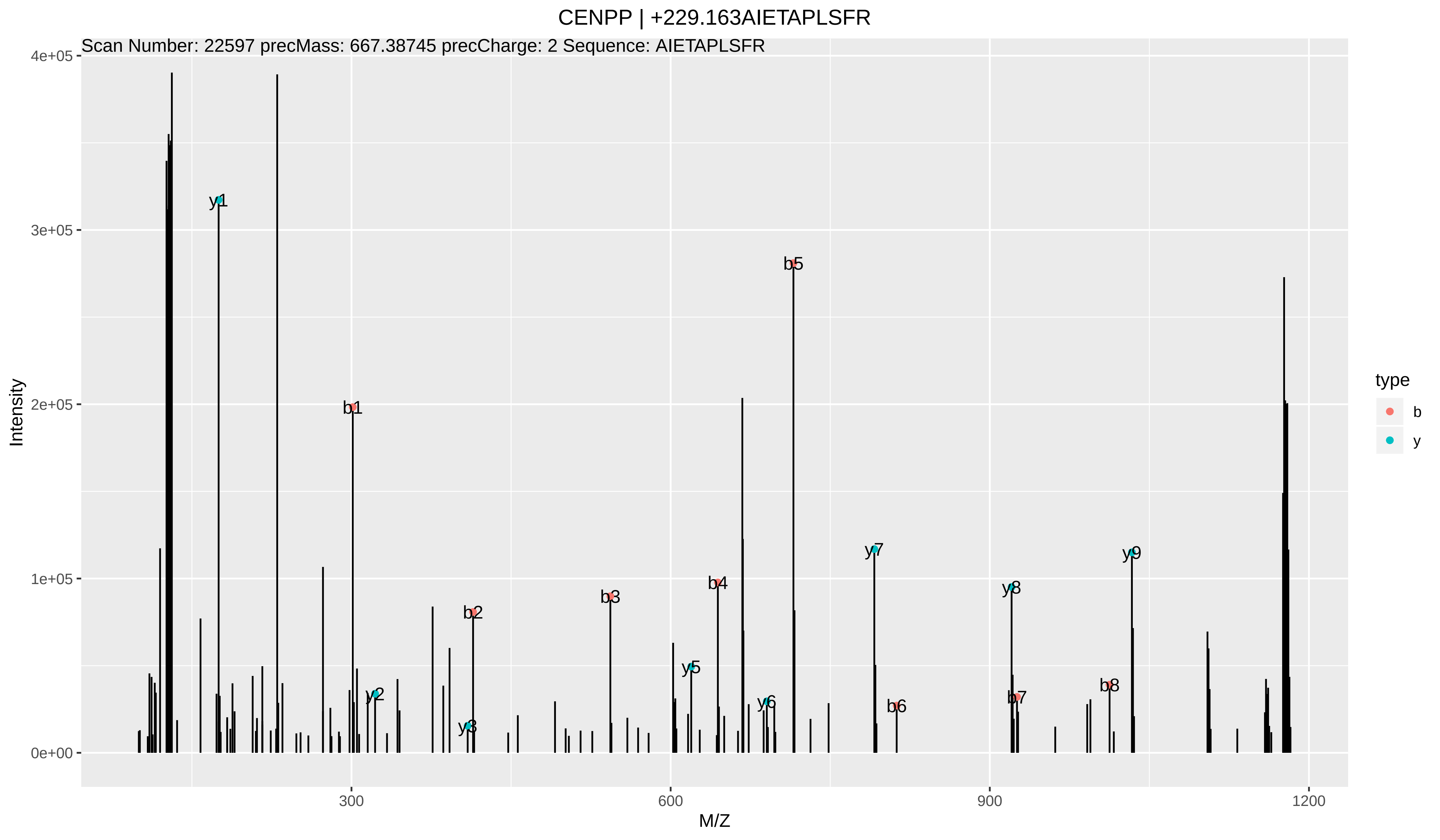

CENPP | +229.163AIETAPLSFR
Scan Number: 22597 precMass: 667.38745 precCharge: 2 Sequence: AIETAPLSFR
Intensity
M/Z
0e+00
1e+05
2e+05
3e+05
4e+05
300
600
900
1200
y1
b1
y2
y3
b2
b3
y5
b4
y6
b5
y7
b6
y8
b7
b8
y9
type
b
y

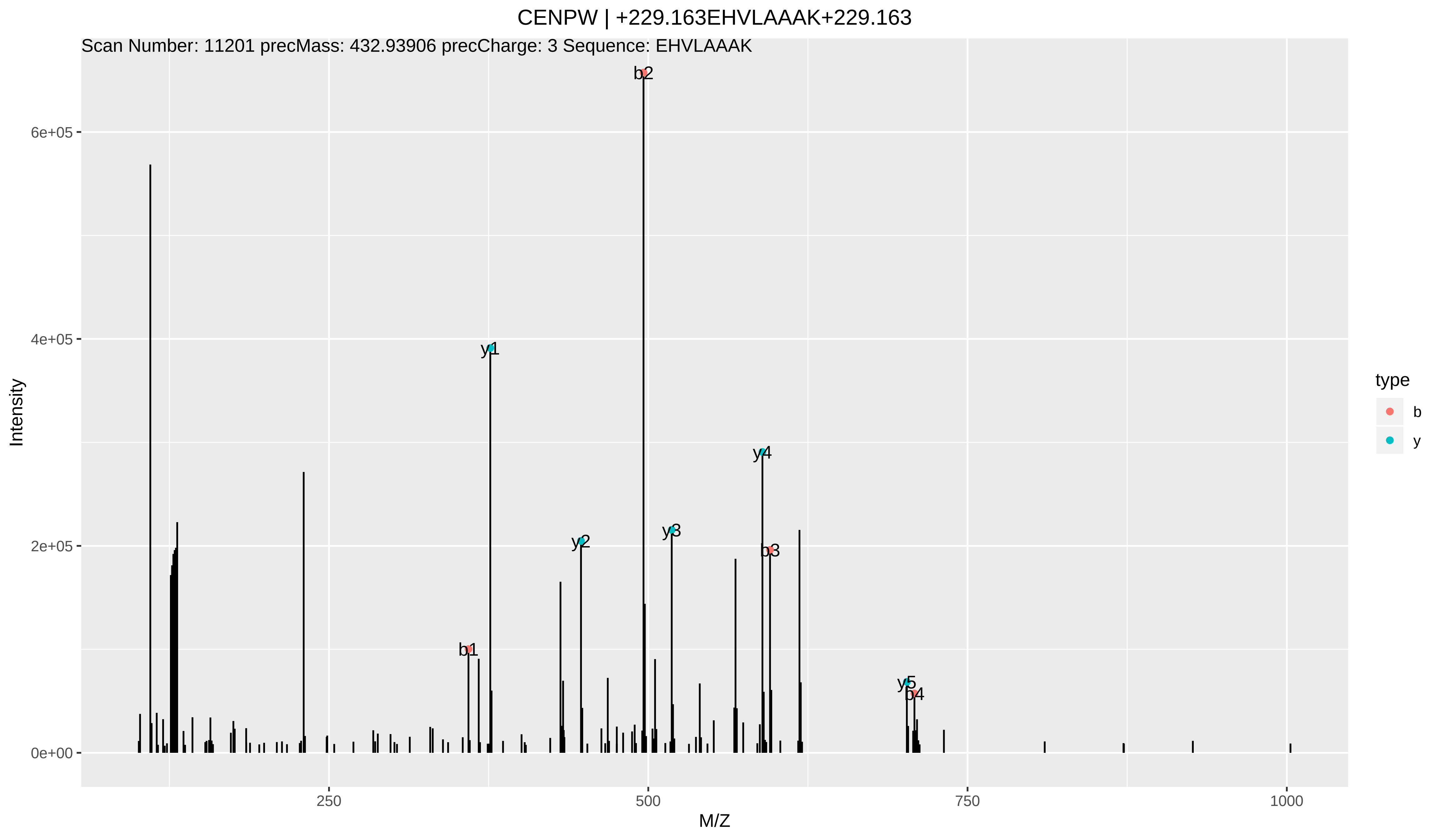

CENPW | +229.163EHVLAAAK+229.163
Scan Number: 11201 precMass: 432.93906 precCharge: 3 Sequence: EHVLAAAK
b2
y1
y4
y3
y2
b3
b1
y5
b4
6e+05
4e+05
2e+05
0e+00
Intensity
250
500
750
1000
M/Z
type
b
y

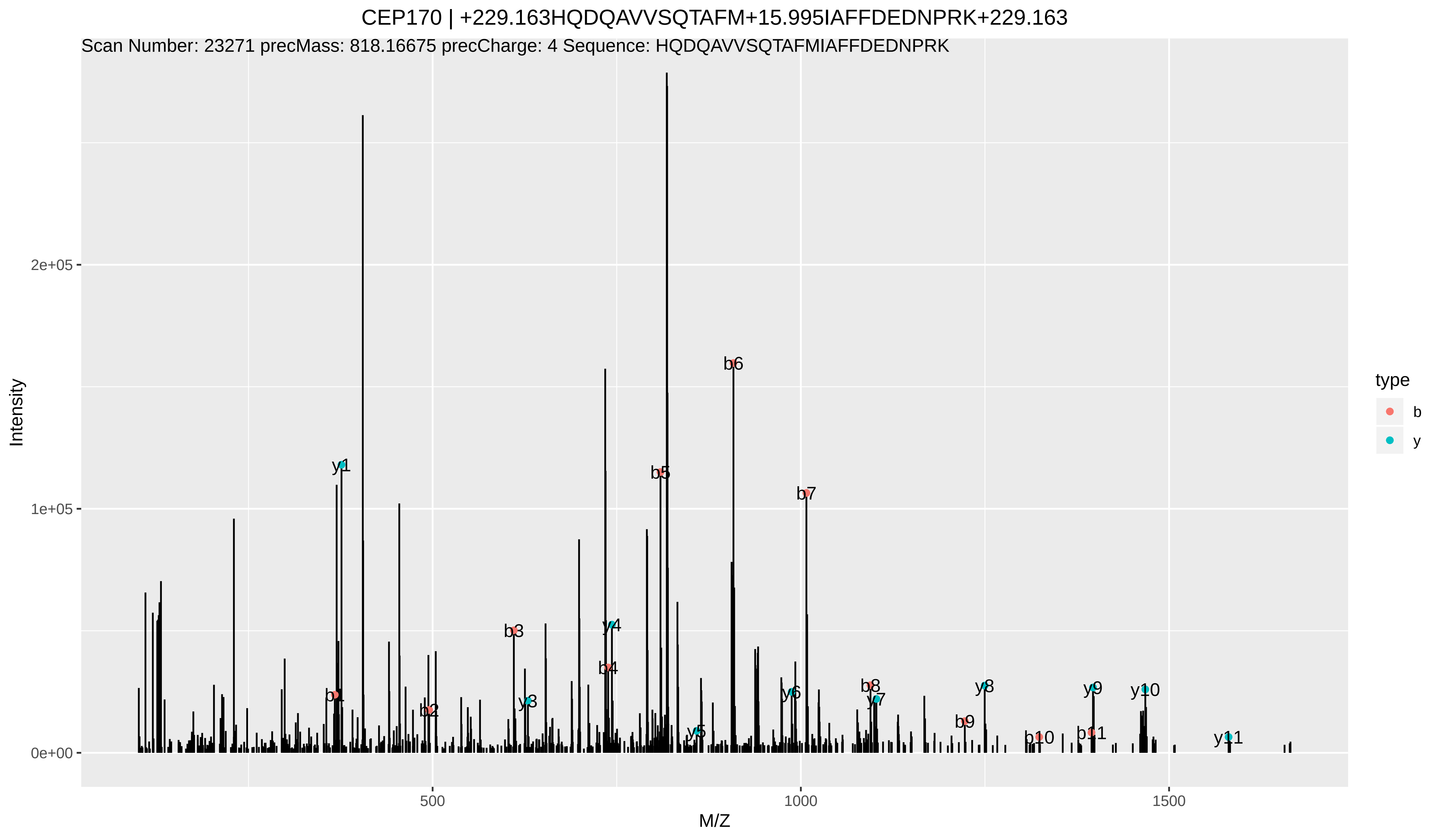

CEP170 | +229.163HQDQAVVSQTAFM+15.995IAFFDEDNPRK+229.163
Scan Number: 23271 precMass: 818.16675 precCharge: 4 Sequence: HQDQAVVSQTAFMIAFFDEDNPRK
Intensity
M/Z
type
b
y
2e+05
1e+05
0e+00
500
1000
1500
y1
b1
b2
b3
y3
y4
b4
b5
y5
b6
y6
b7
b8
y7
b9
y8
b10
b11
y9
y10
y11

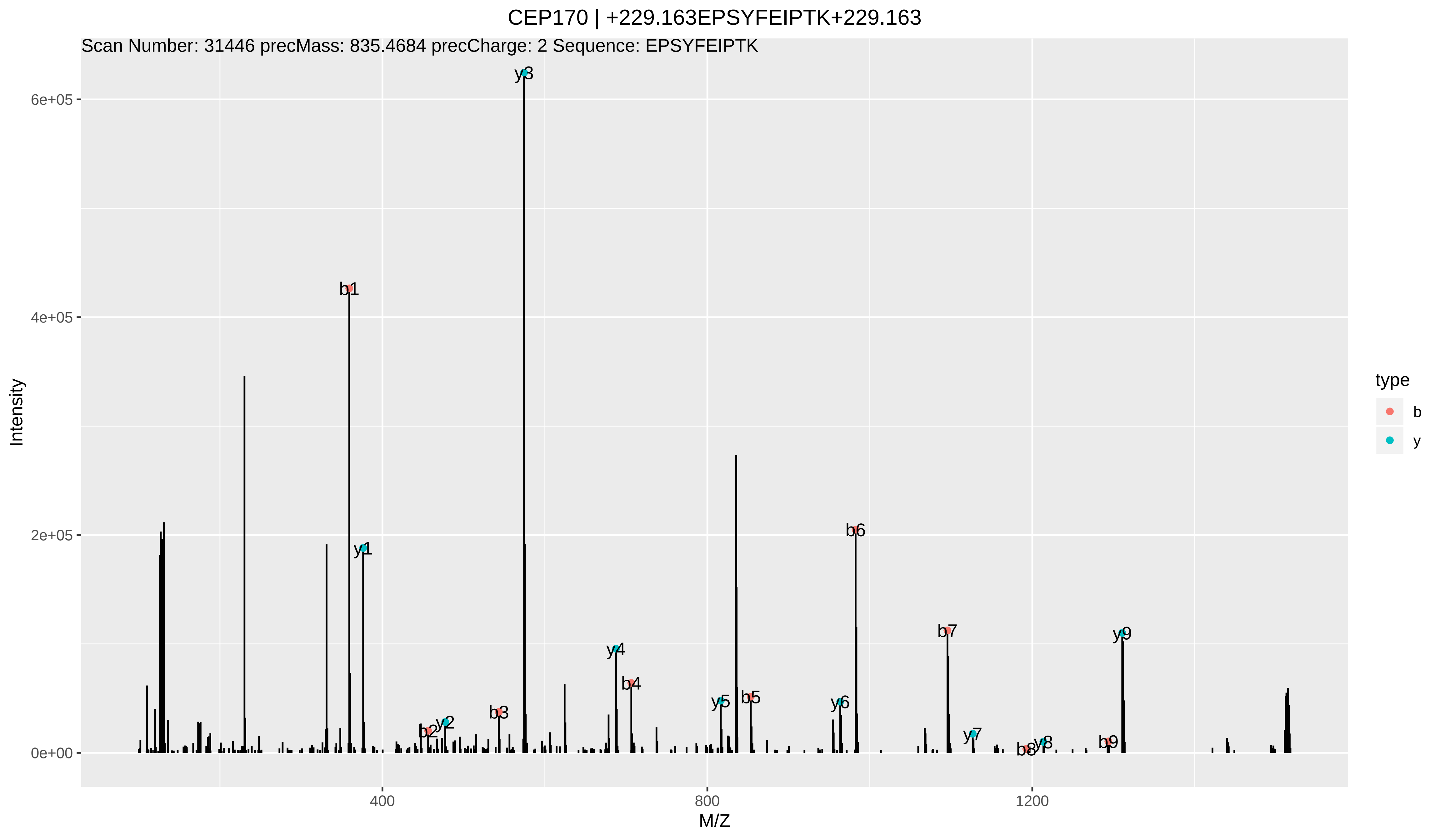

CEP170 | +229.163EPSYFEIPTK+229.163
Scan Number: 31446 precMass: 835.4684 precCharge: 2 Sequence: EPSYFEIPTK
Intensity
M/Z
6e+05
4e+05
2e+05
0e+00
400
800
1200
y3
b1
y1
b6
b7
y9
y4
b4
y5
b5
y6
b3
y2
b2
y7
b8
y8
b9
type
b
y

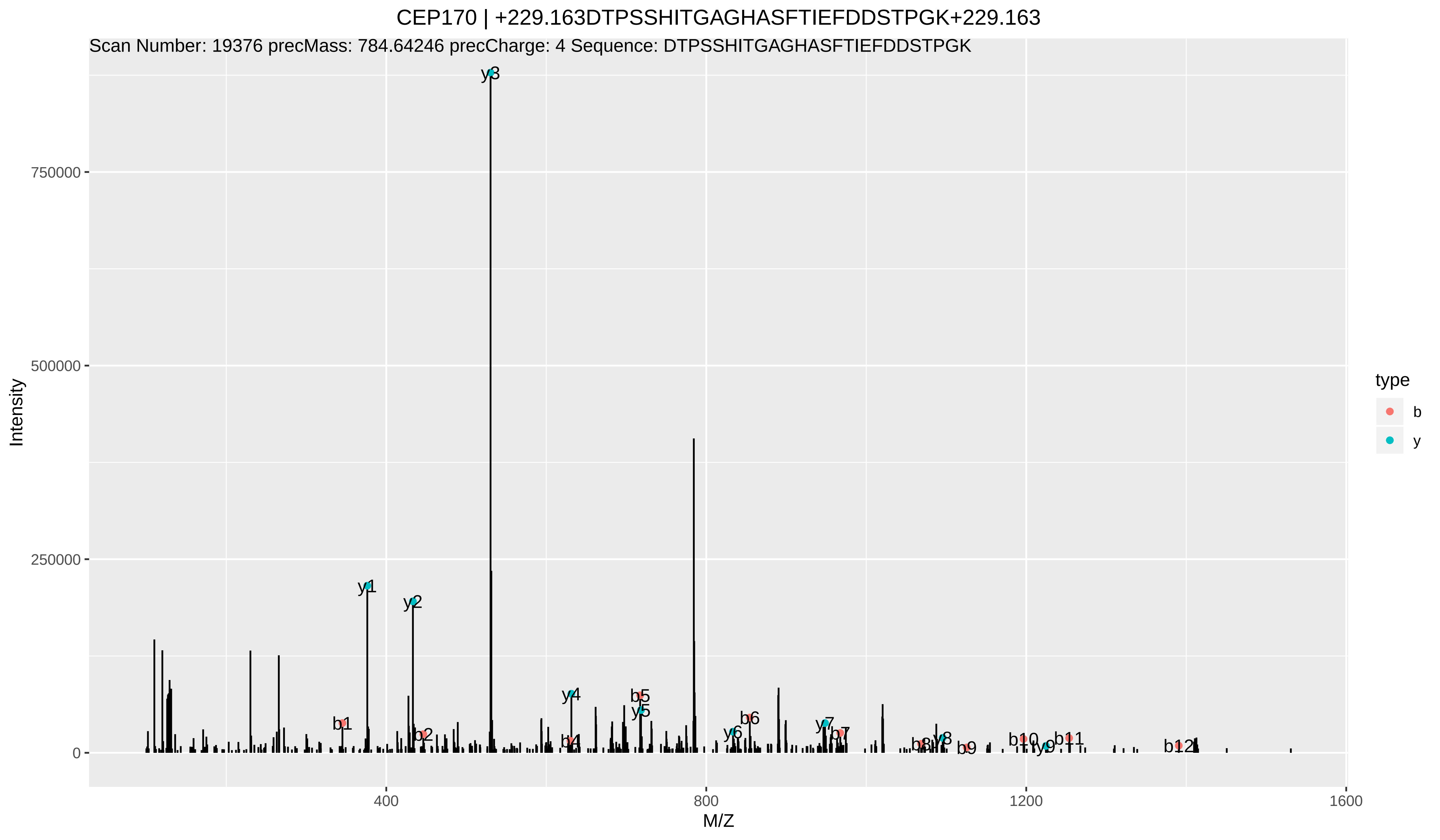

CEP170 | +229.163DTPSSHITGAGHASFTIEFDDSTPGK+229.163
Scan Number: 19376 precMass: 784.64246 precCharge: 4 Sequence: DTPSSHITGAGHASFTIEFDDSTPGK
Intensity
M/Z
type
b
y
750000
500000
250000
0
400
800
1200
1600
y3
y1
y2
b1
b2
y4
b4
b5
y5
y6
b6
y7
b7
b8
y8
b9
b10
y9
b11
b12

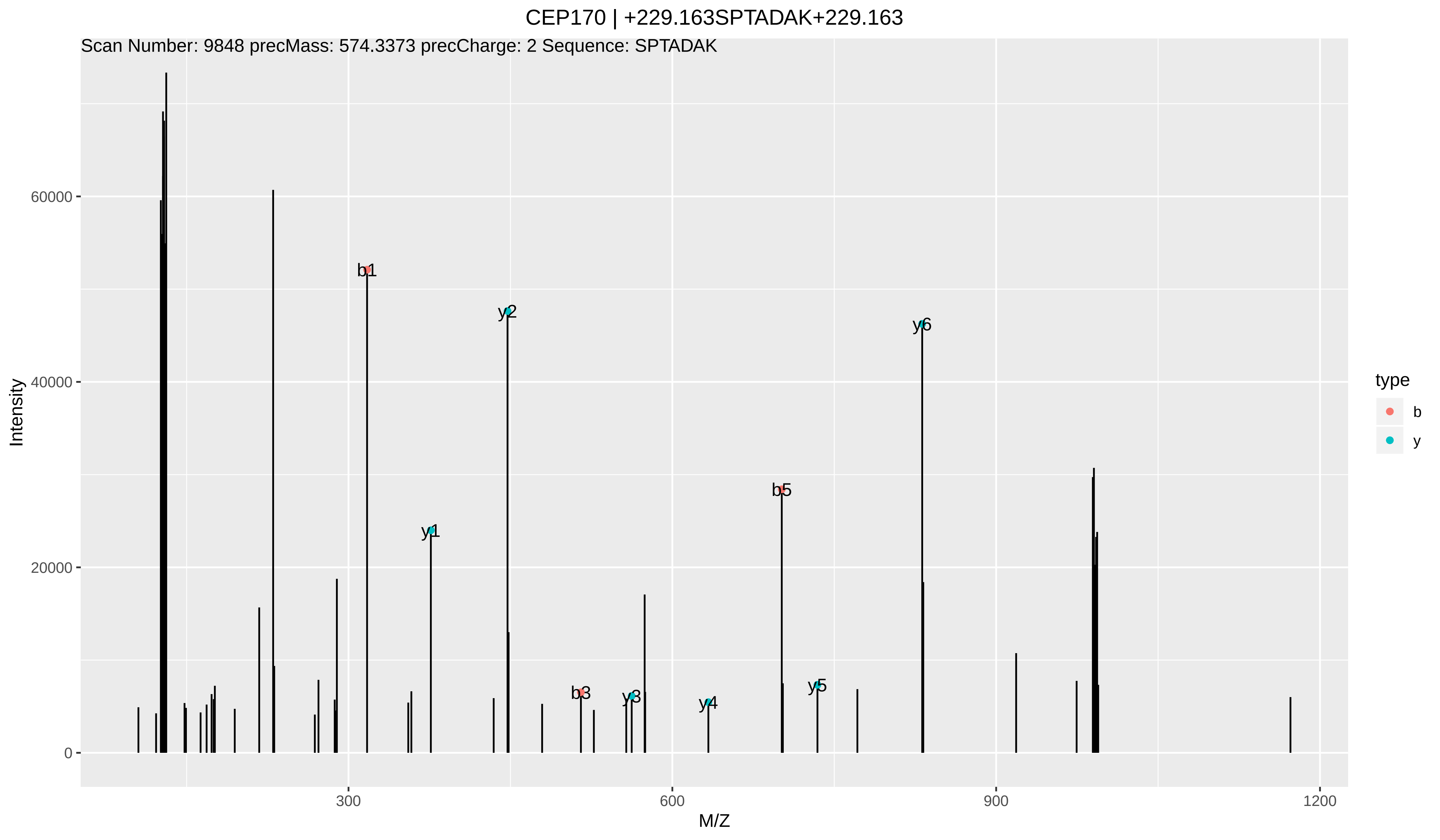

CEP170 | +229.163SPTADAK+229.163
Scan Number: 9848 precMass: 574.3373 precCharge: 2 Sequence: SPTADAK
b1
y1
y2
b3
y3
y4
b5
y5
y6
Intensity
0
20000
40000
60000
M/Z
300
600
900
1200
type
b
y

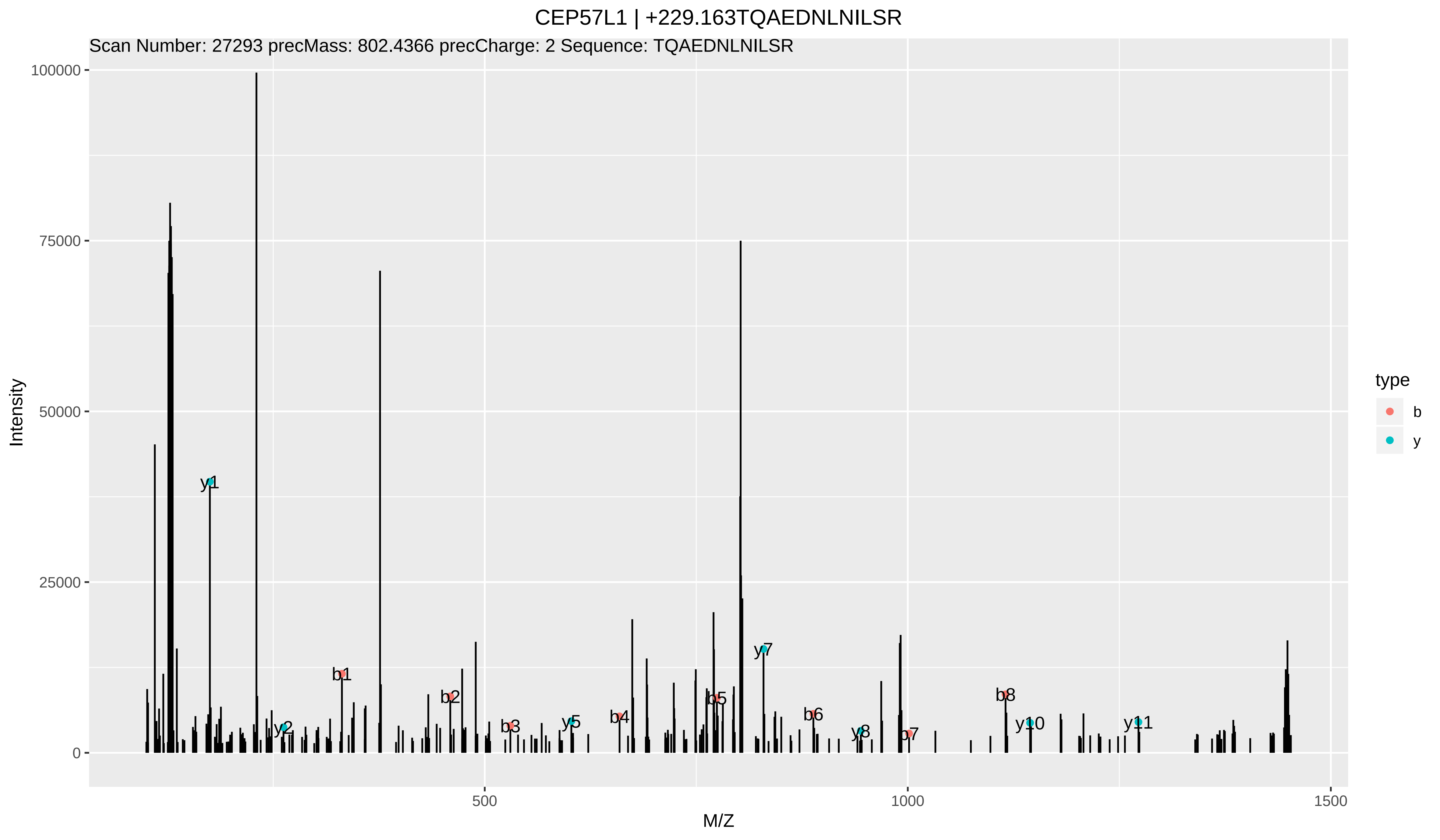

CEP57L1 | +229.163TQAEDNLNILSR
Scan Number: 27293 precMass: 802.4366 precCharge: 2 Sequence: TQAEDNLNILSR
Intensity
M/Z
100000
75000
50000
25000
0
500
1000
1500
y1
y2
b1
b2
b3
y5
b4
b5
y7
b6
y8
b7
b8
y10
y11
type
b
y

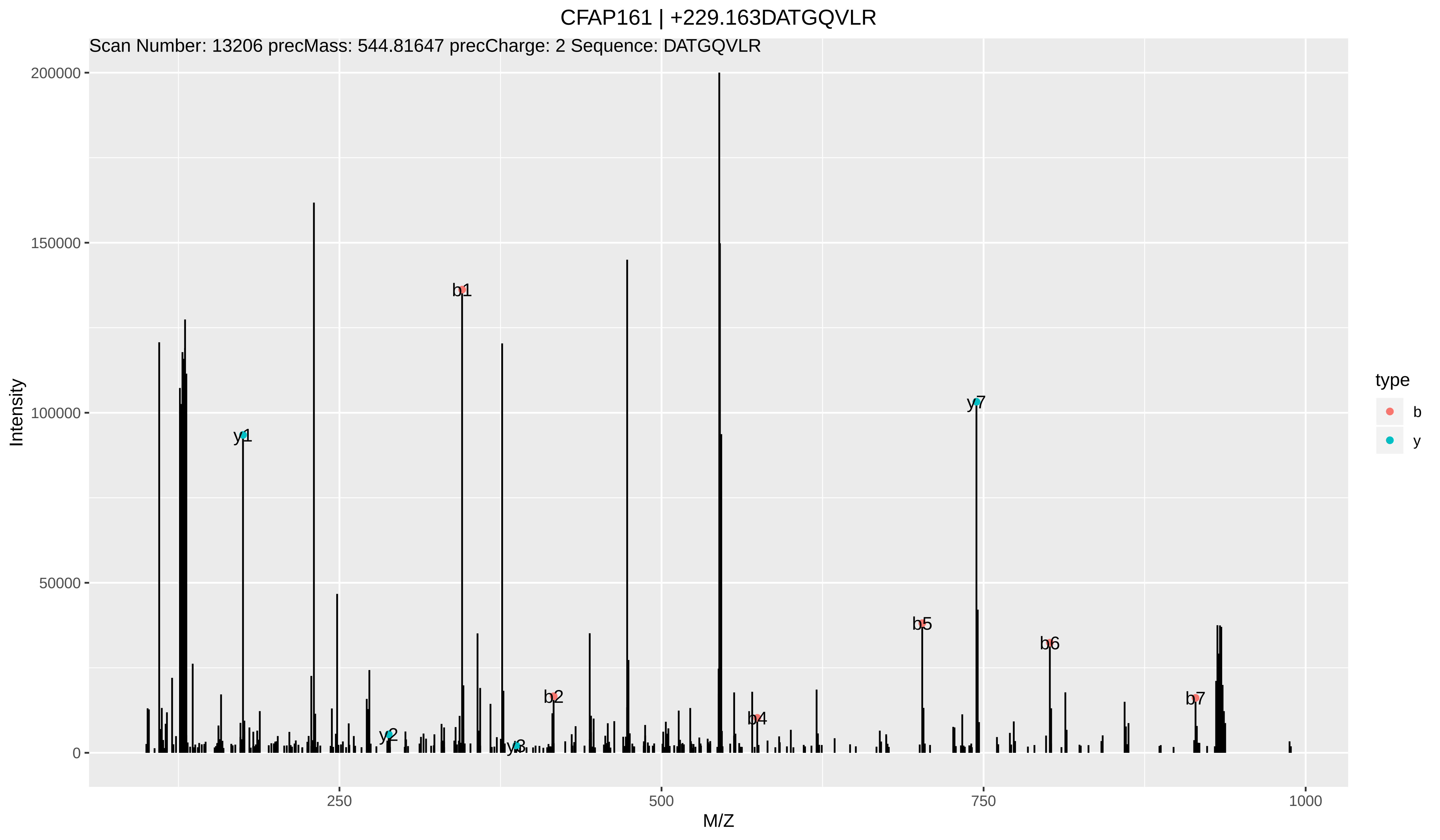

CFAP161 | +229.163DATGQVLR
Scan Number: 13206 precMass: 544.81647 precCharge: 2 Sequence: DATGQVLR
Intensity
200000
150000
100000
50000
0
M/Z
250
500
750
1000
y1
y2
y3
y7
b1
b2
b4
b5
b6
b7
type
b
y

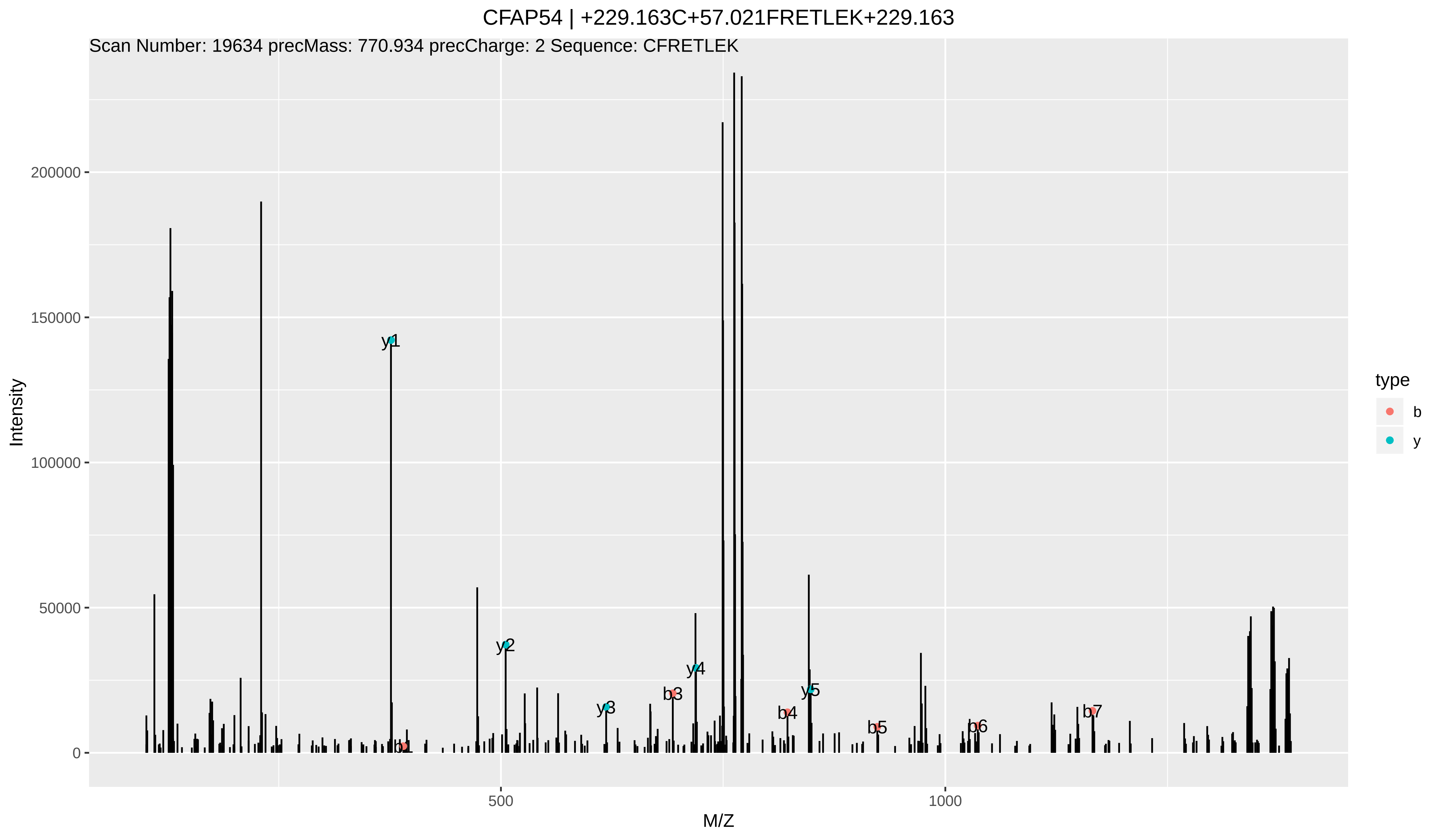

CFAP54 | +229.163C+57.021FRETLEK+229.163
Scan Number: 19634 precMass: 770.934 precCharge: 2 Sequence: CFRETLEK
Intensity
200000
150000
100000
50000
0
M/Z
500
1000
y1
b1
y2
y3
b3
y4
b4
y5
b5
b6
b7
type
b
y

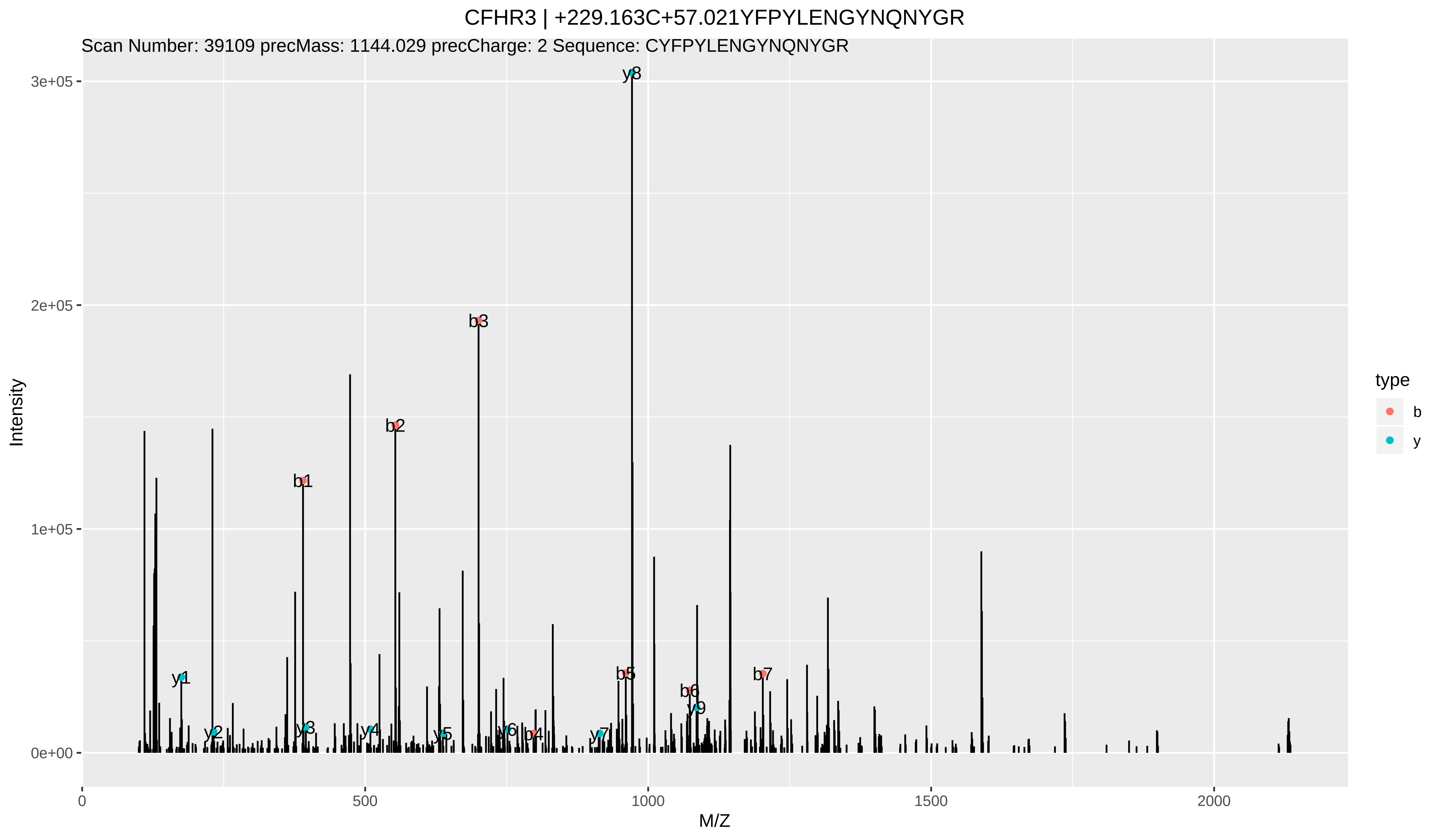

CFHR3 | +229.163C+57.021YFPYLENGYNQNYGR
Scan Number: 39109 precMass: 1144.029 precCharge: 2 Sequence: CYFPYLENGYNQNYGR
Intensity
M/Z
type
b
y
y8
b3
b2
b1
y1
y2
y3
y4
y5
y6
b4
y7
b5
b6
y9
b7

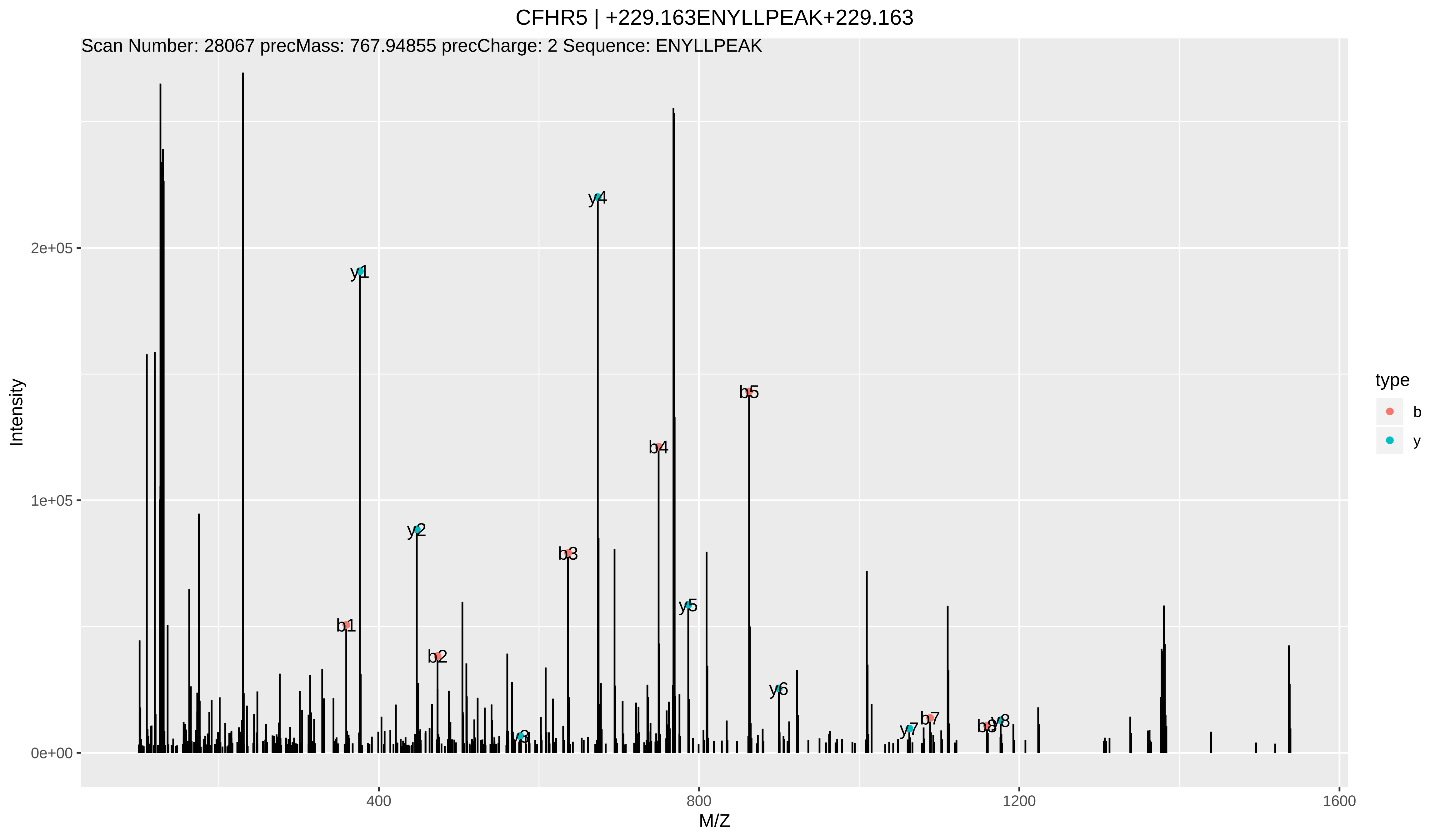
CFHR5 | +229.163ENYLLPEAK+229.163
Scan Number: 28067 precMass: 767.94855 precCharge: 2 Sequence: ENYLLPEAK
Intensity
2e+05
1e+05
0e+00
M/Z
400
800
1200
1600
y1
y2
y3
y4
y5
y6
y7
y8
b1
b2
b3
b4
b5
b7
b8
type
b
y

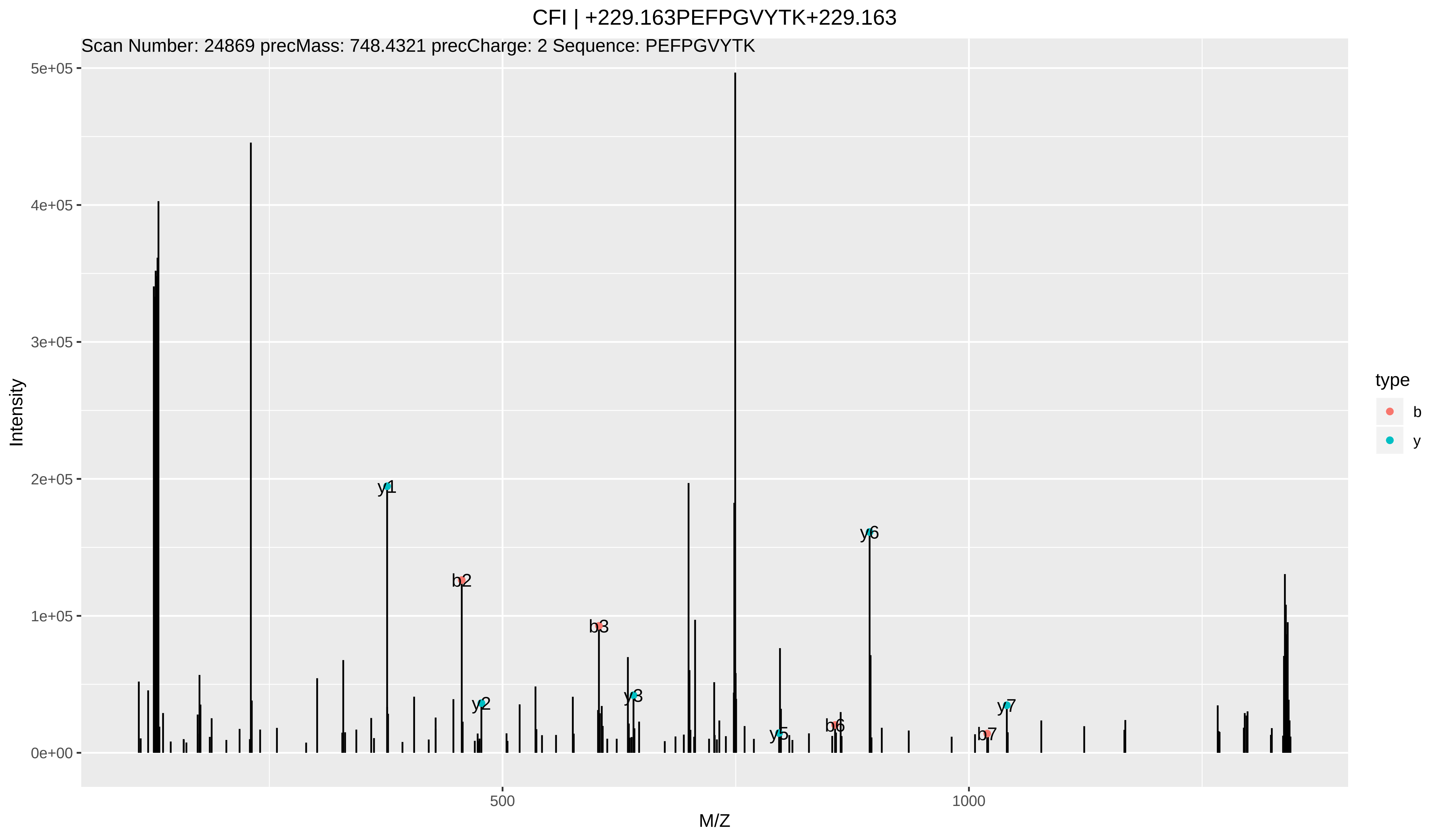

CFI | +229.163PEFPGVYTK+229.163
Scan Number: 24869 precMass: 748.4321 precCharge: 2 Sequence: PEFPGVYTK
5e+05
4e+05
3e+05
2e+05
1e+05
0e+00
Intensity
500
1000
M/Z
y1
b2
y2
b3
y3
y5
b6
y6
b7
y7
type
b
y

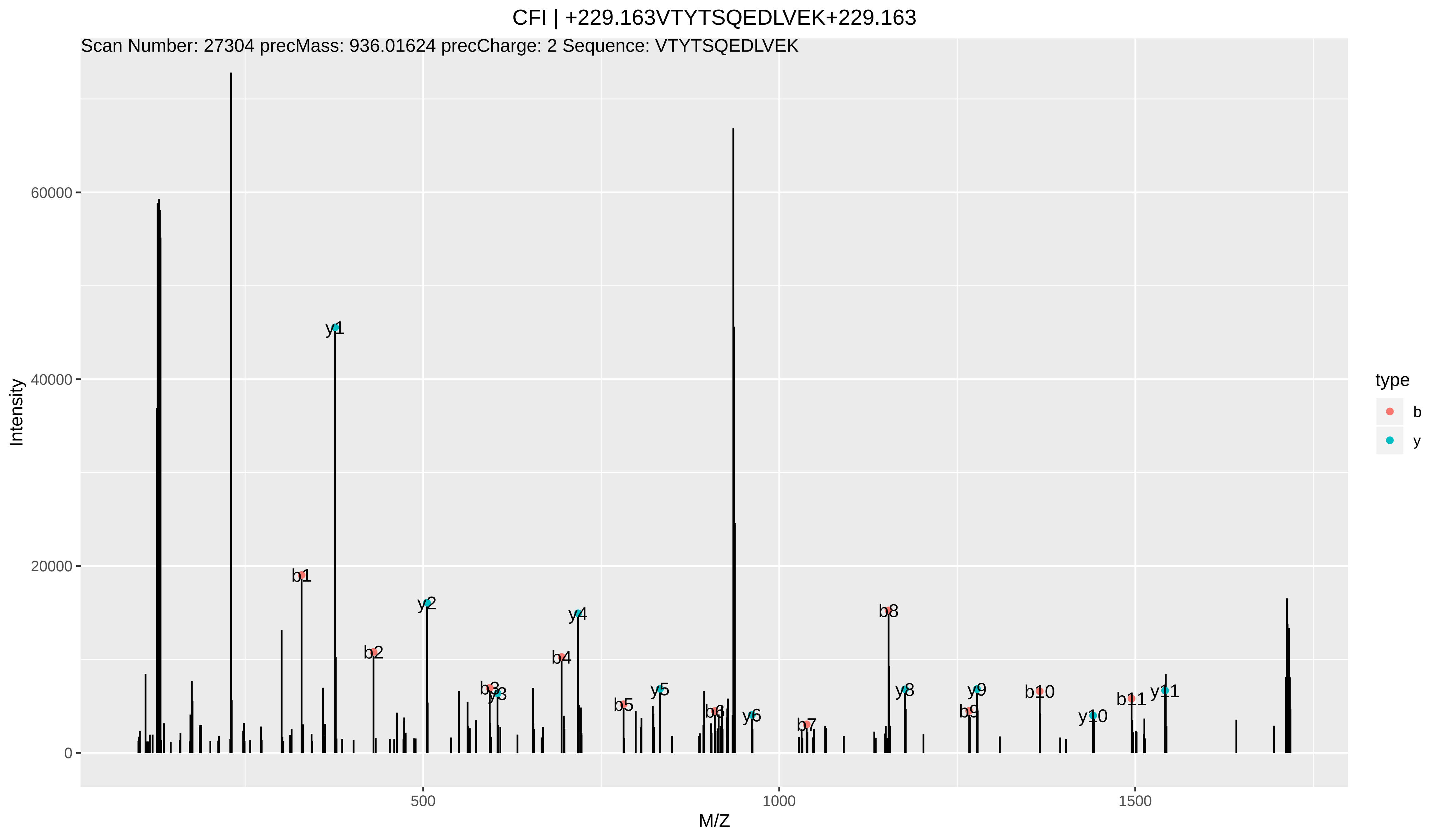

CFI | +229.163VTYTSQEDLVEK+229.163
Scan Number: 27304 precMass: 936.01624 precCharge: 2 Sequence: VTYTSQEDLVEK
Intensity
M/Z
0
20000
40000
60000
500
1000
1500
type
b
y
b1
b2
b3
b4
b5
b6
b7
b8
b9
b10
b11
y1
y2
y3
y4
y5
y6
y8
y9
y10
y11

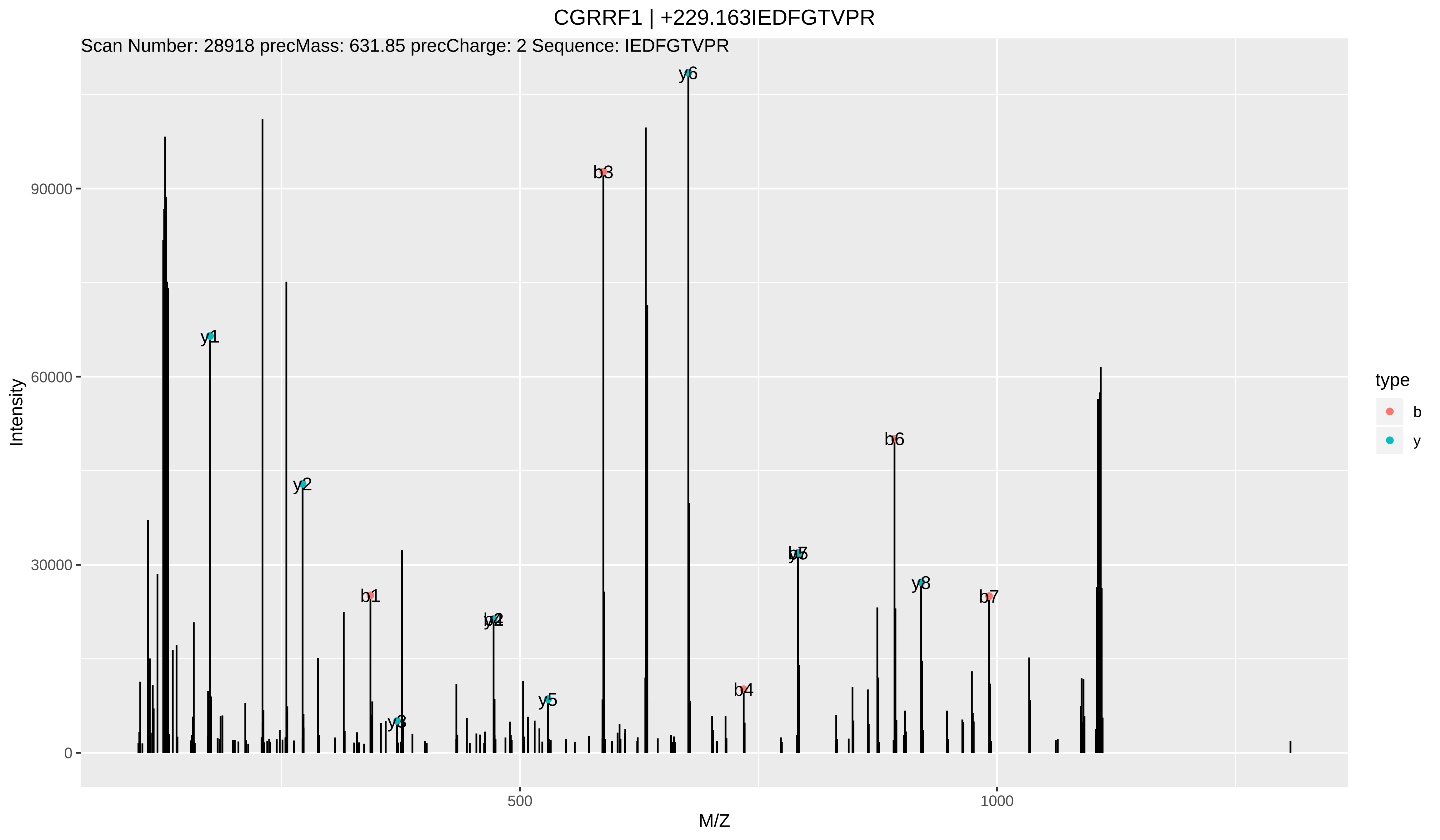
CGRRF1 | +229.163IEDFGTVPR
Scan Number: 28918 precMass: 631.85 precCharge: 2 Sequence: IEDFGTVPR
Intensity
M/Z
0
30000
60000
90000
500
1000
y1
y2
b1
y3
b2
y4
y5
b3
y6
b4
b5
y7
b6
y8
b7
type
b
y

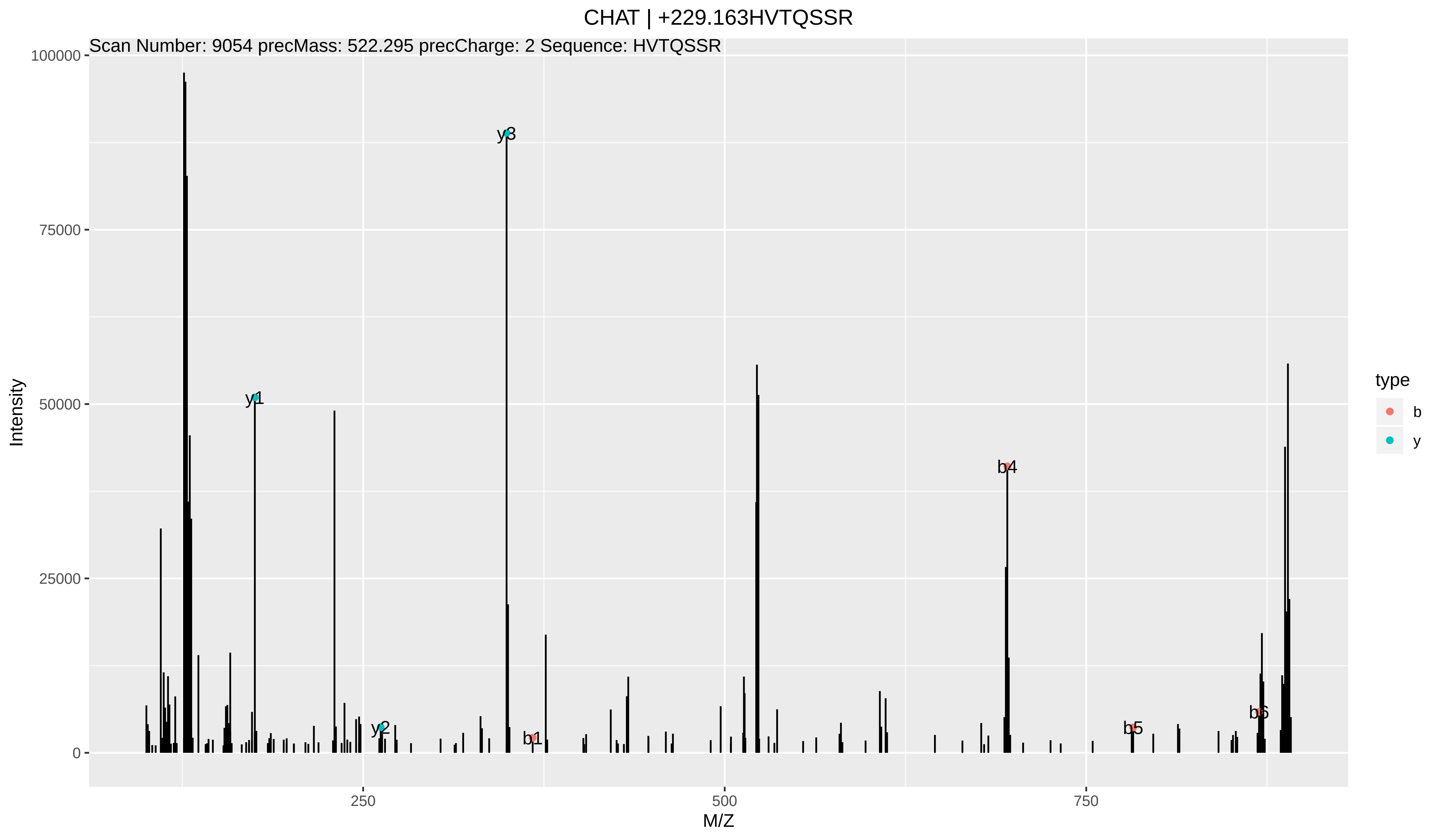

CHAT | +229.163HVTQSSR
Scan Number: 9054 precMass: 522.295 precCharge: 2 Sequence: HVTQSSR
Intensity
100000
75000
50000
25000
0
M/Z
250
500
750
y1
y2
y3
b1
b4
b5
b6
type
b
y

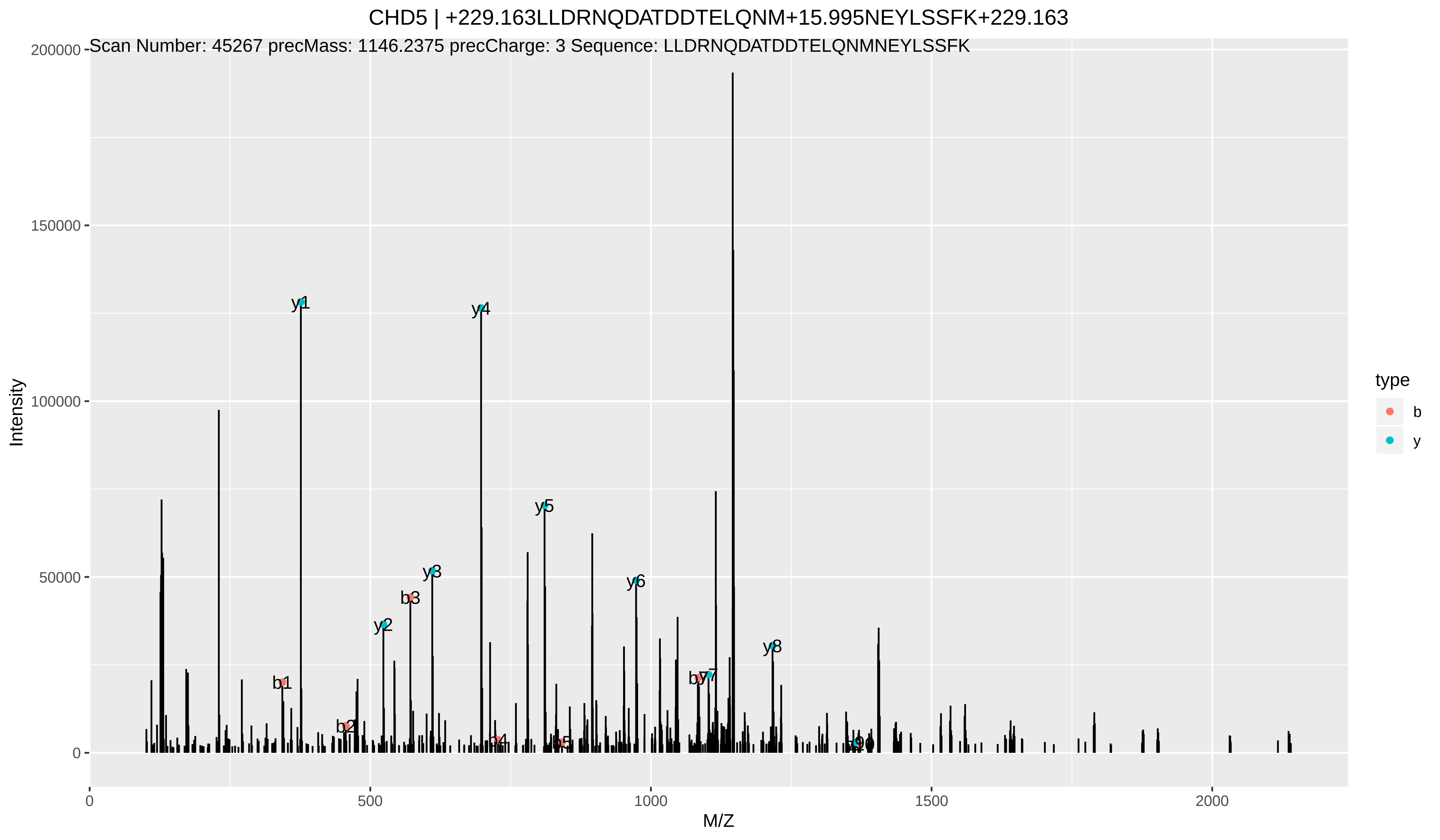

CHD5 | +229.163LLDRNQDATDDTELQNM+15.995NEYLSSFK+229.163
Scan Number: 45267 precMass: 1146.2375 precCharge: 3 Sequence: LLDRNQDATDDTELQNMNEYLSSFK
Intensity
M/Z
0
50000
100000
150000
200000
0
500
1000
1500
2000
type
b
y
y1
y2
y3
y4
y5
y6
y8
b1
b3

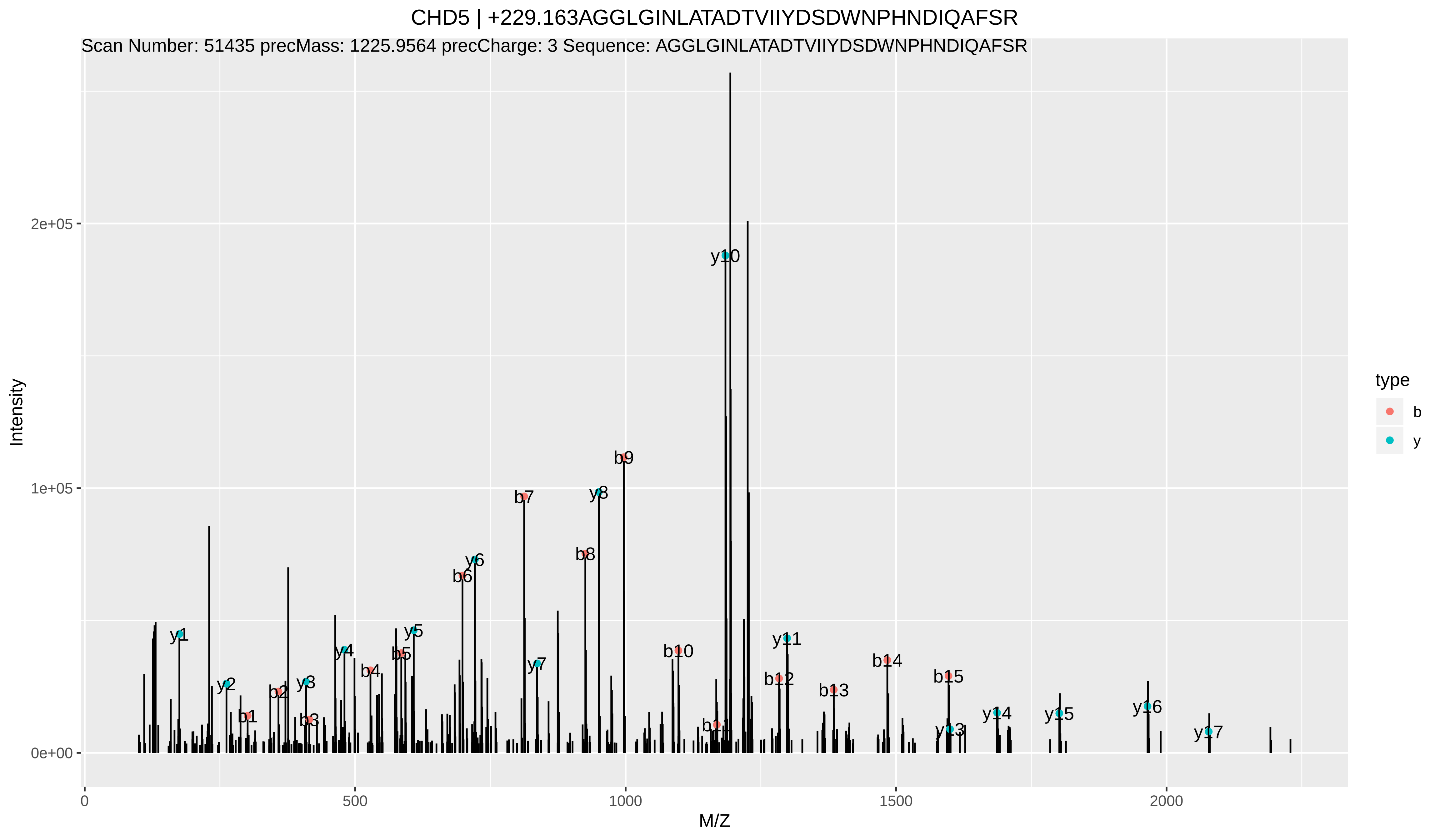

CHD5 | +229.163AGGLGINLATADTVIIYDSDWNPHNDIQAFSR
Scan Number: 51435 precMass: 1225.9564 precCharge: 3 Sequence: AGGLGINLATADTVIIYDSDWNPHNDIQAFSR
Intensity
M/Z
type
b
y

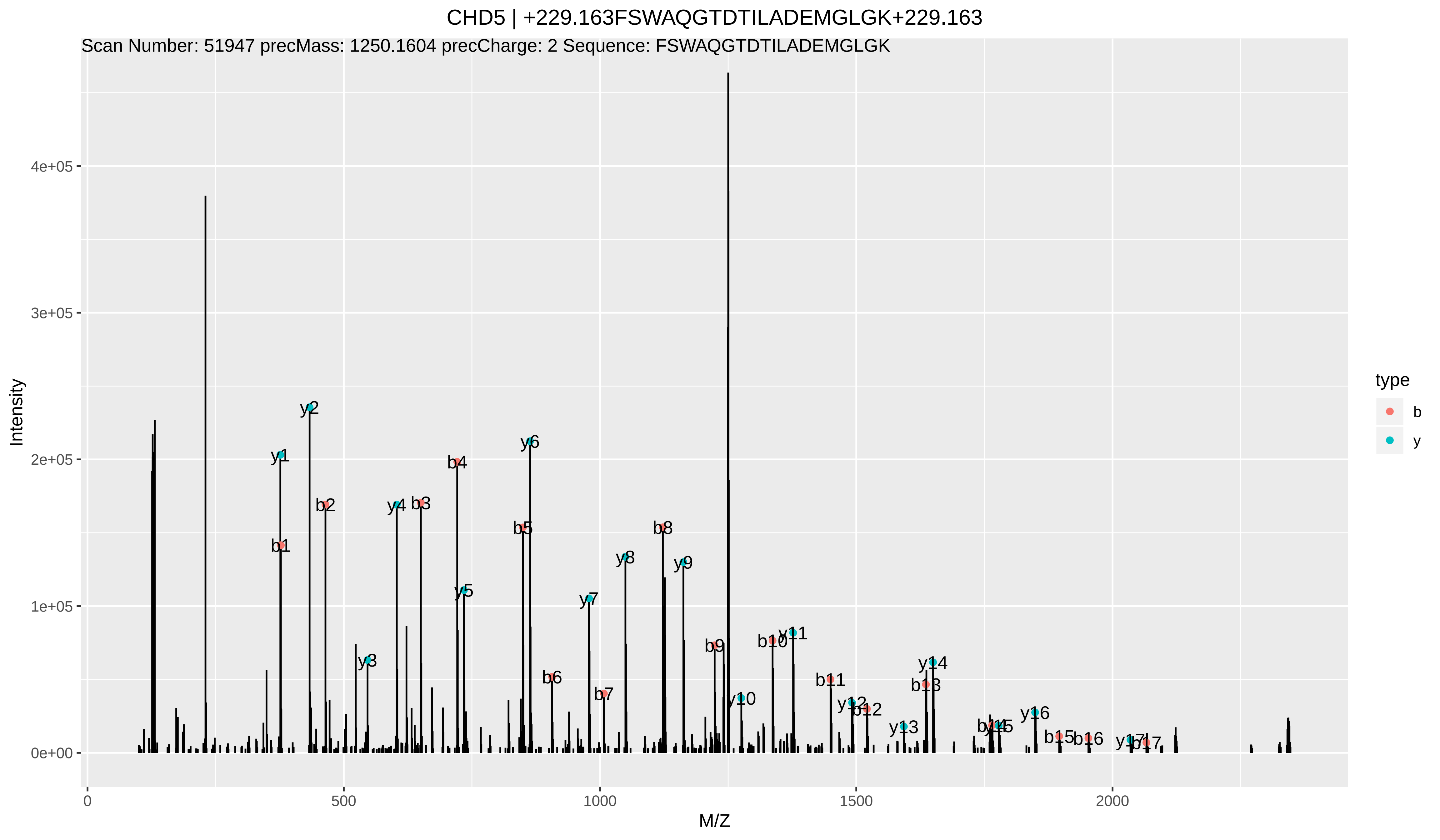

CHD5 | +229.163FSWAQGTDTILADEMGLGK+229.163
Scan Number: 51947 precMass: 1250.1604 precCharge: 2 Sequence: FSWAQGTDTILADEMGLGK
Intensity
M/Z
0e+00
1e+05
2e+05
3e+05
4e+05
0
500
1000
1500
2000
type
b
y
y1
y2
b1
b2
y3
y4
b3
b4
y5
b5
y6
b6
y7
b7
y8
b8
y9
b9
y10
b10
y11
b11
y12
b12
y13
y14
b13
y16
b15
b16

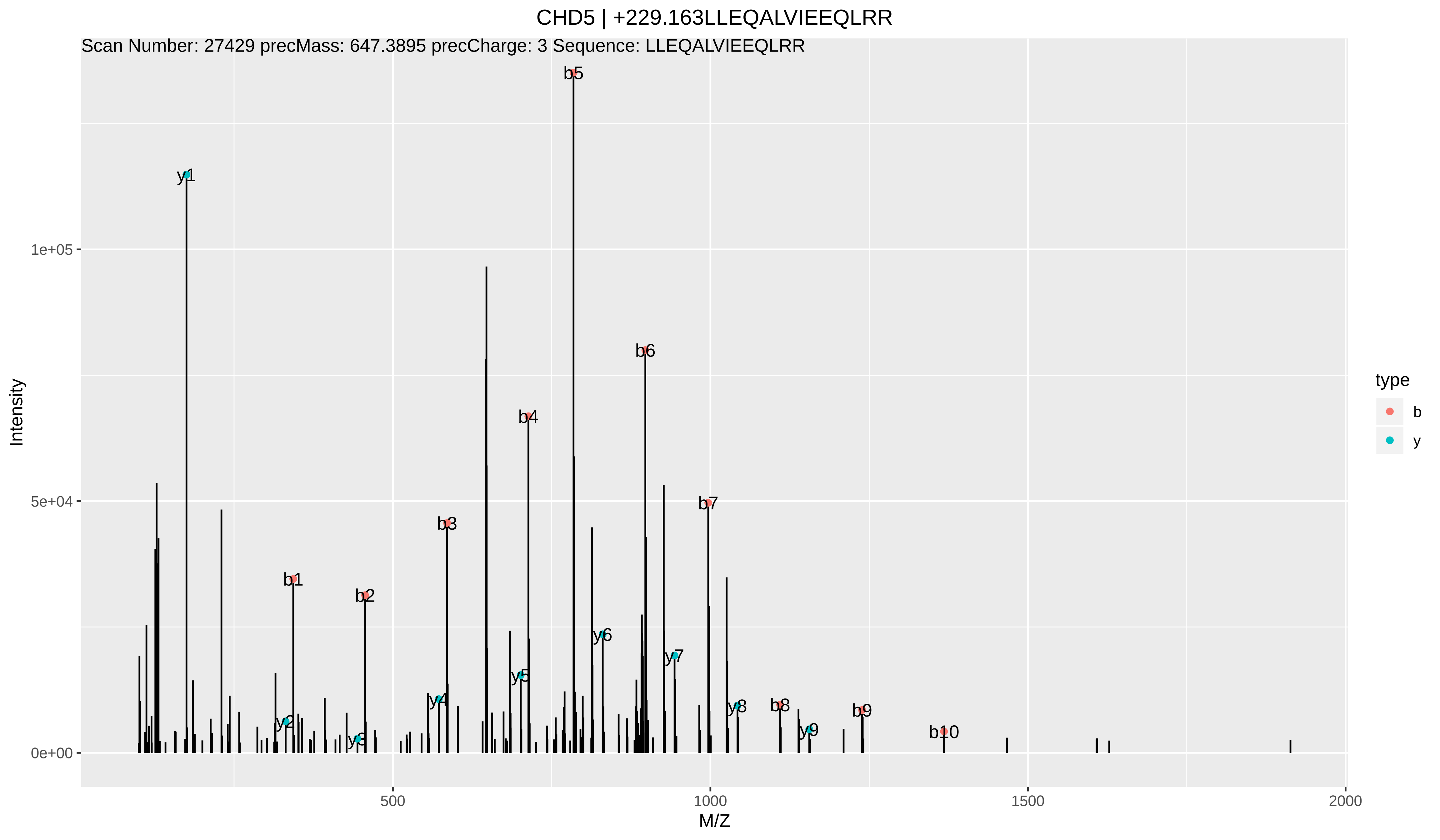
CHD5 | +229.163LLEQALVIEEQLRR
Scan Number: 27429 precMass: 647.3895 precCharge: 3 Sequence: LLEQALVIEEQLRR
Intensity
M/Z
0e+00
5e+04
1e+05
500
1000
1500
2000
b5
y1
b6
b4
b7
b3
b1
b2
y6
y7
y5
y4
y8
b8
b9
y2
y9
b10
y3
type
b
y

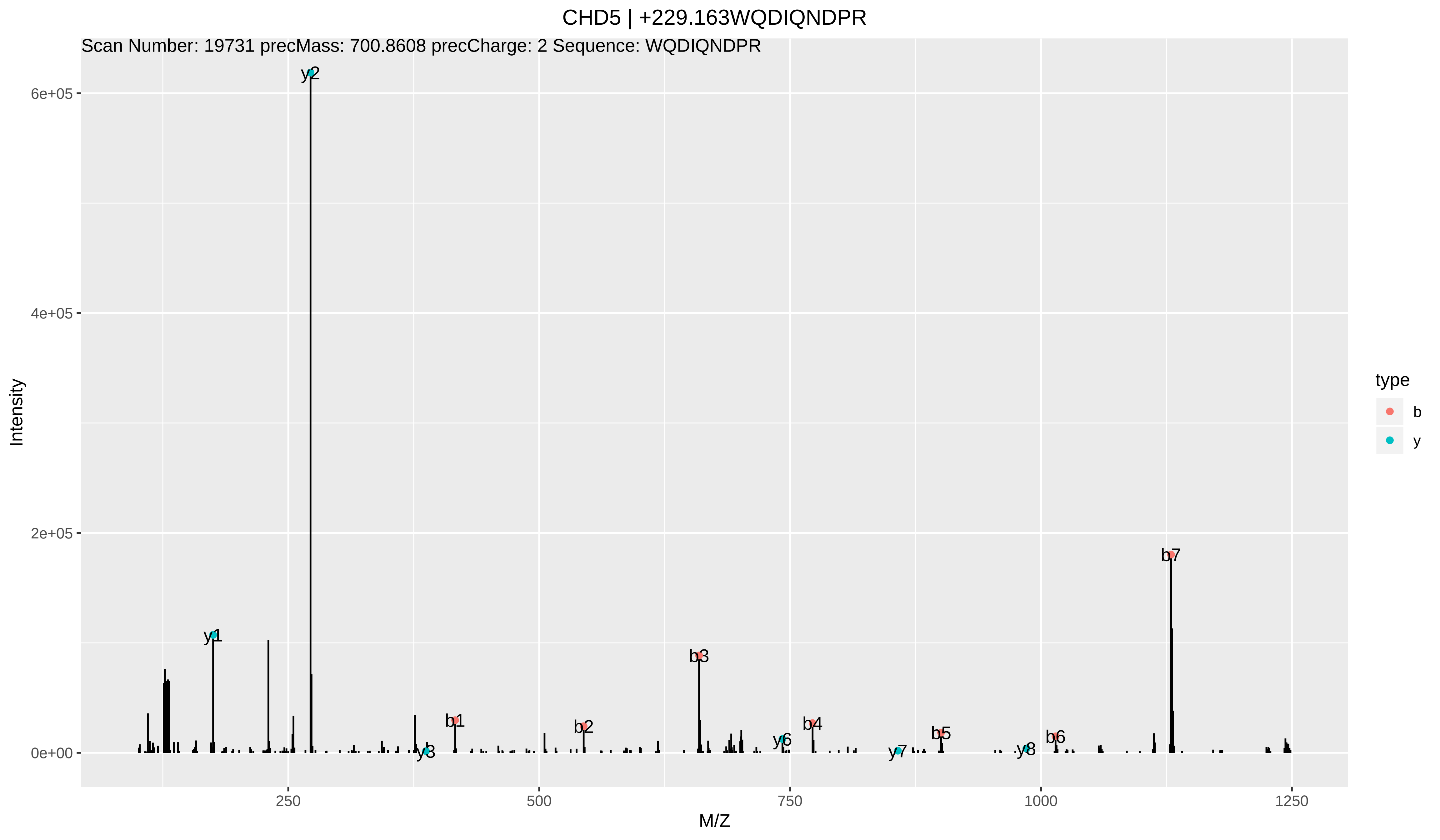

CHD5 | +229.163WQDIQNDPR
Scan Number: 19731 precMass: 700.8608 precCharge: 2 Sequence: WQDIQNDPR
y2
y1
b3
b7
b1
y3
b2
y6
b4
y7
b5
y8
b6
Intensity
6e+05
4e+05
2e+05
0e+00
M/Z
250
500
750
1000
1250
type
b
y

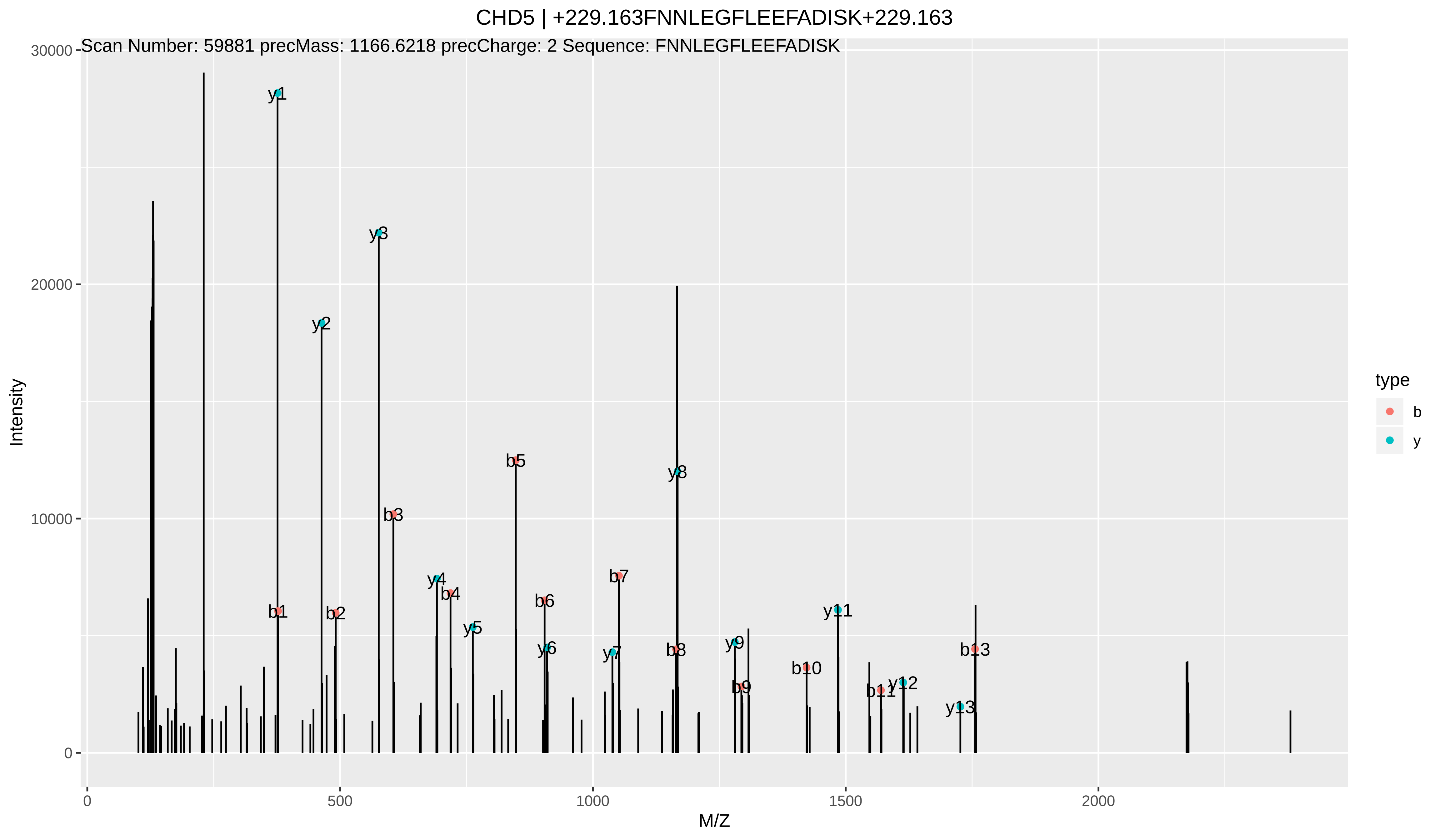

CHD5 | +229.163FNNLEGFLEEFADISK+229.163
Scan Number: 59881 precMass: 1166.6218 precCharge: 2 Sequence: FNNLEGFLEEFADISK
Intensity
M/Z
type
b
y
y1
y2
y3
y4
y5
y6
y7
y8
y9
y11
y12
y13
b1
b2
b3
b4
b5
b6
b7
b8
b9
b10
b11
b13

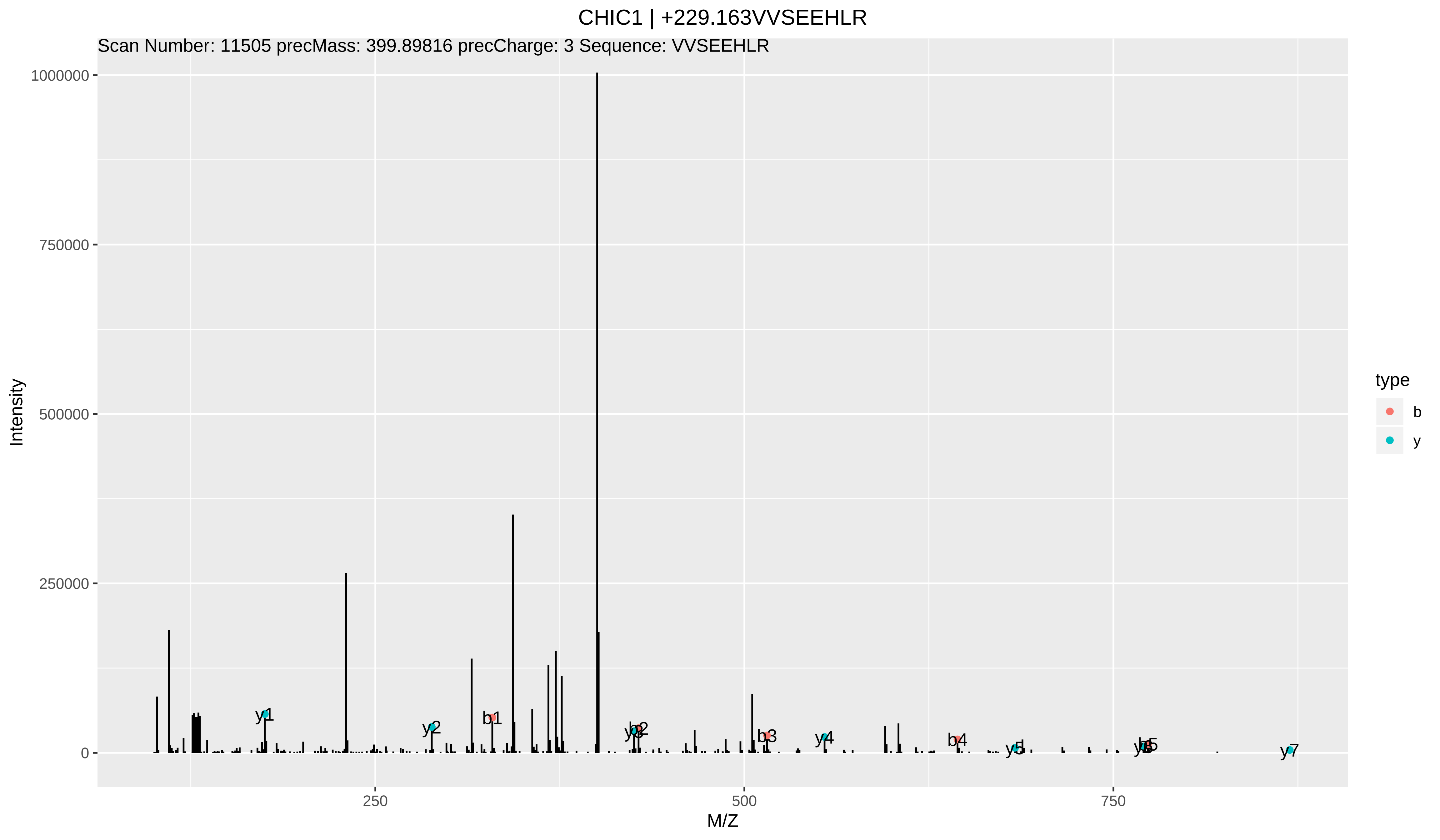
CHIC1 | +229.163VVSEEHLR
Scan Number: 11505 precMass: 399.89816 precCharge: 3 Sequence: VVSEEHLR
Intensity
1000000
750000
500000
250000
0
M/Z
250
500
750
y1
y2
b1
y3
b2
b3
y4
b4
y5
y6
b5
y7
type
b
y

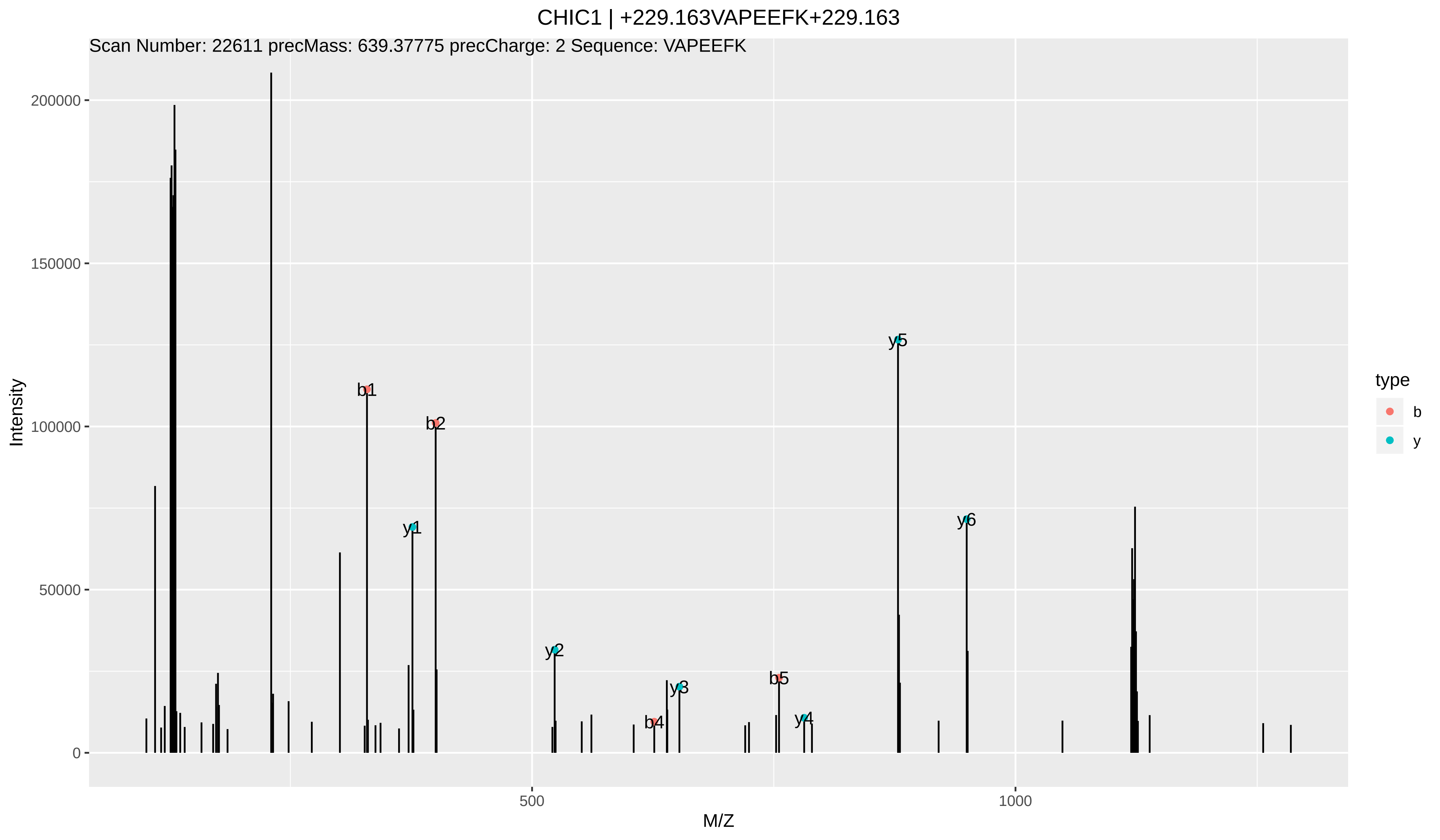
CHIC1 | +229.163VAPEEFK+229.163
Scan Number: 22611 precMass: 639.37775 precCharge: 2 Sequence: VAPEEFK
Intensity
200000
150000
100000
50000
0
M/Z
500
1000
b1
b2
b4
b5
y1
y2
y3
y4
y5
y6
type
b
y

CHL1 | +229.163EDLHPDPEIQSVK+229.163

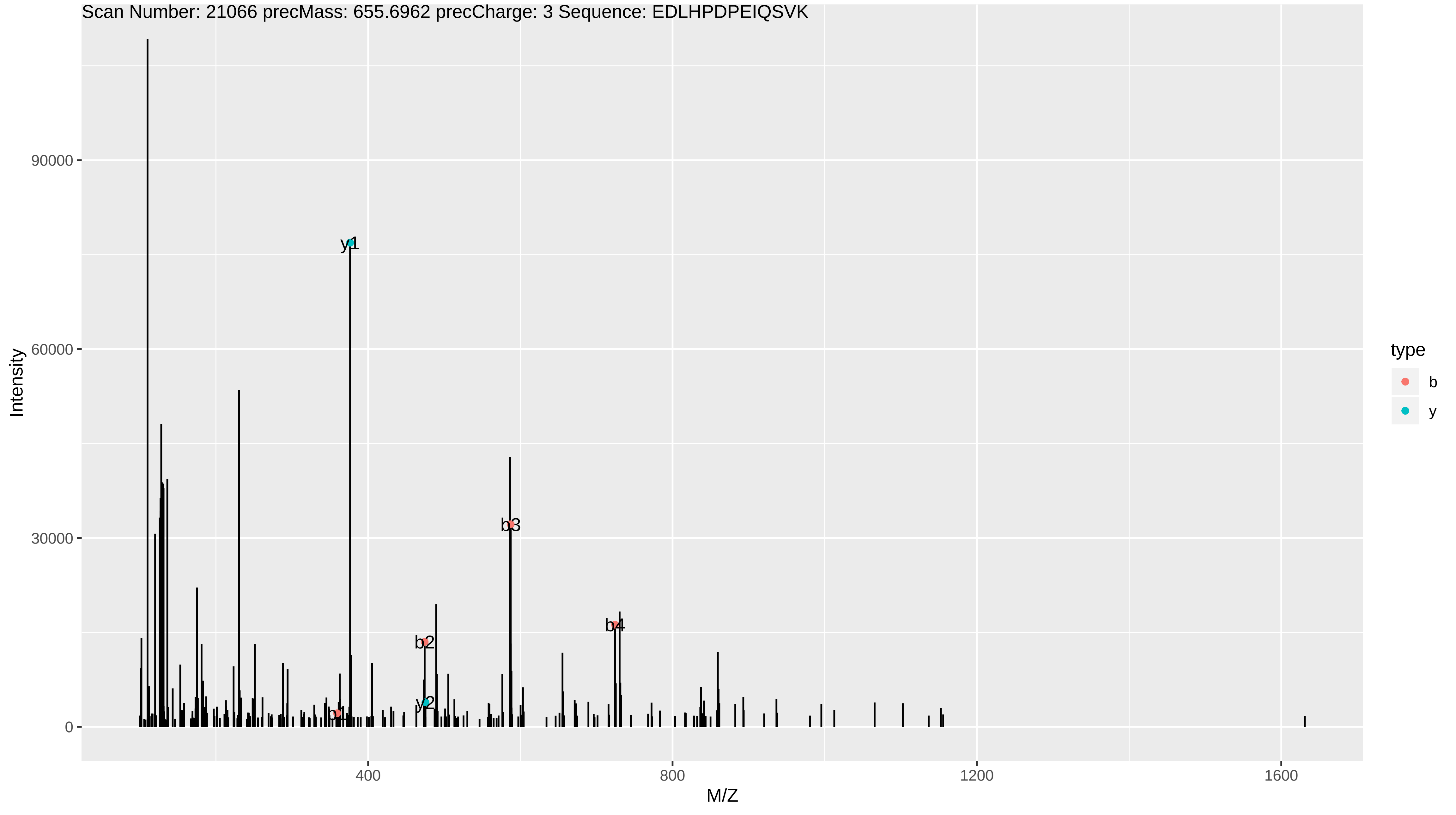

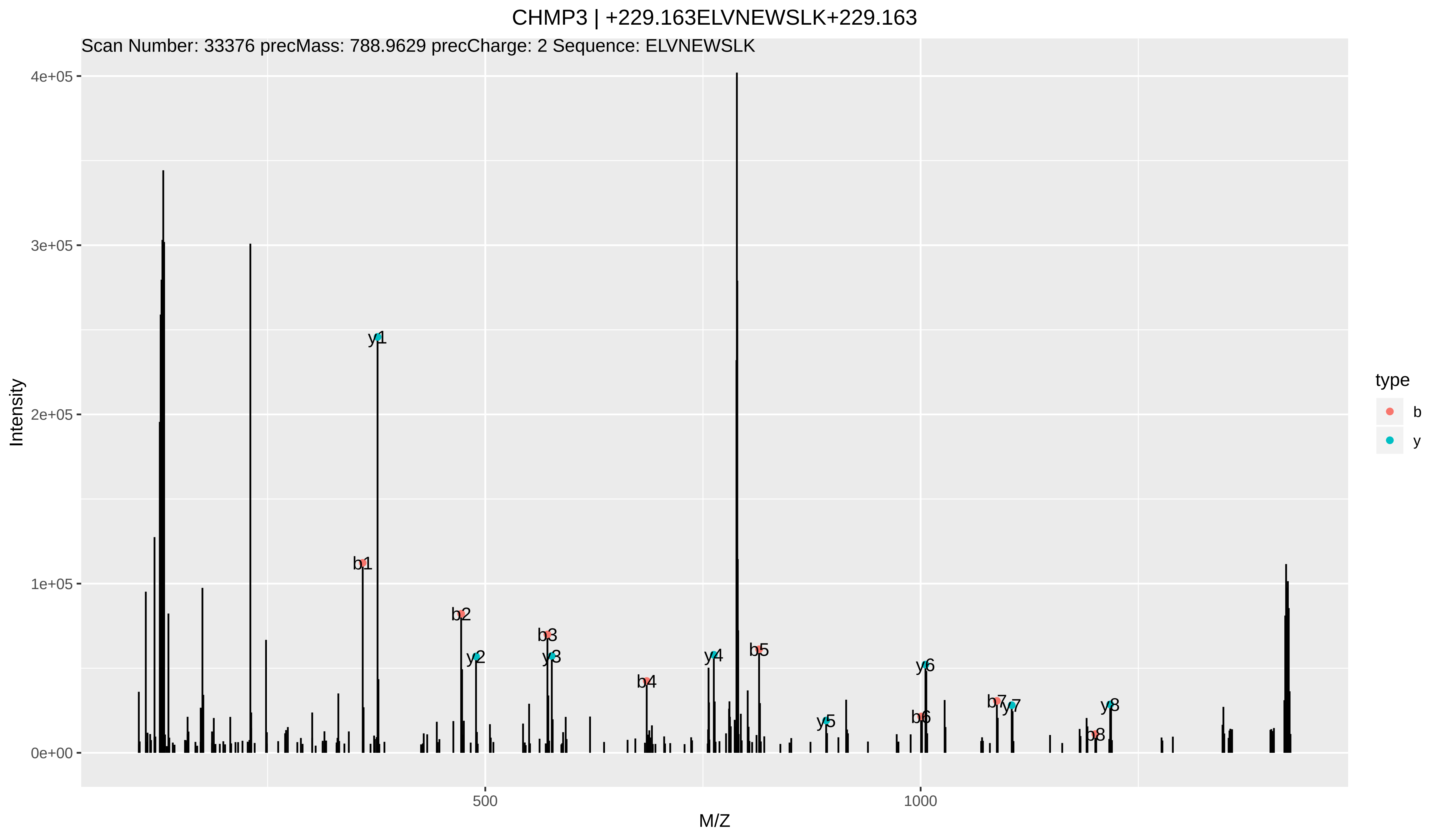
CHMP3 | +229.163ELVNEWSLK+229.163
Scan Number: 33376 precMass: 788.9629 precCharge: 2 Sequence: ELVNEWSLK
Intensity
4e+05
3e+05
2e+05
1e+05
0e+00
M/Z
500
1000
y1
b1
b2
y2
b3
y3
b4
y4
b5
y5
y6
b6
b7
y7
y8
b8
type
b
y

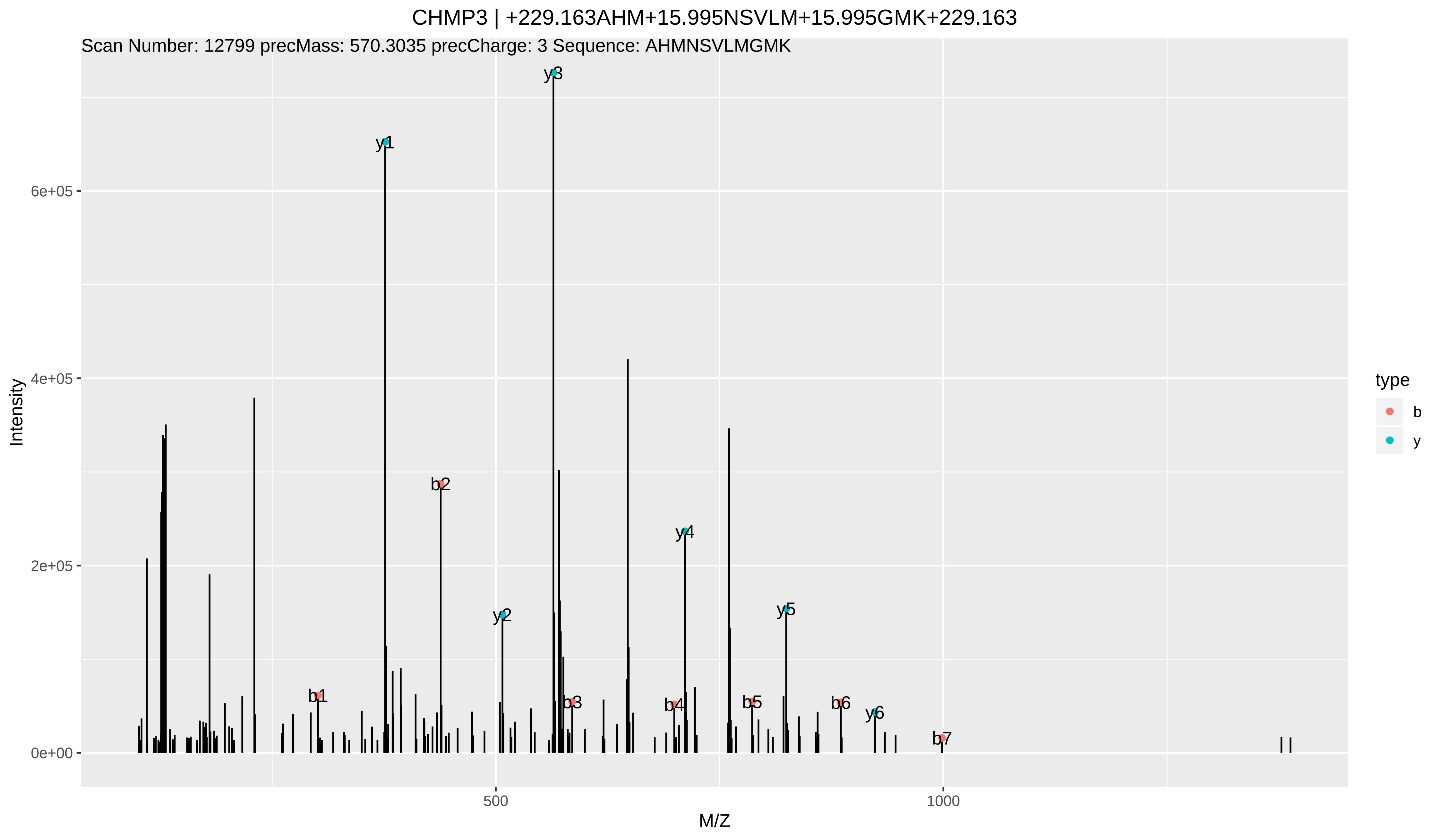

CHMP3 | +229.163AHM+15.995NSVLM+15.995GMK+229.163
Scan Number: 12799 precMass: 570.3035 precCharge: 3 Sequence: AHMNSVLMGMK
y3
y1
b2
y4
y2
y5
b1
b3
b4
b5
b6
y6
b7
Intensity
6e+05
4e+05
2e+05
0e+00
500
1000
M/Z
type
b
y

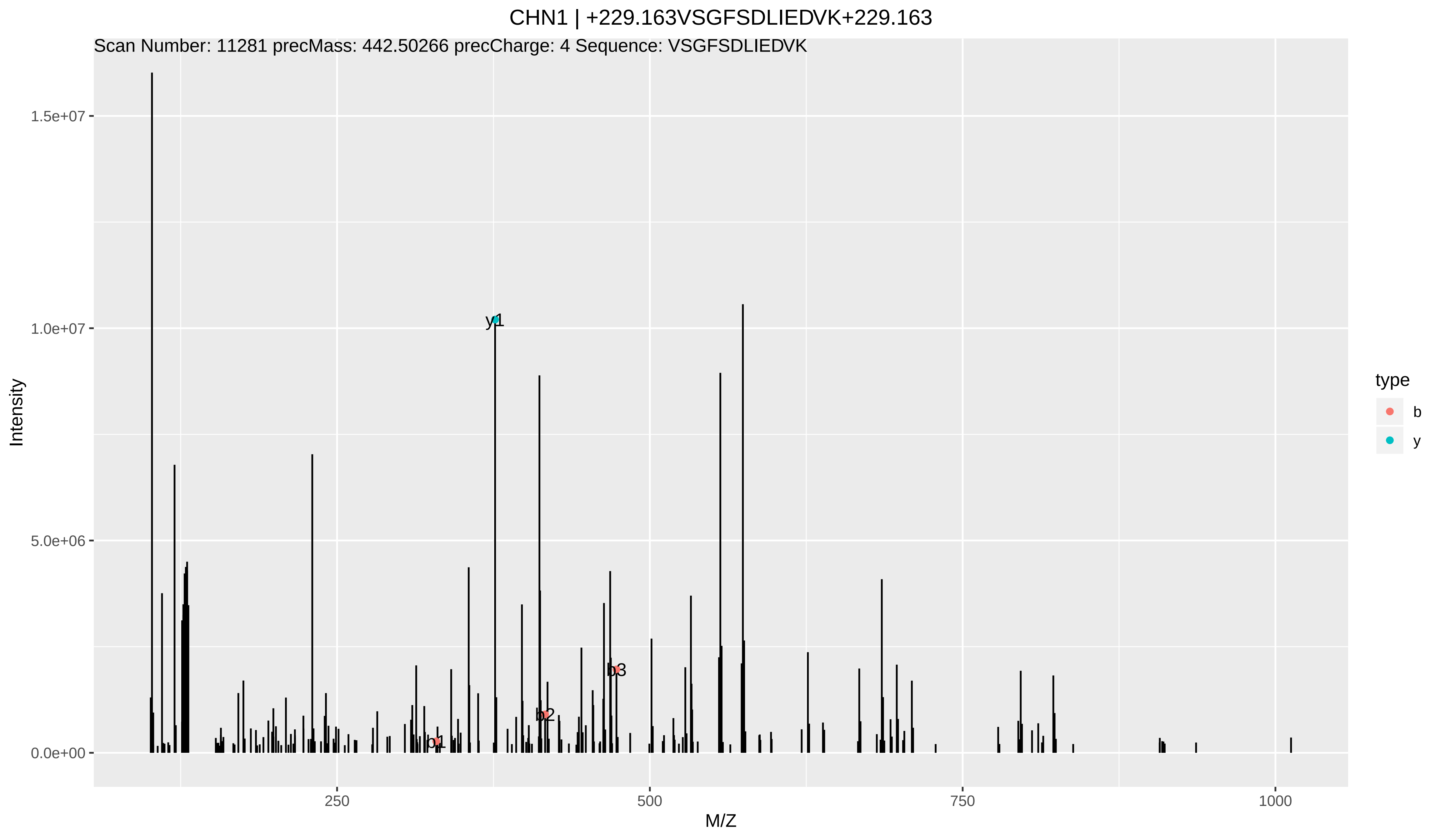

CHN1 | +229.163VSGFSDLIEDVK+229.163
Scan Number: 11281 precMass: 442.50266 precCharge: 4 Sequence: VSGFSDLIEDVK
Intensity
1.5e+07
1.0e+07
5.0e+06
0.0e+00
M/Z
250
500
750
1000
y1
b1
b2
b3
type
b
y

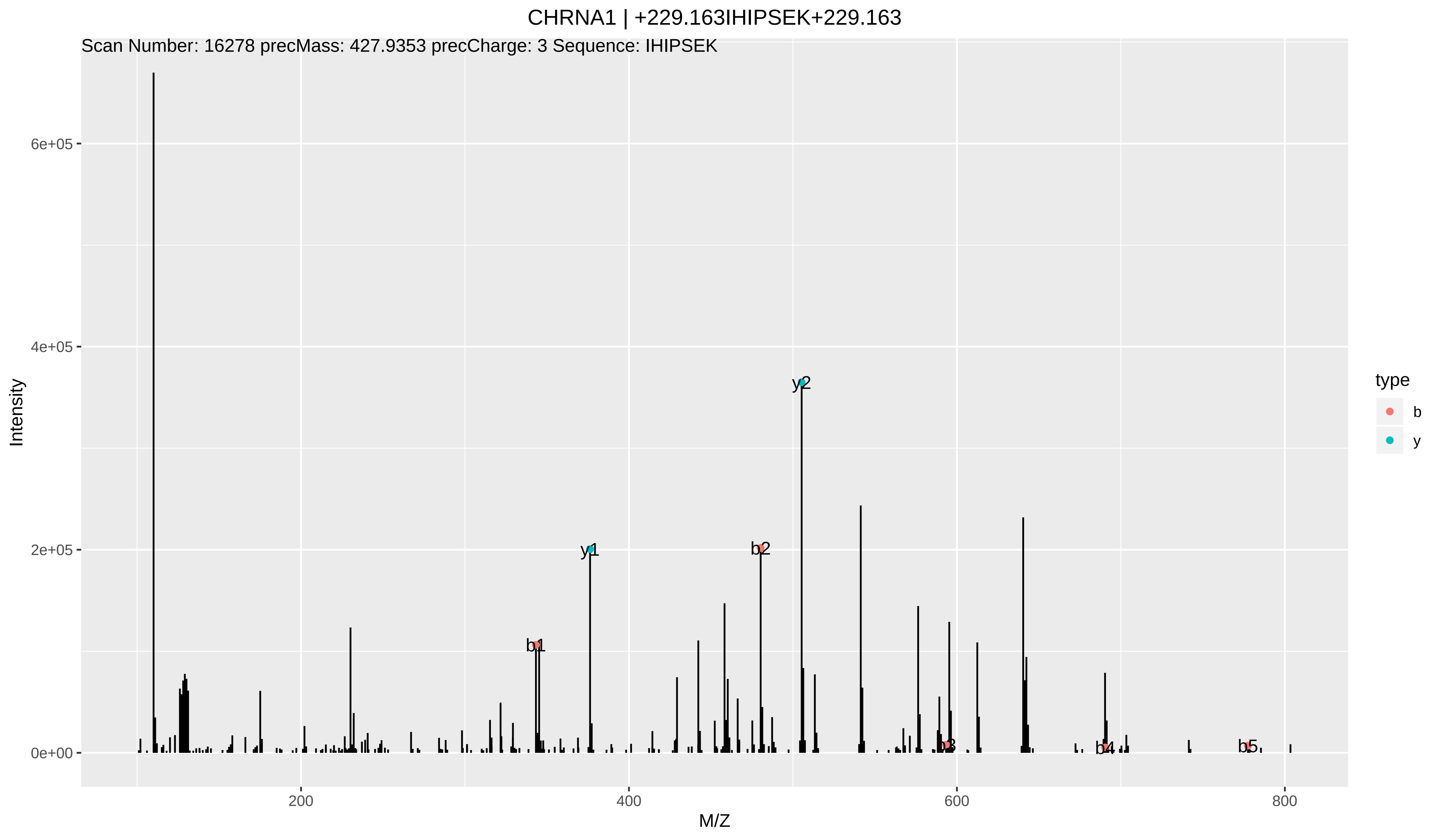

CHRNA1 | +229.163IHIPSEK+229.163
Scan Number: 16278 precMass: 427.9353 precCharge: 3 Sequence: IHIPSEK
Intensity
6e+05
4e+05
2e+05
0e+00
M/Z
200
400
600
800
y2
y1
b2
b1
b3
b4
b5
type
b
y

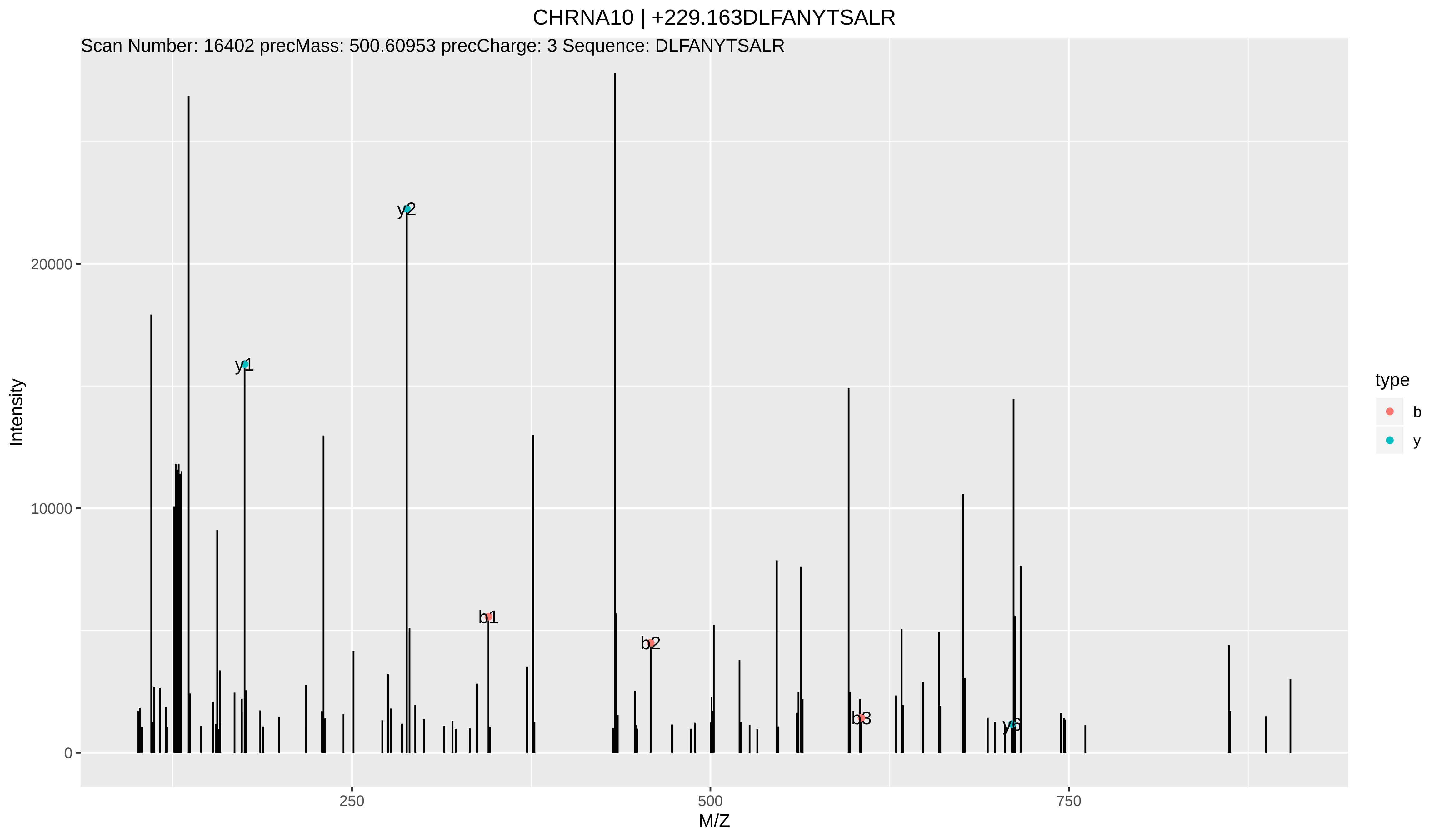

CHRNA10 | +229.163DLFANYTSALR
Scan Number: 16402 precMass: 500.60953 precCharge: 3 Sequence: DLFANYTSALR
Intensity
20000
10000
0
M/Z
250
500
750
y1
y2
b1
b2
b3
y6
type
b
y

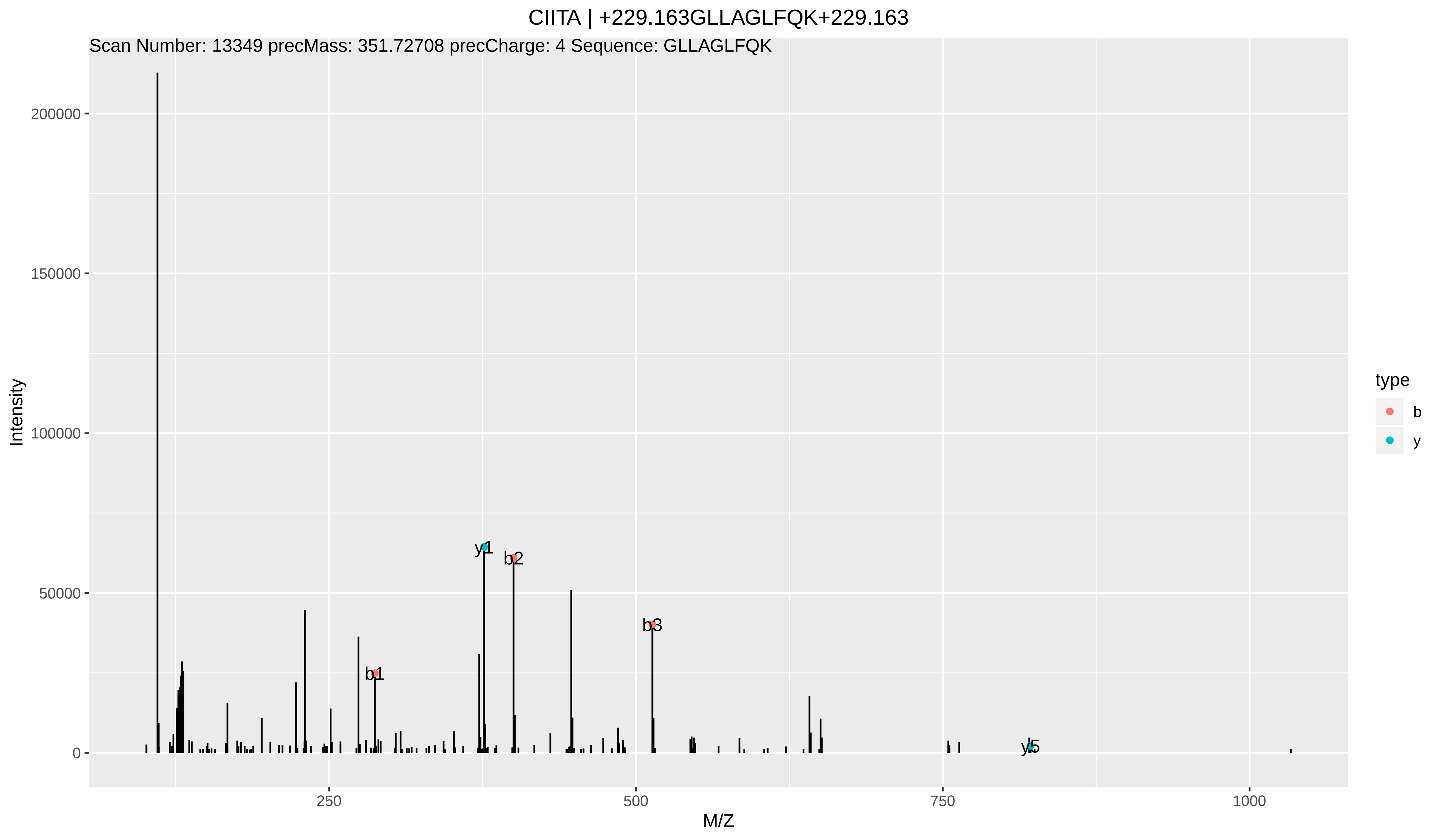
CIITA | +229.163GLLAGLFQK+229.163
Scan Number: 13349 precMass: 351.72708 precCharge: 4 Sequence: GLLAGLFQK
Intensity
200000
150000
100000
50000
0
y1
b2
b3
b1
y5
250
500
750
1000
M/Z
type
b
y

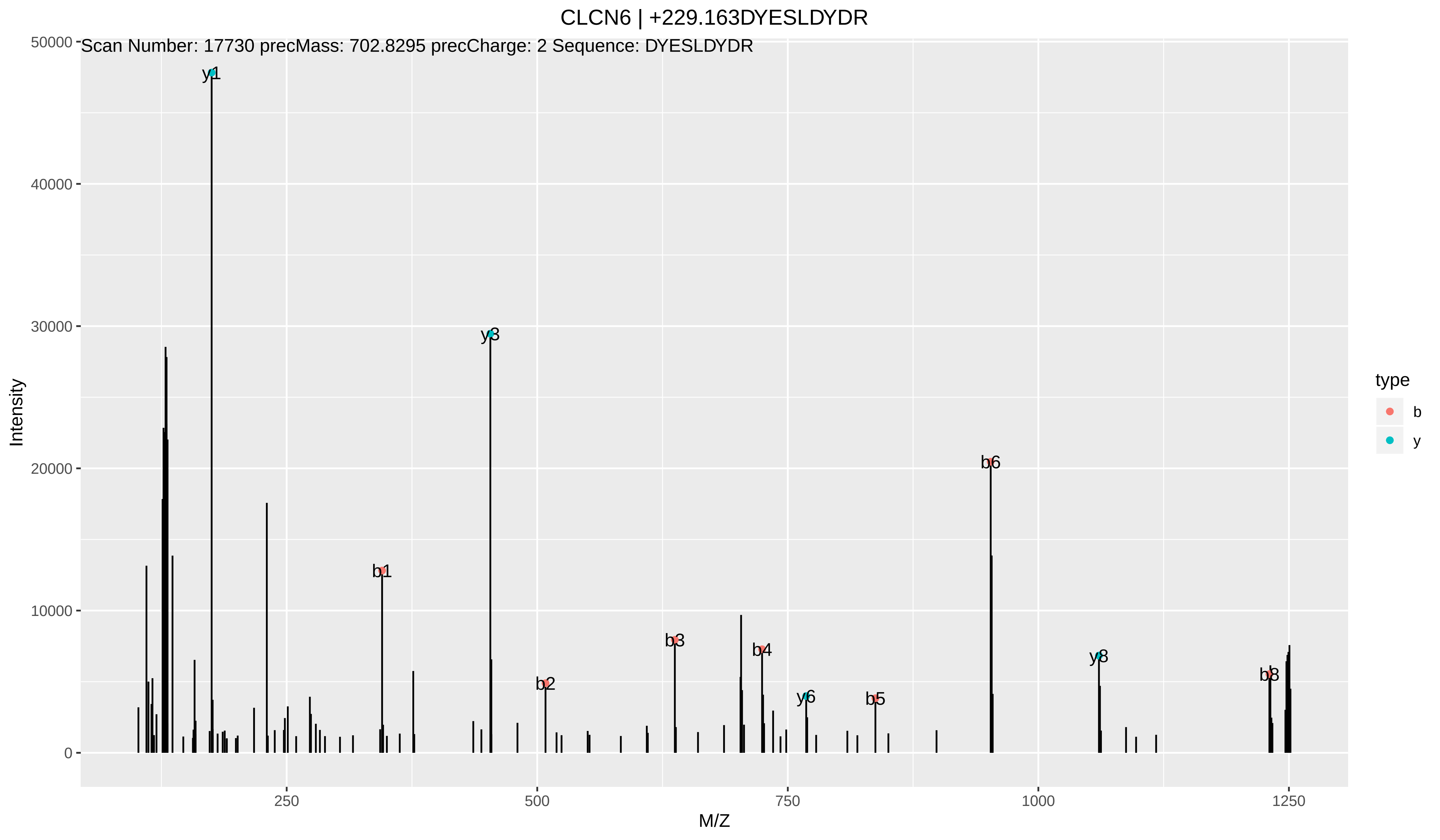
CLCN6 | +229.163DYESLDYDR
Scan Number: 17730 precMass: 702.8295 precCharge: 2 Sequence: DYESLDYDR
Intensity
M/Z
type
b
y
y1
y3
b1
b2
b3
b4
y6
b5
b6
y8
b8
0
10000
20000
30000
40000
50000
250
500
750
1000
1250

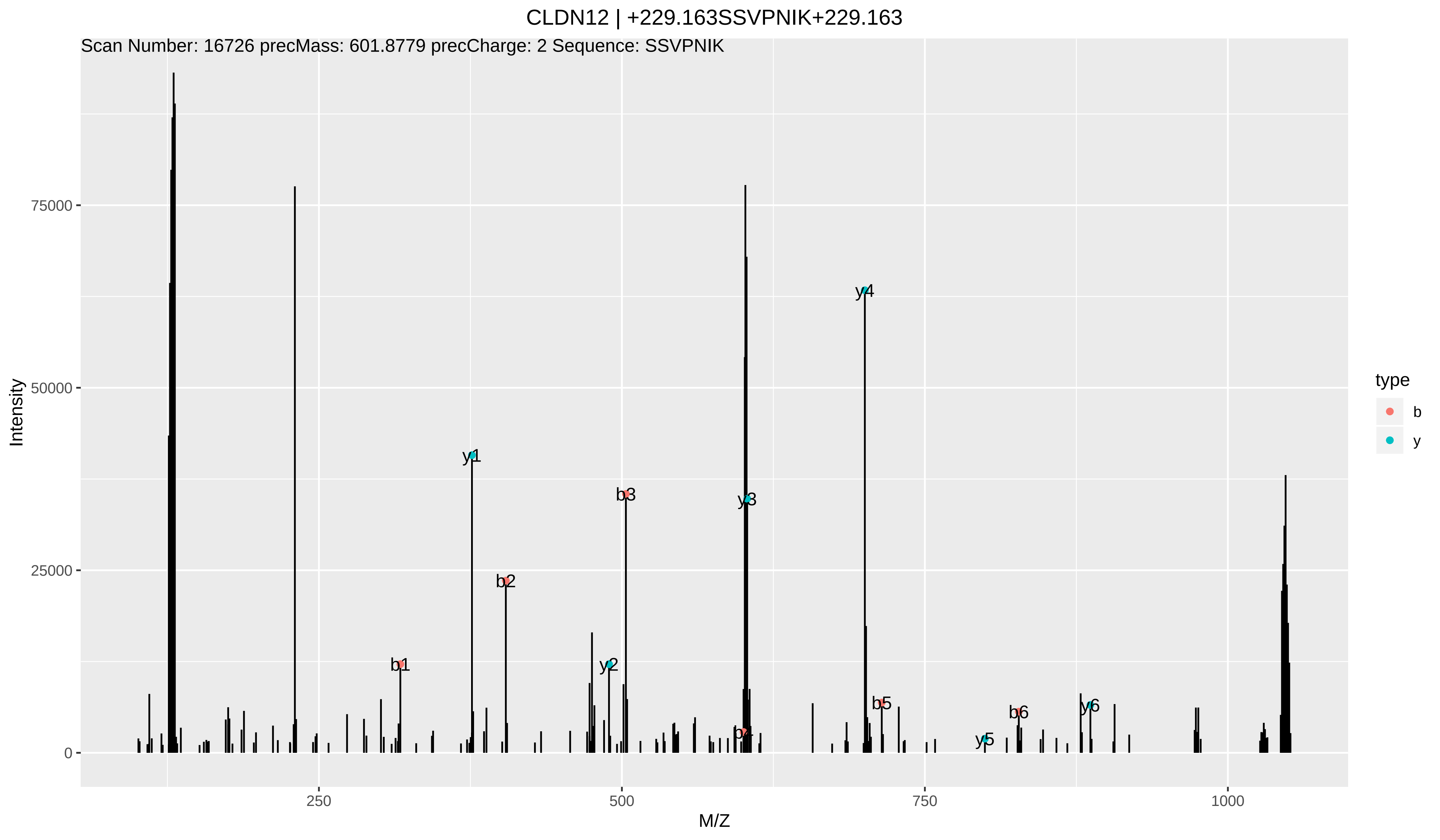

CLDN12 | +229.163SSVPNIK+229.163
Scan Number: 16726 precMass: 601.8779 precCharge: 2 Sequence: SSVPNIK
Intensity
75000
50000
25000
0
M/Z
250
500
750
1000
y1
b3
y3
y4
b2
b1
y2
b5
b6
y6
y5
type
b
y

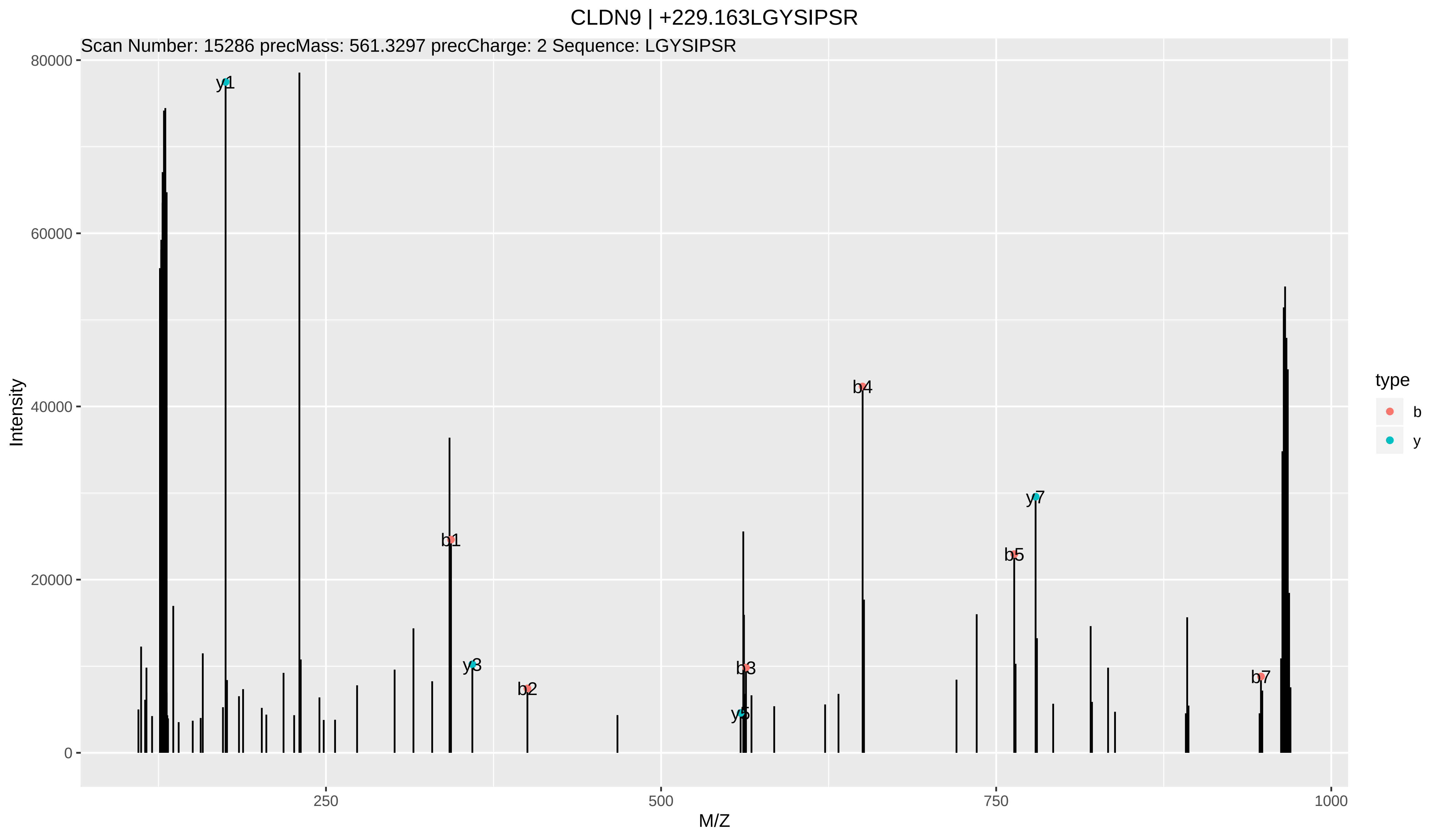
CLDN9 | +229.163LGYSIPSR
Scan Number: 15286 precMass: 561.3297 precCharge: 2 Sequence: LGYSIPSR
Intensity
M/Z
80000
60000
40000
20000
0
250
500
750
1000
y1
b1
y3
b2
b3
y5
b4
b5
y7
b7
type
b
y

CLDN9 | +229.163VYDSLLALPQDLQAAR

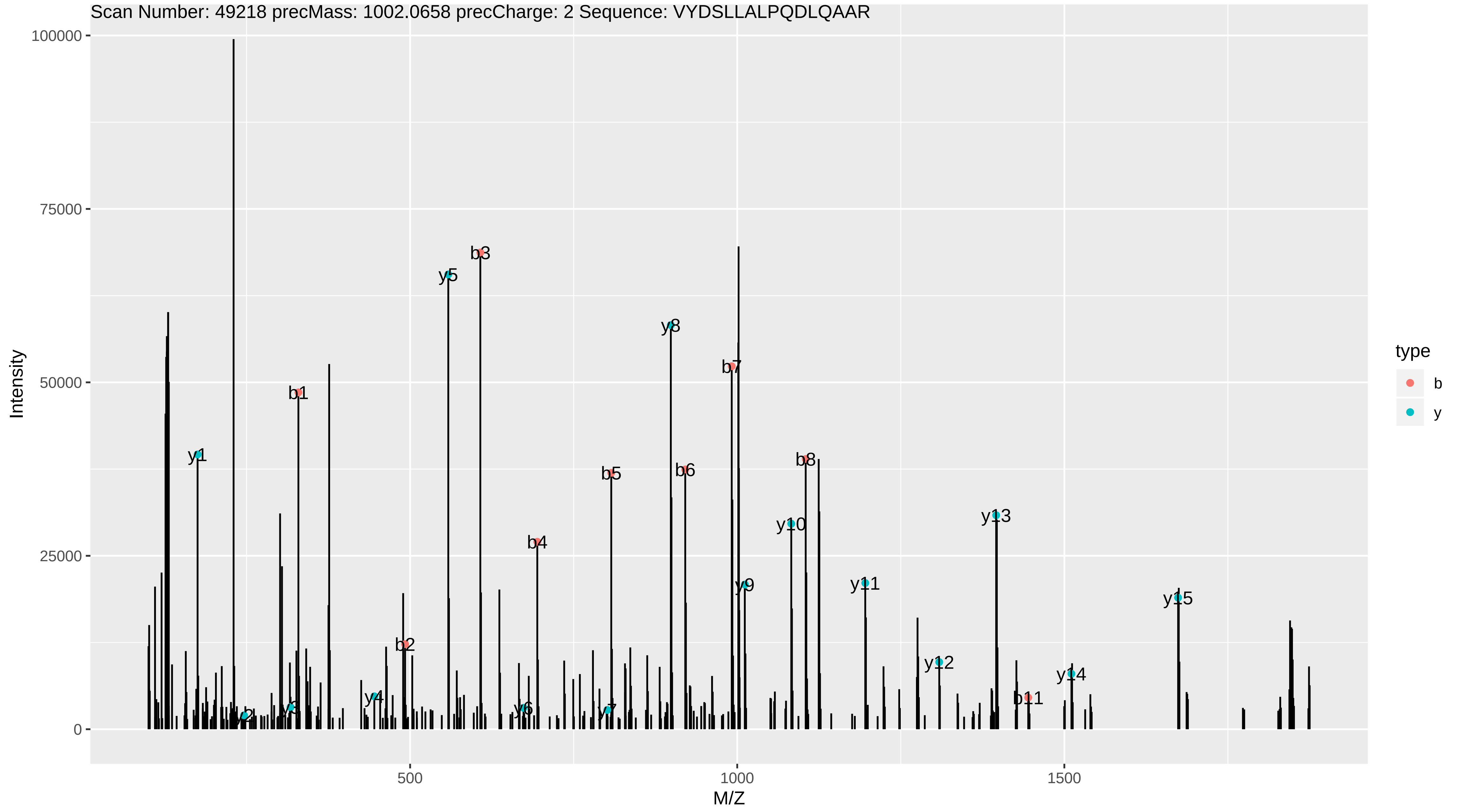

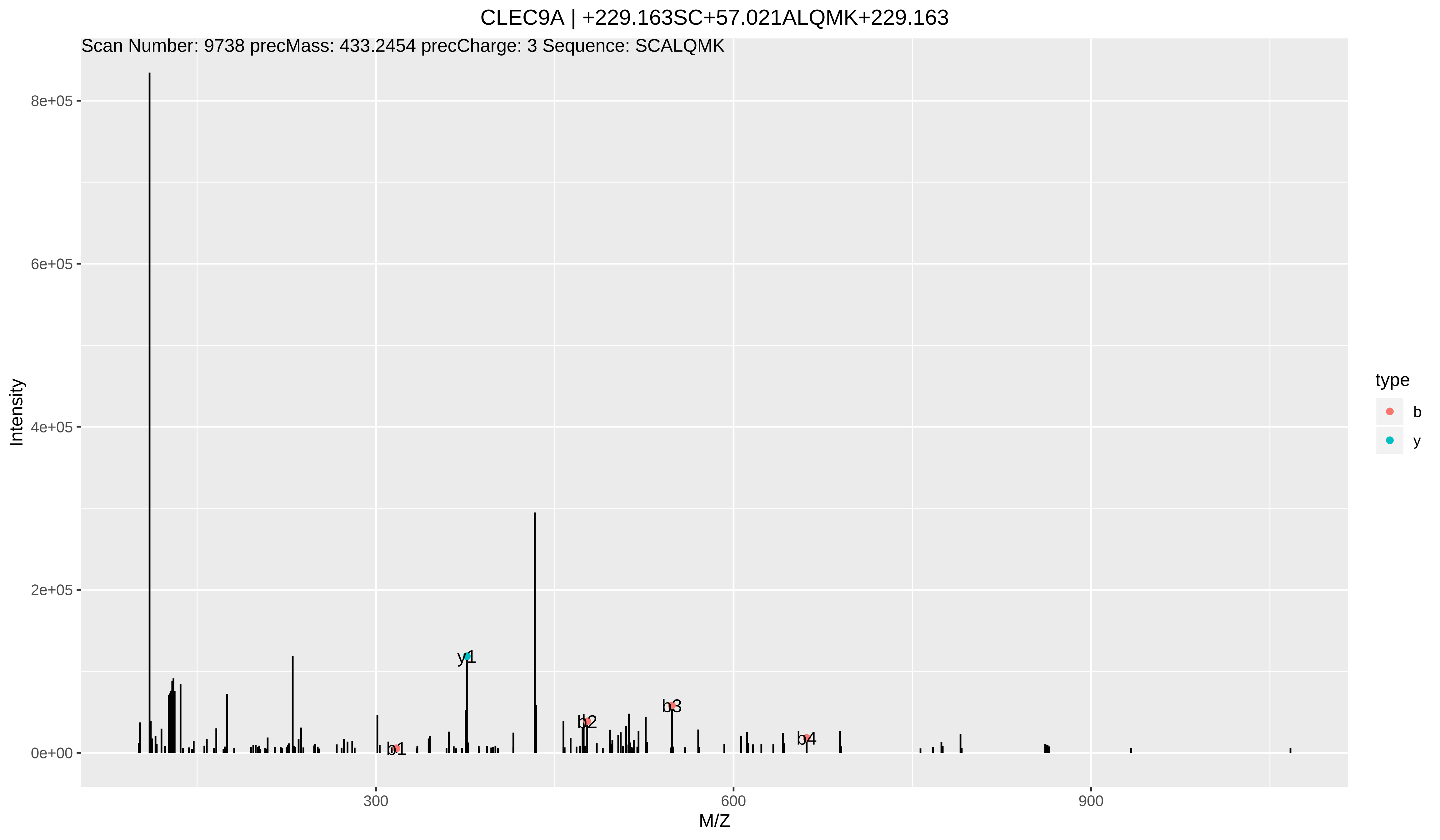

CLEC9A | +229.163SC+57.021ALQMK+229.163
Scan Number: 9738 precMass: 433.2454 precCharge: 3 Sequence: SCALQMK
Intensity
8e+05
6e+05
4e+05
2e+05
0e+00
M/Z
300
600
900
y1
b1
b2
b3
b4
type
b
y

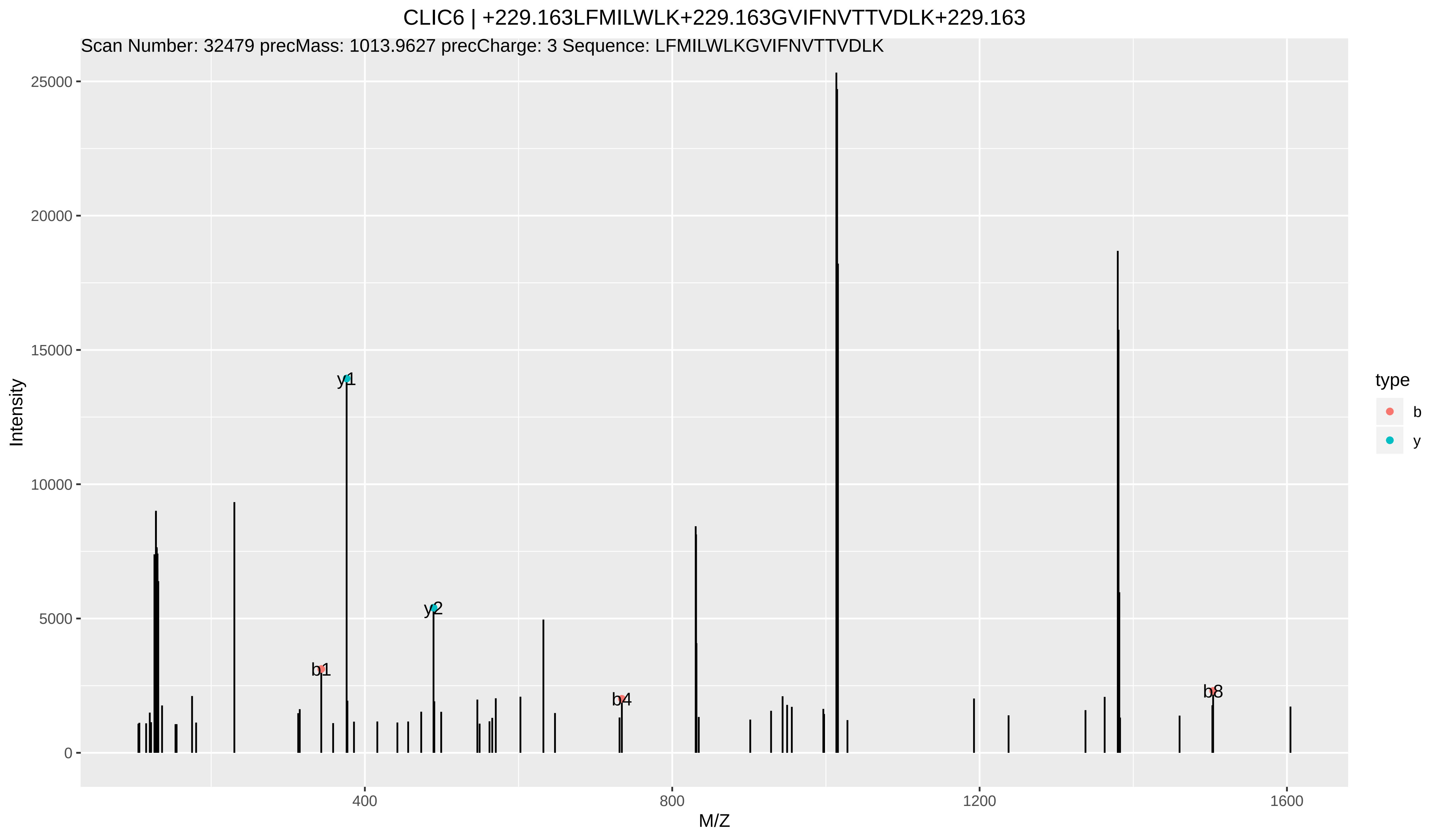

CLIC6 | +229.163LFMILWLK+229.163GVIFNVTTVDLK+229.163
Scan Number: 32479 precMass: 1013.9627 precCharge: 3 Sequence: LFMILWLKGVIFNVTTVDLK
Intensity
25000
20000
15000
10000
5000
0
400
800
1200
1600
M/Z
y1
y2
b1
b4
b8
type
b
y

CMC2 | +229.163LFNPPEESEK+229.163

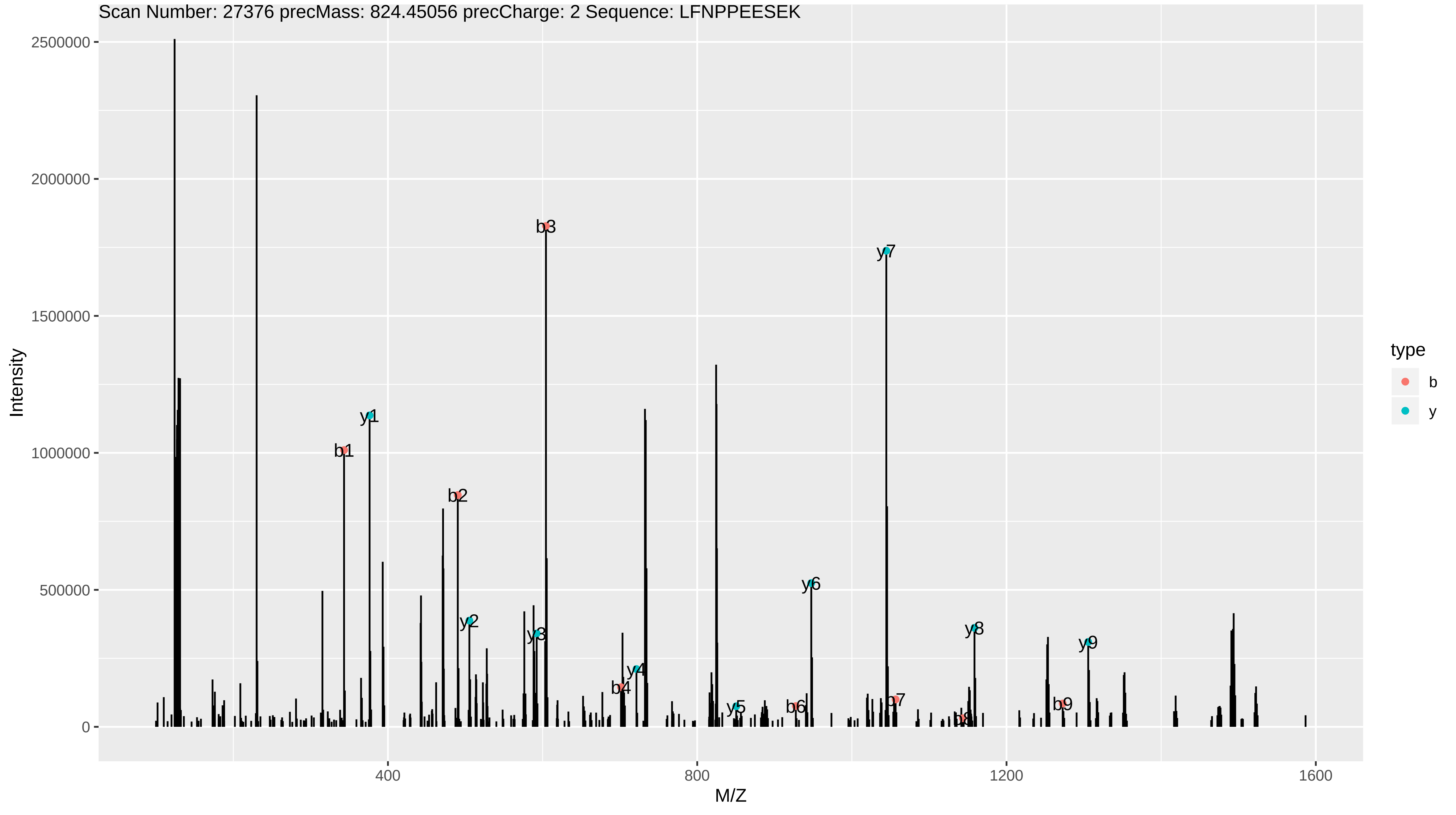

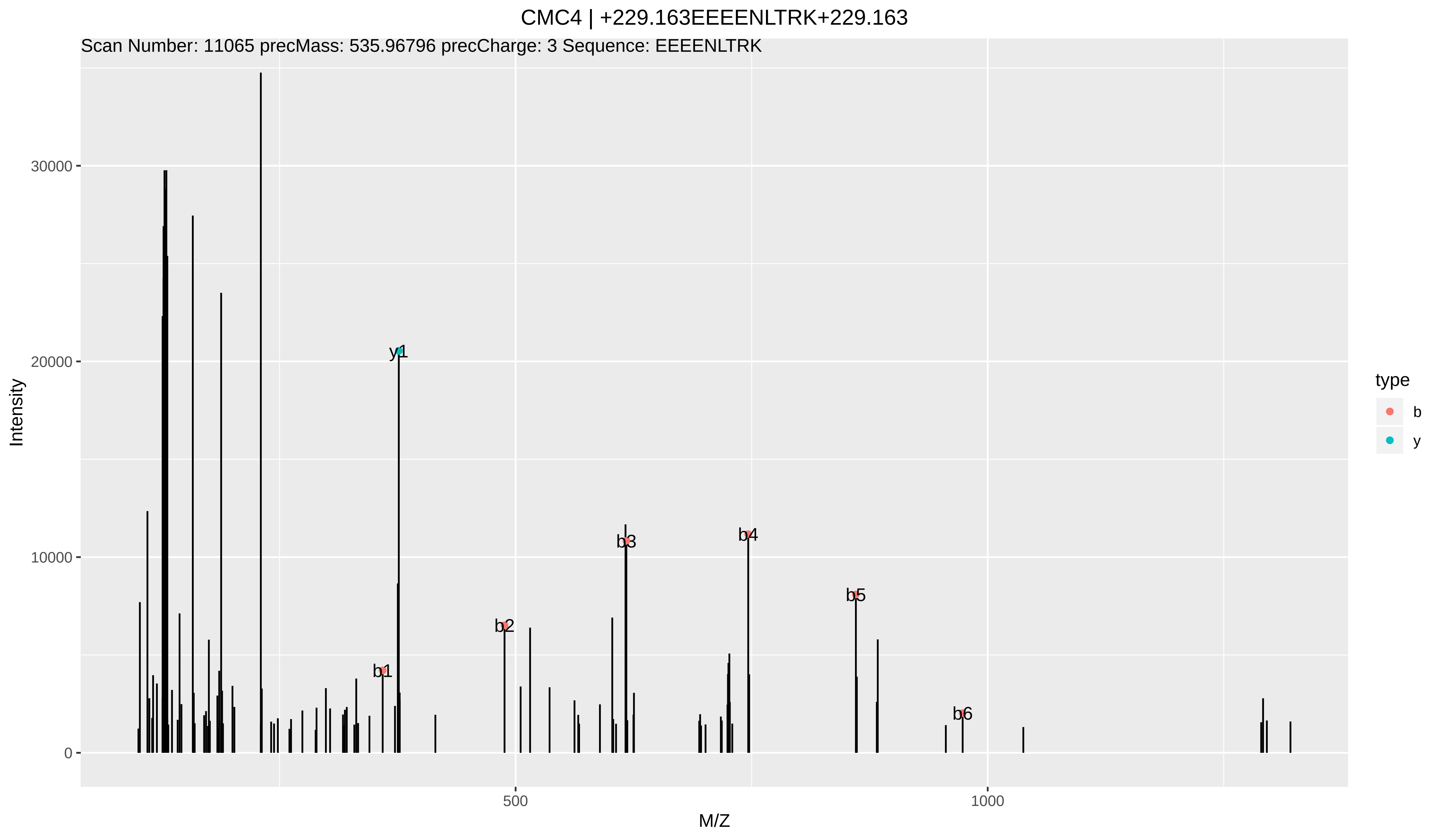

CMC4 | +229.163EEEENLTRK+229.163
Scan Number: 11065 precMass: 535.96796 precCharge: 3 Sequence: EEEENLTRK
Intensity
30000
20000
10000
0
500
1000
M/Z
y1
b1
b2
b3
b4
b5
b6
type
b
y

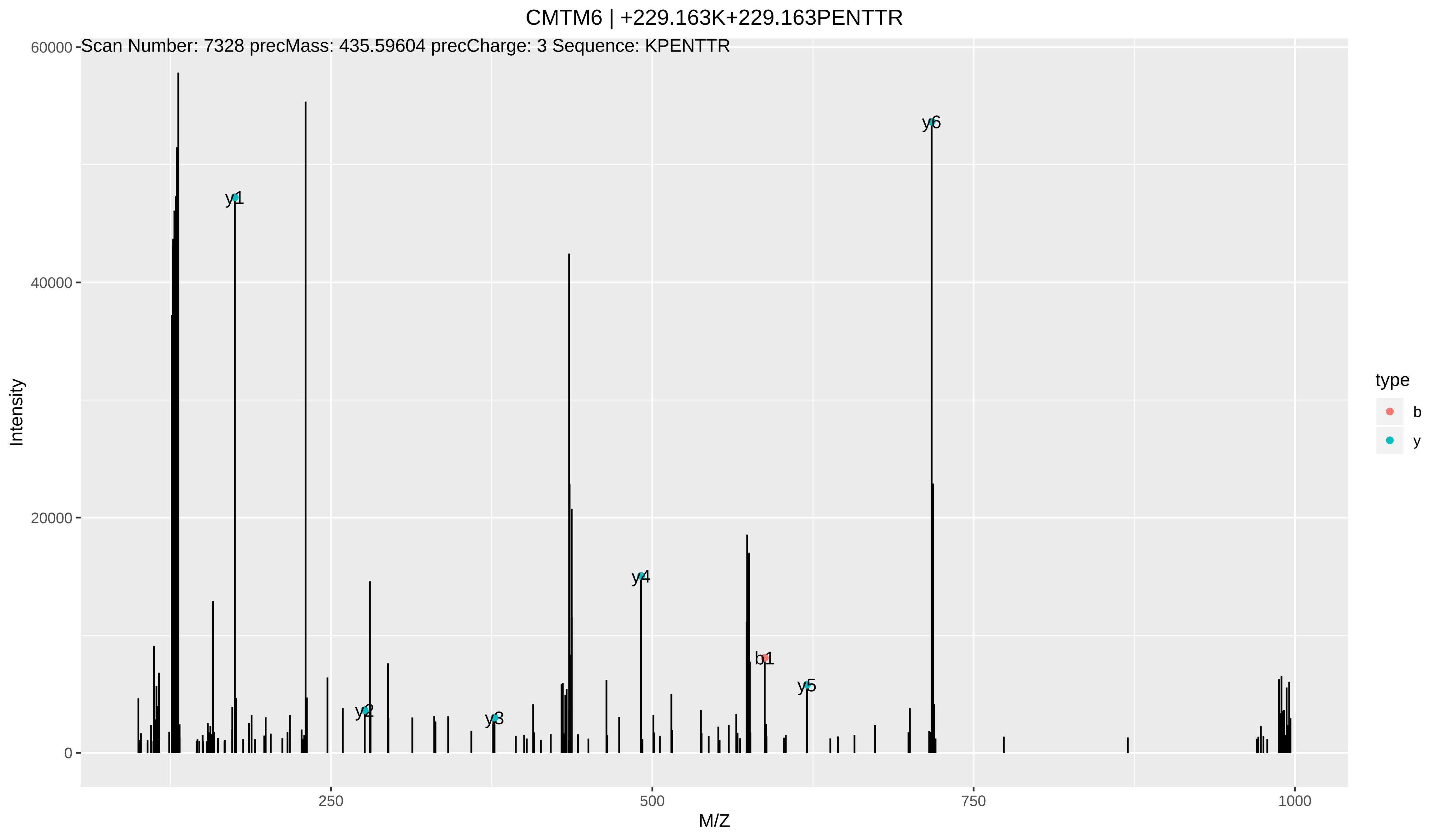

CMTM6 | +229.163K+229.163PENTTR
Scan Number: 7328 precMass: 435.59604 precCharge: 3 Sequence: KPENTTR
Intensity
60000
40000
20000
0
M/Z
250
500
750
1000
y1
y2
y3
y4
b1
y5
y6
type
b
y

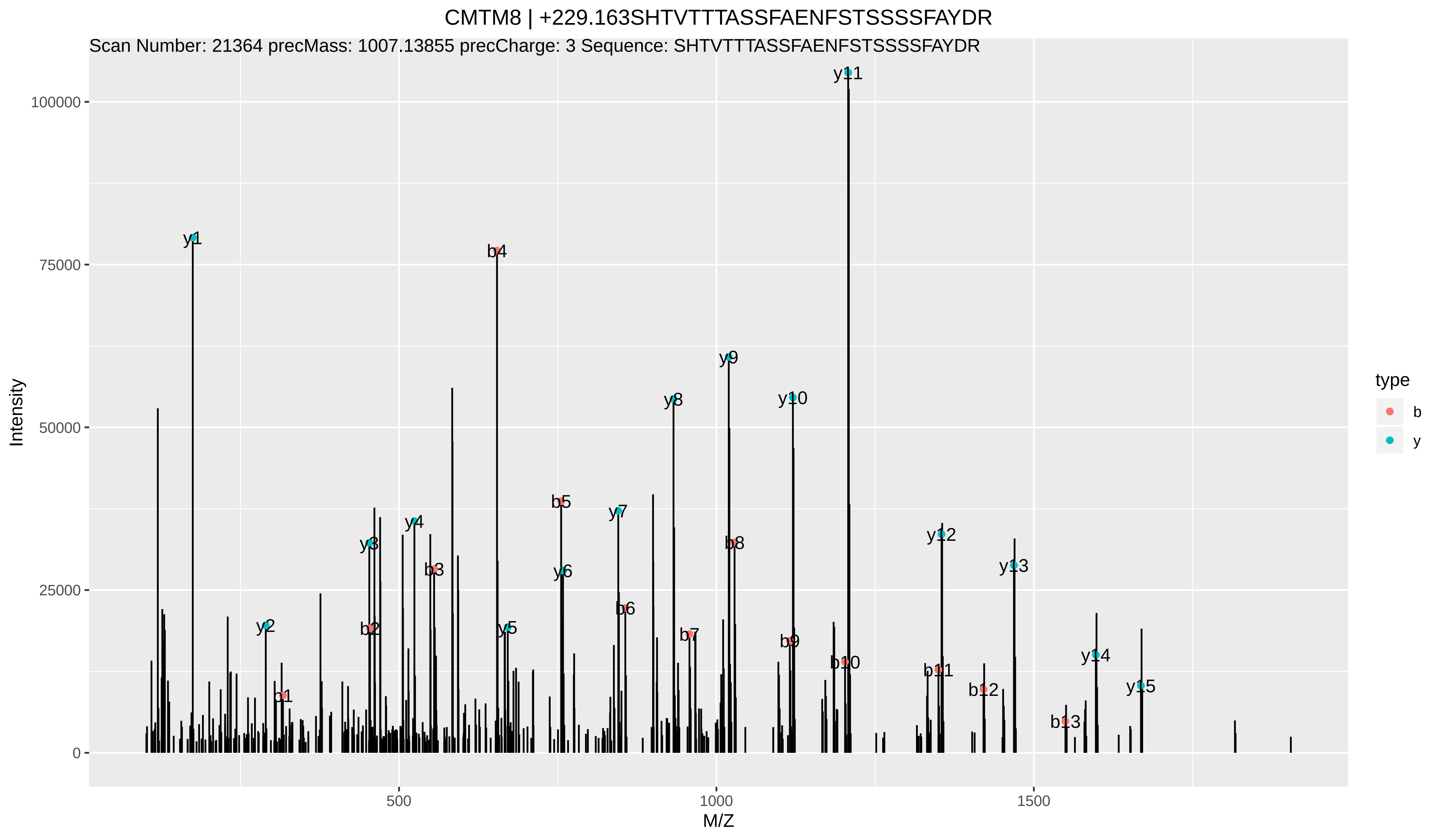

CMTM8 | +229.163SHTVTTTASSFAENFSTSSSSFAYDR
Scan Number: 21364 precMass: 1007.13855 precCharge: 3 Sequence: SHTVTTTASSFAENFSTSSSSFAYDR
Intensity
M/Z
type
b
y
y1
b4
y11
y9
y8
y10
b5
y7
y4
y3
b8
y12
b3
y6
y13
b6
y2
b2
y5
b7
b9
b10
y14
b11
b12
y15
b1
b13
100000
75000
50000
25000
0
500
1000
1500

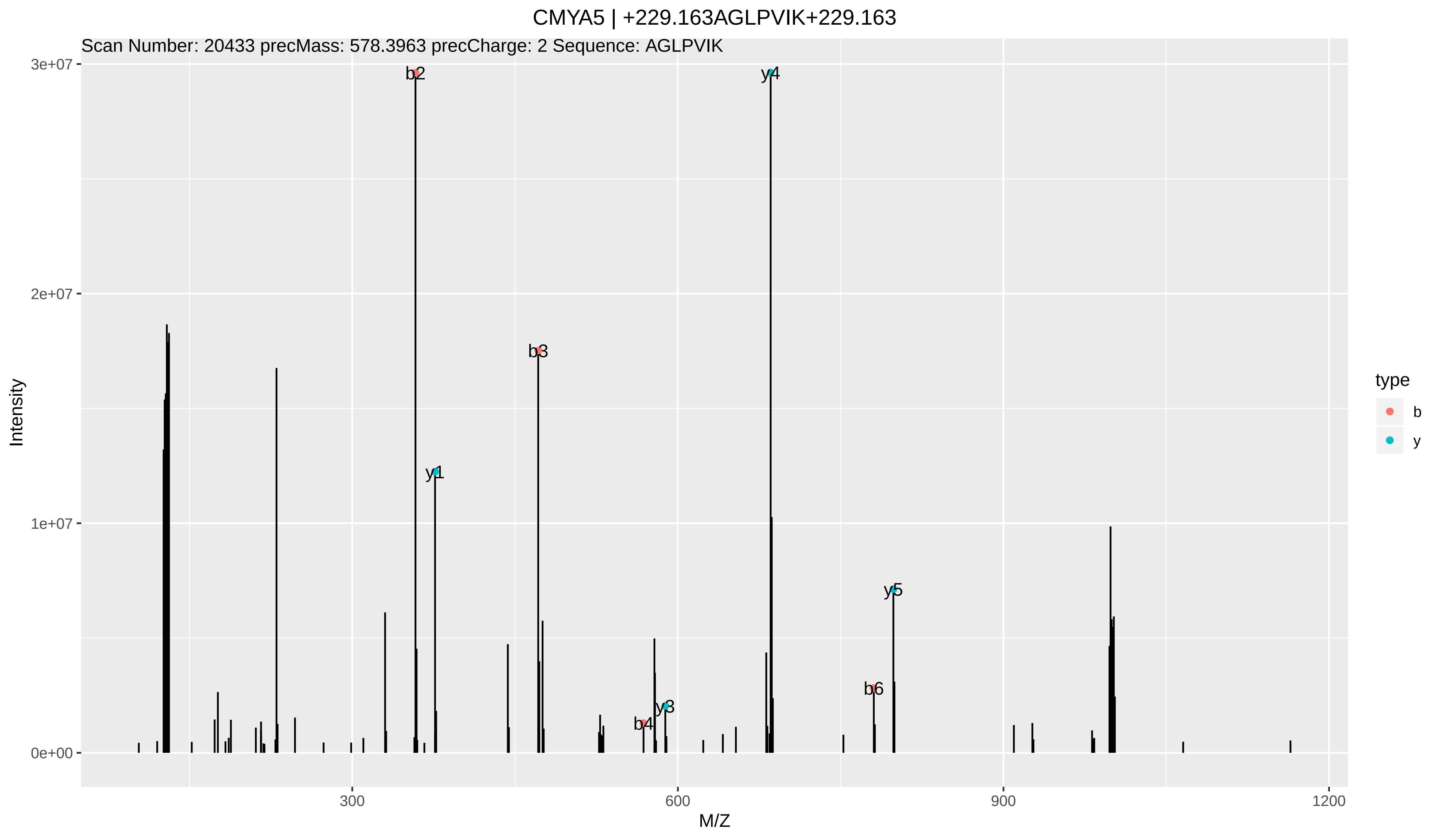
CMYA5 | +229.163AGLPVIK+229.163
Scan Number: 20433 precMass: 578.3963 precCharge: 2 Sequence: AGLPVIK
3e+07
2e+07
1e+07
0e+00
Intensity
300
600
900
1200
M/Z
b2
y4
b3
y1
y5
b6
y3
b4
type
b
y

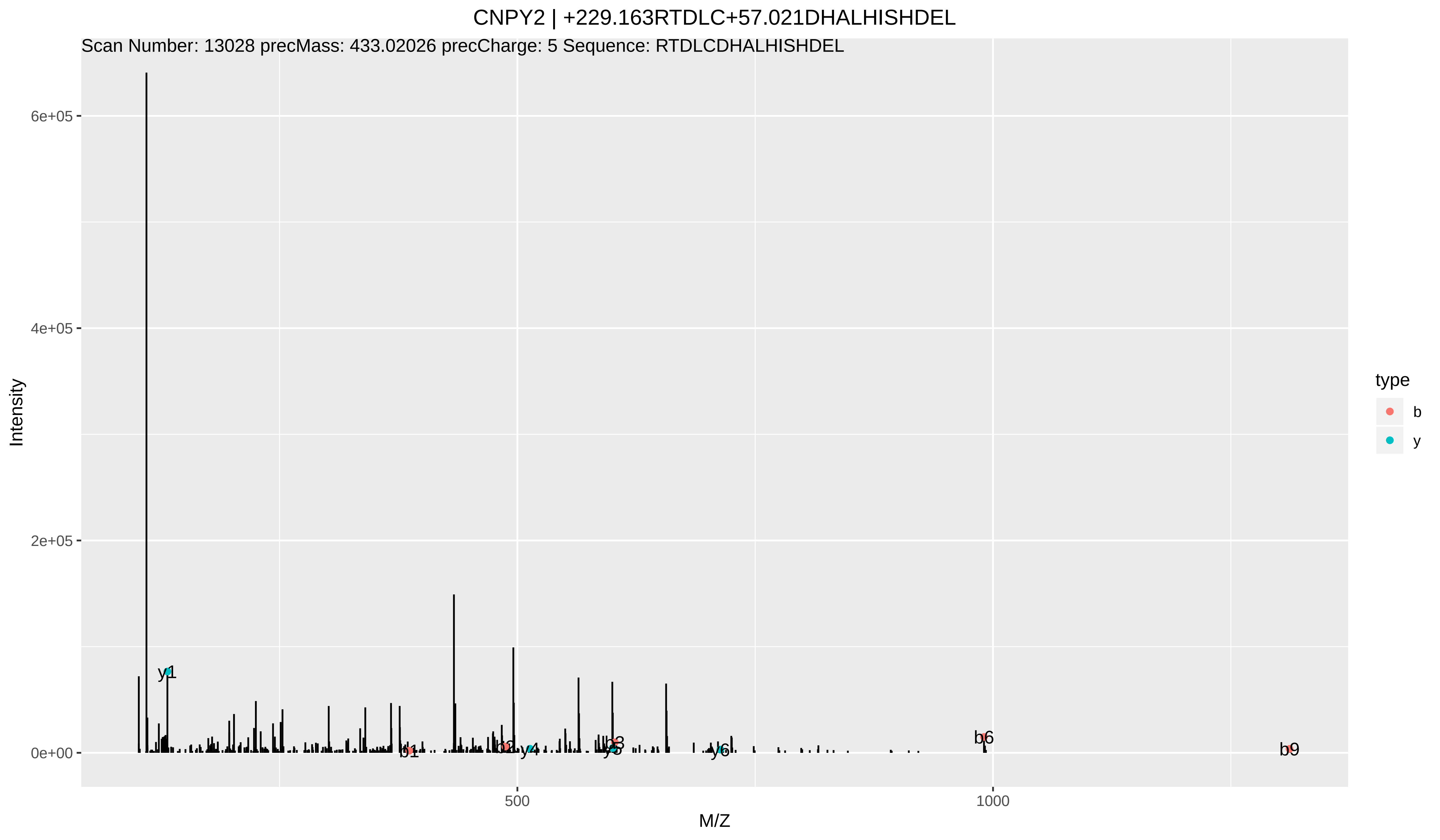

CNPY2 | +229.163RTDLC+57.021DHALHISHDEL
Scan Number: 13028 precMass: 433.02026 precCharge: 5 Sequence: RTDLCDHALHISHDEL
Intensity
6e+05
4e+05
2e+05
0e+00
M/Z
500
1000
y1
b1
b2
y4
b3
y5
y6
b6
b9
type
b
y

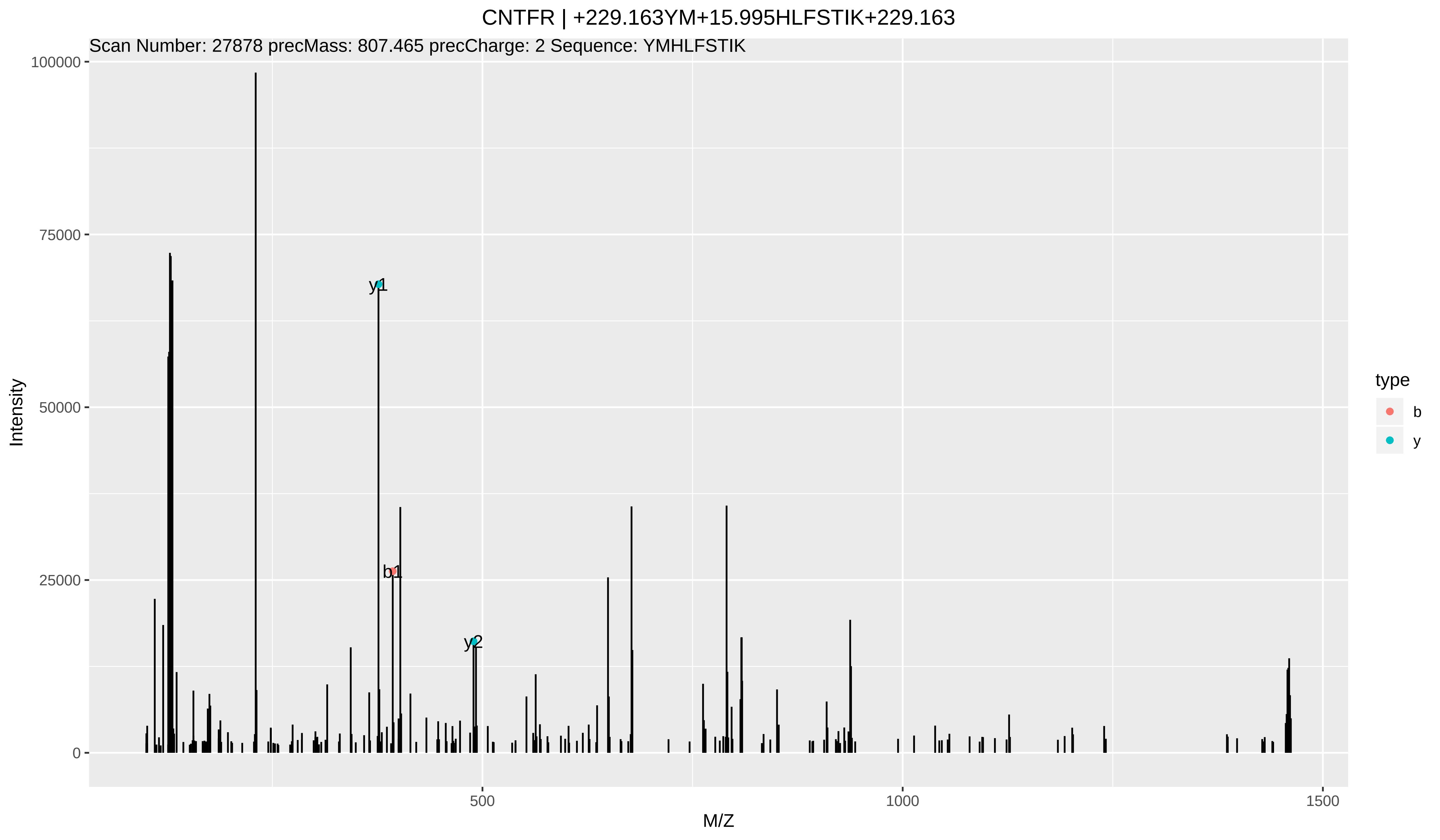

CNTFR | +229.163YM+15.995HLFSTIK+229.163
Scan Number: 27878 precMass: 807.465 precCharge: 2 Sequence: YMHLFSTIK
Intensity
100000
75000
50000
25000
0
M/Z
500
1000
1500
y1
b1
y2
type
b
y

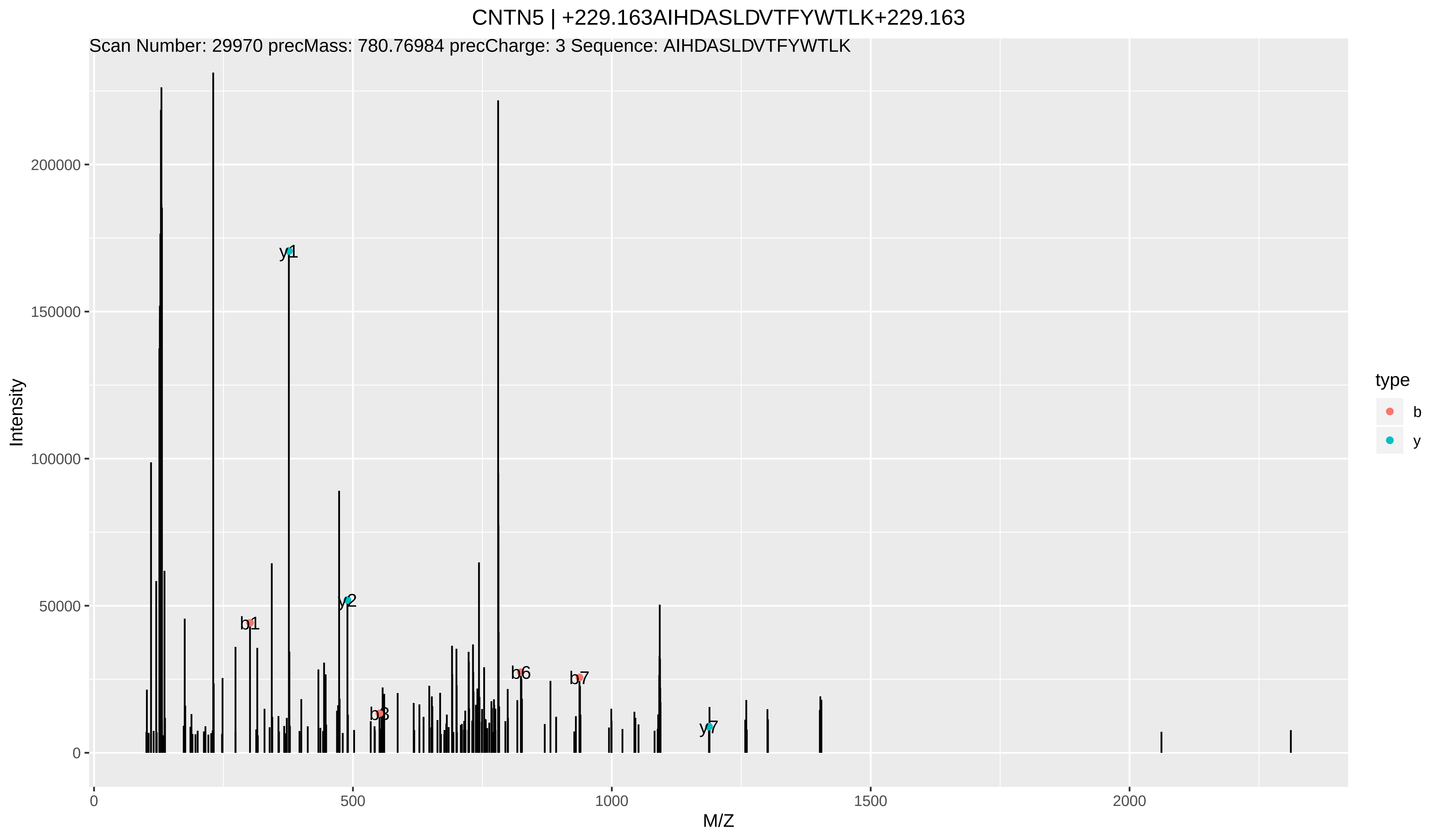

CNTN5 | +229.163AIHDASLDVTFYWTLK+229.163
Scan Number: 29970 precMass: 780.76984 precCharge: 3 Sequence: AIHDASLDVTFYWTLK
Intensity
200000
150000
100000
50000
0
0
500
1000
1500
2000
M/Z
y1
y2
b1
b3
b6
b7
y7
type
b
y

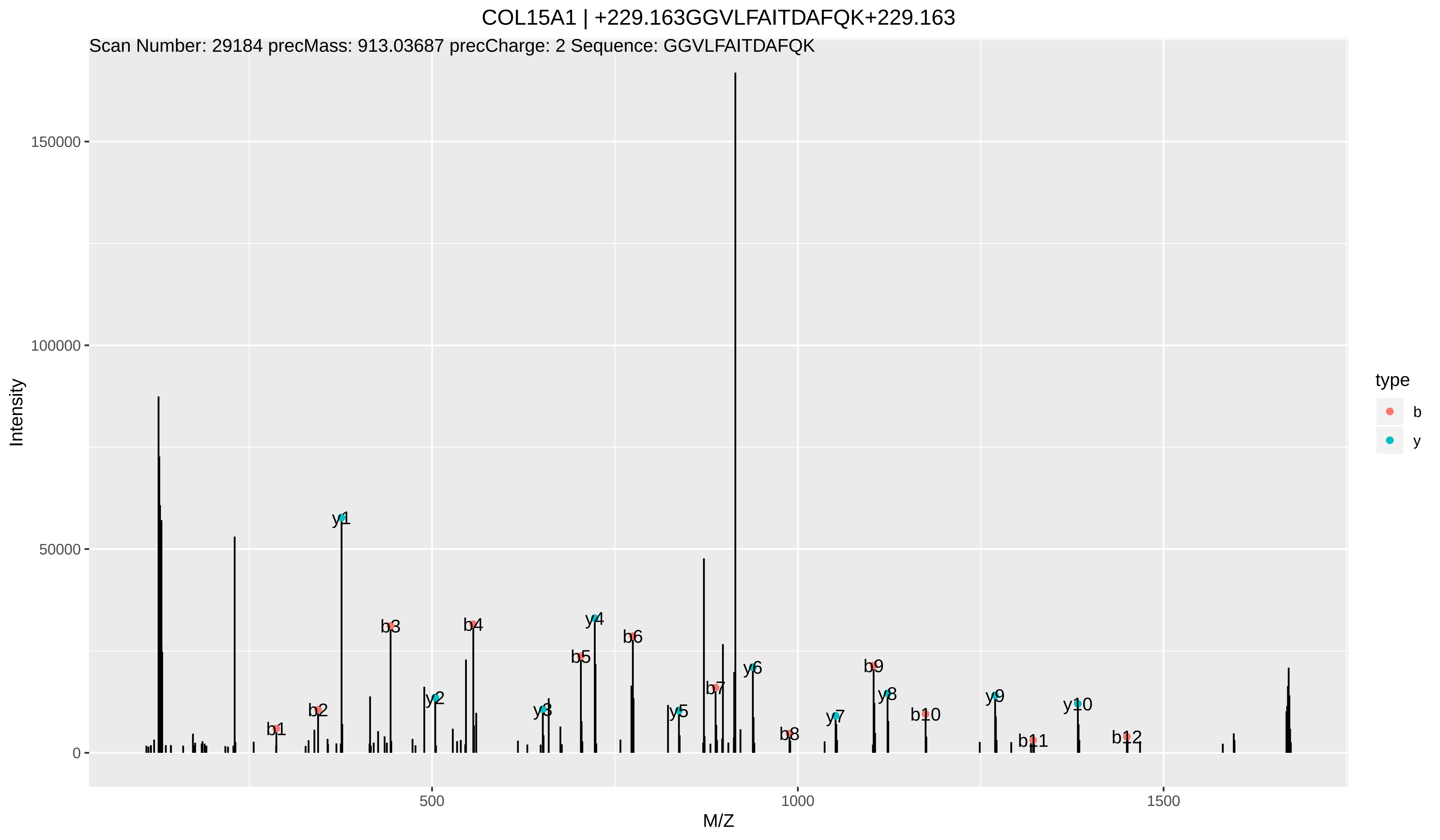

COL15A1 | +229.163GGVLFAITDAFQK+229.163
Scan Number: 29184 precMass: 913.03687 precCharge: 2 Sequence: GGVLFAITDAFQK
Intensity
150000
100000
50000
0
M/Z
500
1000
1500
y1
b3
b4
y4
b6
b5
y6
b9
b7
y8
y2
b2
y3
y5
b10
y9
y10
y7
b1
b8
b11
b12
type
b
y

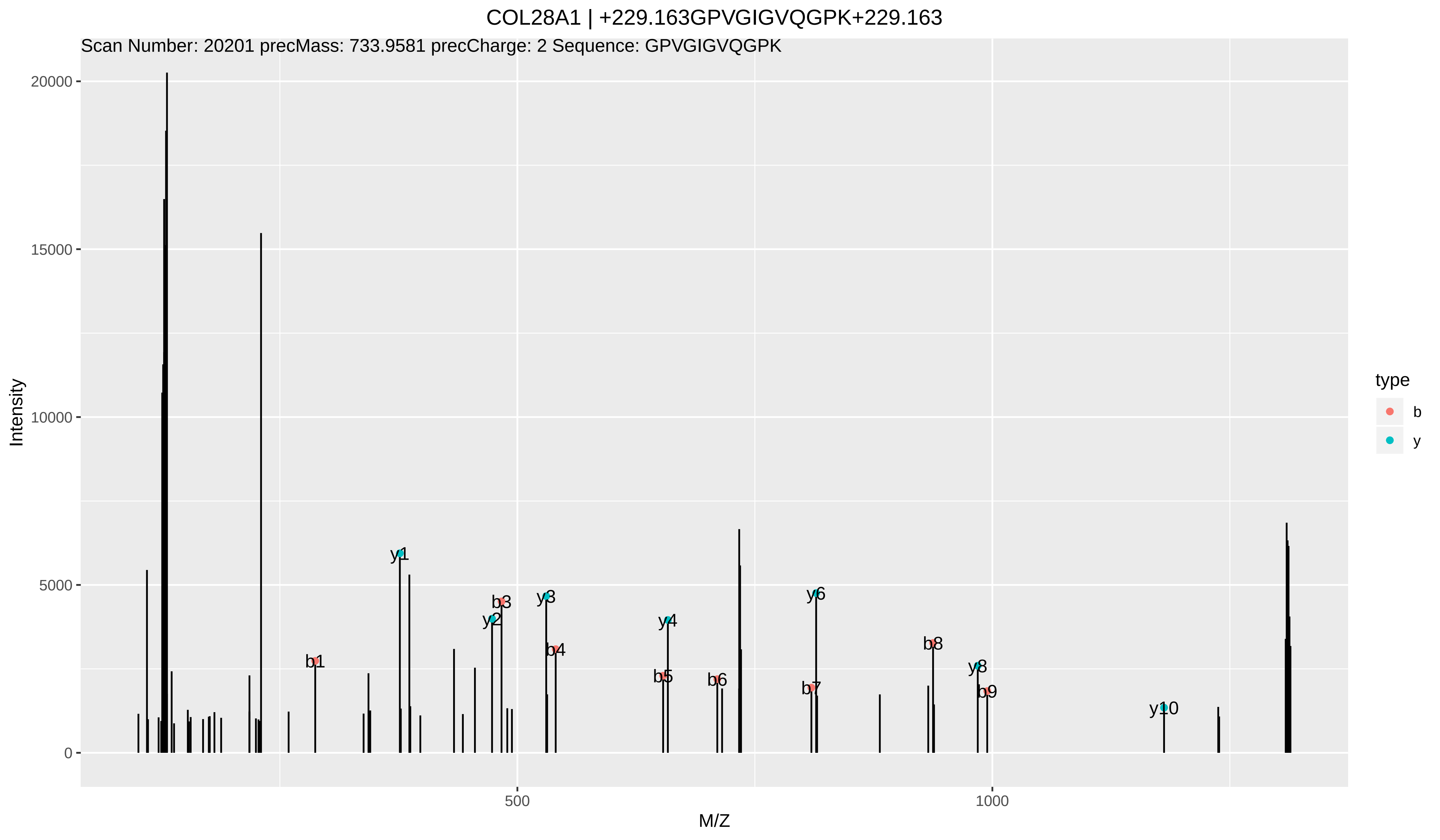

COL28A1 | +229.163GPVGIGVQGPK+229.163
Scan Number: 20201 precMass: 733.9581 precCharge: 2 Sequence: GPVGIGVQGPK
Intensity
0
5000
10000
15000
20000
M/Z
500
1000
y1
y2
b3
y3
b4
b1
b5
y4
b6
y6
b7
b8
y8
b9
y10
type
b
y

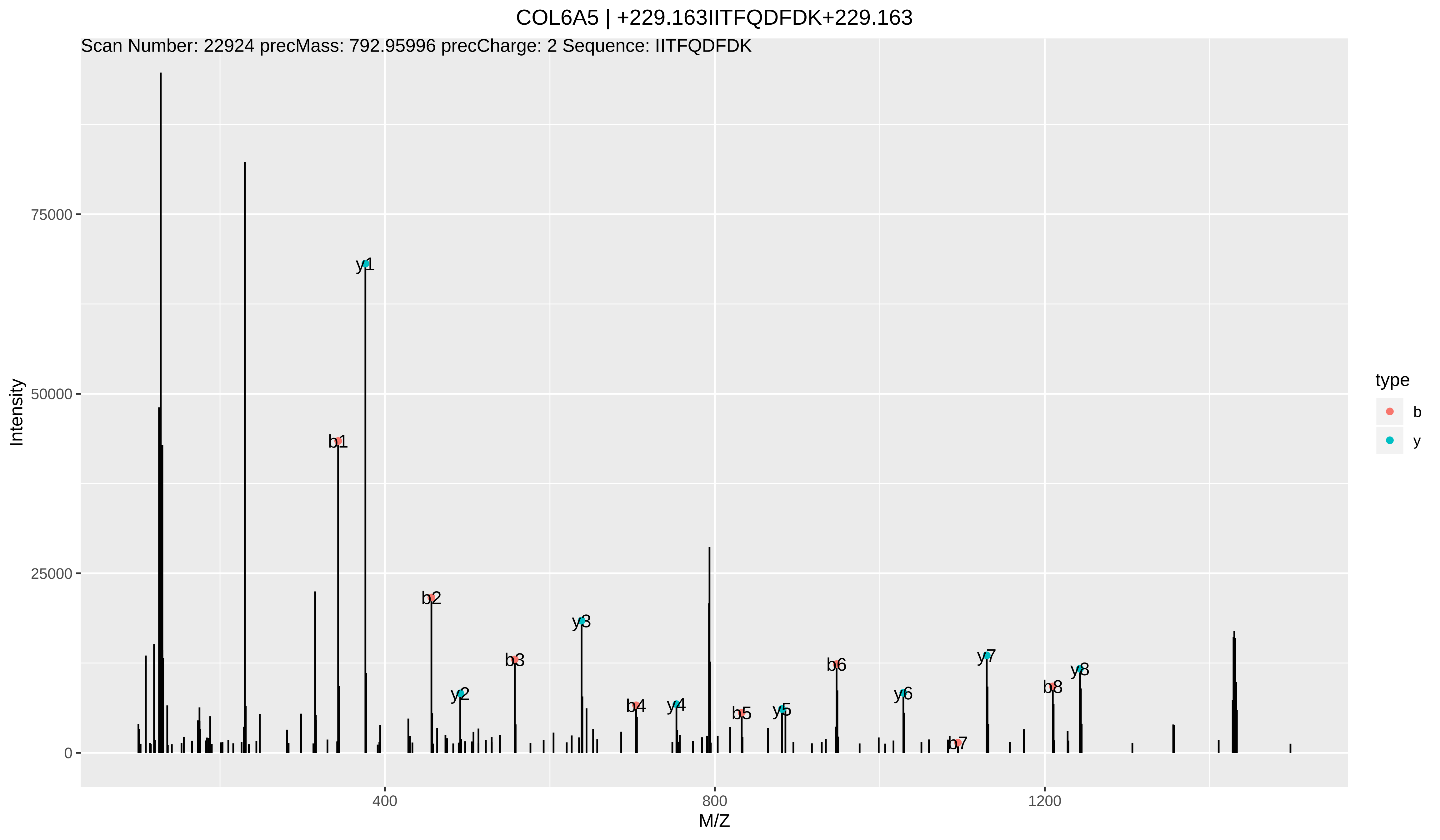
COL6A5 | +229.163IITFQDFDK+229.163
Scan Number: 22924 precMass: 792.95996 precCharge: 2 Sequence: IITFQDFDK
Intensity
75000
50000
25000
0
400
800
1200
M/Z
y1
b1
b2
y2
b3
y3
b4
y4
b5
y5
b6
y6
b7
y7
b8
y8
type
b
y

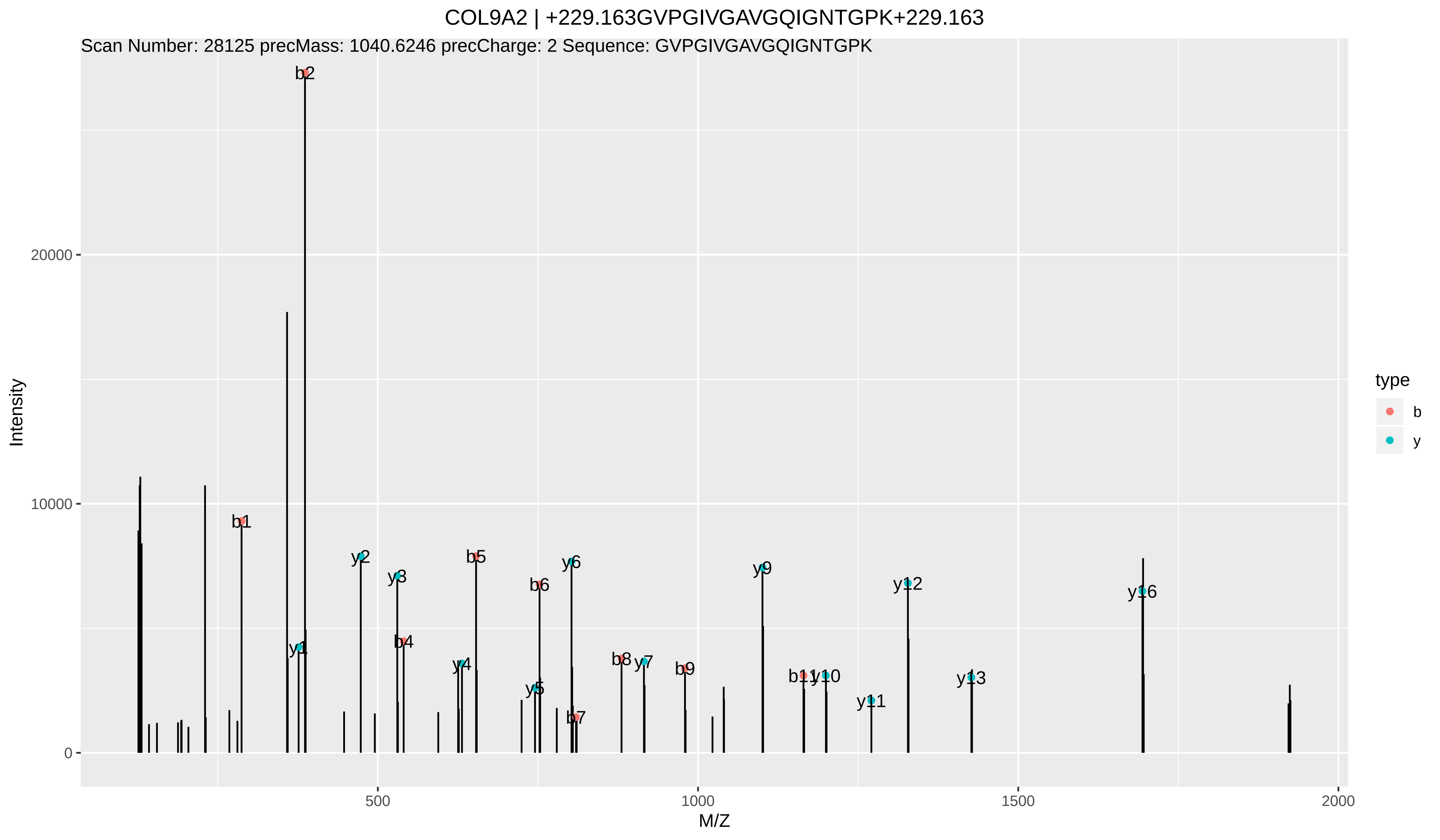
COL9A2 | +229.163GVPGIVGAVGQIGNTGPK+229.163
Scan Number: 28125 precMass: 1040.6246 precCharge: 2 Sequence: GVPGIVGAVGQIGNTGPK
Intensity
M/Z
type
b
y
b2
b1
y1
y2
y3
b4
y4
b5
y5
b6
y6
b7
b8
y7
b9
y9
b11
y10
y11
y12
y13
y16
0
10000
20000
500
1000
1500
2000

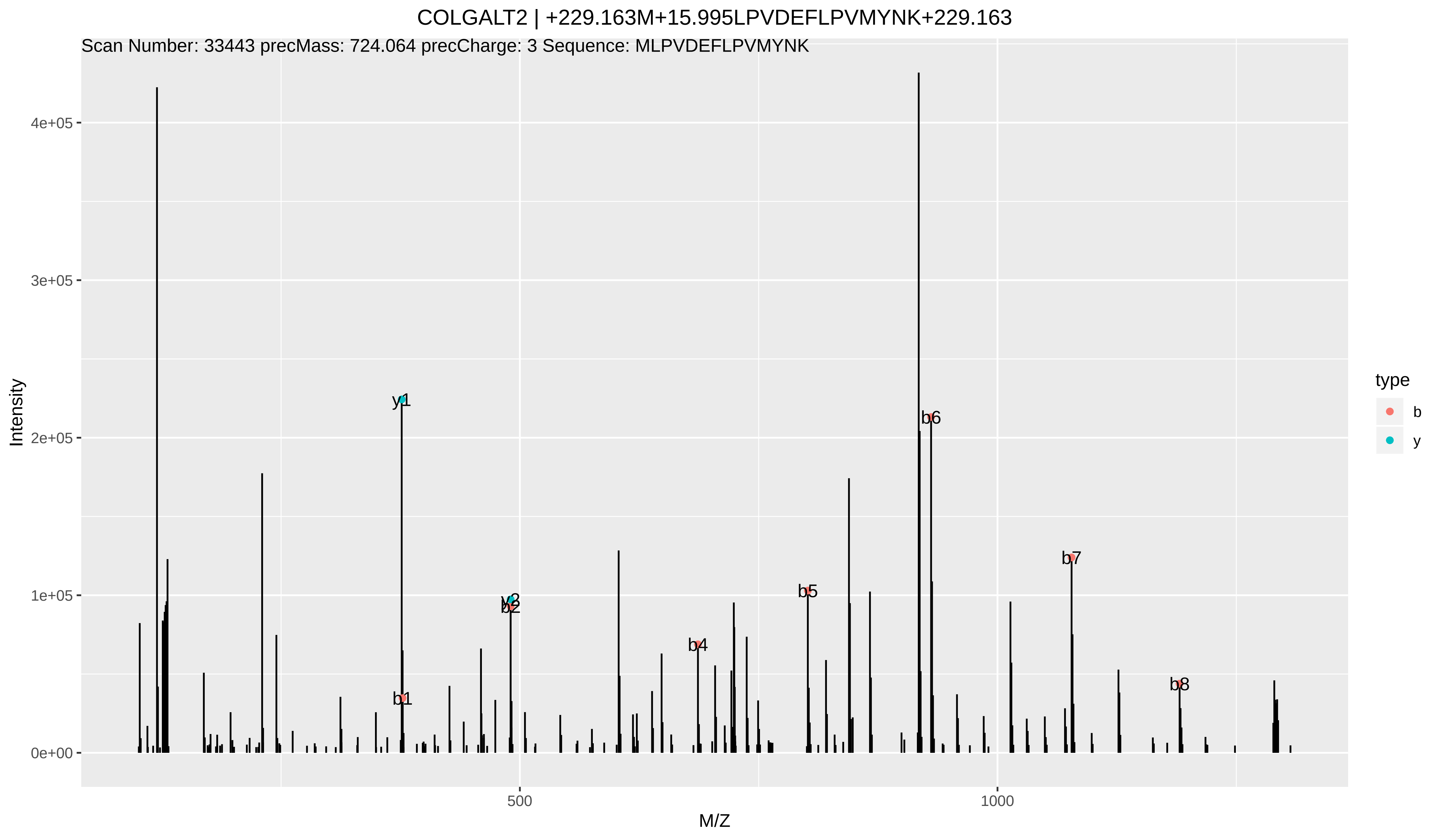
COLGALT2 | +229.163M+15.995LPVDEFLPVMYNK+229.163
Scan Number: 33443 precMass: 724.064 precCharge: 3 Sequence: MLPVDEFLPVMYNK
Intensity
M/Z
4e+05
3e+05
2e+05
1e+05
0e+00
500
1000
y1
b1
y2
b2
b4
b5
b6
b7
b8
type
b
y

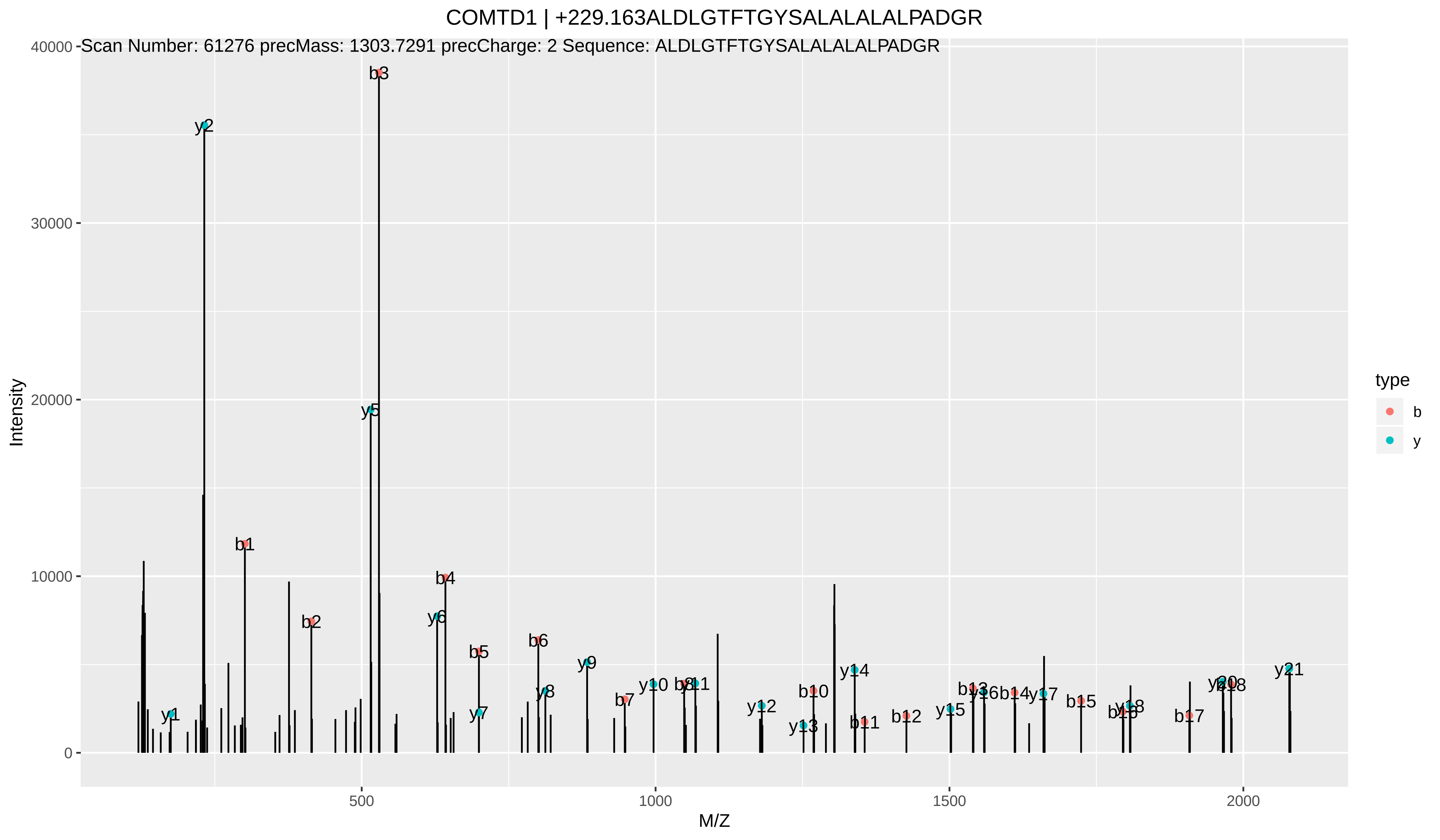

COMTD1 | +229.163ALDLGTFTGYSALALALALPADGR
Scan Number: 61276 precMass: 1303.7291 precCharge: 2 Sequence: ALDLGTFTGYSALALALALPADGR
Intensity
M/Z
type
b
y

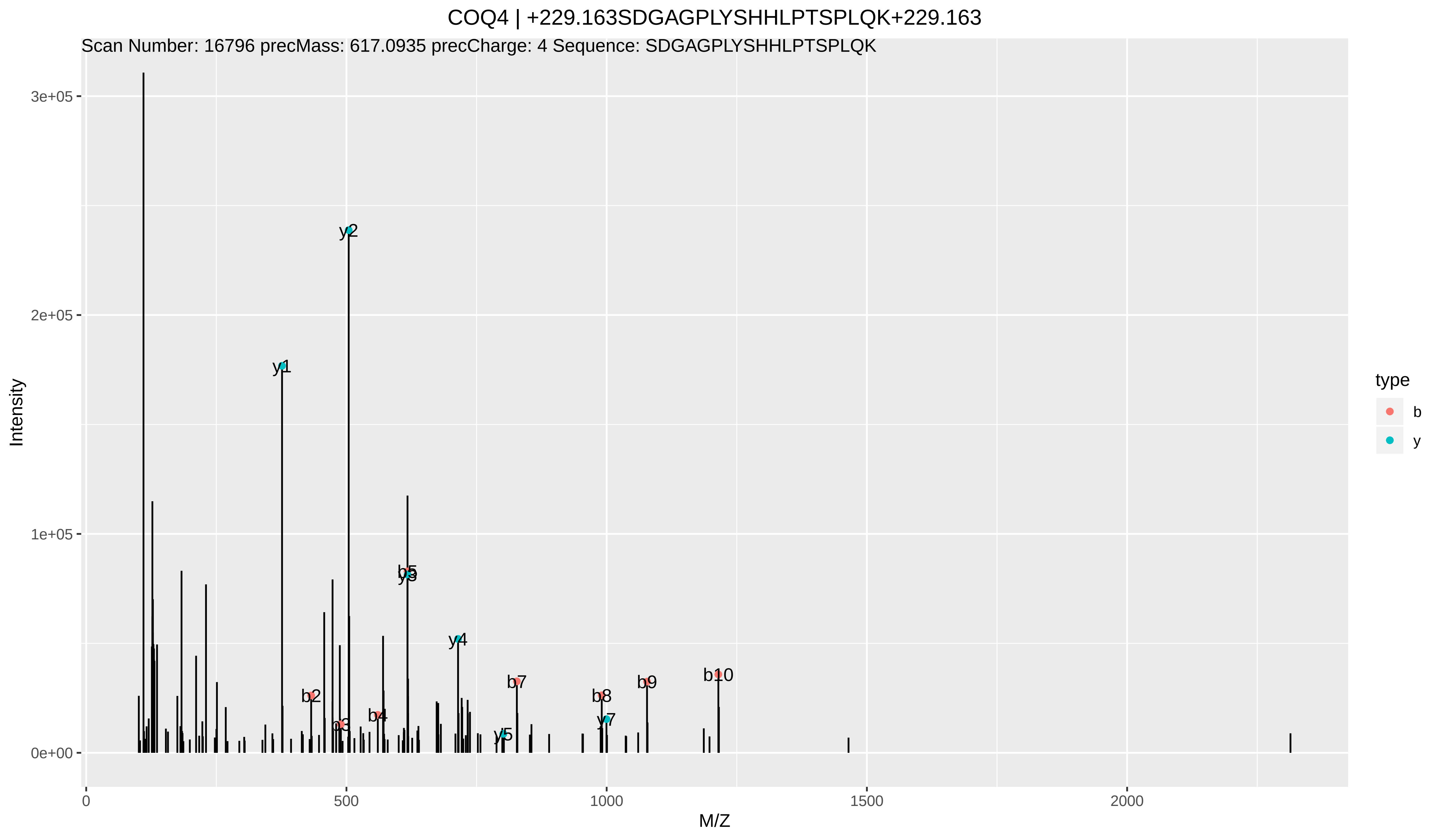
COQ4 | +229.163SDGAGPLYSHHLPTSPLQK+229.163
Scan Number: 16796 precMass: 617.0935 precCharge: 4 Sequence: SDGAGPLYSHHLPTSPLQK
Intensity
M/Z
3e+05
2e+05
1e+05
0e+00
0
500
1000
1500
2000
y2
y1
b5
y3
y4
b2
b3
b4
y5
b7
b8
y7
b9
b10
type
b
y

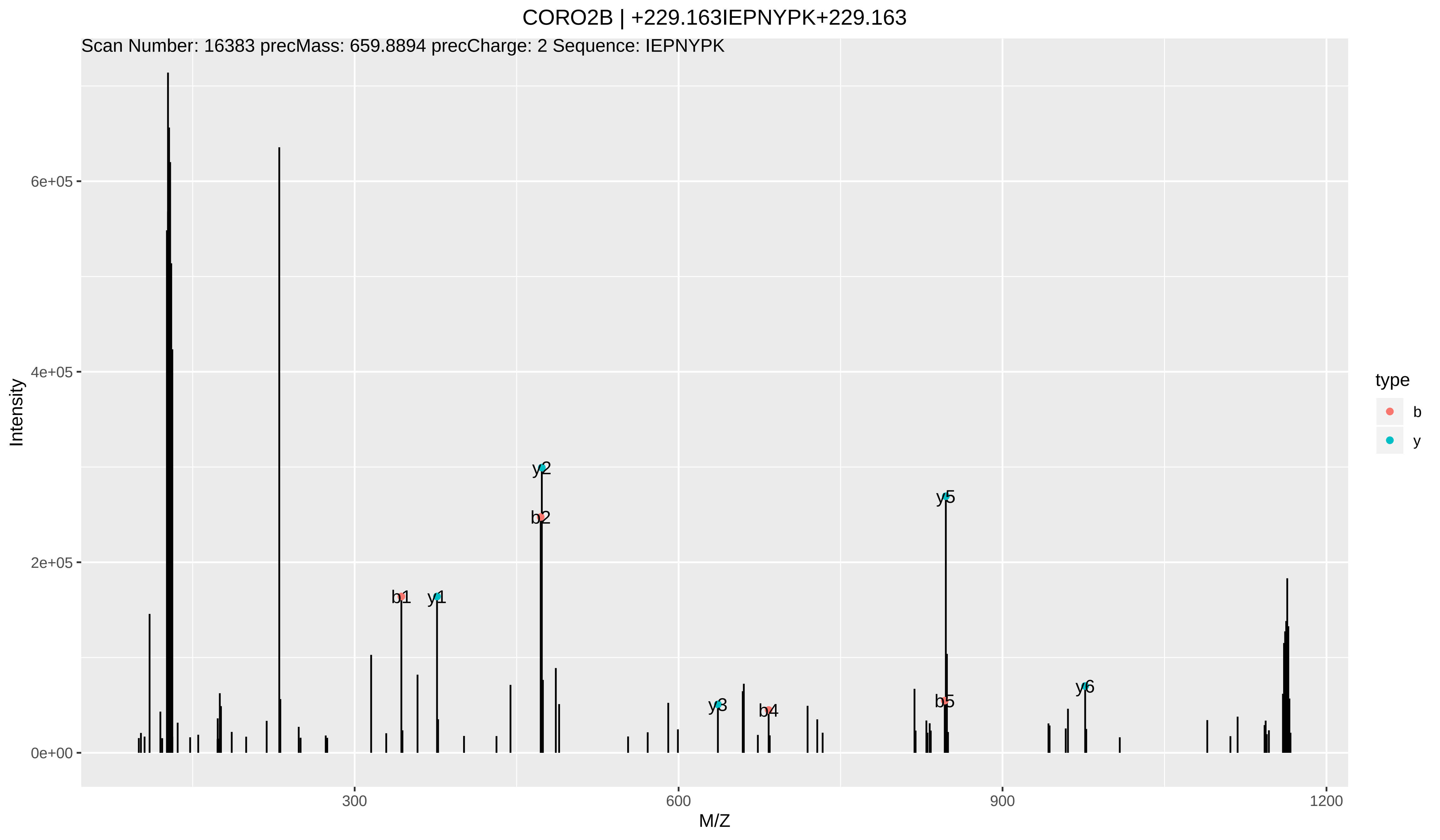

CORO2B | +229.163IEPNYPK+229.163
Scan Number: 16383 precMass: 659.8894 precCharge: 2 Sequence: IEPNYPK
Intensity
6e+05
4e+05
2e+05
0e+00
M/Z
300
600
900
1200
y2
b2
y5
b1
y1
y3
b4
b5
y6
type
b
y

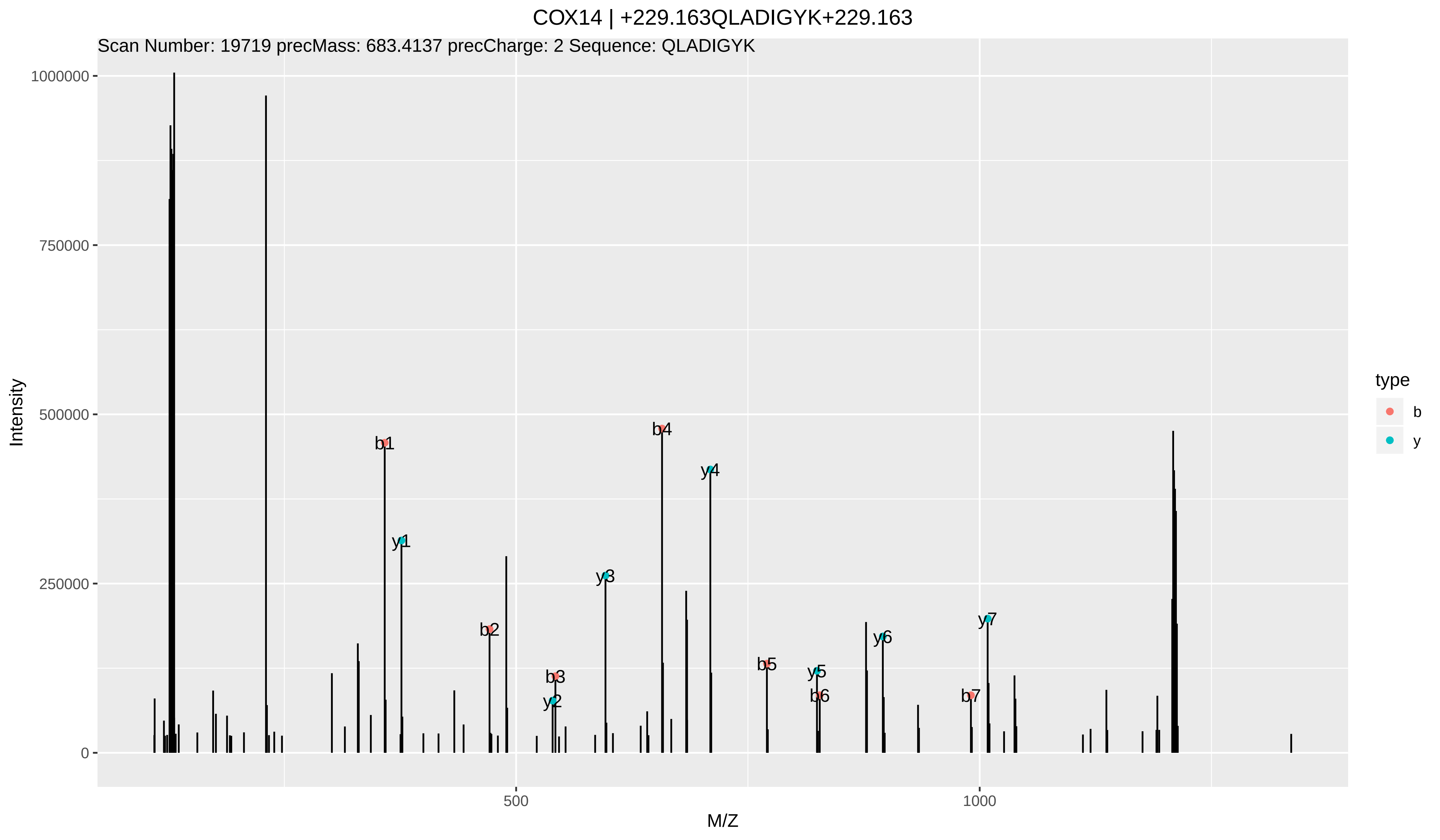
COX14 | +229.163QLADIGYK+229.163
Scan Number: 19719 precMass: 683.4137 precCharge: 2 Sequence: QLADIGYK
Intensity
1000000
750000
500000
250000
0
M/Z
500
1000
b1
y1
b2
b3
y2
y3
b4
y4
b5
y5
b6
y6
b7
y7
type
b
y

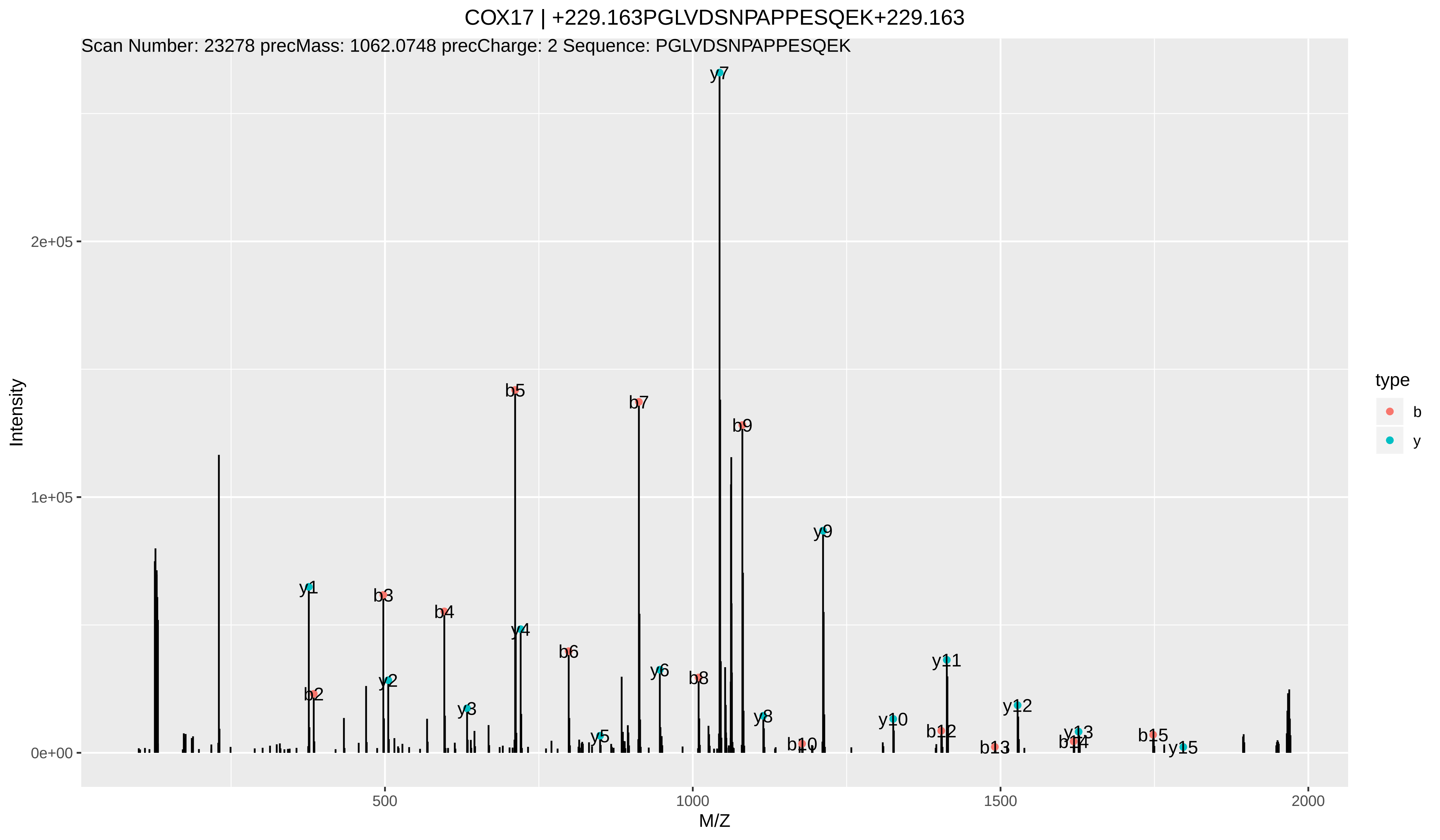

COX17 | +229.163PGLVDSNPAPPESQEK+229.163
Scan Number: 23278 precMass: 1062.0748 precCharge: 2 Sequence: PGLVDSNPAPPESQEK
Intensity
M/Z
0e+00
1e+05
2e+05
500
1000
1500
2000
type
b
y
y7
b5
b7
b9
y9
y1
b3
b4
y4
b6
y6
b8
y2
b2
y3
y5
y8
b10
y10
y11
b12
y12
b13
y13
b14
b15
y15

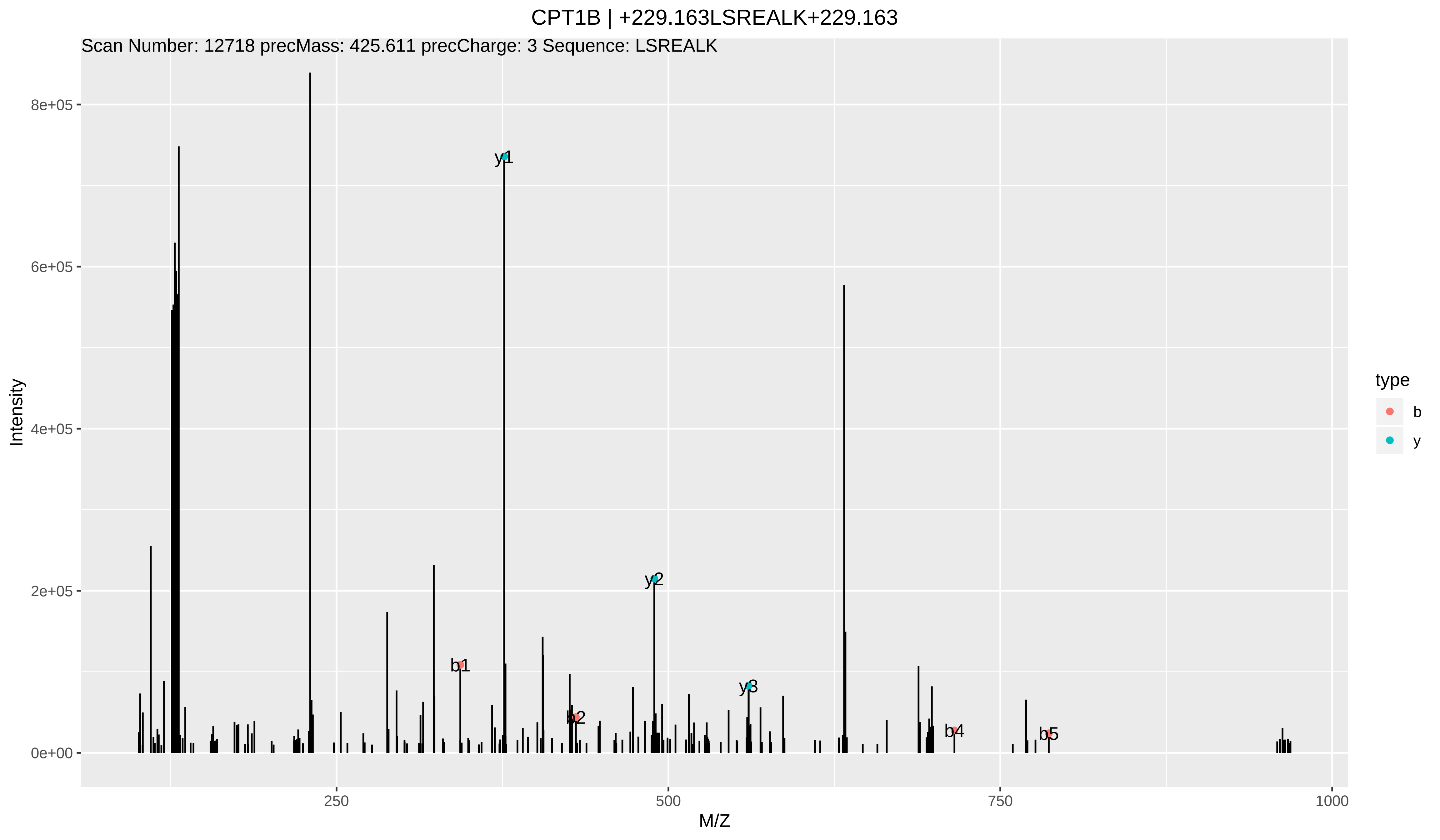

CPT1B | +229.163LSREALK+229.163
Scan Number: 12718 precMass: 425.611 precCharge: 3 Sequence: LSREALK
Intensity
8e+05
6e+05
4e+05
2e+05
0e+00
M/Z
250
500
750
1000
y1
y2
y3
b1
b2
b4
b5
type
b
y

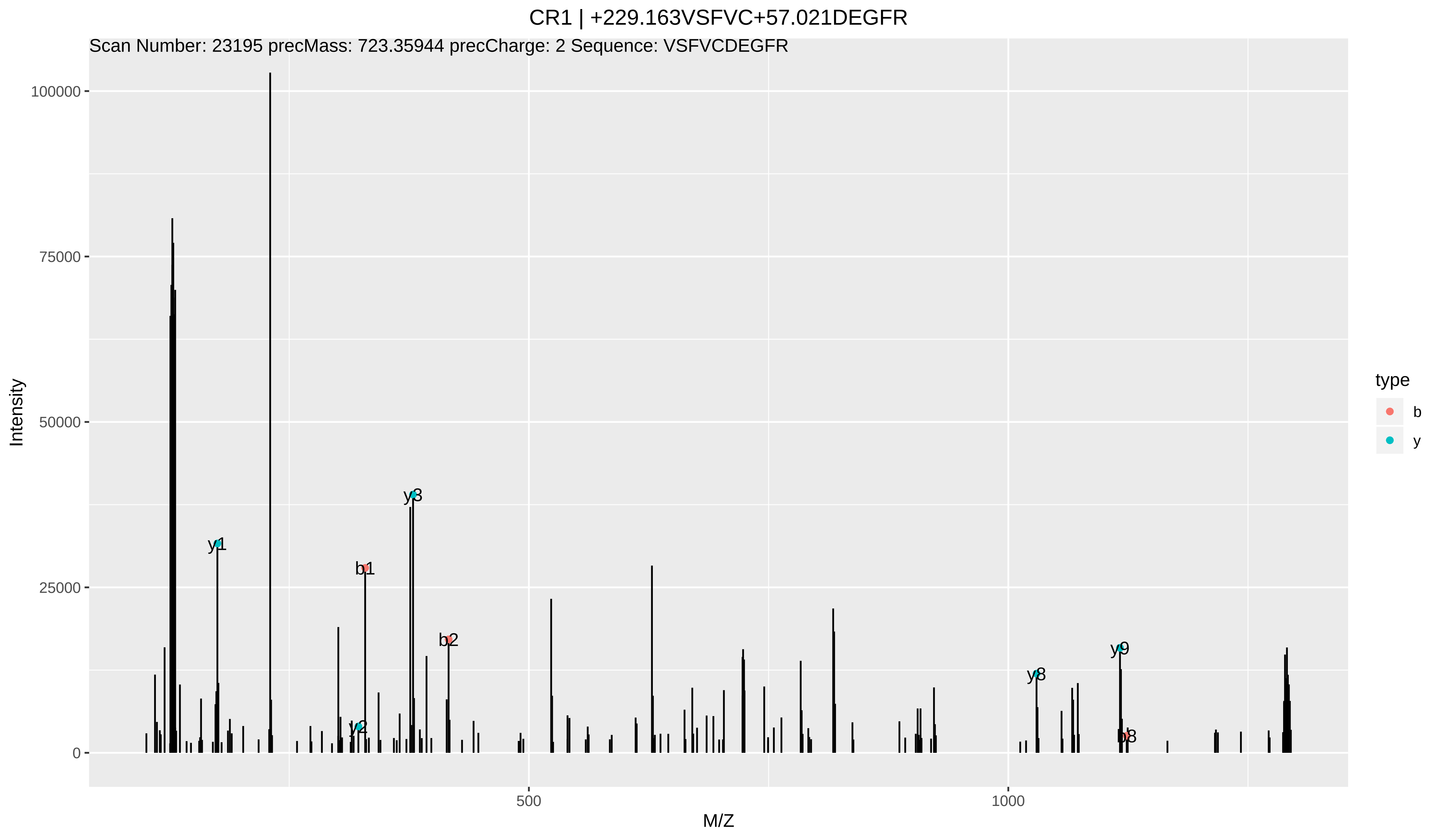

CR1 | +229.163VSFVC+57.021DEGFR
Scan Number: 23195 precMass: 723.35944 precCharge: 2 Sequence: VSFVCDEGFR
Intensity
100000
75000
50000
25000
0
M/Z
500
1000
y1
b1
y2
y3
b2
y8
y9
b8
type
b
y

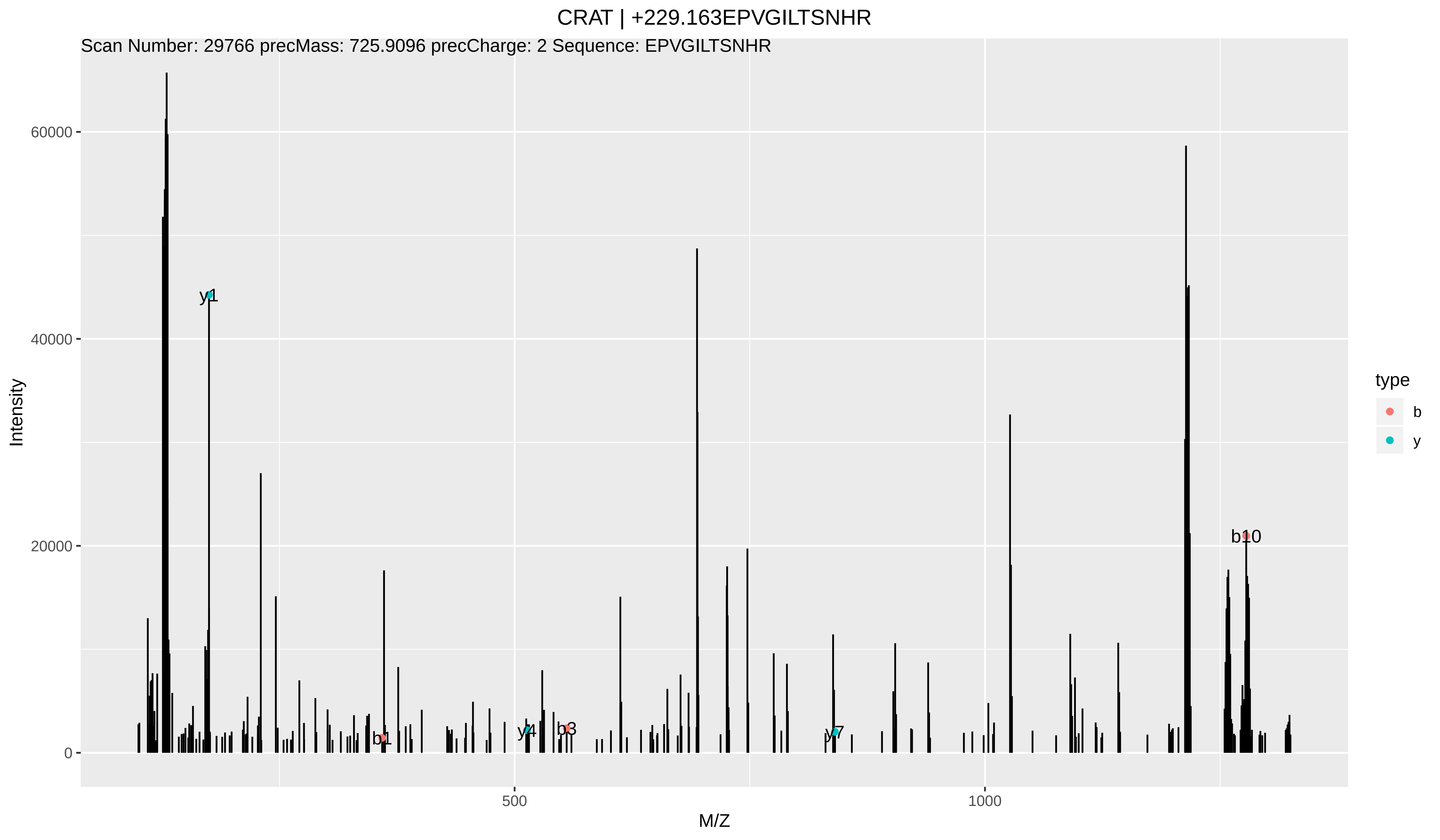

CRAT | +229.163EPVGILTSNHR
Scan Number: 29766 precMass: 725.9096 precCharge: 2 Sequence: EPVGILTSNHR
Intensity
60000
40000
20000
0
M/Z
500
1000
y1
b1
y4
b3
y7
b10
type
b
y

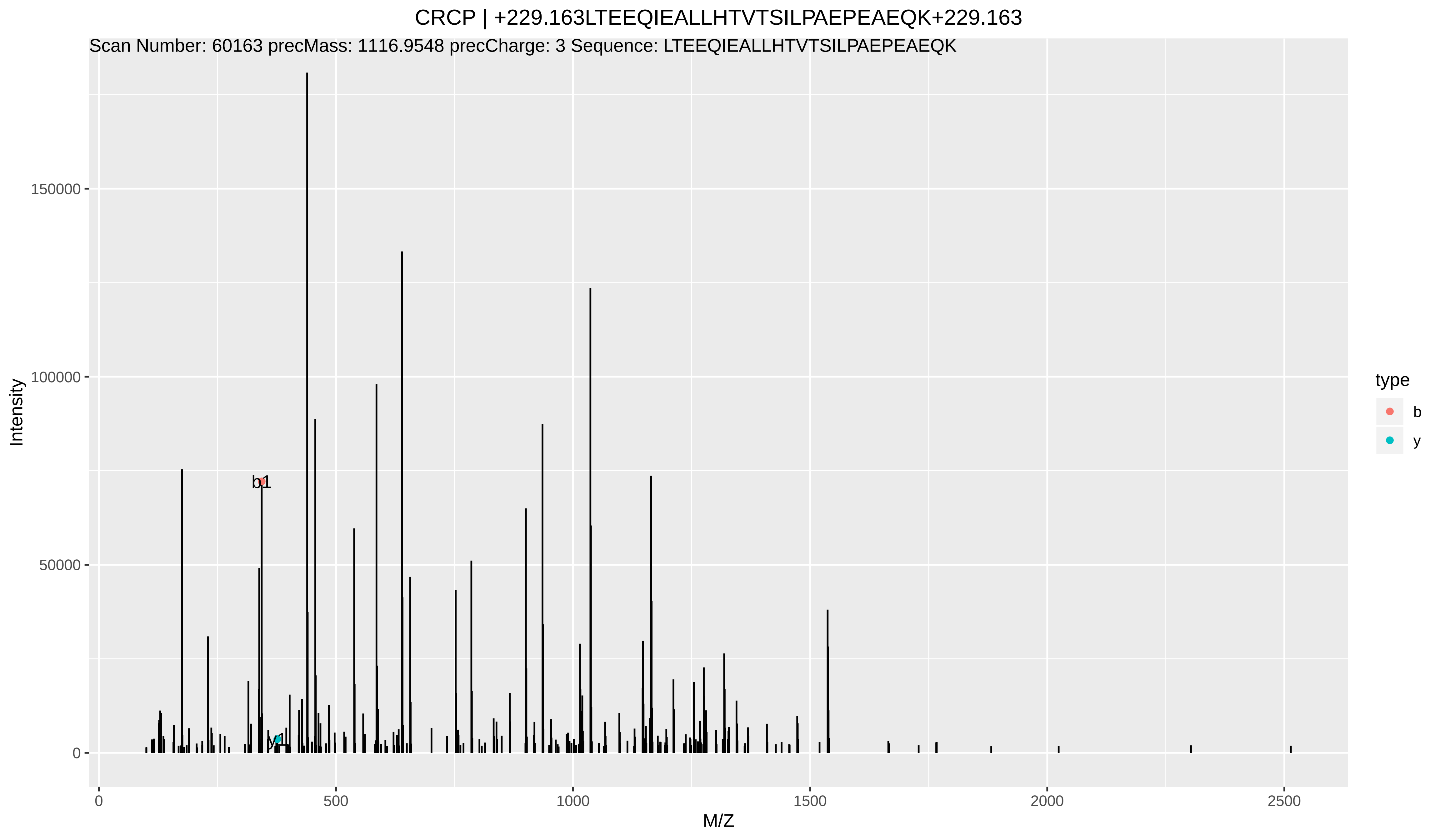

CRCP | +229.163LTEEQIEALLHTVTSILPAEPEAEQK+229.163
Scan Number: 60163 precMass: 1116.9548 precCharge: 3 Sequence: LTEEQIEALLHTVTSILPAEPEAEQK
Intensity
150000
100000
50000
0
M/Z
0
500
1000
1500
2000
2500
b1
y1
type
b
y

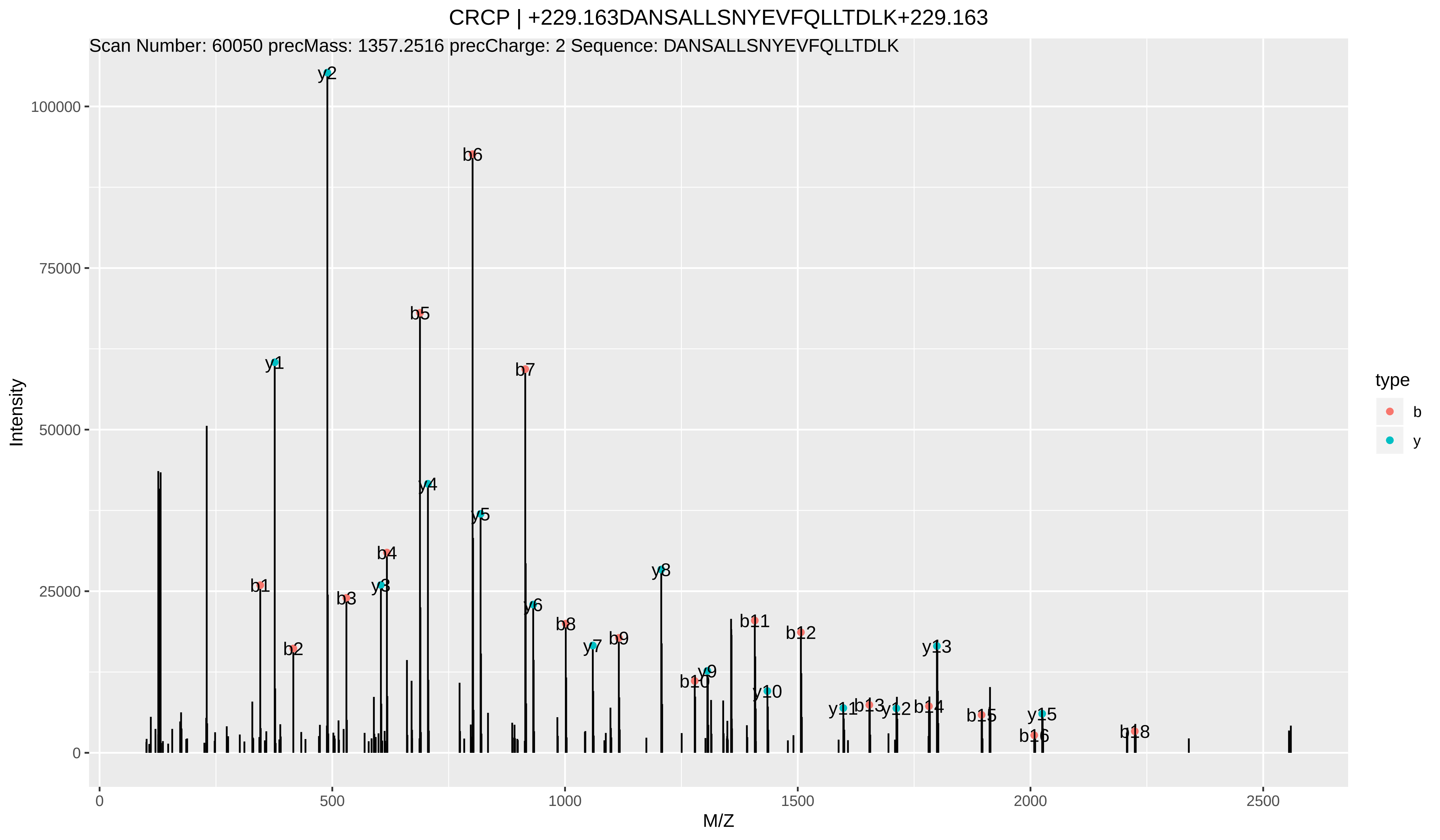

CRCP | +229.163DANSALLSNYEVFQLLTDLK+229.163
Scan Number: 60050 precMass: 1357.2516 precCharge: 2 Sequence: DANSALLSNYEVFQLLTDLK
Intensity
100000
75000
50000
25000
0
0
500
1000
1500
2000
2500
M/Z
y2
b6
b5
y1
b7
y4
y5
b4
b1
y3
b3
y6
y8
b8
b2
y7
b9
b11
b12
y13
y9
b10
y10
y11
b13
y12
b14
b15
y15
b16
b18
type
b
y

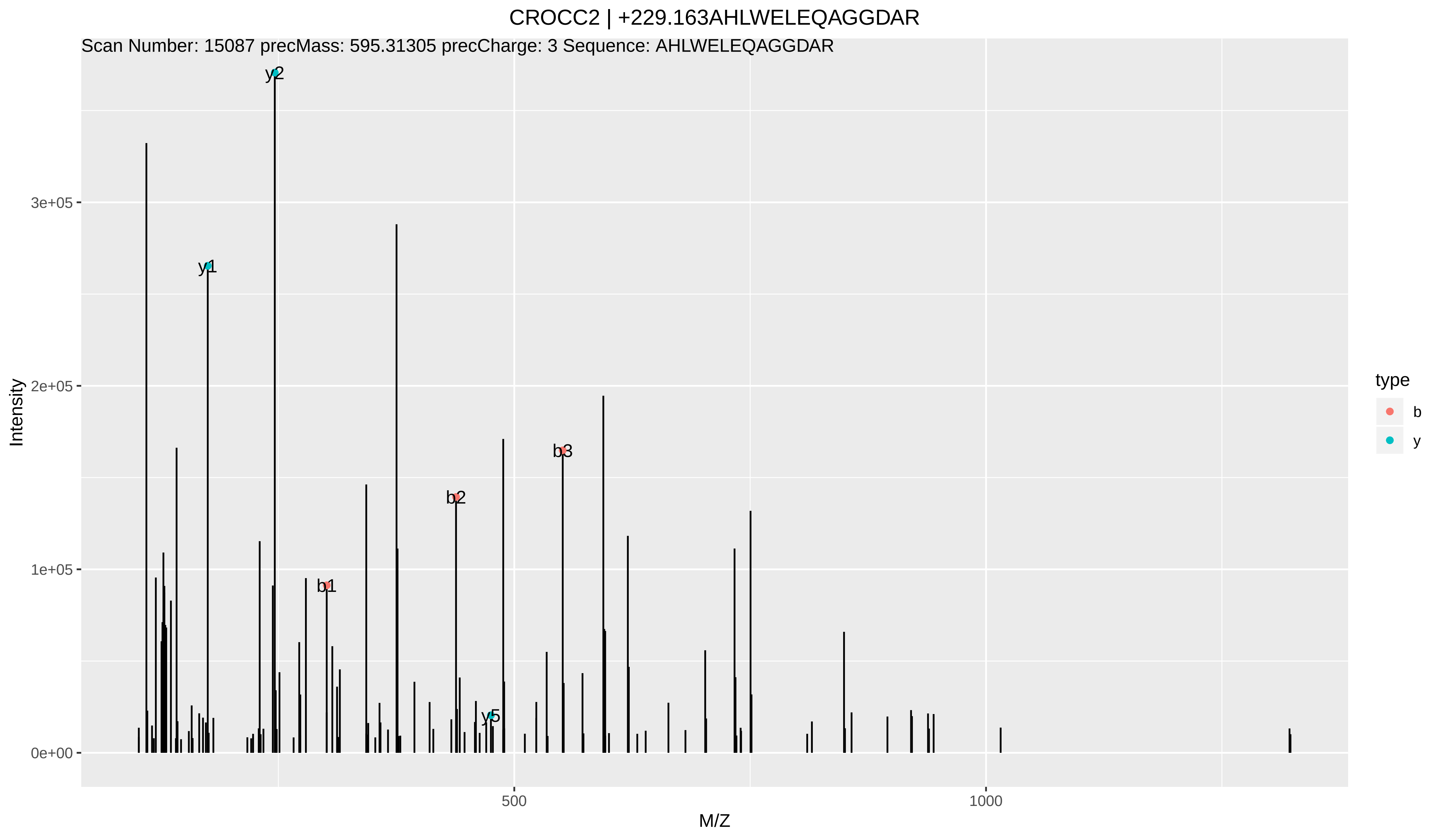

CROCC2 | +229.163AHLWELEQAGGDAR
Scan Number: 15087 precMass: 595.31305 precCharge: 3 Sequence: AHLWELEQAGGDAR
y2
y1
b1
b2
b3
y5
Intensity
M/Z
3e+05
2e+05
1e+05
0e+00
500
1000
type
b
y

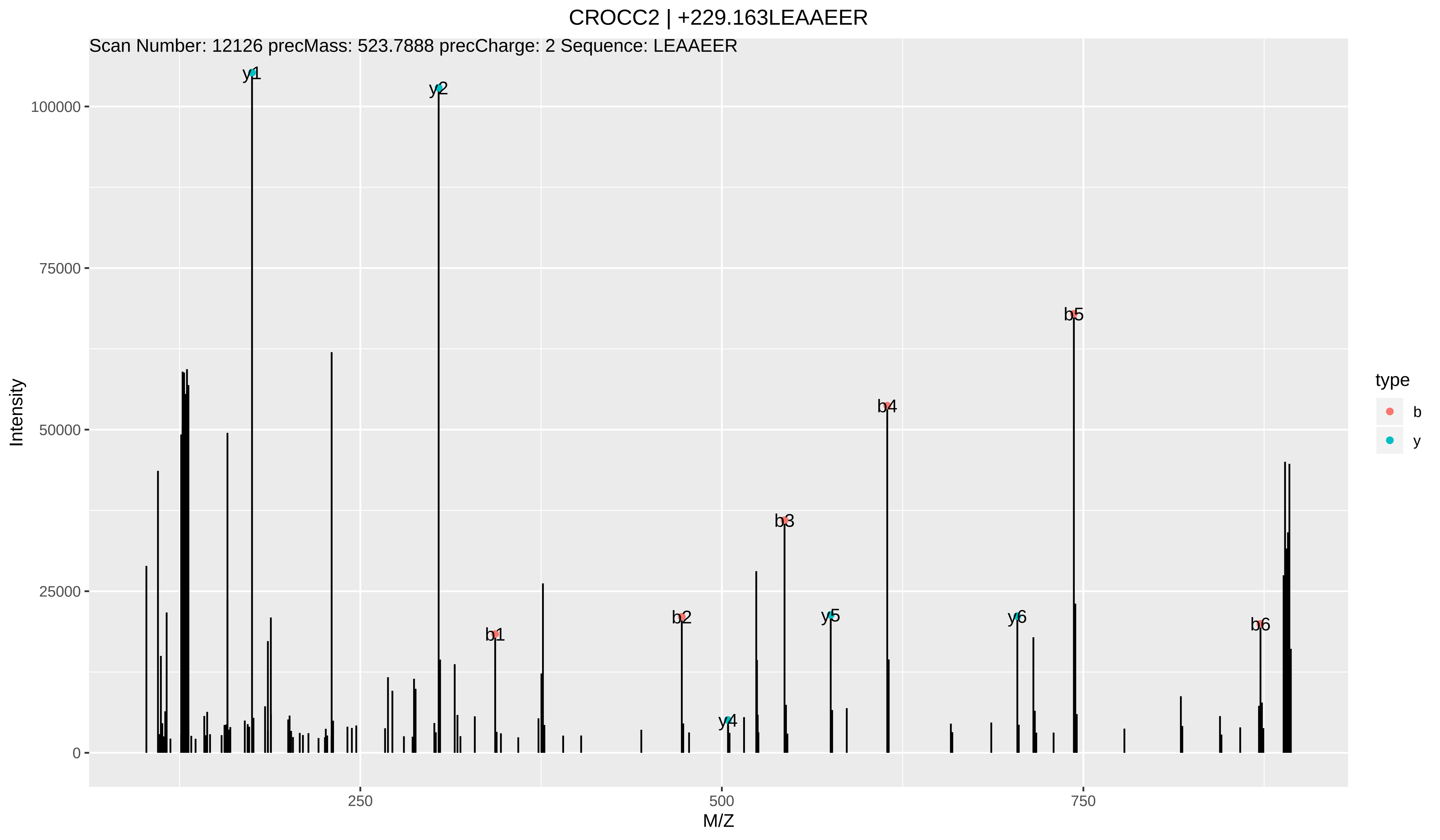

CROCC2 | +229.163LEAAEER
Scan Number: 12126 precMass: 523.7888 precCharge: 2 Sequence: LEAAEER
y1
y2
b1
b2
y4
b3
y5
b4
y6
b5
b6
Intensity
100000
75000
50000
25000
0
250
500
750
M/Z
type
b
y

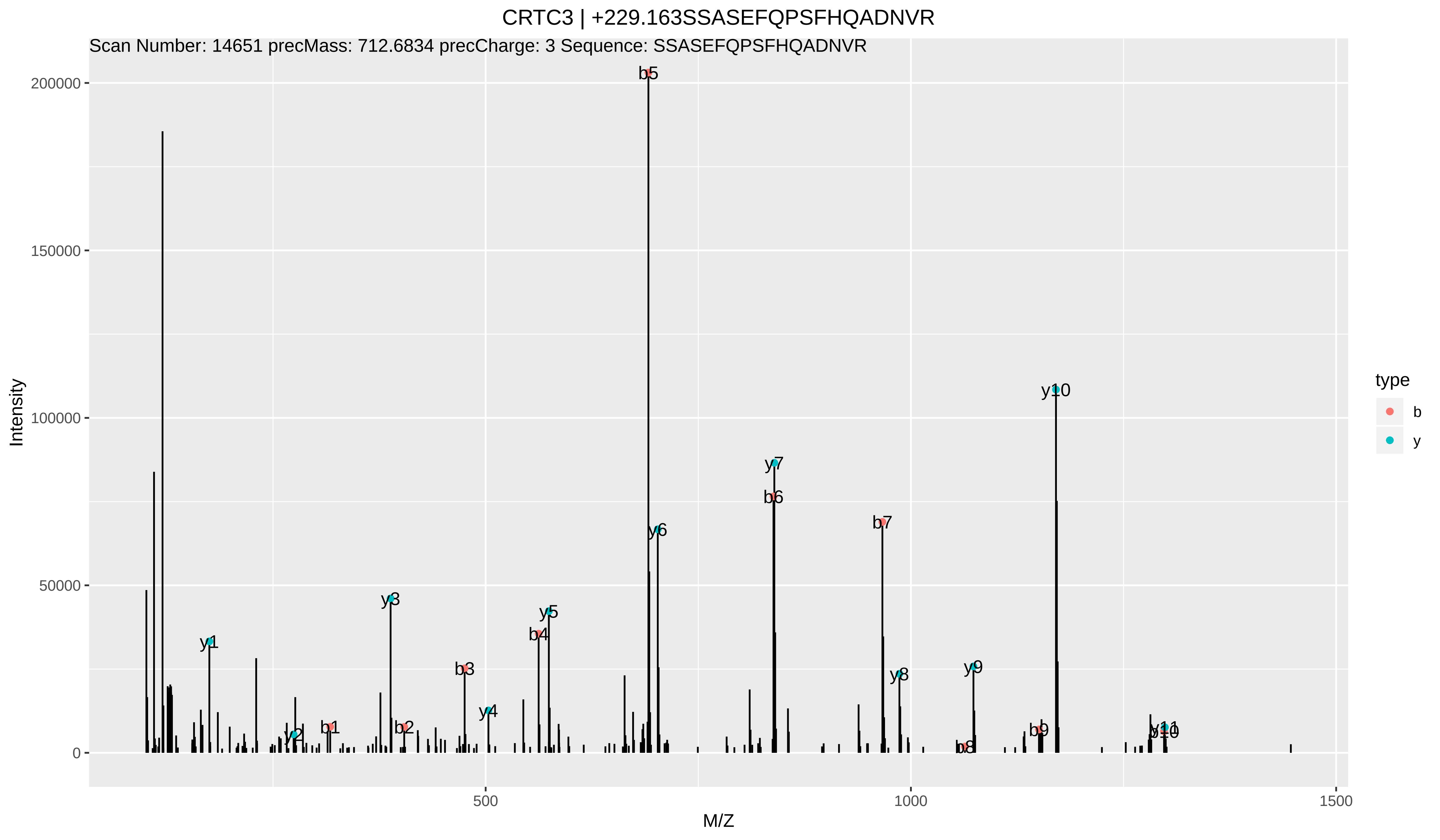

CRTC3 | +229.163SSASEFQPSFHQADNVR
Scan Number: 14651 precMass: 712.6834 precCharge: 3 Sequence: SSASEFQPSFHQADNVR
Intensity
M/Z
0
50000
100000
150000
200000
500
1000
1500
b5
y10
y7
b6
y6
b7
y3
y5
b4
y1
b3
y8
y9
y4
y2
b1
b2
b9
y11
b10
b8
type
b
y

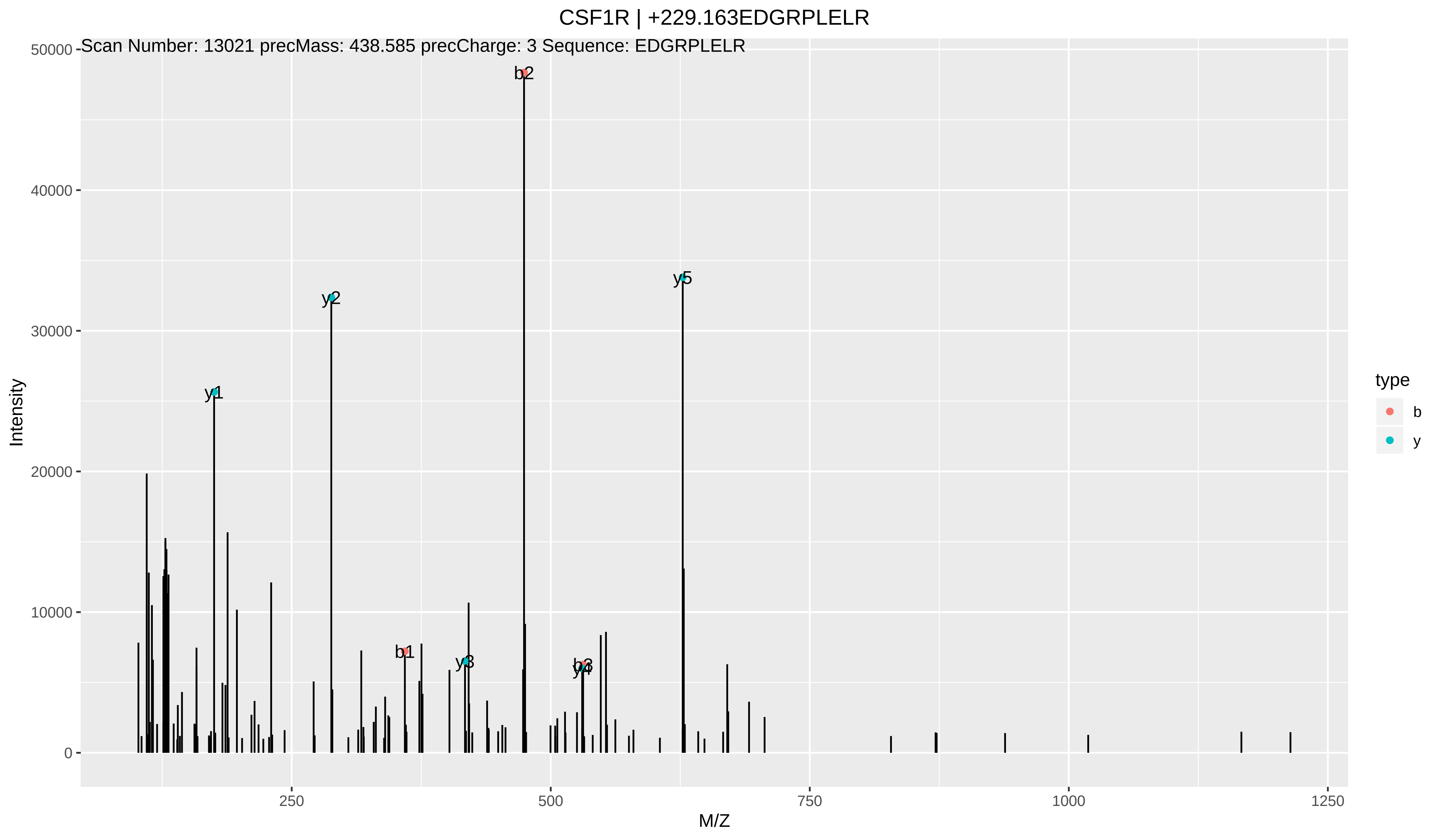

CSF1R | +229.163EDGRPLELR
Scan Number: 13021 precMass: 438.585 precCharge: 3 Sequence: EDGRPLELR
Intensity
M/Z
50000
40000
30000
20000
10000
0
250
500
750
1000
1250
b2
y5
y2
y1
b1
y3
b3
y4
type
b
y

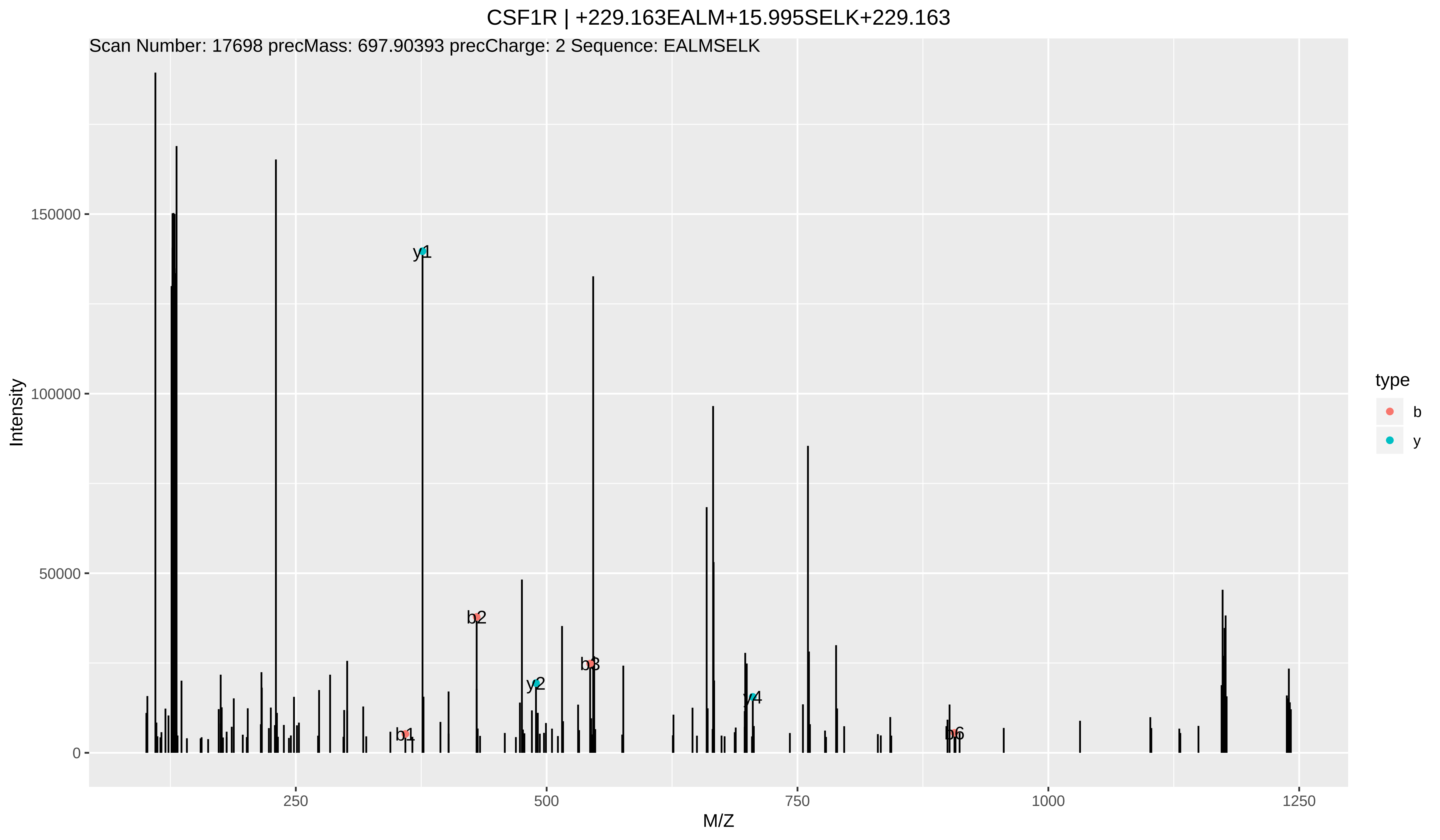

CSF1R | +229.163EALM+15.995SELK+229.163
Scan Number: 17698 precMass: 697.90393 precCharge: 2 Sequence: EALMSELK
Intensity
150000
100000
50000
0
250
500
750
1000
1250
M/Z
y1
b2
b3
y2
y4
b1
b6
type
b
y

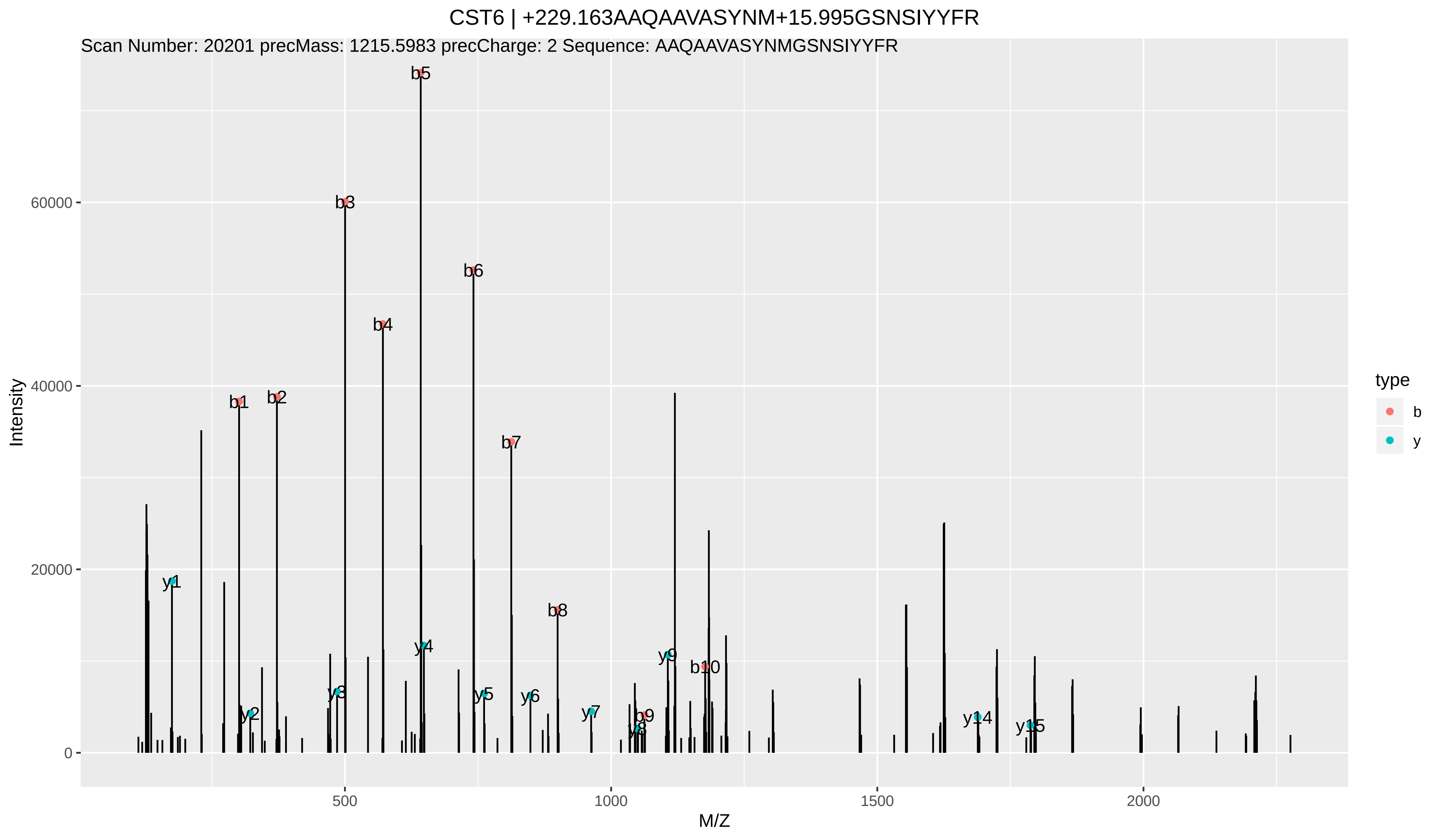

CST6 | +229.163AAQAAVASYNM+15.995GSNSIYYFR
Scan Number: 20201 precMass: 1215.5983 precCharge: 2 Sequence: AAQAAVASYNMGSNSIYYFR
b5
b3
b6
b4
b1
b2
b7
y1
b8
y4
y9
b10
y3
y5
y6
y2
y7
b9
y8
y14
y15
Intensity
60000
40000
20000
0
500
1000
1500
2000
M/Z
type
b
y

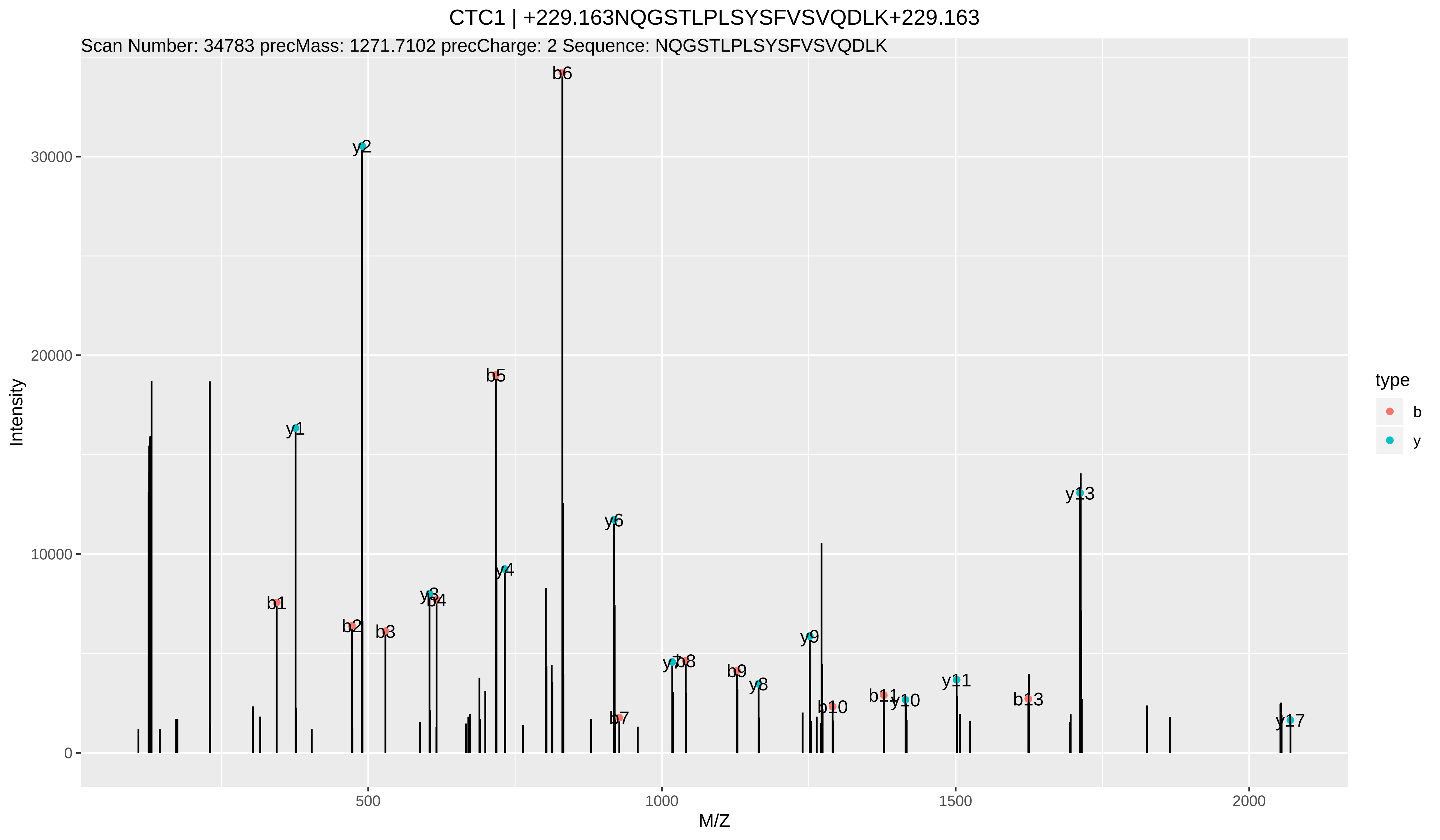

CTC1 | +229.163NQGSTLPLSYSFVSVQDLK+229.163
Scan Number: 34783 precMass: 1271.7102 precCharge: 2 Sequence: NQGSTLPLSYSFVSVQDLK
Intensity
M/Z
type
b
y
b6
y2
b5
y1
y13
y6
y4
y3
b4
b1
b2
b3
y9
y7
b8
b9
y8
y11
b10
b11
y10
b13
b7
y17
0
10000
20000
30000
500
1000
1500
2000

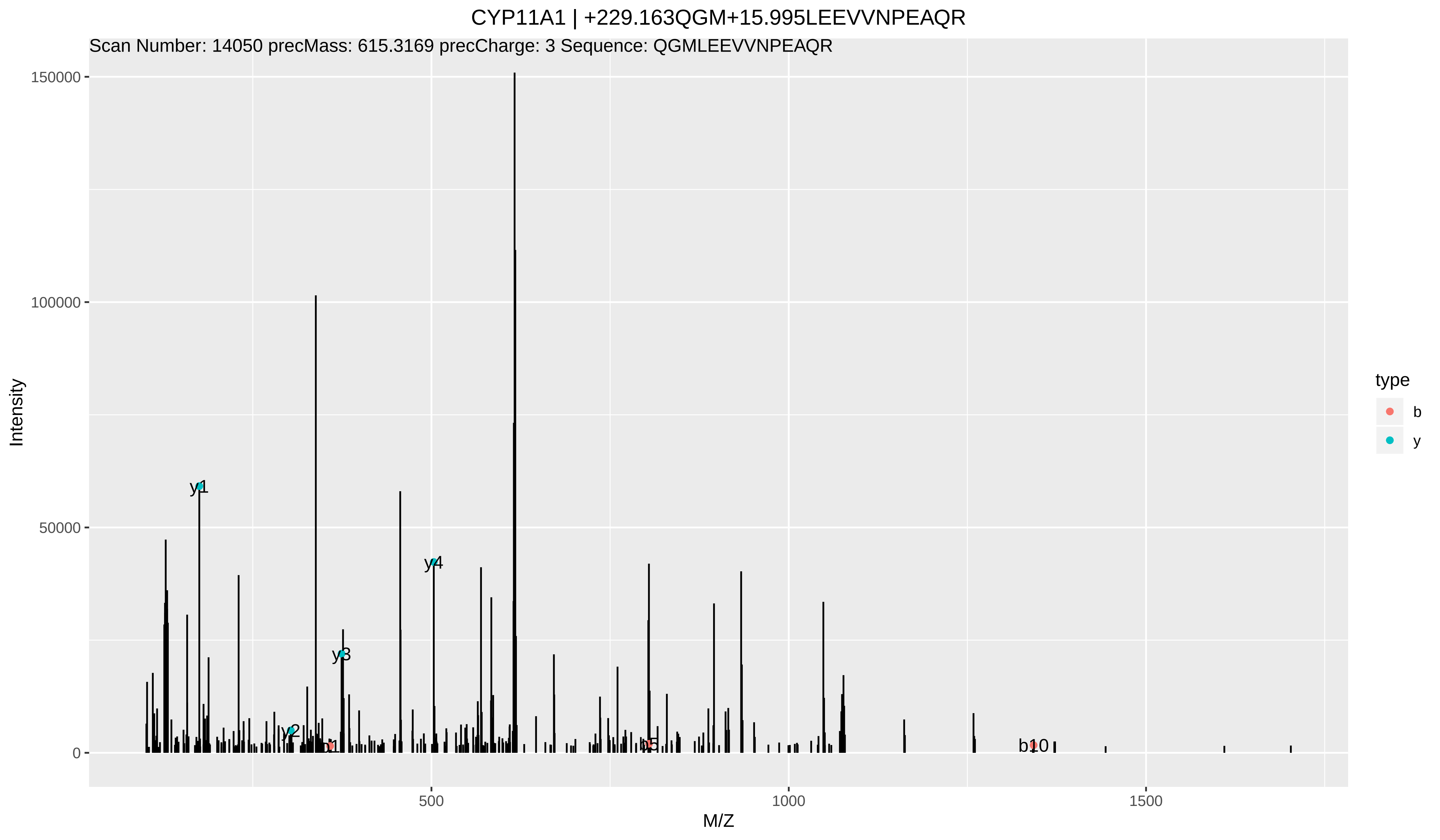

CYP11A1 | +229.163QGM+15.995LEEVVNPEAQR
Scan Number: 14050 precMass: 615.3169 precCharge: 3 Sequence: QGMLEEVVNPEAQR
Intensity
150000
100000
50000
0
M/Z
500
1000
1500
y1
y2
y3
y4
b1
b5
b10
type
b
y

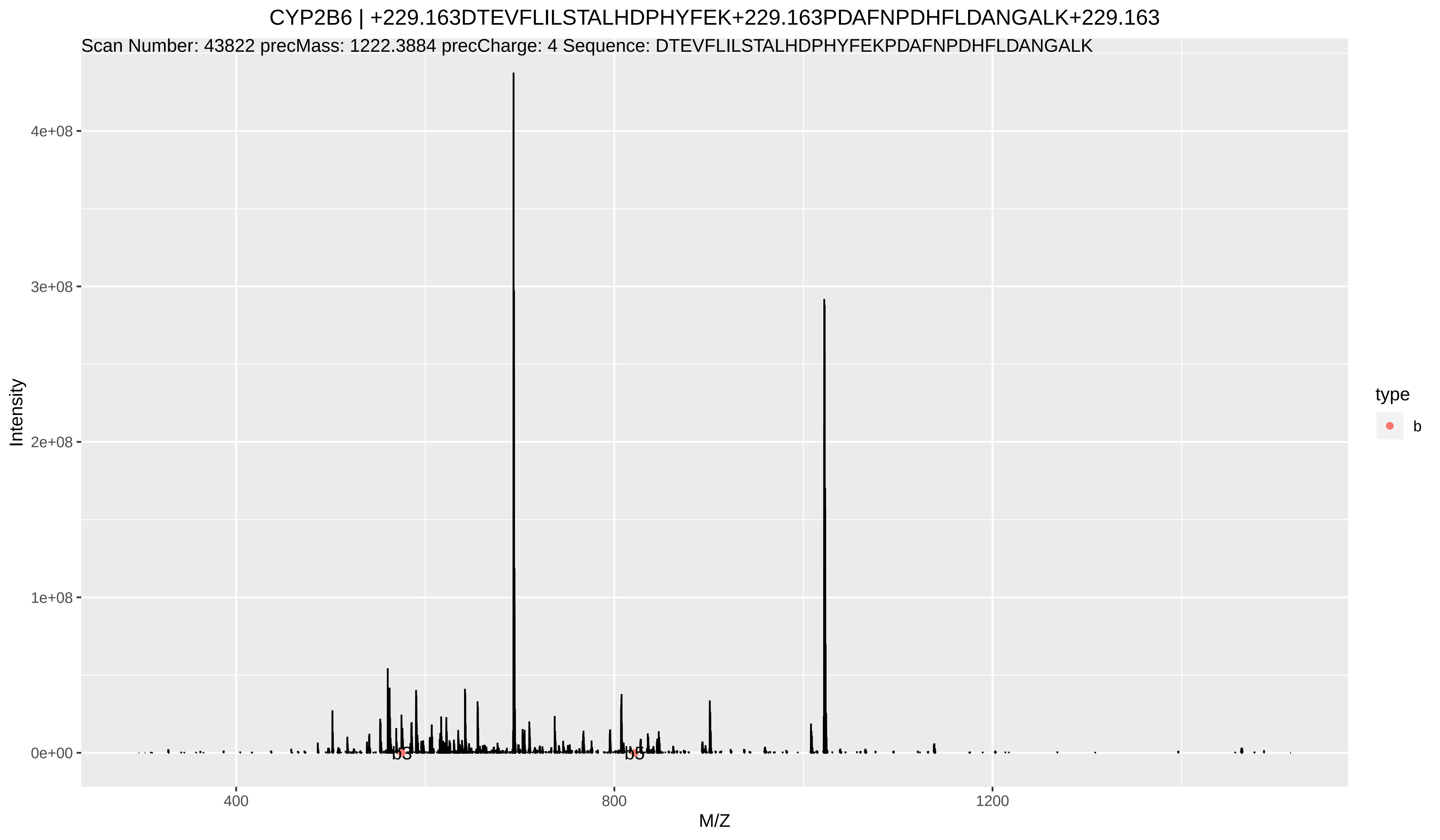

CYP2B6 | +229.163DTEVFLILSTALHDPHYFEK+229.163PDAFNPDHFLDANGALK+229.163
Scan Number: 43822 precMass: 1222.3884 precCharge: 4 Sequence: DTEVFLILSTALHDPHYFEKPDAFNPDHFLDANGALK
Intensity
4e+08
3e+08
2e+08
1e+08
0e+00
400
800
1200
M/Z
b3
b5
type
b

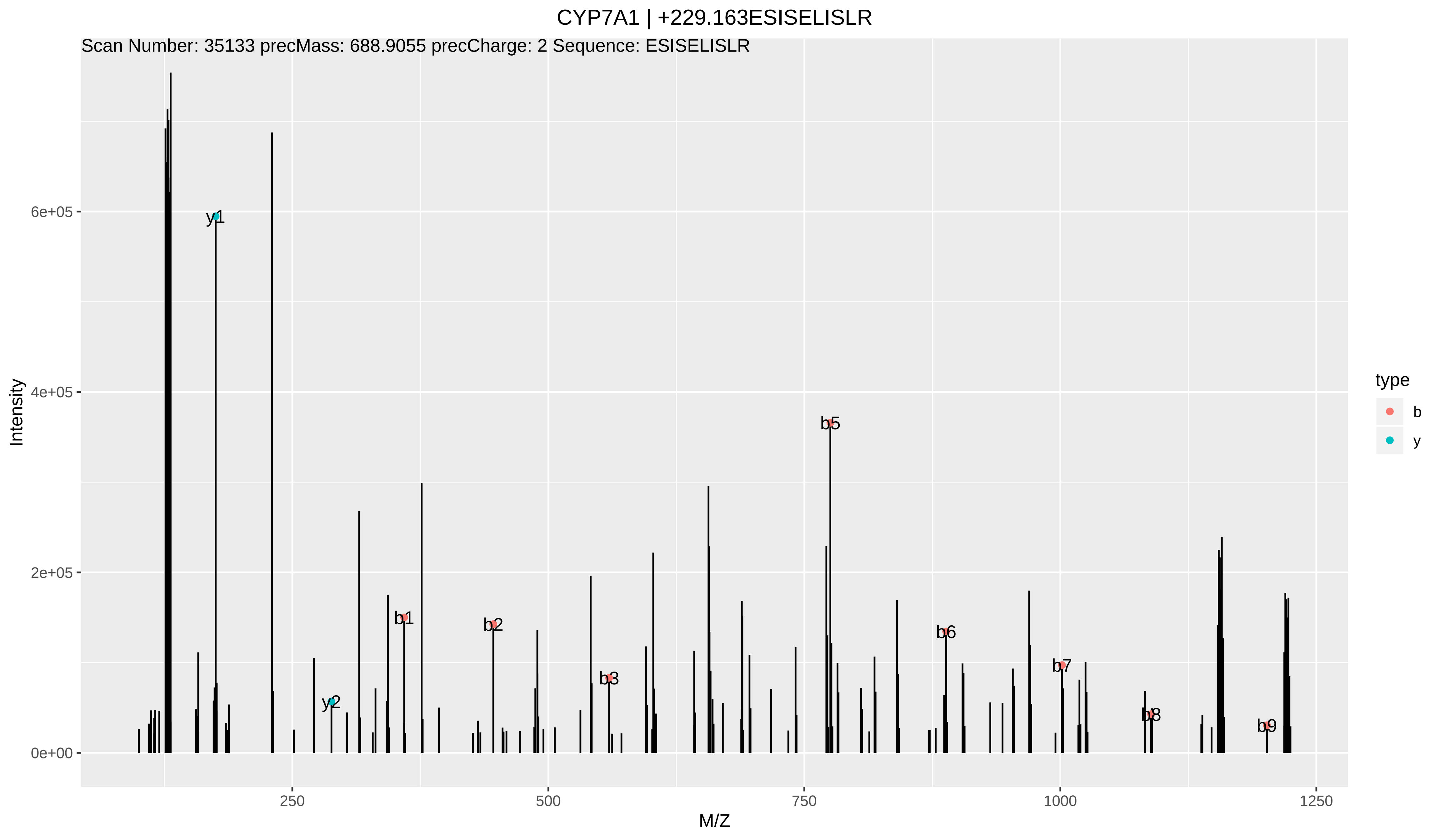

CYP7A1 | +229.163ESISELISLR
Scan Number: 35133 precMass: 688.9055 precCharge: 2 Sequence: ESISELISLR
Intensity
6e+05
4e+05
2e+05
0e+00
M/Z
250
500
750
1000
1250
y1
y2
b1
b2
b3
b5
b6
b7
b8
b9
type
b
y

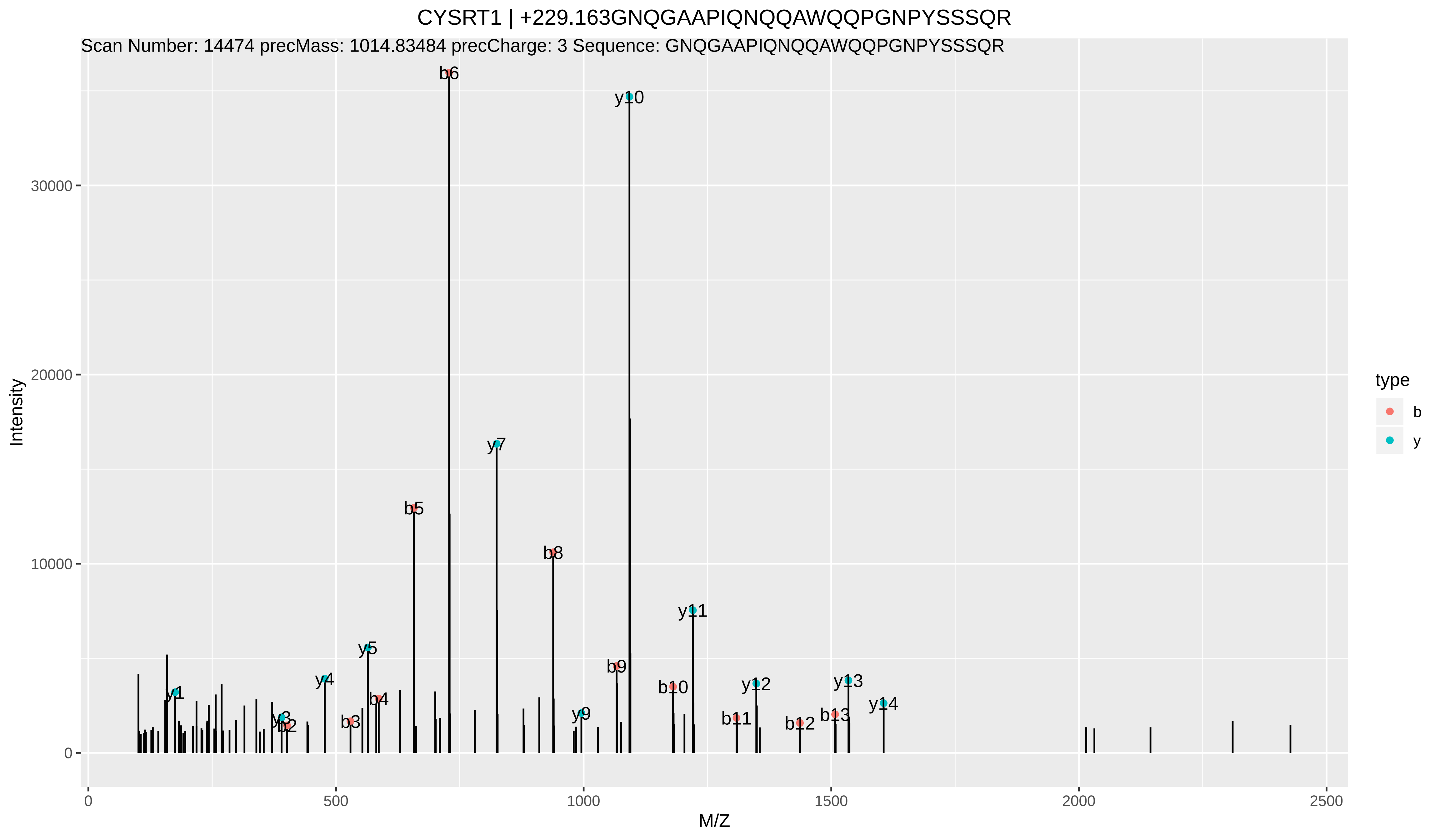
CYSRT1 | +229.163GNQGAAPIQNQQAWQQPGNPYSSSQR
Scan Number: 14474 precMass: 1014.83484 precCharge: 3 Sequence: GNQGAAPIQNQQAWQQPGNPYSSSQR
Intensity
M/Z
type
b
y
b6
y10
y7
b5
b8
y11
y5
b9
y4
b10
y12
y13
y14
y1
b4
y9
b13
b11
b12
y3
b2
b3
0
10000
20000
30000
0
500
1000
1500
2000
2500

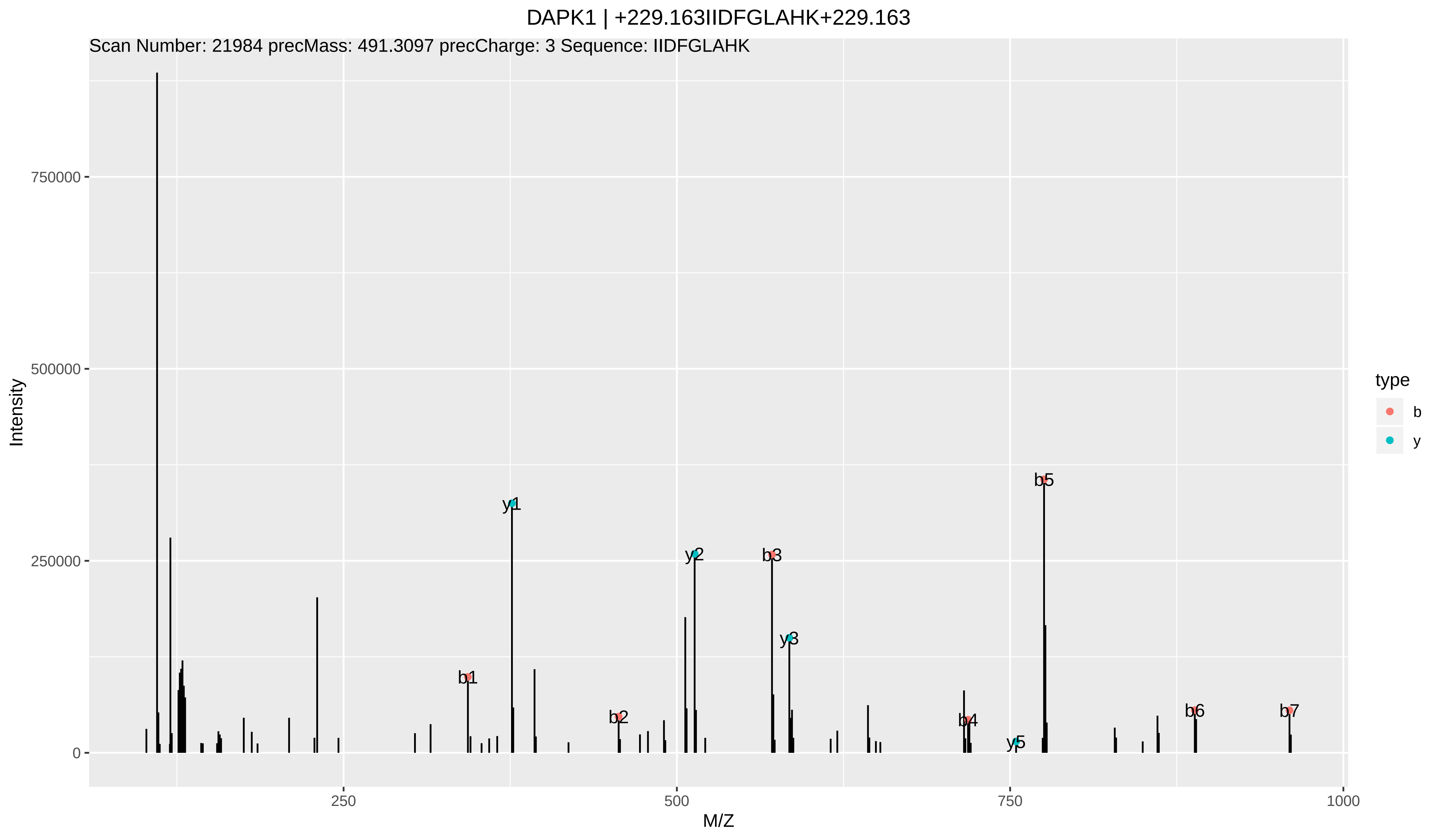

DAPK1 | +229.163IIDFGLAHK+229.163
Scan Number: 21984 precMass: 491.3097 precCharge: 3 Sequence: IIDFGLAHK
Intensity
750000
500000
250000
0
M/Z
250
500
750
1000
y1
y2
b3
y3
b1
b2
b4
y5
b5
b6
b7
type
b
y

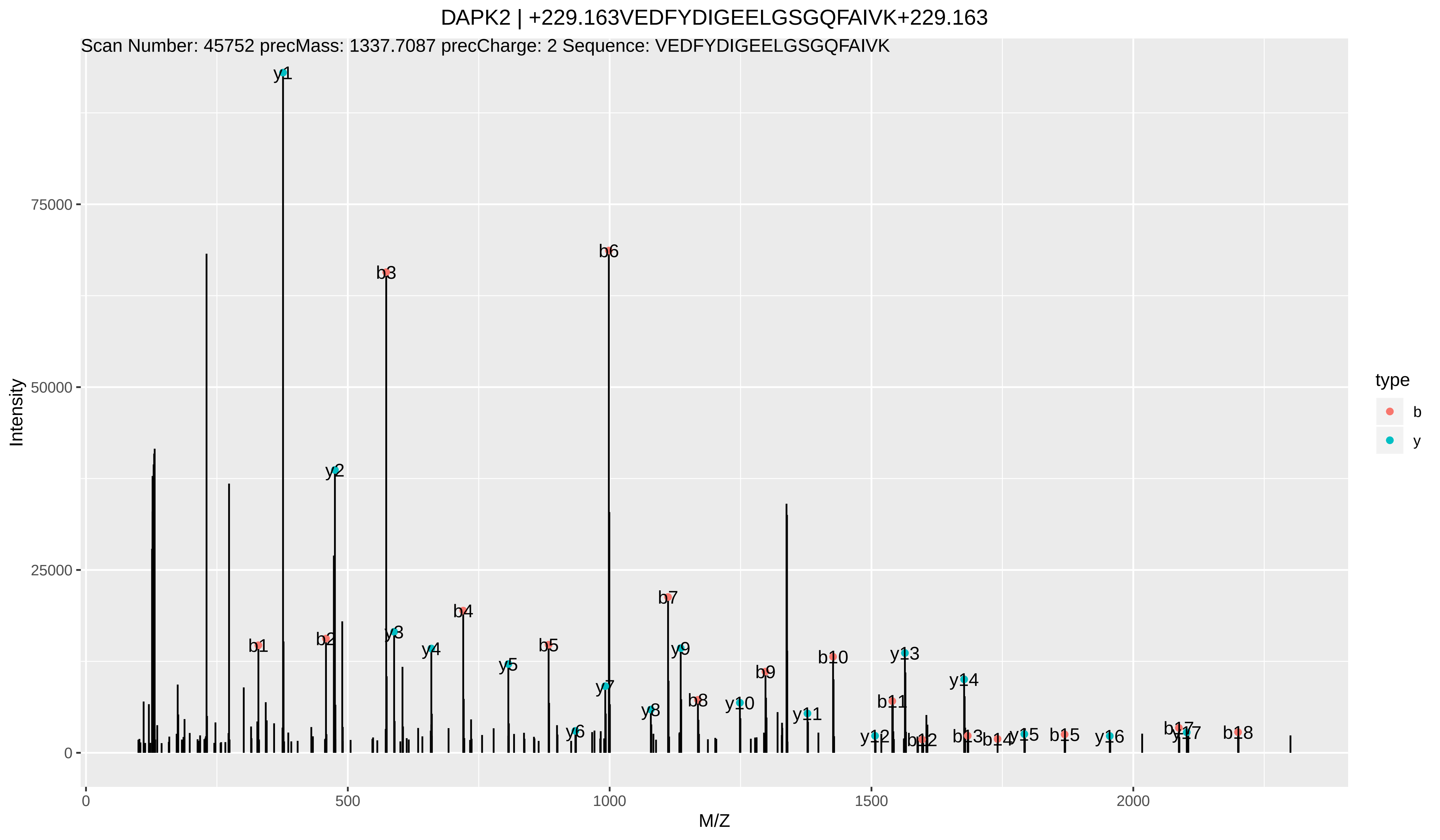

DAPK2 | +229.163VEDFYDIGEELGSGQFAIVK+229.163
Scan Number: 45752 precMass: 1337.7087 precCharge: 2 Sequence: VEDFYDIGEELGSGQFAIVK
Intensity
M/Z
0
25000
50000
75000
0
500
1000
1500
2000
type
b
y
y1
y2
y3
y4
y5
y6
y7
y8
y9
y10
y11
y12
y13
y14
y15
y16
y17
b1
b2
b3
b4
b5
b6
b7
b8
b9
b10
b11
b12
b13
b14
b15
b17
b18

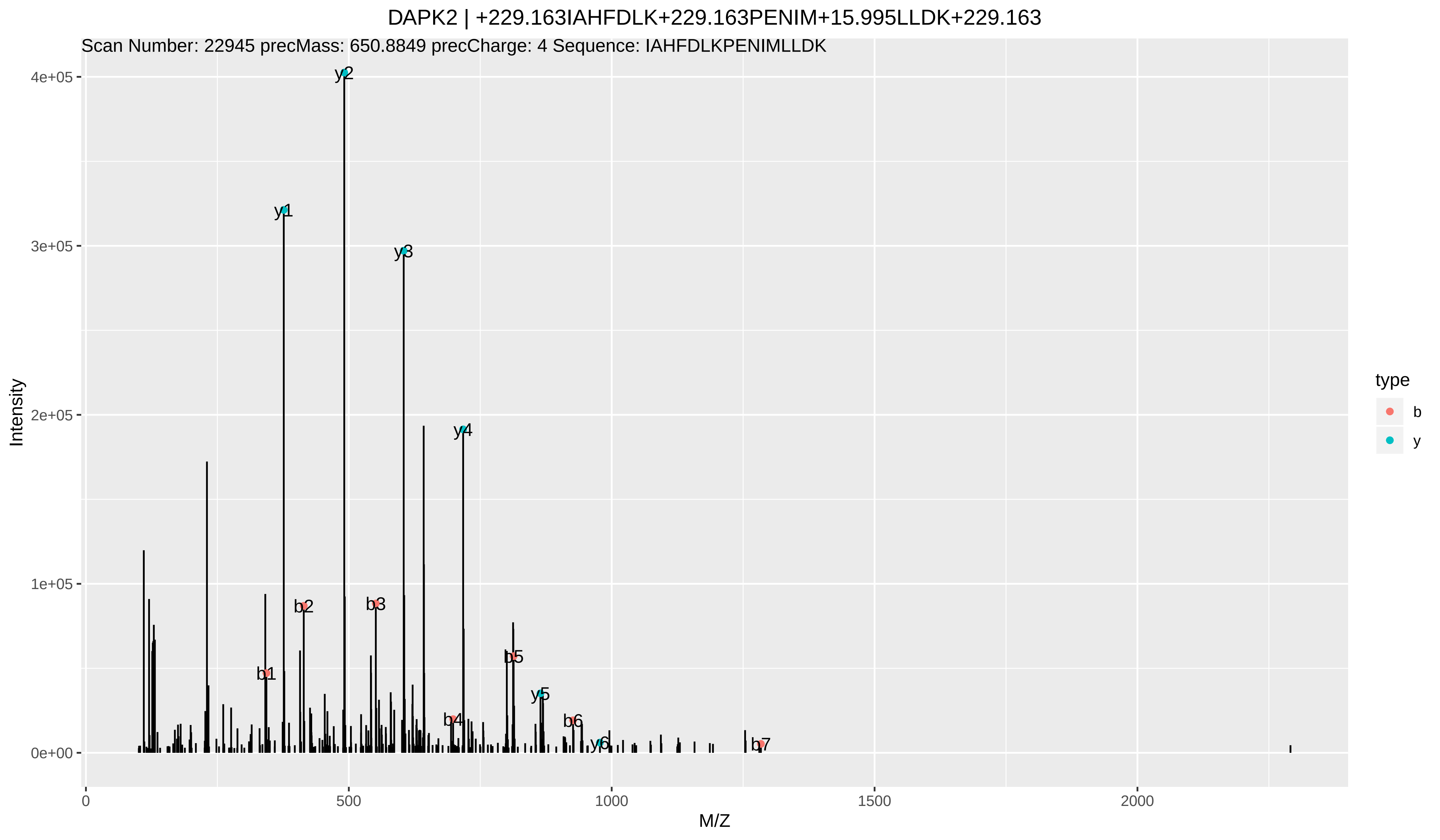

DAPK2 | +229.163IAHFDLK+229.163PENIM+15.995LLDK+229.163
Scan Number: 22945 precMass: 650.8849 precCharge: 4 Sequence: IAHFDLKPENIMLLDK
Intensity
M/Z
4e+05
3e+05
2e+05
1e+05
0e+00
0
500
1000
1500
2000
y2
y1
y3
y4
b1
b2
b3
b5
y5
b4
b6
y6
b7
type
b
y

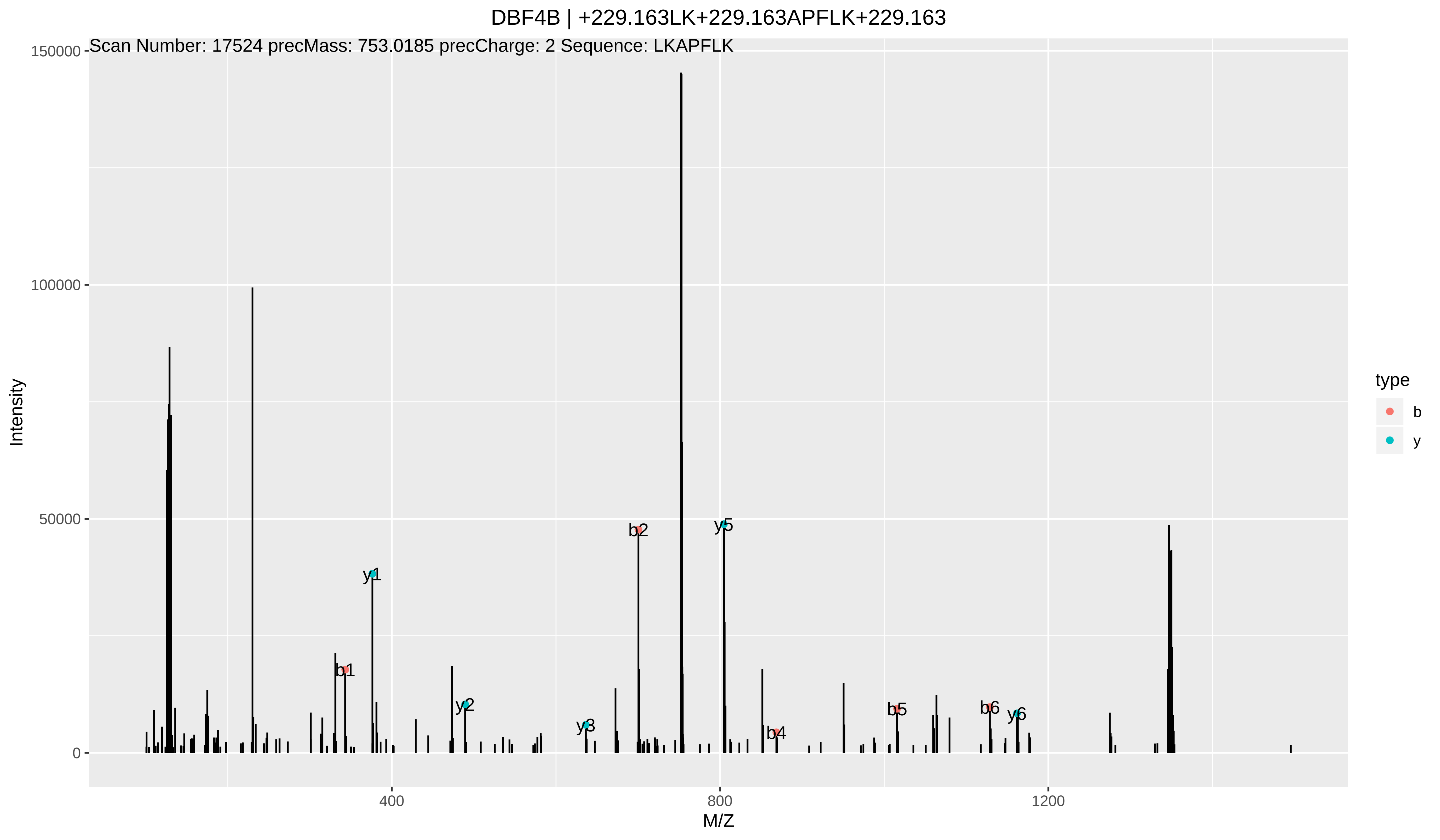

DBF4B | +229.163LK+229.163APFLK+229.163
Scan Number: 17524 precMass: 753.0185 precCharge: 2 Sequence: LKAPFLK
Intensity
150000
100000
50000
0
400
800
1200
M/Z
y1
b1
y2
y3
b2
y5
b4
b5
b6
y6
type
b
y

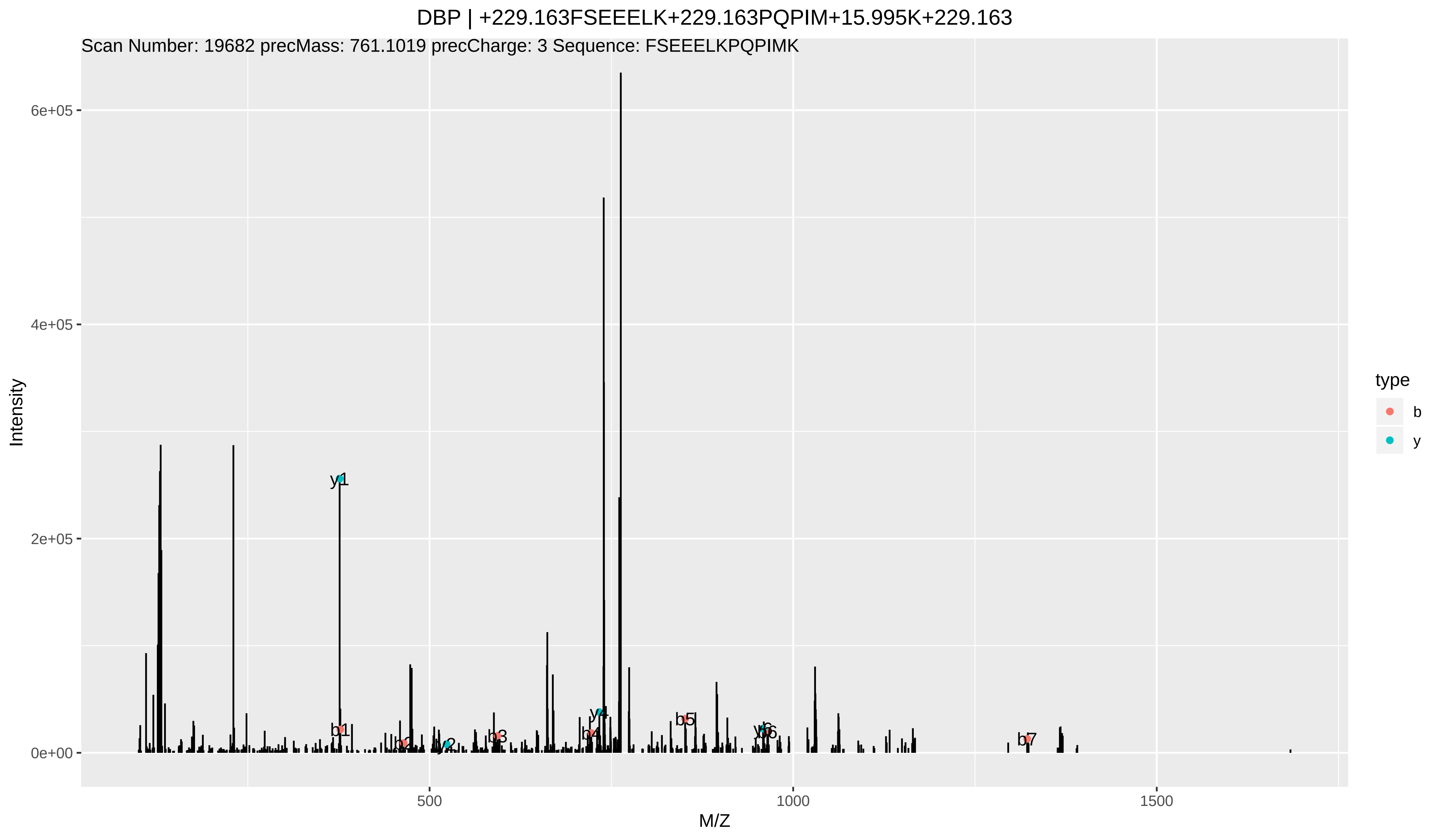

DBP | +229.163FSEEELK+229.163PQPIM+15.995K+229.163
Scan Number: 19682 precMass: 761.1019 precCharge: 3 Sequence: FSEEELKPQPIMK
Intensity
6e+05
4e+05
2e+05
0e+00
M/Z
500
1000
1500
y1
b1
b2
y2
b3
y4
b4
b5
y6
b6
b7
type
b
y

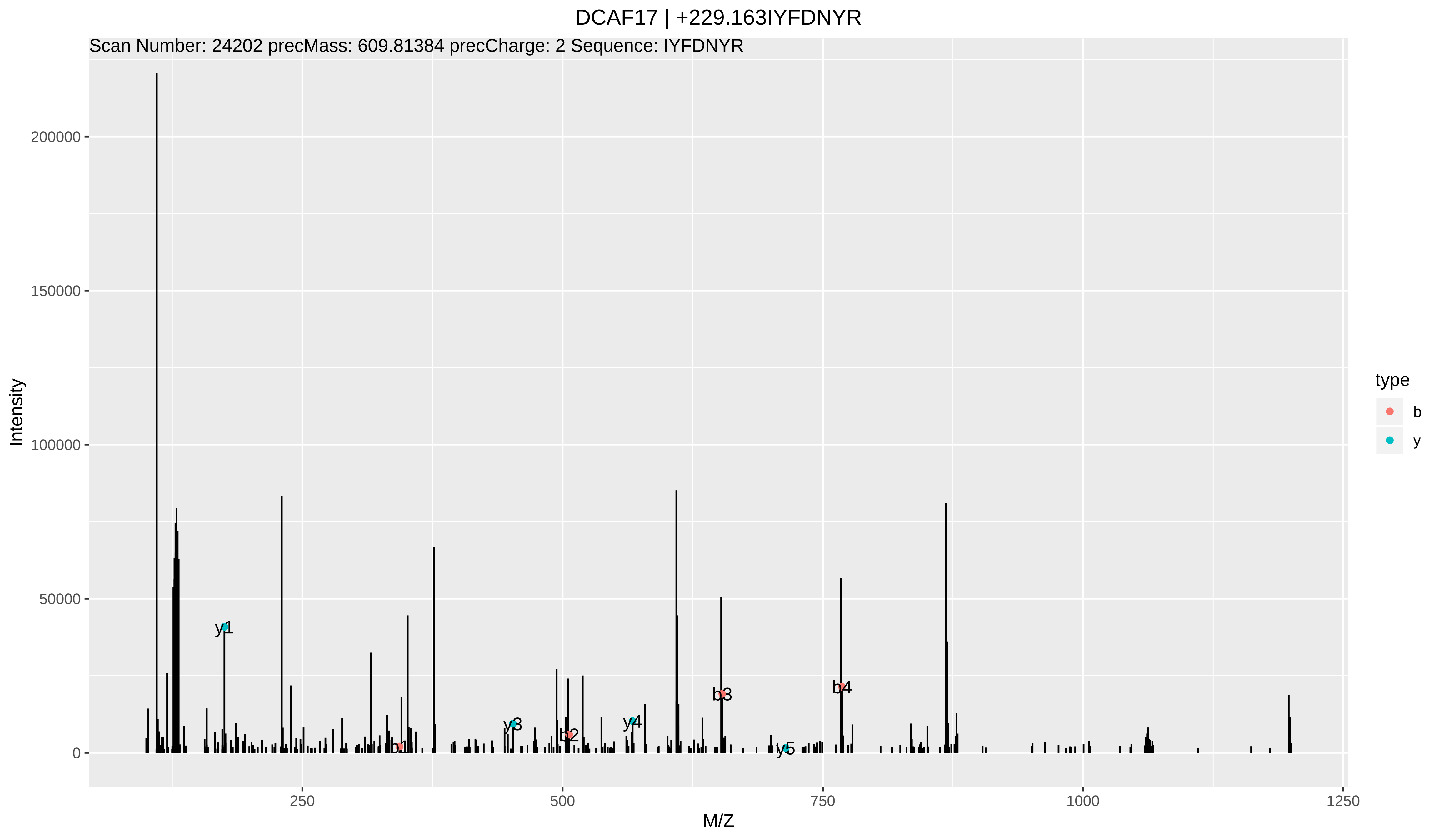

DCAF17 | +229.163IYFDNYR
Scan Number: 24202 precMass: 609.81384 precCharge: 2 Sequence: IYFDNYR
Intensity
200000
150000
100000
50000
0
M/Z
250
500
750
1000
1250
y1
b1
y3
b2
y4
b3
y5
b4
type
b
y

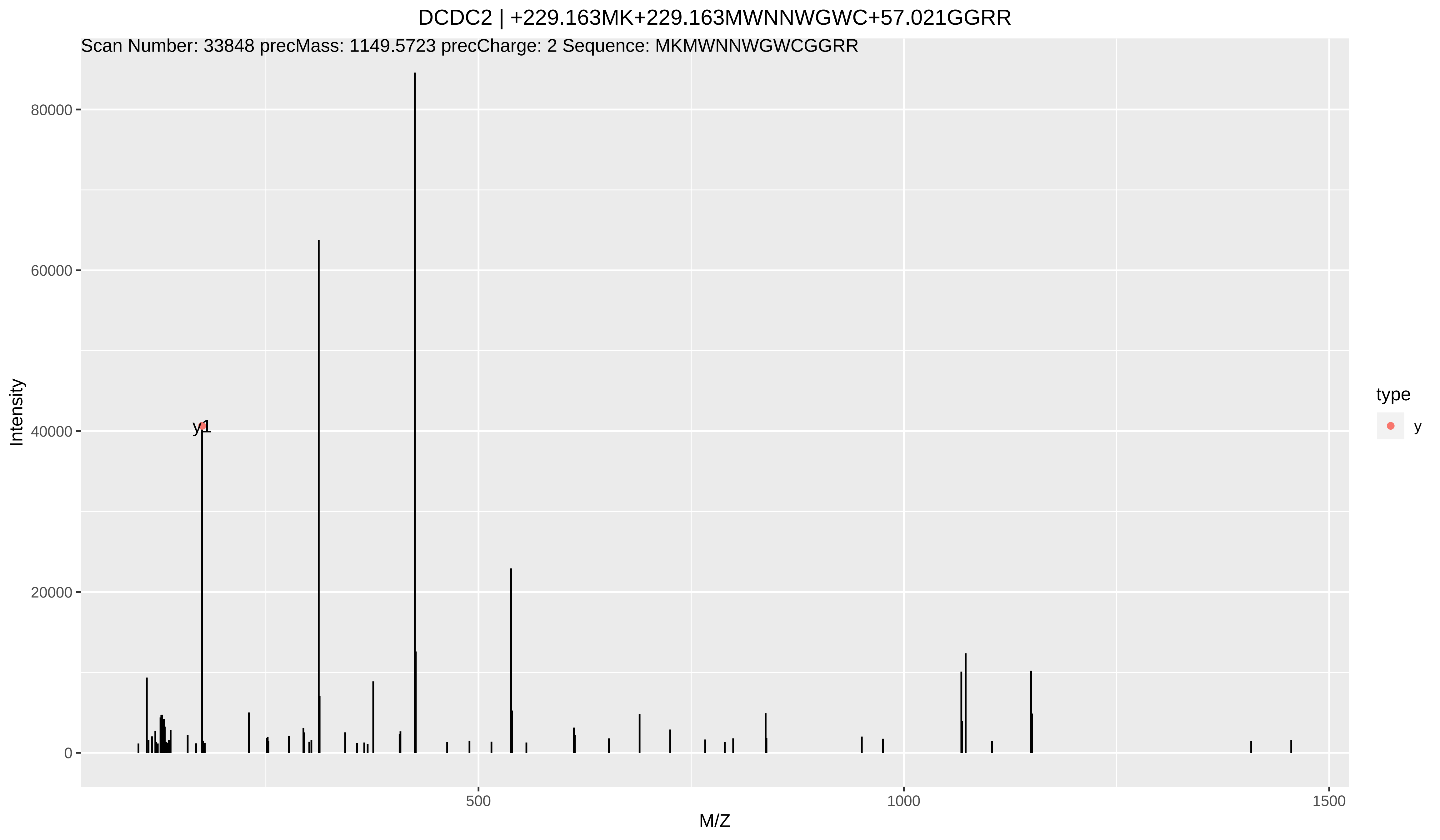

DCDC2 | +229.163MK+229.163MWNNWGWC+57.021GGRR
Scan Number: 33848 precMass: 1149.5723 precCharge: 2 Sequence: MKMWNNWGWCGGRR
Intensity
80000
60000
40000
20000
0
y1
500
1000
1500
M/Z
type
y

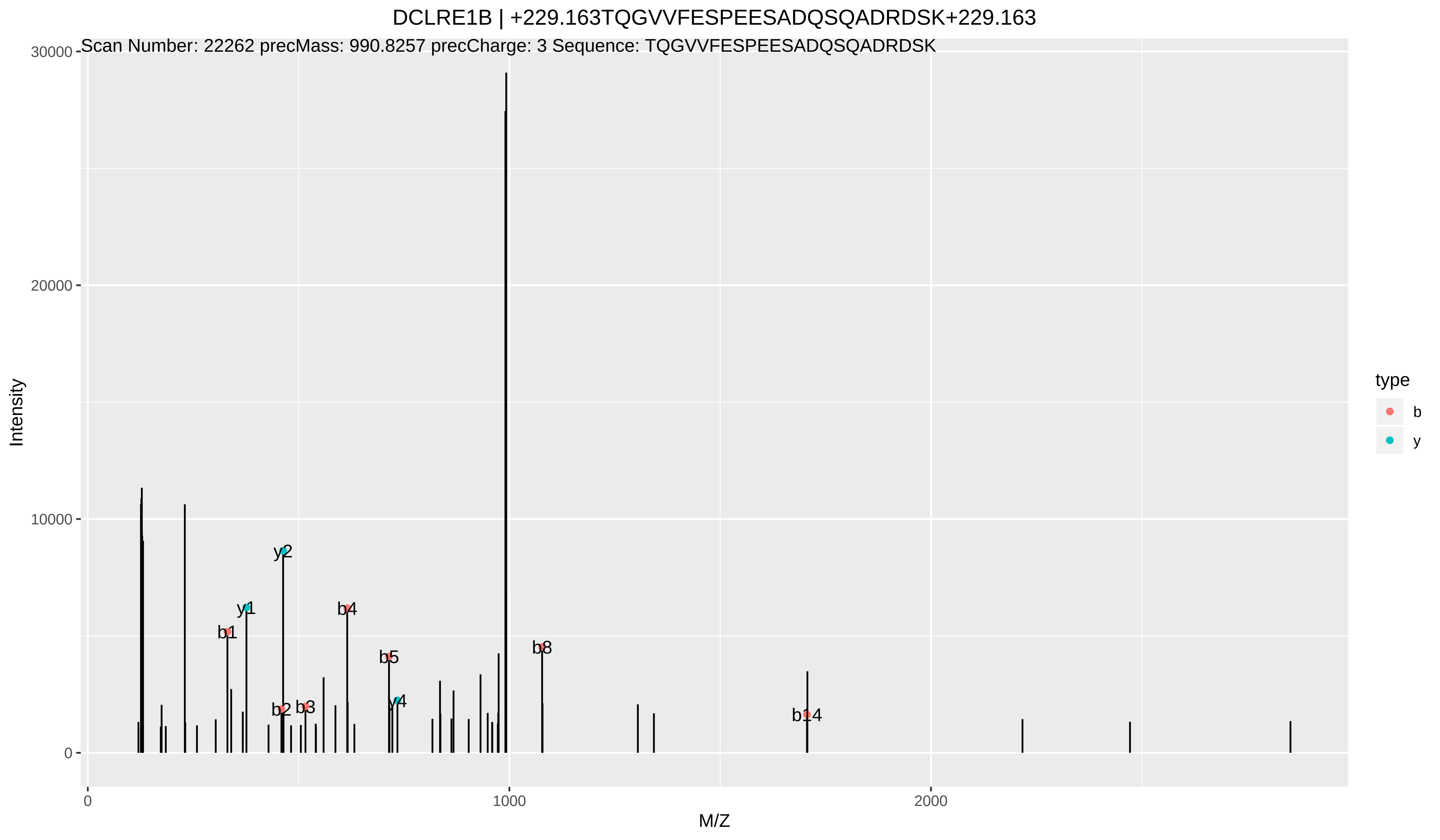
DCLRE1B | +229.163TQGVVFESPEESADQSQADRDSK+229.163
Scan Number: 22262 precMass: 990.8257 precCharge: 3 Sequence: TQGVVFESPEESADQSQADRDSK
Intensity
M/Z
type
b
y
y2
y1
b1
b4
b5
b8
y4
b2
b3
b14
0
10000
20000
30000
0
1000
2000

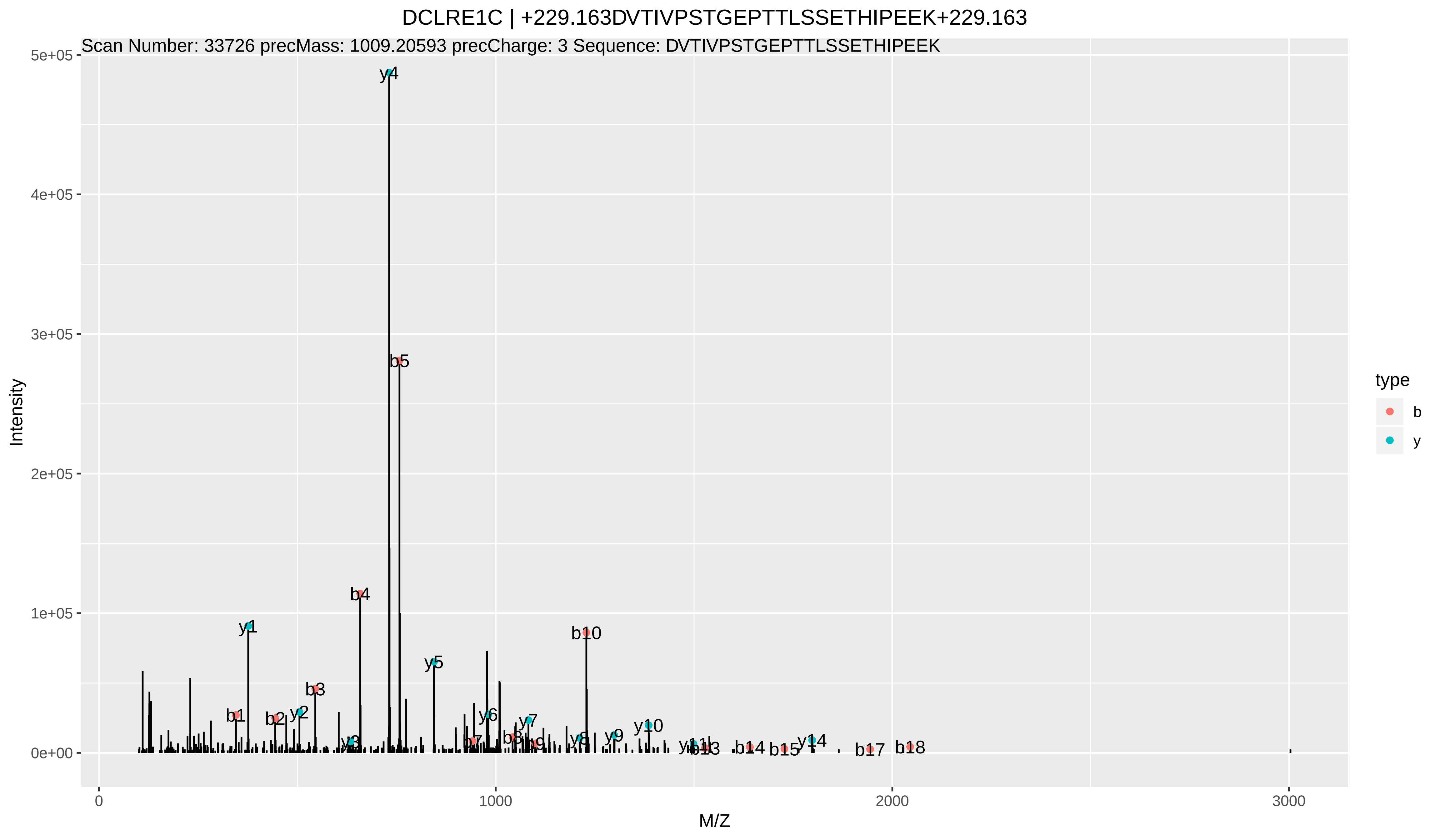
DCLRE1C | +229.163DVTIVPSTGEPTTLSSETHIPEEK+229.163
Scan Number: 33726 precMass: 1009.20593 precCharge: 3 Sequence: DVTIVPSTGEPTTLSSETHIPEEK
Intensity
M/Z
type
b
y
y4
b5
b4
y1
b10
y5
b3
b1
b2
y2
y6
y7
y10
b8
y8
y9
b7
y3
y14
b13
b14
b15
b17
b18
0e+00
1e+05
2e+05
3e+05
4e+05
5e+05
0
1000
2000
3000

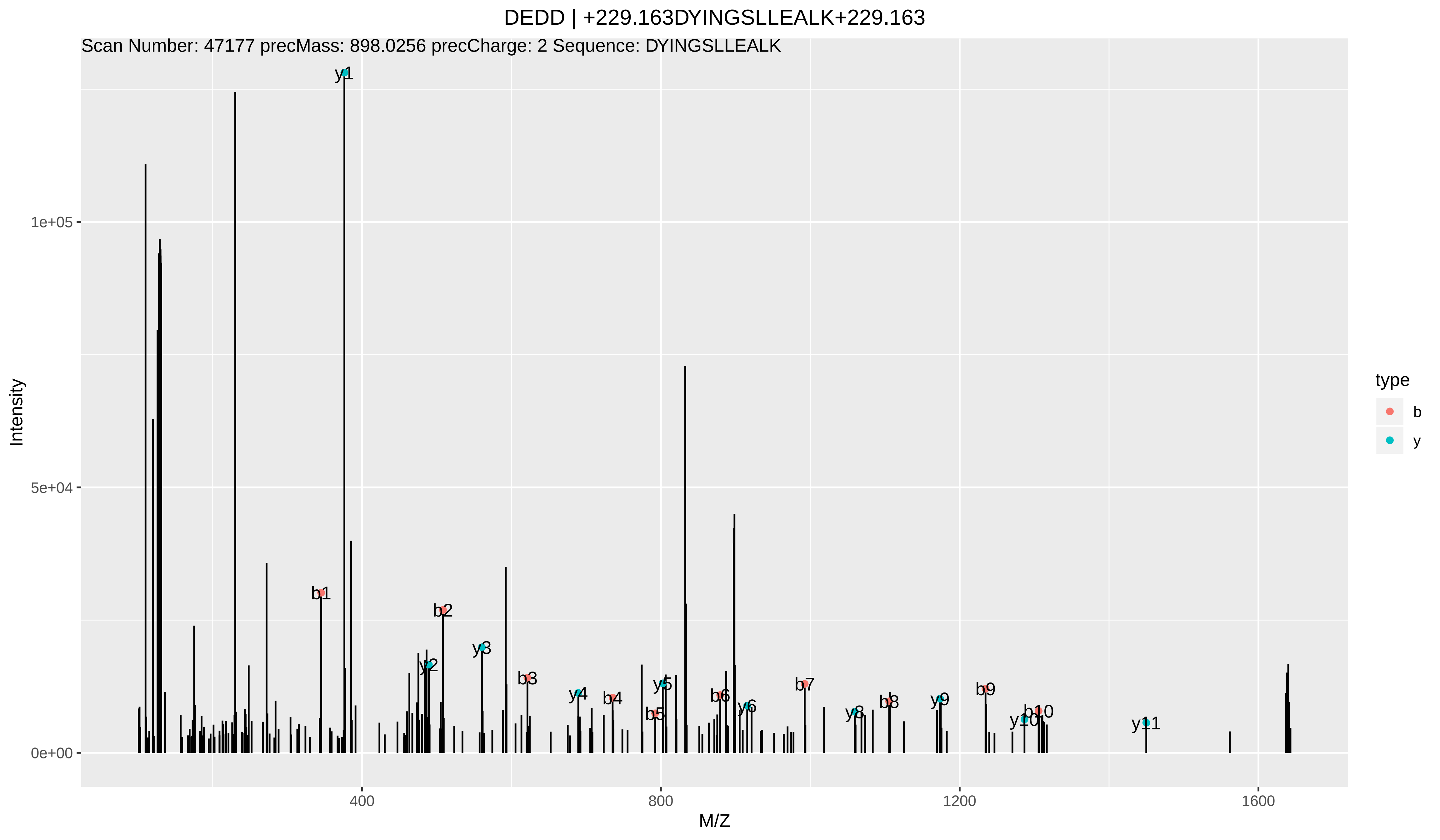

DEDD | +229.163DYINGSLLEALK+229.163
Scan Number: 47177 precMass: 898.0256 precCharge: 2 Sequence: DYINGSLLEALK
Intensity
1e+05
5e+04
0e+00
M/Z
400
800
1200
1600
y1
b1
b2
y2
y3
b3
y4
b4
b5
y5
b6
y6
b7
y8
b8
y9
b9
y10
b10
y11
type
b
y

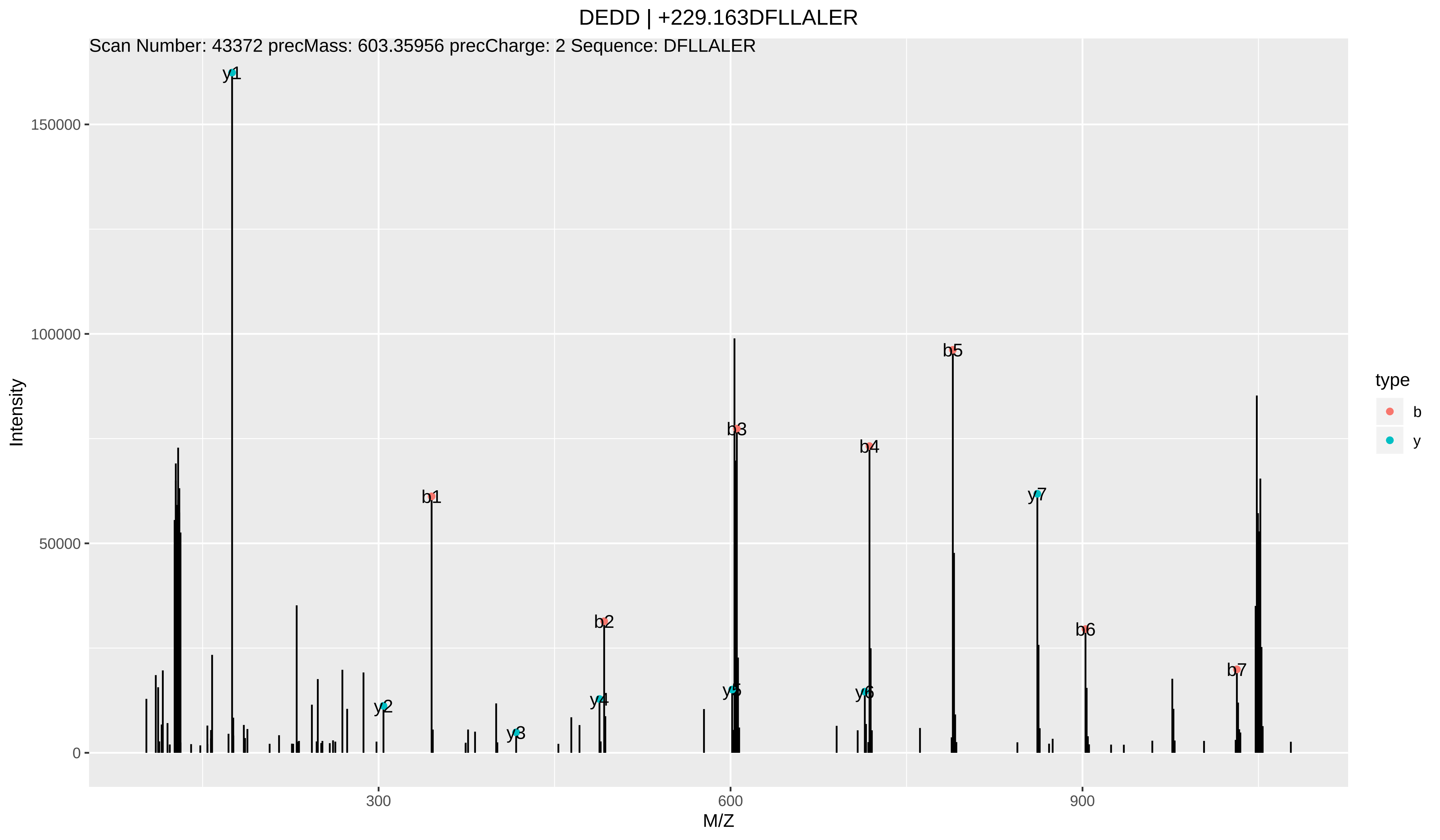
DEDD | +229.163DFLLALER
Scan Number: 43372 precMass: 603.35956 precCharge: 2 Sequence: DFLLALER
Intensity
150000
100000
50000
0
300
600
900
M/Z
y1
b1
y2
y3
b2
y4
b3
y5
b4
y6
b5
y7
b6
b7
type
b
y

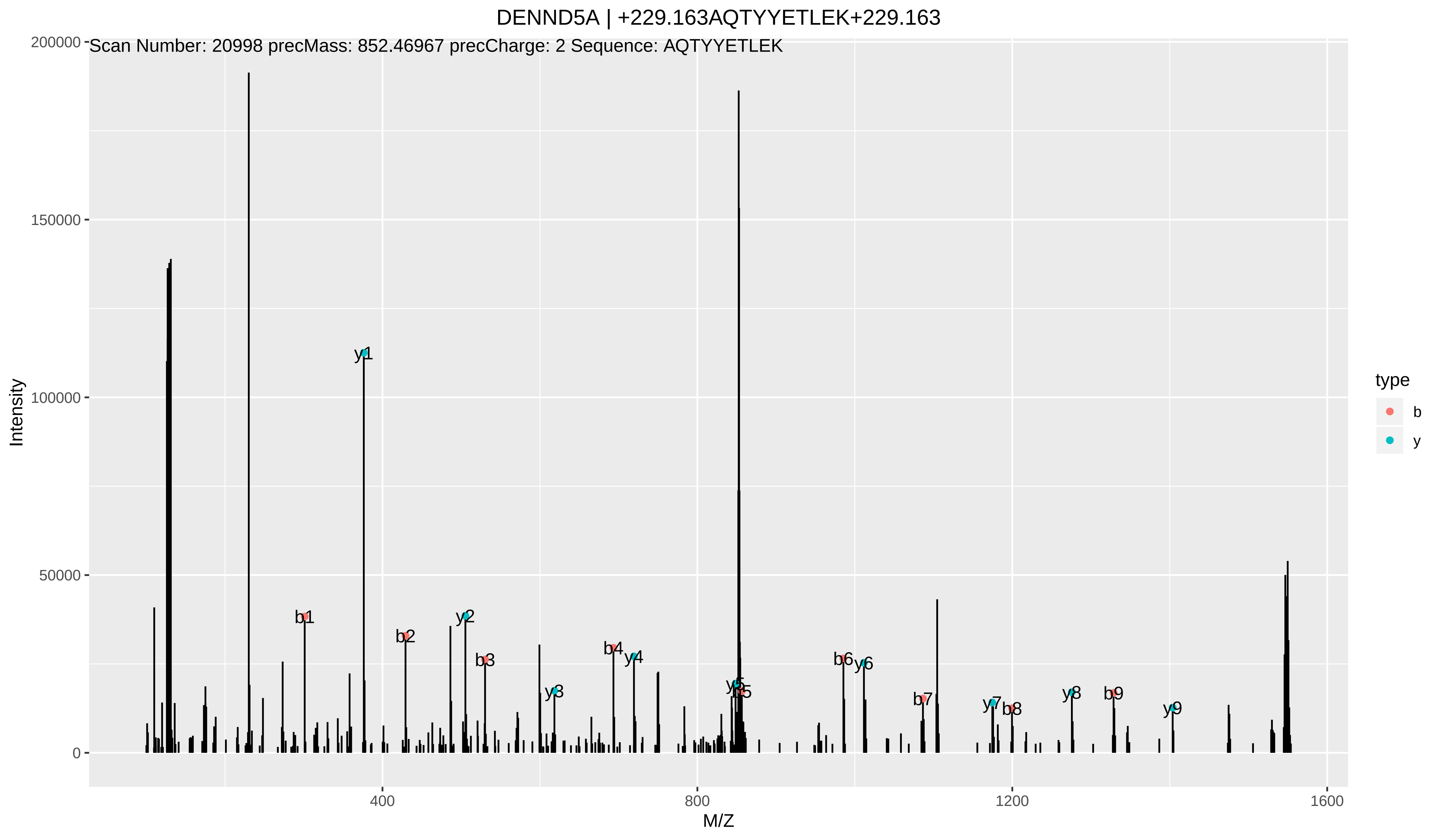

DENND5A | +229.163AQTYYETLEK+229.163
Scan Number: 20998 precMass: 852.46967 precCharge: 2 Sequence: AQTYYETLEK
Intensity
M/Z
0
50000
100000
150000
200000
400
800
1200
1600
b1
b2
b3
b4
b5
b6
b7
b8
b9
y1
y2
y3
y4
y5
y6
y7
y8
y9
type
b
y

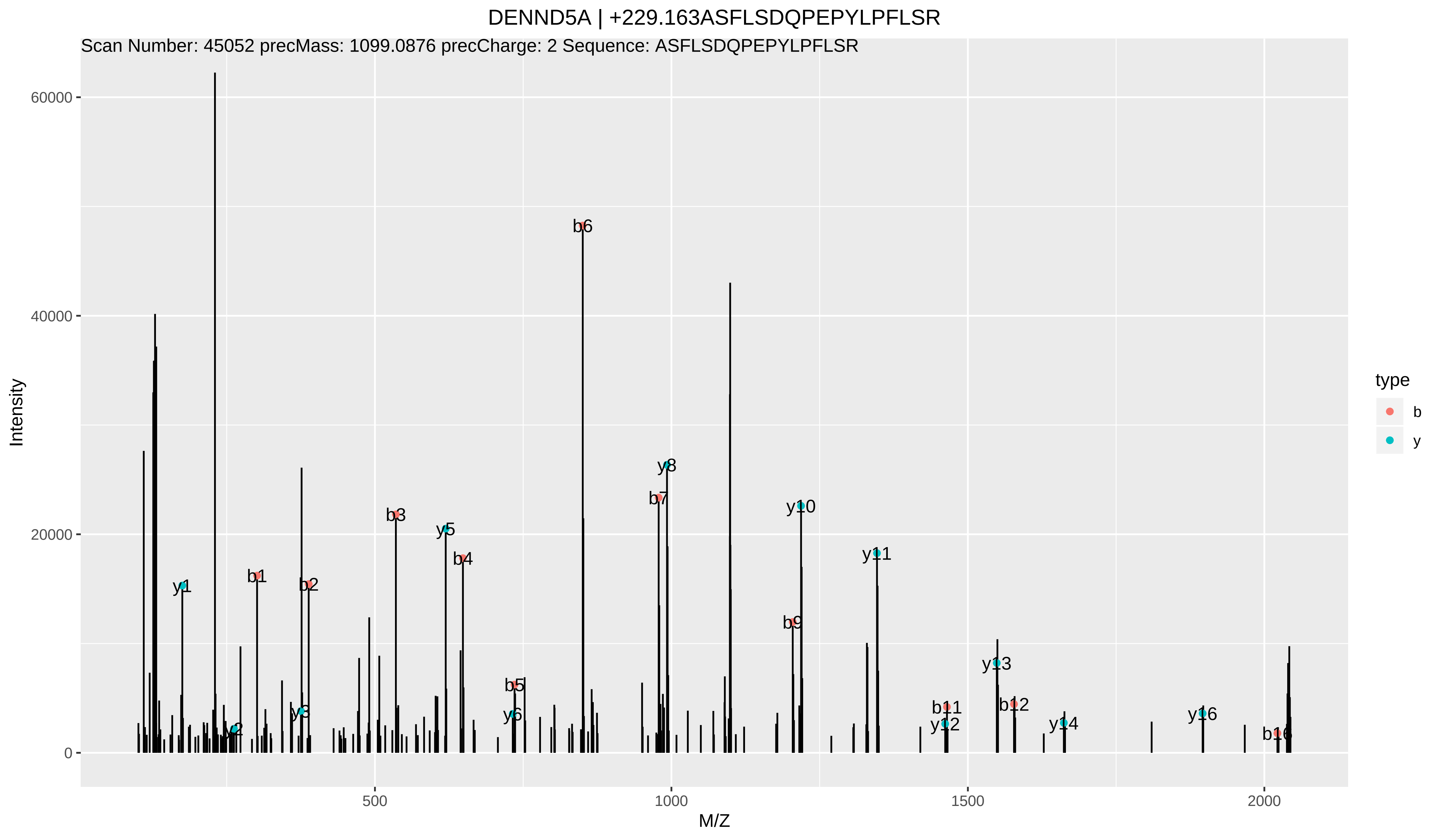

DENND5A | +229.163ASFLSDQPEPYLPFLSR
Scan Number: 45052 precMass: 1099.0876 precCharge: 2 Sequence: ASFLSDQPEPYLPFLSR
Intensity
M/Z
0
20000
40000
60000
500
1000
1500
2000
type
b
y
y1
y2
b1
y3
b2
b3
y5
b4
b5
y6
b6
b7
y8
b9
y10
y11
b11
y12
y13
b12
y14
y16
b16

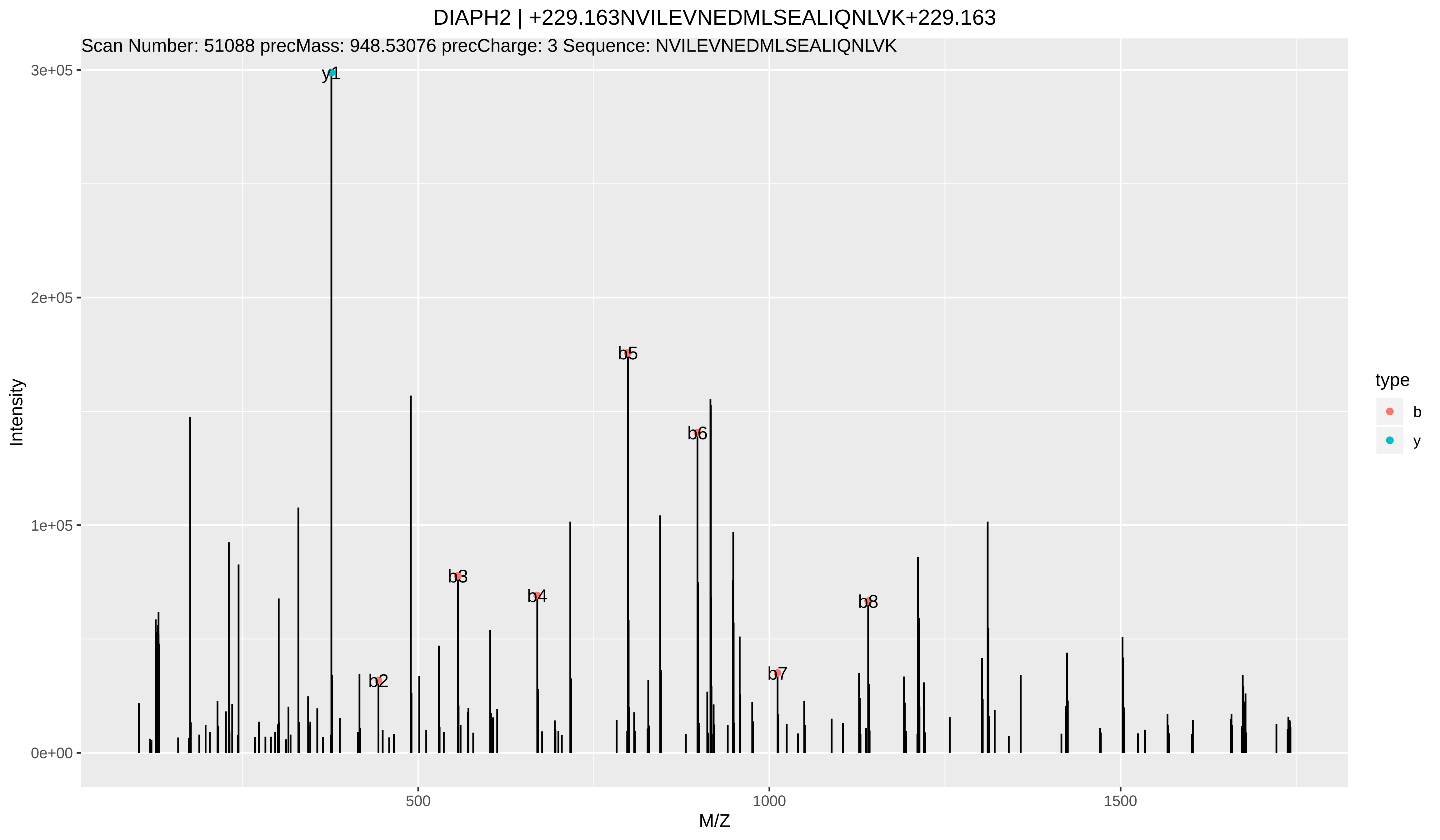
DIAPH2 | +229.163NVILEVNEDMLSEALIQNLVK+229.163
Scan Number: 51088 precMass: 948.53076 precCharge: 3 Sequence: NVILEVNEDMLSEALIQNLVK
Intensity
M/Z
type
b
y
y1
b2
b3
b4
b5
b6
b7
b8
0e+00
1e+05
2e+05
3e+05
500
1000
1500

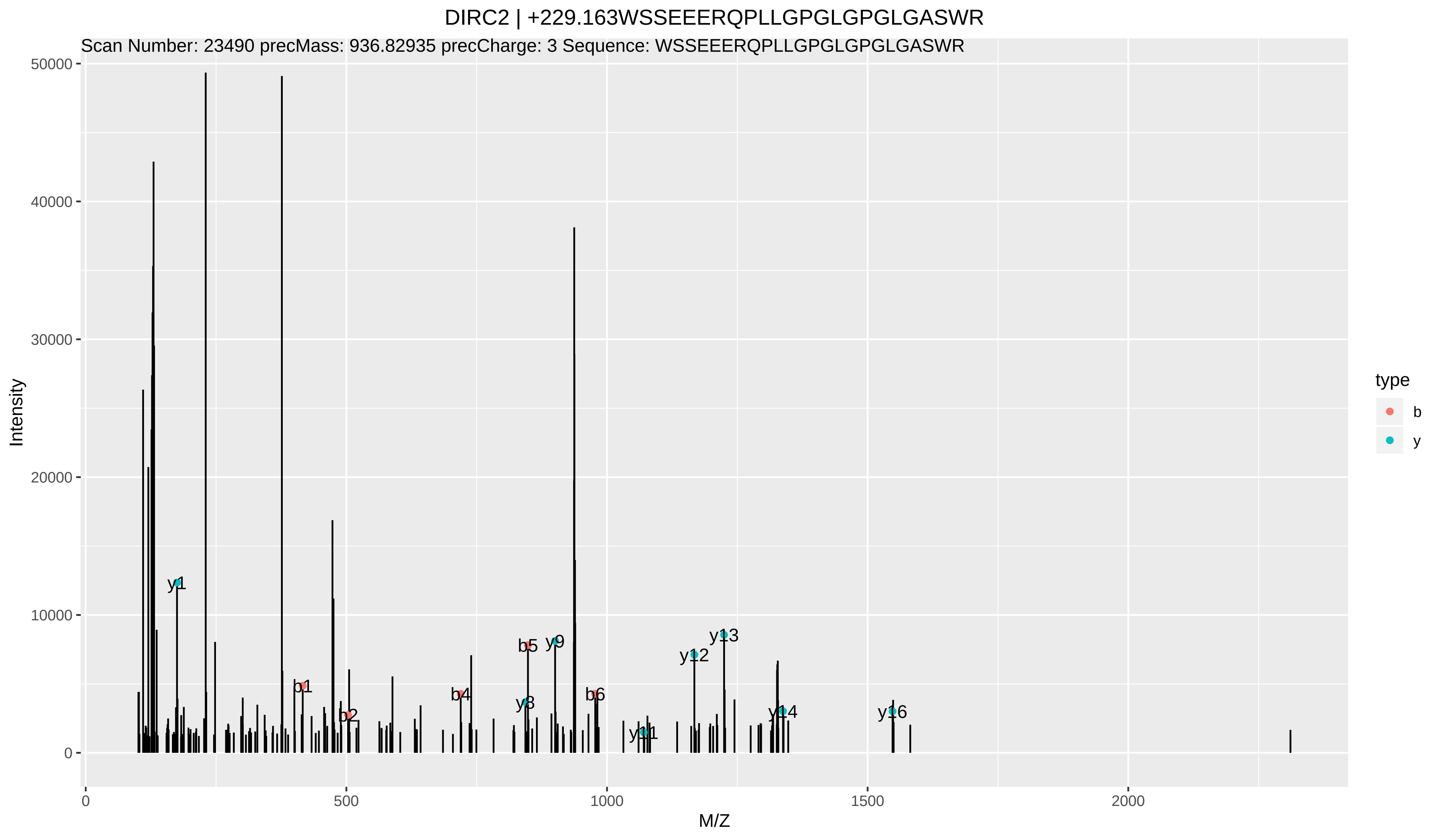

DIRC2 | +229.163WSSEEERQPLLGPGLGPGLGASWR
Scan Number: 23490 precMass: 936.82935 precCharge: 3 Sequence: WSSEEERQPLLGPGLGPGLGASWR
Intensity
M/Z
type
b
y
y1
b1
b2
b4
b5
y8
y9
b6
y11
y12
y13
y14
y16
0
10000
20000
30000
40000
50000
500
1000
1500
2000

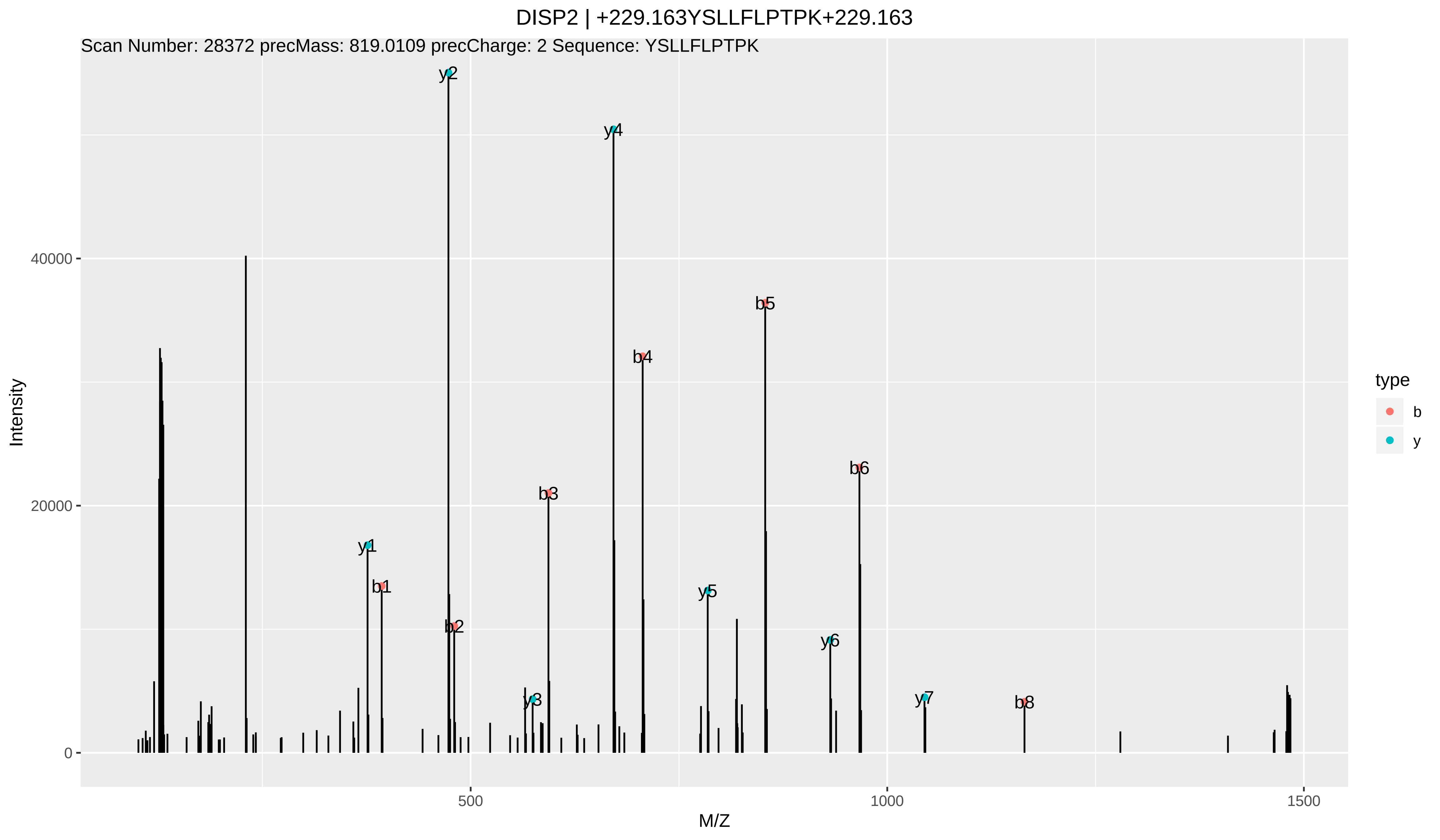

DISP2 | +229.163YSLLFLPTPK+229.163
Scan Number: 28372 precMass: 819.0109 precCharge: 2 Sequence: YSLLFLPTPK
y2
y4
b5
b4
b6
b3
y1
b1
y5
b2
y6
y3
y7
b8
Intensity
40000
20000
0
500
1000
1500
M/Z
type
b
y

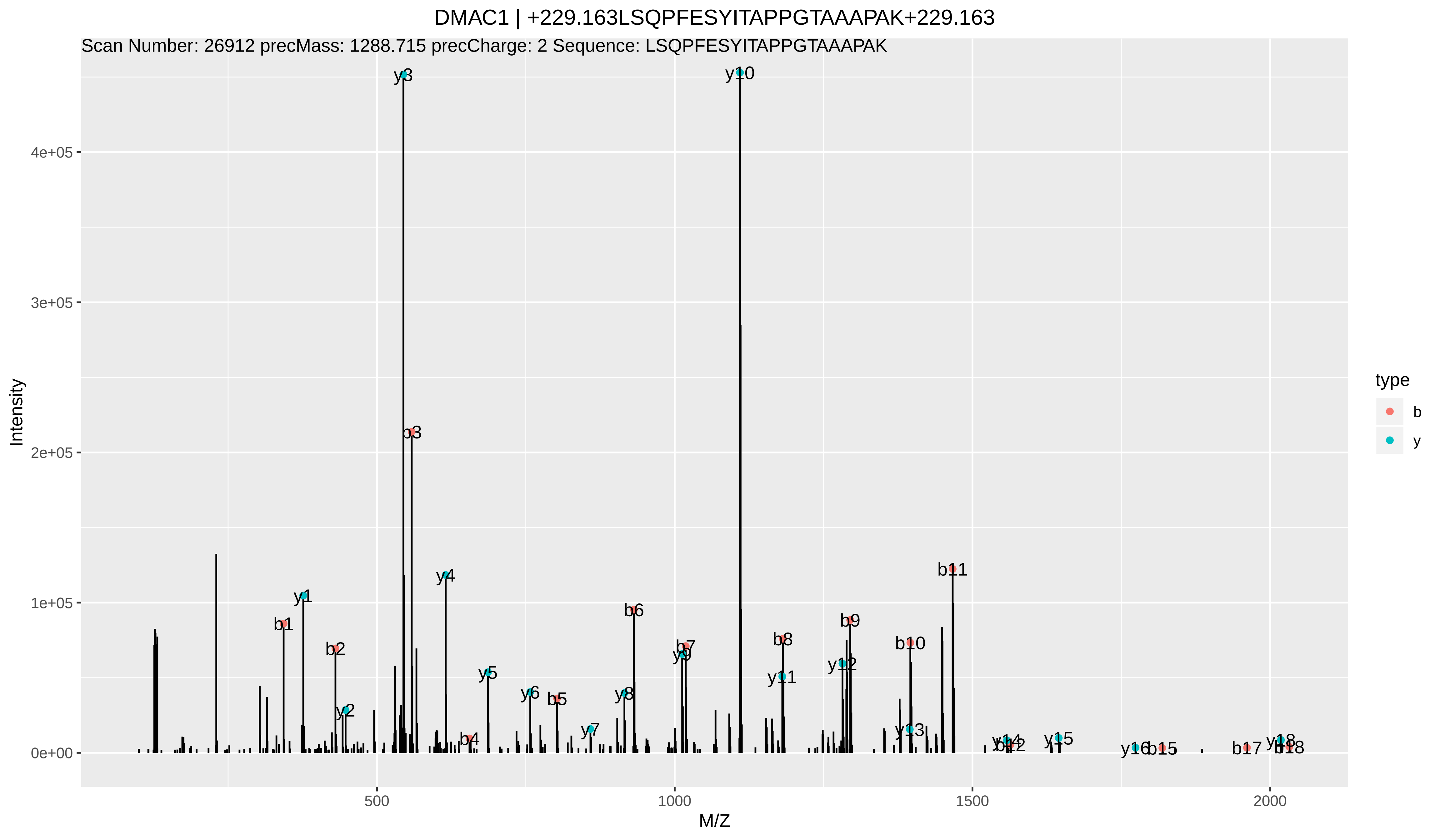

DMAC1 | +229.163LSQPFESYITAPPGTAAAPAK+229.163
Scan Number: 26912 precMass: 1288.715 precCharge: 2 Sequence: LSQPFESYITAPPGTAAAPAK
Intensity
M/Z
type
b
y
0e+00
1e+05
2e+05
3e+05
4e+05
500
1000
1500
2000
y3
y10
b3
y4
b11
y1
b6
b1
b9
b8
b10
b2
b7
y9
y12
y11
y5
y6
b5
y8
y2
y7
y13
b4
y14
b12
y15
y16
b15
b17
y18
b18

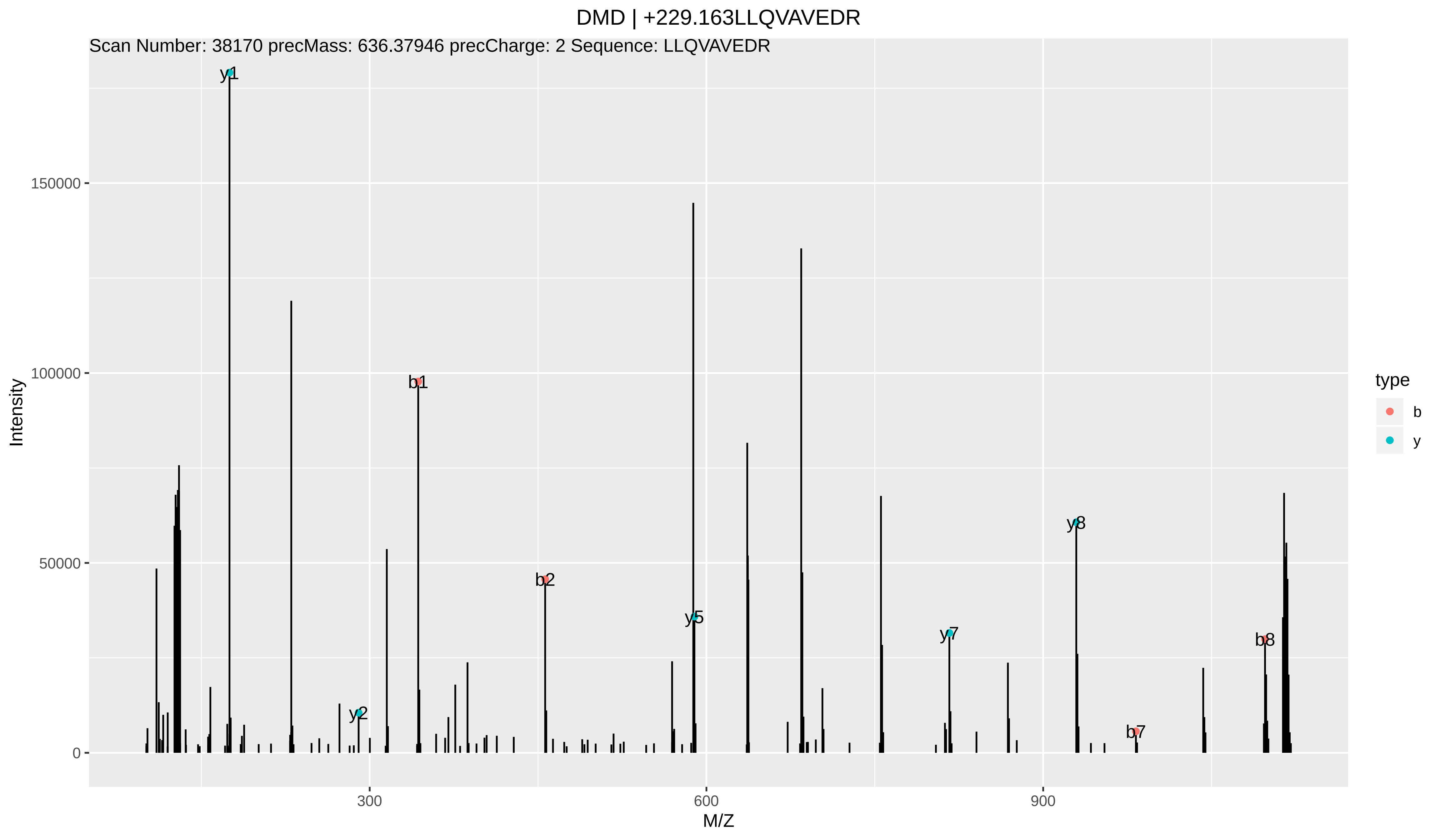

DMD | +229.163LLQVAVEDR
Scan Number: 38170 precMass: 636.37946 precCharge: 2 Sequence: LLQVAVEDR
Intensity
M/Z
0
50000
100000
150000
300
600
900
y1
b1
y2
b2
y5
y7
y8
b7
b8
type
b
y

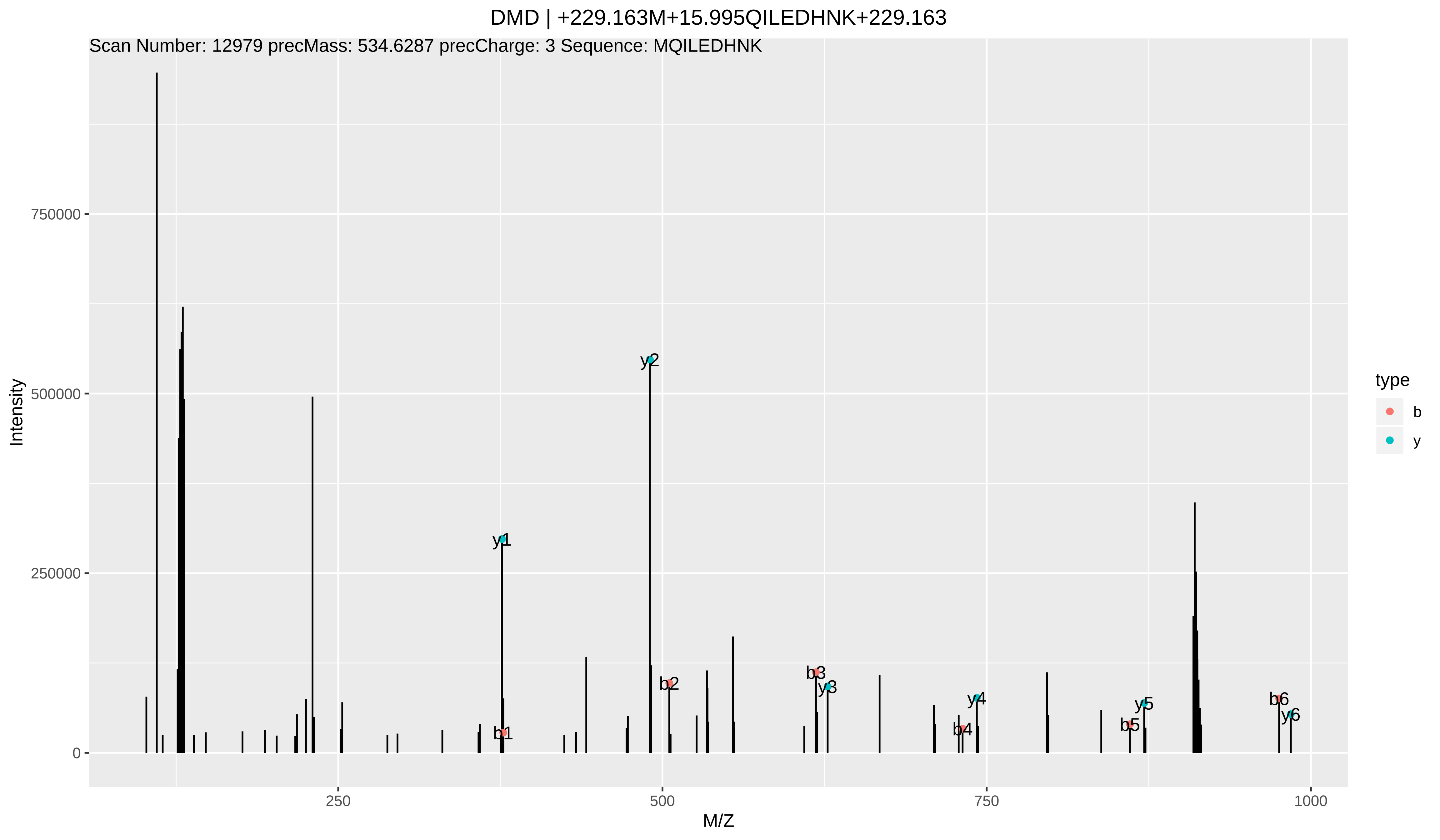
DMD | +229.163M+15.995QILEDHNK+229.163
Scan Number: 12979 precMass: 534.6287 precCharge: 3 Sequence: MQILEDHNK
Intensity
750000
500000
250000
0
M/Z
250
500
750
1000
y1
y2
b1
b2
b3
y3
y4
b4
y5
b5
b6
y6
type
b
y

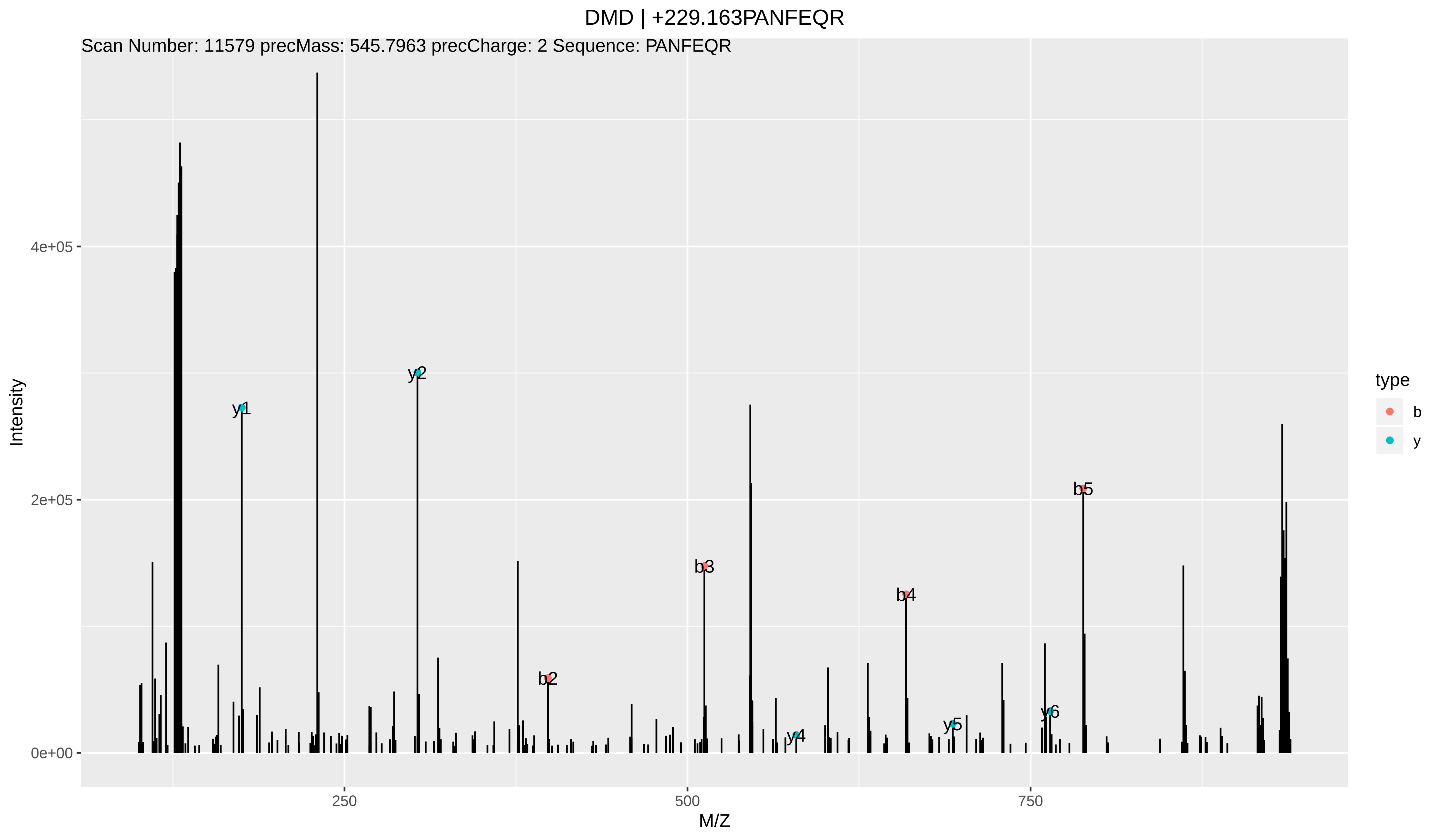

DMD | +229.163PANFEQR
Scan Number: 11579 precMass: 545.7963 precCharge: 2 Sequence: PANFEQR
Intensity
4e+05
2e+05
0e+00
M/Z
250
500
750
y1
y2
b2
b3
y4
b4
y5
y6
b5
type
b
y

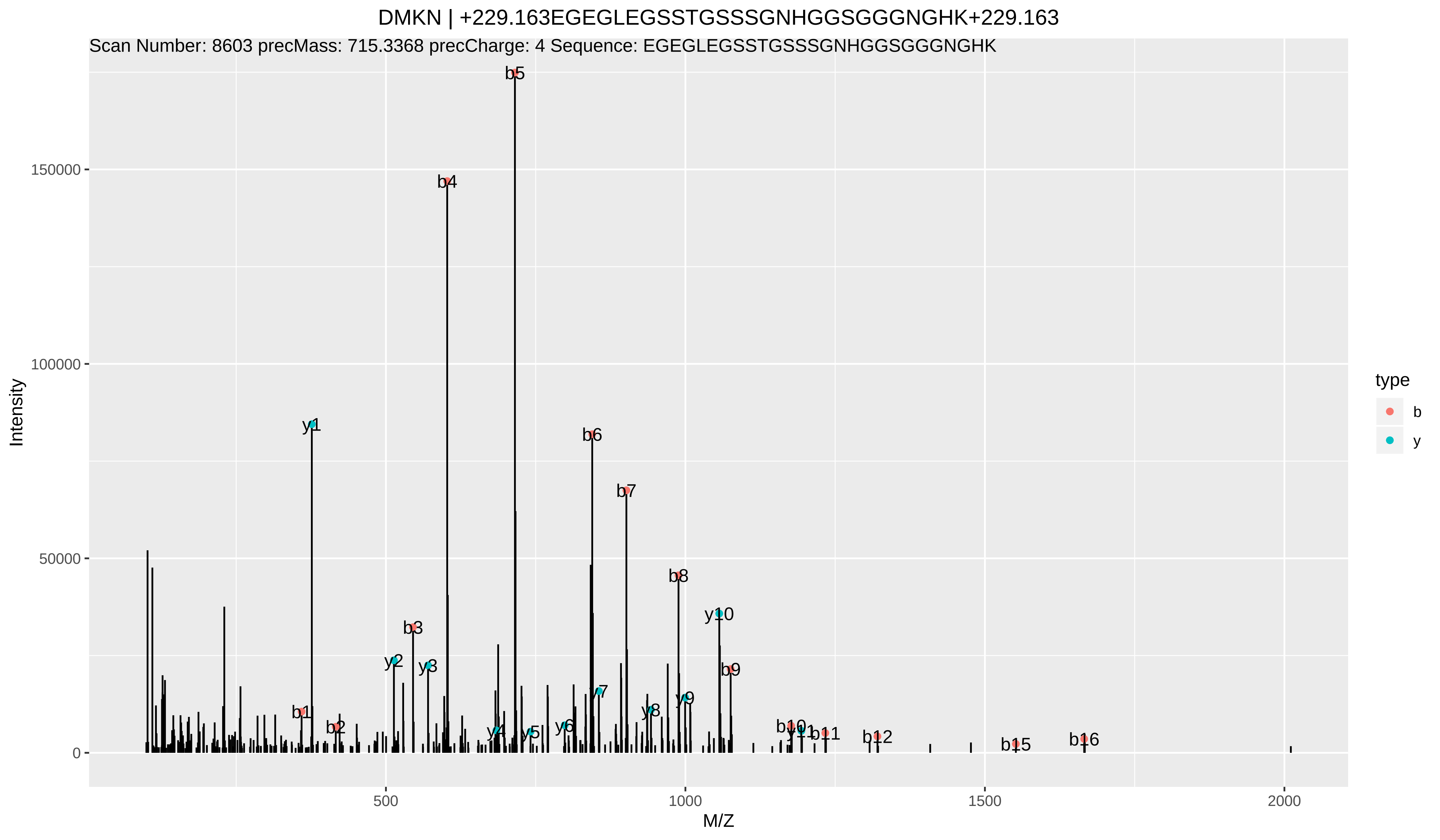

DMKN | +229.163EGEGLEGSSTGSSSGNHGGSGGGNGHK+229.163
Scan Number: 8603 precMass: 715.3368 precCharge: 4 Sequence: EGEGLEGSSTGSSSGNHGGSGGGNGHK
Intensity
M/Z
type
b
y
b5
b4
y1
b6
b7
b8
y10
b3
y2
y3
b9
y7
y9
b1
y8
b2
y4
y5
y6
b10
y11
b11
b12
b15
b16
150000
100000
50000
0
500
1000
1500
2000

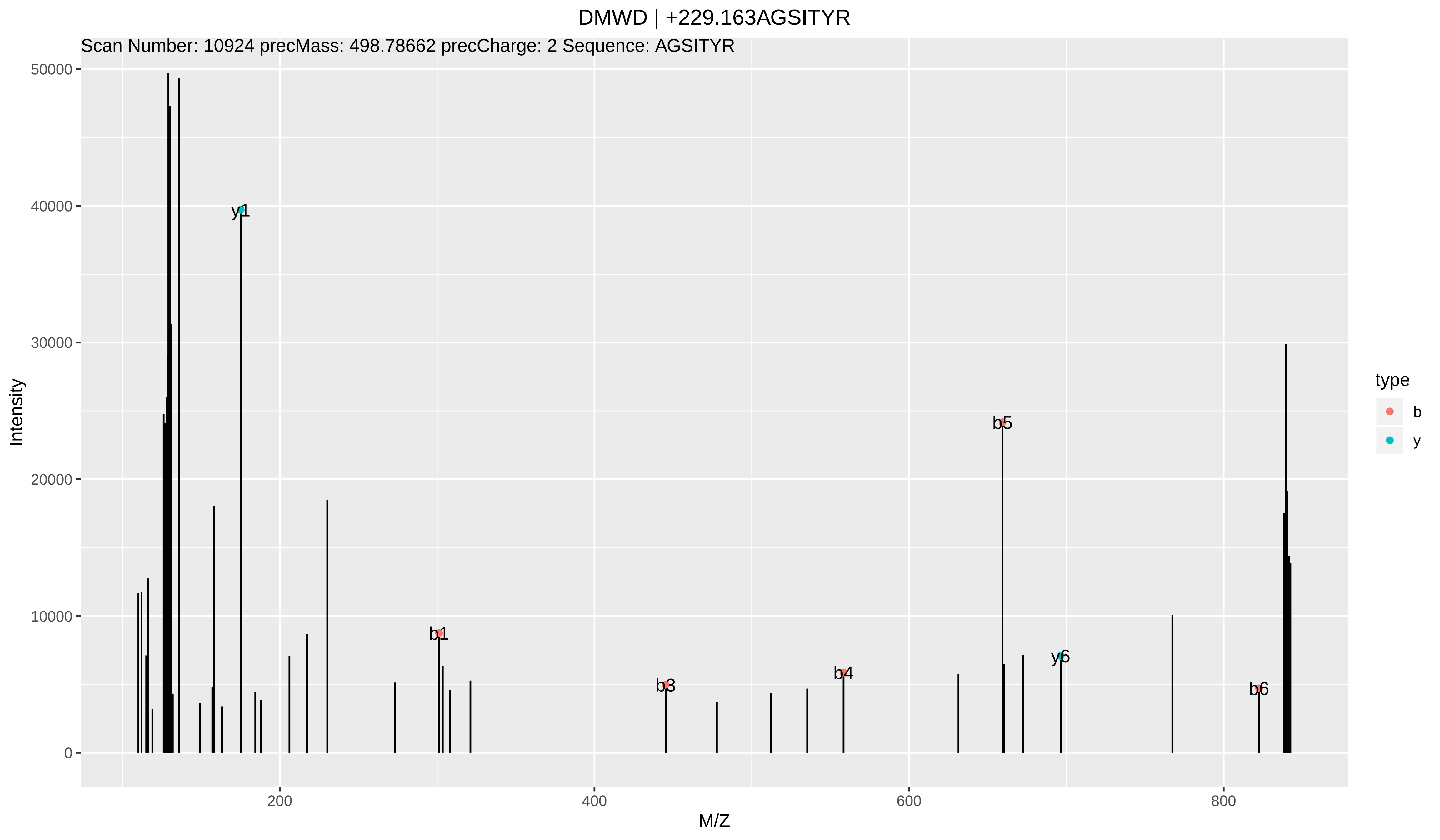

DMWD | +229.163AGSITYR
Scan Number: 10924 precMass: 498.78662 precCharge: 2 Sequence: AGSITYR
Intensity
M/Z
0
10000
20000
30000
40000
50000
200
400
600
800
y1
b1
b3
b4
b5
y6
b6
type
b
y

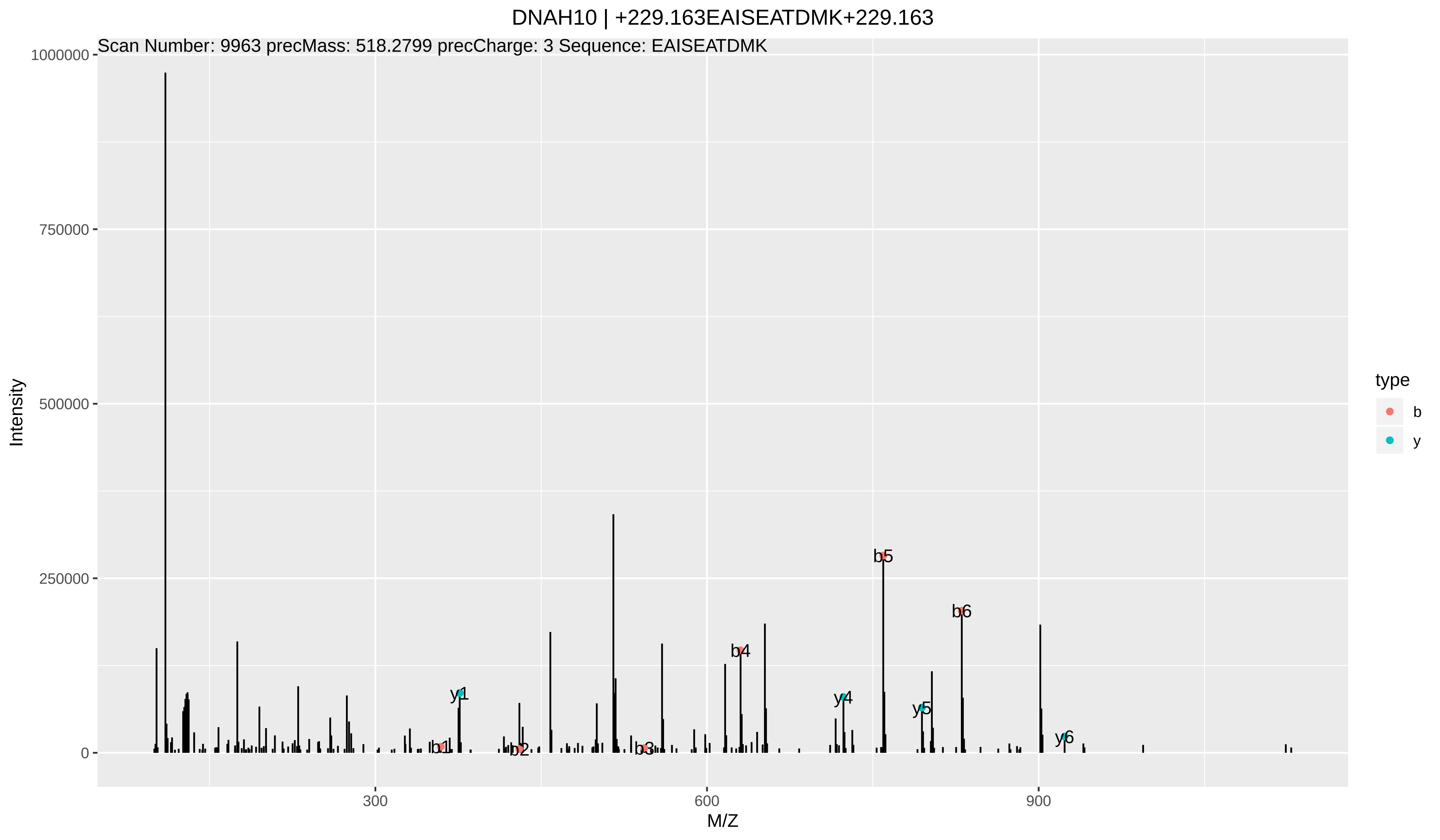
DNAH10 | +229.163EAISEATDMK+229.163
Scan Number: 9963 precMass: 518.2799 precCharge: 3 Sequence: EAISEATDMK
Intensity
1000000
750000
500000
250000
0
M/Z
300
600
900
y1
y4
y5
y6
b1
b2
b3
b4
b5
b6
type
b
y

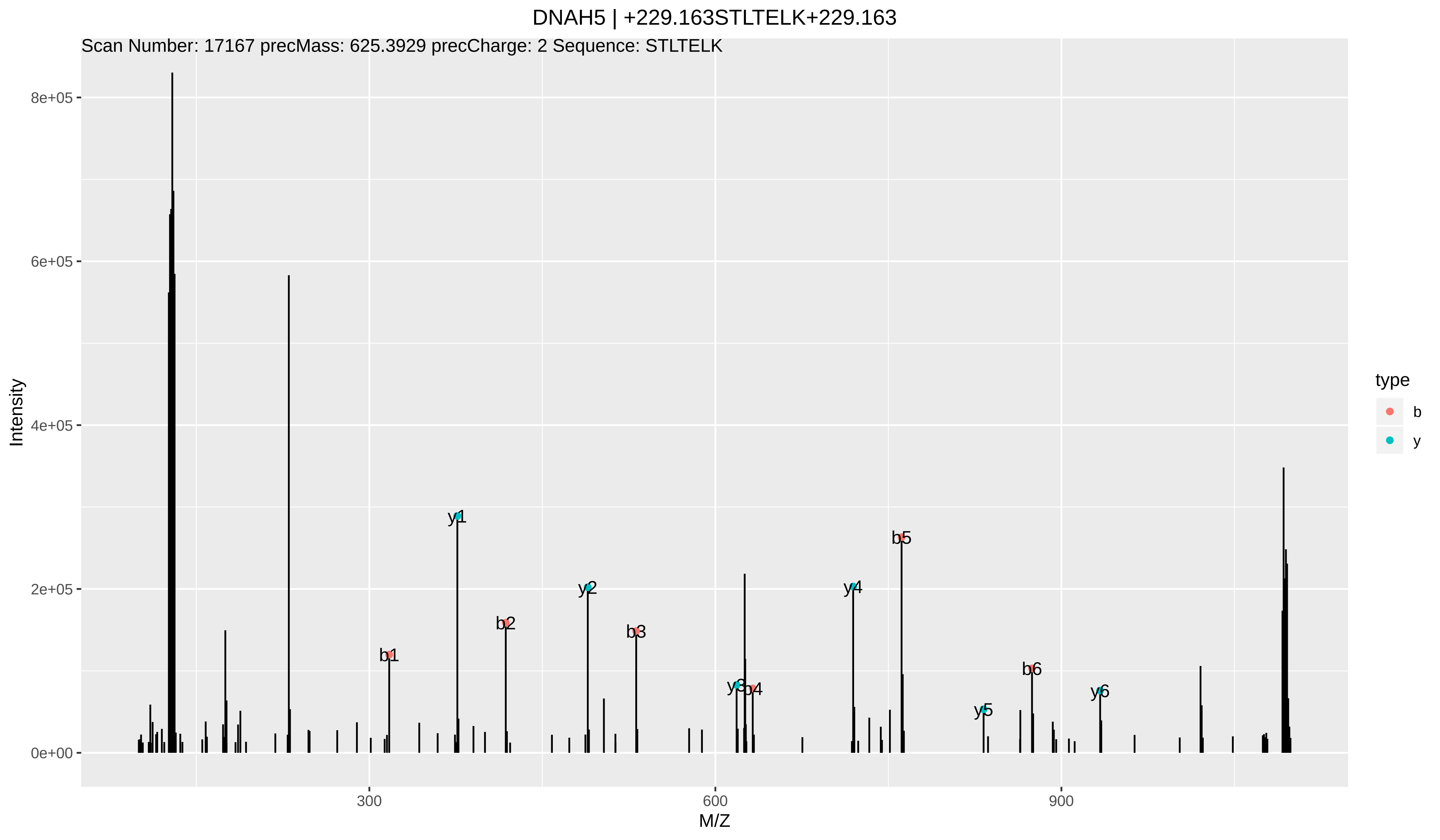

DNAH5 | +229.163STLTELK+229.163
Scan Number: 17167 precMass: 625.3929 precCharge: 2 Sequence: STLTELK
Intensity
8e+05
6e+05
4e+05
2e+05
0e+00
M/Z
300
600
900
y1
b1
b2
y2
b3
y3
b4
y4
b5
y5
b6
y6
type
b
y

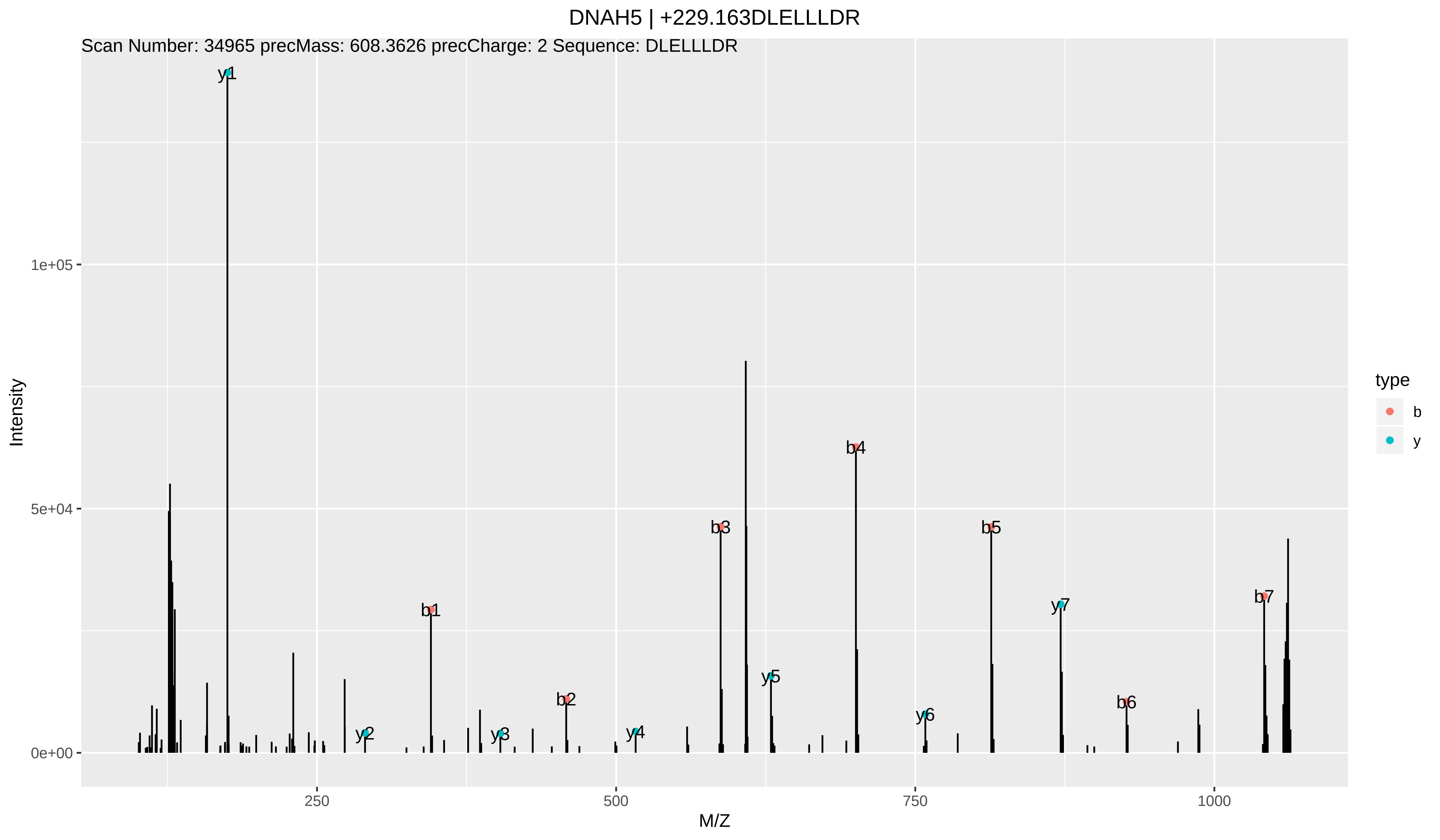

DNAH5 | +229.163DLELLLDR
Scan Number: 34965 precMass: 608.3626 precCharge: 2 Sequence: DLELLLDR
y1
b4
b3
b5
y7
b7
b1
y5
b2
b6
y6
y2
y3
y4
Intensity
1e+05
5e+04
0e+00
250
500
750
1000
M/Z
type
b
y

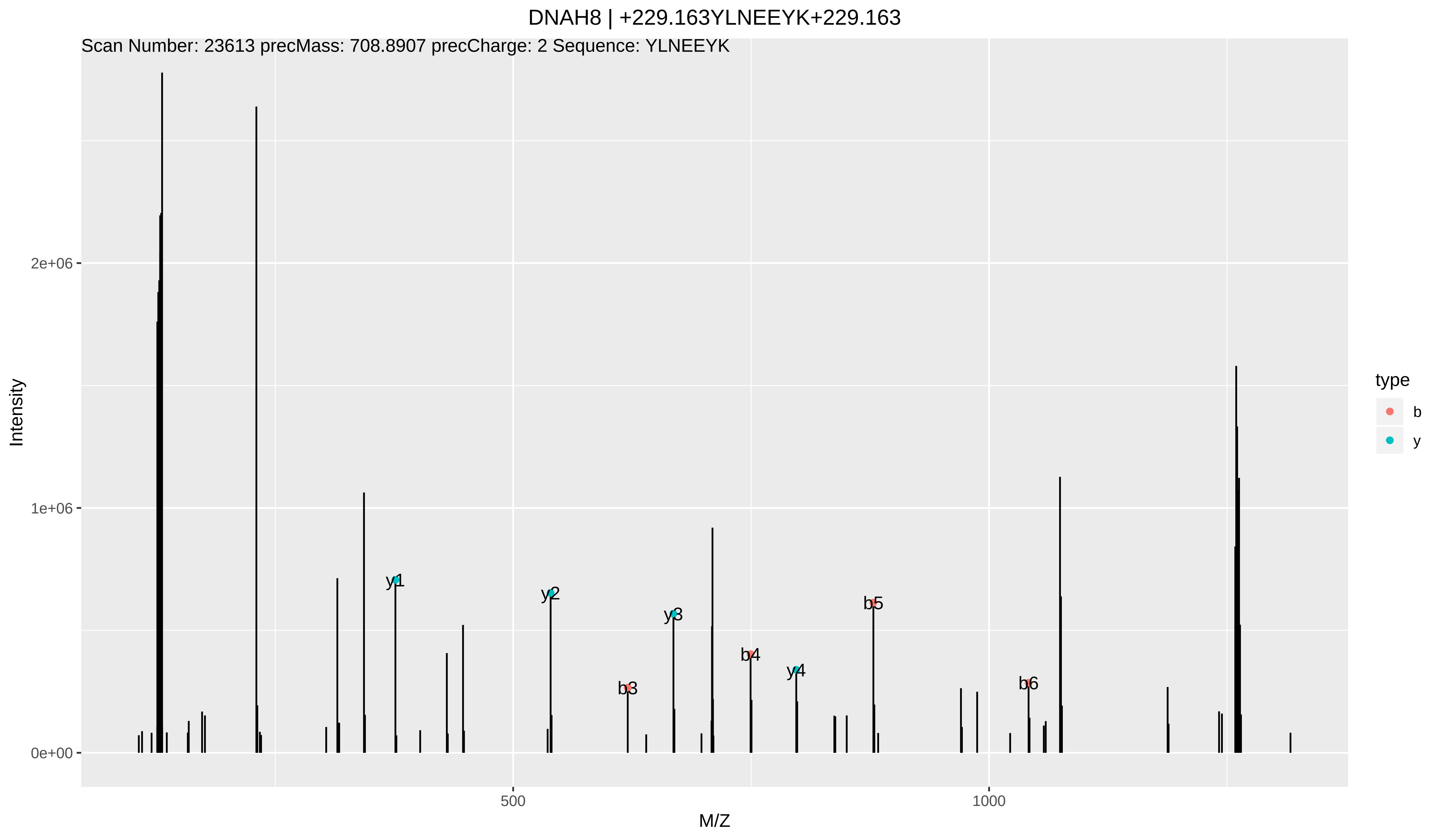
DNAH8 | +229.163YLNEEYK+229.163
Scan Number: 23613 precMass: 708.8907 precCharge: 2 Sequence: YLNEEYK
Intensity
2e+06
1e+06
0e+00
M/Z
500
1000
y1
y2
b3
y3
b4
y4
b5
b6
type
b
y

DNAH9 | +229.163ALAEYLDTK+229.163

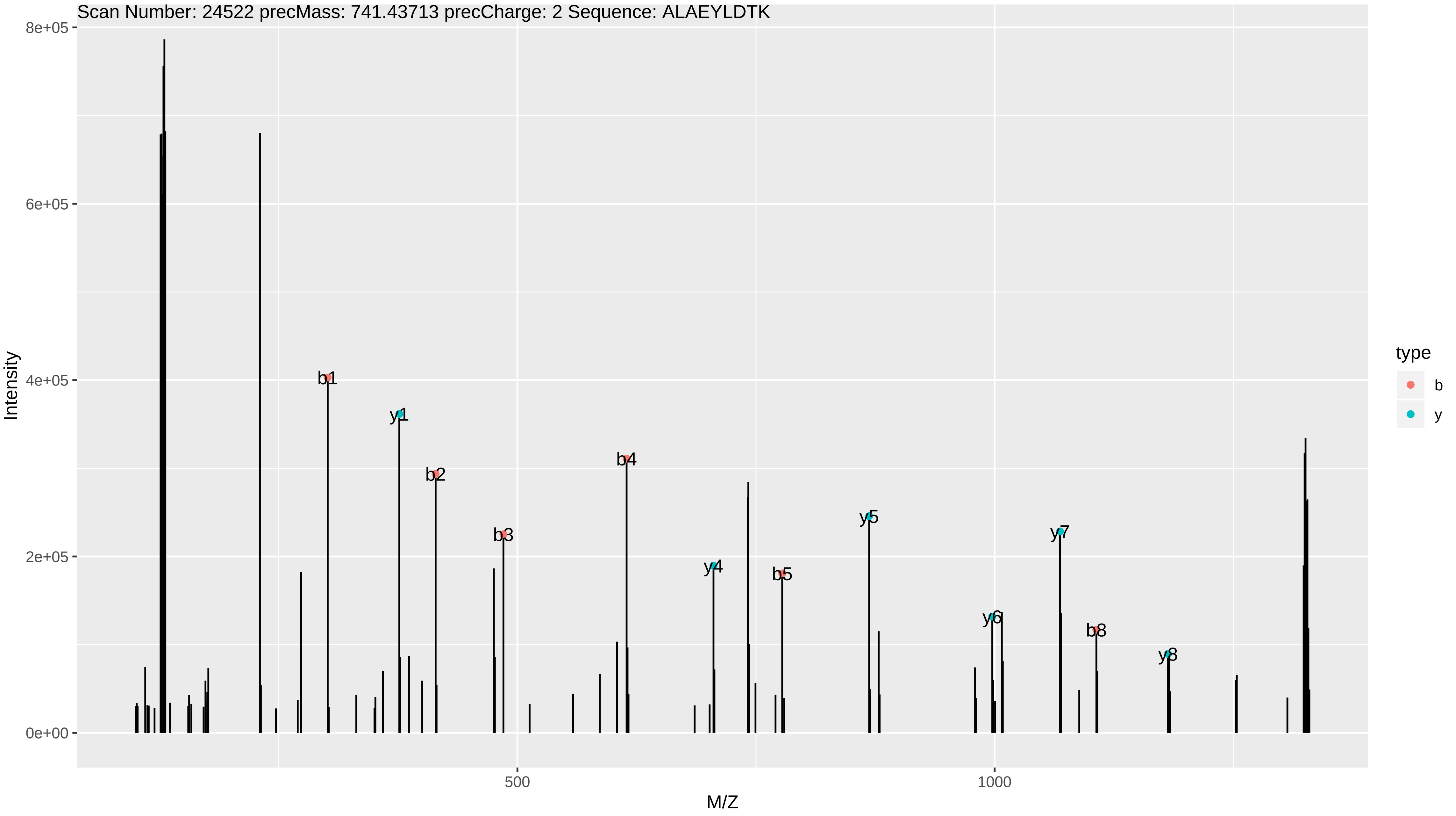

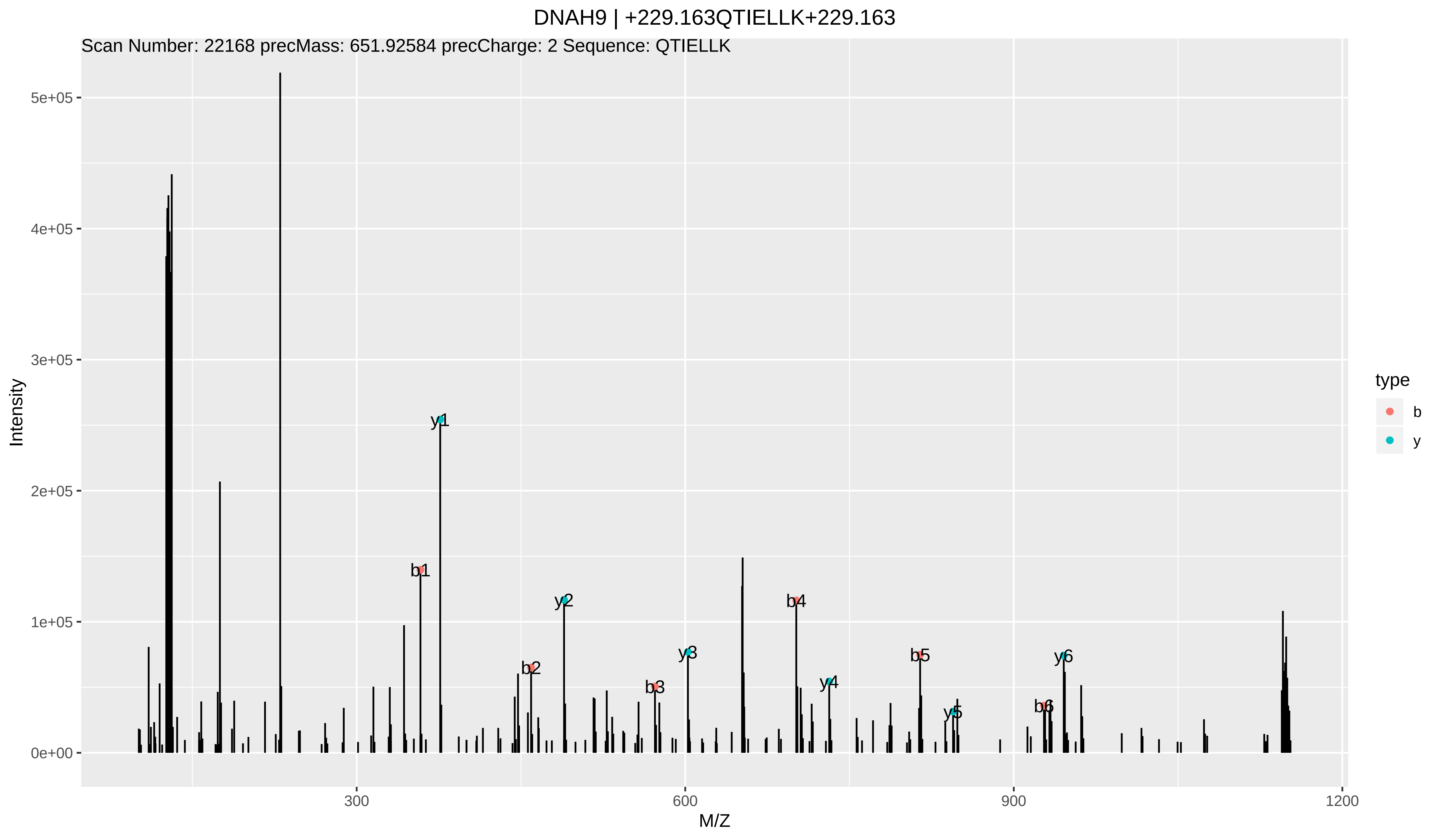
DNAH9 | +229.163QTIELLK+229.163
Scan Number: 22168 precMass: 651.92584 precCharge: 2 Sequence: QTIELLK
Intensity
5e+05
4e+05
3e+05
2e+05
1e+05
0e+00
300
600
900
1200
M/Z
y1
b1
y2
b2
b3
y3
b4
y4
b5
y5
b6
y6
type
b
y

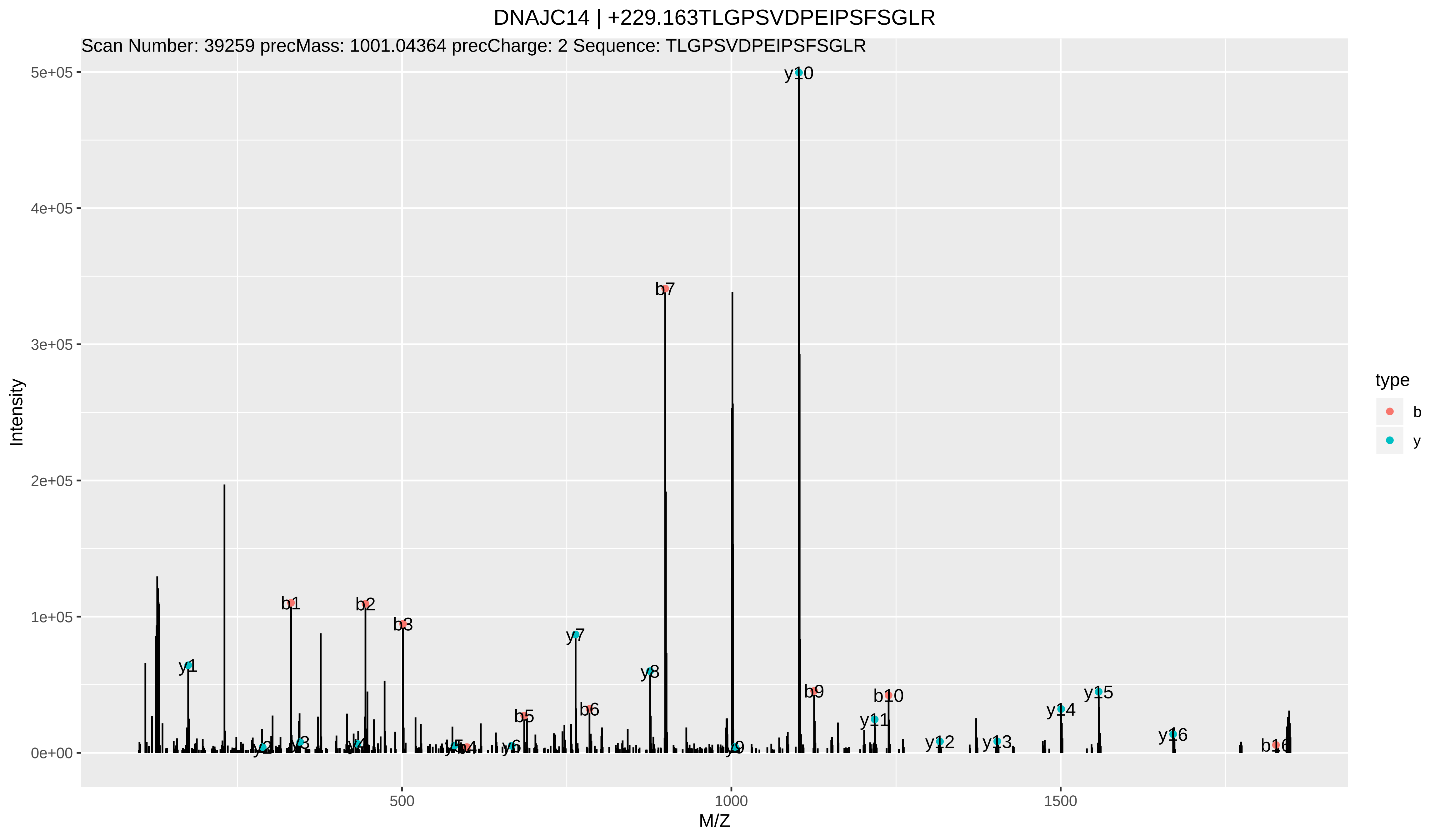
DNAJC14 | +229.163TLGPSVDPEIPSFSGLR
Scan Number: 39259 precMass: 1001.04364 precCharge: 2 Sequence: TLGPSVDPEIPSFSGLR
Intensity
M/Z
0e+00
1e+05
2e+05
3e+05
4e+05
5e+05
500
1000
1500
y10
b7
b1
b2
b3
y1
y7
y8
b9
b10
y11
y14
y15
y16
b5
b6
y12
y13
b16
y2
y3
y4
y5
b4
y6
y9
type
b
y

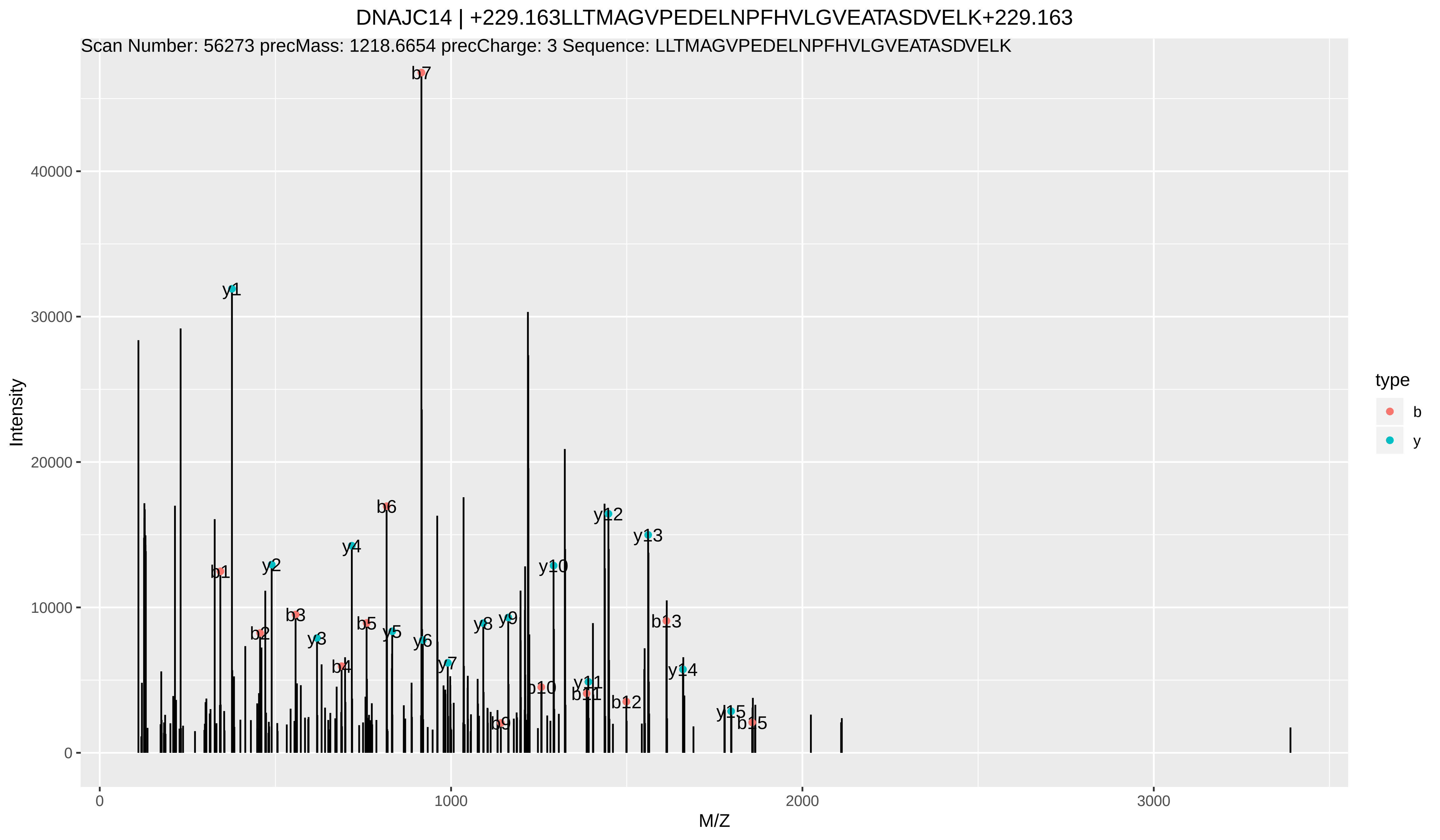

DNAJC14 | +229.163LLTMAGVPEDELNPFHVLGVEATASDVELK+229.163
Scan Number: 56273 precMass: 1218.6654 precCharge: 3 Sequence: LLTMAGVPEDELNPFHVLGVEATASDVELK
Intensity
M/Z
type
b
y
b7
y1
b6
y12
y13
y4
y2
b1
y10
b3
b5
b2
y5
y3
y6
b13
y8
y9
b4
y7
y14
b10
y11
b11
b12
y15
b9
b15
0
10000
20000
30000
40000
1000
2000
3000

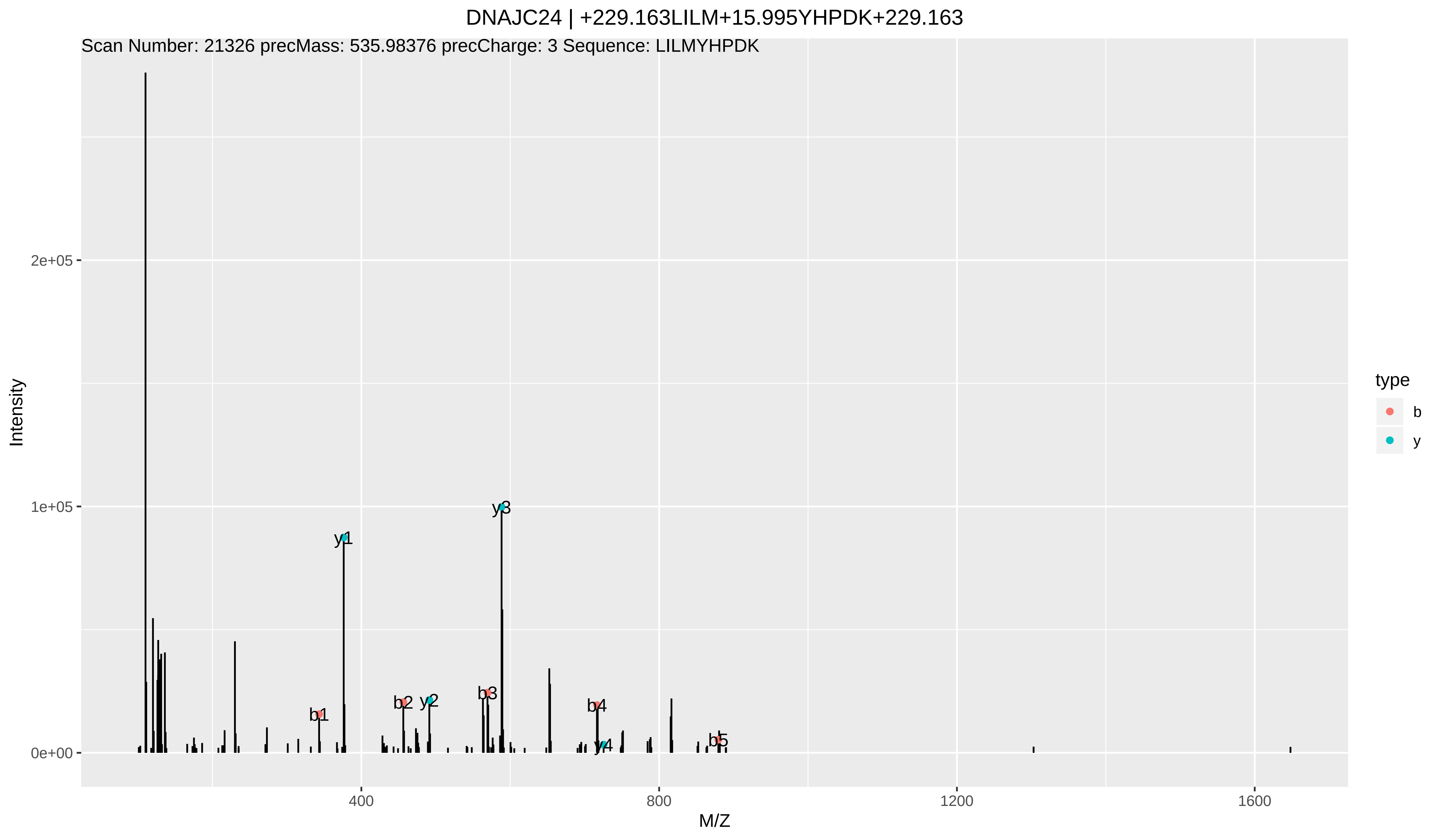

DNAJC24 | +229.163LILM+15.995YHPDK+229.163
Scan Number: 21326 precMass: 535.98376 precCharge: 3 Sequence: LILMYHPDK
Intensity
2e+05
1e+05
0e+00
M/Z
400
800
1200
1600
y1
y2
y3
y4
b1
b2
b3
b4
b5
type
b
y

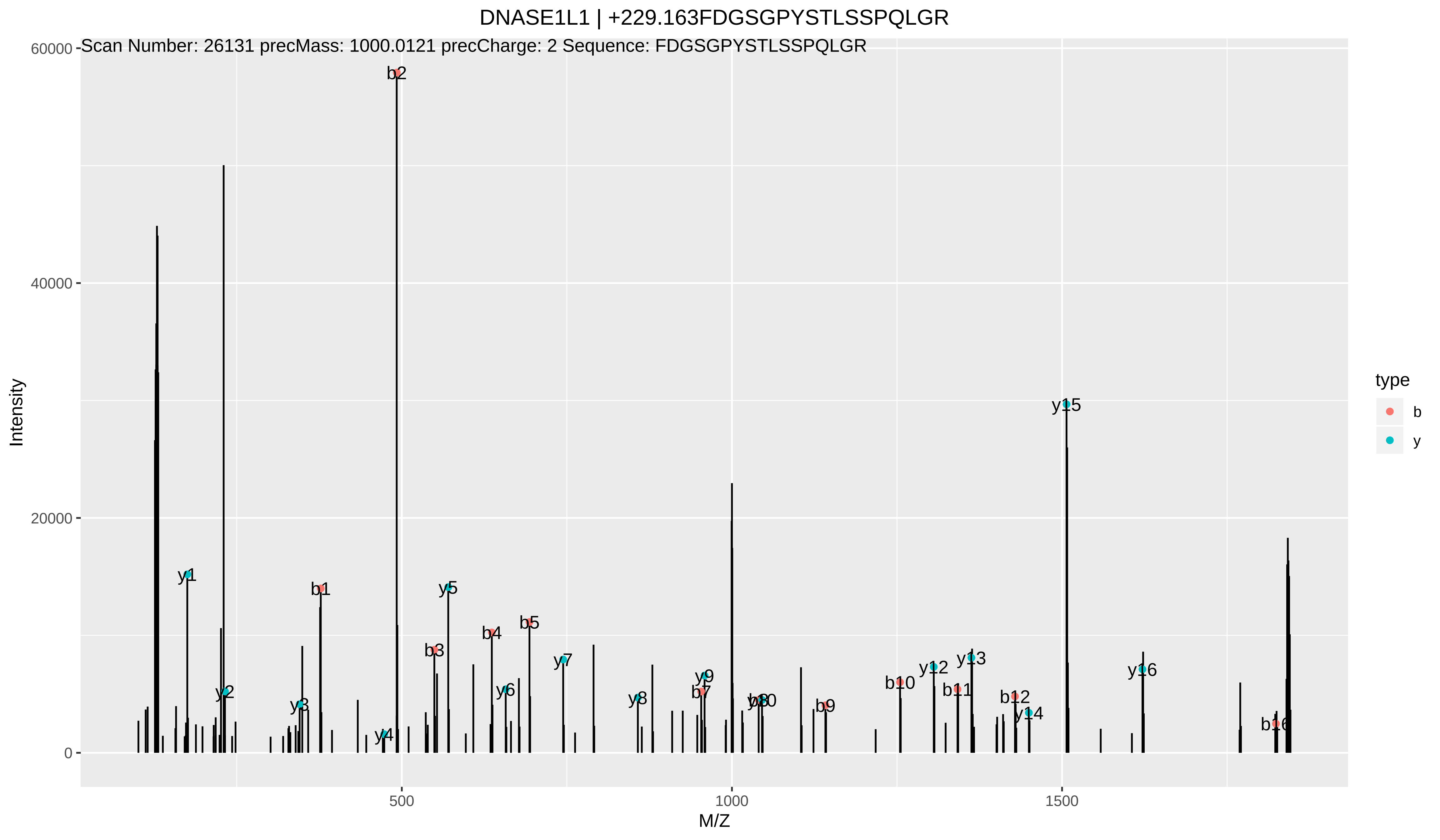

DNASE1L1 | +229.163FDGSGPYSTLSSPQLGR
Scan Number: 26131 precMass: 1000.0121 precCharge: 2 Sequence: FDGSGPYSTLSSPQLGR
Intensity
M/Z
type
b
y

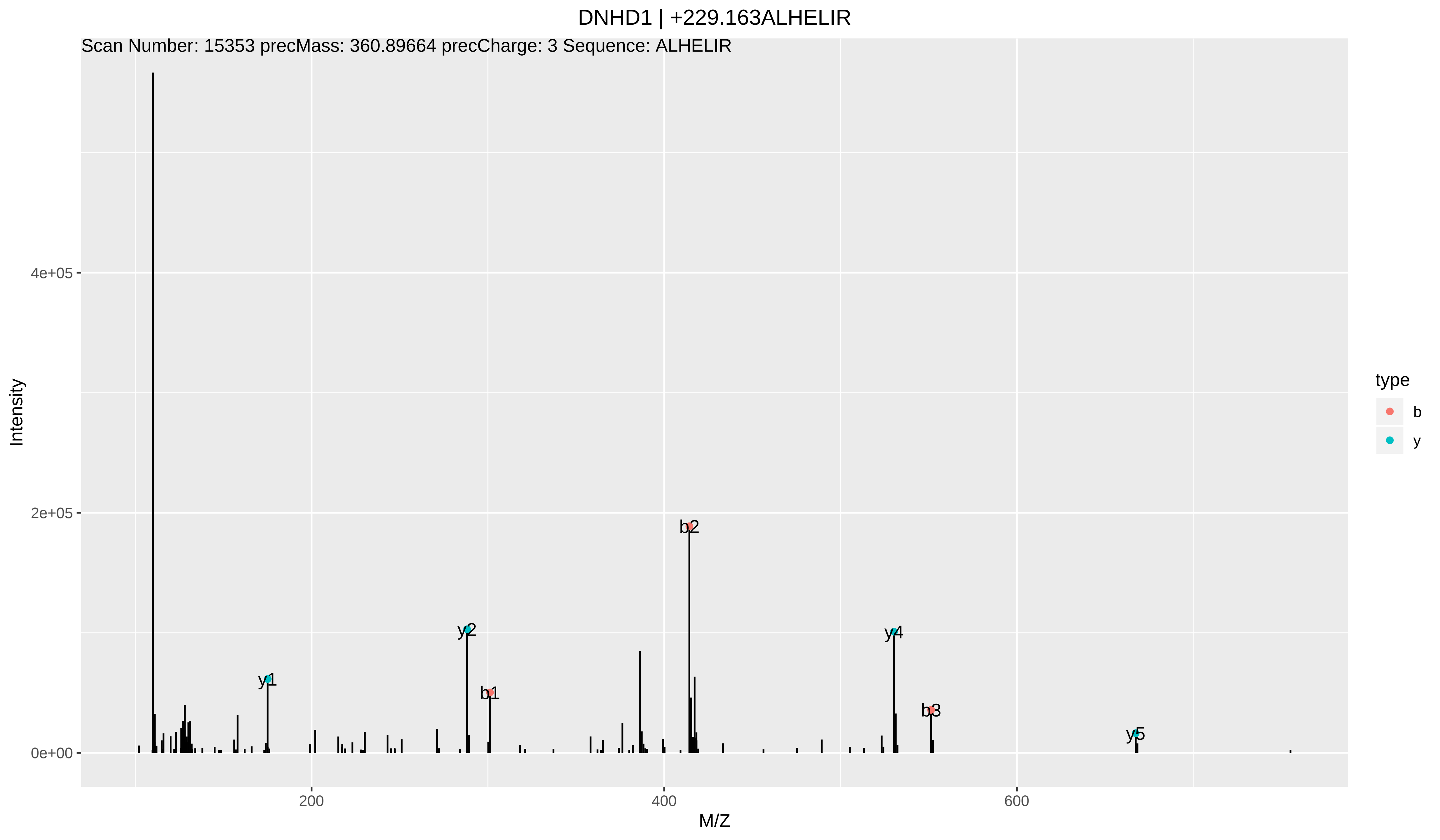
DNHD1 | +229.163ALHELIR
Scan Number: 15353 precMass: 360.89664 precCharge: 3 Sequence: ALHELIR
Intensity
4e+05
2e+05
0e+00
M/Z
200
400
600
y1
y2
b1
b2
y4
b3
y5
type
b
y

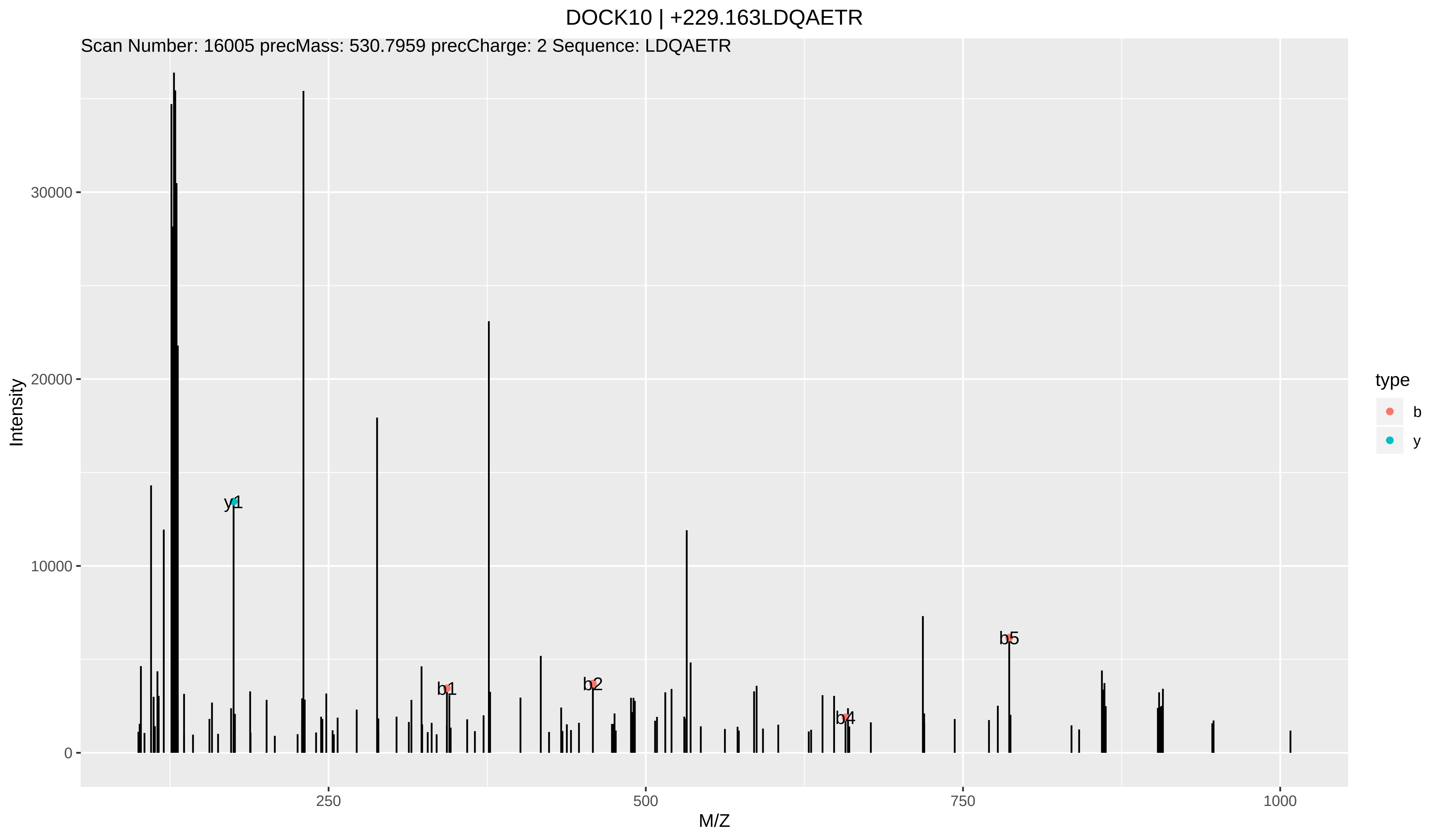

DOCK10 | +229.163LDQAETR
Scan Number: 16005 precMass: 530.7959 precCharge: 2 Sequence: LDQAETR
Intensity
30000
20000
10000
0
250
500
750
1000
M/Z
y1
b1
b2
b4
b5
type
b
y

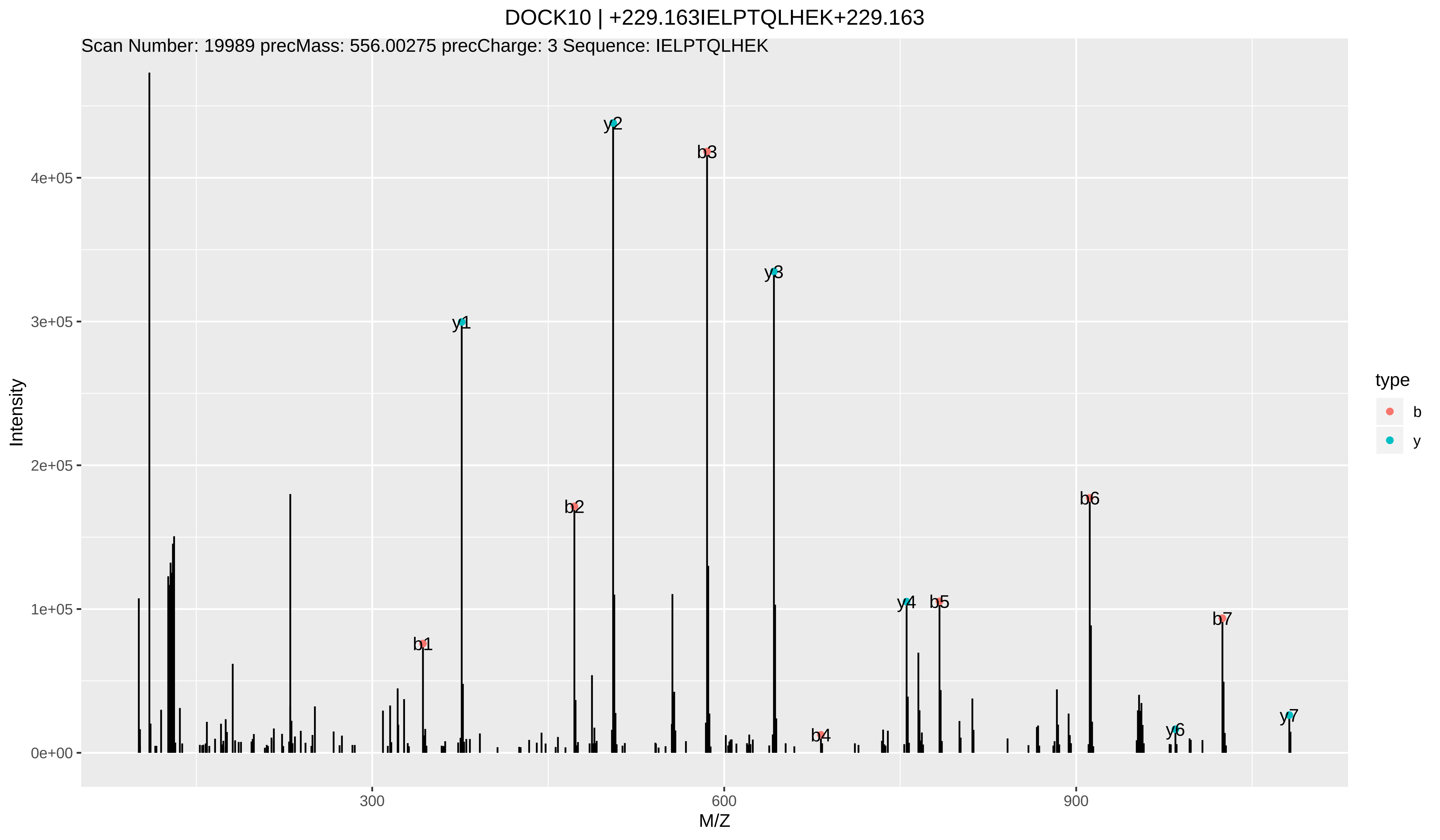

DOCK10 | +229.163IELPTQLHEK+229.163
Scan Number: 19989 precMass: 556.00275 precCharge: 3 Sequence: IELPTQLHEK
Intensity
M/Z
0e+00
1e+05
2e+05
3e+05
4e+05
300
600
900
y2
b3
y3
y1
b2
b6
b1
y4
b5
b7
b4
y6
y7
type
b
y

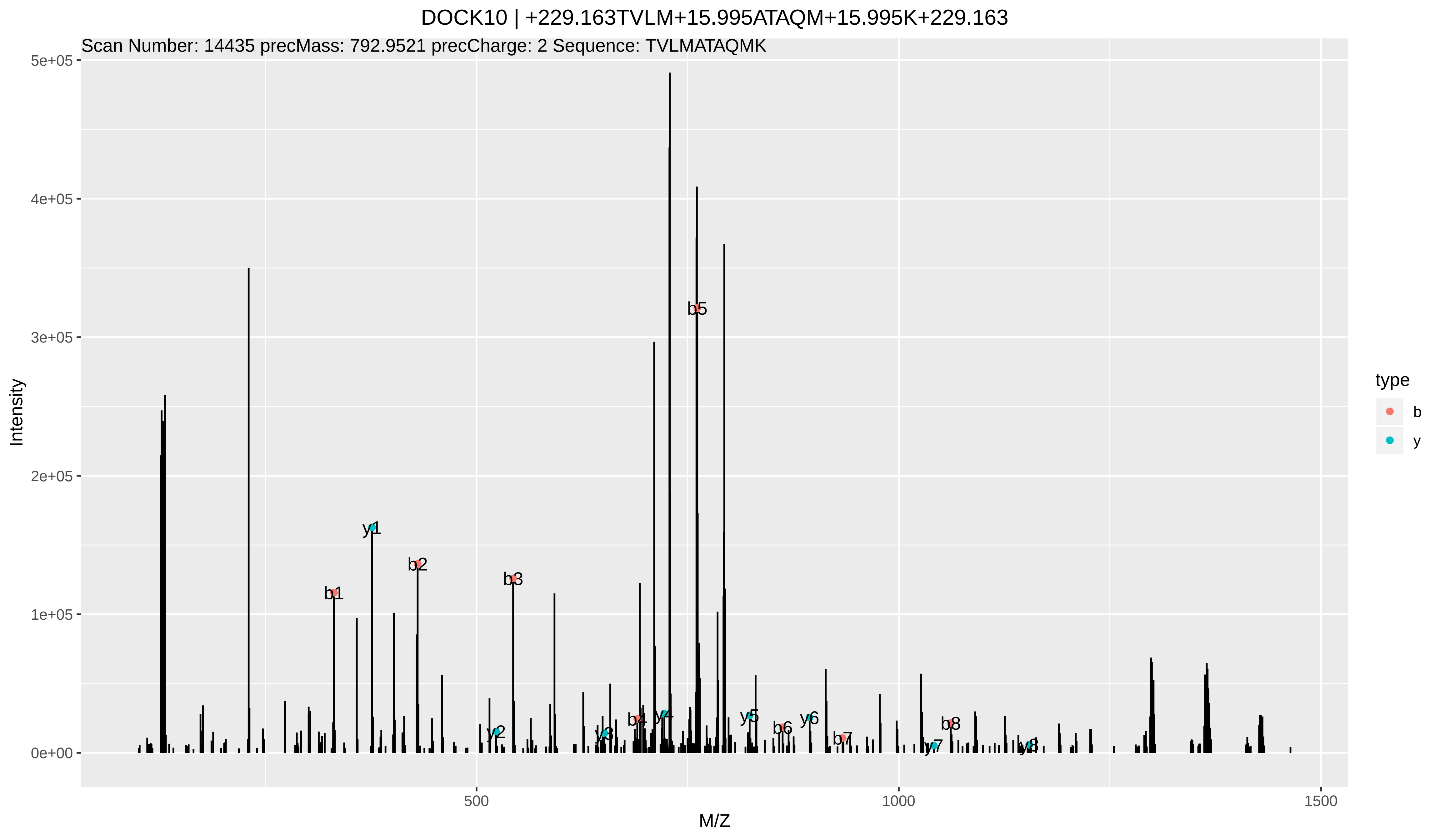

DOCK10 | +229.163TVLM+15.995ATAQM+15.995K+229.163
Scan Number: 14435 precMass: 792.9521 precCharge: 2 Sequence: TVLMATAQMK
Intensity
5e+05
4e+05
3e+05
2e+05
1e+05
0e+00
M/Z
500
1000
1500
b1
b2
b3
b4
b5
b6
b7
b8
y1
y2
y3
y4
y5
y6
y7
y8
type
b
y

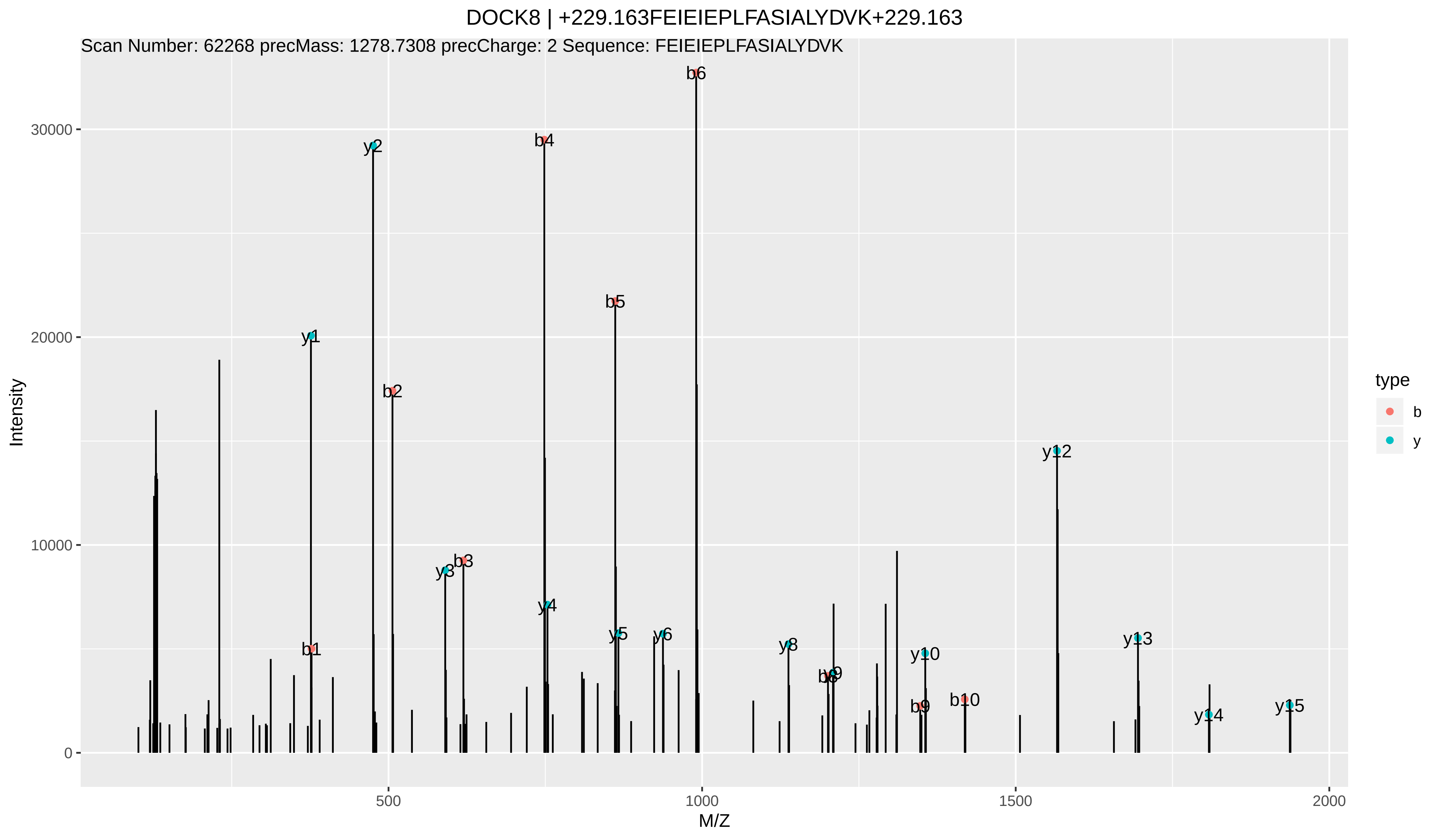

DOCK8 | +229.163FEIEIEPLFASIALYDVK+229.163
Scan Number: 62268 precMass: 1278.7308 precCharge: 2 Sequence: FEIEIEPLFASIALYDVK
Intensity
M/Z
type
b
y
b6
y2
b4
b5
y1
b2
y12
y3
b3
y4
y5
y6
y8
b1
b8
y9
y10
y13
b9
b10
y14
y15
0
10000
20000
30000
500
1000
1500
2000

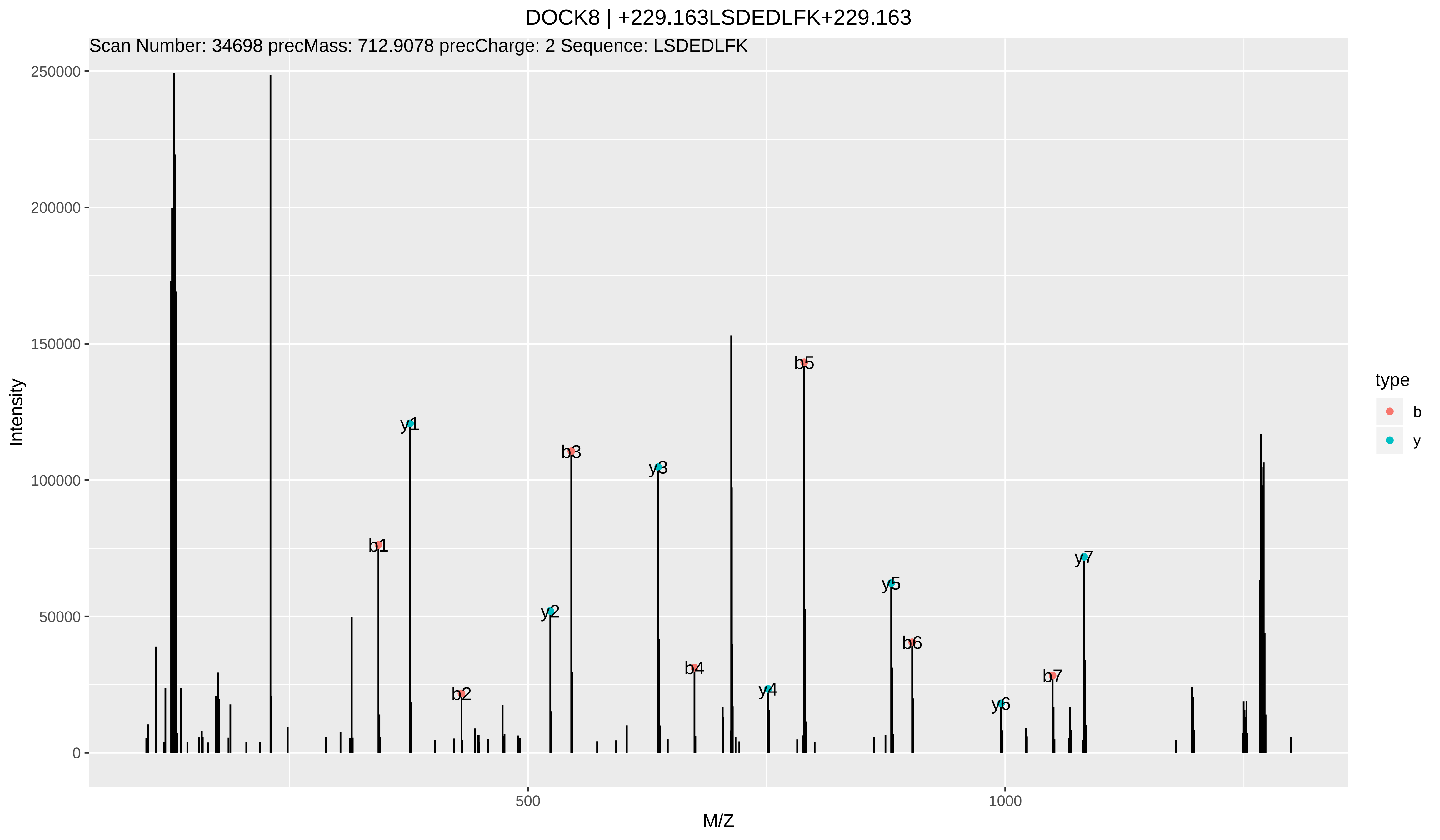

DOCK8 | +229.163LSDEDLFK+229.163
Scan Number: 34698 precMass: 712.9078 precCharge: 2 Sequence: LSDEDLFK
Intensity
250000
200000
150000
100000
50000
0
M/Z
500
1000
b1
y1
b2
y2
b3
y3
b4
y4
b5
y5
b6
y6
b7
y7
type
b
y

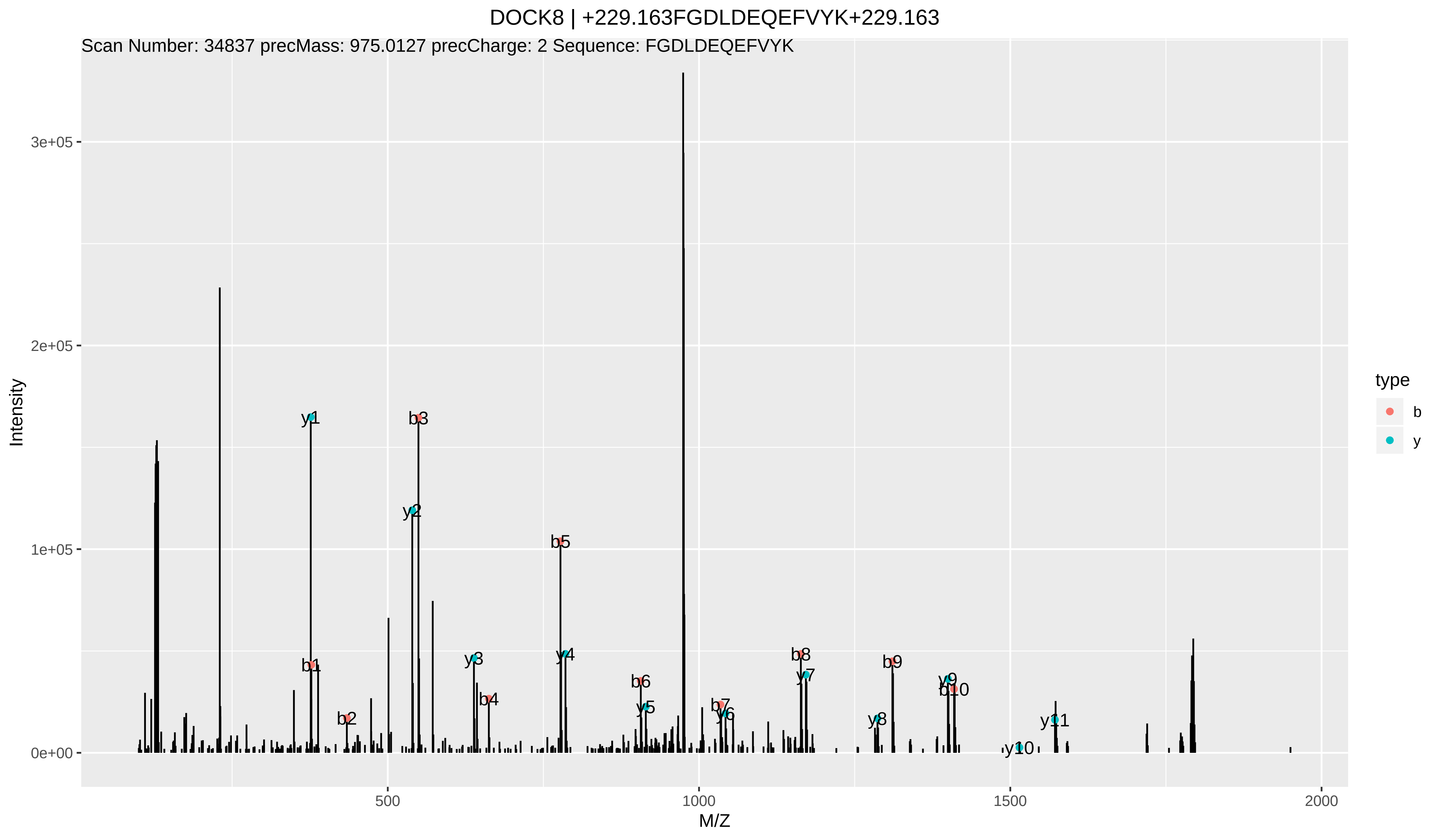

DOCK8 | +229.163FGDLDEQEFVYK+229.163
Scan Number: 34837 precMass: 975.0127 precCharge: 2 Sequence: FGDLDEQEFVYK
Intensity
3e+05
2e+05
1e+05
0e+00
M/Z
500
1000
1500
2000
y1
b3
y2
b5
b1
b2
y3
b4
y4
b6
y5
b7
y6
b8
y7
y8
b9
y9
b10
y10
y11
type
b
y

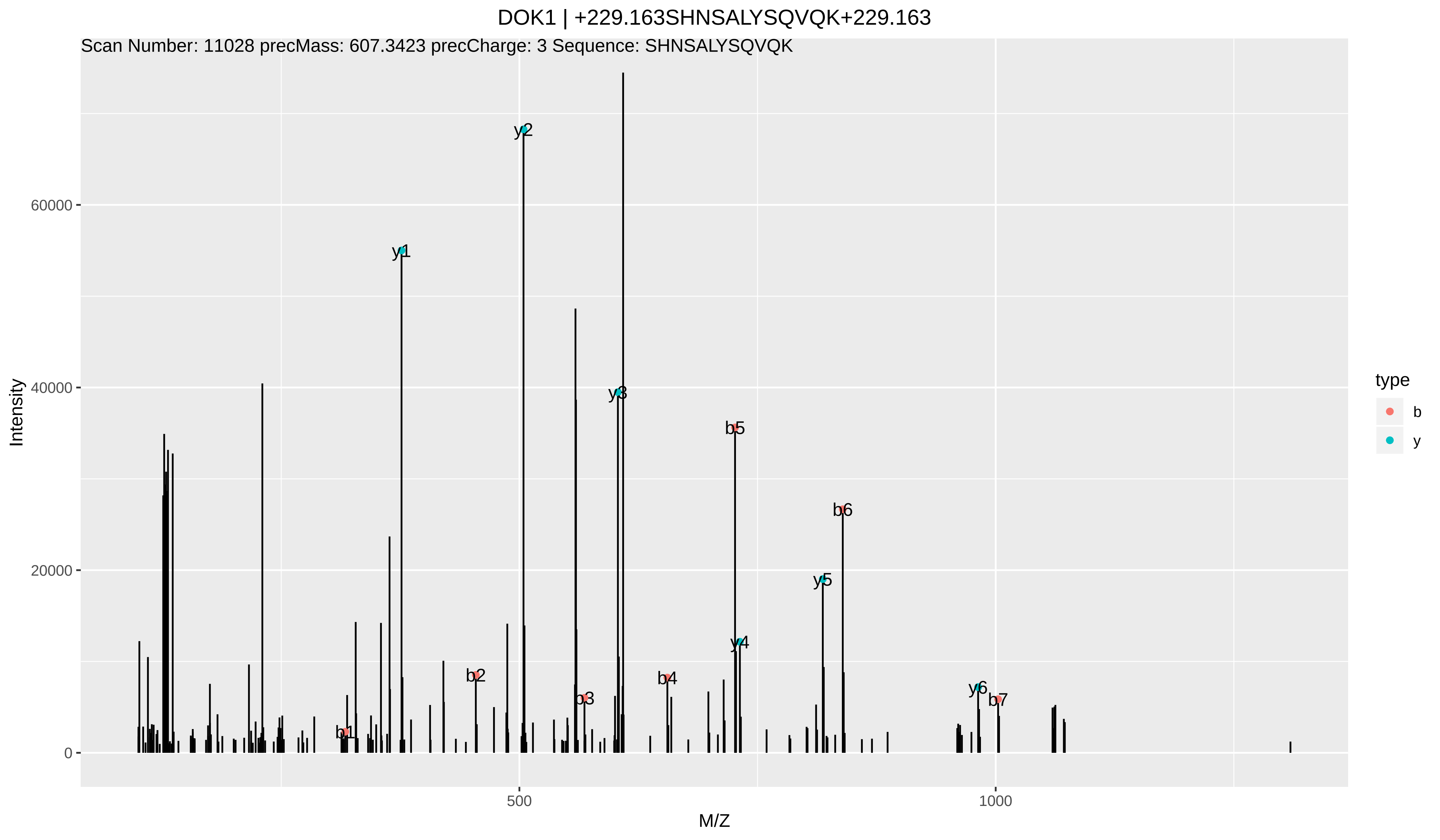
DOK1 | +229.163SHNSALYSQVQK+229.163
Scan Number: 11028 precMass: 607.3423 precCharge: 3 Sequence: SHNSALYSQVQK
Intensity
M/Z
0
20000
40000
60000
500
1000
y1
y2
y3
y4
y5
y6
b1
b2
b3
b4
b5
b6
b7
type
b
y

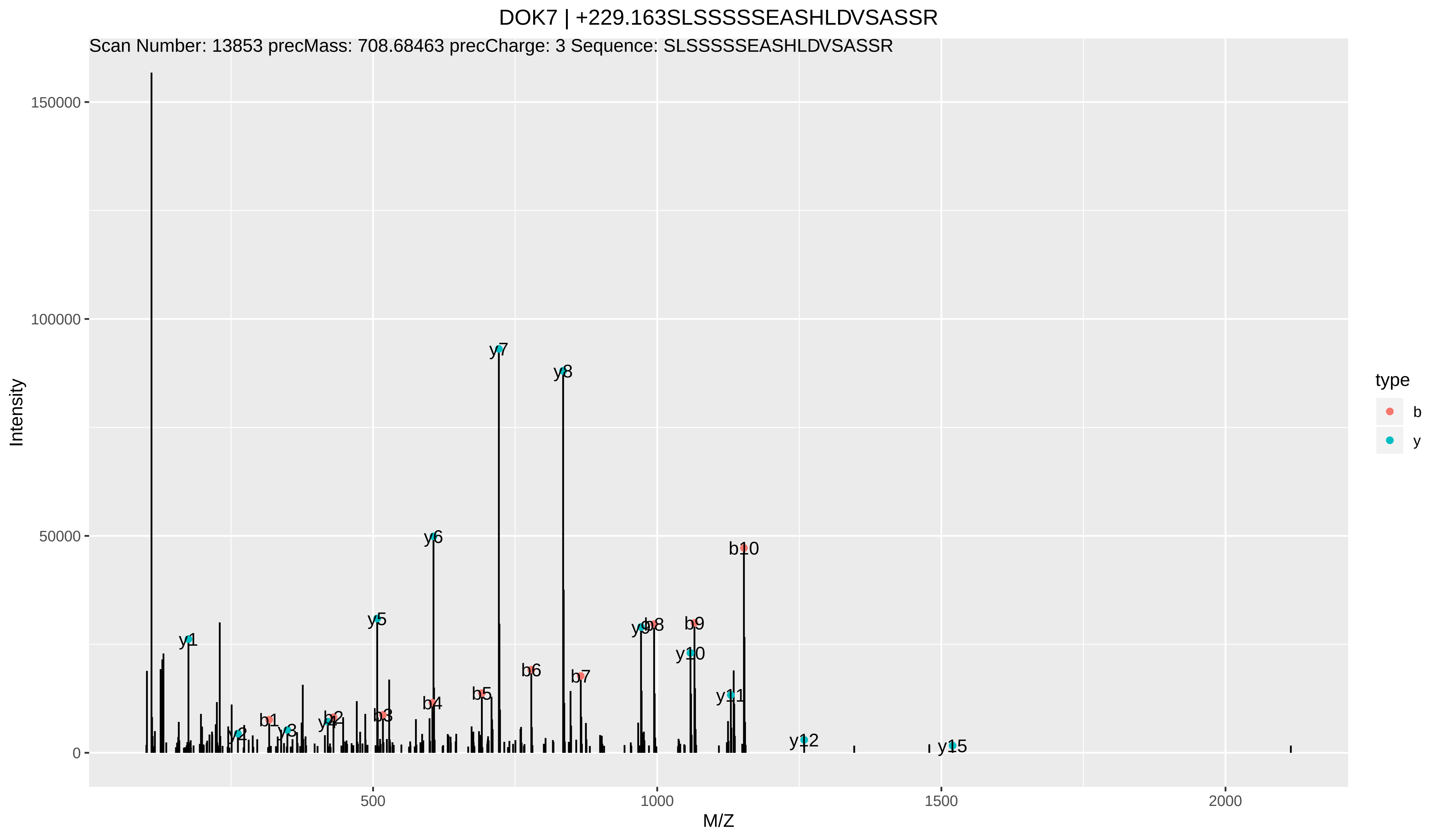
DOK7 | +229.163SLSSSSSEASHLDVSASSR
Scan Number: 13853 precMass: 708.68463 precCharge: 3 Sequence: SLSSSSSEASHLDVSASSR
Intensity
M/Z
0
50000
100000
150000
500
1000
1500
2000
type
b
y
y1
y2
y3
y4
y5
y6
y7
y8
y9
y10
y11
y12
y15
b1
b2
b3
b4
b5
b6
b7
b8
b9
b10

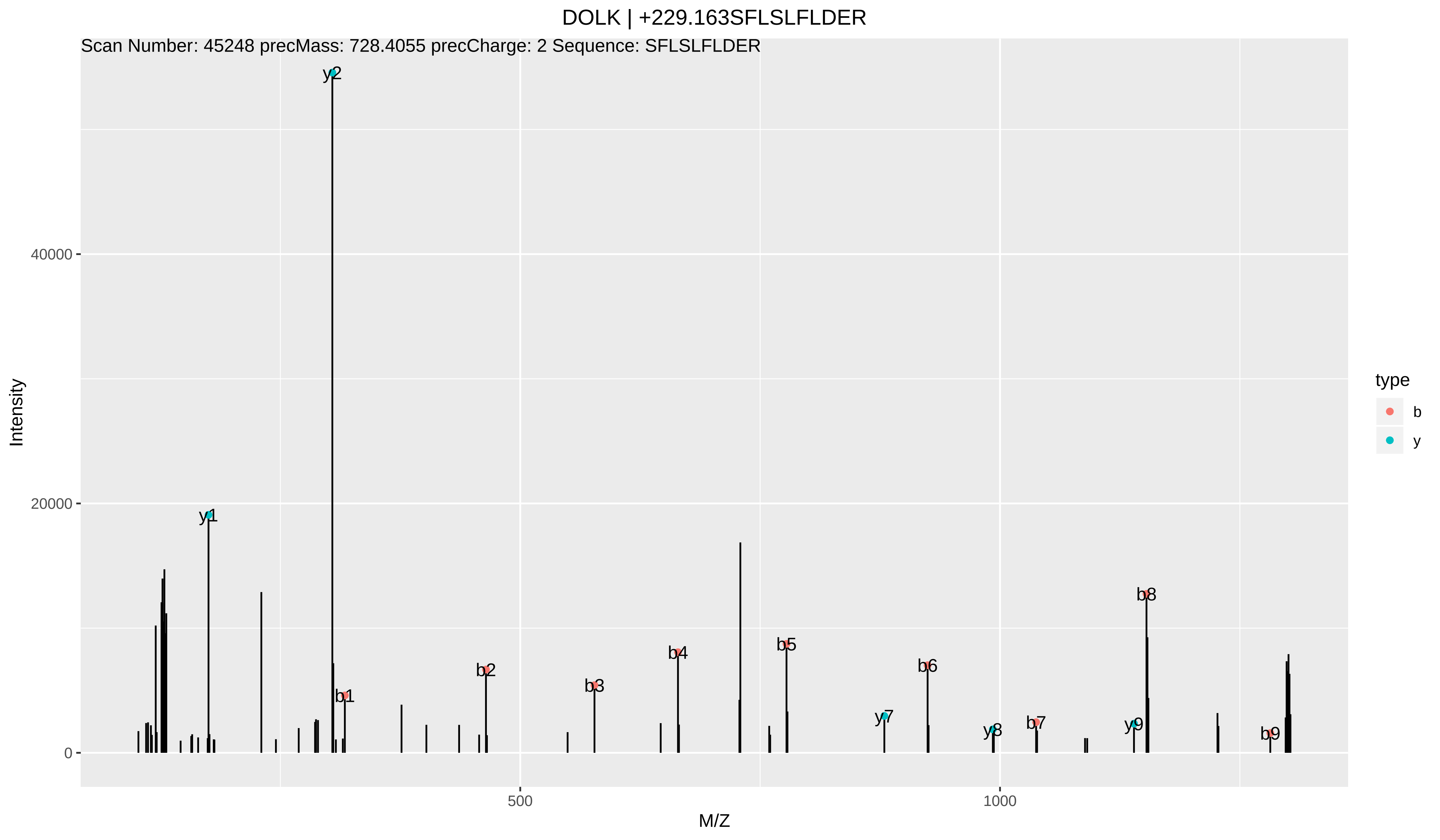
DOLK | +229.163SFLSLFLDER
Scan Number: 45248 precMass: 728.4055 precCharge: 2 Sequence: SFLSLFLDER
Intensity
40000
20000
0
M/Z
500
1000
y2
y1
b1
b2
b3
b4
b5
y7
b6
y8
b7
y9
b8
b9
type
b
y

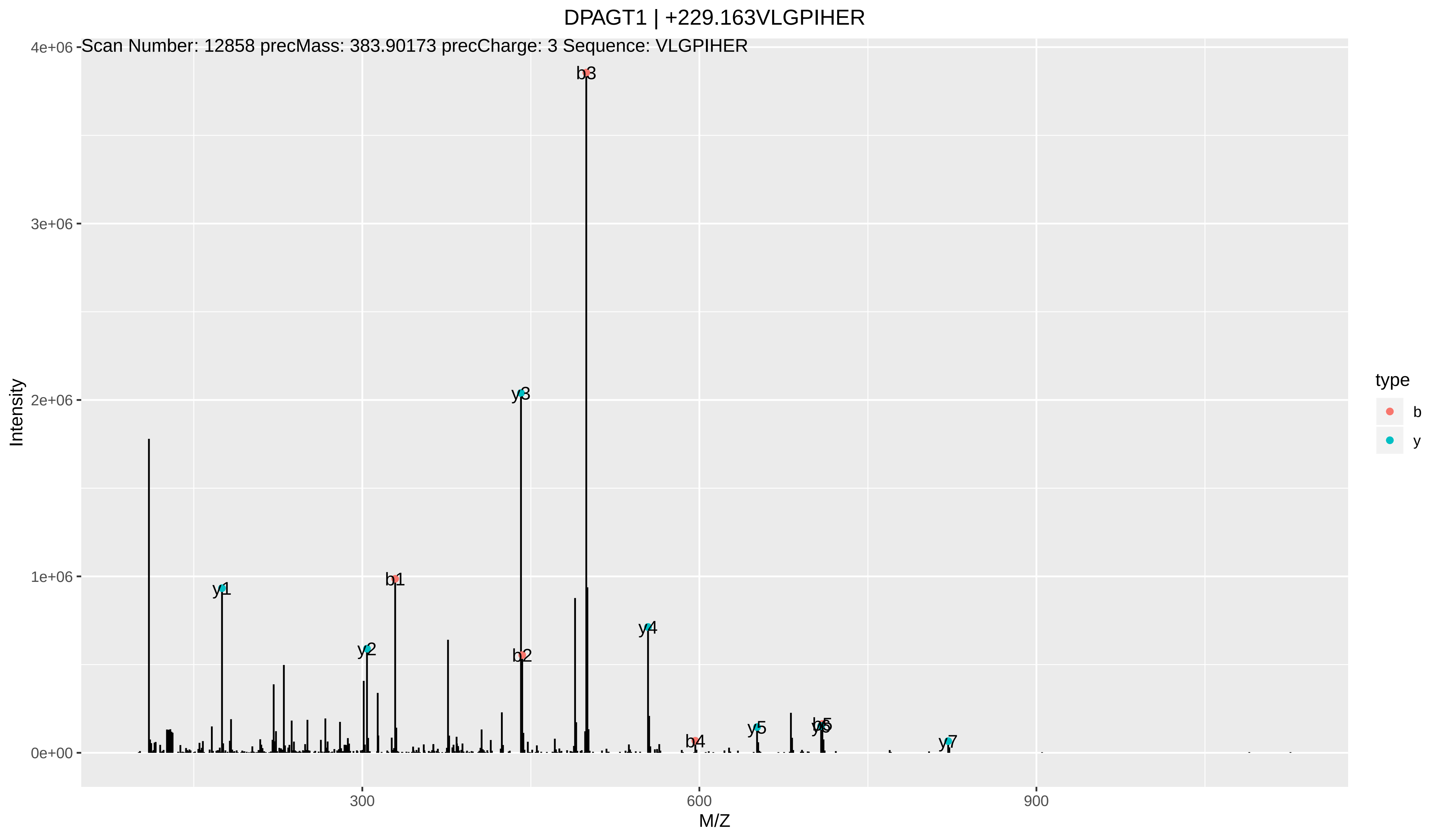

DPAGT1 | +229.163VLGPIHER
Scan Number: 12858 precMass: 383.90173 precCharge: 3 Sequence: VLGPIHER
Intensity
M/Z
4e+06
3e+06
2e+06
1e+06
0e+00
300
600
900
b3
y3
y1
b1
y2
b2
y4
b4
y5
b5
y6
y7
type
b
y

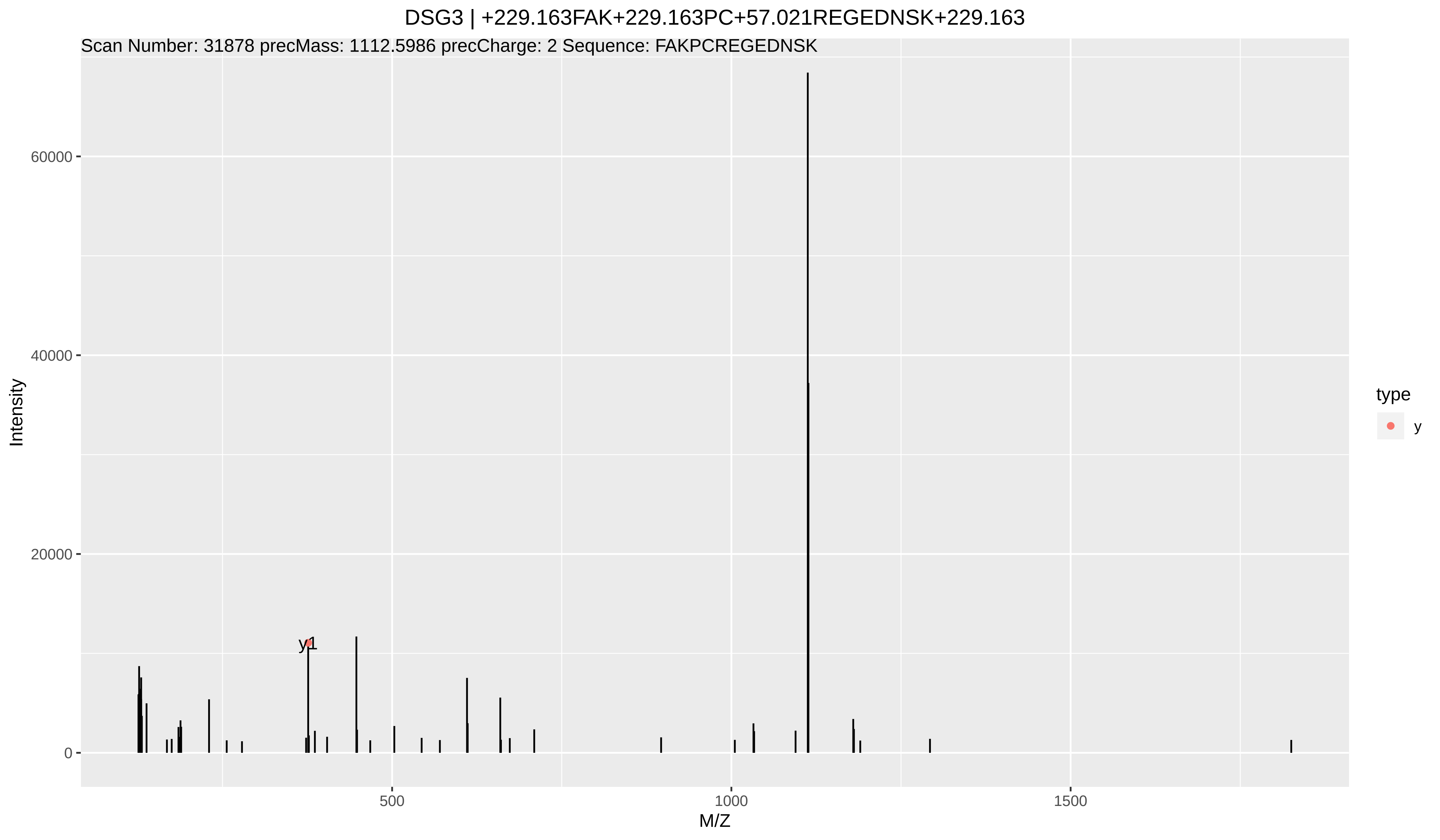

DSG3 | +229.163FAK+229.163PC+57.021REGEDNSK+229.163
Scan Number: 31878 precMass: 1112.5986 precCharge: 2 Sequence: FAKPCREGEDNSK
Intensity
60000
40000
20000
0
500
1000
1500
M/Z
y1
type
y

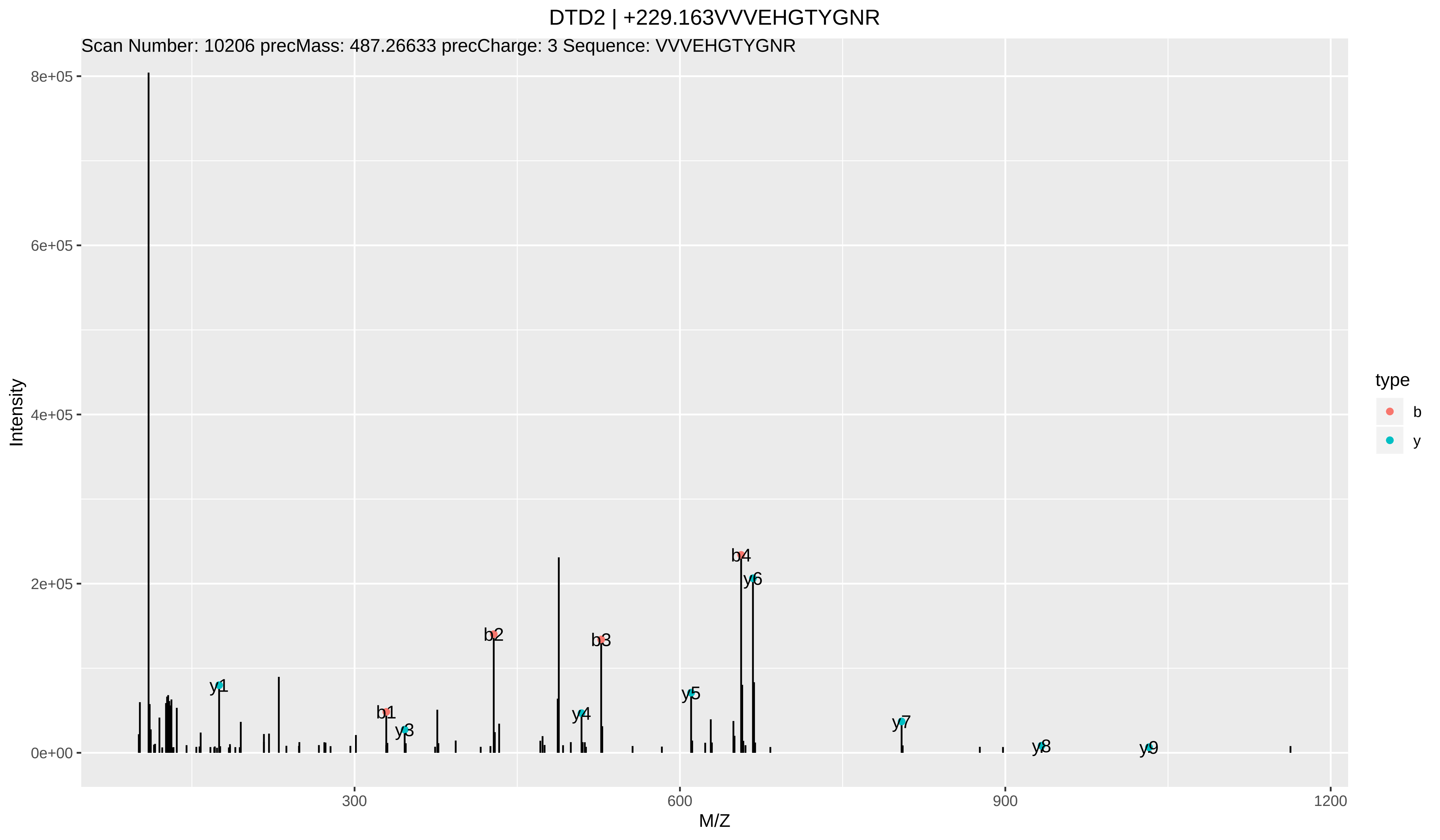

DTD2 | +229.163VVVEHGTYGNR
Scan Number: 10206 precMass: 487.26633 precCharge: 3 Sequence: VVVEHGTYGNR
8e+05
6e+05
4e+05
2e+05
0e+00
Intensity
300
600
900
1200
M/Z
y1
b1
y3
b2
y4
b3
y5
b4
y6
y7
y8
y9
type
b
y

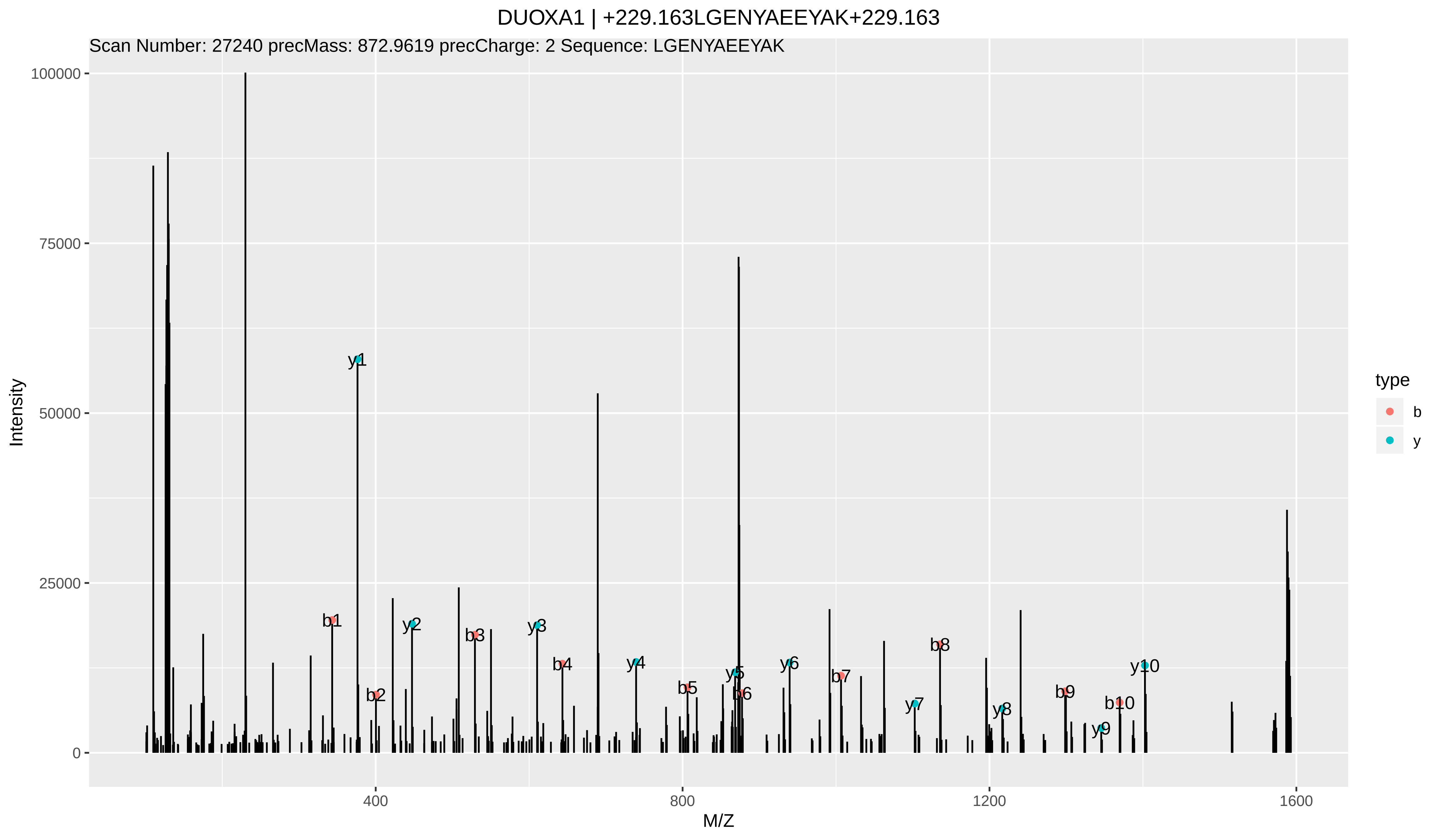

DUOXA1 | +229.163LGENYAEEYAK+229.163
Scan Number: 27240 precMass: 872.9619 precCharge: 2 Sequence: LGENYAEEYAK
Intensity
M/Z
0
25000
50000
75000
100000
400
800
1200
1600
y1
b1
y2
b2
b3
y3
b4
y4
b5
y5
b6
y6
b7
y7
b8
y8
b9
y9
b10
y10
type
b
y

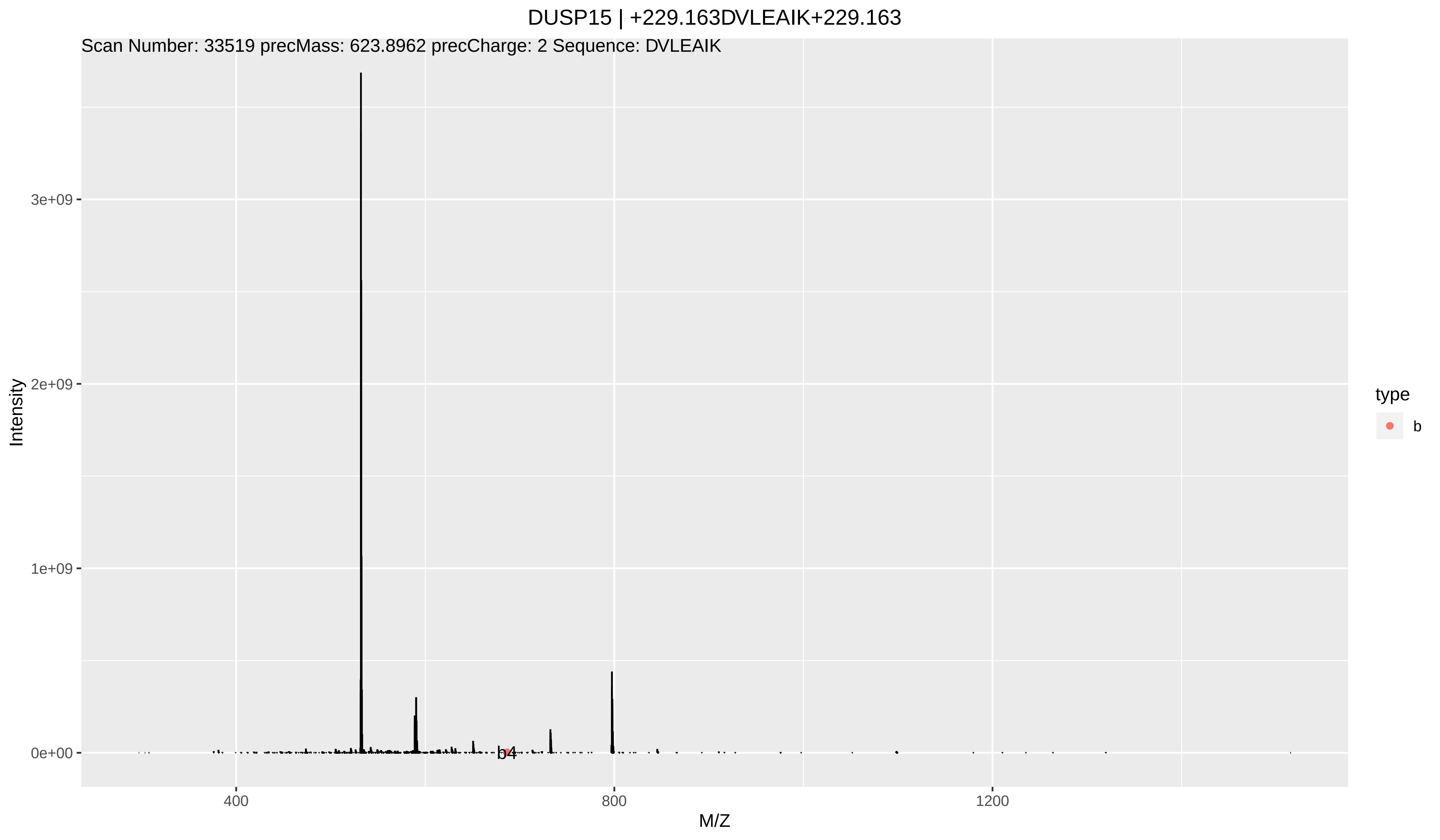

DUSP15 | +229.163DVLEAIK+229.163
Scan Number: 33519 precMass: 623.8962 precCharge: 2 Sequence: DVLEAIK
Intensity
3e+09
2e+09
1e+09
0e+00
b4
400
800
1200
M/Z
type
b

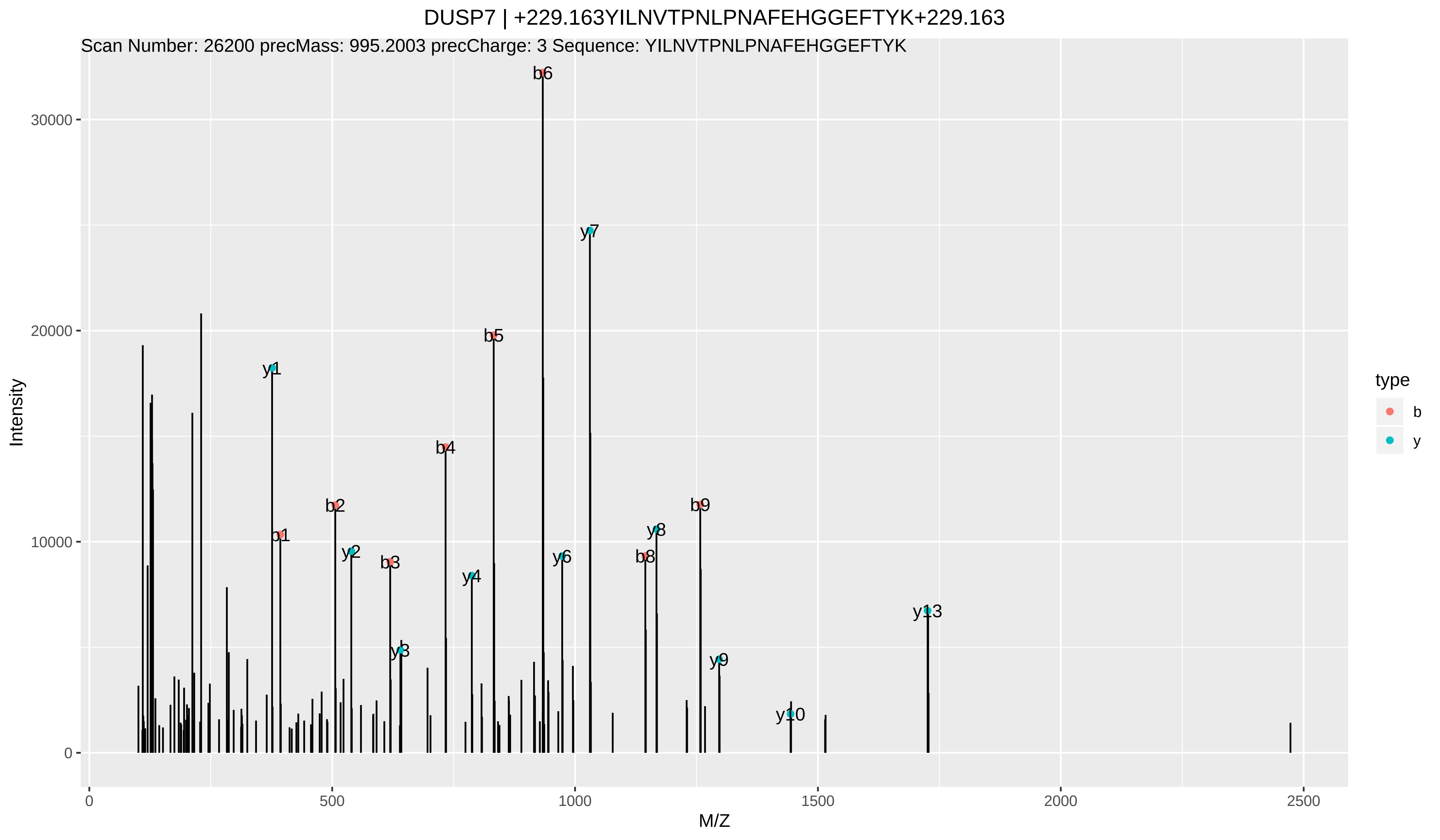
DUSP7 | +229.163YILNVTPNLPNAFEHGGEFTYK+229.163
Scan Number: 26200 precMass: 995.2003 precCharge: 3 Sequence: YILNVTPNLPNAFEHGGEFTYK
Intensity
M/Z
type
b
y
b6
y7
b5
y1
b4
b2
b1
y2
b3
y4
y6
b8
y8
b9
y13
y3
y9
y10
30000
20000
10000
0
0
500
1000
1500
2000
2500

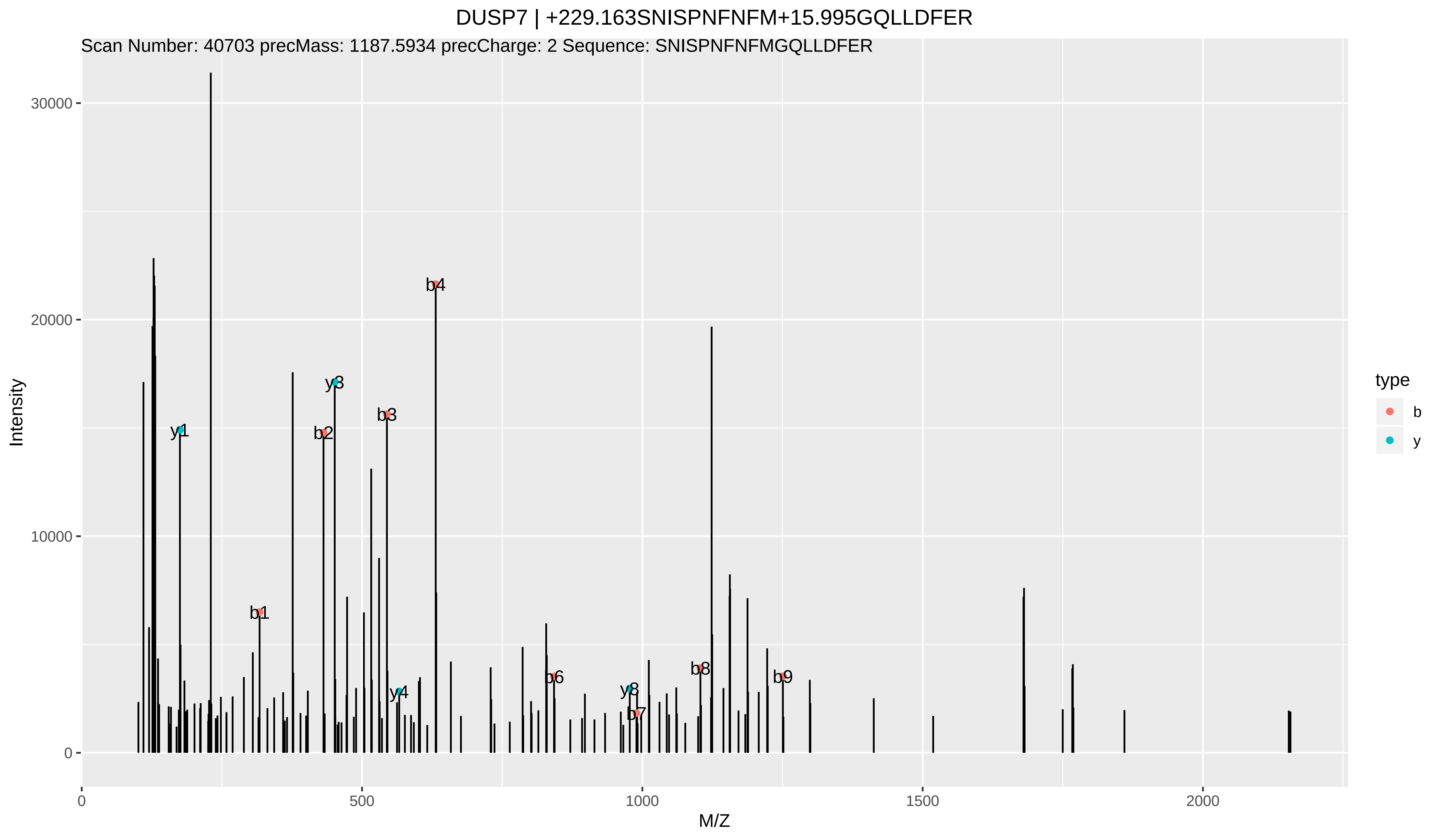

DUSP7 | +229.163SNISPNFNFM+15.995GQLLDFER
Scan Number: 40703 precMass: 1187.5934 precCharge: 2 Sequence: SNISPNFNFMGQLLDFER
Intensity
30000
20000
10000
0
0
500
1000
1500
2000
M/Z
y1
b1
b2
y3
b3
y4
b4
b6
y8
b7
b8
b9
type
b
y

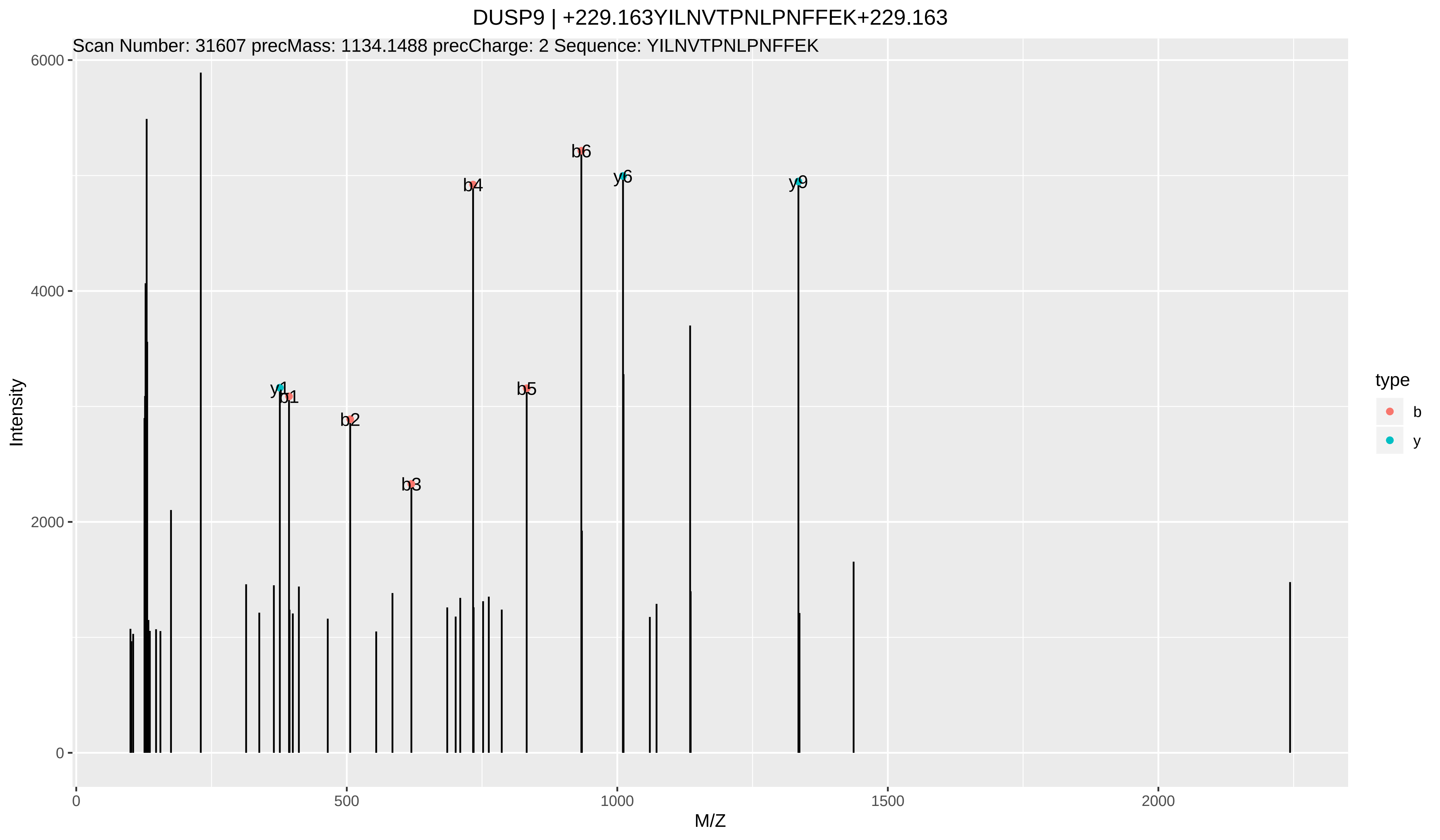
DUSP9 | +229.163YILNVTPNLPNFFEK+229.163
Scan Number: 31607 precMass: 1134.1488 precCharge: 2 Sequence: YILNVTPNLPNFFEK
Intensity
M/Z
0
2000
4000
6000
0
500
1000
1500
2000
y1
b1
b2
b3
b4
b5
b6
y6
y9
type
b
y

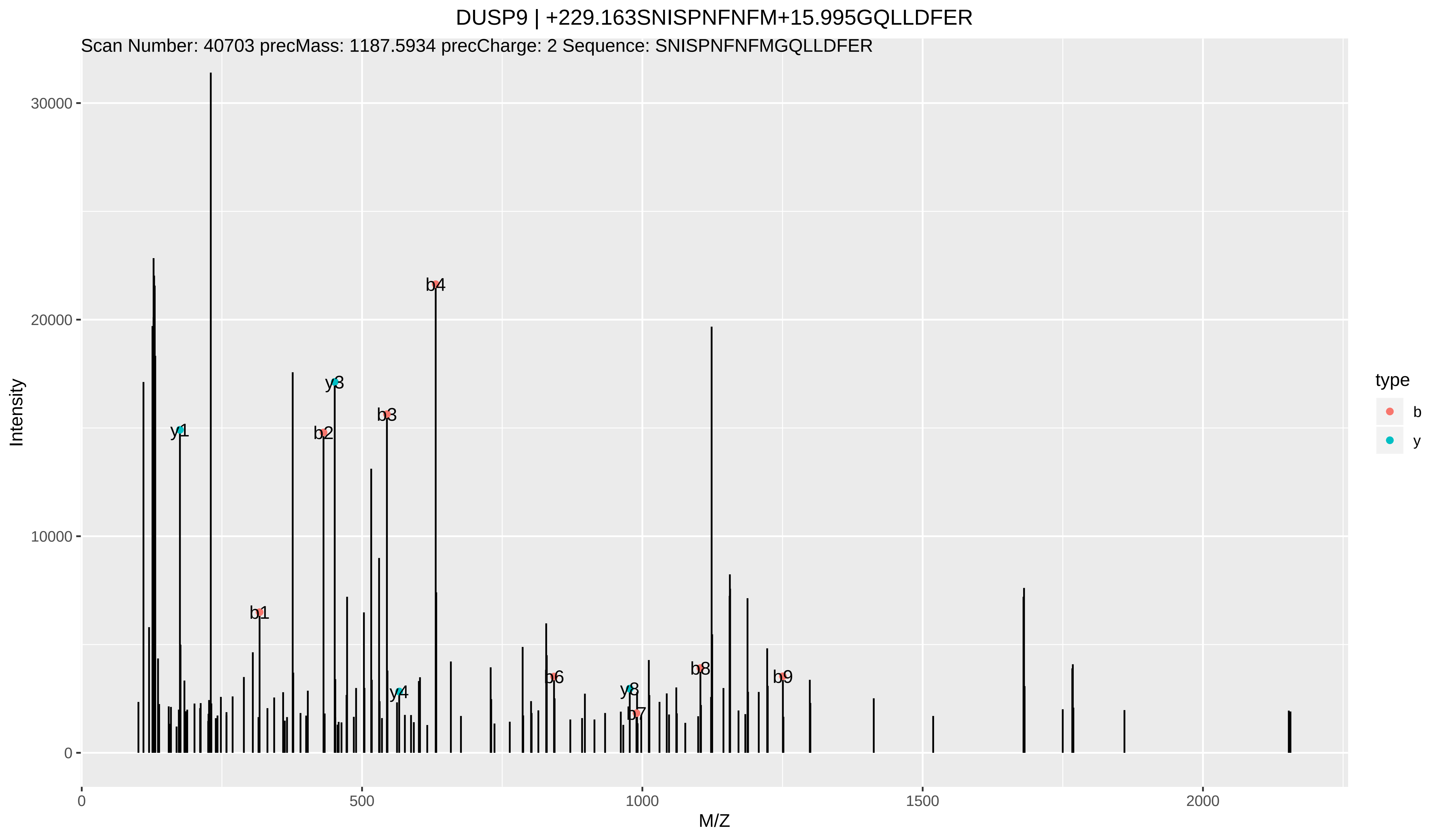
DUSP9 | +229.163SNISPNFNFM+15.995GQLLDFER
Scan Number: 40703 precMass: 1187.5934 precCharge: 2 Sequence: SNISPNFNFMGQLLDFER
Intensity
M/Z
type
b
y
y1
b1
b2
y3
b3
y4
b4
b6
y8
b7
b8
b9
0
10000
20000
30000
0
500
1000
1500
2000

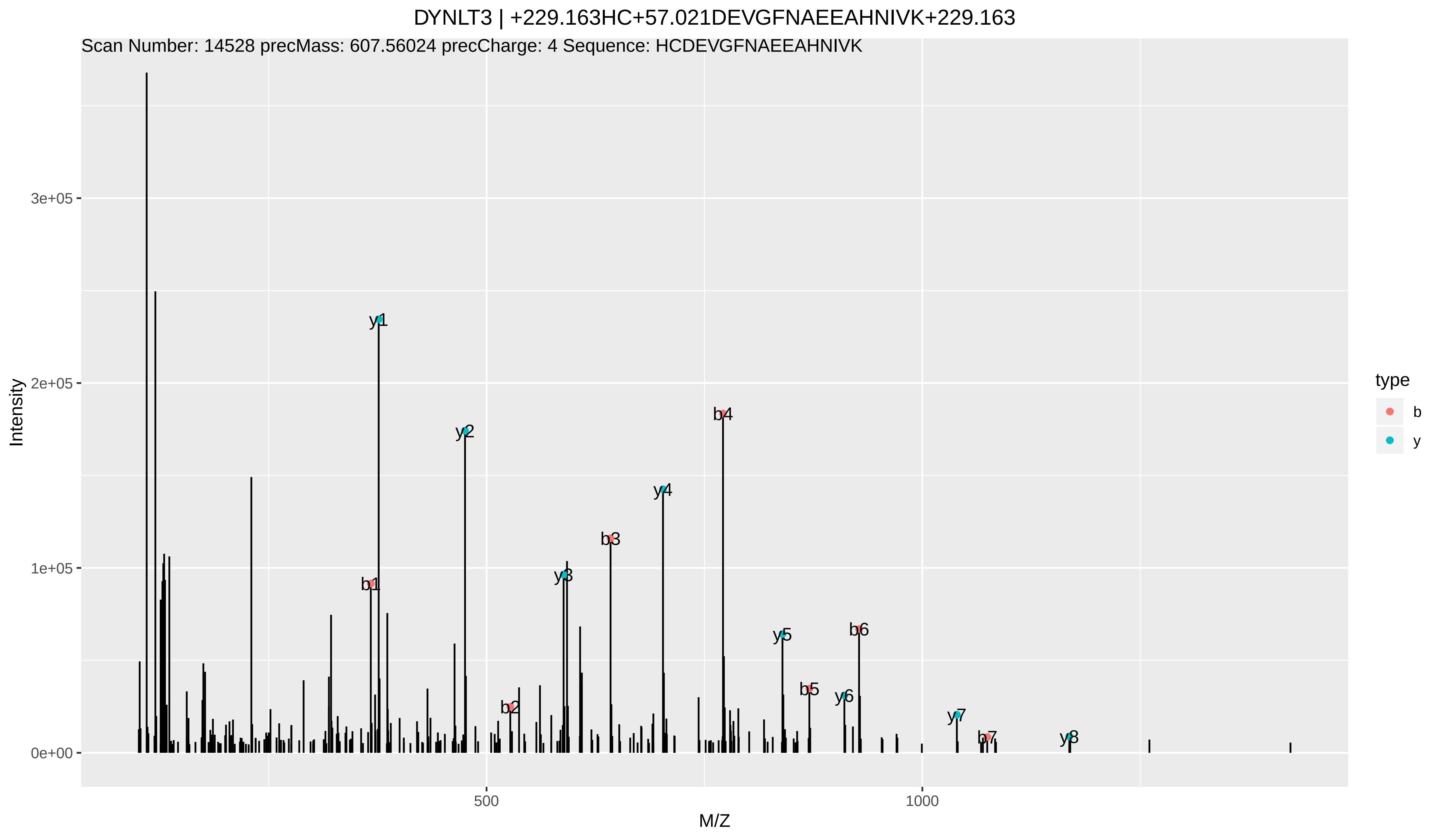

DYNLT3 | +229.163HC+57.021DEVGFNAEEAHNIVK+229.163
Scan Number: 14528 precMass: 607.56024 precCharge: 4 Sequence: HCDEVGFNAEEAHNIVK
Intensity
3e+05
2e+05
1e+05
0e+00
M/Z
500
1000
y1
y2
y3
y4
y5
y6
y7
y8
b1
b2
b3
b4
b5
b6
b7
type
b
y

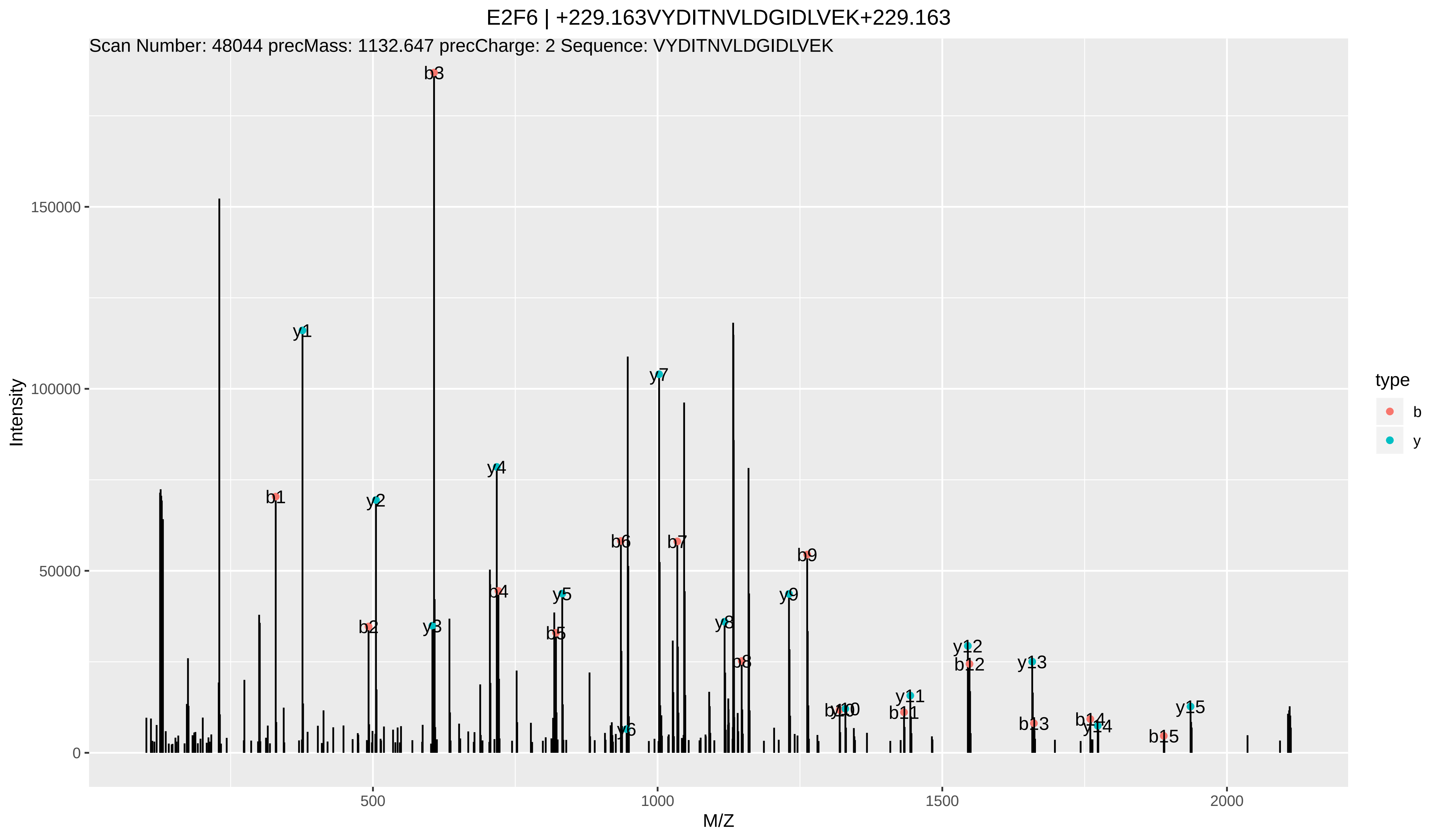

E2F6 | +229.163VYDITNVLDGIDLVEK+229.163
Scan Number: 48044 precMass: 1132.647 precCharge: 2 Sequence: VYDITNVLDGIDLVEK
Intensity
M/Z
type
b
y
0
50000
100000
150000
500
1000
1500
2000
b1
b2
b3
b4
b5
b6
b7
b8
b9
b10
b11
b12
b13
b14
b15
y1
y2
y3
y4
y5
y6
y7
y8
y9
y10
y11
y12
y13
y14
y15

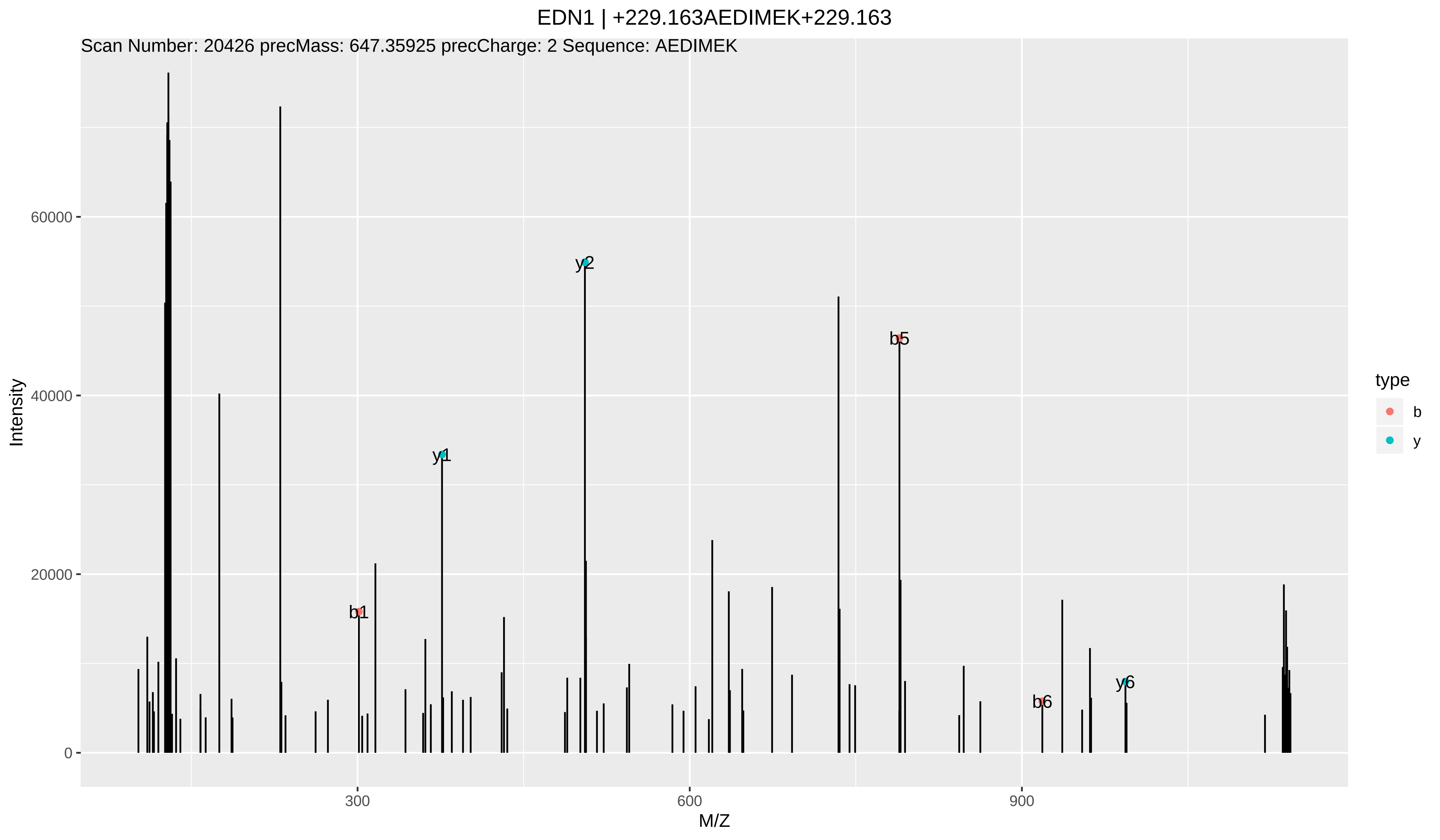

EDN1 | +229.163AEDIMEK+229.163
Scan Number: 20426 precMass: 647.35925 precCharge: 2 Sequence: AEDIMEK
Intensity
60000
40000
20000
0
300
600
900
M/Z
y2
b5
y1
b1
b6
y6
type
b
y

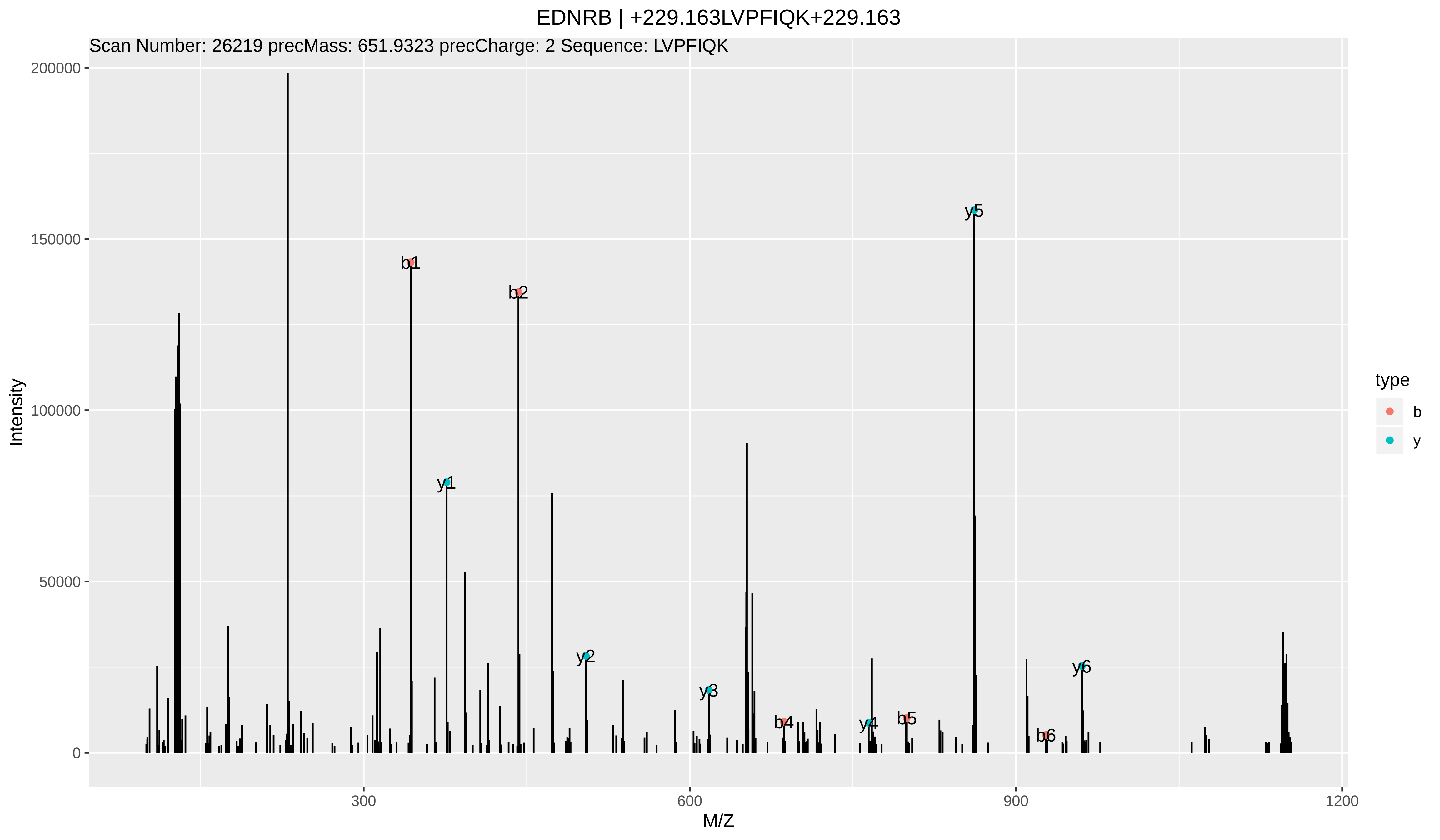
EDNRB | +229.163LVPFIQK+229.163
Scan Number: 26219 precMass: 651.9323 precCharge: 2 Sequence: LVPFIQK
Intensity
M/Z
200000
150000
100000
50000
0
300
600
900
1200
b1
b2
y1
y2
y3
b4
y4
b5
y5
b6
y6
type
b
y

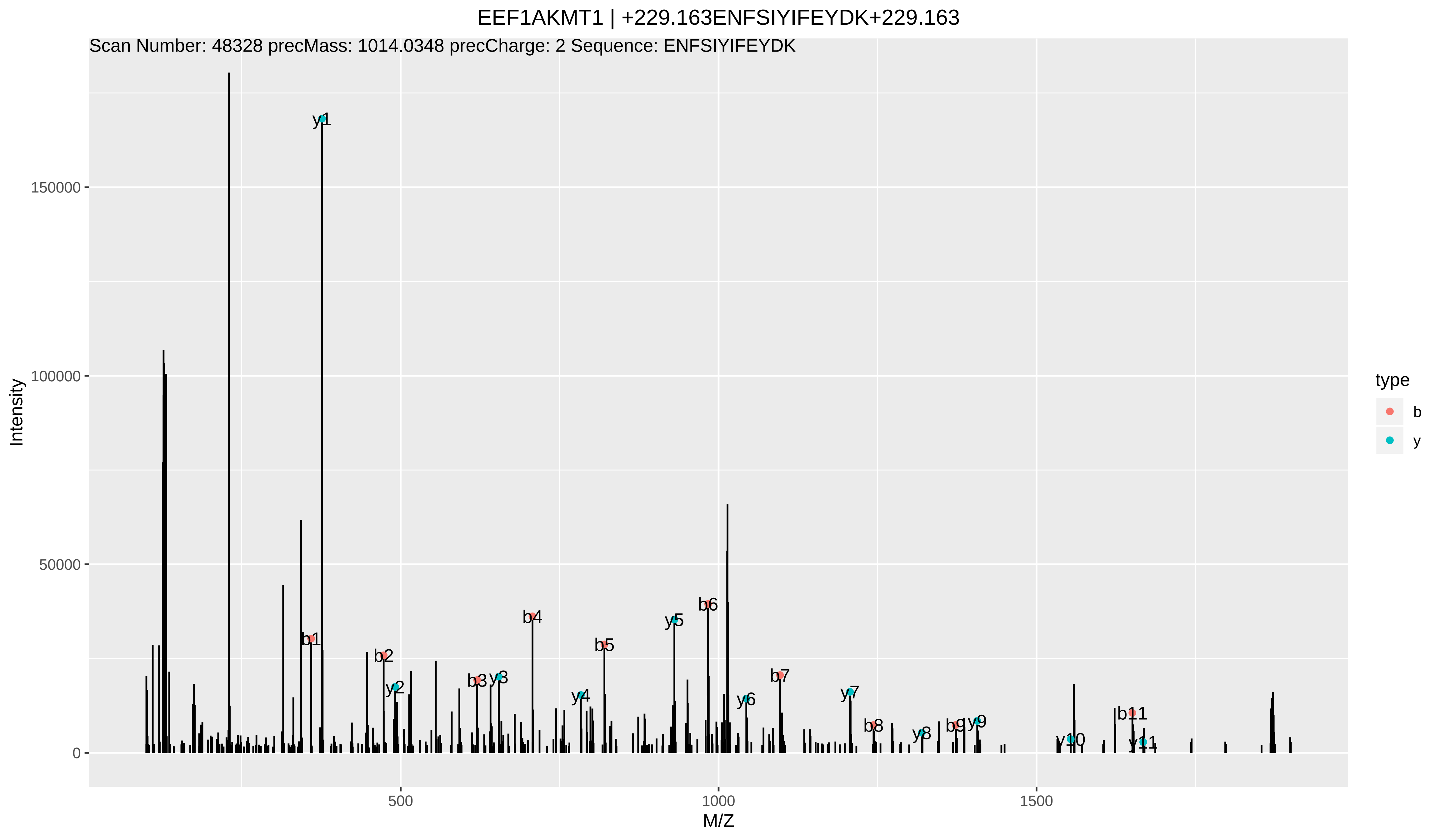
EEF1AKMT1 | +229.163ENFSIYIFEYDK+229.163
Scan Number: 48328 precMass: 1014.0348 precCharge: 2 Sequence: ENFSIYIFEYDK
Intensity
M/Z
0
50000
100000
150000
500
1000
1500
type
b
y
y1
b1
b2
y2
b3
y3
b4
y4
b5
y5
b6
y6
b7
y7
b8
y8
b9
y9
y10
b11
y11

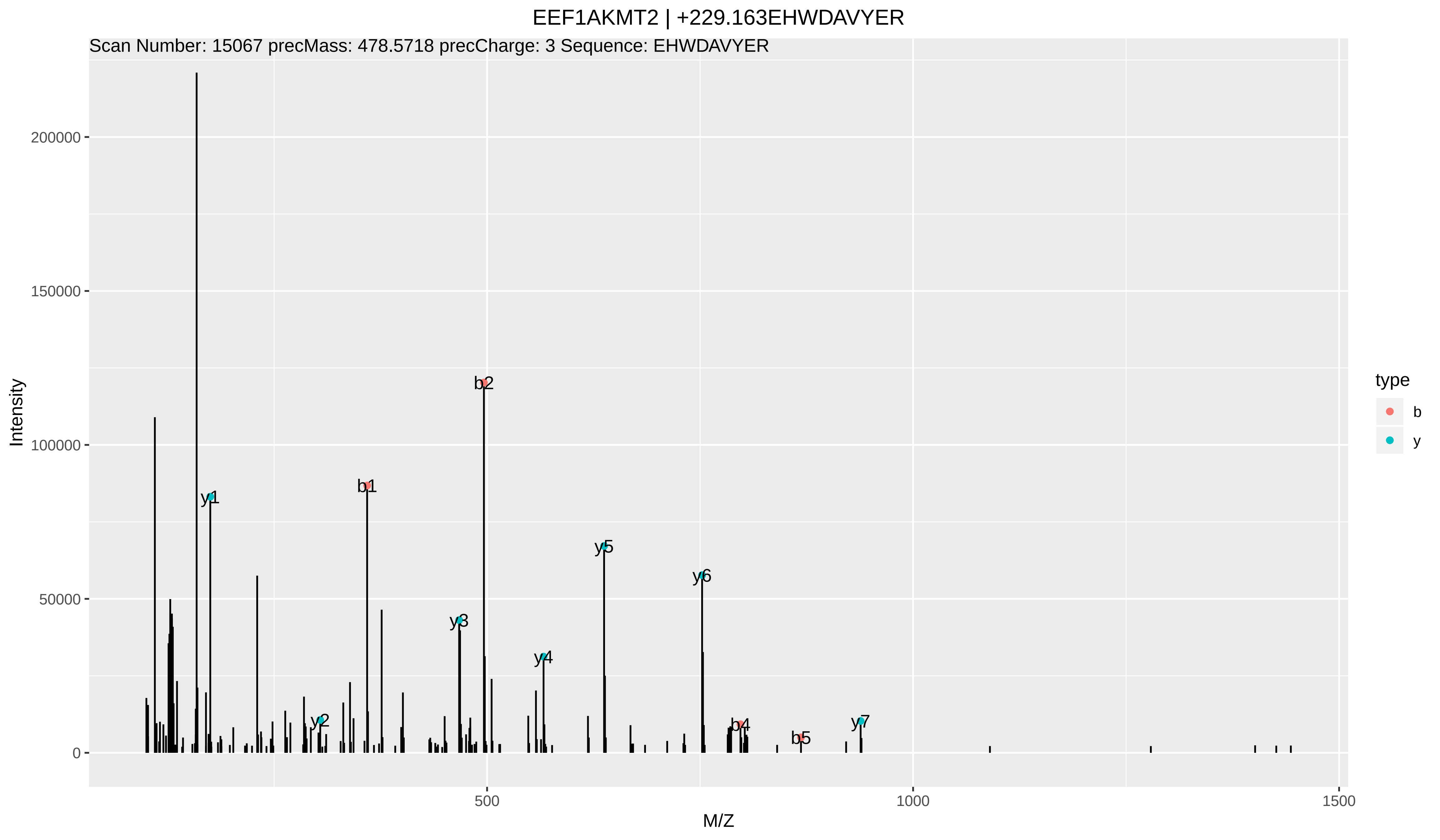
EEF1AKMT2 | +229.163EHWDAVYER
Scan Number: 15067 precMass: 478.5718 precCharge: 3 Sequence: EHWDAVYER
Intensity
M/Z
type
b
y
y1
y2
y3
y4
y5
y6
y7
b1
b2
b4
b5
0
50000
100000
150000
200000
500
1000
1500

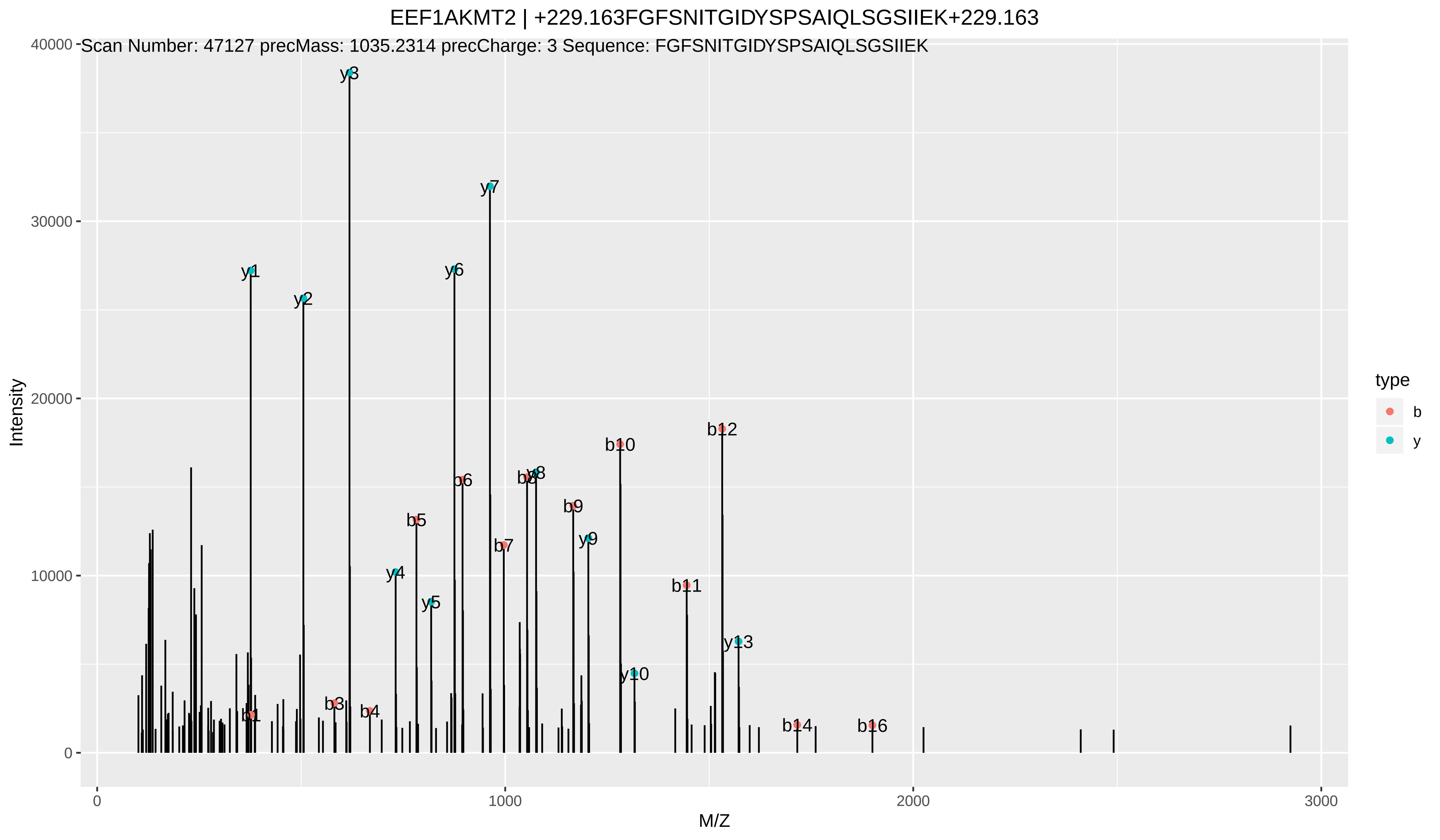

EEF1AKMT2 | +229.163FGFSNITGIDYSPSAIQLSGSIIEK+229.163
Scan Number: 47127 precMass: 1035.2314 precCharge: 3 Sequence: FGFSNITGIDYSPSAIQLSGSIIEK
Intensity
M/Z
0
10000
20000
30000
40000
0
1000
2000
3000
y1
y2
y3
y4
y5
y6
y7
y8
y9
y10
y13
b1
b3
b4
b5
b6
b7
b8
b9
b10
b11
b12
b14
b16
type
b
y

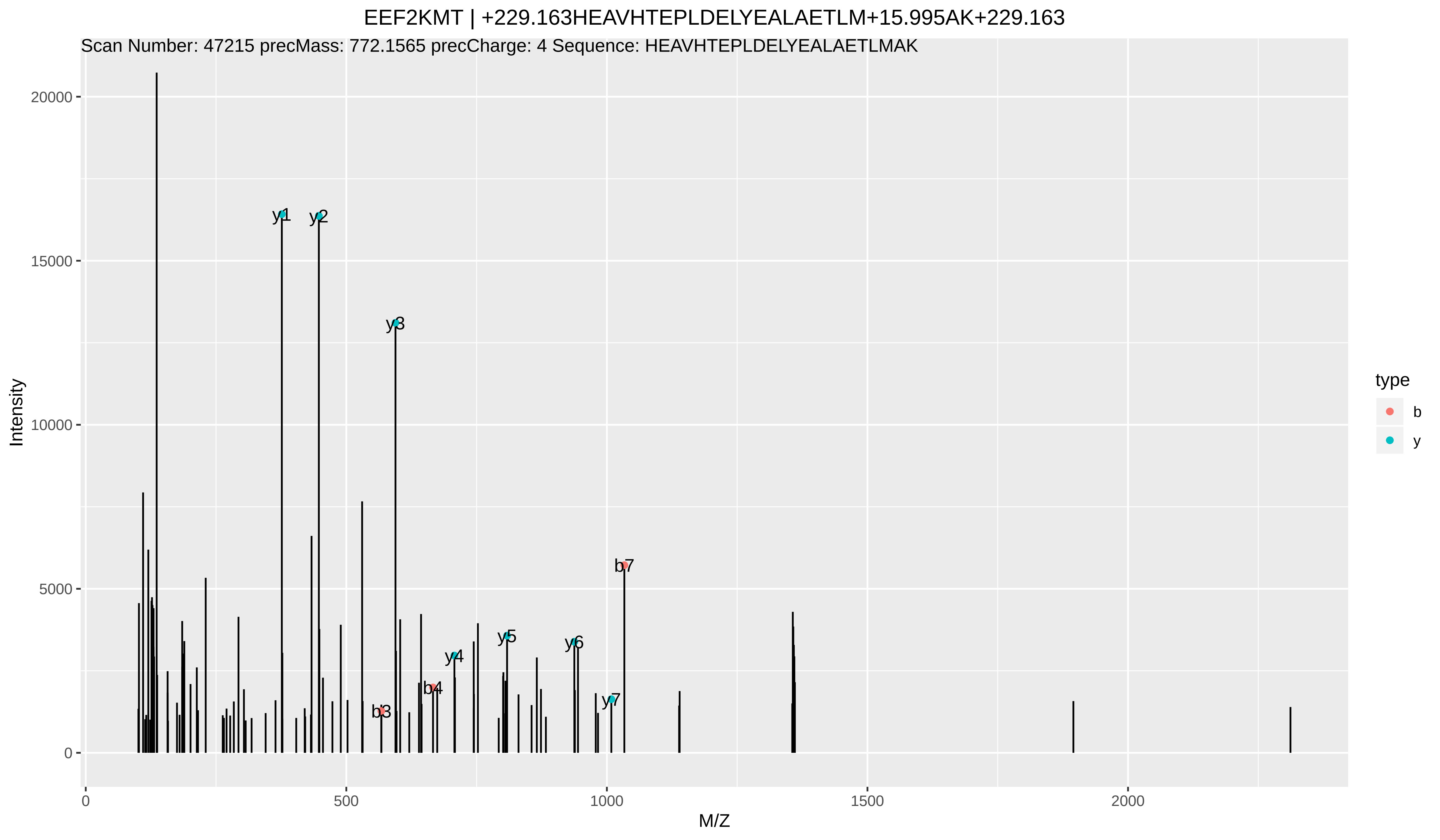
EEF2KMT | +229.163HEAVHTEPLDELYEALAETLM+15.995AK+229.163
Scan Number: 47215 precMass: 772.1565 precCharge: 4 Sequence: HEAVHTEPLDELYEALAETLMAK
Intensity
20000
15000
10000
5000
0
M/Z
0
500
1000
1500
2000
y1
y2
y3
y4
y5
y6
y7
b3
b4
b7
type
b
y

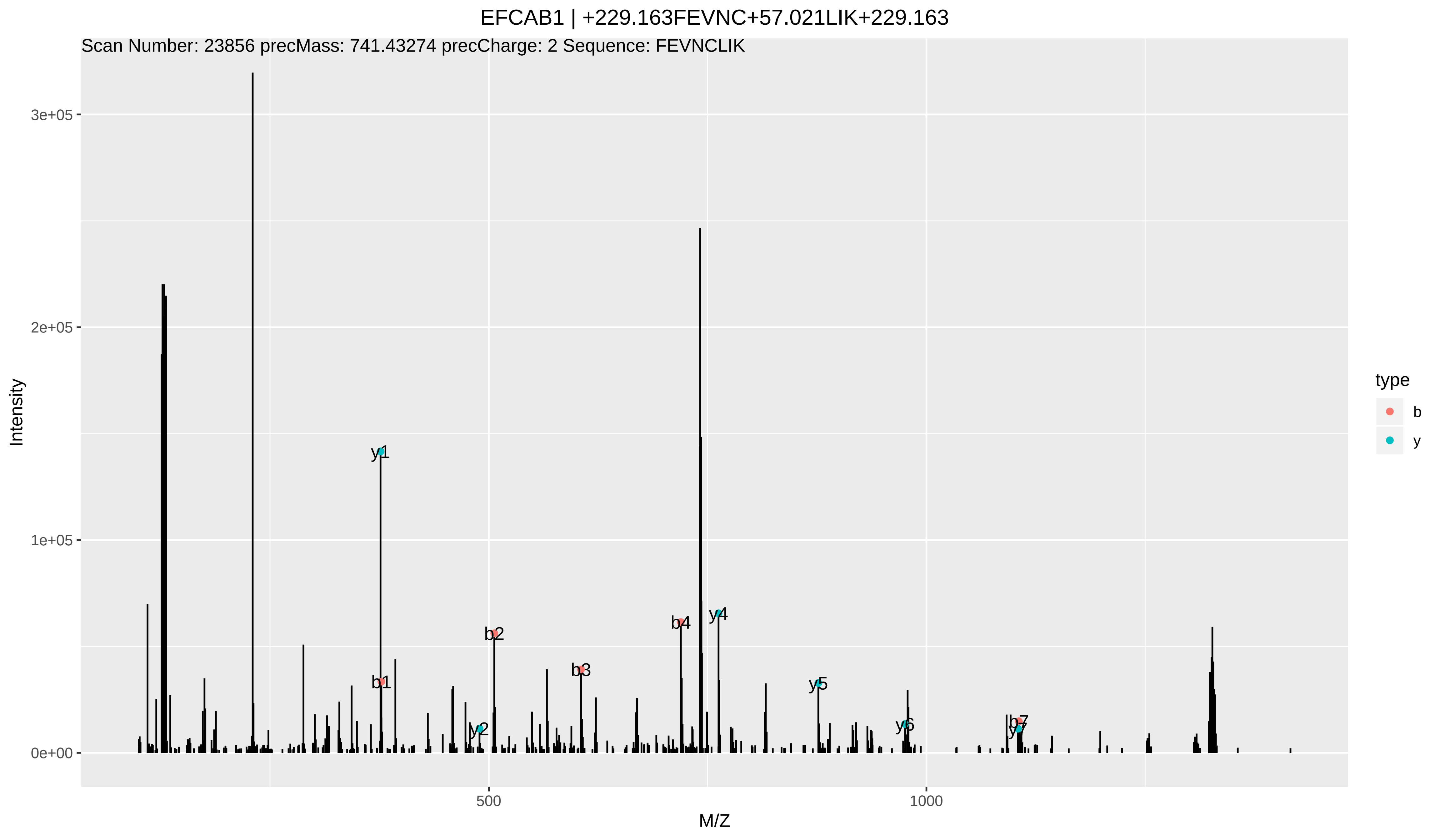

EFCAB1 | +229.163FEVNC+57.021LIK+229.163
Scan Number: 23856 precMass: 741.43274 precCharge: 2 Sequence: FEVNCLIK
Intensity
3e+05
2e+05
1e+05
0e+00
M/Z
500
1000
y1
b1
y2
b2
b3
b4
y4
y5
y6
b7
y7
type
b
y

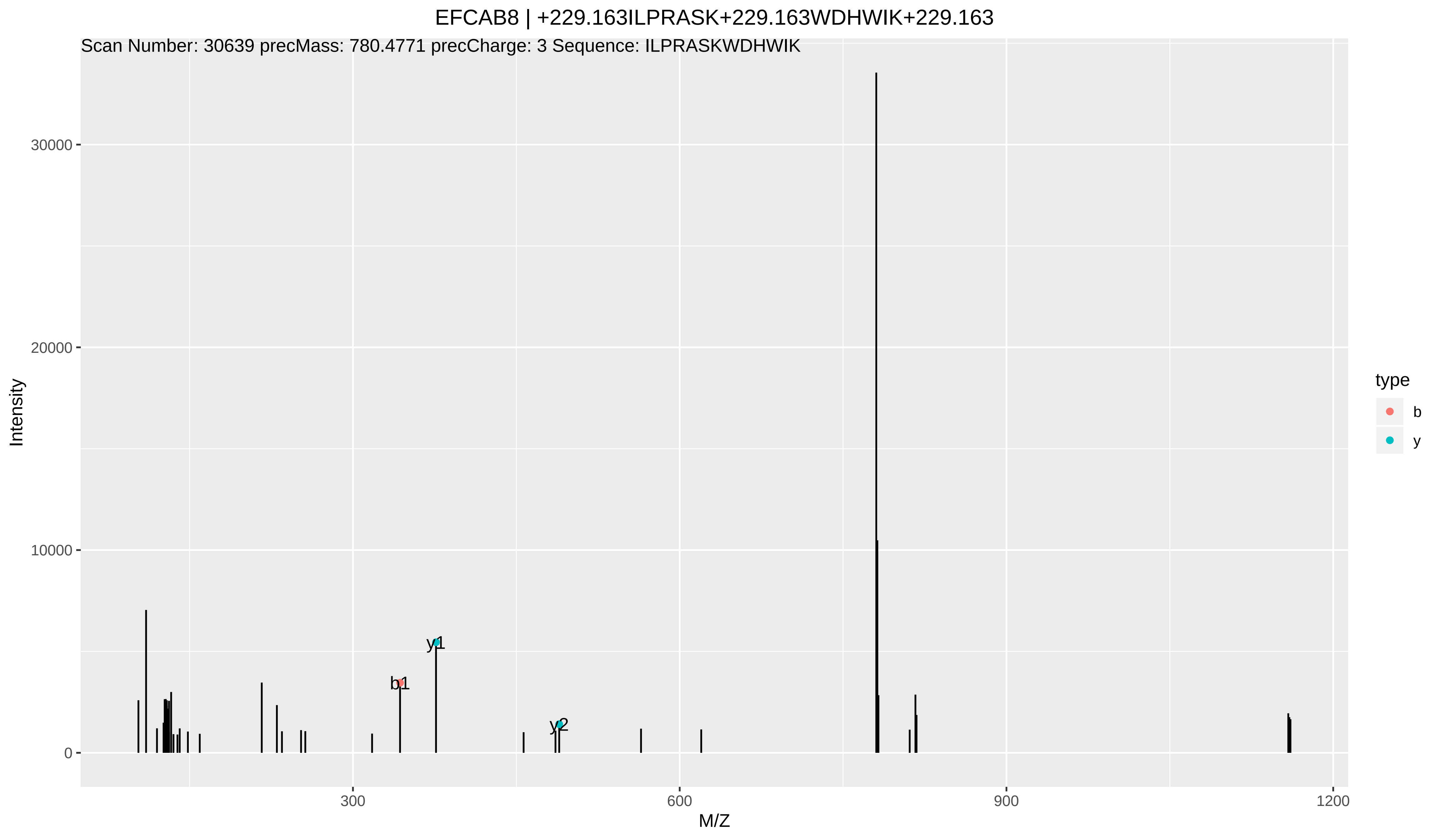

EFCAB8 | +229.163ILPRASK+229.163WDHWIK+229.163
Scan Number: 30639 precMass: 780.4771 precCharge: 3 Sequence: ILPRASKWDHWIK
Intensity
30000
20000
10000
0
300
600
900
1200
M/Z
y1
b1
y2
type
b
y

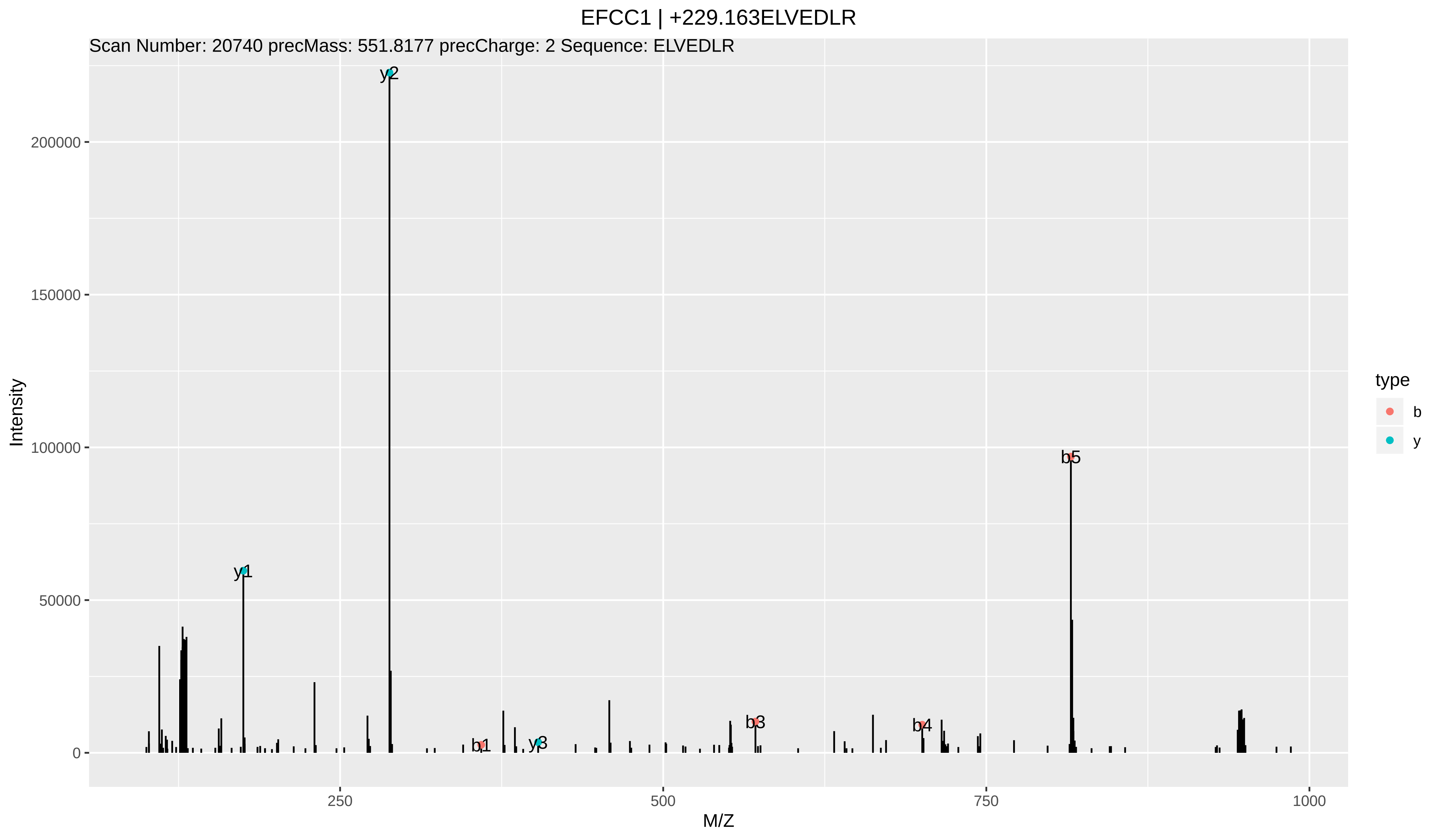

EFCC1 | +229.163ELVEDLR
Scan Number: 20740 precMass: 551.8177 precCharge: 2 Sequence: ELVEDLR
y2
y1
b5
b1
y3
b3
b4
Intensity
200000
150000
100000
50000
0
250
500
750
1000
M/Z
type
b
y

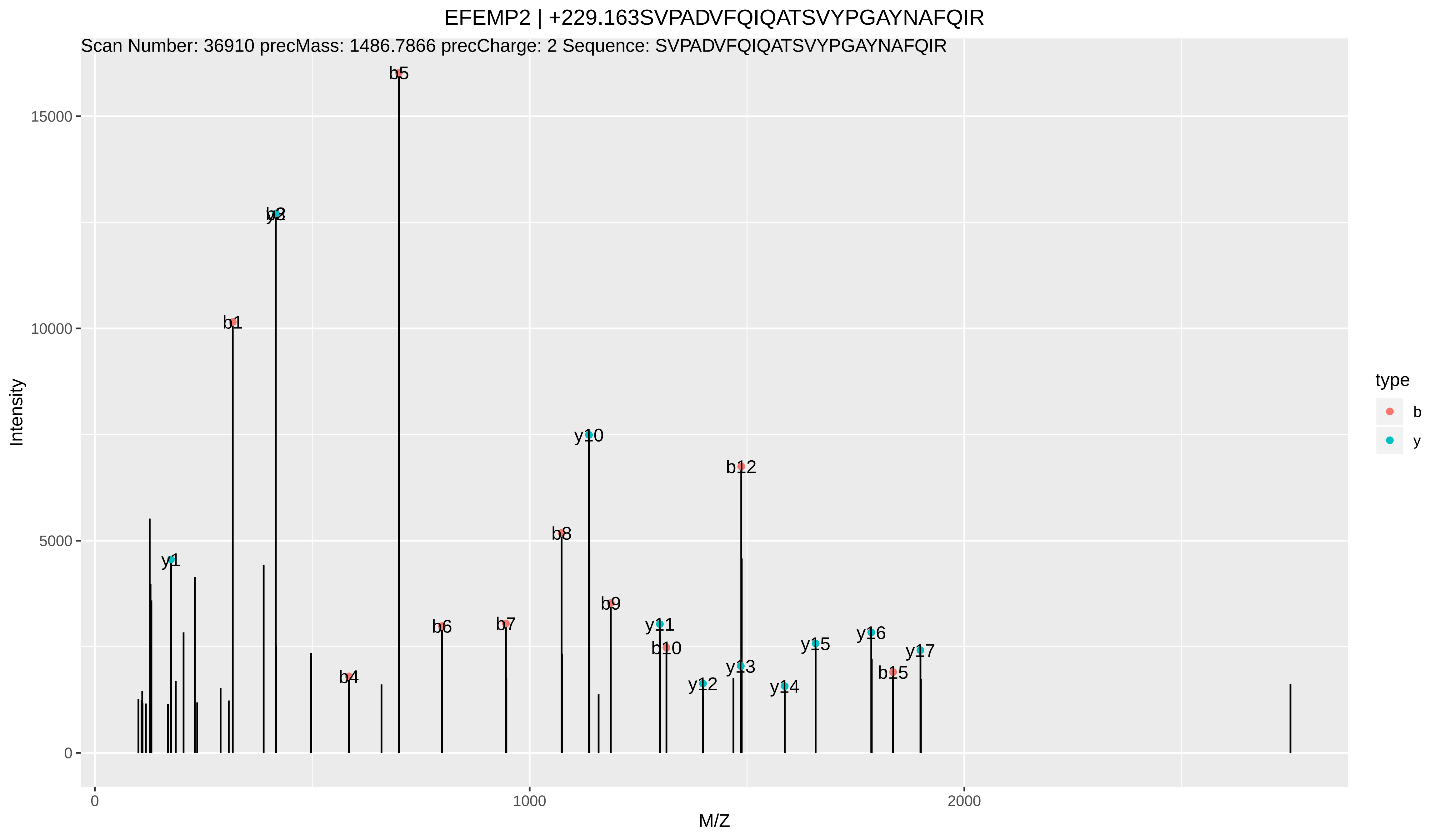

EFEMP2 | +229.163SVPADVFQIQATSVYPGAYNAFQIR
Scan Number: 36910 precMass: 1486.7866 precCharge: 2 Sequence: SVPADVFQIQATSVYPGAYNAFQIR
Intensity
M/Z
type
b
y

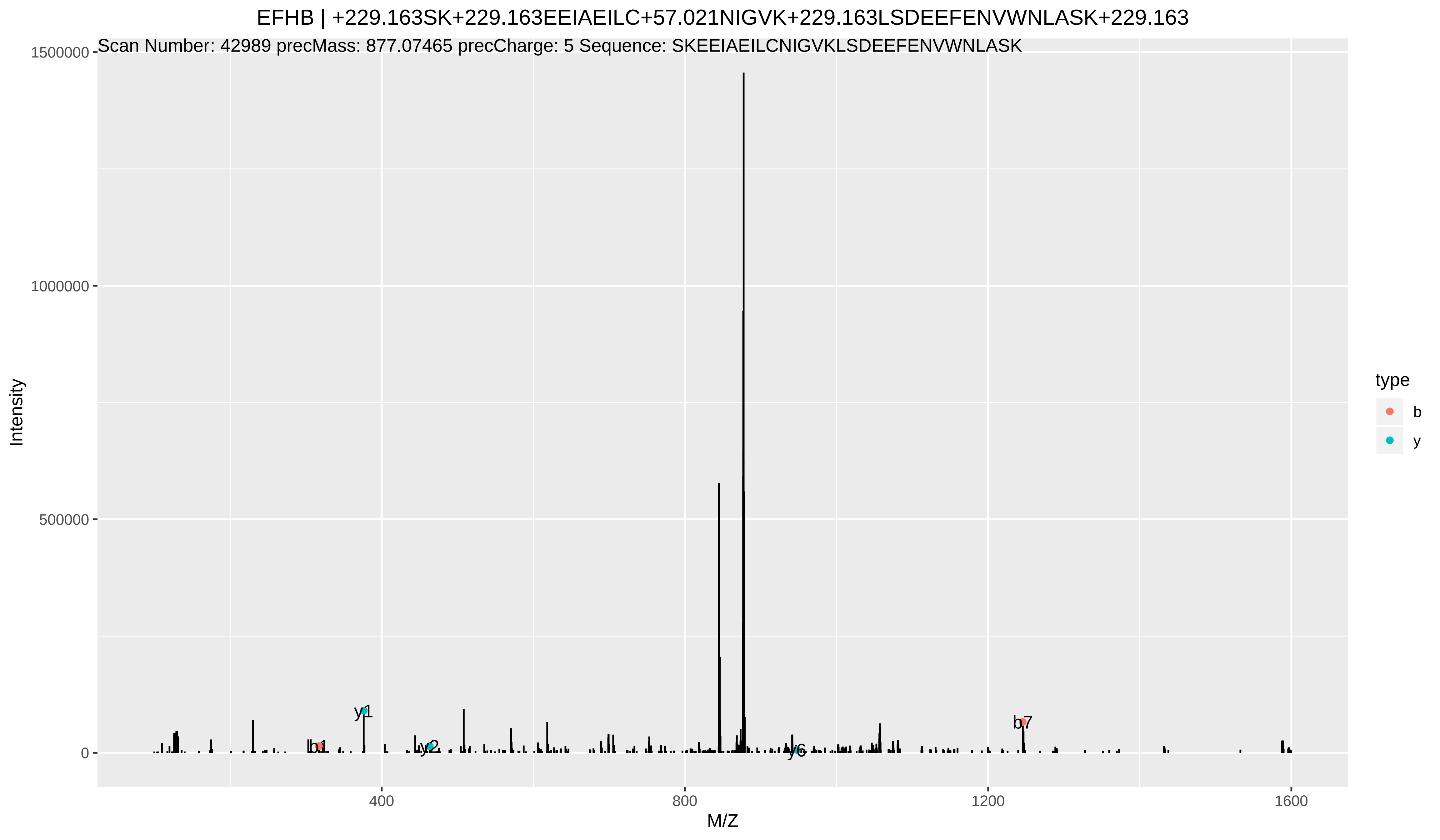

EFHB | +229.163SK+229.163EEIAEILC+57.021NIGVK+229.163LSDEEFENVWNLASK+229.163
Scan Number: 42989 precMass: 877.07465 precCharge: 5 Sequence: SKEEIAEILCNIGVKLSDEEFENVWNLASK
Intensity
1500000
1000000
500000
0
M/Z
400
800
1200
1600
y1
b1
y2
y6
b7
type
b
y

EFHC2 | +229.163LLNADEYTLNYMEQNTDK+229.163YPFSNLK+229.163

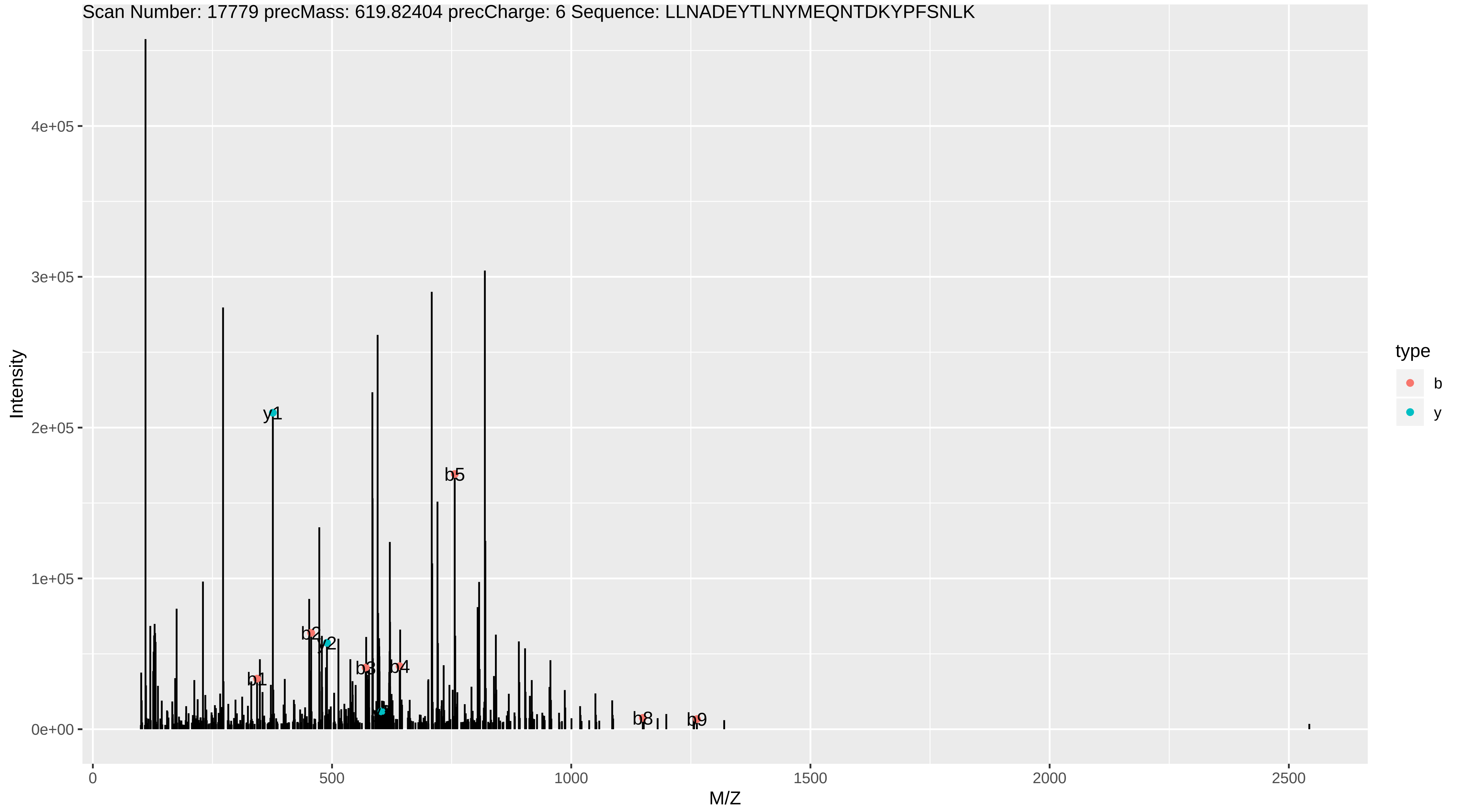

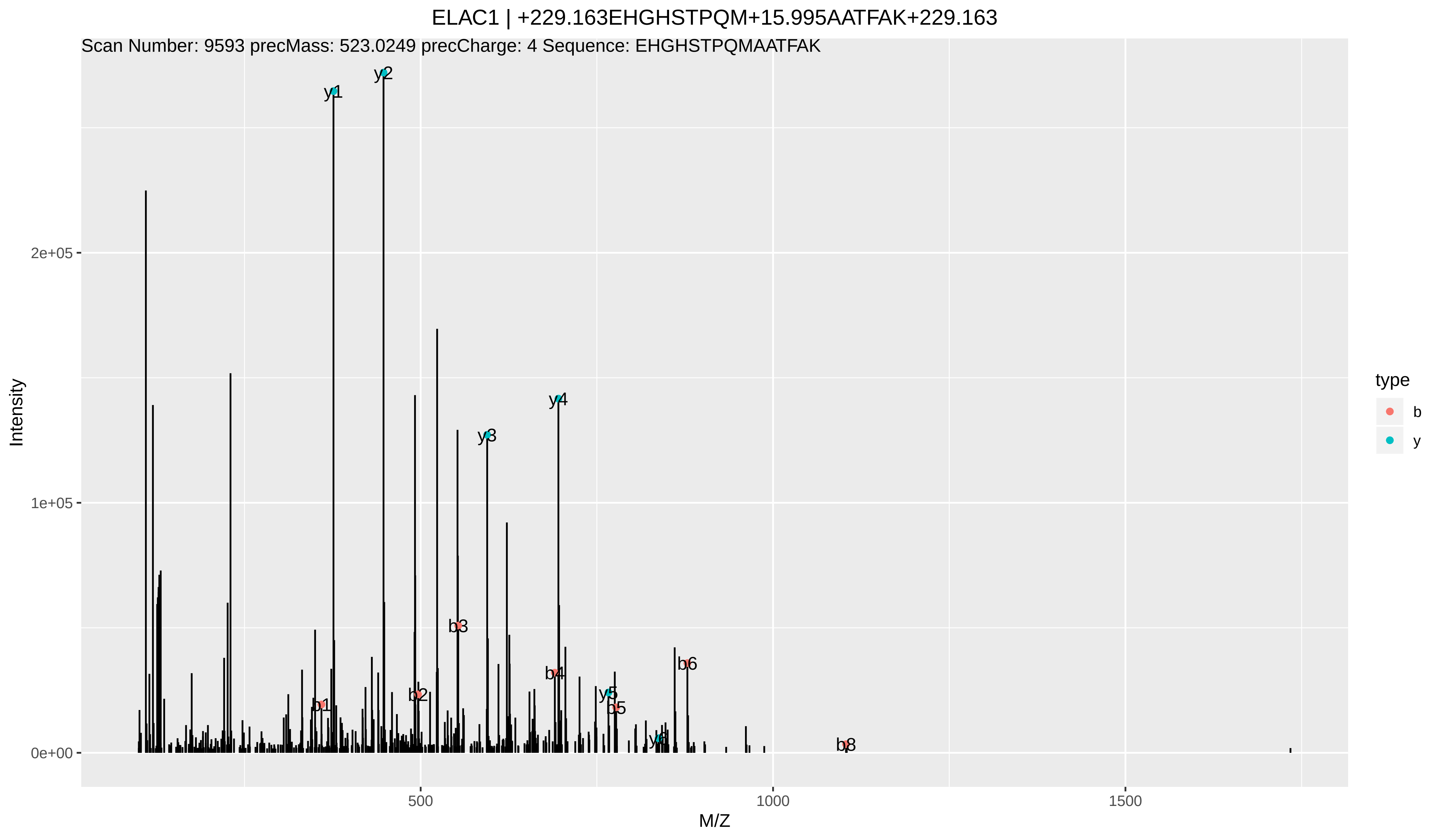

ELAC1 | +229.163EHGHSTPQM+15.995AATFAK+229.163
Scan Number: 9593 precMass: 523.0249 precCharge: 4 Sequence: EHGHSTPQMAATFAK
Intensity
0e+00
1e+05
2e+05
M/Z
500
1000
1500
y1
y2
y3
y4
y5
y6
b1
b2
b3
b4
b5
b6
b8
type
b
y

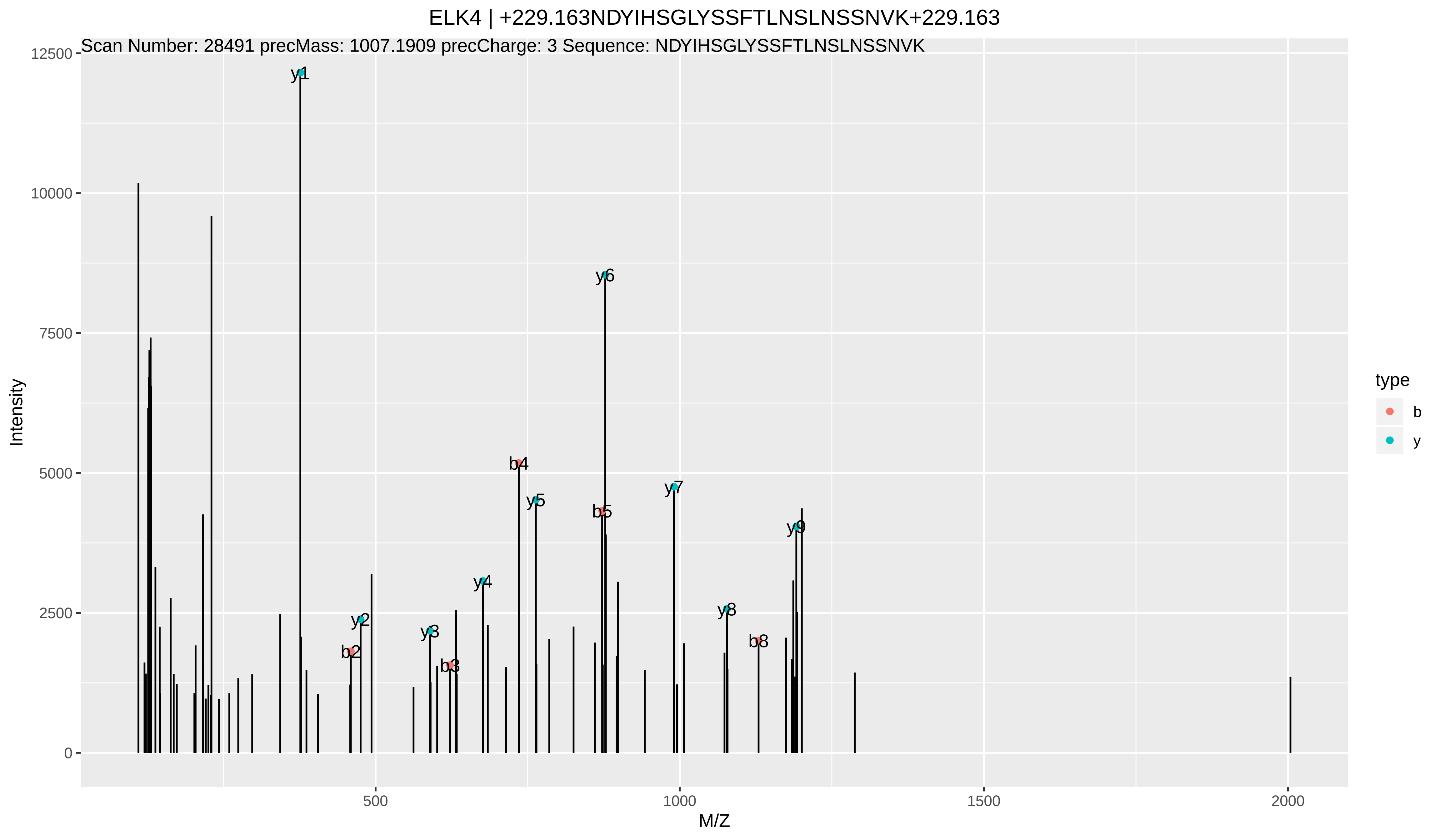

ELK4 | +229.163NDYIHSGLYSSFTLNSLNSSNVK+229.163
Scan Number: 28491 precMass: 1007.1909 precCharge: 3 Sequence: NDYIHSGLYSSFTLNSLNSSNVK
Intensity
M/Z
y1
y2
y3
y4
y5
y6
y7
y8
y9
b2
b3
b4
b5
b8
type
b
y
0
2500
5000
7500
10000
12500
500
1000
1500
2000

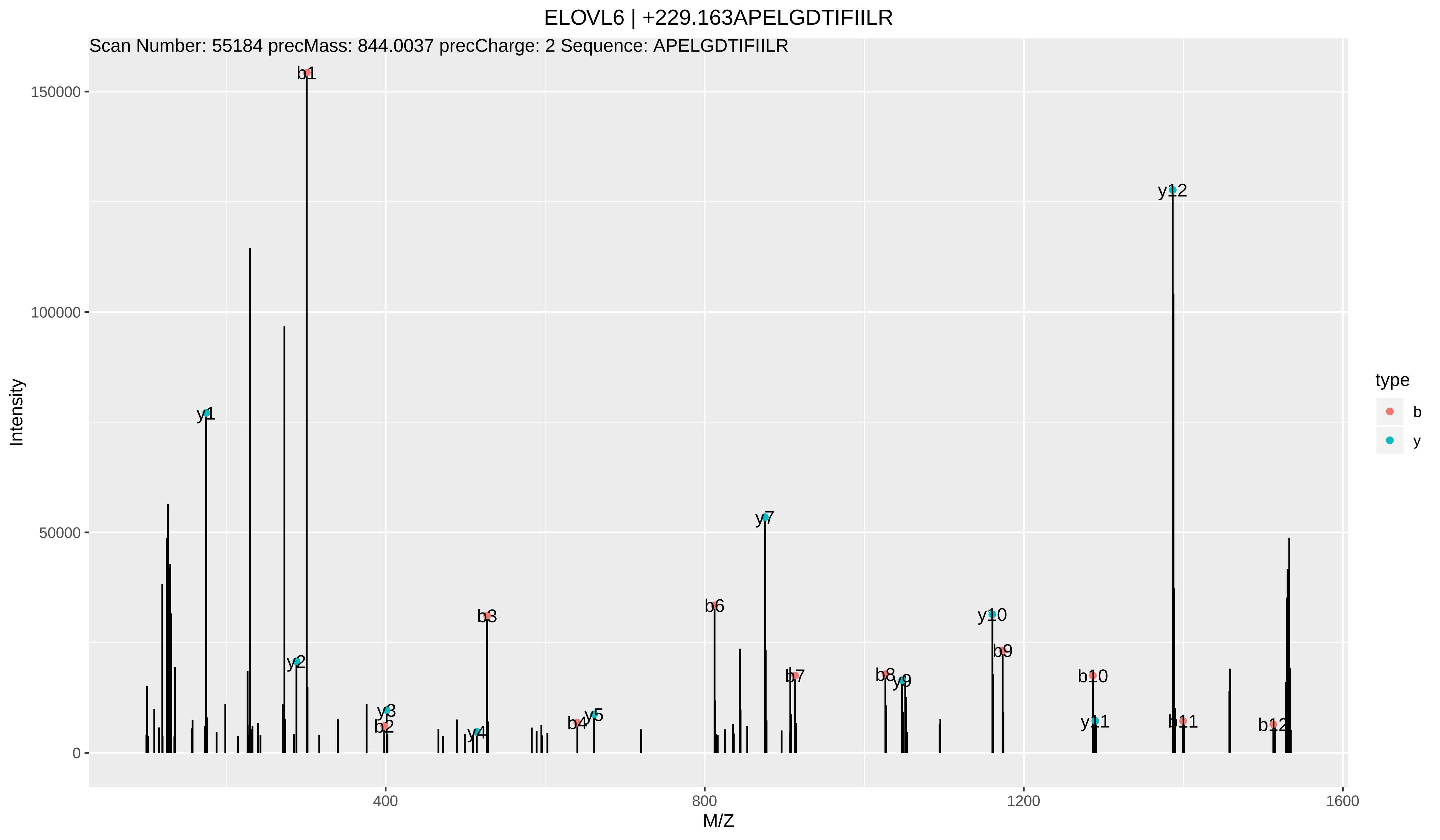

ELOVL6 | +229.163APELGDTIFIILR
Scan Number: 55184 precMass: 844.0037 precCharge: 2 Sequence: APELGDTIFIILR
Intensity
M/Z
0
50000
100000
150000
400
800
1200
1600
b1
y1
y2
y3
b2
y4
b3
b4
y5
b6
y7
b7
b8
y9
y10
b9
b10
y11
y12
b11
b12
type
b
y

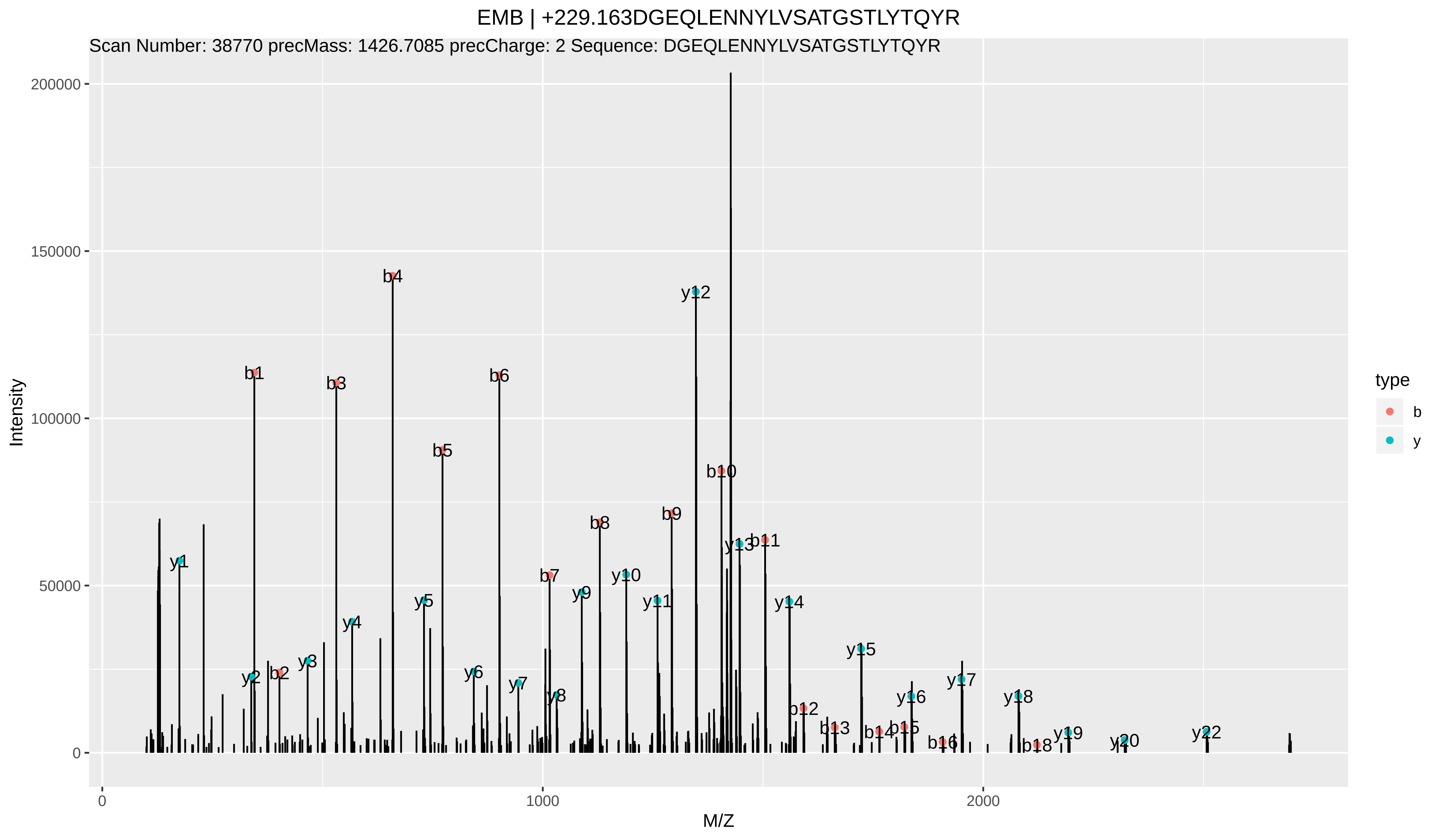

EMB | +229.163DGEQLENNYLVSATGSTLYTQYR
Scan Number: 38770 precMass: 1426.7085 precCharge: 2 Sequence: DGEQLENNYLVSATGSTLYTQYR
Intensity
M/Z
type
b
y
0
50000
100000
150000
200000
1000
2000
b1 b2 b3 b4 b5 b6 b7 b8 b9 b10 b11 b12 b13 b14 b15 b16 b18
y1 y2 y3 y4 y5 y6 y7 y8 y9 y10 y11 y12 y13 y14 y15 y16 y17 y18 y19 y20 y22

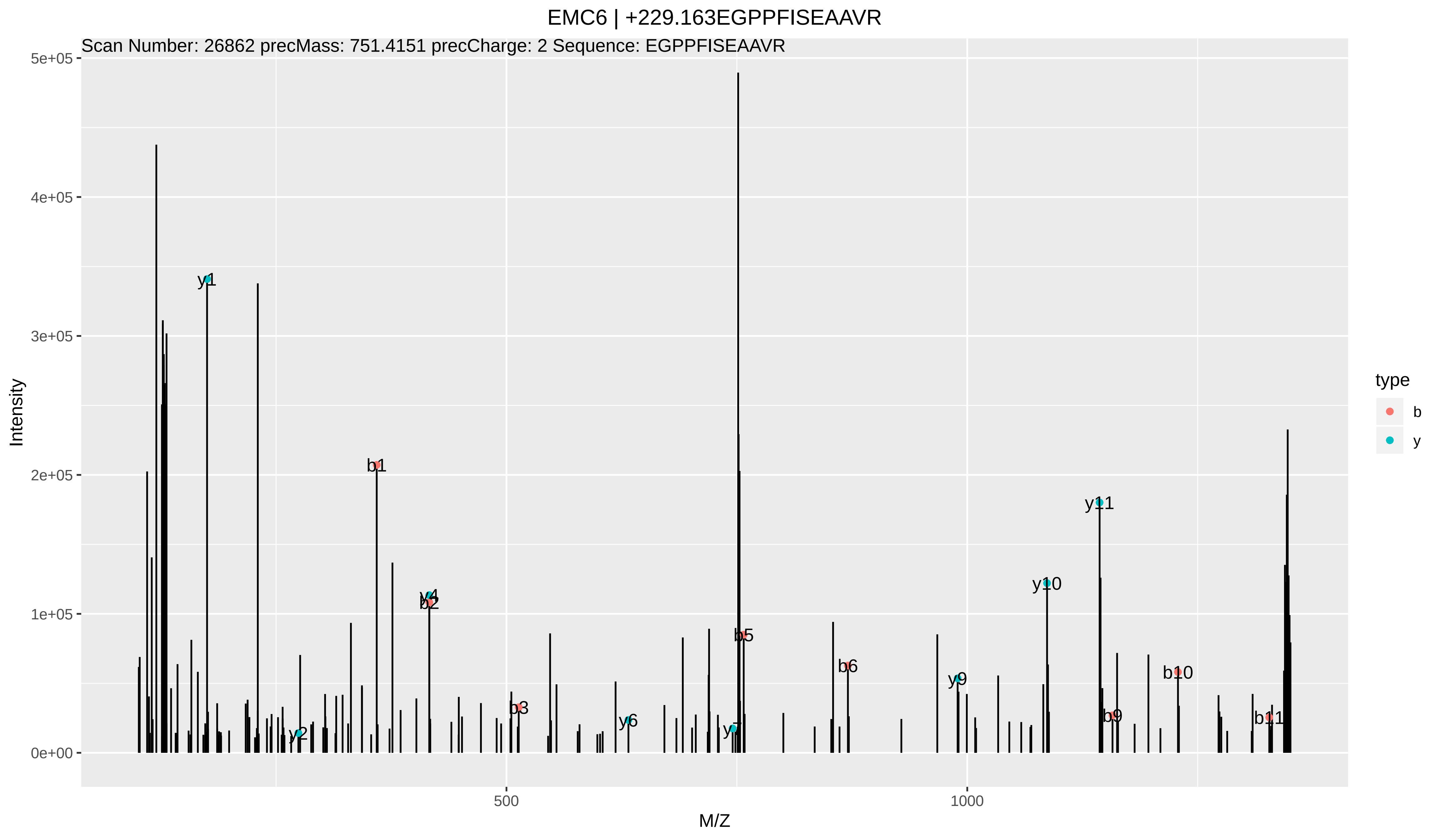

EMC6 | +229.163EGPPFISEAAVR
Scan Number: 26862 precMass: 751.4151 precCharge: 2 Sequence: EGPPFISEAAVR
Intensity
M/Z
0e+00
1e+05
2e+05
3e+05
4e+05
5e+05
500
1000
y1
y2
y4
y6
y7
y9
y10
y11
b1
b2
b3
b5
b6
b9
b10
b11
type
b
y

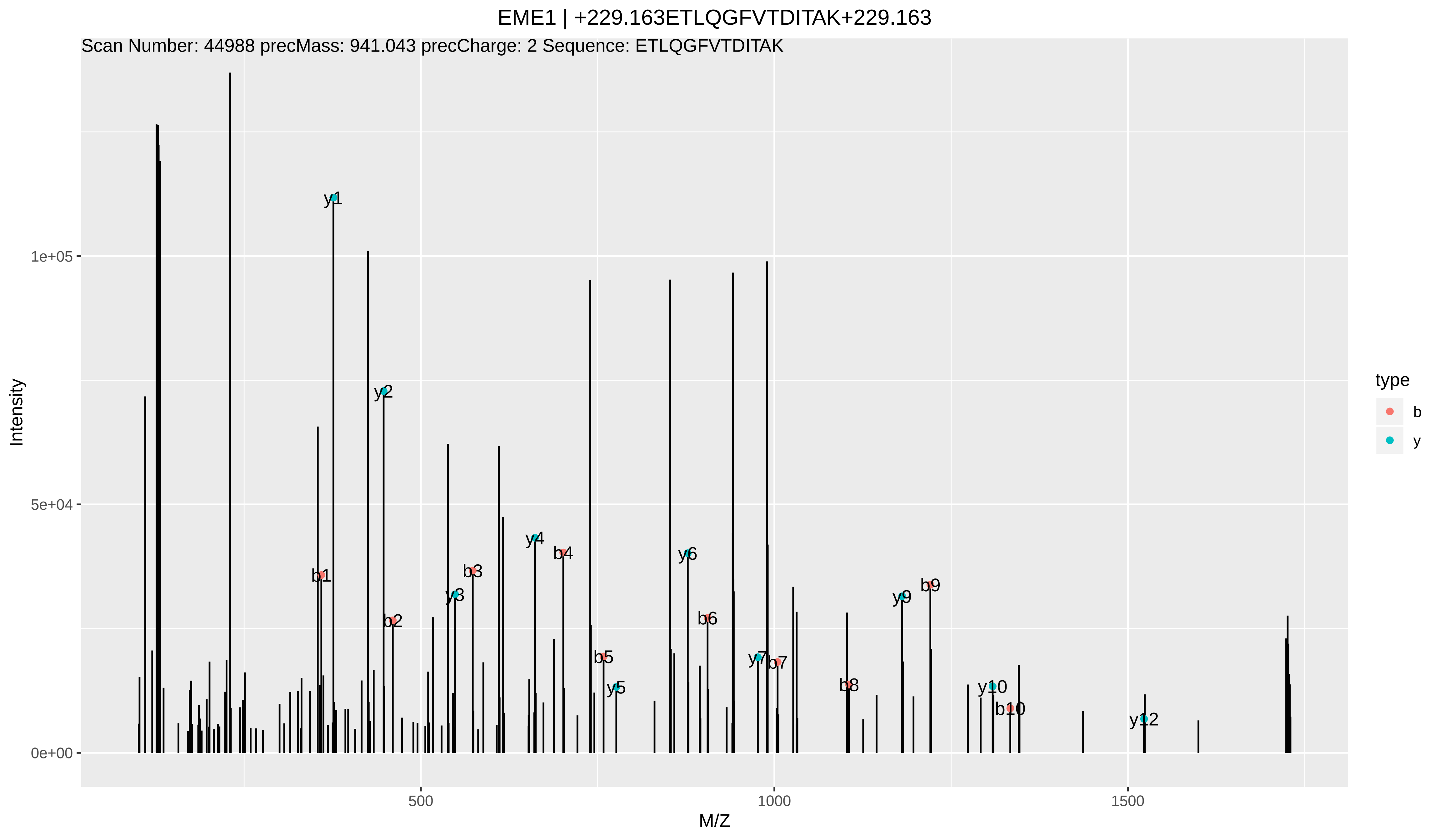

EME1 | +229.163ETLQGFVTDITAK+229.163
Scan Number: 44988 precMass: 941.043 precCharge: 2 Sequence: ETLQGFVTDITAK
Intensity
M/Z
type
b
y
y1
y2
b1
b2
y3
b3
y4
b4
b5
y5
y6
b6
y7
b7
b8
y9
b9
y10
b10
y12

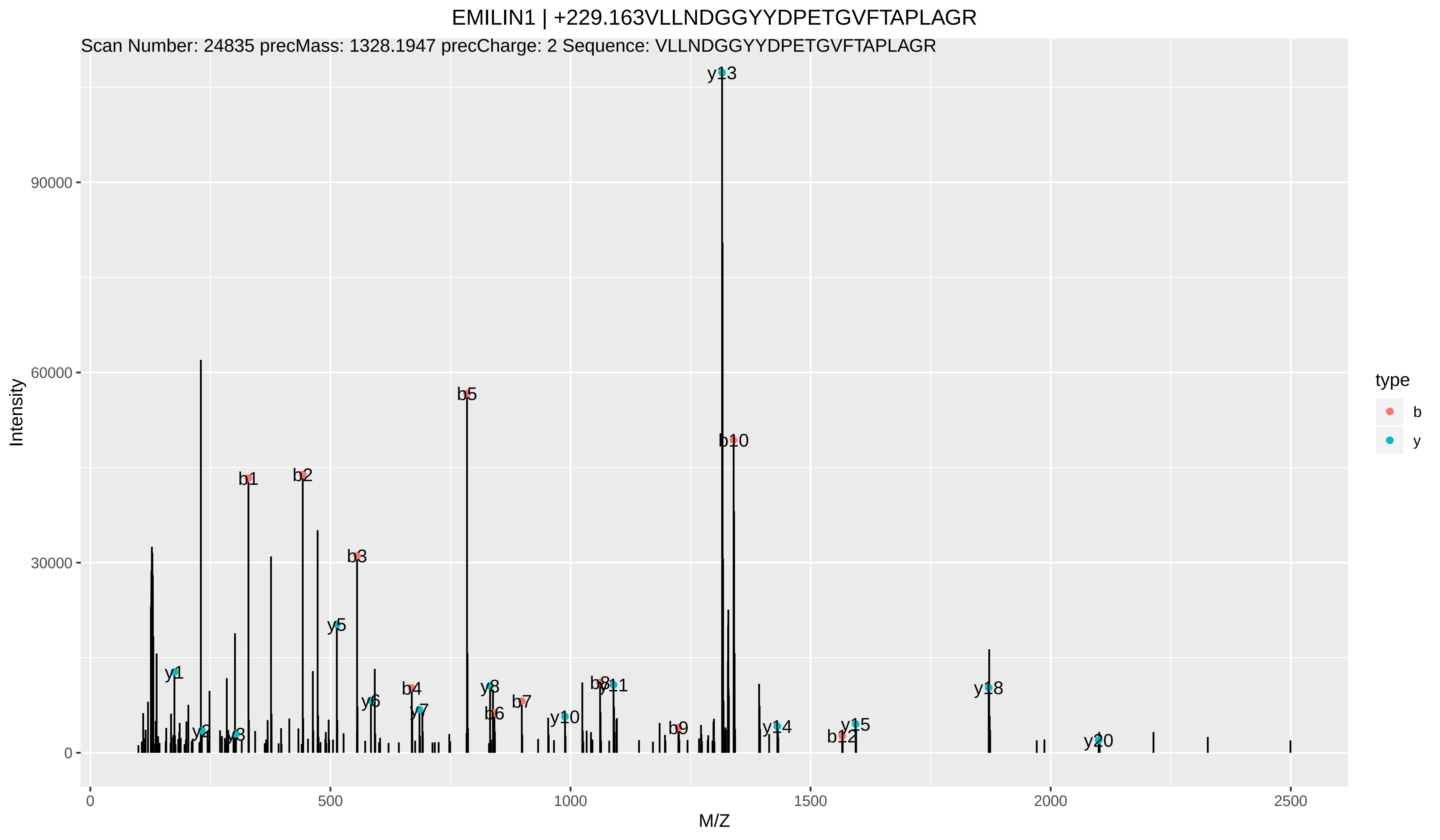
EMILIN1 | +229.163VLLNDGGYYDPETGVFTAPLAGR
Scan Number: 24835 precMass: 1328.1947 precCharge: 2 Sequence: VLLNDGGYYDPETGVFTAPLAGR
Intensity
M/Z
type
b
y
y13
b5
b10
b1
b2
b3
y5
y1
b4
y8
b8
y11
y18
y6
b7
y7
b6
y10
y2
y3
b9
y14
y15
b12
y20
0
30000
60000
90000
0
500
1000
1500
2000
2500

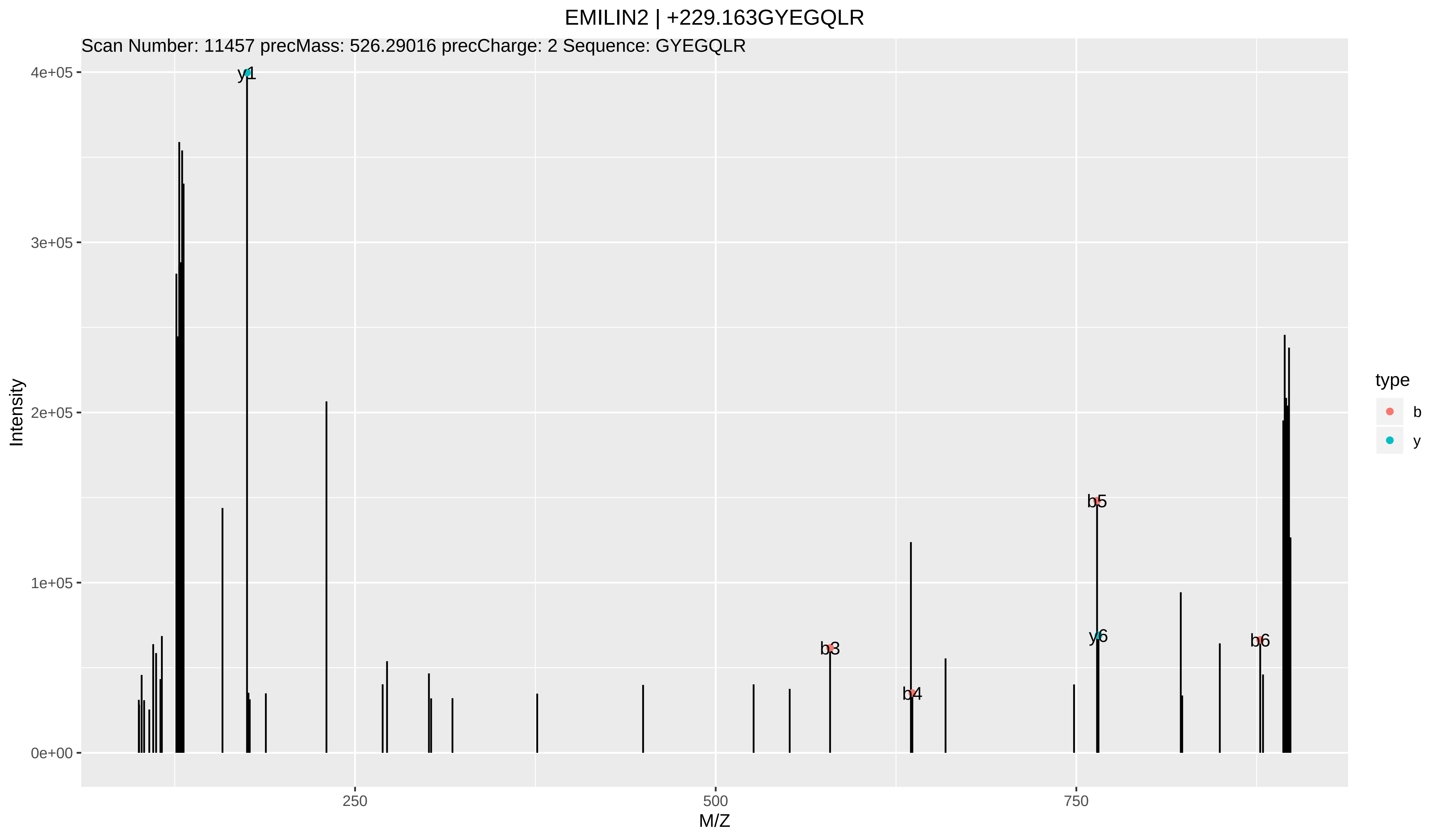

EMILIN2 | +229.163GYEGQLR
Scan Number: 11457 precMass: 526.29016 precCharge: 2 Sequence: GYEGQLR
Intensity
M/Z
4e+05
3e+05
2e+05
1e+05
0e+00
250
500
750
y1
b5
y6
b6
b3
b4
type
b
y

EML1 | +229.163AVTC+57.021IAFSK+229.163

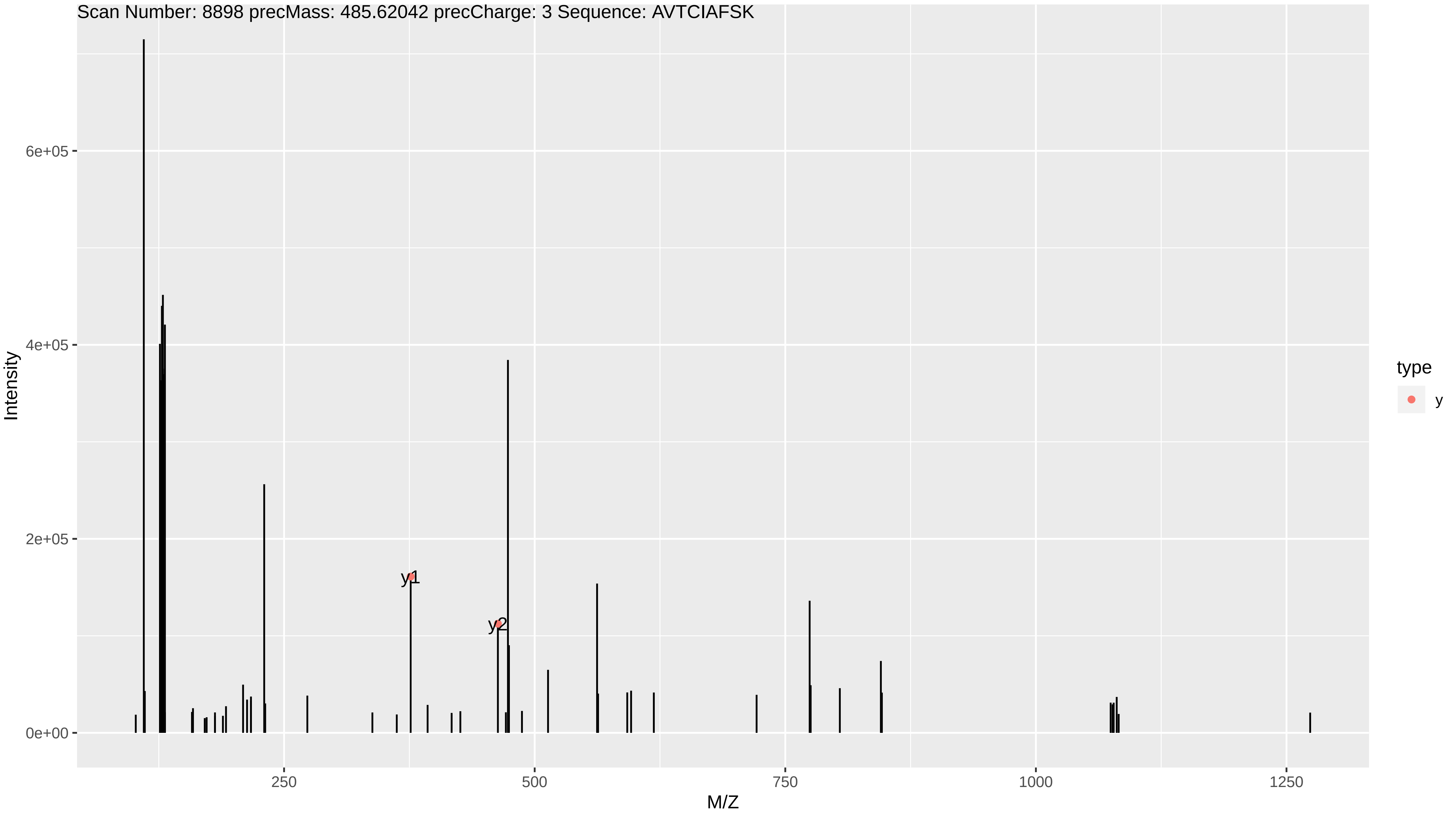

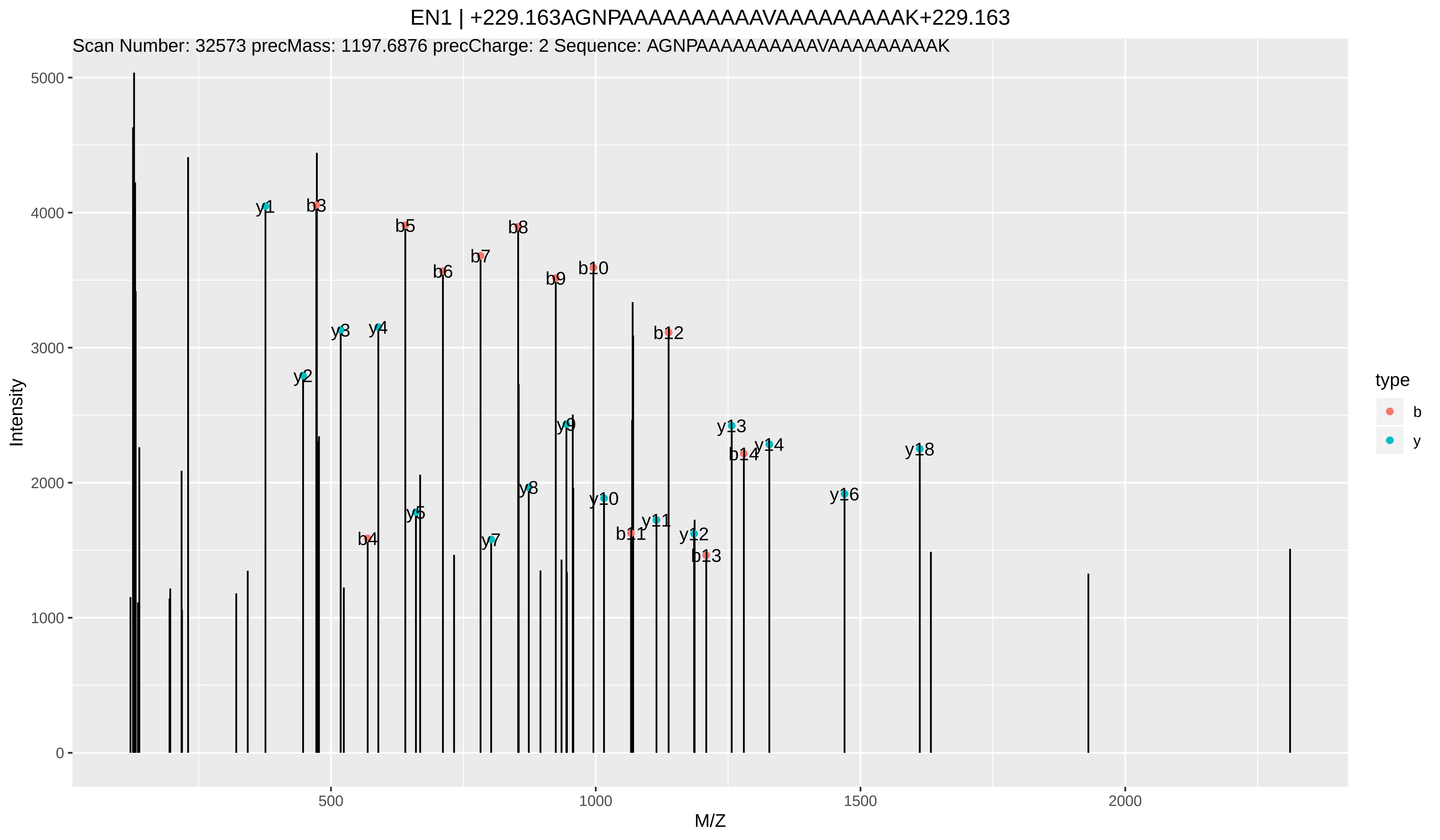

EN1 | +229.163AGNPAAAAAAAAAAVAAAAAAAAAK+229.163
Scan Number: 32573 precMass: 1197.6876 precCharge: 2 Sequence: AGNPAAAAAAAAAAVAAAAAAAAAK
Intensity
M/Z
type
b
y
y1
b3
b5
b8
b7
b6
b10
b9
b12
y3
y4
y2
y9
y13
y14
y18
b14
y8
y10
y16
y5
b4
y7
b11
y11
y12
b13
0
1000
2000
3000
4000
5000
500
1000
1500
2000

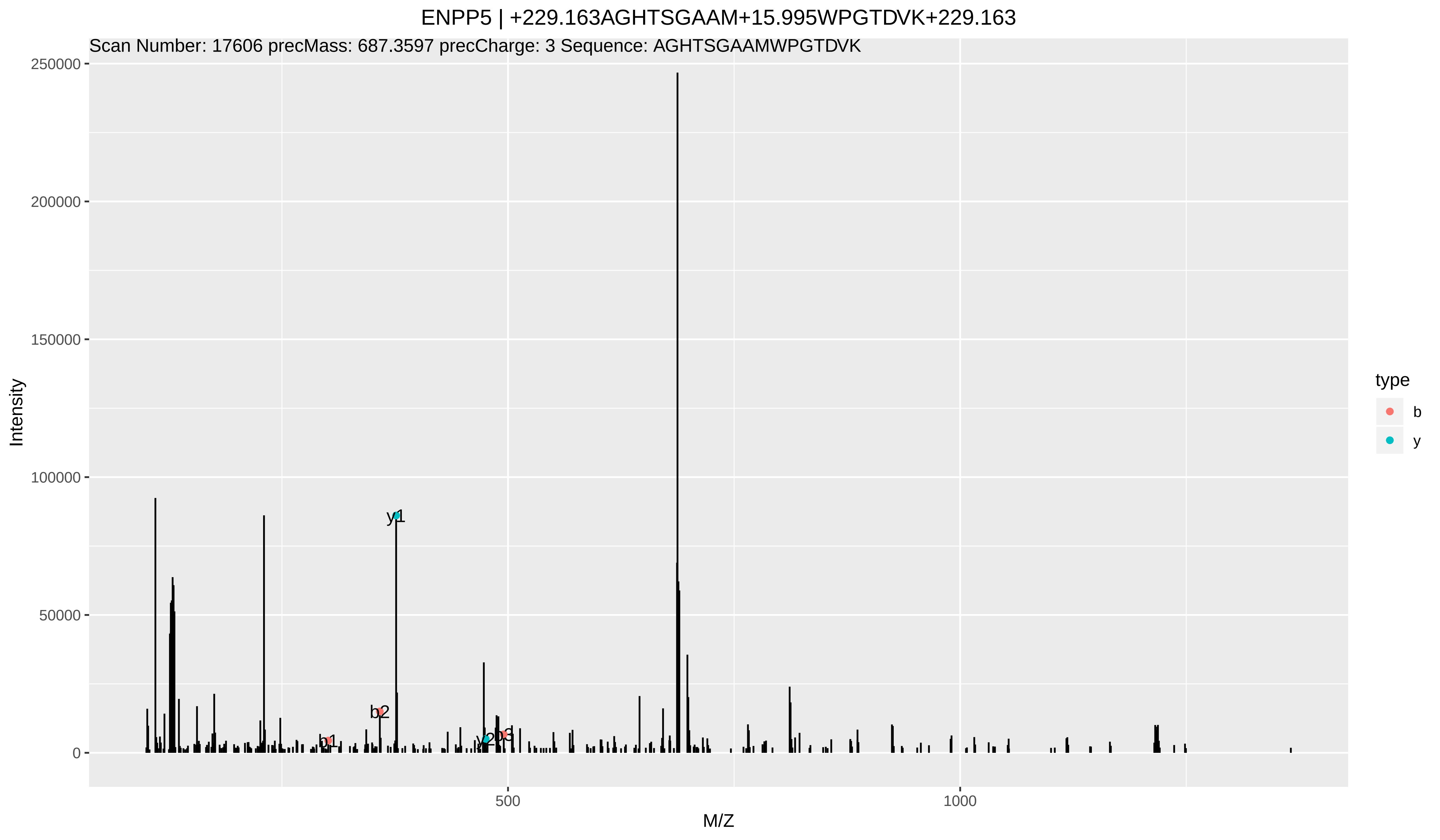

ENPP5 | +229.163AGHTSGAAM+15.995WPGTDVK+229.163
Scan Number: 17606 precMass: 687.3597 precCharge: 3 Sequence: AGHTSGAAMWPGTDVK
Intensity
250000
200000
150000
100000
50000
0
M/Z
500
1000
y1
b2
b1
y2
b3
type
b
y

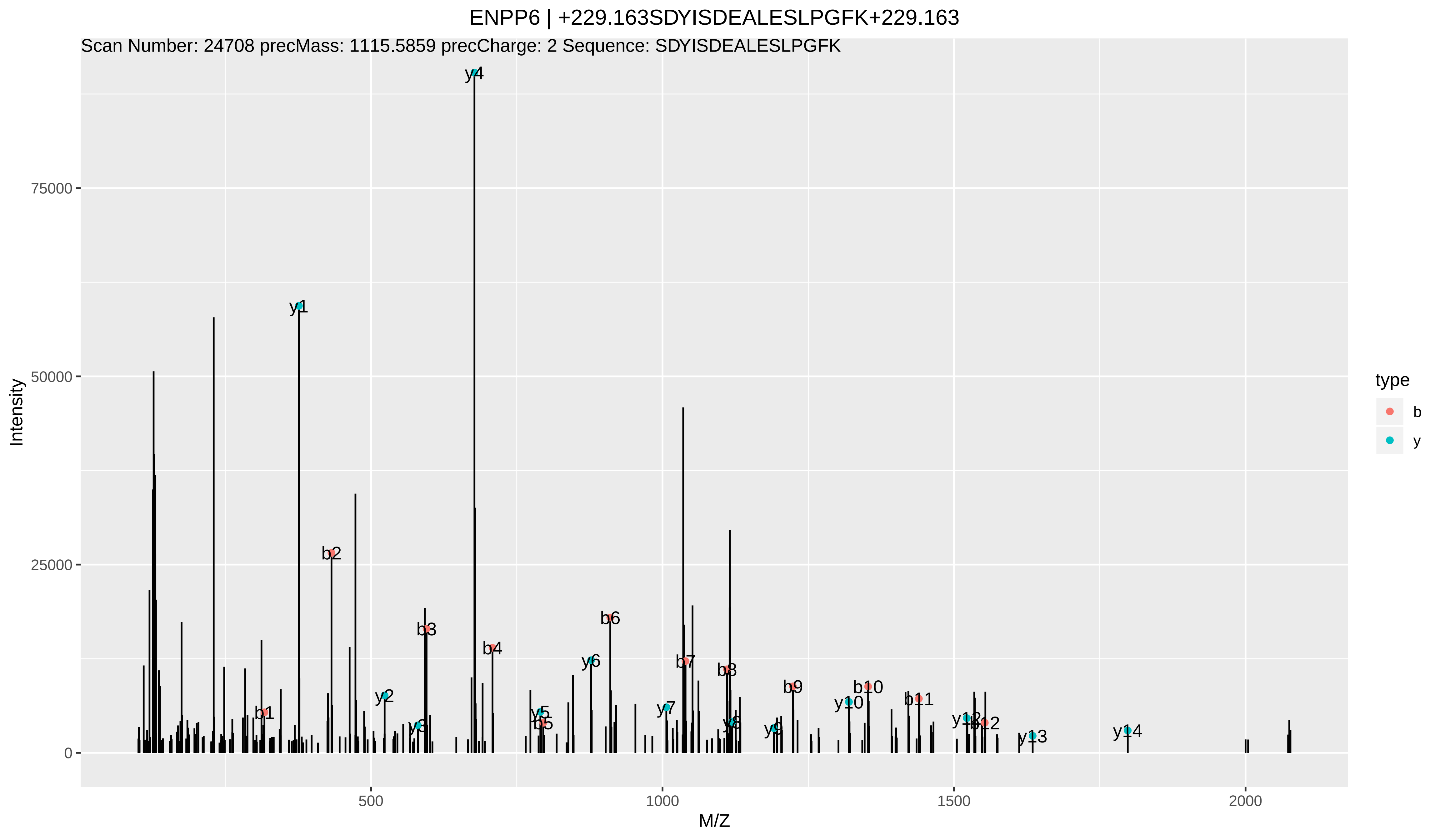

ENPP6 | +229.163SDYISDEALESLPGFK+229.163
Scan Number: 24708 precMass: 1115.5859 precCharge: 2 Sequence: SDYISDEALESLPGFK
Intensity
M/Z
75000
50000
25000
0
500
1000
1500
2000
y4
y1
b2
b3
b4
b6
y6
b7
b8
b9
b10
b11
y10
y2
y3
b1
y5
b5
y7
y8
y9
y12
b12
y13
y14
type
b
y

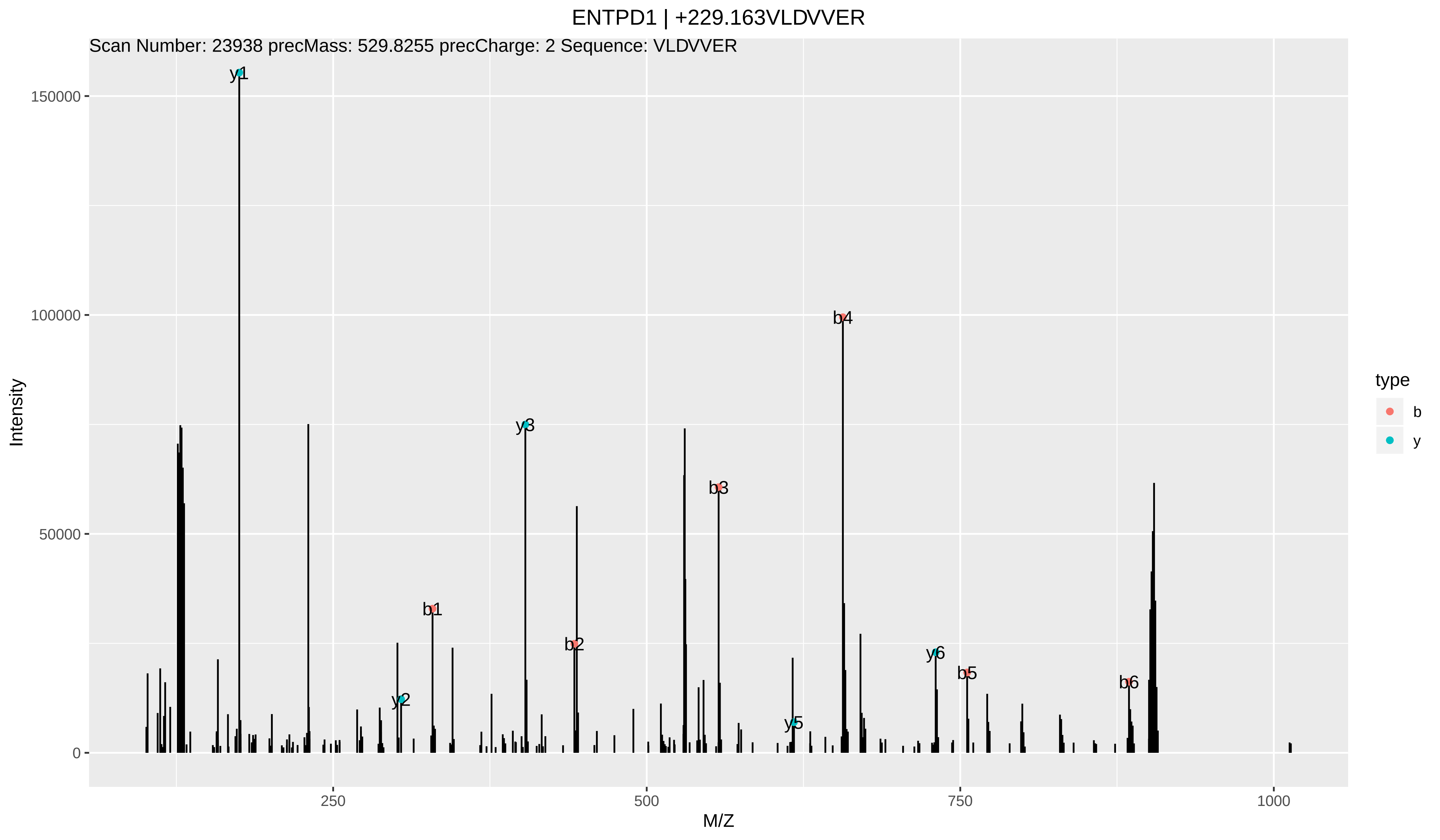

ENTPD1 | +229.163VLDVVER
Scan Number: 23938 precMass: 529.8255 precCharge: 2 Sequence: VLDVVER
y1
y3
b4
b3
b1
b2
y2
y5
y6
b5
b6
Intensity
150000
100000
50000
0
250
500
750
1000
M/Z
type
b
y

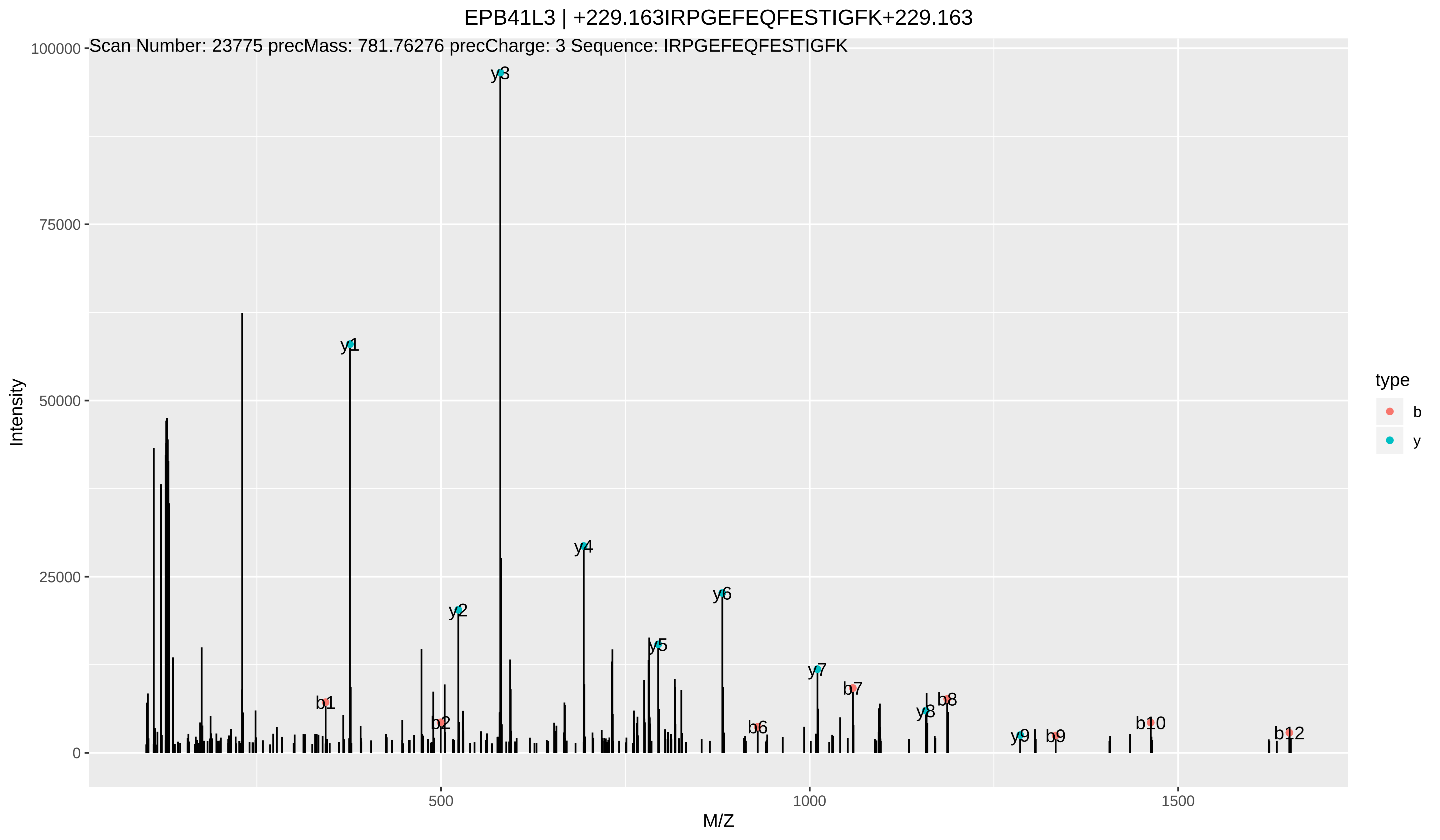
EPB41L3 | +229.163IRPGEFEQFESTIGFK+229.163
Scan Number: 23775 precMass: 781.76276 precCharge: 3 Sequence: IRPGEFEQFESTIGFK
Intensity
M/Z
0
25000
50000
75000
100000
500
1000
1500
y3
y1
y4
y6
y2
y5
y7
b7
b8
y8
b1
b2
b6
y9
b9
b10
b12
type
b
y

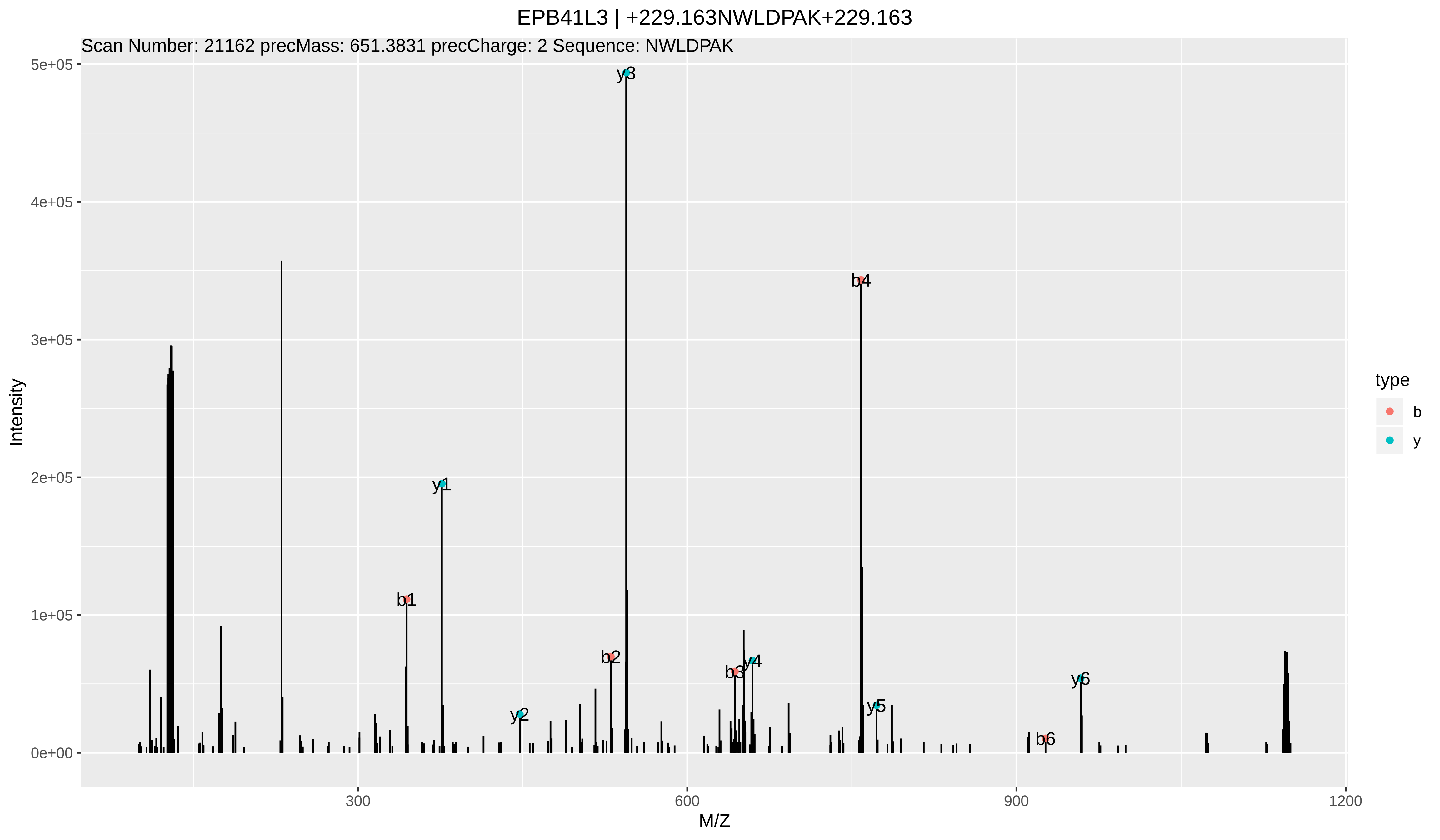

EPB41L3 | +229.163NWLDPAK+229.163
Scan Number: 21162 precMass: 651.3831 precCharge: 2 Sequence: NWLDPAK
Intensity
0e+00
1e+05
2e+05
3e+05
4e+05
5e+05
M/Z
300
600
900
1200
y3
b4
y1
b1
b2
b3
y4
y2
y5
y6
b6
type
b
y

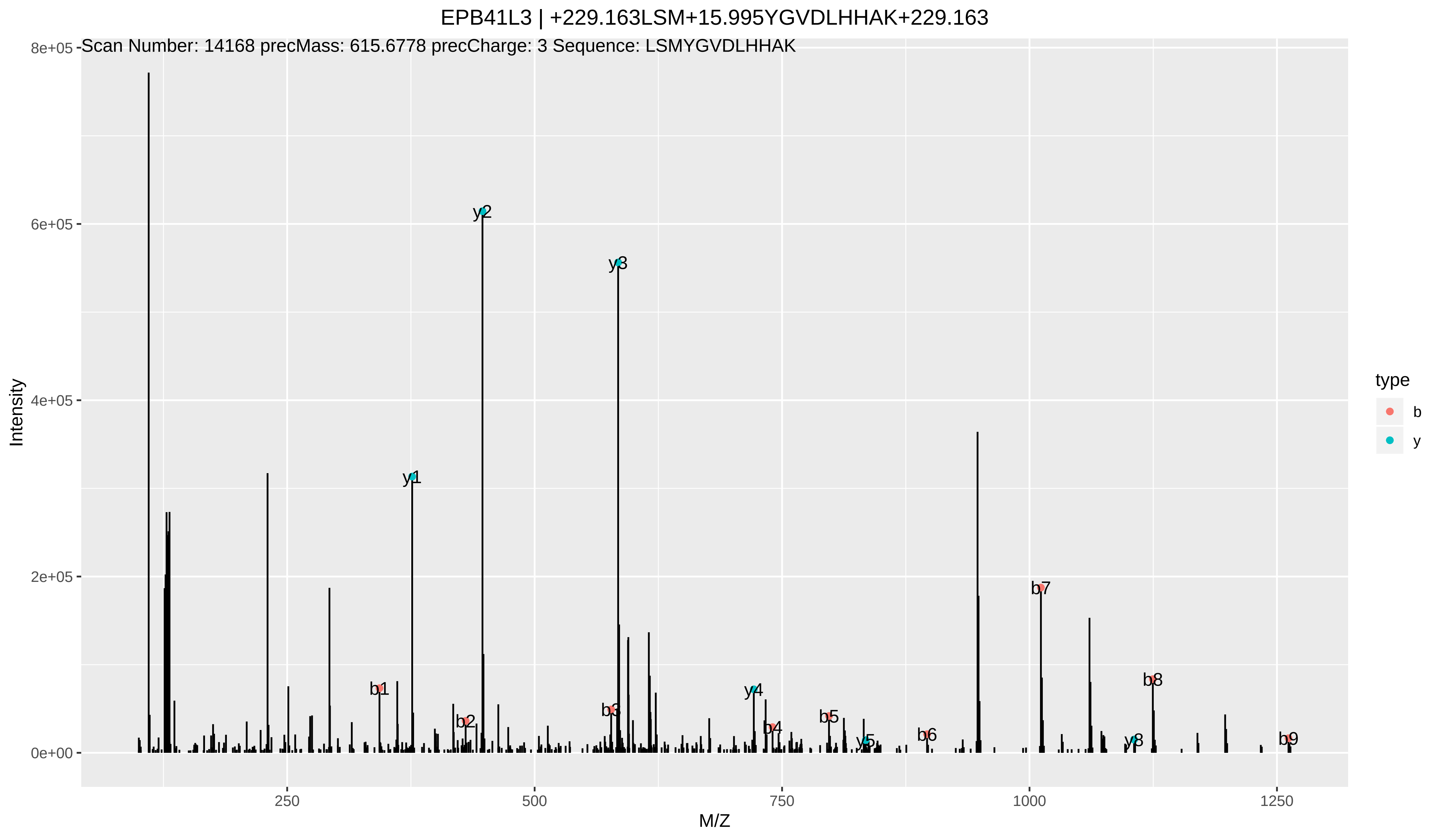
EPB41L3 | +229.163LSM+15.995YGVDLHHAK+229.163
Scan Number: 14168 precMass: 615.6778 precCharge: 3 Sequence: LSMYGVDLHHAK
Intensity
M/Z
0e+00
2e+05
4e+05
6e+05
8e+05
250
500
750
1000
1250
y1
y2
y3
y4
y5
y8
b1
b2
b3
b4
b5
b6
b7
b8
b9
type
b
y

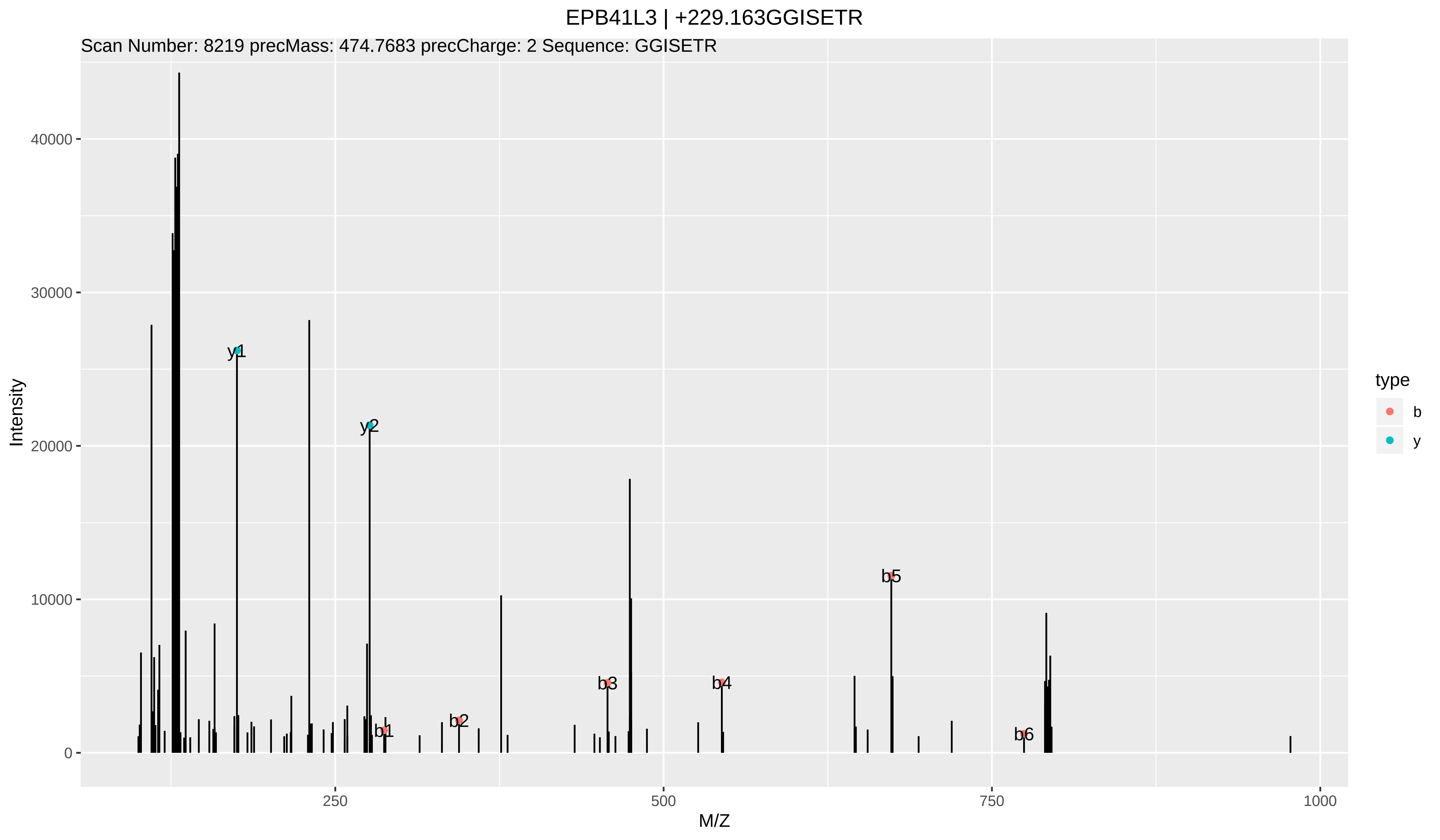
EPB41L3 | +229.163GGISETR
Scan Number: 8219 precMass: 474.7683 precCharge: 2 Sequence: GGISETR
Intensity
M/Z
0
10000
20000
30000
40000
250
500
750
1000
y1
y2
b1
b2
b3
b4
b5
b6
type
b
y

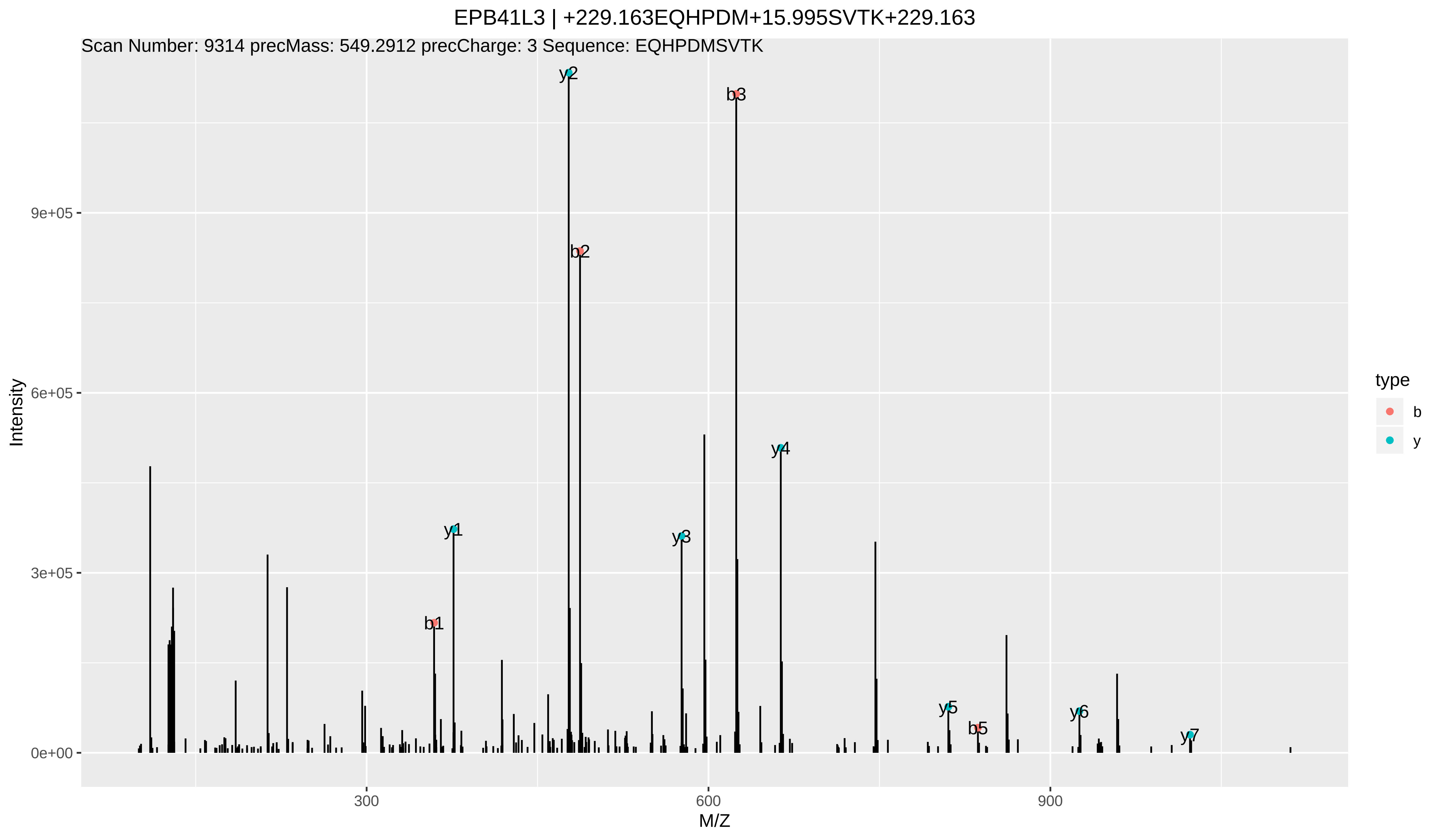
EPB41L3 | +229.163EQHPDM+15.995SVTK+229.163
Scan Number: 9314 precMass: 549.2912 precCharge: 3 Sequence: EQHPDMSVTK
Intensity
M/Z
0e+00
3e+05
6e+05
9e+05
300
600
900
y2
b3
b2
y4
y1
y3
b1
y5
b5
y6
y7
type
b
y

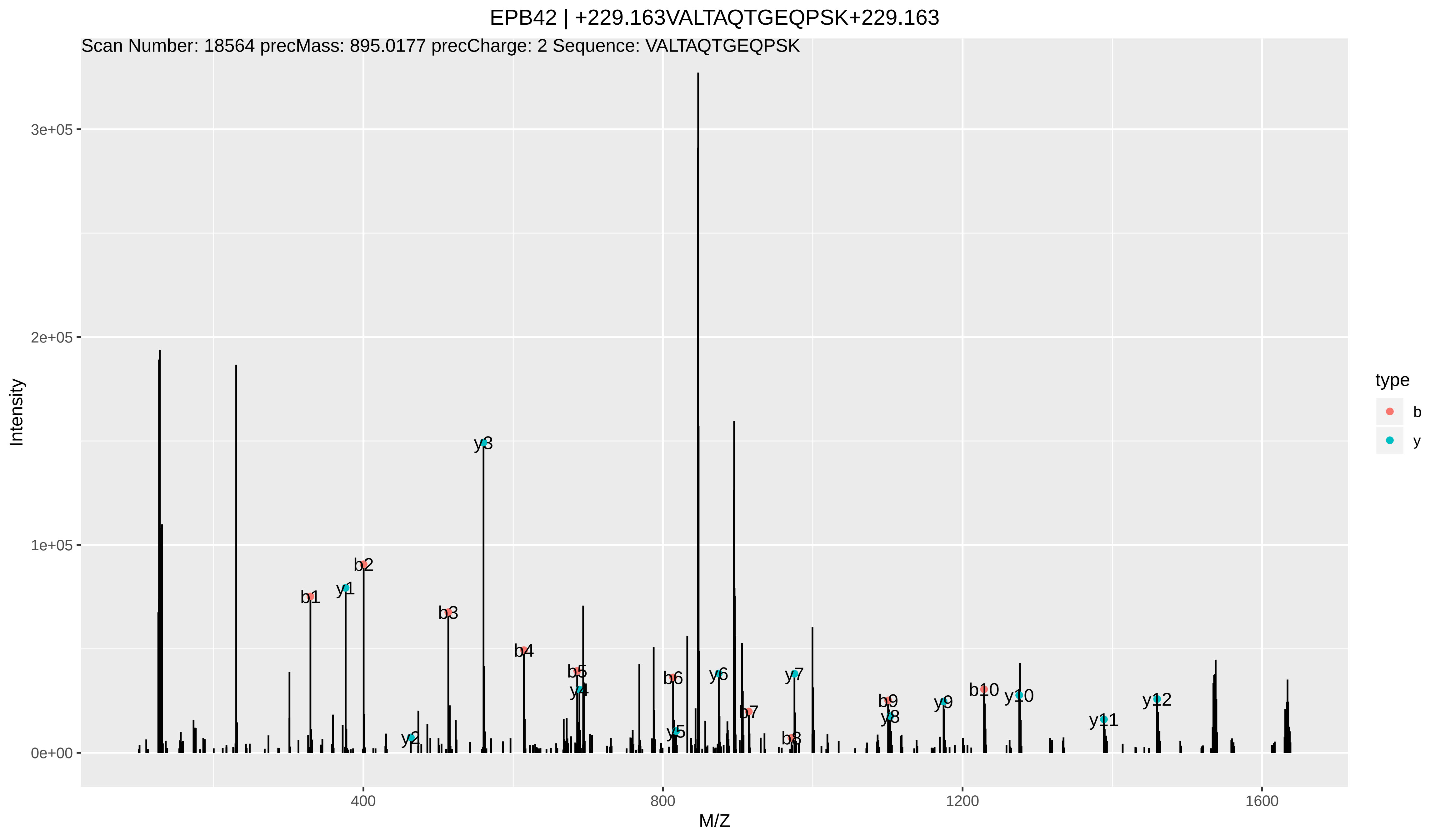
EPB42 | +229.163VALTAQTGEQPSK+229.163
Scan Number: 18564 precMass: 895.0177 precCharge: 2 Sequence: VALTAQTGEQPSK
Intensity
M/Z
3e+05
2e+05
1e+05
0e+00
400
800
1200
1600
b1
y1
b2
b3
y3
y2
b4
b5
y4
b6
y5
y6
b7
y7
b8
b9
y8
y9
b10
y10
y11
y12
type
b
y

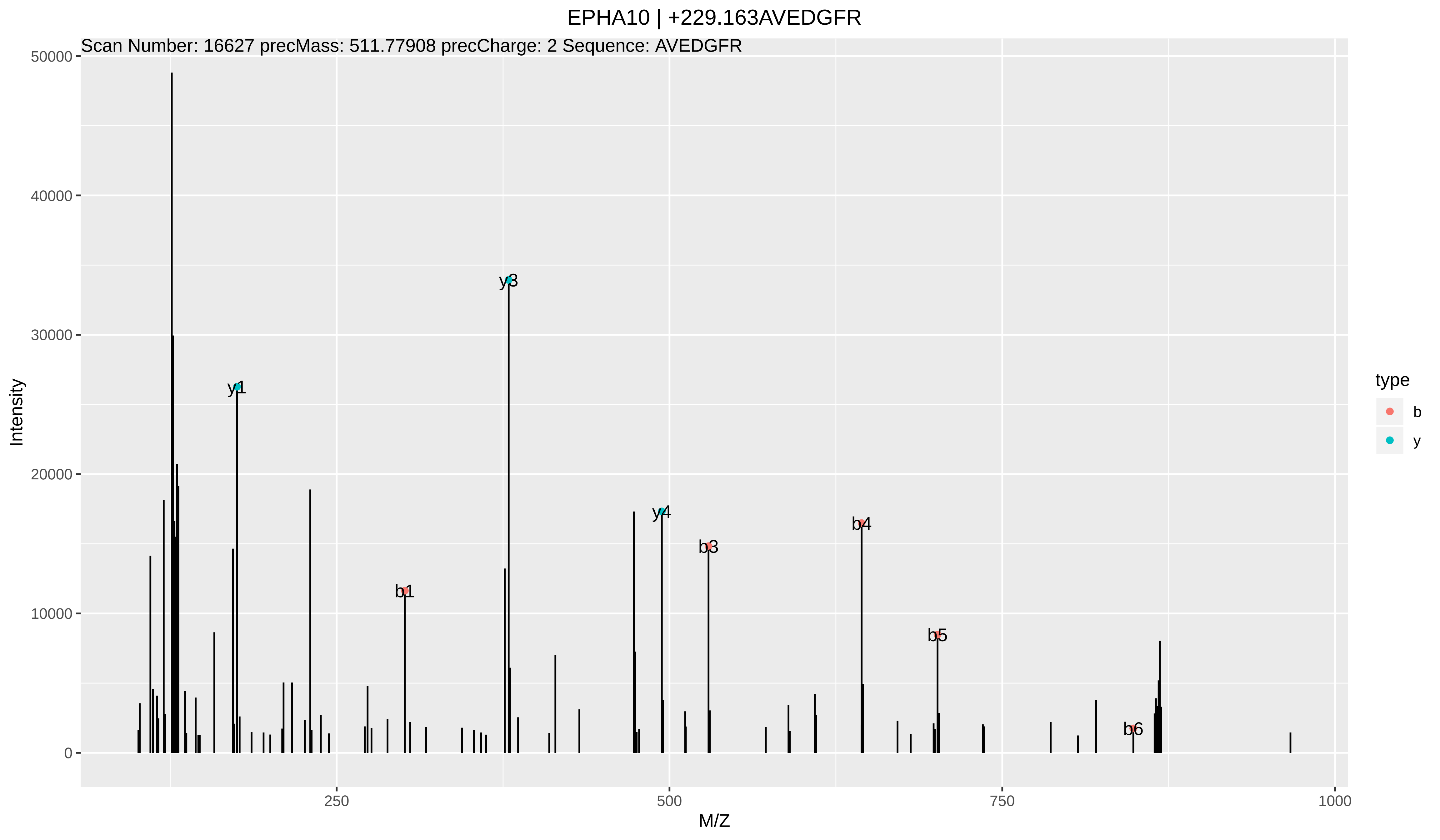

EPHA10 | +229.163AVEDGFR
Scan Number: 16627 precMass: 511.77908 precCharge: 2 Sequence: AVEDGFR
Intensity
M/Z
0
10000
20000
30000
40000
50000
250
500
750
1000
y1
y3
y4
b1
b3
b4
b5
b6
type
b
y

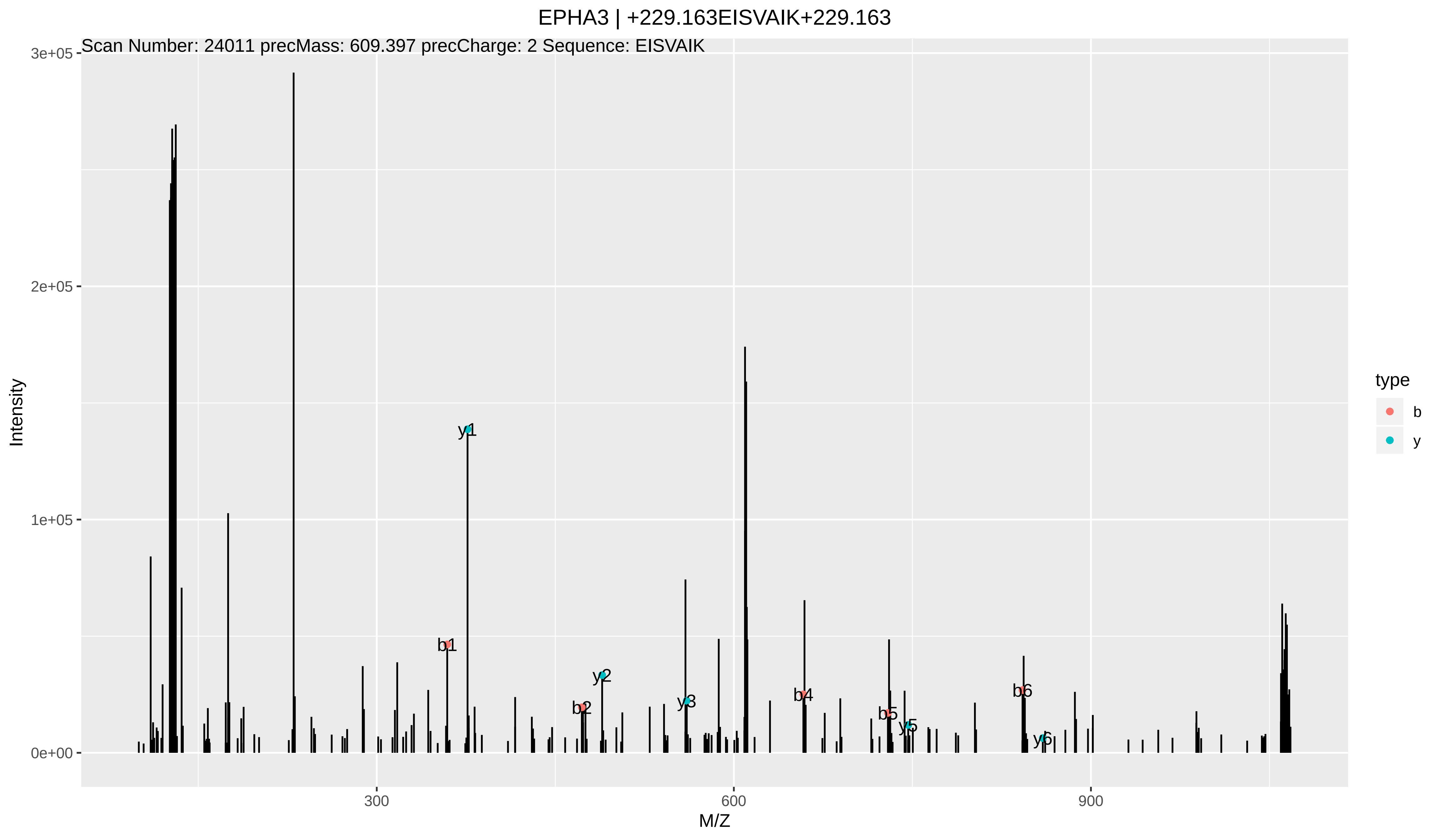
EPHA3 | +229.163EISVAIK+229.163
Scan Number: 24011 precMass: 609.397 precCharge: 2 Sequence: EISVAIK
Intensity
3e+05
2e+05
1e+05
0e+00
300
600
900
M/Z
y1
b1
y2
b2
y3
b4
b5
y5
b6
y6
type
b
y

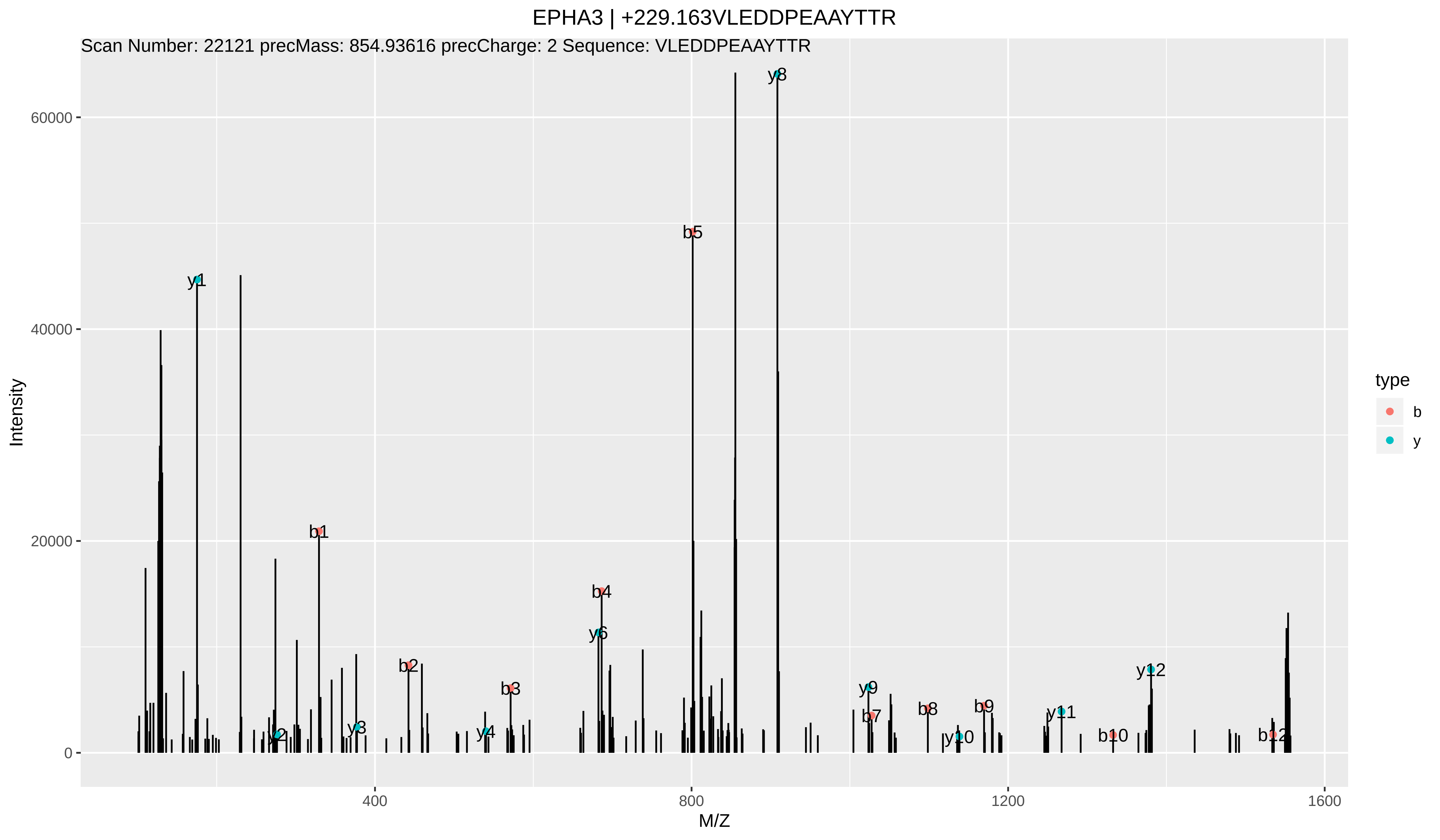

EPHA3 | +229.163VLEDDPEAAYTTR
Scan Number: 22121 precMass: 854.93616 precCharge: 2 Sequence: VLEDDPEAAYTTR
Intensity
M/Z
type
b
y
y1
b1
y2
y3
b2
y4
b3
y6
b4
b5
y8
y9
b7
b8
y10
b9
y11
b10
y12
b12
0
20000
40000
60000
400
800
1200
1600

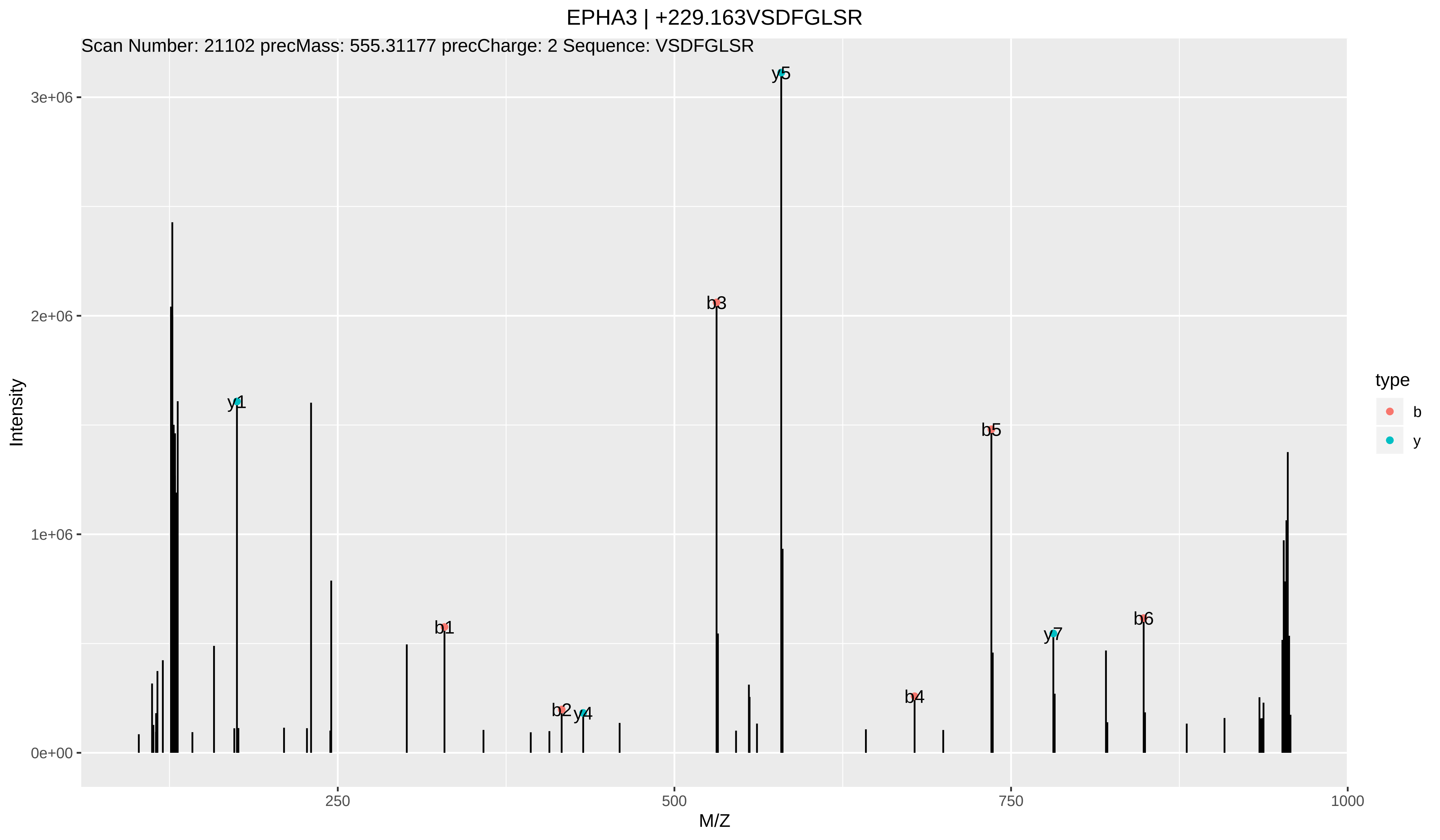
EPHA3 | +229.163VSDFGLSR
Scan Number: 21102 precMass: 555.31177 precCharge: 2 Sequence: VSDFGLSR
y5
b3
y1
b5
b1
b6
y7
b4
b2
y4
3e+06
2e+06
1e+06
0e+00
Intensity
250
500
750
1000
M/Z
type
b
y

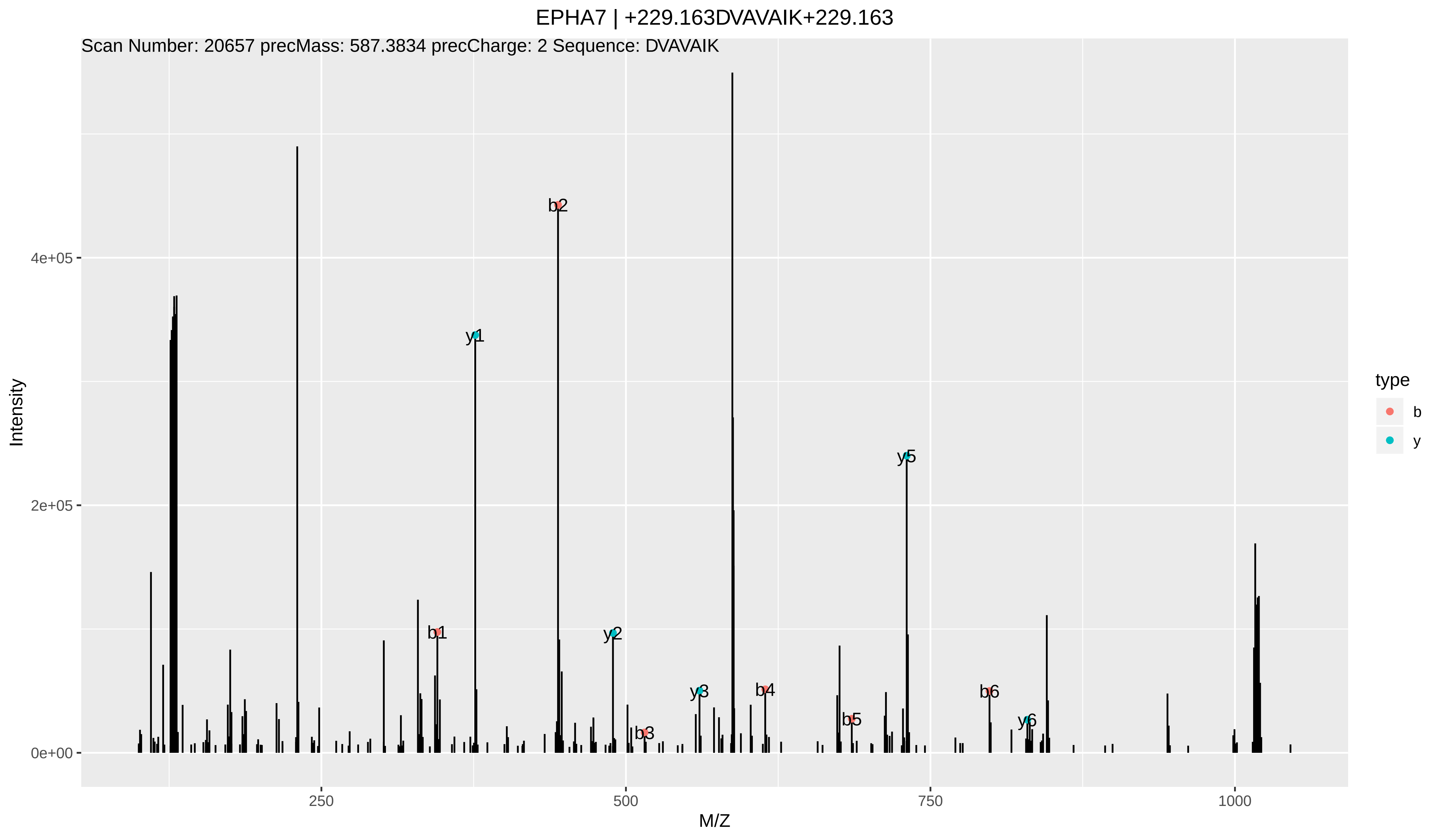
EPHA7 | +229.163DVAVAIK+229.163
Scan Number: 20657 precMass: 587.3834 precCharge: 2 Sequence: DVAVAIK
Intensity
4e+05
2e+05
0e+00
M/Z
250
500
750
1000
b2
y1
y5
b1
y2
y3
b4
b3
b5
b6
y6
type
b
y

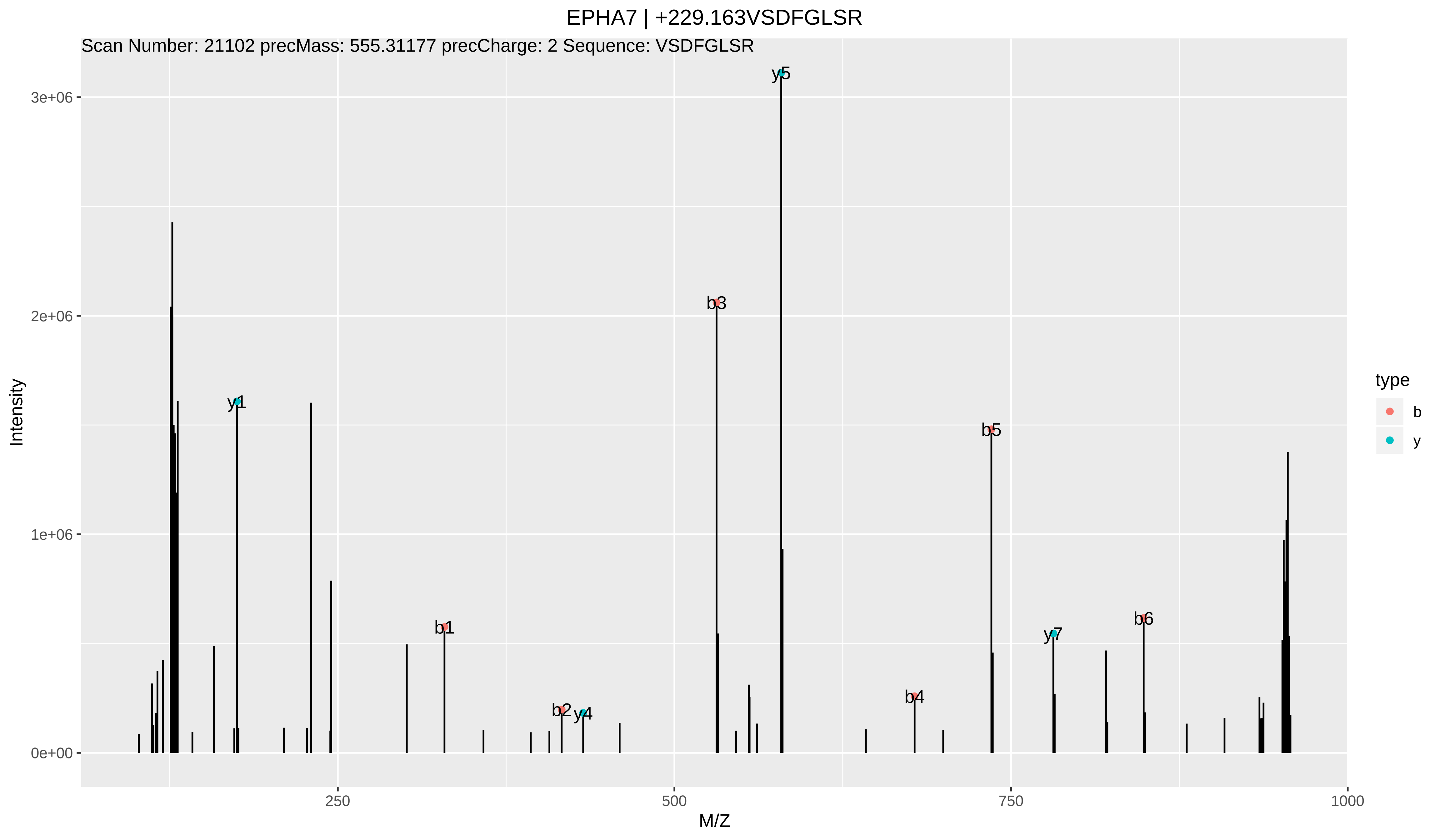

EPHA7 | +229.163VSDFGLSR
Scan Number: 21102 precMass: 555.31177 precCharge: 2 Sequence: VSDFGLSR
Intensity
M/Z
0e+00
1e+06
2e+06
3e+06
250
500
750
1000
y5
b3
y1
b5
b1
b2
y4
b4
y7
b6
type
b
y

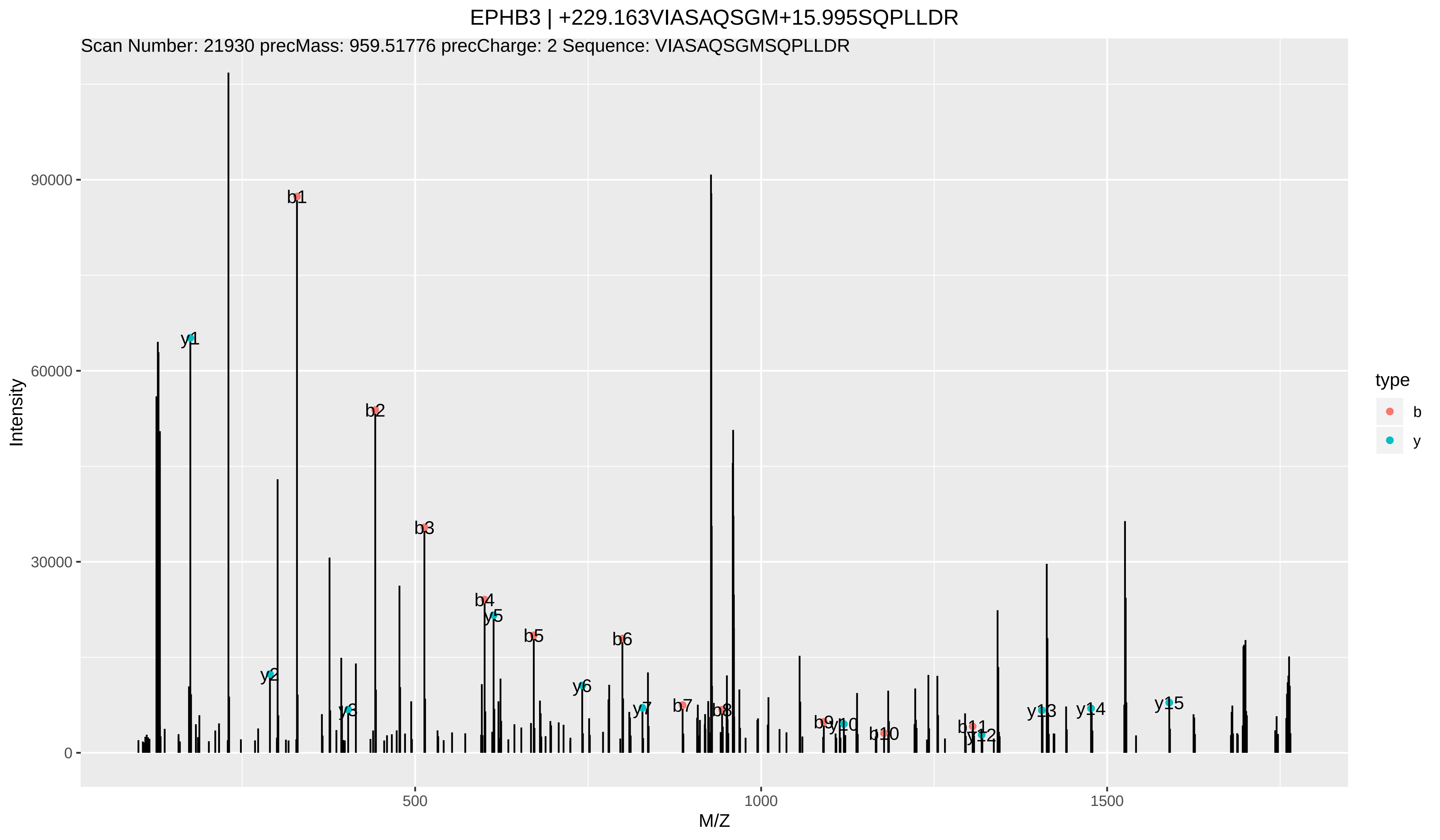
EPHB3 | +229.163VIASAQSGM+15.995SQPLLDR
Scan Number: 21930 precMass: 959.51776 precCharge: 2 Sequence: VIASAQSGMSQPLLDR
Intensity
M/Z
90000
60000
30000
0
500
1000
1500
y1
b1
b2
b3
b4
y5
b5
b6
y2
y3
y6
y7
b7
b8
b9
y10
b10
b11
y12
y13
y14
y15
type
b
y

EPHB3 | +229.163DFLSEASIMGQFDHPNIIR

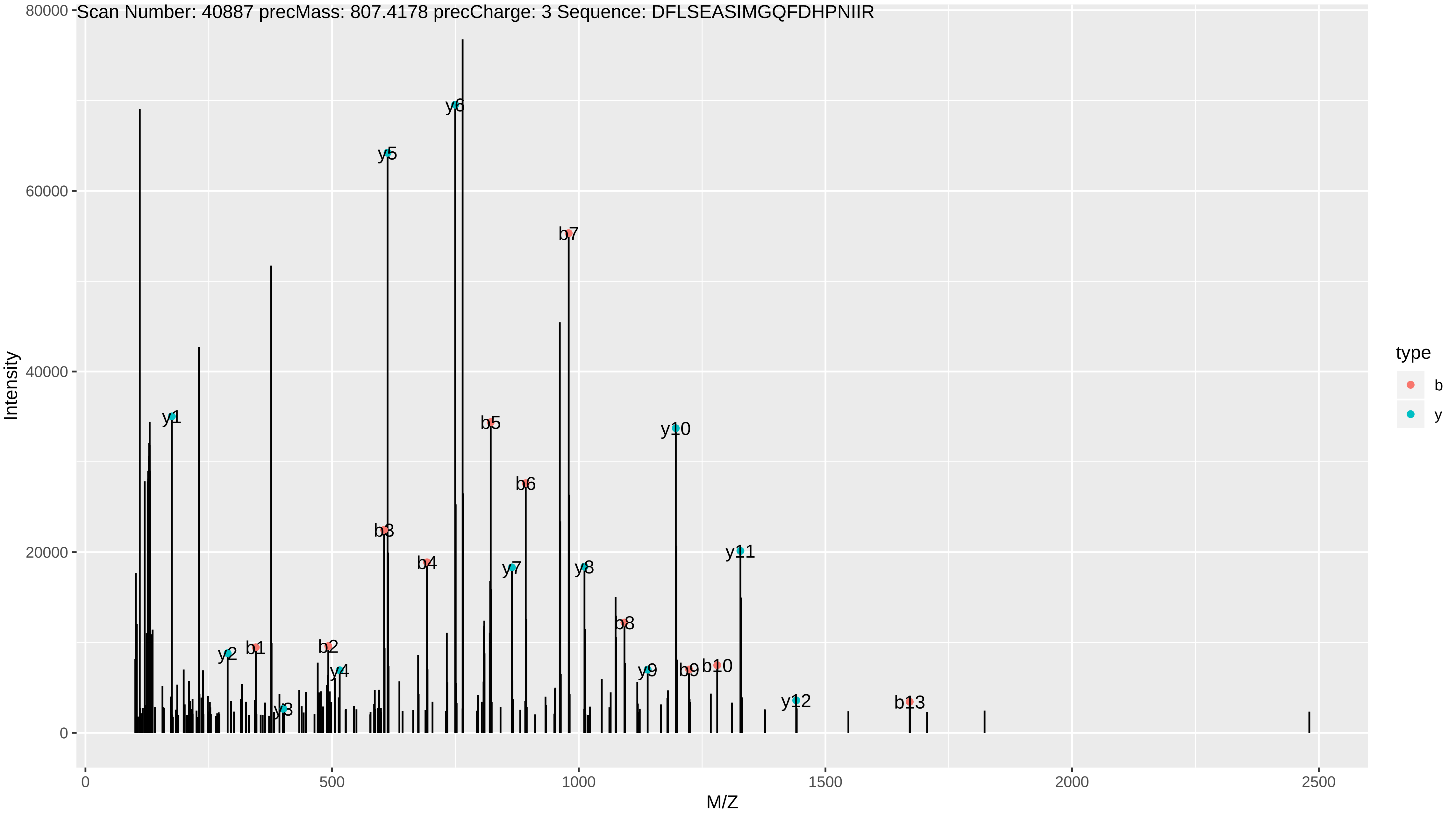

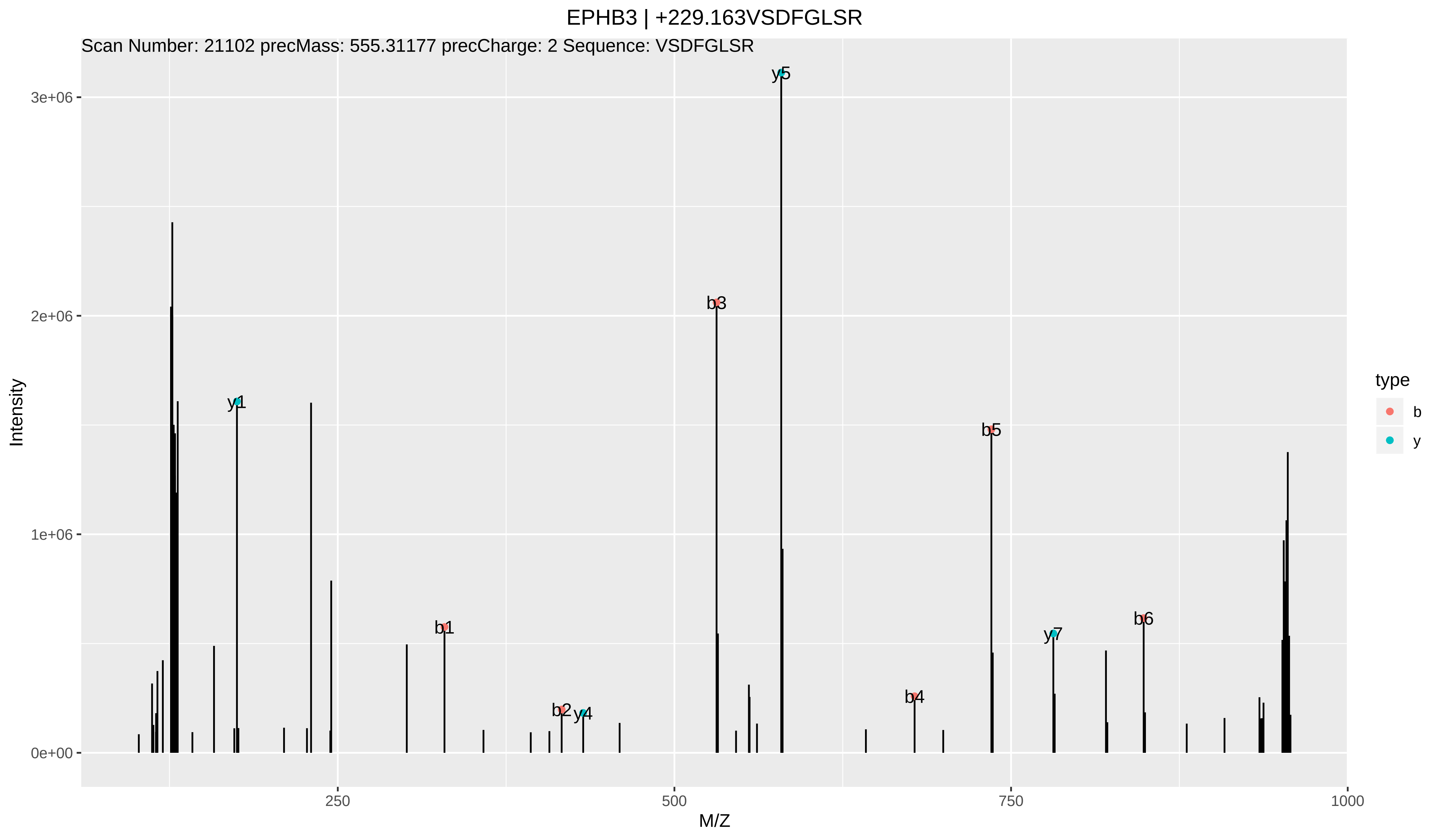
EPHB3 | +229.163VSDFGLSR
Scan Number: 21102 precMass: 555.31177 precCharge: 2 Sequence: VSDFGLSR
Intensity
3e+06
2e+06
1e+06
0e+00
M/Z
250
500
750
1000
y1
b1
b2
y4
b3
y5
b4
b5
y7
b6
type
b
y

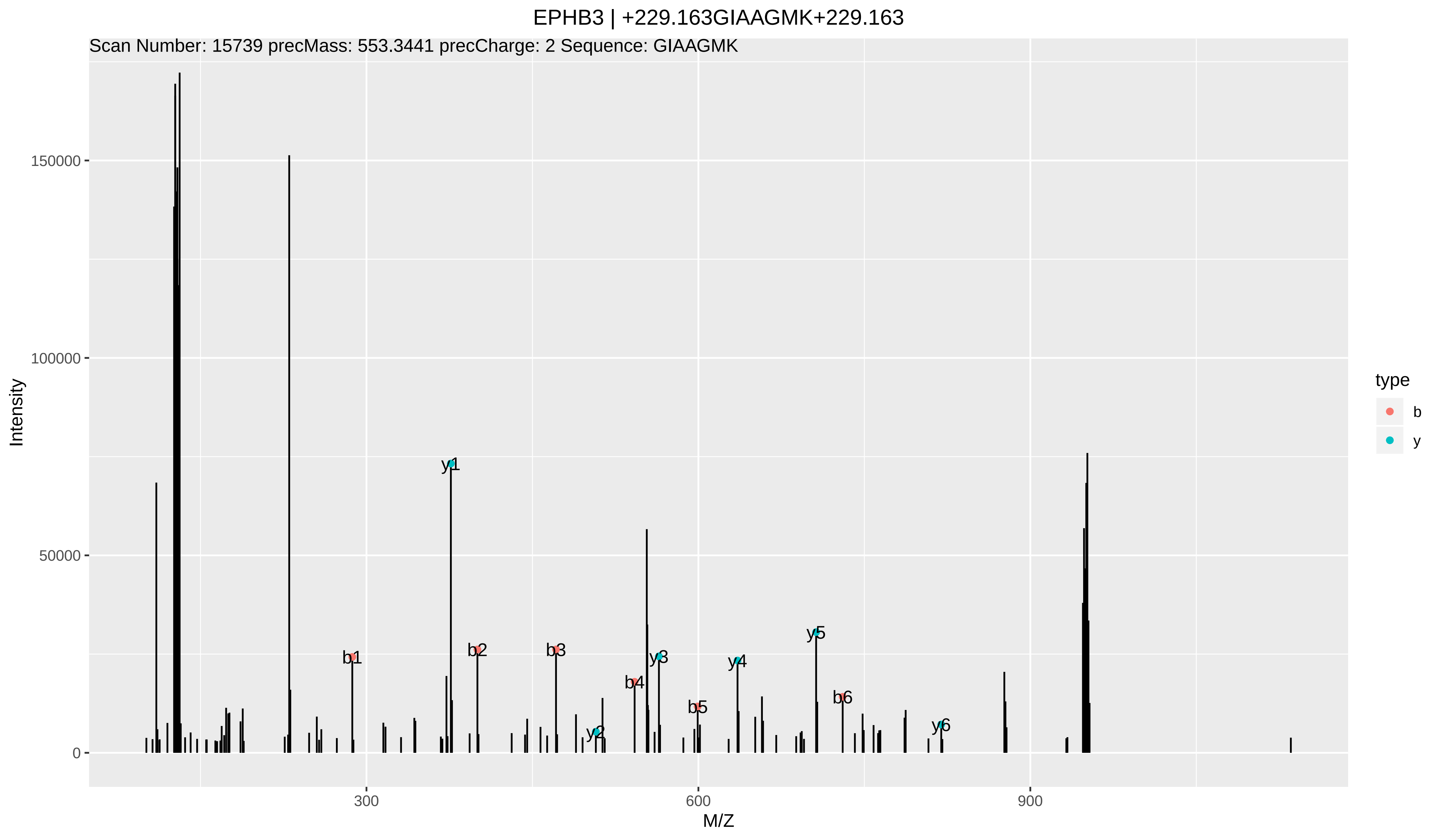

EPHB3 | +229.163GIAAGMK+229.163
Scan Number: 15739 precMass: 553.3441 precCharge: 2 Sequence: GIAAGMK
Intensity
150000
100000
50000
0
300
600
900
M/Z
b1
b2
b3
b4
b5
b6
y1
y2
y3
y4
y5
y6
type
b
y

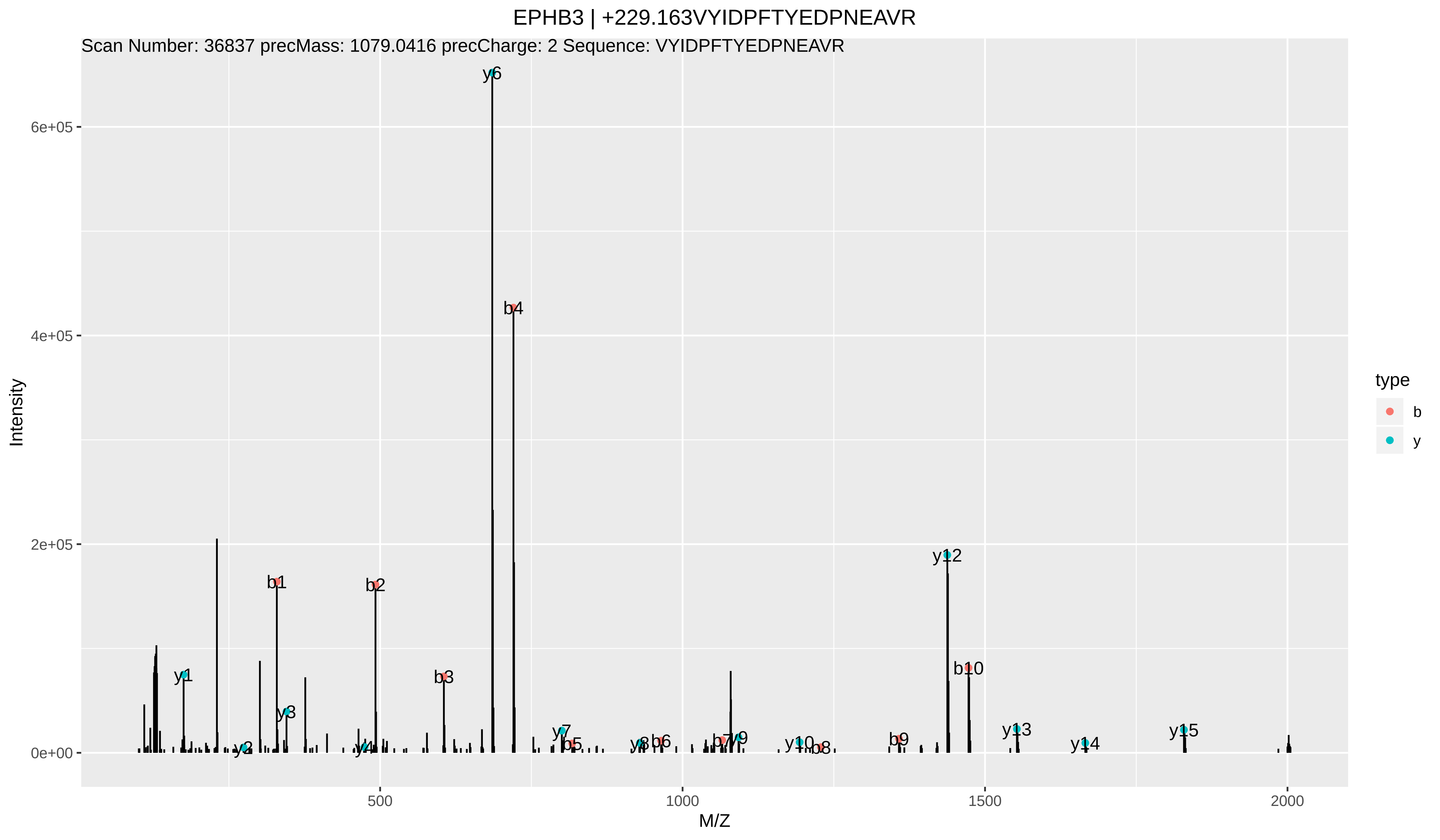
EPHB3 | +229.163VYIDPFTYEDPNEAVR
Scan Number: 36837 precMass: 1079.0416 precCharge: 2 Sequence: VYIDPFTYEDPNEAVR
Intensity
M/Z
type
b
y
6e+05
4e+05
2e+05
0e+00
500
1000
1500
2000
y6
b4
y12
b1
b2
b10
y1
b3
y3
y7
b5
y2
y4
y8
b6
b7
y9
y10
b8
b9
y13
y14
y15

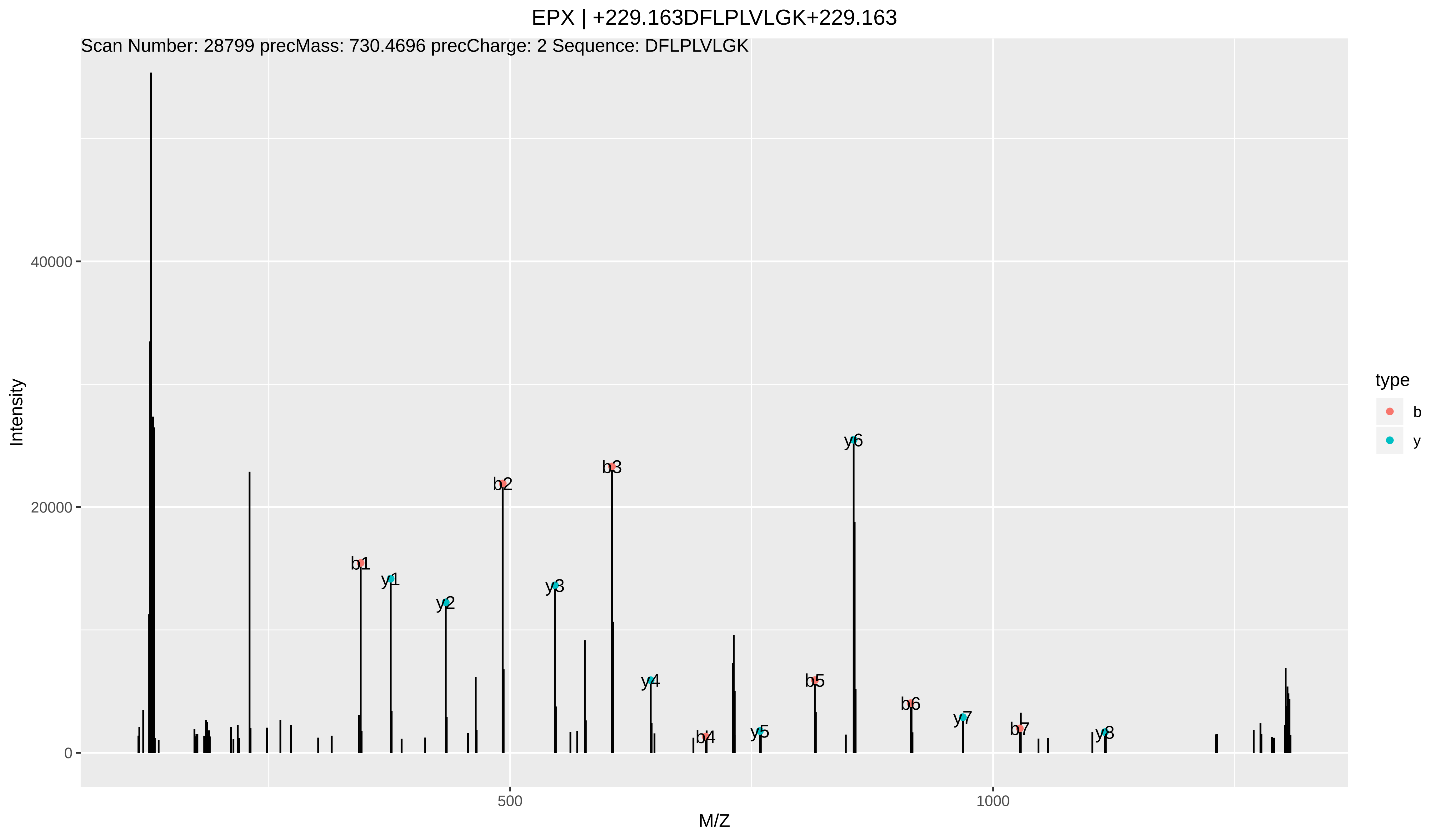

EPX | +229.163DFLPLVLGK+229.163
Scan Number: 28799 precMass: 730.4696 precCharge: 2 Sequence: DFLPLVLGK
Intensity
40000
20000
0
500
1000
M/Z
b1
y1
y2
b2
y3
b3
y4
b4
y5
b5
y6
b6
y7
b7
y8
type
b
y

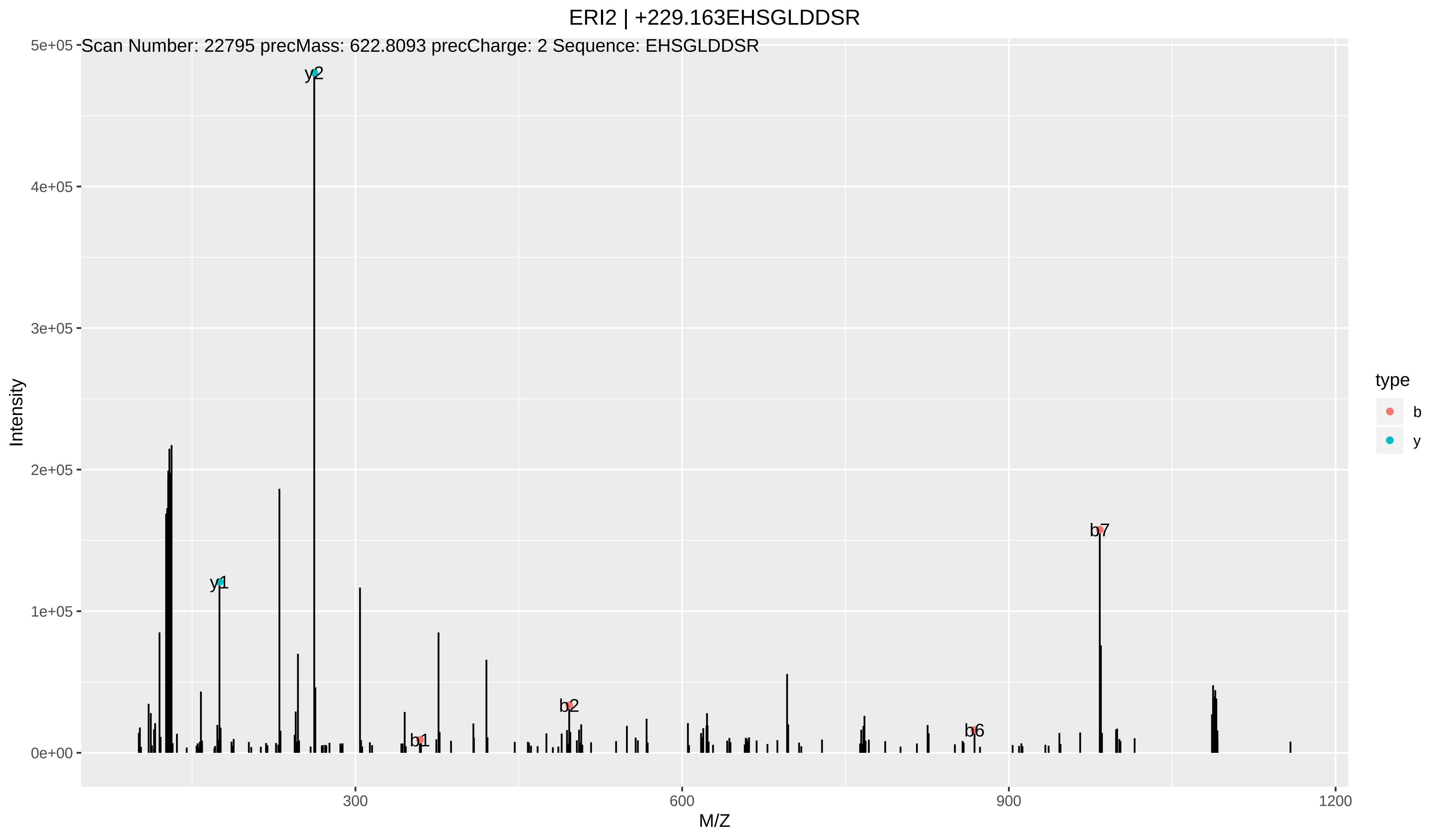

ERI2 | +229.163EHSGLDDSR
Scan Number: 22795 precMass: 622.8093 precCharge: 2 Sequence: EHSGLDDSR
y2
y1
b1
b2
b6
b7
Intensity
5e+05
4e+05
3e+05
2e+05
1e+05
0e+00
300
600
900
1200
M/Z
type
b
y

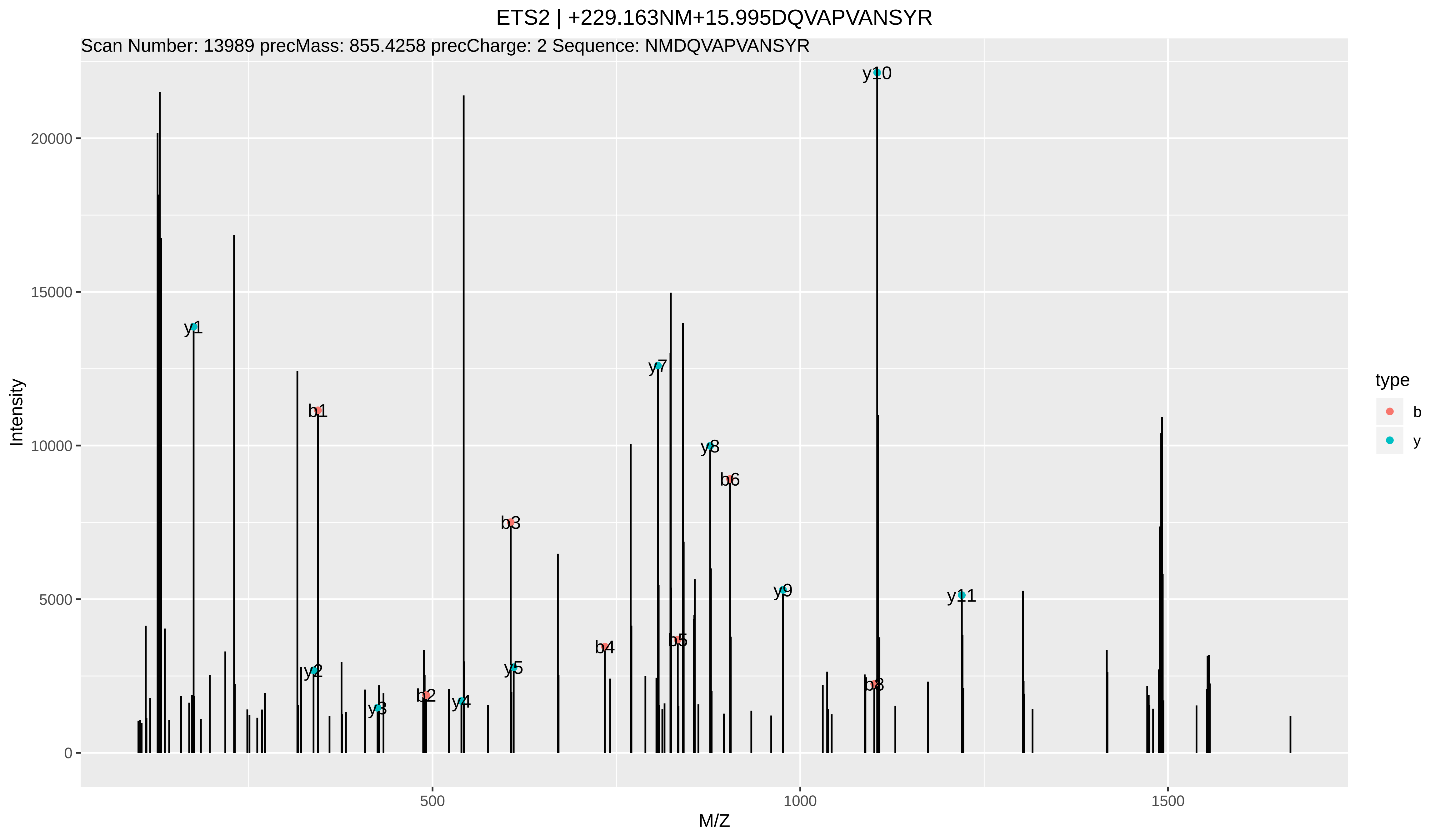

ETS2 | +229.163NM+15.995DQVAPVANSYR
Scan Number: 13989 precMass: 855.4258 precCharge: 2 Sequence: NMDQVAPVANSYR
Intensity
M/Z
0
5000
10000
15000
20000
500
1000
1500
y1
y2
y3
y4
y5
y7
y8
y9
y10
y11
b1
b2
b3
b4
b5
b6
b8
type
b
y

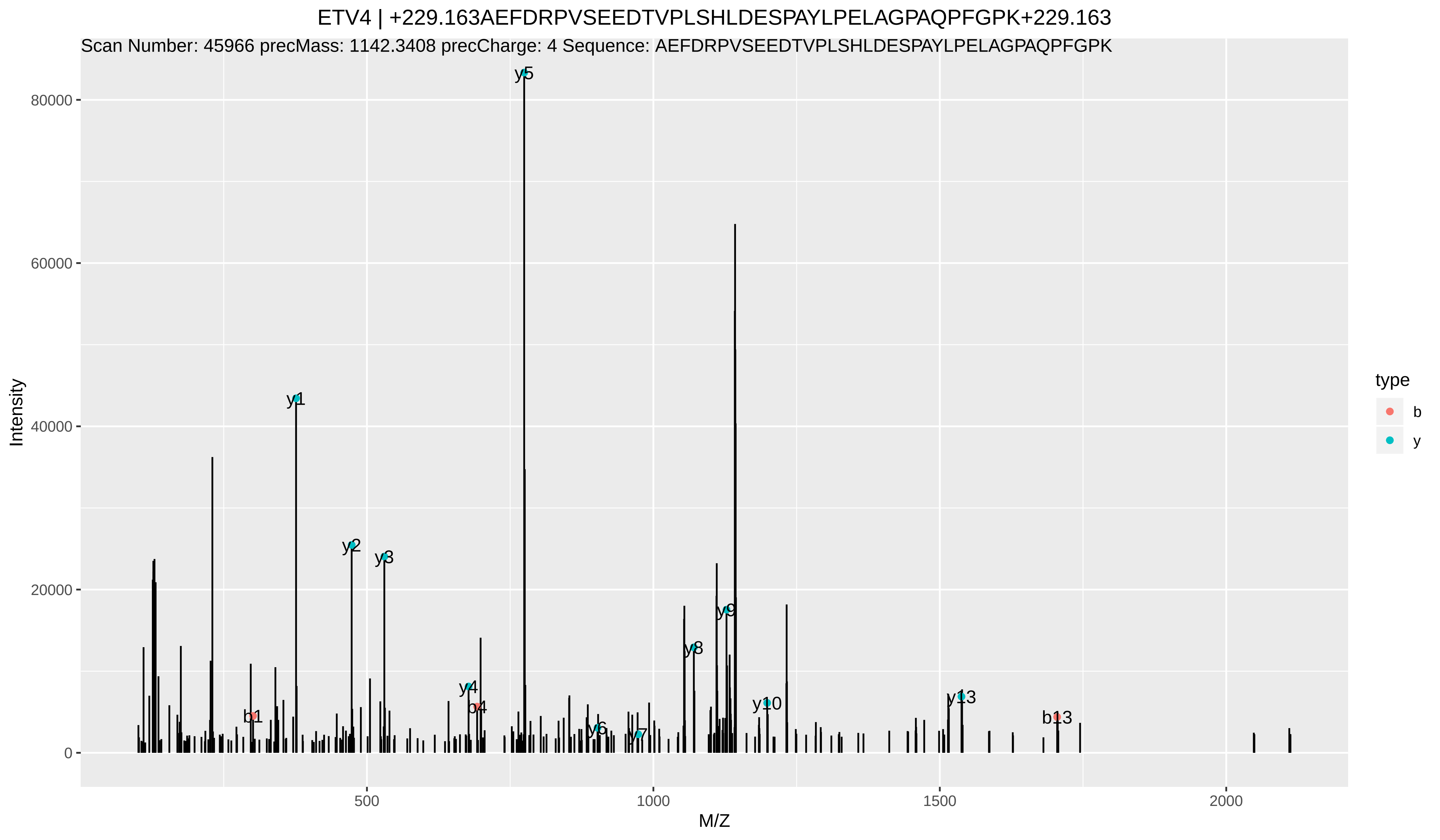

ETV4 | +229.163AEFDRPVSEEDTVPLSHLDESPAYLPELAGPAQPFGPK+229.163
Scan Number: 45966 precMass: 1142.3408 precCharge: 4 Sequence: AEFDRPVSEEDTVPLSHLDESPAYLPELAGPAQPFGPK
Intensity
M/Z
0
20000
40000
60000
80000
500
1000
1500
2000
y5
y1
y2
y3
y4
b4
b1
y6
y7
y8
y9
y10
y13
b13
type
b
y

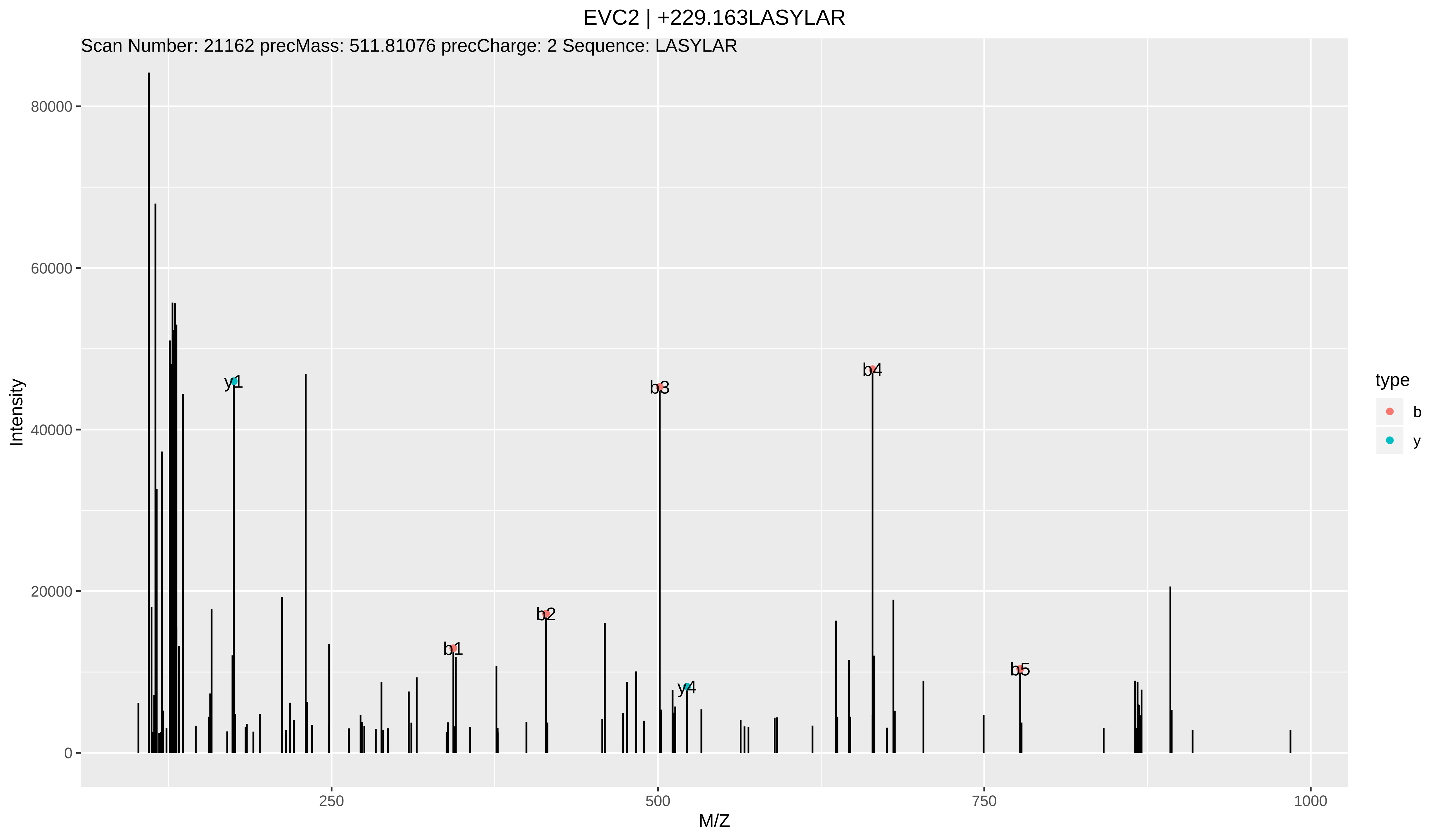

EVC2 | +229.163LASYLAR
Scan Number: 21162 precMass: 511.81076 precCharge: 2 Sequence: LASYLAR
Intensity
80000
60000
40000
20000
0
M/Z
250
500
750
1000
y1
b1
b2
b3
y4
b4
b5
type
b
y

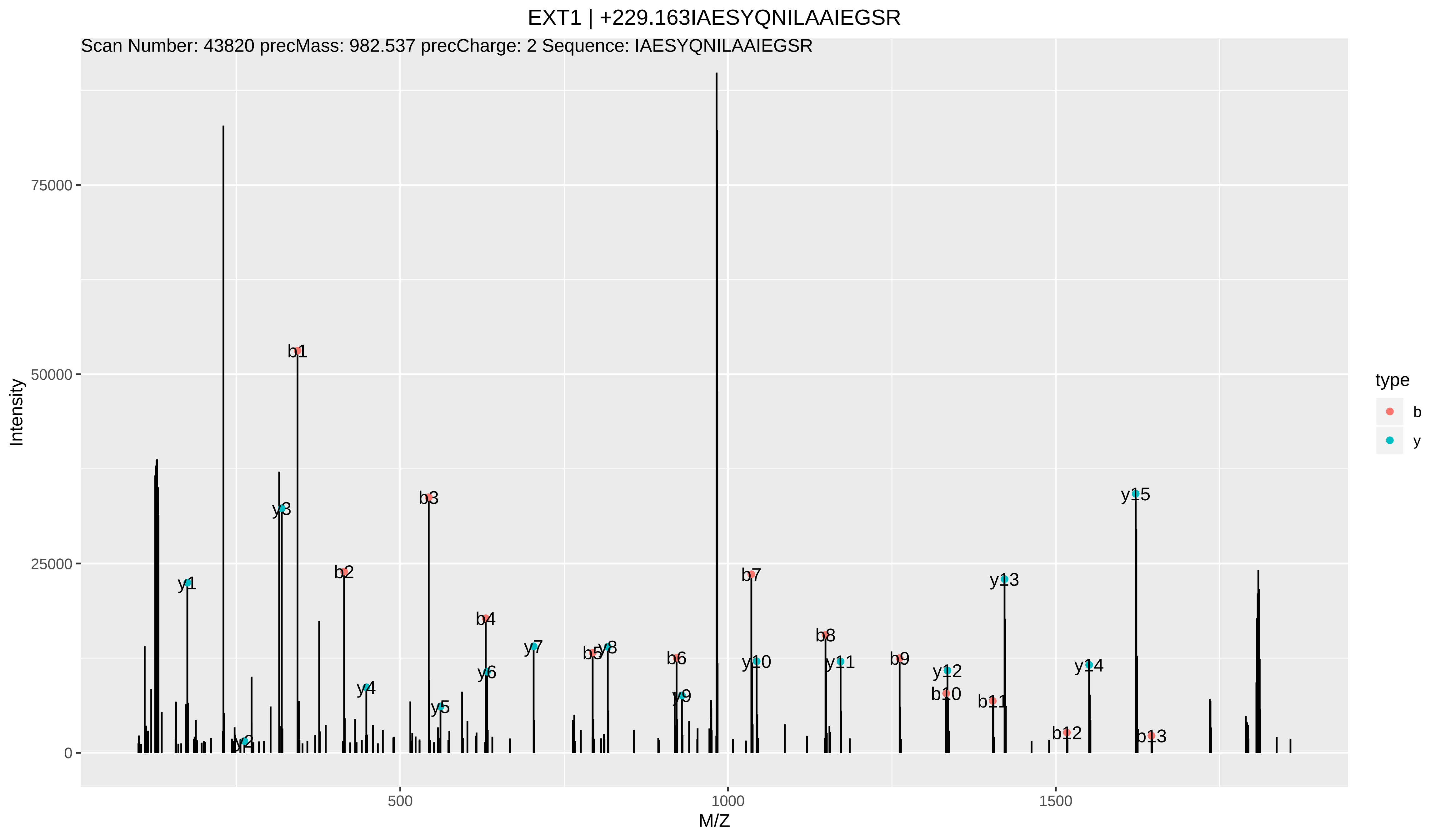
EXT1 | +229.163IAESYQNILAAIEGSR
Scan Number: 43820 precMass: 982.537 precCharge: 2 Sequence: IAESYQNILAAIEGSR
Intensity
M/Z
type
b
y
75000
50000
25000
0
500
1000
1500
b1
b2
b3
b4
b5
b6
b7
b8
b9
b10
b11
b12
b13
y1
y2
y3
y4
y5
y6
y7
y8
y9
y10
y11
y12
y13
y14
y15

EXT1 | +229.163LDPVLFK+229.163

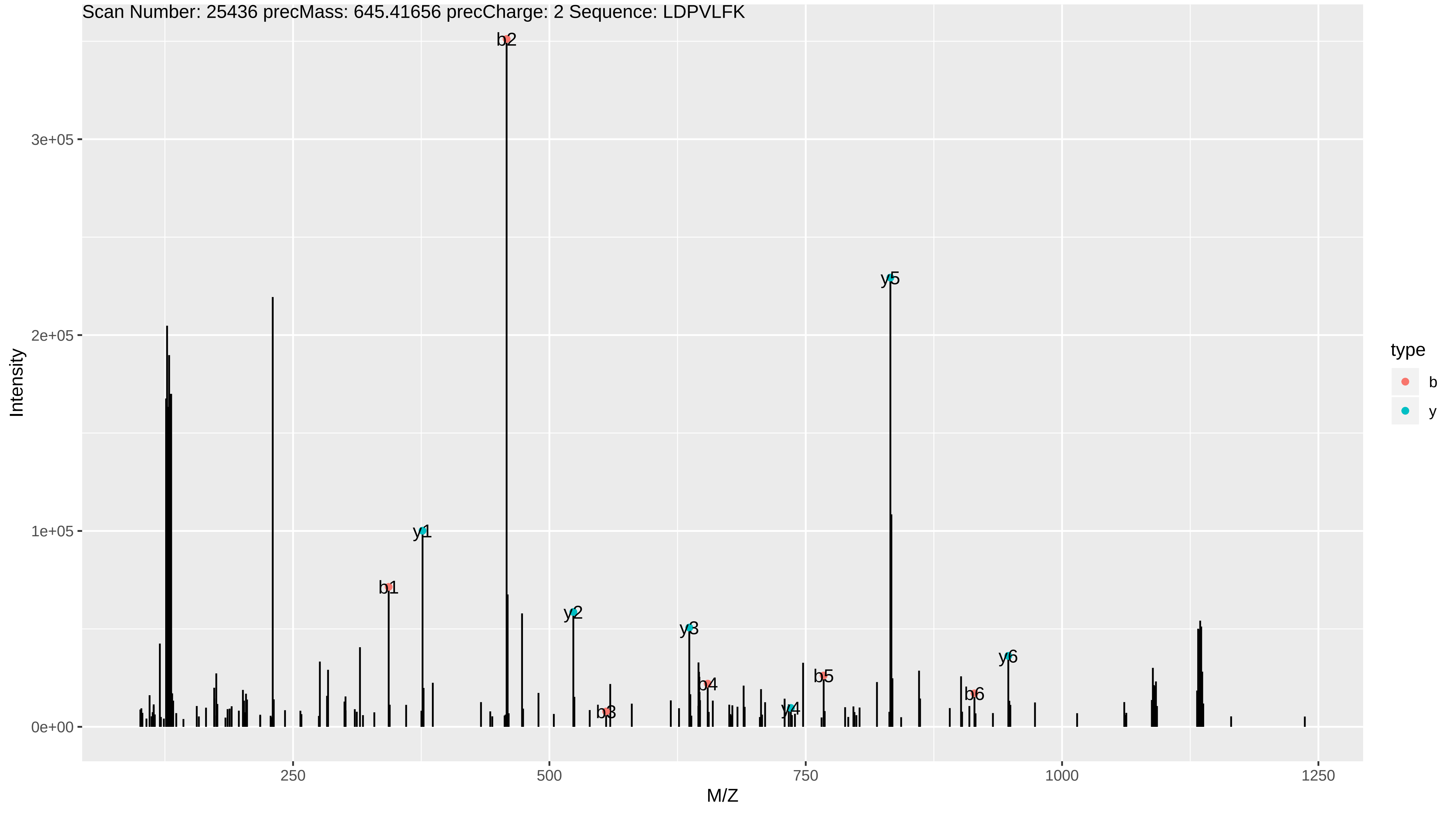

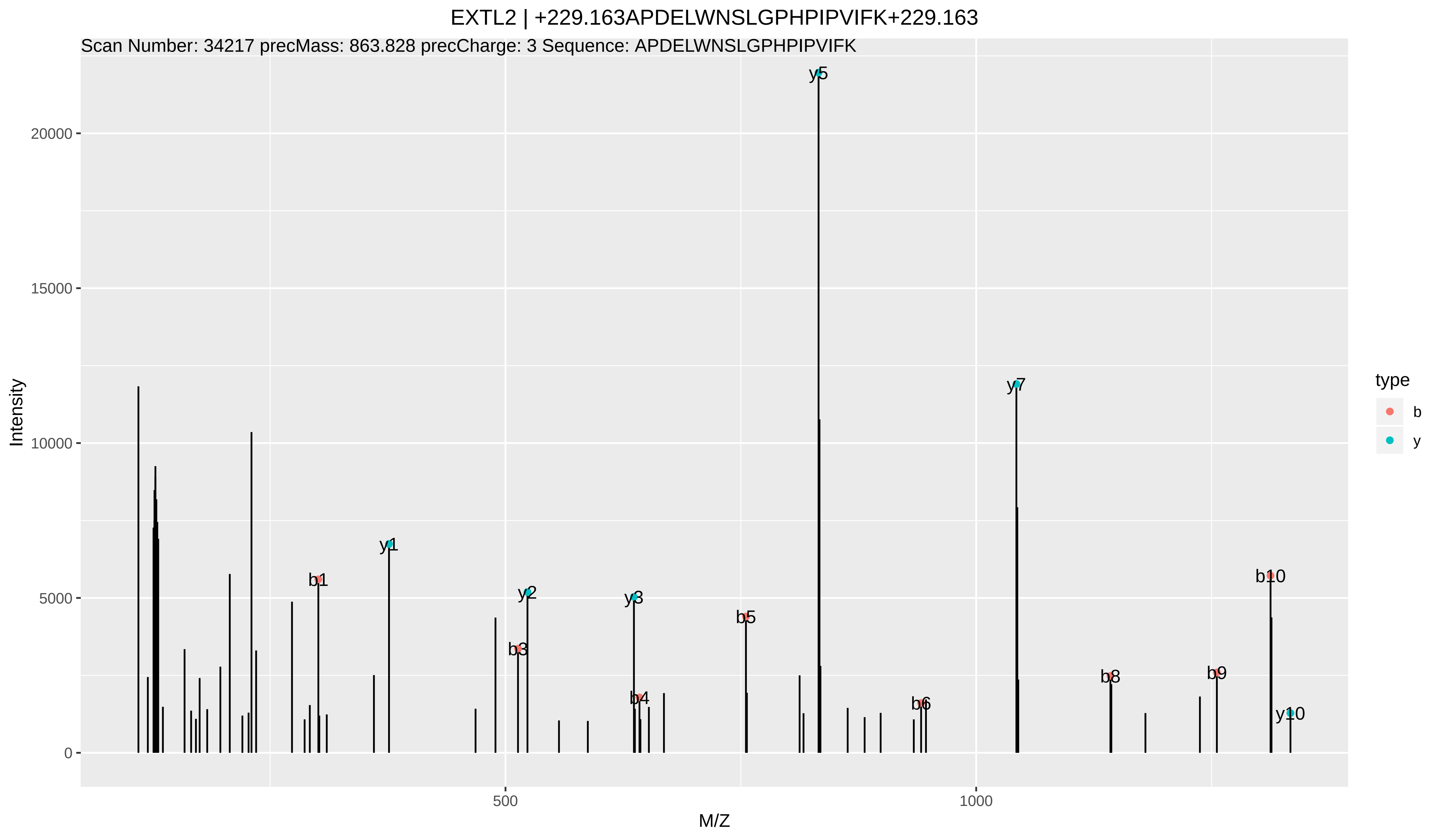
EXTL2 | +229.163APDELWNSLGPHPIPVIFK+229.163
Scan Number: 34217 precMass: 863.828 precCharge: 3 Sequence: APDELWNSLGPHPIPVIFK
Intensity
20000
15000
10000
5000
0
500
1000
M/Z
y5
y7
y1
b1
y2
y3
b3
b5
b4
b6
b8
b9
b10
y10
type
b
y

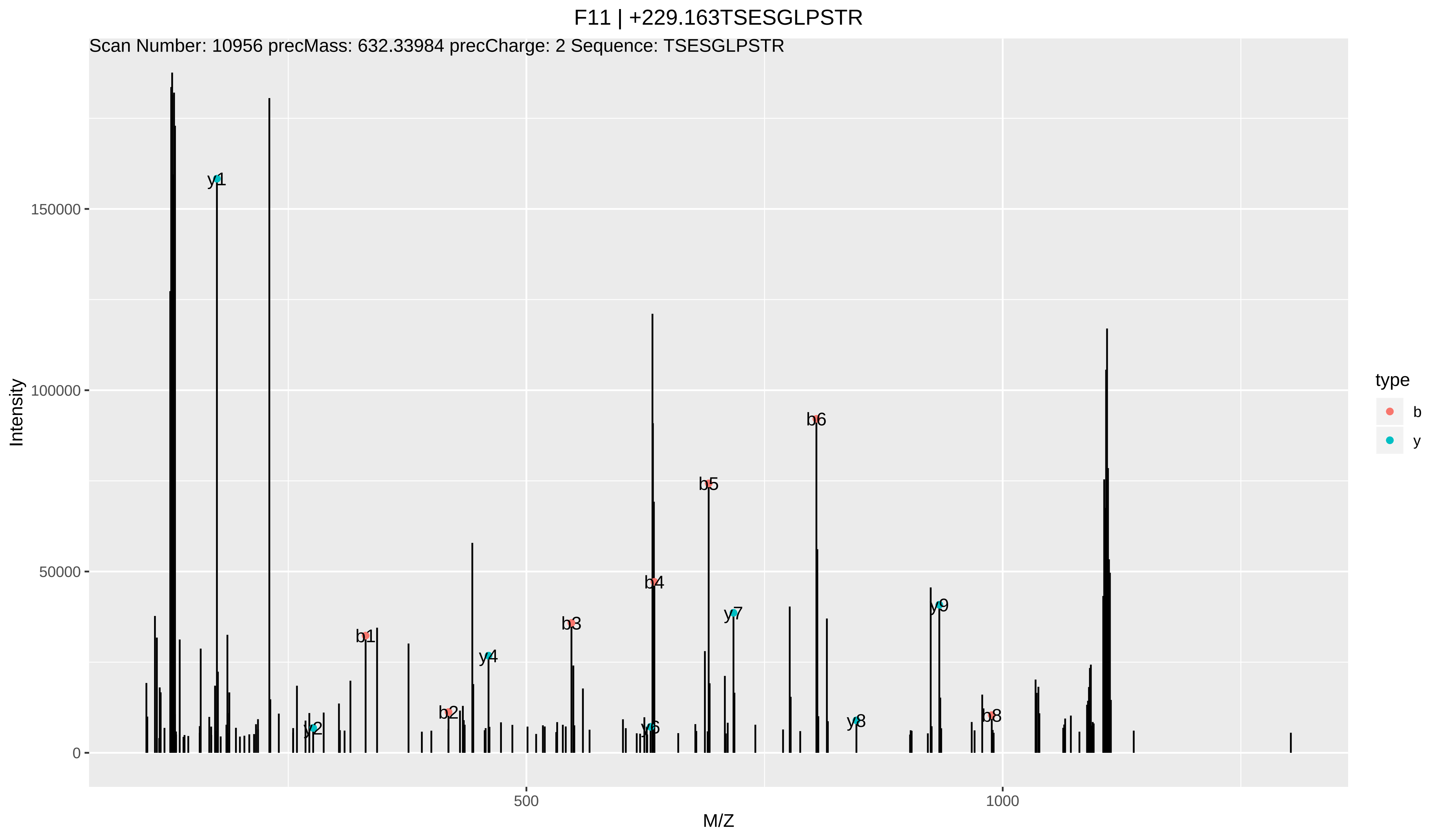
F11 | +229.163TSESGLPSTR
Scan Number: 10956 precMass: 632.33984 precCharge: 2 Sequence: TSESGLPSTR
Intensity
150000
100000
50000
0
M/Z
500
1000
y1
y2
b1
b2
y4
b3
b4
y6
b5
y7
b6
y8
y9
b8
type
b
y

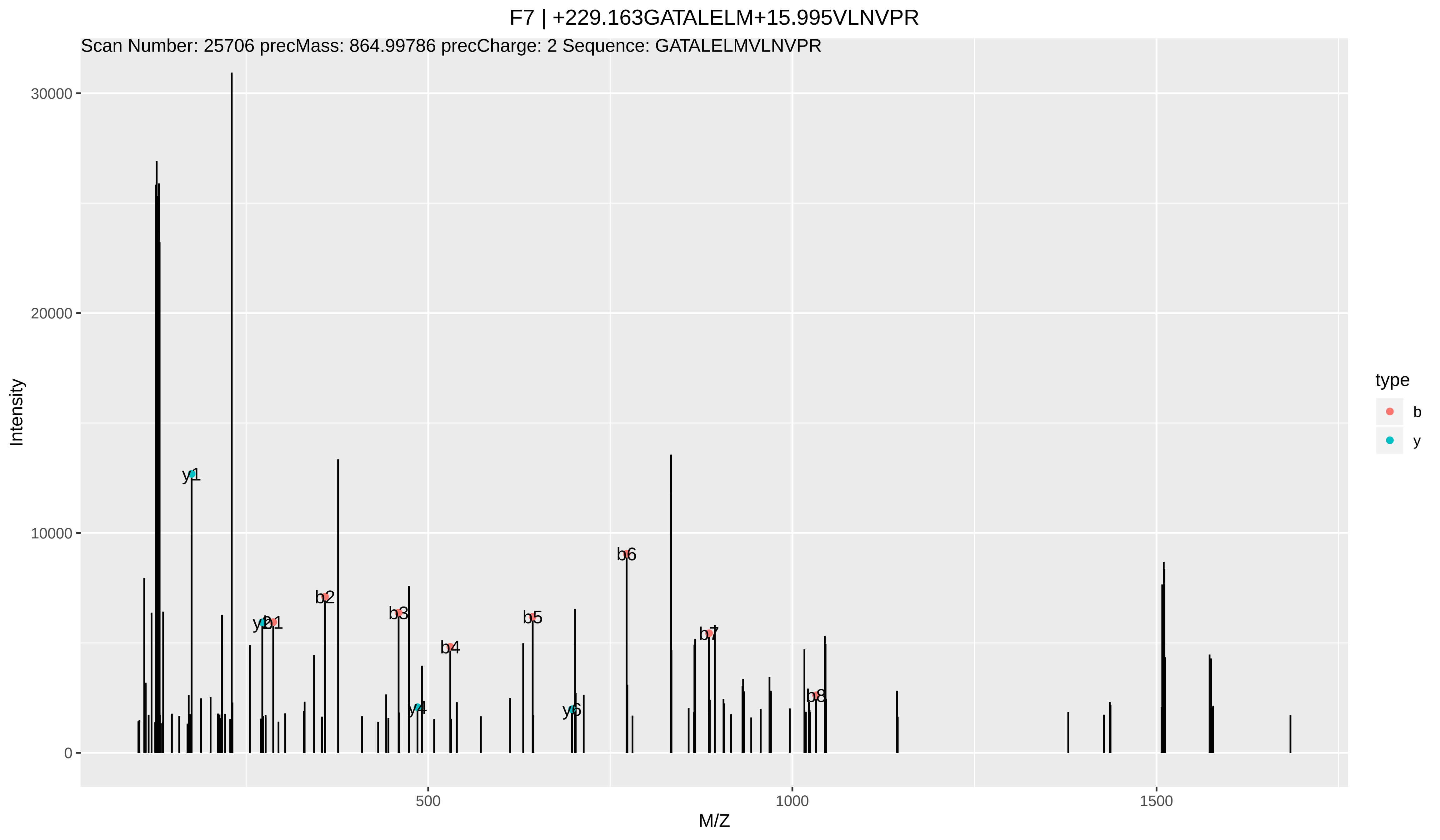
F7 | +229.163GATALELM+15.995VLNVPR
Scan Number: 25706 precMass: 864.99786 precCharge: 2 Sequence: GATALELMVLNVPR
Intensity
M/Z
type
b
y
y1
y2
b1
b2
b3
y4
b4
b5
y6
b6
b7
b8
0
10000
20000
30000
500
1000
1500

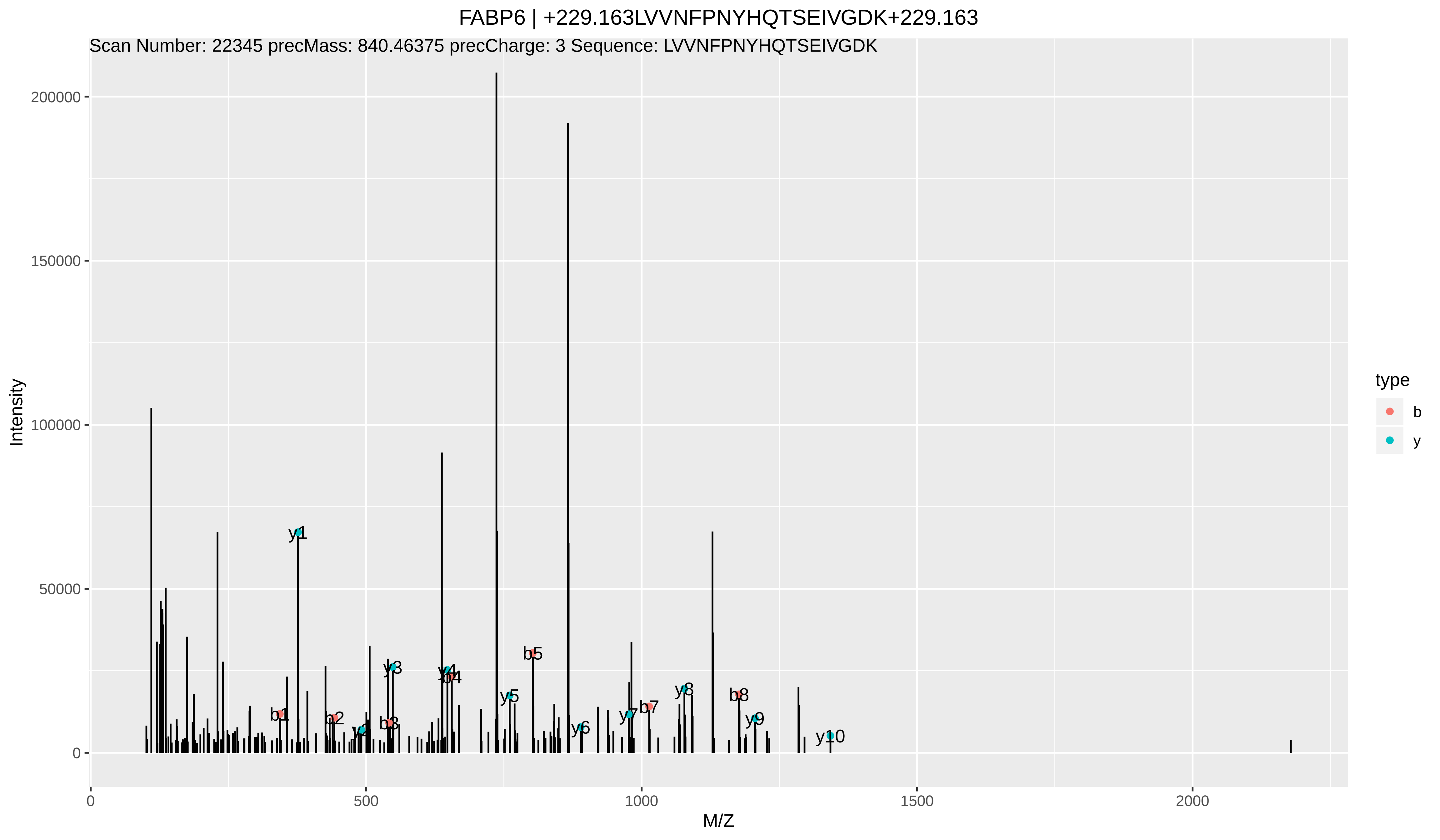

FABP6 | +229.163LVVNFPNYHQTSEIVGDK+229.163
Scan Number: 22345 precMass: 840.46375 precCharge: 3 Sequence: LVVNFPNYHQTSEIVGDK
Intensity
M/Z
0
50000
100000
150000
200000
0
500
1000
1500
2000
type
b
y
y1
b1
b2
y2
b3
y3
y4
b4
b5
y5
y6
y7
b7
y8
b8
y9
y10

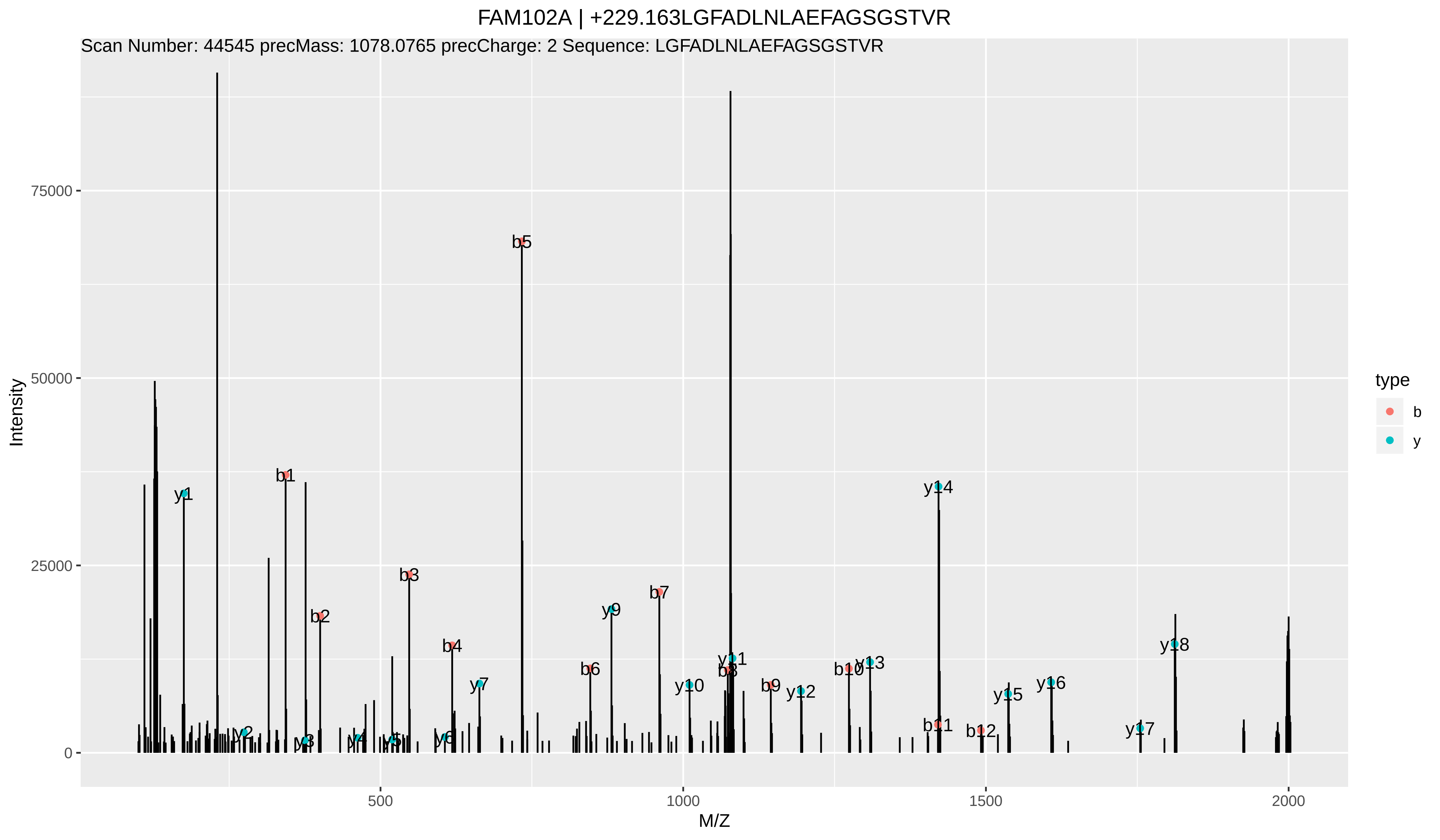

FAM102A | +229.163LGFADLNLAEFAGSGSTVR
Scan Number: 44545 precMass: 1078.0765 precCharge: 2 Sequence: LGFADLNLAEFAGSGSTVR
Intensity
M/Z
75000
50000
25000
0
500
1000
1500
2000
type
b
y
y1
b1
b2
y2
y3
b3
y4
y5
b4
y6
y7
b5
b6
y9
b7
y10
y11
b8
b9
y12
b10
y13
y14
b11
b12
y15
y16
y17
y18

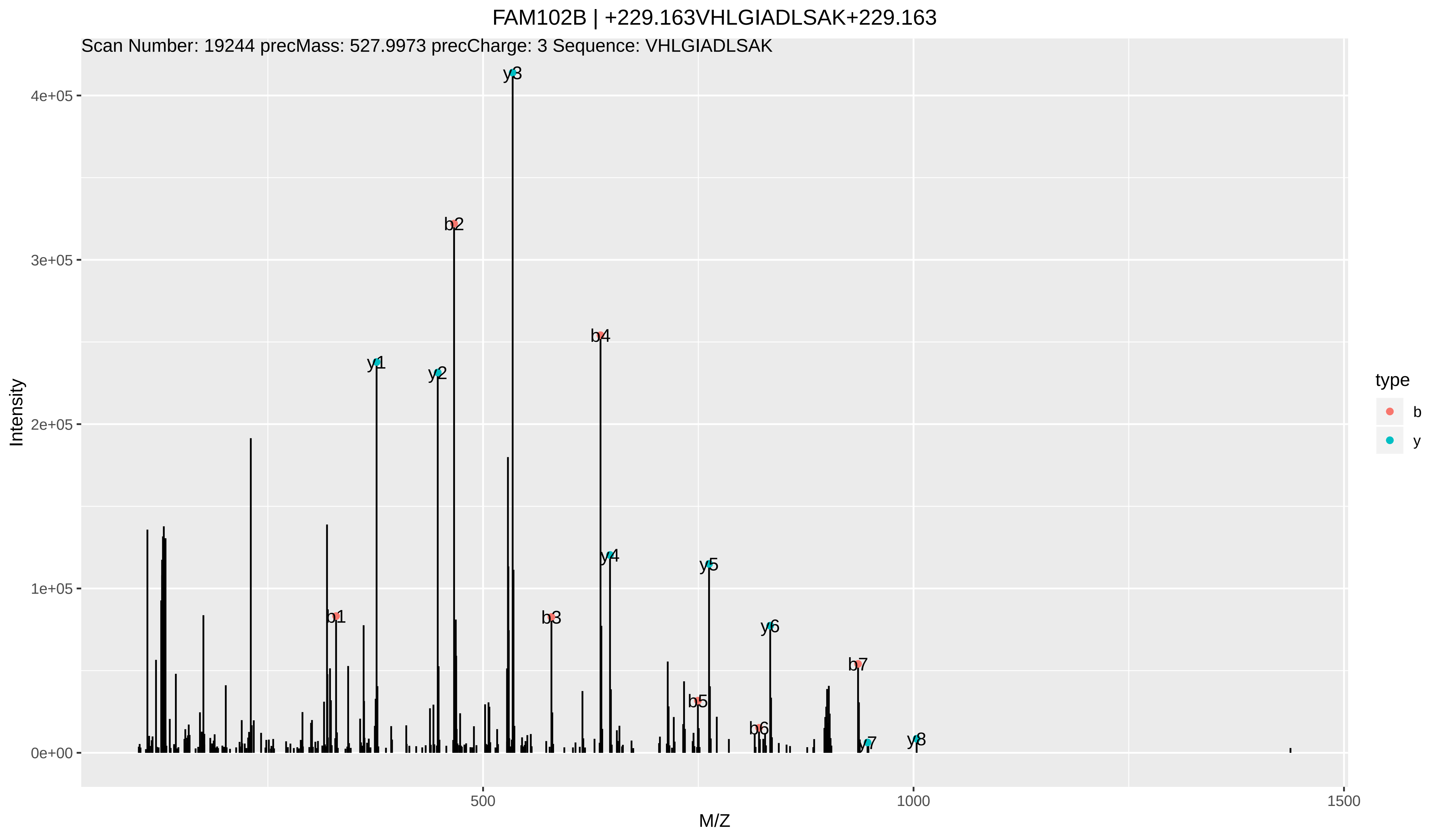

FAM102B | +229.163VHLGIADLSAK+229.163
Scan Number: 19244 precMass: 527.9973 precCharge: 3 Sequence: VHLGIADLSAK
Intensity
M/Z
0e+00
1e+05
2e+05
3e+05
4e+05
500
1000
1500
y3
b2
b4
y1
y2
y4
y5
b1
b3
y6
b7
b5
b6
y7
y8
type
b
y

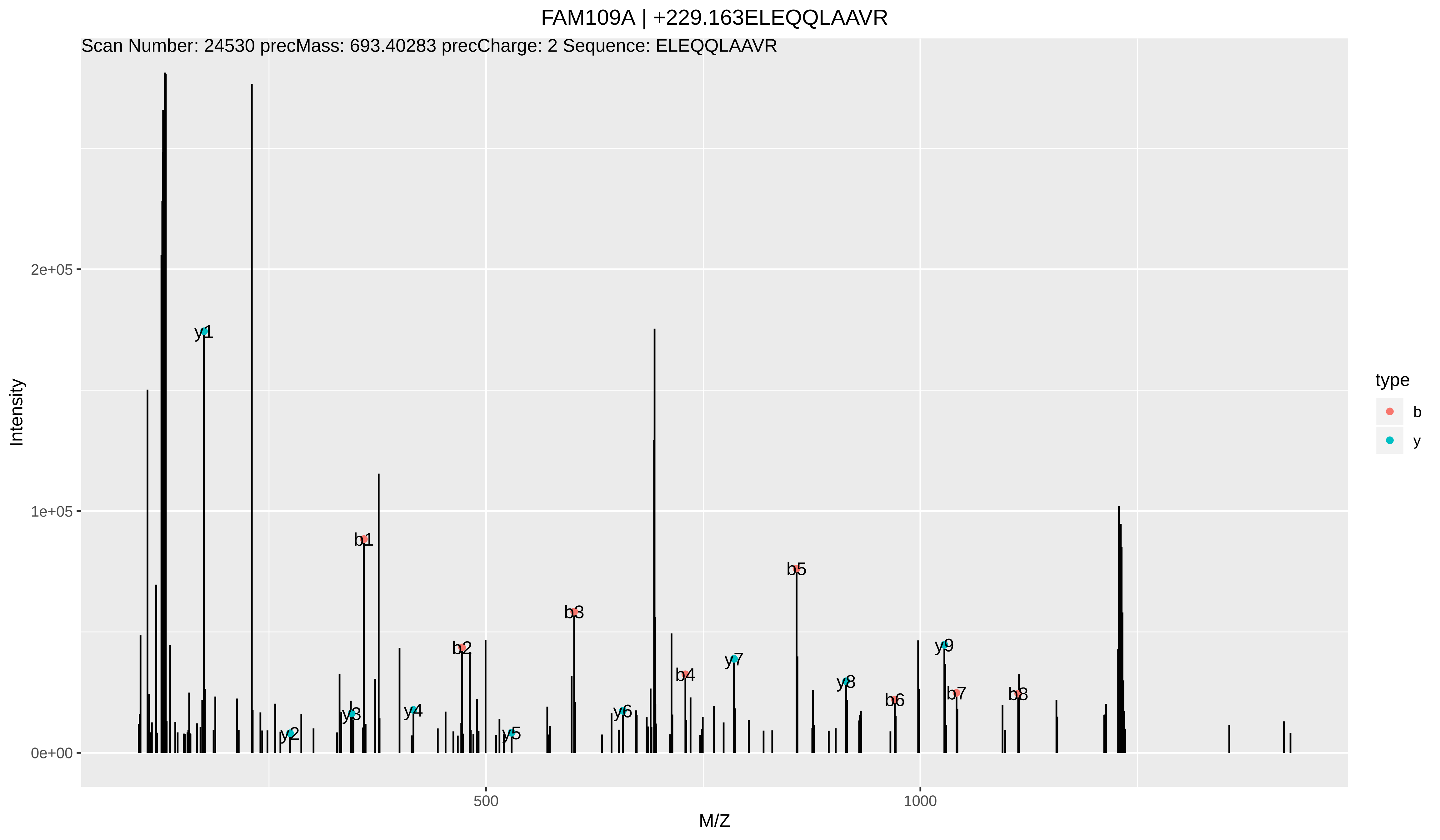
FAM109A | +229.163ELEQQLAAVR
Scan Number: 24530 precMass: 693.40283 precCharge: 2 Sequence: ELEQQLAAVR
Intensity
2e+05
1e+05
0e+00
M/Z
500
1000
y1
y2
y3
y4
y5
y6
y7
y8
y9
b1
b2
b3
b4
b5
b6
b7
b8
type
b
y

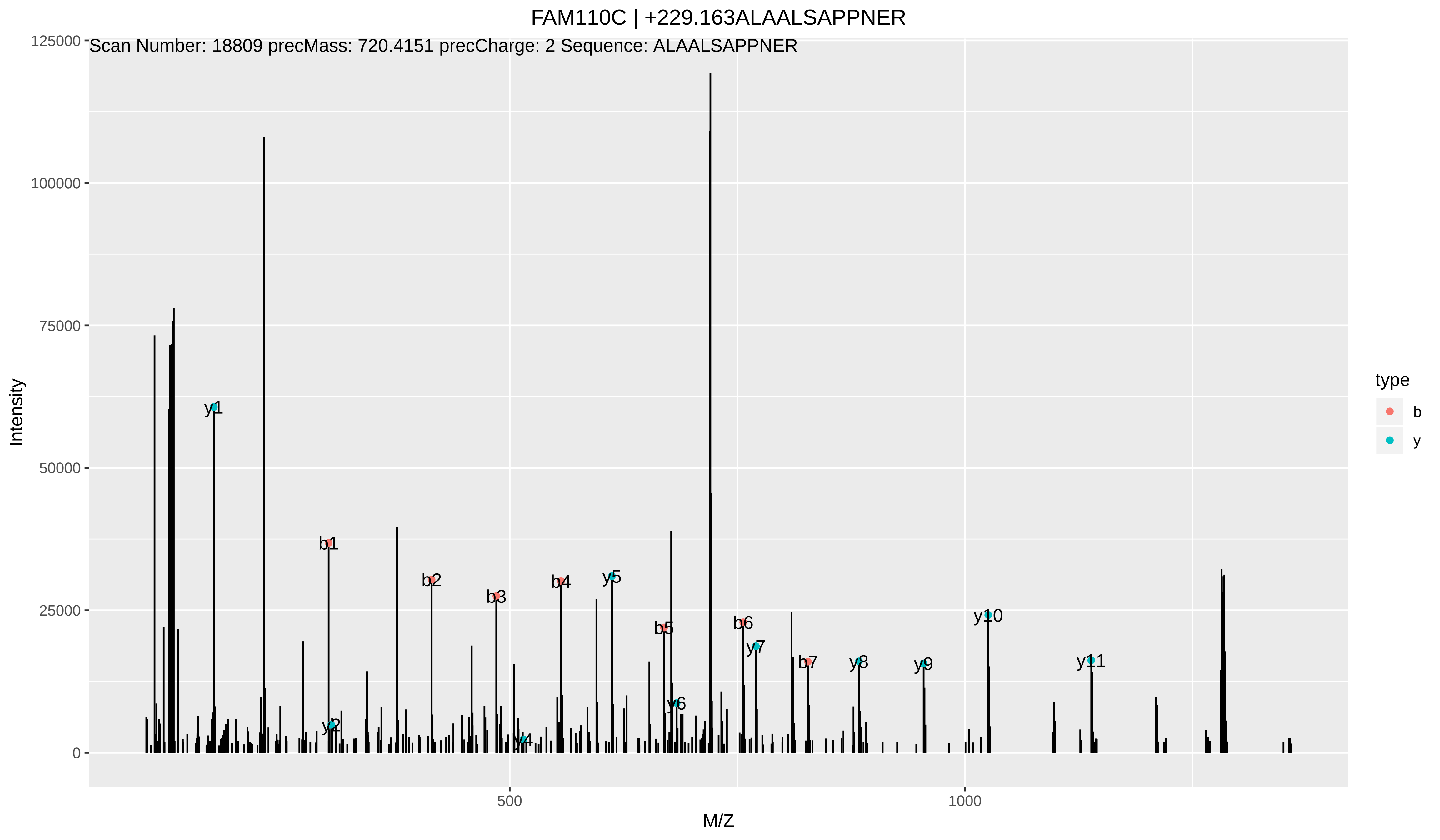

FAM110C | +229.163ALAALSAPPNER
Scan Number: 18809 precMass: 720.4151 precCharge: 2 Sequence: ALAALSAPPNER
Intensity
M/Z
type
b
y
y1
y2
y4
y5
y6
y7
y8
y9
y10
y11
b1
b2
b3
b4
b5
b6
b7
125000
100000
75000
50000
25000
0
500
1000

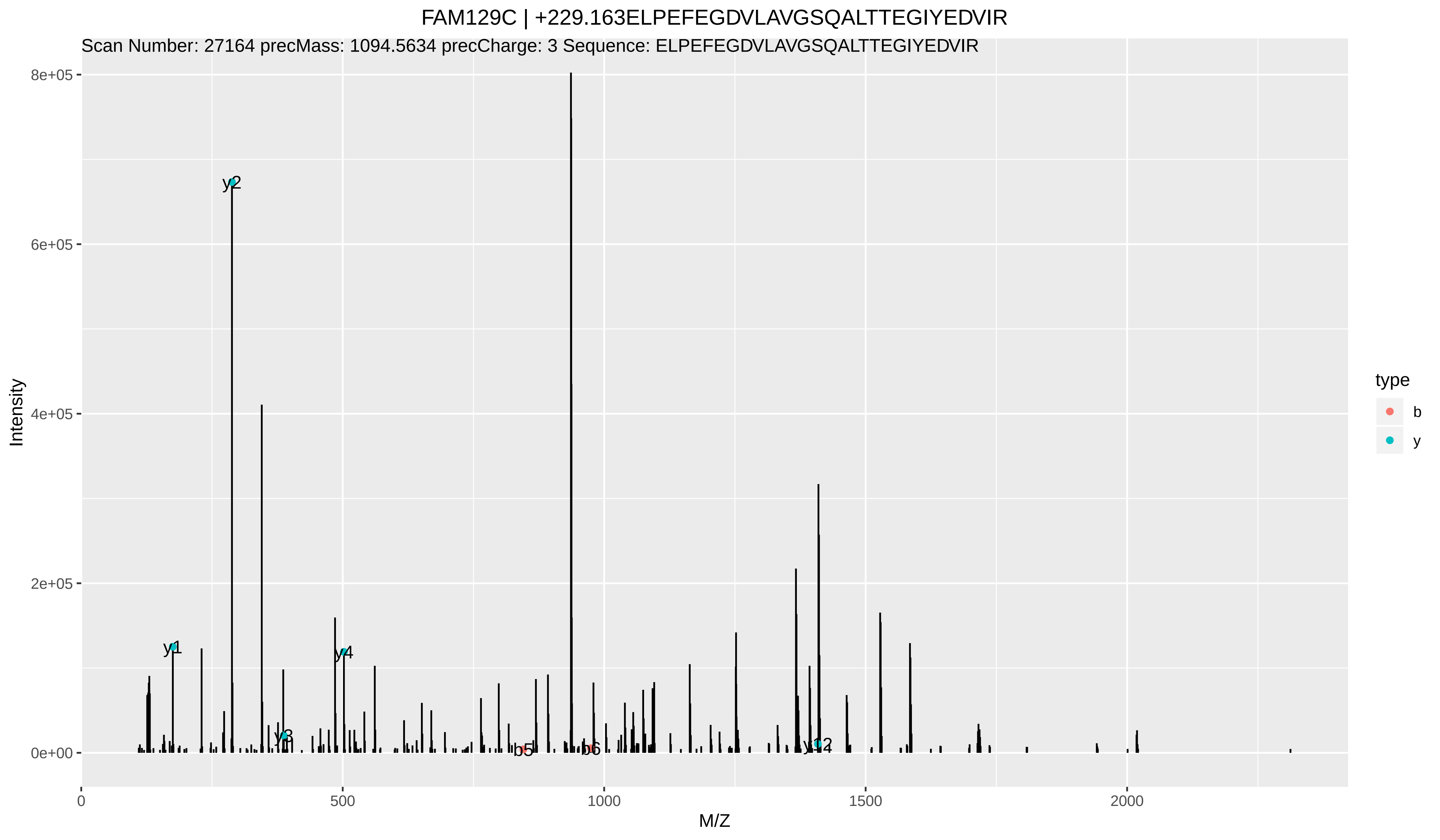
FAM129C | +229.163ELPEFEGDVLAVGSQALTTEGIYEDVIR
Scan Number: 27164 precMass: 1094.5634 precCharge: 3 Sequence: ELPEFEGDVLAVGSQALTTEGIYEDVIR
Intensity
M/Z
y1
y2
y3
y4
b5
b6
y12
type
b
y
0e+00
2e+05
4e+05
6e+05
8e+05
0
500
1000
1500
2000

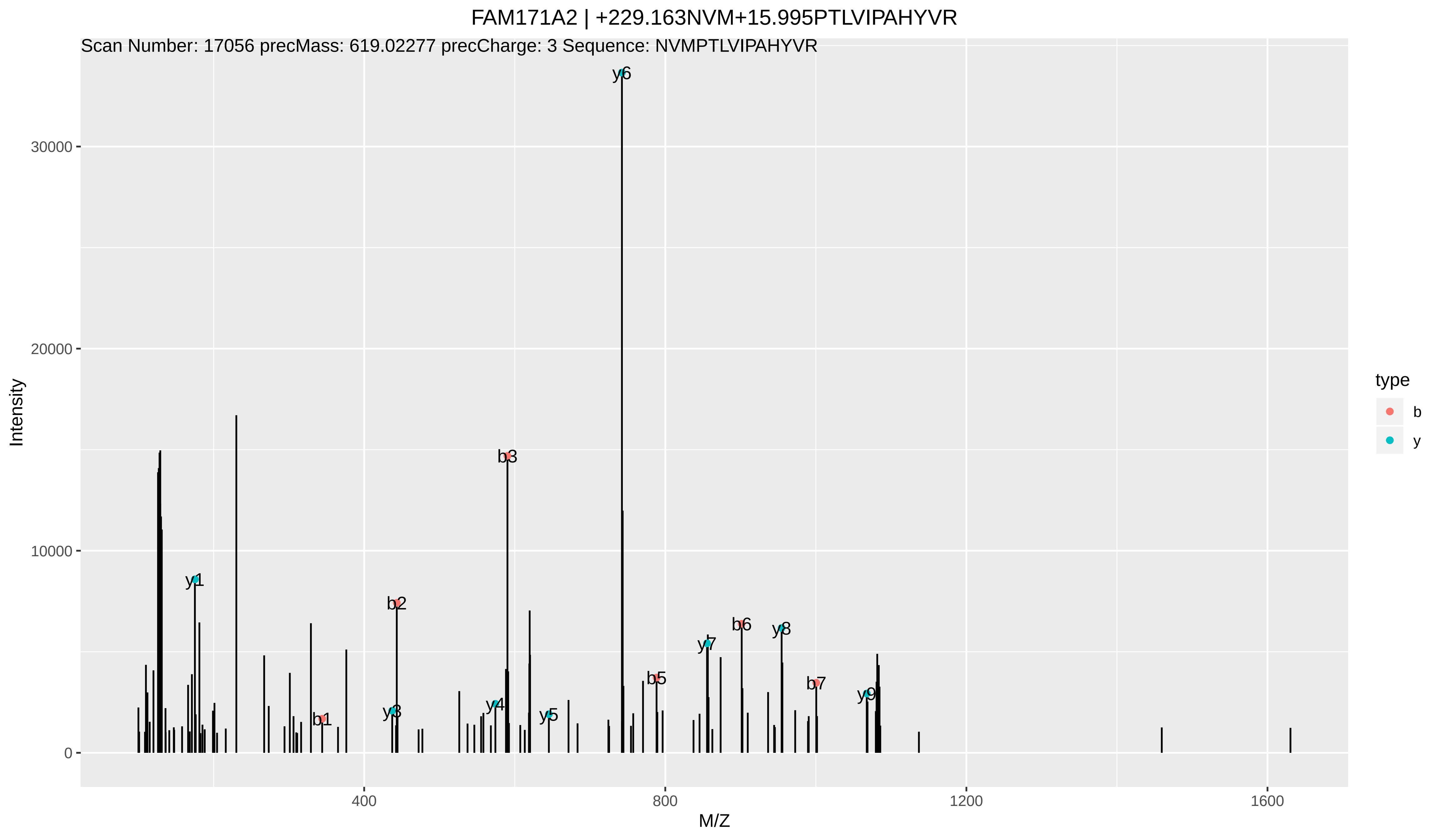

FAM171A2 | +229.163NVM+15.995PTLVIPAHYVR
Scan Number: 17056 precMass: 619.02277 precCharge: 3 Sequence: NVMPTLVIPAHYVR
Intensity
M/Z
0
10000
20000
30000
400
800
1200
1600
y6
b3
y1
b2
b6
y8
y7
b5
b7
y9
y3
y4
y5
b1
type
b
y

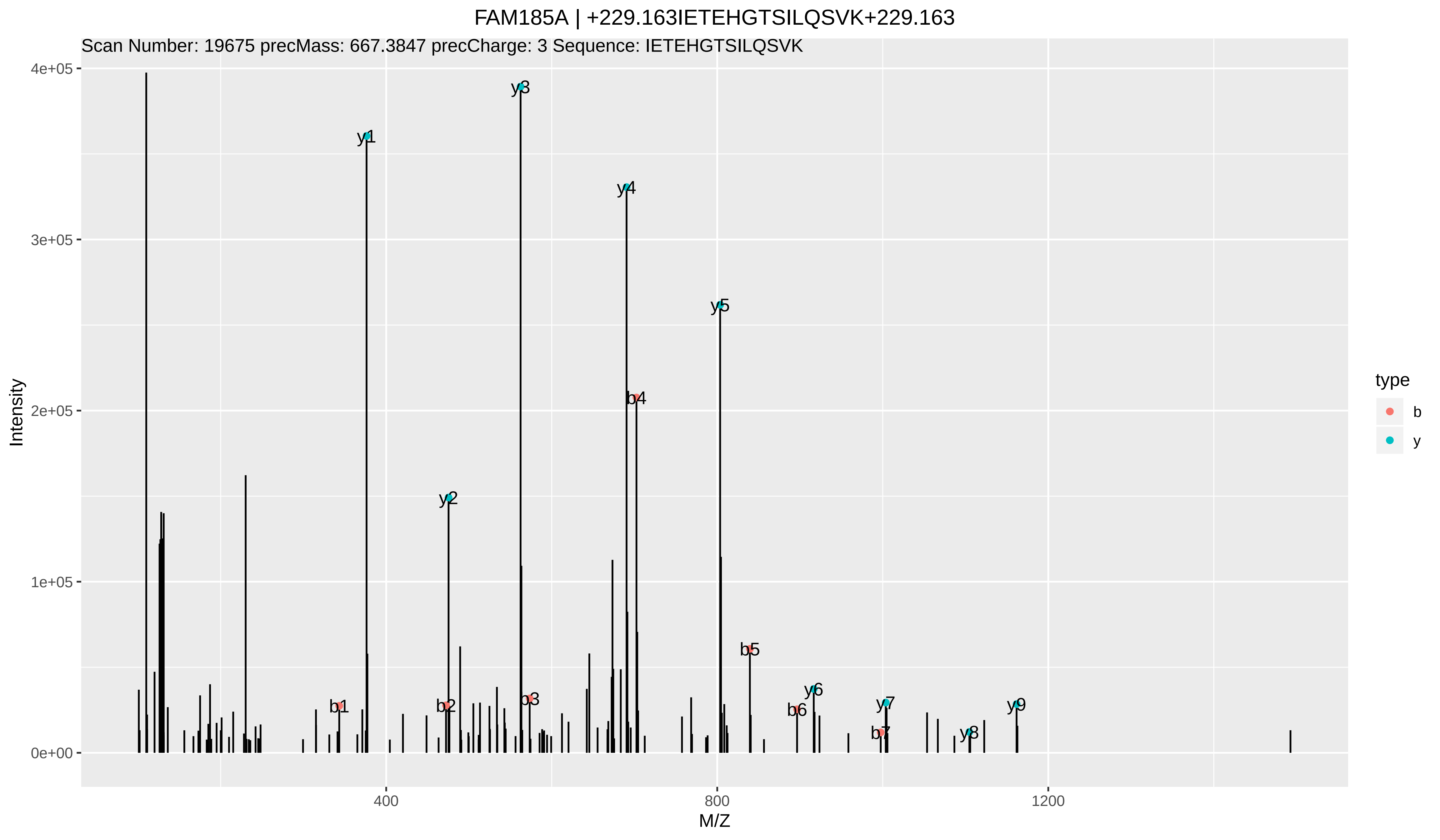
FAM185A | +229.163IETEHGTSILQSVK+229.163
Scan Number: 19675 precMass: 667.3847 precCharge: 3 Sequence: IETEHGTSILQSVK
Intensity
4e+05
3e+05
2e+05
1e+05
0e+00
M/Z
400
800
1200
y1
y2
y3
y4
y5
y6
y7
y8
y9
b1
b2
b3
b4
b5
b6
b7
type
b
y

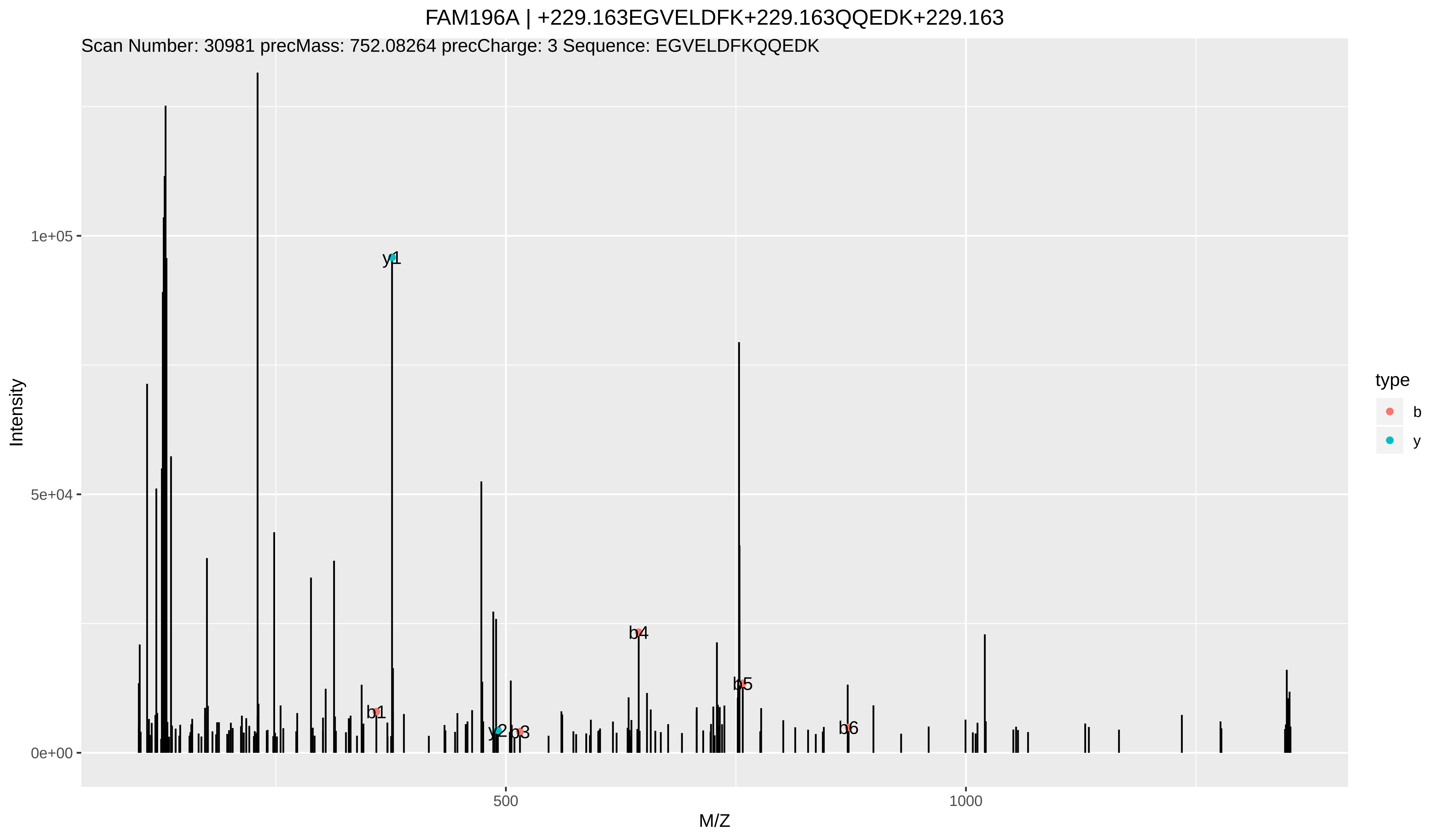
FAM196A | +229.163EGVELDFK+229.163QQEDK+229.163
Scan Number: 30981 precMass: 752.08264 precCharge: 3 Sequence: EGVELDFKQQEDK
Intensity
1e+05
5e+04
0e+00
M/Z
500
1000
y1
y2
b1
b3
b4
b5
b6
type
b
y

FAM20C | +229.163NGLHSVVDDDLDTEHR

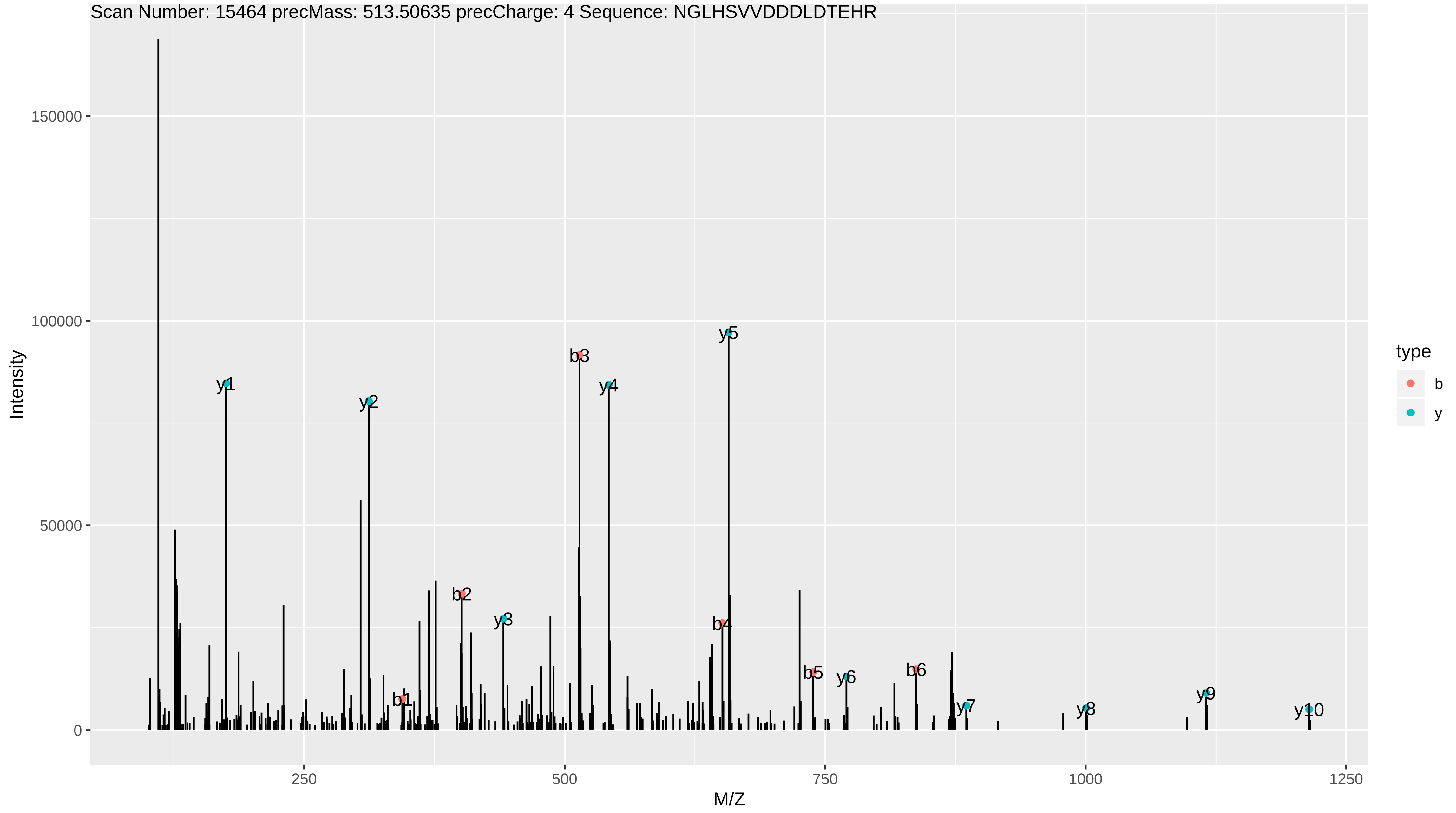

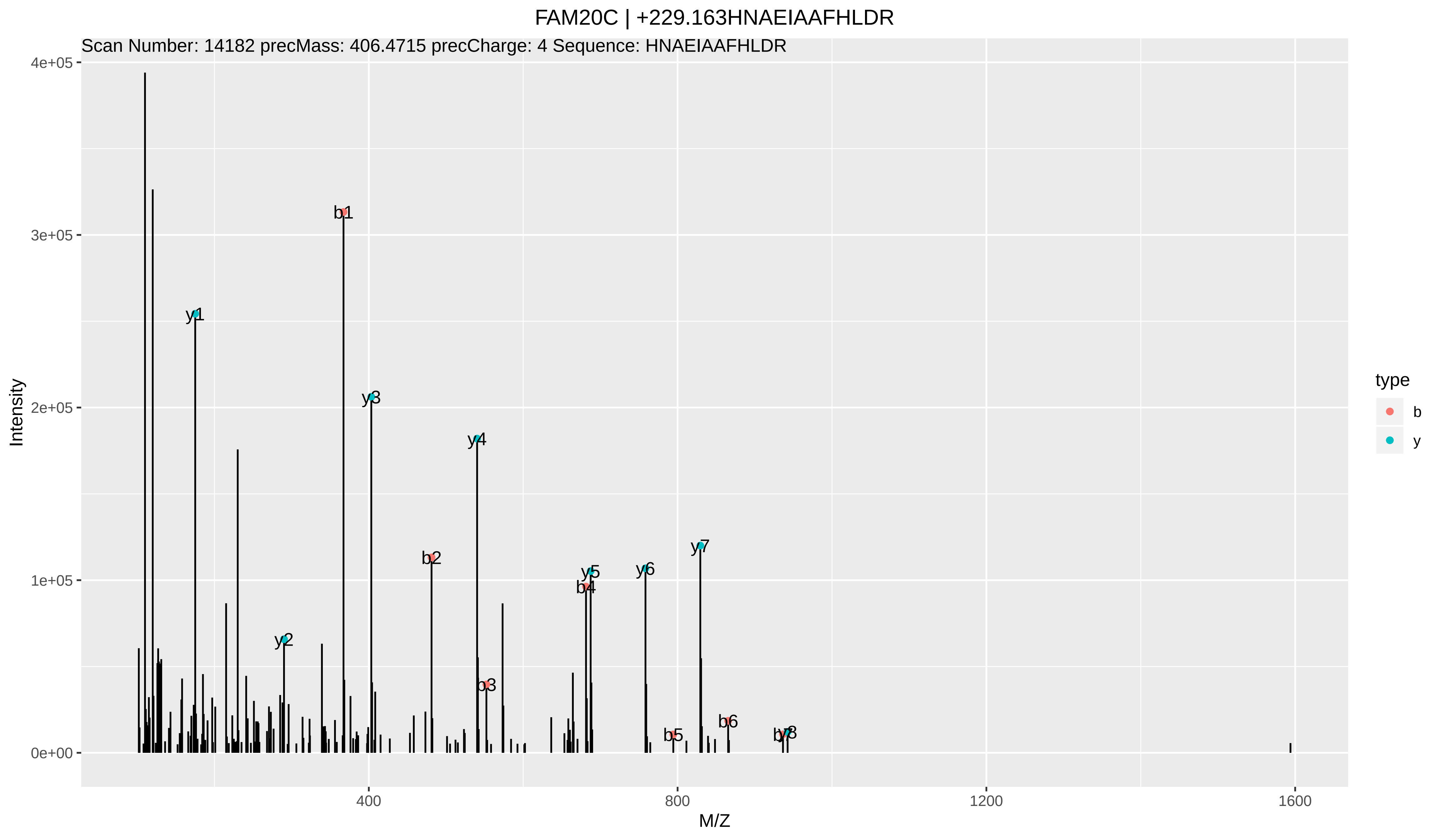

FAM20C | +229.163HNAEIAAFHLDR
Scan Number: 14182 precMass: 406.4715 precCharge: 4 Sequence: HNAEIAAFHLDR
Intensity
M/Z
4e+05
3e+05
2e+05
1e+05
0e+00
400
800
1200
1600
y1
y2
y3
y4
y5
y6
y7
y8
b1
b2
b3
b4
b5
b6
b7
type
b
y

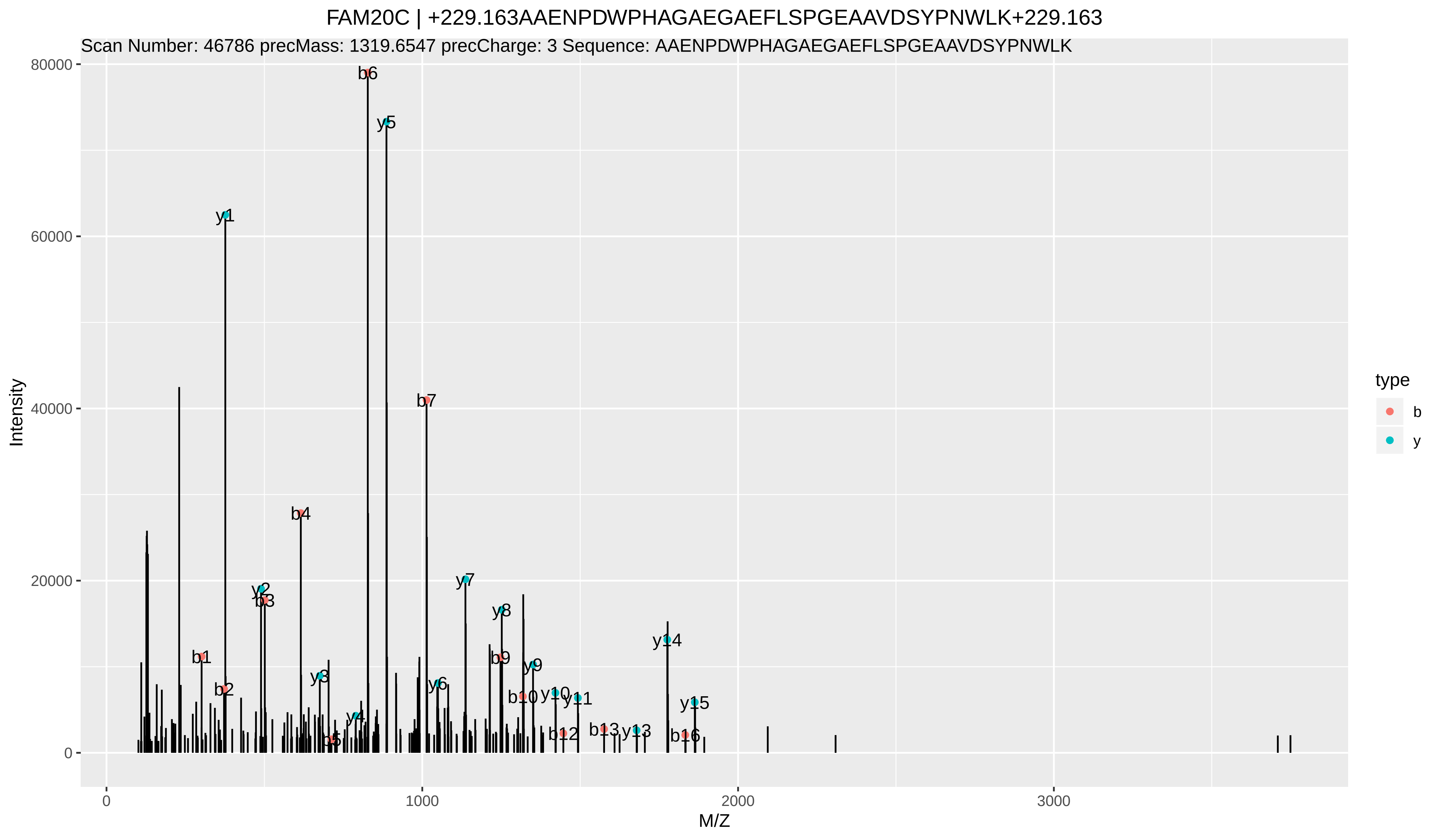

FAM20C | +229.163AAENPDWPHAGAEGAEFLSPGEAAVDSYPNWLK+229.163
Scan Number: 46786 precMass: 1319.6547 precCharge: 3 Sequence: AAENPDWPHAGAEGAEFLSPGEAAVDSYPNWLK
Intensity
M/Z
type
b
y
b1
b2
b3
b4
b5
b6
b7
b9
b10
b12
b13
b16
y1
y2
y3
y4
y5
y6
y7
y8
y9
y10
y11
y13
y14
y15
0
20000
40000
60000
80000
1000
2000
3000

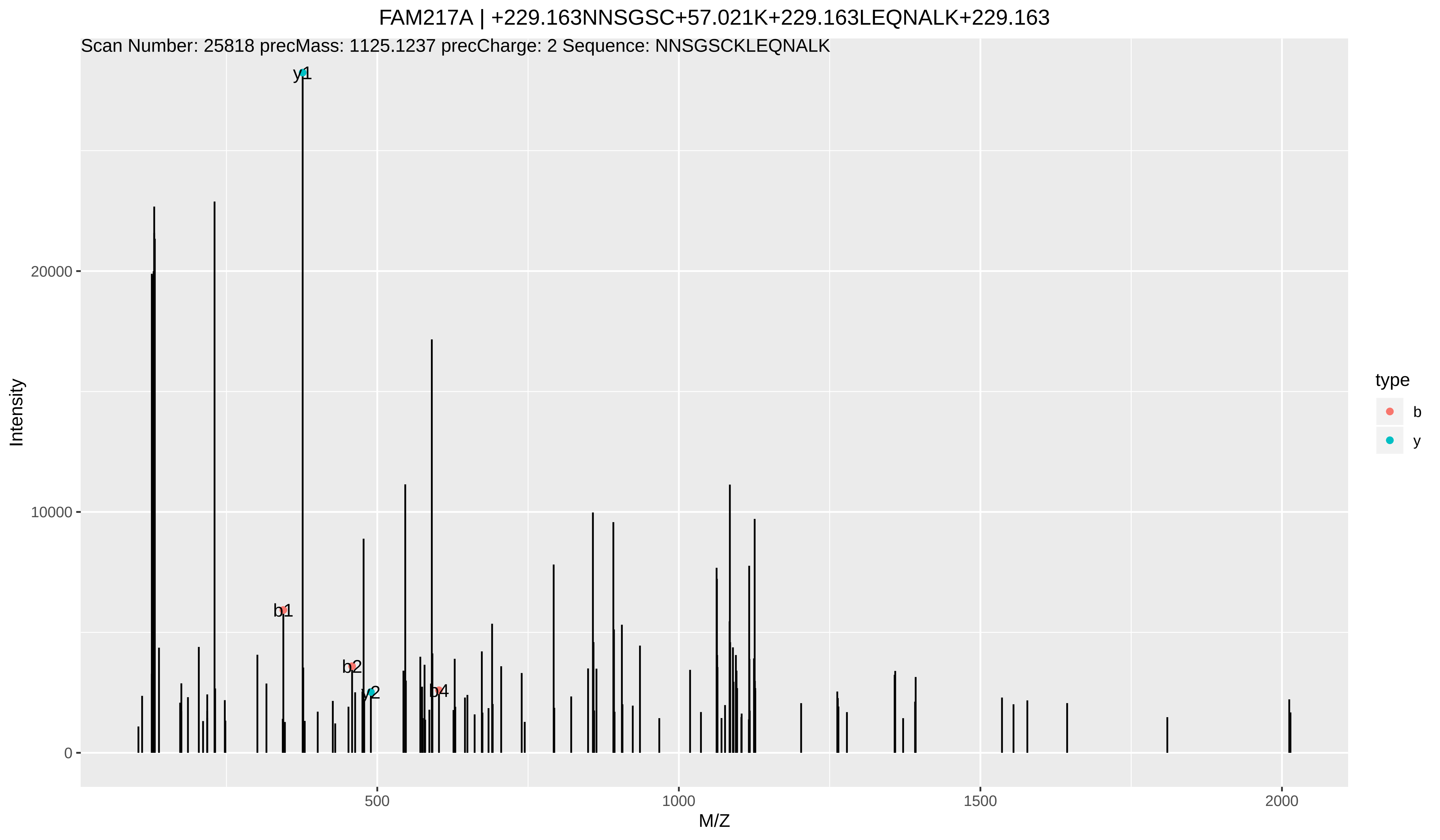

FAM217A | +229.163NNSGSC+57.021K+229.163LEQNALK+229.163
Scan Number: 25818 precMass: 1125.1237 precCharge: 2 Sequence: NNSGSCKLEQNALK
y1
b1
b2
y2
b4
20000
10000
0
500
1000
1500
2000
Intensity
M/Z
type
b
y

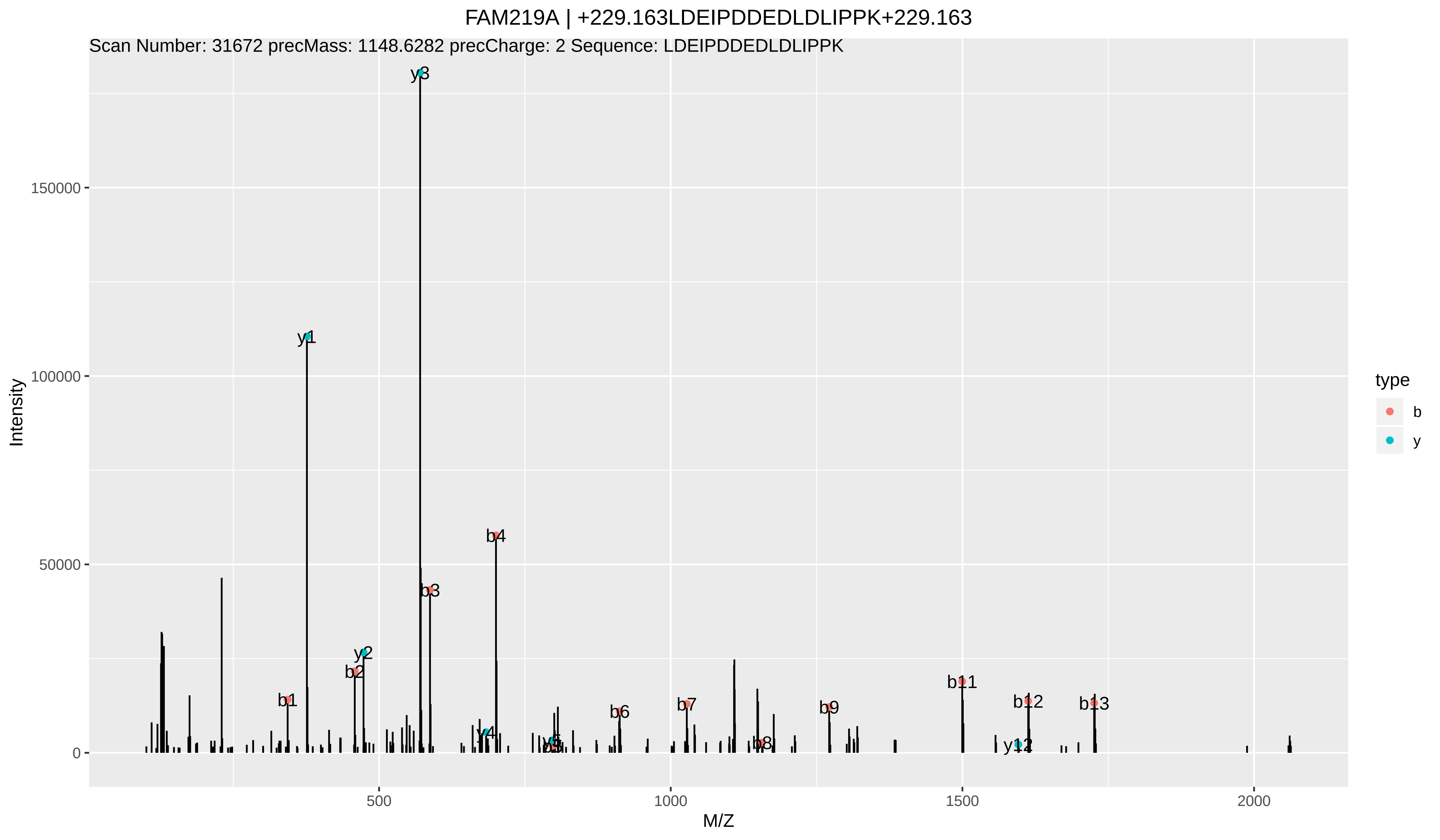

FAM219A | +229.163LDEIPDDEDLDLIPPK+229.163
Scan Number: 31672 precMass: 1148.6282 precCharge: 2 Sequence: LDEIPDDEDLDLIPPK
Intensity
M/Z
150000
100000
50000
0
500
1000
1500
2000
y3
y1
b4
b3
y2
b2
b1
y4
b6
b7
b8
b9
b11
b12
b13
y12
type
b
y

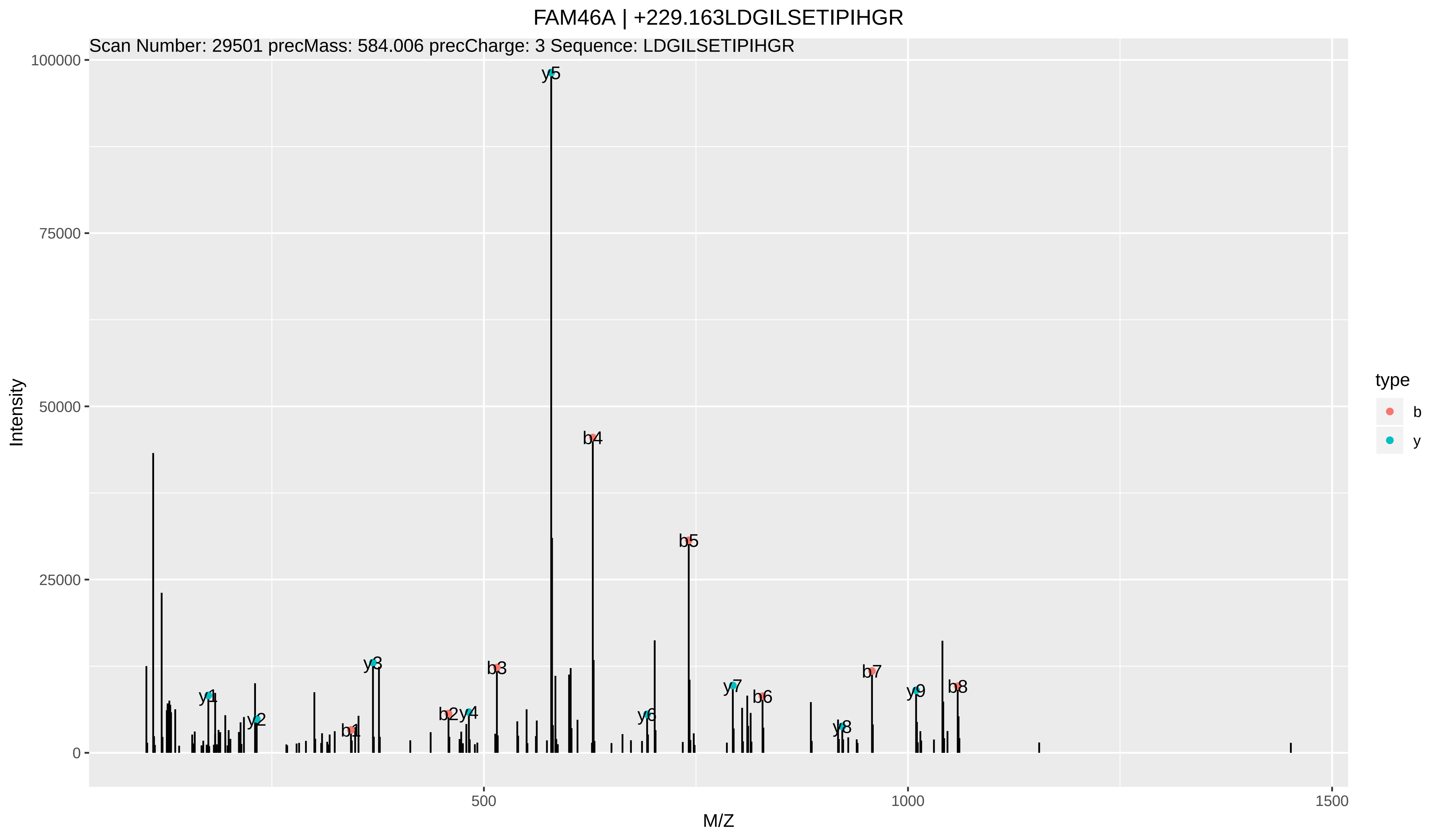

FAM46A | +229.163LDGILSETIPIHGR
Scan Number: 29501 precMass: 584.006 precCharge: 3 Sequence: LDGILSETIPIHGR
Intensity
100000
75000
50000
25000
0
M/Z
500
1000
1500
y5
b4
b5
y3
b3
y1
y2
b1
b2
y4
y6
y7
b6
y8
b7
y9
b8
type
b
y

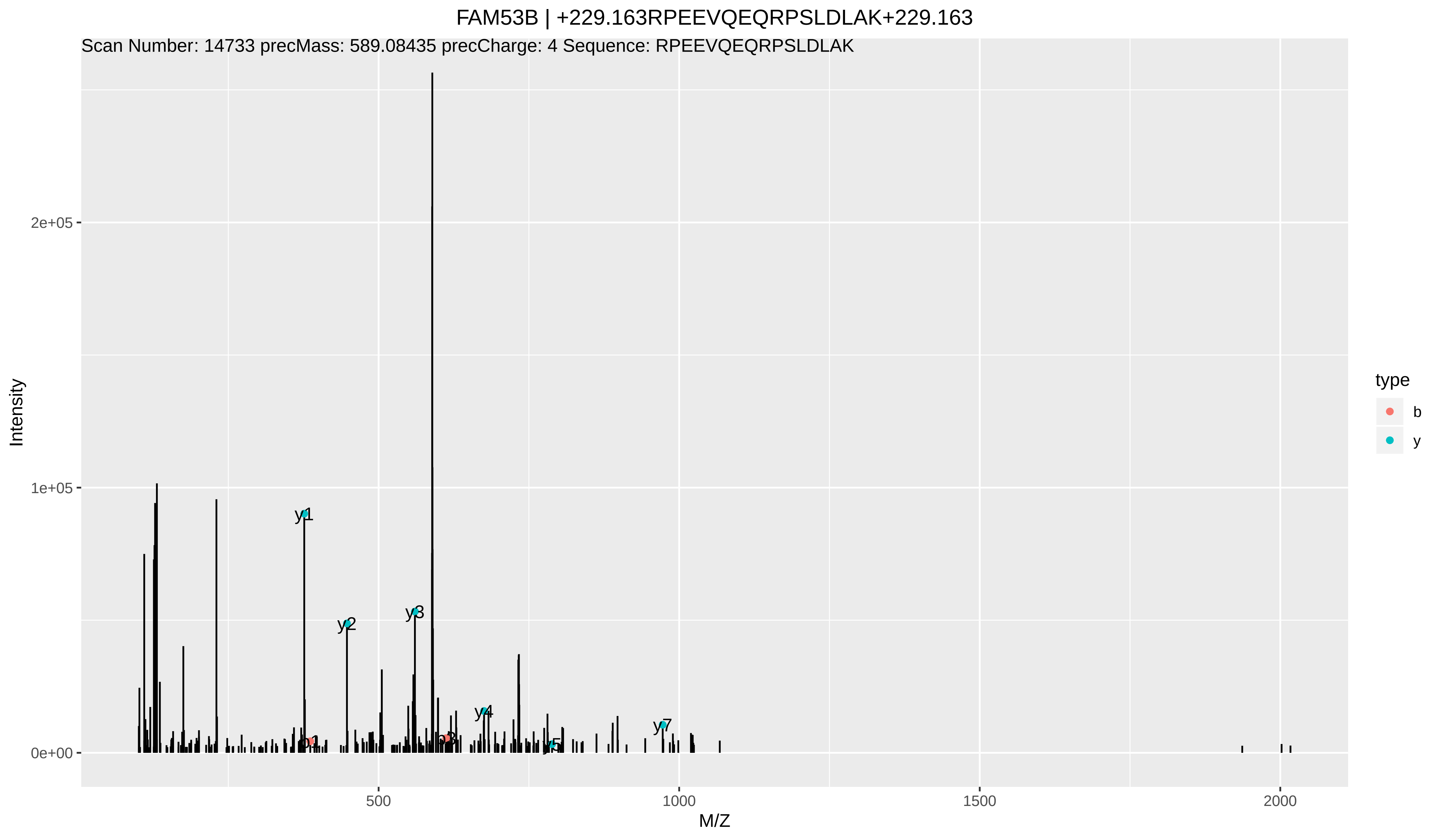
FAM53B | +229.163RPEEVQEQRPSLDLAK+229.163
Scan Number: 14733 precMass: 589.08435 precCharge: 4 Sequence: RPEEVQEQRPSLDLAK
Intensity
2e+05
1e+05
0e+00
M/Z
500
1000
1500
2000
y1
y2
y3
y4
y5
y7
b1
b3
type
b
y

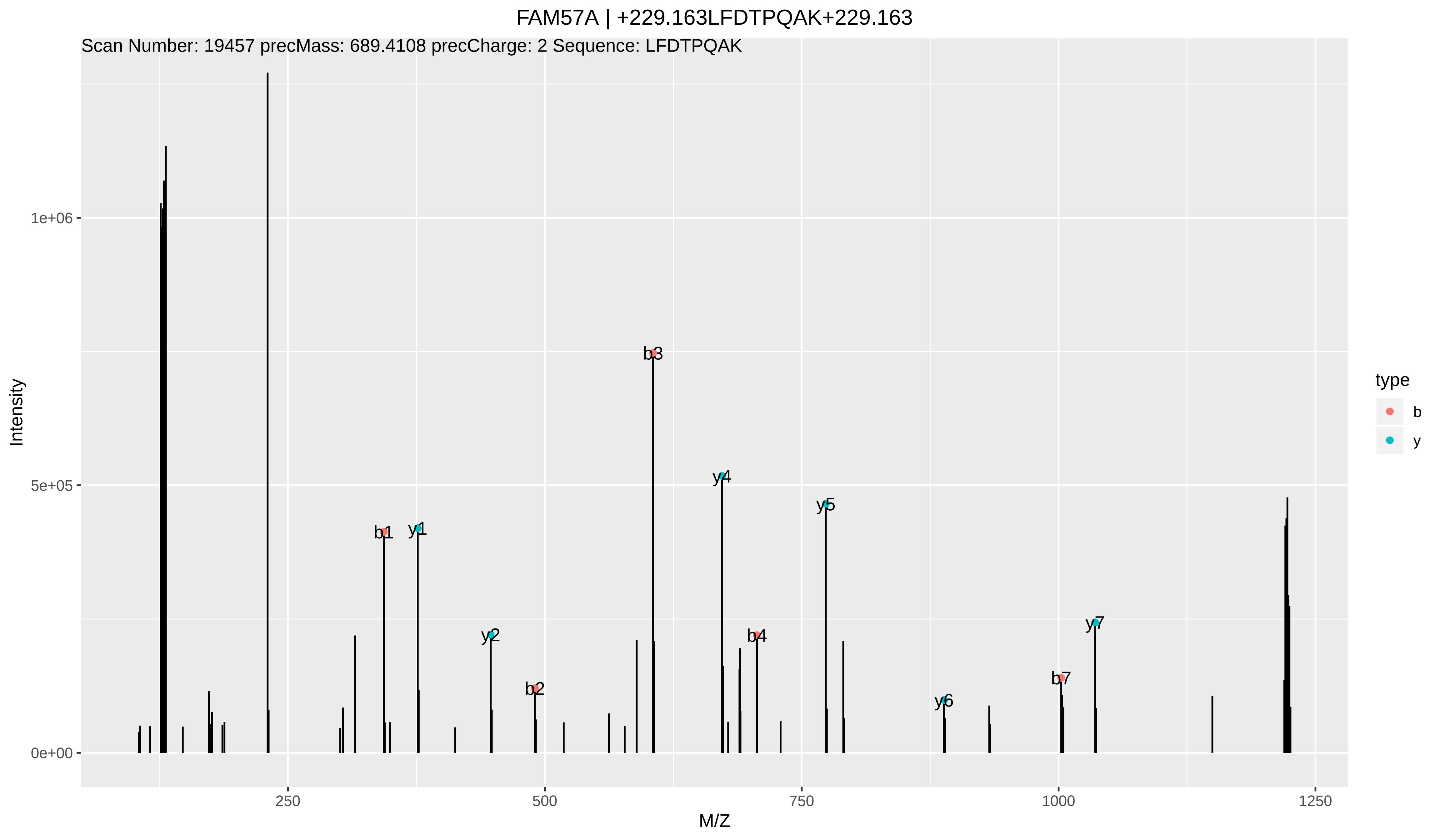

FAM57A | +229.163LFDTPQAK+229.163
Scan Number: 19457 precMass: 689.4108 precCharge: 2 Sequence: LFDTPQAK
Intensity
1e+06
5e+05
0e+00
b1
y1
y2
b2
b3
y4
b4
y5
y6
b7
y7
250
500
750
1000
1250
M/Z
type
b
y

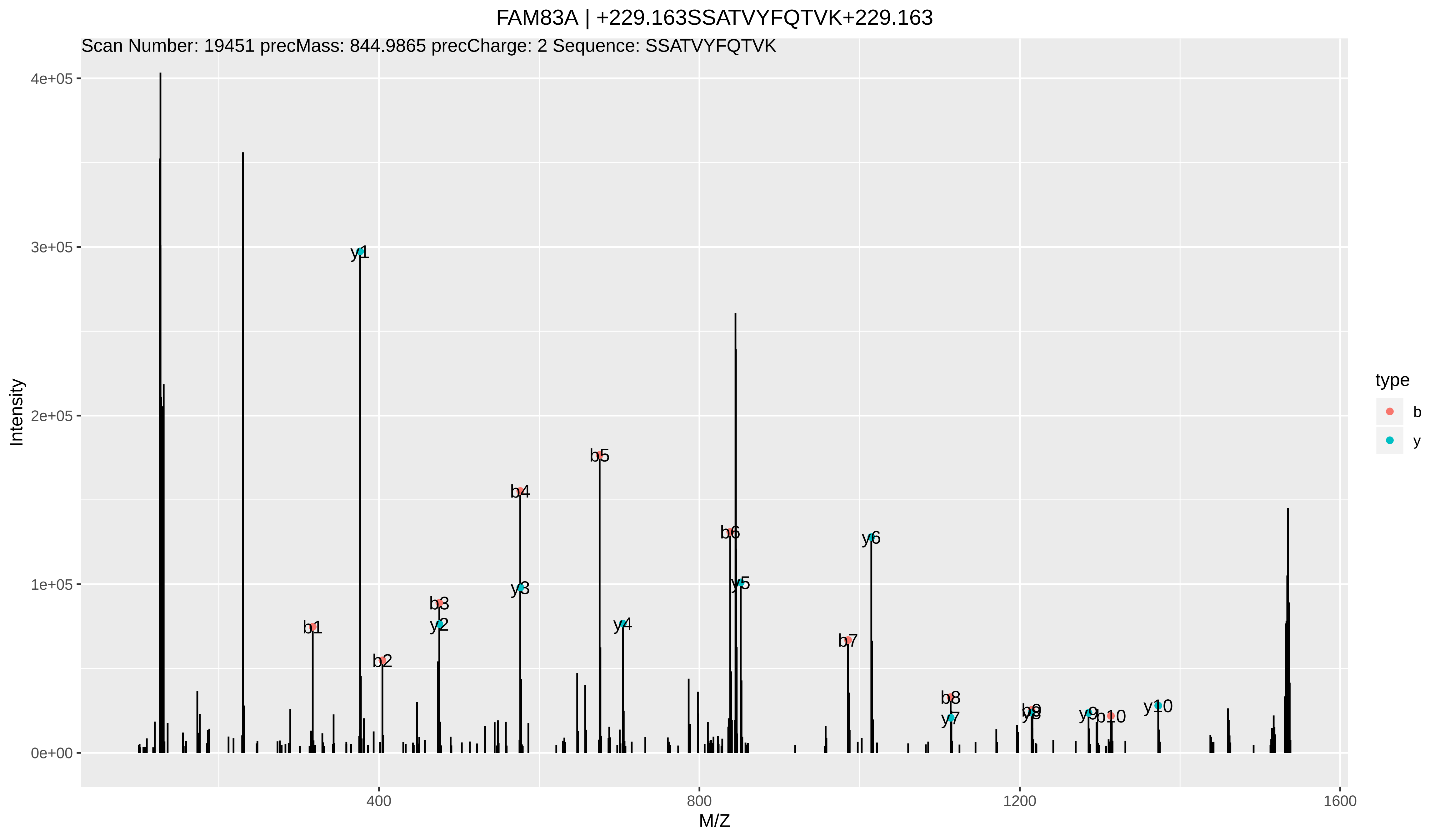
FAM83A | +229.163SSATVYFQTVK+229.163
Scan Number: 19451 precMass: 844.9865 precCharge: 2 Sequence: SSATVYFQTVK
Intensity
M/Z
0e+00
1e+05
2e+05
3e+05
4e+05
400
800
1200
1600
y1
b1
b2
b3
y2
b4
y3
b5
y4
b6
y5
y6
b7
b8
y7
b9
y8
y9
b10
y10
type
b
y

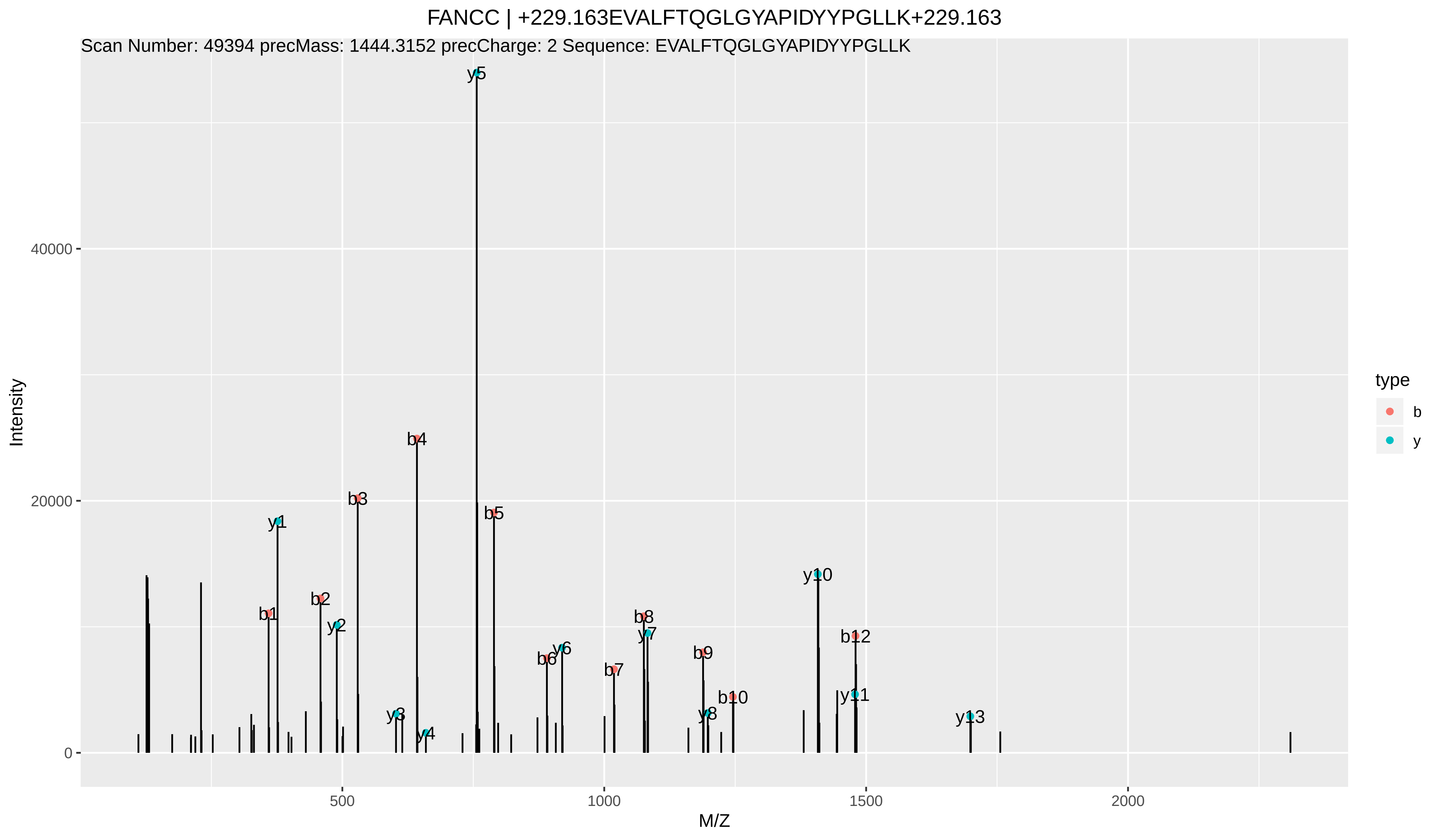

FANCC | +229.163EVALFTQGLGYAPIDYYPGLLK+229.163
Scan Number: 49394 precMass: 1444.3152 precCharge: 2 Sequence: EVALFTQGLGYAPIDYYPGLLK
Intensity
M/Z
0
20000
40000
500
1000
1500
2000
y5
b4
b3
b5
y1
b2
b1
y2
y3
y4
b6
y6
b7
b8
y7
b9
y8
b10
y10
b12
y11
y13
type
b
y

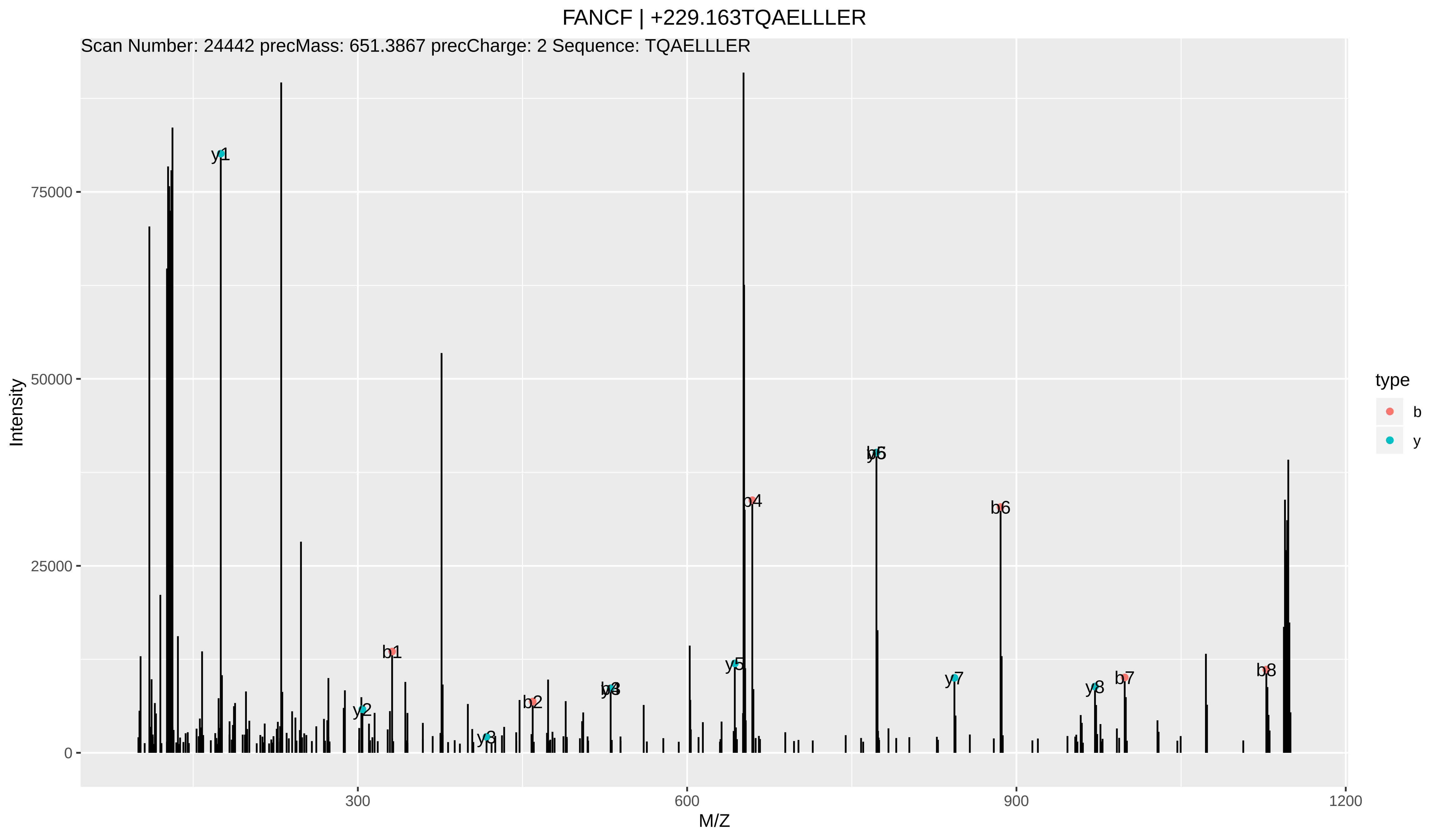

FANCF | +229.163TQAELLLER
Scan Number: 24442 precMass: 651.3867 precCharge: 2 Sequence: TQAELLLER
Intensity
M/Z
type
b
y
y1
b1
y2
y3
b2
b3
y4
y5
b4
b5
y6
b6
y7
y8
b7
b8
0
25000
50000
75000
300
600
900
1200

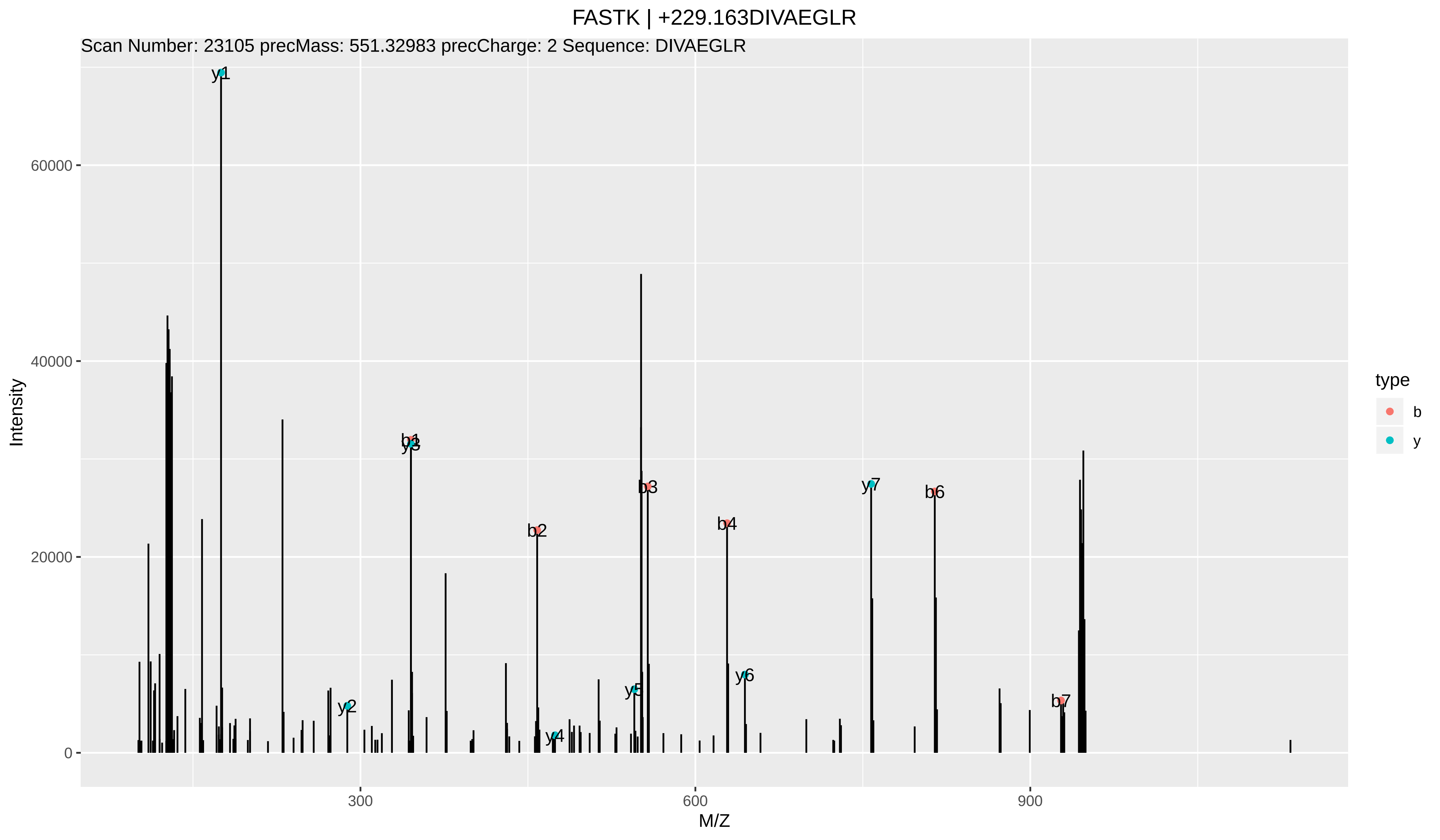

FASTK | +229.163DIVAEGLR
Scan Number: 23105 precMass: 551.32983 precCharge: 2 Sequence: DIVAEGLR
Intensity
M/Z
0
20000
40000
60000
300
600
900
y1
b1
y3
y2
b2
y4
y5
b3
b4
y6
y7
b6
b7
type
b
y

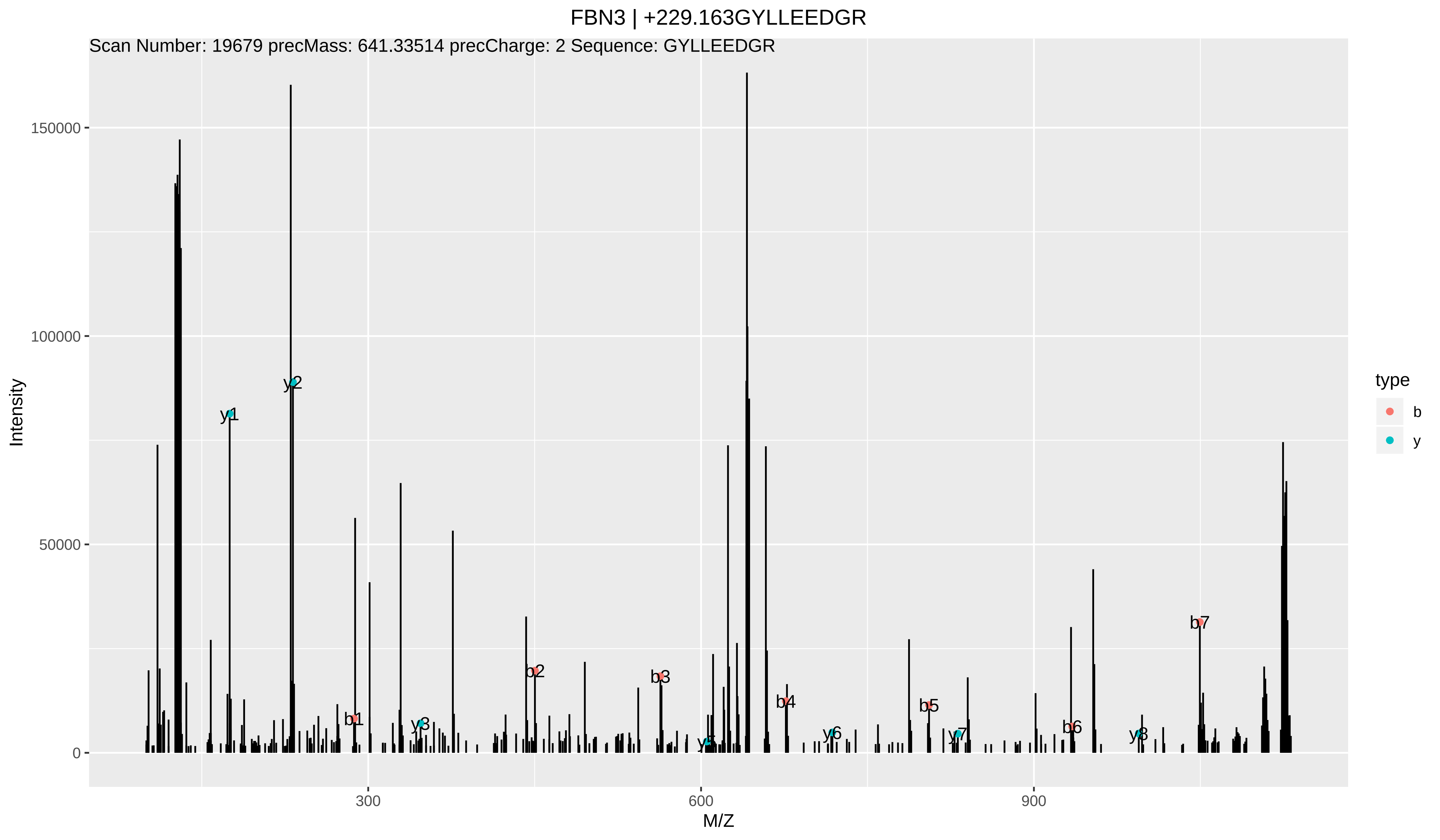

FBN3 | +229.163GYLLEEDGR
Scan Number: 19679 precMass: 641.33514 precCharge: 2 Sequence: GYLLEEDGR
Intensity
150000
100000
50000
0
300
600
900
M/Z
y1
y2
b1
y3
b2
b3
b4
y6
b5
y7
b6
y8
b7
type
b
y

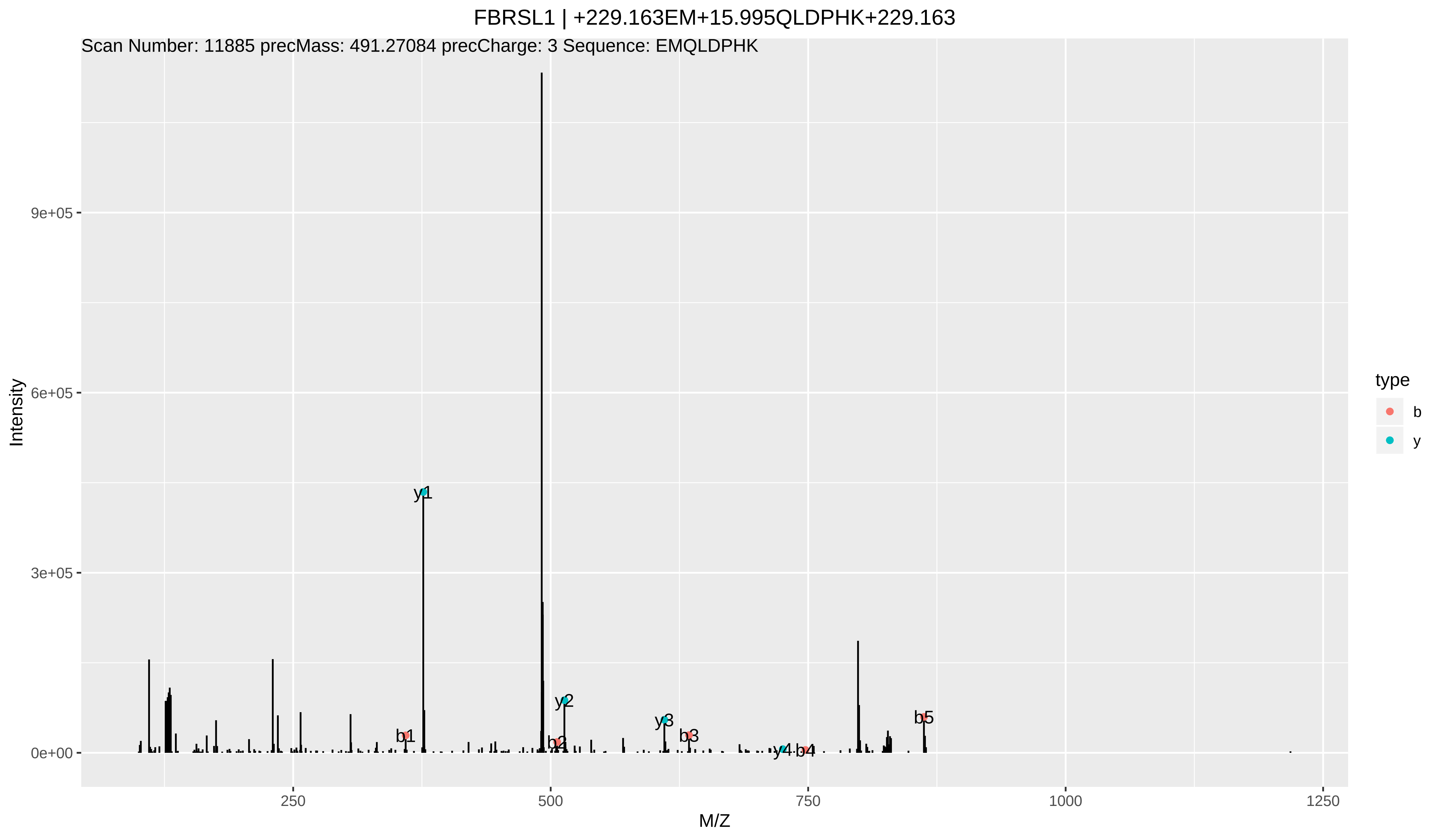

FBRSL1 | +229.163EM+15.995QLDPHK+229.163
Scan Number: 11885 precMass: 491.27084 precCharge: 3 Sequence: EMQLDPHK
Intensity
M/Z
9e+05
6e+05
3e+05
0e+00
250
500
750
1000
1250
y1
y2
y3
y4
b1
b2
b3
b4
b5
type
b
y

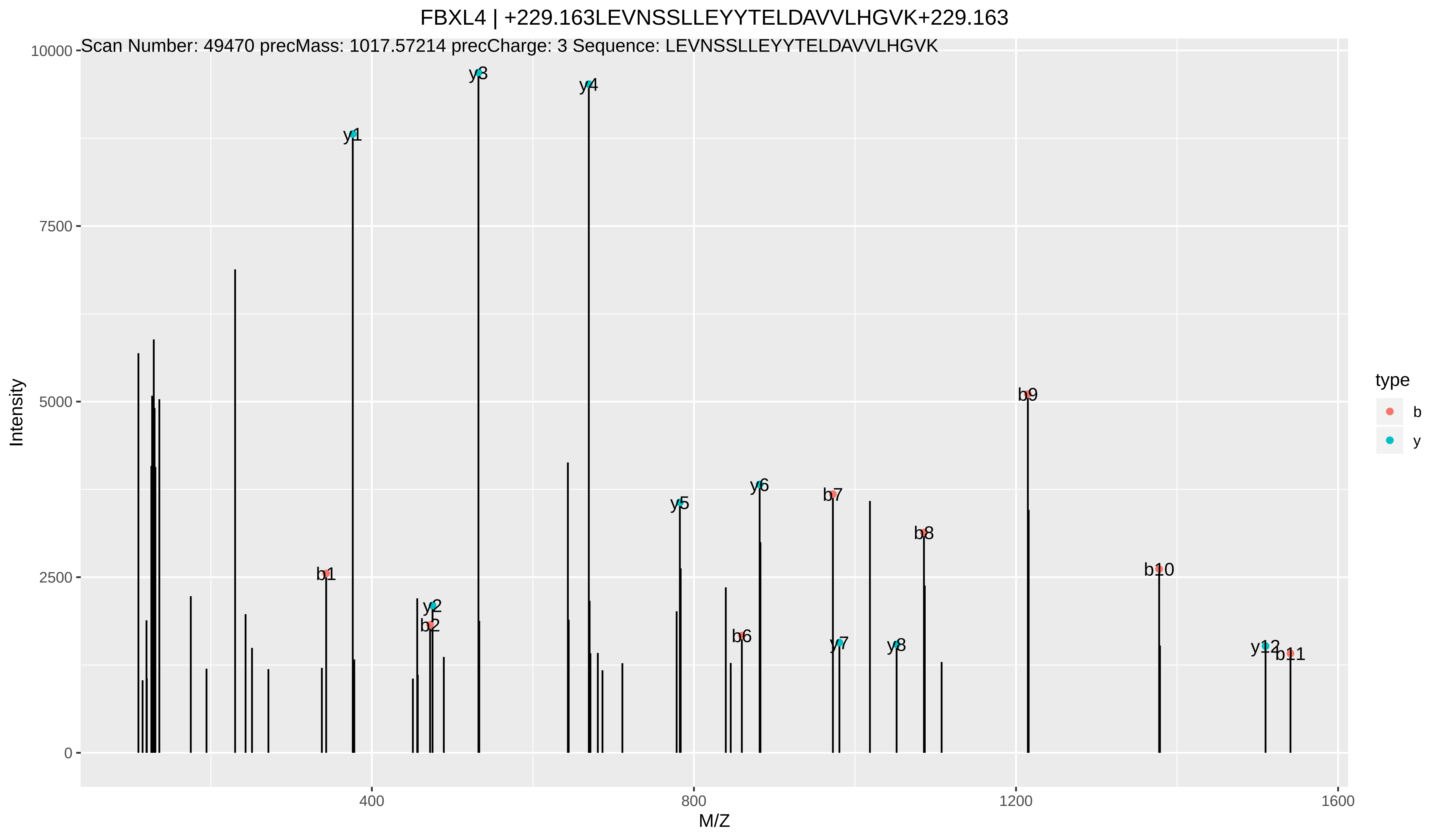

FBXL4 | +229.163LEVNSSLLEYYTELDAVVLHGVK+229.163
Scan Number: 49470 precMass: 1017.57214 precCharge: 3 Sequence: LEVNSSLLEYYTELDAVVLHGVK
Intensity
M/Z
10000
7500
5000
2500
0
400
800
1200
1600
y1
y2
y3
y4
y5
y6
y7
y8
y12
b1
b2
b6
b7
b8
b9
b10
b11
type
b
y

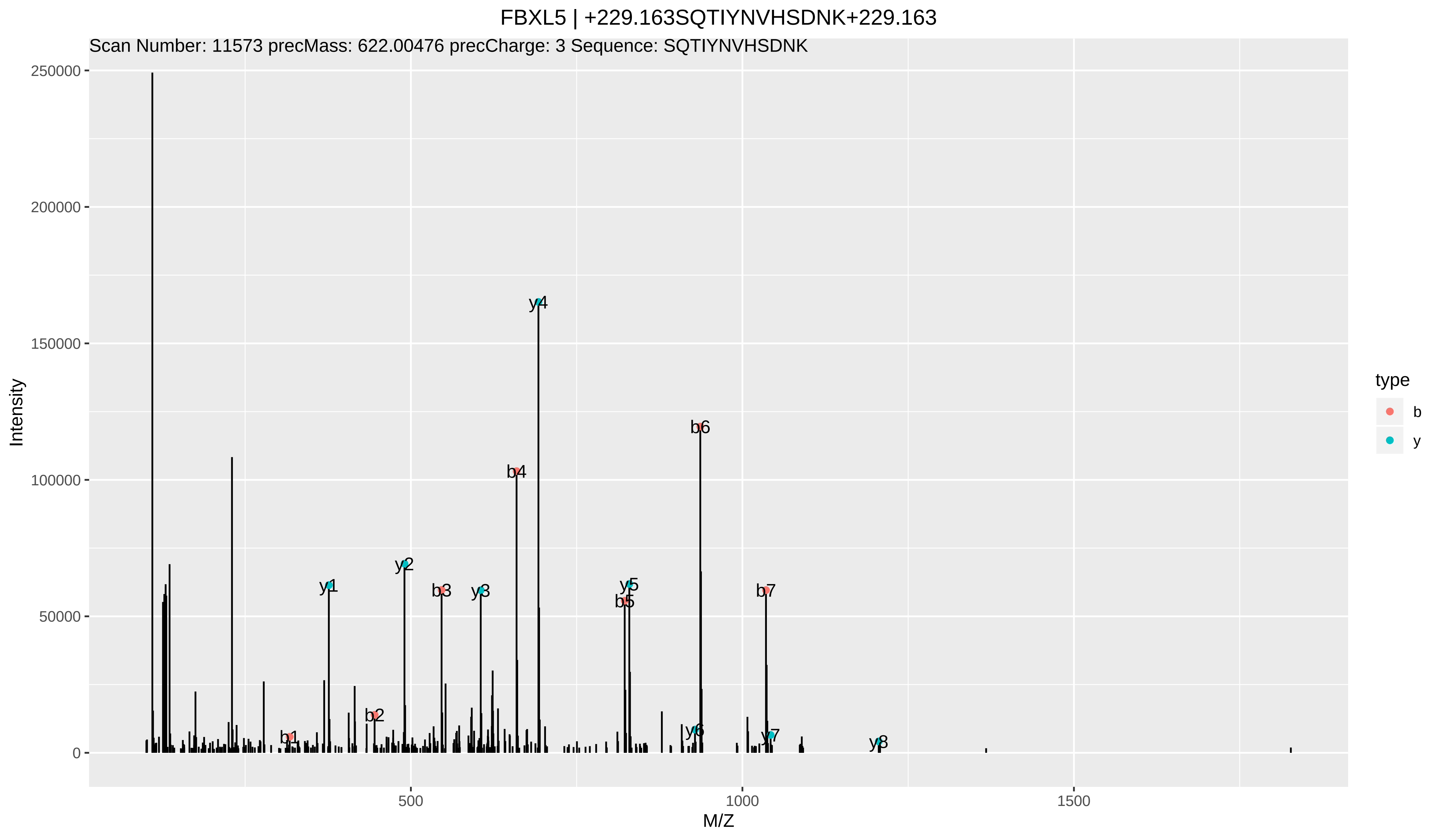
FBXL5 | +229.163SQTIYNVHSDNK+229.163
Scan Number: 11573 precMass: 622.00476 precCharge: 3 Sequence: SQTIYNVHSDNK
Intensity
M/Z
type
b
y
y4
b6
b4
y2
y1
b3
y3
y5
b5
b7
b2
b1
y6
y7
y8
250000
200000
150000
100000
50000
0
500
1000
1500

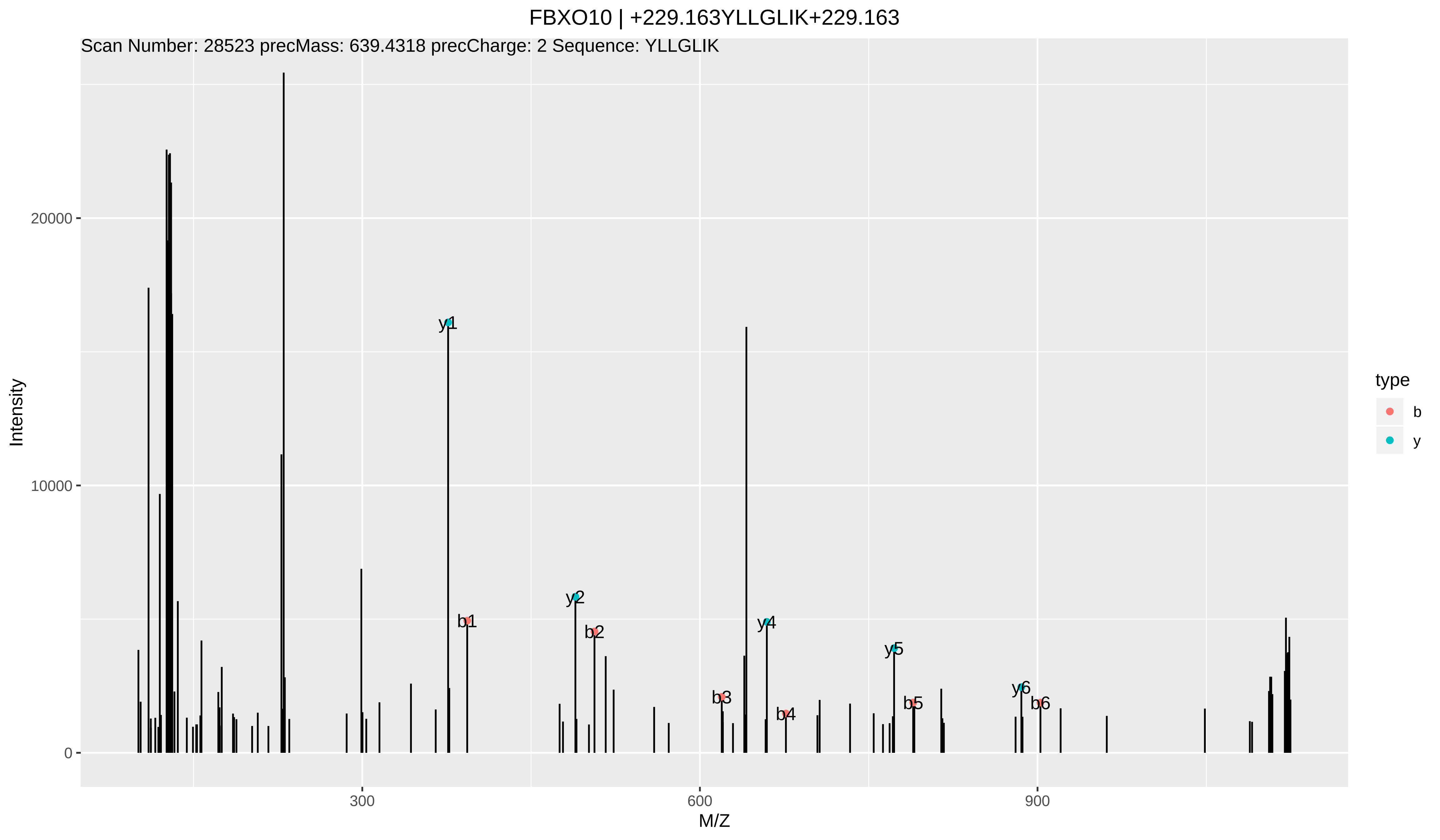

FBXO10 | +229.163YLLGLIK+229.163
Scan Number: 28523 precMass: 639.4318 precCharge: 2 Sequence: YLLGLIK
Intensity
M/Z
0
10000
20000
300
600
900
y1
b1
y2
b2
b3
y4
b4
y5
b5
y6
b6
type
b
y

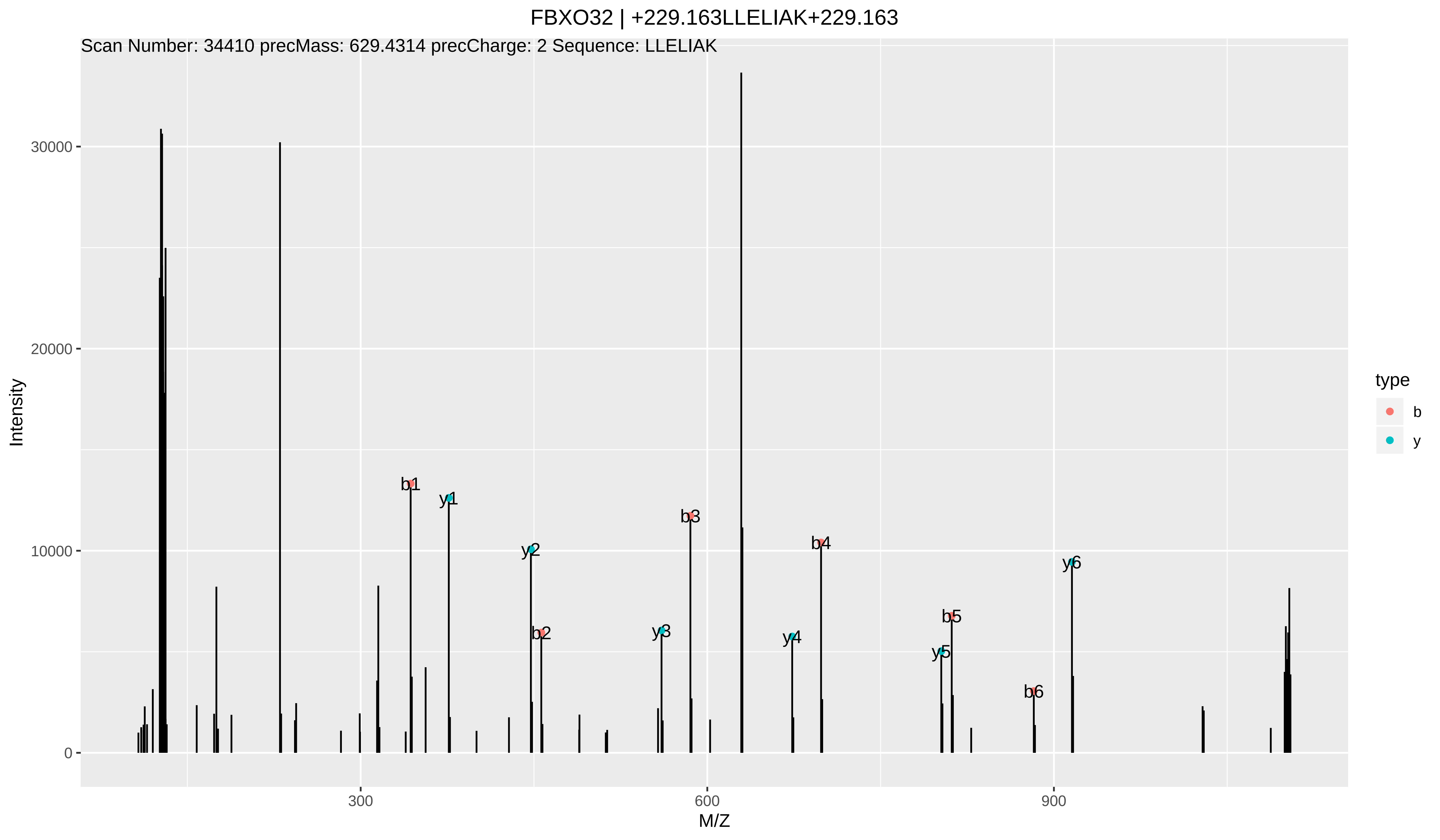

FBXO32 | +229.163LLELIAK+229.163
Scan Number: 34410 precMass: 629.4314 precCharge: 2 Sequence: LLELIAK
Intensity
30000
20000
10000
0
300
600
900
M/Z
b1
y1
y2
b2
y3
b3
y4
b4
y5
b5
b6
y6
type
b
y

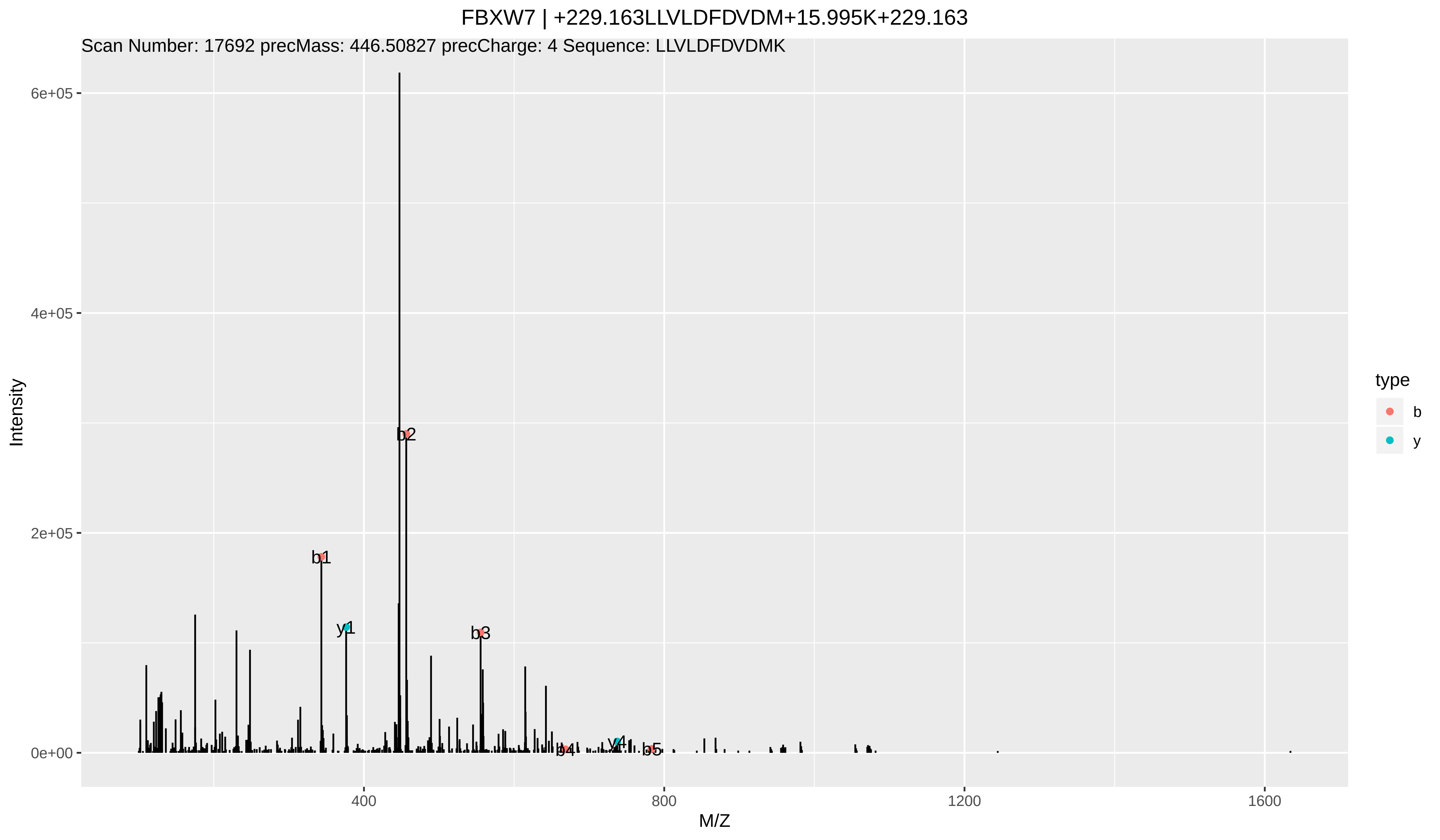

FBXW7 | +229.163LLVLDFDVDM+15.995K+229.163
Scan Number: 17692 precMass: 446.50827 precCharge: 4 Sequence: LLVLDFDVDMK
Intensity
6e+05
4e+05
2e+05
0e+00
M/Z
400
800
1200
1600
b1
b2
b3
b4
b5
y1
y4
type
b
y

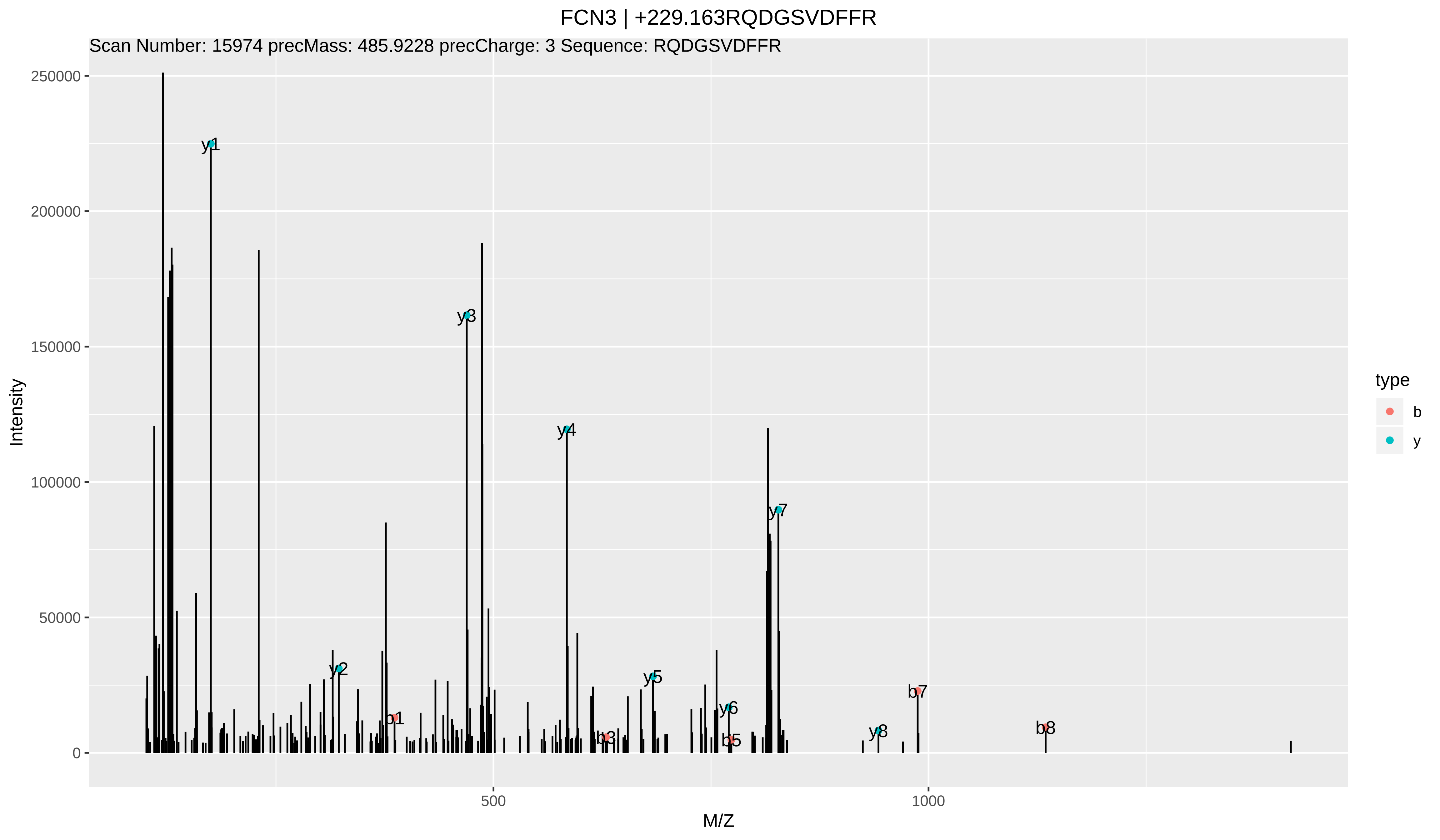
FCN3 | +229.163RQDGSVDFFR
Scan Number: 15974 precMass: 485.9228 precCharge: 3 Sequence: RQDGSVDFFR
Intensity
M/Z
250000
200000
150000
100000
50000
0
500
1000
y1
y2
y3
y4
y5
y6
y7
y8
b1
b3
b5
b7
b8
type
b
y

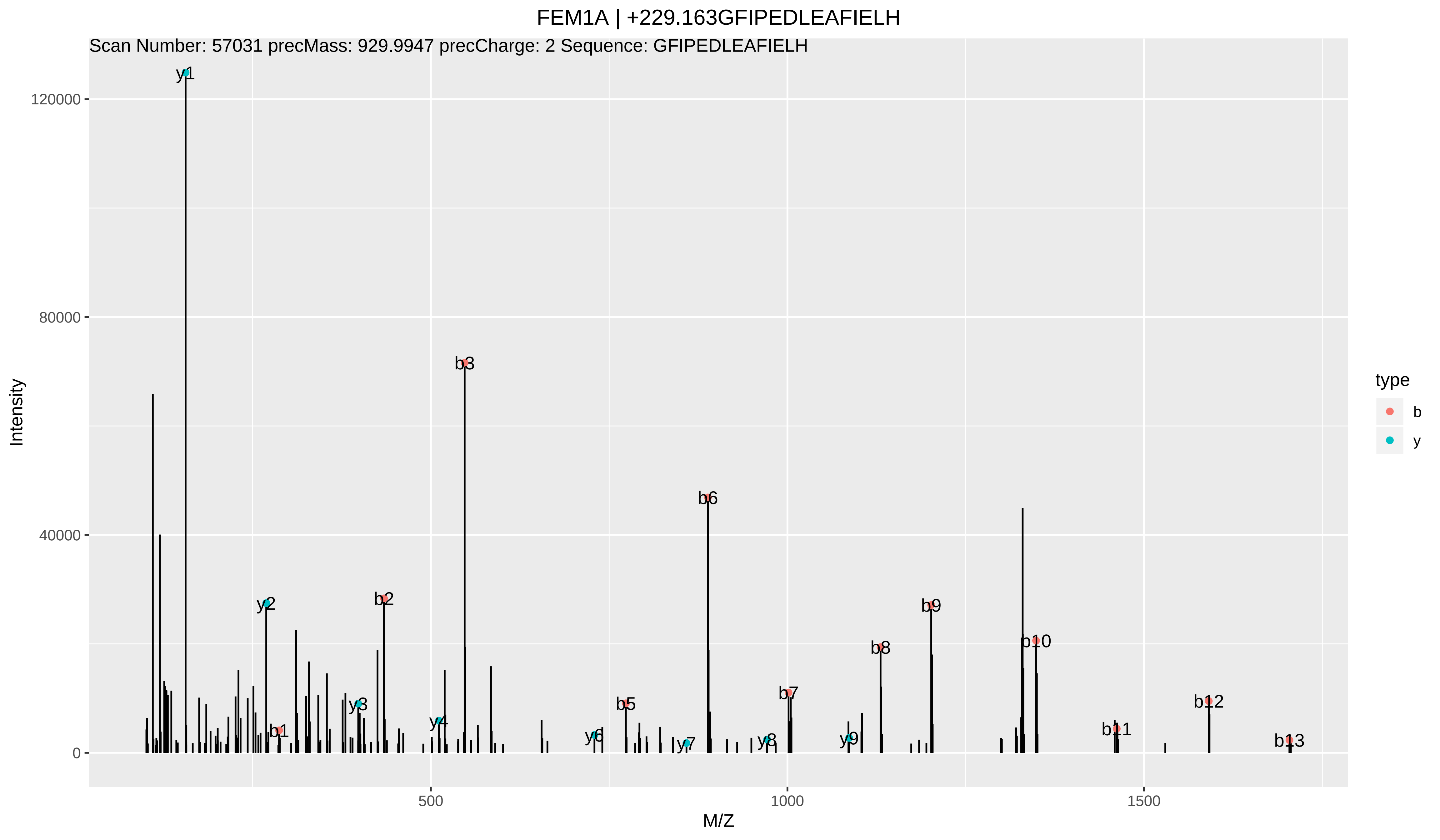
FEM1A | +229.163GFIPEDLEAFIELH
Scan Number: 57031 precMass: 929.9947 precCharge: 2 Sequence: GFIPEDLEAFIELH
Intensity
120000
80000
40000
0
500
1000
1500
M/Z
y1
y2
y3
y4
y6
y7
y8
y9
b1
b2
b3
b5
b6
b7
b8
b9
b10
b11
b12
b13
type
b
y

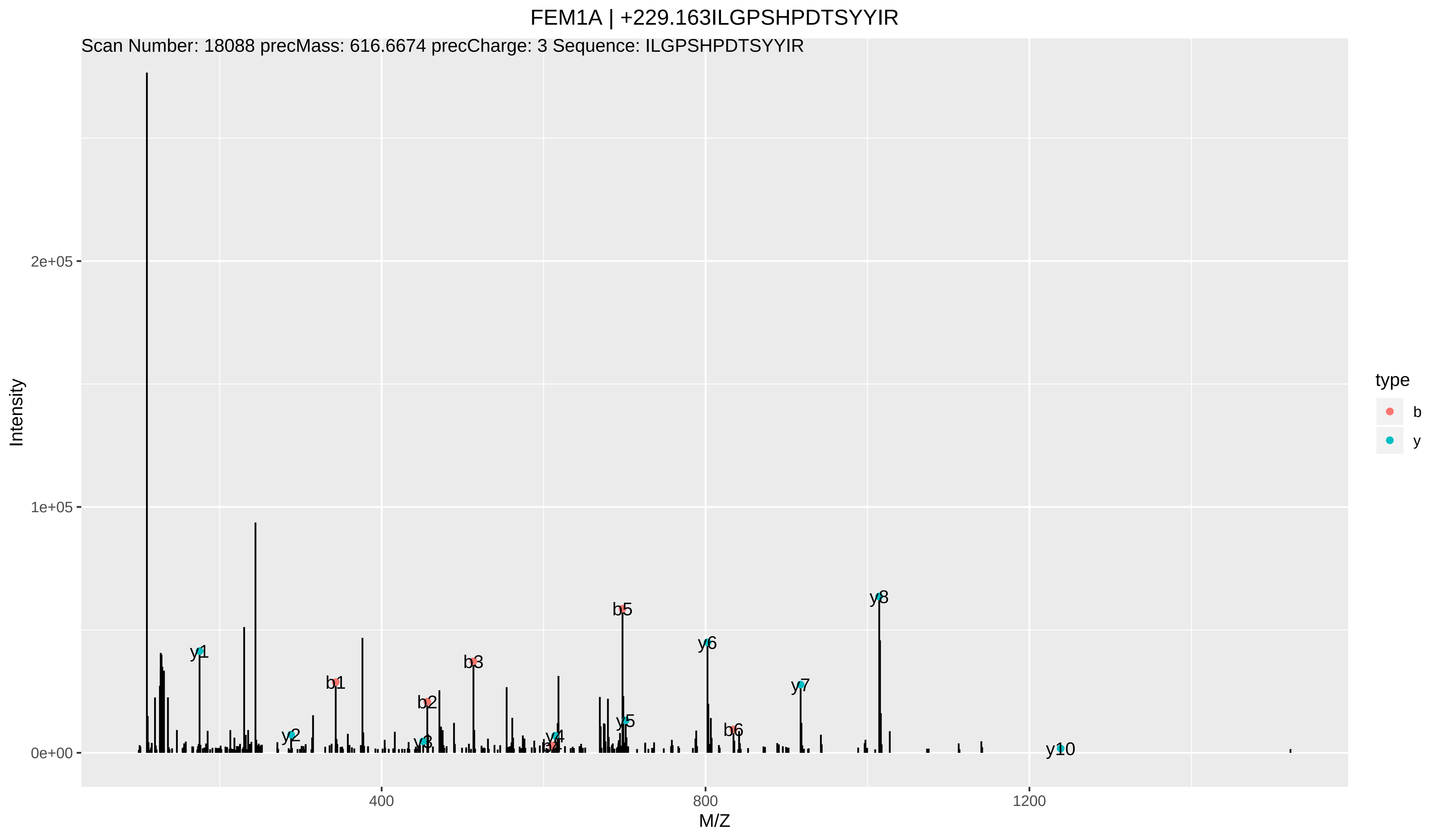

FEM1A | +229.163ILGPSHPDTSYYIR
Scan Number: 18088 precMass: 616.6674 precCharge: 3 Sequence: ILGPSHPDTSYYIR
Intensity
2e+05
1e+05
0e+00
M/Z
400
800
1200
y1
y2
y3
y4
y5
y6
y7
y8
y10
b1
b2
b3
b5
b6
type
b
y

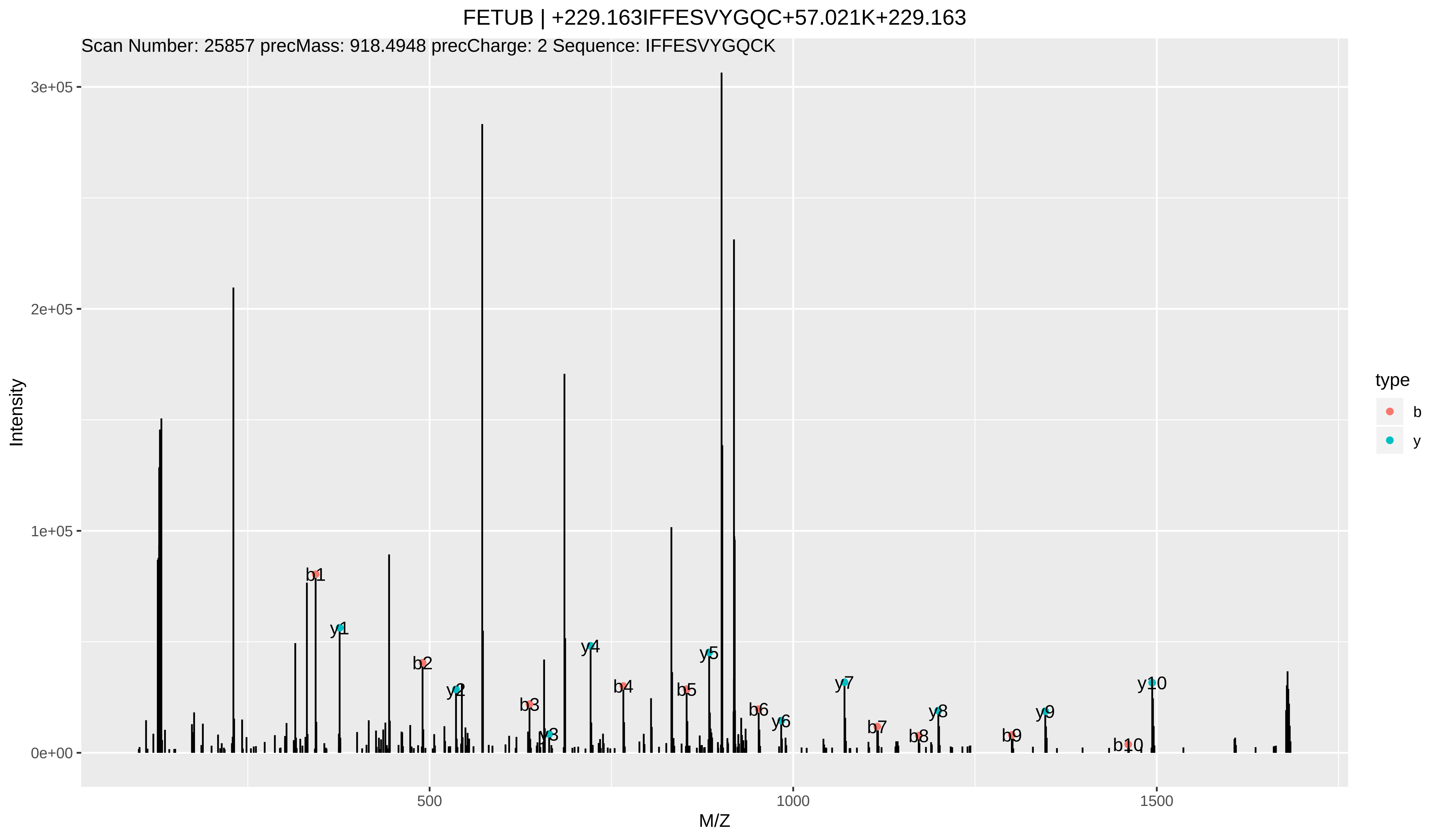

FETUB | +229.163IFFESVYGQC+57.021K+229.163
Scan Number: 25857 precMass: 918.4948 precCharge: 2 Sequence: IFFESVYGQCK
3e+05
2e+05
1e+05
0e+00
Intensity
500
1000
1500
M/Z
type
b
y
b1
y1
b2
y2
b3
y3
y4
b4
b5
y5
b6
y6
y7
b7
b8
y8
b9
y9
y10
b10

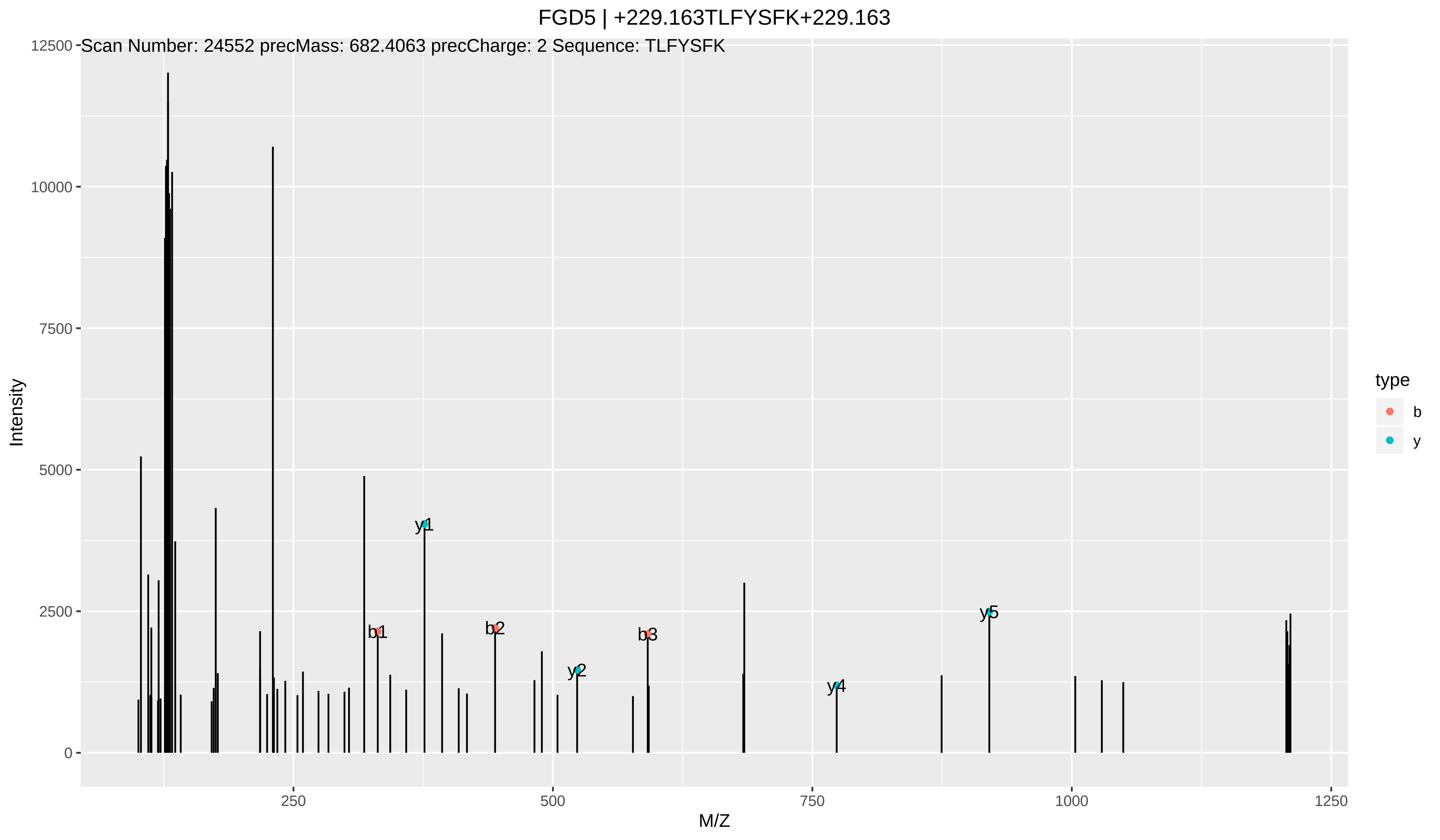

FGD5 | +229.163TLFYSFK+229.163
Scan Number: 24552 precMass: 682.4063 precCharge: 2 Sequence: TLFYSFK
Intensity
M/Z
type
b
y
b1
b2
b3
y1
y2
y4
y5
0
2500
5000
7500
10000
12500
250
500
750
1000
1250

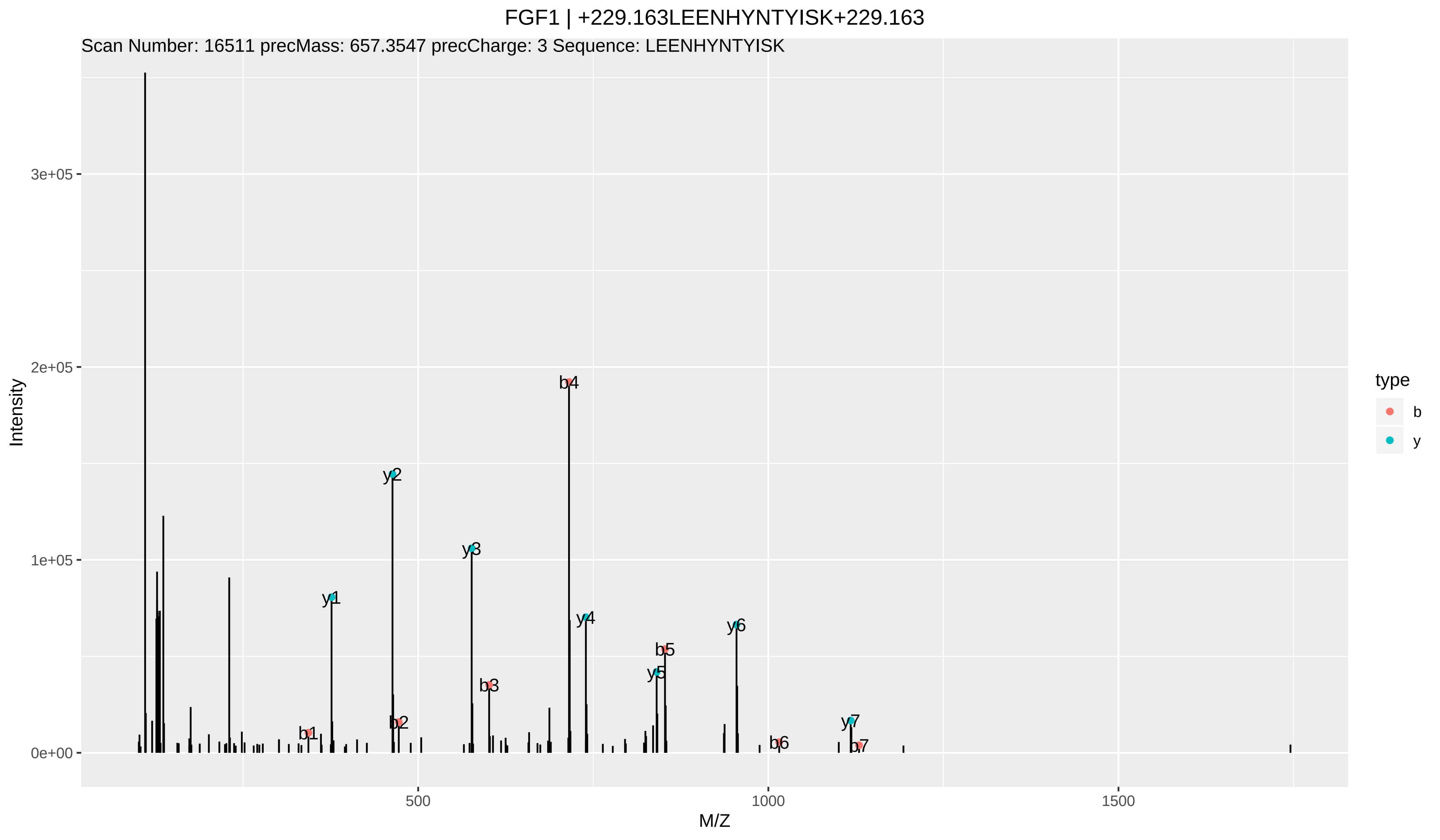

FGF1 | +229.163LEENHYNTYISK+229.163
Scan Number: 16511 precMass: 657.3547 precCharge: 3 Sequence: LEENHYNTYISK
Intensity
3e+05
2e+05
1e+05
0e+00
M/Z
500
1000
1500
b4
y2
y3
y1
y4
y6
b5
y5
b3
b1
b2
y7
b6
b7
type
b
y

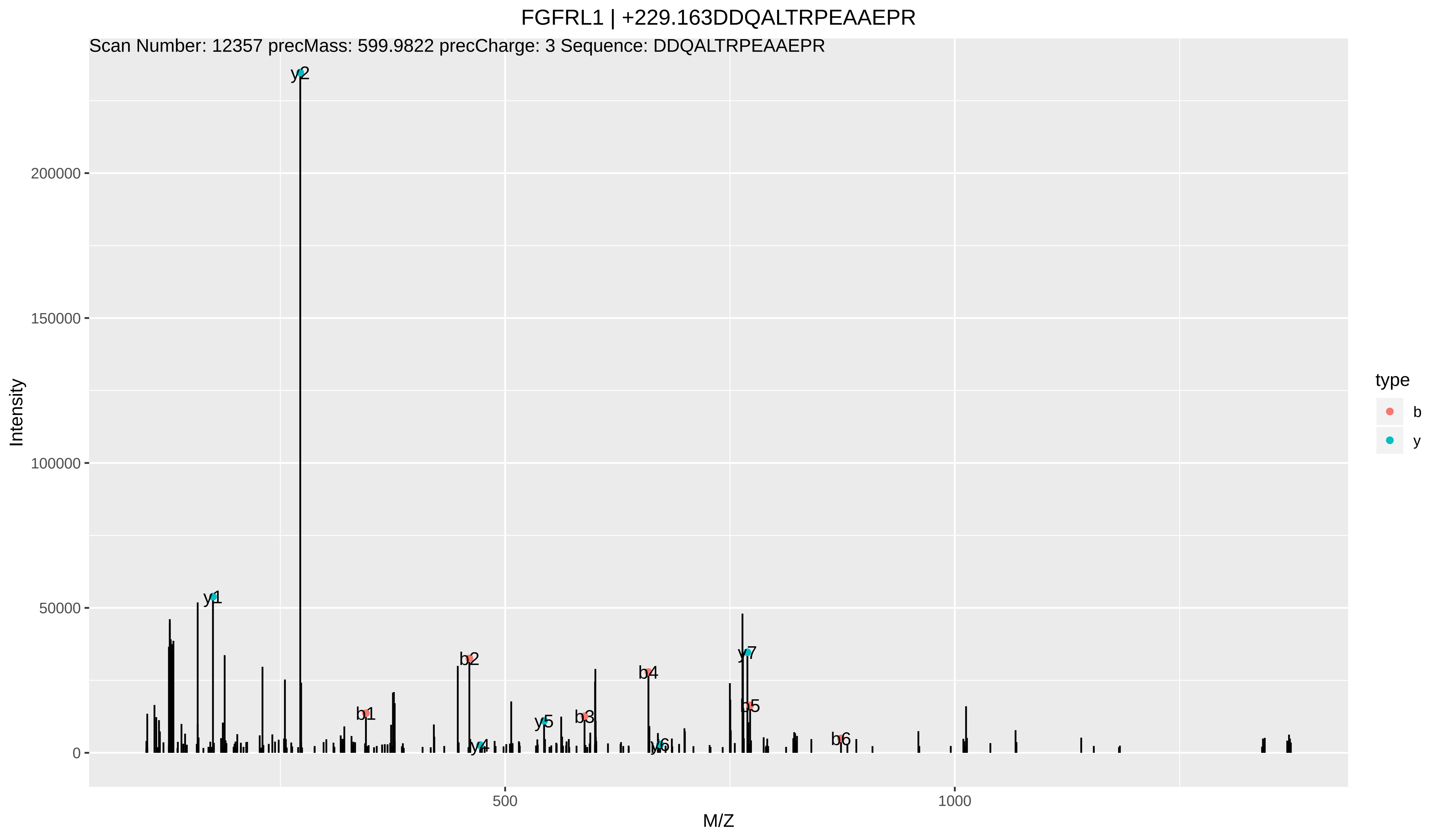

FGFRL1 | +229.163DDQALTRPEAAEPR
Scan Number: 12357 precMass: 599.9822 precCharge: 3 Sequence: DDQALTRPEAAEPR
Intensity
M/Z
type
b
y
y2
y1
b1
b2
y4
y5
b3
b4
y6
y7
b5
b6
200000
150000
100000
50000
0
500
1000

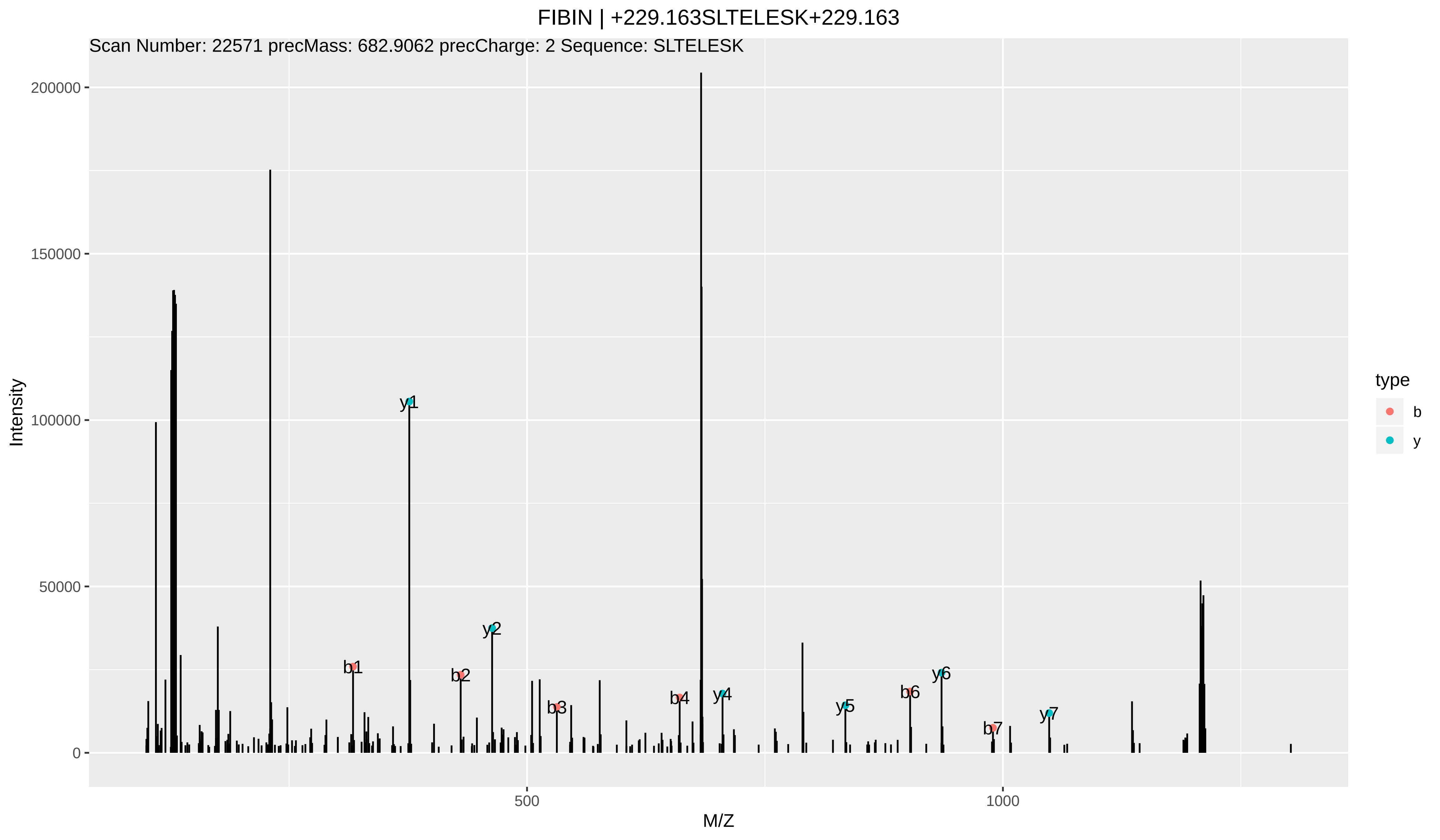

FIBIN | +229.163SLTELESK+229.163
Scan Number: 22571 precMass: 682.9062 precCharge: 2 Sequence: SLTELESK
Intensity
M/Z
0
50000
100000
150000
200000
500
1000
y1
y2
y4
y5
y6
y7
b1
b2
b3
b4
b6
b7
type
b
y

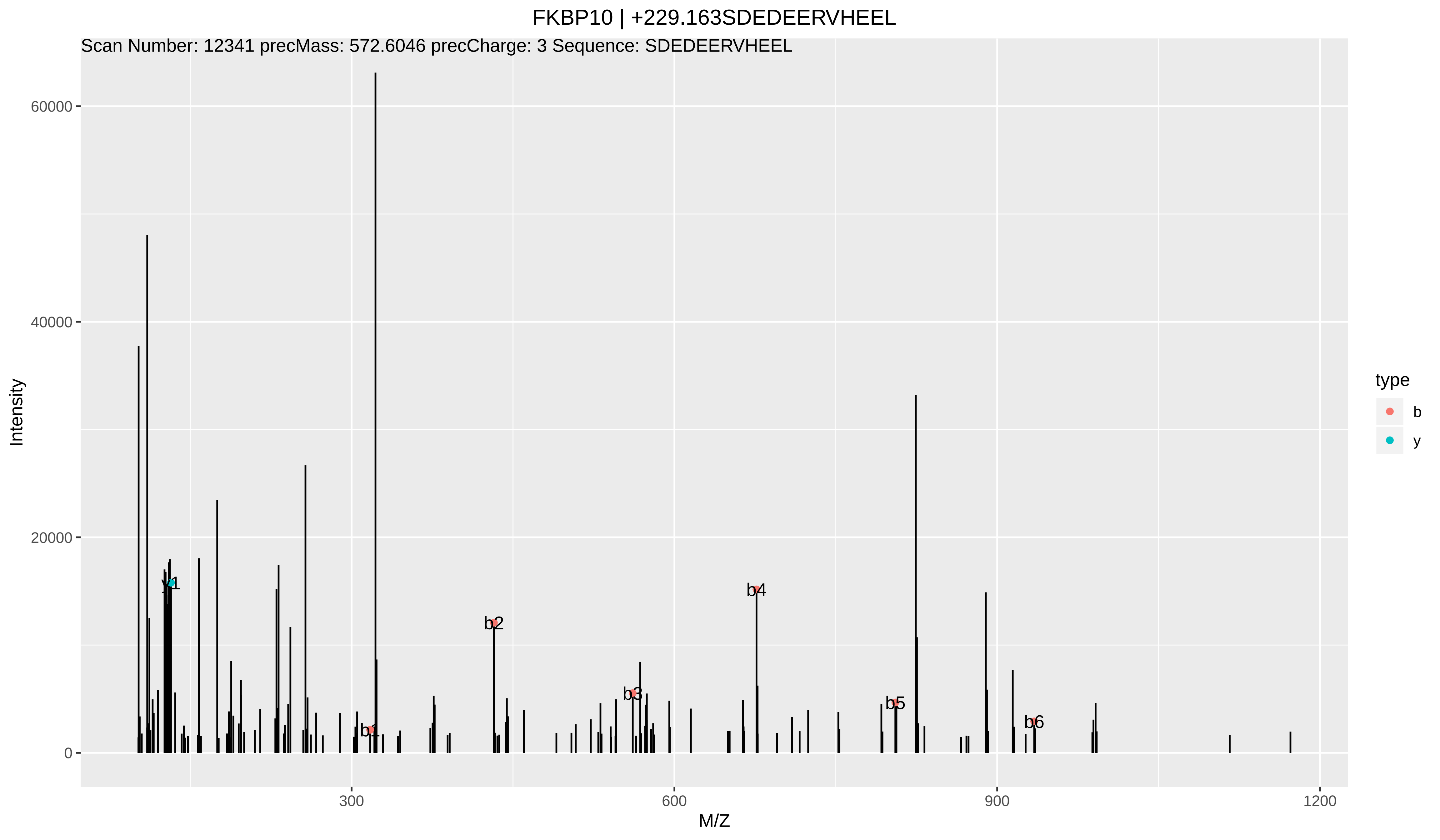

FKBP10 | +229.163SDEDEERVHEEL
Scan Number: 12341 precMass: 572.6046 precCharge: 3 Sequence: SDEDEERVHEEL
Intensity
M/Z
60000
40000
20000
0
300
600
900
1200
y1
b1
b2
b3
b4
b5
b6
type
b
y

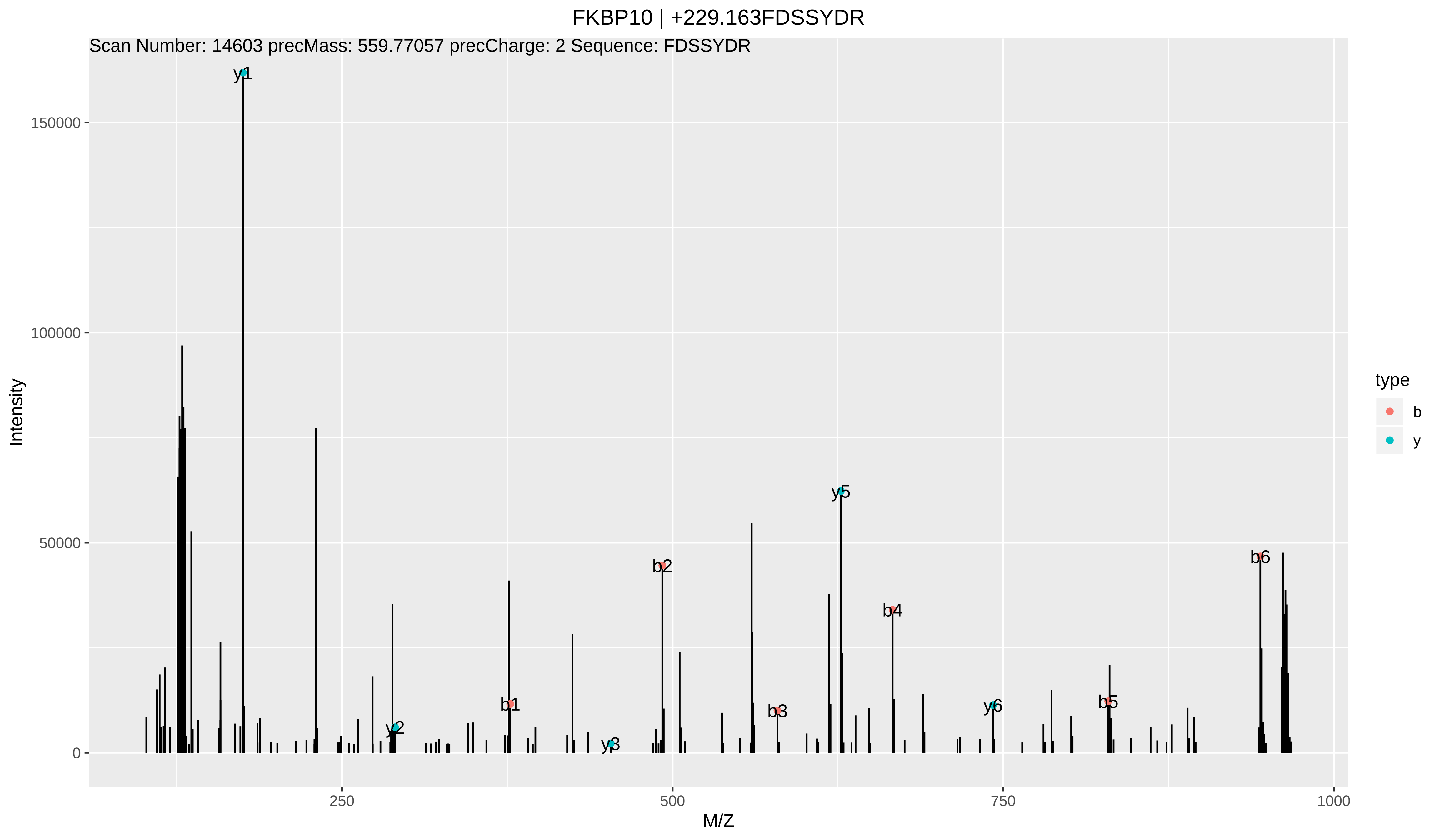
FKBP10 | +229.163FDSSYDR
Scan Number: 14603 precMass: 559.77057 precCharge: 2 Sequence: FDSSYDR
Intensity
M/Z
150000
100000
50000
0
250
500
750
1000
y1
y2
y3
y5
y6
b1
b2
b3
b4
b5
b6
type
b
y

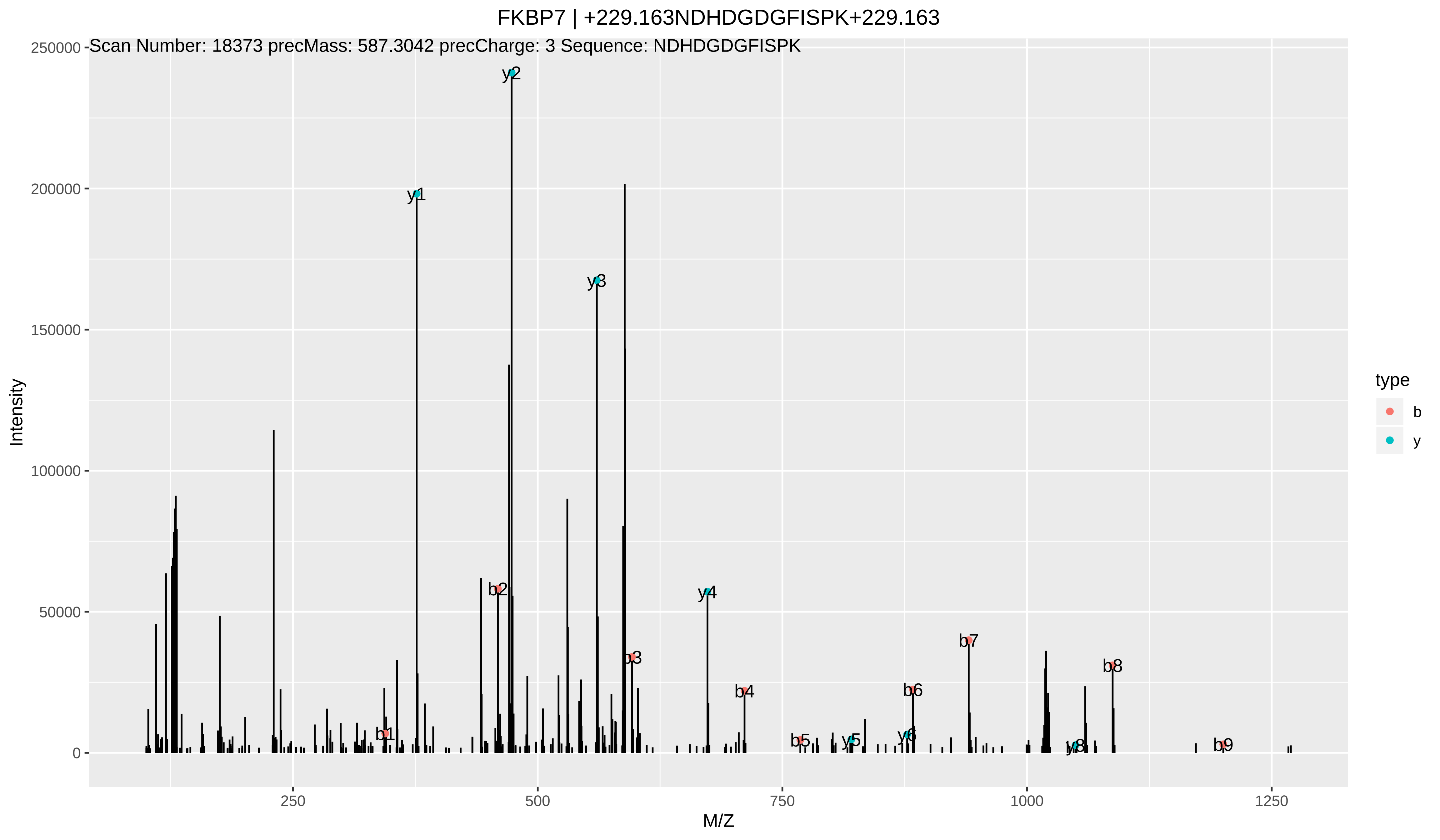
FKBP7 | +229.163NDHDGDGFISPK+229.163
Scan Number: 18373 precMass: 587.3042 precCharge: 3 Sequence: NDHDGDGFISPK
Intensity
M/Z
type
b
y
y1
y2
y3
y4
y5
y6
y8
b1
b2
b3
b4
b5
b6
b7
b8
b9
0
50000
100000
150000
200000
250000
250
500
750
1000
1250

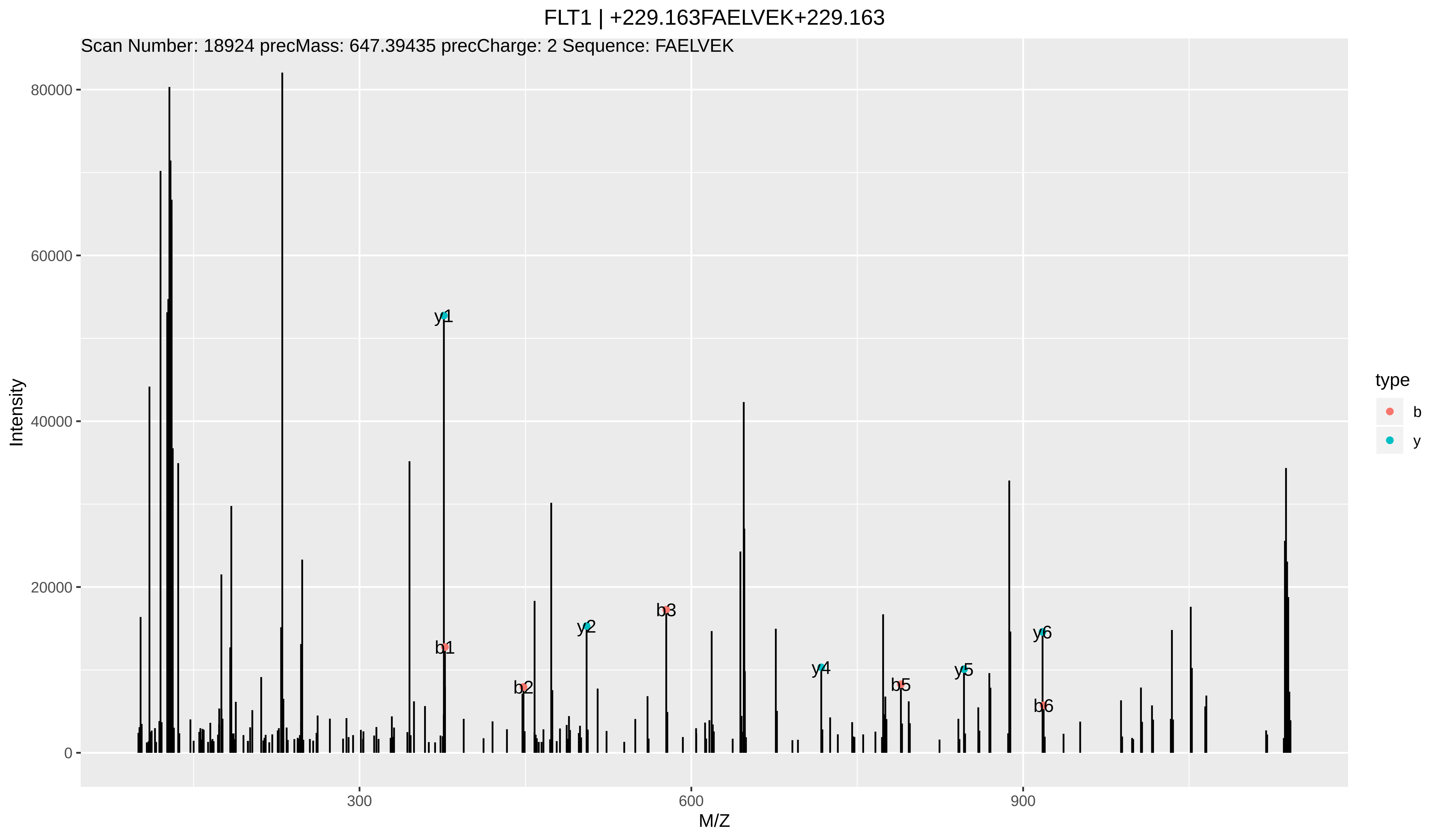
FLT1 | +229.163FAELVEK+229.163
Scan Number: 18924 precMass: 647.39435 precCharge: 2 Sequence: FAELVEK
Intensity
80000
60000
40000
20000
0
300
600
900
M/Z
y1
b1
b2
y2
b3
y4
b5
y5
y6
b6
type
b
y

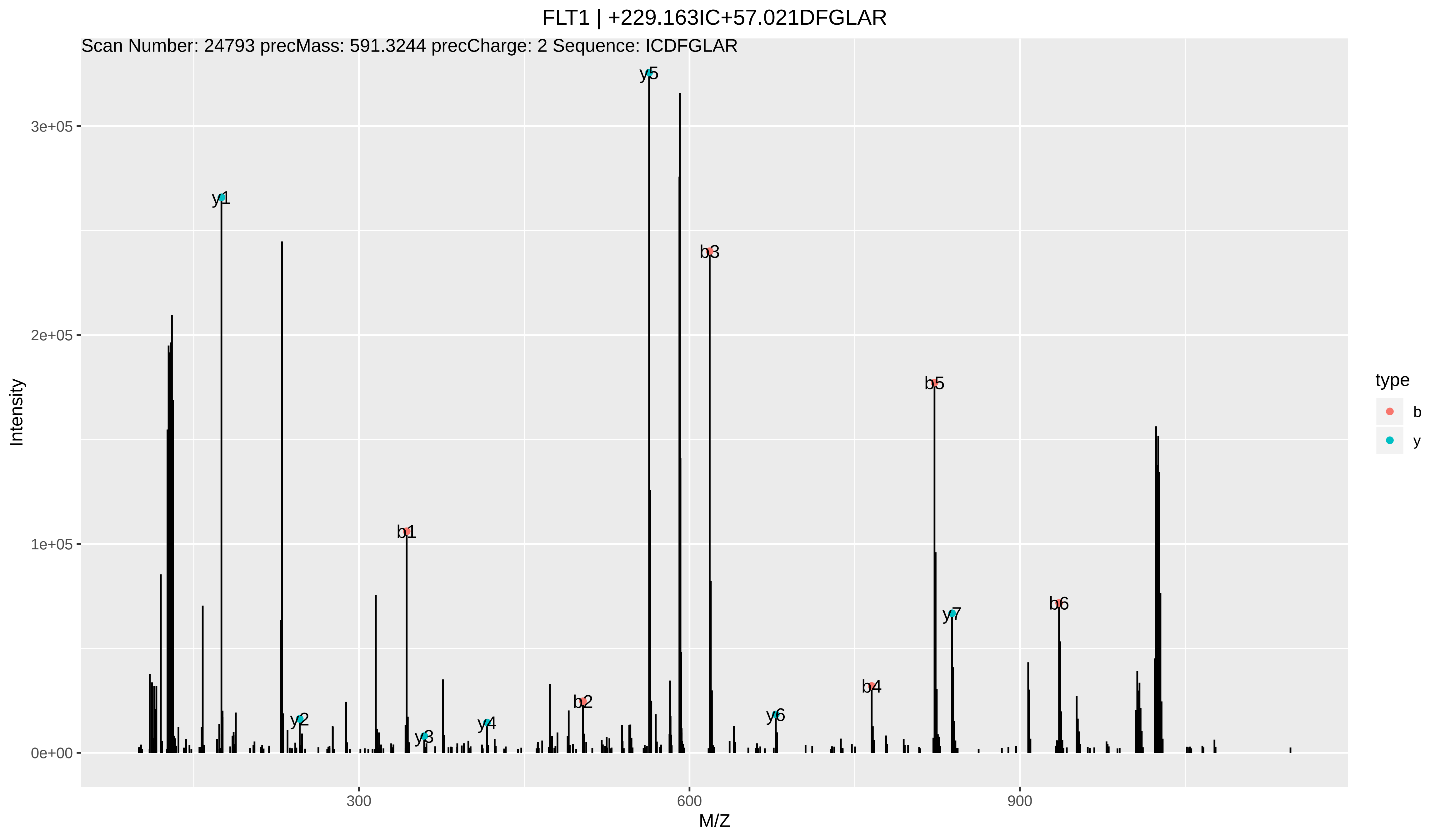
FLT1 | +229.163IC+57.021DFGLAR
Scan Number: 24793 precMass: 591.3244 precCharge: 2 Sequence: ICDFGLAR
Intensity
M/Z
0e+00
1e+05
2e+05
3e+05
300
600
900
y1
y2
y3
y4
y5
y6
y7
b1
b2
b3
b4
b5
b6
type
b
y

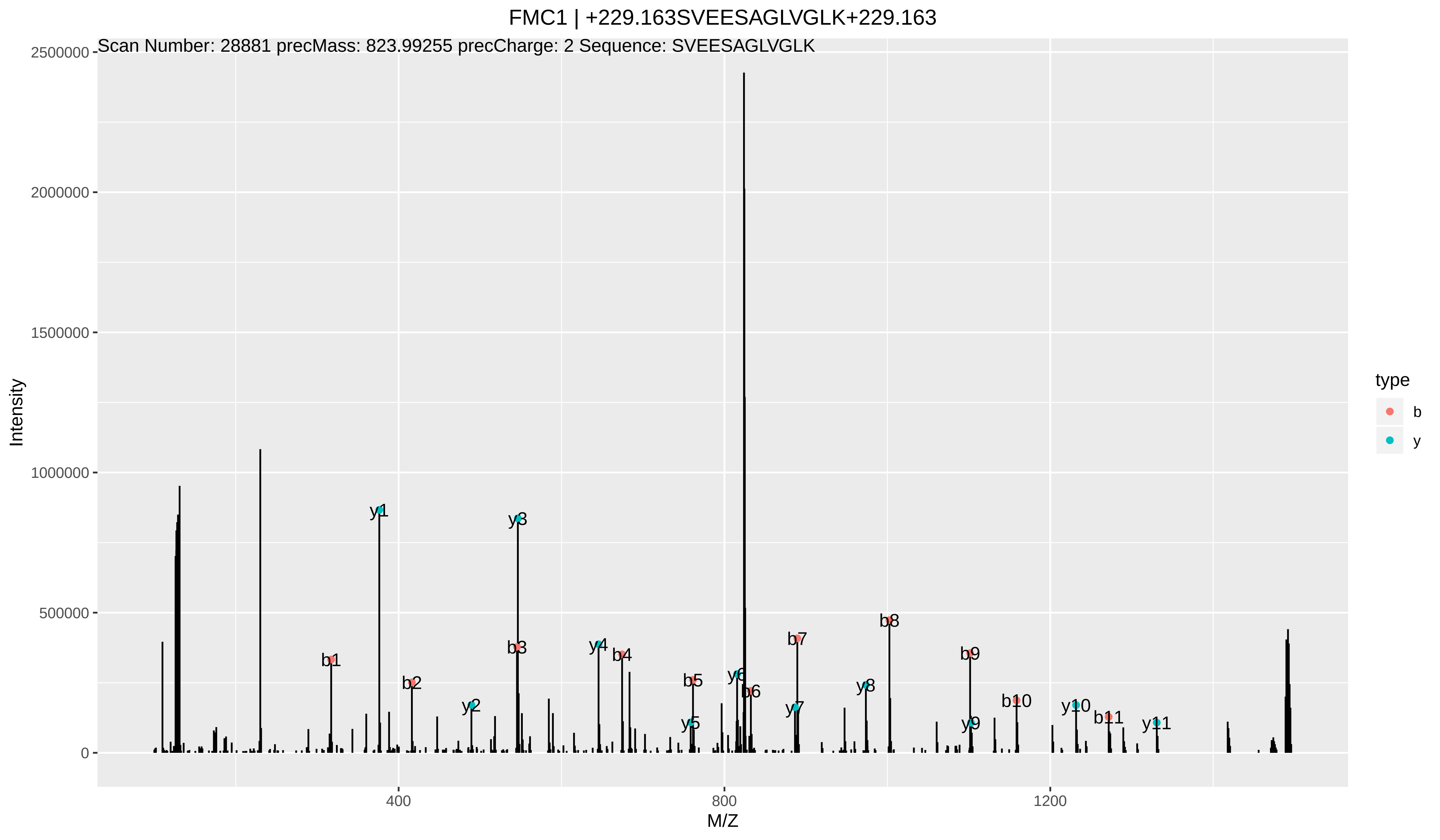

FMC1 | +229.163SVEESAGLVGLK+229.163
Scan Number: 28881 precMass: 823.99255 precCharge: 2 Sequence: SVEESAGLVGLK
Intensity
2500000
2000000
1500000
1000000
500000
0
400
800
1200
M/Z
y1
y3
b1
b2
y2
b3
y4
b4
b5
y5
y6
b6
b7
y7
y8
b8
b9
y9
b10
y10
b11
y11
type
b
y

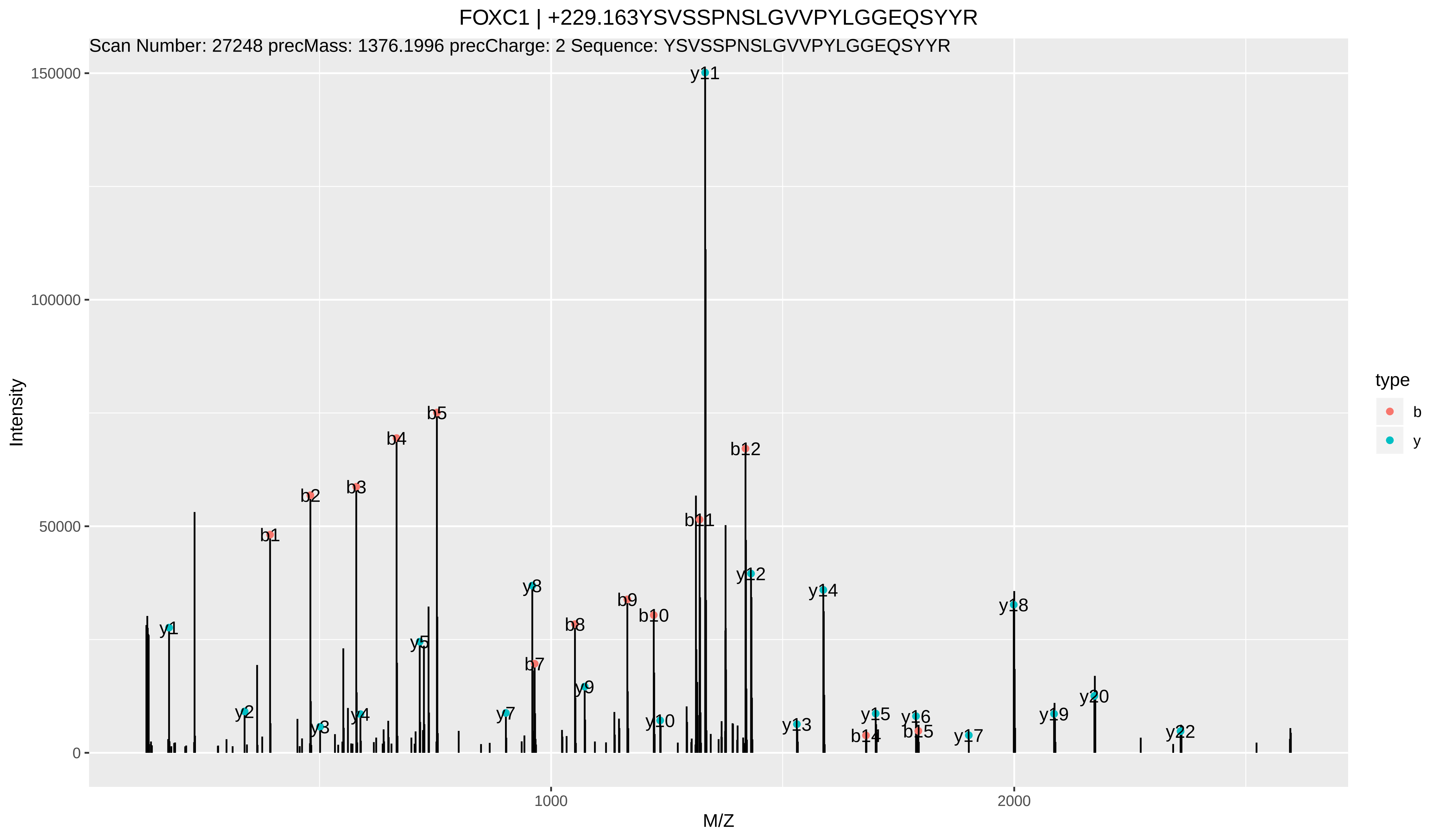
FOXC1 | +229.163YSVSSPNSLGVVPYLGGEQSYYR
Scan Number: 27248 precMass: 1376.1996 precCharge: 2 Sequence: YSVSSPNSLGVVPYLGGEQSYYR
Intensity
M/Z
type
b
y
150000
100000
50000
0
1000
2000
y1
y2
y3
y4
y5
y7
y8
y9
y10
y11
y12
y13
y14
y15
y16
y17
y18
y19
y20
y22
b1
b2
b3
b4
b5
b7
b8
b9
b10
b11
b12
b14
b15

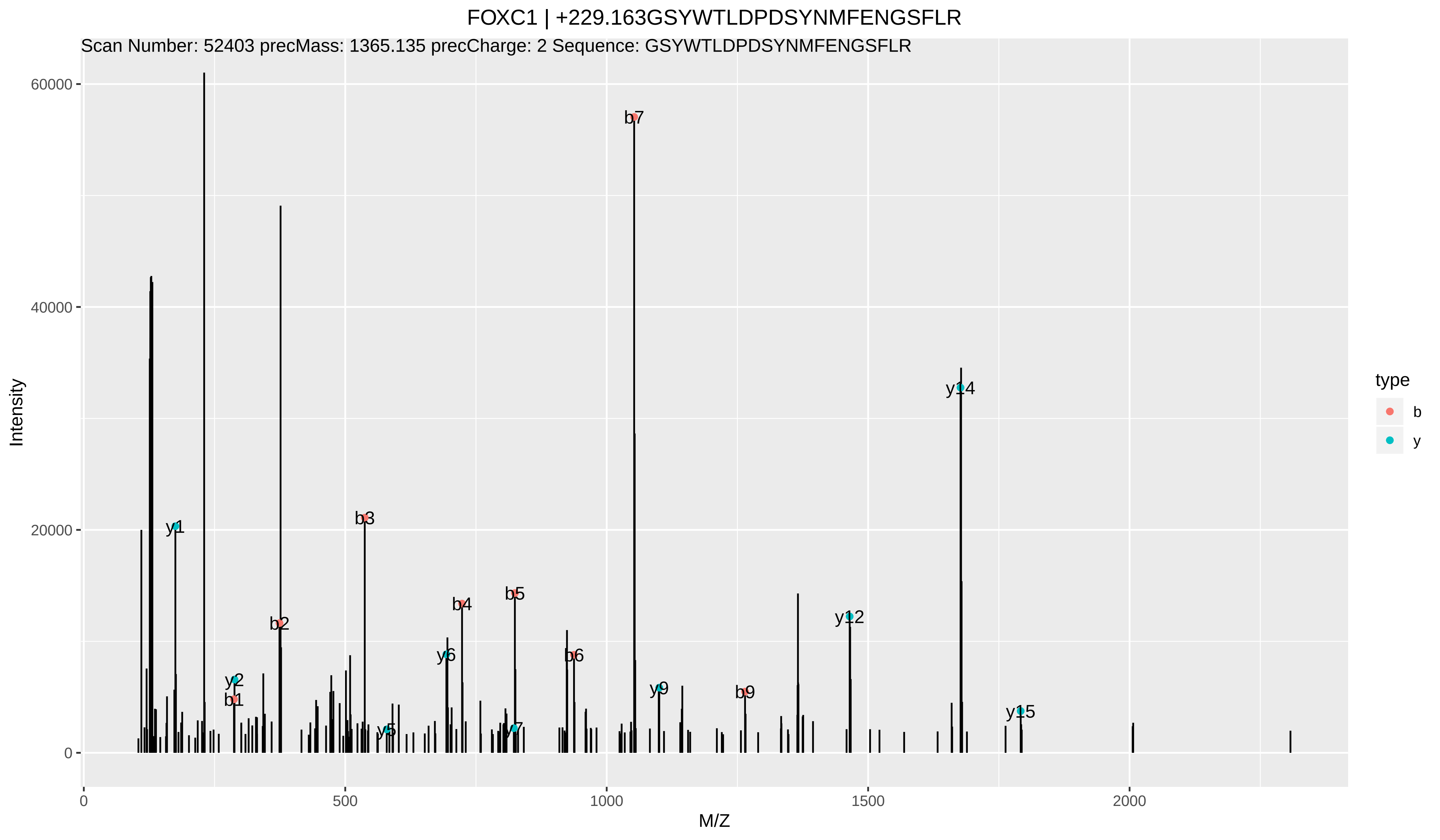
FOXC1 | +229.163GSYWTLDPDSYNMFENGSFLR
Scan Number: 52403 precMass: 1365.135 precCharge: 2 Sequence: GSYWTLDPDSYNMFENGSFLR
Intensity
M/Z
0
20000
40000
60000
0
500
1000
1500
2000
b7
y14
b3
y1
b5
b4
y12
b2
y6
b6
y2
b1
y9
b9
y15
y5
y7
type
b
y

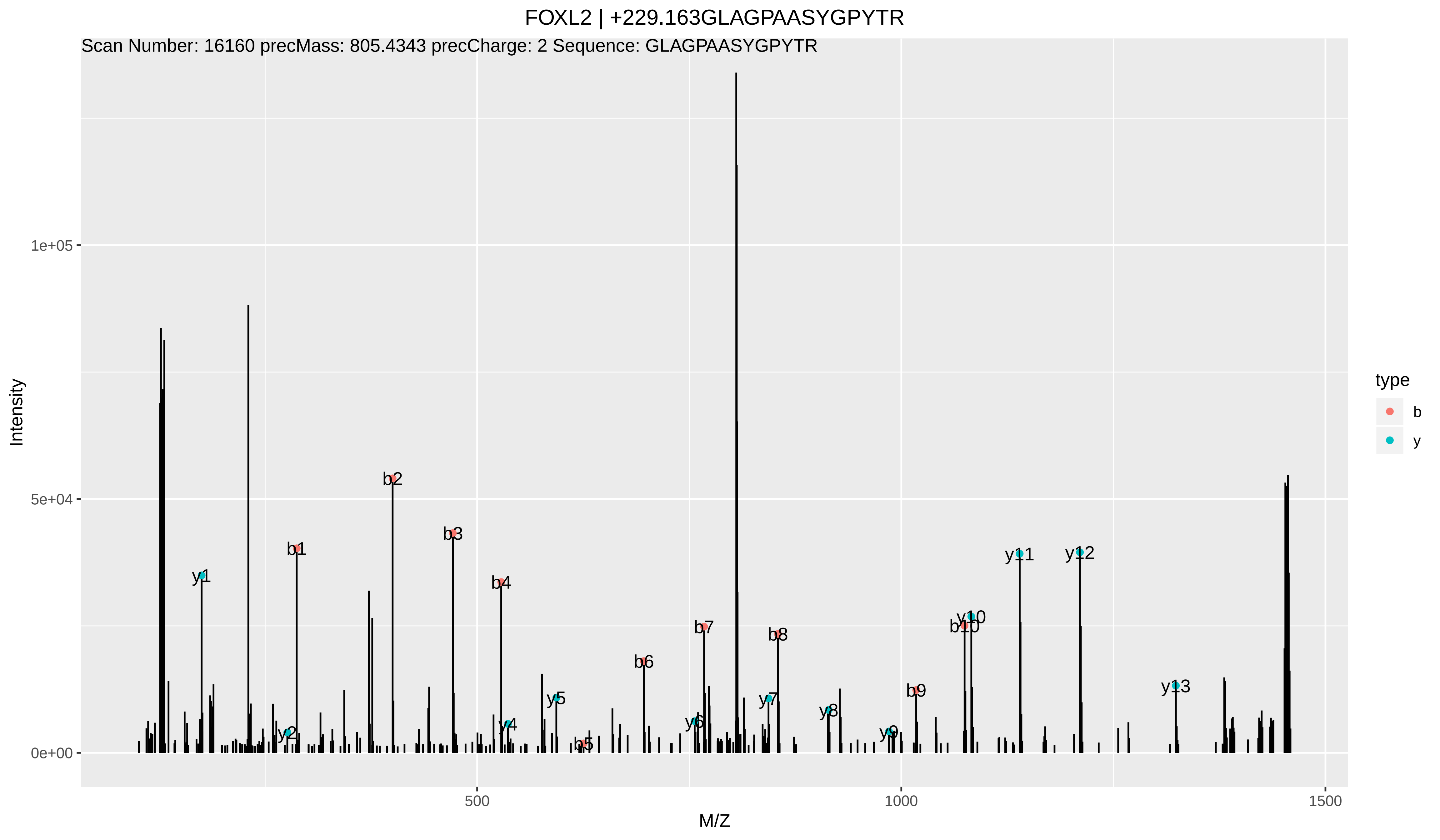

FOXL2 | +229.163GLAGPAASYGPYTR
Scan Number: 16160 precMass: 805.4343 precCharge: 2 Sequence: GLAGPAASYGPYTR
Intensity
M/Z
0e+00
5e+04
1e+05
500
1000
1500
type
b
y
y1
y2
b1
b2
b3
b4
y4
y5
b5
b6
y6
b7
y7
b8
y8
y9
b9
b10
y10
y11
y12
y13

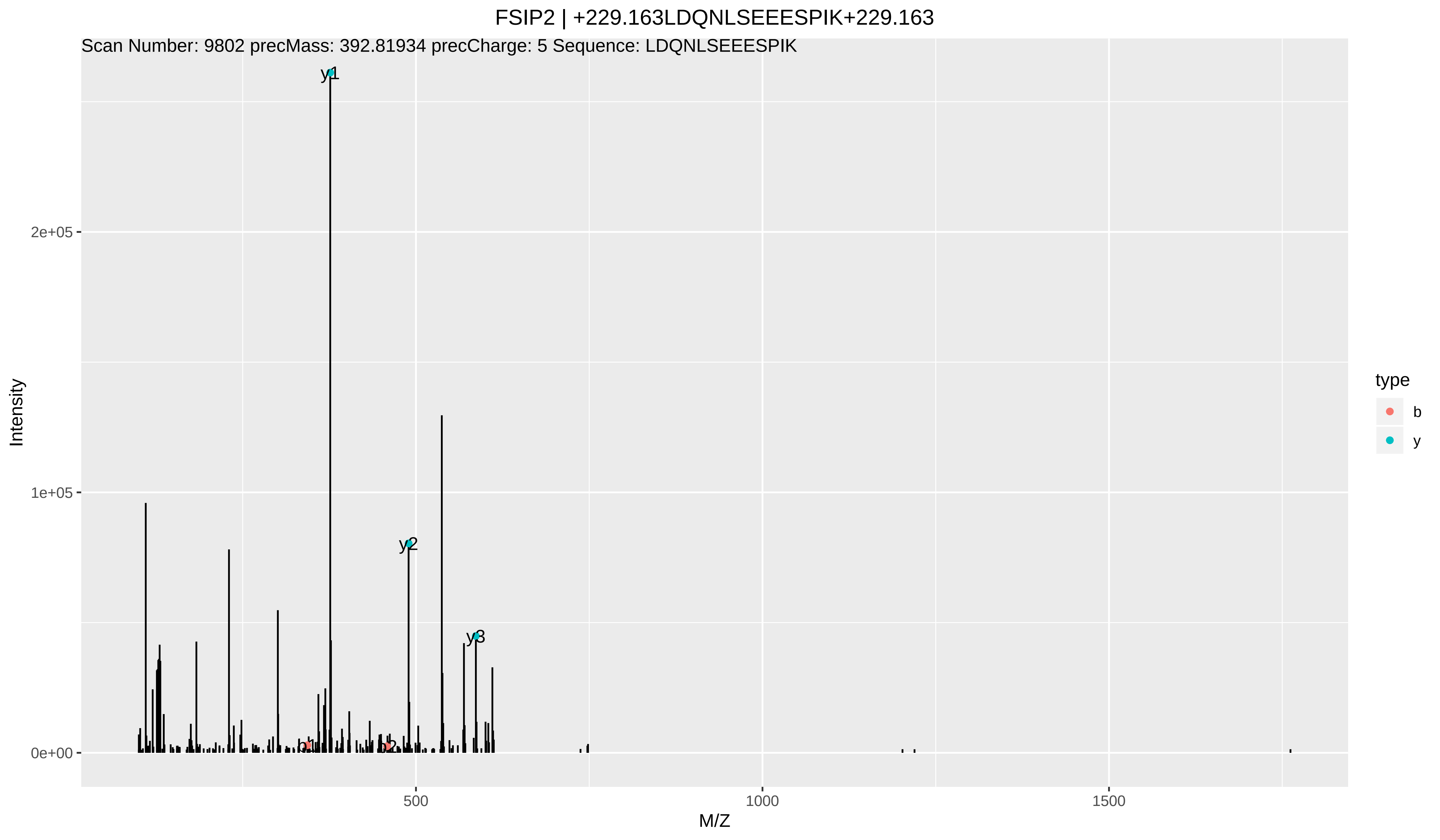
FSIP2 | +229.163LDQNLSEEESPIK+229.163
Scan Number: 9802 precMass: 392.81934 precCharge: 5 Sequence: LDQNLSEEESPIK
y1
y2
y3
b1
b2
Intensity
2e+05
1e+05
0e+00
M/Z
500
1000
1500
type
b
y

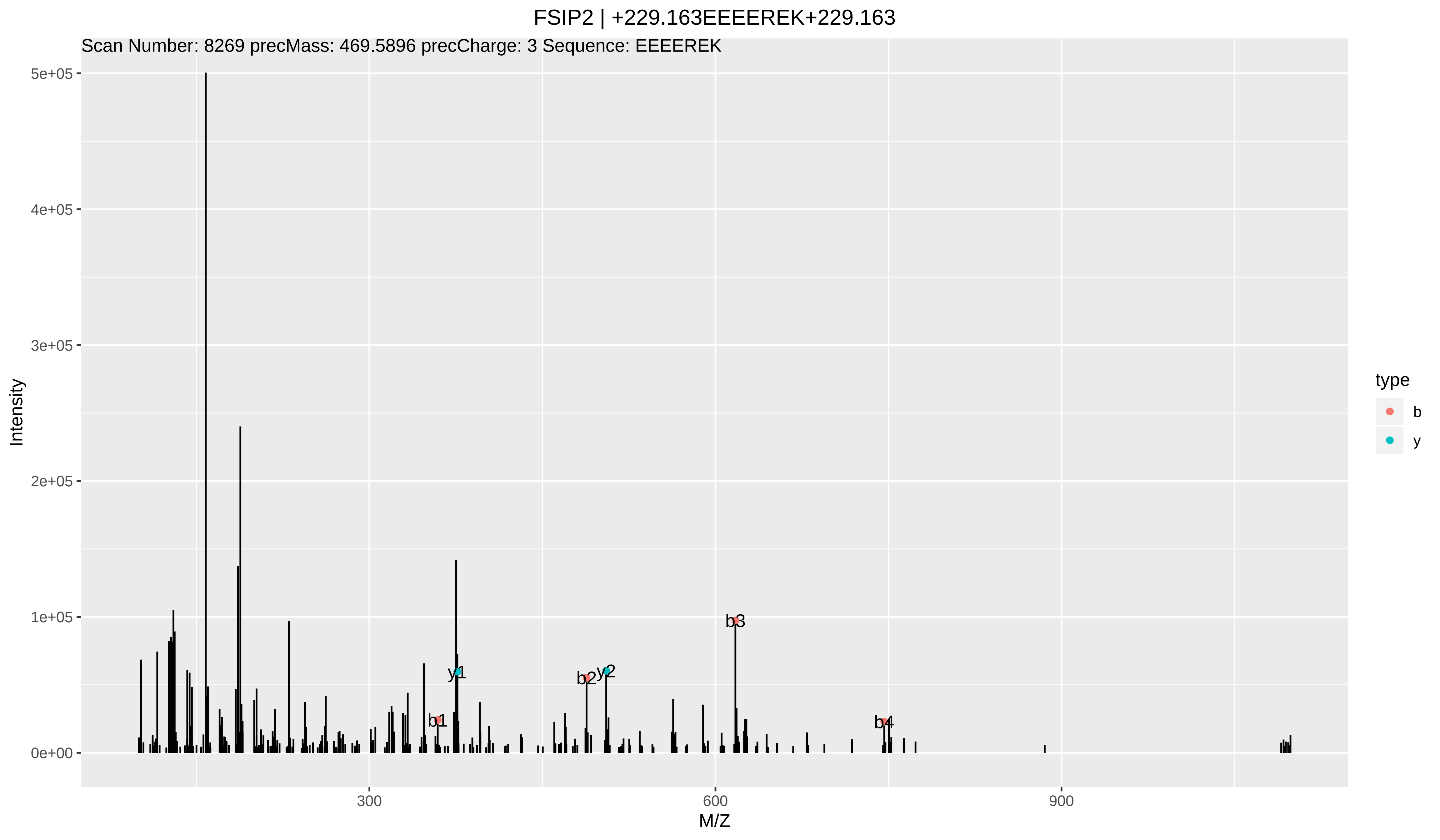

FSIP2 | +229.163EEEEREK+229.163
Scan Number: 8269 precMass: 469.5896 precCharge: 3 Sequence: EEEEREK
Intensity
5e+05
4e+05
3e+05
2e+05
1e+05
0e+00
300
600
900
M/Z
b1
y1
b2
y2
b3
b4
type
b
y

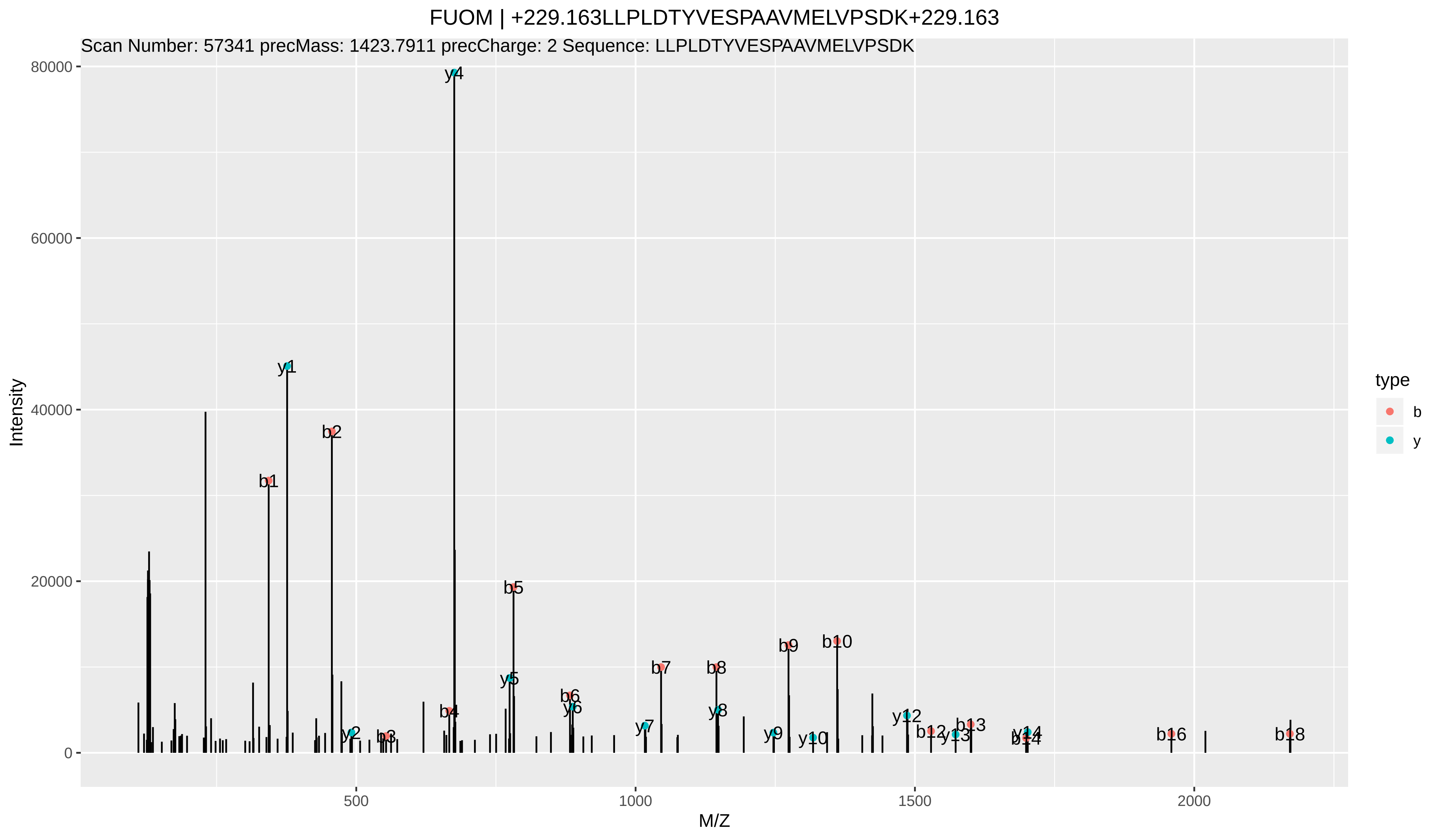
FUOM | +229.163LLPLDTYVESPAAVMELVPSDK+229.163
Scan Number: 57341 precMass: 1423.7911 precCharge: 2 Sequence: LLPLDTYVESPAAVMELVPSDK
Intensity
M/Z
type
b
y
y4
y1
b2
b1
b5
y5
b6
y6
b4
y2
b3
b7
y7
b8
y8
b9
y9
b10
y10
y12
b12
y13
b13
y14
b14
b16
b18
0
20000
40000
60000
80000
500
1000
1500
2000

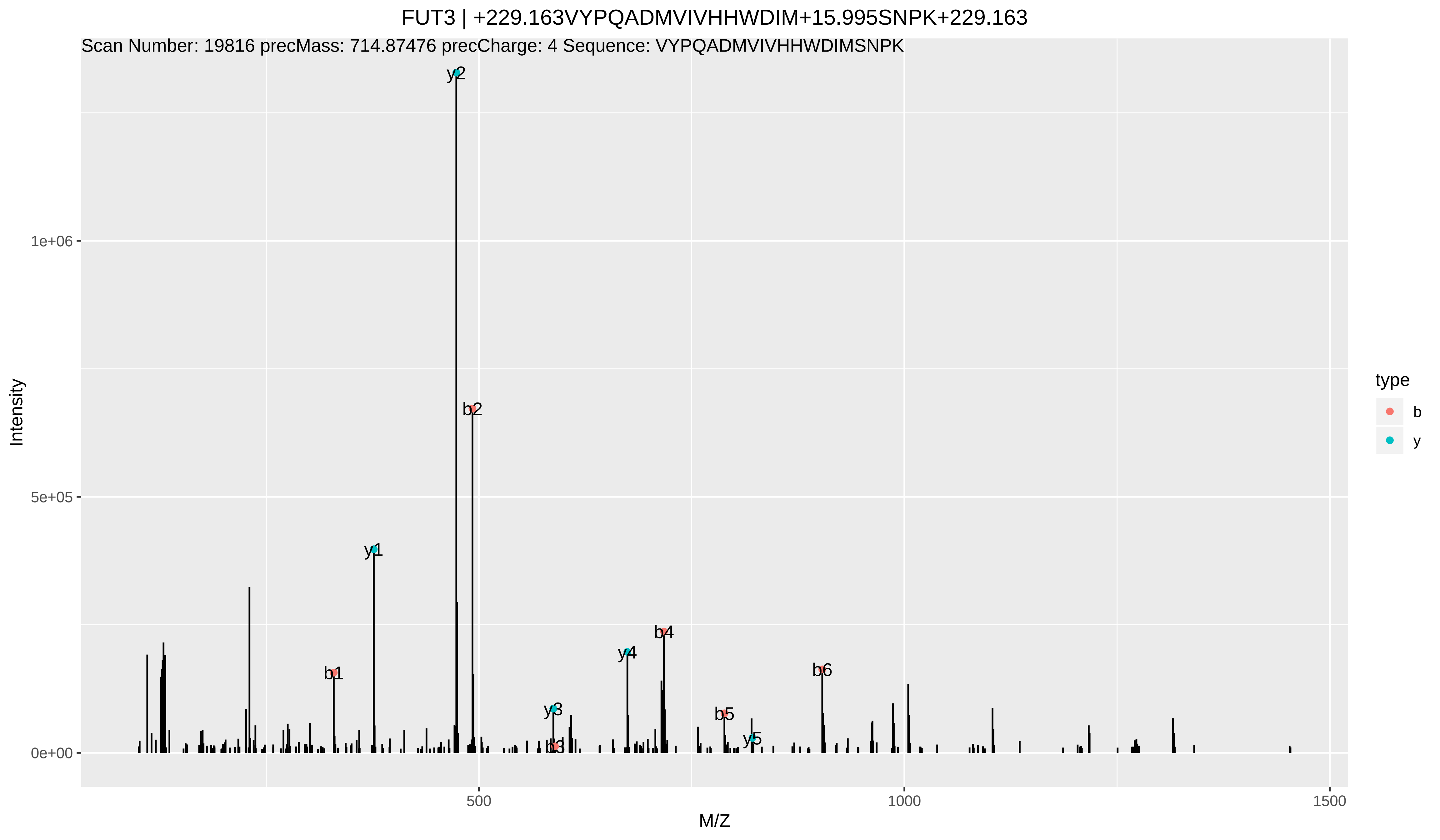

FUT3 | +229.163VYPQADMVIVHHWDIM+15.995SNPK+229.163
Scan Number: 19816 precMass: 714.87476 precCharge: 4 Sequence: VYPQADMVIVHHWDIMSNPK
y2
b2
y1
b1
y3
b3
y4
b4
b5
y5
b6
Intensity
1e+06
5e+05
0e+00
500
1000
1500
M/Z
type
b
y

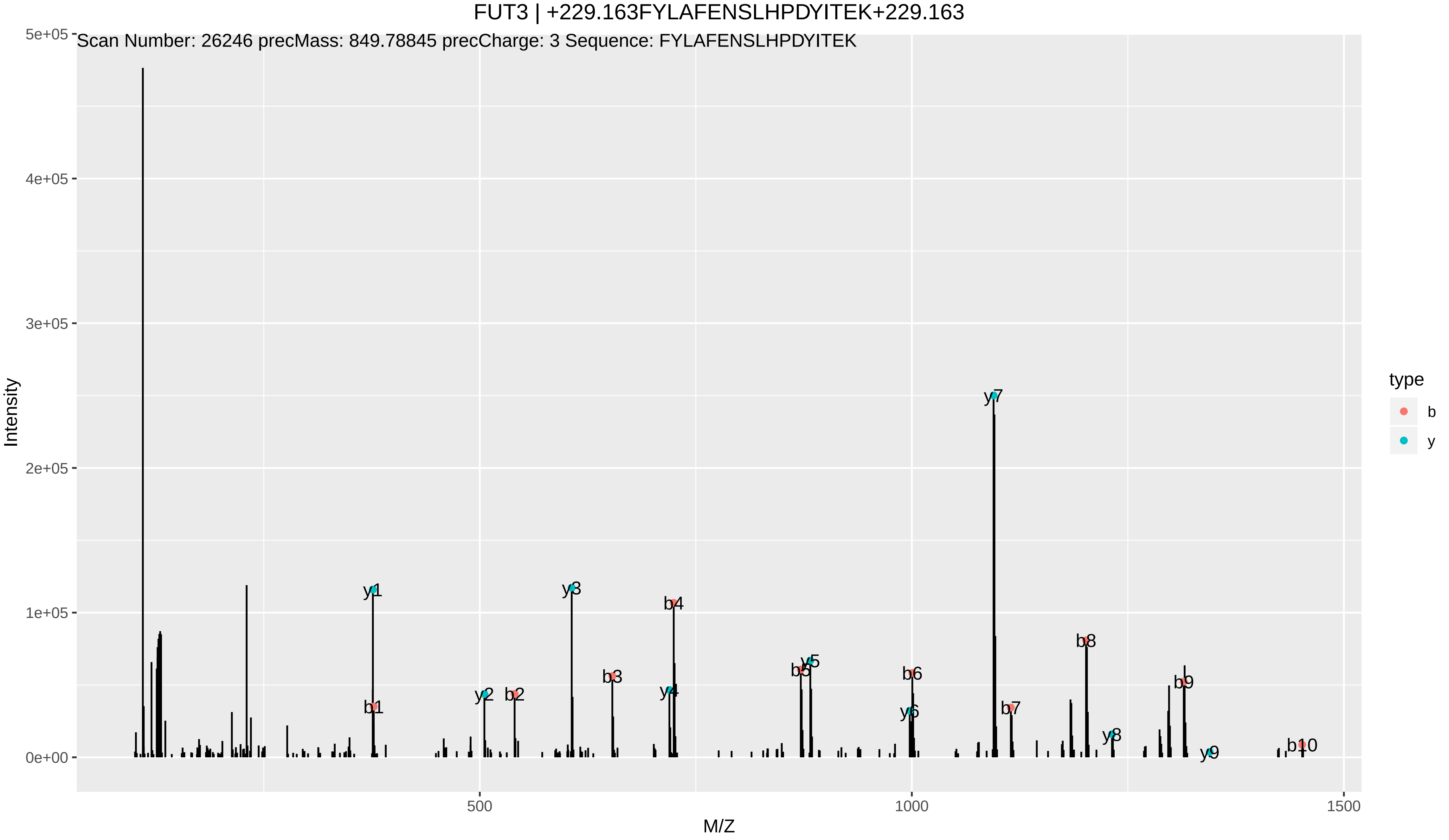
FUT3 | +229.163FYLAFENSLHPDYITEK+229.163
Scan Number: 26246 precMass: 849.78845 precCharge: 3 Sequence: FYLAFENSLHPDYITEK
Intensity
M/Z
0e+00
1e+05
2e+05
3e+05
4e+05
5e+05
500
1000
1500
y1
b1
y2
b2
y3
b3
y4
b4
b5
y5
y6
b6
y7
b7
b8
y8
b9
y9
b10
type
b
y

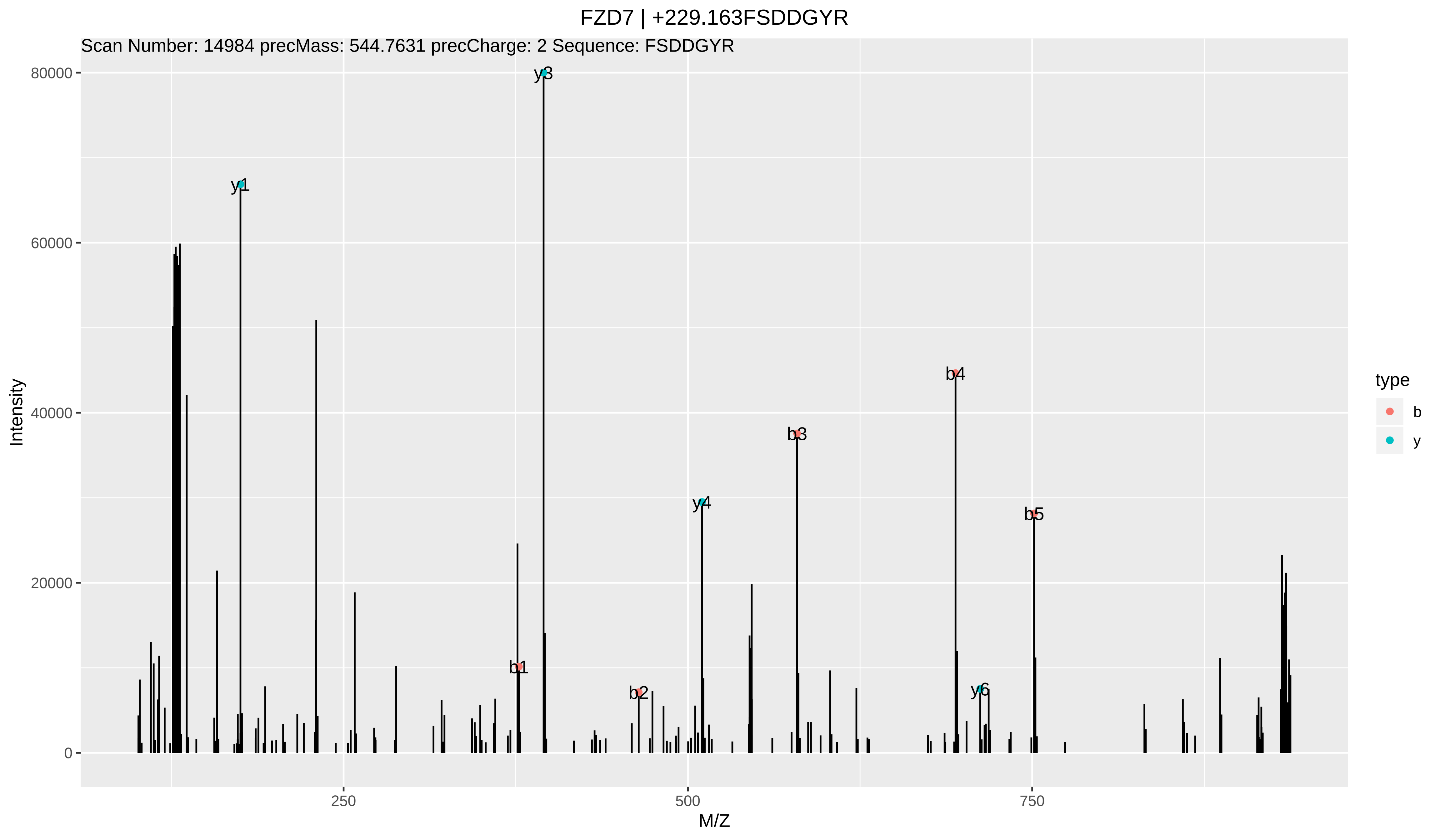

FZD7 | +229.163FSDDGYR
Scan Number: 14984 precMass: 544.7631 precCharge: 2 Sequence: FSDDGYR
Intensity
M/Z
80000
60000
40000
20000
0
250
500
750
y1
y3
y4
y6
b1
b2
b3
b4
b5
type
b
y

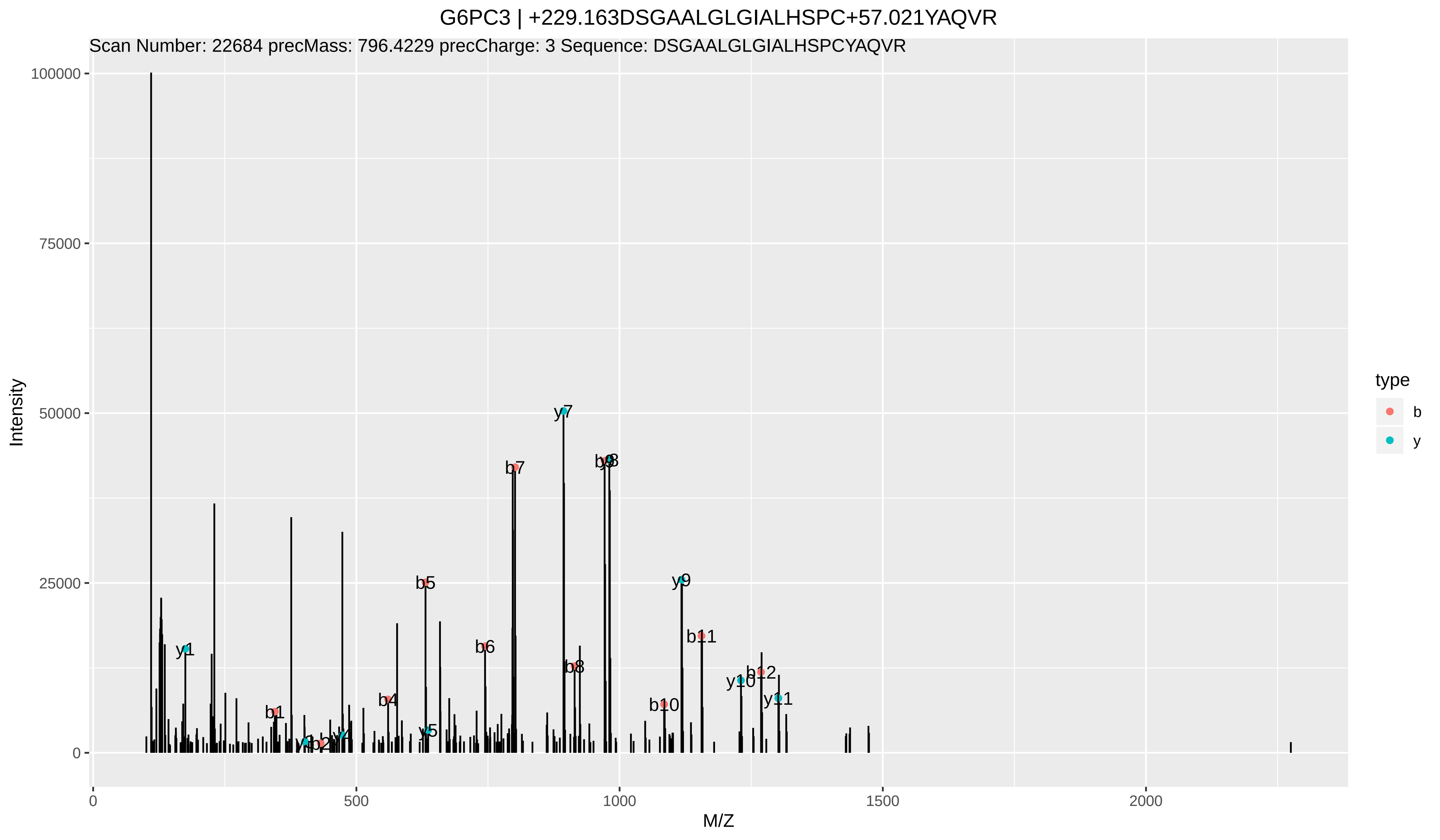
G6PC3 | +229.163DSGAALGLGIALHSPC+57.021YAQVR
Scan Number: 22684 precMass: 796.4229 precCharge: 3 Sequence: DSGAALGLGIALHSPCYAQVR
Intensity
M/Z
type
b
y
100000
75000
50000
25000
0
0
500
1000
1500
2000
y1
b1
b4
b5
y5
b6
b7
y7
b8
b10
y9
b11
y10
b12
y11

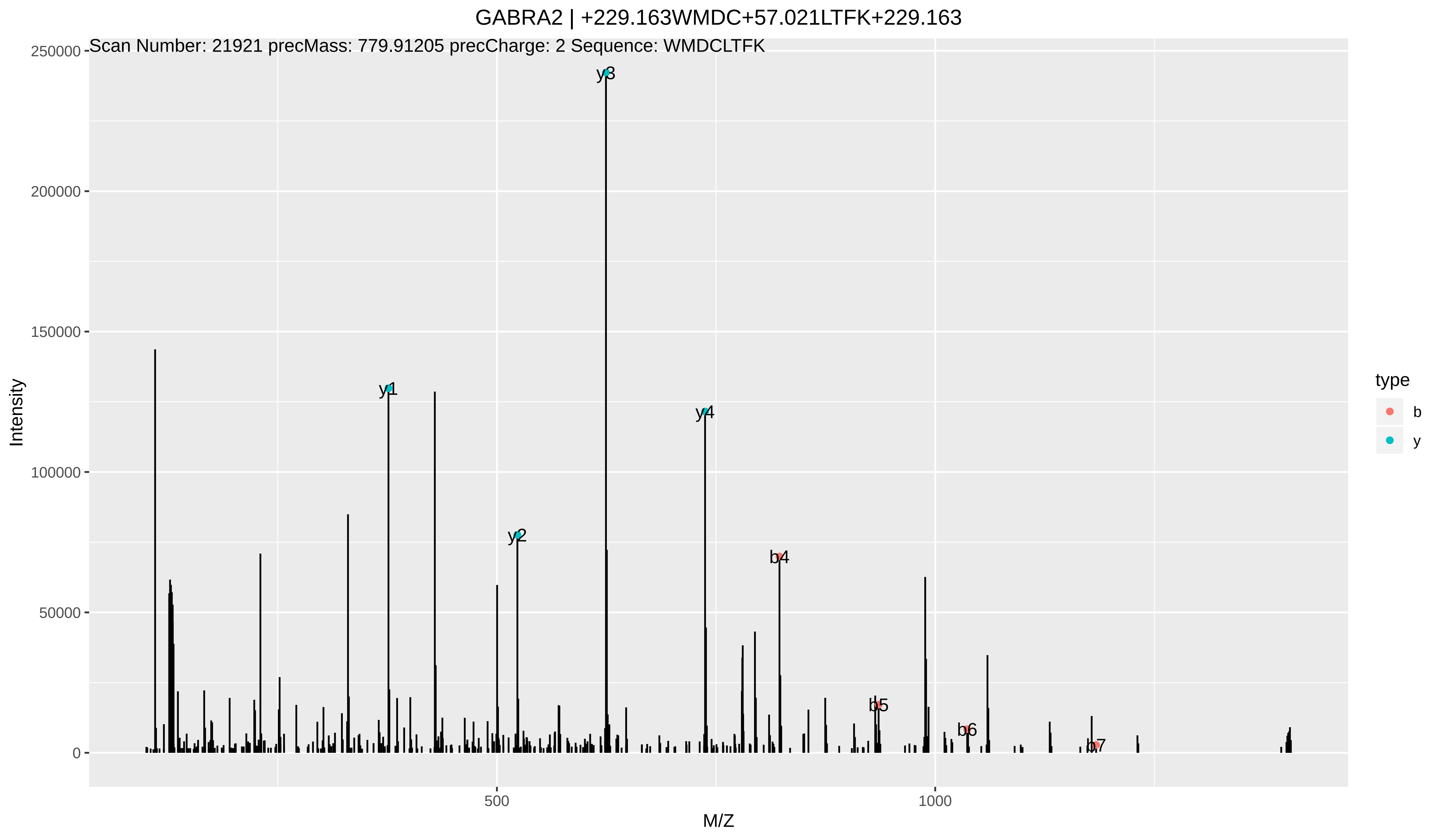

GABRA2 | +229.163WMDC+57.021LTFK+229.163
Scan Number: 21921 precMass: 779.91205 precCharge: 2 Sequence: WMDCLTFK
Intensity
M/Z
0
50000
100000
150000
200000
250000
500
1000
y1
y2
y3
y4
b4
b5
b6
b7
type
b
y

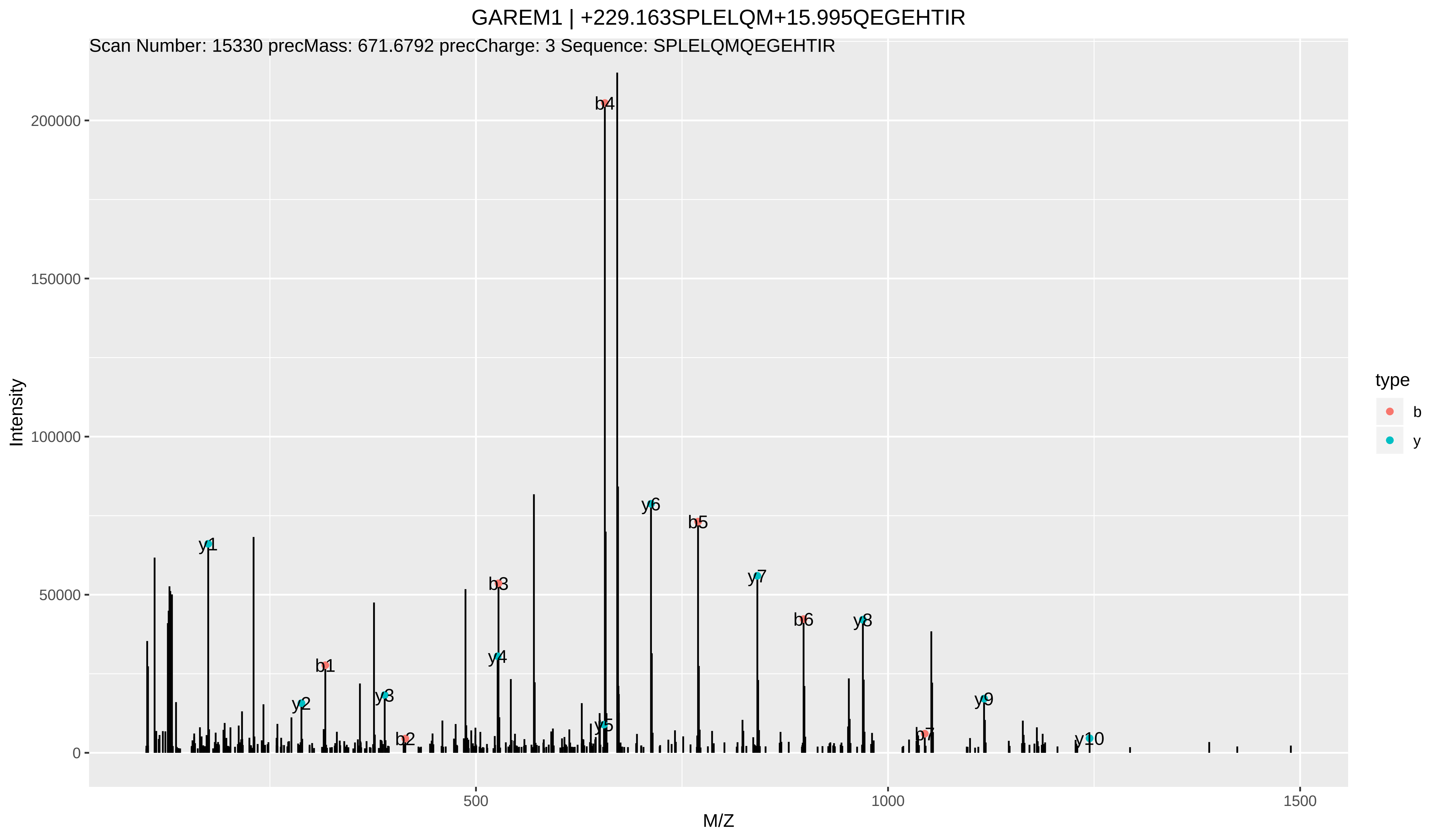

GAREM1 | +229.163SPLELQM+15.995QEGEHTIR
Scan Number: 15330 precMass: 671.6792 precCharge: 3 Sequence: SPLELQMQEGEHTIR
Intensity
M/Z
200000
150000
100000
50000
0
500
1000
1500
b4
y6
b5
y1
y7
b3
b6
y8
y4
b1
y2
y3
b2
y5
y9
b7
y10
type
b
y

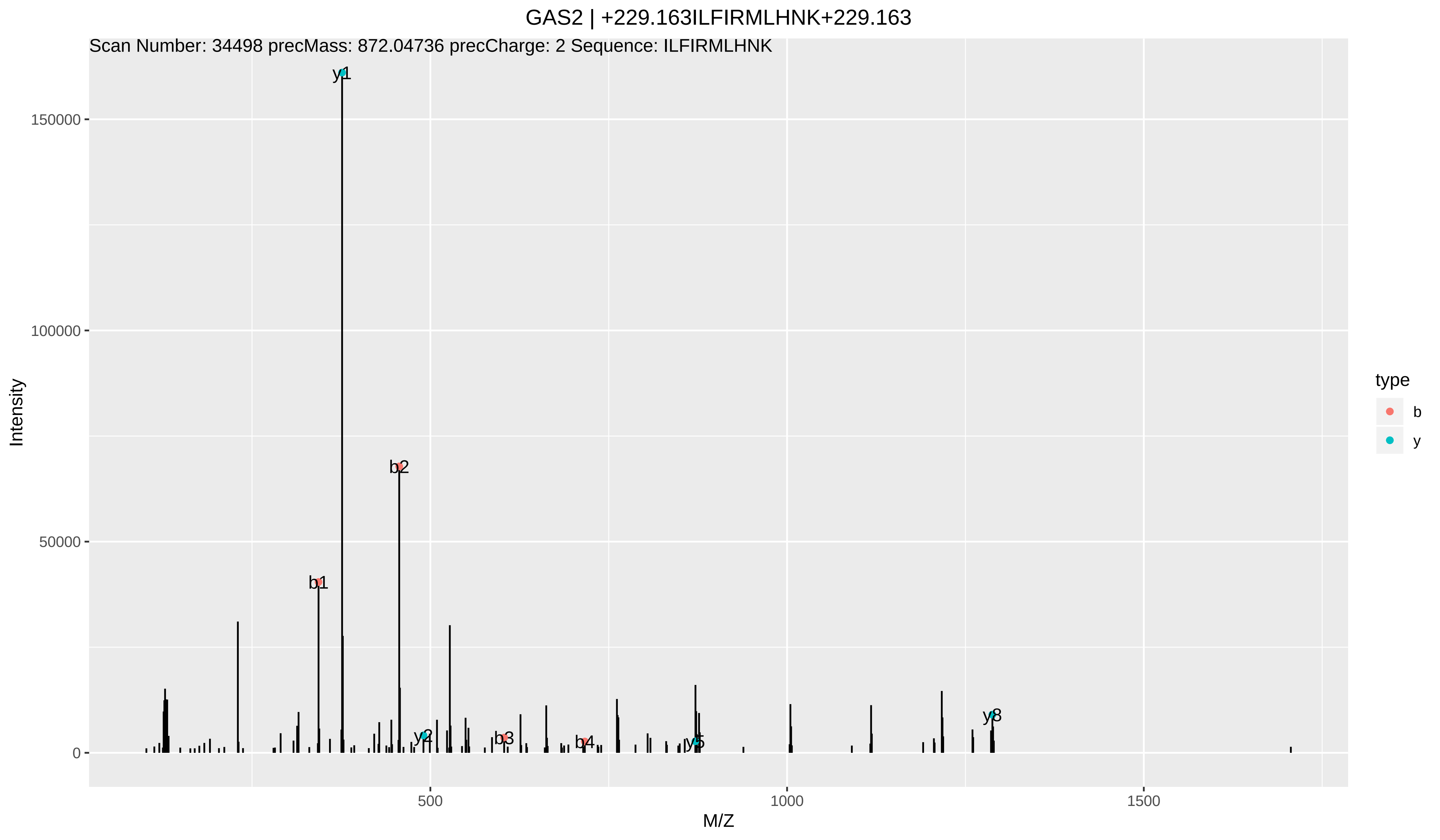

GAS2 | +229.163ILFIRMLHNK+229.163
Scan Number: 34498 precMass: 872.04736 precCharge: 2 Sequence: ILFIRMLHNK
y1
b2
b1
y2
b3
b4
y5
y8
150000
100000
50000
0
500
1000
1500
Intensity
M/Z
type
b
y

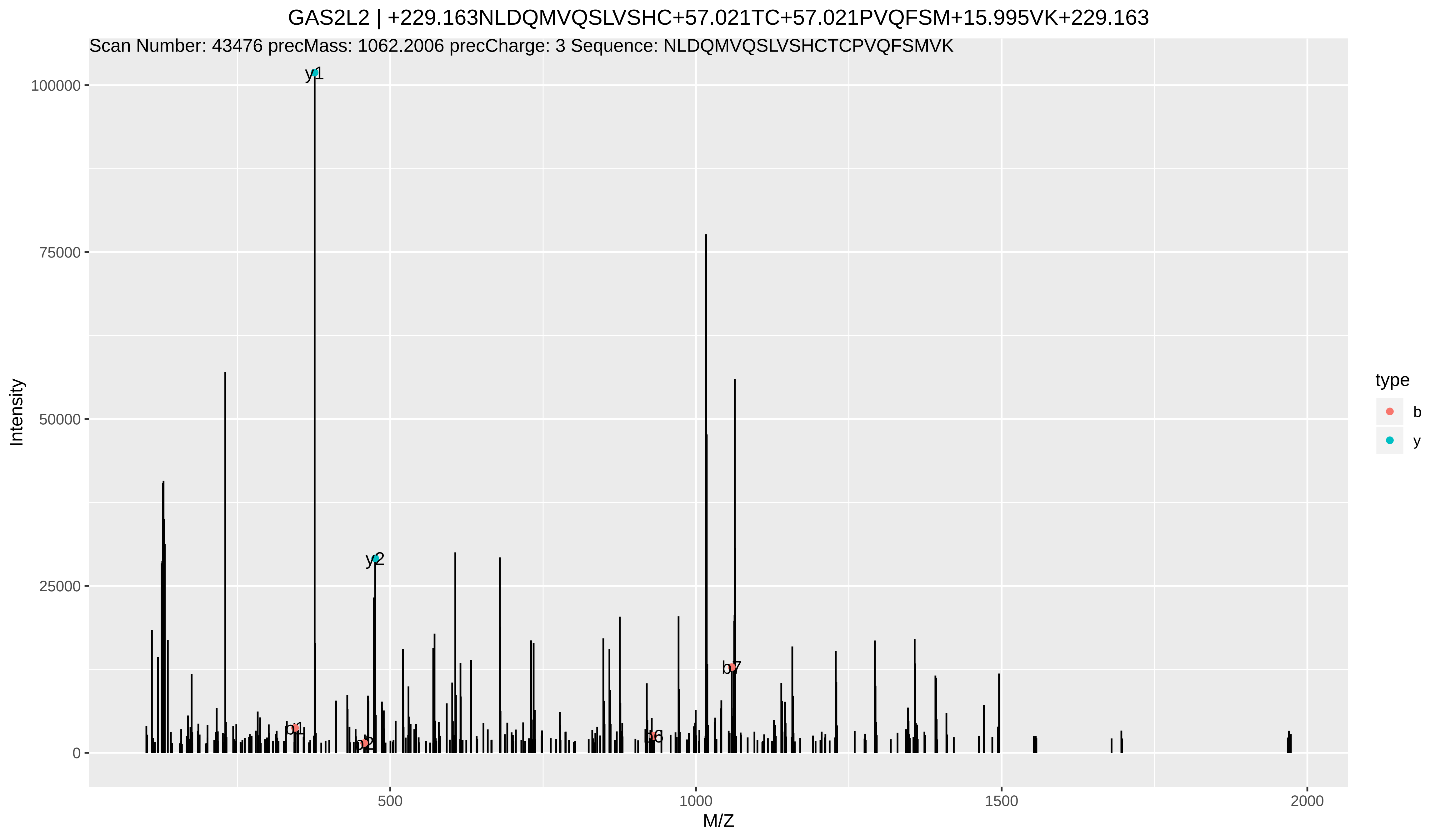

GAS2L2 | +229.163NLDQMVQSLVSHC+57.021TC+57.021PVQFSM+15.995VK+229.163
Scan Number: 43476 precMass: 1062.2006 precCharge: 3 Sequence: NLDQMVQSLVSHCTCPVQFSMVK
Intensity
100000
75000
50000
25000
0
M/Z
500
1000
1500
2000
y1
y2
b1
b2
b6
b7
type
b
y

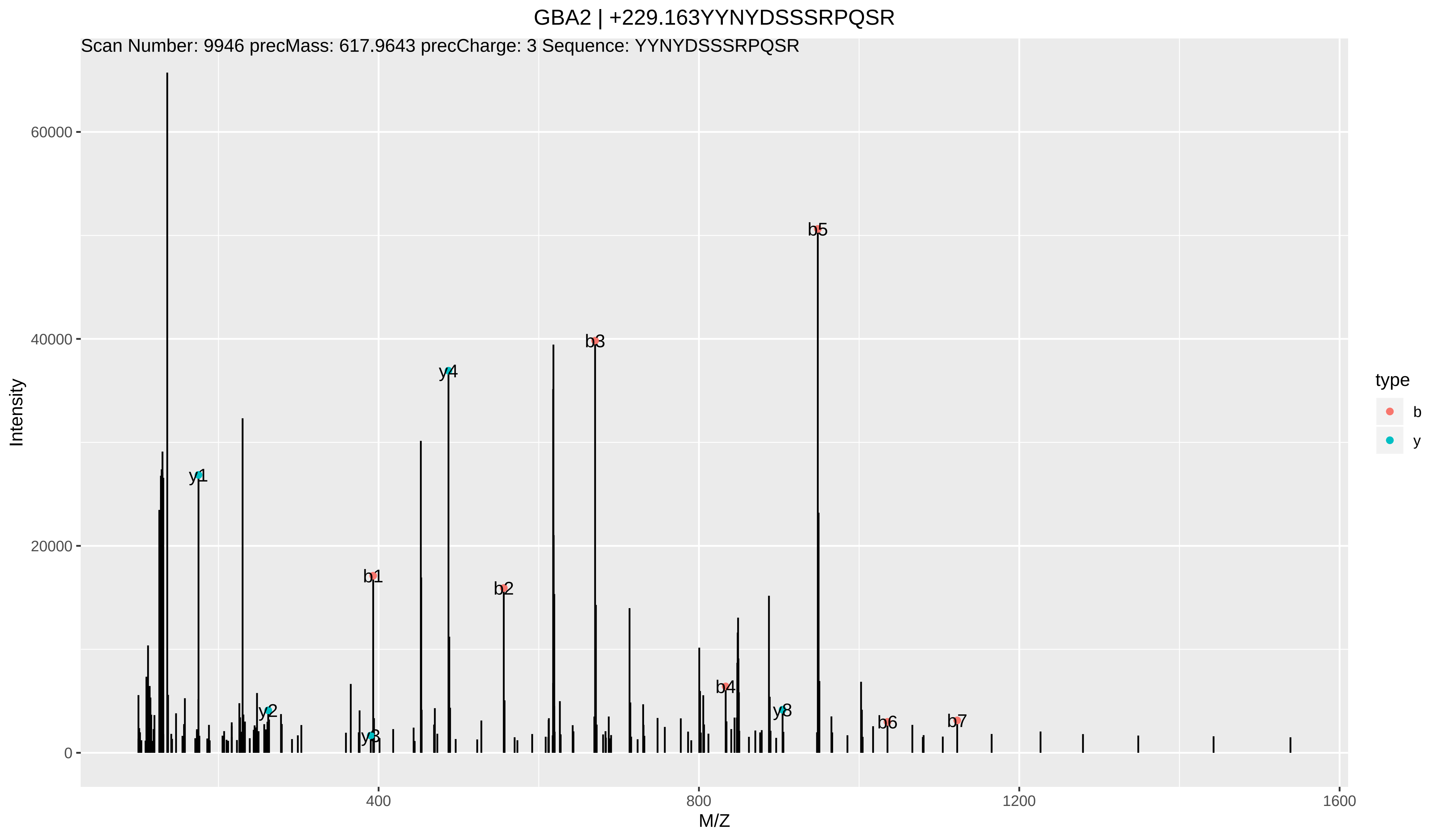
GBA2 | +229.163YYNYDSSSRPQSR
Scan Number: 9946 precMass: 617.9643 precCharge: 3 Sequence: YYNYDSSSRPQSR
Intensity
60000
40000
20000
0
M/Z
400
800
1200
1600
y1
y2
y3
y4
y8
b1
b2
b3
b4
b5
b6
b7
type
b
y

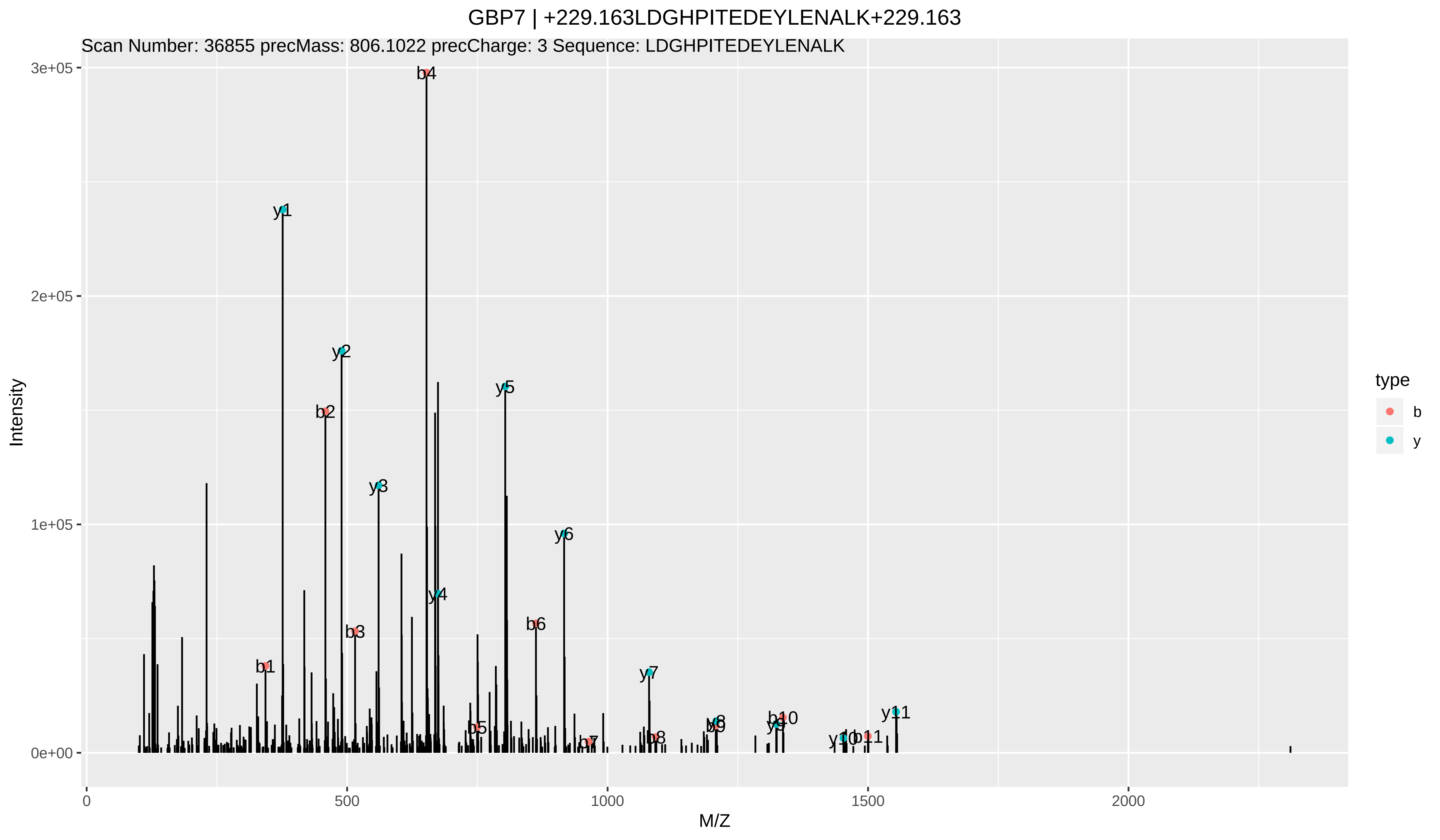

GBP7 | +229.163LDGHPITEDEYLENALK+229.163
Scan Number: 36855 precMass: 806.1022 precCharge: 3 Sequence: LDGHPITEDEYLENALK
Intensity
M/Z
3e+05
2e+05
1e+05
0e+00
0
500
1000
1500
2000
b4
y1
y2
b2
y5
y3
y6
y4
b6
b3
b1
y7
b5
b7
b8
y8
b9
b10
y9
y10
b11
y11
type
b
y

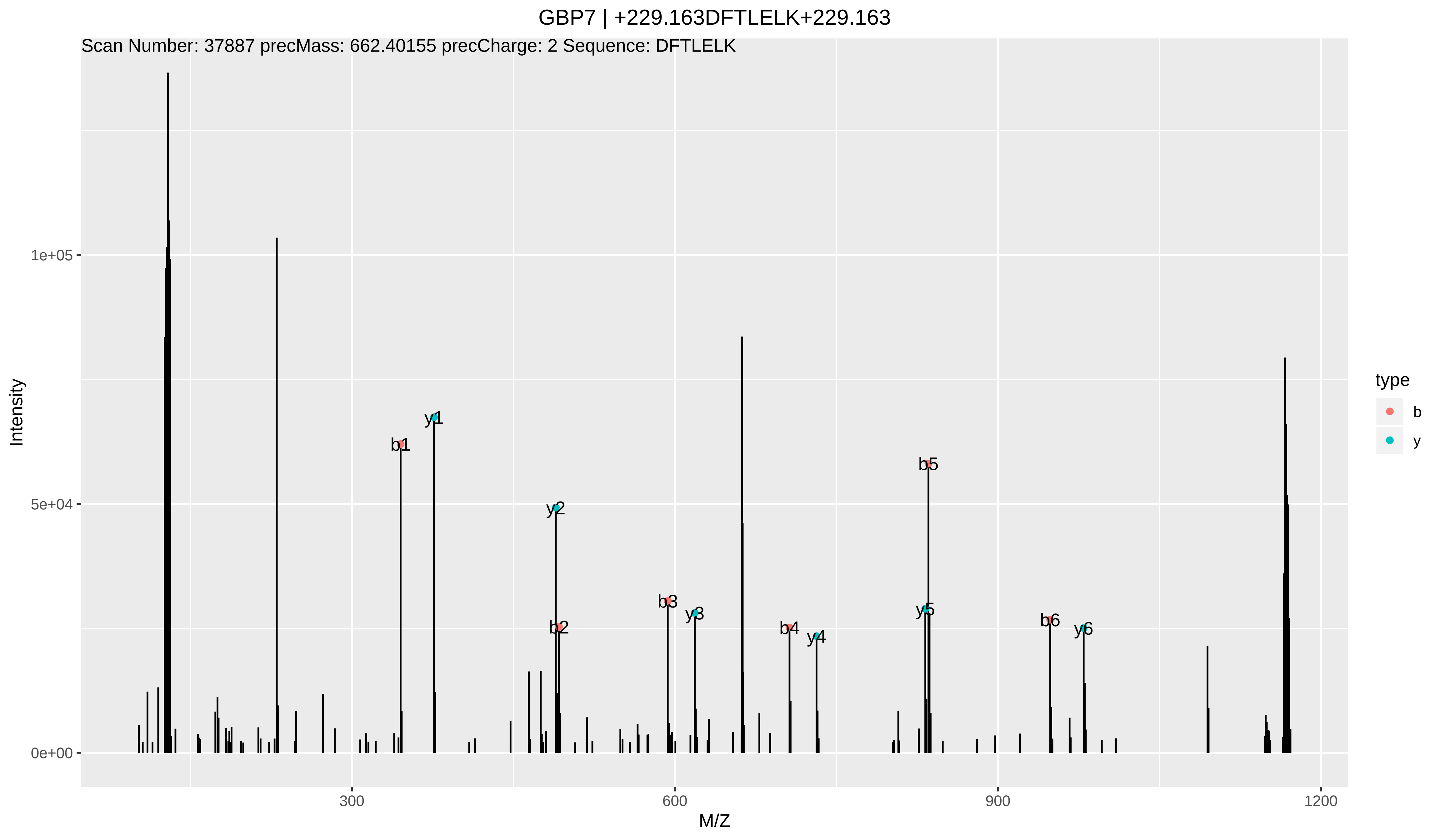

GBP7 | +229.163DFTLELK+229.163
Scan Number: 37887 precMass: 662.40155 precCharge: 2 Sequence: DFTLELK
Intensity
1e+05
5e+04
0e+00
M/Z
300
600
900
1200
y1
b1
y2
b2
b3
y3
b4
y4
b5
y5
b6
y6
type
b
y

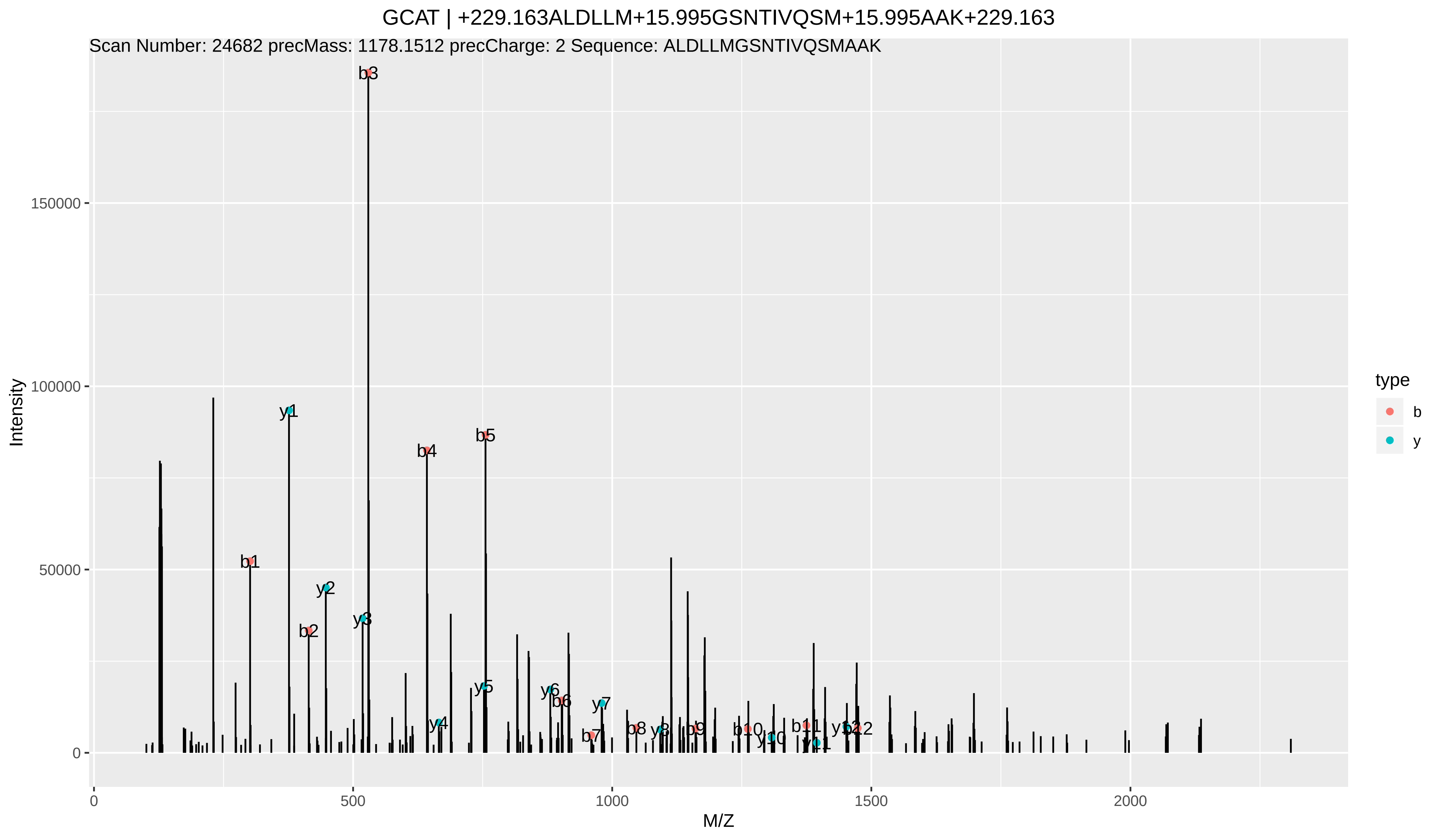

GCAT | +229.163ALDLLM+15.995GSNTIVQSM+15.995AAK+229.163
Scan Number: 24682 precMass: 1178.1512 precCharge: 2 Sequence: ALDLLMGSNTIVQSMAAK
Intensity
M/Z
0
50000
100000
150000
500
1000
1500
2000
type
b
y
b1
b2
b3
b4
b5
b6
b7
b8
b9
b10
b11
b12
y1
y2
y3
y4
y5
y6
y7
y8
y10
y11
y12

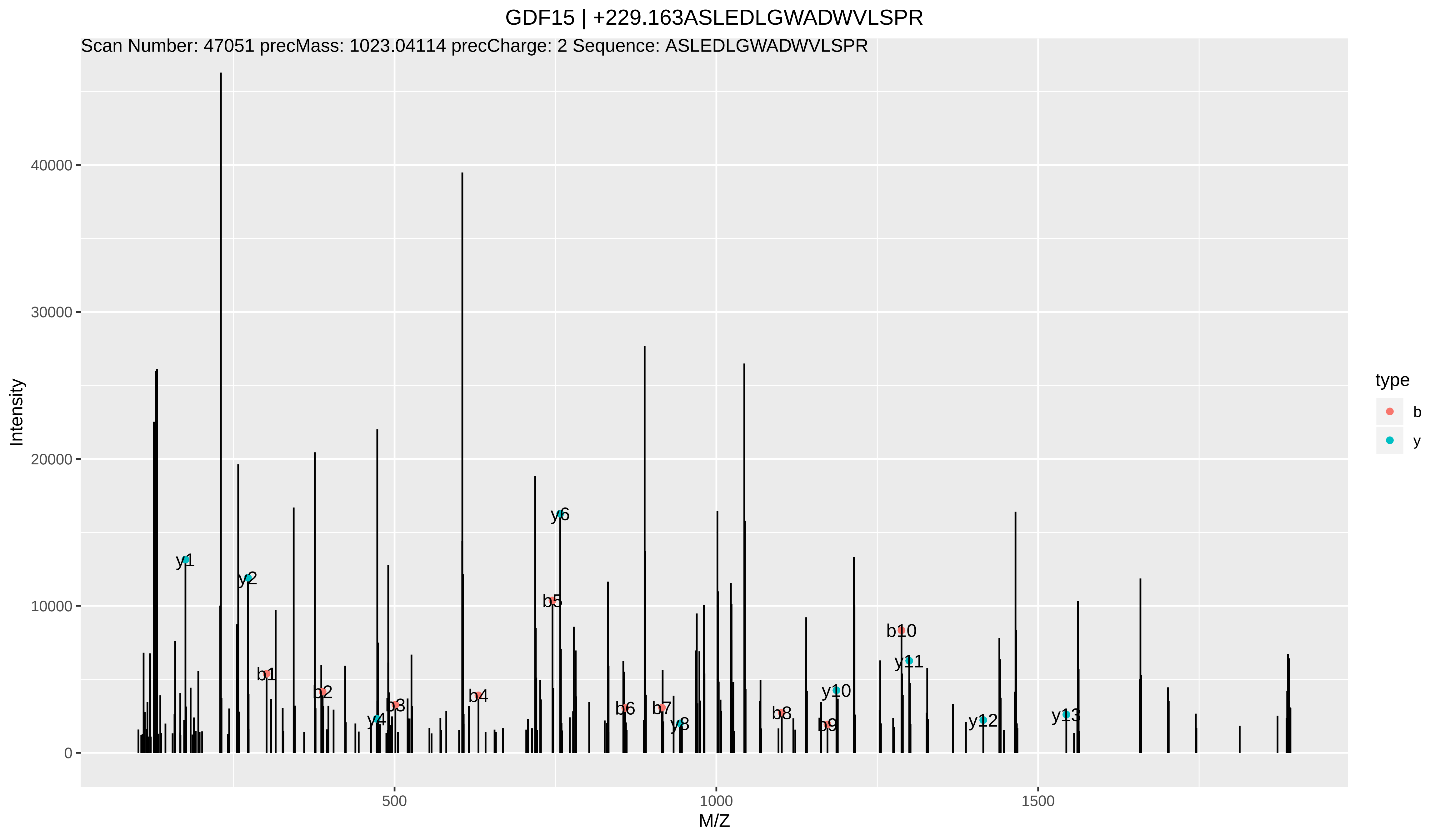
GDF15 | +229.163ASLEDLGWADWVLSPR
Scan Number: 47051 precMass: 1023.04114 precCharge: 2 Sequence: ASLEDLGWADWVLSPR
Intensity
M/Z
0
10000
20000
30000
40000
500
1000
1500
y1
y2
b1
b2
y4
b3
b4
b5
y6
b6
b7
y8
b8
b9
y10
b10
y11
y12
y13
type
b
y

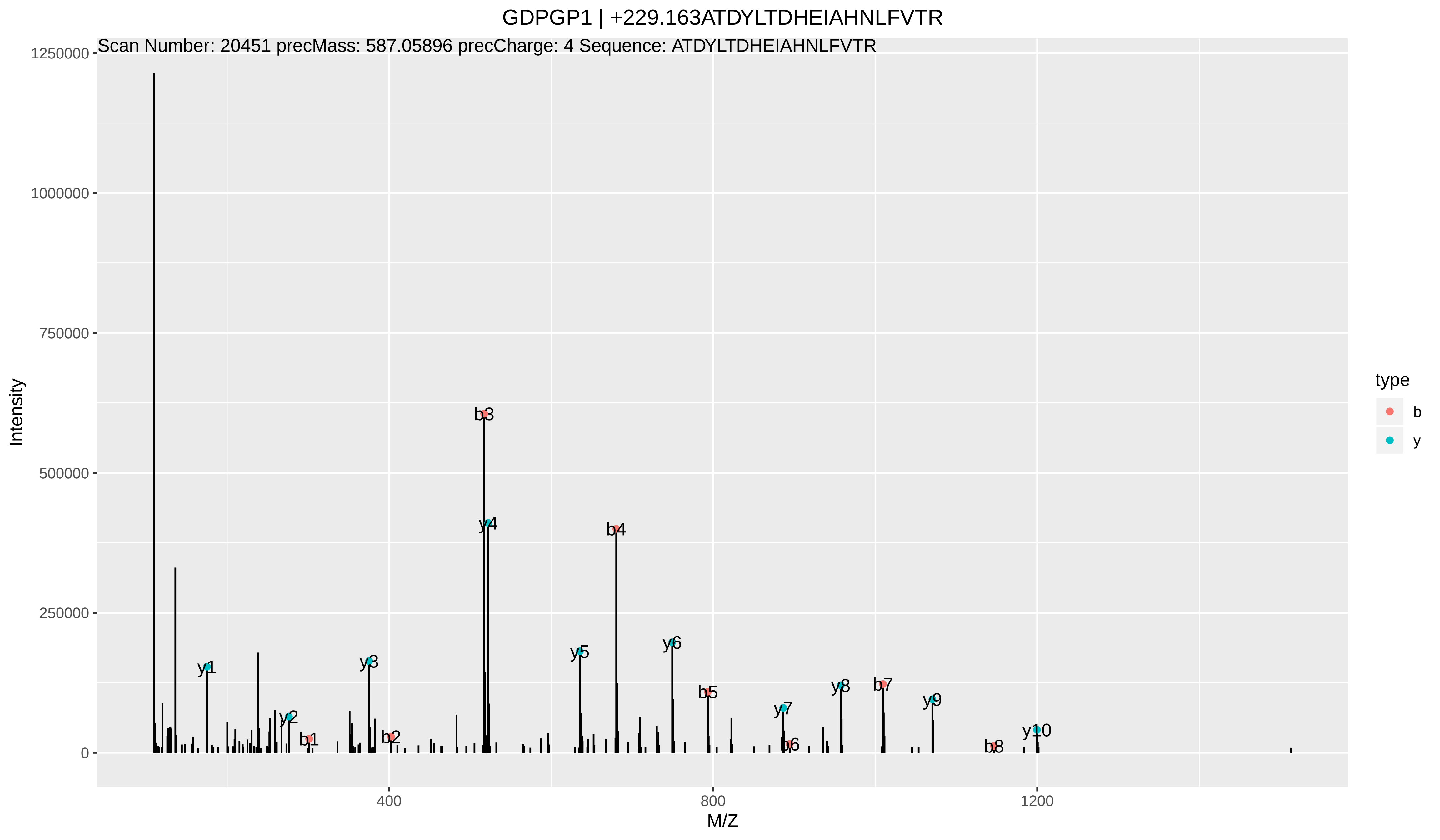

GDPGP1 | +229.163ATDYLTDHEIAHNLFVTR
Scan Number: 20451 precMass: 587.05896 precCharge: 4 Sequence: ATDYLTDHEIAHNLFVTR
Intensity
1250000
1000000
750000
500000
250000
0
M/Z
400
800
1200
b3
y4
b4
y1
y2
b1
y3
b2
y5
y6
b5
y7
b6
y8
b7
y9
b8
y10
type
b
y

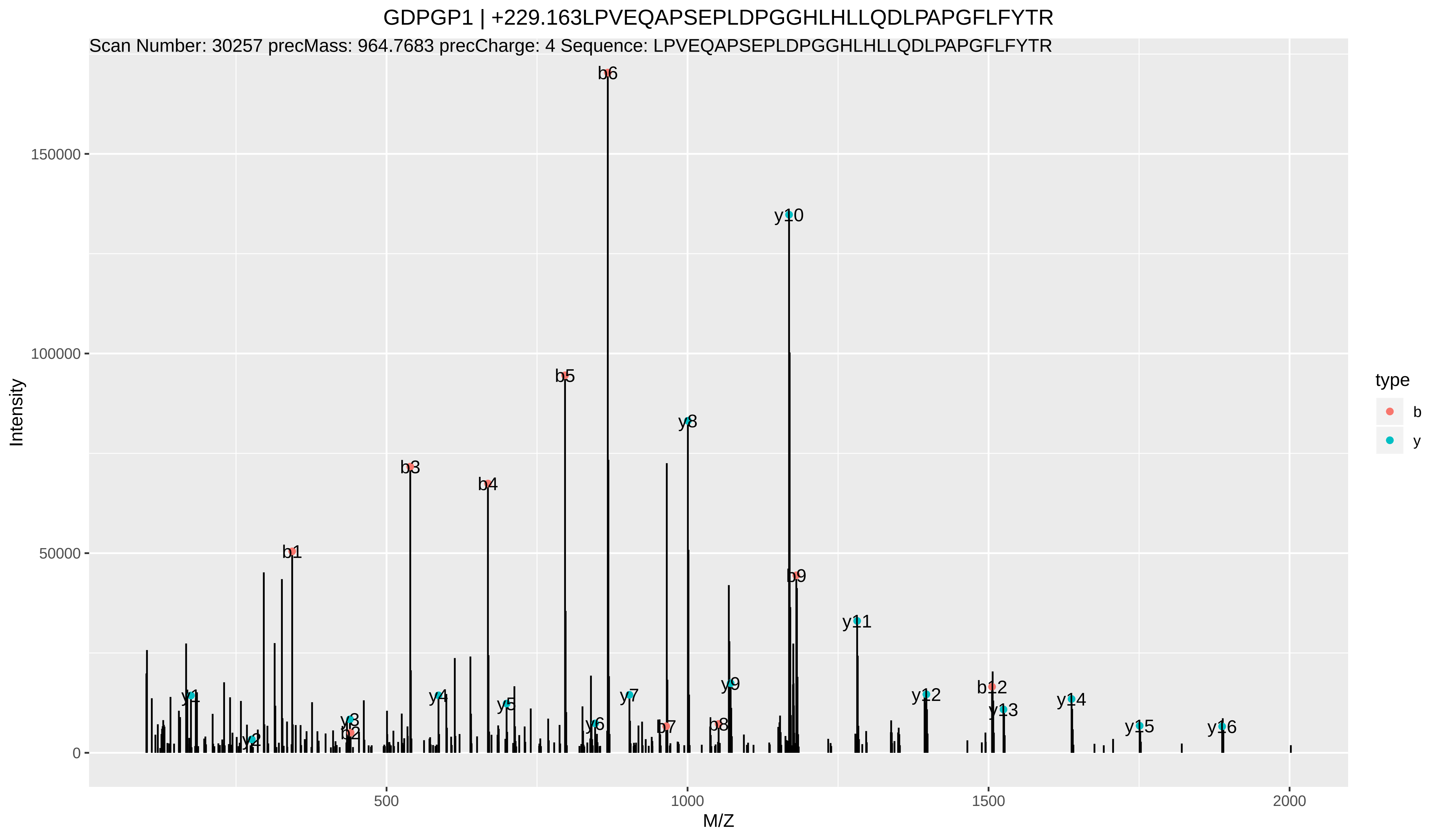
GDPGP1 | +229.163LPVEQAPSEPLDPGGHLHLLQDLPAPGFLFYTR
Scan Number: 30257 precMass: 964.7683 precCharge: 4 Sequence: LPVEQAPSEPLDPGGHLHLLQDLPAPGFLFYTR
Intensity
M/Z
type
b
y
b1
b2
b3
b4
b5
b6
b7
b8
b9
b12
y1
y2
y3
y4
y5
y6
y7
y8
y9
y10
y11
y12
y13
y14
y15
y16
0
50000
100000
150000
500
1000
1500
2000

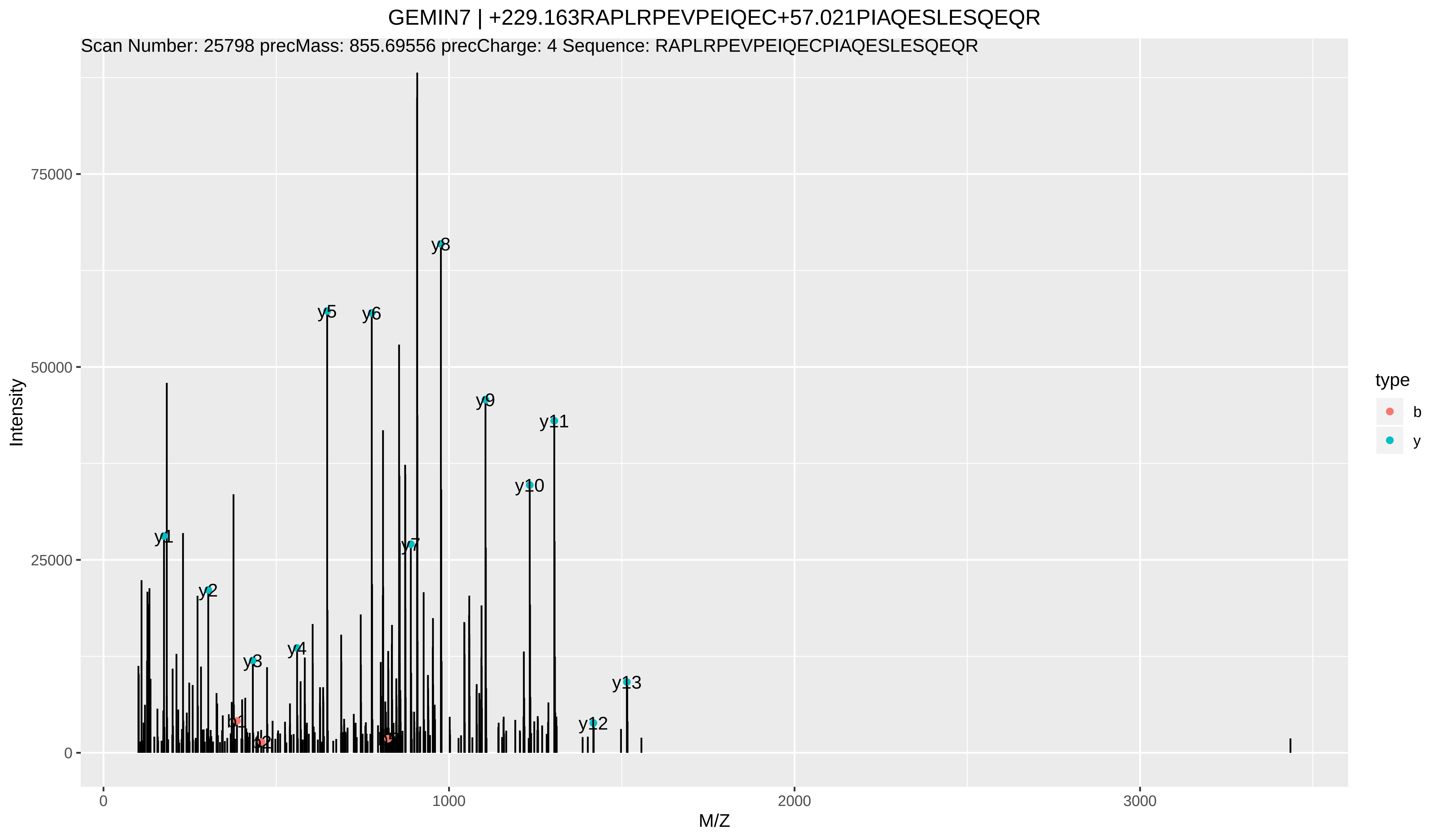

GEMIN7 | +229.163RAPLRPEVPEIQEC+57.021PIAQESLESQEQR
Scan Number: 25798 precMass: 855.69556 precCharge: 4 Sequence: RAPLRPEVPEIQECPIAQESLESQEQR
Intensity
75000
50000
25000
0
0
1000
2000
3000
M/Z
y1
y2
y3
y4
y5
y6
y7
y8
y9
y10
y11
y12
y13
type
b
y

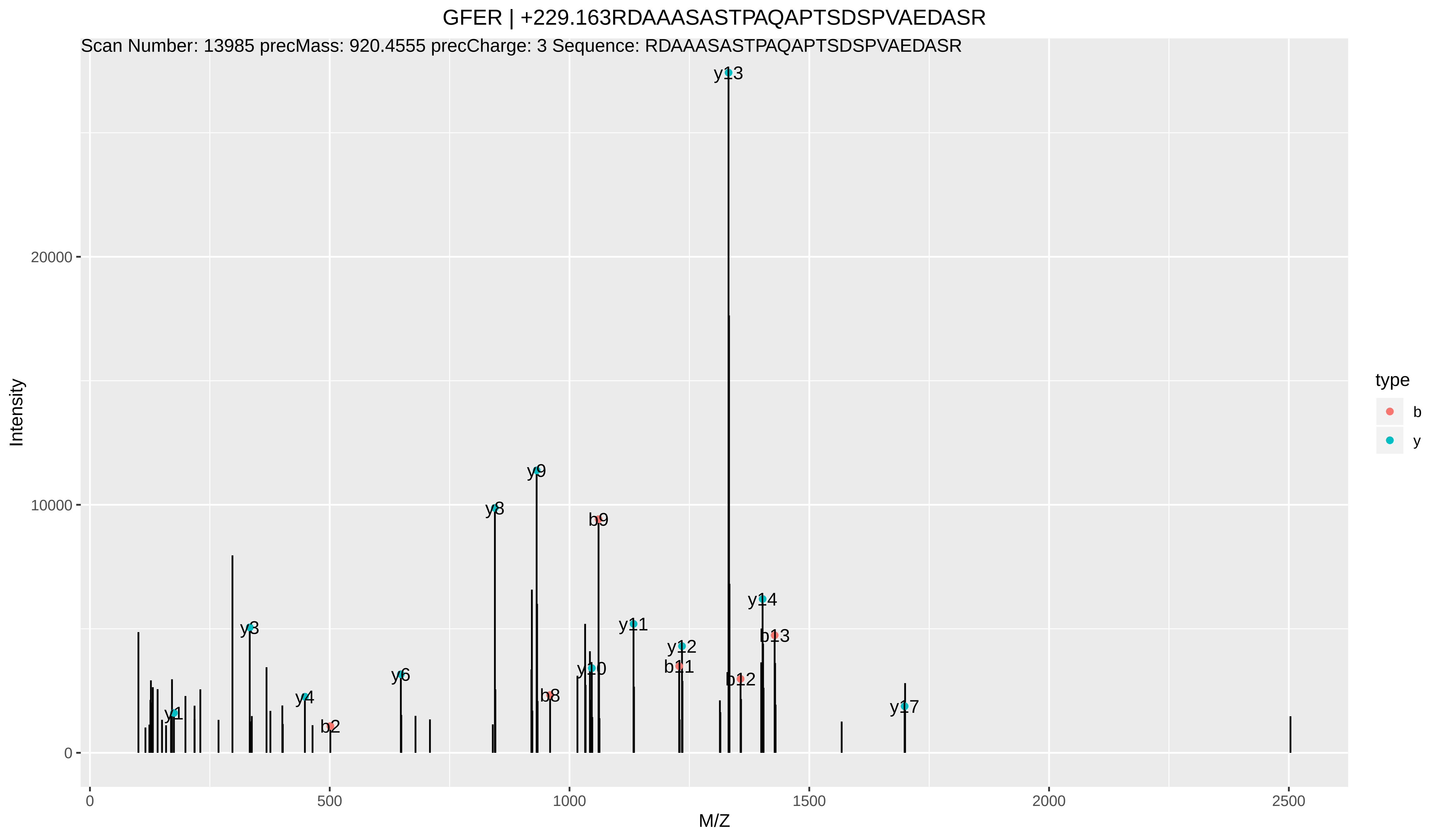
GFER | +229.163RDAAASASTPAQAPTSDSPVAEDASR
Scan Number: 13985 precMass: 920.4555 precCharge: 3 Sequence: RDAAASASTPAQAPTSDSPVAEDASR
y13
y9
y8
b9
y14
y11
b13
y3
y12
b11
y10
y6
b12
b8
y4
y17
y1
b2
Intensity
20000
10000
0
0
500
1000
1500
2000
2500
M/Z
type
b
y

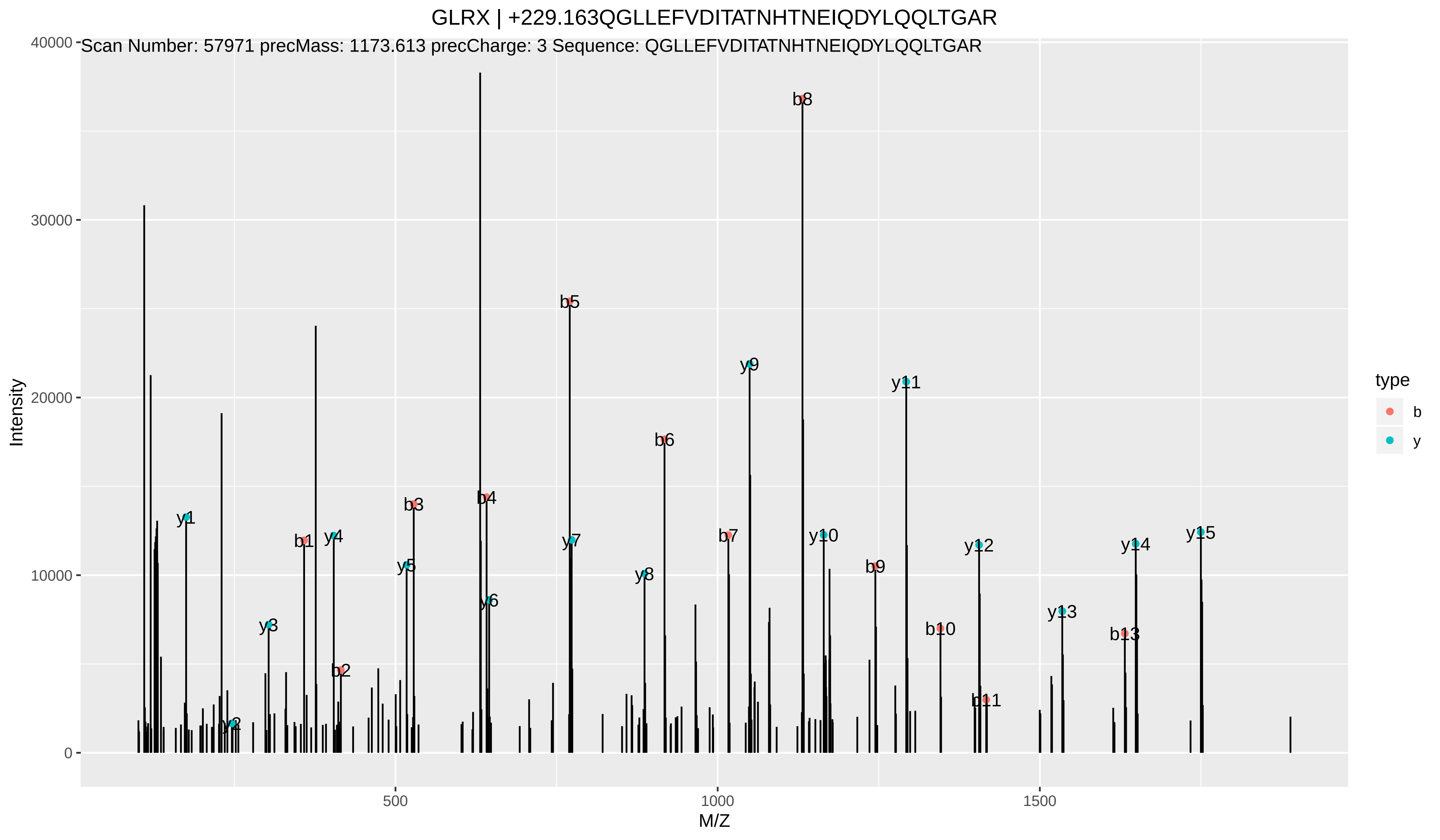
GLRX | +229.163QGLLEFVDITATNHTNEIQDYLQQLTGAR
Scan Number: 57971 precMass: 1173.613 precCharge: 3 Sequence: QGLLEFVDITATNHTNEIQDYLQQLTGAR
Intensity
M/Z
0
10000
20000
30000
40000
500
1000
1500
b8
b5
y9
y11
b6
y1
b1
y4
b3
b4
y7
b7
y10
y15
y12
y14
b9
y5
y8
y6
y3
b10
y13
b13
b2
b11
y2
type
b
y

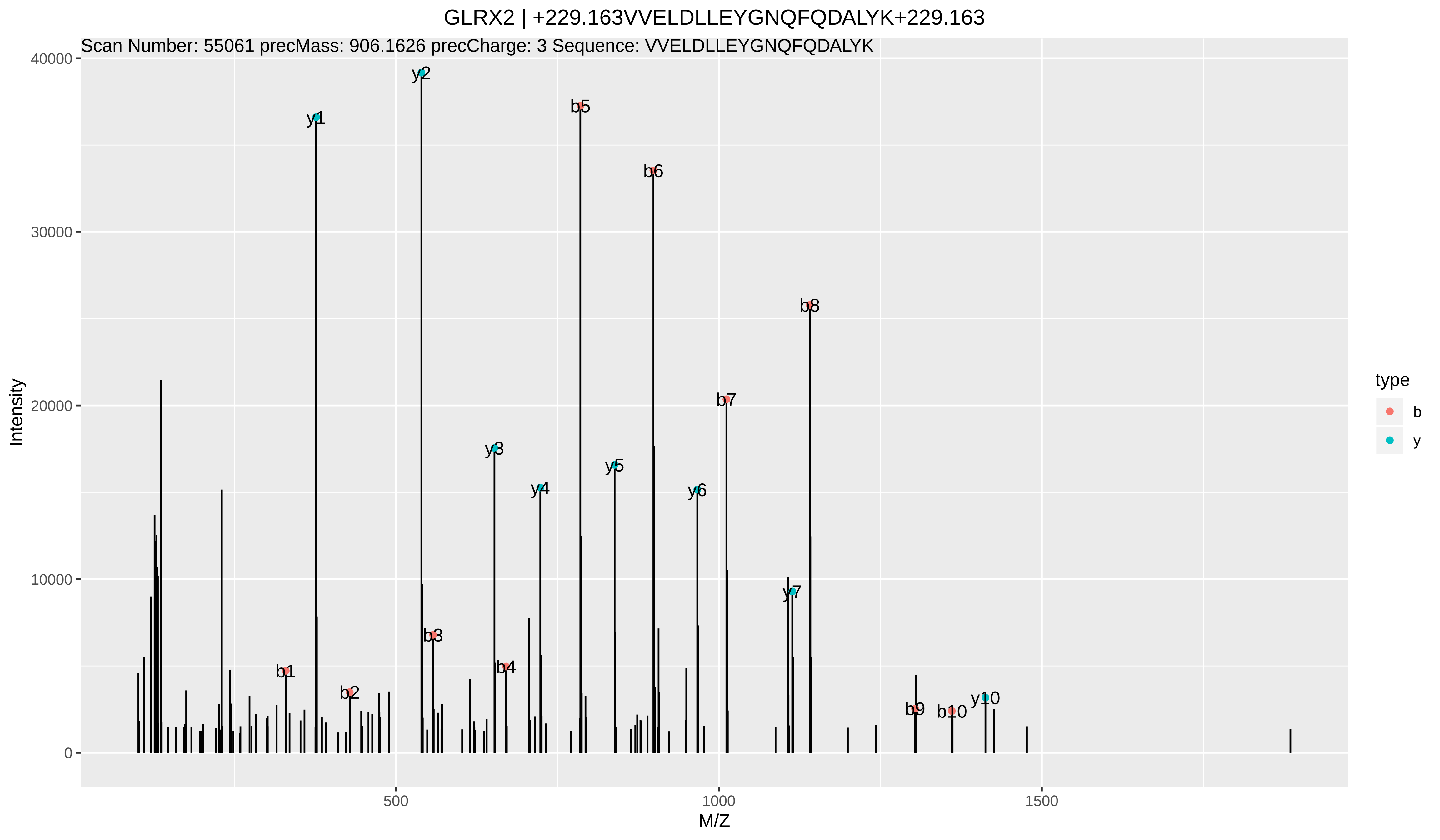

GLRX2 | +229.163VVELDLLEYGNQFQDALYK+229.163
Scan Number: 55061 precMass: 906.1626 precCharge: 3 Sequence: VVELDLLEYGNQFQDALYK
Intensity
M/Z
type
b
y
y1
y2
y3
y4
y5
y6
y7
y10
b1
b2
b3
b4
b5
b6
b7
b8
b9
b10
0
10000
20000
30000
40000
500
1000
1500

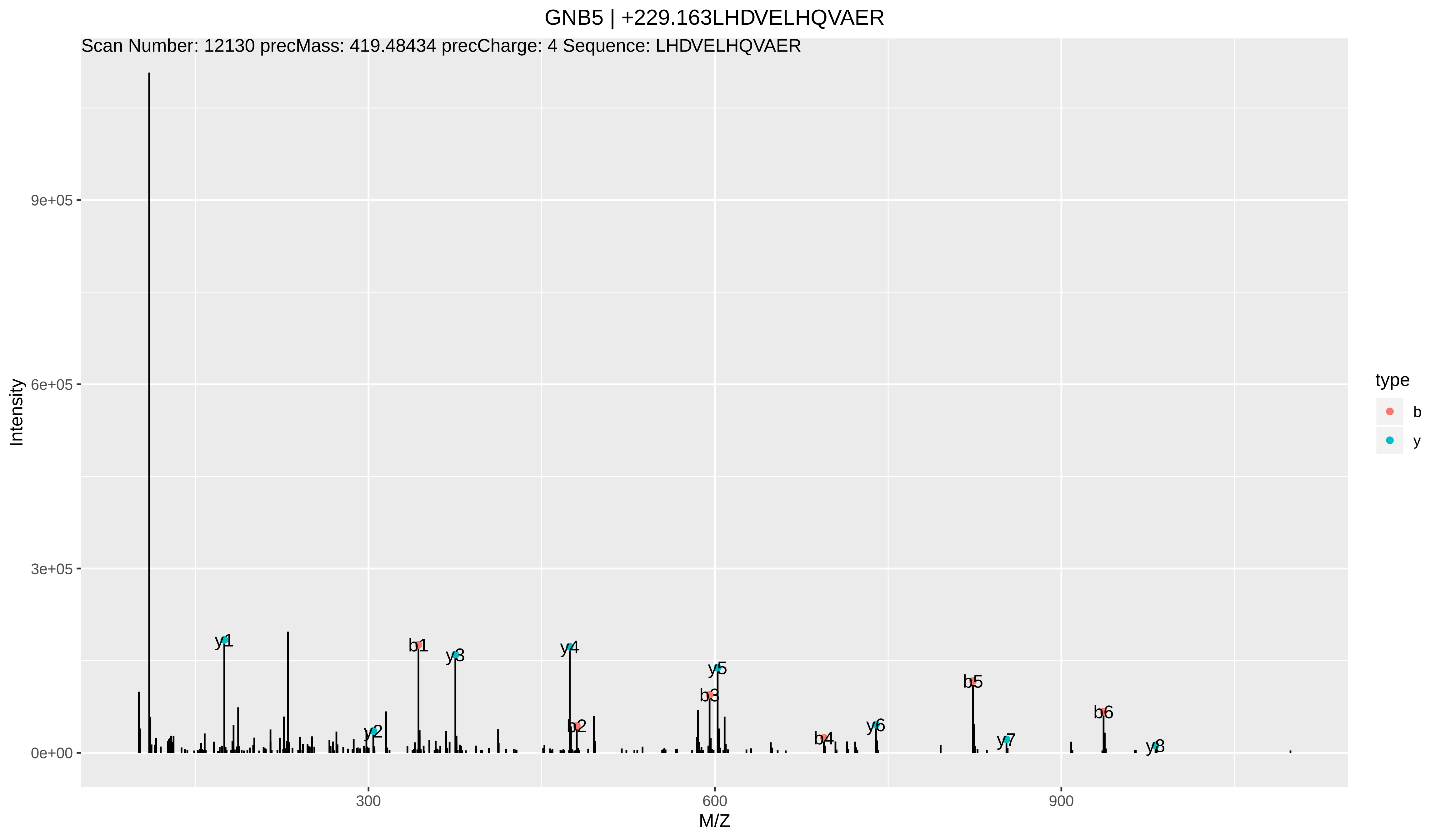
GNB5 | +229.163LHDVELHQVAER
Scan Number: 12130 precMass: 419.48434 precCharge: 4 Sequence: LHDVELHQVAER
Intensity
9e+05
6e+05
3e+05
0e+00
M/Z
300
600
900
y1
b1
y3
y4
y2
b2
y5
b3
b4
y6
b5
y7
b6
y8
type
b
y

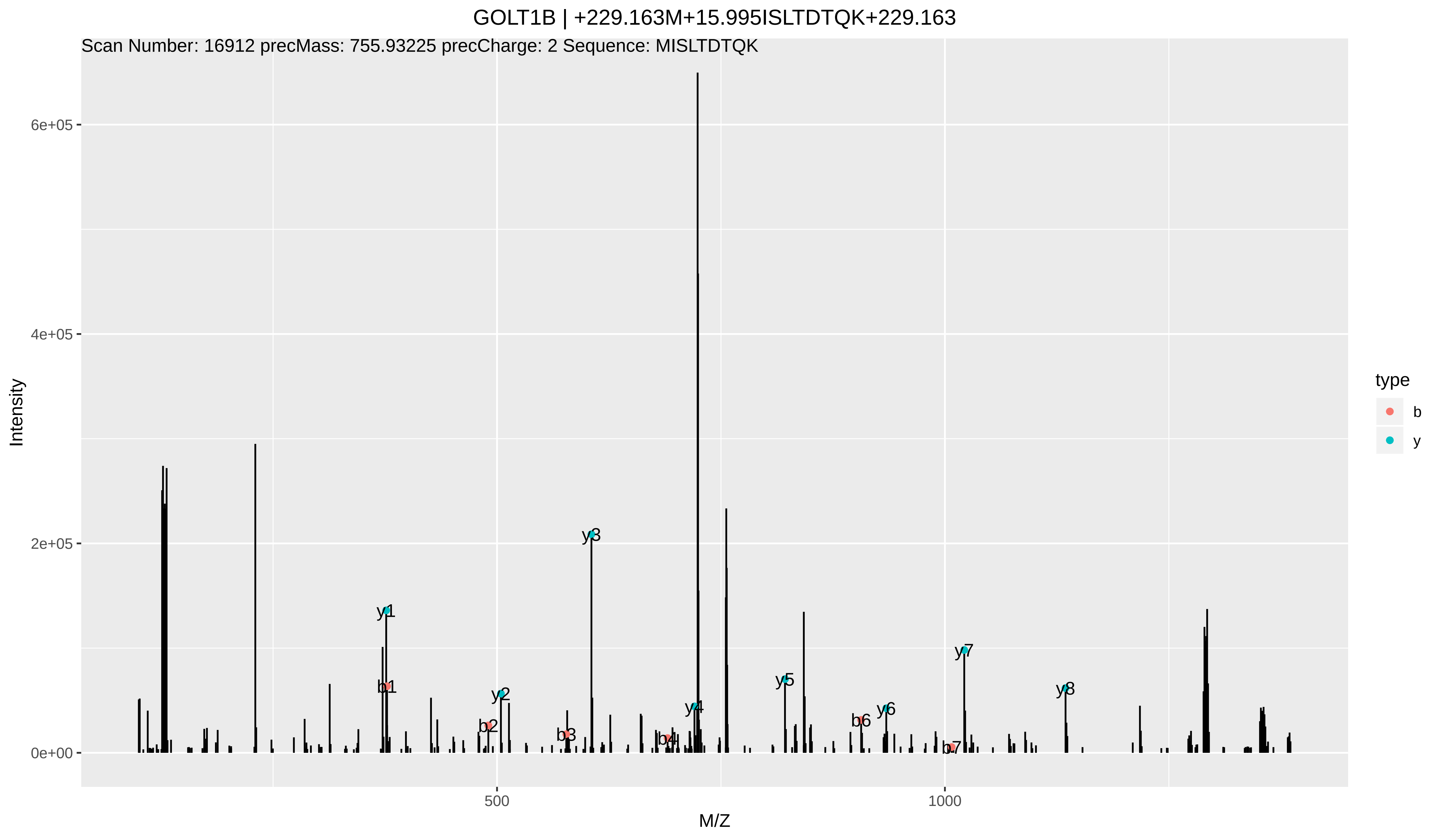

GOLT1B | +229.163M+15.995ISLTDTQK+229.163
Scan Number: 16912 precMass: 755.93225 precCharge: 2 Sequence: MISLTDTQK
Intensity
6e+05
4e+05
2e+05
0e+00
M/Z
500
1000
y1
b1
y2
b2
y3
b3
y4
b4
y5
b6
y6
y7
b7
y8
type
b
y

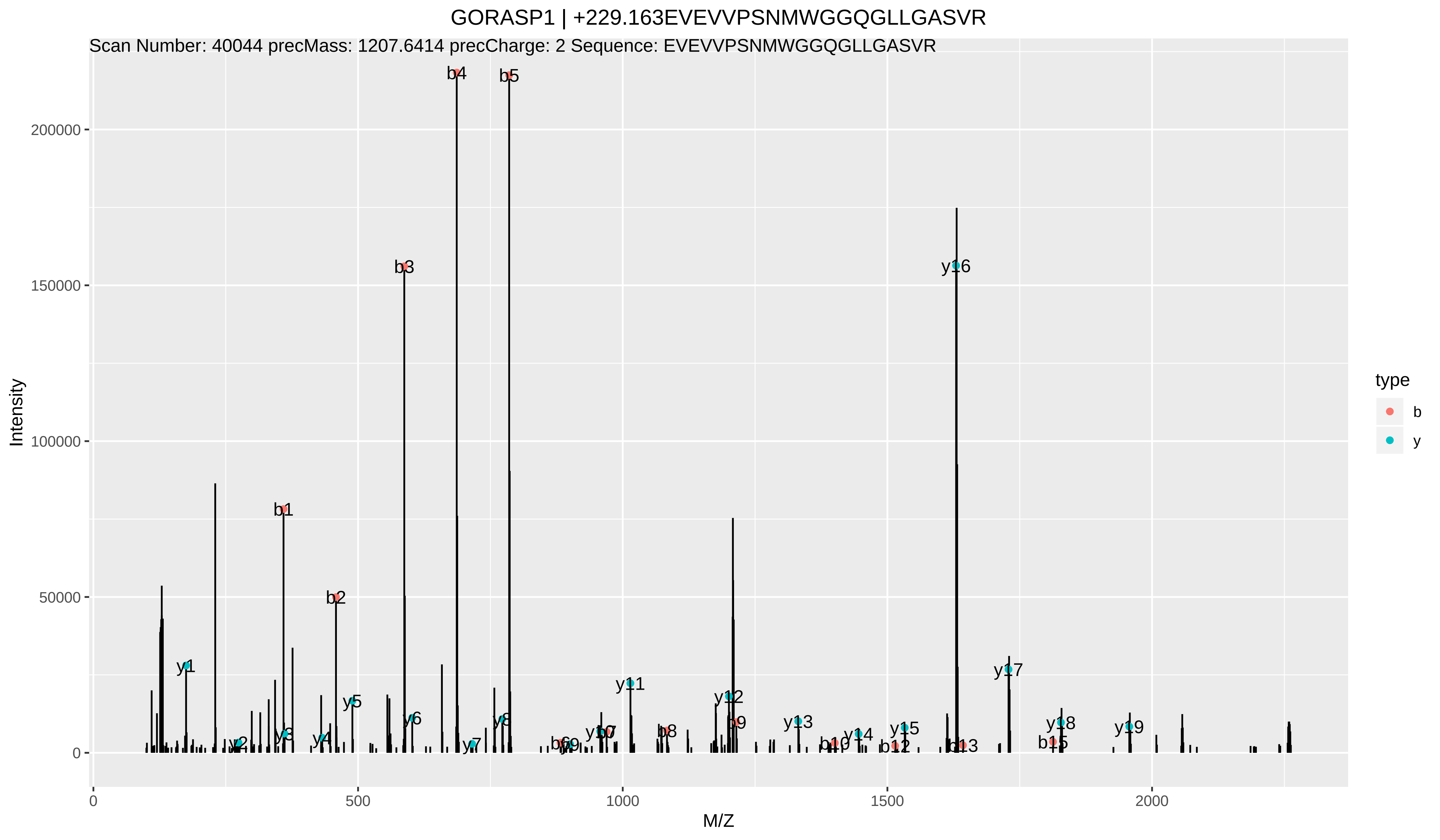
GORASP1 | +229.163EVEVVPSNMWGGQGLLGASVR
Scan Number: 40044 precMass: 1207.6414 precCharge: 2 Sequence: EVEVVPSNMWGGQGLLGASVR
Intensity
M/Z
0
50000
100000
150000
200000
0
500
1000
1500
2000
b1
b2
b3
b4
b5
b8
b10
b12
b13
b15
y1
y3
y4
y5
y6
y8
y11
y12
y13
y14
y15
y16
y17
y18
y19
type
b
y

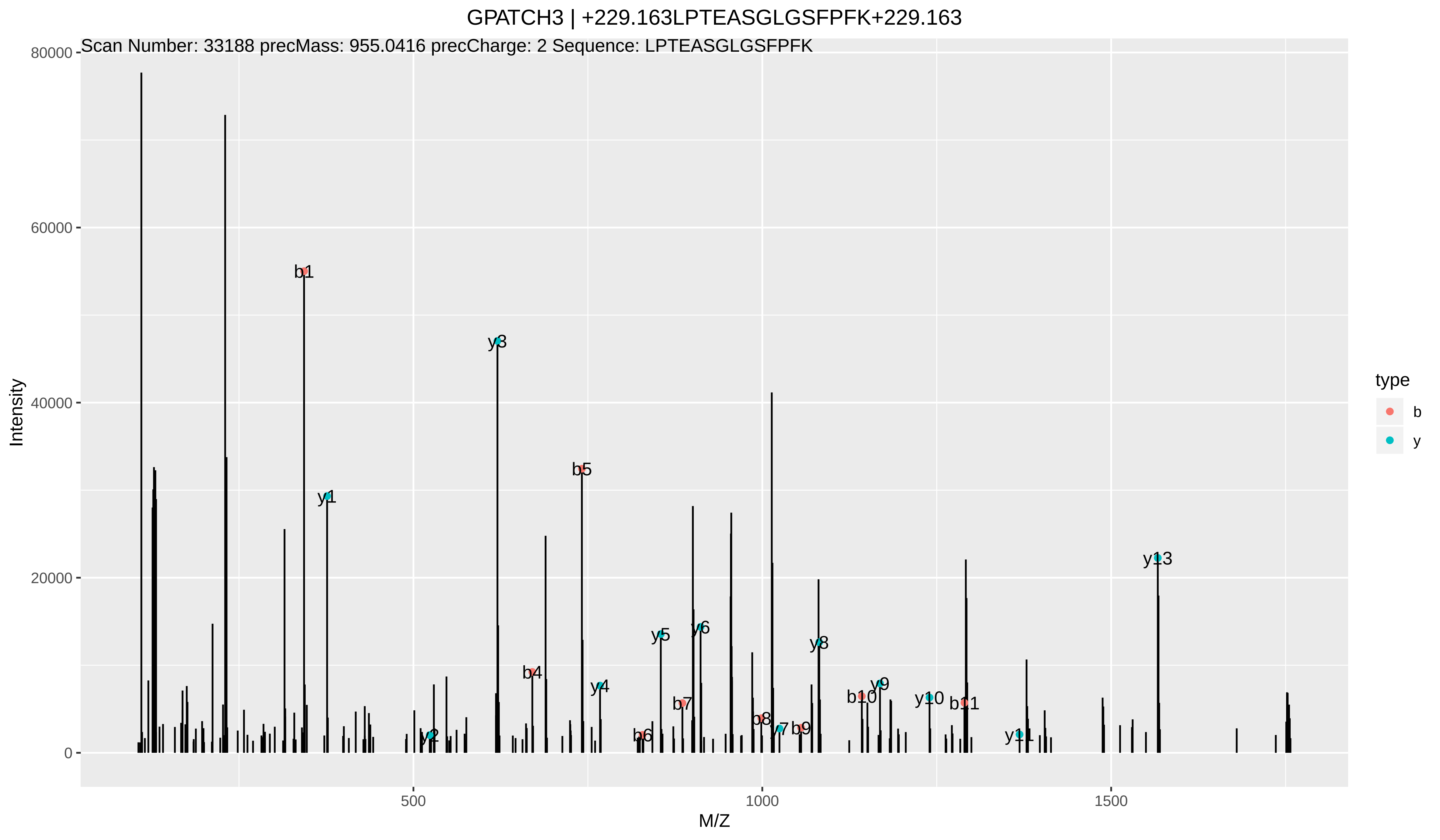

GPATCH3 | +229.163LPTEASGLGSFPFK+229.163
Scan Number: 33188 precMass: 955.0416 precCharge: 2 Sequence: LPTEASGLGSFPFK
Intensity
M/Z
type
b
y
b1
y1
y2
y3
b4
b5
y4
b6
y5
b7
y6
b8
y7
b9
y8
b10
y9
y10
b11
y11
y13

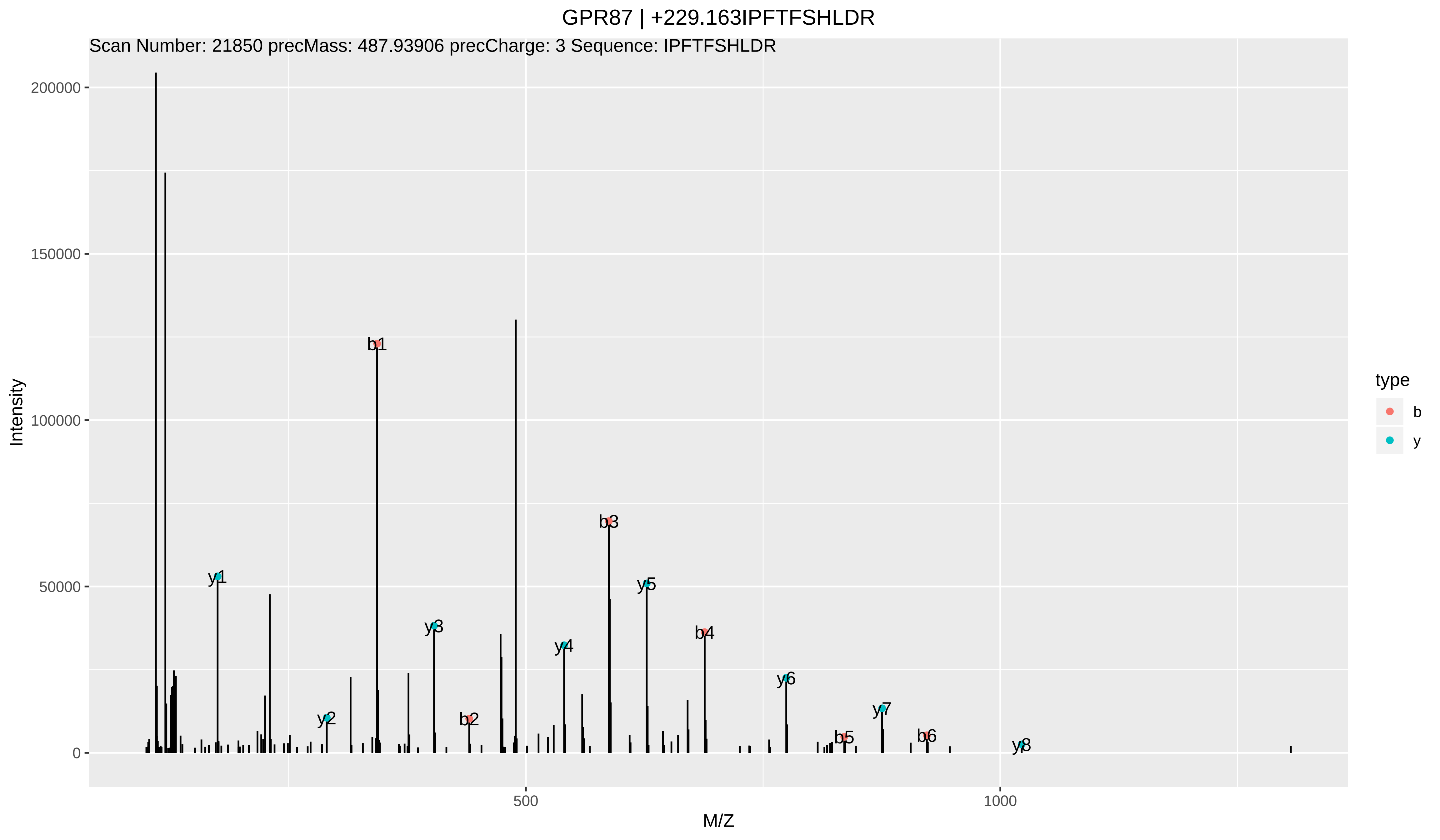

GPR87 | +229.163IPFTFSHLDR
Scan Number: 21850 precMass: 487.93906 precCharge: 3 Sequence: IPFTFSHLDR
Intensity
200000
150000
100000
50000
0
500
1000
M/Z
b1
b2
b3
b4
b5
b6
y1
y2
y3
y4
y5
y6
y7
y8
type
b
y

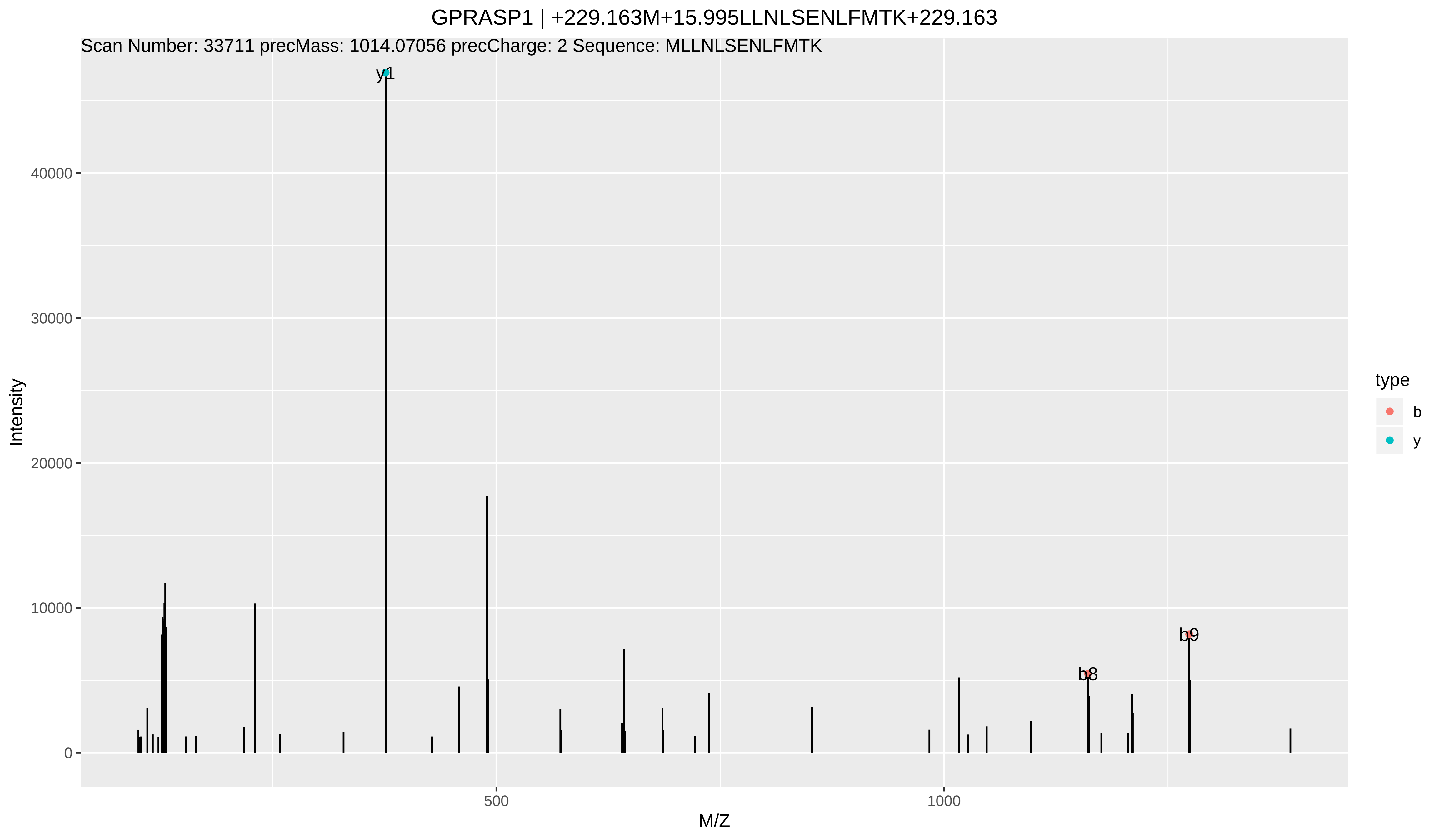

GPRASP1 | +229.163M+15.995LLNLSENLFMTK+229.163
Scan Number: 33711 precMass: 1014.07056 precCharge: 2 Sequence: MLLNLSENLFMTK
y1
b8
b9
Intensity
40000
30000
20000
10000
0
500
1000
M/Z
type
b
y

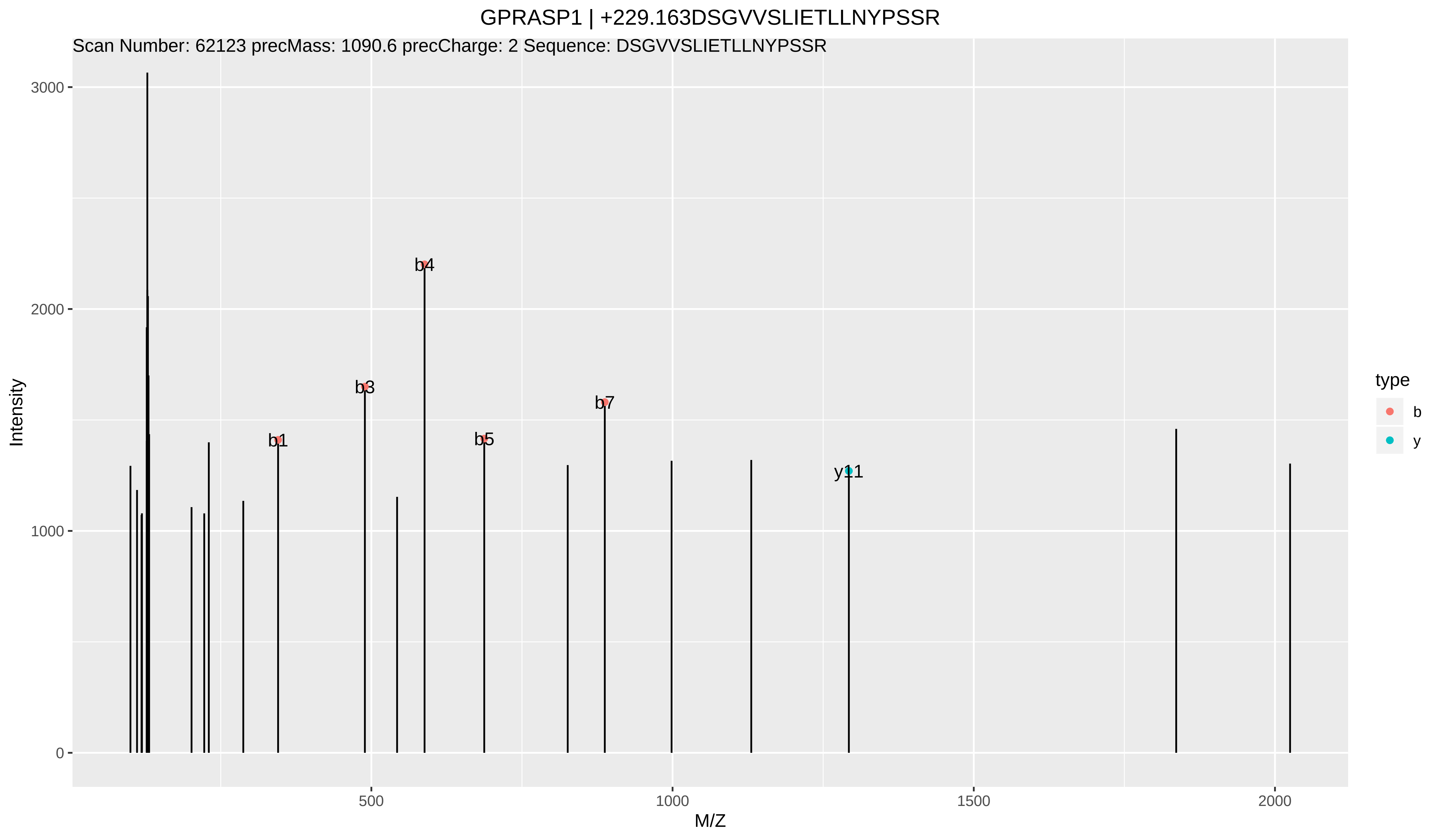

GPRASP1 | +229.163DSGVVSLIETLLNYPSSR
Scan Number: 62123 precMass: 1090.6 precCharge: 2 Sequence: DSGVVSLIETLLNYPSSR
Intensity
3000
2000
1000
0
M/Z
500
1000
1500
2000
b1
b3
b4
b5
b7
y11
type
b
y

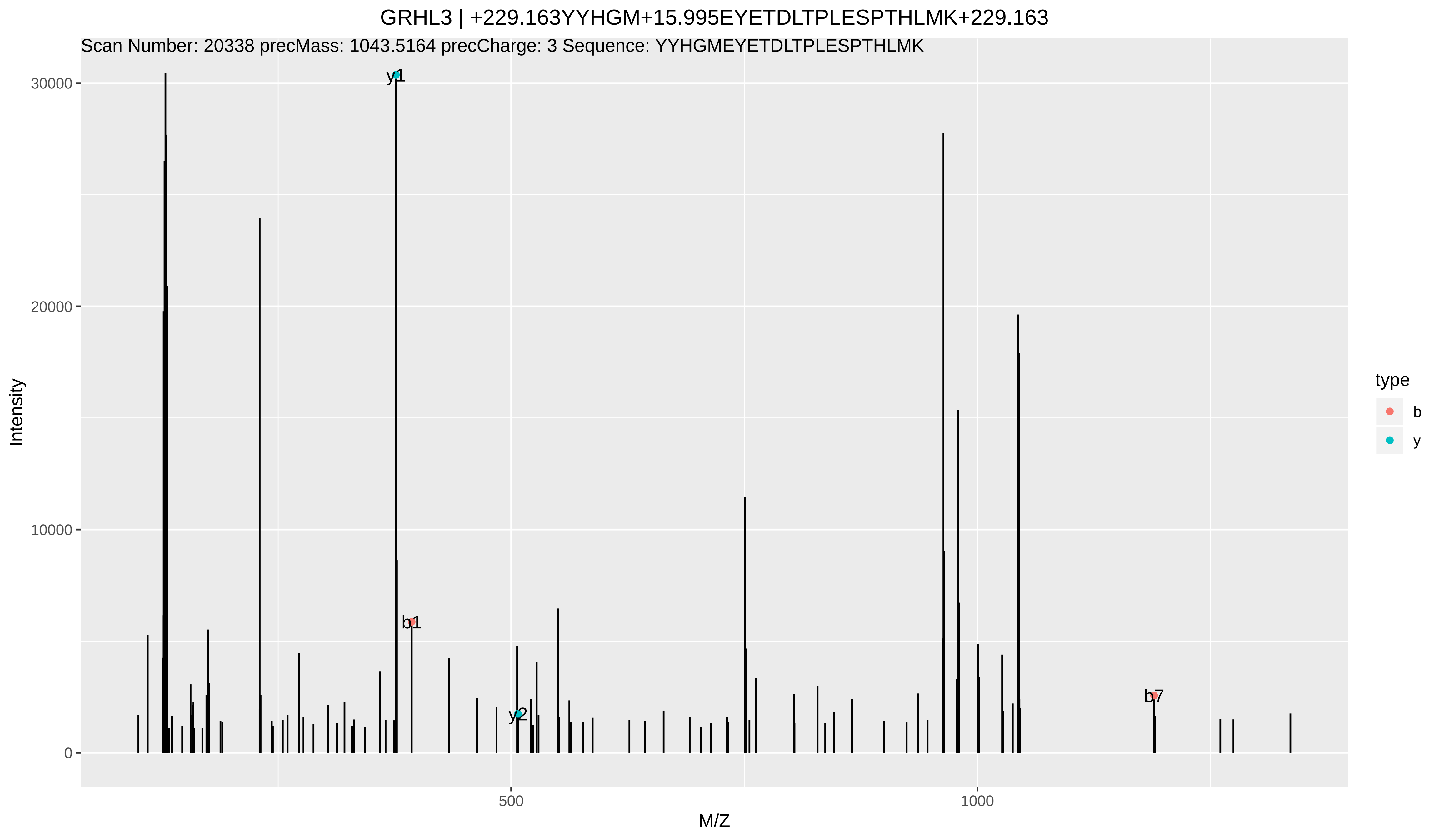
GRHL3 | +229.163YYHGM+15.995EYETDLTPLESPTHLMK+229.163
Scan Number: 20338 precMass: 1043.5164 precCharge: 3 Sequence: YYHGMEYETDLTPLESPTHLMK
y1
b1
y2
b7
30000
20000
10000
0
500
1000
Intensity
M/Z
type
b
y

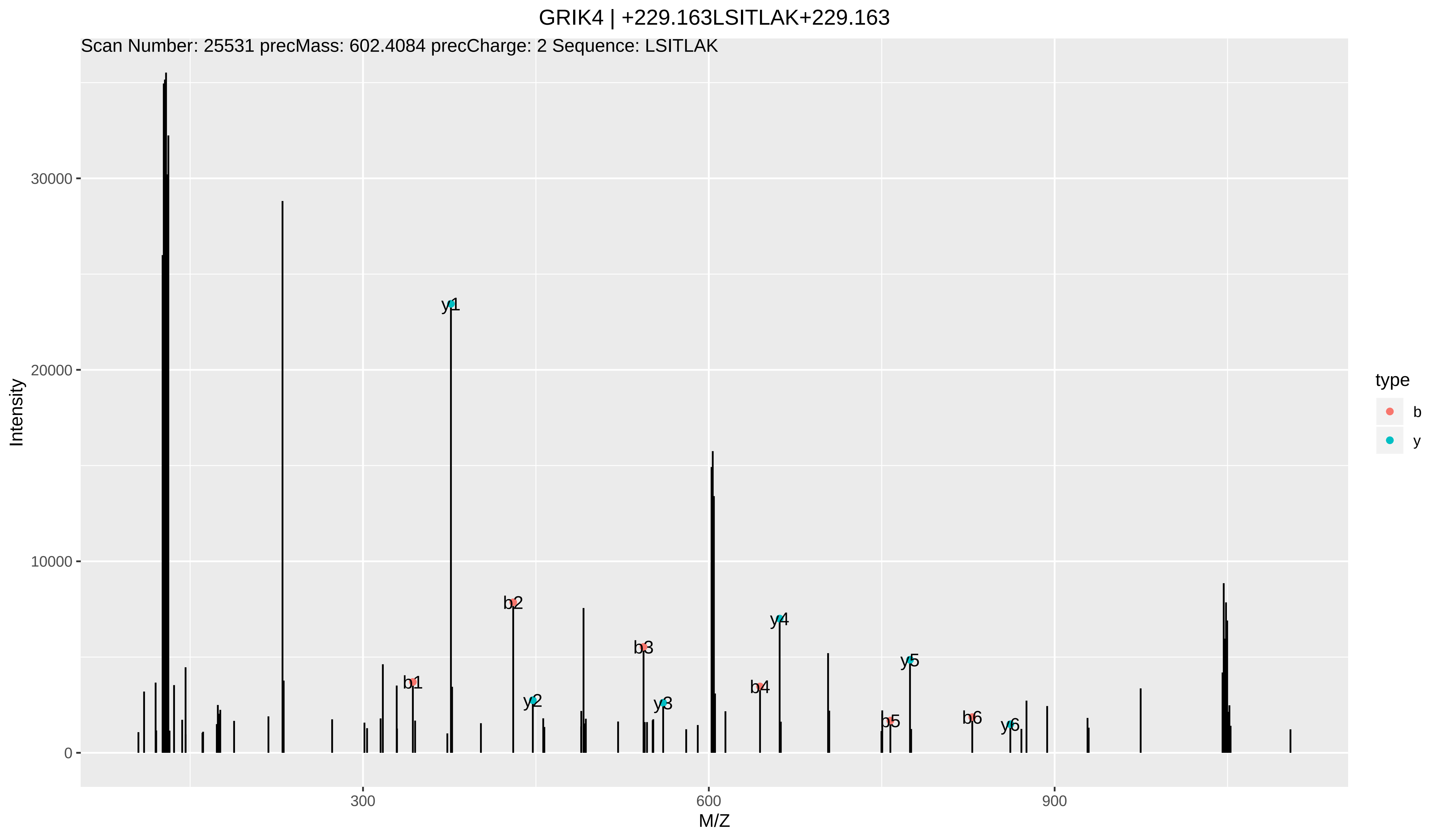

GRIK4 | +229.163LSITLAK+229.163
Scan Number: 25531 precMass: 602.4084 precCharge: 2 Sequence: LSITLAK
Intensity
30000
20000
10000
0
300
600
900
M/Z
y1
b2
y4
b3
y5
b1
b4
y2
y3
b5
b6
y6
type
b
y

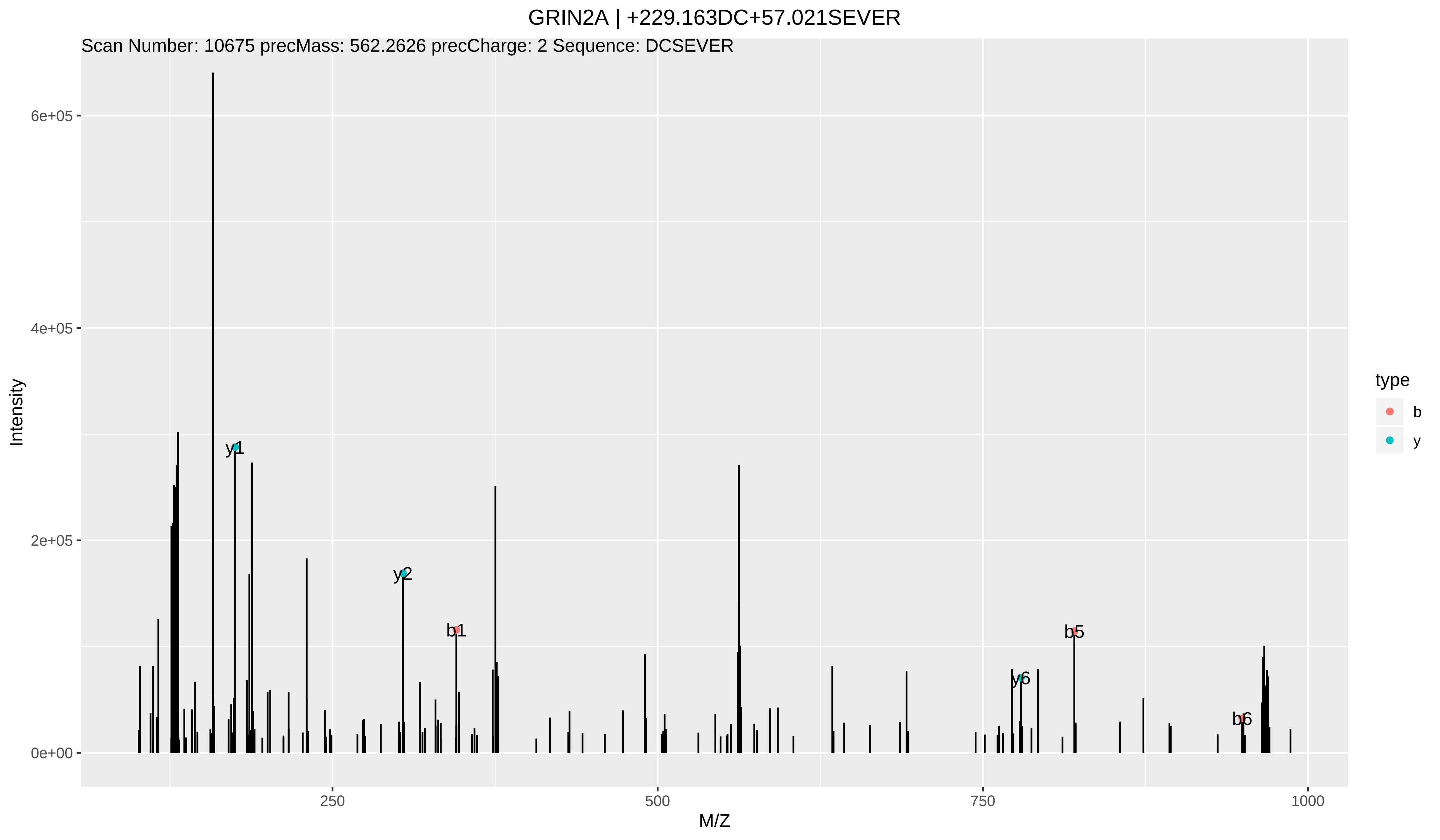

GRIN2A | +229.163DC+57.021SEVER
Scan Number: 10675 precMass: 562.2626 precCharge: 2 Sequence: DCSEVER
Intensity
6e+05
4e+05
2e+05
0e+00
M/Z
250
500
750
1000
y1
y2
b1
y6
b5
b6
type
b
y

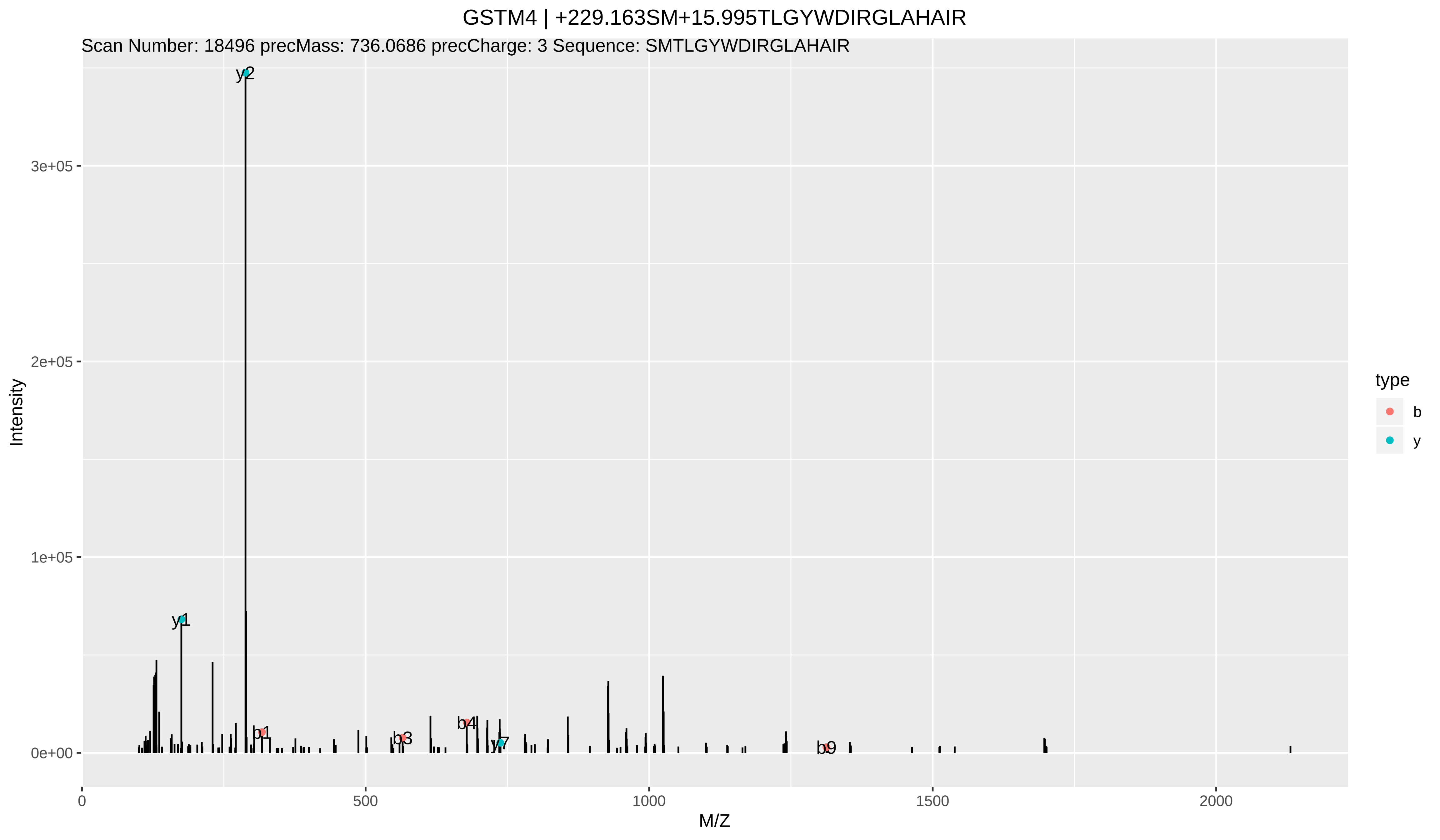

GSTM4 | +229.163SM+15.995TLGYWDIRGLAHAIR
Scan Number: 18496 precMass: 736.0686 precCharge: 3 Sequence: SMTLGYWDIRGLAHAIR
y2
y1
b1
b3
b4
y7
b9
Intensity
3e+05
2e+05
1e+05
0e+00
0
500
1000
1500
2000
M/Z
type
b
y

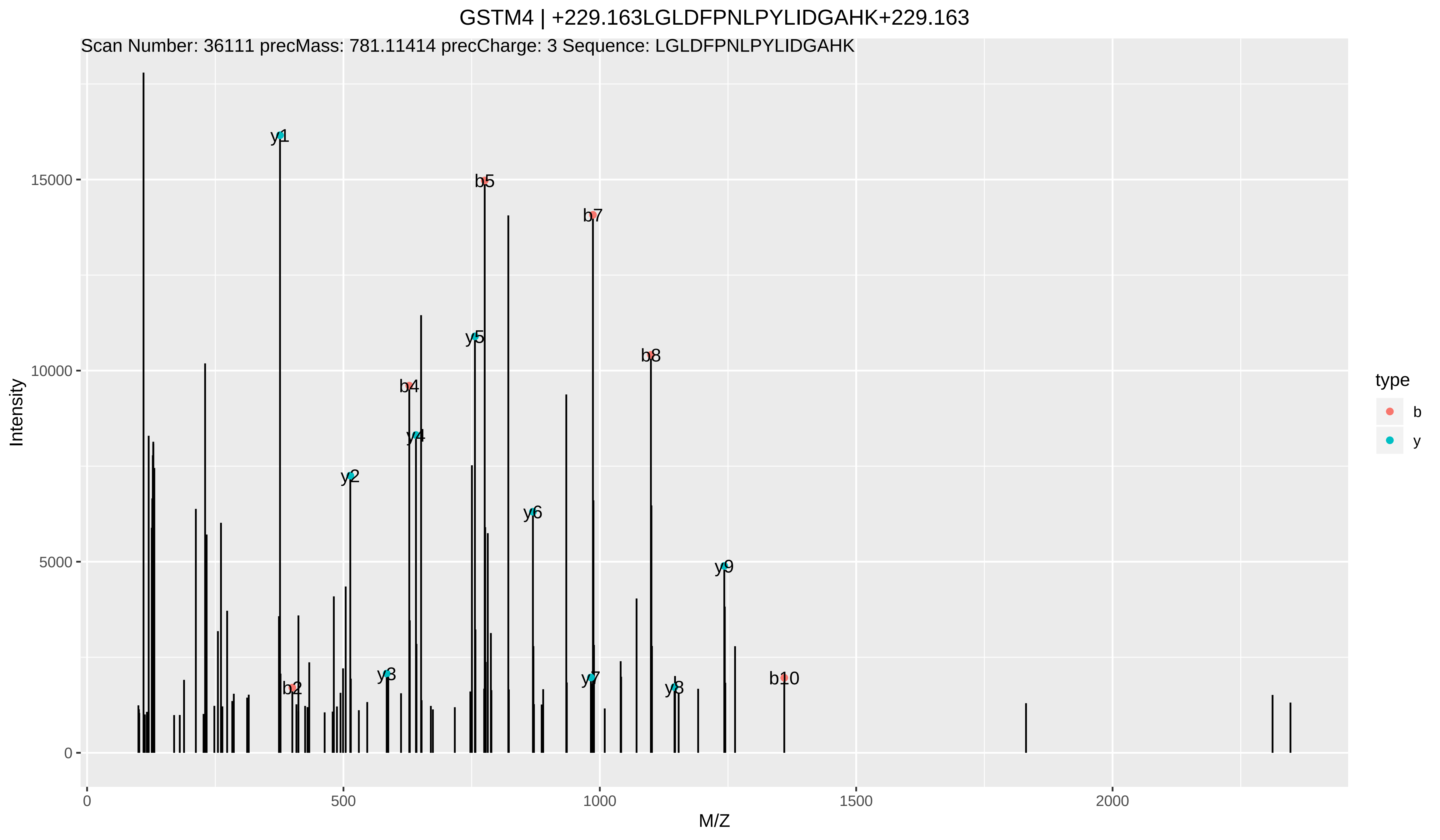

GSTM4 | +229.163LGLDFPNLPYLIDGAHK+229.163
Scan Number: 36111 precMass: 781.11414 precCharge: 3 Sequence: LGLDFPNLPYLIDGAHK
Intensity
M/Z
0
5000
10000
15000
0
500
1000
1500
2000
y1
b5
b7
y5
b8
b4
y4
y2
y6
y9
y3
b2
y7
y8
b10
type
b
y

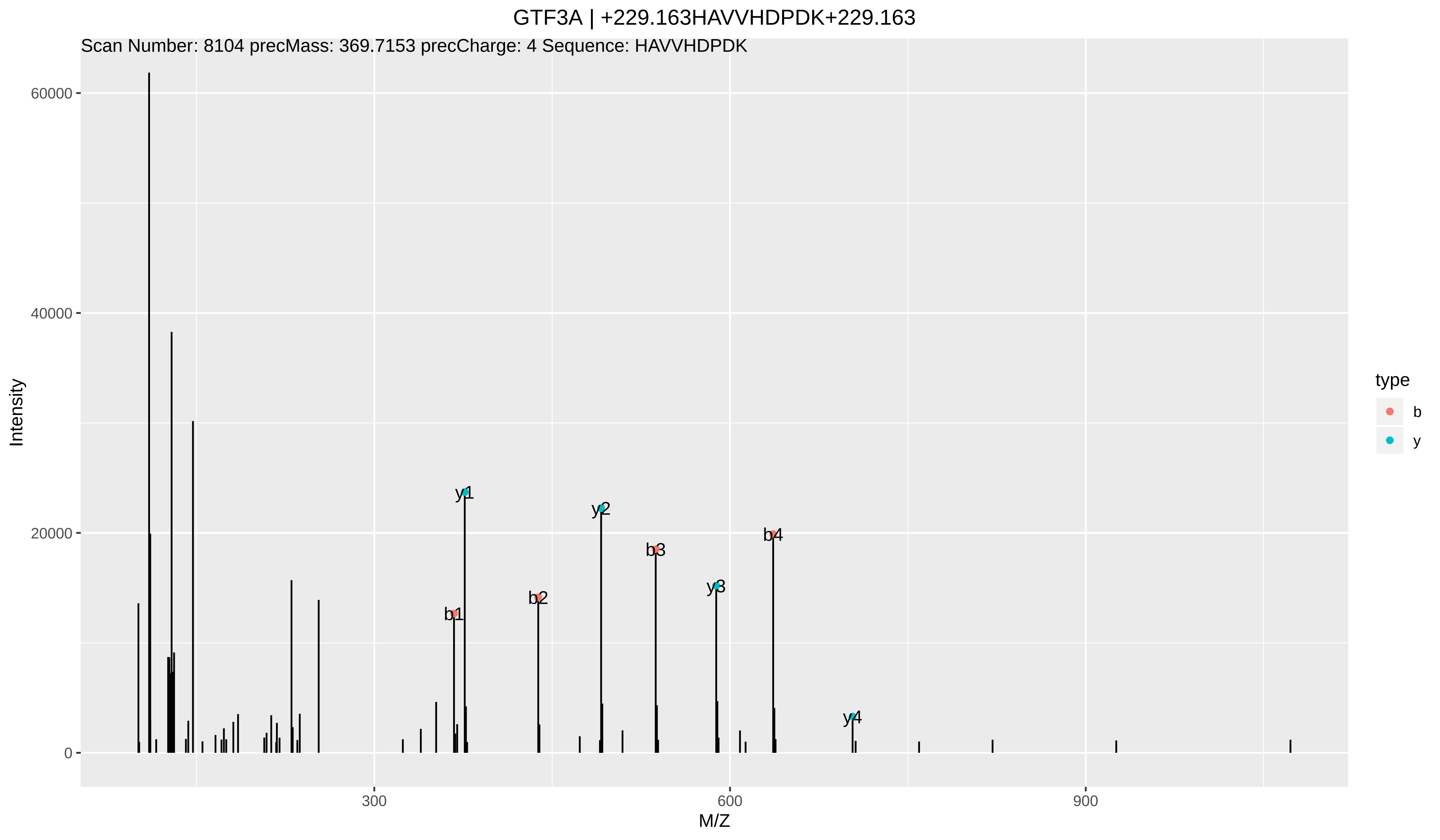

GTF3A | +229.163HAVVHDPDK+229.163
Scan Number: 8104 precMass: 369.7153 precCharge: 4 Sequence: HAVVHDPDK
Intensity
M/Z
60000
40000
20000
0
300
600
900
y1
y2
b3
b4
y3
b2
b1
y4
type
b
y

GTF3C6 | +229.163K+229.163EGEENIGGVEWLQIK+229.163

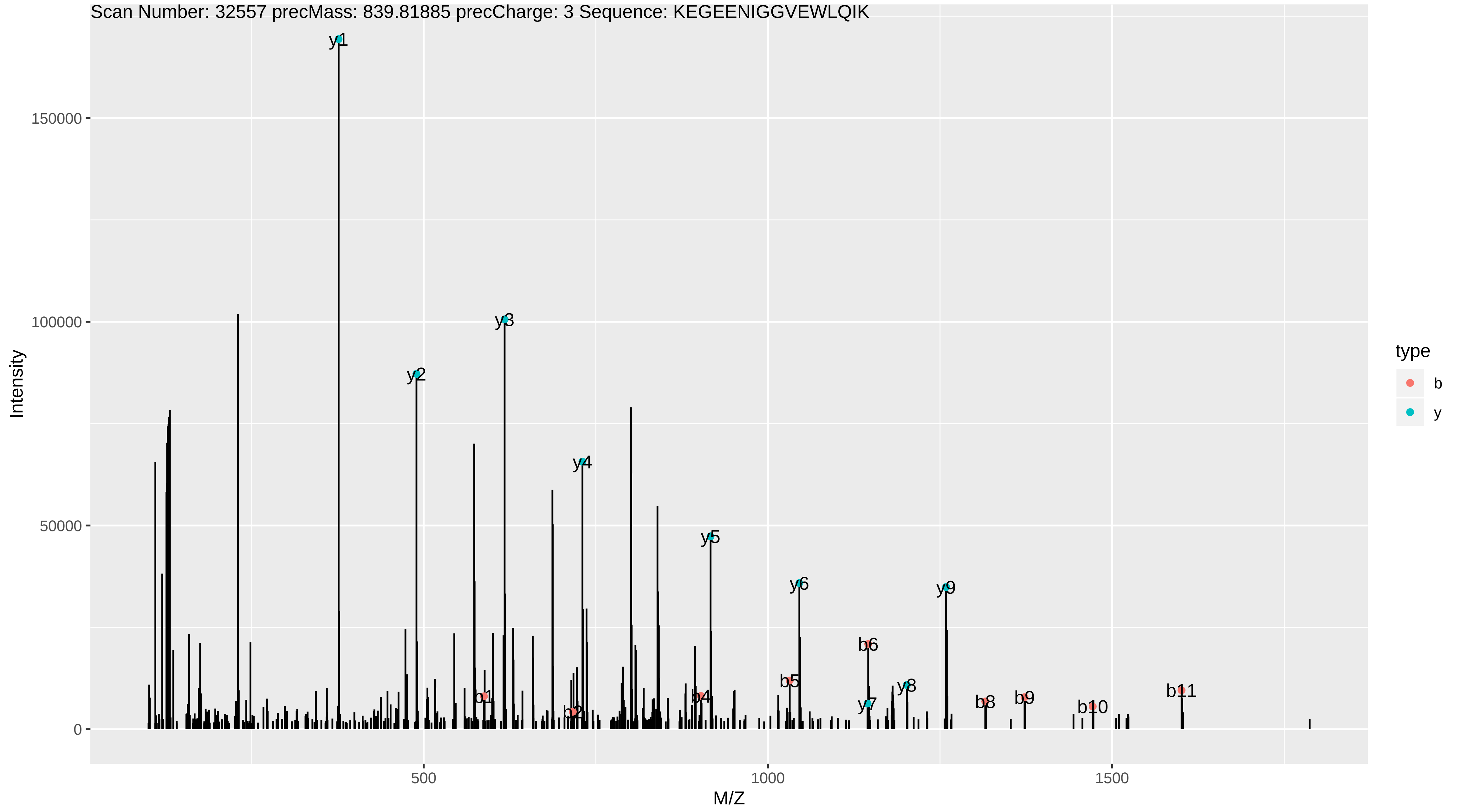

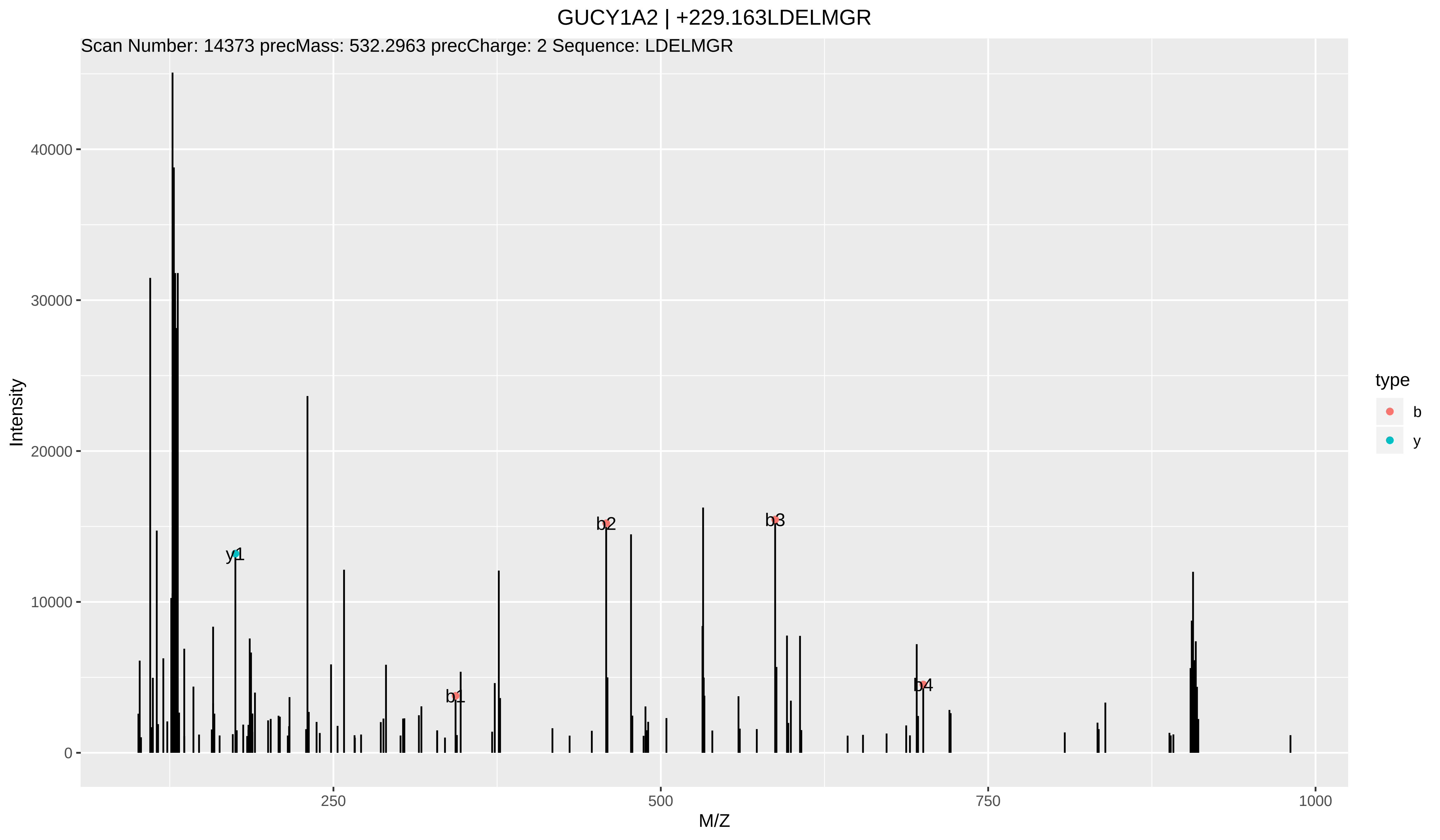

GUCY1A2 | +229.163LDELMGR
Scan Number: 14373 precMass: 532.2963 precCharge: 2 Sequence: LDELMGR
Intensity
M/Z
0
10000
20000
30000
40000
250
500
750
1000
y1
b1
b2
b3
b4
type
b
y

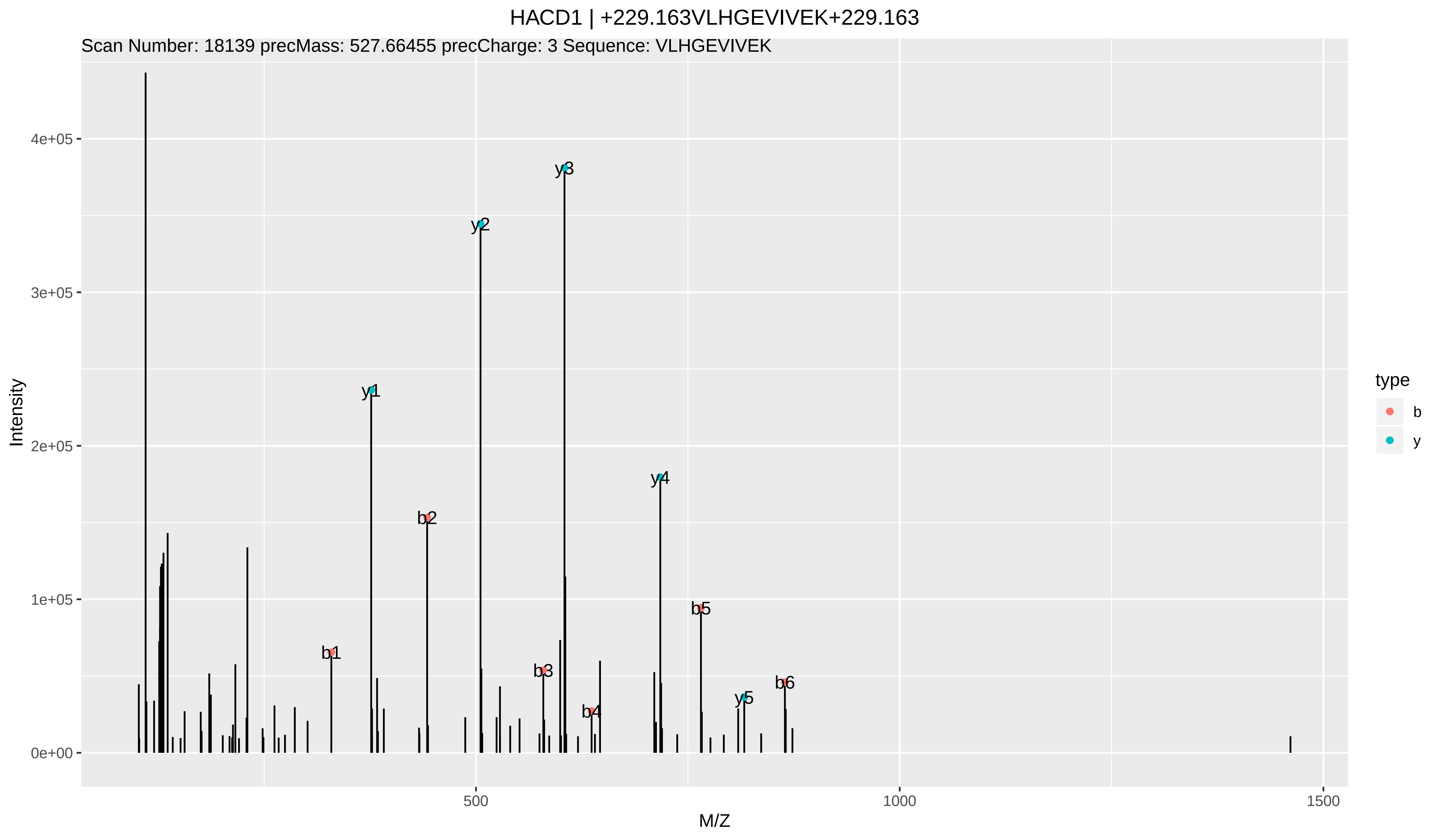

HACD1 | +229.163VLHGEVIVEK+229.163
Scan Number: 18139 precMass: 527.66455 precCharge: 3 Sequence: VLHGEVIVEK
Intensity
M/Z
type
b
y
y1
y2
y3
y4
y5
b1
b2
b3
b4
b5
b6
0e+00
1e+05
2e+05
3e+05
4e+05
500
1000
1500

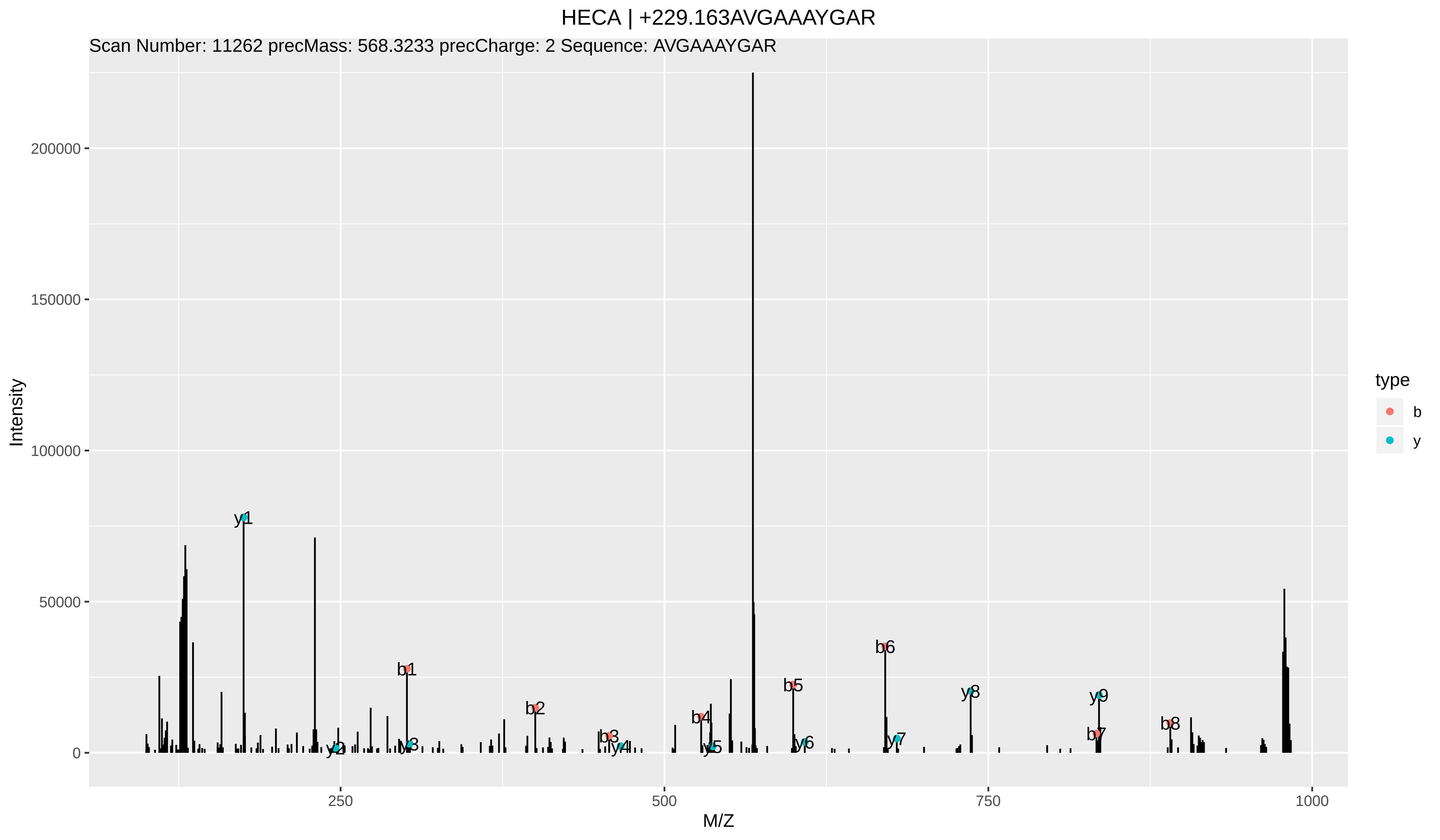

HECA | +229.163AVGAAAYGAR
Scan Number: 11262 precMass: 568.3233 precCharge: 2 Sequence: AVGAAAYGAR
Intensity
0
50000
100000
150000
200000
M/Z
250
500
750
1000
y1
y2
b1
y3
b2
b3
y4
b4
y5
b5
y6
b6
y7
y8
b7
y9
b8
type
b
y

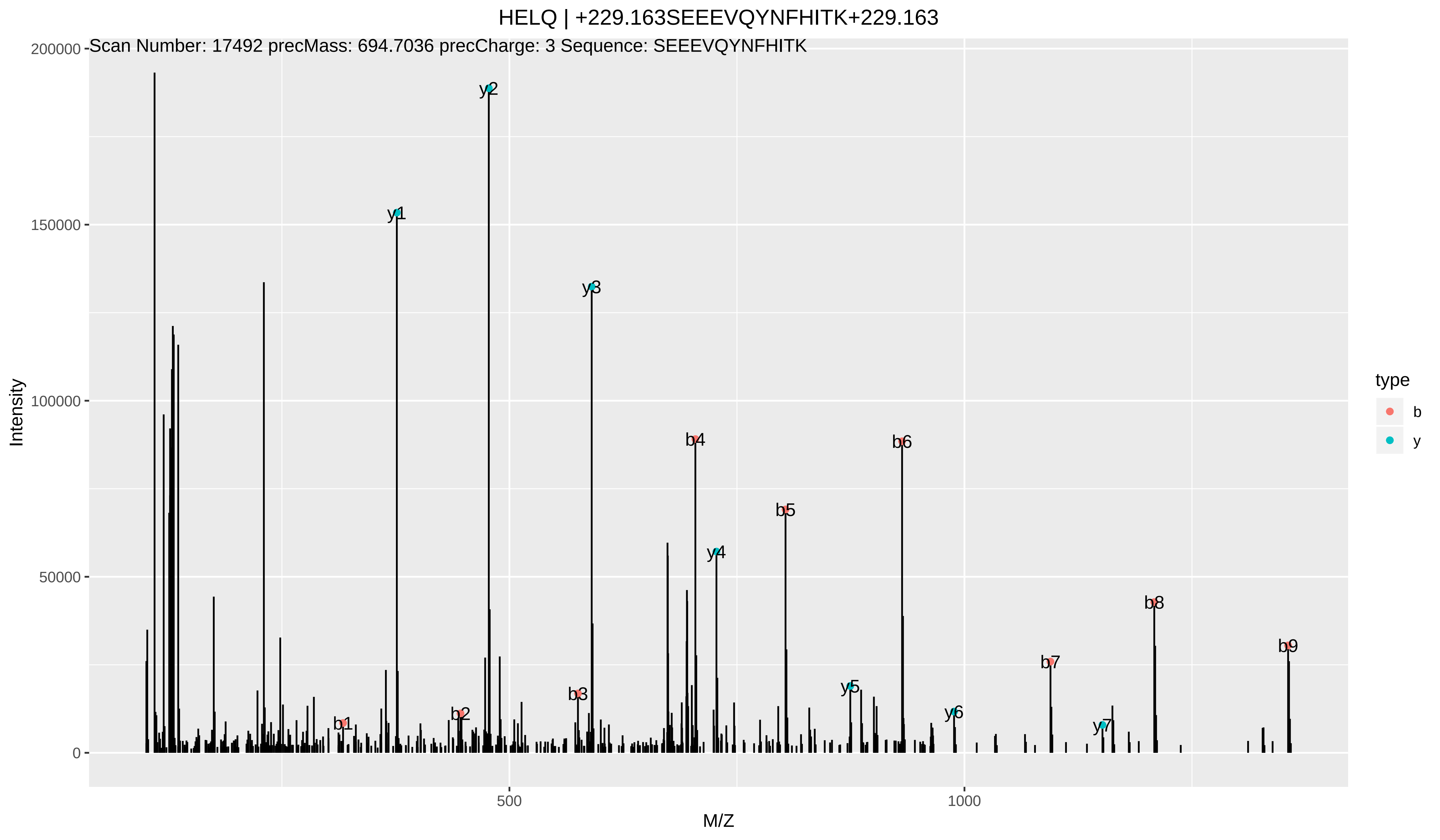

HELQ | +229.163SEEEVQYNFHITK+229.163
Scan Number: 17492 precMass: 694.7036 precCharge: 3 Sequence: SEEEVQYNFHITK
Intensity
200000
150000
100000
50000
0
M/Z
500
1000
y1
y2
y3
y4
y5
y6
y7
b1
b2
b3
b4
b5
b6
b7
b8
b9
type
b
y

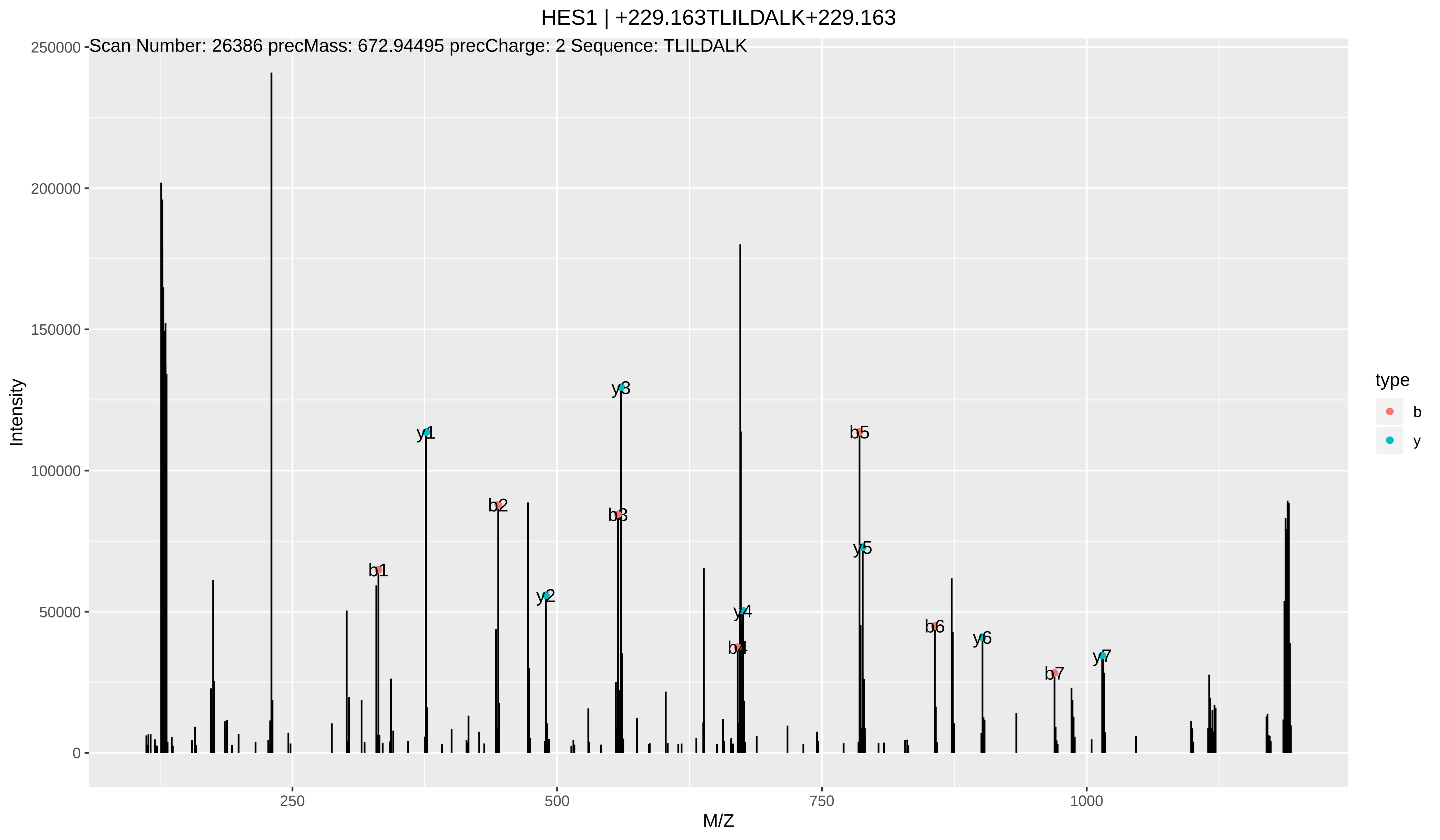
HES1 | +229.163TLILDALK+229.163
Scan Number: 26386 precMass: 672.94495 precCharge: 2 Sequence: TLILDALK
Intensity
M/Z
type
b
y
b1
y1
b2
y2
b3
y3
b4
y4
b5
y5
b6
y6
b7
y7

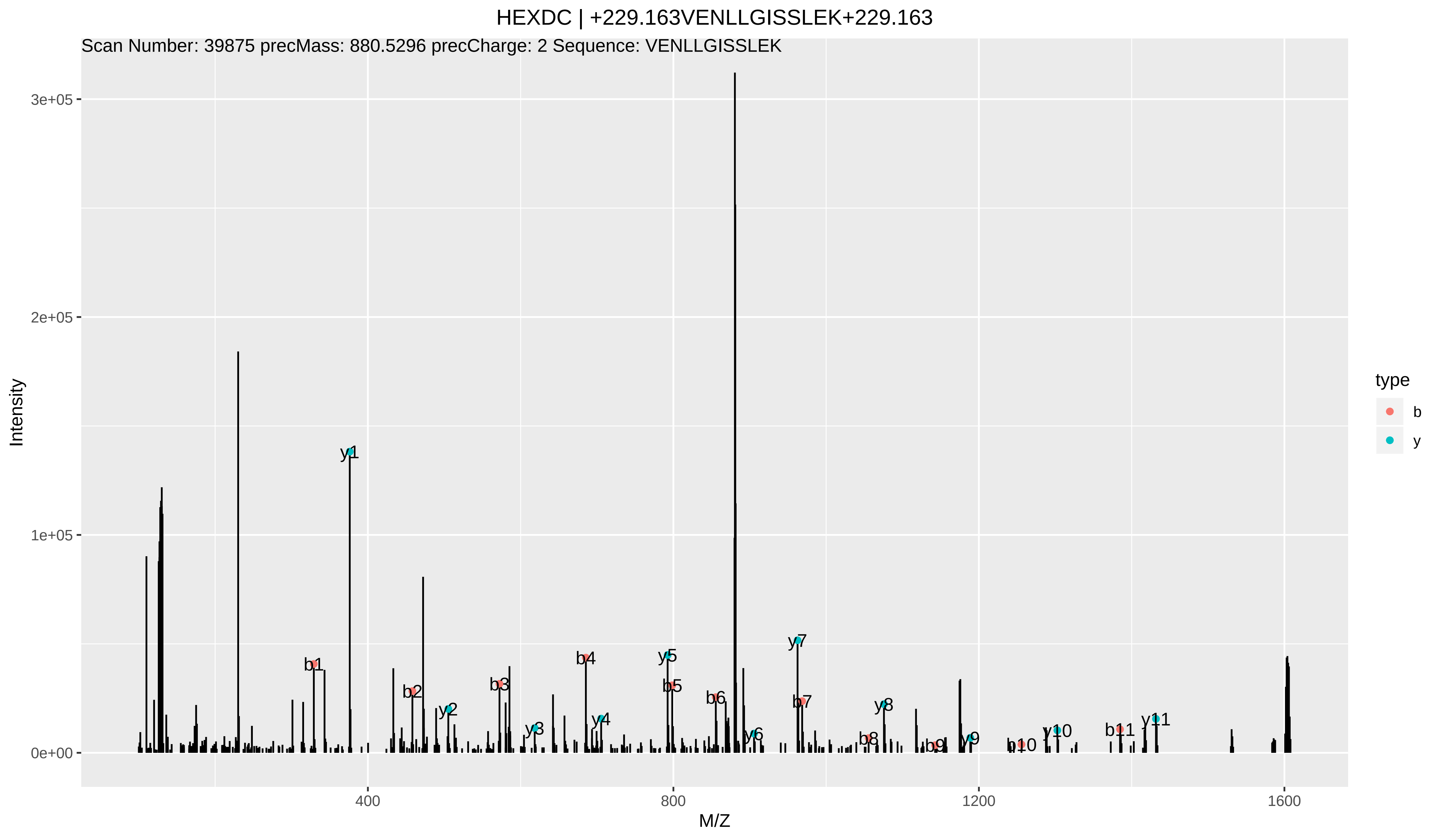
HEXDC | +229.163VENLLGISSLEK+229.163
Scan Number: 39875 precMass: 880.5296 precCharge: 2 Sequence: VENLLGISSLEK
Intensity
M/Z
3e+05
2e+05
1e+05
0e+00
400
800
1200
1600
y1
b1
b2
y2
b3
y3
b4
y4
y5
b5
b6
y6
y7
b7
b8
y8
b9
y9
b10
y10
b11
y11
type
b
y

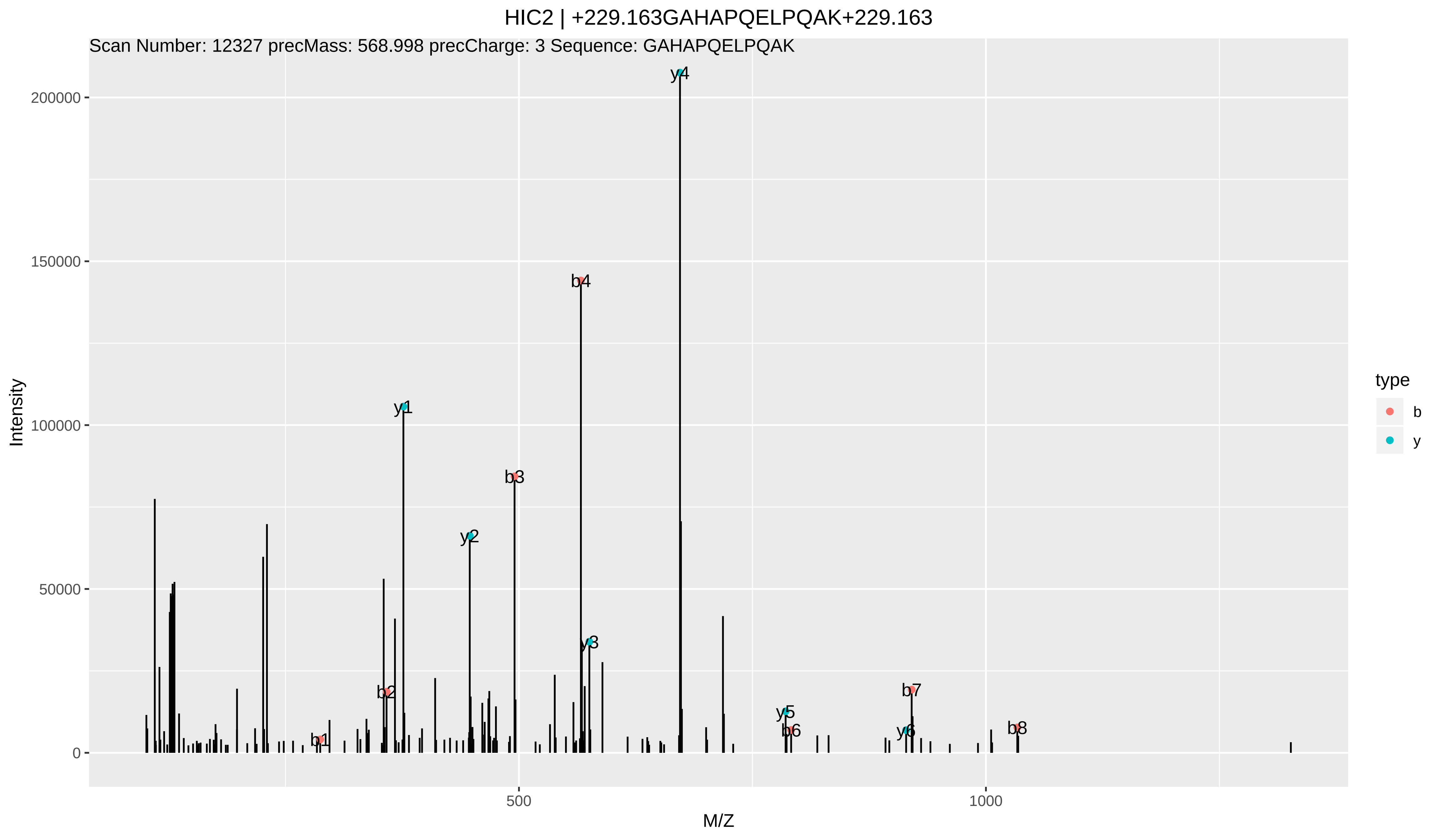

HIC2 | +229.163GAHAPQELPQAK+229.163
Scan Number: 12327 precMass: 568.998 precCharge: 3 Sequence: GAHAPQELPQAK
Intensity
200000
150000
100000
50000
0
500
1000
M/Z
y4
b4
y1
b3
y2
y3
b2
b1
y5
b6
b7
y6
b8
type
b
y

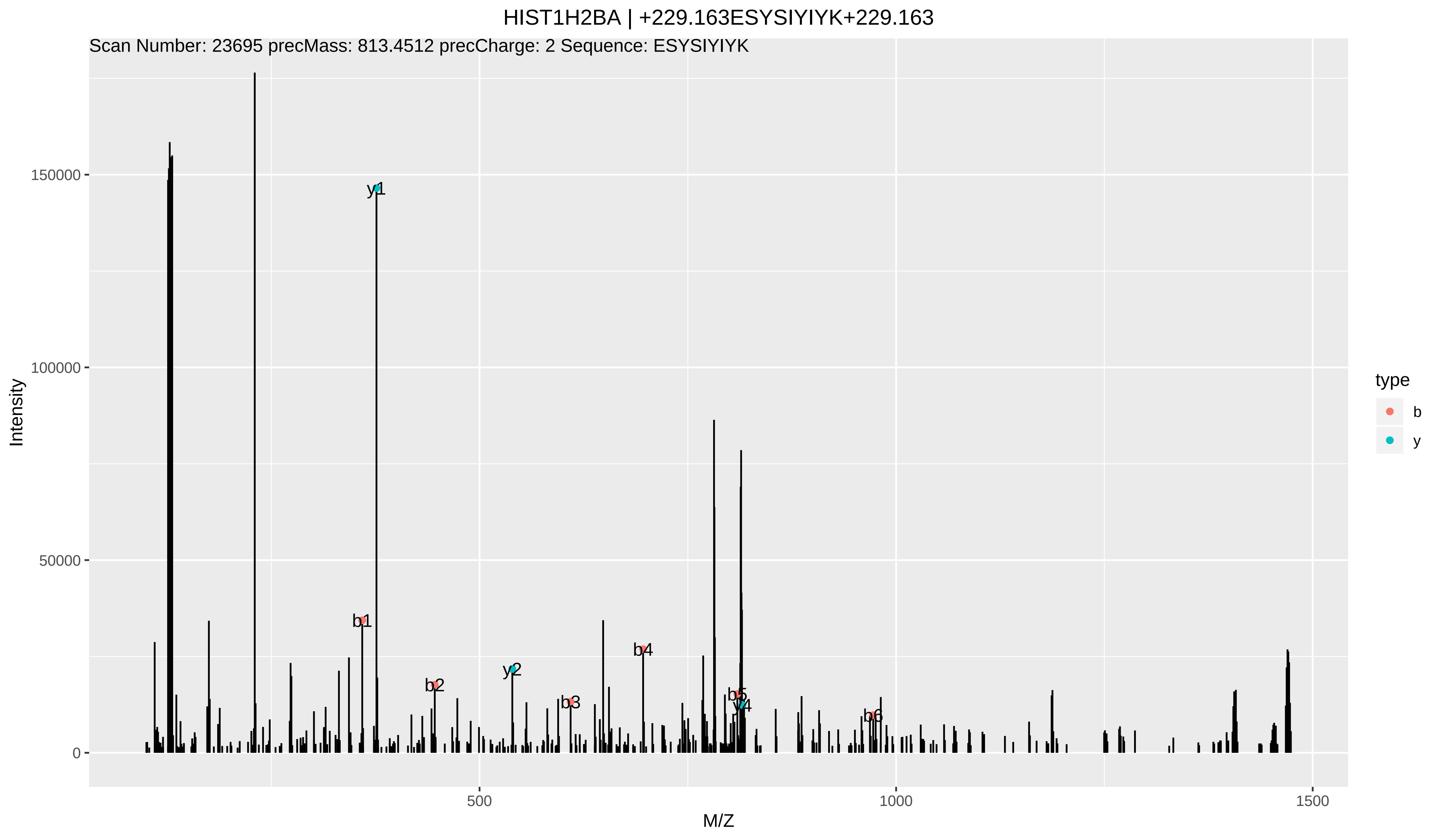

HIST1H2BA | +229.163ESYSIYIYK+229.163
Scan Number: 23695 precMass: 813.4512 precCharge: 2 Sequence: ESYSIYIYK
Intensity
M/Z
0
50000
100000
150000
500
1000
1500
y1
b1
b2
y2
b3
b4
b5
y4
b6
type
b
y

HIST1H2BA | +229.163IASEASR

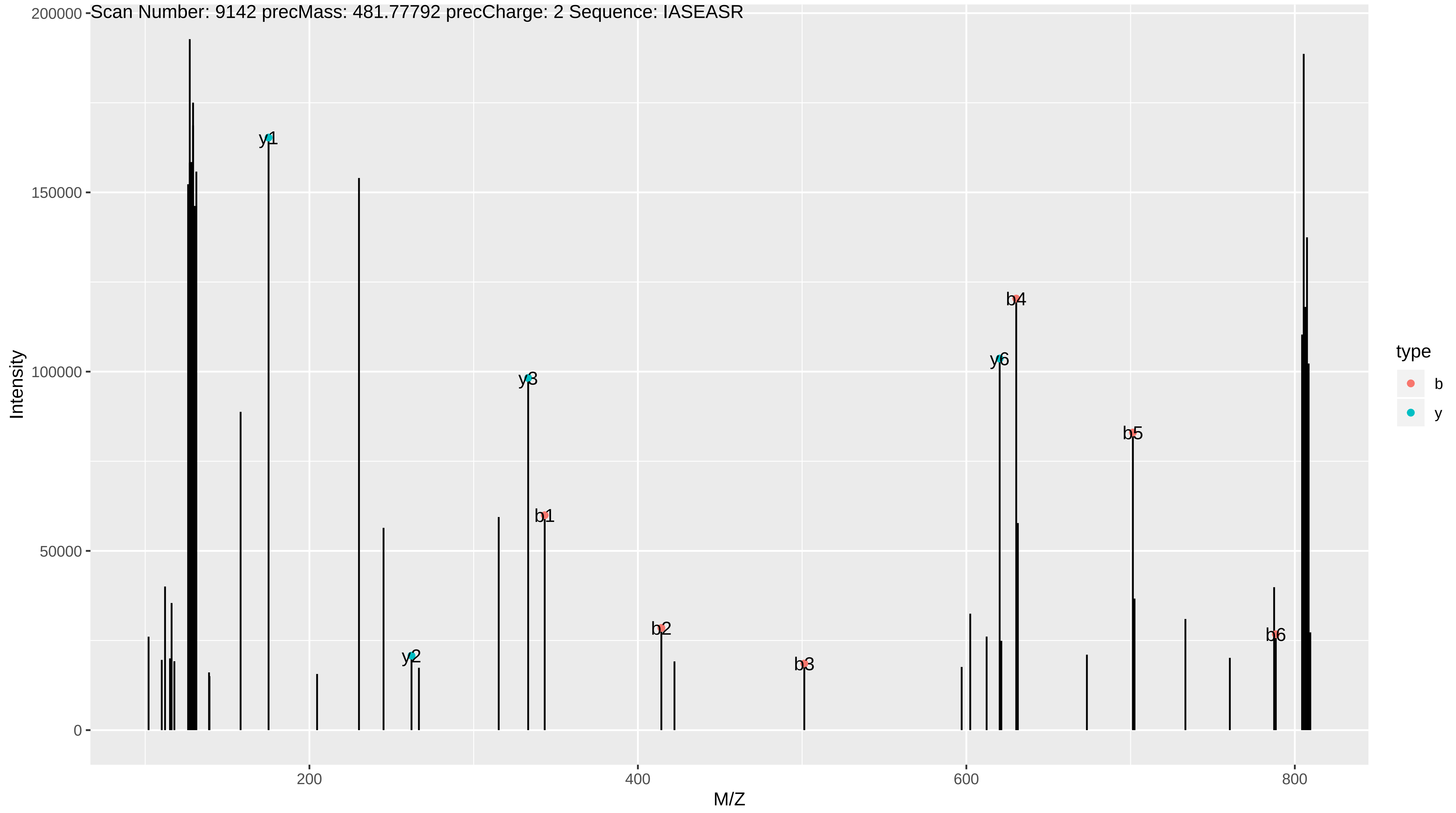

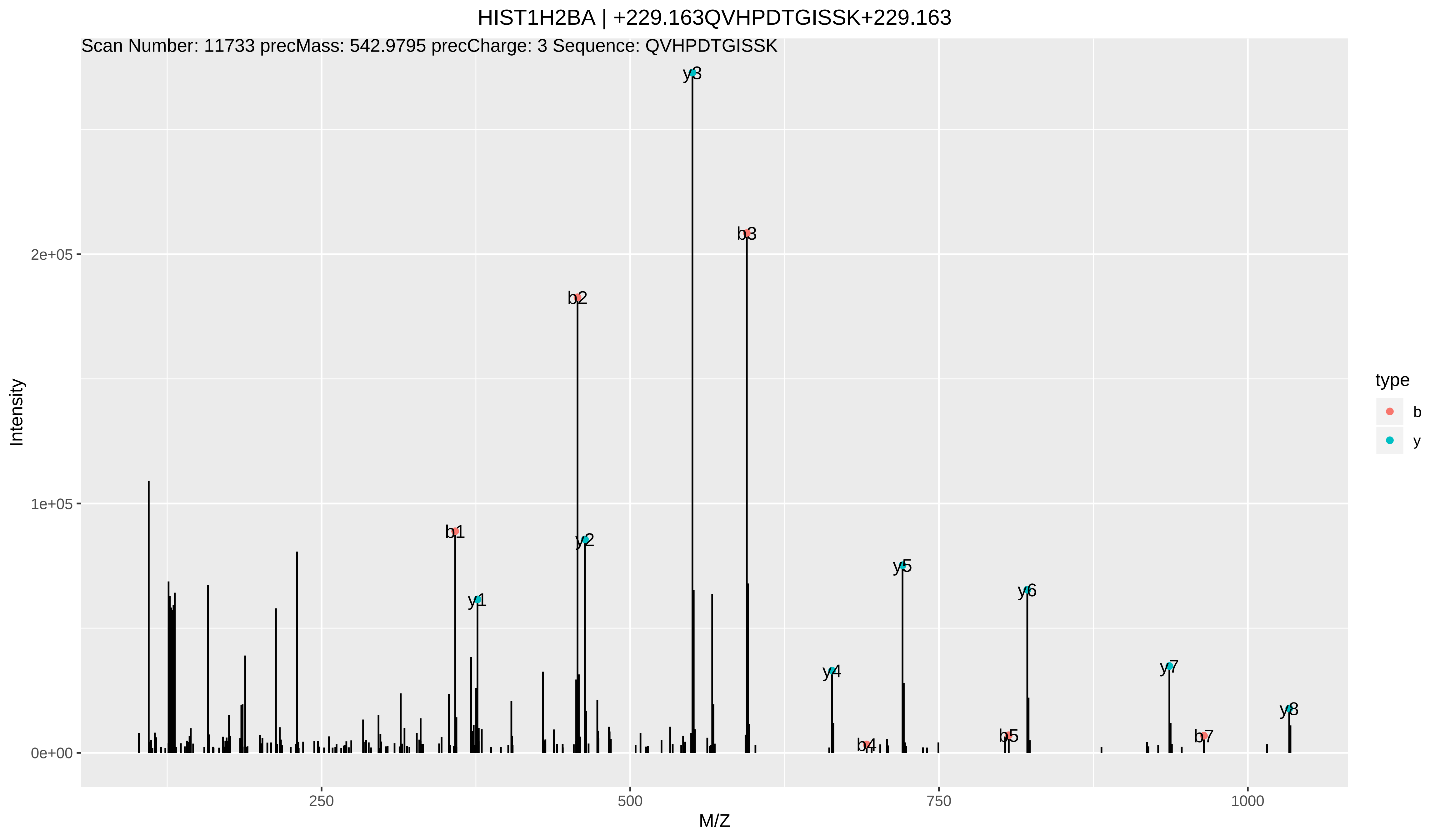

HIST1H2BA | +229.163QVHPDTGISSK+229.163
Scan Number: 11733 precMass: 542.9795 precCharge: 3 Sequence: QVHPDTGISSK
Intensity
M/Z
2e+05
1e+05
0e+00
250
500
750
1000
y3
b3
b2
b1
y2
y1
y5
y6
y4
y7
y8
b4
b5
b7
type
b
y

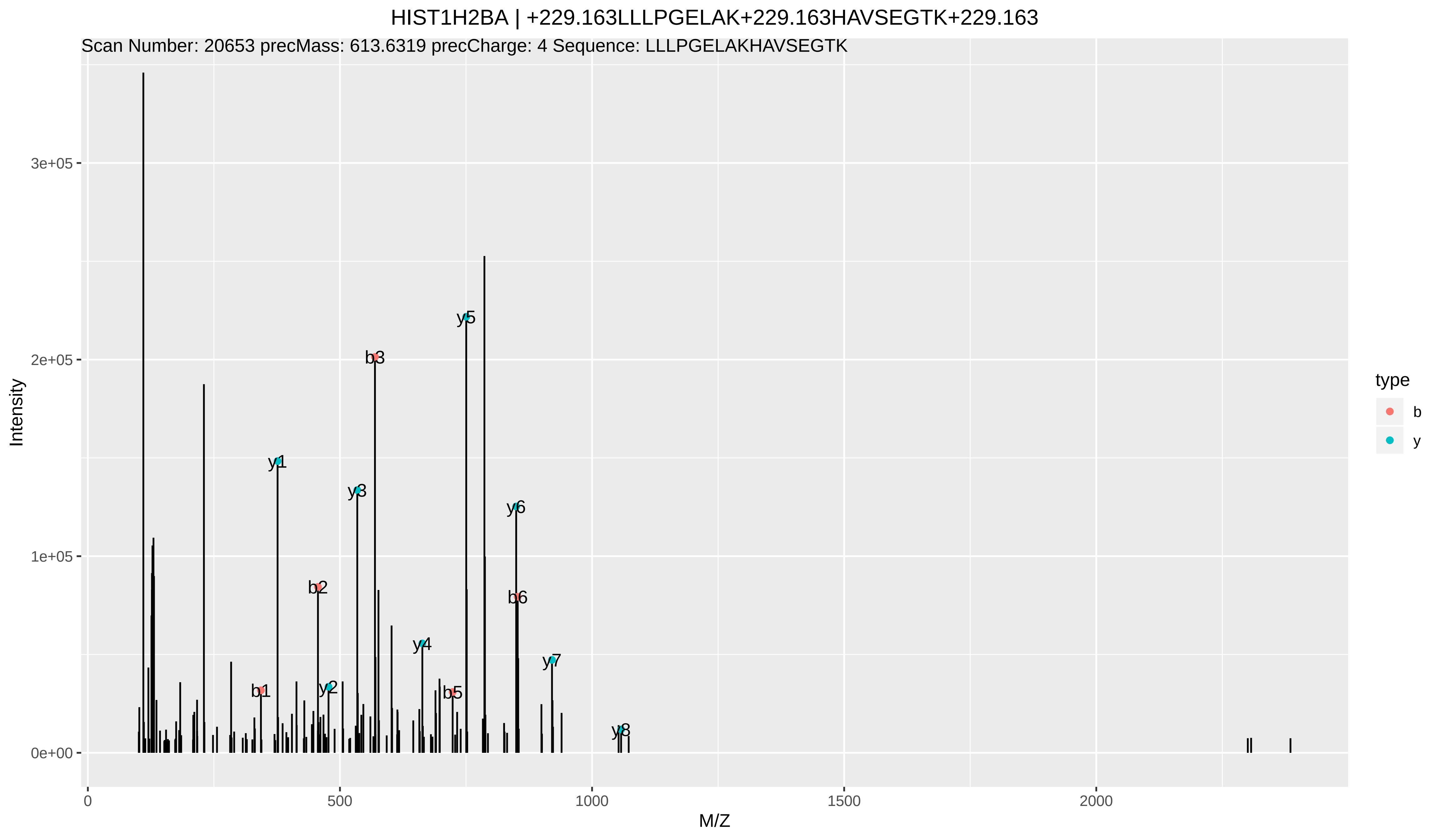
HIST1H2BA | +229.163LLLPGELAK+229.163HAVSEGTK+229.163
Scan Number: 20653 precMass: 613.6319 precCharge: 4 Sequence: LLLPGELAKHAVSEGTK
Intensity
3e+05
2e+05
1e+05
0e+00
M/Z
0
500
1000
1500
2000
y5
b3
y1
y3
y6
b2
b6
y4
y7
b1
y2
b5
y8
type
b
y

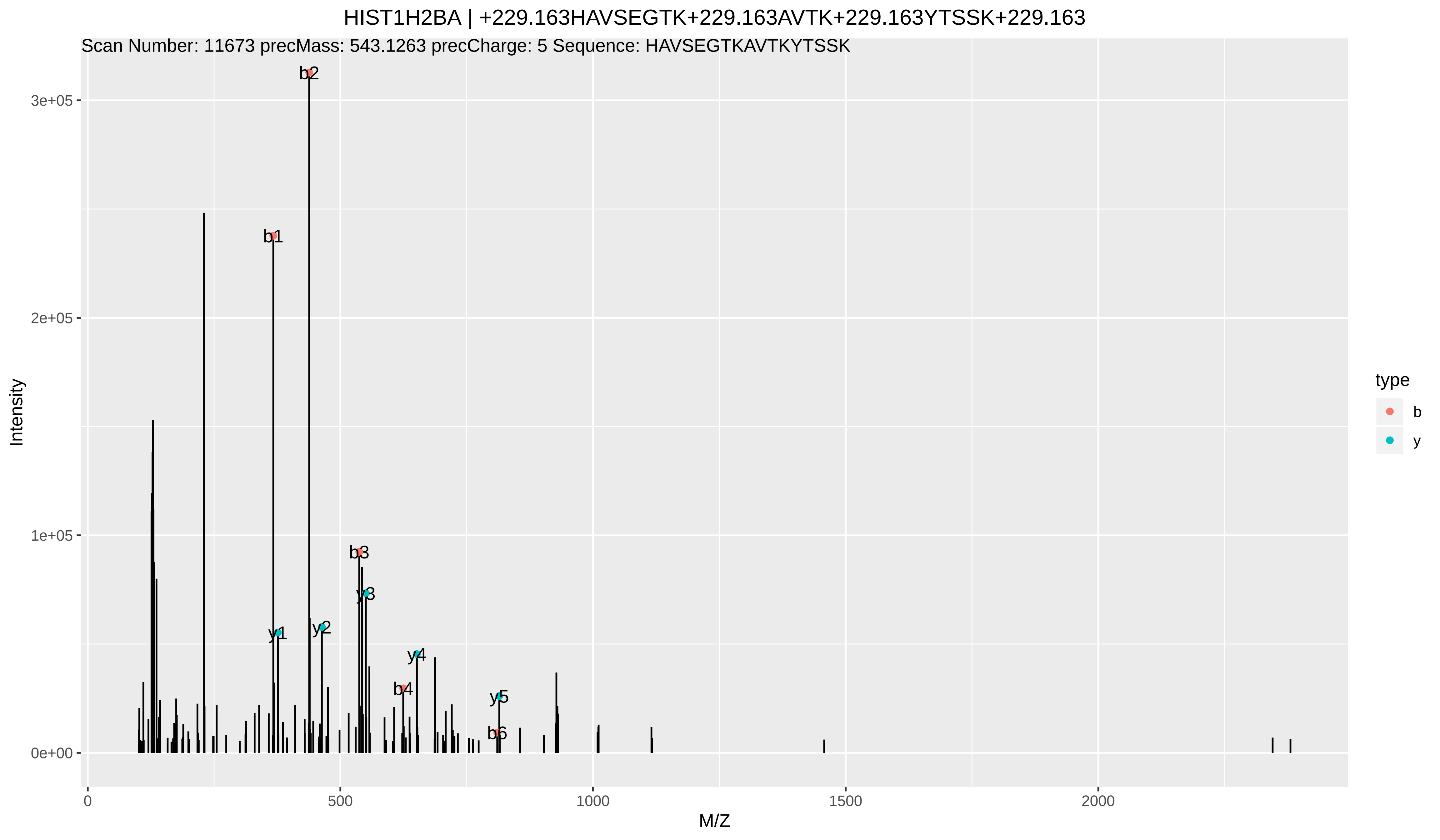
HIST1H2BA | +229.163HAVSEGTK+229.163AVTK+229.163YTSSK+229.163
Scan Number: 11673 precMass: 543.1263 precCharge: 5 Sequence: HAVSEGTKAVTKYTSSK
Intensity
M/Z
type
b
y
b1
b2
b3
b4
b6
y1
y2
y3
y4
y5
3e+05
2e+05
1e+05
0e+00
0
500
1000
1500
2000

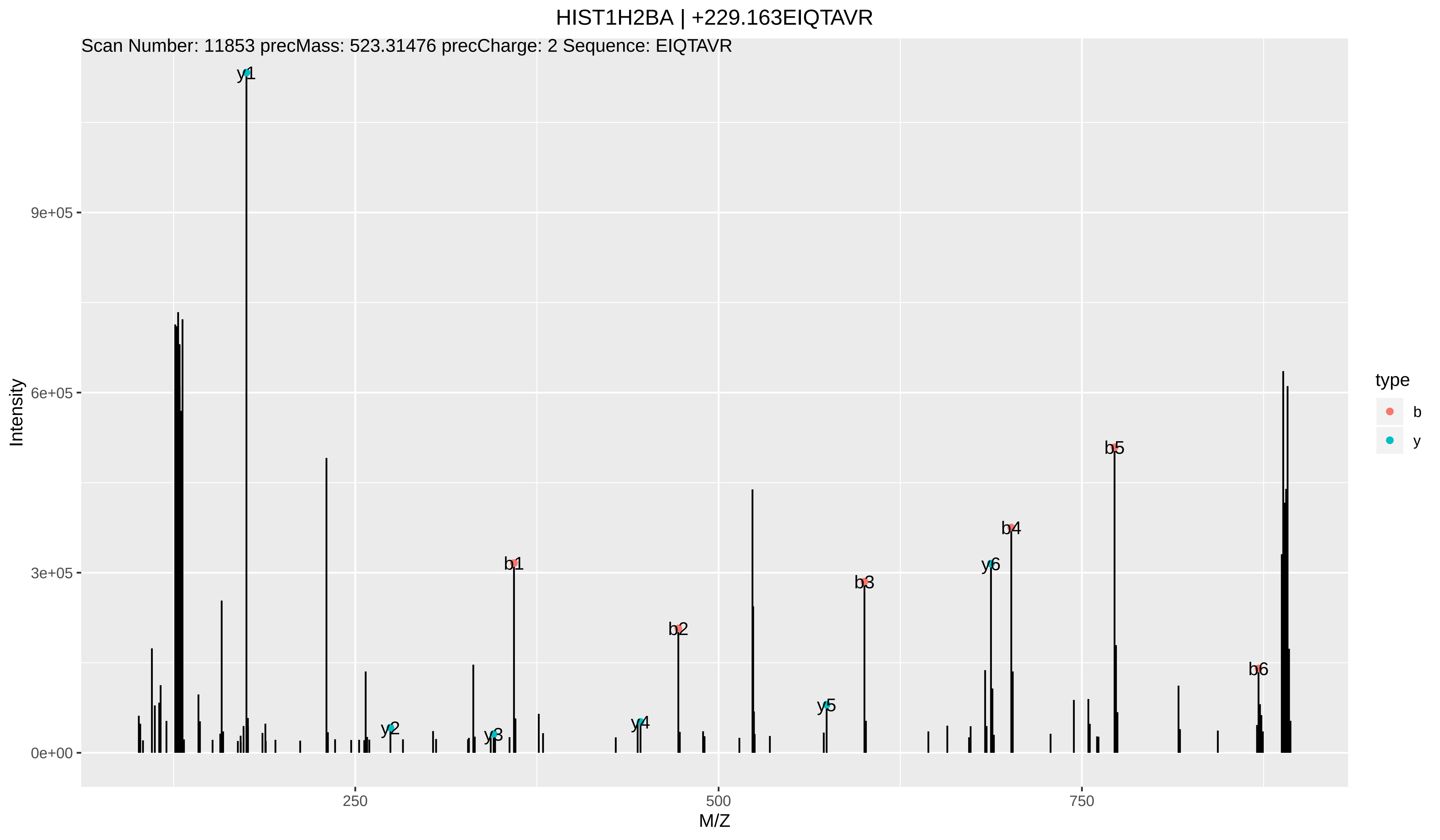

HIST1H2BA | +229.163EIQTAVR
Scan Number: 11853 precMass: 523.31476 precCharge: 2 Sequence: EIQTAVR
y1
b5
b4
b1
y6
b3
b2
b6
y5
y4
y2
y3
Intensity
9e+05
6e+05
3e+05
0e+00
250
500
750
M/Z
type
b
y

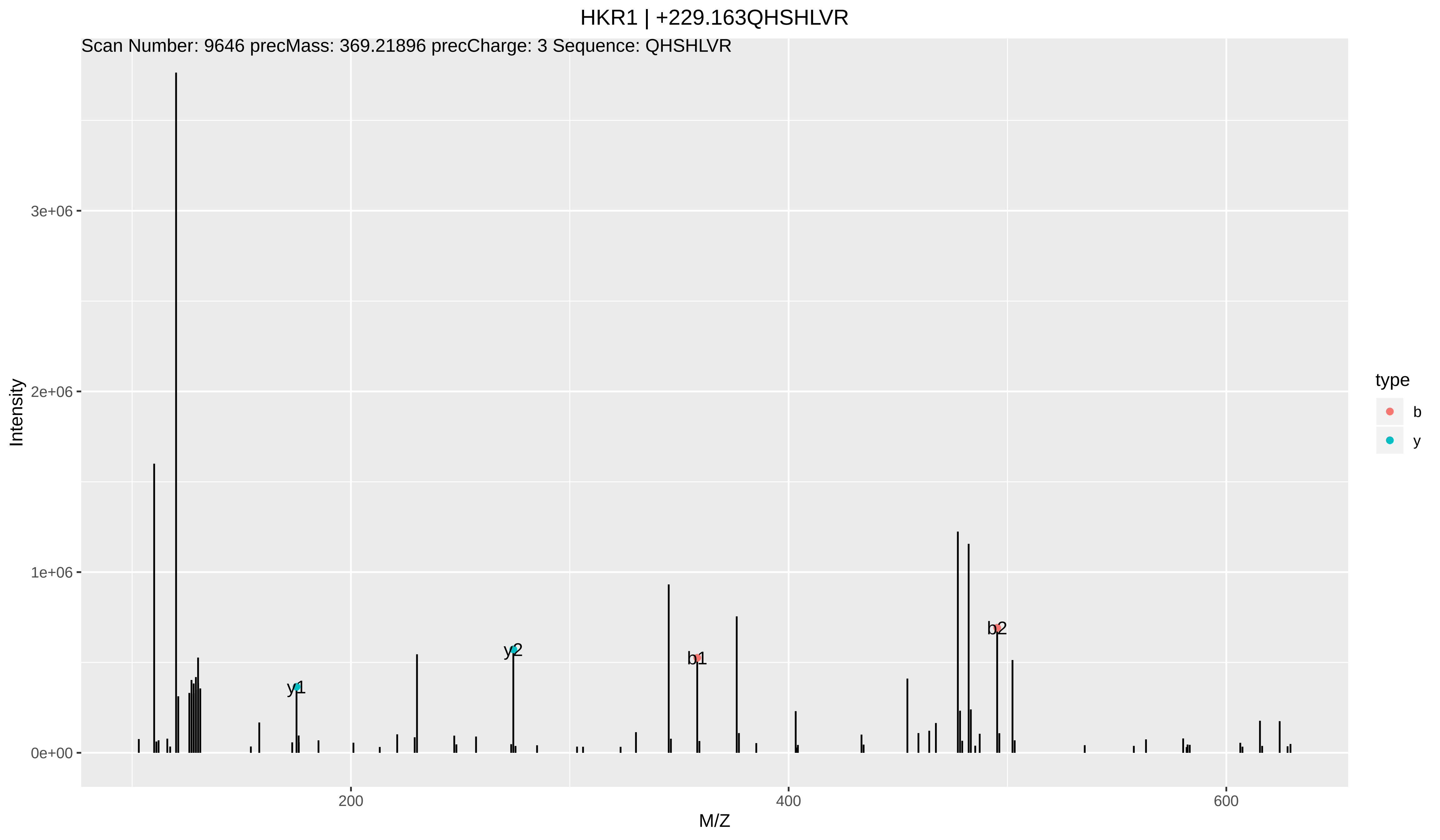

HKR1 | +229.163QHSHLVR
Scan Number: 9646 precMass: 369.21896 precCharge: 3 Sequence: QHSHLVR
Intensity
3e+06
2e+06
1e+06
0e+00
y1
y2
b1
b2
200
400
600
M/Z
type
b
y

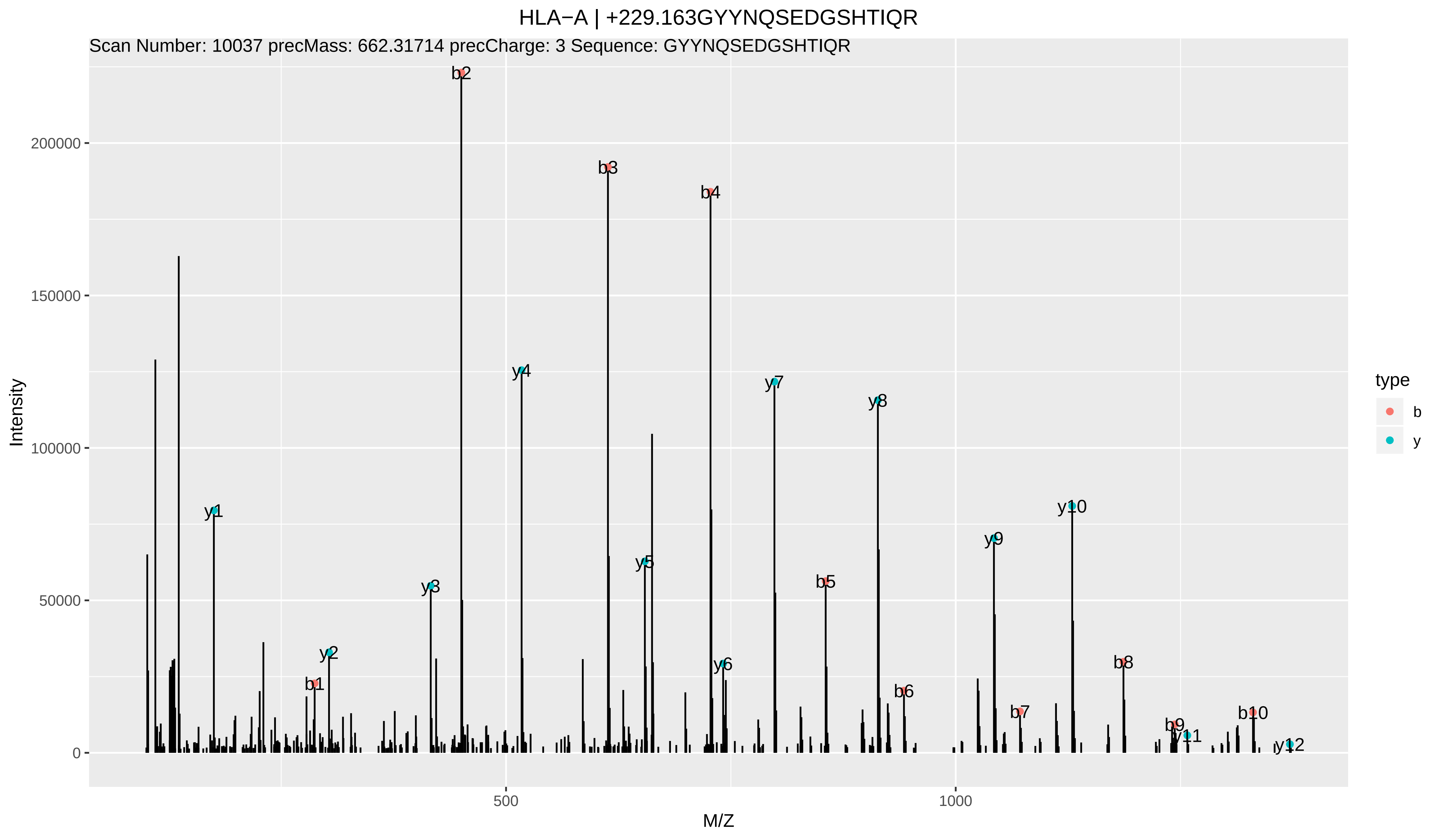

HLA−A | +229.163GYYNQSEDGSHTIQR
Scan Number: 10037 precMass: 662.31714 precCharge: 3 Sequence: GYYNQSEDGSHTIQR
Intensity
M/Z
0
50000
100000
150000
200000
500
1000
b2
b3
b4
y4
y7
y8
y10
y1
y9
y5
b5
y3
y2
y6
b1
b6
b8
b7
b9
y11
b10
y12
type
b
y

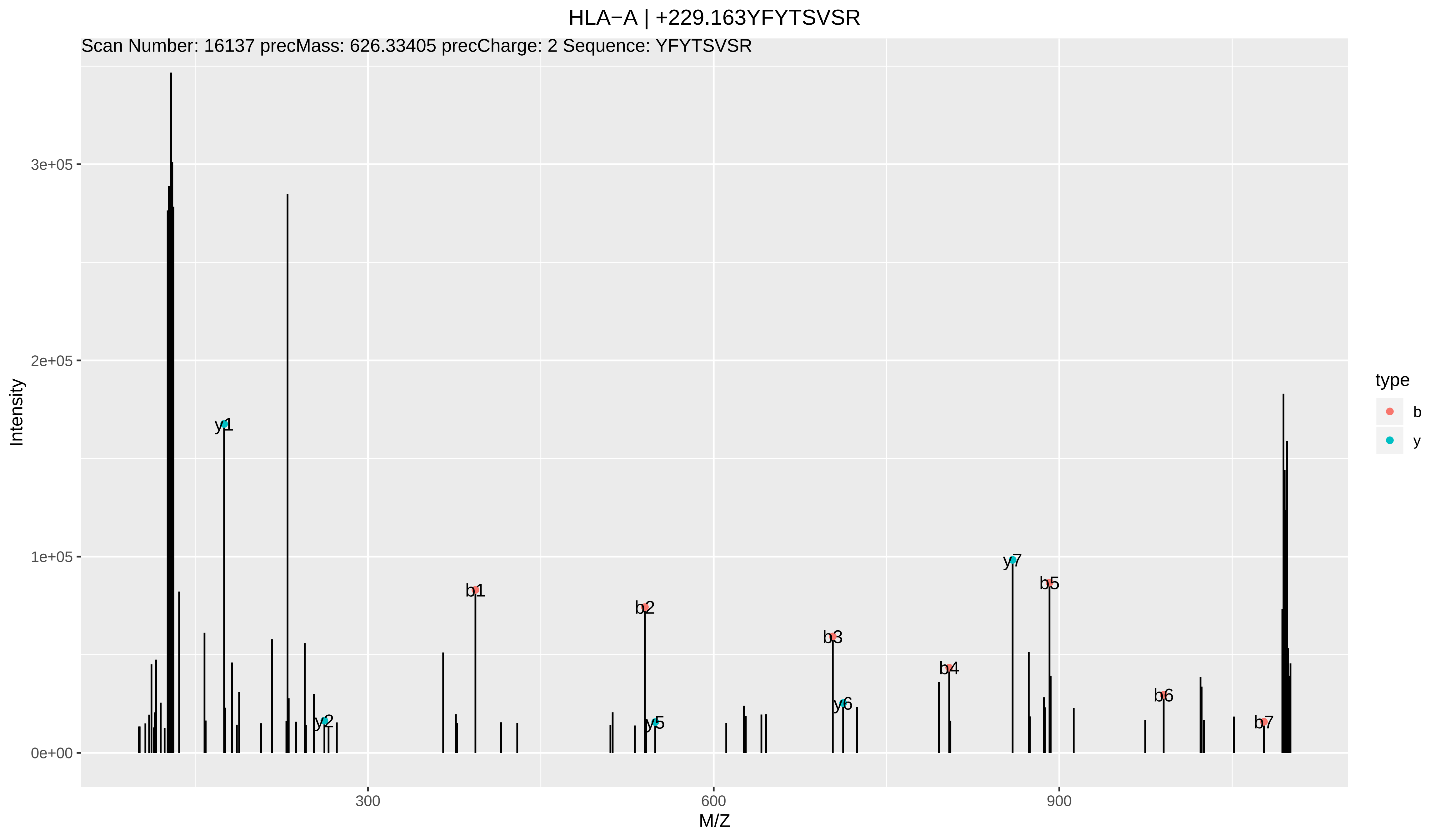
HLA−A | +229.163YFYTSVSR
Scan Number: 16137 precMass: 626.33405 precCharge: 2 Sequence: YFYTSVSR
Intensity
3e+05
2e+05
1e+05
0e+00
M/Z
300
600
900
y1
y2
b1
b2
y5
b3
y6
b4
y7
b5
b6
b7
type
b
y

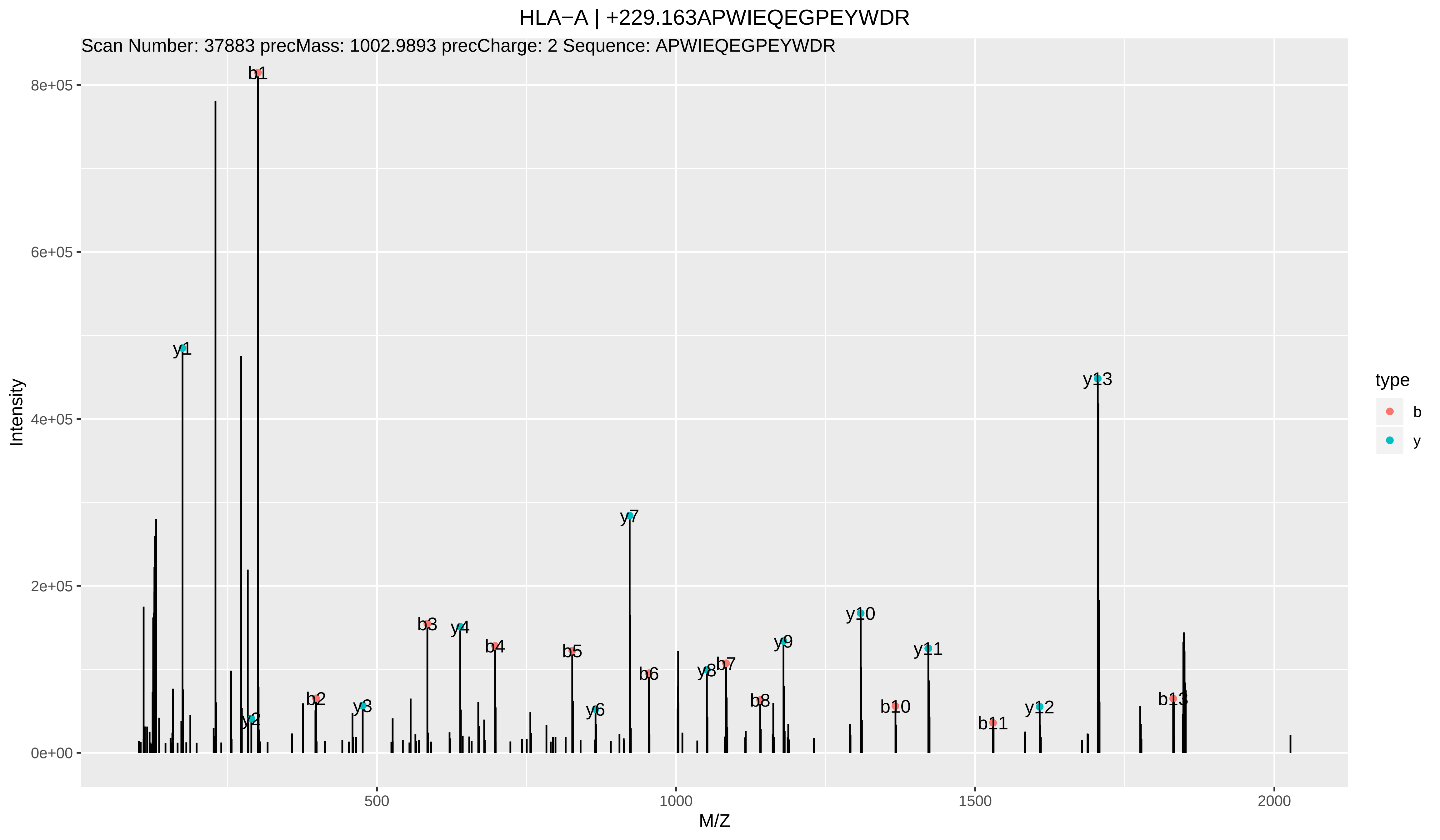

HLA−A | +229.163APWIEQEGPEYWDR
Scan Number: 37883 precMass: 1002.9893 precCharge: 2 Sequence: APWIEQEGPEYWDR
Intensity
8e+05
6e+05
4e+05
2e+05
0e+00
M/Z
500
1000
1500
2000
type
b
y
b1
y1
y2
b2
y3
b3
y4
b4
b5
y6
y7
b6
y8
b7
b8
y9
y10
b10
y11
b11
y12
y13
b13

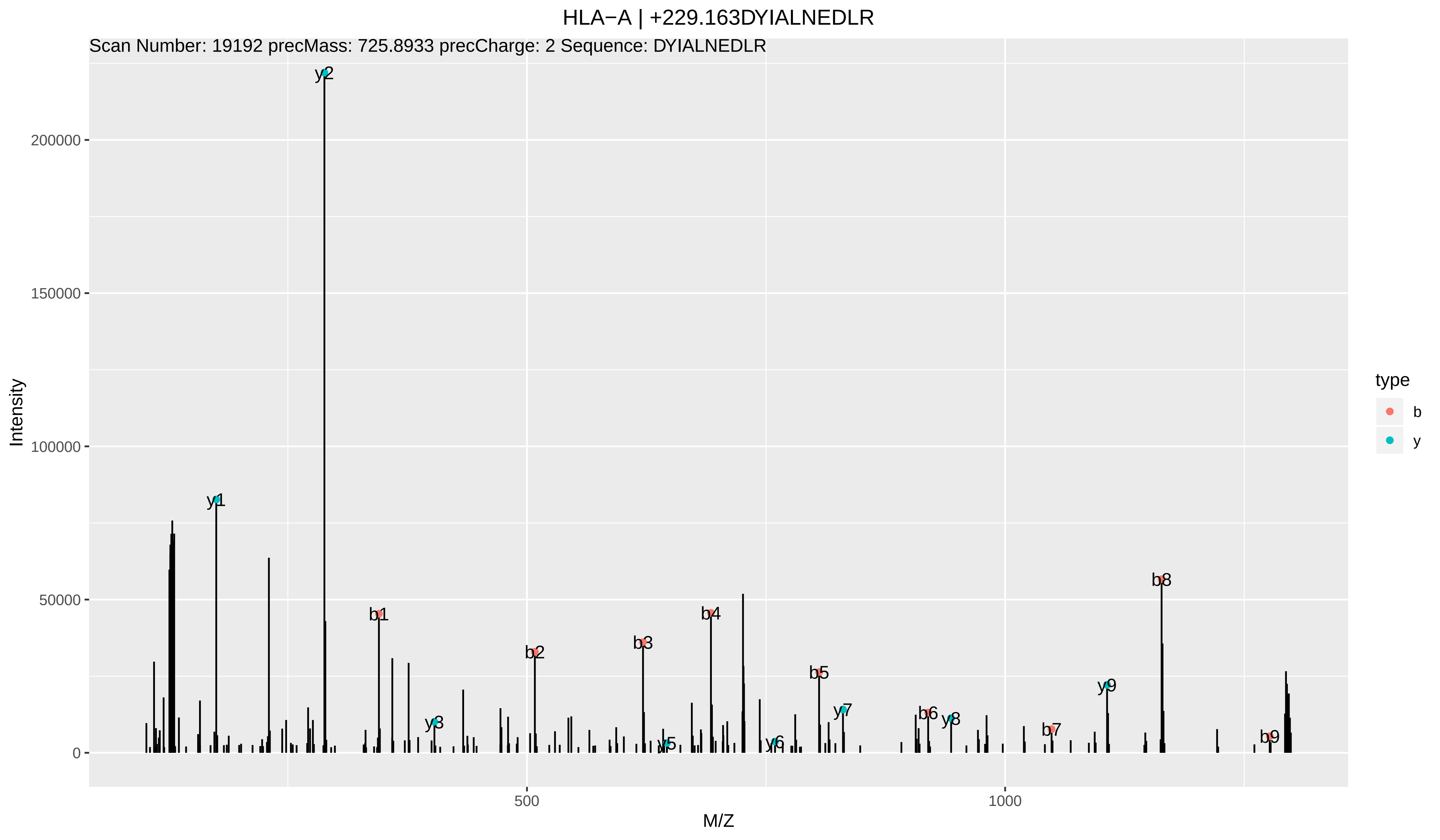

HLA−A | +229.163DYIALNEDLR
Scan Number: 19192 precMass: 725.8933 precCharge: 2 Sequence: DYIALNEDLR
Intensity
M/Z
200000
150000
100000
50000
0
500
1000
y1
y2
y3
y5
y6
y7
y8
y9
b1
b2
b3
b4
b5
b6
b7
b8
b9
type
b
y

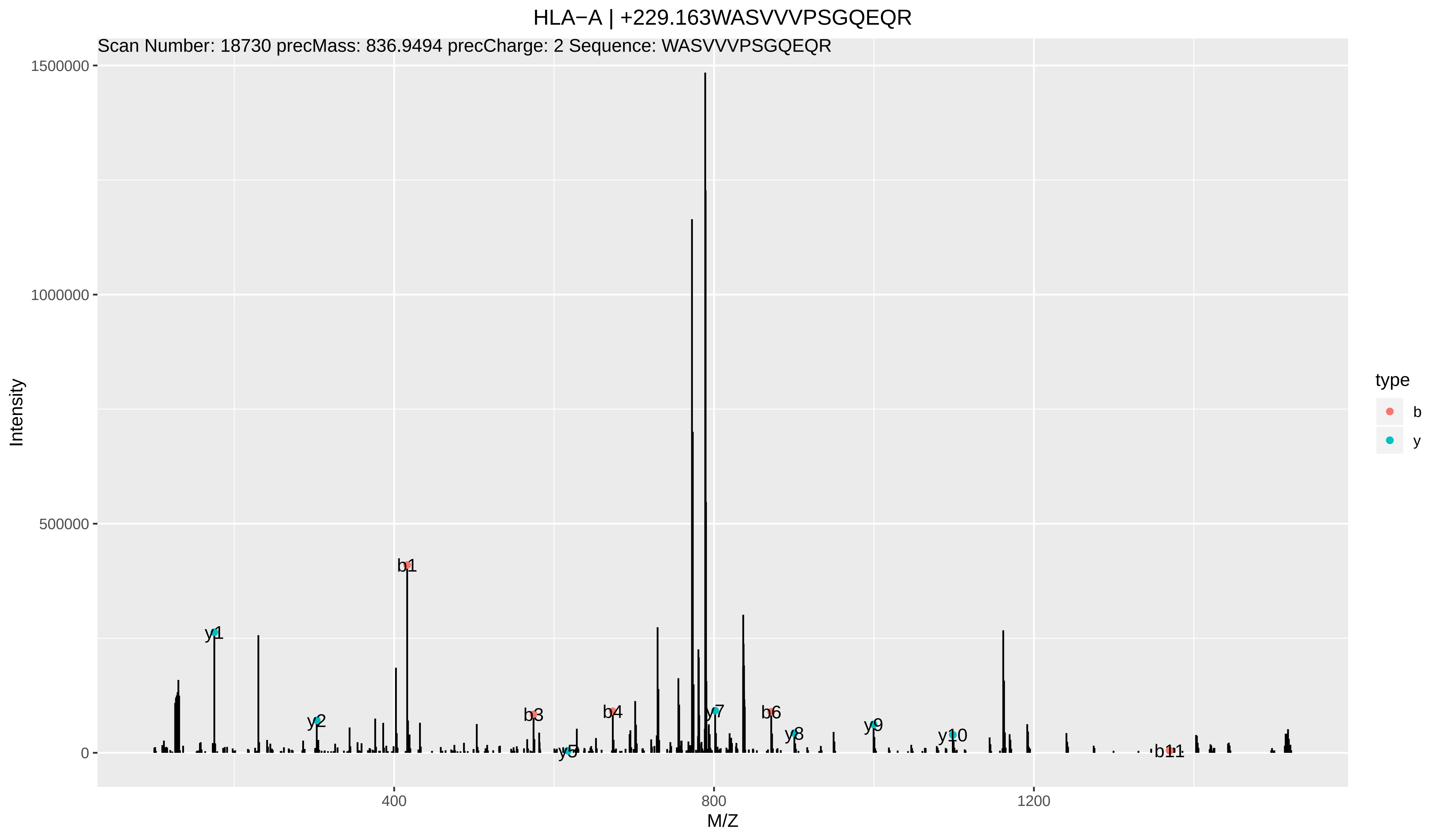

HLA−A | +229.163WASVVVPSGQEQR
Scan Number: 18730 precMass: 836.9494 precCharge: 2 Sequence: WASVVVPSGQEQR
Intensity
1500000
1000000
500000
0
M/Z
400
800
1200
y1
y2
b1
b3
y5
b4
y7
b6
y8
y9
y10
b11
type
b
y

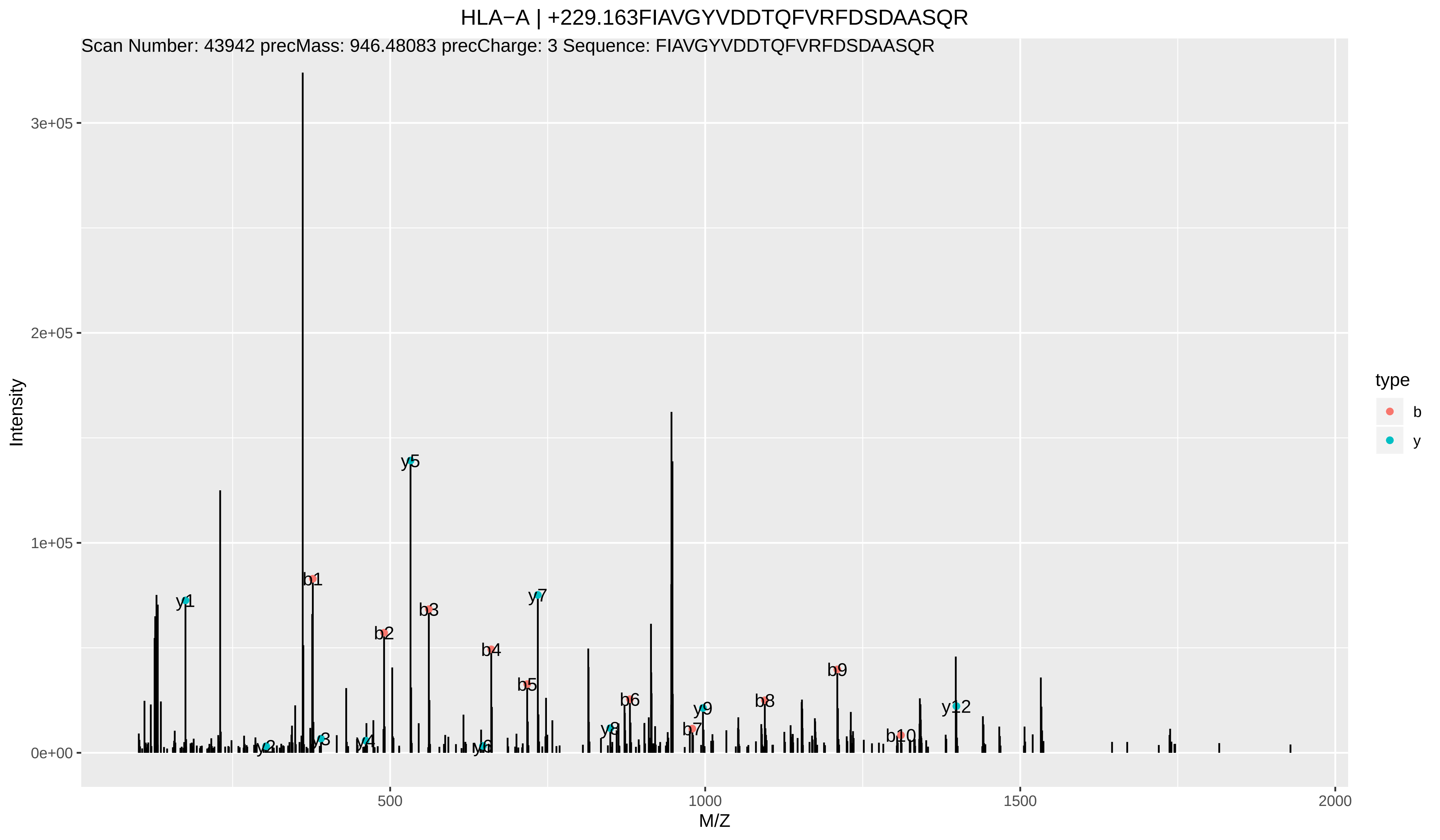

HLA−A | +229.163FIAVGYVDDTQFVRFDSDAASQR
Scan Number: 43942 precMass: 946.48083 precCharge: 3 Sequence: FIAVGYVDDTQFVRFDSDAASQR
Intensity
3e+05
2e+05
1e+05
0e+00
M/Z
500
1000
1500
2000
type
b
y
y1
y2
y3
y4
y5
y6
y7
y8
y9
y12
b1
b2
b3
b4
b5
b6
b7
b8
b9
b10

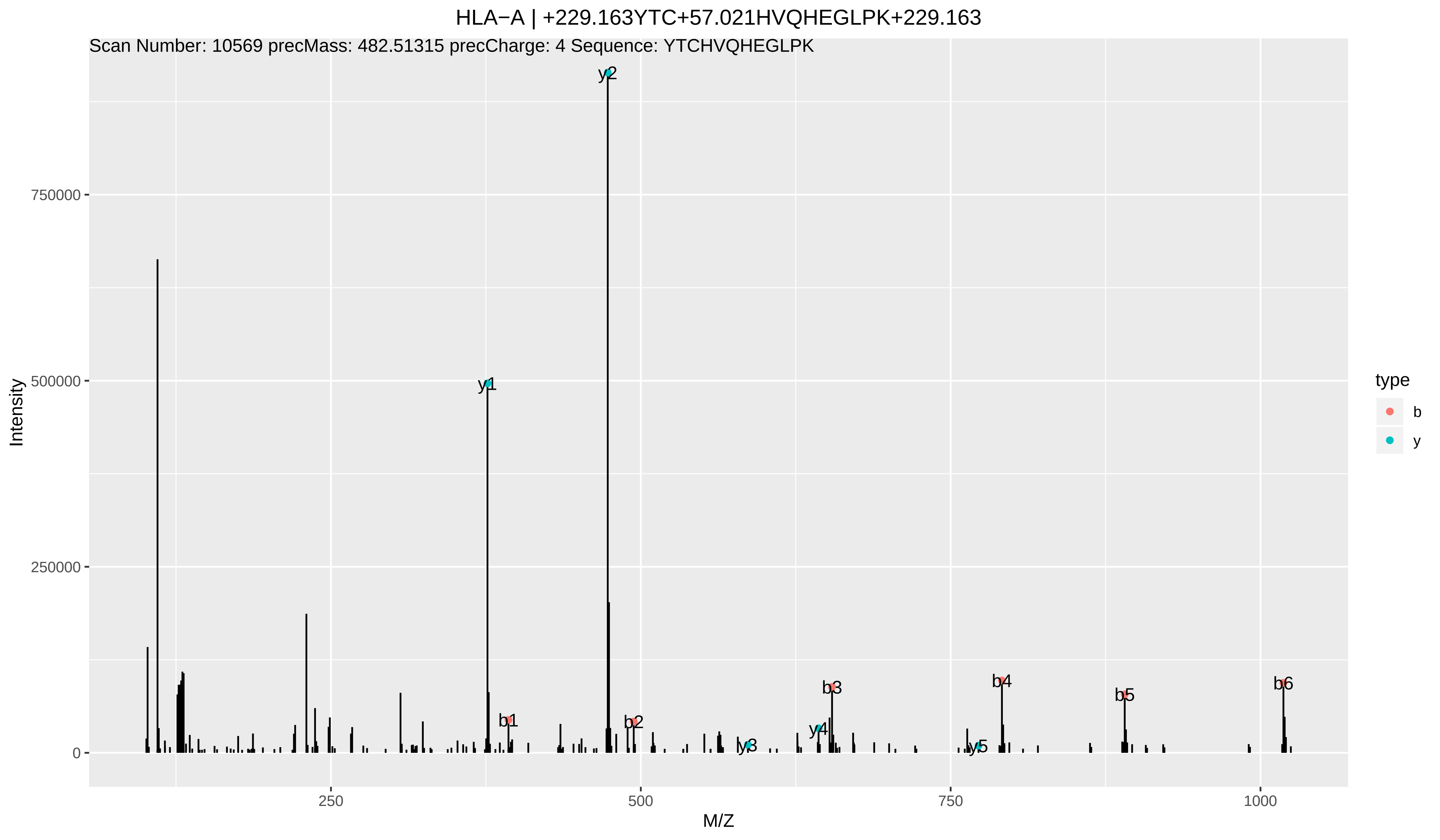

HLA−A | +229.163YTC+57.021HVQHEGLPK+229.163
Scan Number: 10569 precMass: 482.51315 precCharge: 4 Sequence: YTCHVQHEGLPK
Intensity
750000
500000
250000
0
M/Z
250
500
750
1000
y1
y2
y3
y4
y5
b1
b2
b3
b4
b5
b6
type
b
y

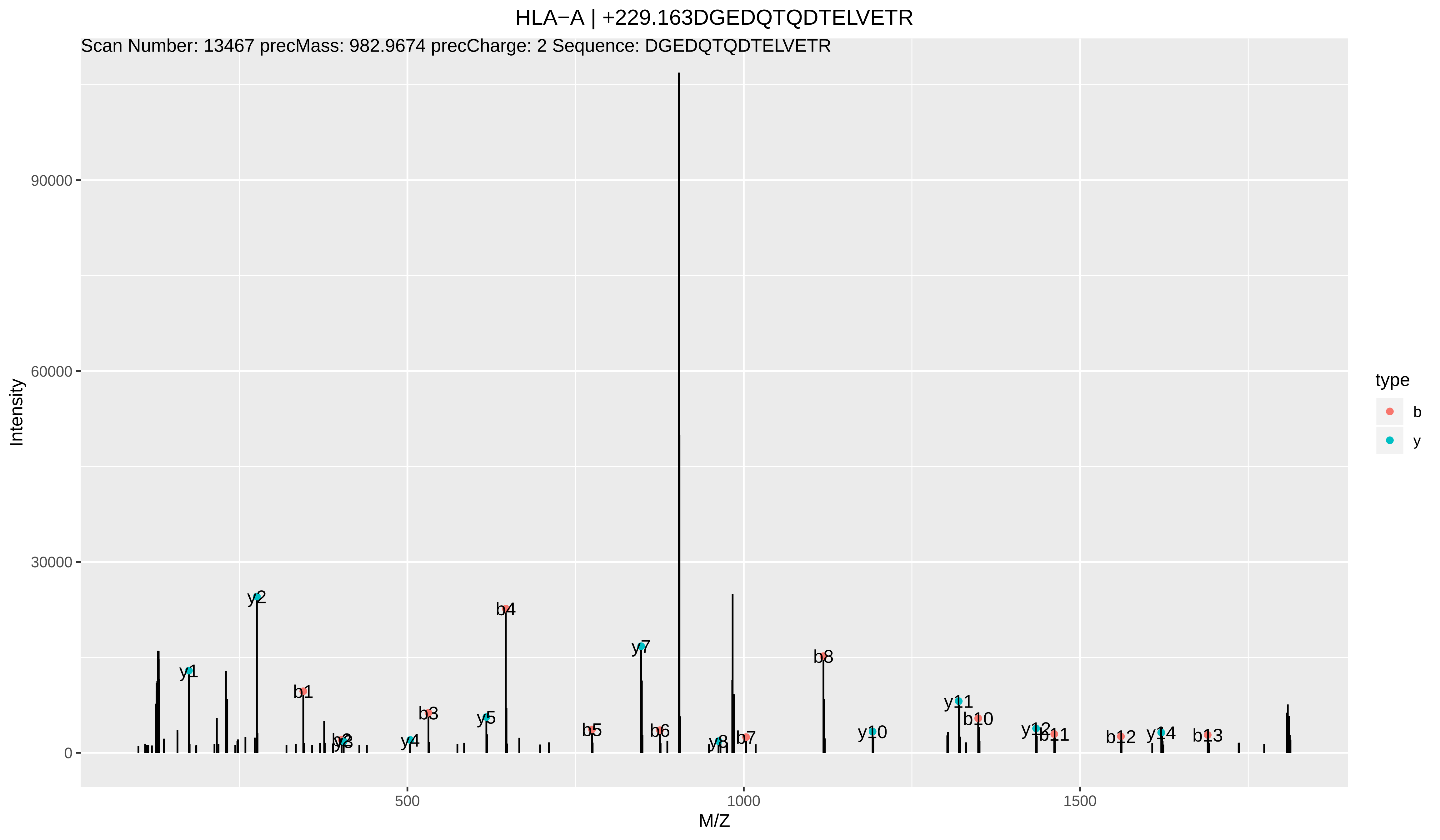
HLA−A | +229.163DGEDQTQDTELVETR
Scan Number: 13467 precMass: 982.9674 precCharge: 2 Sequence: DGEDQTQDTELVETR
Intensity
M/Z
90000
60000
30000
0
500
1000
1500
type
b
y
y1
y2
b1
y4
b3
y5
b4
b5
y7
b6
y8
b7
b8
y10
y11
b10
y12
b11
b12
y14
b13

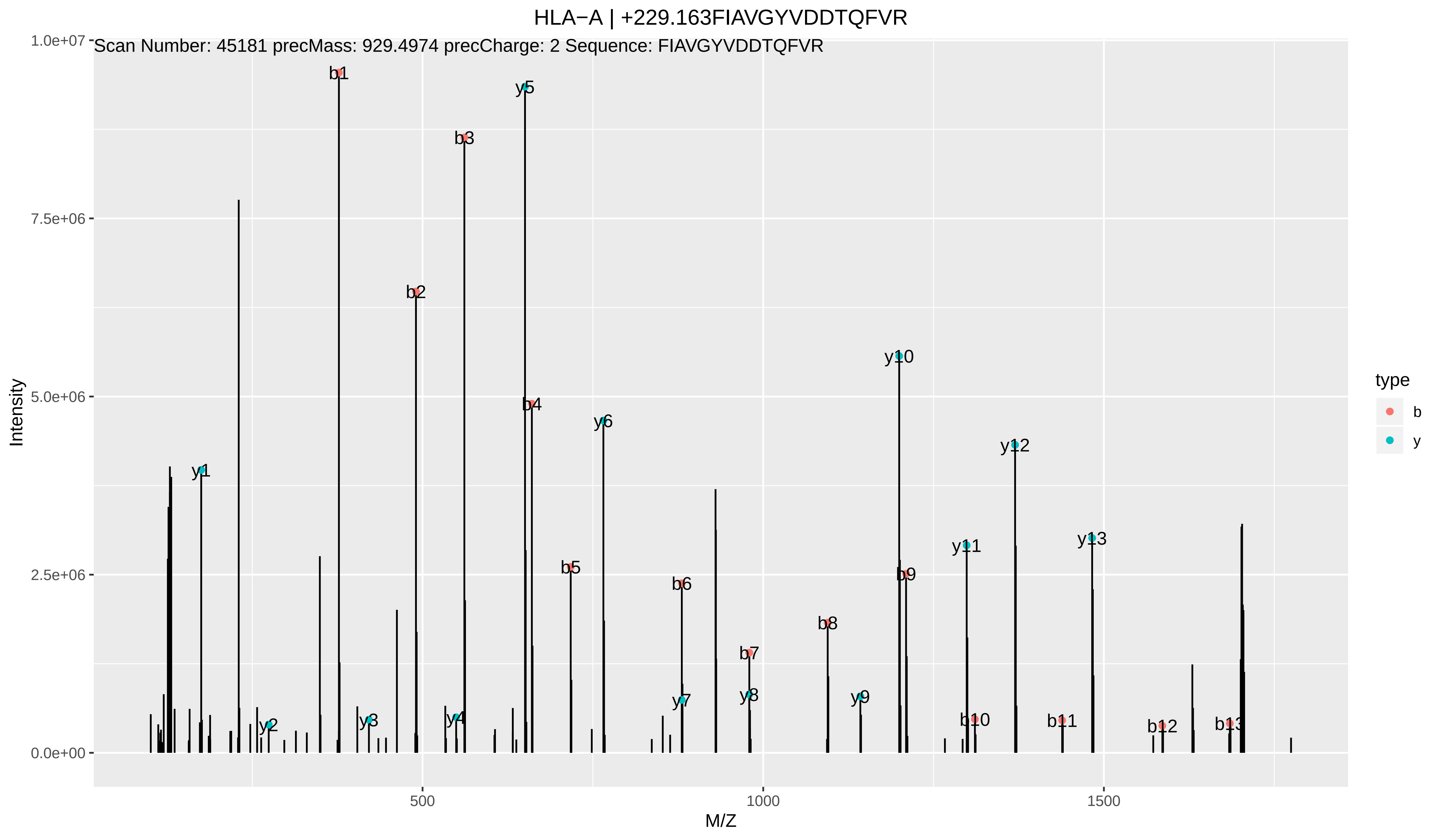

HLA−A | +229.163FIAVGYVDDTQFVR
Scan Number: 45181 precMass: 929.4974 precCharge: 2 Sequence: FIAVGYVDDTQFVR
Intensity
M/Z
0.0e+00
2.5e+06
5.0e+06
7.5e+06
1.0e+07
500
1000
1500
type
b
y
b1
b2
b3
b4
b5
b6
b7
b8
b9
b10
b11
b12
b13
y1
y2
y3
y4
y5
y6
y7
y8
y9
y10
y11
y12
y13

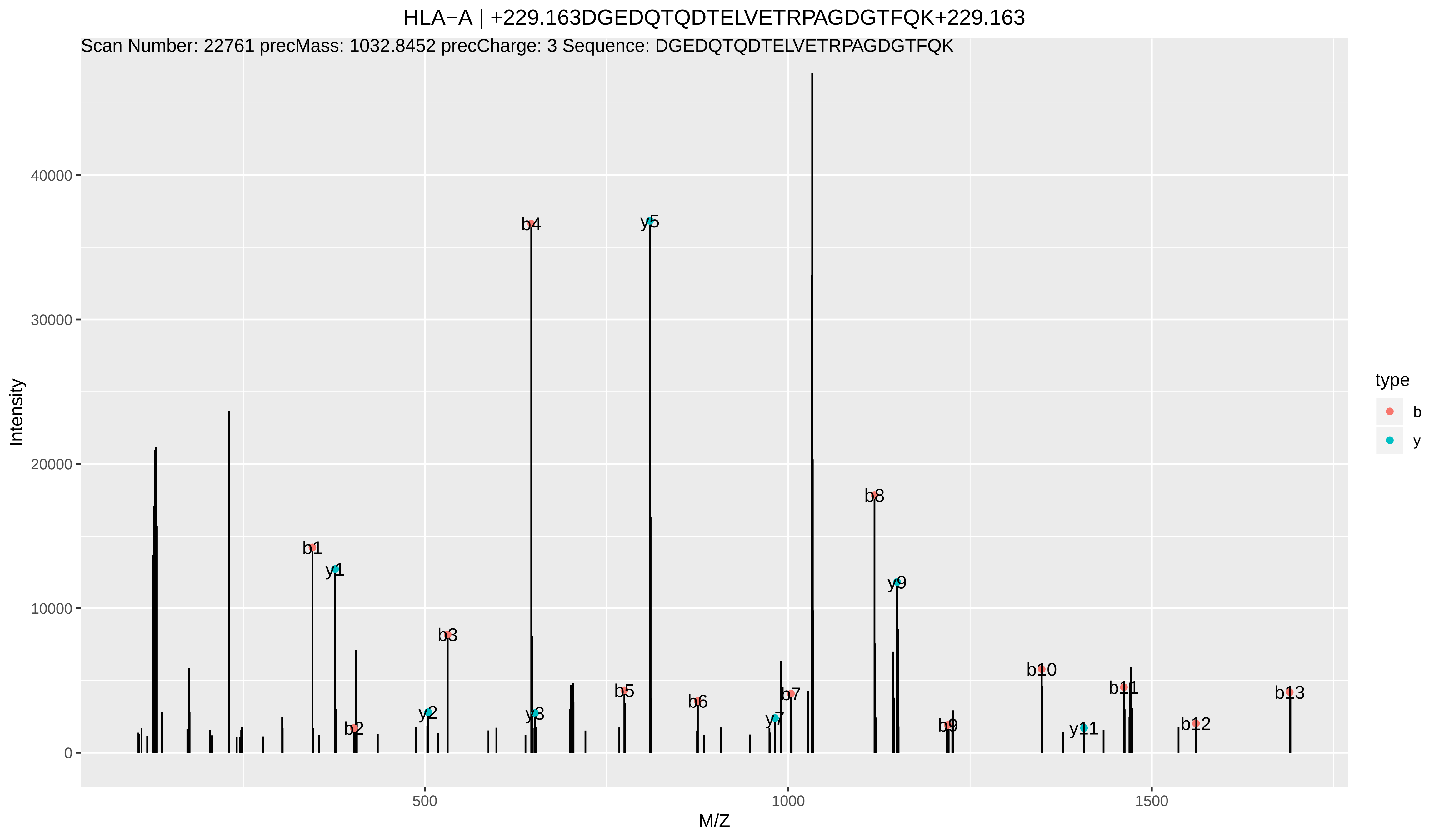

HLA−A | +229.163DGEDQTQDTELVETRPAGDGTFQK+229.163
Scan Number: 22761 precMass: 1032.8452 precCharge: 3 Sequence: DGEDQTQDTELVETRPAGDGTFQK
Intensity
M/Z
0
10000
20000
30000
40000
500
1000
1500
type
b
y
b1
y1
b2
y2
b3
b4
y3
b5
y5
b6
y7
b7
b8
y9
b9
b10
y11
b11
b12
b13

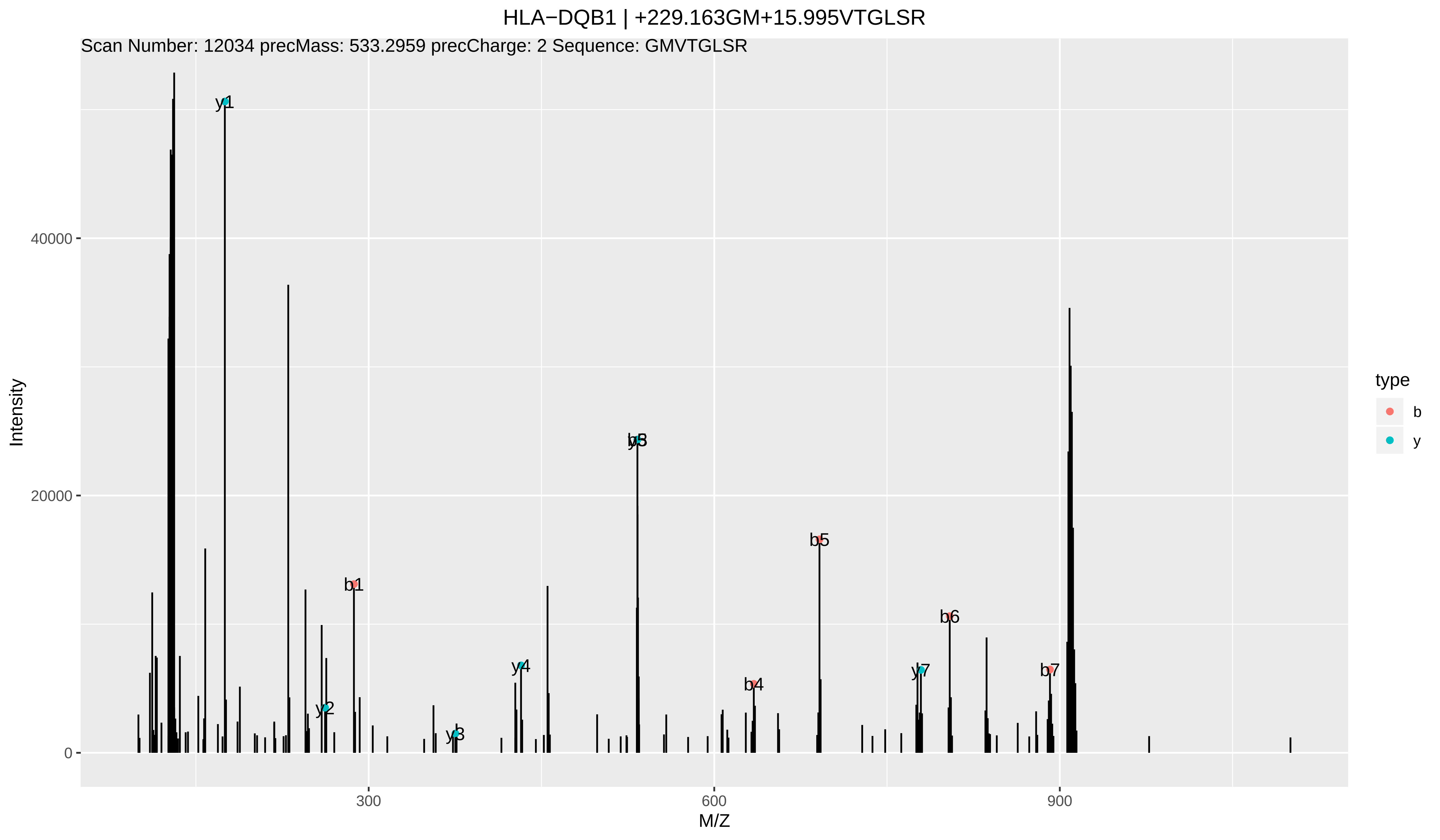
HLA−DQB1 | +229.163GM+15.995VTGLSR
Scan Number: 12034 precMass: 533.2959 precCharge: 2 Sequence: GMVTGLSR
Intensity
0
20000
40000
M/Z
300
600
900
y1
y2
y3
y4
b1
b3
y5
b4
b5
b6
y7
b7
type
b
y

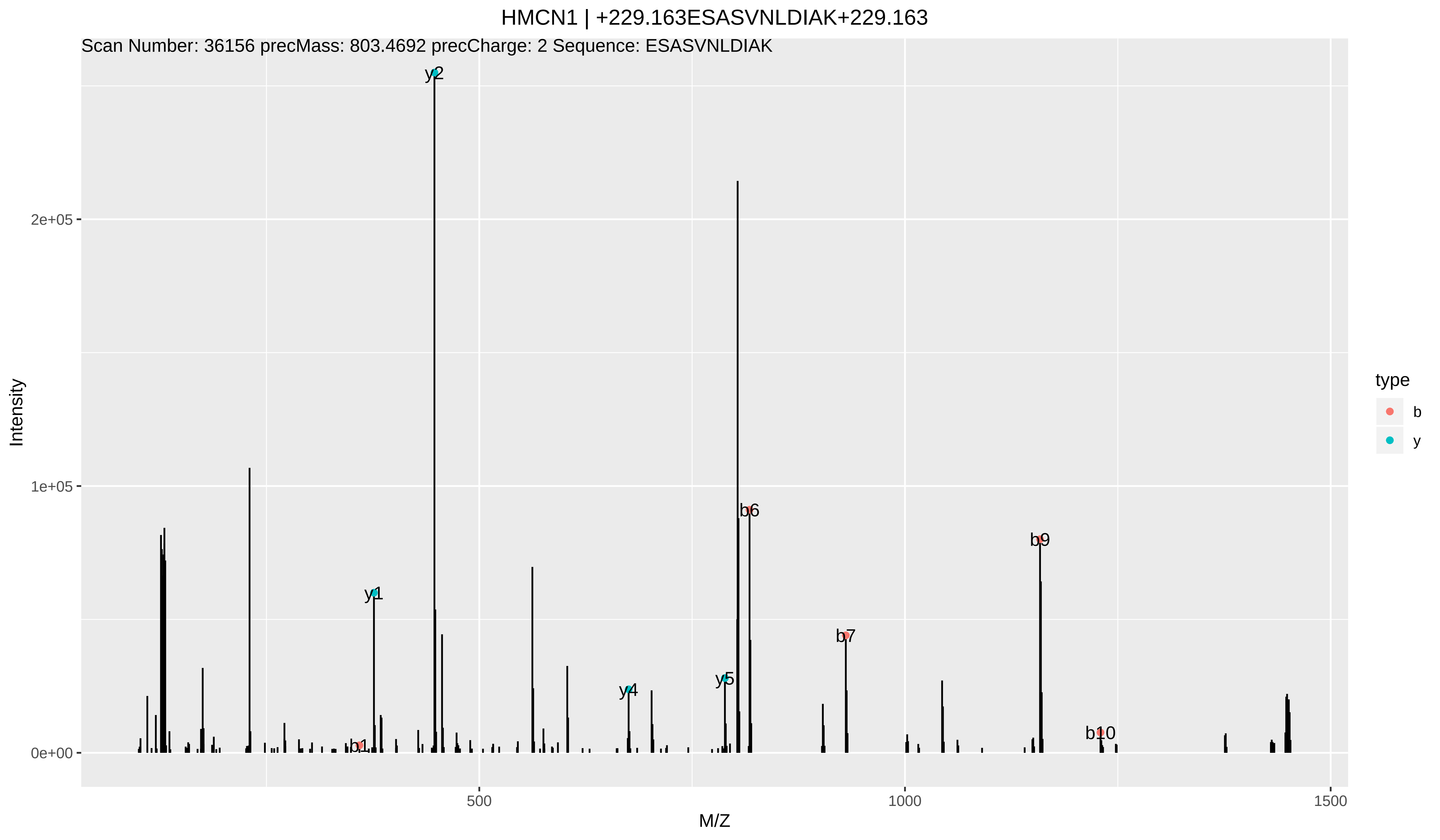

HMCN1 | +229.163ESASVNLDIAK+229.163
Scan Number: 36156 precMass: 803.4692 precCharge: 2 Sequence: ESASVNLDIAK
y2
b6
b9
y1
b7
y5
y4
b10
b1
Intensity
2e+05
1e+05
0e+00
500
1000
1500
M/Z
type
b
y

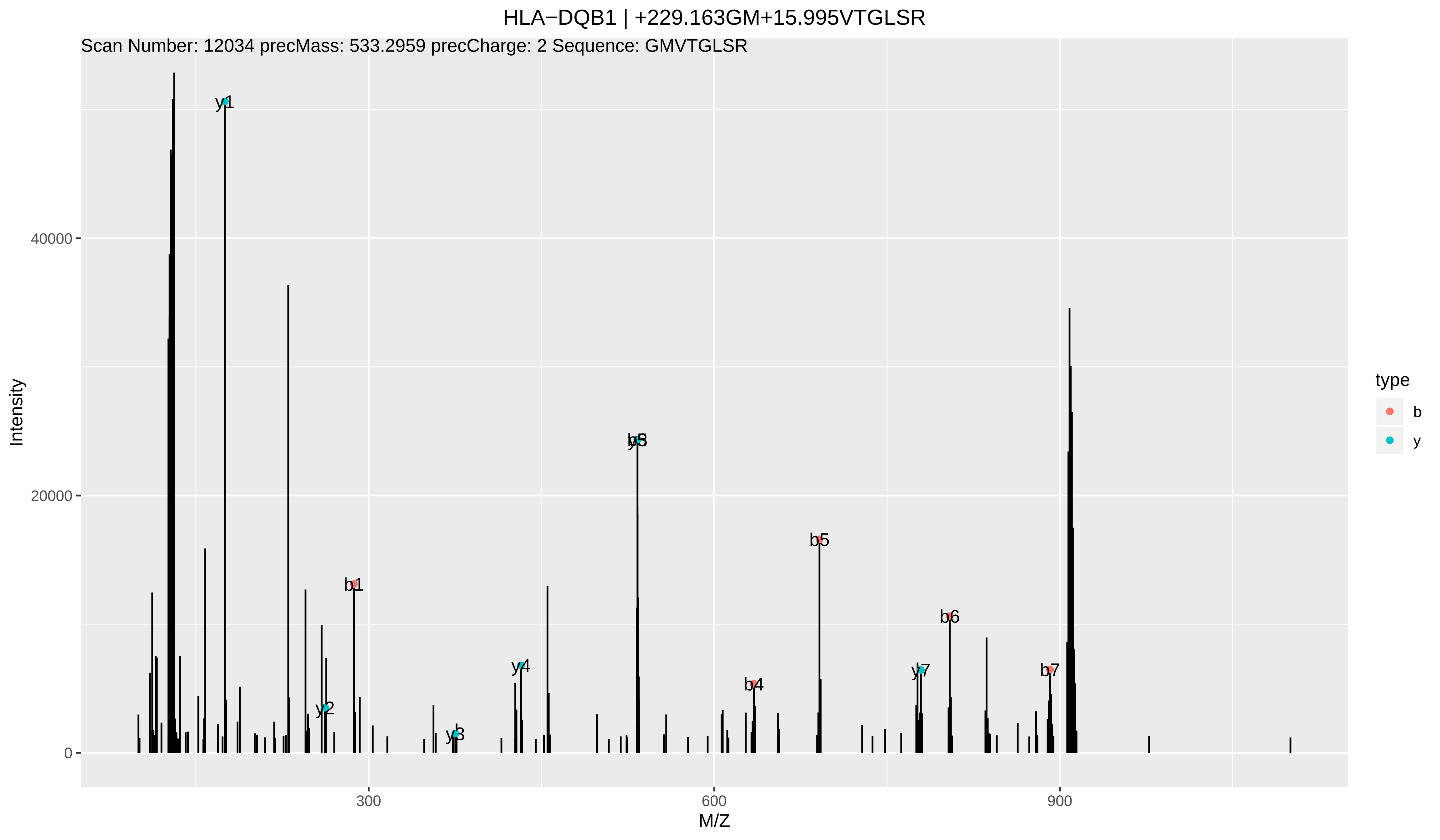
HLA−DQB1 | +229.163GM+15.995VTGLSR
Scan Number: 12034 precMass: 533.2959 precCharge: 2 Sequence: GMVTGLSR
Intensity
M/Z
0
20000
40000
300
600
900
y1
y2
y3
y4
b1
b4
b5
b6
b7
y7
type
b
y

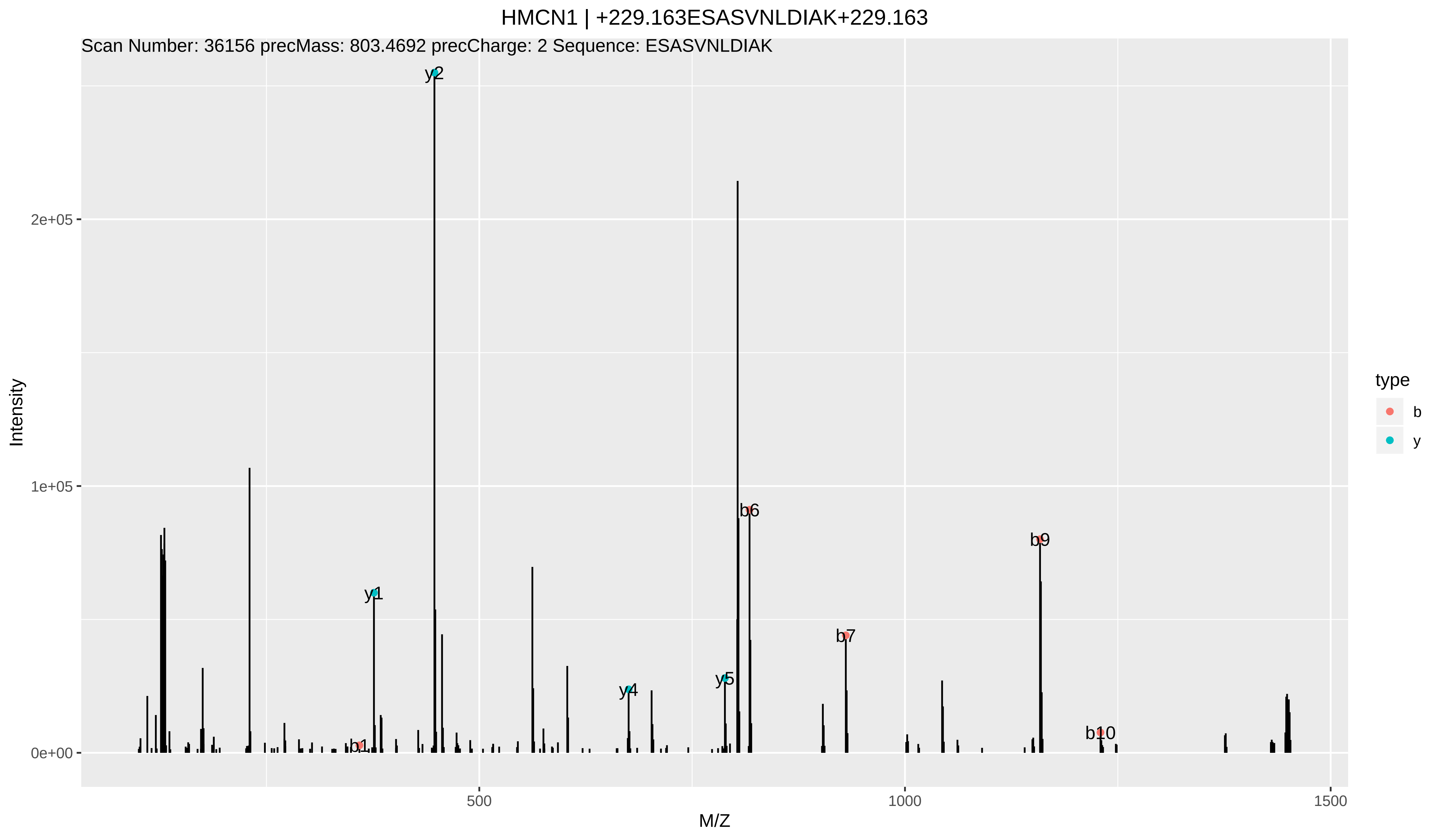

HMCN1 | +229.163ESASVNLDIAK+229.163
Scan Number: 36156 precMass: 803.4692 precCharge: 2 Sequence: ESASVNLDIAK
Intensity
2e+05
1e+05
0e+00
M/Z
500
1000
1500
y2
b6
b9
y1
b7
y5
y4
b10
b1
type
b
y

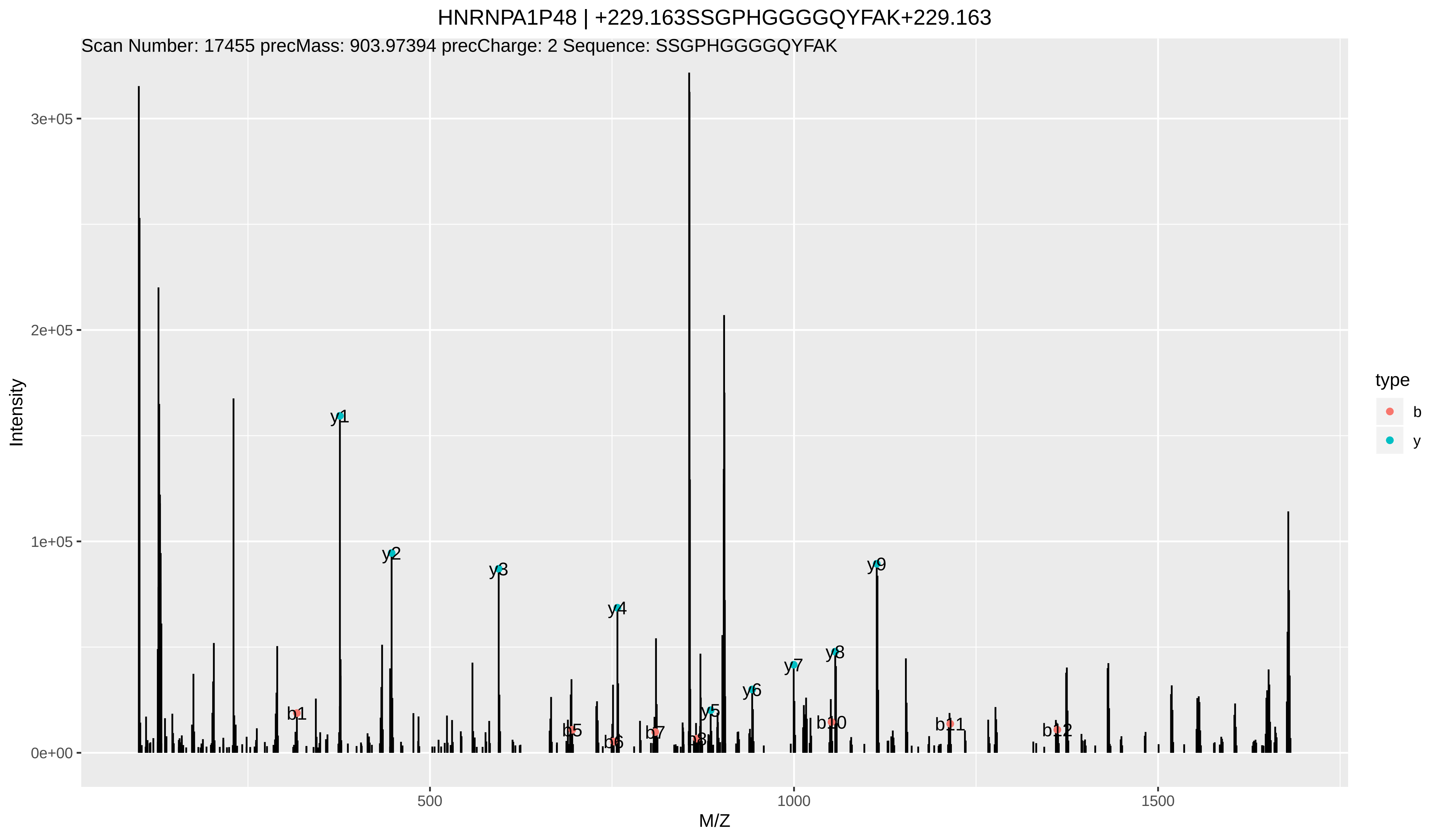

HNRNPA1P48 | +229.163SSGPHGGGGQYFAK+229.163
Scan Number: 17455 precMass: 903.97394 precCharge: 2 Sequence: SSGPHGGGGQYFAK
Intensity
3e+05
2e+05
1e+05
0e+00
M/Z
500
1000
1500
y1
y2
y3
y4
y5
y6
y7
y8
y9
b1
b5
b6
b7
b8
b10
b11
b12
type
b
y

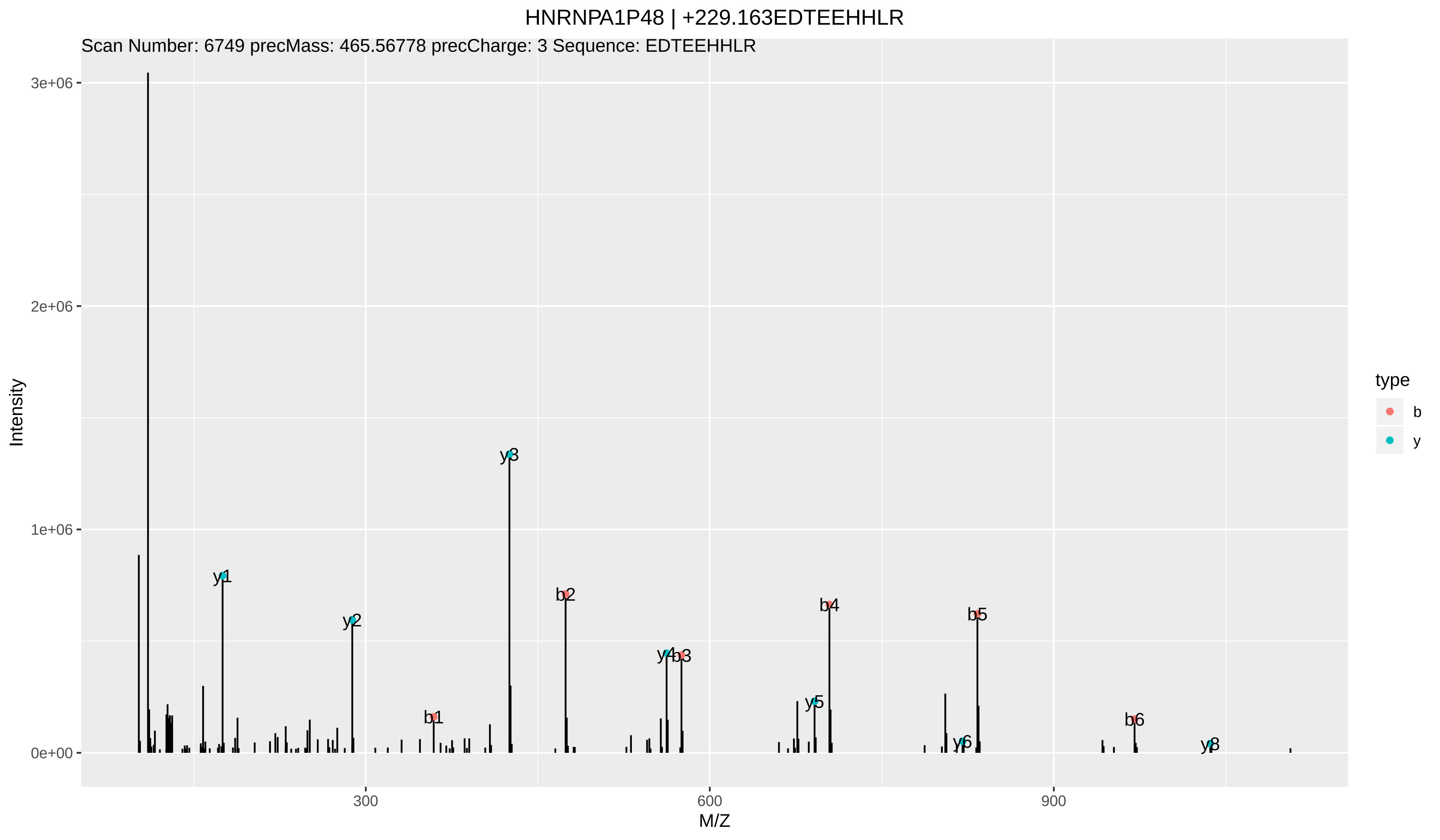
HNRNPA1P48 | +229.163EDTEEHHLR
Scan Number: 6749 precMass: 465.56778 precCharge: 3 Sequence: EDTEEHHLR
Intensity
3e+06
2e+06
1e+06
0e+00
M/Z
300
600
900
y1
y2
y3
b1
b2
y4
b3
y5
b4
b5
y6
b6
y8
type
b
y

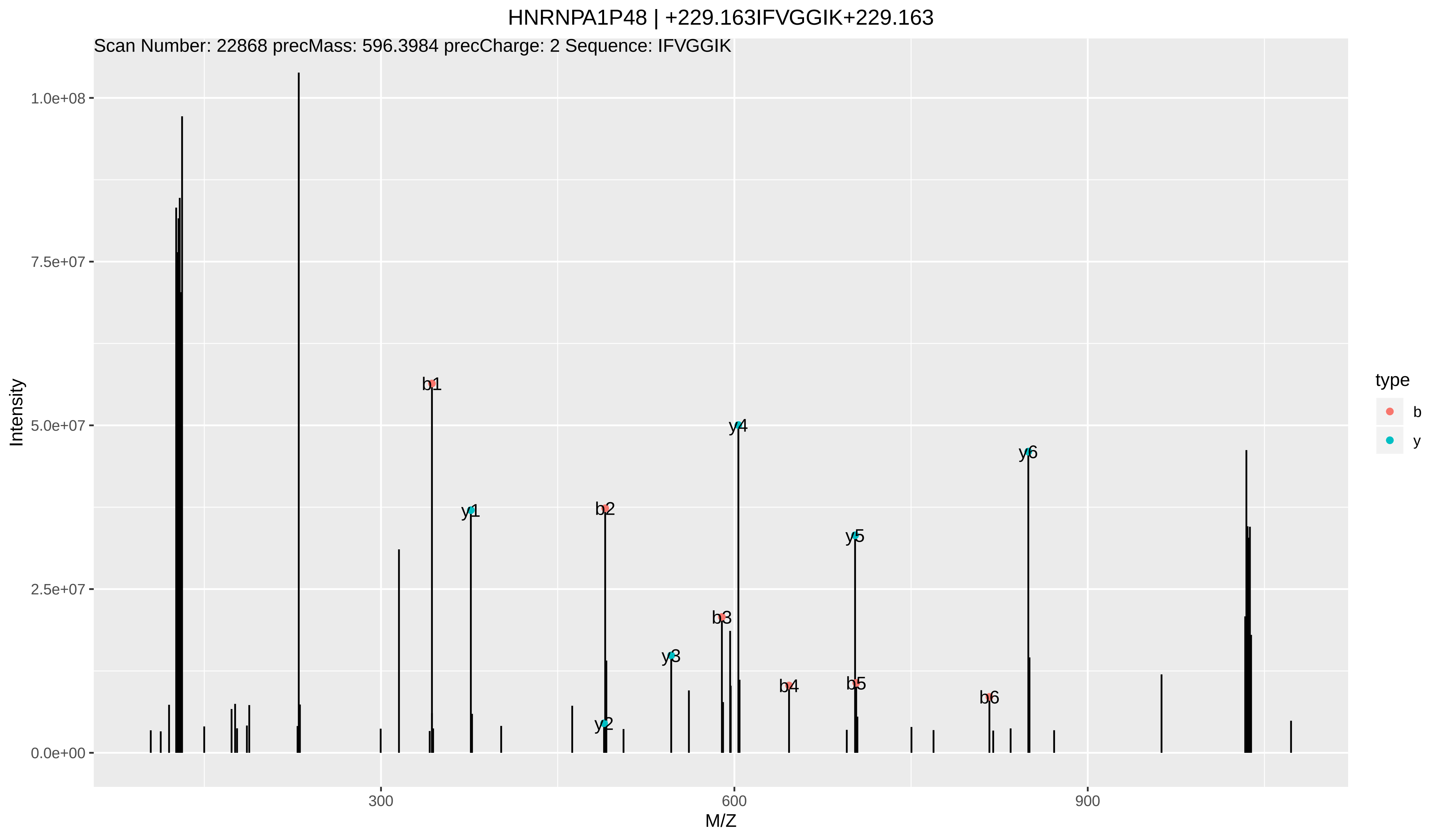

HNRNPA1P48 | +229.163IFVGGIK+229.163
Scan Number: 22868 precMass: 596.3984 precCharge: 2 Sequence: IFVGGIK
Intensity
1.0e+08
7.5e+07
5.0e+07
2.5e+07
0.0e+00
b1
y1
b2
y2
y3
b3
y4
b4
y5
b5
b6
y6
300
600
900
M/Z
type
b
y

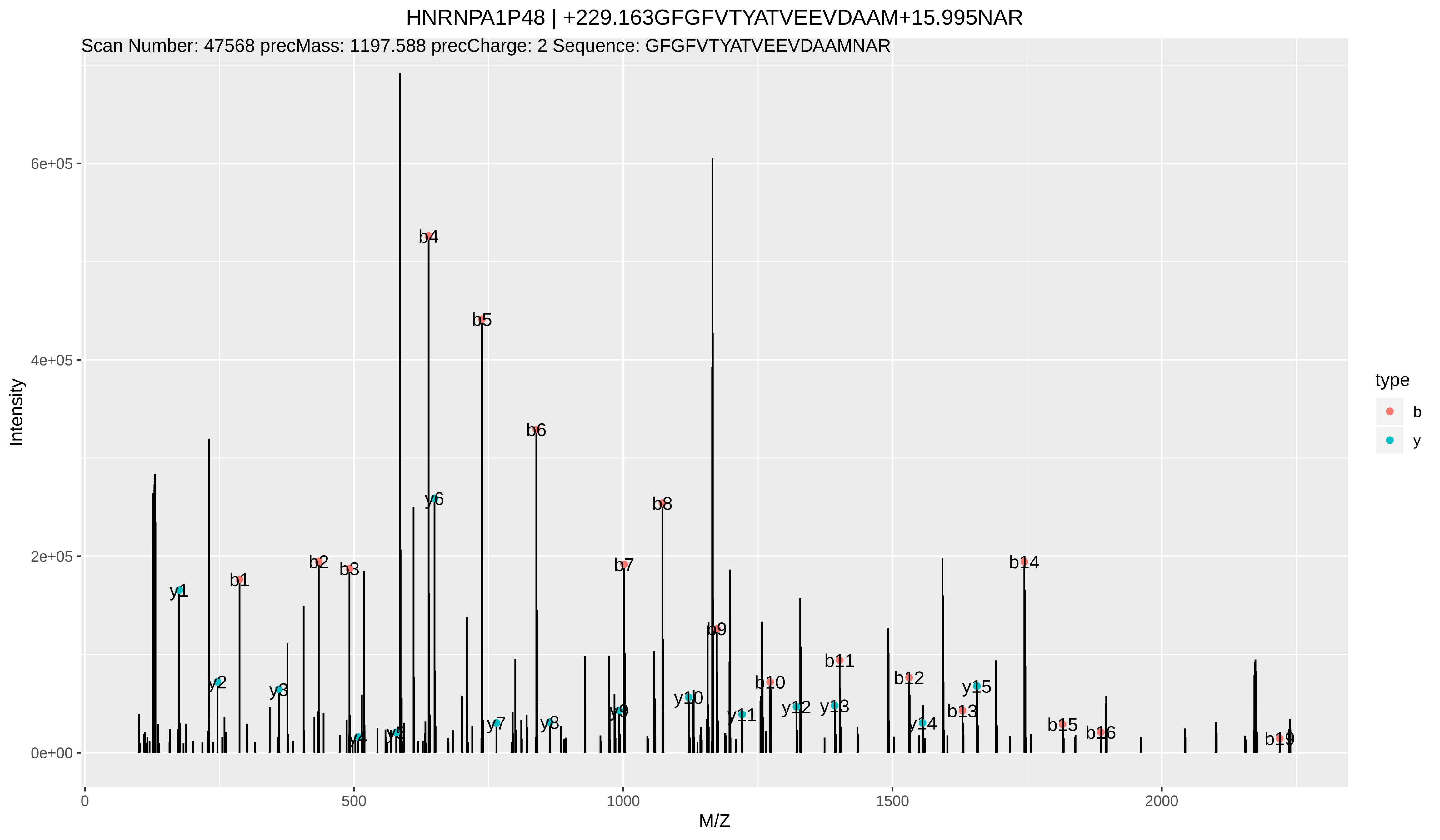

HNRNPA1P48 | +229.163GFGFVTYATVEEVDAAM+15.995NAR
Scan Number: 47568 precMass: 1197.588 precCharge: 2 Sequence: GFGFVTYATVEEVDAAMNAR
Intensity
M/Z
type
b
y
b1
b2
b3
b4
b5
b6
b7
b8
b9
b10
b11
b12
b13
b14
b15
b16
b19
y1
y2
y3
y4
y5
y6
y7
y8
y9
y10
y11
y12
y13
y14
y15
0e+00
2e+05
4e+05
6e+05
0
500
1000
1500
2000

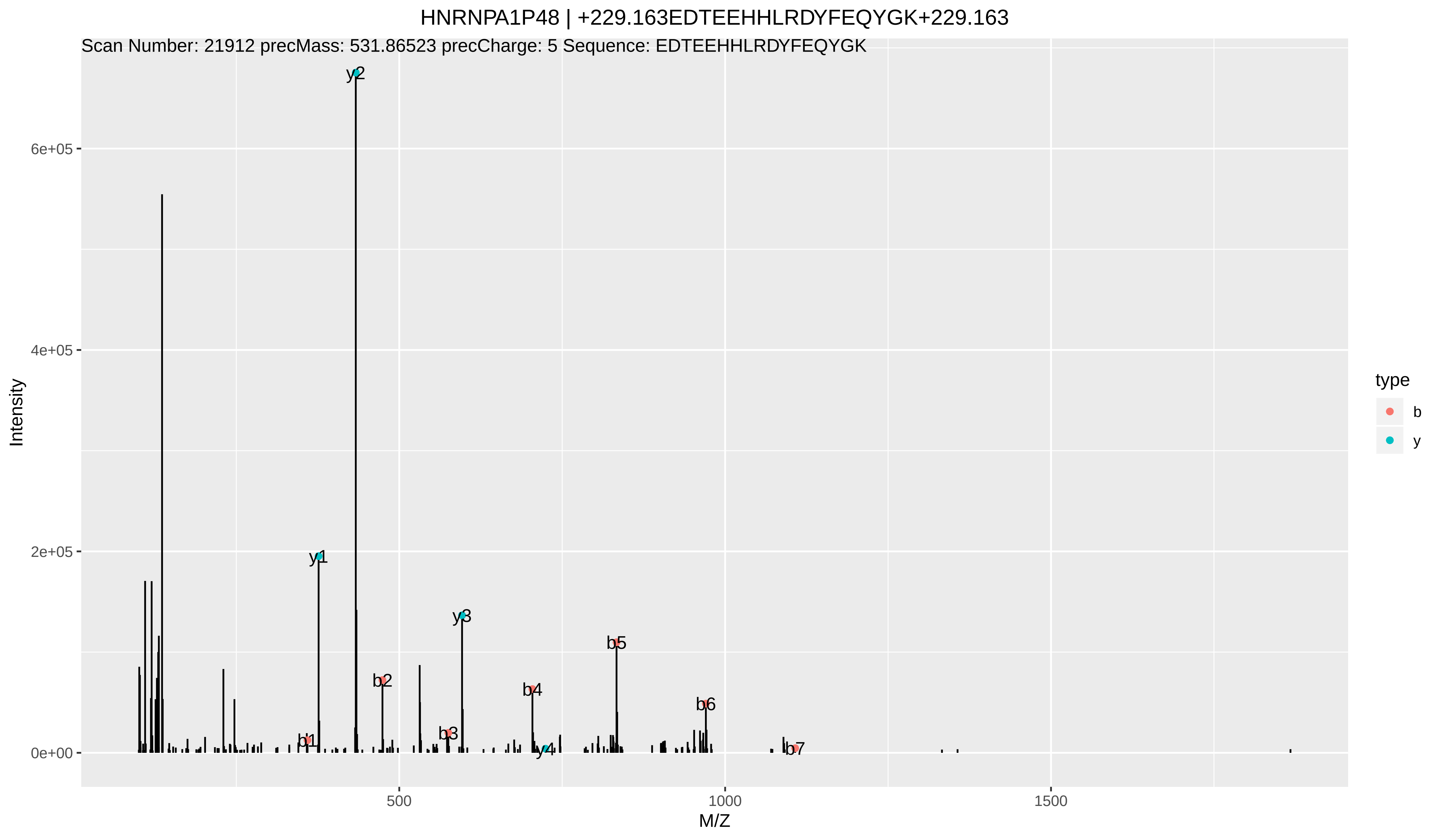
HNRNPA1P48 | +229.163EDTEEHHLRDYFEQYGK+229.163
Scan Number: 21912 precMass: 531.86523 precCharge: 5 Sequence: EDTEEHHLRDYFEQYGK
Intensity
M/Z
0e+00
2e+05
4e+05
6e+05
500
1000
1500
y1
y2
y3
y4
b1
b2
b3
b4
b5
b6
b7
type
b
y

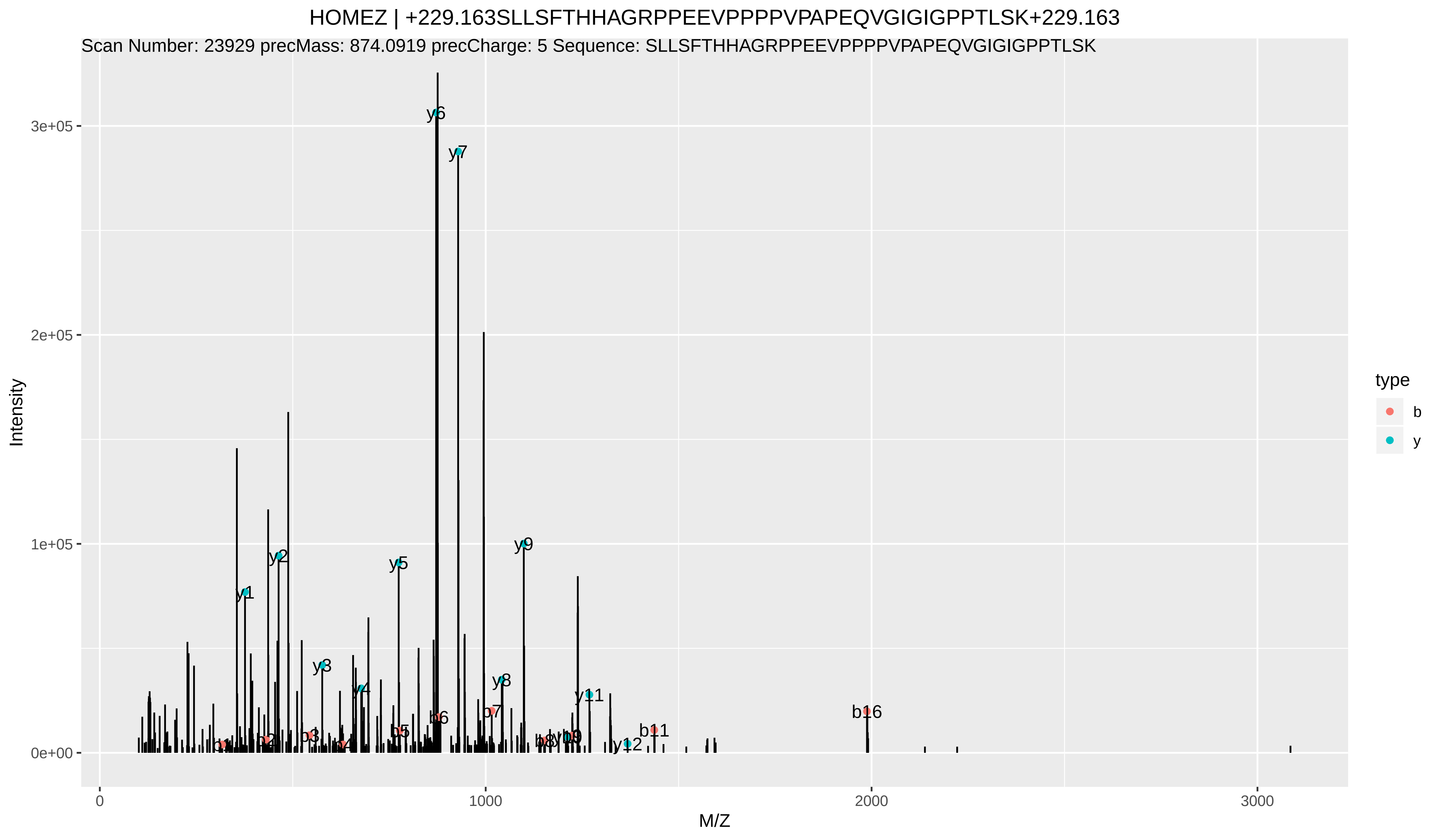
HOMEZ | +229.163SLLSFTHHAGRPPEEVPPPPVPAPEQVGIGIGPPTLSK+229.163
Scan Number: 23929 precMass: 874.0919 precCharge: 5 Sequence: SLLSFTHHAGRPPEEVPPPPVPAPEQVGIGIGPPTLSK
Intensity
M/Z
type
b
y
y6
y7
y9
y2
y5
y1
y3
y4
y8
y11
y12
b6
b7
b11
b16
0e+00
1e+05
2e+05
3e+05
0
1000
2000
3000

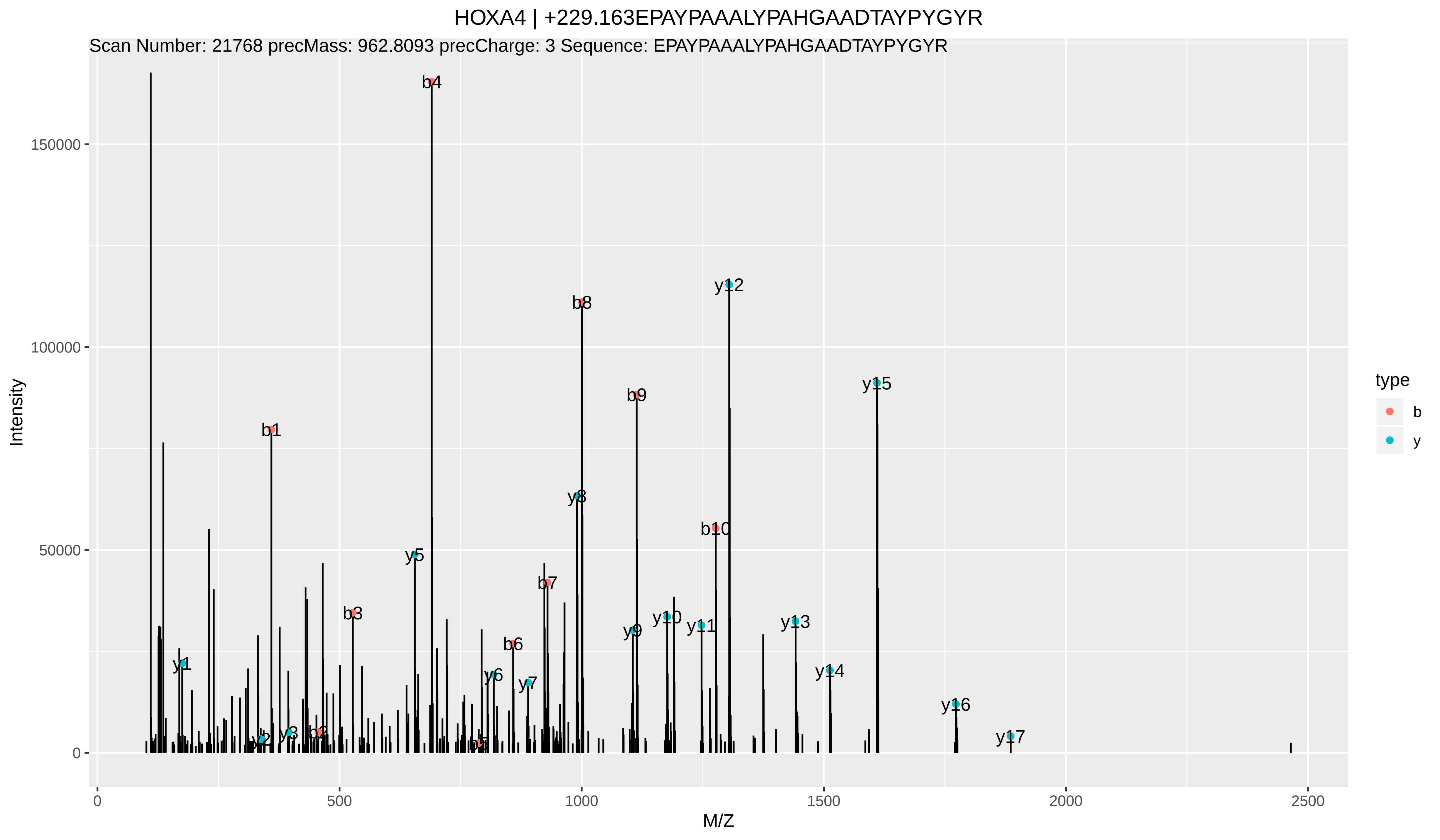

HOXA4 | +229.163EPAYPAAALYPAHGAADTAYPYGYR
Scan Number: 21768 precMass: 962.8093 precCharge: 3 Sequence: EPAYPAAALYPAHGAADTAYPYGYR
Intensity
M/Z
type
b
y
0
50000
100000
150000
500
1000
1500
2000
2500
b1
b3
b4
b6
b7
b8
b9
b10
y1
y2
y3
y5
y6
y7
y8
y9
y10
y11
y12
y13
y14
y15
y16
y17

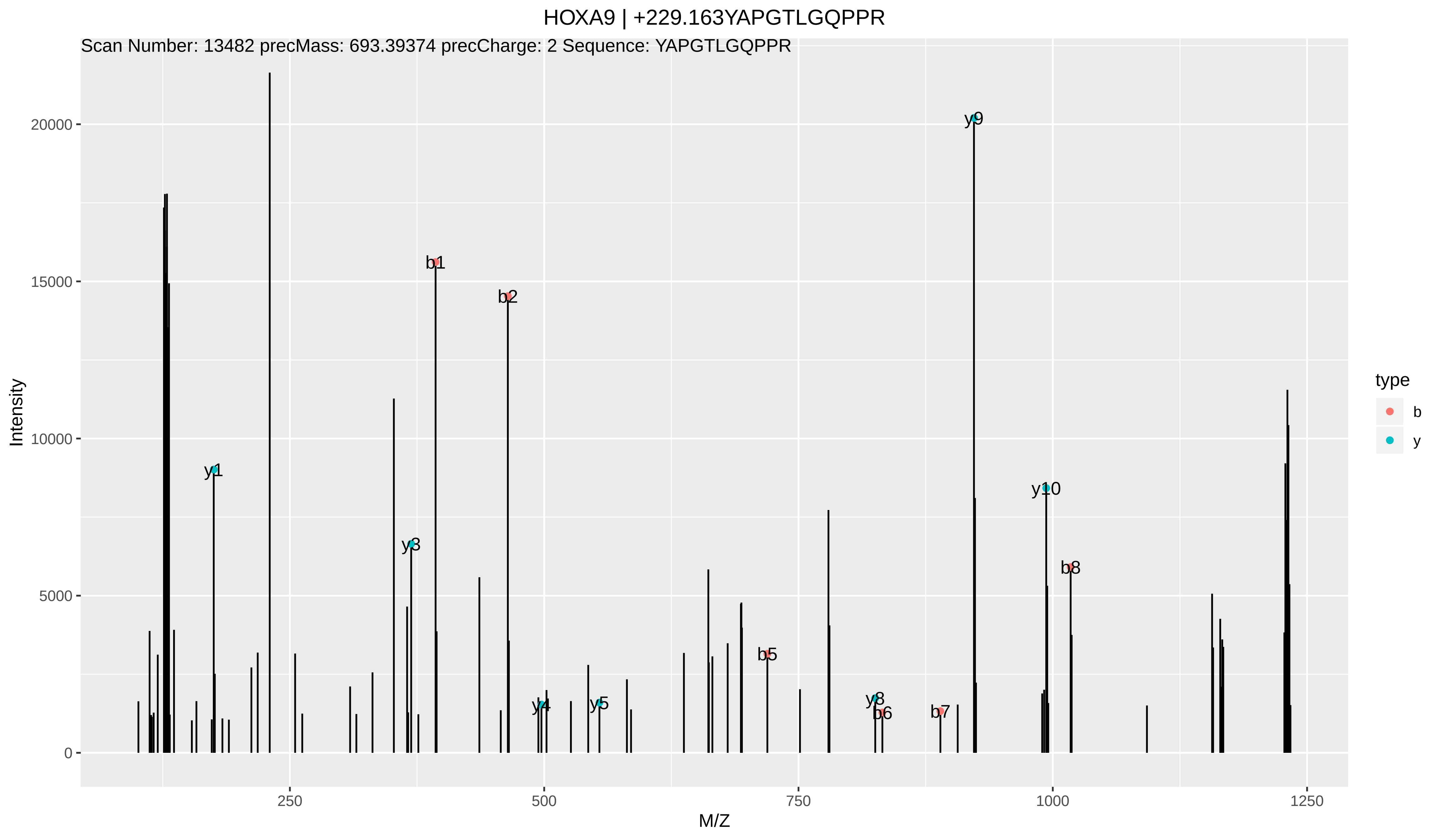
HOXA9 | +229.163YAPGTLGQPPR
Scan Number: 13482 precMass: 693.39374 precCharge: 2 Sequence: YAPGTLGQPPR
Intensity
M/Z
0
5000
10000
15000
20000
250
500
750
1000
1250
y1
y3
b1
b2
y4
y5
b5
y8
b6
b7
y9
y10
b8
type
b
y

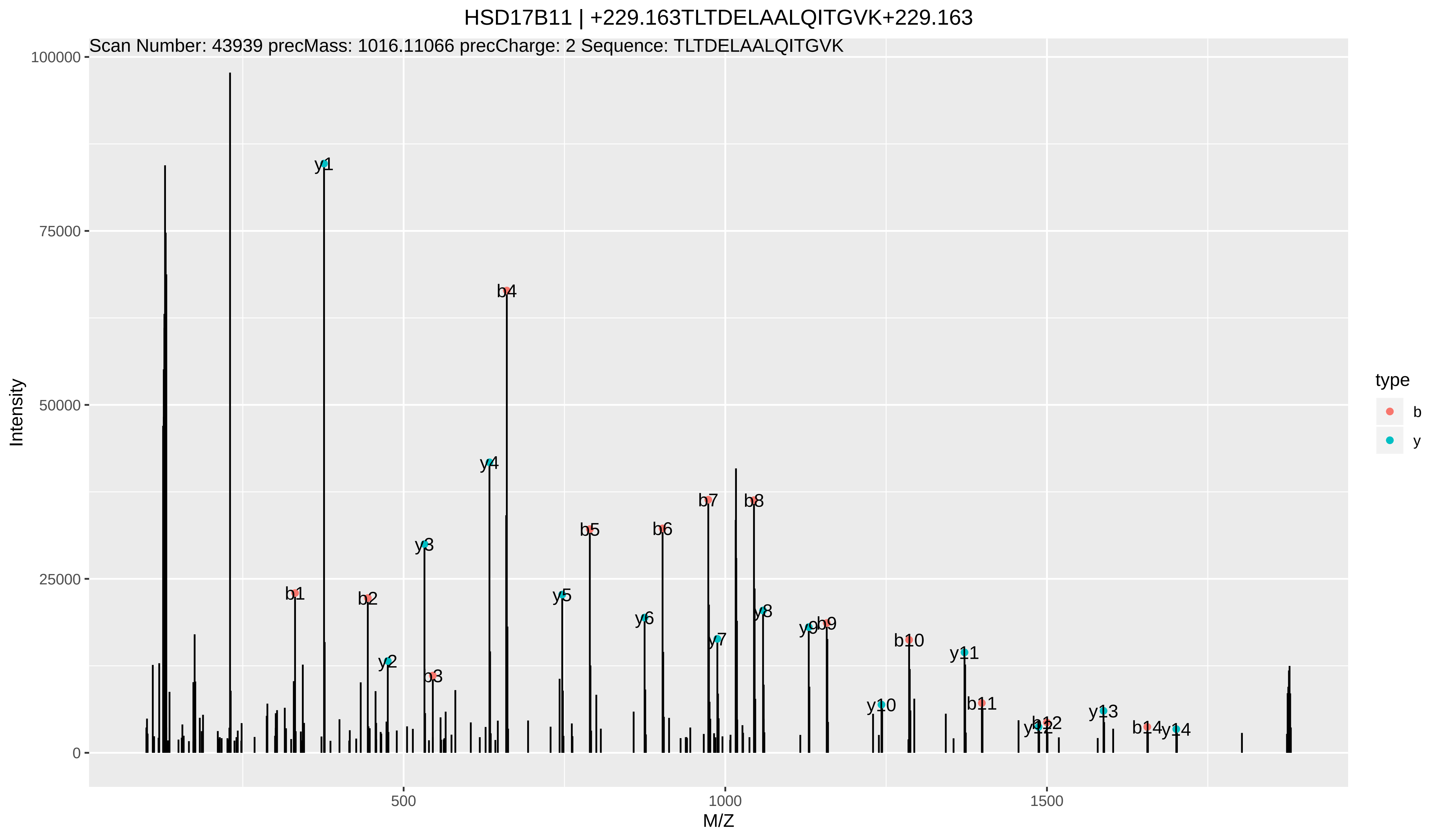

HSD17B11 | +229.163TLTDELAALQITGVK+229.163
Scan Number: 43939 precMass: 1016.11066 precCharge: 2 Sequence: TLTDELAALQITGVK
Intensity
M/Z
100000
75000
50000
25000
0
500
1000
1500
y1
b4
y4
b7
b8
b5
b6
y3
b1
b2
y5
y6
y8
y9
b9
y7
b10
y11
y2
b3
y10
b11
y13
b12
y12
b14
y14
type
b
y

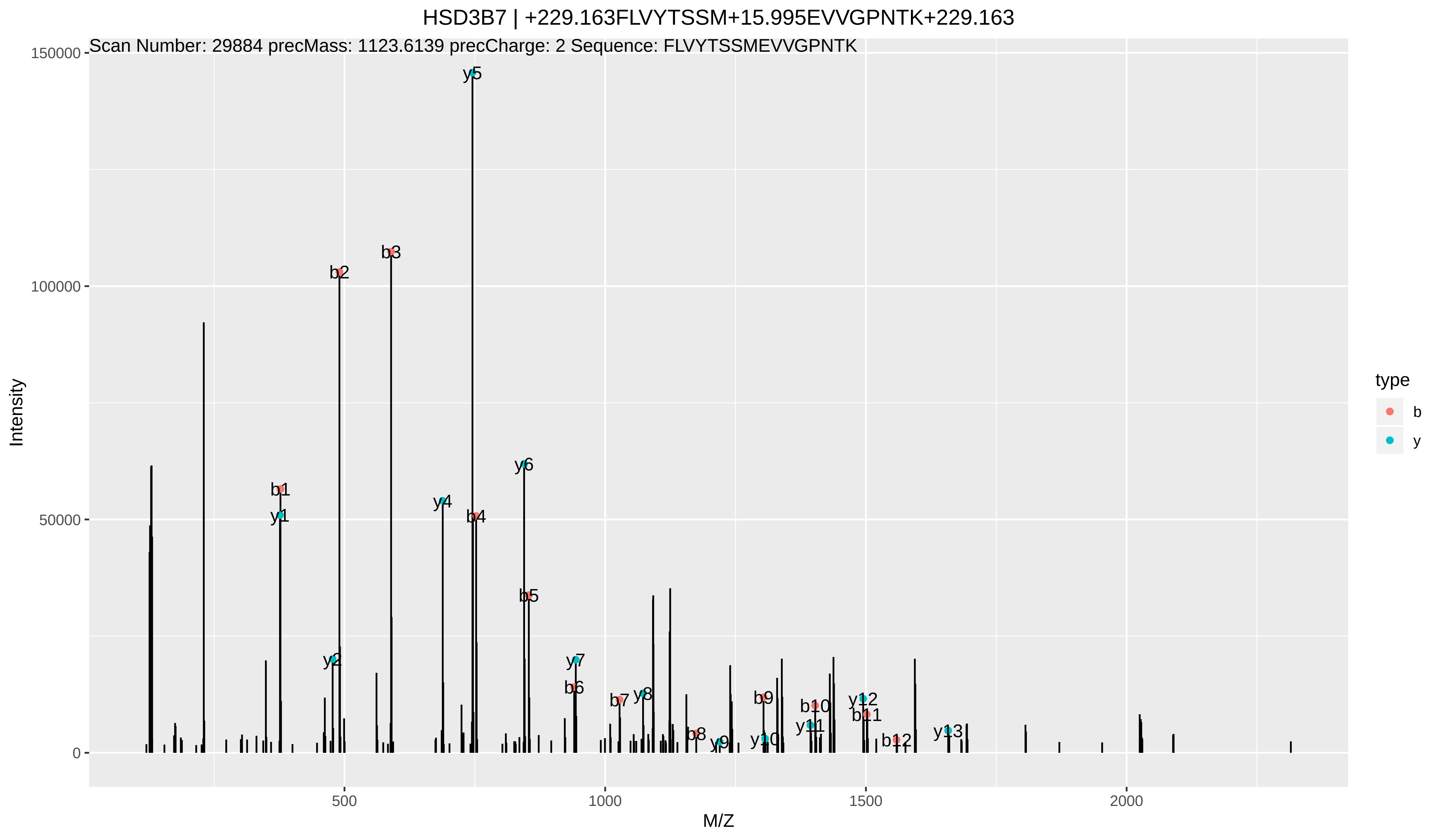

HSD3B7 | +229.163FLVYTSSM+15.995EVVGPNTK+229.163
Scan Number: 29884 precMass: 1123.6139 precCharge: 2 Sequence: FLVYTSSMEVVGPNTK
Intensity
M/Z
150000
100000
50000
0
500
1000
1500
2000
type
b
y
y5
b3
b2
b1
y1
y4
b4
y6
b5
y2
y7
b6
b7
y8
b8
y9
b9
y10
b10
y11
y12
b11
b12
y13

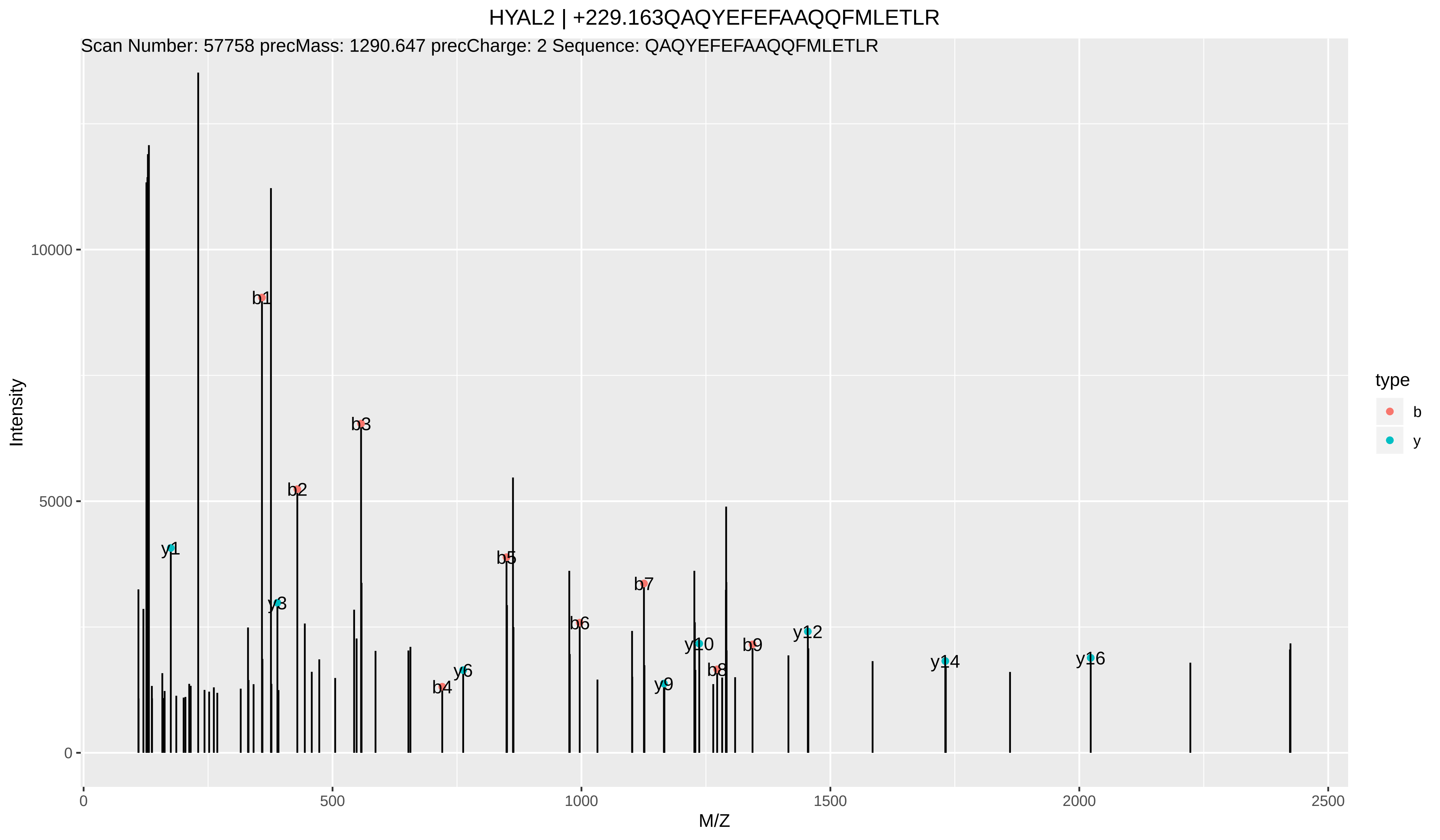

HYAL2 | +229.163QAQYEFEFAAQQFMLETLR
Scan Number: 57758 precMass: 1290.647 precCharge: 2 Sequence: QAQYEFEFAAQQFMLETLR
Intensity
10000
5000
0
0
500
1000
1500
2000
2500
M/Z
y1
b1
b2
y3
b3
b4
y6
b5
b6
b7
y9
y10
b8
b9
y12
y14
y16
type
b
y

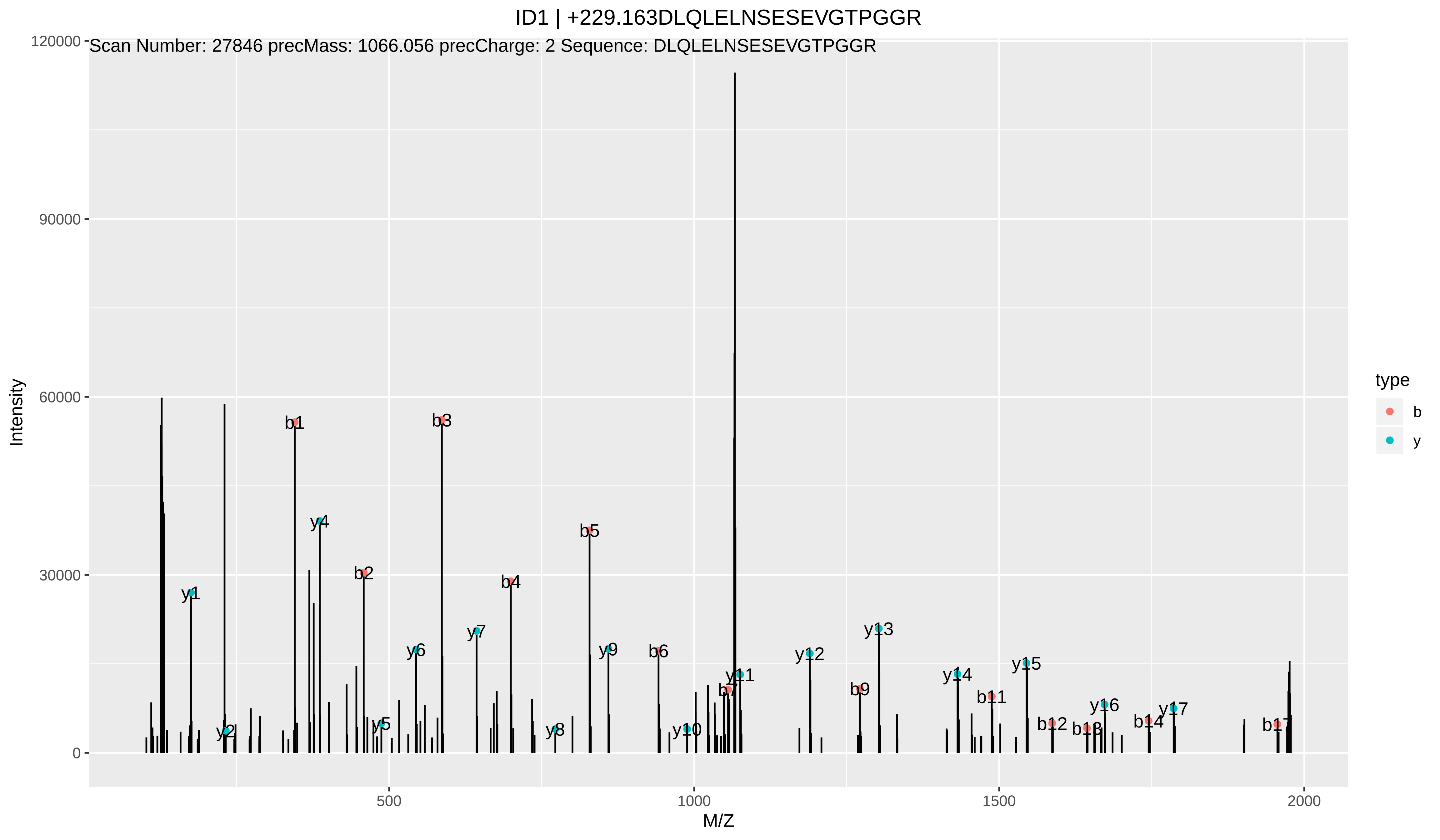

ID1 | +229.163DLQLELNSESEVGTPGGR
Scan Number: 27846 precMass: 1066.056 precCharge: 2 Sequence: DLQLELNSESEVGTPGGR
Intensity
M/Z
type
b
y
b1
b2
b3
b4
b5
b6
b9
b11
b12
b13
b14
b17
y1
y2
y4
y5
y6
y7
y8
y9
y10
y11
y12
y13
y14
y15
y16
y17
0
30000
60000
90000
120000
500
1000
1500
2000

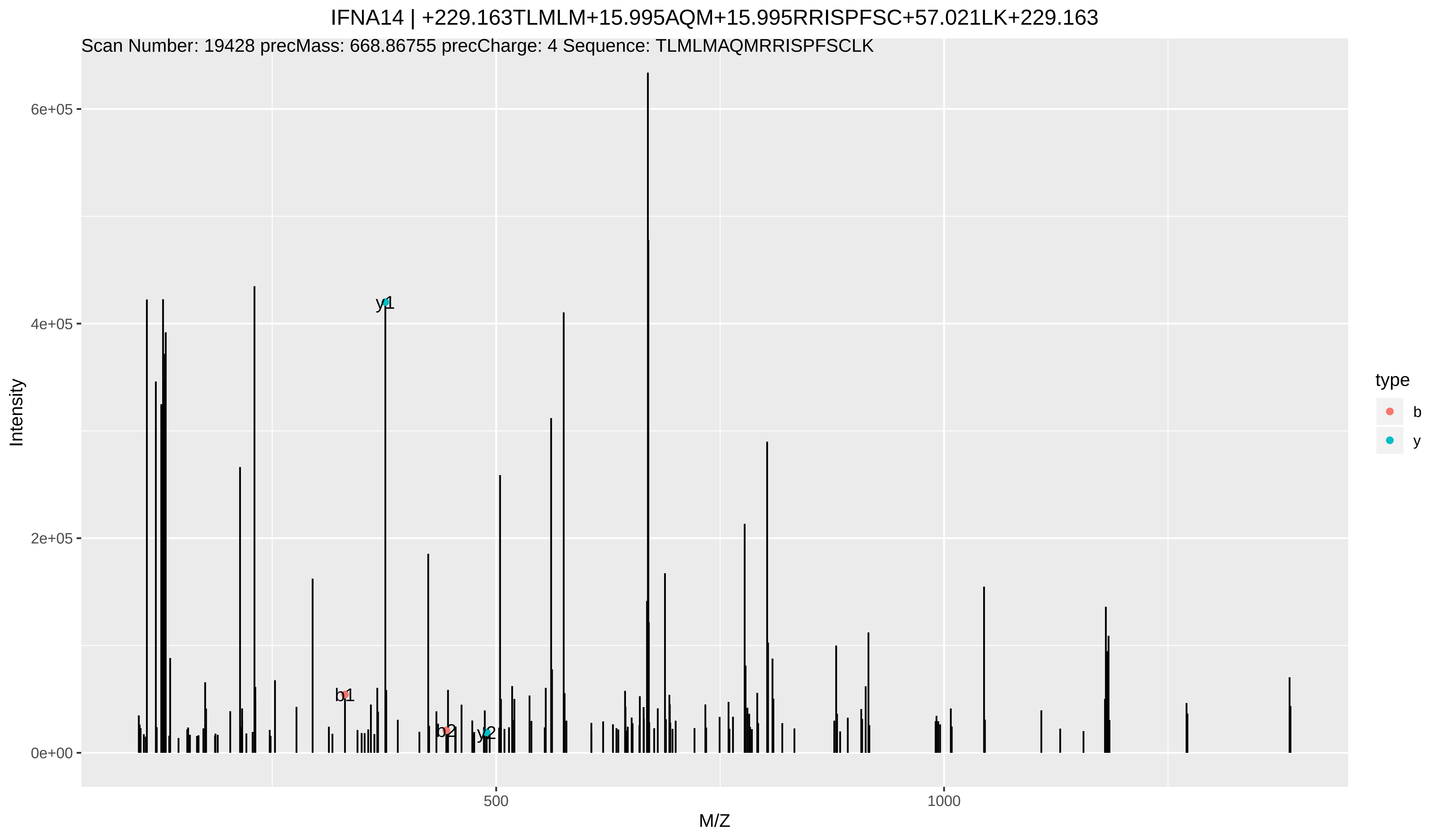

IFNA14 | +229.163TLMLM+15.995AQM+15.995RRISPFSC+57.021LK+229.163
Scan Number: 19428 precMass: 668.86755 precCharge: 4 Sequence: TLMLMAQMRRISPFSCLK
Intensity
M/Z
6e+05
4e+05
2e+05
0e+00
500
1000
y1
b1
b2
y2
type
b
y

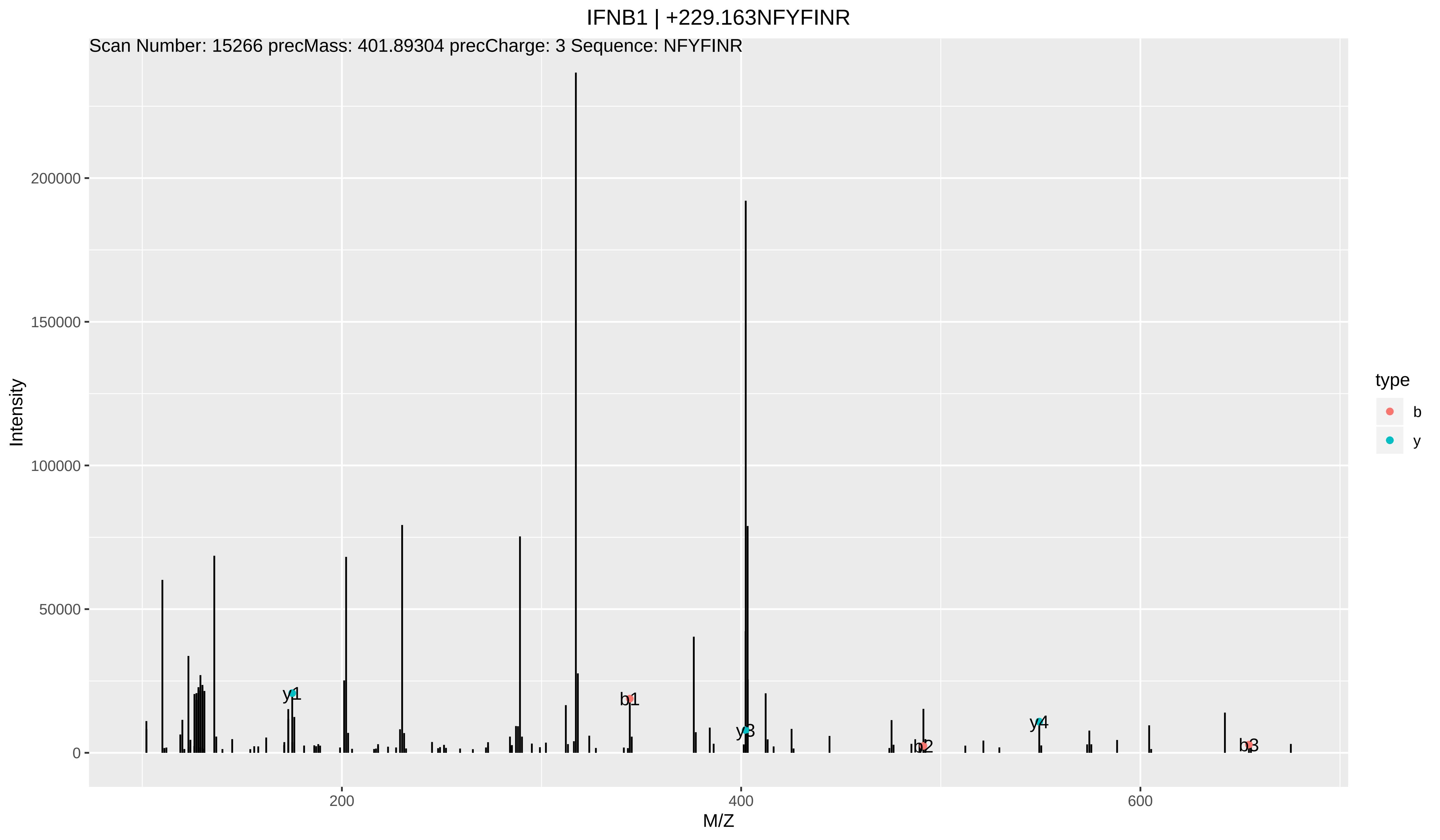
IFNB1 | +229.163NFYFINR
Scan Number: 15266 precMass: 401.89304 precCharge: 3 Sequence: NFYFINR
Intensity
M/Z
0
50000
100000
150000
200000
200
400
600
y1
b1
y3
b2
y4
b3
type
b
y

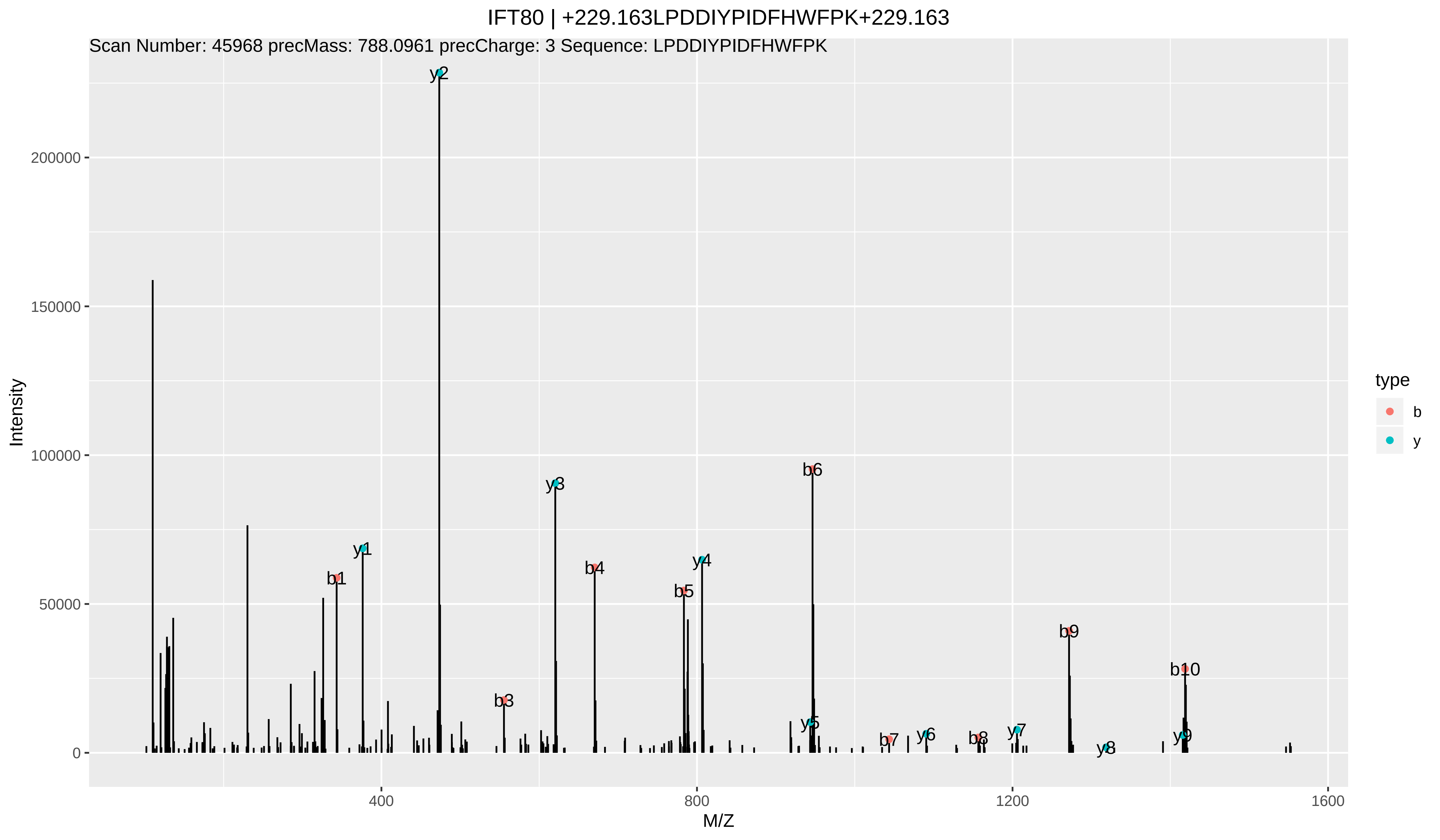

IFT80 | +229.163LPDDIYPIDFHWFPK+229.163
Scan Number: 45968 precMass: 788.0961 precCharge: 3 Sequence: LPDDIYPIDFHWFPK
Intensity
M/Z
type
b
y
y2
y3
b6
y1
y4
b4
b1
b5
b9
b10
b3
y5
b7
y6
b8
y7
y8
y9

IFT80 | +229.163NVLNDAVDLLEFRDR

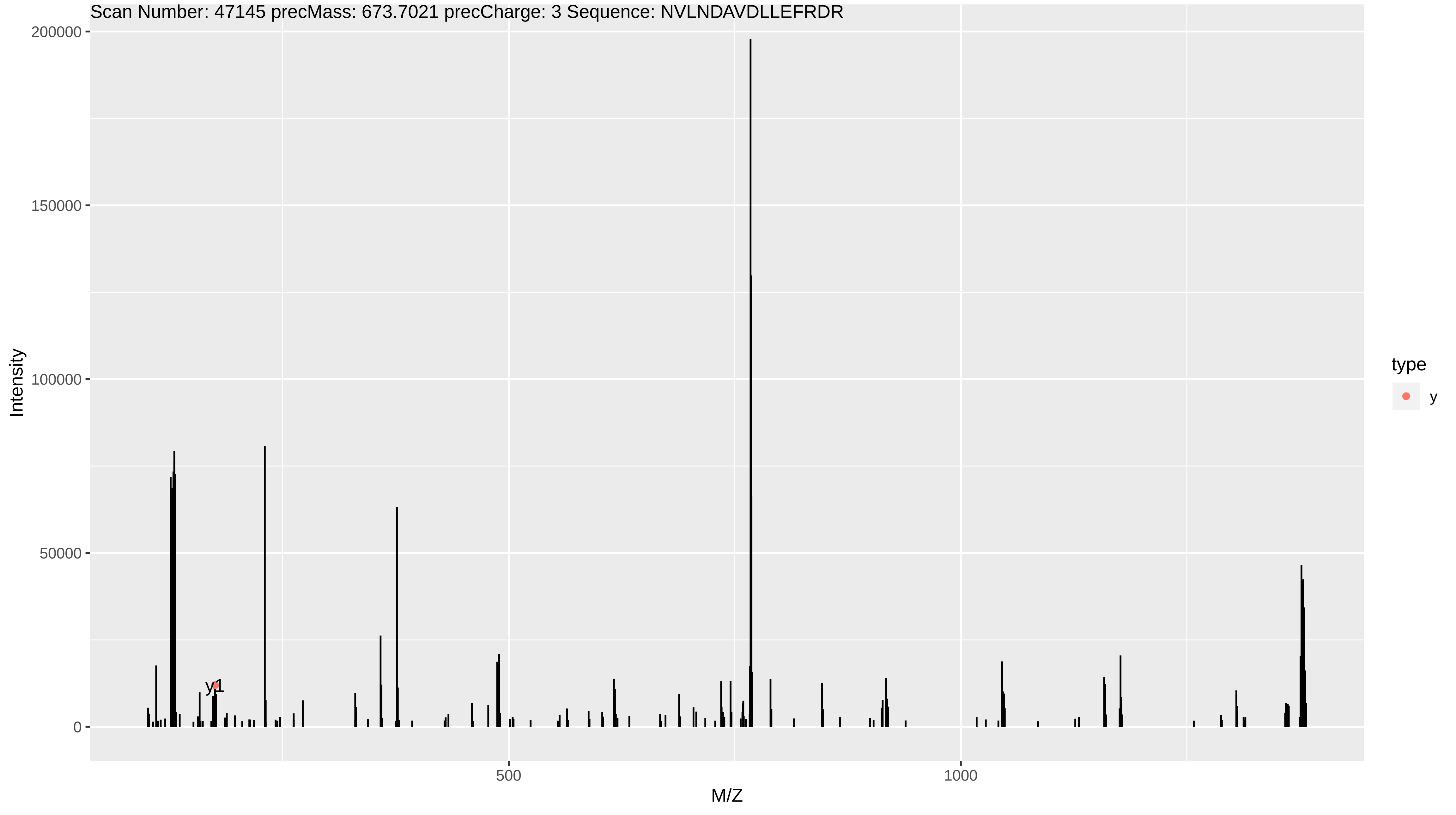

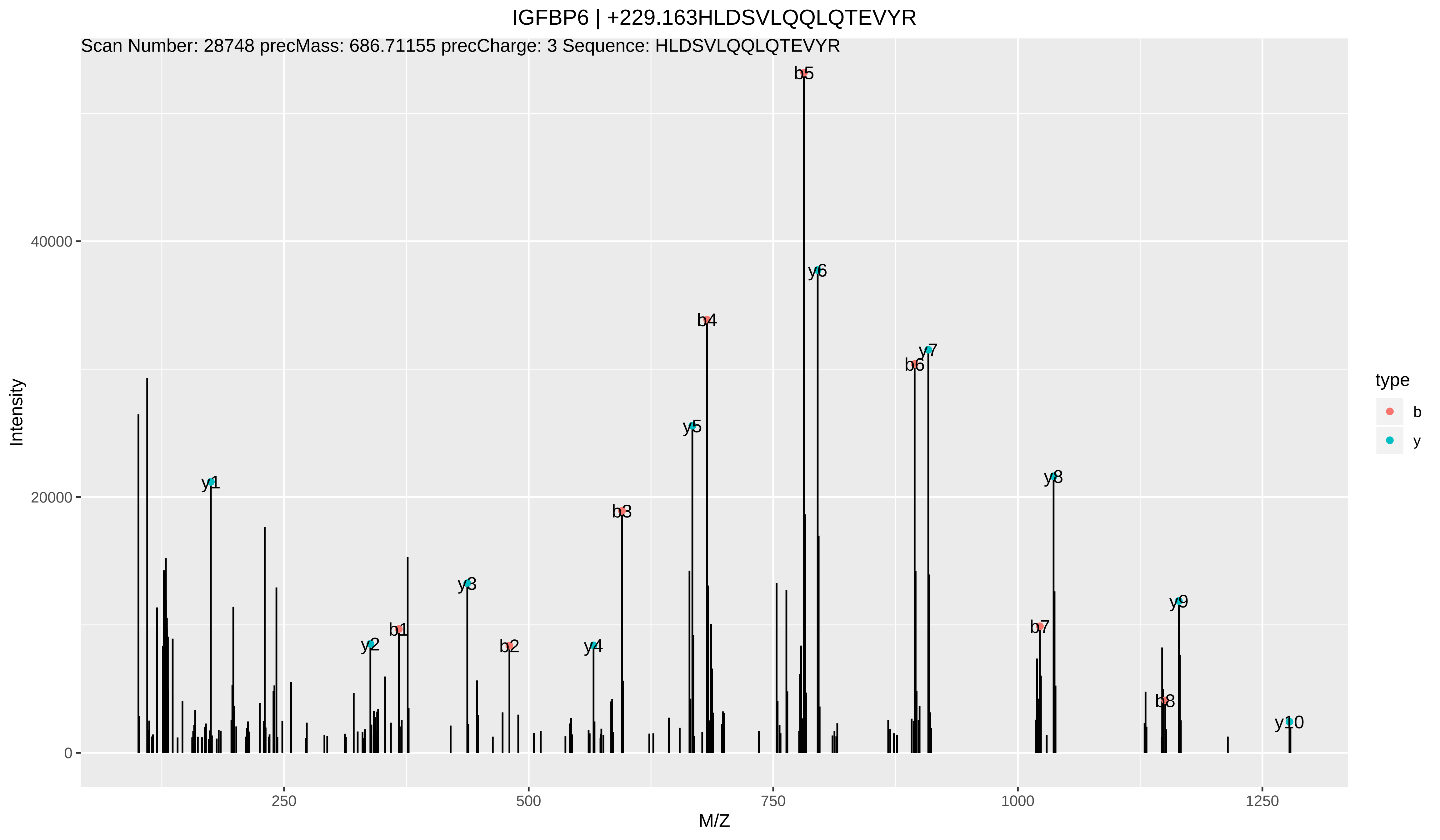
IGFBP6 | +229.163HLDSVLQQLQTEVYR
Scan Number: 28748 precMass: 686.71155 precCharge: 3 Sequence: HLDSVLQQLQTEVYR
Intensity
M/Z
0
20000
40000
250
500
750
1000
1250
b5
y6
b4
y7
b6
y5
y8
y1
b3
y3
y9
b7
b1
y2
b2
y4
b8
y10
type
b
y

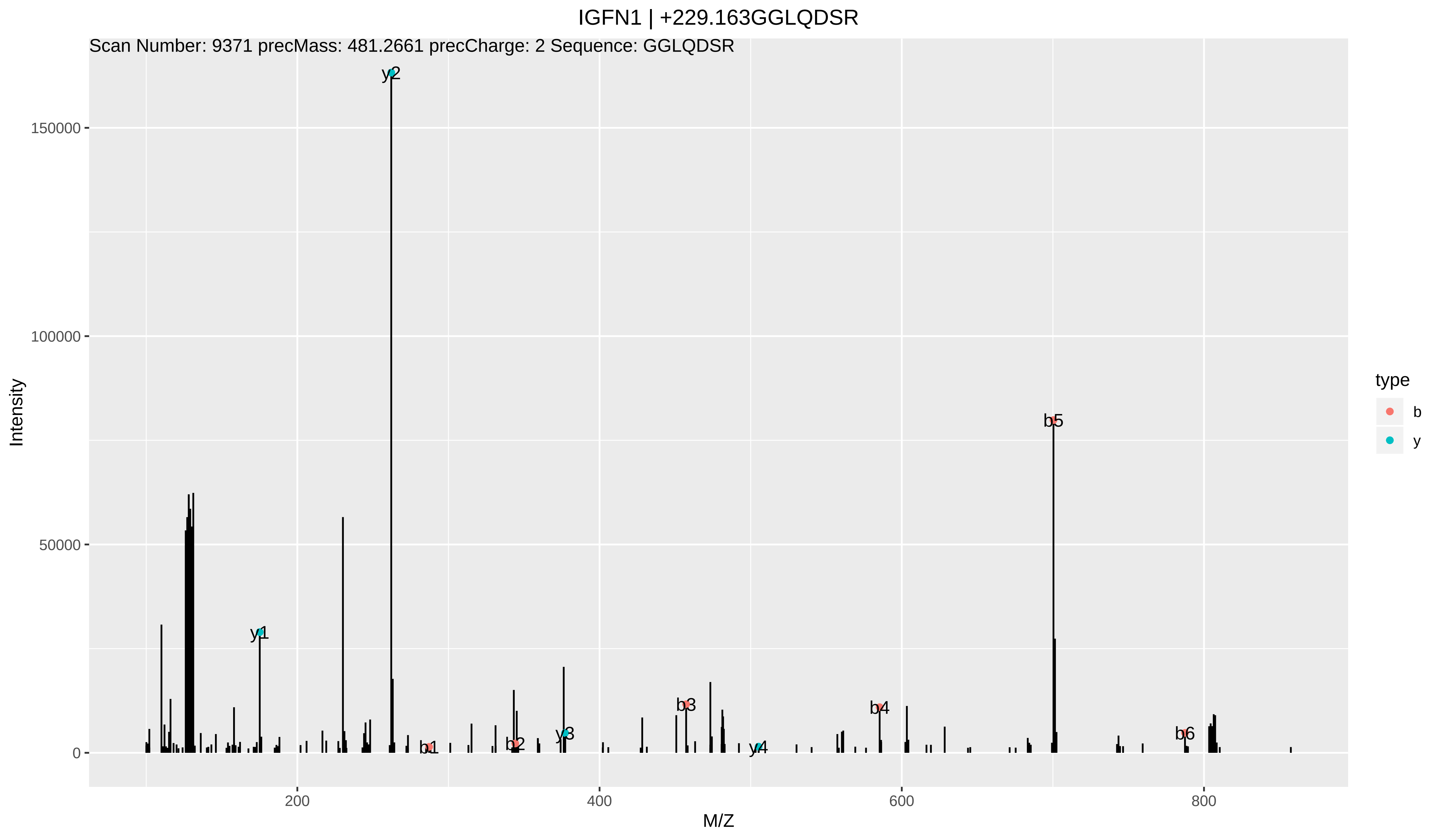
IGFN1 | +229.163GGLQDSR
Scan Number: 9371 precMass: 481.2661 precCharge: 2 Sequence: GGLQDSR
Intensity
150000
100000
50000
0
M/Z
200
400
600
800
y2
y1
b1
b2
y3
b3
y4
b4
b5
b6
type
b
y

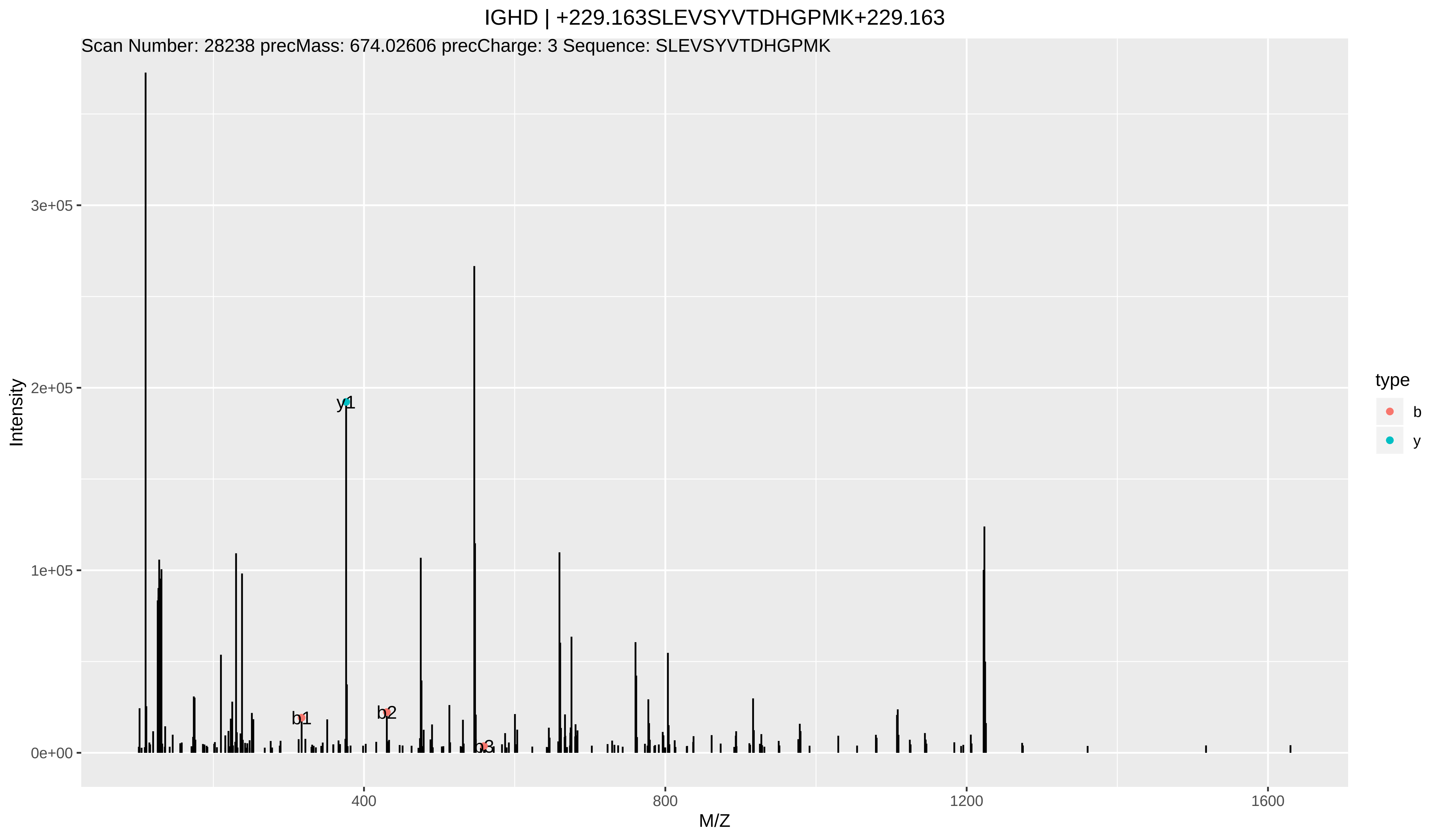

IGHD | +229.163SLEVSYVTDHGPMK+229.163
Scan Number: 28238 precMass: 674.02606 precCharge: 3 Sequence: SLEVSYVTDHGPMK
Intensity
0e+00
1e+05
2e+05
3e+05
M/Z
400
800
1200
1600
y1
b1
b2
b3
type
b
y

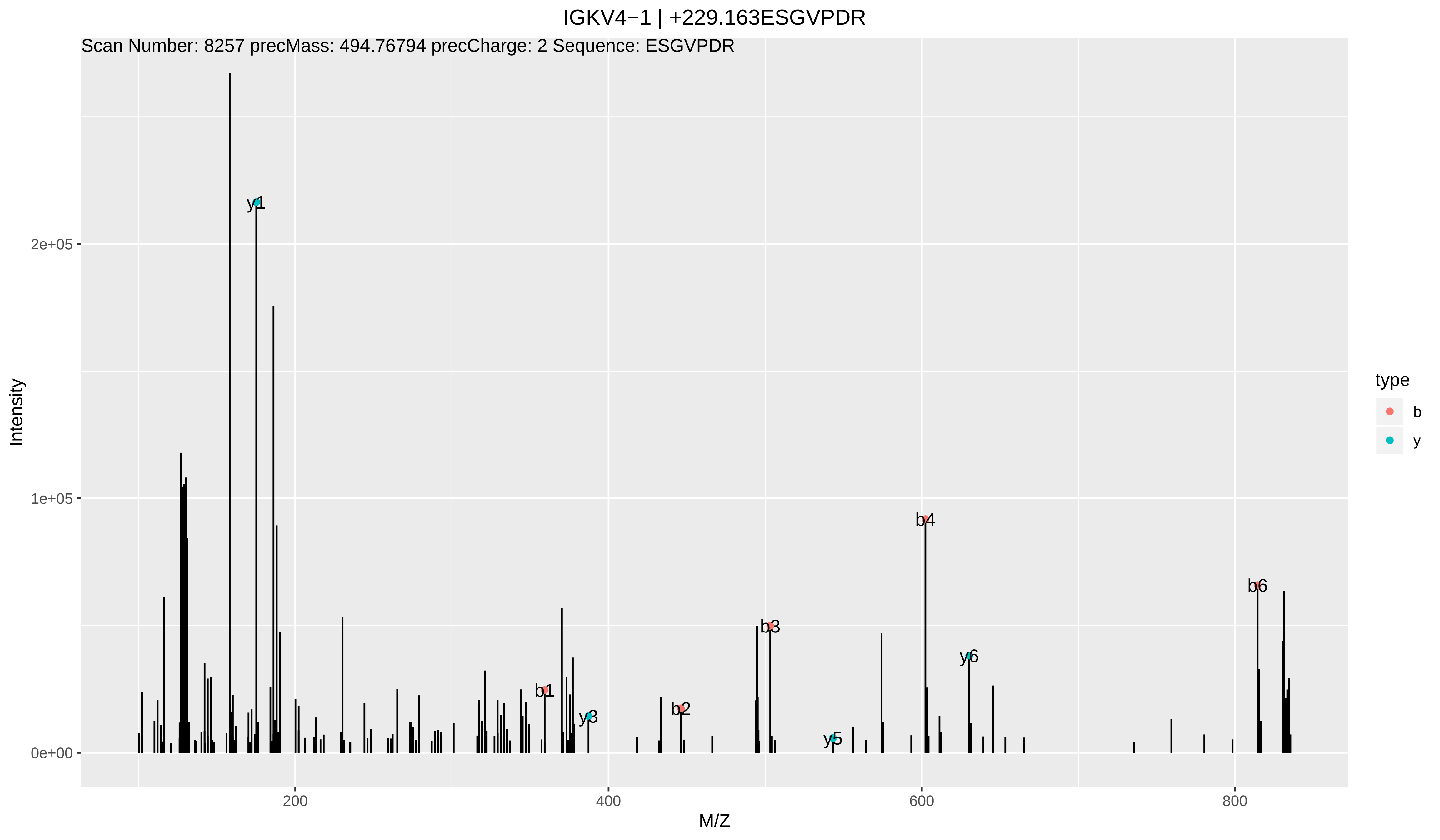
IGKV4−1 | +229.163ESGVPDR
Scan Number: 8257 precMass: 494.76794 precCharge: 2 Sequence: ESGVPDR
Intensity
2e+05
1e+05
0e+00
M/Z
200
400
600
800
y1
b1
y3
b2
b3
y5
b4
y6
b6
type
b
y

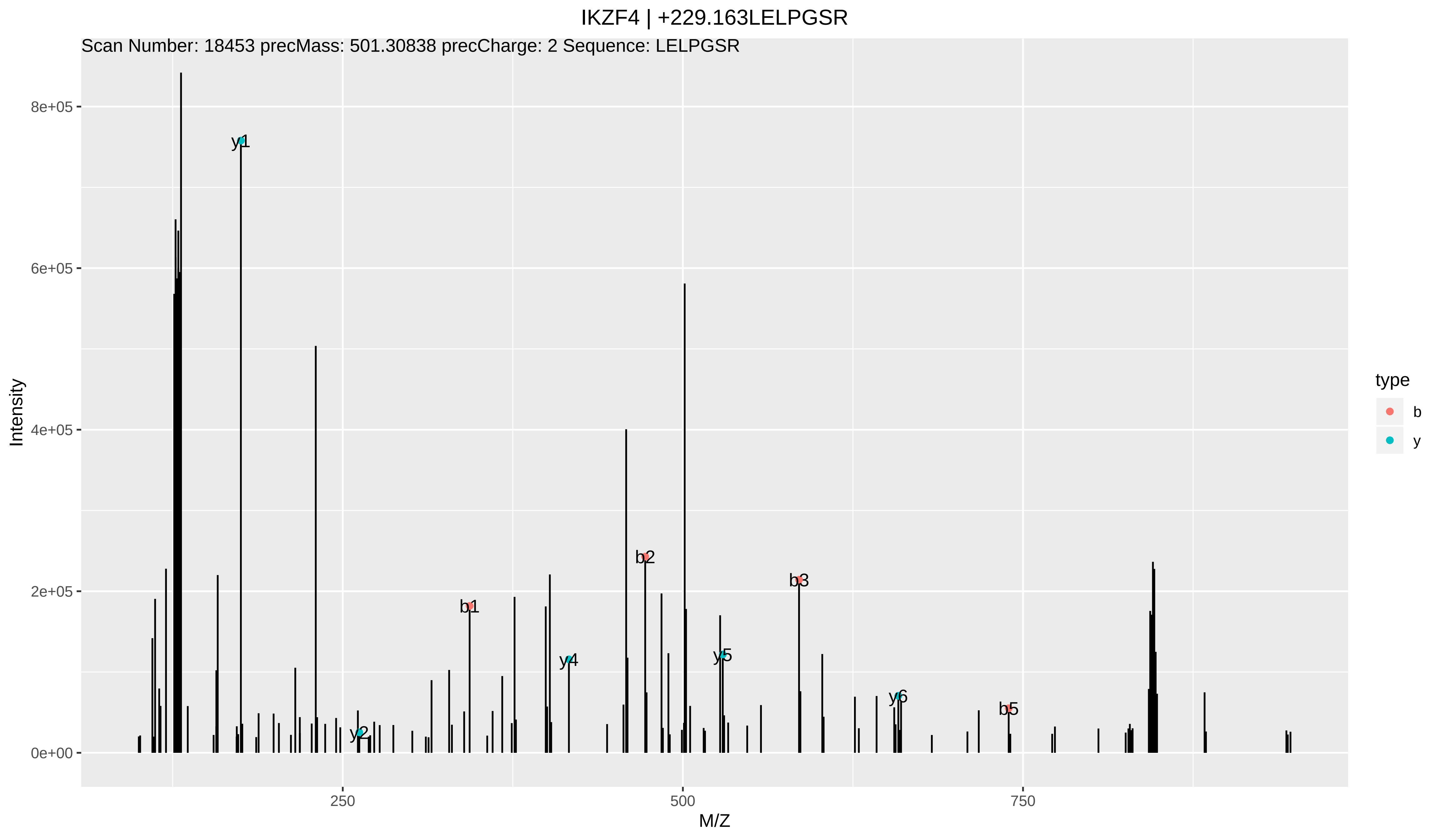

IKZF4 | +229.163LELPGSR
Scan Number: 18453 precMass: 501.30838 precCharge: 2 Sequence: LELPGSR
Intensity
8e+05
6e+05
4e+05
2e+05
0e+00
M/Z
250
500
750
y1
y2
b1
y4
b2
y5
b3
y6
b5
type
b
y

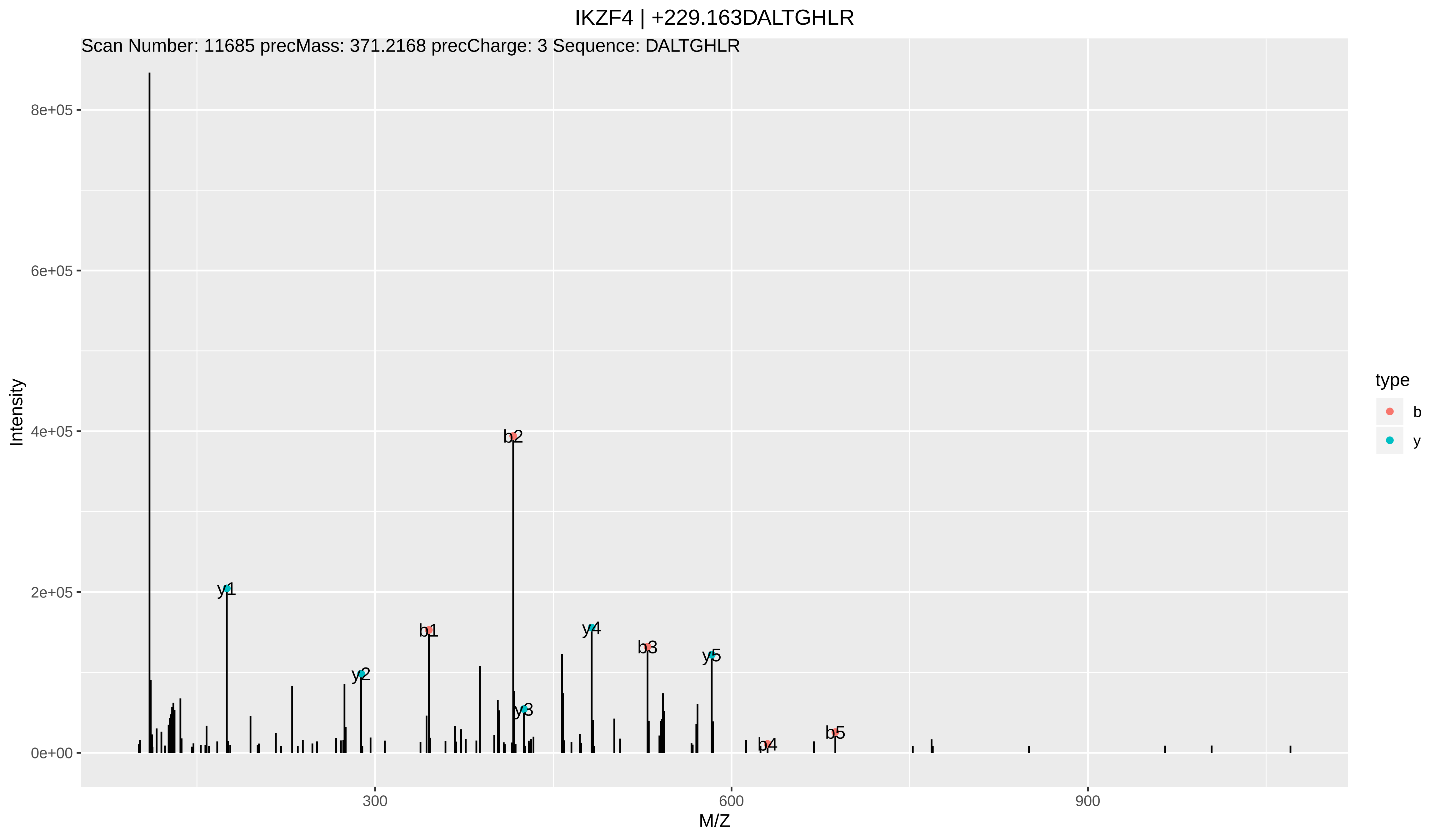

IKZF4 | +229.163DALTGHLR
Scan Number: 11685 precMass: 371.2168 precCharge: 3 Sequence: DALTGHLR
Intensity
8e+05
6e+05
4e+05
2e+05
0e+00
M/Z
300
600
900
y1
y2
b1
b2
y3
y4
b3
y5
b4
b5
type
b
y

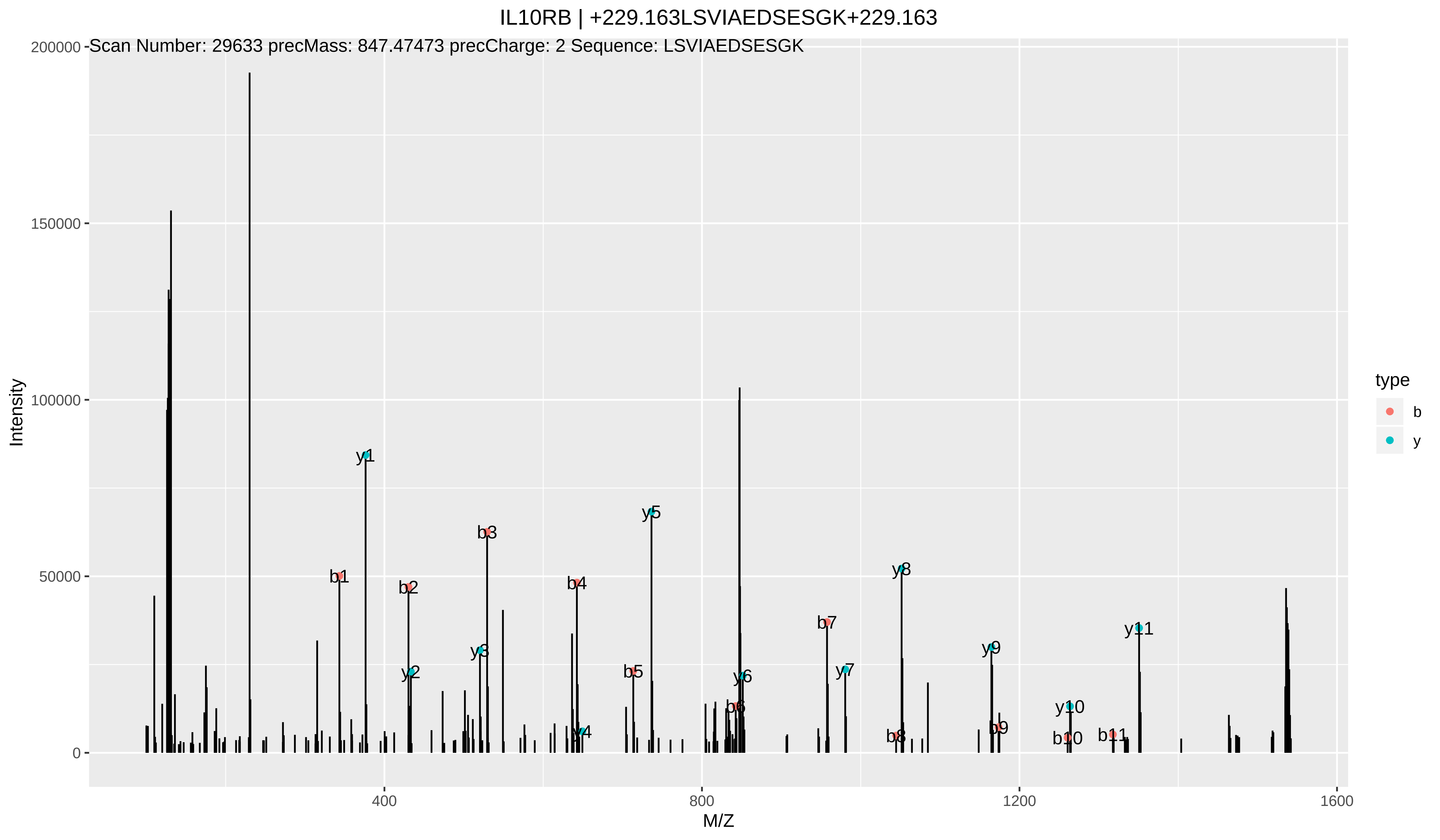

IL10RB | +229.163LSVIAEDSESGK+229.163
Scan Number: 29633 precMass: 847.47473 precCharge: 2 Sequence: LSVIAEDSESGK
Intensity
200000
150000
100000
50000
0
M/Z
400
800
1200
1600
y1
b1
b2
y2
b3
y3
b4
y4
y5
b5
y6
b6
b7
y7
y8
b8
y9
b9
y10
b10
b11
y11
type
b
y

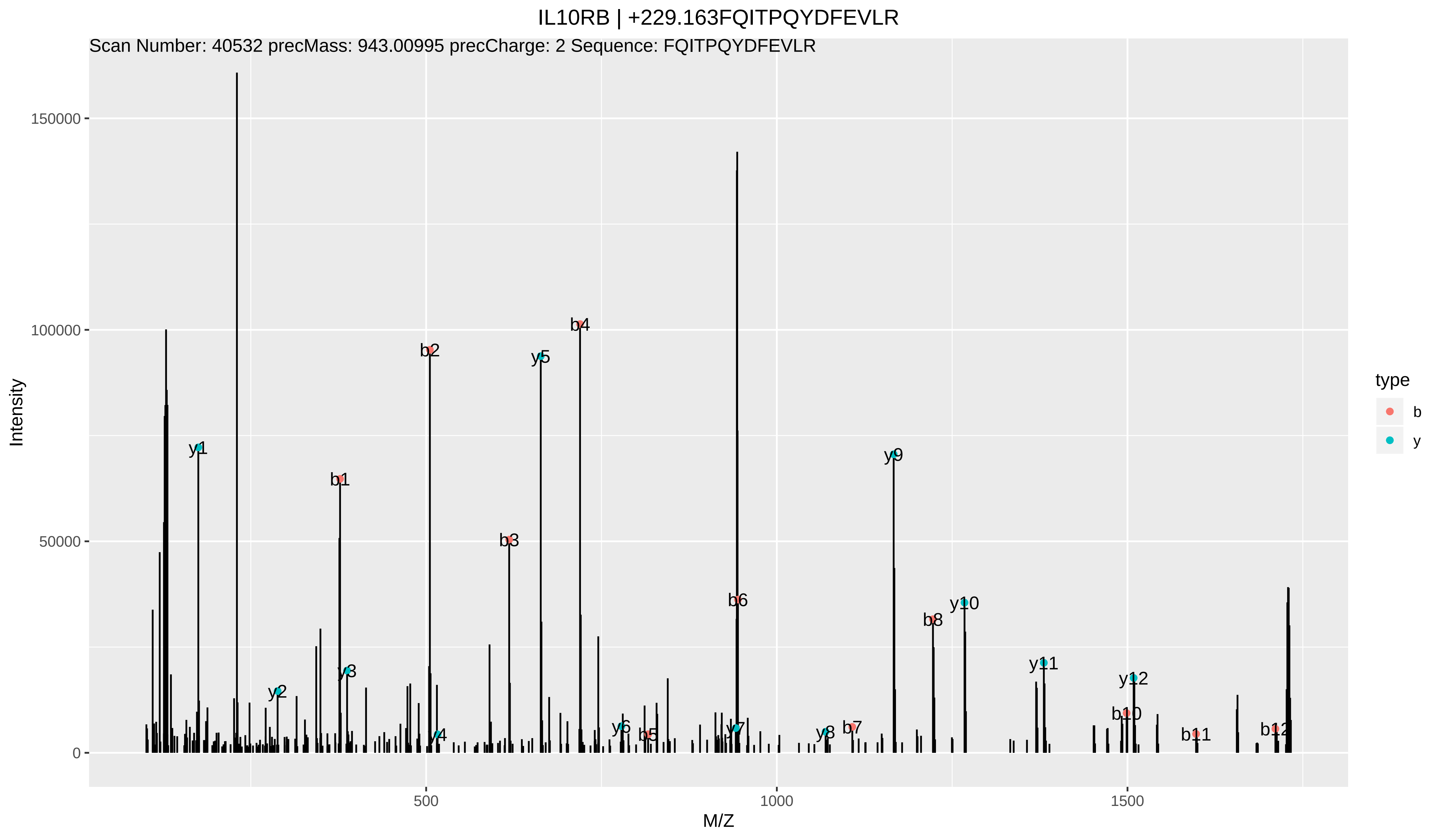
IL10RB | +229.163FQITPQYDFEVLR
Scan Number: 40532 precMass: 943.00995 precCharge: 2 Sequence: FQITPQYDFEVLR
Intensity
150000
100000
50000
0
500
1000
1500
M/Z
y1
b1
y2
y3
b2
y4
b3
y5
b4
y6
b5
b6
y7
y8
b7
y9
b8
y10
y11
y12
b10
b11
b12
type
b
y

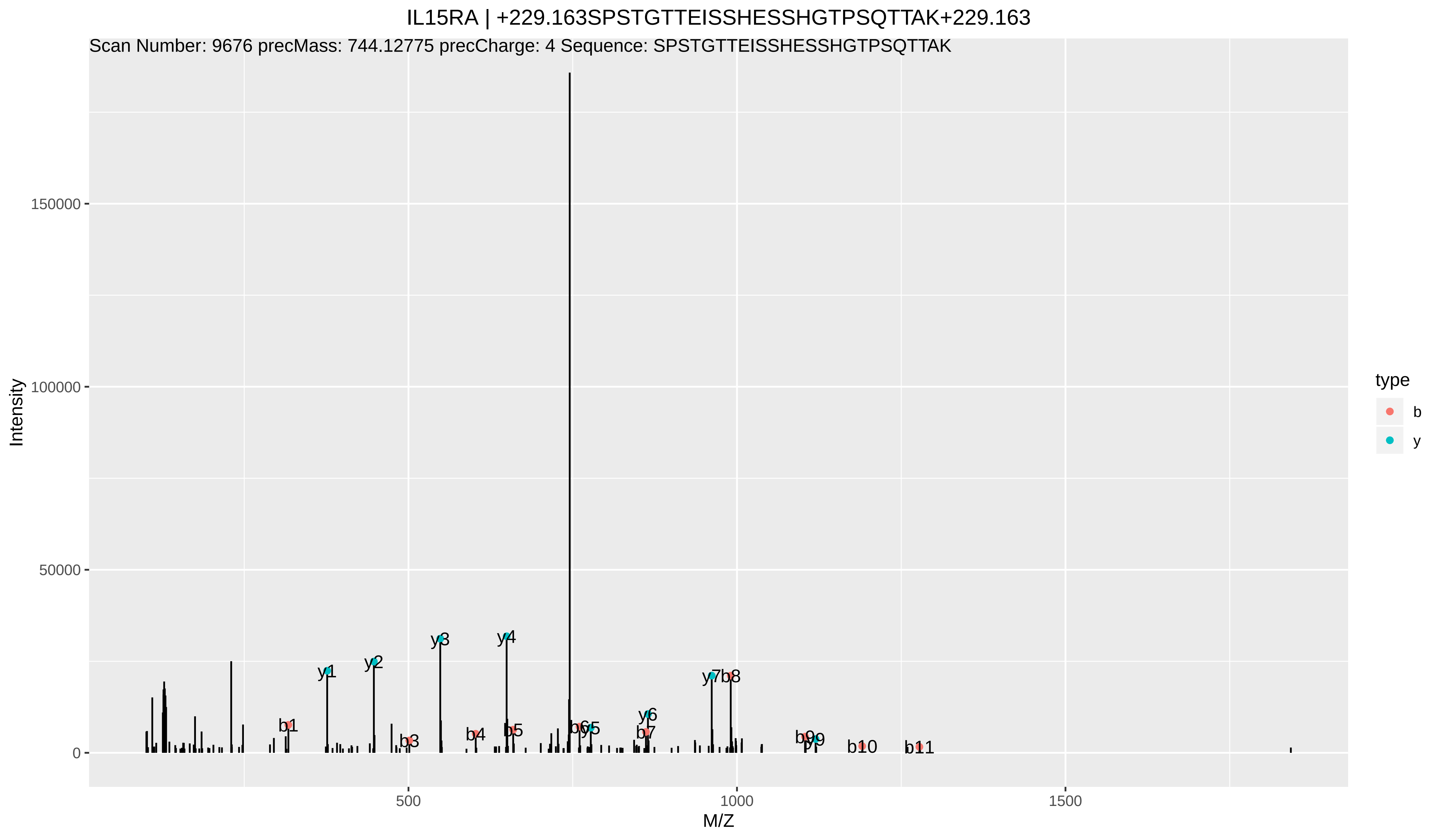
IL15RA | +229.163SPSTGTTEISSHESSHGTPSQTTAK+229.163
Scan Number: 9676 precMass: 744.12775 precCharge: 4 Sequence: SPSTGTTEISSHESSHGTPSQTTAK
Intensity
150000
100000
50000
0
500
1000
1500
M/Z
y1
y2
y3
y4
b1
b3
b4
b5
b6
y5
y6
b7
y7
b8
b9
y9
b10
b11
type
b
y

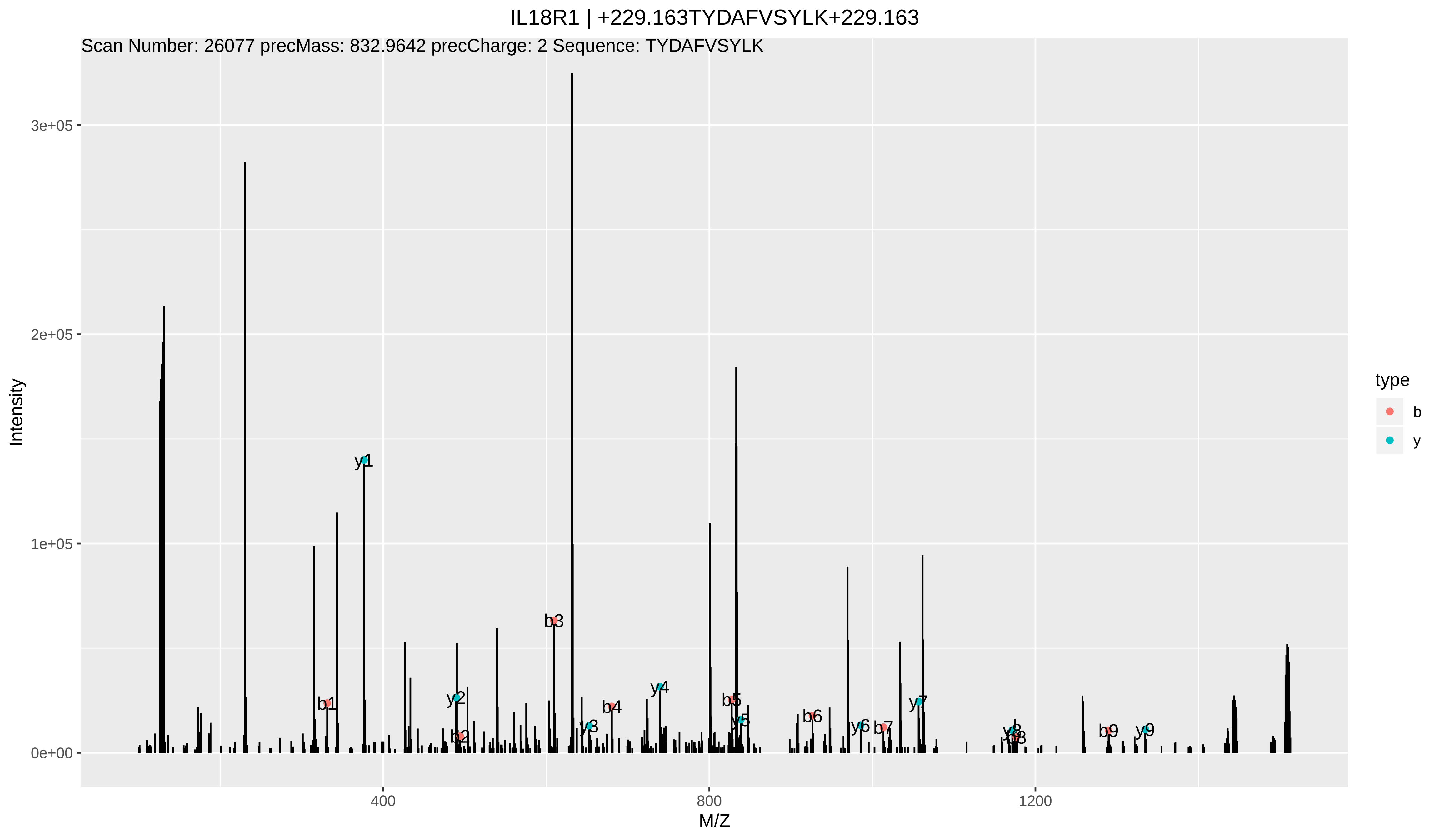

IL18R1 | +229.163TYDAFVSYLK+229.163
Scan Number: 26077 precMass: 832.9642 precCharge: 2 Sequence: TYDAFVSYLK
Intensity
M/Z
3e+05
2e+05
1e+05
0e+00
400
800
1200
type
b
y
y1
y2
y3
y4
y5
y6
y7
y8
y9
b1
b2
b3
b4
b5
b6
b7
b8
b9

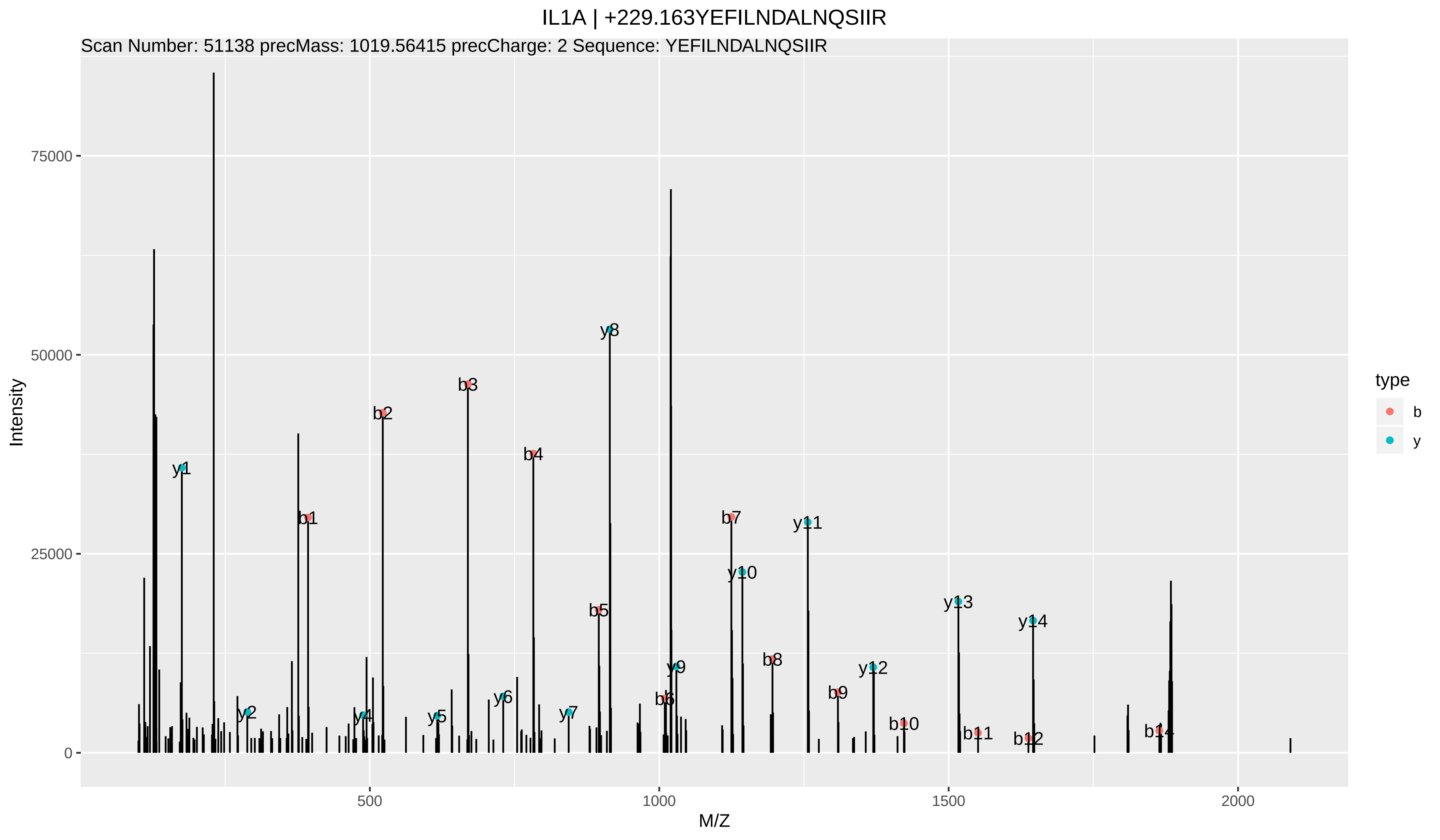

IL1A | +229.163YEFILNDALNQSIIR
Scan Number: 51138 precMass: 1019.56415 precCharge: 2 Sequence: YEFILNDALNQSIIR
Intensity
M/Z
type
b
y
y1
y2
y4
y5
y6
y7
y8
y9
y10
y11
y12
y13
y14
b1
b2
b3
b4
b5
b6
b7
b8
b9
b10
b11
b12
b14

IL23R | +229.163ILLLIPK+229.163

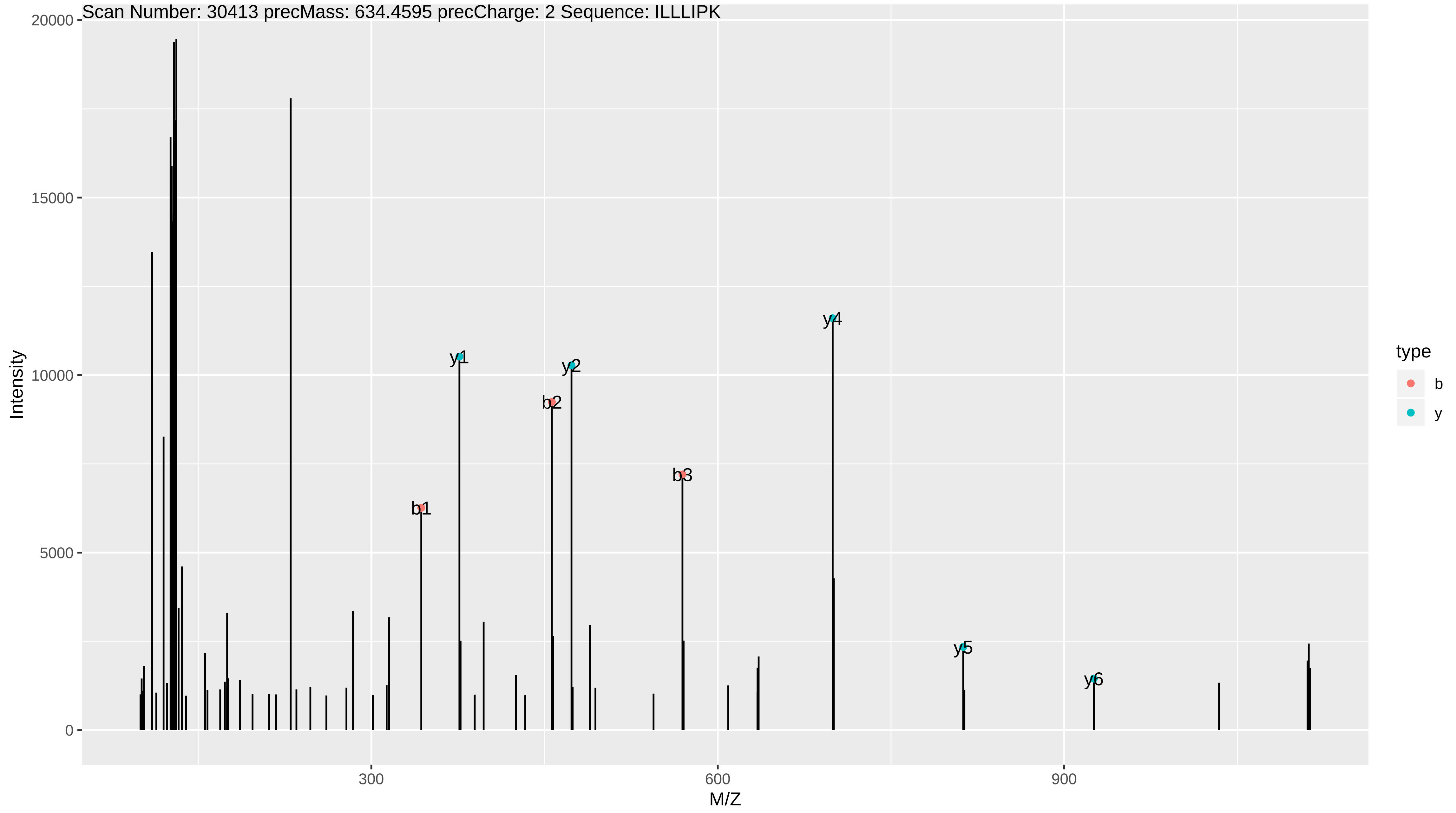

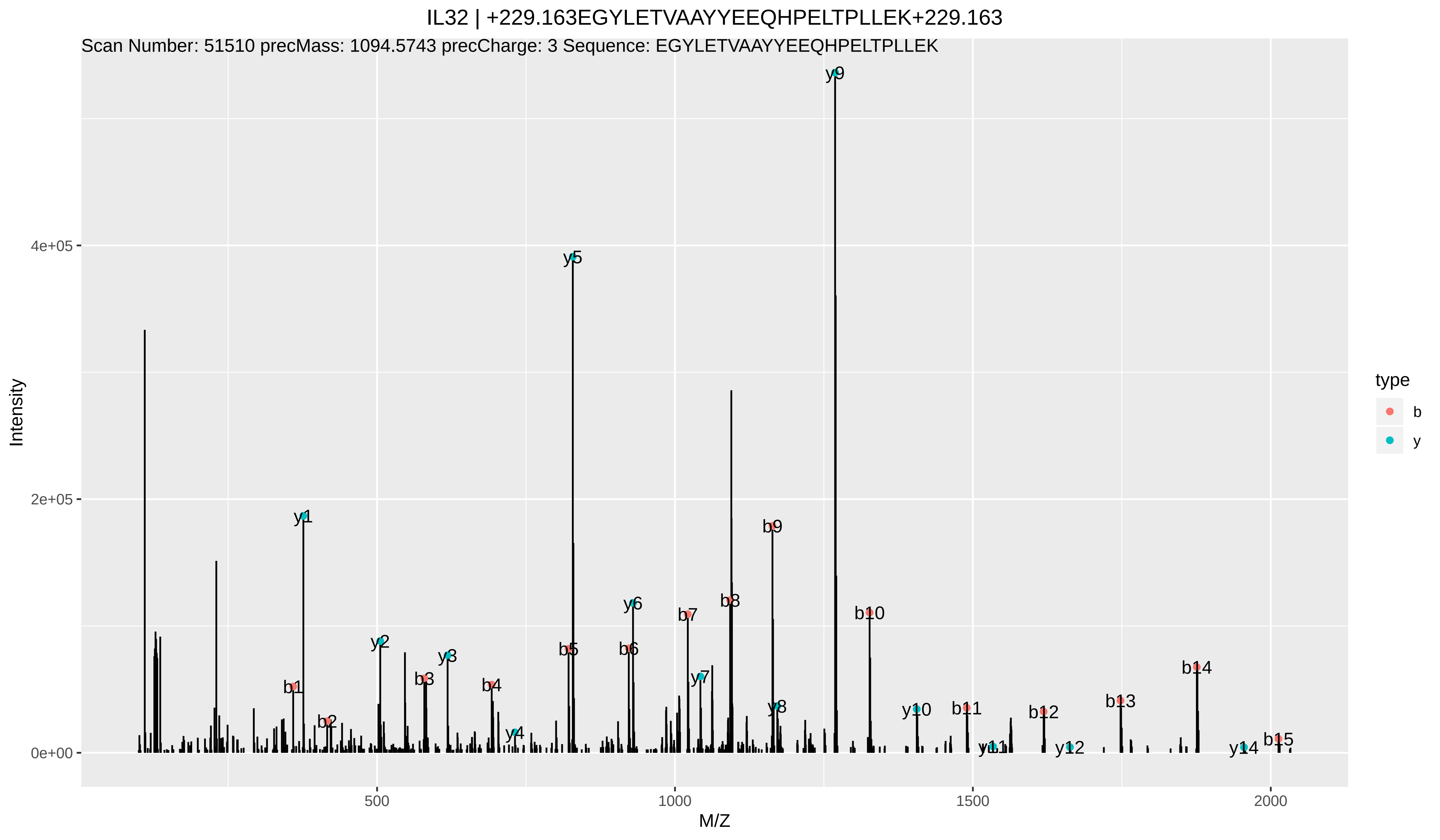
IL32 | +229.163EGYLETVAAYYEEQHPELTPLLEK+229.163
Scan Number: 51510 precMass: 1094.5743 precCharge: 3 Sequence: EGYLETVAAYYEEQHPELTPLLEK
Intensity
4e+05
2e+05
0e+00
M/Z
500
1000
1500
2000
type
b
y
y1
y2
y3
y4
y5
y6
y7
y8
y9
y10
y11
y12
y14
b1
b2
b3
b4
b5
b6
b7
b8
b9
b10
b11
b12
b13
b14
b15

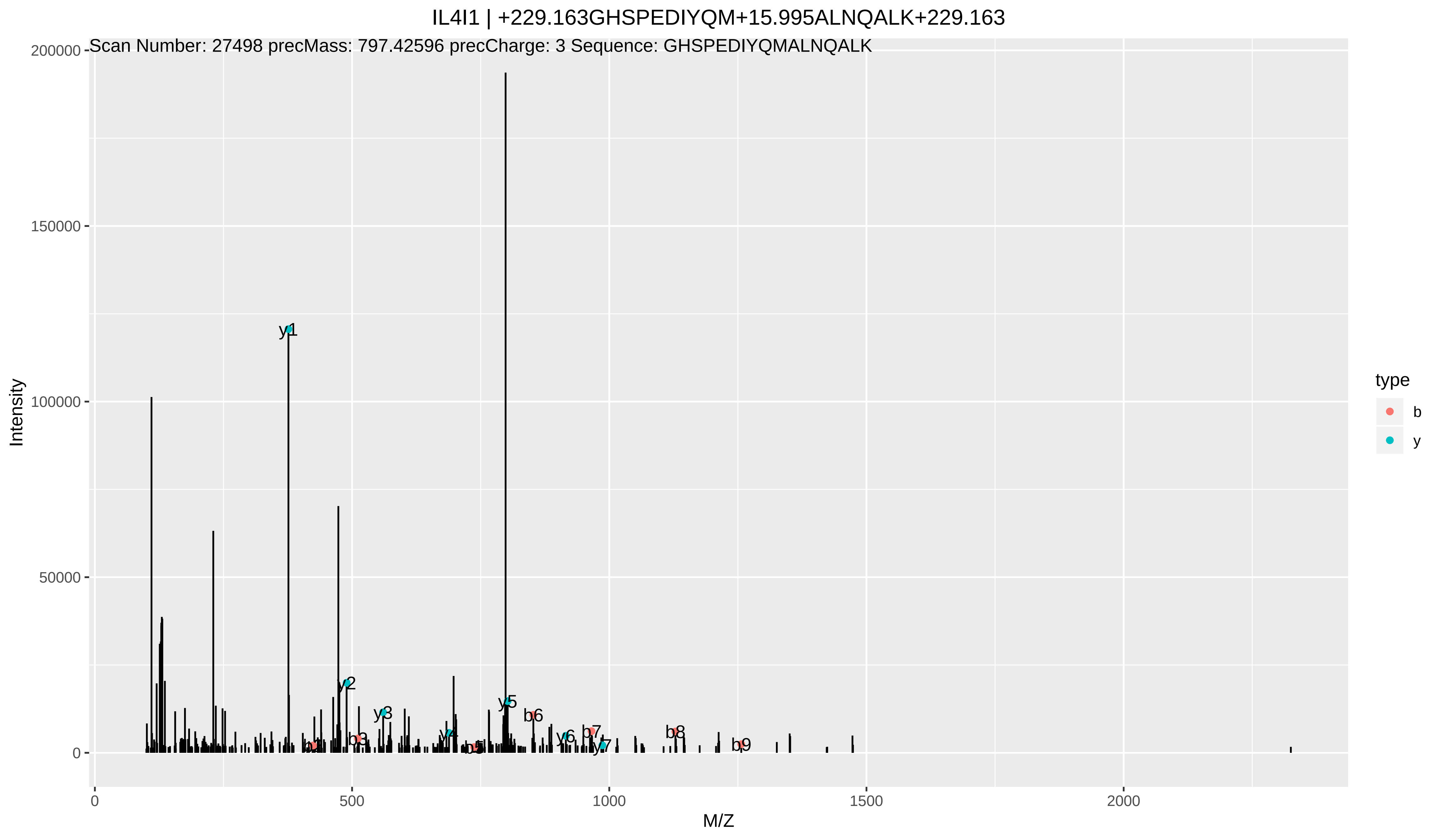
IL4I1 | +229.163GHSPEDIYQM+15.995ALNQALK+229.163
Scan Number: 27498 precMass: 797.42596 precCharge: 3 Sequence: GHSPEDIYQMALNQALK
Intensity
M/Z
type
b
y
y1
y2
y3
y4
y5
y6
y7
b3
b6
b7
b8
b9
200000
150000
100000
50000
0
0
500
1000
1500
2000

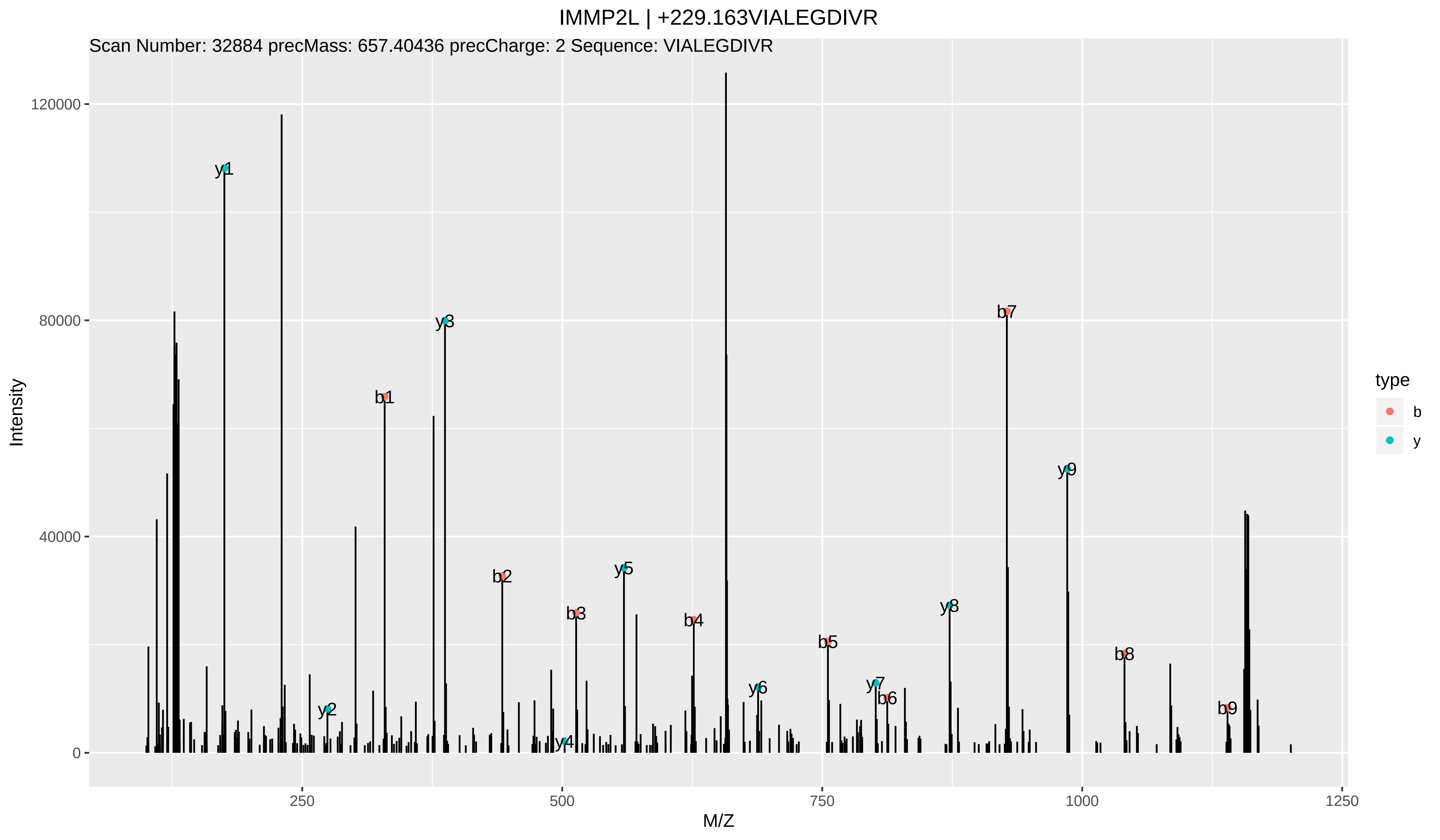
IMMP2L | +229.163VIALEGDIVR
Scan Number: 32884 precMass: 657.40436 precCharge: 2 Sequence: VIALEGDIVR
Intensity
M/Z
120000
80000
40000
0
250
500
750
1000
1250
y1
y2
y3
y4
y5
y6
y7
y8
y9
b1
b2
b3
b4
b5
b6
b7
b8
b9
type
b
y

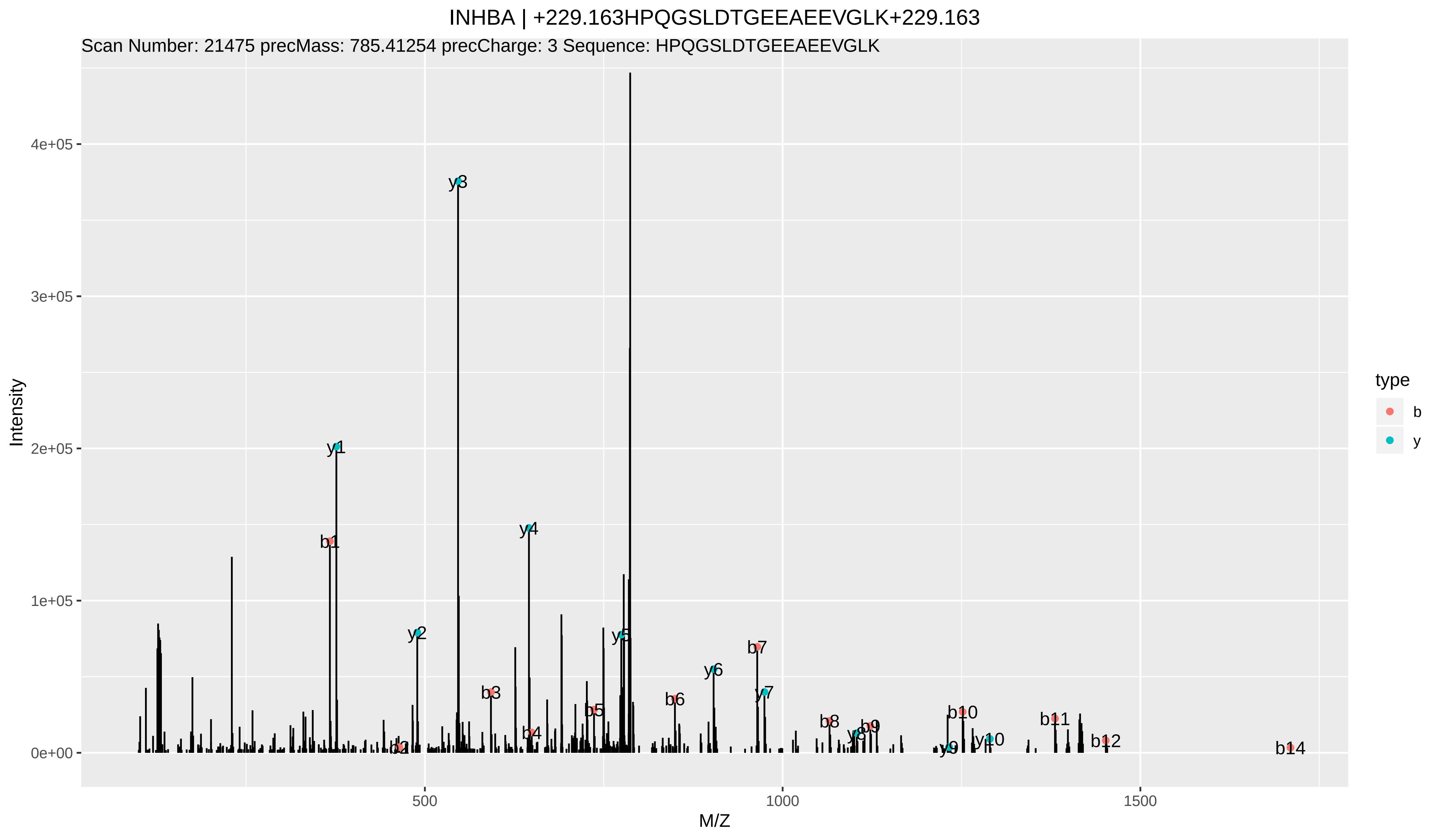
INHBA | +229.163HPQGSLDTGEEAEEVGLK+229.163
Scan Number: 21475 precMass: 785.41254 precCharge: 3 Sequence: HPQGSLDTGEEAEEVGLK
Intensity
4e+05
3e+05
2e+05
1e+05
0e+00
M/Z
500
1000
1500
type
b
y
y1
y2
y3
y4
y5
y6
y7
y8
y9
y10
b1
b2
b3
b4
b5
b6
b7
b8
b9
b10
b11
b12
b14

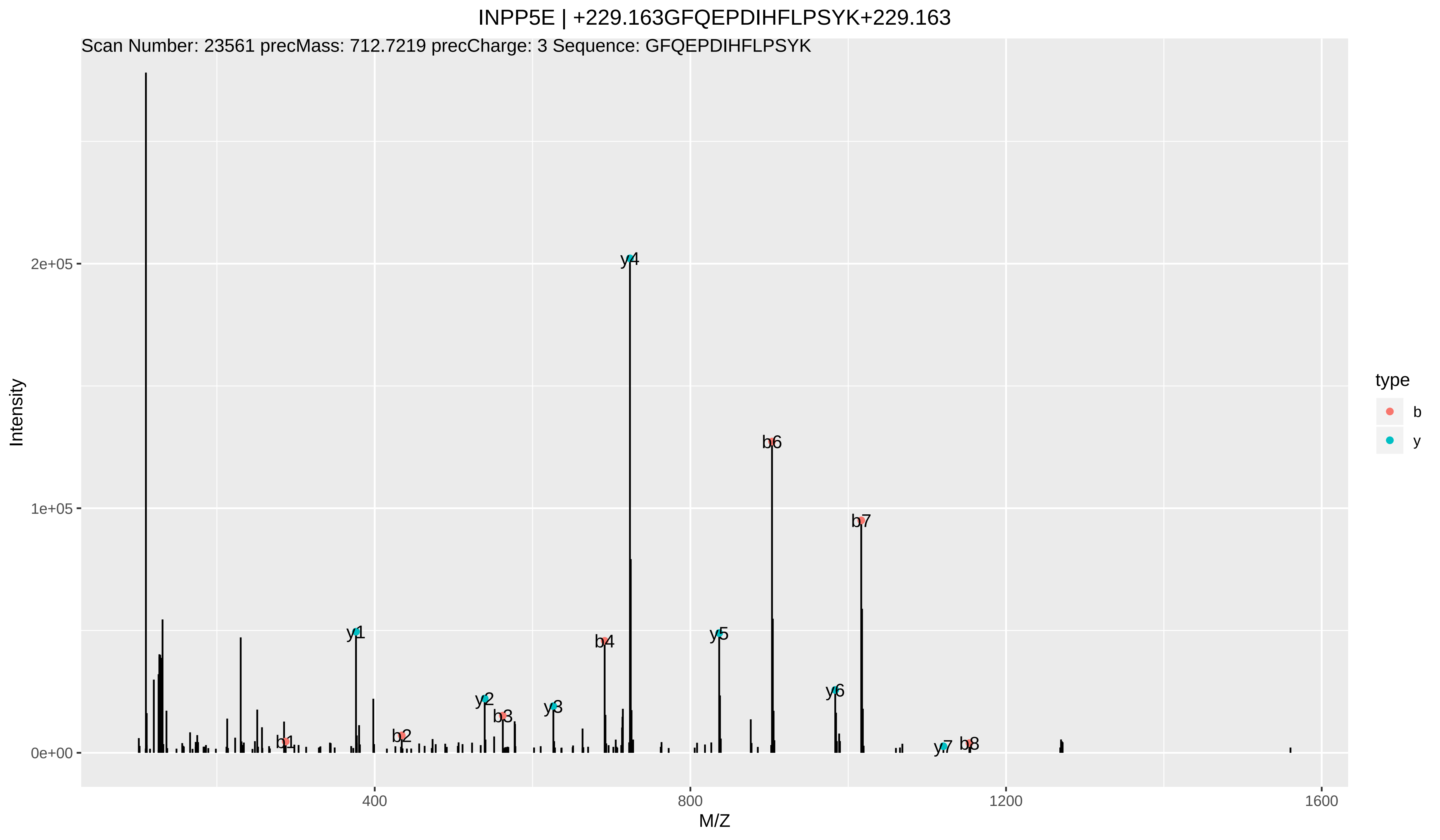

INPP5E | +229.163GFQEPDIHFLPSYK+229.163
Scan Number: 23561 precMass: 712.7219 precCharge: 3 Sequence: GFQEPDIHFLPSYK
Intensity
M/Z
0e+00
1e+05
2e+05
400
800
1200
1600
y4
b6
b7
y1
b4
y5
y6
y2
b3
y3
b2
b1
y7
b8
type
b
y

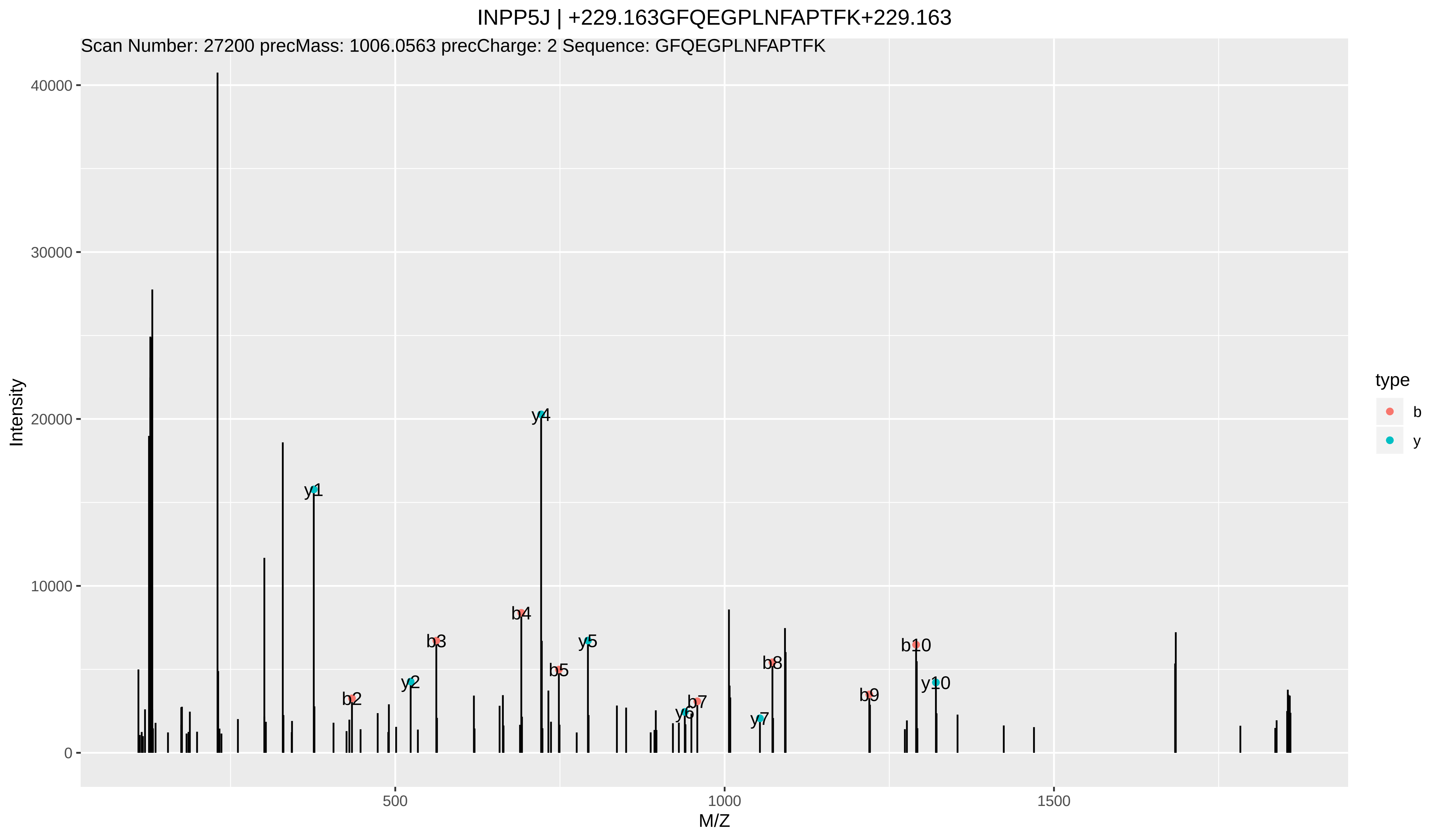

INPP5J | +229.163GFQEGPLNFAPTFK+229.163
Scan Number: 27200 precMass: 1006.0563 precCharge: 2 Sequence: GFQEGPLNFAPTFK
Intensity
40000
30000
20000
10000
0
500
1000
1500
M/Z
y1
y2
y4
y5
y6
y7
y10
b2
b3
b4
b5
b7
b8
b9
b10
type
b
y

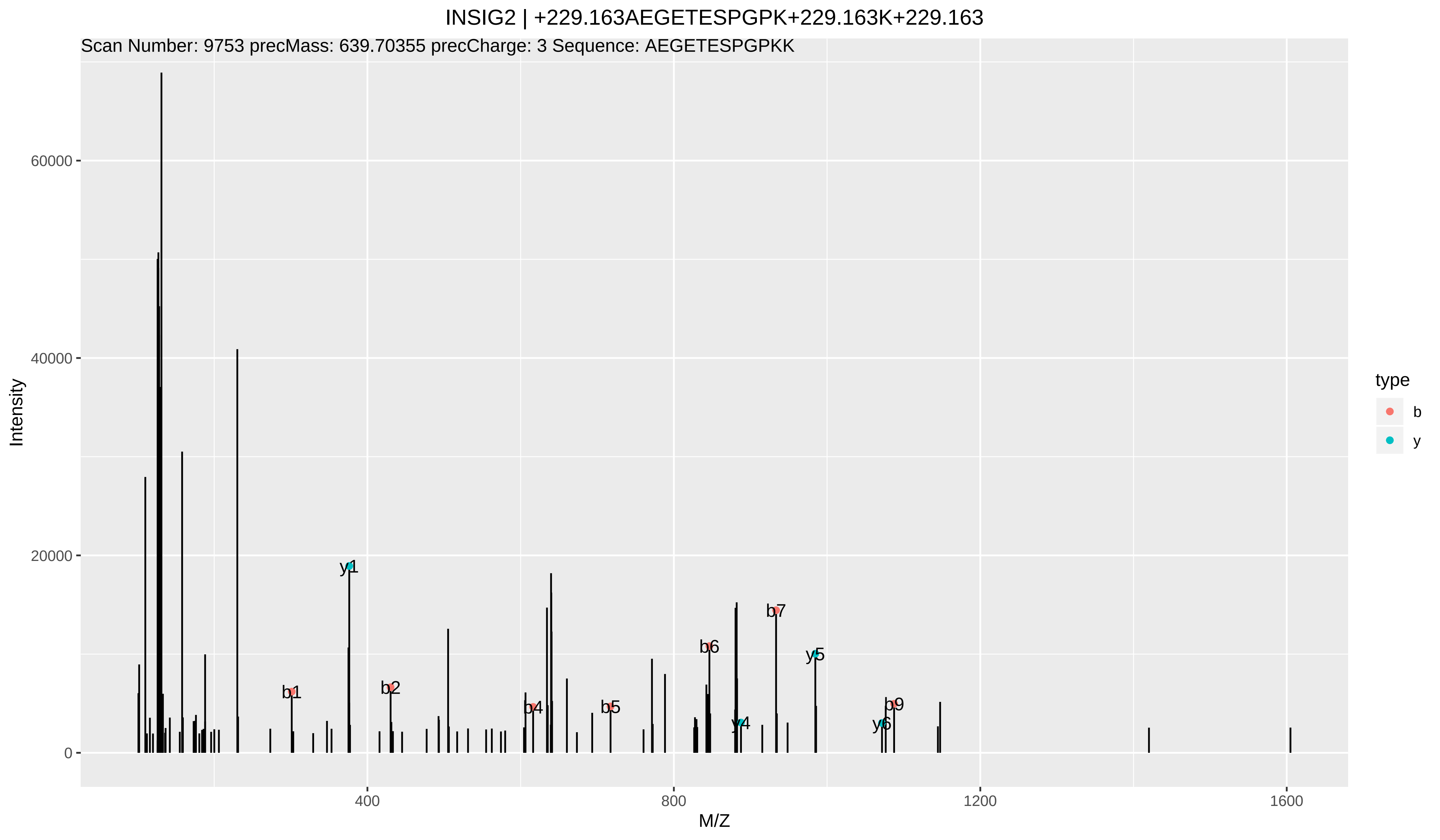
INSIG2 | +229.163AEGETESPGPK+229.163K+229.163
Scan Number: 9753 precMass: 639.70355 precCharge: 3 Sequence: AEGETESPGPKK
Intensity
0
20000
40000
60000
M/Z
400
800
1200
1600
b1
y1
b2
b4
b5
b6
y4
b7
y5
y6
b9
type
b
y

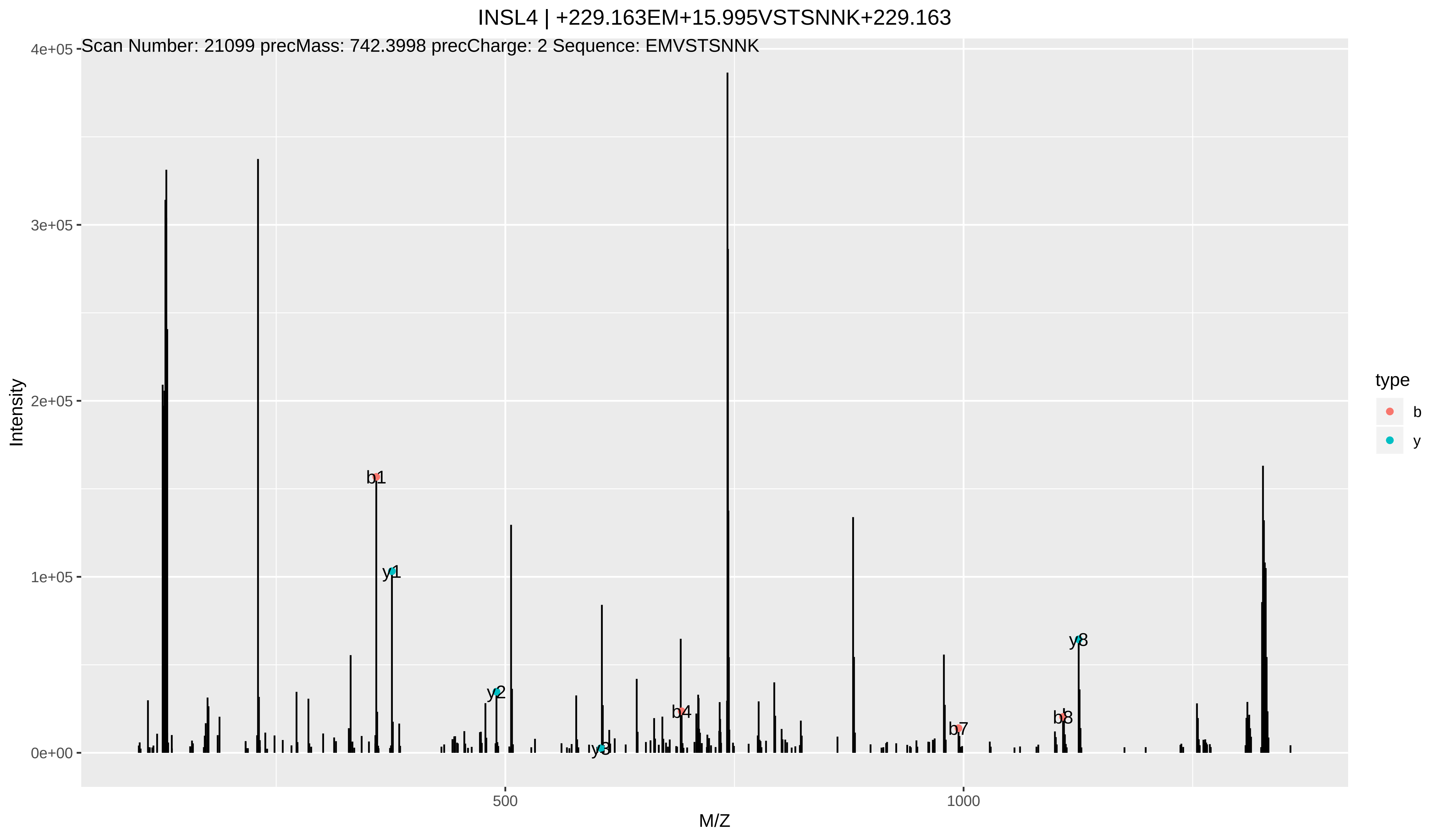

INSL4 | +229.163EM+15.995VSTSNNK+229.163
Scan Number: 21099 precMass: 742.3998 precCharge: 2 Sequence: EMVSTSNNK
Intensity
4e+05
3e+05
2e+05
1e+05
0e+00
M/Z
500
1000
b1
y1
y2
y3
b4
b7
b8
y8
type
b
y

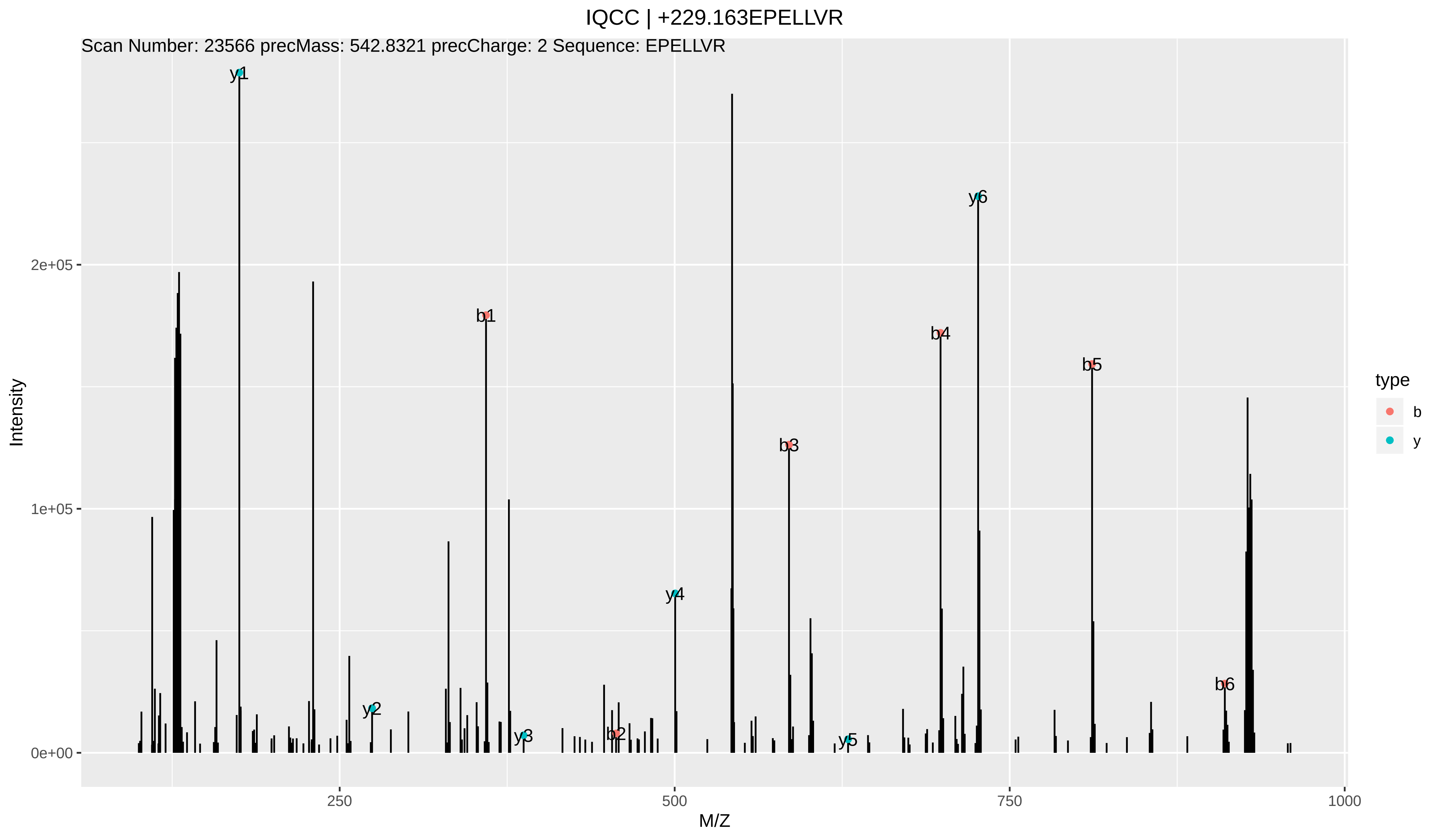
IQCC | +229.163EPELLVR
Scan Number: 23566 precMass: 542.8321 precCharge: 2 Sequence: EPELLVR
y1
y6
b1
b4
b5
b3
y4
y2
y3
b2
y5
b6
Intensity
2e+05
1e+05
0e+00
M/Z
250
500
750
1000
type
b
y

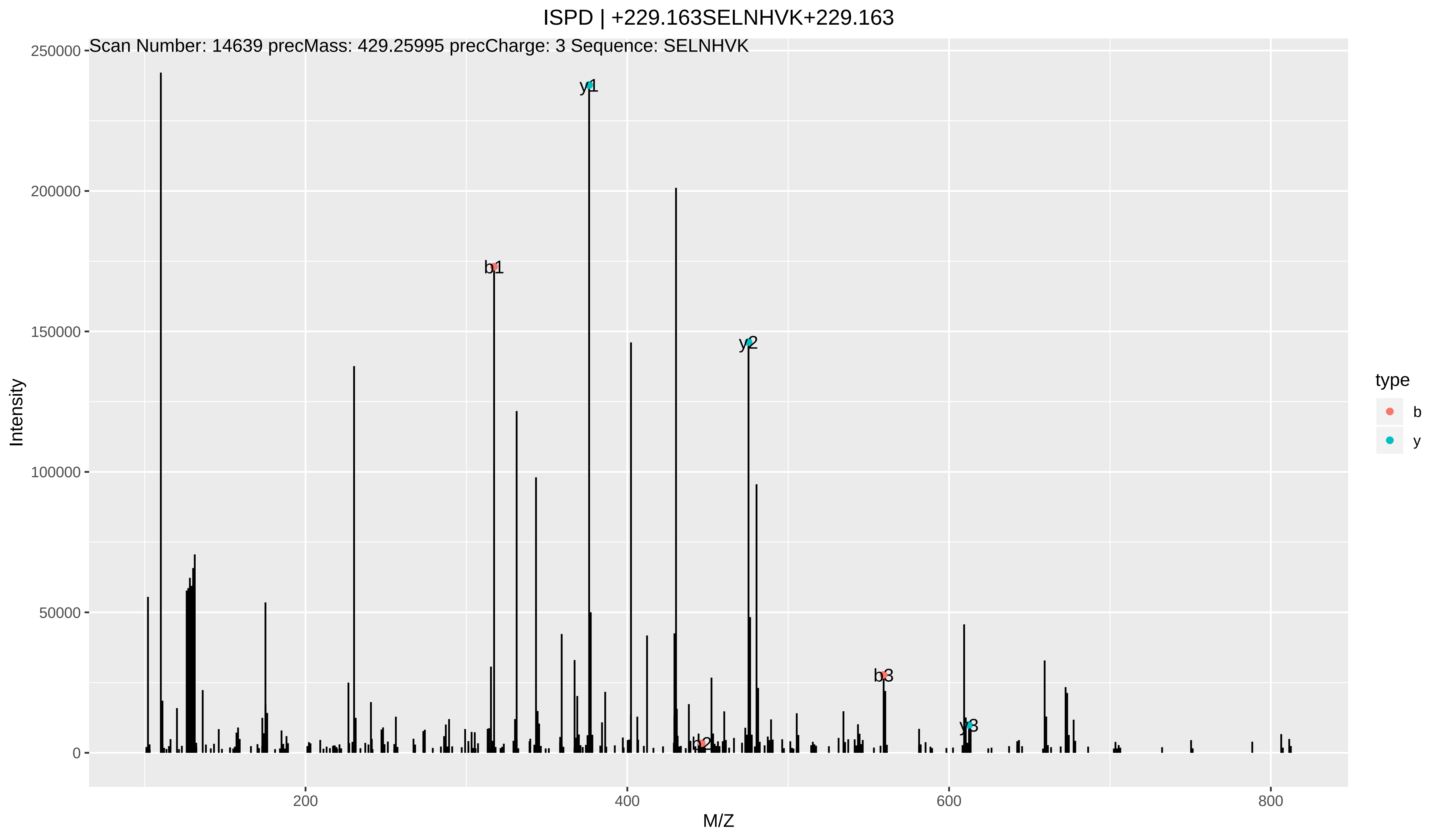
ISPD | +229.163SELNHVK+229.163
Scan Number: 14639 precMass: 429.25995 precCharge: 3 Sequence: SELNHVK
Intensity
M/Z
0
50000
100000
150000
200000
250000
200
400
600
800
y1
b1
y2
b2
b3
y3
type
b
y

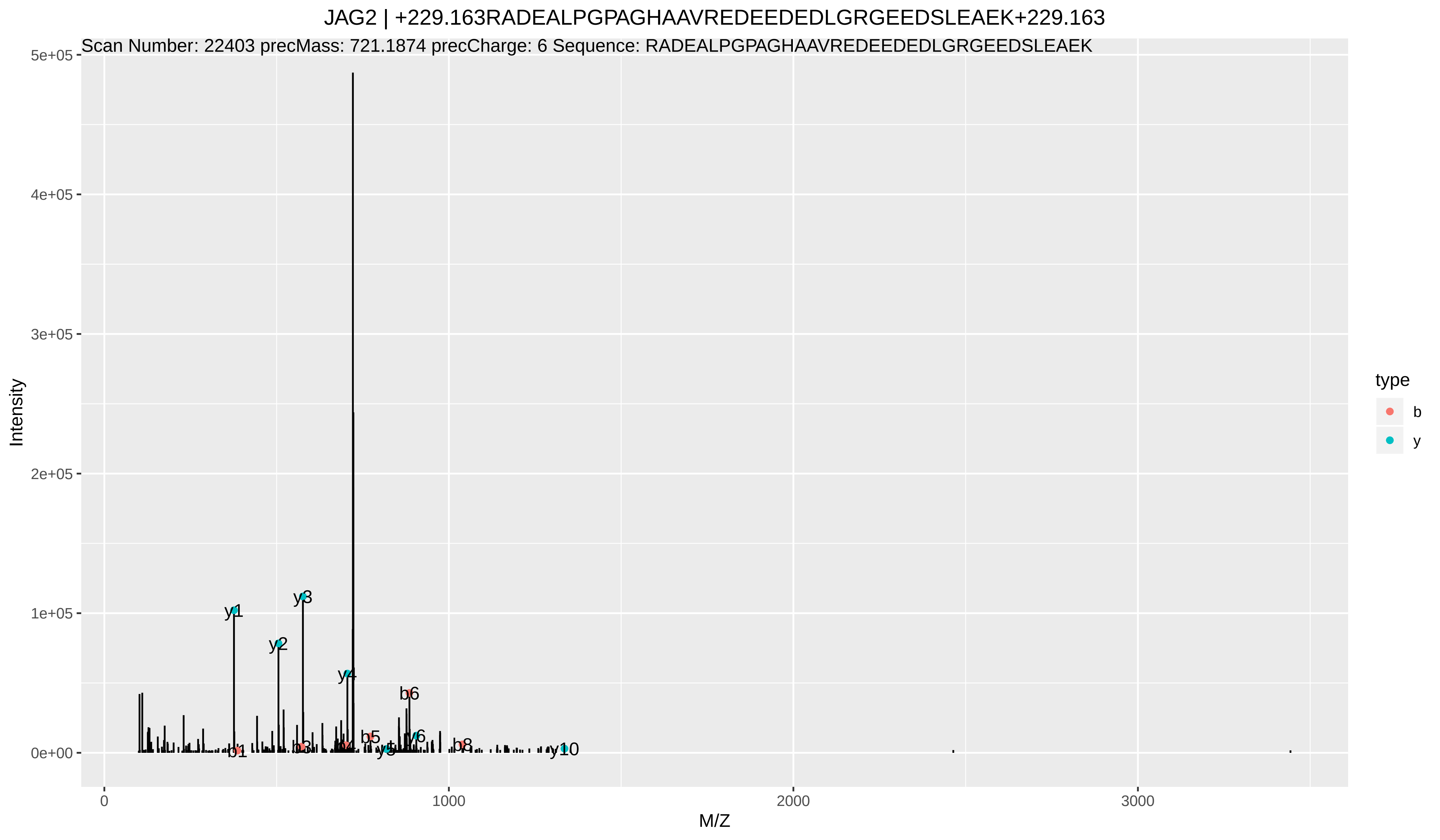

JAG2 | +229.163RADEALPGPAGHAAVREDEEDEDLGRGEEDSLEAEK+229.163
Scan Number: 22403 precMass: 721.1874 precCharge: 6 Sequence: RADEALPGPAGHAAVREDEEDEDLGRGEEDSLEAEK
Intensity
M/Z
type
b
y
0e+00
1e+05
2e+05
3e+05
4e+05
5e+05
0
1000
2000
3000
y1
y2
y3
y4
b6
b1
b3
b5
y5
y6
b8
y10

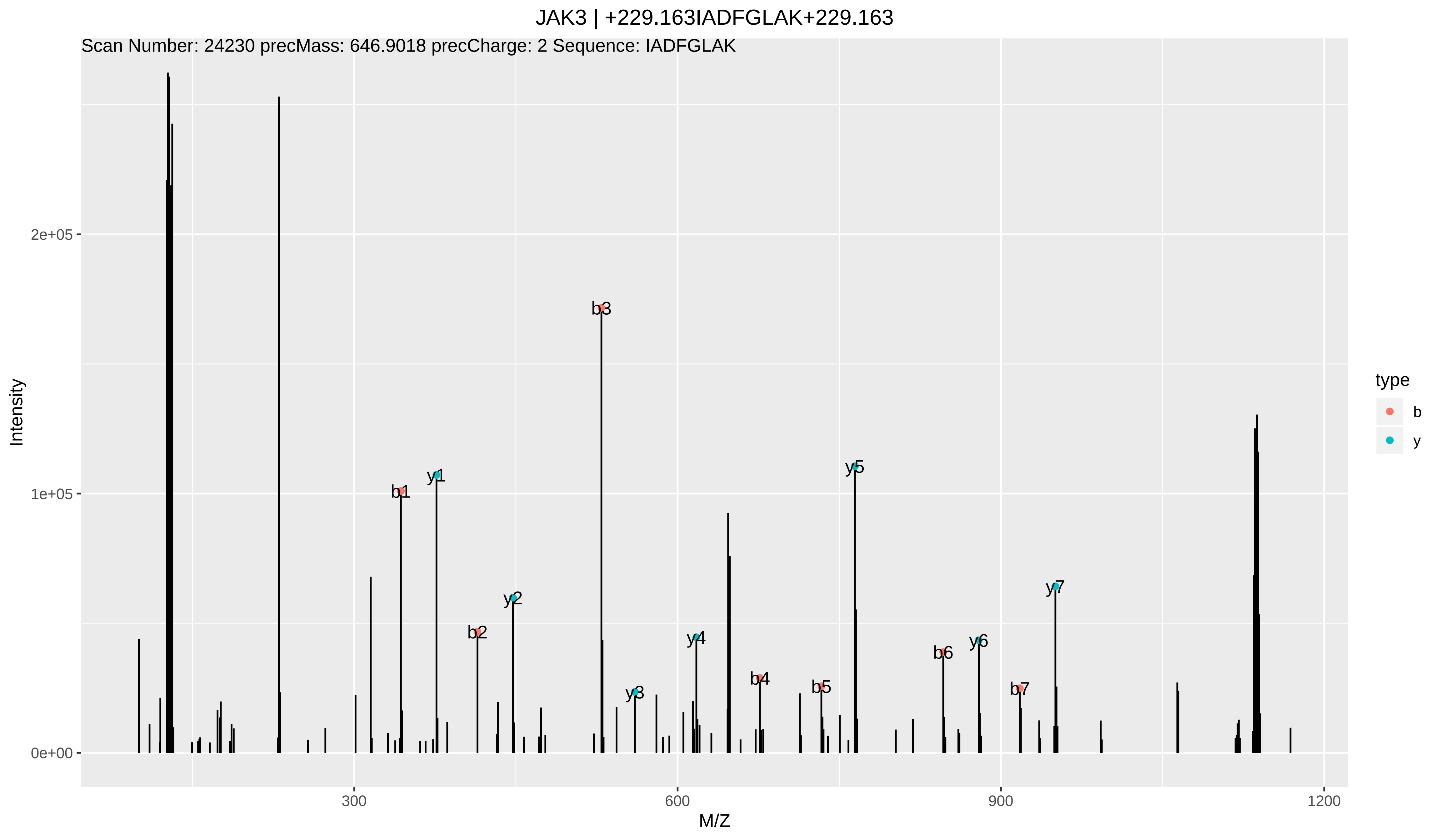

JAK3 | +229.163IADFGLAK+229.163
Scan Number: 24230 precMass: 646.9018 precCharge: 2 Sequence: IADFGLAK
Intensity
2e+05
1e+05
0e+00
M/Z
300
600
900
1200
b3
y1
b1
y5
y7
y2
b2
y4
y6
b6
b4
b5
b7
y3
type
b
y

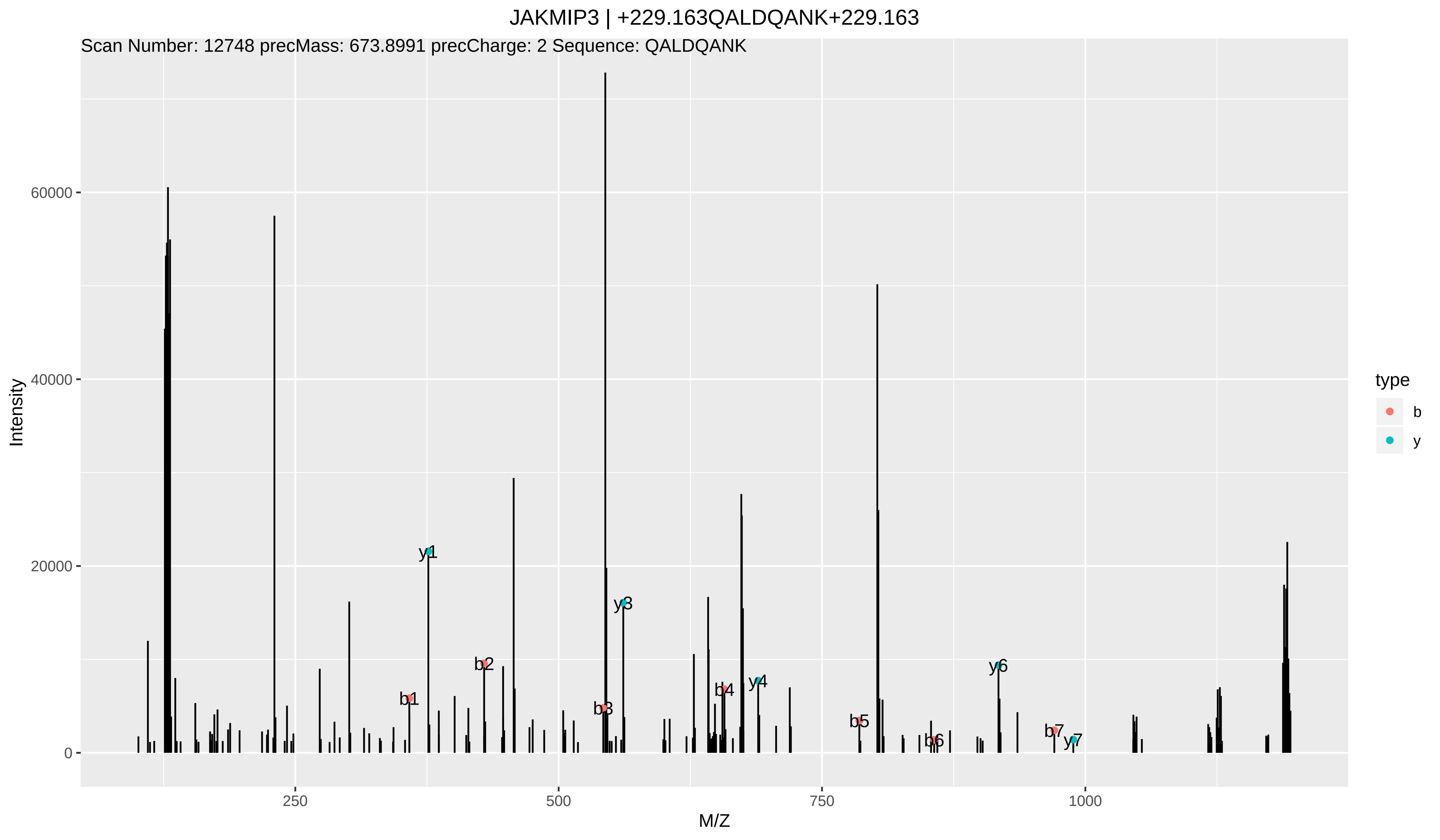

JAKMIP3 | +229.163QALDQANK+229.163
Scan Number: 12748 precMass: 673.8991 precCharge: 2 Sequence: QALDQANK
Intensity
M/Z
60000
40000
20000
0
250
500
750
1000
y1
b1
b2
b3
y3
b4
y4
b5
b6
y6
b7
y7
type
b
y

JDP2 | +229.163NLGAM+15.995IAPLHFLEVK+229.163

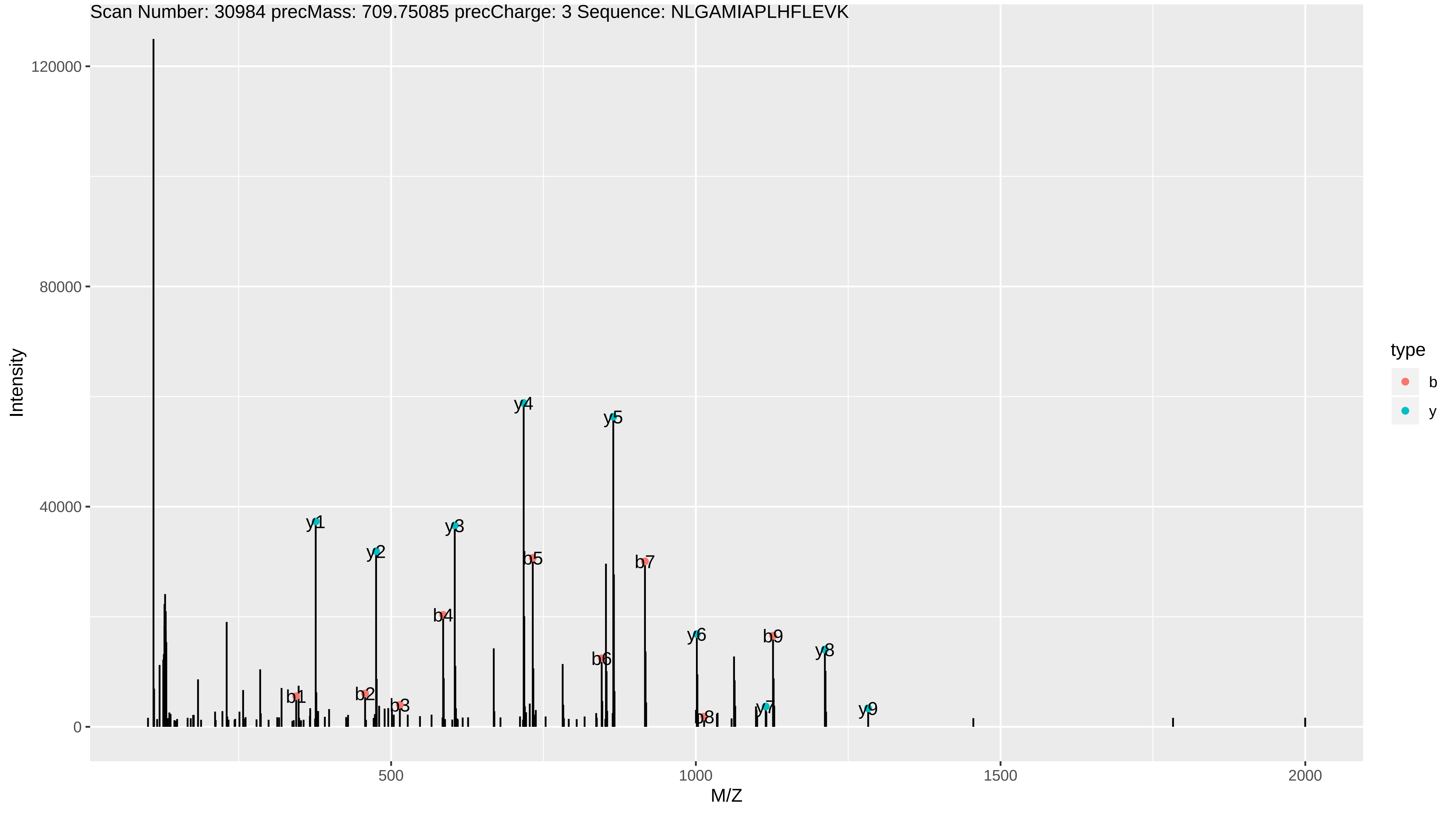

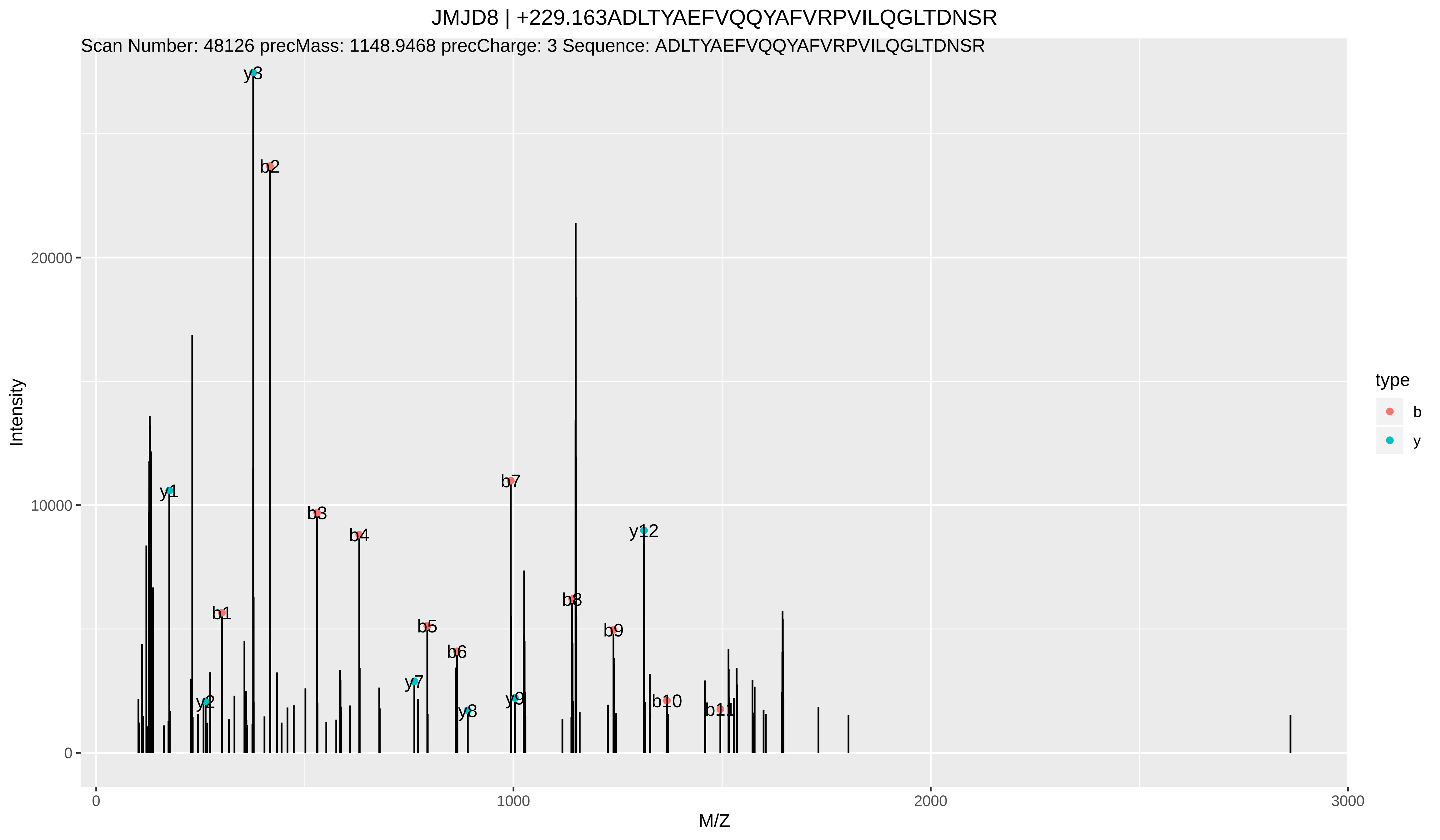

JMJD8 | +229.163ADLTYAEFVQQYAFVRPVILQGLTDNSR
Scan Number: 48126 precMass: 1148.9468 precCharge: 3 Sequence: ADLTYAEFVQQYAFVRPVILQGLTDNSR
y3
b2
y1
b3
b4
b7
y12
b8
b1
b5
b9
b6
y7
y2
y9
b10
y8
b11
Intensity
20000
10000
0
M/Z
0
1000
2000
3000
type
b
y

JOSD1 | +229.163SESLELPQAAPPQIYHEK+229.163

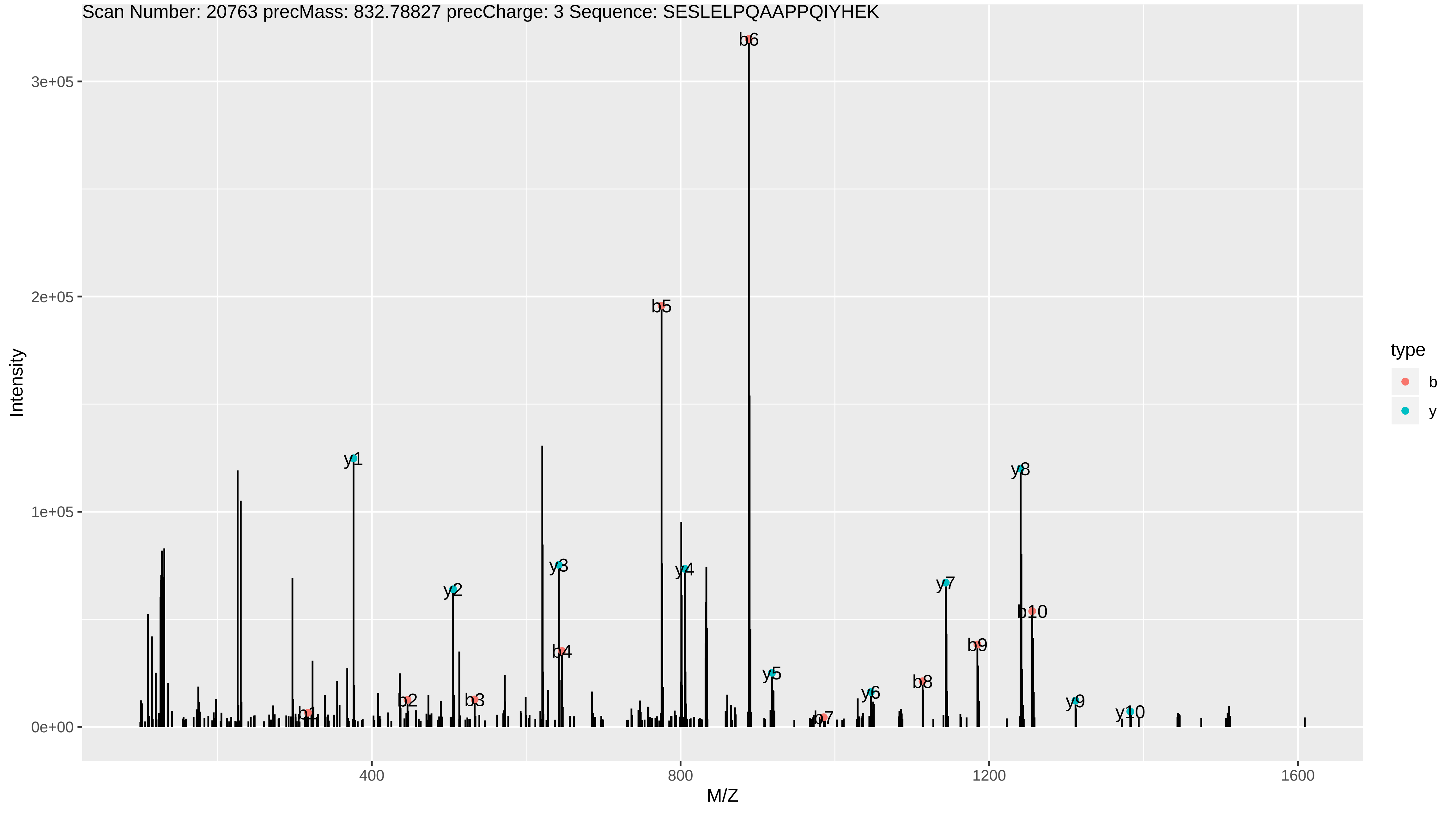

JOSD2 | +229.163QVDGVYYNLDSK+229.163

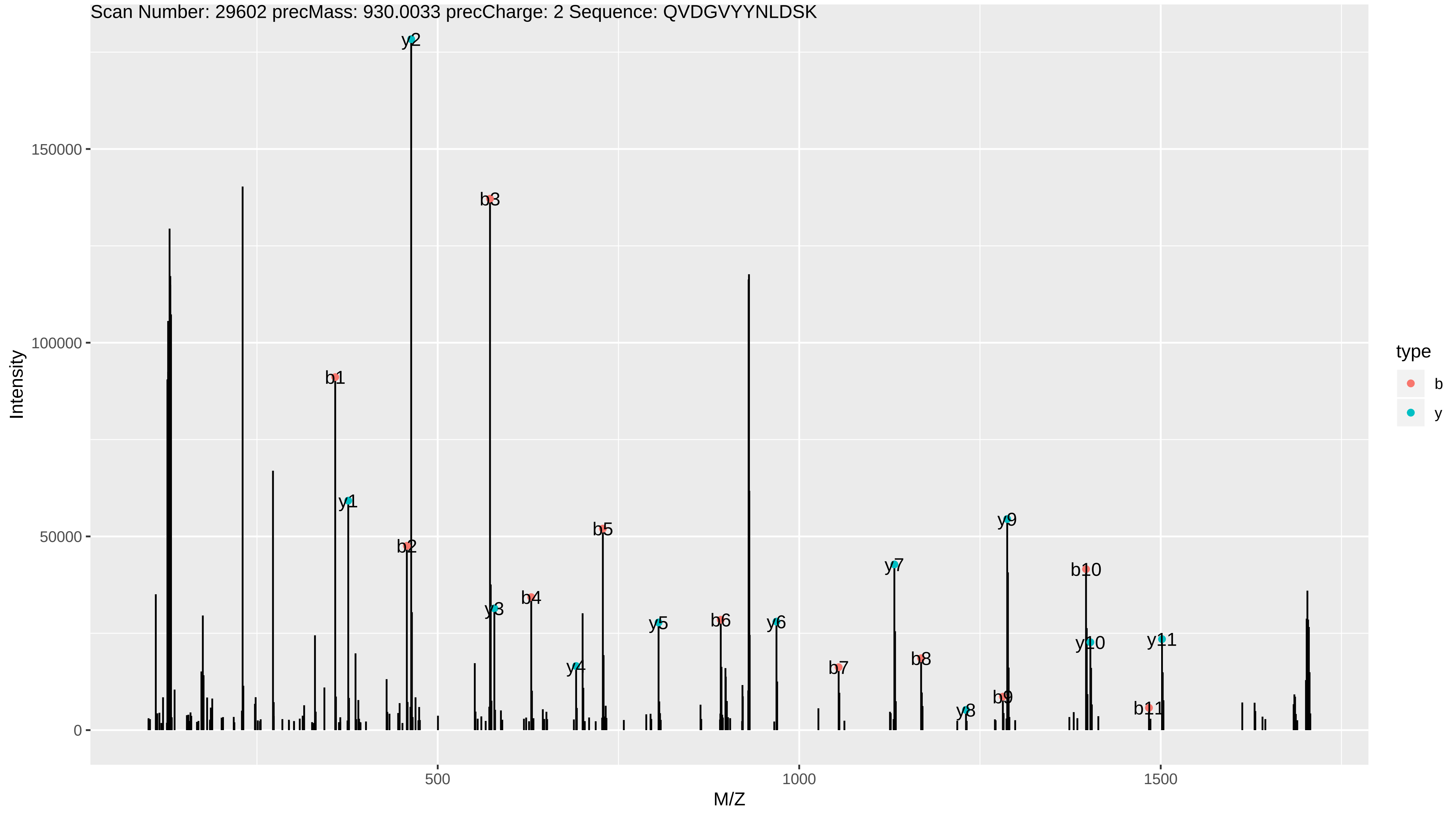

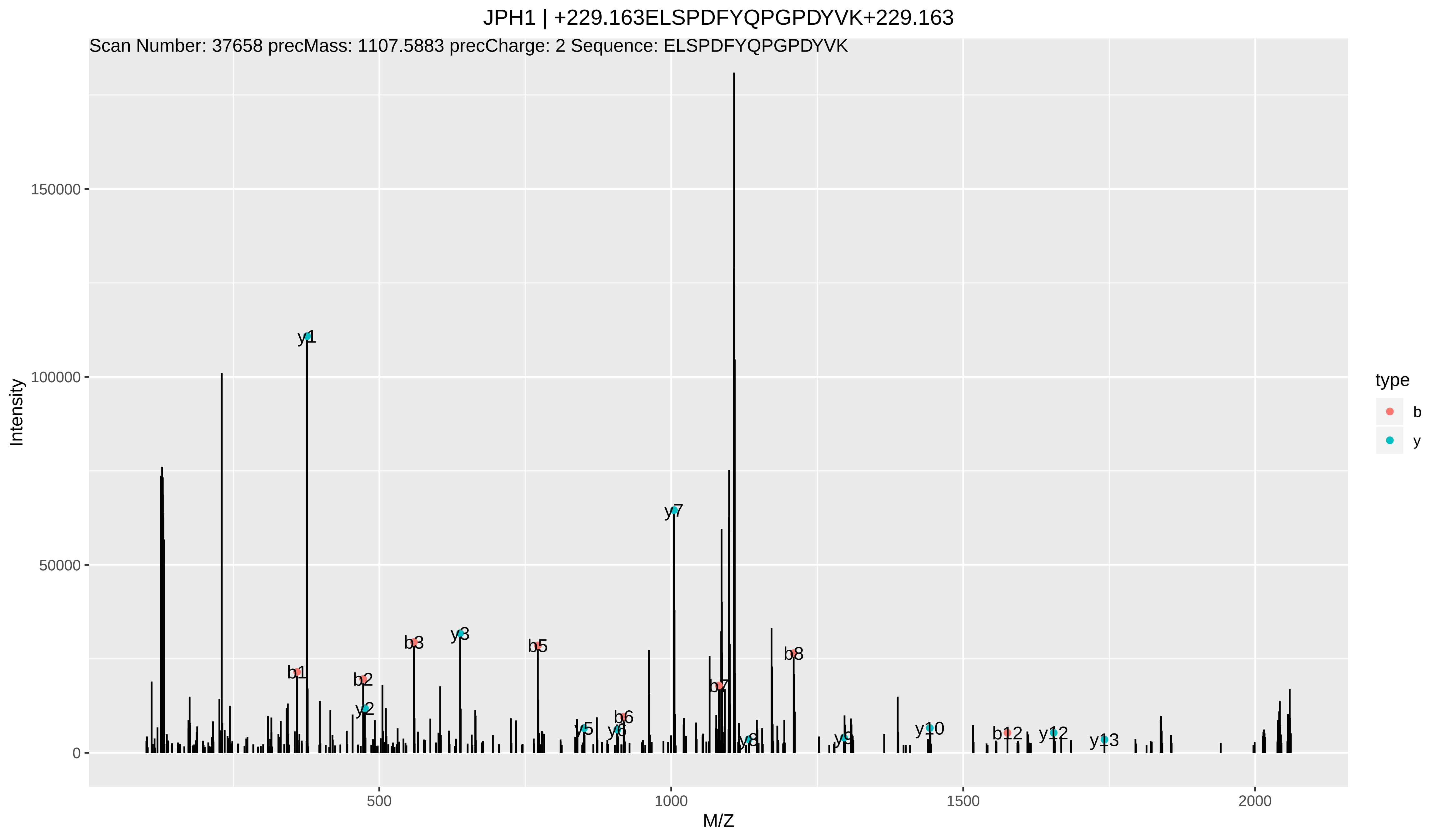
JPH1 | +229.163ELSPDFYQPGPDYVK+229.163
Scan Number: 37658 precMass: 1107.5883 precCharge: 2 Sequence: ELSPDFYQPGPDYVK
Intensity
150000
100000
50000
0
500
1000
1500
2000
M/Z
y1
b1
b2
y2
b3
y3
b5
y5
y6
b6
y7
b7
y8
b8
y9
y10
b12
y12
y13
type
b
y

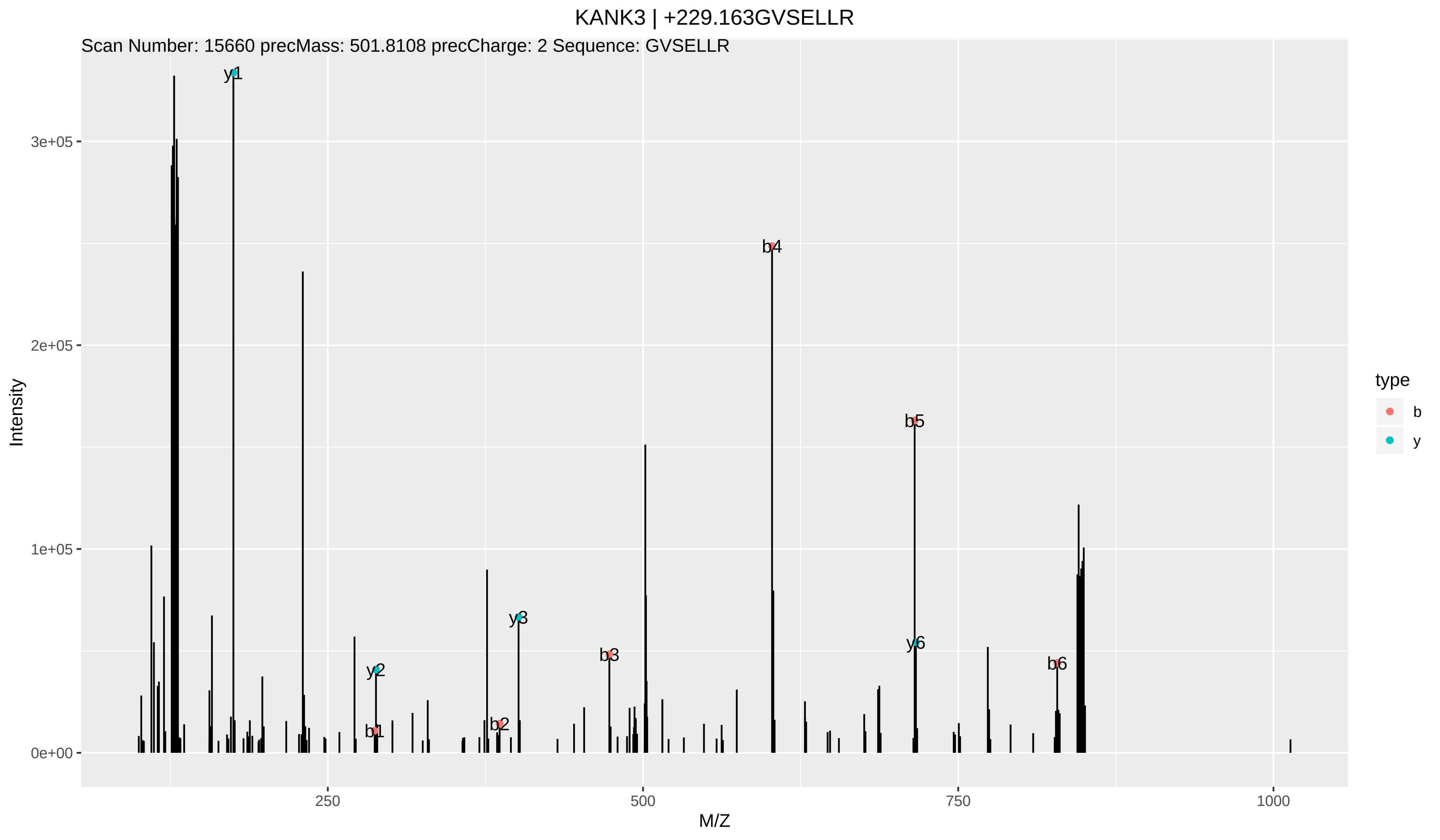
KANK3 | +229.163GVSELLR
Scan Number: 15660 precMass: 501.8108 precCharge: 2 Sequence: GVSELLR
Intensity
M/Z
0e+00
1e+05
2e+05
3e+05
250
500
750
1000
y1
y2
y3
y6
b1
b2
b3
b4
b5
b6
type
b
y

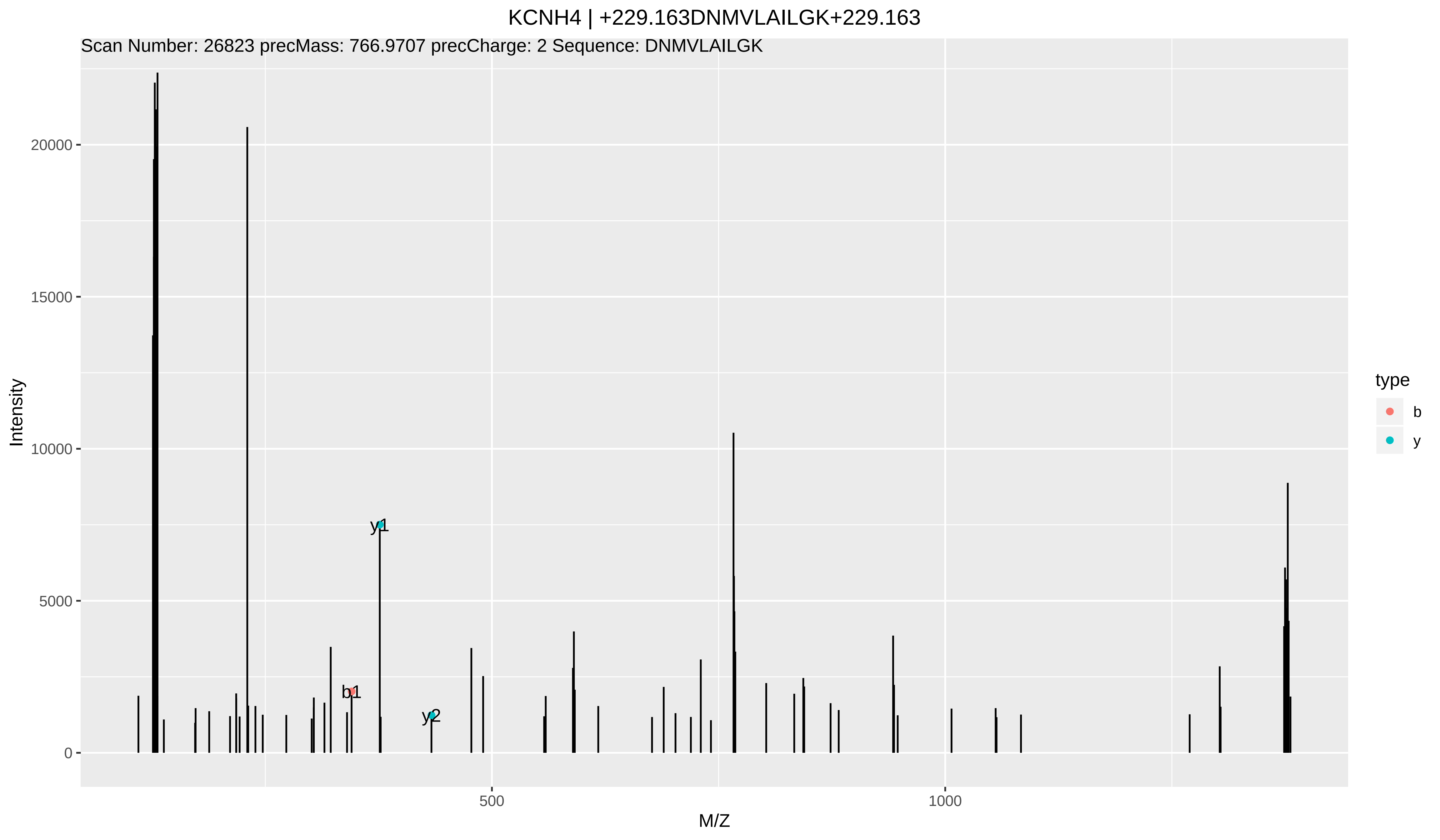

KCNH4 | +229.163DNMVLAILGK+229.163
Scan Number: 26823 precMass: 766.9707 precCharge: 2 Sequence: DNMVLAILGK
Intensity
20000
15000
10000
5000
0
y1
b1
y2
500
1000
M/Z
type
b
y

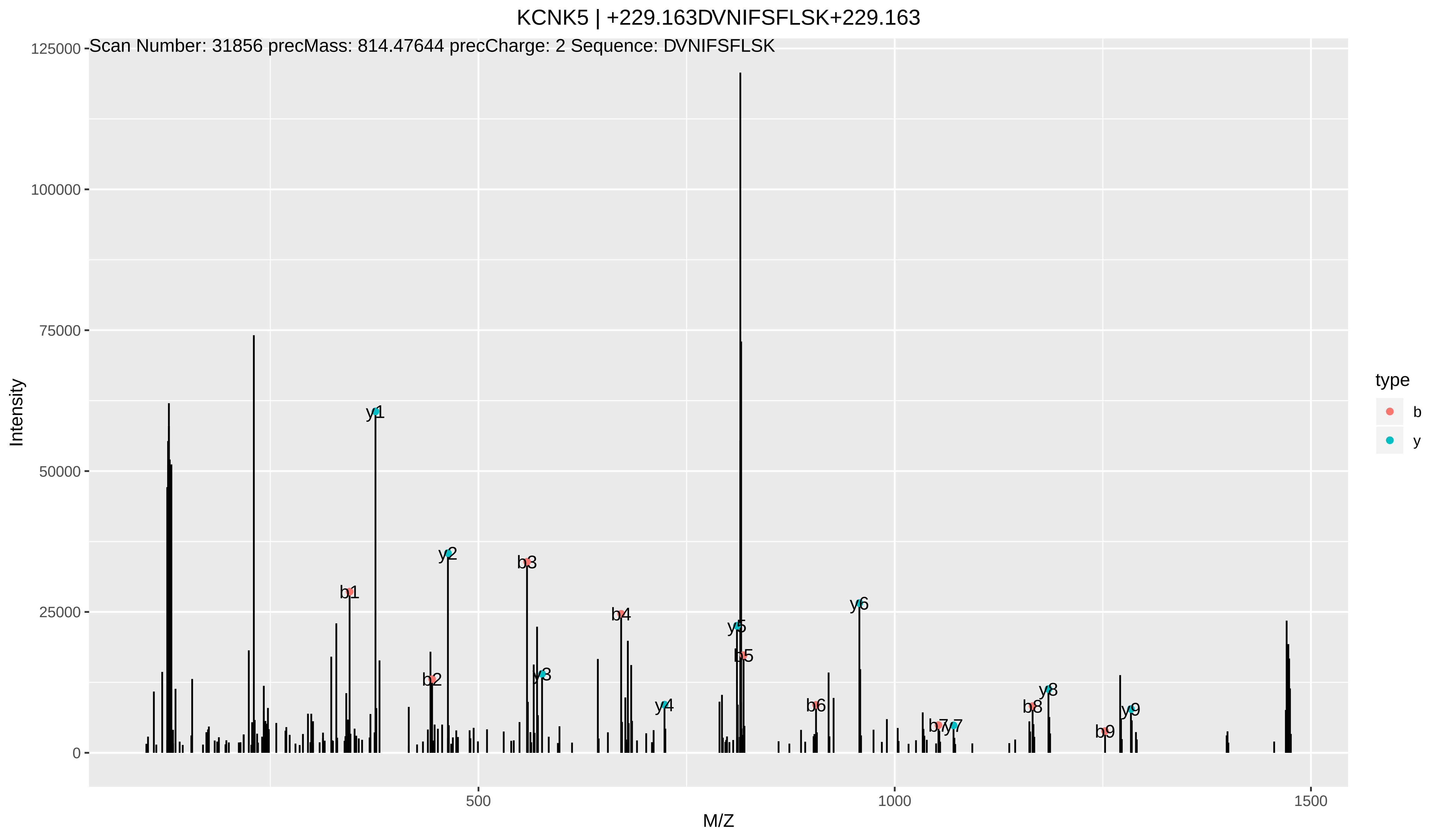

KCNK5 | +229.163DVNIFSFLSK+229.163
Scan Number: 31856 precMass: 814.47644 precCharge: 2 Sequence: DVNIFSFLSK
Intensity
125000
100000
75000
50000
25000
0
500
1000
1500
M/Z
y1
y2
b3
b1
b4
y5
b5
y6
b2
y3
y4
b6
y8
b8
b7
y7
y9
b9
type
b
y

KCNT1 | +229.163TTWWWWTRR

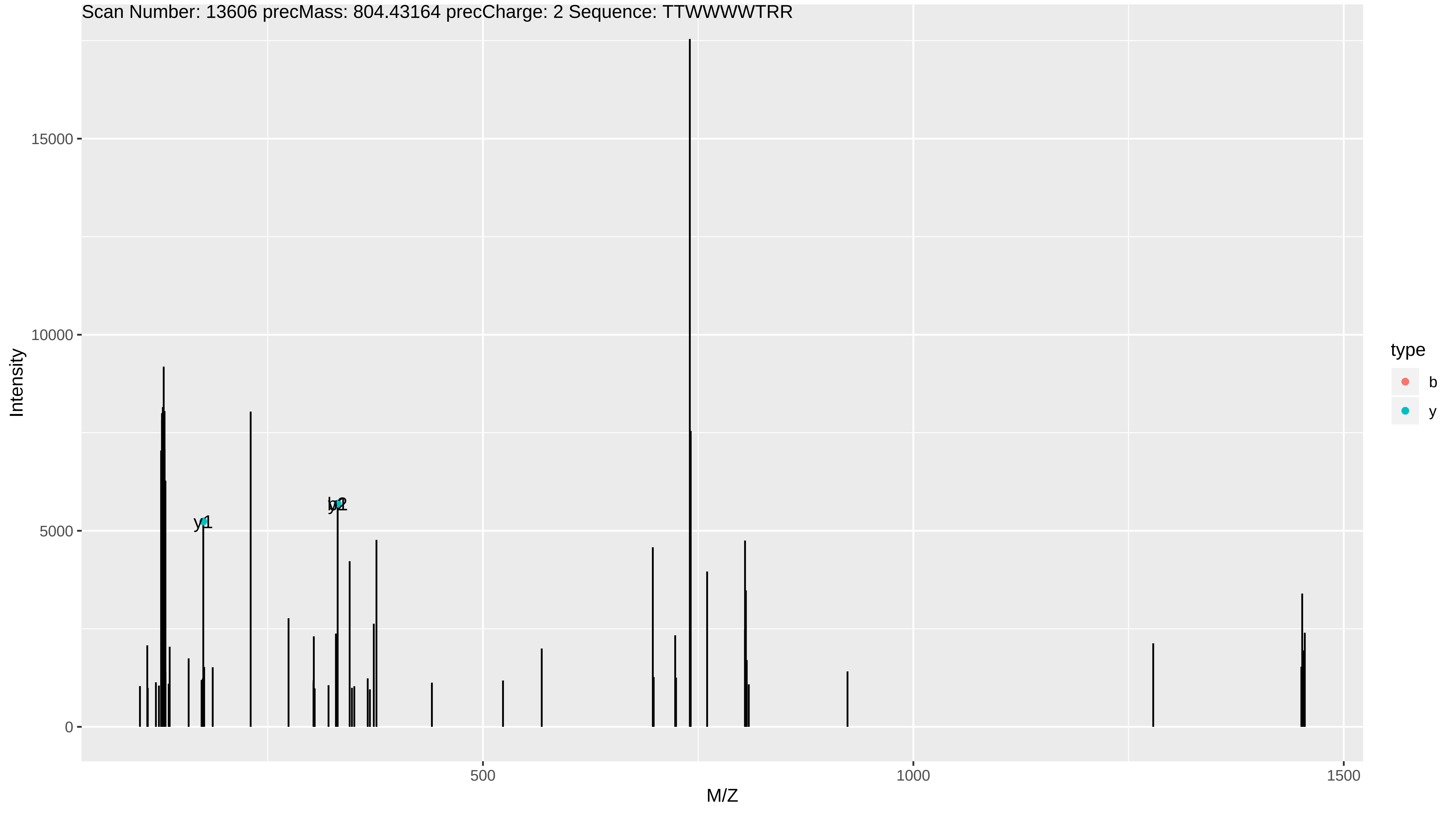

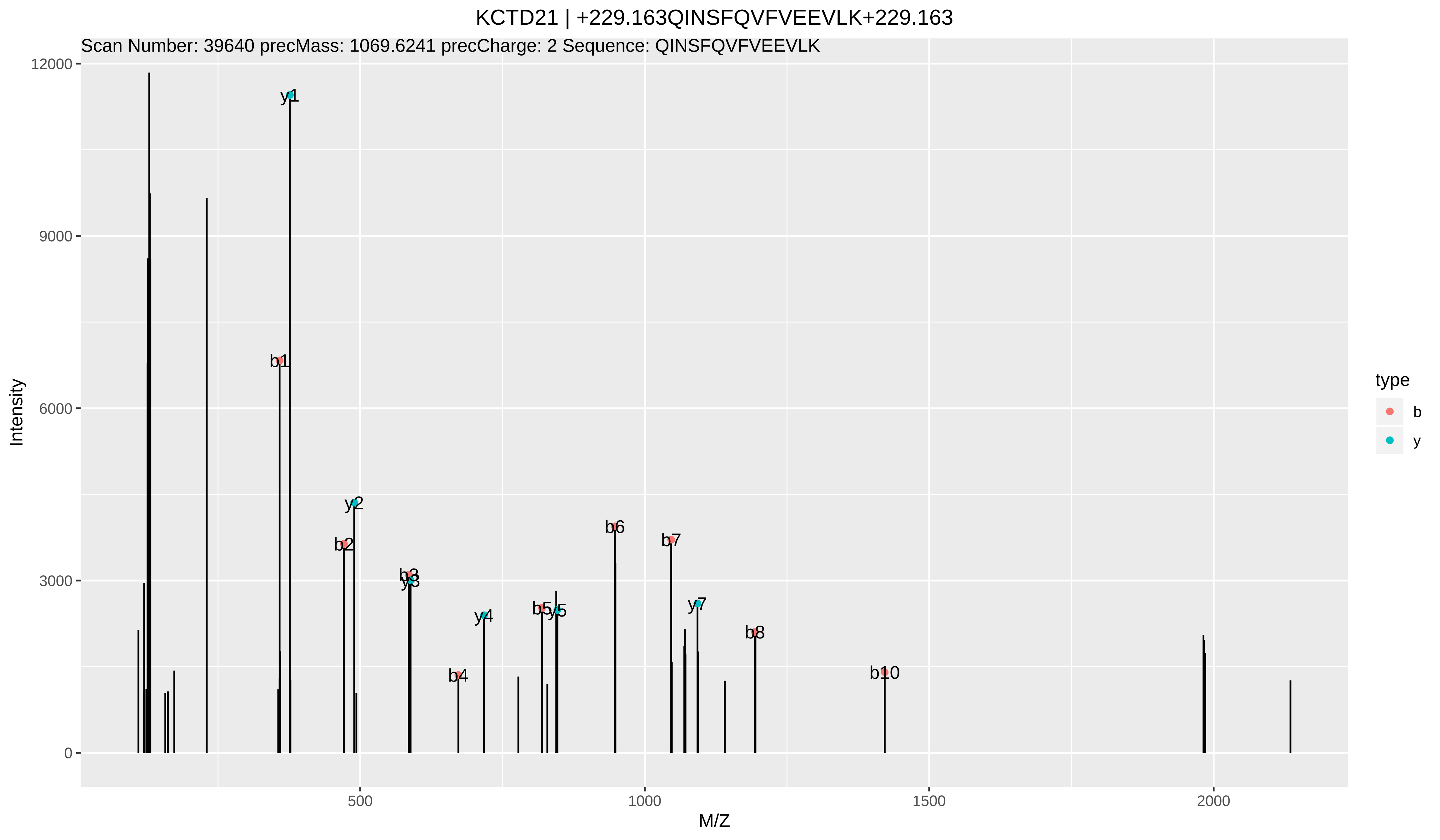

KCTD21 | +229.163QINSFQVFVEEVLK+229.163
Scan Number: 39640 precMass: 1069.6241 precCharge: 2 Sequence: QINSFQVFVEEVLK
Intensity
M/Z
0
3000
6000
9000
12000
500
1000
1500
2000
y1
b1
y2
b2
b3
y3
b4
y4
b5
y5
b6
b7
y7
b8
b10
type
b
y

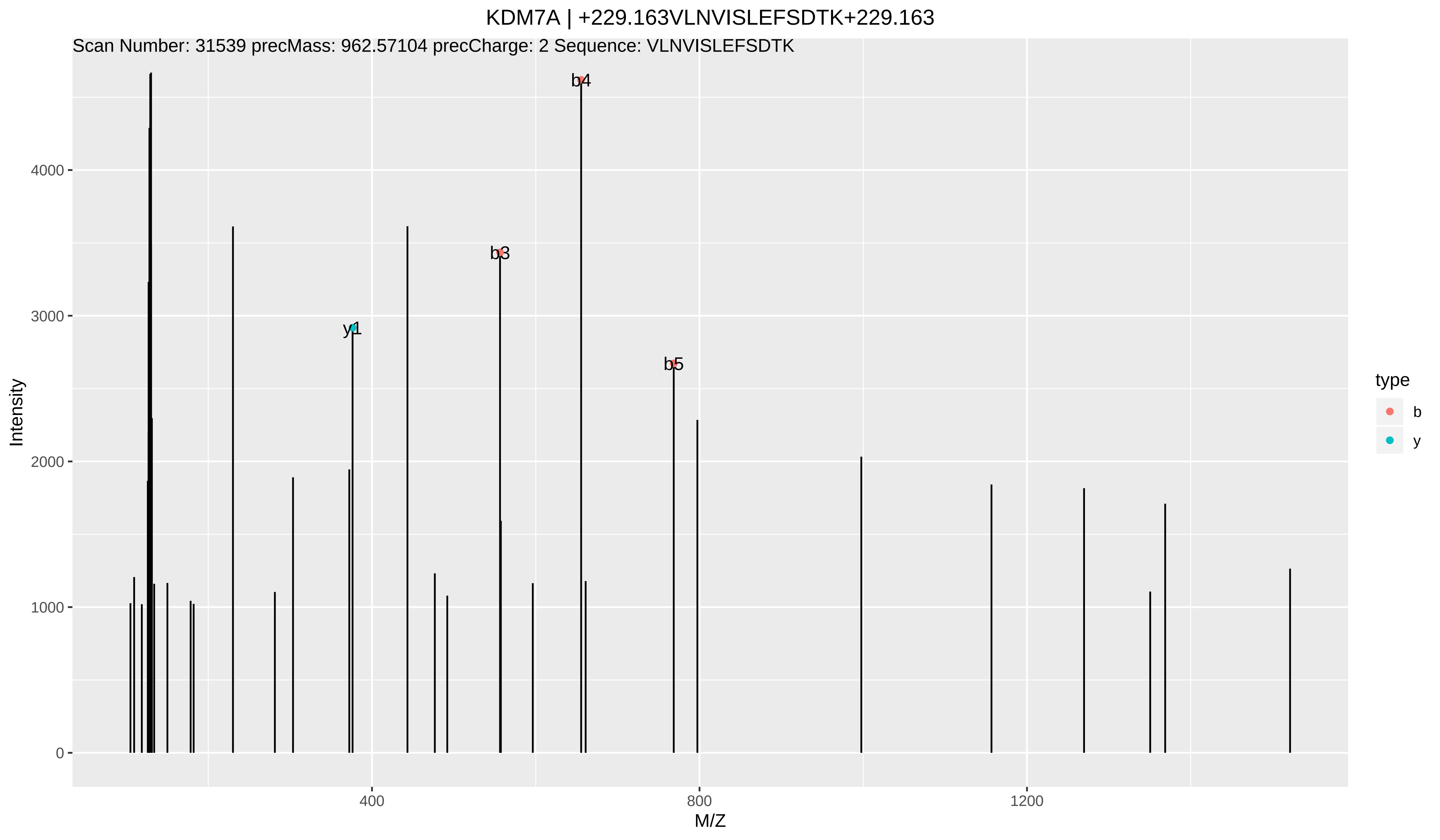

KDM7A | +229.163VLNVISLEFSDTK+229.163
Scan Number: 31539 precMass: 962.57104 precCharge: 2 Sequence: VLNVISLEFSDTK
Intensity
M/Z
y1
b3
b4
b5
0
1000
2000
3000
4000
400
800
1200
type
b
y

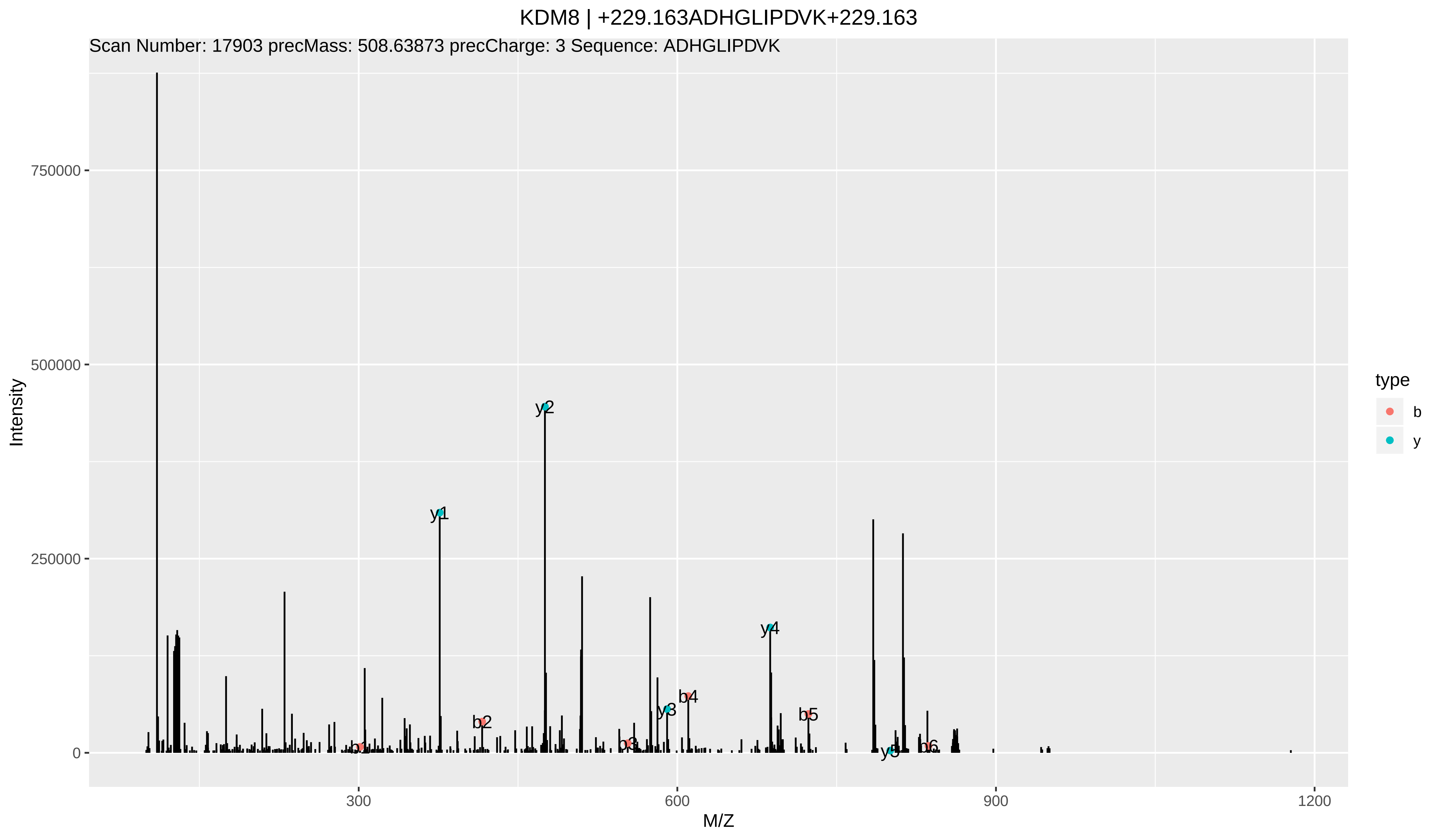

KDM8 | +229.163ADHGLIPDVK+229.163
Scan Number: 17903 precMass: 508.63873 precCharge: 3 Sequence: ADHGLIPDVK
Intensity
750000
500000
250000
0
300
600
900
1200
M/Z
y1
y2
y3
y4
y5
b1
b2
b3
b4
b5
b6
type
b
y

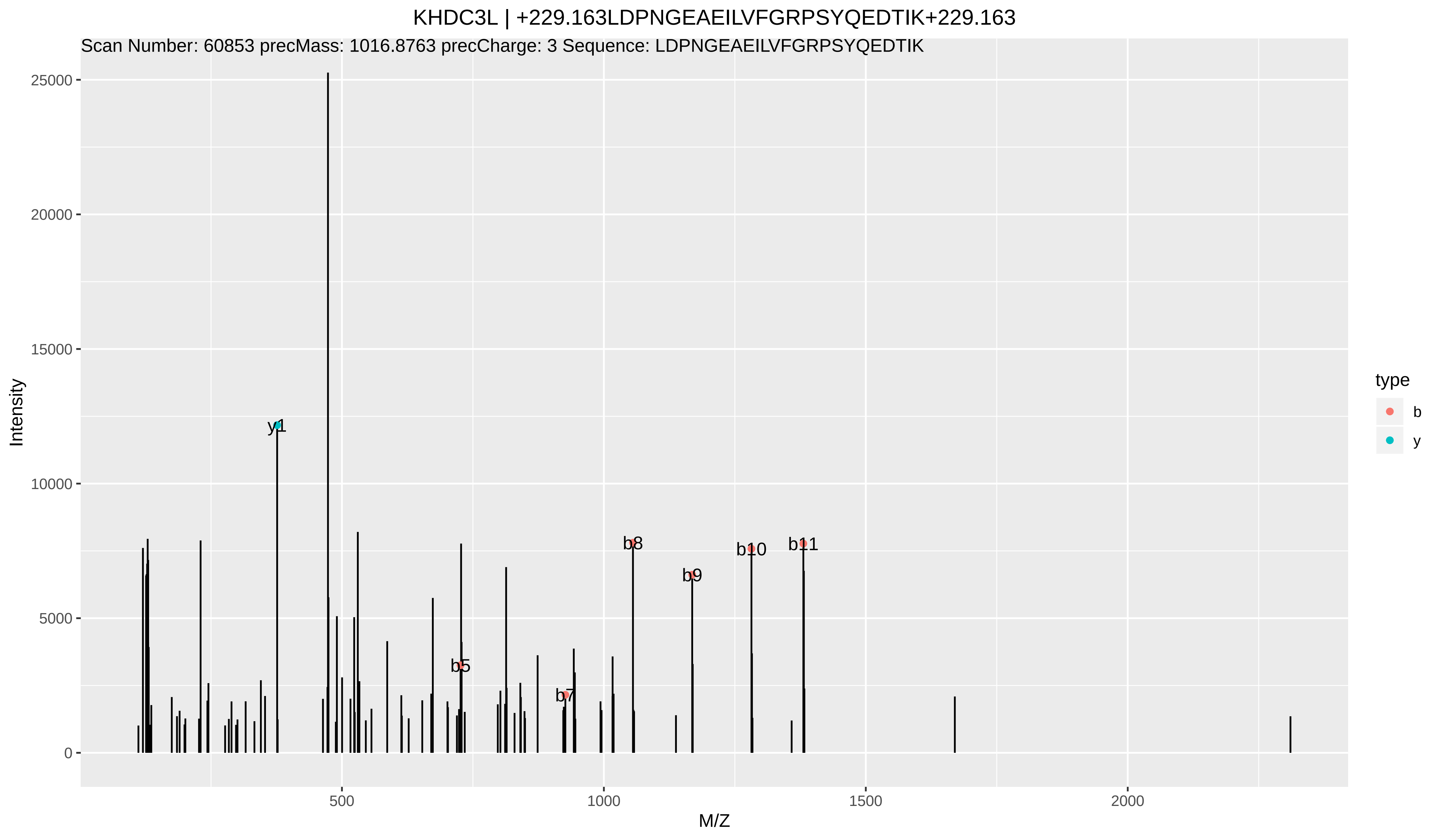

KHDC3L | +229.163LDPNGEAEILVFGRPSYQEDTIK+229.163
Scan Number: 60853 precMass: 1016.8763 precCharge: 3 Sequence: LDPNGEAEILVFGRPSYQEDTIK
Intensity
25000
20000
15000
10000
5000
0
M/Z
500
1000
1500
2000
y1
b5
b7
b8
b9
b10
b11
type
b
y

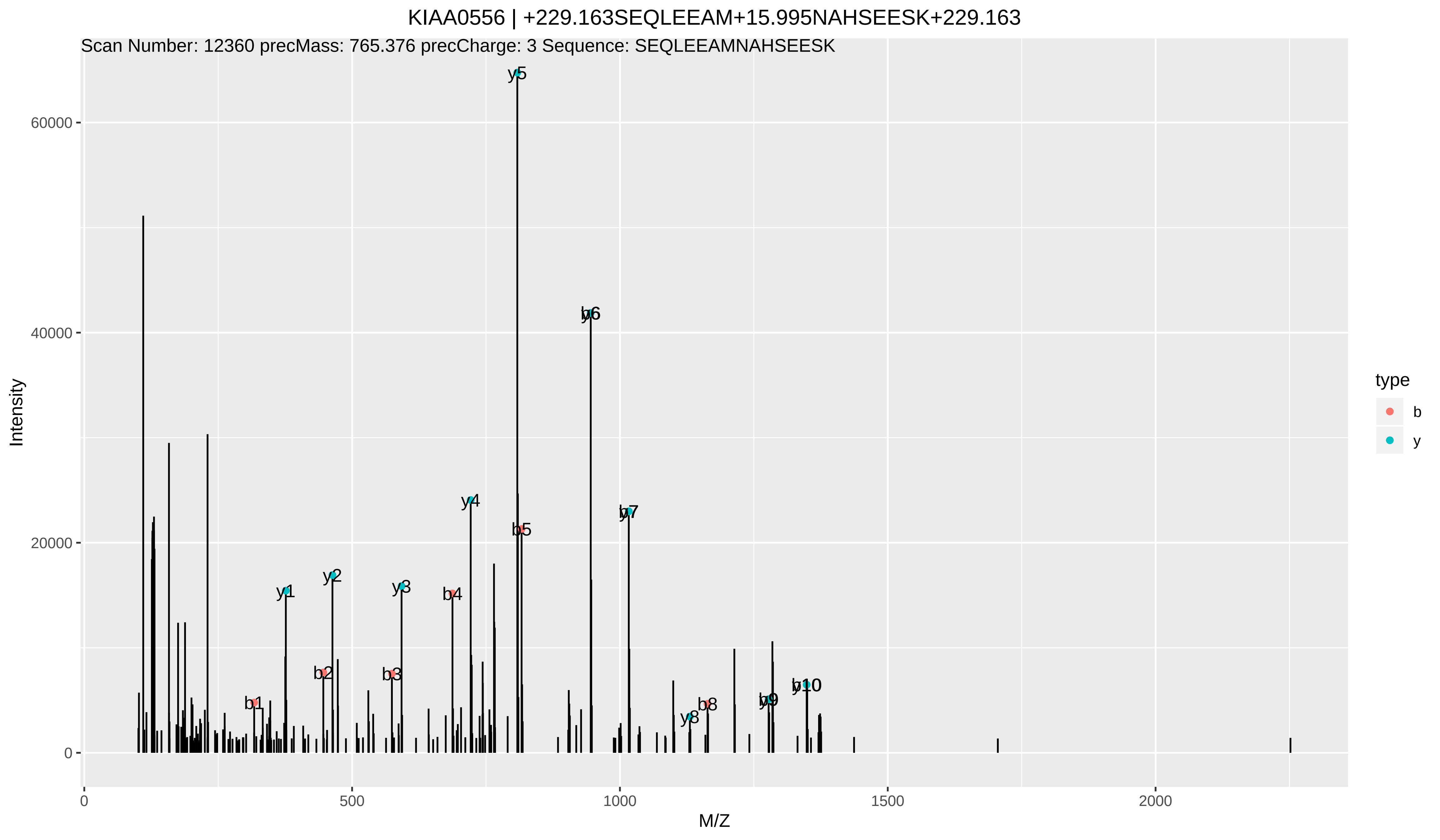

KIAA0556 | +229.163SEQLEEAM+15.995NAHSEESK+229.163
Scan Number: 12360 precMass: 765.376 precCharge: 3 Sequence: SEQLEEAMNAHSEESK
Intensity
M/Z
type
b
y
y5
b6
y6
y4
b5
b7
y7
y1
y2
y3
b4
b2
b3
b1
y8
b8
b9
y9
b10
y10
0
20000
40000
60000
500
1000
1500
2000

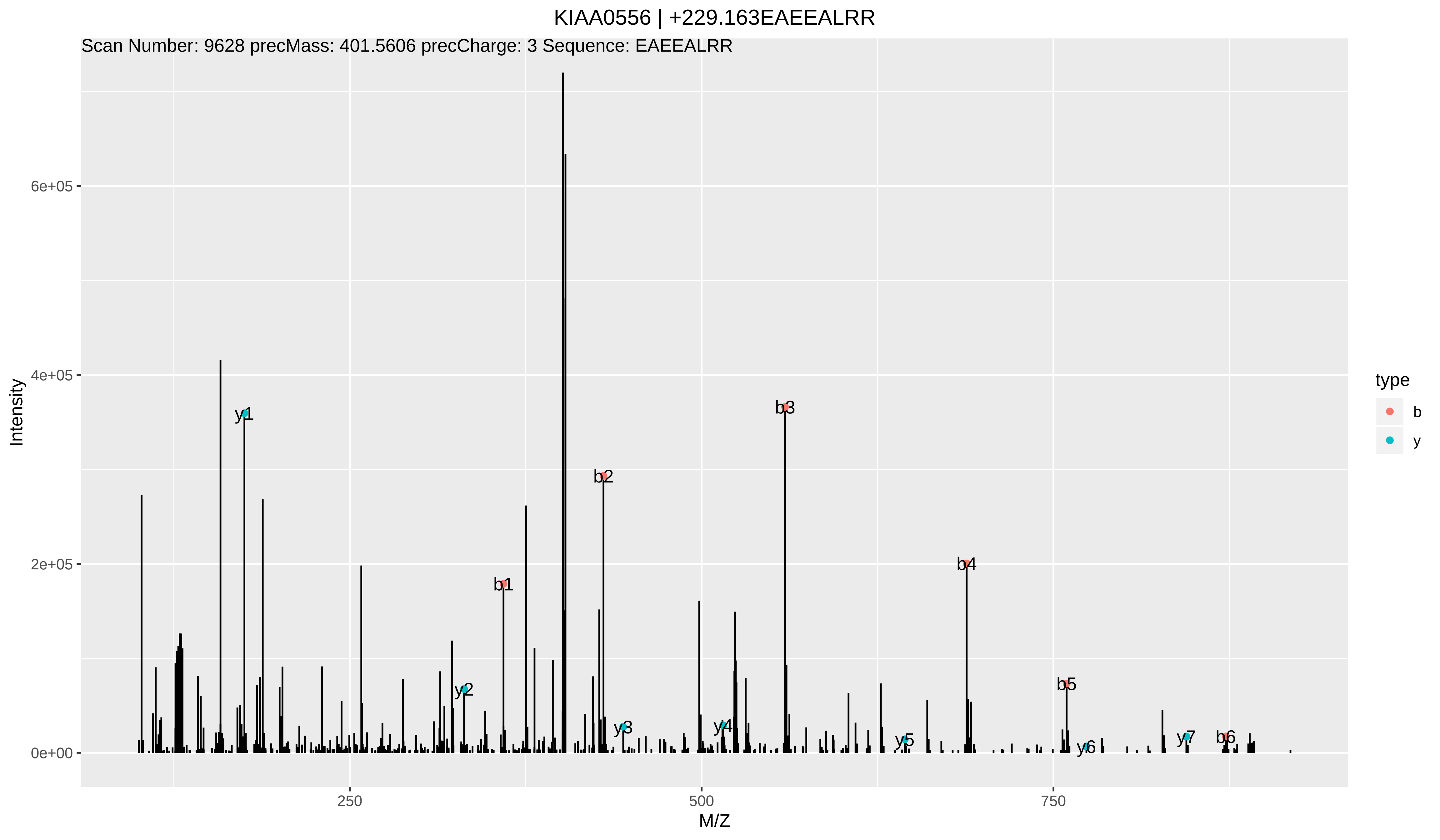

KIAA0556 | +229.163EAEEALRR
Scan Number: 9628 precMass: 401.5606 precCharge: 3 Sequence: EAEEALRR
Intensity
6e+05
4e+05
2e+05
0e+00
250
500
750
M/Z
y1
y2
y3
y4
y5
y6
y7
b1
b2
b3
b4
b5
b6
type
b
y

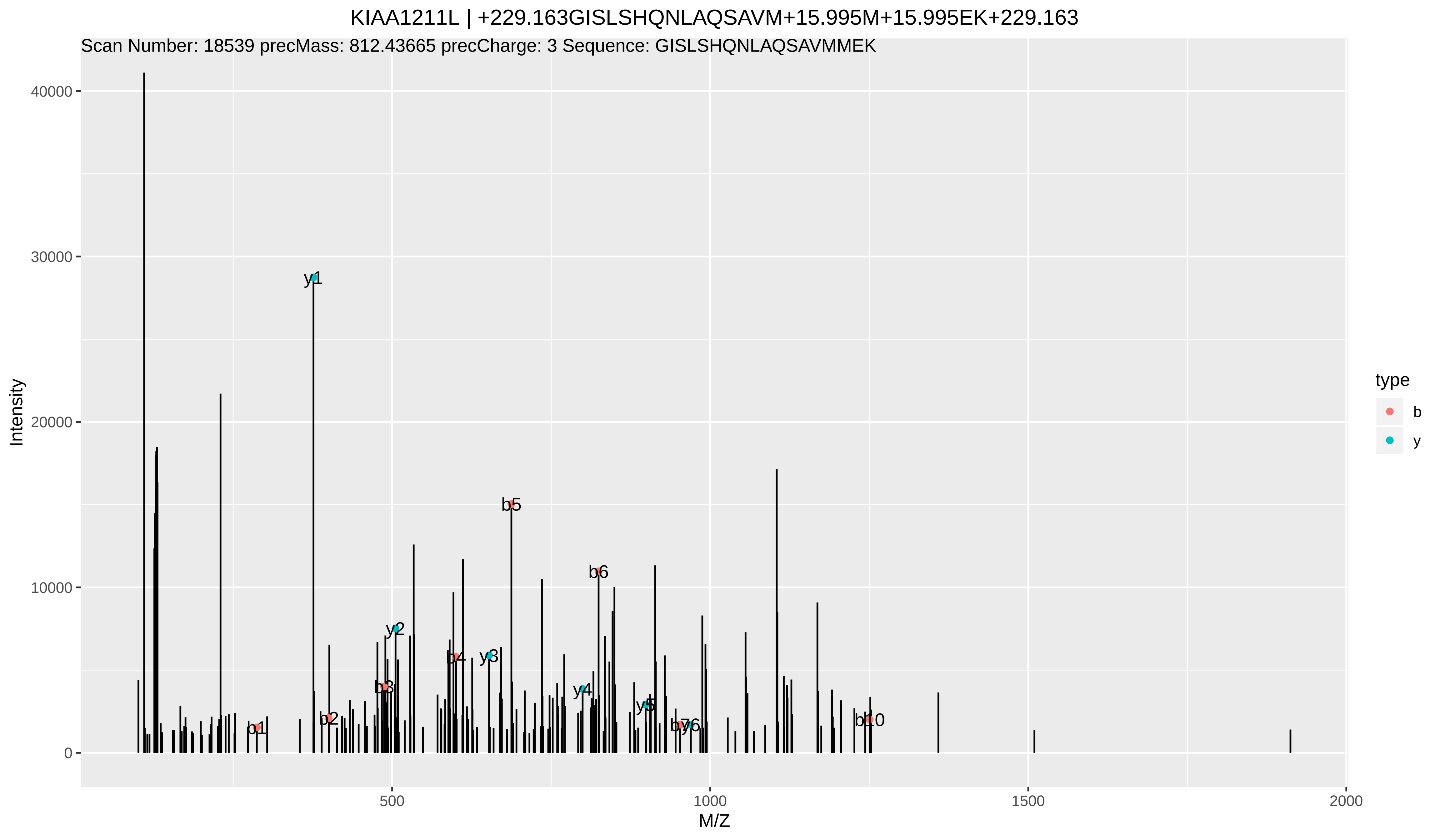
KIAA1211L | +229.163GISLSHQNLAQSAVM+15.995M+15.995EK+229.163
Scan Number: 18539 precMass: 812.43665 precCharge: 3 Sequence: GISLSHQNLAQSAVMMEK
Intensity
M/Z
type
b
y
y1
y2
y3
y4
y5
y6
b1
b2
b3
b4
b5
b6
b7
b10
0
10000
20000
30000
40000
500
1000
1500
2000

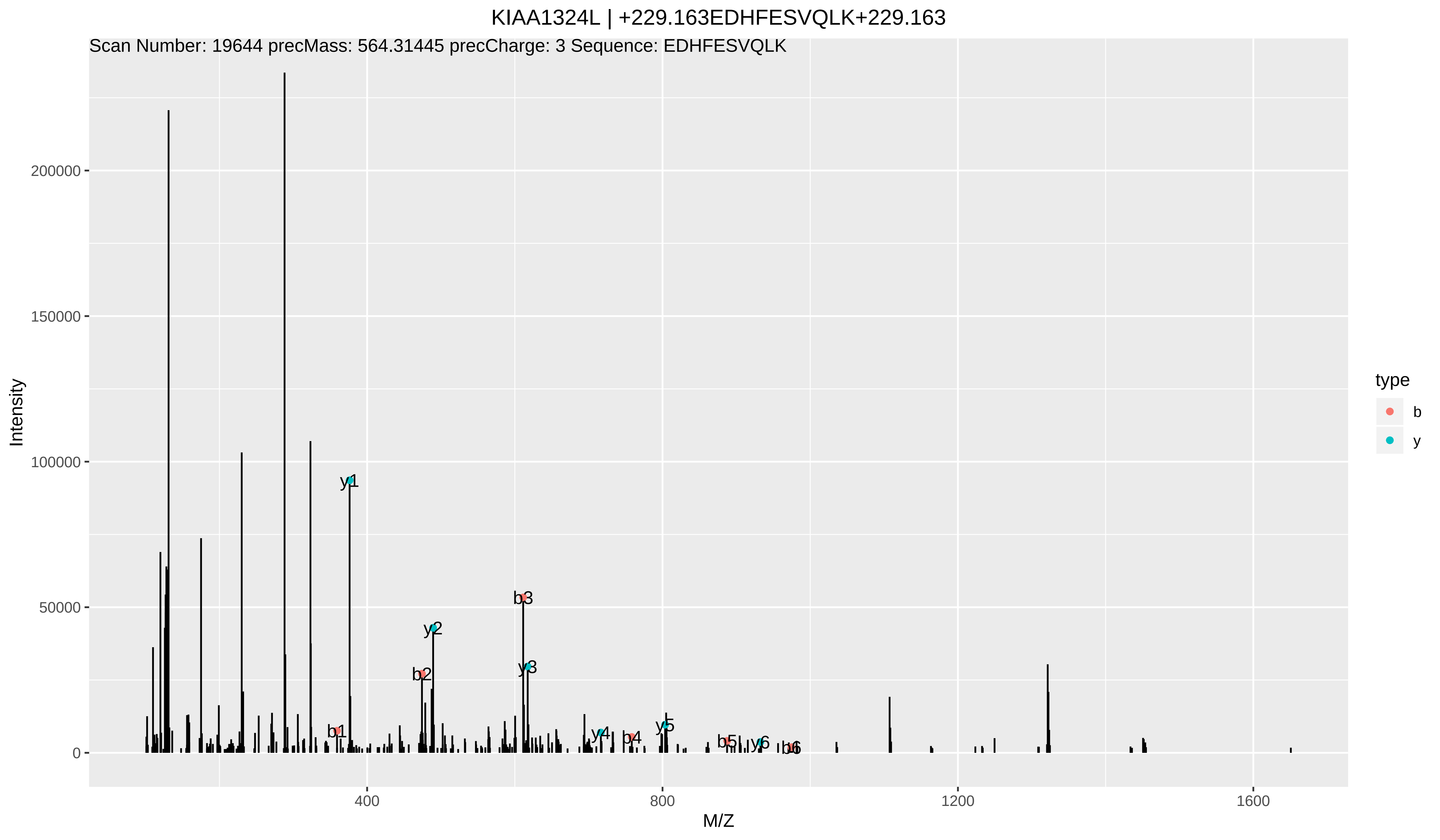
KIAA1324L | +229.163EDHFESVQLK+229.163
Scan Number: 19644 precMass: 564.31445 precCharge: 3 Sequence: EDHFESVQLK
Intensity
M/Z
type
b
y
y1
y2
b2
b3
y3
b1
y4
b4
y5
b5
y6
b6
0
50000
100000
150000
200000
400
800
1200
1600

KIAA1549 | +229.163GLQGPGLGYPTSSTEDLQPGHSSASLIK+229.163

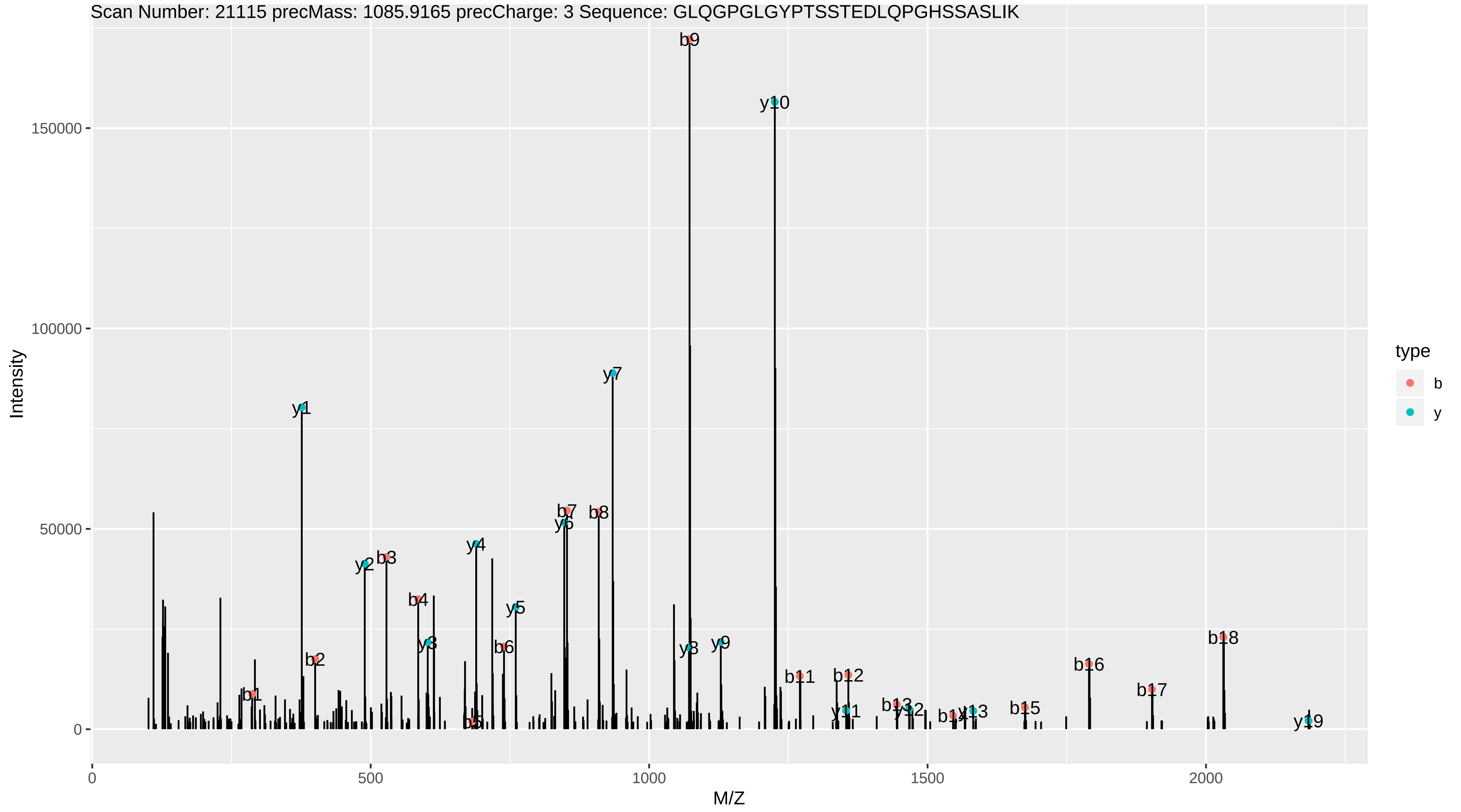

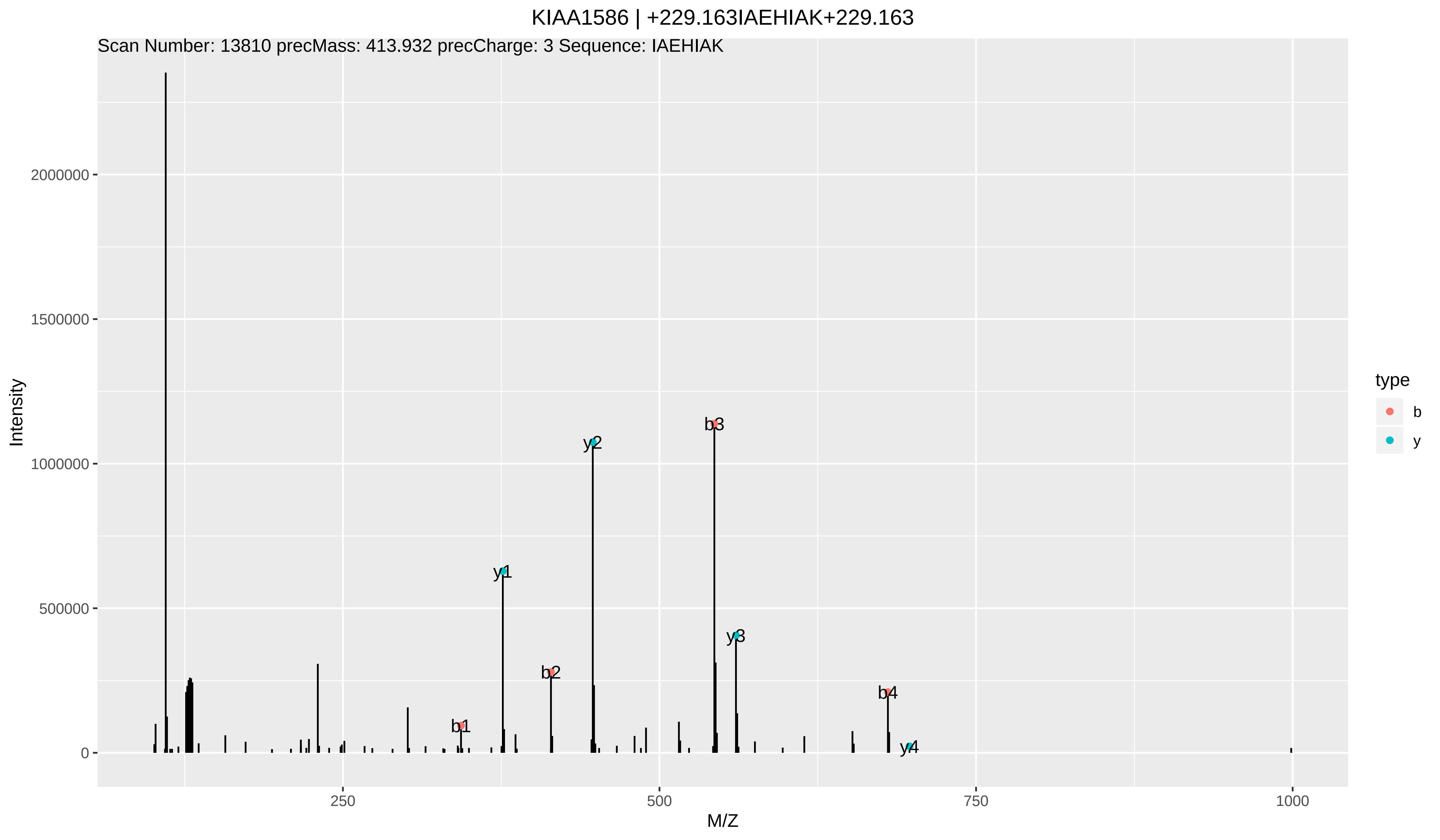
KIAA1586 | +229.163IAEHIAK+229.163
Scan Number: 13810 precMass: 413.932 precCharge: 3 Sequence: IAEHIAK
Intensity
2000000
1500000
1000000
500000
0
M/Z
250
500
750
1000
type
b
y
y1
y2
b3
y3
b2
b4
b1
y4

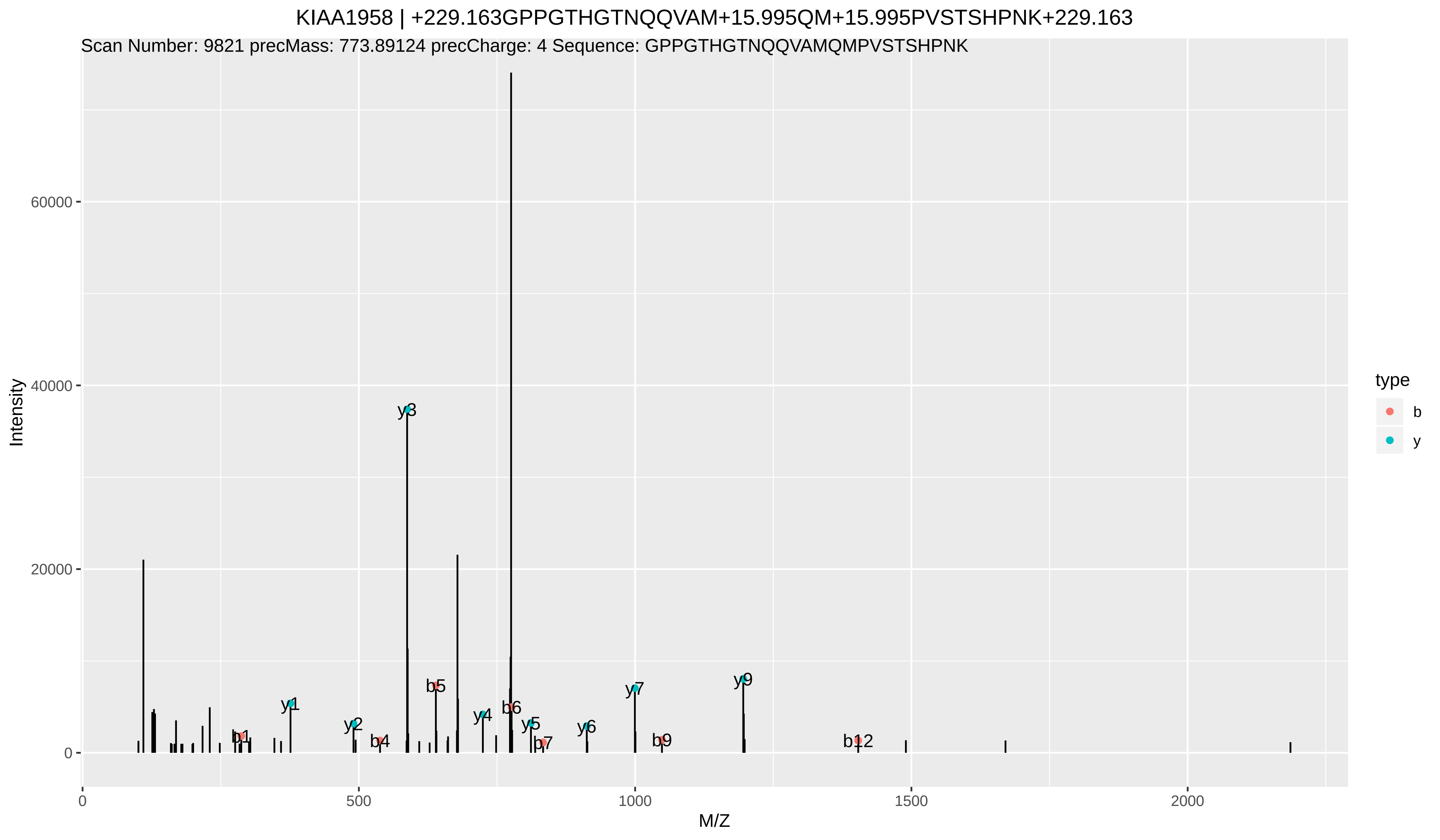

KIAA1958 | +229.163GPPGTHGTNQQVAM+15.995QM+15.995PVSTSHPNK+229.163
Scan Number: 9821 precMass: 773.89124 precCharge: 4 Sequence: GPPGTHGTNQQVAMQMPVSTSHPNK
Intensity
M/Z
60000
40000
20000
0
0
500
1000
1500
2000
y3
b5
y1
y2
b1
b4
y4
b6
y5
b7
y6
y7
b9
y9
b12
type
b
y

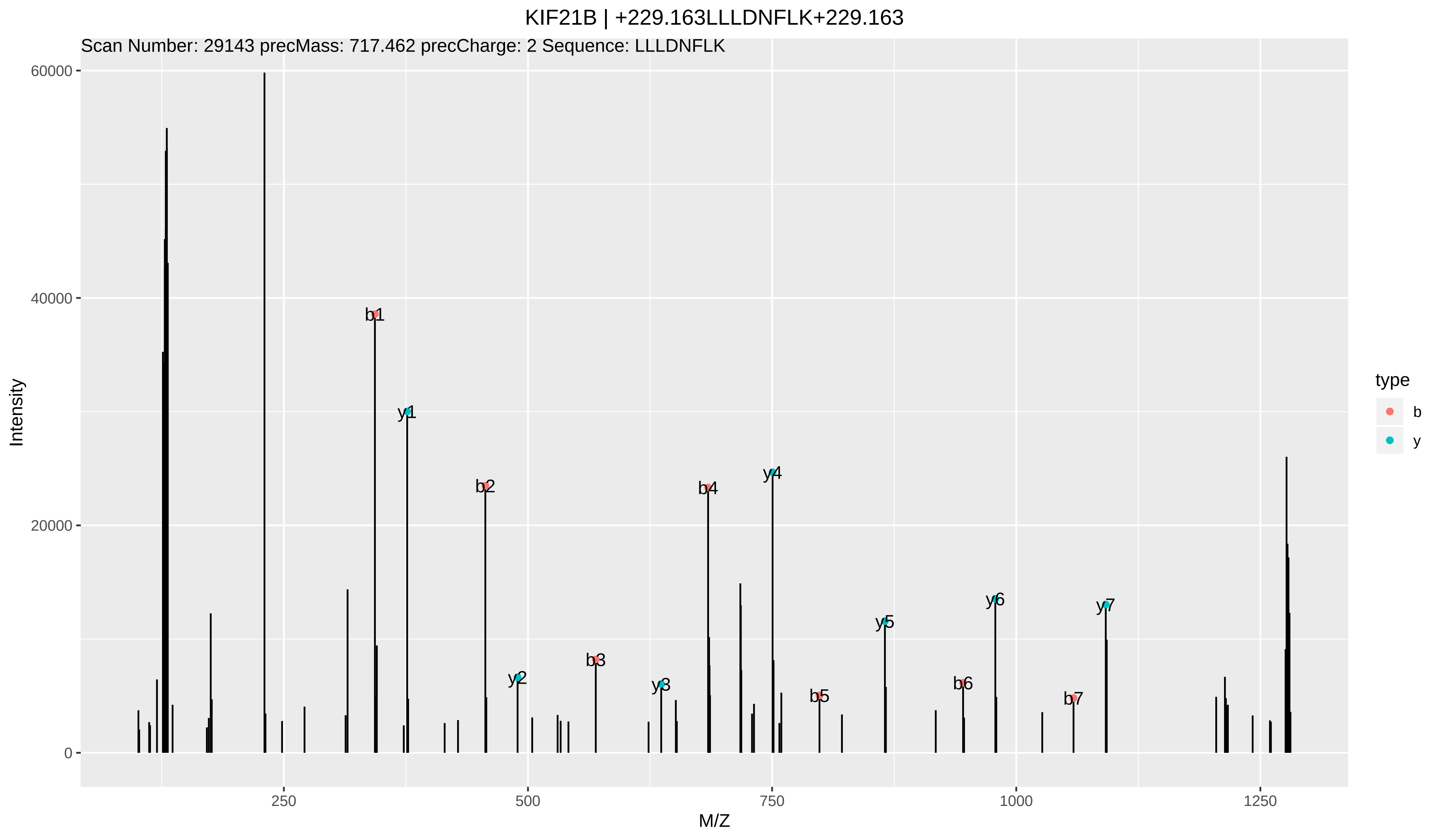

KIF21B | +229.163LLLDNFLK+229.163
Scan Number: 29143 precMass: 717.462 precCharge: 2 Sequence: LLLDNFLK
Intensity
M/Z
60000
40000
20000
0
250
500
750
1000
1250
b1
y1
b2
y2
b3
y3
b4
y4
b5
y5
b6
y6
b7
y7
type
b
y

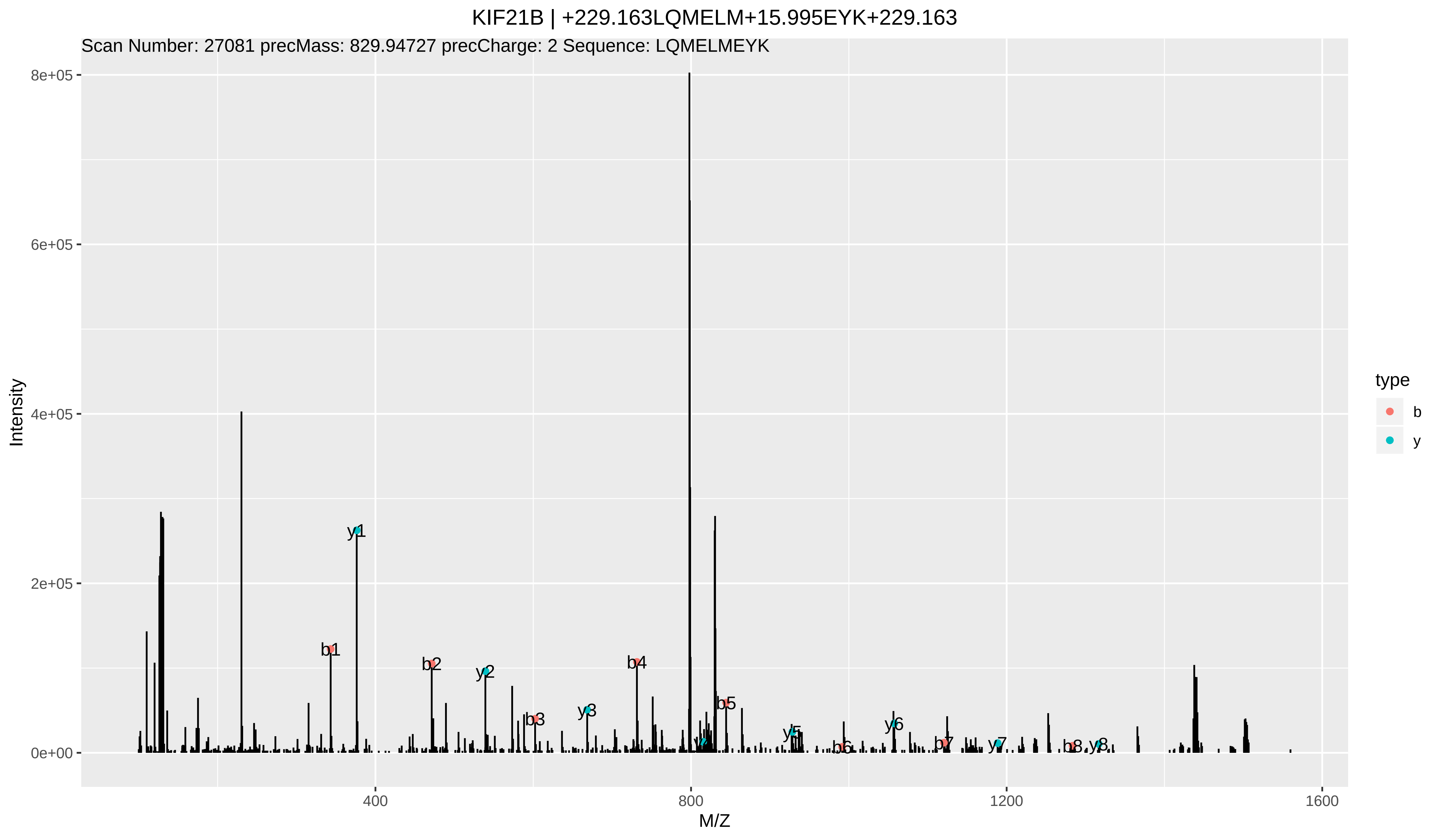
KIF21B | +229.163LQMELM+15.995EYK+229.163
Scan Number: 27081 precMass: 829.94727 precCharge: 2 Sequence: LQMELMEYK
Intensity
M/Z
type
b
y
8e+05
6e+05
4e+05
2e+05
0e+00
400
800
1200
1600
y1
b1
b2
y2
b3
y3
b4
b5
y5
b6
y6
b7
y7
b8
y8

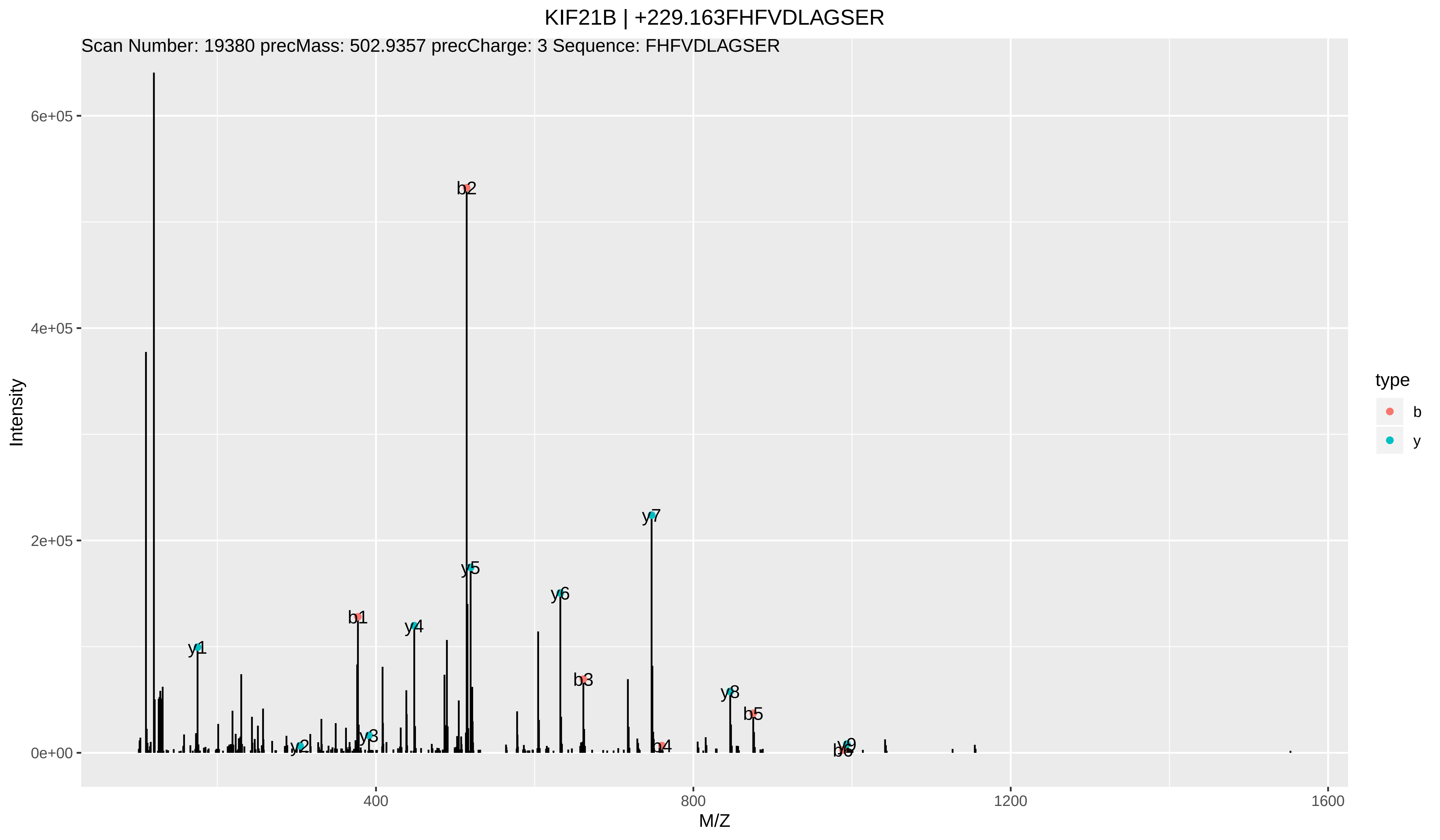

KIF21B | +229.163FHFVDLAGSER
Scan Number: 19380 precMass: 502.9357 precCharge: 3 Sequence: FHFVDLAGSER
Intensity
M/Z
6e+05
4e+05
2e+05
0e+00
400
800
1200
1600
b2
y7
y5
y6
b1
y4
y1
b3
y8
b5
y3
y2
b4
y9
b6
type
b
y

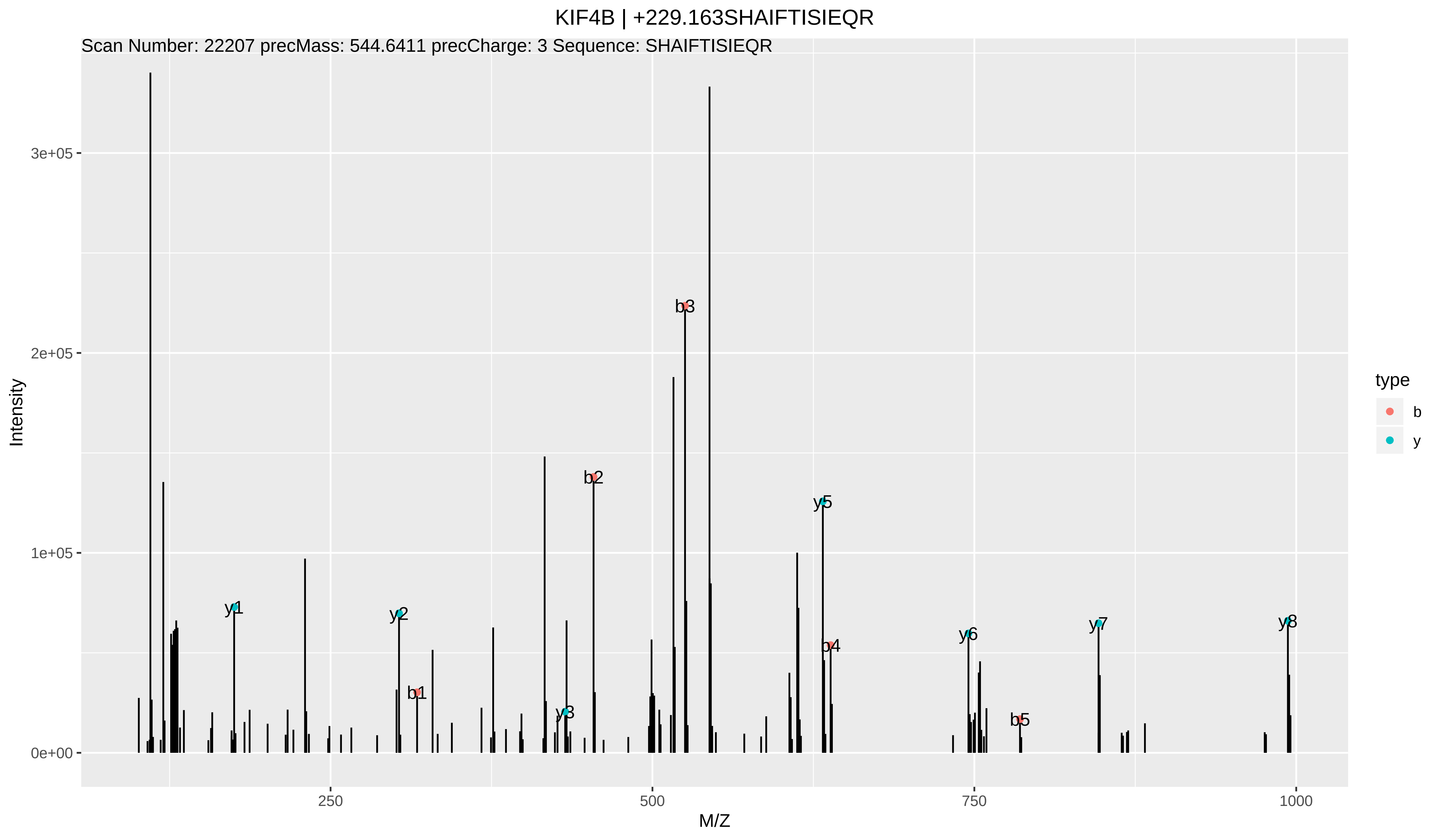

KIF4B | +229.163SHAIFTISIEQR
Scan Number: 22207 precMass: 544.6411 precCharge: 3 Sequence: SHAIFTISIEQR
Intensity
3e+05
2e+05
1e+05
0e+00
M/Z
250
500
750
1000
y1
y2
b1
y3
b2
b3
y5
b4
y6
b5
y7
y8
type
b
y

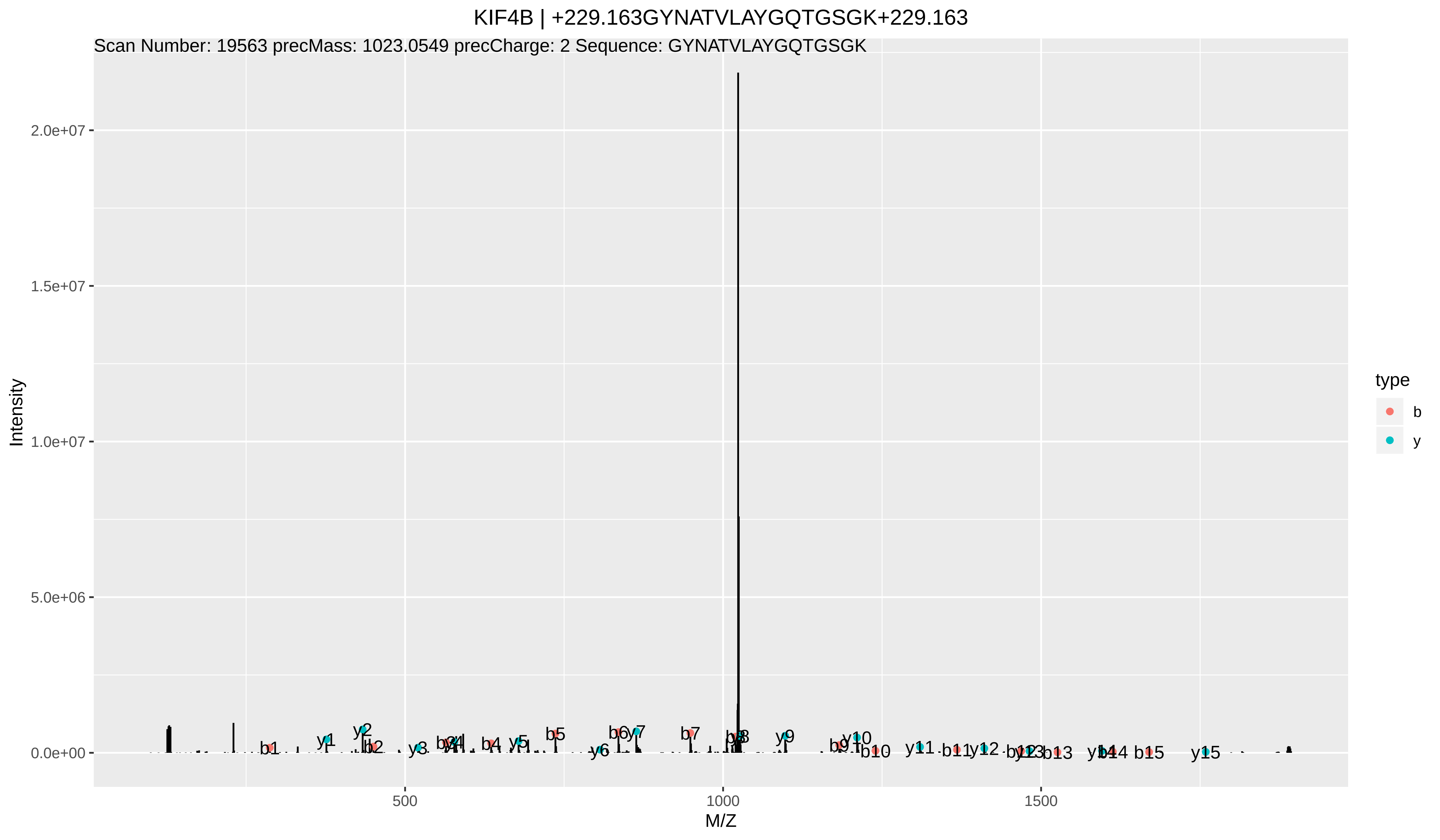
KIF4B | +229.163GYNATVLAYGQTGSGK+229.163
Scan Number: 19563 precMass: 1023.0549 precCharge: 2 Sequence: GYNATVLAYGQTGSGK
Intensity
M/Z
type
b
y
0.0e+00
5.0e+06
1.0e+07
1.5e+07
2.0e+07
500
1000
1500

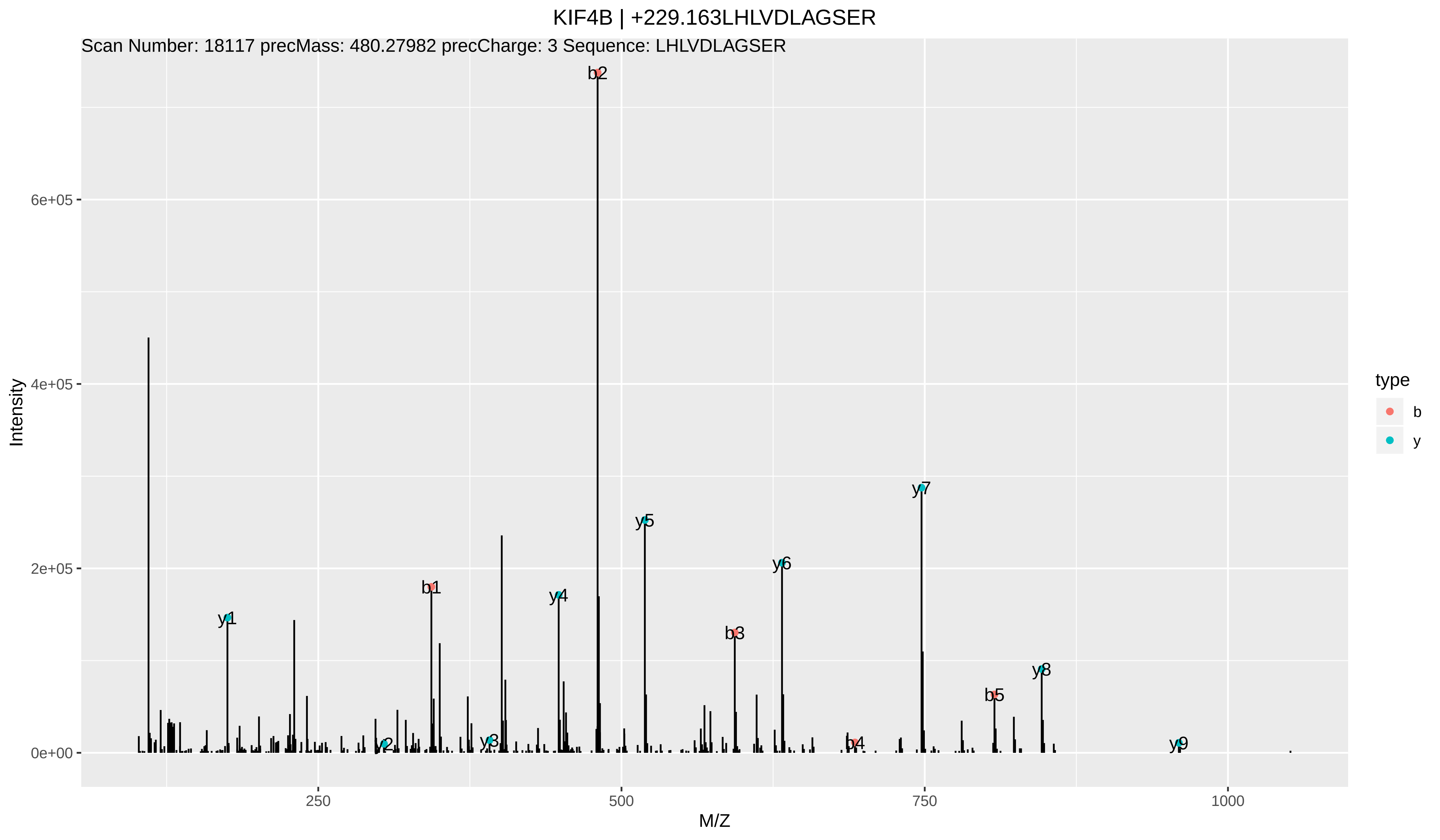

KIF4B | +229.163LHLVDLAGSER
Scan Number: 18117 precMass: 480.27982 precCharge: 3 Sequence: LHLVDLAGSER
Intensity
M/Z
0e+00
2e+05
4e+05
6e+05
250
500
750
1000
b2
y7
y5
y6
b1
y4
y1
b3
y8
b5
y2
y3
b4
y9
type
b
y

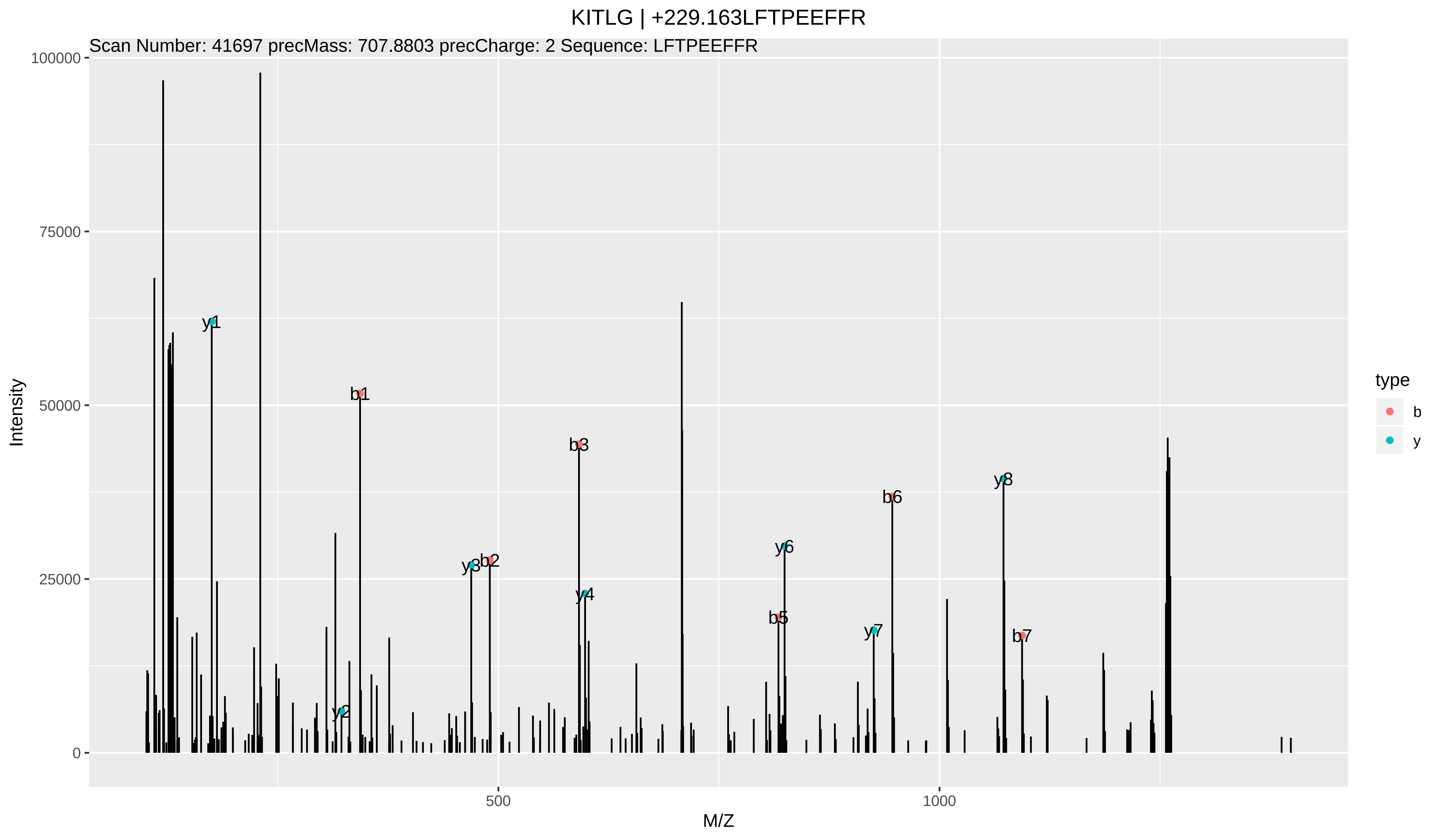
KITLG | +229.163LFTPEEFFR
Scan Number: 41697 precMass: 707.8803 precCharge: 2 Sequence: LFTPEEFFR
Intensity
M/Z
100000
75000
50000
25000
0
500
1000
y1
b1
b3
y8
b6
y6
y3
b2
y4
b5
y7
b7
y2
type
b
y

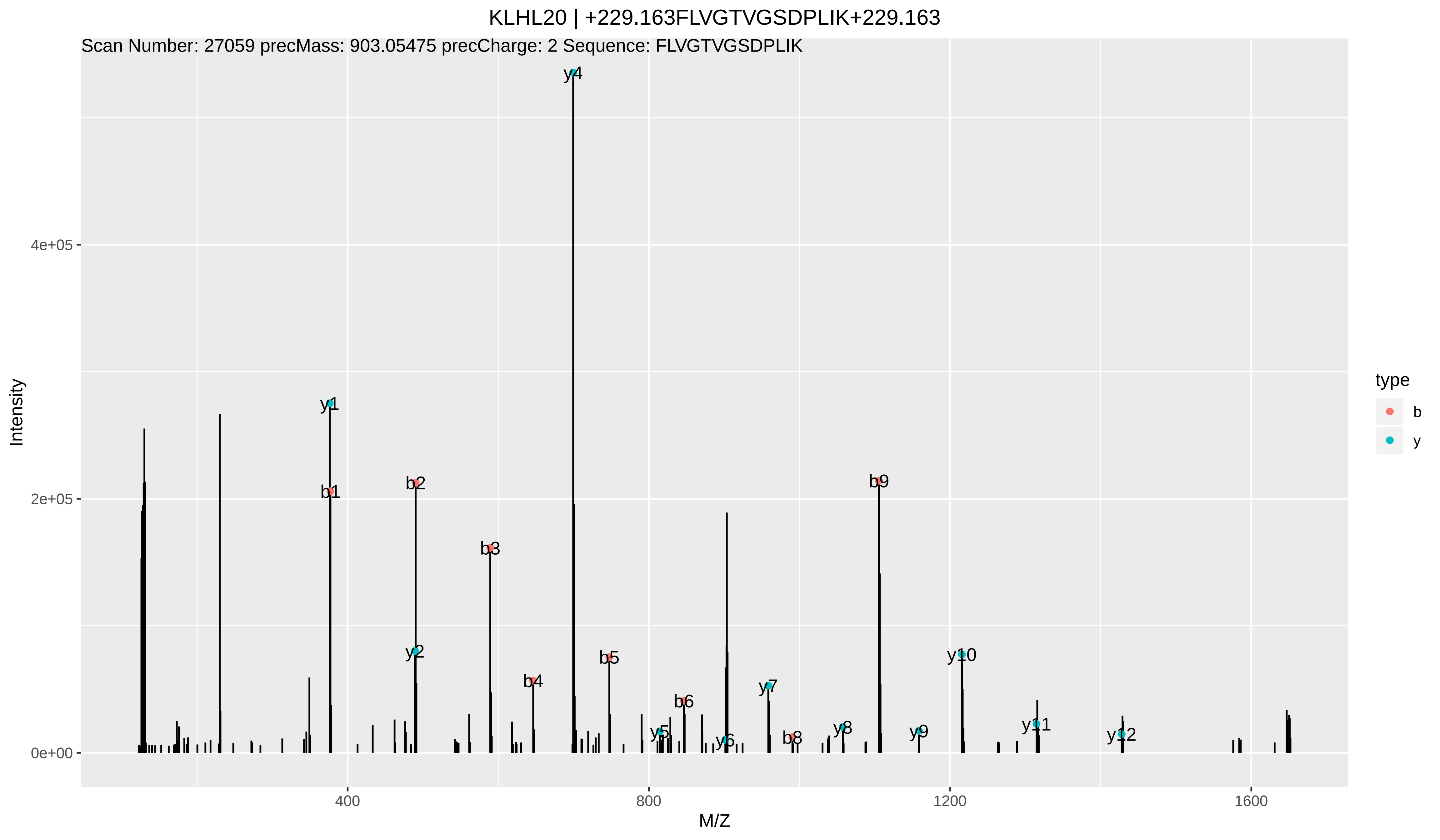

KLHL20 | +229.163FLVGTVGSDPLIK+229.163
Scan Number: 27059 precMass: 903.05475 precCharge: 2 Sequence: FLVGTVGSDPLIK
y4
y1
b1
b2
b9
b3
y2
b5
y10
b4
y7
b6
y11
y5
y8
y9
y6
b8
y12
4e+05
2e+05
0e+00
Intensity
400
800
1200
1600
M/Z
type
b
y

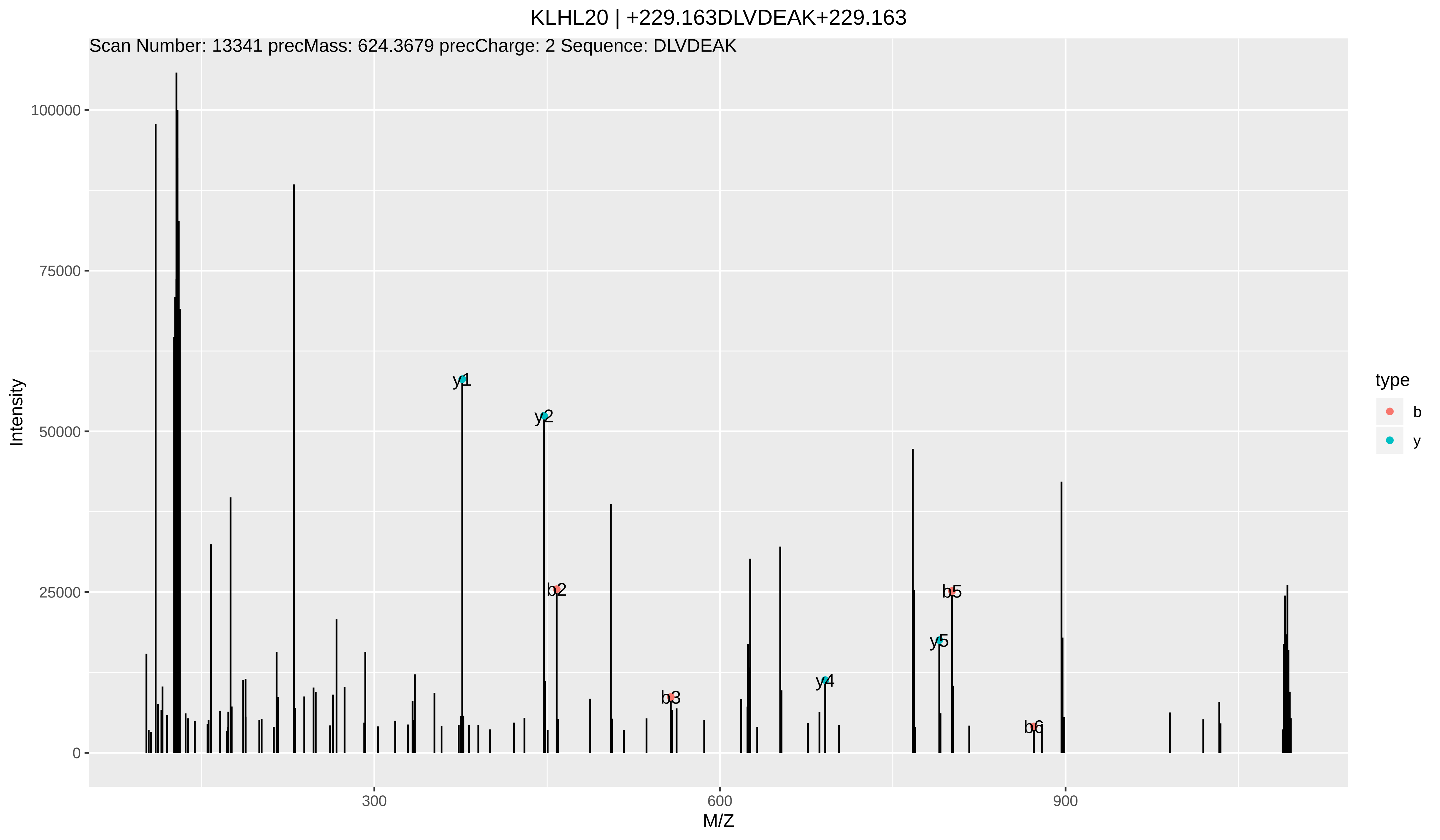

KLHL20 | +229.163DLVDEAK+229.163
Scan Number: 13341 precMass: 624.3679 precCharge: 2 Sequence: DLVDEAK
Intensity
100000
75000
50000
25000
0
300
600
900
M/Z
y1
y2
b2
b3
y4
y5
b5
b6
type
b
y

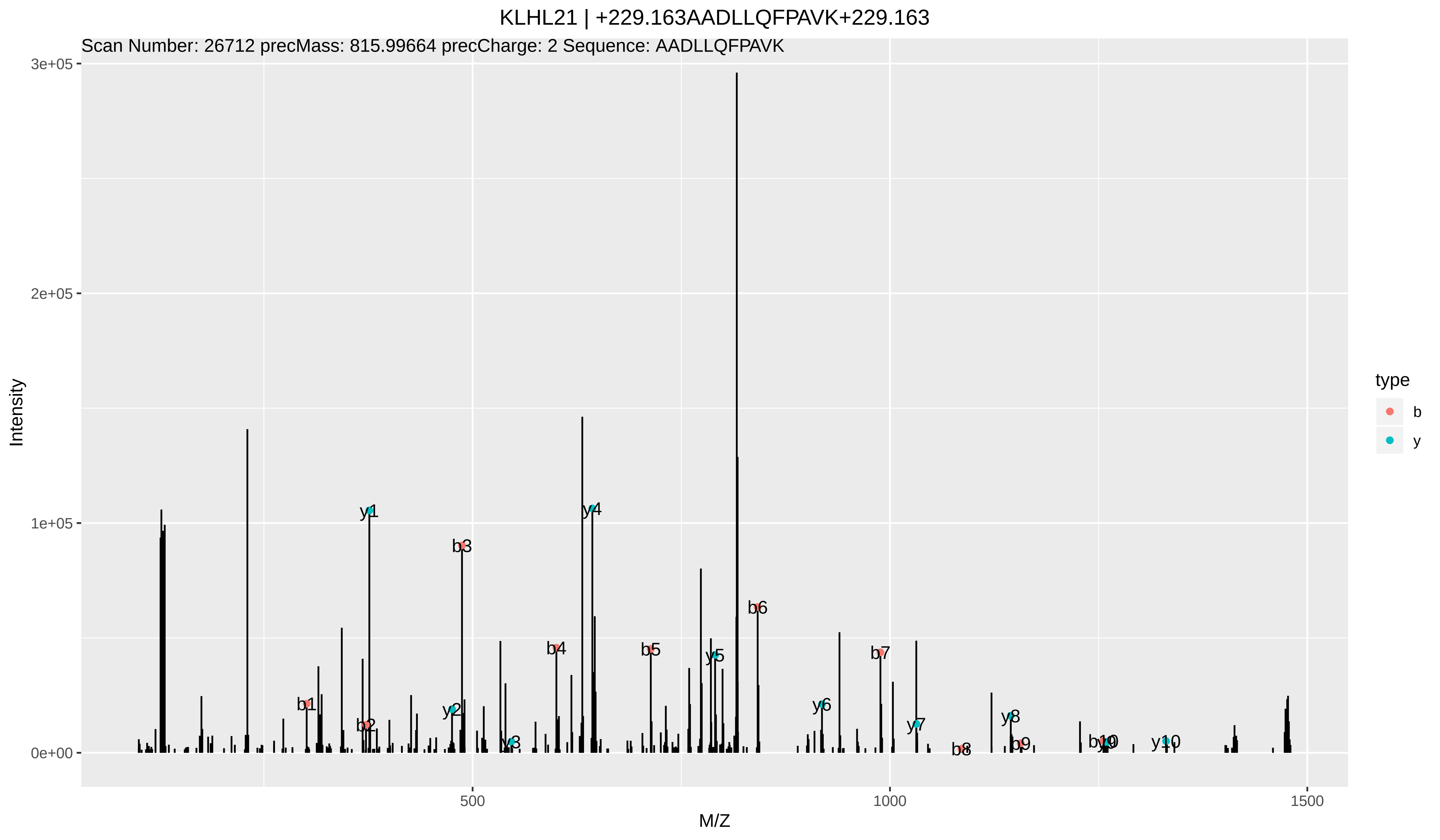

KLHL21 | +229.163AADLLQFPAVK+229.163
Scan Number: 26712 precMass: 815.99664 precCharge: 2 Sequence: AADLLQFPAVK
Intensity
3e+05
2e+05
1e+05
0e+00
M/Z
500
1000
1500
type
b
y
y1
y2
y3
y4
y5
y6
y7
y8
y10
b1
b2
b3
b4
b5
b6
b7
b8
b9
b10

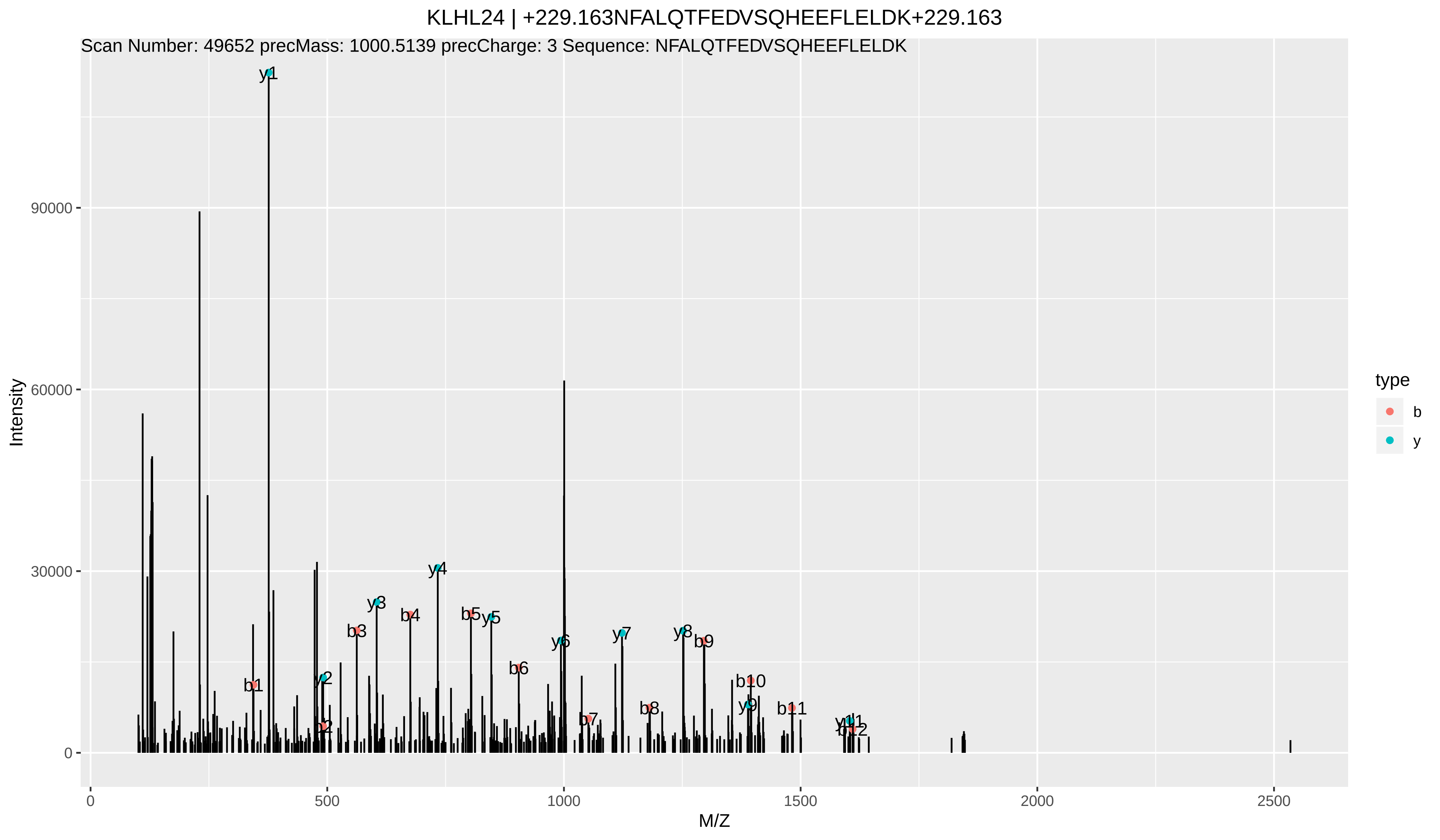

KLHL24 | +229.163NFALQTFEDVSQHEEFLELDK+229.163
Scan Number: 49652 precMass: 1000.5139 precCharge: 3 Sequence: NFALQTFEDVSQHEEFLELDK
Intensity
90000
60000
30000
0
M/Z
0
500
1000
1500
2000
2500
y1
y2
y3
y4
y5
y6
y7
y8
y9
y11
b1
b2
b3
b4
b5
b6
b7
b8
b9
b10
b11
b12
type
b
y

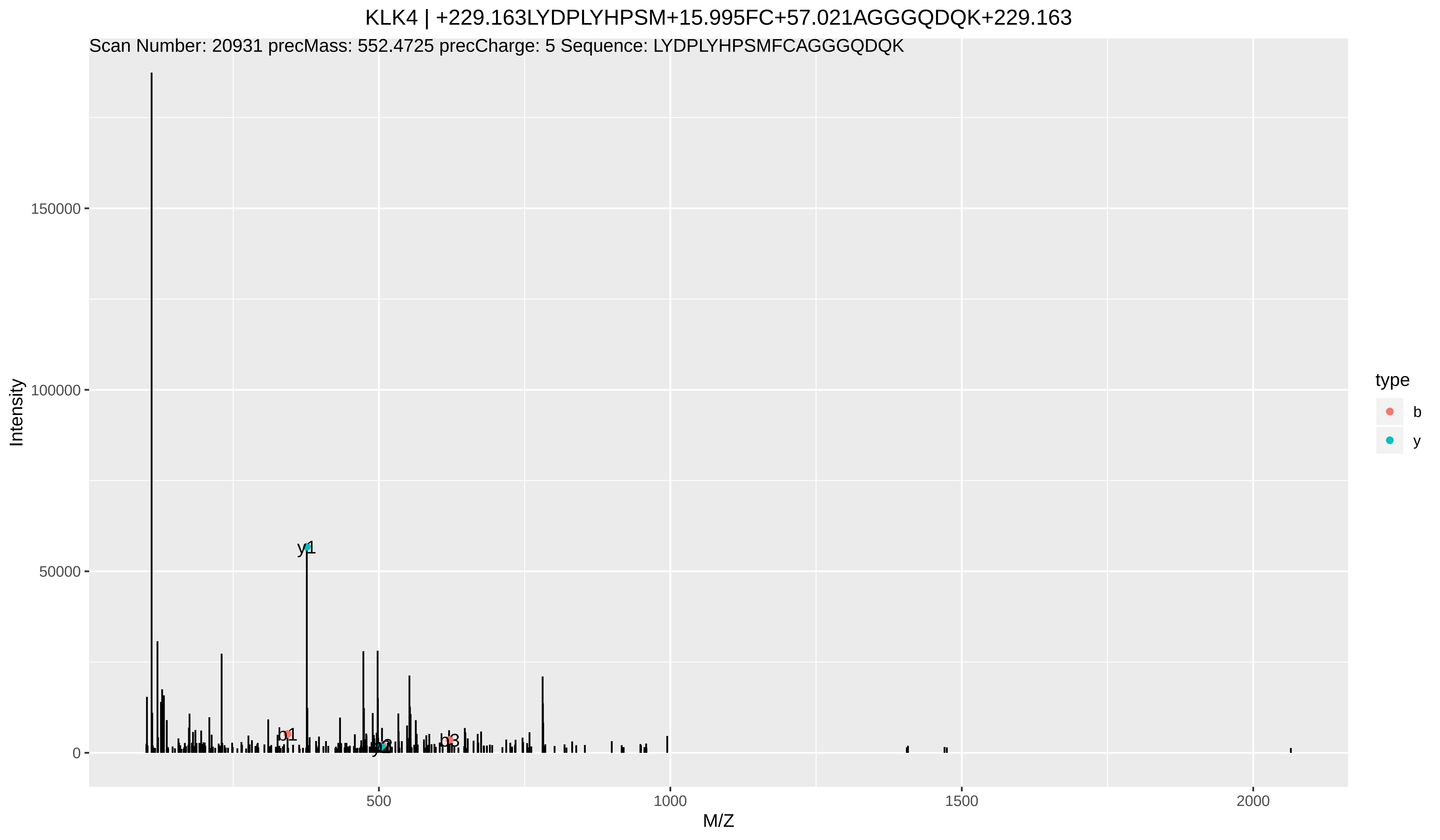

KLK4 | +229.163LYDPLYHPSM+15.995FC+57.021AGGGQDQK+229.163
Scan Number: 20931 precMass: 552.4725 precCharge: 5 Sequence: LYDPLYHPSMFCAGGGQDQK
Intensity
150000
100000
50000
0
500
1000
1500
2000
M/Z
y1
b1
y2
b3
type
b
y

KNDC1 | +229.163IAEVM+15.995EELK+229.163

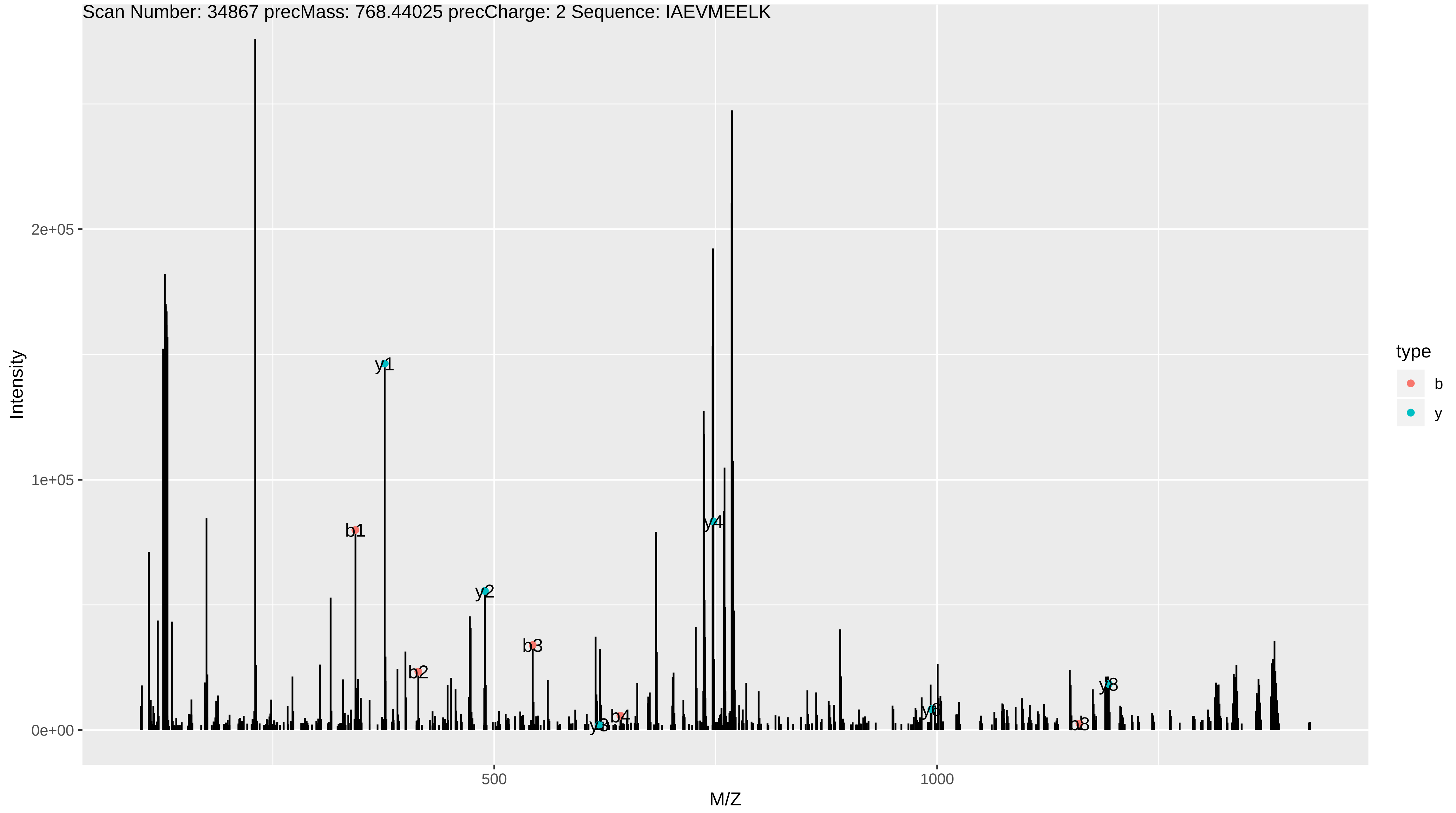

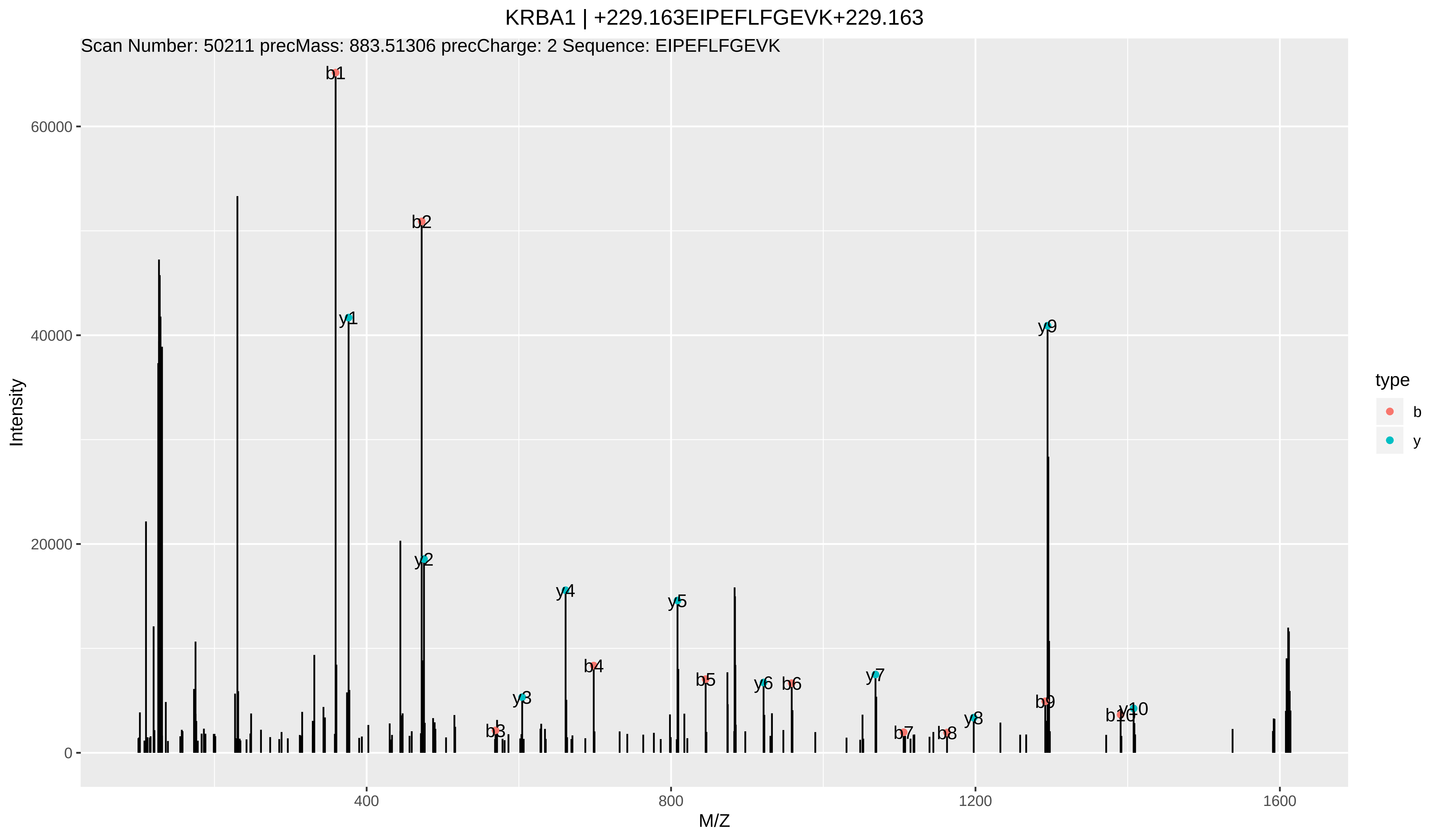

KRBA1 | +229.163EIPEFLFGEVK+229.163
Scan Number: 50211 precMass: 883.51306 precCharge: 2 Sequence: EIPEFLFGEVK
b1
b2
y1
y9
y2
y4
y5
b4
b5
y6
b6
y7
y3
b9
b10
y10
b3
y8
b7
b8
Intensity
60000
40000
20000
0
400
800
1200
1600
M/Z
type
b
y

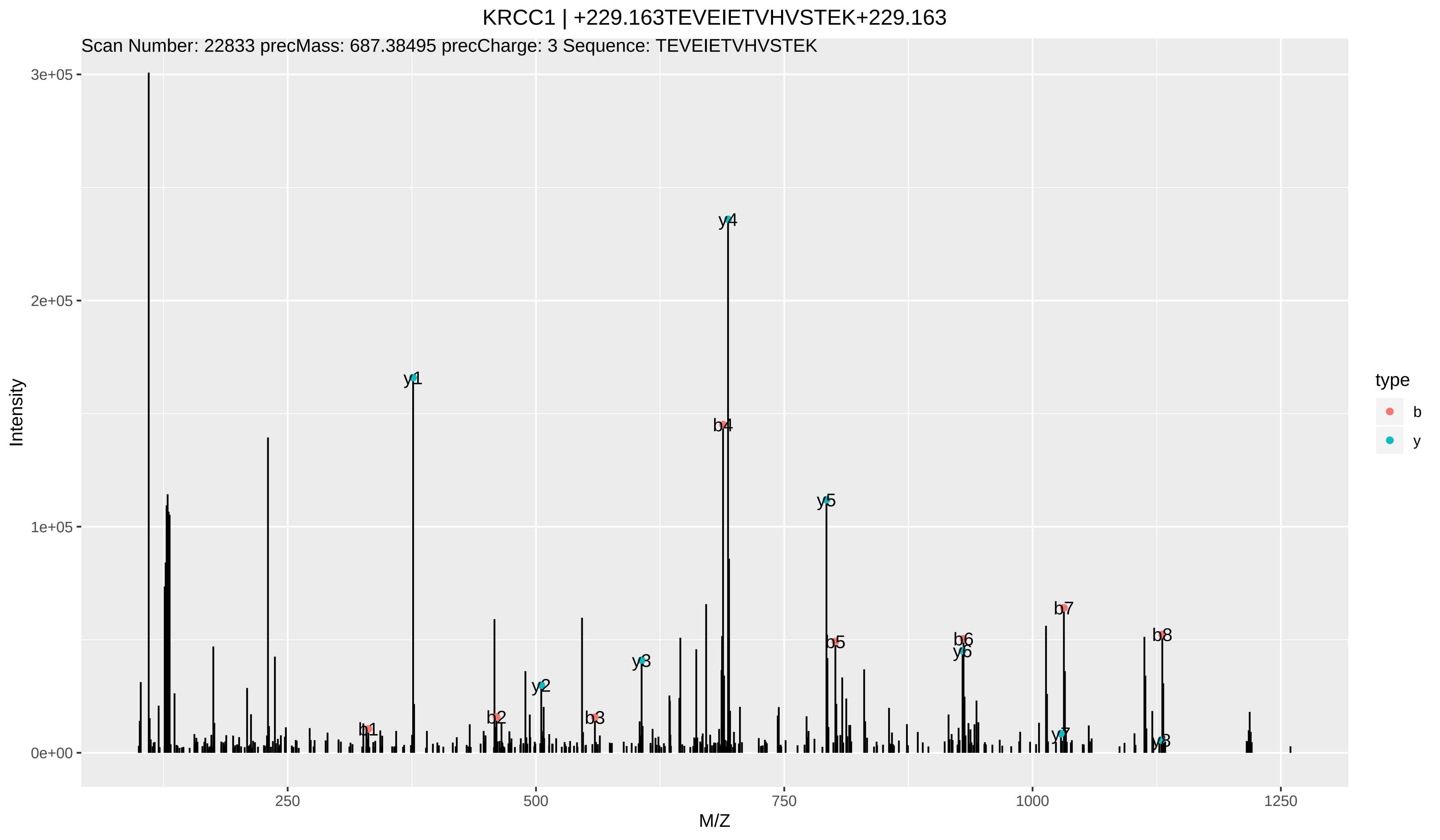

KRCC1 | +229.163TEVEIETVHVSTEK+229.163
Scan Number: 22833 precMass: 687.38495 precCharge: 3 Sequence: TEVEIETVHVSTEK
Intensity
3e+05
2e+05
1e+05
0e+00
M/Z
250
500
750
1000
1250
type
b
y
y1
y2
y3
y4
y5
y6
y7
y8
b1
b2
b3
b4
b5
b6
b7
b8

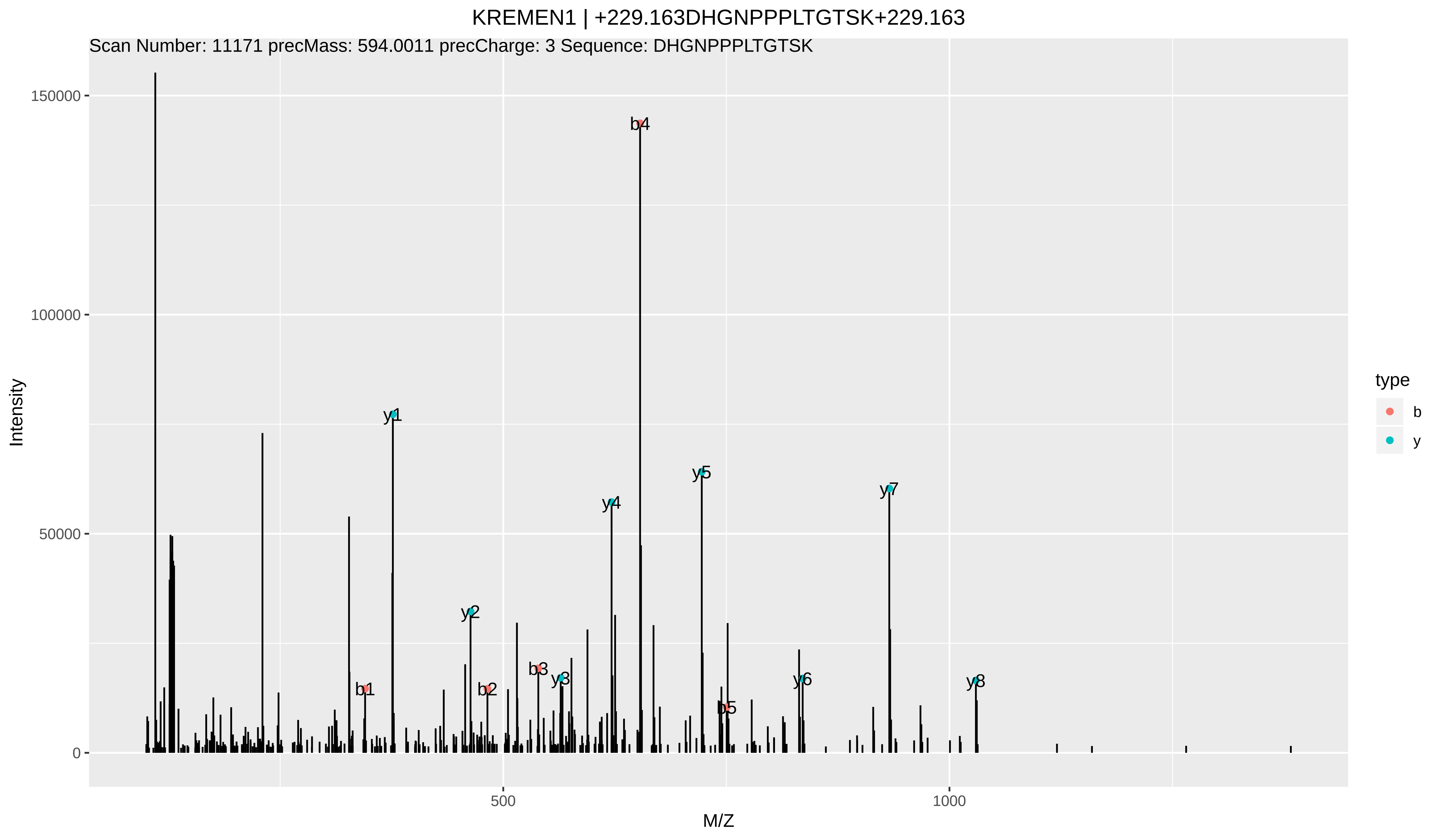

KREMEN1 | +229.163DHGNPPPLTGTSK+229.163
Scan Number: 11171 precMass: 594.0011 precCharge: 3 Sequence: DHGNPPPLTGTSK
Intensity
150000
100000
50000
0
500
1000
M/Z
b4
y1
y5
y7
y4
y2
b3
y3
b1
b2
y6
y8
b5
type
b
y

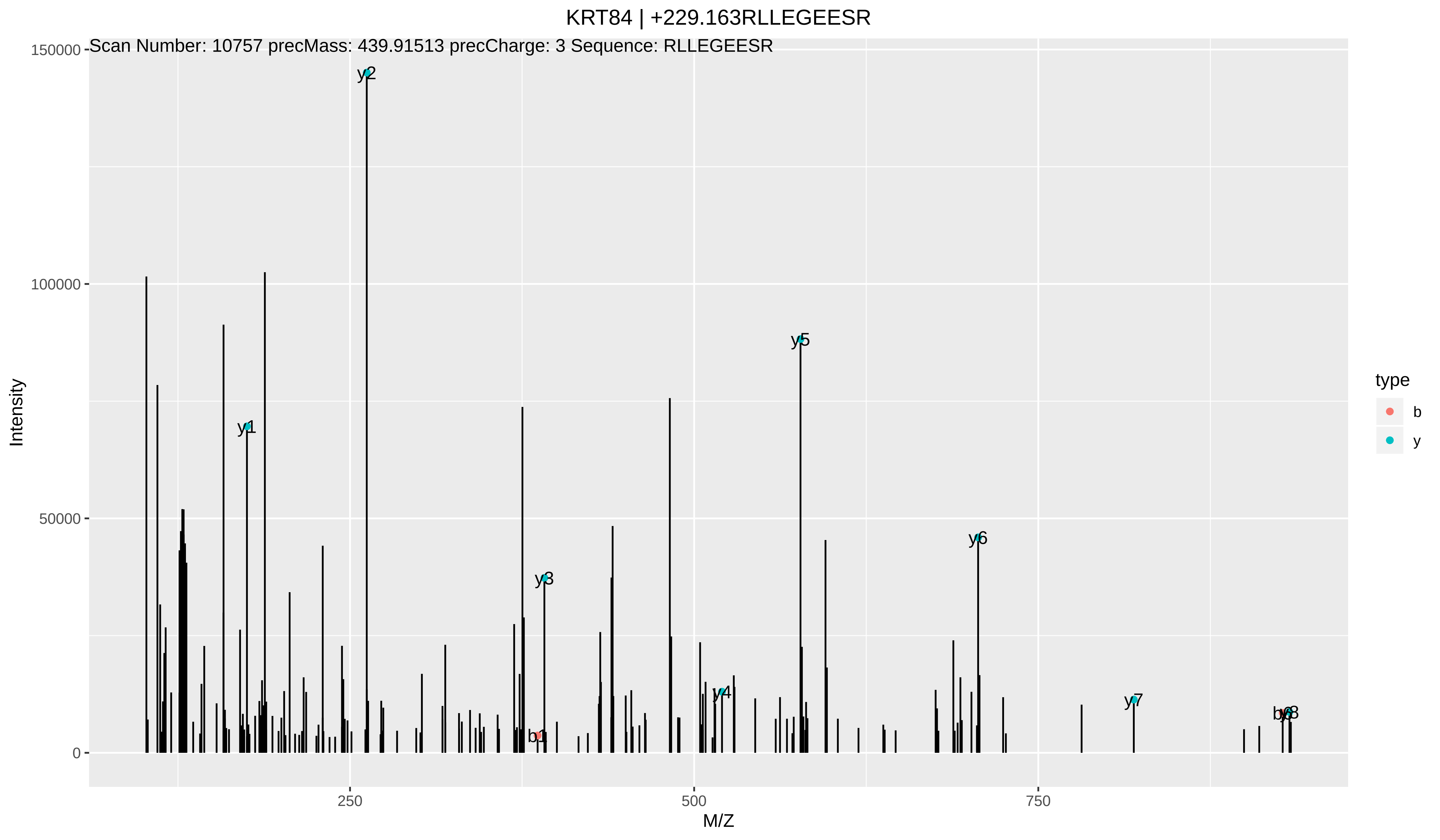

KRT84 | +229.163RLLEGEESR
Scan Number: 10757 precMass: 439.91513 precCharge: 3 Sequence: RLLEGEESR
Intensity
M/Z
150000
100000
50000
0
250
500
750
y1
y2
y3
b1
y4
y5
y6
y7
b8
y8
type
b
y

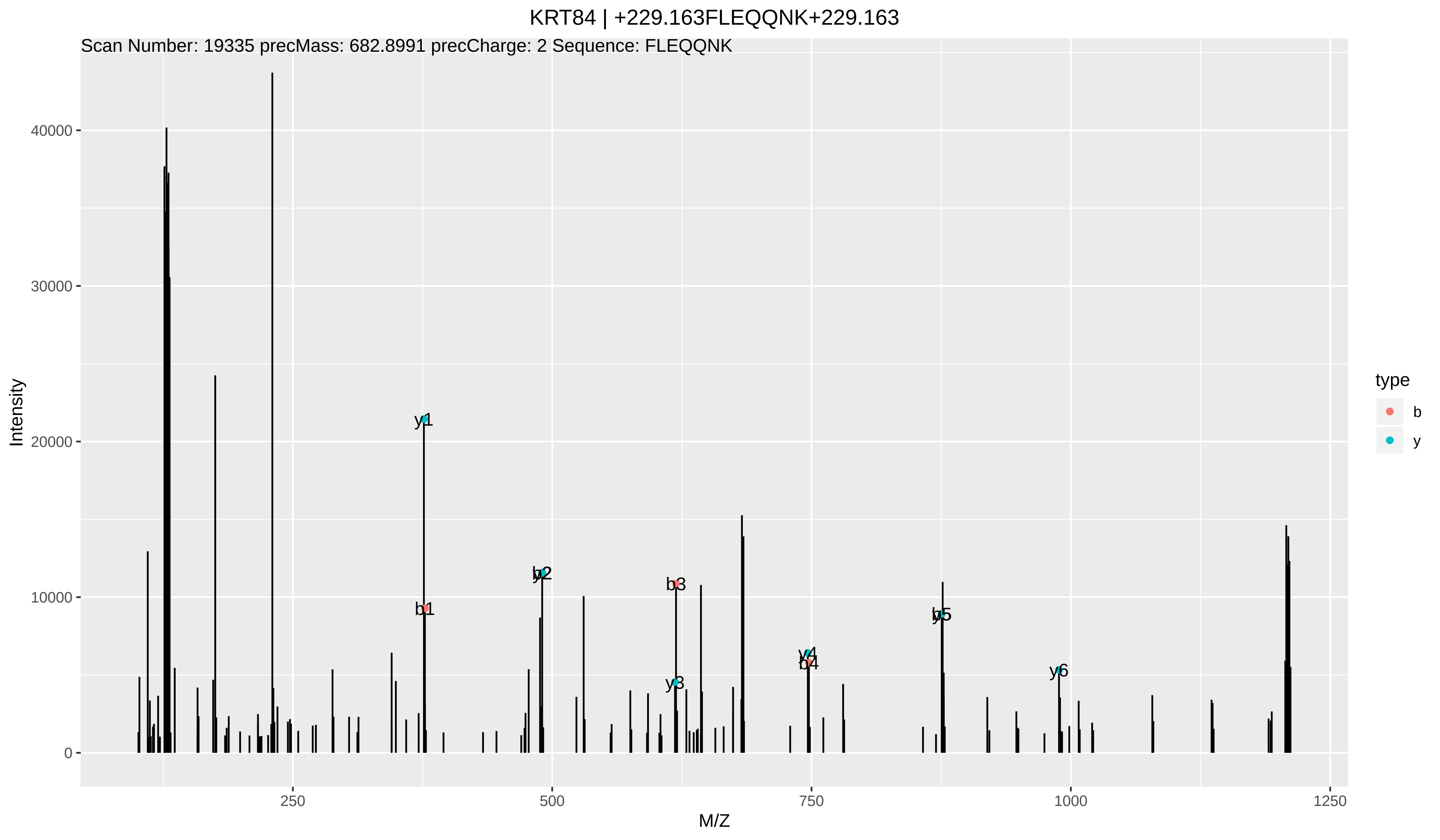

KRT84 | +229.163FLEQQNK+229.163
Scan Number: 19335 precMass: 682.8991 precCharge: 2 Sequence: FLEQQNK
Intensity
40000
30000
20000
10000
0
250
500
750
1000
1250
M/Z
y1
b1
y2
b3
y3
y4
b4
y5
y6
type
b
y

KRT84 | +229.163LLEGEESR

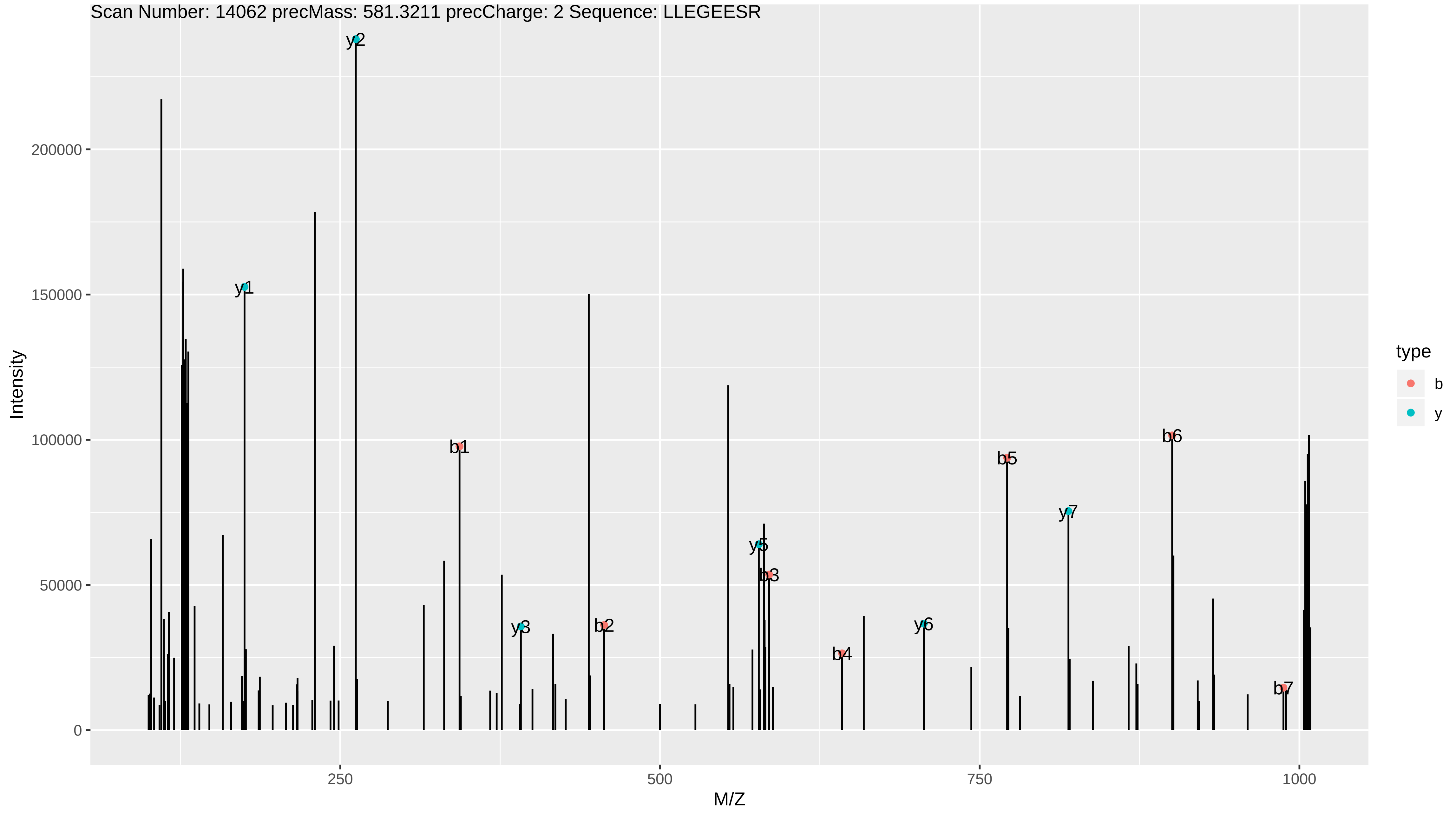

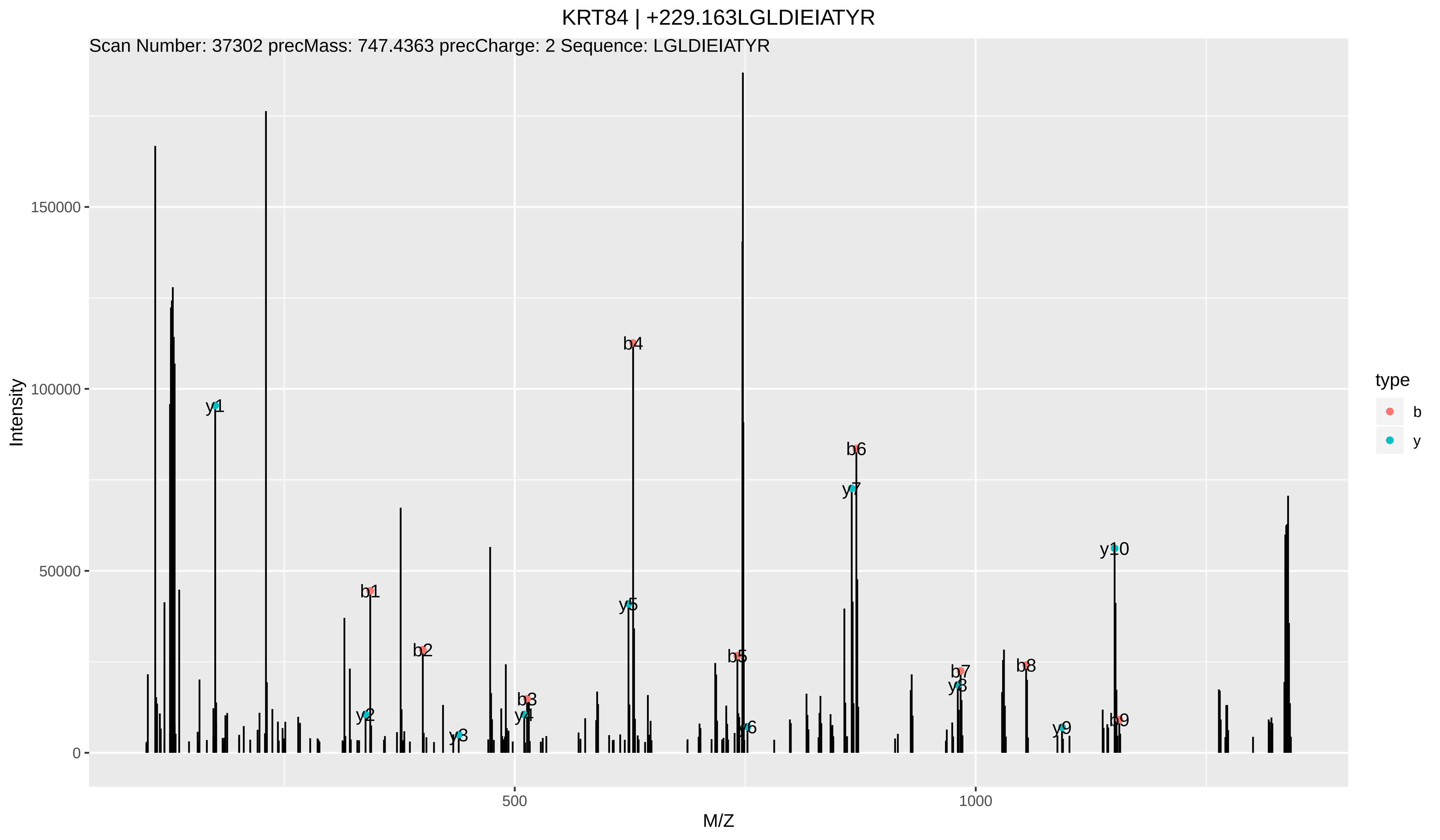

KRT84 | +229.163LGLDIEIATYR
Scan Number: 37302 precMass: 747.4363 precCharge: 2 Sequence: LGLDIEIATYR
Intensity
150000
100000
50000
0
M/Z
500
1000
type
b
y
y1
b1
y2
b2
y3
b3
y4
b4
y5
b5
y6
b6
y7
b7
y8
b8
y9
b9
y10

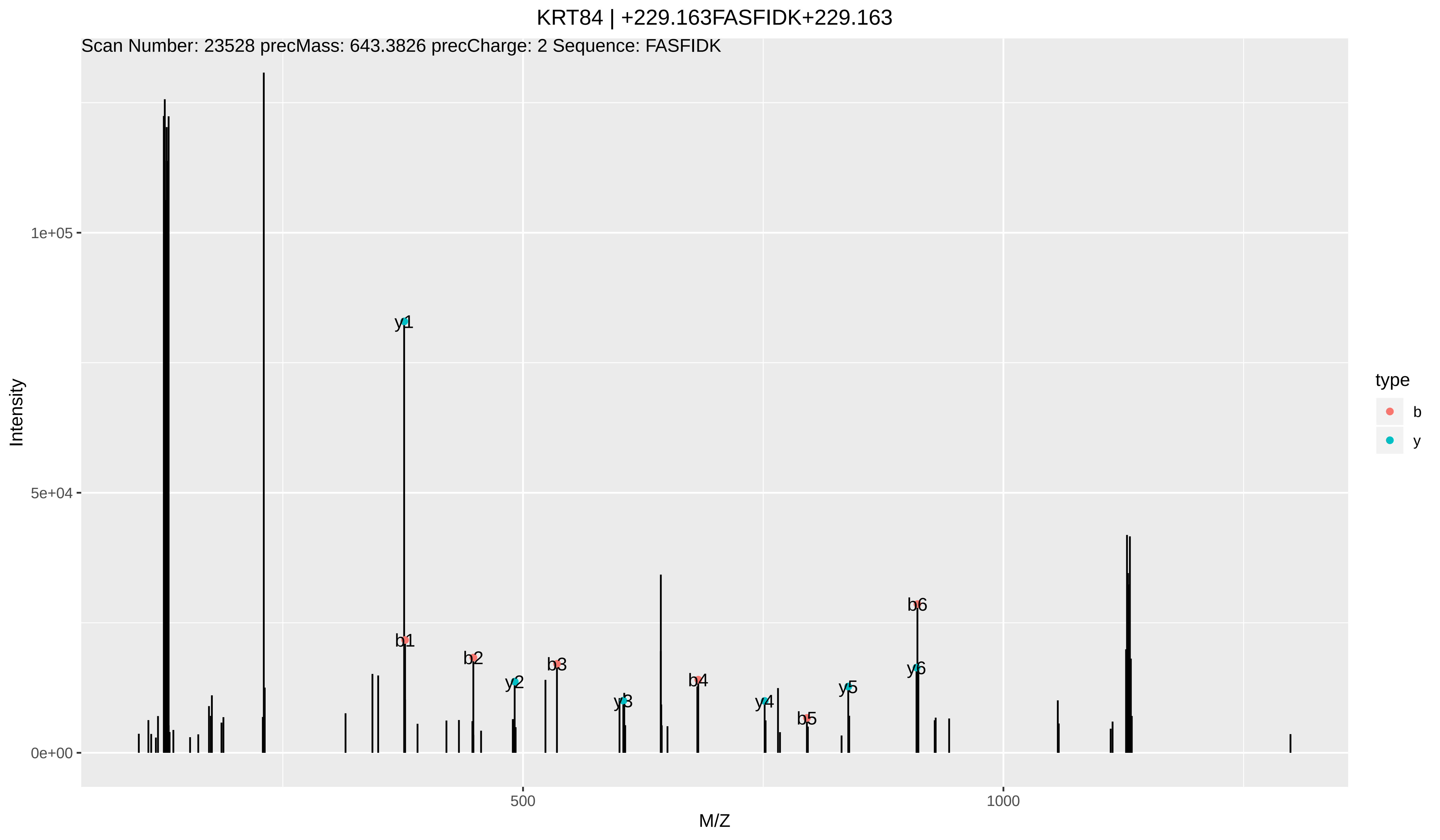
KRT84 | +229.163FASFIDK+229.163
Scan Number: 23528 precMass: 643.3826 precCharge: 2 Sequence: FASFIDK
Intensity
1e+05
5e+04
0e+00
M/Z
500
1000
y1
b1
b2
y2
b3
y3
b4
y4
b5
y5
b6
y6
type
b
y

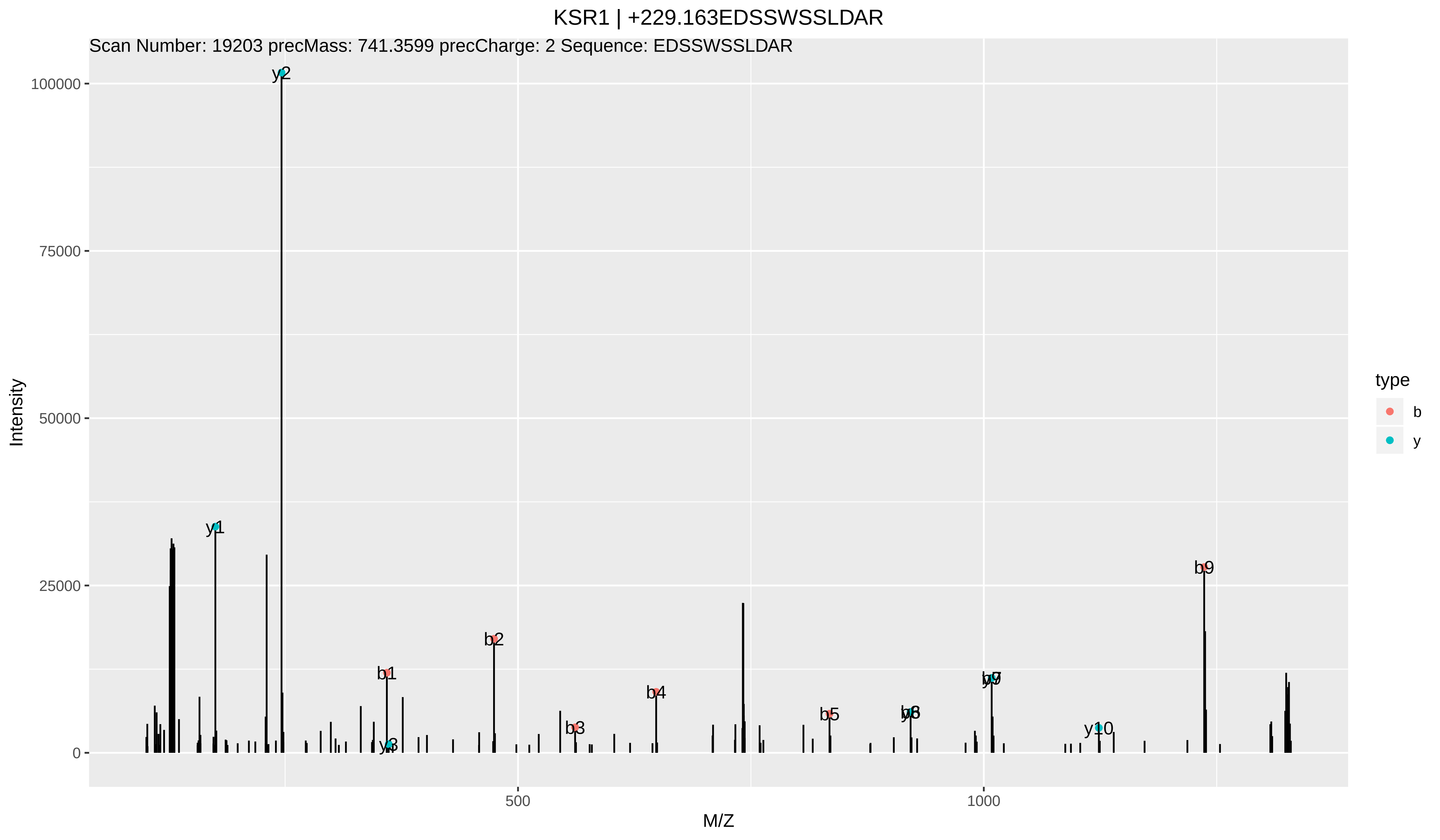

KSR1 | +229.163EDSSWSSLDAR
Scan Number: 19203 precMass: 741.3599 precCharge: 2 Sequence: EDSSWSSLDAR
Intensity
M/Z
100000
75000
50000
25000
0
500
1000
y2
y1
b9
b2
b1
b4
y9
y7
b3
y3
b5
b6
y8
y10
type
b
y

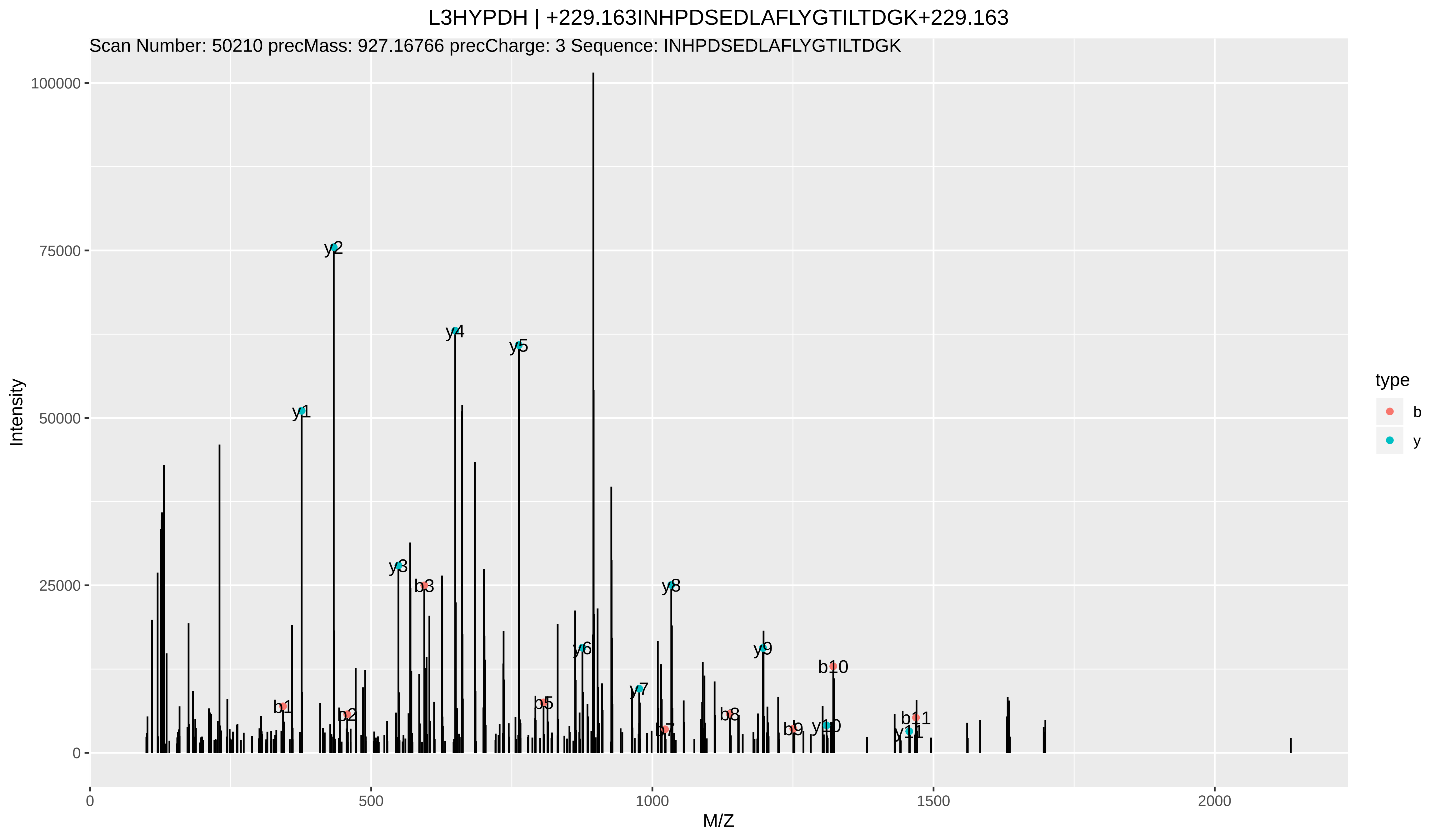
L3HYPDH | +229.163INHPDSEDLAFLYGTILTDGK+229.163
Scan Number: 50210 precMass: 927.16766 precCharge: 3 Sequence: INHPDSEDLAFLYGTILTDGK
Intensity
M/Z
type
b
y
y1
y2
y3
y4
y5
y6
y7
y8
y9
y10
y11
b1
b2
b3
b5
b7
b8
b9
b10
b11
0
25000
50000
75000
100000
500
1000
1500
2000

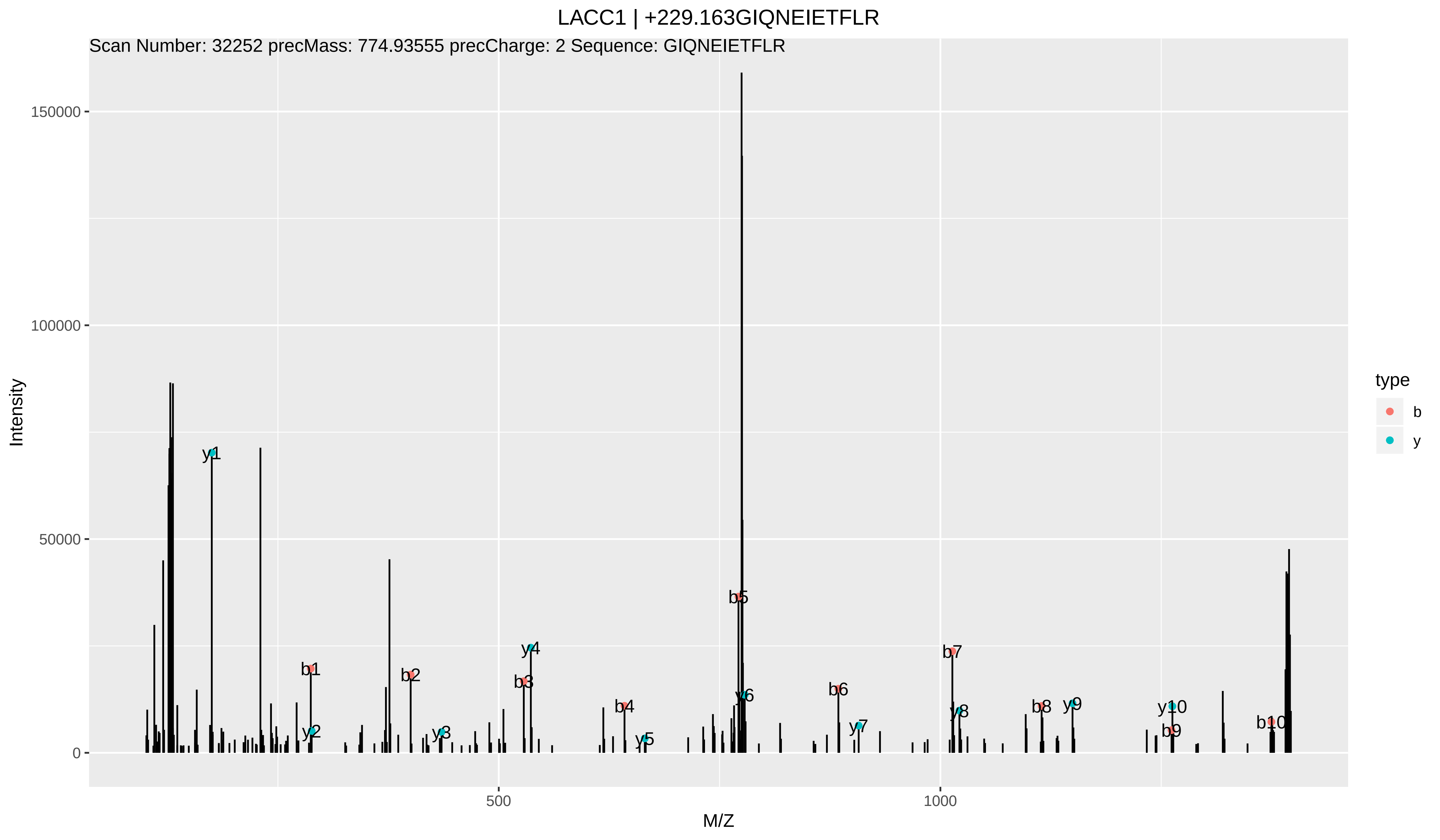

LACC1 | +229.163GIQNEIETFLR
Scan Number: 32252 precMass: 774.93555 precCharge: 2 Sequence: GIQNEIETFLR
Intensity
150000
100000
50000
0
M/Z
500
1000
y1
b1
y2
b2
y3
b3
y4
b4
y5
b5
y6
b6
y7
b7
y8
b8
y9
b9
y10
b10
type
b
y

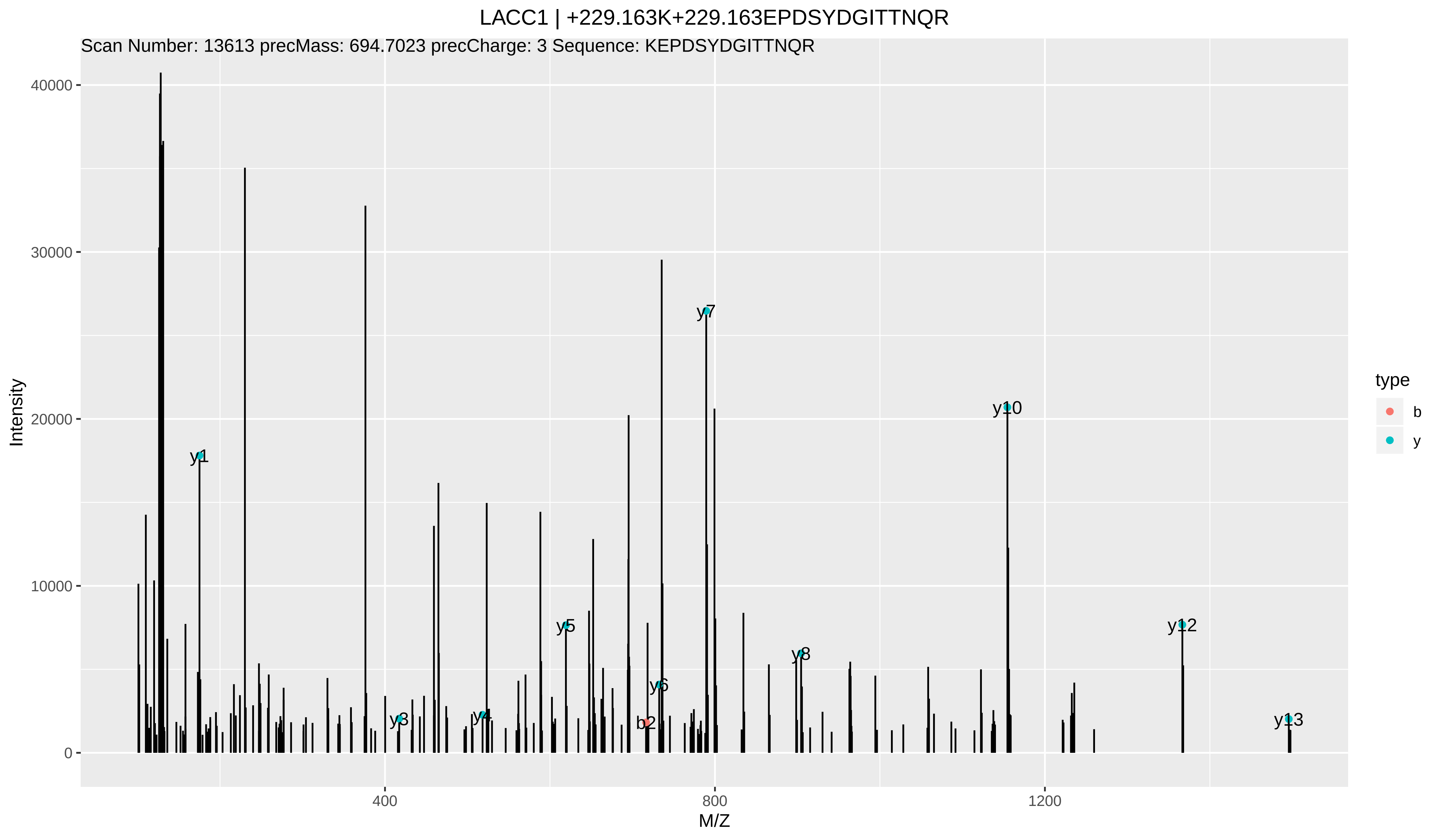

LACC1 | +229.163K+229.163EPDSYDGITTNQR
Scan Number: 13613 precMass: 694.7023 precCharge: 3 Sequence: KEPDSYDGITTNQR
Intensity
M/Z
0
10000
20000
30000
40000
400
800
1200
y1
y3
y4
y5
b2
y6
y7
y8
y10
y12
y13
type
b
y

LAMP3 | +229.163AEM+15.995GIQLIVQDK+229.163

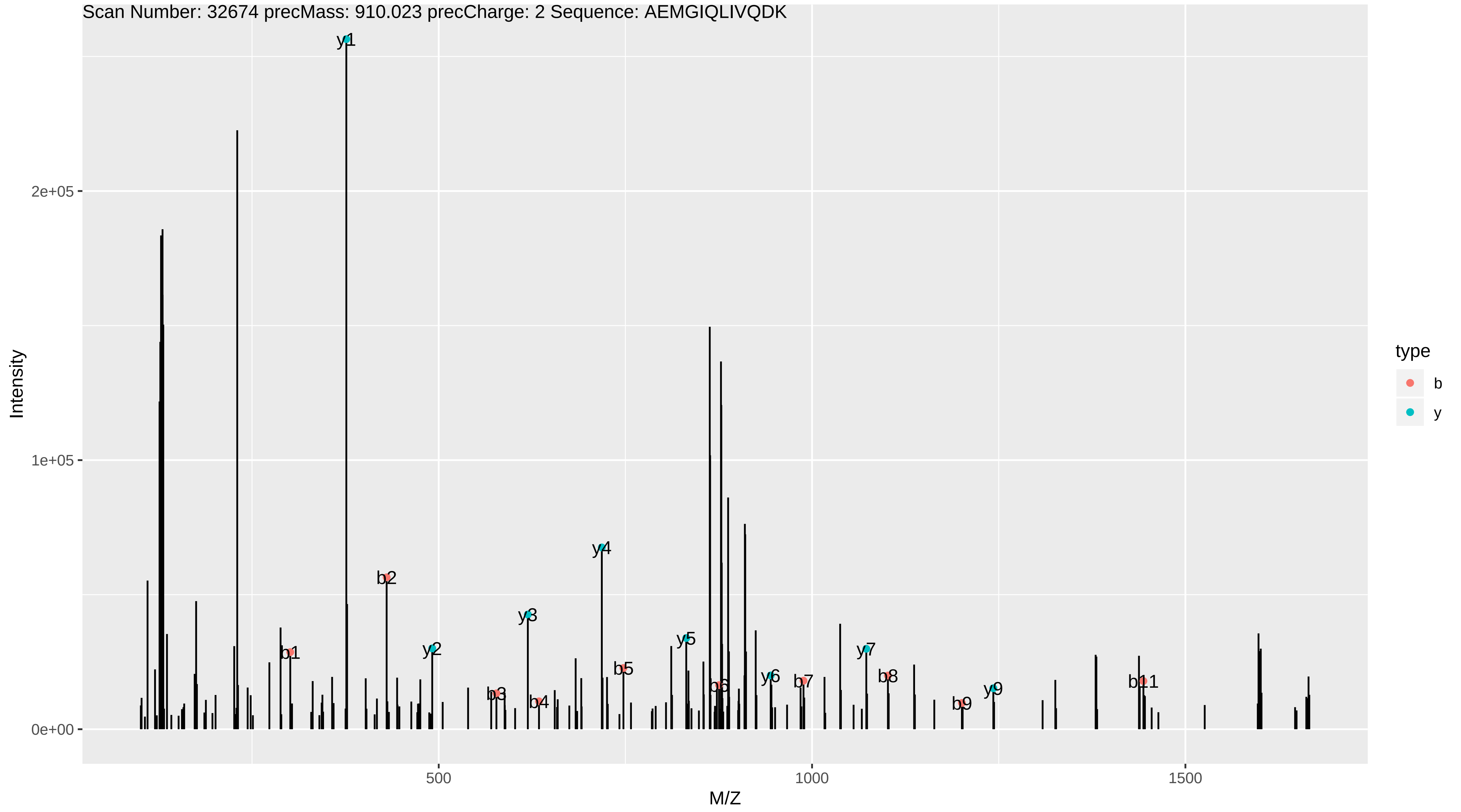

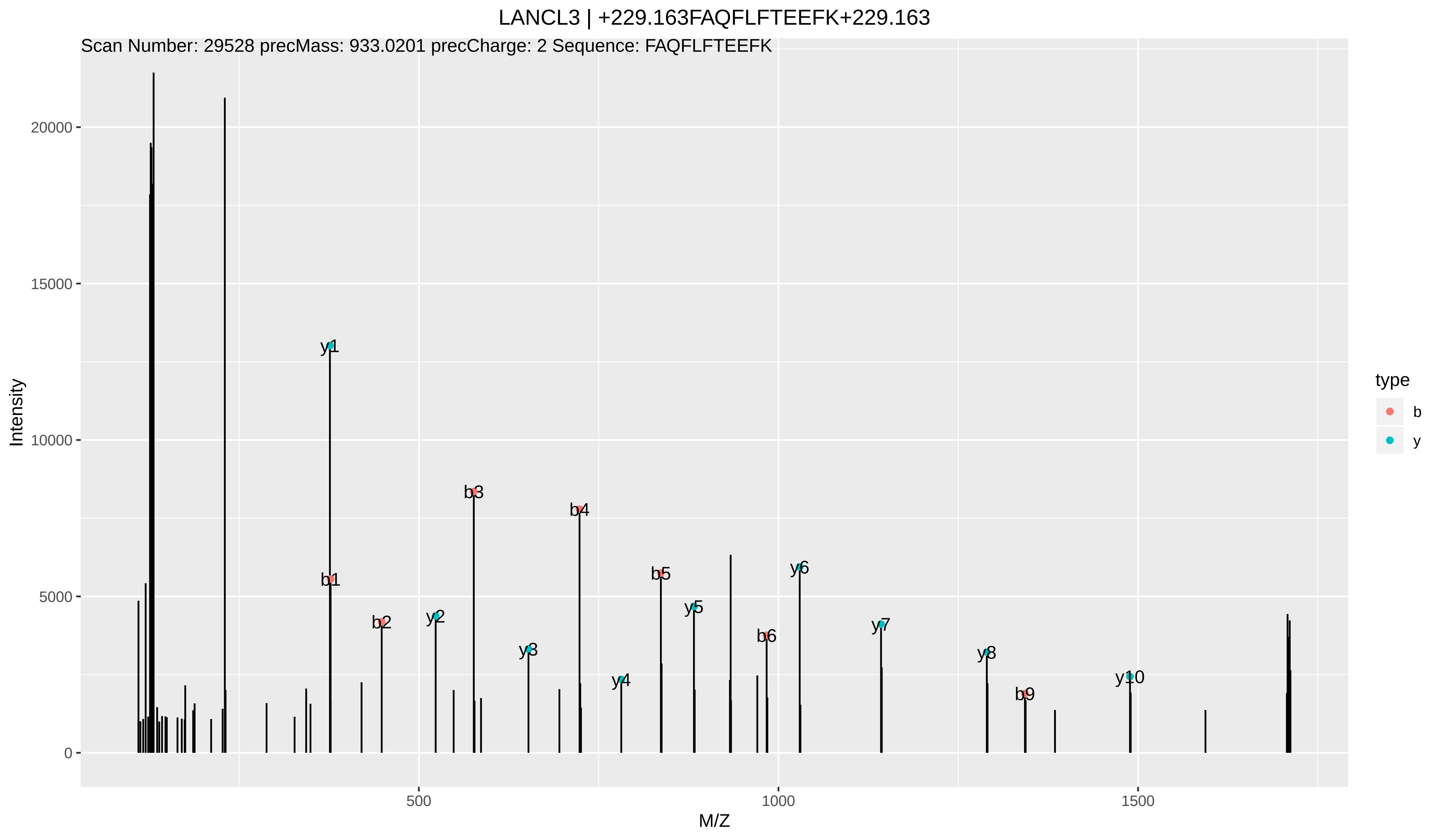

LANCL3 | +229.163FAQFLFTEEFK+229.163
Scan Number: 29528 precMass: 933.0201 precCharge: 2 Sequence: FAQFLFTEEFK
Intensity
20000
15000
10000
5000
0
M/Z
500
1000
1500
y1
b1
b2
y2
b3
y3
b4
y4
b5
y5
b6
y6
y7
y8
b9
y10
type
b
y

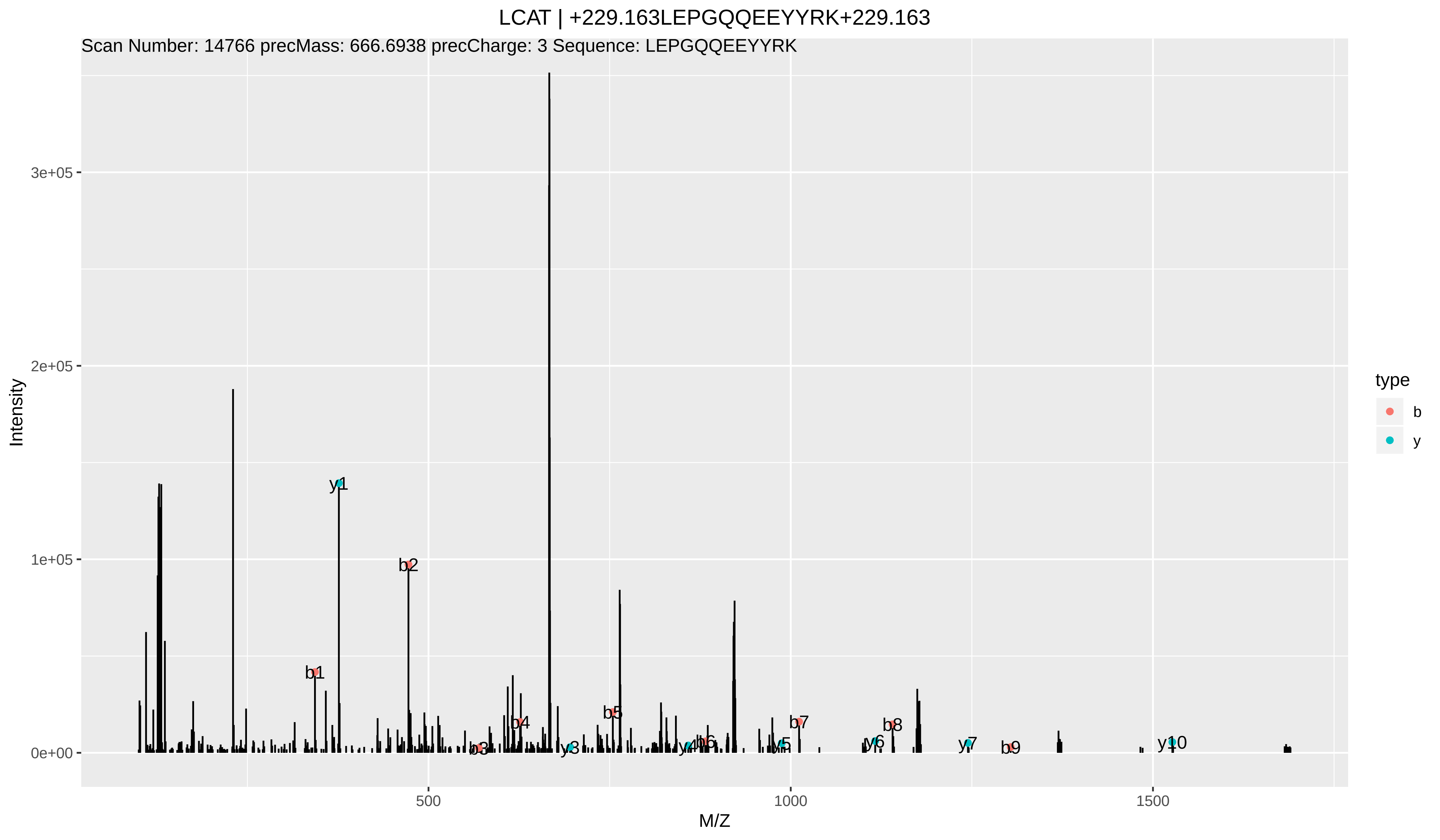

LCAT | +229.163LEPGQQEEYYRK+229.163
Scan Number: 14766 precMass: 666.6938 precCharge: 3 Sequence: LEPGQQEEYYRK
Intensity
M/Z
3e+05
2e+05
1e+05
0e+00
500
1000
1500
y1
b1
b2
b3
b4
y3
b5
y4
b6
y5
b7
y6
b8
y7
b9
y10
type
b
y

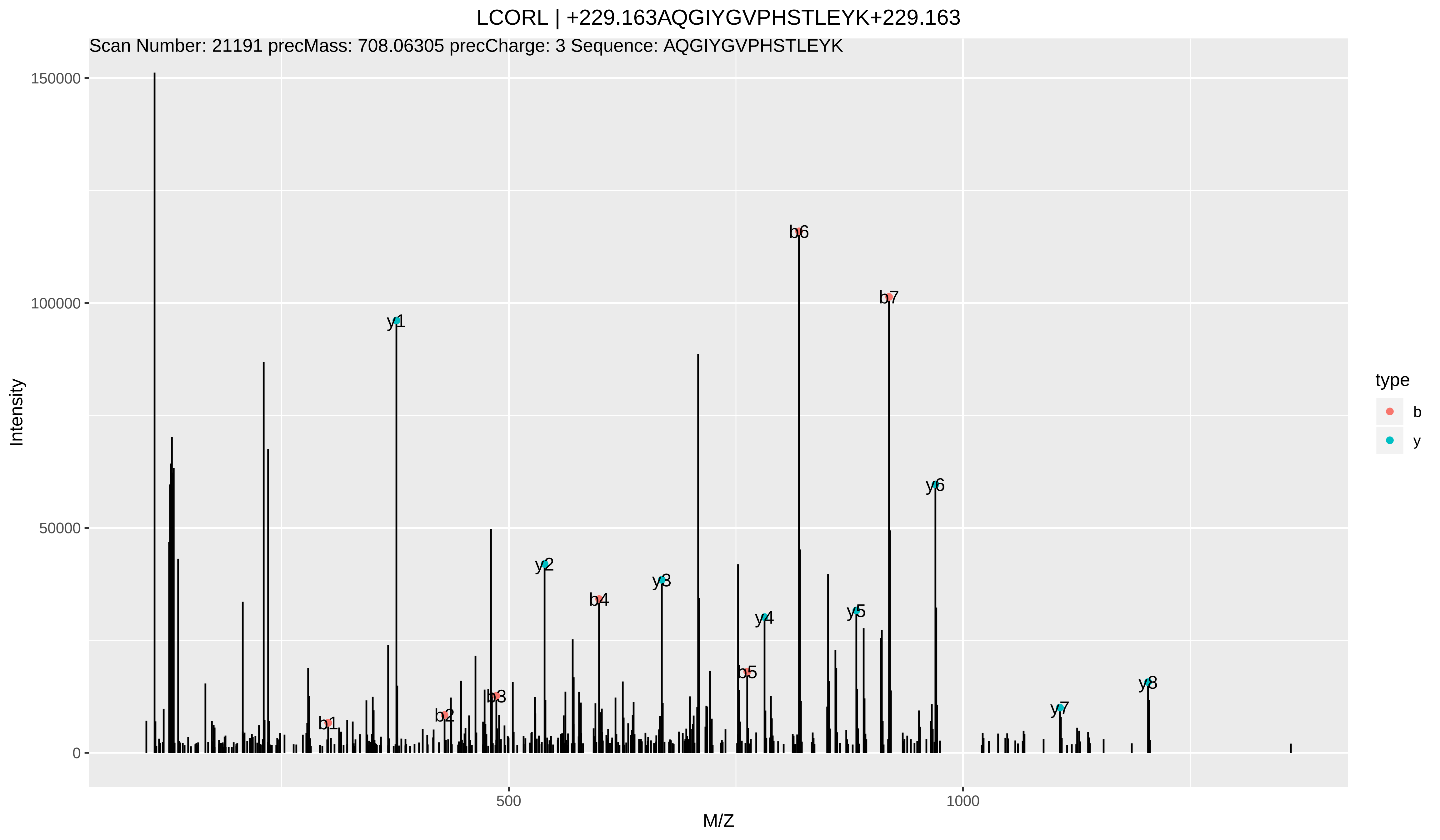

LCORL | +229.163AQGIYGVPHSTLEYK+229.163
Scan Number: 21191 precMass: 708.06305 precCharge: 3 Sequence: AQGIYGVPHSTLEYK
Intensity
150000
100000
50000
0
M/Z
500
1000
y1
y2
y3
y4
y5
y6
y7
y8
b1
b2
b3
b4
b5
b6
b7
type
b
y

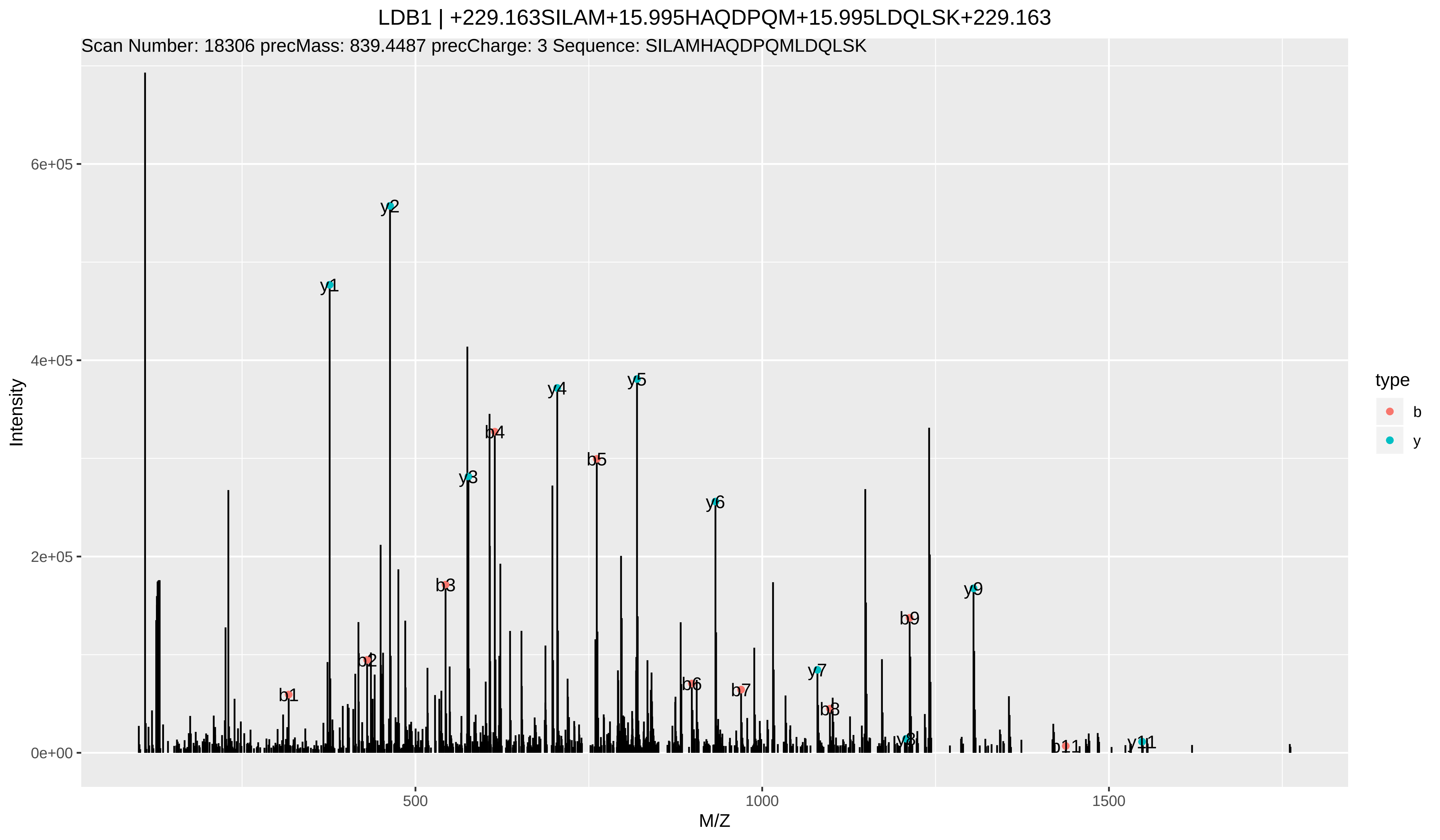

LDB1 | +229.163SILAM+15.995HAQDPQM+15.995LDQLSK+229.163
Scan Number: 18306 precMass: 839.4487 precCharge: 3 Sequence: SILAMHAQDPQMLDQLSK
Intensity
M/Z
6e+05
4e+05
2e+05
0e+00
500
1000
1500
y1
y2
y3
y4
y5
y6
y7
y8
y9
y11
b1
b2
b3
b4
b5
b6
b7
b8
b9
b11
type
b
y

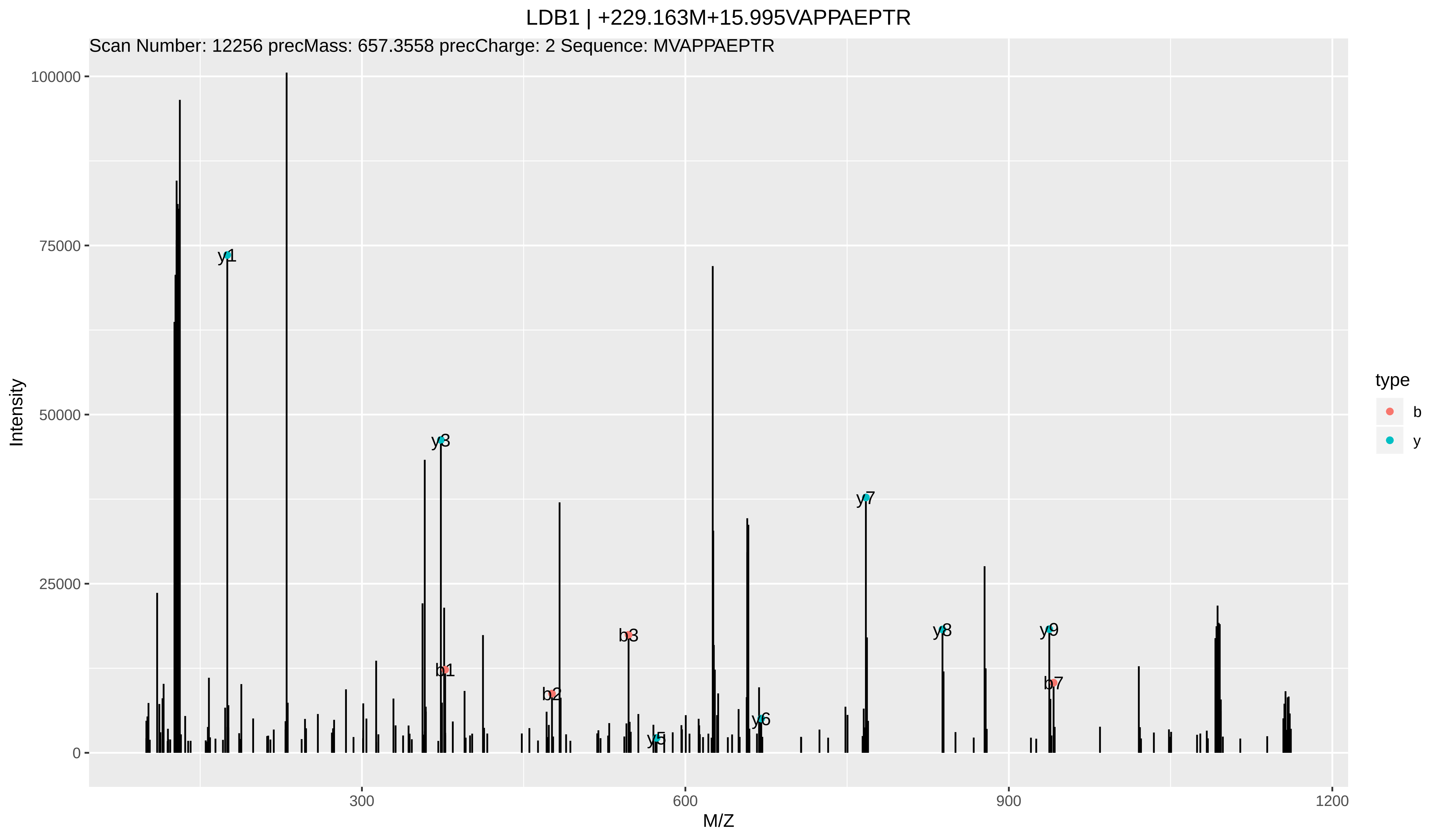

LDB1 | +229.163M+15.995VAPPAEPTR
Scan Number: 12256 precMass: 657.3558 precCharge: 2 Sequence: MVAPPAEPTR
Intensity
100000
75000
50000
25000
0
300
600
900
1200
M/Z
y1
y3
b1
b2
b3
y5
y6
y7
y8
y9
b7
type
b
y

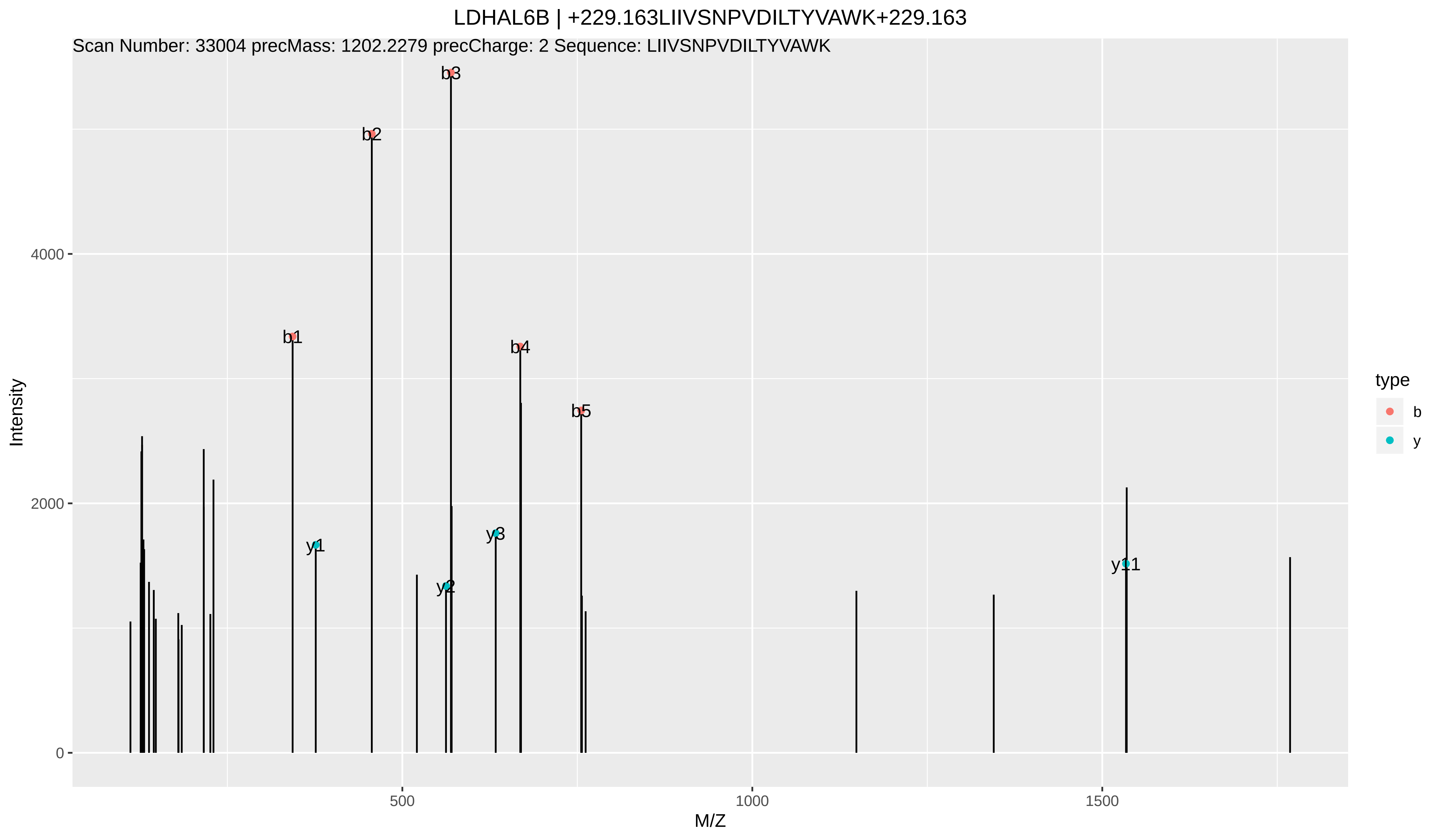
LDHAL6B | +229.163LIIVSNPVDILTYVAWK+229.163
Scan Number: 33004 precMass: 1202.2279 precCharge: 2 Sequence: LIIVSNPVDILTYVAWK
b3
b2
b1
b4
b5
y1
y2
y3
y11
Intensity
0
2000
4000
M/Z
500
1000
1500
type
b
y

LEPR | +229.163VHLLYVLPEVLEDSPLVPQK+229.163

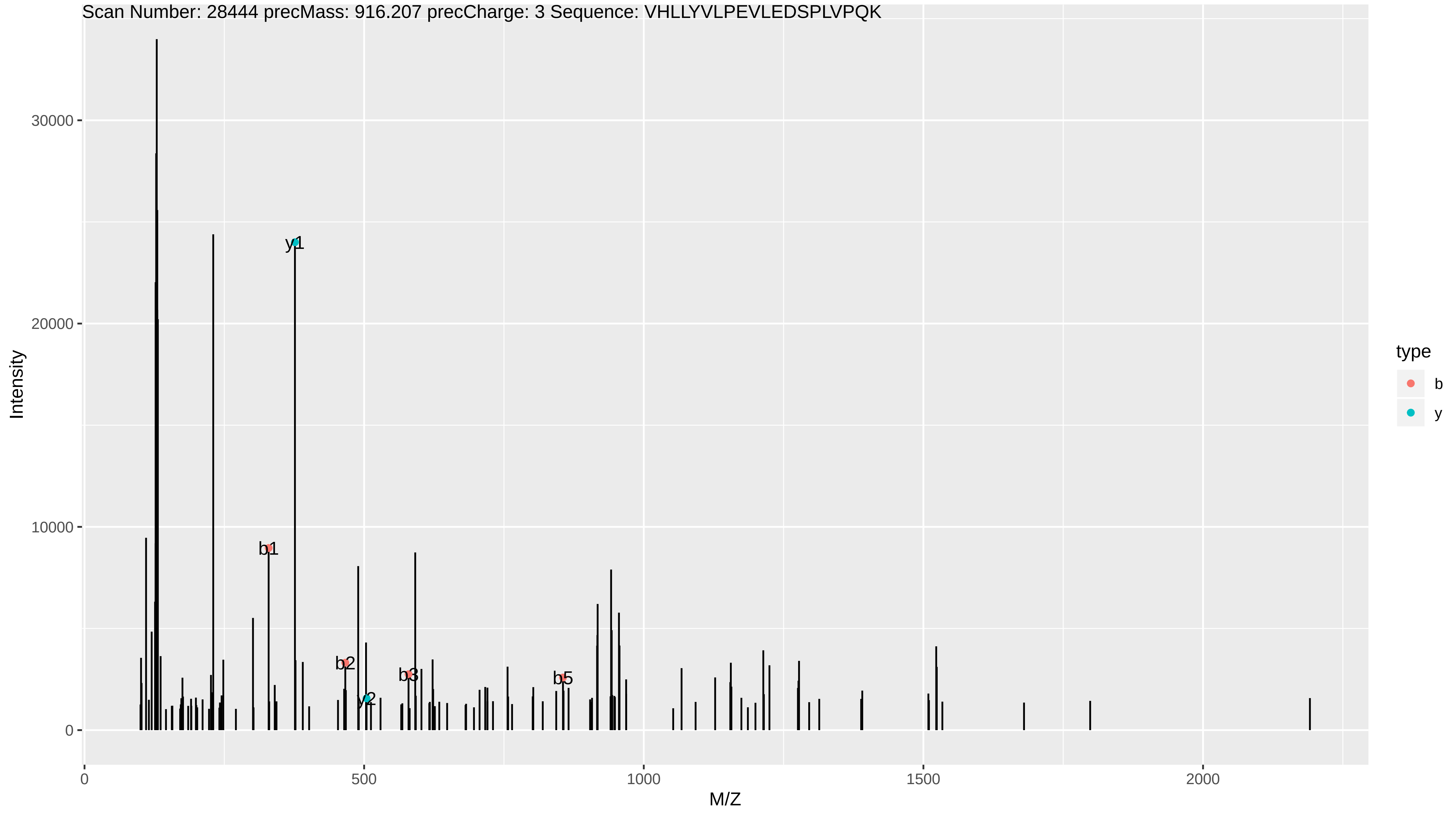

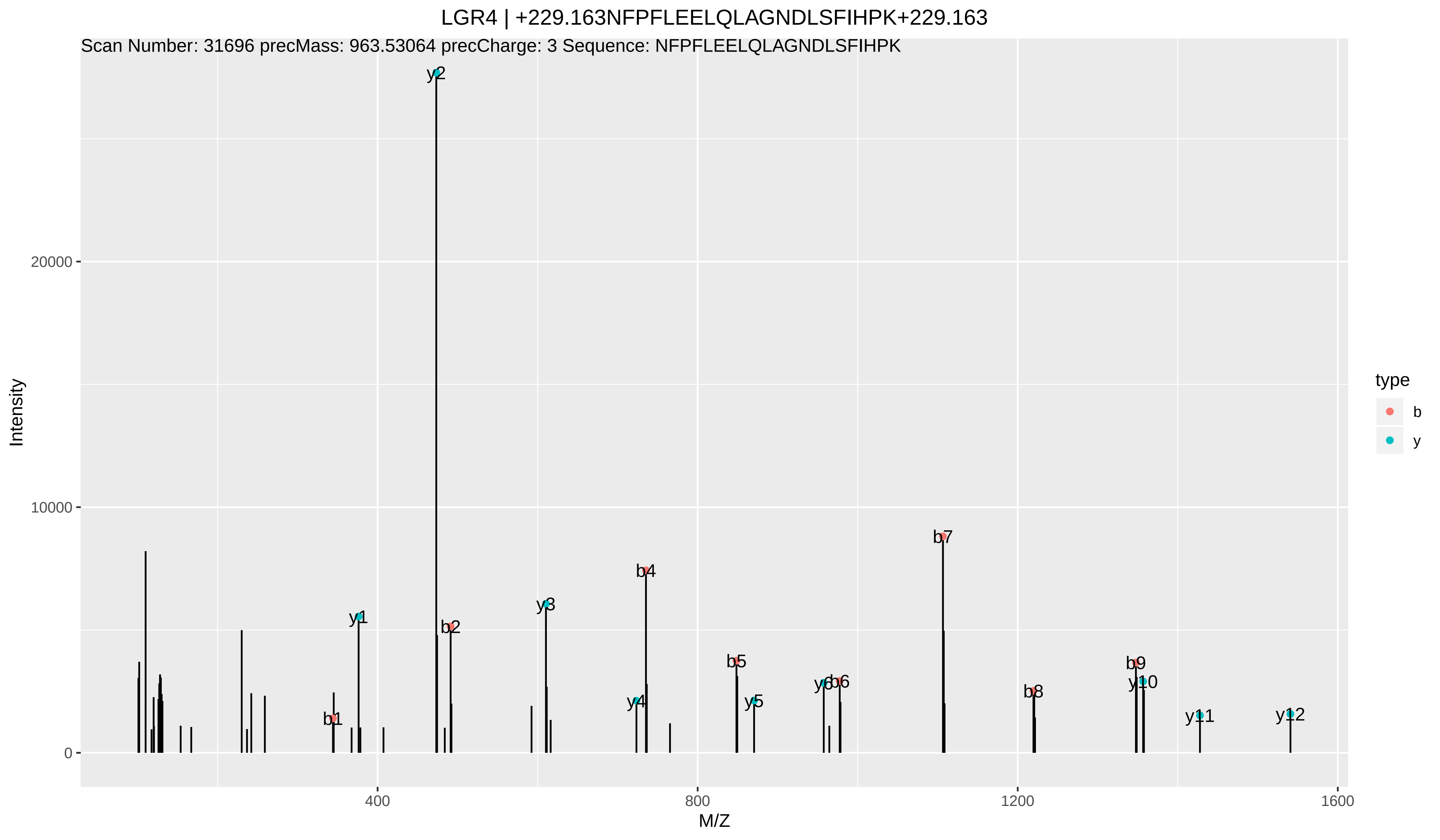

LGR4 | +229.163NFPFLEELQLAGNDLSFIHPK+229.163
Scan Number: 31696 precMass: 963.53064 precCharge: 3 Sequence: NFPFLEELQLAGNDLSFIHPK
Intensity
M/Z
20000
10000
0
400
800
1200
1600
y2
y1
b1
b2
y3
b4
y4
b5
y5
y6
b6
b7
b8
b9
y10
y11
y12
type
b
y

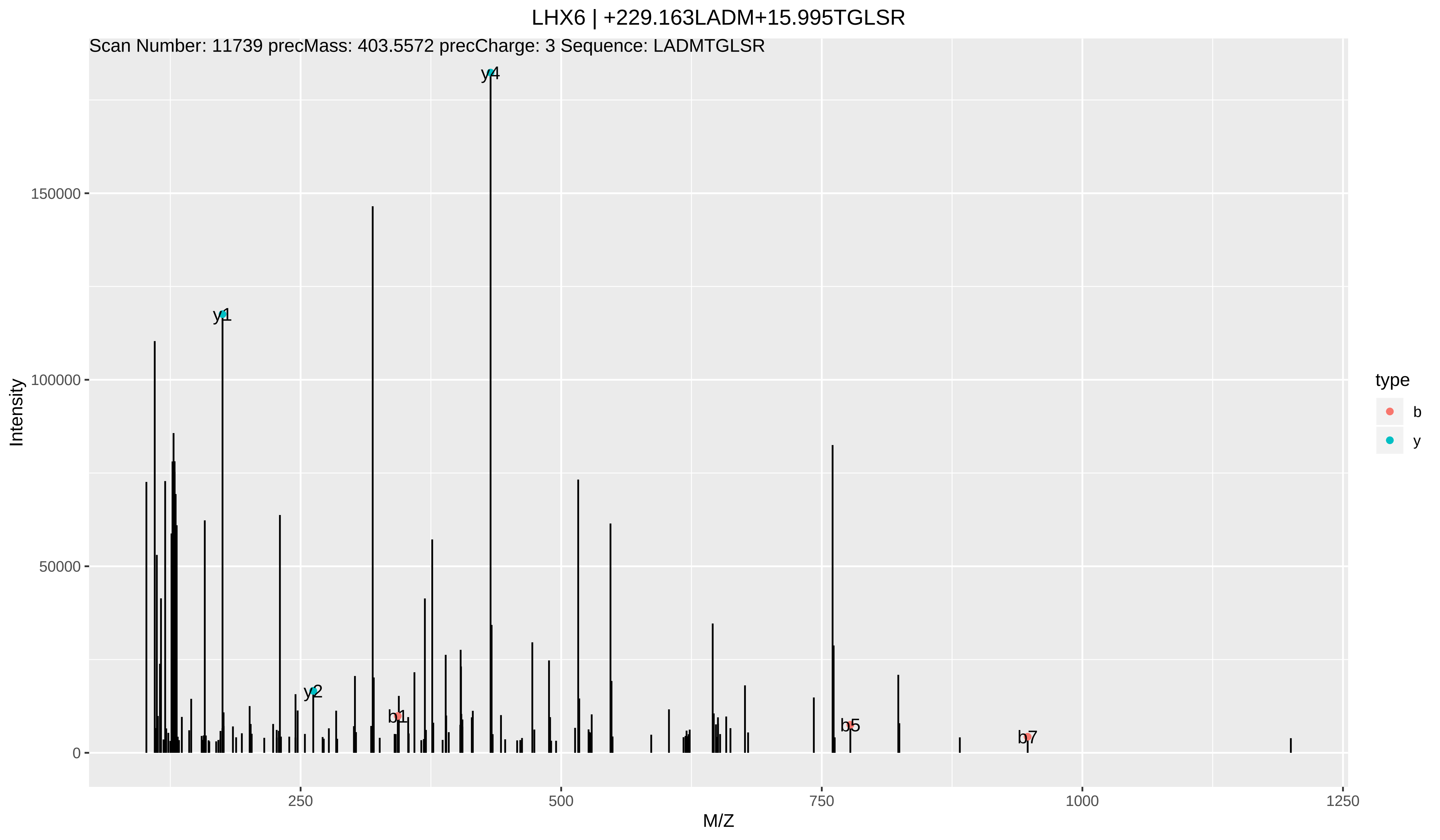

LHX6 | +229.163LADM+15.995TGLSR
Scan Number: 11739 precMass: 403.5572 precCharge: 3 Sequence: LADMTGLSR
y4
y1
y2
b1
b5
b7
Intensity
150000
100000
50000
0
250
500
750
1000
1250
M/Z
type
b
y

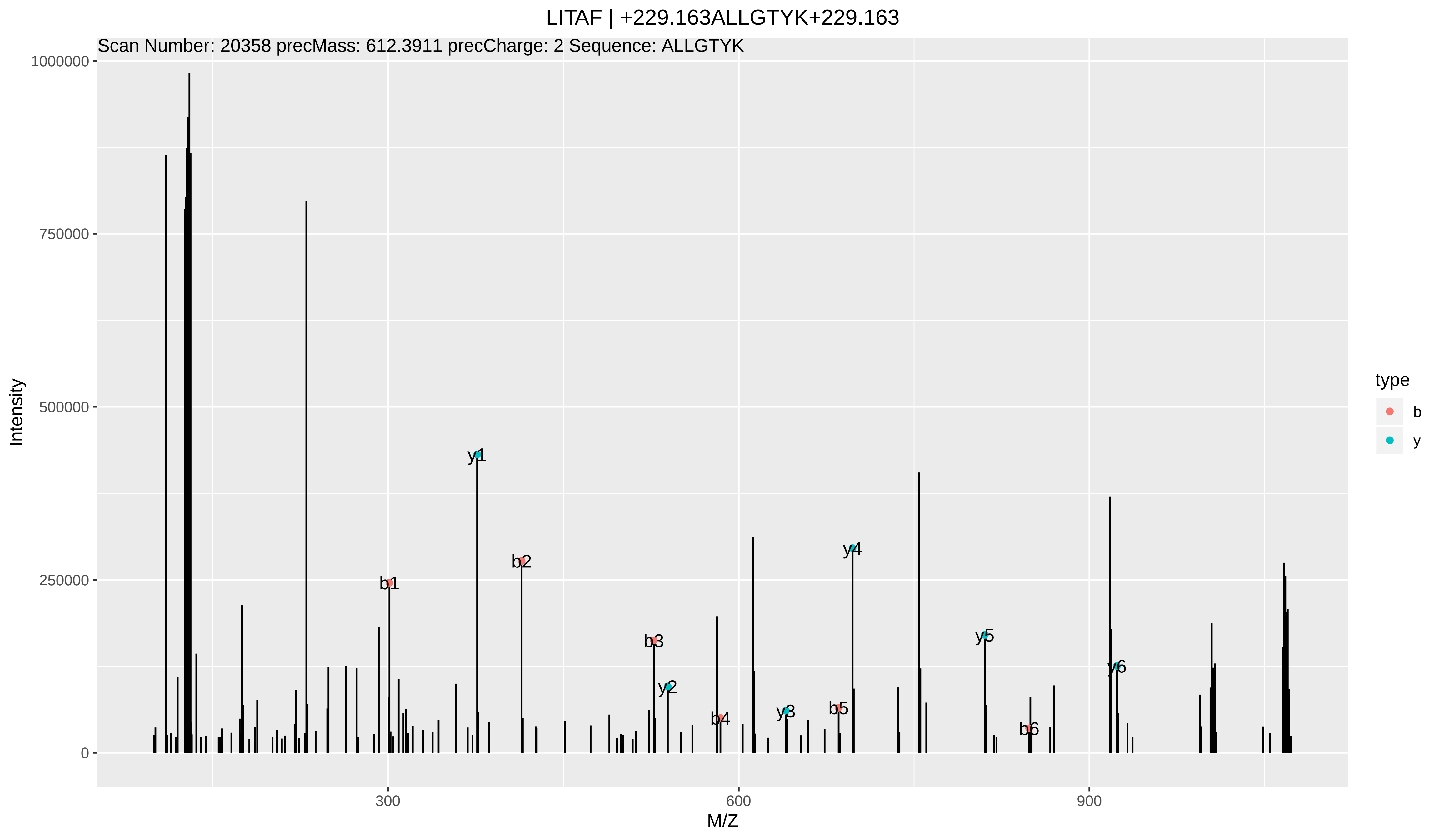

LITAF | +229.163ALLGTYK+229.163
Scan Number: 20358 precMass: 612.3911 precCharge: 2 Sequence: ALLGTYK
Intensity
1000000
750000
500000
250000
0
M/Z
300
600
900
type
b
y
y1
b1
b2
b3
y2
b4
y3
b5
y4
y5
b6
y6

LNX2 | +229.163LLAGDQILQVNNYNISNVSHNYAR

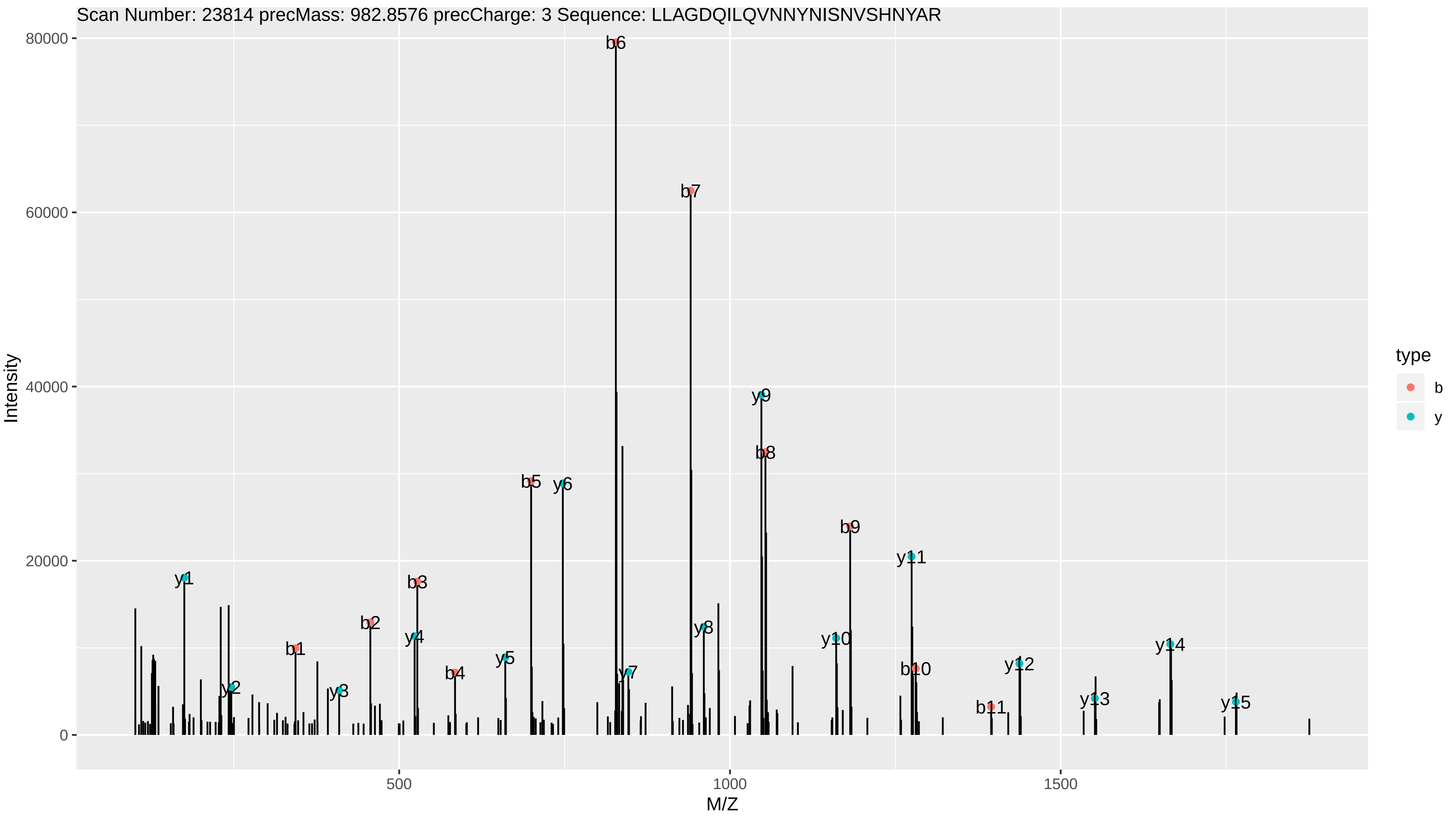

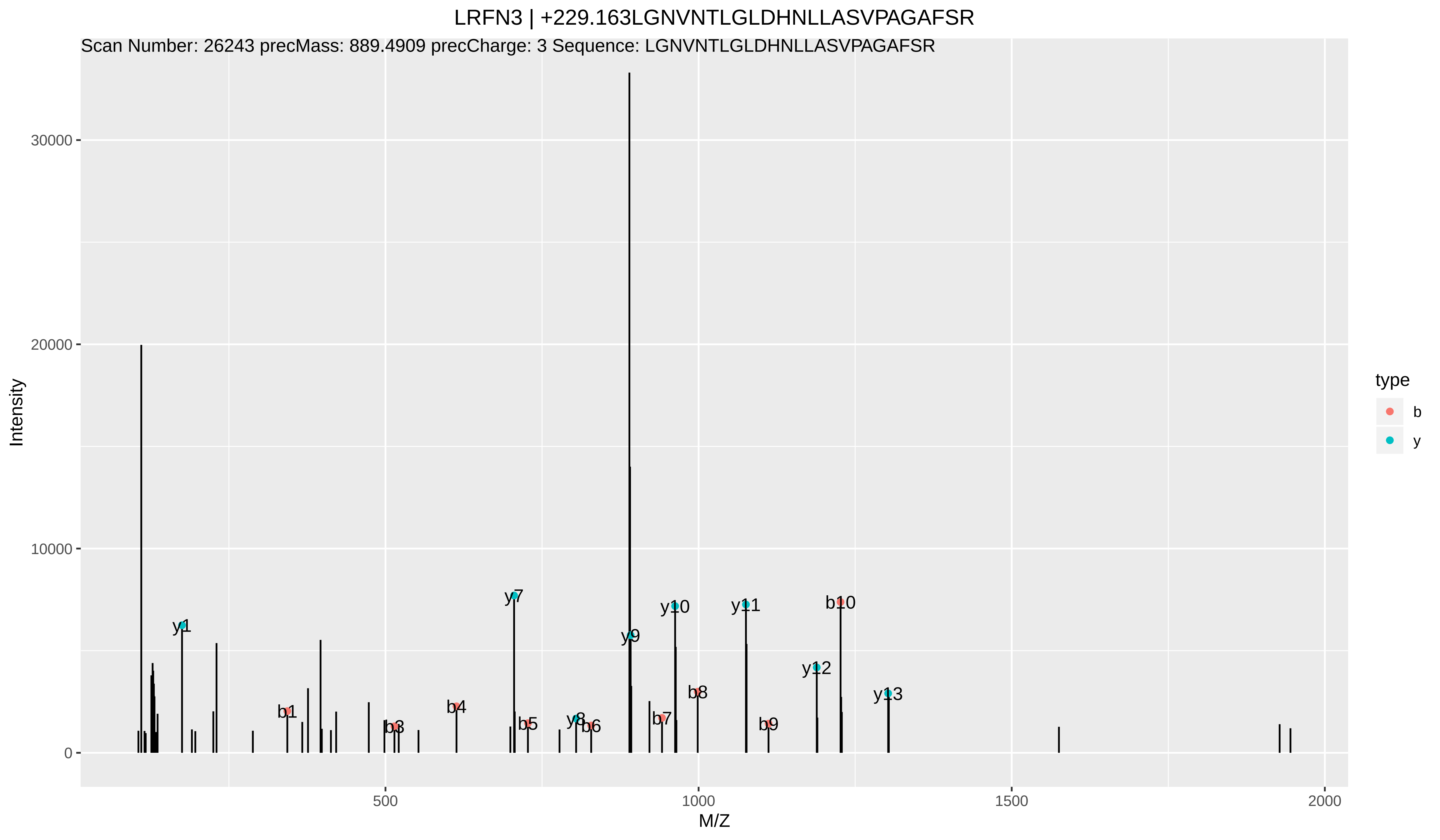

LRFN3 | +229.163LGNVNTLGLDHNLLASVPAGAFSR
Scan Number: 26243 precMass: 889.4909 precCharge: 3 Sequence: LGNVNTLGLDHNLLASVPAGAFSR
Intensity
M/Z
type
b
y
y1
b1
b3
b4
y7
b5
y8
b6
y9
b7
y10
b8
y11
b9
y12
b10
y13
0
10000
20000
30000
500
1000
1500
2000

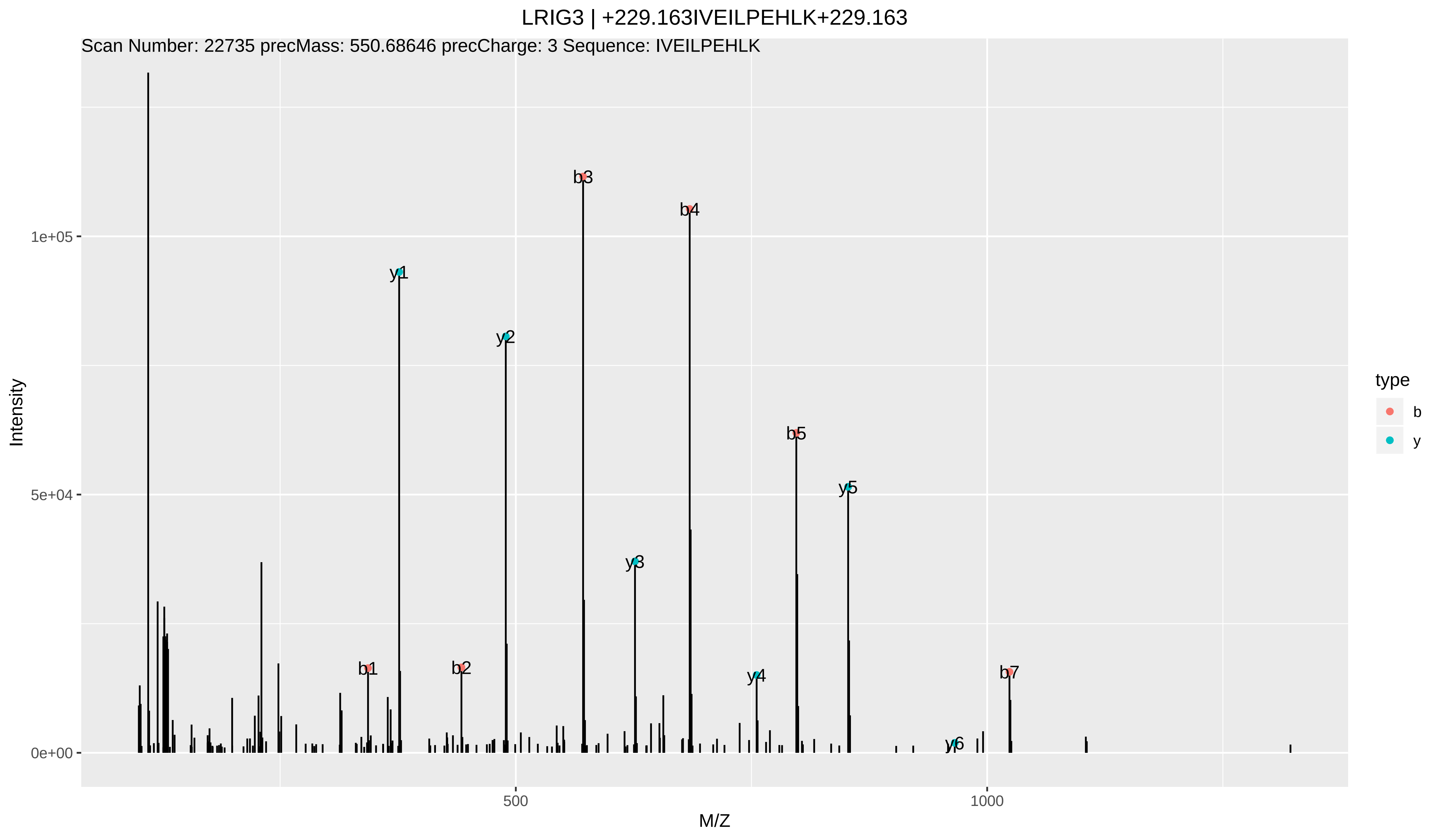
LRIG3 | +229.163IVEILPEHLK+229.163
Scan Number: 22735 precMass: 550.68646 precCharge: 3 Sequence: IVEILPEHLK
Intensity
1e+05
5e+04
0e+00
M/Z
500
1000
b1
y1
b2
y2
b3
y3
b4
y4
b5
y5
y6
b7
type
b
y

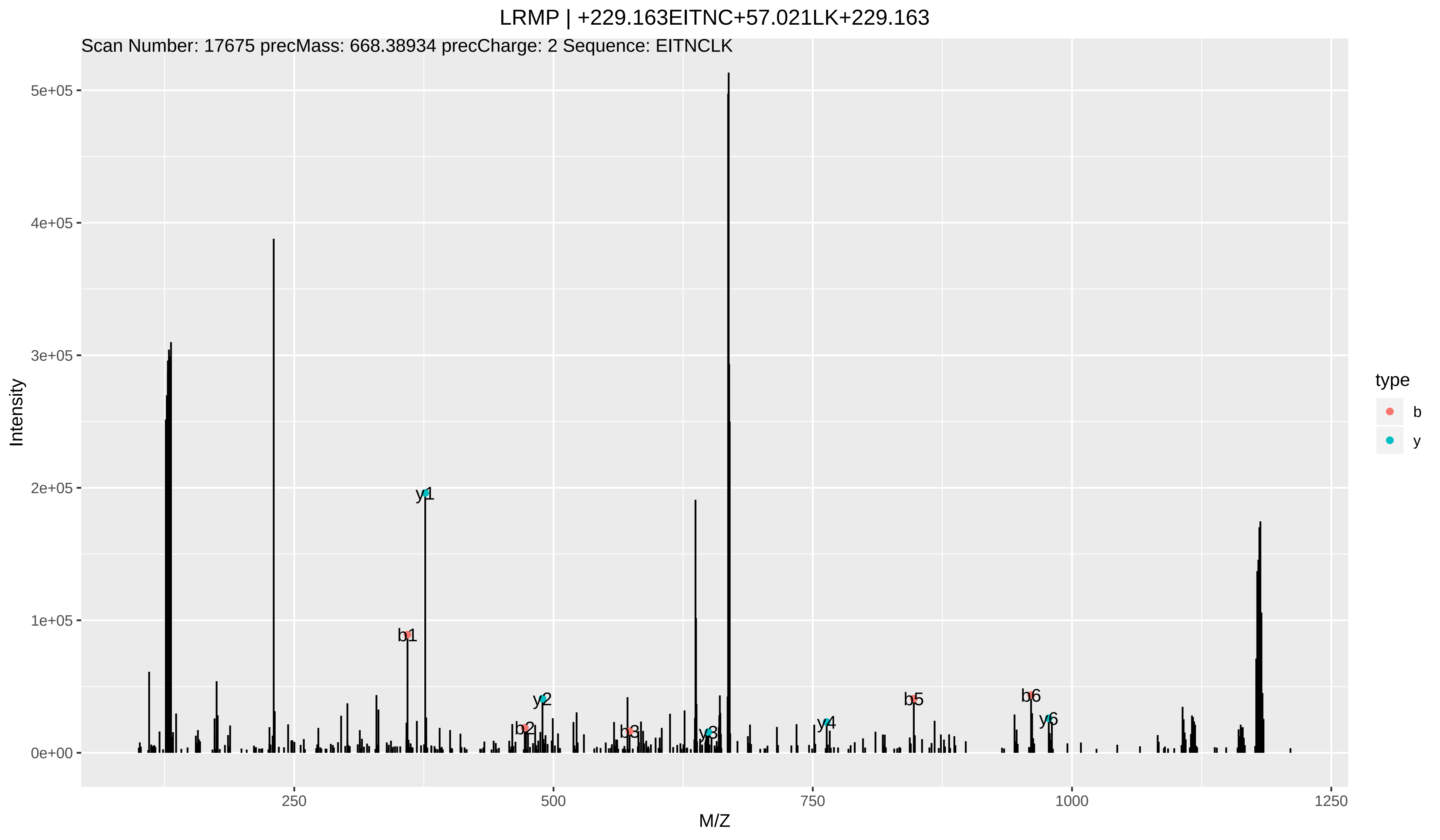
LRMP | +229.163EITNC+57.021LK+229.163
Scan Number: 17675 precMass: 668.38934 precCharge: 2 Sequence: EITNCLK
Intensity
M/Z
0e+00
1e+05
2e+05
3e+05
4e+05
5e+05
250
500
750
1000
1250
b1
y1
y2
b2
b3
y3
y4
b5
b6
y6
type
b
y

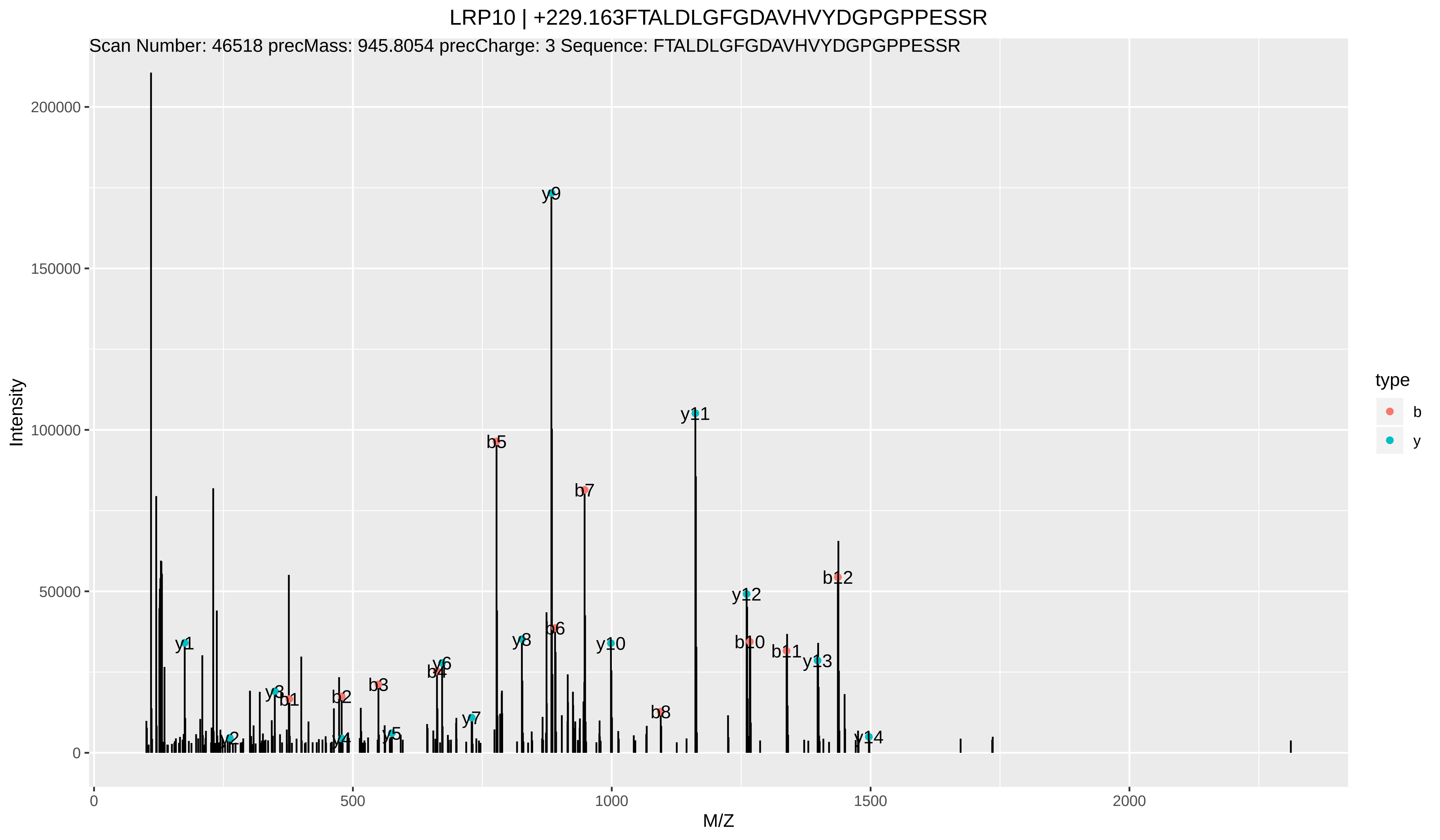

LRP10 | +229.163FTALDLGFGDAVHVYDGPGPPESSR
Scan Number: 46518 precMass: 945.8054 precCharge: 3 Sequence: FTALDLGFGDAVHVYDGPGPPESSR
Intensity
M/Z
type
b
y
y9
y11
b5
b7
b12
y12
b6
y8
y10
b10
b11
y13
y1
y6
b4
b3
y3
b1
b2
b8
y7
y5
y2
y4
y14
200000
150000
100000
50000
0
0
500
1000
1500
2000

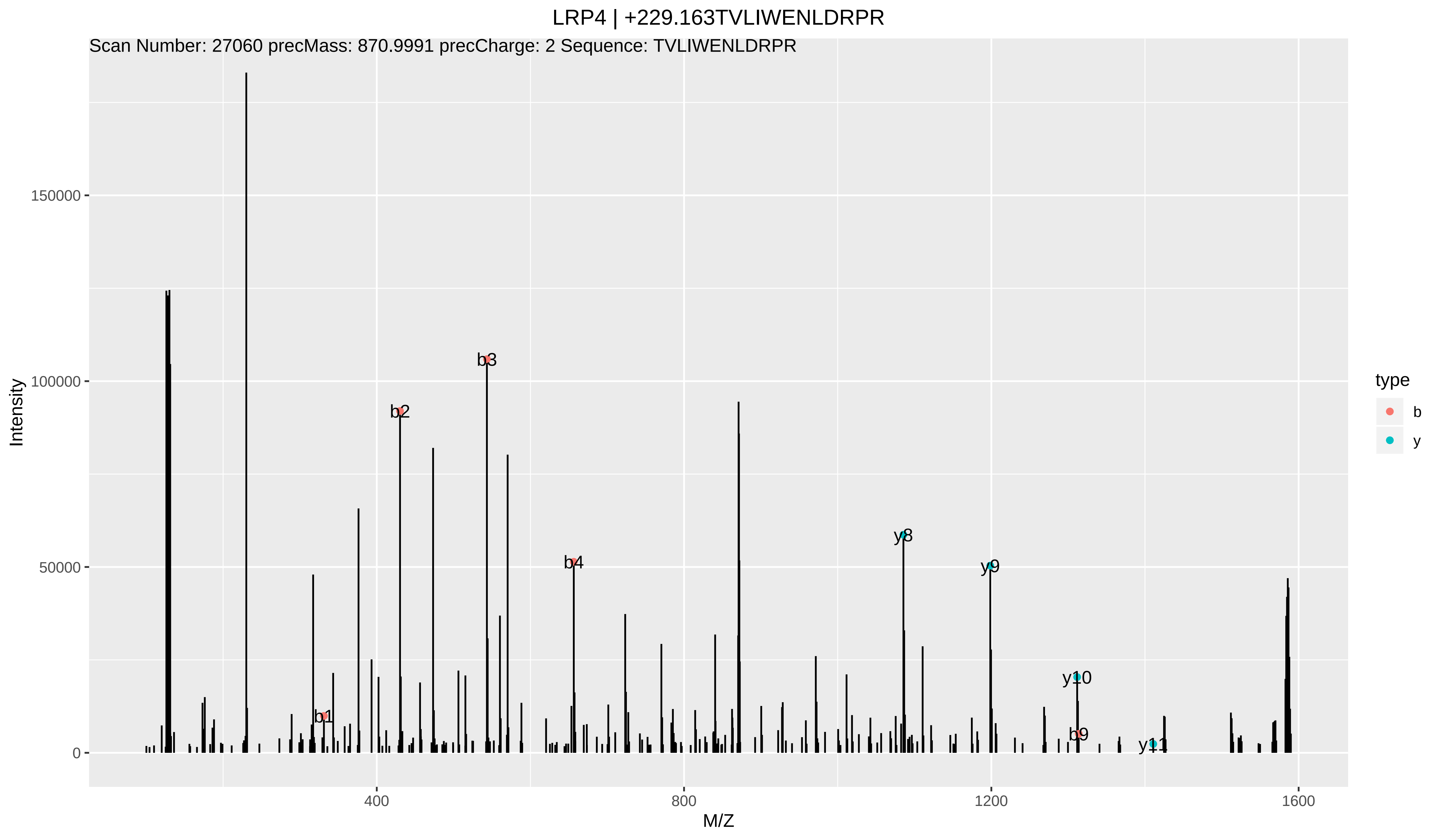
LRP4 | +229.163TVLIWENLDRPR
Scan Number: 27060 precMass: 870.9991 precCharge: 2 Sequence: TVLIWENLDRPR
Intensity
150000
100000
50000
0
M/Z
400
800
1200
1600
b1
b2
b3
b4
b9
y8
y9
y10
y11
type
b
y

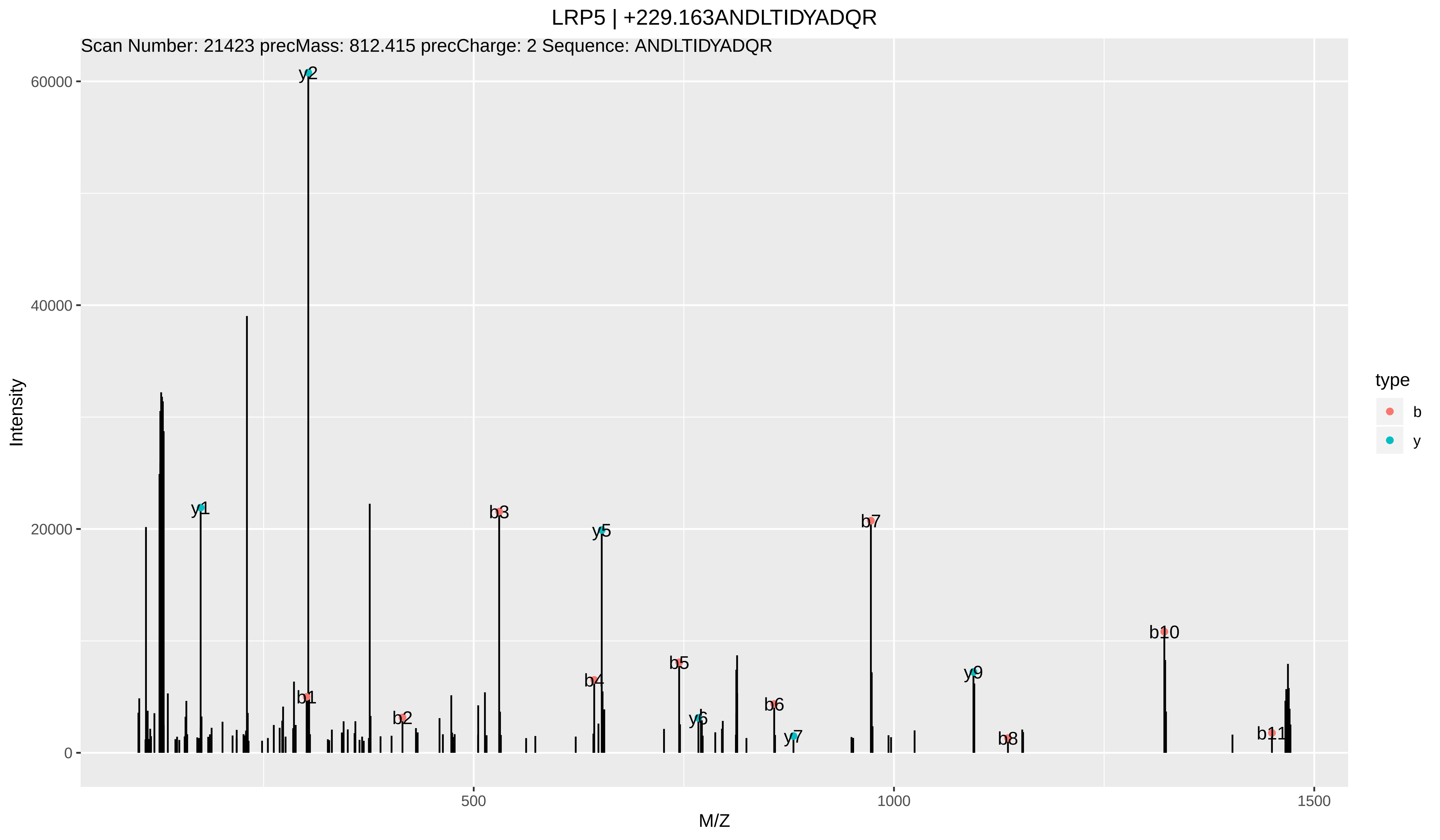
LRP5 | +229.163ANDLTIDYADQR
Scan Number: 21423 precMass: 812.415 precCharge: 2 Sequence: ANDLTIDYADQR
Intensity
60000
40000
20000
0
500
1000
1500
M/Z
y2
y1
b3
y5
b7
b10
b5
b4
y9
b1
b6
b2
y6
y7
b8
b11
type
b
y

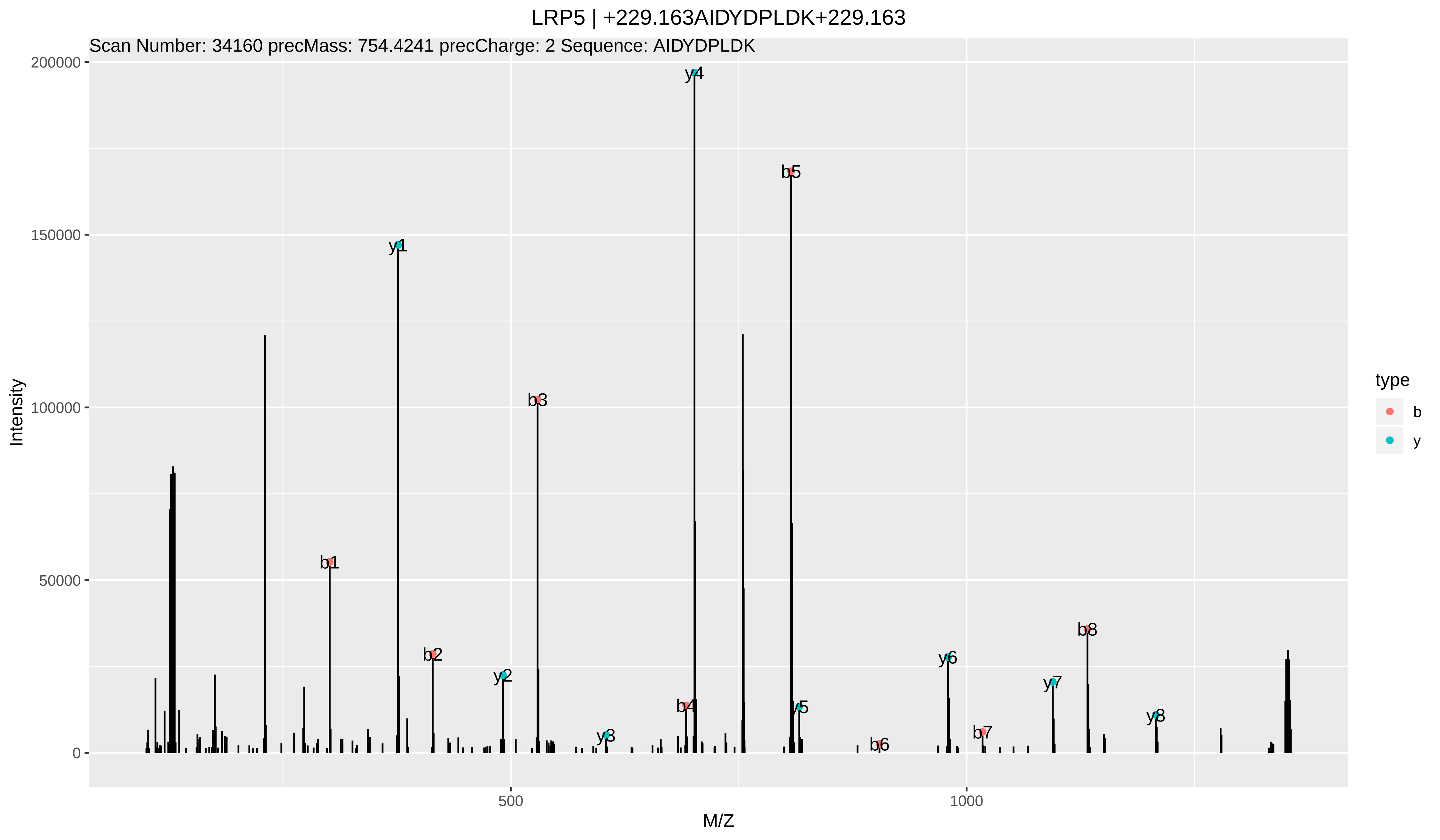

LRP5 | +229.163AIDYDPLDK+229.163
Scan Number: 34160 precMass: 754.4241 precCharge: 2 Sequence: AIDYDPLDK
Intensity
M/Z
200000
150000
100000
50000
0
500
1000
y4
b5
y1
b3
b1
b2
y2
y3
b4
y5
b6
y6
b7
y7
b8
y8
type
b
y

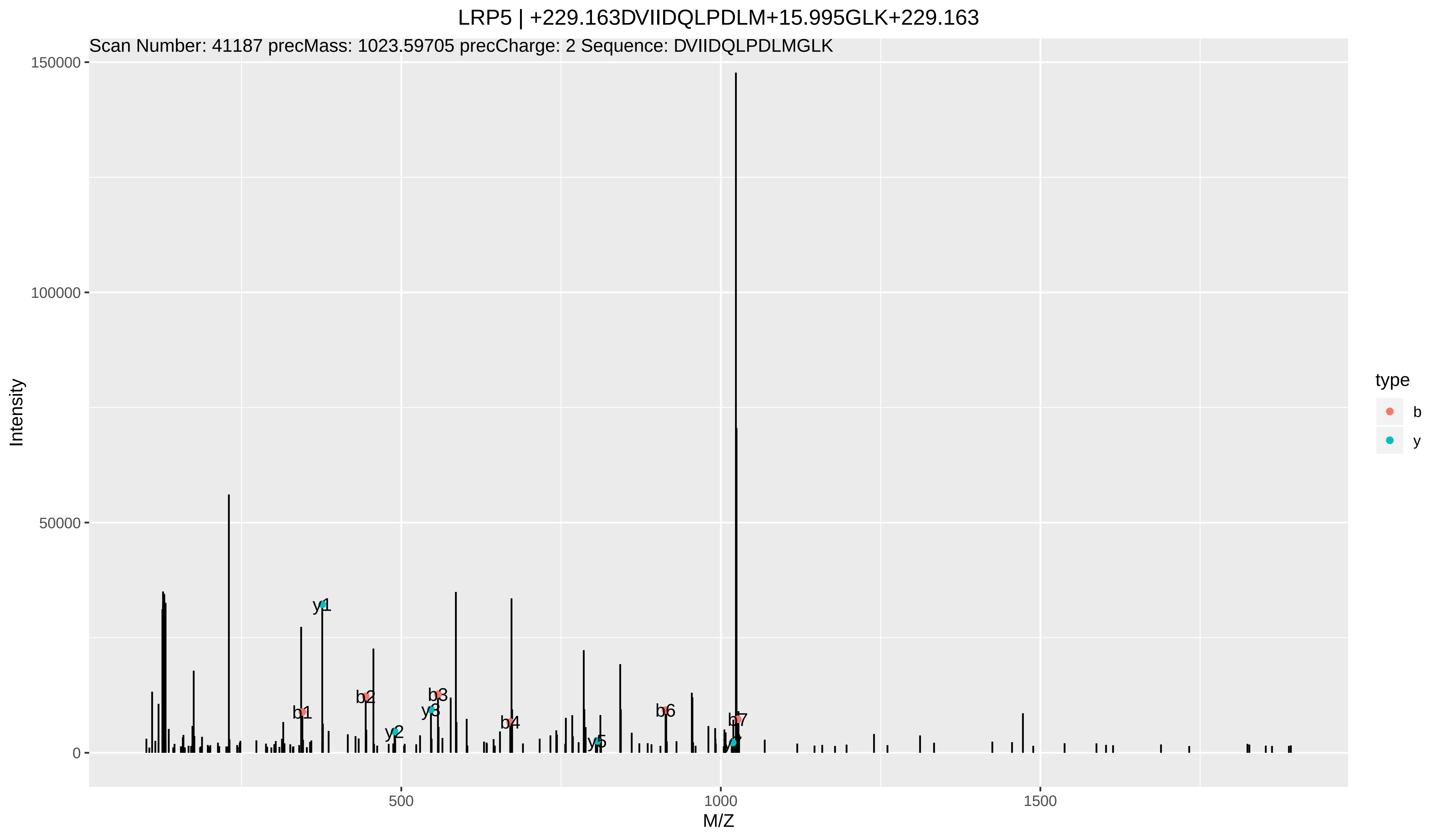

LRP5 | +229.163DVIIDQLPDLM+15.995GLK+229.163
Scan Number: 41187 precMass: 1023.59705 precCharge: 2 Sequence: DVIIDQLPDLMGLK
Intensity
M/Z
150000
100000
50000
0
500
1000
1500
y1
b1
b2
y2
y3
b3
b4
y5
b6
b7
type
b
y

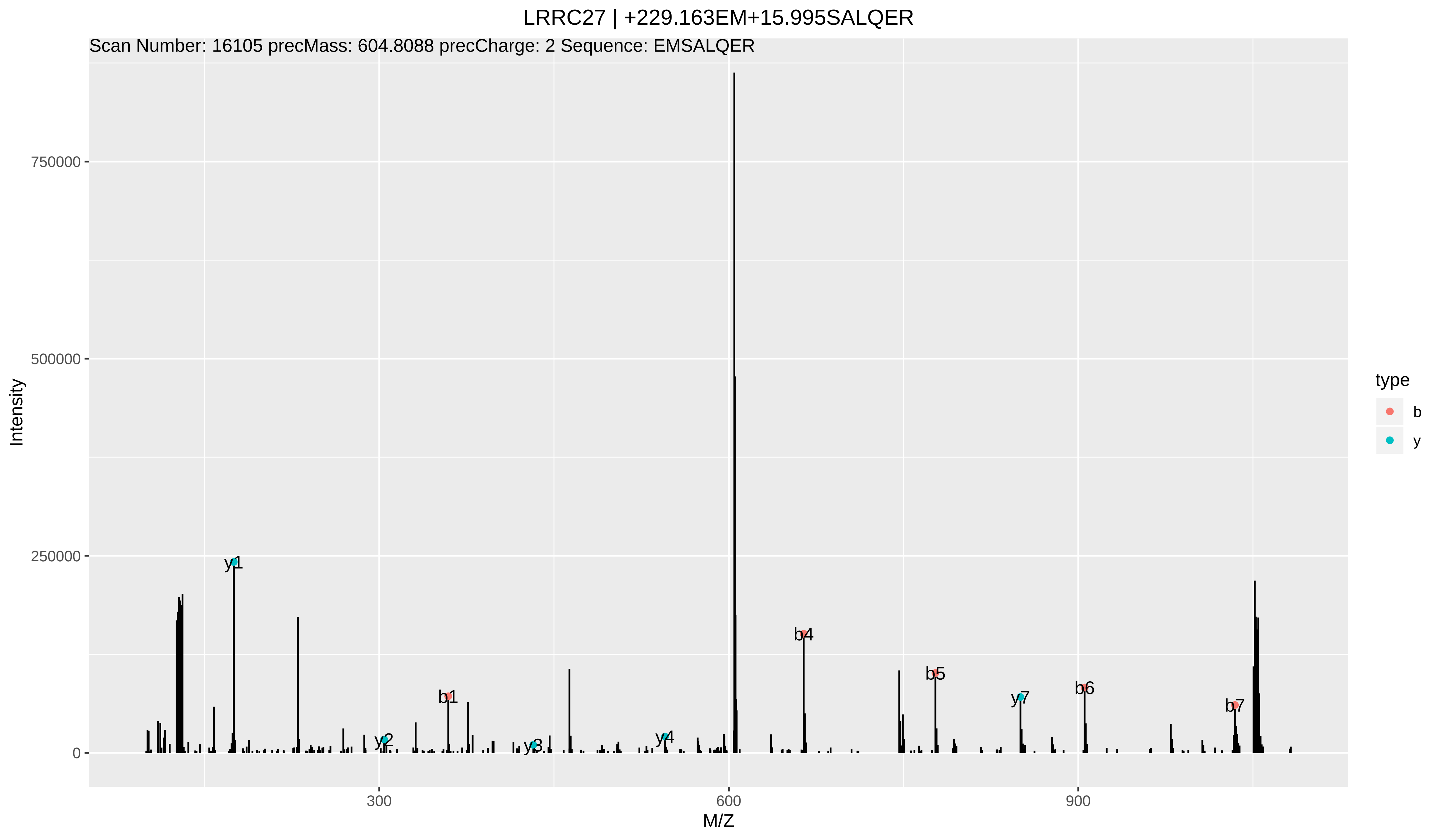

LRRC27 | +229.163EM+15.995SALQER
Scan Number: 16105 precMass: 604.8088 precCharge: 2 Sequence: EMSALQER
Intensity
M/Z
750000
500000
250000
0
300
600
900
y1
y2
b1
y3
y4
b4
b5
y7
b6
b7
type
b
y

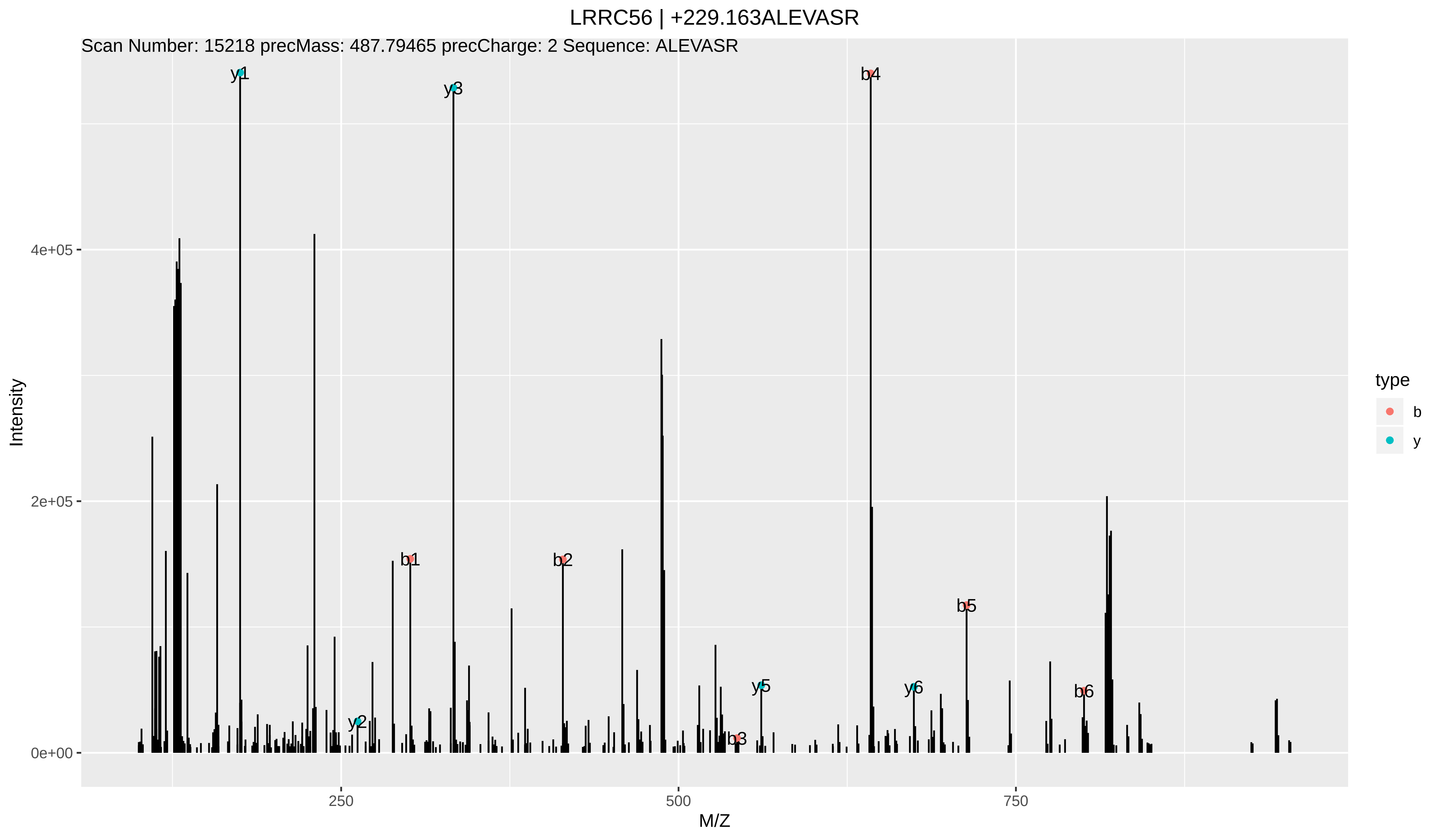

LRRC56 | +229.163ALEVASR
Scan Number: 15218 precMass: 487.79465 precCharge: 2 Sequence: ALEVASR
y1
y3
b4
b1
b2
b5
y2
y5
y6
b6
b3
4e+05
2e+05
0e+00
Intensity
250
500
750
M/Z
type
b
y

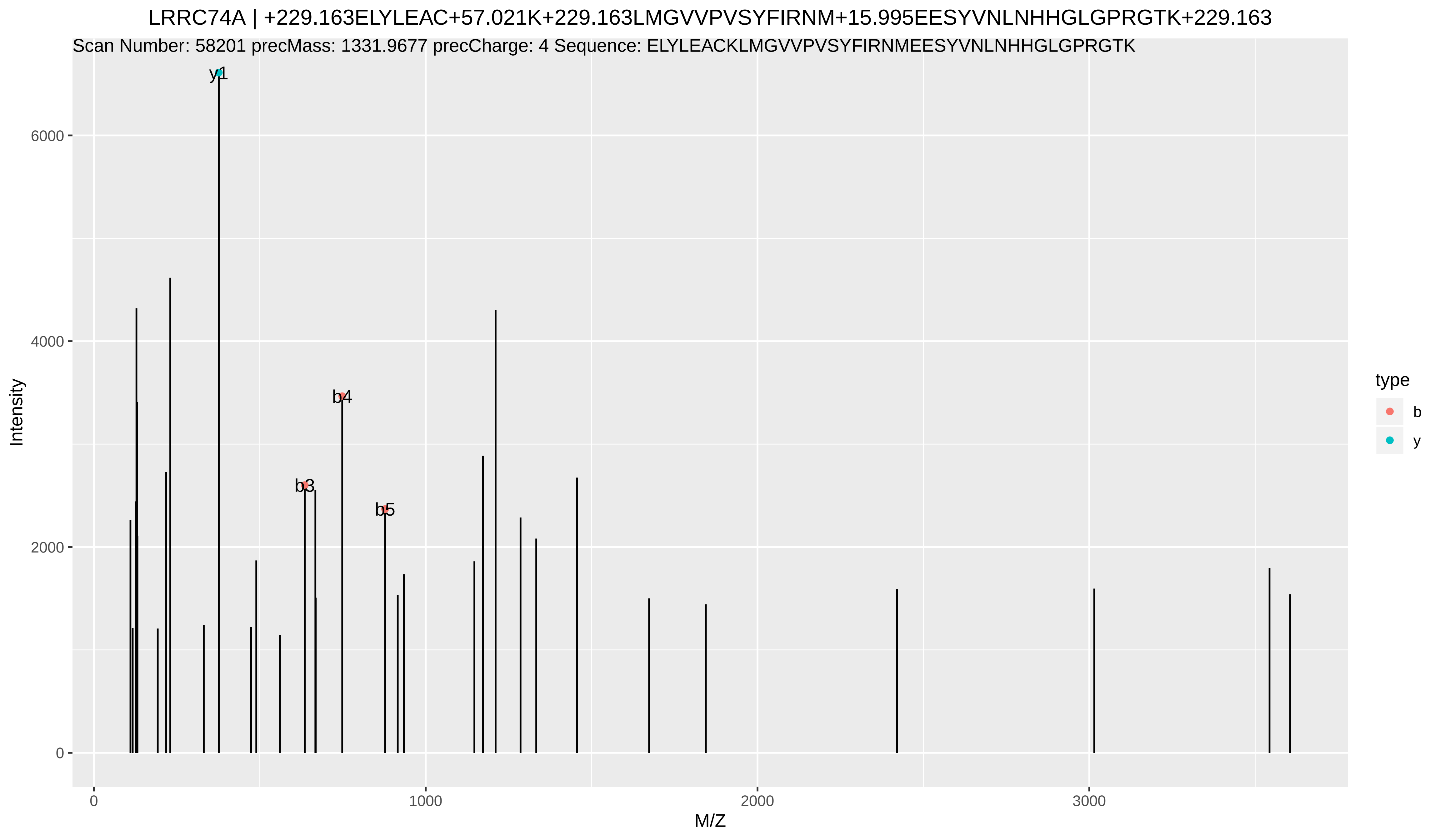

LRRC74A | +229.163ELYLEAC+57.021K+229.163LMGVVPVSYFIRNM+15.995EESYVNLNHHGLGPRGTK+229.163
Scan Number: 58201 precMass: 1331.9677 precCharge: 4 Sequence: ELYLEACKLMGVVPVSYFIRNMEESYVNLNHHGLGPRGTK
y1
b4
b3
b5
Intensity
6000
4000
2000
0
0
1000
2000
3000
M/Z
type
b
y

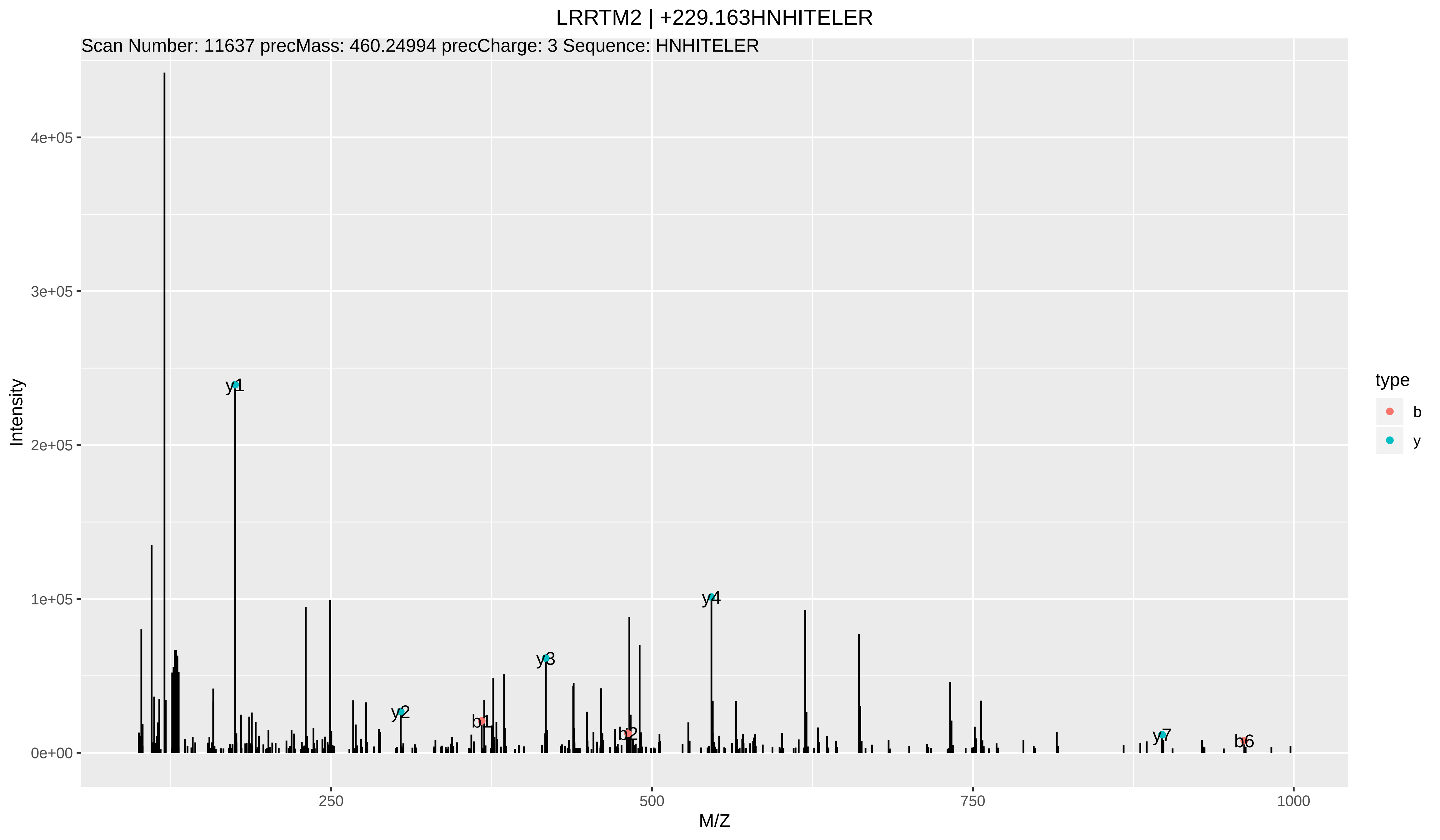

LRRTM2 | +229.163HNHITELER
Scan Number: 11637 precMass: 460.24994 precCharge: 3 Sequence: HNHITELER
Intensity
4e+05
3e+05
2e+05
1e+05
0e+00
y1
y2
b1
y3
b2
y4
y7
b6
250
500
750
1000
M/Z
type
b
y

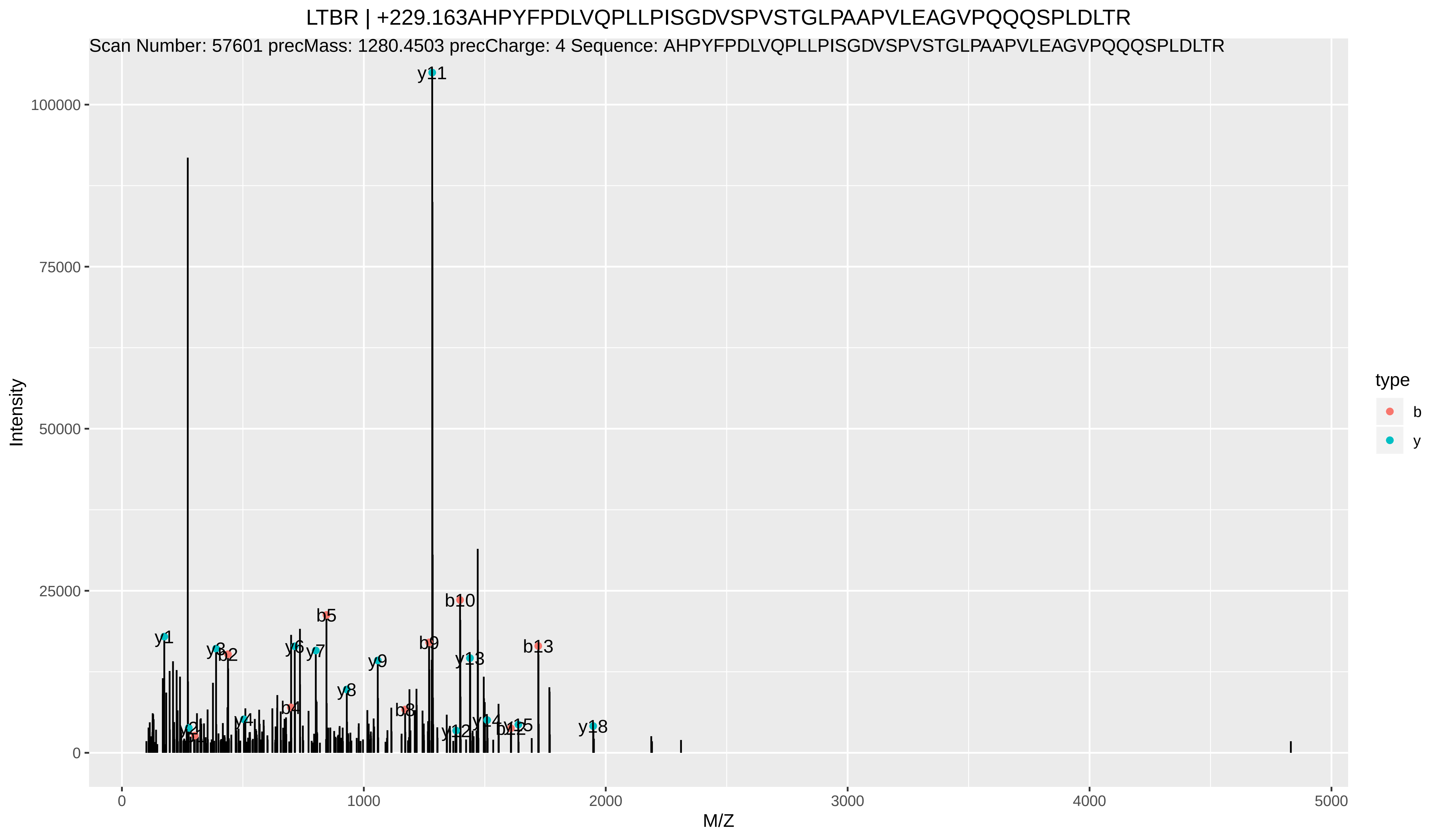
LTBR | +229.163AHPYFPDLVQPLLPISGDVSPVSTGLPAAPVLEAGVPQQQSPLDLTR
Scan Number: 57601 precMass: 1280.4503 precCharge: 4 Sequence: AHPYFPDLVQPLLPISGDVSPVSTGLPAAPVLEAGVPQQQSPLDLTR
Intensity
M/Z
type
b
y
y11
b10
b5
y1
y3
b2
y6
y7
b9
y13
b13
y9
y8
b4
b8
y4
y2
y12
y14
b12
y15
y18
100000
75000
50000
25000
0
1000
2000
3000
4000
5000

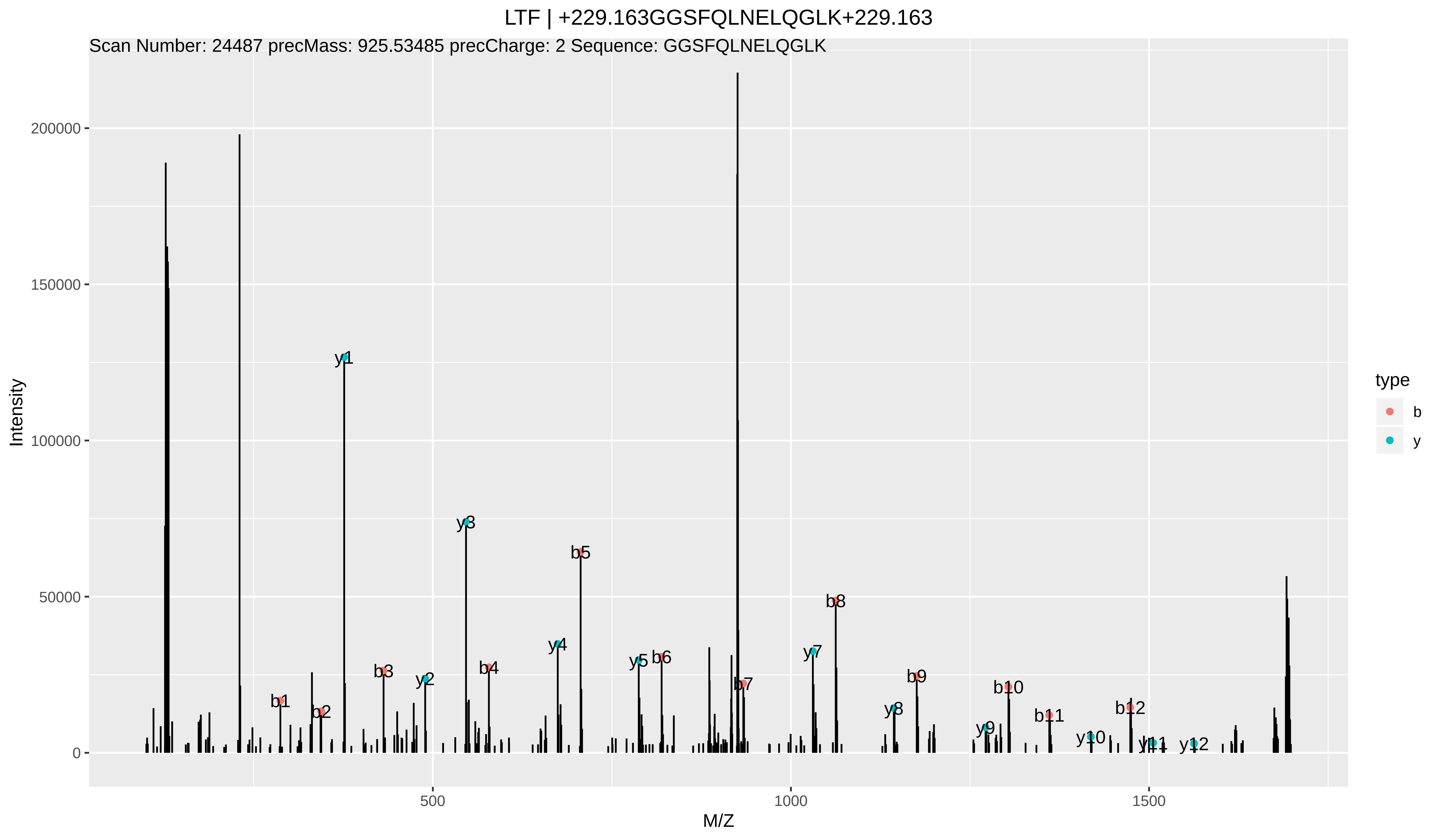
LTF | +229.163GGSFQLNELQGLK+229.163
Scan Number: 24487 precMass: 925.53485 precCharge: 2 Sequence: GGSFQLNELQGLK
Intensity
M/Z
0
50000
100000
150000
200000
500
1000
1500
y1
y2
y3
y4
y5
y7
y8
y9
y10
y11
y12
b1
b2
b3
b4
b5
b6
b7
b8
b9
b10
b11
b12
type
b
y

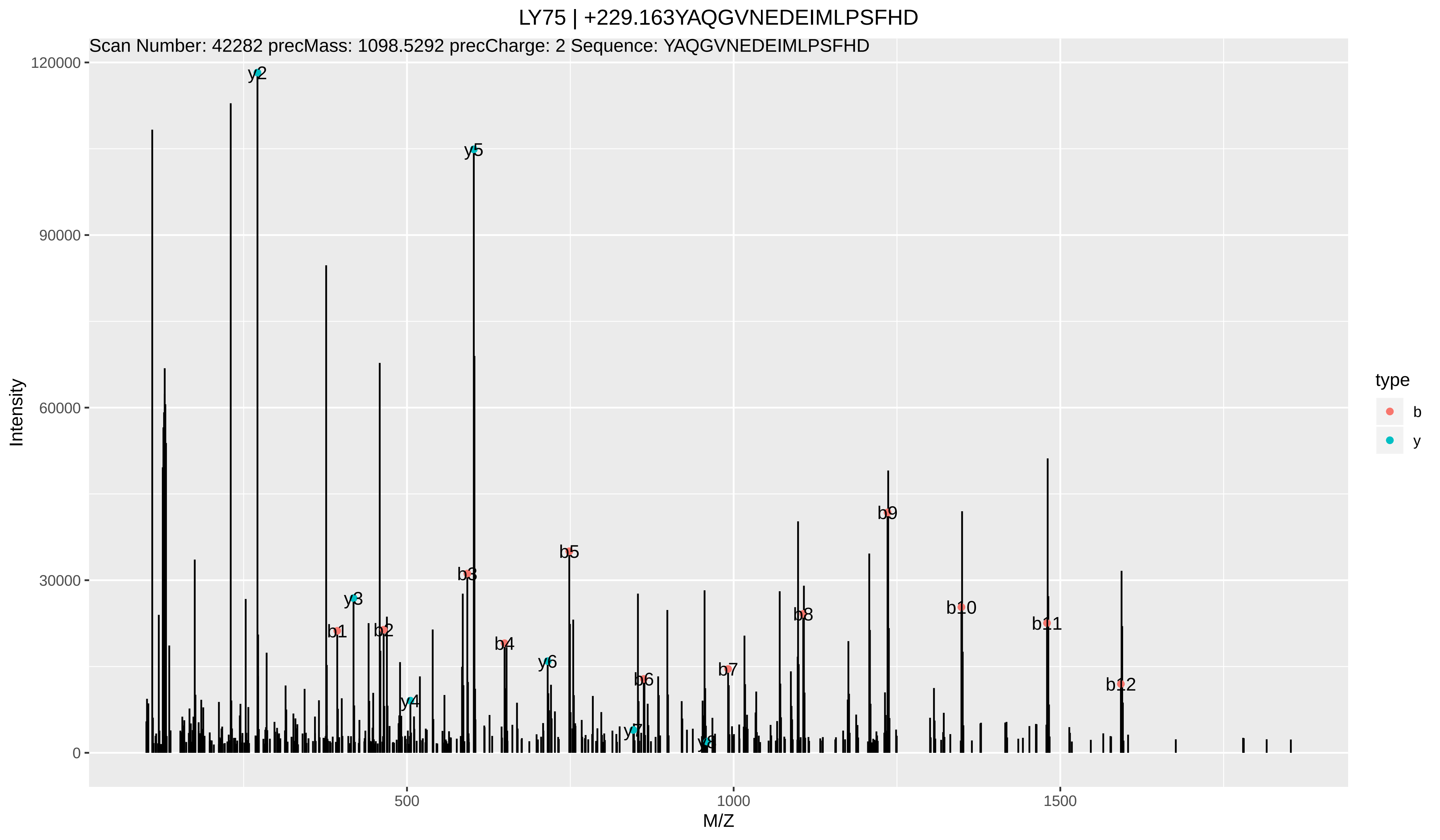
LY75 | +229.163YAQGVNEDEIMLPSFHD
Scan Number: 42282 precMass: 1098.5292 precCharge: 2 Sequence: YAQGVNEDEIMLPSFHD
Intensity
M/Z
type
b
y
y2
y5
y3
y4
y6
y7
b1
b2
b3
b4
b5
b6
b7
b8
b9
b10
b11
b12
120000
90000
60000
30000
0
500
1000
1500

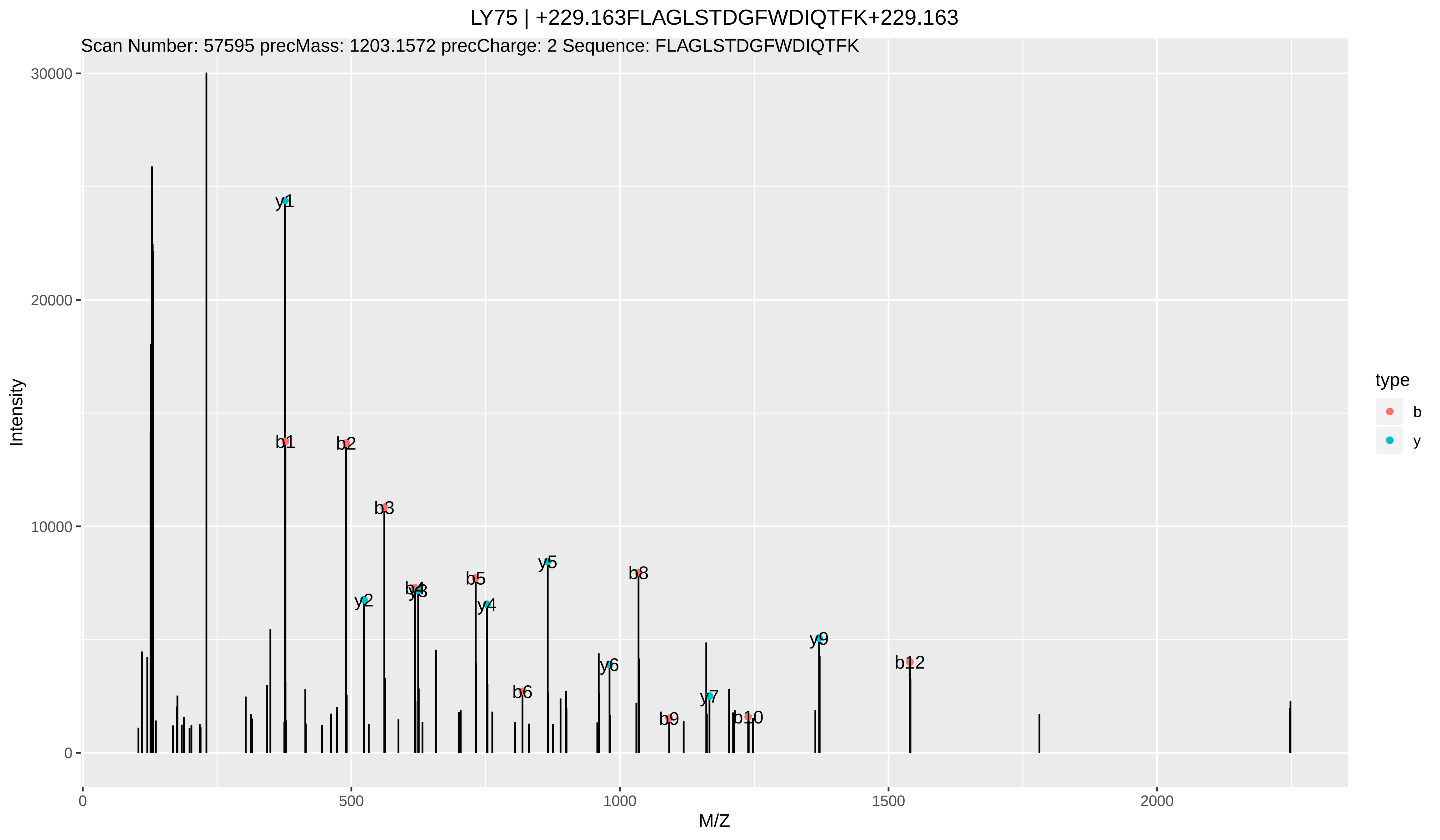

LY75 | +229.163FLAGLSTDGFWDIQTFK+229.163
Scan Number: 57595 precMass: 1203.1572 precCharge: 2 Sequence: FLAGLSTDGFWDIQTFK
Intensity
M/Z
type
b
y
y1
b1
b2
b3
y2
b4
y3
b5
y4
y5
b6
b8
y6
b9
y7
b10
y9
b12
30000
20000
10000
0
0
500
1000
1500
2000

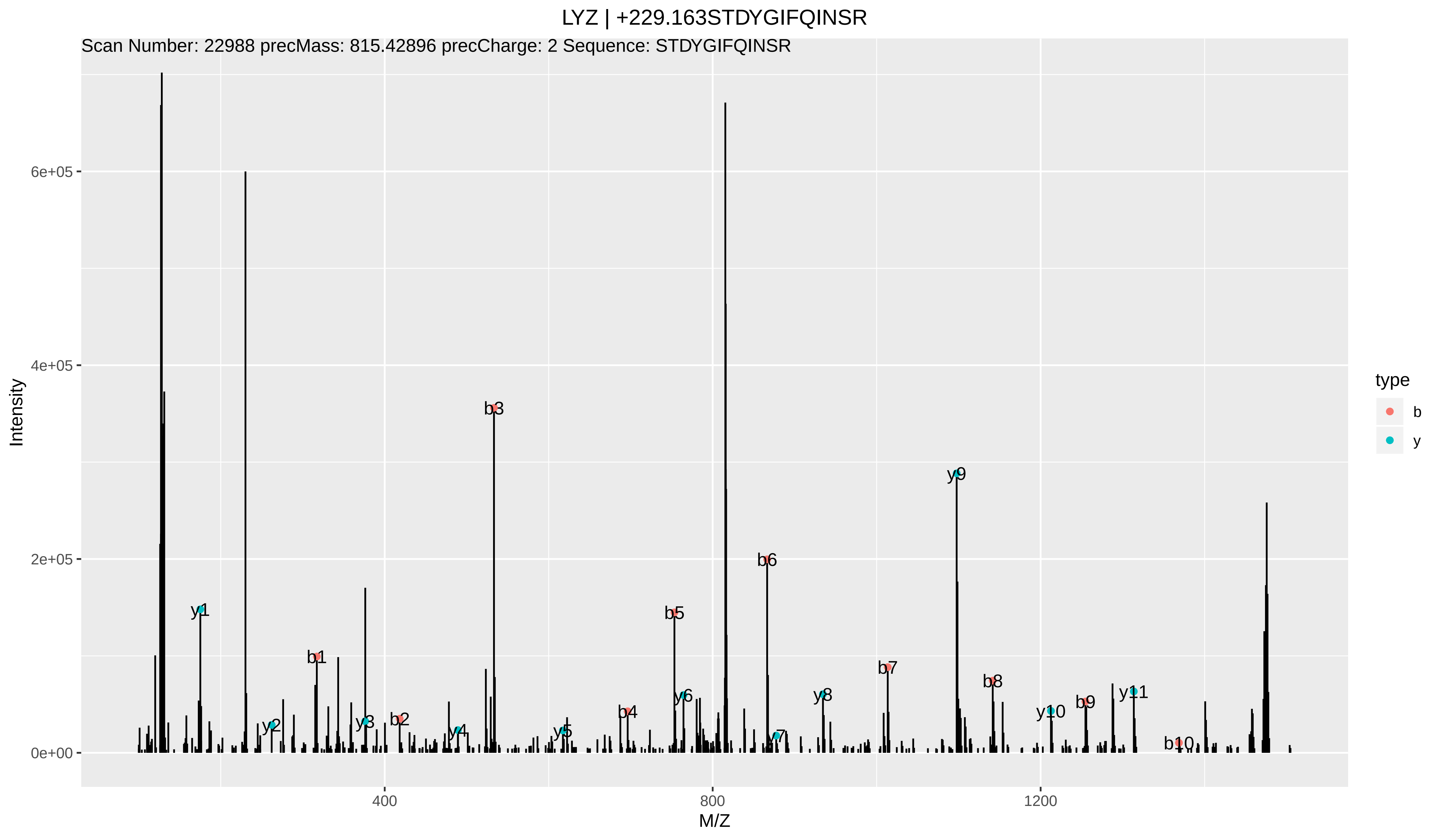

LYZ | +229.163STDYGIFQINSR
Scan Number: 22988 precMass: 815.42896 precCharge: 2 Sequence: STDYGIFQINSR
Intensity
6e+05
4e+05
2e+05
0e+00
M/Z
400
800
1200
y1
y2
b1
y3
b2
y4
b3
y5
b4
b5
y6
b6
y7
y8
b7
y9
b8
y10
b9
y11
b10
type
b
y

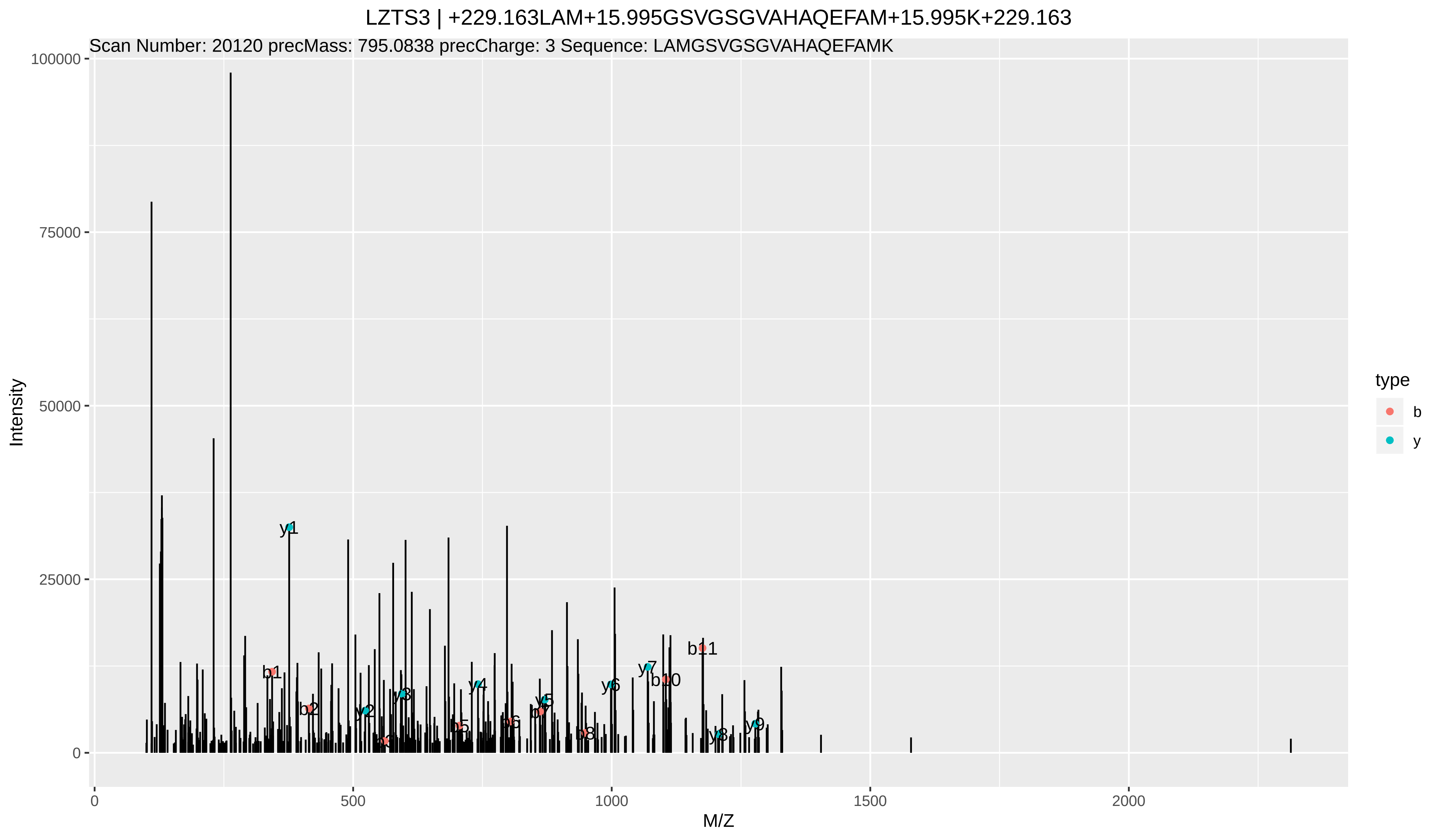

LZTS3 | +229.163LAM+15.995GSVGSGVAHAQEFAM+15.995K+229.163
Scan Number: 20120 precMass: 795.0838 precCharge: 3 Sequence: LAMGSVGSGVAHAQEFAMK
Intensity
M/Z
type
b
y
100000
75000
50000
25000
0
0
500
1000
1500
2000
y1
b1
b2
y2
y3
y4
b5
b6
b7
y5
y6
y7
b8
b10
b11
y8
y9

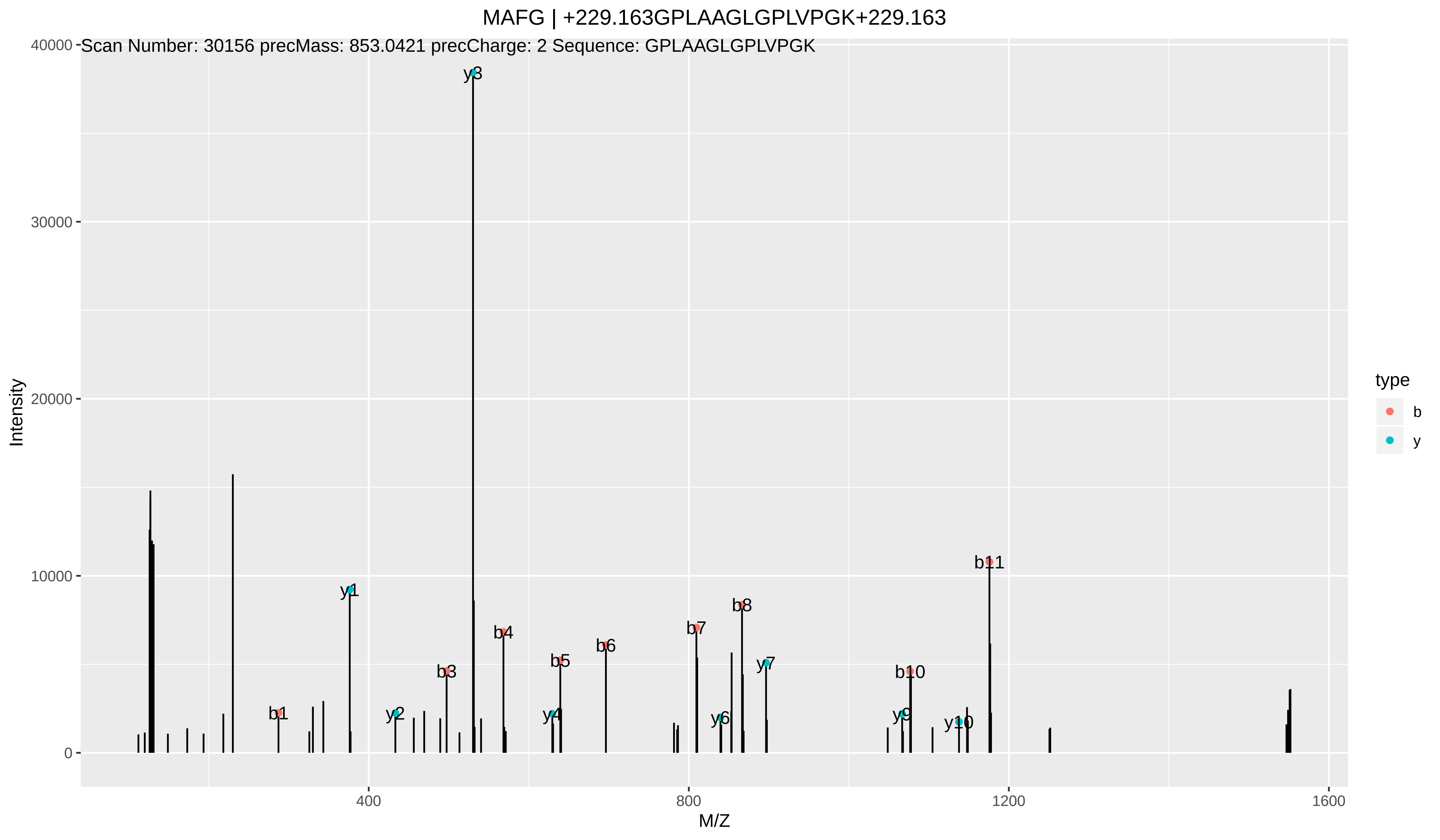

MAFG | +229.163GPLAAGLGPLVPGK+229.163
Scan Number: 30156 precMass: 853.0421 precCharge: 2 Sequence: GPLAAGLGPLVPGK
Intensity
M/Z
type
b
y
y3
y1
b11
b8
b7
b4
b6
b5
b3
y7
b10
b1
y2
y4
y6
y9
y10
0
10000
20000
30000
40000
400
800
1200
1600

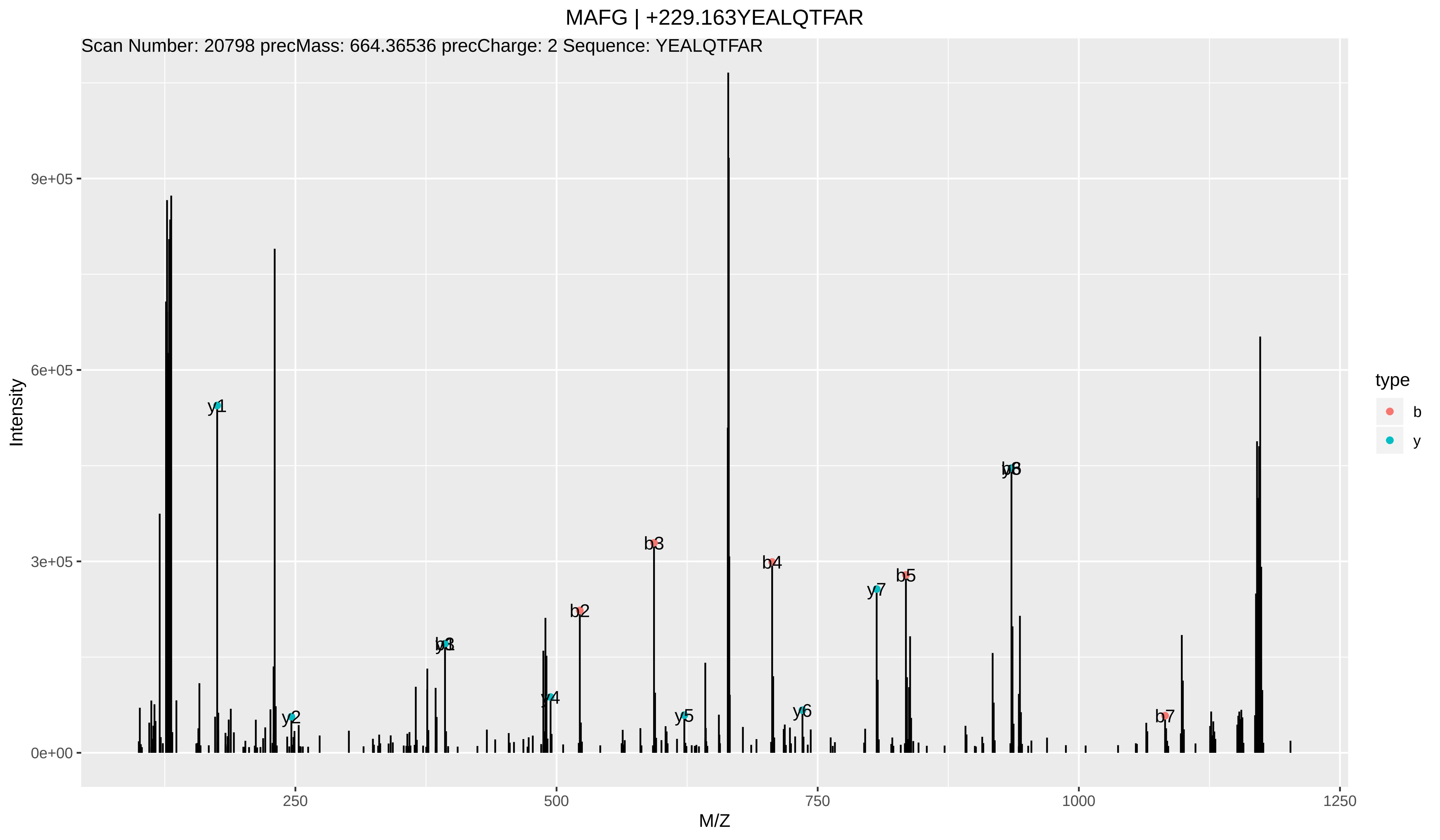

MAFG | +229.163YEALQTFAR
Scan Number: 20798 precMass: 664.36536 precCharge: 2 Sequence: YEALQTFAR
Intensity
M/Z
0e+00
3e+05
6e+05
9e+05
250
500
750
1000
1250
y1
y2
b1
y3
y4
b2
b3
y5
b4
y6
y7
b5
b6
y8
b7
type
b
y

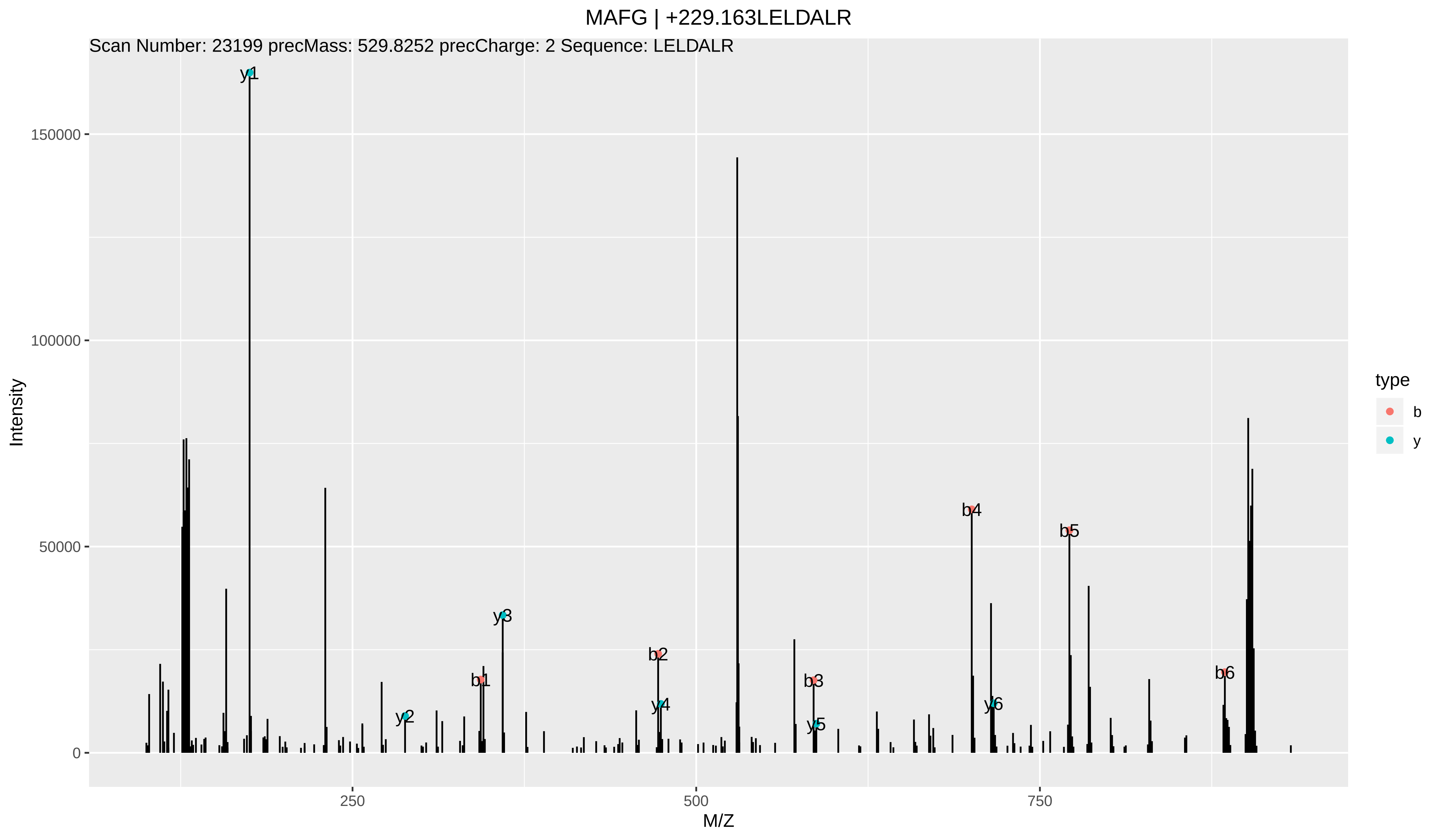
MAFG | +229.163LELDALR
Scan Number: 23199 precMass: 529.8252 precCharge: 2 Sequence: LELDALR
y1
y2
y3
y4
y5
y6
b1
b2
b3
b4
b5
b6
Intensity
150000
100000
50000
0
250
500
750
M/Z
type
b
y

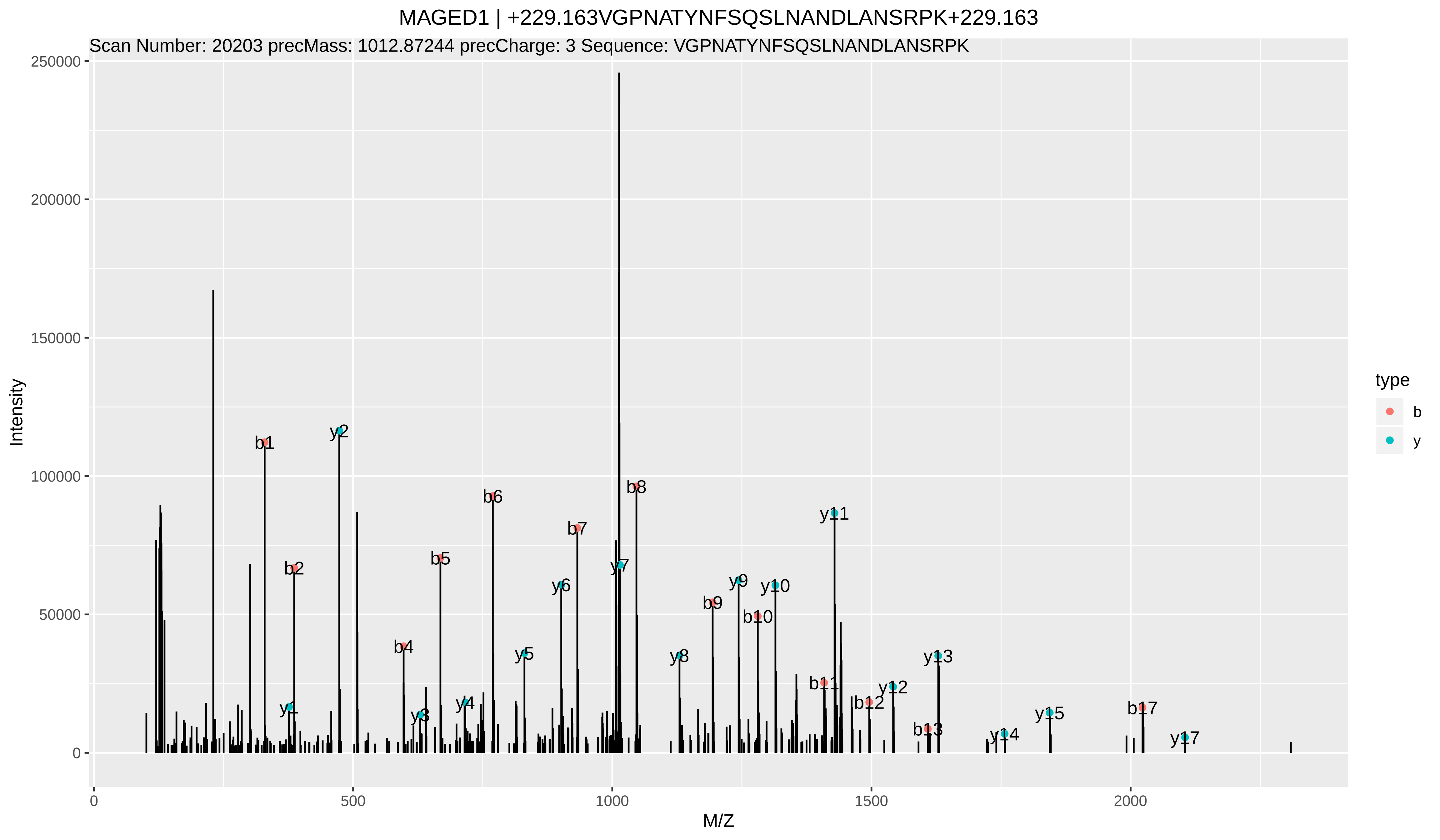

MAGED1 | +229.163VGPNATYNFSQSLNANDLANSRPK+229.163
Scan Number: 20203 precMass: 1012.87244 precCharge: 3 Sequence: VGPNATYNFSQSLNANDLANSRPK
Intensity
M/Z
type
b
y
0
50000
100000
150000
200000
250000
0
500
1000
1500
2000
b1
b2
b4
b5
b6
b7
b8
b9
b10
b11
b12
b13
b17
y1
y2
y3
y4
y5
y6
y7
y8
y9
y10
y11
y12
y13
y14
y15
y17

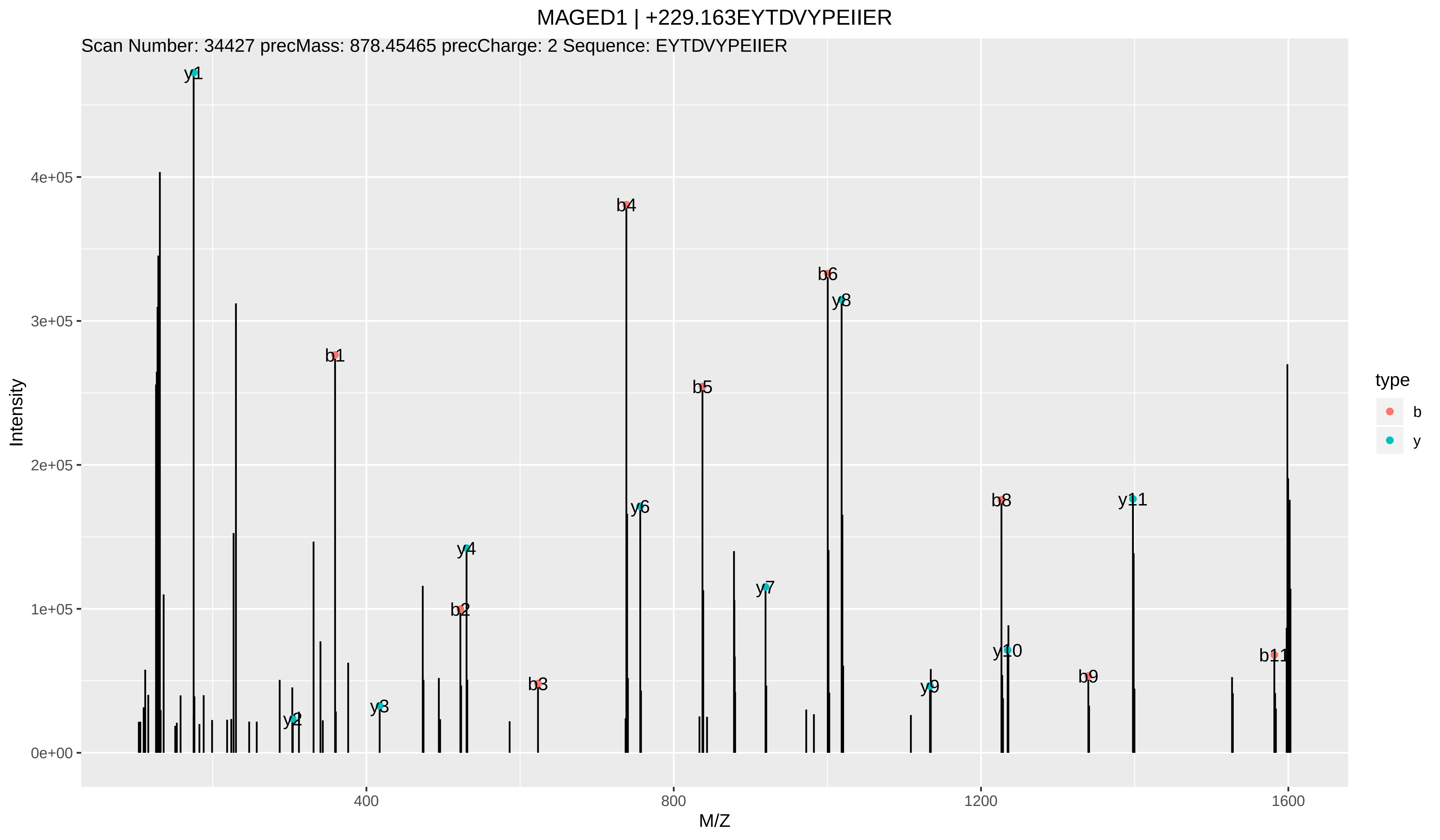
MAGED1 | +229.163EYTDVYPEIIER
Scan Number: 34427 precMass: 878.45465 precCharge: 2 Sequence: EYTDVYPEIIER
Intensity
M/Z
4e+05
3e+05
2e+05
1e+05
0e+00
400
800
1200
1600
y1
b4
b6
y8
b1
b5
y6
b8
y11
y4
y7
b2
y10
b11
b3
b9
y9
y3
y2
type
b
y

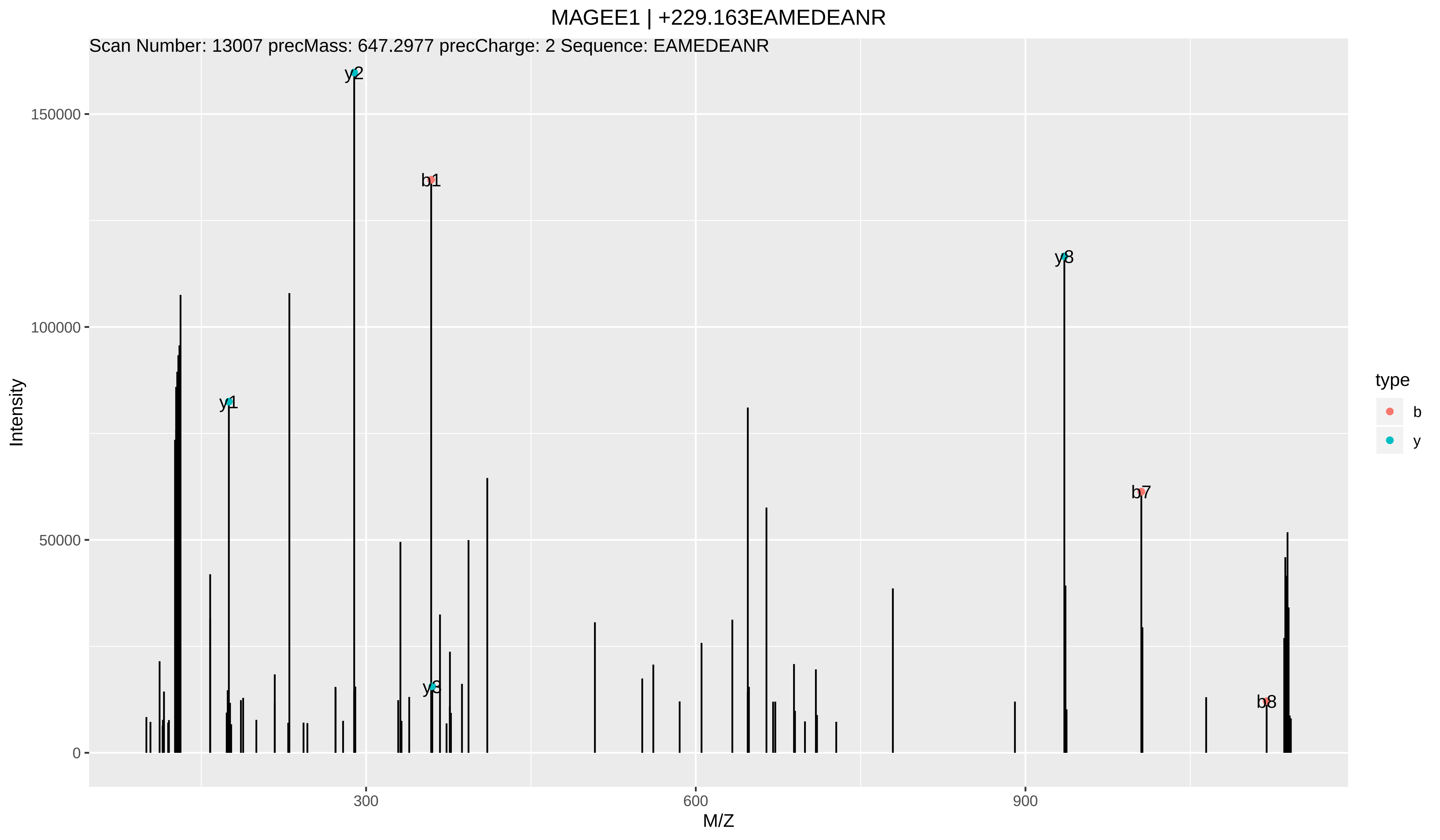
MAGEE1 | +229.163EAMEDEANR
Scan Number: 13007 precMass: 647.2977 precCharge: 2 Sequence: EAMEDEANR
y2
b1
y8
y1
b7
y3
b8
Intensity
150000
100000
50000
0
300
600
900
M/Z
type
b
y

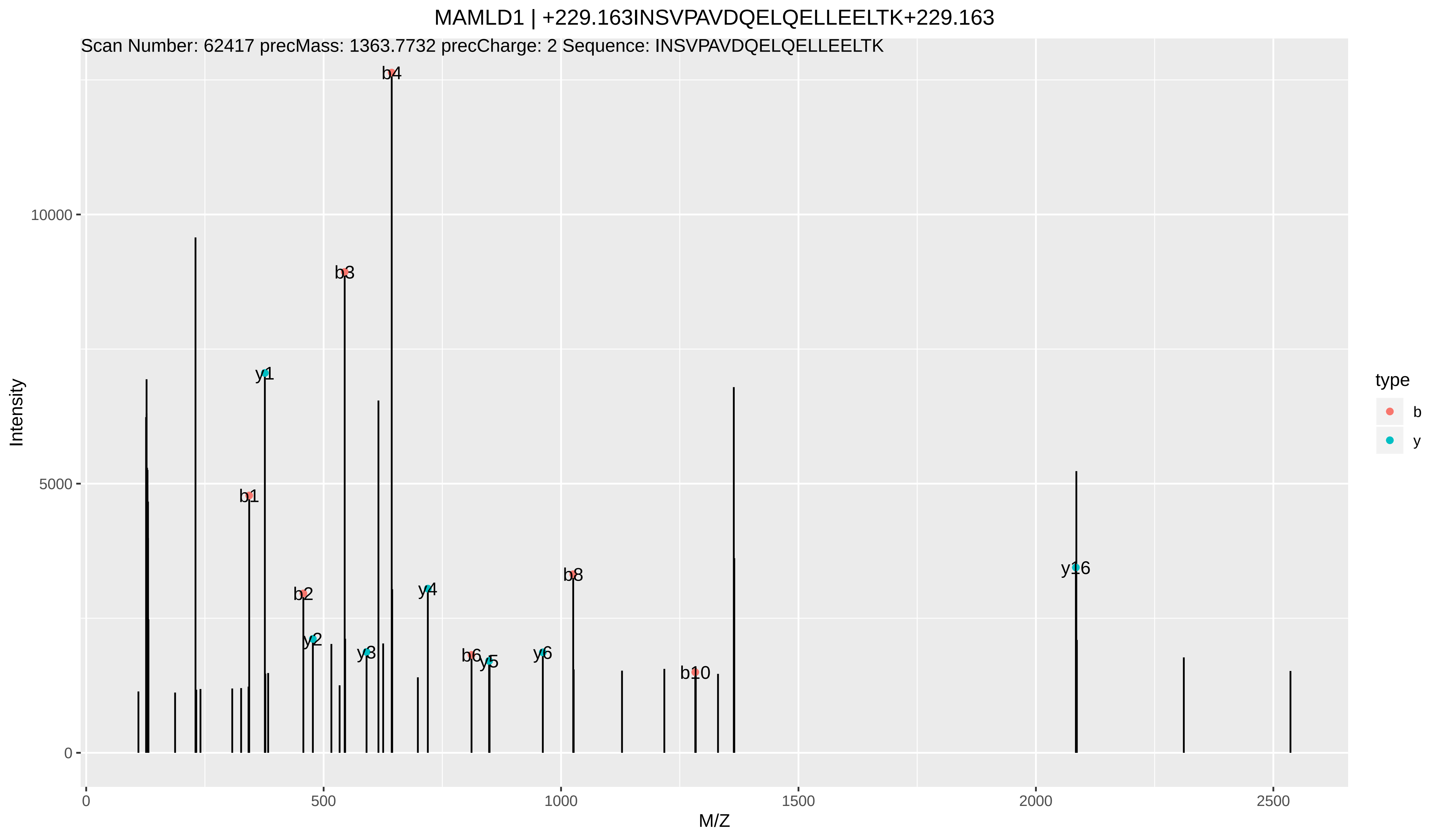
MAMLD1 | +229.163INSVPAVDQELQELLEELTK+229.163
Scan Number: 62417 precMass: 1363.7732 precCharge: 2 Sequence: INSVPAVDQELQELLEELTK
b4
b3
y1
b1
b2
y2
y3
b6
y5
y6
y4
b8
b10
y16
Intensity
10000
5000
0
0
500
1000
1500
2000
2500
M/Z
type
b
y

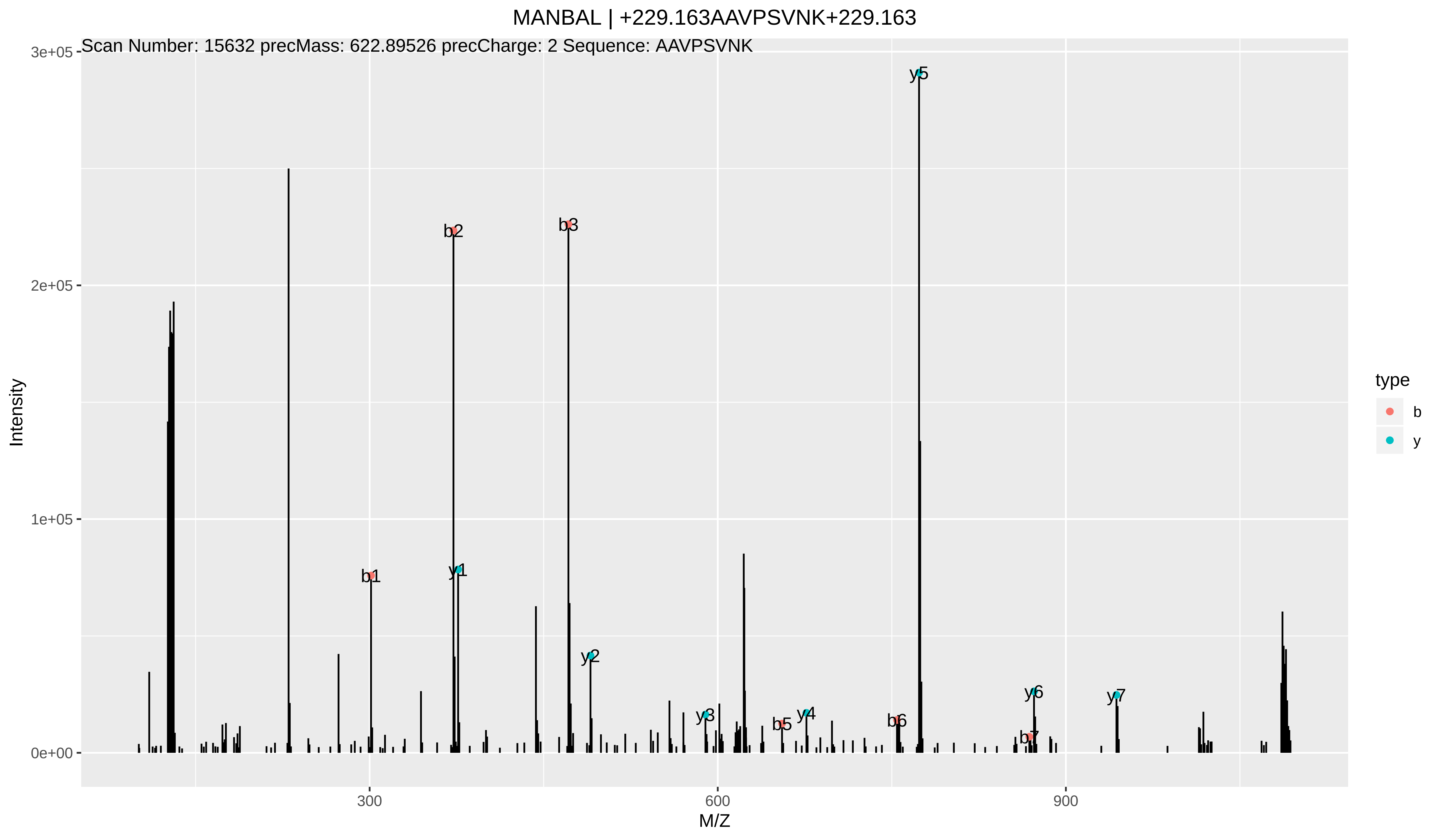

MANBAL | +229.163AAVPSVNK+229.163
Scan Number: 15632 precMass: 622.89526 precCharge: 2 Sequence: AAVPSVNK
3e+05
2e+05
1e+05
0e+00
Intensity
300
600
900
M/Z
y5
b2
b3
b1
y1
y2
y3
b5
y4
b6
b7
y6
y7
type
b
y

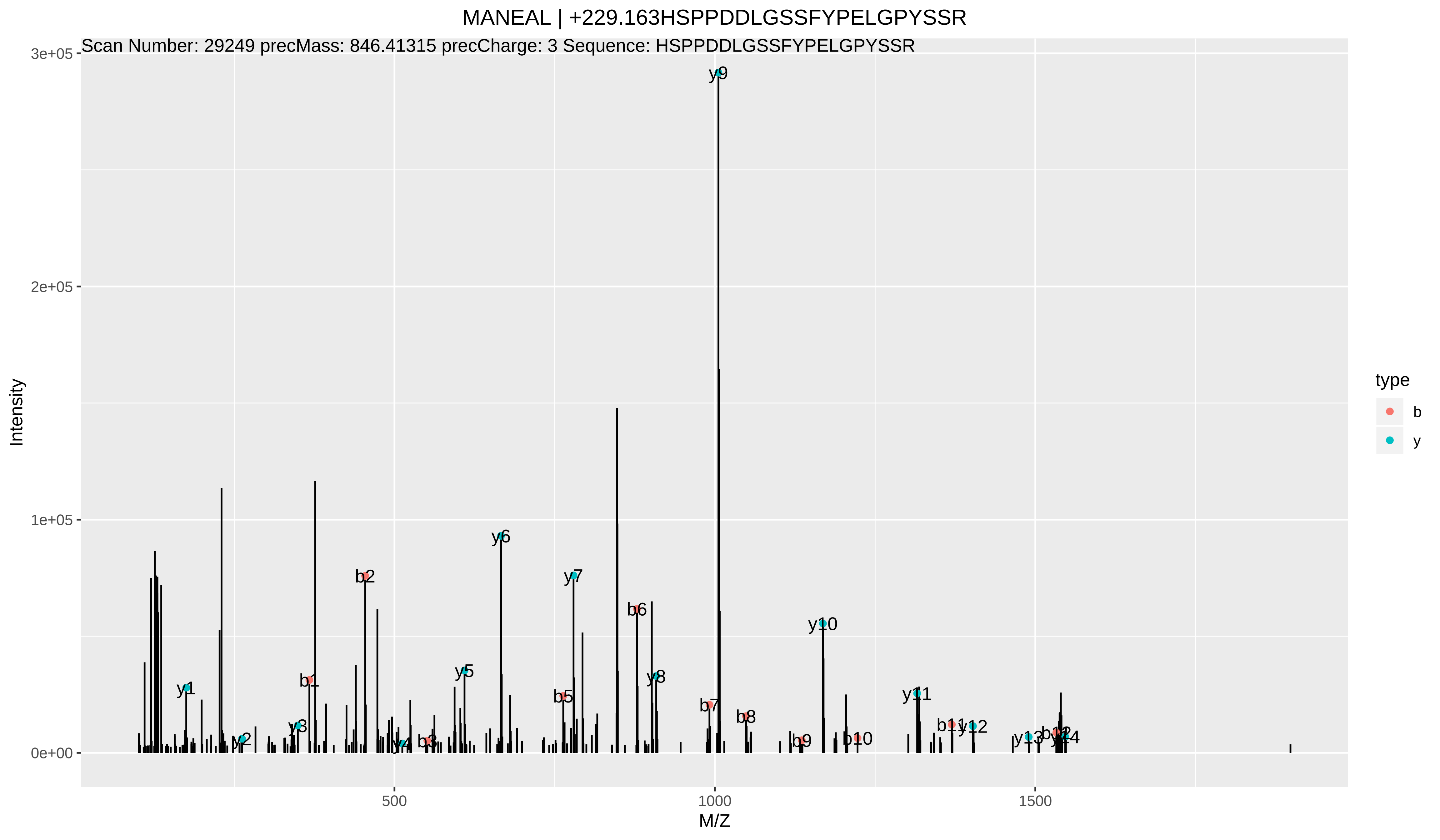

MANEAL | +229.163HSPPDDLGSSFYPELGPYSSR
Scan Number: 29249 precMass: 846.41315 precCharge: 3 Sequence: HSPPDDLGSSFYPELGPYSSR
Intensity
M/Z
3e+05
2e+05
1e+05
0e+00
500
1000
1500
y9
y6
b2
y7
b6
y10
y5
y8
y1
b1
b5
b7
y11
b8
y3
y2
y4
b3
b9
b10
b11
y12
y13
b12
y14
type
b
y

MAOB | +229.163YVDLGGSYVGPTQNR

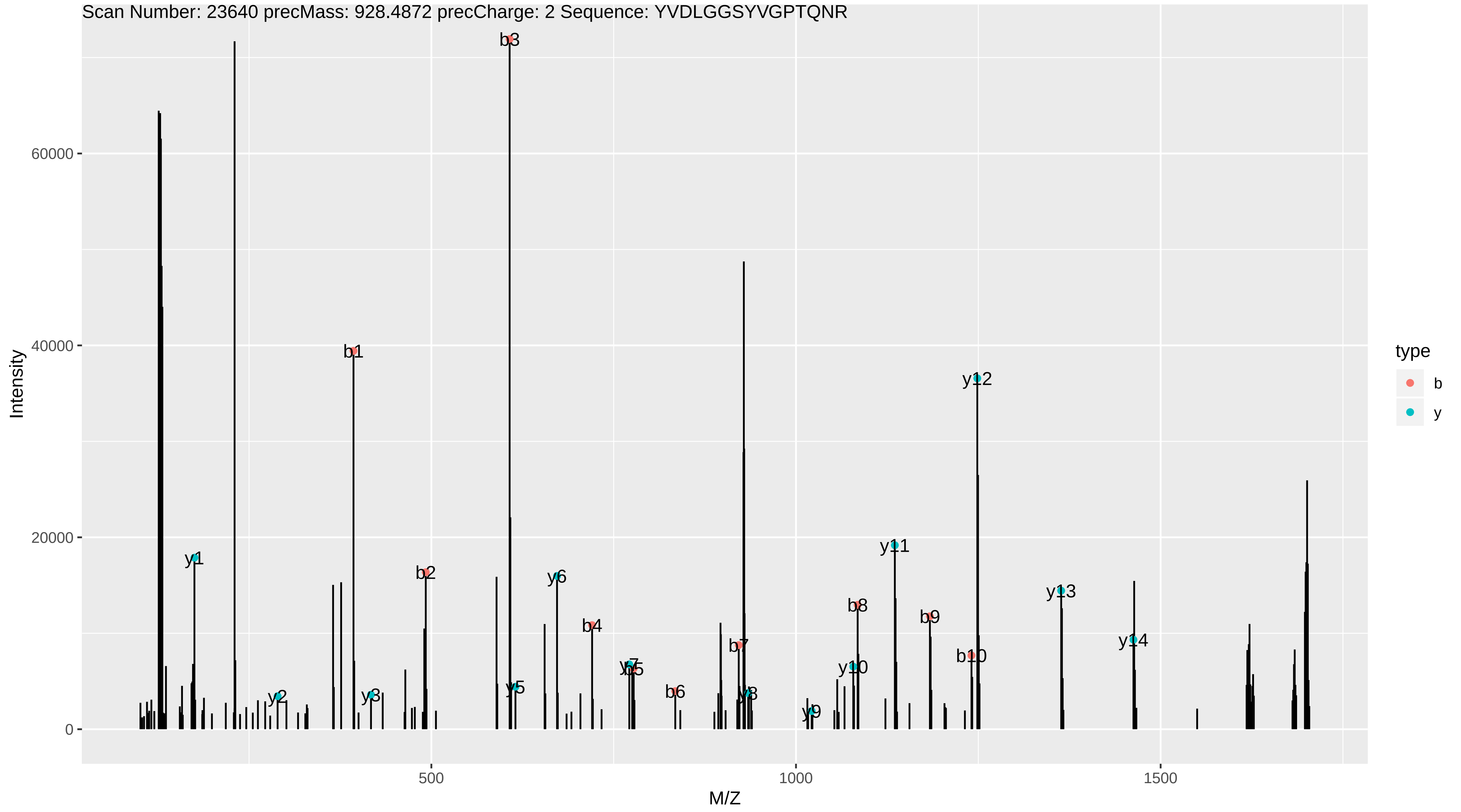

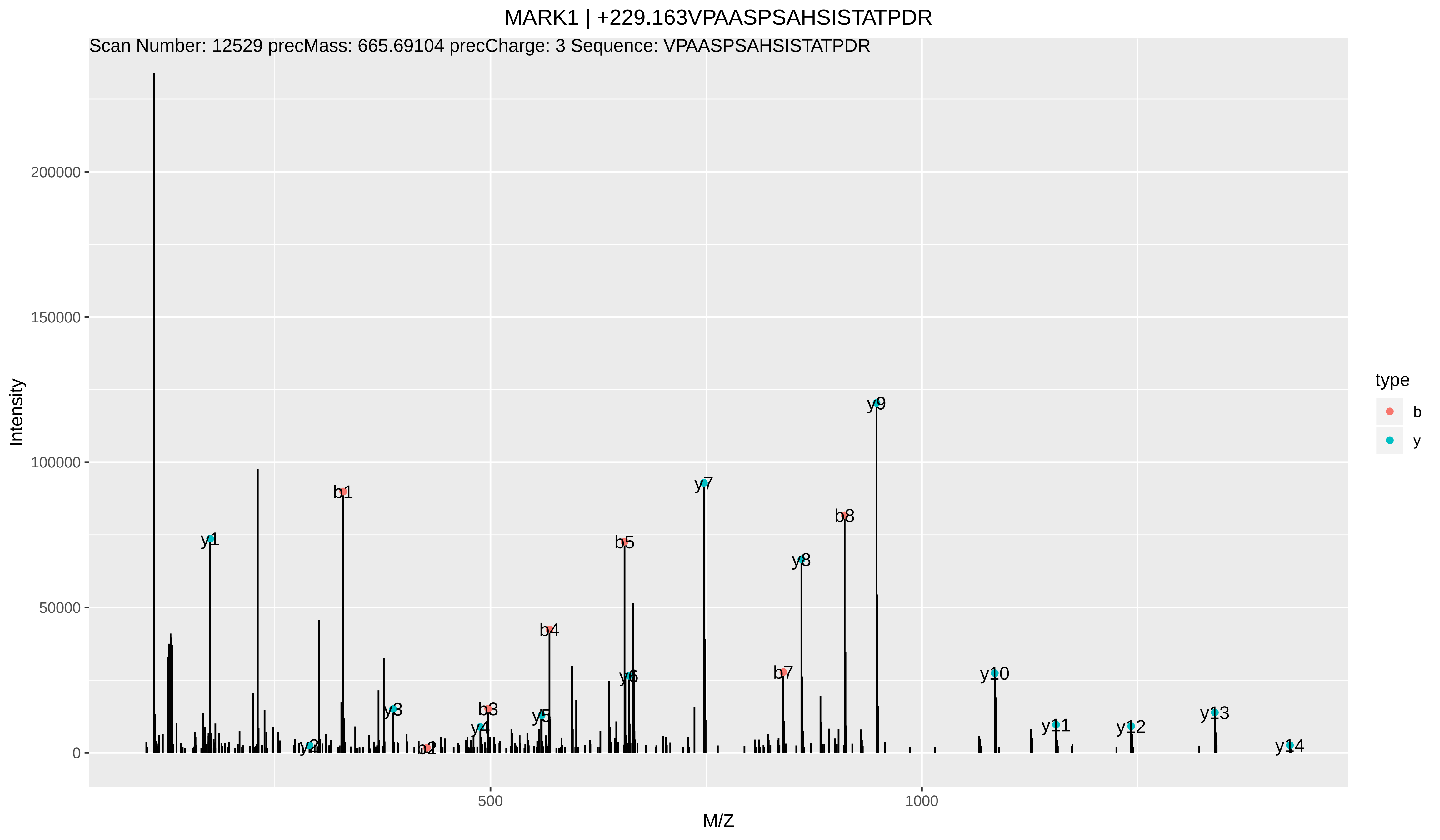

MARK1 | +229.163VPAASPSAHSISTATPDR
Scan Number: 12529 precMass: 665.69104 precCharge: 3 Sequence: VPAASPSAHSISTATPDR
Intensity
200000
150000
100000
50000
0
M/Z
500
1000
type
b
y
y1
y2
y3
y4
y5
y6
y7
y8
y9
y10
y11
y12
y13
y14
b1
b2
b3
b4
b5
b7
b8

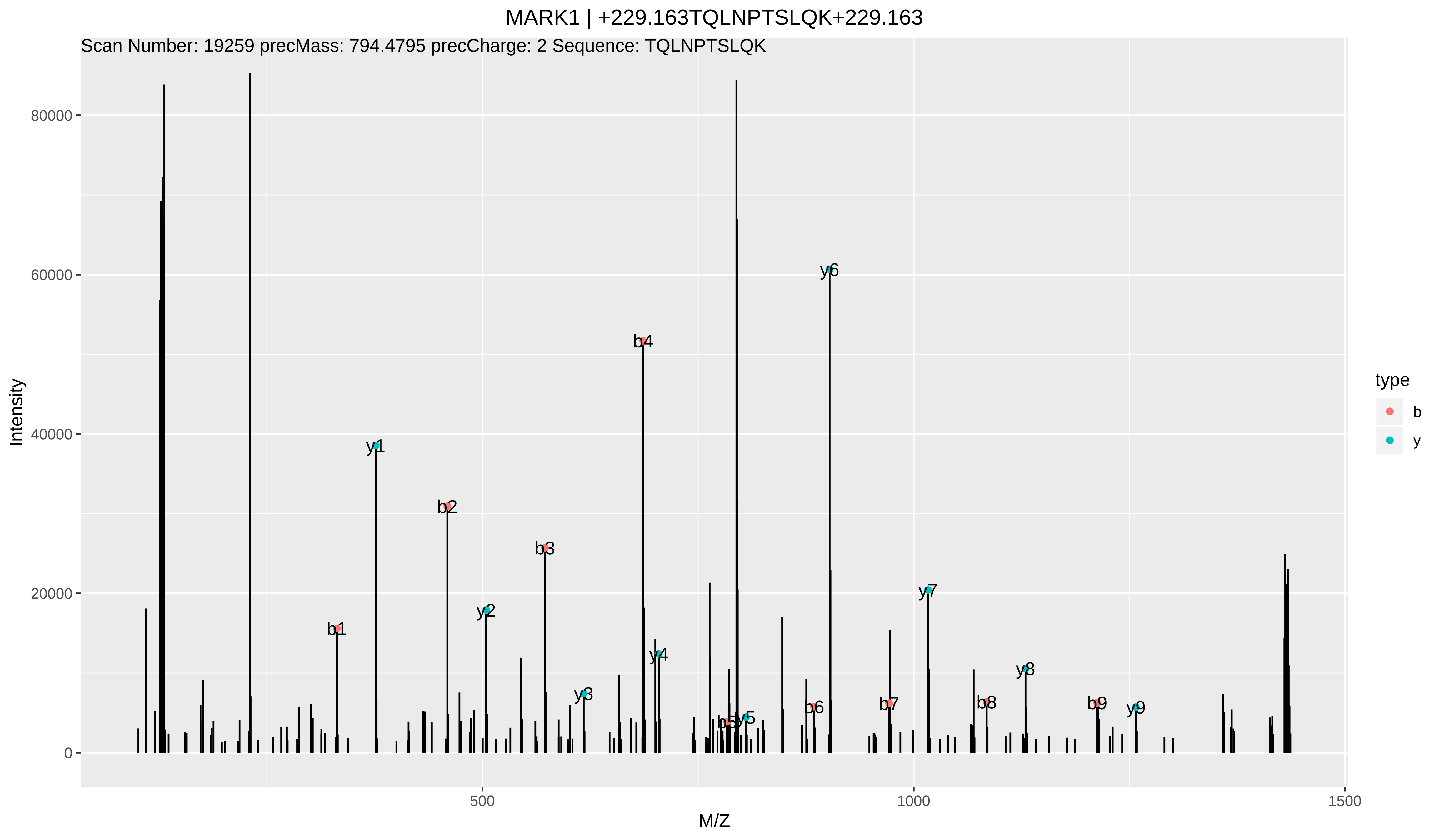
MARK1 | +229.163TQLNPTSLQK+229.163
Scan Number: 19259 precMass: 794.4795 precCharge: 2 Sequence: TQLNPTSLQK
Intensity
80000
60000
40000
20000
0
M/Z
500
1000
1500
b1
y1
b2
y2
b3
y3
b4
y4
b5
y5
b6
y6
b7
y7
b8
y8
b9
y9
type
b
y

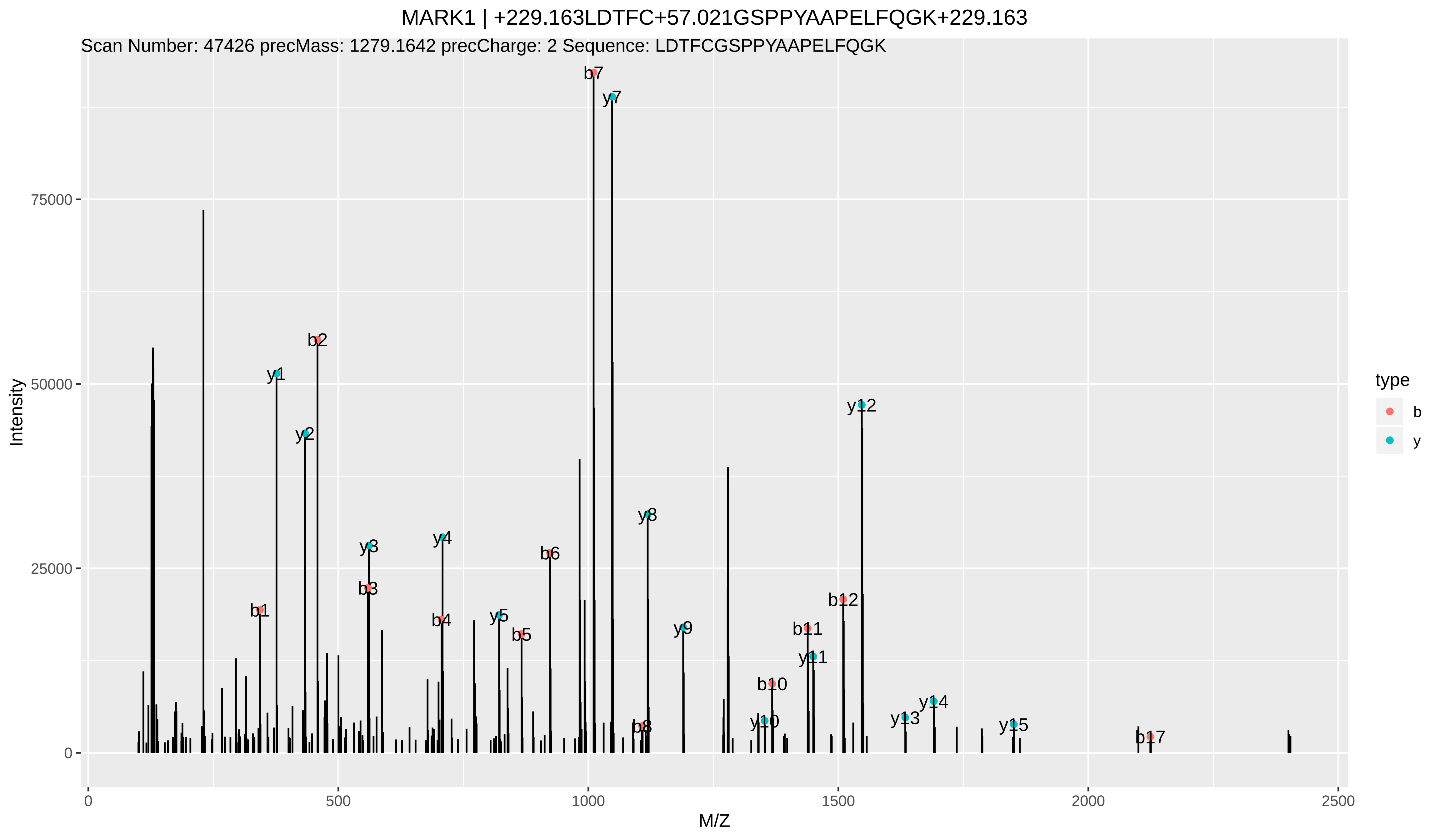

MARK1 | +229.163LDTFC+57.021GSPPYAAPELFQGK+229.163
Scan Number: 47426 precMass: 1279.1642 precCharge: 2 Sequence: LDTFCGSPPYAAPELFQGK
Intensity
M/Z
0
25000
50000
75000
0
500
1000
1500
2000
2500
b1
b2
b3
b4
b5
b6
b7
b8
b10
b11
b12
b17
y1
y2
y3
y4
y5
y7
y8
y9
y10
y11
y12
y13
y14
y15
type
b
y

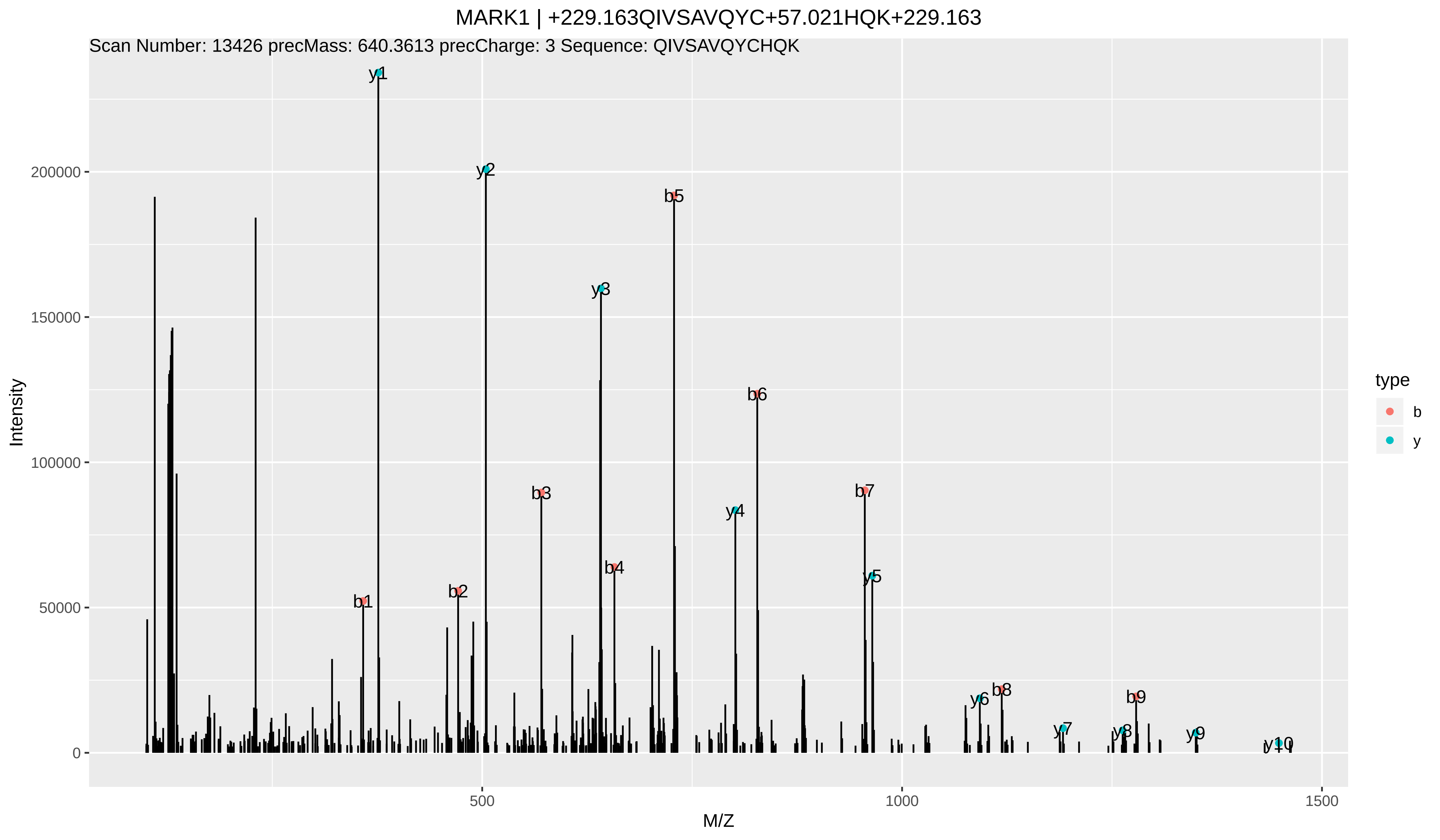

MARK1 | +229.163QIVSAVQYC+57.021HQK+229.163
Scan Number: 13426 precMass: 640.3613 precCharge: 3 Sequence: QIVSAVQYCHQK
Intensity
M/Z
type
b
y
y1
y2
y3
y4
y5
y6
y7
y8
y9
y10
b1
b2
b3
b4
b5
b6
b7
b8
b9
0
50000
100000
150000
200000
500
1000
1500

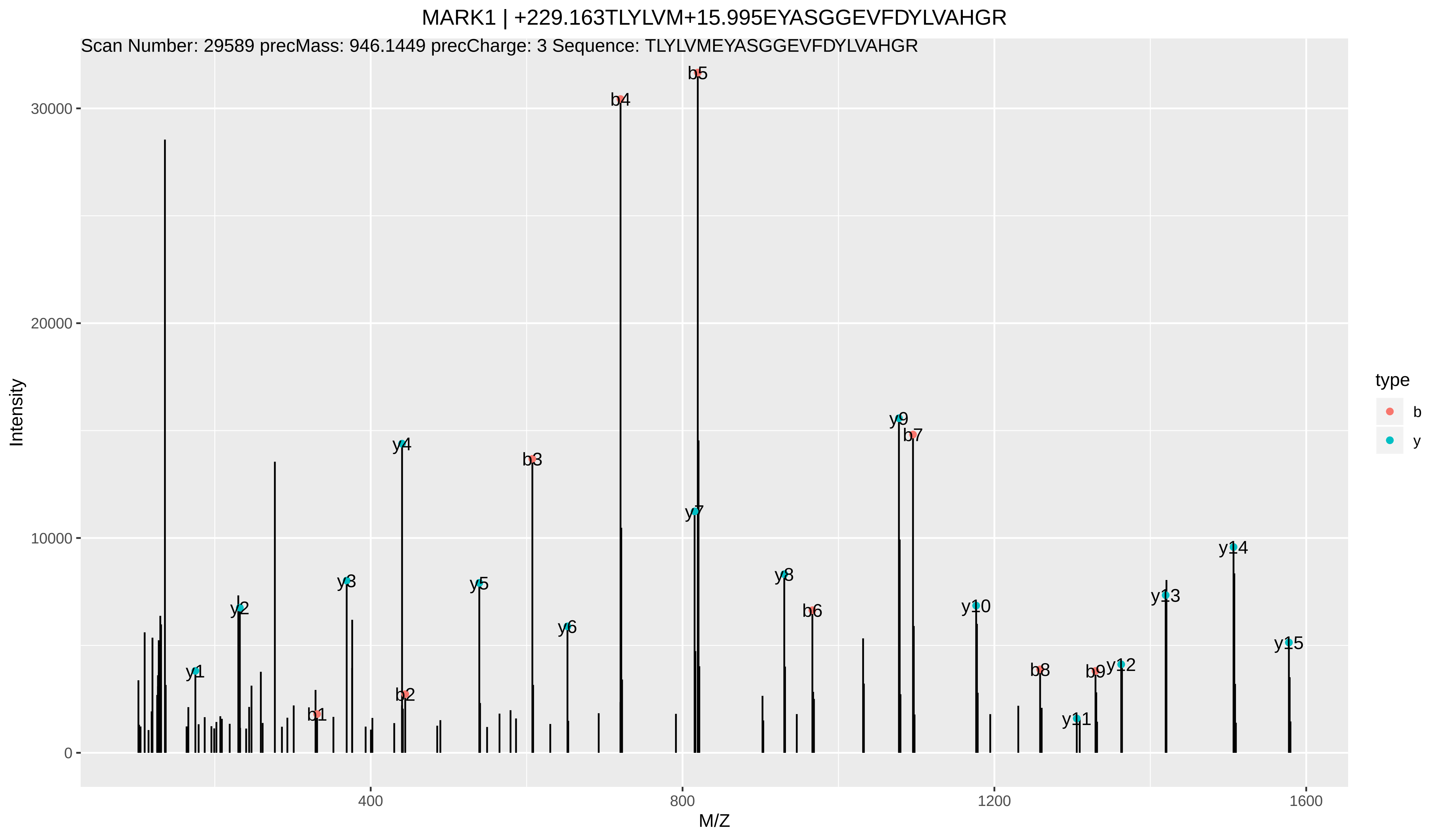

MARK1 | +229.163TLYLVM+15.995EYASGGEVFDYLVAHGR
Scan Number: 29589 precMass: 946.1449 precCharge: 3 Sequence: TLYLVMEYASGGEVFDYLVAHGR
Intensity
M/Z
type
b
y
b1
b2
b3
b4
b5
b6
b7
b8
b9
y1
y2
y3
y4
y5
y6
y7
y8
y9
y10
y11
y12
y13
y14
y15
0
10000
20000
30000
400
800
1200
1600

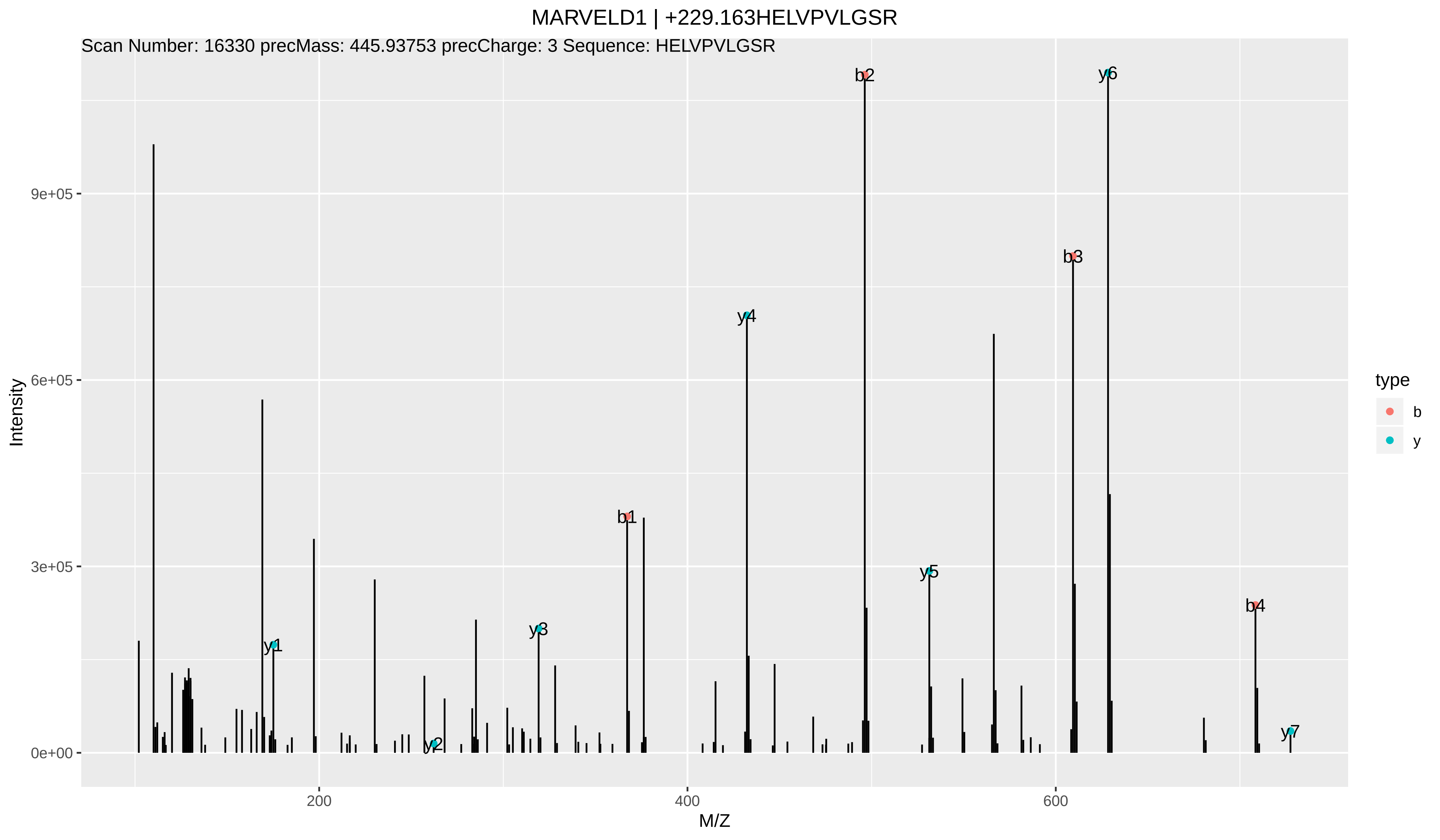

MARVELD1 | +229.163HELVPVLGSR
Scan Number: 16330 precMass: 445.93753 precCharge: 3 Sequence: HELVPVLGSR
Intensity
9e+05
6e+05
3e+05
0e+00
M/Z
200
400
600
b2
y6
b3
y4
b1
y5
b4
y1
y3
y2
y7
type
b
y

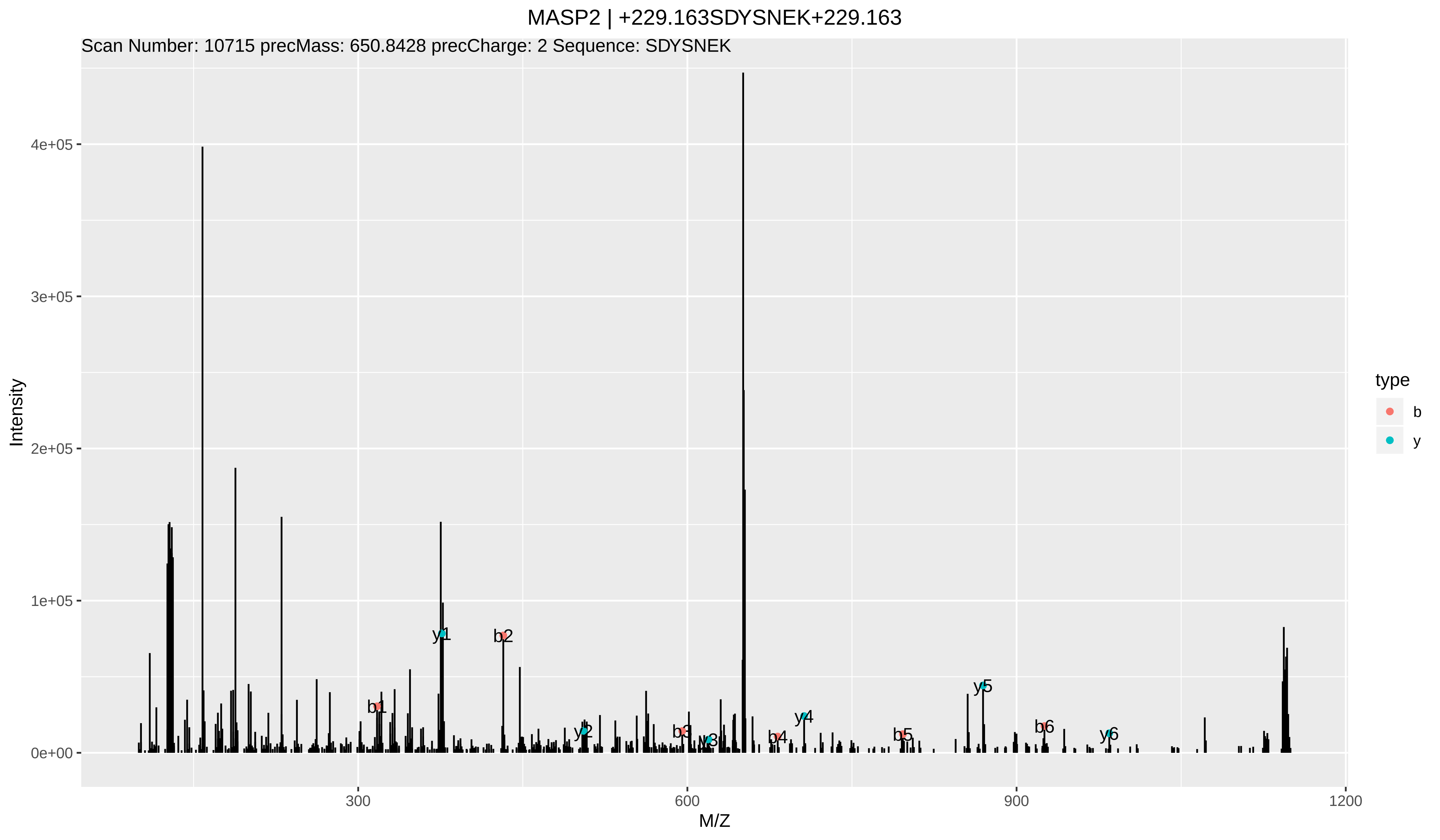

MASP2 | +229.163SDYSNEK+229.163
Scan Number: 10715 precMass: 650.8428 precCharge: 2 Sequence: SDYSNEK
Intensity
4e+05
3e+05
2e+05
1e+05
0e+00
M/Z
300
600
900
1200
y1
b2
b1
y2
b3
y3
b4
y4
b5
y5
b6
y6
type
b
y

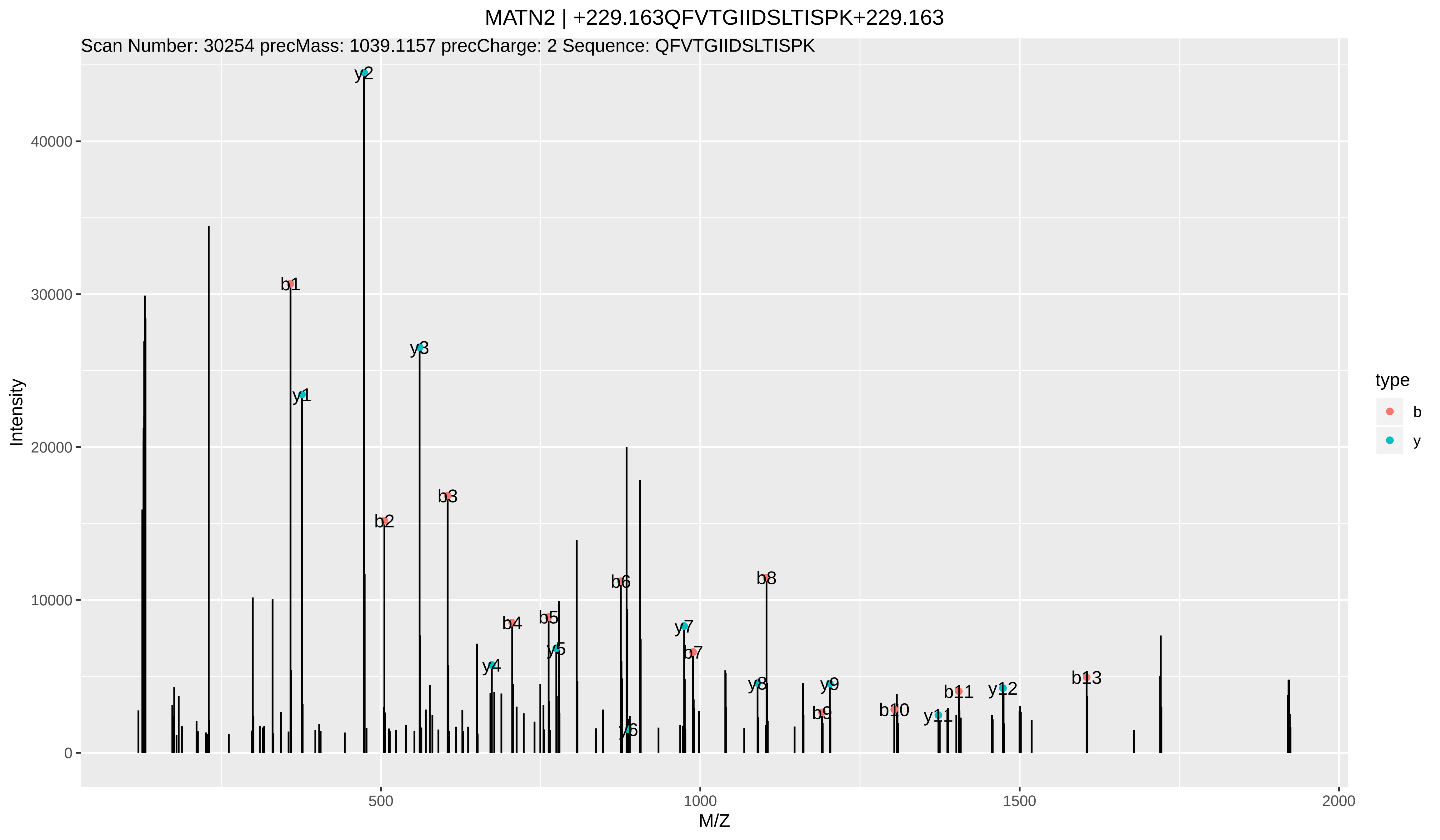
MATN2 | +229.163QFVTGIIDSLTISPK+229.163
Scan Number: 30254 precMass: 1039.1157 precCharge: 2 Sequence: QFVTGIIDSLTISPK
Intensity
M/Z
type
b
y
y2
b1
y1
y3
b3
b2
b6
b8
b4
b5
y7
y5
b7
y4
y8
y9
b13
b11
y12
b10
b9
y11
y6
0
10000
20000
30000
40000
500
1000
1500
2000

MBLAC1 | +229.163YLPHGLGEGQPLR

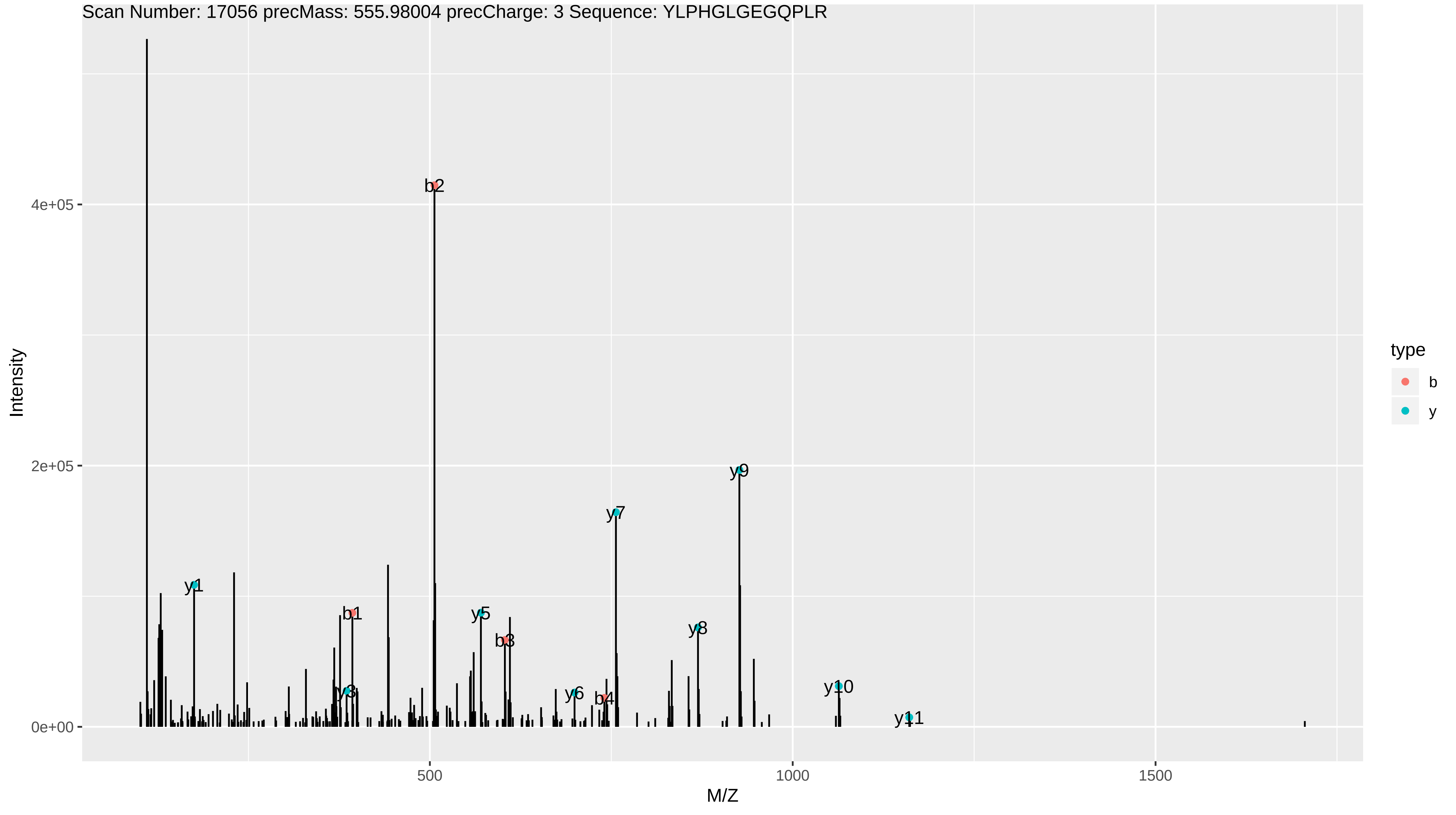

MCM10 | +229.163FLQSSSEVESPAVPSSSR

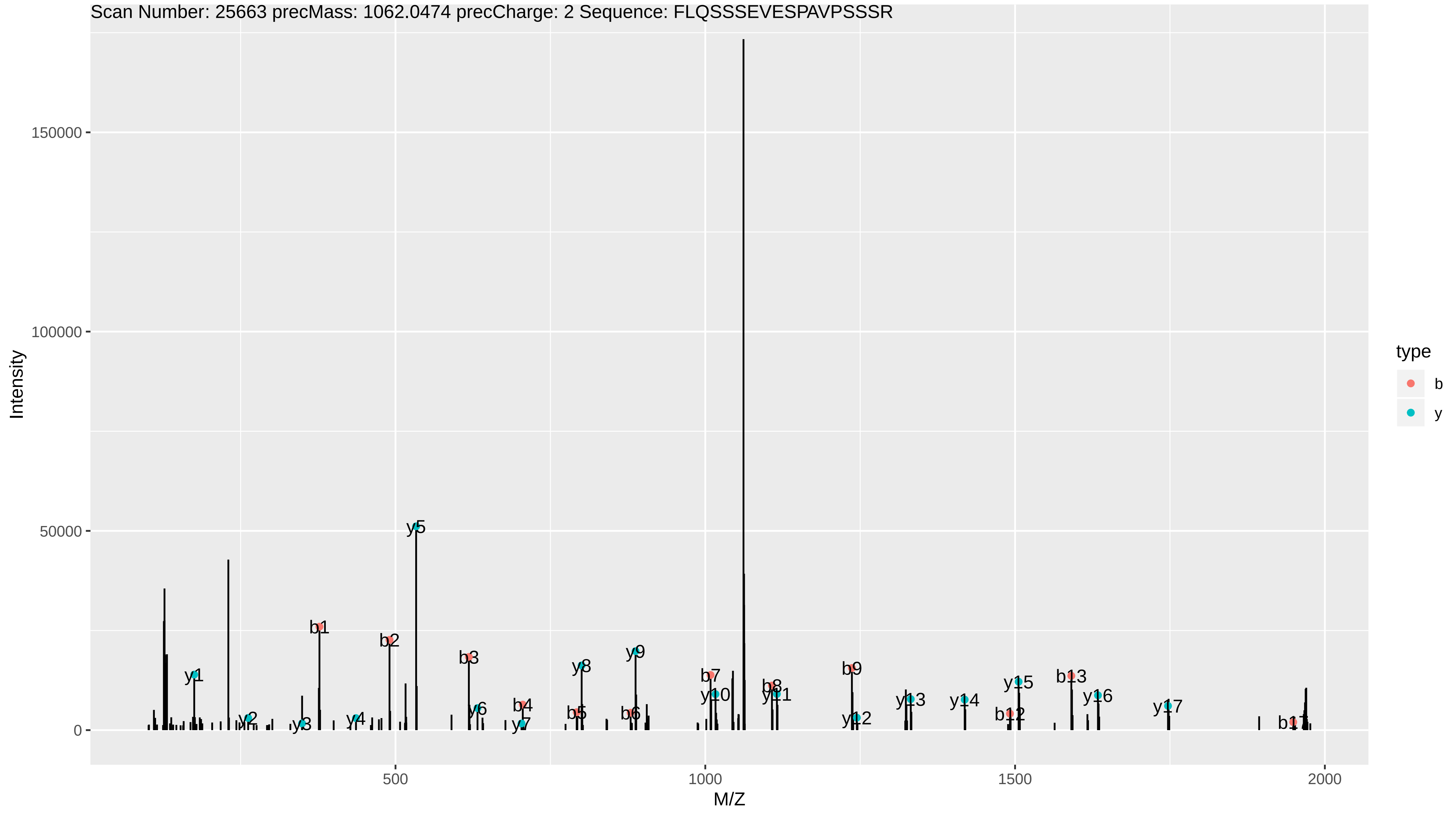

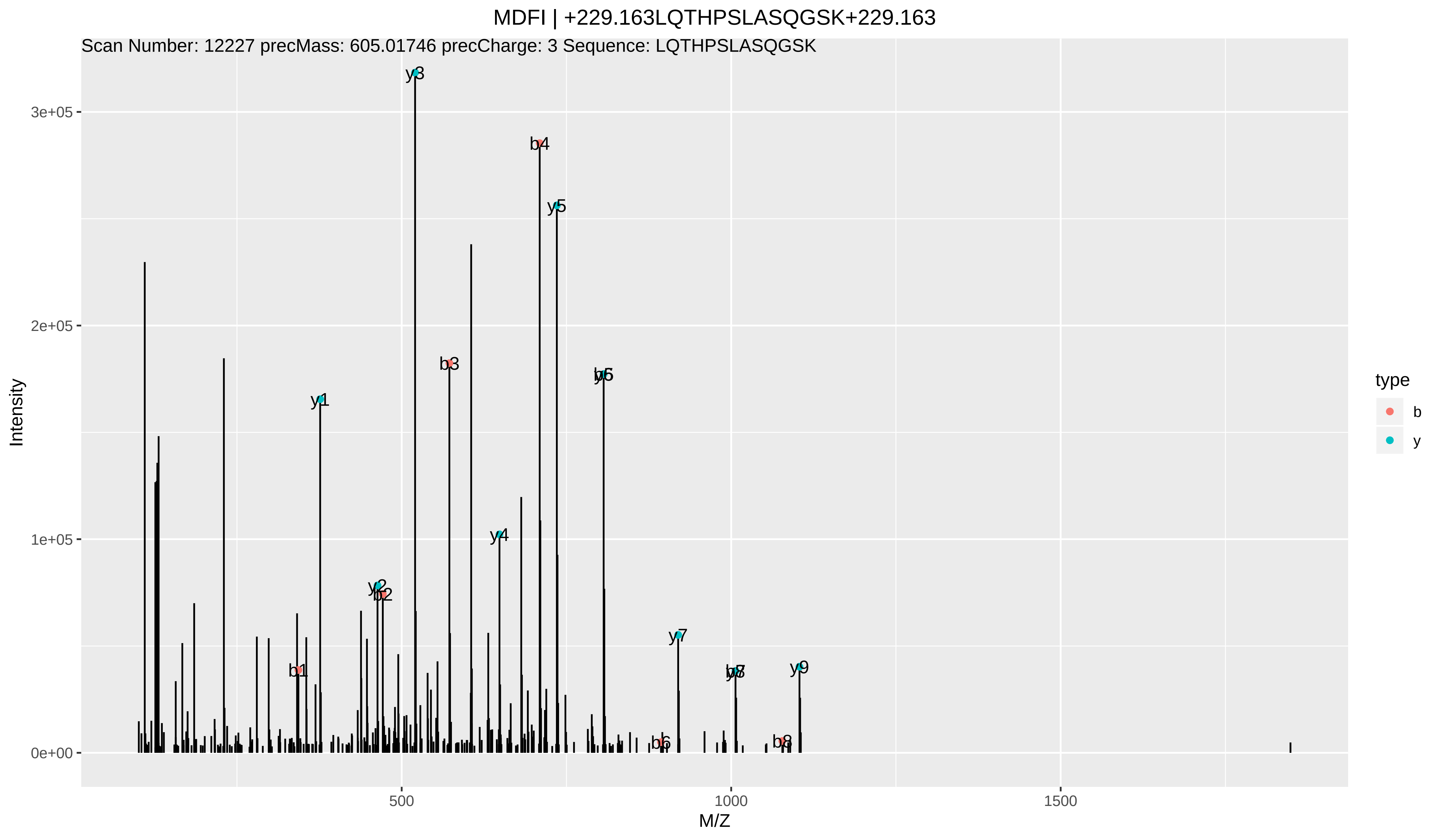

MDFI | +229.163LQTHPSLASQGSK+229.163
Scan Number: 12227 precMass: 605.01746 precCharge: 3 Sequence: LQTHPSLASQGSK
Intensity
M/Z
3e+05
2e+05
1e+05
0e+00
500
1000
1500
y3
b4
y5
b3
b5
y6
y1
y4
y2
b2
y7
b1
b7
y8
y9
b6
b8
type
b
y

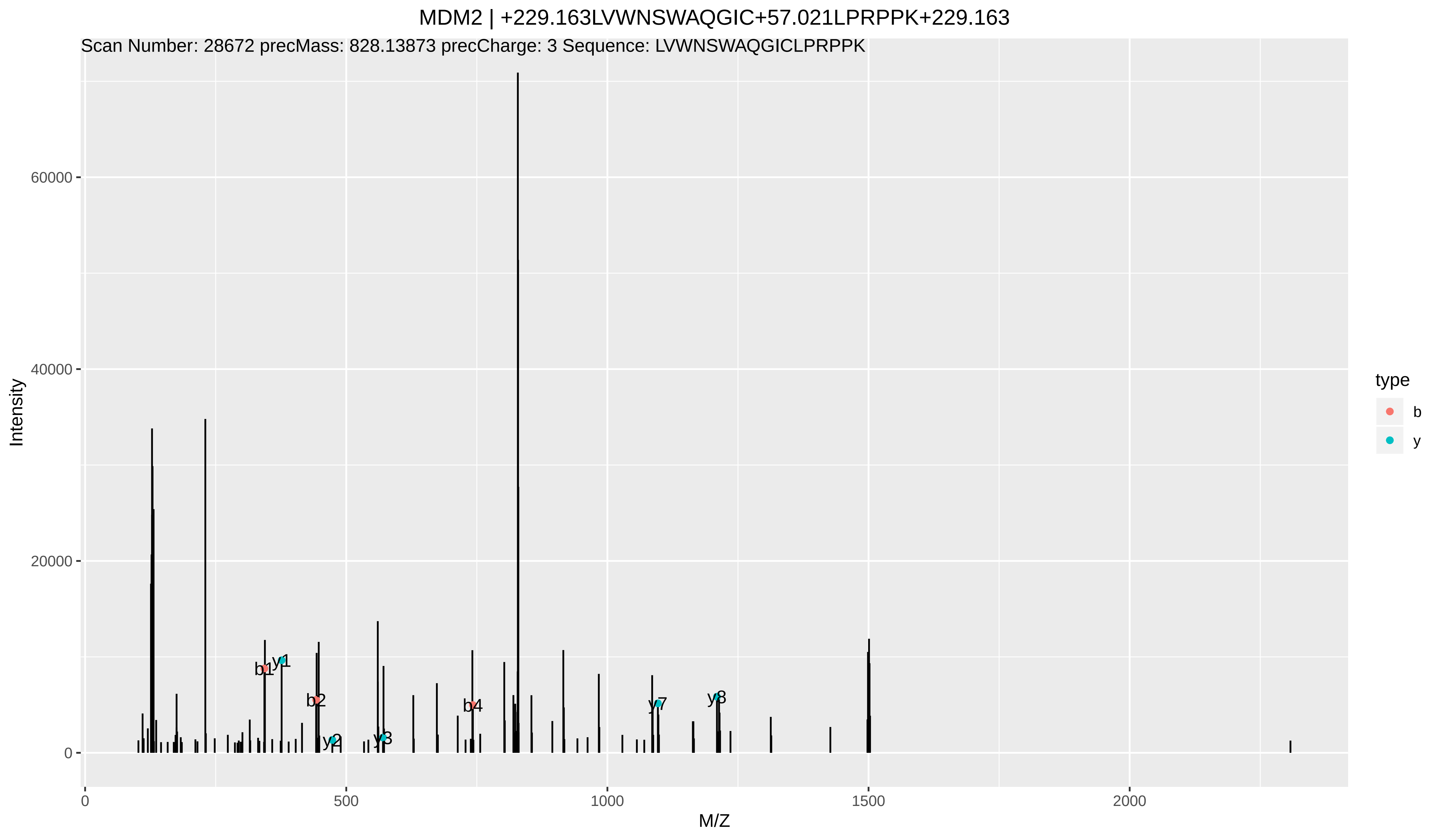

MDM2 | +229.163LVWNSWAQGIC+57.021LPRPPK+229.163
Scan Number: 28672 precMass: 828.13873 precCharge: 3 Sequence: LVWNSWAQGICLPRPPK
Intensity
60000
40000
20000
0
M/Z
0
500
1000
1500
2000
b1
y1
b2
y2
y3
b4
y7
y8
type
b
y

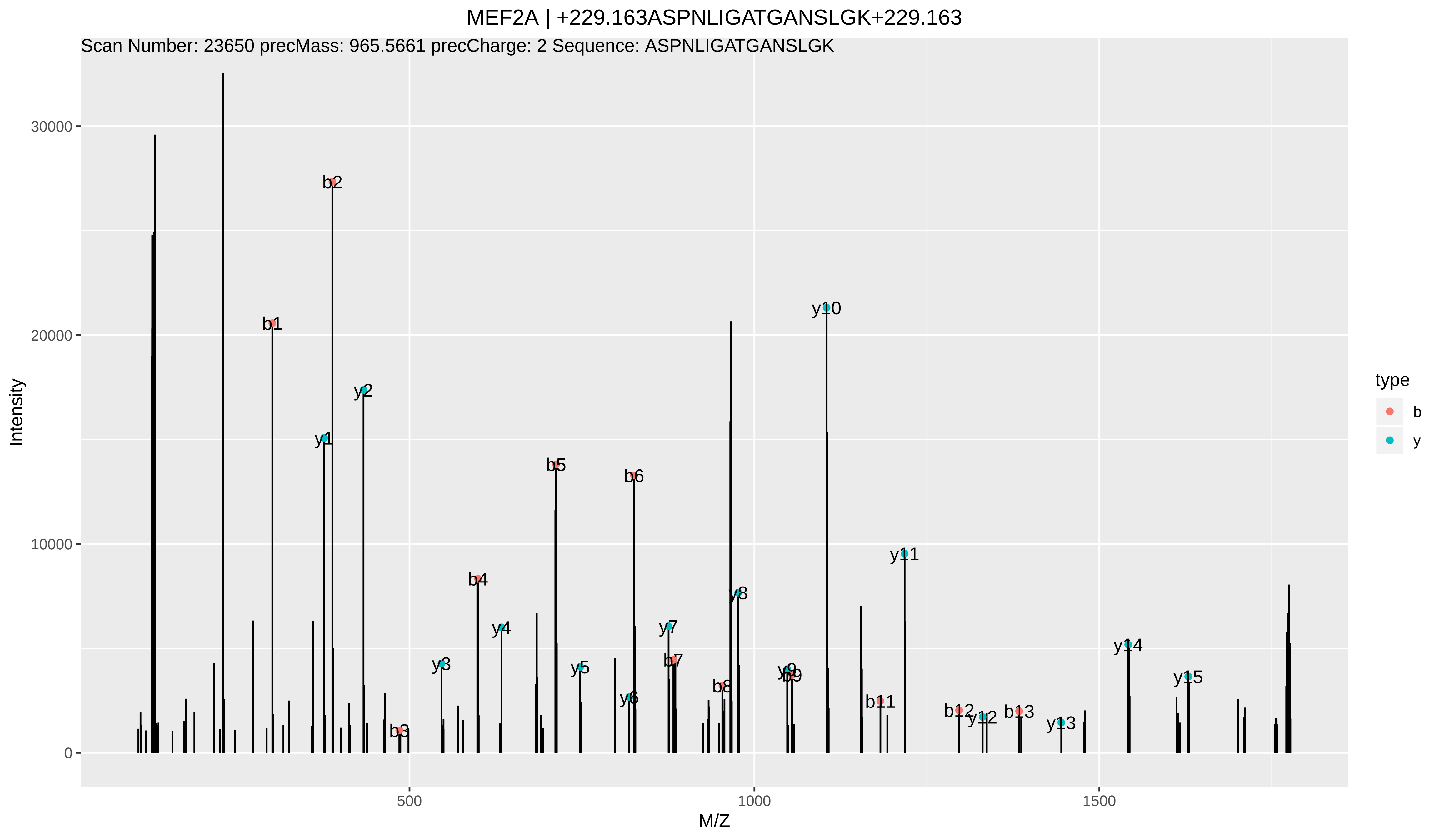

MEF2A | +229.163ASPNLIGATGANSLGK+229.163
Scan Number: 23650 precMass: 965.5661 precCharge: 2 Sequence: ASPNLIGATGANSLGK
Intensity
M/Z
30000
20000
10000
0
500
1000
1500
b1
b2
b3
b4
b5
b6
b7
b8
b9
b11
b12
b13
y1
y2
y3
y4
y5
y6
y7
y8
y9
y10
y11
y12
y13
y14
y15
type
b
y

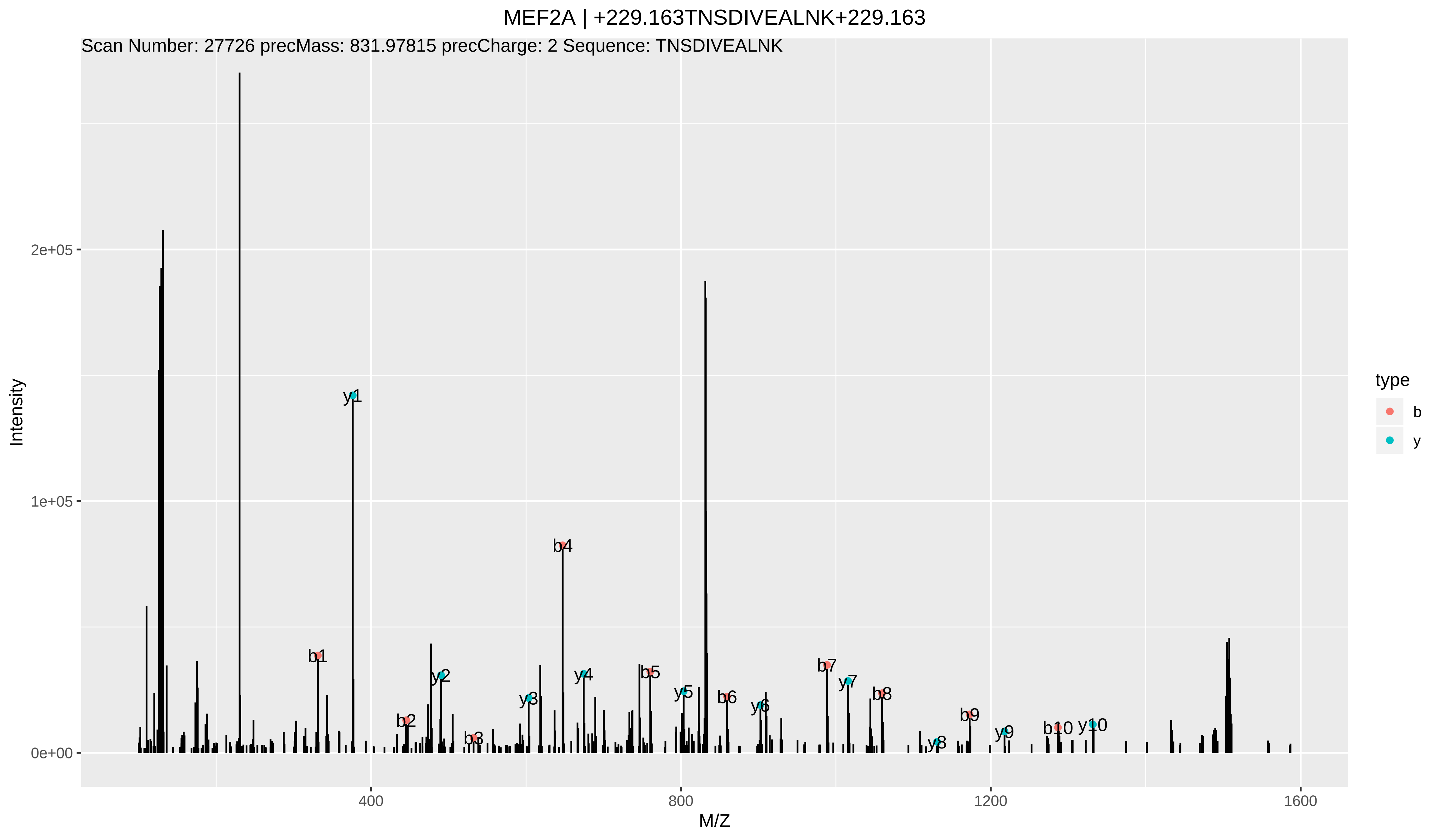

MEF2A | +229.163TNSDIVEALNK+229.163
Scan Number: 27726 precMass: 831.97815 precCharge: 2 Sequence: TNSDIVEALNK
Intensity
2e+05
1e+05
0e+00
M/Z
400
800
1200
1600
y1
b1
b2
y2
b3
y3
b4
y4
b5
y5
b6
y6
b7
y7
b8
y8
b9
y9
b10
y10
type
b
y

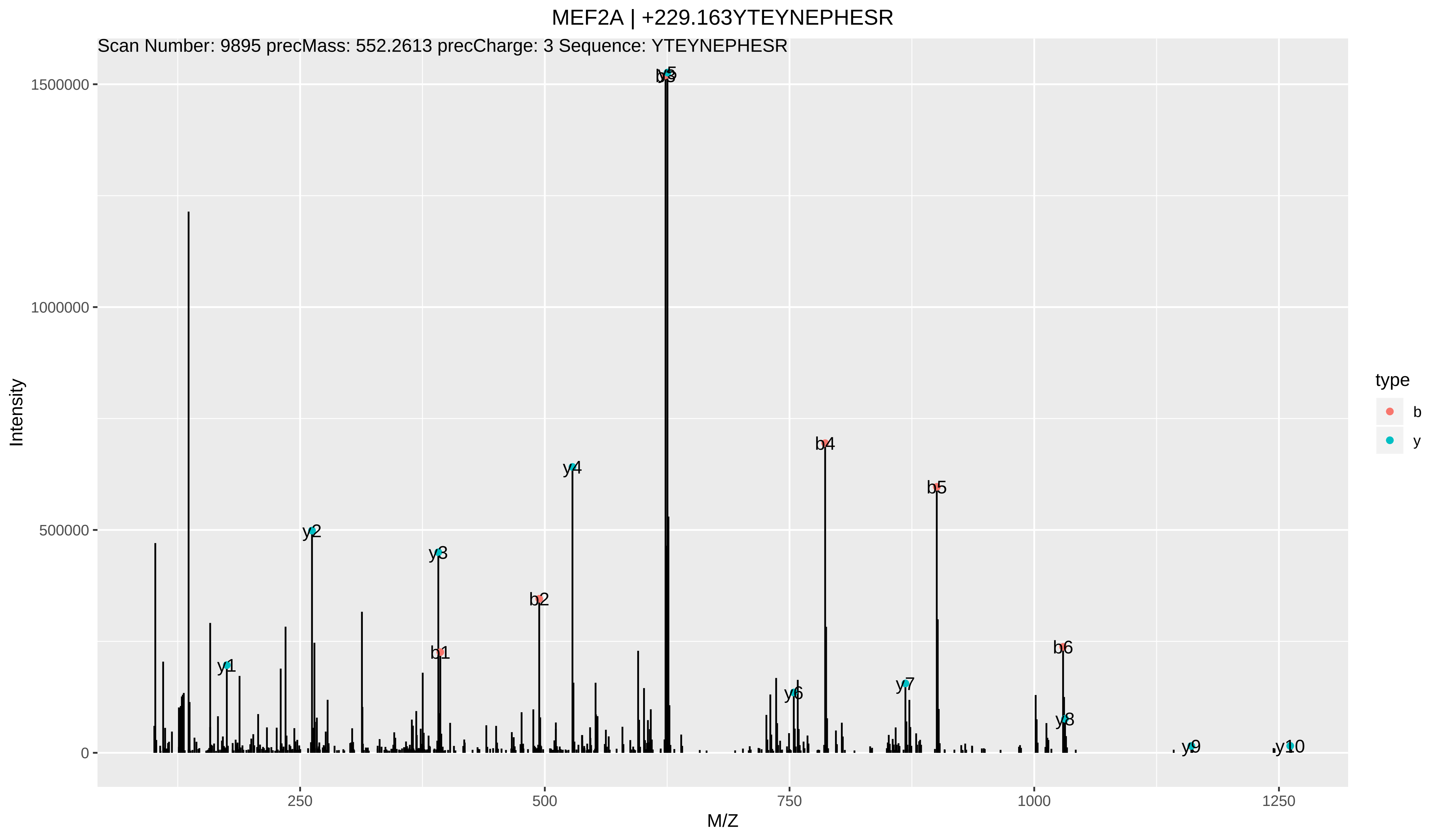

MEF2A | +229.163YTEYNEPHESR
Scan Number: 9895 precMass: 552.2613 precCharge: 3 Sequence: YTEYNEPHESR
Intensity
M/Z
0
500000
1000000
1500000
250
500
750
1000
1250
type
b
y
y1
y2
y3
y4
y5
y6
y7
y8
y9
y10
b1
b2
b3
b4
b5
b6

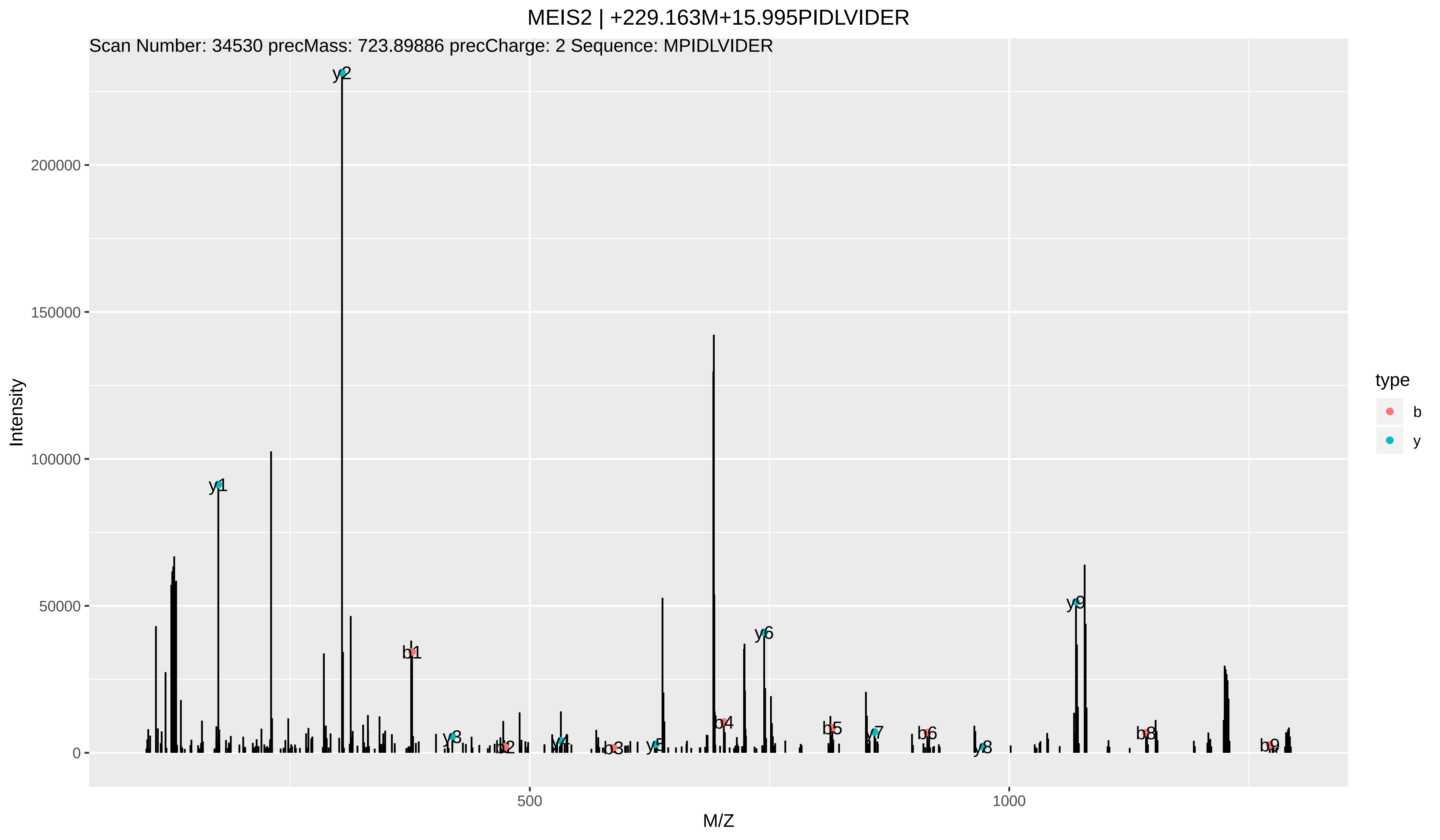

MEIS2 | +229.163M+15.995PIDLVIDER
Scan Number: 34530 precMass: 723.89886 precCharge: 2 Sequence: MPIDLVIDER
Intensity
M/Z
0
50000
100000
150000
200000
500
1000
y1
y2
y3
y4
y5
y6
y7
y8
y9
b1
b2
b3
b4
b5
b6
b8
b9
type
b
y

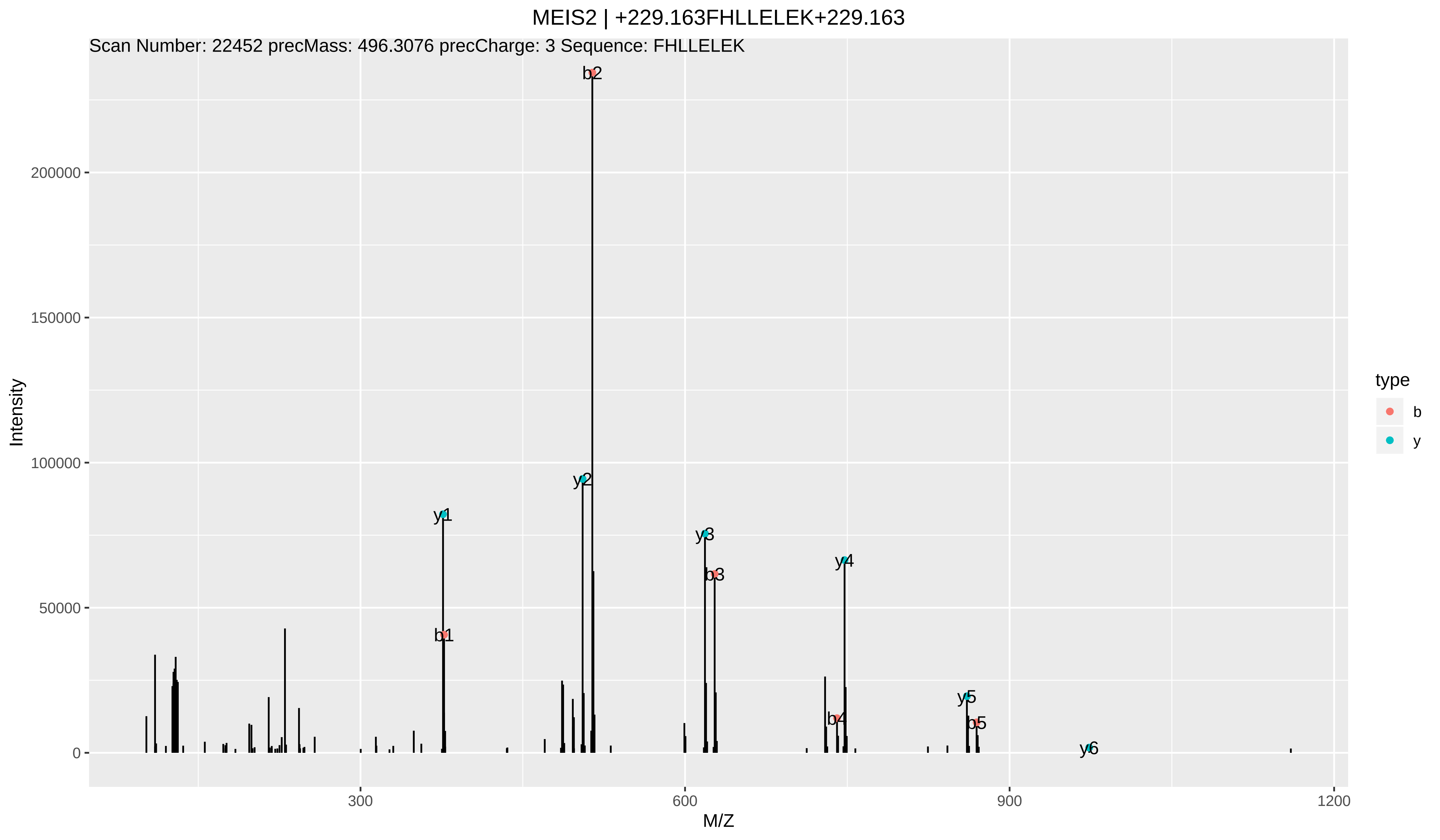

MEIS2 | +229.163FHLLELEK+229.163
Scan Number: 22452 precMass: 496.3076 precCharge: 3 Sequence: FHLLELEK
Intensity
M/Z
0
50000
100000
150000
200000
300
600
900
1200
b2
y2
y1
b1
y3
b3
y4
b4
y5
b5
y6
type
b
y

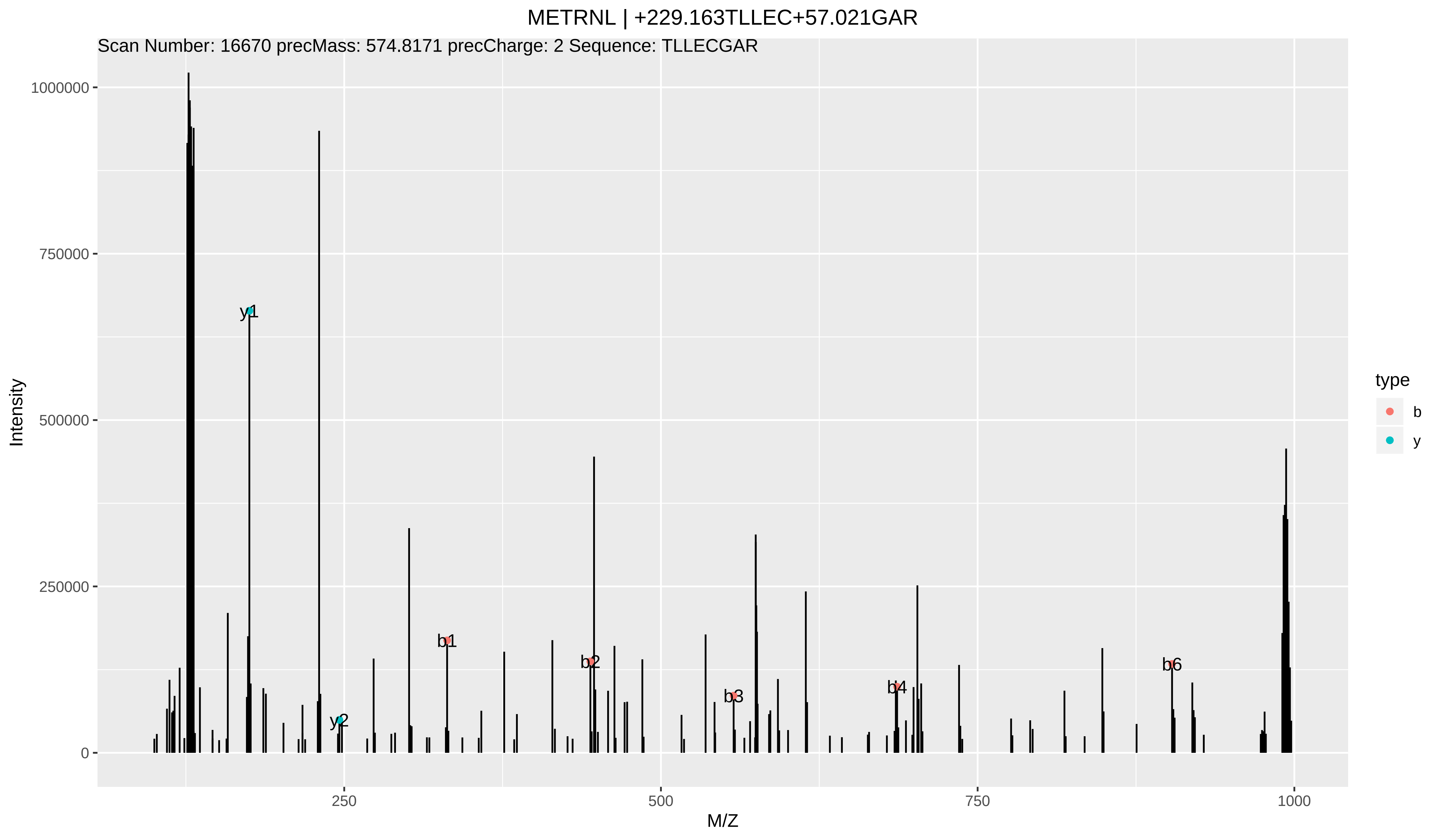

METRNL | +229.163TLLEC+57.021GAR
Scan Number: 16670 precMass: 574.8171 precCharge: 2 Sequence: TLLECGAR
Intensity
M/Z
1000000
750000
500000
250000
0
250
500
750
1000
y1
y2
b1
b2
b3
b4
b6
type
b
y

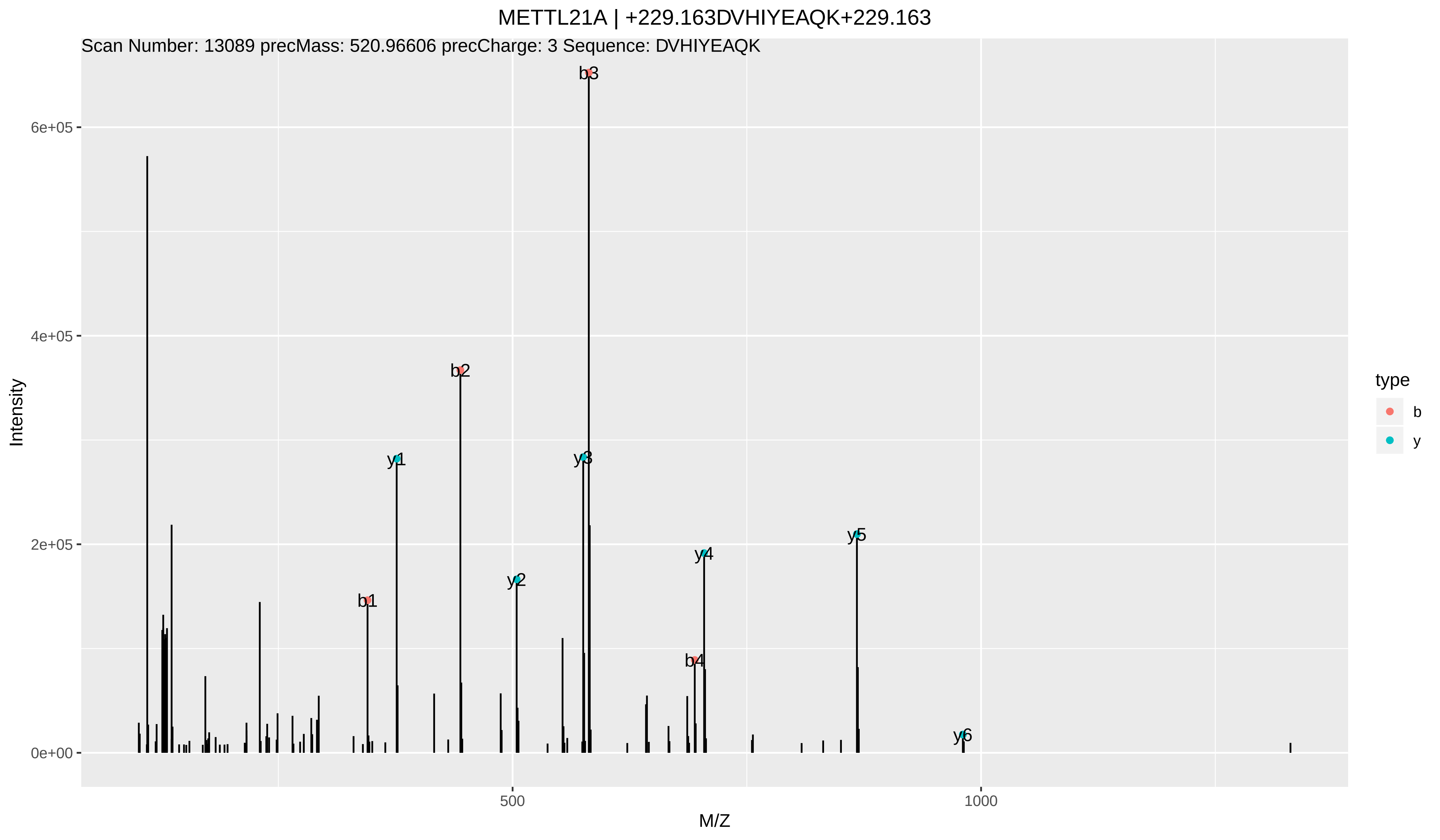

METTL21A | +229.163DVHIYEAQK+229.163
Scan Number: 13089 precMass: 520.96606 precCharge: 3 Sequence: DVHIYEAQK
b3
b2
y1
y3
y5
y4
y2
b1
b4
y6
6e+05
4e+05
2e+05
0e+00
Intensity
500
1000
M/Z
type
b
y

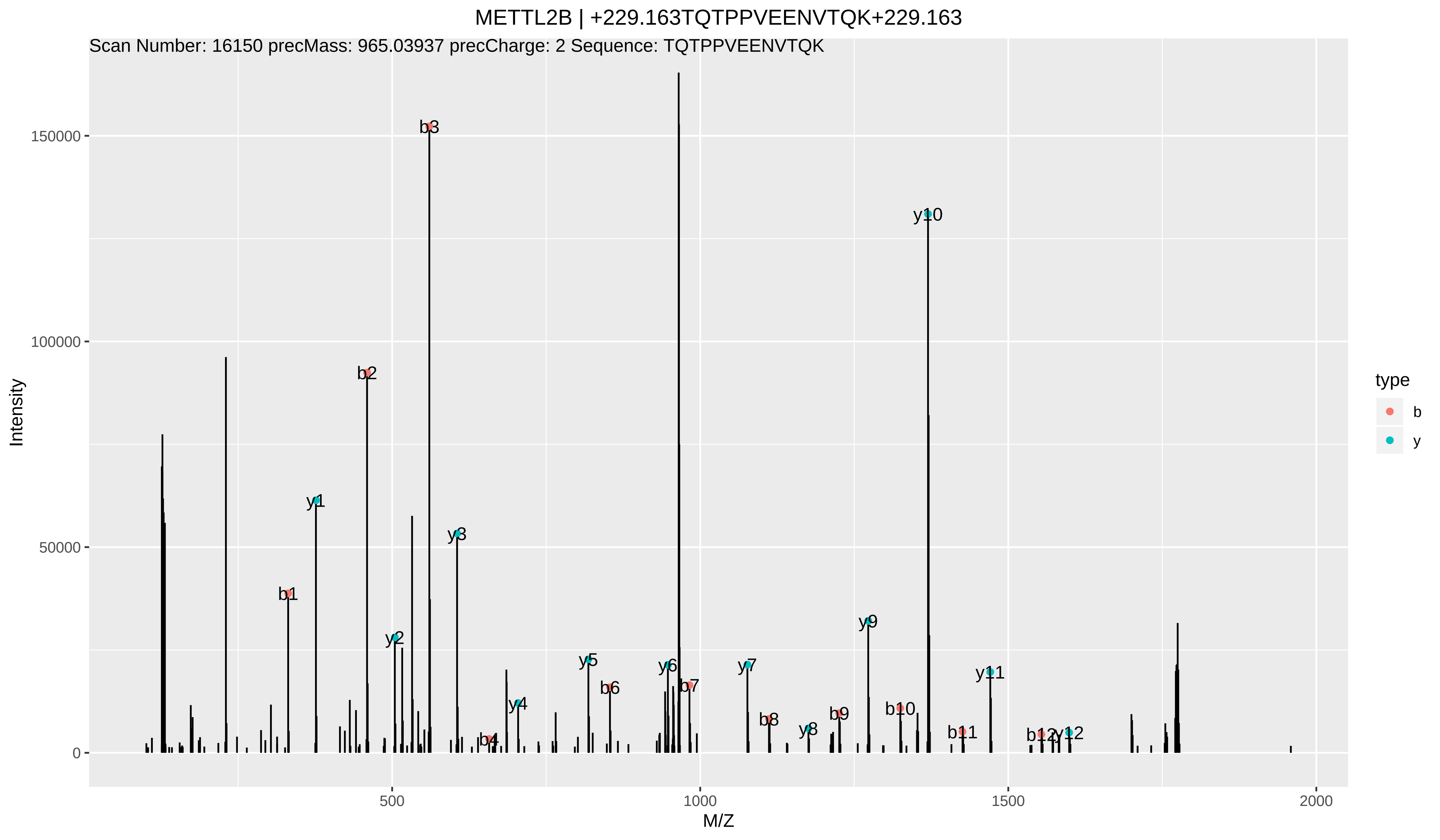
METTL2B | +229.163TQTPPVEENVTQK+229.163
Scan Number: 16150 precMass: 965.03937 precCharge: 2 Sequence: TQTPPVEENVTQK
Intensity
M/Z
type
b
y
b1
b2
b3
b4
b6
b7
b8
b9
b10
b11
b12
y1
y2
y3
y4
y5
y6
y7
y8
y9
y10
y11
y12

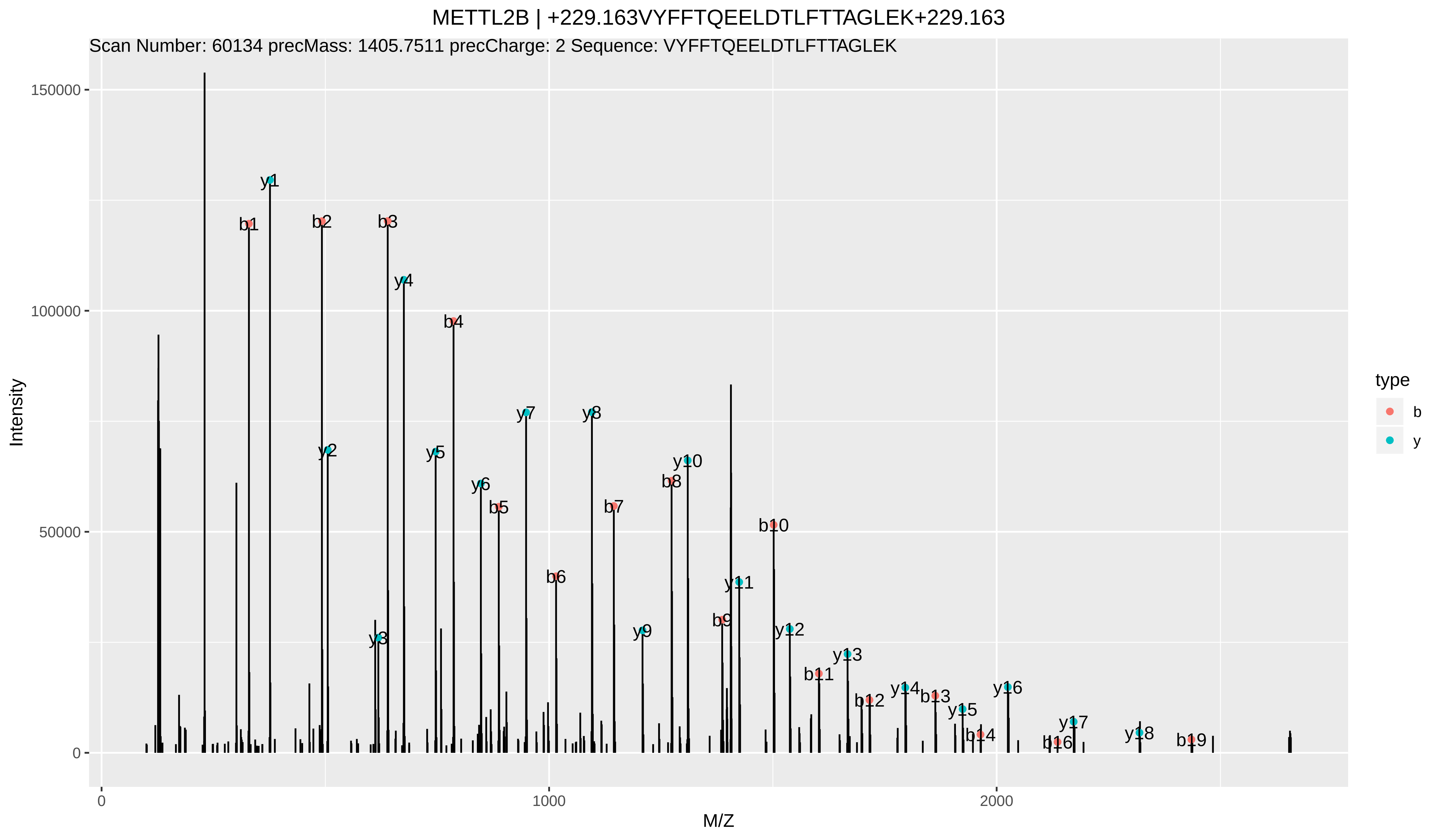
METTL2B | +229.163VYFFTQEELDTLFTTAGLEK+229.163
Scan Number: 60134 precMass: 1405.7511 precCharge: 2 Sequence: VYFFTQEELDTLFTTAGLEK
Intensity
M/Z
type
b
y
150000
100000
50000
0
1000
2000
y1
b1
b2
b3
y4
b4
y2
y5
y6
b5
y7
y8
b7
b8
y10
b10
b6
y11
b9
y9
y3
y12
y13
b11
y14
b12
b13
y16
y15
b14
y17
y18
b16
b19

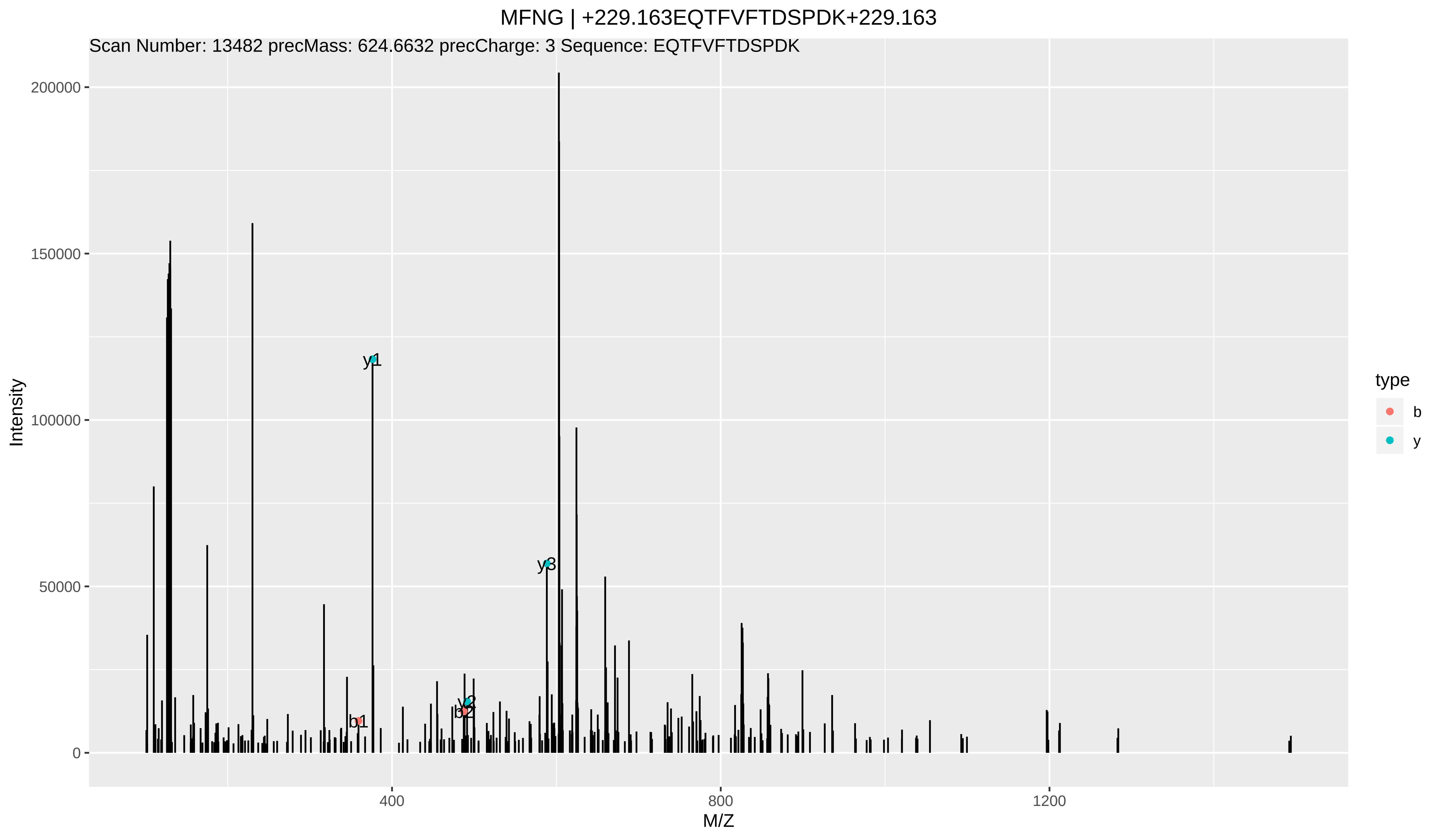
MFNG | +229.163EQTFVFTDSPDK+229.163
Scan Number: 13482 precMass: 624.6632 precCharge: 3 Sequence: EQTFVFTDSPDK
Intensity
200000
150000
100000
50000
0
M/Z
400
800
1200
y1
y3
y2
b2
b1
type
b
y

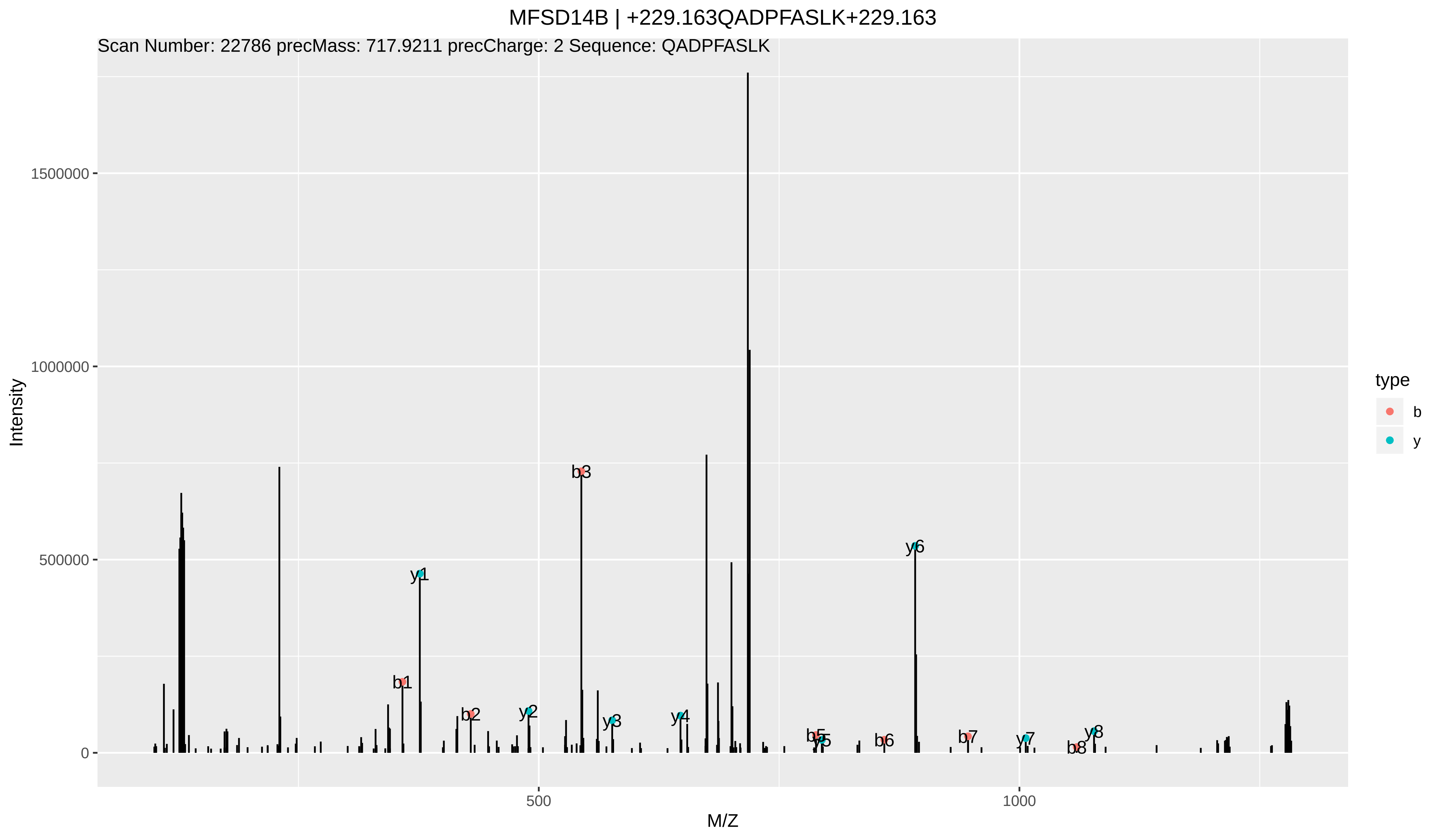

MFSD14B | +229.163QADPFASLK+229.163
Scan Number: 22786 precMass: 717.9211 precCharge: 2 Sequence: QADPFASLK
Intensity
M/Z
1500000
1000000
500000
0
500
1000
b1
b2
b3
b5
b6
b7
b8
y1
y2
y3
y4
y5
y6
y7
y8
type
b
y

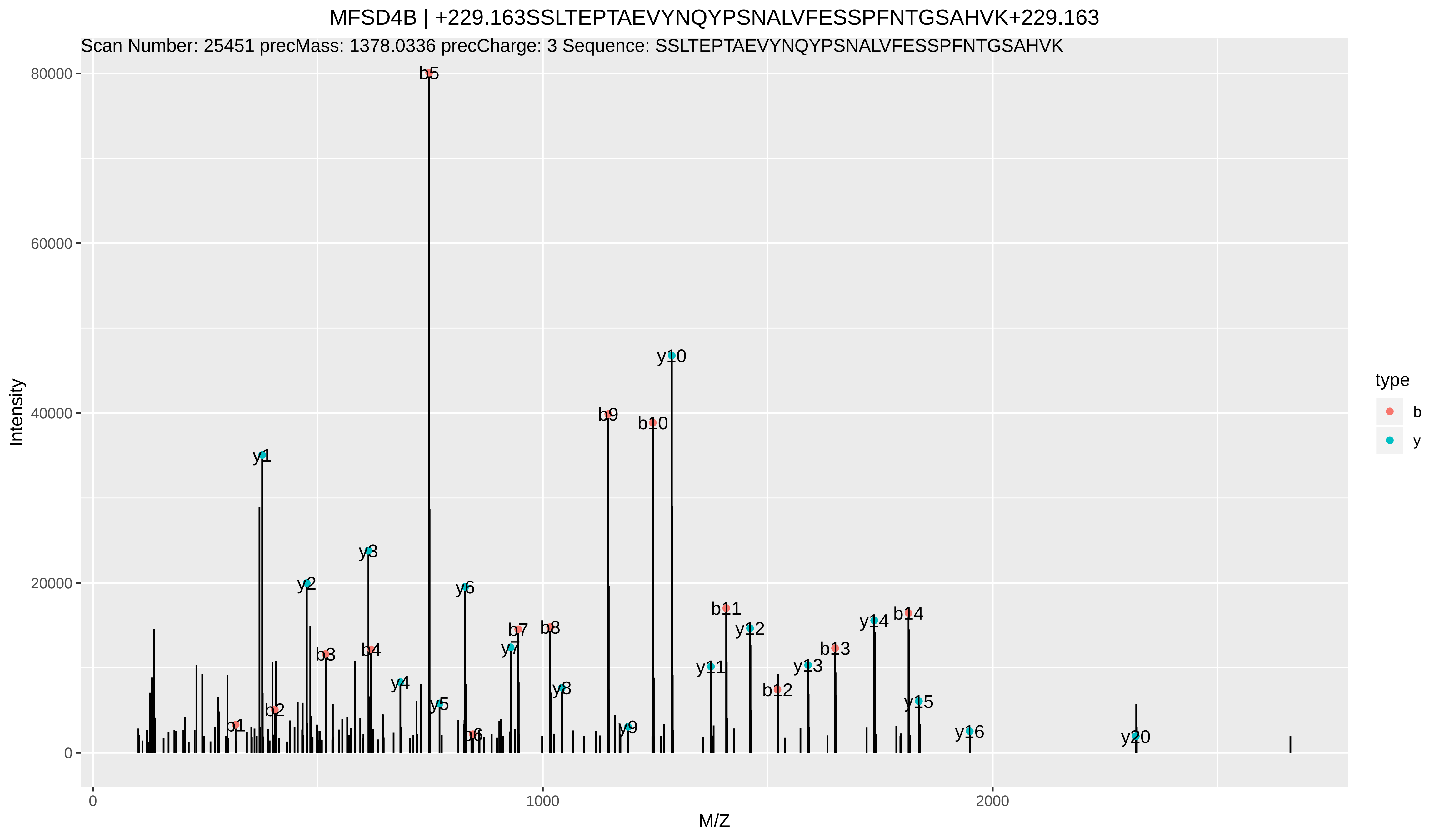
MFSD4B | +229.163SSLTEPTAEVYNQYPSNALVFESSPFNTGSAHVK+229.163
Scan Number: 25451 precMass: 1378.0336 precCharge: 3 Sequence: SSLTEPTAEVYNQYPSNALVFESSPFNTGSAHVK
Intensity
M/Z
type
b
y
b5
y10
b9
b10
y1
y3
y2
y6
b11
y14
b14
y12
b8
b7
y7
b4
b3
b13
y11
y13
y4
y8
b12
y5
y15
b2
b1
y9
y16
b6
y20
0
20000
40000
60000
80000
1000
2000

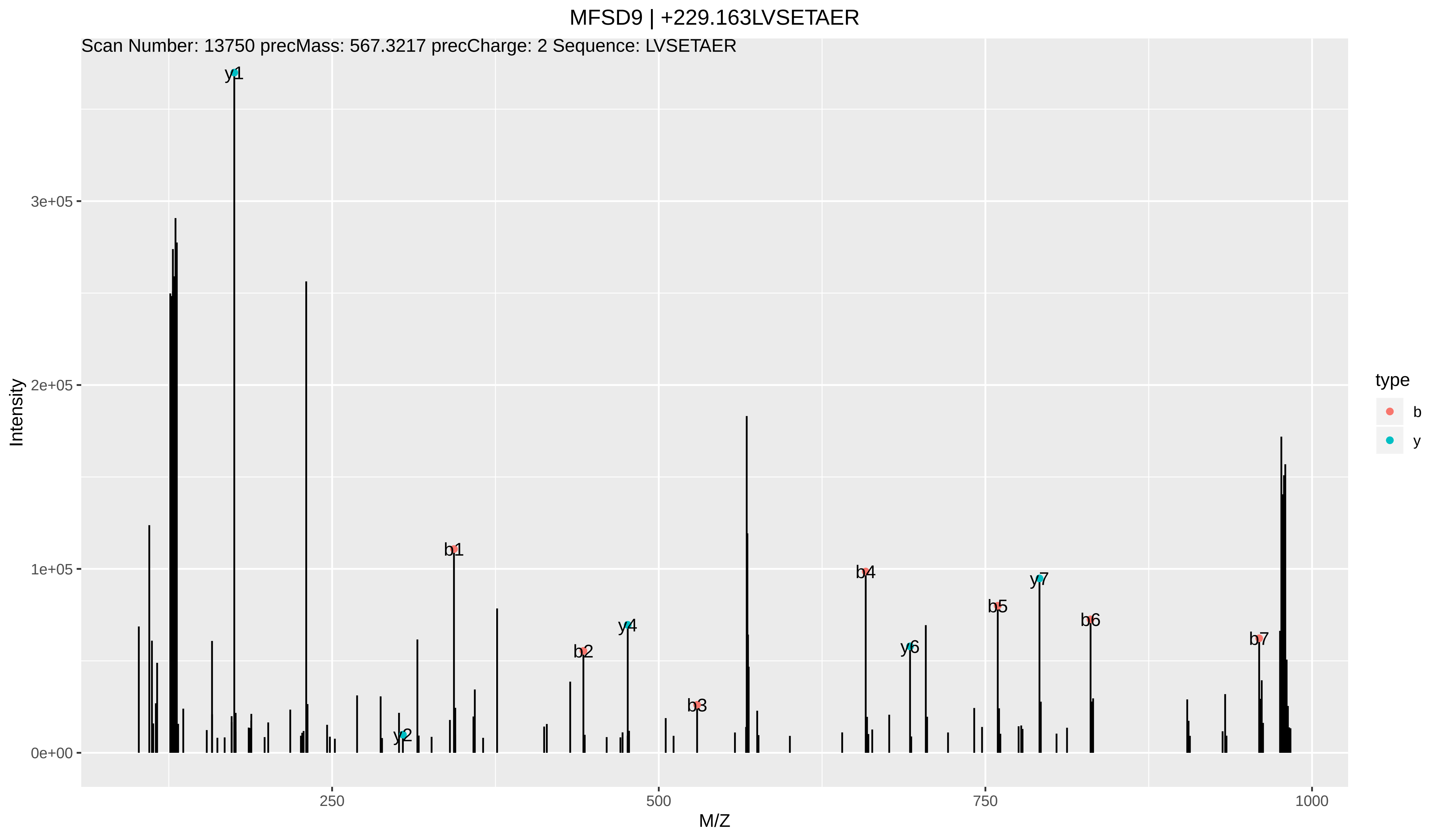

MFSD9 | +229.163LVSETAER
Scan Number: 13750 precMass: 567.3217 precCharge: 2 Sequence: LVSETAER
y1
b1
b2
y4
b3
b4
y6
b5
y7
b6
b7
y2
Intensity
3e+05
2e+05
1e+05
0e+00
250
500
750
1000
M/Z
type
b
y

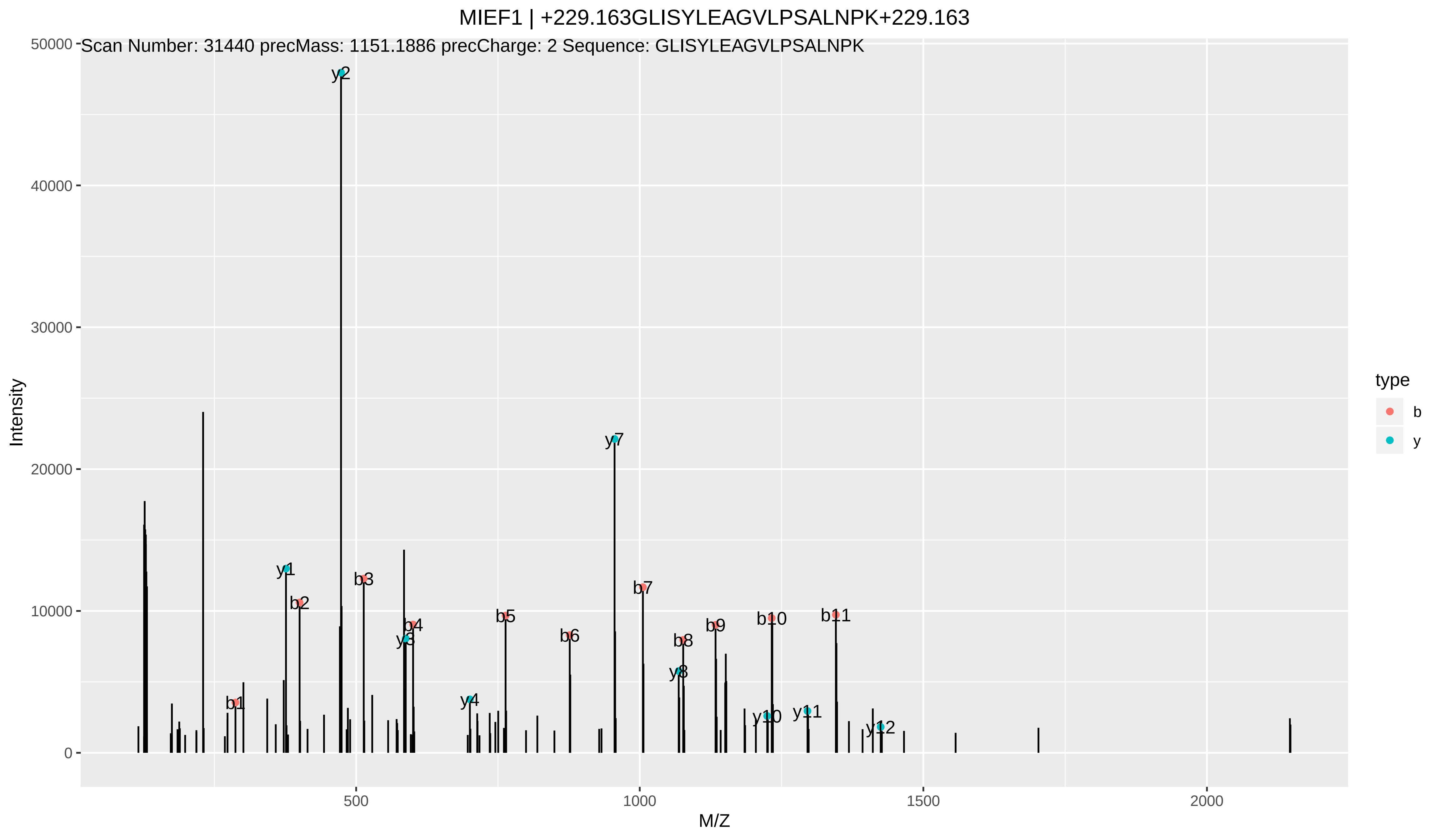

MIEF1 | +229.163GLISYLEAGVLPSALNPK+229.163
Scan Number: 31440 precMass: 1151.1886 precCharge: 2 Sequence: GLISYLEAGVLPSALNPK
Intensity
M/Z
0
10000
20000
30000
40000
50000
500
1000
1500
2000
y2
y7
y1
b3
b2
b7
b5
b11
b10
b9
b4
y3
b6
b8
y8
b1
y4
y11
y10
y12
type
b
y

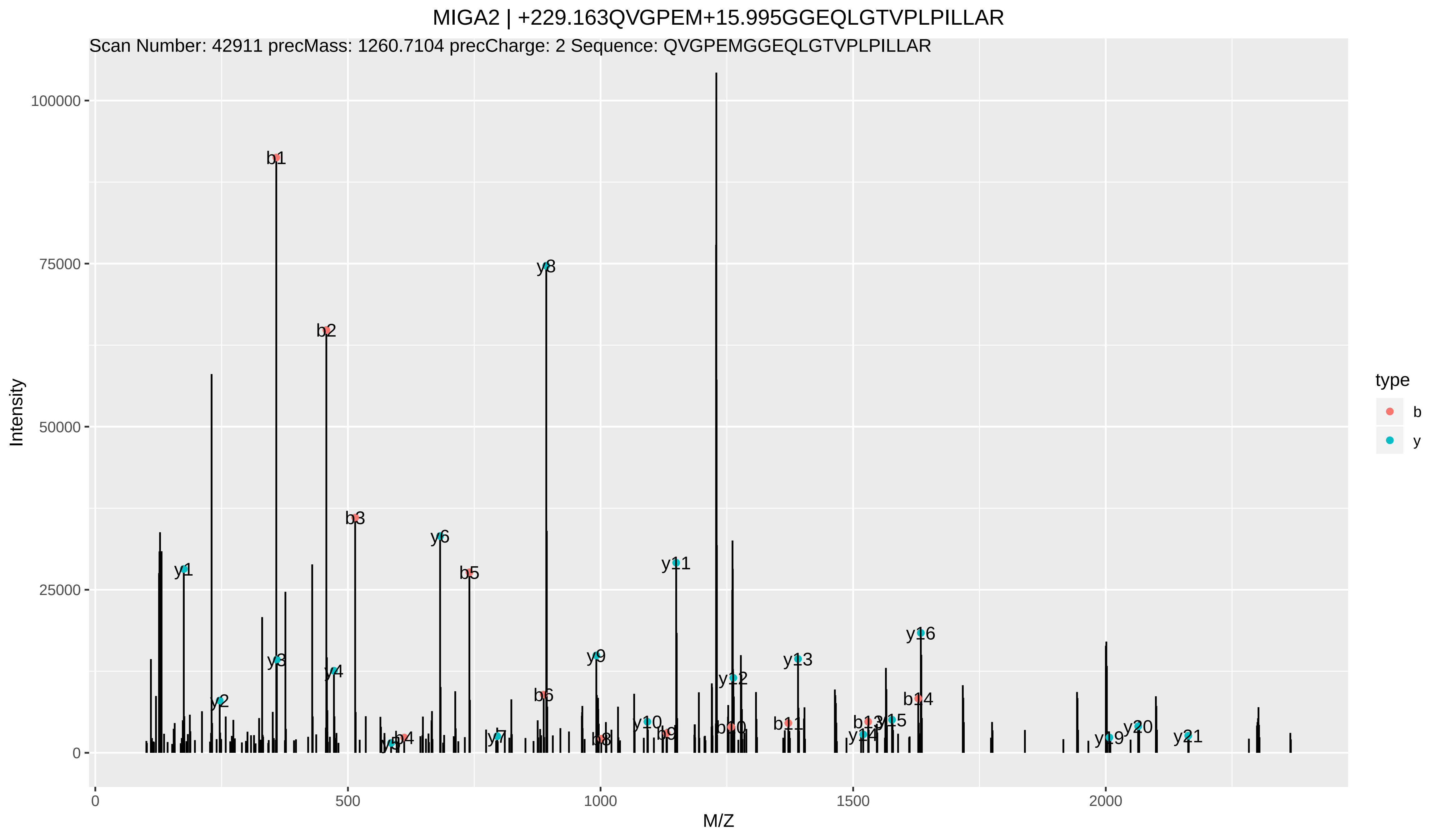
MIGA2 | +229.163QVGPEM+15.995GGEQLGTVPLPILLAR
Scan Number: 42911 precMass: 1260.7104 precCharge: 2 Sequence: QVGPEMGGEQLGTVPLPILLAR
Intensity
M/Z
type
b
y
0
25000
50000
75000
100000
500
1000
1500
2000
b1
b2
b3
b4
b5
b6
b8
b9
b10
b11
b13
b14
y1
y2
y3
y4
y5
y6
y7
y8
y9
y10
y11
y12
y13
y14
y15
y16
y19
y20
y21

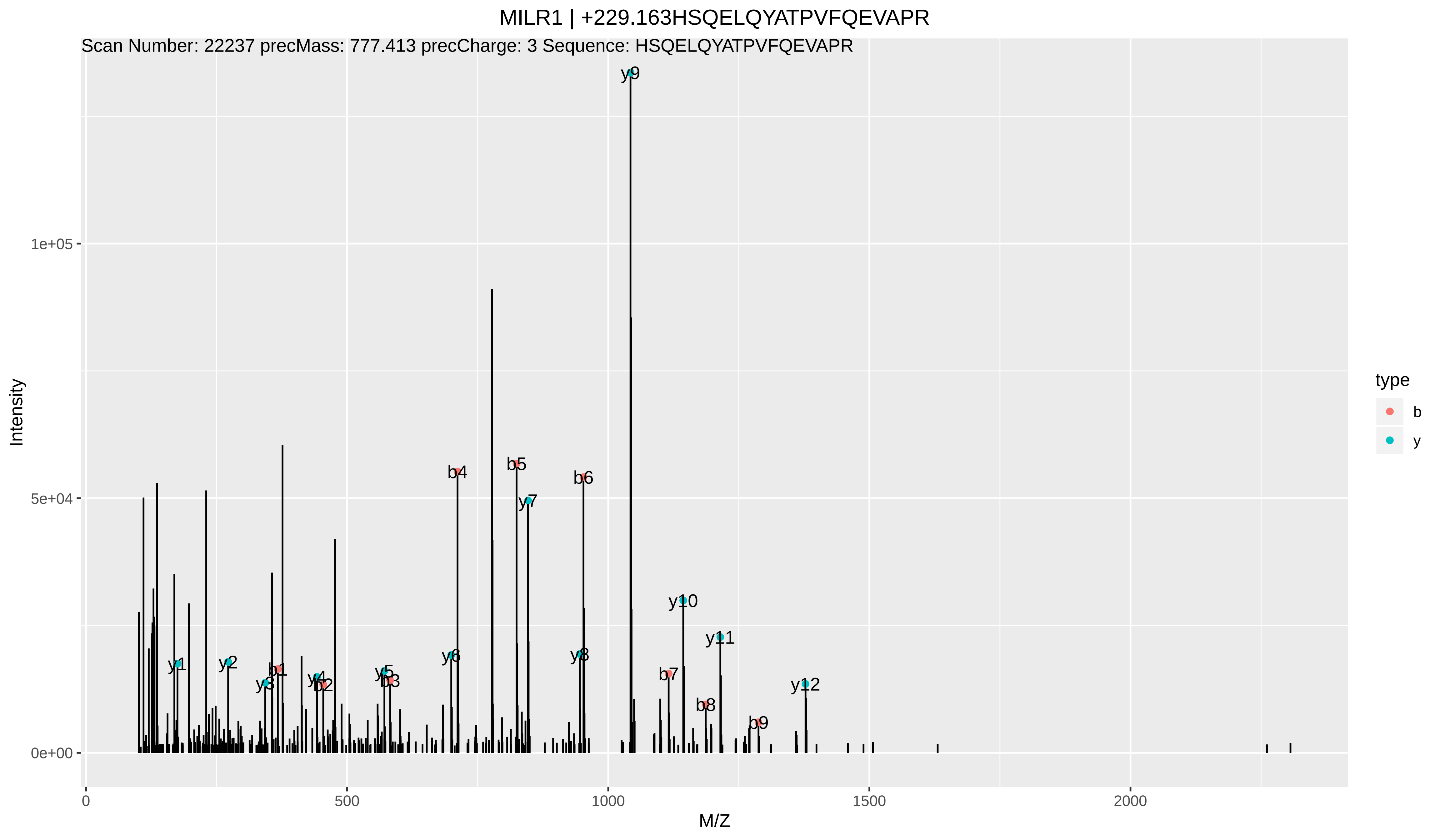

MILR1 | +229.163HSQELQYATPVFQEVAPR
Scan Number: 22237 precMass: 777.413 precCharge: 3 Sequence: HSQELQYATPVFQEVAPR
Intensity
M/Z
1e+05
5e+04
0e+00
0
500
1000
1500
2000
y9
b4
b5
b6
y7
y10
y11
y1
y2
y3
b1
y4
b2
y5
b3
y6
y8
b7
b8
b9
y12
type
b
y

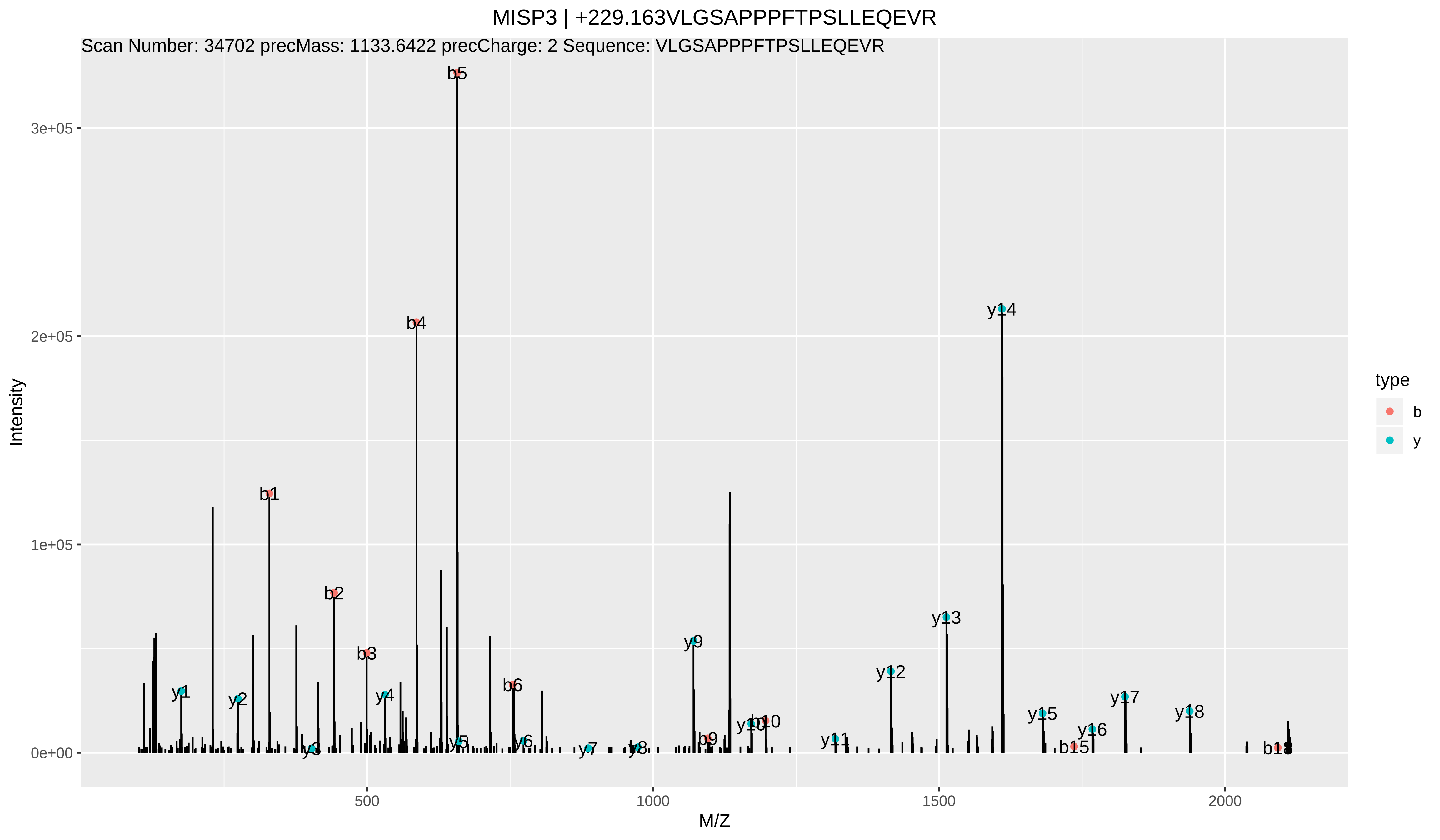
MISP3 | +229.163VLGSAPPPFTPSLLEQEVR
Scan Number: 34702 precMass: 1133.6422 precCharge: 2 Sequence: VLGSAPPPFTPSLLEQEVR
Intensity
M/Z
0e+00
1e+05
2e+05
3e+05
500
1000
1500
2000
type
b
y
b1
b2
b3
b4
b5
b6
b9
b10
b15
b18
y1
y2
y3
y4
y5
y6
y7
y8
y9
y10
y11
y12
y13
y14
y15
y16
y17
y18

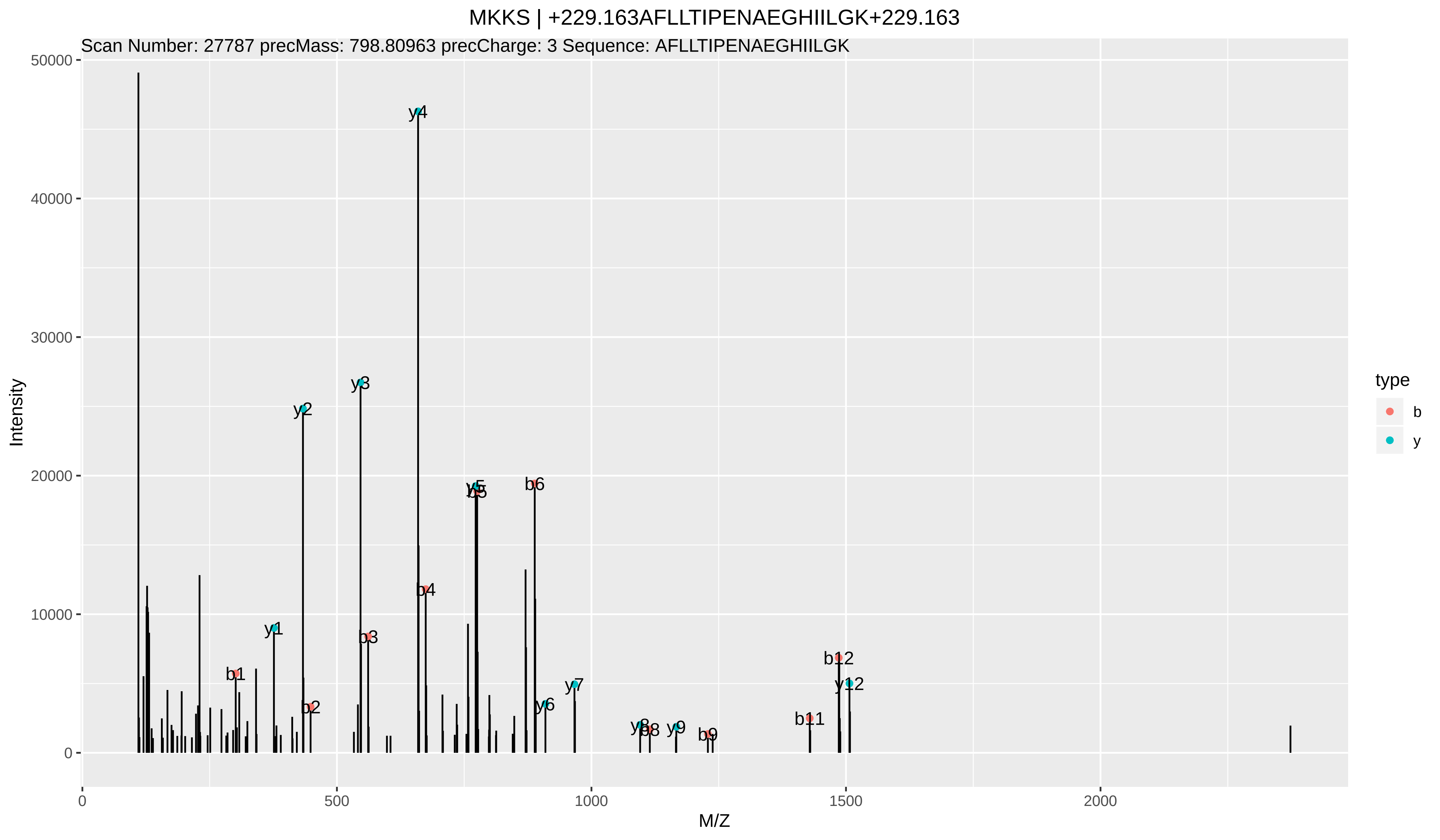
MKKS | +229.163AFLLTIPENAEGHIILGK+229.163
Scan Number: 27787 precMass: 798.80963 precCharge: 3 Sequence: AFLLTIPENAEGHIILGK
Intensity
M/Z
type
b
y
y4
y3
y2
y1
b1
b2
b3
b4
y5
b5
b6
y6
y7
y8
b8
y9
b9
b11
b12
y12
0
10000
20000
30000
40000
50000
500
1000
1500
2000

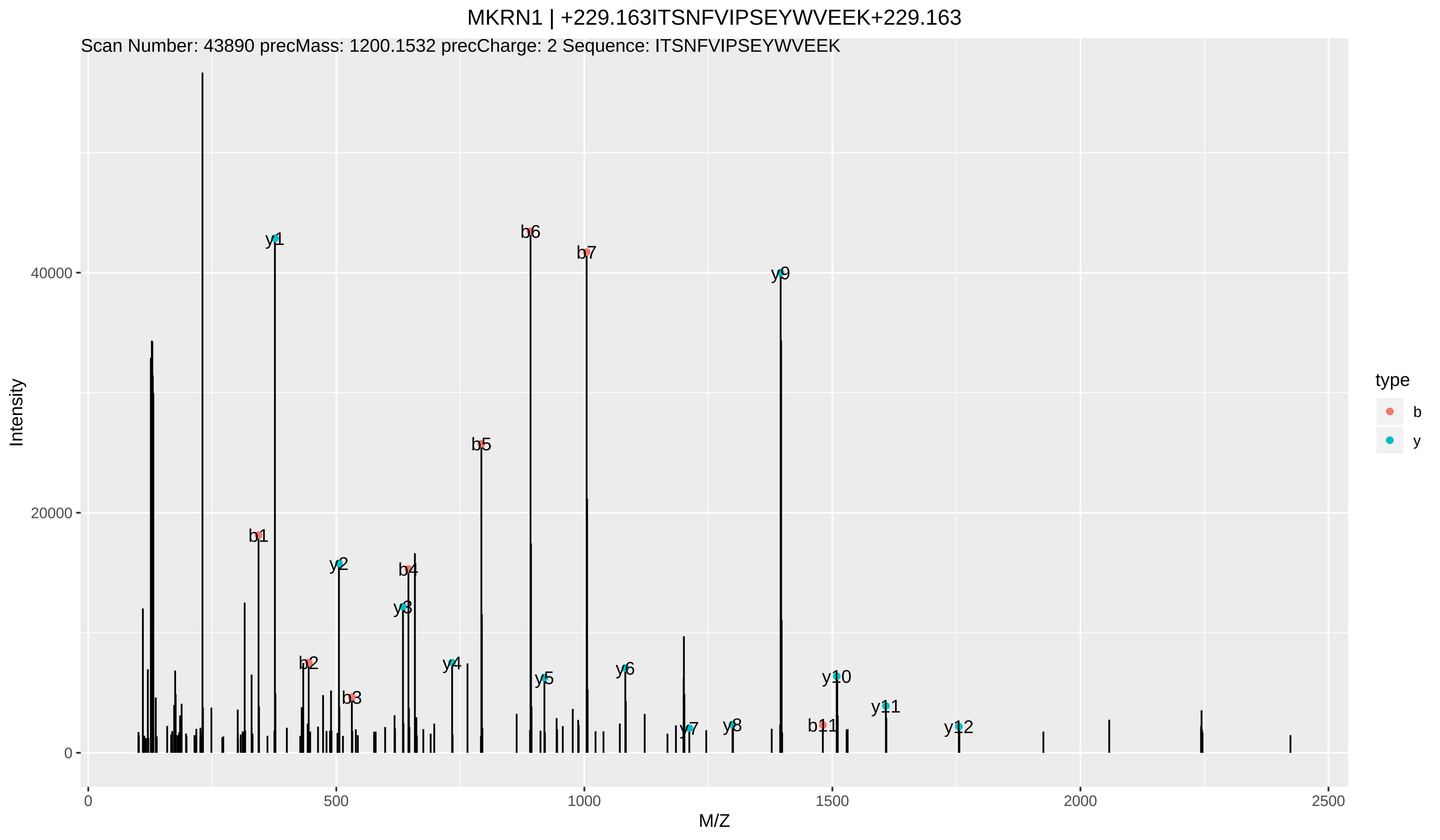
MKRN1 | +229.163ITSNFVIPSEYWVEEK+229.163
Scan Number: 43890 precMass: 1200.1532 precCharge: 2 Sequence: ITSNFVIPSEYWVEEK
Intensity
M/Z
0
20000
40000
0
500
1000
1500
2000
2500
y1
b6
b7
y9
b5
b1
y2
b4
y3
b2
y4
y6
y5
b3
y10
y11
y7
y8
b11
y12
type
b
y

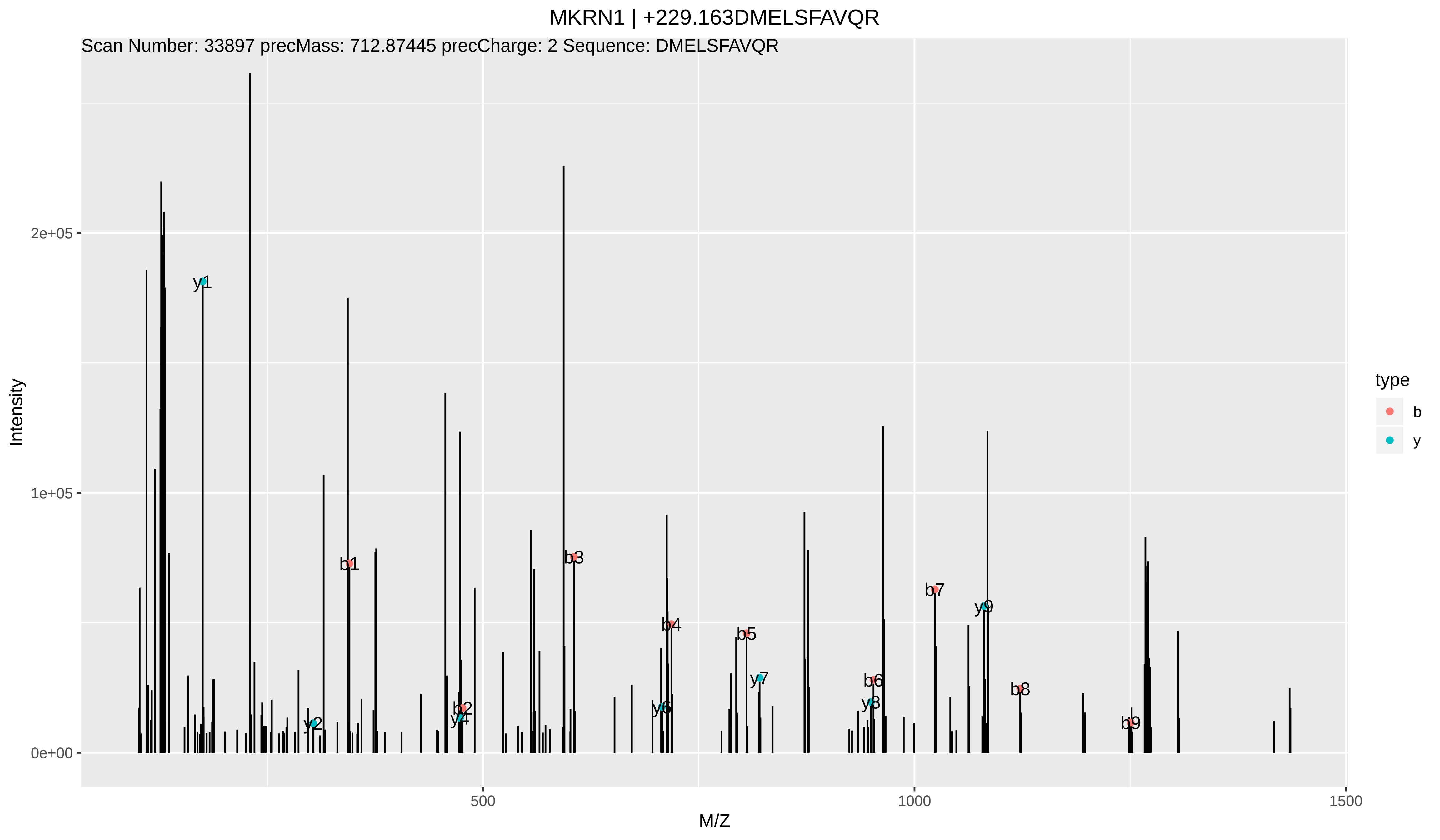

MKRN1 | +229.163DMELSFAVQR
Scan Number: 33897 precMass: 712.87445 precCharge: 2 Sequence: DMELSFAVQR
Intensity
2e+05
1e+05
0e+00
M/Z
500
1000
1500
y1
y2
b1
b2
y4
b3
y6
b4
b5
y7
b6
y8
b7
y9
b8
b9
type
b
y

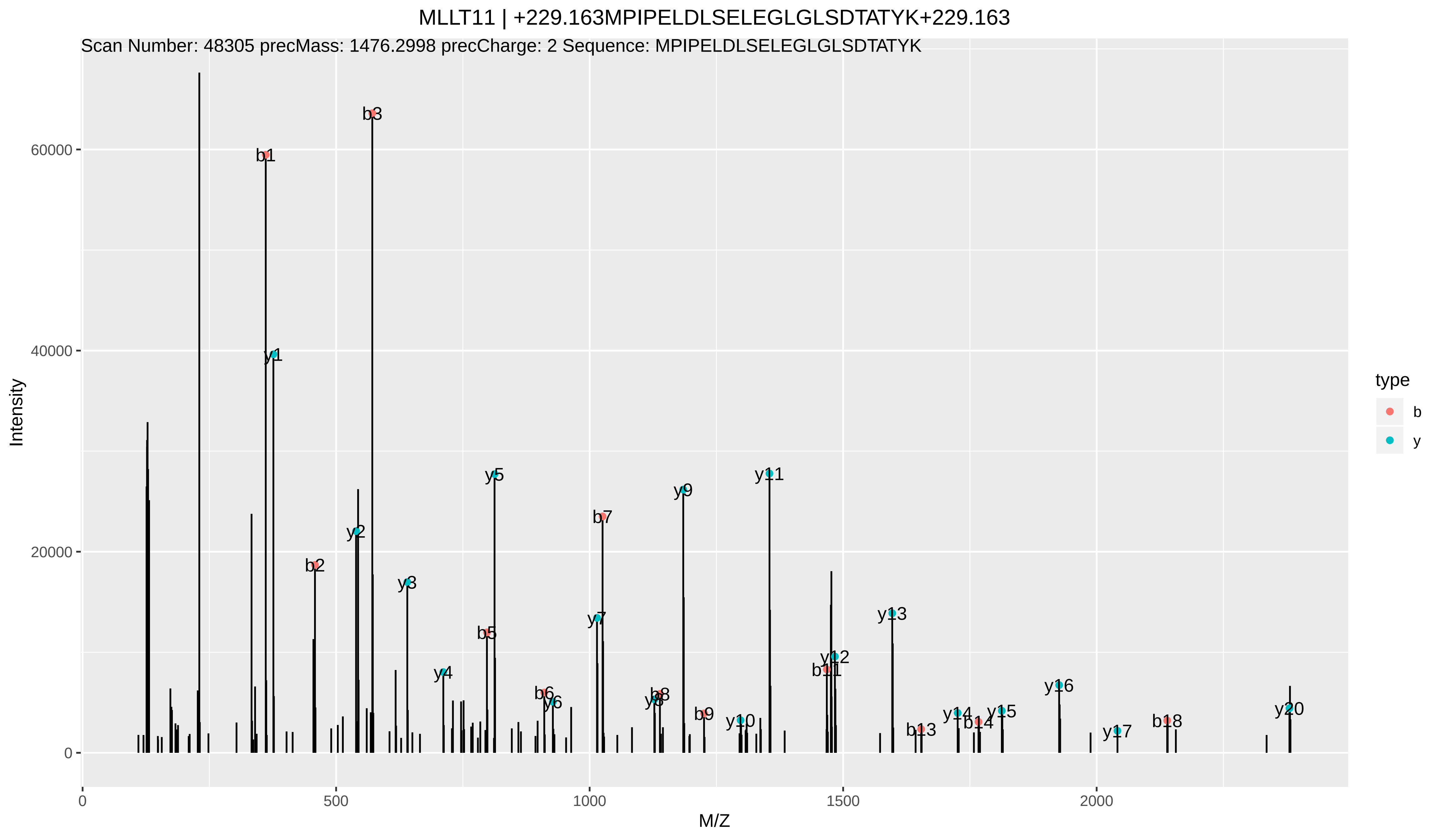

MLLT11 | +229.163MPIPELDLSELEGLGLSDTATYK+229.163
Scan Number: 48305 precMass: 1476.2998 precCharge: 2 Sequence: MPIPELDLSELEGLGLSDTATYK
Intensity
M/Z
0
20000
40000
60000
0
500
1000
1500
2000
type
b
y
b1
b2
b3
b5
b6
b7
b8
b9
b11
b13
b14
b18
y1
y2
y3
y4
y5
y6
y7
y8
y9
y10
y11
y12
y13
y14
y15
y16
y17
y20

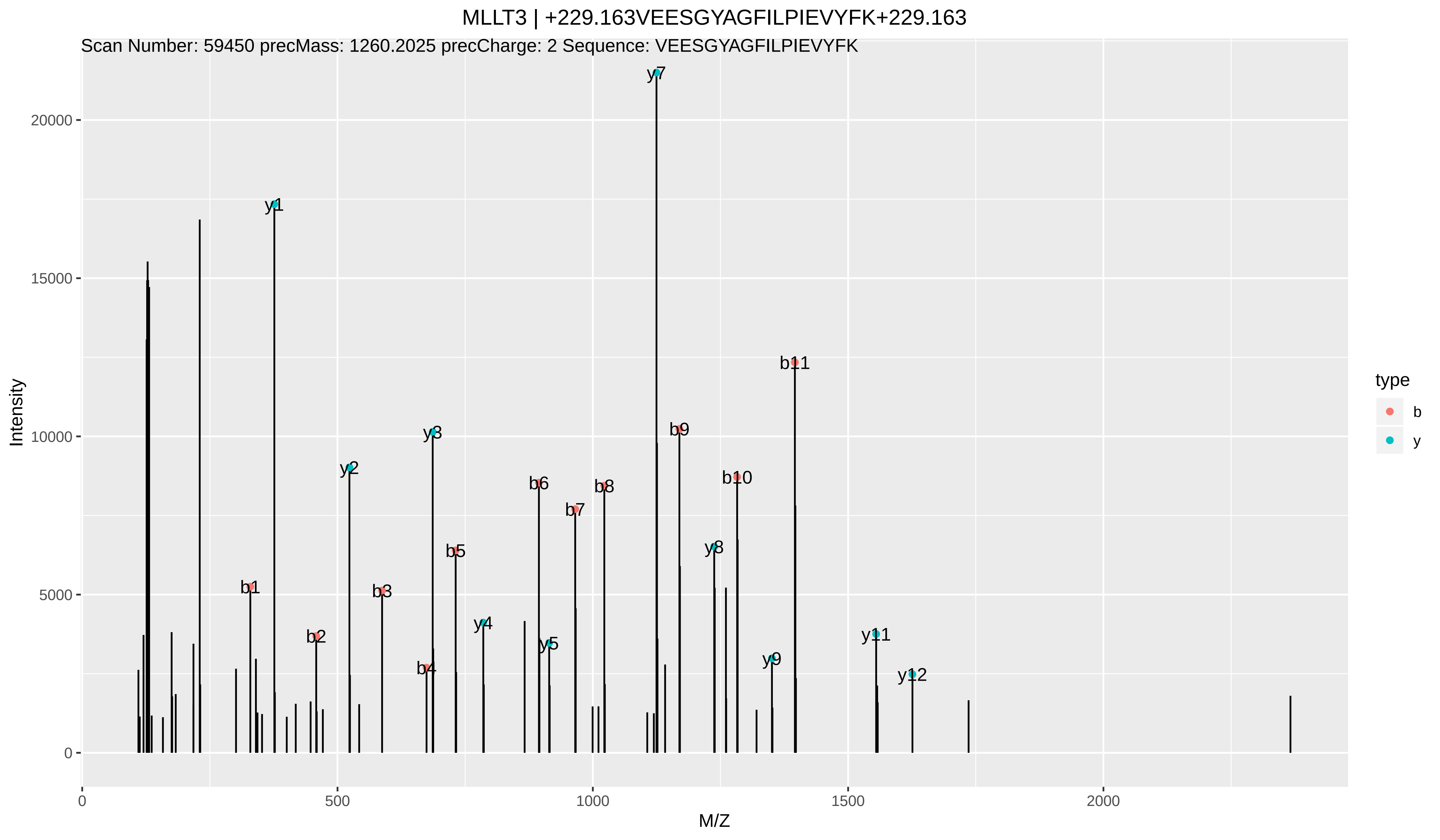
MLLT3 | +229.163VEESGYAGFILPIEVYFK+229.163
Scan Number: 59450 precMass: 1260.2025 precCharge: 2 Sequence: VEESGYAGFILPIEVYFK
Intensity
M/Z
type
b
y
y1
y2
y3
y4
y5
y7
y8
y9
y11
y12
b1
b2
b3
b4
b5
b6
b7
b8
b9
b10
b11
0
5000
10000
15000
20000
0
500
1000
1500
2000

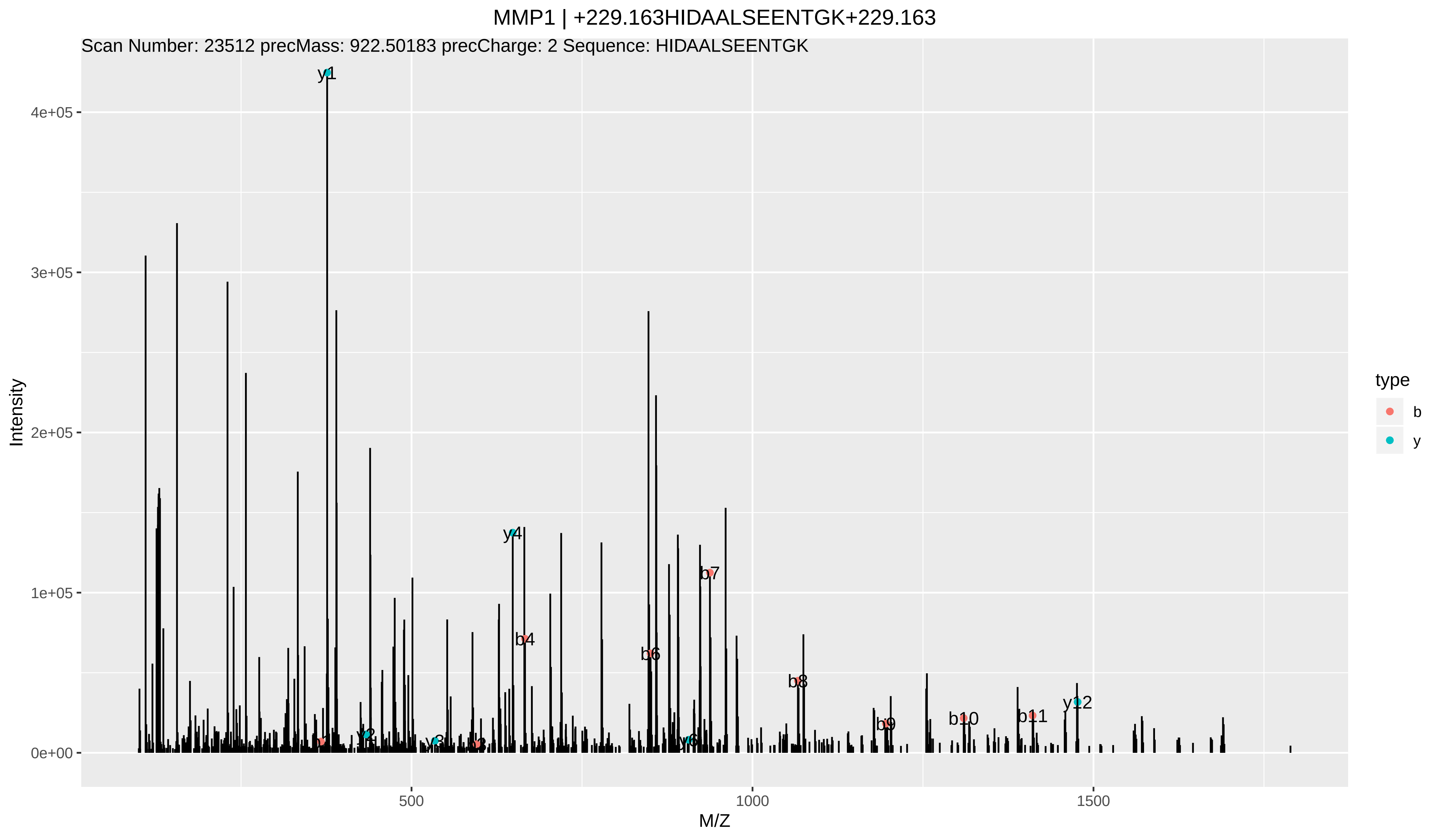

MMP1 | +229.163HIDAALSEENTGK+229.163
Scan Number: 23512 precMass: 922.50183 precCharge: 2 Sequence: HIDAALSEENTGK
y1
y4
b7
b4
b6
b8
y12
b10
b11
b9
y2
y3
y6
Intensity
4e+05
3e+05
2e+05
1e+05
0e+00
500
1000
1500
M/Z
type
b
y

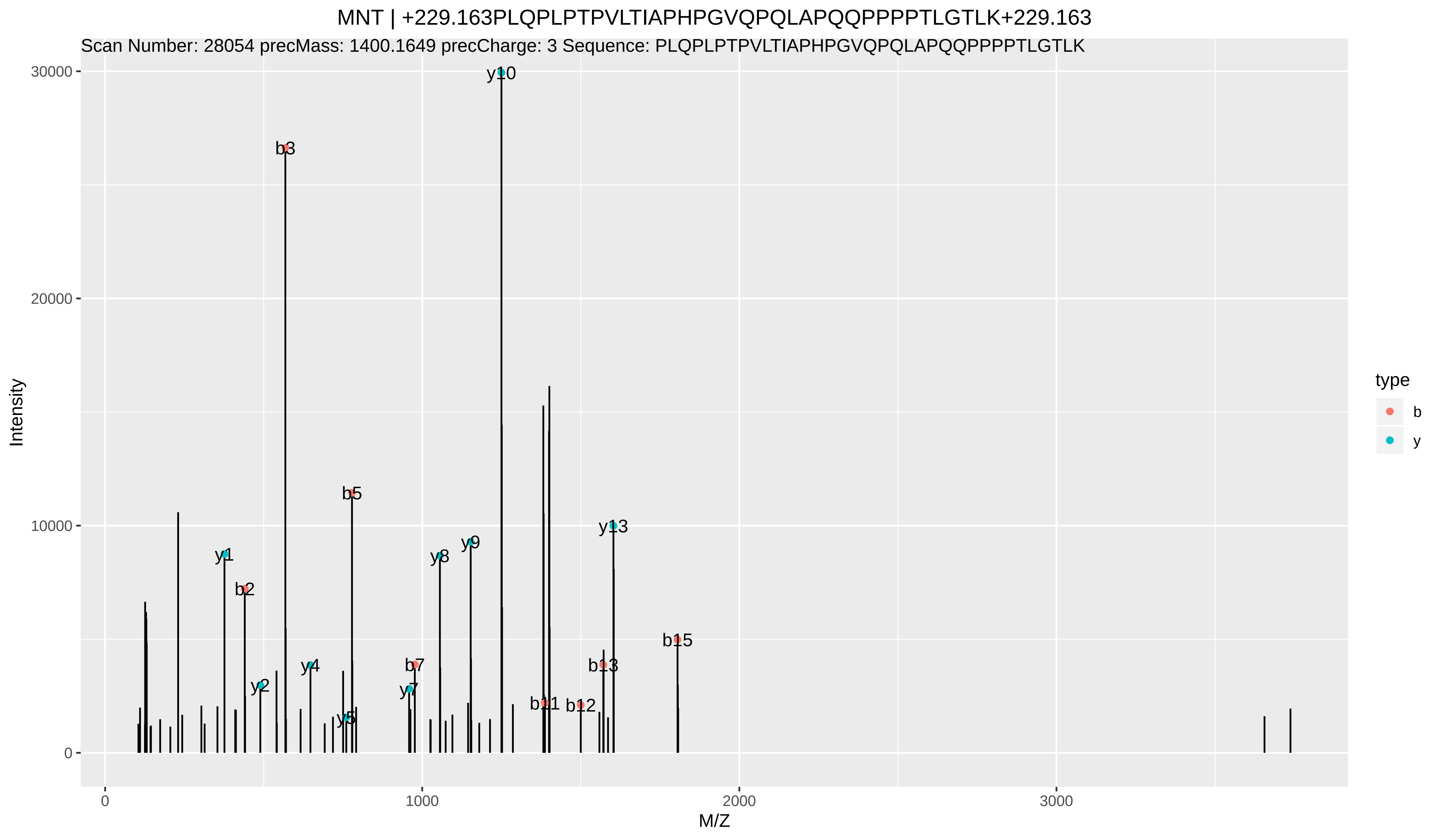

MNT | +229.163PLQPLPTPVLTIAPHPGVQPQLAPQQPPPPTLGTLK+229.163
Scan Number: 28054 precMass: 1400.1649 precCharge: 3 Sequence: PLQPLPTPVLTIAPHPGVQPQLAPQQPPPPTLGTLK
Intensity
M/Z
0
10000
20000
30000
0
1000
2000
3000
y10
b3
b5
y13
y9
y8
y1
b2
b15
y4
b7
b13
y2
y7
b11
b12
y5
type
b
y

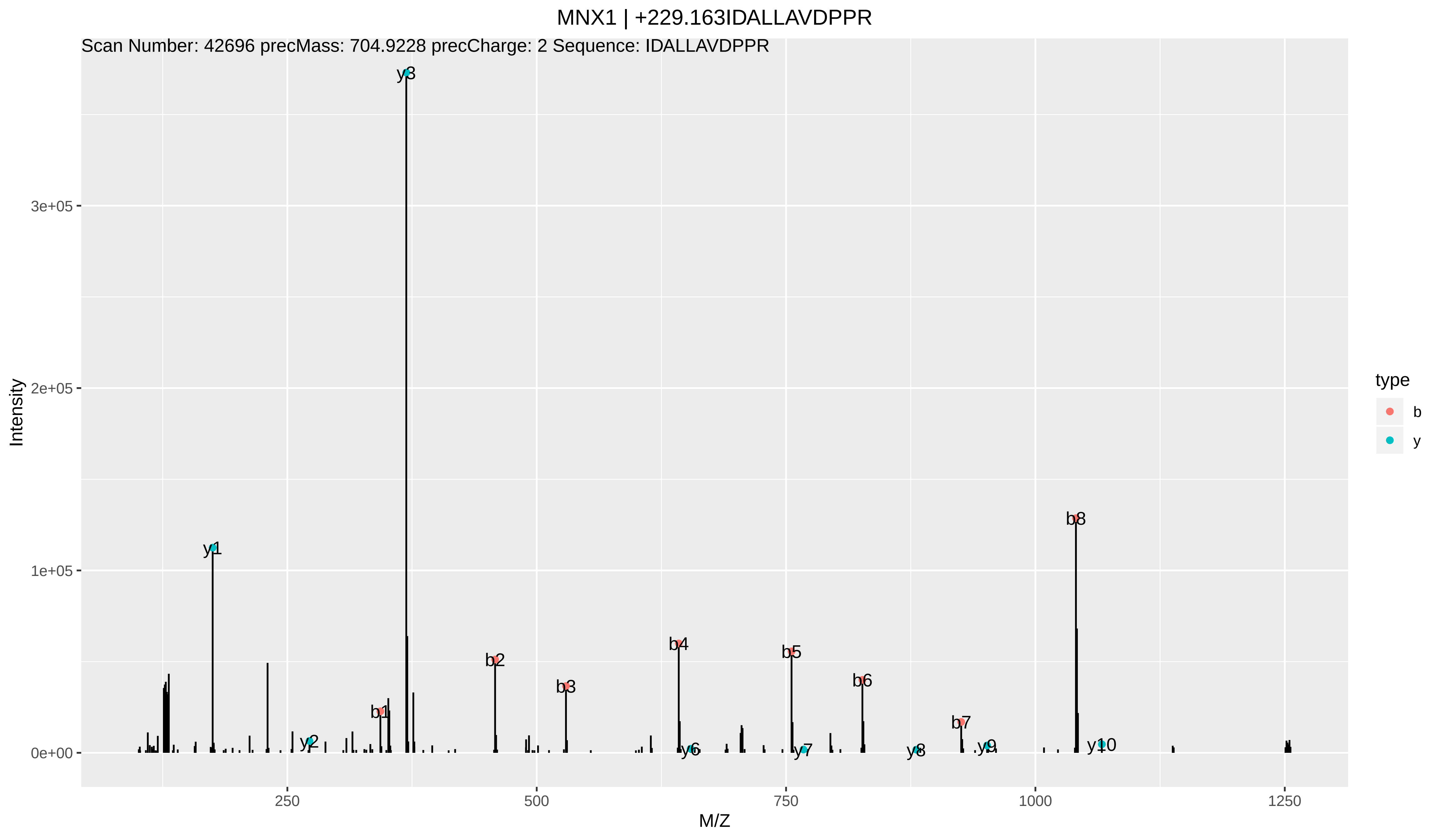

MNX1 | +229.163IDALLAVDPPR
Scan Number: 42696 precMass: 704.9228 precCharge: 2 Sequence: IDALLAVDPPR
Intensity
M/Z
type
b
y
y1
y2
y3
y6
y7
y8
y9
y10
b1
b2
b3
b4
b5
b6
b7
b8
3e+05
2e+05
1e+05
0e+00
250
500
750
1000
1250

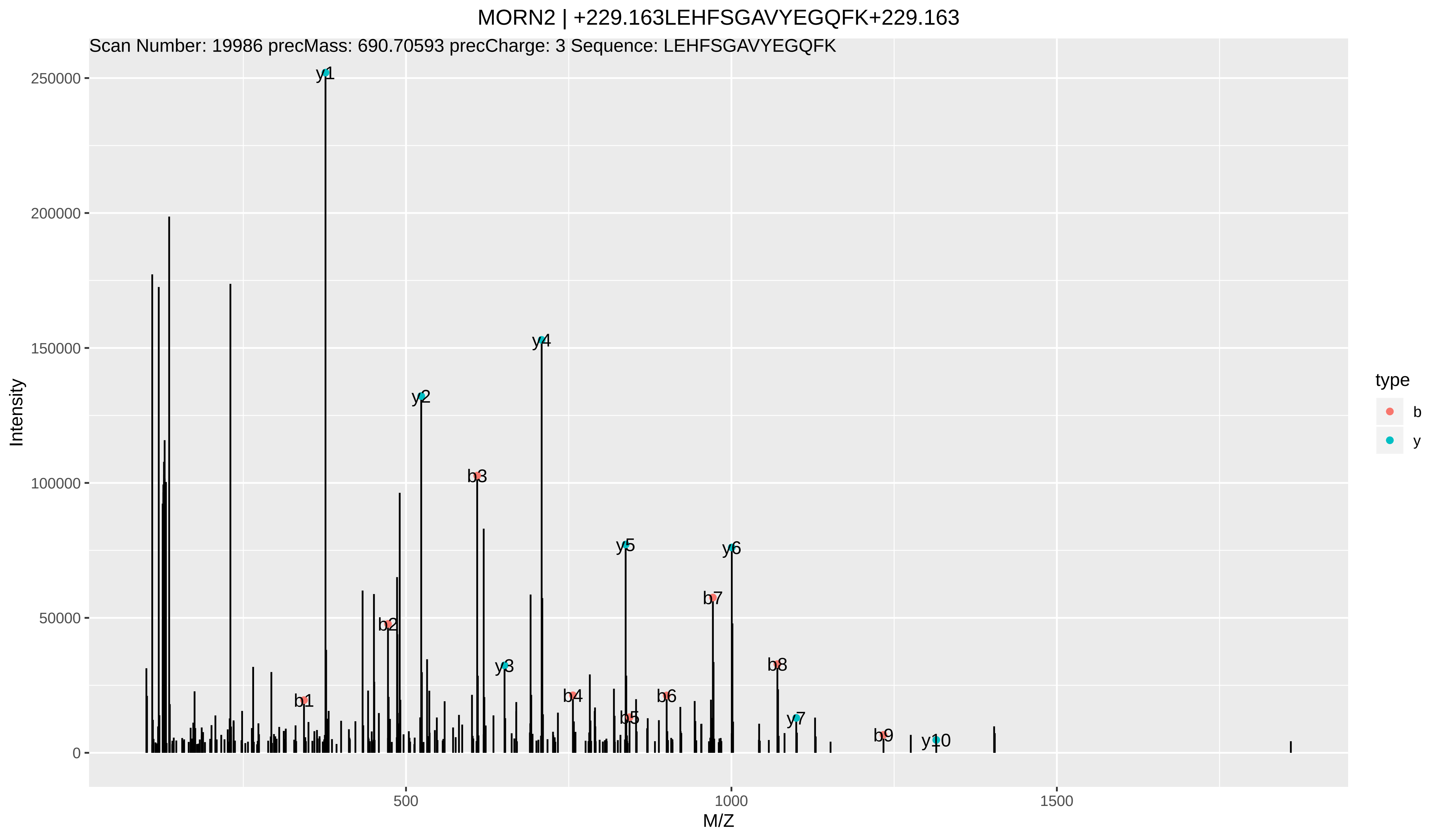
MORN2 | +229.163LEHFSGAVYEGQFK+229.163
Scan Number: 19986 precMass: 690.70593 precCharge: 3 Sequence: LEHFSGAVYEGQFK
Intensity
250000
200000
150000
100000
50000
0
M/Z
500
1000
1500
y1
y2
y4
b3
y5
y6
b7
b2
y3
b8
b1
b4
b6
b5
y7
b9
y10
type
b
y

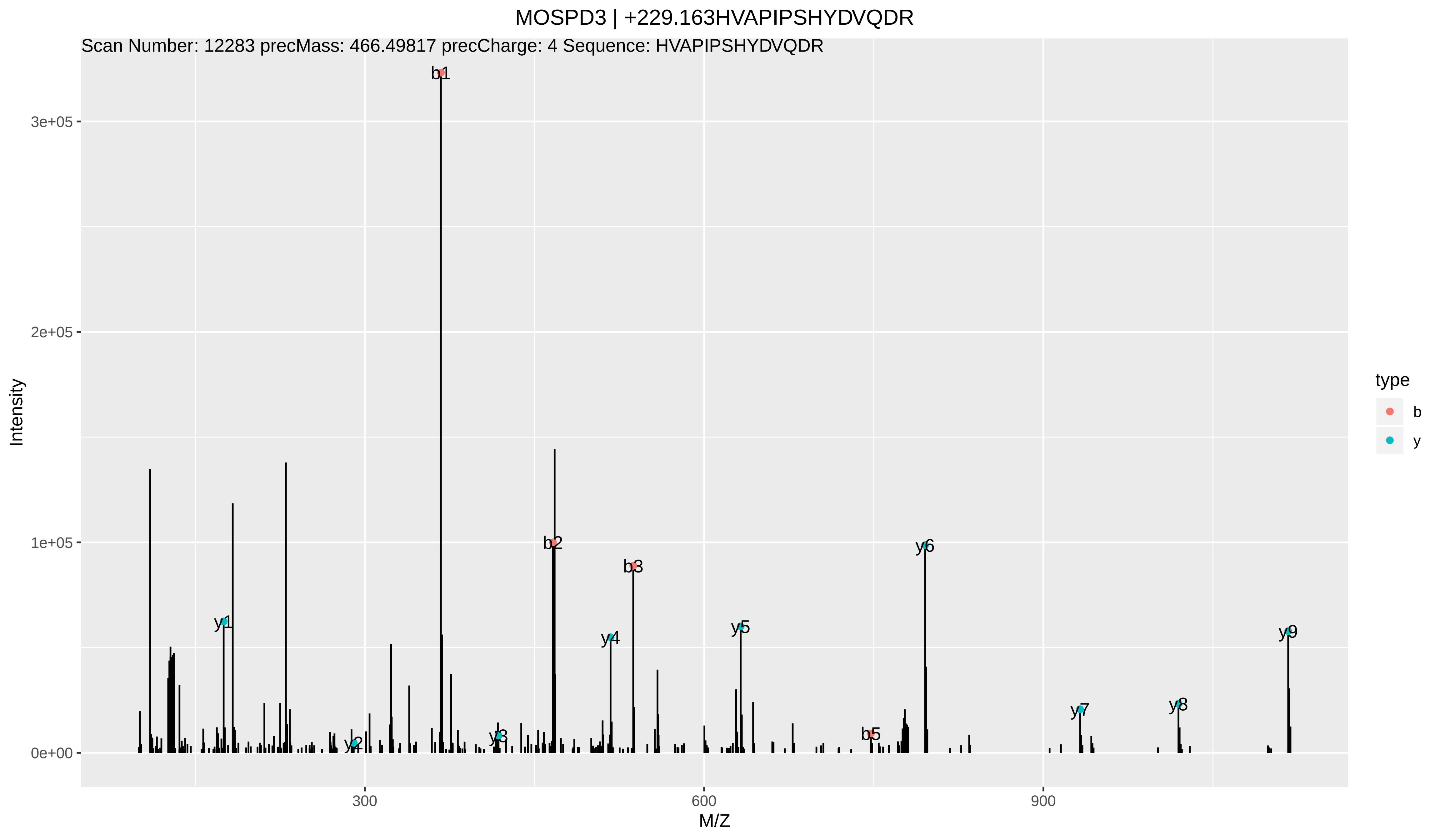
MOSPD3 | +229.163HVAPIPSHYDVQDR
Scan Number: 12283 precMass: 466.49817 precCharge: 4 Sequence: HVAPIPSHYDVQDR
Intensity
M/Z
0e+00
1e+05
2e+05
3e+05
300
600
900
b1
b2
b3
b5
y1
y2
y3
y4
y5
y6
y7
y8
y9
type
b
y

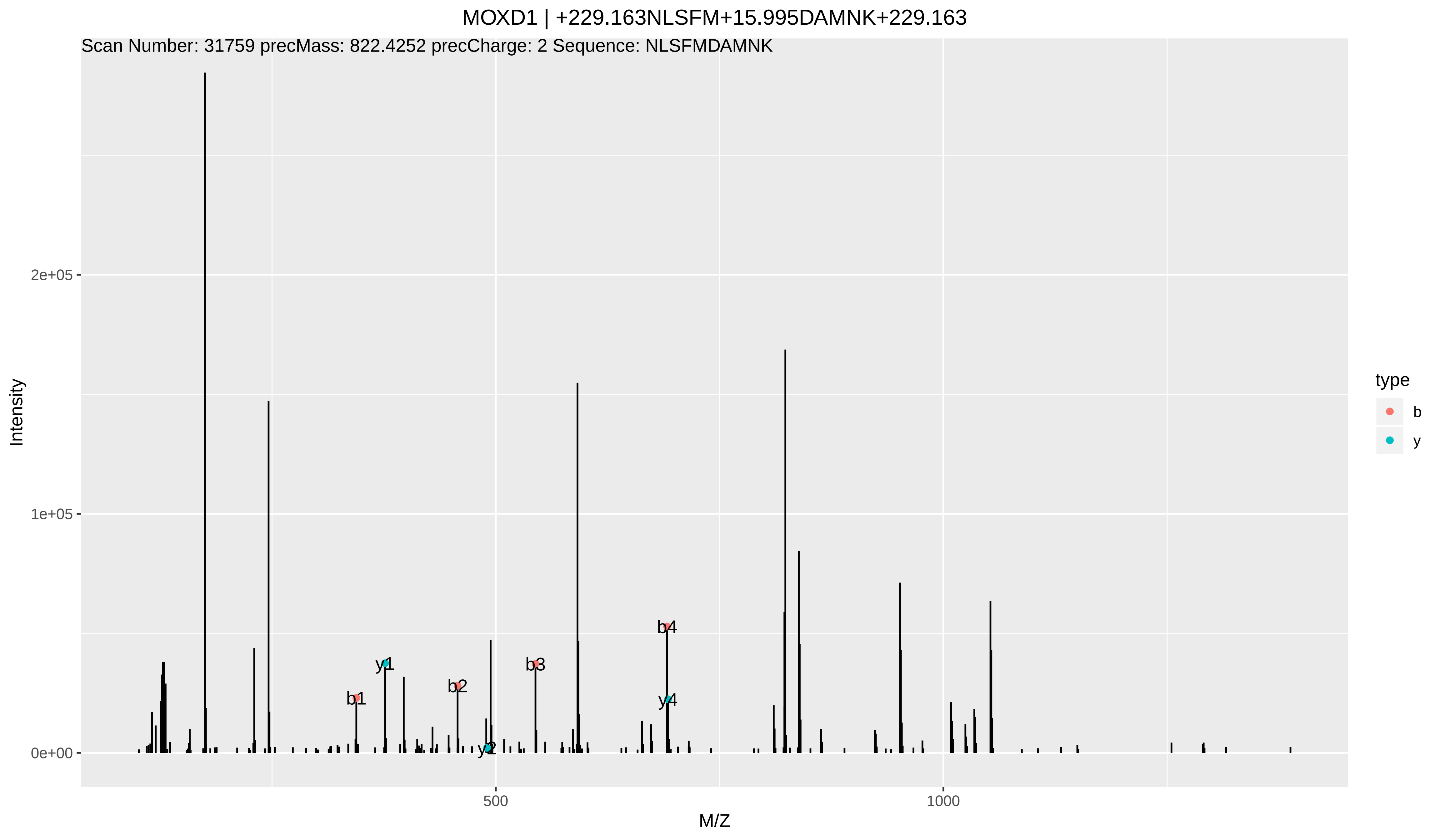
MOXD1 | +229.163NLSFM+15.995DAMNK+229.163
Scan Number: 31759 precMass: 822.4252 precCharge: 2 Sequence: NLSFMDAMNK
Intensity
2e+05
1e+05
0e+00
M/Z
500
1000
b1
y1
b2
y2
b3
b4
y4
type
b
y

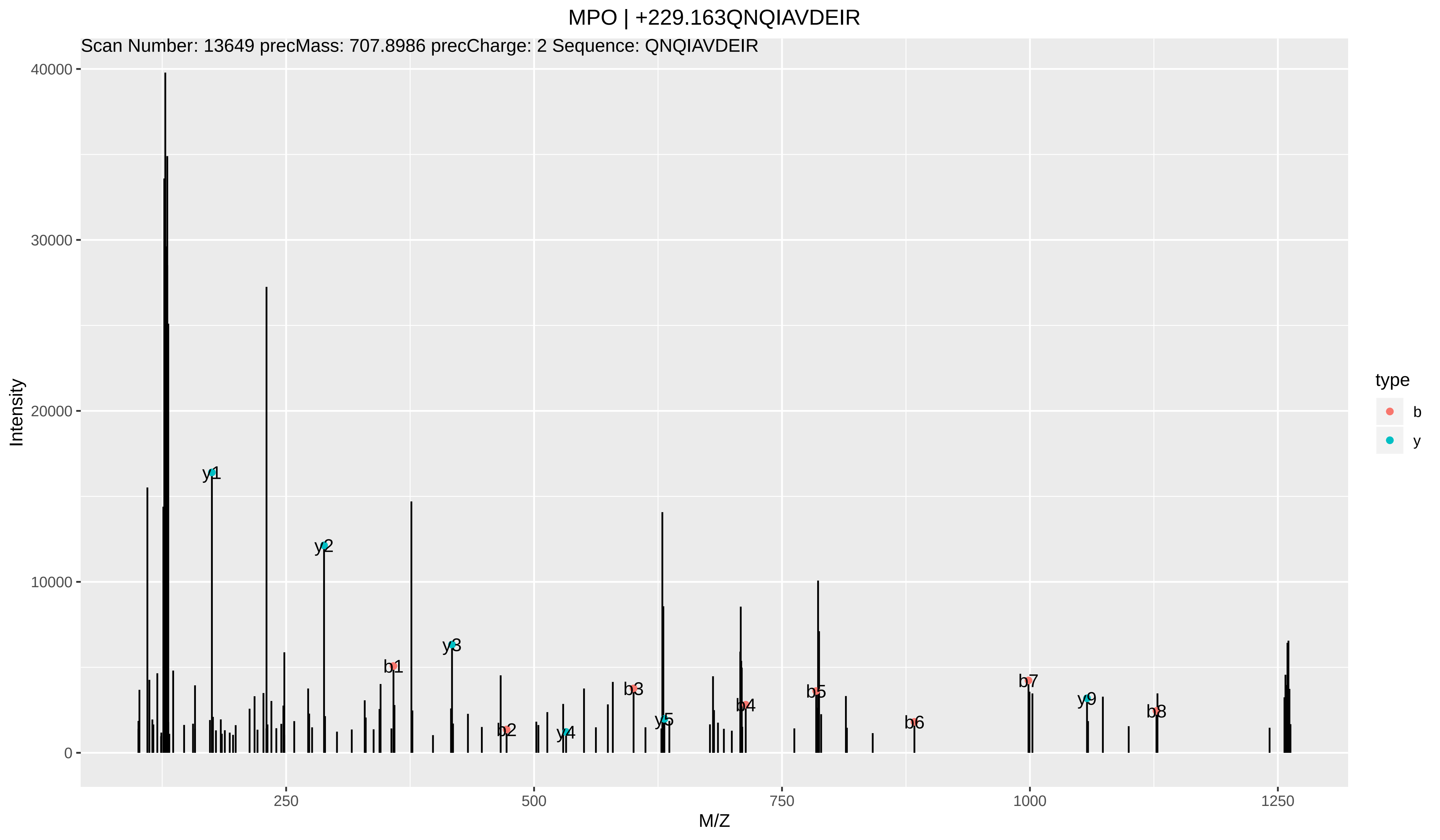

MPO | +229.163QNQIAVDEIR
Scan Number: 13649 precMass: 707.8986 precCharge: 2 Sequence: QNQIAVDEIR
Intensity
40000
30000
20000
10000
0
250
500
750
1000
1250
M/Z
y1
y2
b1
y3
b2
y4
b3
y5
b4
b5
b6
b7
y9
b8
type
b
y

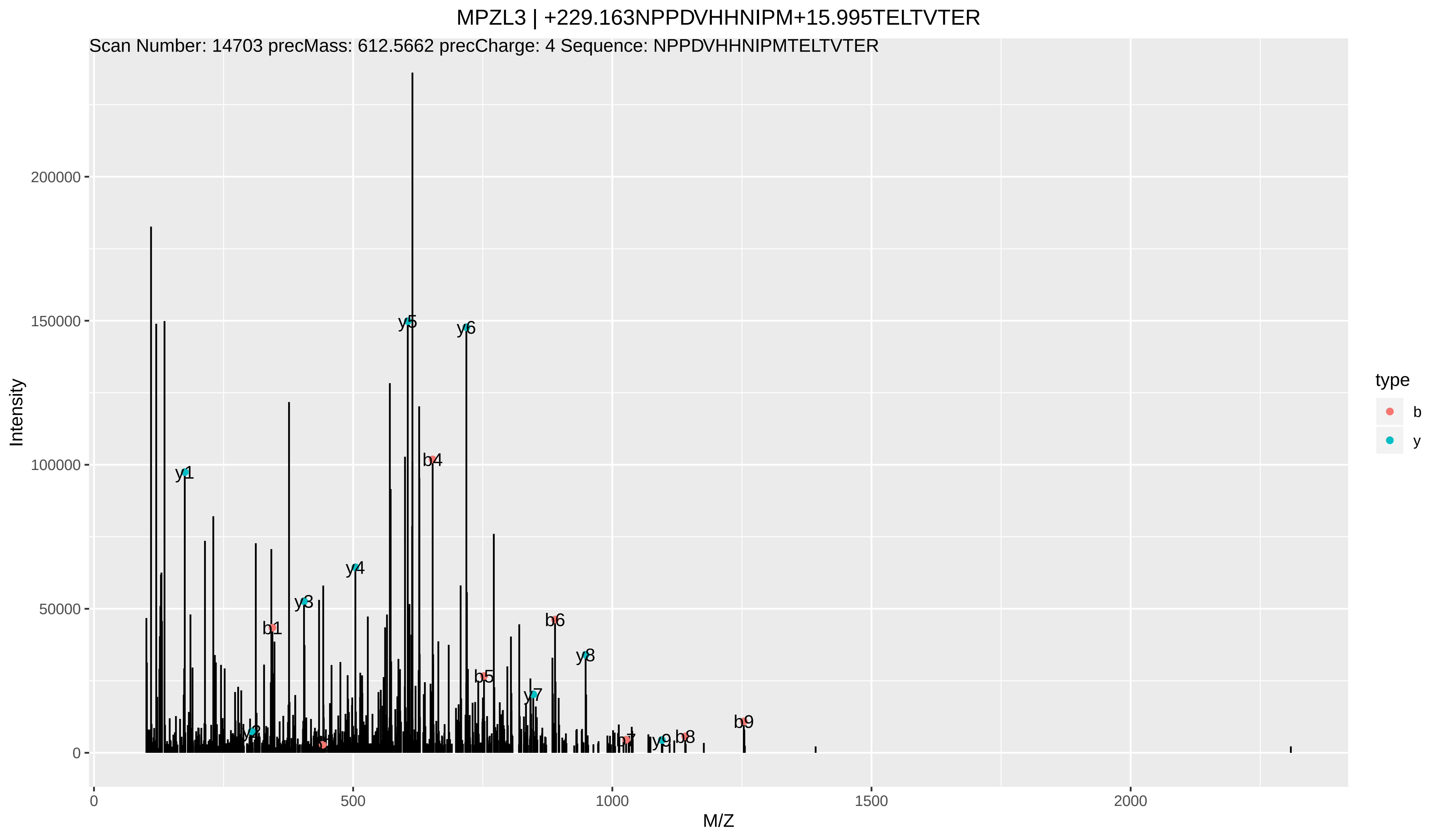

MPZL3 | +229.163NPPDVHHNIPM+15.995TELTVTER
Scan Number: 14703 precMass: 612.5662 precCharge: 4 Sequence: NPPDVHHNIPMTELTVTER
Intensity
M/Z
0
50000
100000
150000
200000
0
500
1000
1500
2000
y1
y2
y3
y4
y5
y6
y7
y8
y9
b1
b4
b5
b6
b7
b8
b9
type
b
y

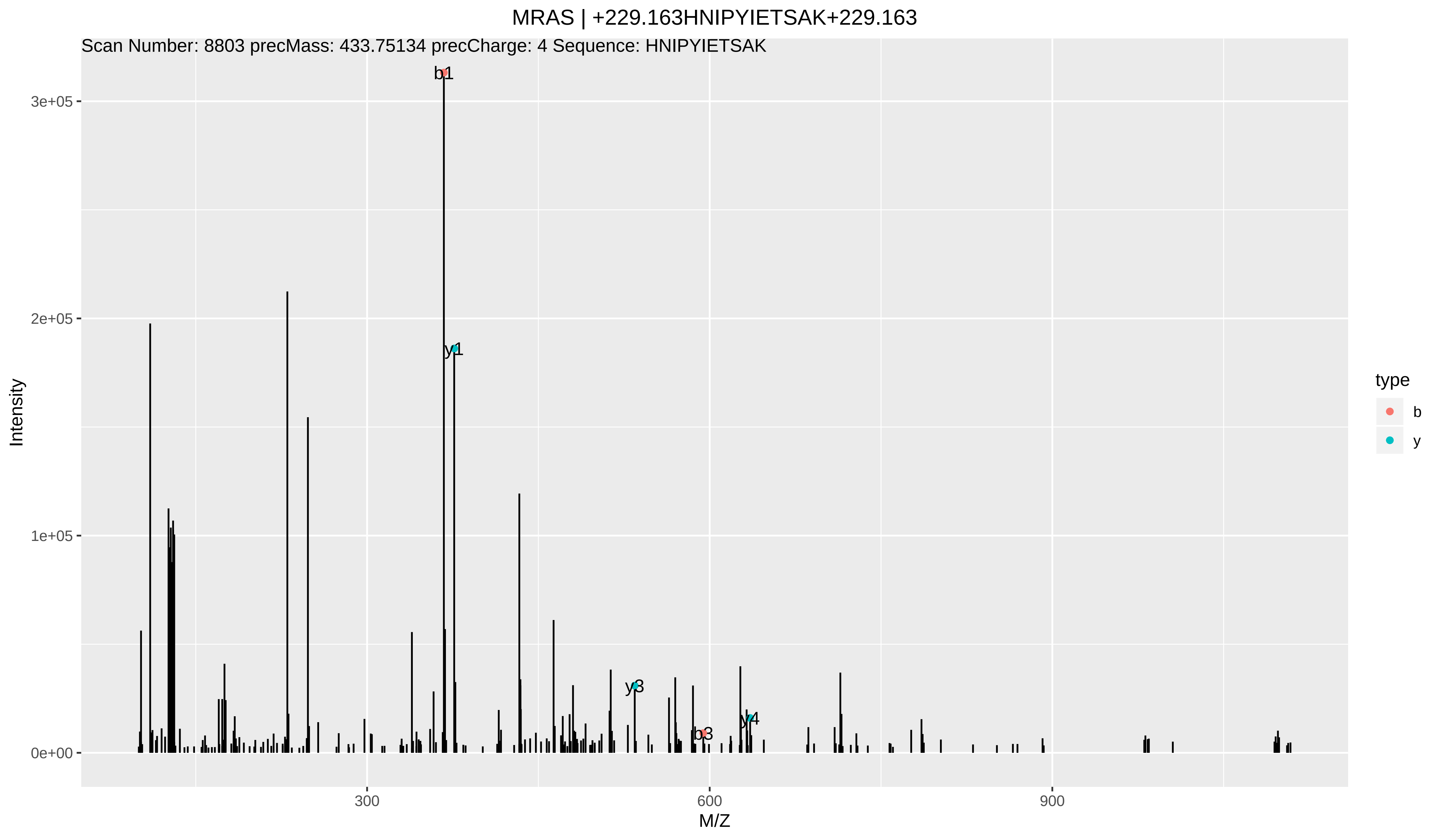

MRAS | +229.163HNIPYIETSAK+229.163
Scan Number: 8803 precMass: 433.75134 precCharge: 4 Sequence: HNIPYIETSAK
b1
y1
y3
b3
y4
Intensity
3e+05
2e+05
1e+05
0e+00
300
600
900
M/Z
type
b
y

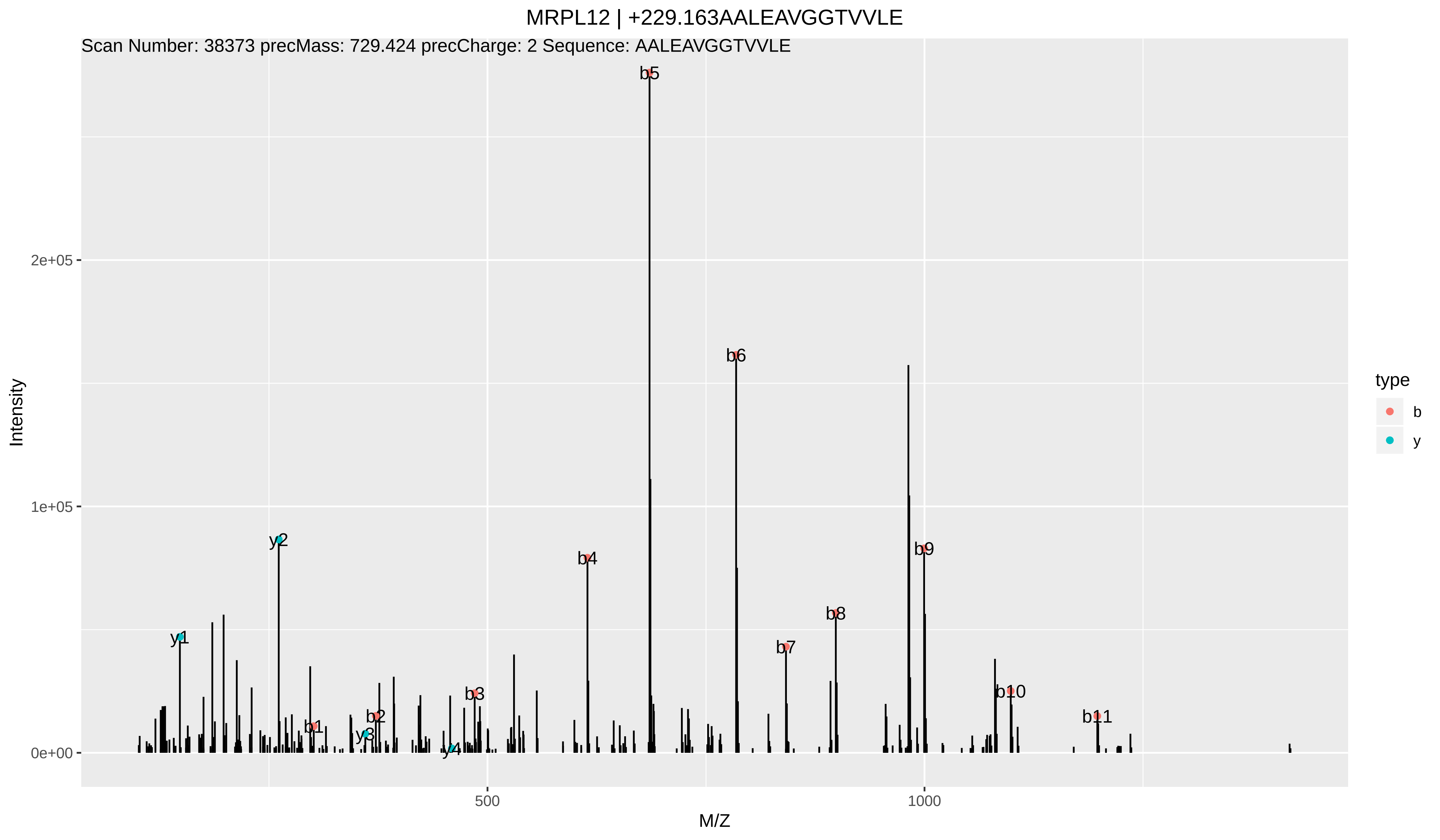

MRPL12 | +229.163AALEAVGGTVVLE
Scan Number: 38373 precMass: 729.424 precCharge: 2 Sequence: AALEAVGGTVVLE
Intensity
M/Z
type
b
y
2e+05
1e+05
0e+00
500
1000
b5
b6
b9
b4
y2
b8
y1
b7
b3
b10
b2
b11
b1
y3
y4

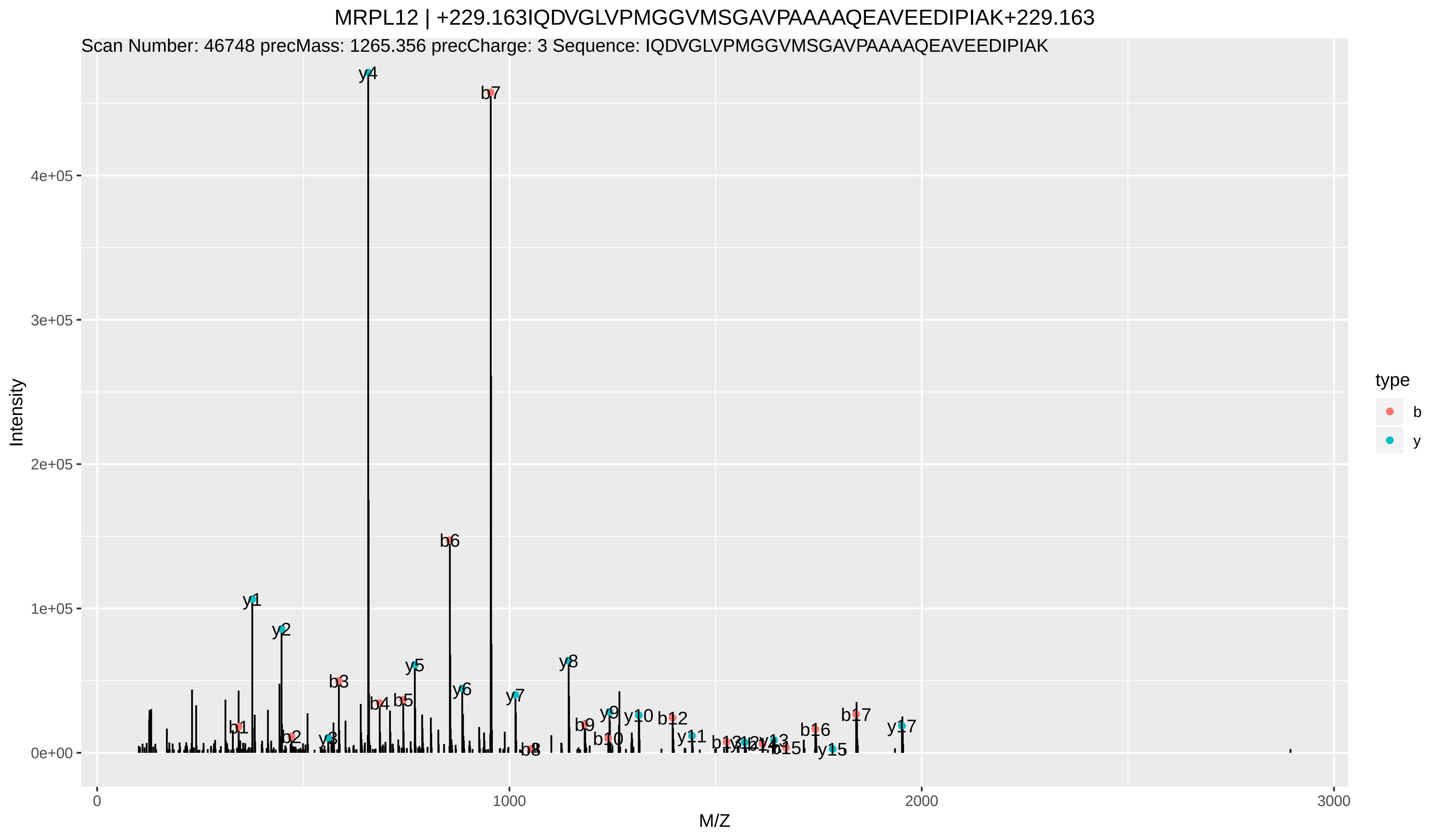
MRPL12 | +229.163IQDVGLVPMGGVMSGAVPAAAAQEAVEEDIPIAK+229.163
Scan Number: 46748 precMass: 1265.356 precCharge: 3 Sequence: IQDVGLVPMGGVMSGAVPAAAAQEAVEEDIPIAK
Intensity
M/Z
type
b
y
y4
b7
b6
y1
y2
y5
y8
b3
y6
y7
b4
b5
y9
y10
b12
b1
b2
y3
b9
b10
y11
b16
b17
y17
b15
y15
b8
0e+00
1e+05
2e+05
3e+05
4e+05
0
1000
2000
3000

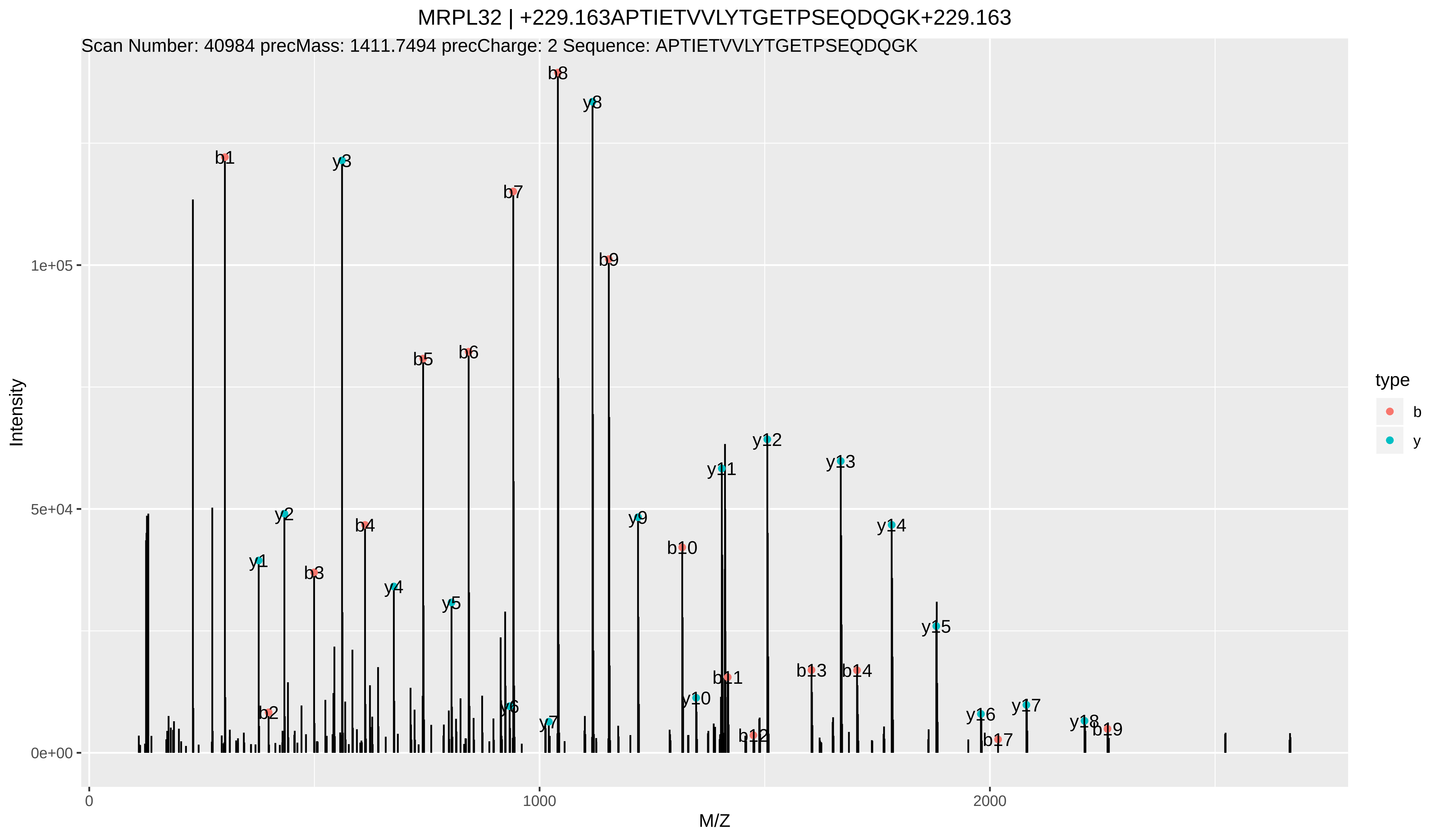

MRPL32 | +229.163APTIETVVLYTGETPSEQDQGK+229.163
Scan Number: 40984 precMass: 1411.7494 precCharge: 2 Sequence: APTIETVVLYTGETPSEQDQGK
Intensity
M/Z
0e+00
5e+04
1e+05
0
1000
2000
type
b
y
b1
b2
b3
b4
b5
b6
b7
b8
b9
b10
b11
b12
b13
b14
b17
b19
y1
y2
y3
y4
y5
y6
y7
y8
y9
y10
y11
y12
y13
y14
y15
y16
y17
y18

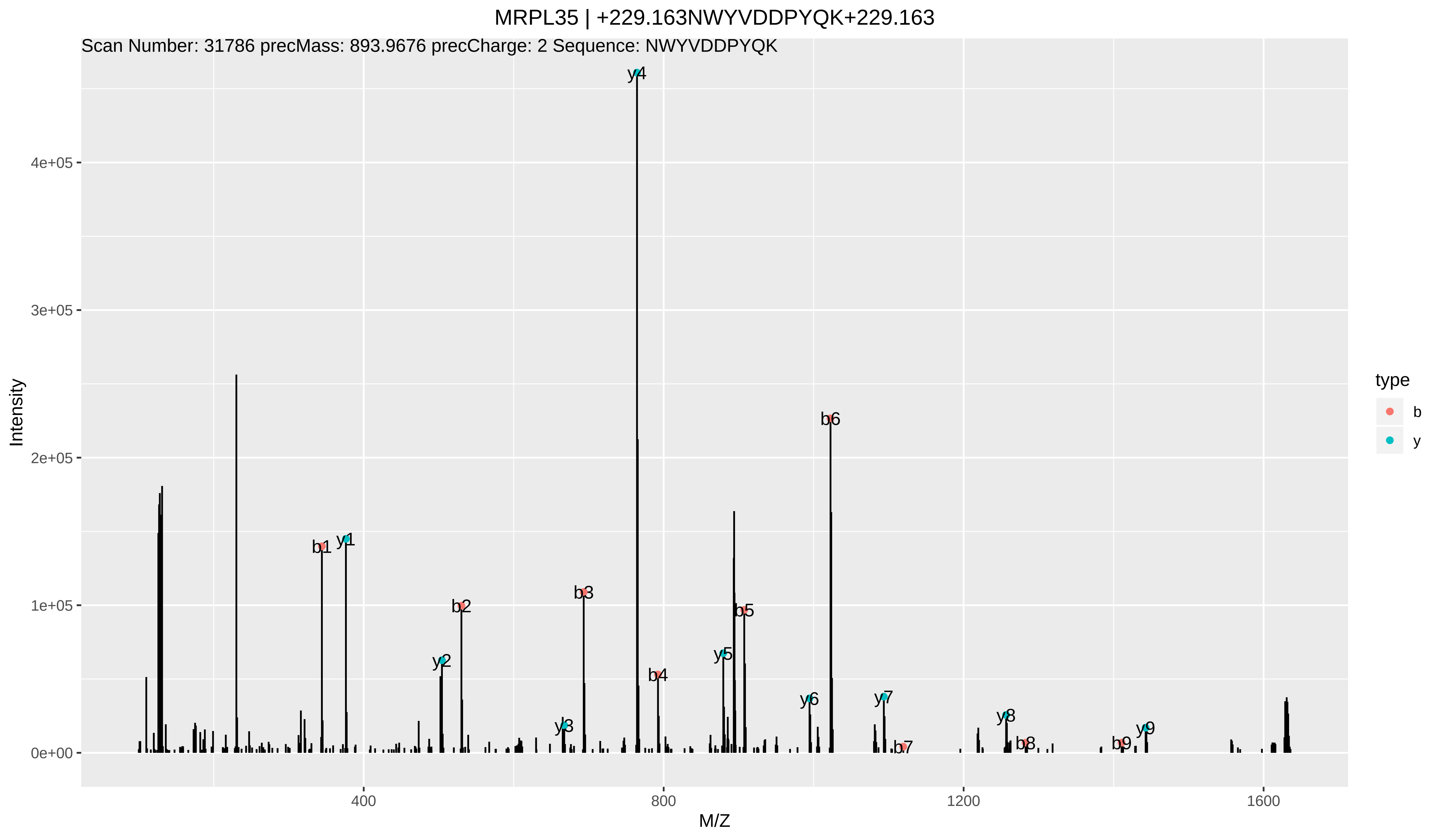

MRPL35 | +229.163NWYVDDPYQK+229.163
Scan Number: 31786 precMass: 893.9676 precCharge: 2 Sequence: NWYVDDPYQK
Intensity
M/Z
type
b
y
y4
b6
b1
y1
b2
b3
b5
y2
y5
b4
y6
y7
y3
y8
y9
b8
b9
b7
4e+05
3e+05
2e+05
1e+05
0e+00
400
800
1200
1600

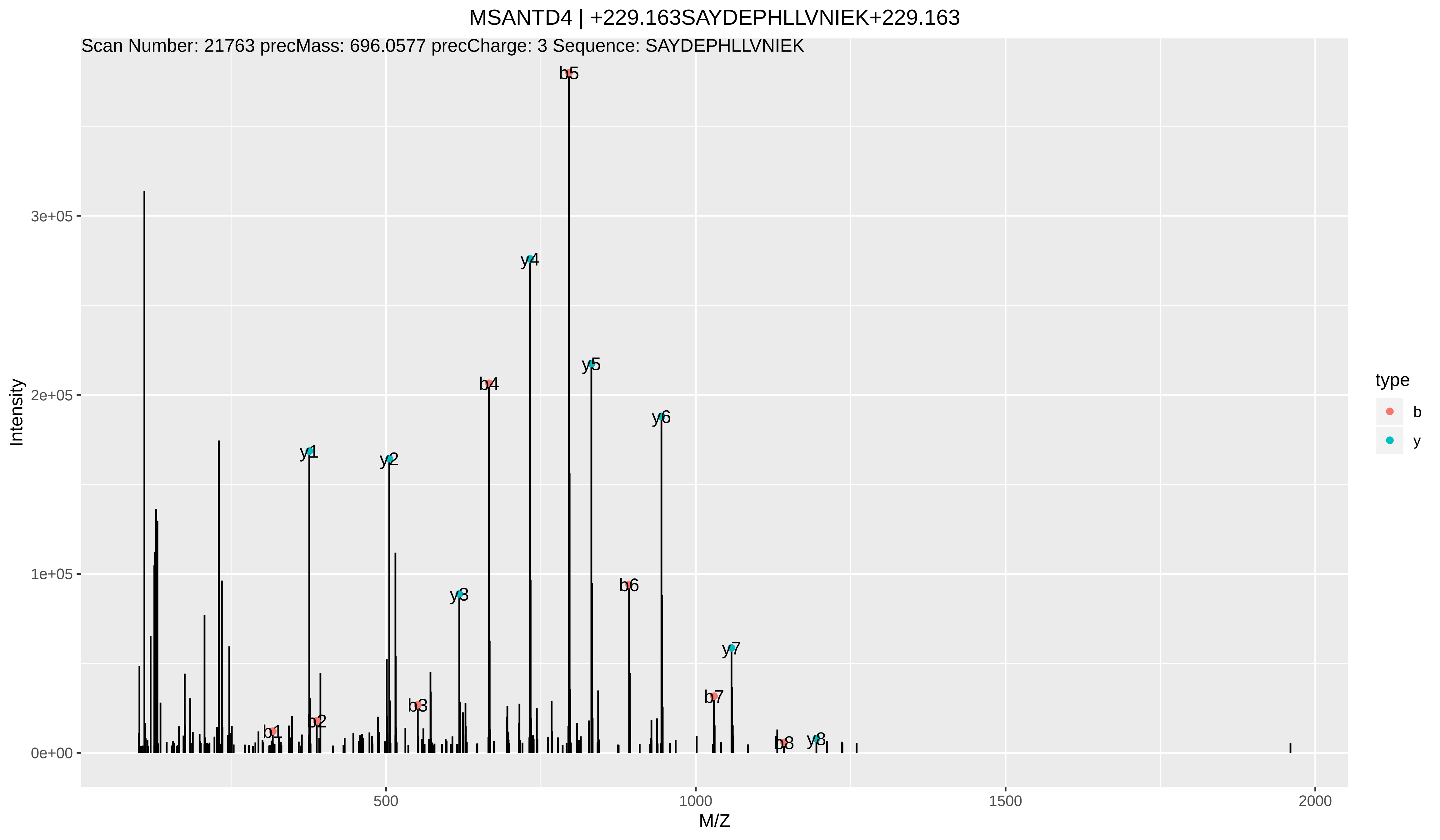
MSANTD4 | +229.163SAYDEPHLLVNIEK+229.163
Scan Number: 21763 precMass: 696.0577 precCharge: 3 Sequence: SAYDEPHLLVNIEK
Intensity
M/Z
type
b
y
b1
b2
b3
b4
b5
b6
b7
b8
y1
y2
y3
y4
y5
y6
y7
y8
0e+00
1e+05
2e+05
3e+05
500
1000
1500
2000

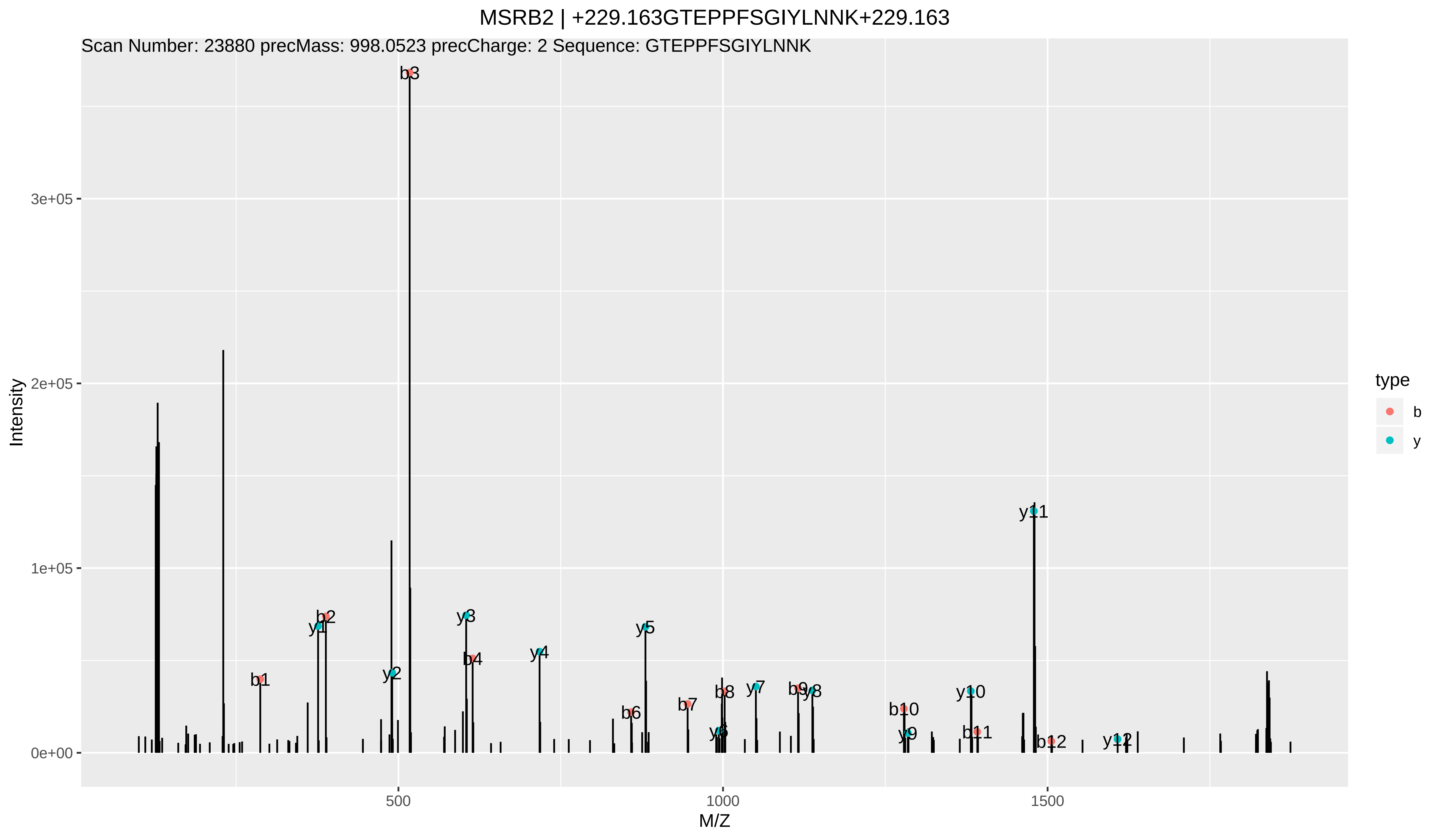
MSRB2 | +229.163GTEPPFSGIYLNNK+229.163
Scan Number: 23880 precMass: 998.0523 precCharge: 2 Sequence: GTEPPFSGIYLNNK
Intensity
3e+05
2e+05
1e+05
0e+00
500
1000
1500
M/Z
b3
b1
y1
b2
y2
y3
b4
y4
y5
b6
b7
y6
b8
y7
b9
y8
b10
y9
y10
b11
y11
b12
y12
type
b
y

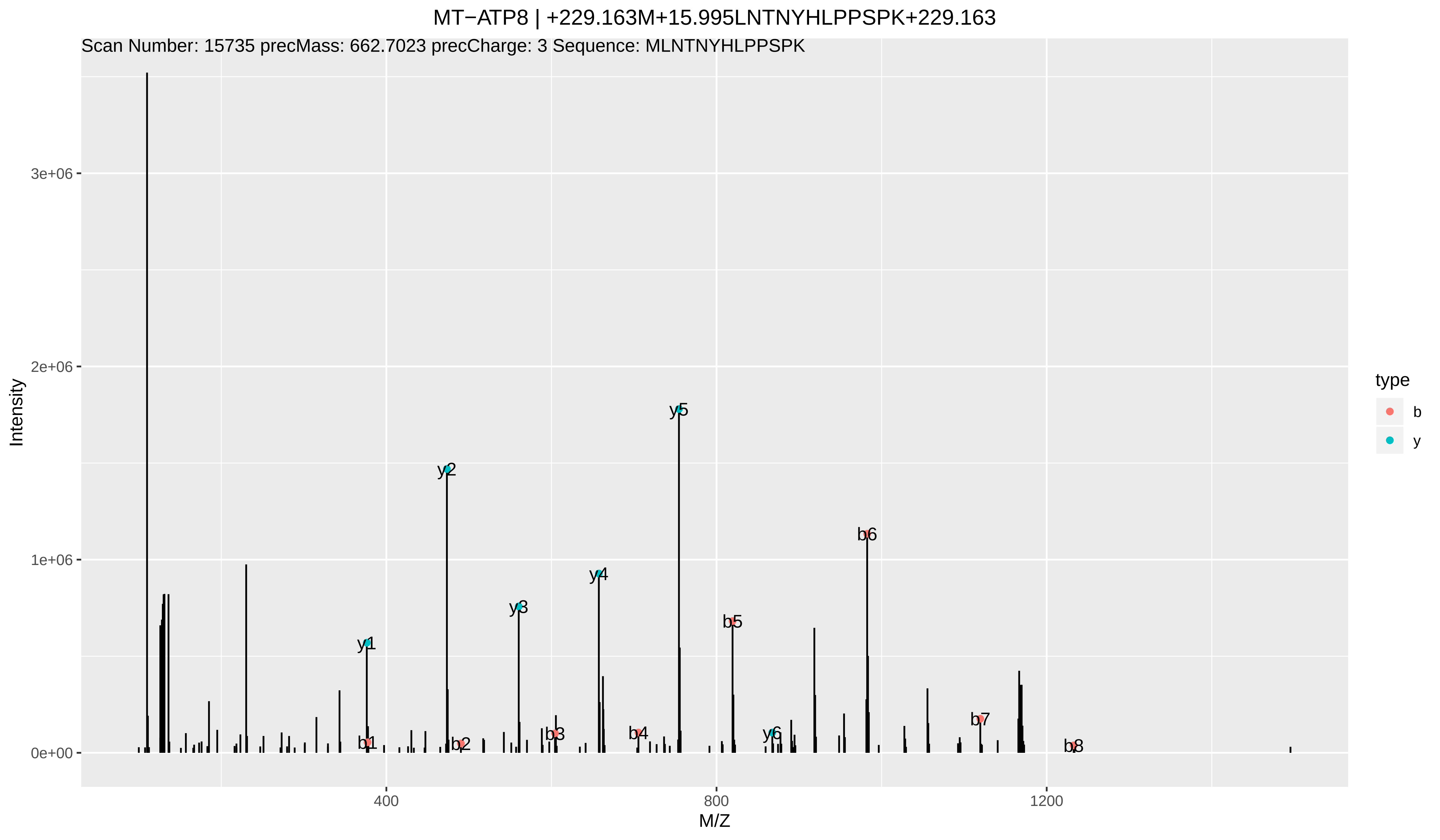

MT−ATP8 | +229.163M+15.995LNTNYHLPPSPK+229.163
Scan Number: 15735 precMass: 662.7023 precCharge: 3 Sequence: MLNTNYHLPPSPK
Intensity
3e+06
2e+06
1e+06
0e+00
M/Z
400
800
1200
y1
y2
y3
y4
y5
y6
b1
b2
b3
b4
b5
b6
b7
b8
type
b
y

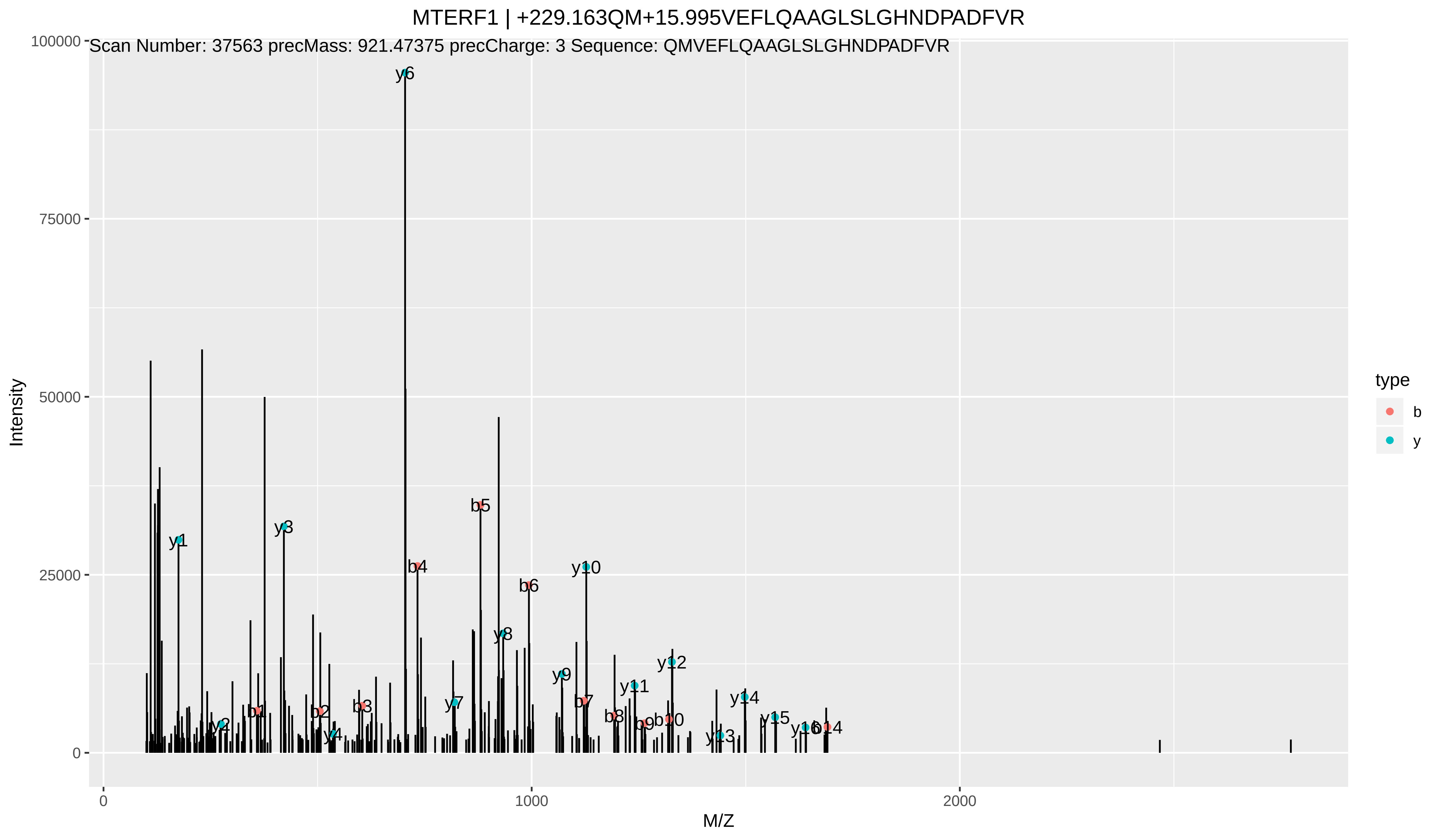
MTERF1 | +229.163QM+15.995VEFLQAAGLSLGHNDPADFVR
Scan Number: 37563 precMass: 921.47375 precCharge: 3 Sequence: QMVEFLQAAGLSLGHNDPADFVR
Intensity
M/Z
0
25000
50000
75000
100000
0
1000
2000
type
b
y
y6
b5
y3
y1
b4
y10
b6
y8
y12
y9
y11
b7
y14
y7
b3
b8
b2
b1
y15
b9
b10
y16
b14
y2
y4
y13

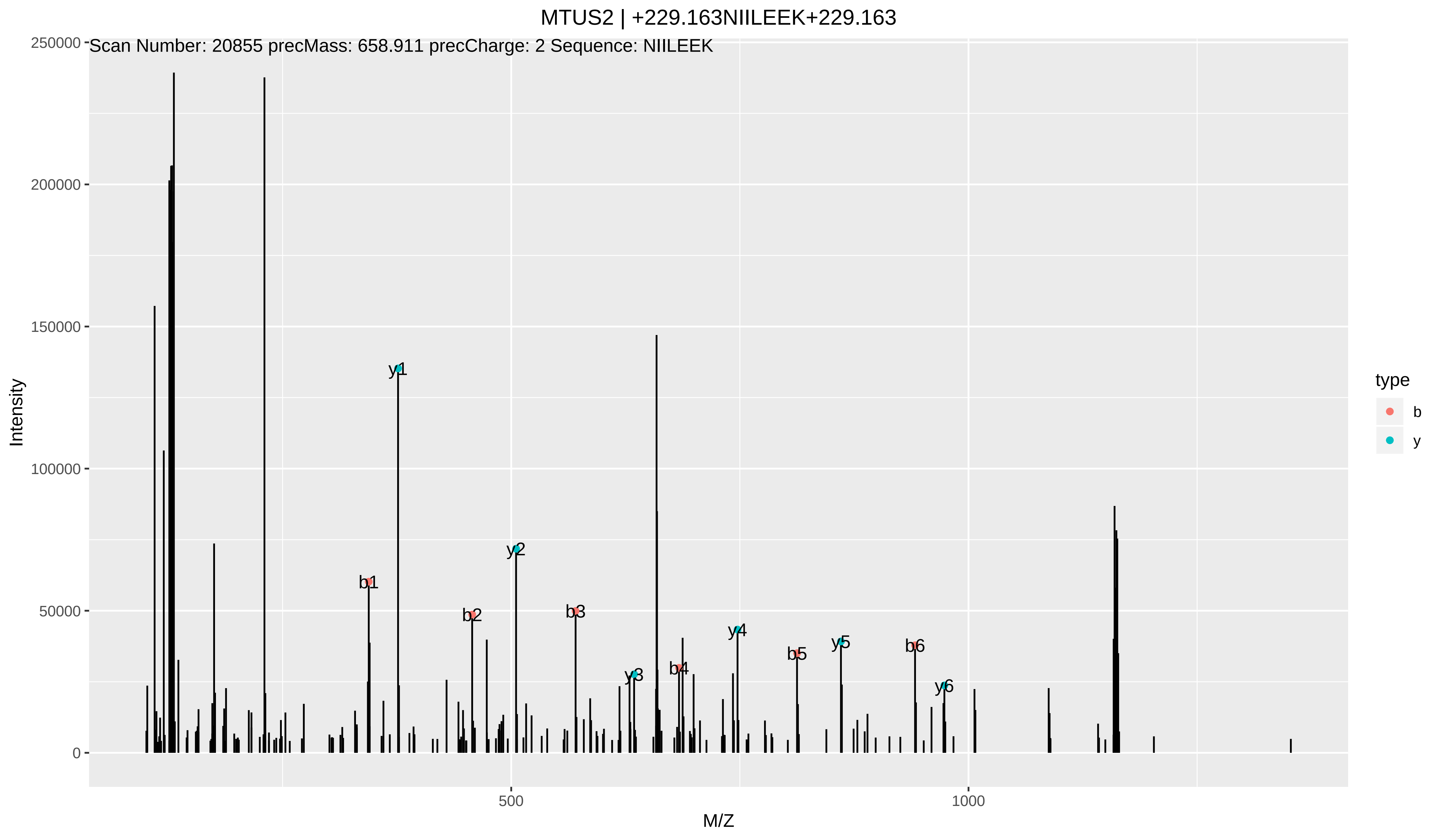

MTUS2 | +229.163NIILEEK+229.163
Scan Number: 20855 precMass: 658.911 precCharge: 2 Sequence: NIILEEK
Intensity
250000
200000
150000
100000
50000
0
M/Z
500
1000
y1
y2
b1
b2
b3
y3
b4
y4
b5
y5
b6
y6
type
b
y

MUC20 | +229.163ASTPAGPVPEAETR

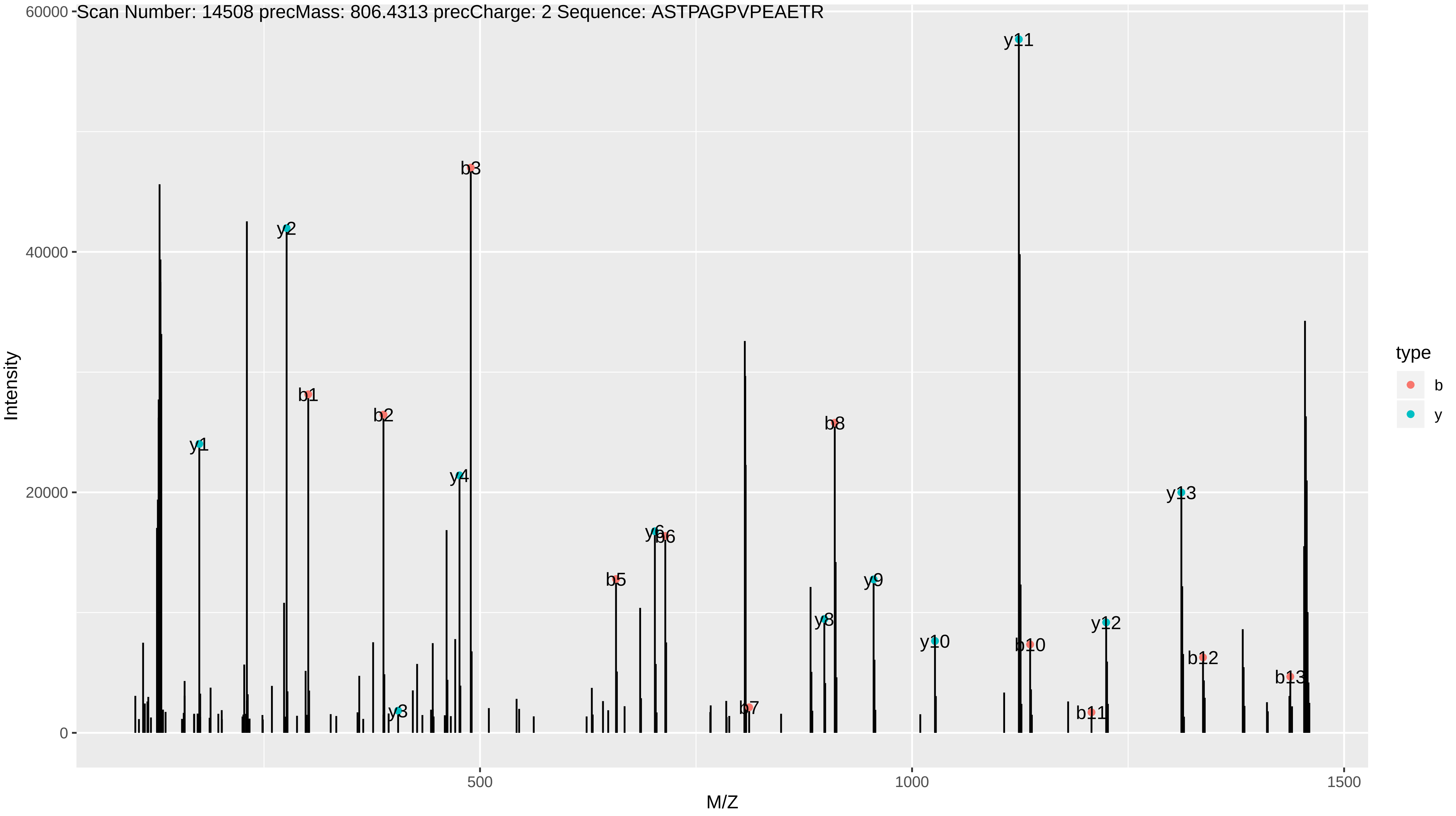

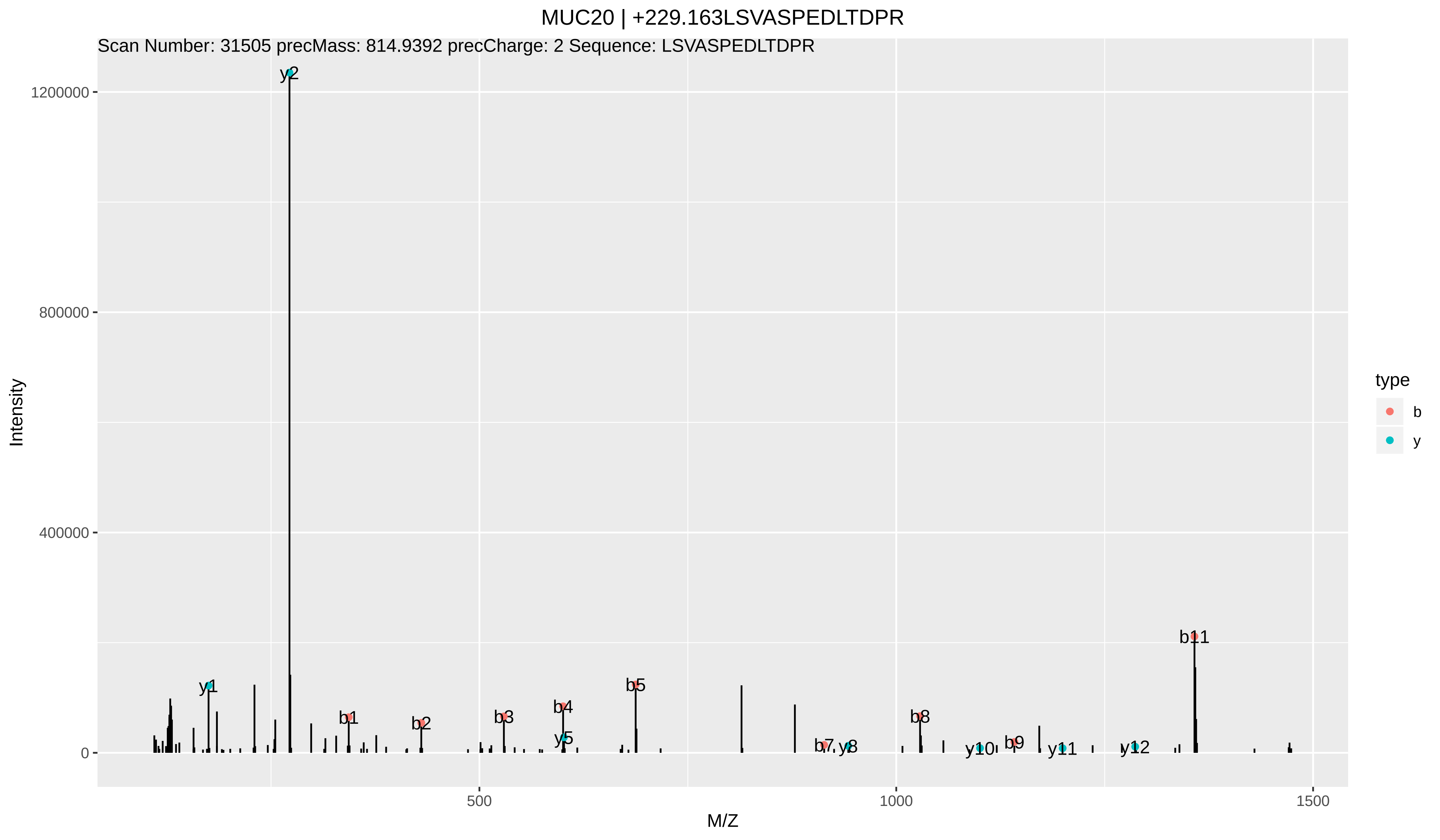
MUC20 | +229.163LSVASPEDLTDPR
Scan Number: 31505 precMass: 814.9392 precCharge: 2 Sequence: LSVASPEDLTDPR
Intensity
M/Z
type
b
y
y1
y2
b1
b2
b3
b4
y5
b5
b7
y8
b8
y10
b9
y11
y12
b11
0
400000
800000
1200000
500
1000
1500

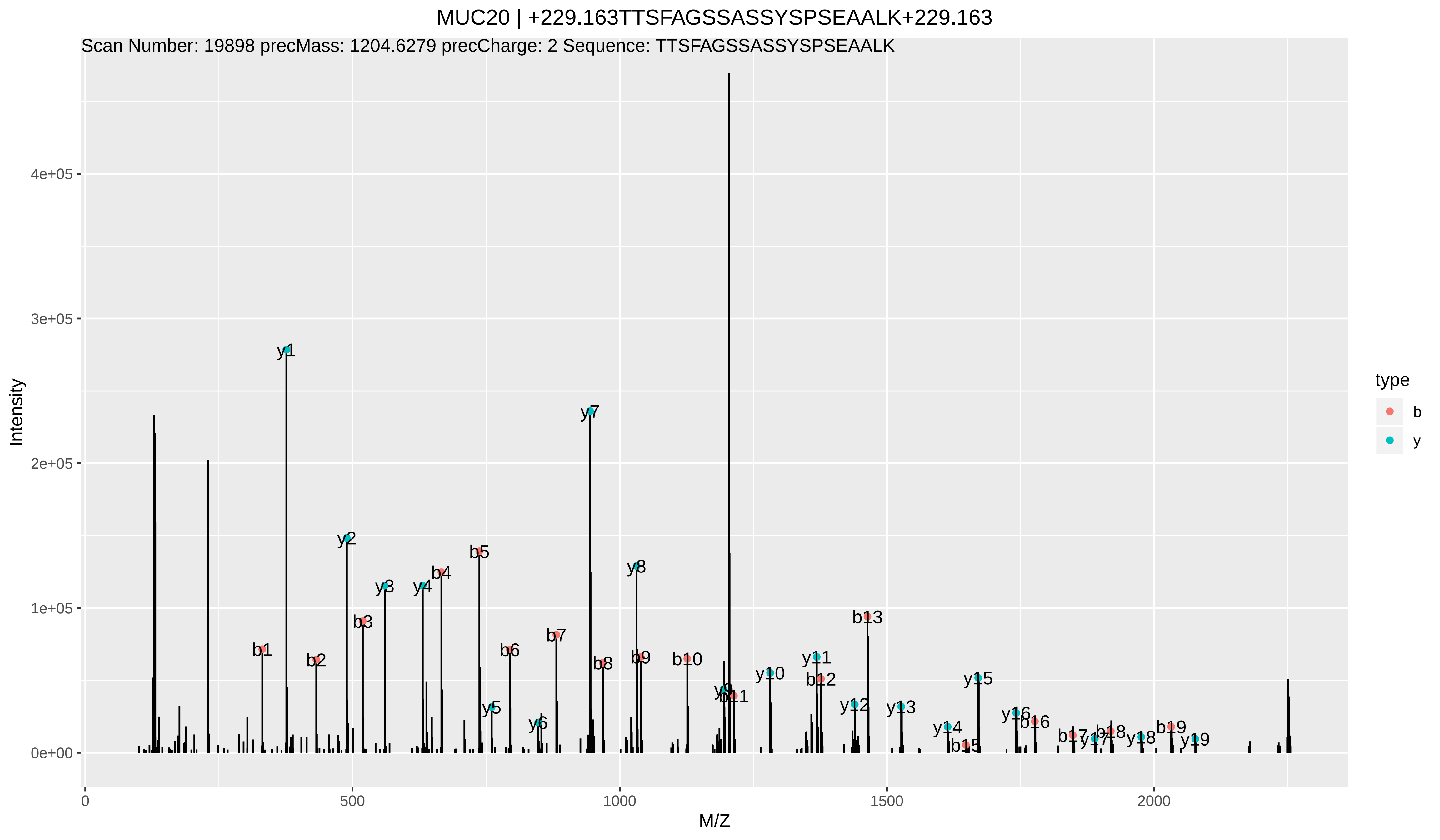

MUC20 | +229.163TTSFAGSSASSYSPSEAALK+229.163
Scan Number: 19898 precMass: 1204.6279 precCharge: 2 Sequence: TTSFAGSSASSYSPSEAALK
Intensity
M/Z
type
b
y
0e+00
1e+05
2e+05
3e+05
4e+05
0
500
1000
1500
2000
y1
y2
y3
y4
y5
y6
y7
y8
y9
y10
y11
y12
y13
y14
y15
y16
y17
y18
y19
b1
b2
b3
b4
b5
b6
b7
b8
b9
b10
b11
b12
b13
b15
b16
b17
b18
b19

MUC20 | +229.163GLSSESSASSDGPHPVITPSR

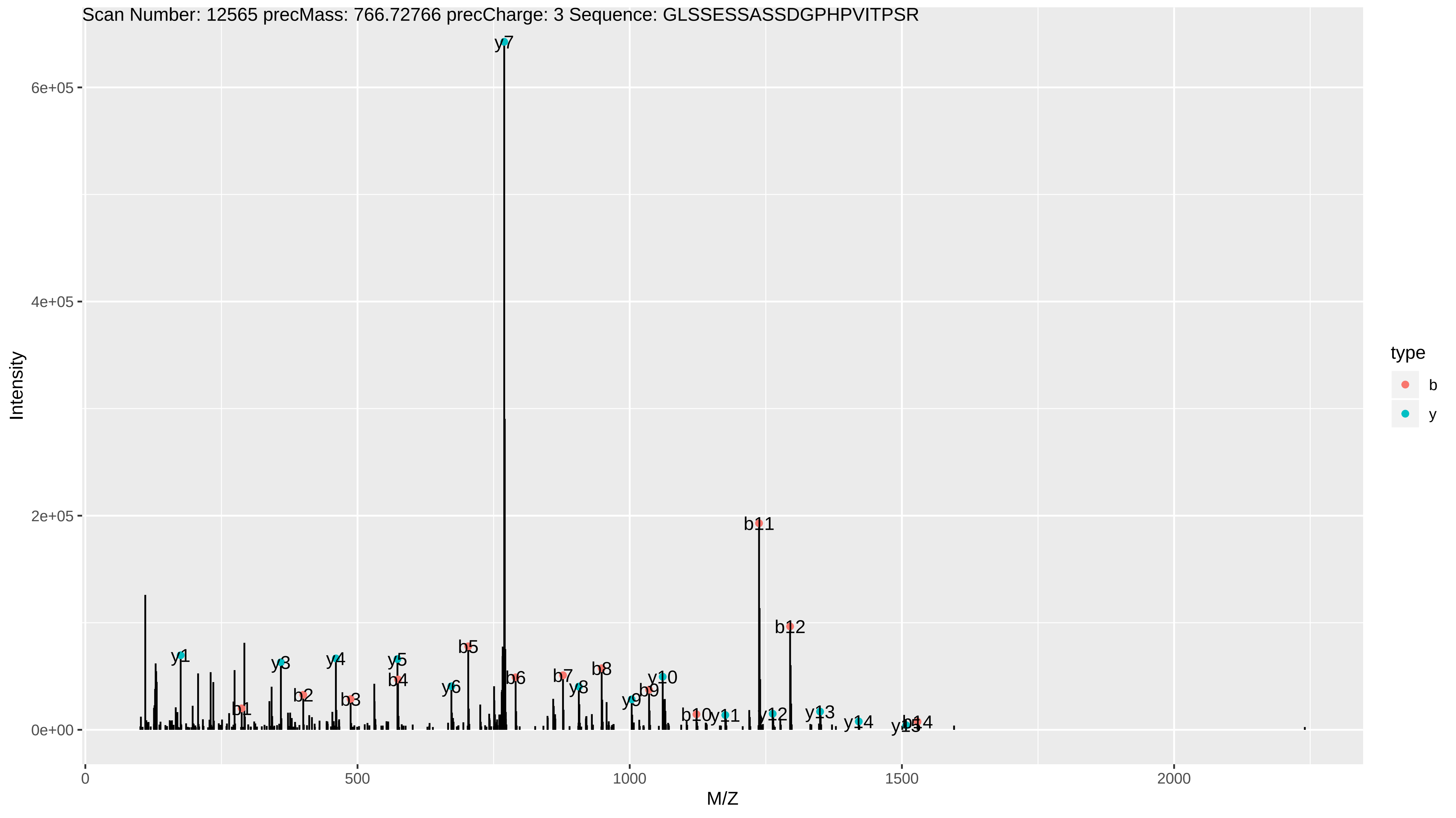

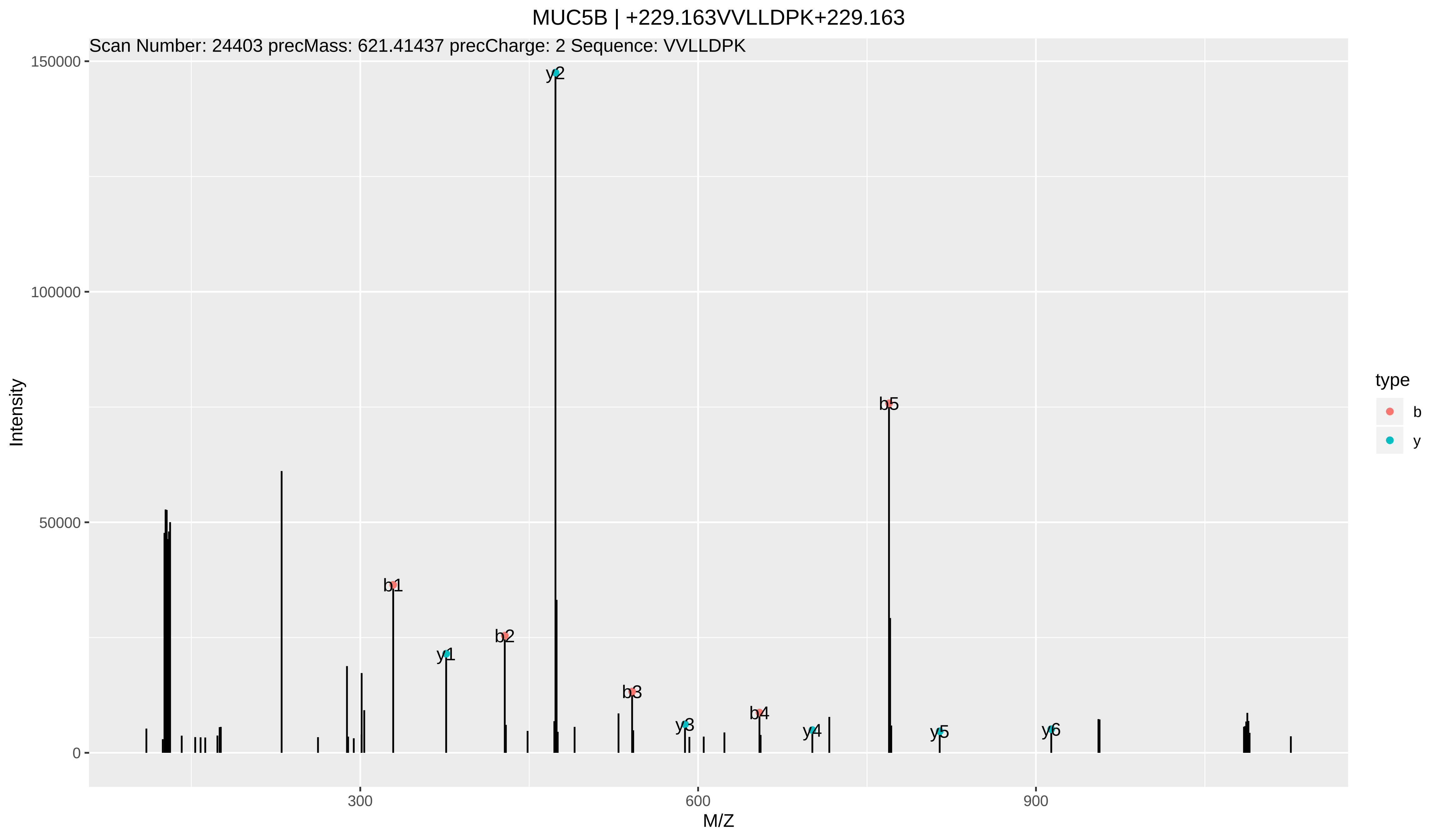

MUC5B | +229.163VVLLDPK+229.163
Scan Number: 24403 precMass: 621.41437 precCharge: 2 Sequence: VVLLDPK
Intensity
150000
100000
50000
0
300
600
900
M/Z
y2
b5
b1
b2
y1
b3
y3
b4
y4
y5
y6
type
b
y

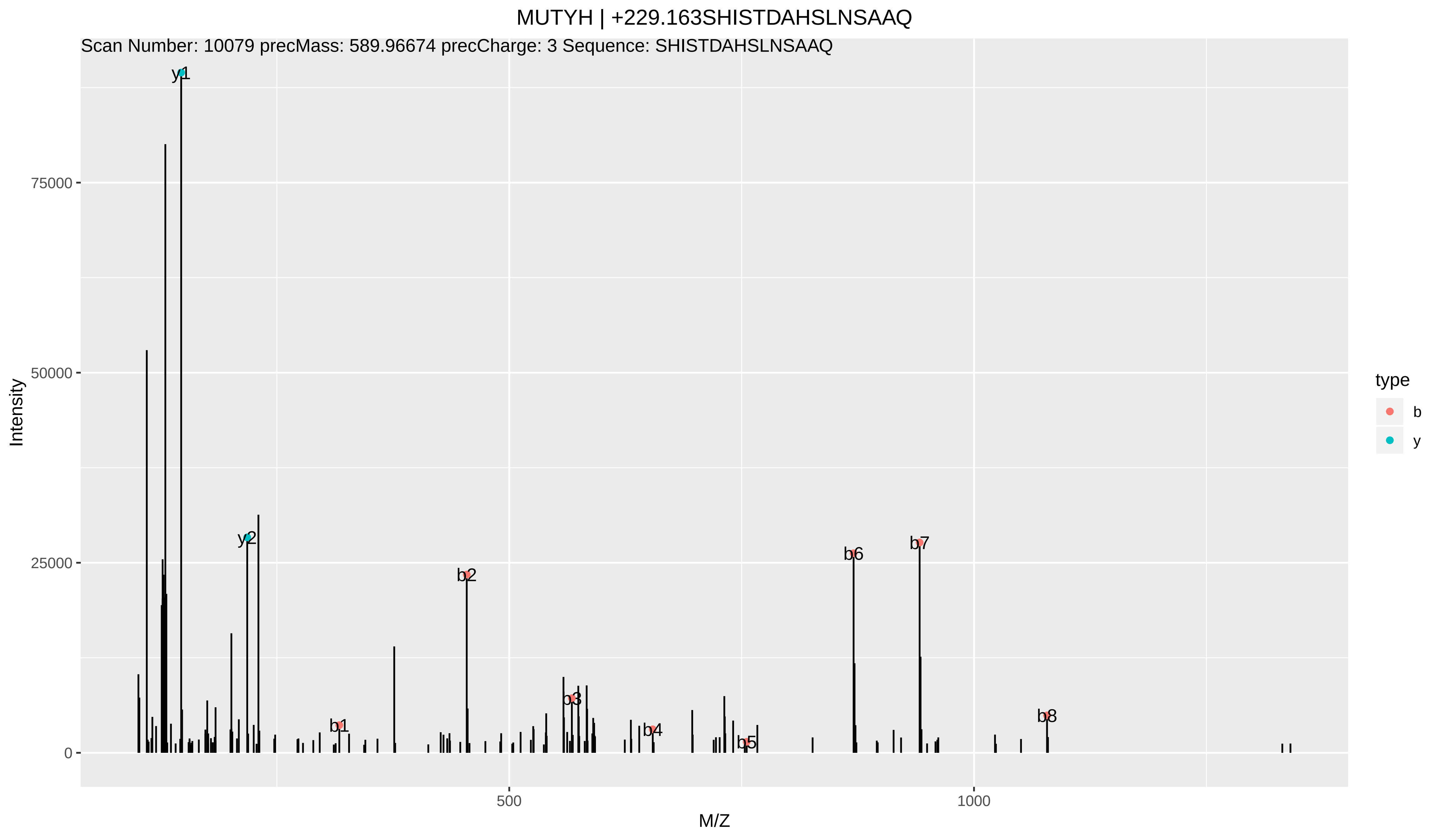

MUTYH | +229.163SHISTDAHSLNSAAQ
Scan Number: 10079 precMass: 589.96674 precCharge: 3 Sequence: SHISTDAHSLNSAAQ
y1
y2
b1
b2
b3
b4
b5
b6
b7
b8
Intensity
0
25000
50000
75000
M/Z
500
1000
type
b
y

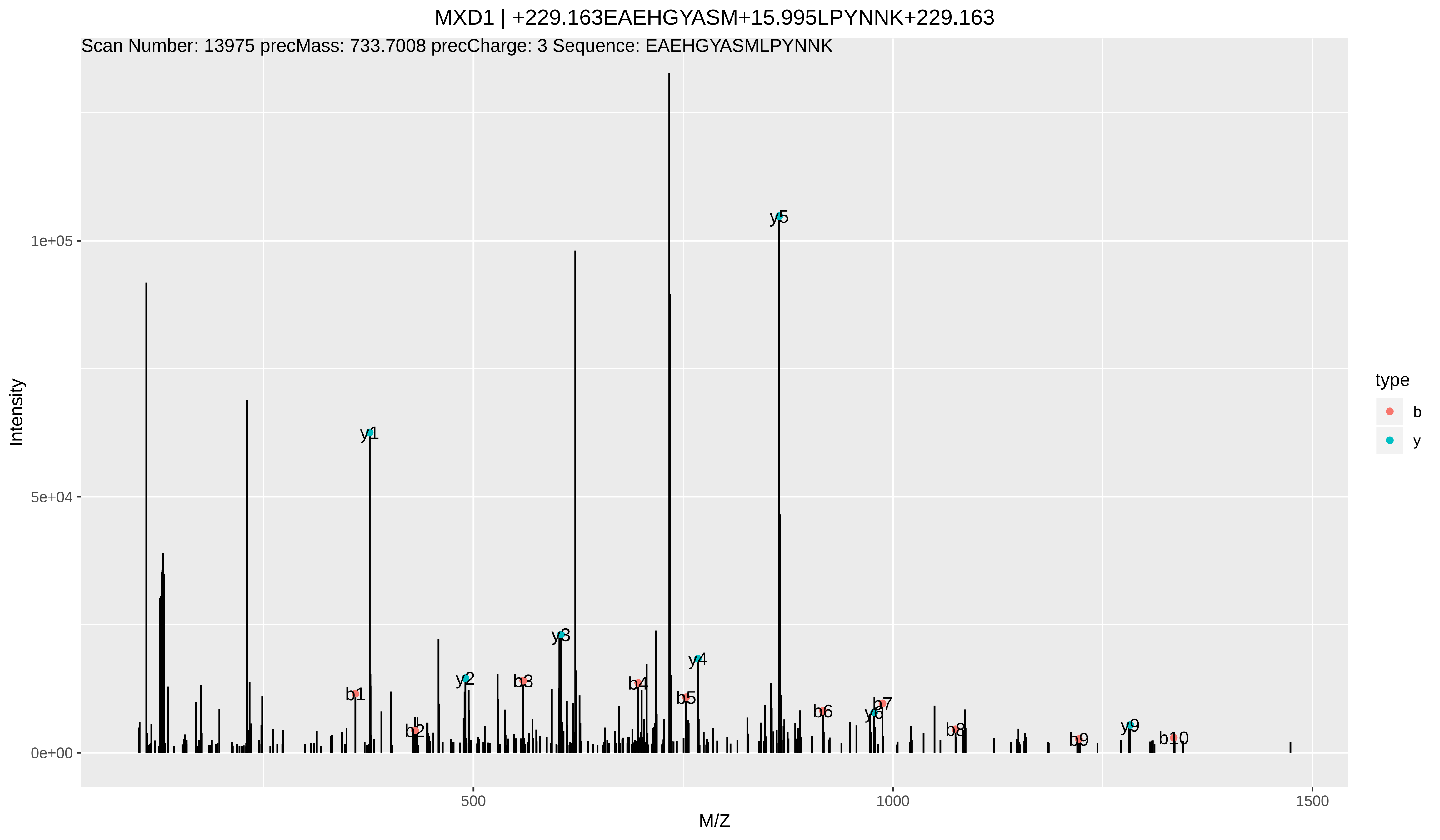

MXD1 | +229.163EAEHGYASM+15.995LPYNNK+229.163
Scan Number: 13975 precMass: 733.7008 precCharge: 3 Sequence: EAEHGYASMLPYNNK
Intensity
1e+05
5e+04
0e+00
M/Z
500
1000
1500
y1
y2
y3
y4
y5
y6
y9
b1
b2
b3
b4
b5
b6
b7
b8
b9
b10
type
b
y

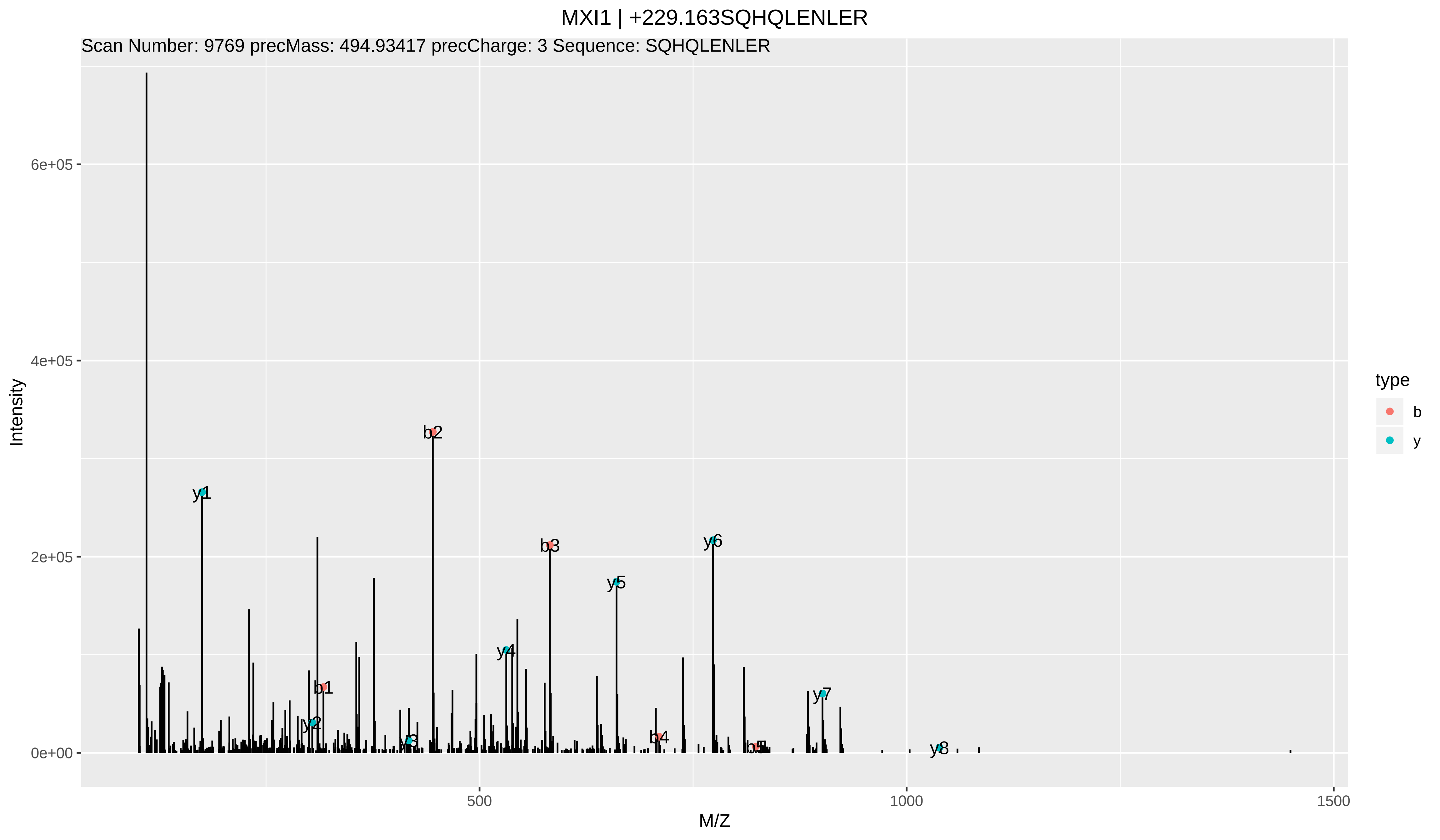
MXI1 | +229.163SQHQLENLER
Scan Number: 9769 precMass: 494.93417 precCharge: 3 Sequence: SQHQLENLER
Intensity
6e+05
4e+05
2e+05
0e+00
M/Z
500
1000
1500
type
b
y
b1
b2
b3
b4
y1
y2
y4
y5
y6
y7
y8

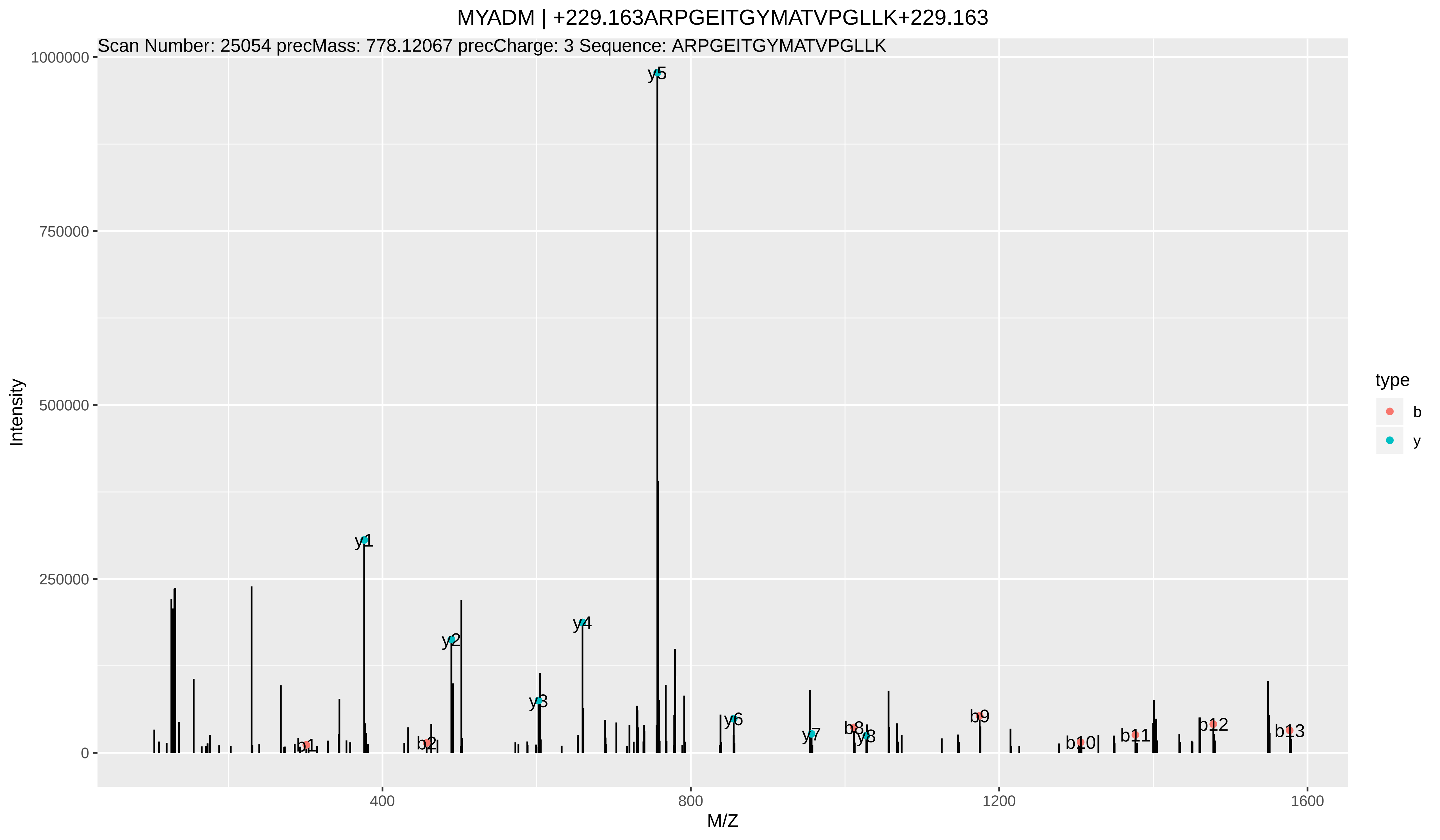

MYADM | +229.163ARPGEITGYMATVPGLLK+229.163
Scan Number: 25054 precMass: 778.12067 precCharge: 3 Sequence: ARPGEITGYMATVPGLLK
Intensity
1000000
750000
500000
250000
0
M/Z
400
800
1200
1600
y5
y1
y2
y3
y4
y6
y7
y8
b1
b2
b8
b9
b10
b11
b12
b13
type
b
y

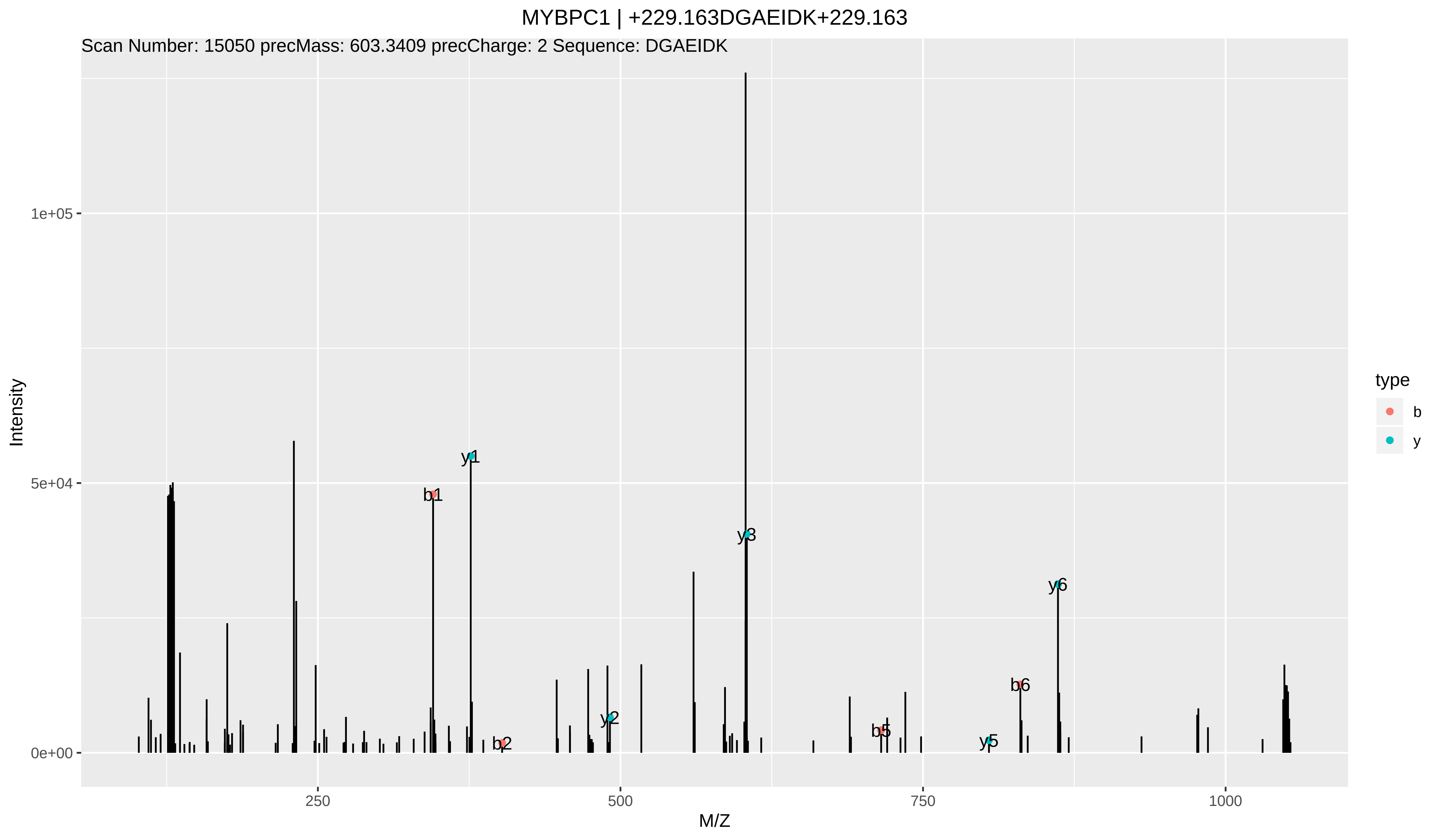
MYBPC1 | +229.163DGAEIDK+229.163
Scan Number: 15050 precMass: 603.3409 precCharge: 2 Sequence: DGAEIDK
Intensity
1e+05
5e+04
0e+00
M/Z
250
500
750
1000
y1
b1
y3
y6
b6
y2
b2
b5
y5
type
b
y

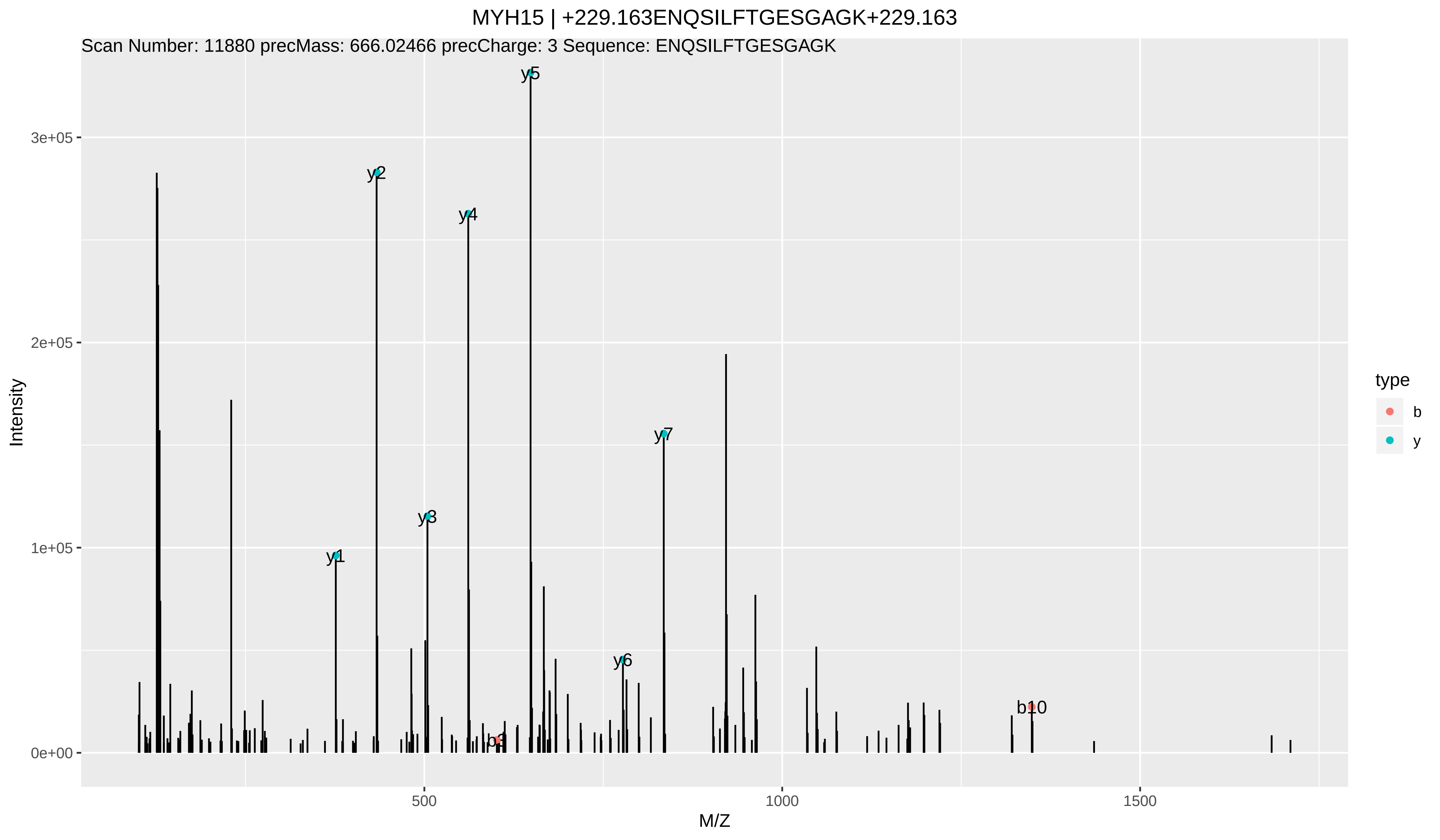

MYH15 | +229.163ENQSILFTGESGAGK+229.163
Scan Number: 11880 precMass: 666.02466 precCharge: 3 Sequence: ENQSILFTGESGAGK
Intensity
M/Z
type
b
y
y1
y2
y3
y4
y5
y6
y7
b10
3e+05
2e+05
1e+05
0e+00
500
1000
1500

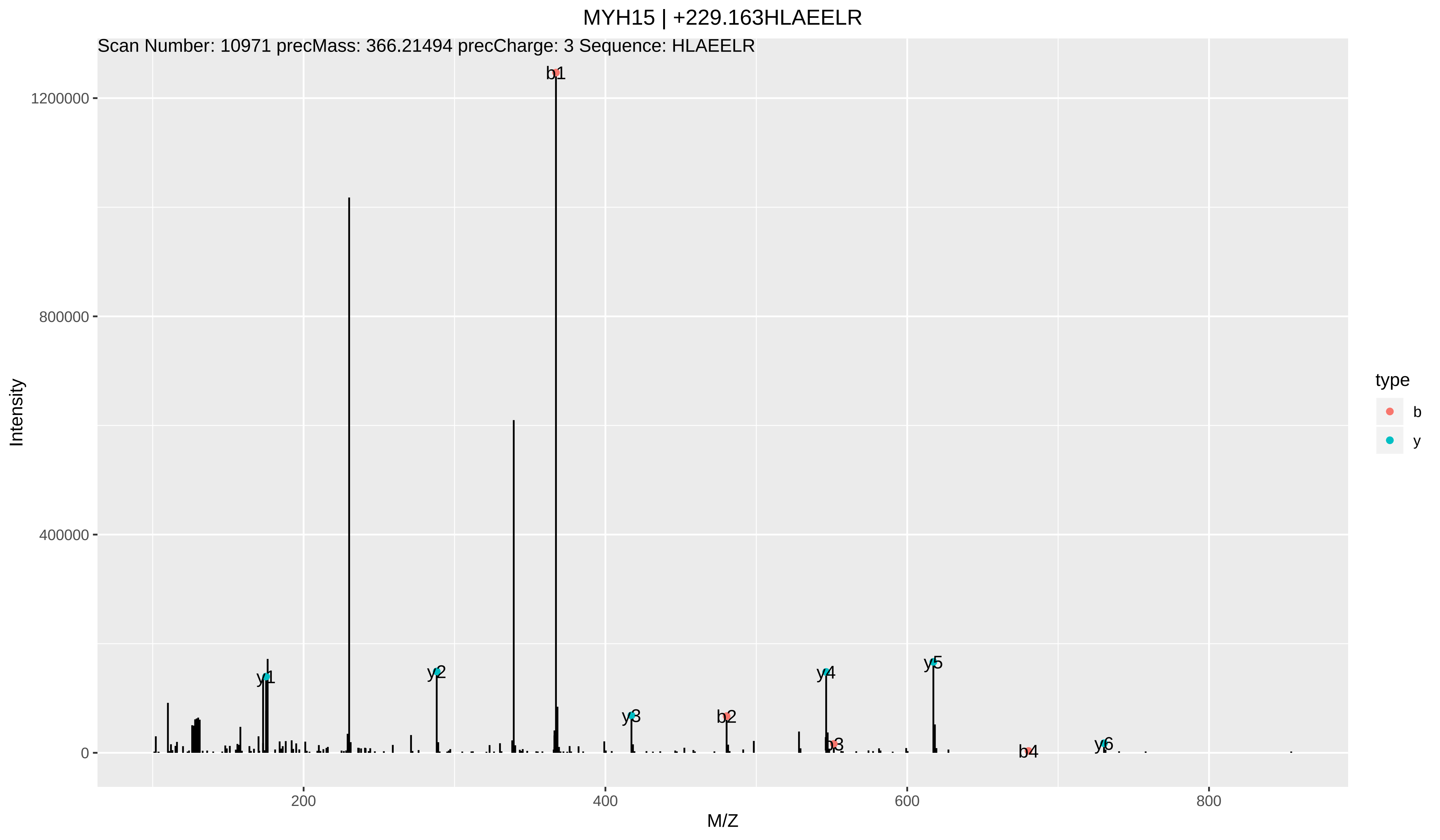

MYH15 | +229.163HLAEELR
Scan Number: 10971 precMass: 366.21494 precCharge: 3 Sequence: HLAEELR
Intensity
1200000
800000
400000
0
200
400
600
800
M/Z
b1
y1
y2
y3
b2
y4
b3
y5
b4
y6
type
b
y

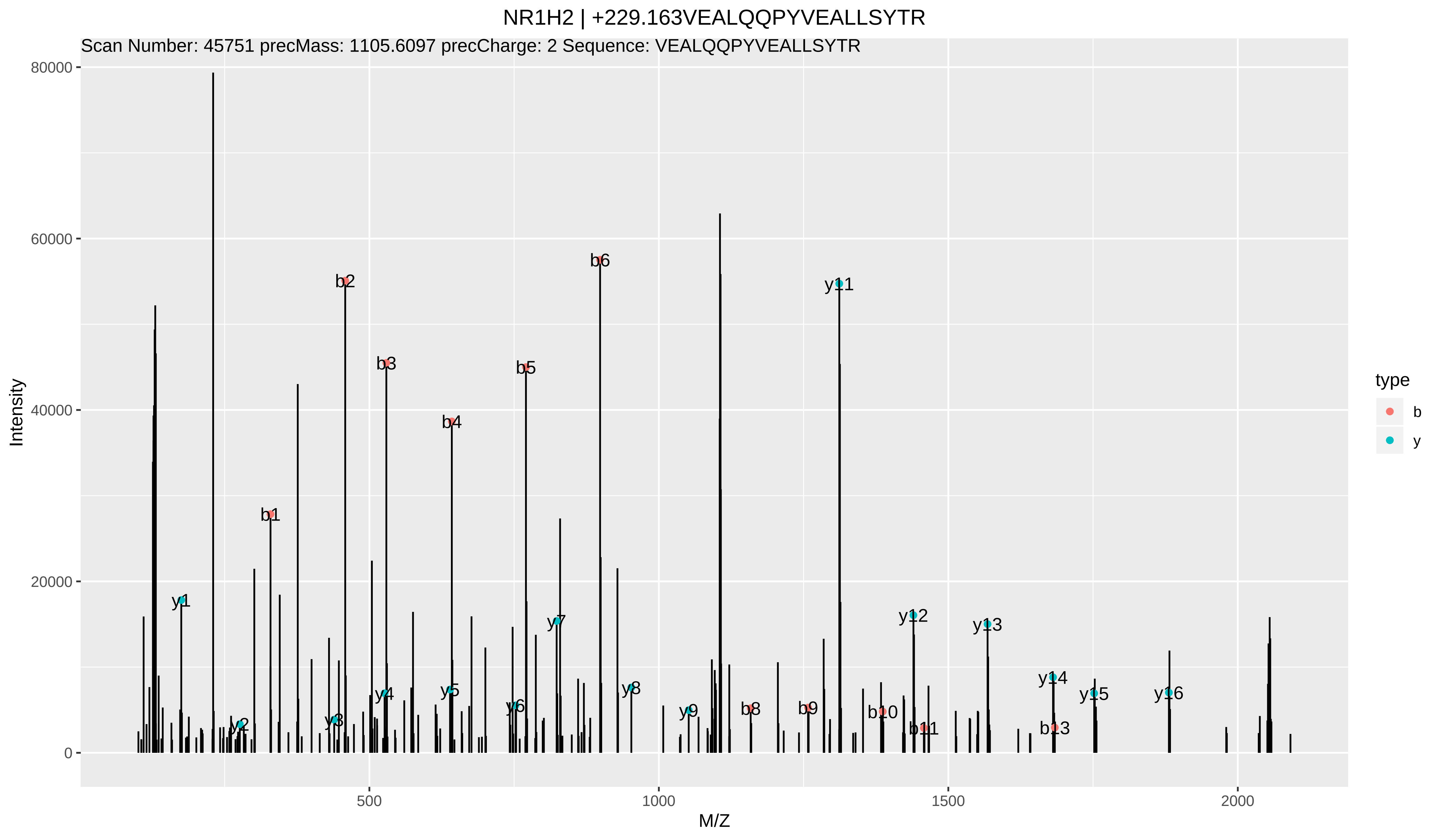
NR1H2 | +229.163VEALQQPYVEALLSYTR
Scan Number: 45751 precMass: 1105.6097 precCharge: 2 Sequence: VEALQQPYVEALLSYTR
Intensity
M/Z
0
20000
40000
60000
80000
500
1000
1500
2000
type
b
y
b1
b2
b3
b4
b5
b6
b8
b9
b10
b11
b13
y1
y2
y3
y4
y5
y6
y7
y8
y9
y11
y12
y13
y14
y15
y16

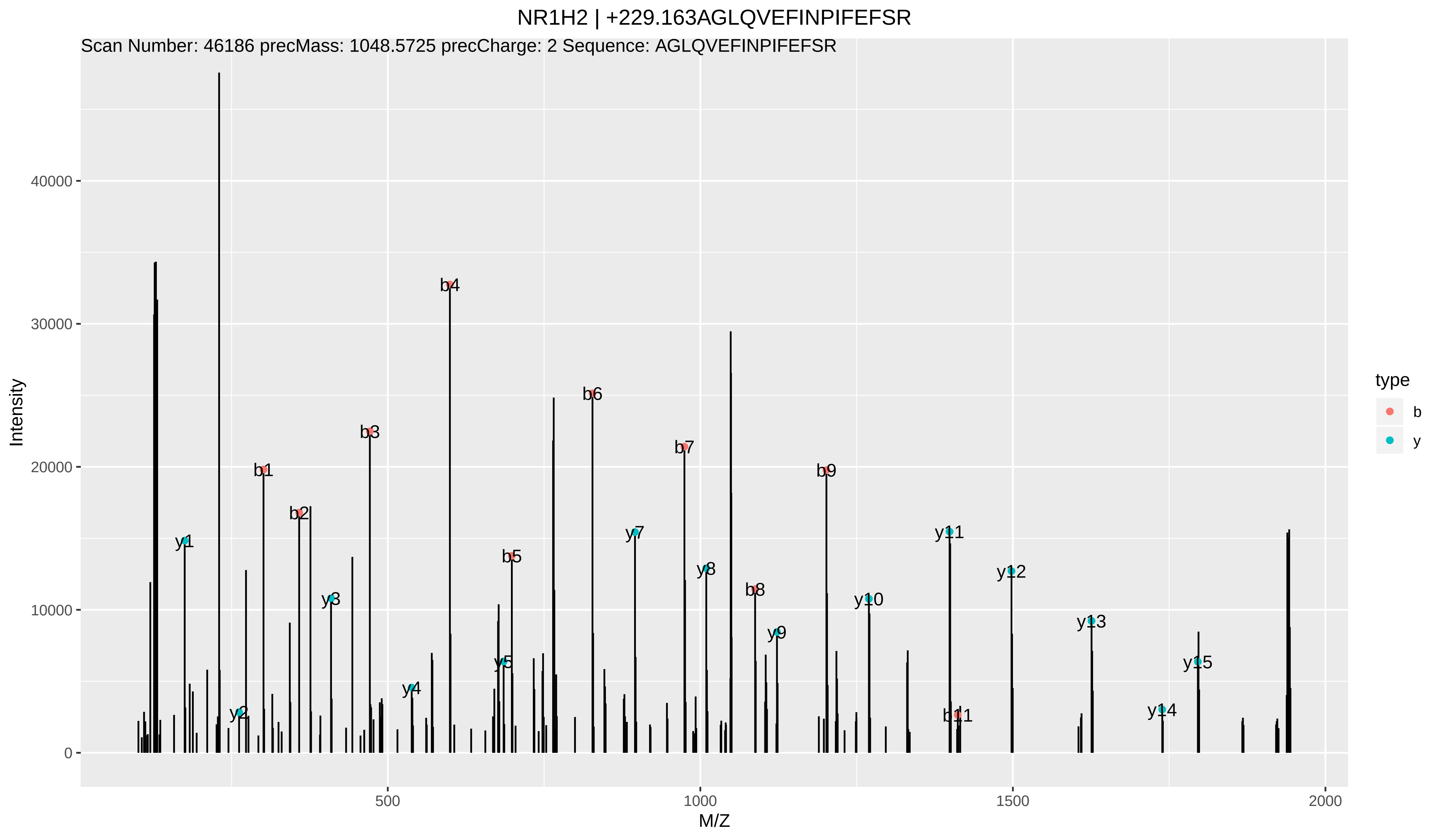
NR1H2 | +229.163AGLQVEFINPIFEFSR
Scan Number: 46186 precMass: 1048.5725 precCharge: 2 Sequence: AGLQVEFINPIFEFSR
Intensity
M/Z
type
b
y
b1
b2
b3
b4
b5
b6
b7
b8
b9
b11
y1
y2
y3
y4
y5
y7
y8
y9
y10
y11
y12
y13
y14
y15
0
10000
20000
30000
40000
500
1000
1500
2000

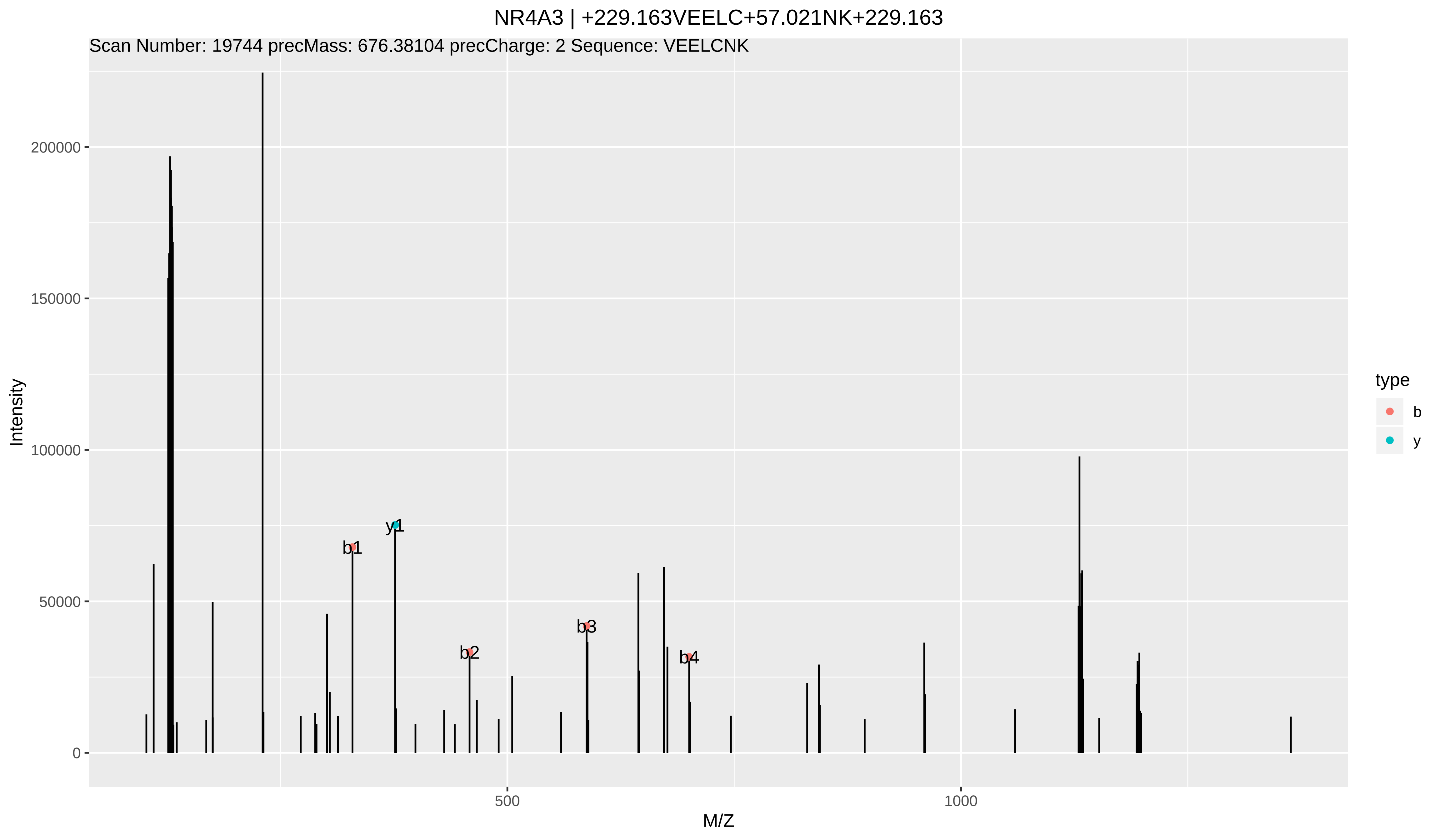

NR4A3 | +229.163VEELC+57.021NK+229.163
Scan Number: 19744 precMass: 676.38104 precCharge: 2 Sequence: VEELCNK
Intensity
200000
150000
100000
50000
0
y1
b1
b2
b3
b4
500
1000
M/Z
type
b
y

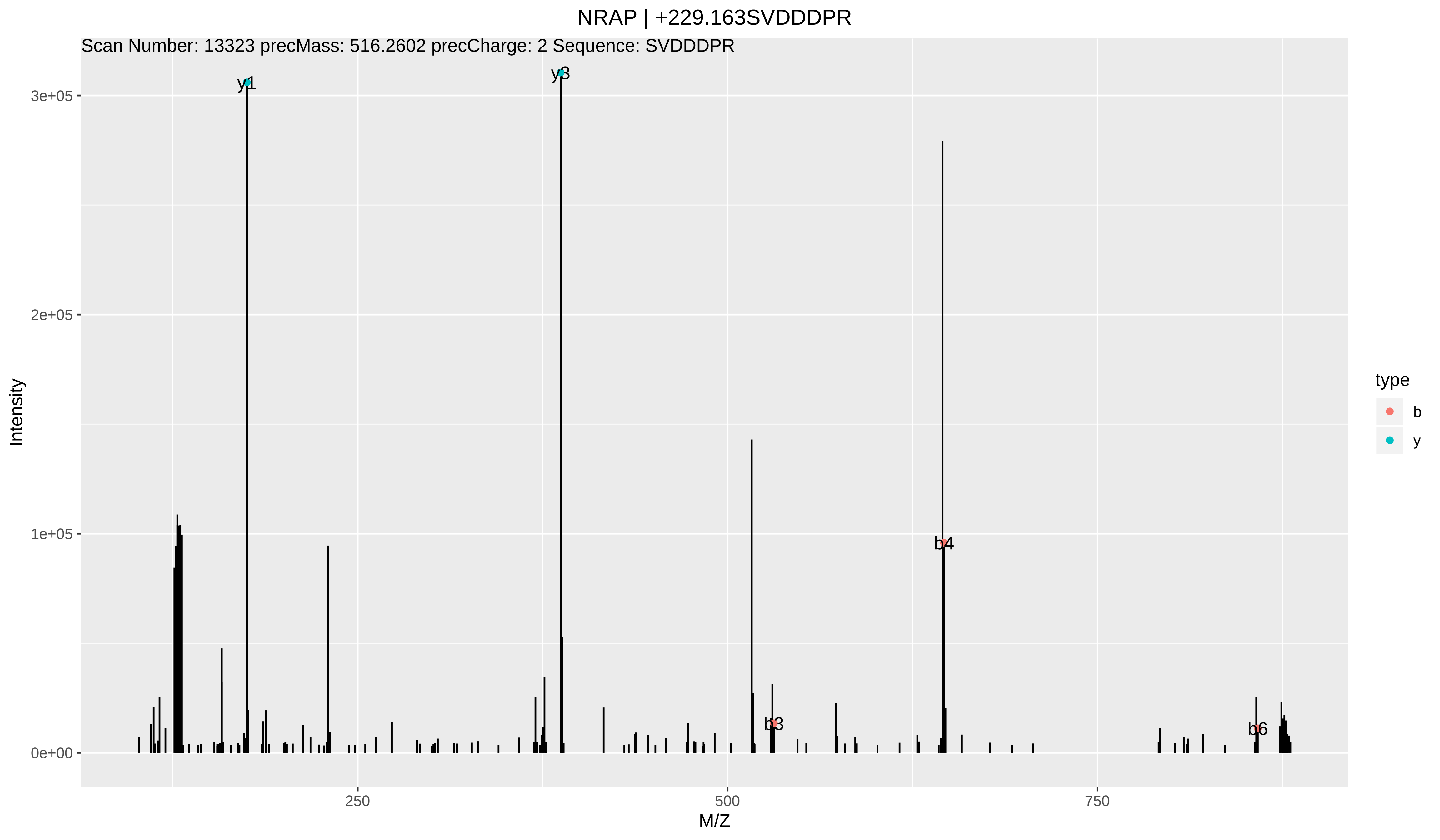

NRAP | +229.163SVDDDPR
Scan Number: 13323 precMass: 516.2602 precCharge: 2 Sequence: SVDDDPR
y1
y3
b4
b3
b6
3e+05
2e+05
1e+05
0e+00
Intensity
250
500
750
M/Z
type
b
y

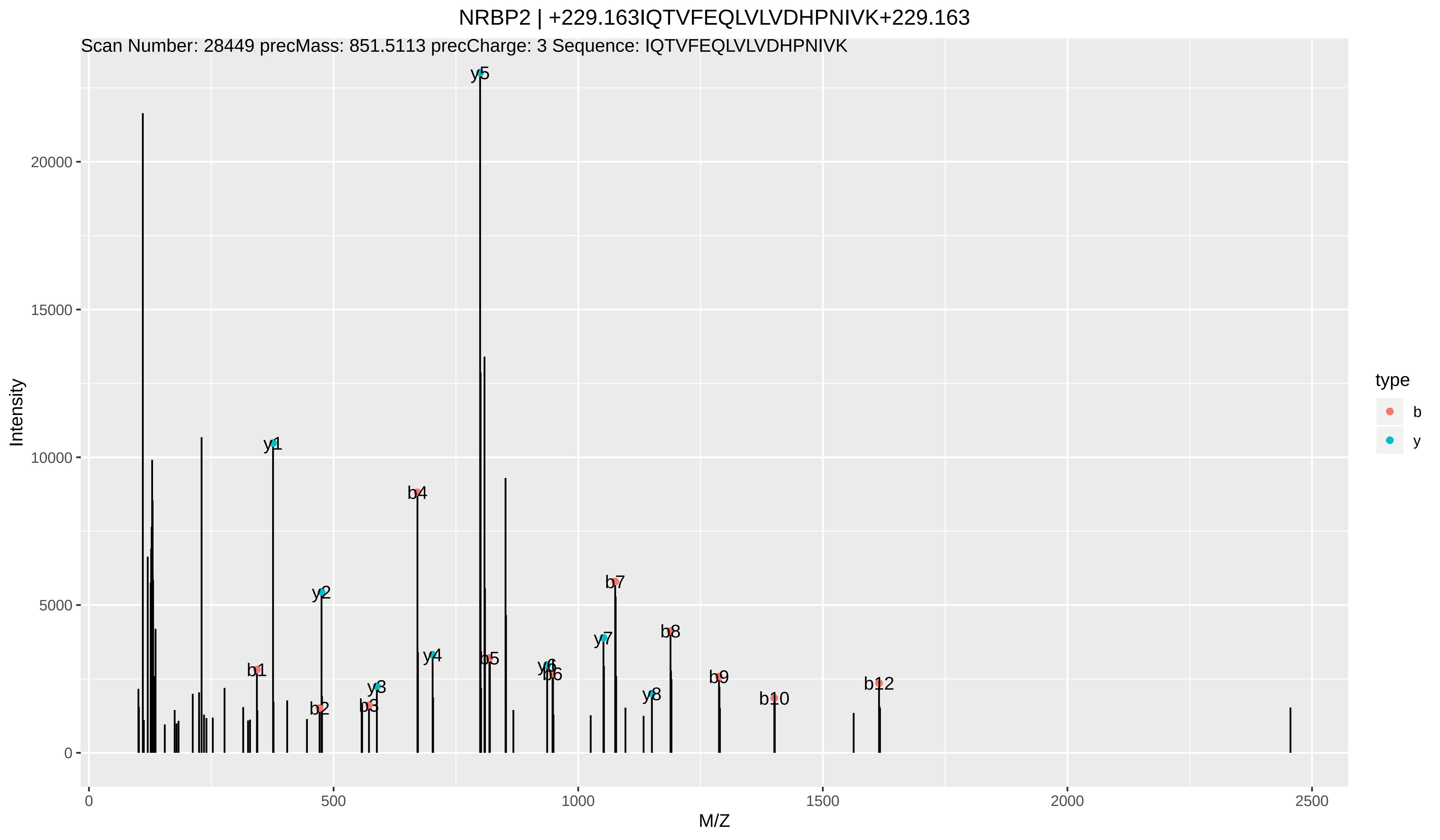
NRBP2 | +229.163IQTVFEQLVLVDHPNIVK+229.163
Scan Number: 28449 precMass: 851.5113 precCharge: 3 Sequence: IQTVFEQLVLVDHPNIVK
Intensity
M/Z
type
b
y
y5
y1
b4
y2
b7
b8
y7
y4
b5
y6
b6
b1
y3
b9
b12
y8
b10
b2
b3
0
5000
10000
15000
20000
0
500
1000
1500
2000
2500

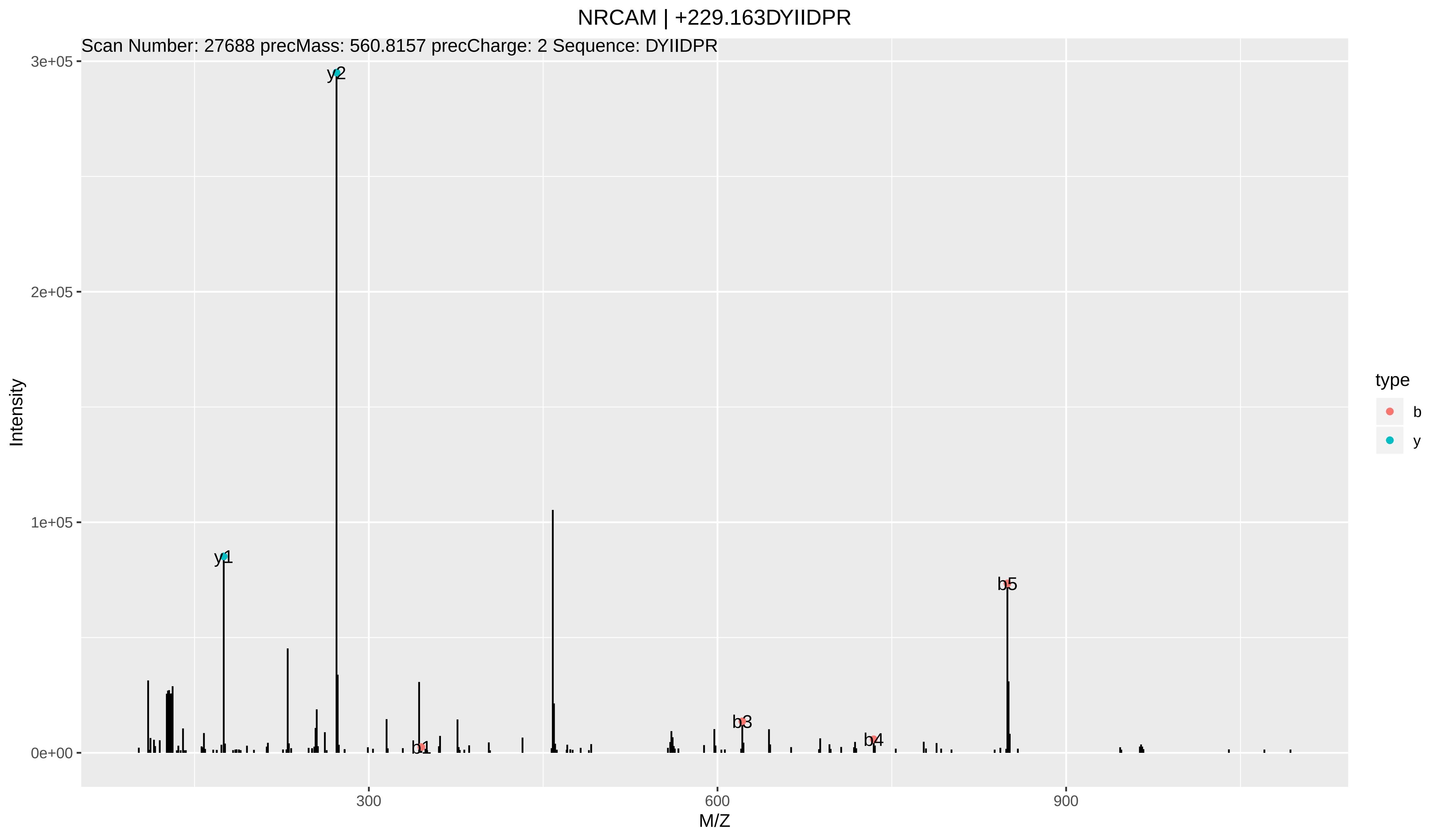

NRCAM | +229.163DYIIDPR
Scan Number: 27688 precMass: 560.8157 precCharge: 2 Sequence: DYIIDPR
Intensity
M/Z
3e+05
2e+05
1e+05
0e+00
300
600
900
y2
y1
b1
b3
b4
b5
type
b
y

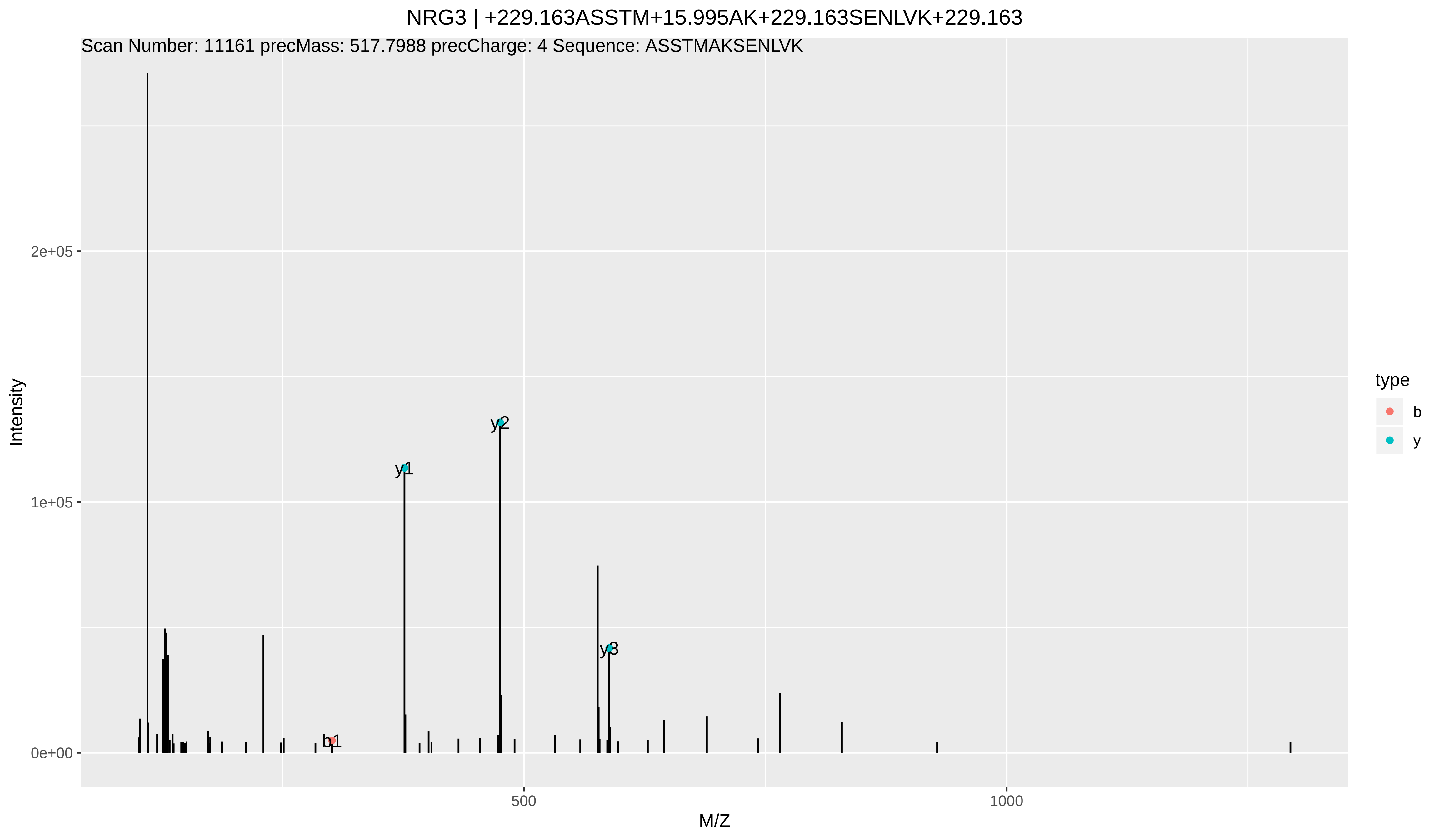

NRG3 | +229.163ASSTM+15.995AK+229.163SENLVK+229.163
Scan Number: 11161 precMass: 517.7988 precCharge: 4 Sequence: ASSTMAKSENLVK
Intensity
2e+05
1e+05
0e+00
M/Z
500
1000
y1
y2
y3
b1
type
b
y

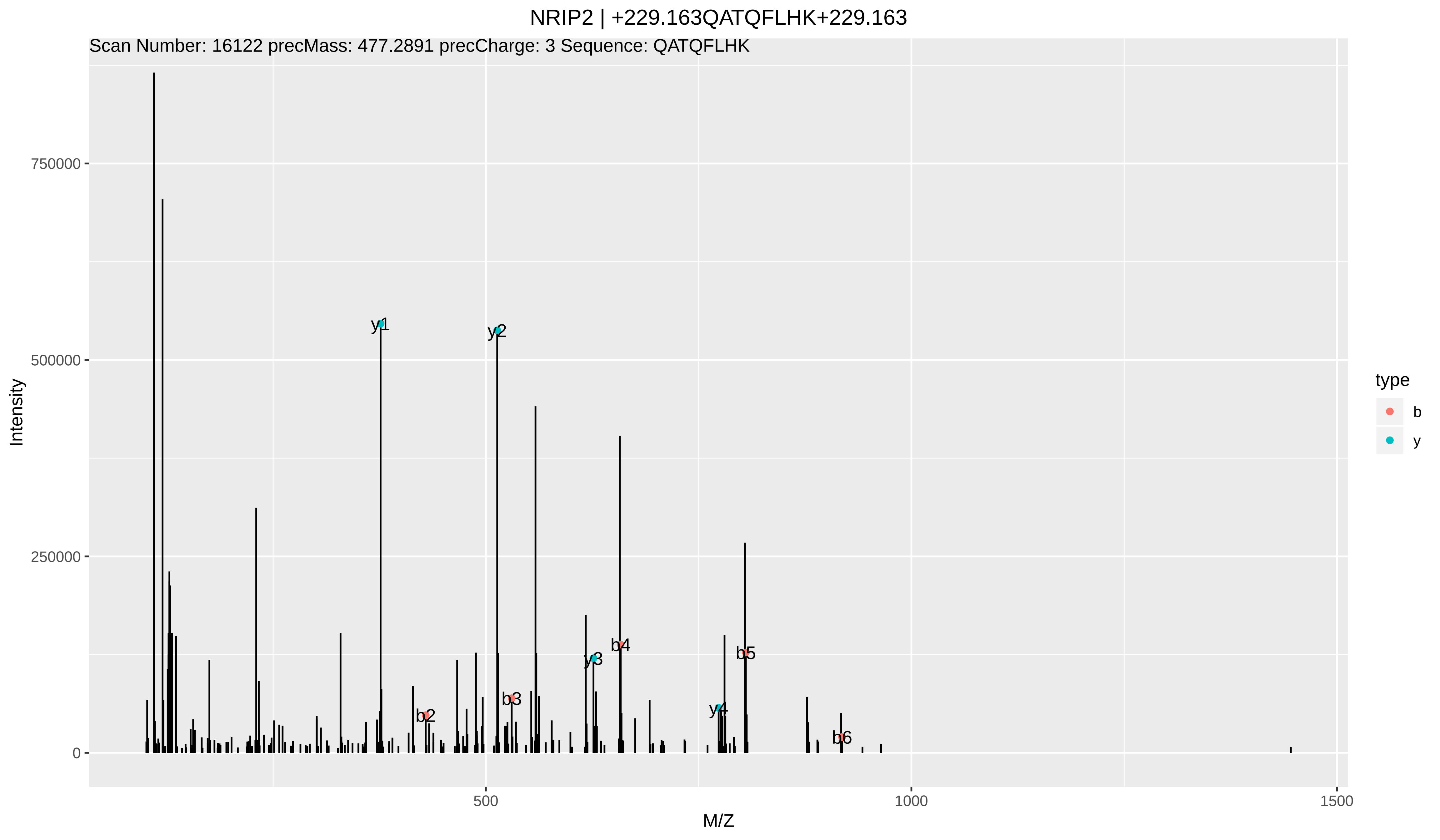
NRIP2 | +229.163QATQFLHK+229.163
Scan Number: 16122 precMass: 477.2891 precCharge: 3 Sequence: QATQFLHK
Intensity
750000
500000
250000
0
M/Z
500
1000
1500
y1
y2
b2
b3
y3
b4
y4
b5
b6
type
b
y

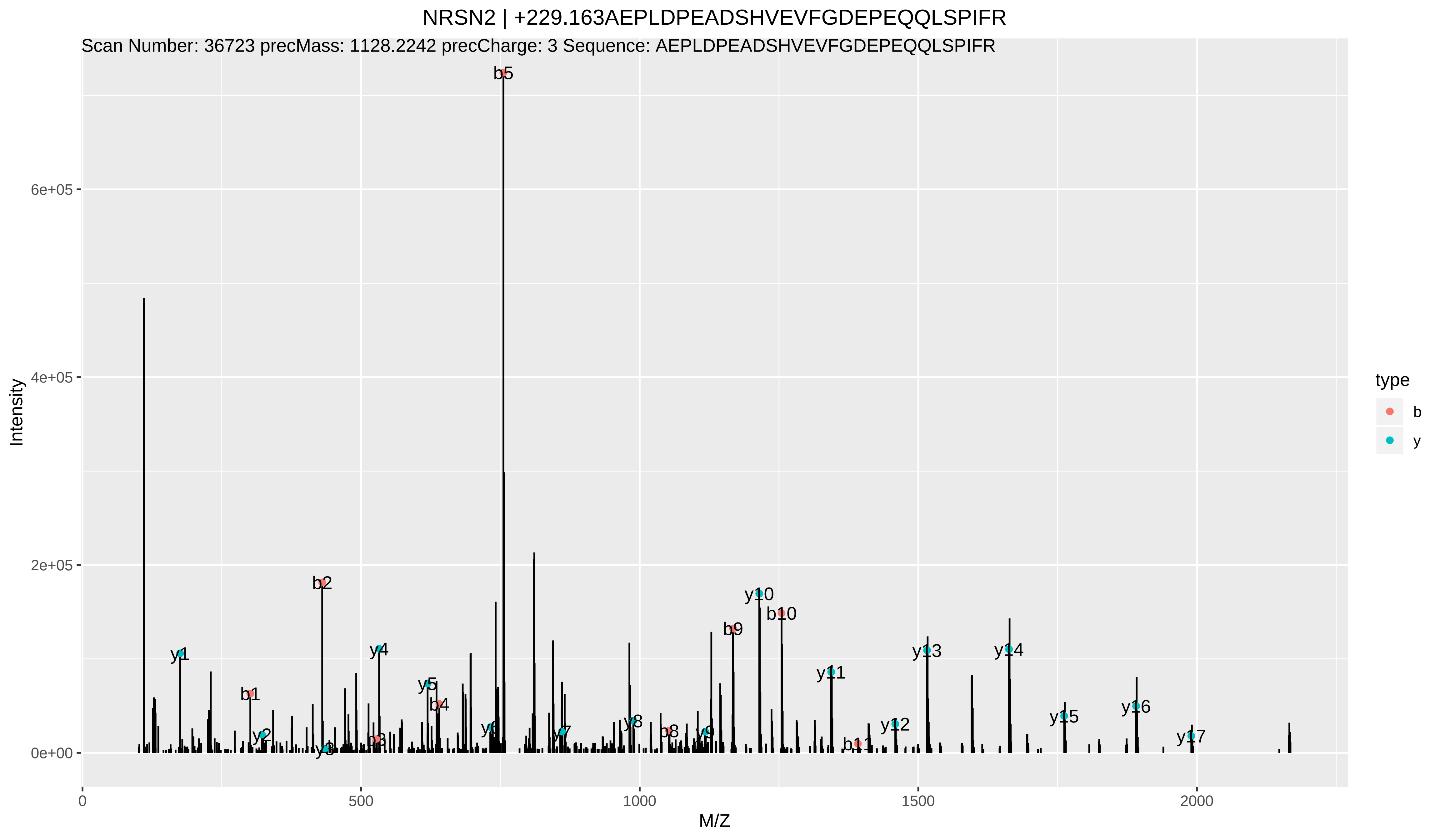
NRSN2 | +229.163AEPLDPEADSHVEVFGDEPEQQLSPIFR
Scan Number: 36723 precMass: 1128.2242 precCharge: 3 Sequence: AEPLDPEADSHVEVFGDEPEQQLSPIFR
Intensity
6e+05
4e+05
2e+05
0e+00
M/Z
0
500
1000
1500
2000
b5
b2
y10
b10
b9
y1
y4
y13
y14
y11
b1
y5
b4
y15
y16
y6
y7
y8
b8
y9
y12
y2
b3
y3
b11
y17
type
b
y

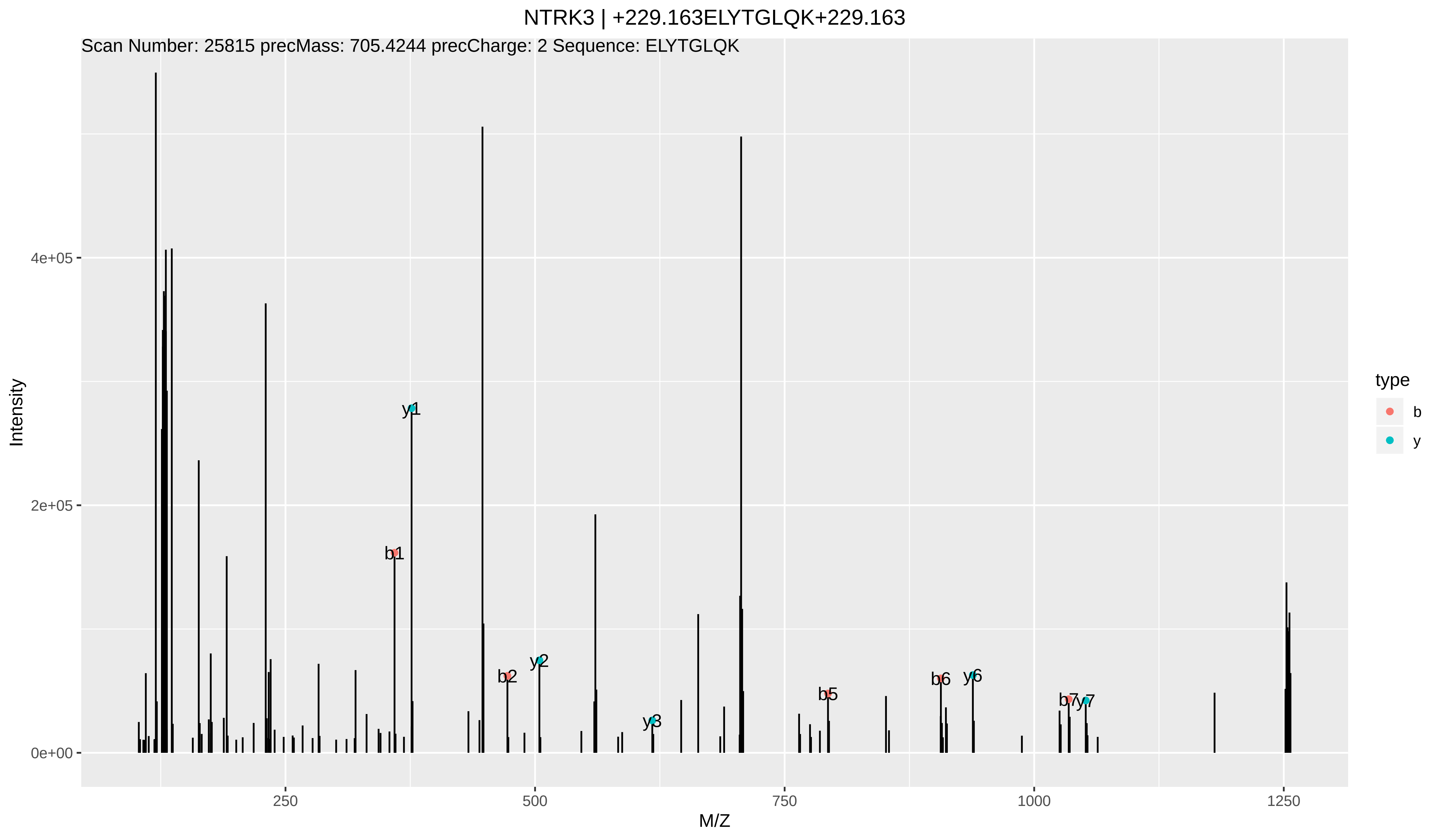

NTRK3 | +229.163ELYTGLQK+229.163
Scan Number: 25815 precMass: 705.4244 precCharge: 2 Sequence: ELYTGLQK
Intensity
4e+05
2e+05
0e+00
M/Z
250
500
750
1000
1250
y1
b1
b2
y2
y3
b5
b6
y6
b7
y7
type
b
y

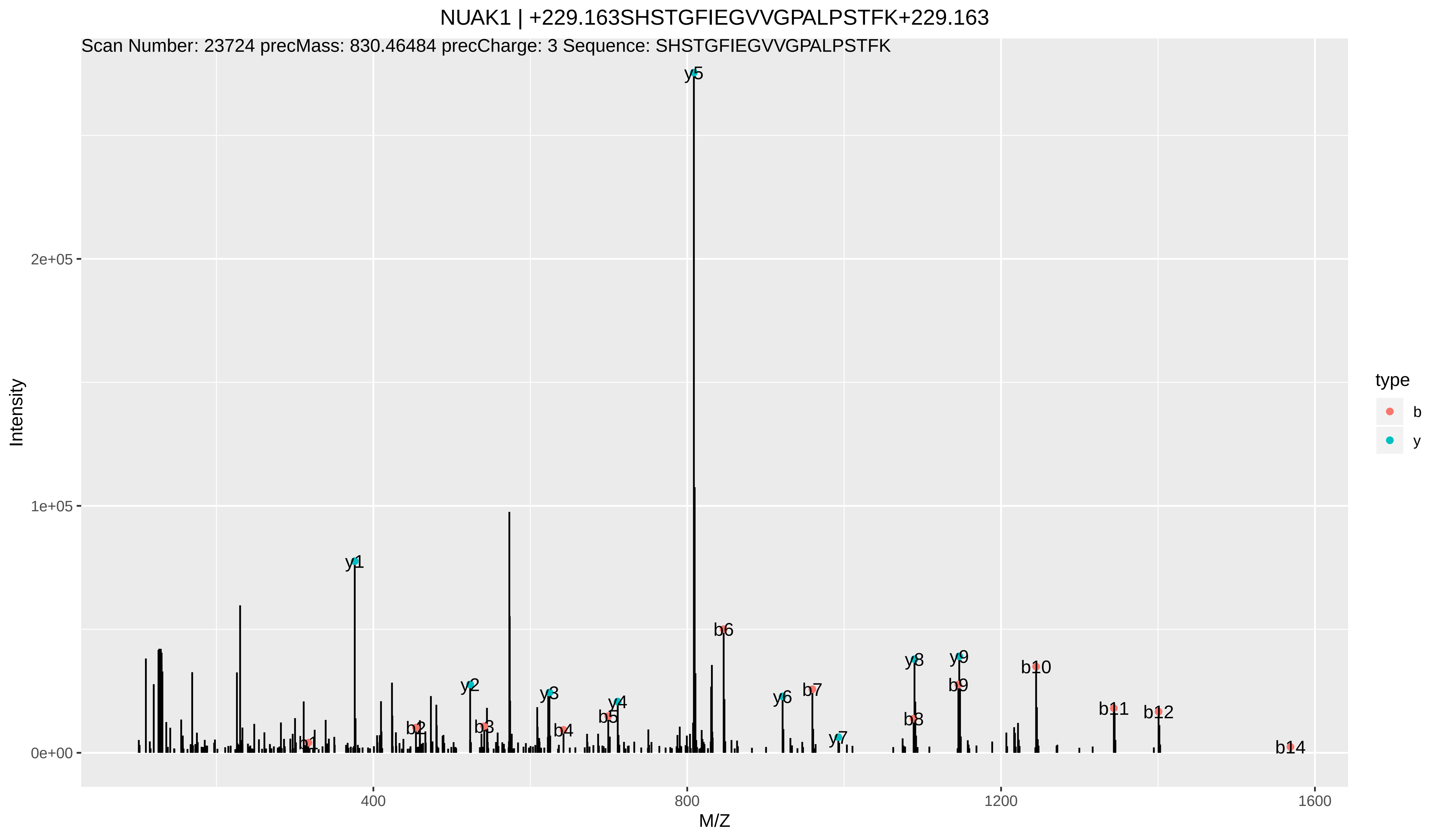

NUAK1 | +229.163SHSTGFIEGVVGPALPSTFK+229.163
Scan Number: 23724 precMass: 830.46484 precCharge: 3 Sequence: SHSTGFIEGVVGPALPSTFK
Intensity
M/Z
0e+00
1e+05
2e+05
400
800
1200
1600
y5
y1
y2
y3
y4
y6
y7
y8
y9
b1
b2
b3
b4
b5
b6
b7
b8
b9
b10
b11
b12
b14
type
b
y

NUDT18 | +229.163VMEEDLQSQLLQR

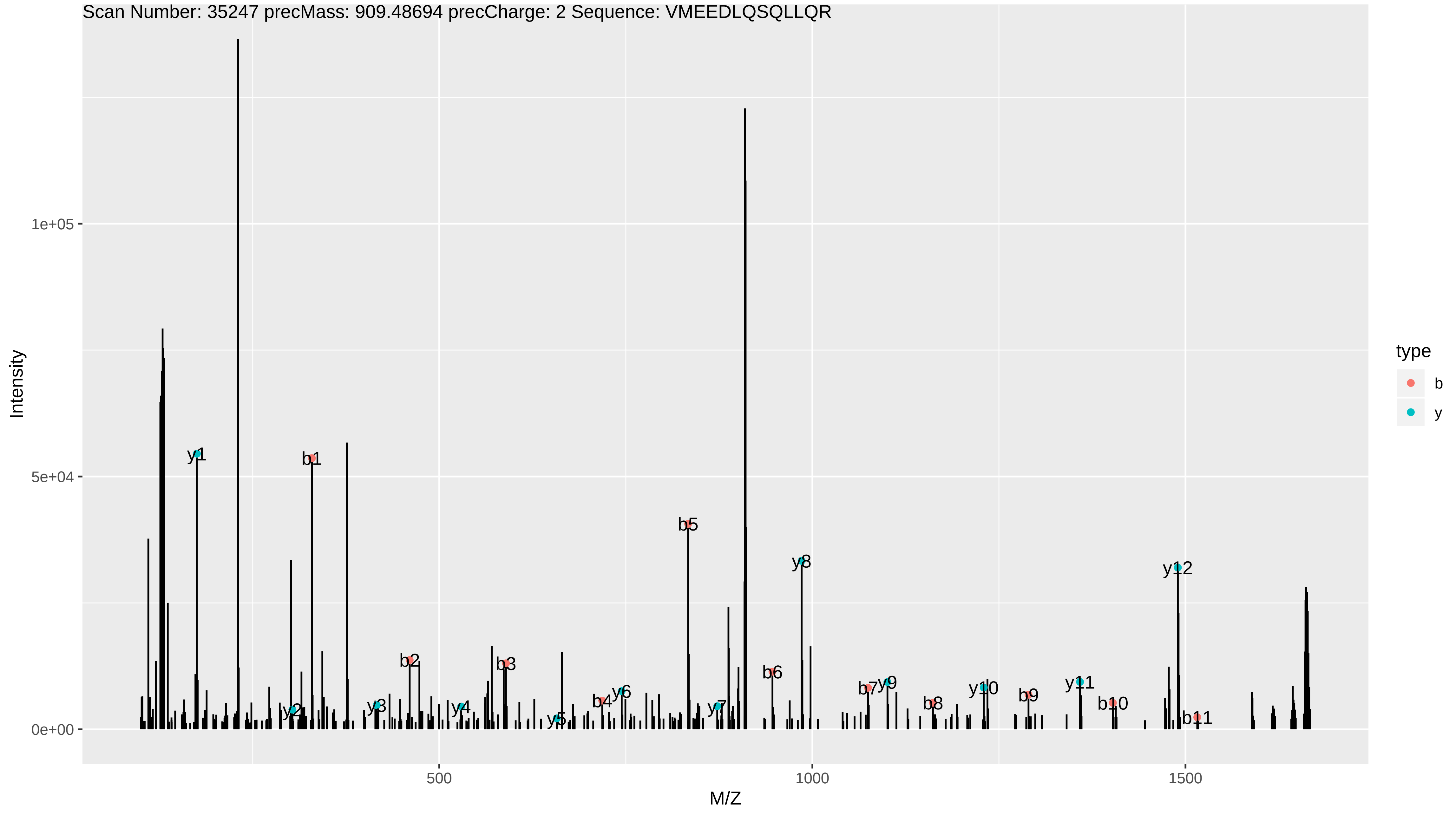

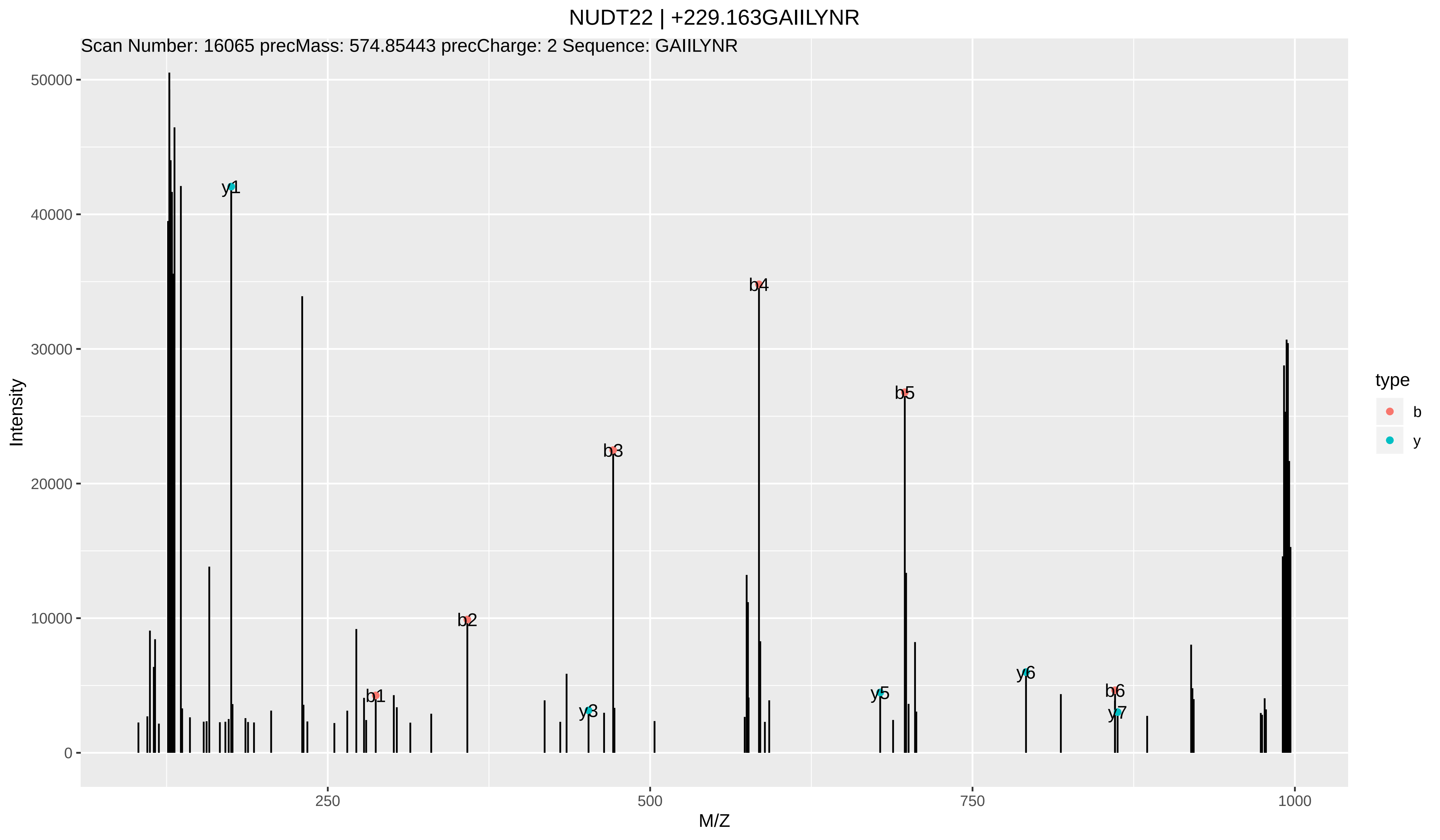

NUDT22 | +229.163GAIILYNR
Scan Number: 16065 precMass: 574.85443 precCharge: 2 Sequence: GAIILYNR
Intensity
M/Z
type
b
y
y1
b1
b2
y3
b3
b4
y5
b5
y6
b6
y7
0
10000
20000
30000
40000
50000
250
500
750
1000

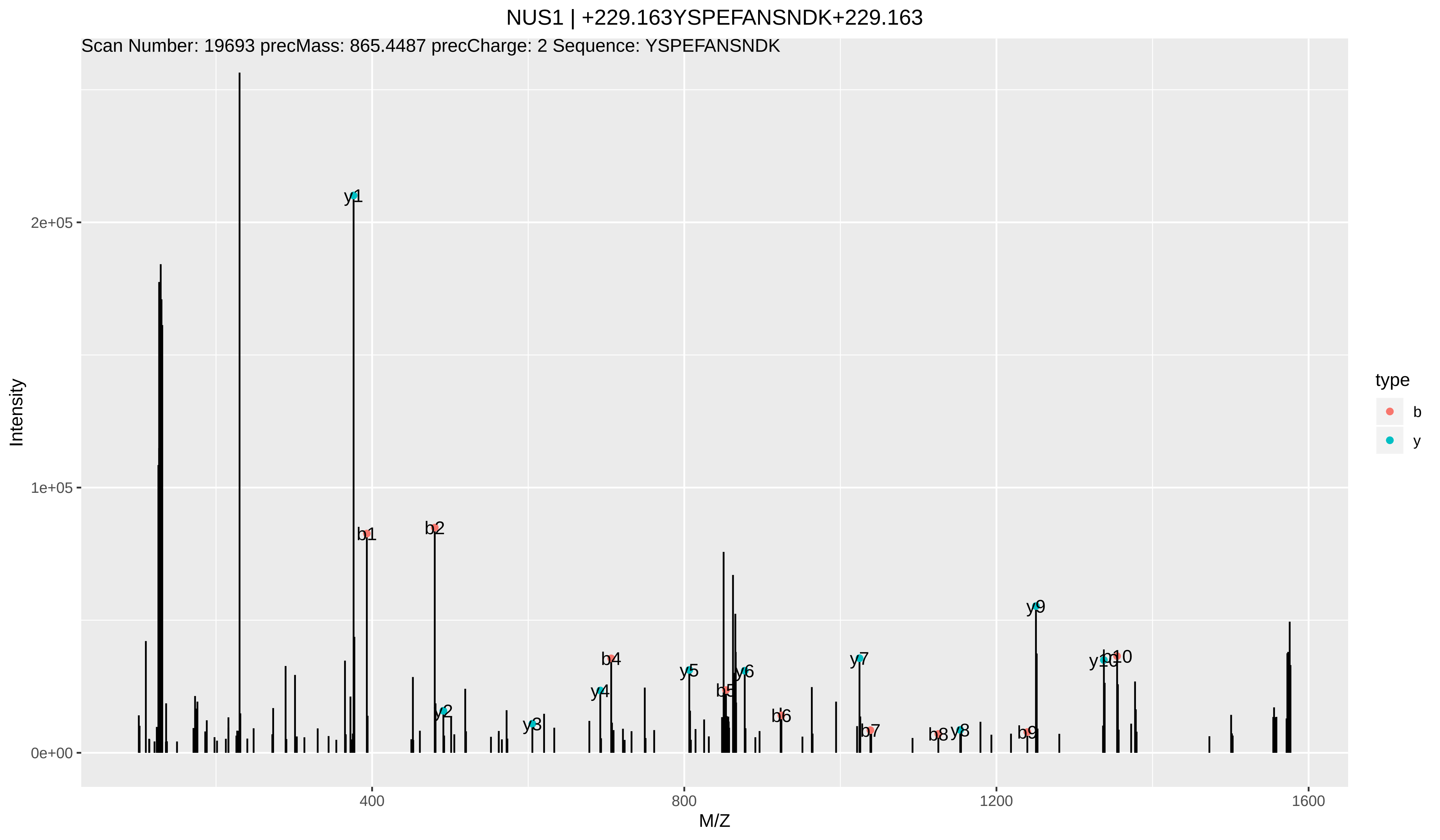

NUS1 | +229.163YSPEFANSNDK+229.163
Scan Number: 19693 precMass: 865.4487 precCharge: 2 Sequence: YSPEFANSNDK
Intensity
2e+05
1e+05
0e+00
M/Z
400
800
1200
1600
type
b
y
y1
b1
b2
y2
y3
b4
y4
y5
b5
y6
b6
y7
b7
b8
y8
y9
b9
y10
b10

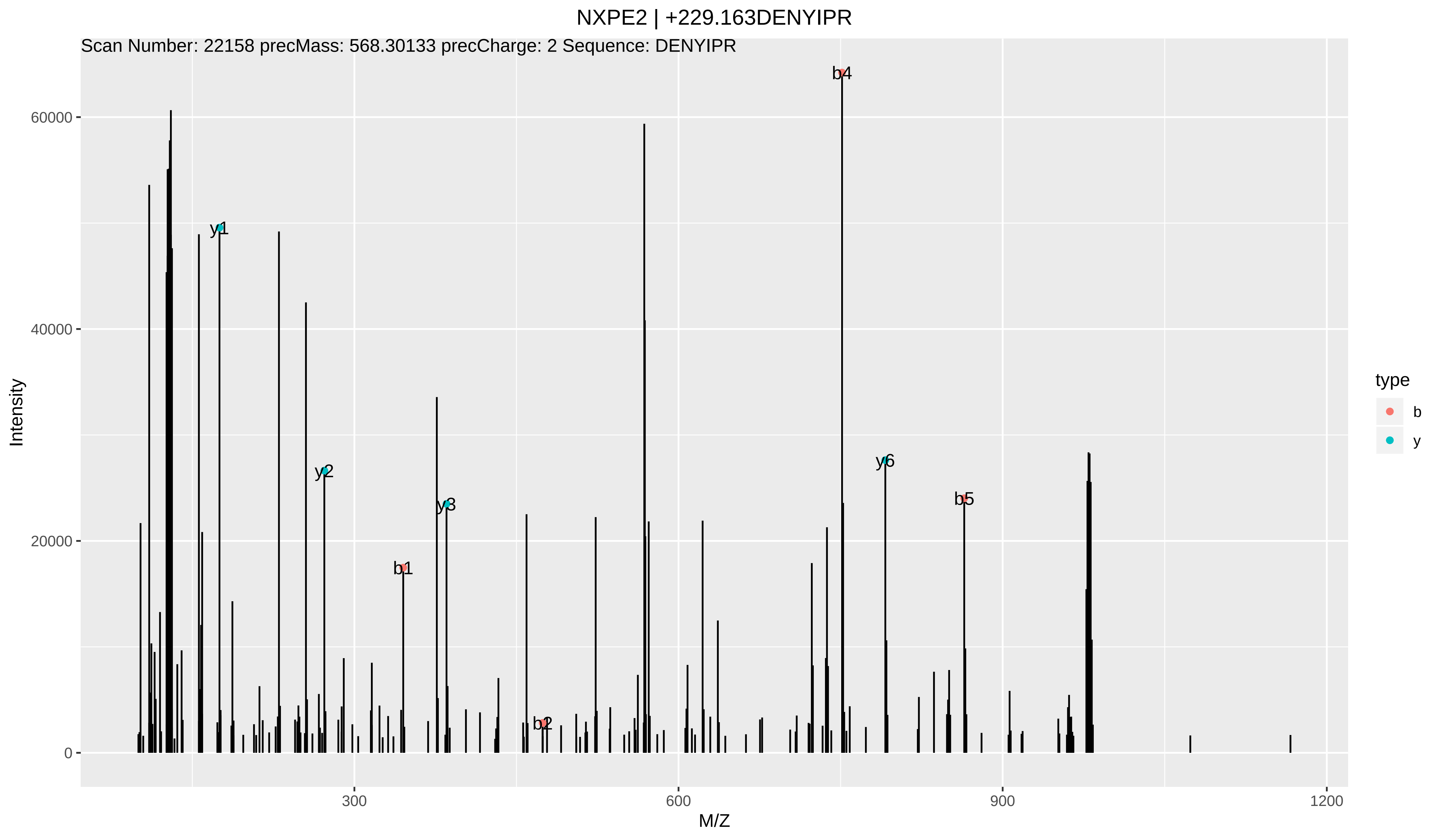
NXPE2 | +229.163DENYIPR
Scan Number: 22158 precMass: 568.30133 precCharge: 2 Sequence: DENYIPR
Intensity
M/Z
0
20000
40000
60000
300
600
900
1200
y1
y2
y3
b1
b2
b4
y6
b5
type
b
y

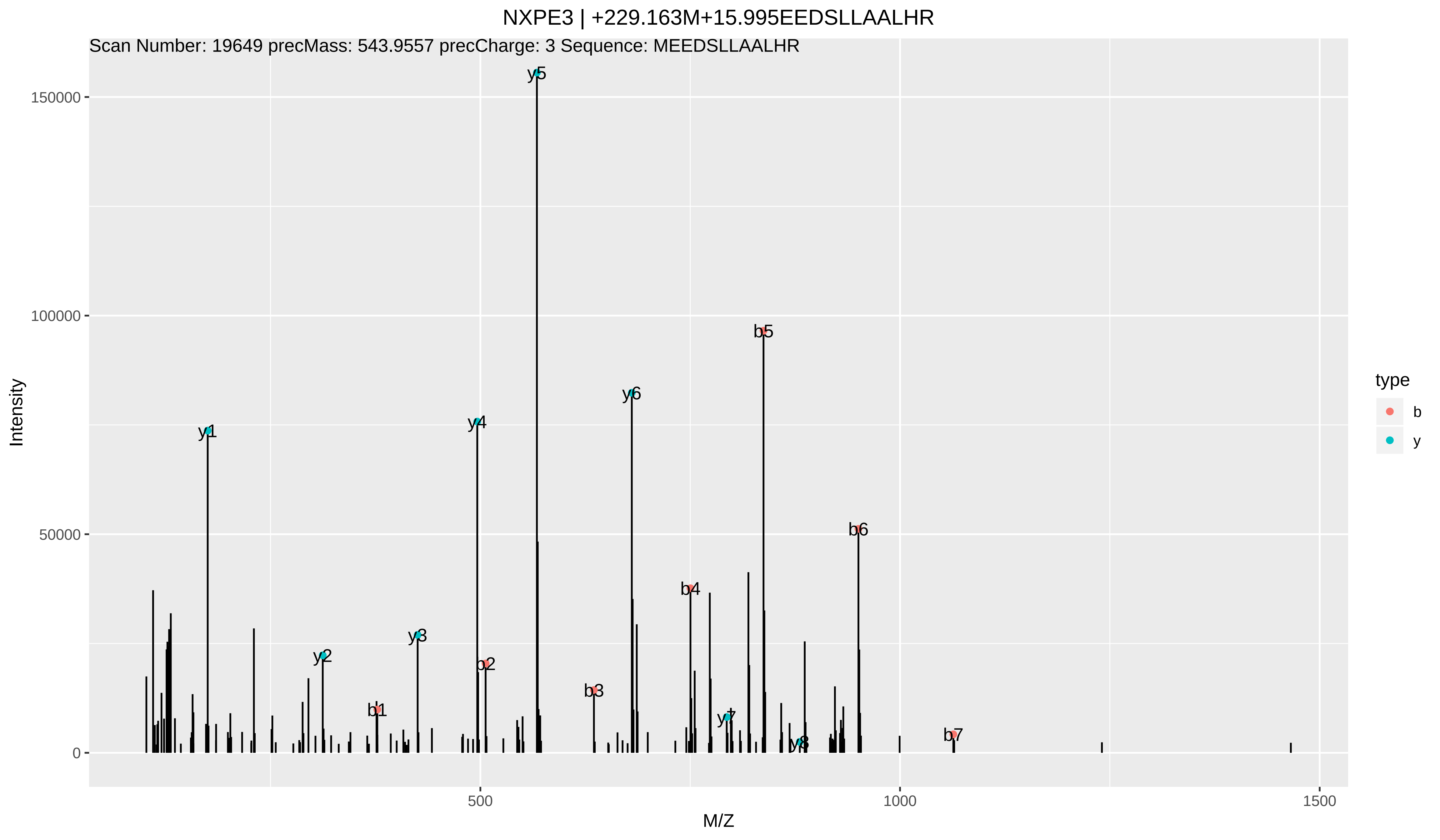

NXPE3 | +229.163M+15.995EEDSLLAALHR
Scan Number: 19649 precMass: 543.9557 precCharge: 3 Sequence: MEEDSLLAALHR
Intensity
M/Z
type
b
y
y1
y2
y3
y4
y5
y6
y7
y8
b1
b2
b3
b4
b5
b6
b7
0
50000
100000
150000
500
1000
1500

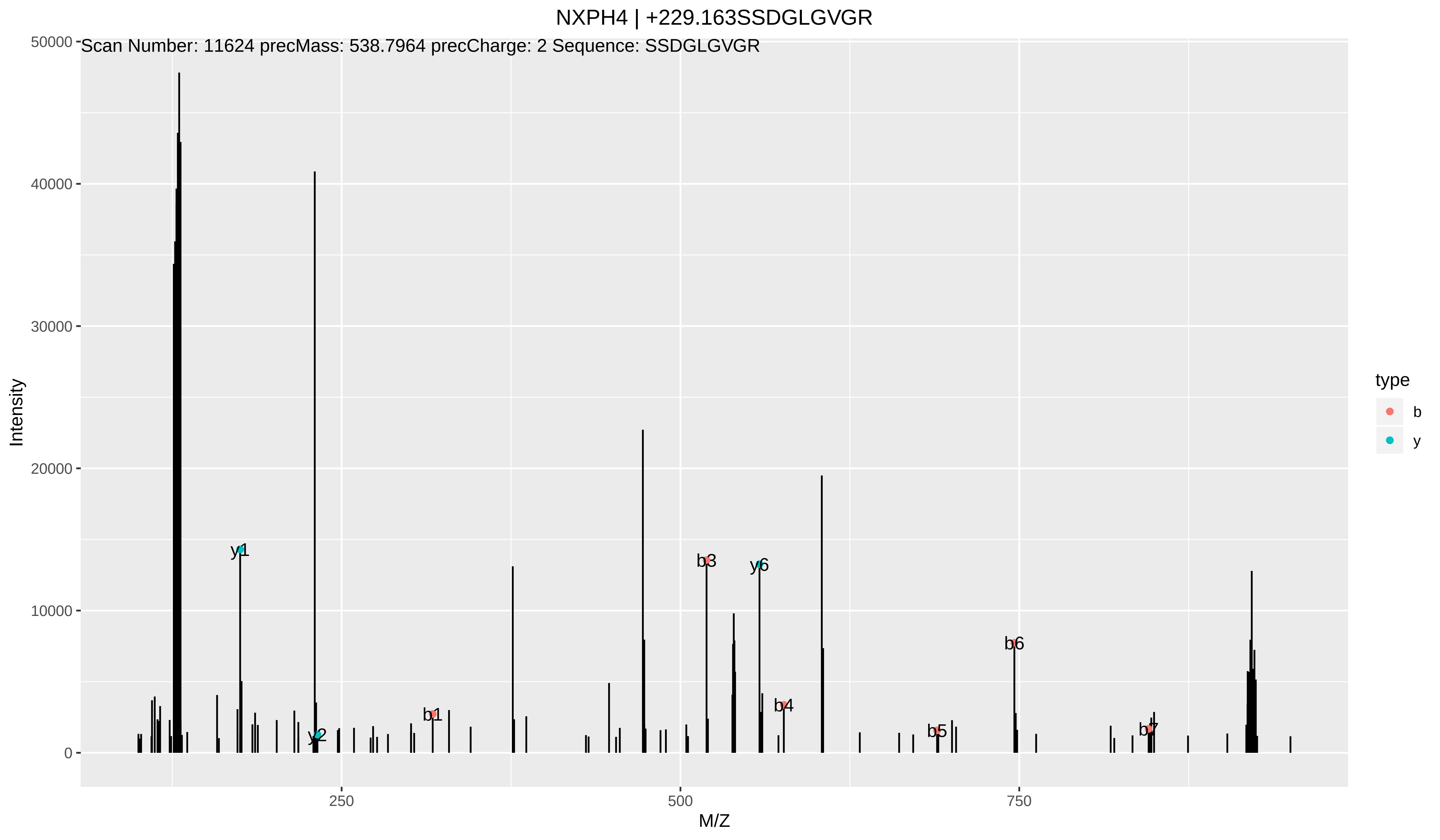

NXPH4 | +229.163SSDGLGVGR
Scan Number: 11624 precMass: 538.7964 precCharge: 2 Sequence: SSDGLGVGR
Intensity
M/Z
0
10000
20000
30000
40000
50000
250
500
750
y1
y2
b1
b3
y6
b4
b5
b6
b7
type
b
y

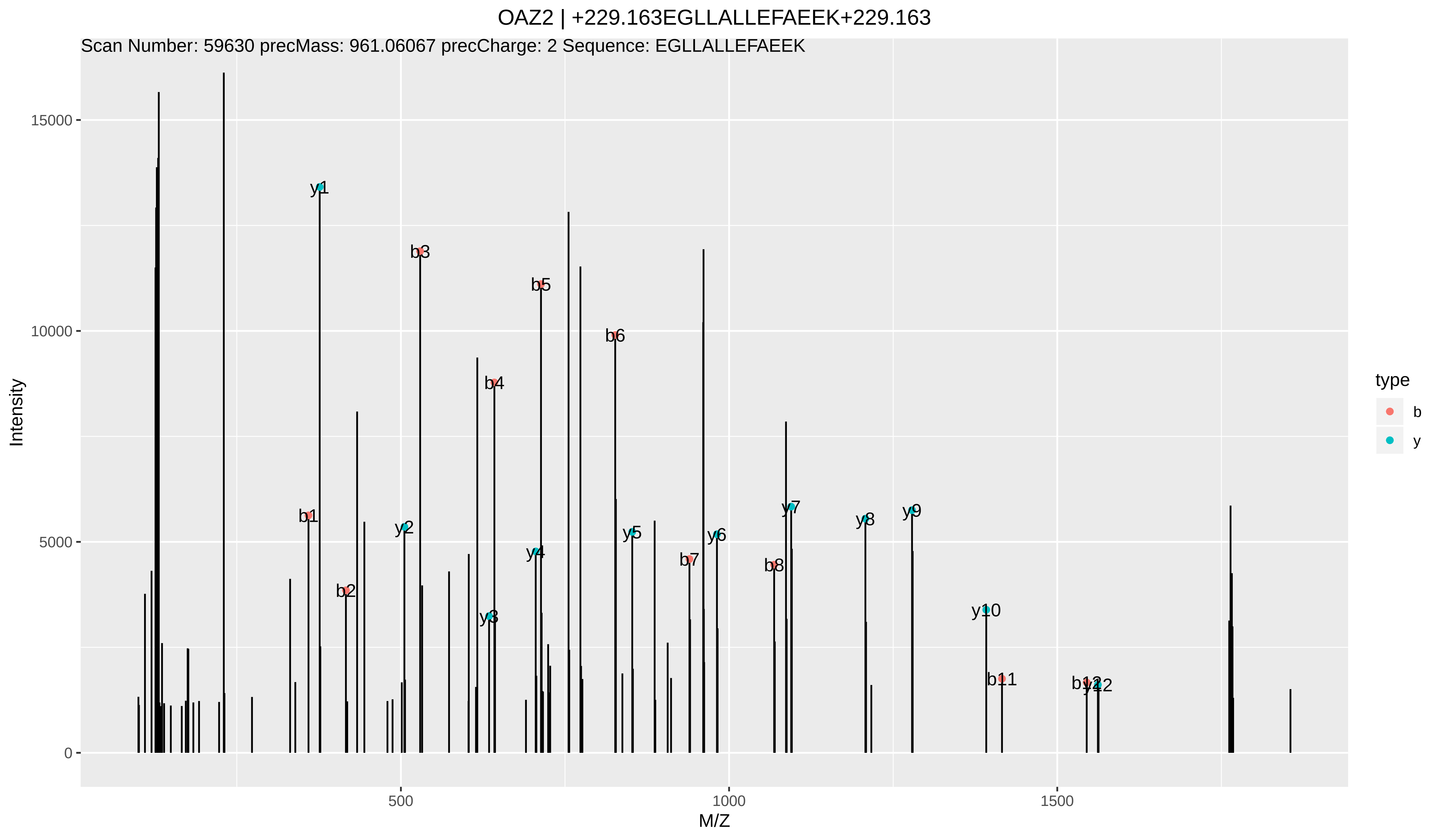
OAZ2 | +229.163EGLLALLEFAEEK+229.163
Scan Number: 59630 precMass: 961.06067 precCharge: 2 Sequence: EGLLALLEFAEEK
Intensity
M/Z
0
5000
10000
15000
500
1000
1500
type
b
y
y1
b1
b2
y2
b3
y3
b4
y4
b5
b6
y5
b7
y6
b8
y7
y8
y9
y10
b11
b12
y12

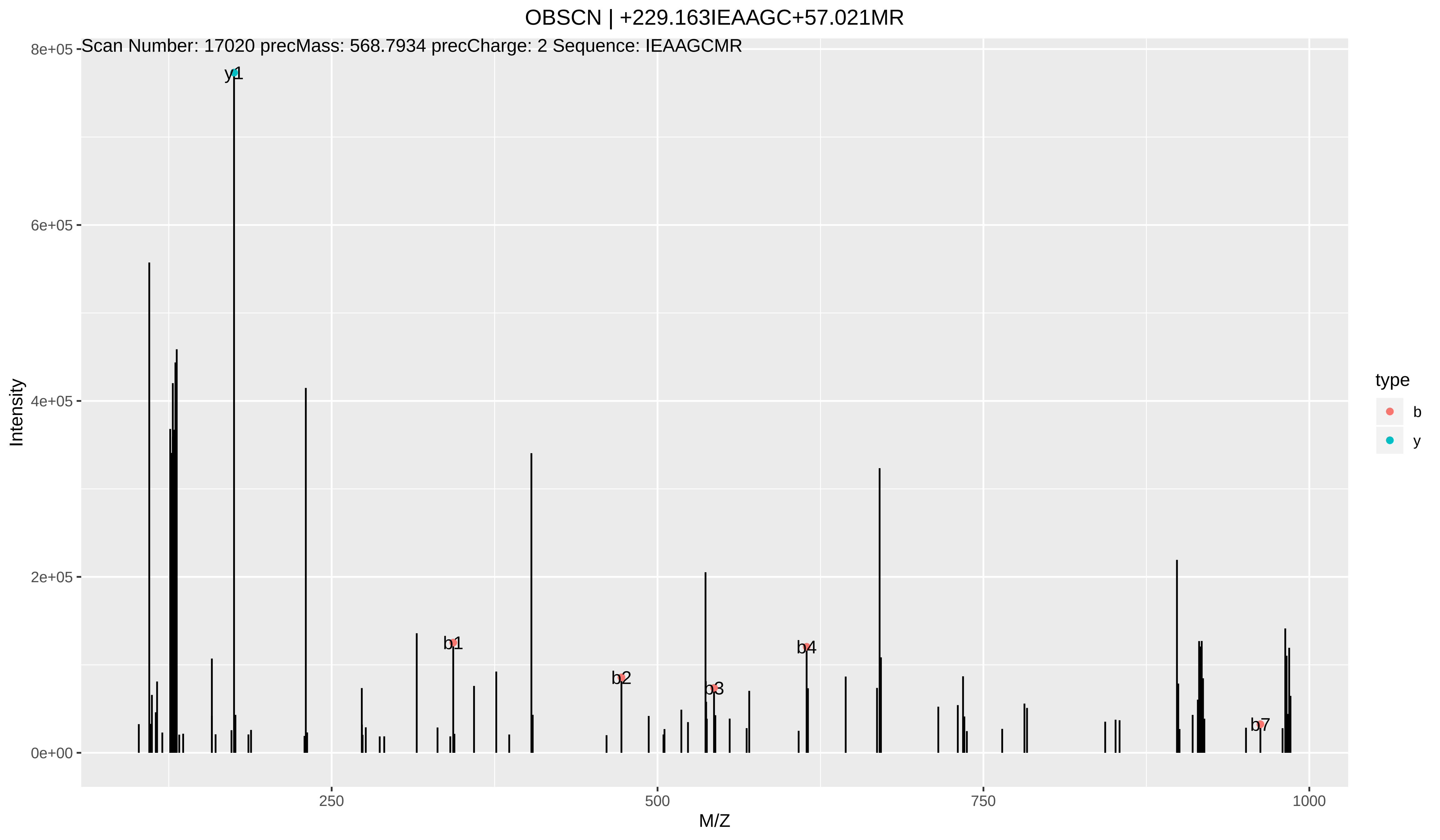
OBSCN | +229.163IEAAGC+57.021MR
Scan Number: 17020 precMass: 568.7934 precCharge: 2 Sequence: IEAAGCMR
y1
b1
b2
b3
b4
b7
Intensity
8e+05
6e+05
4e+05
2e+05
0e+00
M/Z
250
500
750
1000
type
b
y

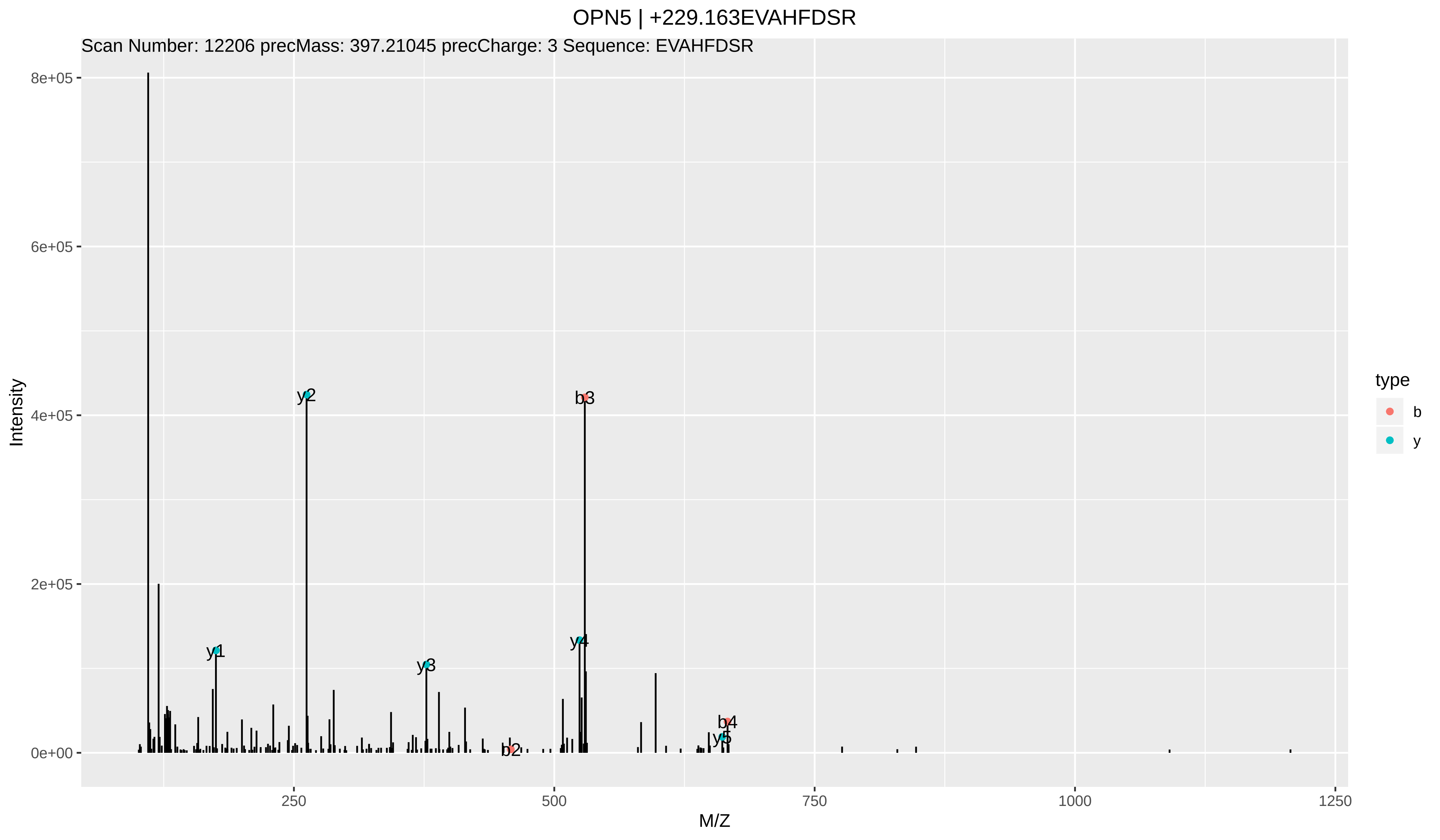

OPN5 | +229.163EVAHFDSR
Scan Number: 12206 precMass: 397.21045 precCharge: 3 Sequence: EVAHFDSR
Intensity
8e+05
6e+05
4e+05
2e+05
0e+00
y1
y2
y3
y4
y5
b2
b3
b4
M/Z
250
500
750
1000
1250
type
b
y

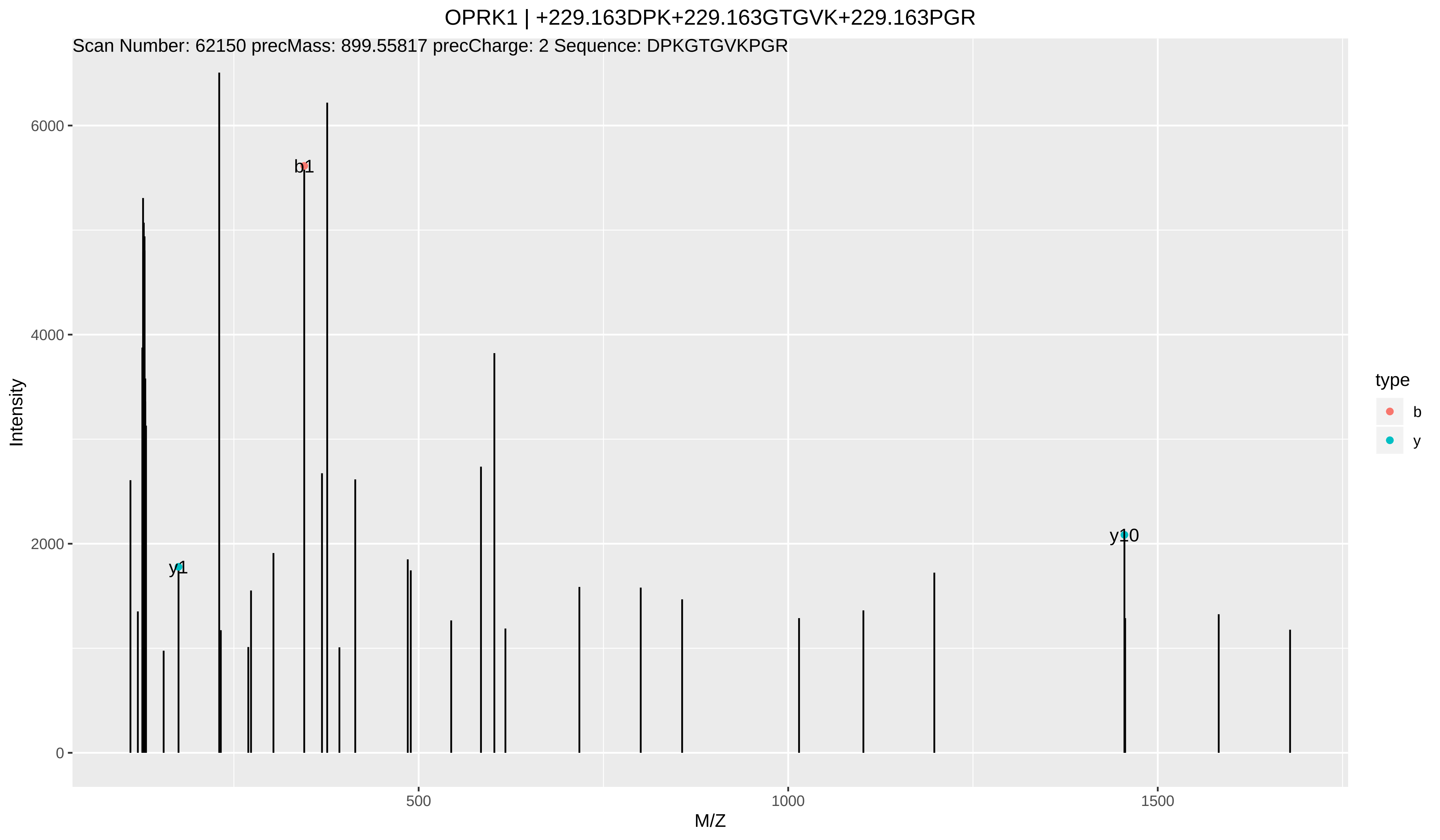
OPRK1 | +229.163DPK+229.163GTGVK+229.163PGR
Scan Number: 62150 precMass: 899.55817 precCharge: 2 Sequence: DPKGTGVKPGR
b1
y1
y10
Intensity
6000
4000
2000
0
500
1000
1500
M/Z
type
b
y

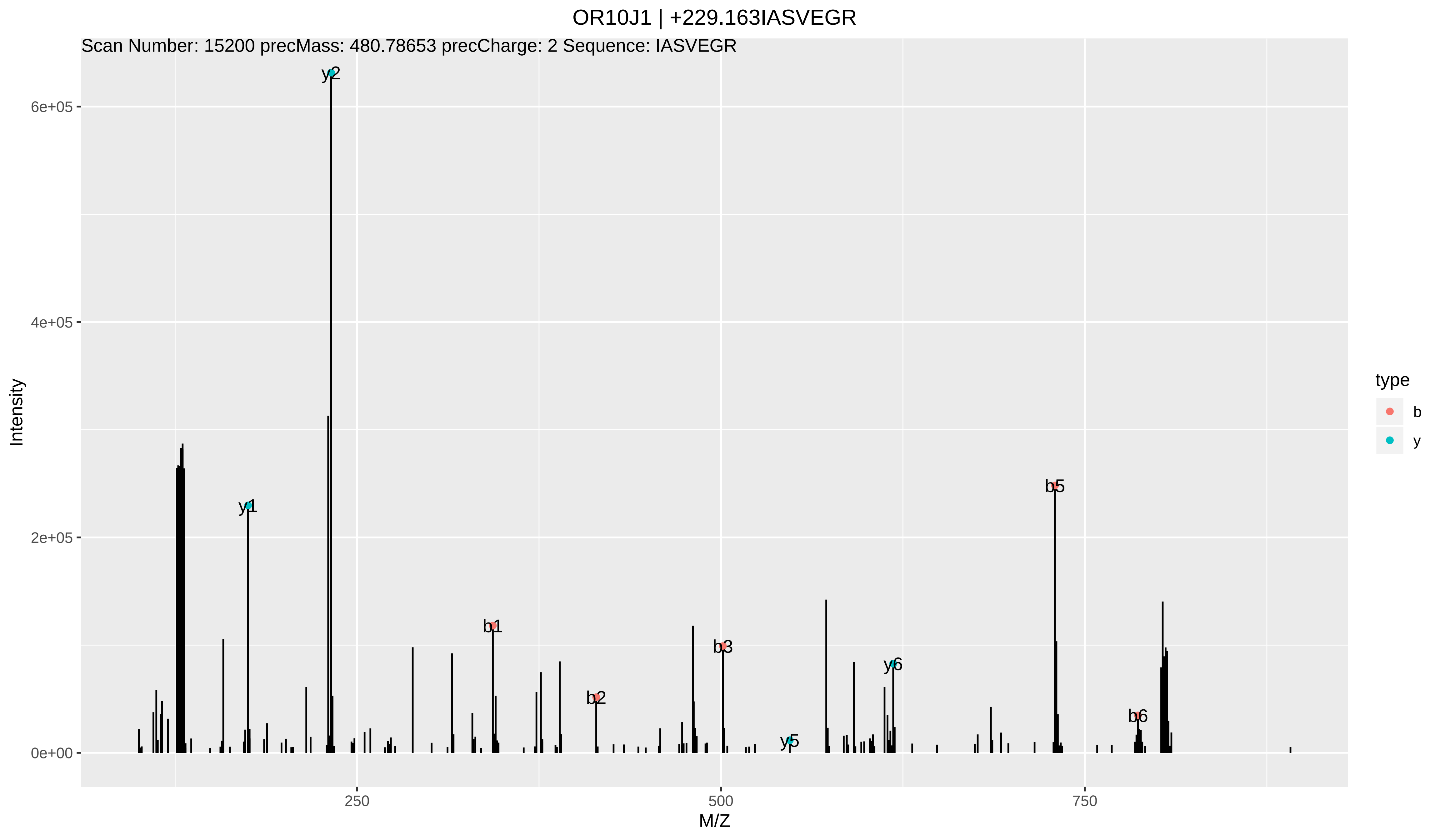
OR10J1 | +229.163IASVEGR
Scan Number: 15200 precMass: 480.78653 precCharge: 2 Sequence: IASVEGR
Intensity
M/Z
6e+05
4e+05
2e+05
0e+00
250
500
750
y2
y1
b1
b2
b3
y5
y6
b5
b6
type
b
y

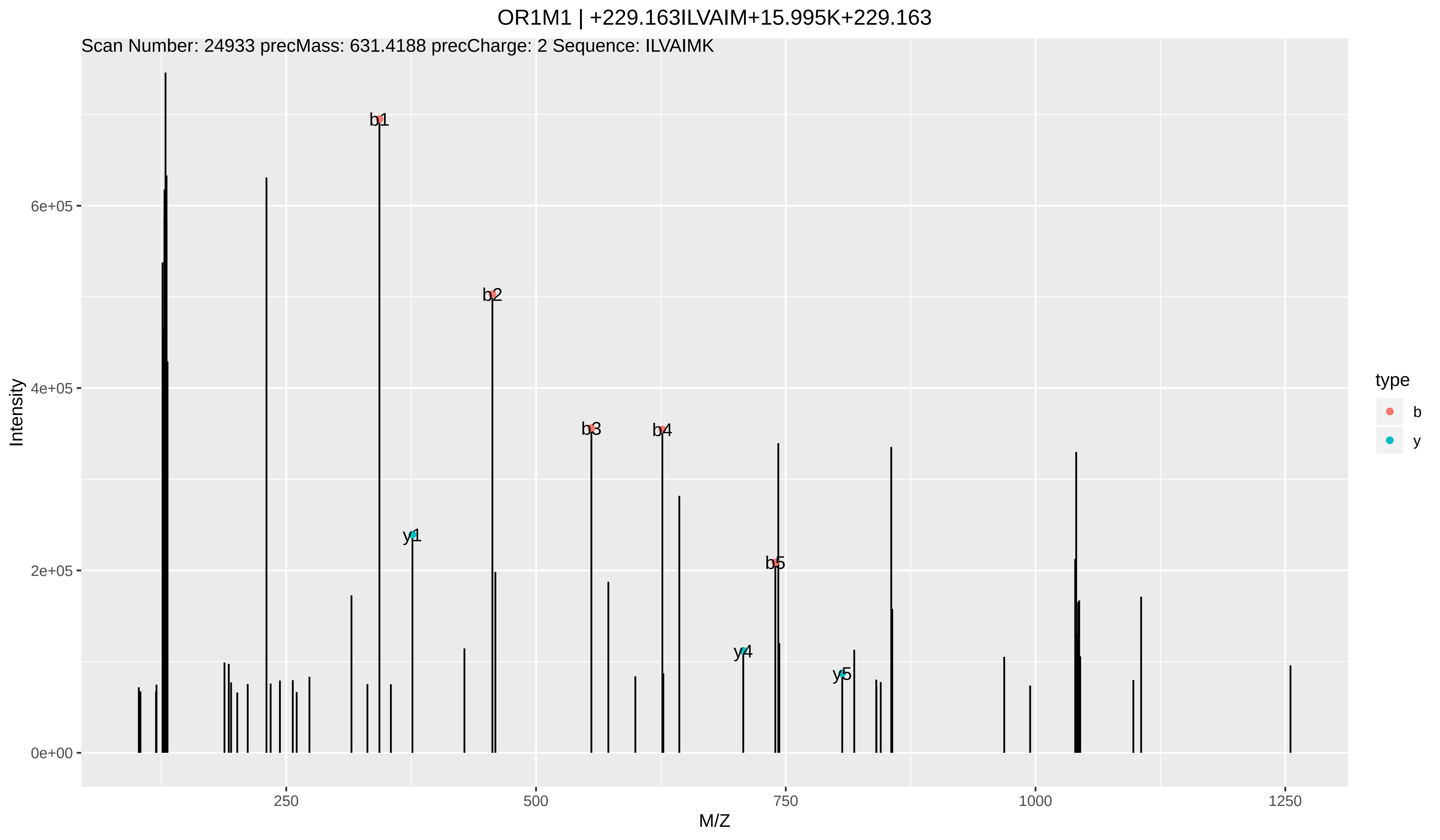

OR1M1 | +229.163ILVAIM+15.995K+229.163
Scan Number: 24933 precMass: 631.4188 precCharge: 2 Sequence: ILVAIMK
Intensity
6e+05
4e+05
2e+05
0e+00
M/Z
250
500
750
1000
1250
b1
b2
b3
b4
b5
y1
y4
y5
type
b
y

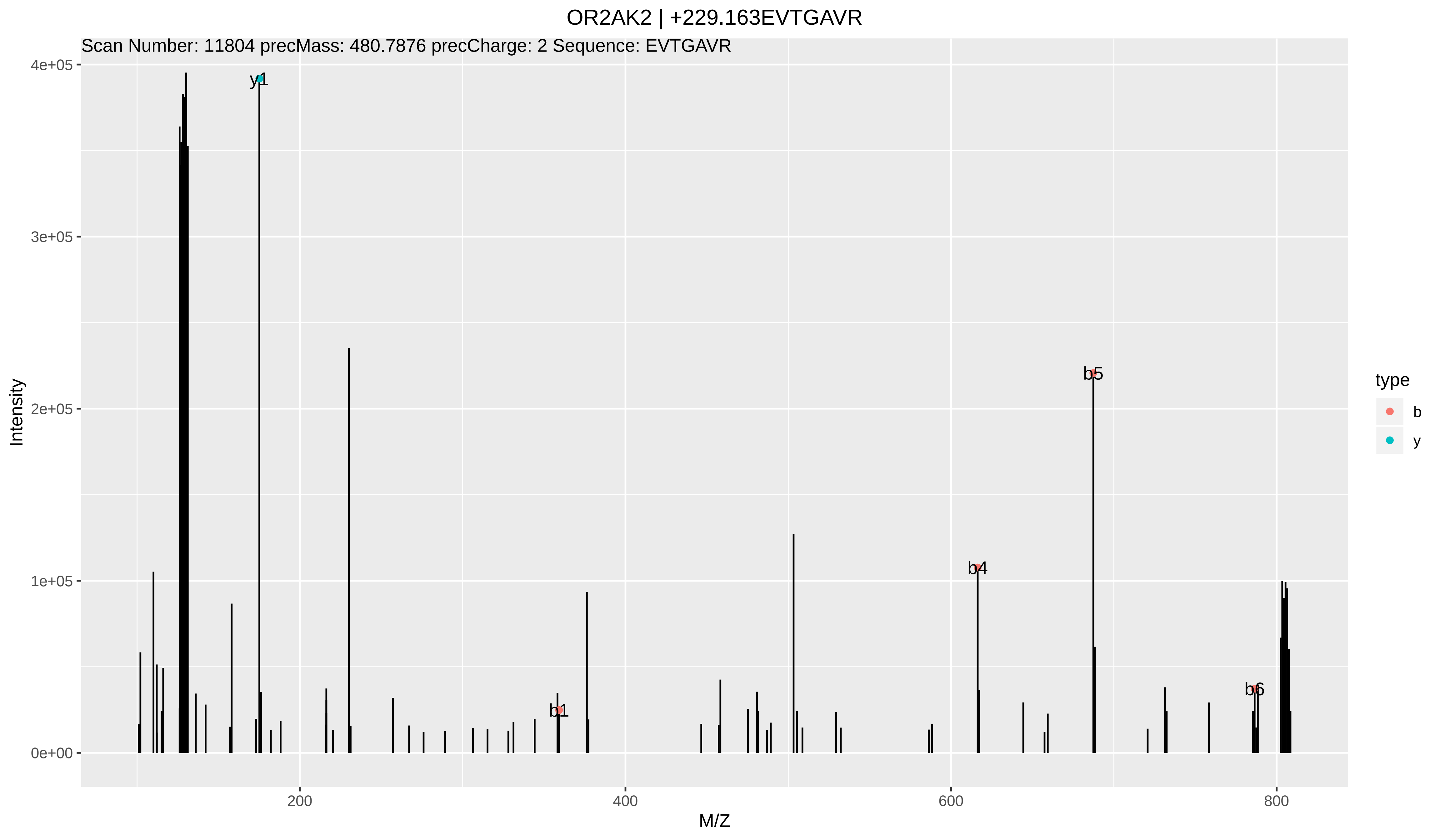

OR2AK2 | +229.163EVTGAVR
Scan Number: 11804 precMass: 480.7876 precCharge: 2 Sequence: EVTGAVR
Intensity
M/Z
4e+05
3e+05
2e+05
1e+05
0e+00
200
400
600
800
y1
b1
b4
b5
b6
type
b
y

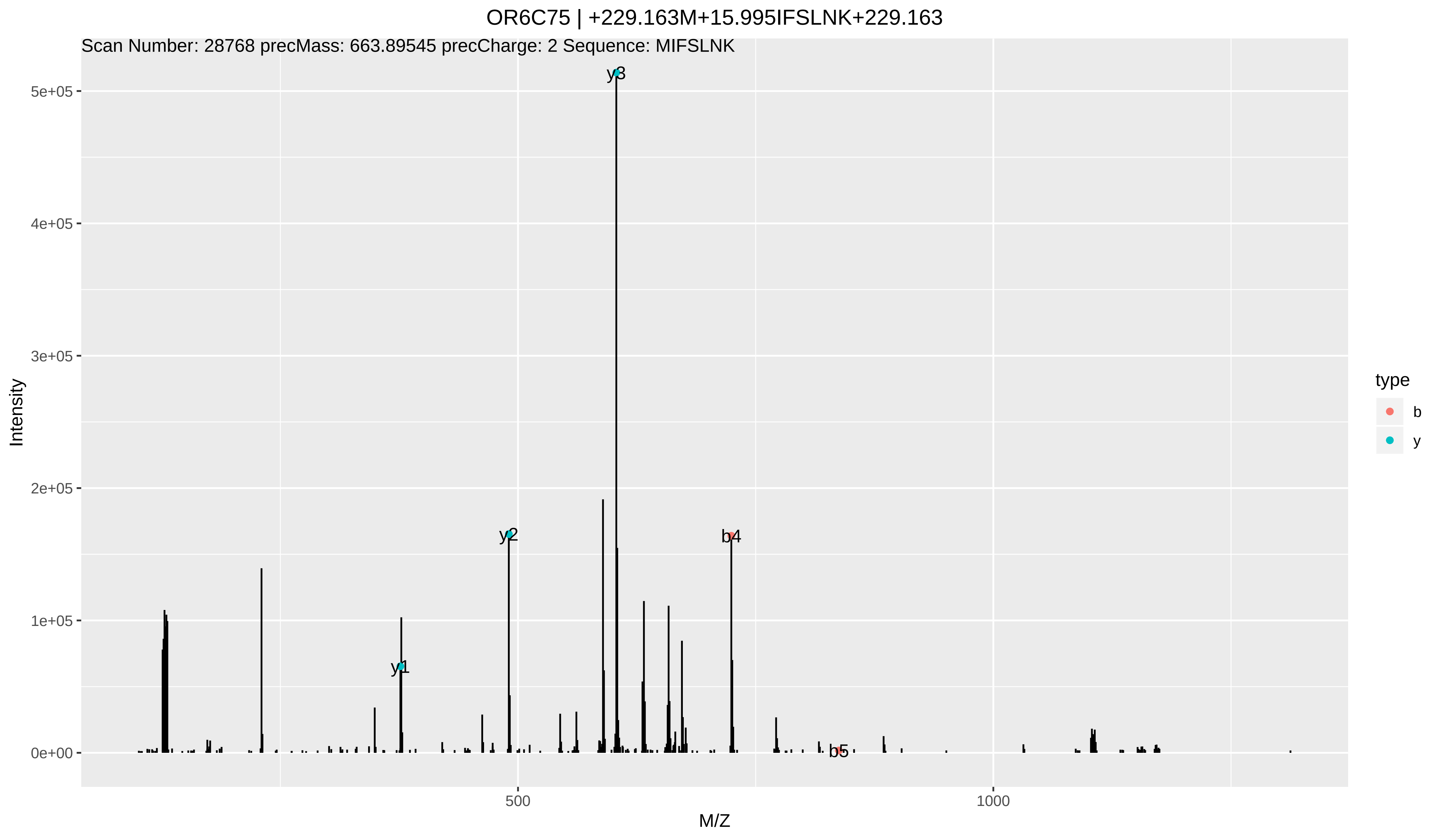

OR6C75 | +229.163M+15.995IFSLNK+229.163
Scan Number: 28768 precMass: 663.89545 precCharge: 2 Sequence: MIFSLNK
y3
y2
b4
y1
b5
5e+05
4e+05
3e+05
2e+05
1e+05
0e+00
Intensity
500
1000
M/Z
type
b
y

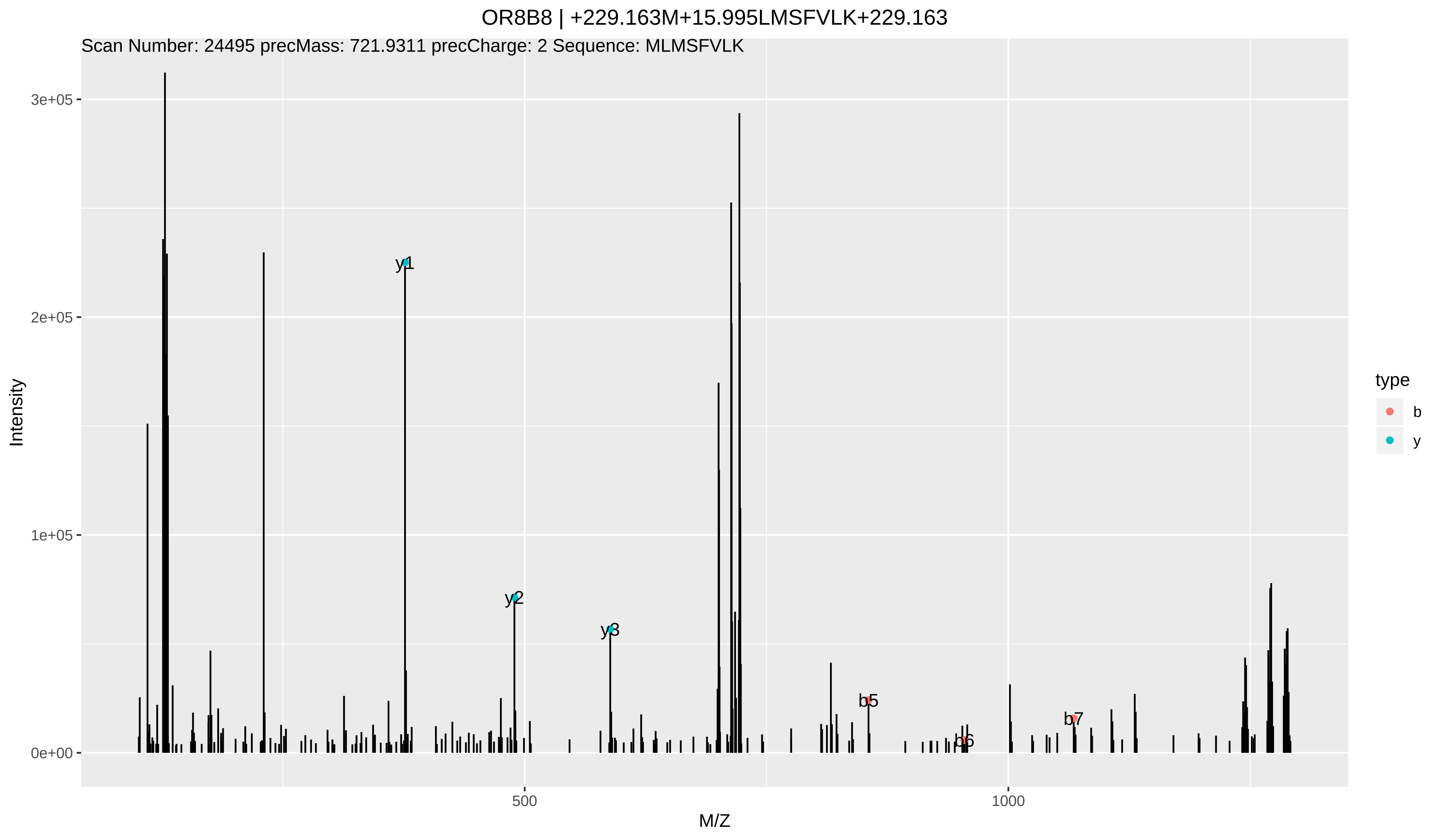

OR8B8 | +229.163M+15.995LMSFVLK+229.163
Scan Number: 24495 precMass: 721.9311 precCharge: 2 Sequence: MLMSFVLK
Intensity
3e+05
2e+05
1e+05
0e+00
500
1000
M/Z
y1
y2
y3
b5
b6
b7
type
b
y

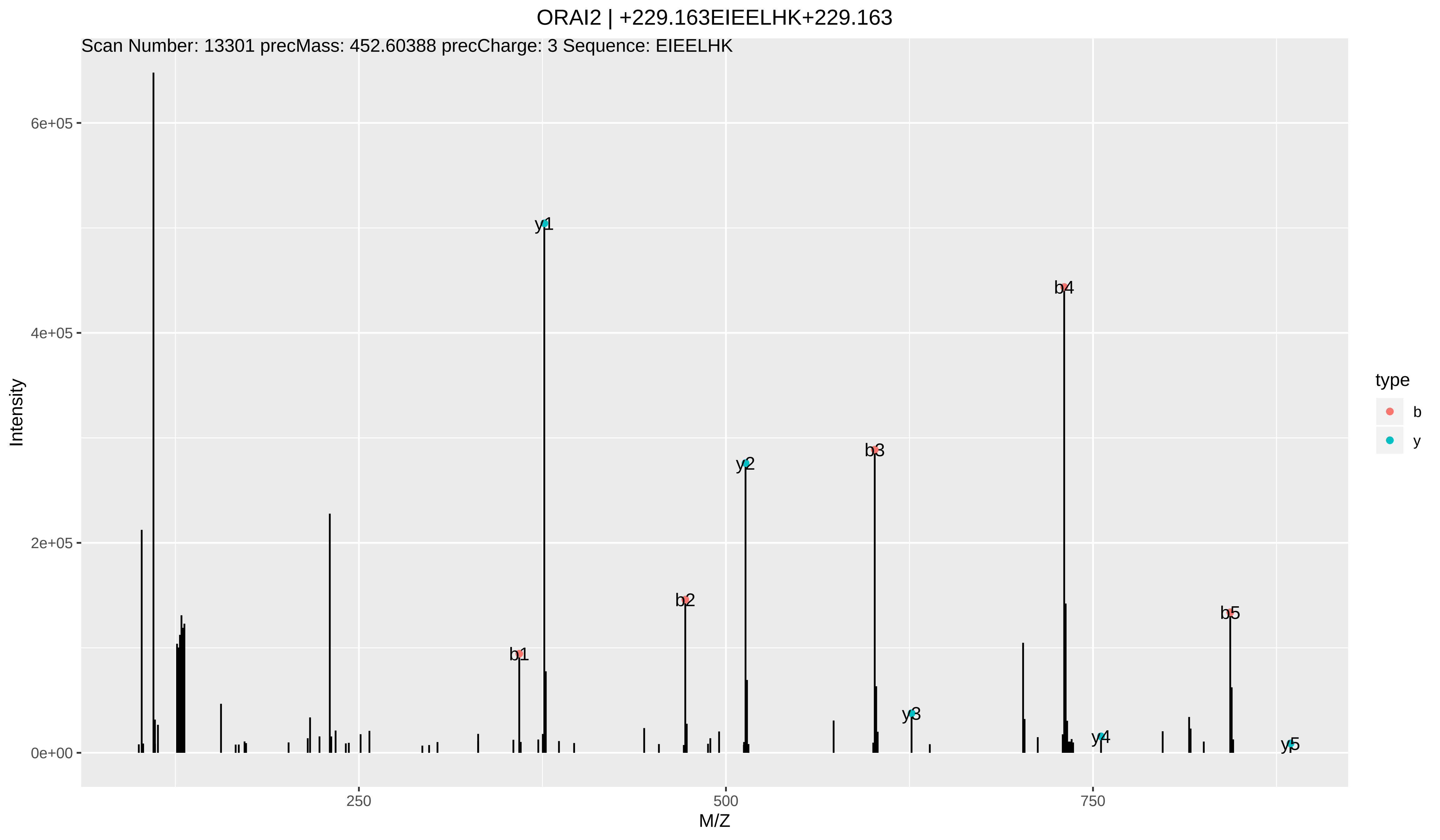
ORAI2 | +229.163EIEELHK+229.163
Scan Number: 13301 precMass: 452.60388 precCharge: 3 Sequence: EIEELHK
Intensity
M/Z
6e+05
4e+05
2e+05
0e+00
250
500
750
y1
b4
y2
b3
b2
b5
b1
y3
y4
y5
type
b
y

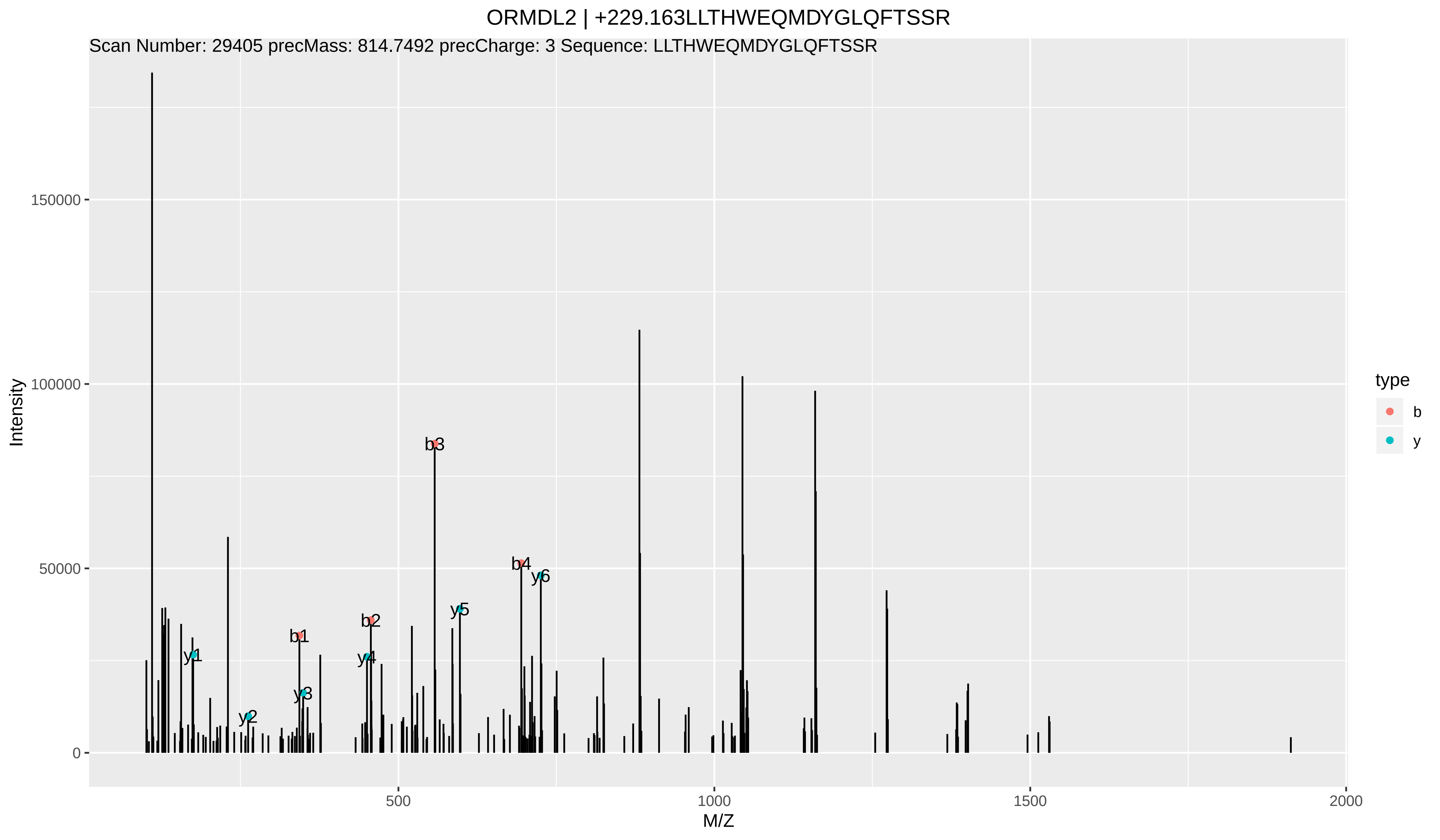

ORMDL2 | +229.163LLTHWEQMDYGLQFTSSR
Scan Number: 29405 precMass: 814.7492 precCharge: 3 Sequence: LLTHWEQMDYGLQFTSSR
Intensity
M/Z
0
50000
100000
150000
500
1000
1500
2000
b1
b2
b3
b4
y1
y2
y3
y4
y5
y6
type
b
y

ORMDL2 | +229.163M+15.995NVGVAHSEVNPNTR

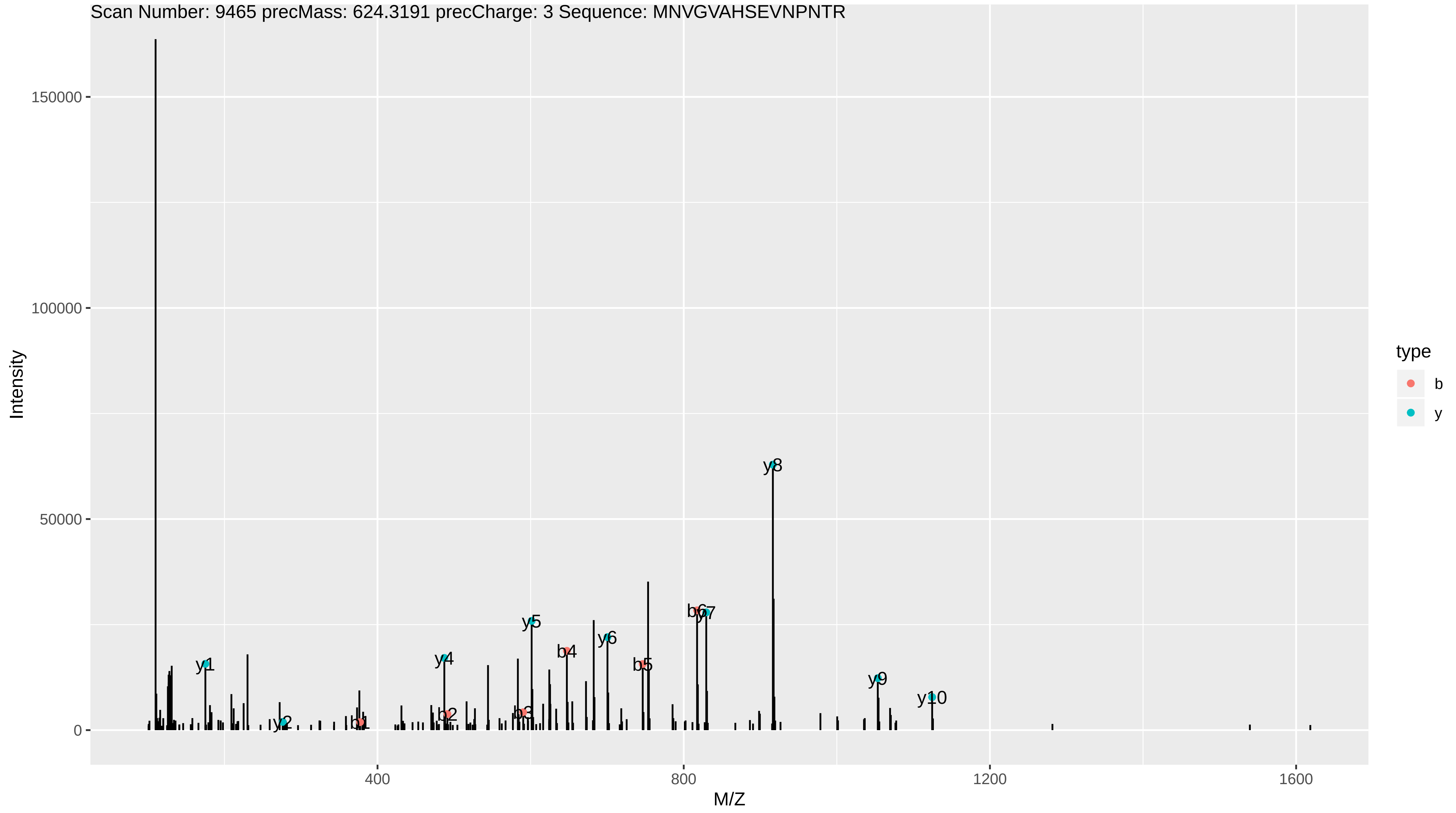

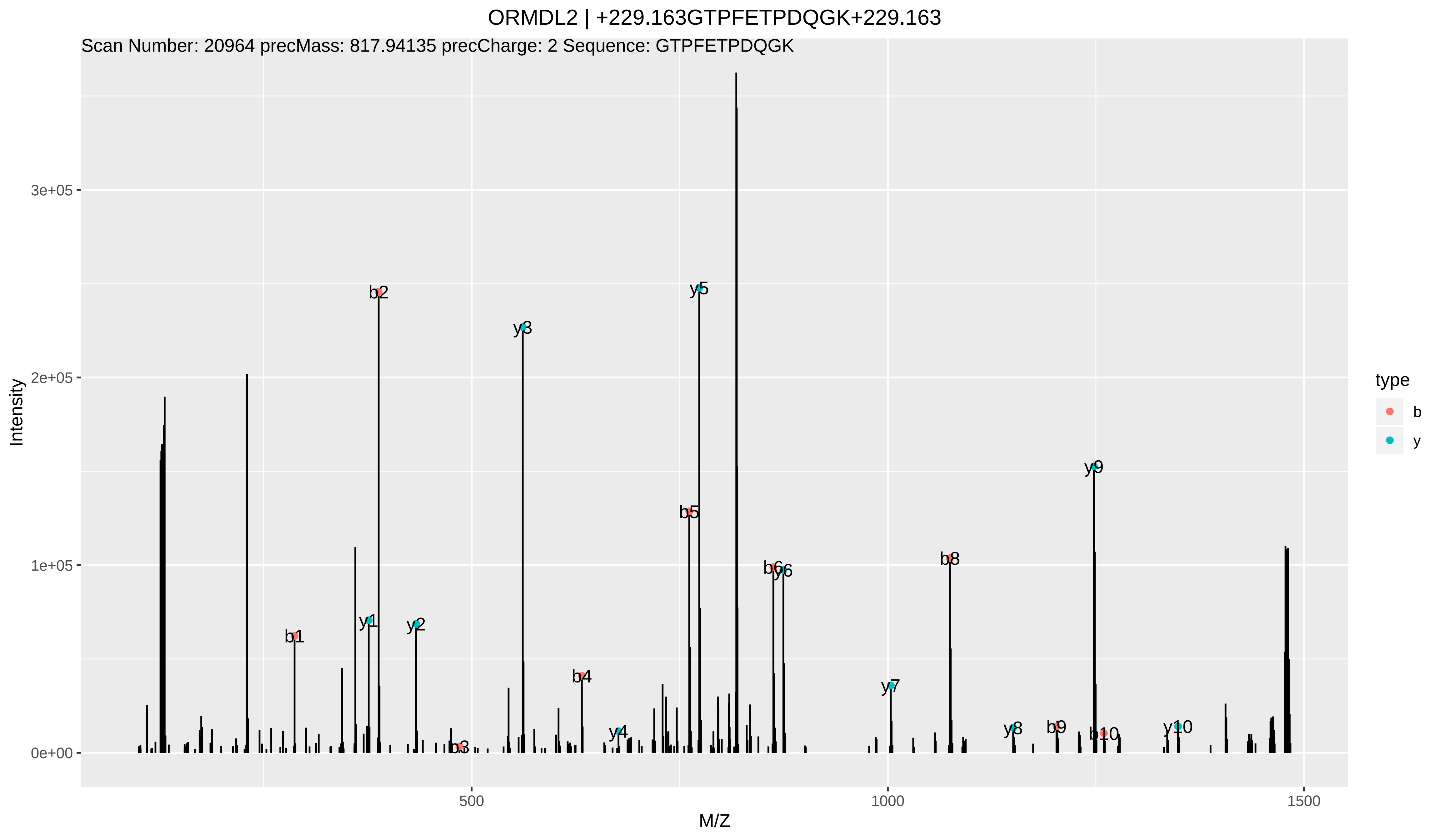

ORMDL2 | +229.163GTPFETPDQGK+229.163
Scan Number: 20964 precMass: 817.94135 precCharge: 2 Sequence: GTPFETPDQGK
Intensity
M/Z
type
b
y
b1
b2
b3
b4
b5
b6
b8
b9
b10
y1
y2
y3
y4
y5
y6
y7
y8
y9
y10
0e+00
1e+05
2e+05
3e+05
500
1000
1500

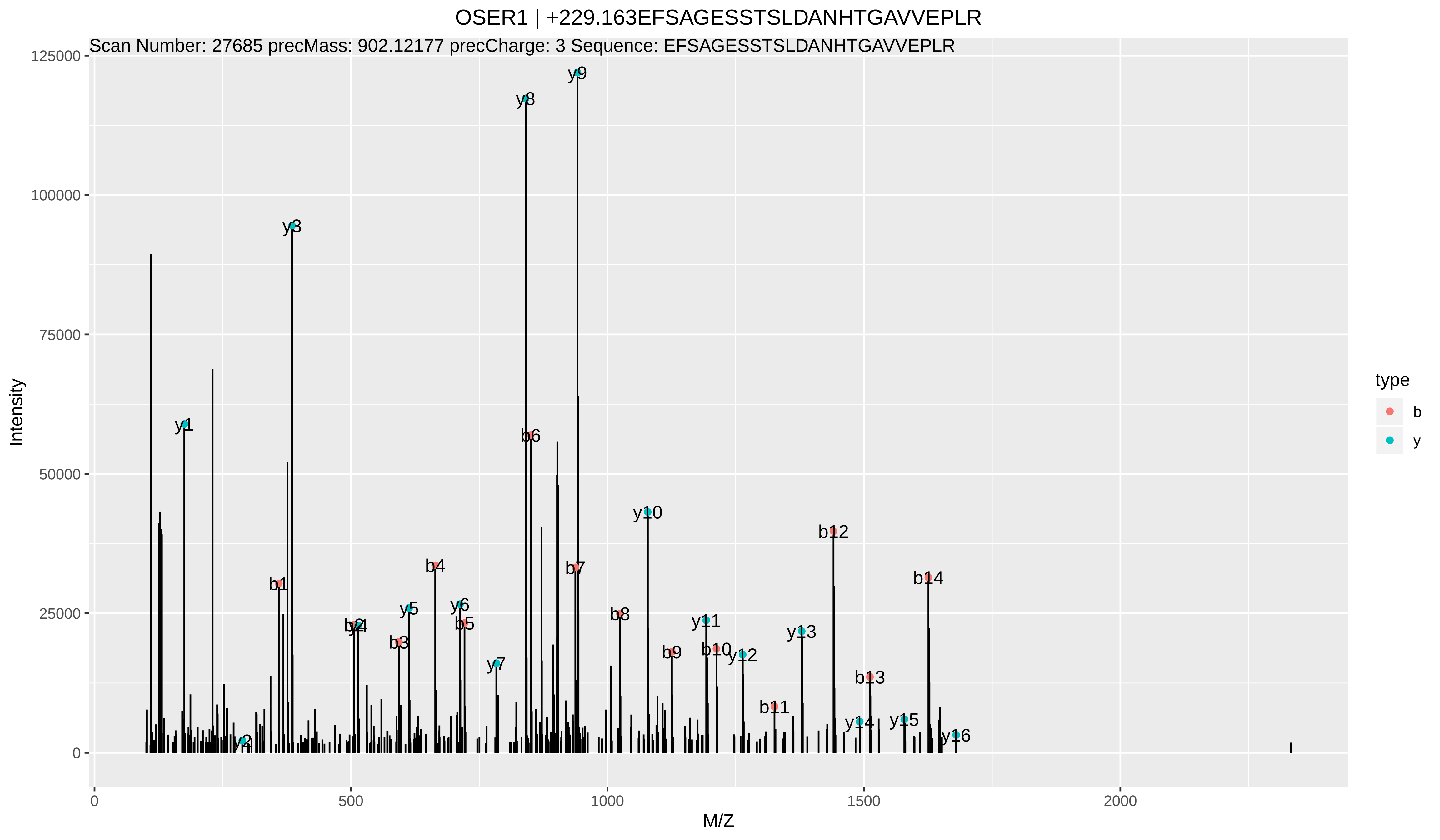

OSER1 | +229.163EFSAGESSTSLDANHTGAVVEPLR
Scan Number: 27685 precMass: 902.12177 precCharge: 3 Sequence: EFSAGESSTSLDANHTGAVVEPLR
Intensity
M/Z
type
b
y
y9
y8
y3
y1
b6
y10
b12
b7
b4
b1
b14
y6
y5
b8
b2
y4
b5
y11
y13
b3
b10
b9
y12
y7
b13
b11
y14
y15
y16
y2
125000
100000
75000
50000
25000
0
0
500
1000
1500
2000

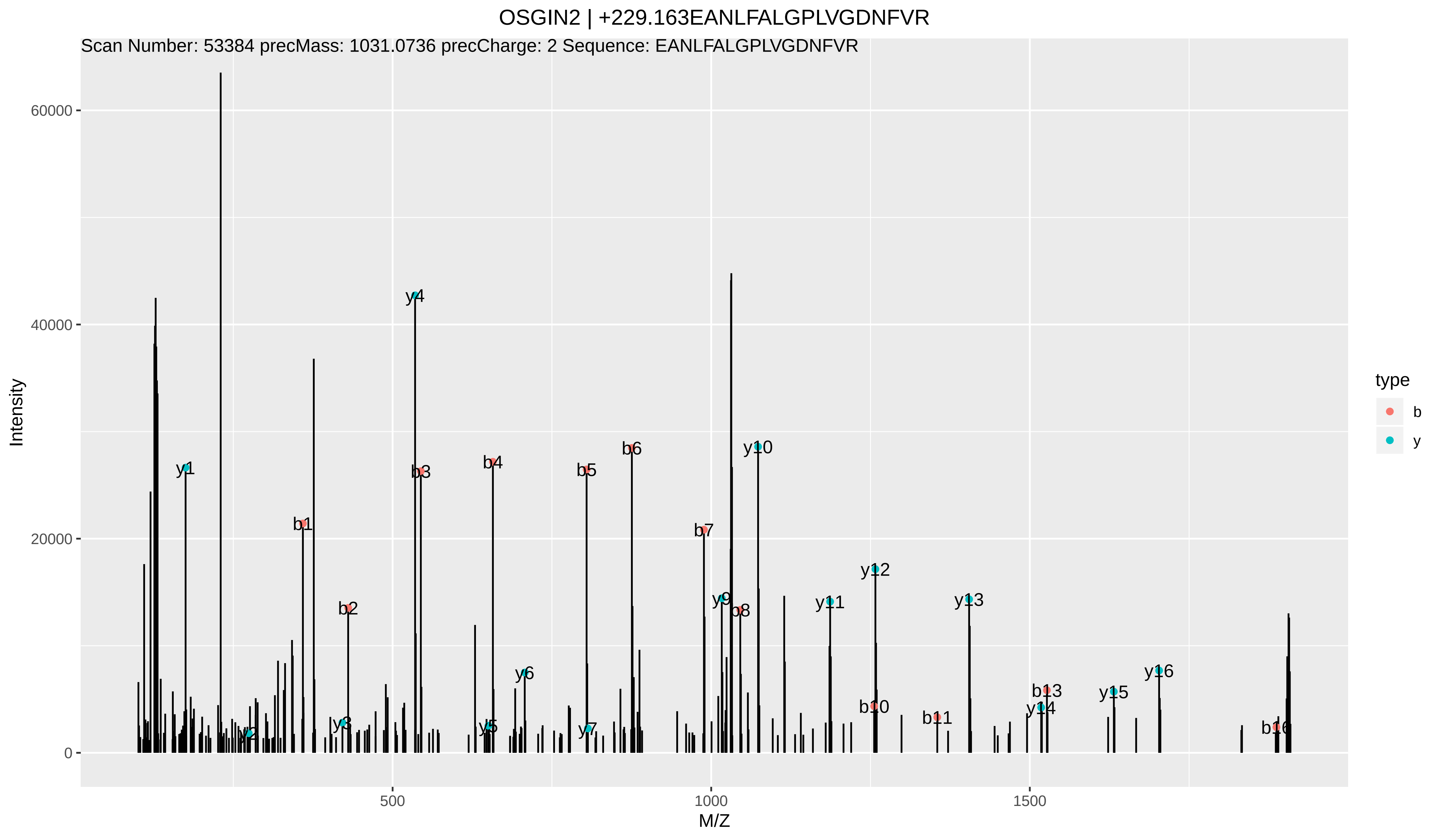

OSGIN2 | +229.163EANLFALGPLVGDNFVR
Scan Number: 53384 precMass: 1031.0736 precCharge: 2 Sequence: EANLFALGPLVGDNFVR
Intensity
M/Z
0
20000
40000
60000
500
1000
1500
type
b
y
y1
y2
y3
y4
y5
y6
y7
y9
y10
y11
y12
y13
y14
y15
y16
b1
b2
b3
b4
b5
b6
b7
b8
b10
b11
b13
b16

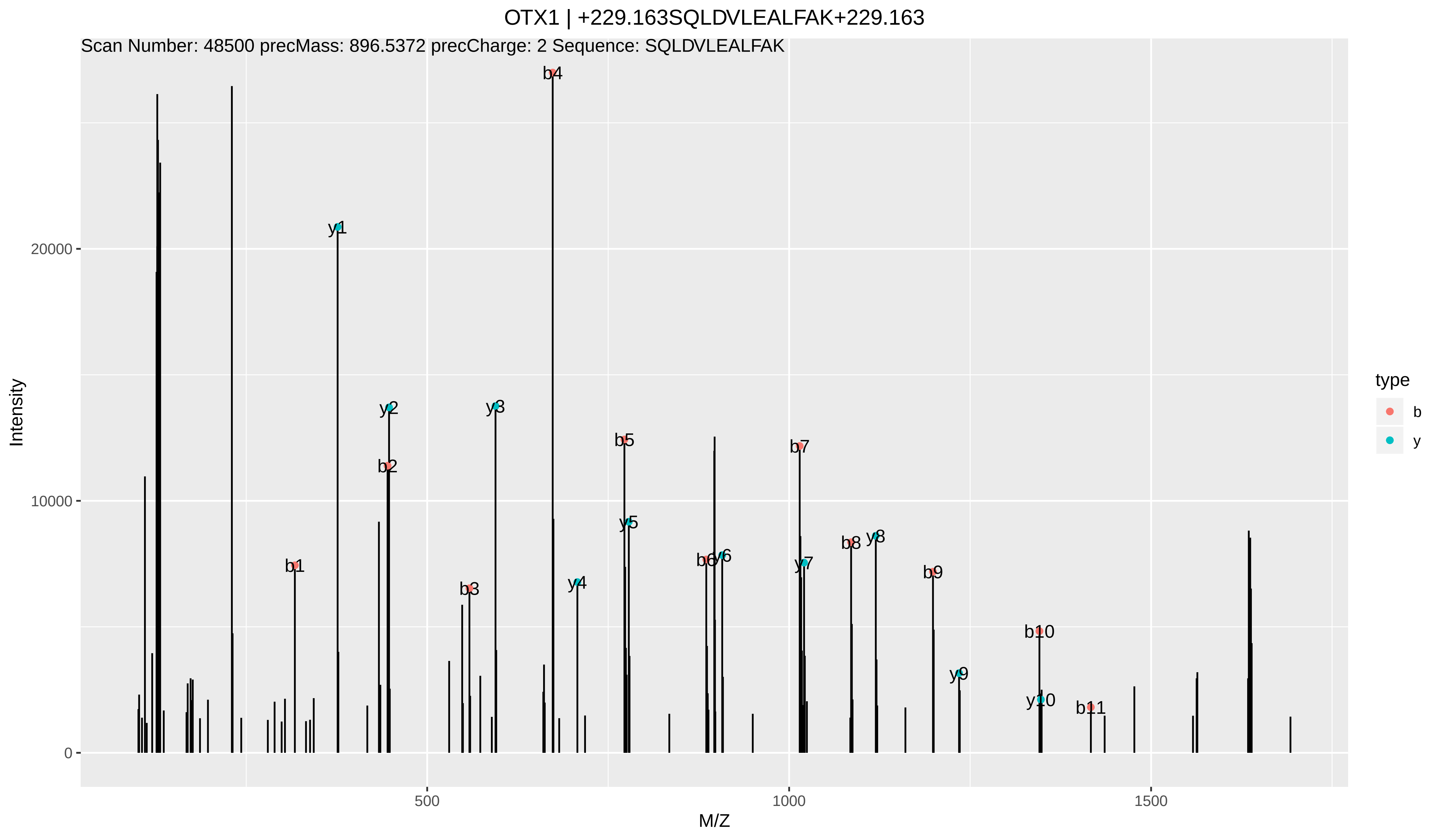
OTX1 | +229.163SQLDVLEALFAK+229.163
Scan Number: 48500 precMass: 896.5372 precCharge: 2 Sequence: SQLDVLEALFAK
Intensity
M/Z
0
10000
20000
500
1000
1500
b1
b2
b3
b4
b5
b6
b7
b8
b9
b10
b11
y1
y2
y3
y4
y5
y6
y7
y8
y9
y10
type
b
y

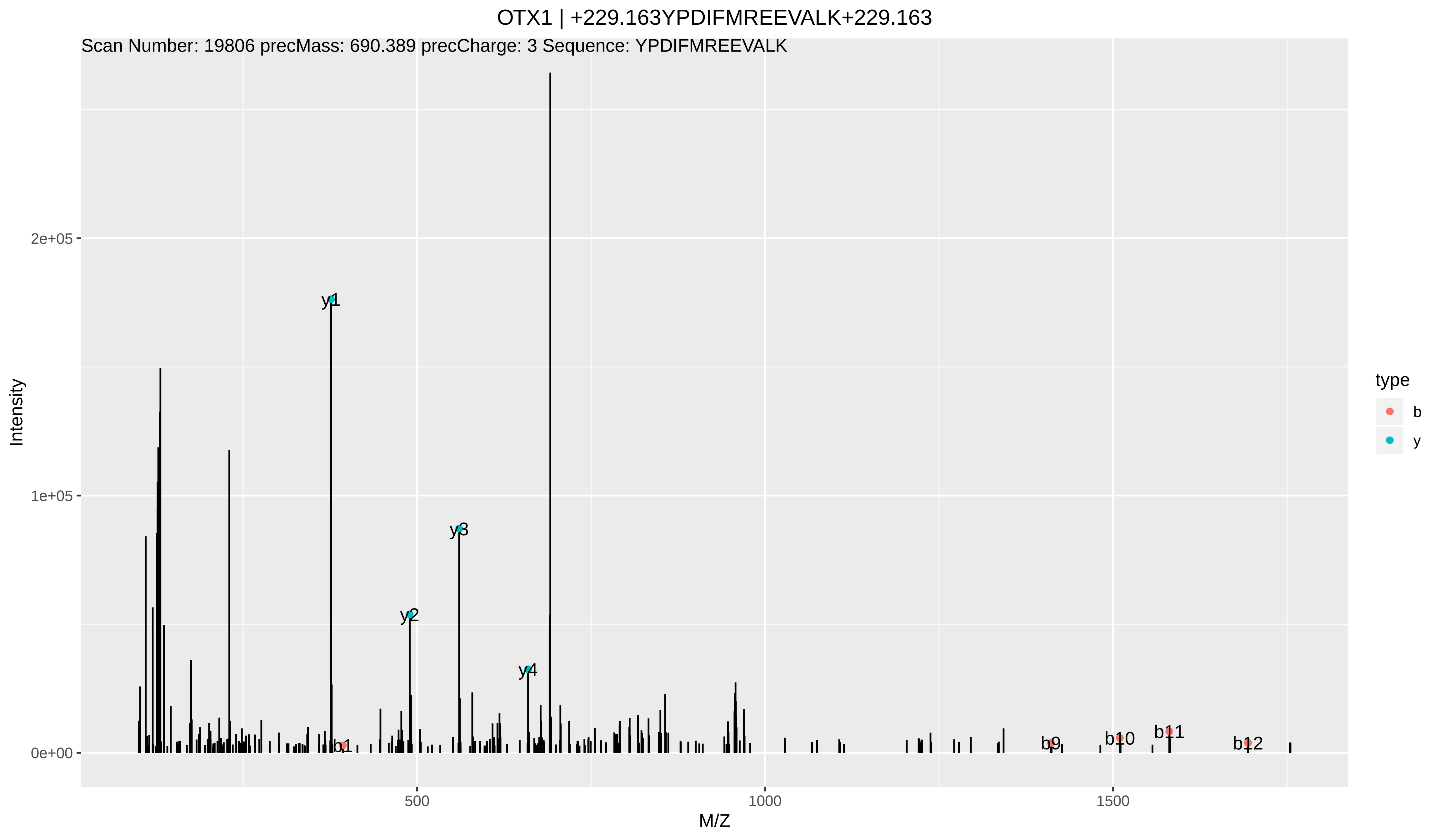

OTX1 | +229.163YPDIFMREEVALK+229.163
Scan Number: 19806 precMass: 690.389 precCharge: 3 Sequence: YPDIFMREEVALK
Intensity
2e+05
1e+05
0e+00
M/Z
500
1000
1500
y1
y2
y3
y4
b1
b9
b10
b11
b12
type
b
y

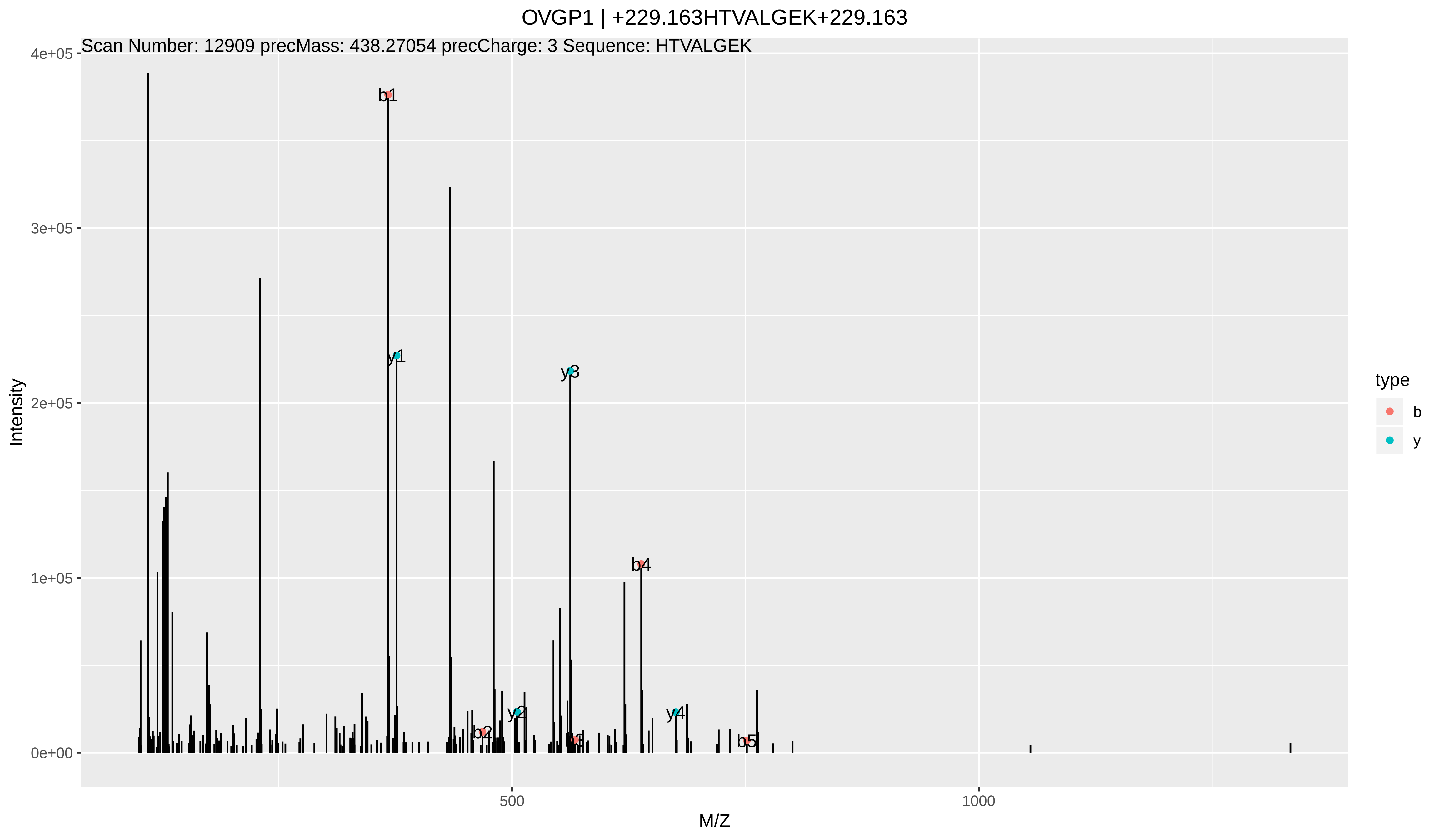

OVGP1 | +229.163HTVALGEK+229.163
Scan Number: 12909 precMass: 438.27054 precCharge: 3 Sequence: HTVALGEK
Intensity
4e+05
3e+05
2e+05
1e+05
0e+00
M/Z
500
1000
b1
y1
y3
b4
y2
b2
y4
b5
type
b
y

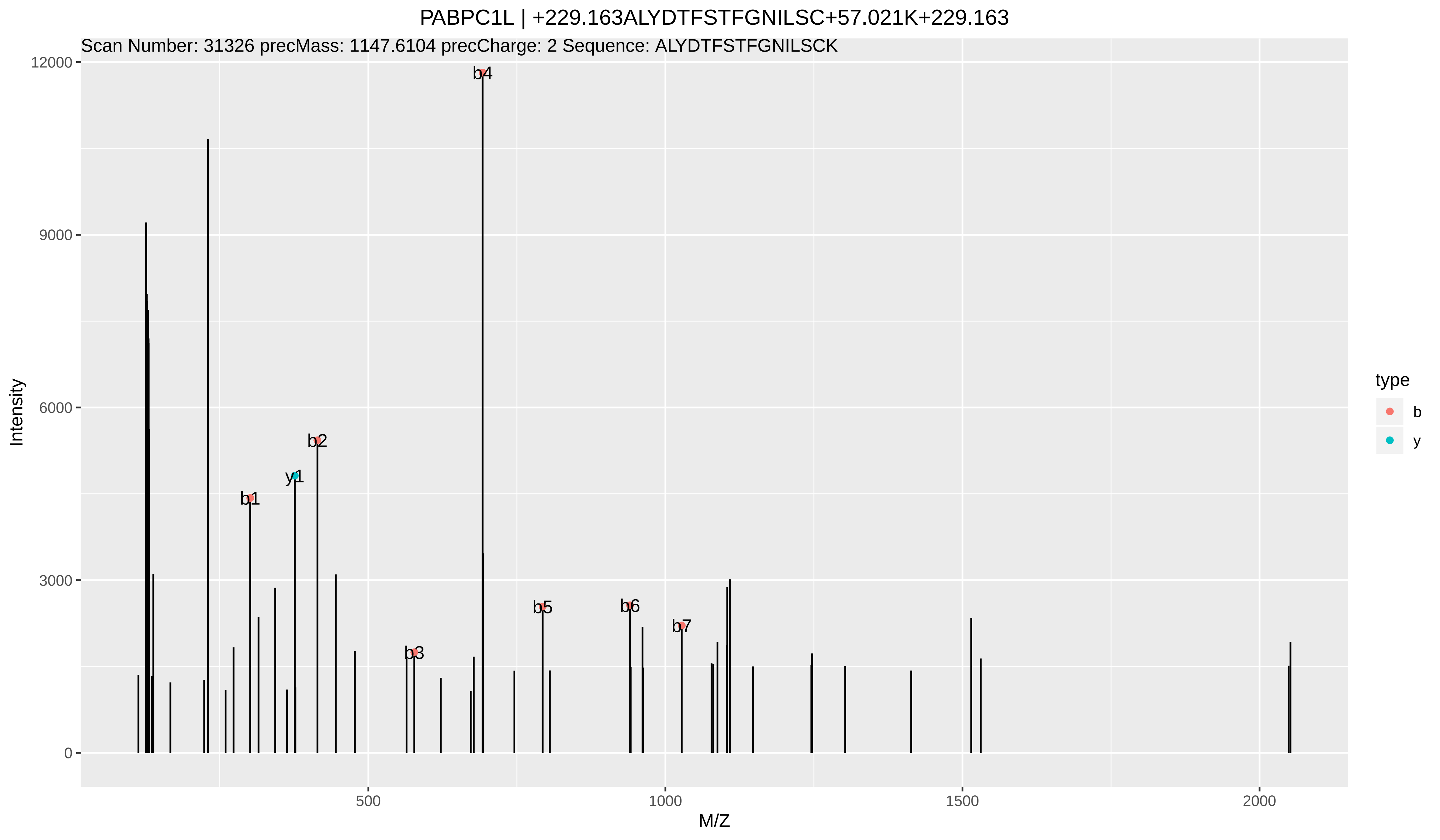

PABPC1L | +229.163ALYDTFSTFGNILSC+57.021K+229.163
Scan Number: 31326 precMass: 1147.6104 precCharge: 2 Sequence: ALYDTFSTFGNILSCK
Intensity
M/Z
type
b
y
b1
b2
b3
b4
b5
b6
b7
y1
0
3000
6000
9000
12000
500
1000
1500
2000

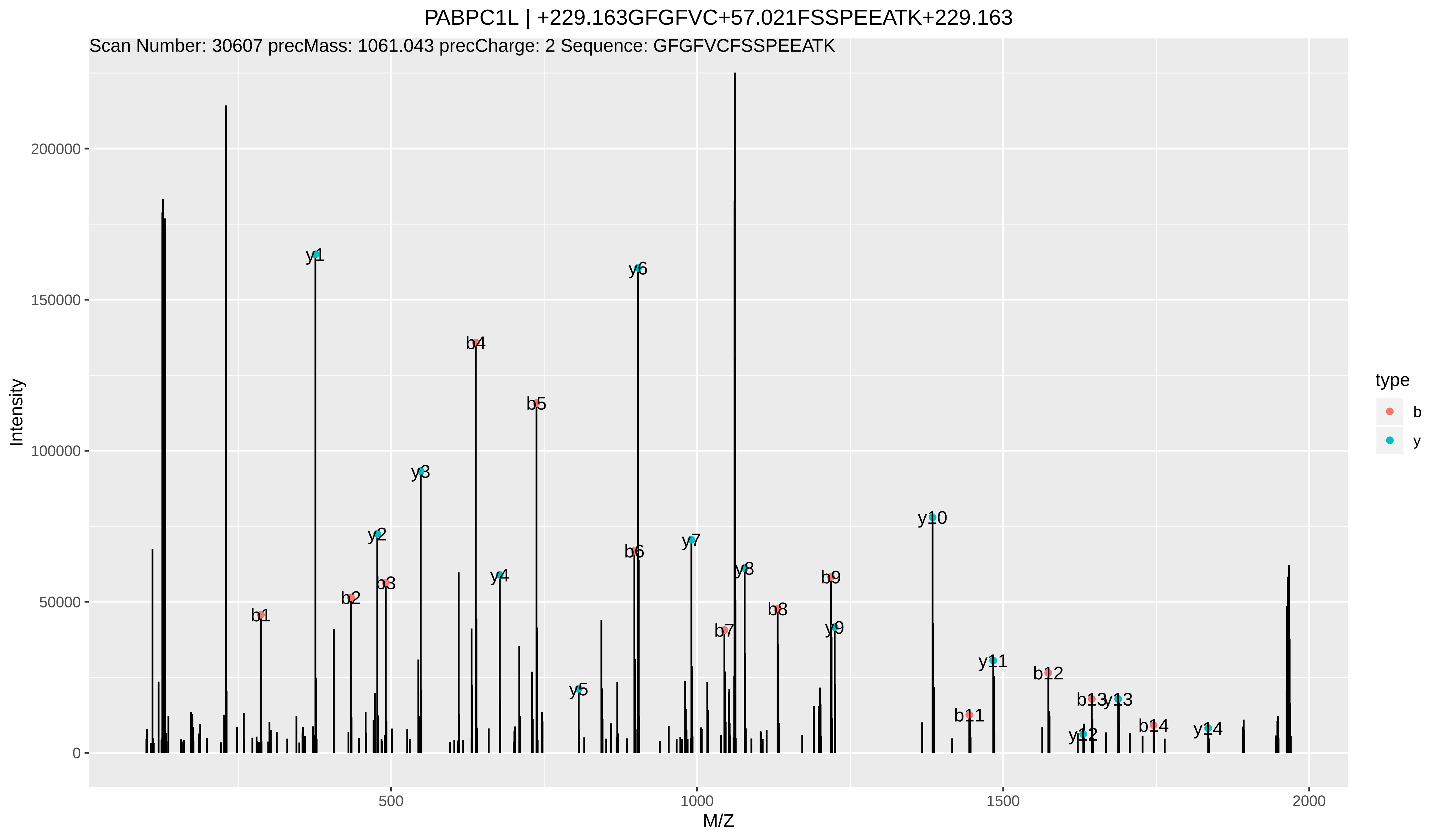

PABPC1L | +229.163GFGFVC+57.021FSSPEEATK+229.163
Scan Number: 30607 precMass: 1061.043 precCharge: 2 Sequence: GFGFVCFSSPEEATK
Intensity
M/Z
type
b
y
0
50000
100000
150000
200000
500
1000
1500
2000
y1
y2
y3
y4
y5
y6
y7
y8
y9
y10
y11
y12
y13
y14
b1
b2
b3
b4
b5
b6
b7
b8
b9
b11
b12
b13
b14

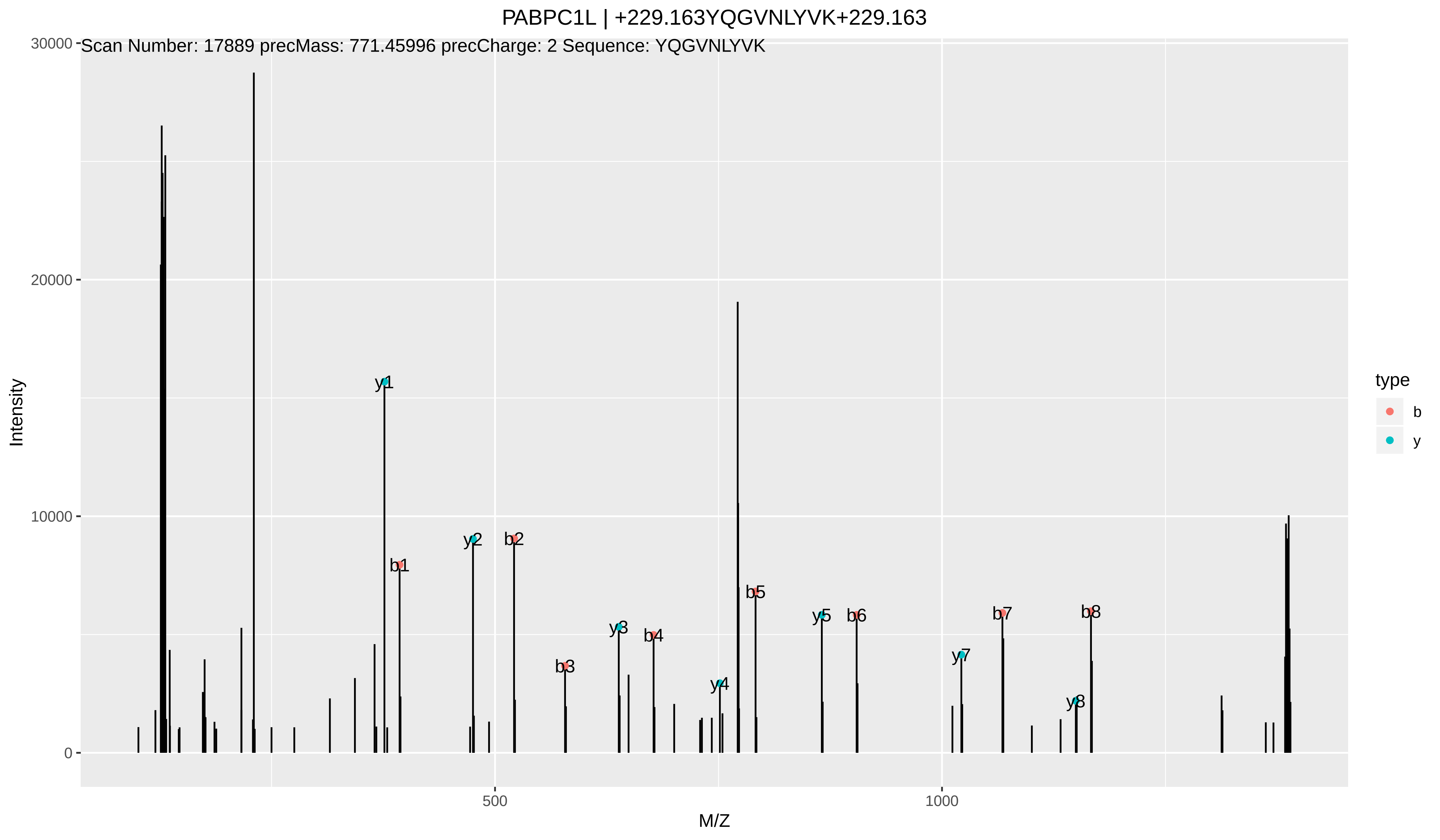

PABPC1L | +229.163YQGVNLYVK+229.163
Scan Number: 17889 precMass: 771.45996 precCharge: 2 Sequence: YQGVNLYVK
Intensity
M/Z
0
10000
20000
30000
500
1000
y1
b1
y2
b2
b3
y3
b4
y4
b5
y5
b6
y7
b7
y8
b8
type
b
y

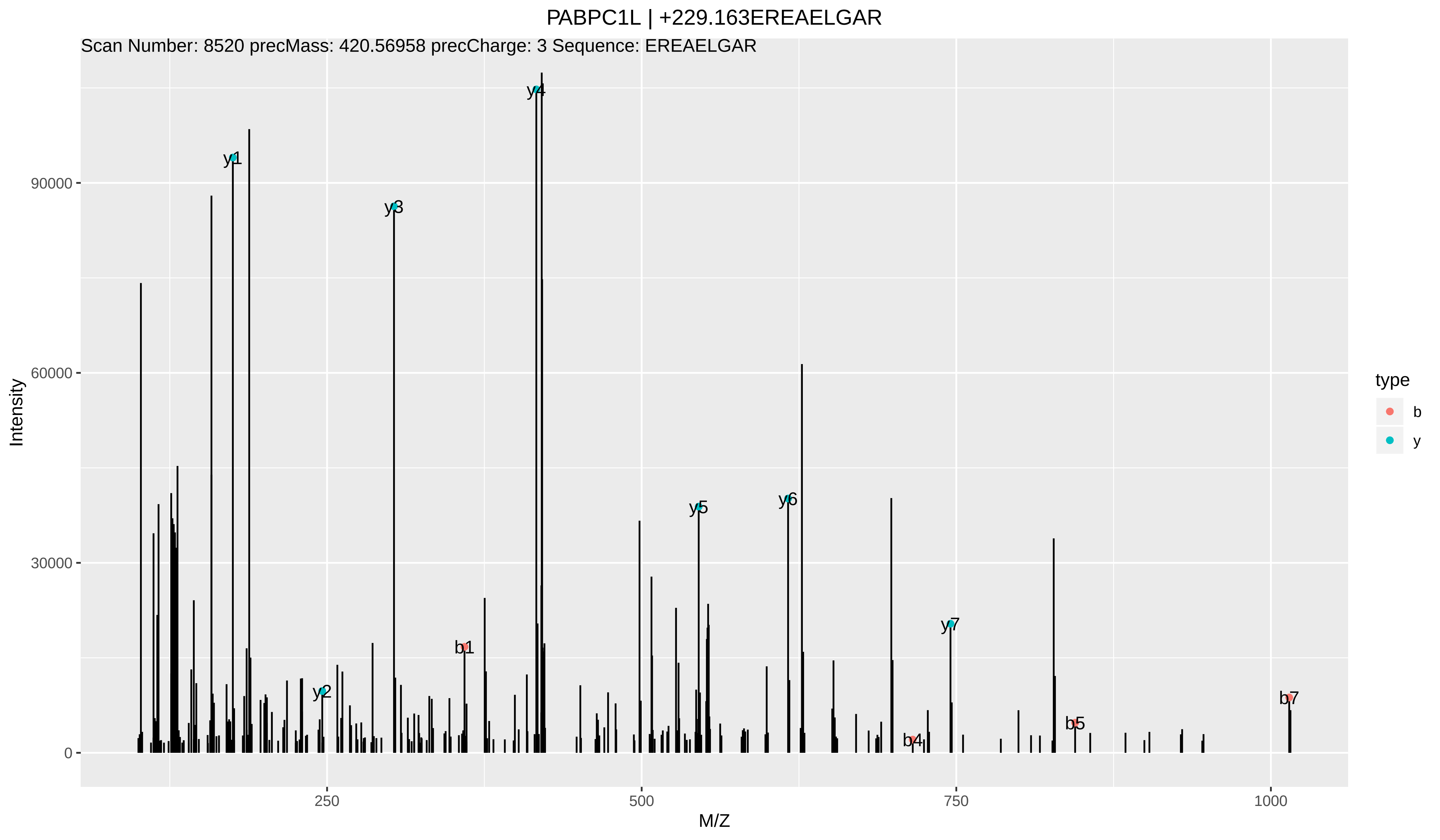
PABPC1L | +229.163EREAELGAR
Scan Number: 8520 precMass: 420.56958 precCharge: 3 Sequence: EREAELGAR
Intensity
M/Z
type
b
y
y1
y2
y3
y4
y5
y6
y7
b1
b4
b5
b7
0
30000
60000
90000
250
500
750
1000

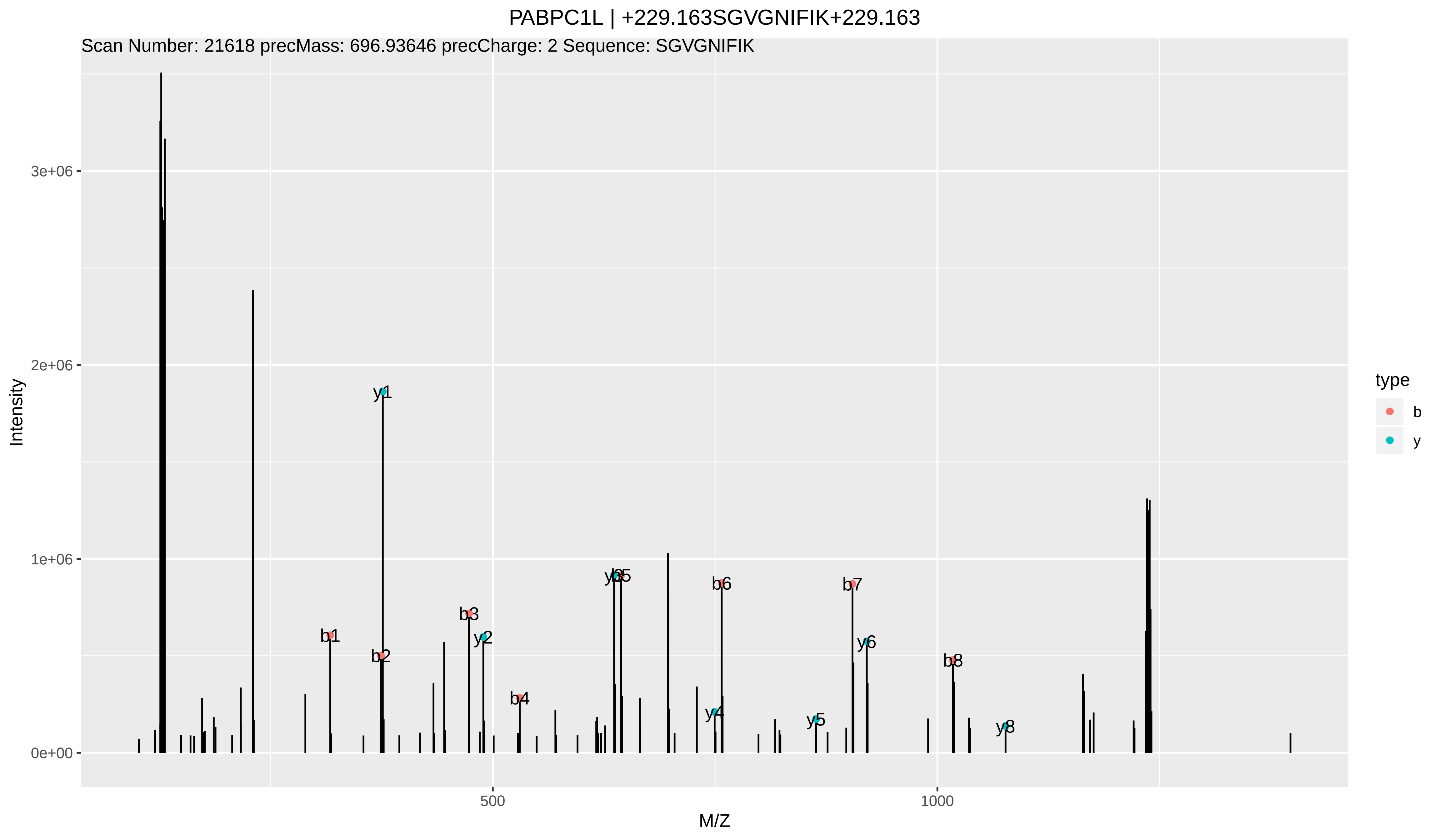

PABPC1L | +229.163SGVGNIFIK+229.163
Scan Number: 21618 precMass: 696.93646 precCharge: 2 Sequence: SGVGNIFIK
Intensity
3e+06
2e+06
1e+06
0e+00
M/Z
500
1000
y1
b1
b2
b3
y2
b4
y3
b5
b6
y4
y5
b7
y6
b8
y8
type
b
y

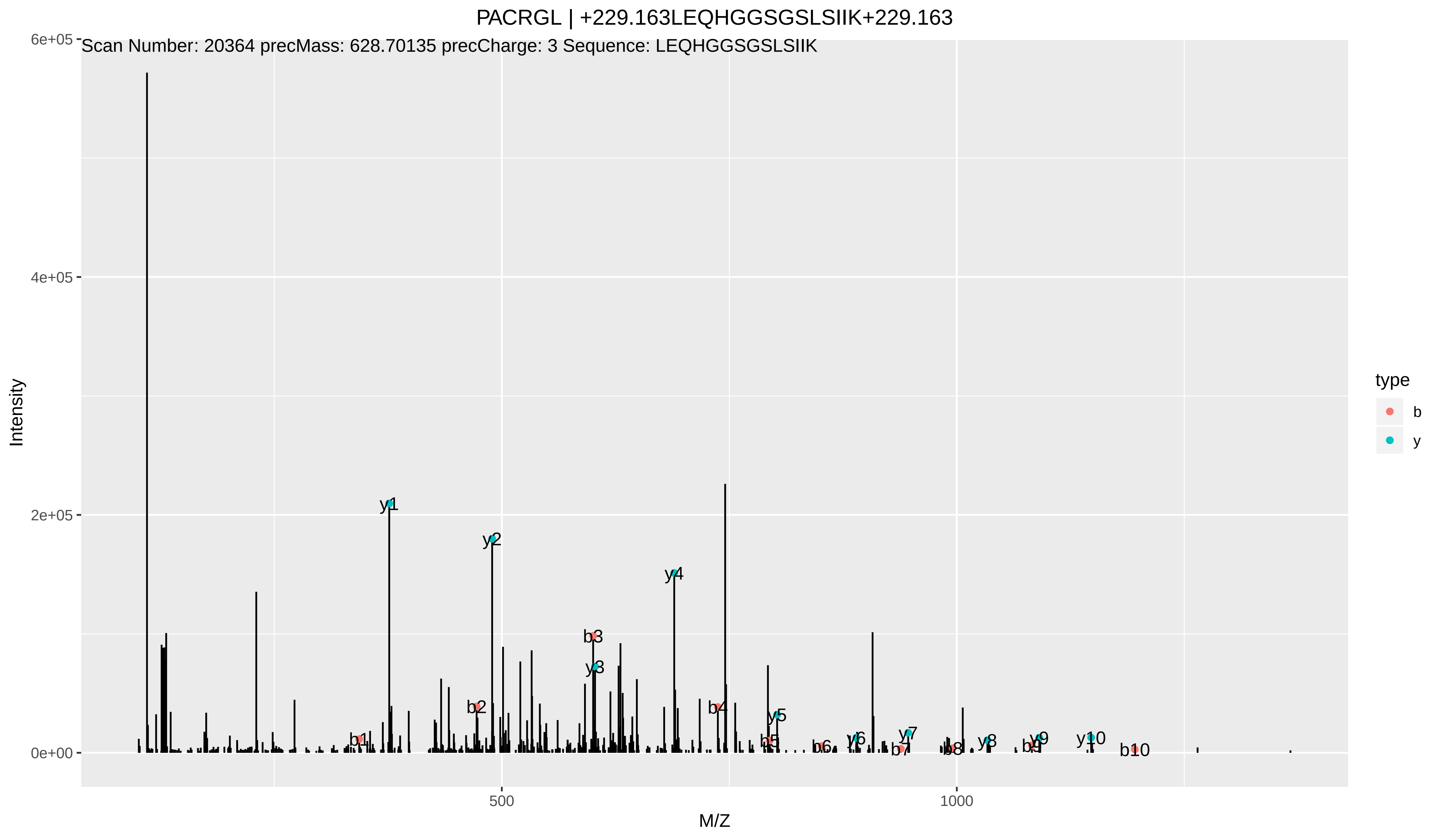

PACRGL | +229.163LEQHGGSGSLSIIK+229.163
Scan Number: 20364 precMass: 628.70135 precCharge: 3 Sequence: LEQHGGSGSLSIIK
Intensity
6e+05
4e+05
2e+05
0e+00
M/Z
500
1000
y1
y2
y3
y4
y5
y6
y7
y8
y9
y10
b1
b2
b3
b4
b5
b6
b7
b8
b9
b10
type
b
y

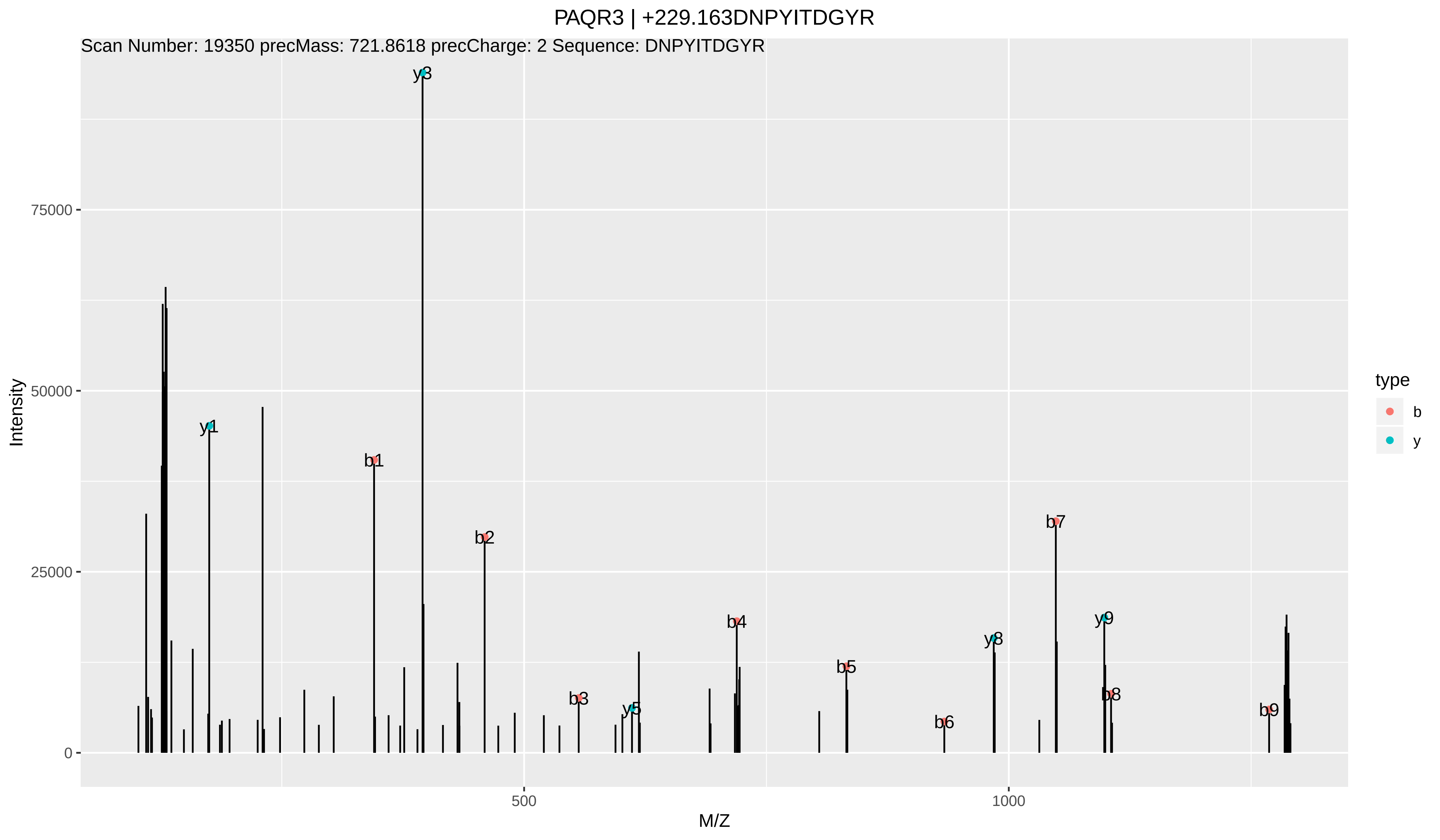

PAQR3 | +229.163DNPYITDGYR
Scan Number: 19350 precMass: 721.8618 precCharge: 2 Sequence: DNPYITDGYR
Intensity
M/Z
0
25000
50000
75000
500
1000
y3
y1
b1
b2
b3
y5
b4
b5
b6
y8
b7
y9
b8
b9
type
b
y

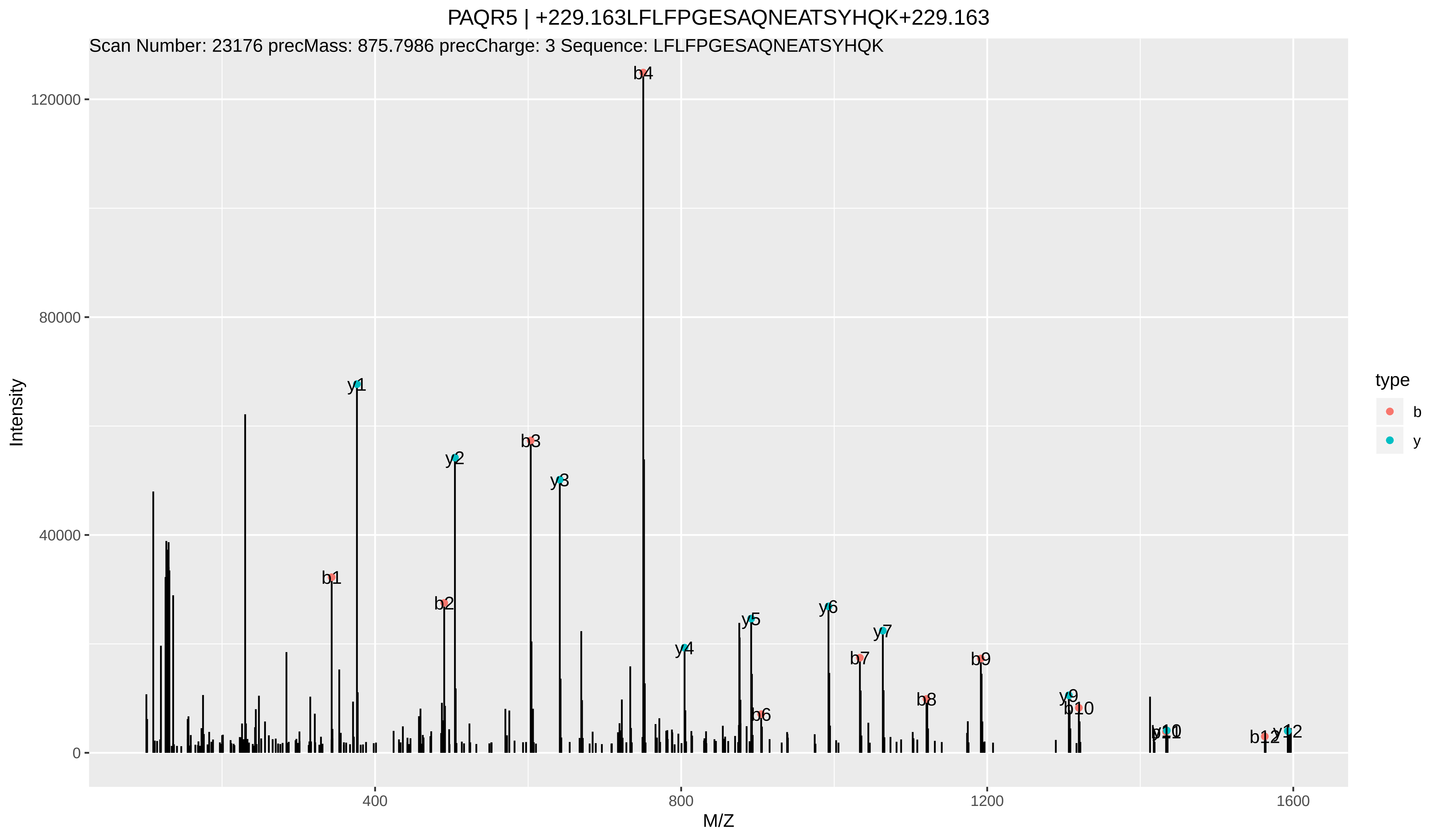
PAQR5 | +229.163LFLFPGESAQNEATSYHQK+229.163
Scan Number: 23176 precMass: 875.7986 precCharge: 3 Sequence: LFLFPGESAQNEATSYHQK
Intensity
M/Z
0
40000
80000
120000
400
800
1200
1600
type
b
y
b1
b2
b3
b4
b6
b7
b8
b9
b10
b12
y1
y2
y3
y4
y5
y6
y7
y9
y10
y11
y12

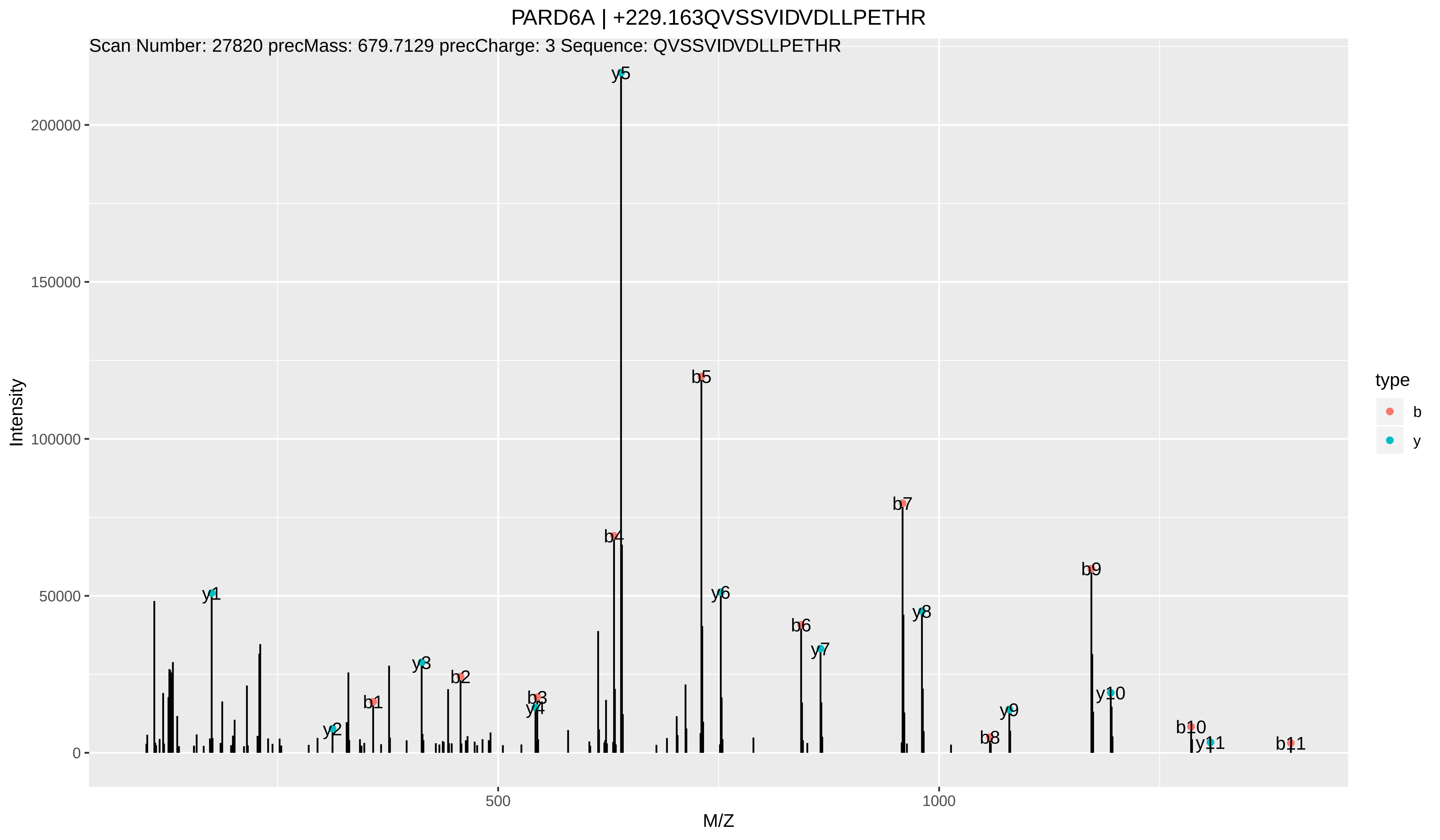
PARD6A | +229.163QVSSVIDVDLLPETHR
Scan Number: 27820 precMass: 679.7129 precCharge: 3 Sequence: QVSSVIDVDLLPETHR
Intensity
M/Z
type
b
y
y1
y2
y3
y4
y5
y6
y7
y8
y9
y10
y11
b1
b2
b3
b4
b5
b6
b7
b8
b9
b10
b11
0
50000
100000
150000
200000
500
1000

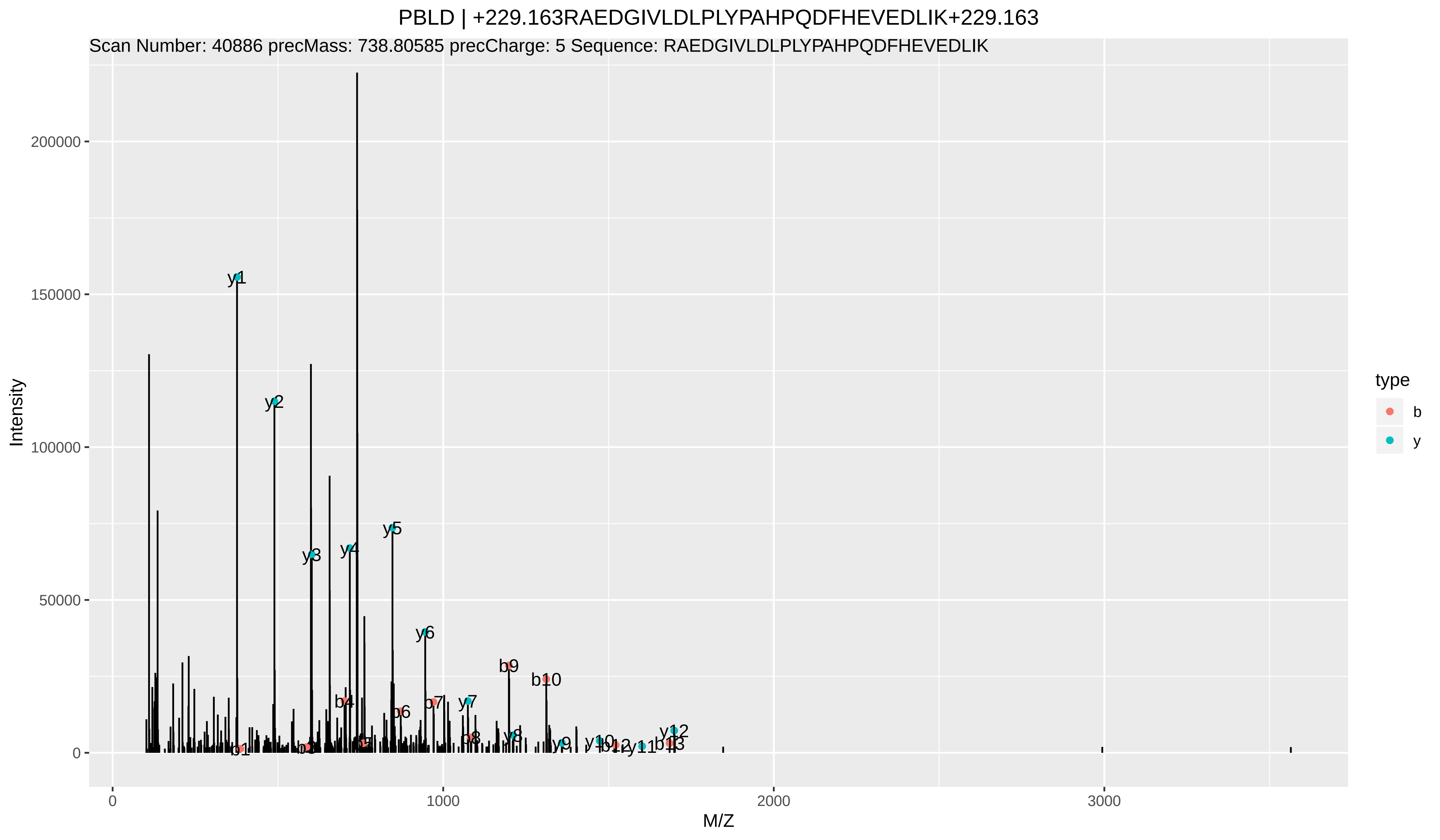

PBLD | +229.163RAEDGIVLDLPLYPAHPQDFHEVEDLIK+229.163
Scan Number: 40886 precMass: 738.80585 precCharge: 5 Sequence: RAEDGIVLDLPLYPAHPQDFHEVEDLIK
Intensity
M/Z
0
50000
100000
150000
200000
1000
2000
3000
y1
y2
y3
y4
y5
y6
y7
y8
y9
y10
y11
y12
b1
b3
b4
b5
b6
b7
b8
b9
b10
b12
b13
type
b
y

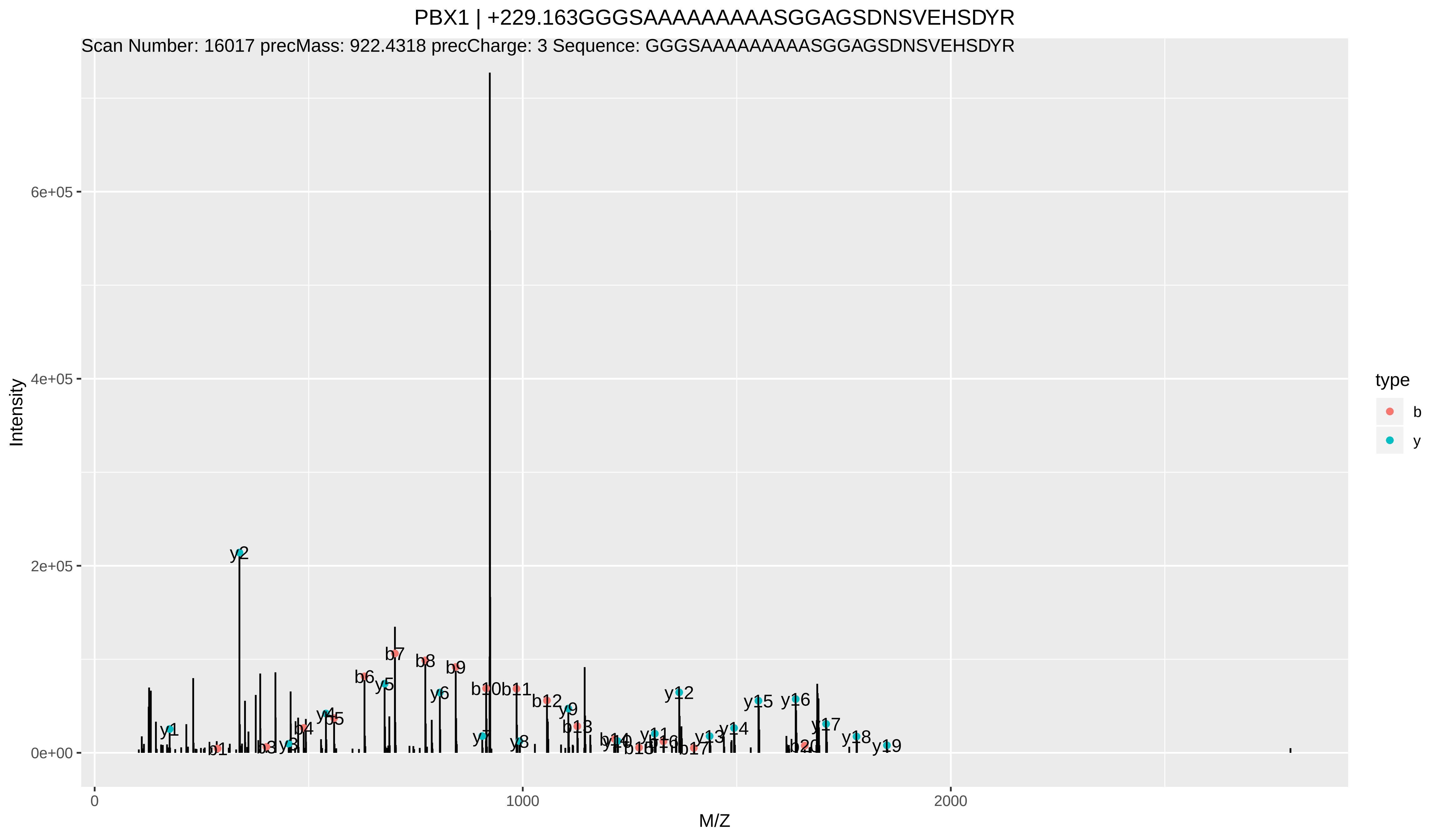
PBX1 | +229.163GGGSAAAAAAAAASGGAGSDNSVEHSDYR
Scan Number: 16017 precMass: 922.4318 precCharge: 3 Sequence: GGGSAAAAAAAAASGGAGSDNSVEHSDYR
Intensity
M/Z
0e+00
2e+05
4e+05
6e+05
0
1000
2000
type
b
y
y1
y2
y3
y4
y5
y6
y7
y8
y9
y11
y12
y13
y14
y15
y16
y17
y18
y19
b1
b3
b4
b5
b6
b7
b8
b9
b10
b11
b12
b13
b17
b20

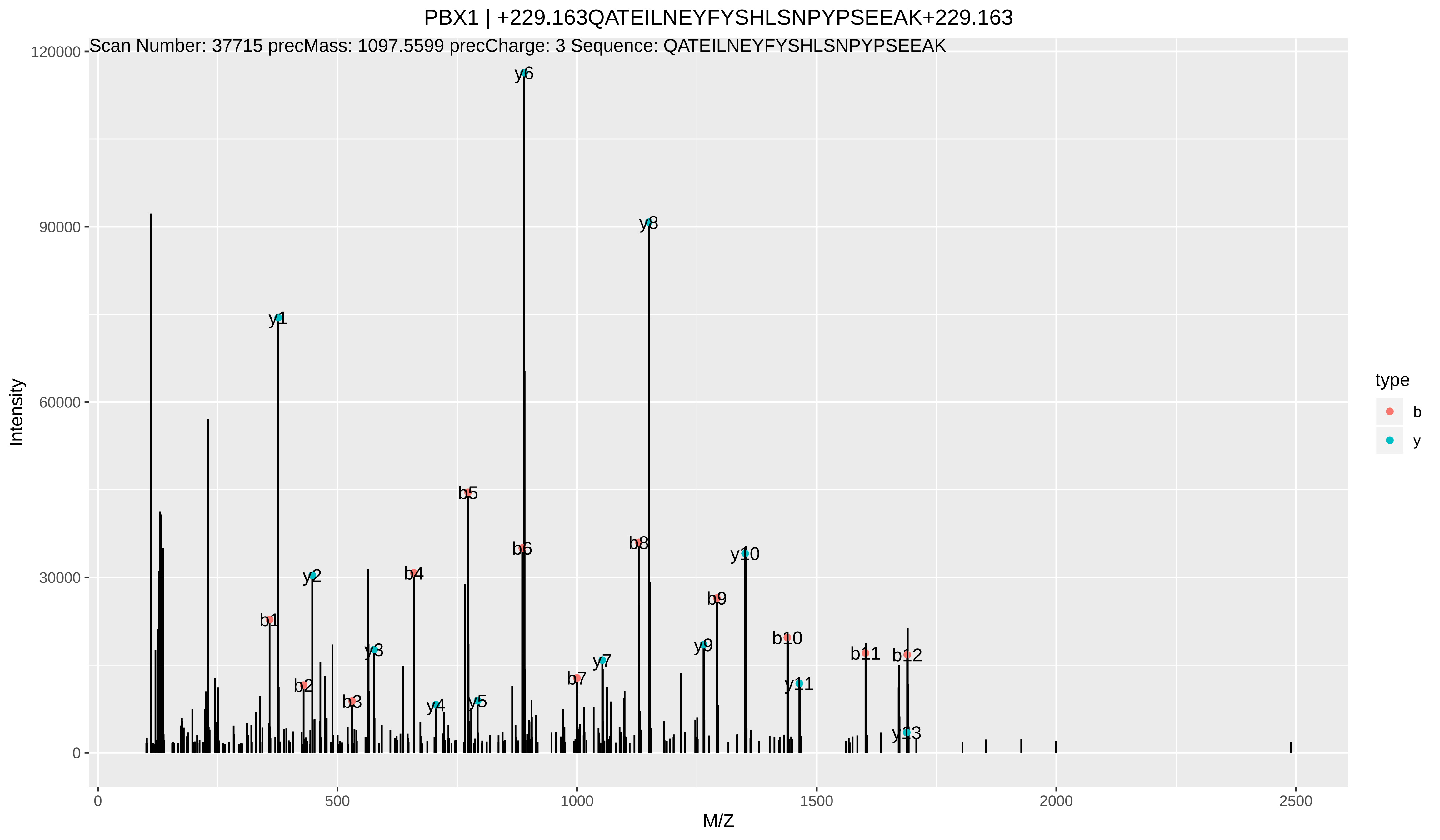
PBX1 | +229.163QATEILNEYFYSHLSNPYPSEEAK+229.163
Scan Number: 37715 precMass: 1097.5599 precCharge: 3 Sequence: QATEILNEYFYSHLSNPYPSEEAK
Intensity
M/Z
0
30000
60000
90000
120000
0
500
1000
1500
2000
2500
y6
y8
y1
b5
b6
b8
y10
y2
b4
b9
b1
b10
y3
y9
b11
b12
y7
b7
y11
b2
b3
y4
y5
y13
type
b
y

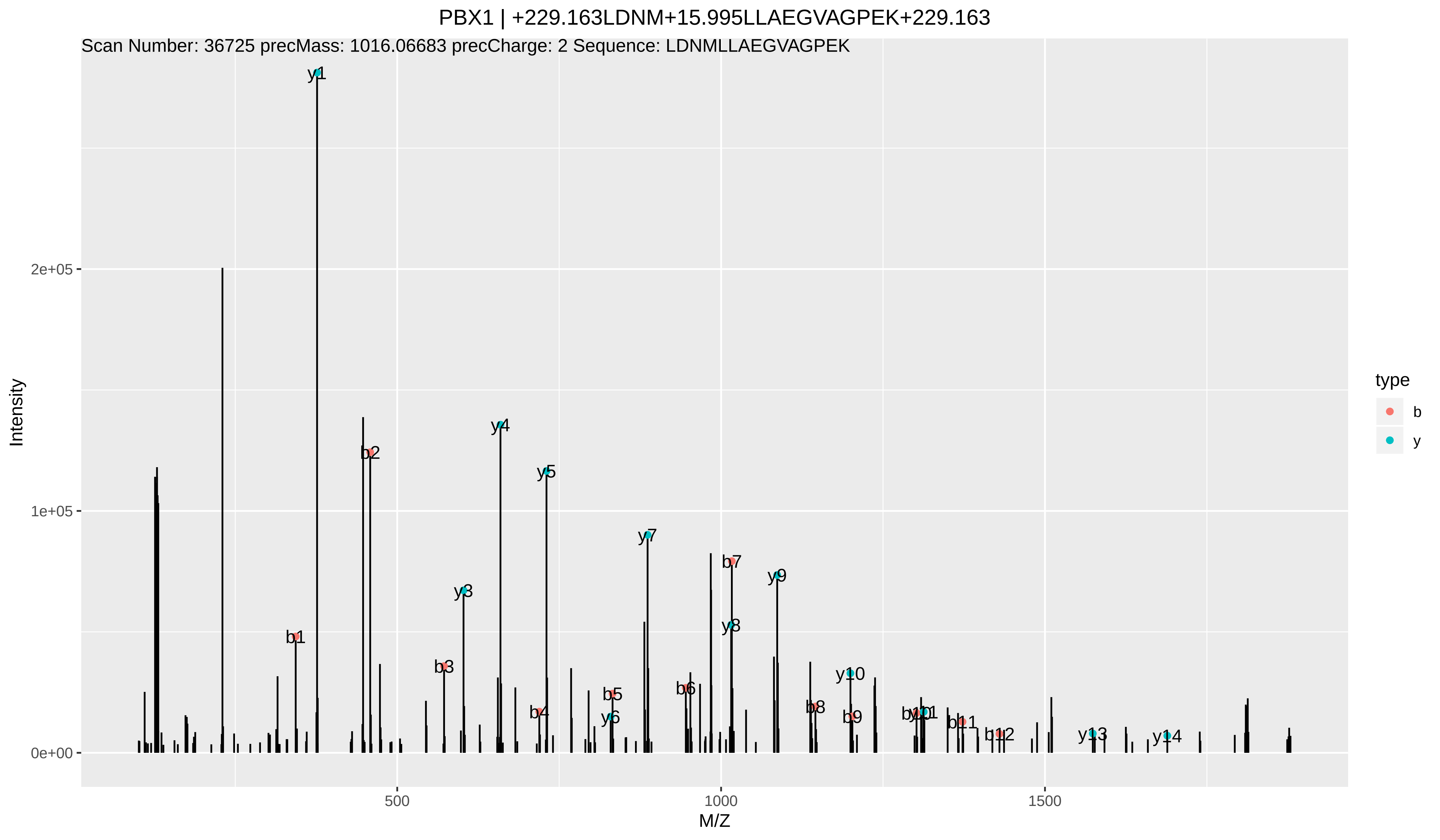
PBX1 | +229.163LDNM+15.995LLAEGVAGPEK+229.163
Scan Number: 36725 precMass: 1016.06683 precCharge: 2 Sequence: LDNMLLAEGVAGPEK
Intensity
M/Z
0e+00
1e+05
2e+05
500
1000
1500
y1
b1
b2
b3
b4
b5
b6
b7
b8
b9
b10
b11
b12
y3
y4
y5
y6
y7
y8
y9
y10
y11
y13
y14
type
b
y

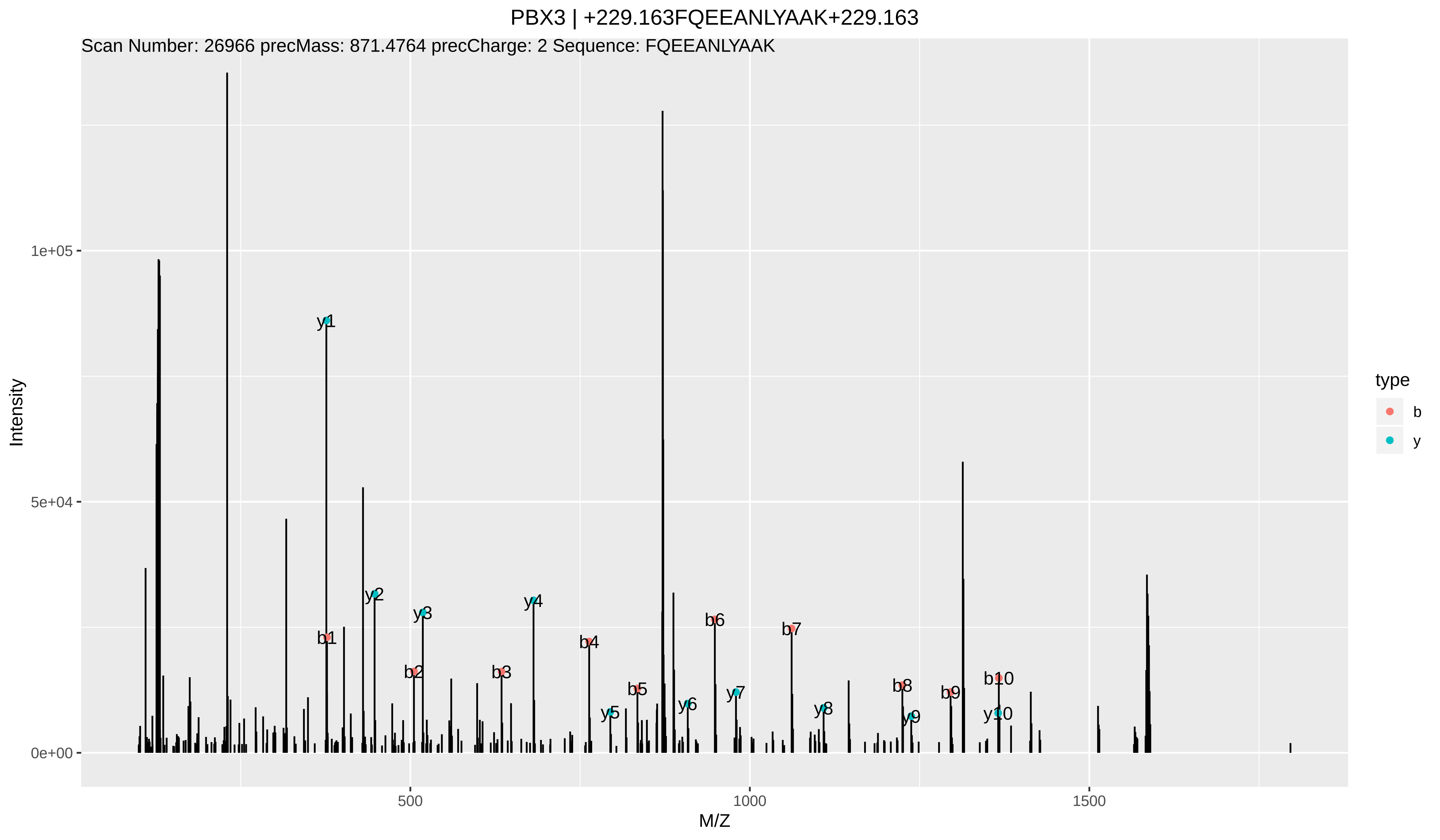

PBX3 | +229.163FQEEANLYAAK+229.163
Scan Number: 26966 precMass: 871.4764 precCharge: 2 Sequence: FQEEANLYAAK
Intensity
1e+05
5e+04
0e+00
M/Z
500
1000
1500
y1
y2
y3
y4
y5
y6
y7
y8
y9
y10
b1
b2
b3
b4
b5
b6
b7
b8
b9
b10
type
b
y

PBX3 | +229.163QATEILNEYFYSHLSNPYPSEEAK+229.163

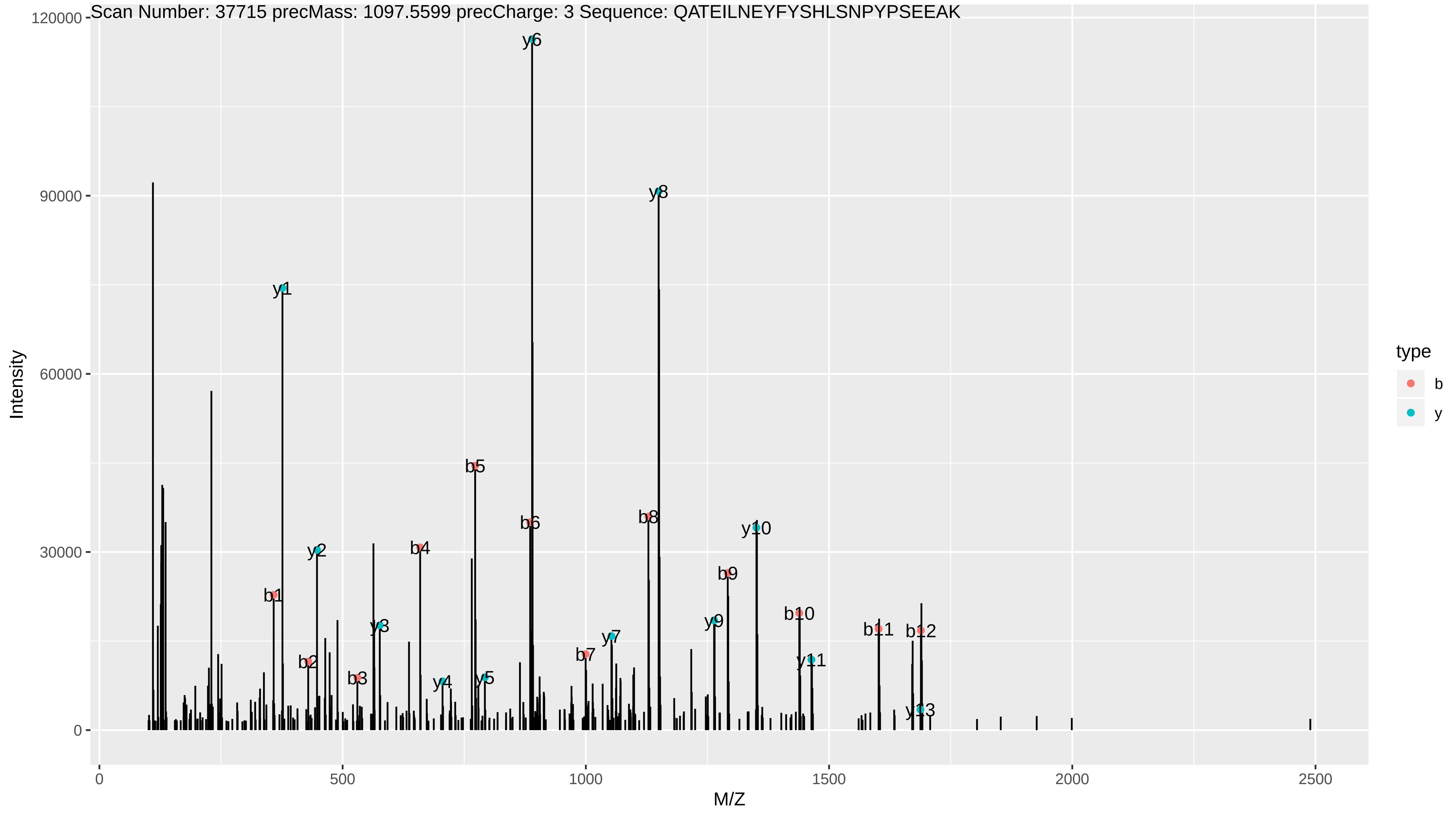

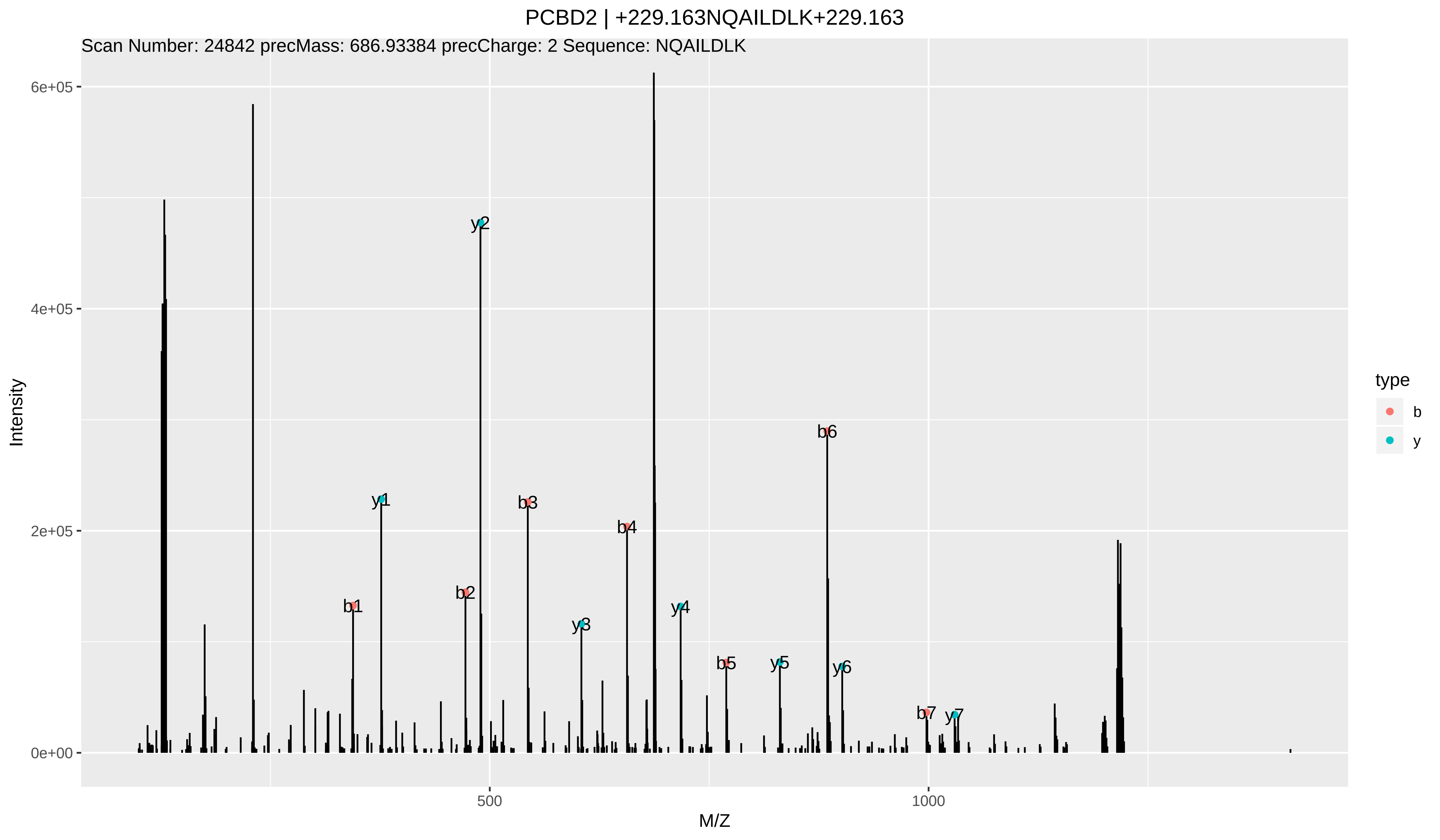

PCBD2 | +229.163NQAILDLK+229.163
Scan Number: 24842 precMass: 686.93384 precCharge: 2 Sequence: NQAILDLK
Intensity
M/Z
6e+05
4e+05
2e+05
0e+00
500
1000
b1
y1
b2
y2
b3
y3
b4
y4
b5
y5
b6
y6
b7
y7
type
b
y

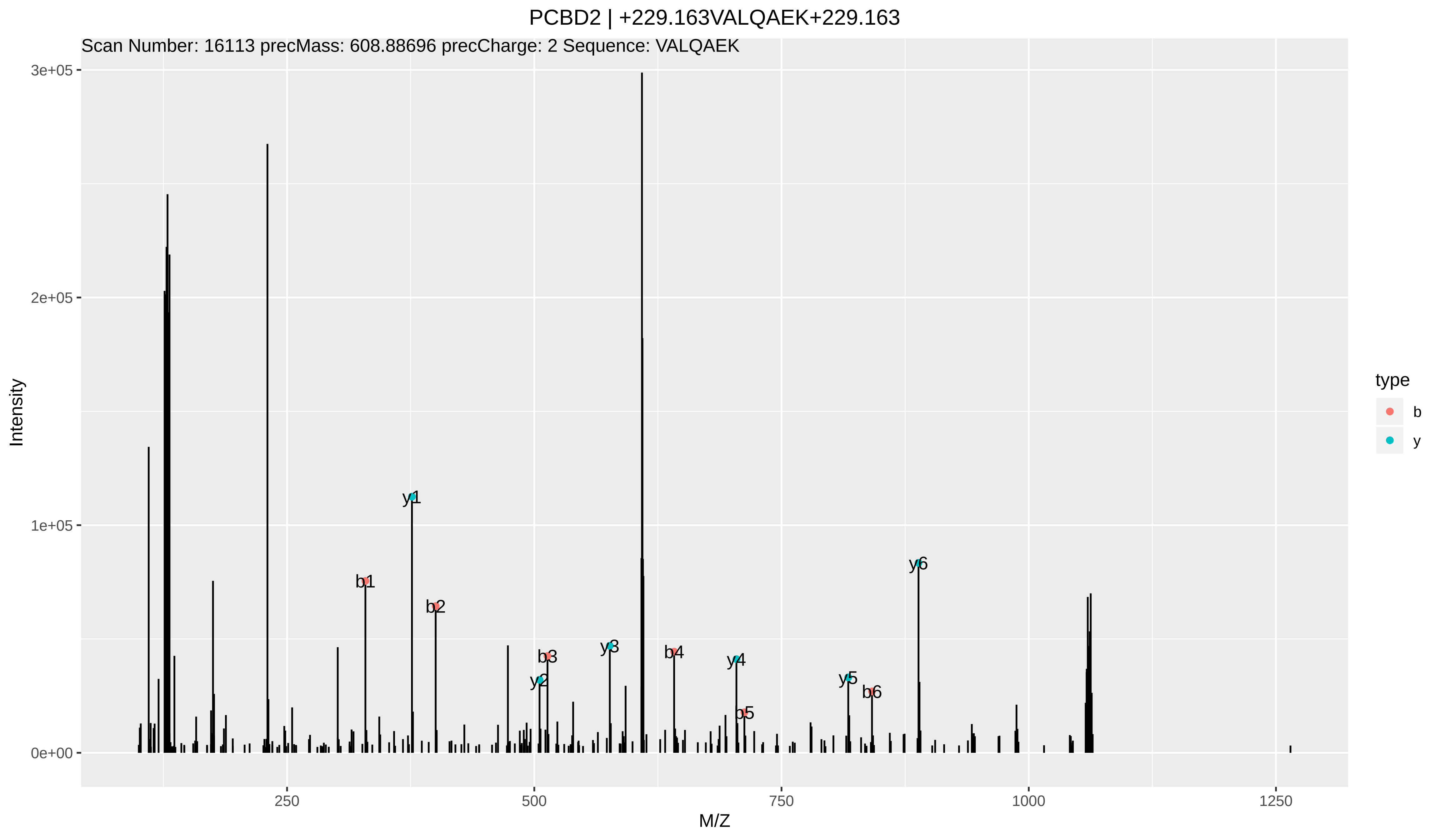
PCBD2 | +229.163VALQAEK+229.163
Scan Number: 16113 precMass: 608.88696 precCharge: 2 Sequence: VALQAEK
Intensity
3e+05
2e+05
1e+05
0e+00
M/Z
250
500
750
1000
1250
y1
b1
b2
y2
b3
y3
b4
y4
b5
y5
b6
y6
type
b
y

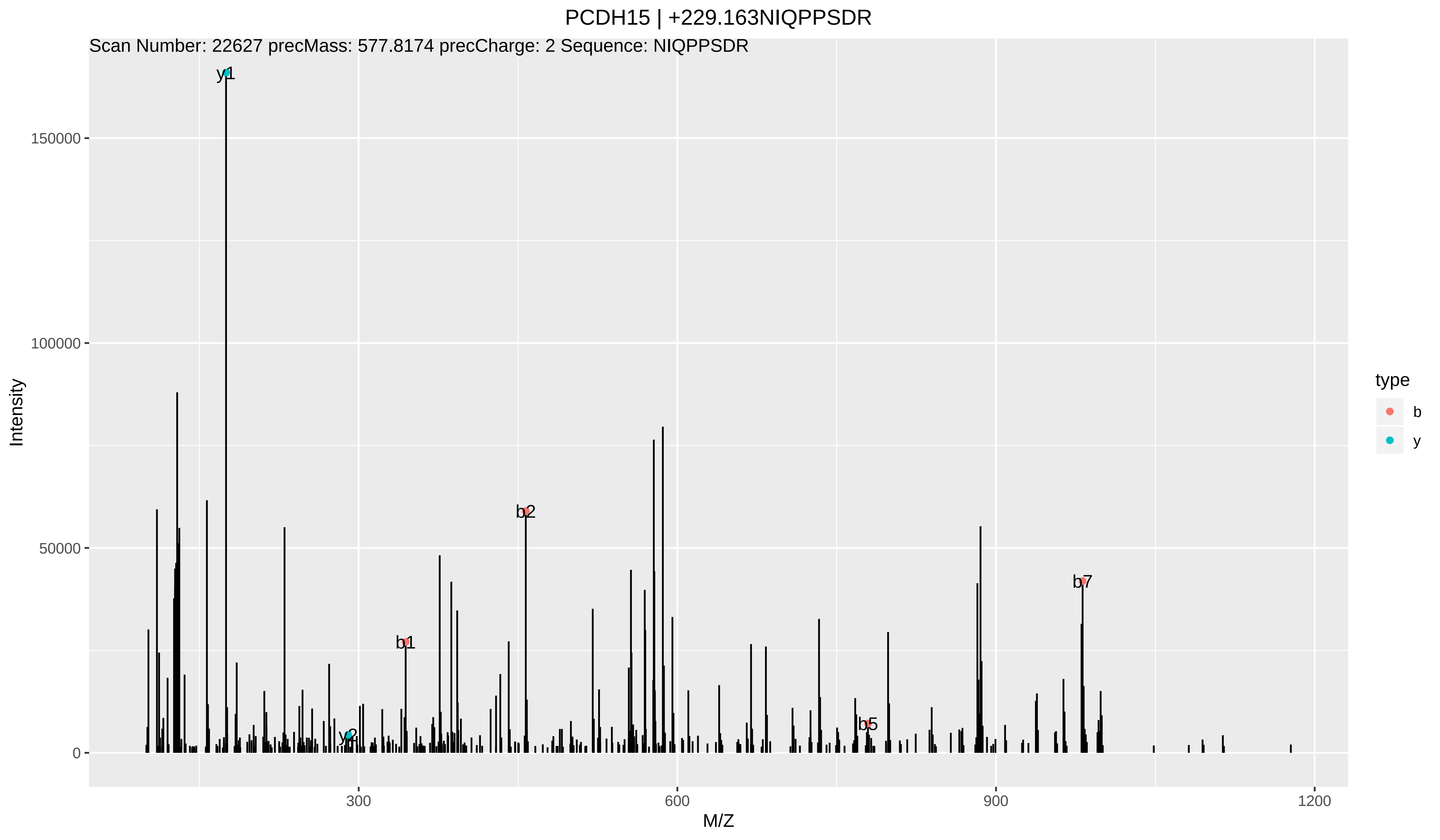
PCDH15 | +229.163NIQPPSDR
Scan Number: 22627 precMass: 577.8174 precCharge: 2 Sequence: NIQPPSDR
y1
y2
b1
b2
b5
b7
Intensity
150000
100000
50000
0
300
600
900
1200
M/Z
type
b
y

PDE1C | +229.163DHSNIGNDSK+229.163

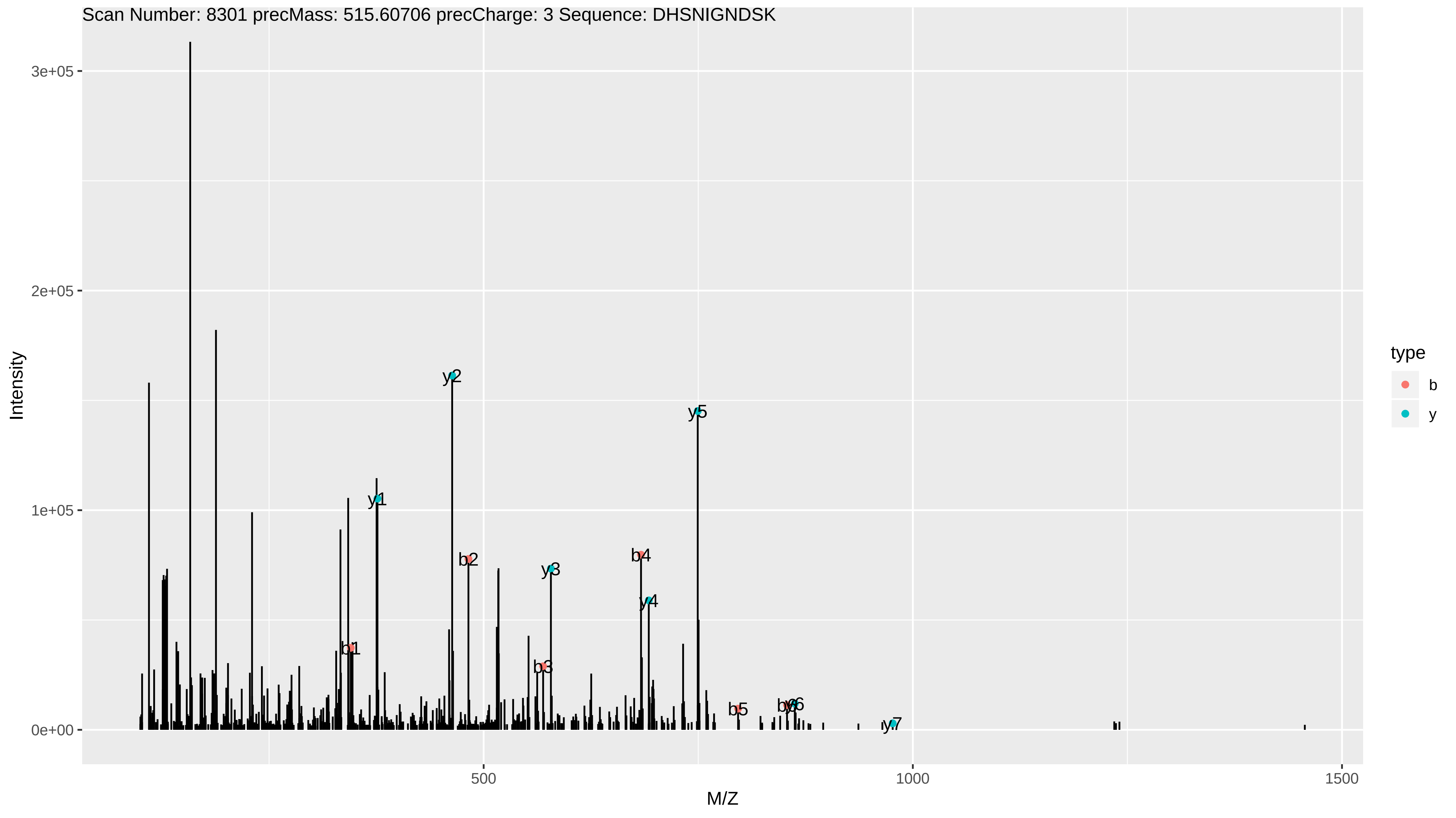

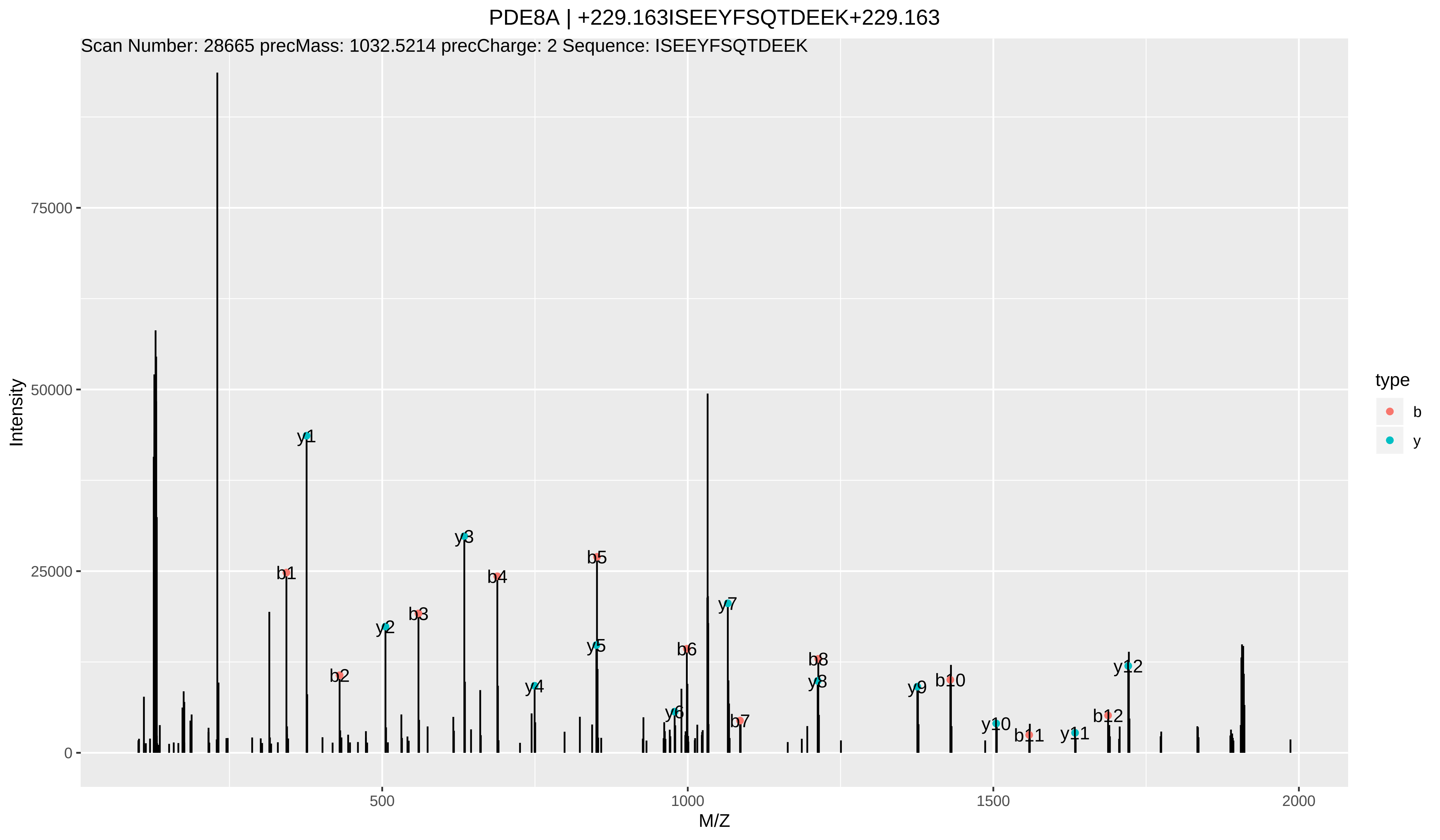

PDE8A | +229.163ISEEYFSQTDEEK+229.163
Scan Number: 28665 precMass: 1032.5214 precCharge: 2 Sequence: ISEEYFSQTDEEK
Intensity
M/Z
type
b
y
y1
b1
b2
y2
b3
y3
b4
y4
b5
y5
y6
b6
y7
b7
b8
y8
y9
b10
y10
b11
y11
b12
y12
0
25000
50000
75000
500
1000
1500
2000

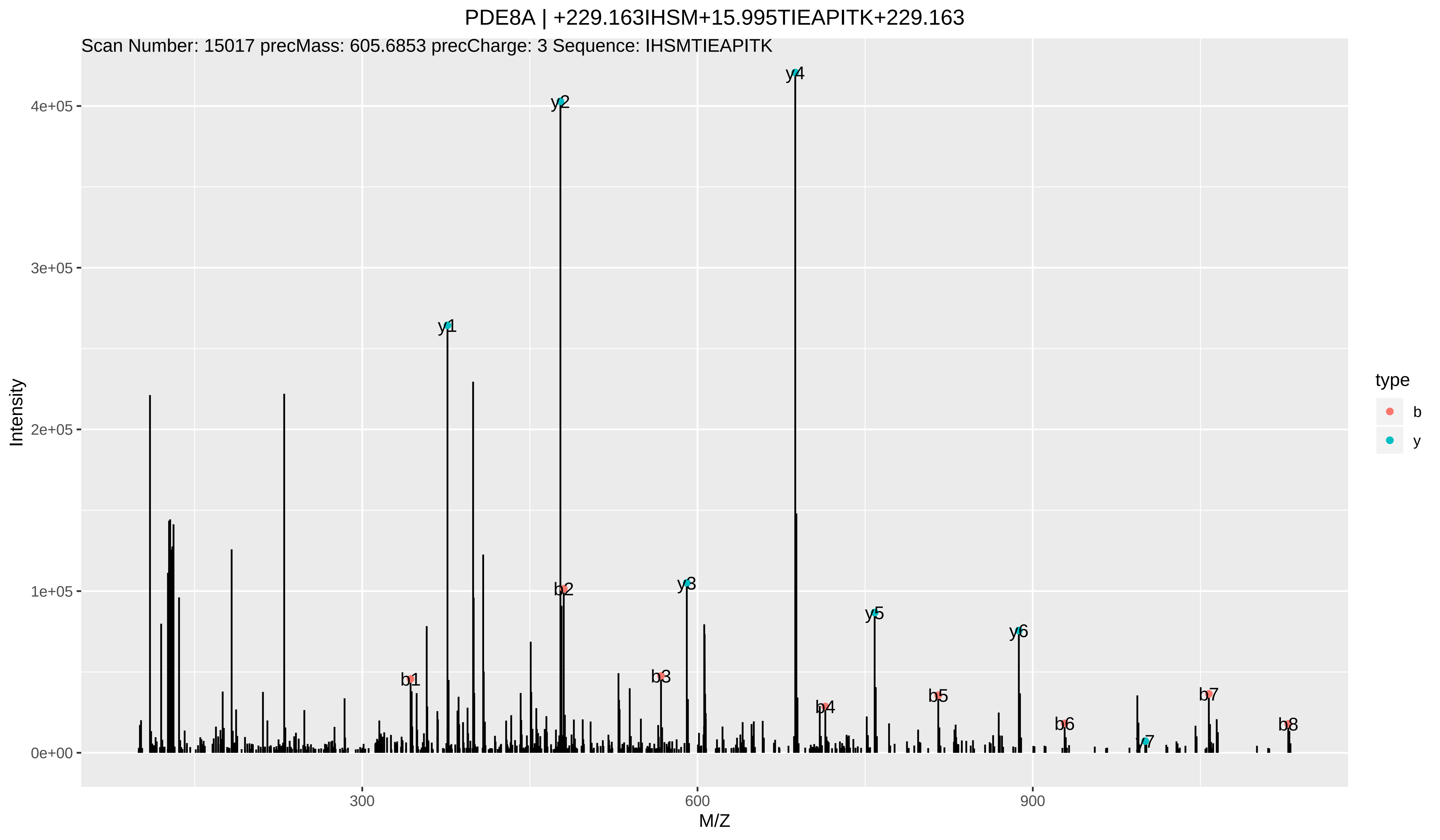

PDE8A | +229.163IHSM+15.995TIEAPITK+229.163
Scan Number: 15017 precMass: 605.6853 precCharge: 3 Sequence: IHSMTIEAPITK
Intensity
M/Z
type
b
y
y1
y2
y3
y4
y5
y6
y7
b1
b2
b3
b4
b5
b6
b7
b8

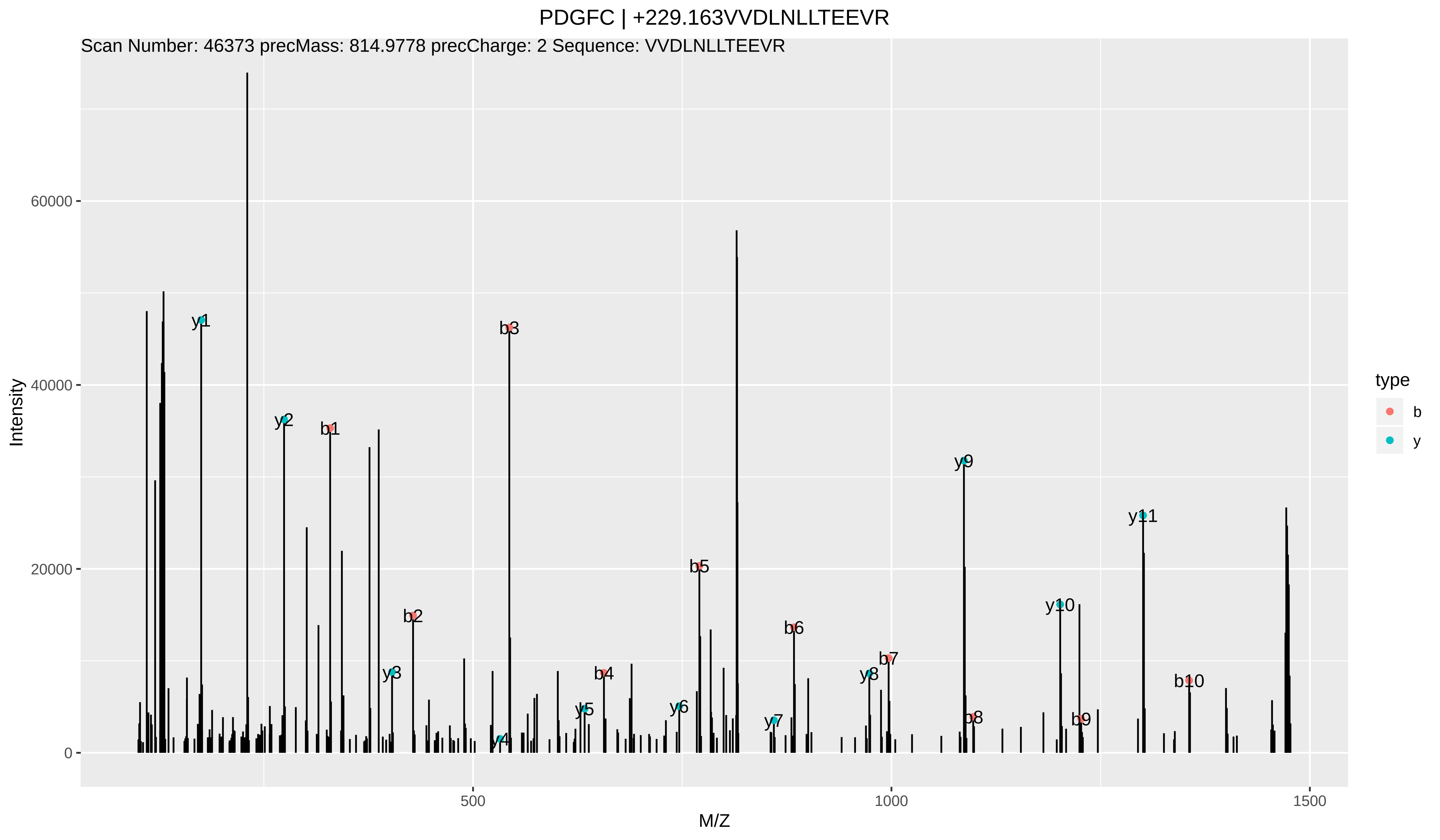
PDGFC | +229.163VVDLNLLTEEVR
Scan Number: 46373 precMass: 814.9778 precCharge: 2 Sequence: VVDLNLLTEEVR
Intensity
M/Z
type
b
y
0
20000
40000
60000
500
1000
1500
y1
y2
b1
b3
b2
y3
y4
y5
b4
y6
b5
y7
b6
y8
b7
y9
b8
y10
b9
y11
b10

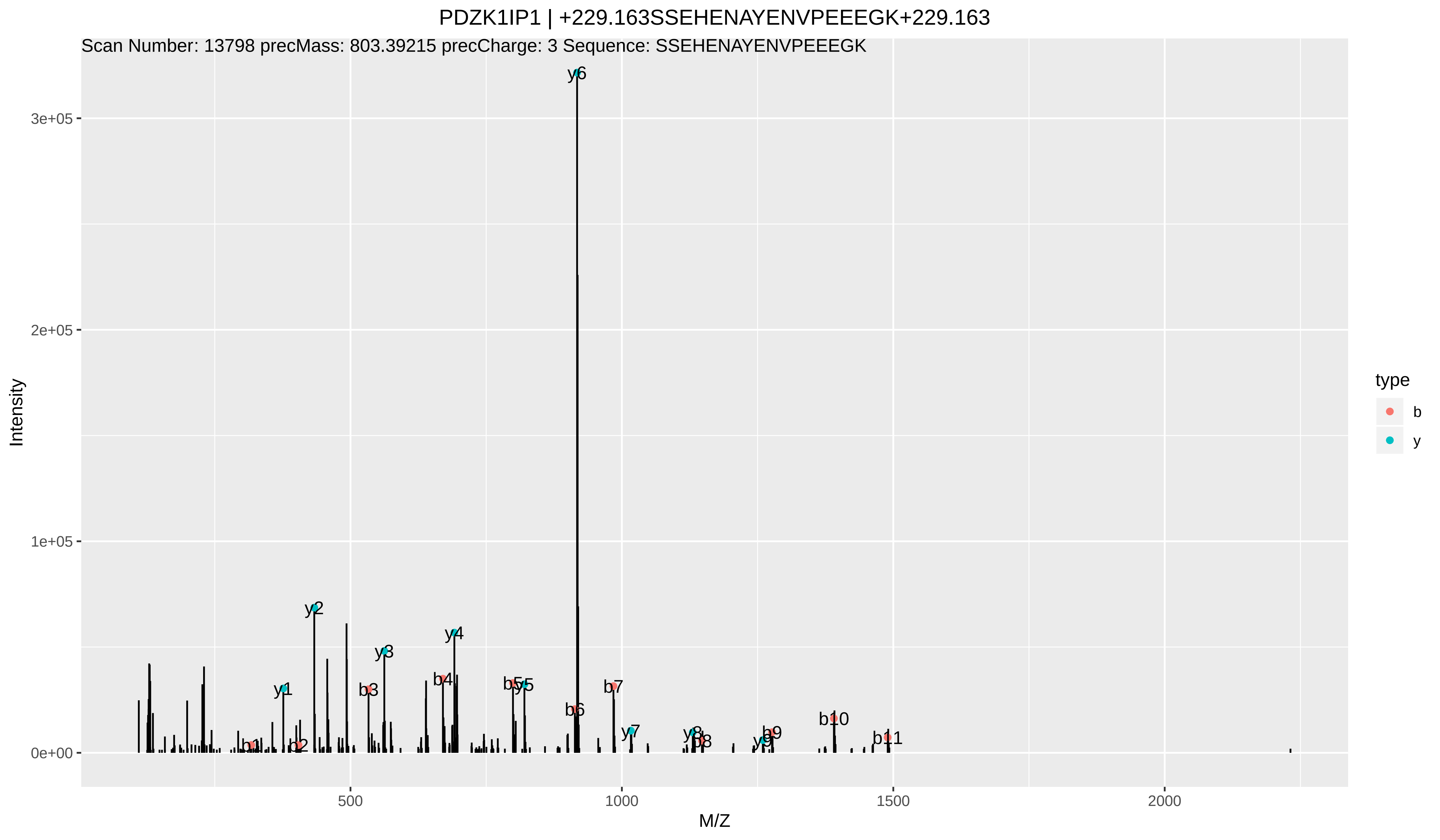
PDZK1IP1 | +229.163SSEHENAYENVPEEEGK+229.163
Scan Number: 13798 precMass: 803.39215 precCharge: 3 Sequence: SSEHENAYENVPEEEGK
Intensity
3e+05
2e+05
1e+05
0e+00
M/Z
500
1000
1500
2000
type
b
y
y6
y2
y4
y3
y1
b3
b4
b5
y5
b6
b7
y7
y8
b8
y9
b9
b10
b11
b1
b2

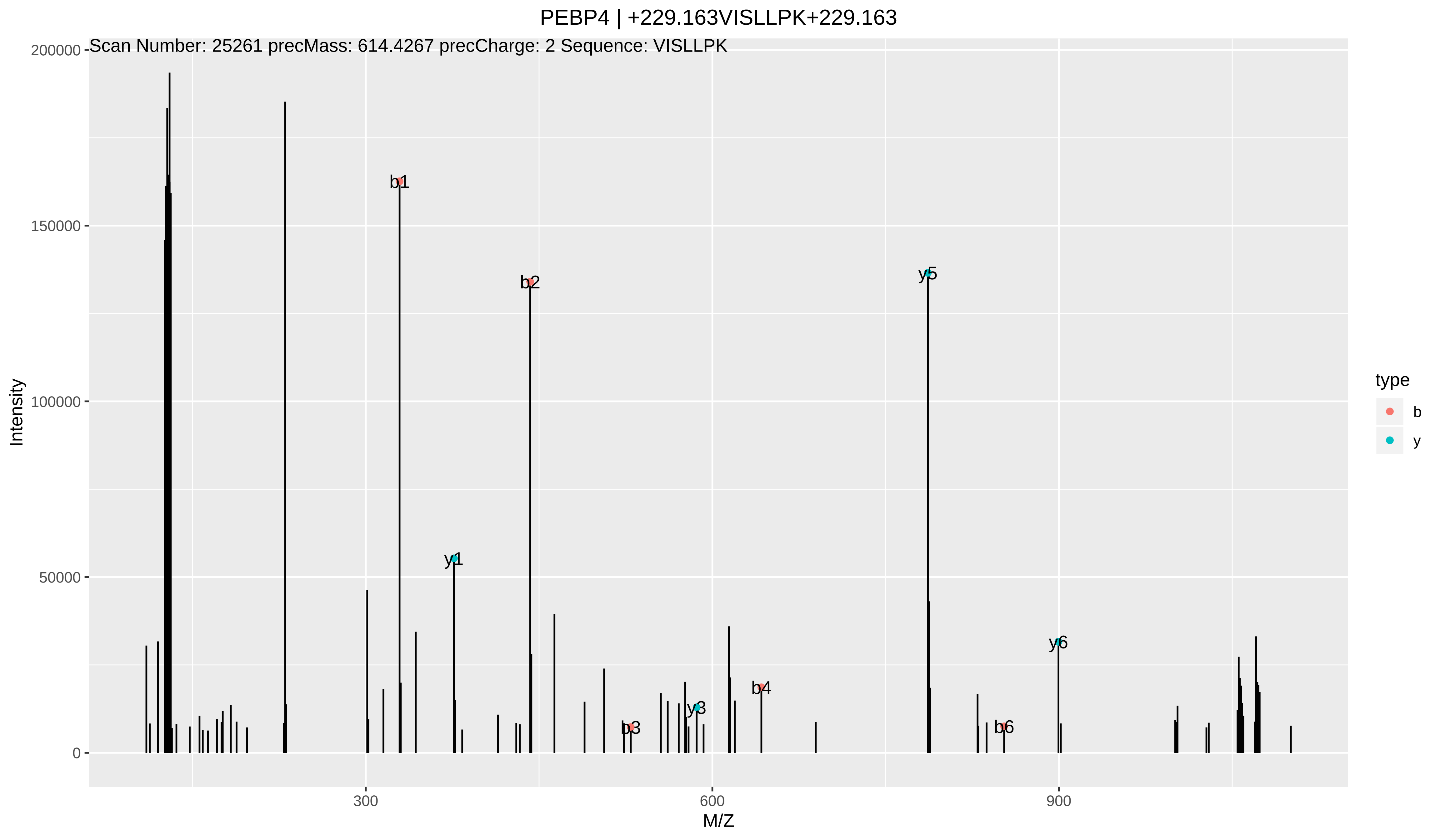
PEBP4 | +229.163VISLLPK+229.163
Scan Number: 25261 precMass: 614.4267 precCharge: 2 Sequence: VISLLPK
Intensity
200000
150000
100000
50000
0
300
600
900
M/Z
b1
b2
y1
y5
y6
b4
y3
b3
b6
type
b
y

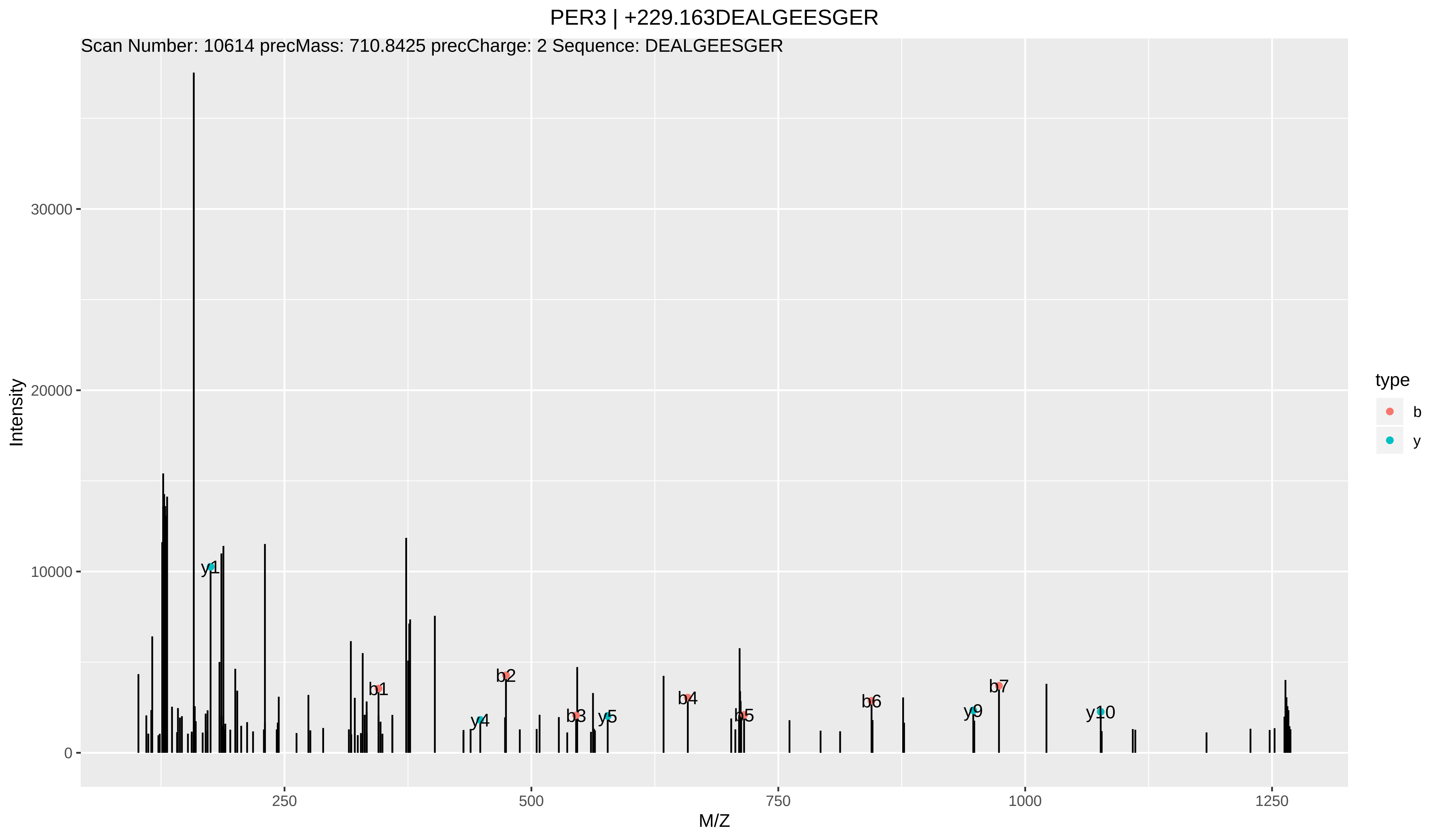
PER3 | +229.163DEALGEESGER
Scan Number: 10614 precMass: 710.8425 precCharge: 2 Sequence: DEALGEESGER
Intensity
M/Z
type
b
y
y1
b1
y4
b2
b3
y5
b4
b5
b6
y9
b7
y10
0
10000
20000
30000
250
500
750
1000
1250

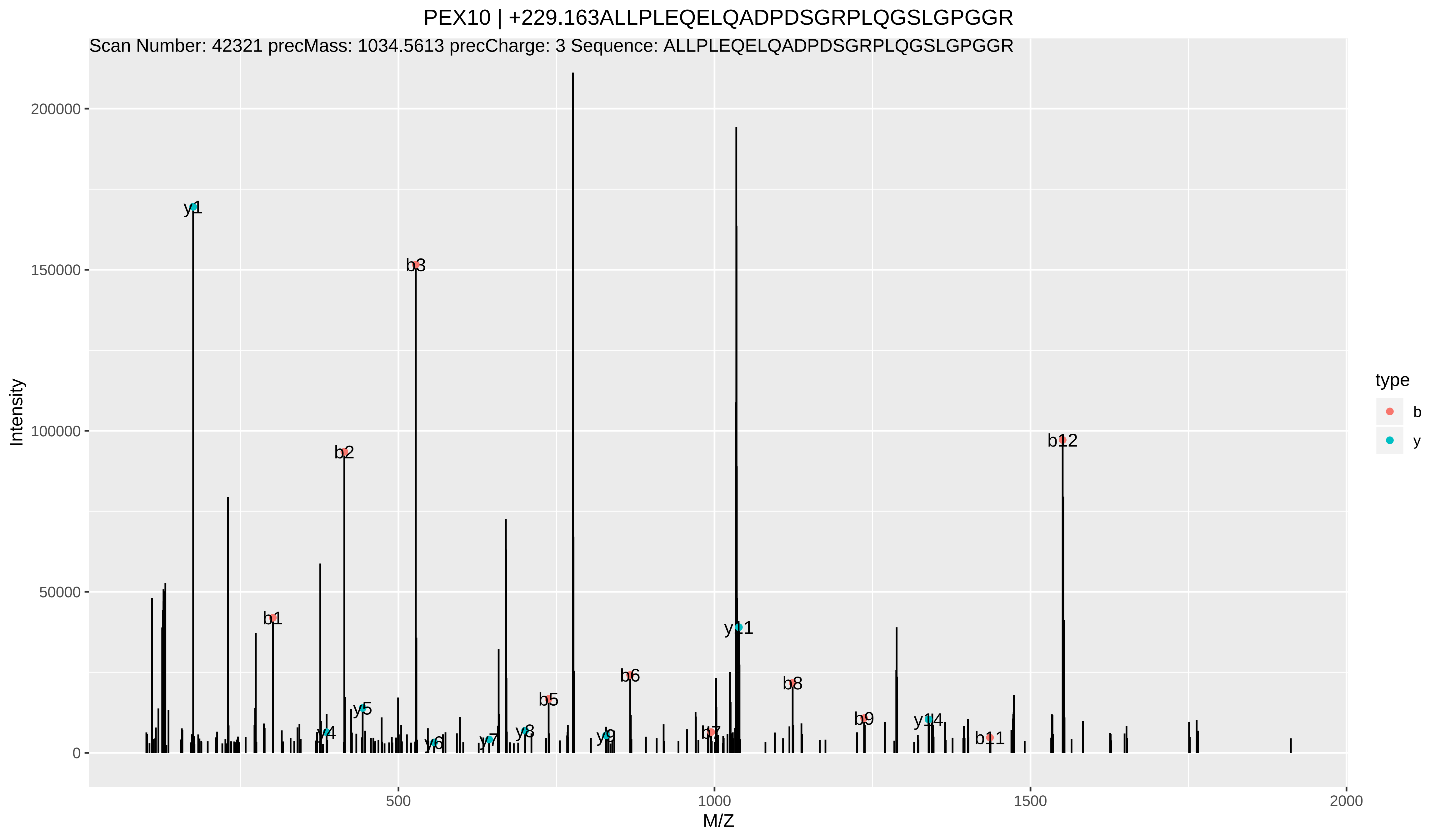
PEX10 | +229.163ALLPLEQELQADPDSGRPLQGSLGPGGR
Scan Number: 42321 precMass: 1034.5613 precCharge: 3 Sequence: ALLPLEQELQADPDSGRPLQGSLGPGGR
Intensity
M/Z
type
b
y
y1
b3
b2
b12
b1
y11
b6
b8
b5
y5
b9
y14
y4
b7
y8
y9
y6
y7
b11
0
50000
100000
150000
200000
500
1000
1500
2000

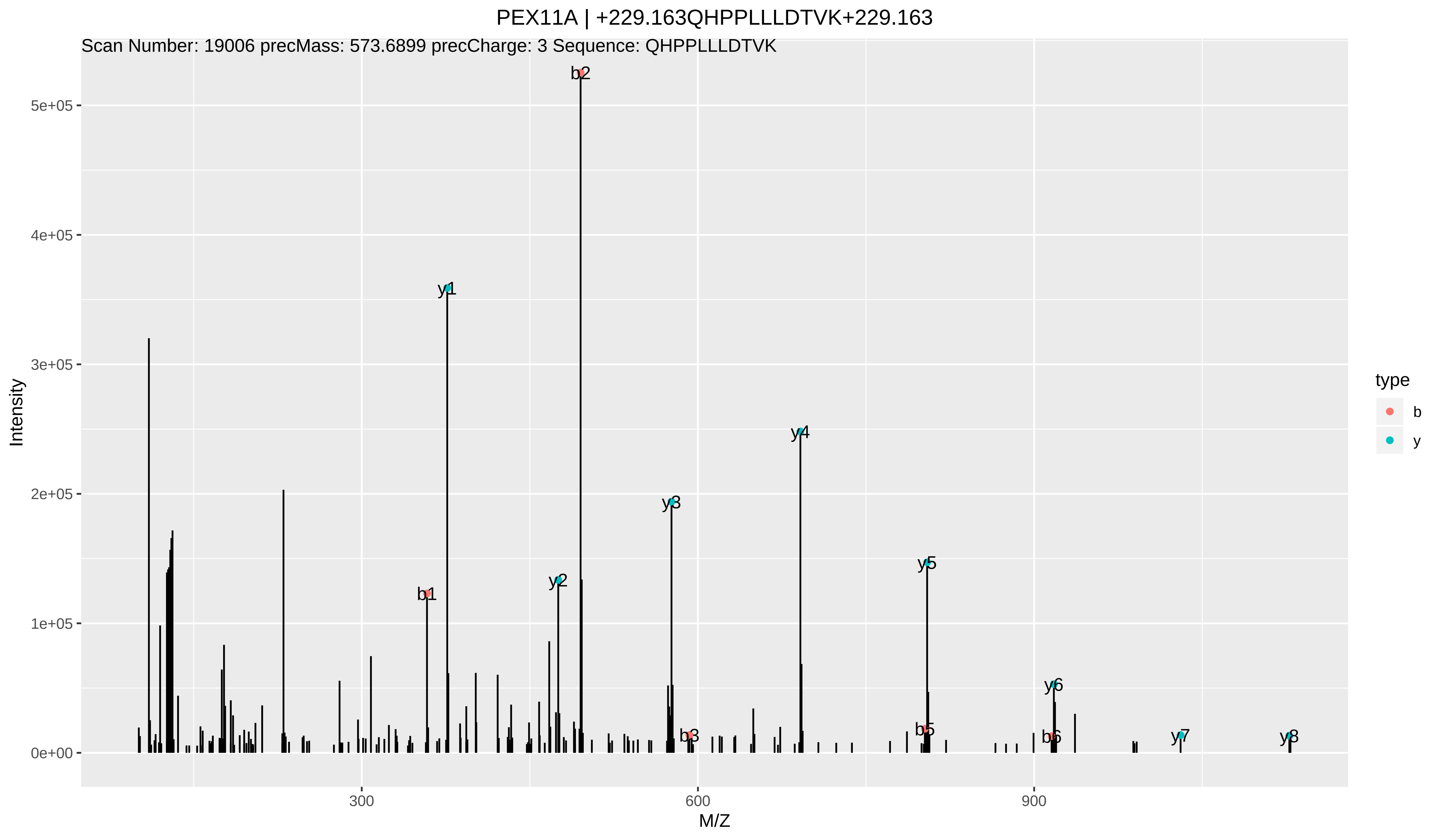

PEX11A | +229.163QHPPLLLDTVK+229.163
Scan Number: 19006 precMass: 573.6899 precCharge: 3 Sequence: QHPPLLLDTVK
Intensity
M/Z
0e+00
1e+05
2e+05
3e+05
4e+05
5e+05
300
600
900
b2
y1
y4
y3
y5
y2
b1
y6
b5
b3
b6
y7
y8
type
b
y

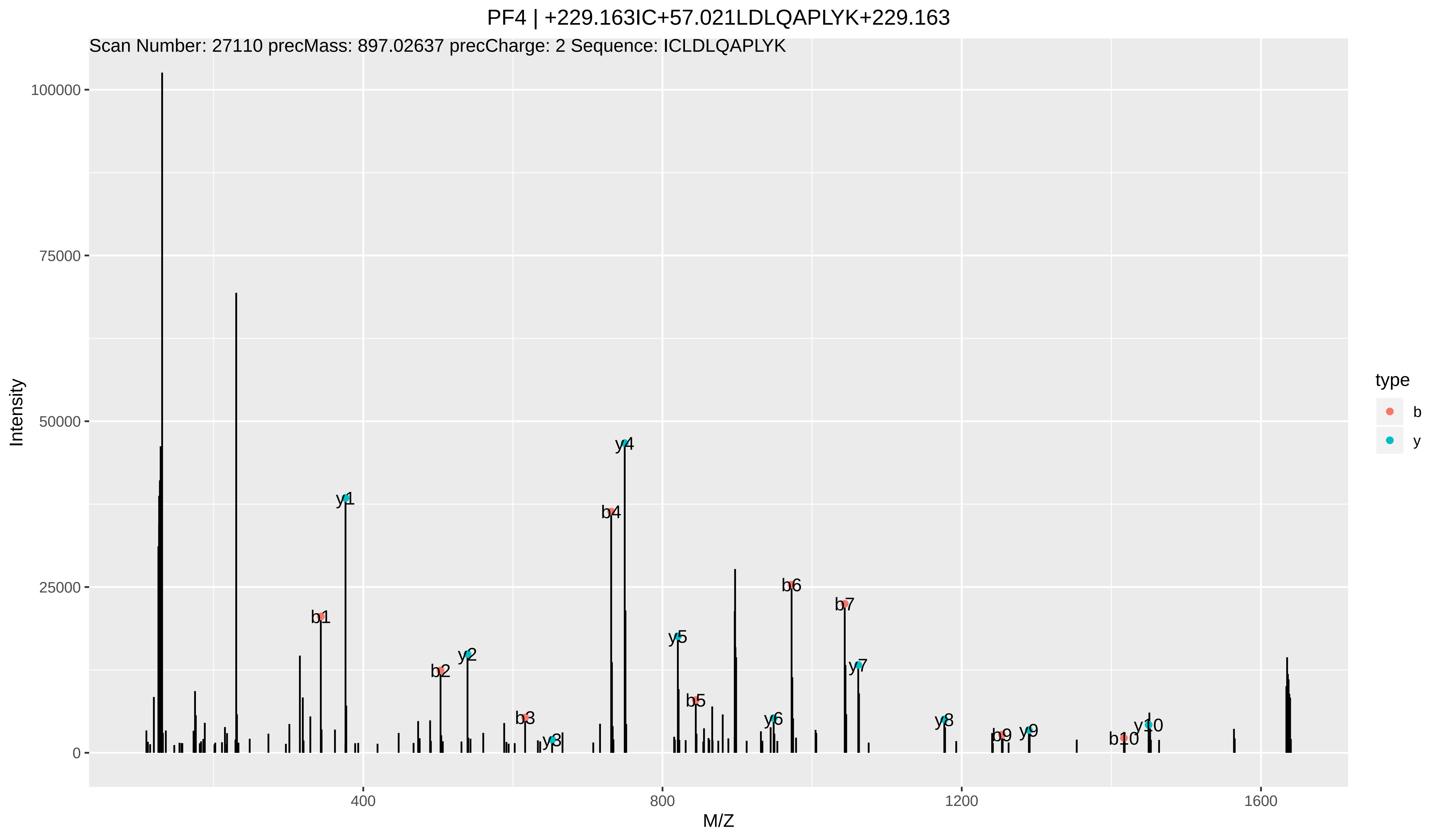

PF4 | +229.163IC+57.021LDLQAPLYK+229.163
Scan Number: 27110 precMass: 897.02637 precCharge: 2 Sequence: ICLDLQAPLYK
Intensity
100000
75000
50000
25000
0
400
800
1200
1600
M/Z
b1
y1
b2
y2
b3
y3
b4
y4
y5
b5
y6
b6
b7
y7
y8
b9
y9
b10
y10
type
b
y

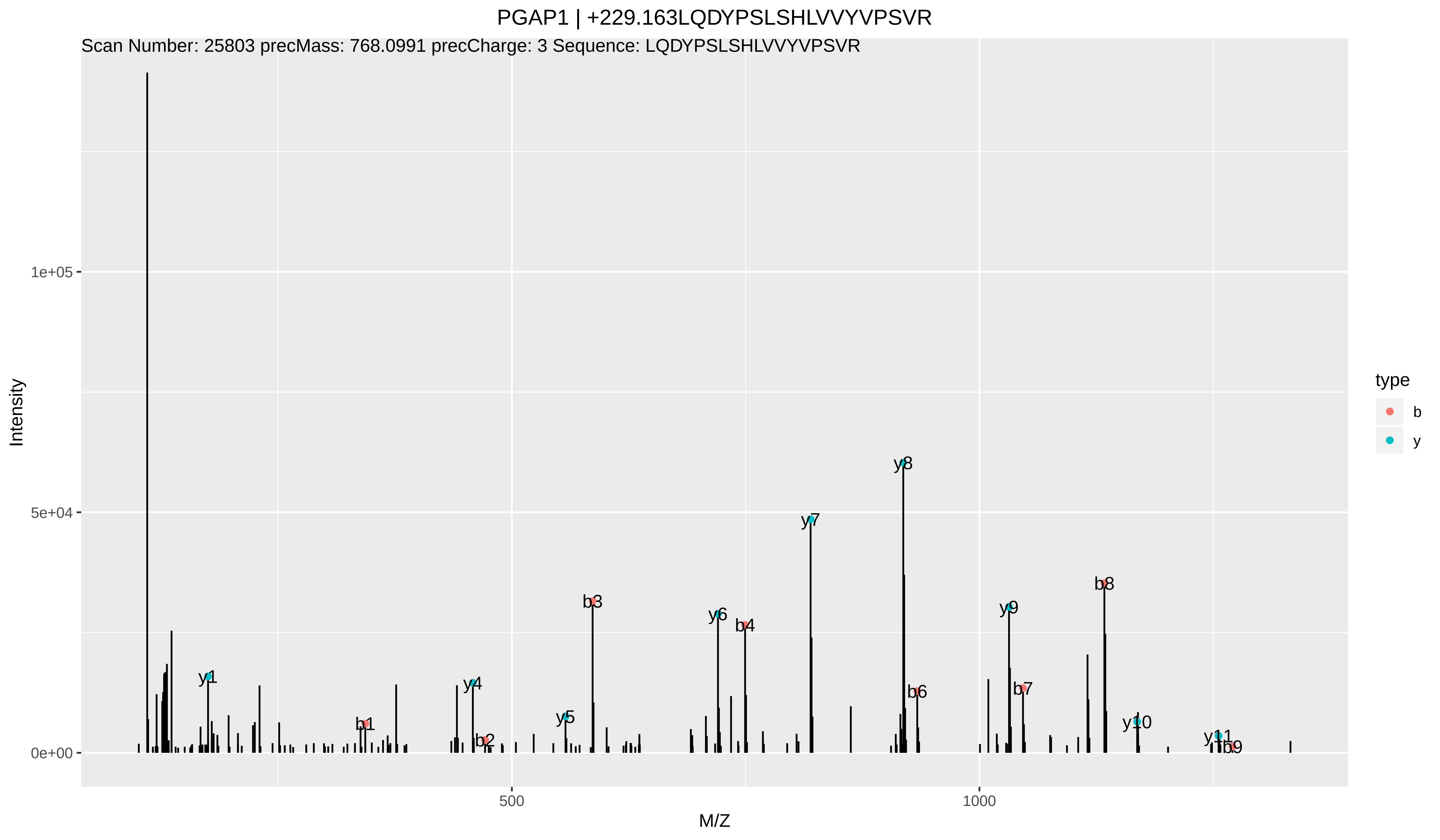
PGAP1 | +229.163LQDYPSLSHLVVYVPSVR
Scan Number: 25803 precMass: 768.0991 precCharge: 3 Sequence: LQDYPSLSHLVVYVPSVR
Intensity
1e+05
5e+04
0e+00
M/Z
500
1000
type
b
y
y1
b1
y4
b2
y5
b3
y6
b4
y7
y8
b6
y9
b7
b8
y10
y11
b9

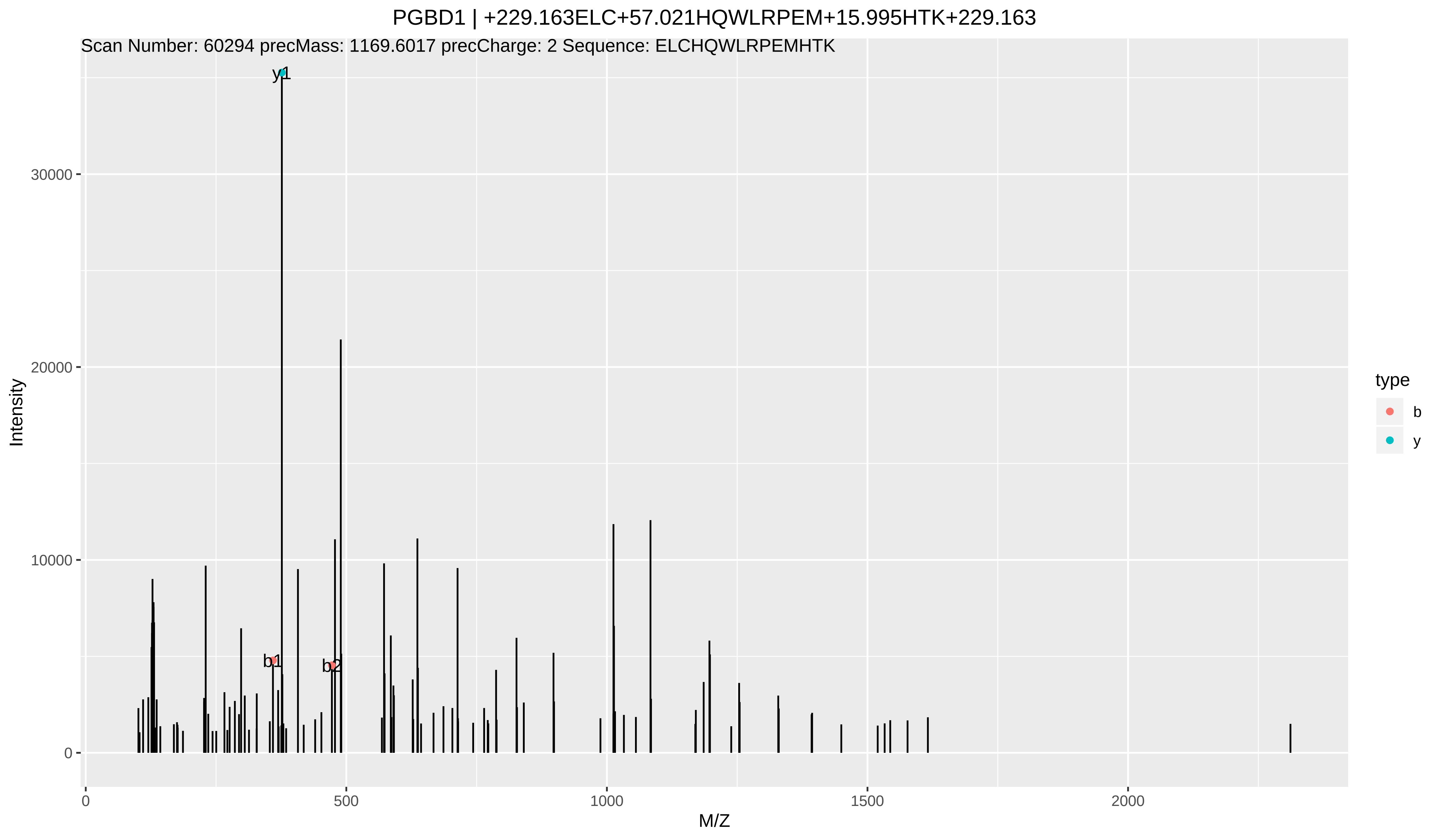

PGBD1 | +229.163ELC+57.021HQWLRPEM+15.995HTK+229.163
Scan Number: 60294 precMass: 1169.6017 precCharge: 2 Sequence: ELCHQWLRPEMHTK
y1
b1
b2
Intensity
30000
20000
10000
0
0
500
1000
1500
2000
M/Z
type
b
y

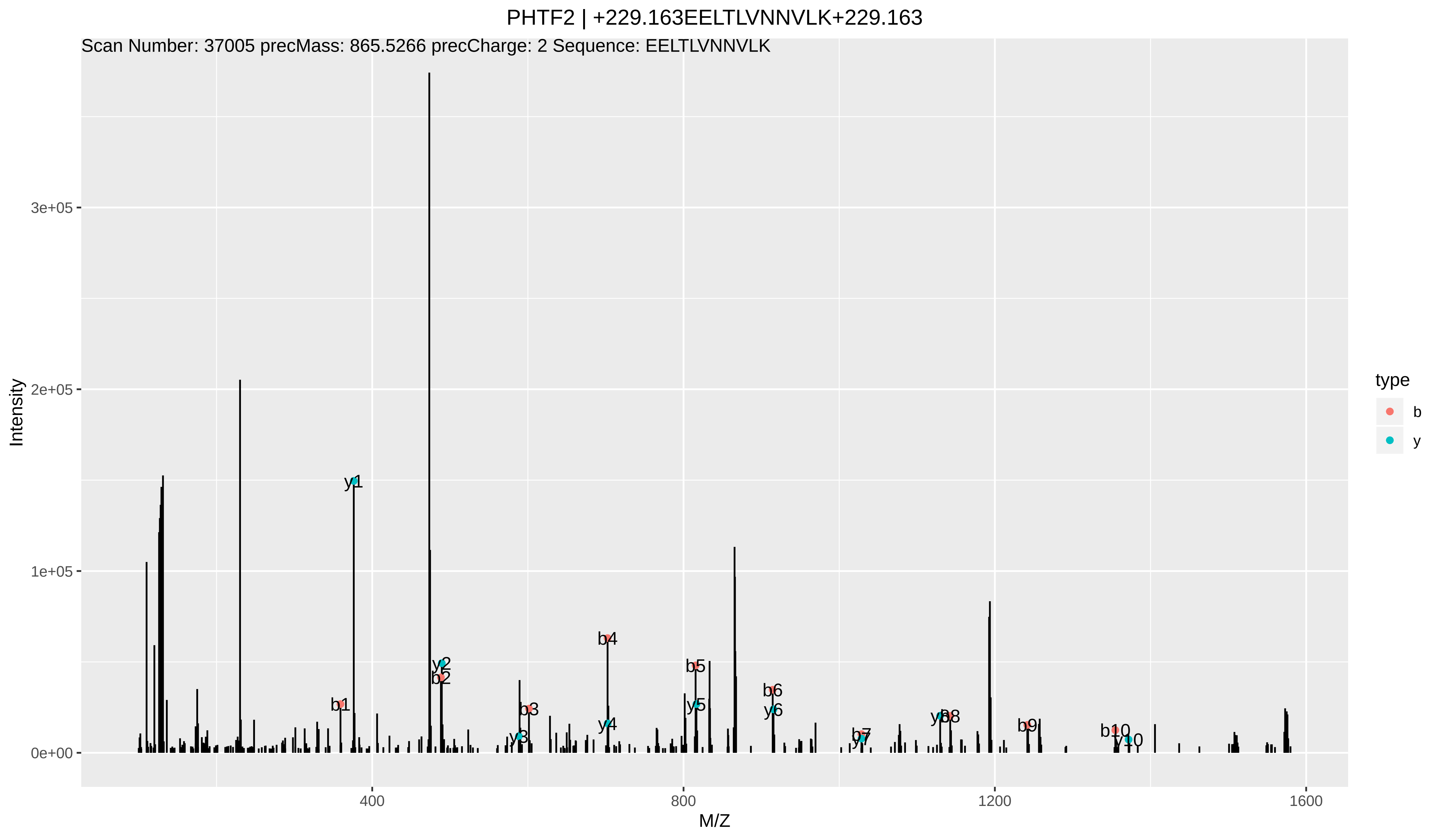
PHTF2 | +229.163EELTLVNNVLK+229.163
Scan Number: 37005 precMass: 865.5266 precCharge: 2 Sequence: EELTLVNNVLK
Intensity
M/Z
type
b
y
y1
y2
b2
b1
b3
y3
b4
y4
b5
y5
b6
y6
b7
y7
y8
b8
b9
b10
y10
3e+05
2e+05
1e+05
0e+00
400
800
1200
1600

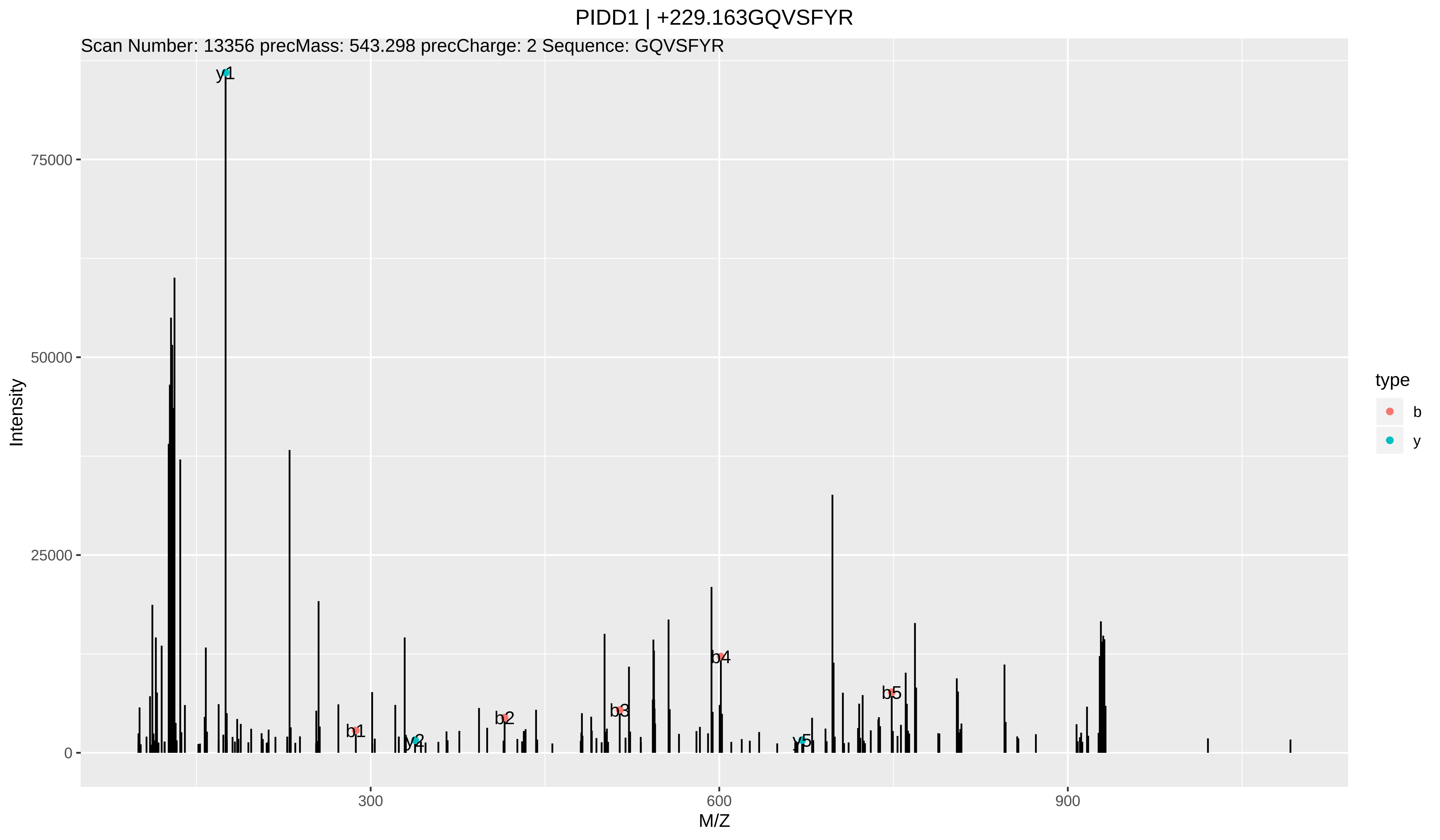
PIDD1 | +229.163GQVSFYR
Scan Number: 13356 precMass: 543.298 precCharge: 2 Sequence: GQVSFYR
y1
y2
y5
b1
b2
b3
b4
b5
Intensity
75000
50000
25000
0
300
600
900
M/Z
type
b
y

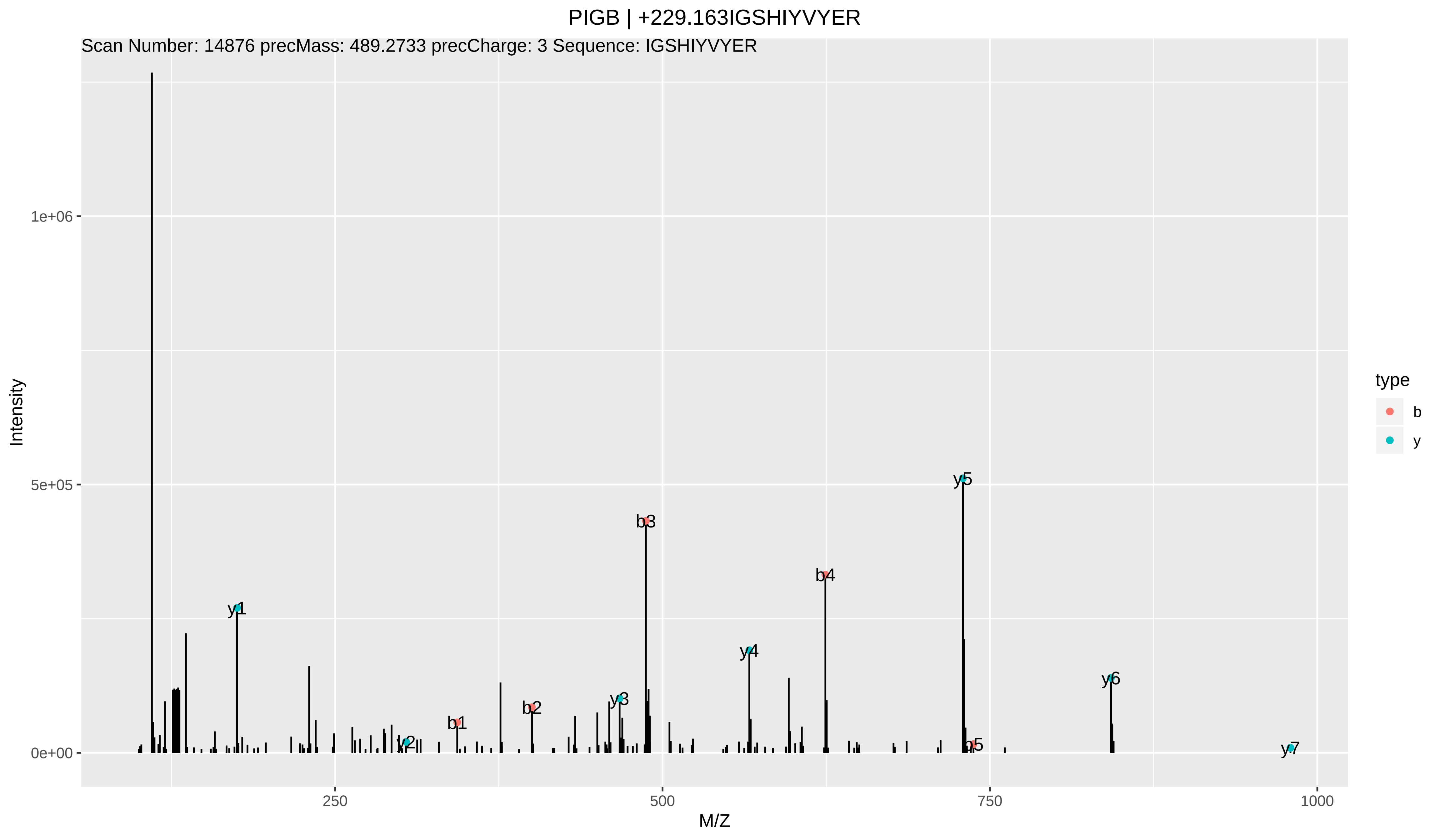

PIGB | +229.163IGSHIYVYER
Scan Number: 14876 precMass: 489.2733 precCharge: 3 Sequence: IGSHIYVYER
Intensity
1e+06
5e+05
0e+00
M/Z
250
500
750
1000
y1
y2
b1
b2
y3
b3
y4
b4
y5
b5
y6
y7
type
b
y

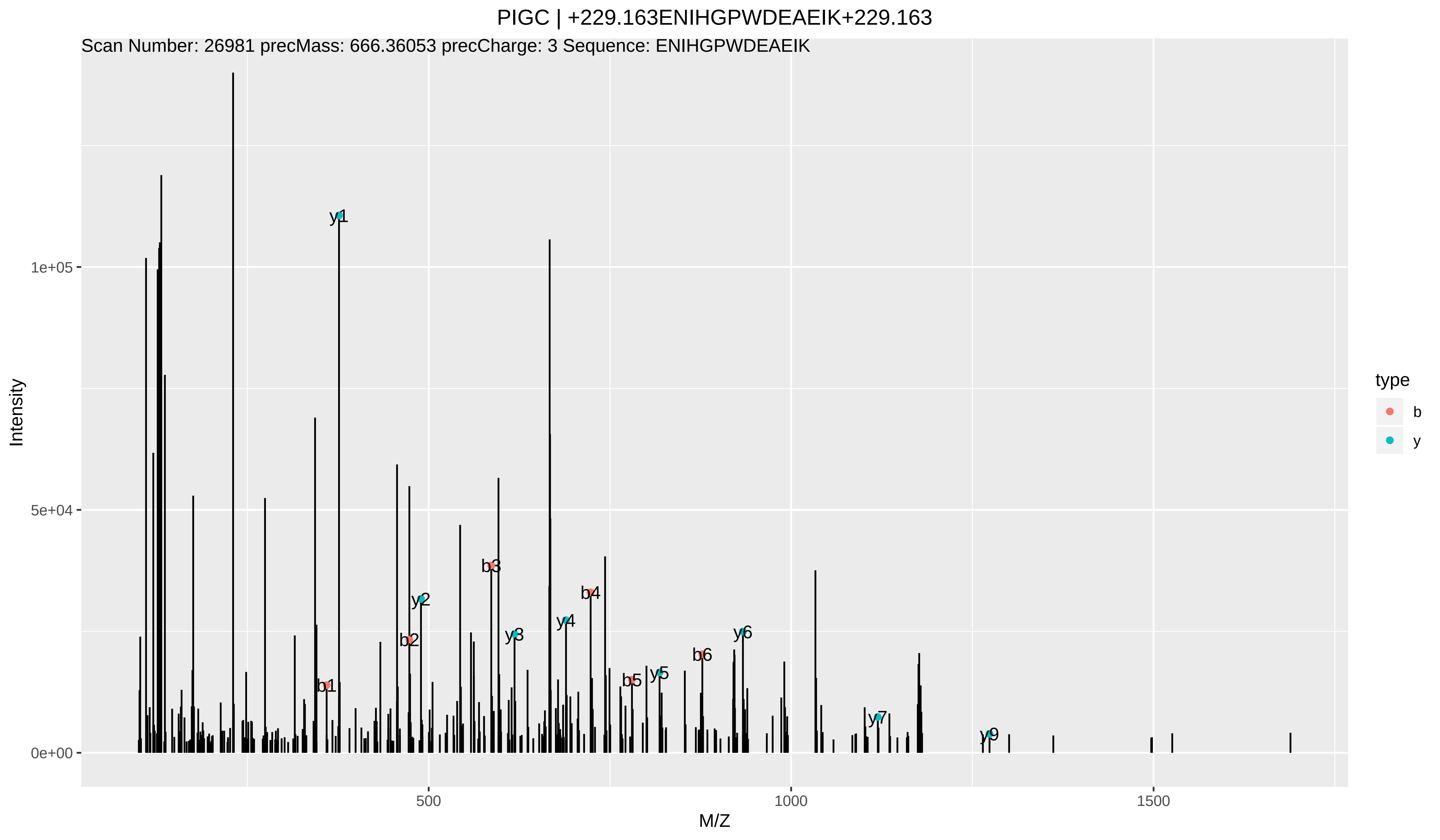

PIGC | +229.163ENIHGPWDEAEIK+229.163
Scan Number: 26981 precMass: 666.36053 precCharge: 3 Sequence: ENIHGPWDEAEIK
Intensity
1e+05
5e+04
0e+00
M/Z
500
1000
1500
type
b
y
y1
y2
y3
y4
y5
y6
y7
y9
b1
b2
b3
b4
b5
b6

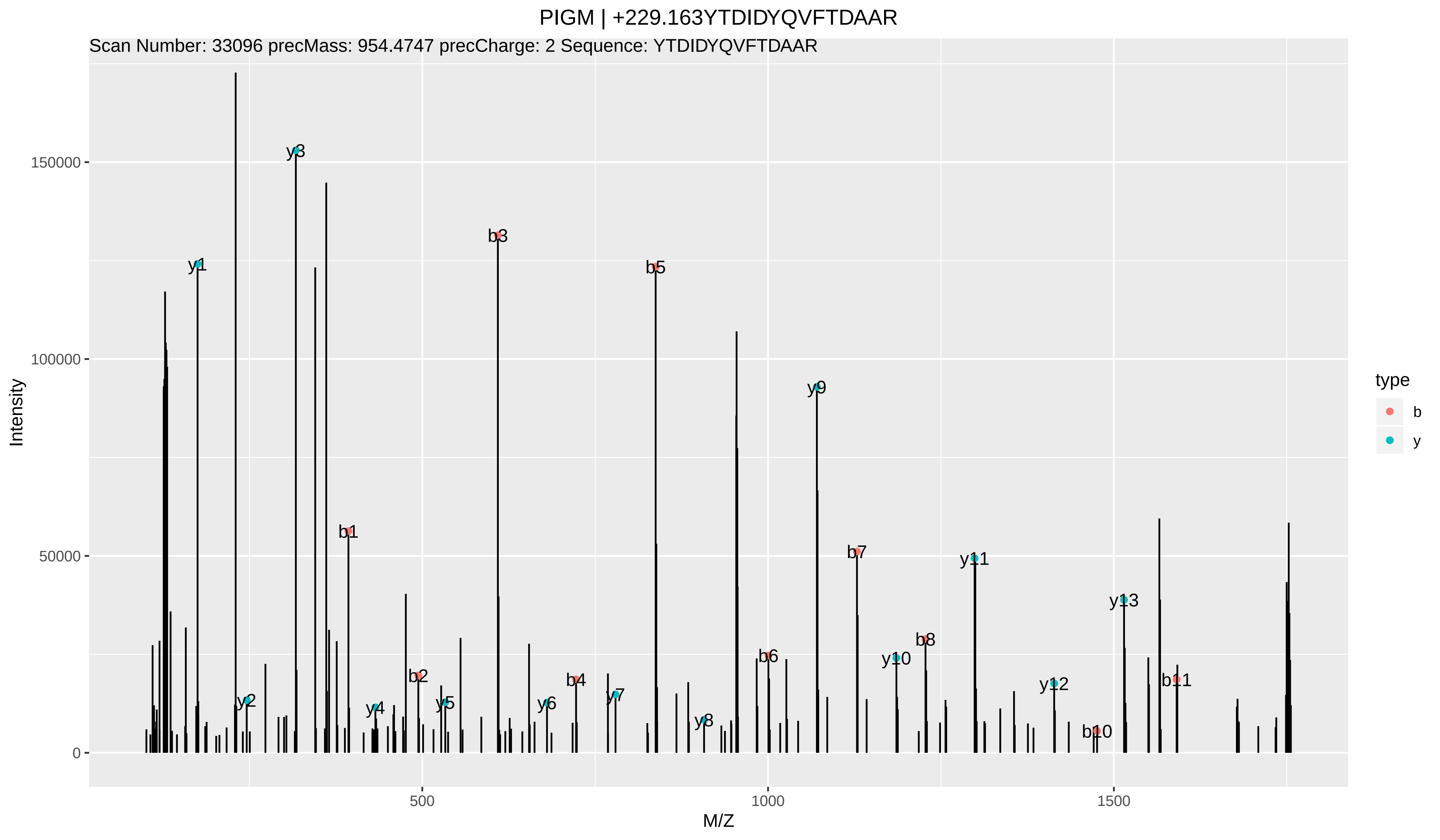
PIGM | +229.163YTDIDYQVFTDAAR
Scan Number: 33096 precMass: 954.4747 precCharge: 2 Sequence: YTDIDYQVFTDAAR
Intensity
150000
100000
50000
0
M/Z
500
1000
1500
y1
y2
y3
b1
y4
b2
y5
b3
y6
b4
y7
b5
y8
b6
y9
b7
y10
b8
y11
y12
b10
y13
b11
type
b
y

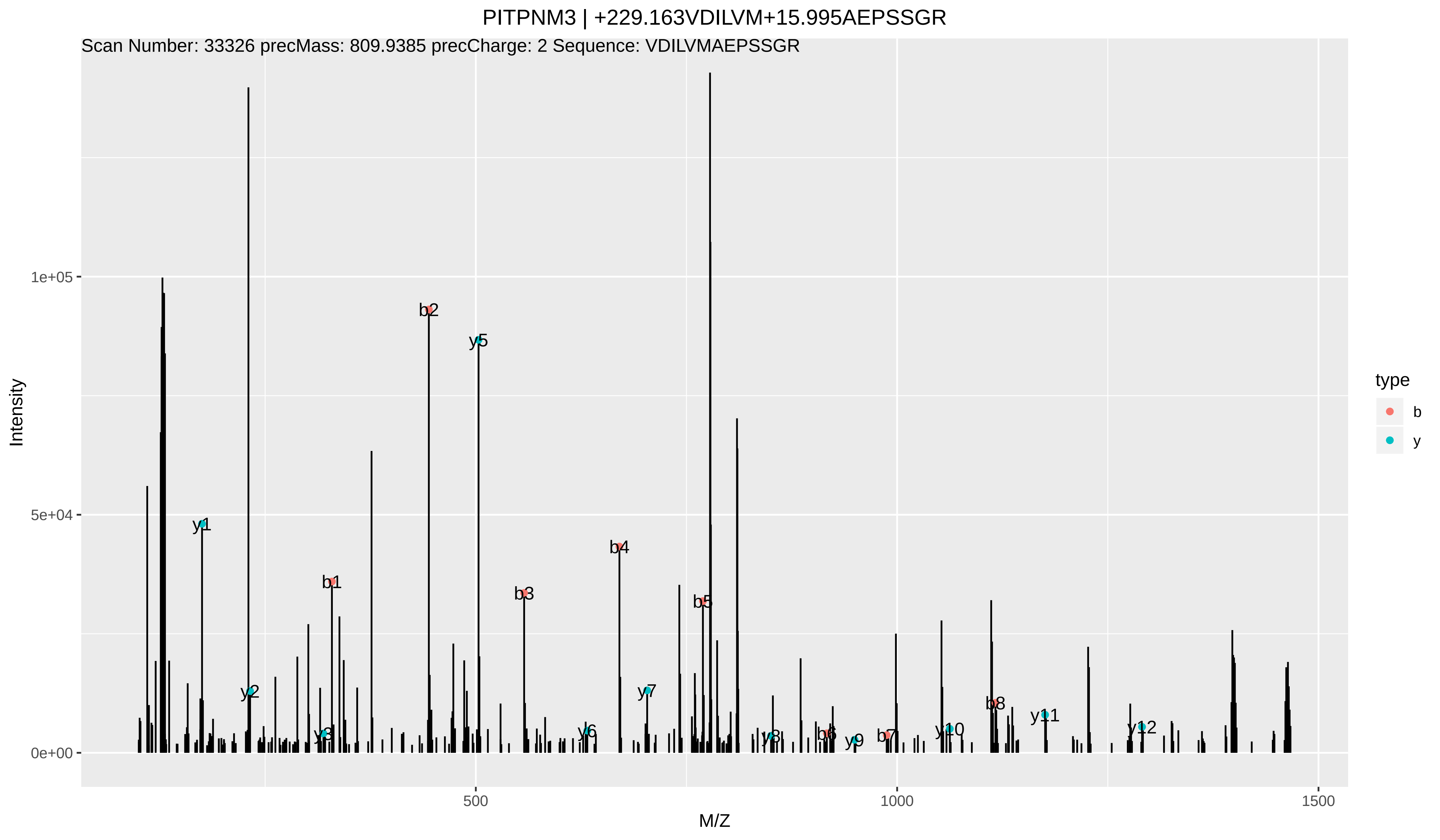

PITPNM3 | +229.163VDILVM+15.995AEPSSGR
Scan Number: 33326 precMass: 809.9385 precCharge: 2 Sequence: VDILVMAEPSSGR
Intensity
1e+05
5e+04
0e+00
M/Z
500
1000
1500
y1
y2
y3
b1
b2
y5
b3
b4
y6
y7
b5
y8
b6
y9
b7
y10
b8
y11
y12
type
b
y

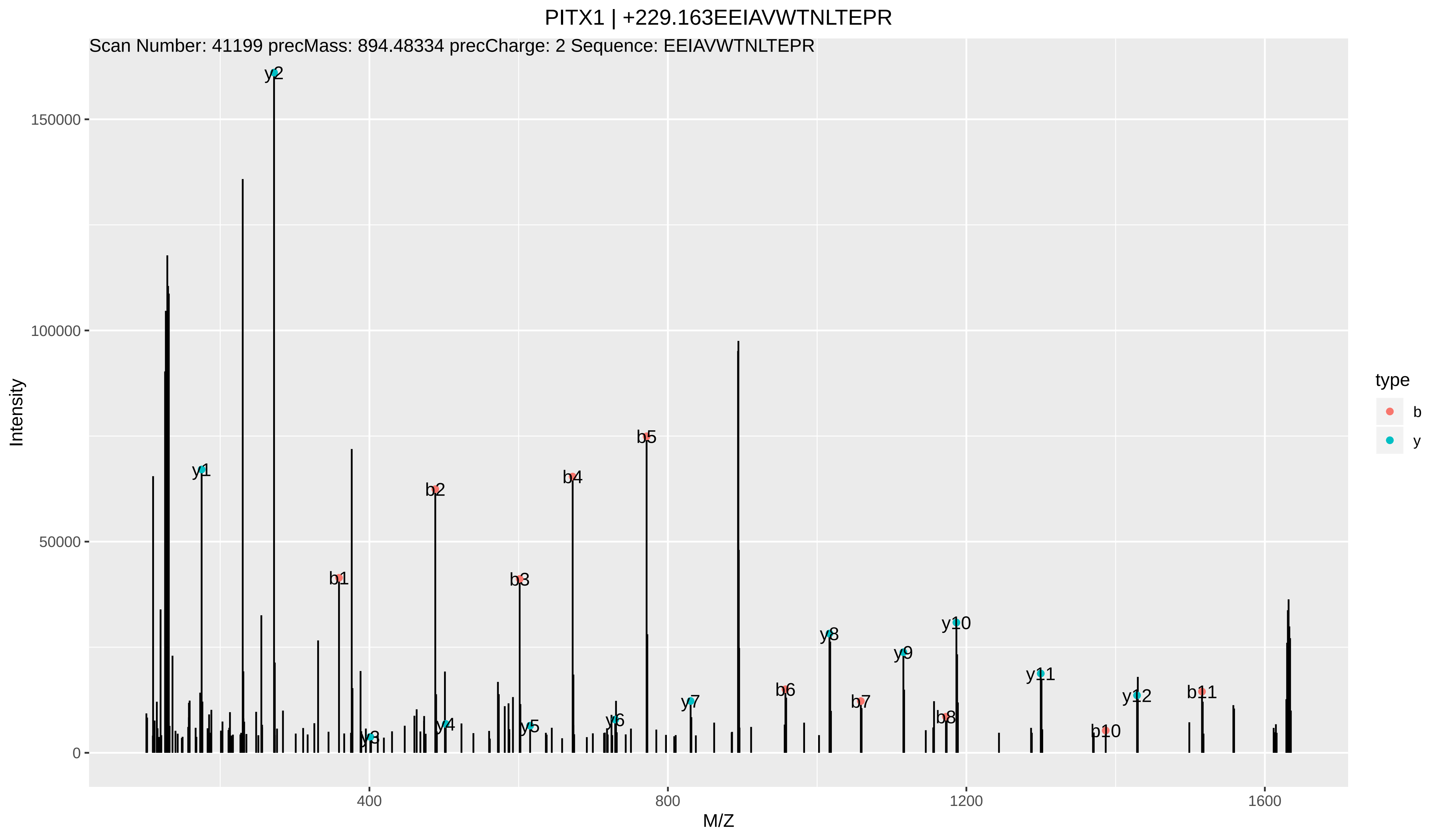

PITX1 | +229.163EEIAVWTNLTEPR
Scan Number: 41199 precMass: 894.48334 precCharge: 2 Sequence: EEIAVWTNLTEPR
Intensity
150000
100000
50000
0
M/Z
400
800
1200
1600
y1
y2
y3
y4
y5
y6
y7
y8
y9
y10
y11
y12
b1
b2
b3
b4
b5
b6
b7
b8
b10
b11
type
b
y

PITX1 | +229.163THFTSQQLQELEATFQR

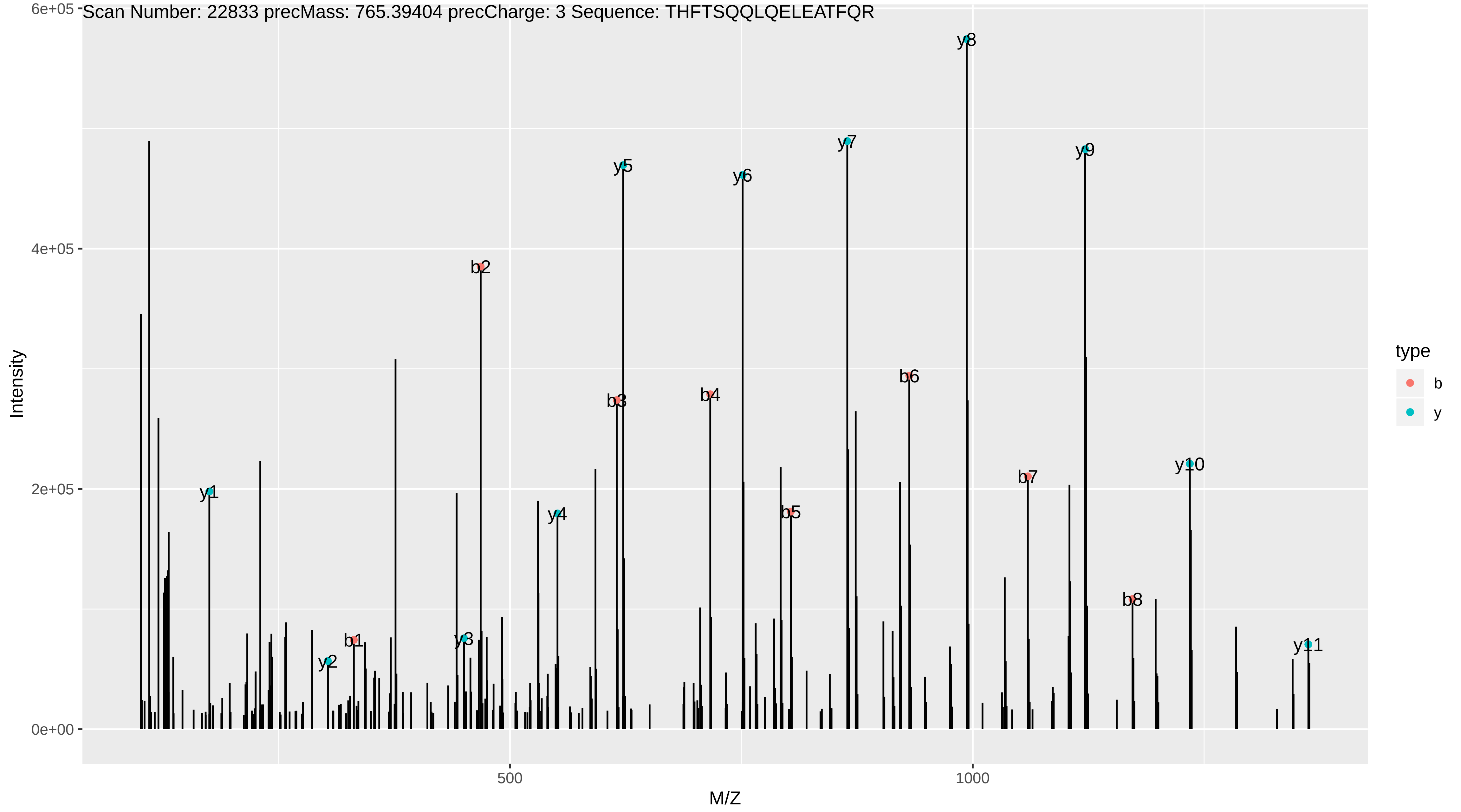

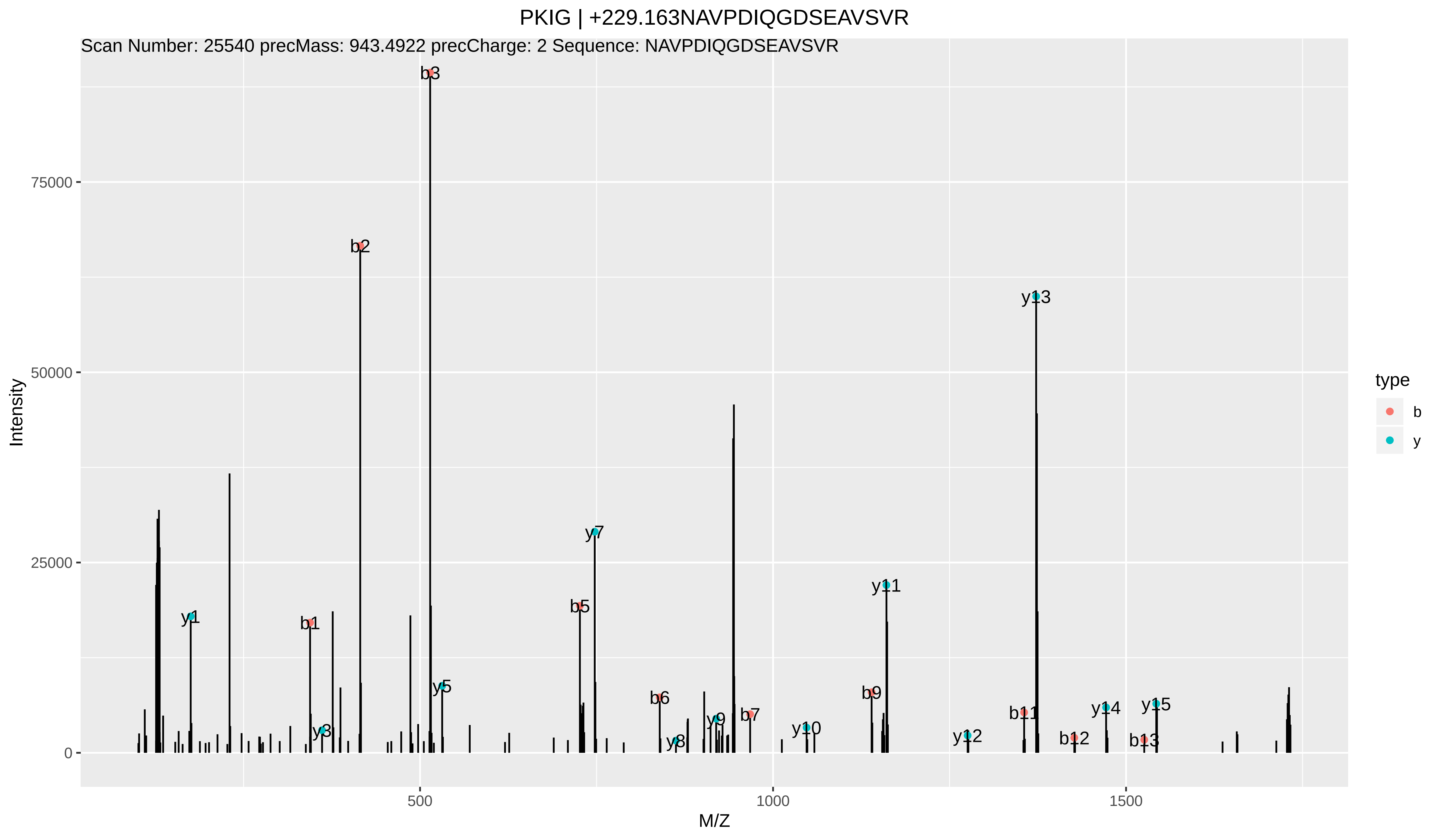
PKIG | +229.163NAVPDIQGDSEAVSVR
Scan Number: 25540 precMass: 943.4922 precCharge: 2 Sequence: NAVPDIQGDSEAVSVR
Intensity
M/Z
0
25000
50000
75000
500
1000
1500
type
b
y
b1
b2
b3
b5
b6
b7
b9
b11
b12
b13
y1
y3
y5
y7
y8
y9
y10
y11
y12
y13
y14
y15

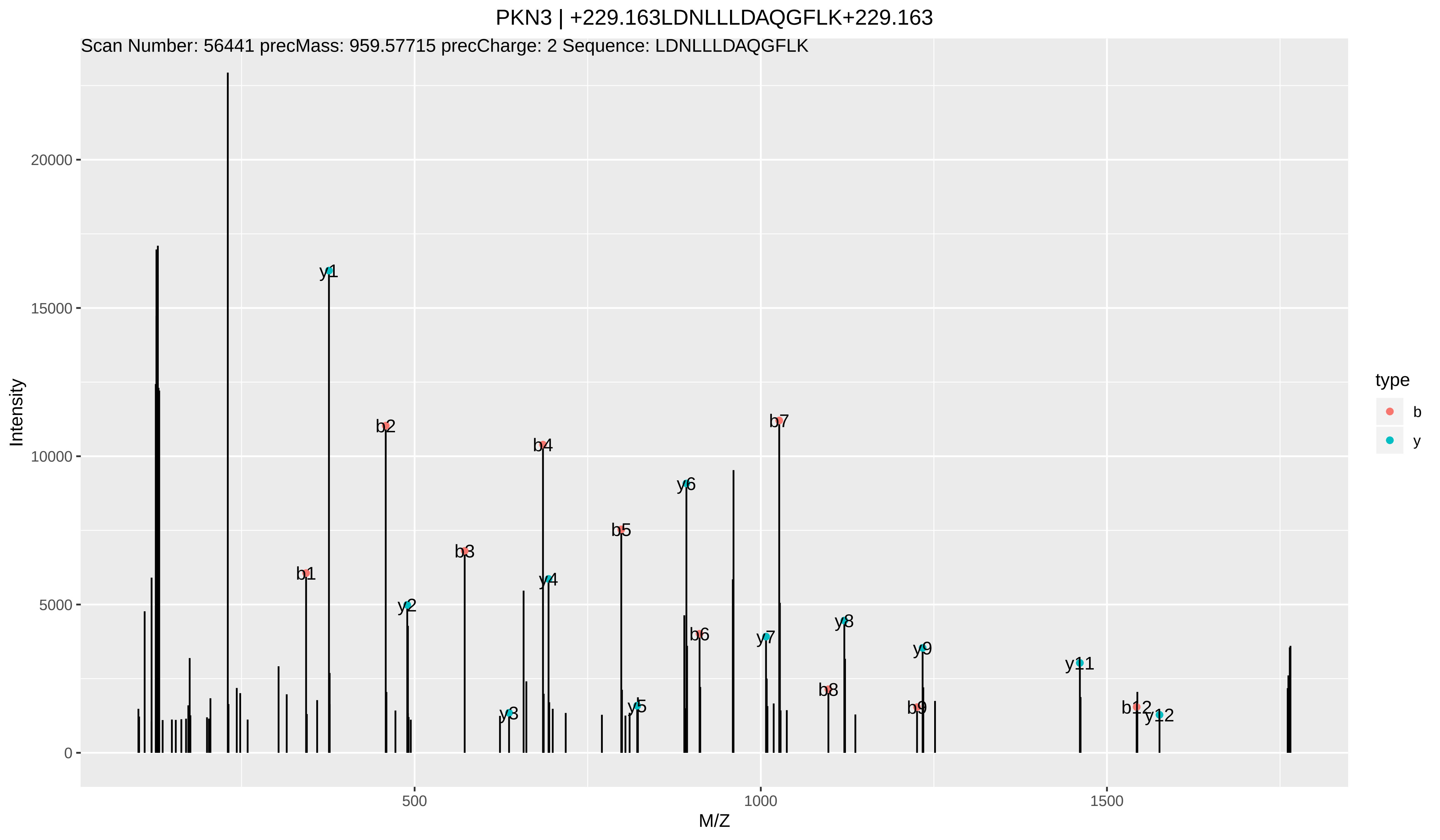

PKN3 | +229.163LDNLLLDAQGFLK+229.163
Scan Number: 56441 precMass: 959.57715 precCharge: 2 Sequence: LDNLLLDAQGFLK
Intensity
M/Z
0
5000
10000
15000
20000
500
1000
1500
y1
b1
b2
y2
b3
y3
b4
y4
b5
y5
y6
b6
y7
b7
b8
y8
b9
y9
y11
b12
y12
type
b
y

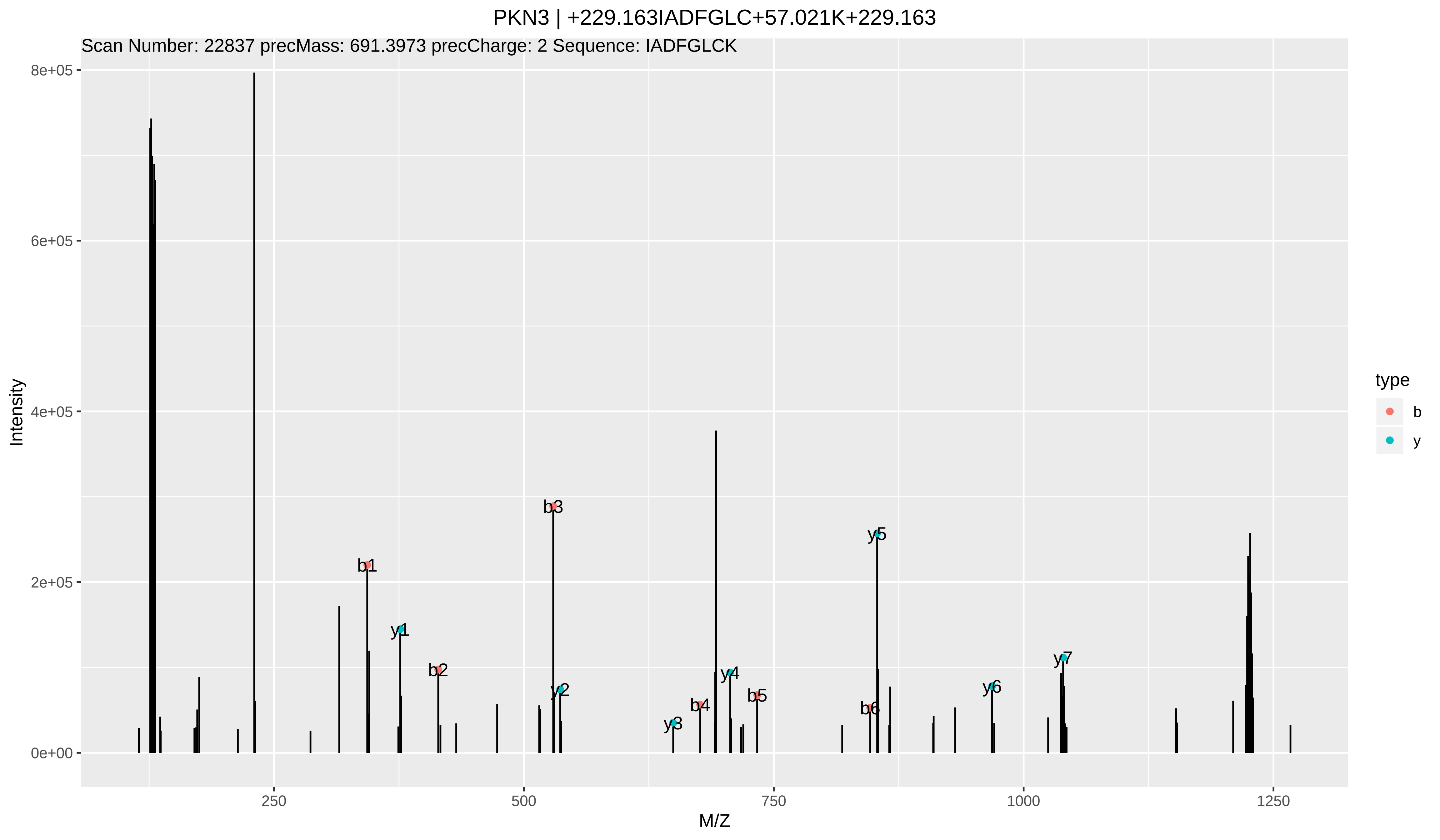

PKN3 | +229.163IADFGLC+57.021K+229.163
Scan Number: 22837 precMass: 691.3973 precCharge: 2 Sequence: IADFGLCK
Intensity
M/Z
0e+00
2e+05
4e+05
6e+05
8e+05
250
500
750
1000
1250
b1
b2
b3
b4
b5
b6
y1
y2
y3
y4
y5
y6
y7
type
b
y

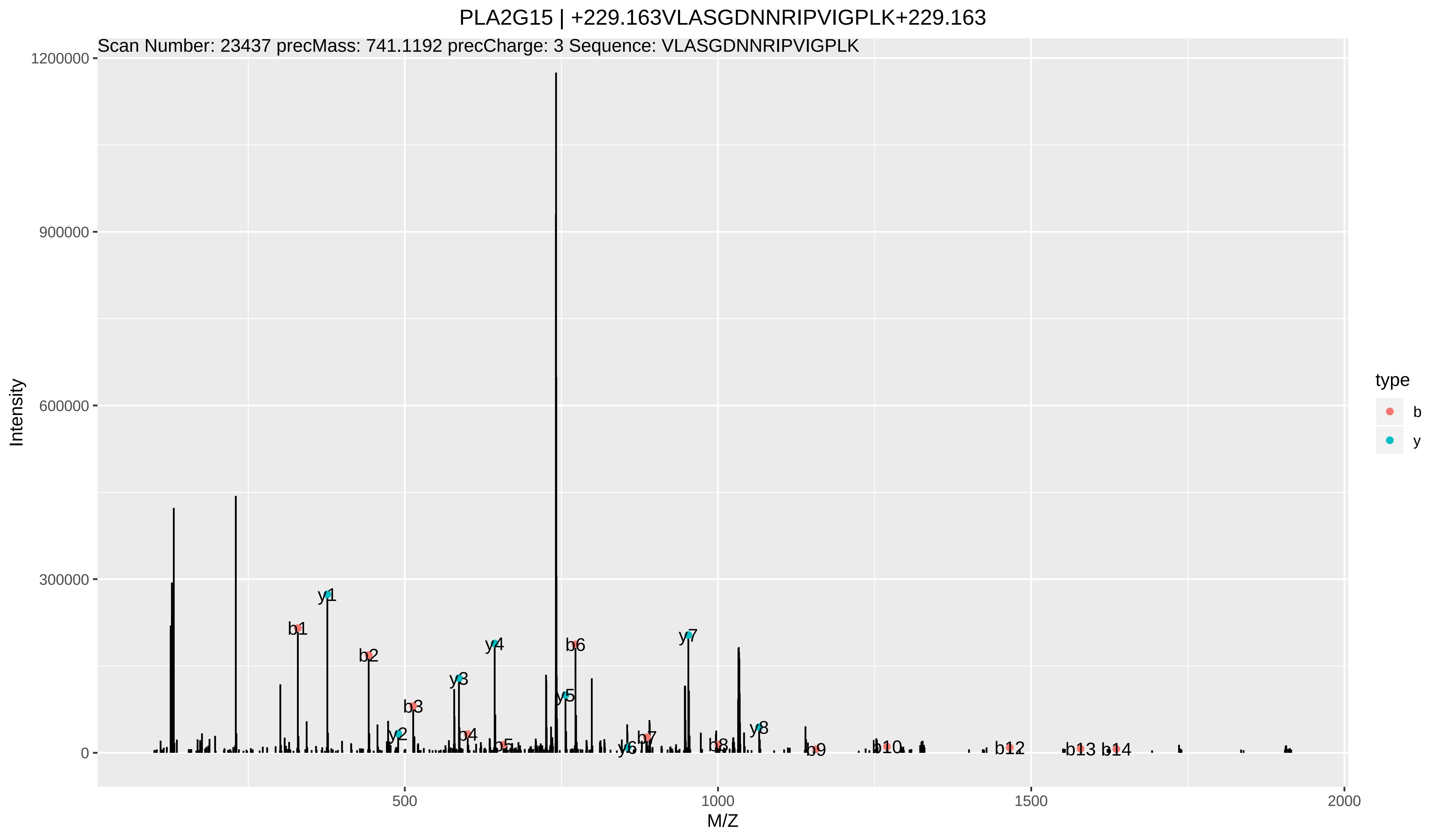

PLA2G15 | +229.163VLASGDNNRIPVIGPLK+229.163
Scan Number: 23437 precMass: 741.1192 precCharge: 3 Sequence: VLASGDNNRIPVIGPLK
Intensity
1200000
900000
600000
300000
0
M/Z
500
1000
1500
2000
type
b
y
y1
b1
b2
y2
b3
y3
b4
y4
b5
y5
b6
y6
b7
y7
b8
y8
b9
b10
b12
b13
b14

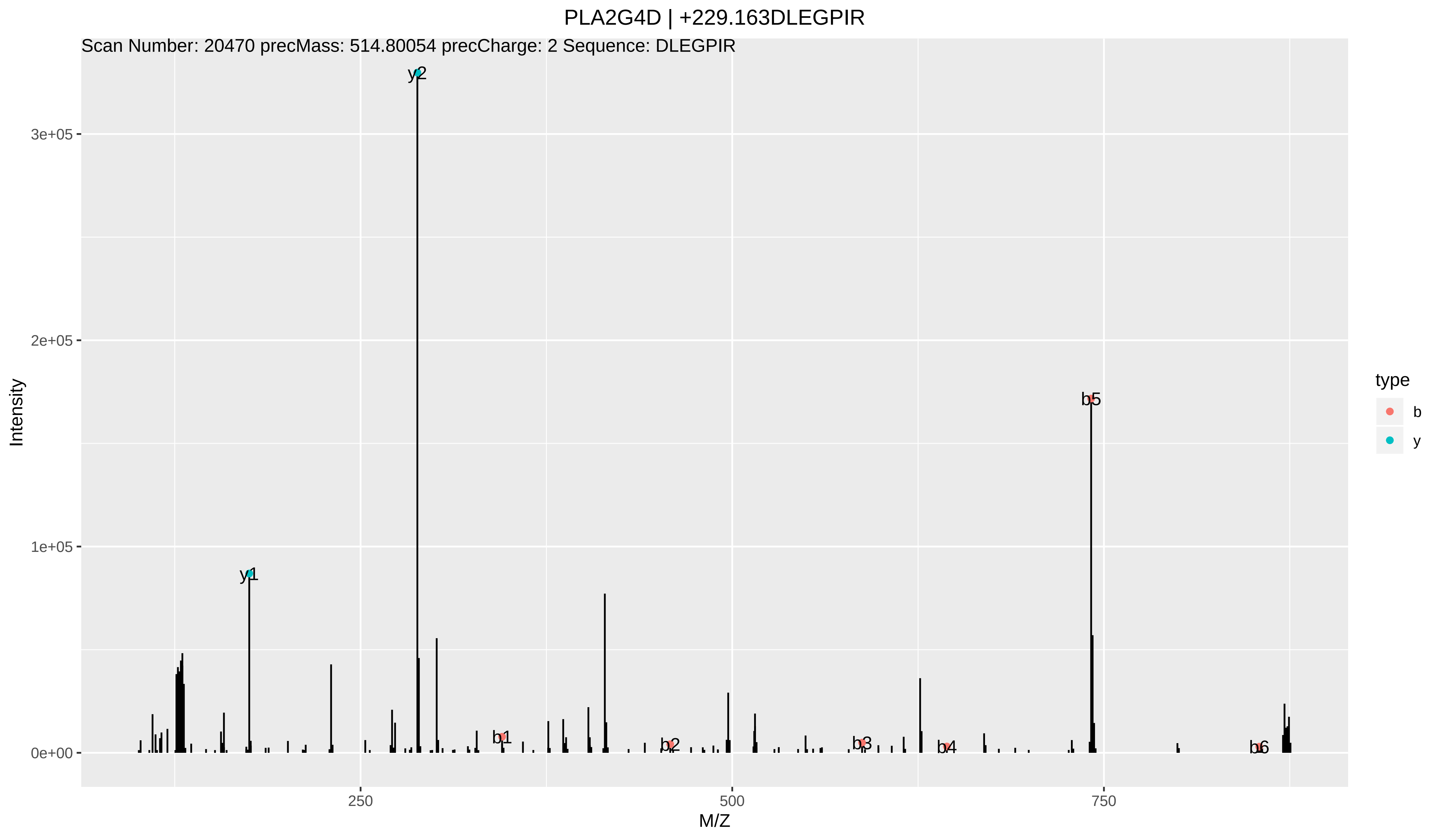
PLA2G4D | +229.163DLEGPIR
Scan Number: 20470 precMass: 514.80054 precCharge: 2 Sequence: DLEGPIR
y2
y1
b1
b2
b3
b4
b5
b6
Intensity
3e+05
2e+05
1e+05
0e+00
250
500
750
M/Z
type
b
y

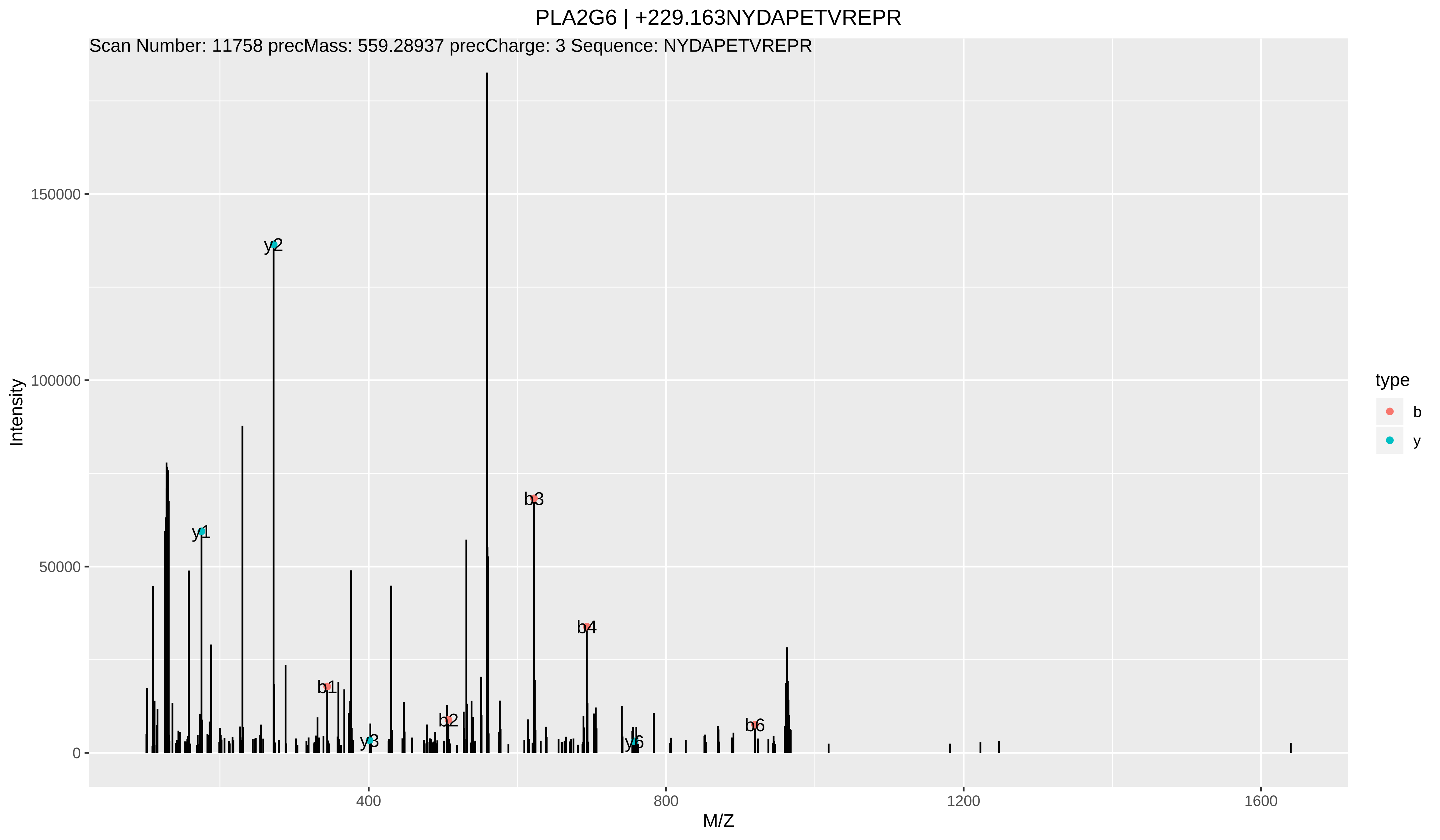

PLA2G6 | +229.163NYDAPETVREPR
Scan Number: 11758 precMass: 559.28937 precCharge: 3 Sequence: NYDAPETVREPR
Intensity
150000
100000
50000
0
M/Z
400
800
1200
1600
y2
y1
b3
b4
b1
b2
y3
y6
b6
type
b
y

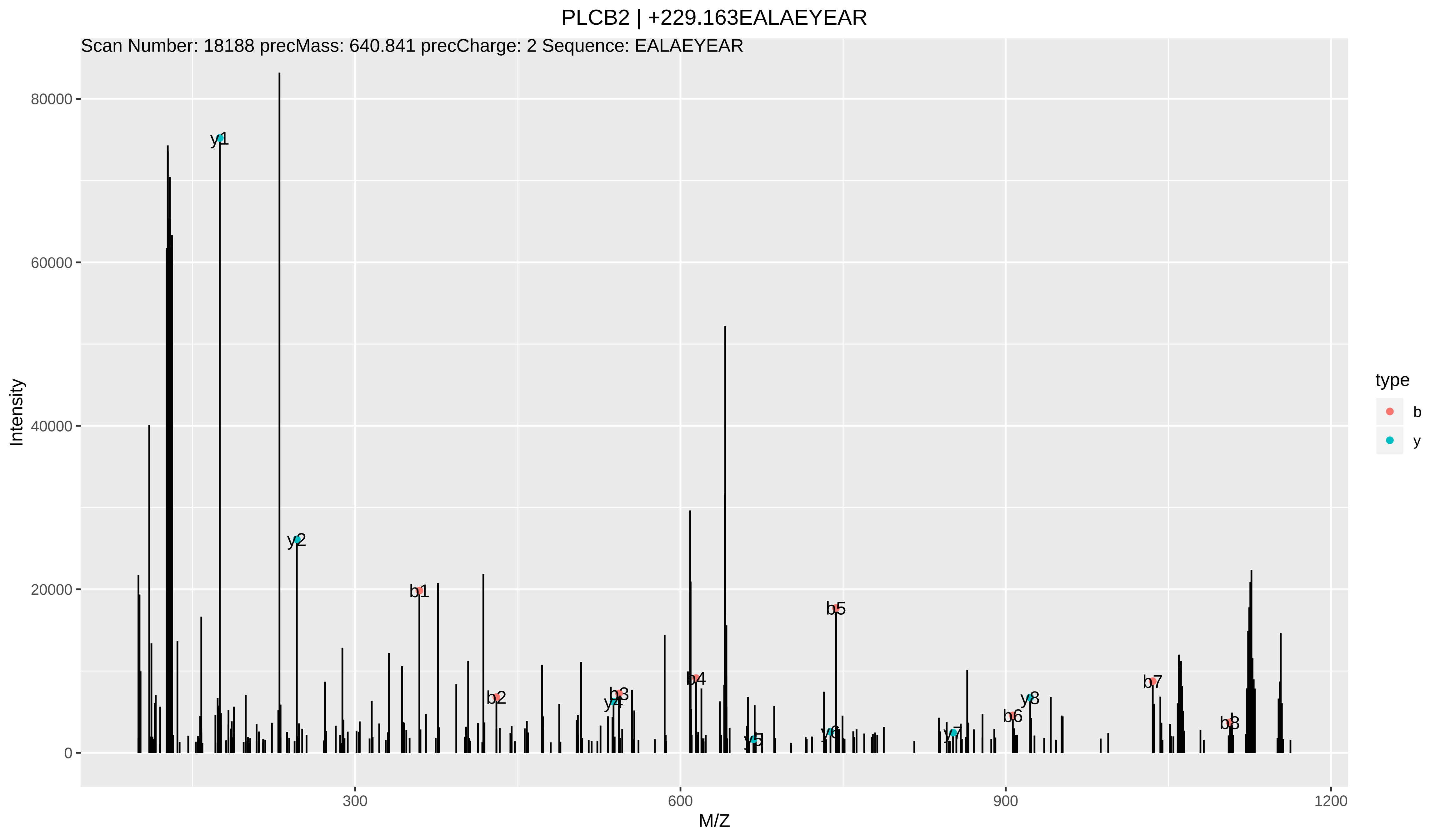
PLCB2 | +229.163EALAEYEAR
Scan Number: 18188 precMass: 640.841 precCharge: 2 Sequence: EALAEYEAR
Intensity
M/Z
80000
60000
40000
20000
0
300
600
900
1200
y1
y2
b1
b2
b3
y4
b4
y5
b5
y6
y7
b6
y8
b7
b8
type
b
y

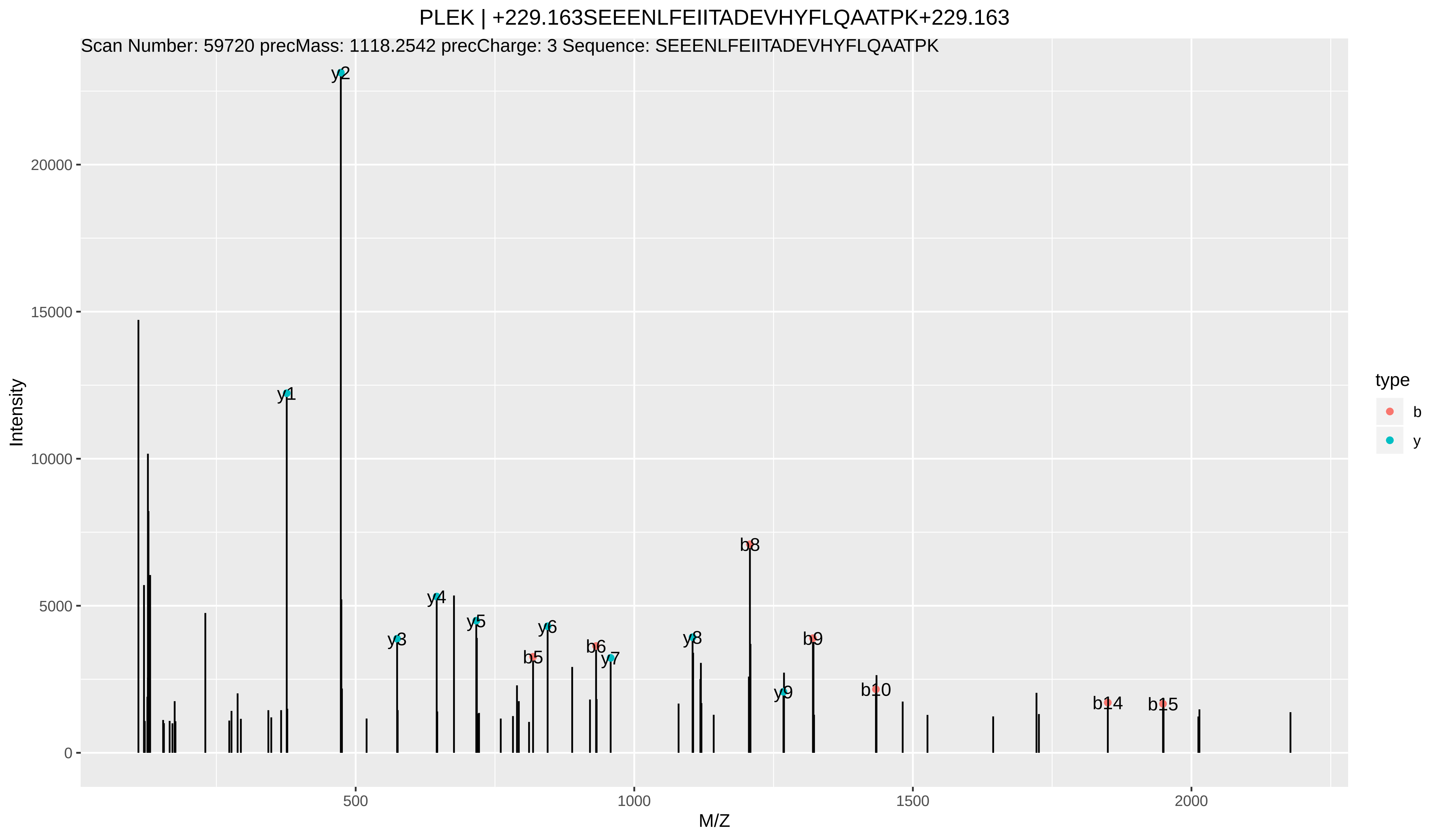

PLEK | +229.163SEEENLFEIITADEVHYFLQAATPK+229.163
Scan Number: 59720 precMass: 1118.2542 precCharge: 3 Sequence: SEEENLFEIITADEVHYFLQAATPK
Intensity
M/Z
0
5000
10000
15000
20000
500
1000
1500
2000
y1
y2
y3
y4
y5
y6
y7
y8
y9
b5
b6
b8
b9
b10
b14
b15
type
b
y

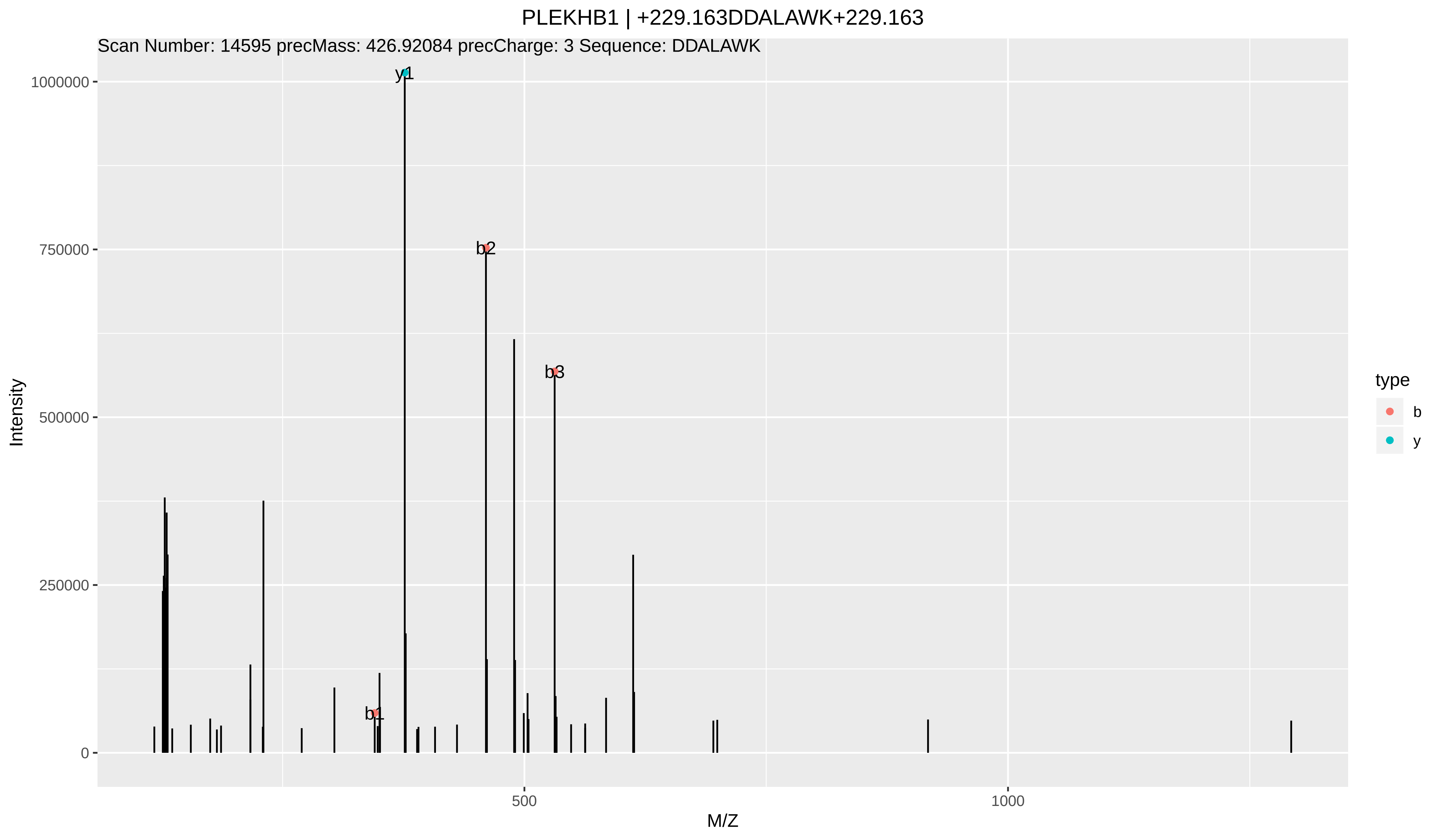
PLEKHB1 | +229.163DDALAWK+229.163
Scan Number: 14595 precMass: 426.92084 precCharge: 3 Sequence: DDALAWK
y1
b2
b3
b1
1000000
750000
500000
250000
0
500
1000
M/Z
Intensity
type
b
y

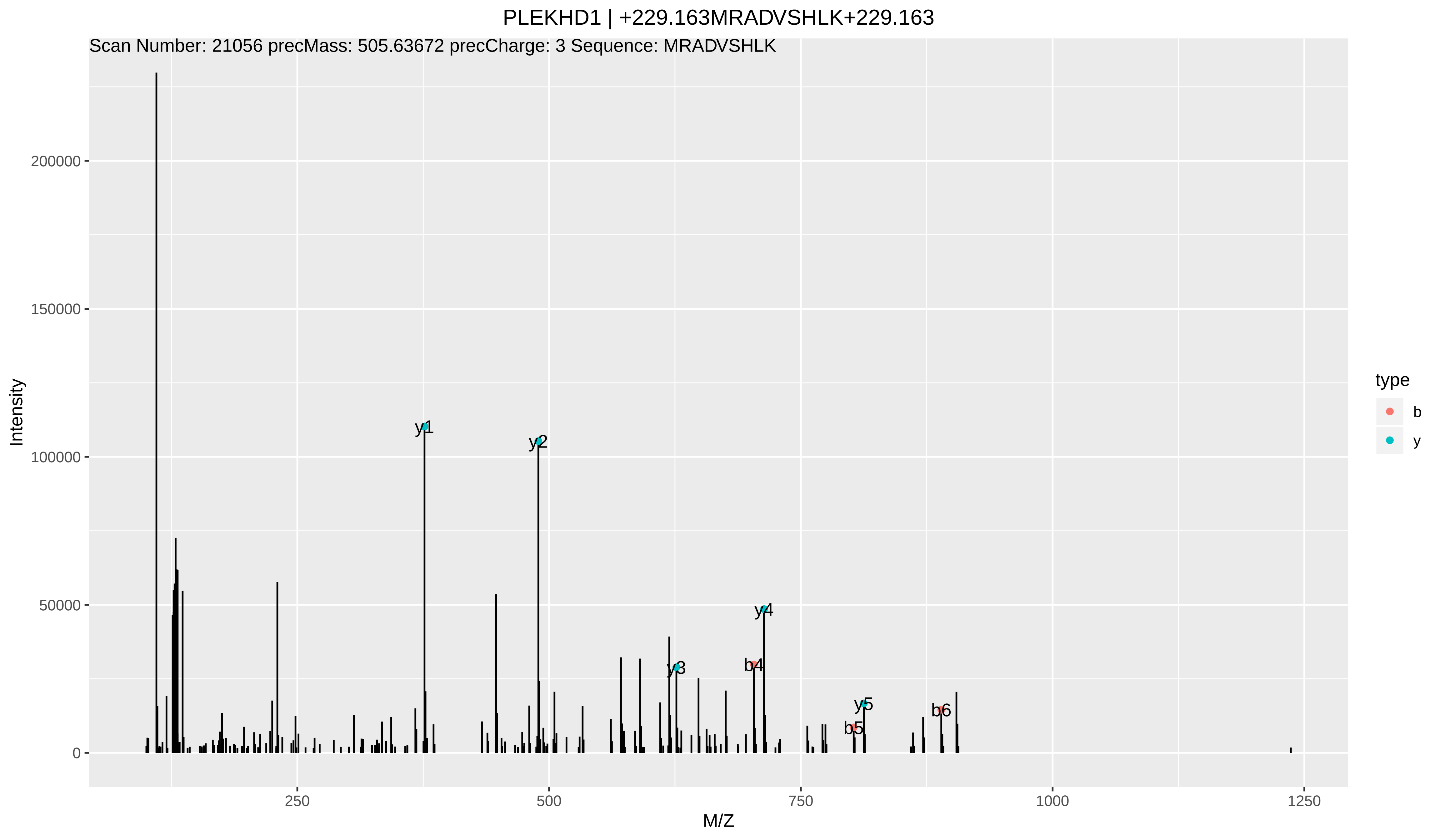

PLEKHD1 | +229.163MRADVSHLK+229.163
Scan Number: 21056 precMass: 505.63672 precCharge: 3 Sequence: MRADVSHLK
Intensity
200000
150000
100000
50000
0
250
500
750
1000
1250
M/Z
y1
y2
y3
y4
y5
b4
b5
b6
type
b
y

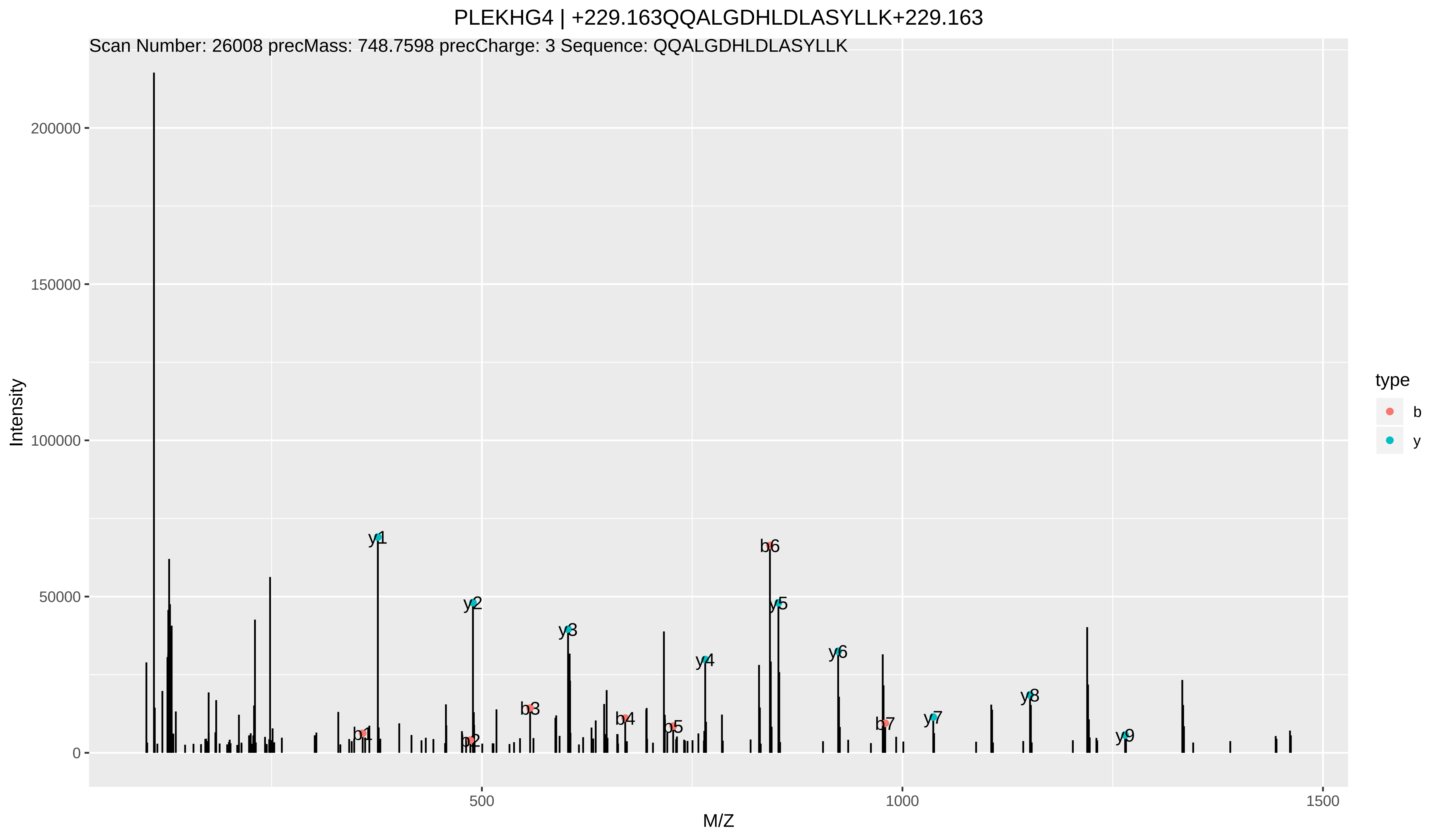

PLEKHG4 | +229.163QQALGDHLDLASYLLK+229.163
Scan Number: 26008 precMass: 748.7598 precCharge: 3 Sequence: QQALGDHLDLASYLLK
Intensity
200000
150000
100000
50000
0
M/Z
500
1000
1500
y1
y2
y3
y4
y5
y6
y7
y8
y9
b1
b2
b3
b4
b5
b6
b7
type
b
y

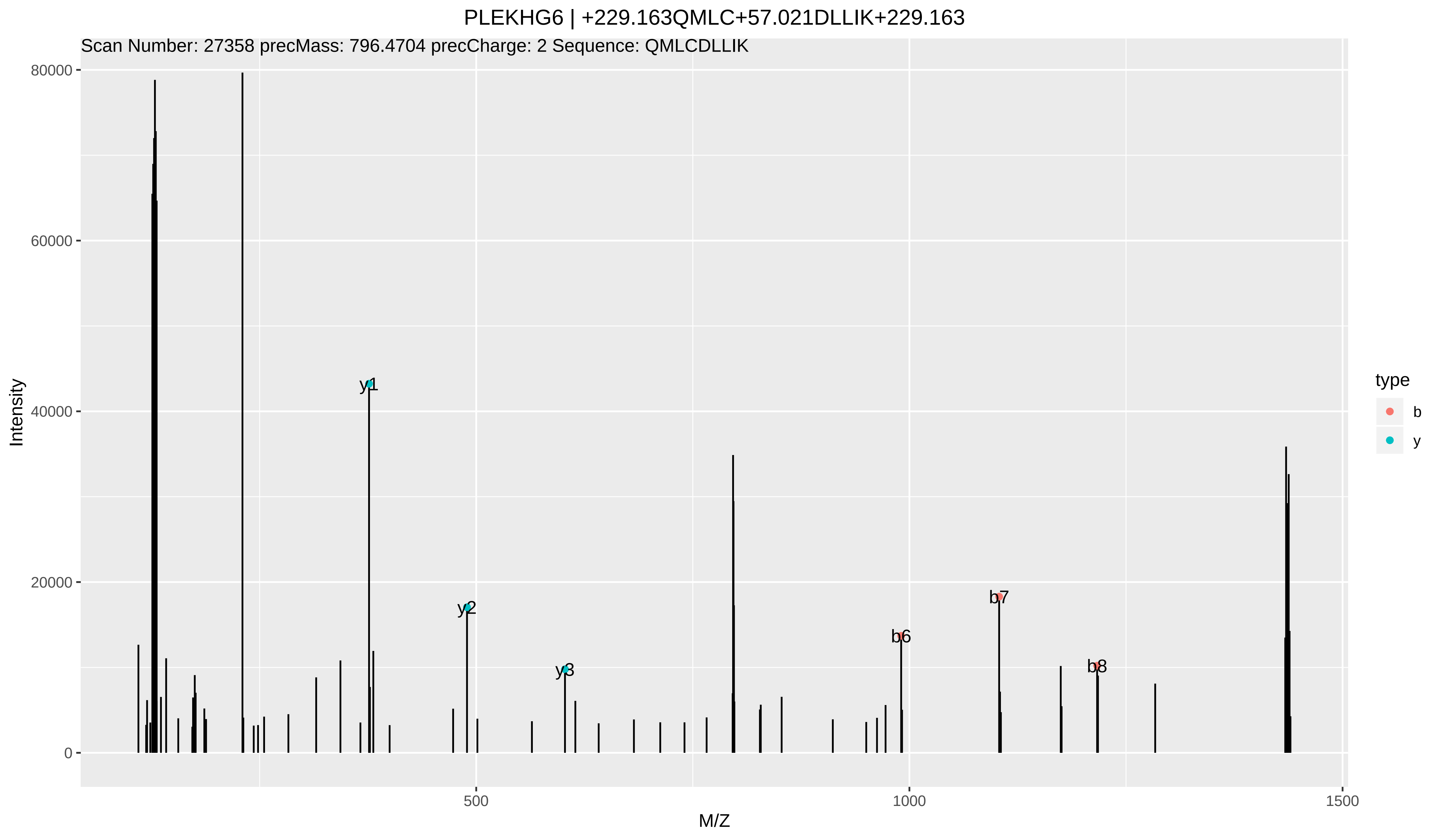

PLEKHG6 | +229.163QMLC+57.021DLLIK+229.163
Scan Number: 27358 precMass: 796.4704 precCharge: 2 Sequence: QMLCDLLIK
Intensity
0
20000
40000
60000
80000
M/Z
500
1000
1500
y1
y2
y3
b6
b7
b8
type
b
y

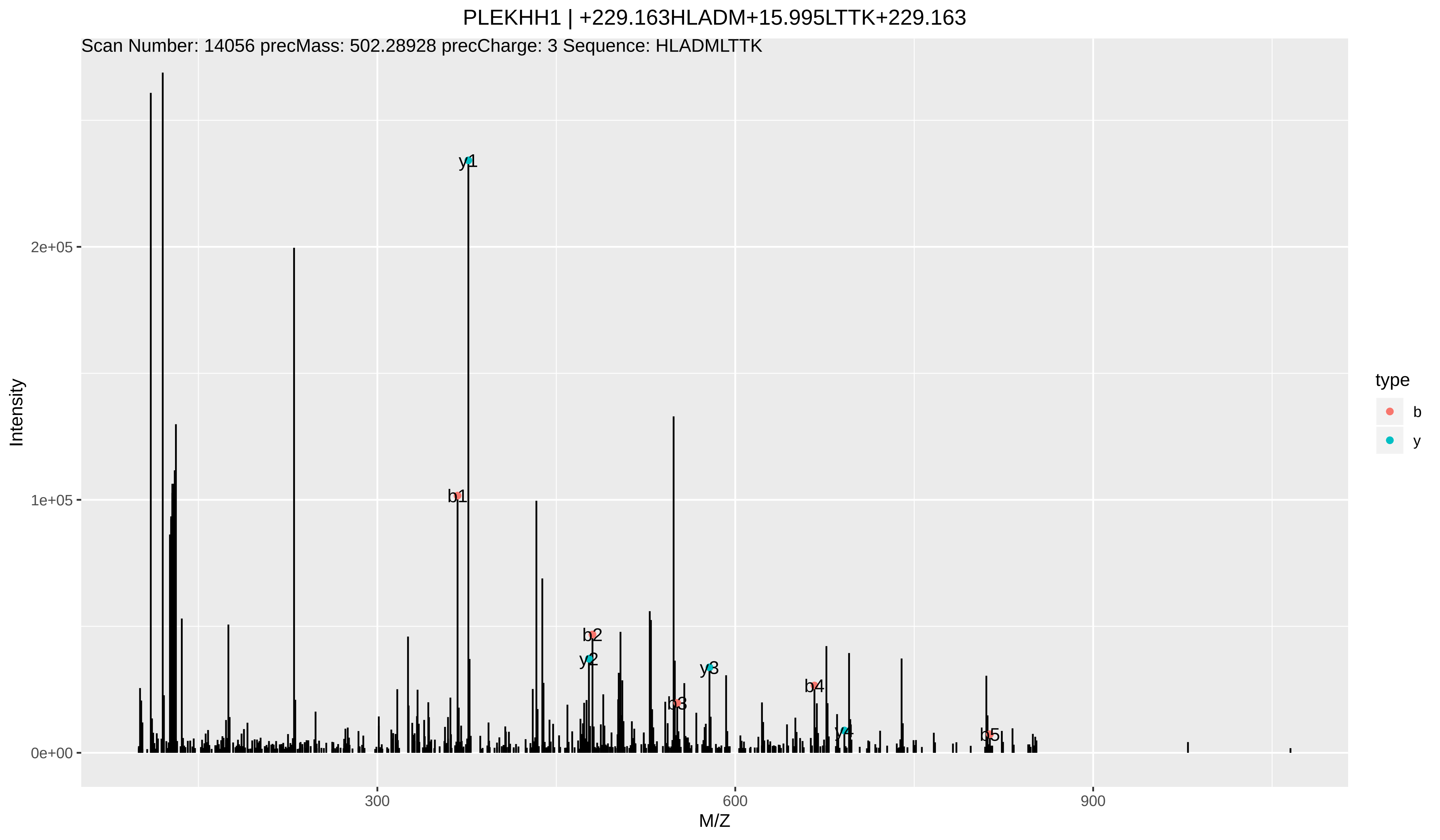

PLEKHH1 | +229.163HLADM+15.995LTTK+229.163
Scan Number: 14056 precMass: 502.28928 precCharge: 3 Sequence: HLADMLTTK
Intensity
2e+05
1e+05
0e+00
M/Z
300
600
900
y1
b1
b2
y2
y3
b3
b4
y4
b5
type
b
y

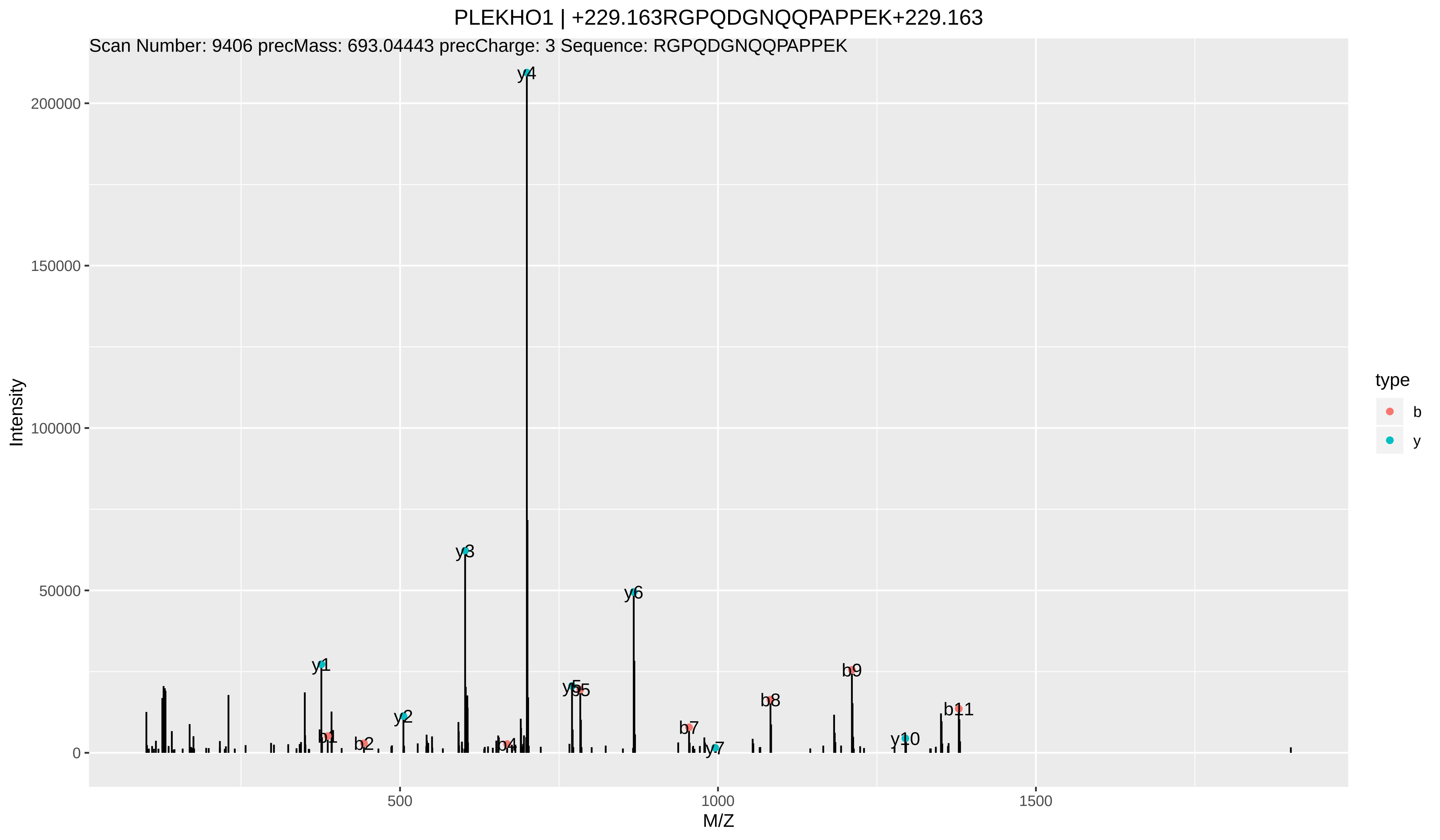
PLEKHO1 | +229.163RGPQDGNQQPAPPEK+229.163
Scan Number: 9406 precMass: 693.04443 precCharge: 3 Sequence: RGPQDGNQQPAPPEK
Intensity
200000
150000
100000
50000
0
500
1000
1500
M/Z
y4
y3
y6
y1
y5
b5
b9
b8
b11
y2
b7
b1
b2
b4
y7
y10
type
b
y

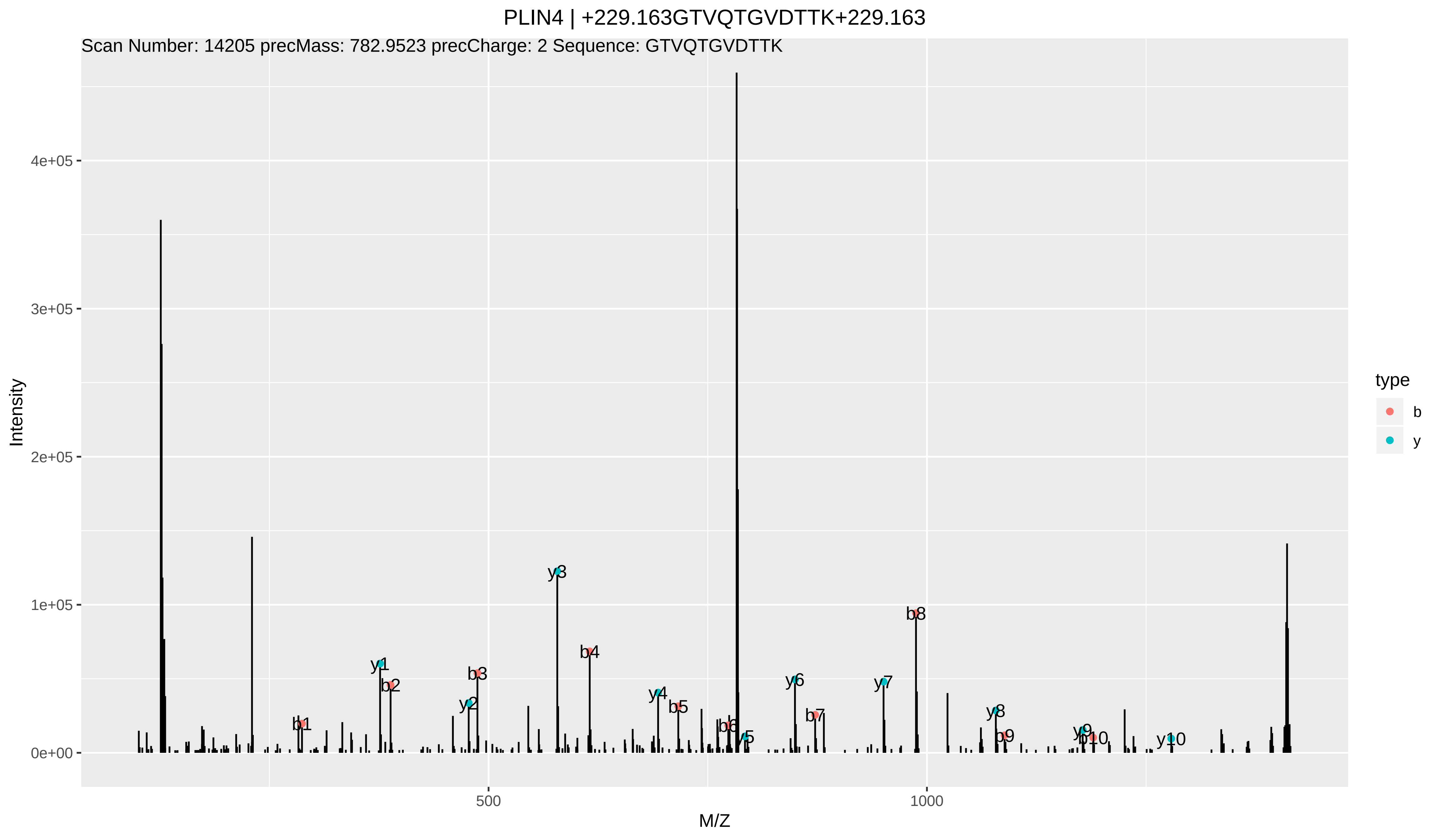

PLIN4 | +229.163GTVQTGVDTTK+229.163
Scan Number: 14205 precMass: 782.9523 precCharge: 2 Sequence: GTVQTGVDTTK
Intensity
0e+00
1e+05
2e+05
3e+05
4e+05
M/Z
500
1000
b1
y1
b2
y2
b3
y3
b4
y4
b5
b6
y5
y6
b7
y7
b8
y8
b9
y9
b10
y10
type
b
y

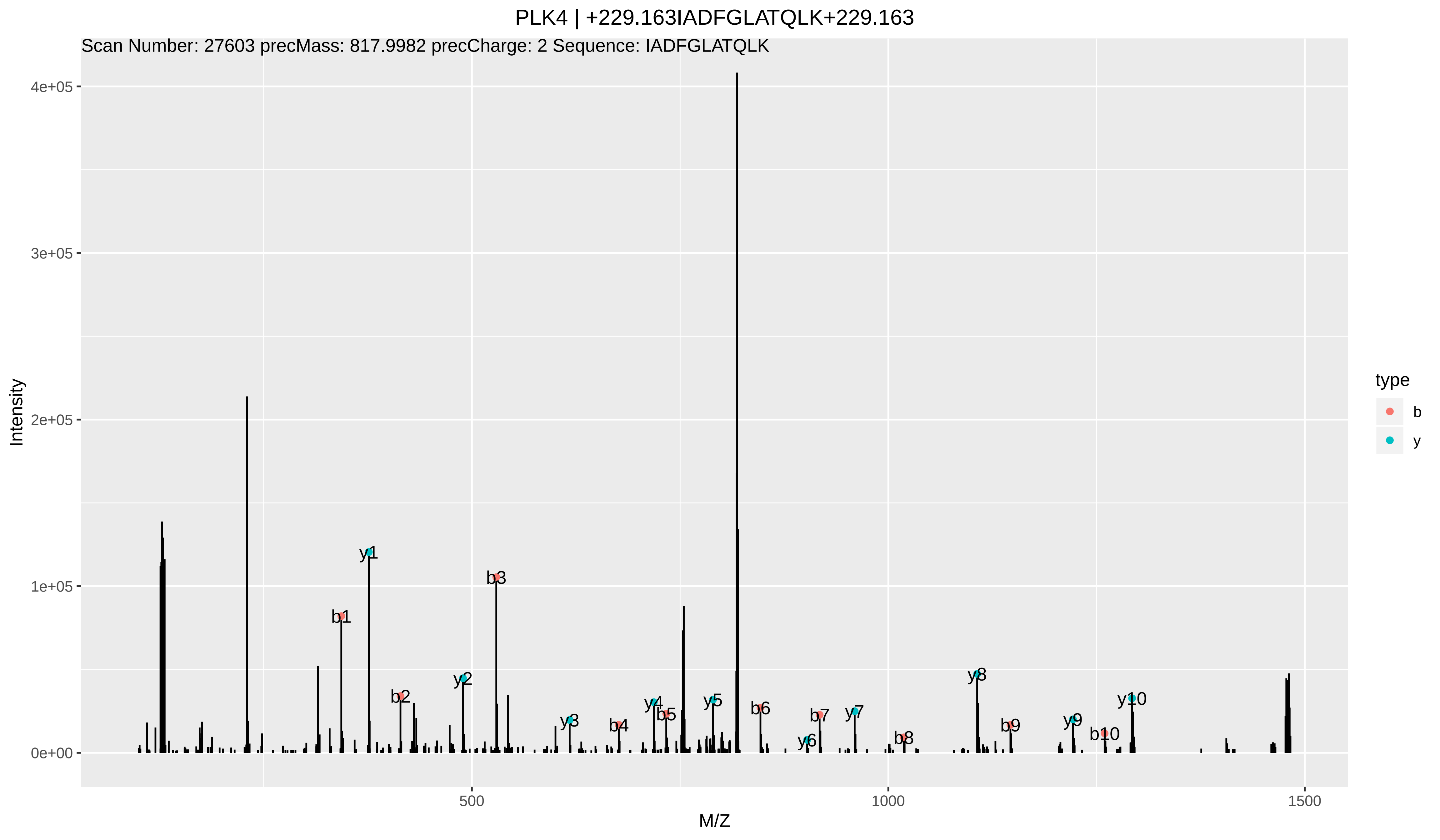

PLK4 | +229.163IADFGLATQLK+229.163
Scan Number: 27603 precMass: 817.9982 precCharge: 2 Sequence: IADFGLATQLK
Intensity
4e+05
3e+05
2e+05
1e+05
0e+00
M/Z
500
1000
1500
b1
y1
b2
y2
b3
y3
b4
y4
b5
y5
b6
y6
b7
y7
b8
y8
b9
y9
b10
y10
type
b
y

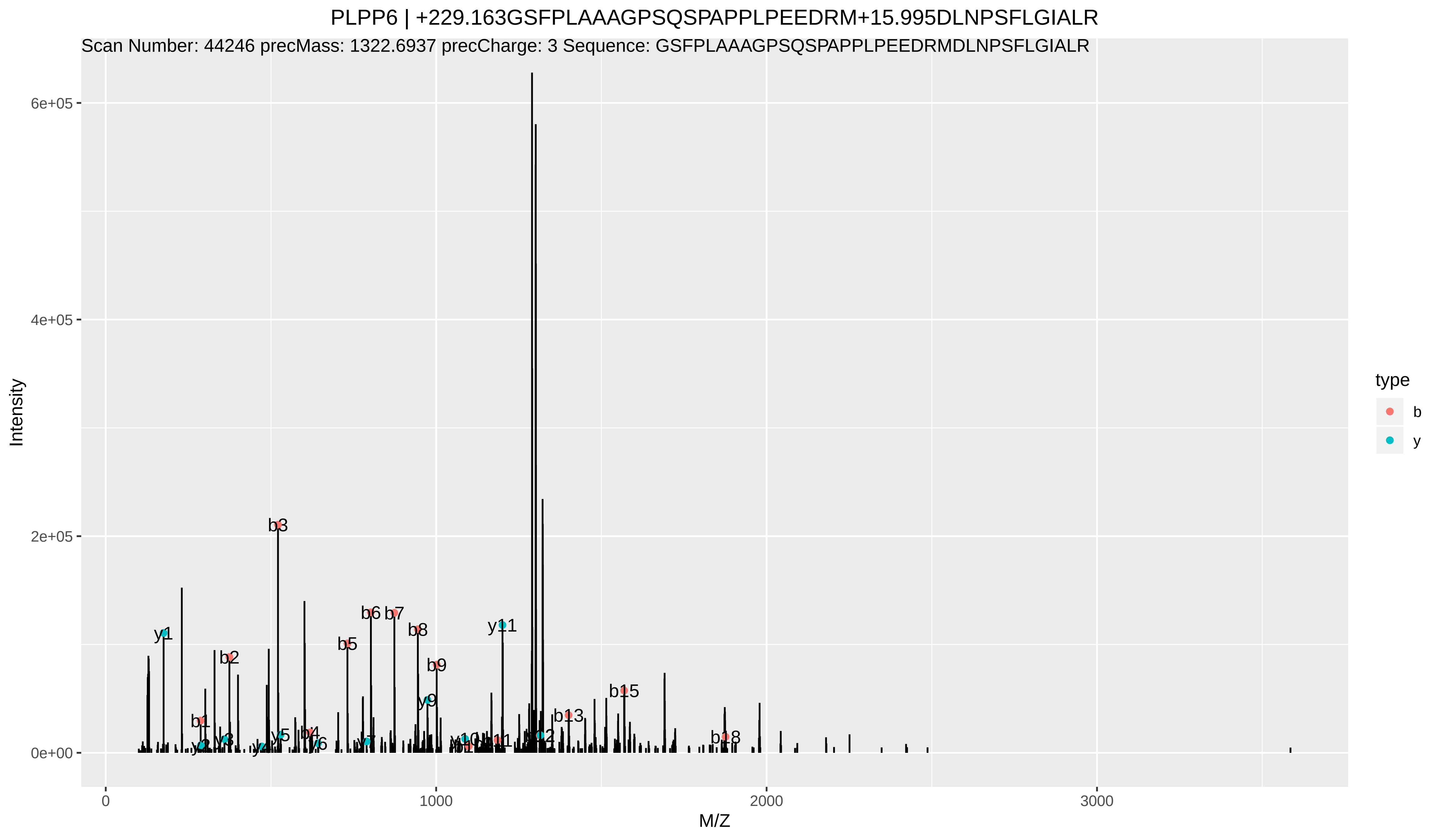

PLPP6 | +229.163GSFPLAAAGPSQSPAPPLPEEDRM+15.995DLNPSFLGIALR
Scan Number: 44246 precMass: 1322.6937 precCharge: 3 Sequence: GSFPLAAAGPSQSPAPPLPEEDRMDLNPSFLGIALR
Intensity
M/Z
type
b
y

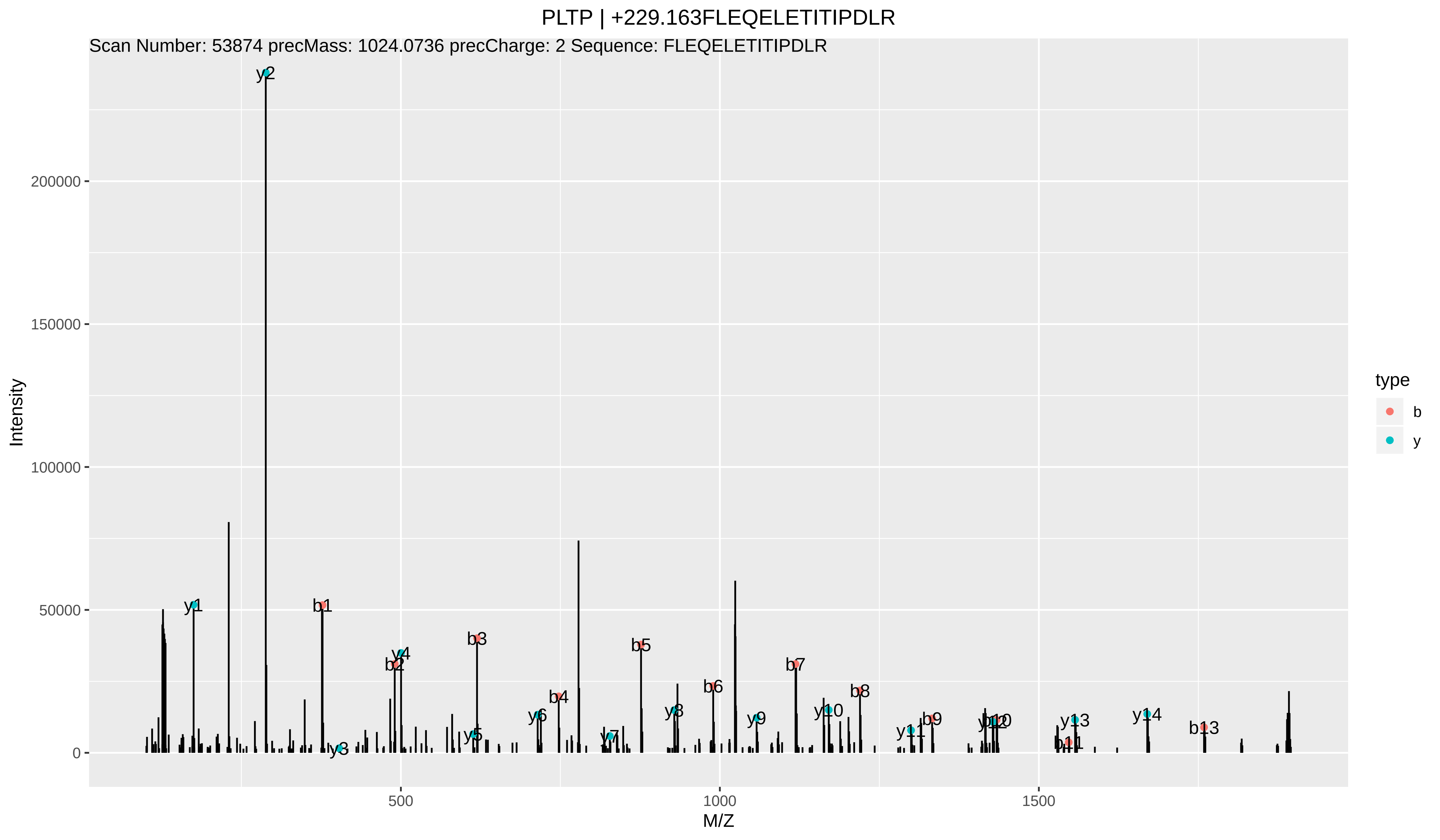

PLTP | +229.163FLEQELETITIPDLR
Scan Number: 53874 precMass: 1024.0736 precCharge: 2 Sequence: FLEQELETITIPDLR
Intensity
M/Z
0
50000
100000
150000
200000
500
1000
1500
type
b
y
y1
y2
y3
y4
y5
y6
y7
y8
y9
y10
y11
y12
y13
y14
b1
b2
b3
b4
b5
b6
b7
b8
b9
b10
b11
b13

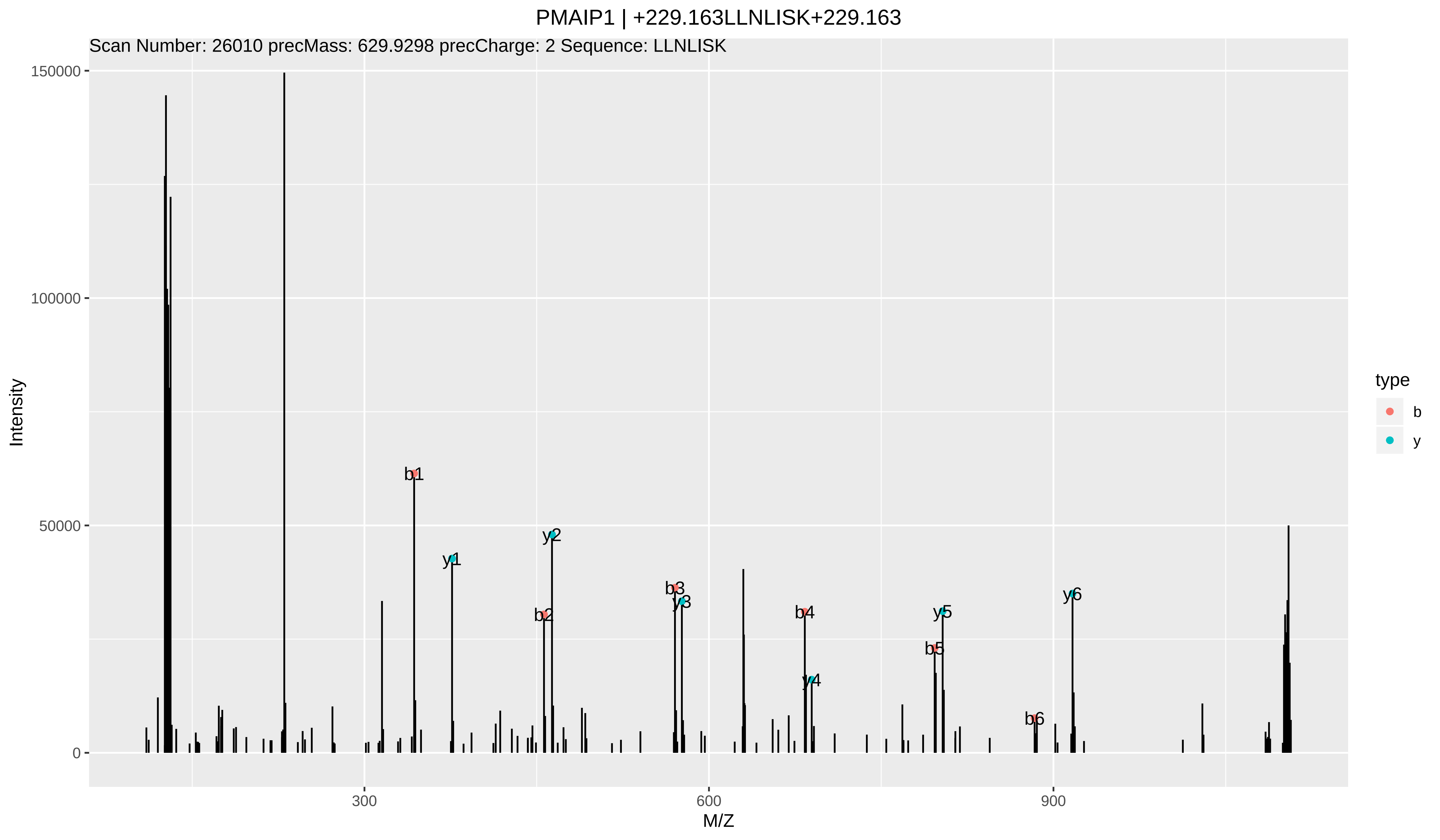

PMAIP1 | +229.163LLNLISK+229.163
Scan Number: 26010 precMass: 629.9298 precCharge: 2 Sequence: LLNLISK
Intensity
150000
100000
50000
0
300
600
900
M/Z
b1
y1
y2
b2
b3
y3
b4
y4
b5
y5
b6
y6
type
b
y

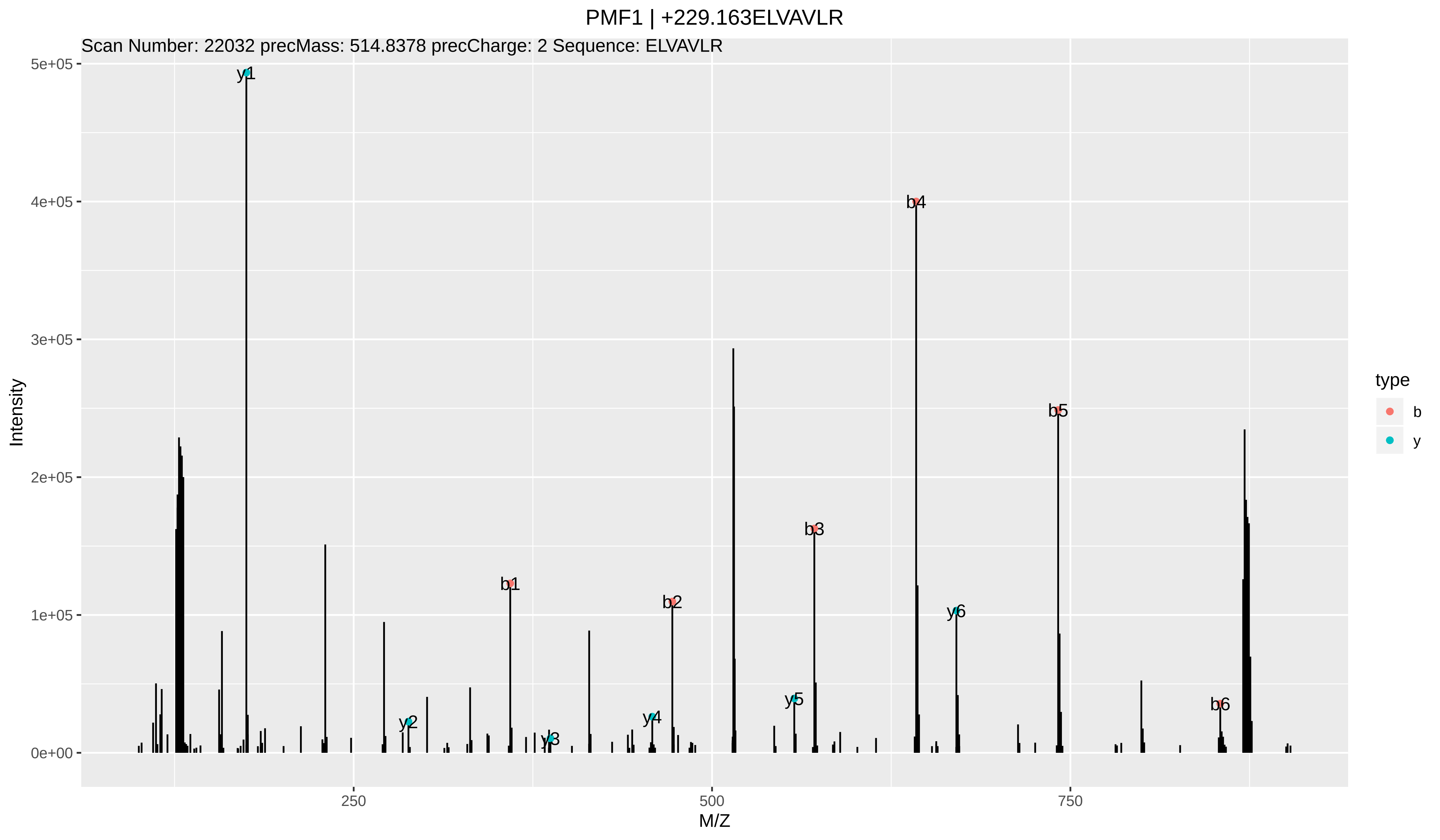

PMF1 | +229.163ELVAVLR
Scan Number: 22032 precMass: 514.8378 precCharge: 2 Sequence: ELVAVLR
Intensity
M/Z
0e+00
1e+05
2e+05
3e+05
4e+05
5e+05
250
500
750
y1
y2
y3
y4
y5
y6
b1
b2
b3
b4
b5
b6
type
b
y

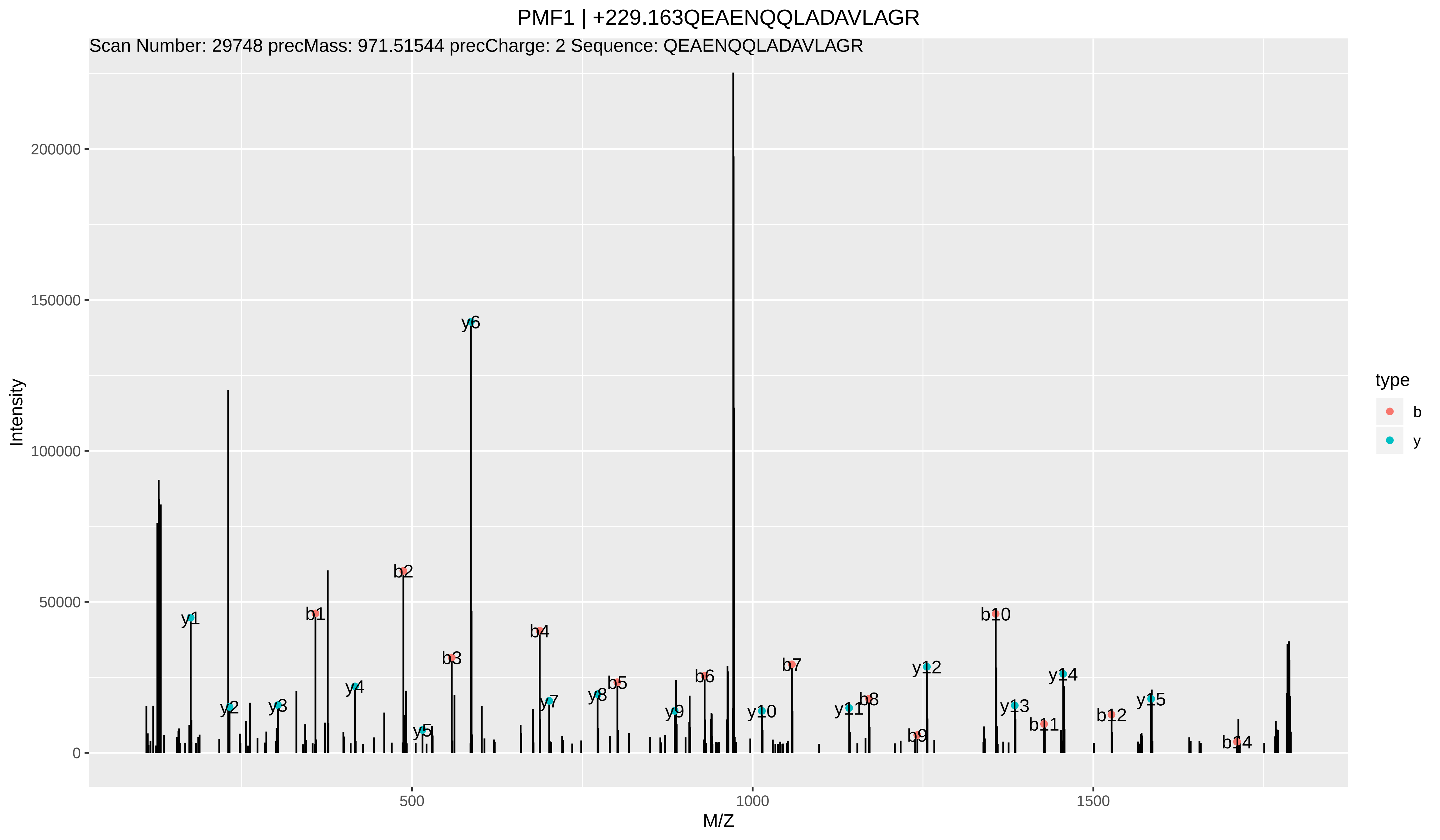

PMF1 | +229.163QEAENQQLADAVLAGR
Scan Number: 29748 precMass: 971.51544 precCharge: 2 Sequence: QEAENQQLADAVLAGR
Intensity
M/Z
0
50000
100000
150000
200000
500
1000
1500
type
b
y
y1
y2
y3
y4
y5
y6
y7
y8
y9
y10
y11
y12
y13
y14
y15
b1
b2
b3
b4
b5
b6
b7
b8
b9
b10
b11
b12
b14

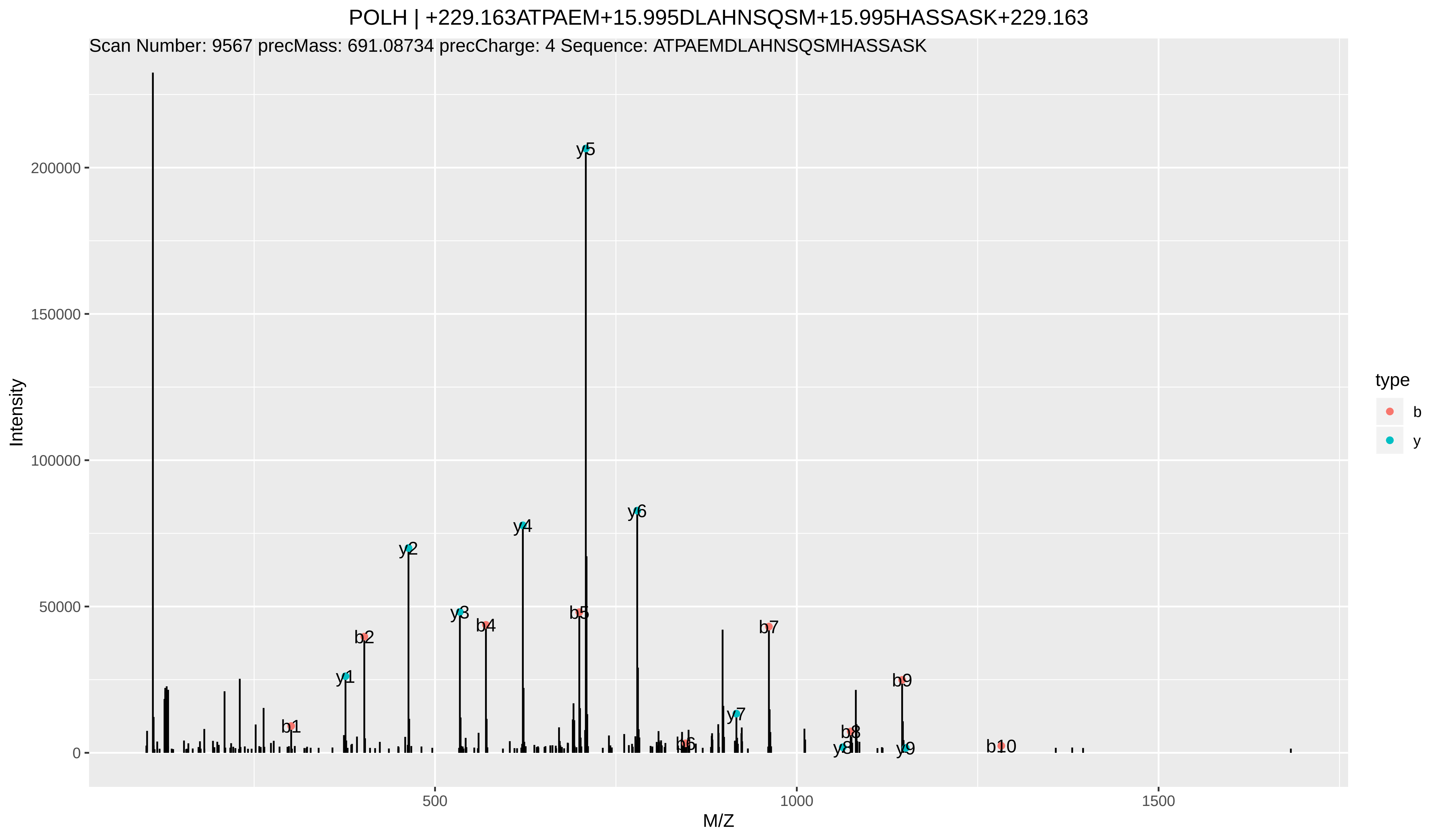

POLH | +229.163ATPAEM+15.995DLAHNSQSM+15.995HASSASK+229.163
Scan Number: 9567 precMass: 691.08734 precCharge: 4 Sequence: ATPAEMDLAHNSQSMHASSASK
Intensity
M/Z
200000
150000
100000
50000
0
500
1000
1500
y5
y6
y4
y2
y3
b4
b5
b7
b2
y1
b9
y7
b1
b8
b6
y8
y9
b10
type
b
y

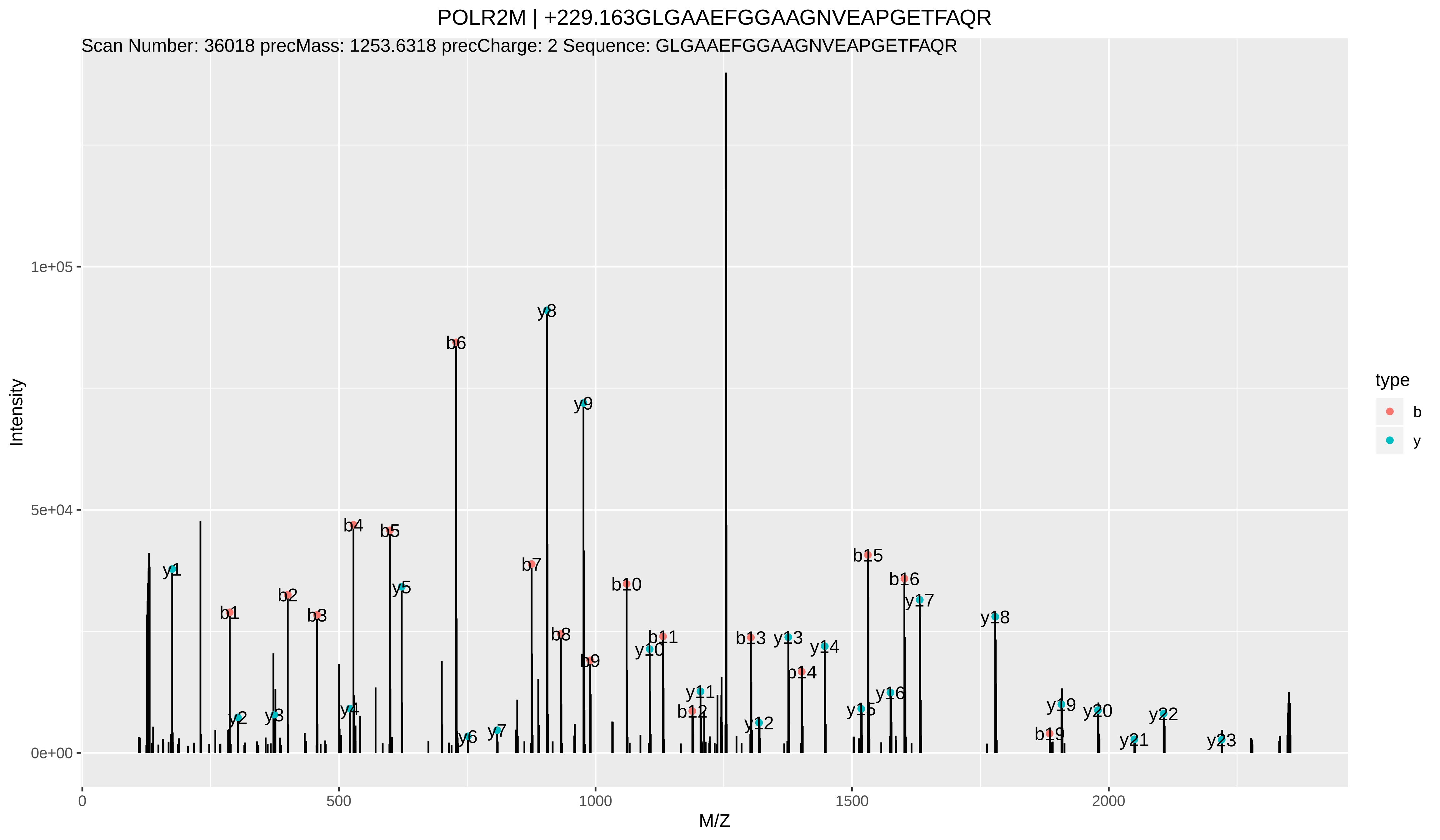

POLR2M | +229.163GLGAAEFGGAAGNVEAPGETFAQR
Scan Number: 36018 precMass: 1253.6318 precCharge: 2 Sequence: GLGAAEFGGAAGNVEAPGETFAQR
Intensity
M/Z
1e+05
5e+04
0e+00
0
500
1000
1500
2000
type
b
y
b1
b2
b3
b4
b5
b6
b7
b8
b9
b10
b11
b12
b13
b14
b15
b16
b19
y1
y2
y3
y4
y5
y6
y7
y8
y9
y10
y11
y12
y13
y14
y15
y16
y17
y18
y19
y20
y21
y22
y23

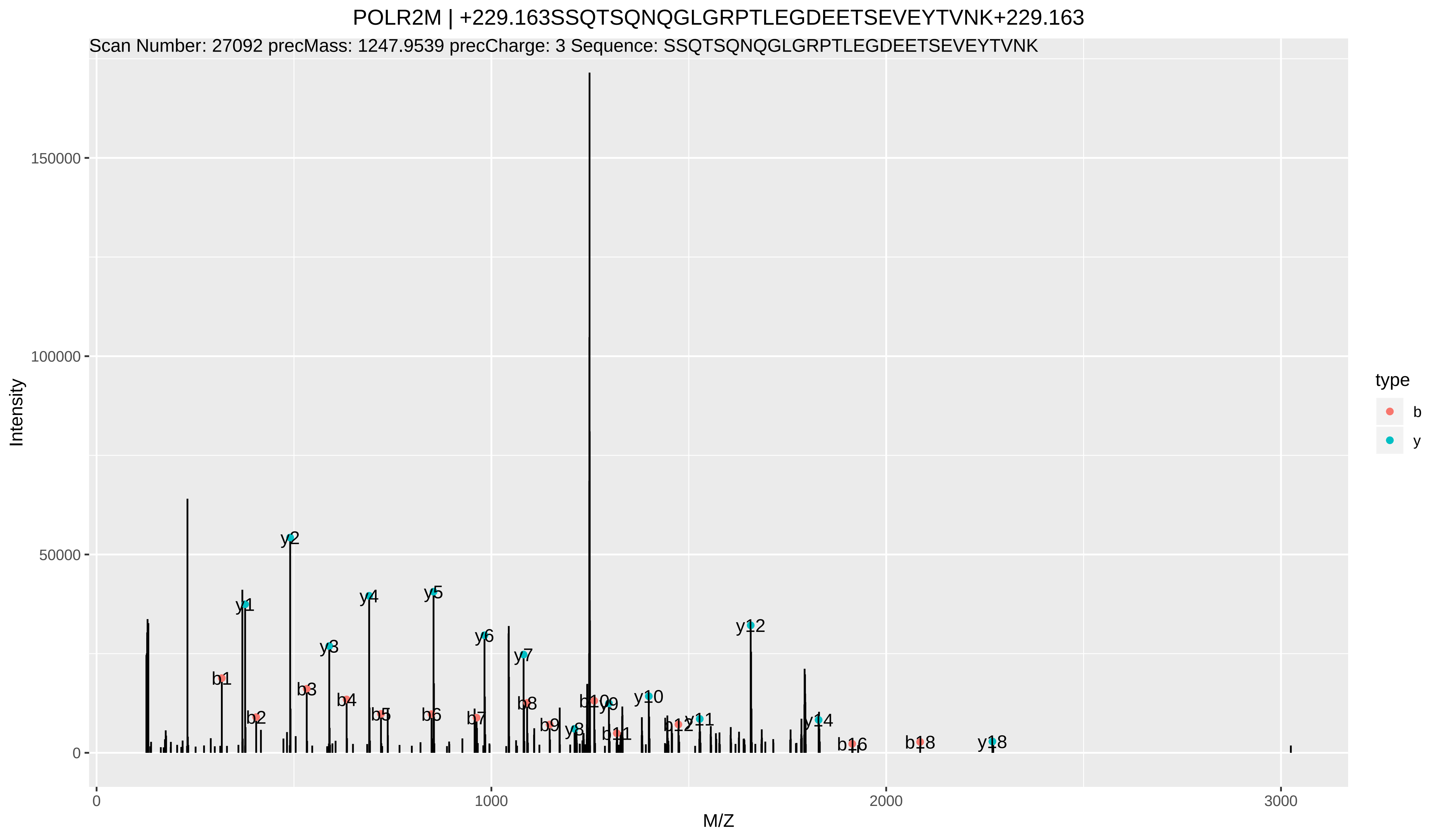
POLR2M | +229.163SSQTSQNQGLGRPTLEGDEETSEVEYTVNK+229.163
Scan Number: 27092 precMass: 1247.9539 precCharge: 3 Sequence: SSQTSQNQGLGRPTLEGDEETSEVEYTVNK
Intensity
M/Z
type
b
y
y1
y2
y3
y4
y5
y6
y7
y8
y9
y10
y11
y12
y14
y18
b1
b2
b3
b4
b5
b6
b7
b8
b9
b10
b11
b12
b16
b18
0
50000
100000
150000
0
1000
2000
3000

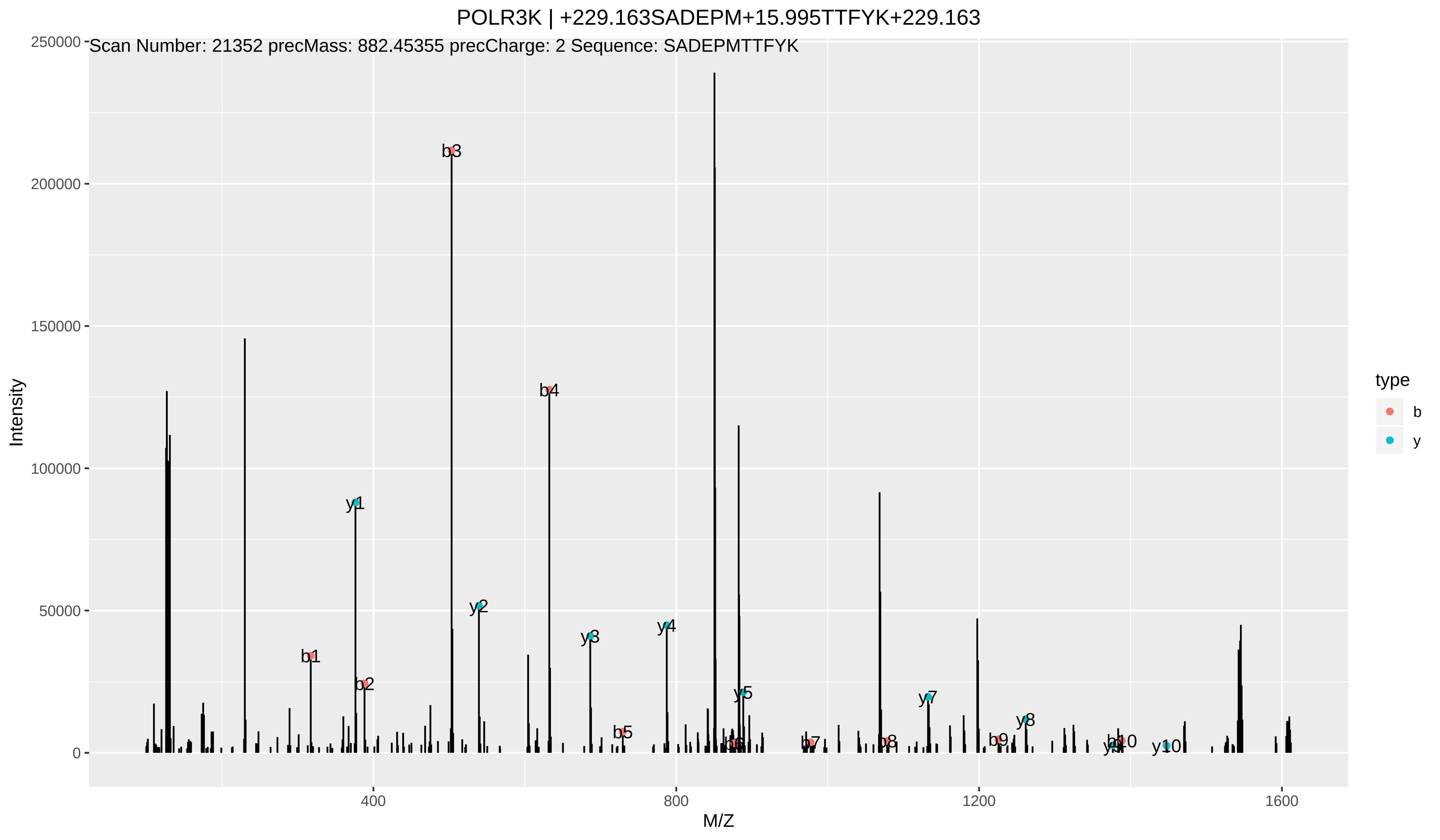
POLR3K | +229.163SADEPM+15.995TTFYK+229.163
Scan Number: 21352 precMass: 882.45355 precCharge: 2 Sequence: SADEPMTTFYK
Intensity
M/Z
type
b
y
b1
b2
b3
b4
b5
b6
b7
b8
b9
b10
y1
y2
y3
y4
y5
y7
y8
y9
y10
250000
200000
150000
100000
50000
0
400
800
1200
1600

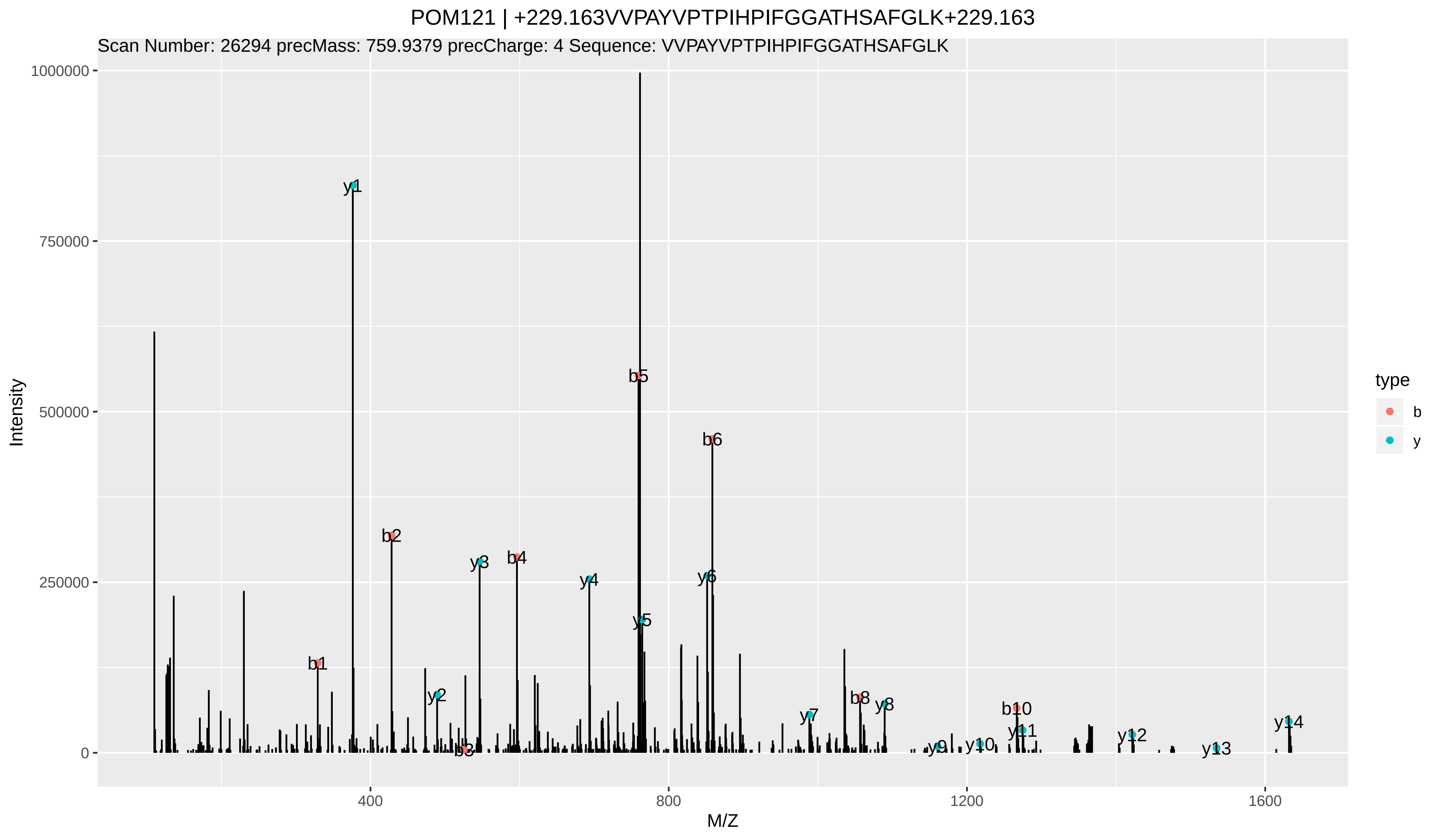

POM121 | +229.163VVPAYVPTPIHPIFGGATHSAFGLK+229.163
Scan Number: 26294 precMass: 759.9379 precCharge: 4 Sequence: VVPAYVPTPIHPIFGGATHSAFGLK
Intensity
1000000
750000
500000
250000
0
400
800
1200
1600
M/Z
type
b
y
y1
b5
b6
b2
y3
b4
y4
y6
y5
b1
y2
b3
y7
b8
y8
b10
y11
y12
y14
y9
y10
y13

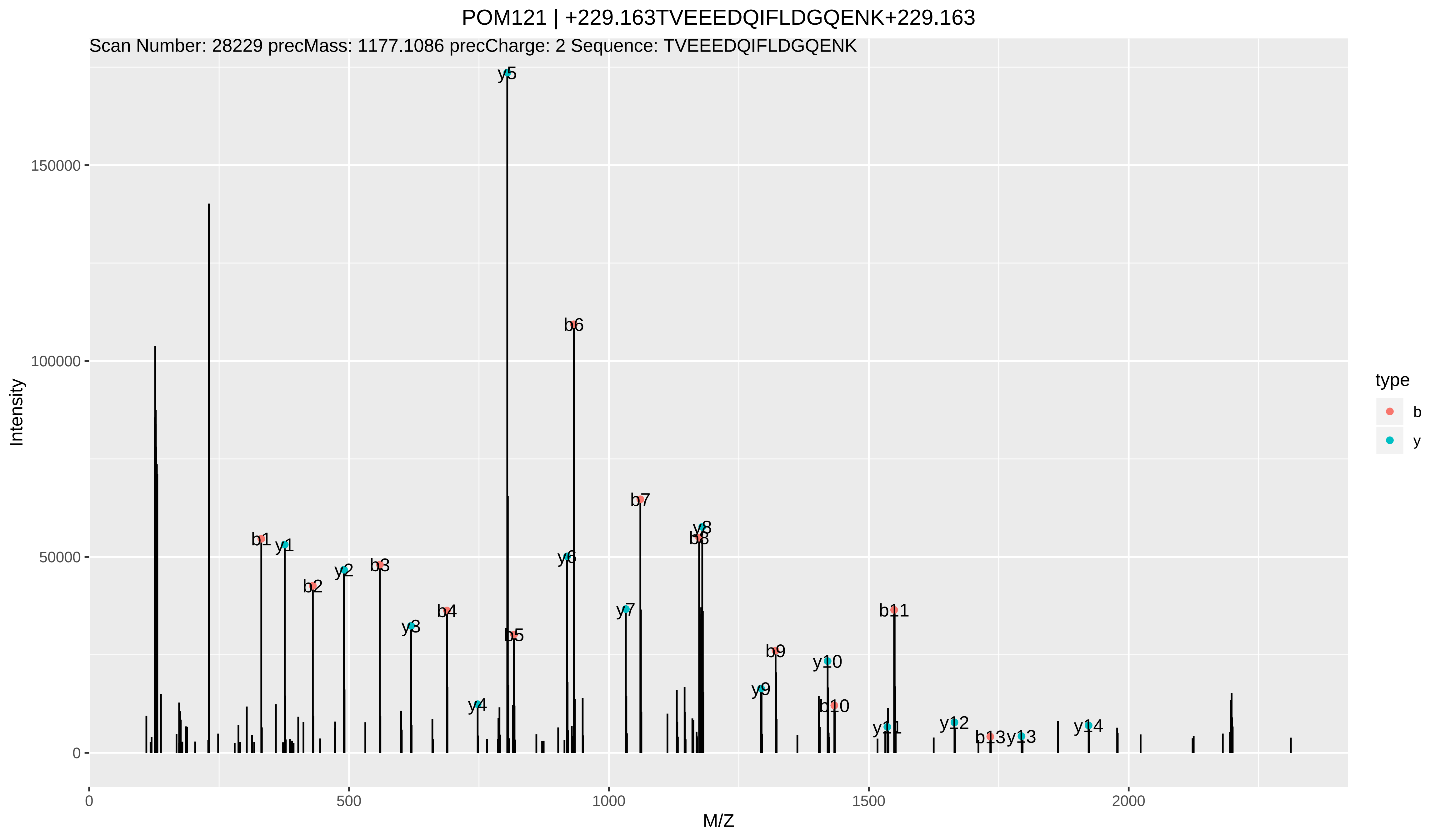
POM121 | +229.163TVEEEDQIFLDGQENK+229.163
Scan Number: 28229 precMass: 1177.1086 precCharge: 2 Sequence: TVEEEDQIFLDGQENK
Intensity
M/Z
0
50000
100000
150000
0
500
1000
1500
2000
y5
b6
b7
y8
b8
b1
y1
y2
b2
b3
y6
y3
b4
y7
b11
b5
b9
y10
y9
b10
y4
y11
y12
b13
y13
y14
type
b
y

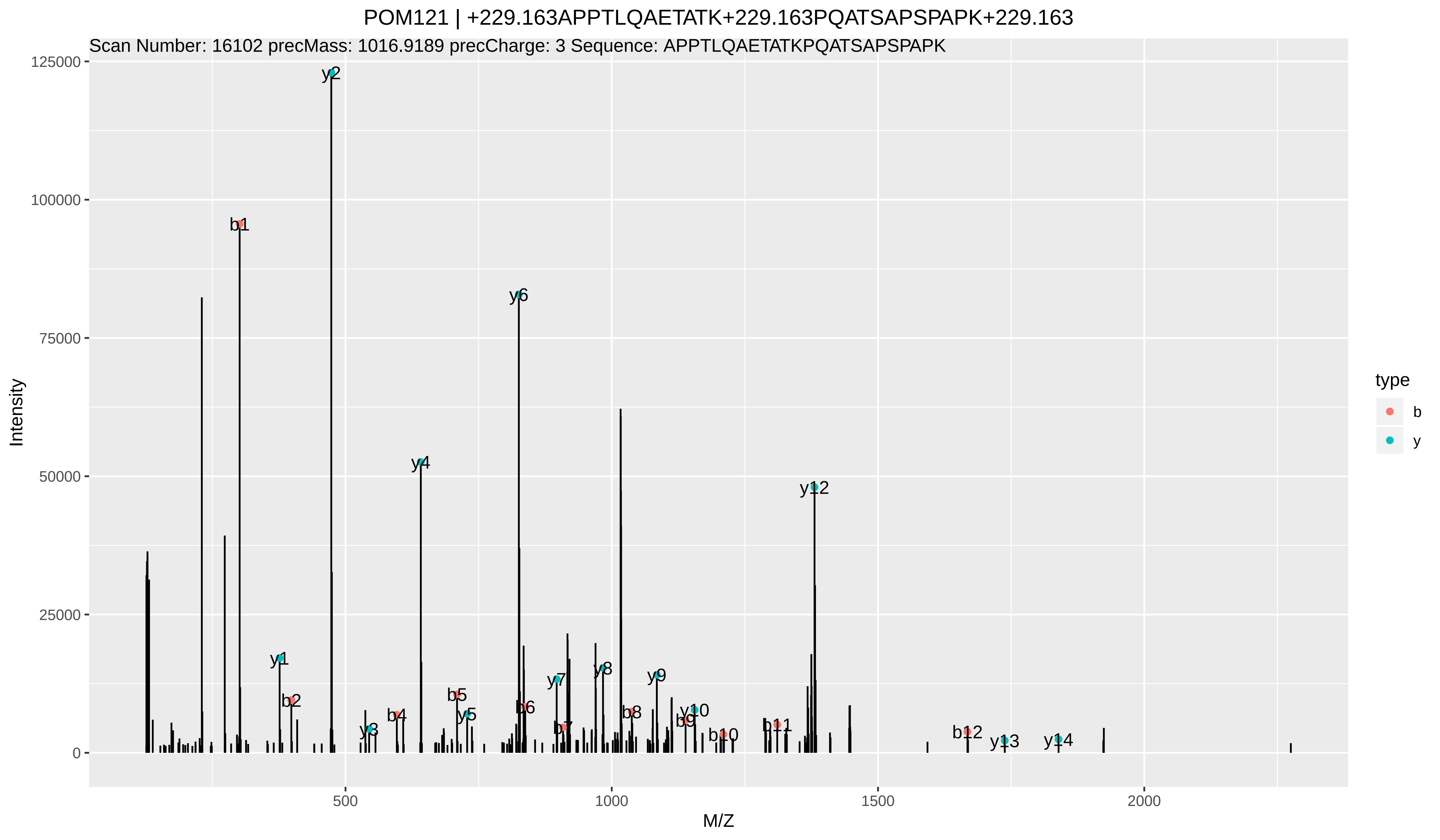

POM121 | +229.163APPTLQAETATK+229.163PQATSAPSPAPK+229.163
Scan Number: 16102 precMass: 1016.9189 precCharge: 3 Sequence: APPTLQAETATKPQATSAPSPAPK
Intensity
M/Z
0
25000
50000
75000
100000
125000
500
1000
1500
2000
y2
b1
y6
y4
y12
y1
b2
y3
b4
b5
y5
b6
b7
y7
y8
y9
b8
b9
y10
b10
b11
b12
y13
y14
type
b
y

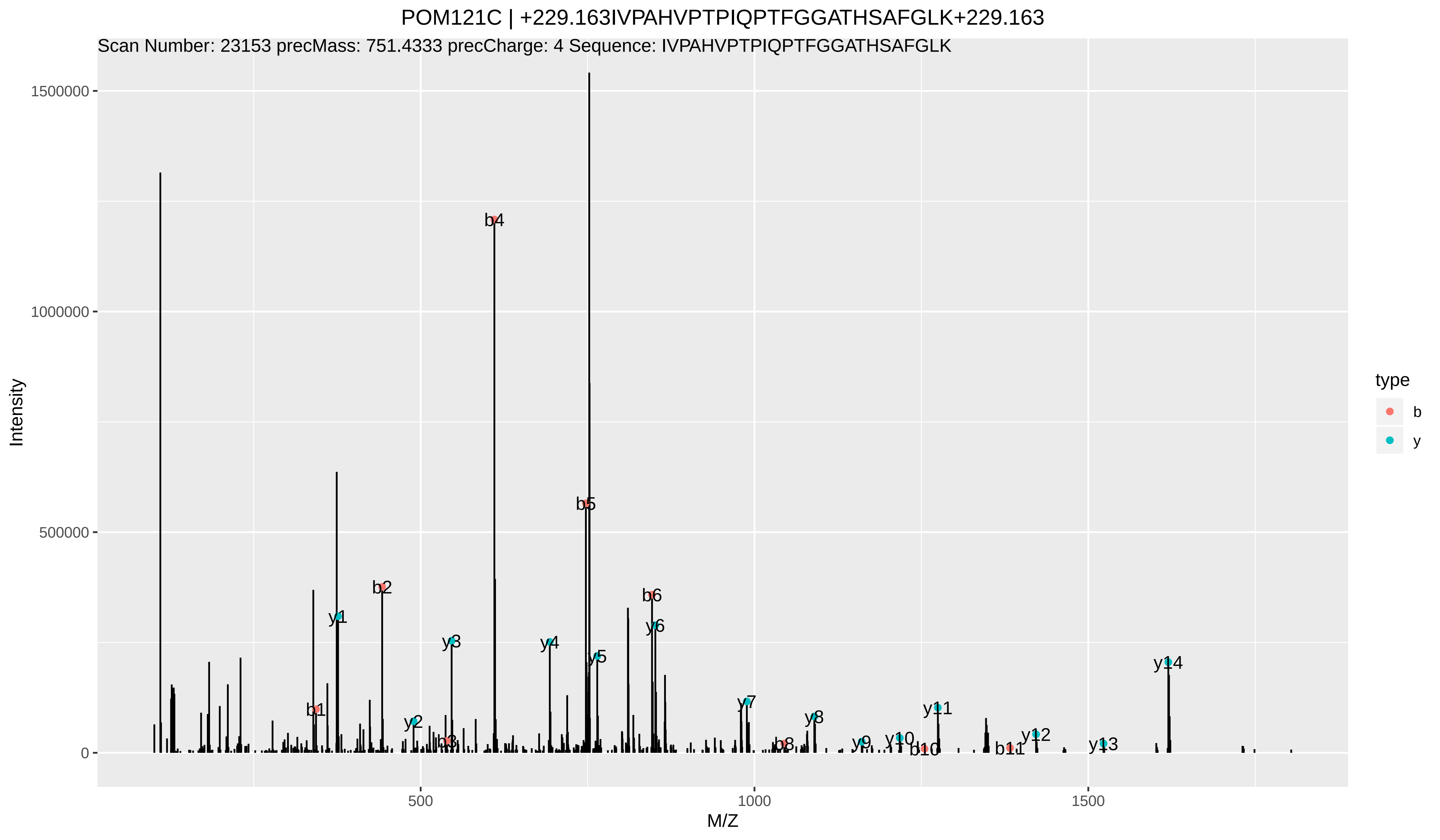
POM121C | +229.163IVPAHVPTPIQPTFGGATHSAFGLK+229.163
Scan Number: 23153 precMass: 751.4333 precCharge: 4 Sequence: IVPAHVPTPIQPTFGGATHSAFGLK
Intensity
1500000
1000000
500000
0
M/Z
500
1000
1500
type
b
y
b1
b2
b3
b4
b5
b6
b8
b10
b11
y1
y2
y3
y4
y5
y6
y7
y8
y9
y10
y11
y12
y13
y14

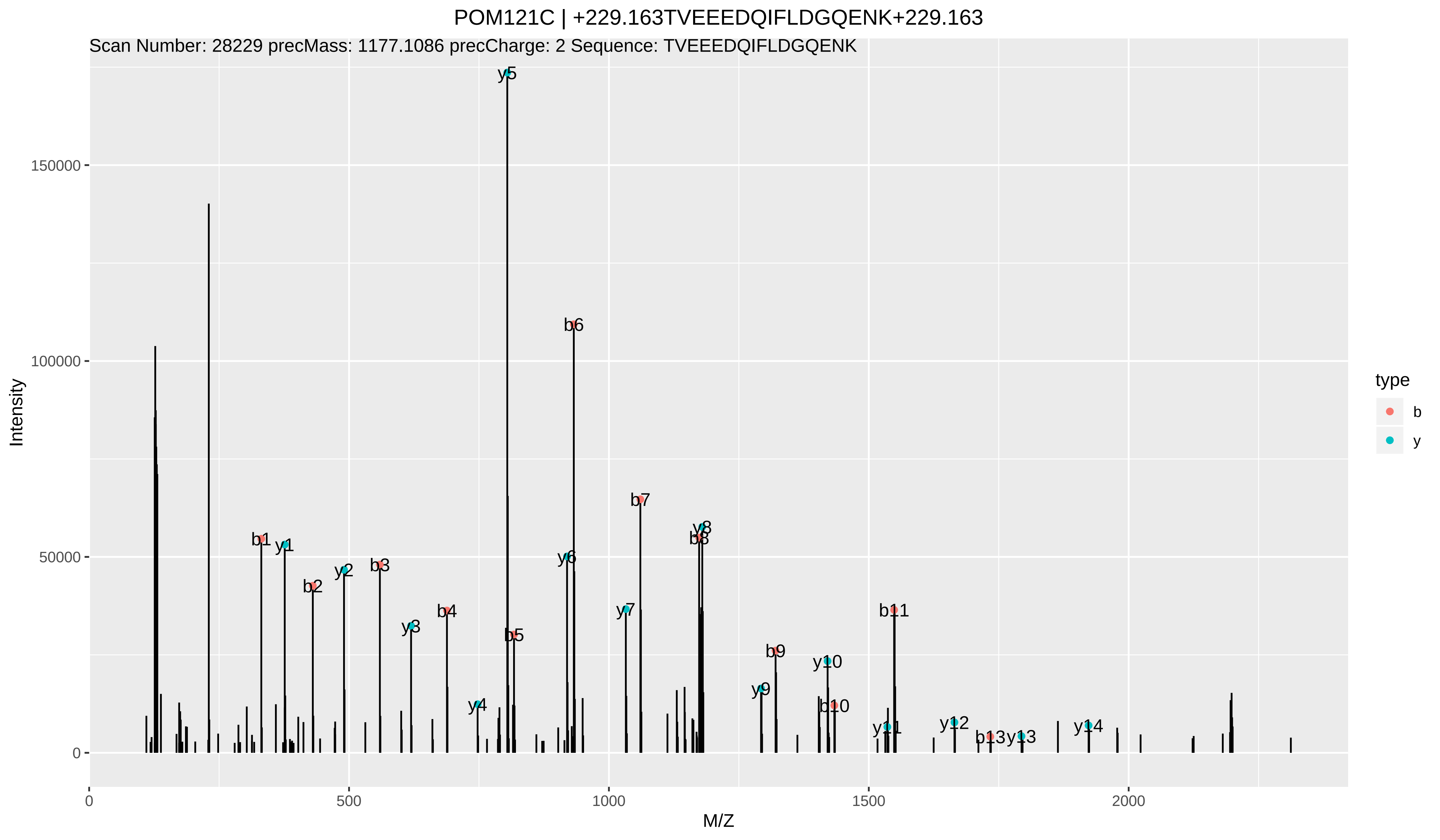
POM121C | +229.163TVEEEDQIFLDGQENK+229.163
Scan Number: 28229 precMass: 1177.1086 precCharge: 2 Sequence: TVEEEDQIFLDGQENK
Intensity
M/Z
0
50000
100000
150000
0
500
1000
1500
2000
type
b
y
y5
b6
b7
y8
b8
b1
y1
b2
y2
b3
y3
b4
b5
y4
y6
y7
b9
y9
y10
b10
b11
y11
y12
b13
y13
y14

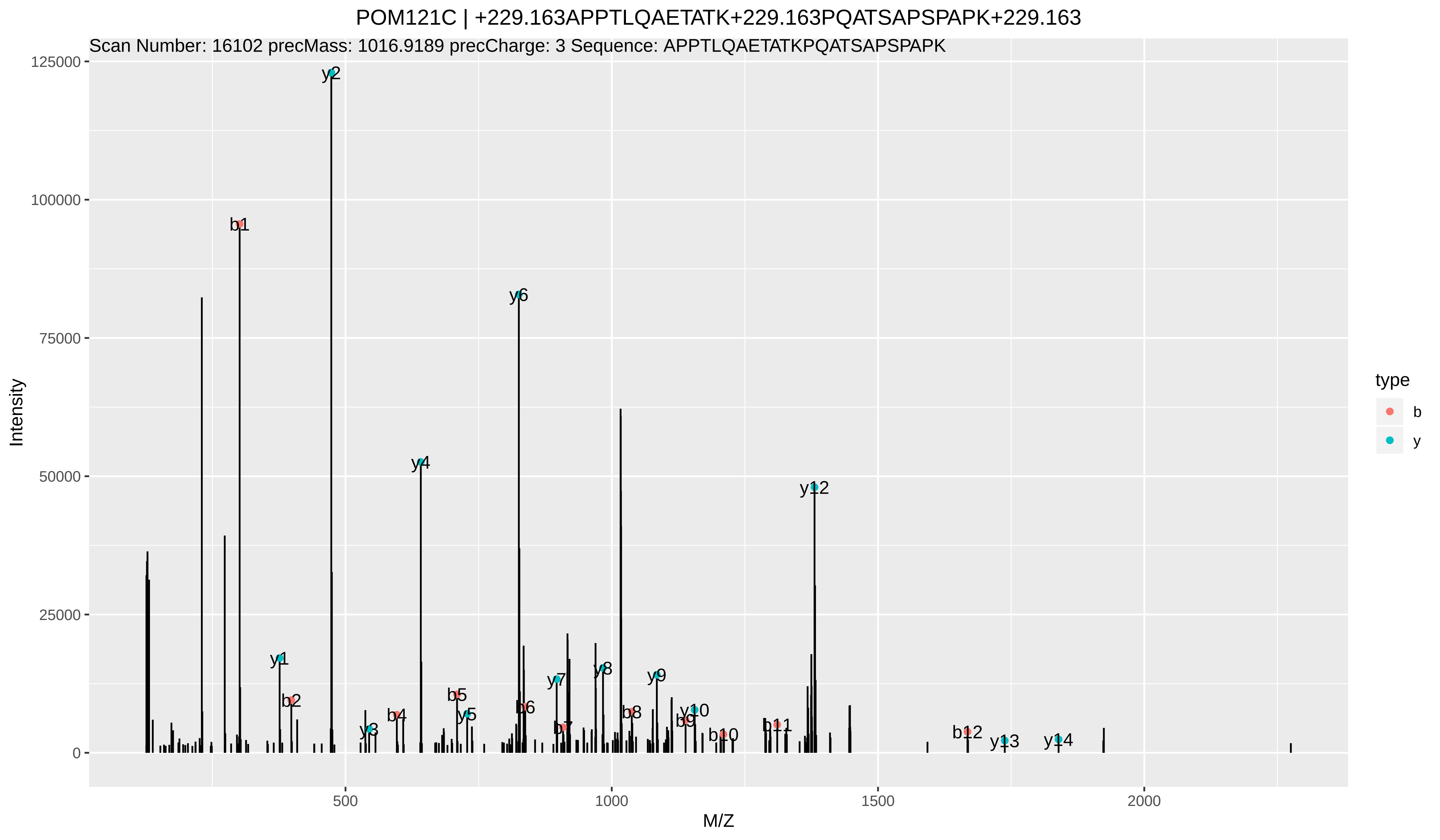

POM121C | +229.163APPTLQAETATK+229.163PQATSAPSPAPK+229.163
Scan Number: 16102 precMass: 1016.9189 precCharge: 3 Sequence: APPTLQAETATKPQATSAPSPAPK
Intensity
M/Z
type
b
y
y2
b1
y6
y4
y12
y1
b2
y3
b4
b5
y5
b6
y7
b7
y8
b8
y9
b9
y10
b10
b11
b12
y13
y14

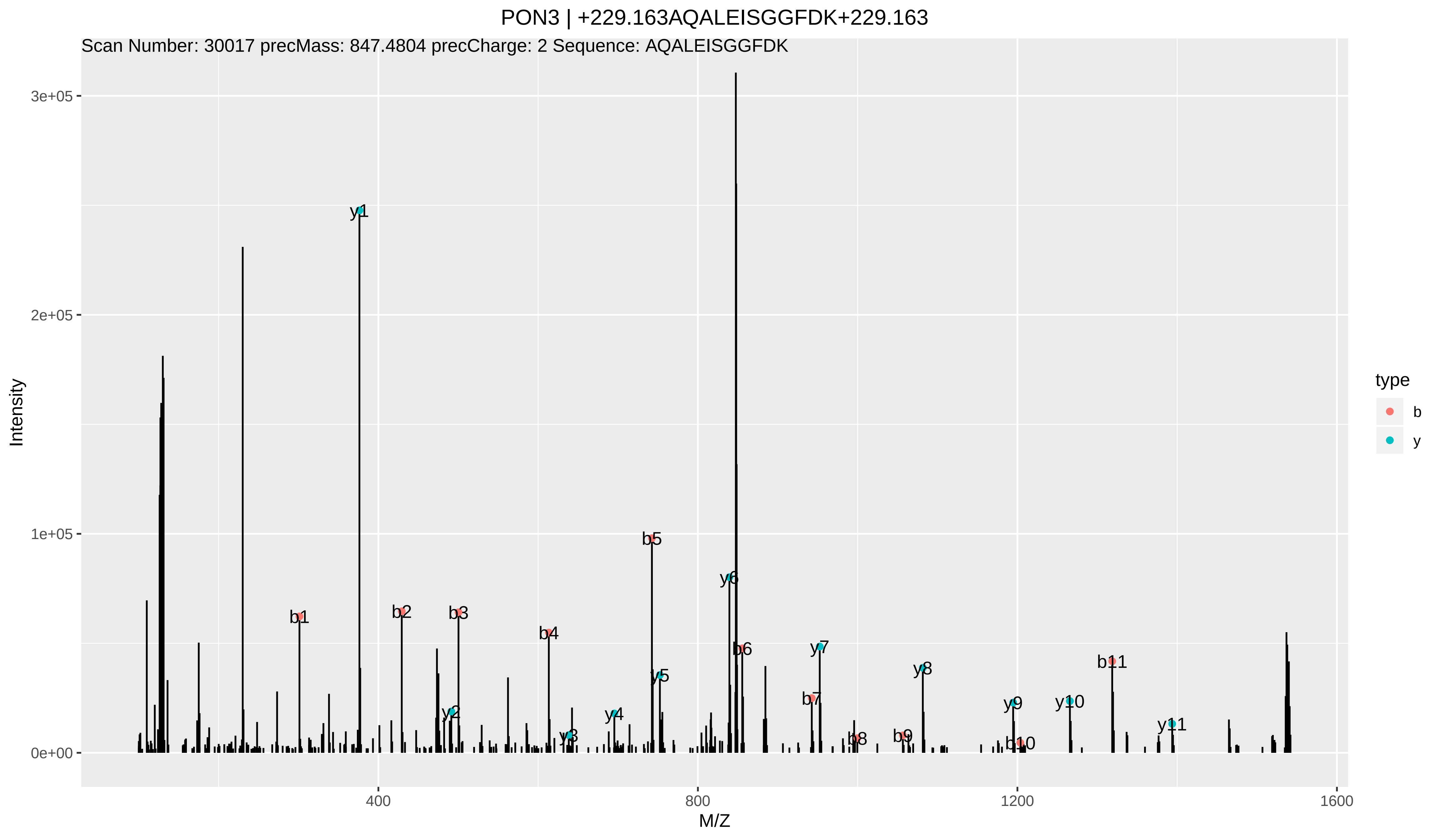

PON3 | +229.163AQALEISGGFDK+229.163
Scan Number: 30017 precMass: 847.4804 precCharge: 2 Sequence: AQALEISGGFDK
Intensity
3e+05
2e+05
1e+05
0e+00
M/Z
400
800
1200
1600
y1
b1
b2
b3
y2
b4
y3
y4
b5
y5
y6
b6
b7
y7
b8
b9
y8
y9
b10
y10
b11
y11
type
b
y

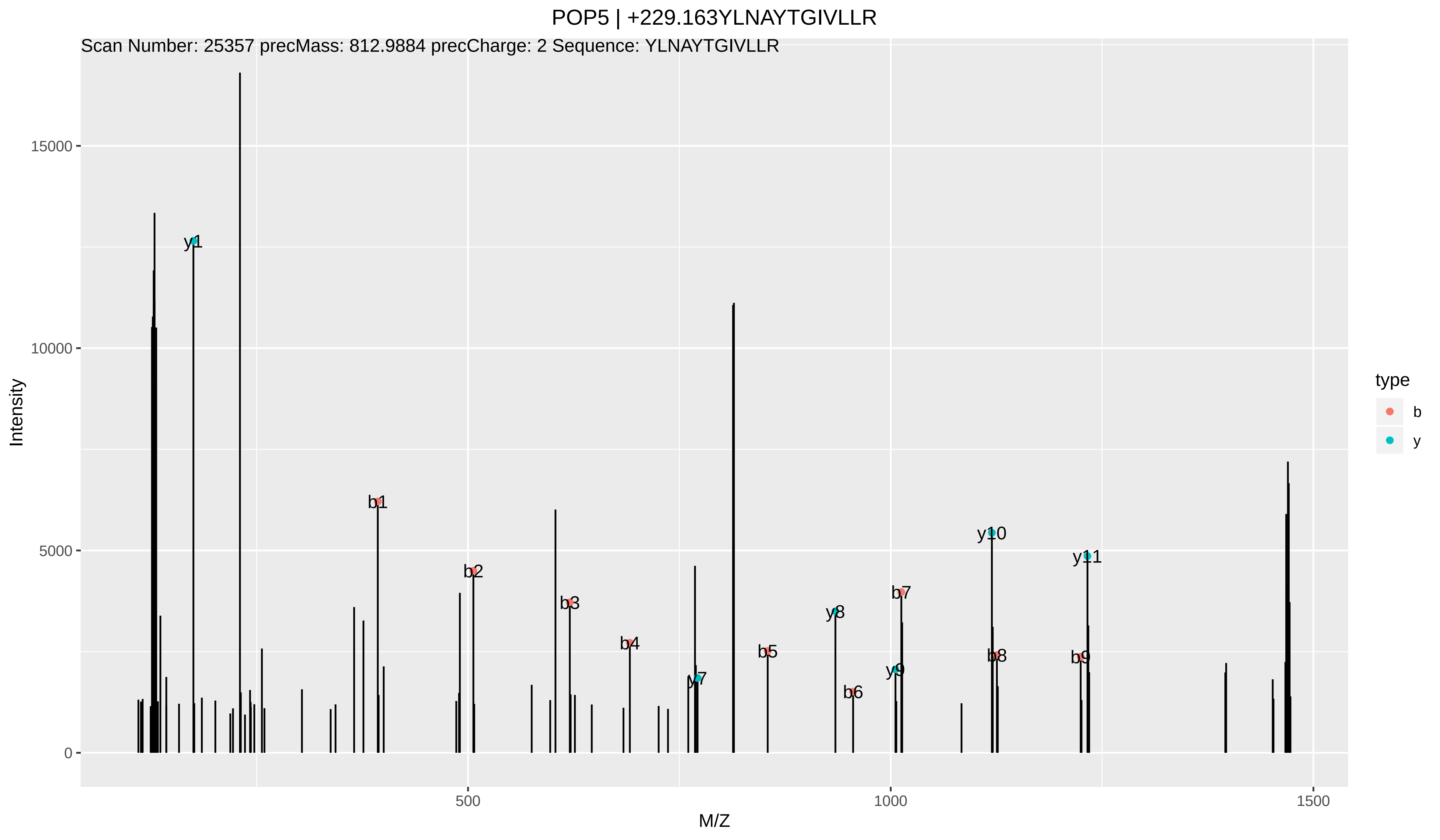

POP5 | +229.163YLNAYTGIVLLR
Scan Number: 25357 precMass: 812.9884 precCharge: 2 Sequence: YLNAYTGIVLLR
Intensity
M/Z
0
5000
10000
15000
500
1000
1500
y1
b1
b2
b3
b4
y7
b5
y8
b6
y9
b7
y10
b8
y11
b9
type
b
y

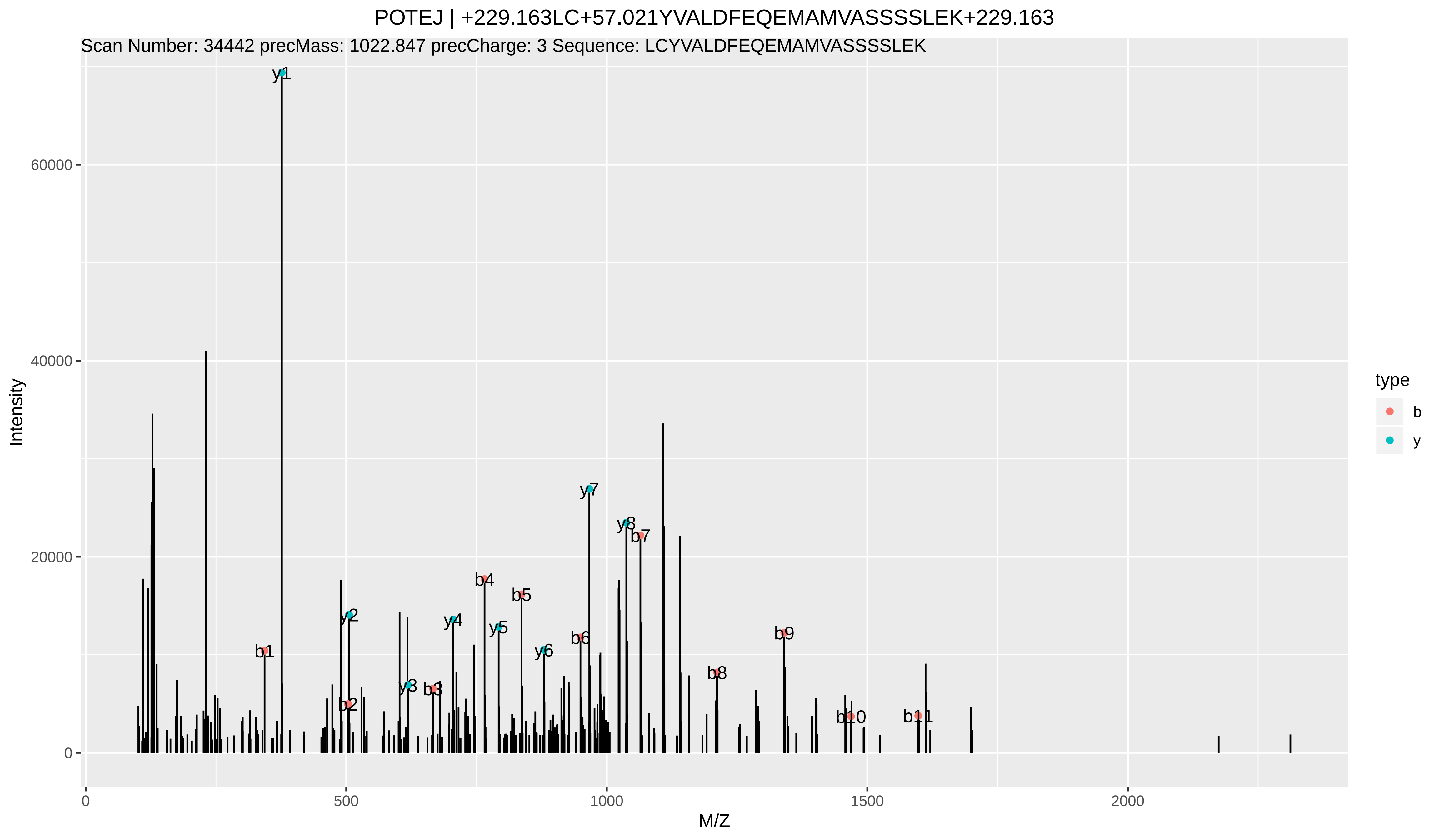
POTEJ | +229.163LC+57.021YVALDFEQEMAMVASSSSLEK+229.163
Scan Number: 34442 precMass: 1022.847 precCharge: 3 Sequence: LCYVALDFEQEMAMVASSSSLEK
Intensity
M/Z
0
20000
40000
60000
0
500
1000
1500
2000
type
b
y
y1
y2
y3
y4
y5
y6
y7
y8
b1
b2
b3
b4
b5
b6
b7
b8
b9
b10
b11

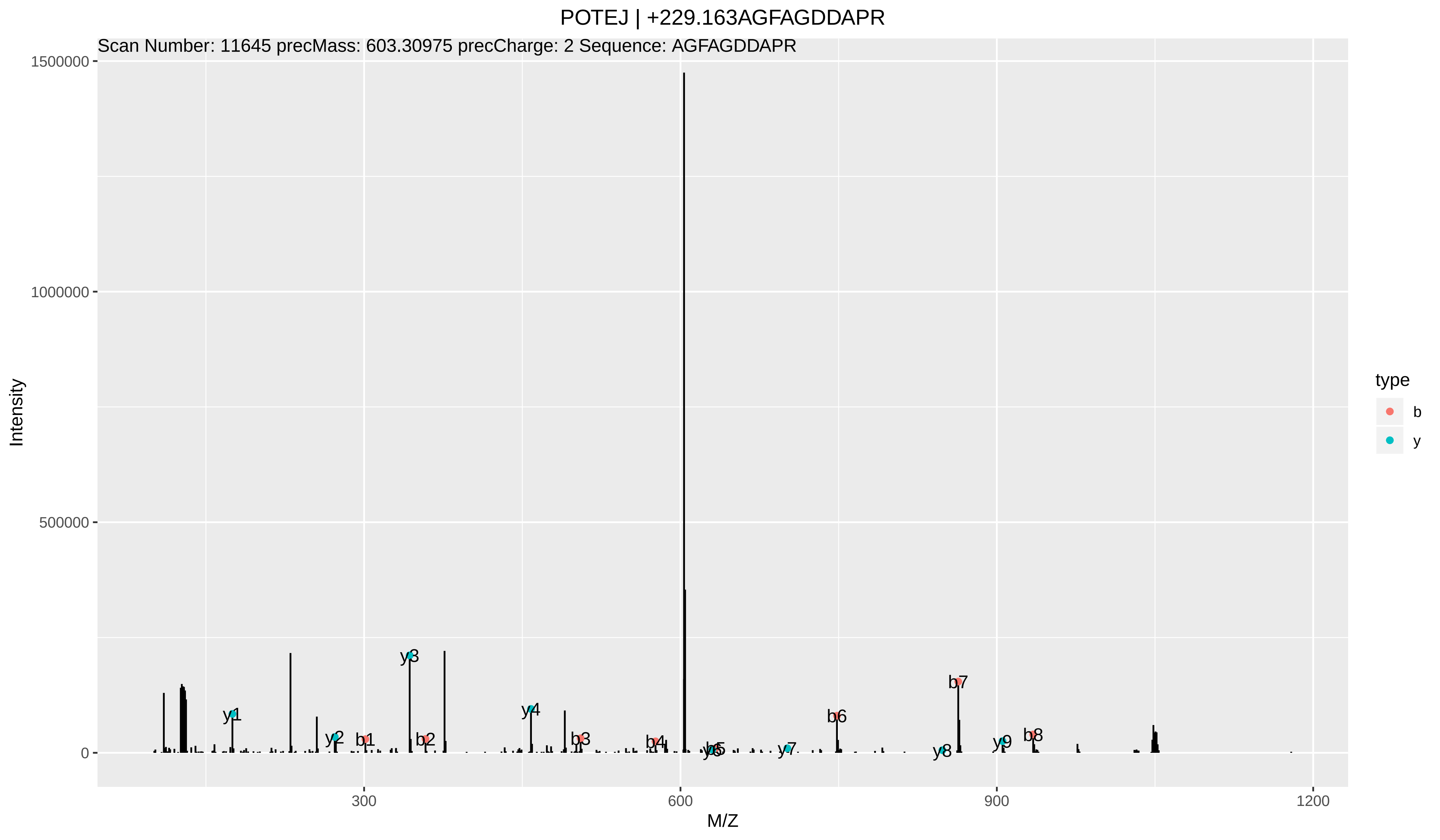
POTEJ | +229.163AGFAGDDAPR
Scan Number: 11645 precMass: 603.30975 precCharge: 2 Sequence: AGFAGDDAPR
Intensity
M/Z
1500000
1000000
500000
0
300
600
900
1200
y1
y2
b1
y3
b2
y4
b3
b4
y6
b5
y7
b6
b7
y8
y9
b8
type
b
y

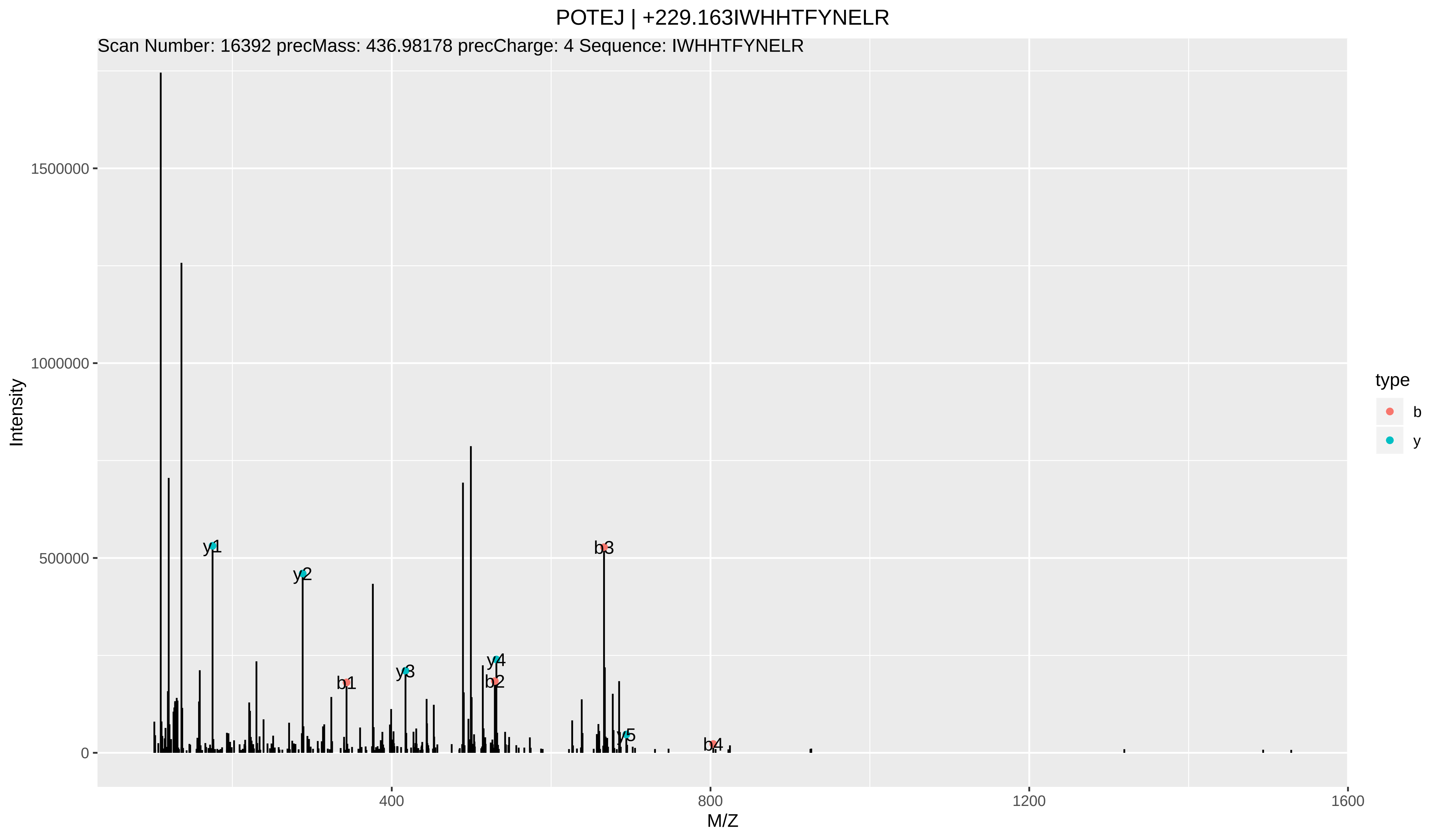

POTEJ | +229.163IWHHTFYNELR
Scan Number: 16392 precMass: 436.98178 precCharge: 4 Sequence: IWHHTFYNELR
Intensity
1500000
1000000
500000
0
M/Z
400
800
1200
1600
y1
y2
b1
y3
y4
b2
b3
y5
b4
type
b
y

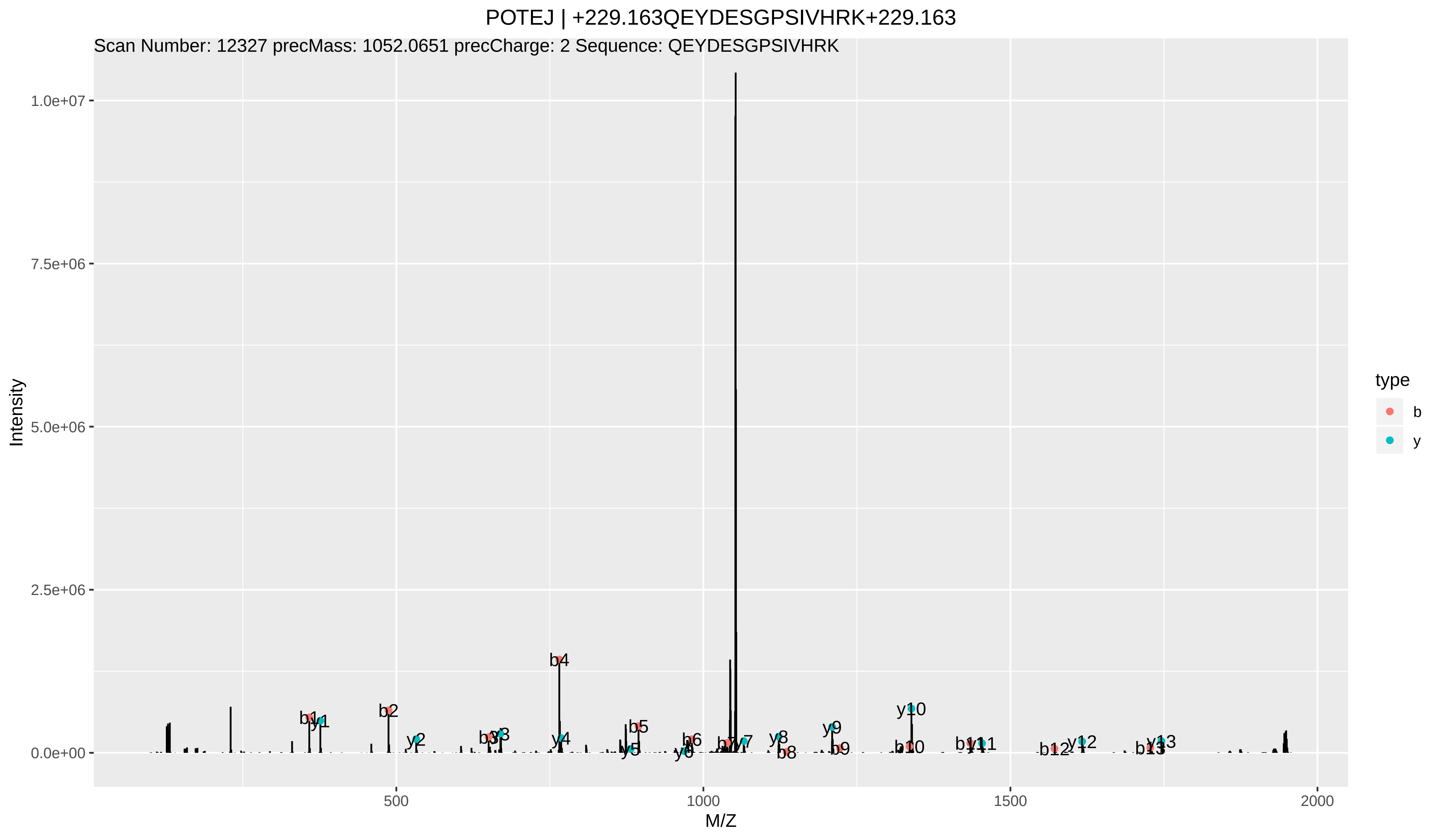

POTEJ | +229.163QEYDESGPSIVHRK+229.163
Scan Number: 12327 precMass: 1052.0651 precCharge: 2 Sequence: QEYDESGPSIVHRK
Intensity
M/Z
0.0e+00
2.5e+06
5.0e+06
7.5e+06
1.0e+07
500
1000
1500
2000
type
b
y
b1
y1
b2
y2
b3
y3
b4
y4
b5
y5
b6
y6
b7
y7
y8
b8
y9
b9
y10
b10
b11
y11
b12
y12
b13
y13

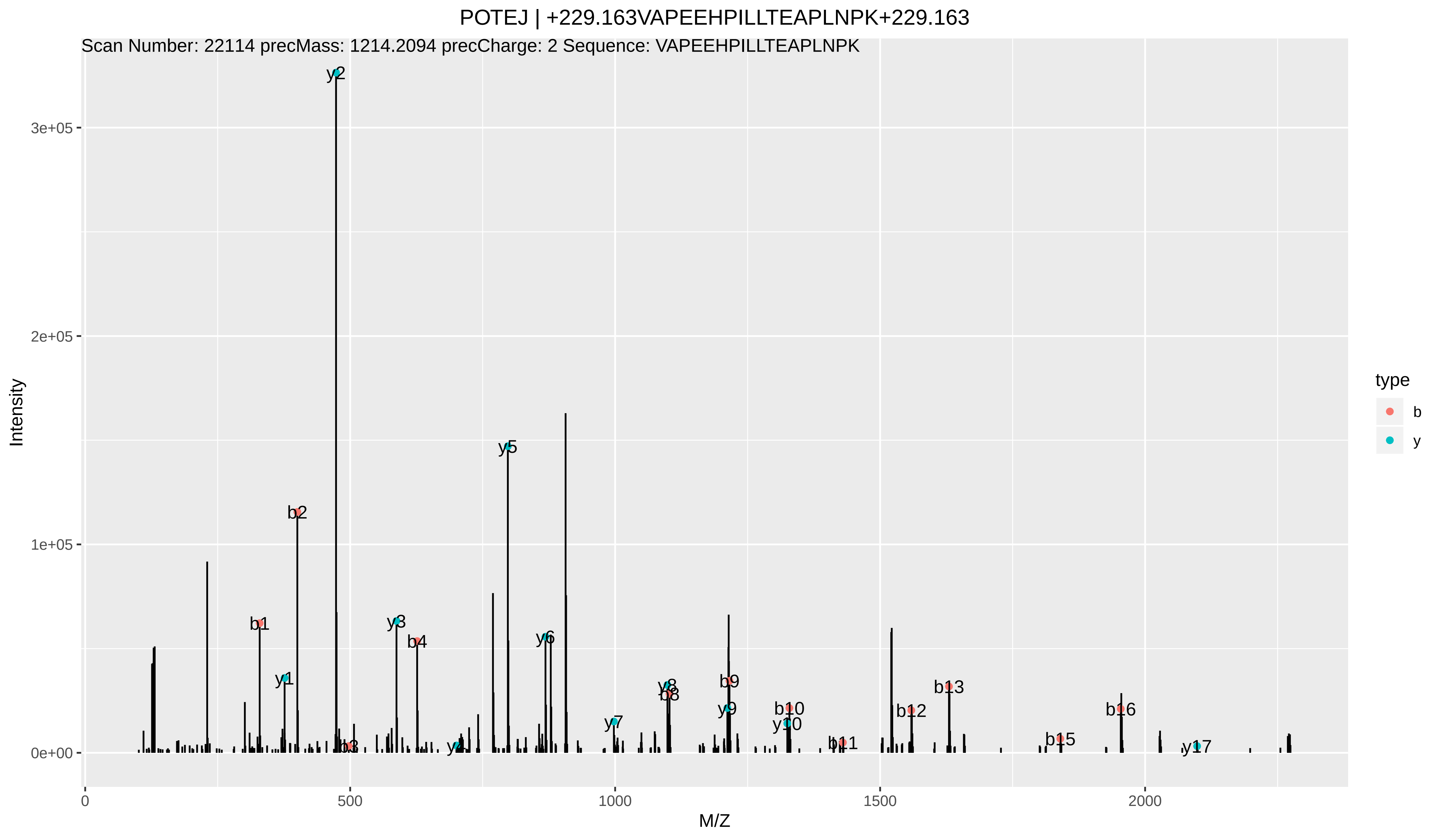
POTEJ | +229.163VAPEEHPILLTEAPLNPK+229.163
Scan Number: 22114 precMass: 1214.2094 precCharge: 2 Sequence: VAPEEHPILLTEAPLNPK
Intensity
M/Z
3e+05
2e+05
1e+05
0e+00
0
500
1000
1500
2000
y2
b2
y5
b1
y1
y3
b4
y6
b3
y4
y7
y8
b8
b9
y9
b10
y10
b11
b12
b13
b15
b16
y17
type
b
y

POTEJ | +229.163C+57.021PEALFQPC+57.021FLGMESC+57.021GIHETTFNSIMK+229.163

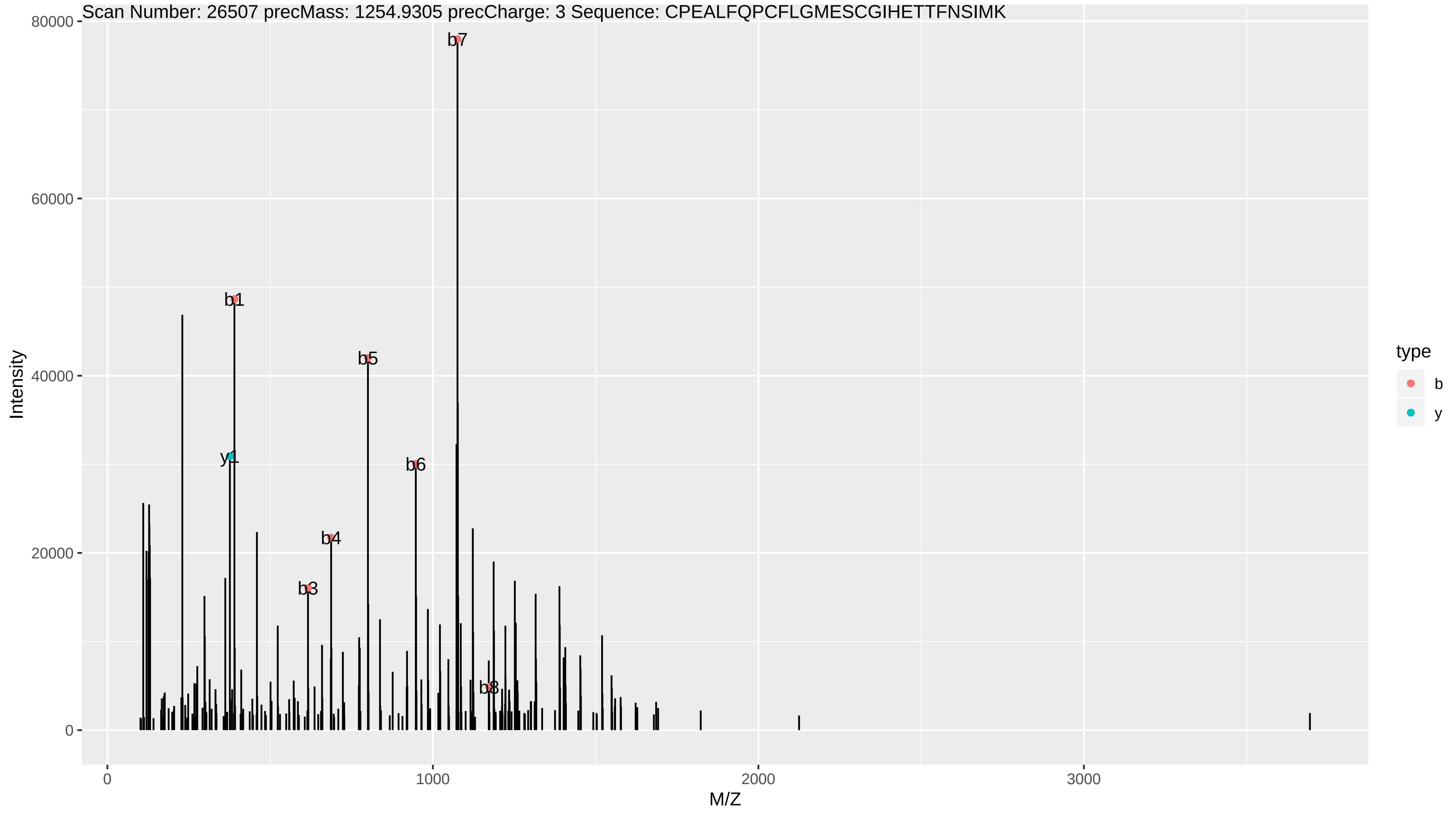

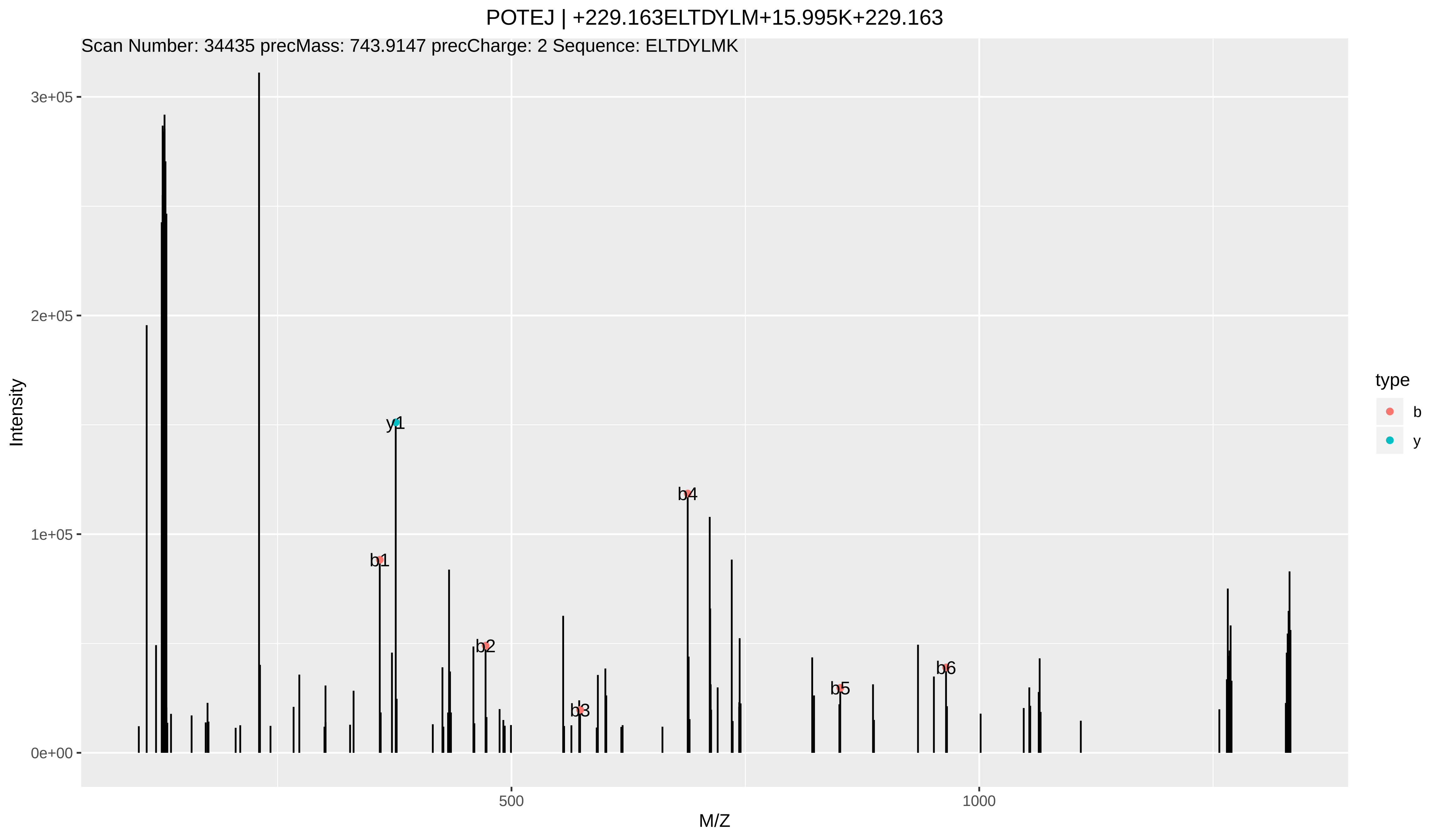
POTEJ | +229.163ELTDYLM+15.995K+229.163
Scan Number: 34435 precMass: 743.9147 precCharge: 2 Sequence: ELTDYLMK
Intensity
M/Z
3e+05
2e+05
1e+05
0e+00
500
1000
y1
b1
b2
b3
b4
b5
b6
type
b
y

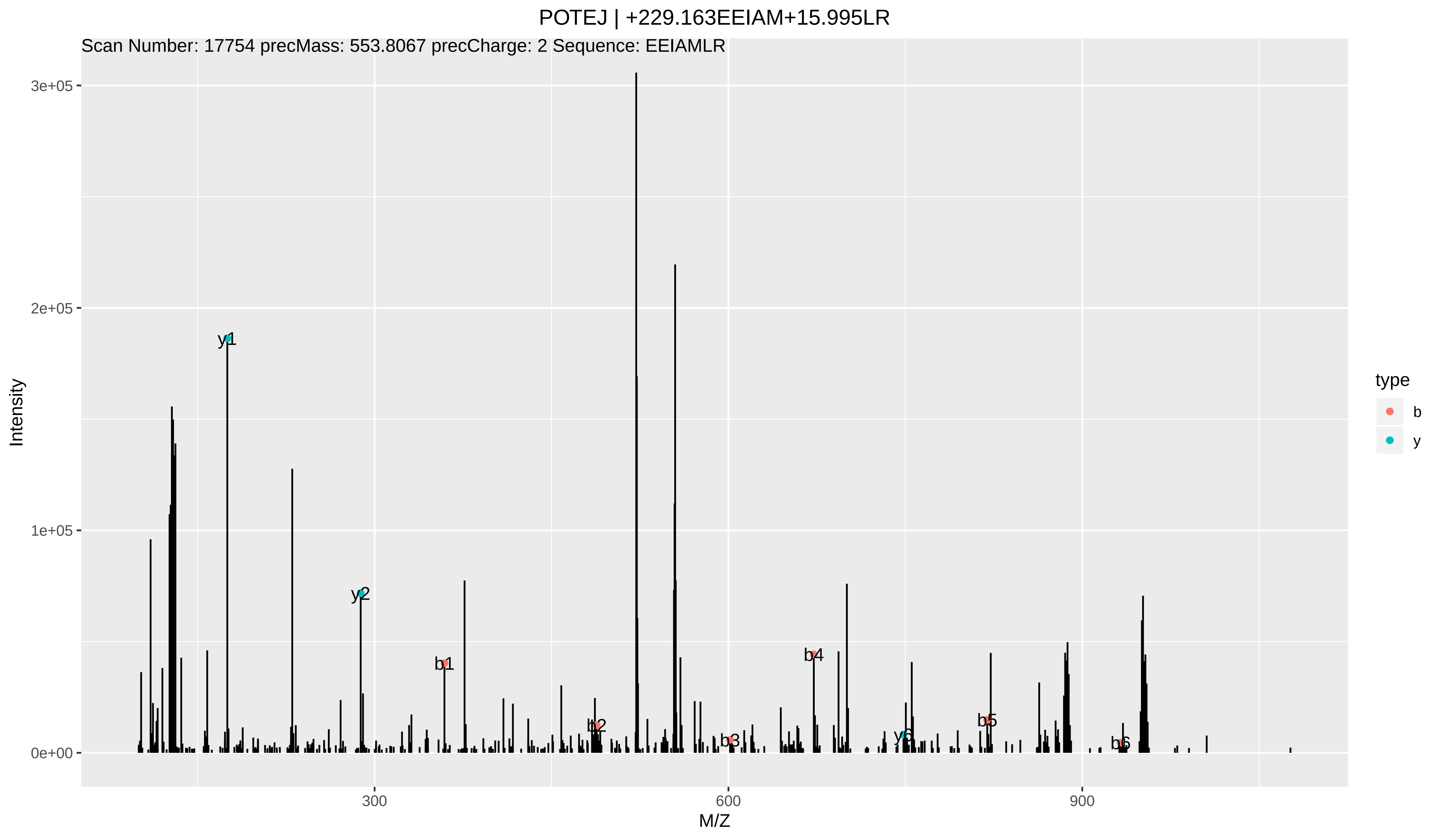

POTEJ | +229.163EEIAM+15.995LR
Scan Number: 17754 precMass: 553.8067 precCharge: 2 Sequence: EEIAMLR
Intensity
3e+05
2e+05
1e+05
0e+00
M/Z
300
600
900
y1
y2
b1
b2
b3
b4
y6
b5
b6
type
b
y

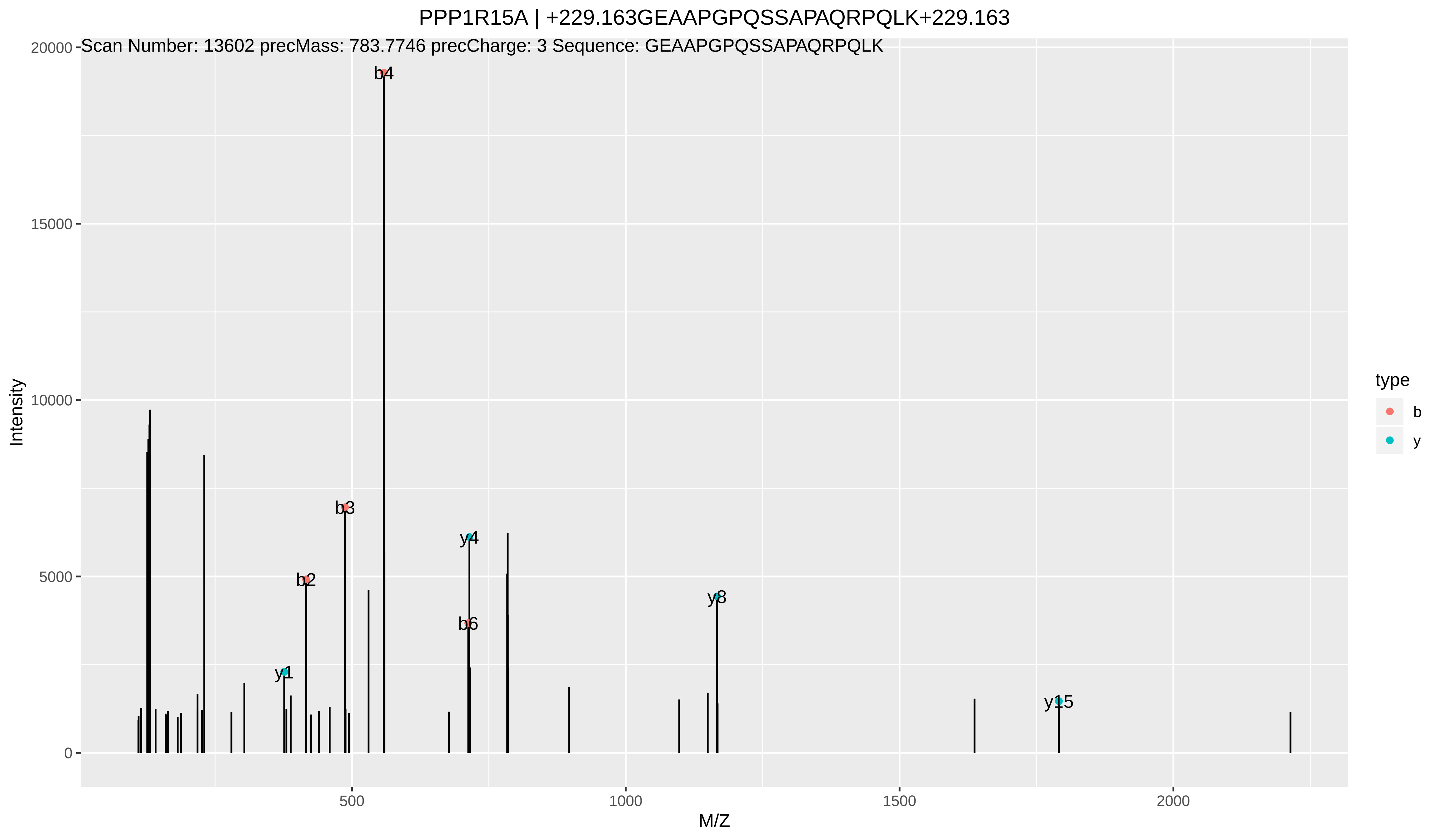

PPP1R15A | +229.163GEAAPGPQSSAPAQRPQLK+229.163
Scan Number: 13602 precMass: 783.7746 precCharge: 3 Sequence: GEAAPGPQSSAPAQRPQLK
Intensity
0
5000
10000
15000
20000
M/Z
500
1000
1500
2000
b4
b3
b2
b6
y1
y4
y8
y15
type
b
y

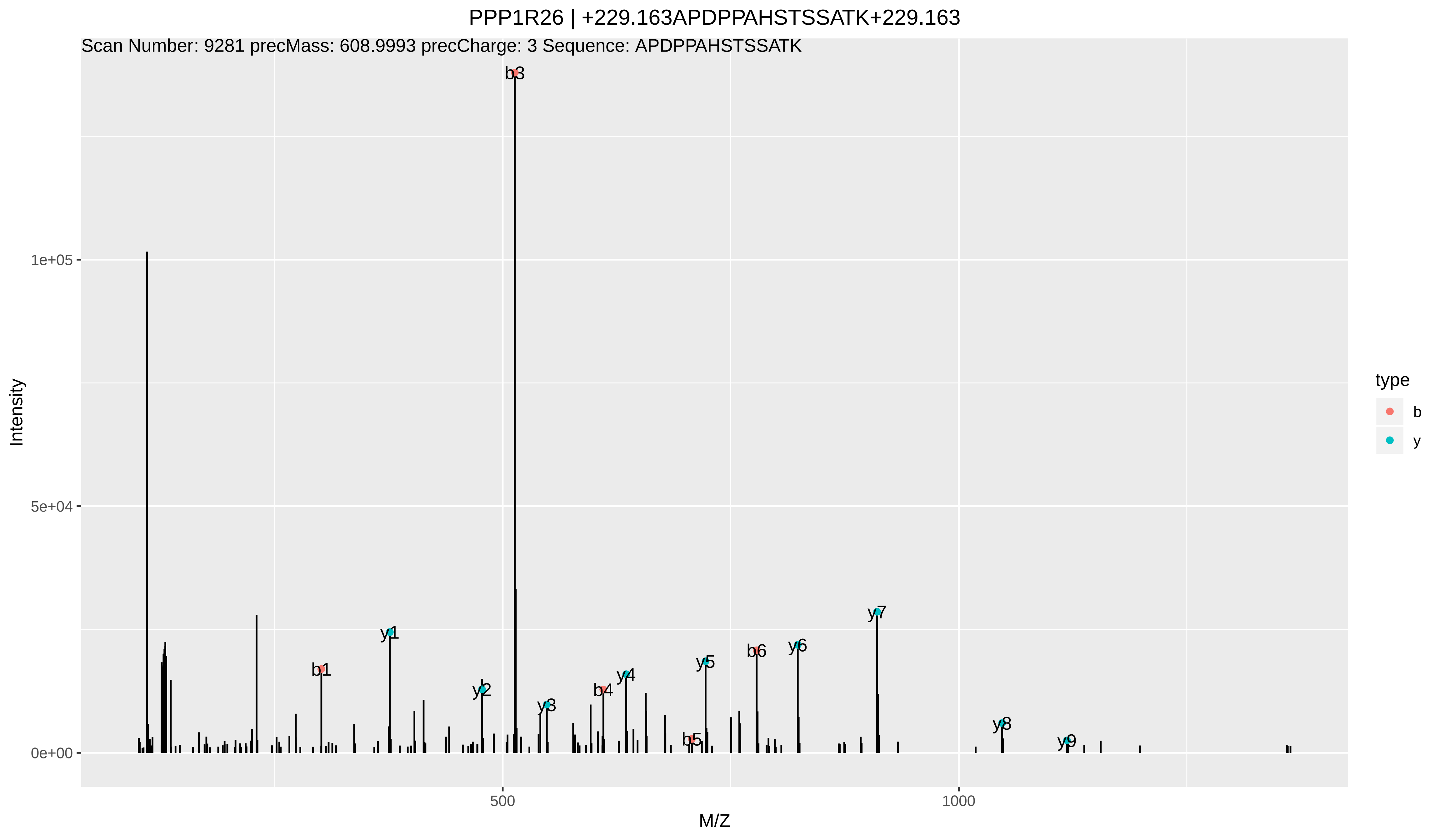

PPP1R26 | +229.163APDPPAHSTSSATK+229.163
Scan Number: 9281 precMass: 608.9993 precCharge: 3 Sequence: APDPPAHSTSSATK
b3
y1
b1
y2
y3
b4
y4
b5
y5
b6
y6
y7
y8
y9
Intensity
1e+05
5e+04
0e+00
M/Z
500
1000
type
b
y

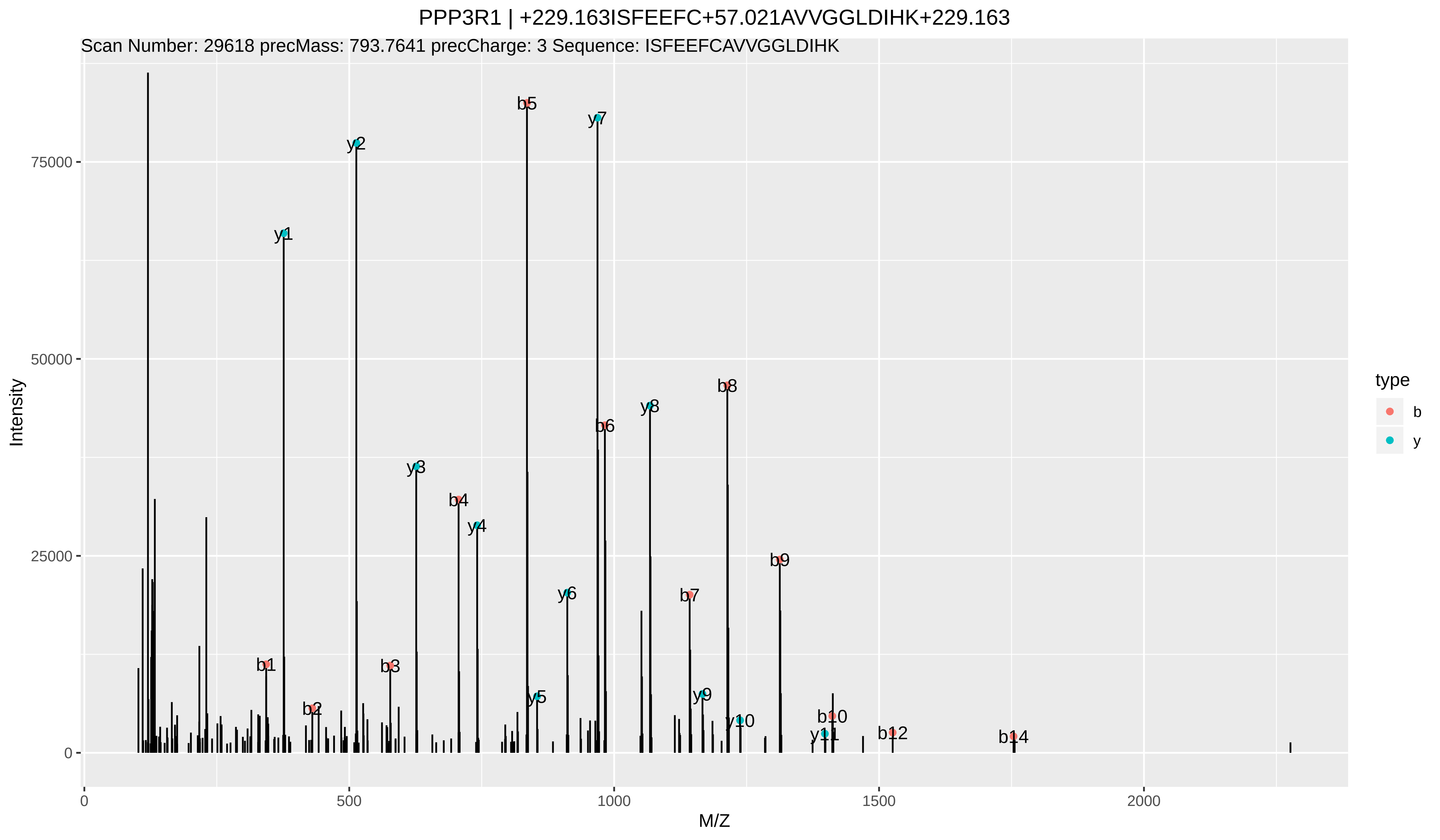
PPP3R1 | +229.163ISFEEFC+57.021AVVGGLDIHK+229.163
Scan Number: 29618 precMass: 793.7641 precCharge: 3 Sequence: ISFEEFCAVVGGLDIHK
Intensity
M/Z
type
b
y
b1
b2
b3
b4
b5
b6
b7
b8
b9
b10
b12
b14
y1
y2
y3
y4
y5
y6
y7
y8
y9
y10
y11
0
25000
50000
75000
500
1000
1500
2000

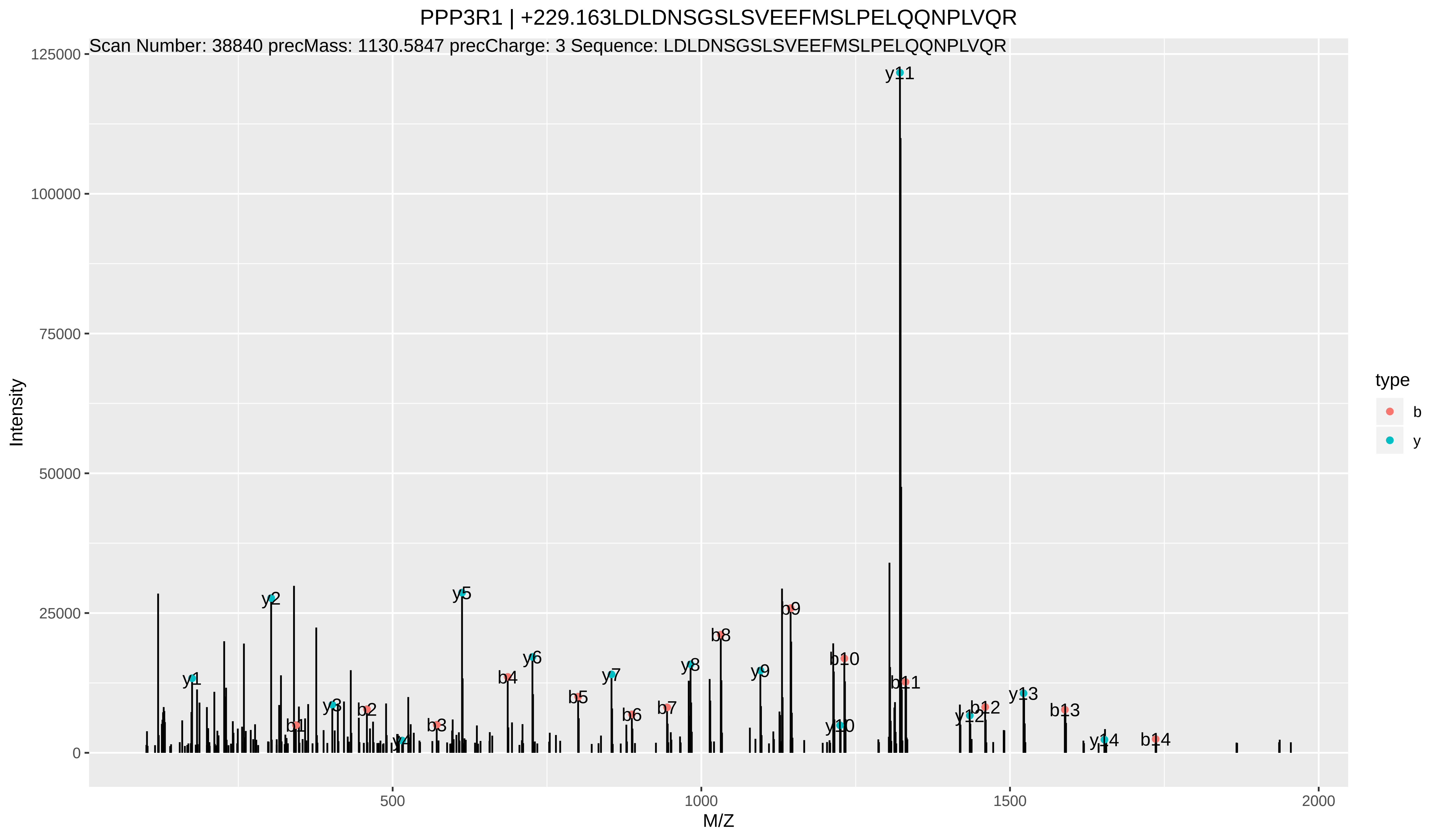
PPP3R1 | +229.163LDLDNSGSLSVEEFMSLPELQQNPLVQR
Scan Number: 38840 precMass: 1130.5847 precCharge: 3 Sequence: LDLDNSGSLSVEEFMSLPELQQNPLVQR
Intensity
M/Z
type
b
y
y1
y2
y3
y4
y5
y6
y7
y8
y9
y10
y11
y12
y13
y14
b1
b2
b3
b4
b5
b6
b7
b8
b9
b10
b11
b12
b13
b14
0
25000
50000
75000
100000
125000
500
1000
1500
2000

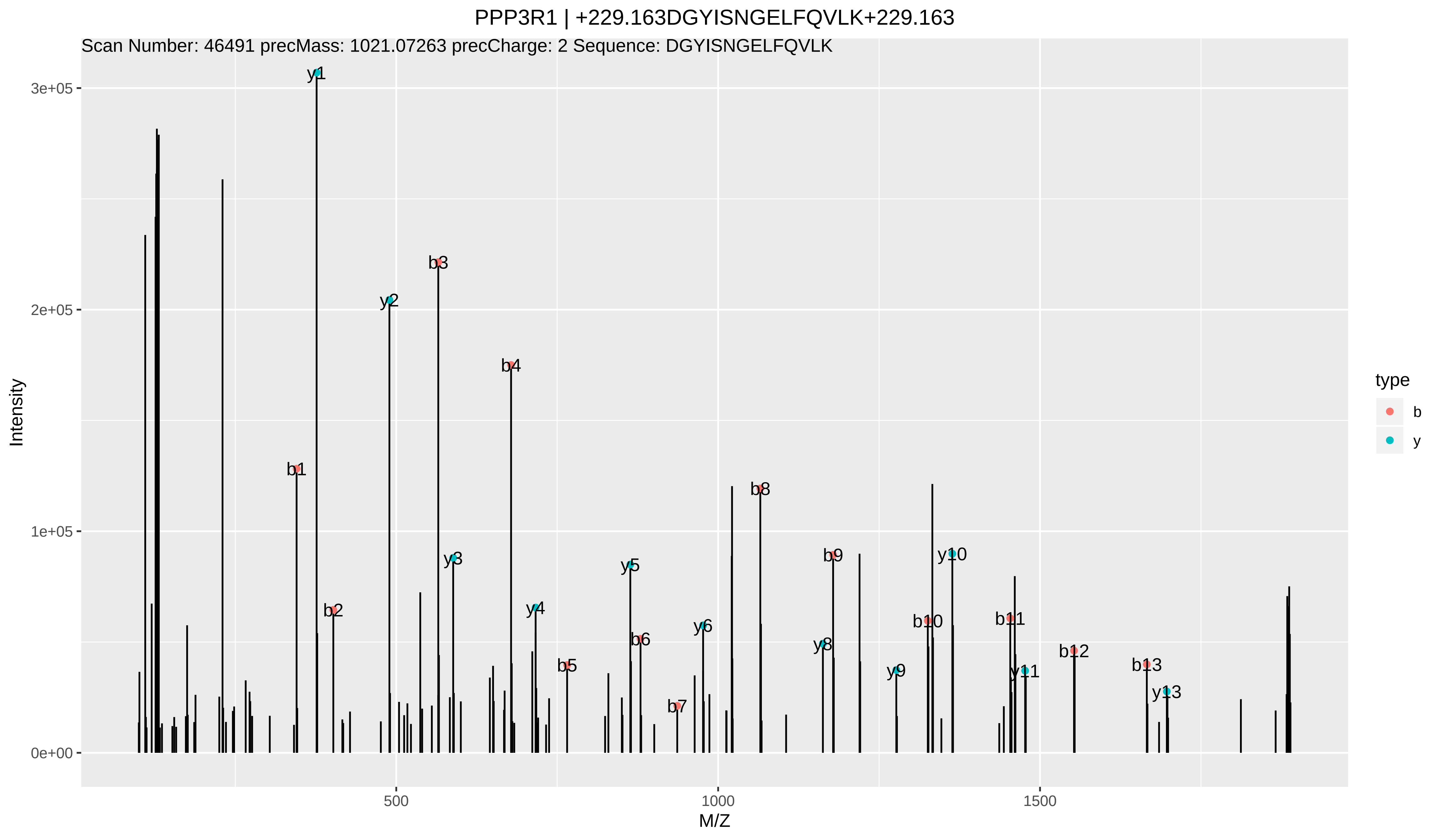
PPP3R1 | +229.163DGYISNGELFQVLK+229.163
Scan Number: 46491 precMass: 1021.07263 precCharge: 2 Sequence: DGYISNGELFQVLK
Intensity
3e+05
2e+05
1e+05
0e+00
M/Z
500
1000
1500
y1
y2
b3
b4
b1
b2
y3
y4
b5
y5
b6
b7
y6
b8
y8
b9
y9
b10
y10
b11
y11
b12
b13
y13
type
b
y

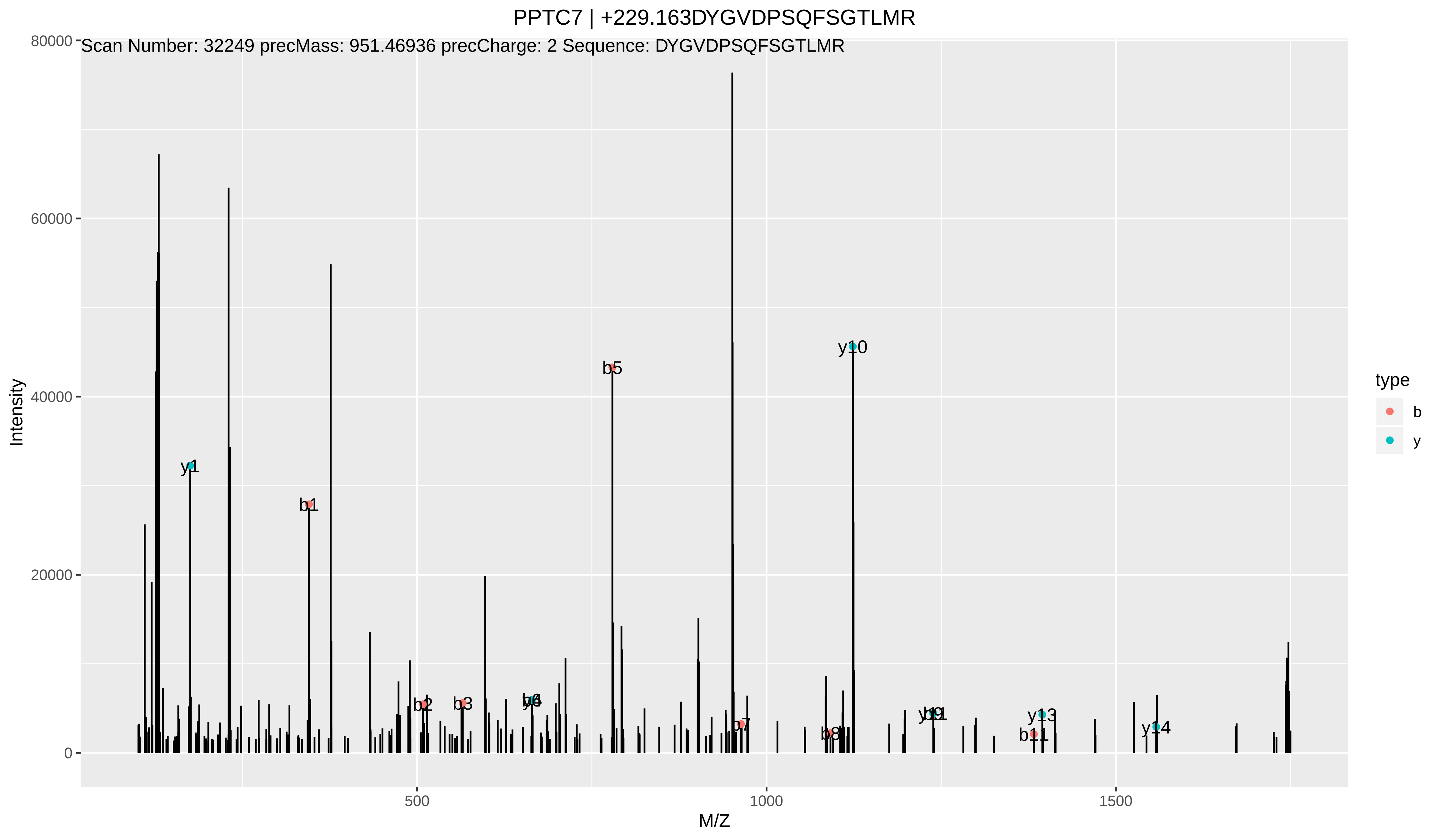
PPTC7 | +229.163DYGVDPSQFSGTLMR
Scan Number: 32249 precMass: 951.46936 precCharge: 2 Sequence: DYGVDPSQFSGTLMR
Intensity
M/Z
type
b
y
y1
b1
b2
b3
b5
y10
b7
b8
y13
b11
y14
0
20000
40000
60000
80000
500
1000
1500

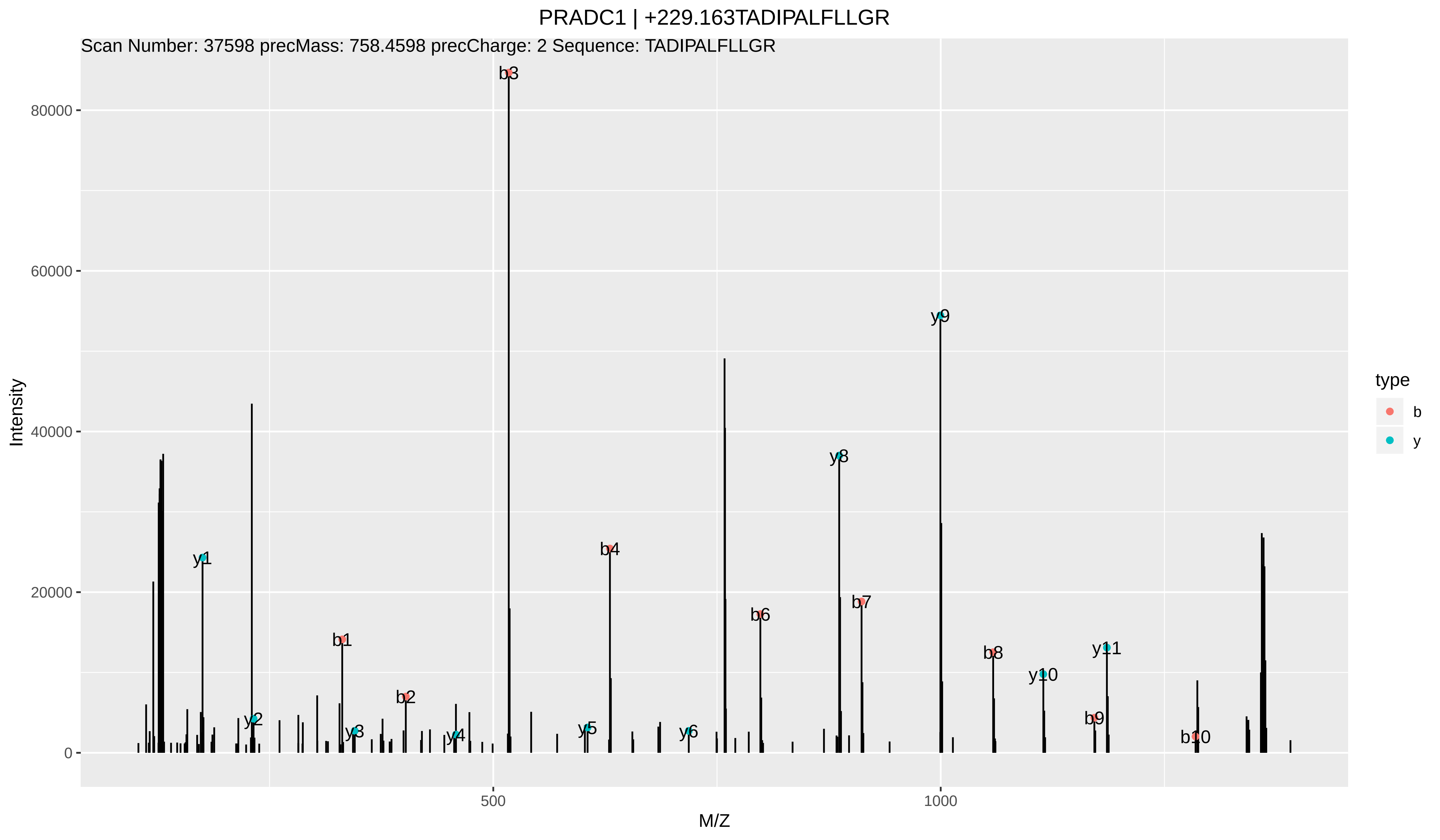

PRADC1 | +229.163TADIPALFLLGR
Scan Number: 37598 precMass: 758.4598 precCharge: 2 Sequence: TADIPALFLLGR
Intensity
M/Z
0
20000
40000
60000
80000
500
1000
b3
y9
y8
b4
y1
b7
b6
b1
b8
y11
y10
b2
y2
b9
y3
y5
y6
y4
b10
type
b
y

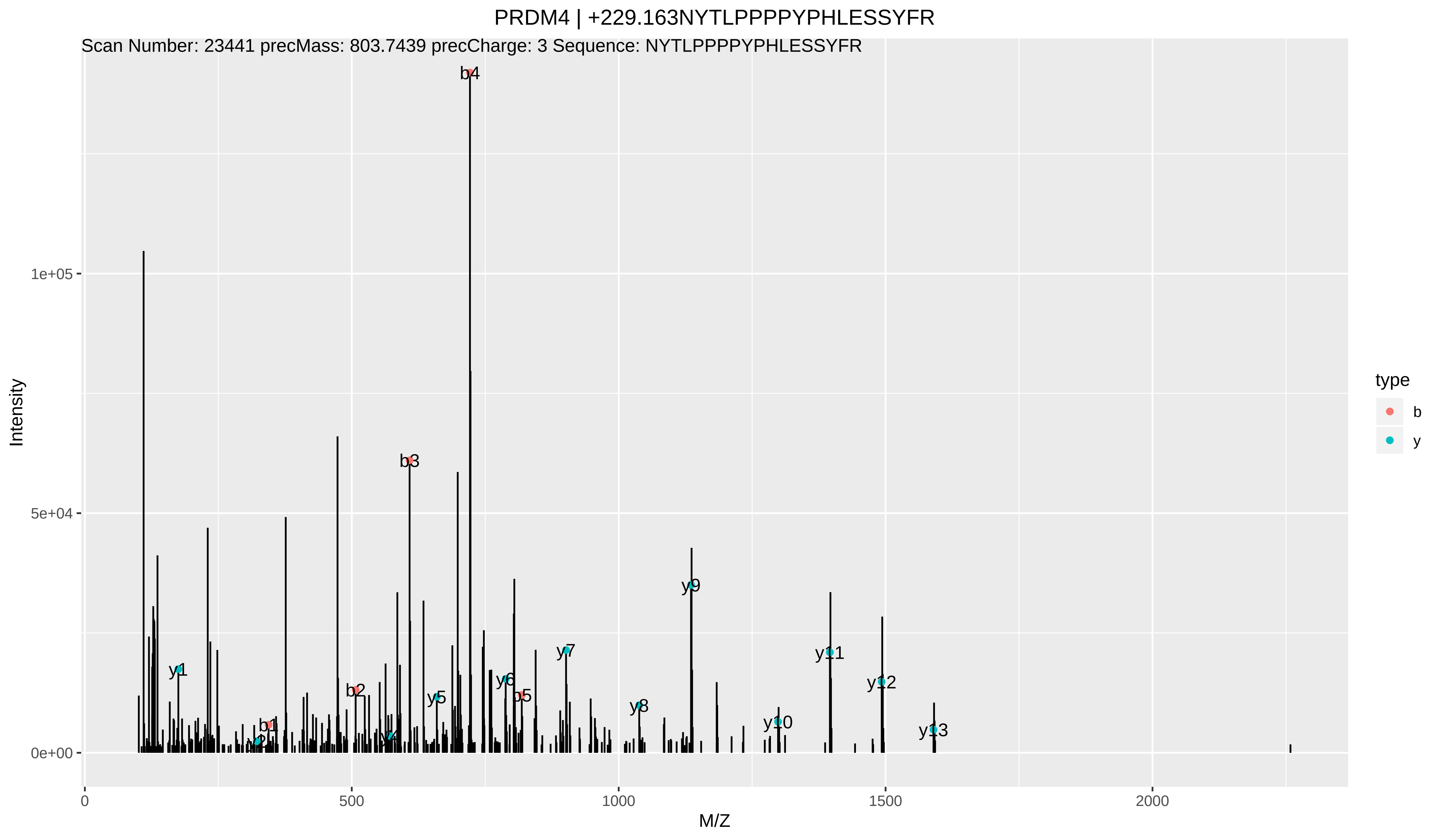

PRDM4 | +229.163NYTLPPPPYPHLESSYFR
Scan Number: 23441 precMass: 803.7439 precCharge: 3 Sequence: NYTLPPPPYPHLESSYFR
b4
b3
y9
y7
y11
y12
y1
y6
b2
b5
y5
y8
y10
y13
b1
y4
Intensity
1e+05
5e+04
0e+00
0
500
1000
1500
2000
M/Z
type
b
y

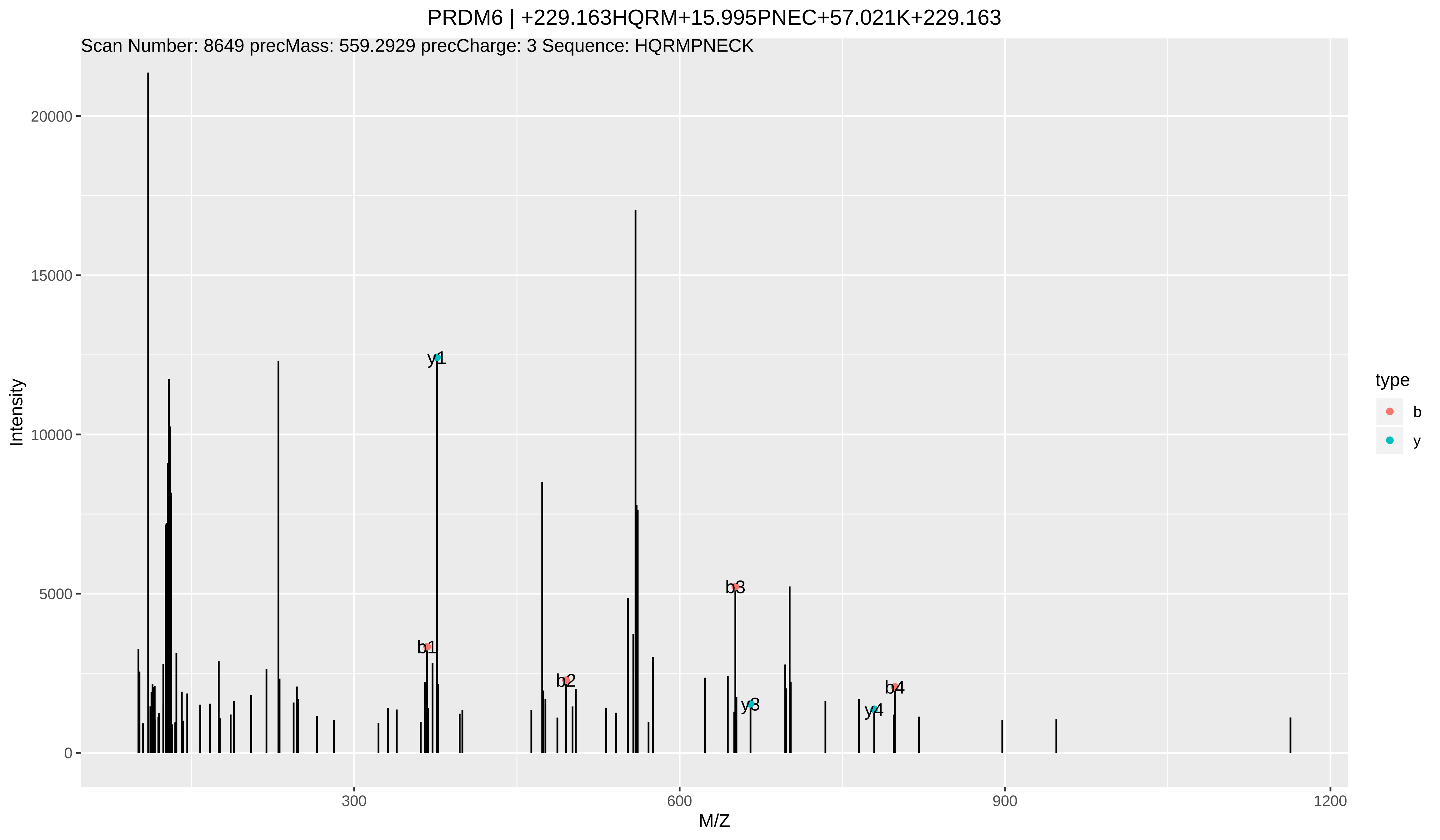

PRDM6 | +229.163HQRM+15.995PNEC+57.021K+229.163
Scan Number: 8649 precMass: 559.2929 precCharge: 3 Sequence: HQRMPNECK
Intensity
20000
15000
10000
5000
0
300
600
900
1200
M/Z
y1
b1
b2
b3
y3
y4
b4
type
b
y

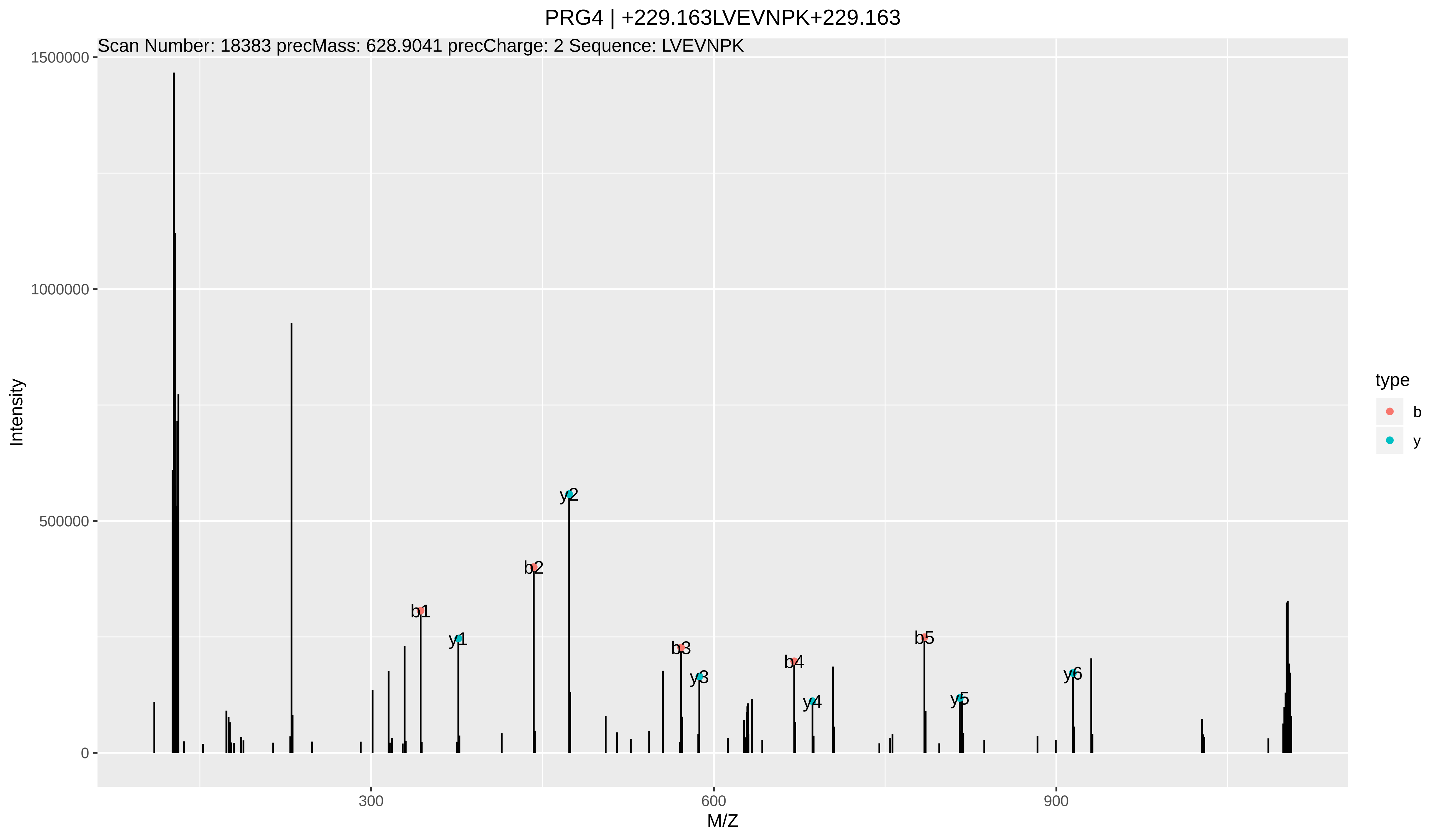
PRG4 | +229.163LVEVNPK+229.163
Scan Number: 18383 precMass: 628.9041 precCharge: 2 Sequence: LVEVNPK
Intensity
1500000
1000000
500000
0
M/Z
300
600
900
b1
y1
b2
y2
b3
y3
b4
y4
b5
y5
y6
type
b
y

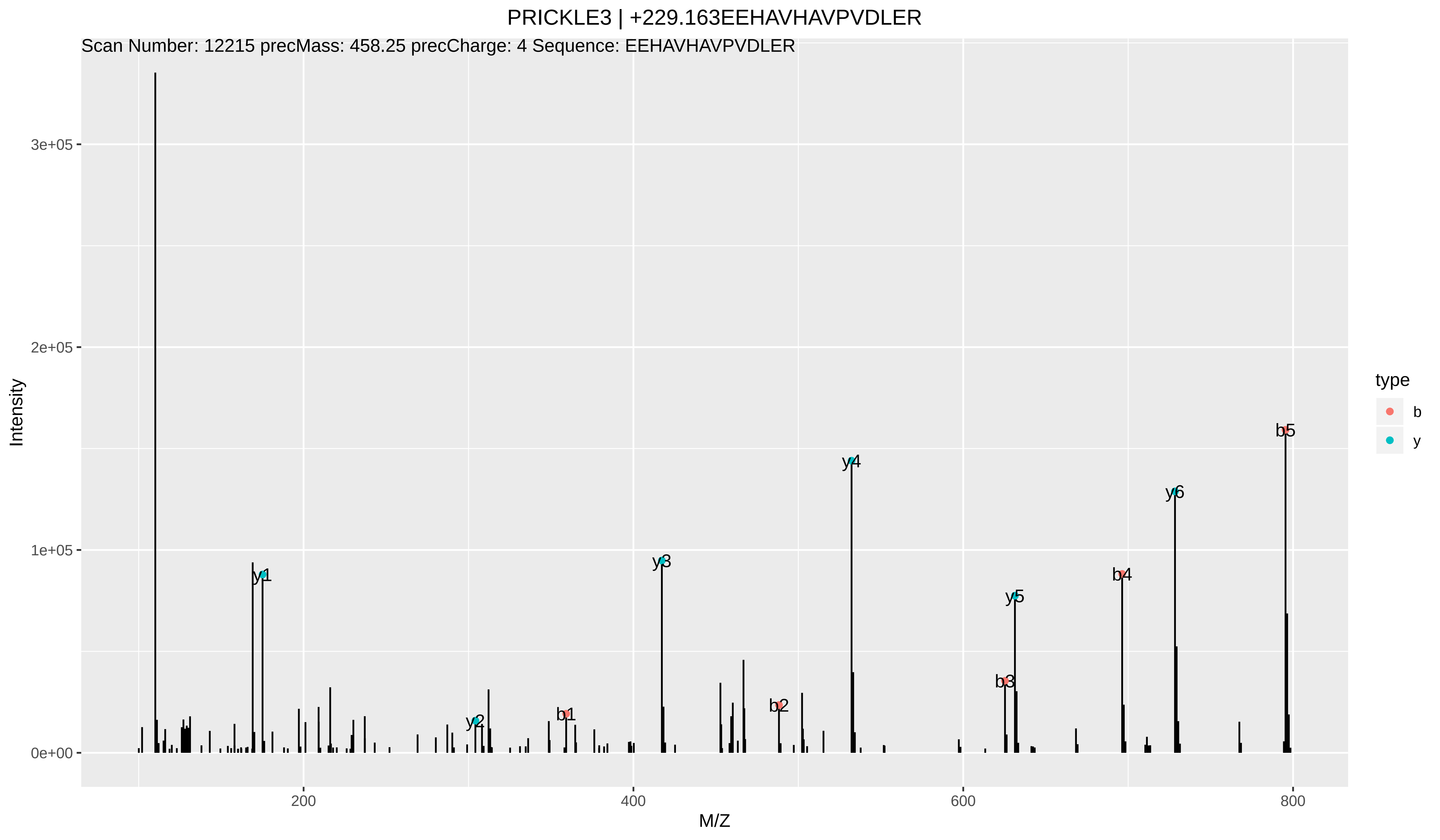
PRICKLE3 | +229.163EEHAVHAVPVDLER
Scan Number: 12215 precMass: 458.25 precCharge: 4 Sequence: EEHAVHAVPVDLER
Intensity
3e+05
2e+05
1e+05
0e+00
y1
y2
b1
y3
b2
y4
b3
y5
b4
y6
b5
200
400
600
800
M/Z
type
b
y

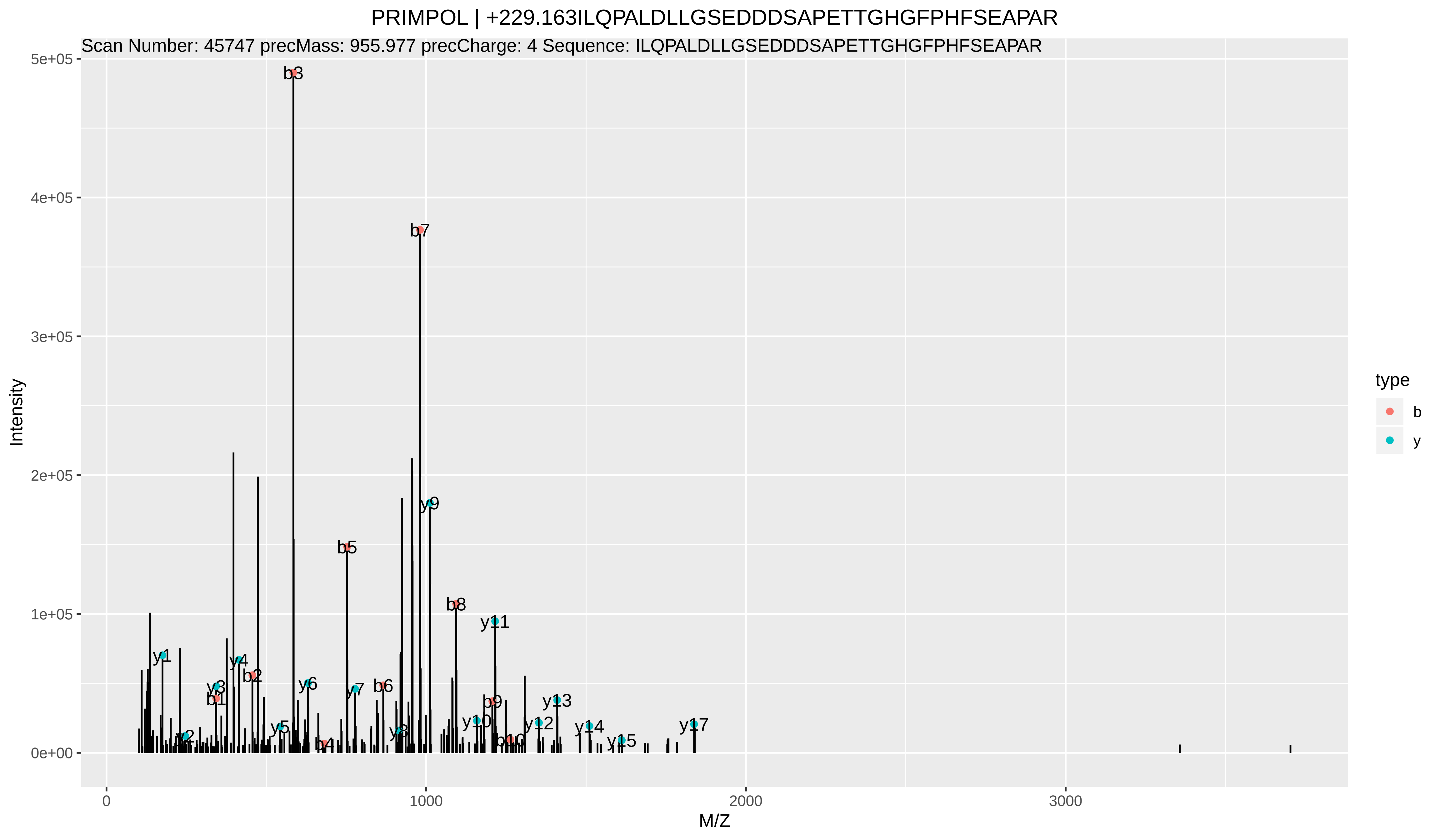

PRIMPOL | +229.163ILQPALDLLGSEDDDSAPETTGHGFPHFSEAPAR
Scan Number: 45747 precMass: 955.977 precCharge: 4 Sequence: ILQPALDLLGSEDDDSAPETTGHGFPHFSEAPAR
Intensity
M/Z
type
b
y
b3
b7
y9
b5
b8
y11
y1
y4
b2
y3
b1
y6
y7
b6
b9
y13
y10
y12
y14
y17
y15
b10
y2
y5
b4
y8
0e+00
1e+05
2e+05
3e+05
4e+05
5e+05
0
1000
2000
3000

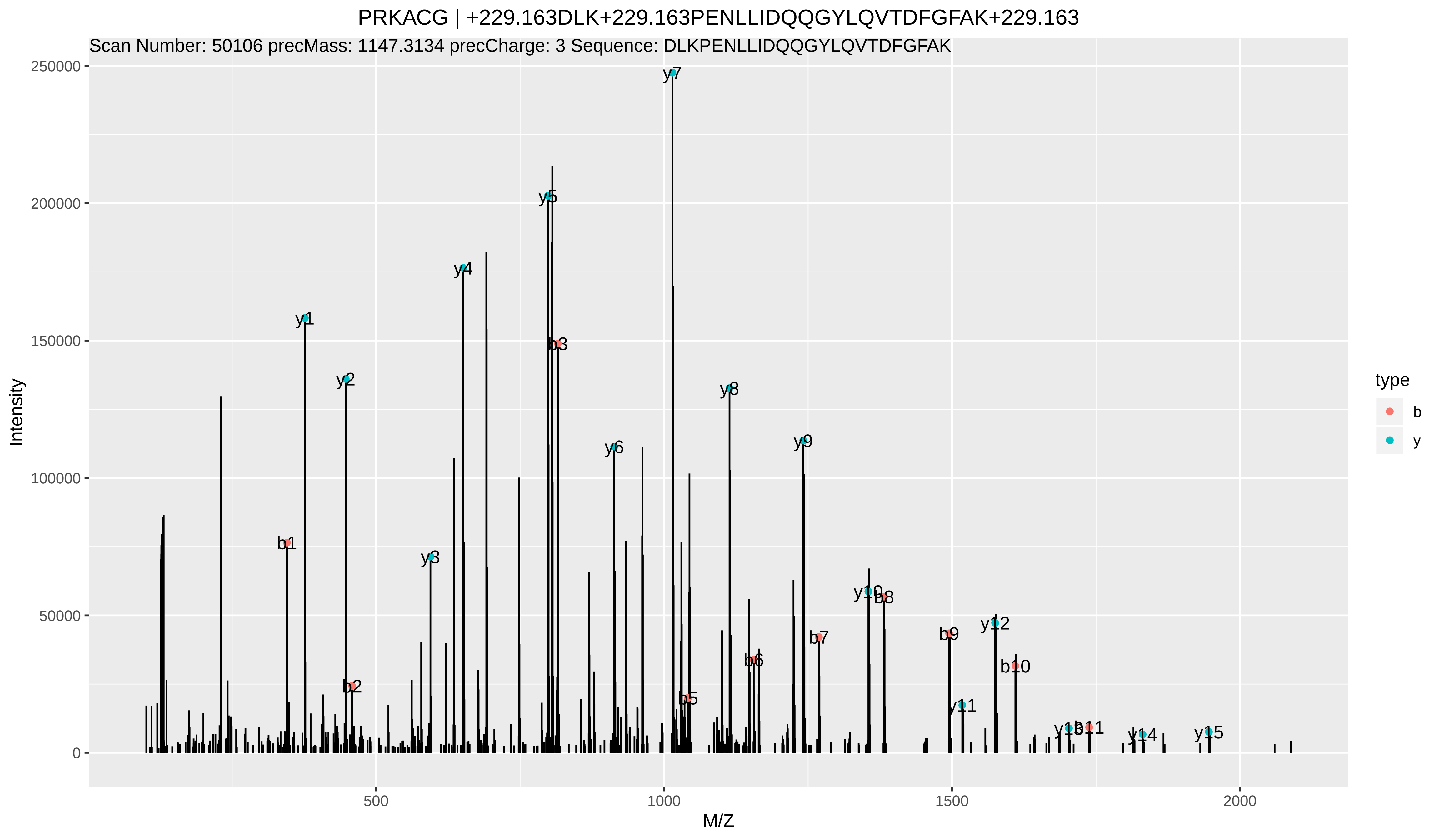

PRKACG | +229.163DLK+229.163PENLLIDQQGYLQVTDFGFAK+229.163
Scan Number: 50106 precMass: 1147.3134 precCharge: 3 Sequence: DLKPENLLIDQQGYLQVTDFGFAK
Intensity
M/Z
type
b
y
y7
y5
y4
y1
b3
y2
y8
y6
y9
b1
y3
y10
b8
b7
b9
y12
b6
b10
b2
b5
y11
y13
b11
y14
y15

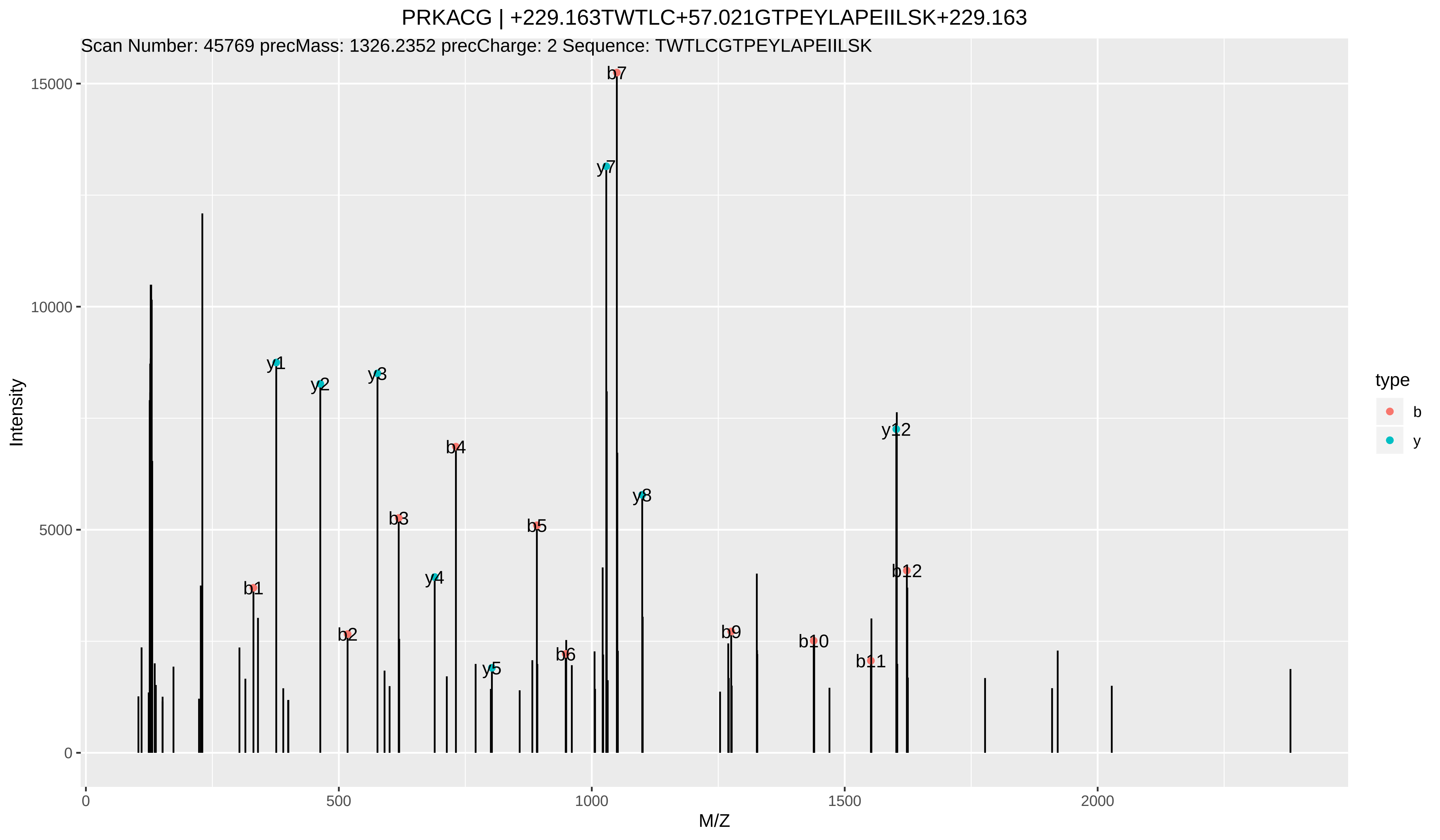
PRKACG | +229.163TWTLC+57.021GTPEYLAPEIILSK+229.163
Scan Number: 45769 precMass: 1326.2352 precCharge: 2 Sequence: TWTLCGTPEYLAPEIILSK
Intensity
M/Z
type
b
y
b7
y7
y1
y2
y3
b4
y12
y8
b3
b5
b12
y4
b1
b2
b9
b10
b6
b11
y5
15000
10000
5000
0
0
500
1000
1500
2000

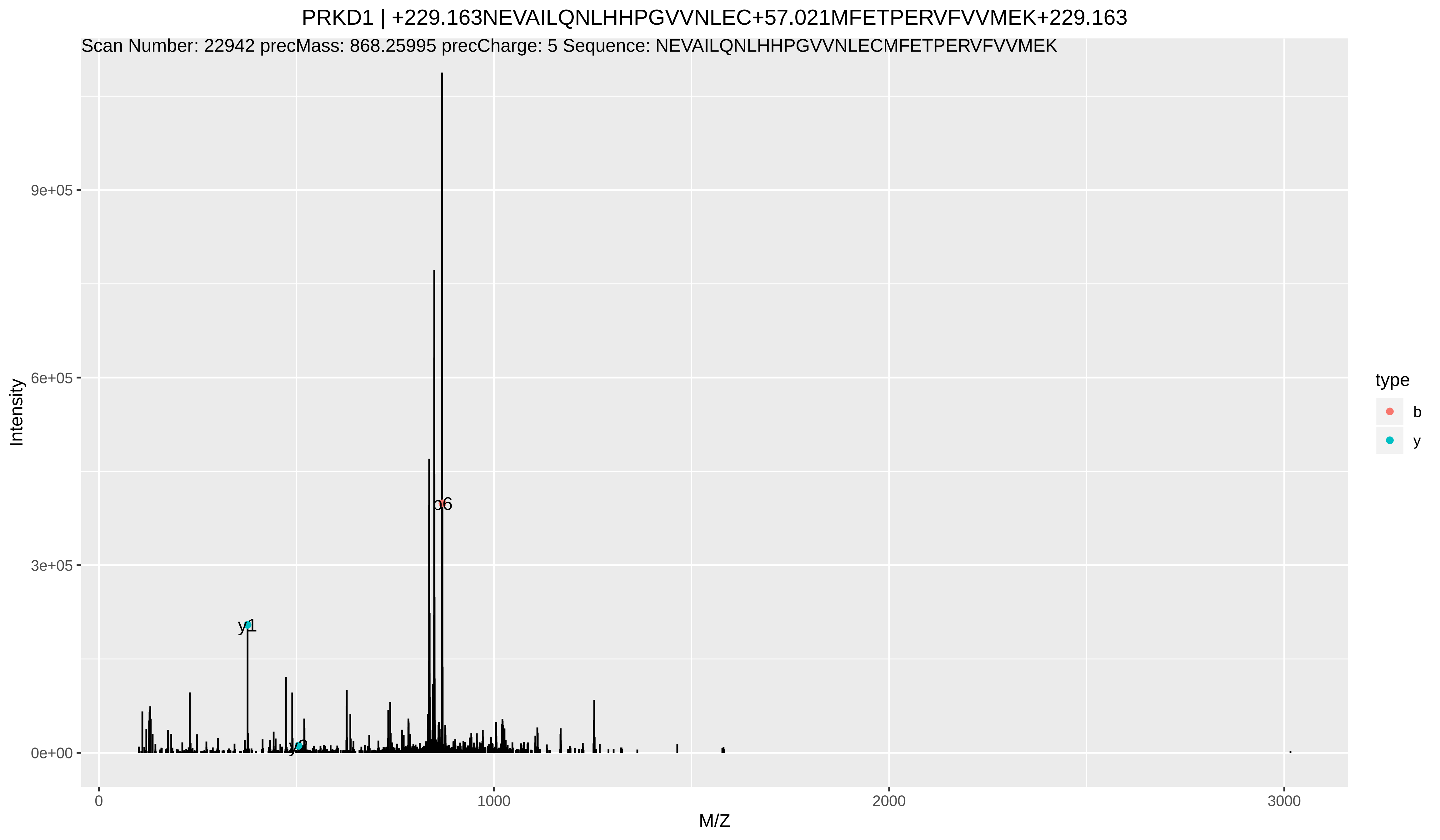
PRKD1 | +229.163NEVAILQNLHHPGVVNLEC+57.021MFETPERVFVVMEK+229.163
Scan Number: 22942 precMass: 868.25995 precCharge: 5 Sequence: NEVAILQNLHHPGVVNLECMFETPERVFVVMEK
Intensity
9e+05
6e+05
3e+05
0e+00
0
1000
2000
3000
M/Z
b6
y1
y2
type
b
y

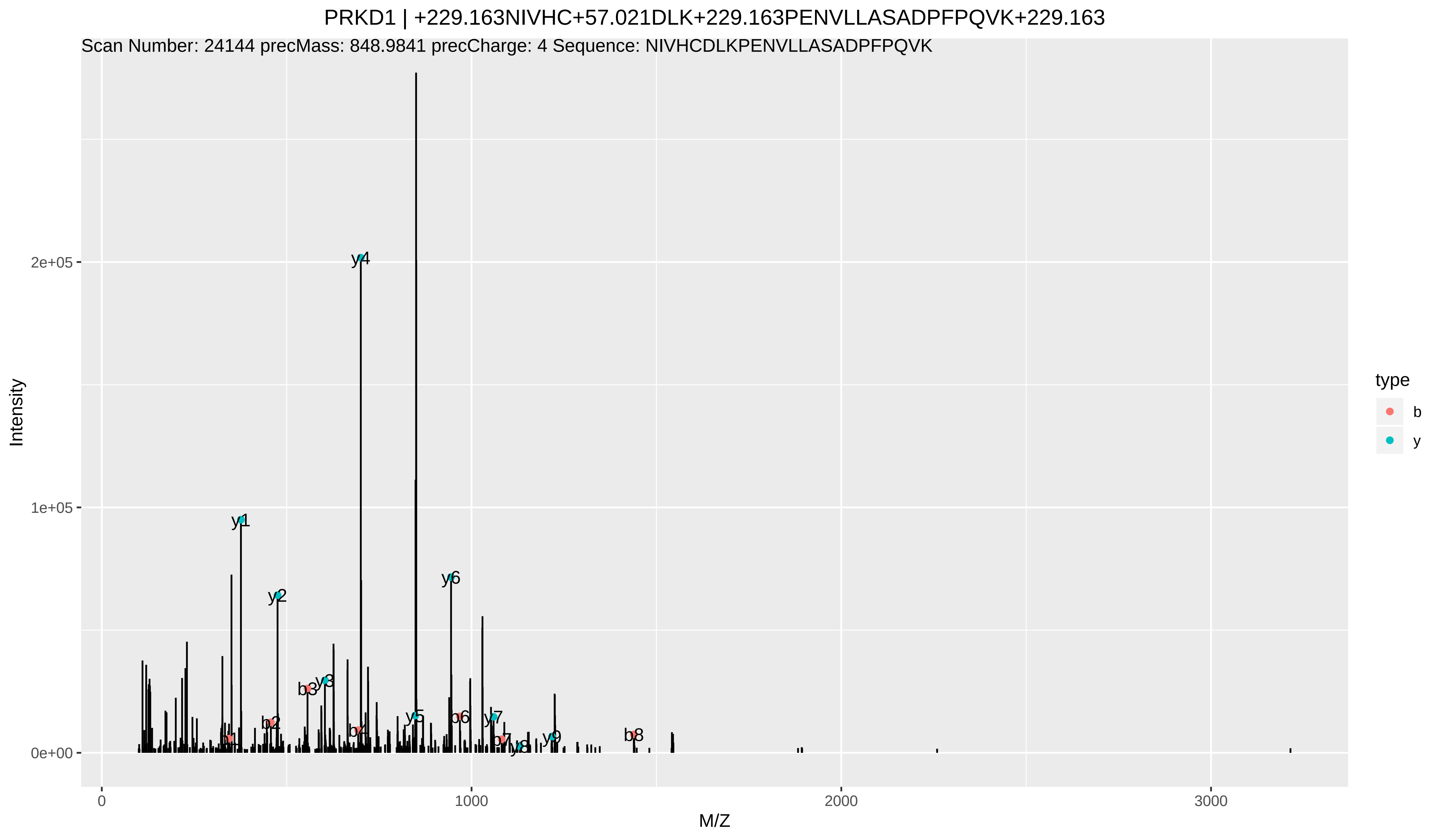
PRKD1 | +229.163NIVHC+57.021DLK+229.163PENVLLASADPFPQVK+229.163
Scan Number: 24144 precMass: 848.9841 precCharge: 4 Sequence: NIVHCDLKPENVLLASADPFPQVK
Intensity
M/Z
0e+00
1e+05
2e+05
0
1000
2000
3000
type
b
y
y1
y2
y3
y4
y5
y6
y7
y8
y9
b1
b2
b3
b4
b6
b7
b8

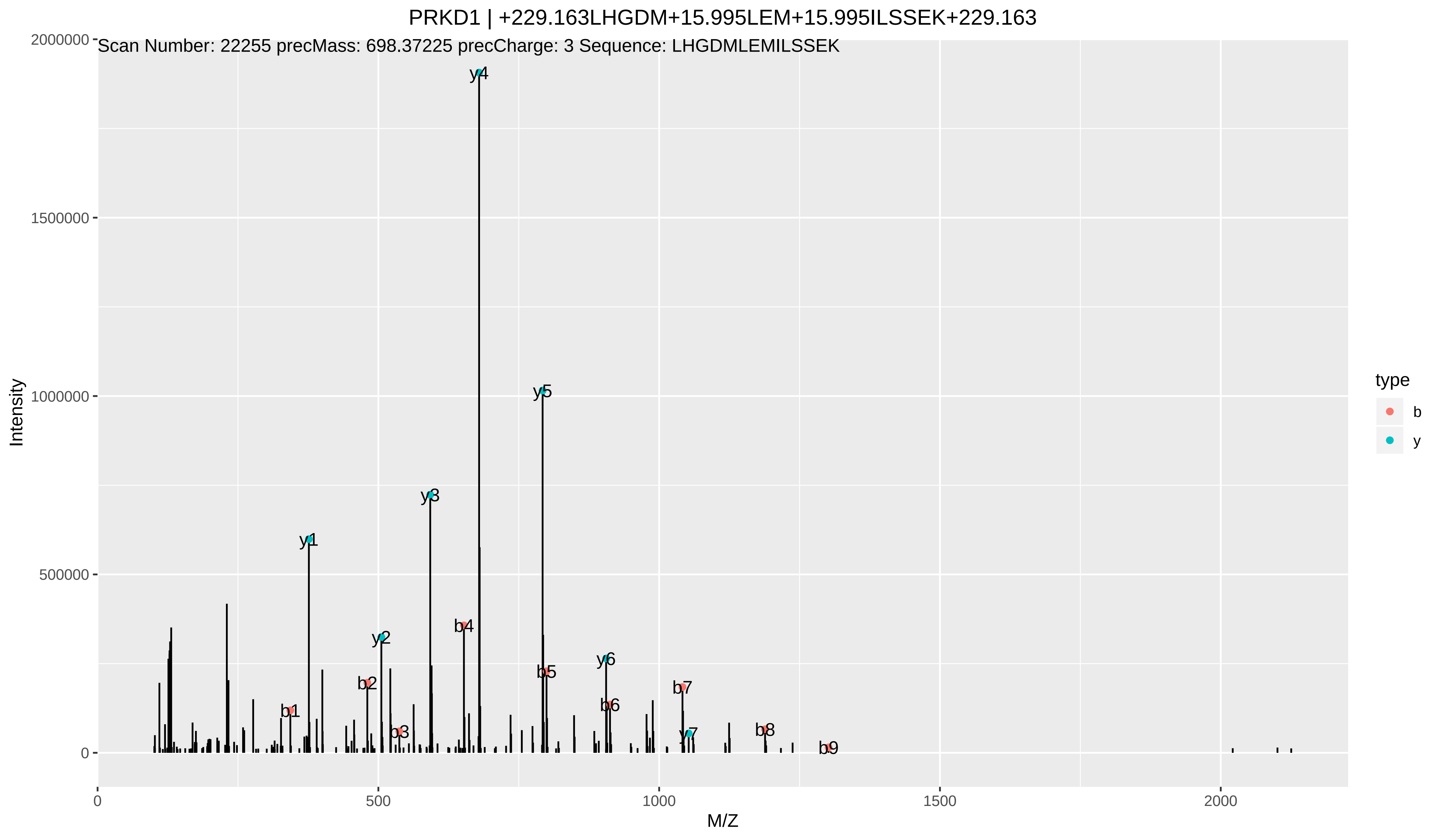

PRKD1 | +229.163LHGDM+15.995LEM+15.995ILSSEK+229.163
Scan Number: 22255 precMass: 698.37225 precCharge: 3 Sequence: LHGDMLEMILSSEK
Intensity
2000000
1500000
1000000
500000
0
M/Z
0
500
1000
1500
2000
y4
y5
y3
y1
b4
y2
y6
b5
b2
b7
b6
b1
b3
y7
b8
b9
type
b
y

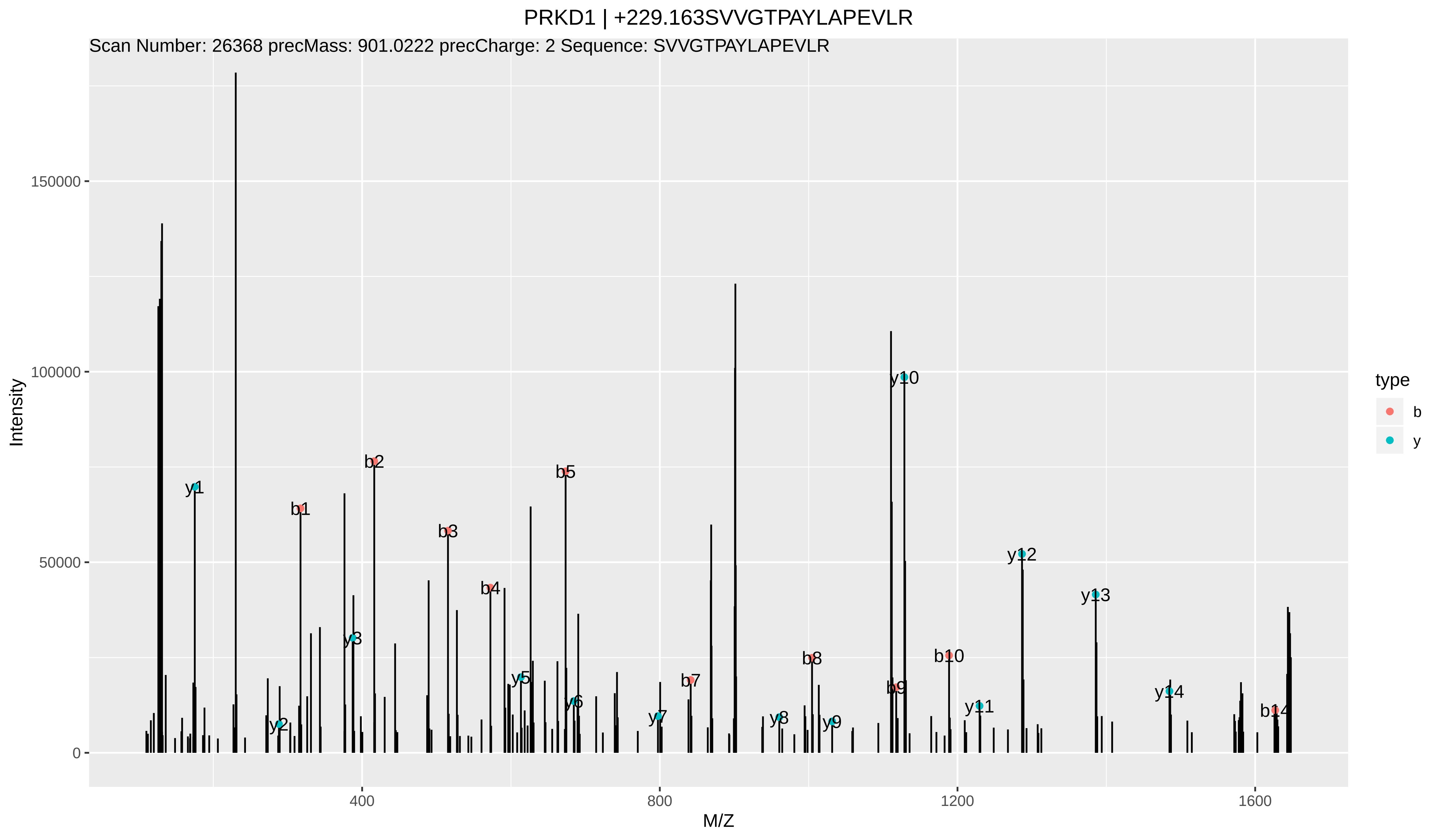

PRKD1 | +229.163SVVGTPAYLAPEVLR
Scan Number: 26368 precMass: 901.0222 precCharge: 2 Sequence: SVVGTPAYLAPEVLR
Intensity
150000
100000
50000
0
M/Z
400
800
1200
1600
y1
b1
b2
y2
y3
b3
b4
y5
b5
y6
y7
b7
y8
b8
y9
b9
y10
b10
y11
y12
y13
y14
b14
type
b
y

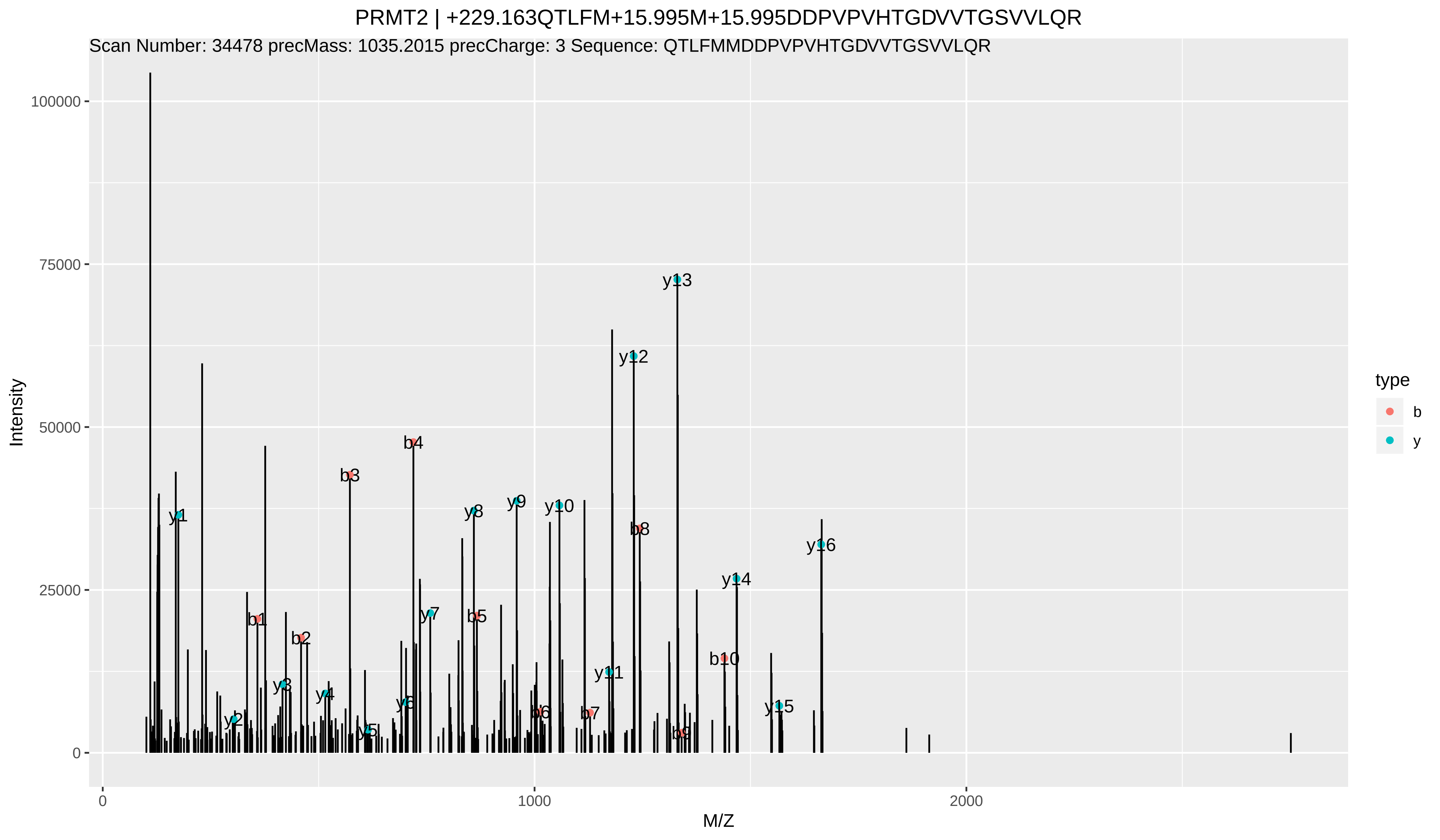
PRMT2 | +229.163QTLFM+15.995M+15.995DDPVPVHTGDVVTGSVVLQR
Scan Number: 34478 precMass: 1035.2015 precCharge: 3 Sequence: QTLFMMDDPVPVHTGDVVTGSVVLQR
Intensity
M/Z
type
b
y
0
25000
50000
75000
100000
0
1000
2000
y1
y2
y3
y4
y5
y6
y7
y8
y9
y10
y11
y12
y13
y14
y15
y16
b1
b2
b3
b4
b5
b6
b7
b8
b9
b10

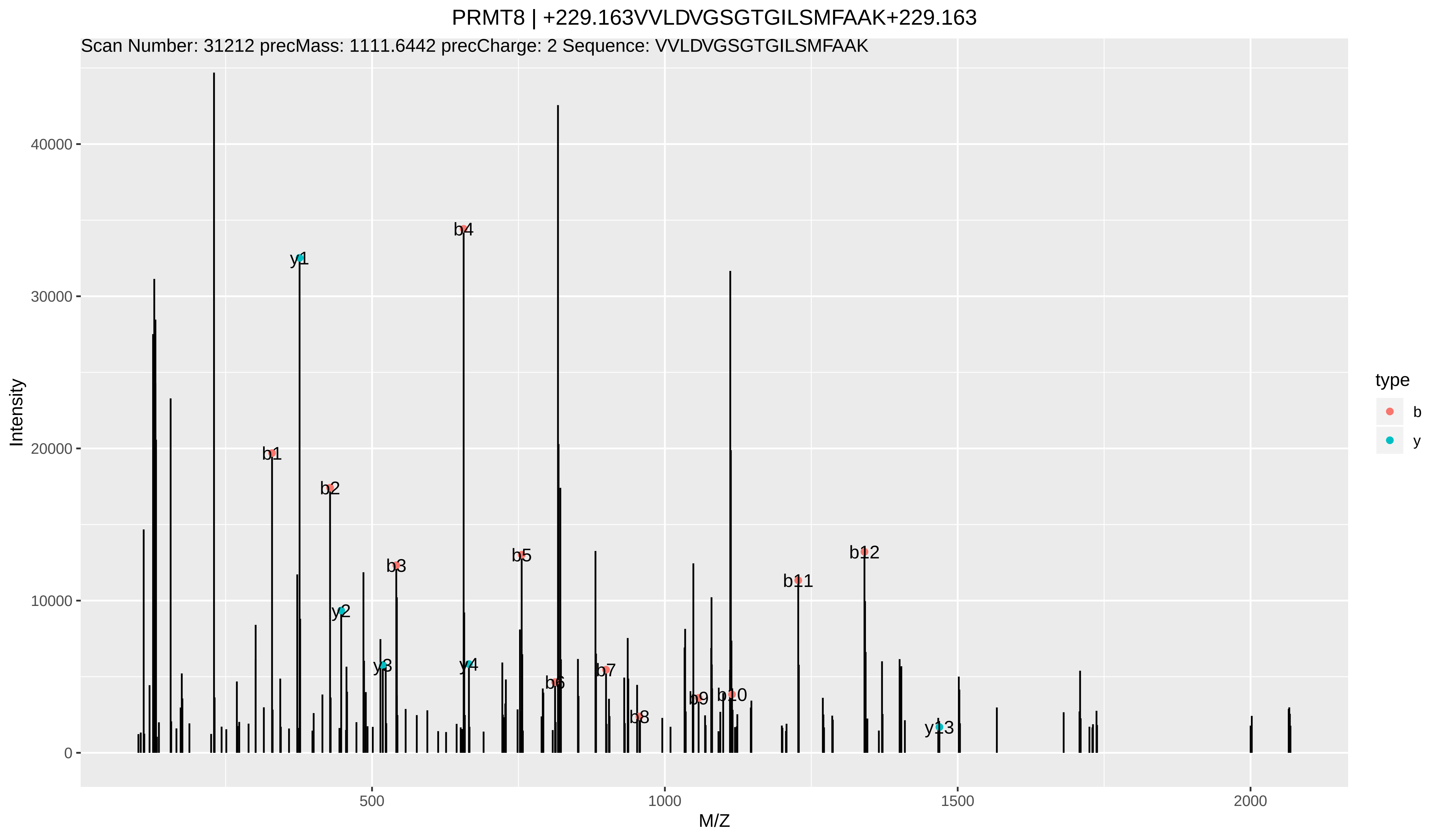

PRMT8 | +229.163VVLDVGSGTGILSMFAAK+229.163
Scan Number: 31212 precMass: 1111.6442 precCharge: 2 Sequence: VVLDVGSGTGILSMFAAK
Intensity
M/Z
0
10000
20000
30000
40000
500
1000
1500
2000
b1
b2
b3
b4
b5
b6
b7
b8
b9
b10
b11
b12
y1
y2
y3
y4
y13
type
b
y

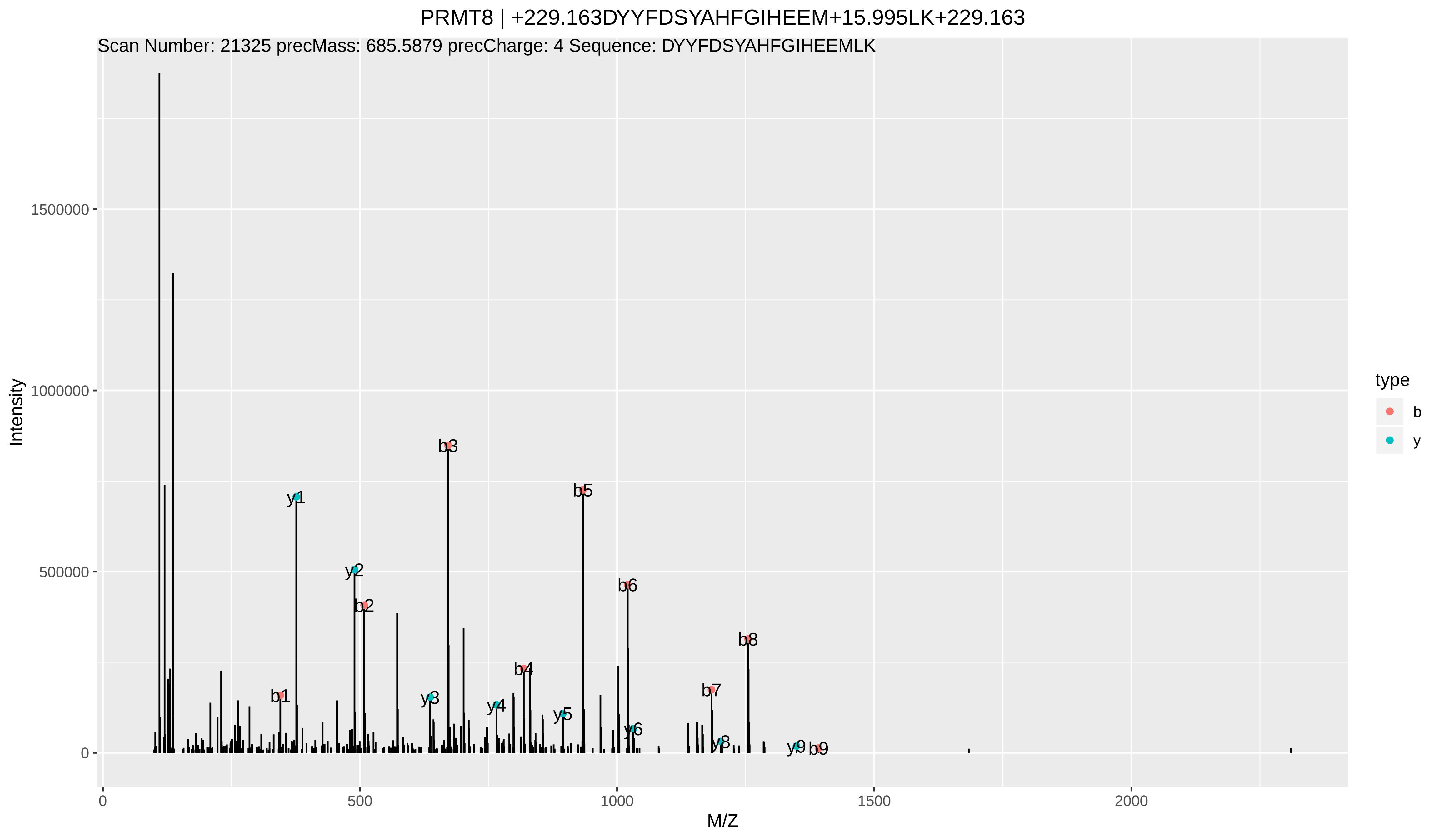
PRMT8 | +229.163DYYFDSYAHFGIHEEM+15.995LK+229.163
Scan Number: 21325 precMass: 685.5879 precCharge: 4 Sequence: DYYFDSYAHFGIHEEMLK
Intensity
1500000
1000000
500000
0
M/Z
0
500
1000
1500
2000
b1
b2
b3
b4
b5
b6
b7
b8
b9
y1
y2
y3
y4
y5
y6
y8
y9
type
b
y

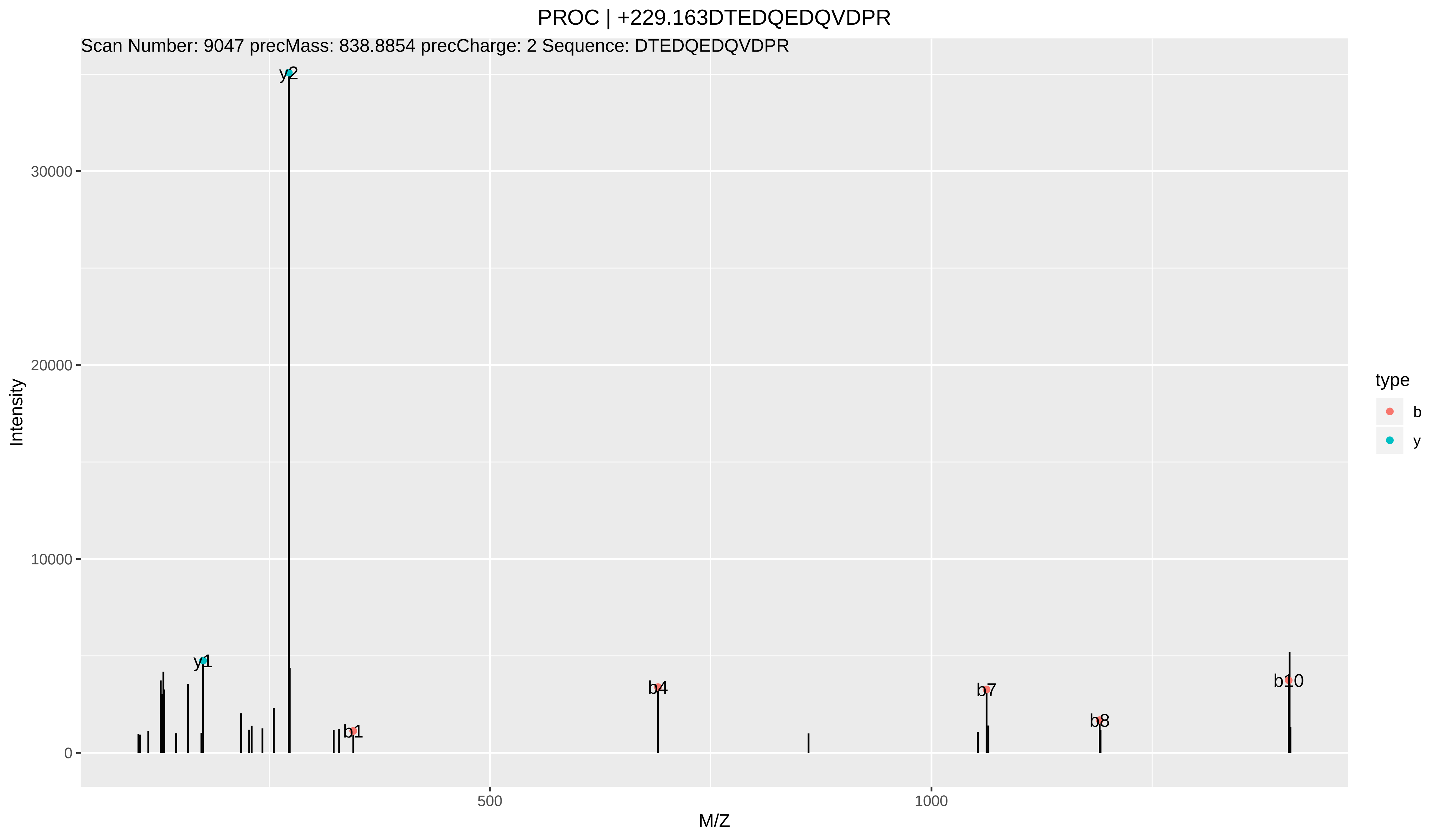

PROC | +229.163DTEDQEDQVDPR
Scan Number: 9047 precMass: 838.8854 precCharge: 2 Sequence: DTEDQEDQVDPR
Intensity
30000
20000
10000
0
M/Z
500
1000
y2
y1
b1
b4
b7
b8
b10
type
b
y

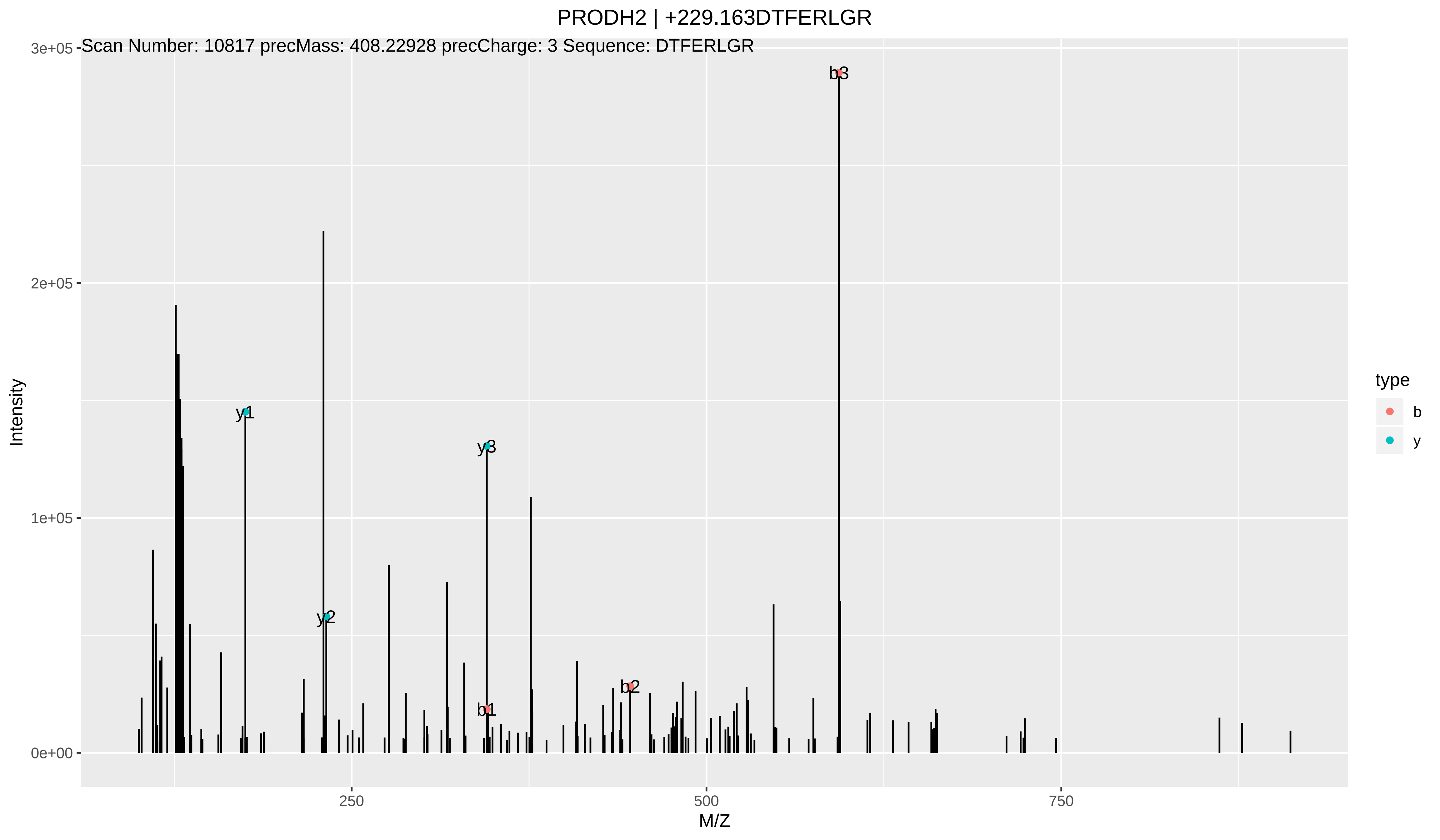

PRODH2 | +229.163DTFERLGR
Scan Number: 10817 precMass: 408.22928 precCharge: 3 Sequence: DTFERLGR
Intensity
3e+05
2e+05
1e+05
0e+00
M/Z
250
500
750
b3
y1
y3
y2
b2
b1
type
b
y

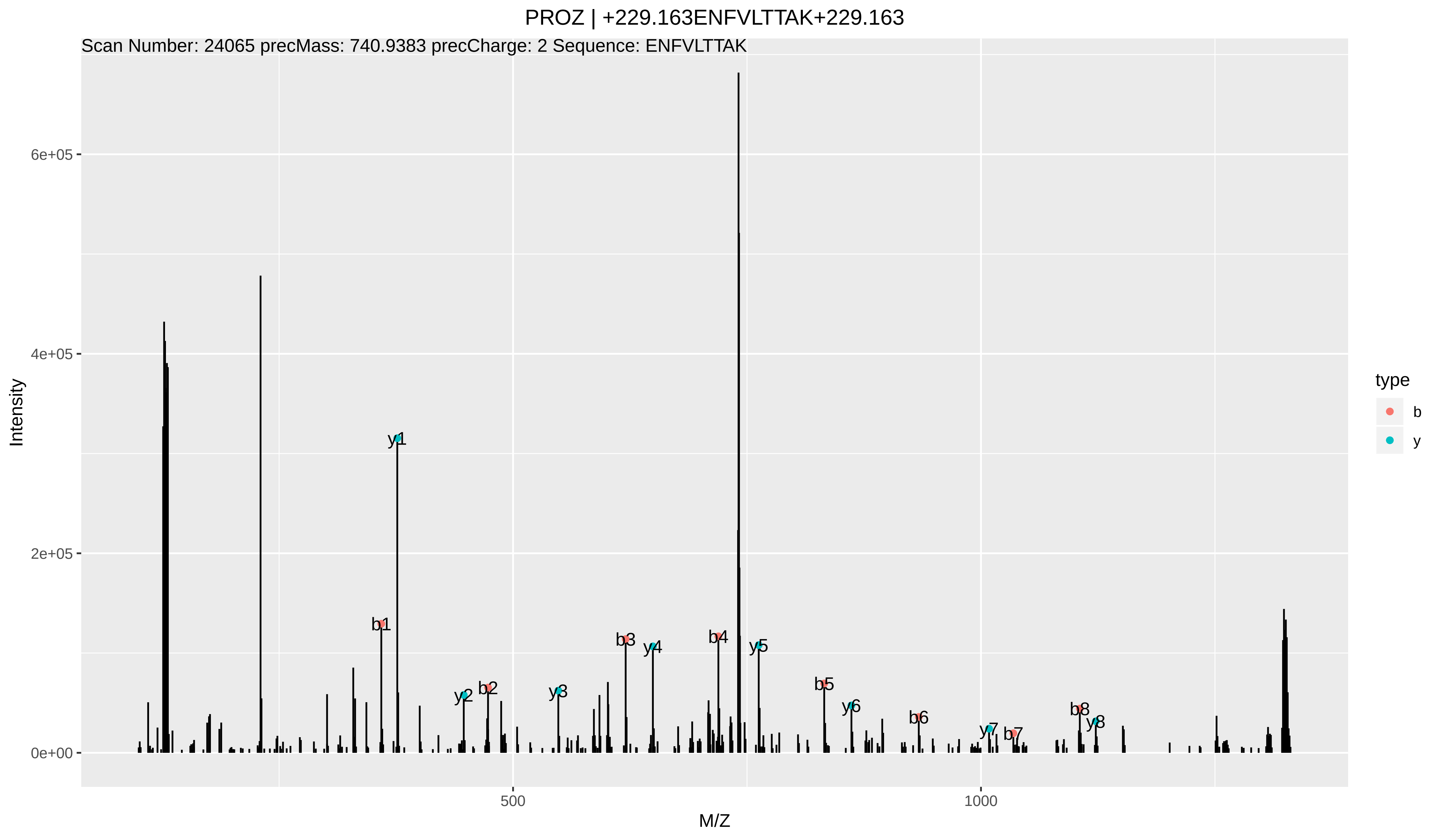

PROZ | +229.163ENFVLTTAK+229.163
Scan Number: 24065 precMass: 740.9383 precCharge: 2 Sequence: ENFVLTTAK
Intensity
6e+05
4e+05
2e+05
0e+00
M/Z
500
1000
y1
b1
y2
b2
y3
b3
y4
b4
y5
b5
y6
b6
y7
b7
b8
y8
type
b
y

PRR5 | +229.163SYNTPLLNPVQEHEAEGAAAGGTSIR

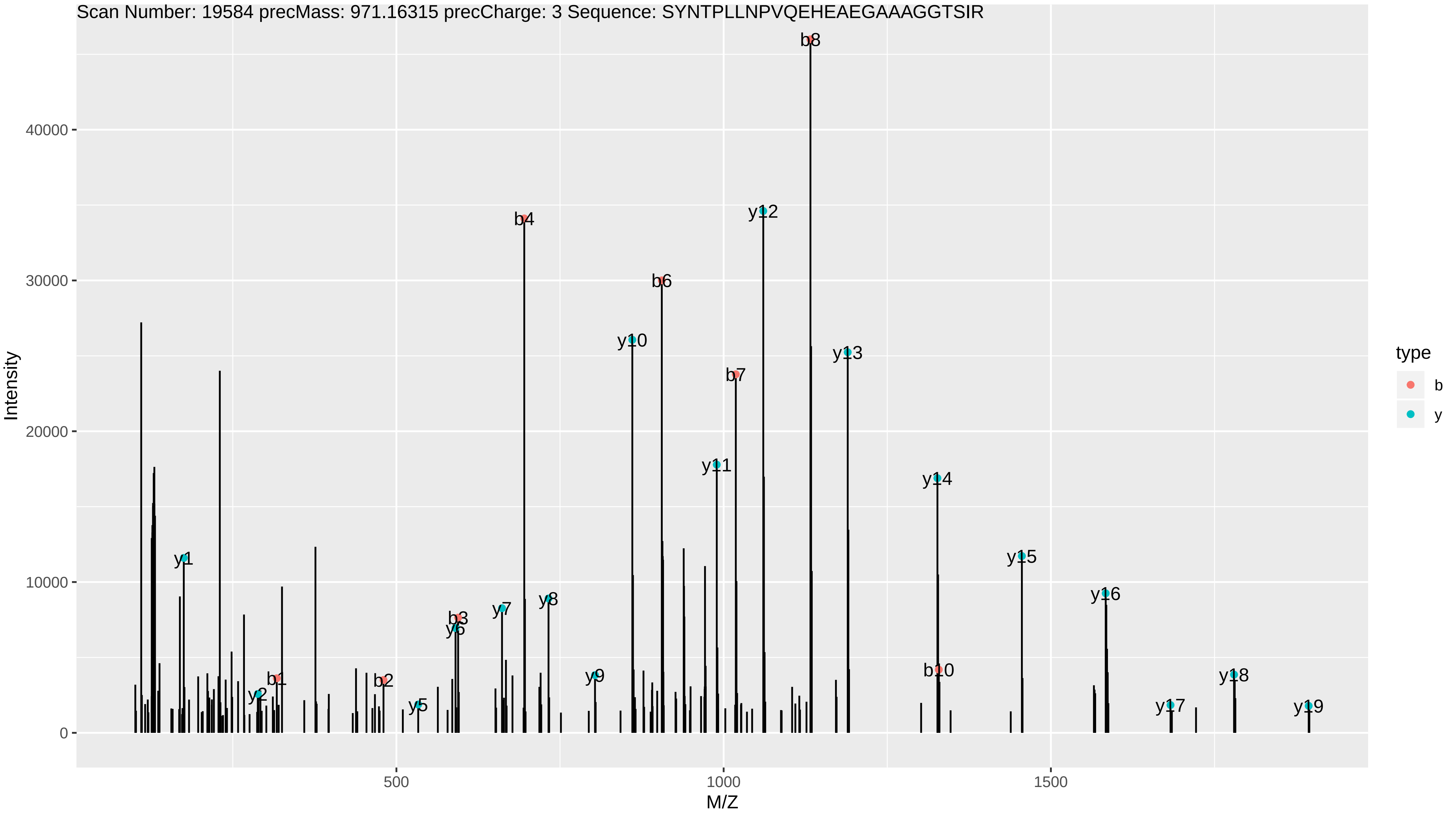

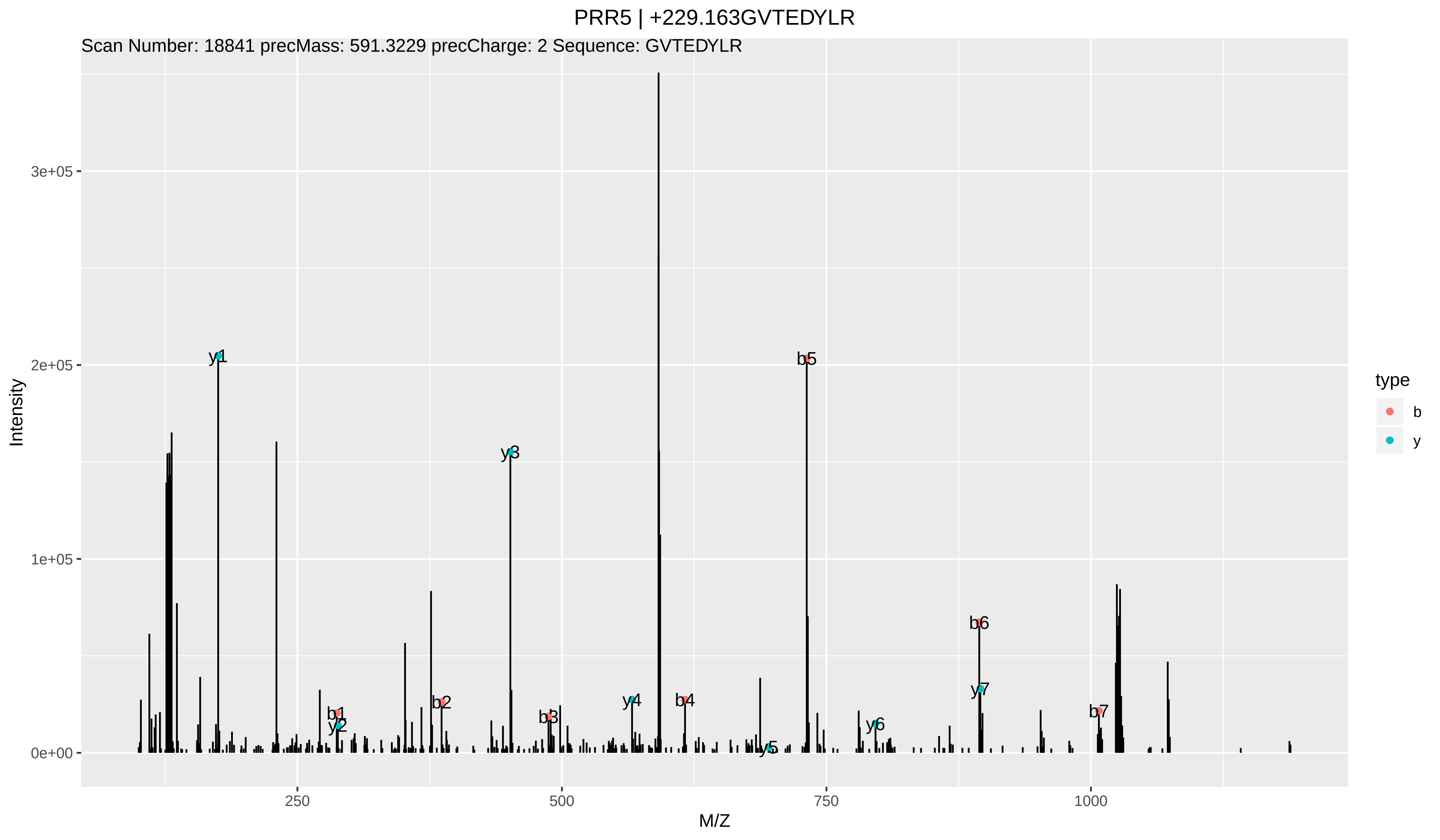
PRR5 | +229.163GVTEDYLR
Scan Number: 18841 precMass: 591.3229 precCharge: 2 Sequence: GVTEDYLR
Intensity
M/Z
3e+05
2e+05
1e+05
0e+00
250
500
750
1000
y1
y2
y3
y4
y5
y6
y7
b1
b2
b3
b4
b5
b6
b7
type
b
y

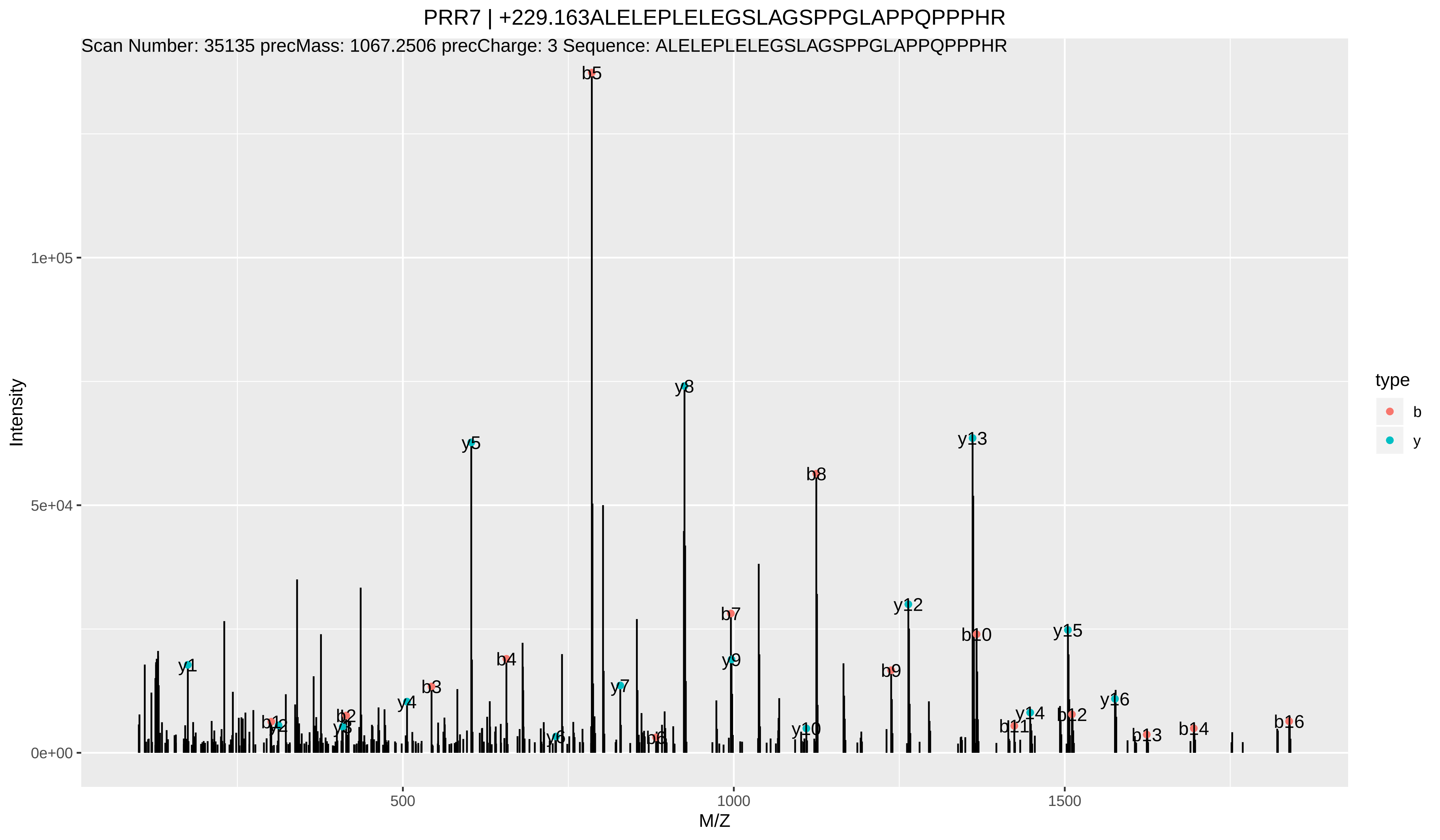
PRR7 | +229.163ALELEPLELEGSLAGSPPGLAPPQPPPHR
Scan Number: 35135 precMass: 1067.2506 precCharge: 3 Sequence: ALELEPLELEGSLAGSPPGLAPPQPPPHR
Intensity
M/Z
0e+00
5e+04
1e+05
500
1000
1500
type
b
y
b5
y8
y5
y13
b8
y12
b7
b10
y15
y1
b4
y9
b9
b3
y7
y4
y16
b2
b1
y2
y3
y6
b6
y10
b11
y14
b12
b13
b14
b16

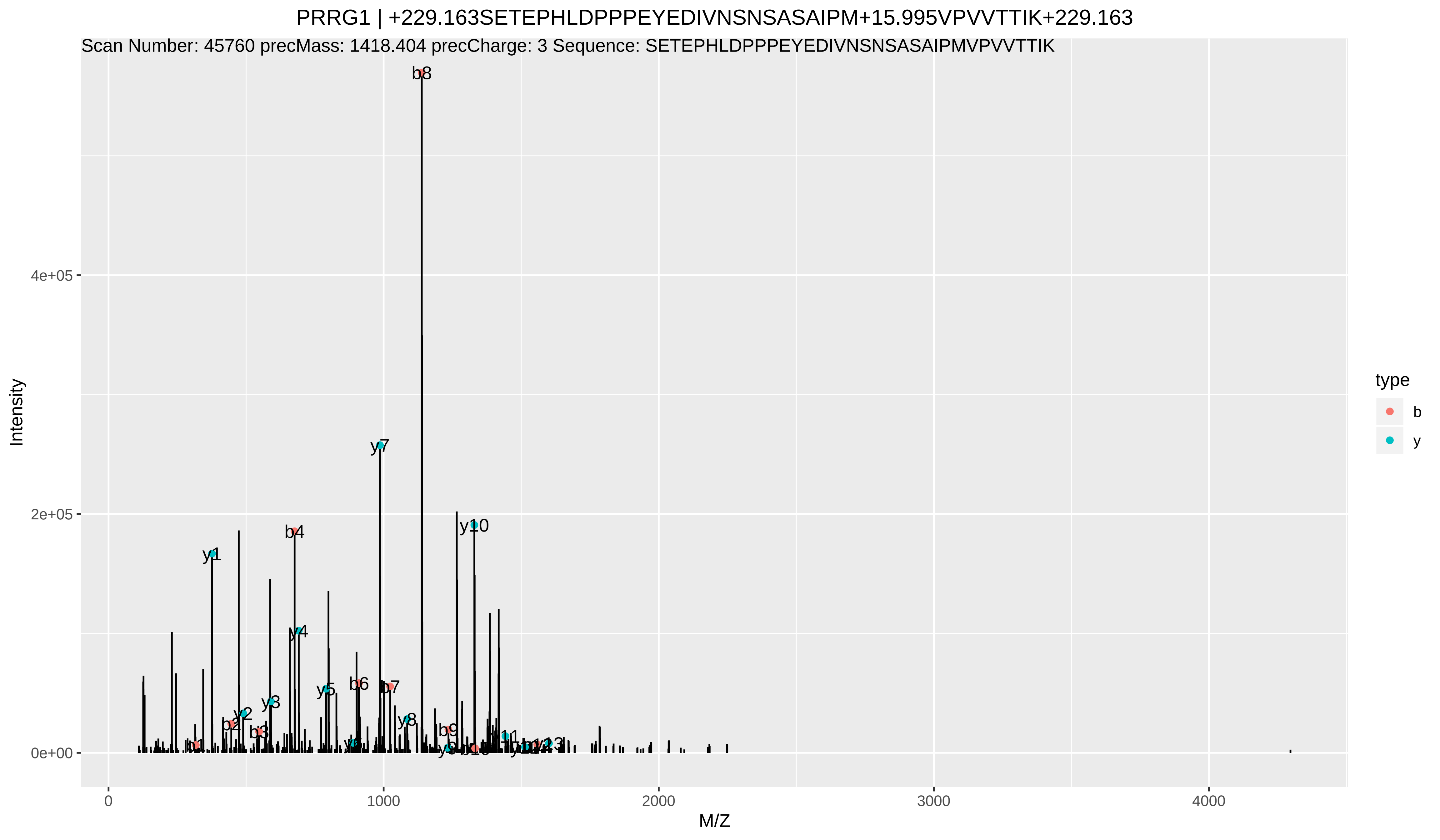

PRRG1 | +229.163SETEPHLDPPPEYEDIVNSNSASAIPM+15.995VPVVTTIK+229.163
Scan Number: 45760 precMass: 1418.404 precCharge: 3 Sequence: SETEPHLDPPPEYEDIVNSNSASAIPMVPVVTTIK
Intensity
M/Z
type
b
y

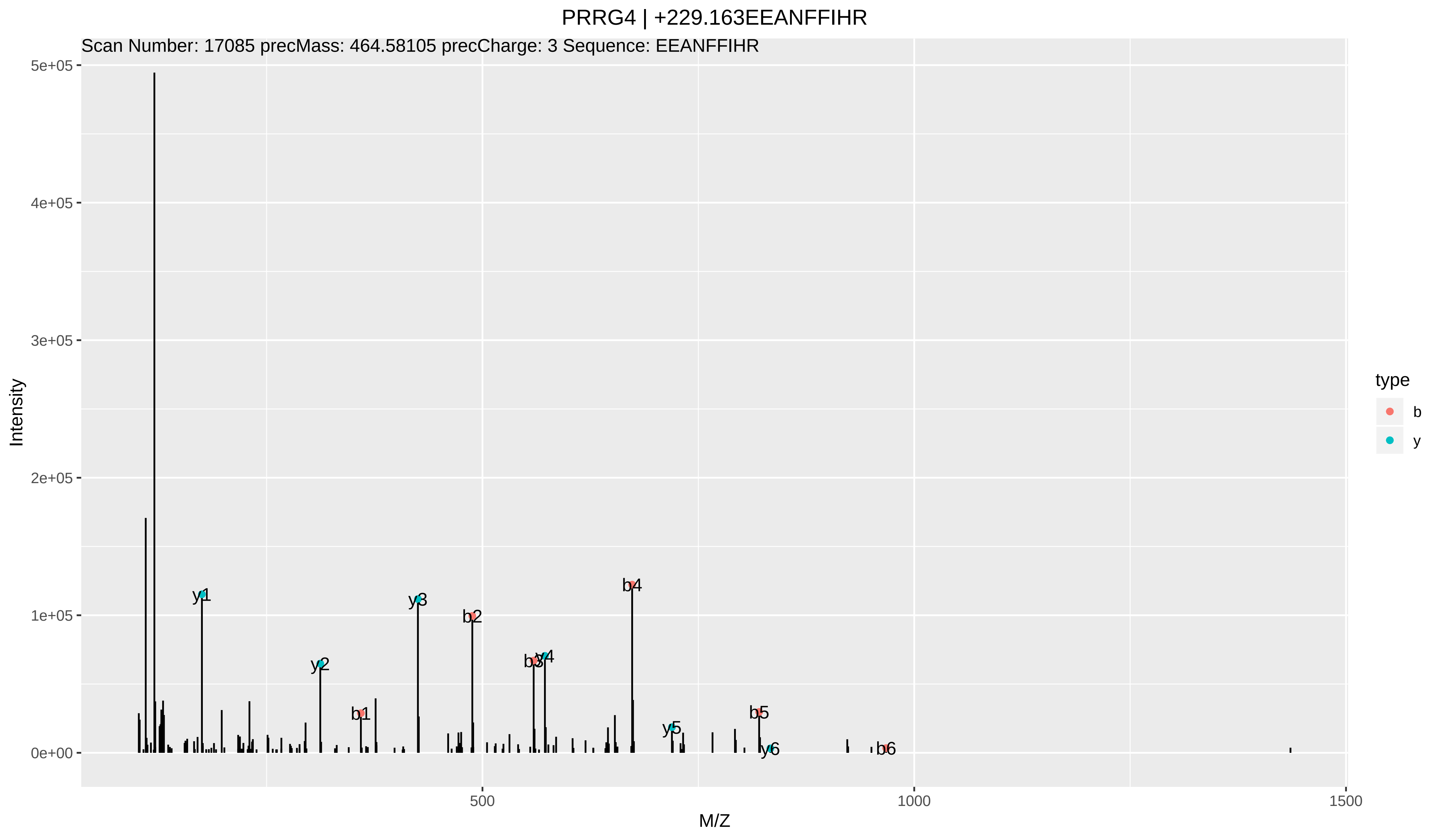

PRRG4 | +229.163EEANFFIHR
Scan Number: 17085 precMass: 464.58105 precCharge: 3 Sequence: EEANFFIHR
Intensity
5e+05
4e+05
3e+05
2e+05
1e+05
0e+00
y1
y2
b1
y3
b2
b3
y4
b4
y5
b5
y6
b6
500
1000
1500
M/Z
type
b
y

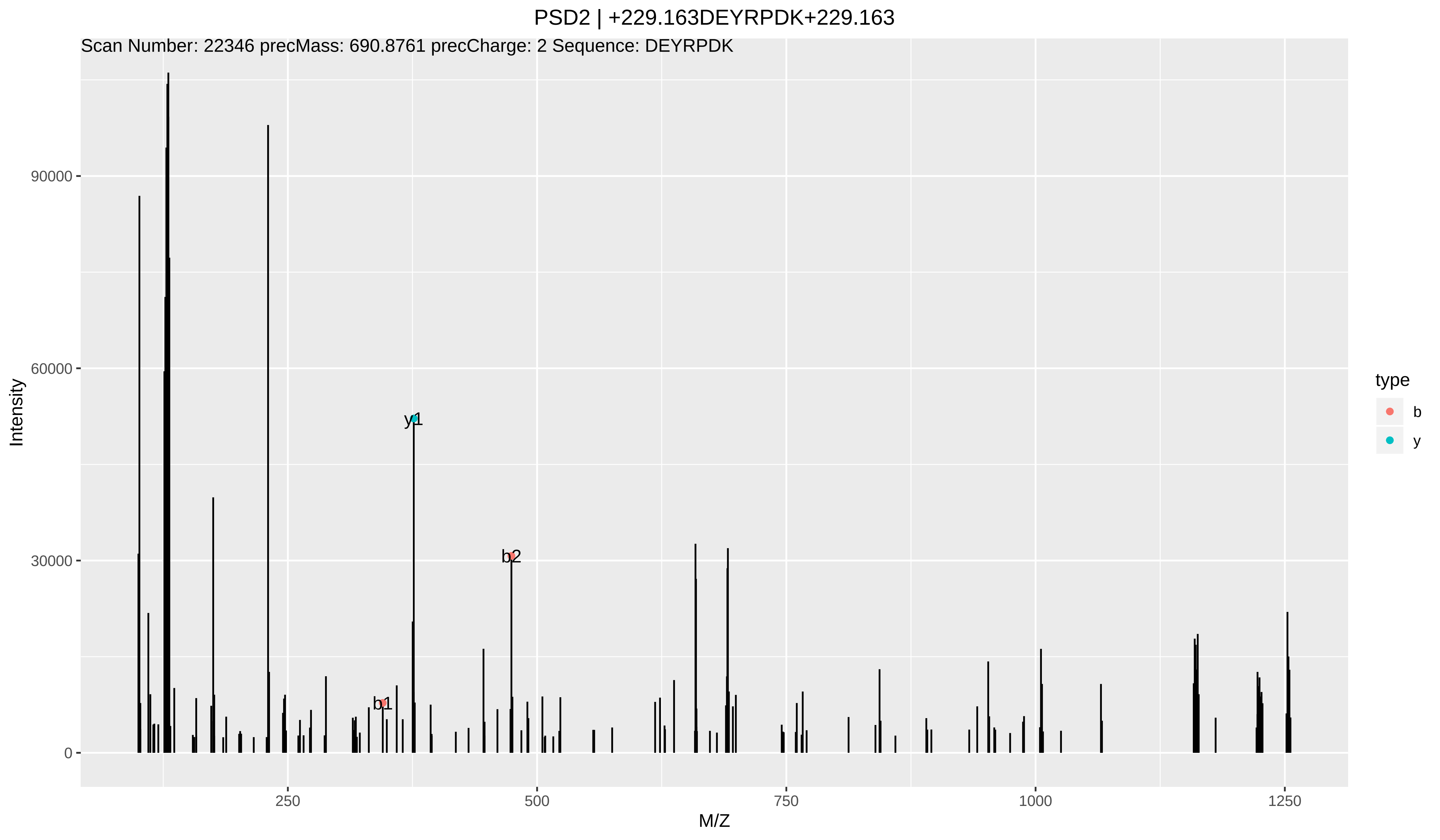
PSD2 | +229.163DEYRPDK+229.163
Scan Number: 22346 precMass: 690.8761 precCharge: 2 Sequence: DEYRPDK
y1
b2
b1
Intensity
90000
60000
30000
0
250
500
750
1000
1250
M/Z
type
b
y

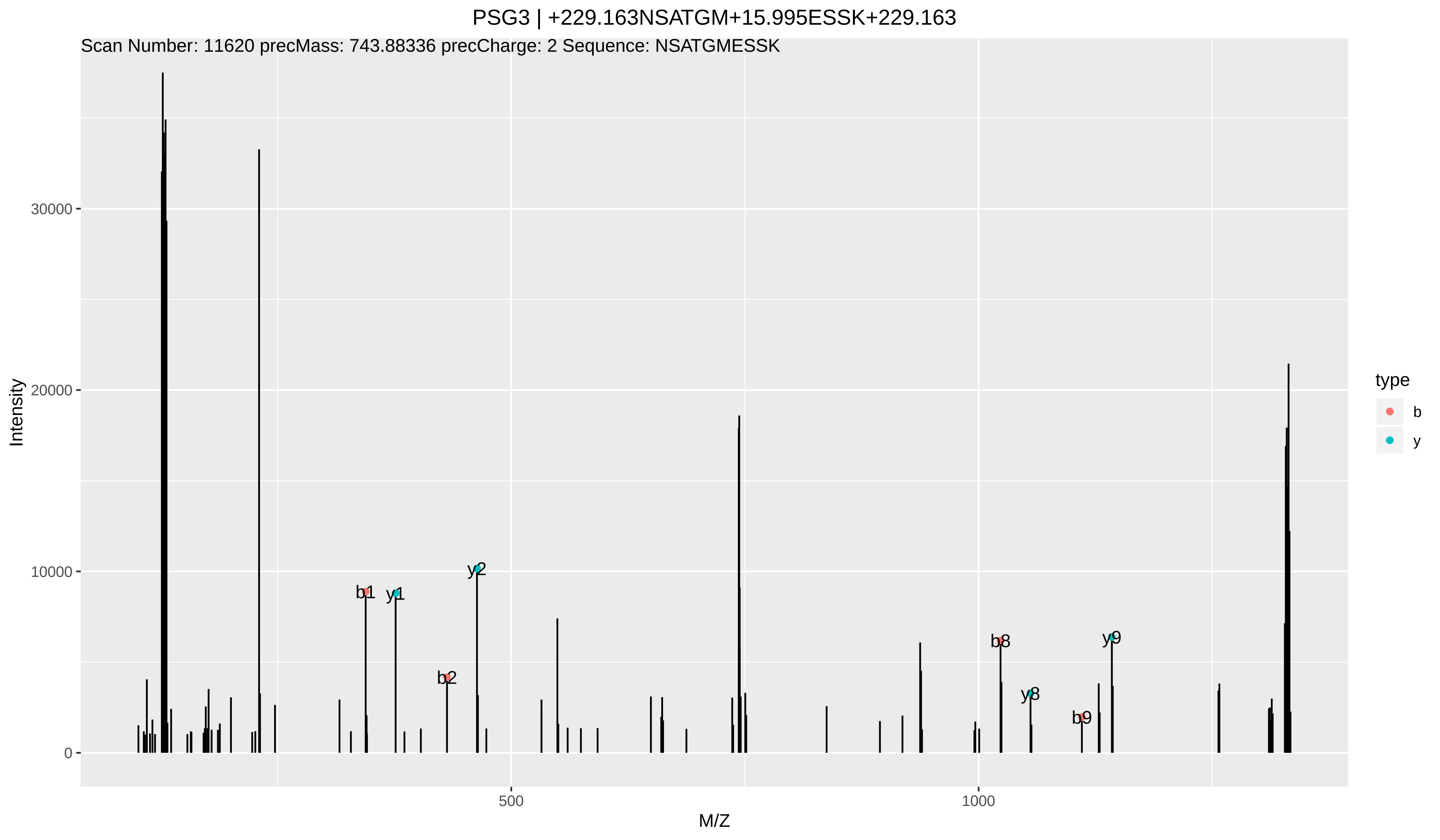
PSG3 | +229.163NSATGM+15.995ESSK+229.163
Scan Number: 11620 precMass: 743.88336 precCharge: 2 Sequence: NSATGMESSK
Intensity
30000
20000
10000
0
M/Z
500
1000
b1
y1
y2
b2
b8
y8
b9
y9
type
b
y

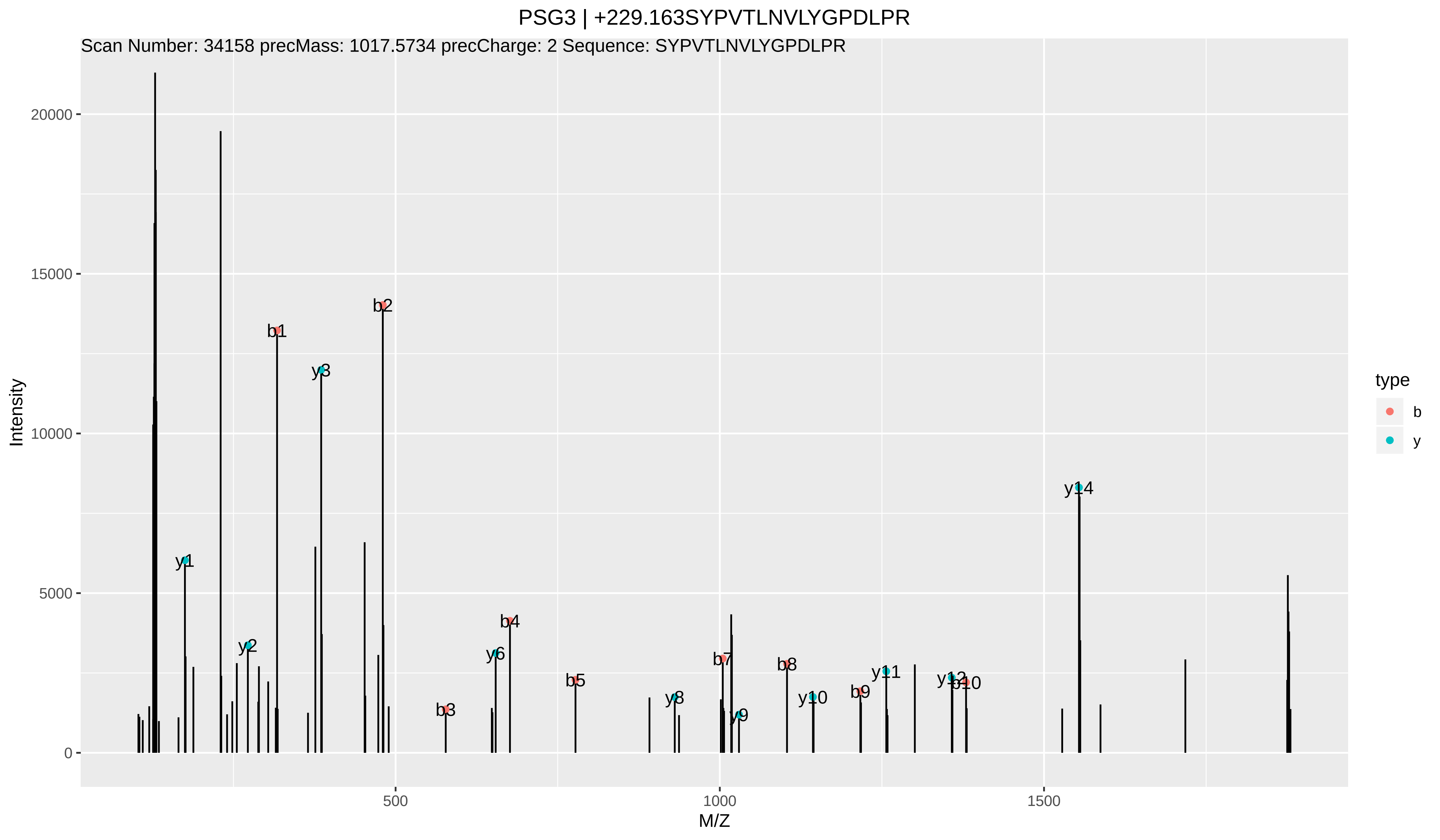

PSG3 | +229.163SYPVTLNVLYGPDLPR
Scan Number: 34158 precMass: 1017.5734 precCharge: 2 Sequence: SYPVTLNVLYGPDLPR
Intensity
M/Z
0
5000
10000
15000
20000
500
1000
1500
b1
b2
b3
b4
b5
b7
b8
b9
b10
y1
y2
y3
y6
y8
y9
y10
y11
y12
y14
type
b
y

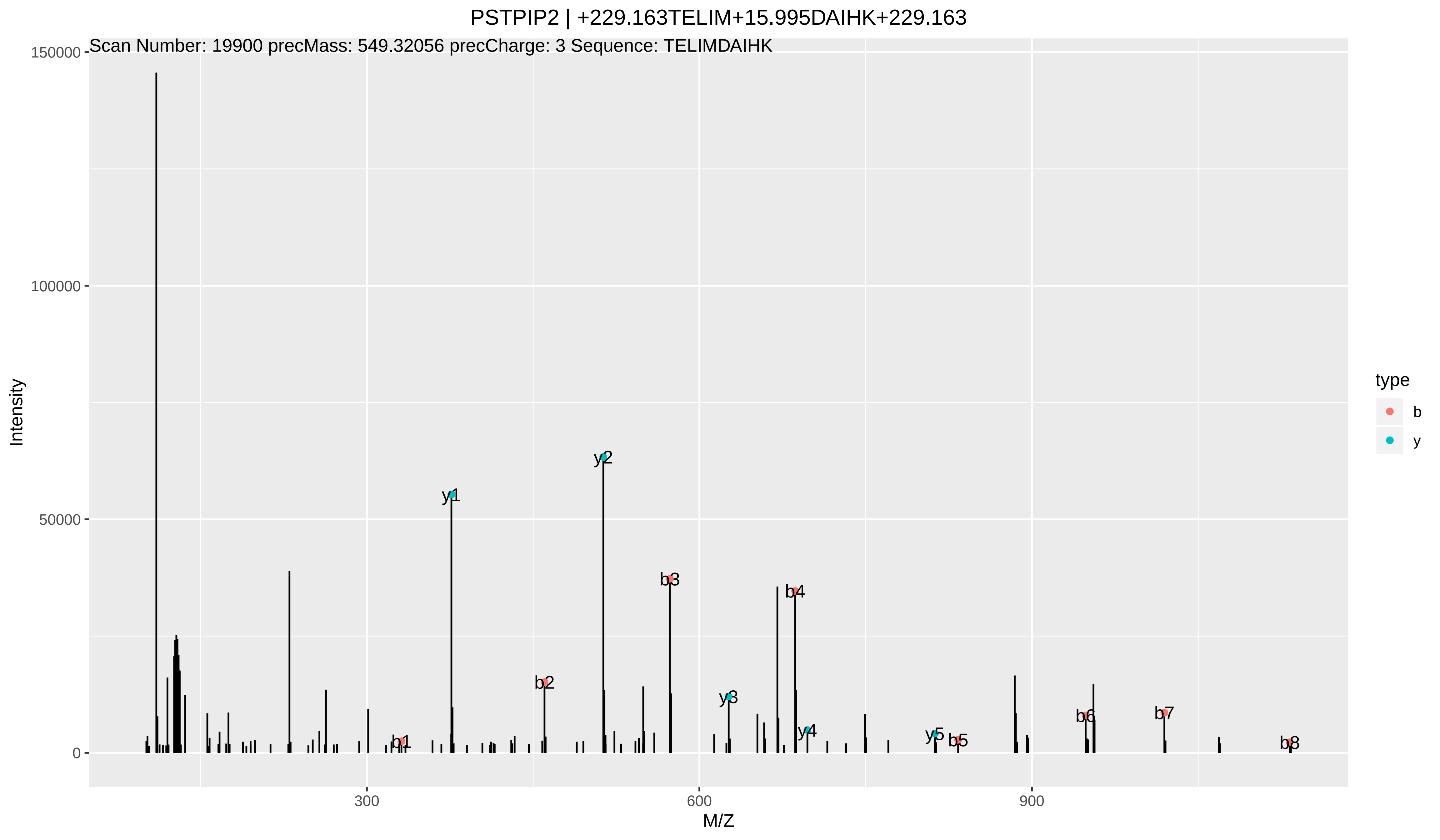

PSTPIP2 | +229.163TELIM+15.995DAIHK+229.163
Scan Number: 19900 precMass: 549.32056 precCharge: 3 Sequence: TELIMDAIHK
Intensity
150000
100000
50000
0
M/Z
300
600
900
y1
y2
b1
b2
b3
b4
y3
y4
y5
b5
b6
b7
b8
type
b
y

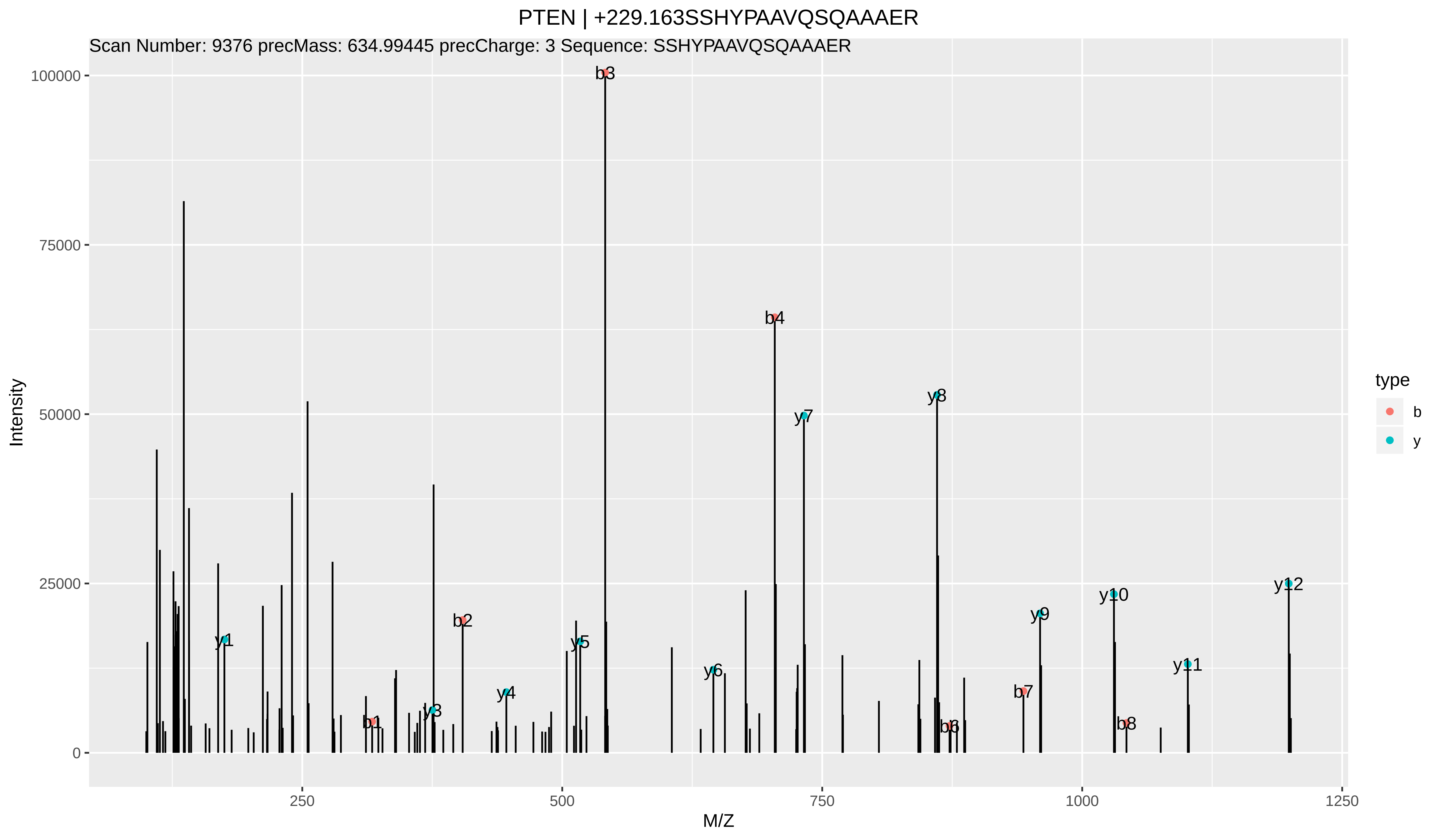
PTEN | +229.163SSHYPAAVQSQAAAER
Scan Number: 9376 precMass: 634.99445 precCharge: 3 Sequence: SSHYPAAVQSQAAAER
Intensity
M/Z
100000
75000
50000
25000
0
250
500
750
1000
1250
b3
b4
y8
y7
y12
y10
y9
b2
y1
y5
y6
y11
y4
b7
y3
b1
b6
b8
type
b
y

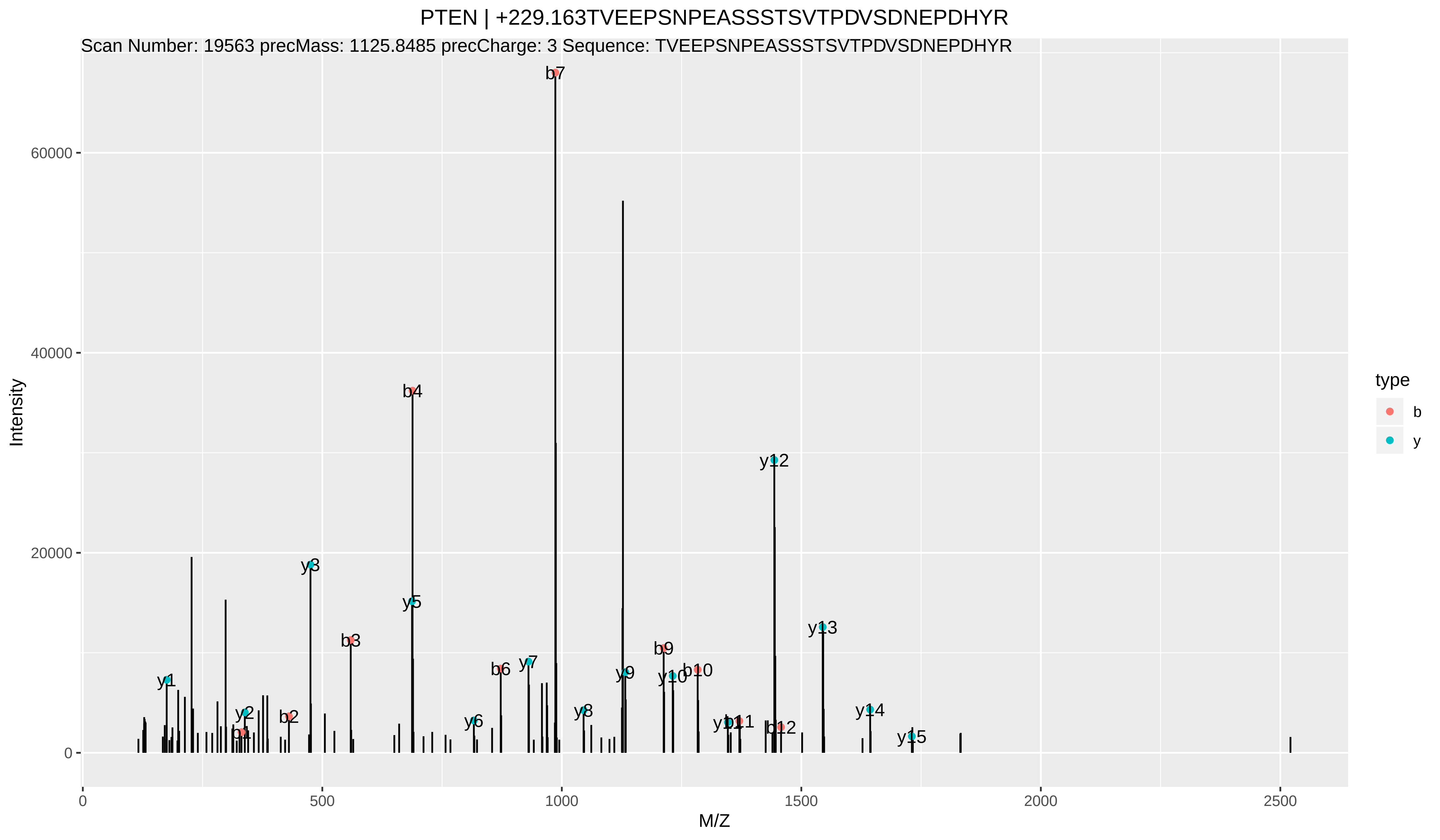

PTEN | +229.163TVEEPSNPEASSSTSVTPDVSDNEPDHYR
Scan Number: 19563 precMass: 1125.8485 precCharge: 3 Sequence: TVEEPSNPEASSSTSVTPDVSDNEPDHYR
Intensity
M/Z
type
b
y
b7
b4
y12
y3
y5
y13
b3
b9
y7
b6
b10
y1
y9
y10
y8
y14
y2
b2
y6
y11
b11
b12
b1
y15
0
20000
40000
60000
0
500
1000
1500
2000
2500

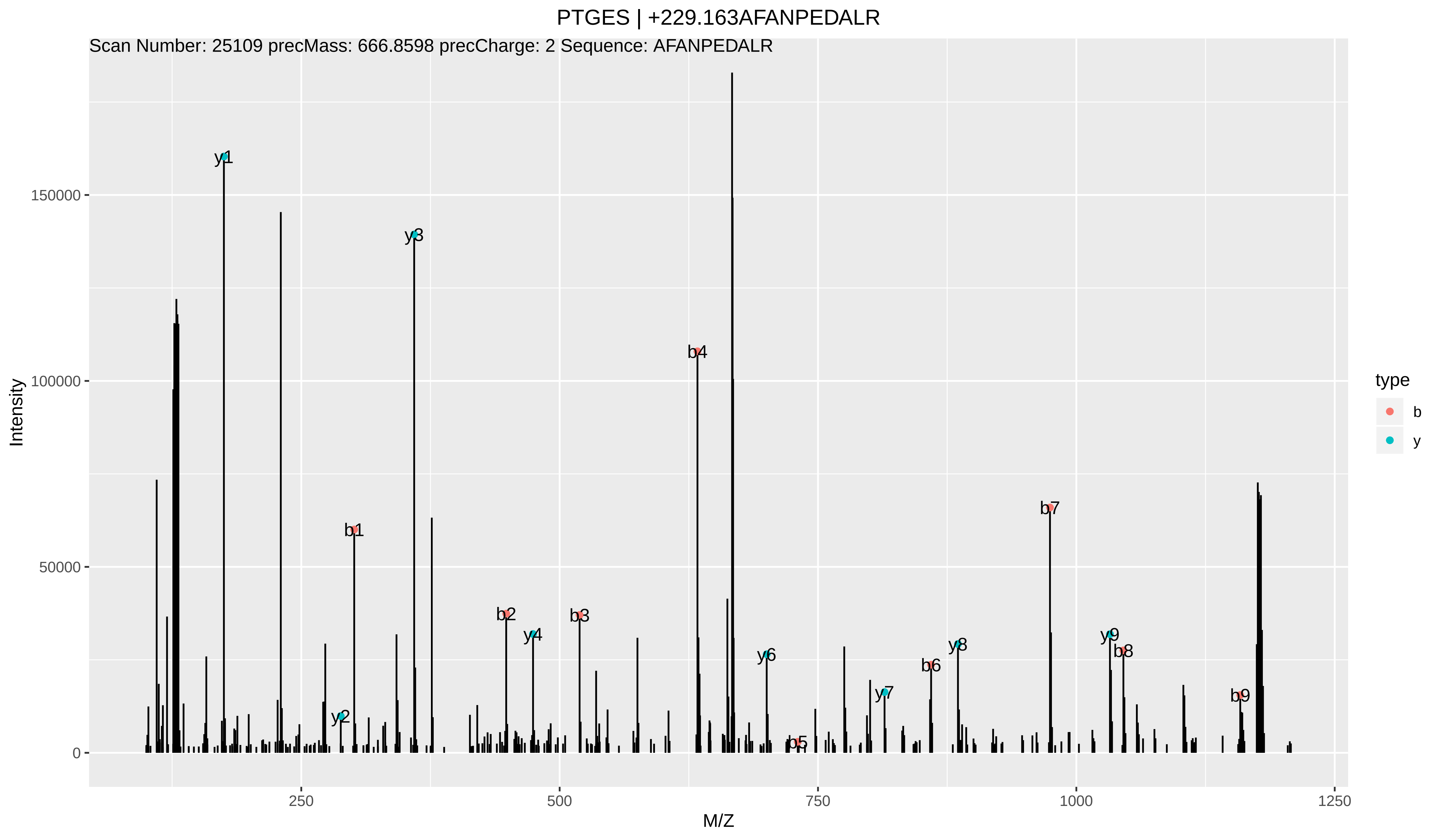

PTGES | +229.163AFANPEDALR
Scan Number: 25109 precMass: 666.8598 precCharge: 2 Sequence: AFANPEDALR
Intensity
M/Z
0
50000
100000
150000
250
500
750
1000
1250
y1
y3
b4
b1
b7
b2
b3
y4
y6
y8
y9
b8
b6
y7
b9
y2
b5
type
b
y

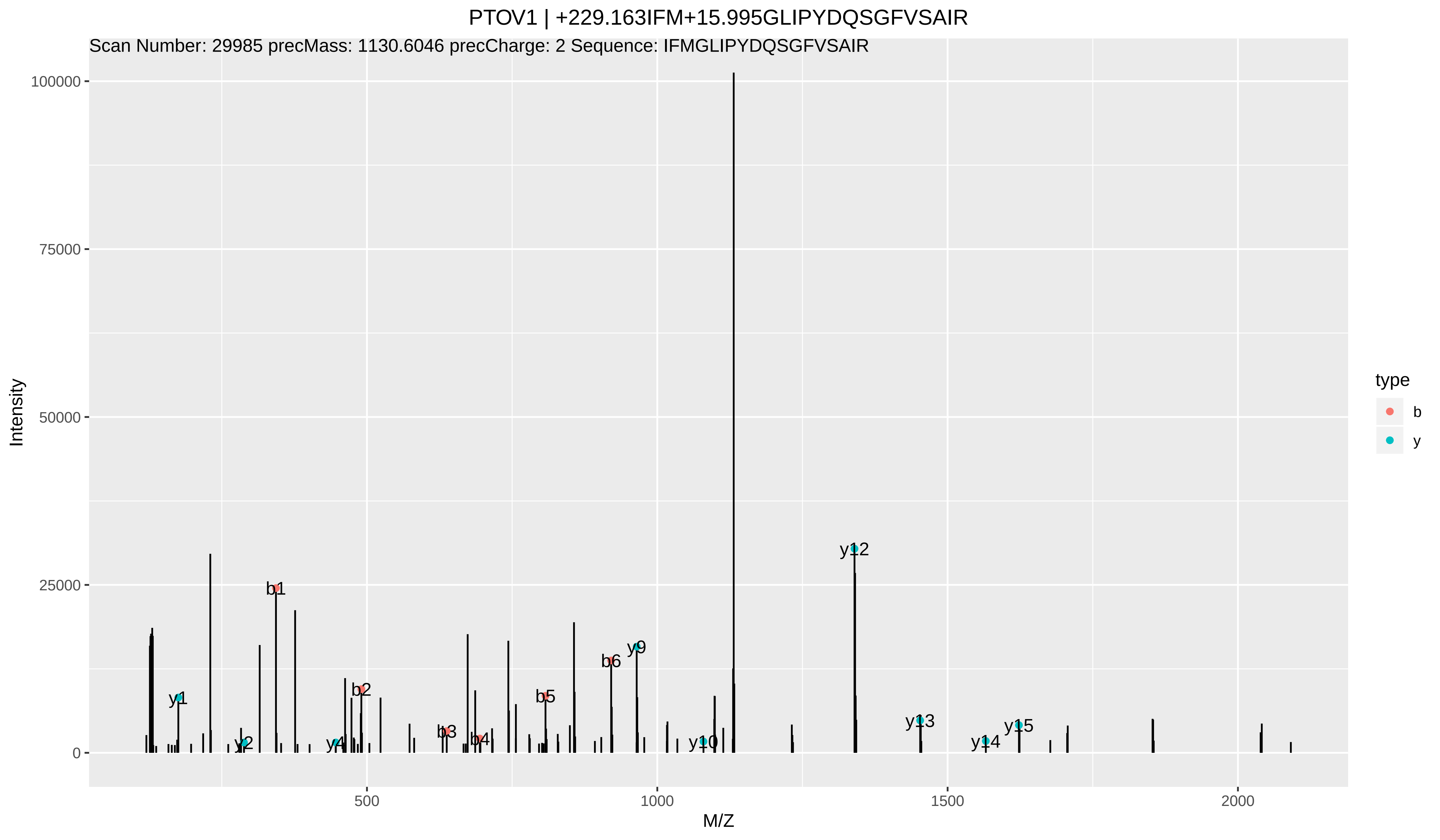
PTOV1 | +229.163IFM+15.995GLIPYDQSGFVSAIR
Scan Number: 29985 precMass: 1130.6046 precCharge: 2 Sequence: IFMGLIPYDQSGFVSAIR
Intensity
100000
75000
50000
25000
0
500
1000
1500
2000
M/Z
y1
y2
b1
y4
b2
b3
b4
b5
b6
y9
y10
y12
y13
y14
y15
type
b
y

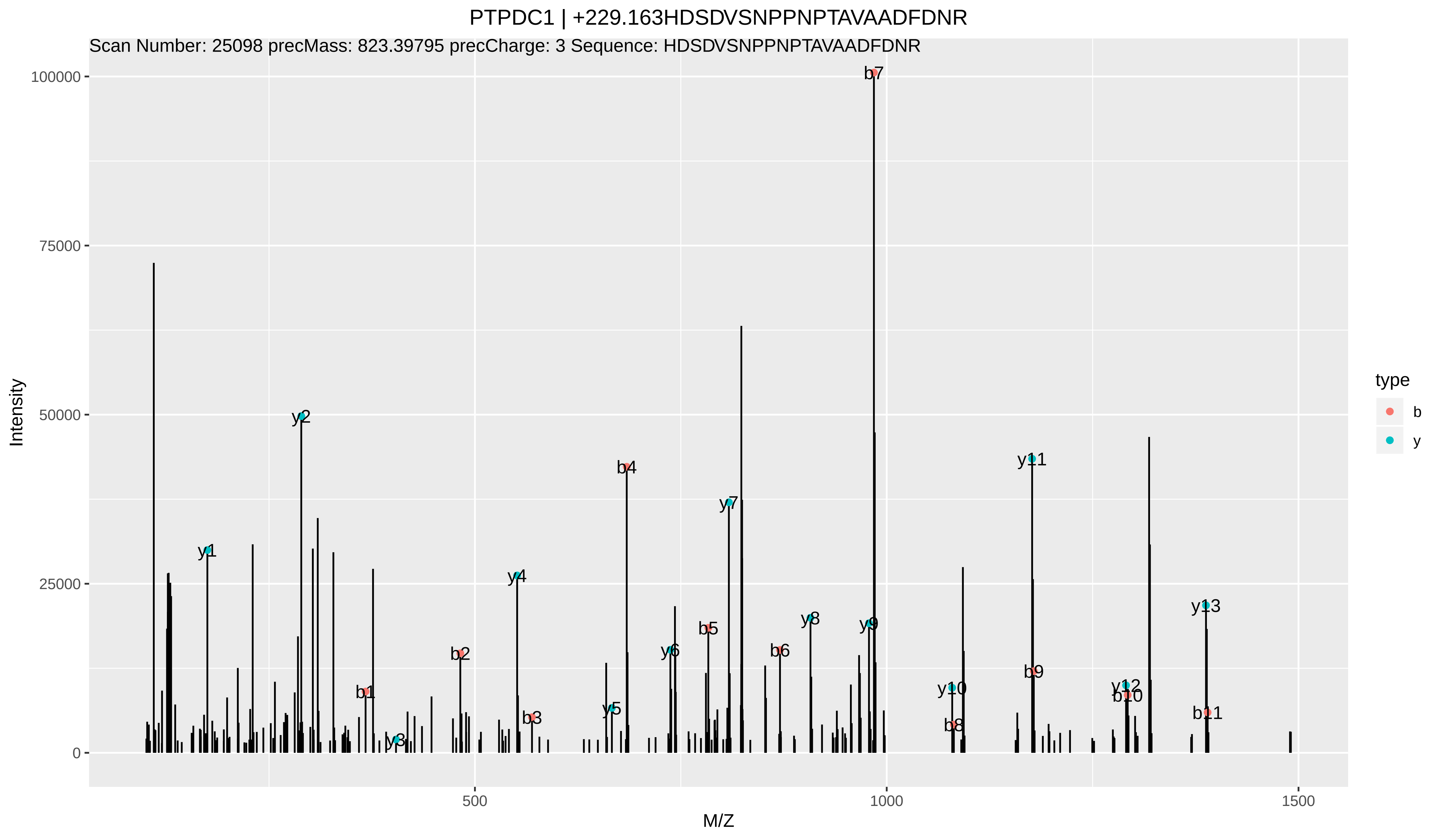

PTPDC1 | +229.163HDSDVSNPPNPTAVAADFDNR
Scan Number: 25098 precMass: 823.39795 precCharge: 3 Sequence: HDSDVSNPPNPTAVAADFDNR
Intensity
M/Z
0
25000
50000
75000
100000
500
1000
1500
b7
y2
y11
b4
y7
y1
y4
y13
b5
y8
y9
b2
b6
y6
b9
y10
y12
b10
b1
b3
y5
b11
b8
y3
type
b
y

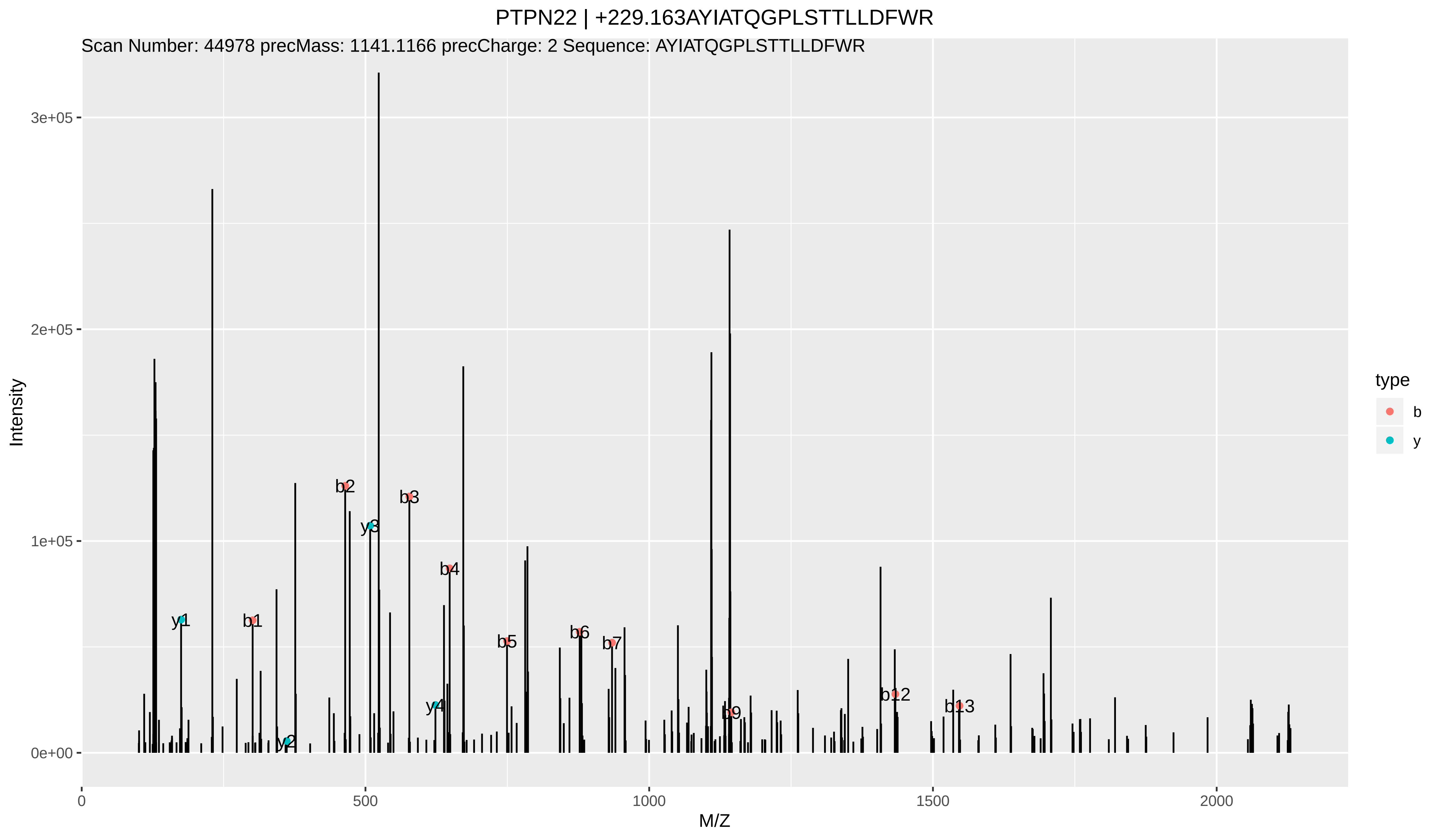

PTPN22 | +229.163AYIATQGPLSTTLLDFWR
Scan Number: 44978 precMass: 1141.1166 precCharge: 2 Sequence: AYIATQGPLSTTLLDFWR
Intensity
M/Z
type
b
y
y1
b1
y2
b2
y3
b3
y4
b4
b5
b6
b7
b9
b12
b13
3e+05
2e+05
1e+05
0e+00
0
500
1000
1500
2000

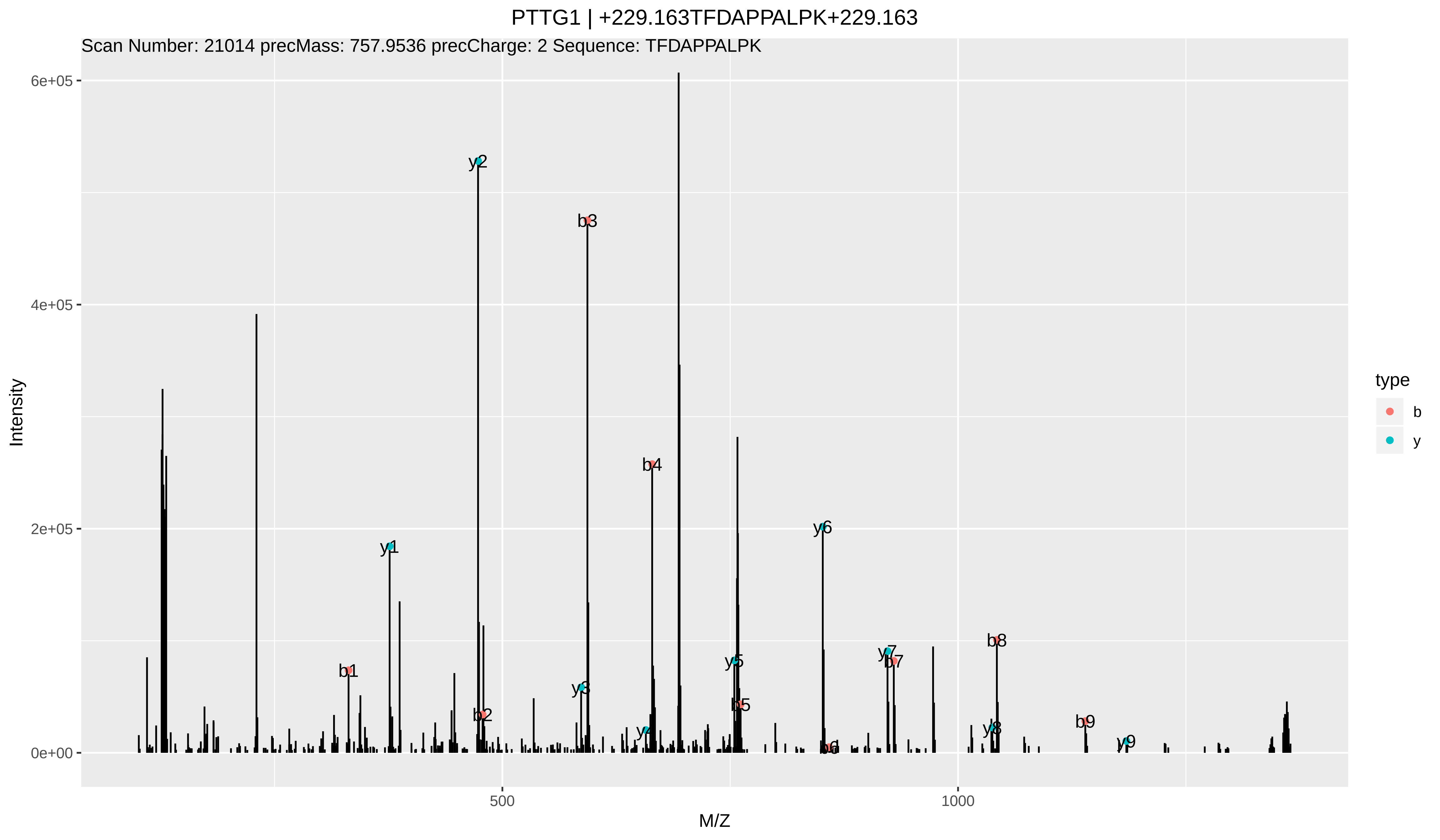

PTTG1 | +229.163TFDAPPALPK+229.163
Scan Number: 21014 precMass: 757.9536 precCharge: 2 Sequence: TFDAPPALPK
Intensity
M/Z
6e+05
4e+05
2e+05
0e+00
500
1000
y2
b3
b4
y6
y1
b1
y3
b2
y5
b5
y7
b7
b8
y8
b9
y9
type
b
y

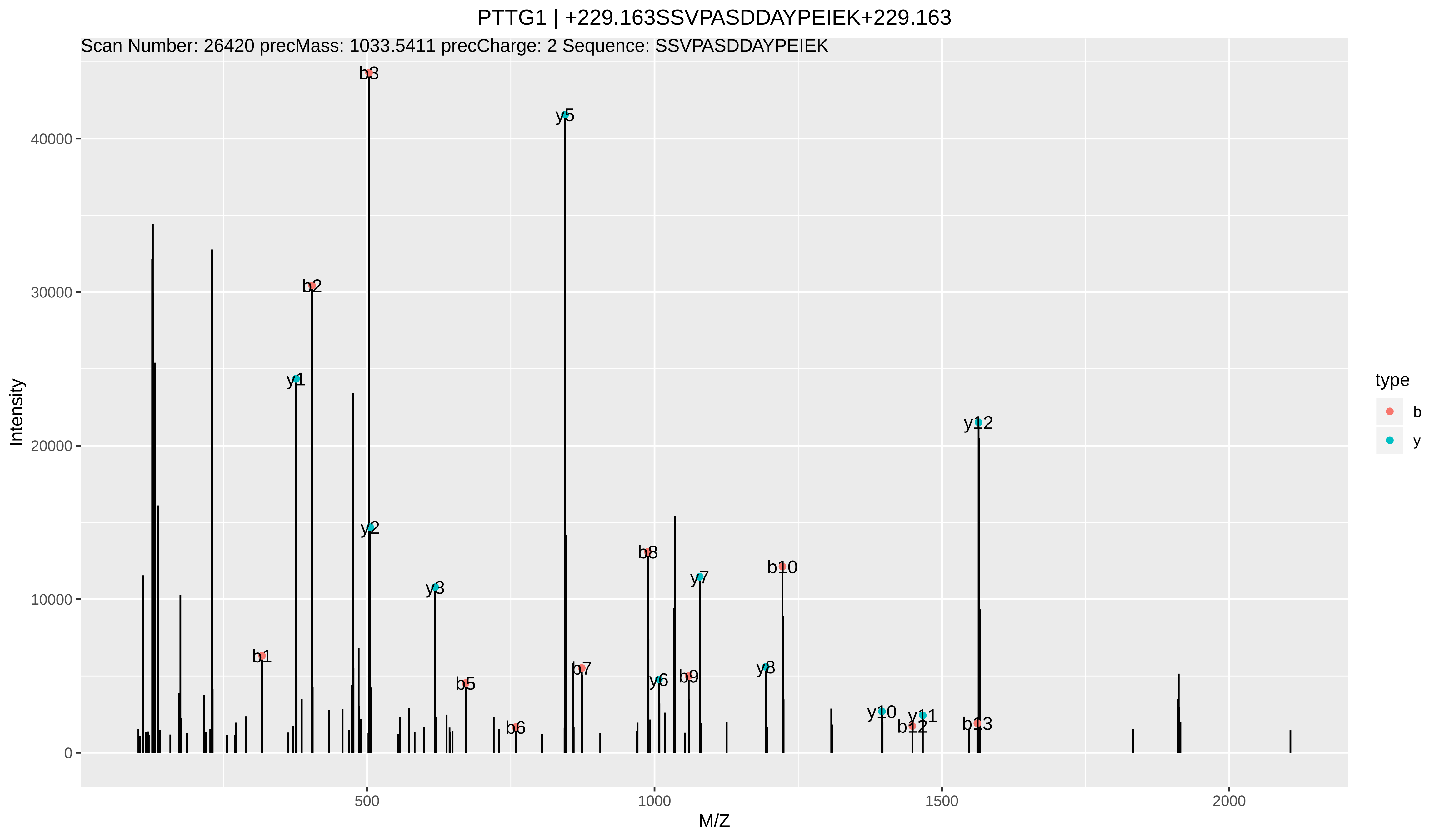
PTTG1 | +229.163SSVPASDDAYPEIEK+229.163
Scan Number: 26420 precMass: 1033.5411 precCharge: 2 Sequence: SSVPASDDAYPEIEK
Intensity
M/Z
0
10000
20000
30000
40000
500
1000
1500
2000
b3
y5
b2
y1
y2
b8
y7
b10
y3
b1
b5
b7
y6
b9
y8
b6
y10
y11
b12
b13
y12
type
b
y

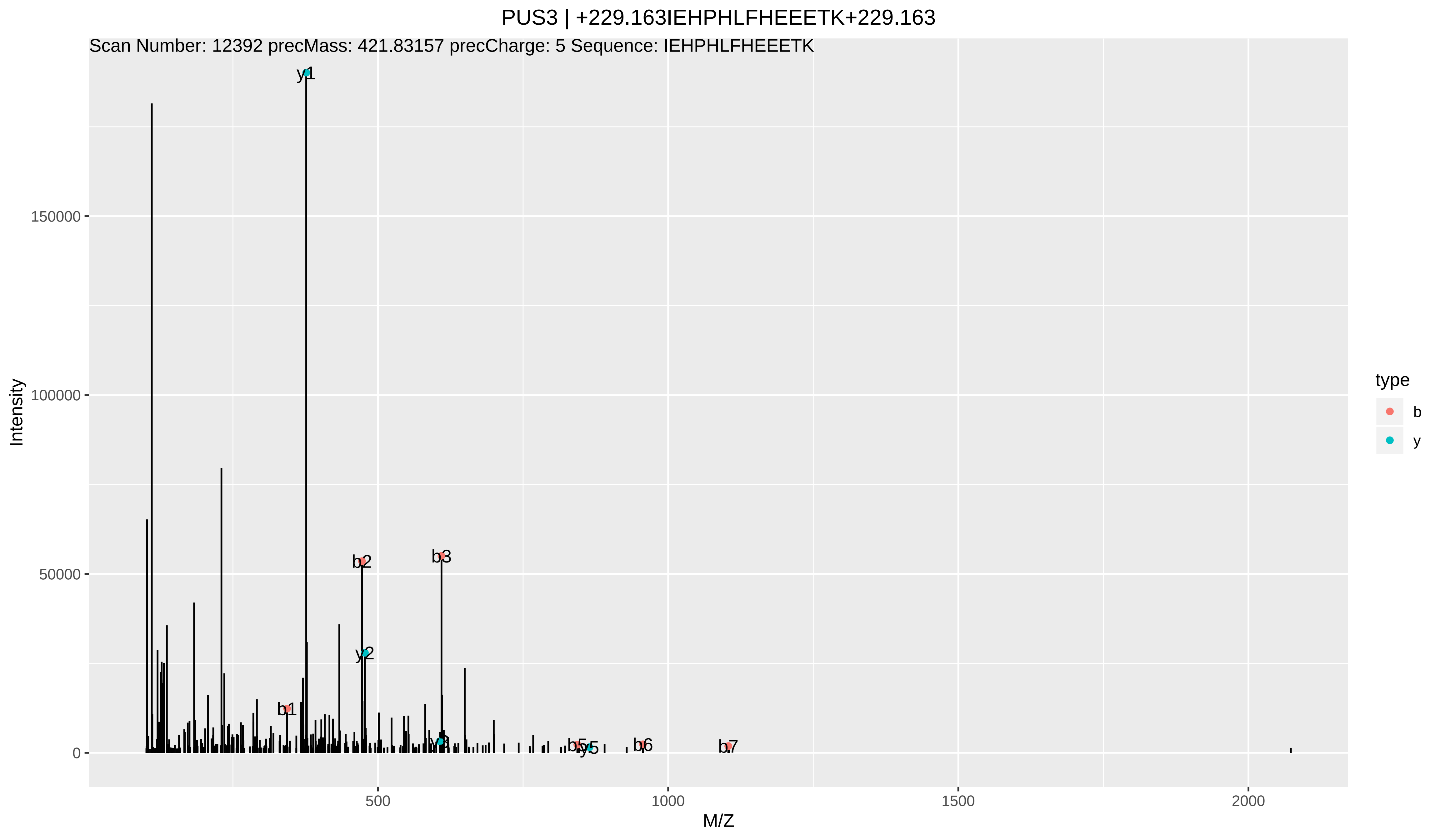

PUS3 | +229.163IEHPHLFHEEETK+229.163
Scan Number: 12392 precMass: 421.83157 precCharge: 5 Sequence: IEHPHLFHEEETK
y1
b2
b3
y2
b1
y3
b5
y5
b6
b7
150000
100000
50000
0
500
1000
1500
2000
M/Z
Intensity
type
b
y

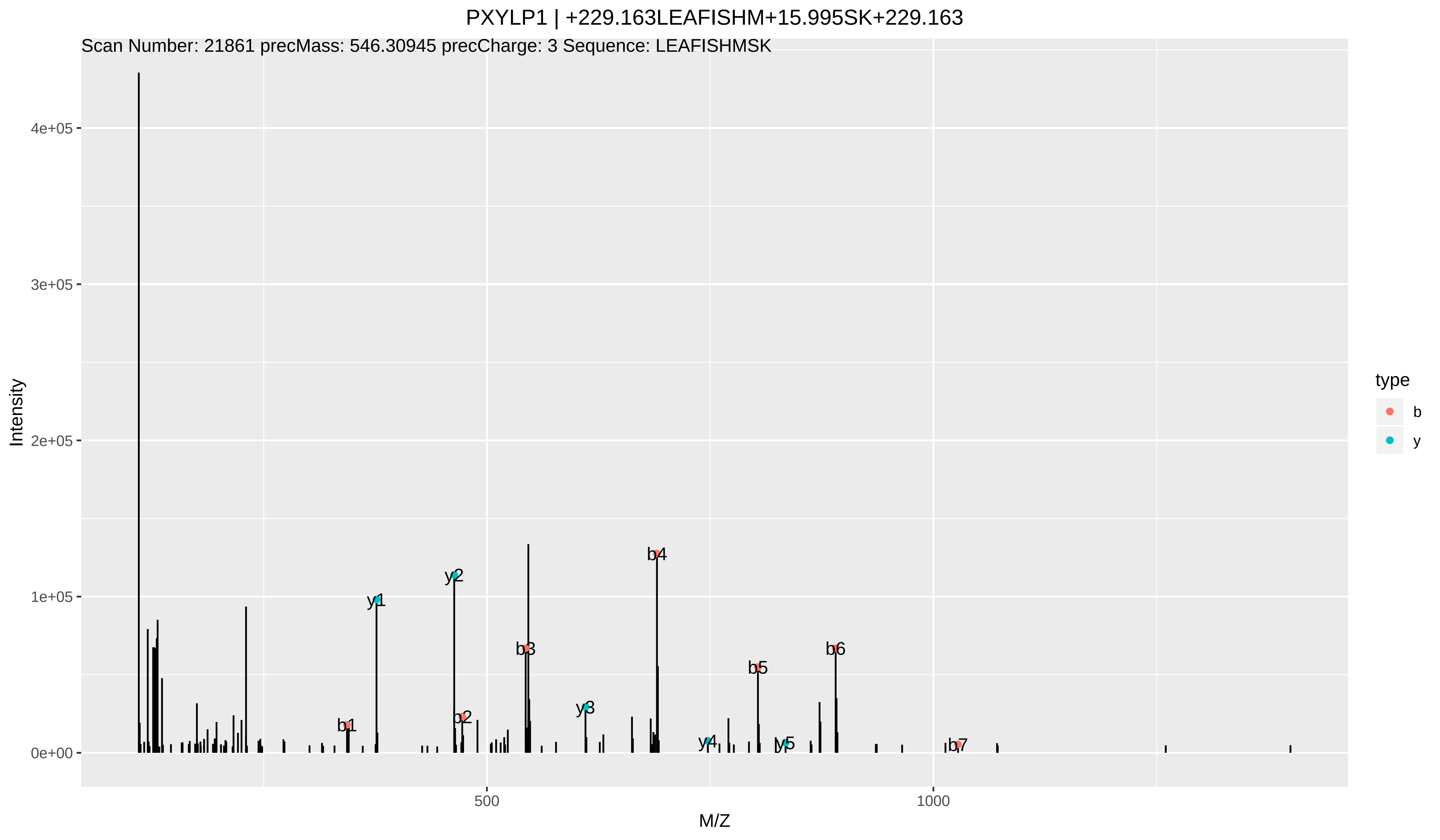
PXYLP1 | +229.163LEAFISHM+15.995SK+229.163
Scan Number: 21861 precMass: 546.30945 precCharge: 3 Sequence: LEAFISHMSK
Intensity
4e+05
3e+05
2e+05
1e+05
0e+00
M/Z
500
1000
y1
y2
b4
b3
b6
b5
y3
b2
b1
y4
y5
b7
type
b
y

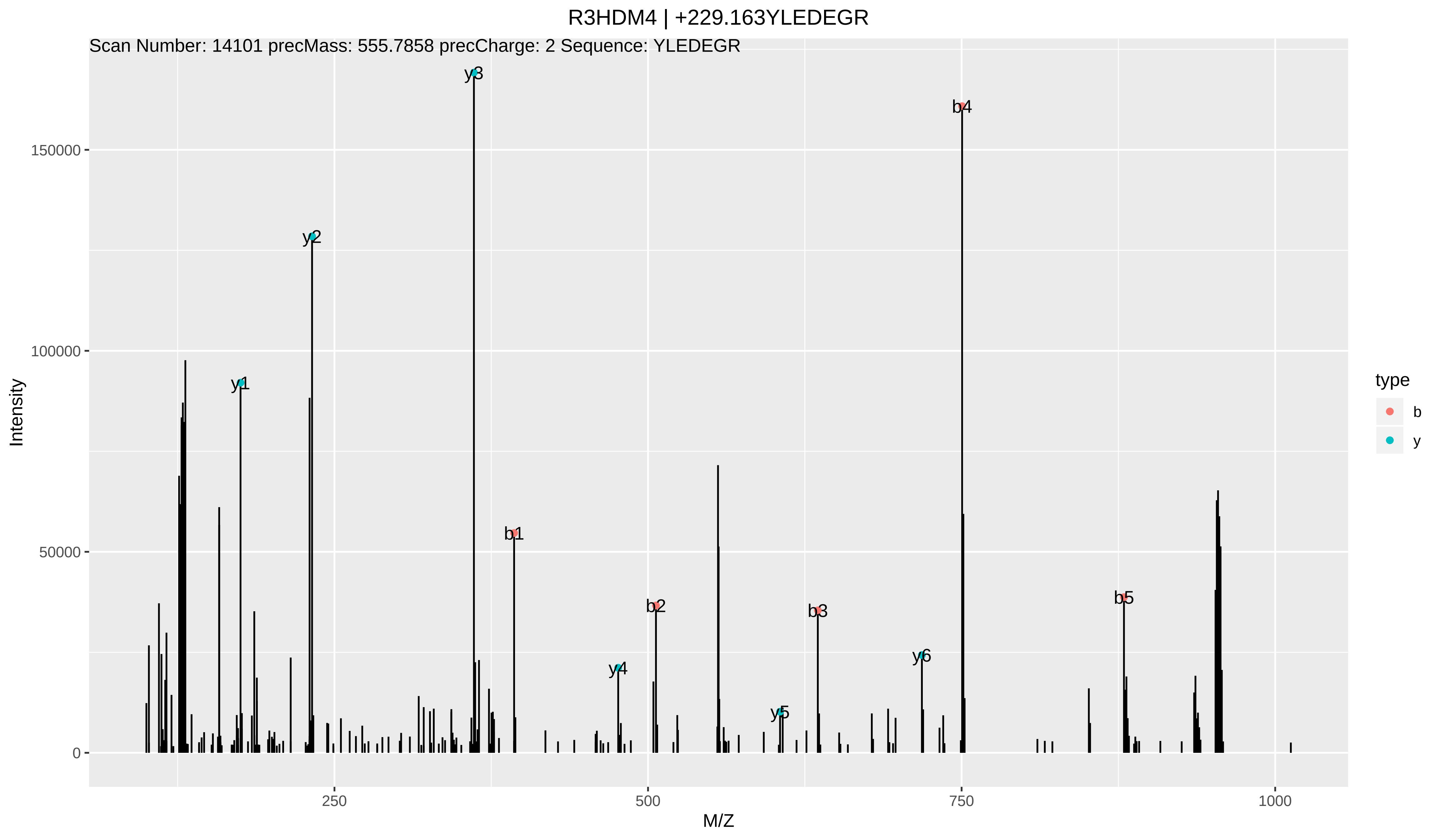

R3HDM4 | +229.163YLEDEGR
Scan Number: 14101 precMass: 555.7858 precCharge: 2 Sequence: YLEDEGR
Intensity
M/Z
150000
100000
50000
0
250
500
750
1000
y1
y2
y3
y4
y5
y6
b1
b2
b3
b4
b5
type
b
y

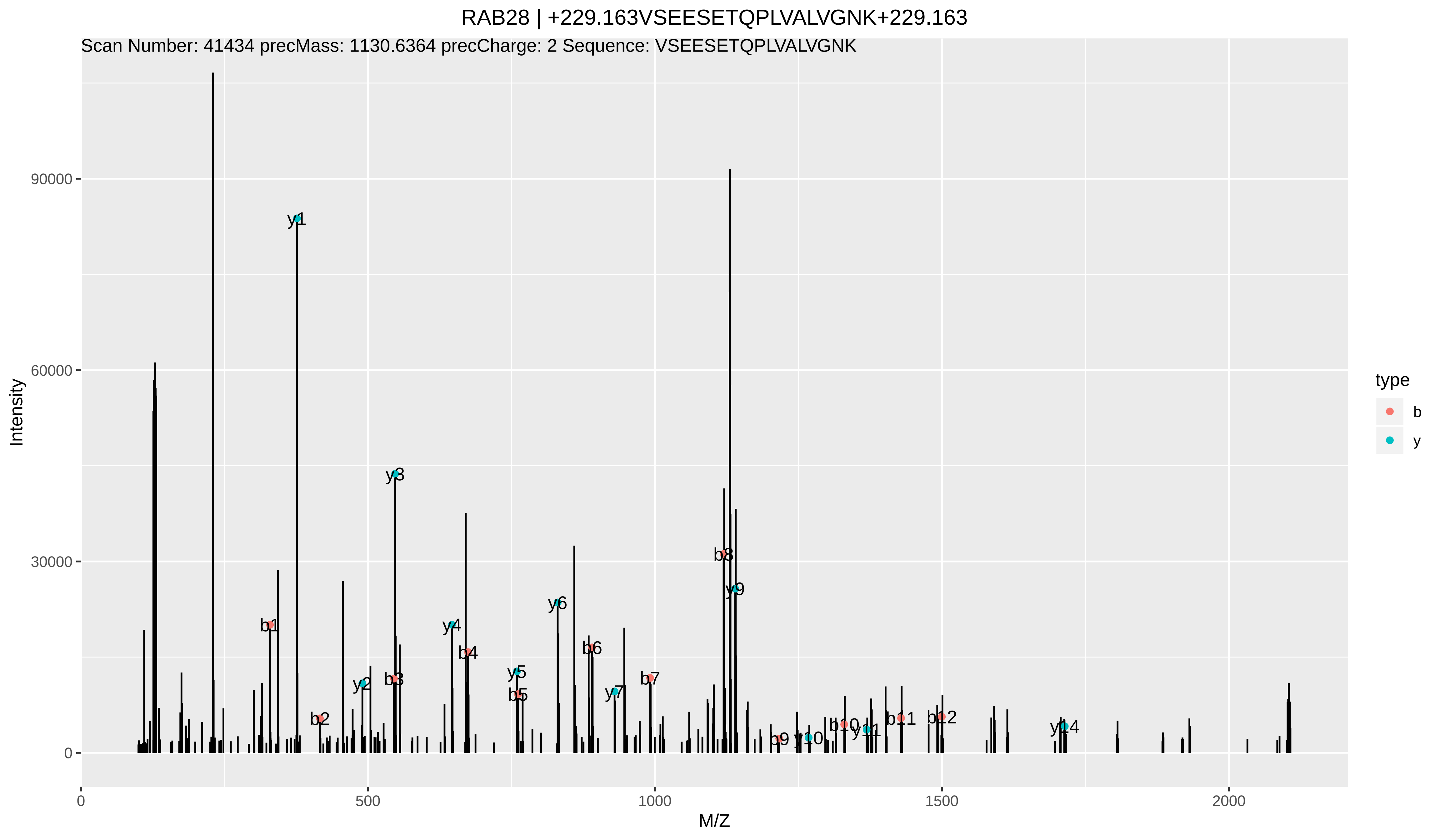
RAB28 | +229.163VSEESETQPLVALVGNK+229.163
Scan Number: 41434 precMass: 1130.6364 precCharge: 2 Sequence: VSEESETQPLVALVGNK
Intensity
M/Z
type
b
y
y1
y2
y3
y4
y5
y6
y7
y9
y10
y11
y14
b1
b2
b3
b4
b5
b6
b7
b8
b9
b10
b11
b12
0
30000
60000
90000
0
500
1000
1500
2000

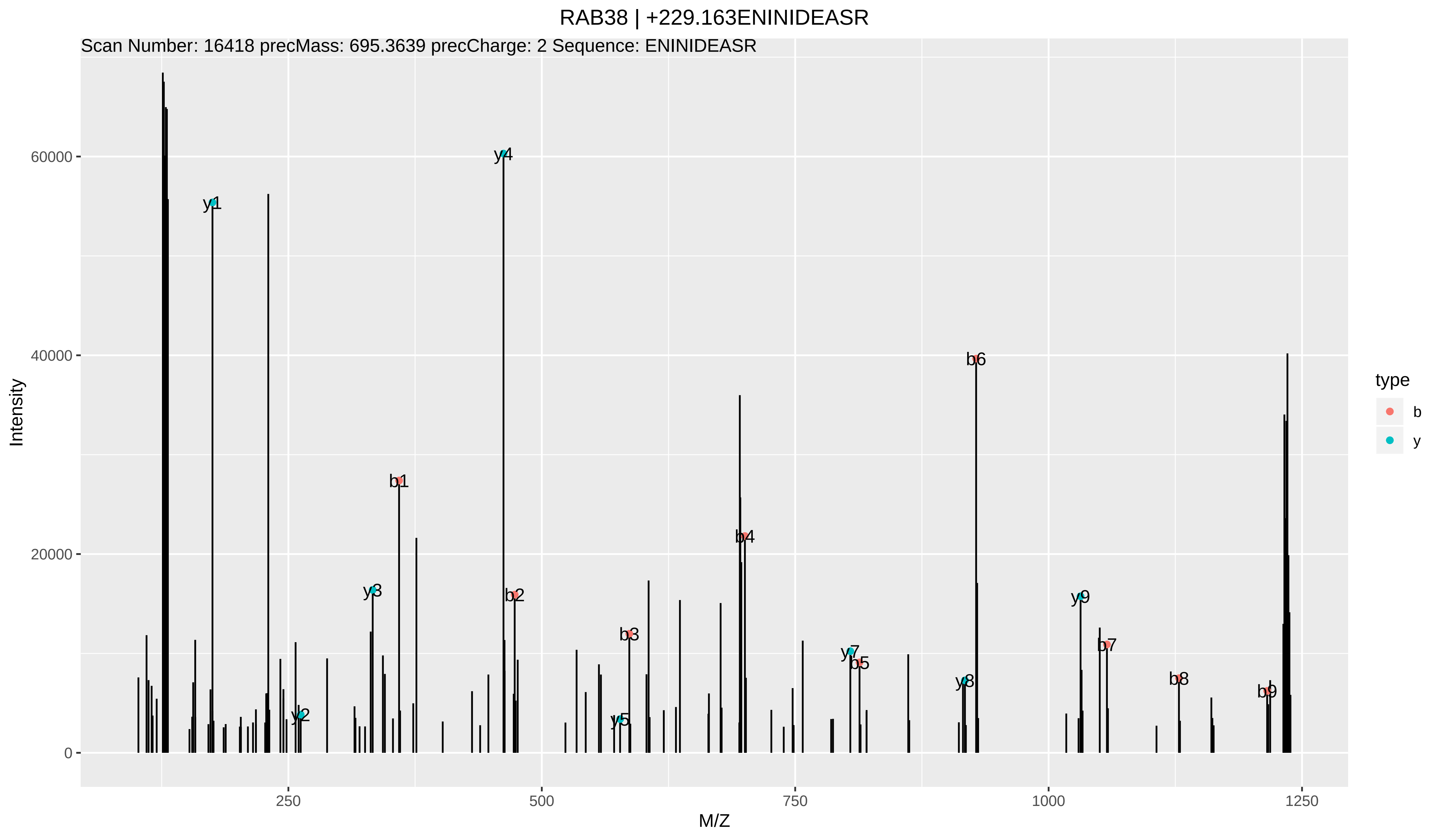
RAB38 | +229.163ENINIDEASR
Scan Number: 16418 precMass: 695.3639 precCharge: 2 Sequence: ENINIDEASR
Intensity
M/Z
type
b
y
y1
y2
y3
y4
y5
y7
y8
y9
b1
b2
b3
b4
b5
b6
b7
b8
b9
0
20000
40000
60000
250
500
750
1000
1250

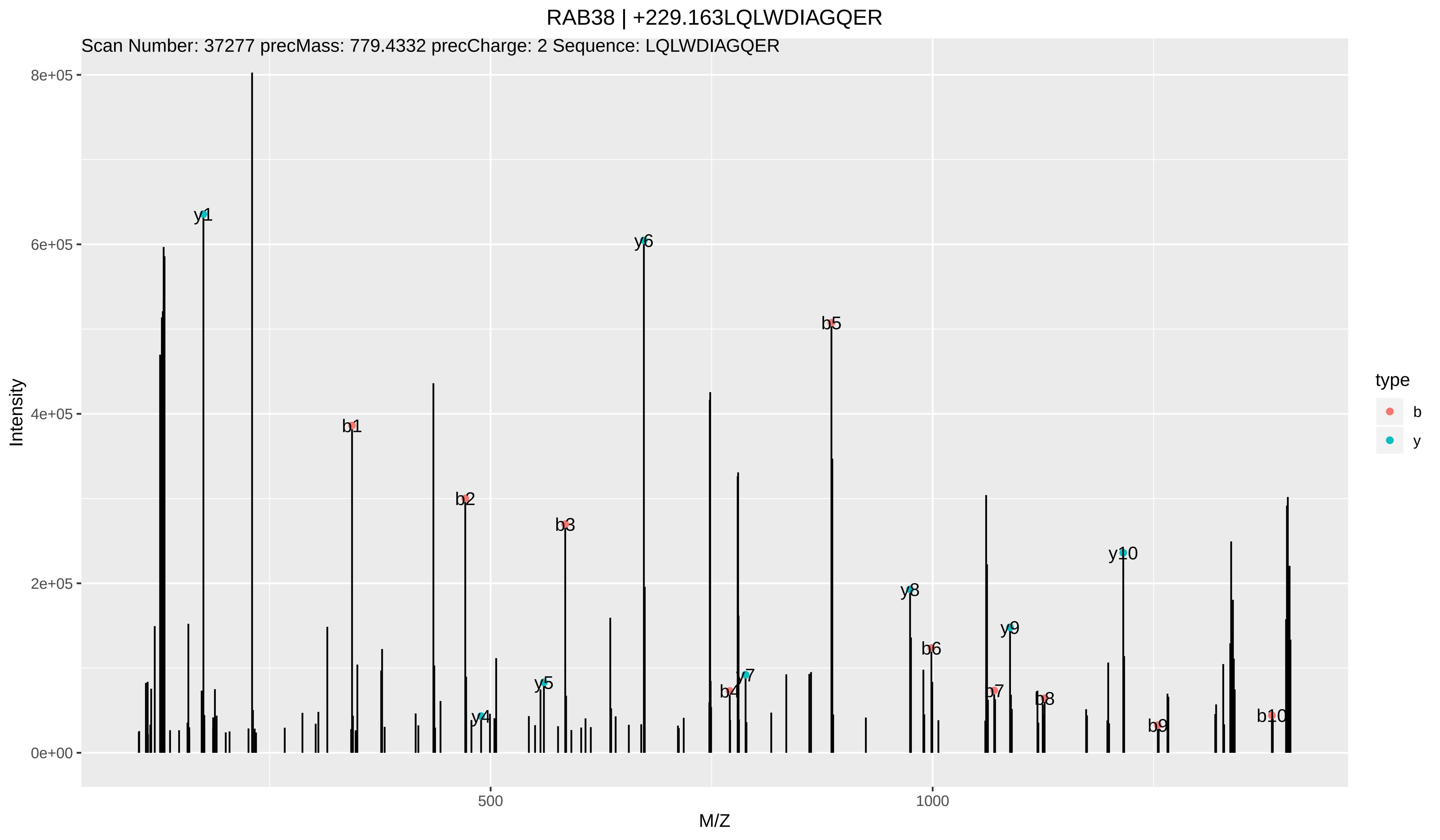

RAB38 | +229.163LQLWDIAGQER
Scan Number: 37277 precMass: 779.4332 precCharge: 2 Sequence: LQLWDIAGQER
Intensity
M/Z
8e+05
6e+05
4e+05
2e+05
0e+00
500
1000
y1
y4
y5
y6
y7
y8
y9
y10
b1
b2
b3
b4
b5
b6
b7
b8
b9
b10
type
b
y

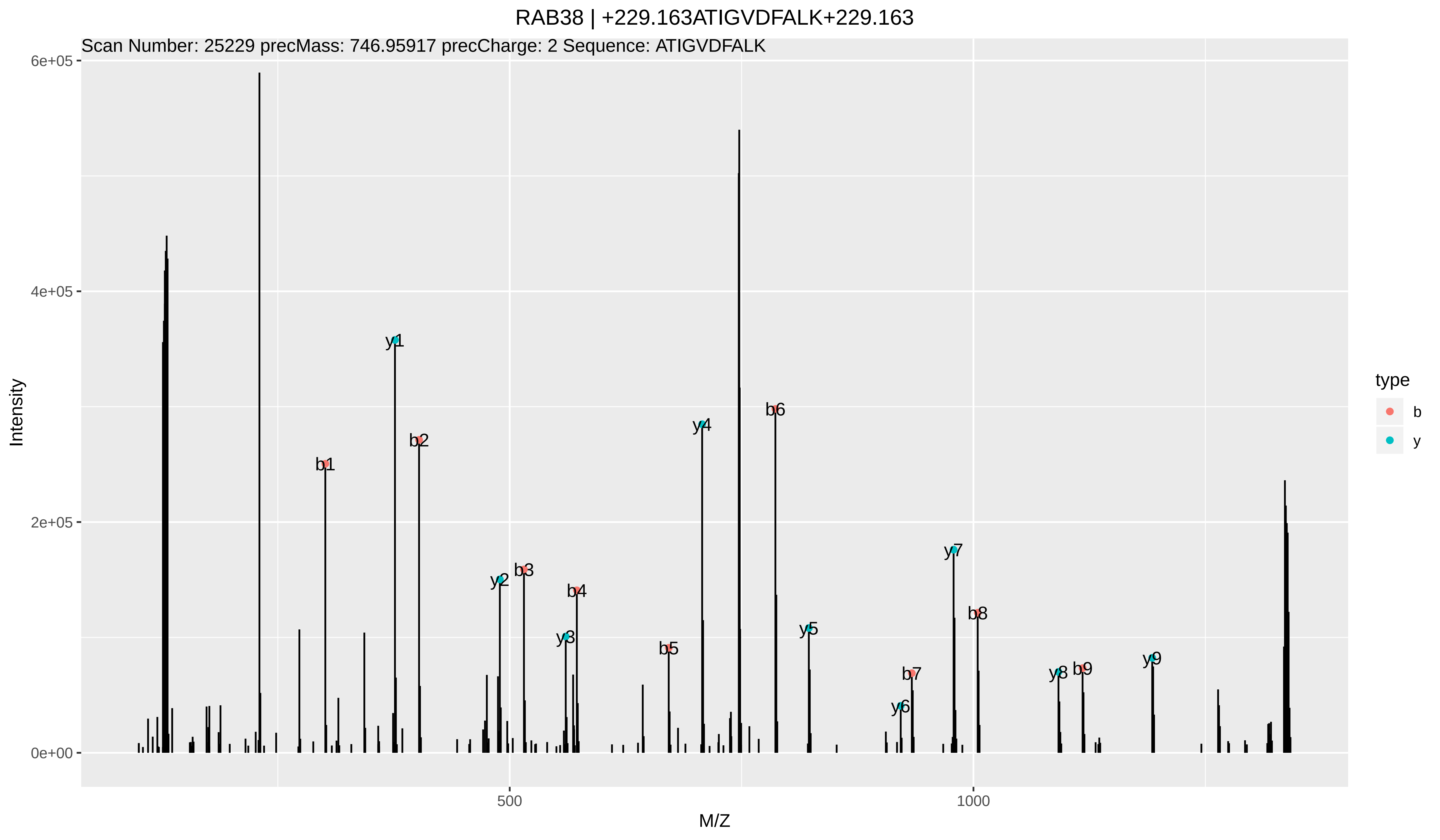

RAB38 | +229.163ATIGVDFALK+229.163
Scan Number: 25229 precMass: 746.95917 precCharge: 2 Sequence: ATIGVDFALK
Intensity
6e+05
4e+05
2e+05
0e+00
M/Z
500
1000
y1
b1
b2
y2
b3
b4
y3
b5
y4
b6
y5
y6
b7
y7
b8
y8
b9
y9
type
b
y

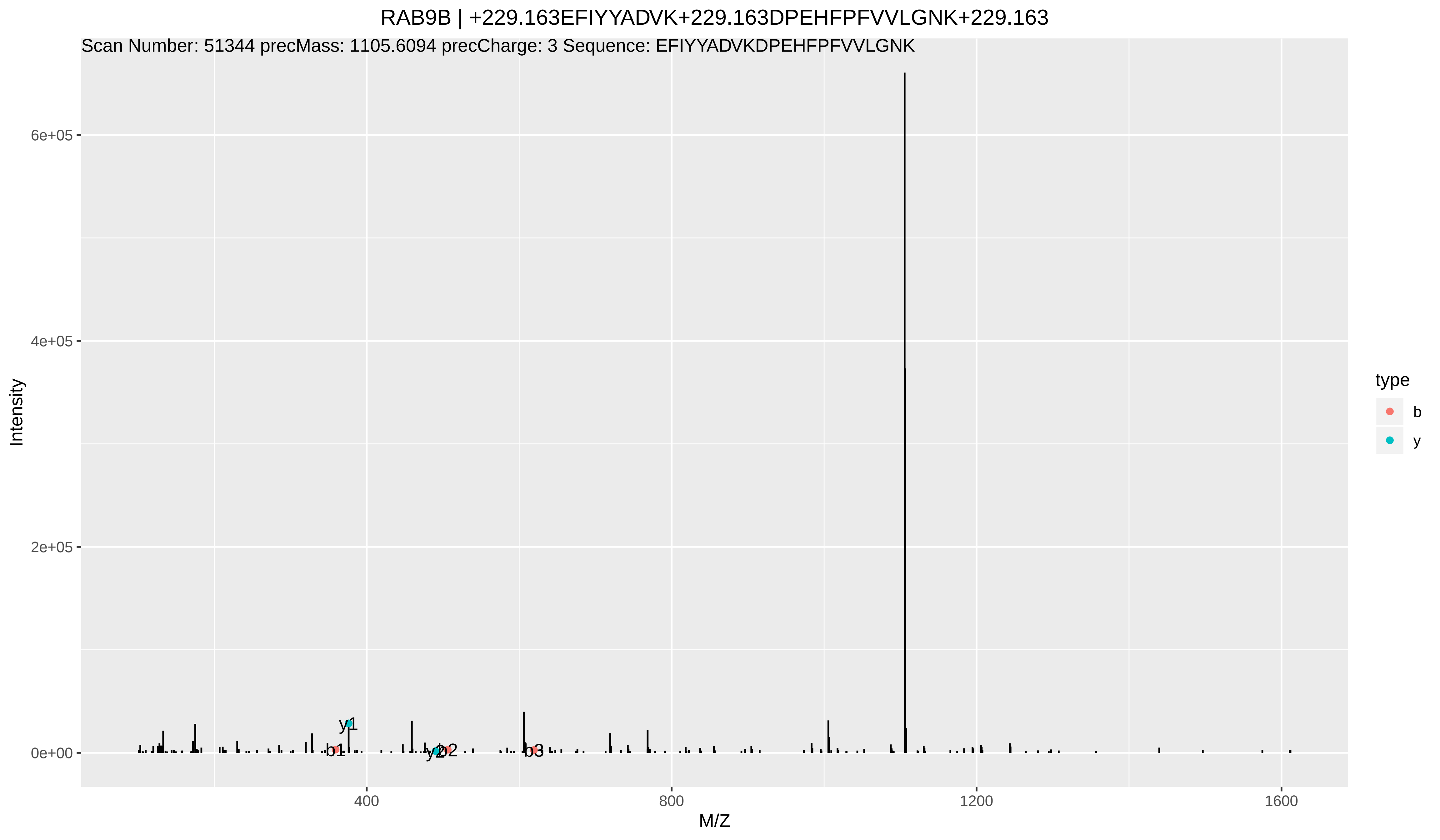

RAB9B | +229.163EFIYYADVK+229.163DPEHFPFVVLGNK+229.163
Scan Number: 51344 precMass: 1105.6094 precCharge: 3 Sequence: EFIYYADVKDPEHFPFVVLGNK
Intensity
6e+05
4e+05
2e+05
0e+00
y1
b1
y2
b2
b3
400
800
1200
1600
M/Z
type
b
y

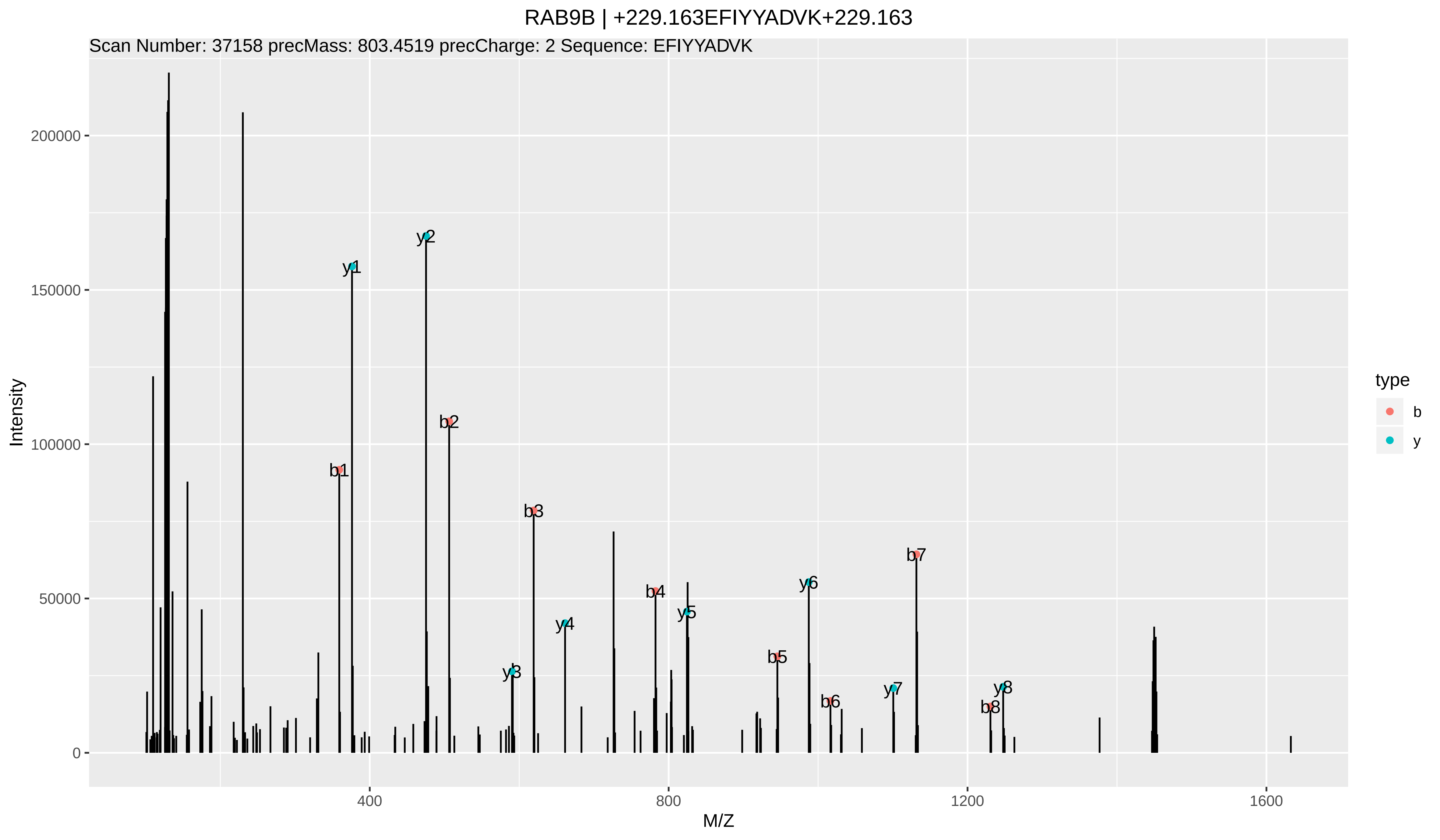
RAB9B | +229.163EFIYYADVK+229.163
Scan Number: 37158 precMass: 803.4519 precCharge: 2 Sequence: EFIYYADVK
Intensity
M/Z
200000
150000
100000
50000
0
400
800
1200
1600
y1
y2
b1
b2
b3
y3
y4
b4
y5
b5
y6
b6
y7
b7
b8
y8
type
b
y

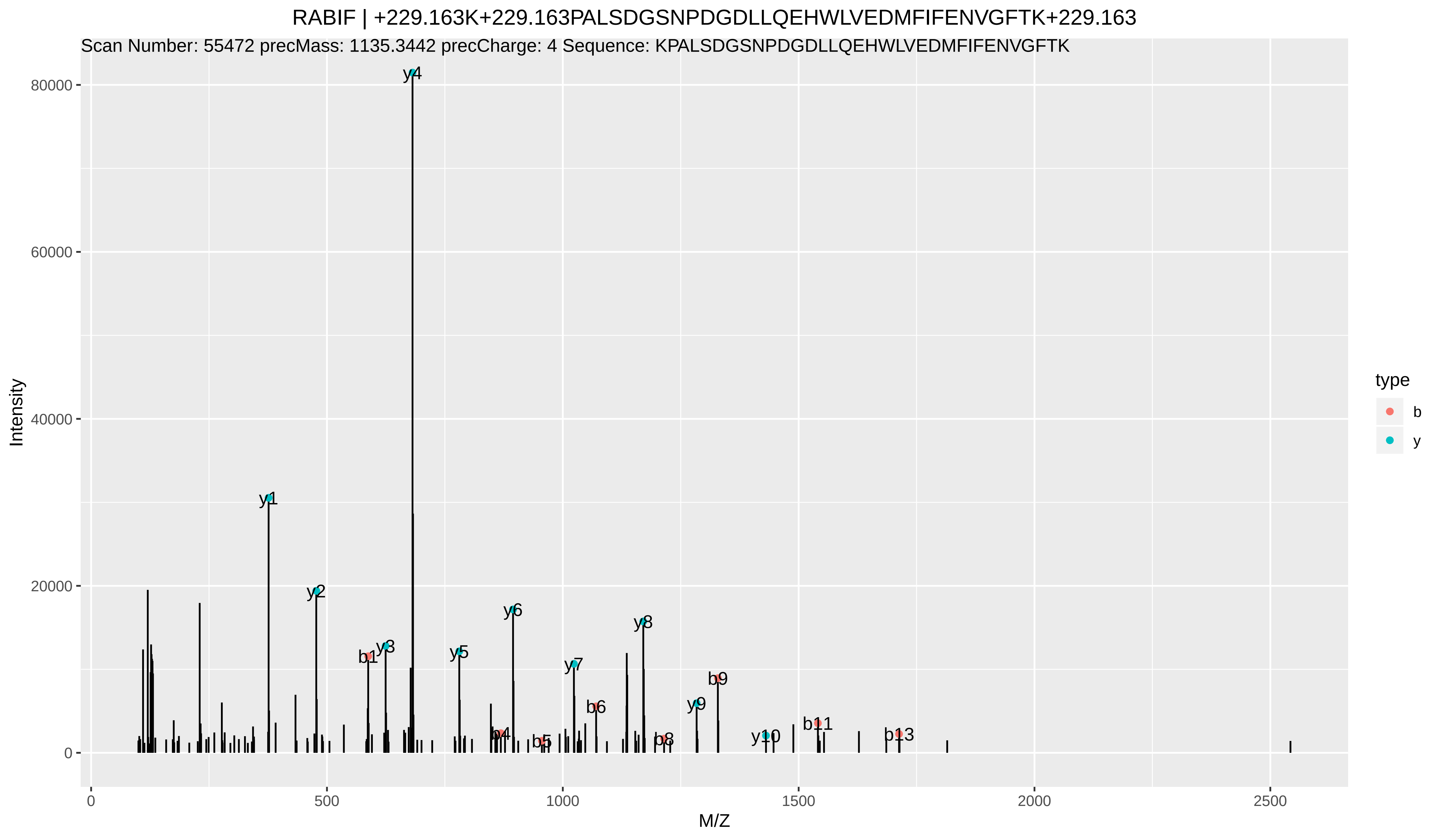
RABIF | +229.163K+229.163PALSDGSNPDGDLLQEHWLVEDMFIFENVGFTK+229.163
Scan Number: 55472 precMass: 1135.3442 precCharge: 4 Sequence: KPALSDGSNPDGDLLQEHWLVEDMFIFENVGFTK
Intensity
80000
60000
40000
20000
0
M/Z
0
500
1000
1500
2000
2500
y4
y1
y2
b1
y3
y5
y6
b4
b5
y7
b6
y8
b8
y9
b9
y10
b11
b13
type
b
y

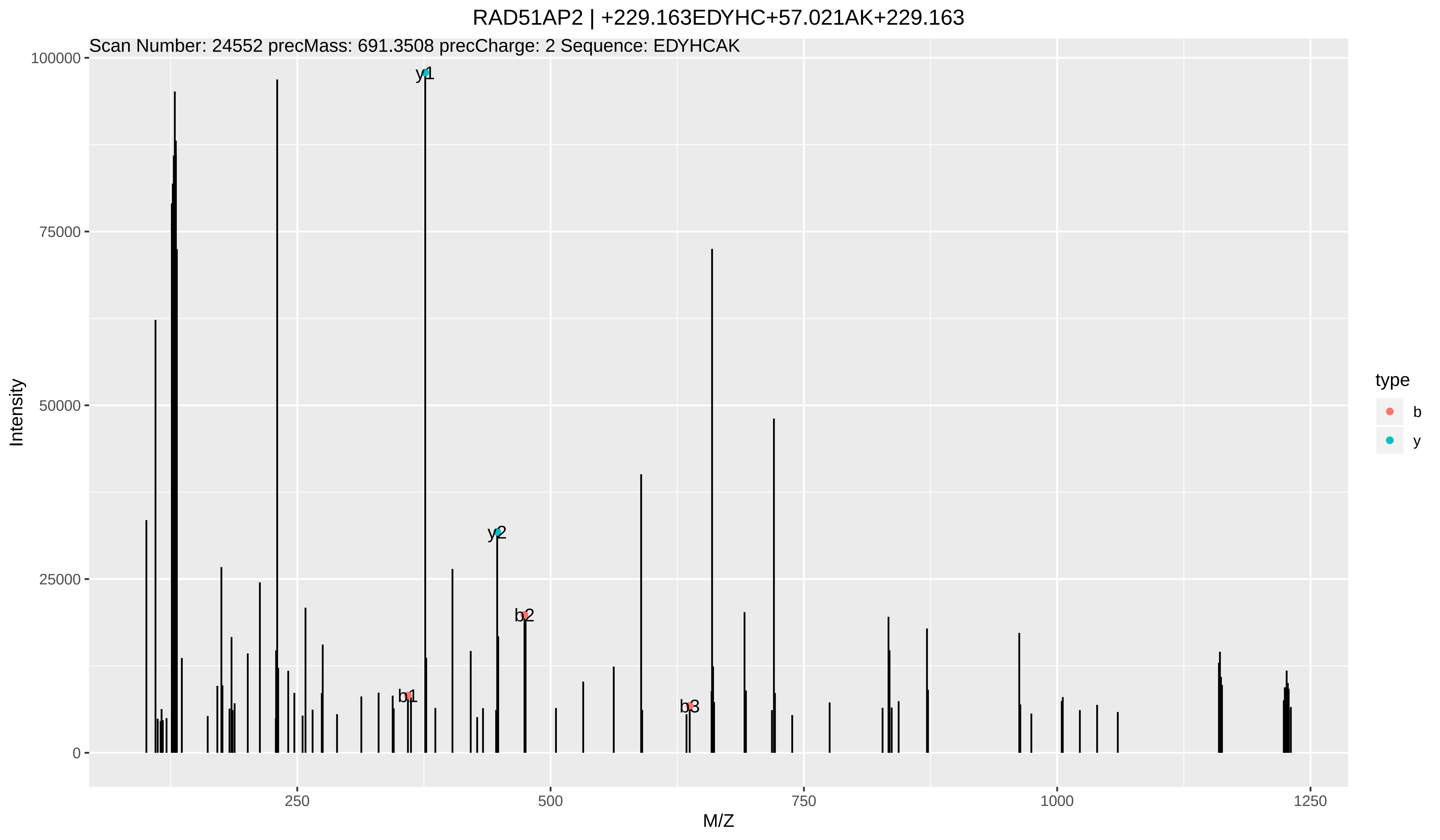
RAD51AP2 | +229.163EDYHC+57.021AK+229.163
Scan Number: 24552 precMass: 691.3508 precCharge: 2 Sequence: EDYHCAK
y1
y2
b1
b2
b3
Intensity
100000
75000
50000
25000
0
250
500
750
1000
1250
M/Z
type
b
y

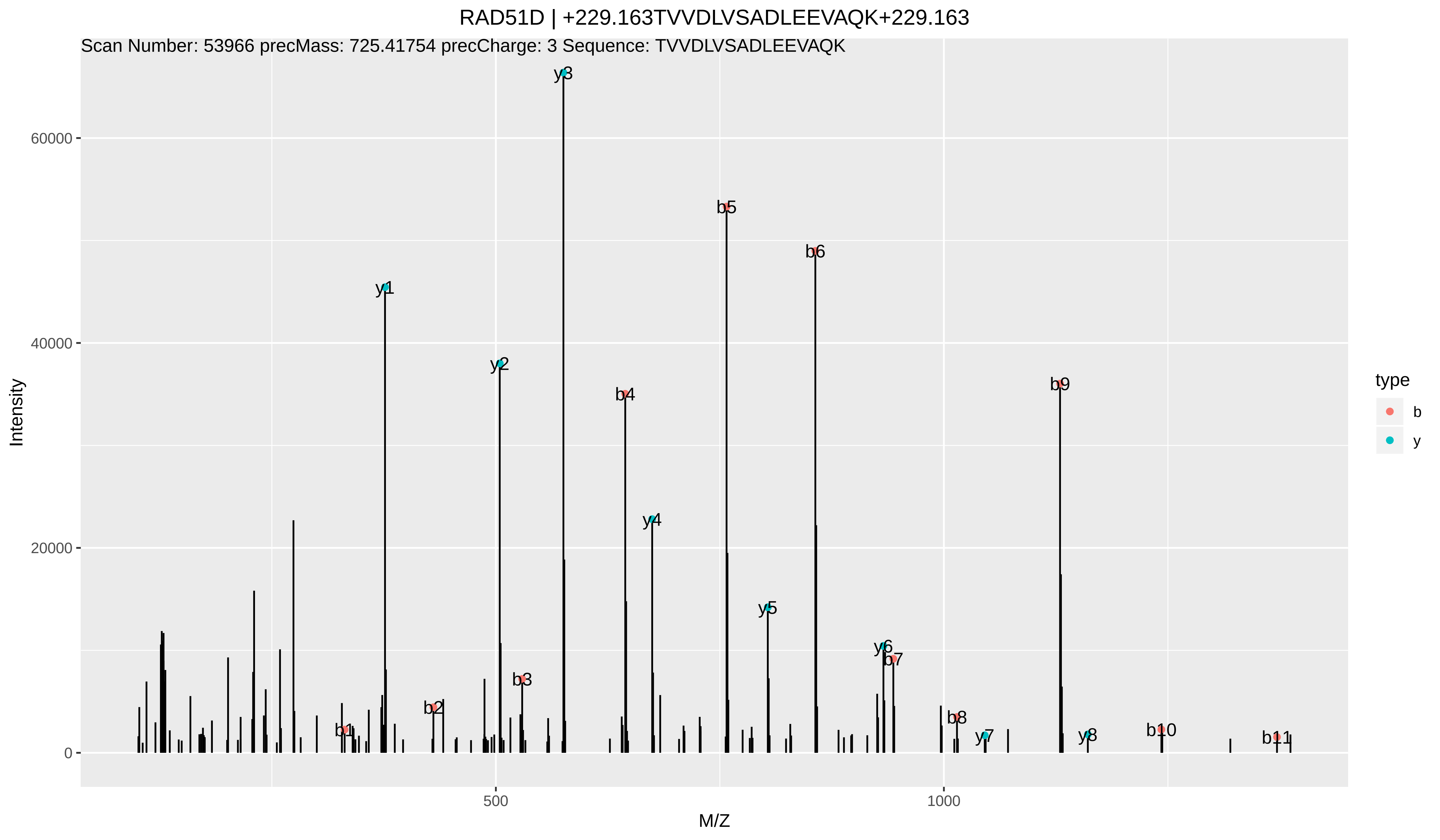

RAD51D | +229.163TVVDLVSADLEEVAQK+229.163
Scan Number: 53966 precMass: 725.41754 precCharge: 3 Sequence: TVVDLVSADLEEVAQK
Intensity
M/Z
0
20000
40000
60000
500
1000
y1
y2
y3
y4
y5
y6
y7
y8
b1
b2
b3
b4
b5
b6
b7
b8
b9
b10
b11
type
b
y

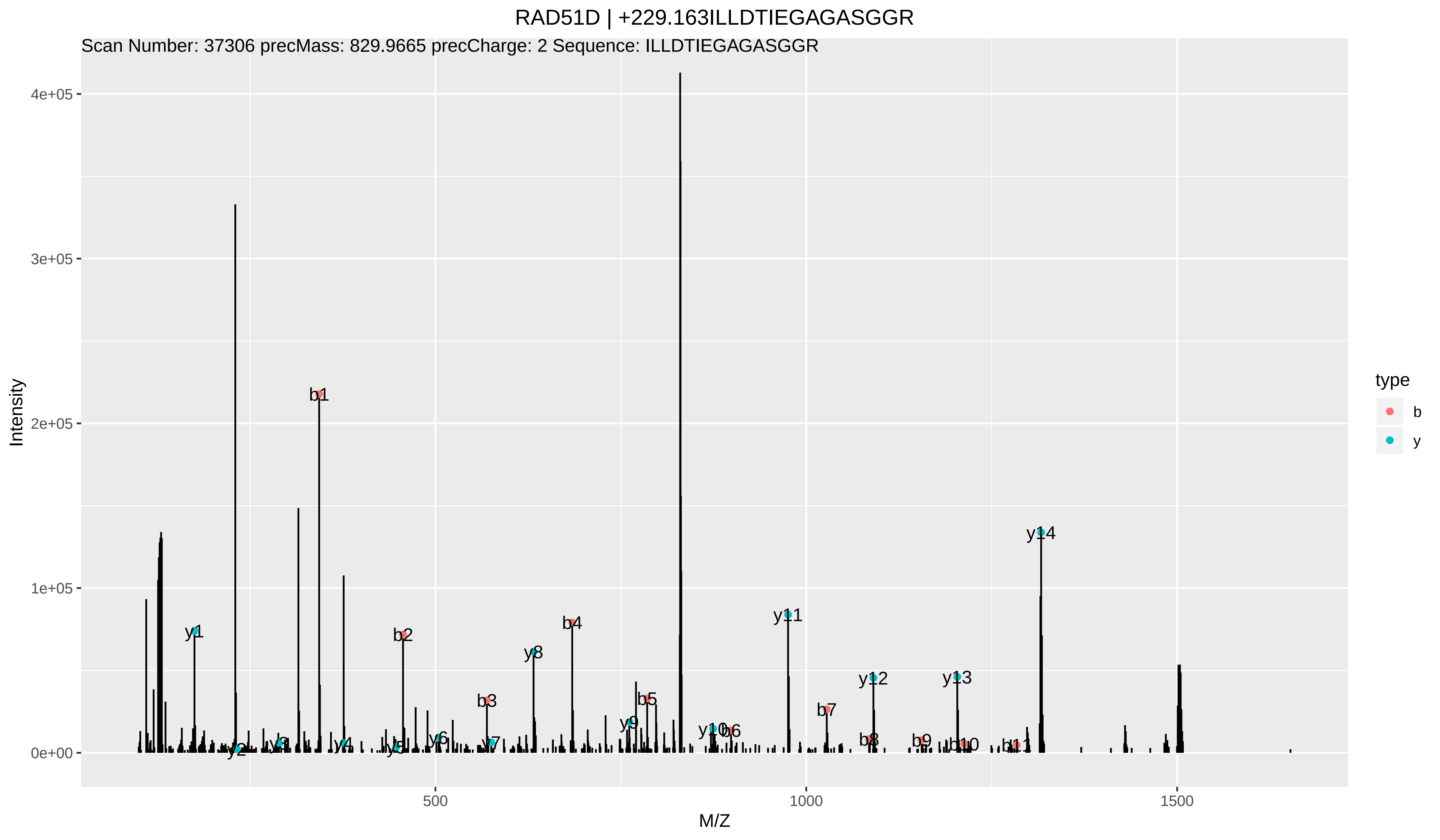

RAD51D | +229.163ILLDTIEGAGASGGR
Scan Number: 37306 precMass: 829.9665 precCharge: 2 Sequence: ILLDTIEGAGASGGR
Intensity
M/Z
0e+00
1e+05
2e+05
3e+05
4e+05
500
1000
1500
type
b
y
b1
b2
b3
b4
b5
b6
b7
b8
b9
b10
b11
y1
y2
y3
y4
y5
y6
y7
y8
y9
y10
y11
y12
y13
y14

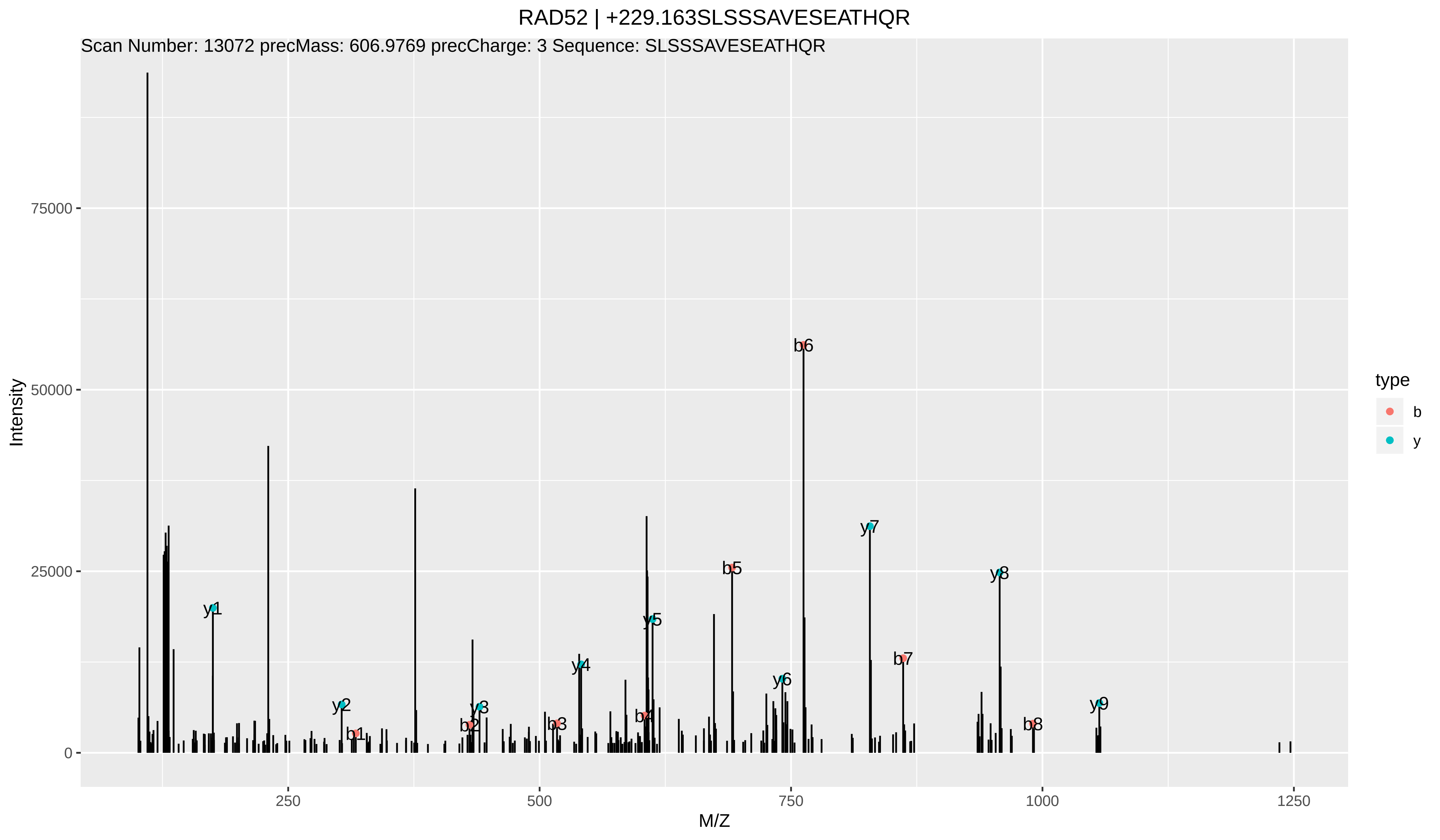

RAD52 | +229.163SLSSSAVESEATHQR
Scan Number: 13072 precMass: 606.9769 precCharge: 3 Sequence: SLSSSAVESEATHQR
Intensity
M/Z
0
25000
50000
75000
250
500
750
1000
1250
y1
y2
b1
b2
y3
b3
y4
b4
y5
b5
y6
b6
y7
b7
y8
b8
y9
type
b
y

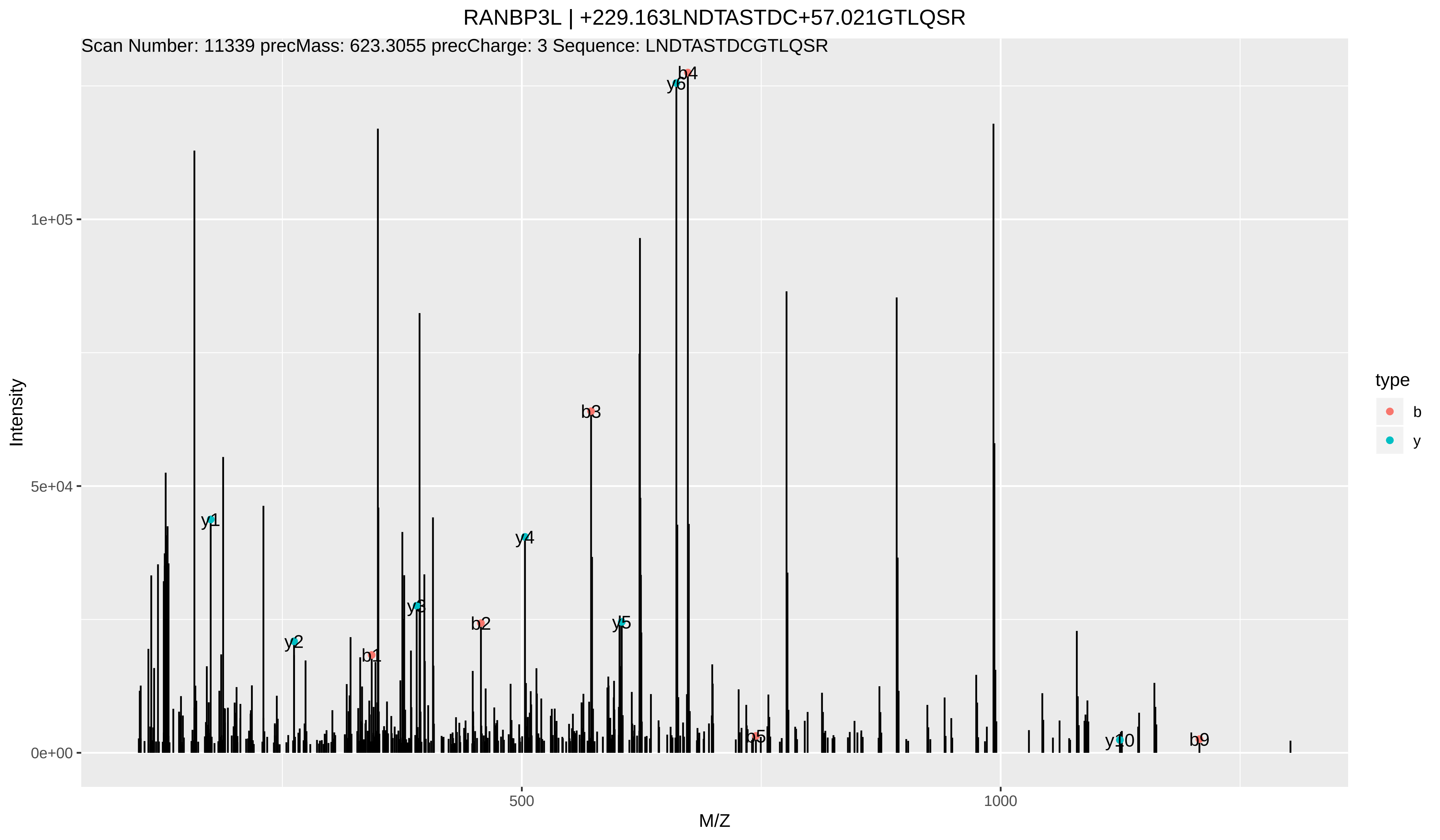

RANBP3L | +229.163LNDTASTDC+57.021GTLQSR
Scan Number: 11339 precMass: 623.3055 precCharge: 3 Sequence: LNDTASTDCGTLQSR
Intensity
1e+05
5e+04
0e+00
M/Z
500
1000
b4
y6
b3
y1
y4
y3
b2
y5
y2
b1
b5
y10
b9
type
b
y

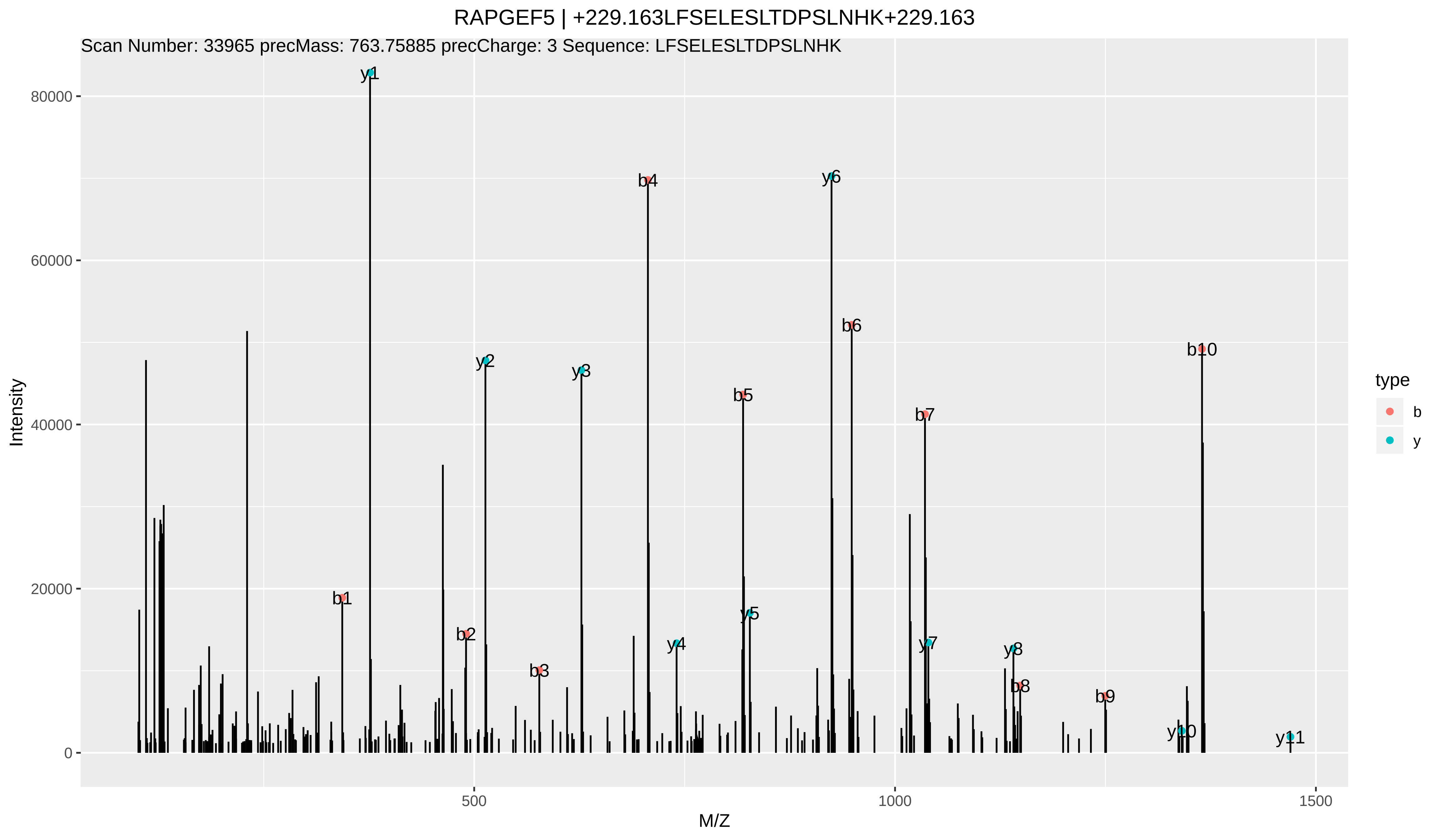

RAPGEF5 | +229.163LFSELESLTDPSLNHK+229.163
Scan Number: 33965 precMass: 763.75885 precCharge: 3 Sequence: LFSELESLTDPSLNHK
Intensity
M/Z
0
20000
40000
60000
80000
500
1000
1500
y1
b4
y6
b6
b10
y2
y3
b5
b7
b1
y5
b2
y4
y7
y8
b3
b8
b9
y10
y11
type
b
y

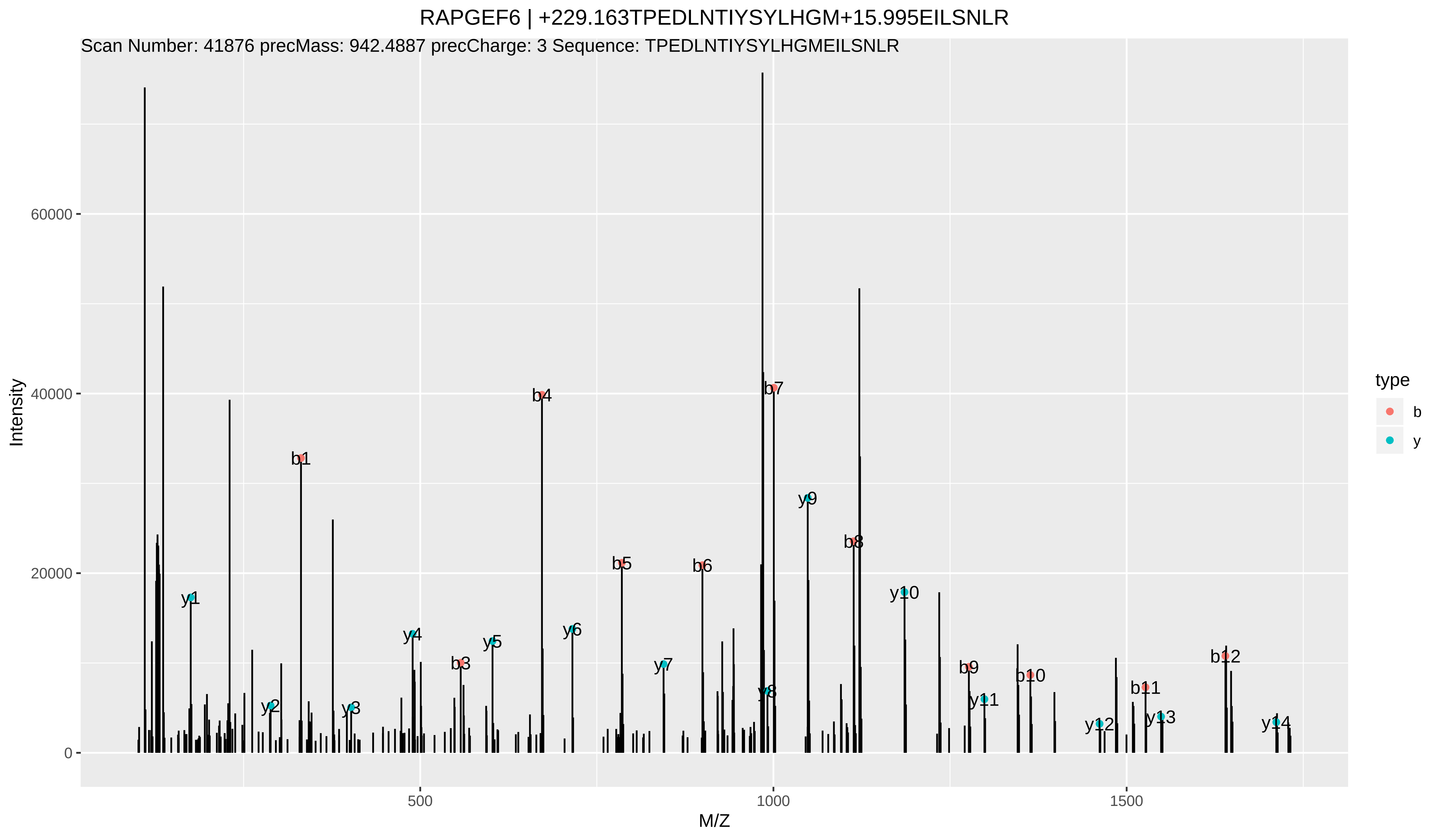

RAPGEF6 | +229.163TPEDLNTIYSYLHGM+15.995EILSNLR
Scan Number: 41876 precMass: 942.4887 precCharge: 3 Sequence: TPEDLNTIYSYLHGMEILSNLR
Intensity
60000
40000
20000
0
M/Z
500
1000
1500
b1
b3
b4
b5
b6
b7
b8
b9
b10
b11
b12
y1
y2
y3
y4
y5
y6
y7
y8
y9
y10
y11
y12
y13
y14
type
b
y

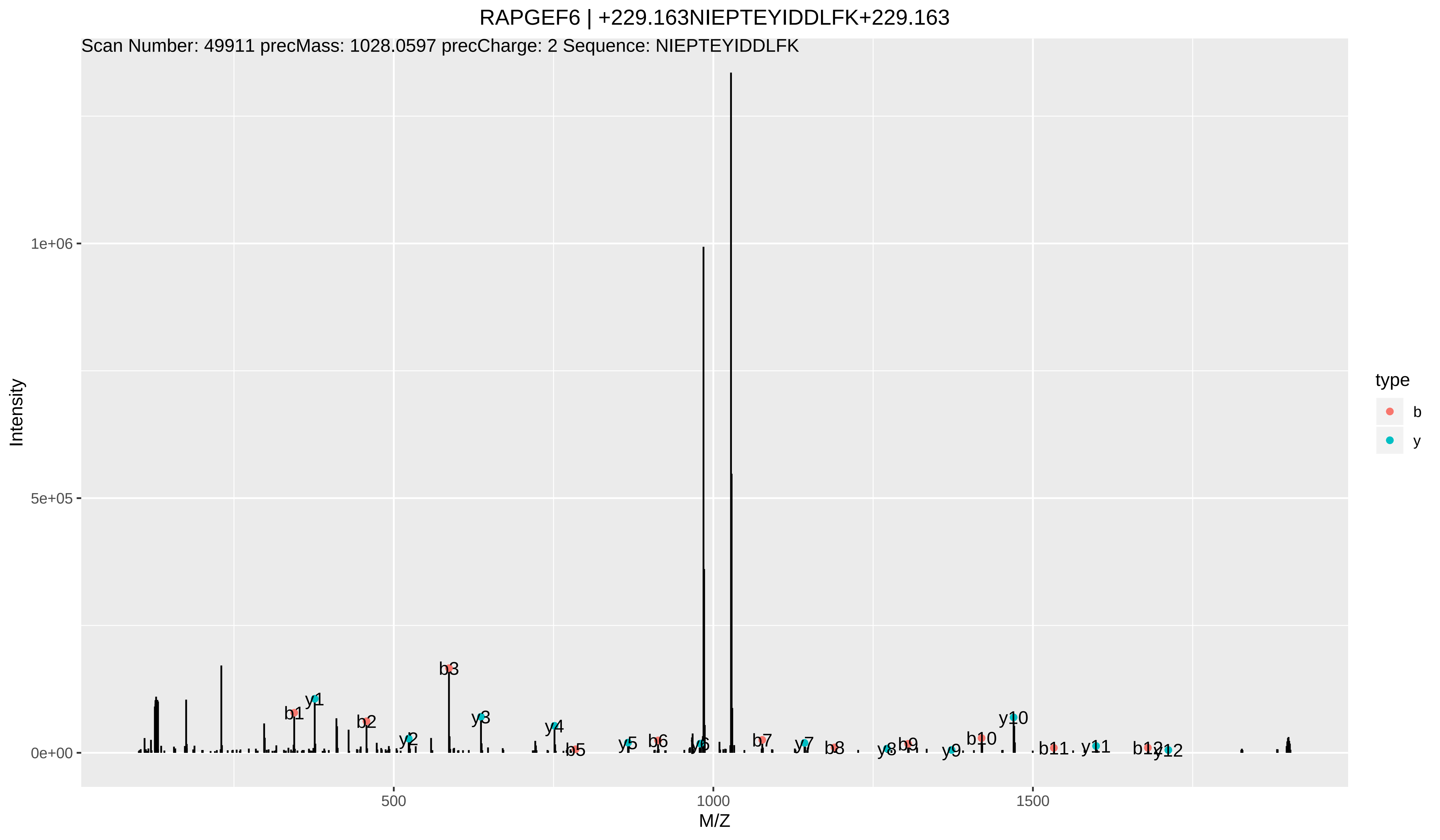

RAPGEF6 | +229.163NIEPTEYIDDLFK+229.163
Scan Number: 49911 precMass: 1028.0597 precCharge: 2 Sequence: NIEPTEYIDDLFK
Intensity
1e+06
5e+05
0e+00
500
1000
1500
M/Z
type
b
y
b1
y1
b2
y2
b3
y3
y4
b5
y5
b6
y6
b7
y7
b8
y8
b9
y9
b10
y10
b11
y11
b12
y12

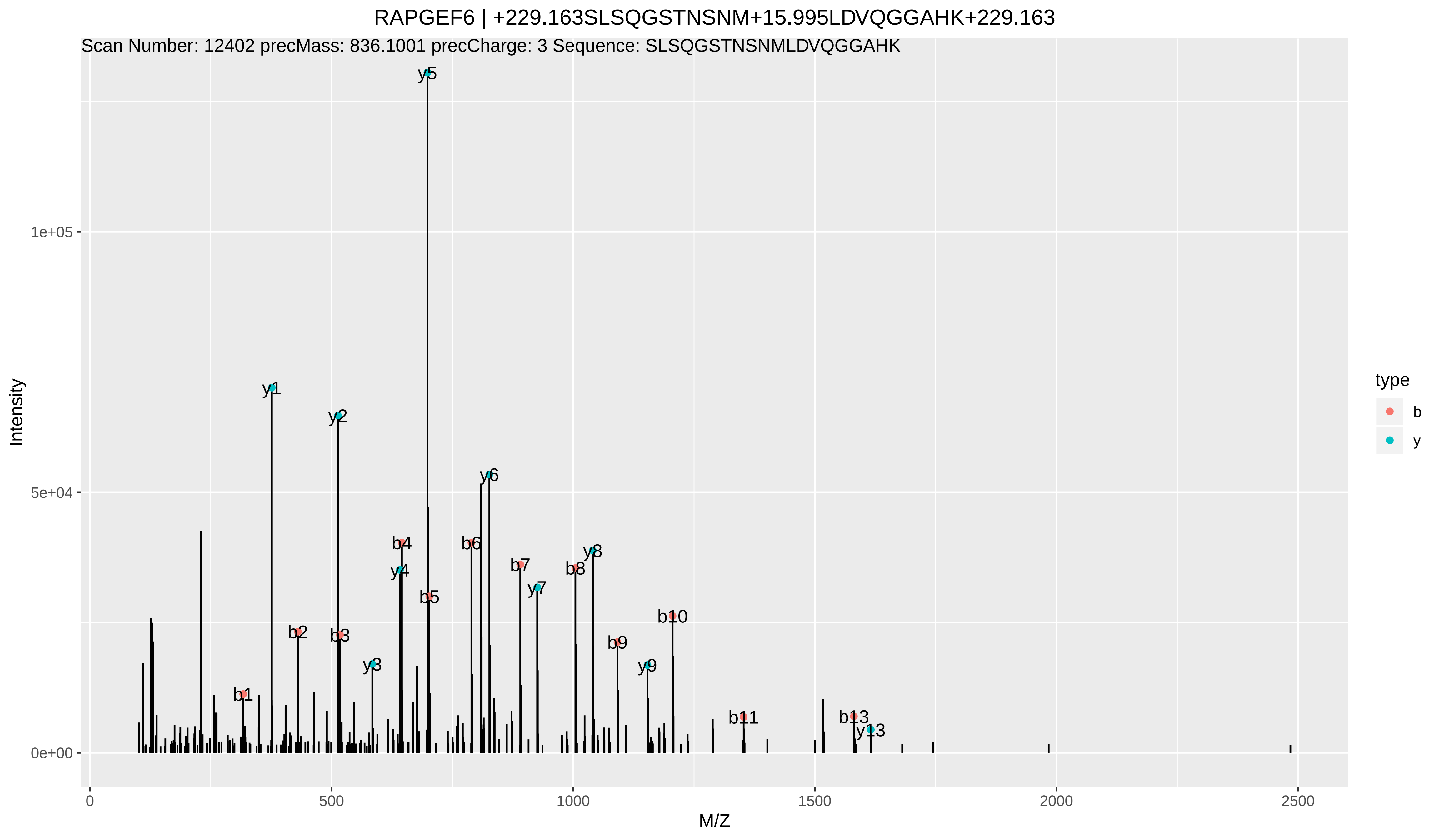

RAPGEF6 | +229.163SLSQGSTNSNM+15.995LDVQGGAHK+229.163
Scan Number: 12402 precMass: 836.1001 precCharge: 3 Sequence: SLSQGSTNSNMLDVQGGAHK
Intensity
1e+05
5e+04
0e+00
M/Z
0
500
1000
1500
2000
2500
y5
y1
y2
y6
b4
b6
y8
b7
b8
y4
y7
b5
b10
b2
b3
b9
y3
y9
b1
b11
b13
y13
type
b
y

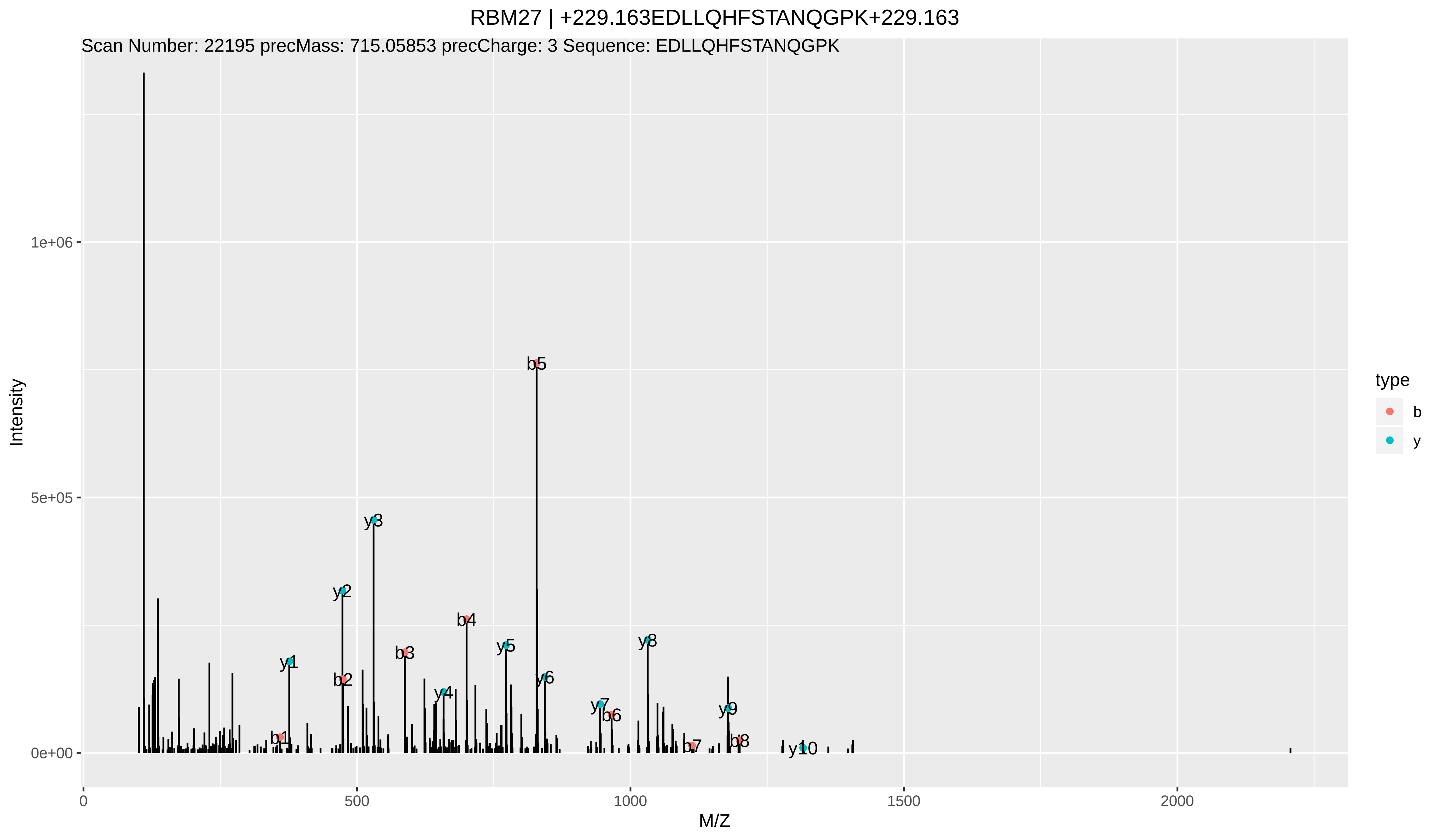

RBM27 | +229.163EDLLQHFSTANQGPK+229.163
Scan Number: 22195 precMass: 715.05853 precCharge: 3 Sequence: EDLLQHFSTANQGPK
Intensity
1e+06
5e+05
0e+00
M/Z
0
500
1000
1500
2000
b5
y3
y2
b4
y8
b3
y5
y1
y6
b2
y4
y7
b6
y9
b1
b7
b8
y10
type
b
y

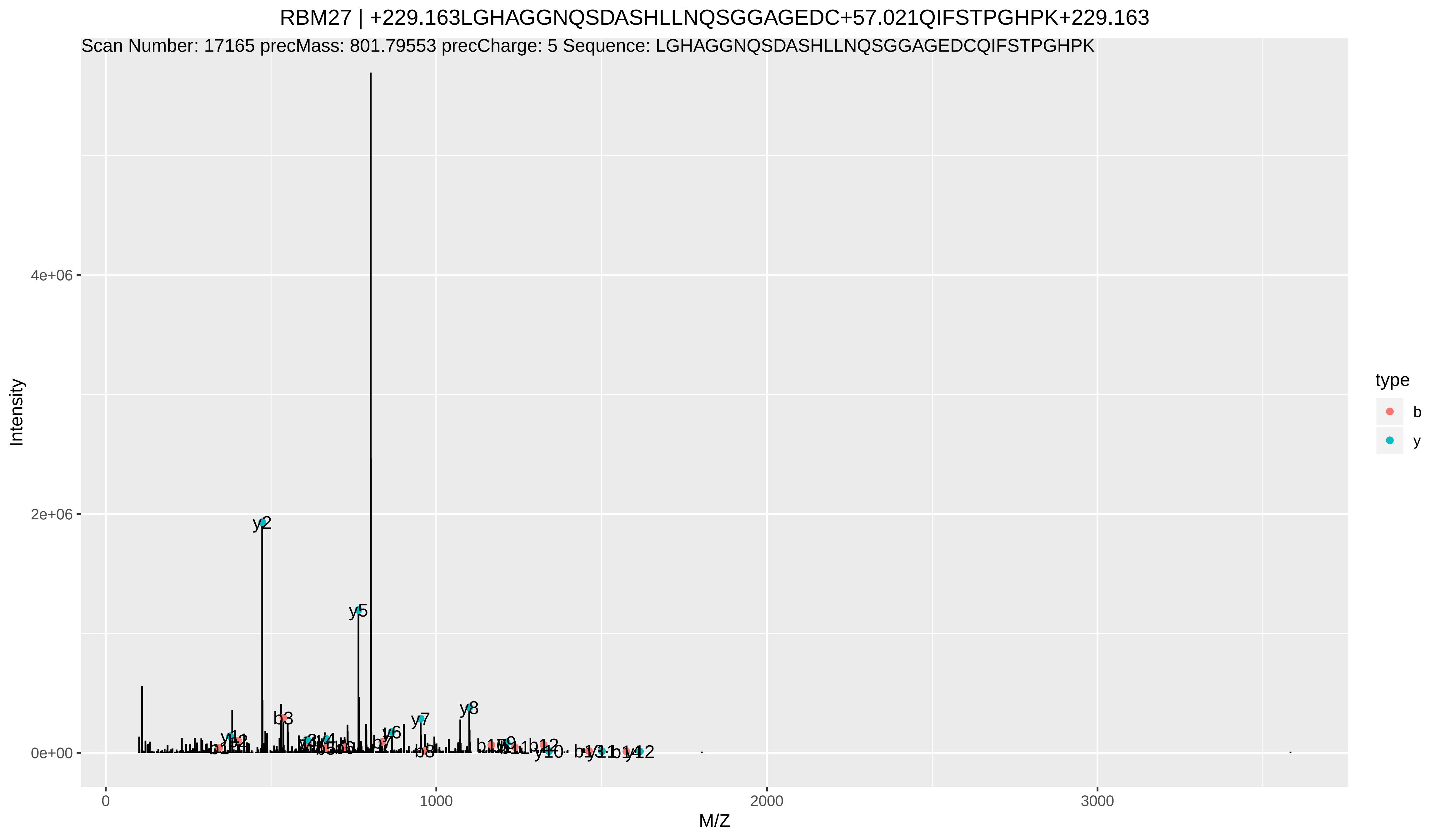

RBM27 | +229.163LGHAGGNQSDASHLLNQSGGAGEDC+57.021QIFSTPGHPK+229.163
Scan Number: 17165 precMass: 801.79553 precCharge: 5 Sequence: LGHAGGNQSDASHLLNQSGGAGEDCQIFSTPGHPK
Intensity
4e+06
2e+06
0e+00
M/Z
0
1000
2000
3000
type
b
y
y2
y5
y7
y8
y6
b3
b8
y10

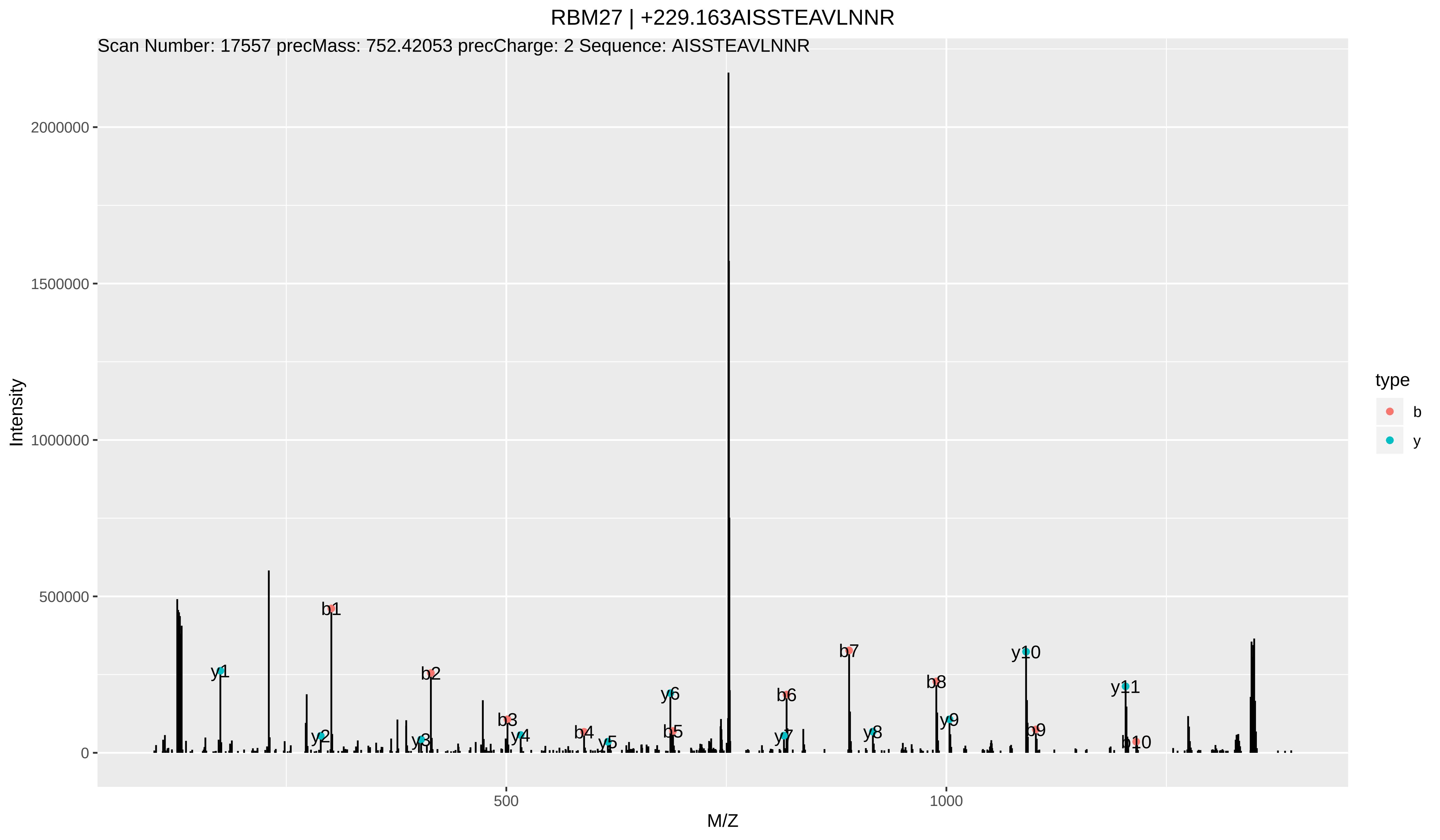

RBM27 | +229.163AISSTEAVLNNR
Scan Number: 17557 precMass: 752.42053 precCharge: 2 Sequence: AISSTEAVLNNR
Intensity
M/Z
type
b
y
y1
b1
y2
b2
y3
b3
y4
b4
y5
y6
b5
y7
b6
b7
y8
b8
y9
y10
b9
y11
b10

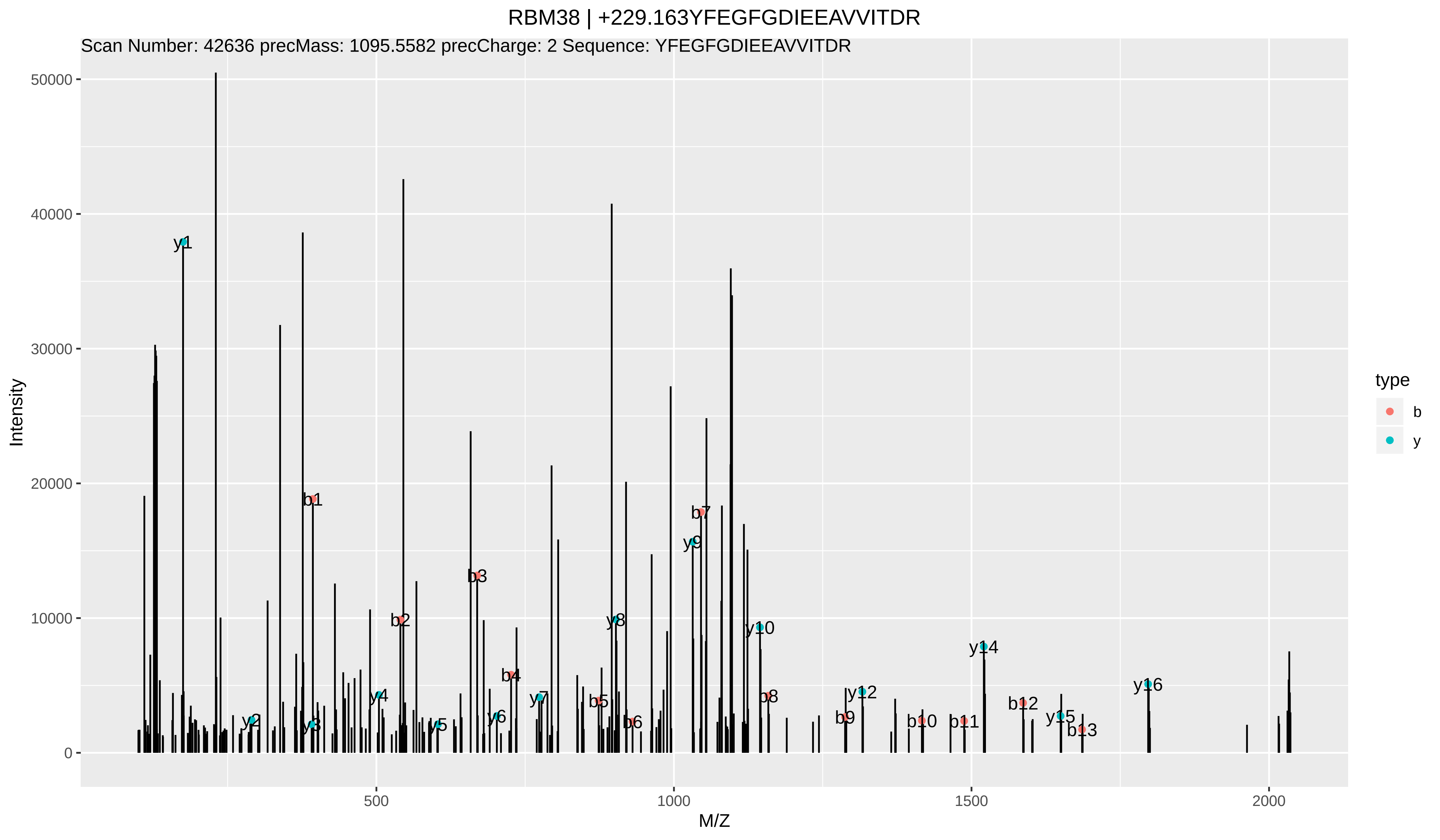

RBM38 | +229.163YFEGFGDIEEAVVITDR
Scan Number: 42636 precMass: 1095.5582 precCharge: 2 Sequence: YFEGFGDIEEAVVITDR
Intensity
M/Z
type
b
y
y1
b1
b2
b3
b4
b5
b6
b7
b8
b9
b10
b11
b12
b13
y2
y3
y4
y5
y6
y7
y8
y9
y10
y12
y14
y15
y16

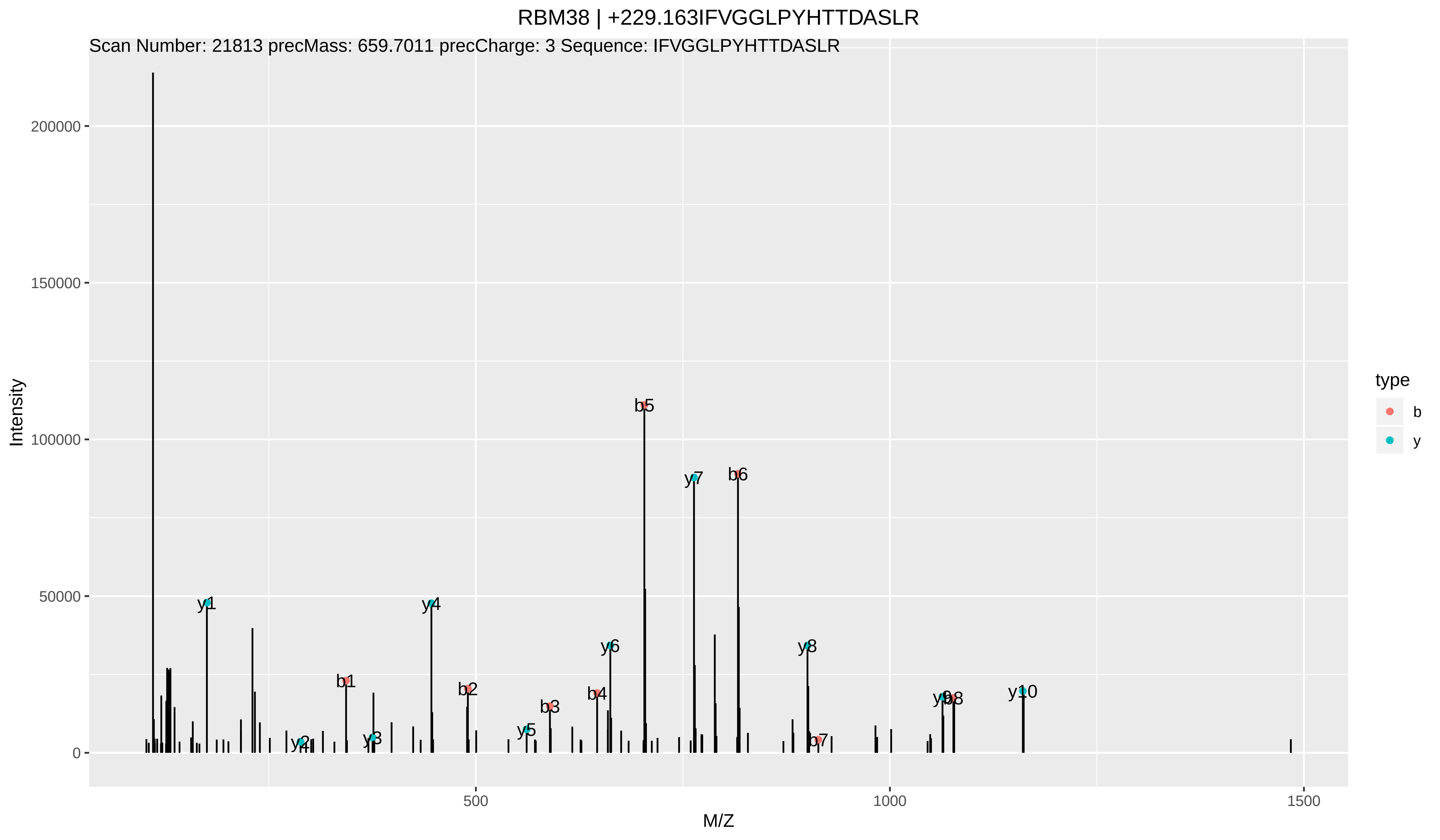

RBM38 | +229.163IFVGGLPYHTTDASLR
Scan Number: 21813 precMass: 659.7011 precCharge: 3 Sequence: IFVGGLPYHTTDASLR
Intensity
200000
150000
100000
50000
0
M/Z
500
1000
1500
b5
y7
b6
y1
y4
y6
y8
b1
b2
b3
b4
y2
y3
y5
b7
y9
b8
y10
type
b
y

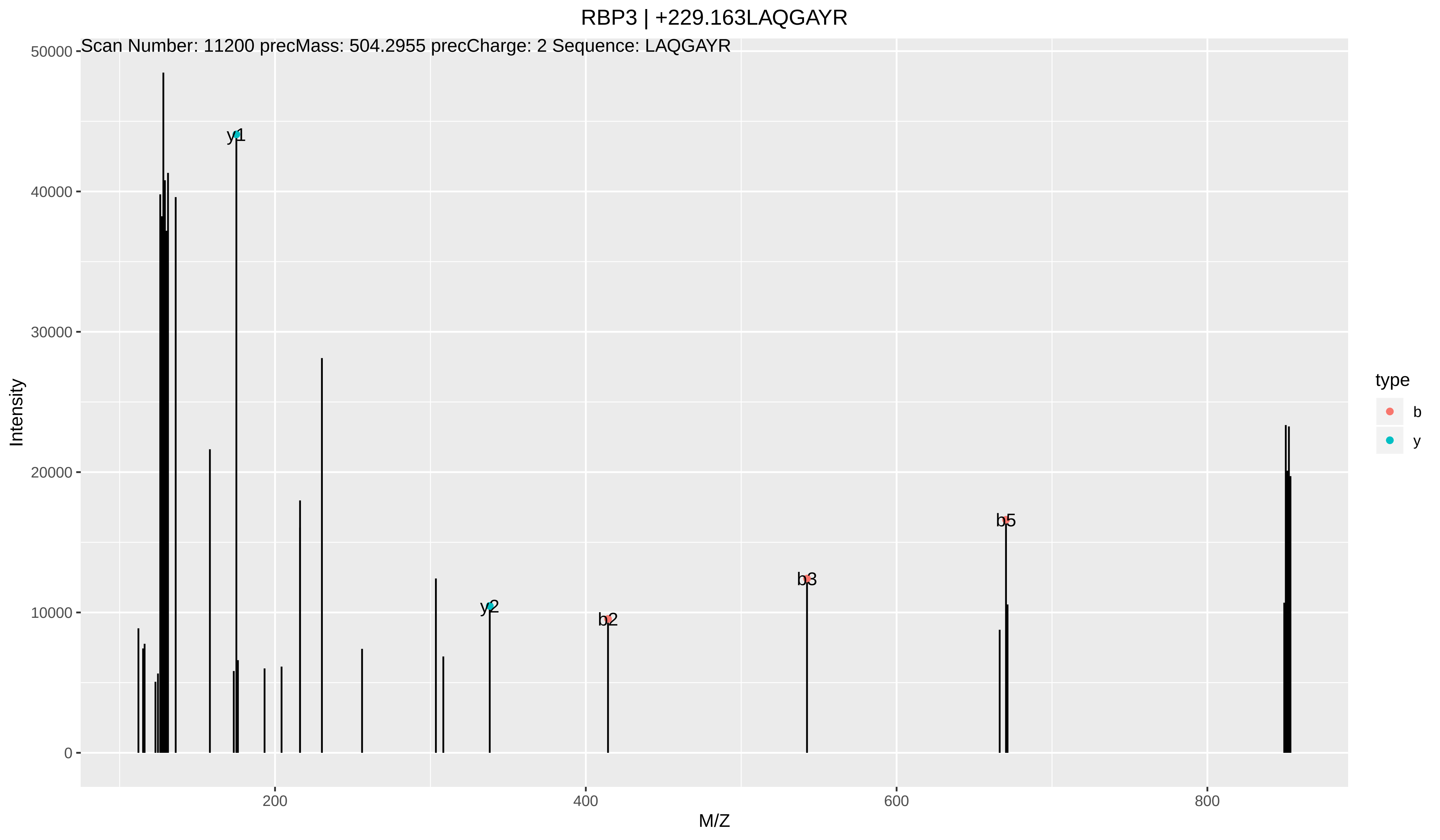
RBP3 | +229.163LAQGAYR
Scan Number: 11200 precMass: 504.2955 precCharge: 2 Sequence: LAQGAYR
Intensity
50000
40000
30000
20000
10000
0
M/Z
200
400
600
800
y1
y2
b2
b3
b5
type
b
y

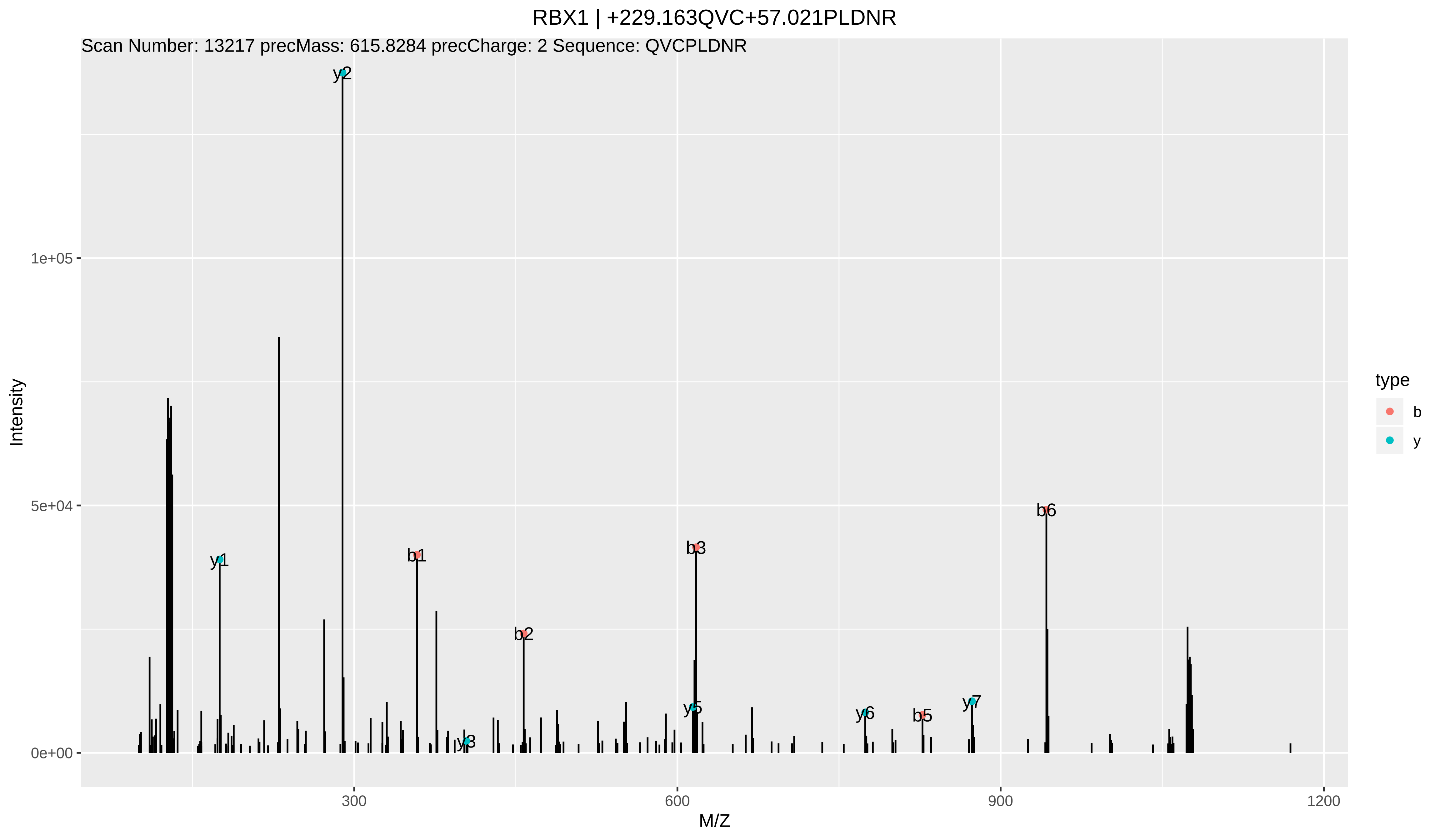

RBX1 | +229.163QVC+57.021PLDNR
Scan Number: 13217 precMass: 615.8284 precCharge: 2 Sequence: QVCPLDNR
Intensity
M/Z
type
b
y
y2
y1
b1
y3
b2
b3
y5
y6
b5
y7
b6
0e+00
5e+04
1e+05
300
600
900
1200

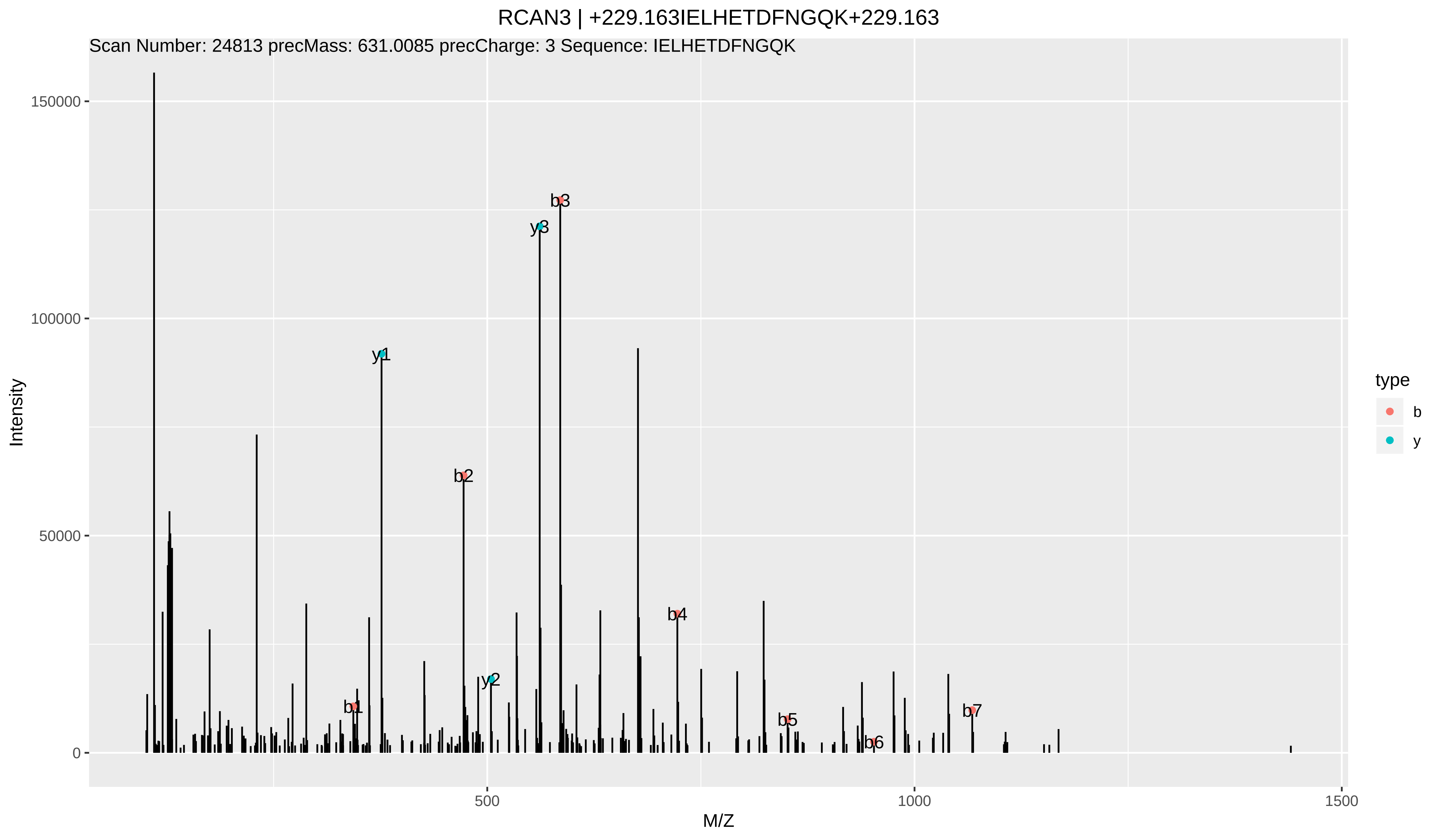

RCAN3 | +229.163IELHETDFNGQK+229.163
Scan Number: 24813 precMass: 631.0085 precCharge: 3 Sequence: IELHETDFNGQK
Intensity
M/Z
0
50000
100000
150000
500
1000
1500
b1
y1
b2
y2
y3
b3
b4
b5
b6
b7
type
b
y

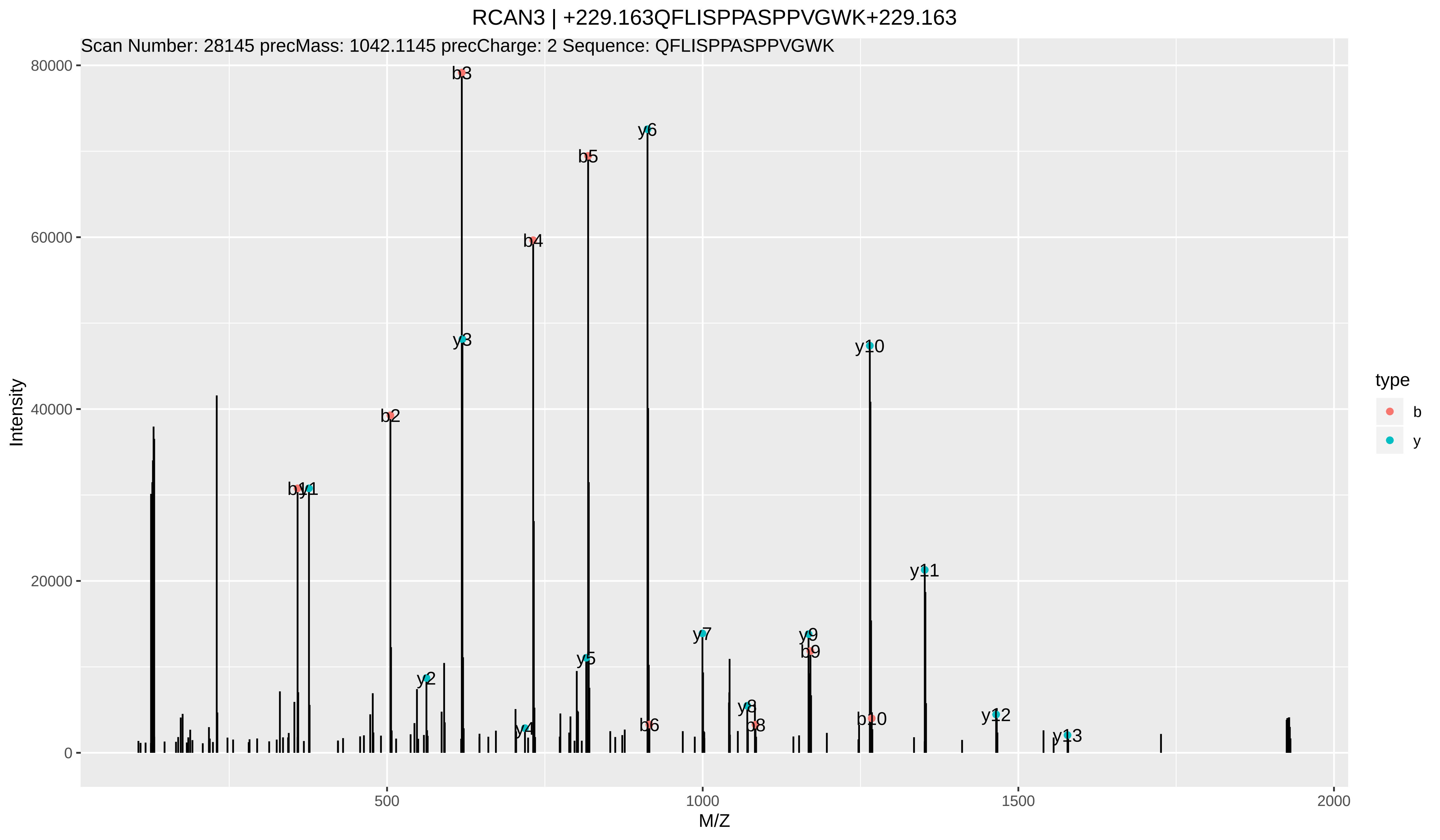

RCAN3 | +229.163QFLISPPASPPVGWK+229.163
Scan Number: 28145 precMass: 1042.1145 precCharge: 2 Sequence: QFLISPPASPPVGWK
Intensity
M/Z
type
b
y
b3
y6
b5
b4
y3
y10
b2
b1
y1
y11
y7
y9
b9
y5
y2
y8
b8
b6
b10
y12
y4
y13
0
20000
40000
60000
80000
500
1000
1500
2000

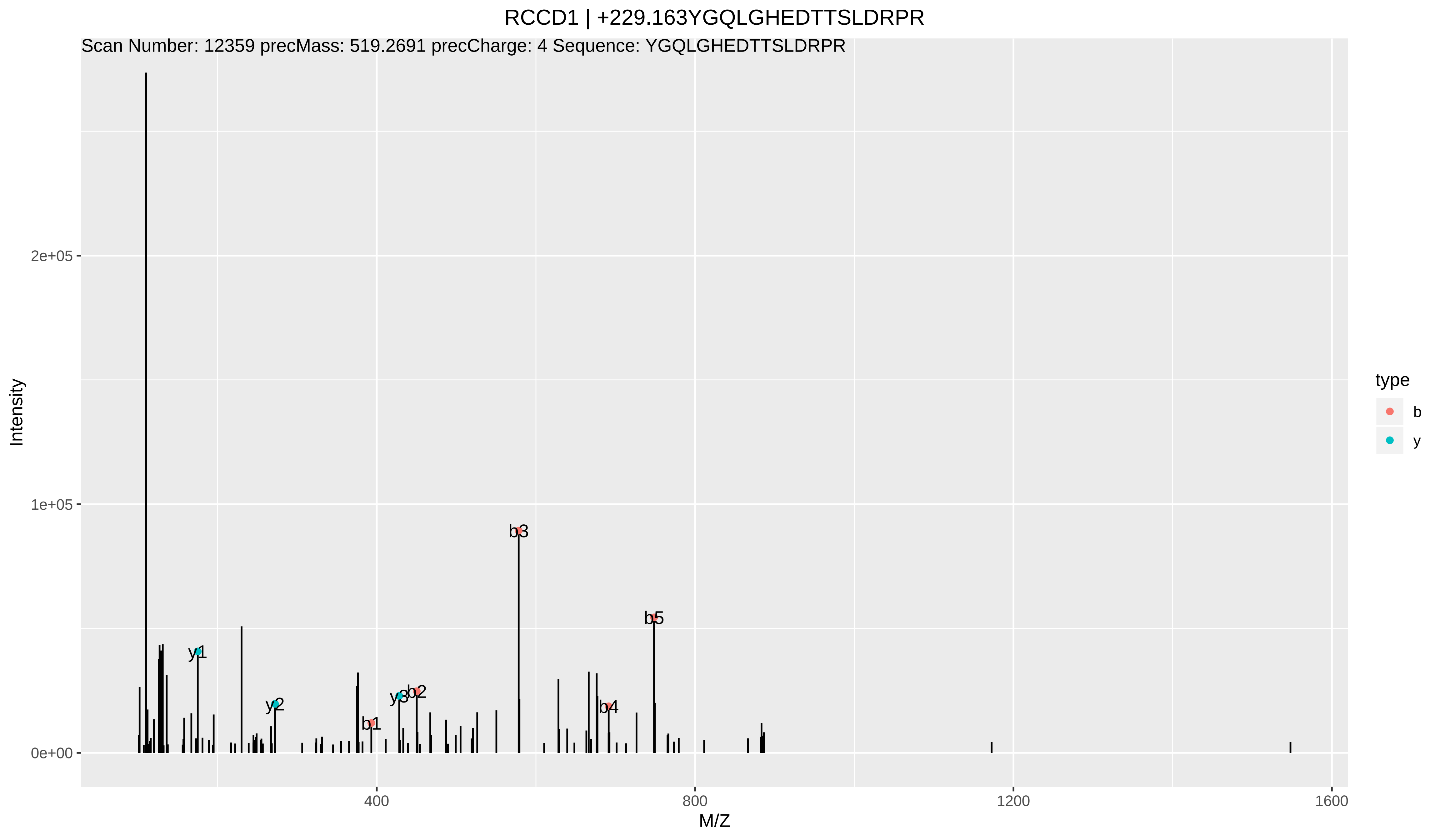

RCCD1 | +229.163YGQLGHEDTTSLDRPR
Scan Number: 12359 precMass: 519.2691 precCharge: 4 Sequence: YGQLGHEDTTSLDRPR
Intensity
2e+05
1e+05
0e+00
M/Z
400
800
1200
1600
y1
y2
y3
b1
b2
b3
b4
b5
type
b
y

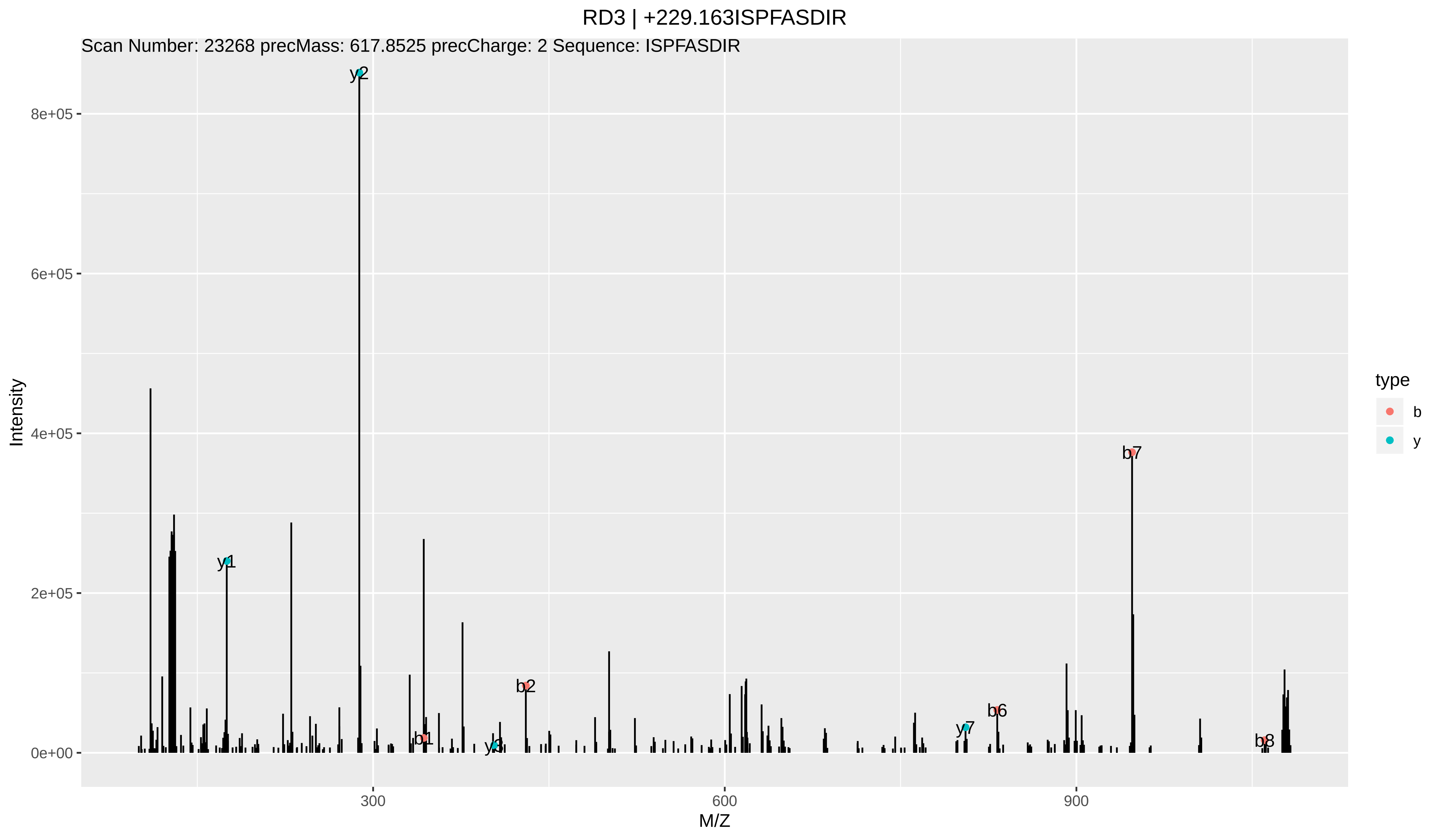
RD3 | +229.163ISPFASDIR
Scan Number: 23268 precMass: 617.8525 precCharge: 2 Sequence: ISPFASDIR
y2
y1
b7
b2
b6
y7
b1
y3
b8
Intensity
8e+05
6e+05
4e+05
2e+05
0e+00
300
600
900
M/Z
type
b
y

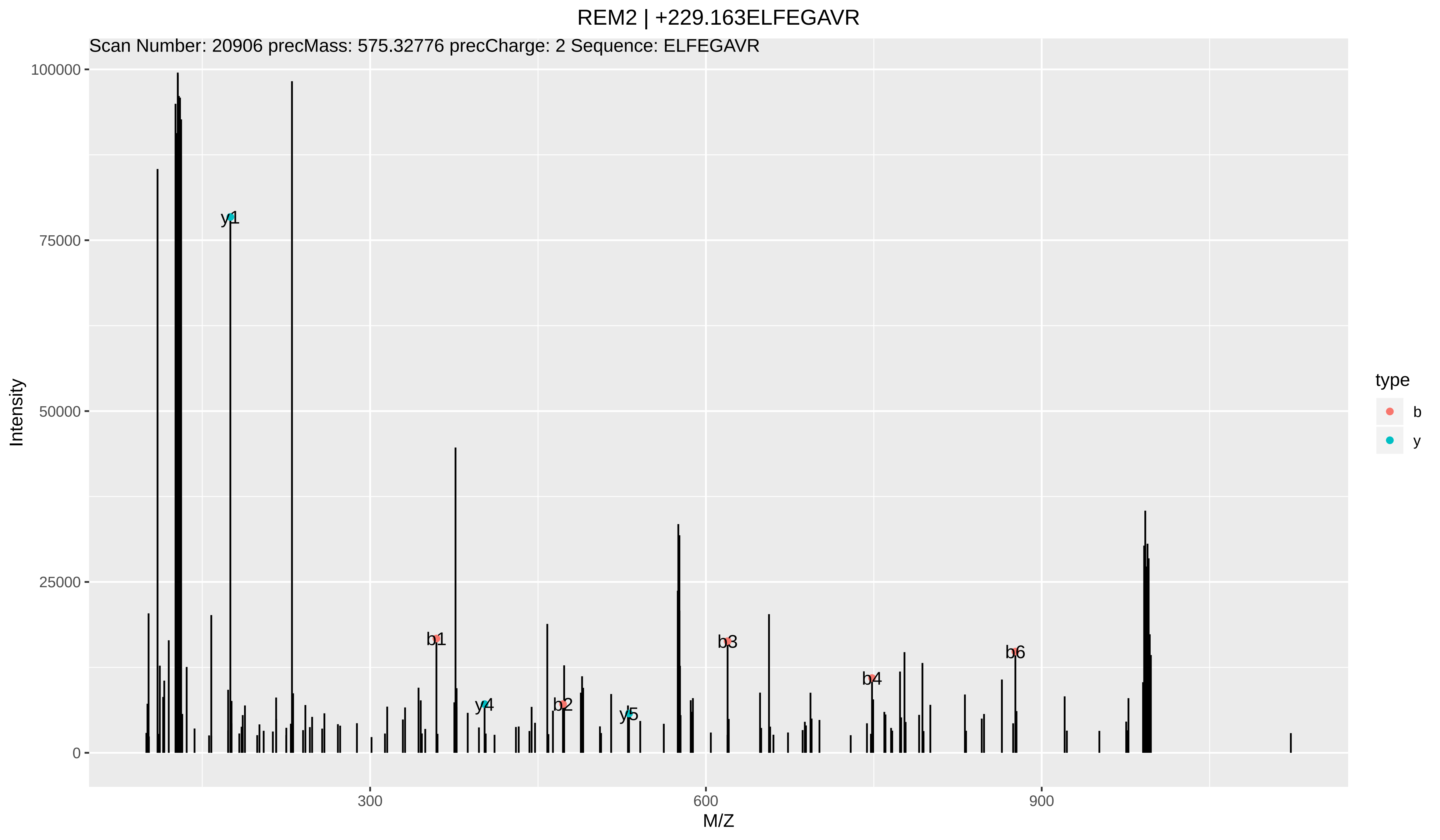

REM2 | +229.163ELFEGAVR
Scan Number: 20906 precMass: 575.32776 precCharge: 2 Sequence: ELFEGAVR
100000
75000
50000
25000
0
Intensity
300
600
900
M/Z
y1
b1
y4
b2
y5
b3
b4
b6
type
b
y

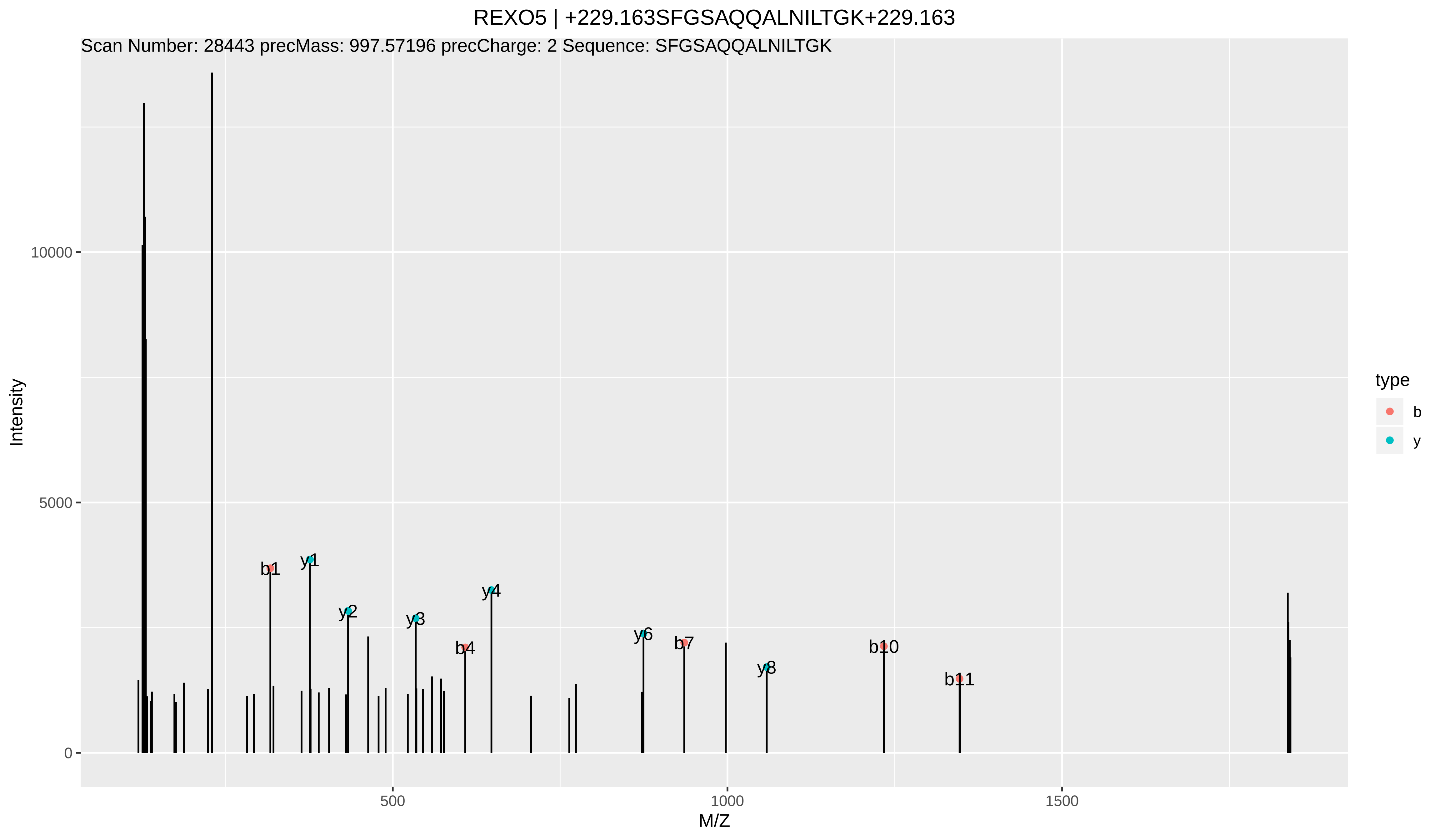
REXO5 | +229.163SFGSAQQALNILTGK+229.163
Scan Number: 28443 precMass: 997.57196 precCharge: 2 Sequence: SFGSAQQALNILTGK
Intensity
10000
5000
0
500
1000
1500
M/Z
b1
y1
y2
y3
b4
y4
y6
b7
y8
b10
b11
type
b
y

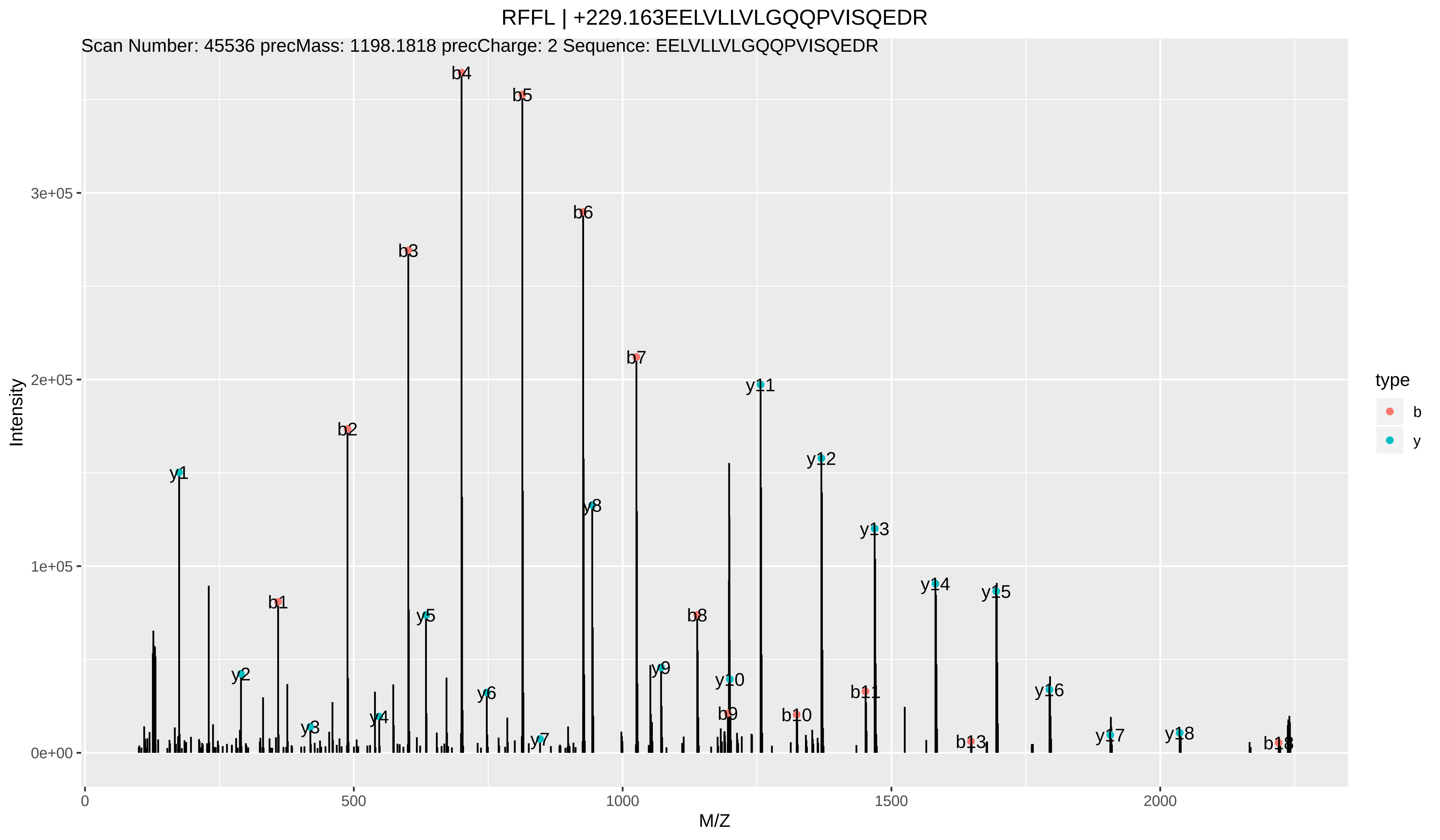

RFFL | +229.163EELVLLVLGQQPVISQEDR
Scan Number: 45536 precMass: 1198.1818 precCharge: 2 Sequence: EELVLLVLGQQPVISQEDR
Intensity
M/Z
type
b
y

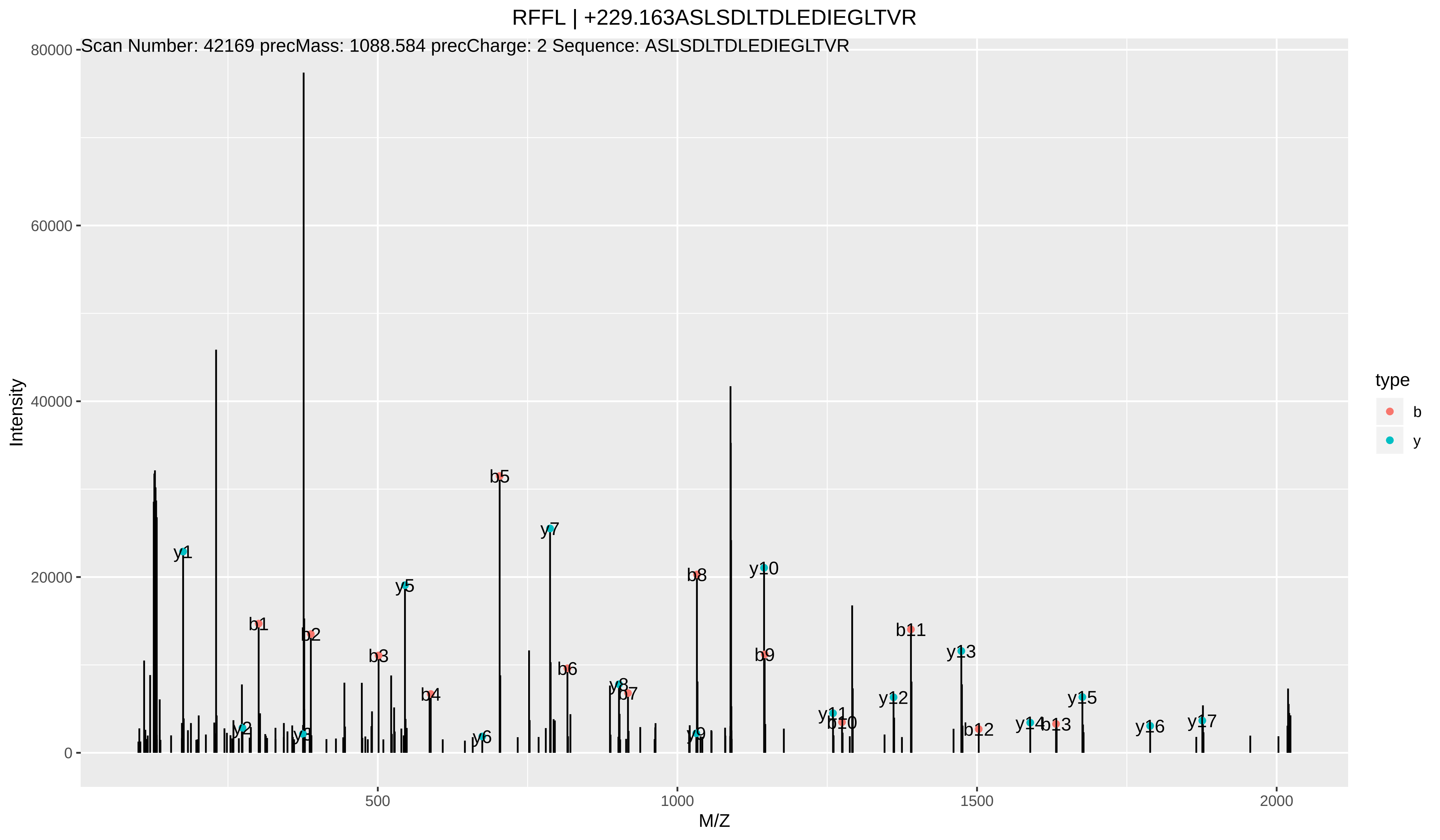
RFFL | +229.163ASLSDLTDLEDIEGLTVR
Scan Number: 42169 precMass: 1088.584 precCharge: 2 Sequence: ASLSDLTDLEDIEGLTVR
Intensity
M/Z
0
20000
40000
60000
80000
500
1000
1500
2000
type
b
y
y1
y2
y3
y5
y6
y7
y8
y9
y10
y11
y12
y13
y14
y15
y16
y17
b1
b2
b3
b4
b5
b6
b7
b8
b9
b10
b11
b12
b13

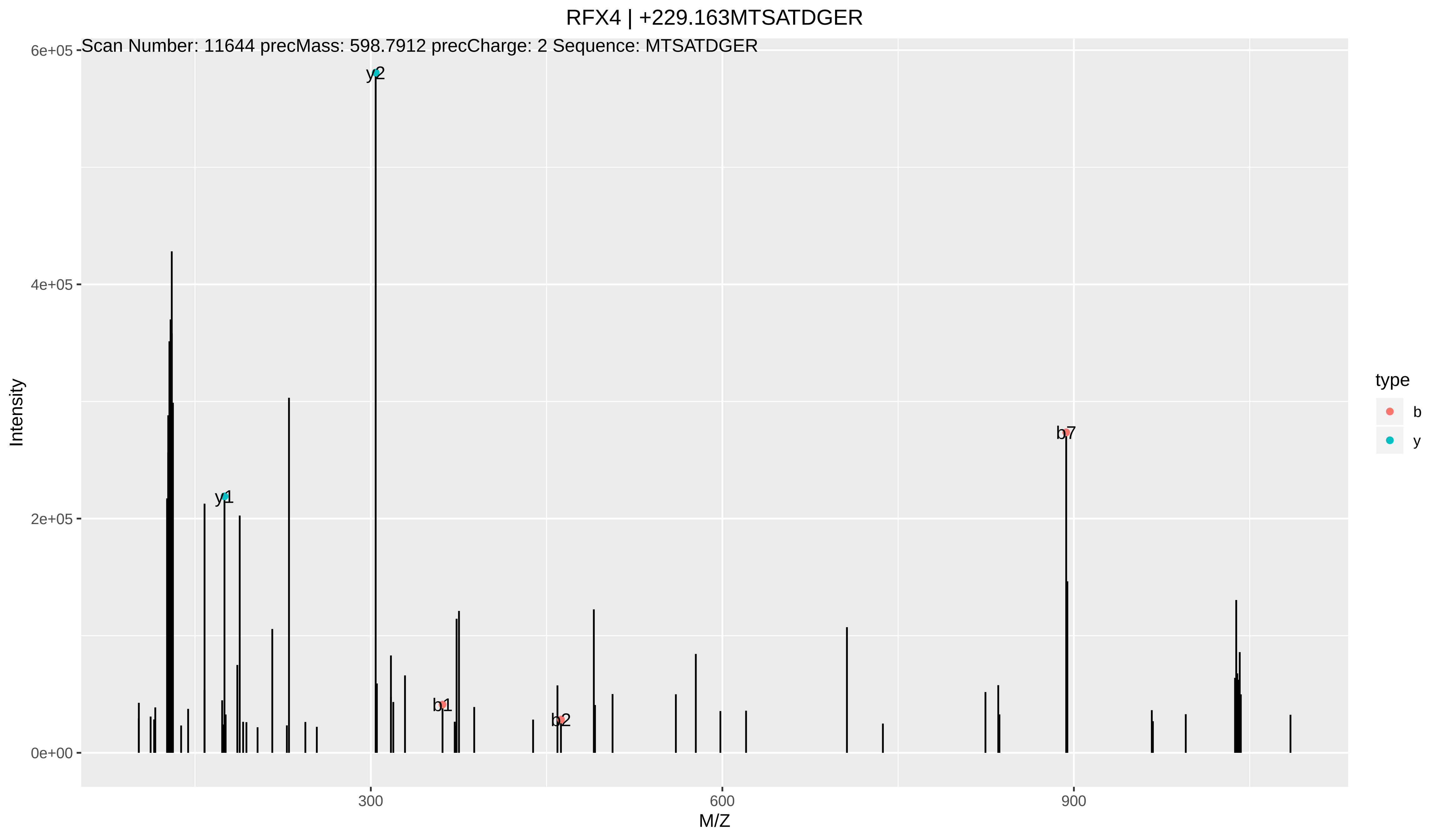

RFX4 | +229.163MTSATDGER
Scan Number: 11644 precMass: 598.7912 precCharge: 2 Sequence: MTSATDGER
y2
y1
b1
b2
b7
Intensity
M/Z
type
b
y

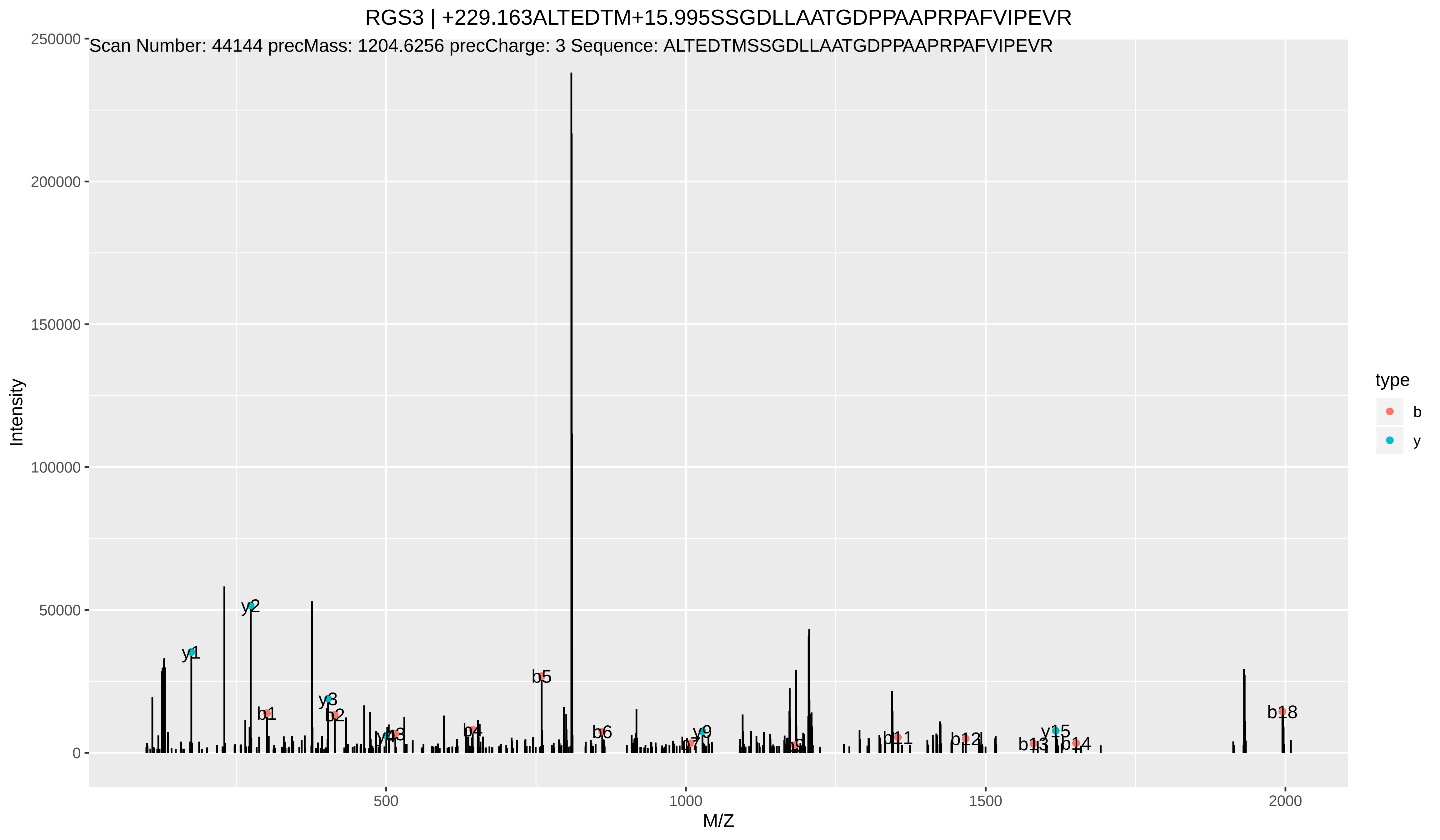

RGS3 | +229.163ALTEDTM+15.995SSGDLLAATGDPPAAPRPAFVIPEVR
Scan Number: 44144 precMass: 1204.6256 precCharge: 3 Sequence: ALTEDTMSSGDLLAATGDPPAAPRPAFVIPEVR
Intensity
M/Z
type
b
y

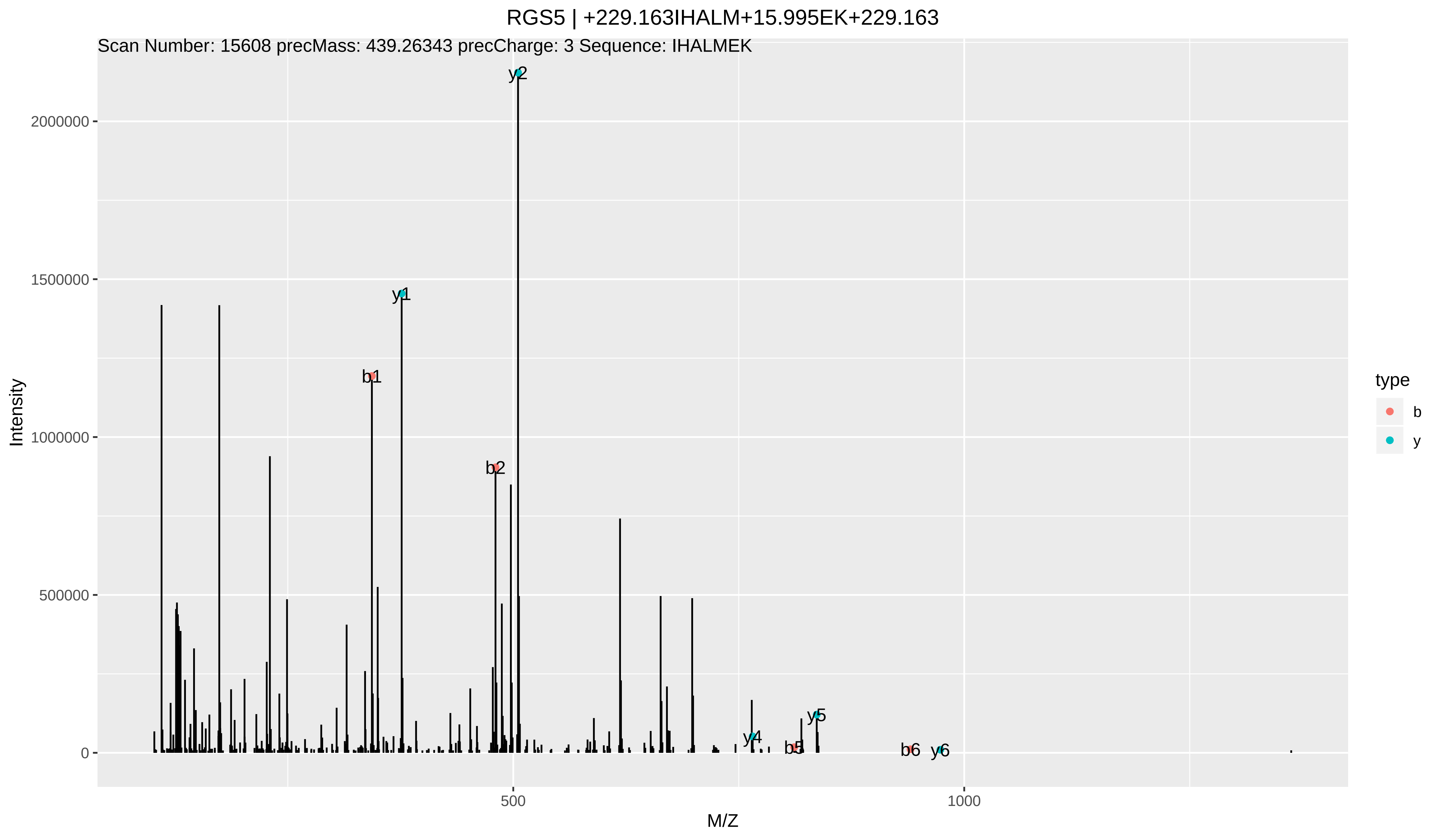

RGS5 | +229.163IHALM+15.995EK+229.163
Scan Number: 15608 precMass: 439.26343 precCharge: 3 Sequence: IHALMEK
Intensity
2000000
1500000
1000000
500000
0
500
1000
M/Z
y2
y1
b1
b2
y5
y4
b5
b6
y6
type
b
y

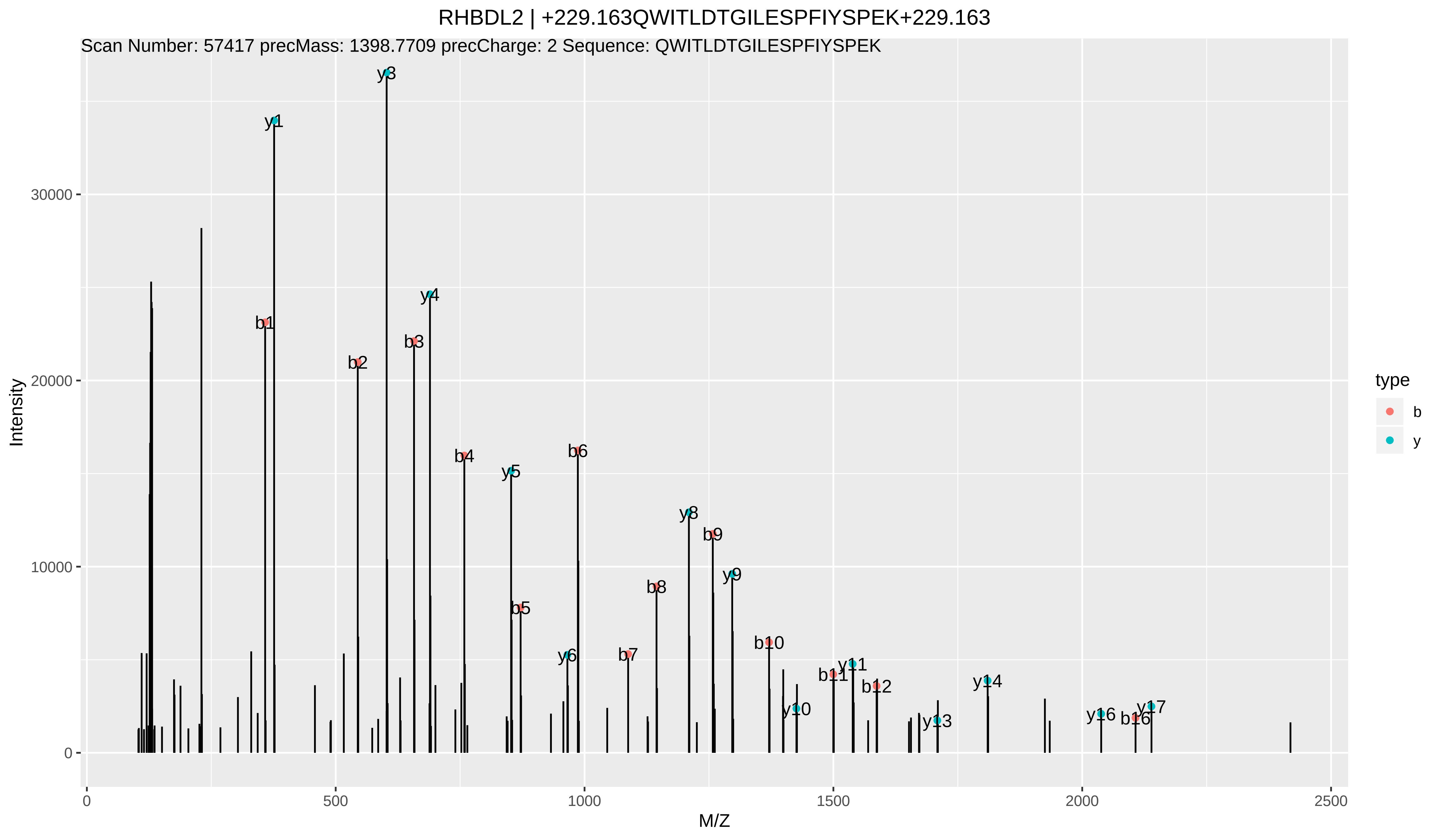

RHBDL2 | +229.163QWITLDTGILESPFIYSPEK+229.163
Scan Number: 57417 precMass: 1398.7709 precCharge: 2 Sequence: QWITLDTGILESPFIYSPEK
Intensity
M/Z
type
b
y
y1
y3
y4
y5
y6
y8
y9
y10
y11
y13
y14
y16
y17
b1
b2
b3
b4
b5
b6
b7
b8
b9
b10
b11
b12
b16
0
10000
20000
30000
500
1000
1500
2000
2500

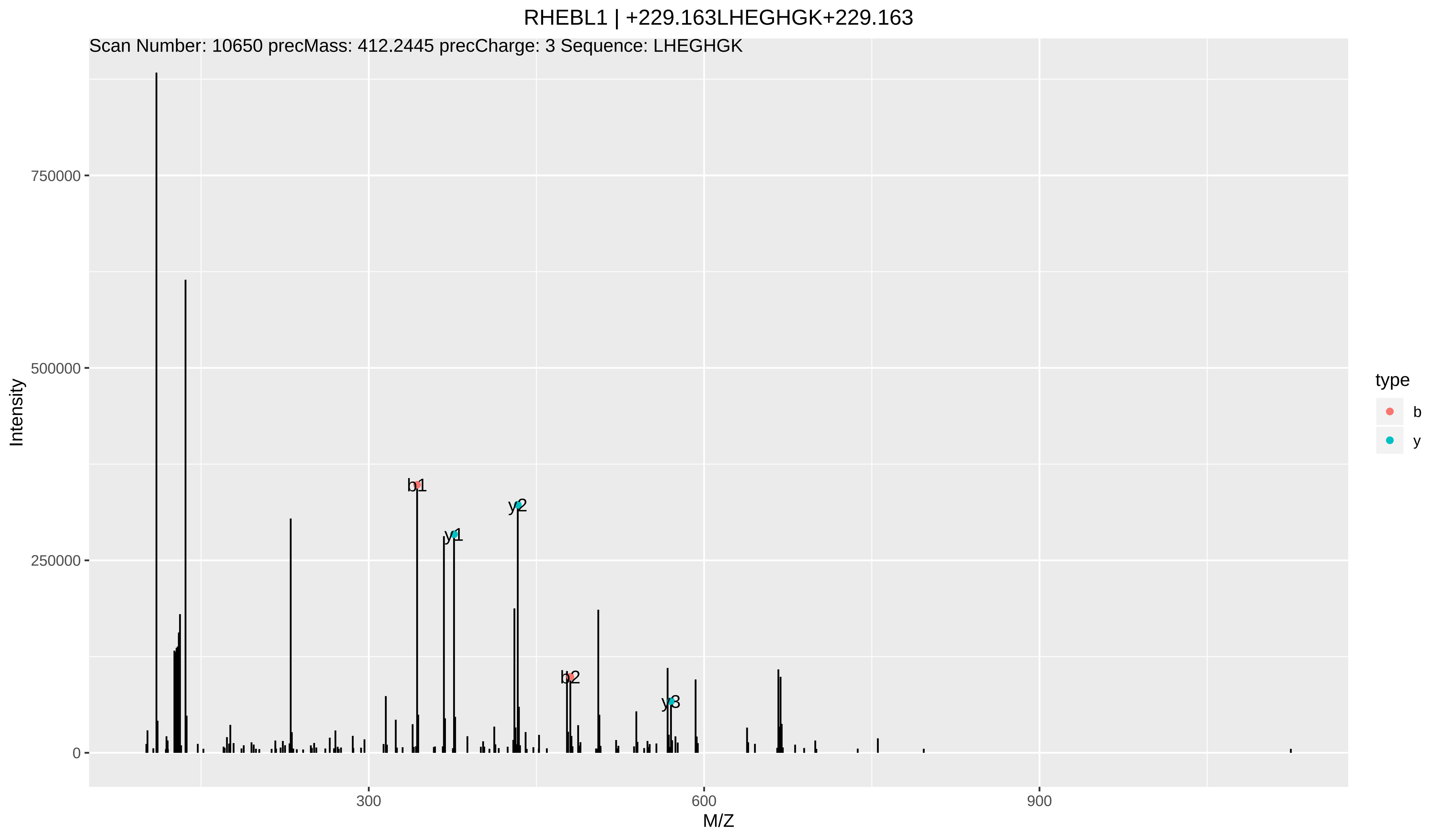

RHEBL1 | +229.163LHEGHGK+229.163
Scan Number: 10650 precMass: 412.2445 precCharge: 3 Sequence: LHEGHGK
Intensity
750000
500000
250000
0
300
600
900
M/Z
b1
y1
y2
b2
y3
type
b
y

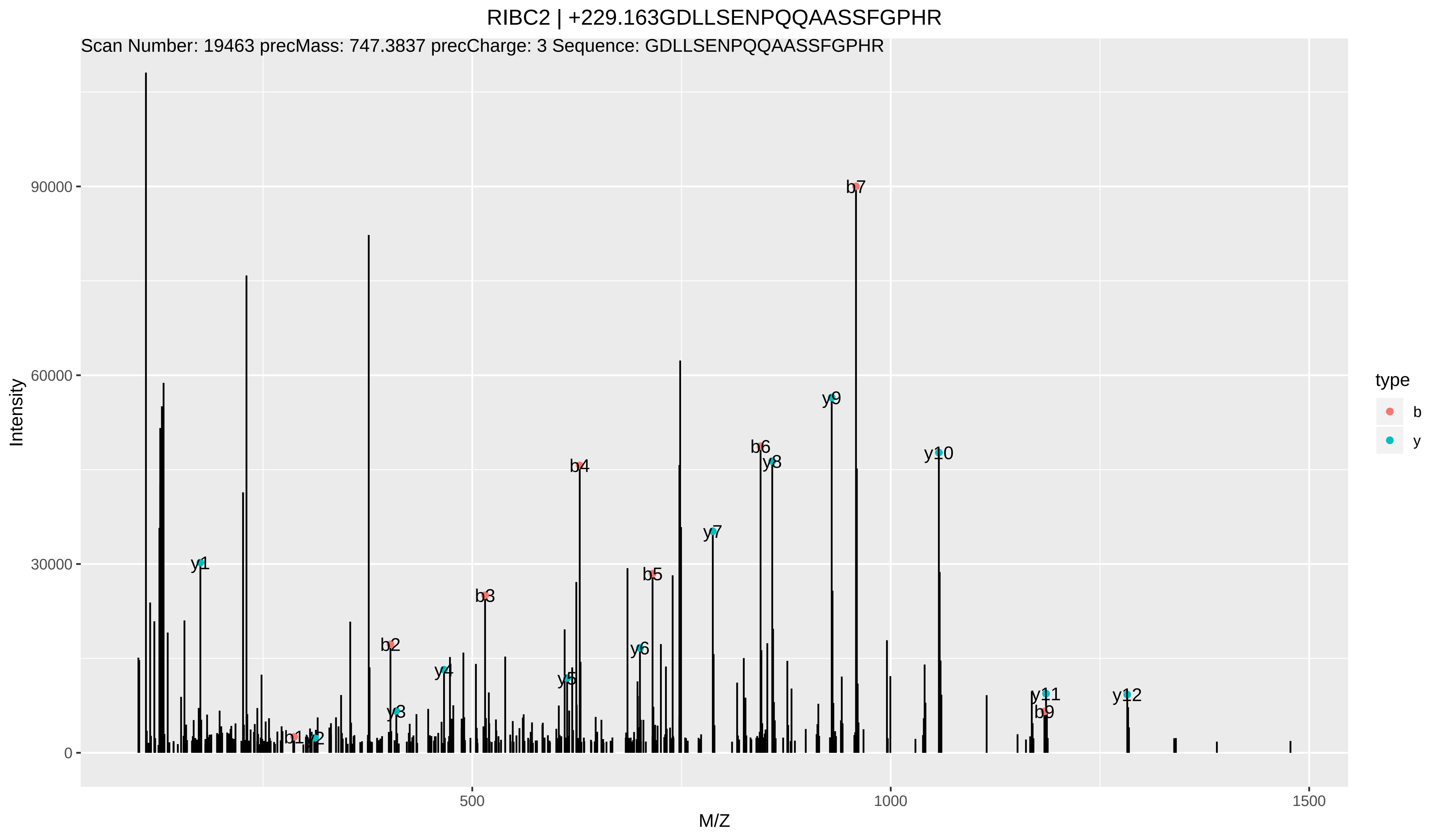
RIBC2 | +229.163GDLLSENPQQAASSFGPHR
Scan Number: 19463 precMass: 747.3837 precCharge: 3 Sequence: GDLLSENPQQAASSFGPHR
Intensity
90000
60000
30000
0
500
1000
1500
M/Z
type
b
y
y1
b1
y2
b2
y3
y4
b3
y5
b4
y6
b5
y7
b6
y8
y9
b7
y10
y11
b9
y12

RILPL2 | +229.163SPFQLTAEDVYDISYLLGR

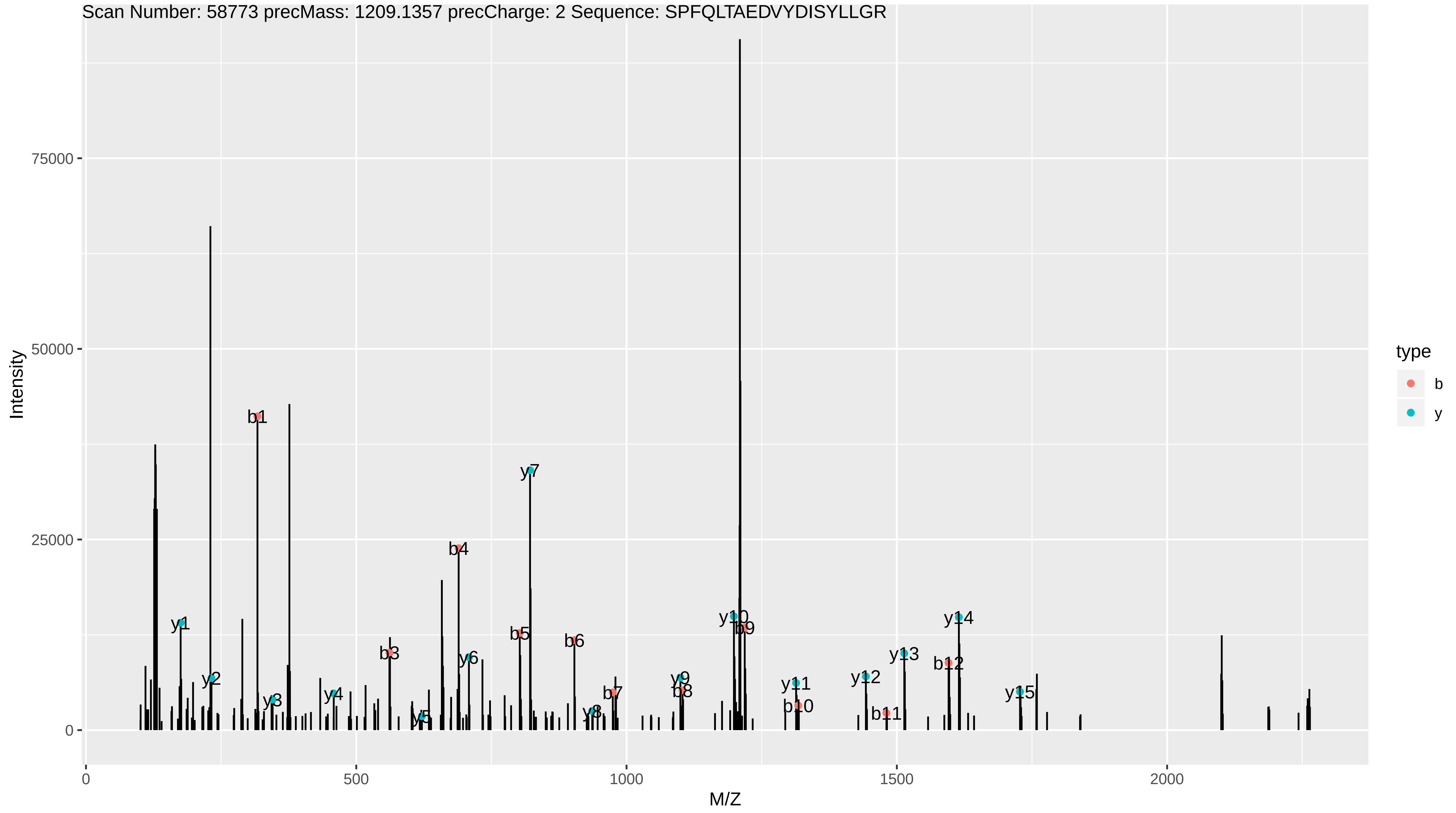

RIMBP2 | +229.163LNMAAC+57.021TYR

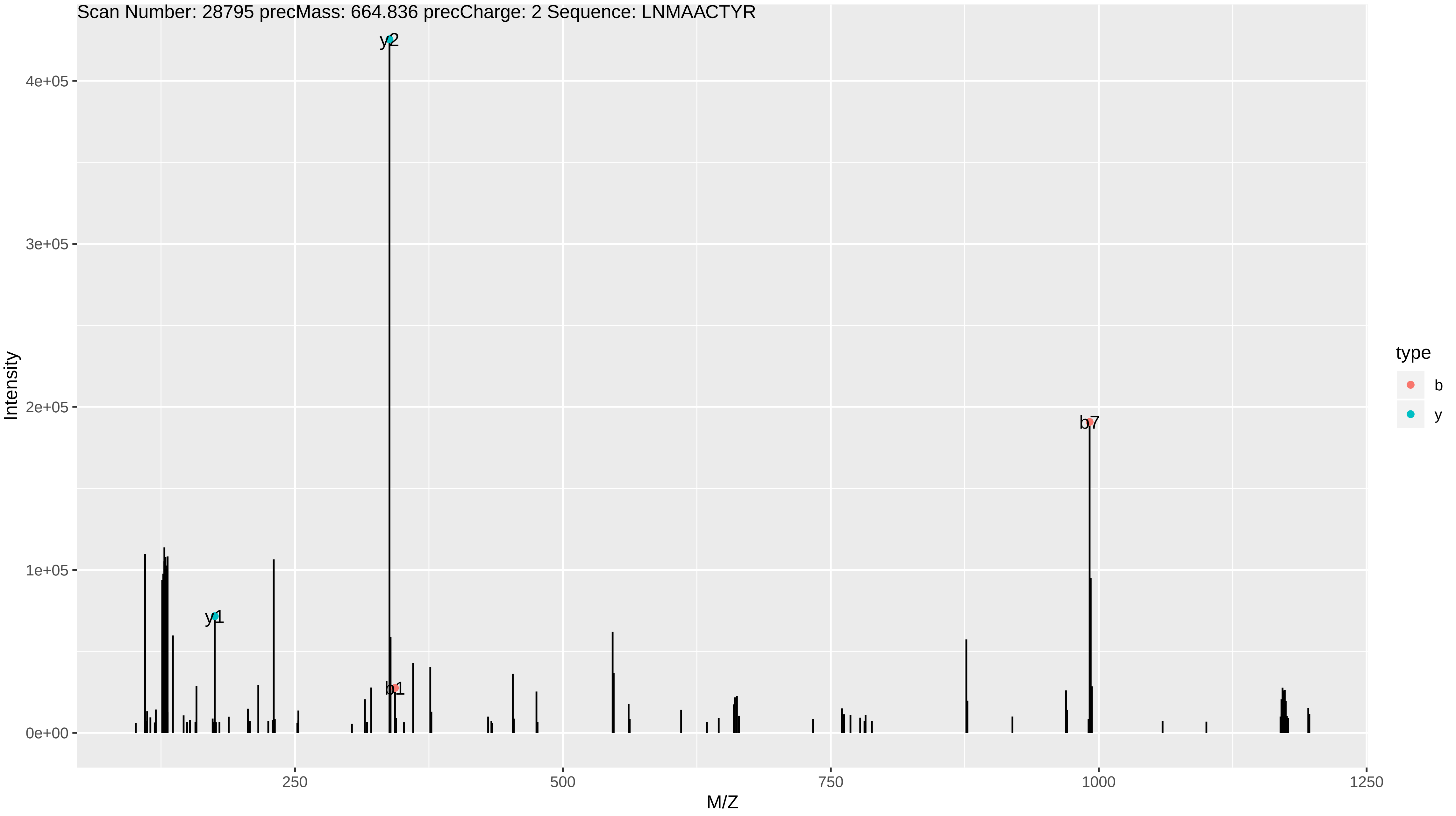

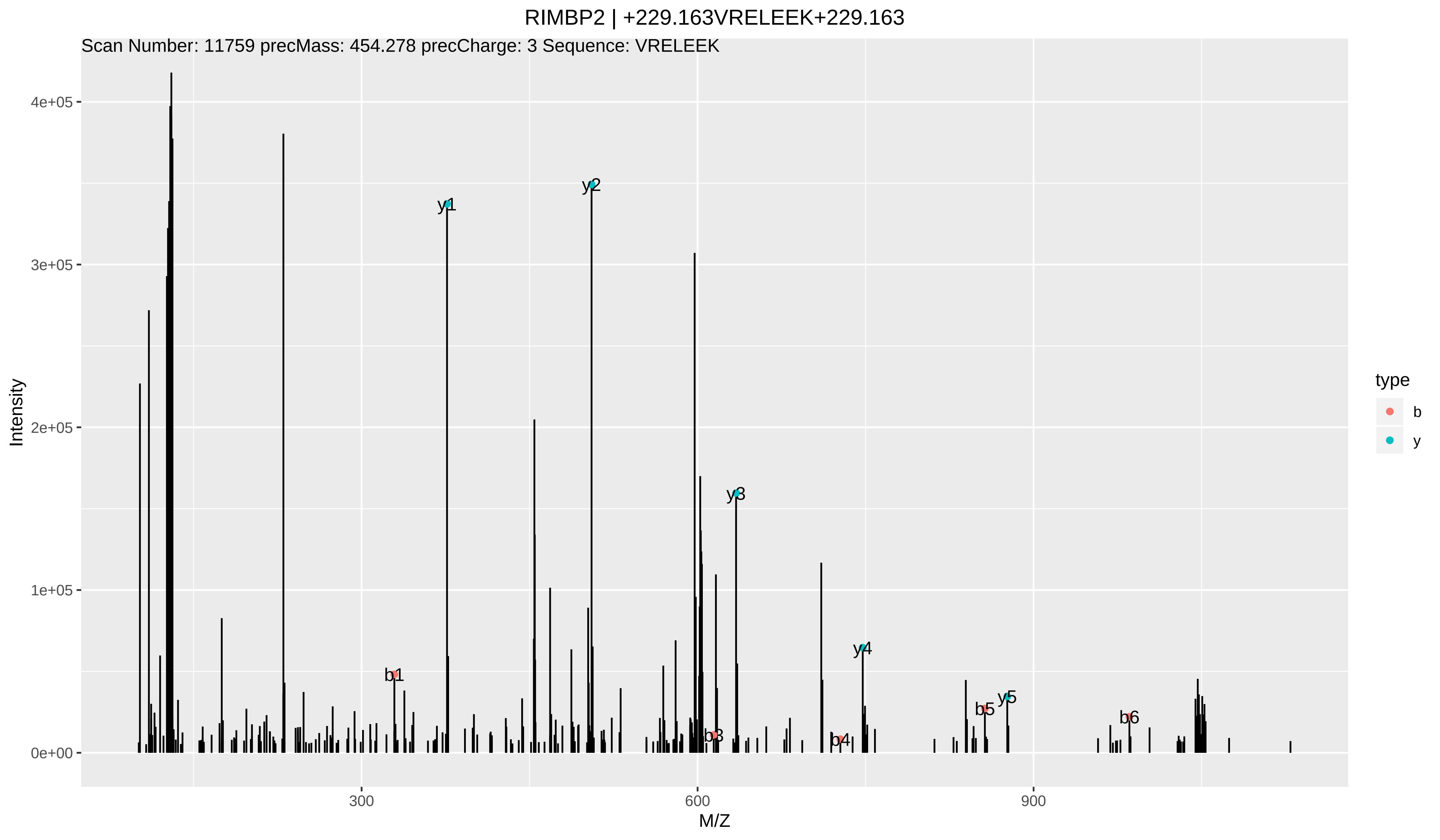

RIMBP2 | +229.163VRELEEK+229.163
Scan Number: 11759 precMass: 454.278 precCharge: 3 Sequence: VRELEEK
Intensity
4e+05
3e+05
2e+05
1e+05
0e+00
M/Z
300
600
900
y1
y2
y3
y4
y5
b1
b3
b4
b5
b6
type
b
y

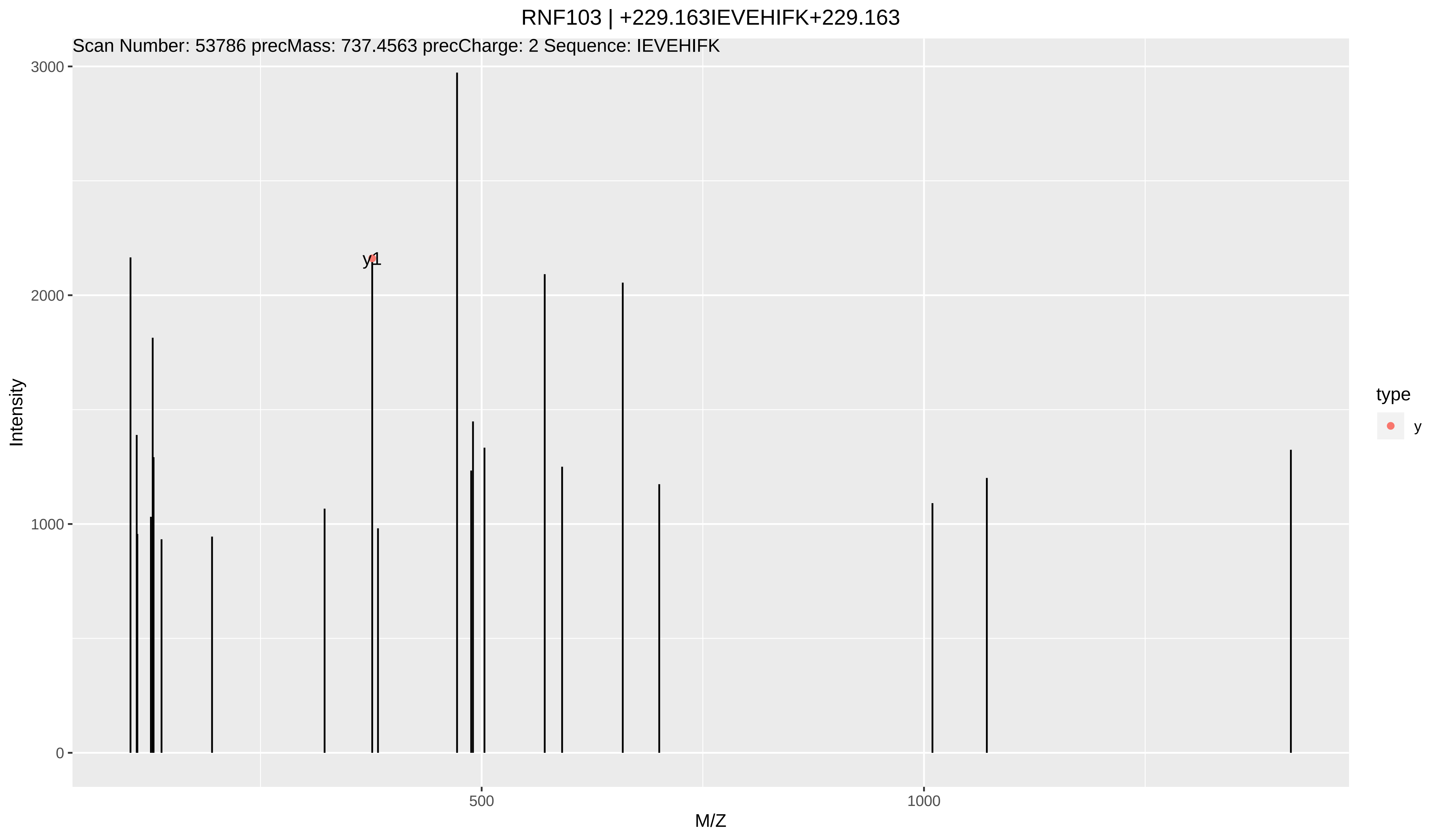
RNF103 | +229.163IEVEHIFK+229.163
Scan Number: 53786 precMass: 737.4563 precCharge: 2 Sequence: IEVEHIFK
Intensity
3000
2000
1000
0
M/Z
500
1000
y1
type
y

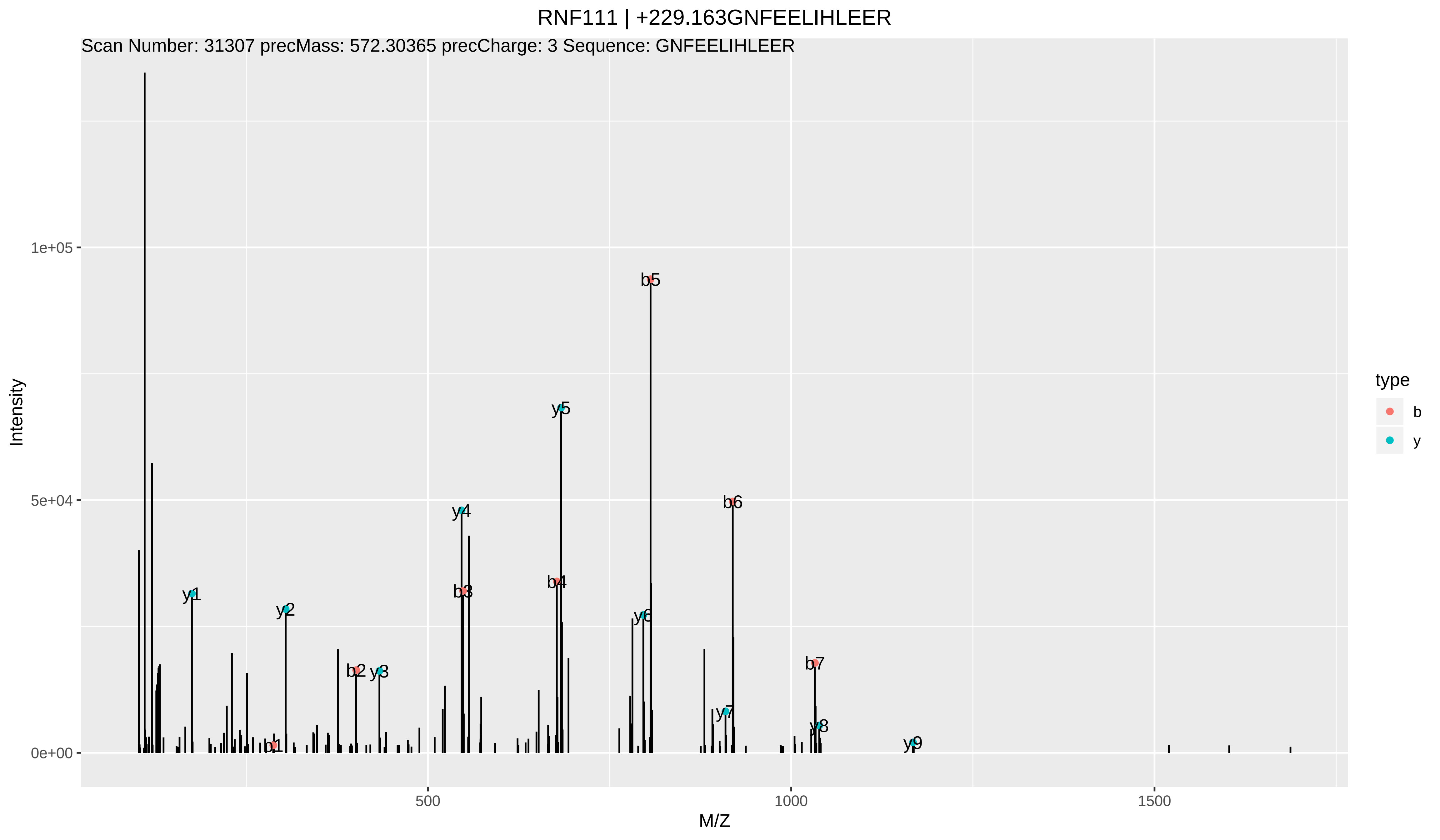

RNF111 | +229.163GNFEELIHLEER
Scan Number: 31307 precMass: 572.30365 precCharge: 3 Sequence: GNFEELIHLEER
Intensity
1e+05
5e+04
0e+00
M/Z
500
1000
1500
b5
y5
y4
b6
b4
b3
y1
y2
y6
b2
y3
b7
y7
y8
b1
y9
type
b
y

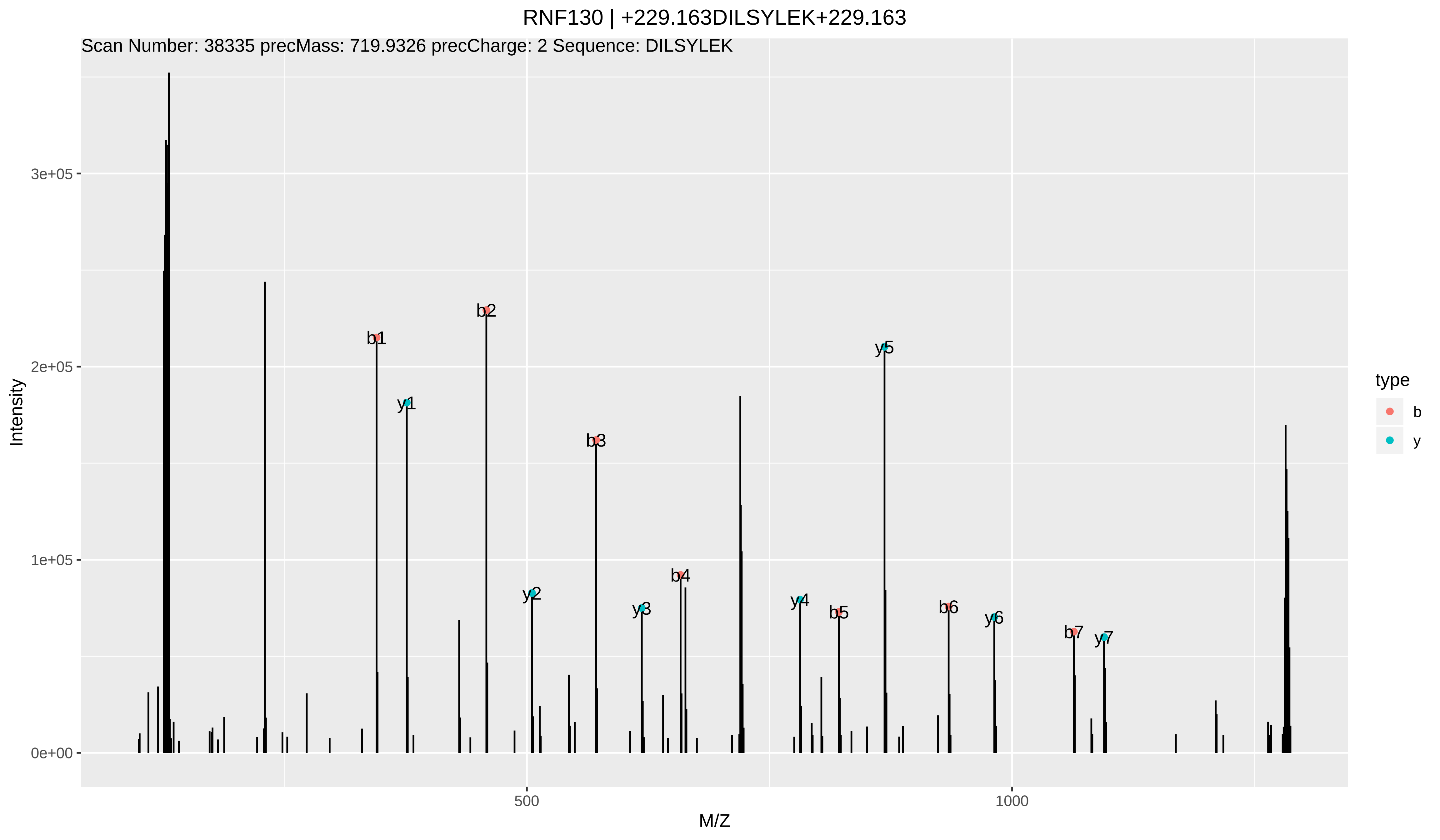
RNF130 | +229.163DILSYLEK+229.163
Scan Number: 38335 precMass: 719.9326 precCharge: 2 Sequence: DILSYLEK
Intensity
3e+05
2e+05
1e+05
0e+00
M/Z
500
1000
b1
y1
b2
y2
b3
y3
b4
y4
b5
y5
b6
y6
b7
y7
type
b
y

RNF138 | +229.163SETSTSDNTETYQENTSSSGHPTFK+229.163

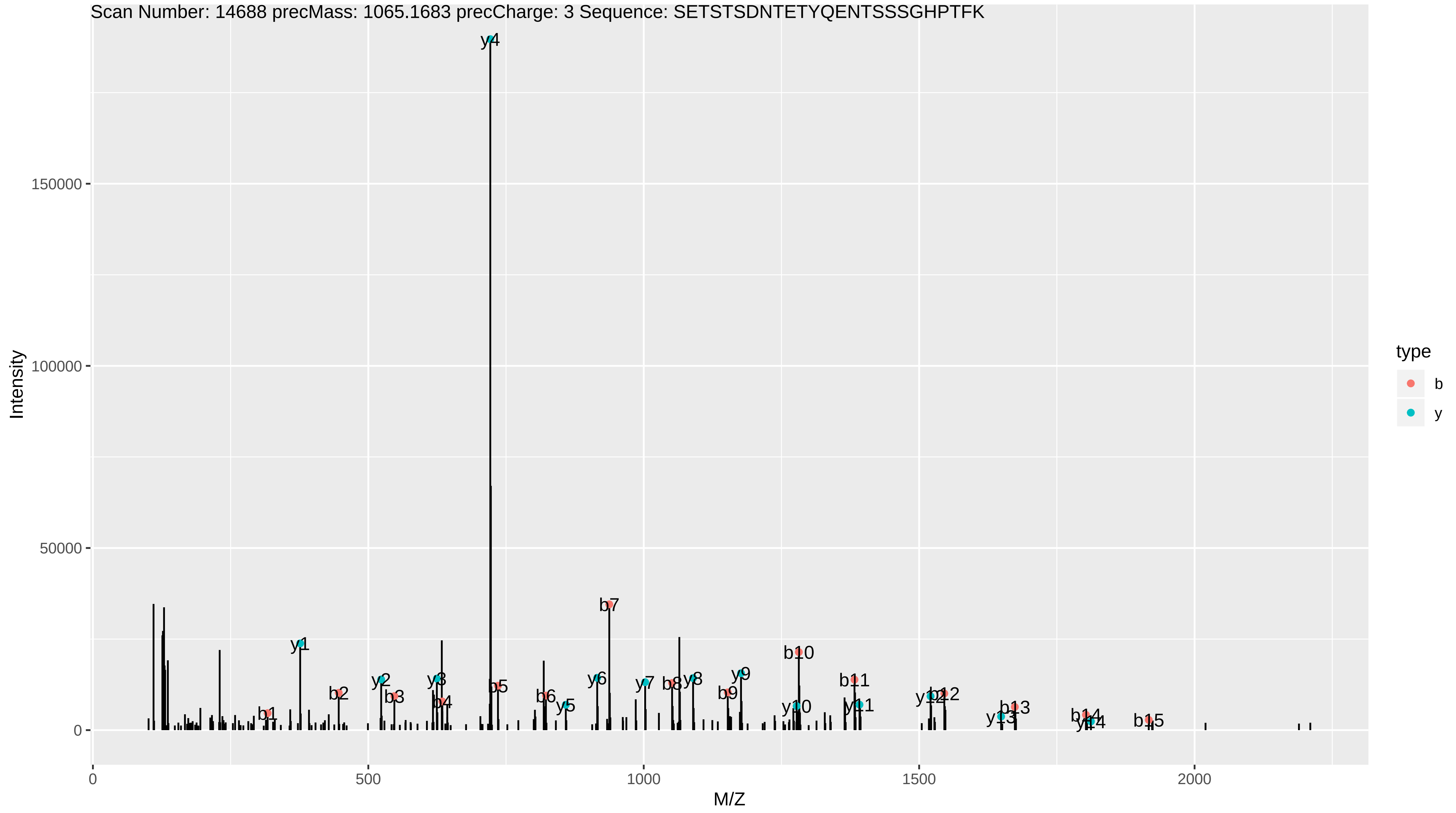

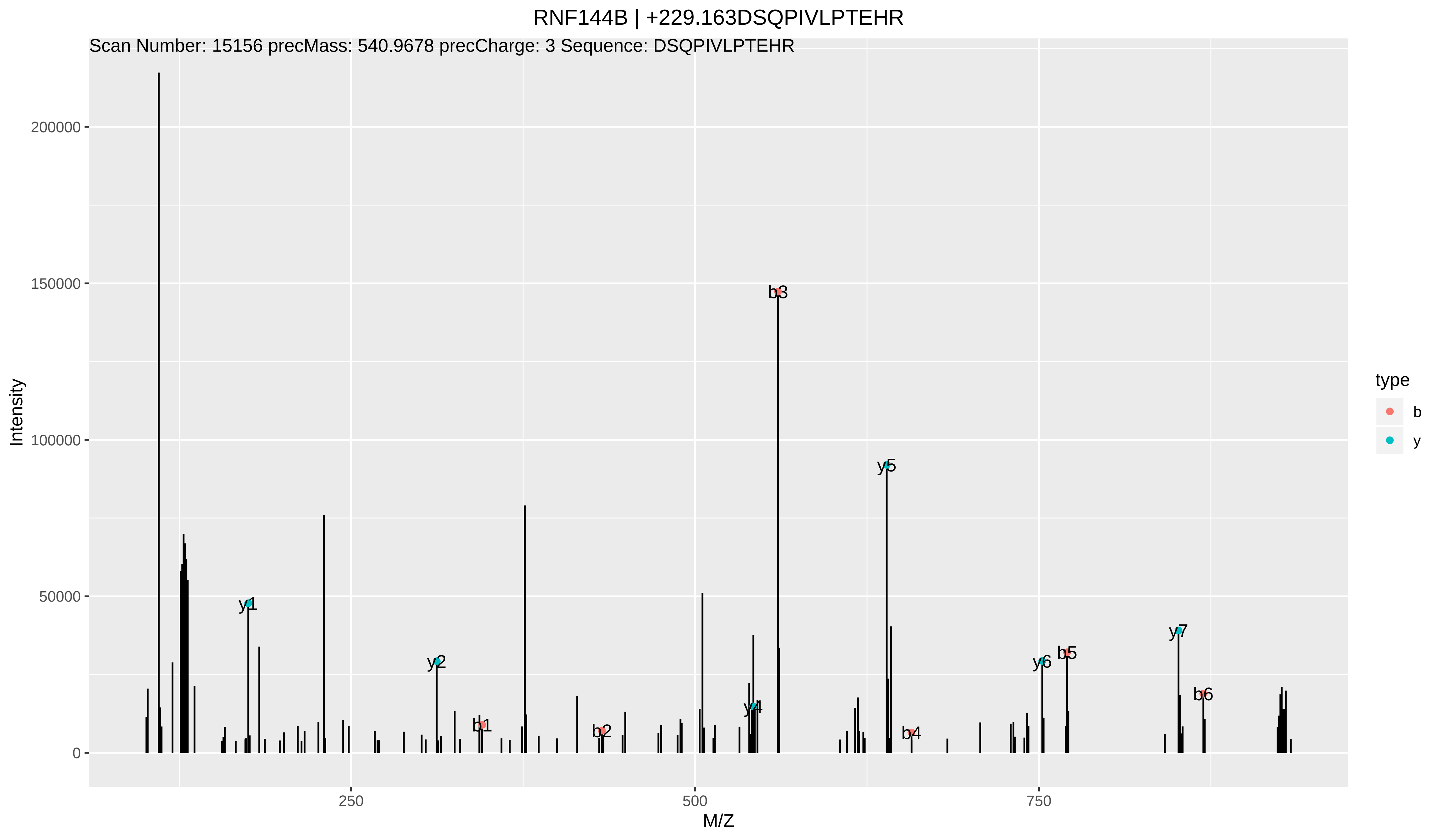

RNF144B | +229.163DSQPIVLPTEHR
Scan Number: 15156 precMass: 540.9678 precCharge: 3 Sequence: DSQPIVLPTEHR
Intensity
M/Z
0
50000
100000
150000
200000
250
500
750
y1
y2
b1
b2
y4
b3
y5
b4
y6
b5
y7
b6
type
b
y

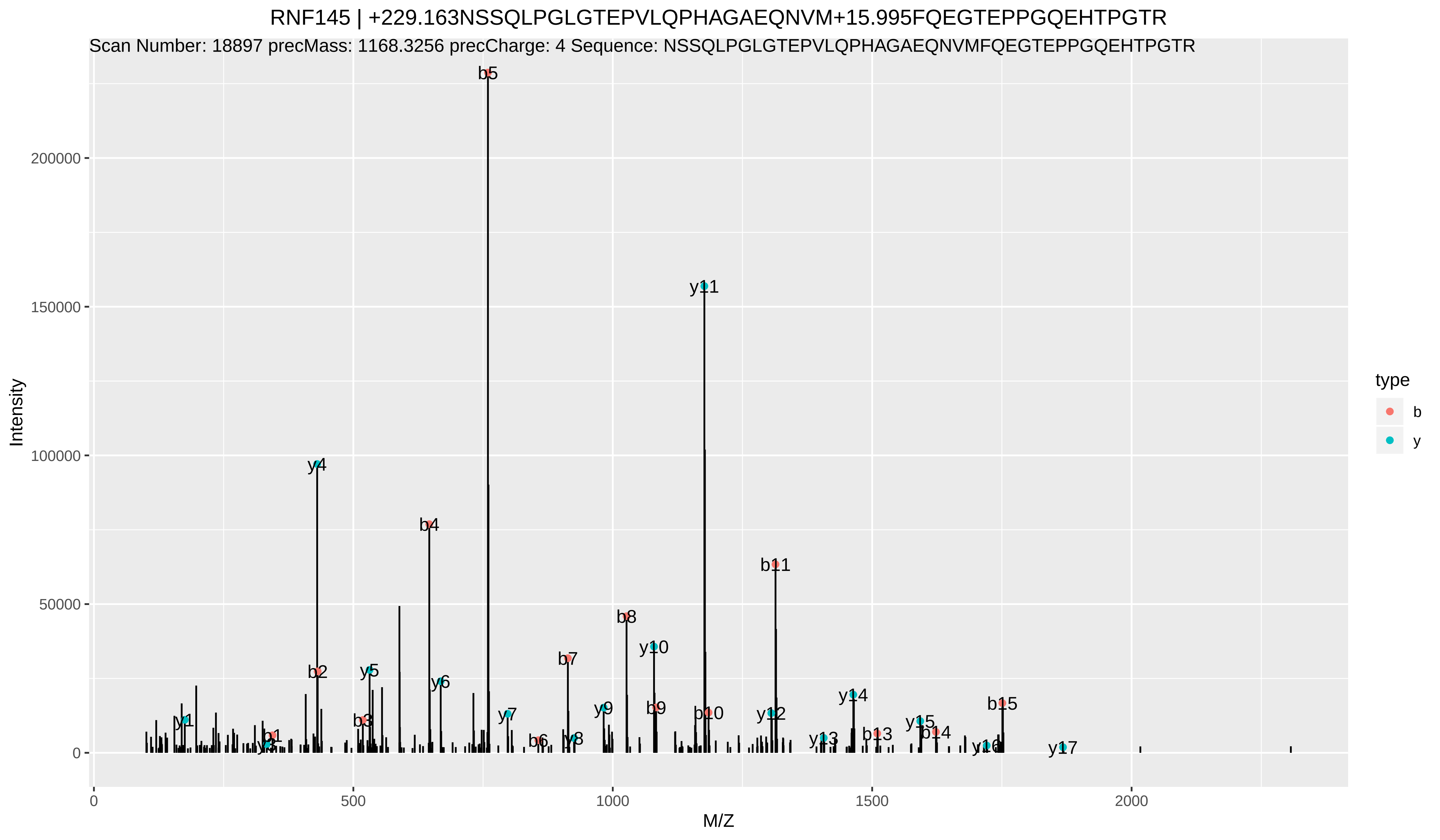

RNF145 | +229.163NSSQLPGLGTEPVLQPHAGAEQNVM+15.995FQEGTEPPGQEHTPGTR
Scan Number: 18897 precMass: 1168.3256 precCharge: 4 Sequence: NSSQLPGLGTEPVLQPHAGAEQNVMFQEGTEPPGQEHTPGTR
Intensity
M/Z
type
b
y
0
50000
100000
150000
200000
500
1000
1500
2000
b5
y11
y4
b4
b11
b8
y10
b7
b2
y5
y6
y14
b15
y7
y9
b9
b10
y12
y1
b3
b1
y3
b6
y8
y13
b13
y15
b14
y16
y17

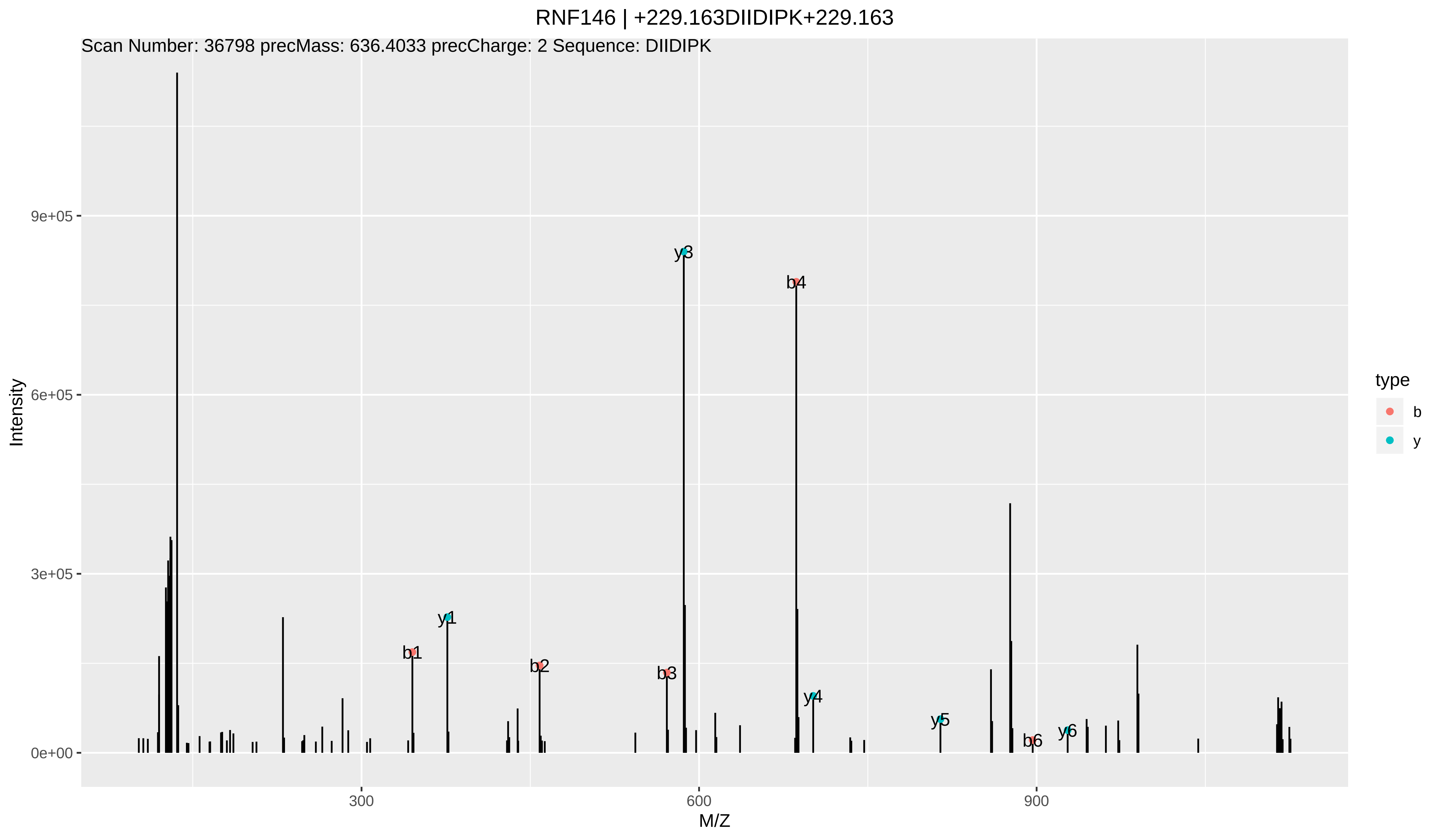
RNF146 | +229.163DIIDIPK+229.163
Scan Number: 36798 precMass: 636.4033 precCharge: 2 Sequence: DIIDIPK
Intensity
9e+05
6e+05
3e+05
0e+00
M/Z
300
600
900
y3
b4
y1
b1
b2
b3
y4
y5
b6
y6
type
b
y

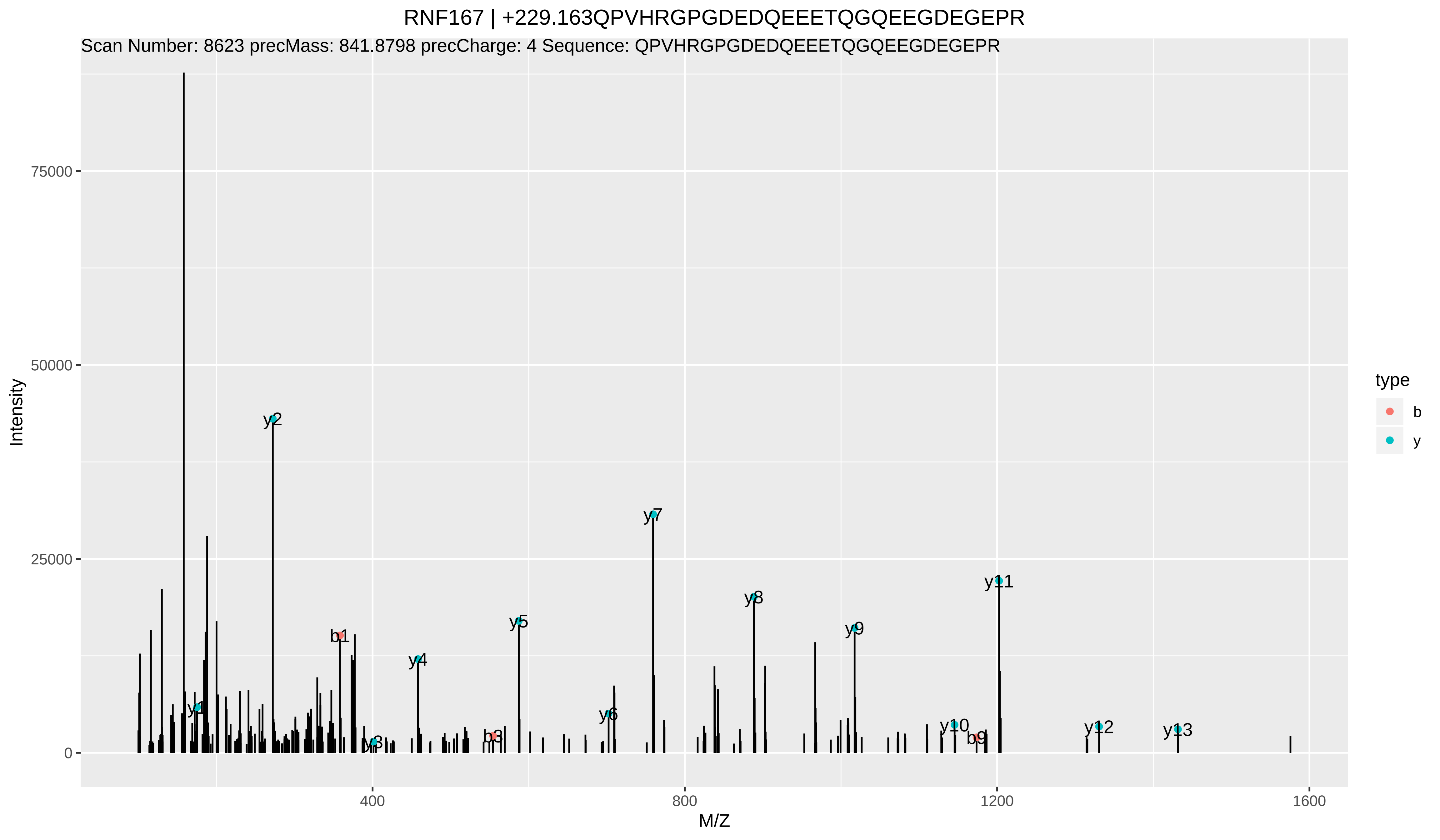

RNF167 | +229.163QPVHRGPGDEDQEEETQGQEEGDEGEPR
Scan Number: 8623 precMass: 841.8798 precCharge: 4 Sequence: QPVHRGPGDEDQEEETQGQEEGDEGEPR
Intensity
75000
50000
25000
0
M/Z
400
800
1200
1600
y2
y7
y11
y8
b1
y5
y9
y4
y1
y6
y3
b3
y10
b9
y12
y13
type
b
y

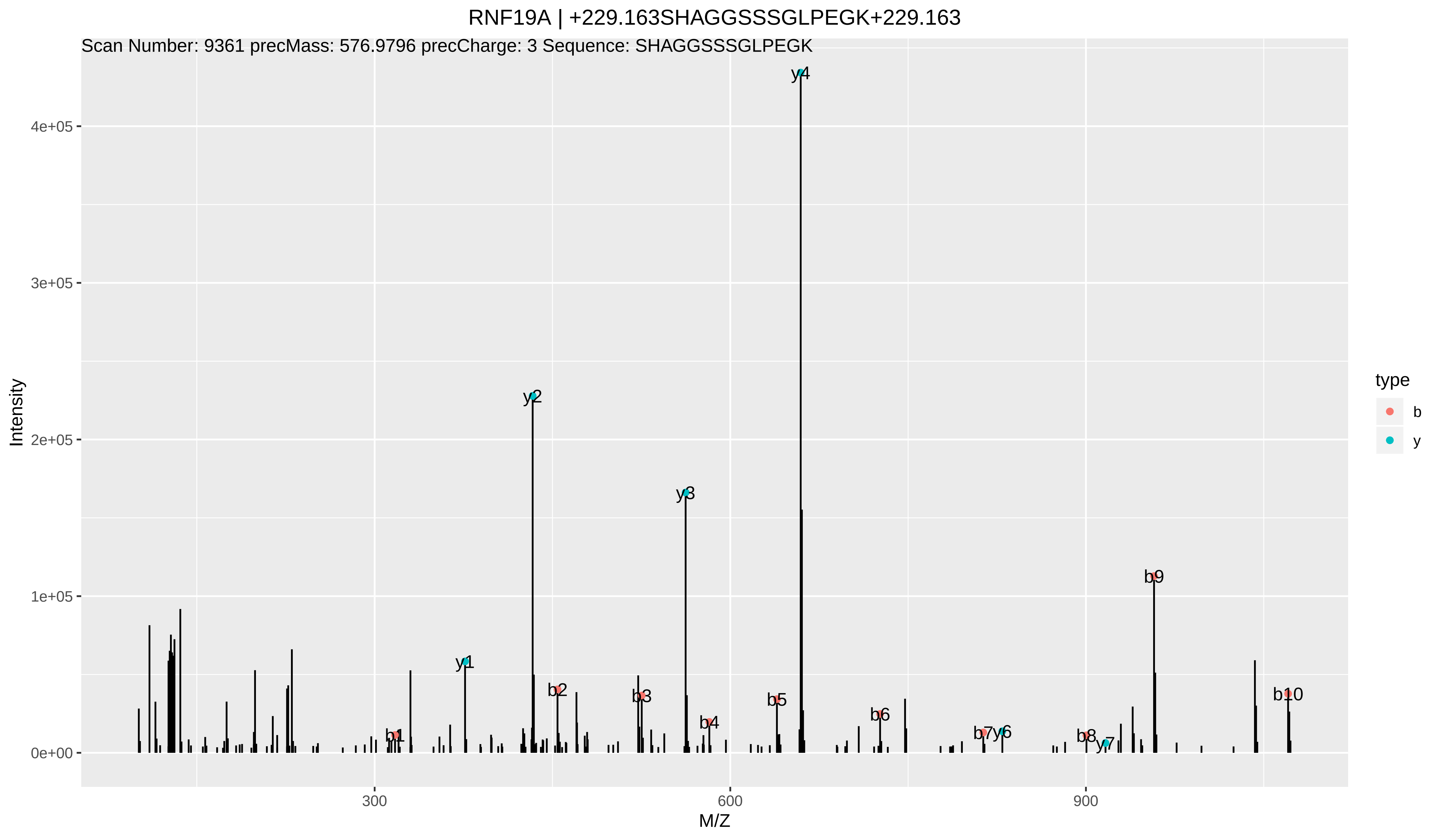

RNF19A | +229.163SHAGGSSSGLPEGK+229.163
Scan Number: 9361 precMass: 576.9796 precCharge: 3 Sequence: SHAGGSSSGLPEGK
Intensity
M/Z
4e+05
3e+05
2e+05
1e+05
0e+00
300
600
900
y1
y2
y3
y4
y6
y7
b1
b2
b3
b4
b5
b6
b7
b8
b9
b10
type
b
y

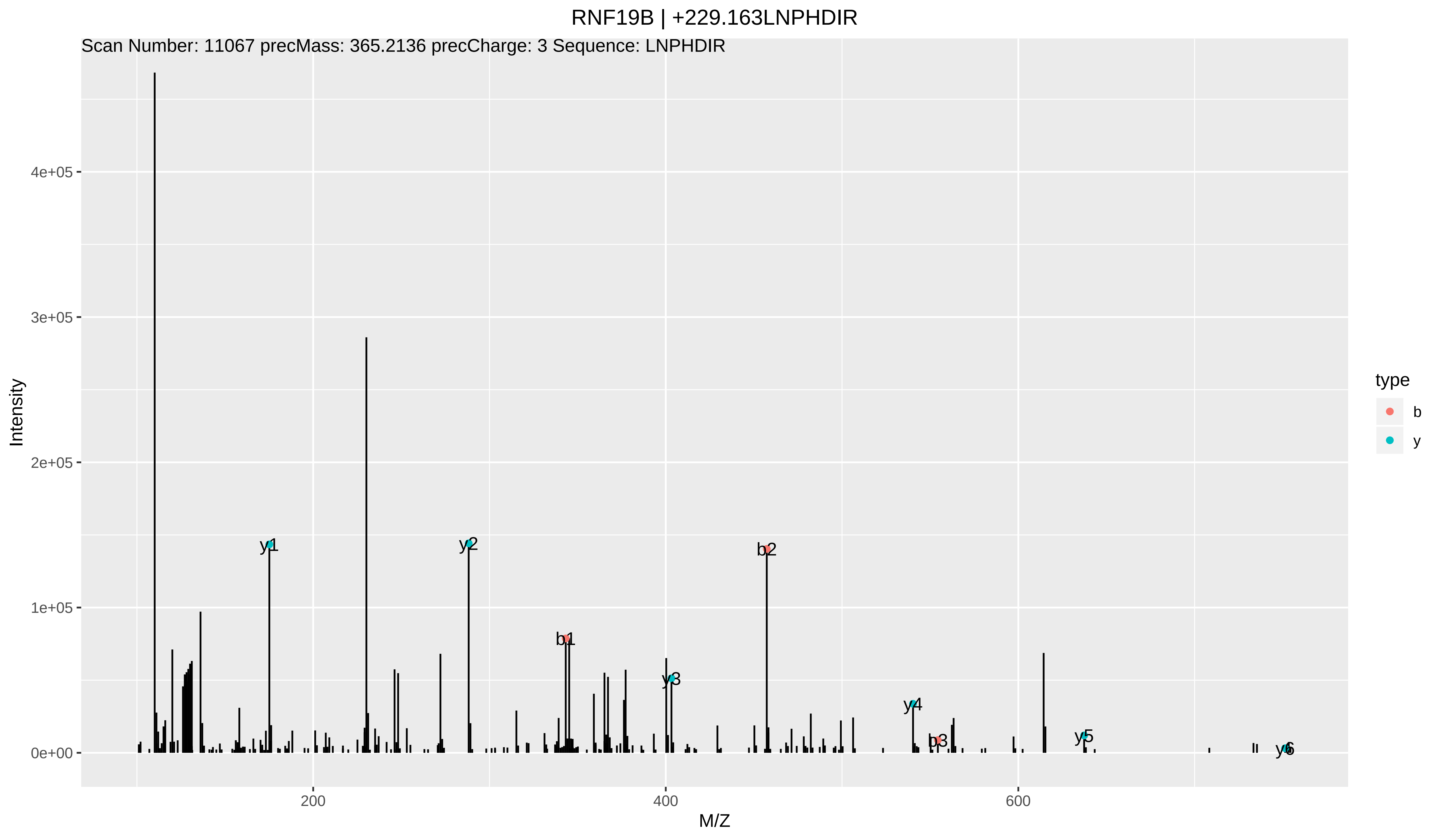
RNF19B | +229.163LNPHDIR
Scan Number: 11067 precMass: 365.2136 precCharge: 3 Sequence: LNPHDIR
Intensity
M/Z
4e+05
3e+05
2e+05
1e+05
0e+00
200
400
600
y1
y2
b2
b1
y3
y4
b3
y5
y6
type
b
y

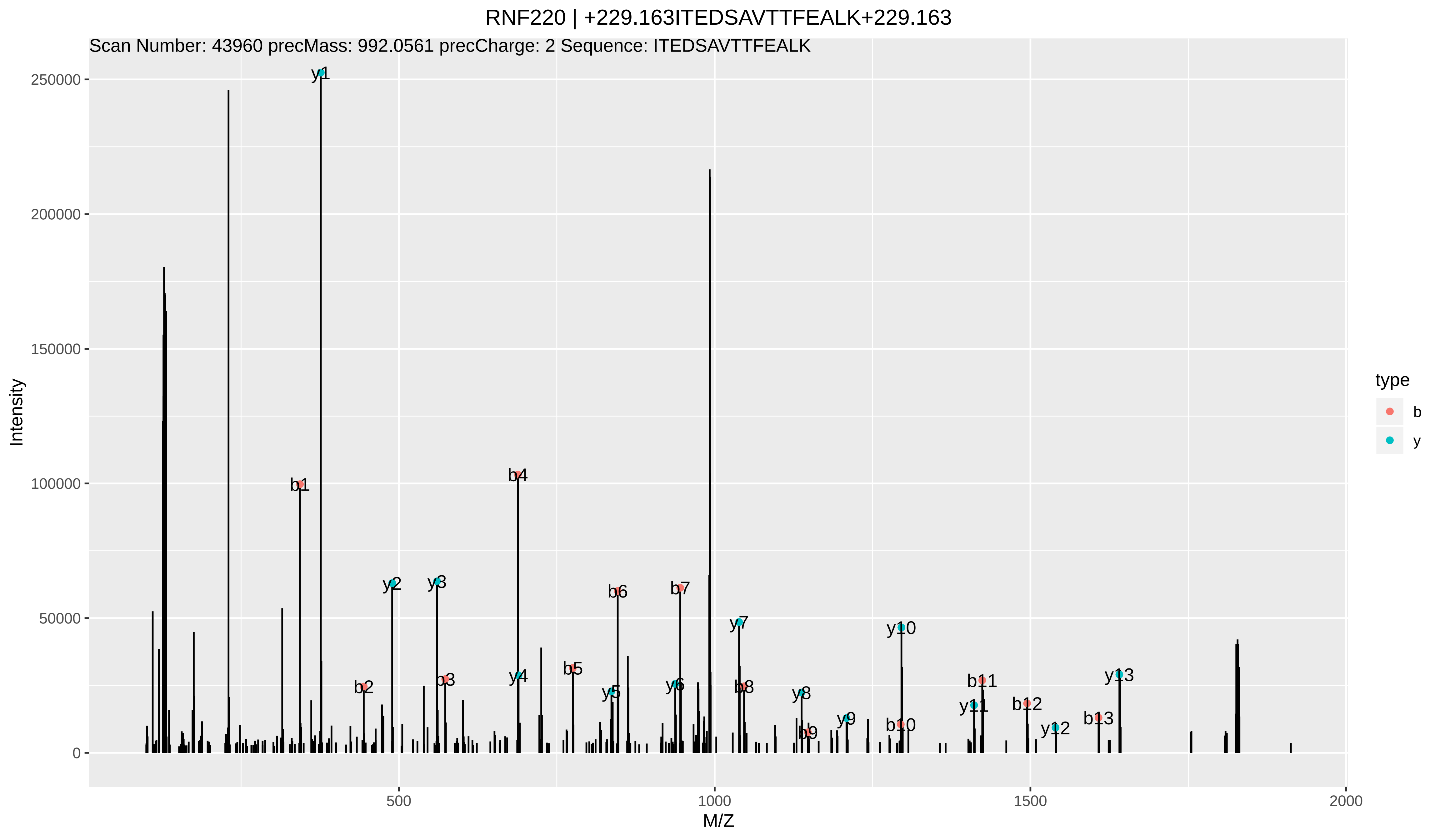
RNF220 | +229.163ITEDSAVTTFEALK+229.163
Scan Number: 43960 precMass: 992.0561 precCharge: 2 Sequence: ITEDSAVTTFEALK
Intensity
M/Z
type
b
y
y1
b1
b4
y2
y3
b6
b7
y7
y10
b2
b3
y4
b5
y5
y6
b8
y8
b9
y9
b10
y11
b11
b12
y12
b13
y13
0
50000
100000
150000
200000
250000
500
1000
1500
2000

RNF41 | +229.163YYENYVAK+229.163

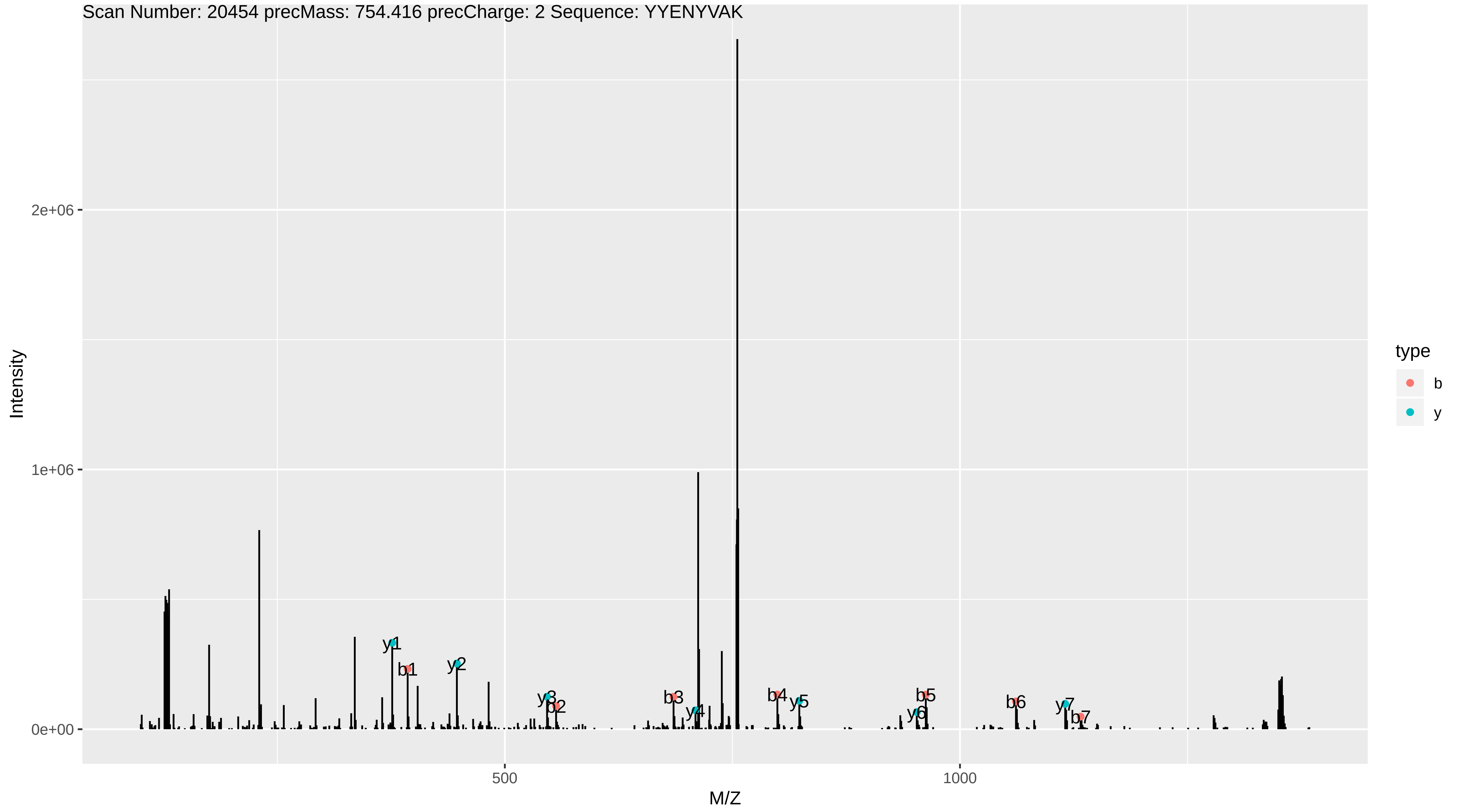

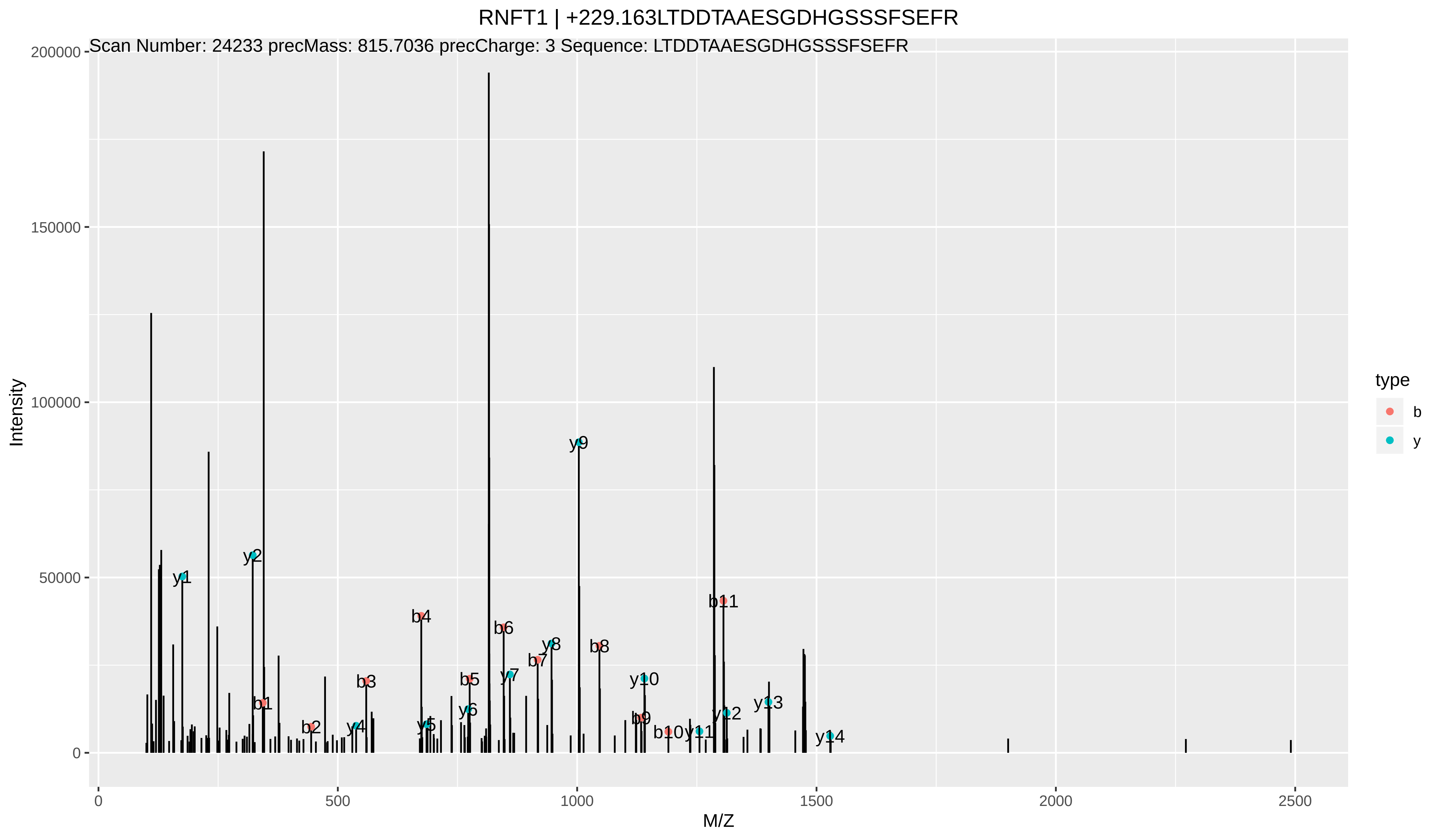

RNFT1 | +229.163LTDDTAAESGDHGSSSFSEFR
Scan Number: 24233 precMass: 815.7036 precCharge: 3 Sequence: LTDDTAAESGDHGSSSFSEFR
Intensity
M/Z
0
50000
100000
150000
200000
0
500
1000
1500
2000
2500
y1
y2
b1
b2
y4
b3
b4
y5
y6
b5
b6
y7
b7
y8
y9
b8
y10
b9
b10
y11
b11
y12
y13
y14
type
b
y

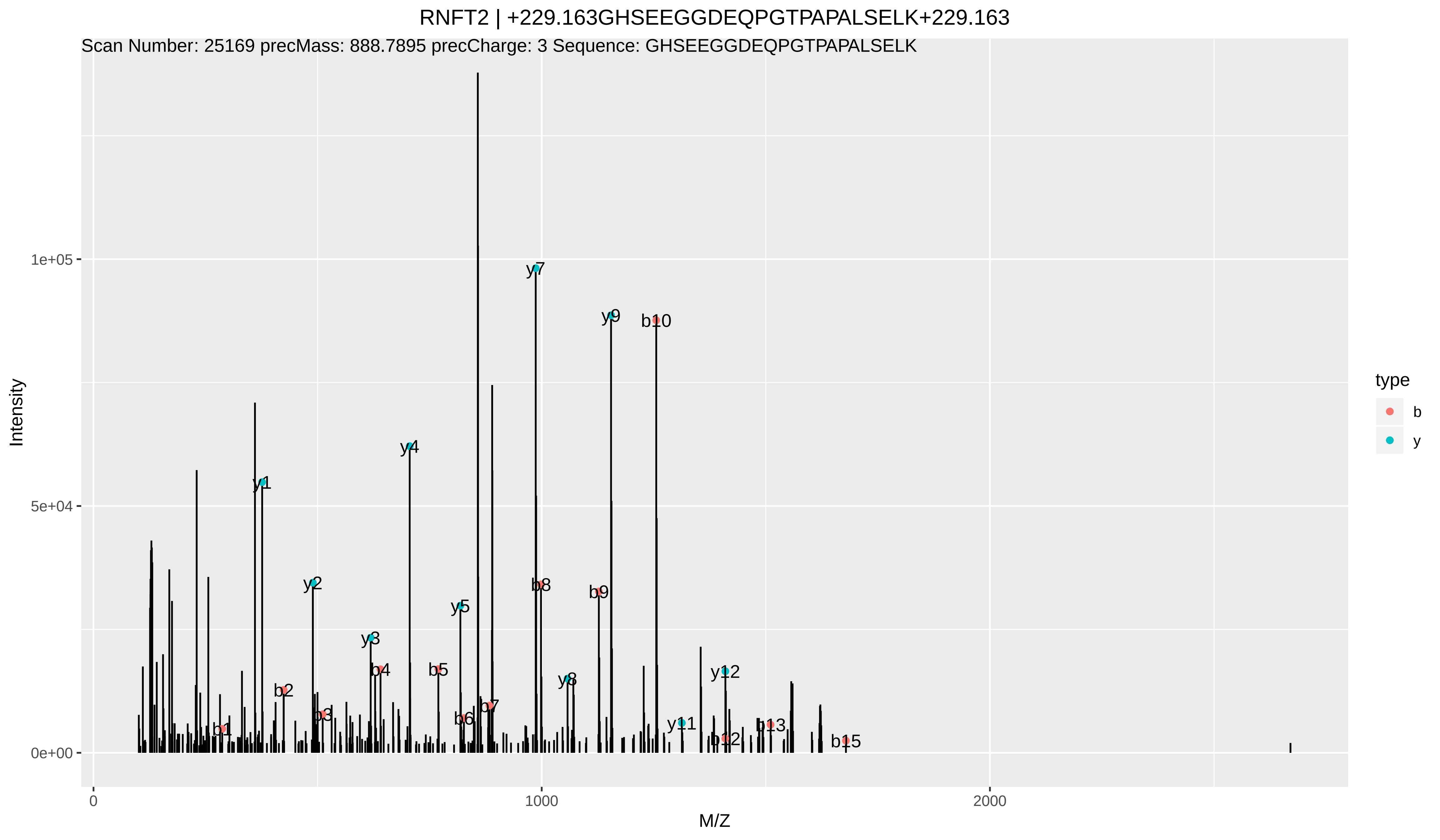
RNFT2 | +229.163GHSEEGGDEQPGTPAPALSELK+229.163
Scan Number: 25169 precMass: 888.7895 precCharge: 3 Sequence: GHSEEGGDEQPGTPAPALSELK
Intensity
M/Z
0e+00
5e+04
1e+05
0
1000
2000
type
b
y
y1
y2
y3
y4
y5
y7
y8
y9
y11
y12
b1
b2
b3
b4
b5
b6
b7
b8
b9
b10
b12
b13
b15

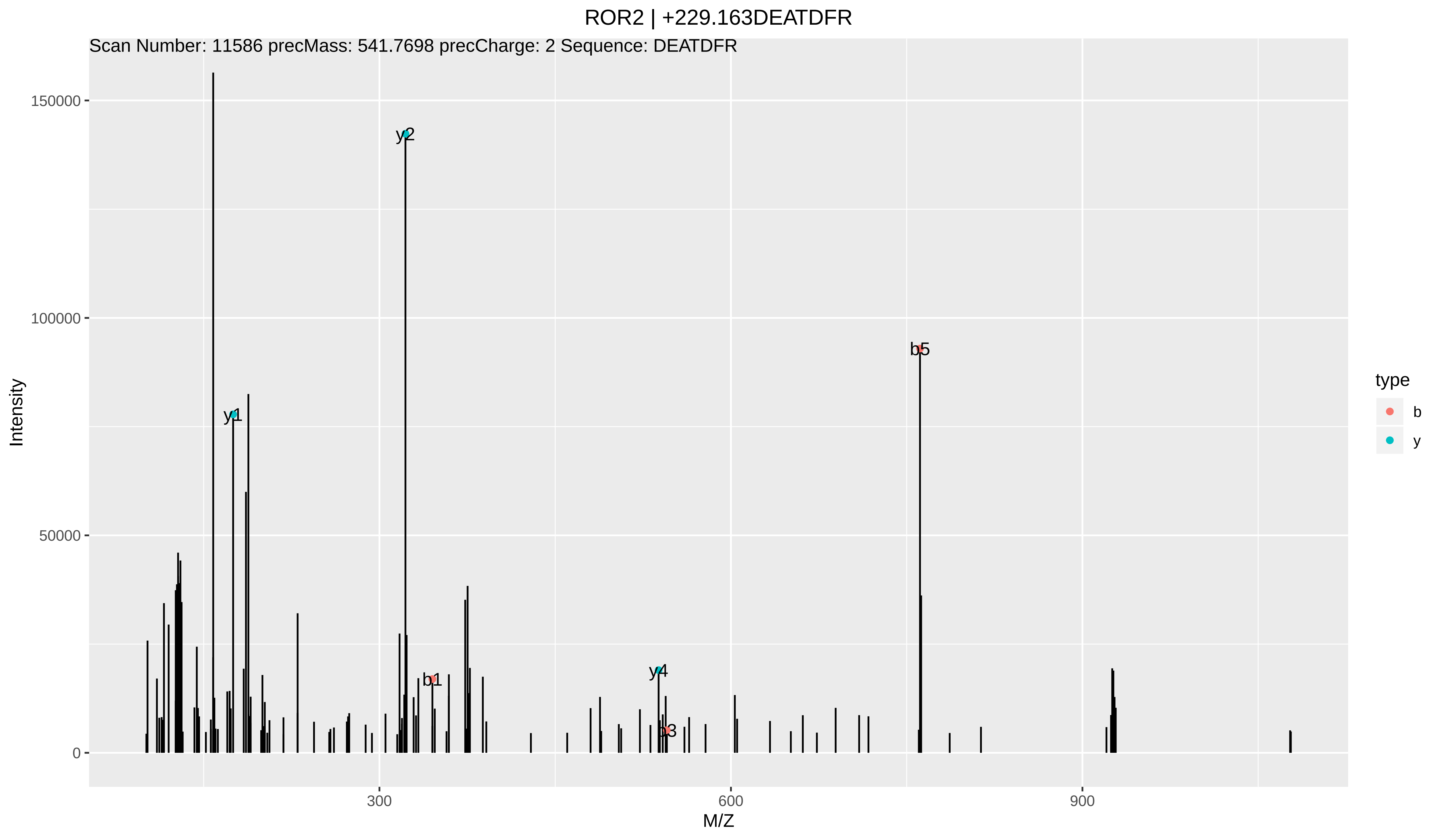

ROR2 | +229.163DEATDFR
Scan Number: 11586 precMass: 541.7698 precCharge: 2 Sequence: DEATDFR
Intensity
150000
100000
50000
0
300
600
900
M/Z
y2
y1
b5
b1
y4
b3
type
b
y

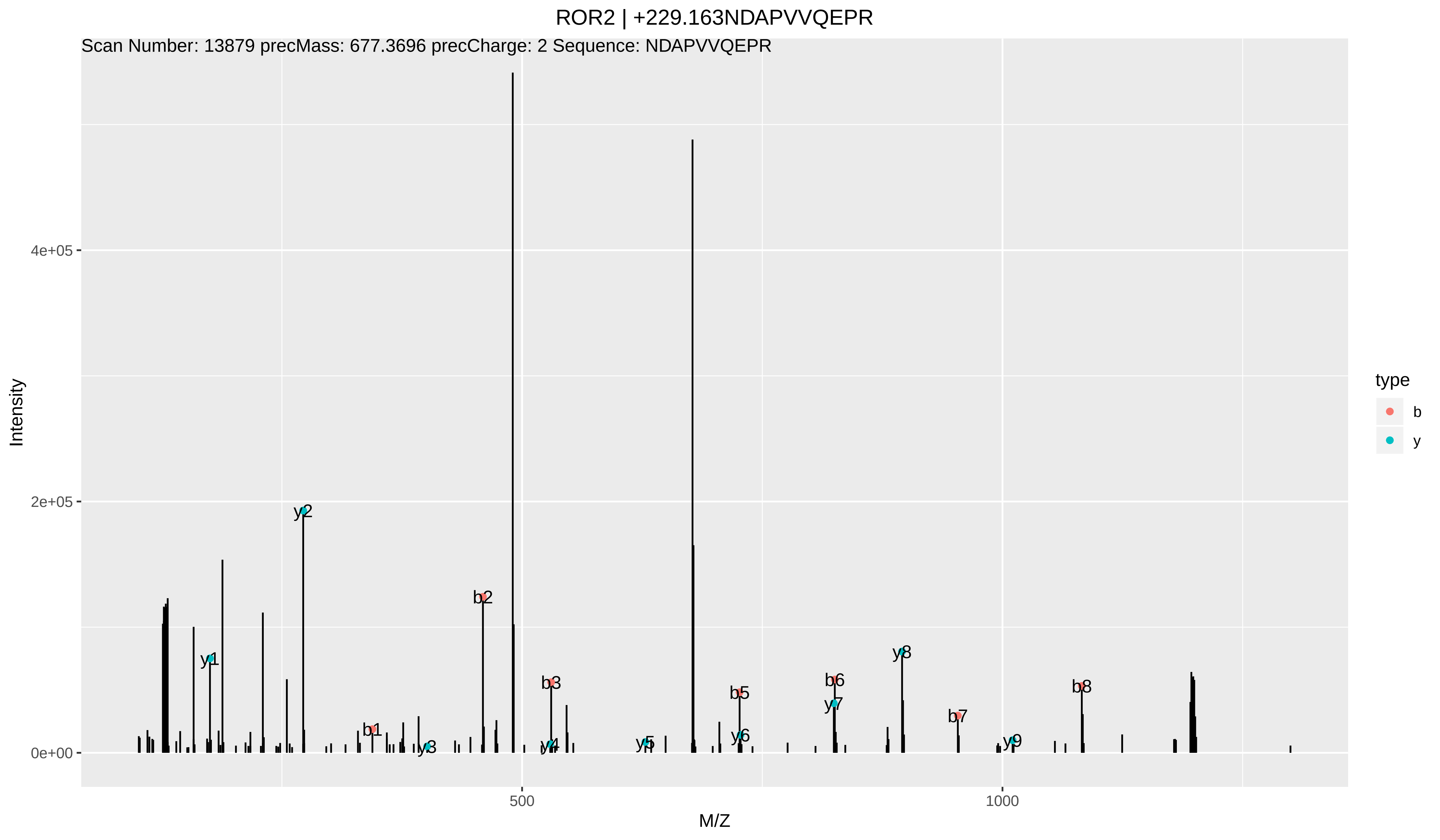

ROR2 | +229.163NDAPVVQEPR
Scan Number: 13879 precMass: 677.3696 precCharge: 2 Sequence: NDAPVVQEPR
Intensity
4e+05
2e+05
0e+00
M/Z
500
1000
y1
y2
y3
y4
y5
y6
y7
y8
y9
b1
b2
b3
b5
b6
b7
b8
type
b
y

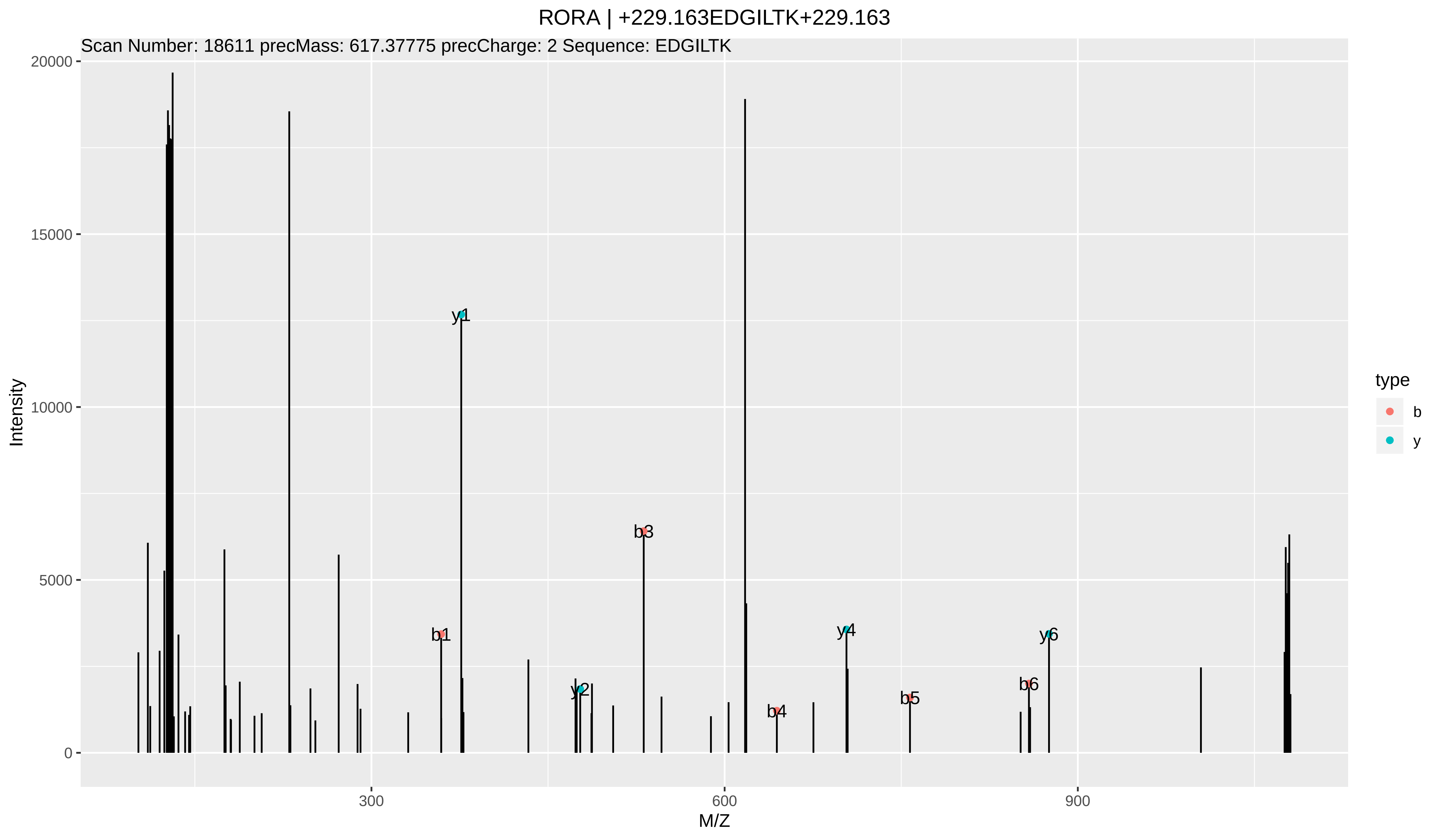

RORA | +229.163EDGILTK+229.163
Scan Number: 18611 precMass: 617.37775 precCharge: 2 Sequence: EDGILTK
Intensity
20000
15000
10000
5000
0
M/Z
300
600
900
y1
b1
y2
b3
b4
y4
b5
b6
y6
type
b
y

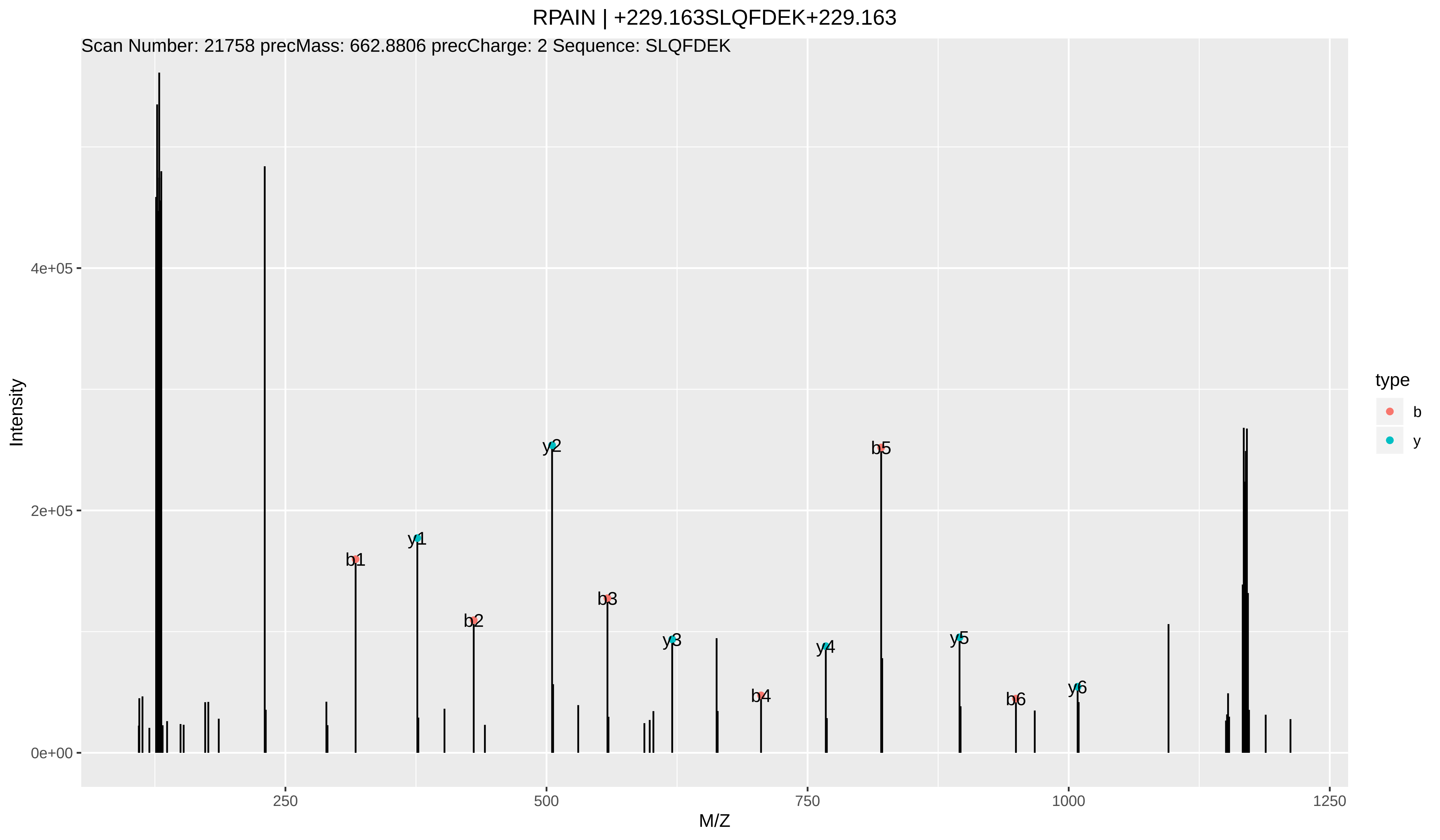
RPAIN | +229.163SLQFDEK+229.163
Scan Number: 21758 precMass: 662.8806 precCharge: 2 Sequence: SLQFDEK
Intensity
4e+05
2e+05
0e+00
M/Z
250
500
750
1000
1250
b1
y1
b2
y2
b3
y3
b4
y4
b5
y5
b6
y6
type
b
y

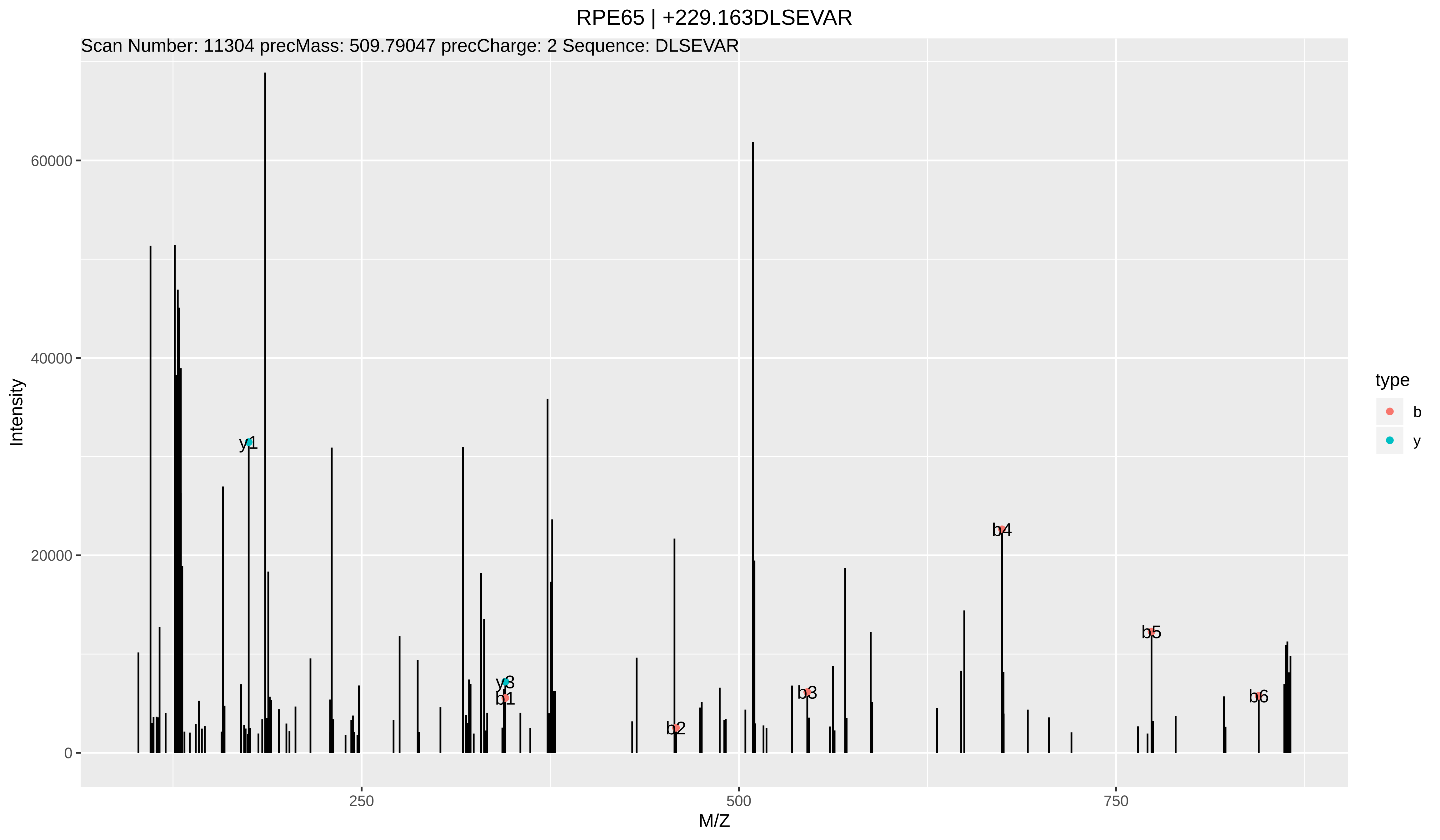

RPE65 | +229.163DLSEVAR
Scan Number: 11304 precMass: 509.79047 precCharge: 2 Sequence: DLSEVAR
Intensity
60000
40000
20000
0
250
500
750
M/Z
y1
y3
b1
b2
b3
b4
b5
b6
type
b
y

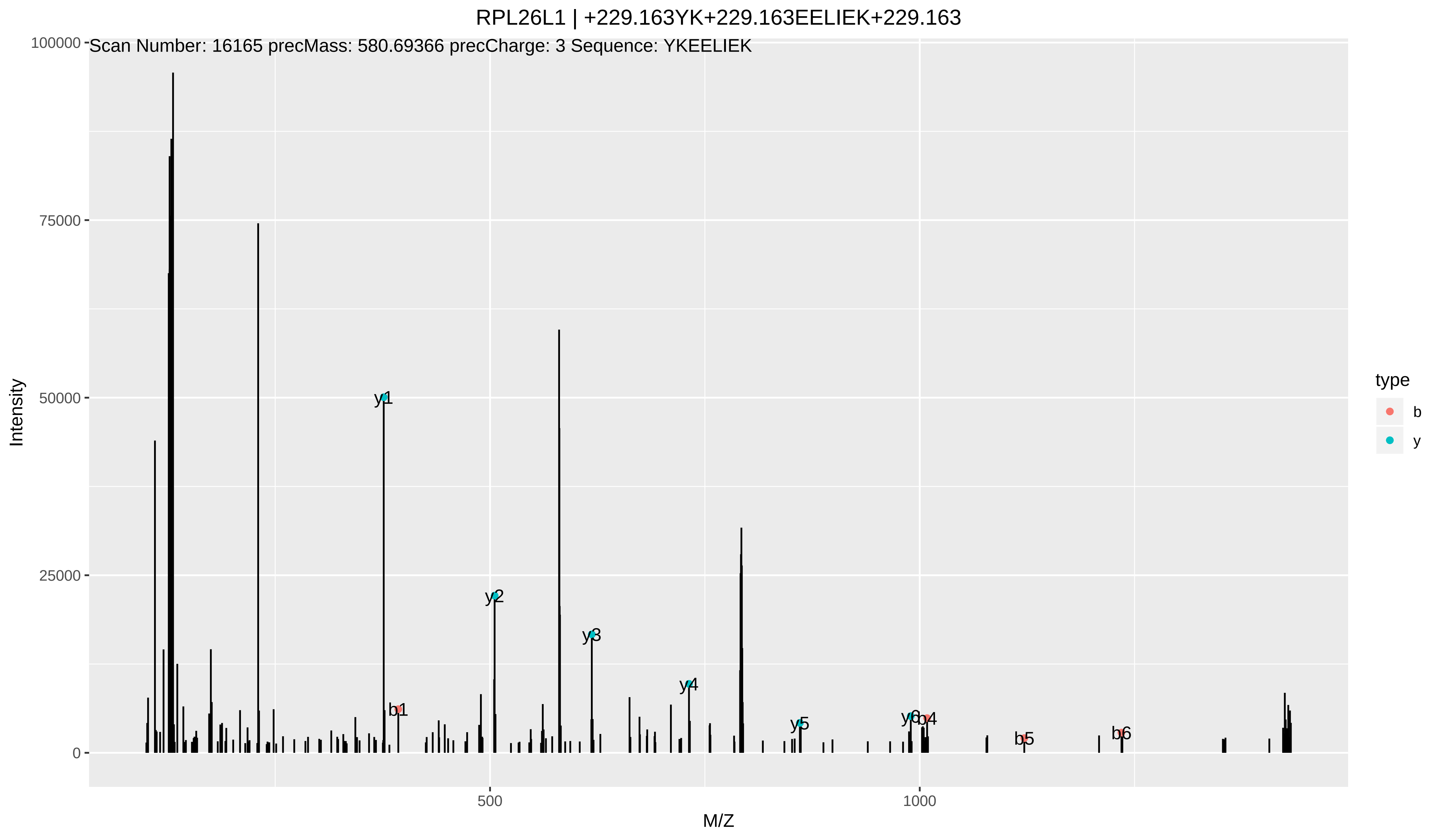

RPL26L1 | +229.163YK+229.163EELIEK+229.163
Scan Number: 16165 precMass: 580.69366 precCharge: 3 Sequence: YKEELIEK
Intensity
100000
75000
50000
25000
0
M/Z
500
1000
y1
b1
y2
y3
y4
y5
y6
b4
b5
b6
type
b
y

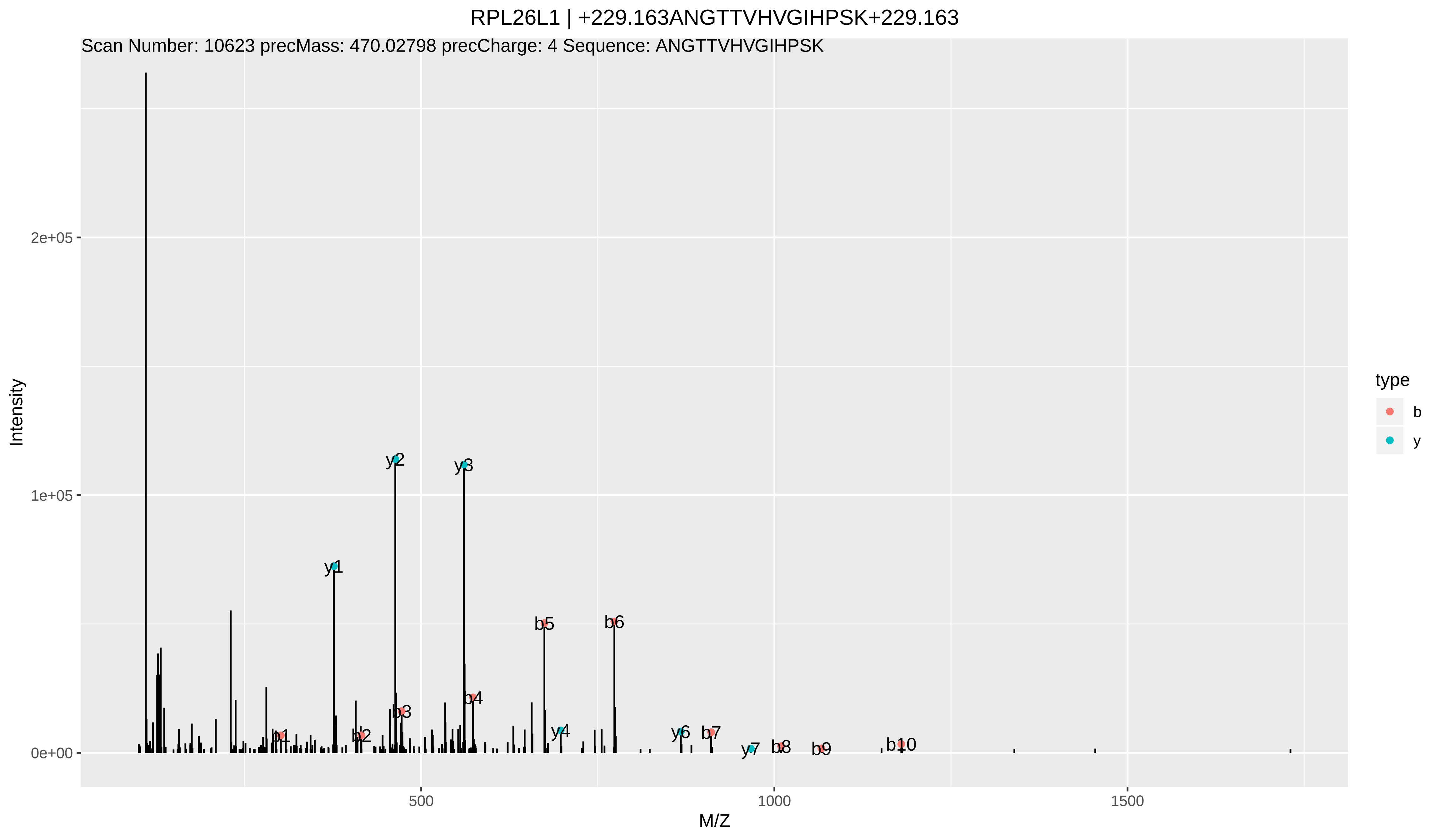

RPL26L1 | +229.163ANGTTVHVGIHPSK+229.163
Scan Number: 10623 precMass: 470.02798 precCharge: 4 Sequence: ANGTTVHVGIHPSK
Intensity
M/Z
0e+00
1e+05
2e+05
500
1000
1500
y1
y2
y3
y4
y6
y7
b1
b2
b3
b4
b5
b6
b7
b8
b9
b10
type
b
y

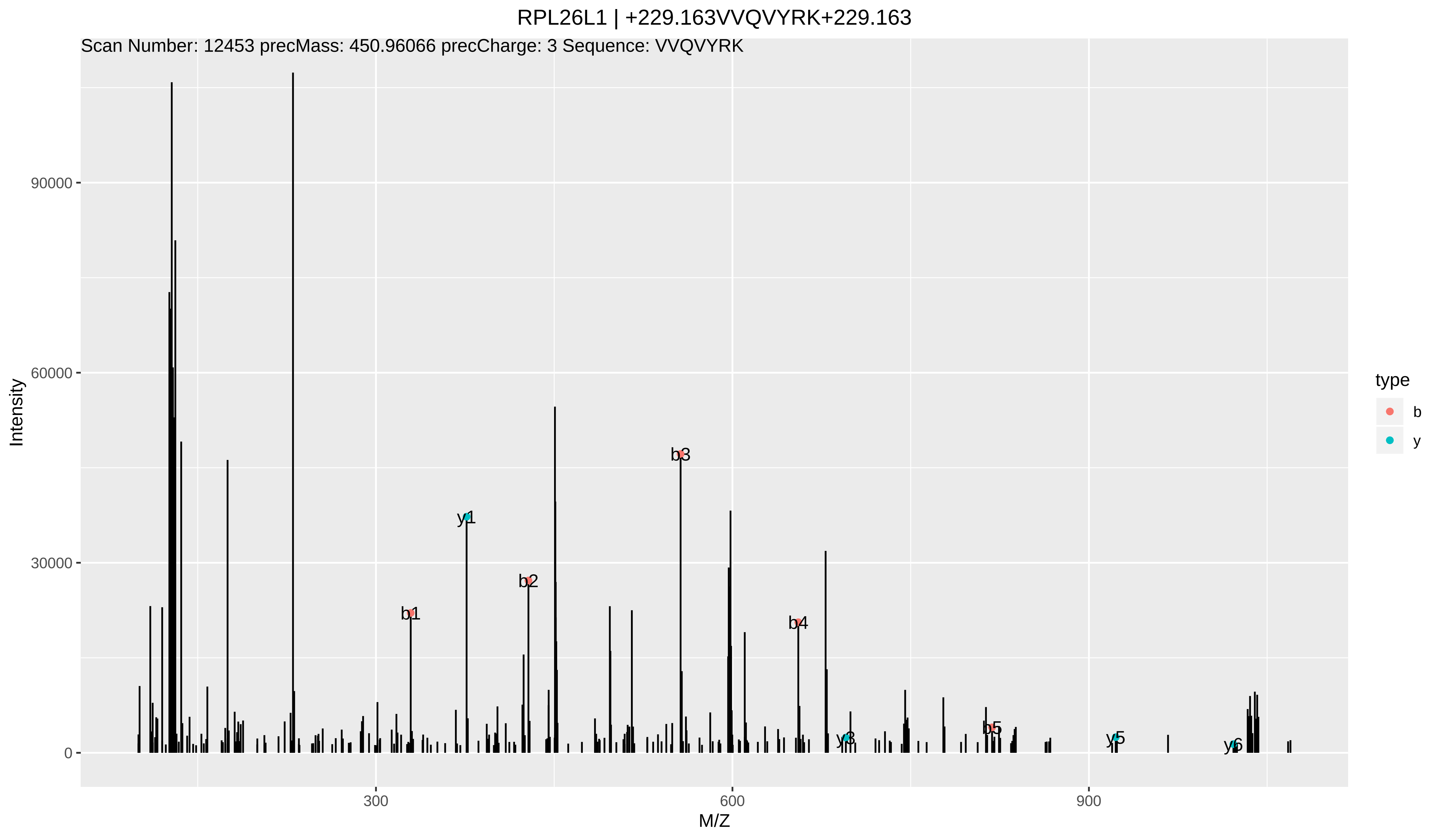
RPL26L1 | +229.163VVQVYRK+229.163
Scan Number: 12453 precMass: 450.96066 precCharge: 3 Sequence: VVQVYRK
Intensity
0
30000
60000
90000
M/Z
300
600
900
b1
y1
b2
b3
b4
y3
b5
y5
y6
type
b
y

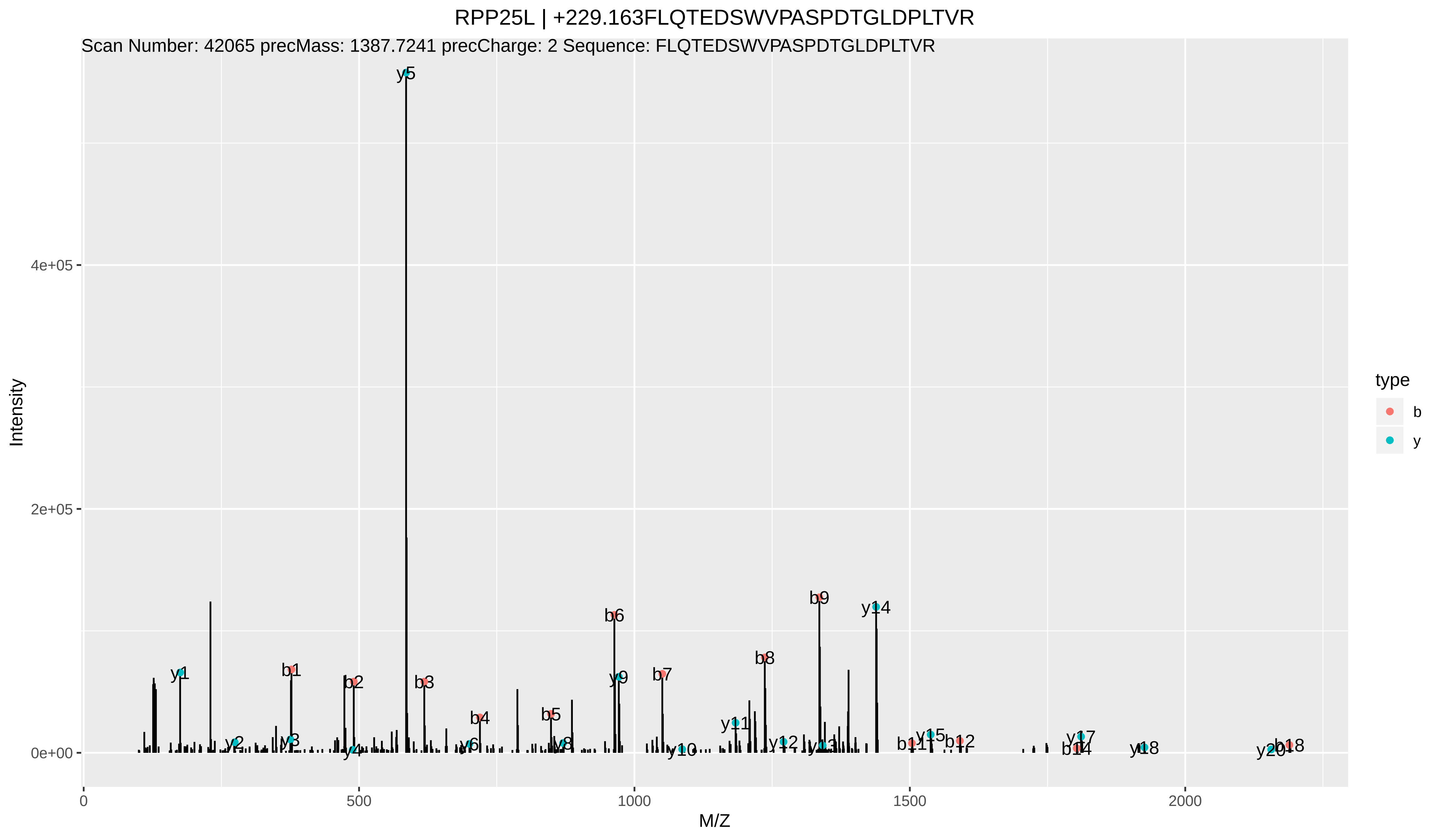
RPP25L | +229.163FLQTEDSWVPASPDTGLDPLTVR
Scan Number: 42065 precMass: 1387.7241 precCharge: 2 Sequence: FLQTEDSWVPASPDTGLDPLTVR
Intensity
M/Z
type
b
y
4e+05
2e+05
0e+00
0
500
1000
1500
2000
y5
b9
y14
b6
b8
y1
b1
b2
b3
y9
b7
b4
b5
y11
y2
y3
y4
y6
y8
y10
y12
b11
y15
b12
y17
b14
y18
y20
b18

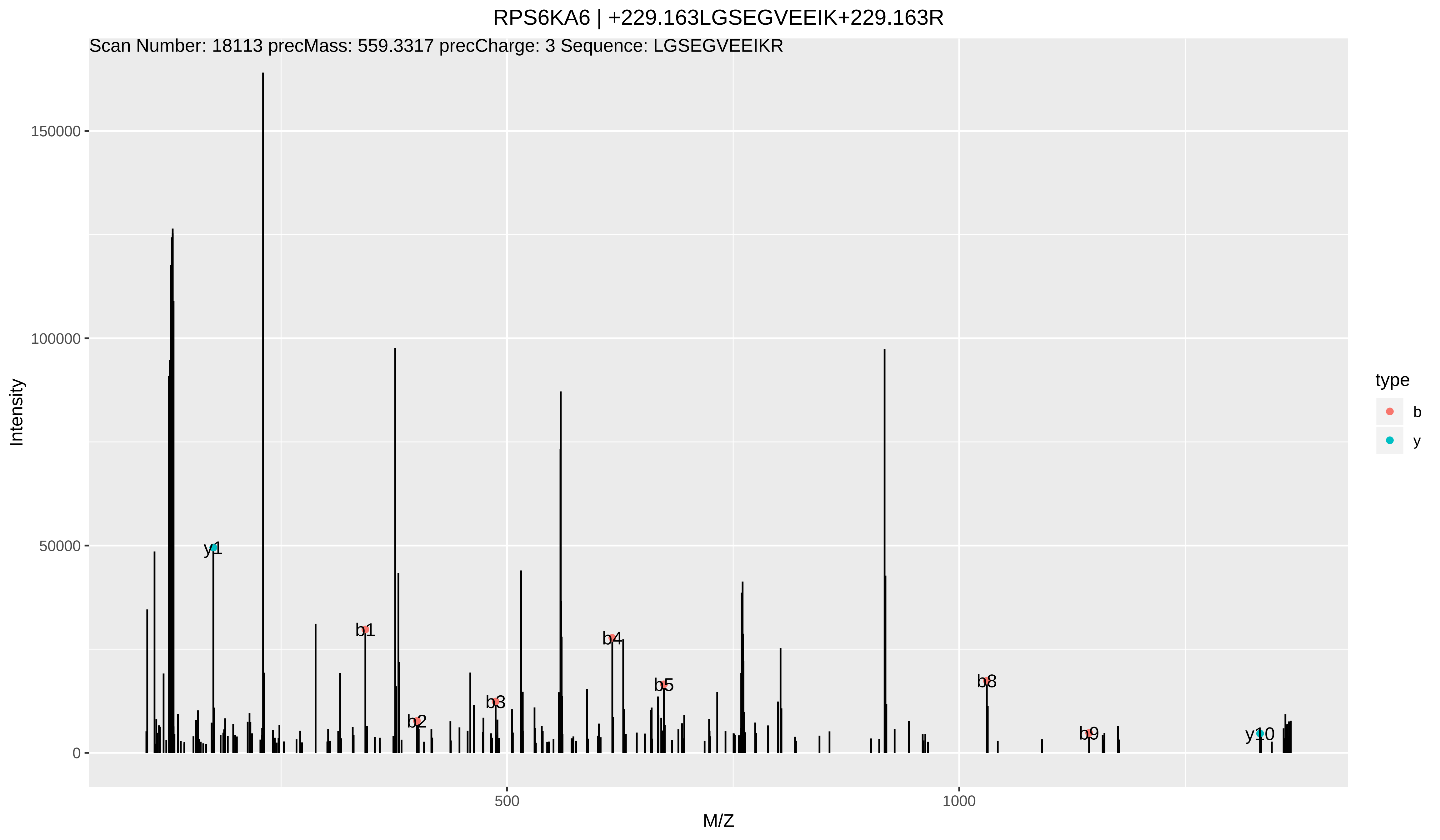

RPS6KA6 | +229.163LGSEGVEEIK+229.163R
Scan Number: 18113 precMass: 559.3317 precCharge: 3 Sequence: LGSEGVEEIKR
Intensity
150000
100000
50000
0
500
1000
M/Z
y1
b1
b2
b3
b4
b5
b8
b9
y10
type
b
y

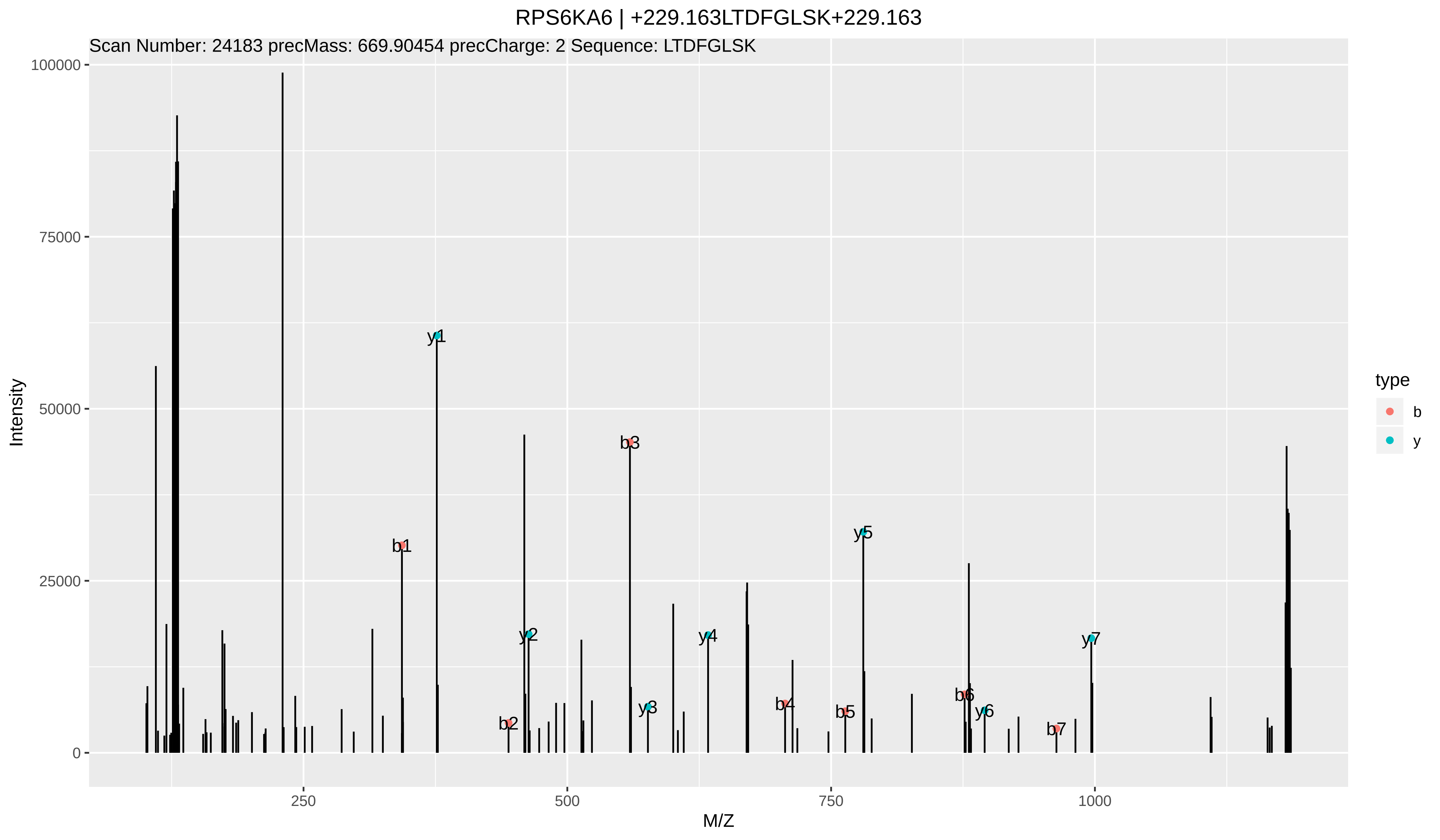

RPS6KA6 | +229.163LTDFGLSK+229.163
Scan Number: 24183 precMass: 669.90454 precCharge: 2 Sequence: LTDFGLSK
Intensity
M/Z
100000
75000
50000
25000
0
250
500
750
1000
y1
b3
b1
y5
y2
y4
y7
b6
y3
b4
y6
b5
b2
b7
type
b
y

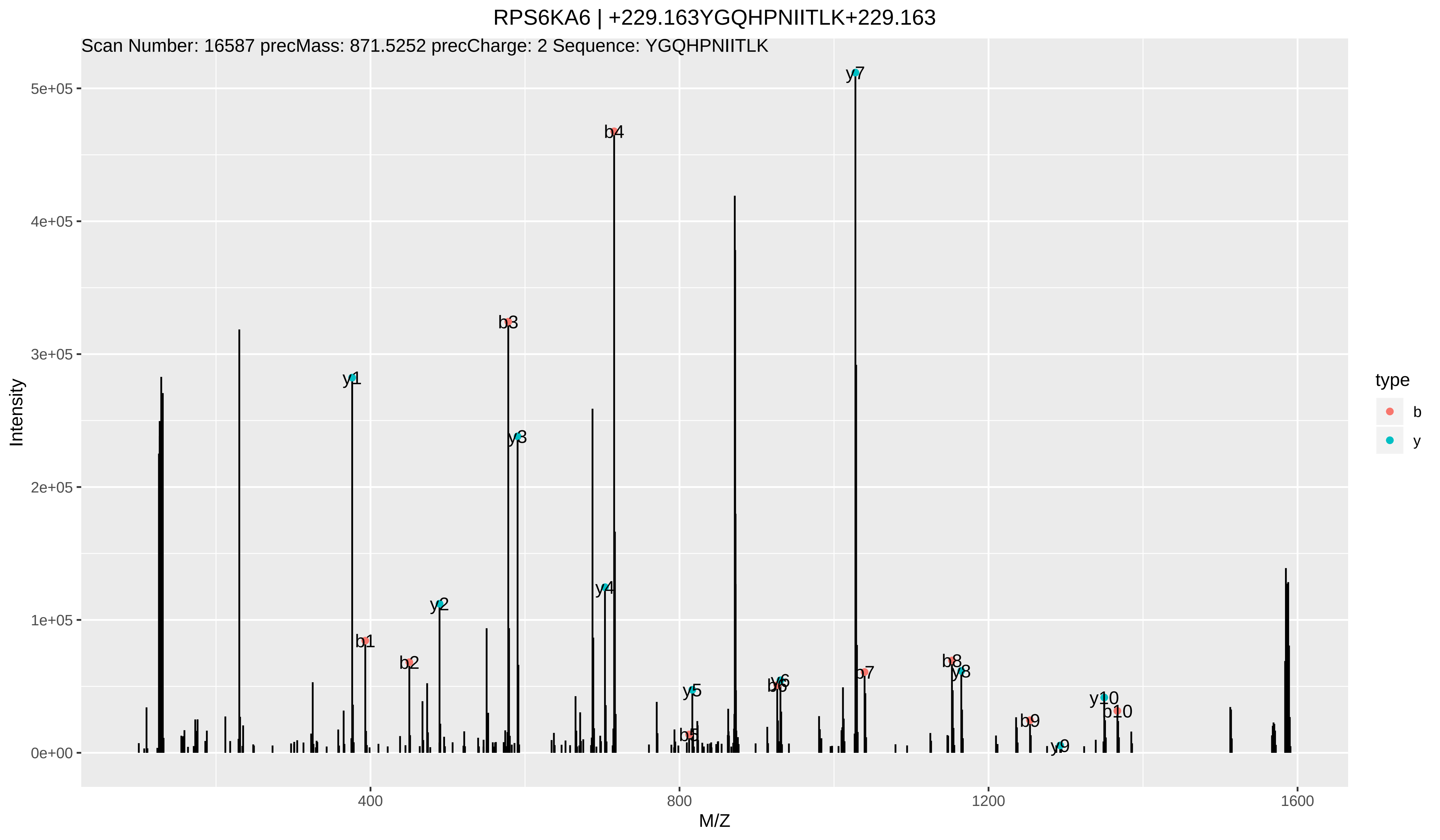
RPS6KA6 | +229.163YGQHPNIITLK+229.163
Scan Number: 16587 precMass: 871.5252 precCharge: 2 Sequence: YGQHPNIITLK
Intensity
M/Z
5e+05
4e+05
3e+05
2e+05
1e+05
0e+00
400
800
1200
1600
y7
b4
b3
y1
y3
y4
y2
b1
b2
y5
b5
y6
b6
b7
b8
y8
b9
y9
y10
b10
type
b
y

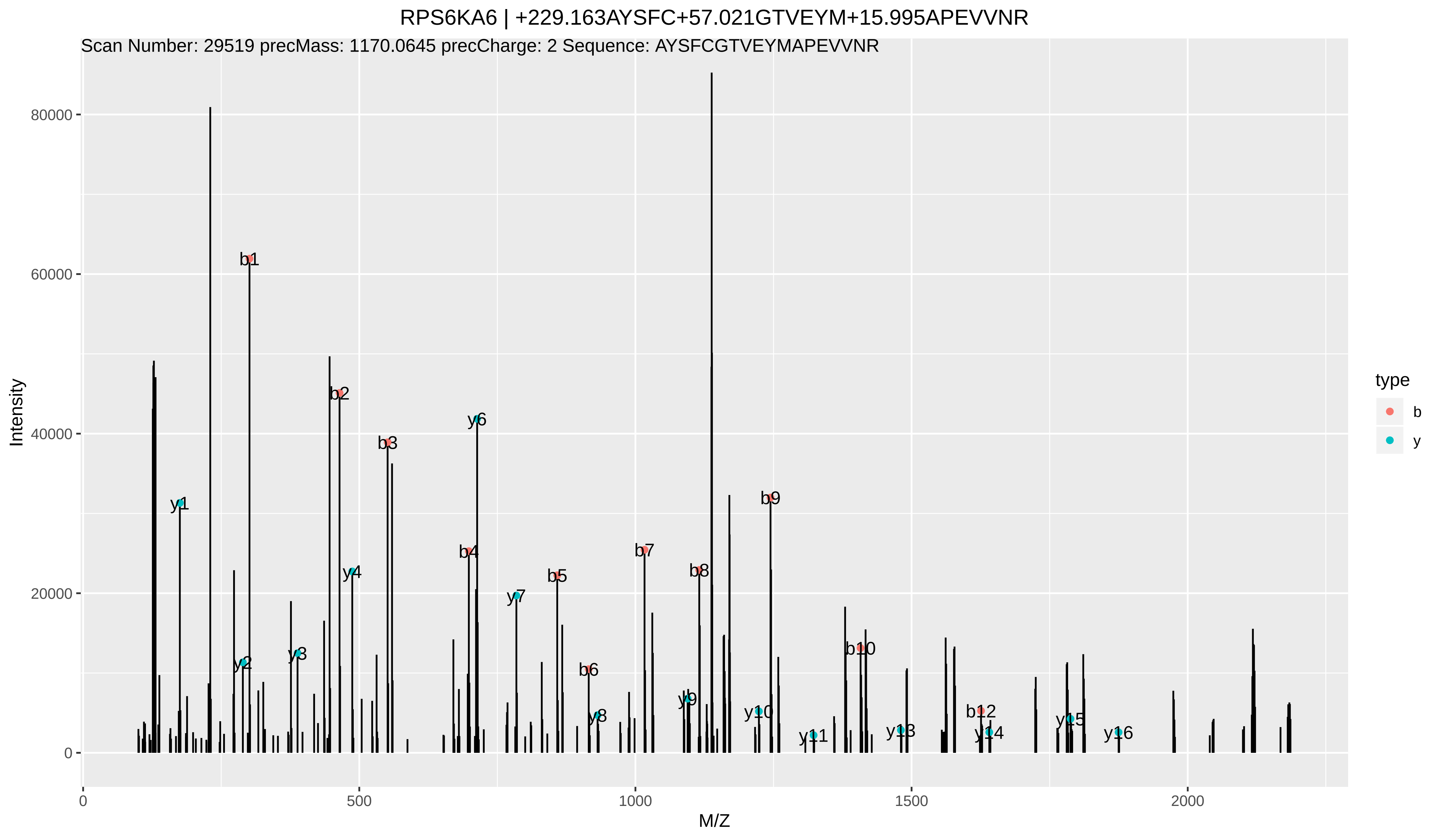

RPS6KA6 | +229.163AYSFC+57.021GTVEYM+15.995APEVVNR
Scan Number: 29519 precMass: 1170.0645 precCharge: 2 Sequence: AYSFCGTVEYMAPEVVNR
Intensity
M/Z
type
b
y
b1
b2
b3
b4
b5
b6
b7
b8
b9
b10
b12
y1
y2
y3
y4
y6
y7
y8
y9
y10
y11
y13
y14
y15
y16
0
20000
40000
60000
80000
500
1000
1500
2000

RPS6KA6 | +229.163DILVEVNHPFIVK+229.163

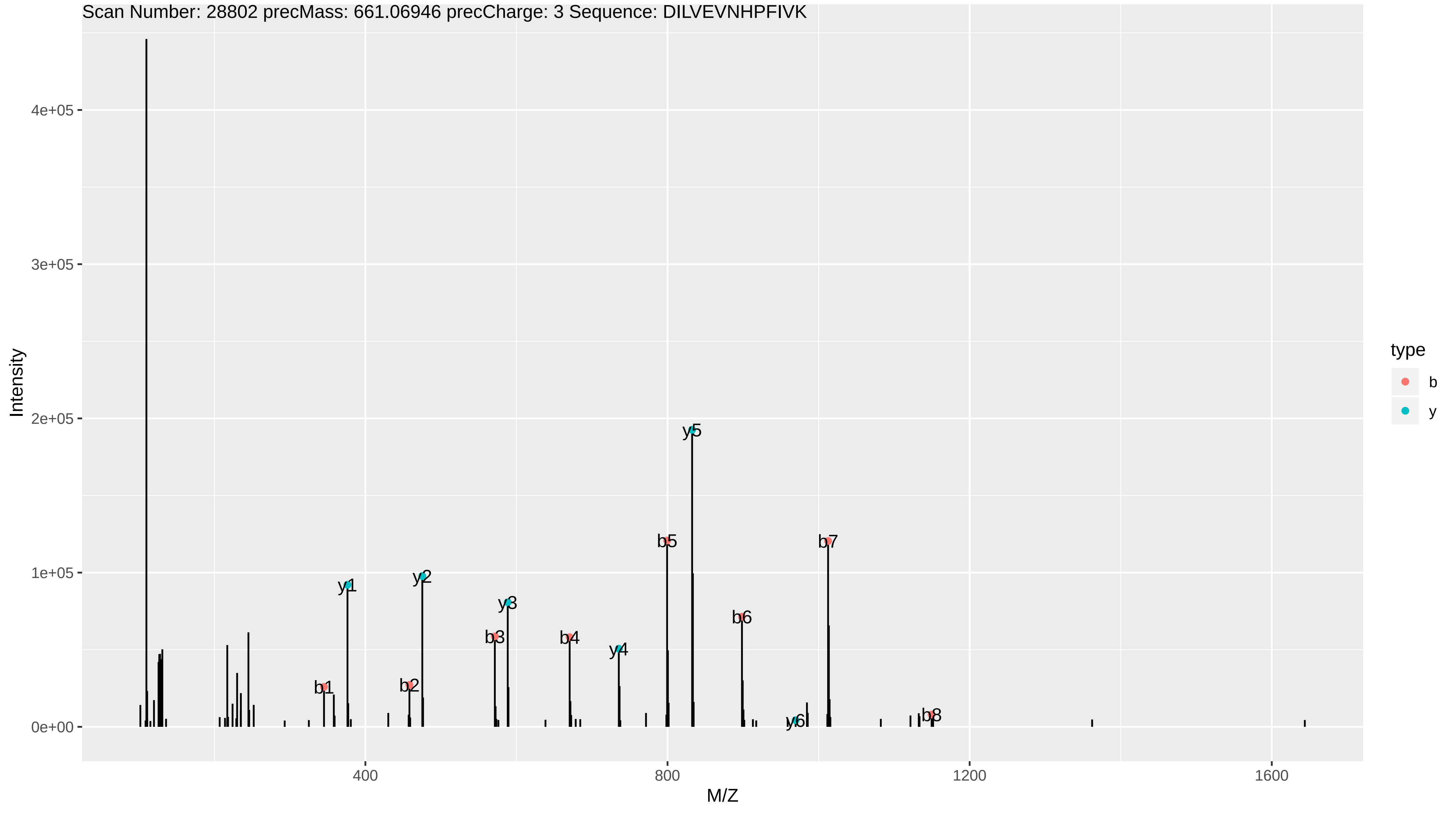

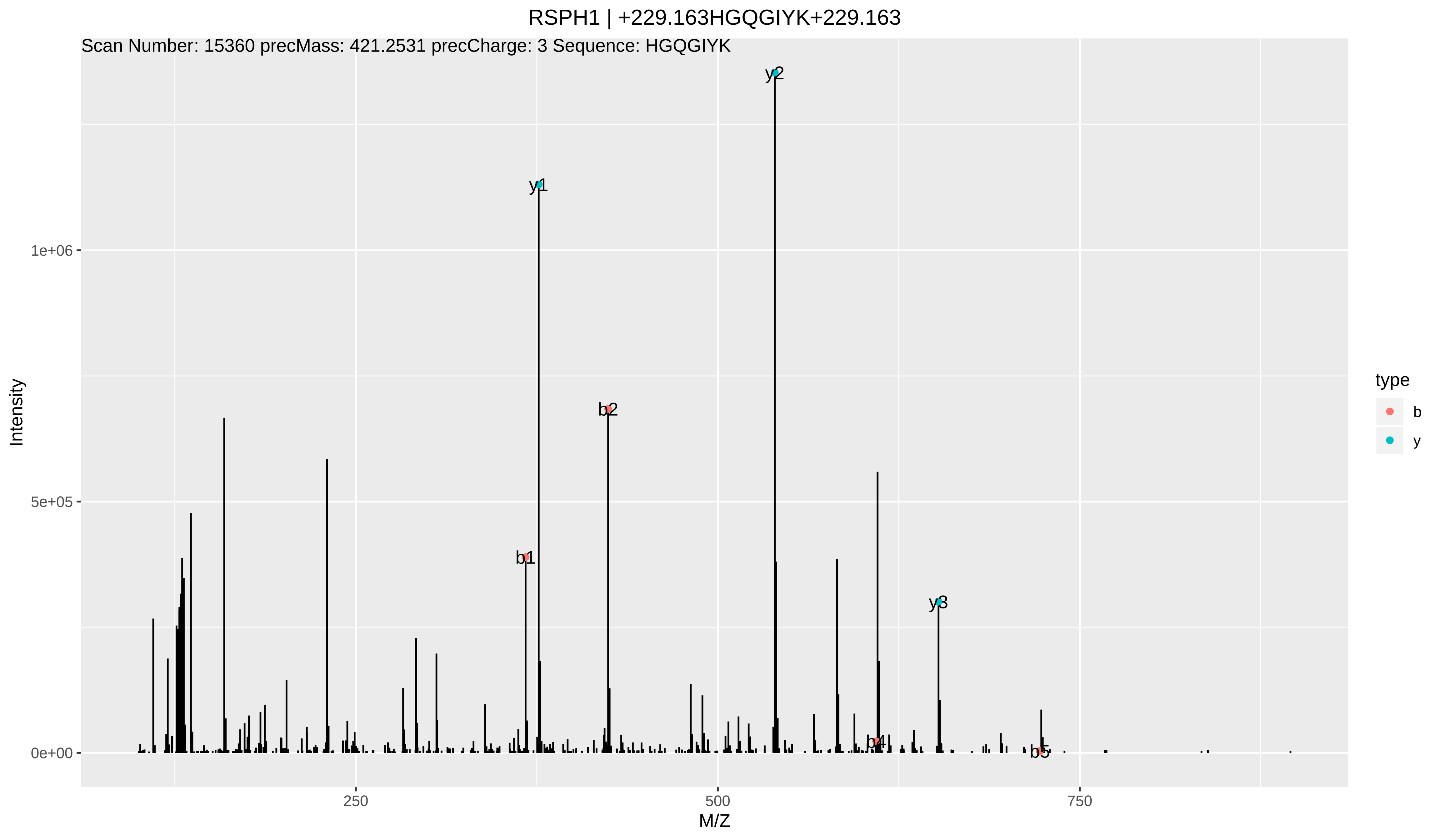

RSPH1 | +229.163HGQGIYK+229.163
Scan Number: 15360 precMass: 421.2531 precCharge: 3 Sequence: HGQGIYK
Intensity
1e+06
5e+05
0e+00
M/Z
250
500
750
y2
y1
b2
b1
y3
b4
b5
type
b
y

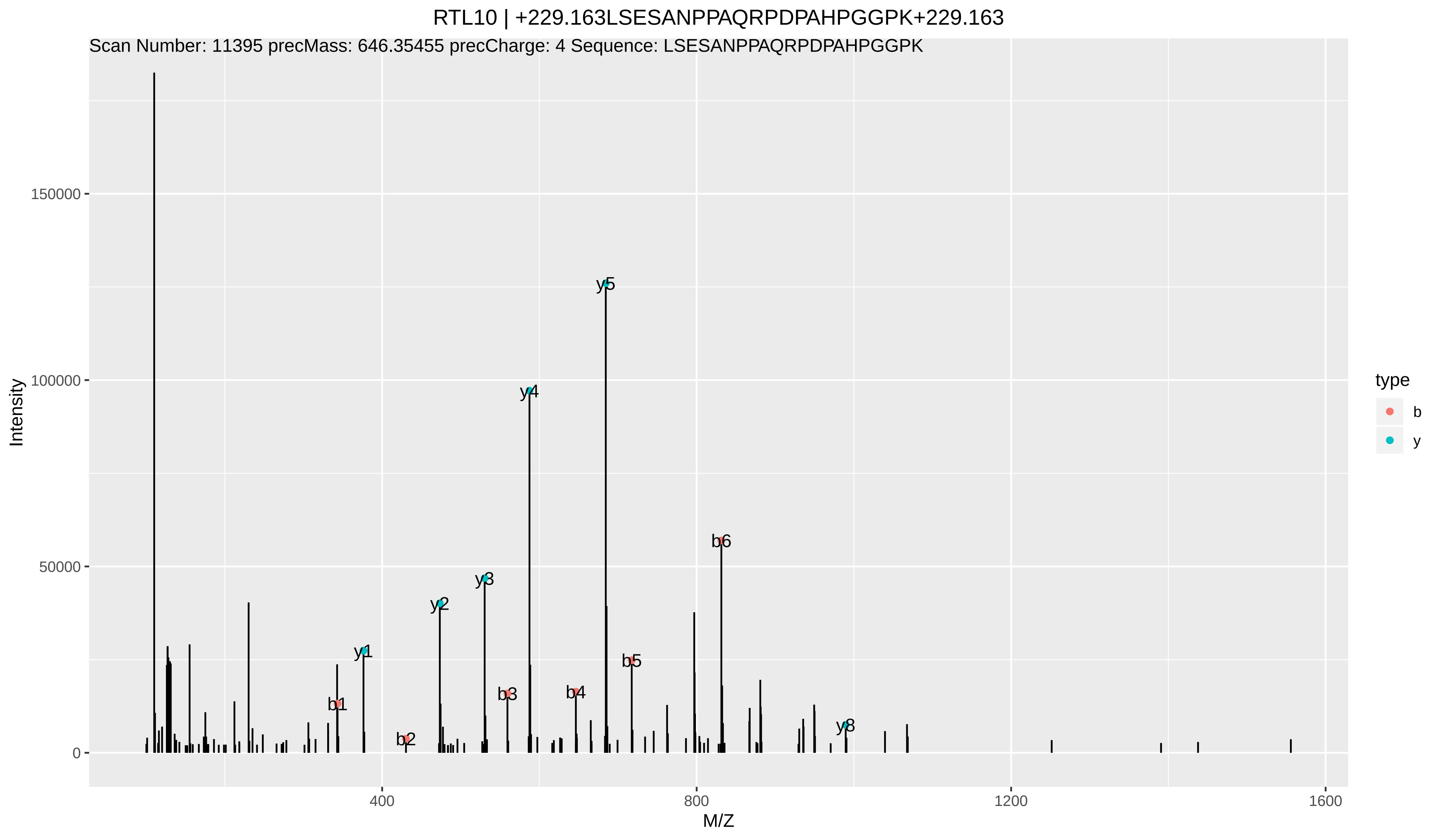

RTL10 | +229.163LSESANPPAQRPDPAHPGGPK+229.163
Scan Number: 11395 precMass: 646.35455 precCharge: 4 Sequence: LSESANPPAQRPDPAHPGGPK
Intensity
150000
100000
50000
0
400
800
1200
1600
M/Z
y5
y4
b6
y3
y2
y1
b5
b3
b4
b1
y8
b2
type
b
y

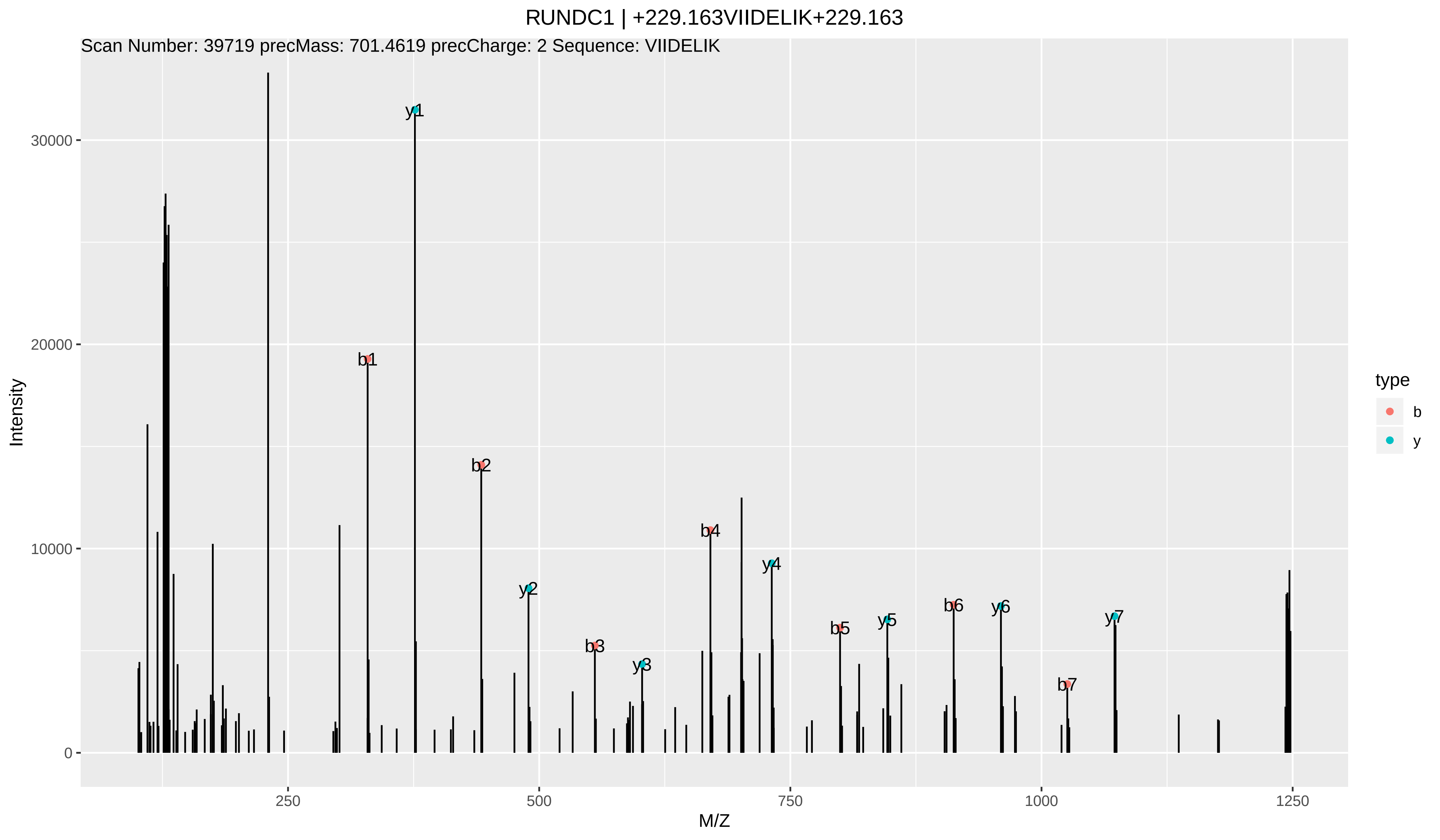

RUNDC1 | +229.163VIIDELIK+229.163
Scan Number: 39719 precMass: 701.4619 precCharge: 2 Sequence: VIIDELIK
Intensity
M/Z
0
10000
20000
30000
250
500
750
1000
1250
y1
b1
b2
y2
b3
y3
b4
y4
b5
y5
b6
y6
b7
y7
type
b
y

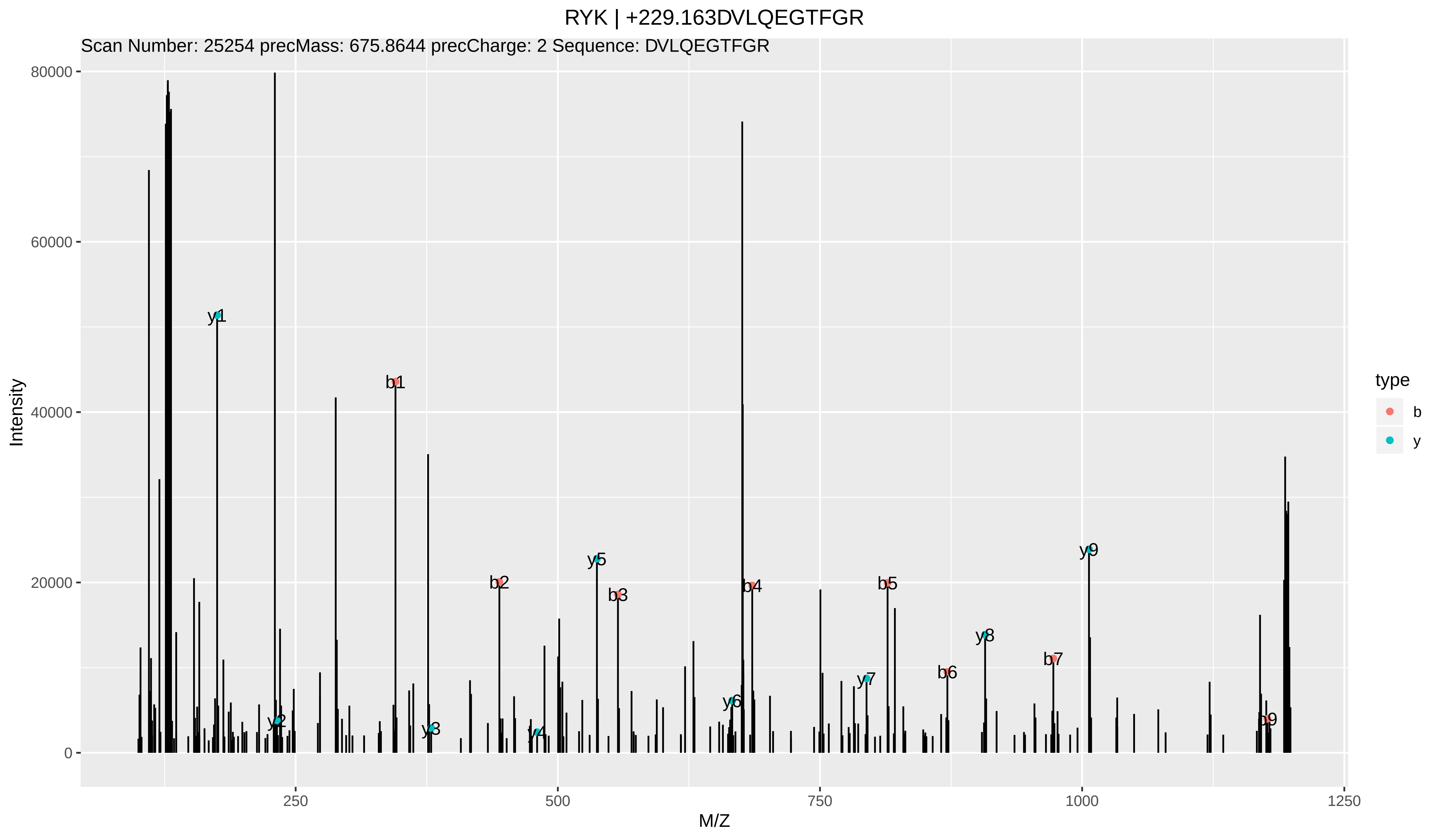
RYK | +229.163DVLQEGTFGR
Scan Number: 25254 precMass: 675.8644 precCharge: 2 Sequence: DVLQEGTFGR
Intensity
M/Z
type
b
y
y1
b1
y2
y3
b2
y4
y5
b3
y6
b4
y7
b5
b6
y8
b7
y9
b9
0
20000
40000
60000
80000
250
500
750
1000
1250

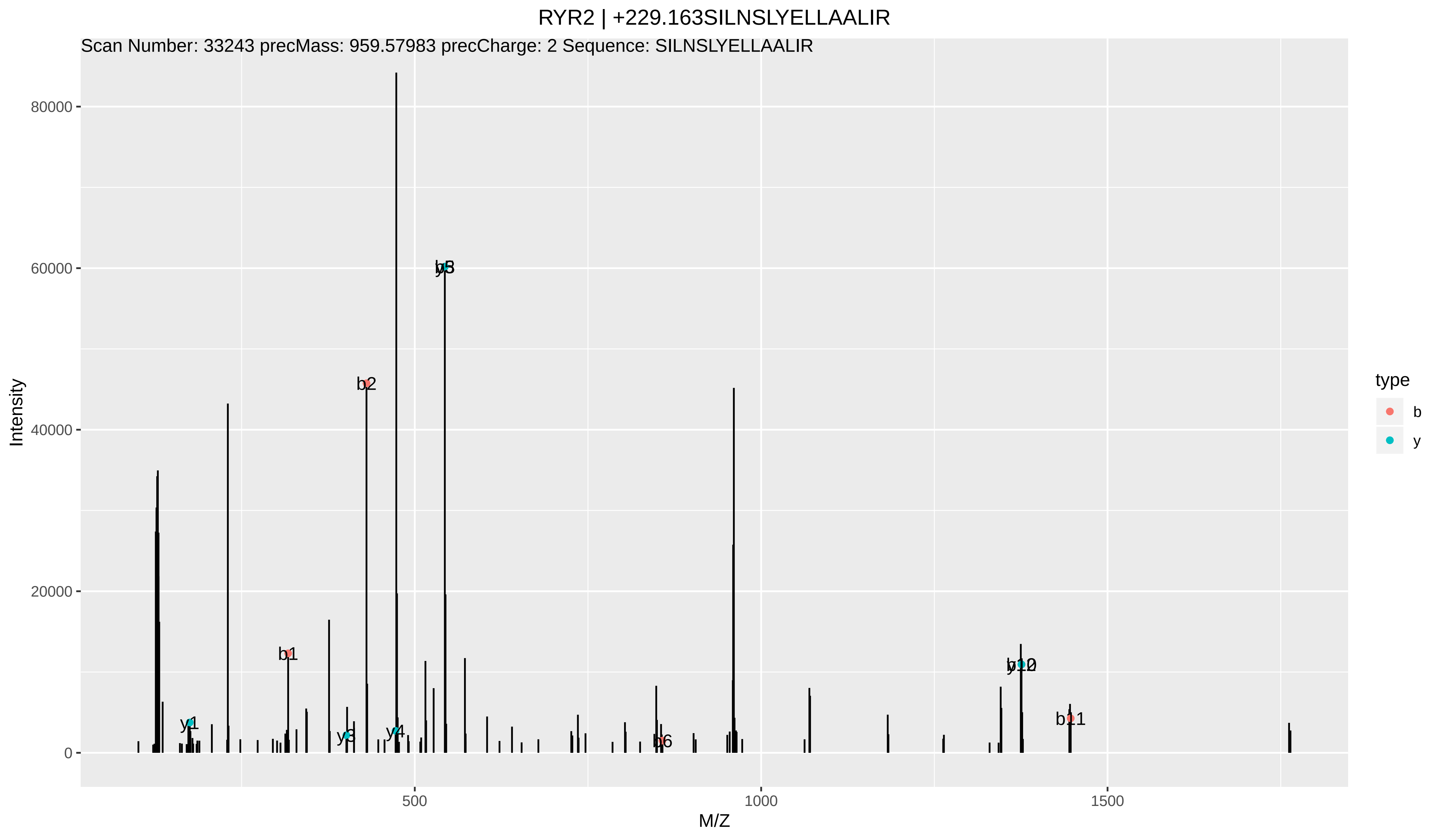

RYR2 | +229.163SILNSLYELLAALIR
Scan Number: 33243 precMass: 959.57983 precCharge: 2 Sequence: SILNSLYELLAALIR
Intensity
M/Z
80000
60000
40000
20000
0
500
1000
1500
b1
b2
b6
b11
y1
y3
y4
y12
type
b
y

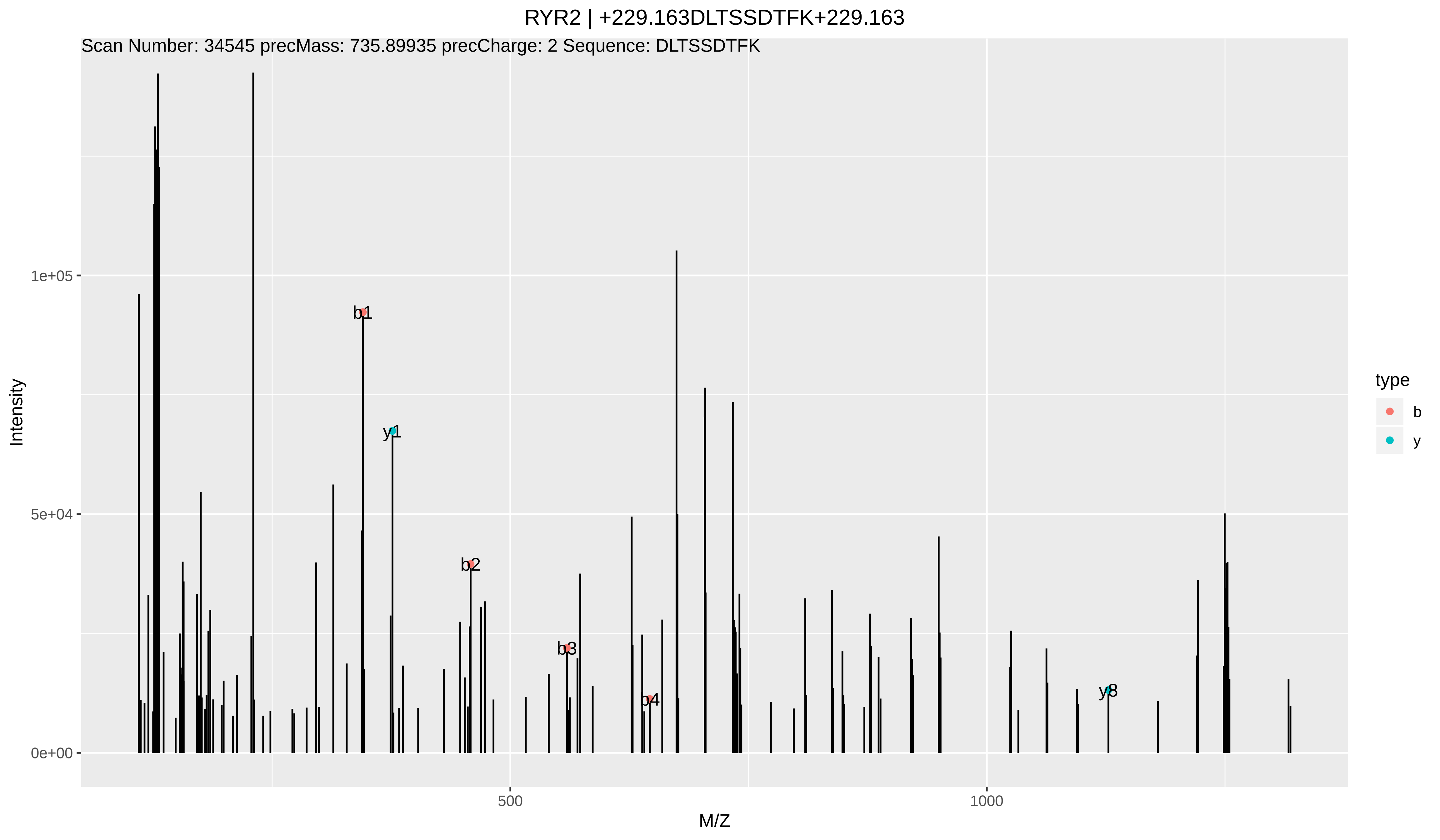

RYR2 | +229.163DLTSSDTFK+229.163
Scan Number: 34545 precMass: 735.89935 precCharge: 2 Sequence: DLTSSDTFK
Intensity
1e+05
5e+04
0e+00
M/Z
500
1000
b1
y1
b2
b3
b4
y8
type
b
y

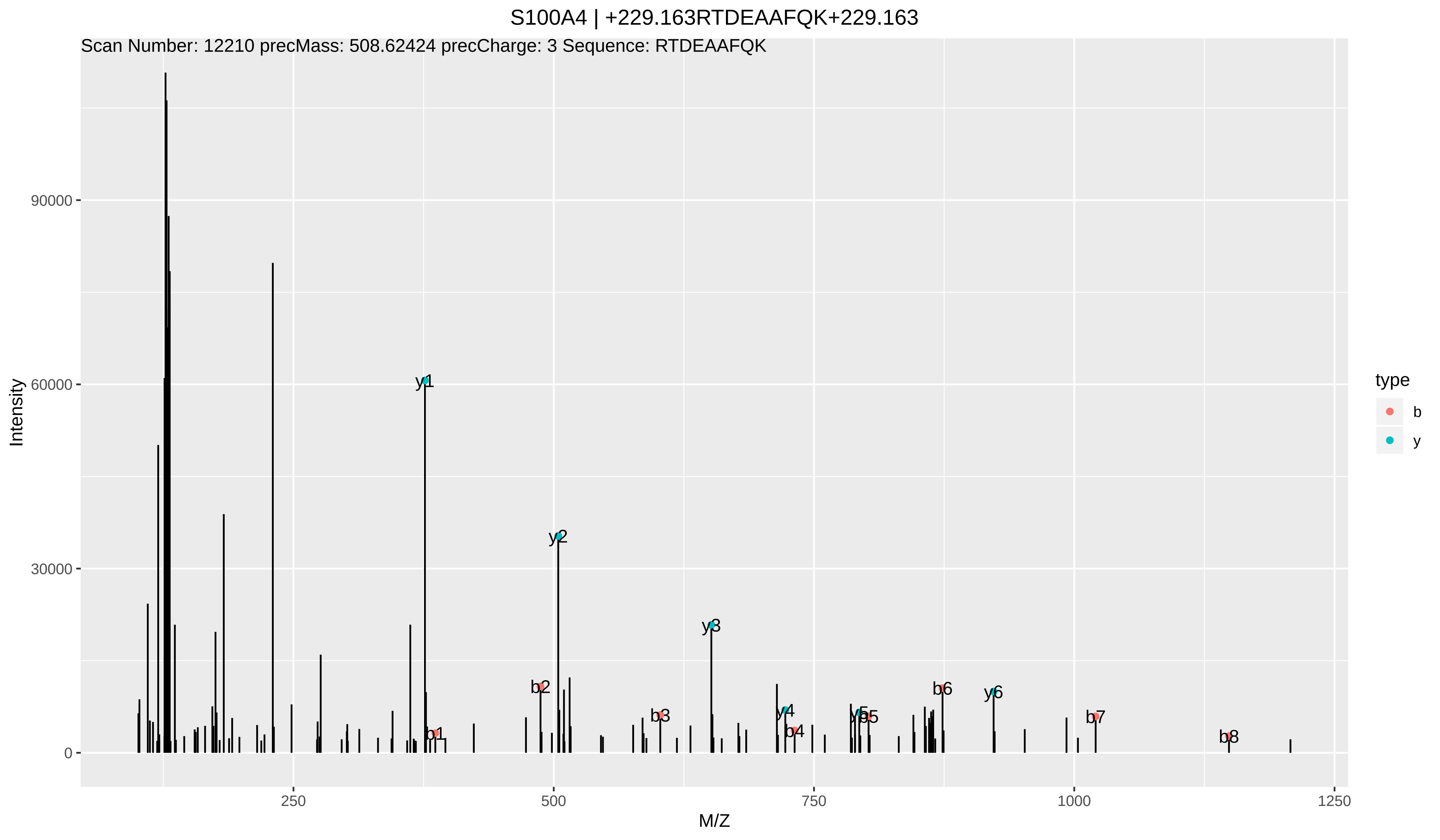

S100A4 | +229.163RTDEAAFQK+229.163
Scan Number: 12210 precMass: 508.62424 precCharge: 3 Sequence: RTDEAAFQK
Intensity
90000
60000
30000
0
M/Z
250
500
750
1000
1250
y1
y2
y3
y4
y5
y6
b1
b2
b3
b4
b5
b6
b7
b8
type
b
y

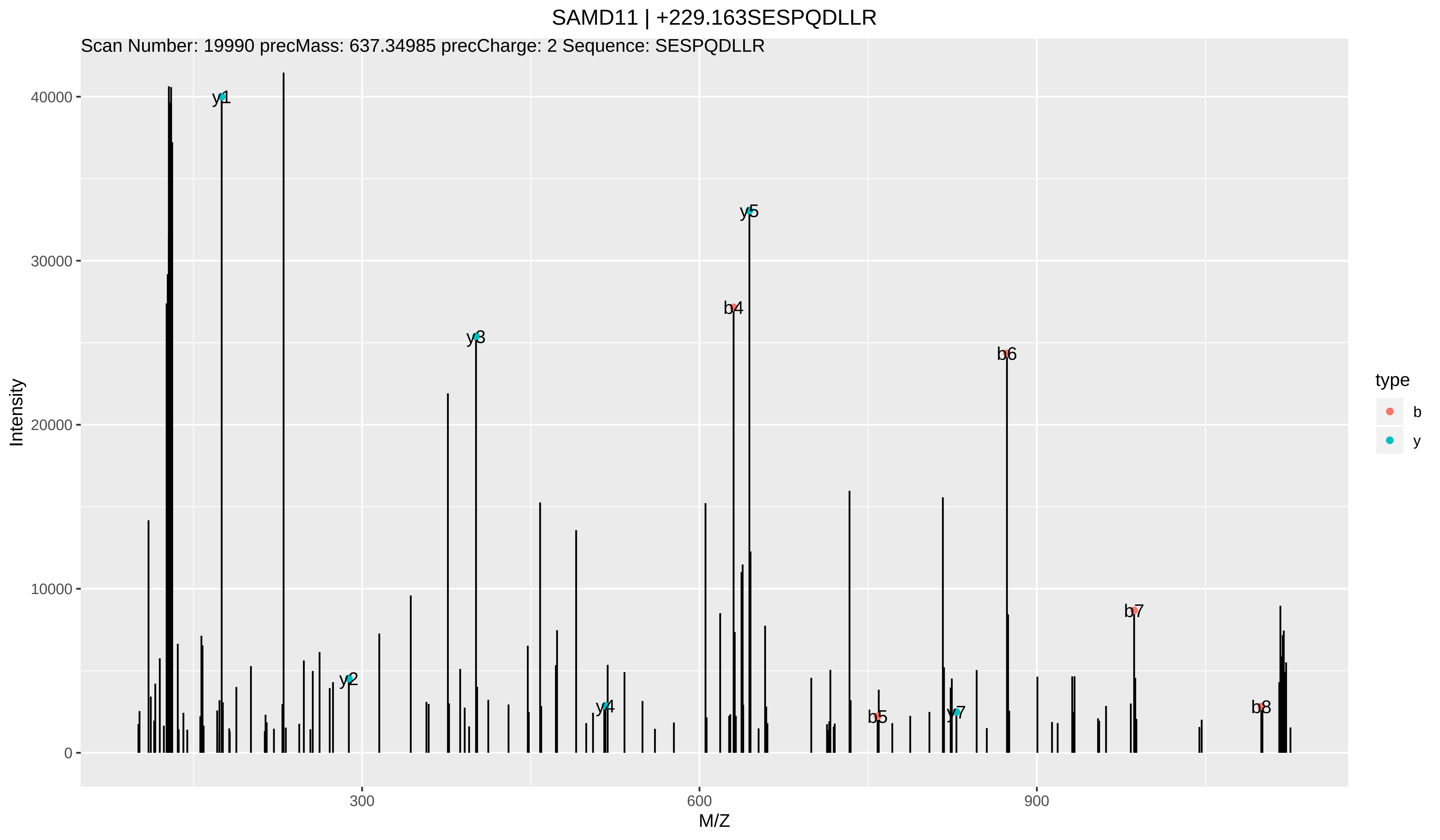

SAMD11 | +229.163SESPQDLLR
Scan Number: 19990 precMass: 637.34985 precCharge: 2 Sequence: SESPQDLLR
Intensity
M/Z
type
b
y
y1
y2
y3
y4
y5
y7
b4
b5
b6
b7
b8
0
10000
20000
30000
40000
300
600
900

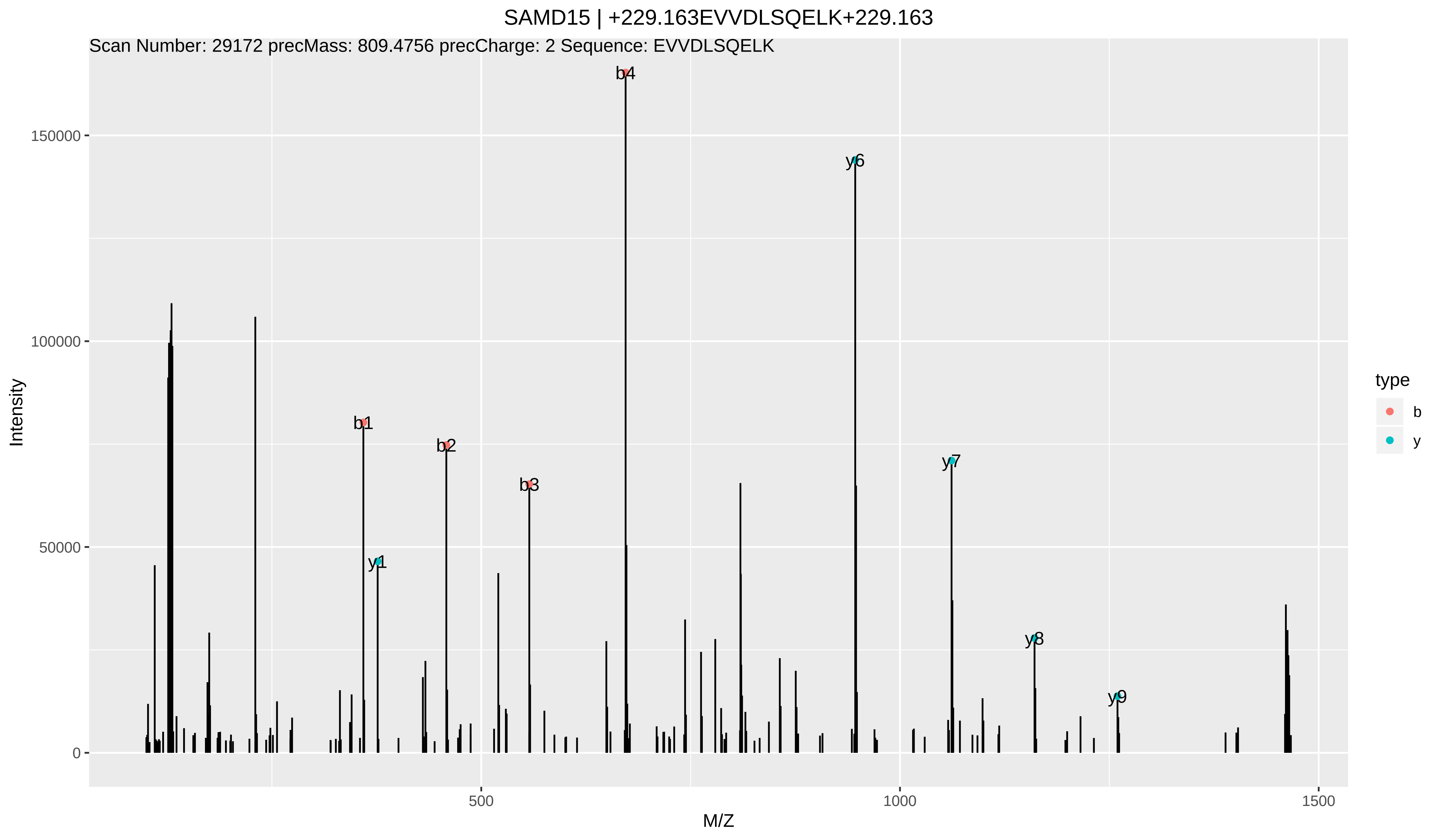

SAMD15 | +229.163EVVDLSQELK+229.163
Scan Number: 29172 precMass: 809.4756 precCharge: 2 Sequence: EVVDLSQELK
b4
y6
b1
b2
y7
b3
y1
y8
y9
150000
100000
50000
0
500
1000
1500
M/Z
Intensity
type
b
y

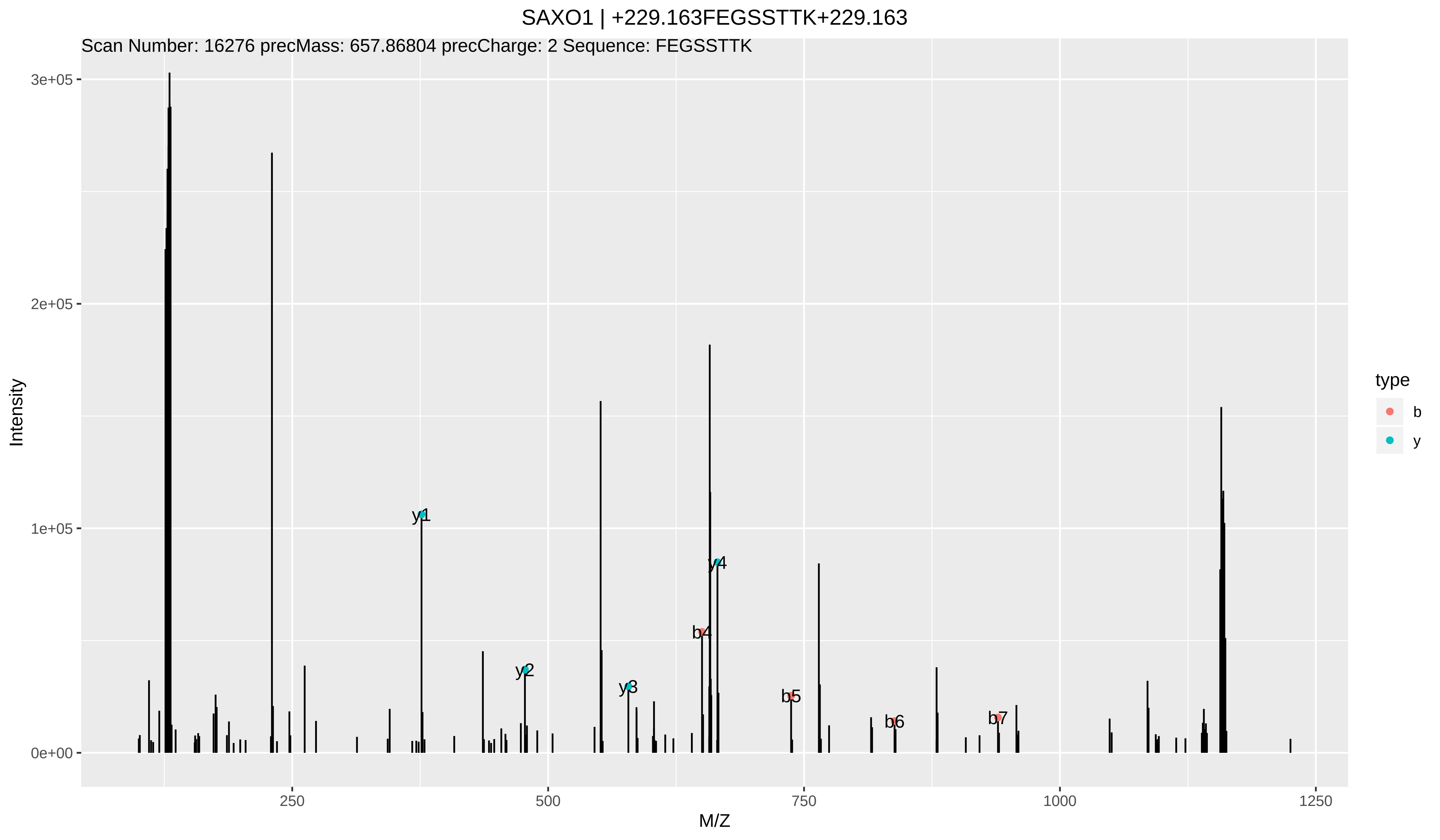
SAXO1 | +229.163FEGSSTTK+229.163
Scan Number: 16276 precMass: 657.86804 precCharge: 2 Sequence: FEGSSTTK
Intensity
3e+05
2e+05
1e+05
0e+00
M/Z
250
500
750
1000
1250
y1
y2
y3
y4
b4
b5
b6
b7
type
b
y

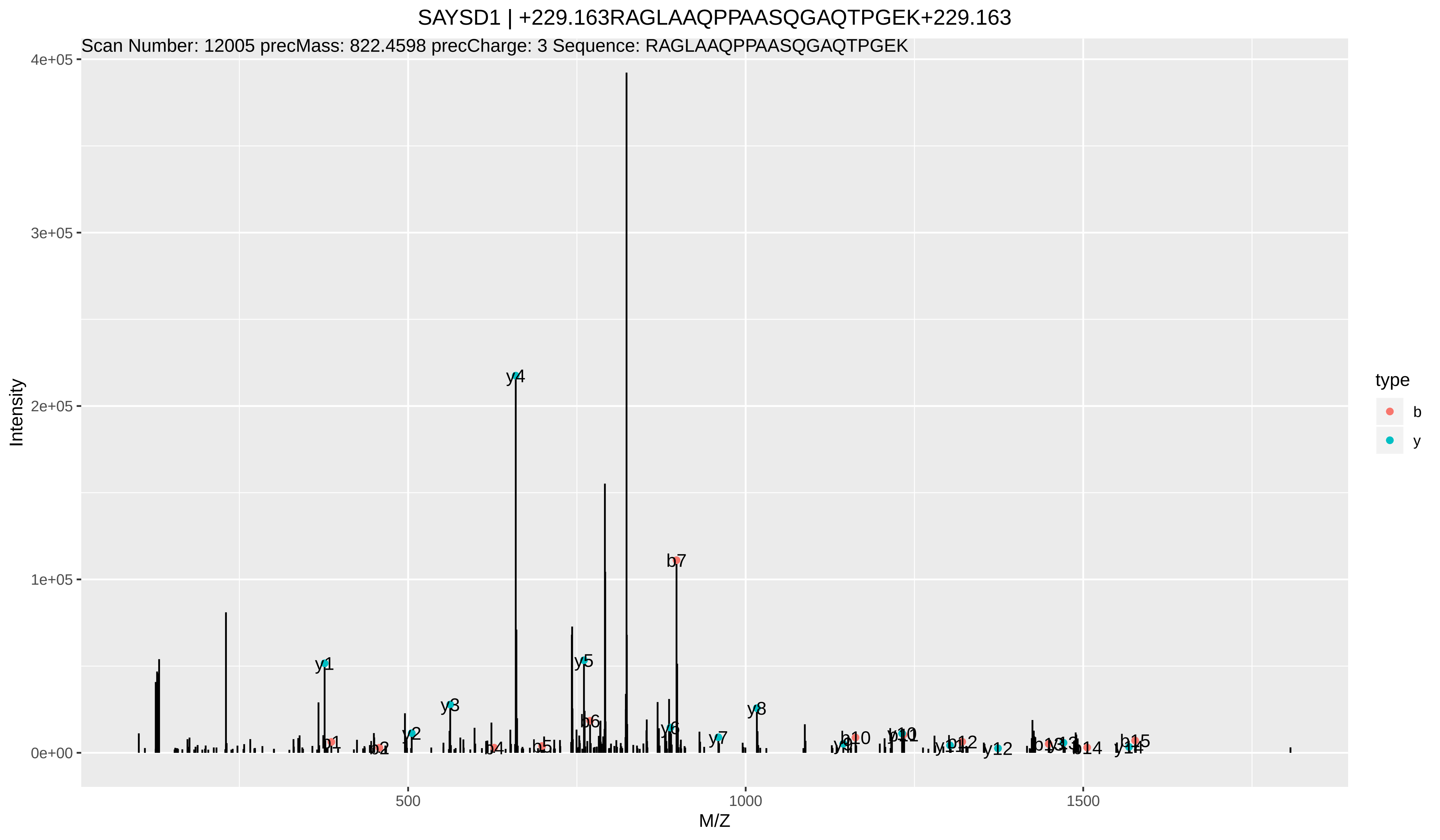

SAYSD1 | +229.163RAGLAAQPPAASQGAQTPGEK+229.163
Scan Number: 12005 precMass: 822.4598 precCharge: 3 Sequence: RAGLAAQPPAASQGAQTPGEK
Intensity
M/Z
0e+00
1e+05
2e+05
3e+05
4e+05
500
1000
1500
type
b
y
y1
y2
y3
y4
y5
y6
y7
y8
y12
b1
b2
b4
b5
b6
b7
b10
b12
b14
b15

SCMH1 | +229.163EADPQLGPHADLFR

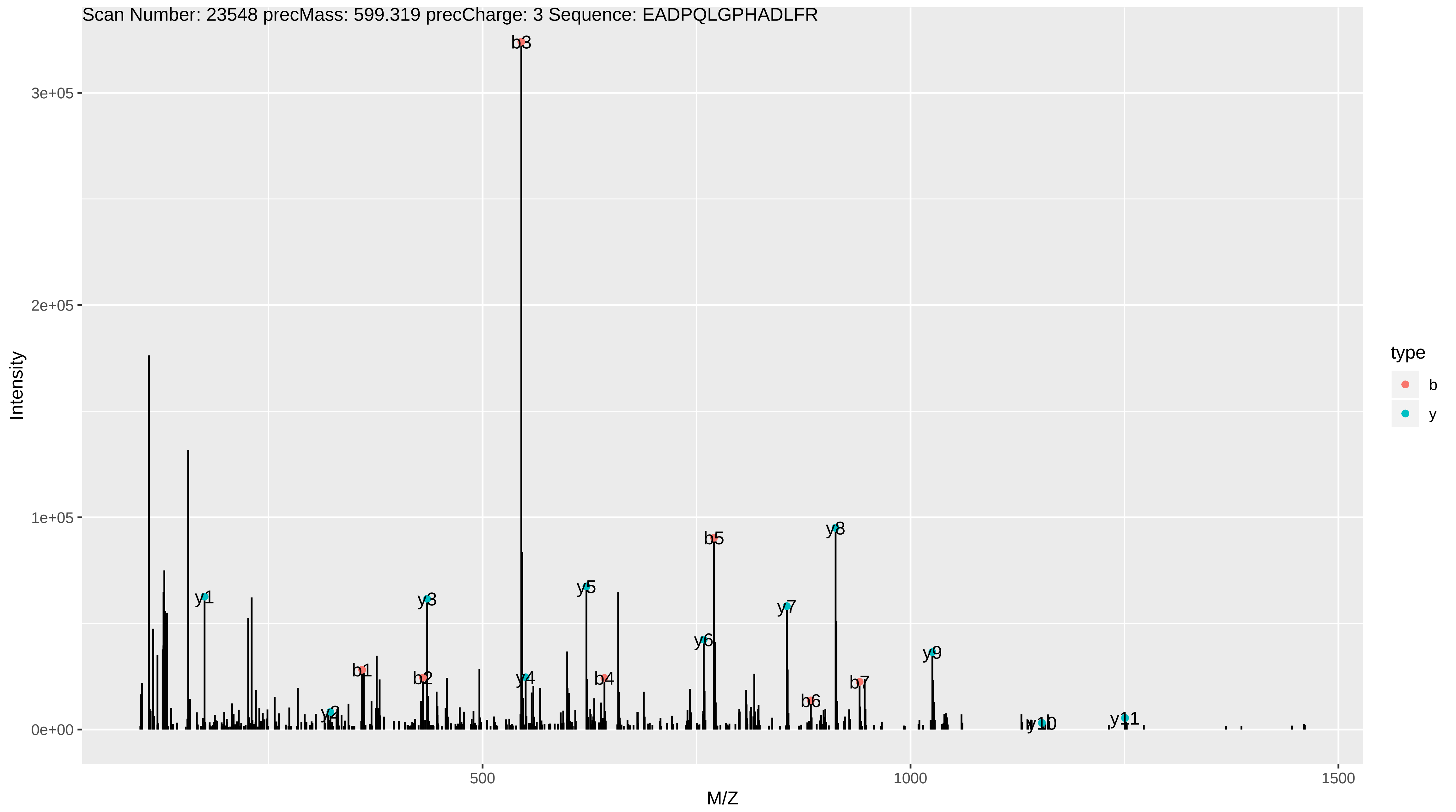

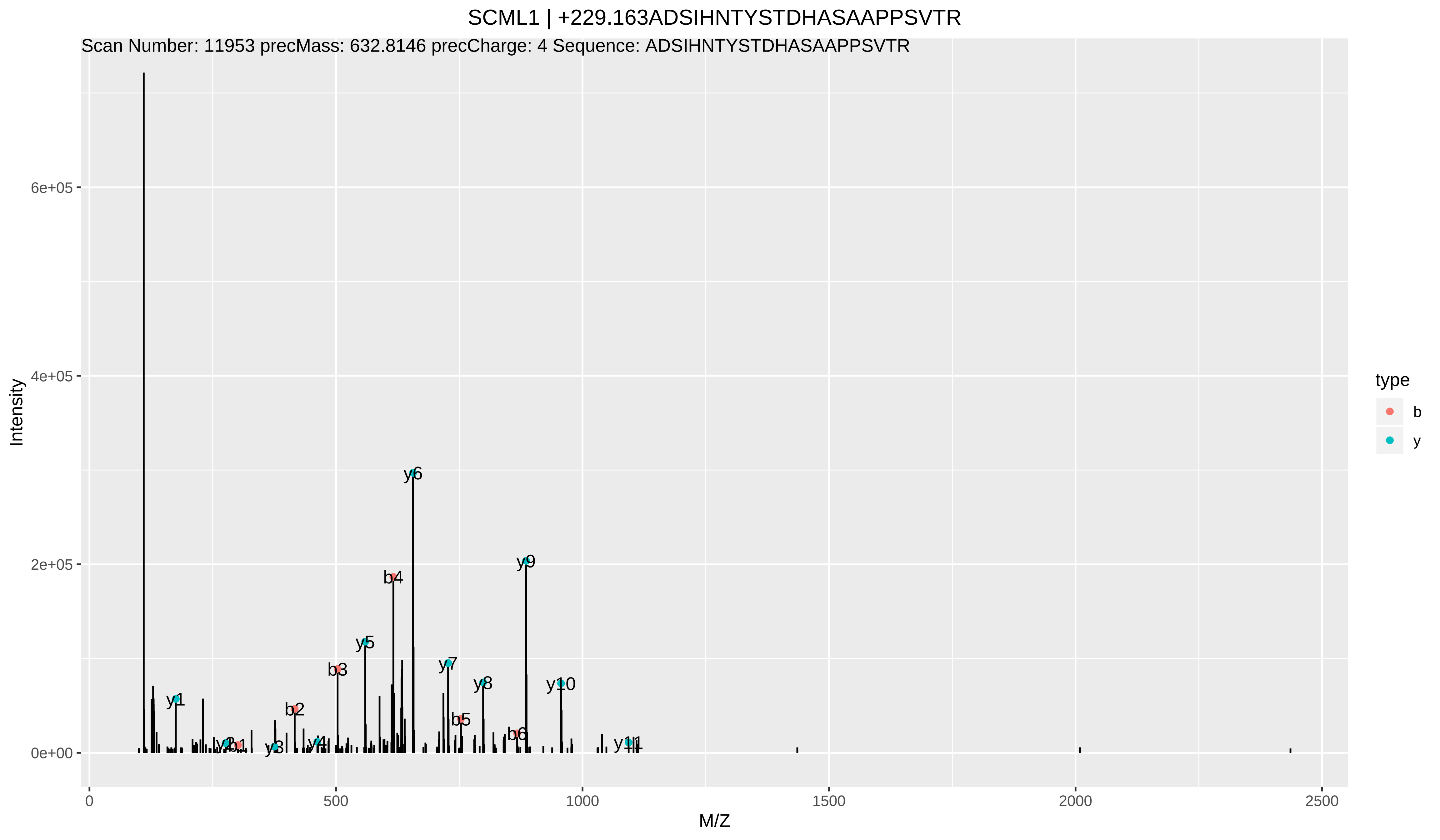

SCML1 | +229.163ADSIHNTYSTDHASAAPPSVTR
Scan Number: 11953 precMass: 632.8146 precCharge: 4 Sequence: ADSIHNTYSTDHASAAPPSVTR
Intensity
0e+00
2e+05
4e+05
6e+05
M/Z
0
500
1000
1500
2000
2500
y1
y2
b1
y3
b2
y4
b3
y5
b4
y6
y7
b5
y8
b6
y9
y10
y11
type
b
y

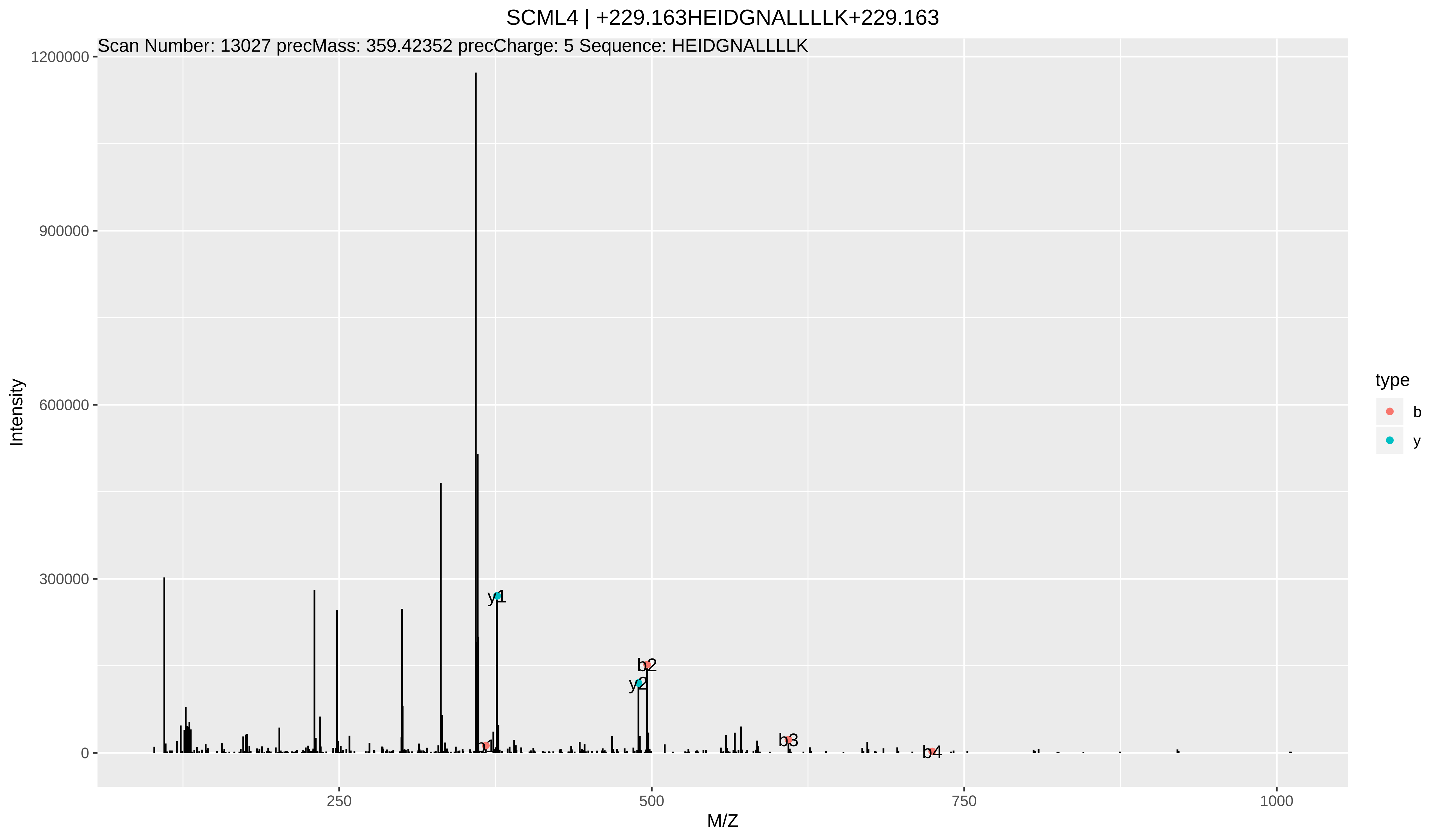

SCML4 | +229.163HEIDGNALLLLK+229.163
Scan Number: 13027 precMass: 359.42352 precCharge: 5 Sequence: HEIDGNALLLLK
Intensity
1200000
900000
600000
300000
0
250
500
750
1000
M/Z
y1
b2
y2
b3
b4
type
b
y

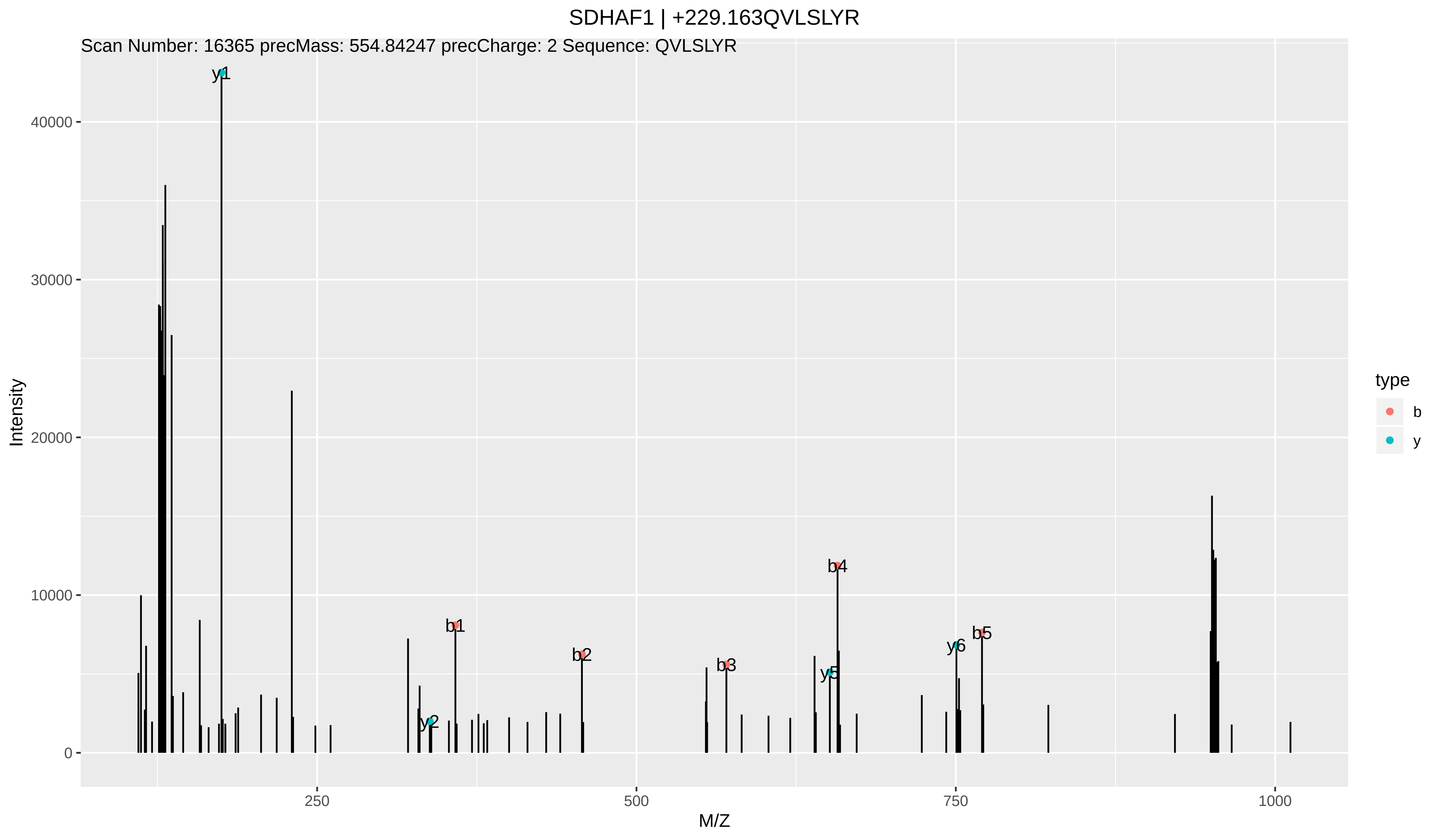
SDHAF1 | +229.163QVLSLYR
Scan Number: 16365 precMass: 554.84247 precCharge: 2 Sequence: QVLSLYR
y1
b1
b2
b3
b4
b5
y2
y5
y6
Intensity
40000
30000
20000
10000
0
250
500
750
1000
M/Z
type
b
y

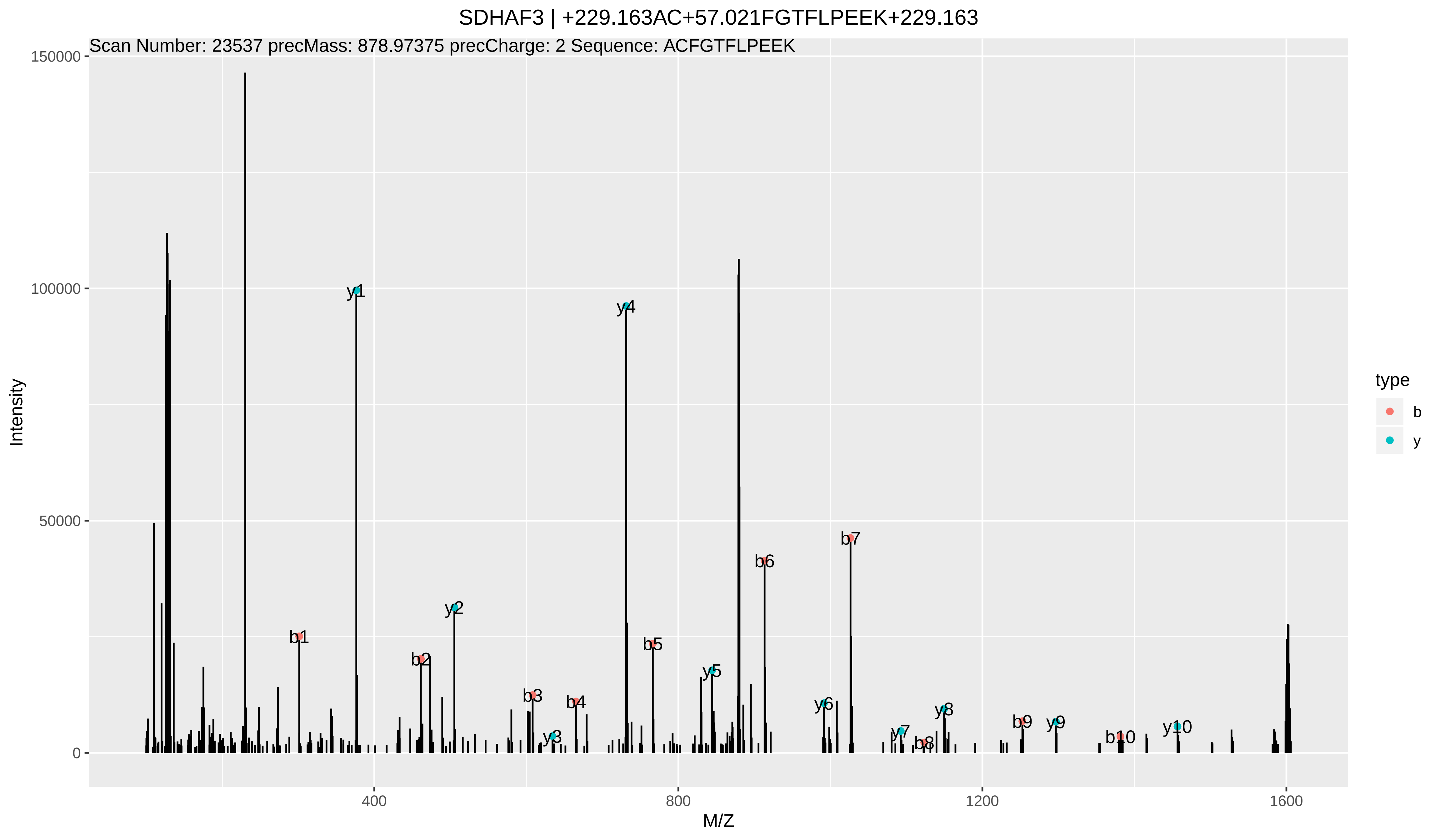
SDHAF3 | +229.163AC+57.021FGTFLPEEK+229.163
Scan Number: 23537 precMass: 878.97375 precCharge: 2 Sequence: ACFGTFLPEEK
Intensity
M/Z
150000
100000
50000
0
400
800
1200
1600
type
b
y
b1
b2
b3
b4
b5
b6
b7
b8
b9
b10
y1
y2
y3
y4
y5
y6
y7
y8
y9
y10

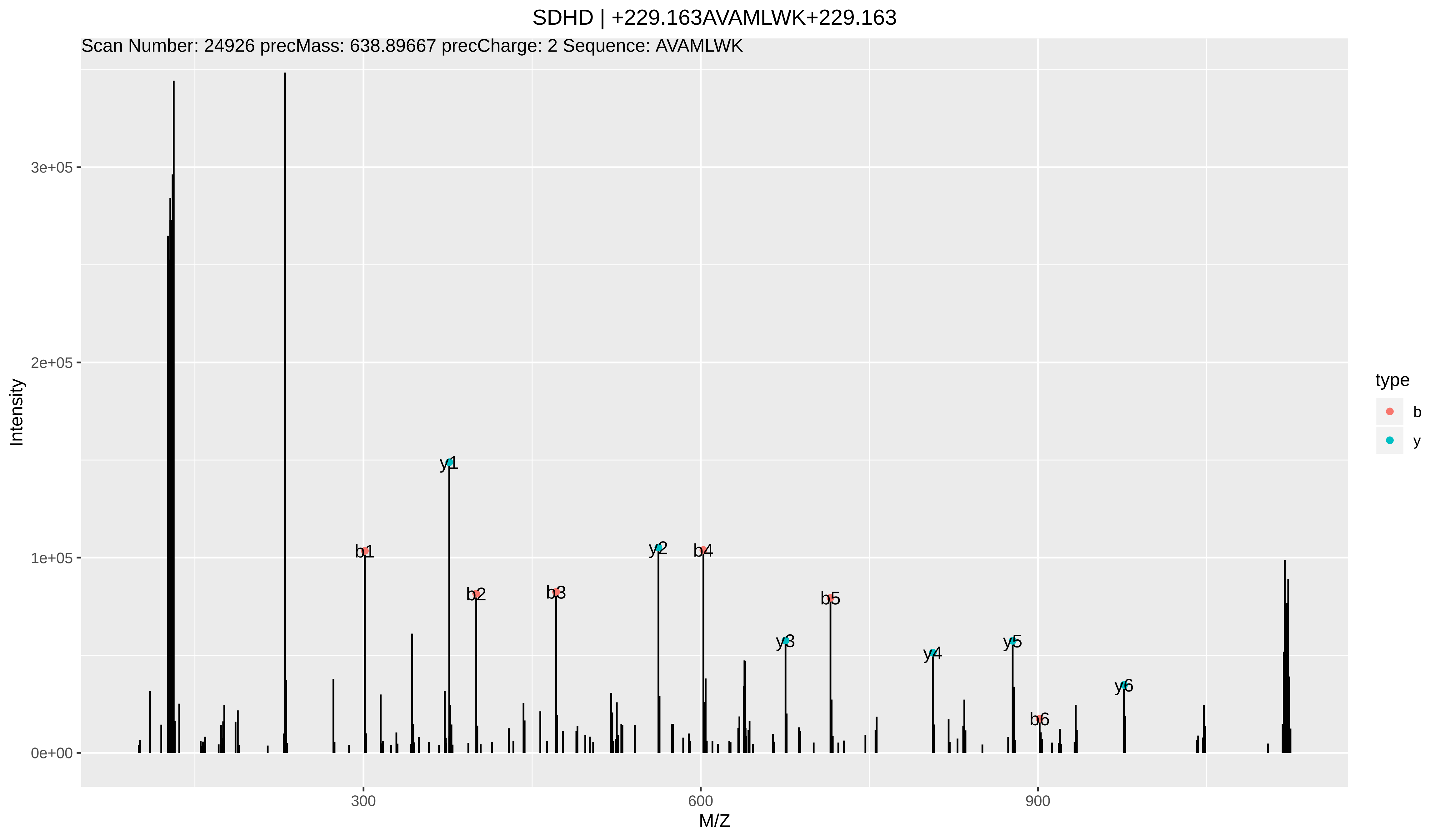

SDHD | +229.163AVAMLWK+229.163
Scan Number: 24926 precMass: 638.89667 precCharge: 2 Sequence: AVAMLWK
Intensity
3e+05
2e+05
1e+05
0e+00
M/Z
300
600
900
y1
b1
b2
b3
y2
b4
y3
b5
y4
y5
b6
y6
type
b
y

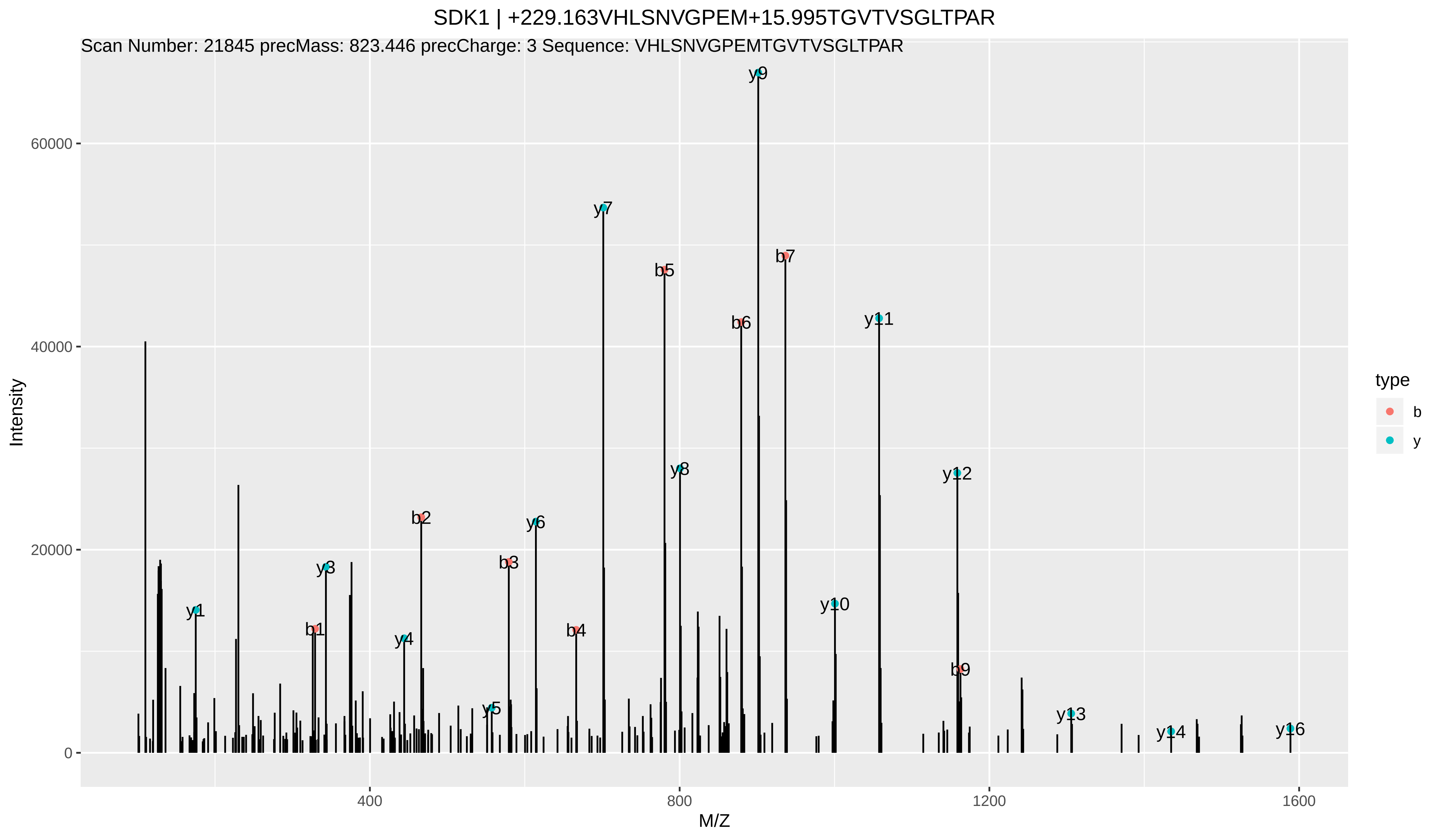
SDK1 | +229.163VHLSNVGPEM+15.995TGVTVSGLTPAR
Scan Number: 21845 precMass: 823.446 precCharge: 3 Sequence: VHLSNVGPEMTGVTVSGLTPAR
Intensity
M/Z
type
b
y
y1
b1
y3
y4
b2
y5
b3
y6
b4
y7
b5
y8
b6
y9
b7
y10
y11
y12
b9
y13
y14
y16
0
20000
40000
60000
400
800
1200
1600

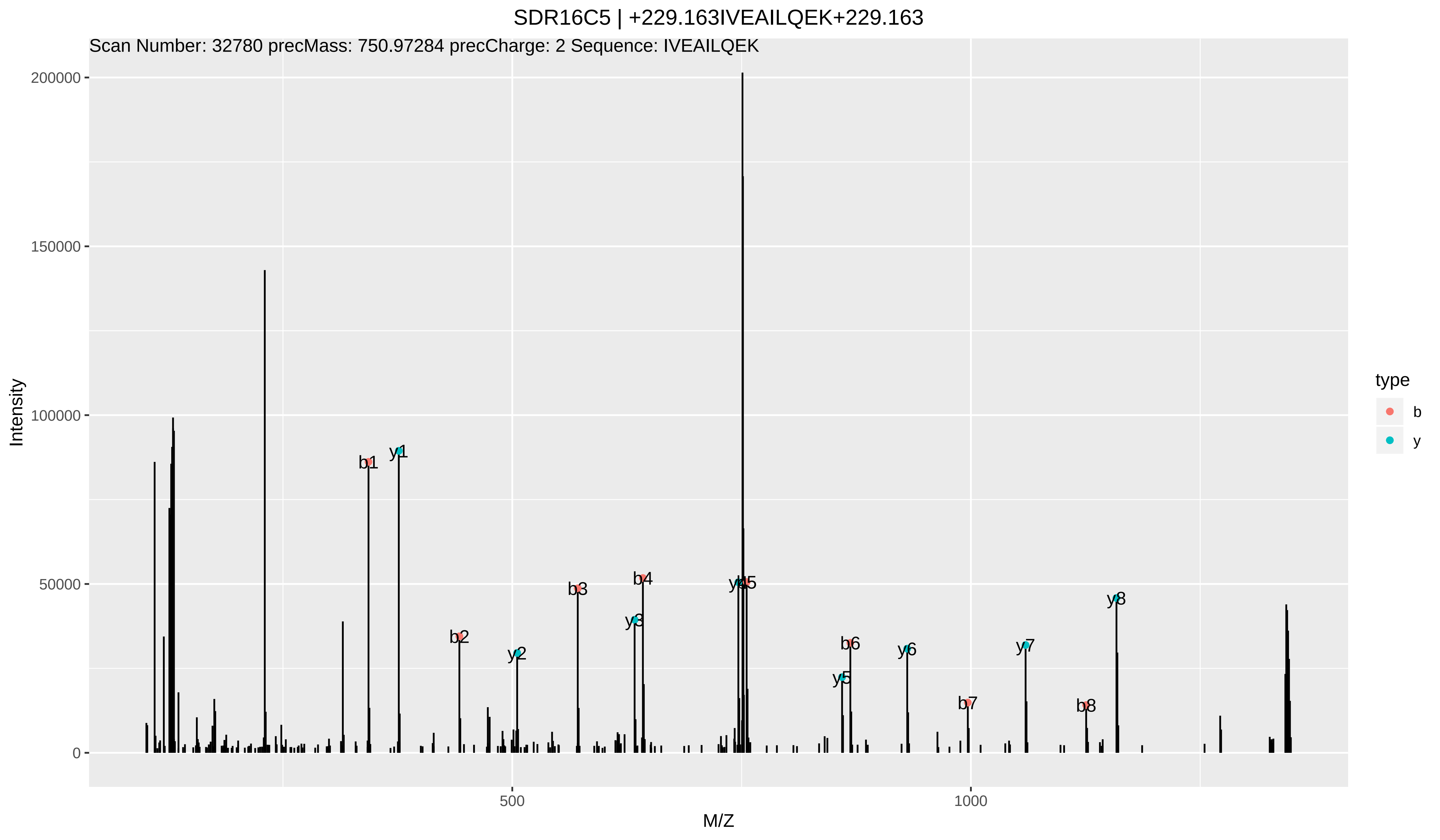

SDR16C5 | +229.163IVEAILQEK+229.163
Scan Number: 32780 precMass: 750.97284 precCharge: 2 Sequence: IVEAILQEK
Intensity
200000
150000
100000
50000
0
500
1000
M/Z
b1
y1
b2
y2
b3
y3
b4
b5
y5
b6
y6
b7
y7
b8
y8
type
b
y

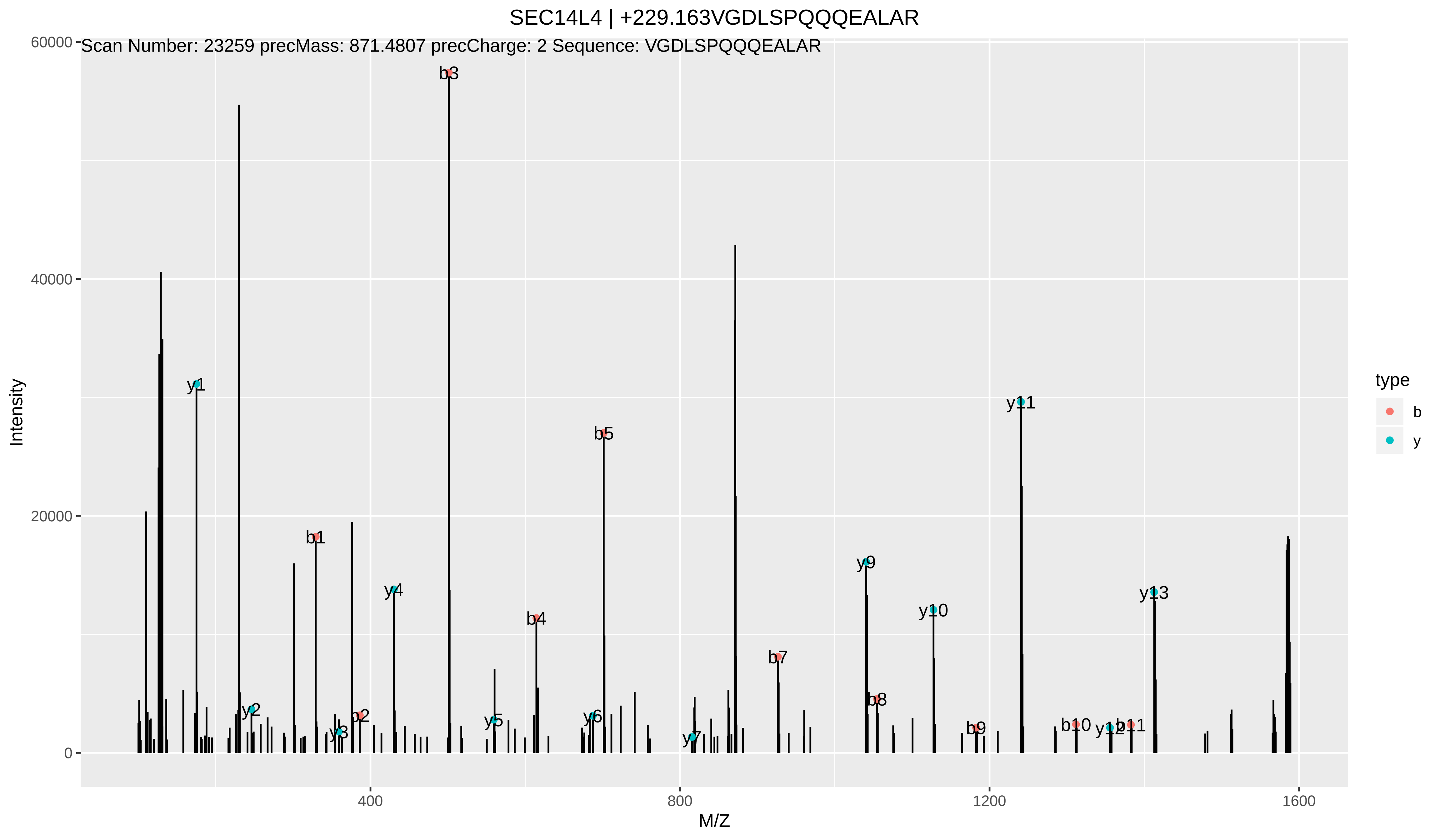

SEC14L4 | +229.163VGDLSPQQQEALAR
Scan Number: 23259 precMass: 871.4807 precCharge: 2 Sequence: VGDLSPQQQEALAR
Intensity
M/Z
60000
40000
20000
0
400
800
1200
1600
b3
y1
b5
y11
b1
y9
y4
y13
y10
b4
b7
b8
y2
b2
y5
y6
y3
b9
b10
y12
b11
y7
type
b
y

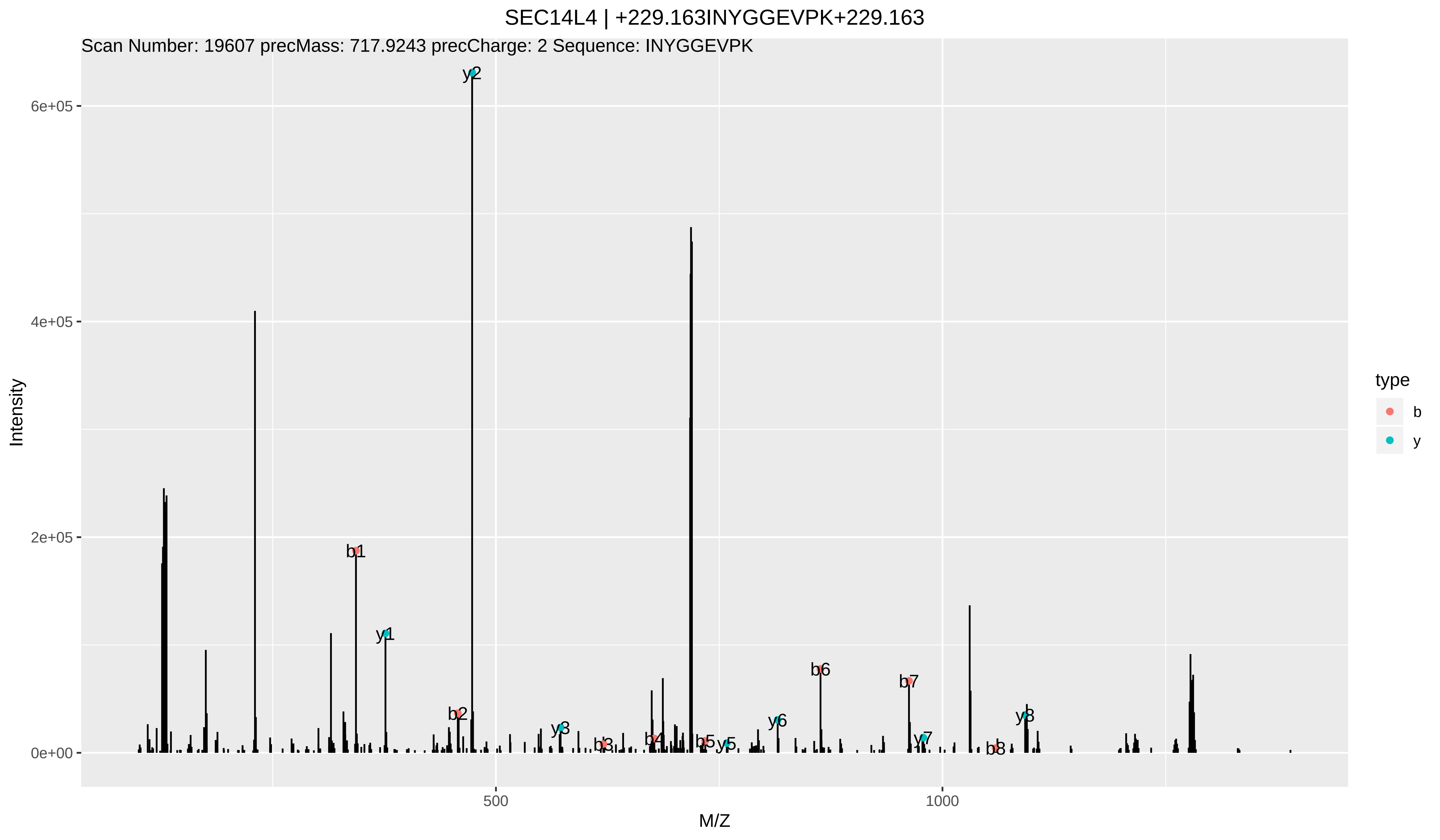

SEC14L4 | +229.163INYGGEVPK+229.163
Scan Number: 19607 precMass: 717.9243 precCharge: 2 Sequence: INYGGEVPK
Intensity
M/Z
6e+05
4e+05
2e+05
0e+00
500
1000
y2
b1
y1
b2
y3
b3
b4
b5
y5
y6
b6
b7
y7
b8
y8
type
b
y

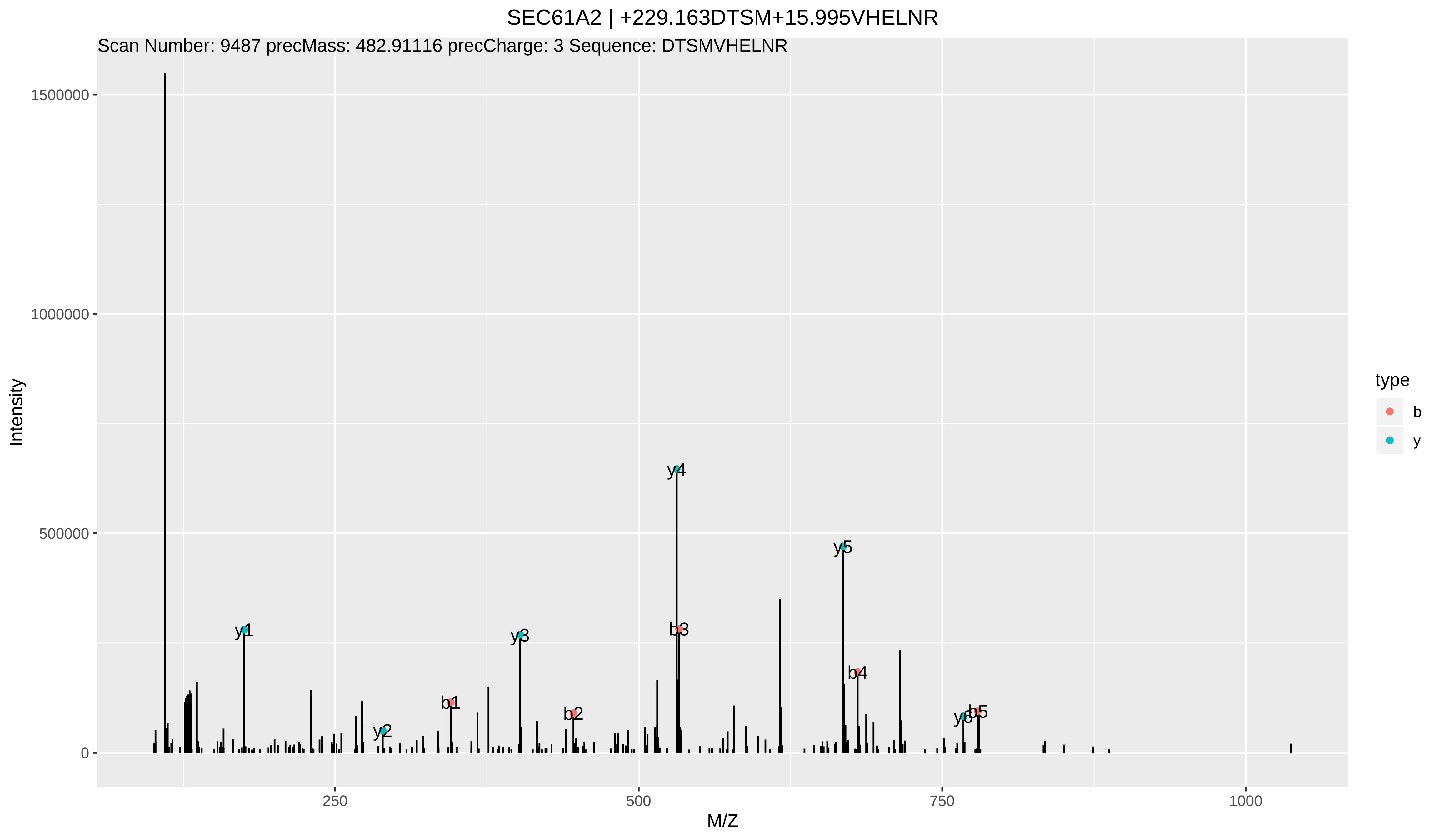

SEC61A2 | +229.163DTSM+15.995VHELNR
Scan Number: 9487 precMass: 482.91116 precCharge: 3 Sequence: DTSMVHELNR
Intensity
M/Z
0
500000
1000000
1500000
250
500
750
1000
y1
y2
b1
y3
b2
y4
b3
y5
b4
y6
b5
type
b
y

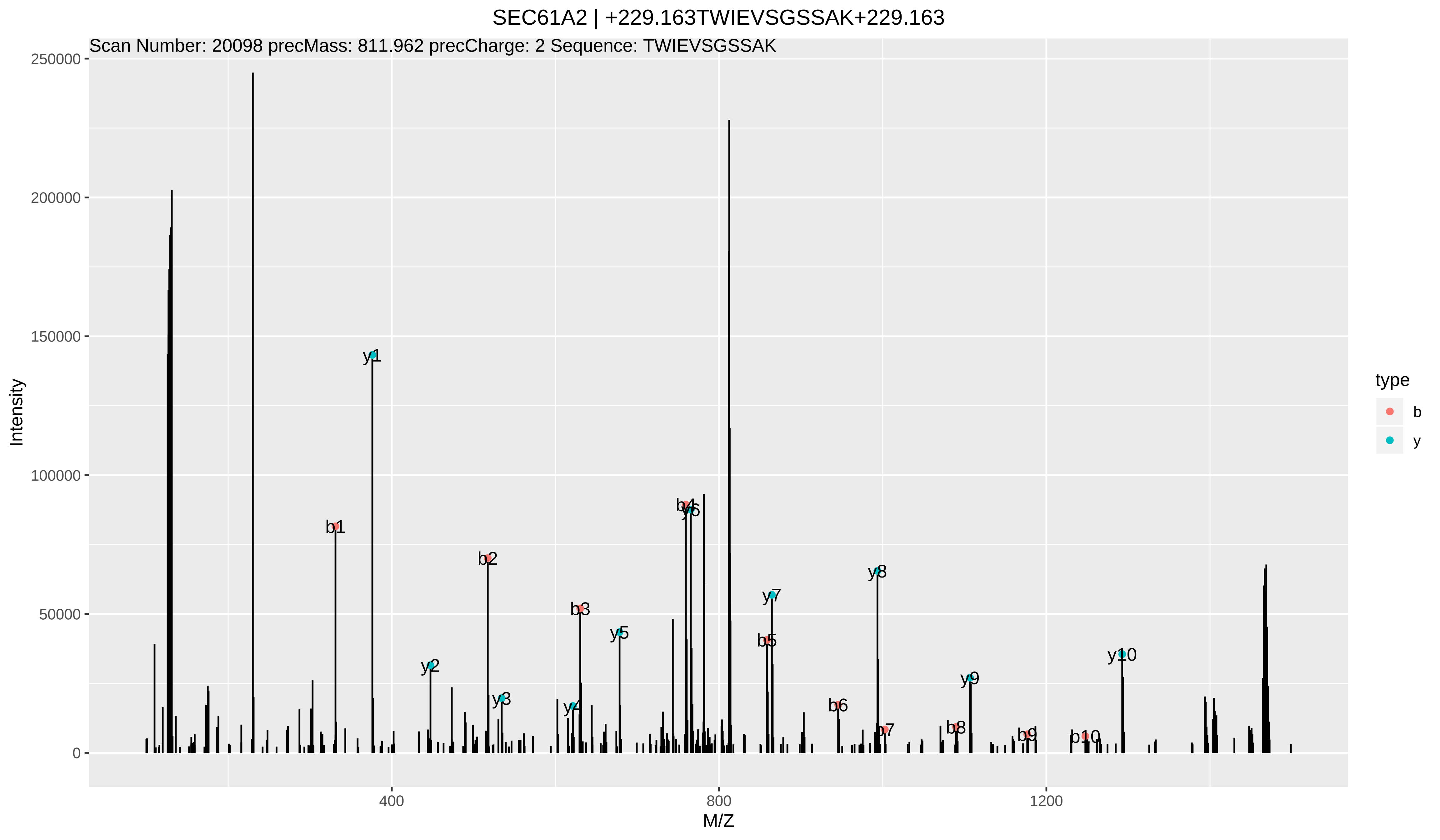

SEC61A2 | +229.163TWIEVSGSSAK+229.163
Scan Number: 20098 precMass: 811.962 precCharge: 2 Sequence: TWIEVSGSSAK
Intensity
250000
200000
150000
100000
50000
0
400
800
1200
M/Z
y1
b1
b2
y2
y3
b3
y4
y5
b4
y6
y7
b5
y8
b6
b7
b8
y9
b9
b10
y10
type
b
y

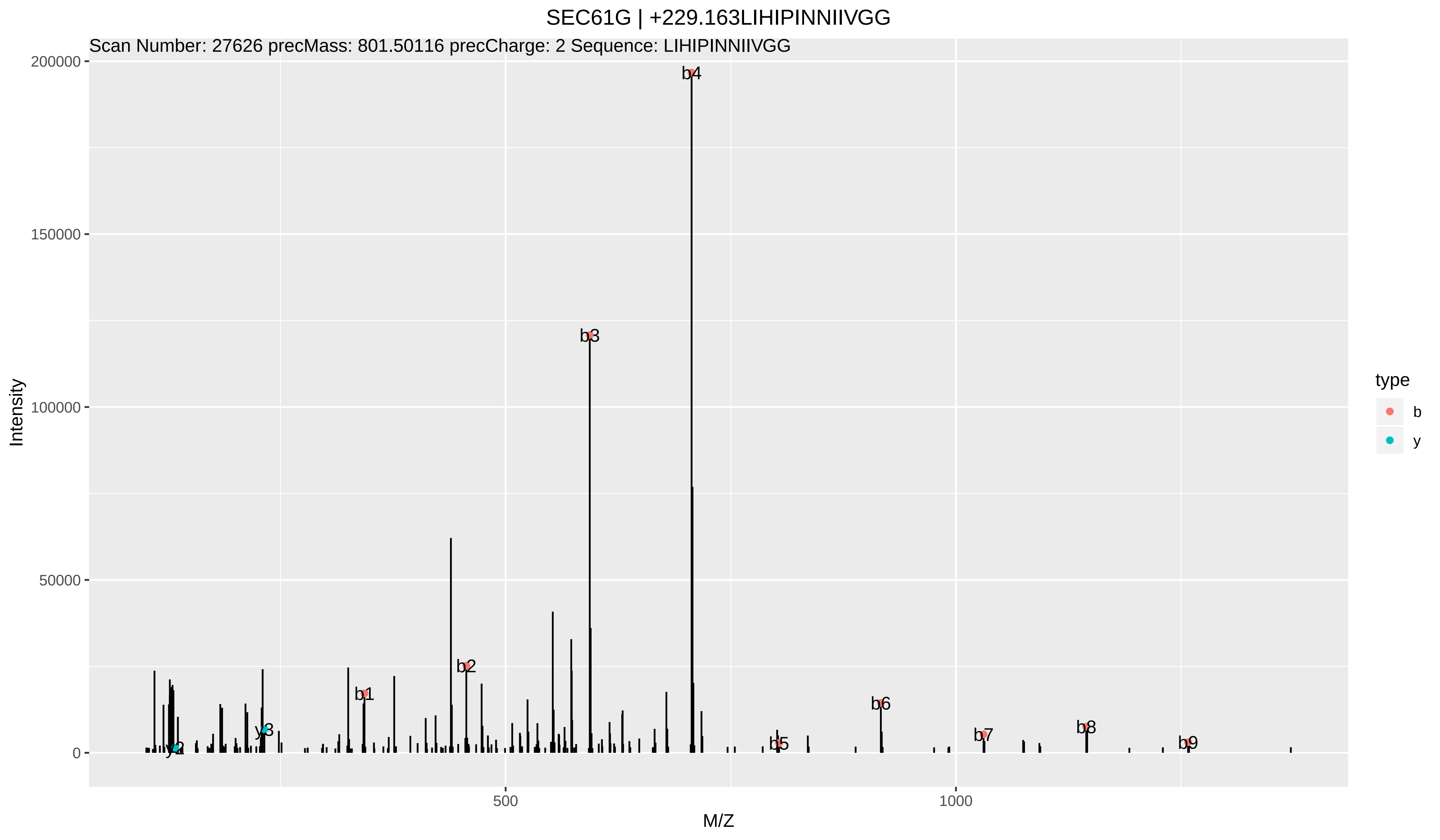

SEC61G | +229.163LIHIPINNIIVGG
Scan Number: 27626 precMass: 801.50116 precCharge: 2 Sequence: LIHIPINNIIVGG
Intensity
200000
150000
100000
50000
0
M/Z
500
1000
b4
b3
b2
b1
b5
b6
b7
b8
b9
y1
y2
y3
type
b
y

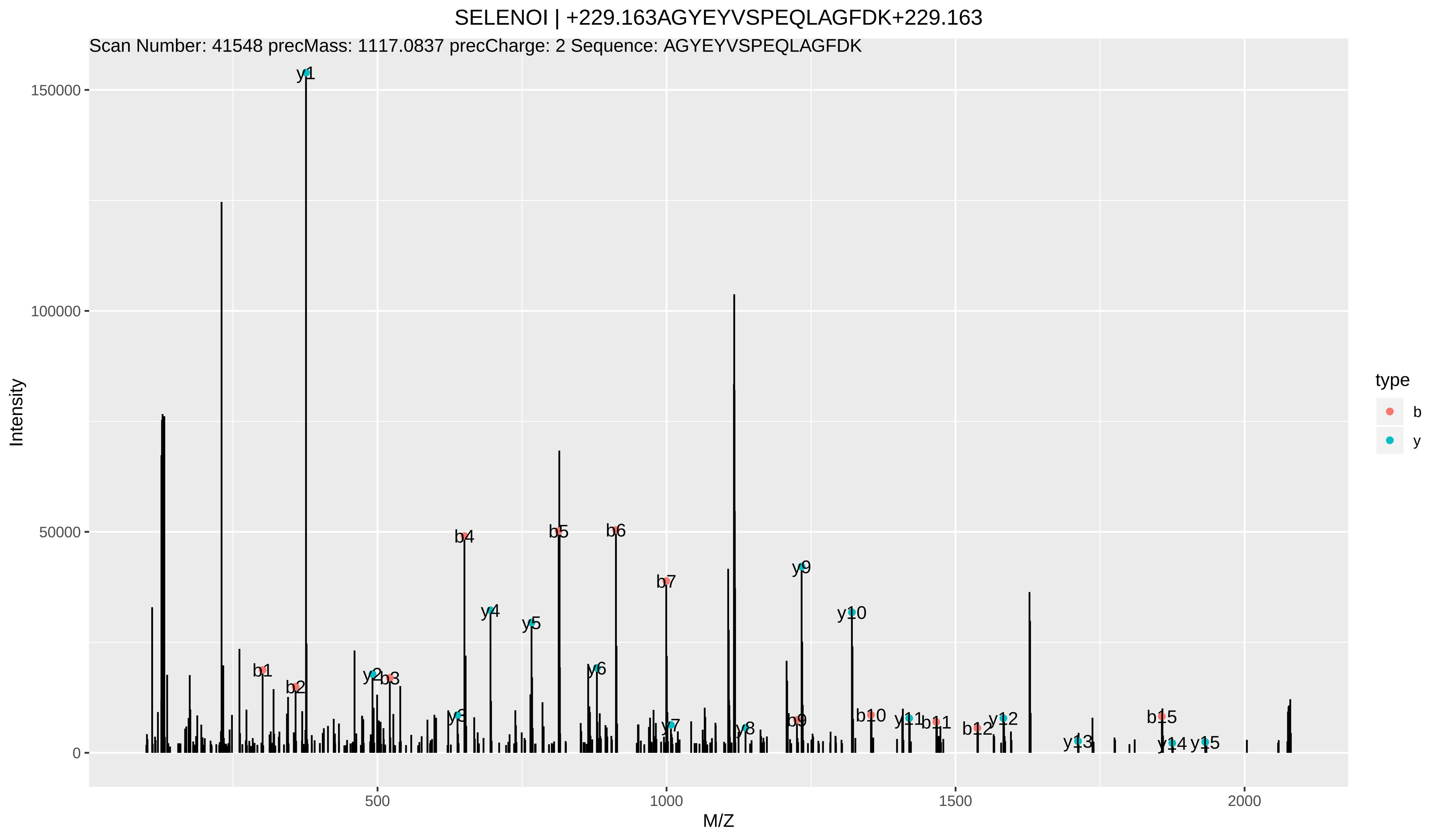

SELENOI | +229.163AGYEYVSPEQLAGFDK+229.163
Scan Number: 41548 precMass: 1117.0837 precCharge: 2 Sequence: AGYEYVSPEQLAGFDK
Intensity
150000
100000
50000
0
M/Z
500
1000
1500
2000
type
b
y
y1
b1
b2
y2
b3
y3
b4
y4
y5
b5
b6
y6
b7
y7
y8
y9
b9
y10
b10
y11
b11
b12
y12
y13
b15
y14
y15

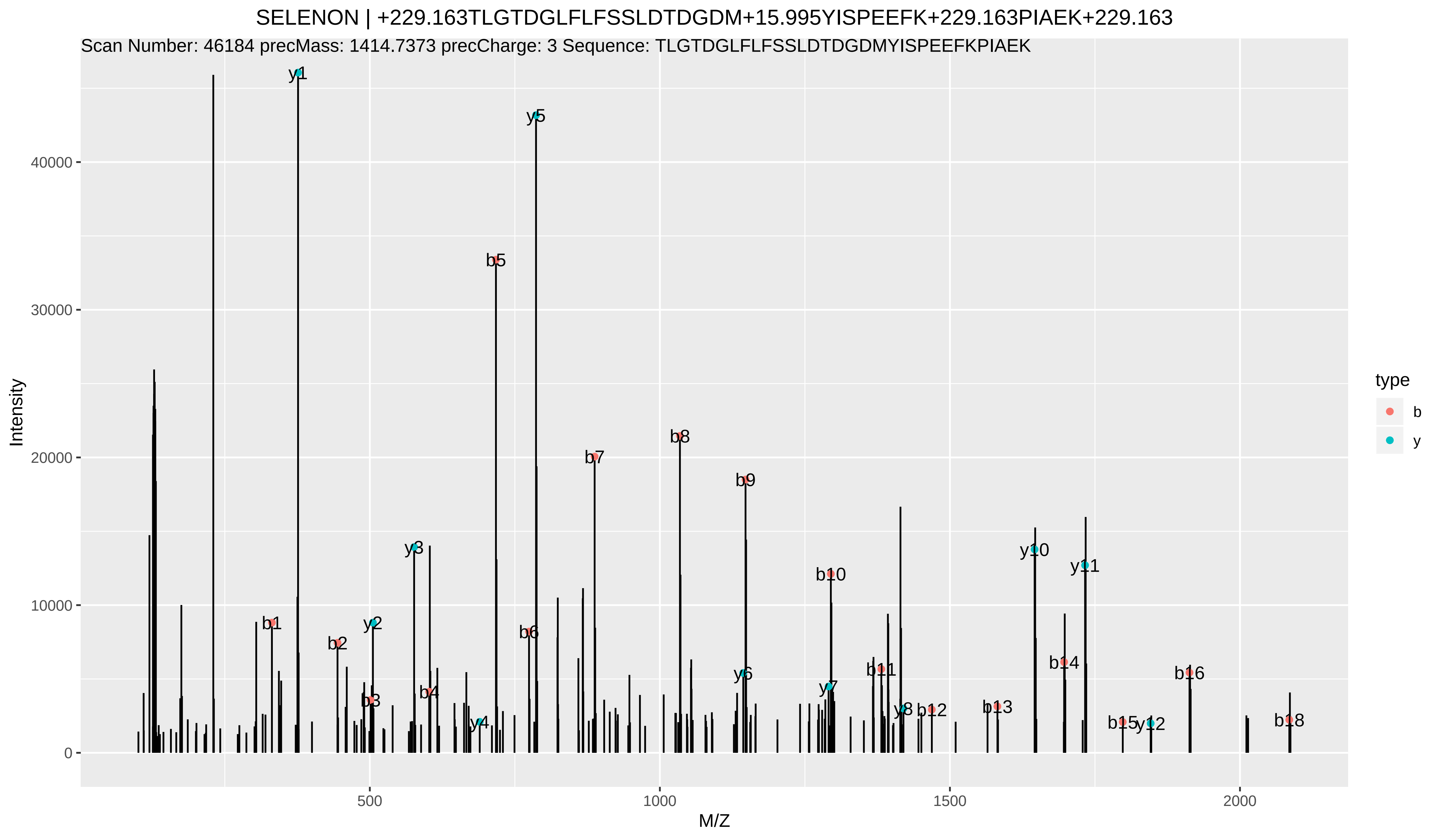

SELENON | +229.163TLGTDGLFLFSSLDTDGDM+15.995YISPEEFK+229.163PIAEK+229.163
Scan Number: 46184 precMass: 1414.7373 precCharge: 3 Sequence: TLGTDGLFLFSSLDTDGDMYISPEEFKPIAEK
Intensity
M/Z
type
b
y
y1
y5
b5
b8
b7
b9
y3
y10
y11
b10
y2
b1
b2
b6
b14
b11
y6
b16
y7
b4
b3
b13
y8
b12
y4
b15
y12
b18
0
10000
20000
30000
40000
500
1000
1500
2000

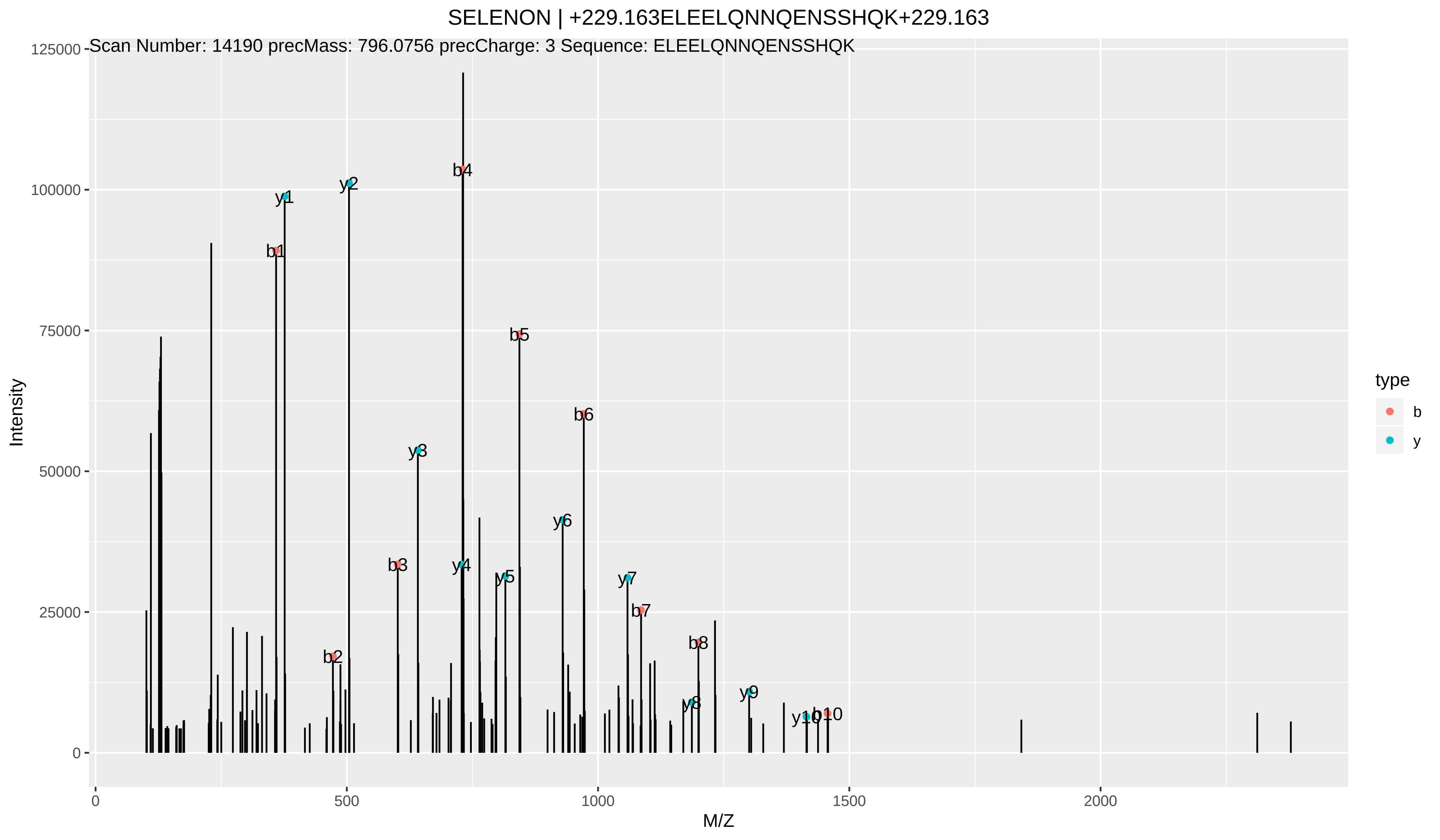

SELENON | +229.163ELELEELQNNQENSSHQK+229.163
Scan Number: 14190 precMass: 796.0756 precCharge: 3 Sequence: ELEELQNNQENSSHQK
Intensity
M/Z
type
b
y
b1
b2
b3
b4
b5
b6
b7
b8
b10
y1
y2
y3
y4
y5
y6
y7
y8
y9
y10

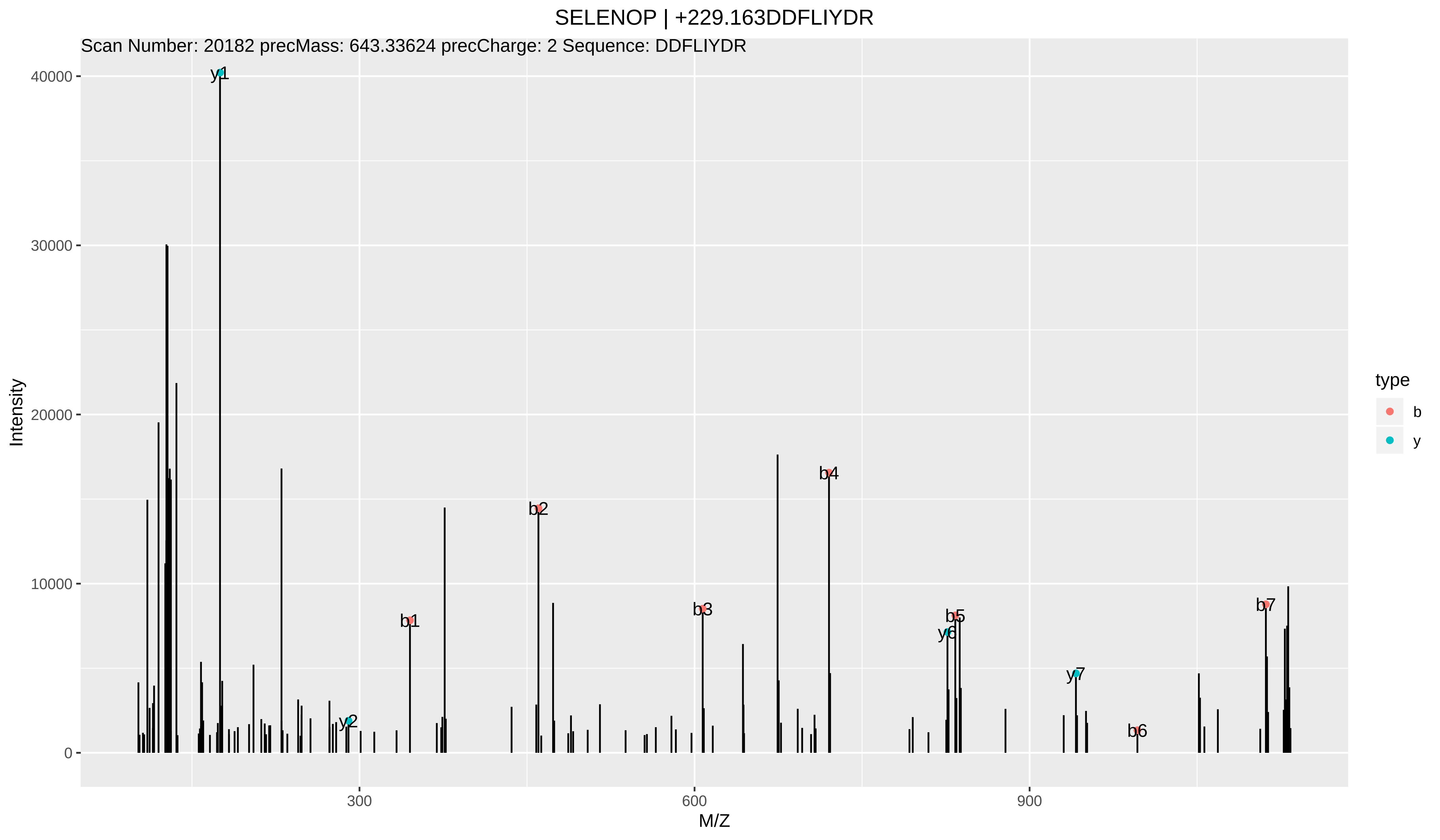
SELENOP | +229.163DDFLIYDR
Scan Number: 20182 precMass: 643.33624 precCharge: 2 Sequence: DDFLIYDR
Intensity
40000
30000
20000
10000
0
M/Z
300
600
900
y1
y2
b1
b2
b3
b4
b5
y6
y7
b6
b7
type
b
y

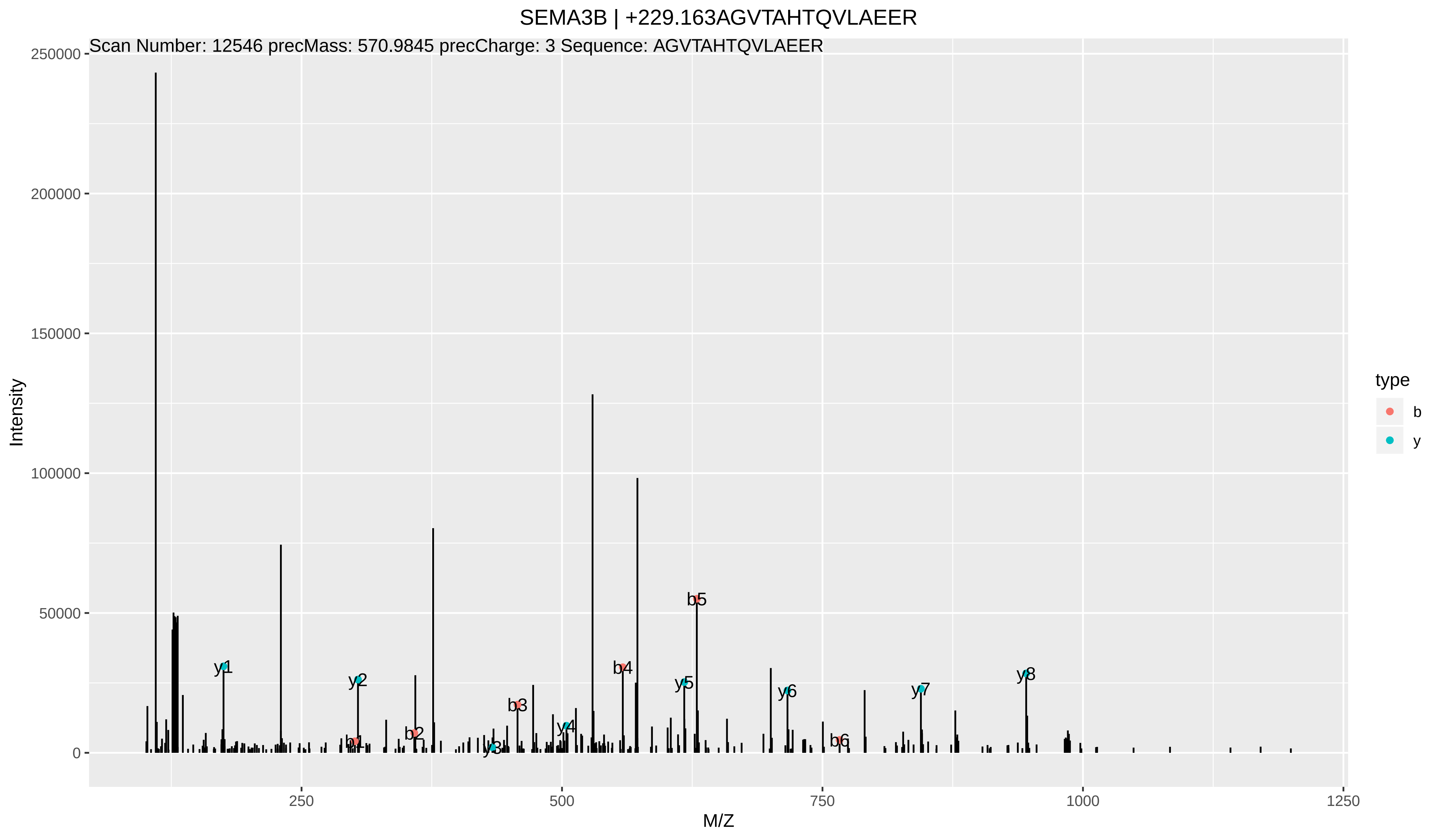

SEMA3B | +229.163AGVTAHTQVLAEER
Scan Number: 12546 precMass: 570.9845 precCharge: 3 Sequence: AGVTAHTQVLAEER
Intensity
M/Z
type
b
y
y1
y2
y3
y4
y5
y6
y7
y8
b1
b2
b3
b4
b5
b6
0
50000
100000
150000
200000
250000
250
500
750
1000
1250

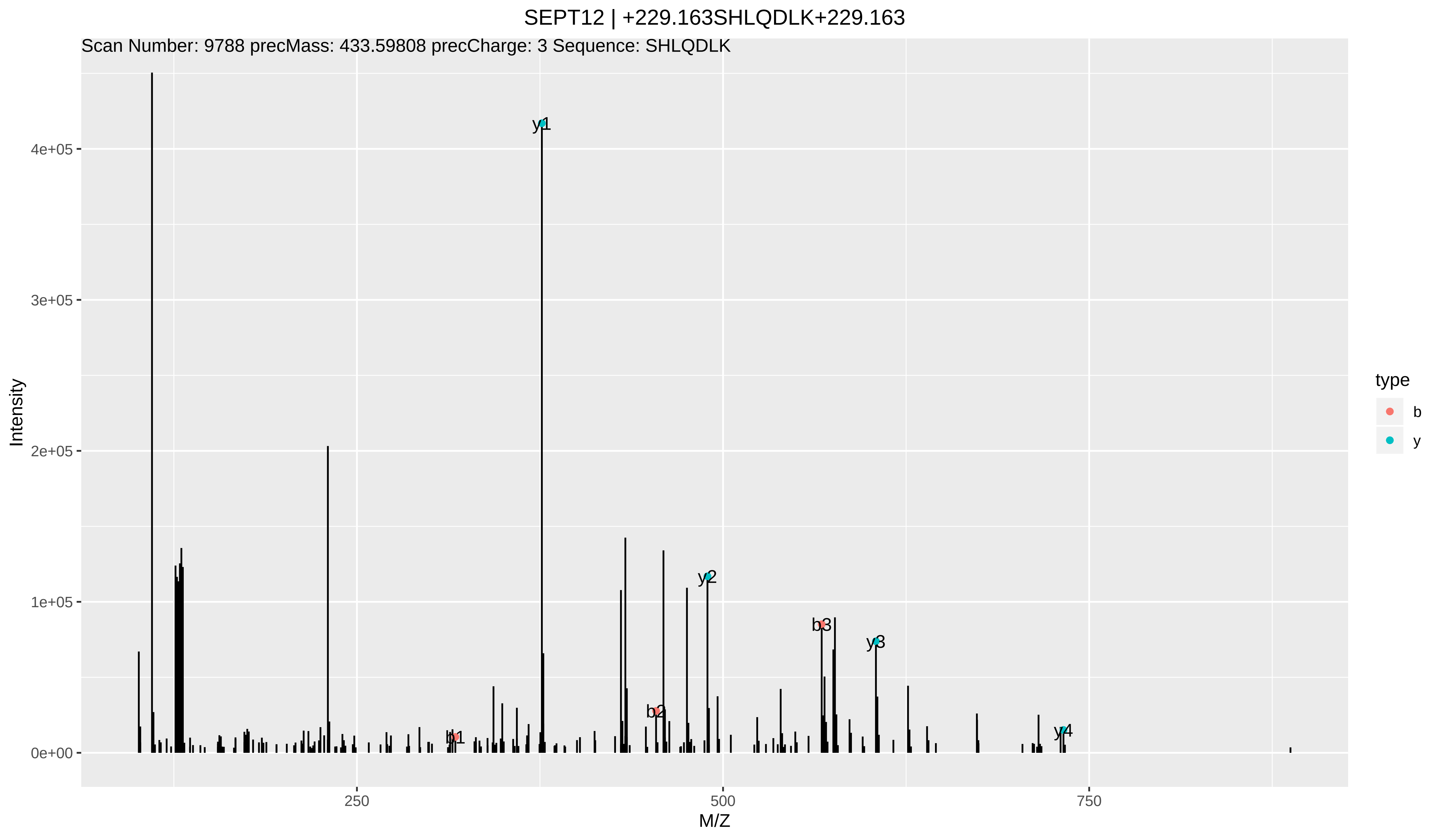

SEPT12 | +229.163SHLQDLK+229.163
Scan Number: 9788 precMass: 433.59808 precCharge: 3 Sequence: SHLQDLK
Intensity
0e+00
1e+05
2e+05
3e+05
4e+05
M/Z
250
500
750
y1
y2
y3
y4
b1
b2
b3
type
b
y

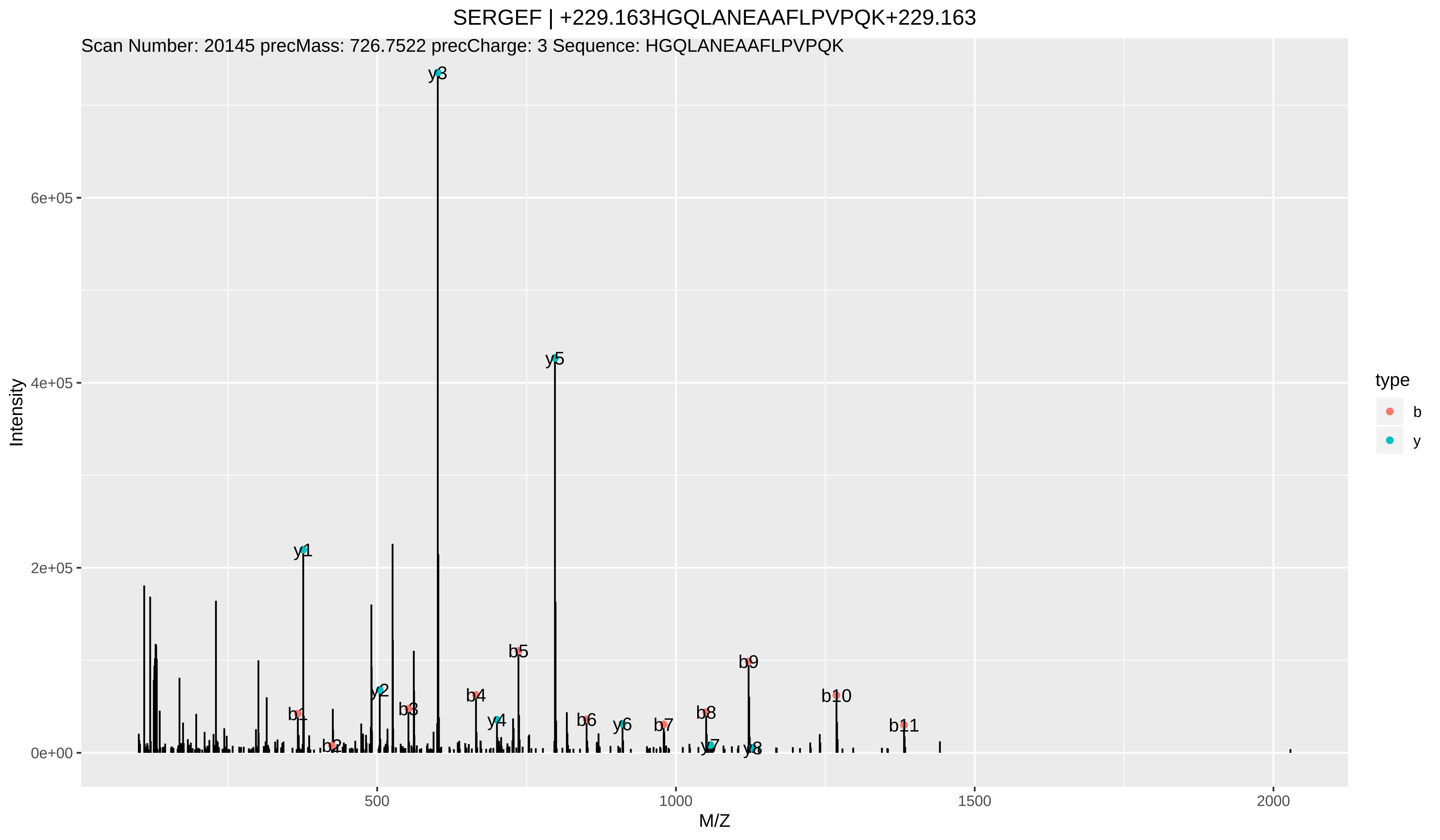

SERGEF | +229.163HGQLANEAAFLPVPQK+229.163
Scan Number: 20145 precMass: 726.7522 precCharge: 3 Sequence: HGQLANEAAFLPVPQK
Intensity
6e+05
4e+05
2e+05
0e+00
500
1000
1500
2000
M/Z
type
b
y
y3
y5
y1
y2
b1
b2
b3
b4
y4
b5
b6
y6
b7
b8
y7
b9
y8
b10
b11

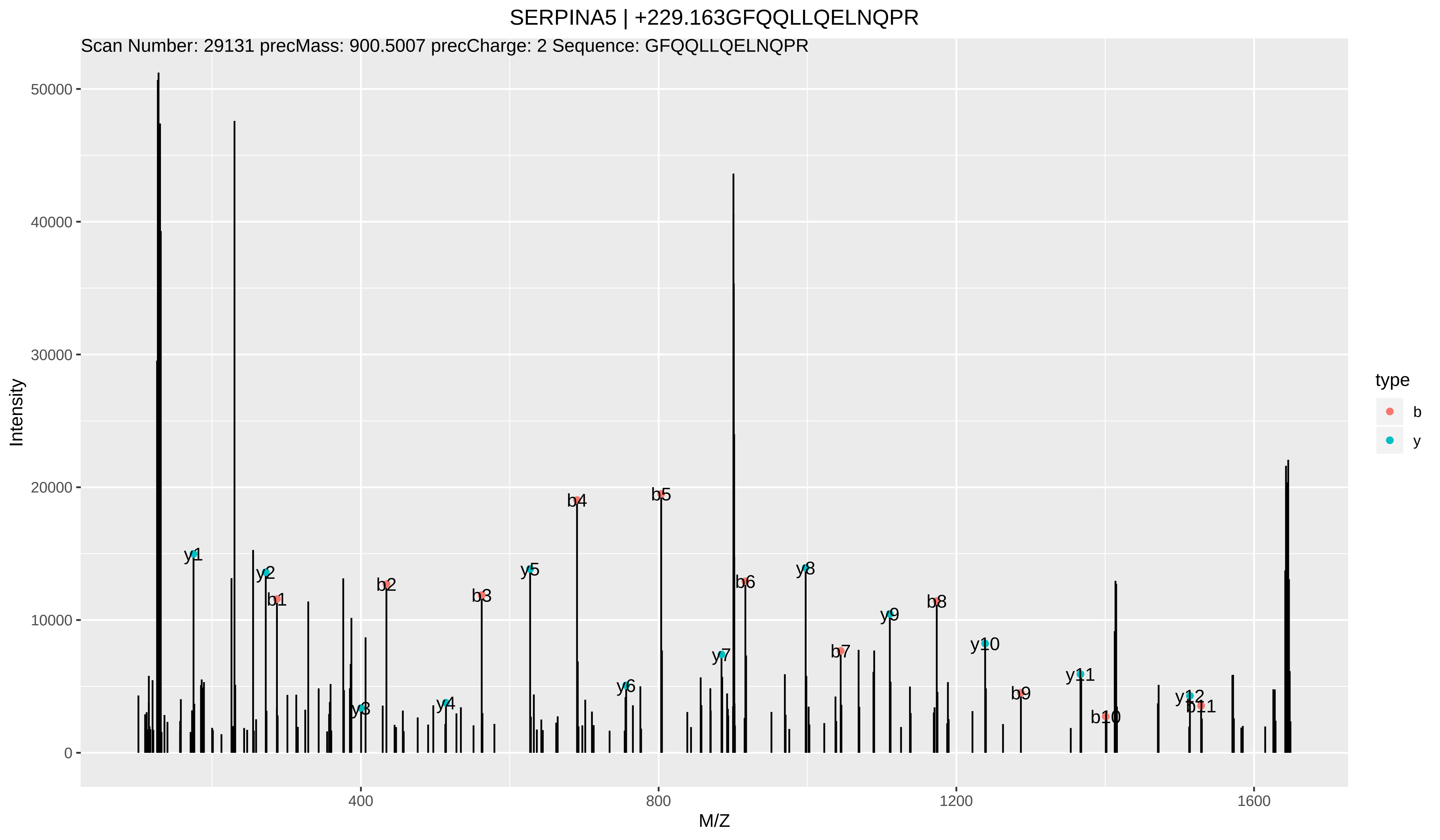
SERPINA5 | +229.163GFQQLLQELNQPR
Scan Number: 29131 precMass: 900.5007 precCharge: 2 Sequence: GFQQLLQELNQPR
Intensity
M/Z
0
10000
20000
30000
40000
50000
400
800
1200
1600
y1
y2
b1
y3
b2
y4
b3
y5
b4
y6
b5
y7
b6
y8
b7
y9
b8
y10
b9
y11
b10
y12
b11
type
b
y

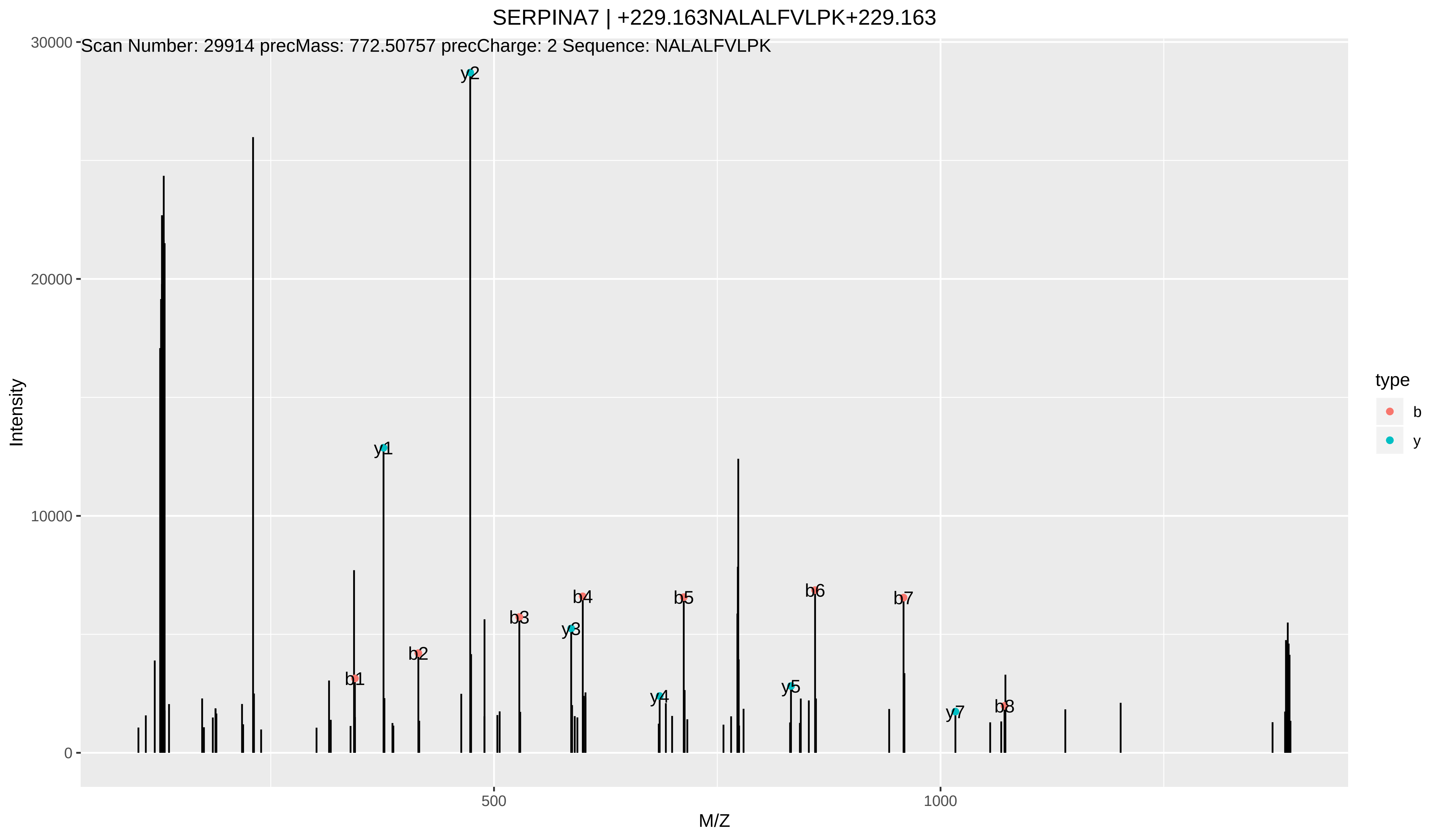

SERPINA7 | +229.163NALALFVLPK+229.163
Scan Number: 29914 precMass: 772.50757 precCharge: 2 Sequence: NALALFVLPK
Intensity
M/Z
30000
20000
10000
0
500
1000
y2
y1
b1
b2
b3
y3
b4
y4
b5
y5
b6
b7
y7
b8
type
b
y

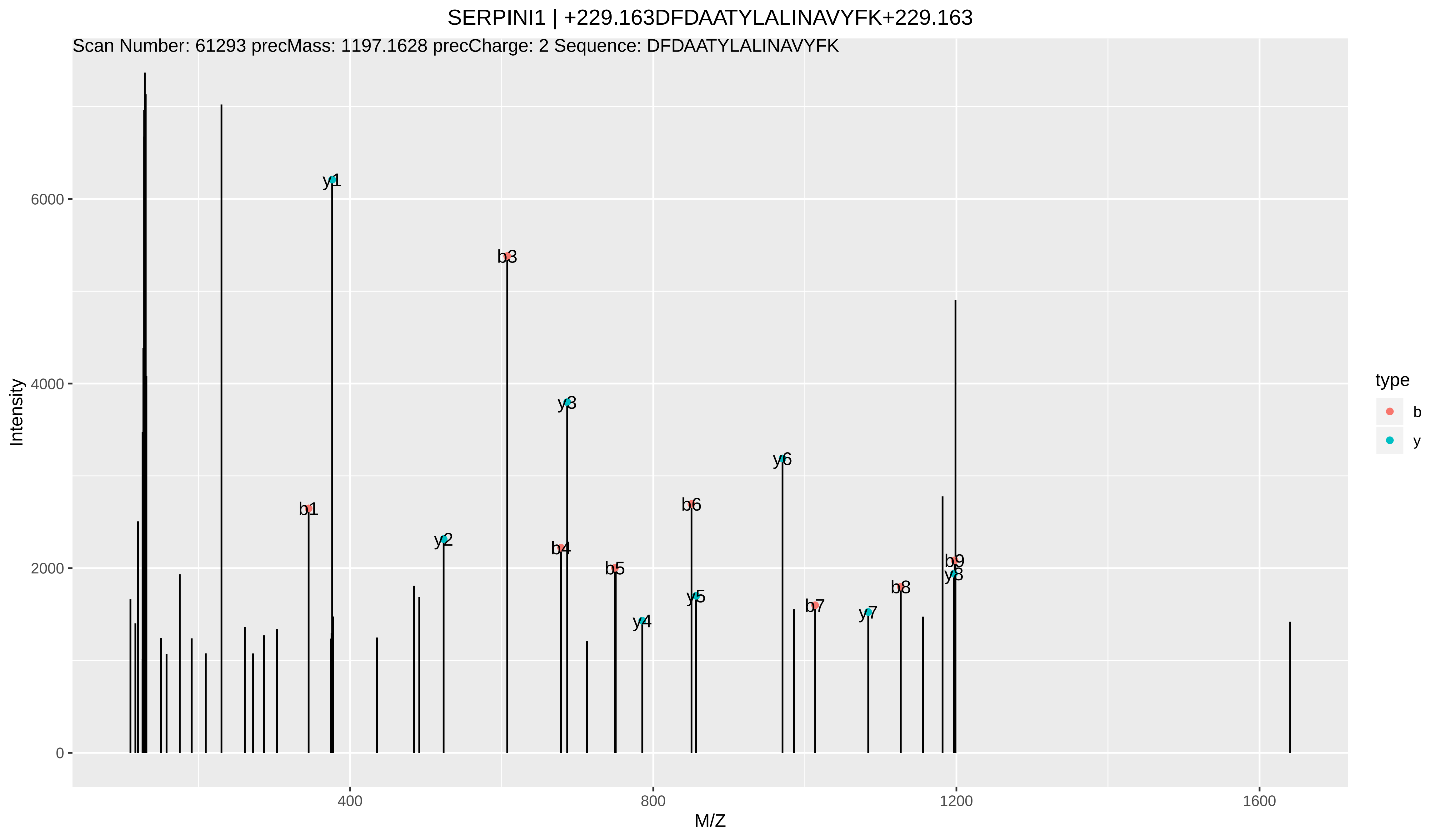

SERPINI1 | +229.163DFDAATYLALINAVYFK+229.163
Scan Number: 61293 precMass: 1197.1628 precCharge: 2 Sequence: DFDAATYLALINAVYFK
Intensity
M/Z
0
2000
4000
6000
400
800
1200
1600
y1
b3
y3
y6
b6
b1
y2
b4
b5
y5
y4
b7
y7
b8
b9
y8
type
b
y

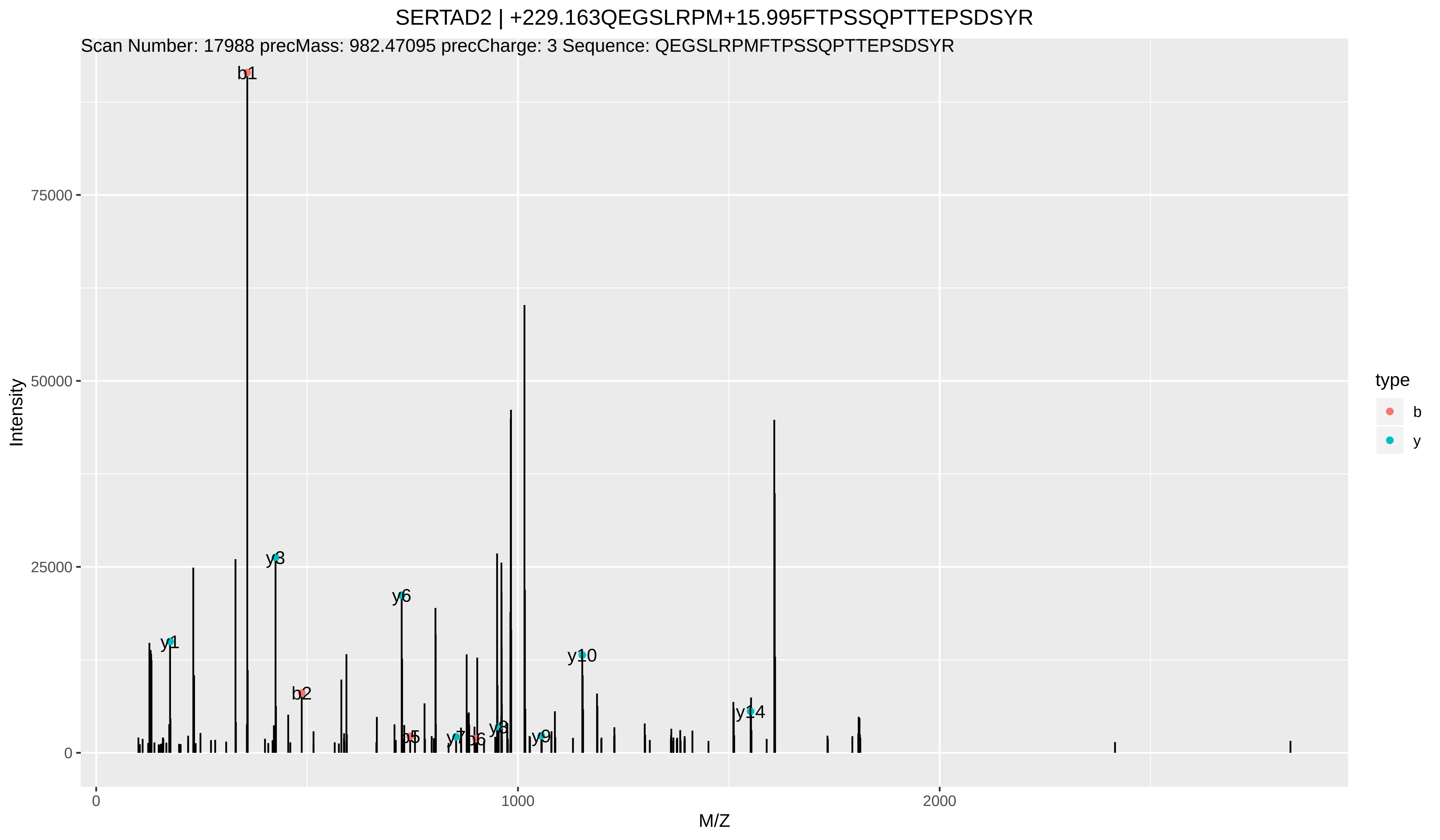

SERTAD2 | +229.163QEGSLRPM+15.995FTPSSQPTTEPSDSYR
Scan Number: 17988 precMass: 982.47095 precCharge: 3 Sequence: QEGSLRPMFTPSSQPTTEPSDSYR
Intensity
75000
50000
25000
0
M/Z
0
1000
2000
b1
b2
b5
b6
y1
y3
y6
y7
y8
y9
y10
y14
type
b
y

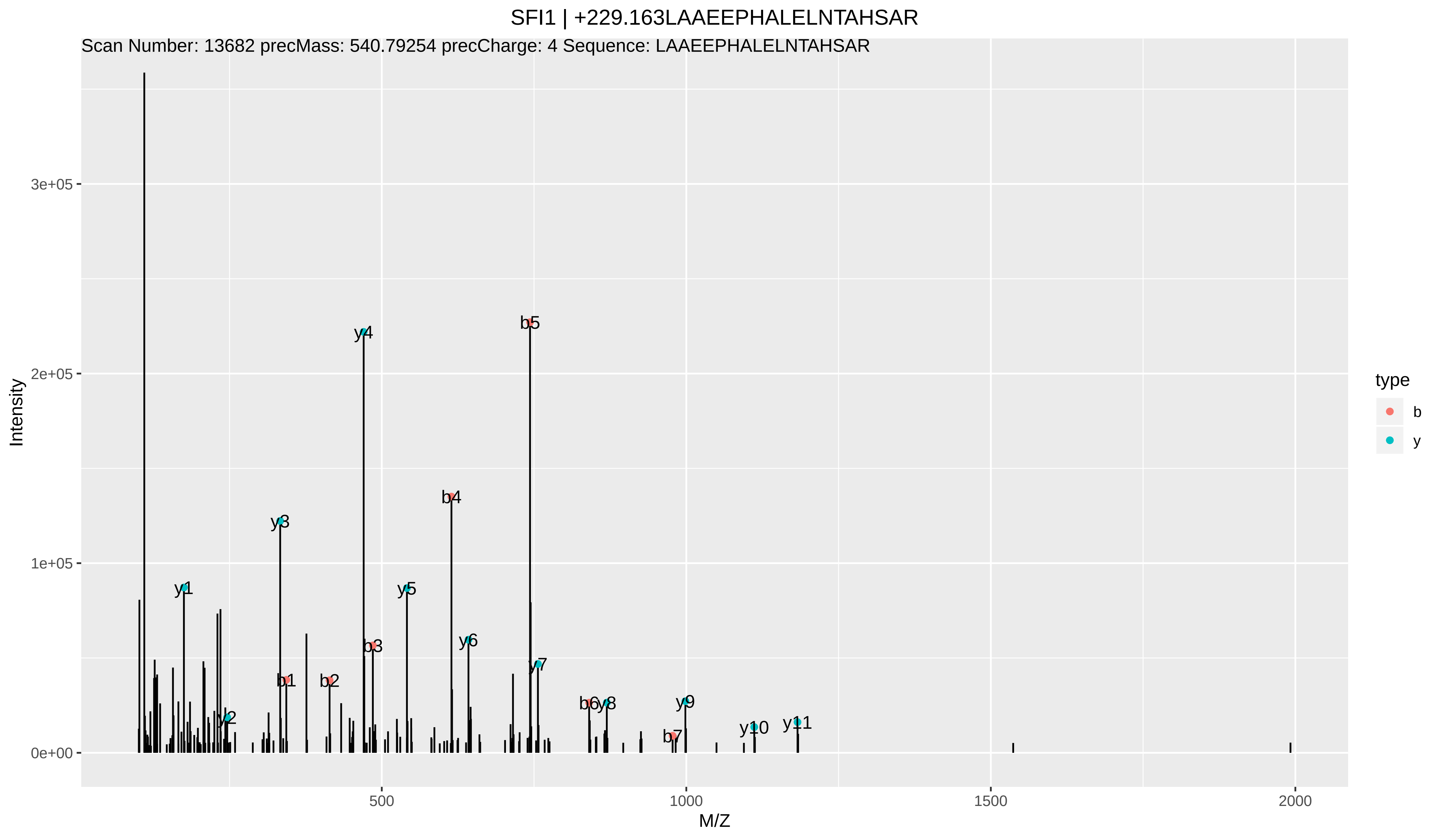
SFI1 | +229.163LAAEEPHALELNTAHSAR
Scan Number: 13682 precMass: 540.79254 precCharge: 4 Sequence: LAAEEPHALELNTAHSAR
Intensity
M/Z
3e+05
2e+05
1e+05
0e+00
500
1000
1500
2000
y1
y2
y3
b1
b2
y4
b3
y5
b4
y6
b5
y7
b6
y8
b7
y9
y10
y11
type
b
y

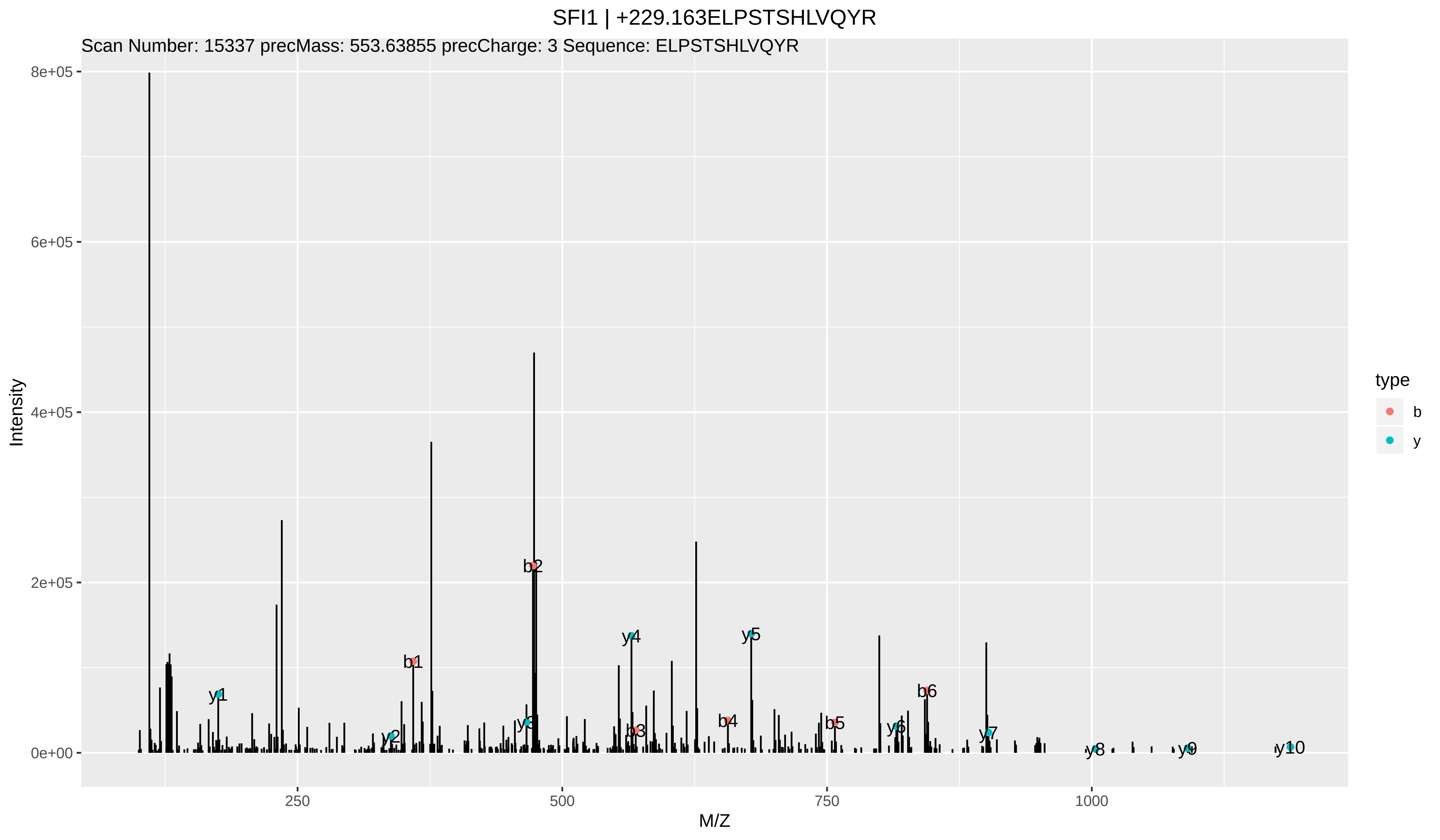

SFI1 | +229.163ELPSTSHLVQYR
Scan Number: 15337 precMass: 553.63855 precCharge: 3 Sequence: ELPSTSHLVQYR
Intensity
8e+05
6e+05
4e+05
2e+05
0e+00
M/Z
250
500
750
1000
y1
y2
y3
y4
y5
y6
y7
y8
y9
y10
b1
b2
b3
b4
b5
b6
type
b
y

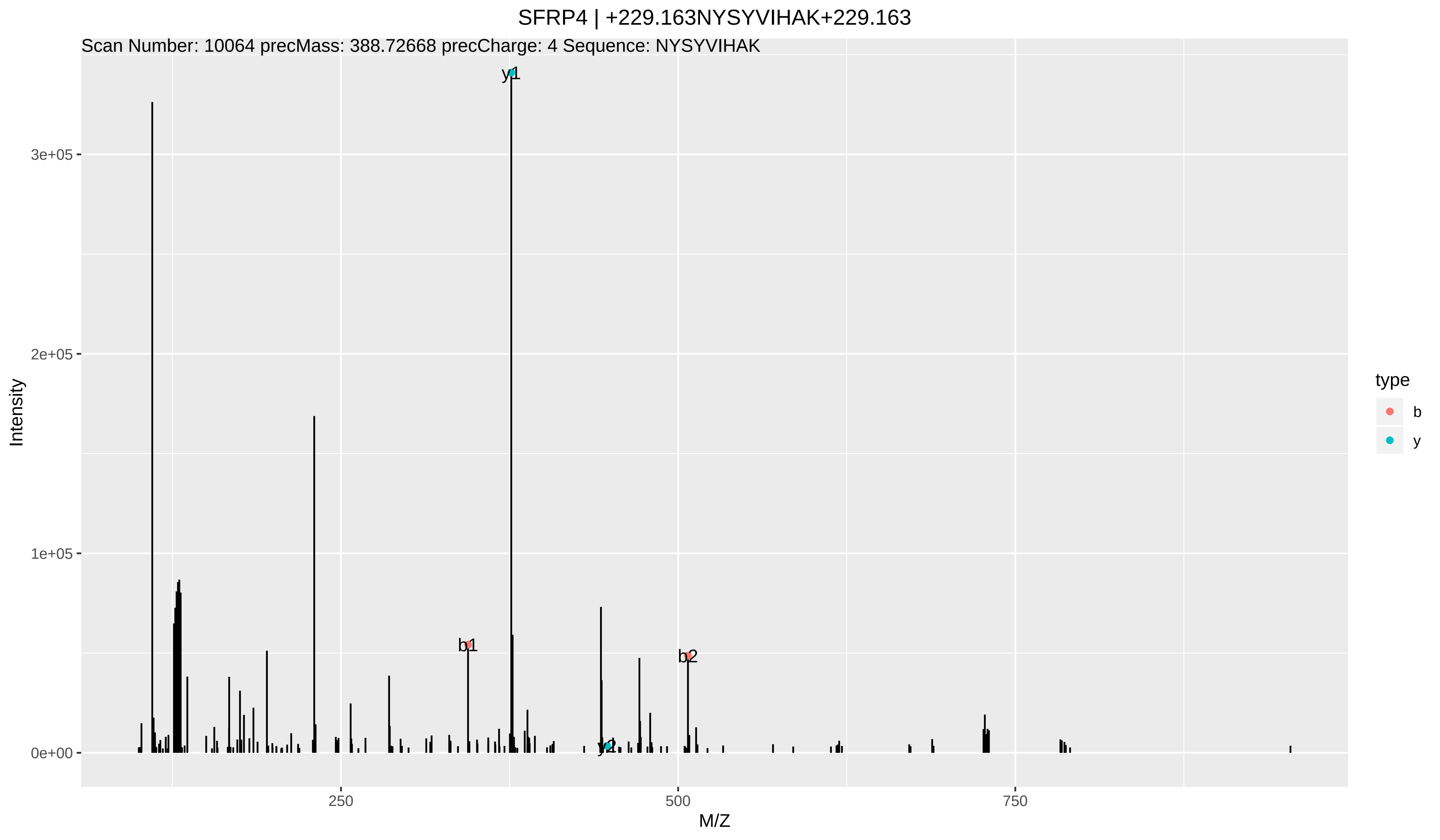

SFRP4 | +229.163NYSYVIHAK+229.163
Scan Number: 10064 precMass: 388.72668 precCharge: 4 Sequence: NYSYVIHAK
y1
b1
b2
y2
Intensity
3e+05
2e+05
1e+05
0e+00
M/Z
250
500
750
type
b
y

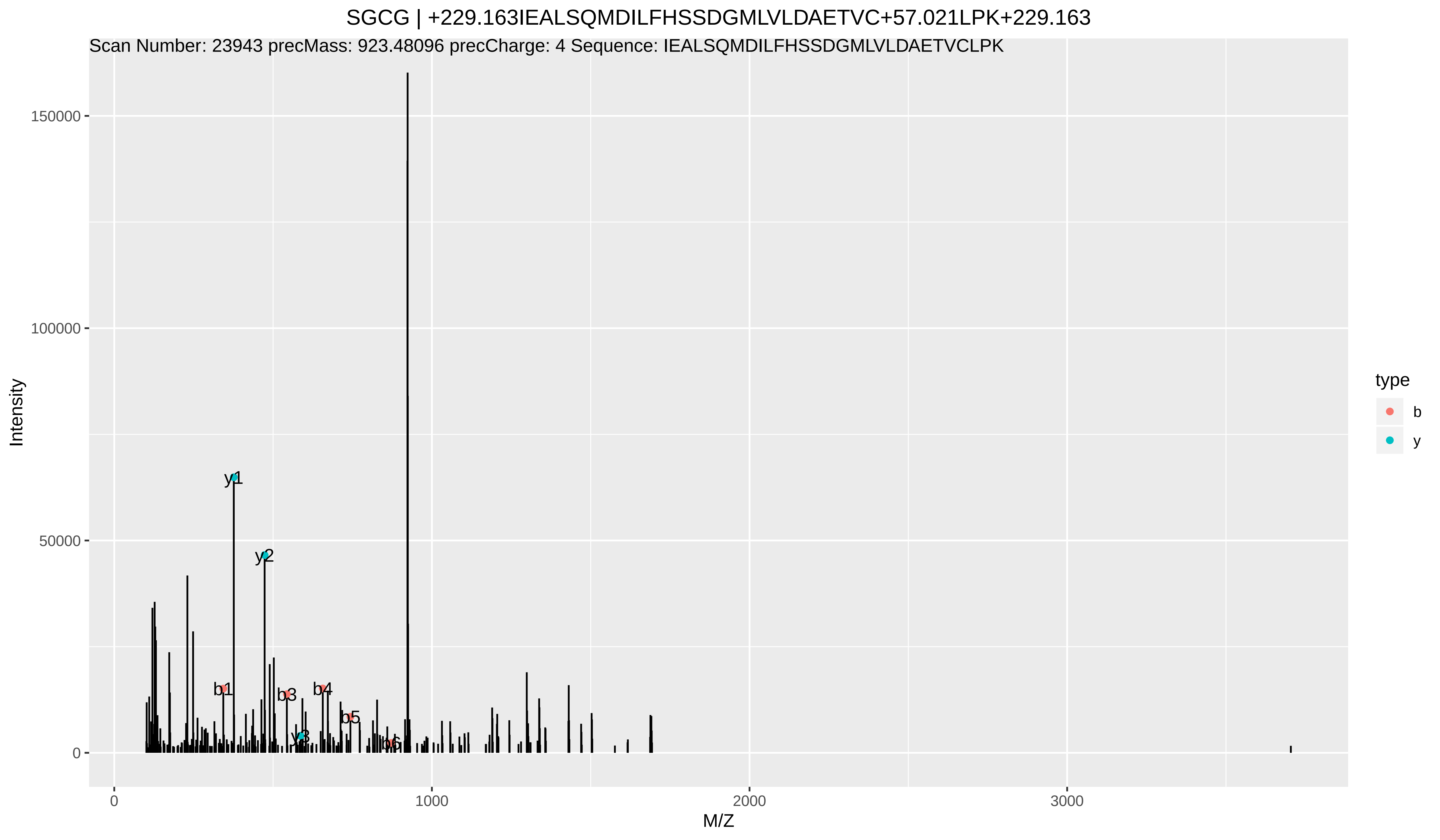

SGCG | +229.163IEALSQMDILFHSSDGMLVLDAETVC+57.021LPK+229.163
Scan Number: 23943 precMass: 923.48096 precCharge: 4 Sequence: IEALSQMDILFHSSDGMLVLDAETVCLPK
Intensity
150000
100000
50000
0
0
1000
2000
3000
M/Z
y1
y2
y3
b1
b3
b4
b5
b6
type
b
y

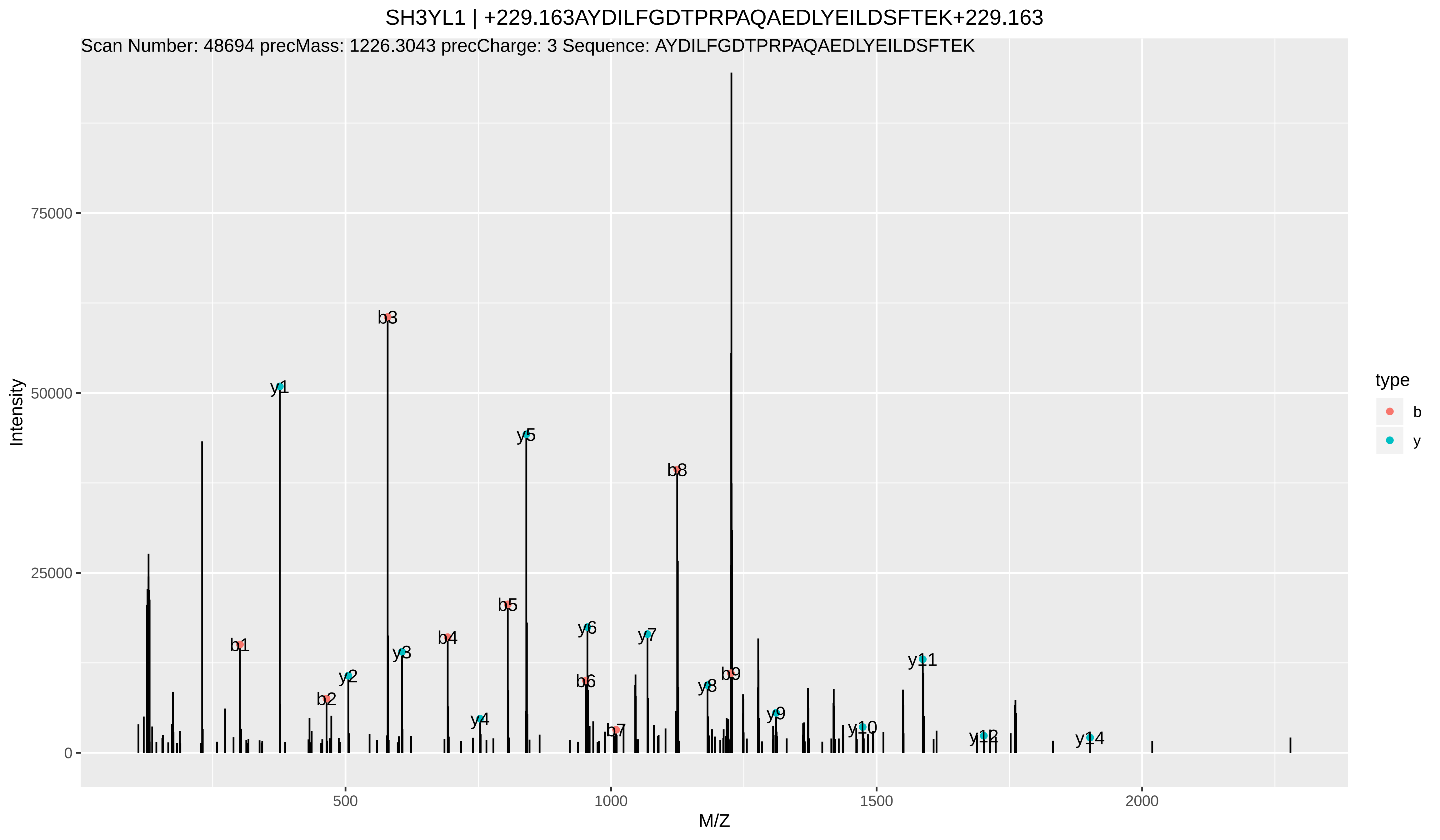
SH3YL1 | +229.163AYDILFGDTPRPAQAEDLYEILDSFTEK+229.163
Scan Number: 48694 precMass: 1226.3043 precCharge: 3 Sequence: AYDILFGDTPRPAQAEDLYEILDSFTEK
Intensity
M/Z
type
b
y
b1
b2
b3
b4
b5
b6
b7
b8
b9
y1
y2
y3
y4
y5
y6
y7
y8
y9
y10
y11
y12
y14
0
25000
50000
75000
500
1000
1500
2000

SHB | +229.163VTIPALAAQFNGNEK+229.163

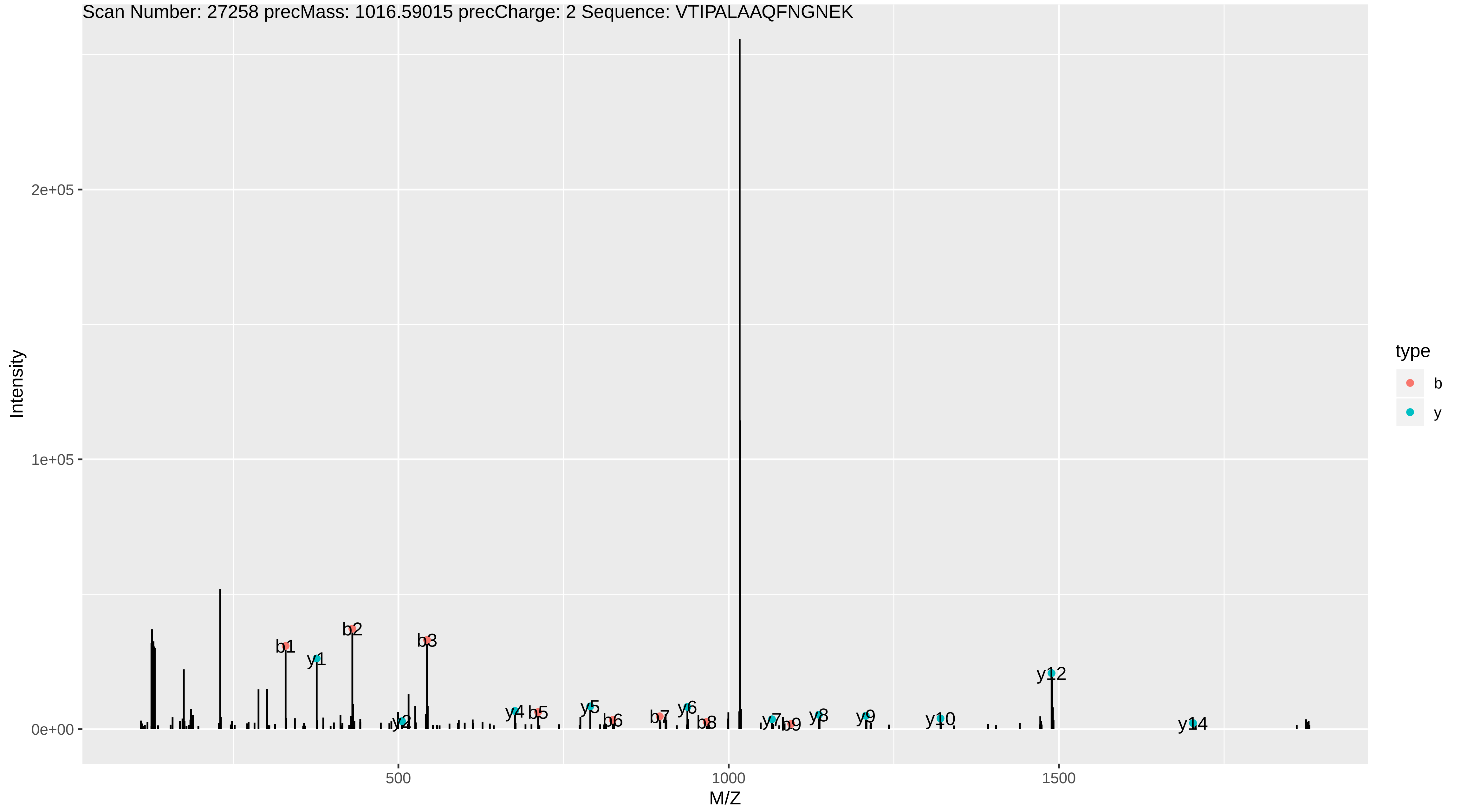

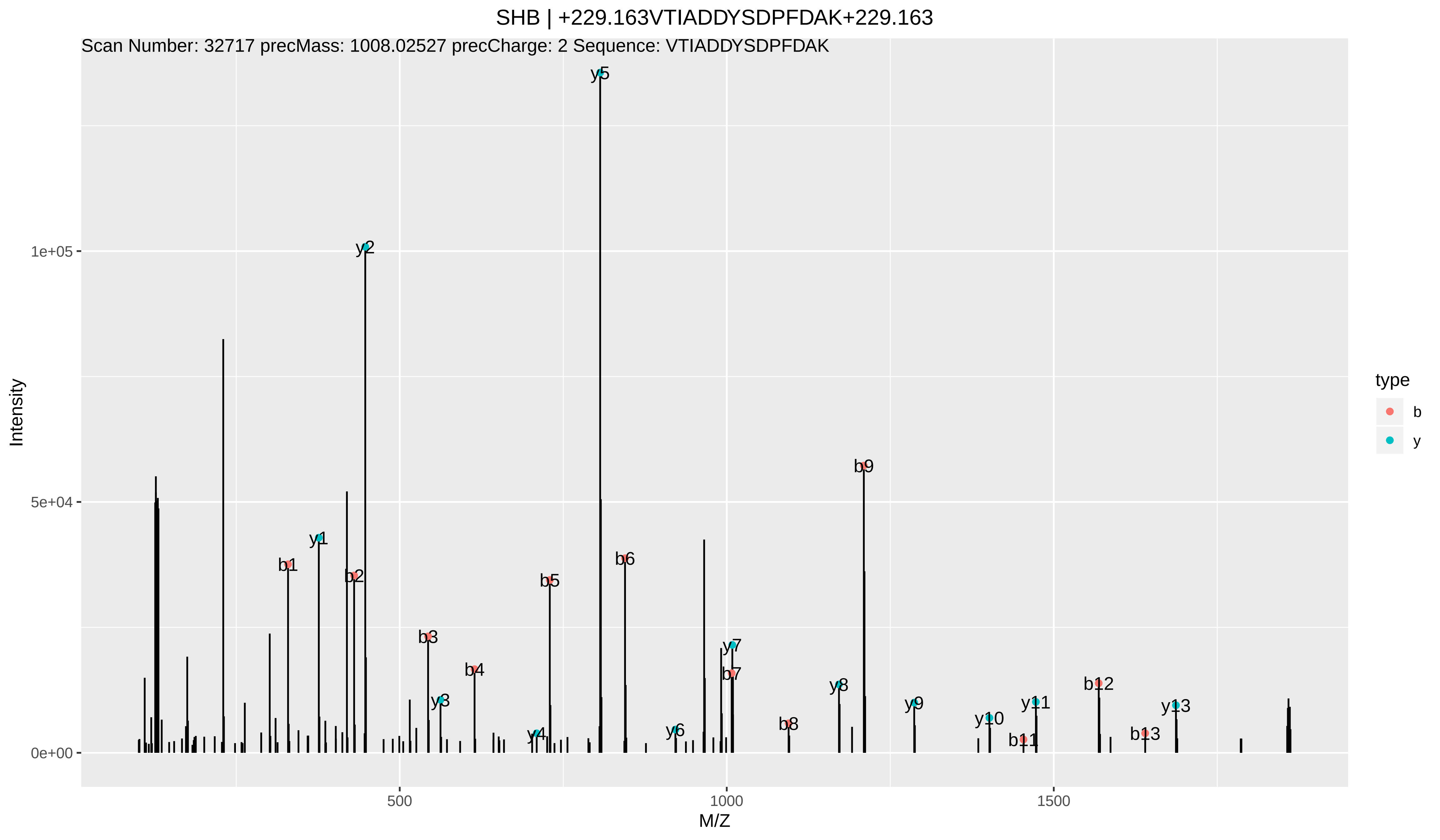

SHB | +229.163VTIADDYSDPFDAK+229.163
Scan Number: 32717 precMass: 1008.02527 precCharge: 2 Sequence: VTIADDYSDPFDAK
Intensity
1e+05
5e+04
0e+00
M/Z
500
1000
1500
y5
y2
b9
y1
b1
b2
b6
b5
b3
y7
b4
b7
y8
b12
y3
y9
y11
y13
y10
y4
y6
b8
b13
b11
type
b
y

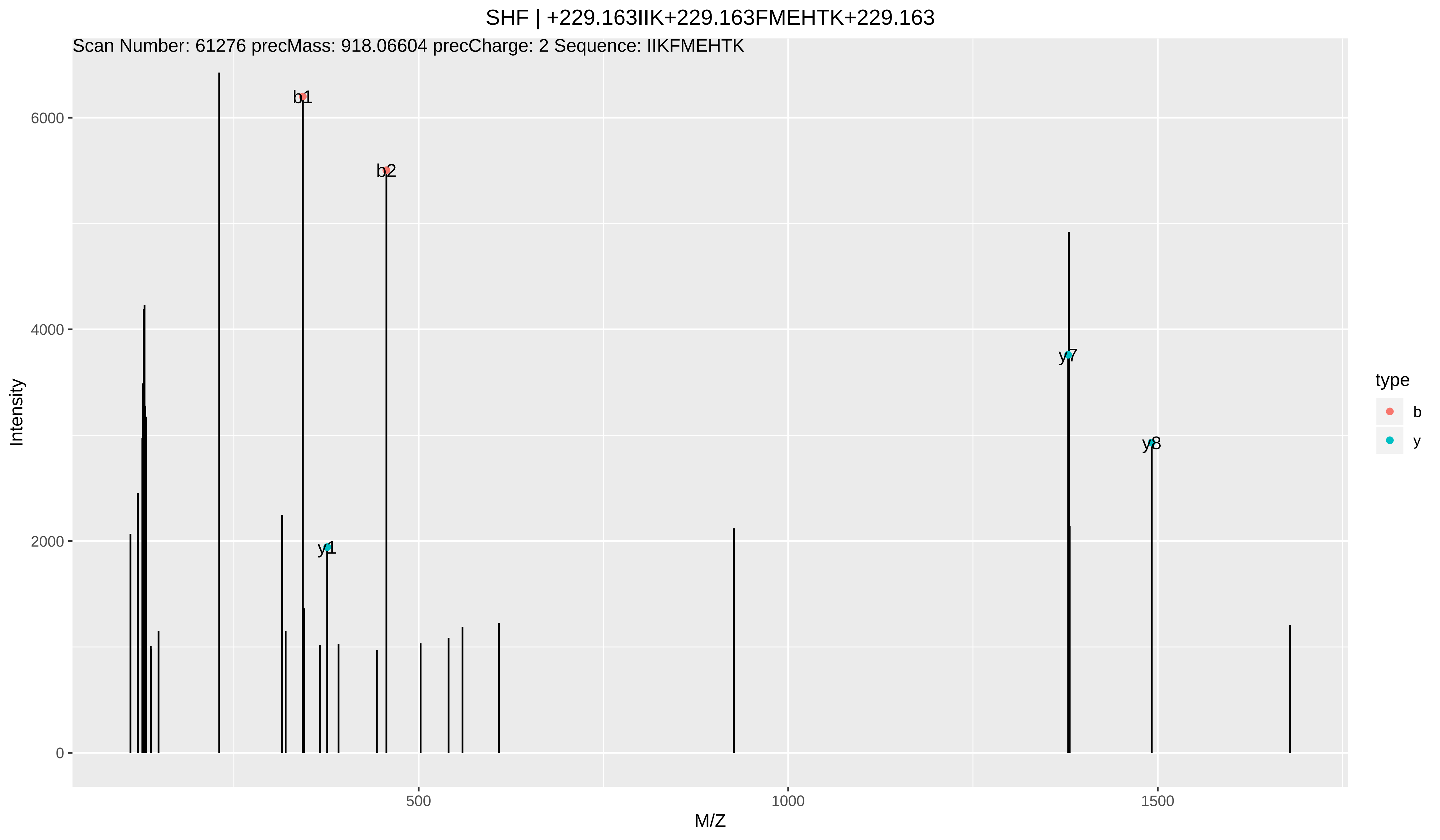

SHF | +229.163IIK+229.163FMEHTK+229.163
Scan Number: 61276 precMass: 918.06604 precCharge: 2 Sequence: IIKFMEHTK
b1
b2
y1
y7
y8
Intensity
0
2000
4000
6000
M/Z
500
1000
1500
type
b
y

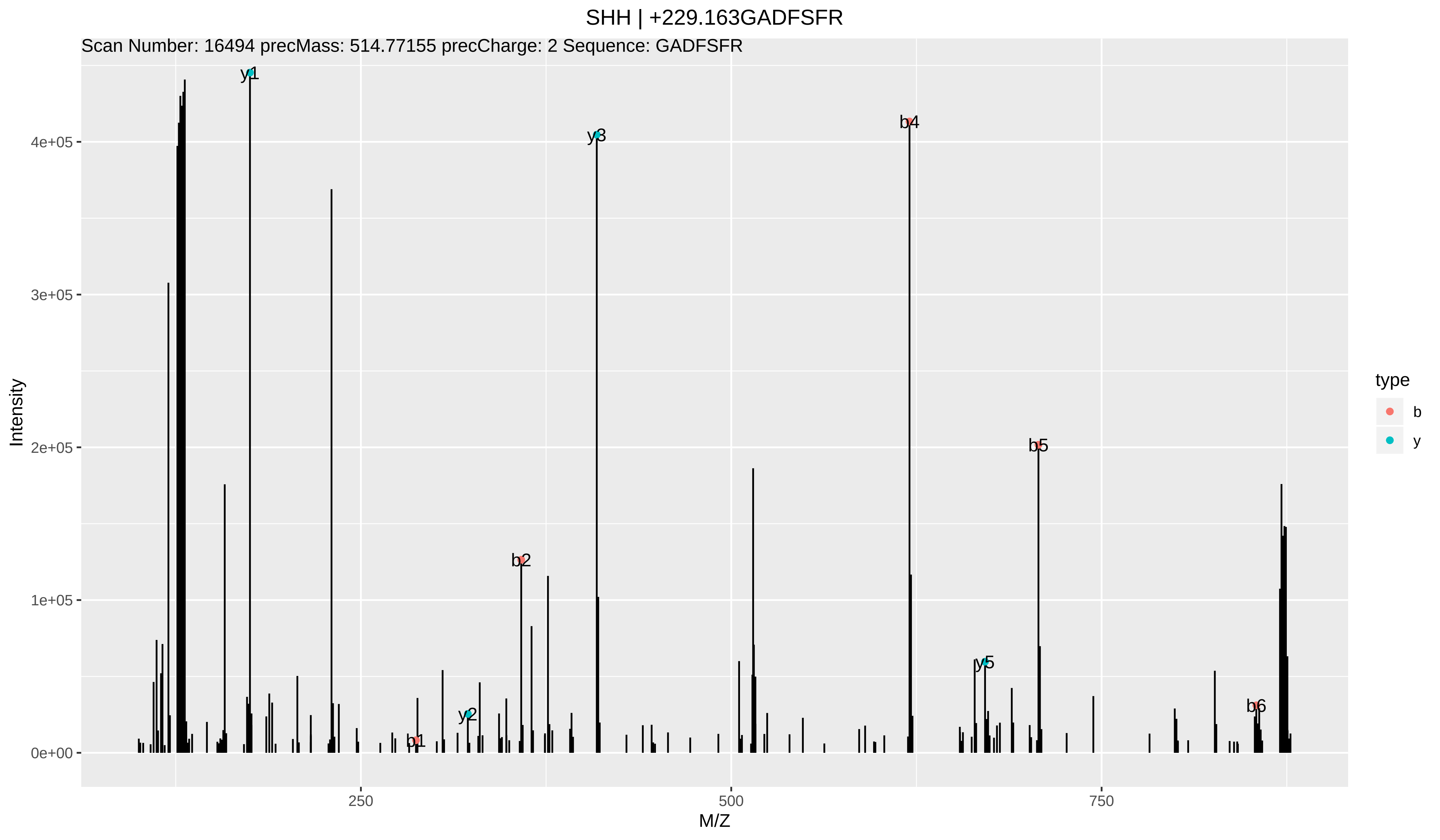

SHH | +229.163GADFSFR
Scan Number: 16494 precMass: 514.77155 precCharge: 2 Sequence: GADFSFR
y1
y3
b4
b5
b2
y5
y2
b1
b6
4e+05
3e+05
2e+05
1e+05
0e+00
Intensity
250
500
750
M/Z
type
b
y

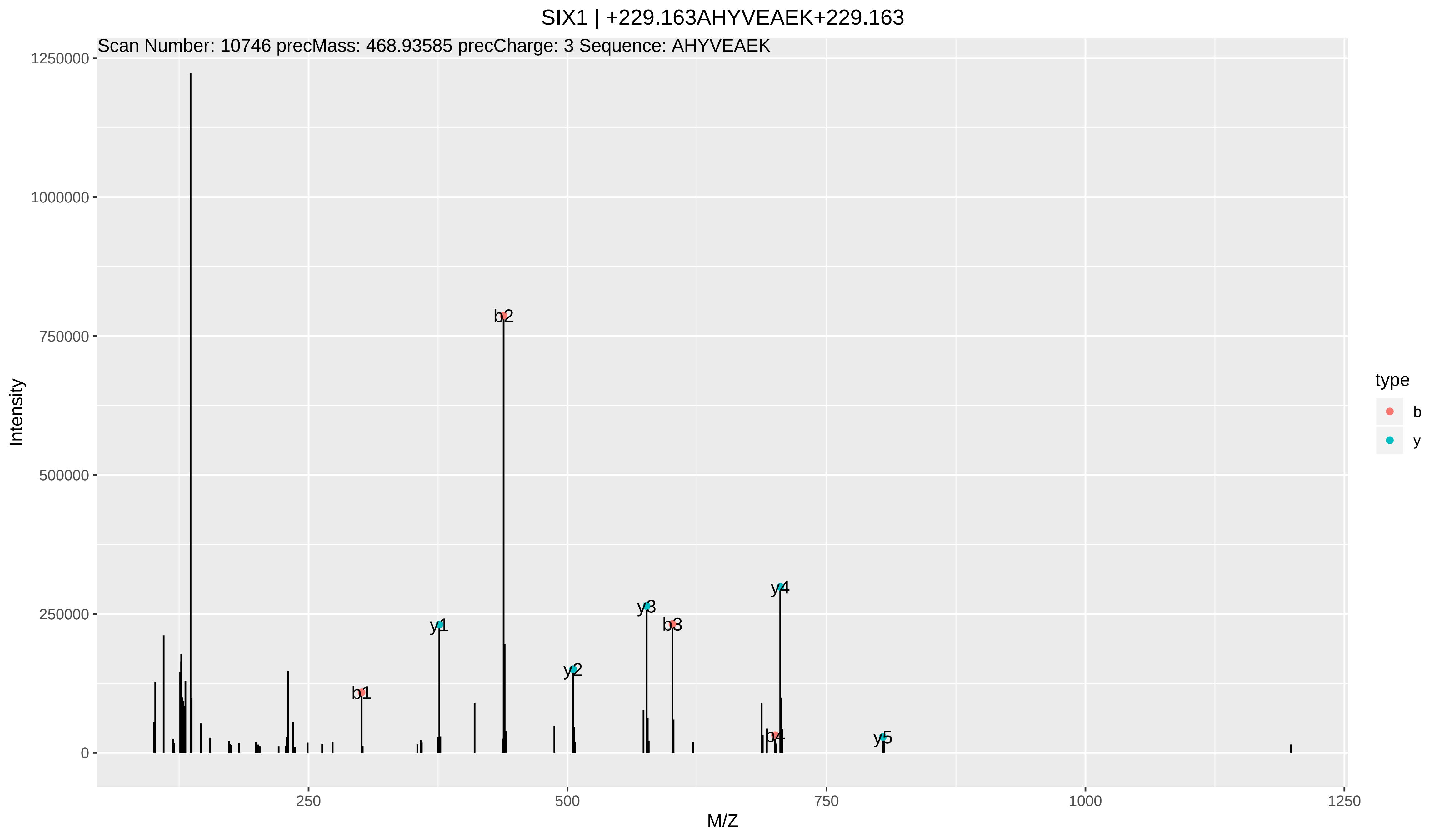
SIX1 | +229.163AHYVEAEK+229.163
Scan Number: 10746 precMass: 468.93585 precCharge: 3 Sequence: AHYVEAEK
Intensity
M/Z
type
b
y
b1
b2
b3
b4
y1
y2
y3
y4
y5
1250000
1000000
750000
500000
250000
0
250
500
750
1000
1250

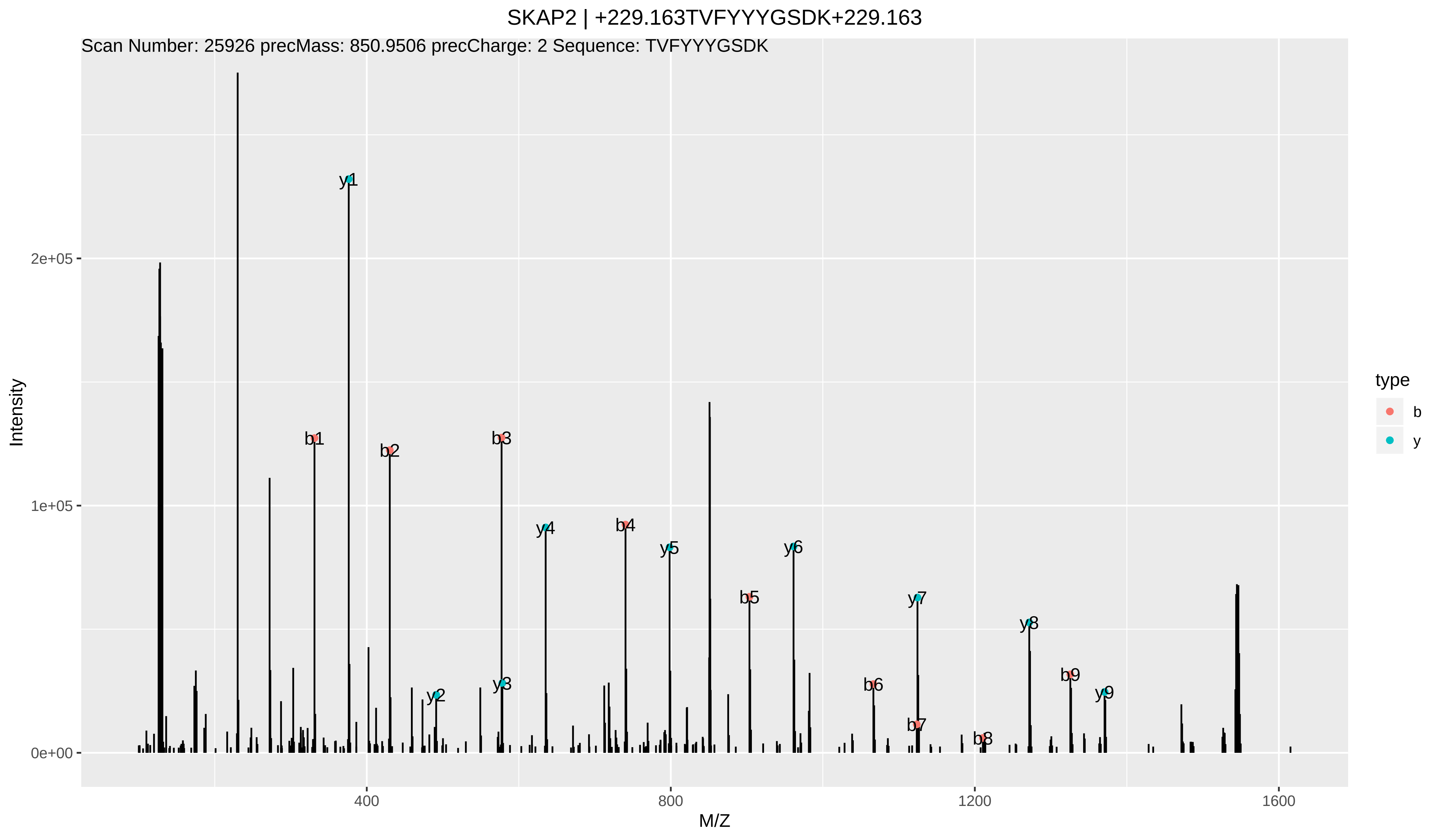

SKAP2 | +229.163TVFYYYGSDK+229.163
Scan Number: 25926 precMass: 850.9506 precCharge: 2 Sequence: TVFYYYGSDK
Intensity
M/Z
0e+00
1e+05
2e+05
400
800
1200
1600
y1
b1
b2
b3
y2
y3
y4
b4
y5
y6
b5
y7
b6
b7
b8
y8
b9
y9
type
b
y

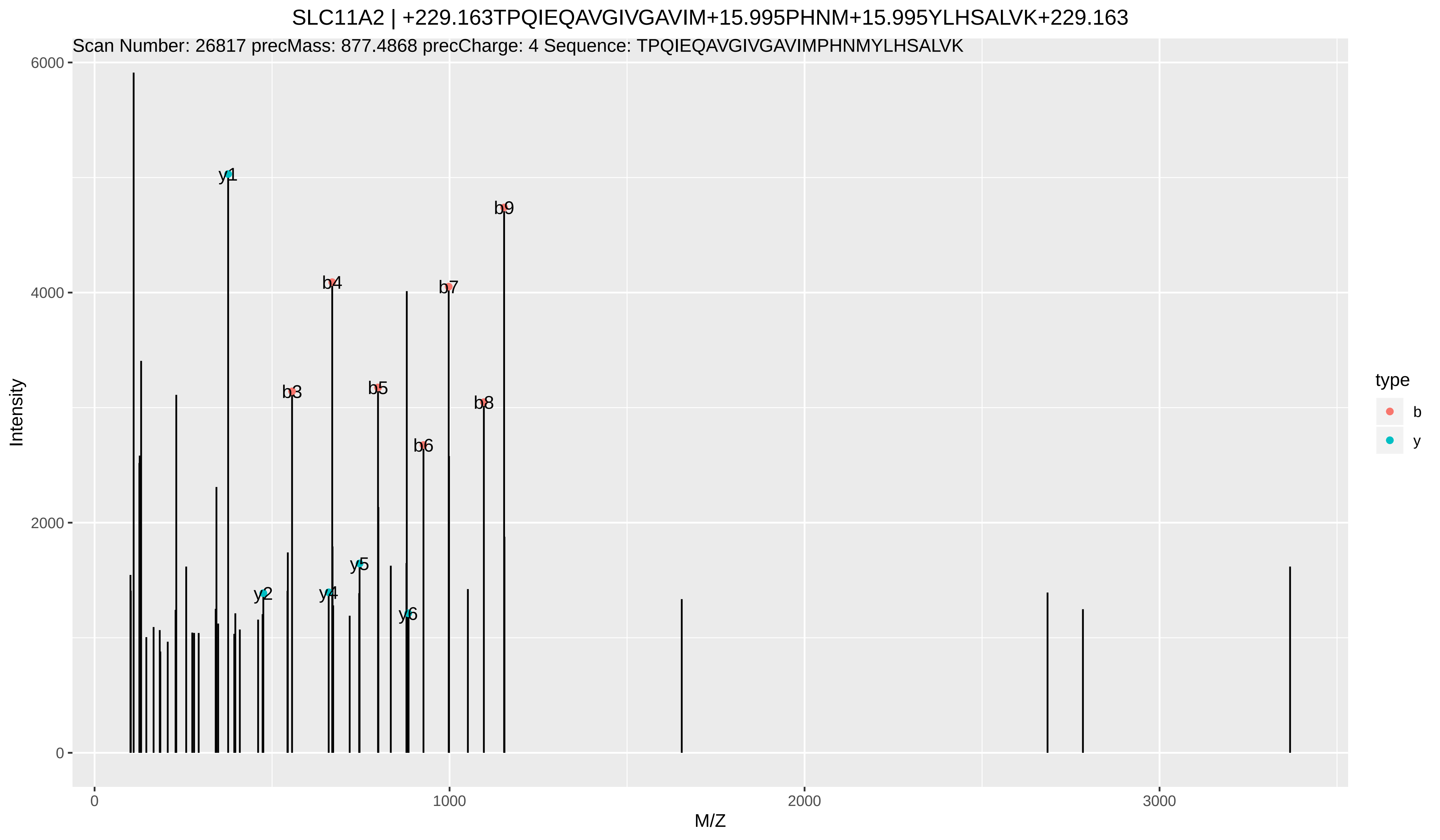

SLC11A2 | +229.163TPQIEQAVGIVGAVIM+15.995PHNM+15.995YLHSALVK+229.163
Scan Number: 26817 precMass: 877.4868 precCharge: 4 Sequence: TPQIEQAVGIVGAVIMPHNMYLHSALVK
Intensity
M/Z
0
2000
4000
6000
0
1000
2000
3000
y1
b9
b4
b7
b3
b5
b8
b6
y5
y2
y4
y6
type
b
y

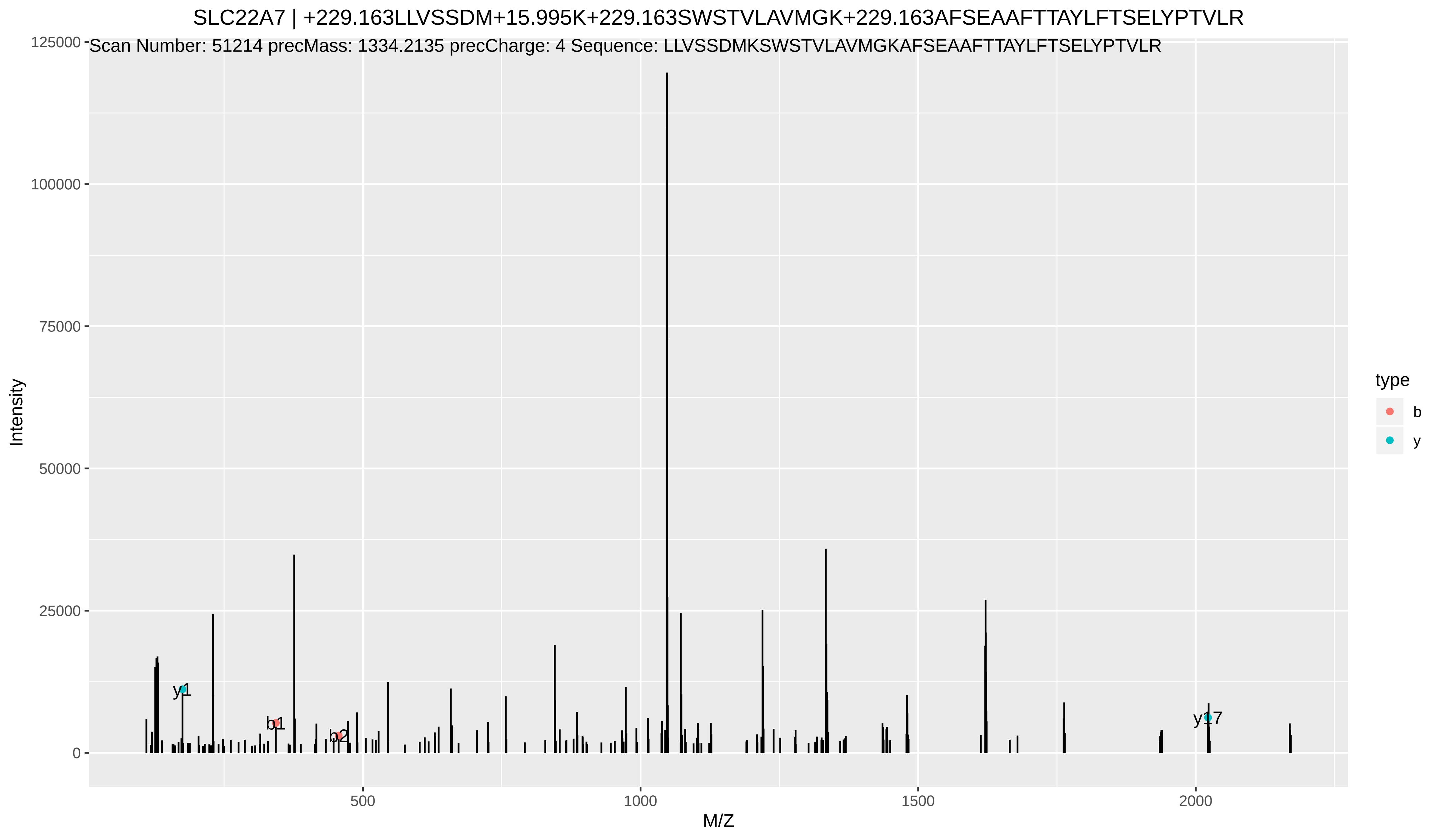

SLC22A7 | +229.163LLVSSDM+15.995K+229.163SWSTVLAVMGK+229.163AFSEAAFTTAYLFTSELYPTVLR
Scan Number: 51214 precMass: 1334.2135 precCharge: 4 Sequence: LLVSSDMKSWSTVLAVMGKAFSEAAFTTAYLFTSELYPTVLR
Intensity
125000
100000
75000
50000
25000
0
M/Z
500
1000
1500
2000
y1
b1
b2
y17
type
b
y

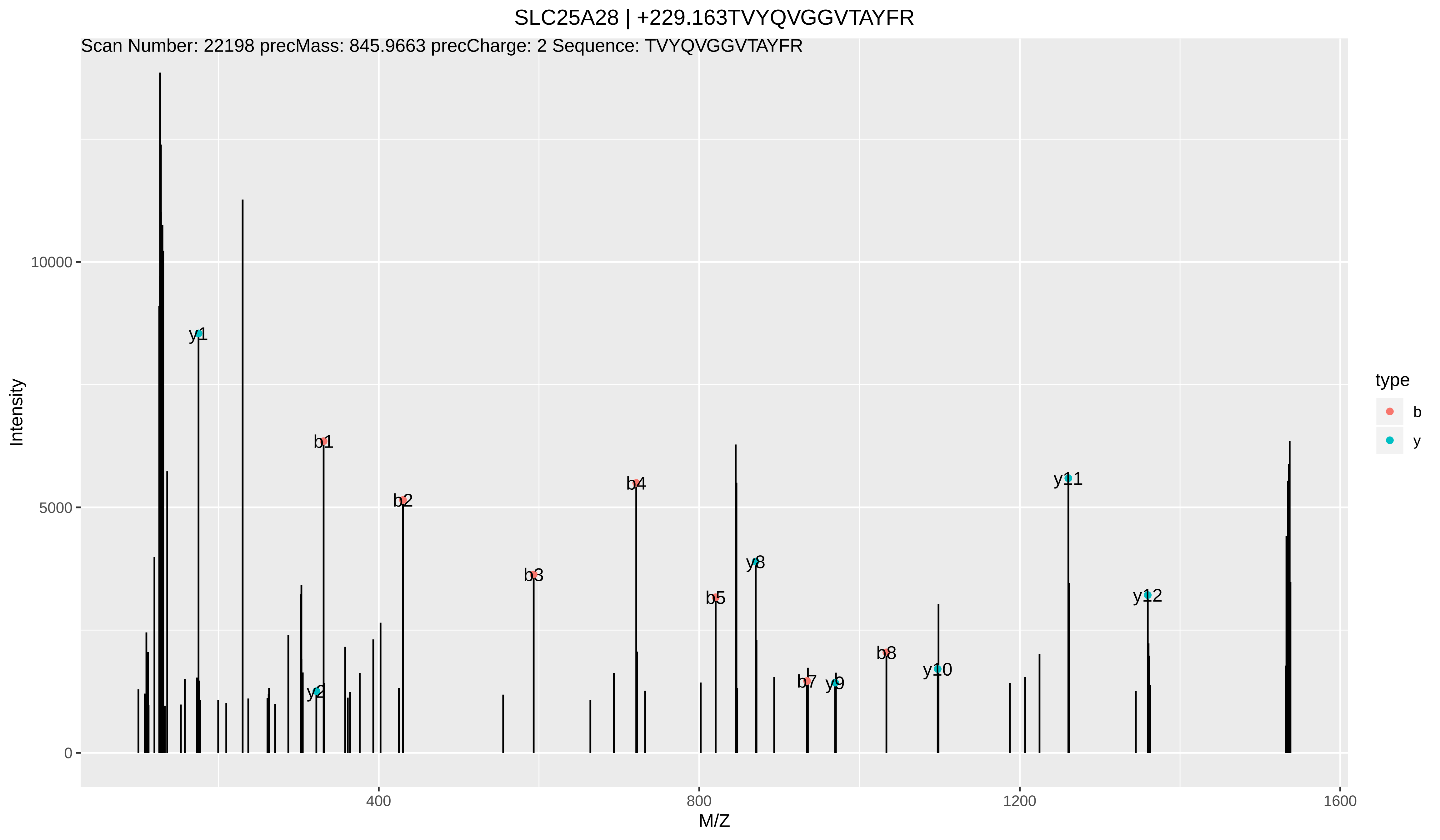

SLC25A28 | +229.163TVYQVGGVTAYFR
Scan Number: 22198 precMass: 845.9663 precCharge: 2 Sequence: TVYQVGGVTAYFR
Intensity
10000
5000
0
400
800
1200
1600
M/Z
y1
y2
b1
b2
b3
b4
b5
y8
b7
y9
b8
y10
y11
y12
type
b
y

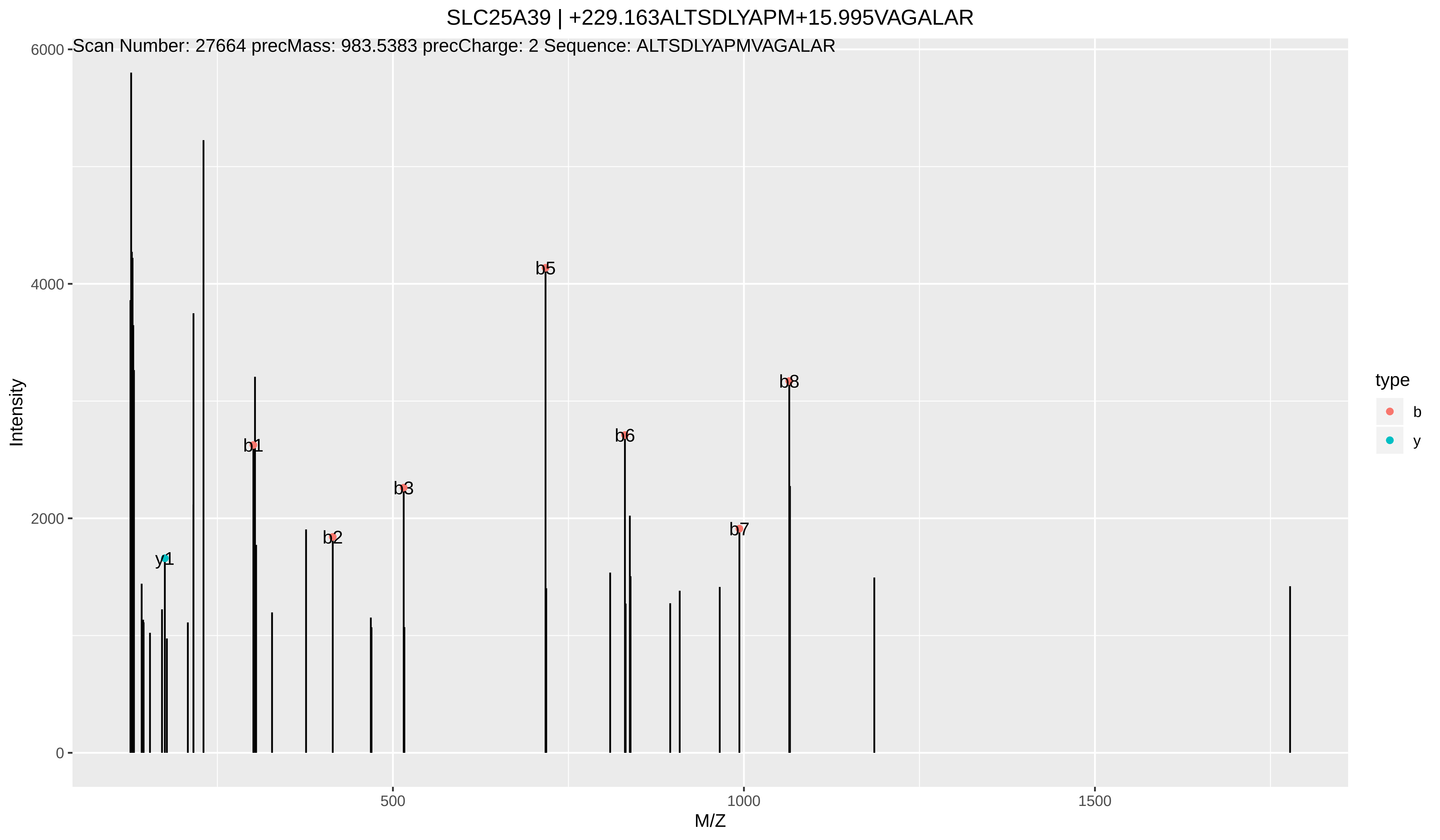
SLC25A39 | +229.163ALTSDLYAPM+15.995VAGALAR
Scan Number: 27664 precMass: 983.5383 precCharge: 2 Sequence: ALTSDLYAPMVAGALAR
Intensity
M/Z
0
2000
4000
6000
500
1000
1500
y1
b1
b2
b3
b5
b6
b7
b8
type
b
y

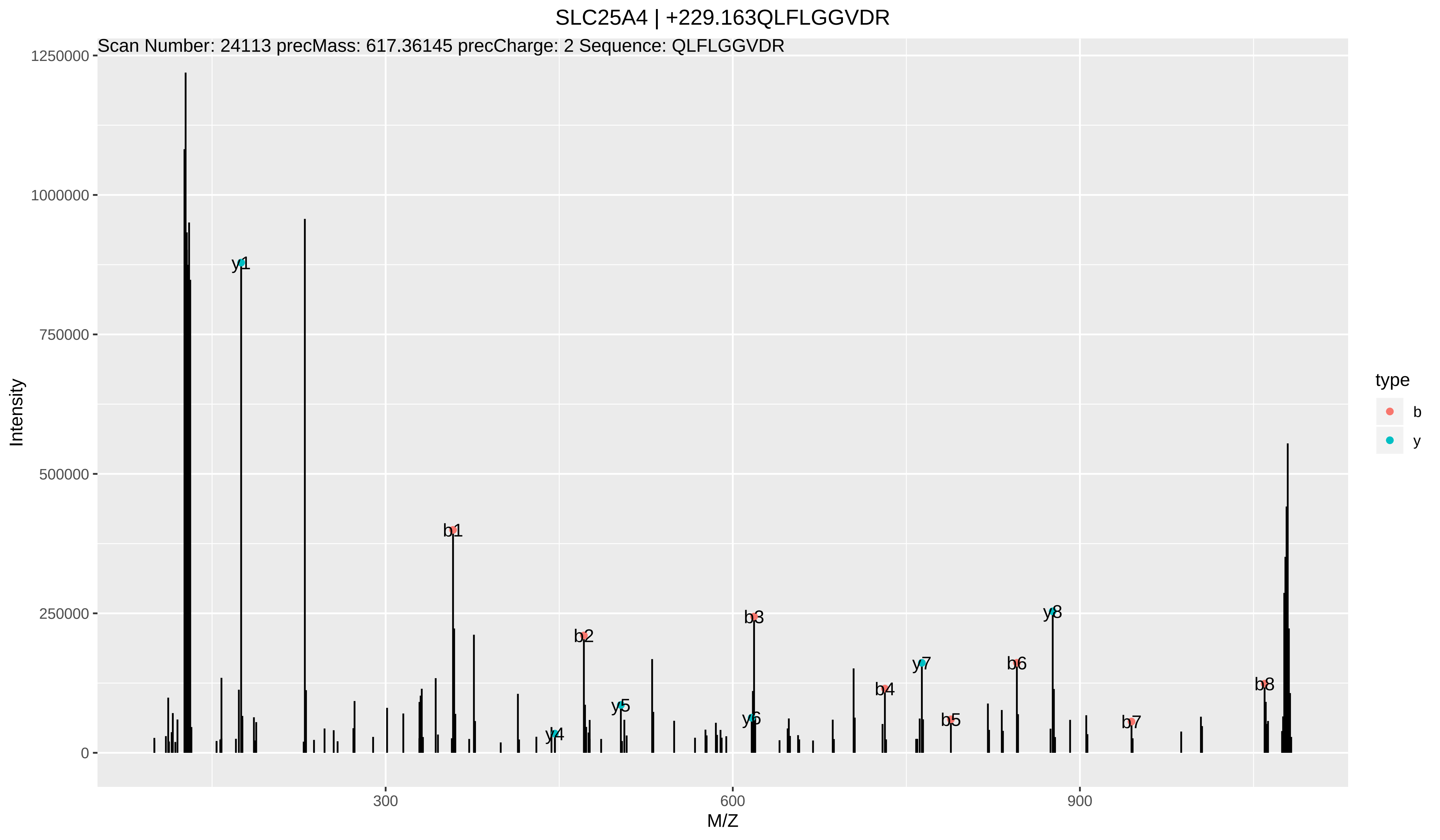

SLC25A4 | +229.163QLFLGGVDR
Scan Number: 24113 precMass: 617.36145 precCharge: 2 Sequence: QLFLGGVDR
Intensity
M/Z
0
250000
500000
750000
1000000
1250000
300
600
900
y1
b1
b2
y4
y5
b3
y6
b4
y7
b5
b6
y8
b7
b8
type
b
y

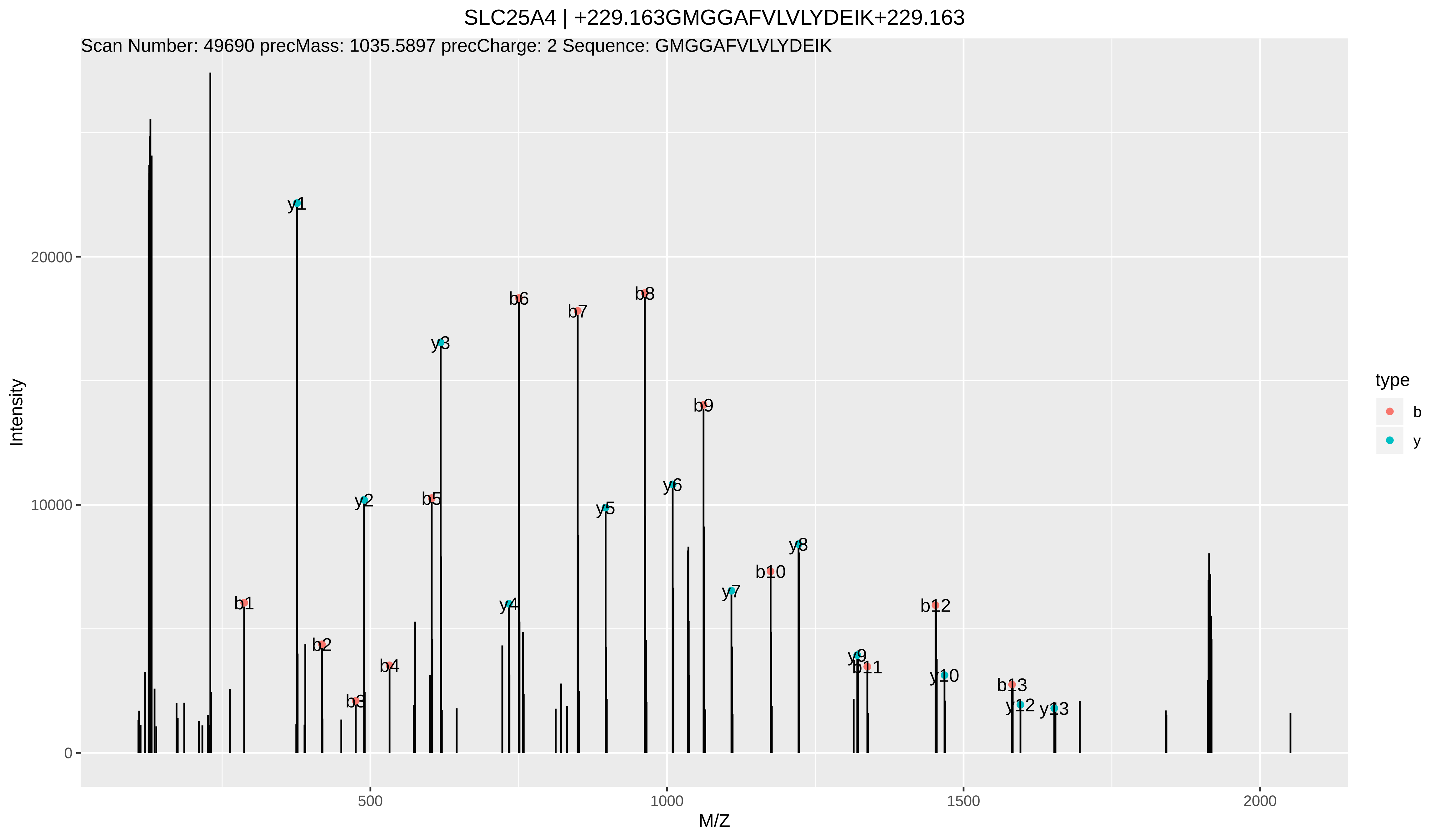
SLC25A4 | +229.163GMGGAFVLVLYDEIK+229.163
Scan Number: 49690 precMass: 1035.5897 precCharge: 2 Sequence: GMGGAFVLVLYDEIK
Intensity
M/Z
0
10000
20000
500
1000
1500
2000
y1
b6
b8
b7
y3
b9
y6
y2
b5
y5
y8
b10
y7
b1
y4
b12
b2
y9
b4
b11
y10
b13
b3
y12
y13
type
b
y

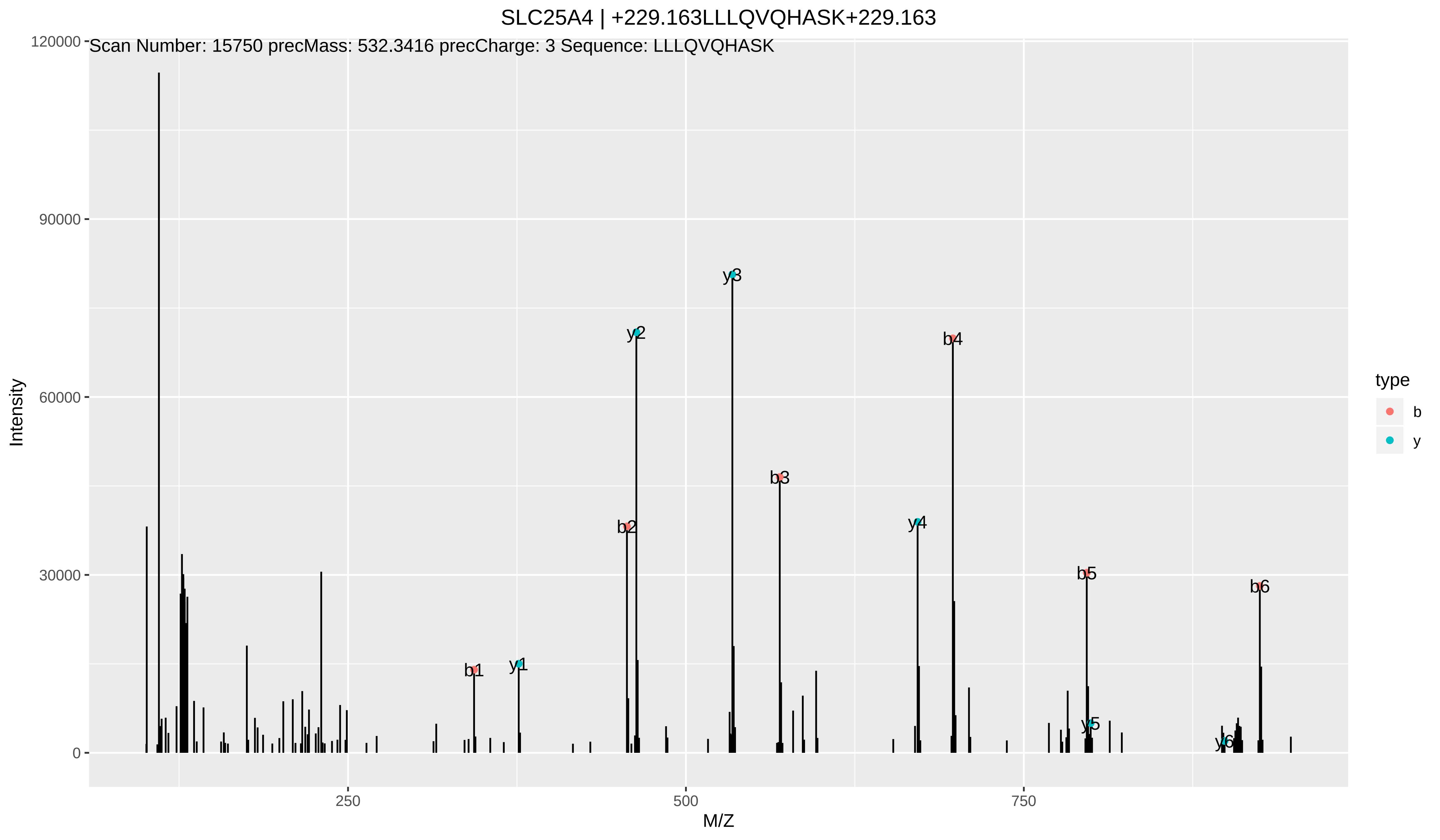

SLC25A4 | +229.163LLLQVQHASK+229.163
Scan Number: 15750 precMass: 532.3416 precCharge: 3 Sequence: LLLQVQHASK
Intensity
120000
90000
60000
30000
0
M/Z
250
500
750
b1
y1
b2
y2
y3
b3
y4
b4
b5
y5
y6
b6
type
b
y

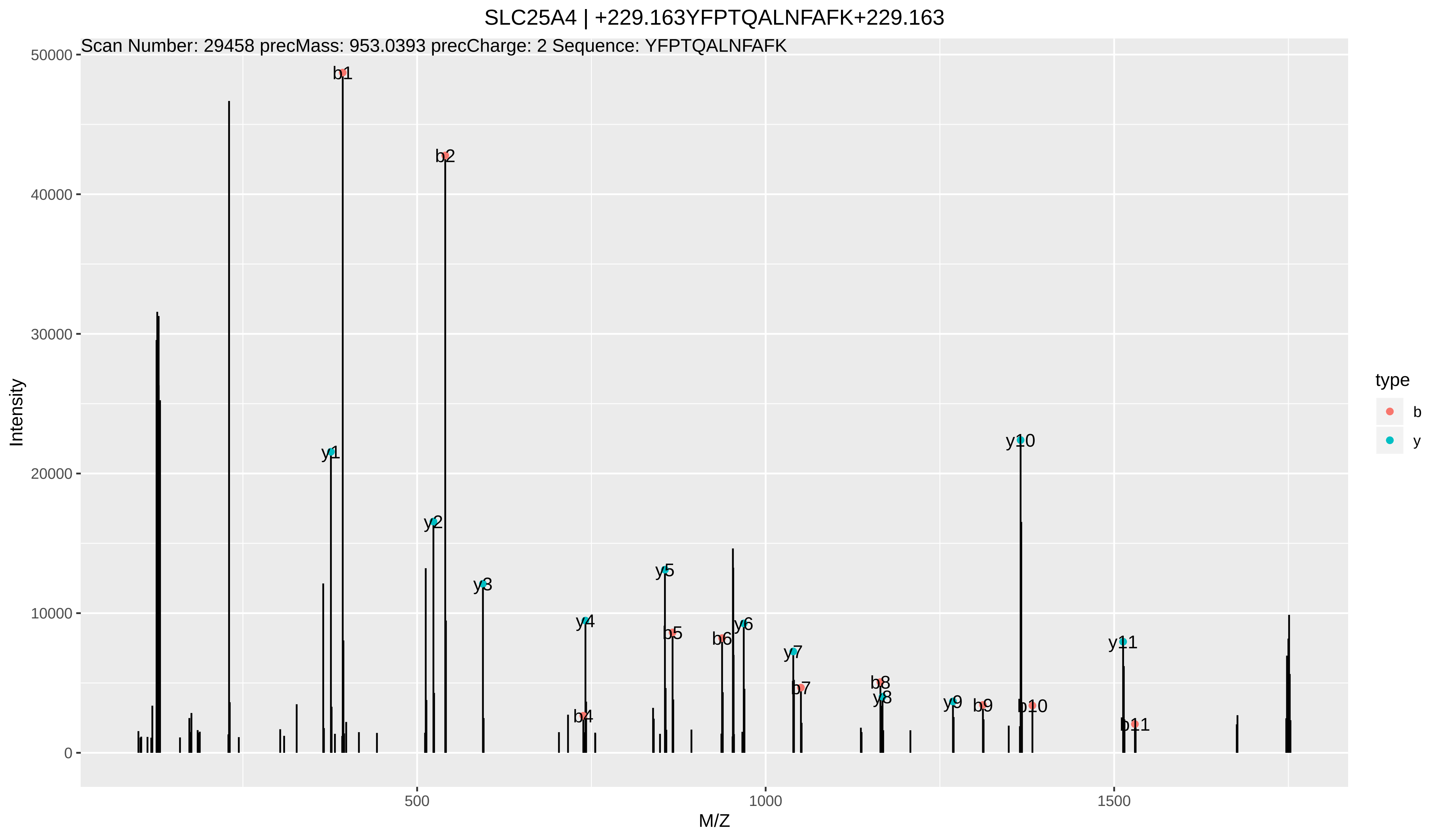

SLC25A4 | +229.163YFPTQALNFAFK+229.163
Scan Number: 29458 precMass: 953.0393 precCharge: 2 Sequence: YFPTQALNFAFK
Intensity
0
10000
20000
30000
40000
50000
M/Z
500
1000
1500
b1
b2
y1
y2
y3
y4
b4
y5
b5
b6
y6
y7
b7
b8
y8
y9
b9
y10
b10
y11
b11
type
b
y

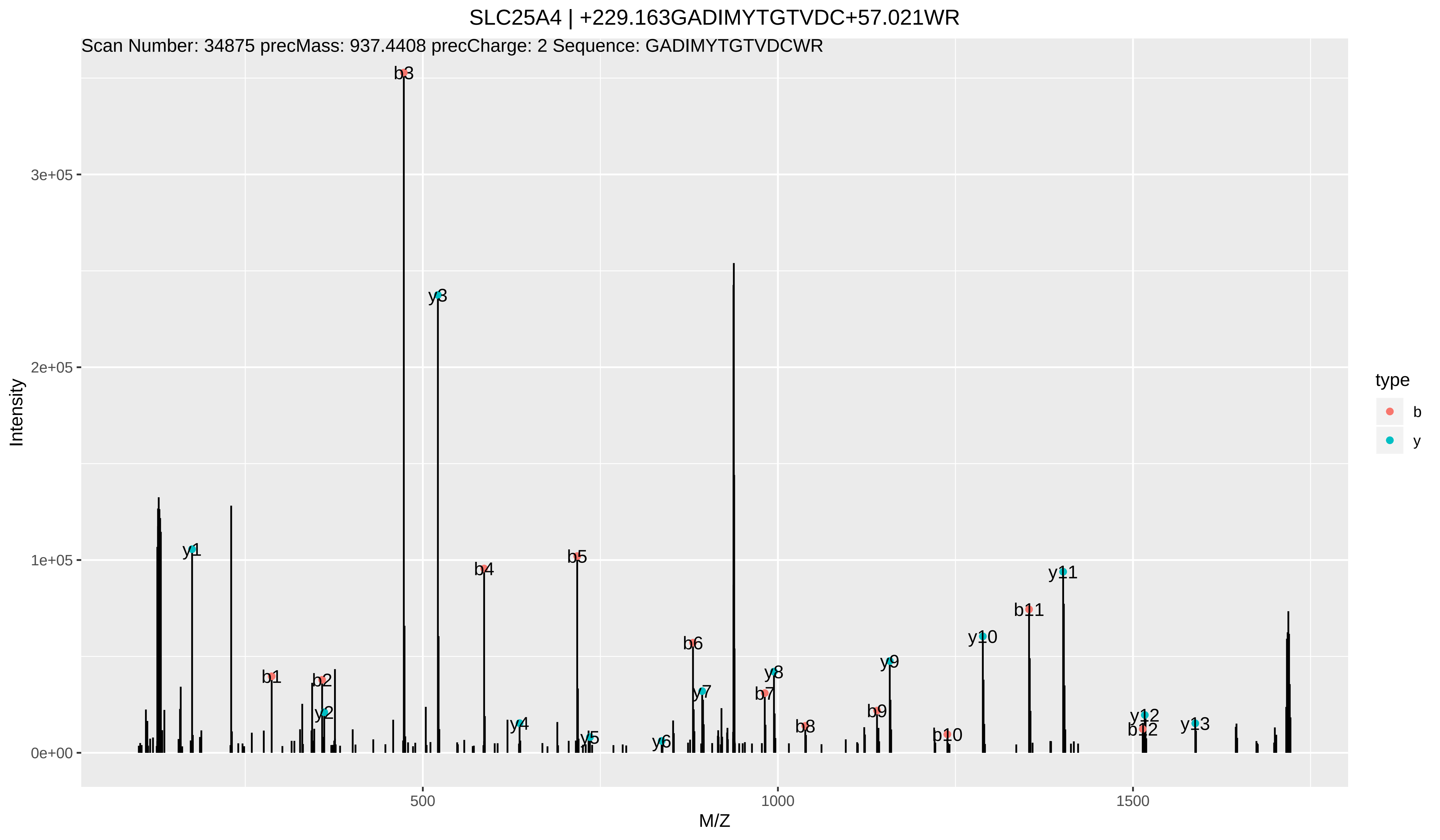

SLC25A4 | +229.163GADIMYTGTVDC+57.021WR
Scan Number: 34875 precMass: 937.4408 precCharge: 2 Sequence: GADIMYTGTVDCWR
Intensity
3e+05
2e+05
1e+05
0e+00
M/Z
500
1000
1500
b3
y3
y1
b1
b2
y2
b4
y4
b5
y5
y6
b6
y7
b7
y8
b8
b9
y9
b10
y10
b11
y11
y12
b12
y13
type
b
y

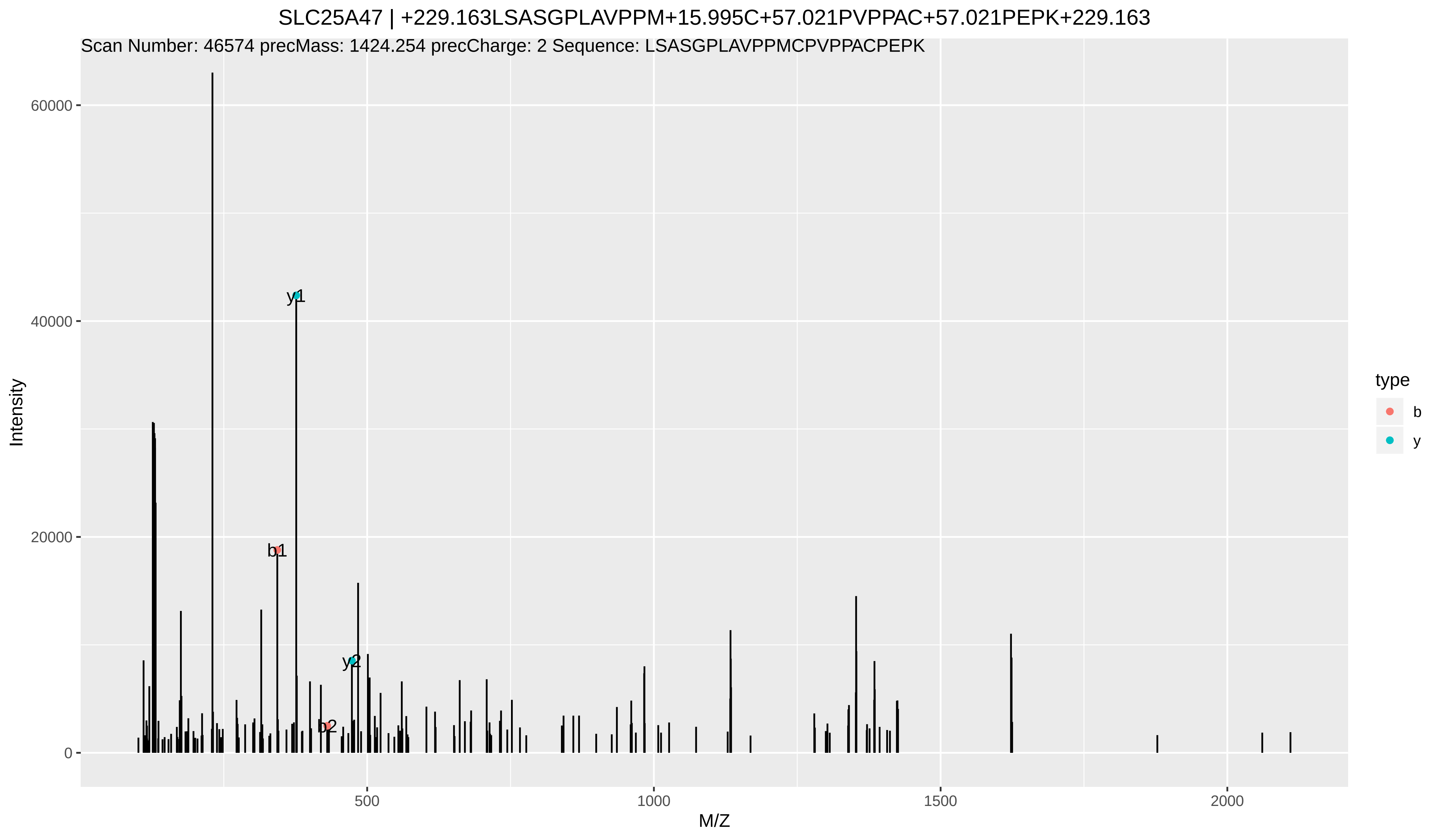

SLC25A47 | +229.163LSASGPLAVPPM+15.995C+57.021PVPPAC+57.021PEPK+229.163
Scan Number: 46574 precMass: 1424.254 precCharge: 2 Sequence: LSASGPLAVPPMCPVPPACPEPK
Intensity
60000
40000
20000
0
M/Z
500
1000
1500
2000
y1
b1
y2
b2
type
b
y

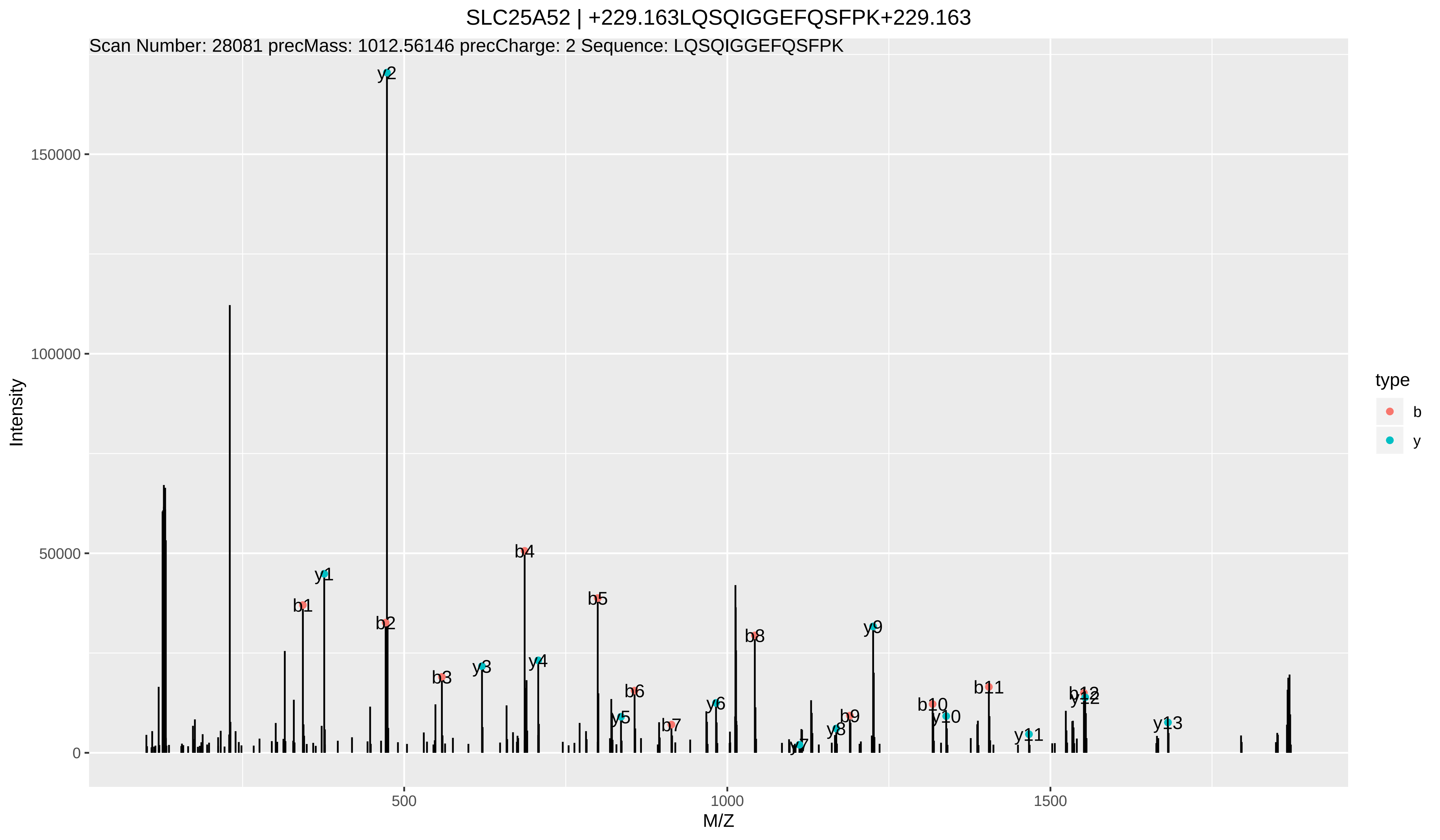

SLC25A52 | +229.163LQSQIGGEFQSFPK+229.163
Scan Number: 28081 precMass: 1012.56146 precCharge: 2 Sequence: LQSQIGGEFQSFPK
Intensity
M/Z
150000
100000
50000
0
500
1000
1500
type
b
y
y2
y1
b1
b2
b3
y3
b4
y4
b5
y5
b6
b7
y6
y7
y8
b8
b9
y9
b10
y10
b11
y11
b12
y12
y13

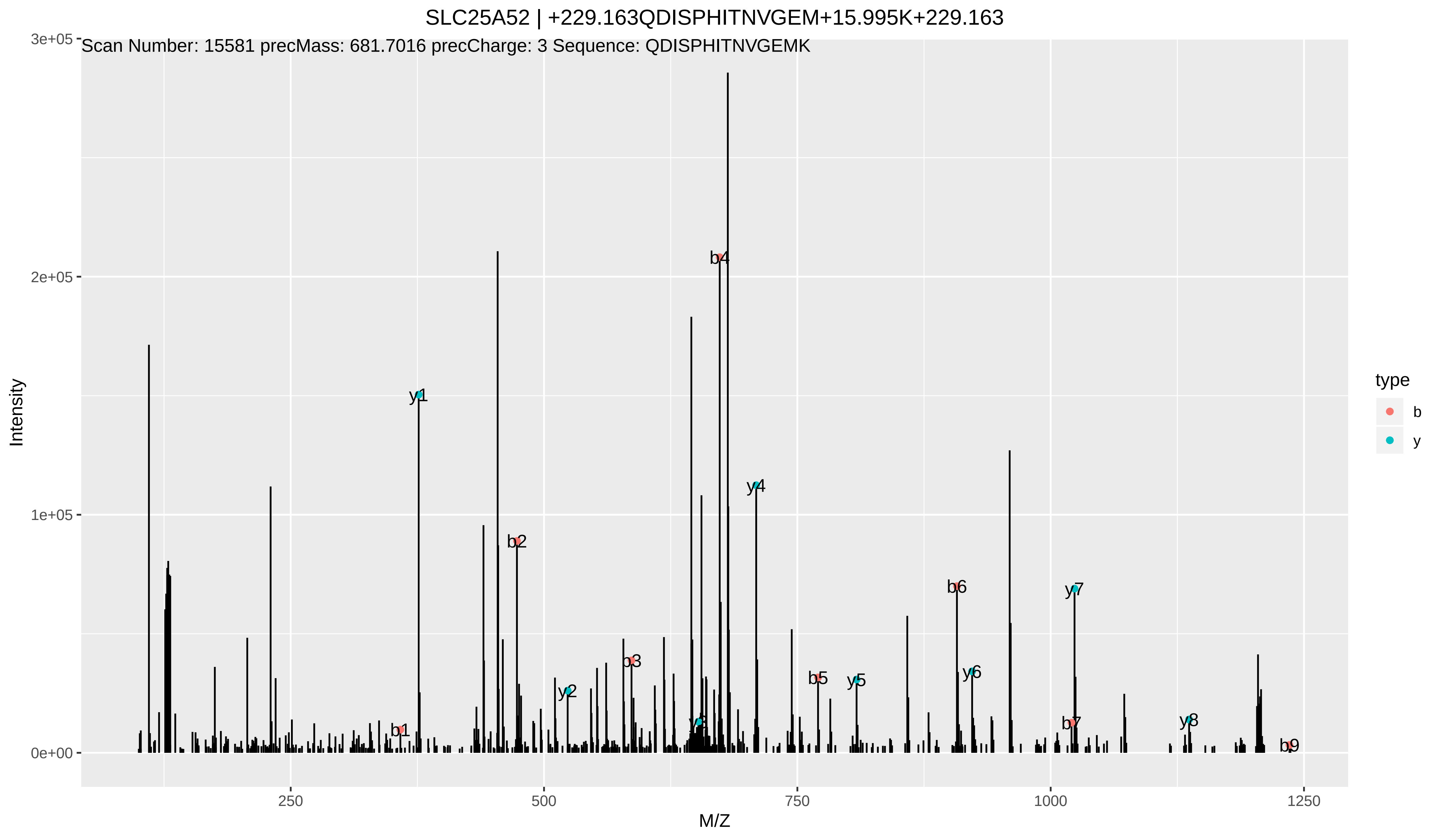

SLC25A52 | +229.163QDISPHITNVGEM+15.995K+229.163
Scan Number: 15581 precMass: 681.7016 precCharge: 3 Sequence: QDISPHITNVGEMK
Intensity
M/Z
3e+05
2e+05
1e+05
0e+00
250
500
750
1000
1250
y1
b1
b2
y2
b3
y3
b4
y4
b5
y5
b6
y6
b7
y7
y8
b9
type
b
y

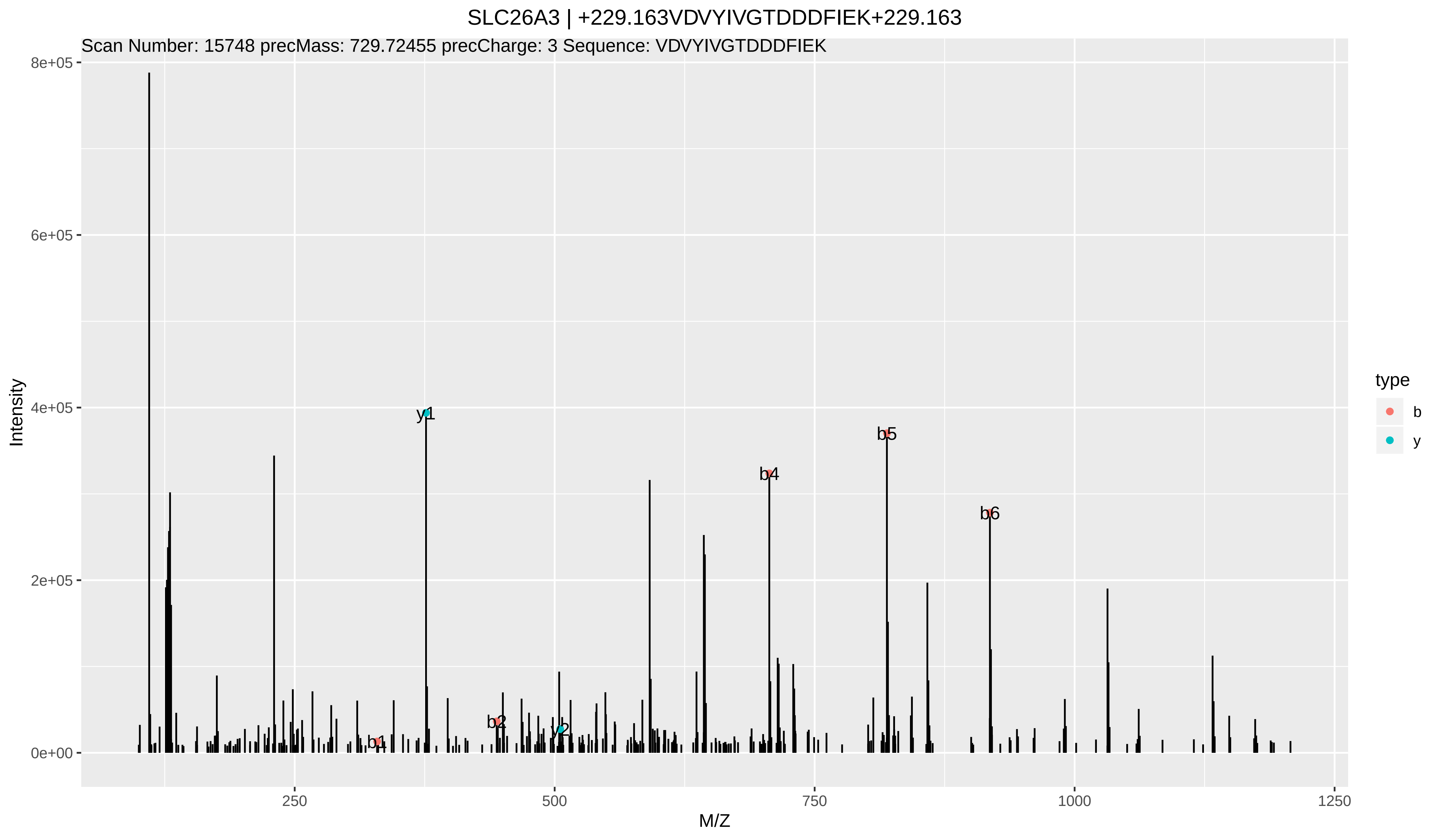

SLC26A3 | +229.163VDVYIVGTDDDFIEK+229.163
Scan Number: 15748 precMass: 729.72455 precCharge: 3 Sequence: VDVYIVGTDDDFIEK
Intensity
8e+05
6e+05
4e+05
2e+05
0e+00
M/Z
250
500
750
1000
1250
y1
b5
b4
b6
b2
y2
b1
type
b
y

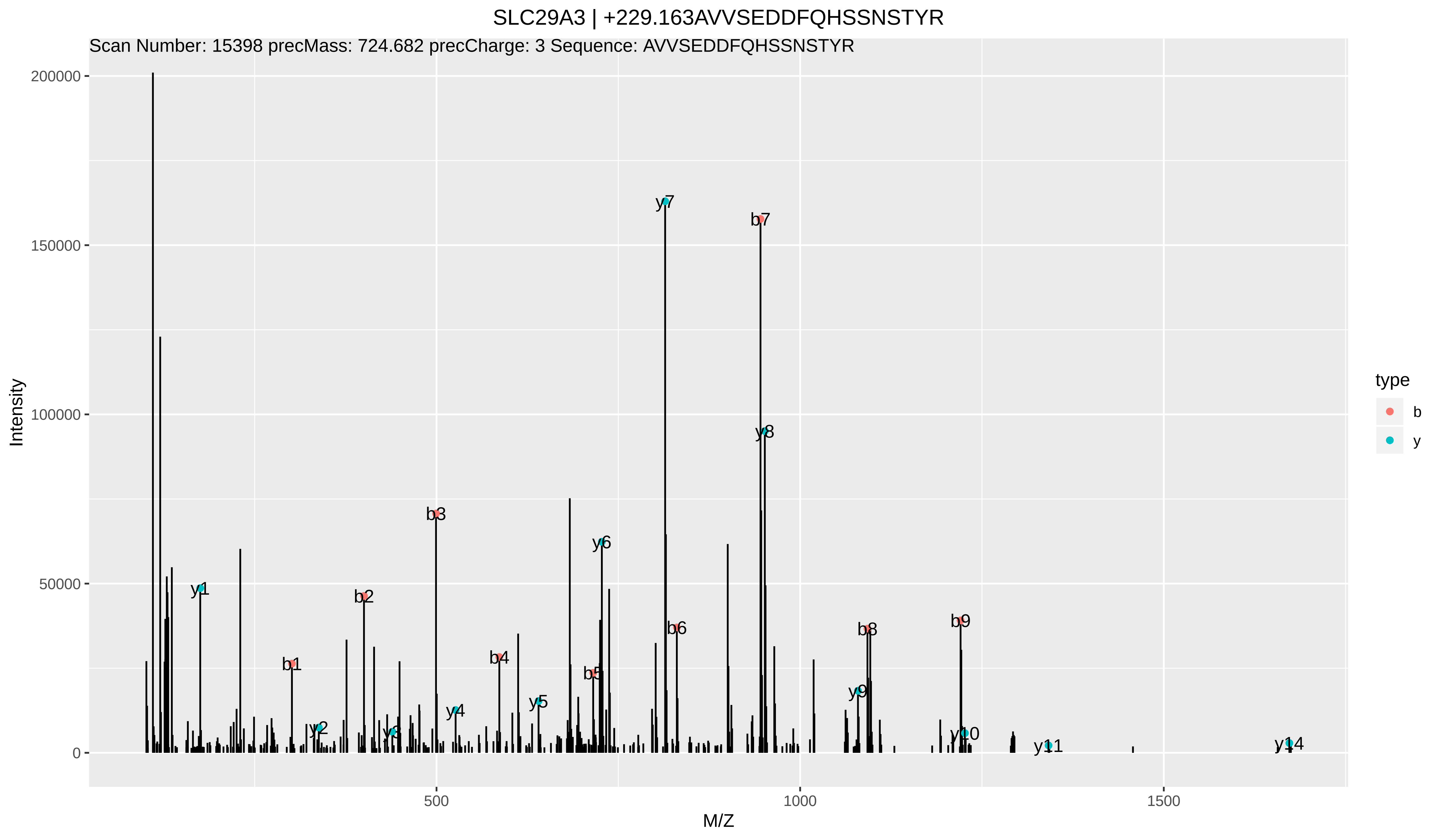
SLC29A3 | +229.163AVVSEDDFQHSSNSTYR
Scan Number: 15398 precMass: 724.682 precCharge: 3 Sequence: AVVSEDDFQHSSNSTYR
Intensity
M/Z
200000
150000
100000
50000
0
500
1000
1500
y7
b7
y8
b3
y6
y1
b2
b6
b8
b9
b1
b4
b5
y9
y5
y4
y2
y3
y10
y11
y14
type
b
y

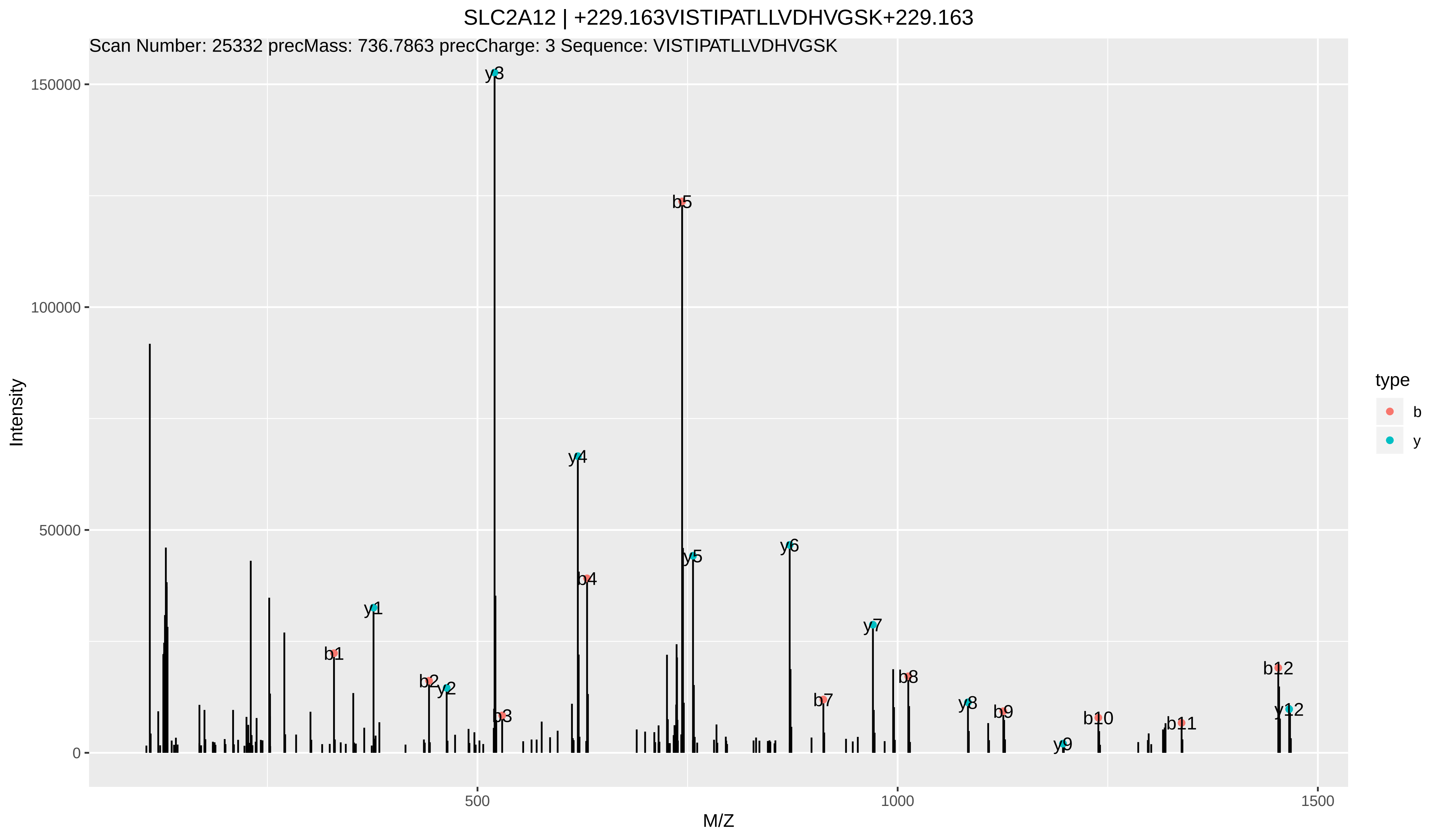

SLC2A12 | +229.163VISTIPATLLVDHVGSK+229.163
Scan Number: 25332 precMass: 736.7863 precCharge: 3 Sequence: VISTIPATLLVDHVGSK
Intensity
M/Z
type
b
y
y3
b5
y4
y6
y5
b4
y1
y7
b1
b12
b8
b2
y2
b7
y8
y12
b9
b3
b10
b11
y9
150000
100000
50000
0
500
1000
1500

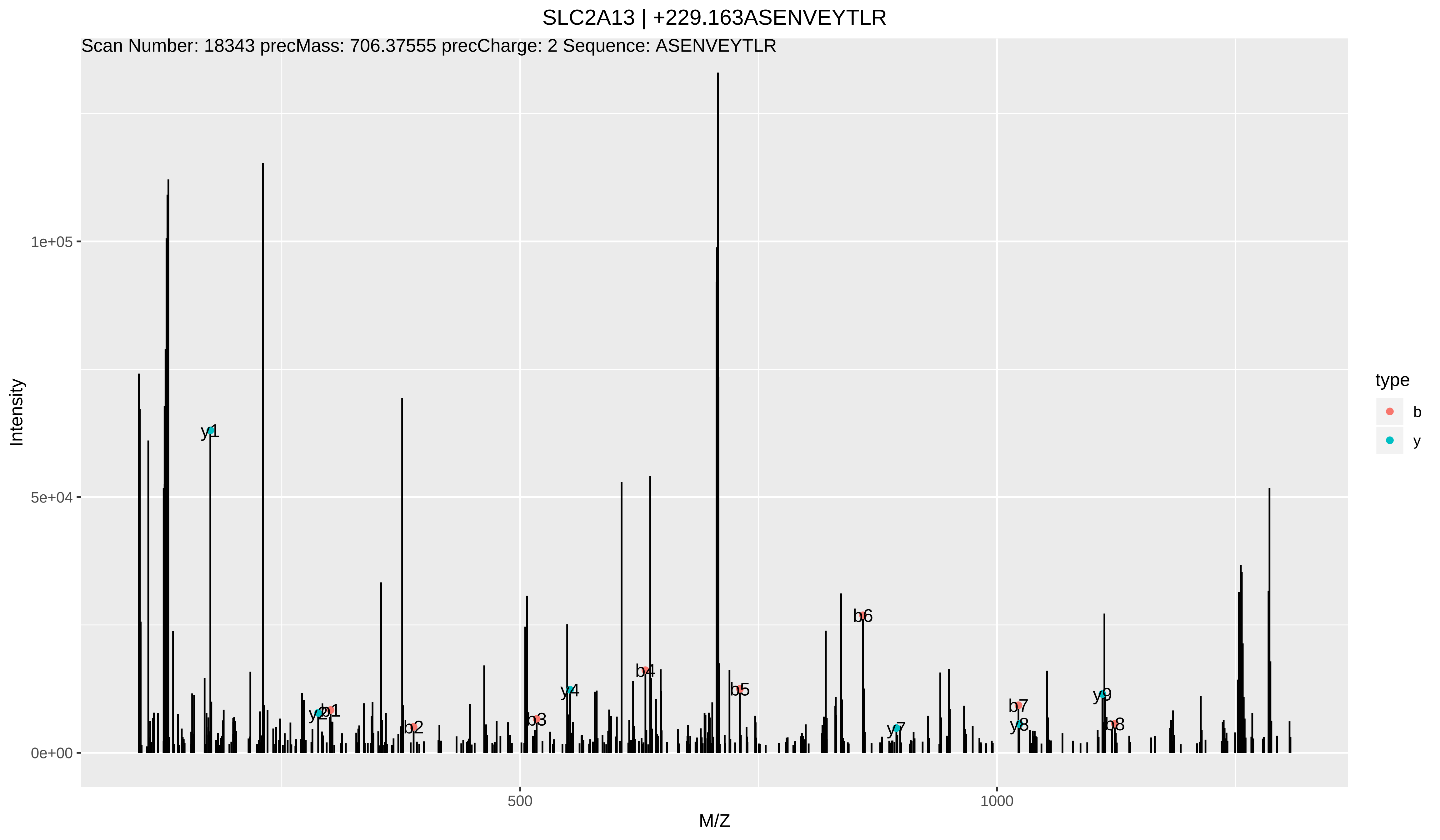
SLC2A13 | +229.163ASENVEYTLR
Scan Number: 18343 precMass: 706.37555 precCharge: 2 Sequence: ASENVEYTLR
Intensity
1e+05
5e+04
0e+00
M/Z
500
1000
y1
y2
b1
b2
b3
y4
b4
b5
b6
y7
b7
y8
y9
b8
type
b
y

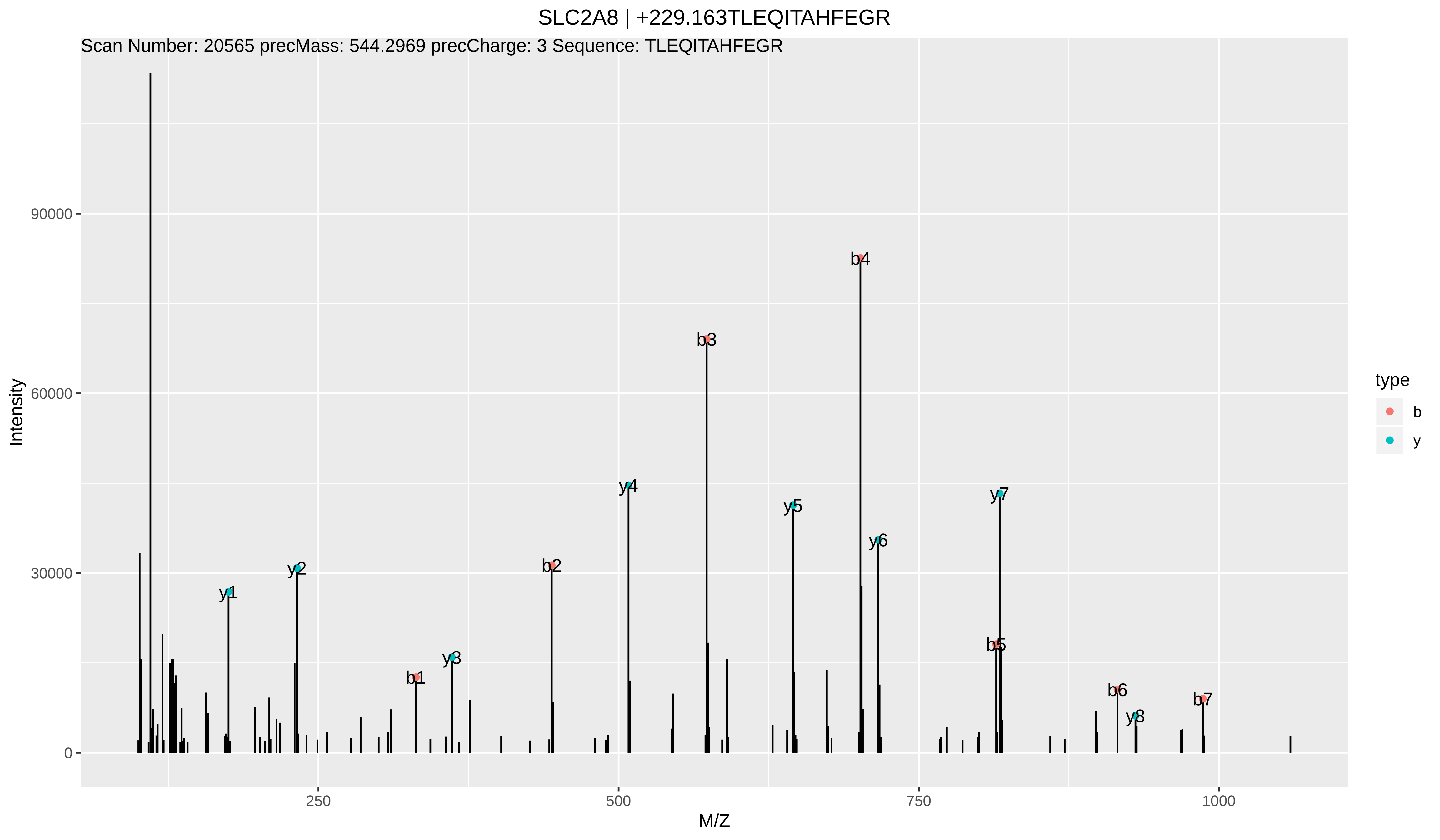

SLC2A8 | +229.163TLEQITAHFEGR
Scan Number: 20565 precMass: 544.2969 precCharge: 3 Sequence: TLEQITAHFEGR
Intensity
90000
60000
30000
0
M/Z
250
500
750
1000
y1
y2
b1
y3
b2
y4
b3
y5
b4
y6
y7
b5
b6
y8
b7
type
b
y

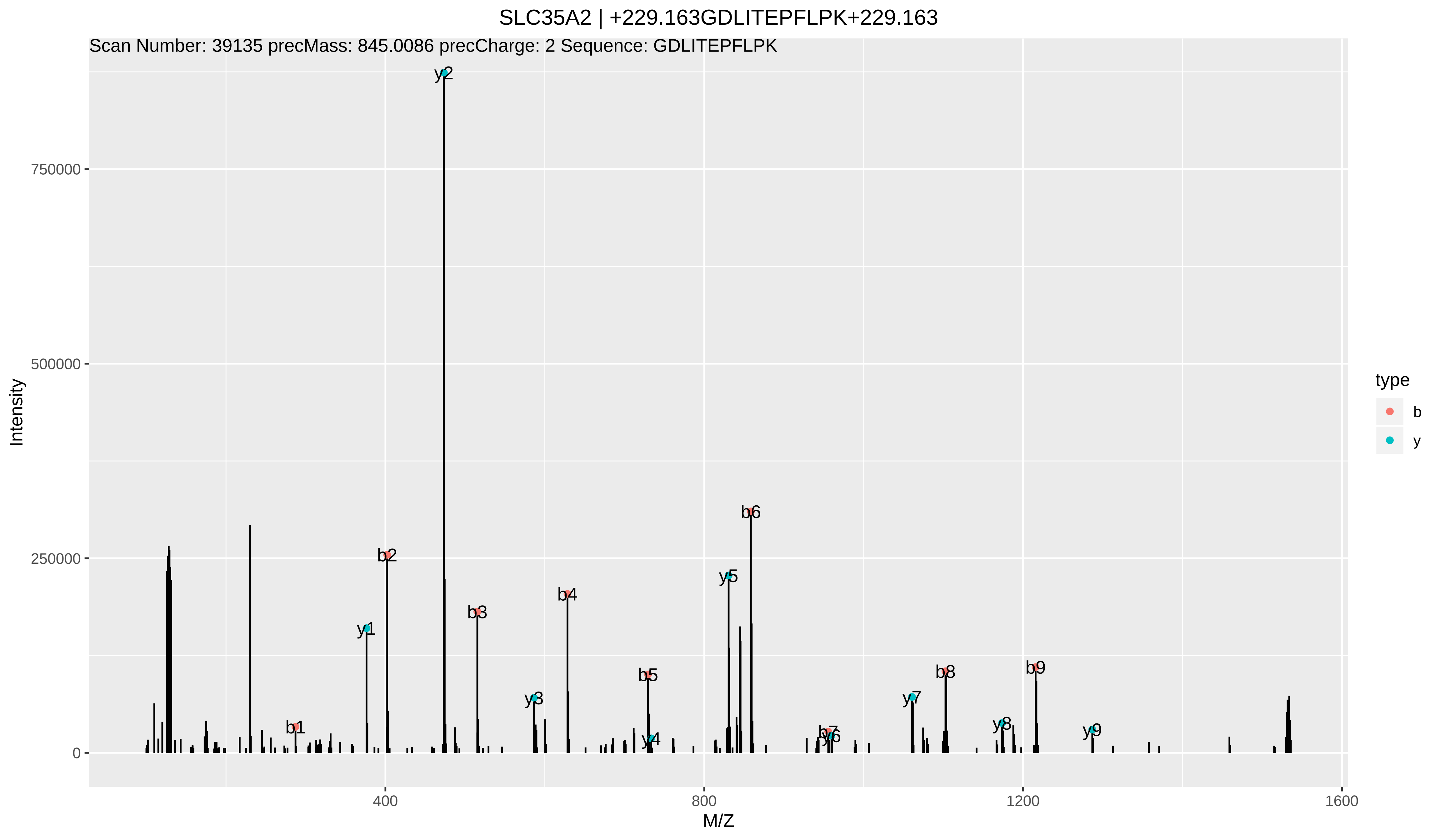

SLC35A2 | +229.163GDLITEPFLPK+229.163
Scan Number: 39135 precMass: 845.0086 precCharge: 2 Sequence: GDLITEPFLPK
y2
b6
b2
y5
b4
b3
y1
b5
y3
y7
b8
b9
b1
y4
b7
y6
y8
y9
750000
500000
250000
0
Intensity
400
800
1200
1600
M/Z
type
b
y

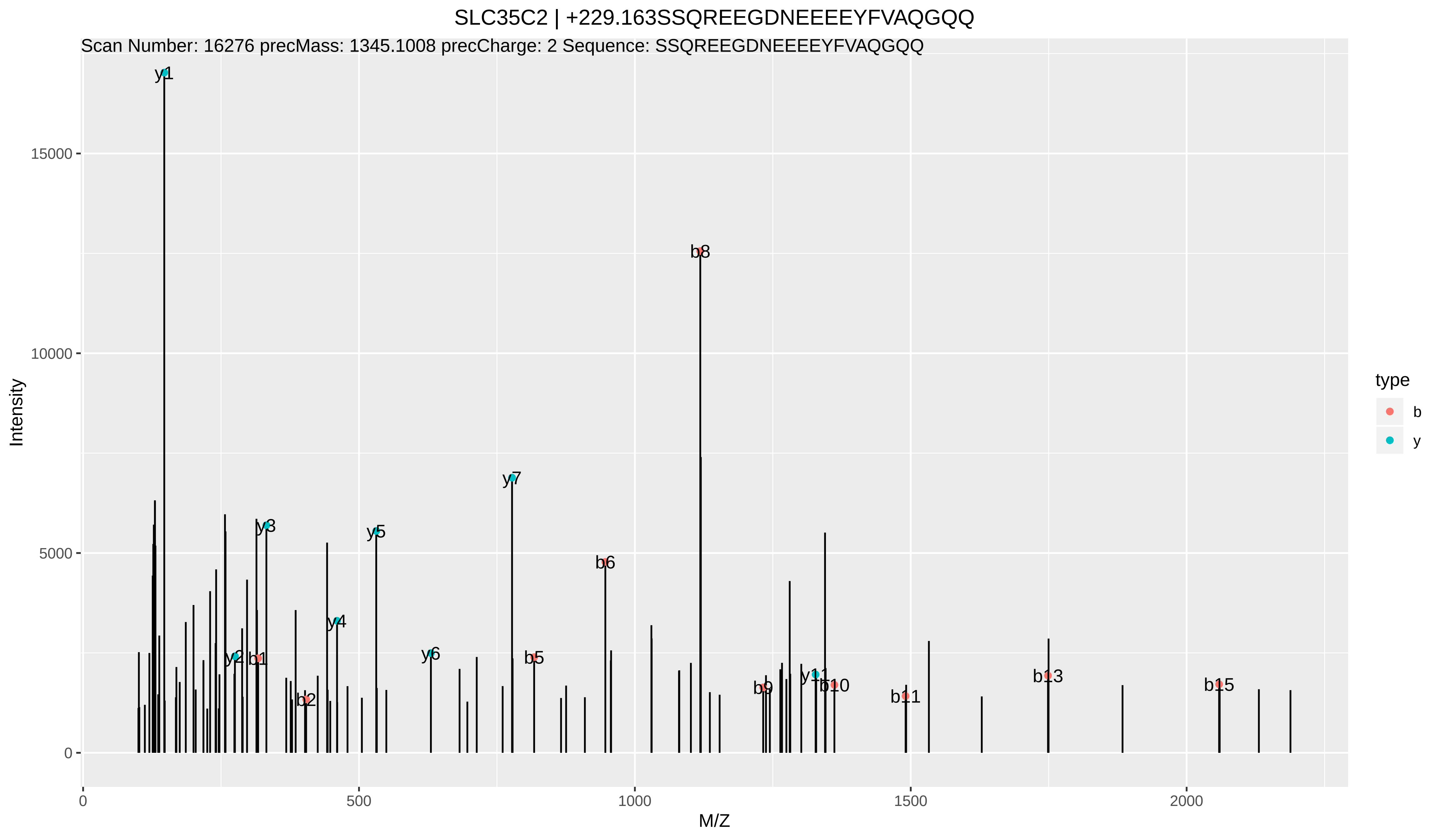
SLC35C2 | +229.163SSQREEGDNEEEEYFVAQGQQ
Scan Number: 16276 precMass: 1345.1008 precCharge: 2 Sequence: SSQREEGDNEEEEYFVAQGQQ
Intensity
M/Z
type
b
y
y1
b8
y7
y3
y5
b6
y4
y2
b1
y6
b5
b2
b9
y11
b10
b11
b13
b15
0
5000
10000
15000
500
1000
1500
2000

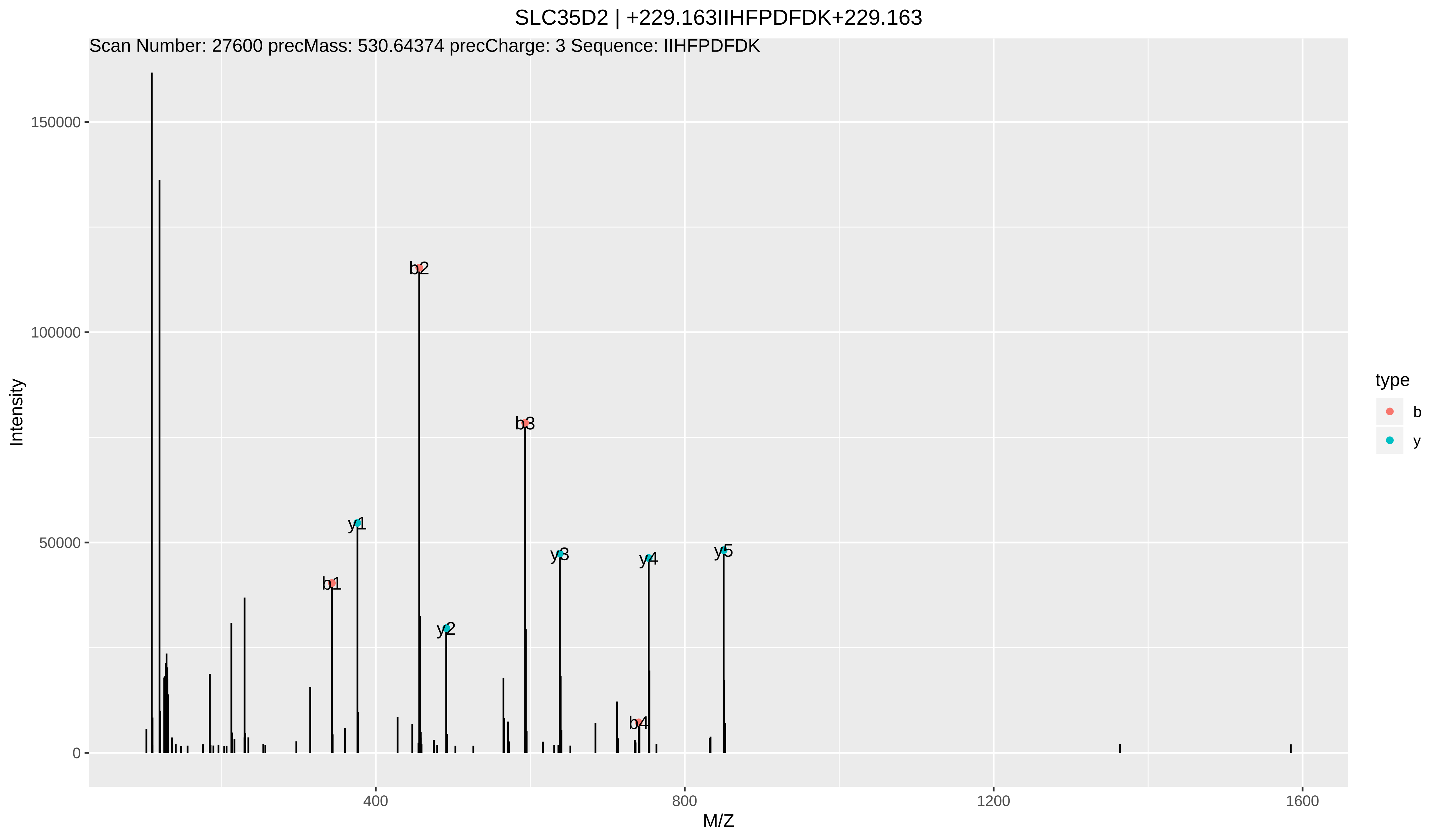

SLC35D2 | +229.163IIHFPDFDK+229.163
Scan Number: 27600 precMass: 530.64374 precCharge: 3 Sequence: IIHFPDFDK
Intensity
150000
100000
50000
0
M/Z
400
800
1200
1600
b1
b2
b3
b4
y1
y2
y3
y4
y5
type
b
y

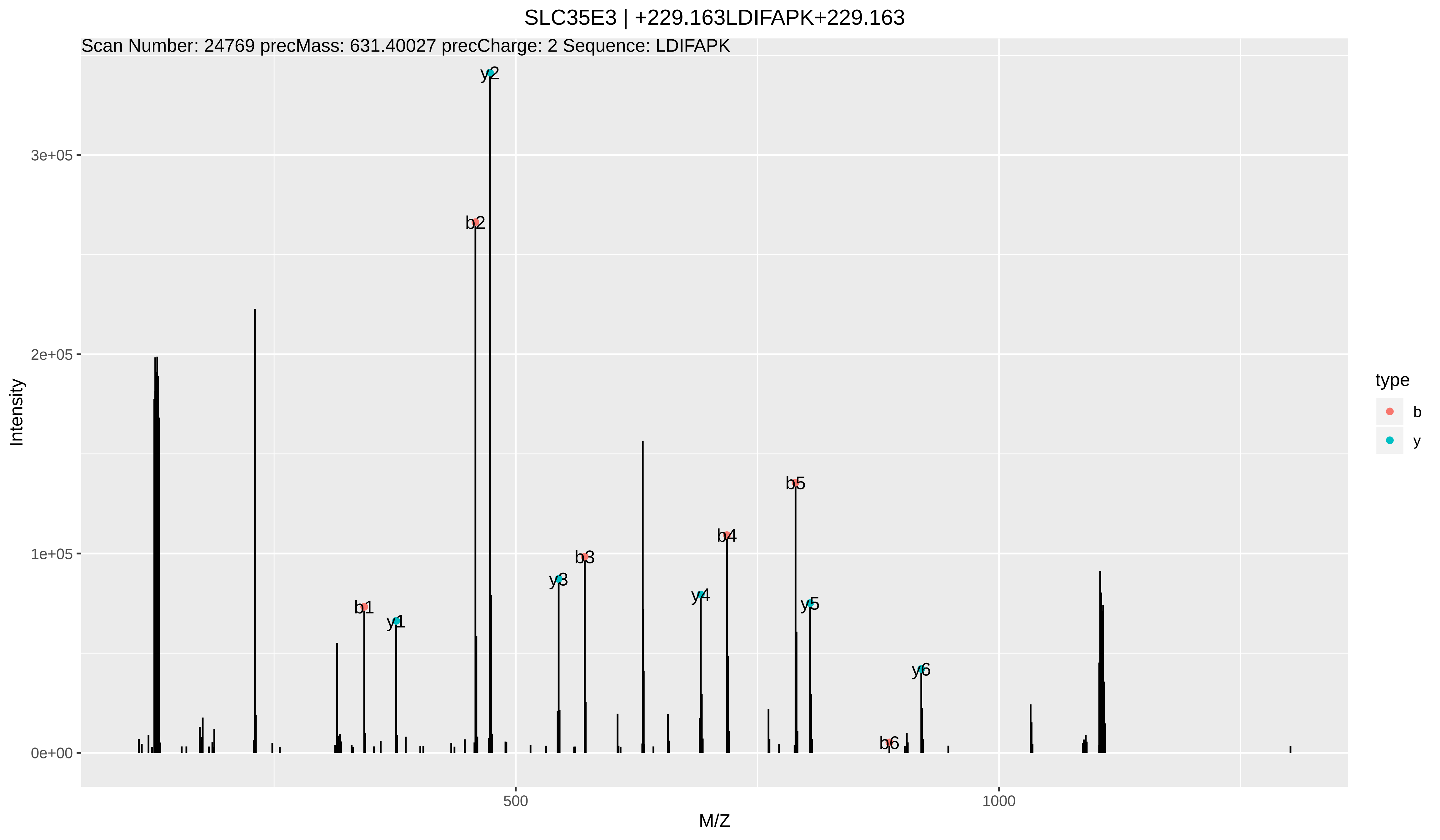
SLC35E3 | +229.163LDIFAPK+229.163
Scan Number: 24769 precMass: 631.40027 precCharge: 2 Sequence: LDIFAPK
Intensity
M/Z
3e+05
2e+05
1e+05
0e+00
500
1000
y2
b2
b5
b4
b3
y3
y4
y5
b1
y1
y6
b6
type
b
y

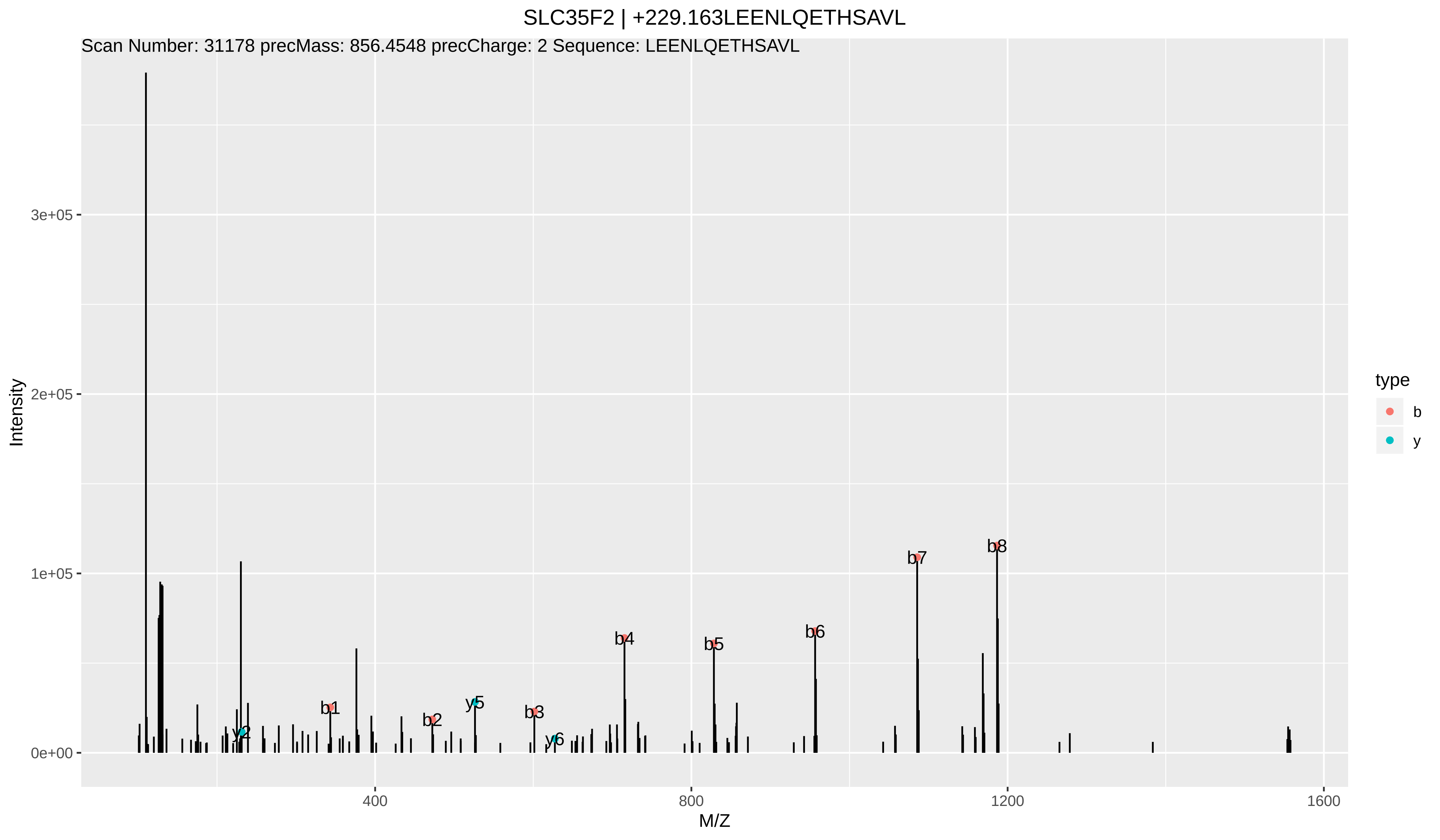

SLC35F2 | +229.163LEENLQETHSAVL
Scan Number: 31178 precMass: 856.4548 precCharge: 2 Sequence: LEENLQETHSAVL
Intensity
M/Z
3e+05
2e+05
1e+05
0e+00
400
800
1200
1600
b1
b2
b3
b4
b5
b6
b7
b8
y2
y5
y6
type
b
y

SLC35F5 | +229.163QLPSSHALEAK+229.163

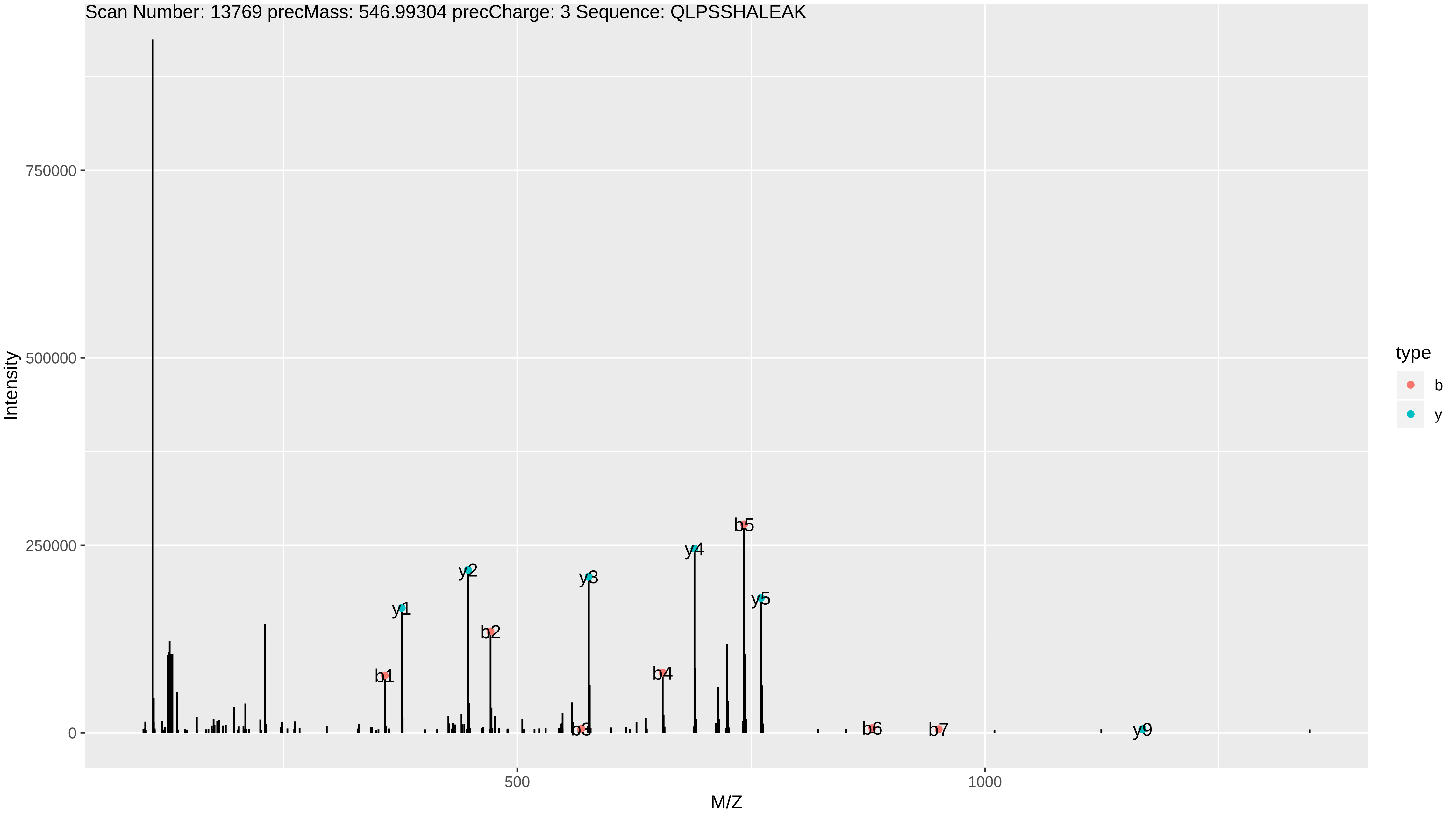

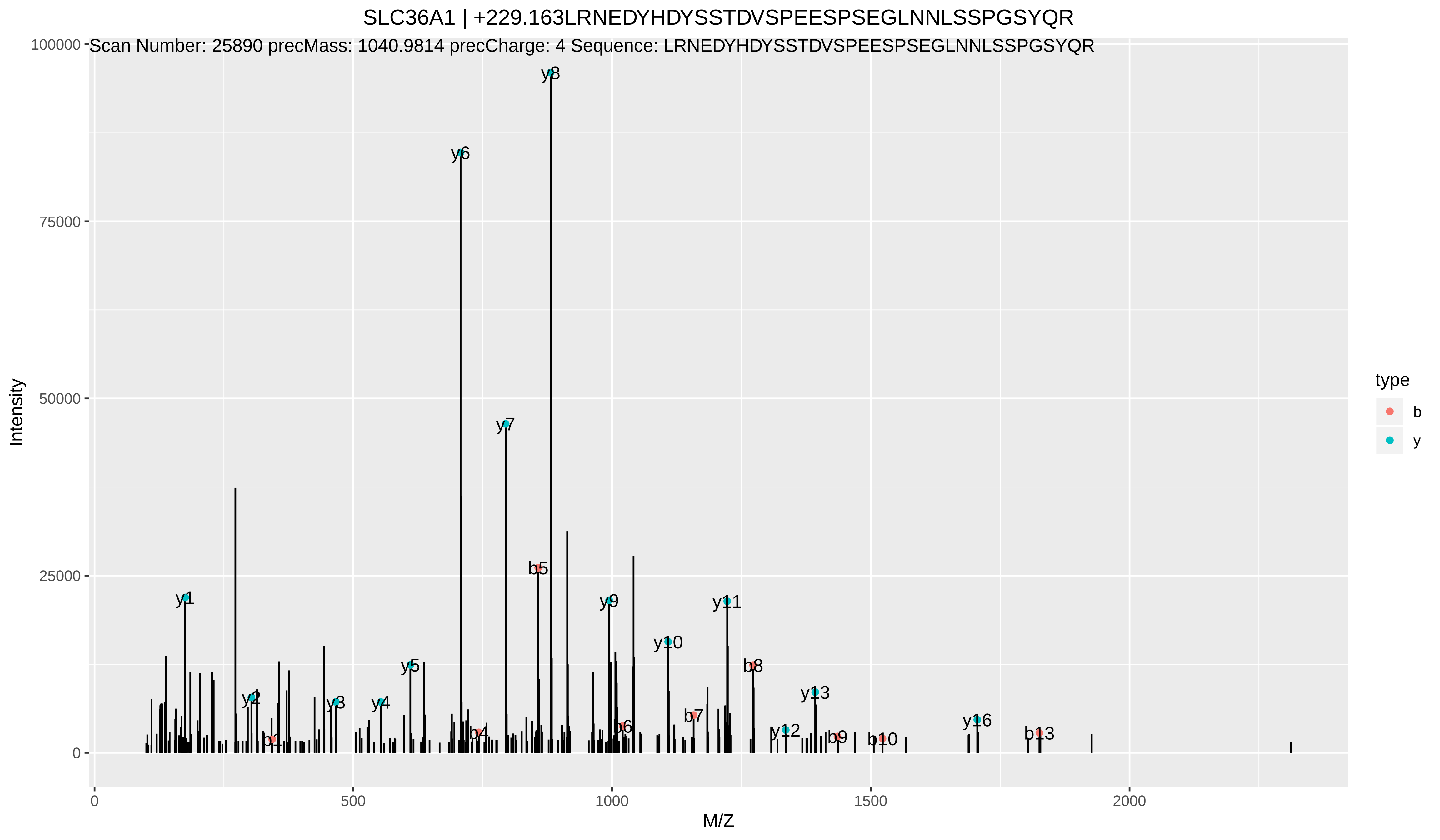

SLC36A1 | +229.163LRNEDYHDYSSTDVSPEESPSEGLNNLSSPGSYQR
Scan Number: 25890 precMass: 1040.9814 precCharge: 4 Sequence: LRNEDYHDYSSTDVSPEESPSEGLNNLSSPGSYQR
Intensity
M/Z
type
b
y
y1
y2
y3
y4
y5
y6
y7
y8
y9
y10
y11
y12
y13
y16
b1
b4
b5
b6
b7
b8
b9
b10
b13
100000
75000
50000
25000
0
500
1000
1500
2000

SLC36A4 | +229.163GNIGTGLLGLPLAIK+229.163

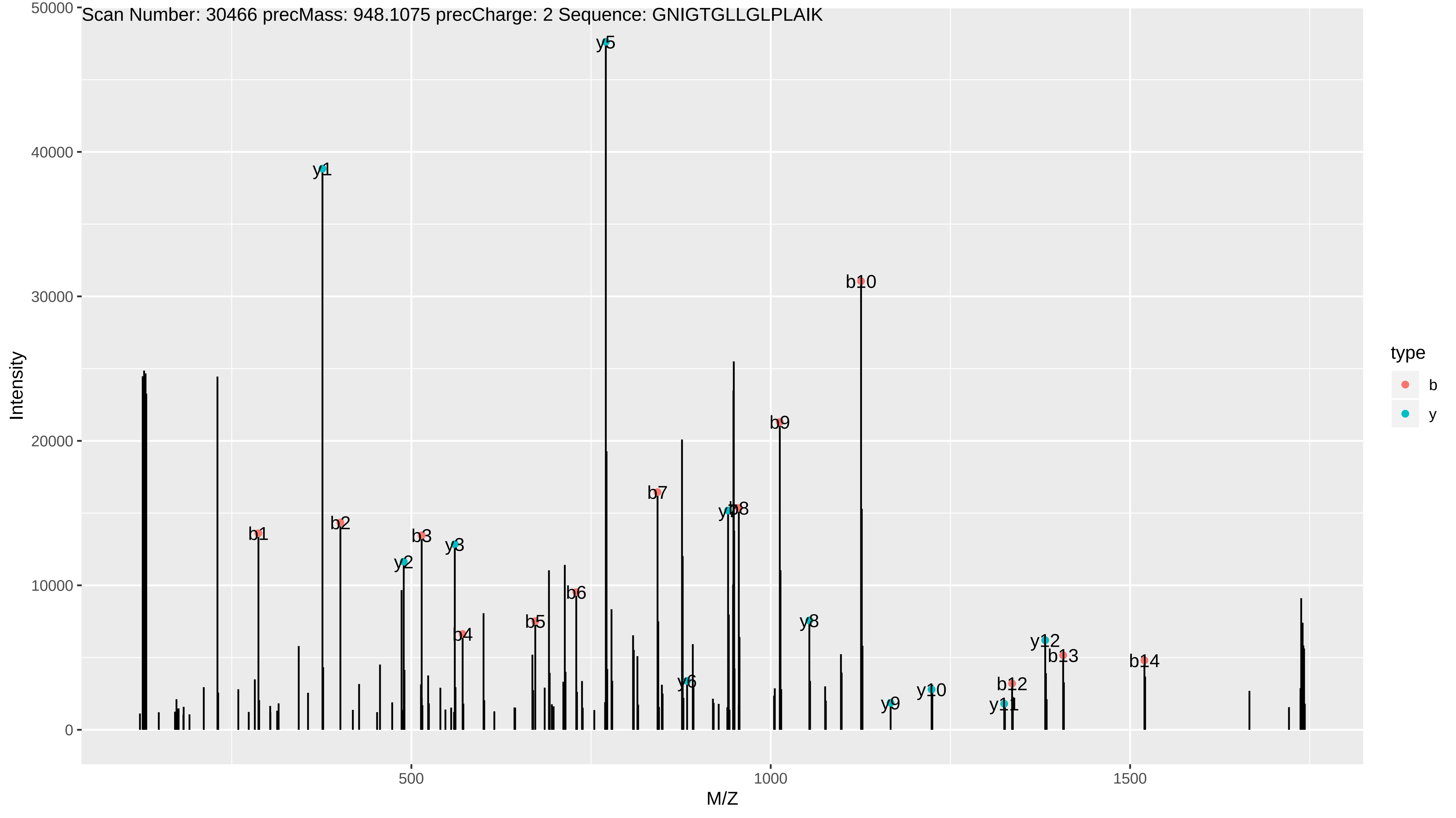

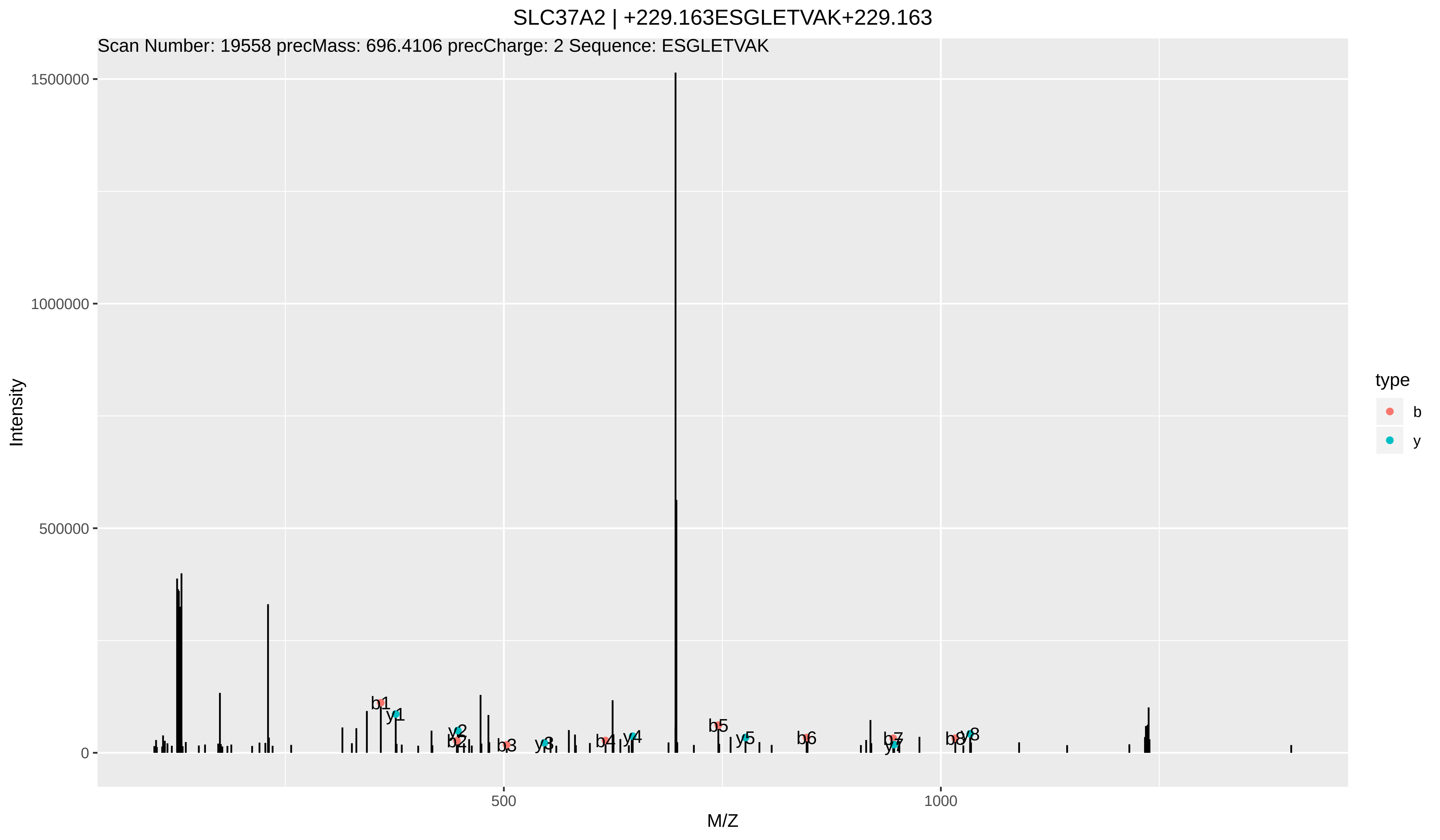
SLC37A2 | +229.163ESGLETVAK+229.163
Scan Number: 19558 precMass: 696.4106 precCharge: 2 Sequence: ESGLETVAK
Intensity
M/Z
1500000
1000000
500000
0
500
1000
b1
y1
y2
b2
b3
y3
b4
y4
b5
y5
b6
b7
y7
b8
y8
type
b
y

SLC37A2 | +229.163K+229.163PISIVK+229.163

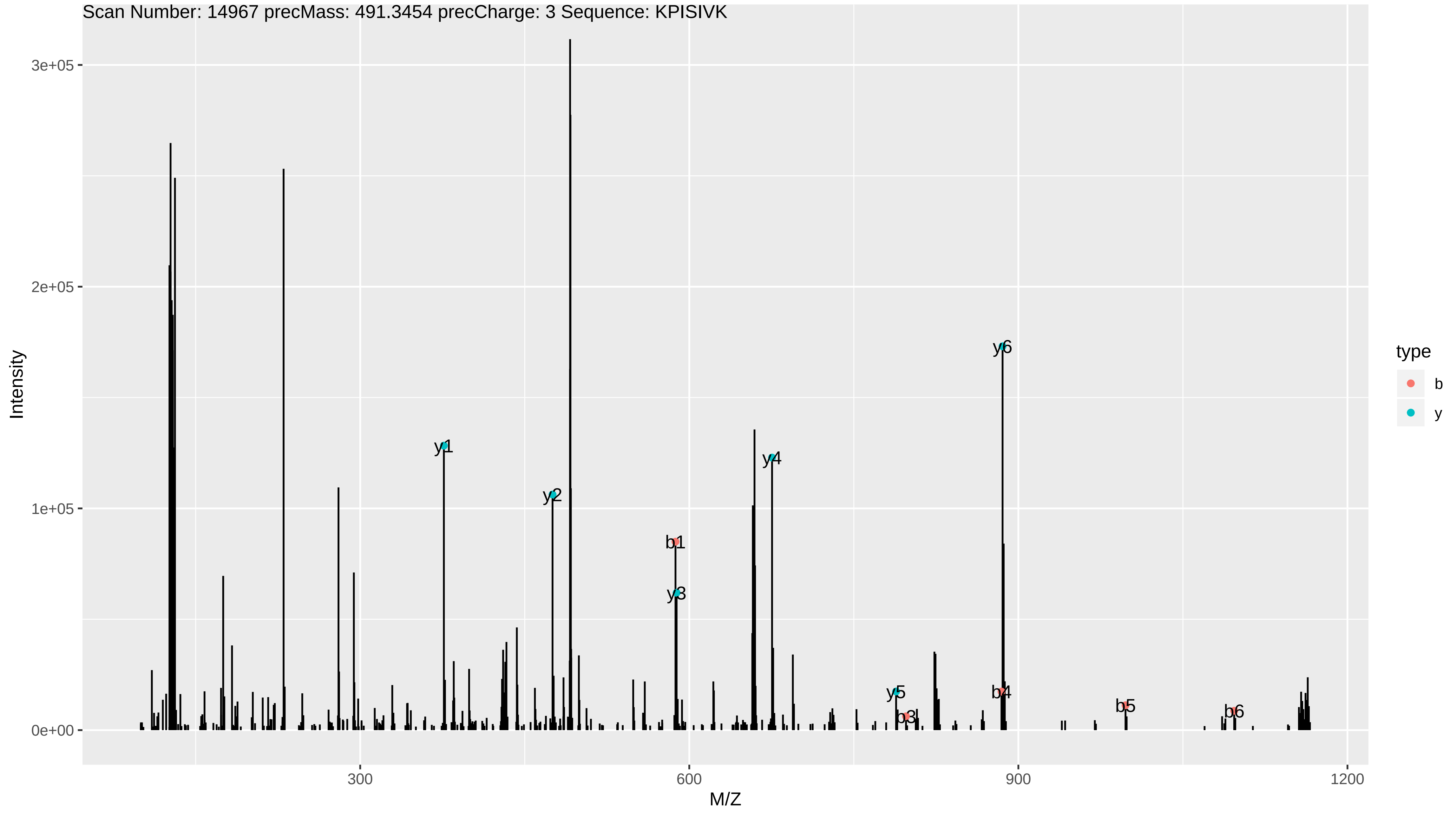

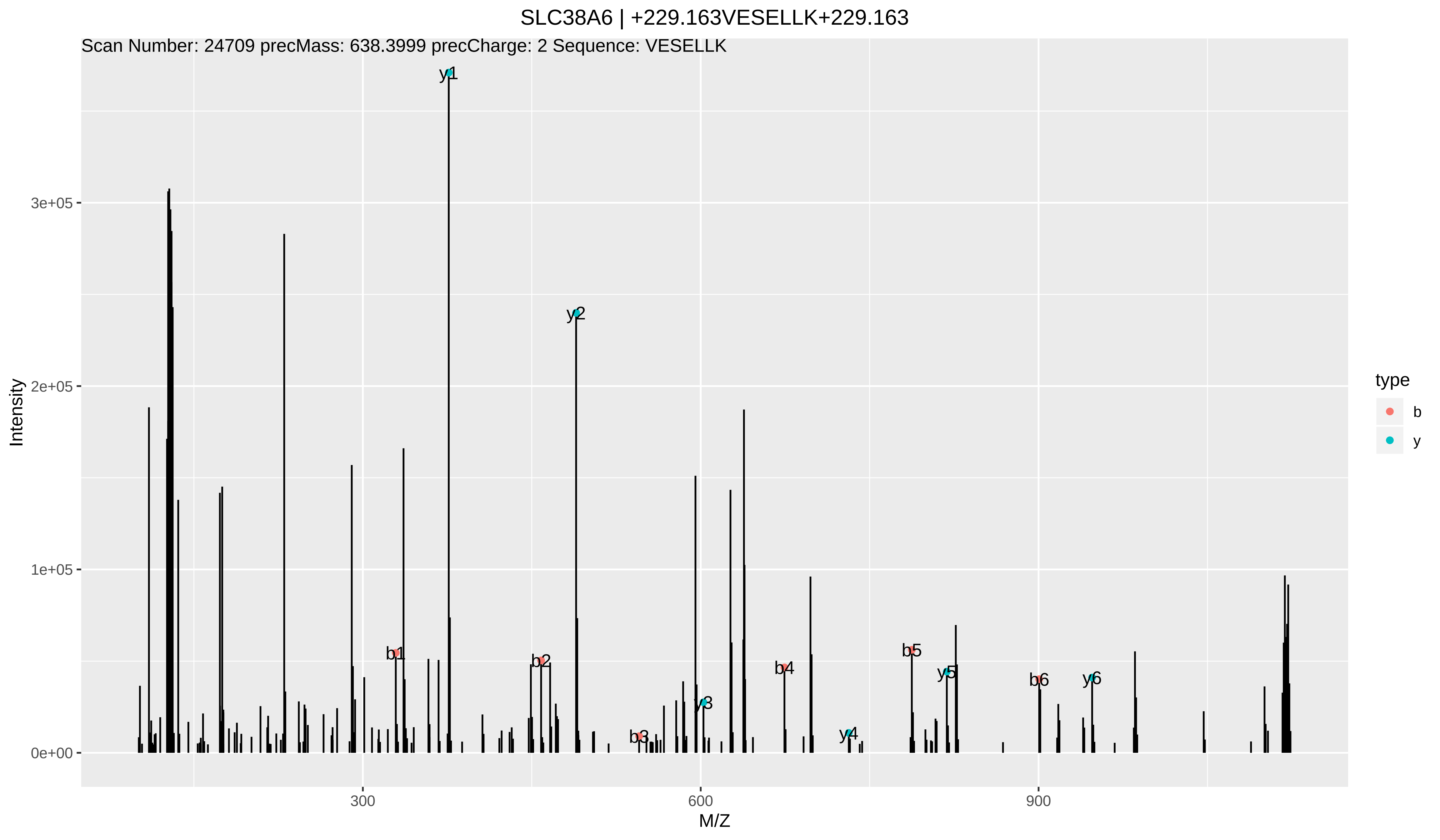

SLC38A6 | +229.163VESELLK+229.163
Scan Number: 24709 precMass: 638.3999 precCharge: 2 Sequence: VESELLK
y1
y2
b1
b2
b3
y3
b4
y4
b5
y5
b6
y6
3e+05
2e+05
1e+05
0e+00
Intensity
300
600
900
M/Z
type
b
y

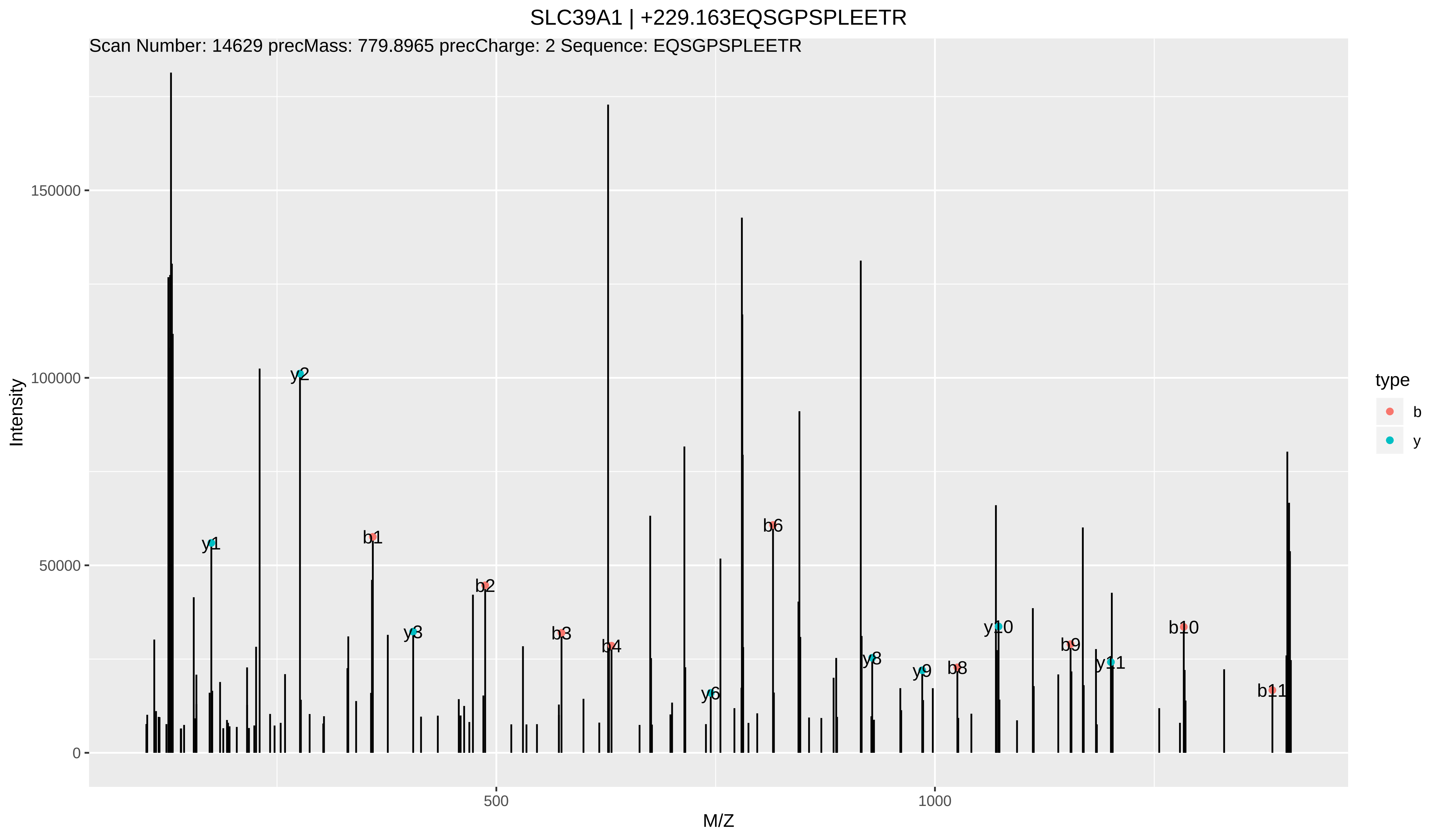
SLC39A1 | +229.163EQSGPSPLEETR
Scan Number: 14629 precMass: 779.8965 precCharge: 2 Sequence: EQSGPSPLEETR
Intensity
150000
100000
50000
0
M/Z
500
1000
y1
y2
b1
y3
b2
b3
b4
y6
b6
y8
y9
b8
y10
b9
y11
b10
b11
type
b
y

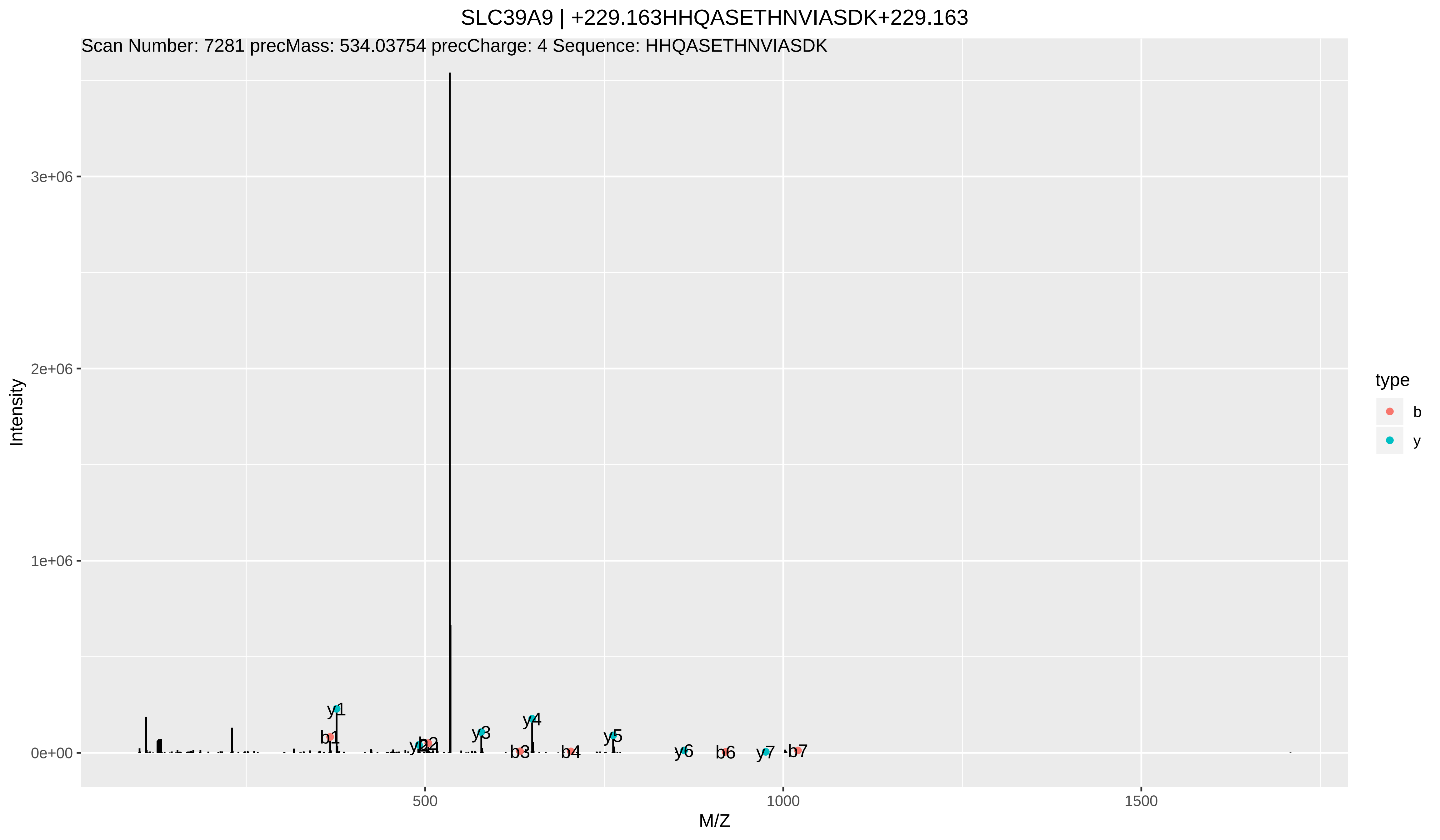
SLC39A9 | +229.163HHQASETHNVIASDK+229.163
Scan Number: 7281 precMass: 534.03754 precCharge: 4 Sequence: HHQASETHNVIASDK
Intensity
3e+06
2e+06
1e+06
0e+00
M/Z
500
1000
1500
y1
b1
y2
b2
y3
b3
y4
b4
y5
y6
b6
y7
b7
type
b
y

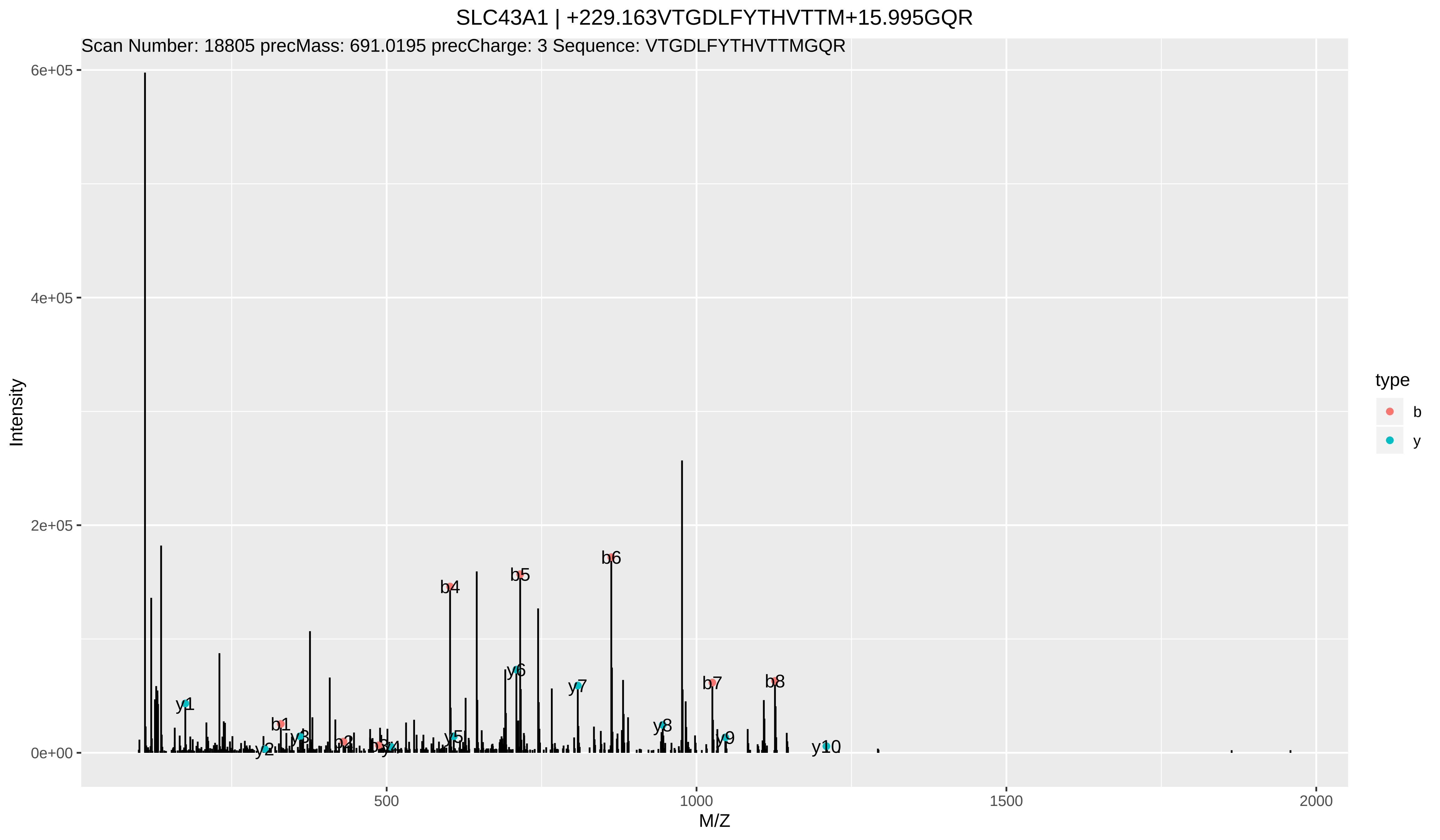
SLC43A1 | +229.163VTGDLFYTHVTTM+15.995GQR
Scan Number: 18805 precMass: 691.0195 precCharge: 3 Sequence: VTGDLFYTHVTTMGQR
Intensity
M/Z
6e+05
4e+05
2e+05
0e+00
500
1000
1500
2000
type
b
y
b1
b2
b3
b4
b5
b6
b7
b8
y1
y2
y3
y4
y5
y6
y7
y8
y9
y10

SLC44A3 | +229.163NEEGTELQAIVR

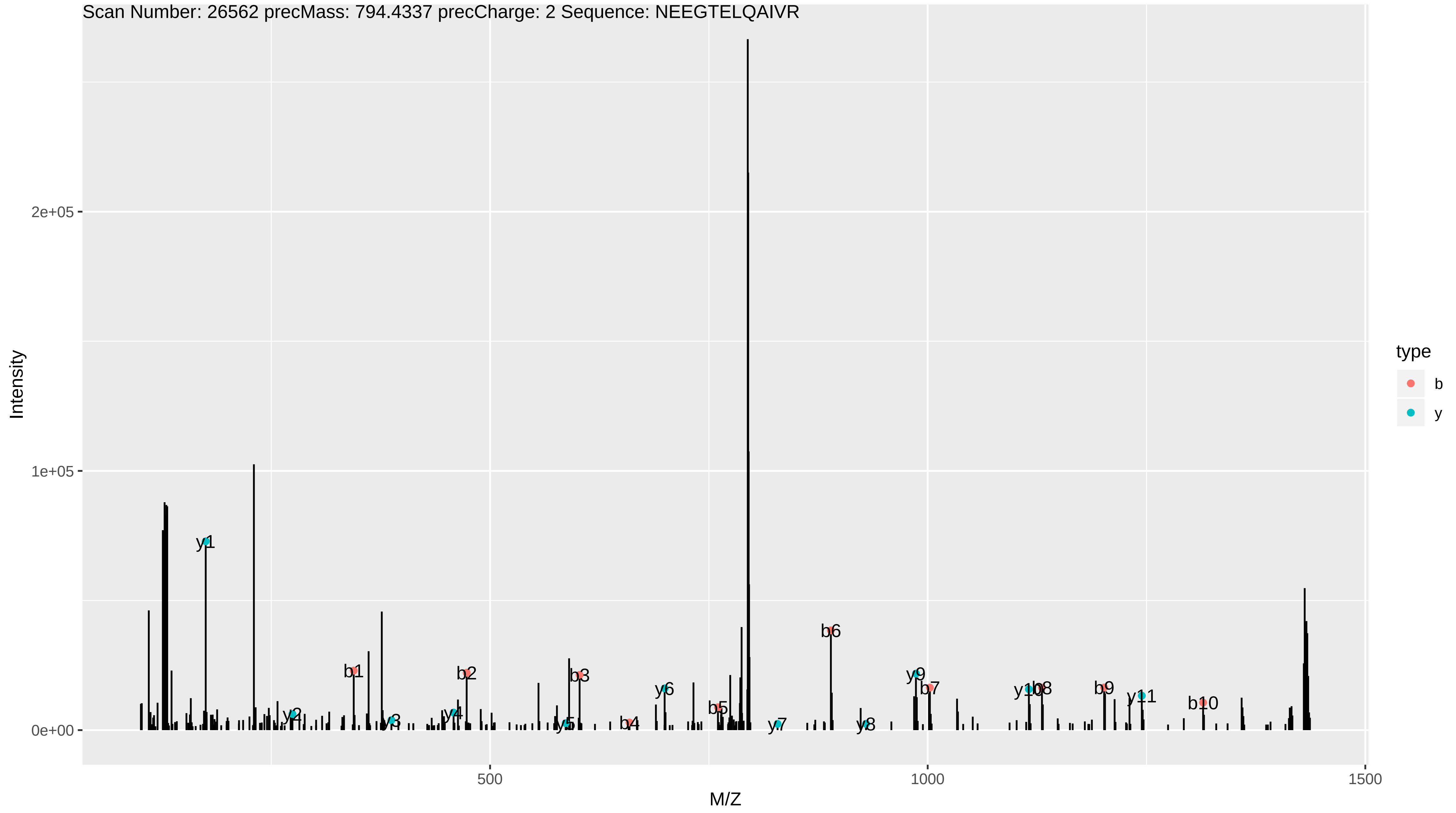

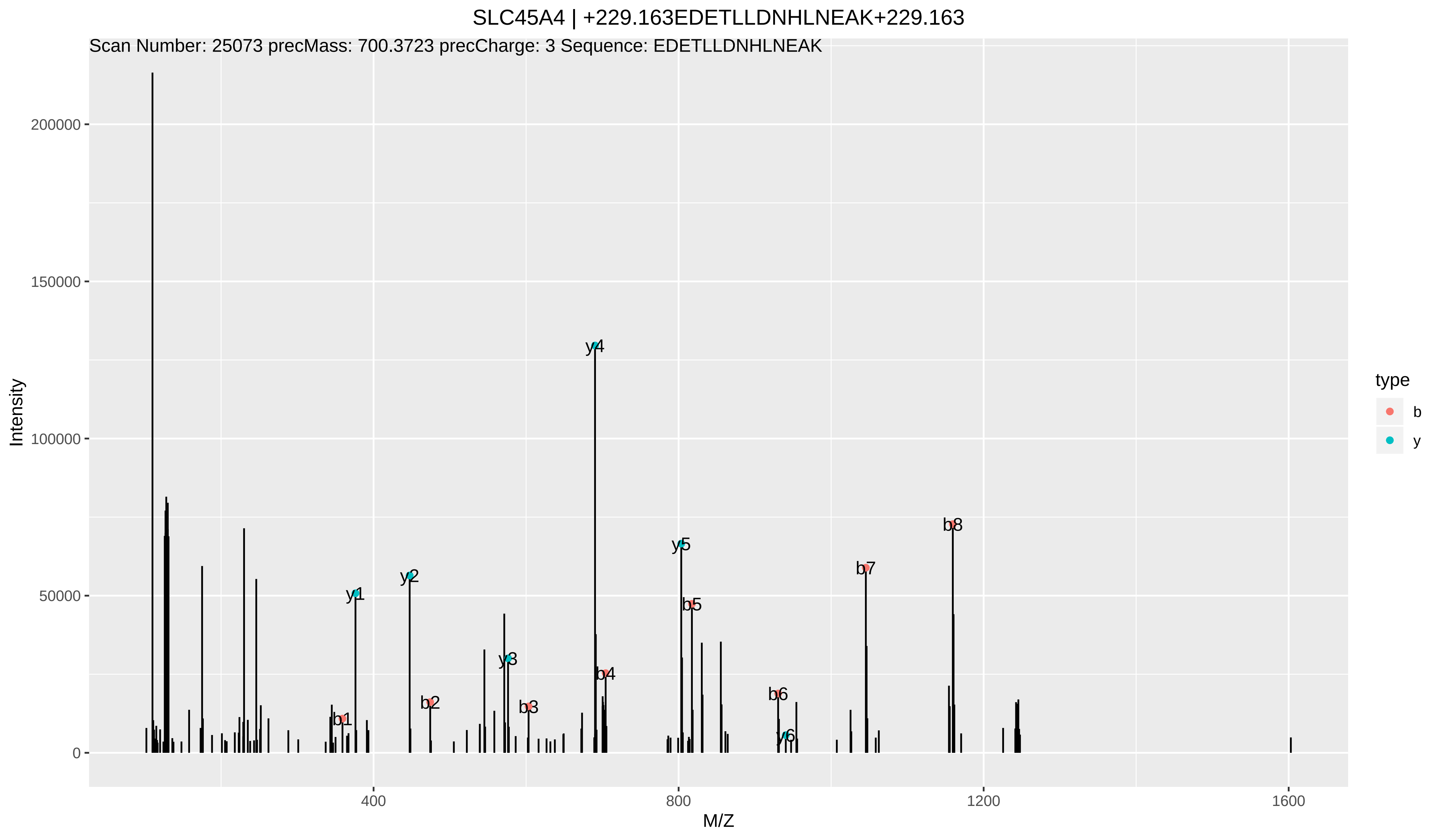
SLC45A4 | +229.163EDETLLDNHLNEAK+229.163
Scan Number: 25073 precMass: 700.3723 precCharge: 3 Sequence: EDETLLDNHLNEAK
Intensity
200000
150000
100000
50000
0
M/Z
400
800
1200
1600
y1
y2
y3
y4
y5
y6
b1
b2
b3
b4
b5
b6
b7
b8
type
b
y

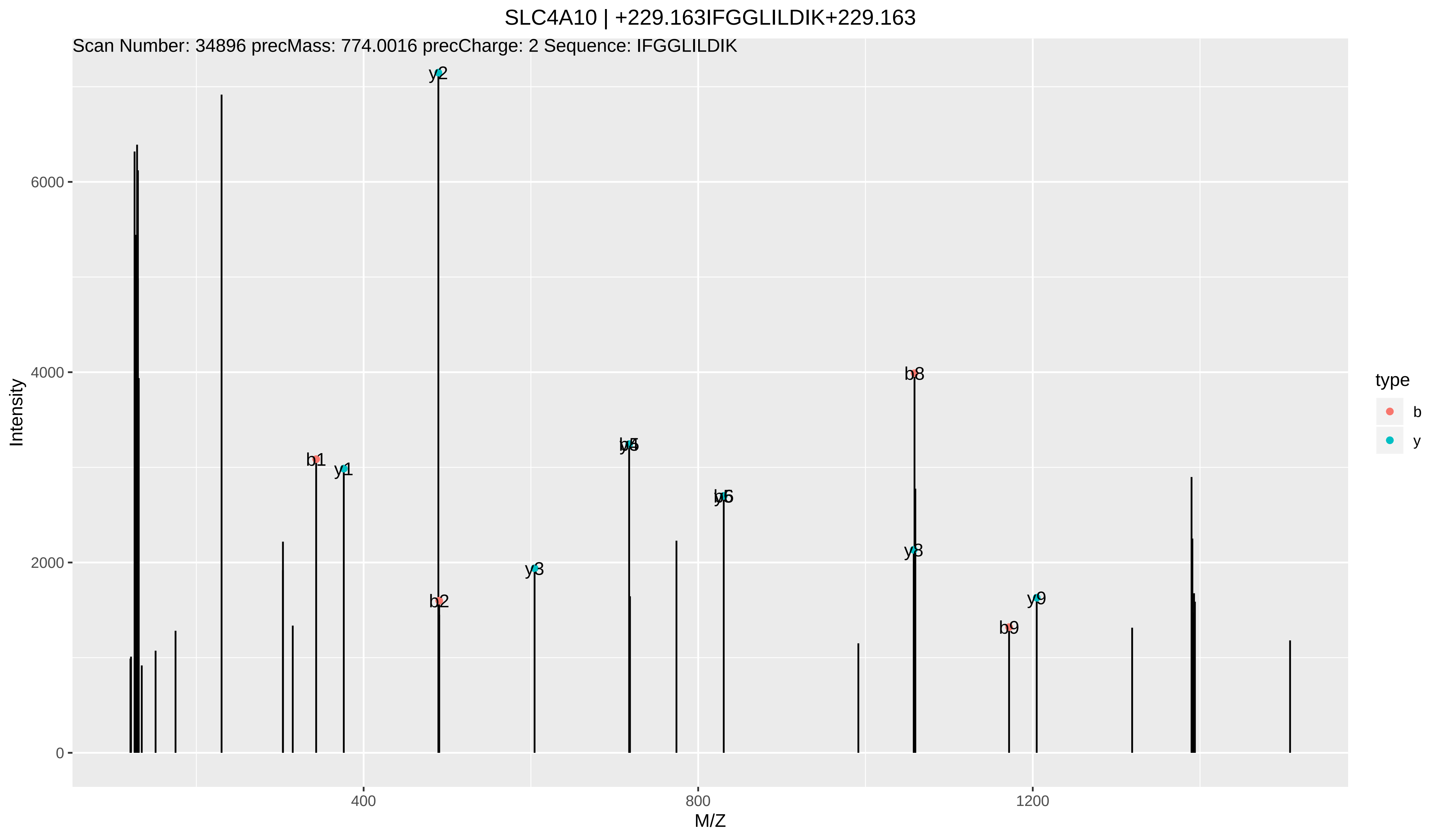
SLC4A10 | +229.163IFGGLILDIK+229.163
Scan Number: 34896 precMass: 774.0016 precCharge: 2 Sequence: IFGGLILDIK
Intensity
M/Z
type
b
y
y2
b1
y1
b2
y3
b5
y5
b6
y6
b8
y8
y9
b9
0
2000
4000
6000
400
800
1200

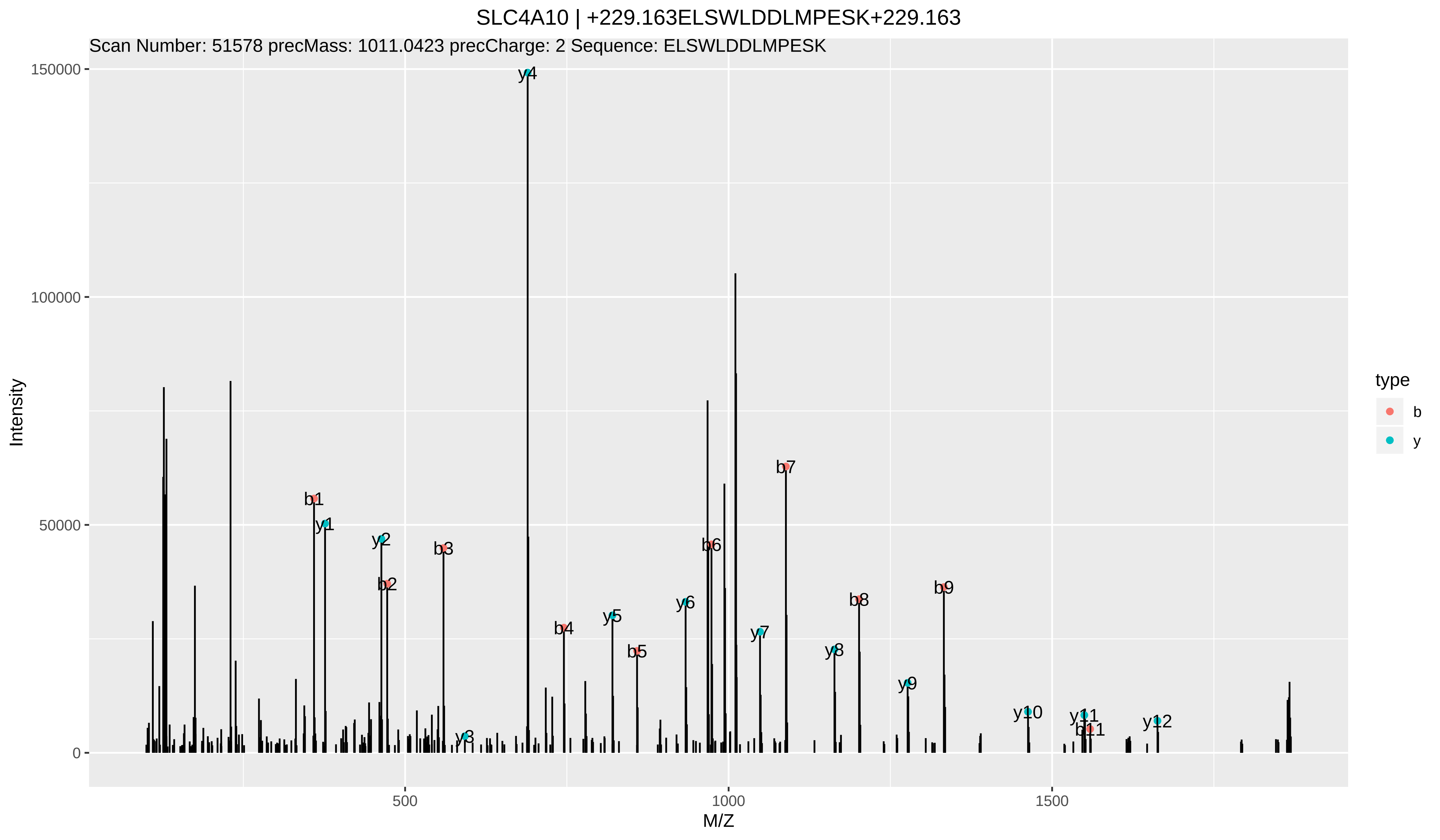
SLC4A10 | +229.163ELSWLDDLMPESK+229.163
Scan Number: 51578 precMass: 1011.0423 precCharge: 2 Sequence: ELSWLDDLMPESK
Intensity
150000
100000
50000
0
M/Z
500
1000
1500
y4
b1
y1
y2
b2
b3
y3
b4
y5
b5
y6
b6
y7
b7
y8
b8
y9
b9
y10
y11
b11
y12
type
b
y

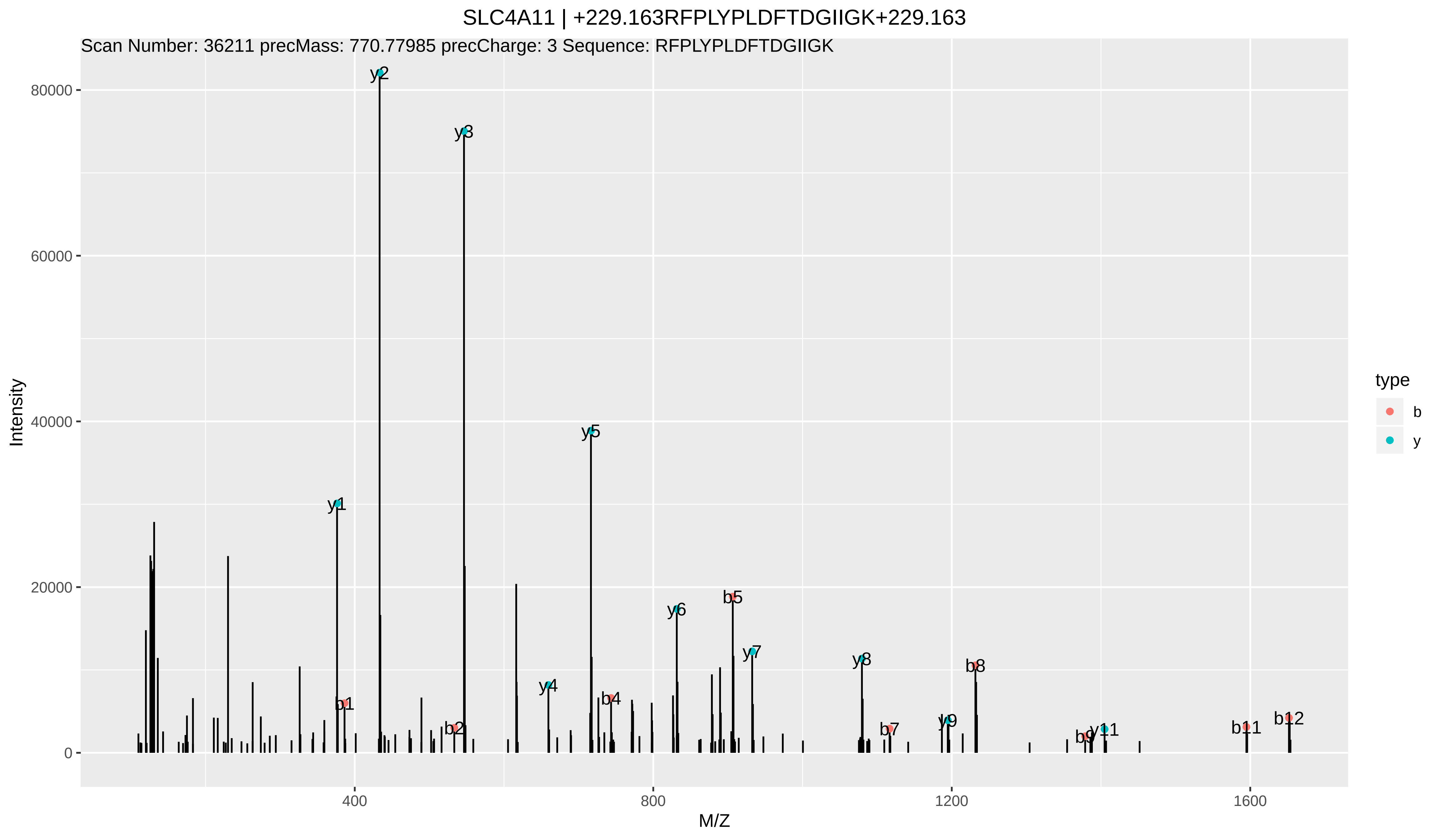
SLC4A11 | +229.163RFPLYPLDFTDGIIGK+229.163
Scan Number: 36211 precMass: 770.77985 precCharge: 3 Sequence: RFPLYPLDFTDGIIGK
Intensity
M/Z
type
b
y
y1
y2
y3
y4
y5
y6
y7
y8
y9
y11
b1
b2
b4
b5
b7
b8
b9
b11
b12
0
20000
40000
60000
80000
400
800
1200
1600

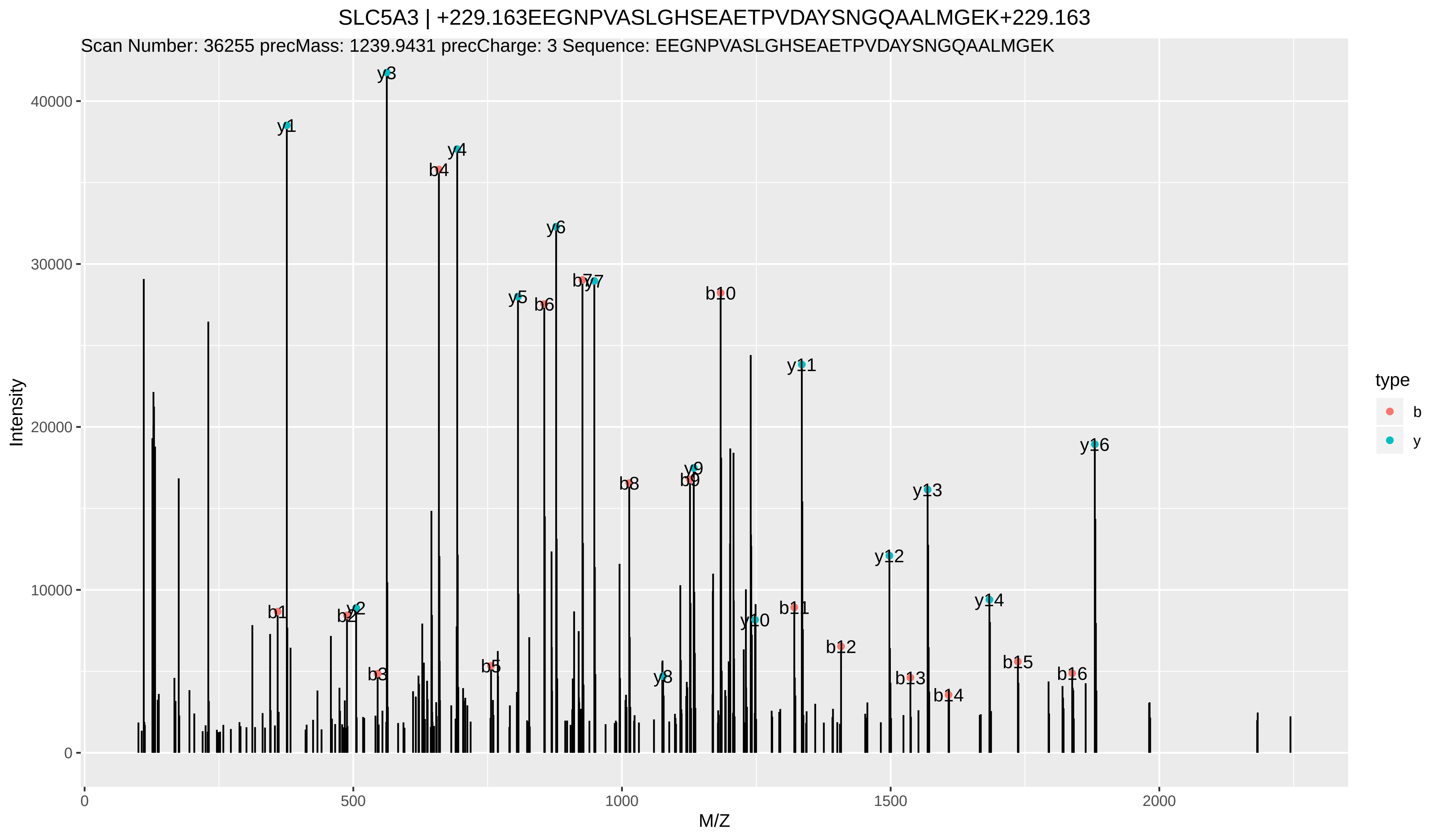

SLC5A3 | +229.163EEGNPVASLGHSEAETPVDAYSNGQAALMGEK+229.163
Scan Number: 36255 precMass: 1239.9431 precCharge: 3 Sequence: EEGNPVASLGHSEAETPVDAYSNGQAALMGEK
Intensity
M/Z
type
b
y
y1
y2
y3
y4
y5
y6
y7
y8
y9
y10
y11
y12
y13
y14
y16
b1
b2
b3
b4
b5
b6
b7
b8
b9
b10
b11
b12
b13
b14
b15
b16
0
10000
20000
30000
40000
500
1000
1500
2000

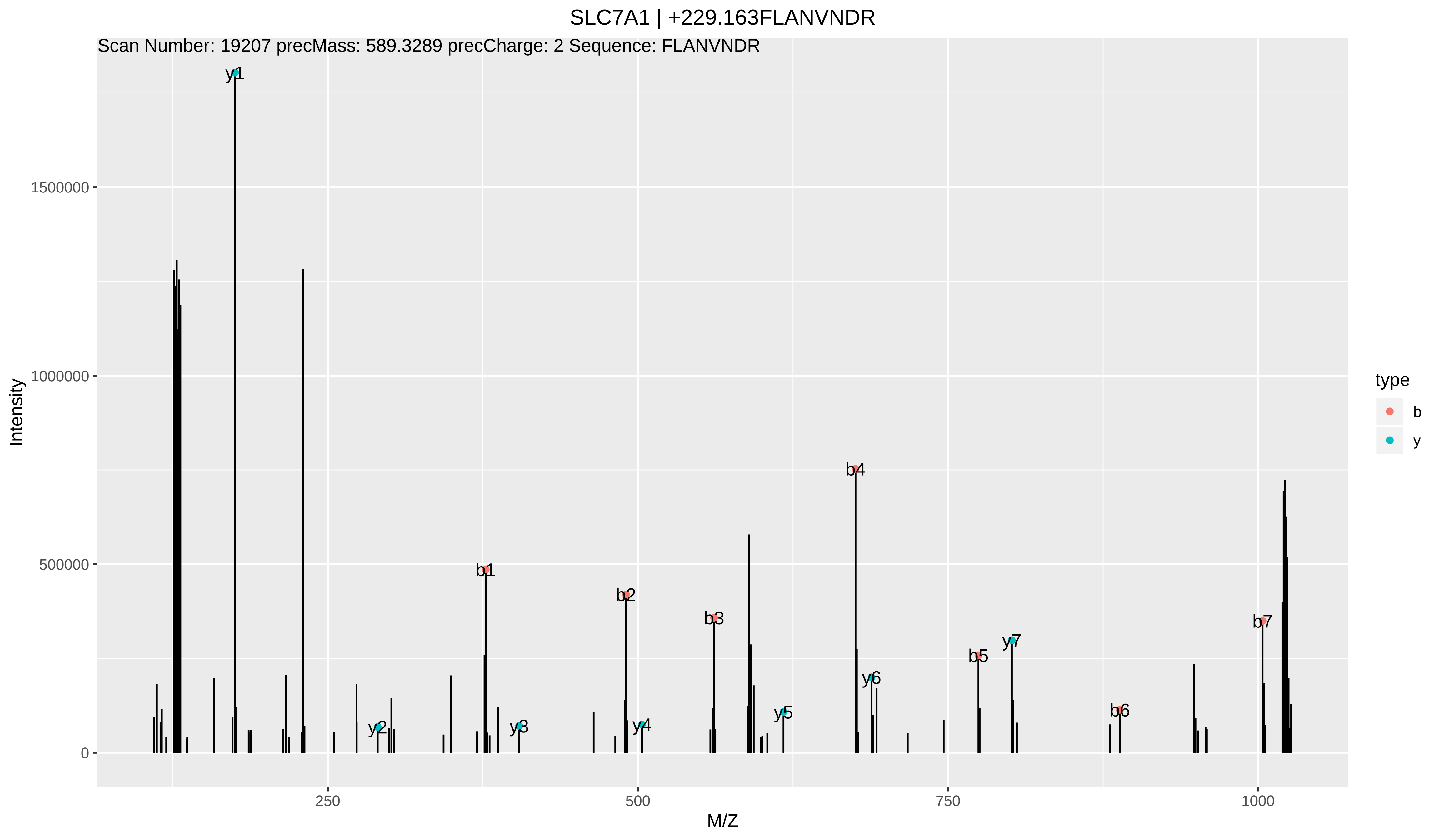
SLC7A1 | +229.163FLANVNDR
Scan Number: 19207 precMass: 589.3289 precCharge: 2 Sequence: FLANVNDR
Intensity
M/Z
type
b
y
y1
b1
y2
y3
b2
y4
b3
y5
b4
y6
b5
y7
b6
b7
0
500000
1000000
1500000
250
500
750
1000

SLC7A1 | +229.163VIYAM+15.995AEDGLLFK+229.163

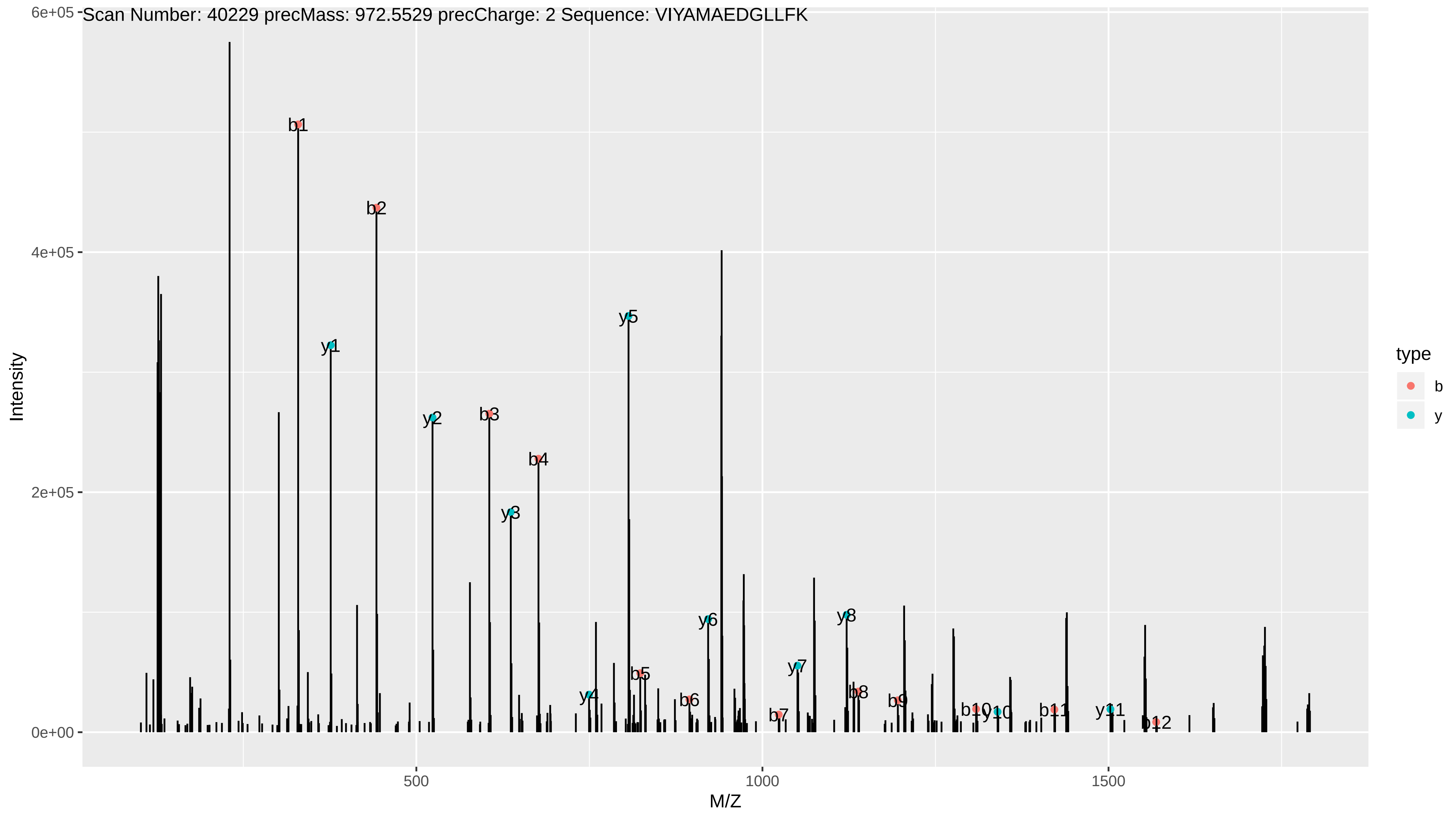

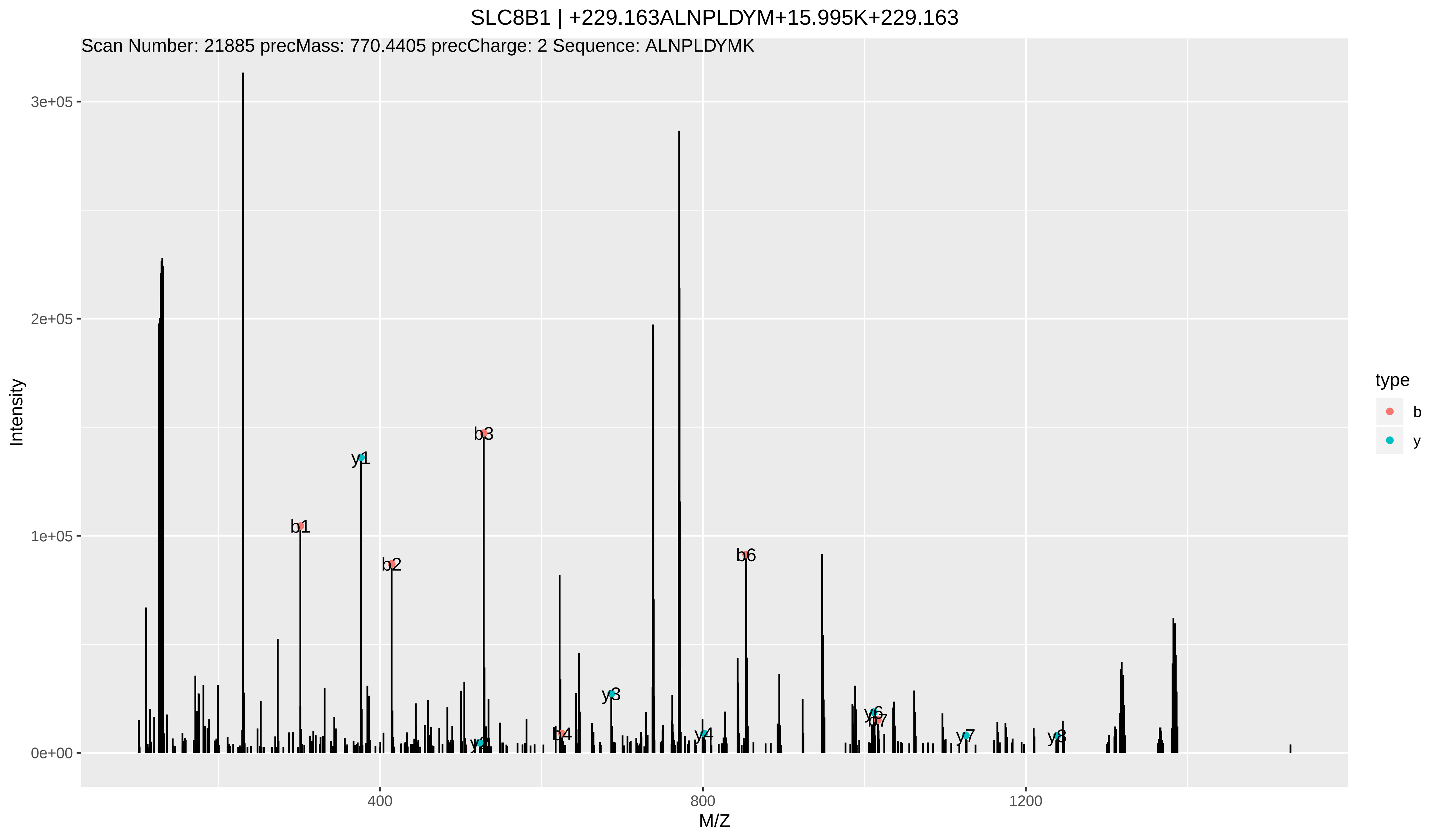
SLC8B1 | +229.163ALNPLDYM+15.995K+229.163
Scan Number: 21885 precMass: 770.4405 precCharge: 2 Sequence: ALNPLDYMK
Intensity
3e+05
2e+05
1e+05
0e+00
M/Z
400
800
1200
b1
b2
b3
b4
b6
b7
y1
y3
y4
y6
y7
y8
type
b
y

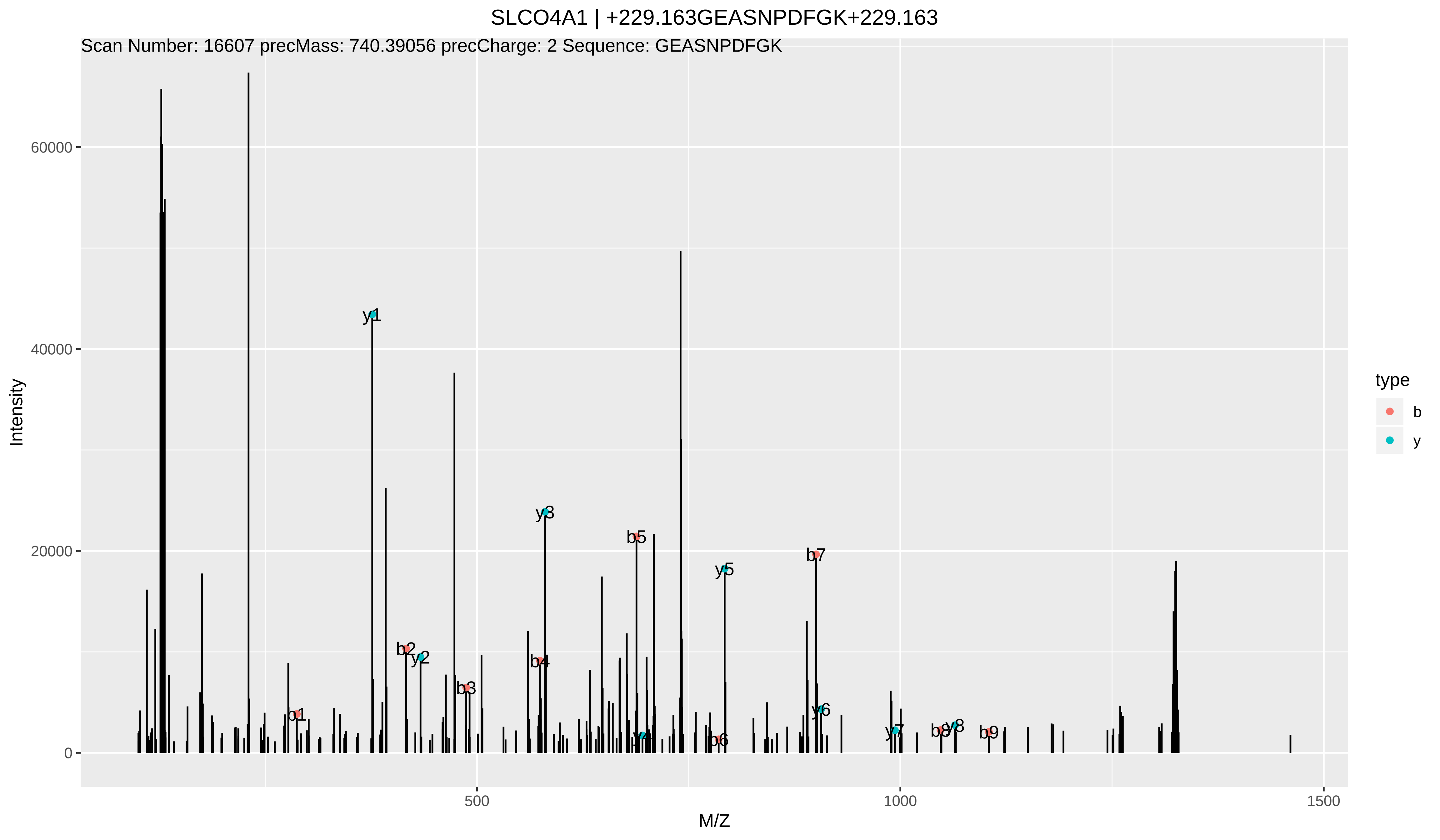
SLCO4A1 | +229.163GEASNPDFGK+229.163
Scan Number: 16607 precMass: 740.39056 precCharge: 2 Sequence: GEASNPDFGK
Intensity
60000
40000
20000
0
500
1000
1500
M/Z
type
b
y
y1
b2
y2
b3
y3
b4
b5
y5
b6
b7
y6
y7
b8
y8
b9
b1

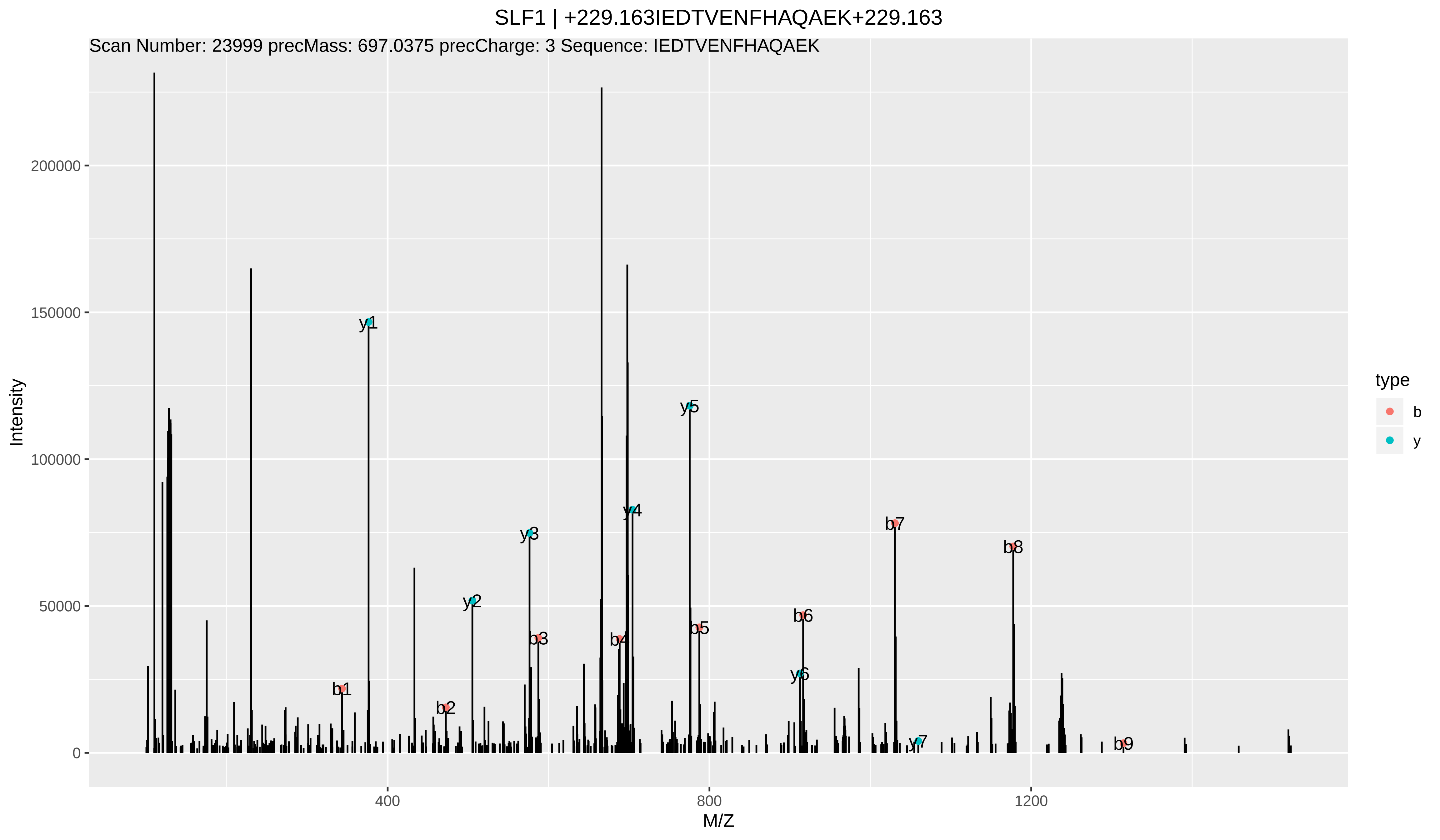
SLF1 | +229.163IEDTVENFHAQAEK+229.163
Scan Number: 23999 precMass: 697.0375 precCharge: 3 Sequence: IEDTVENFHAQAEK
Intensity
200000
150000
100000
50000
0
M/Z
400
800
1200
y1
y2
y3
y4
y5
y6
y7
b1
b2
b3
b4
b5
b6
b7
b8
b9
type
b
y

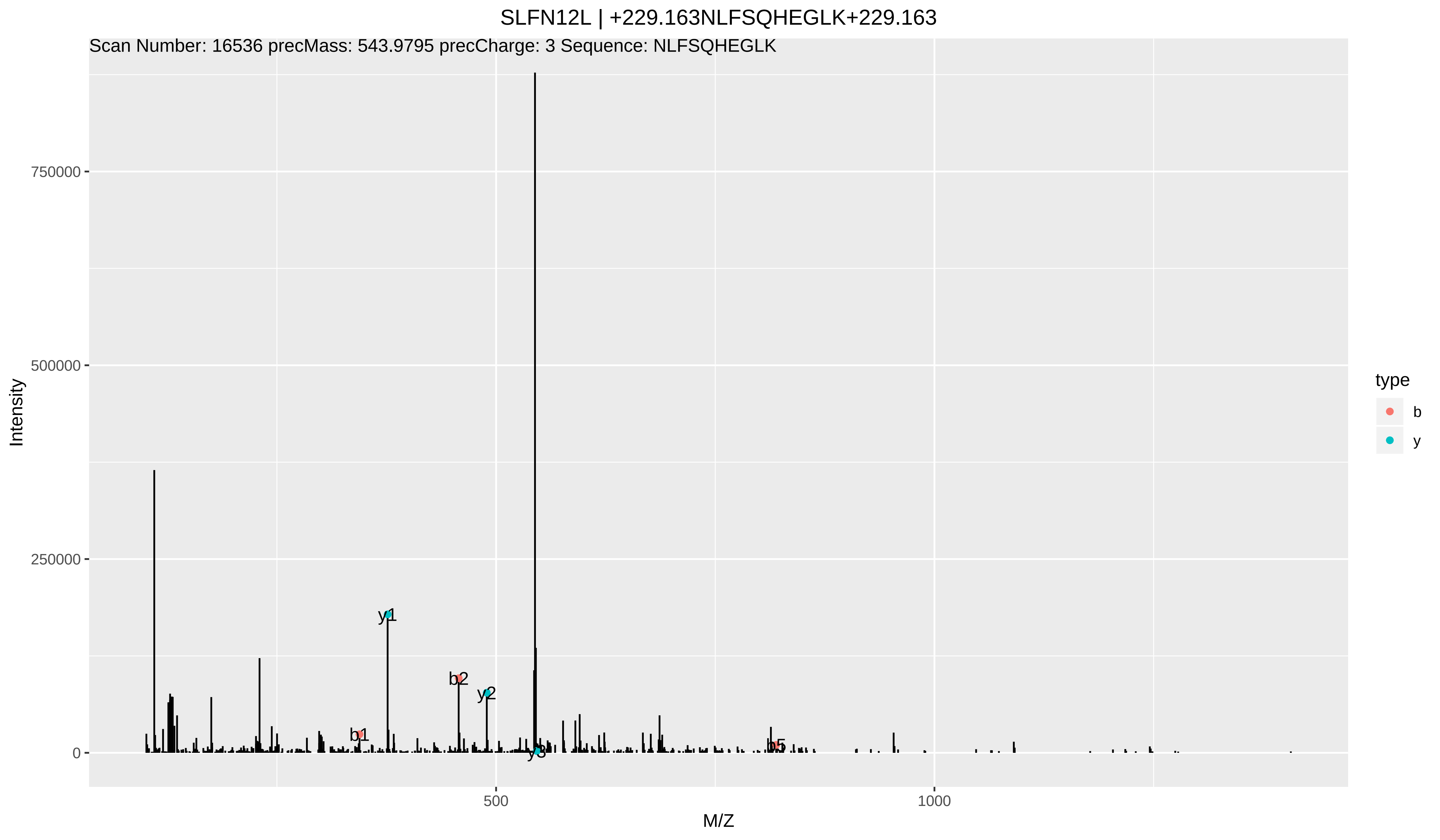

SLFN12L | +229.163NLFSQHEGLK+229.163
Scan Number: 16536 precMass: 543.9795 precCharge: 3 Sequence: NLFSQHEGLK
Intensity
750000
500000
250000
0
M/Z
500
1000
y1
b2
y2
b1
y3
b5
type
b
y

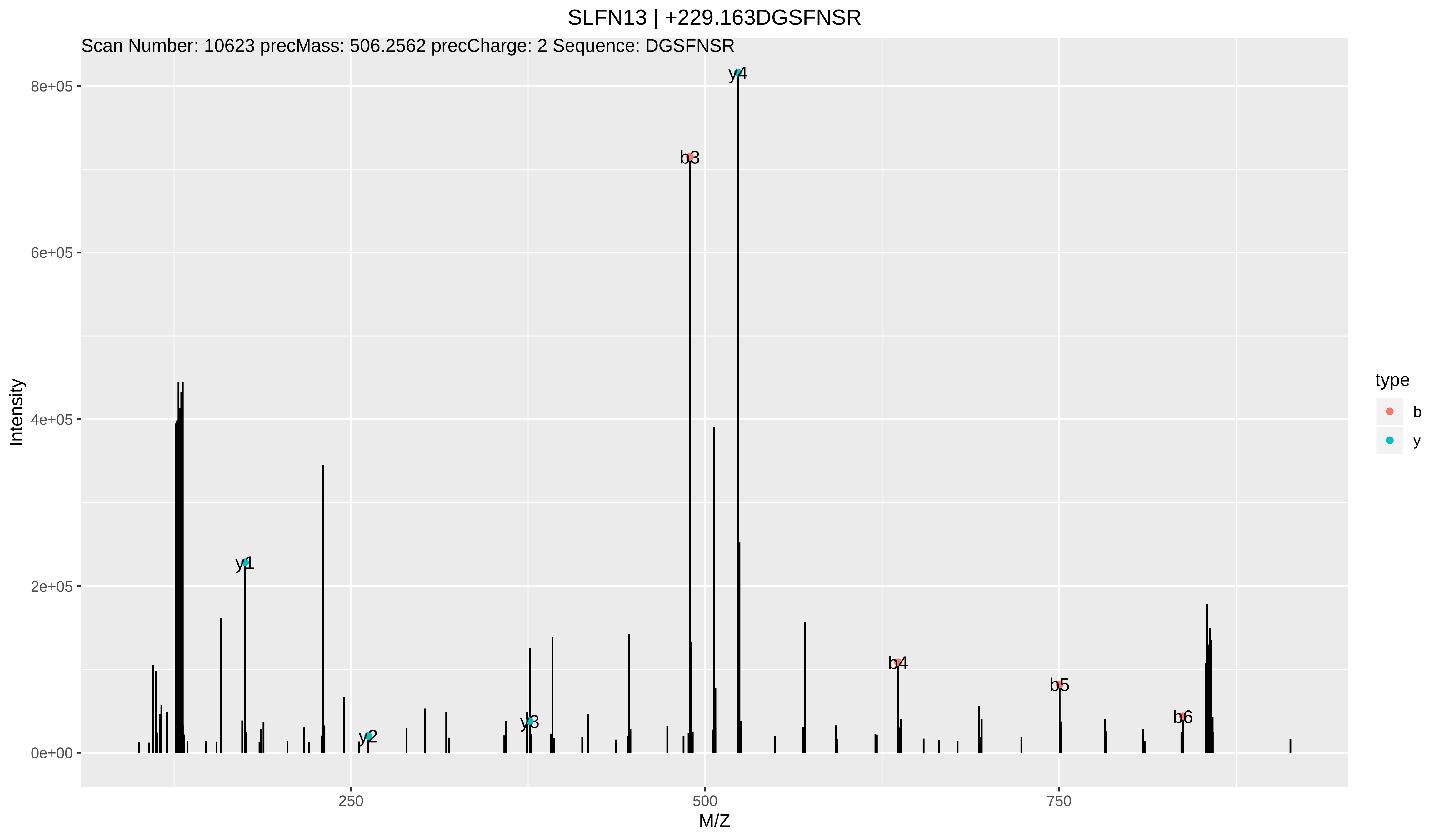
SLFN13 | +229.163DGSFNSR
Scan Number: 10623 precMass: 506.2562 precCharge: 2 Sequence: DGSFNSR
Intensity
8e+05
6e+05
4e+05
2e+05
0e+00
M/Z
250
500
750
y1
y2
y3
b3
y4
b4
b5
b6
type
b
y

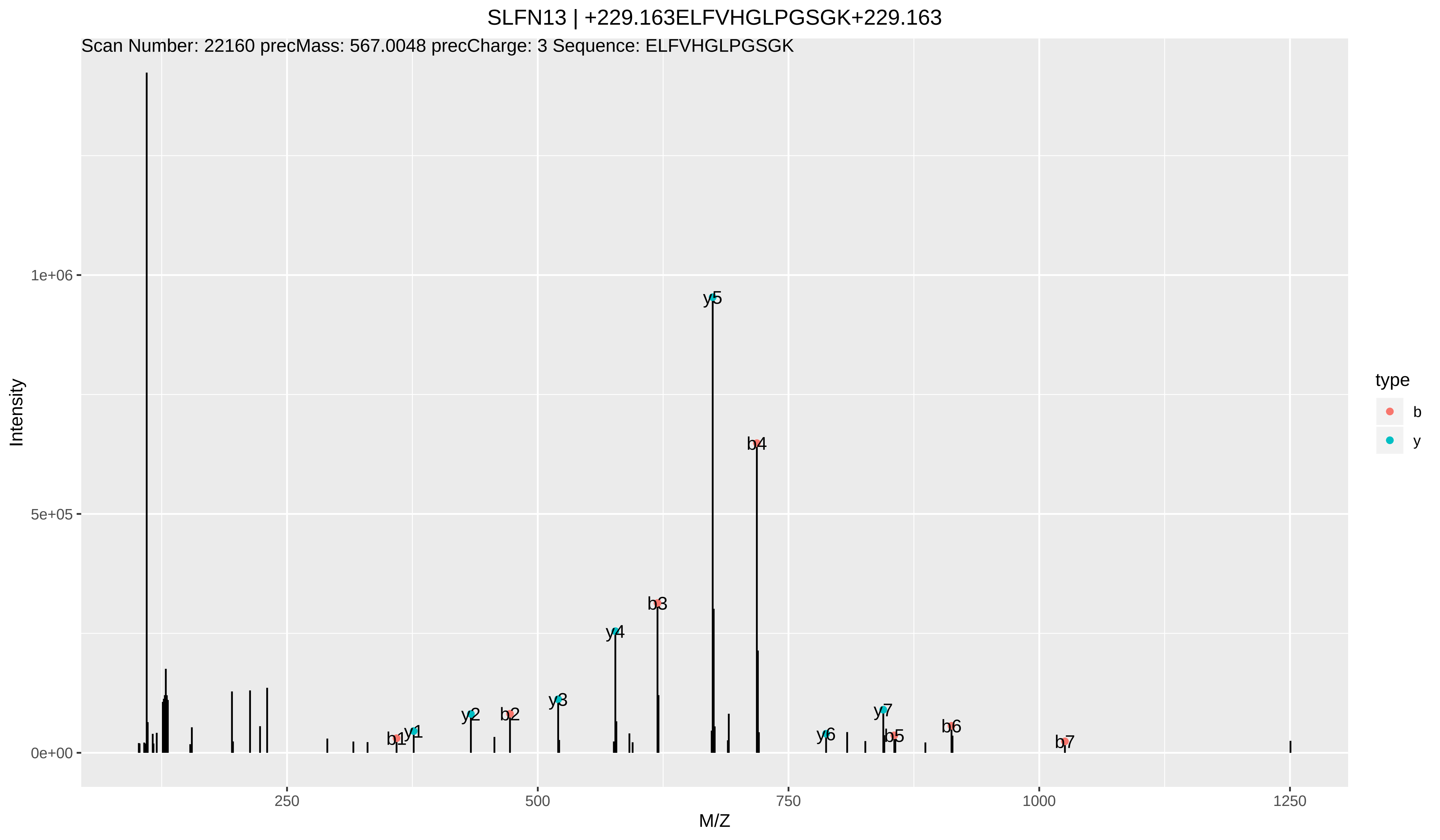
SLFN13 | +229.163ELFVHGLPGSGK+229.163
Scan Number: 22160 precMass: 567.0048 precCharge: 3 Sequence: ELFVHGLPGSGK
Intensity
0e+00
5e+05
1e+06
M/Z
250
500
750
1000
1250
y5
b4
b3
y4
y3
y2
b2
b1
y1
y6
y7
b5
b6
b7
type
b
y

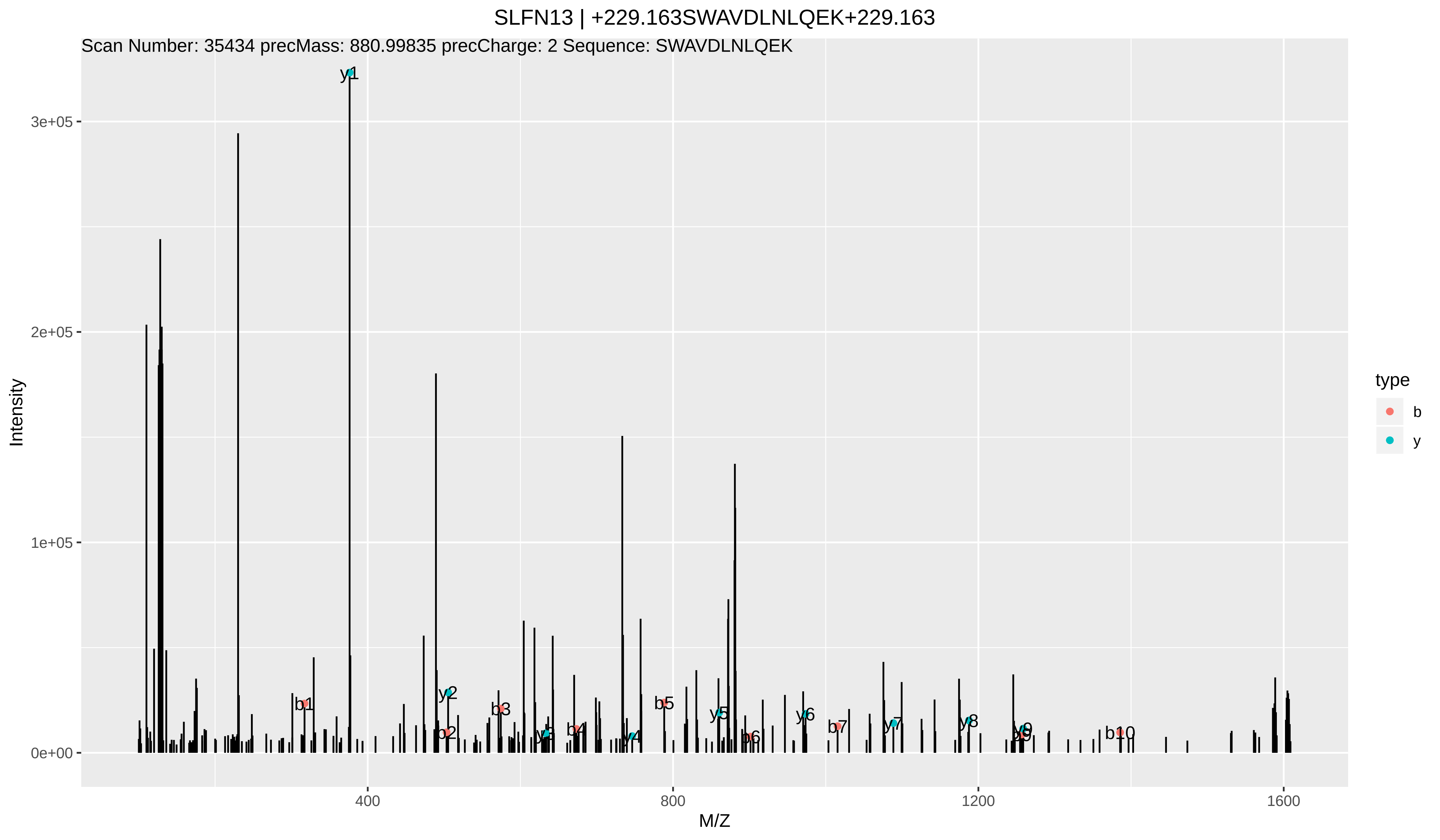

SLFN13 | +229.163SWAVDLNLQEK+229.163
Scan Number: 35434 precMass: 880.99835 precCharge: 2 Sequence: SWAVDLNLQEK
Intensity
M/Z
3e+05
2e+05
1e+05
0e+00
400
800
1200
1600
y1
b1
y2
b2
b3
y3
b4
y4
b5
y5
b6
y6
b7
y7
y8
y9
b9
b10
type
b
y

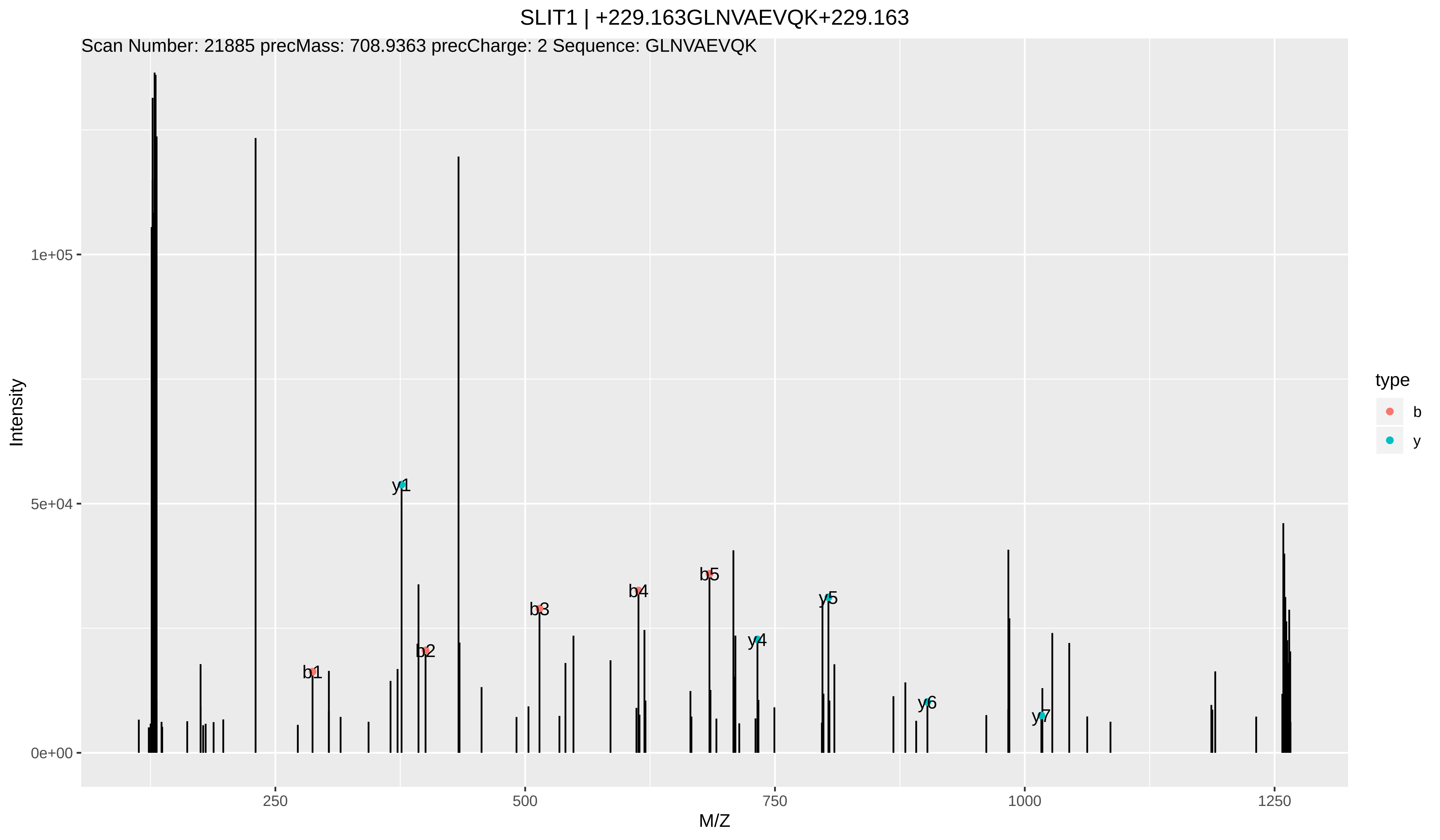

SLIT1 | +229.163GLNVAEVQK+229.163
Scan Number: 21885 precMass: 708.9363 precCharge: 2 Sequence: GLNVAEVQK
Intensity
1e+05
5e+04
0e+00
M/Z
250
500
750
1000
1250
y1
b1
b2
b3
b4
b5
y4
y5
y6
y7
type
b
y

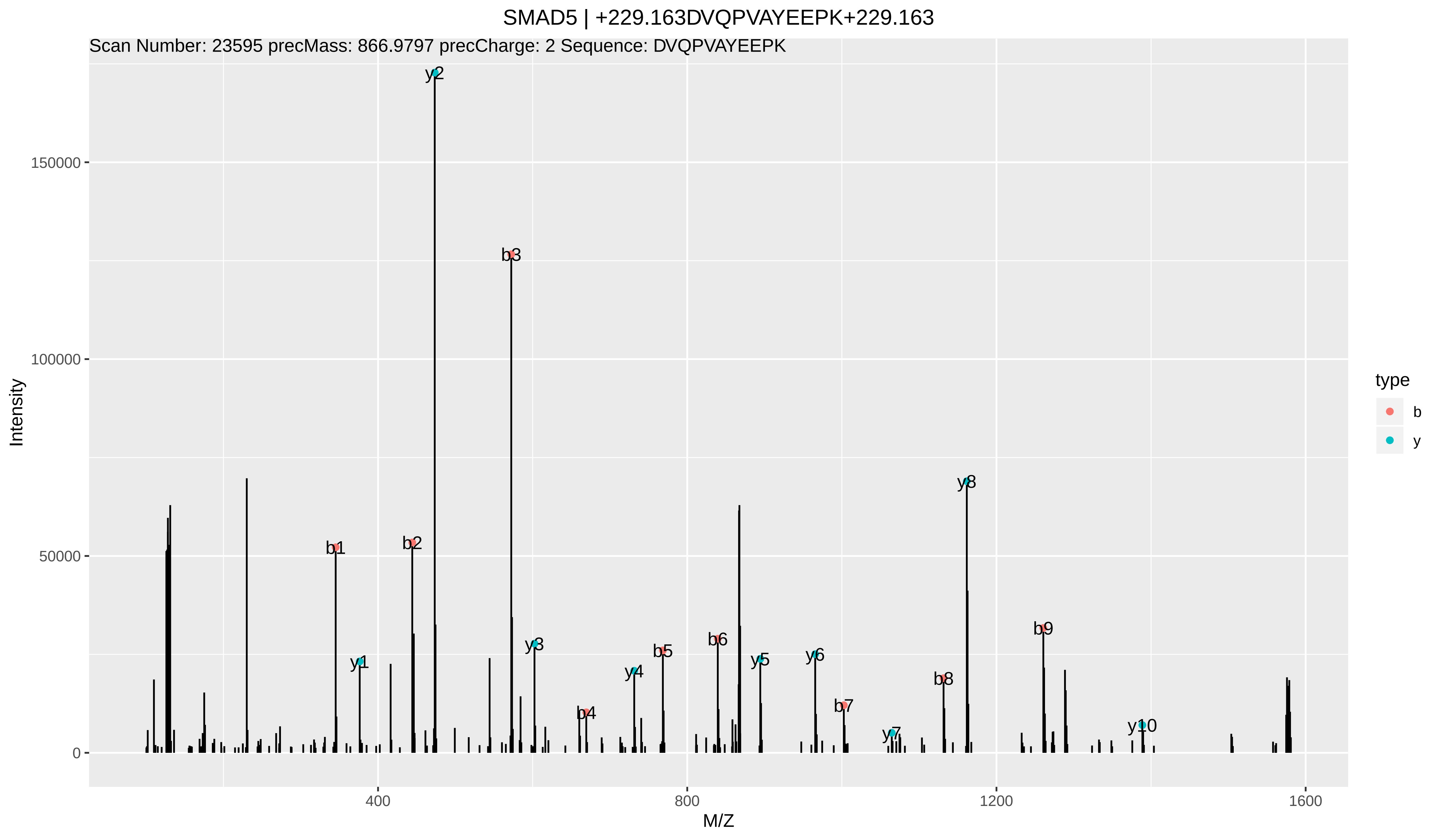
SMAD5 | +229.163DVQPVAYEEPK+229.163
Scan Number: 23595 precMass: 866.9797 precCharge: 2 Sequence: DVQPVAYEEPK
Intensity
M/Z
type
b
y
y2
b3
y8
b1
b2
b9
y3
b6
b5
y1
y5
y6
y4
b8
b7
b4
y10
y7
0
50000
100000
150000
400
800
1200
1600

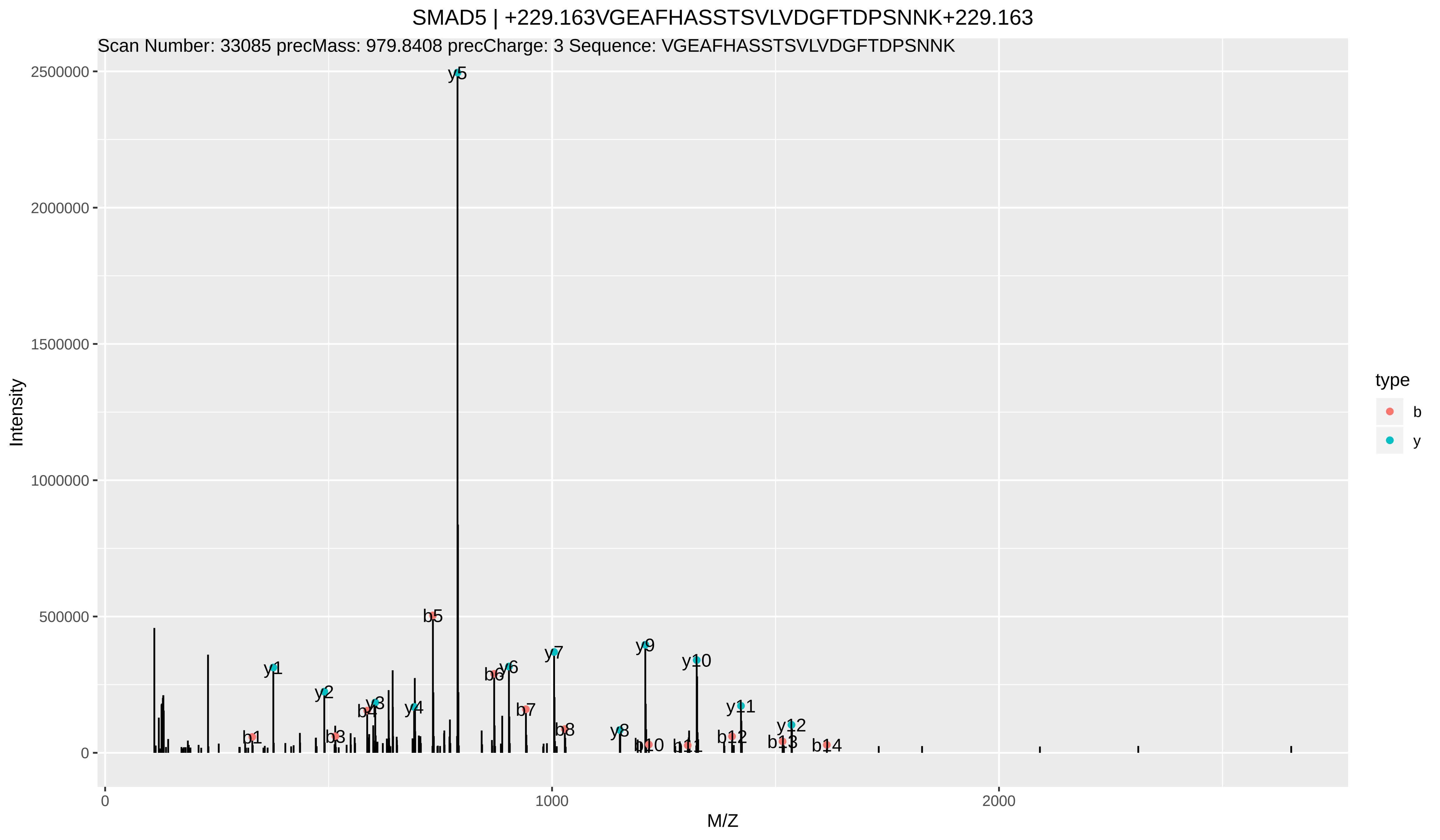

SMAD5 | +229.163VGEAFHASSTSVLVDGFTDPSNNK+229.163
Scan Number: 33085 precMass: 979.8408 precCharge: 3 Sequence: VGEAFHASSTSVLVDGFTDPSNNK
Intensity
M/Z
type
b
y

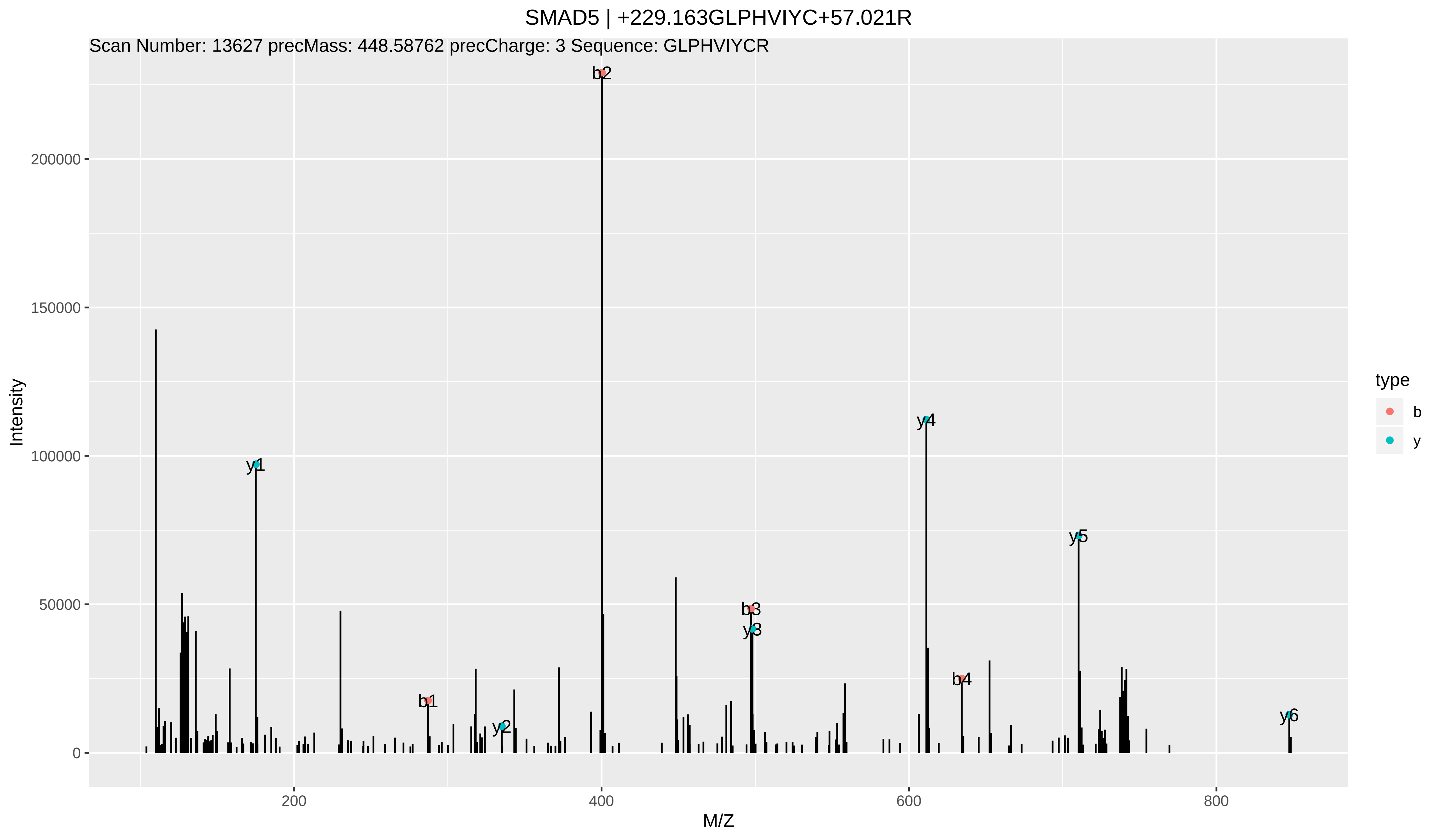
SMAD5 | +229.163GLPHVIYC+57.021R
Scan Number: 13627 precMass: 448.58762 precCharge: 3 Sequence: GLPHVIYCR
Intensity
M/Z
type
b
y
b1
b2
b3
b4
y1
y2
y3
y4
y5
y6
0
50000
100000
150000
200000
200
400
600
800

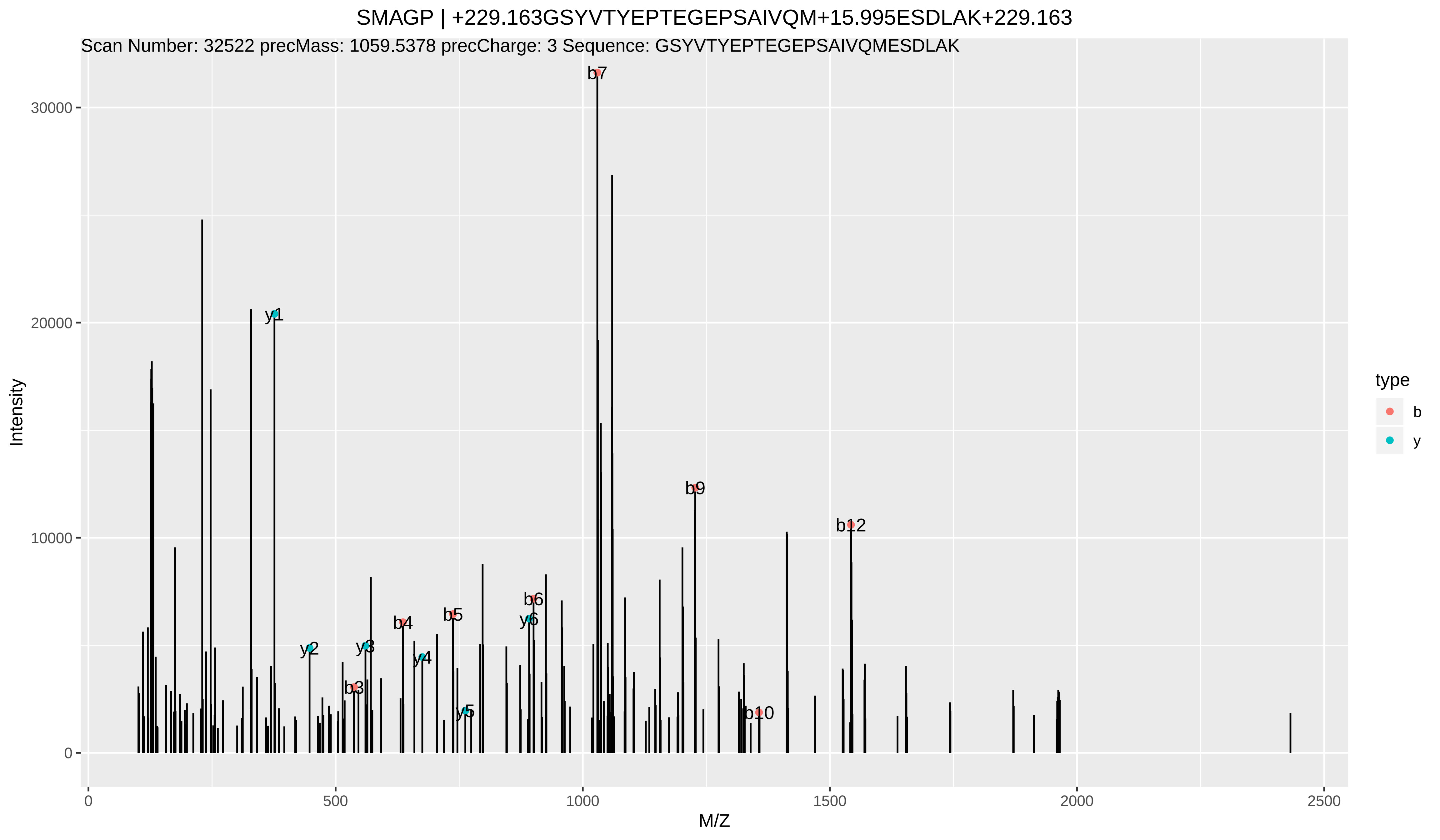
SMAGP | +229.163GSYVTYEPTEGEPSAIVQM+15.995ESDLAK+229.163
Scan Number: 32522 precMass: 1059.5378 precCharge: 3 Sequence: GSYVTYEPTEGEPSAIVQMESDLAK
Intensity
M/Z
0
10000
20000
30000
0
500
1000
1500
2000
2500
b7
y1
b9
b12
b6
y6
b5
b4
y2
y3
y4
b3
y5
b10
type
b
y

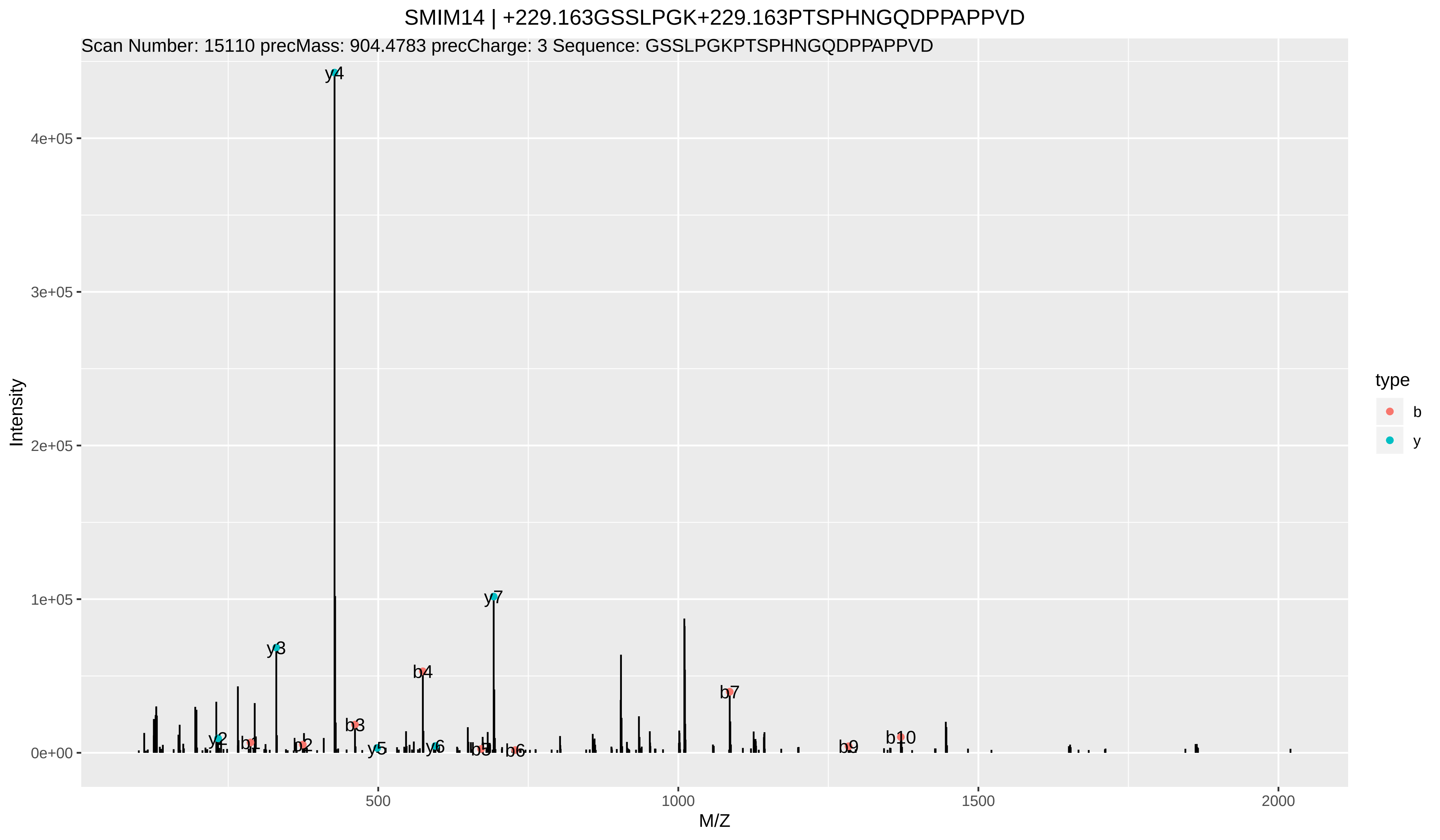
SMIM14 | +229.163GSSLPGK+229.163PTSPHNGQDPPAPPVD
Scan Number: 15110 precMass: 904.4783 precCharge: 3 Sequence: GSSLPGKPTSPHNGQDPPAPPVD
y4
y7
y3
b4
b7
b3
y2
b1
b2
y5
y6
b5
b6
b9
b10
Intensity
M/Z
type
b
y

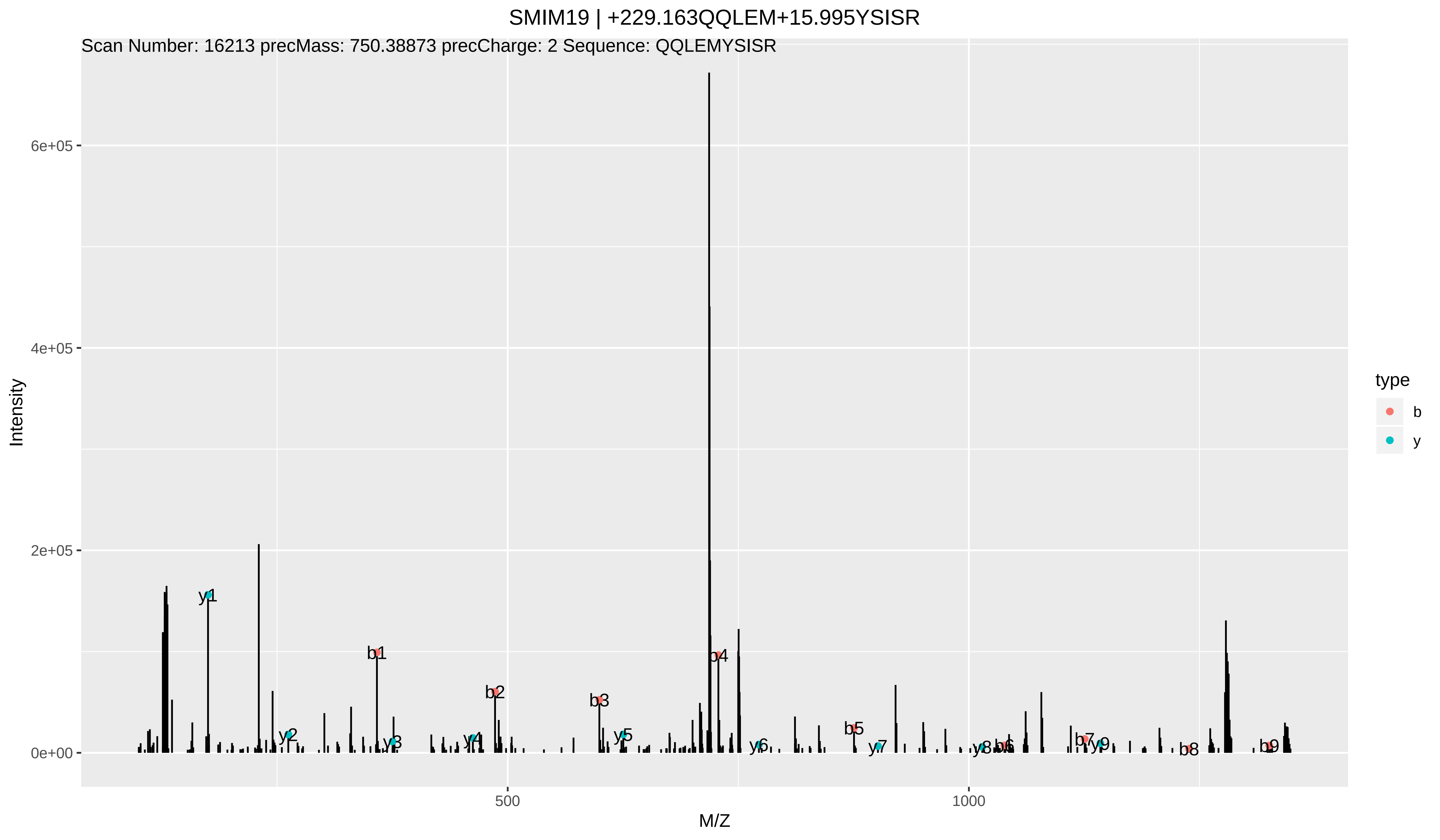
SMIM19 | +229.163QQLEM+15.995YSISR
Scan Number: 16213 precMass: 750.38873 precCharge: 2 Sequence: QQLEMYSISR
Intensity
6e+05
4e+05
2e+05
0e+00
M/Z
500
1000
y1
y2
y3
y4
y5
y6
y7
y8
y9
b1
b2
b3
b4
b5
b6
b7
b8
b9
type
b
y

SMIM20 | +229.163AGIVQEDVQPPGLK+229.163

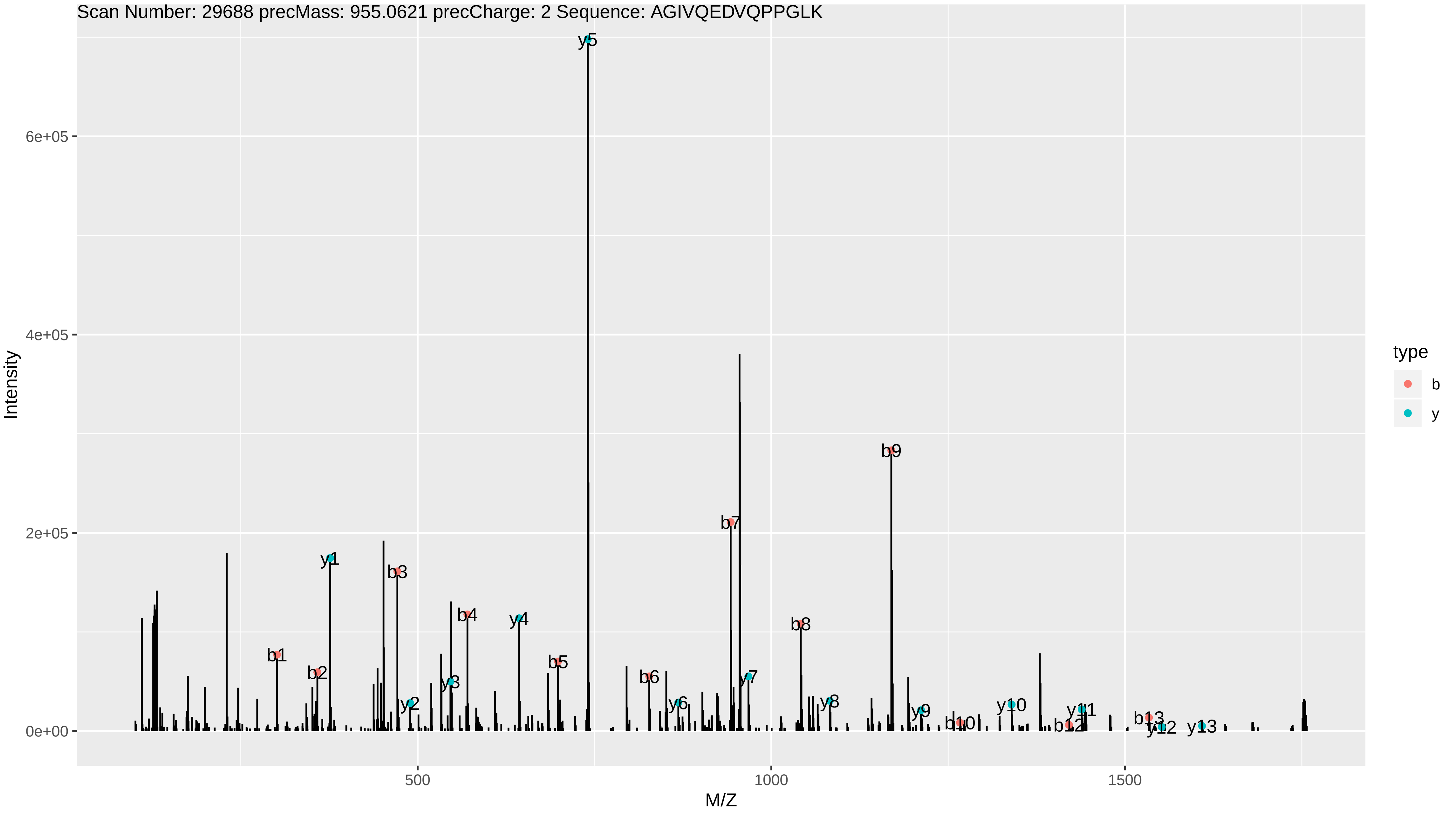

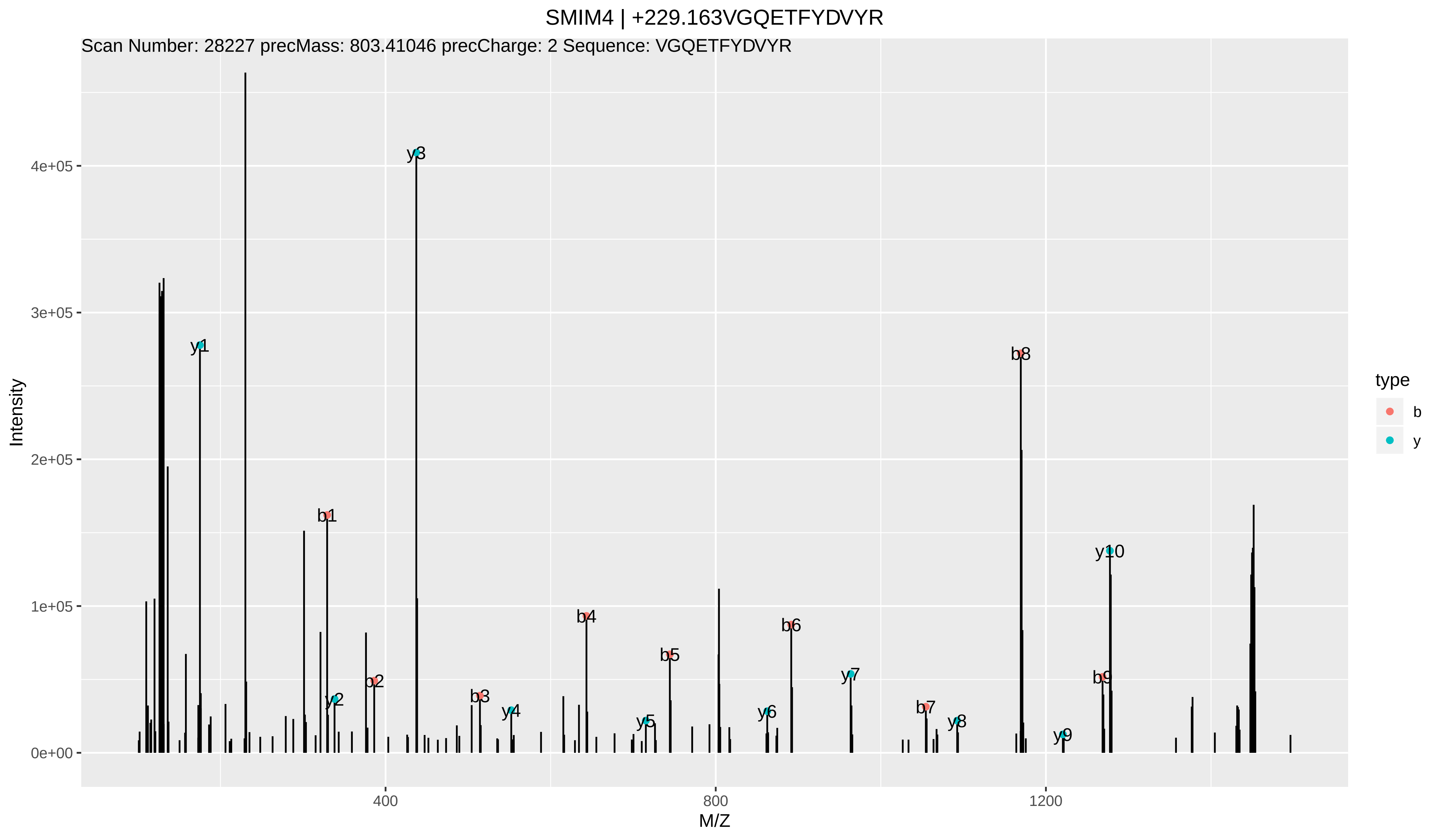
SMIM4 | +229.163VGQETFYDVYR
Scan Number: 28227 precMass: 803.41046 precCharge: 2 Sequence: VGQETFYDVYR
Intensity
0e+00
1e+05
2e+05
3e+05
4e+05
M/Z
400
800
1200
y1
y2
y3
y4
y5
y6
y7
y8
y9
y10
b1
b2
b3
b4
b5
b6
b7
b8
b9
type
b
y

SMIM8 | +229.163AVNPELFIK+229.163

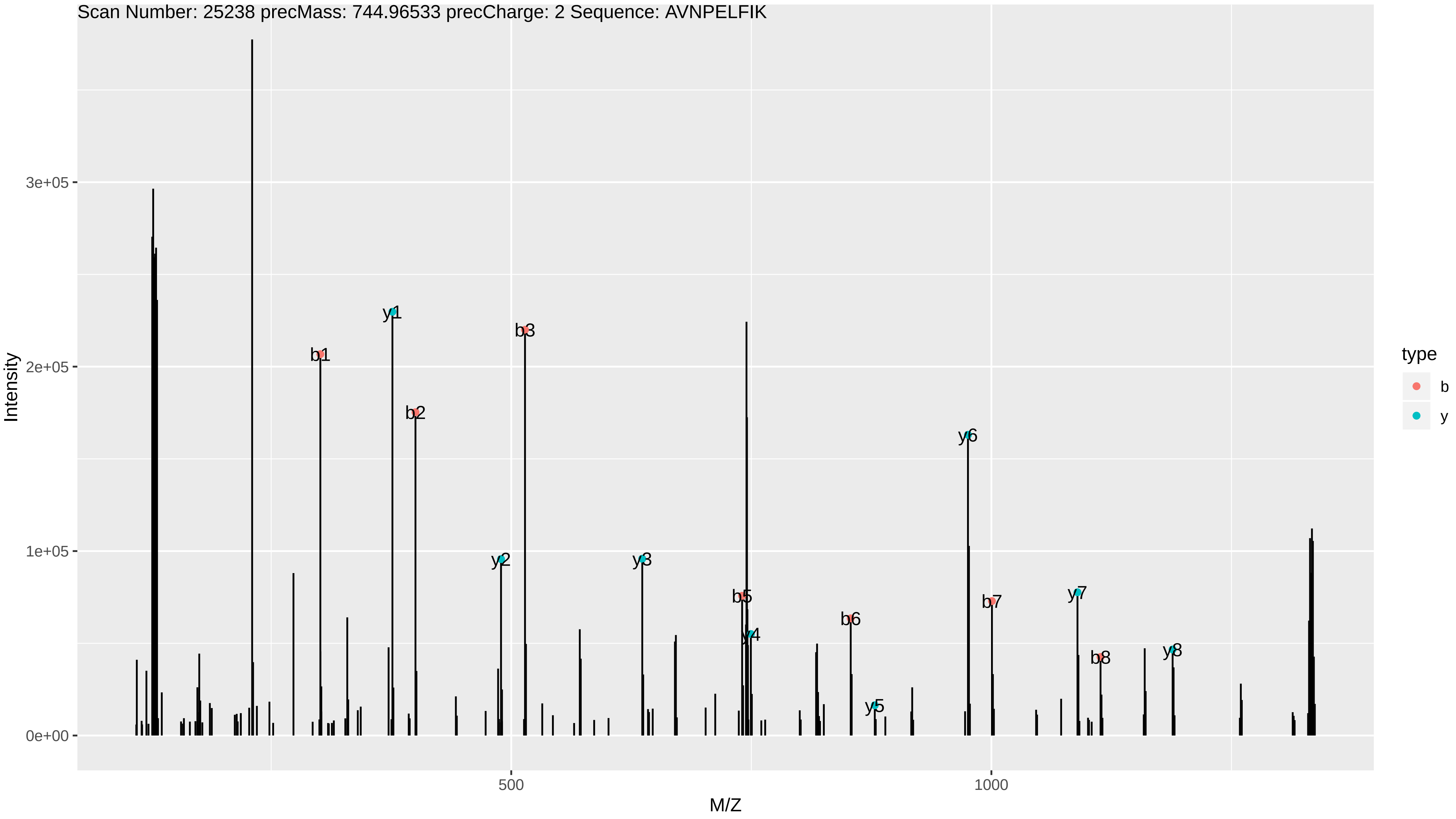

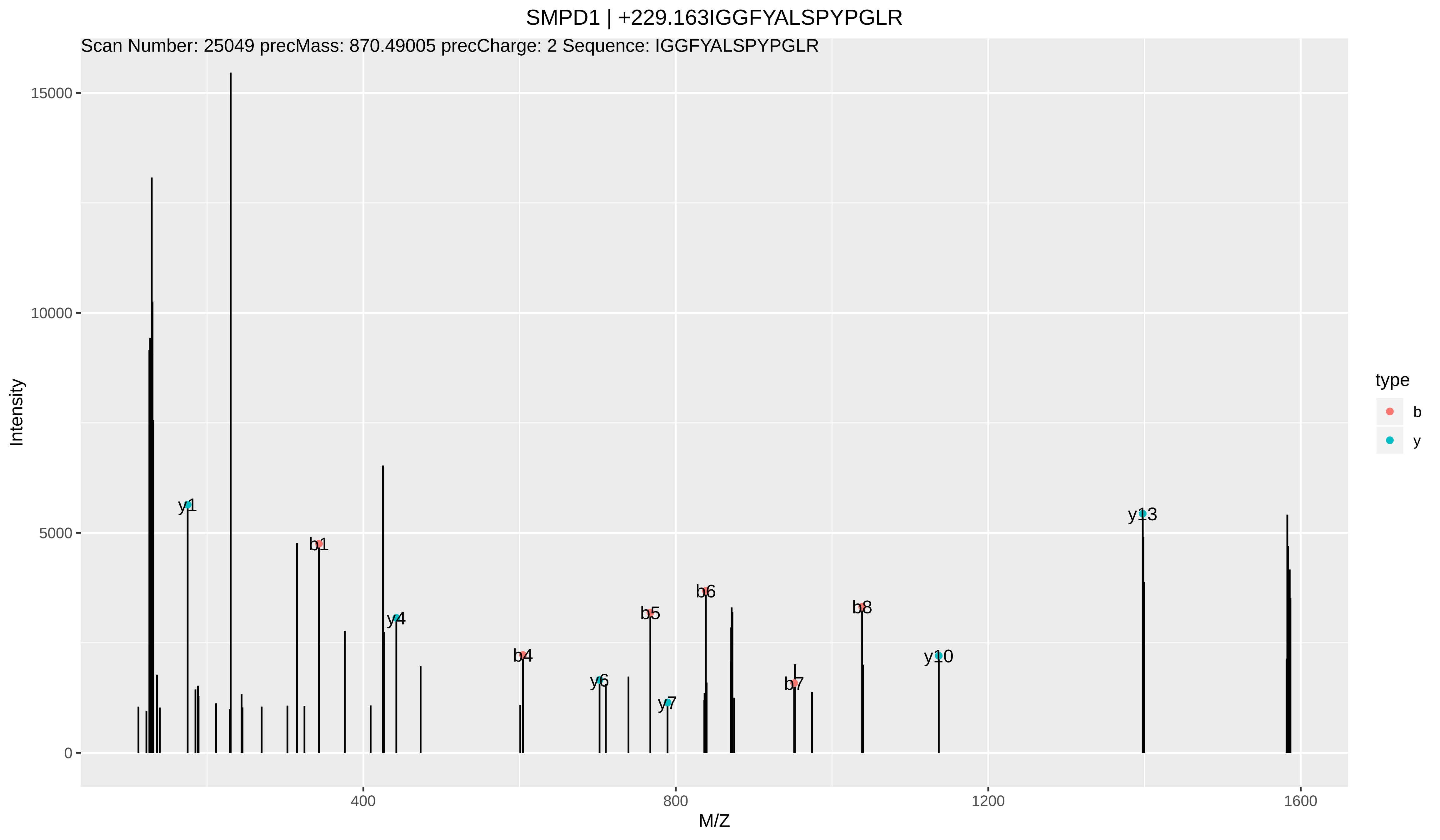

SMPD1 | +229.163IGGFYALSPYPGLR
Scan Number: 25049 precMass: 870.49005 precCharge: 2 Sequence: IGGFYALSPYPGLR
Intensity
15000
10000
5000
0
M/Z
400
800
1200
1600
y1
b1
y4
b4
y6
b5
y7
b6
b7
b8
y10
y13
type
b
y

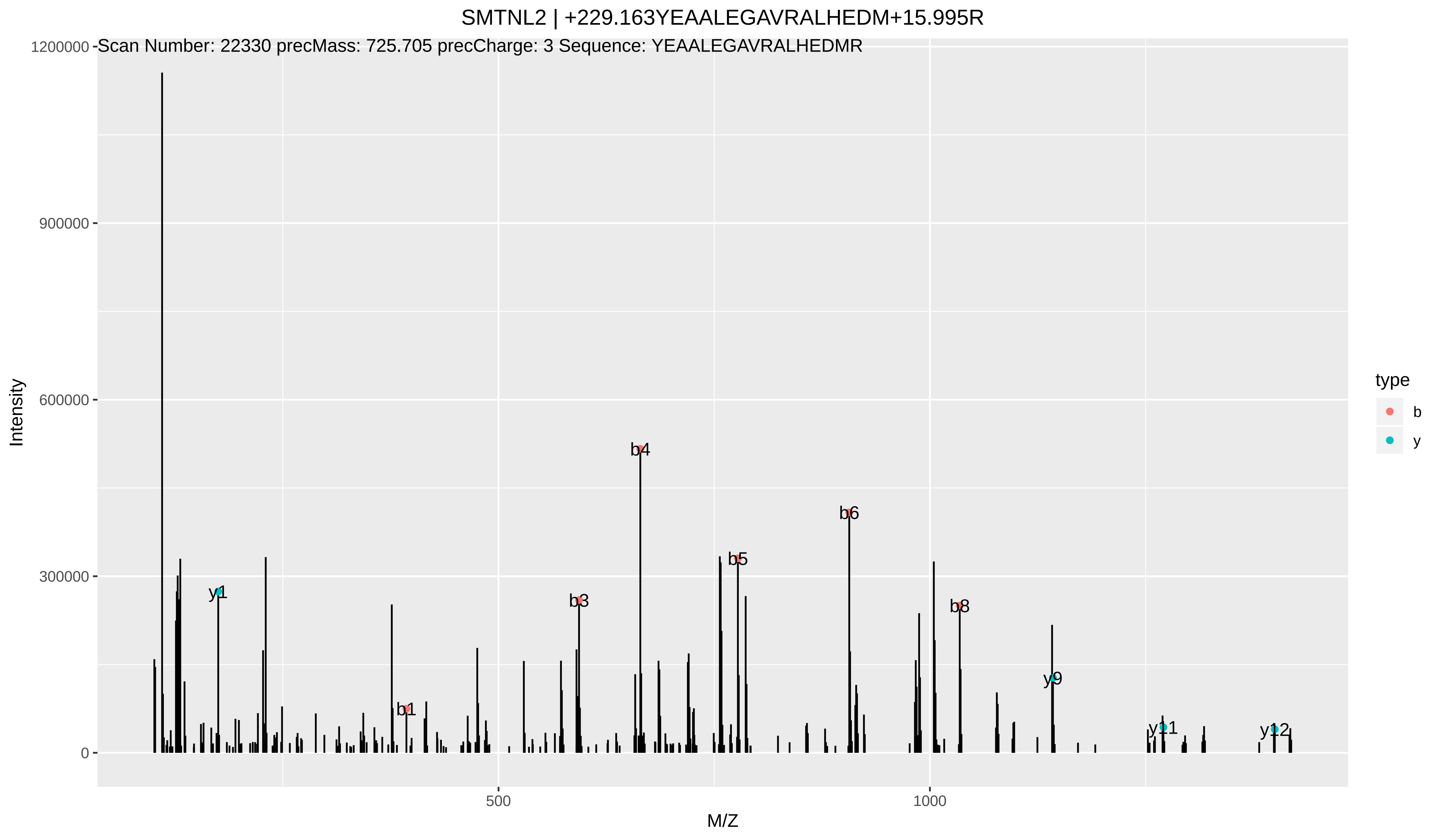

SMTNL2 | +229.163YEAALEGAVRALHEDM+15.995R
Scan Number: 22330 precMass: 725.705 precCharge: 3 Sequence: YEAALEGAVRALHEDMR
Intensity
1200000
900000
600000
300000
0
M/Z
500
1000
type
b
y
y1
b1
b3
b4
b5
b6
b8
y9
y11
y12

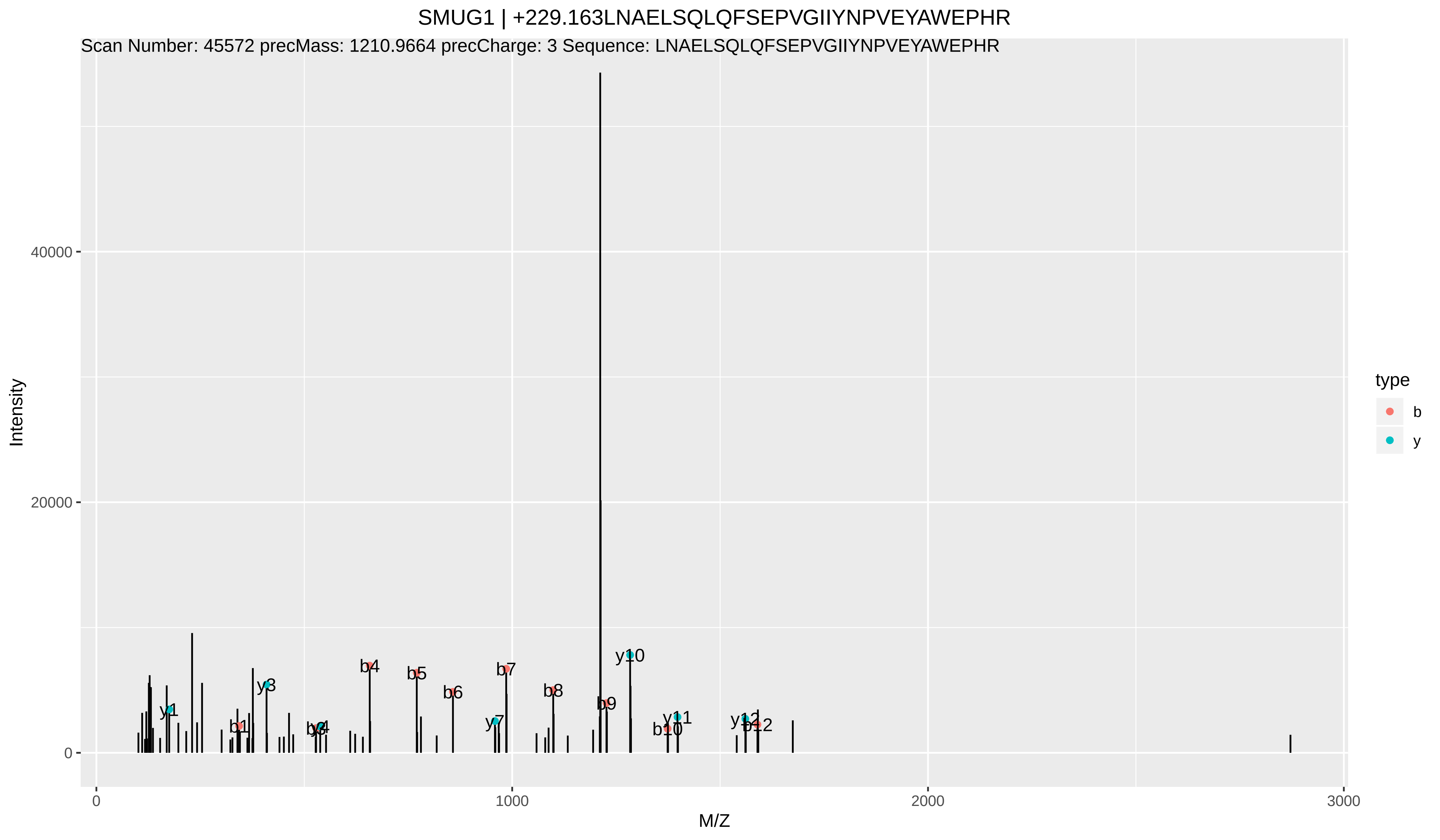
SMUG1 | +229.163LNAELSQLQFSEPVGIIYNPVEYAWEPHR
Scan Number: 45572 precMass: 1210.9664 precCharge: 3 Sequence: LNAELSQLQFSEPVGIIYNPVEYAWEPHR
Intensity
M/Z
type
b
y
y1
b1
y3
b4
b5
b6
y7
b7
b8
b9
y10
y11
b10
y12
b12
0
20000
40000
1000
2000
3000

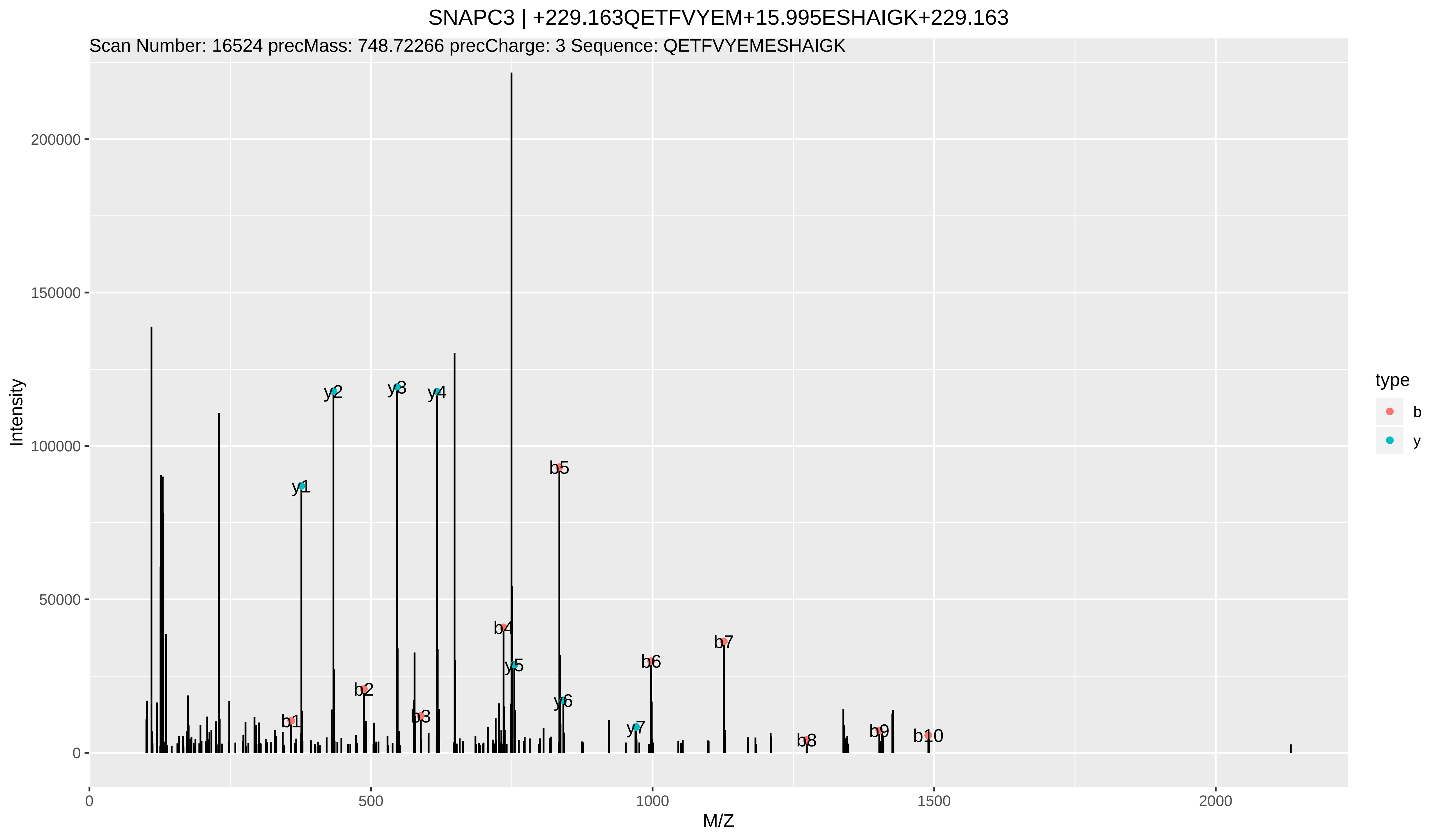
SNAPC3 | +229.163QETFVYEM+15.995ESHAIGK+229.163
Scan Number: 16524 precMass: 748.72266 precCharge: 3 Sequence: QETFVYEMESHAIGK
Intensity
M/Z
type
b
y
y1
y2
y3
y4
y5
y6
y7
b1
b2
b3
b4
b5
b6
b7
b8
b9
b10
0
50000
100000
150000
200000
500
1000
1500
2000

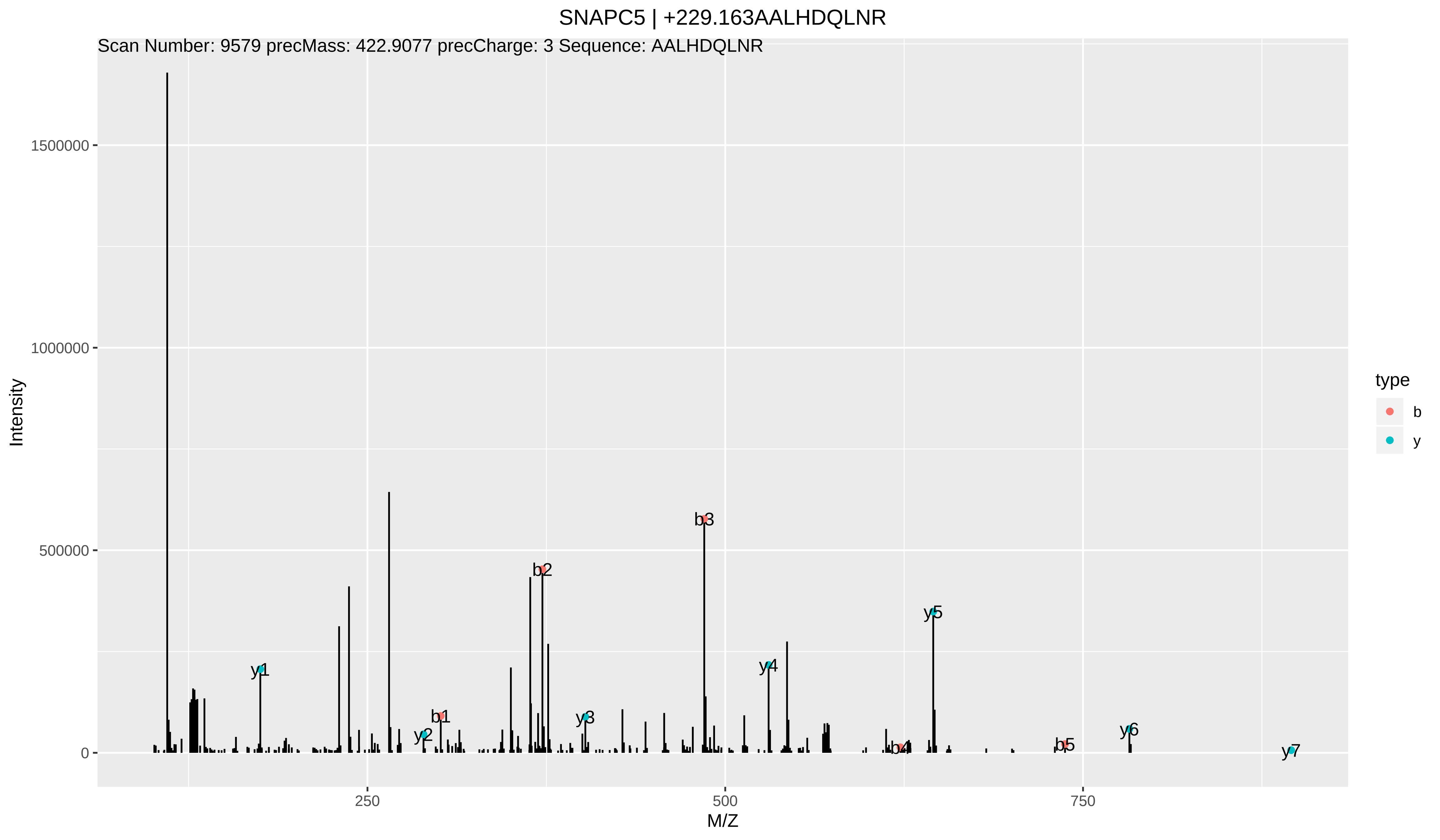
SNAPC5 | +229.163AALHDQLNR
Scan Number: 9579 precMass: 422.9077 precCharge: 3 Sequence: AALHDQLNR
Intensity
1500000
1000000
500000
0
M/Z
250
500
750
y1
y2
b1
b2
y3
b3
y4
b4
y5
b5
y6
y7
type
b
y

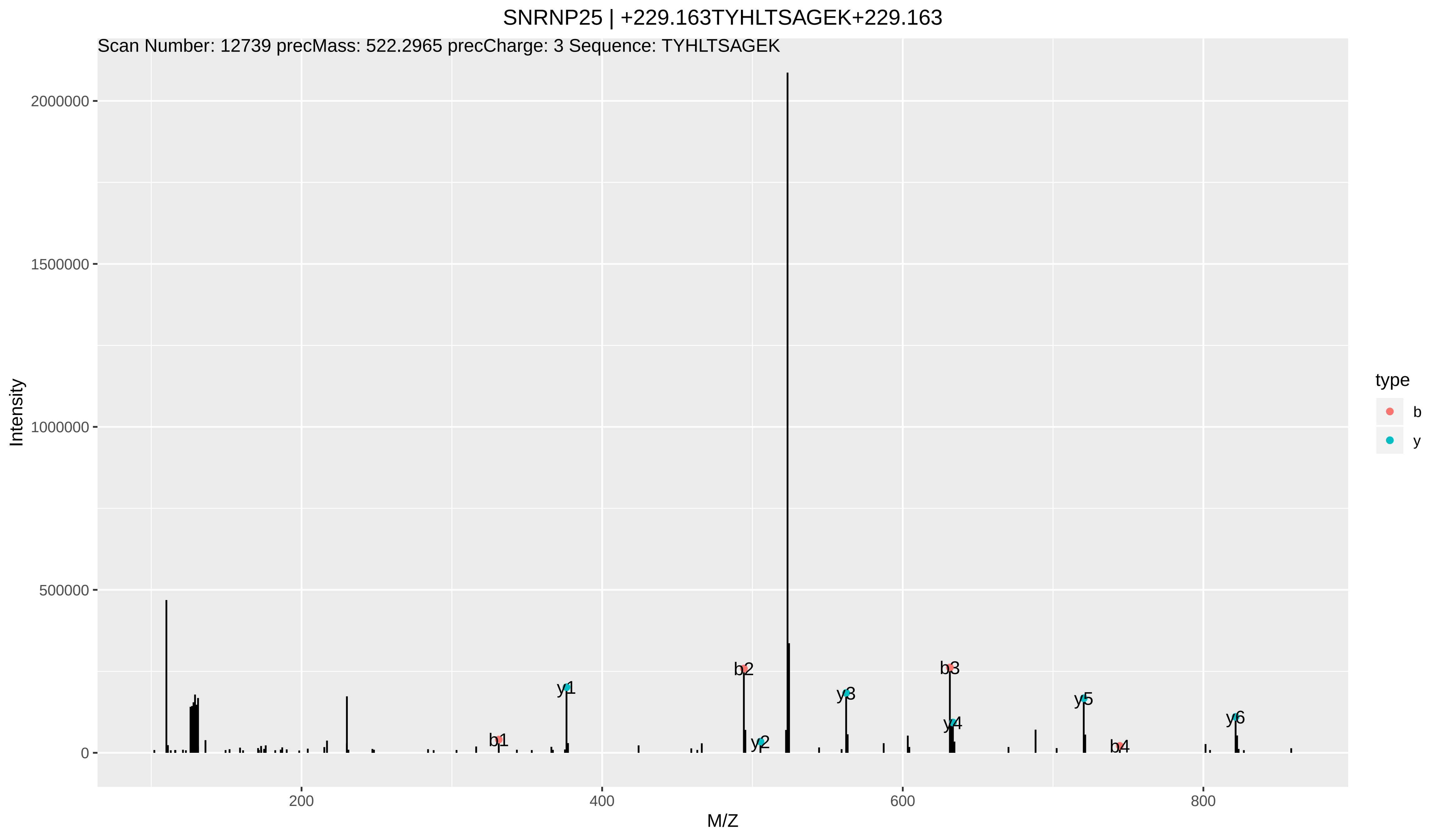

SNRNP25 | +229.163TYHLTSAGEK+229.163
Scan Number: 12739 precMass: 522.2965 precCharge: 3 Sequence: TYHLTSAGEK
Intensity
2000000
1500000
1000000
500000
0
M/Z
200
400
600
800
b1
y1
b2
y2
y3
b3
y4
y5
b4
y6
type
b
y

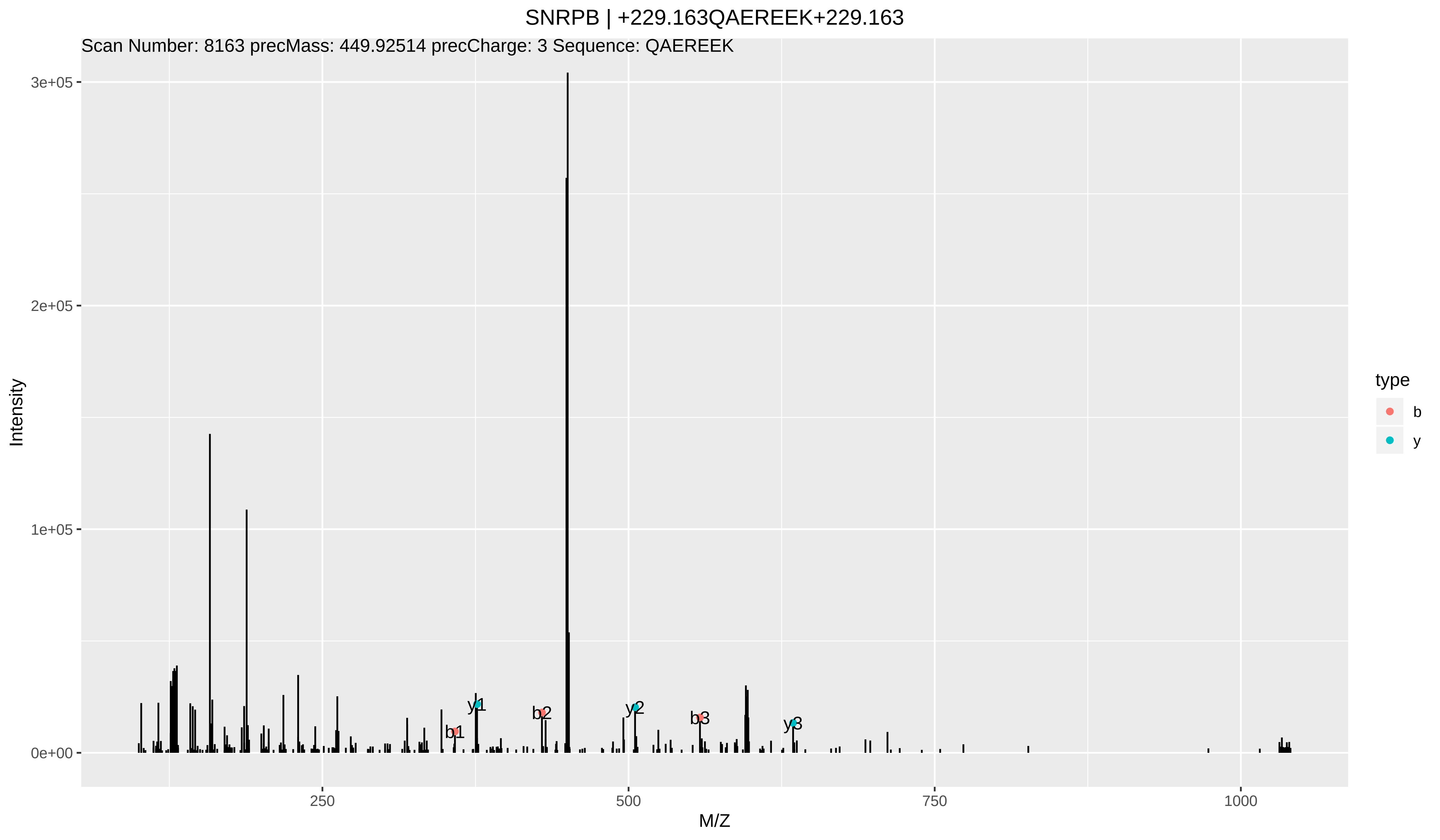
SNRPB | +229.163QAEREEK+229.163
Scan Number: 8163 precMass: 449.92514 precCharge: 3 Sequence: QAEREEK
Intensity
3e+05
2e+05
1e+05
0e+00
M/Z
250
500
750
1000
y1
b1
b2
y2
b3
y3
type
b
y

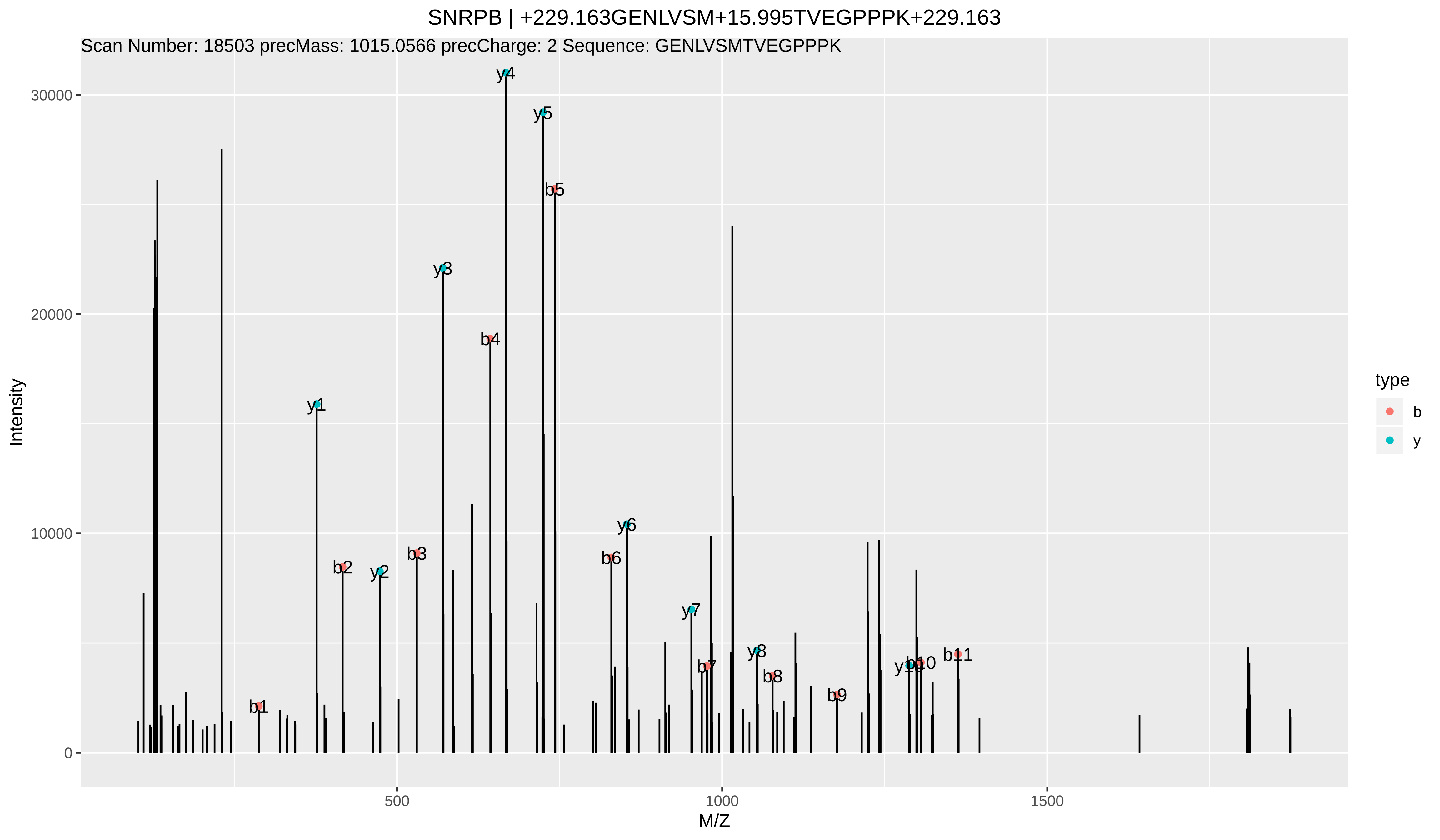

SNRPB | +229.163GENLVSM+15.995TVEGPPPK+229.163
Scan Number: 18503 precMass: 1015.0566 precCharge: 2 Sequence: GENLVSMTVEGPPPK
Intensity
M/Z
0
10000
20000
30000
500
1000
1500
type
b
y
y1
y2
y3
y4
y5
y6
y7
y8
y10
b1
b2
b3
b4
b5
b6
b7
b8
b9
b10
b11

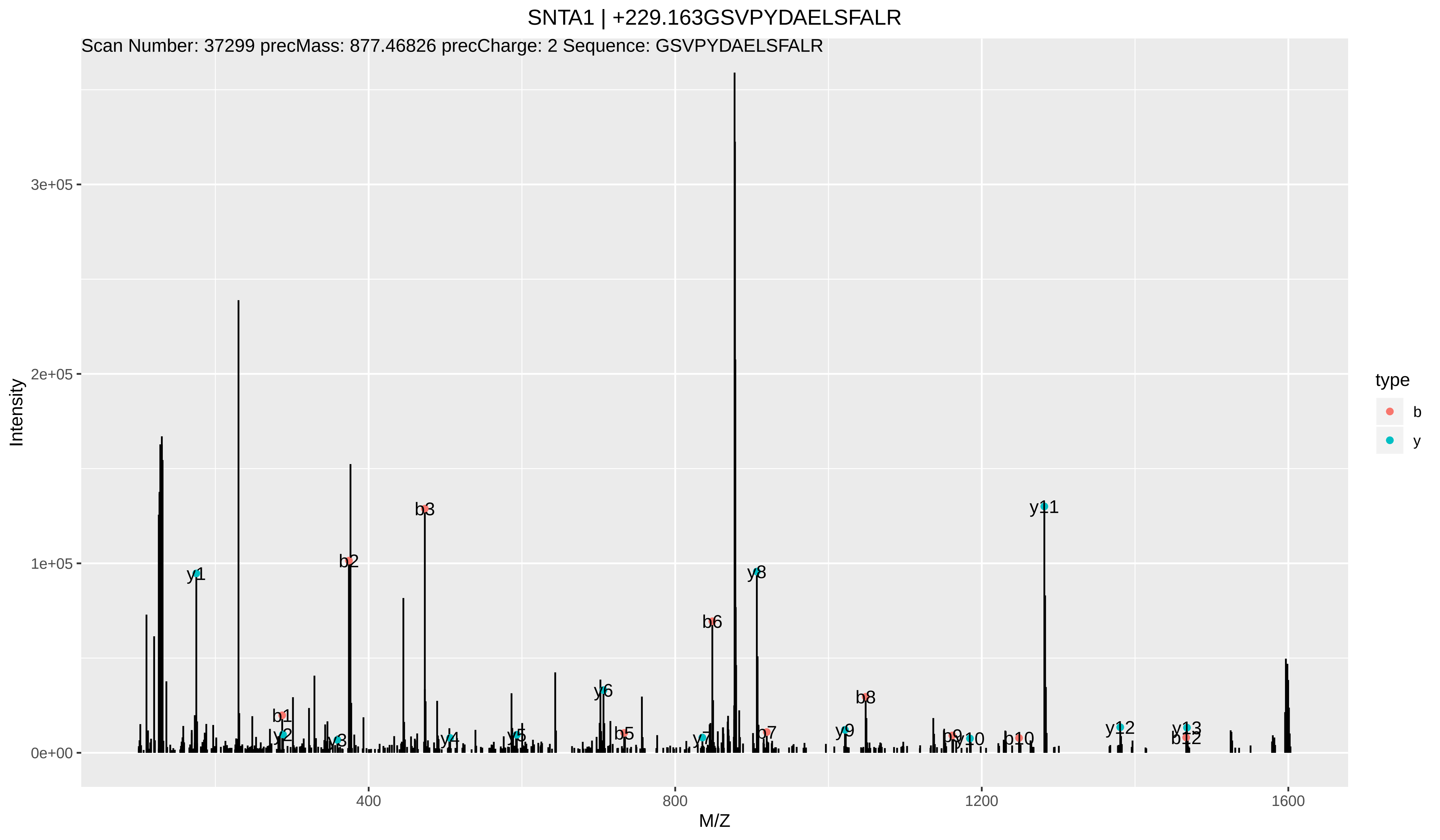

SNTA1 | +229.163GSVPYDAELSFALR
Scan Number: 37299 precMass: 877.46826 precCharge: 2 Sequence: GSVPYDAELSFALR
Intensity
M/Z
3e+05
2e+05
1e+05
0e+00
400
800
1200
1600
y1
b1
y2
y3
b2
b3
y4
y5
y6
b5
y7
b6
y8
b7
y9
b8
b9
y10
b10
y11
y12
y13
b12
type
b
y

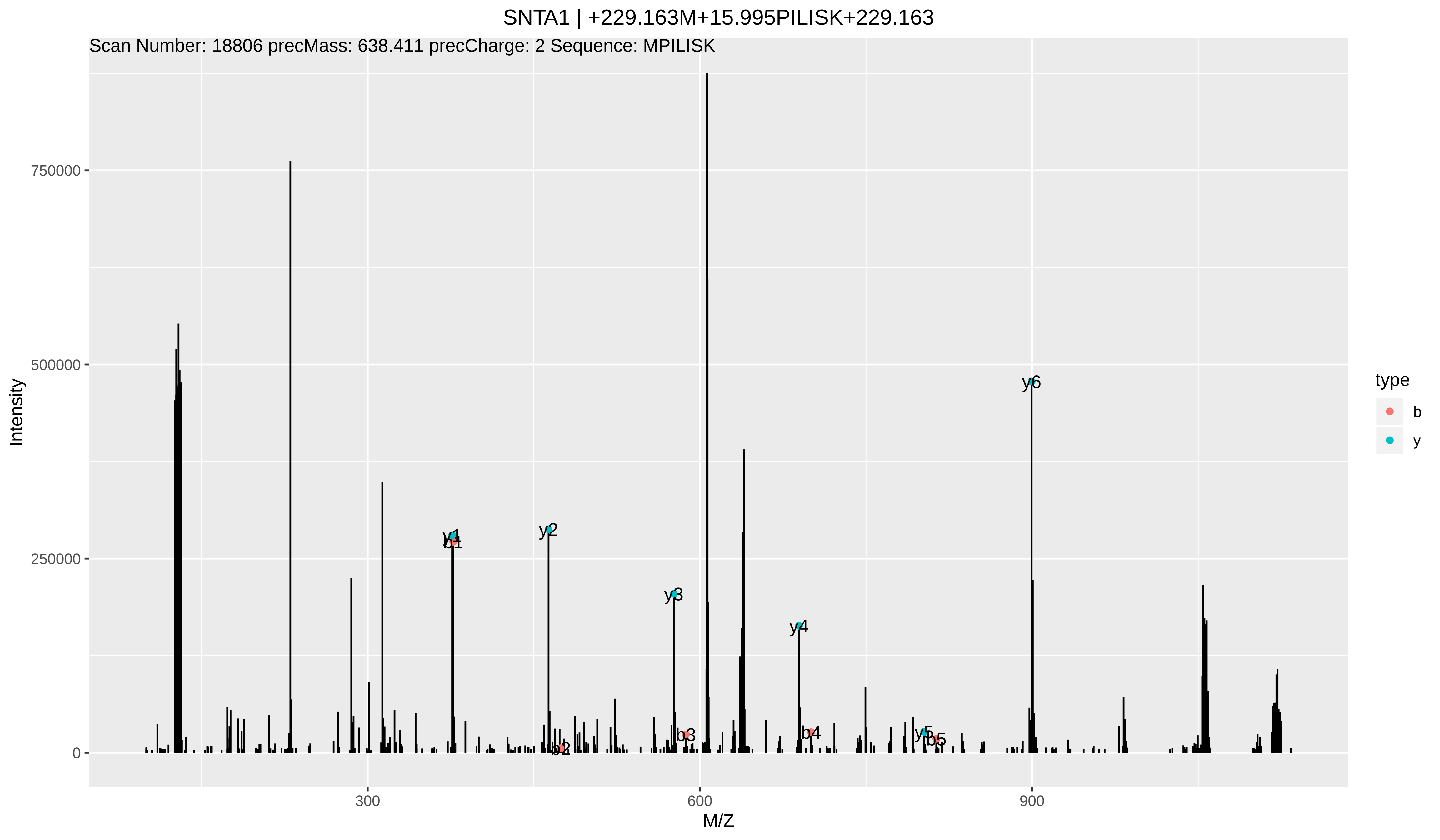
SNTA1 | +229.163M+15.995PILISK+229.163
Scan Number: 18806 precMass: 638.411 precCharge: 2 Sequence: MPILISK
Intensity
750000
500000
250000
0
M/Z
300
600
900
y1
b1
y2
b2
y3
b3
y4
b4
y5
b5
y6
type
b
y

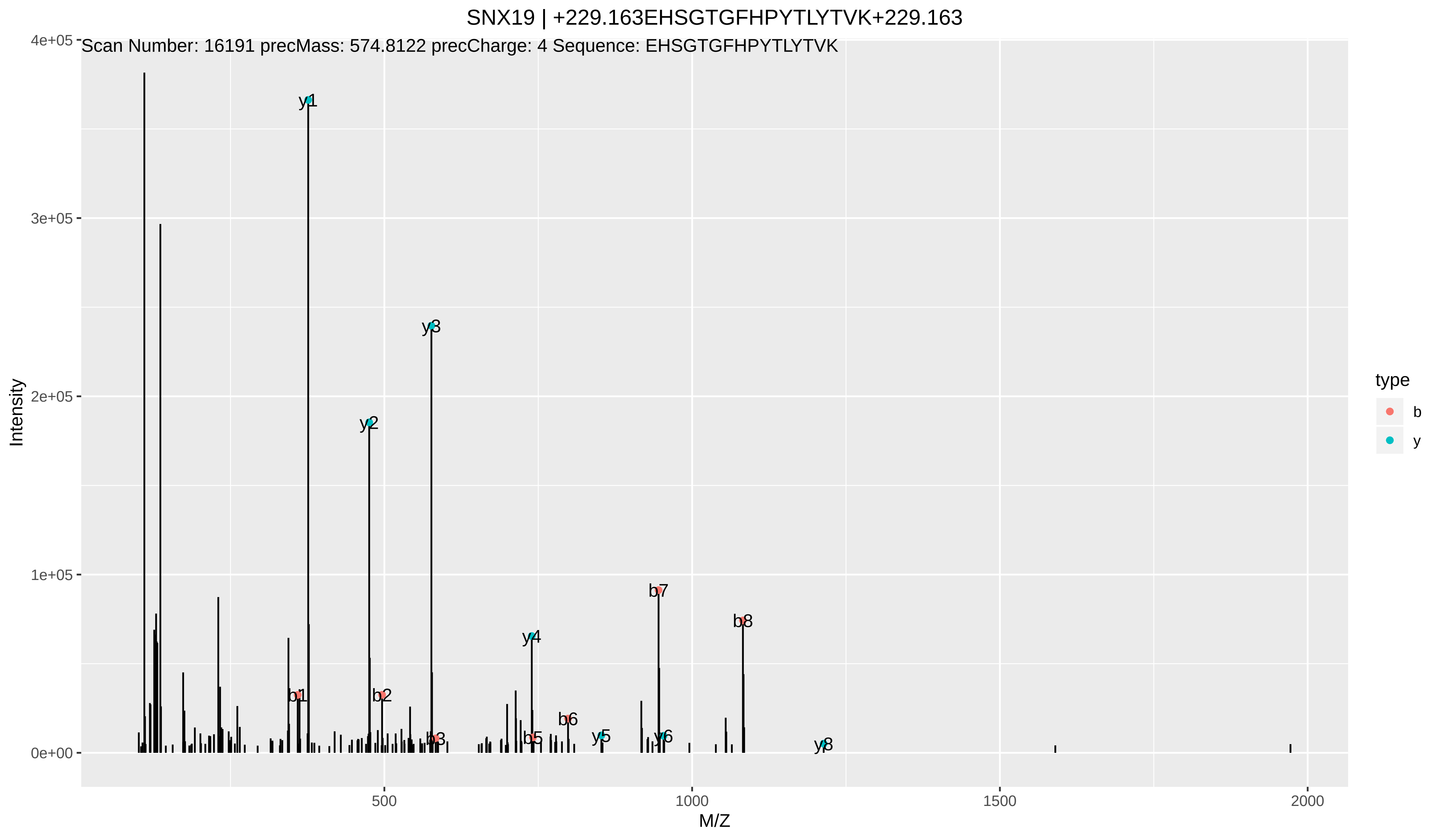
SNX19 | +229.163EHSGTGFHPYTLYTVK+229.163
Scan Number: 16191 precMass: 574.8122 precCharge: 4 Sequence: EHSGTGFHPYTLYTVK
Intensity
M/Z
4e+05
3e+05
2e+05
1e+05
0e+00
500
1000
1500
2000
y1
y2
y3
y4
y5
y6
y8
b1
b2
b3
b5
b6
b7
b8
type
b
y

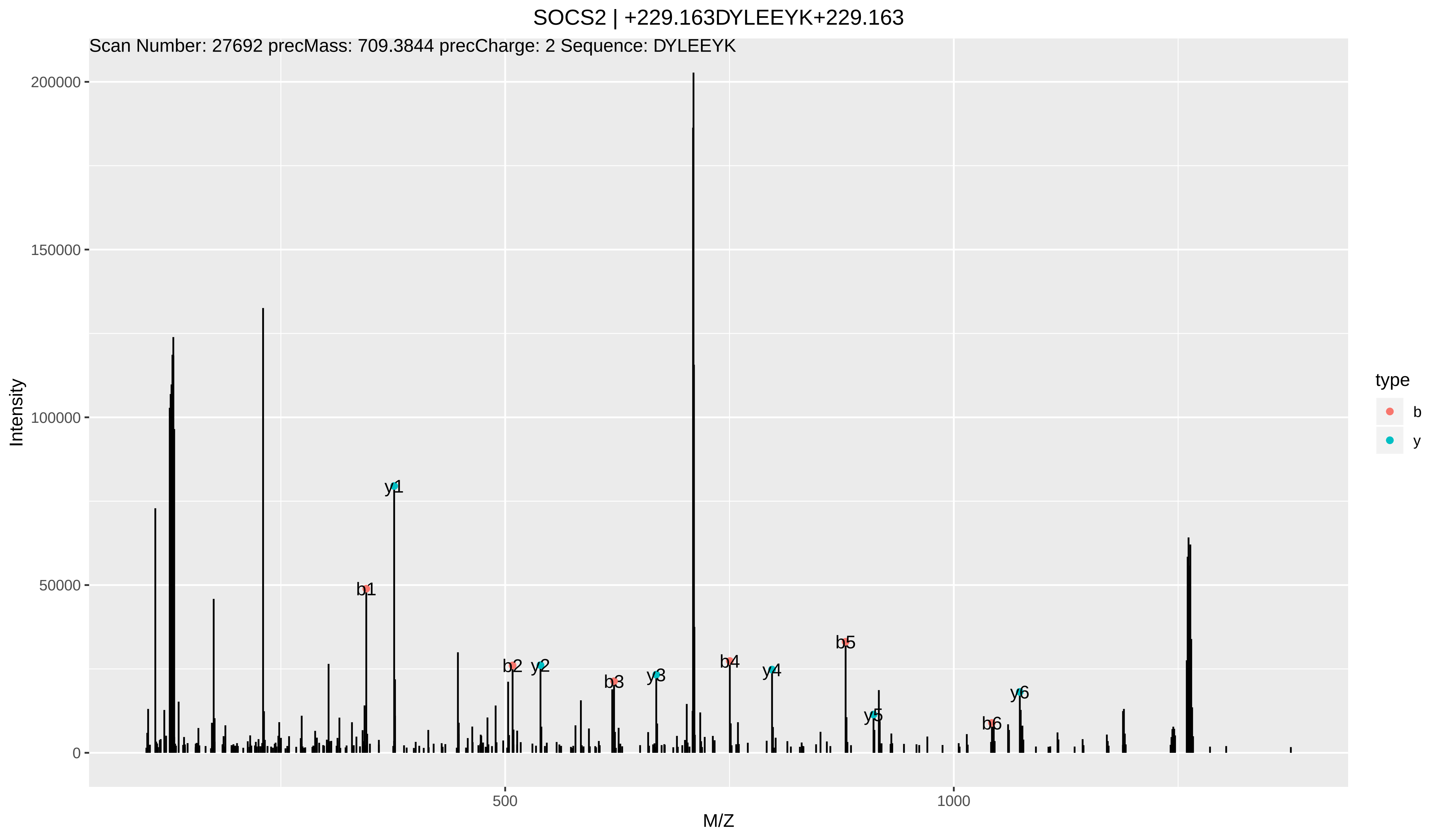
SOCS2 | +229.163DYLEEYK+229.163
Scan Number: 27692 precMass: 709.3844 precCharge: 2 Sequence: DYLEEYK
Intensity
200000
150000
100000
50000
0
M/Z
500
1000
y1
b1
b2
y2
b3
y3
b4
y4
b5
y5
b6
y6
type
b
y

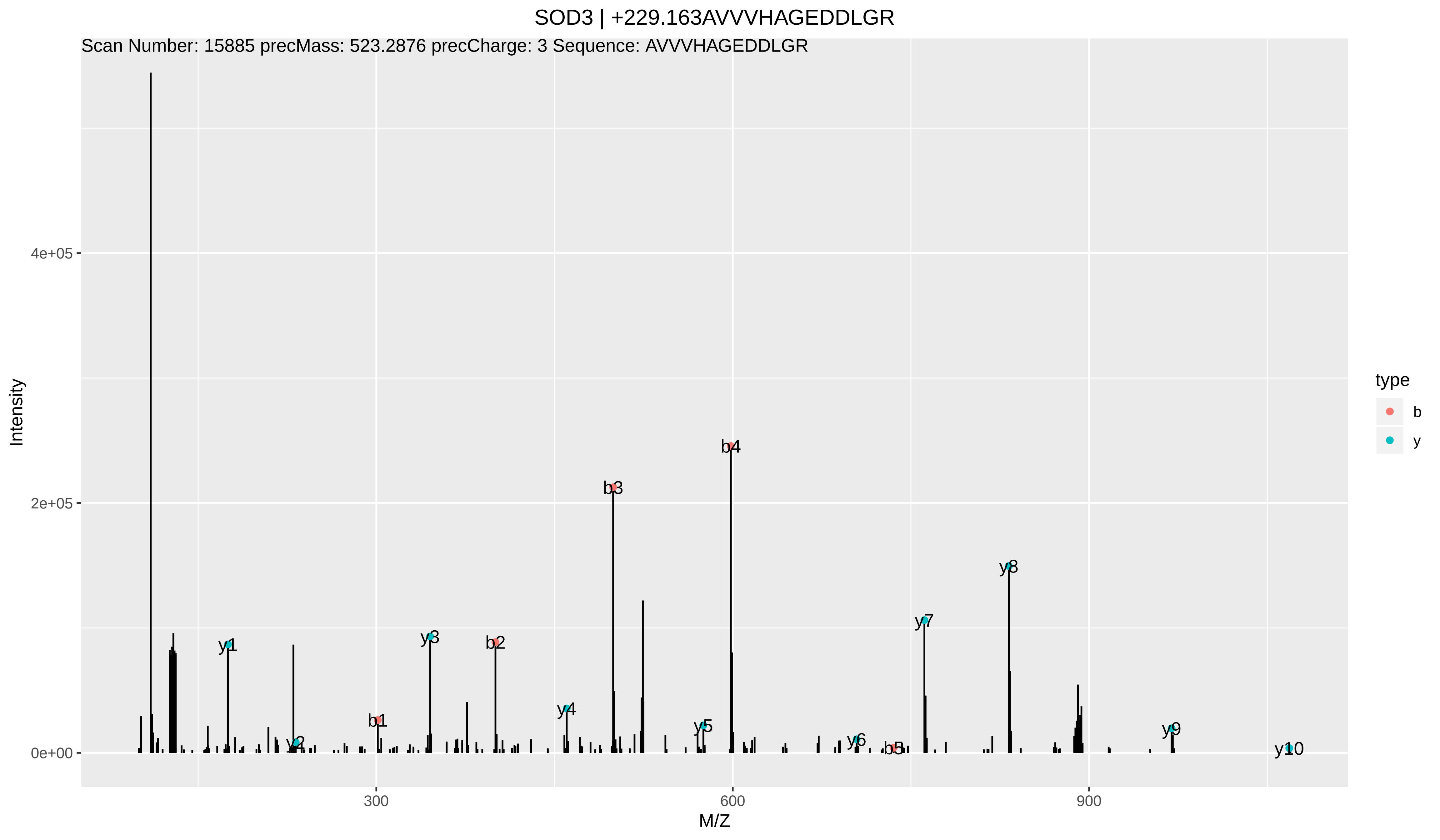

SOD3 | +229.163AVVVHAGEDDLGR
Scan Number: 15885 precMass: 523.2876 precCharge: 3 Sequence: AVVVHAGEDDLGR
Intensity
4e+05
2e+05
0e+00
M/Z
300
600
900
y1
y2
b1
y3
b2
y4
b3
b4
y5
y6
b5
y7
y8
y9
y10
type
b
y

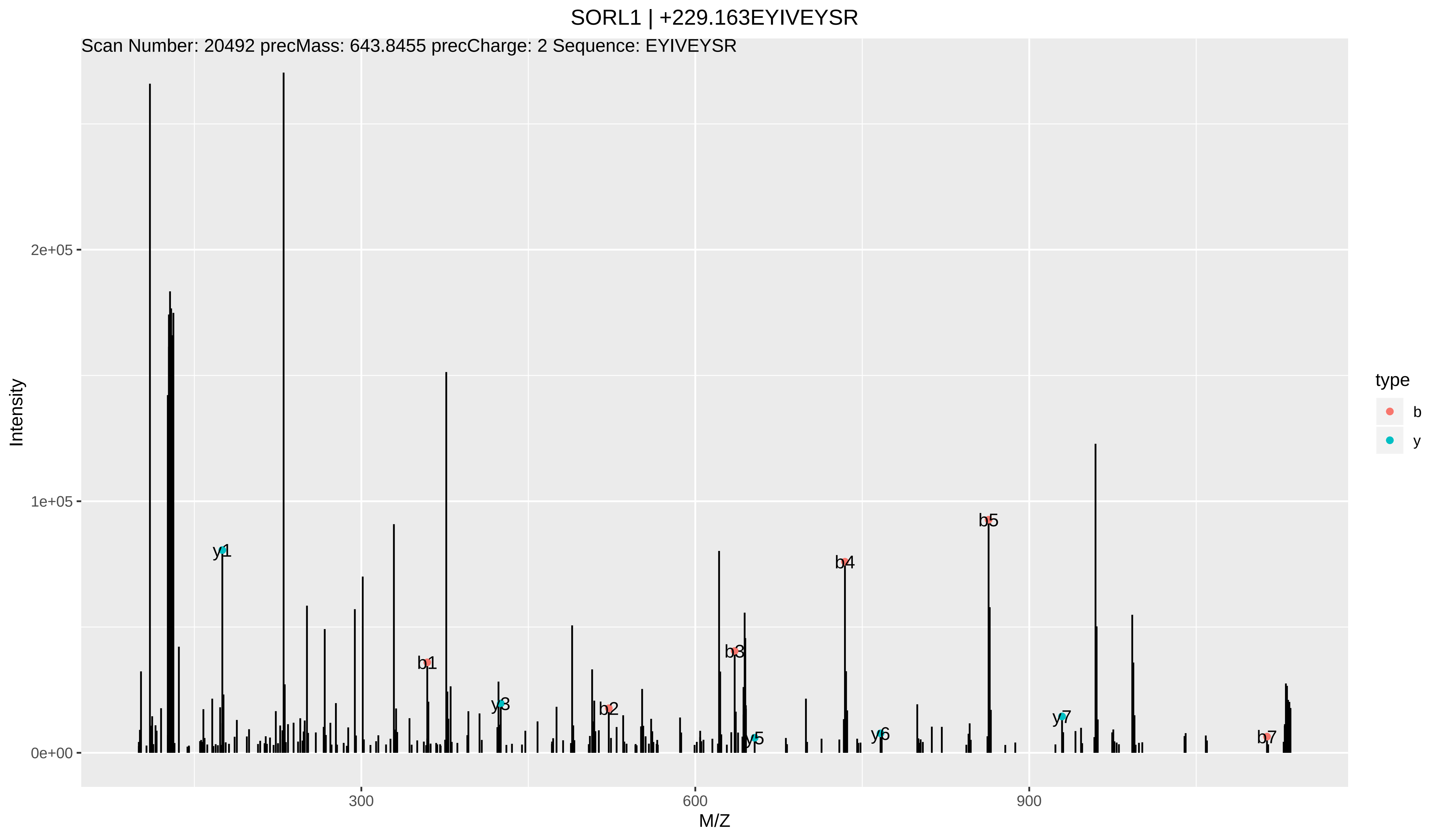
SORL1 | +229.163EYIVEYSR
Scan Number: 20492 precMass: 643.8455 precCharge: 2 Sequence: EYIVEYSR
Intensity
M/Z
0e+00
1e+05
2e+05
300
600
900
y1
b1
y3
b2
b3
y5
b4
y6
b5
y7
b7
type
b
y

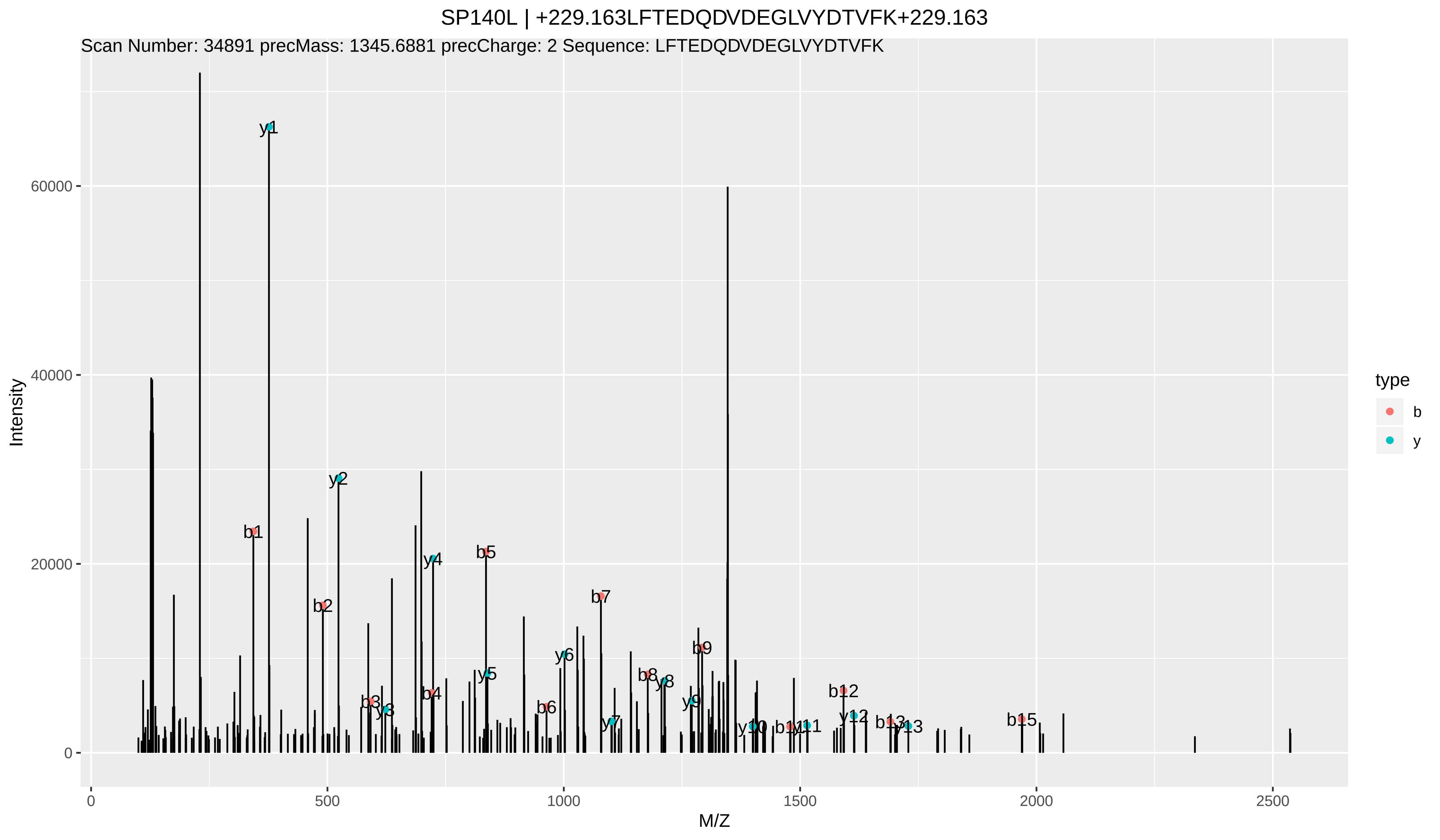

SP140L | +229.163LFTEDQDVDEGLVYDTVFK+229.163
Scan Number: 34891 precMass: 1345.6881 precCharge: 2 Sequence: LFTEDQDVDEGLVYDTVFK
Intensity
M/Z
0
20000
40000
60000
0
500
1000
1500
2000
2500
y1
y2
b1
b2
b3
y3
b4
y4
b5
y5
b6
y6
b7
y7
b8
y8
b9
y9
y10
b11
y11
b12
y12
b13
y13
b15
type
b
y

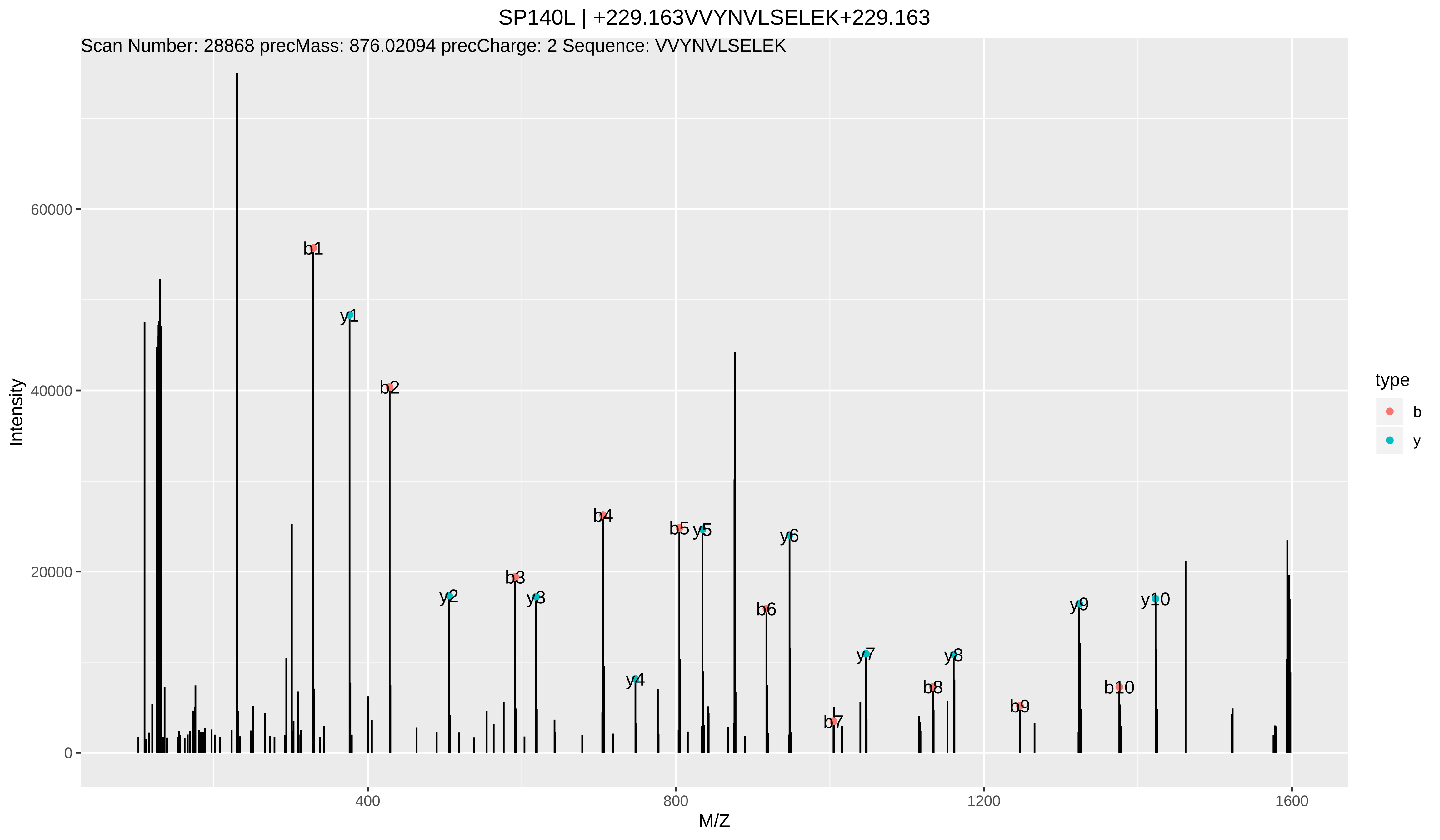

SP140L | +229.163VVYNVLSELEK+229.163
Scan Number: 28868 precMass: 876.02094 precCharge: 2 Sequence: VVYNVLSELEK
Intensity
M/Z
0
20000
40000
60000
400
800
1200
1600
b1
y1
b2
y2
b3
y3
b4
y4
b5
y5
b6
y6
b7
y7
b8
y8
b9
y9
b10
y10
type
b
y

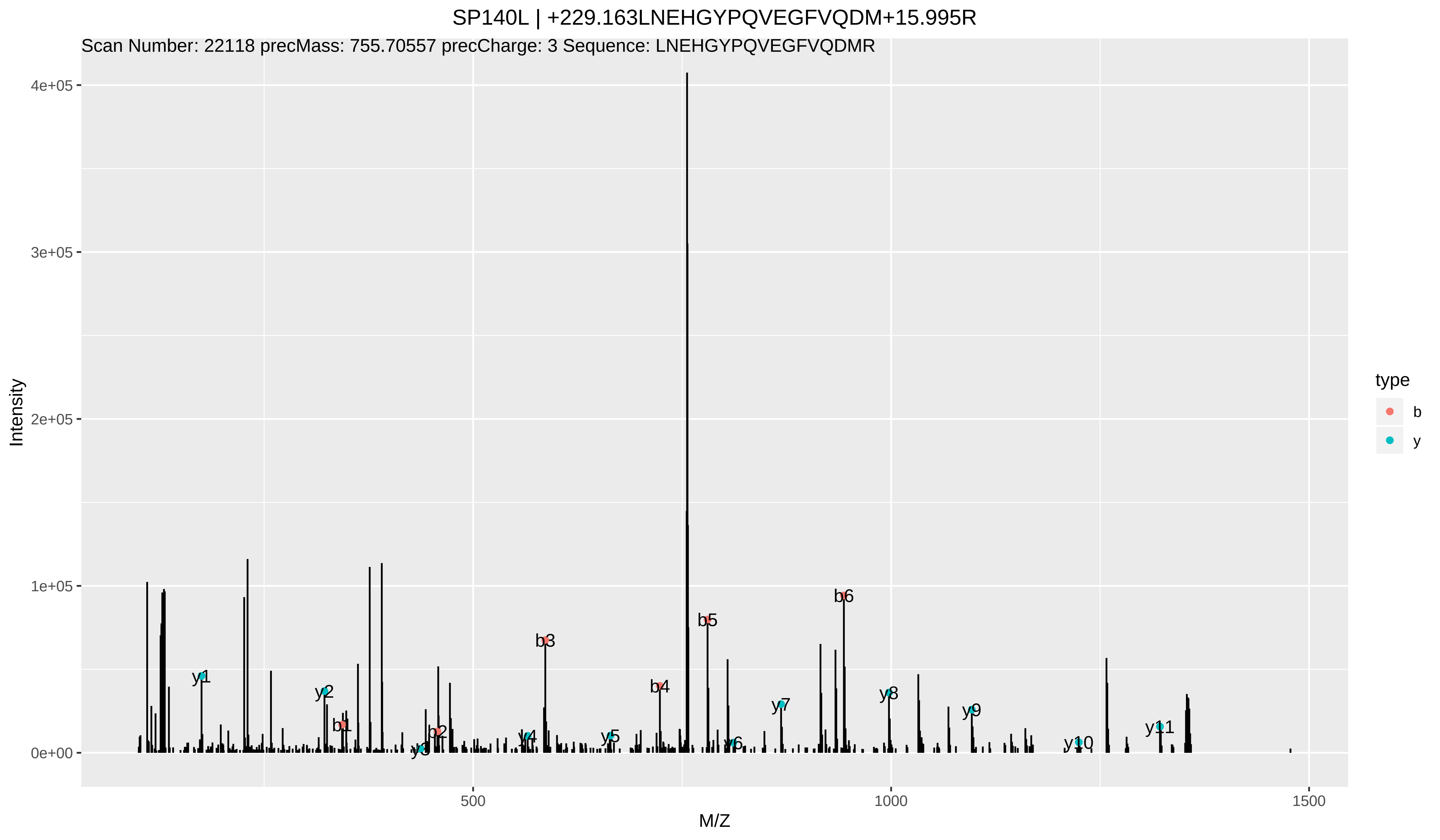

SP140L | +229.163LNEHGYPQVEGFVQDM+15.995R
Scan Number: 22118 precMass: 755.70557 precCharge: 3 Sequence: LNEHGYPQVEGFVQDMR
Intensity
M/Z
type
b
y
0e+00
1e+05
2e+05
3e+05
4e+05
500
1000
1500
y1
y2
b1
y3
b2
y4
b3
y5
b4
b5
y6
y7
b6
y8
y9
y10
y11

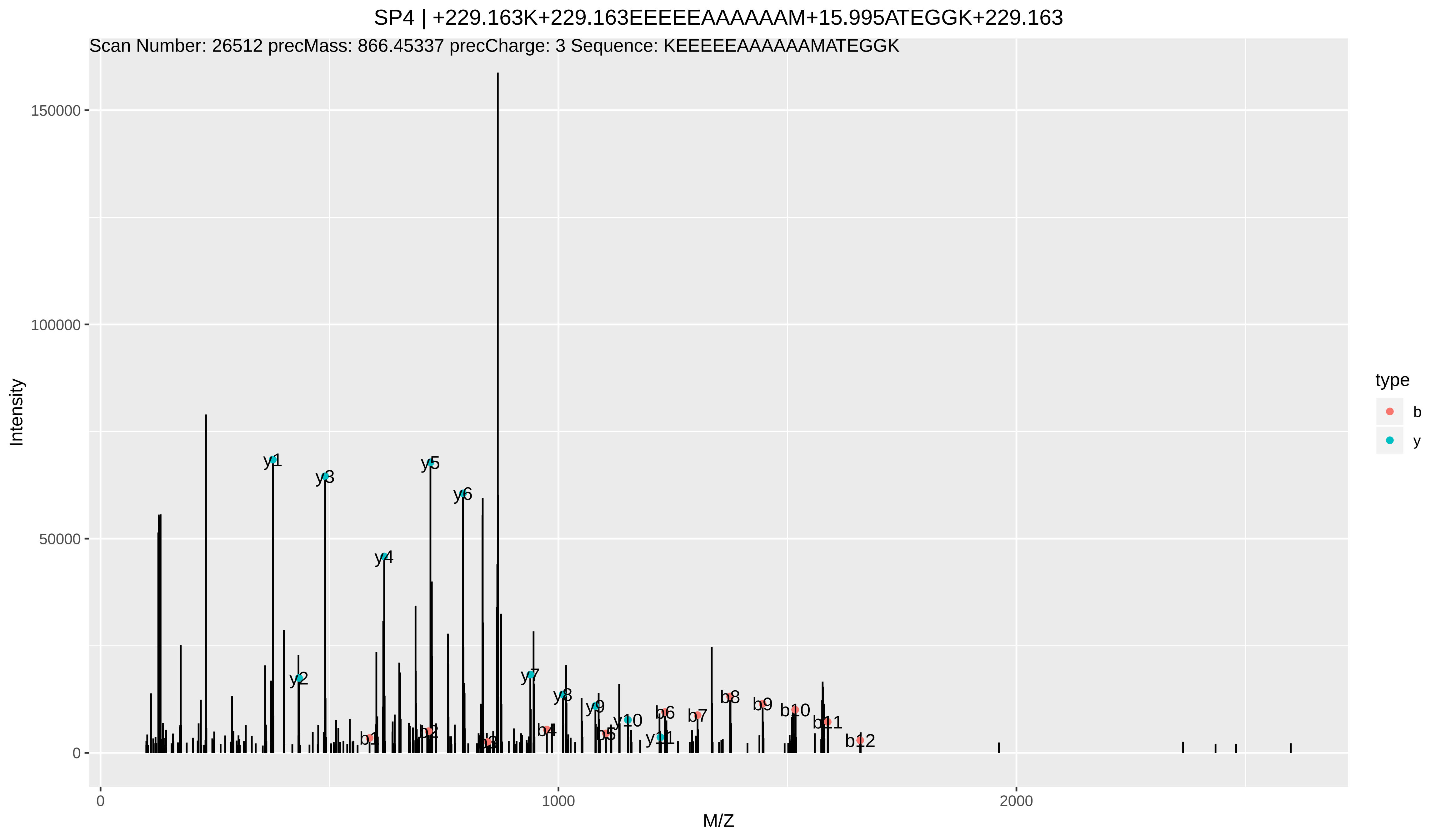
SP4 | +229.163K+229.163EEEEEAAAAAM+15.995ATEGGK+229.163
Scan Number: 26512 precMass: 866.45337 precCharge: 3 Sequence: KEEEEEAAAAAMATEGGK
Intensity
M/Z
type
b
y
0
50000
100000
150000
0
1000
2000
y1
y2
y3
y4
y5
y6
y7
y8
y9
y10
y11
b1
b2
b4
b5
b6
b7
b8
b9
b10
b11
b12

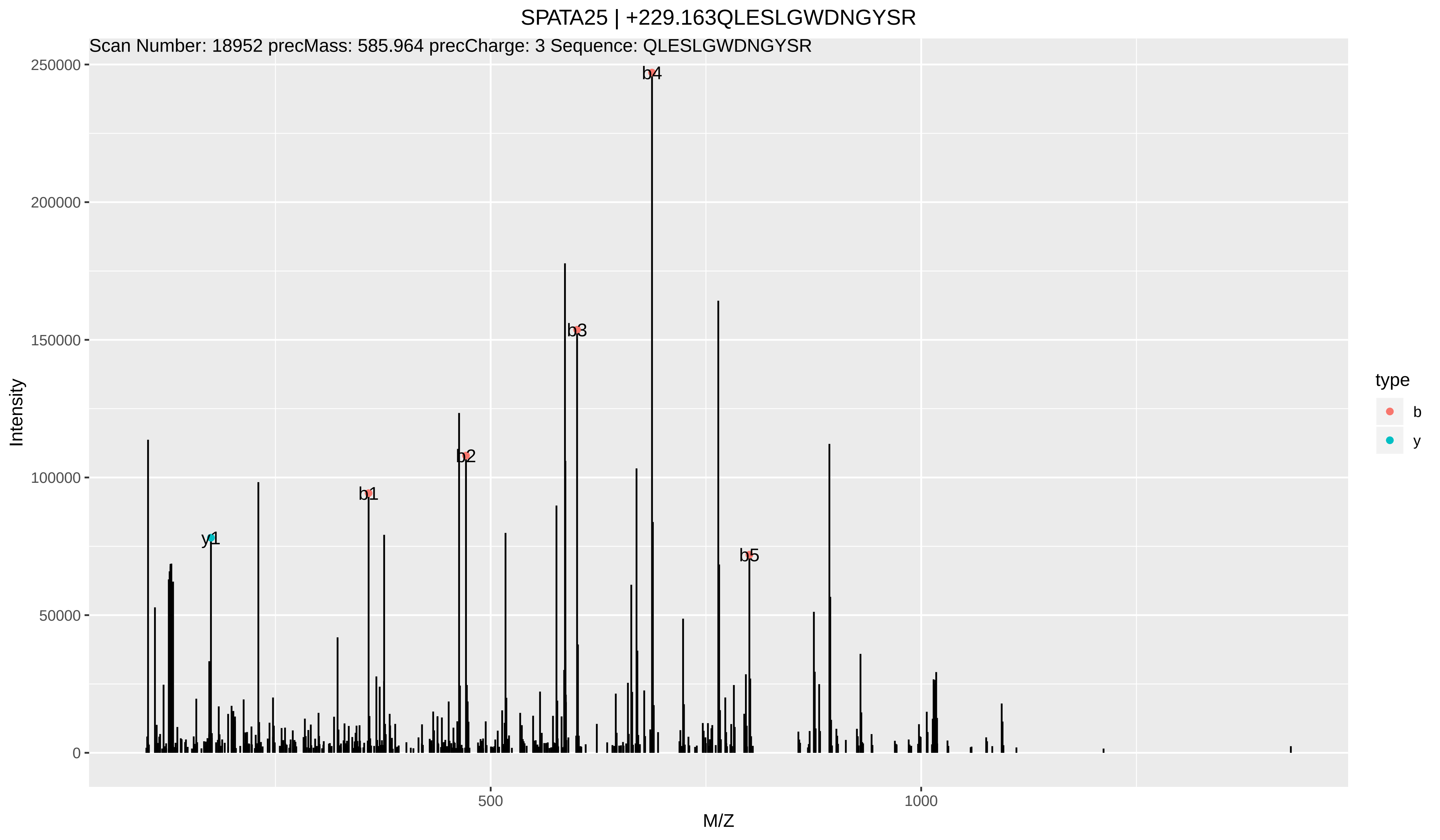

SPATA25 | +229.163QLESLGWDNGYSR
Scan Number: 18952 precMass: 585.964 precCharge: 3 Sequence: QLESLGWDNGYSR
Intensity
M/Z
250000
200000
150000
100000
50000
0
500
1000
b4
b3
b2
b1
y1
b5
type
b
y

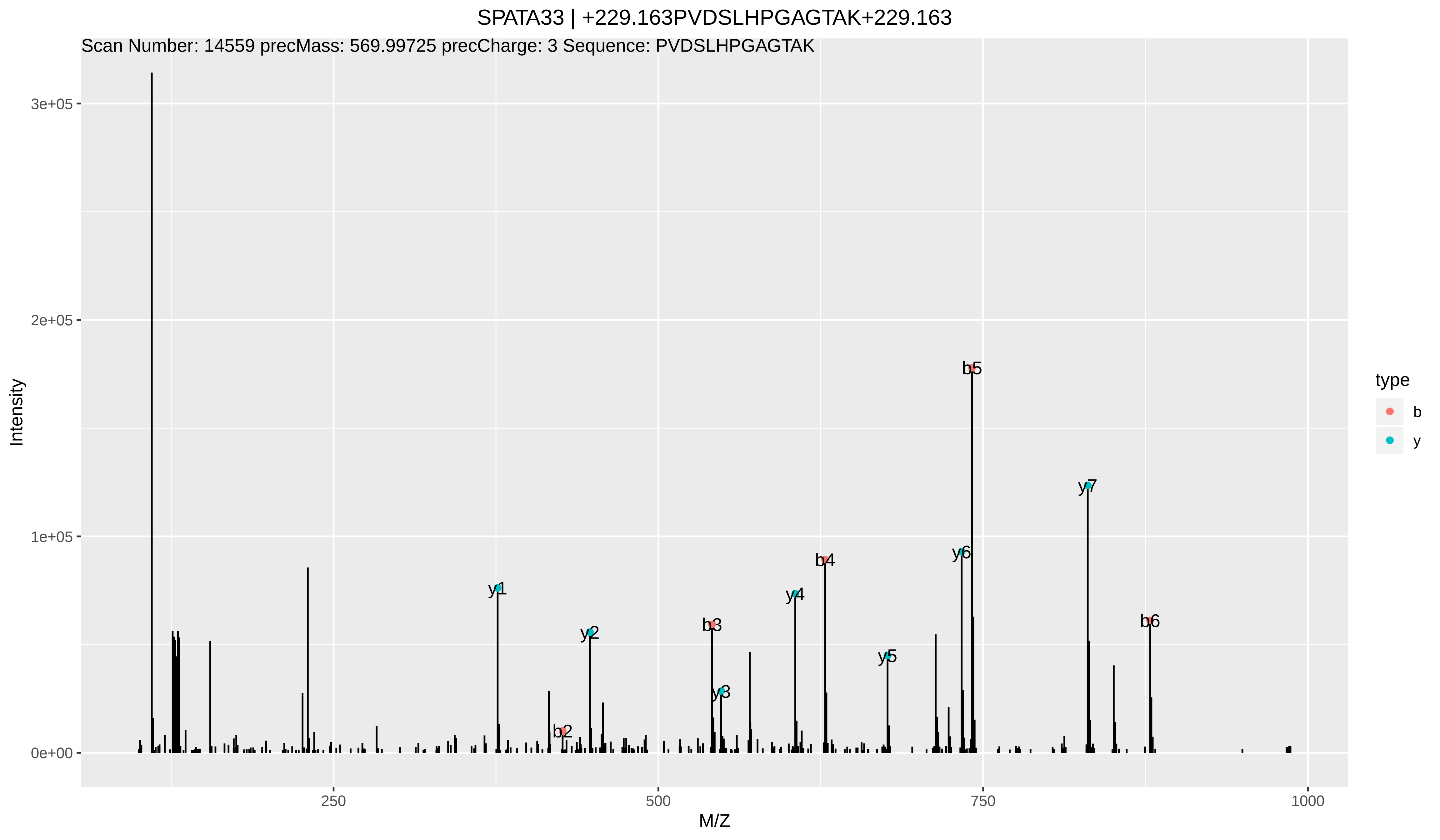

SPATA33 | +229.163PVDSLHPGAGTAK+229.163
Scan Number: 14559 precMass: 569.99725 precCharge: 3 Sequence: PVDSLHPGAGTAK
Intensity
3e+05
2e+05
1e+05
0e+00
M/Z
250
500
750
1000
y1
b2
y2
b3
y3
y4
b4
y5
y6
b5
y7
b6
type
b
y

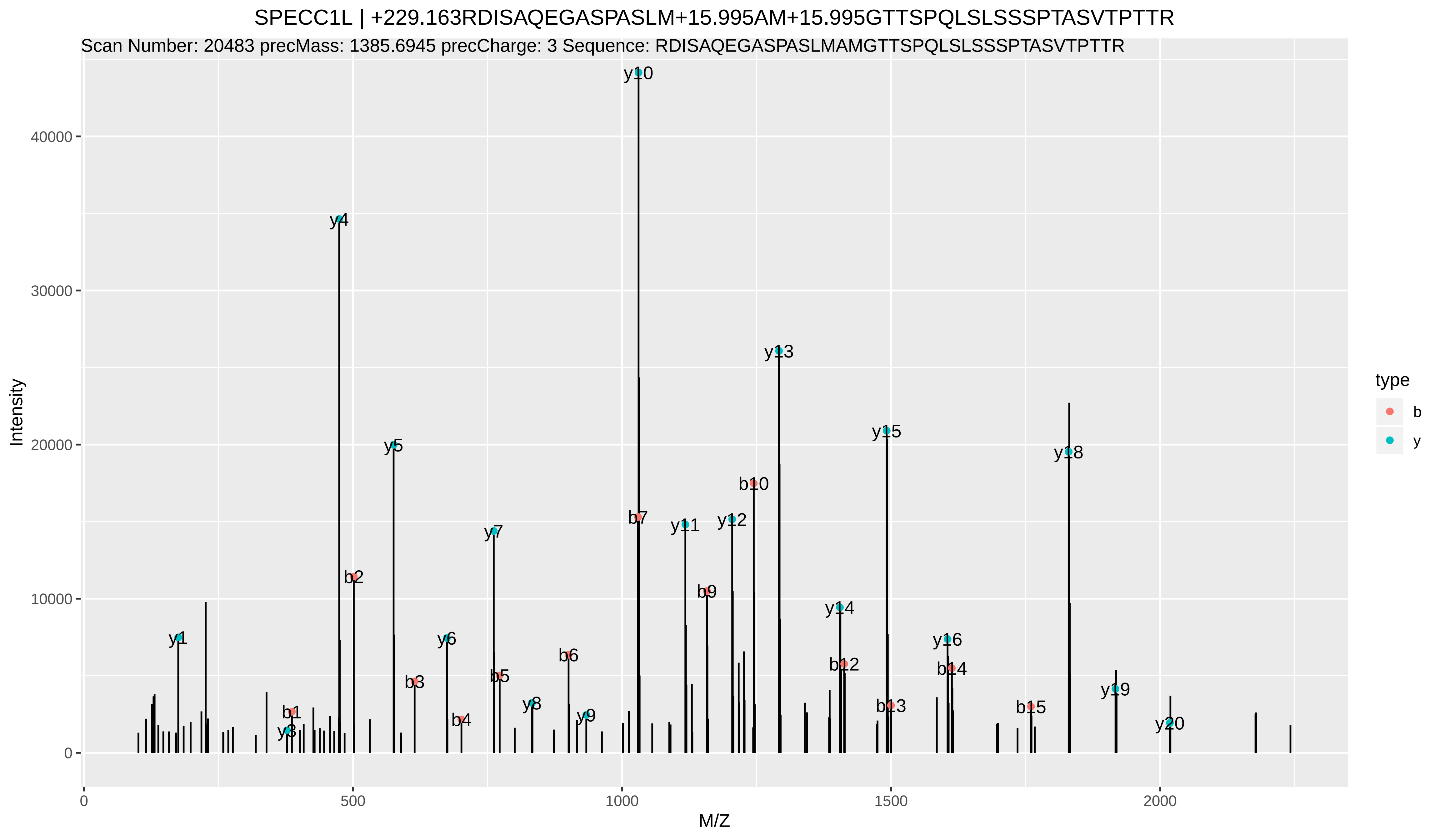
SPECC1L | +229.163RDISAQEGASPASLM+15.995AM+15.995GTTSPQLSLSSSPTASVTPTTR
Scan Number: 20483 precMass: 1385.6945 precCharge: 3 Sequence: RDISAQEGASPASLMAMGTTSPQLSLSSSPTASVTPTTR
Intensity
M/Z
0
10000
20000
30000
40000
500
1000
1500
2000
type
b
y
y1
y3
y4
y5
y6
y7
y8
y9
y10
y11
y12
y13
y14
y15
y16
y18
y19
y20
b1
b2
b3
b4
b5
b6
b7
b9
b10
b12
b13
b14
b15

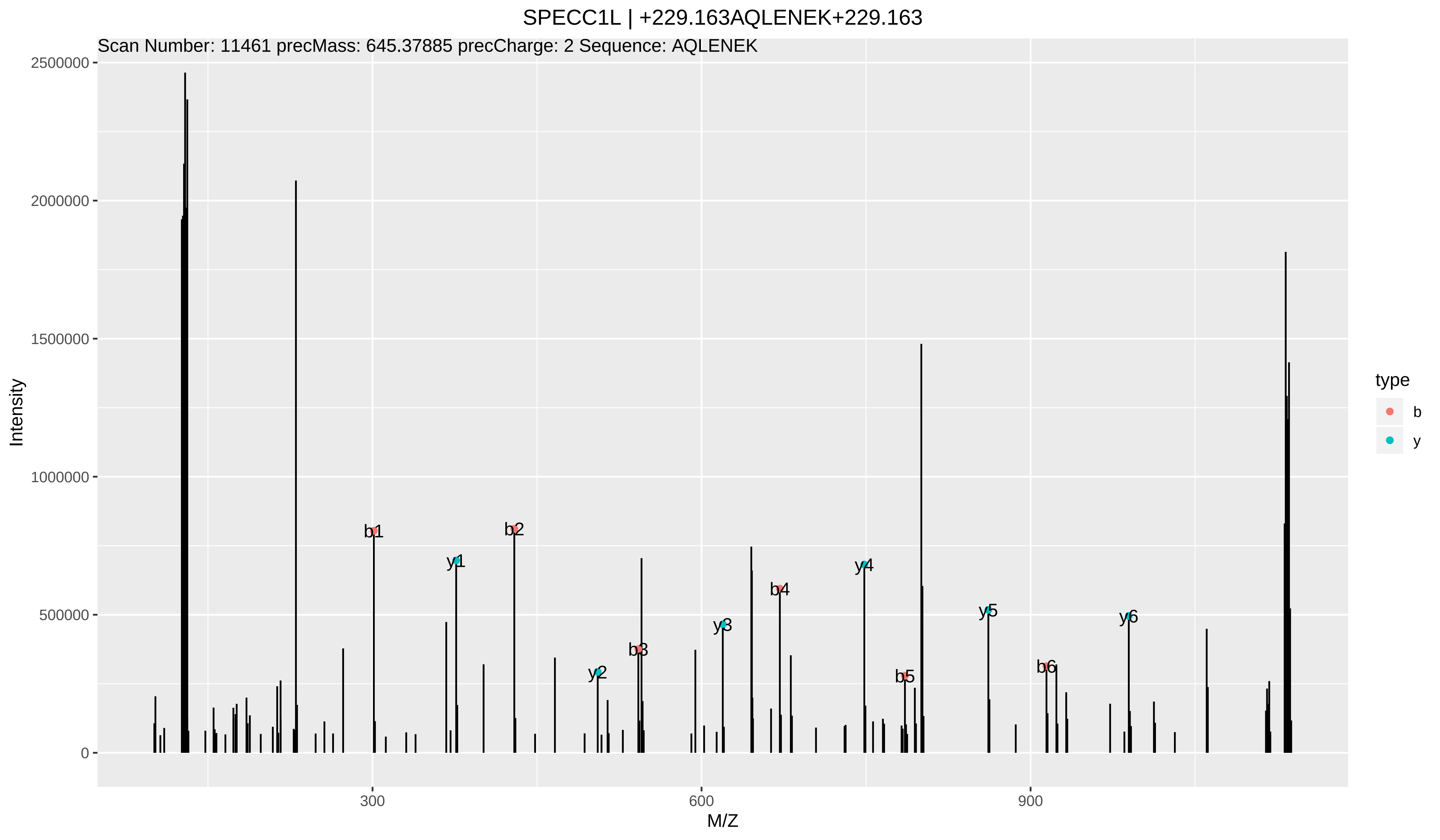

SPECC1L | +229.163AQLENEK+229.163
Scan Number: 11461 precMass: 645.37885 precCharge: 2 Sequence: AQLENEK
Intensity
2500000
2000000
1500000
1000000
500000
0
M/Z
300
600
900
b1
y1
b2
y2
b3
y3
b4
y4
b5
y5
b6
y6
type
b
y

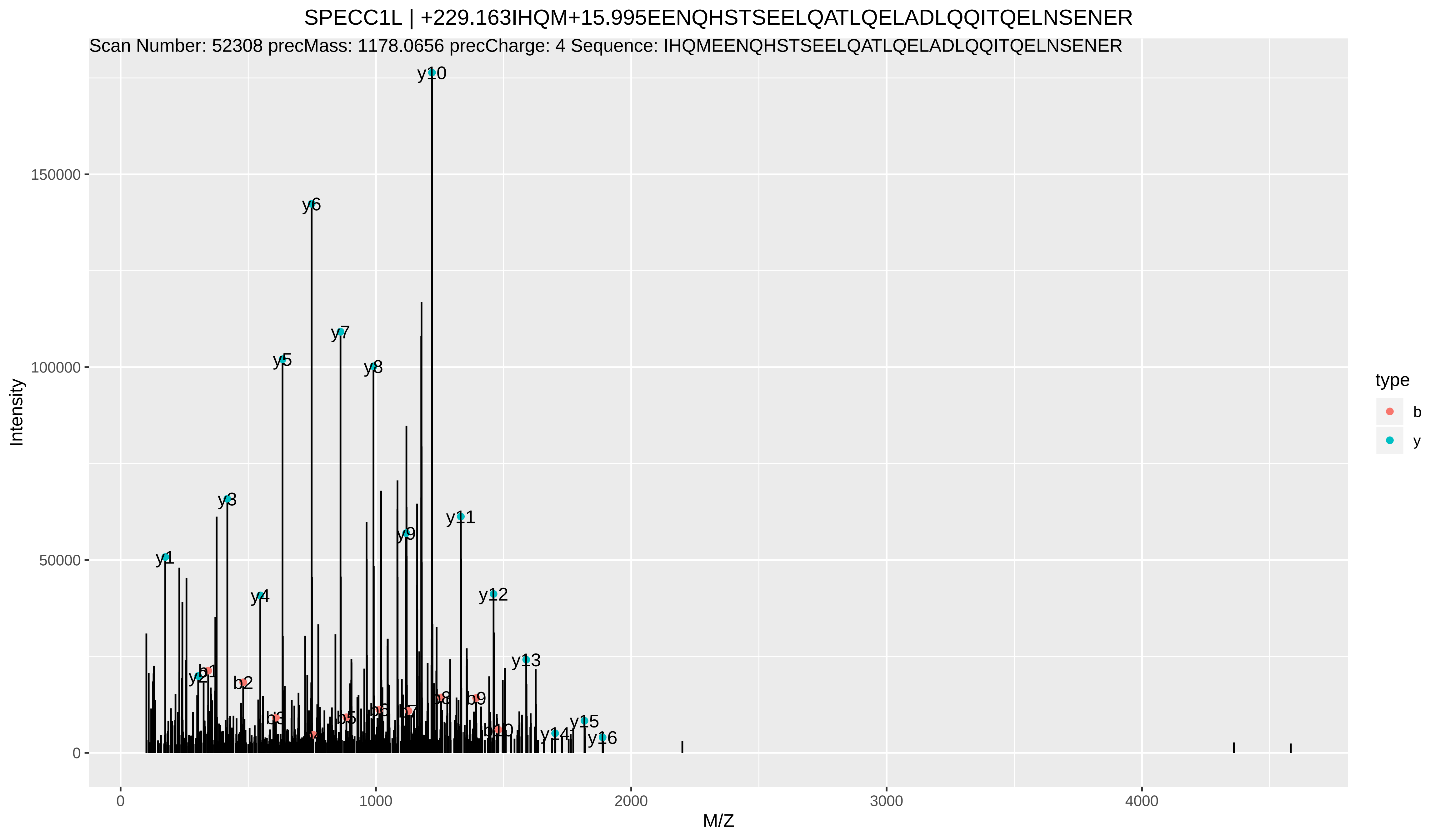

SPECC1L | +229.163IHQM+15.995EENQHSTSEELQATLQELADLQQITQELNSENER
Scan Number: 52308 precMass: 1178.0656 precCharge: 4 Sequence: IHQMEENQHSTSEELQATLQELADLQQITQELNSENER
Intensity
M/Z
type
b
y
y1
y2
b1
b2
y3
y4
y5
y6
y7
y8
y9
y10
y11
y12
y13
y14
y15
y16
b8
b9
b10
0
50000
100000
150000
1000
2000
3000
4000

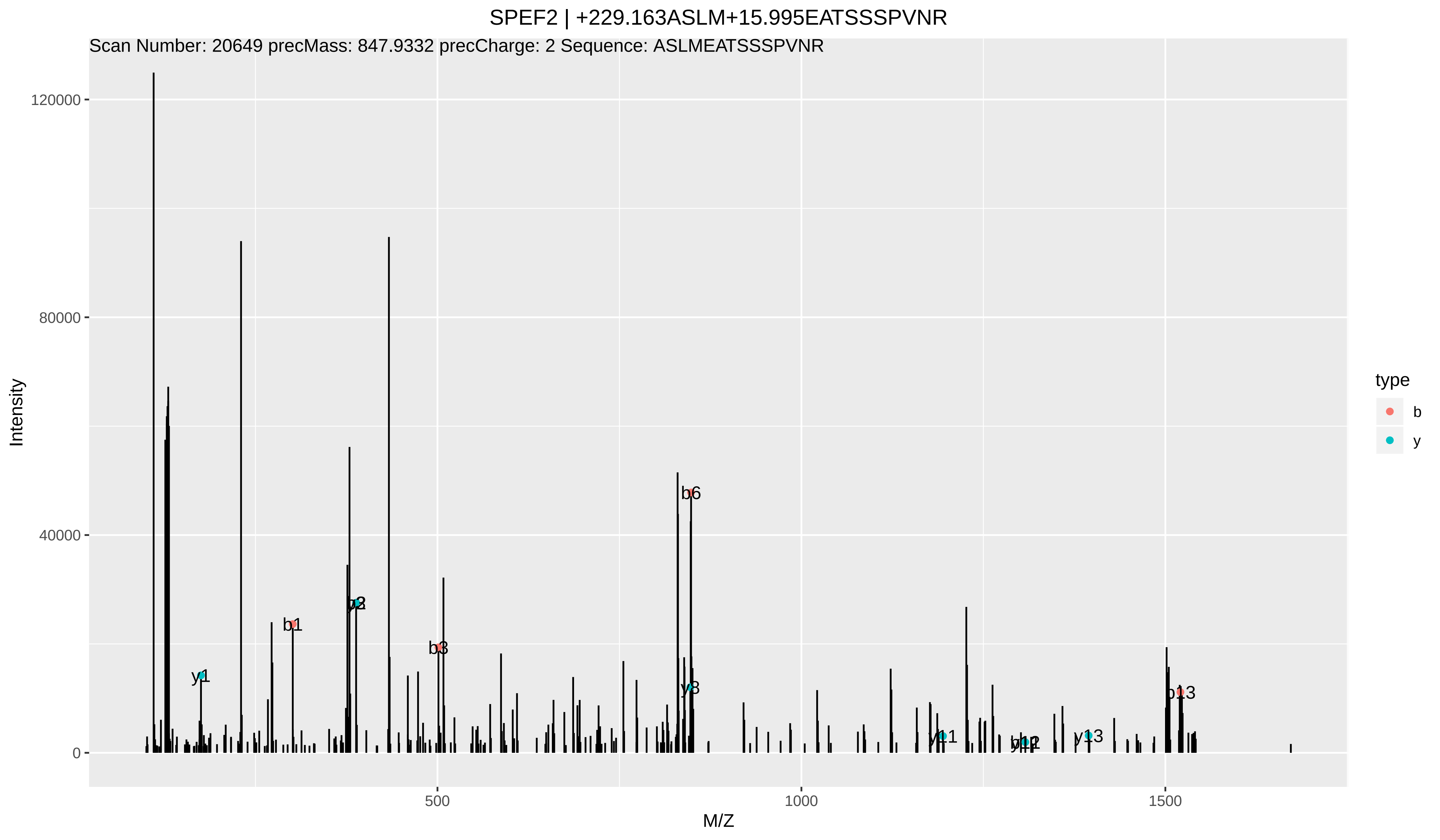

SPEF2 | +229.163ASLM+15.995EATSSSPVNR
Scan Number: 20649 precMass: 847.9332 precCharge: 2 Sequence: ASLMEATSSSPVNR
Intensity
120000
80000
40000
0
M/Z
500
1000
1500
y1
b1
b2
y2
b3
b6
y8
y11
b12
y12
y13
b13
type
b
y

SPEG | +229.163REEPDLQPQLASEAPR

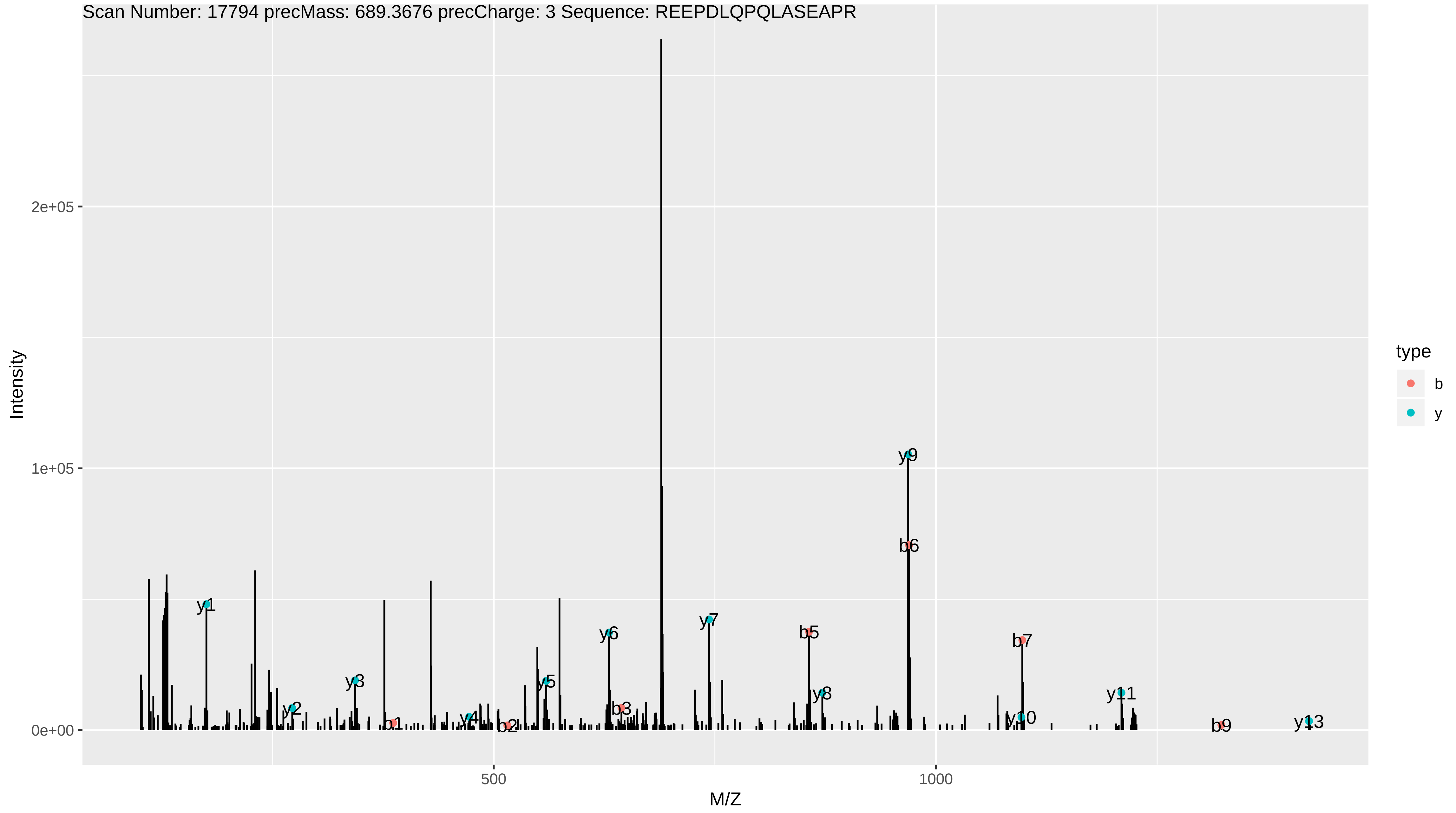

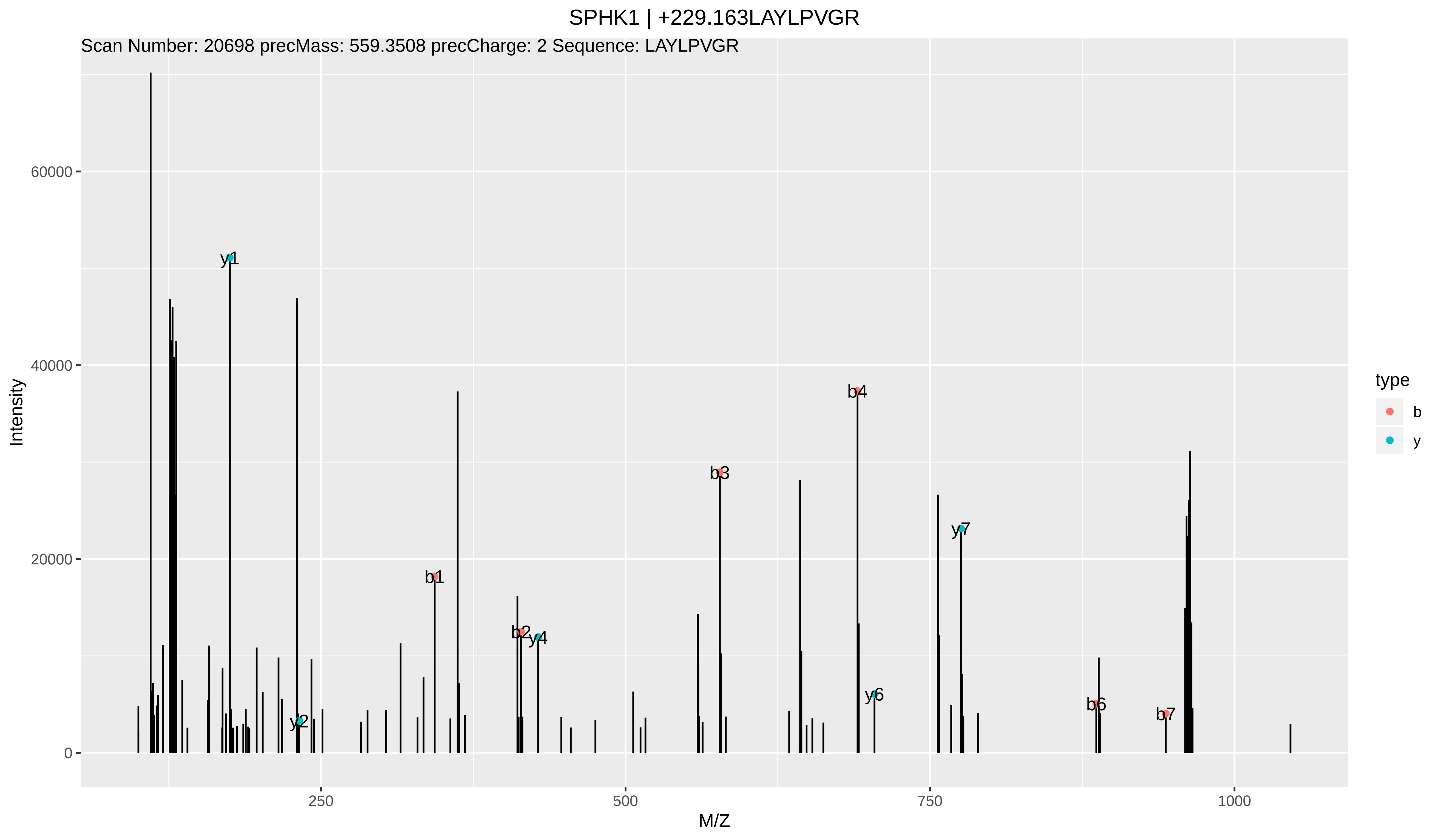
SPHK1 | +229.163LAYLPVGR
Scan Number: 20698 precMass: 559.3508 precCharge: 2 Sequence: LAYLPVGR
Intensity
M/Z
0
20000
40000
60000
250
500
750
1000
y1
y2
b1
b2
y4
b3
b4
y6
y7
b6
b7
type
b
y

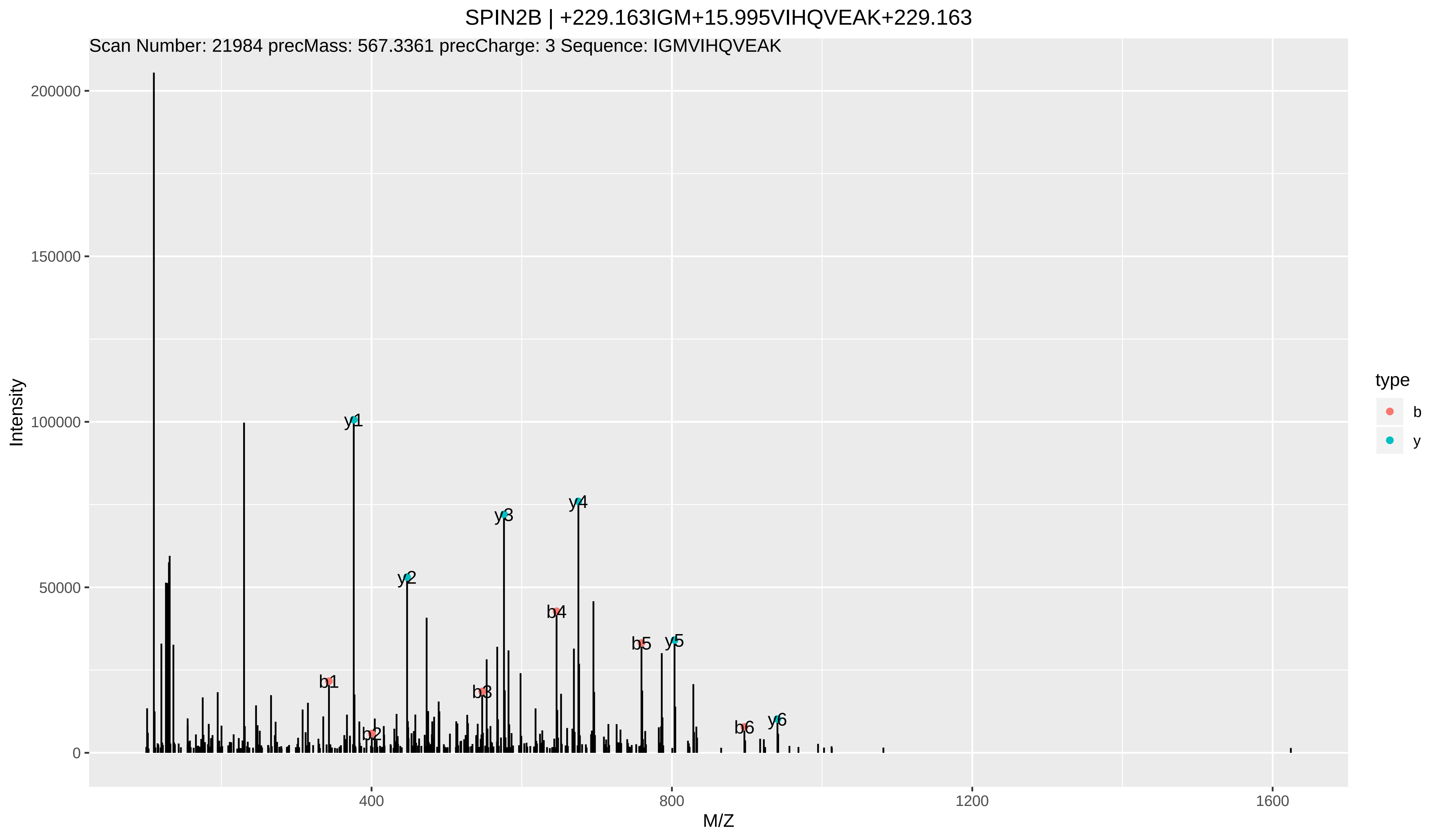
SPIN2B | +229.163IGM+15.995VIHQVEAK+229.163
Scan Number: 21984 precMass: 567.3361 precCharge: 3 Sequence: IGMVIHQVEAK
Intensity
M/Z
200000
150000
100000
50000
0
400
800
1200
1600
y1
y2
y3
y4
y5
y6
b1
b2
b3
b4
b5
b6
type
b
y

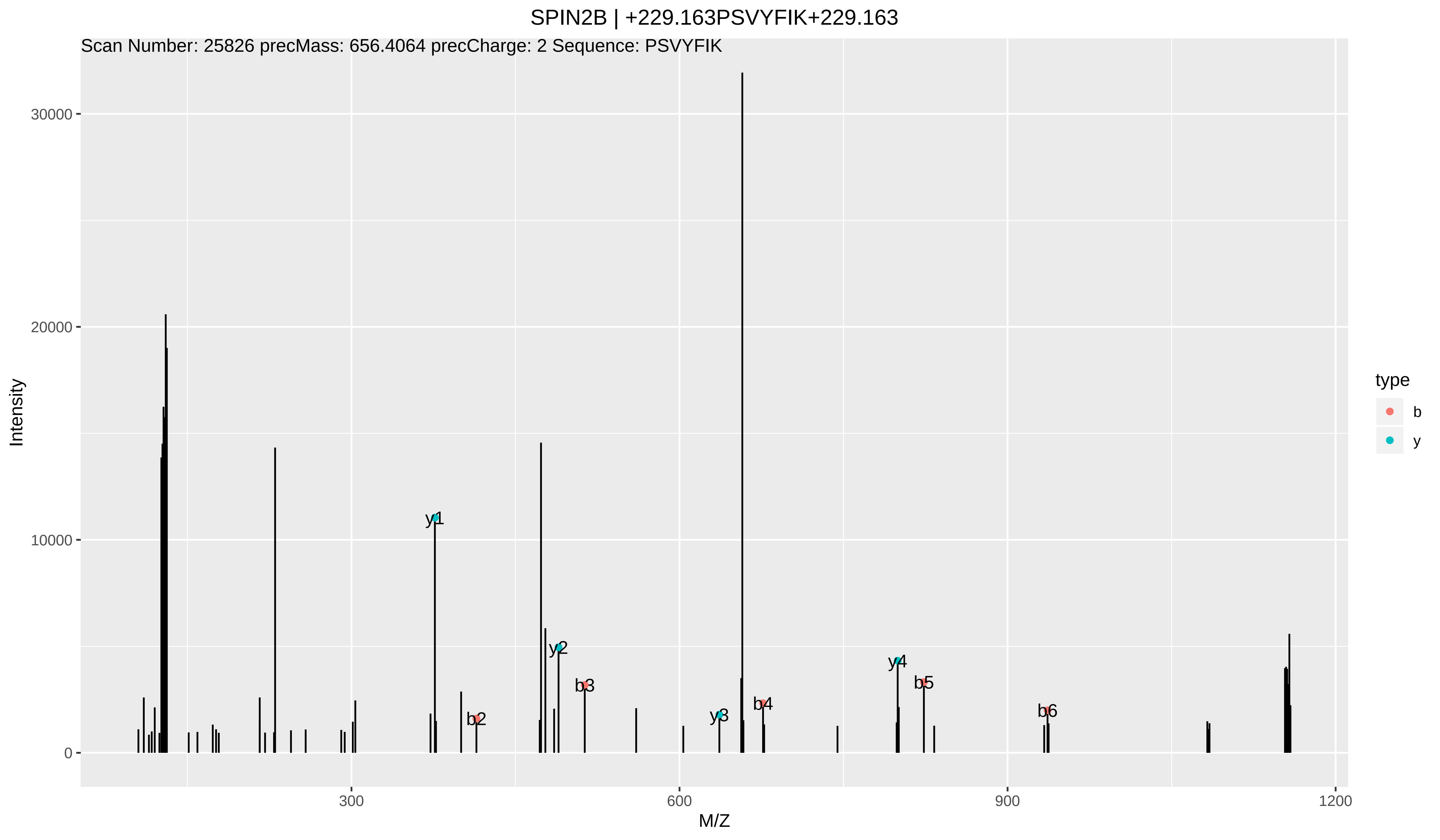
SPIN2B | +229.163PSVYFIK+229.163
Scan Number: 25826 precMass: 656.4064 precCharge: 2 Sequence: PSVYFIK
Intensity
30000
20000
10000
0
M/Z
300
600
900
1200
y1
b2
y2
b3
y3
b4
y4
b5
b6
type
b
y

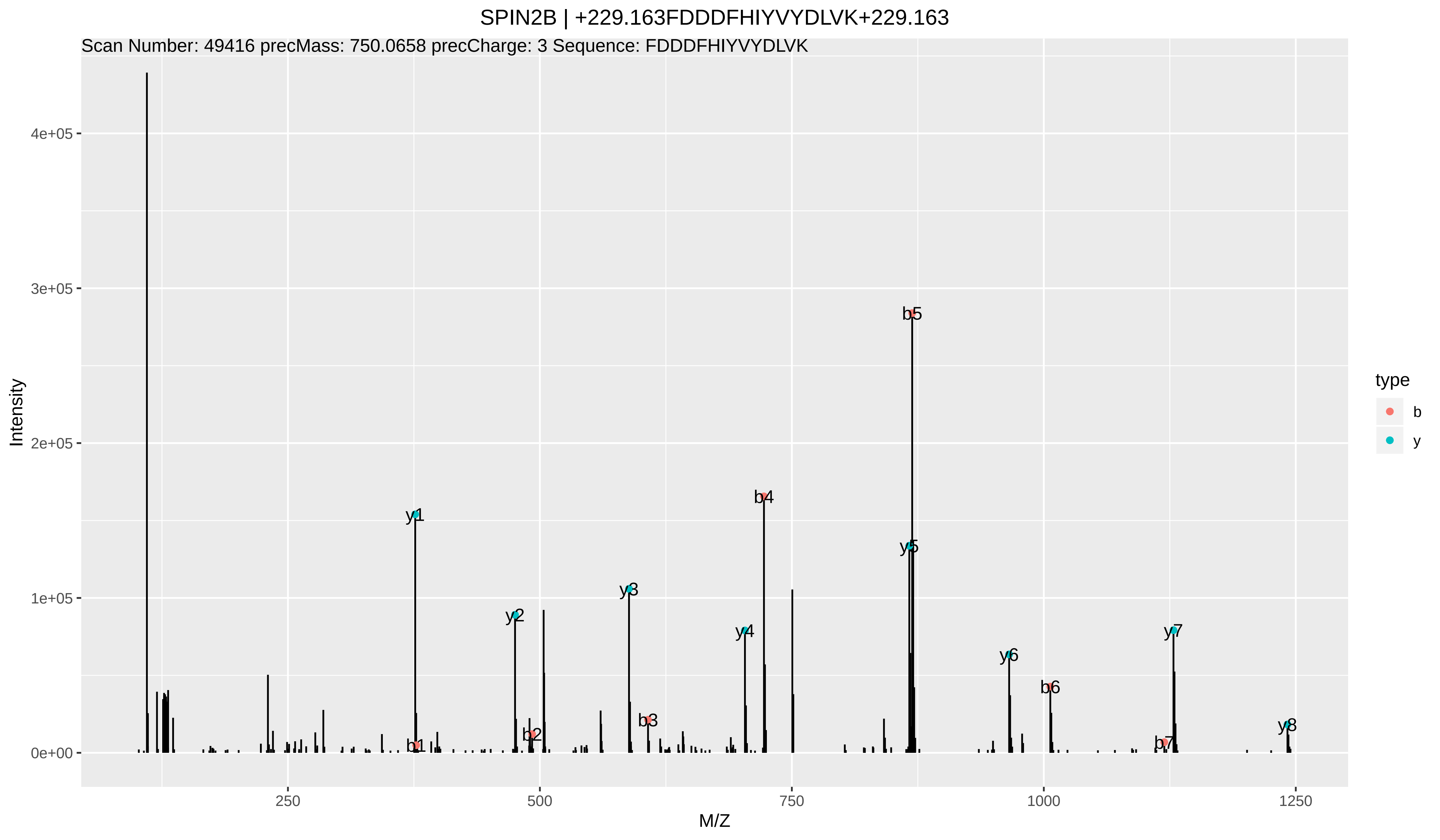

SPIN2B | +229.163FDDDFHIYVYDLVK+229.163
Scan Number: 49416 precMass: 750.0658 precCharge: 3 Sequence: FDDDFHIYVYDLVK
Intensity
4e+05
3e+05
2e+05
1e+05
0e+00
M/Z
250
500
750
1000
1250
y1
b1
y2
b2
y3
b3
y4
b4
y5
b5
y6
b6
y7
b7
y8
type
b
y

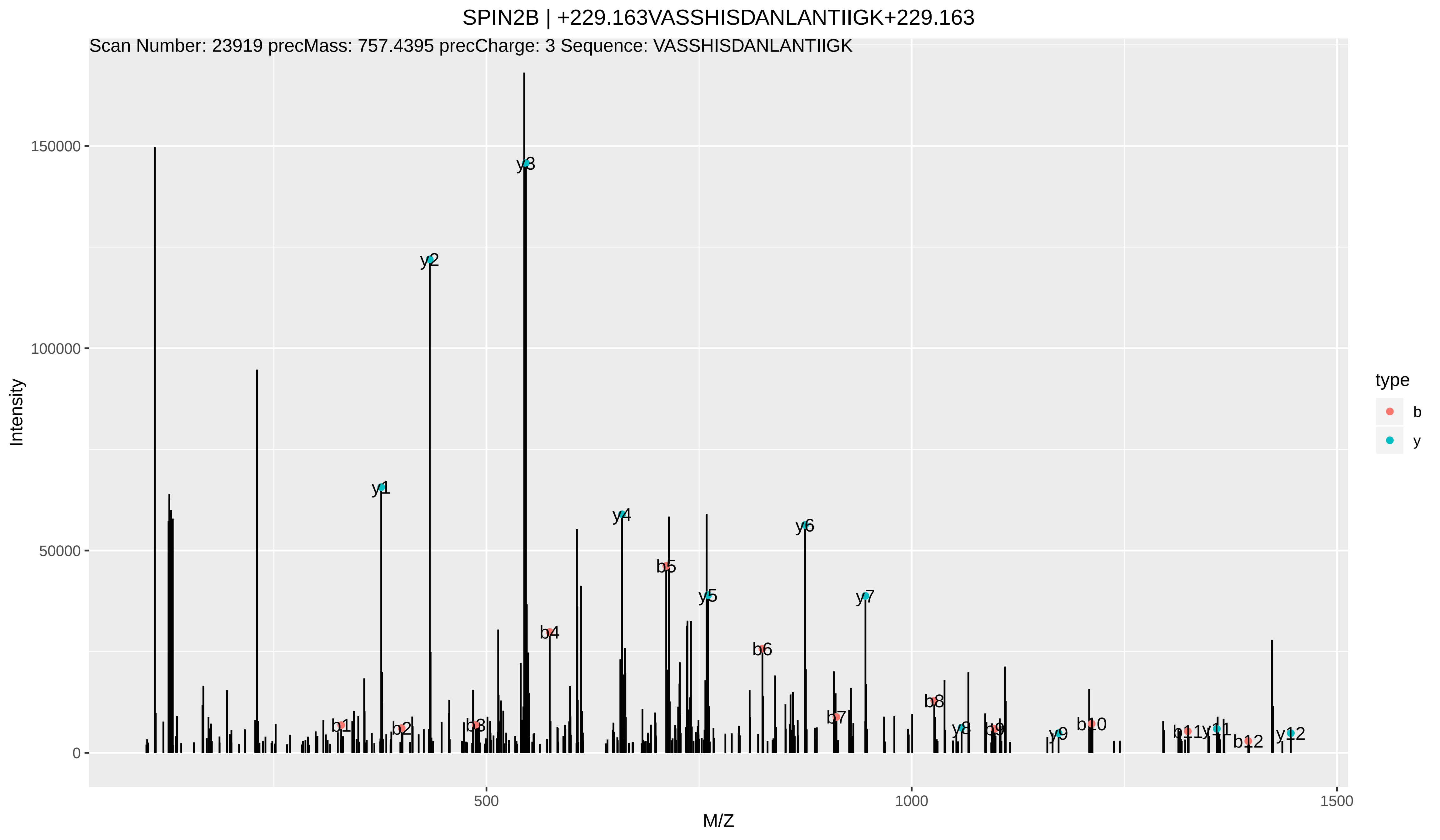
SPIN2B | +229.163VASSHISDANLANTIIGK+229.163
Scan Number: 23919 precMass: 757.4395 precCharge: 3 Sequence: VASSHISDANLANTIIGK
Intensity
M/Z
150000
100000
50000
0
500
1000
1500
type
b
y
y1
y2
y3
y4
y5
y6
y7
y8
y9
y11
y12
b1
b2
b3
b4
b5
b6
b7
b8
b9
b10
b11
b12

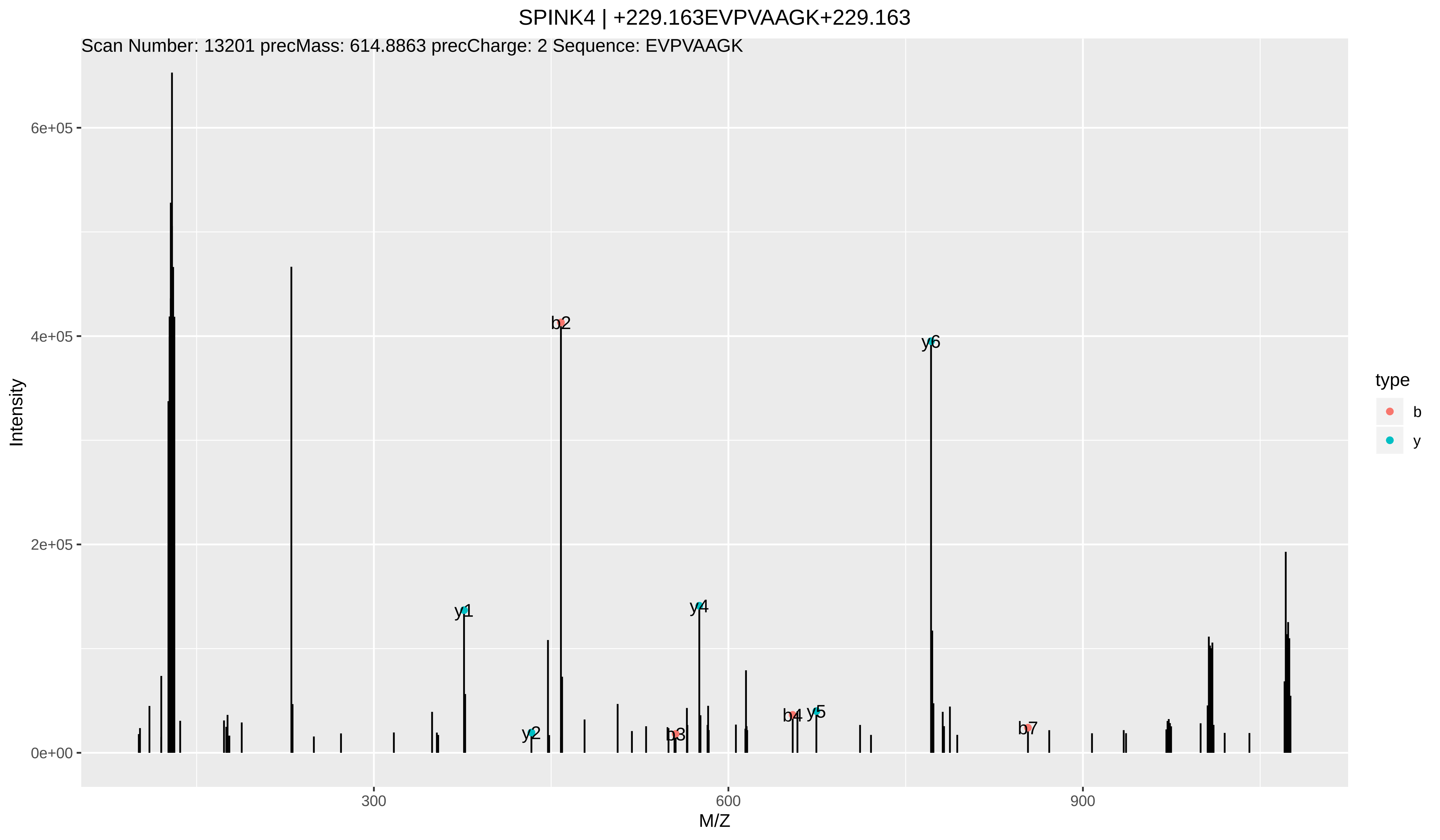

SPINK4 | +229.163EVPVAAGK+229.163
Scan Number: 13201 precMass: 614.8863 precCharge: 2 Sequence: EVPVAAGK
Intensity
6e+05
4e+05
2e+05
0e+00
M/Z
300
600
900
b2
y6
y1
y4
y2
b3
b4
y5
b7
type
b
y

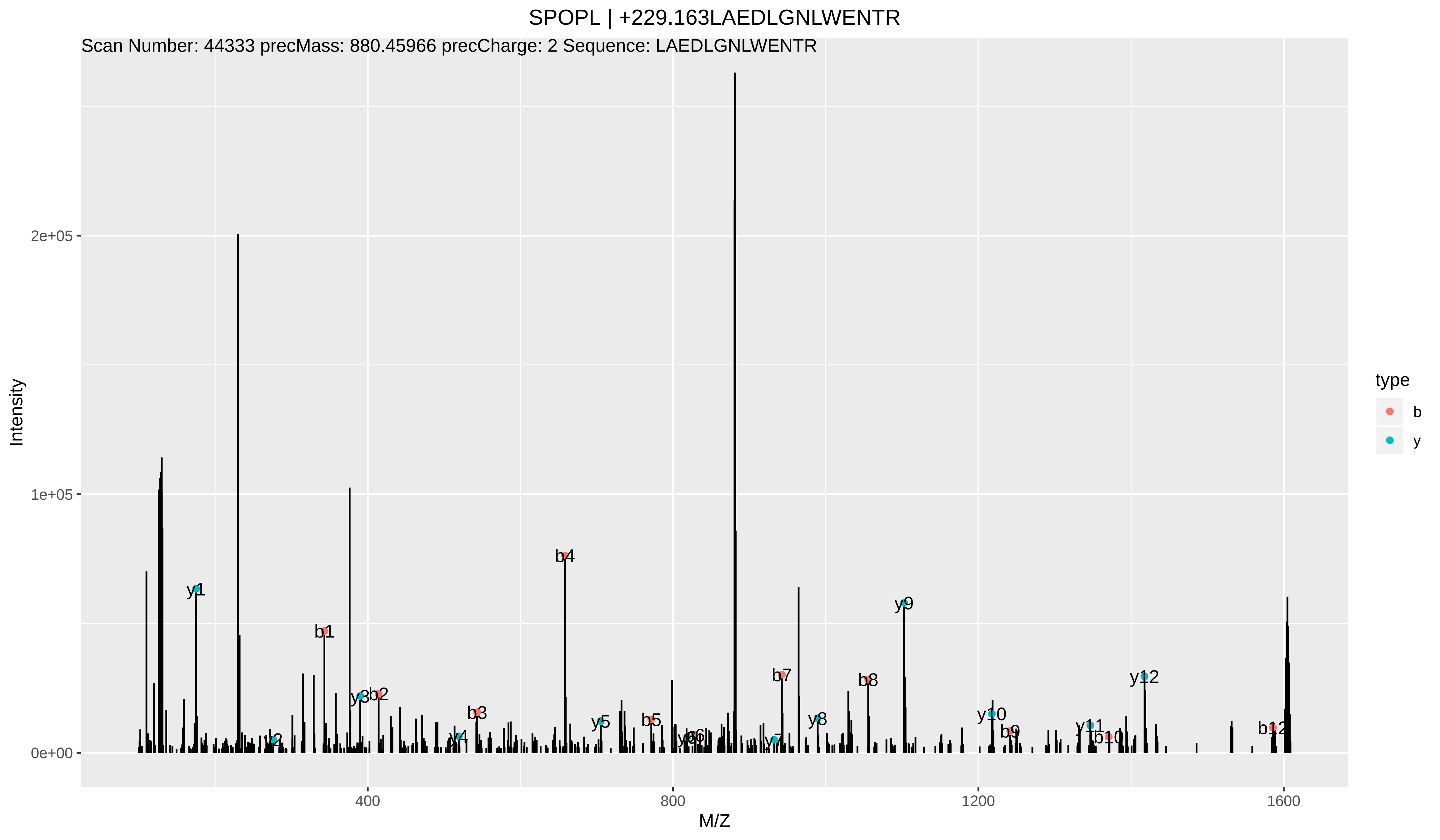

SPOPL | +229.163LAEDLGNLWENTR
Scan Number: 44333 precMass: 880.45966 precCharge: 2 Sequence: LAEDLGNLWENTR
Intensity
M/Z
2e+05
1e+05
0e+00
400
800
1200
1600
y1
b1
y3
b2
b3
b4
y5
b5
y7
b7
y8
b8
y9
y10
b9
y11
b10
y12
b12
type
b
y

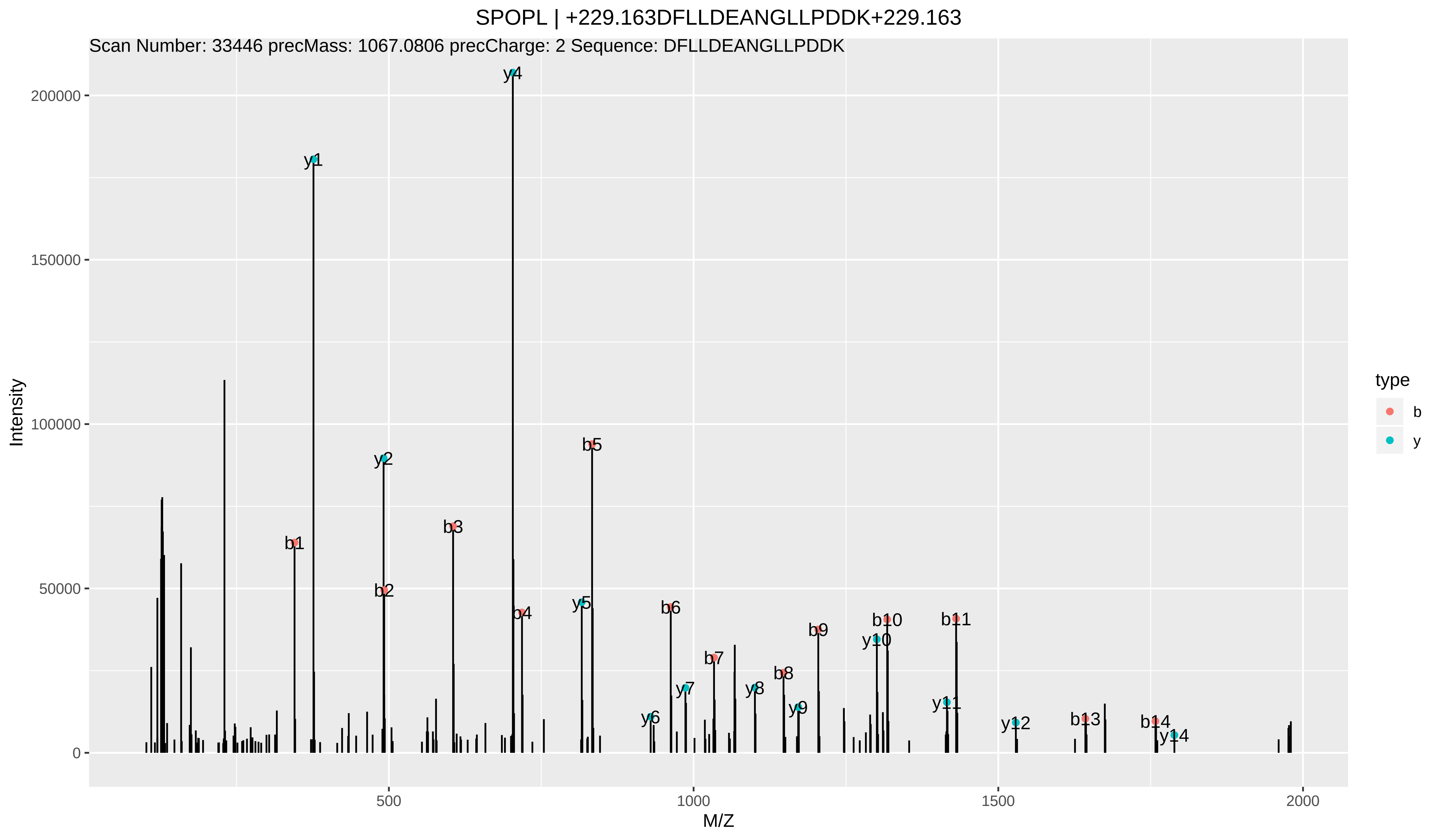

SPOPL | +229.163DFLLDEANGLLPDDK+229.163
Scan Number: 33446 precMass: 1067.0806 precCharge: 2 Sequence: DFLLDEANGLLPDDK
Intensity
M/Z
0
50000
100000
150000
200000
500
1000
1500
2000
type
b
y
y1
y2
y4
y5
y6
y7
y8
y9
y10
y11
y12
y14
b1
b2
b3
b4
b5
b6
b7
b8
b9
b10
b11
b13
b14

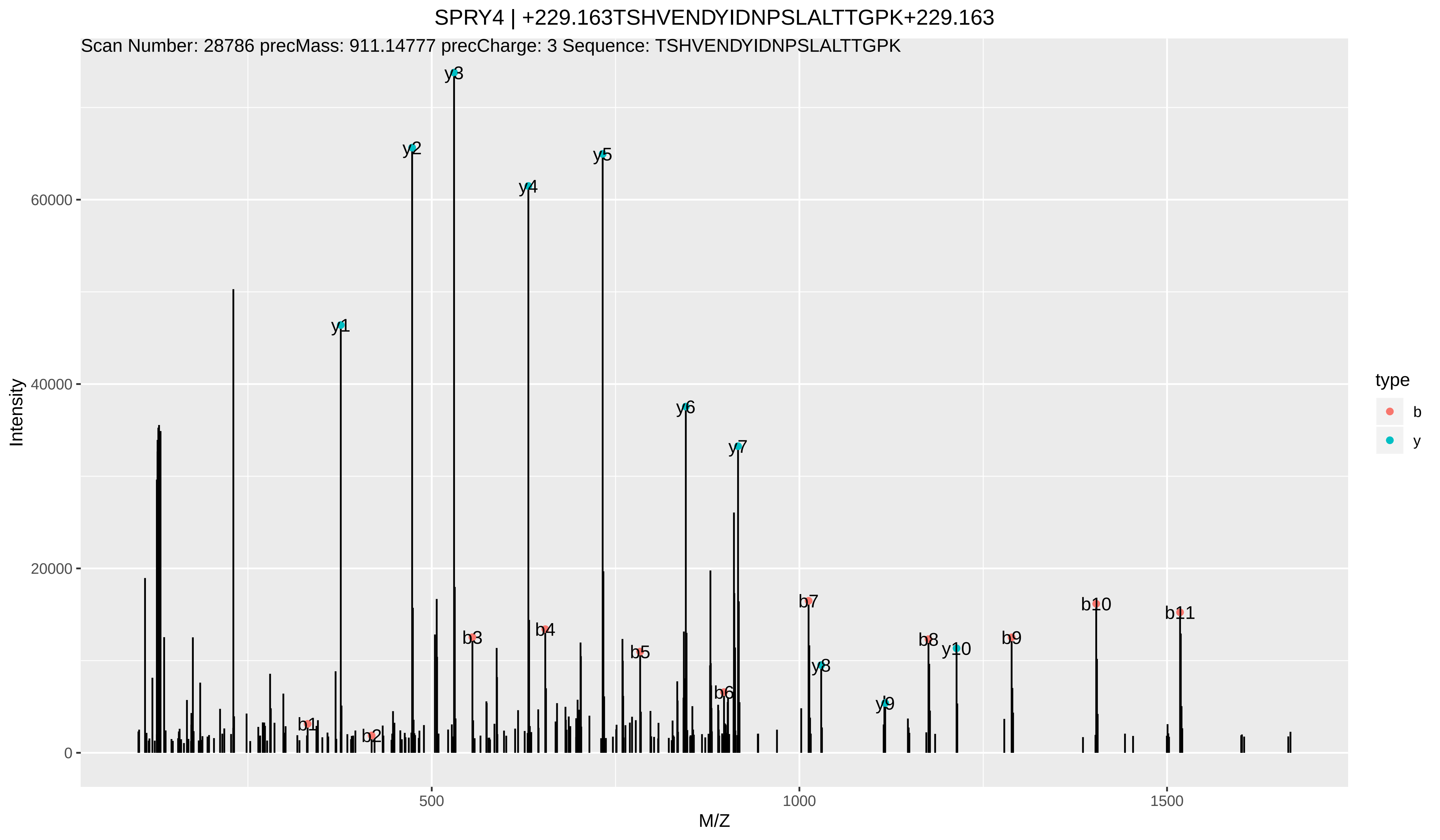

SPRY4 | +229.163TSHVENDYIDNPSLALTTGPK+229.163
Scan Number: 28786 precMass: 911.14777 precCharge: 3 Sequence: TSHVENDYIDNPSLALTTGPK
Intensity
M/Z
0
20000
40000
60000
500
1000
1500
y1
y2
y3
y4
y5
y6
y7
y8
y9
y10
b1
b2
b3
b4
b5
b6
b7
b8
b9
b10
b11
type
b
y

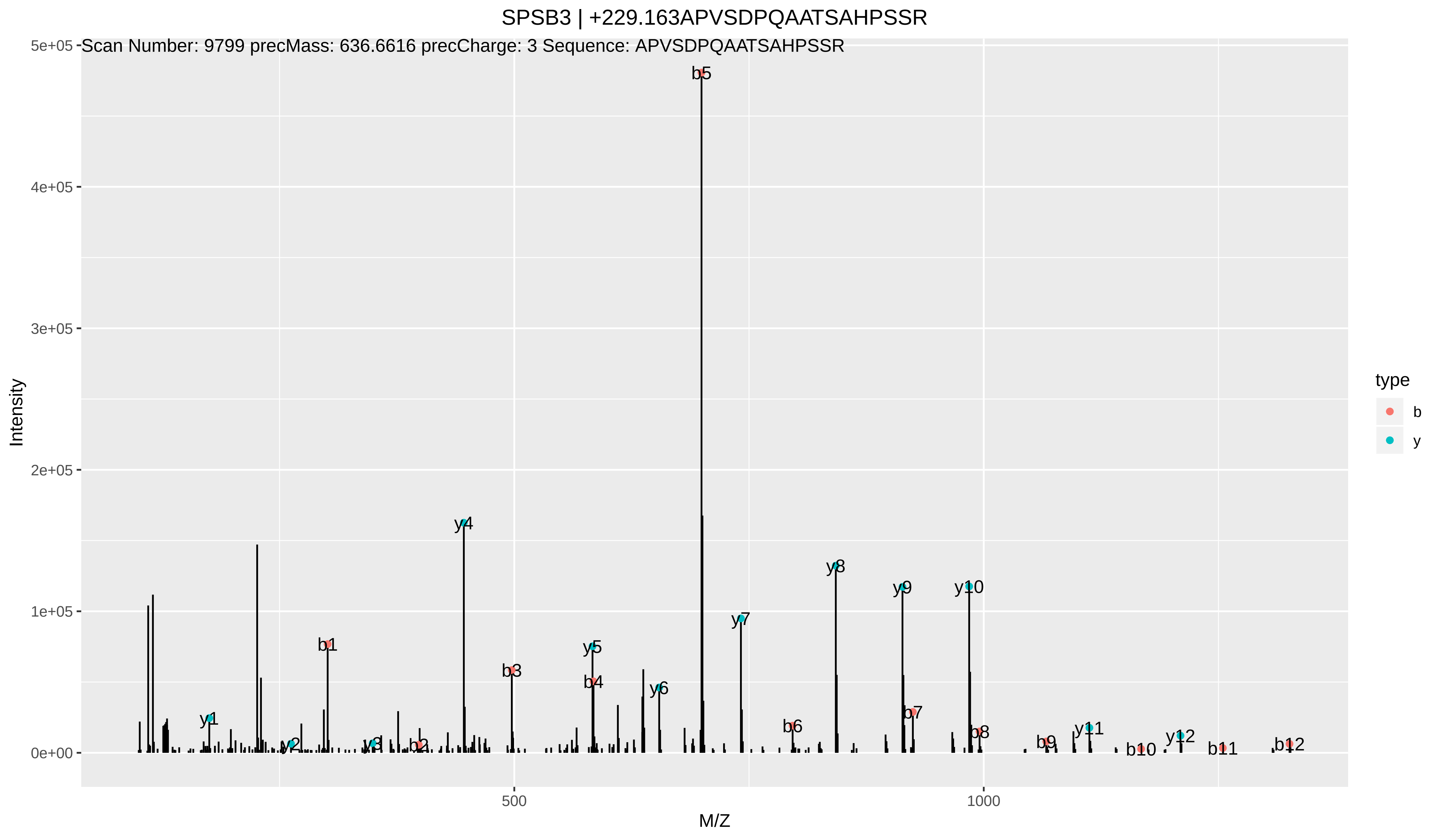

SPSB3 | +229.163APVSDPQAATSAHPSSR
Scan Number: 9799 precMass: 636.6616 precCharge: 3 Sequence: APVSDPQAATSAHPSSR
Intensity
M/Z
0e+00
1e+05
2e+05
3e+05
4e+05
5e+05
500
1000
type
b
y
b1
b2
b3
b4
b5
b6
b7
b8
b9
b10
b11
b12
y1
y2
y3
y4
y5
y6
y7
y8
y9
y10
y11
y12

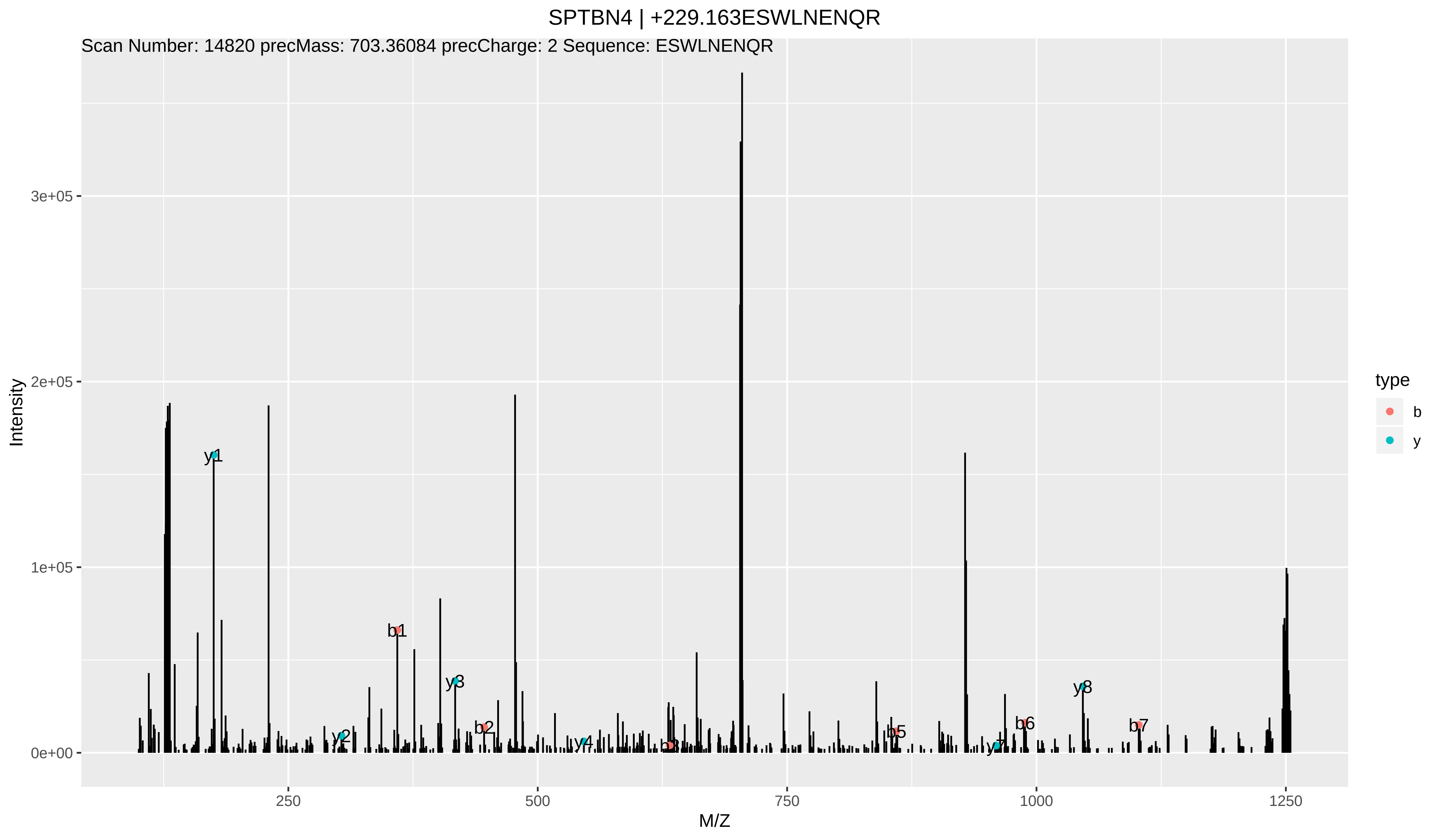

SPTBN4 | +229.163ESWLNENQR
Scan Number: 14820 precMass: 703.36084 precCharge: 2 Sequence: ESWLNENQR
Intensity
3e+05
2e+05
1e+05
0e+00
y1
y2
b1
y3
b2
y4
b3
b5
y7
b6
y8
b7
250
500
750
1000
1250
M/Z
type
b
y

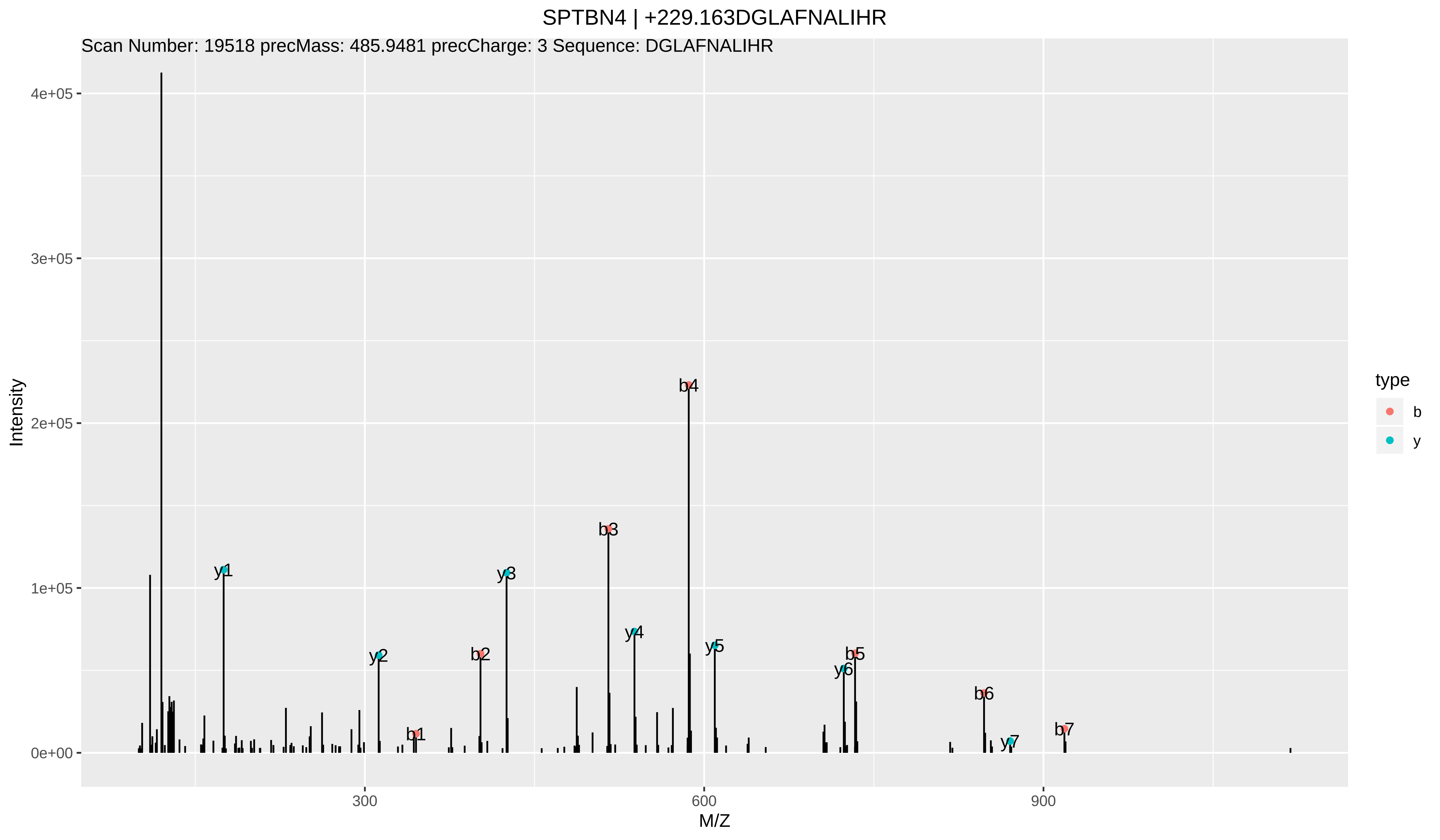
SPTBN4 | +229.163DGLAFNALIHR
Scan Number: 19518 precMass: 485.9481 precCharge: 3 Sequence: DGLAFNALIHR
Intensity
4e+05
3e+05
2e+05
1e+05
0e+00
M/Z
300
600
900
y1
y2
b1
b2
y3
b3
y4
b4
y5
y6
b5
b6
y7
b7
type
b
y

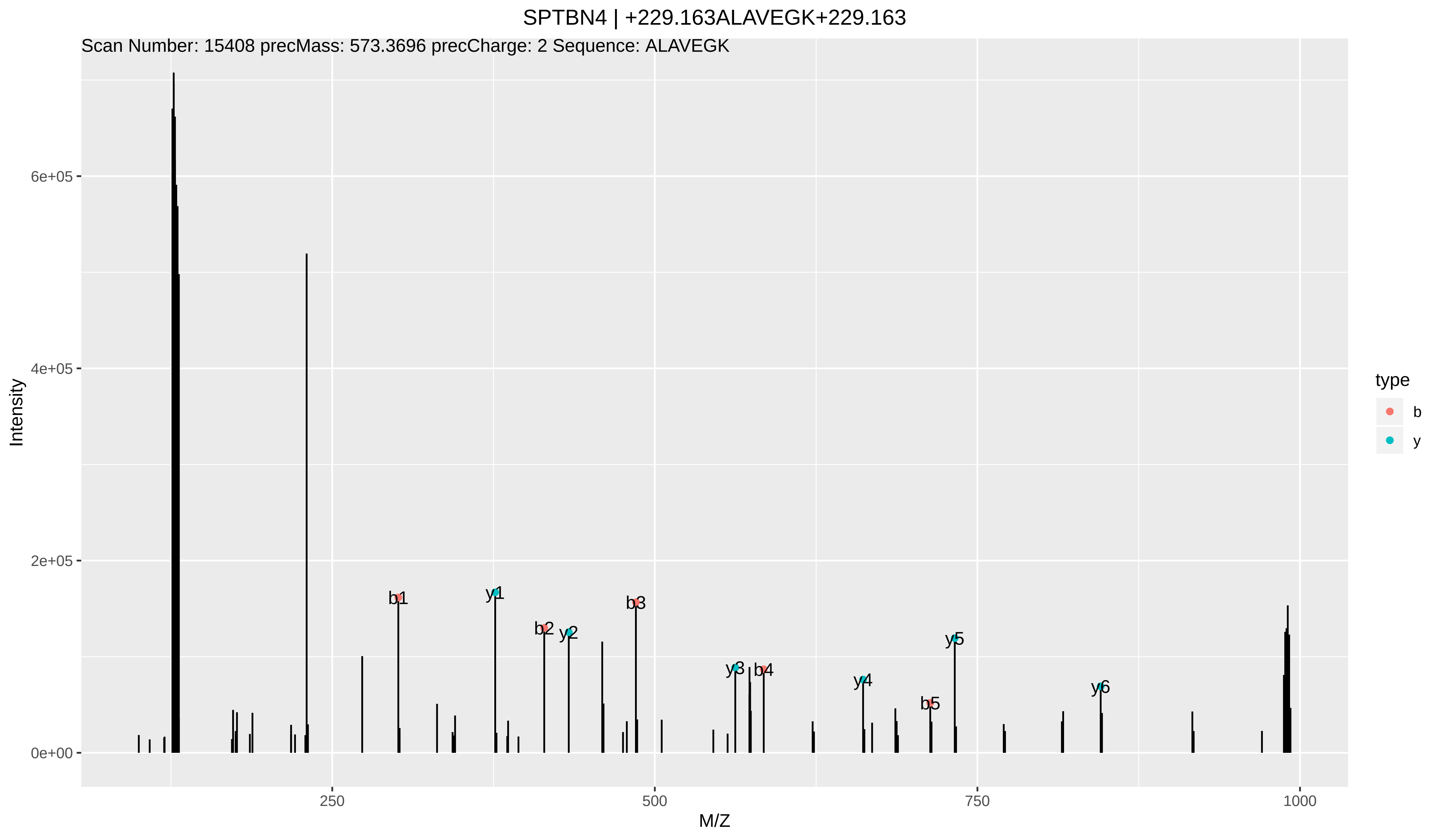

SPTBN4 | +229.163ALAVEGK+229.163
Scan Number: 15408 precMass: 573.3696 precCharge: 2 Sequence: ALAVEGK
Intensity
6e+05
4e+05
2e+05
0e+00
M/Z
250
500
750
1000
b1
y1
b2
y2
b3
y3
b4
y4
b5
y5
y6
type
b
y

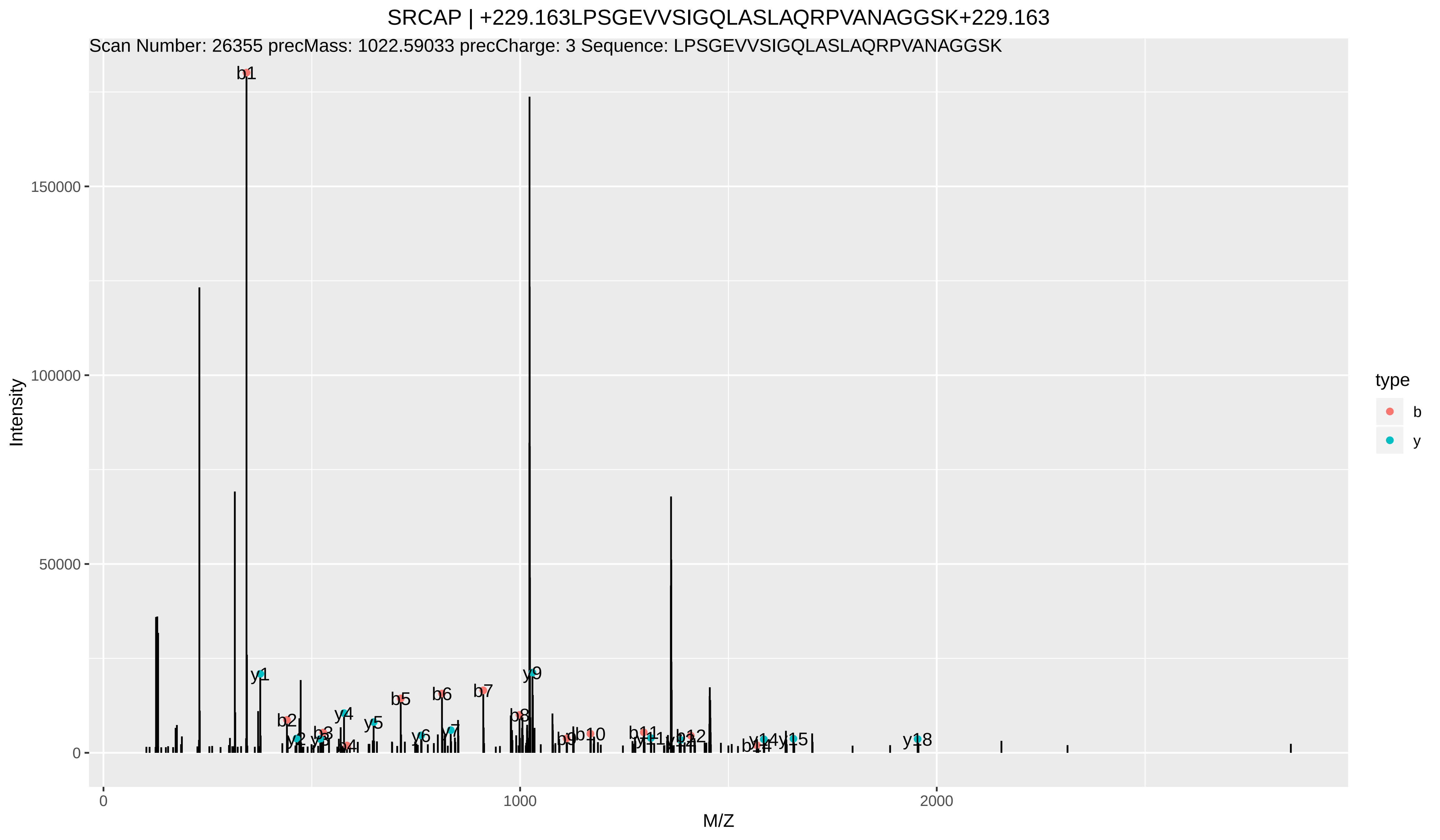
SRCAP | +229.163LPSGEVVSIGQLASLAQRPVANAGGSK+229.163
Scan Number: 26355 precMass: 1022.59033 precCharge: 3 Sequence: LPSGEVVSIGQLASLAQRPVANAGGSK
Intensity
M/Z
150000
100000
50000
0
1000
2000
type
b
y
b1
y1
b2
y2
b3
y3
y4
b4
b5
y5
y6
b6
y7
b7
b8
y9
b9
b10
b11
y11
b12
y12
b14
y14
y15
y18

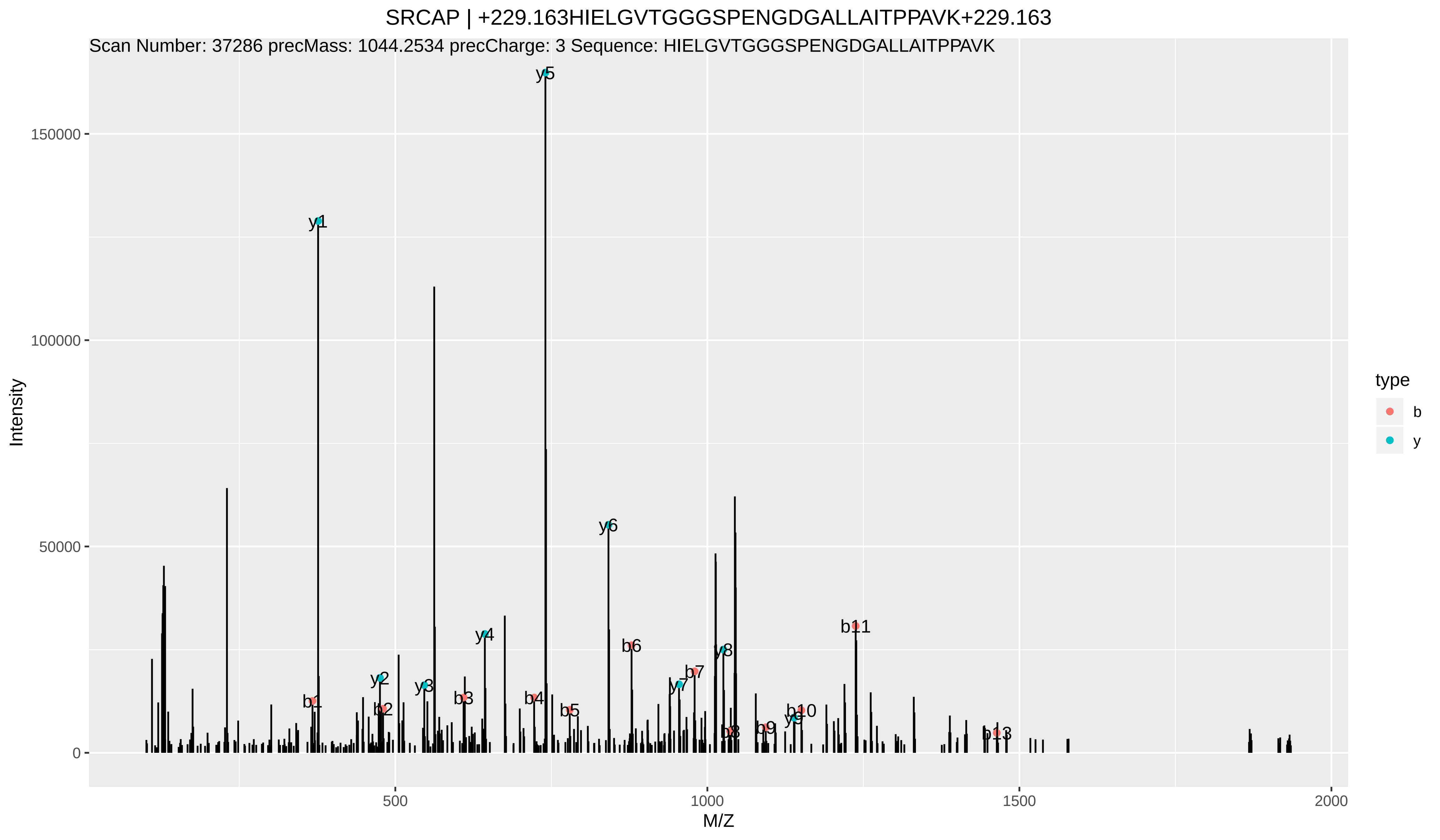
SRCAP | +229.163HIELGVTGGGSPENGDGALLAITPPAVK+229.163
Scan Number: 37286 precMass: 1044.2534 precCharge: 3 Sequence: HIELGVTGGGSPENGDGALLAITPPAVK
Intensity
M/Z
type
b
y
y5
y1
y6
y4
y8
y2
y3
y7
y9
b1
b2
b3
b4
b5
b6
b7
b8
b9
b10
b11
b13
0
50000
100000
150000
500
1000
1500
2000

SRF | +229.163LQPM+15.995ITSETGK+229.163

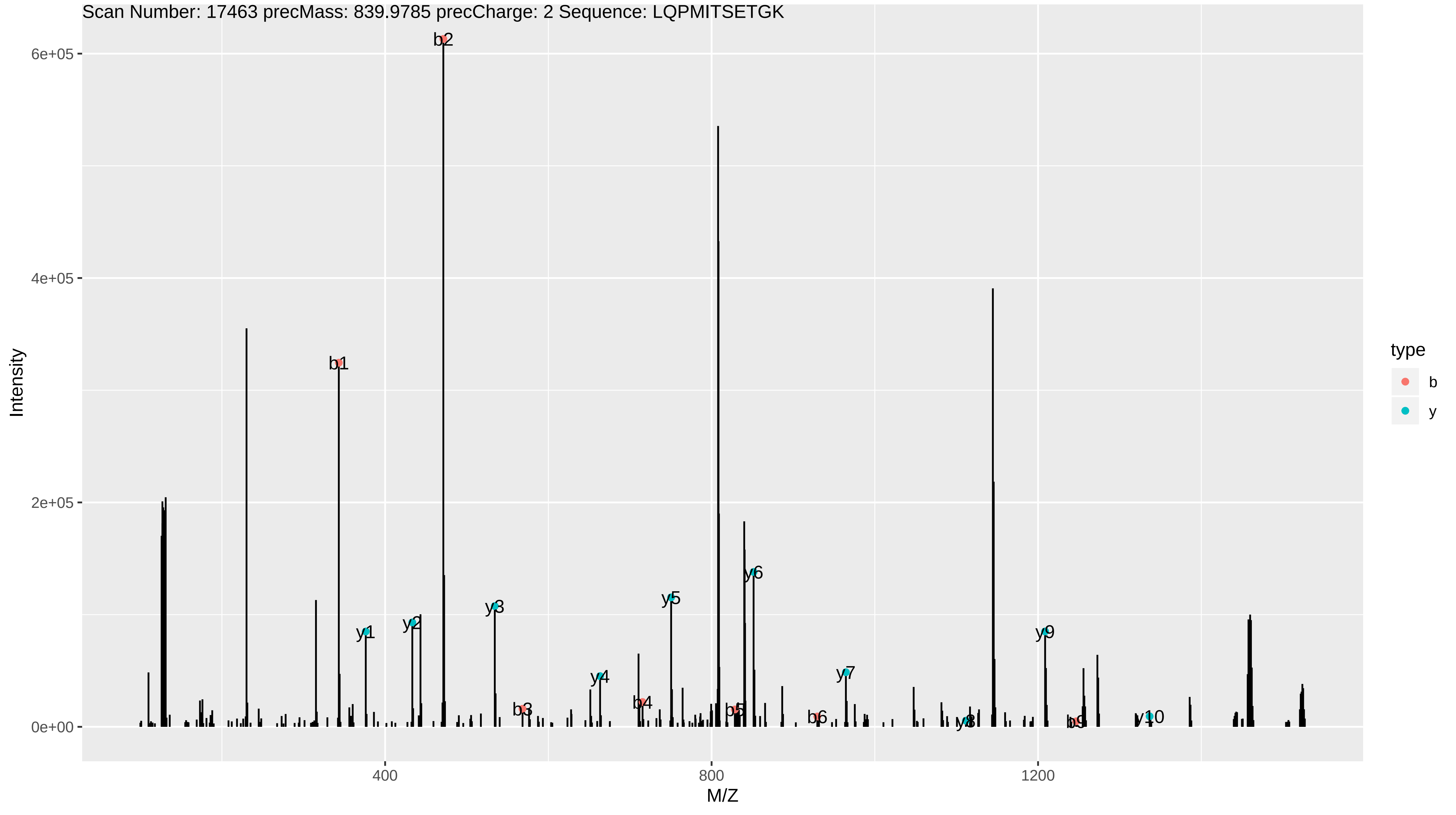

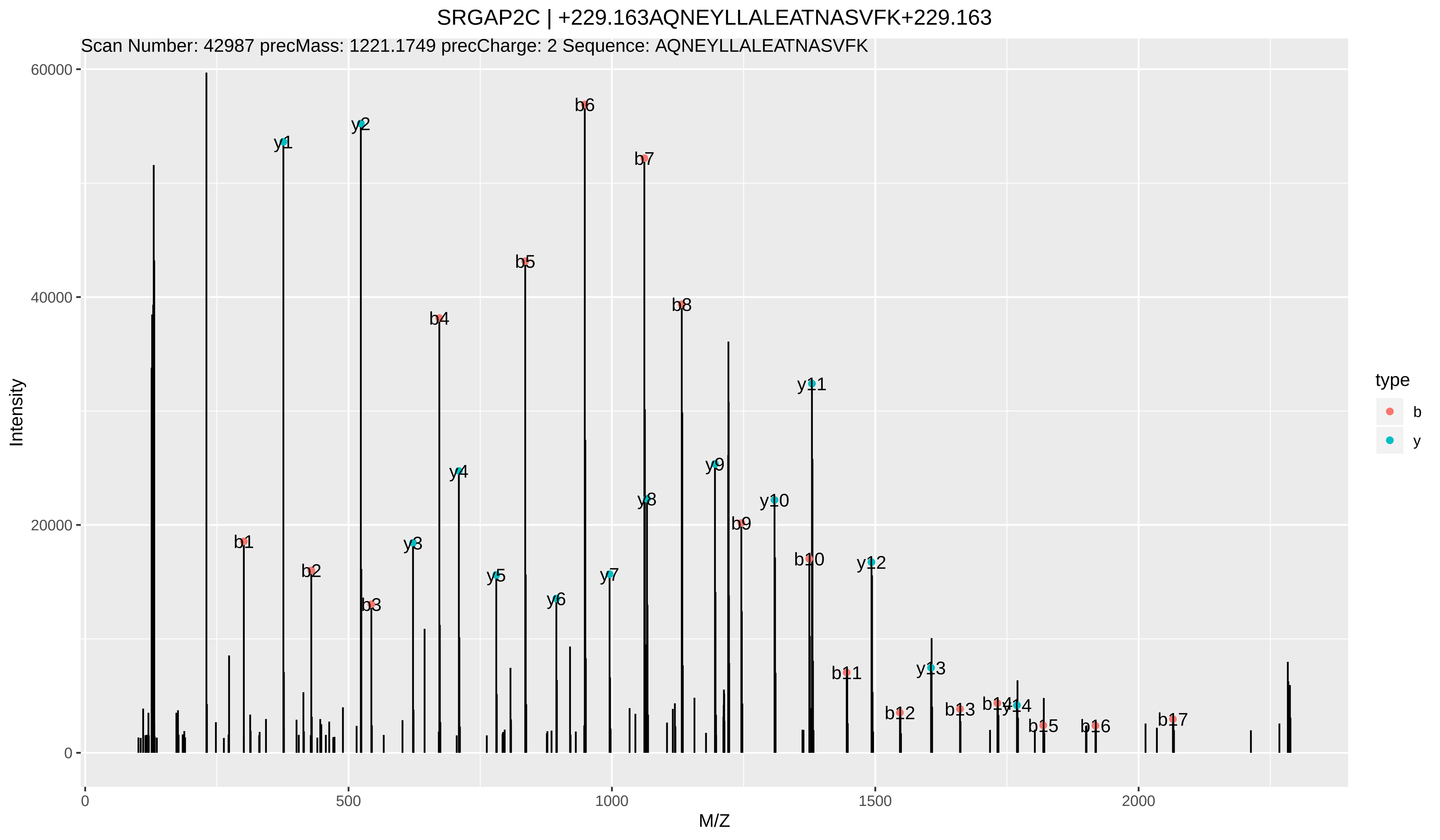
SRGAP2C | +229.163AQNEYLLALEATNASVFK+229.163
Scan Number: 42987 precMass: 1221.1749 precCharge: 2 Sequence: AQNEYLLALEATNASVFK
Intensity
M/Z
type
b
y
b1
b2
b3
b4
b5
b6
b7
b8
b9
b10
b11
b12
b13
b14
b15
b16
b17
y1
y2
y3
y4
y5
y6
y7
y8
y9
y10
y11
y12
y13
y14
0
20000
40000
60000
500
1000
1500
2000

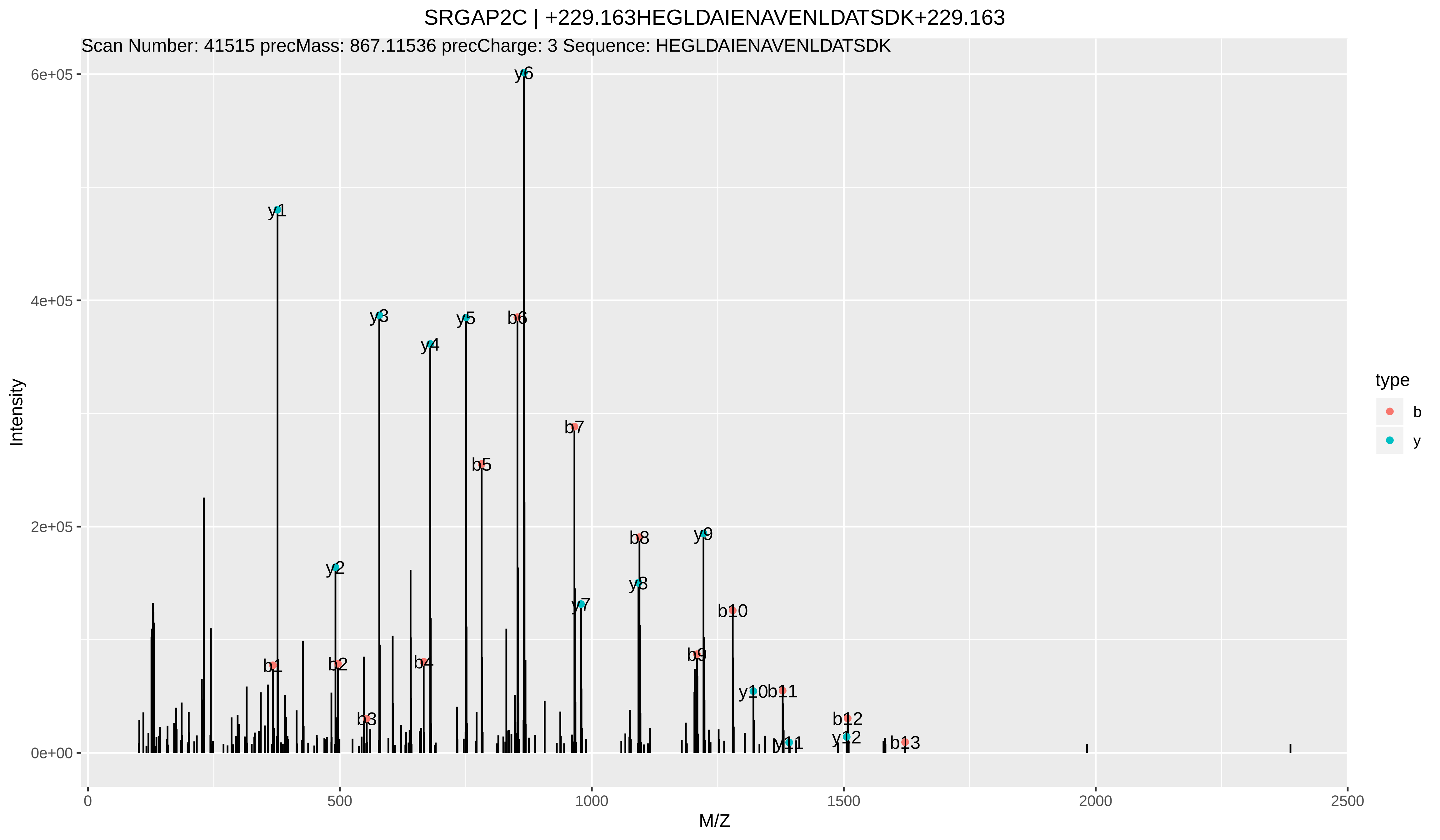
SRGAP2C | +229.163HEGLDAIENAVENLDATSDK+229.163
Scan Number: 41515 precMass: 867.11536 precCharge: 3 Sequence: HEGLDAIENAVENLDATSDK
Intensity
6e+05
4e+05
2e+05
0e+00
M/Z
0
500
1000
1500
2000
2500
y6
y1
y3
y5
b6
y4
b7
b5
b8
y9
y2
y8
y7
b10
b9
b1
b2
b4
y10
b11
b3
b12
y12
b13
type
b
y

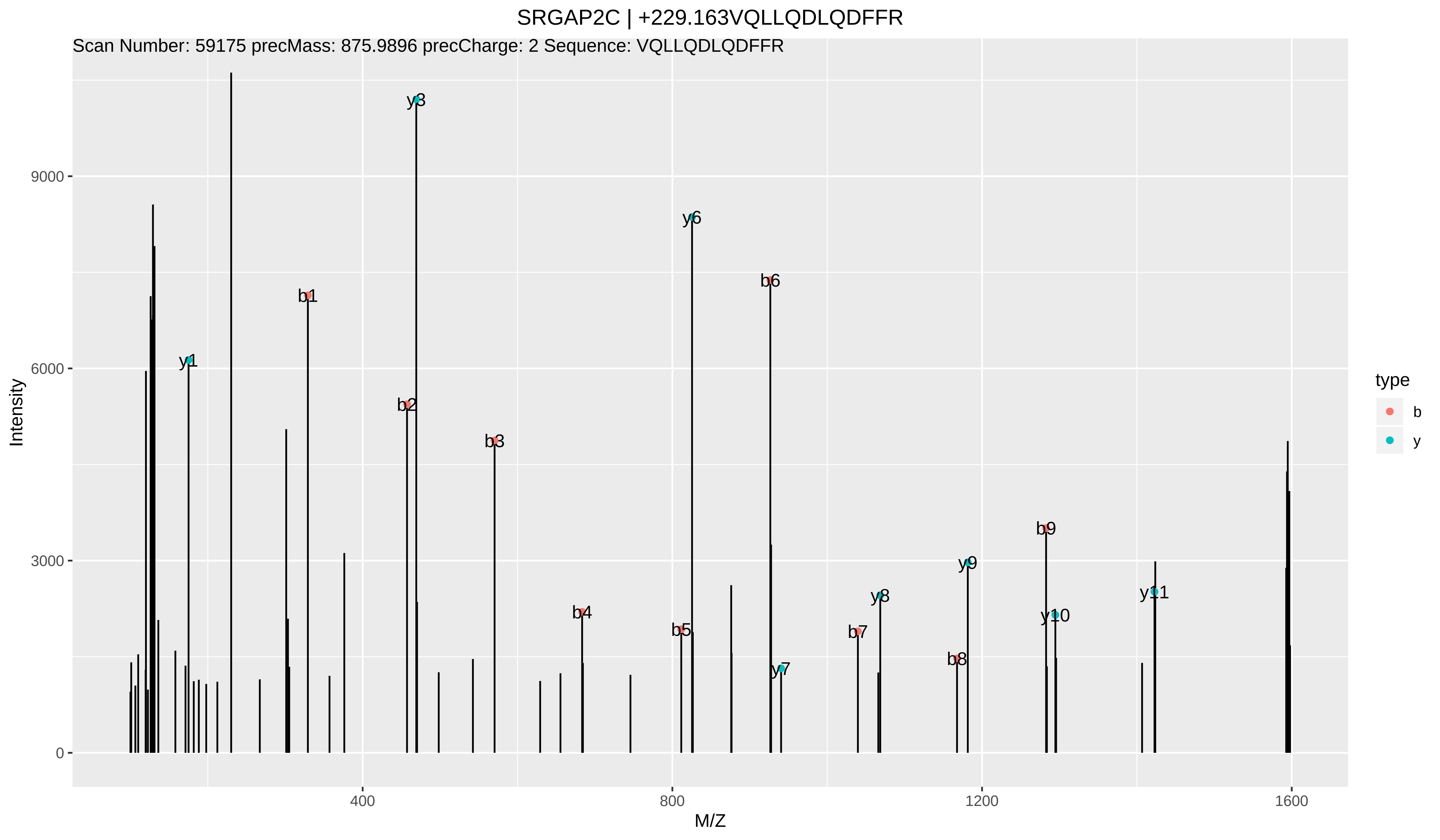

SRGAP2C | +229.163VQLLQDLQDFFR
Scan Number: 59175 precMass: 875.9896 precCharge: 2 Sequence: VQLLQDLQDFFR
Intensity
9000
6000
3000
0
M/Z
400
800
1200
1600
y3
y6
b6
b1
y1
b2
b3
b9
y9
y11
y8
y10
b4
b5
b7
y7
b8
type
b
y

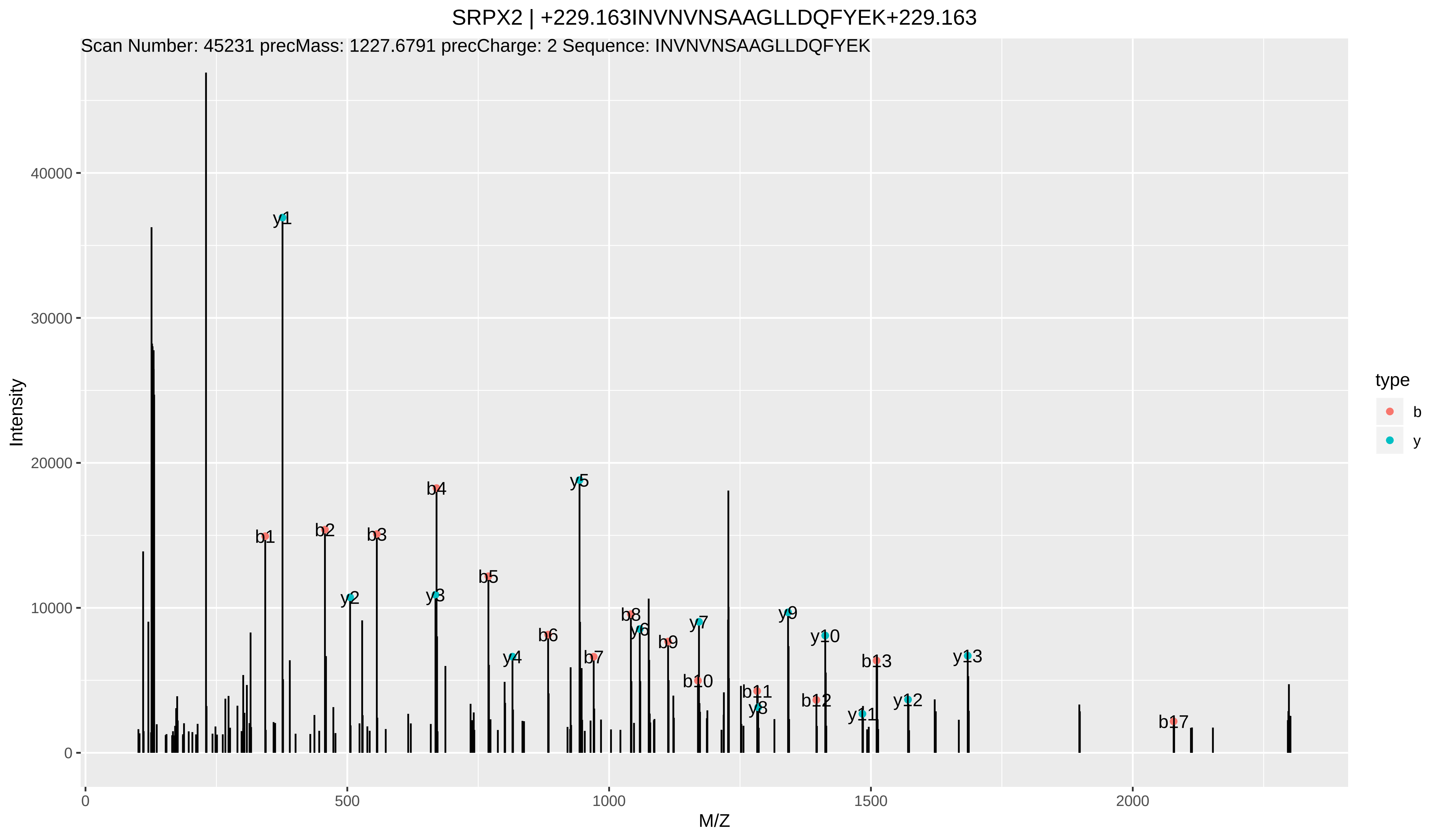
SRPX2 | +229.163INVNVNSAAGLLDQFYEK+229.163
Scan Number: 45231 precMass: 1227.6791 precCharge: 2 Sequence: INVNVNSAAGLLDQFYEK
Intensity
M/Z
0
10000
20000
30000
40000
0
500
1000
1500
2000
y1
b1
b2
b3
b4
y2
y3
b5
y4
b6
y5
b7
b8
y6
b9
y7
b10
b11
y8
y9
y10
b12
b13
y11
y12
y13
b17
type
b
y

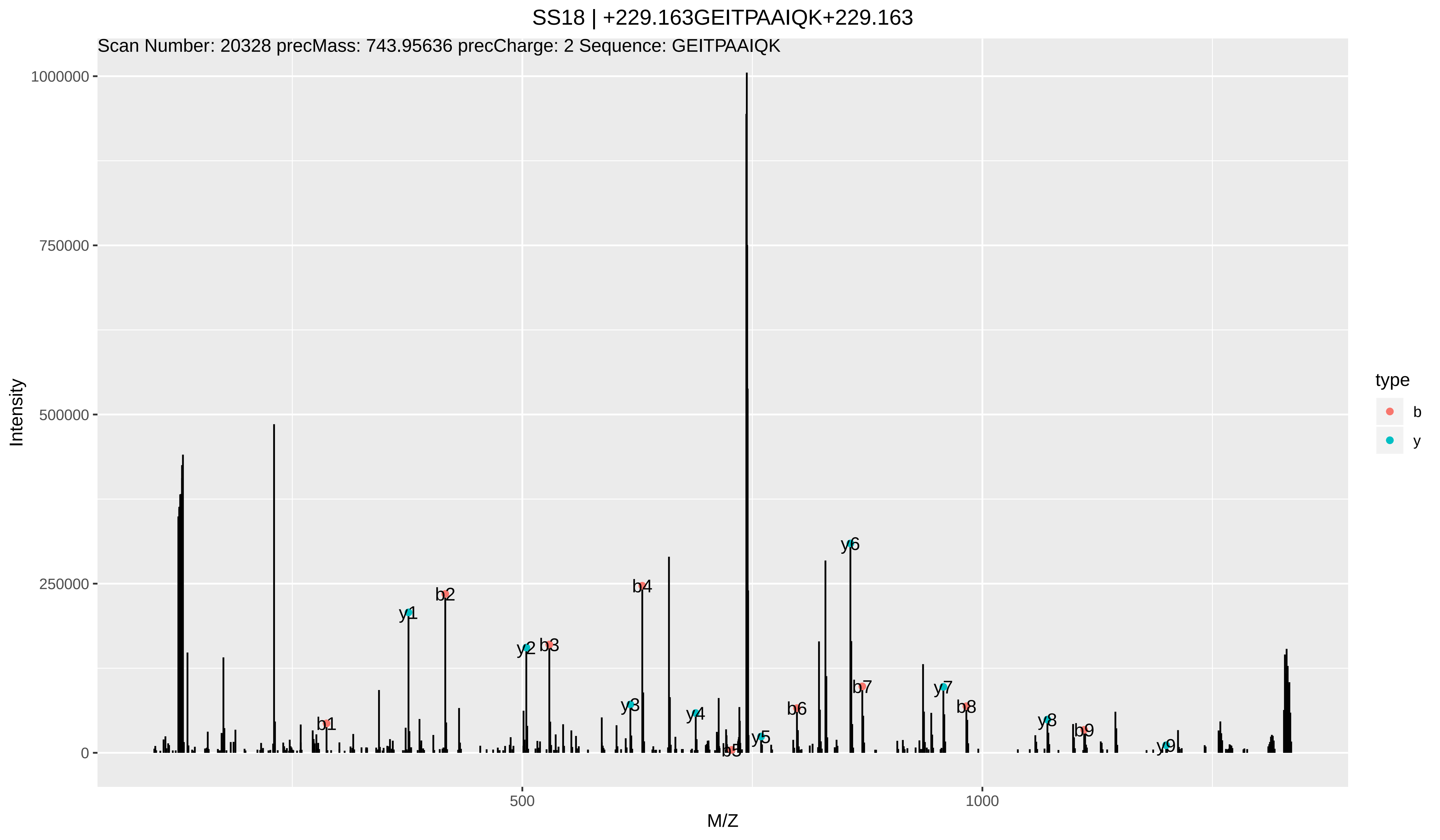

SS18 | +229.163GEITPAAIQK+229.163
Scan Number: 20328 precMass: 743.95636 precCharge: 2 Sequence: GEITPAAIQK
Intensity
1000000
750000
500000
250000
0
500
1000
M/Z
y1
b2
y2
b3
y3
b4
y4
b5
y5
b6
y6
b7
y7
b8
y8
b9
y9
b1
type
b
y

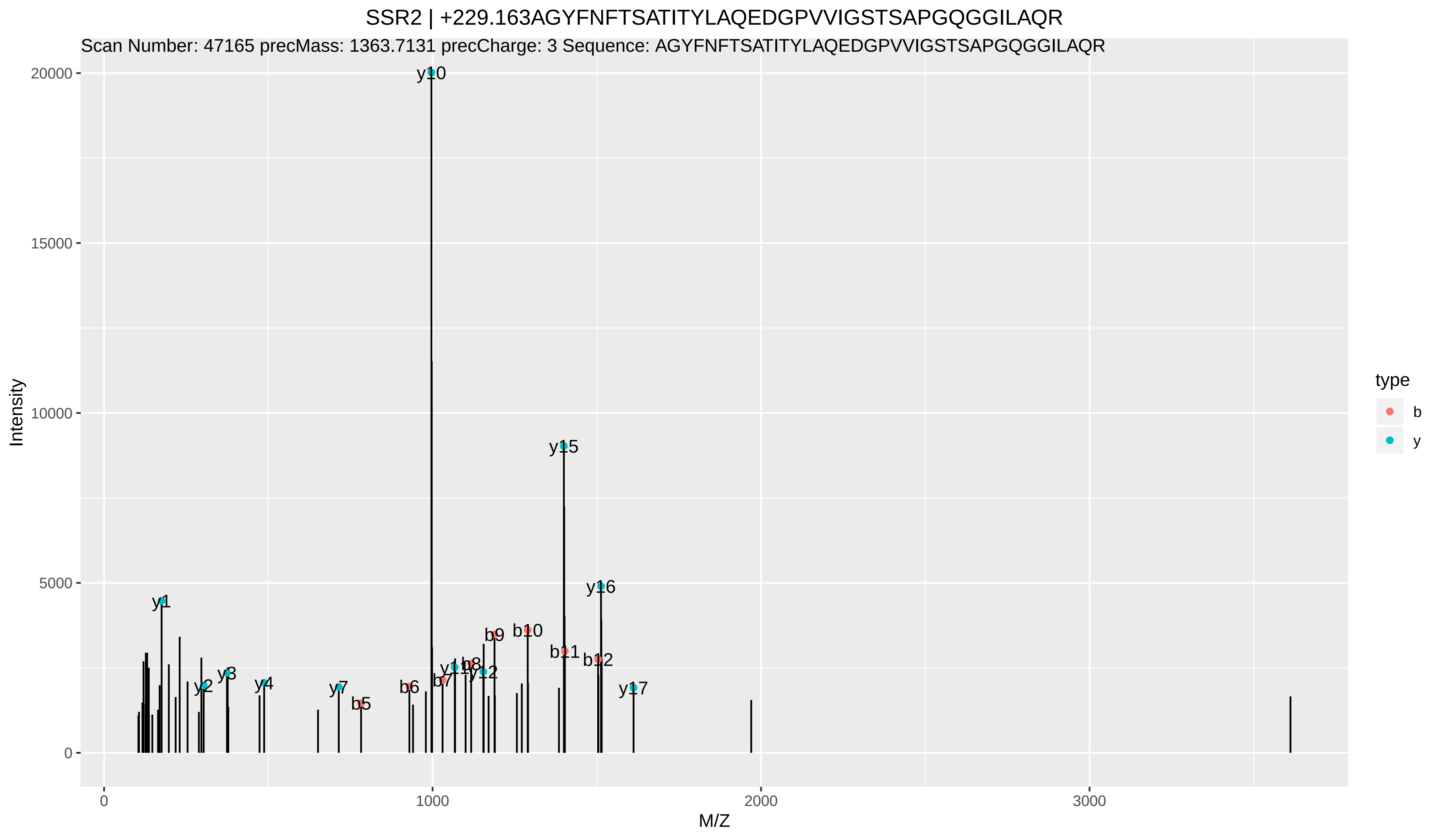

SSR2 | +229.163AGYFNFTSATITYLAQEDGPVVIGSTSAPGQGGILAQR
Scan Number: 47165 precMass: 1363.7131 precCharge: 3 Sequence: AGYFNFTSATITYLAQEDGPVVIGSTSAPGQGGILAQR
Intensity
M/Z
0
5000
10000
15000
20000
0
1000
2000
3000
y10
y15
y16
y1
b9
b10
b11
b12
y2
y3
y4
y7
b5
b6
b7
b8
y12
y17
type
b
y

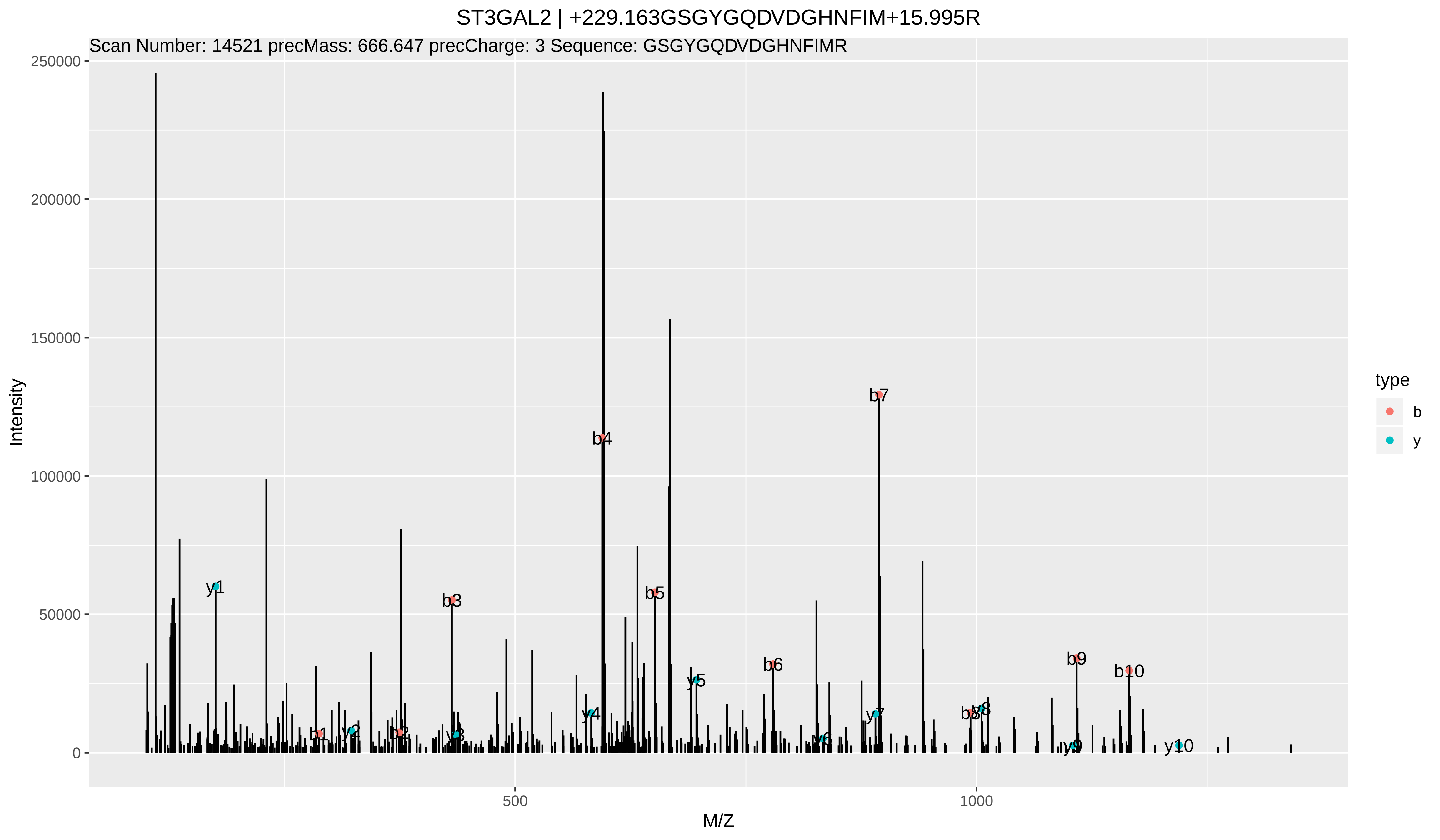
ST3GAL2 | +229.163GSGYGQDVDGHNFIM+15.995R
Scan Number: 14521 precMass: 666.647 precCharge: 3 Sequence: GSGYGQDVDGHNFIMR
Intensity
250000
200000
150000
100000
50000
0
M/Z
500
1000
type
b
y
y1
b1
y2
b2
b3
y3
b4
y4
b5
y5
b6
y6
b7
y7
b8
y8
b9
y9
b10
y10

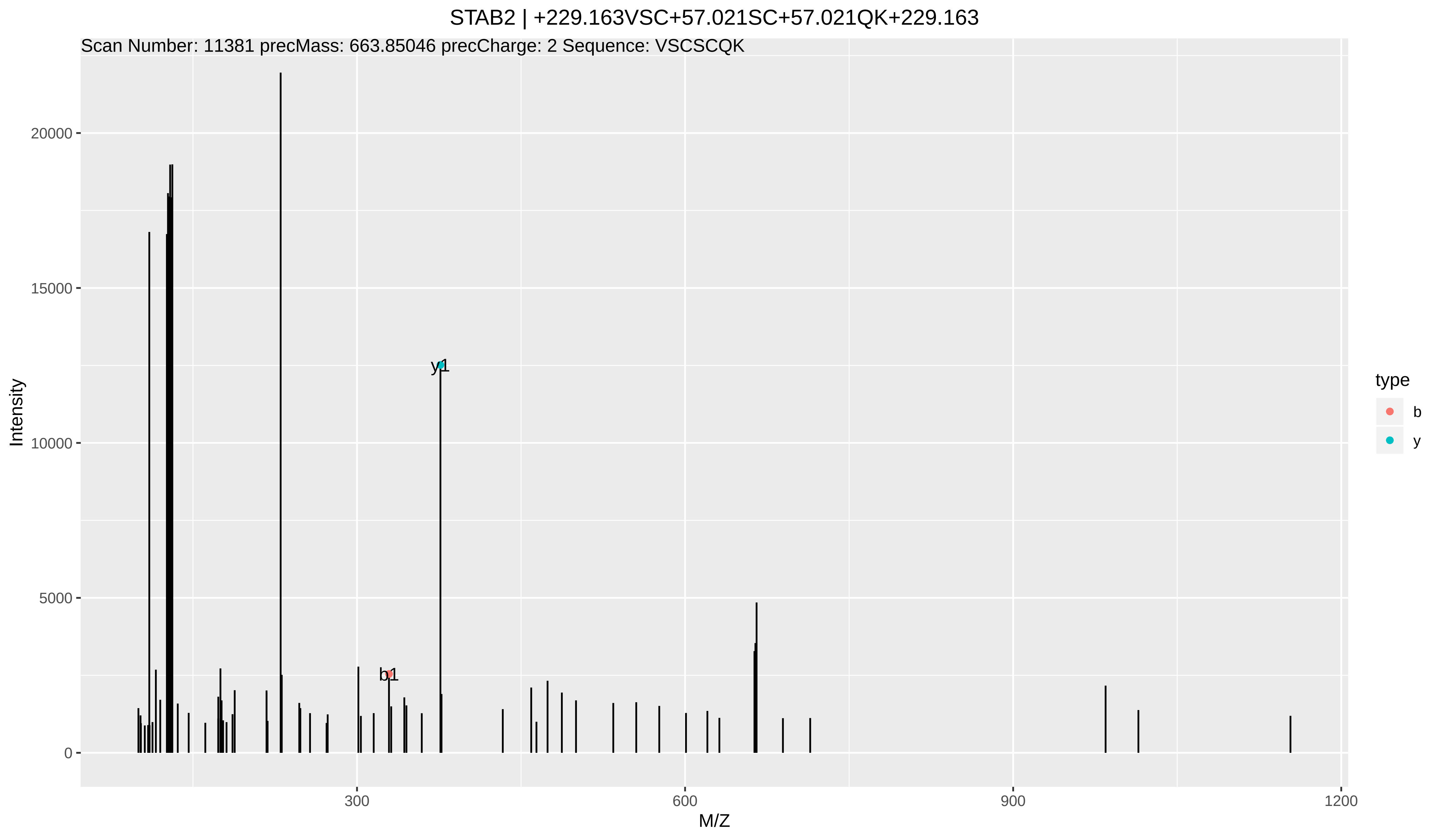
STAB2 | +229.163VSC+57.021SC+57.021QK+229.163
Scan Number: 11381 precMass: 663.85046 precCharge: 2 Sequence: VSCSCQK
Intensity
20000
15000
10000
5000
0
300
600
900
1200
M/Z
y1
b1
type
b
y

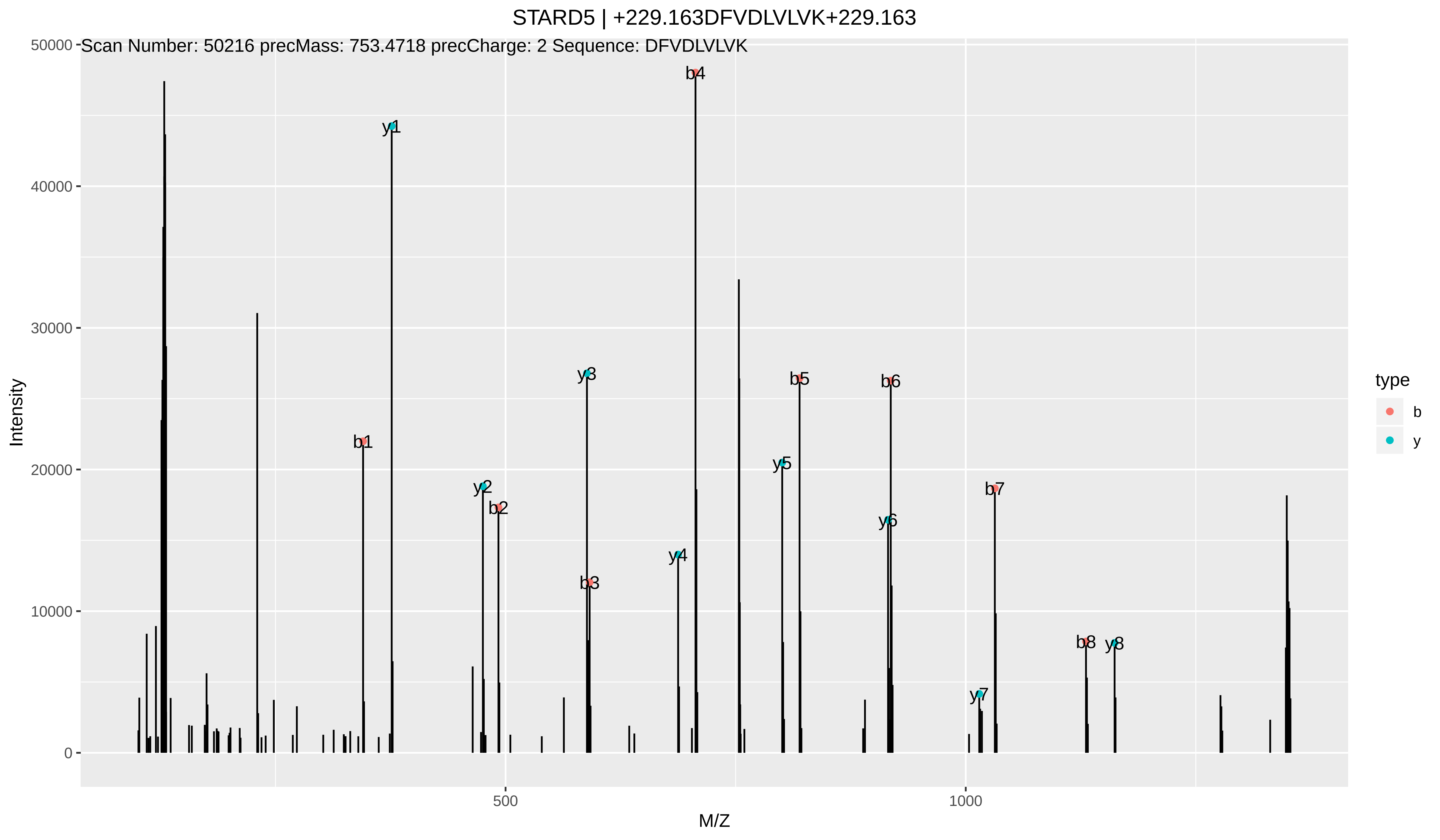

STARD5 | +229.163DFVDLVLVK+229.163
Scan Number: 50216 precMass: 753.4718 precCharge: 2 Sequence: DFVDLVLVK
Intensity
M/Z
0
10000
20000
30000
40000
50000
500
1000
b1
y1
y2
b2
y3
b3
y4
b4
y5
b5
y6
b6
y7
b7
b8
y8
type
b
y

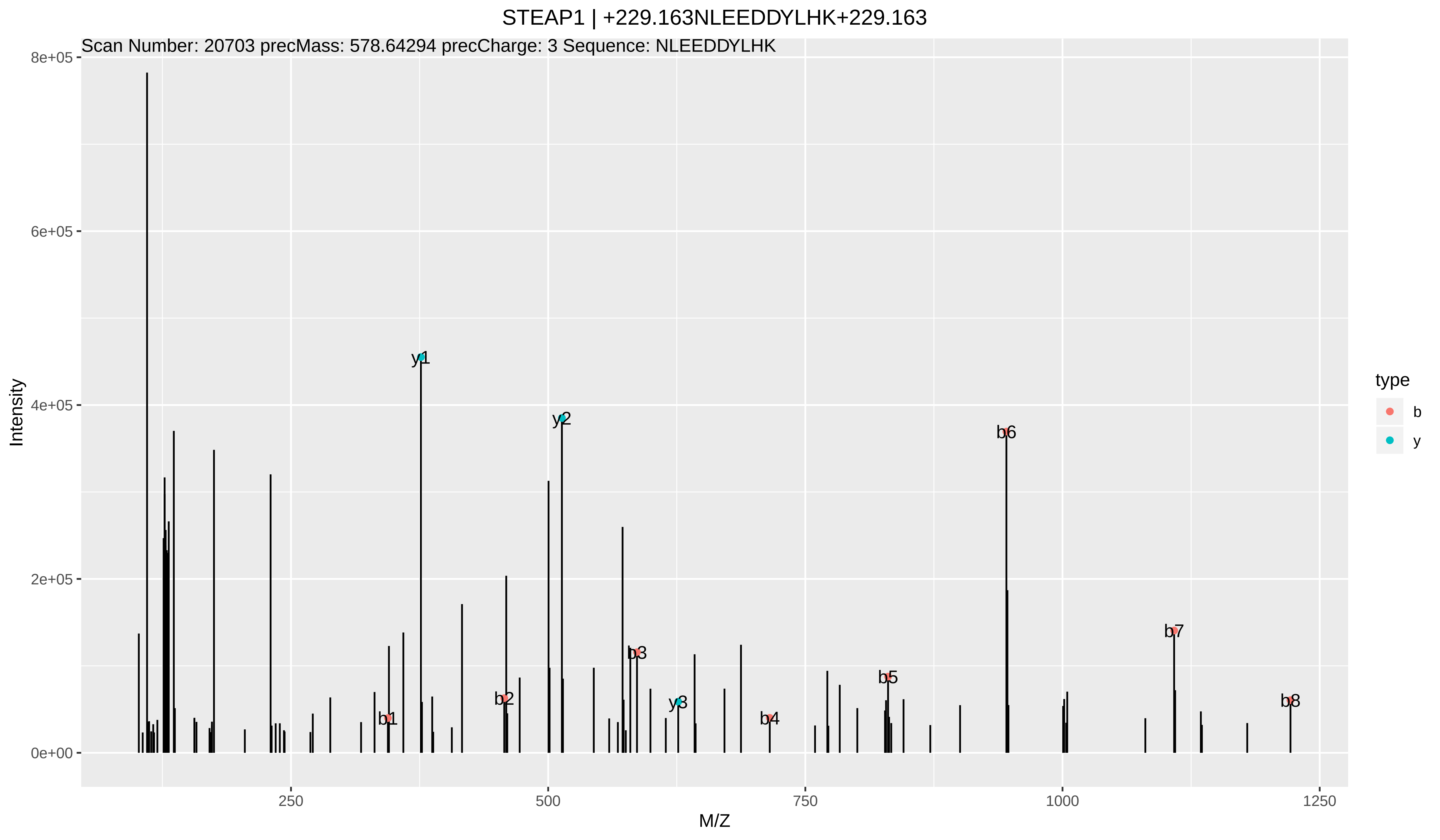
STEAP1 | +229.163NLEEDDYLHK+229.163
Scan Number: 20703 precMass: 578.64294 precCharge: 3 Sequence: NLEEDDYLHK
Intensity
8e+05
6e+05
4e+05
2e+05
0e+00
M/Z
250
500
750
1000
1250
y1
y2
y3
b1
b2
b3
b4
b5
b6
b7
b8
type
b
y

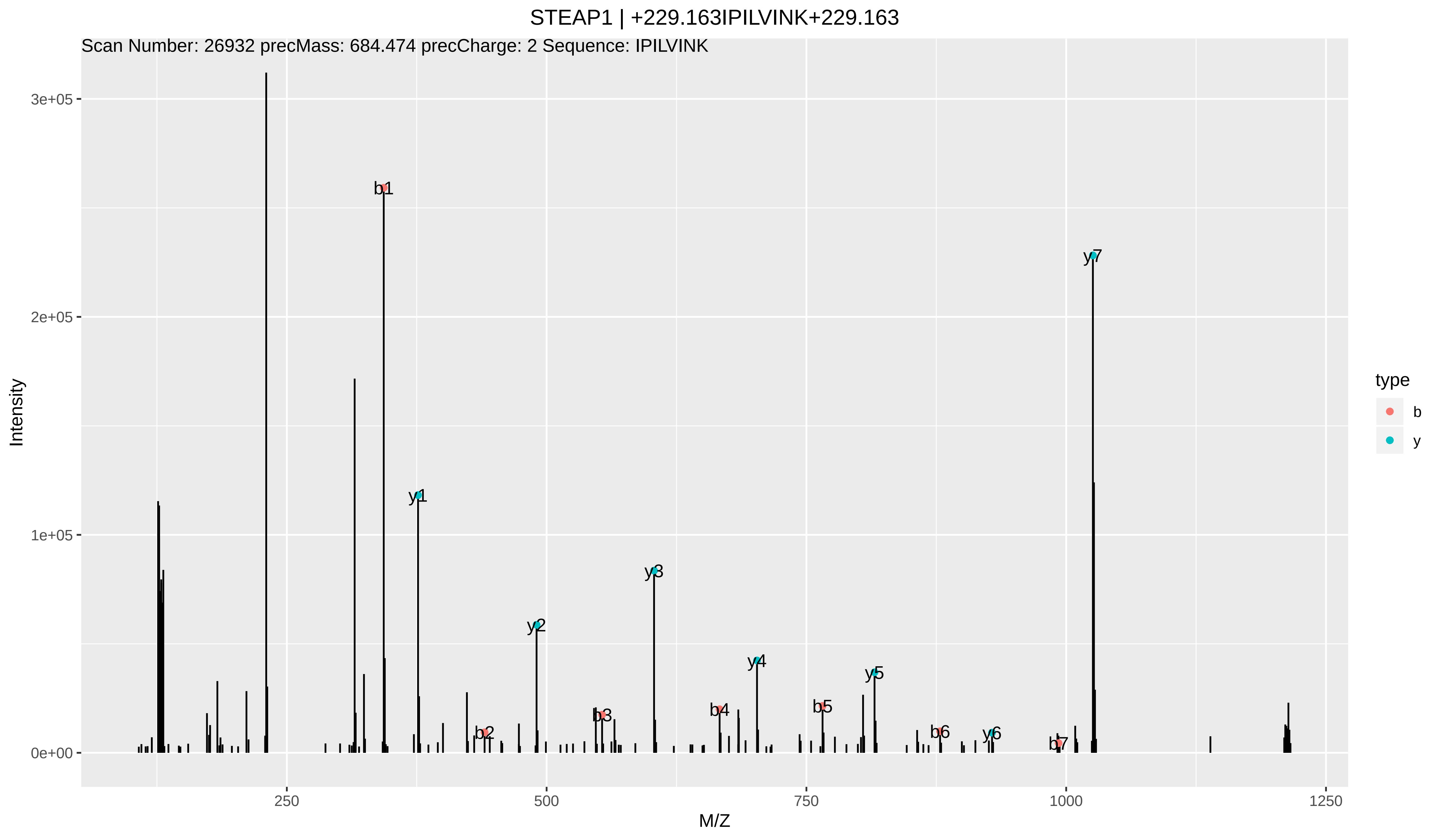
STEAP1 | +229.163IPILVINK+229.163
Scan Number: 26932 precMass: 684.474 precCharge: 2 Sequence: IPILVINK
Intensity
3e+05
2e+05
1e+05
0e+00
M/Z
250
500
750
1000
1250
b1
y1
y2
b2
b3
y3
b4
y4
b5
y5
b6
y6
b7
y7
type
b
y

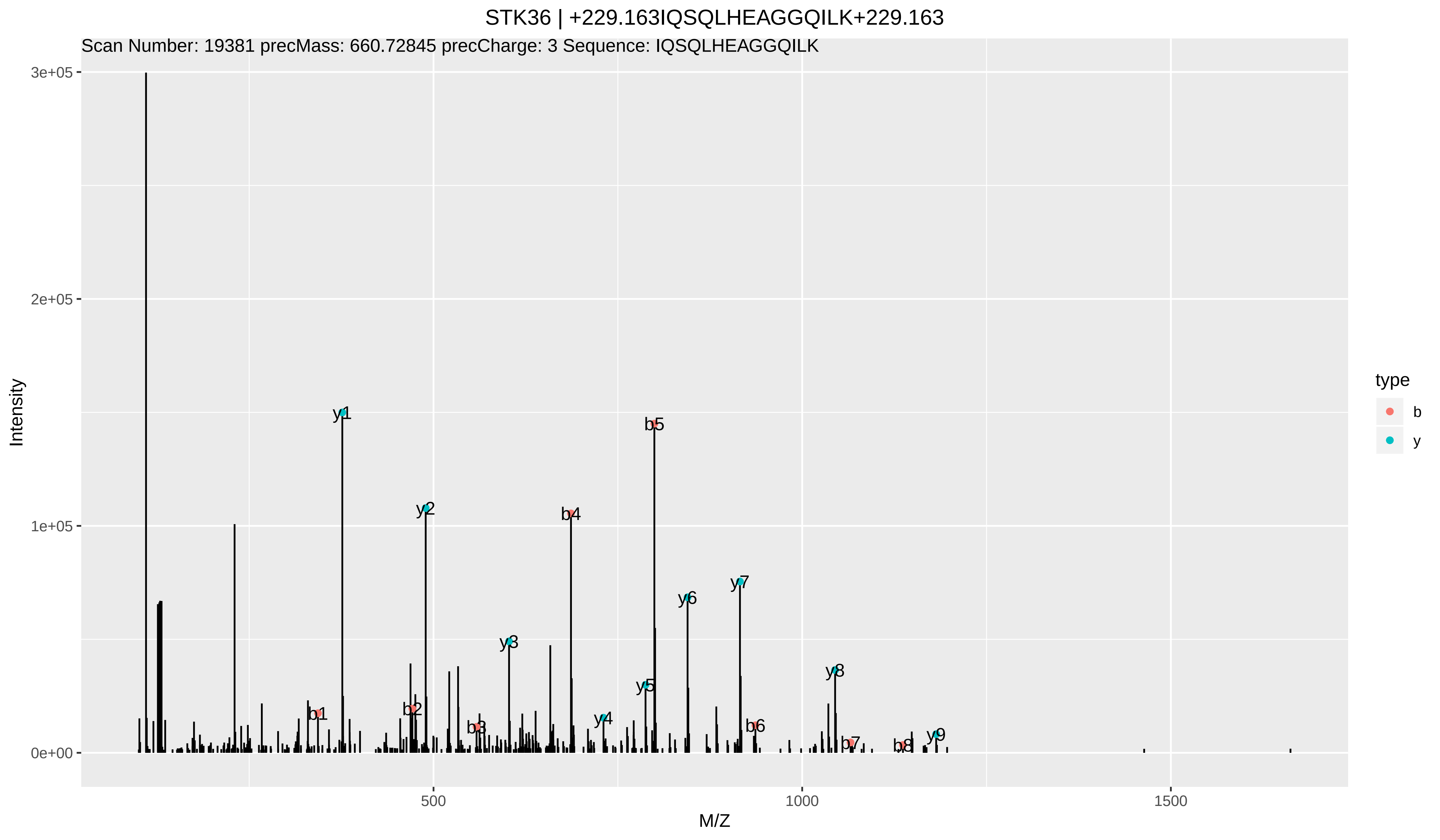
STK36 | +229.163IQSQLHEAGGQILK+229.163
Scan Number: 19381 precMass: 660.72845 precCharge: 3 Sequence: IQSQLHEAGGQILK
Intensity
3e+05
2e+05
1e+05
0e+00
M/Z
500
1000
1500
y1
y2
y3
y4
y5
y6
y7
y8
y9
b1
b2
b3
b4
b5
b6
b7
b8
type
b
y

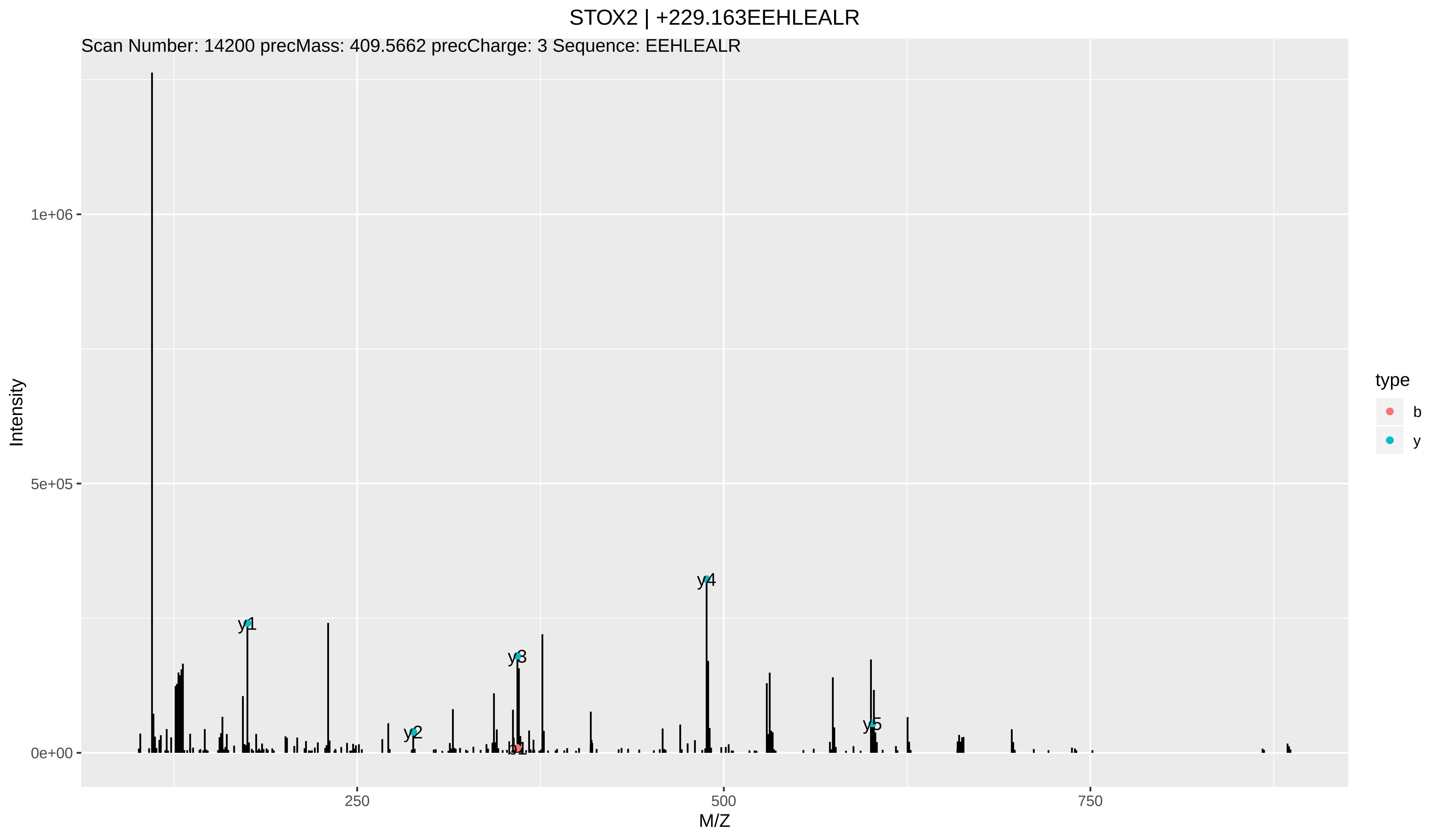
STOX2 | +229.163EEHLEALR
Scan Number: 14200 precMass: 409.5662 precCharge: 3 Sequence: EEHLEALR
Intensity
1e+06
5e+05
0e+00
M/Z
250
500
750
y1
y2
y3
b1
y4
y5
type
b
y

STX11 | +229.163ELSEAAEAQHGPHSAVAR

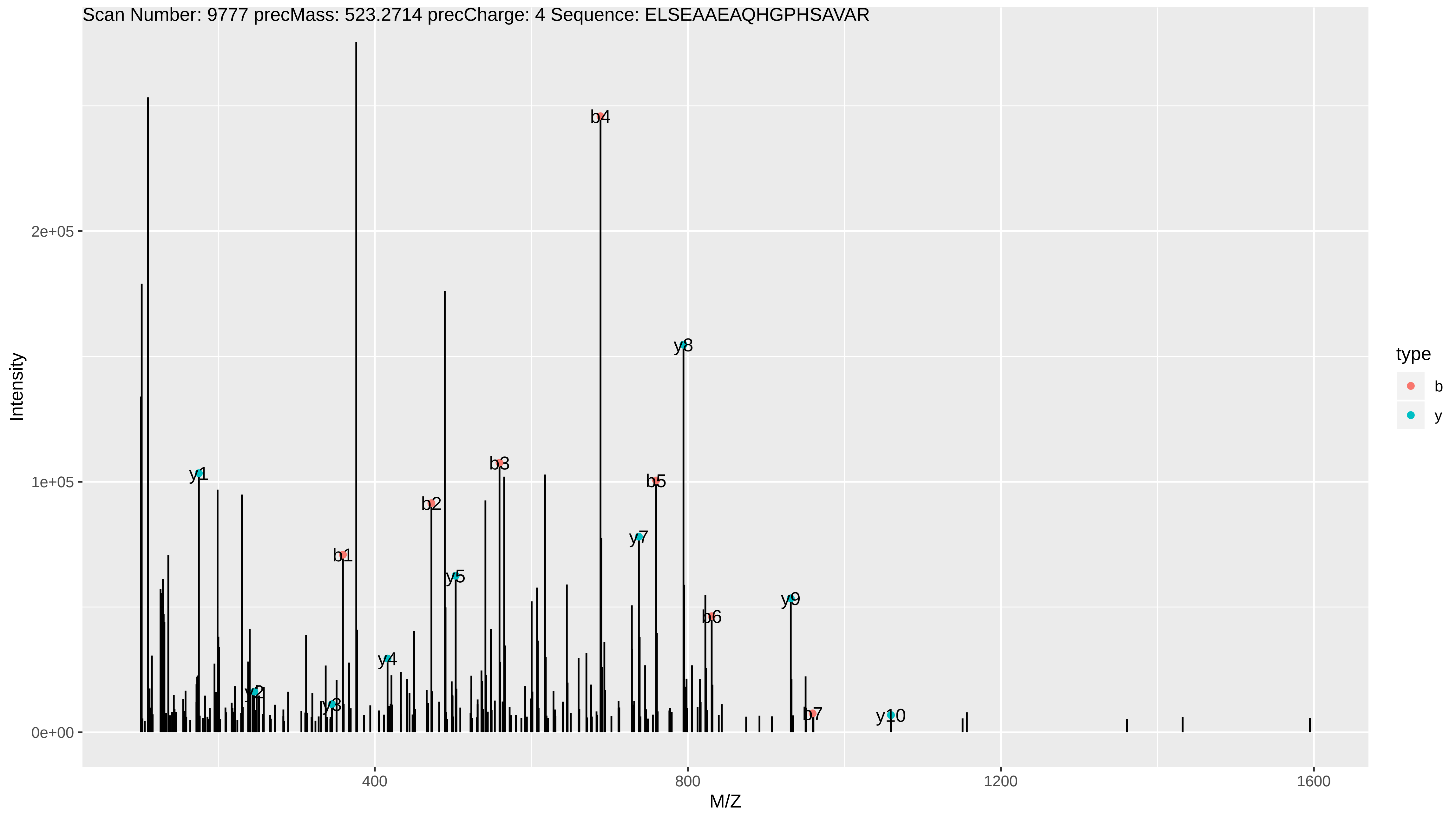

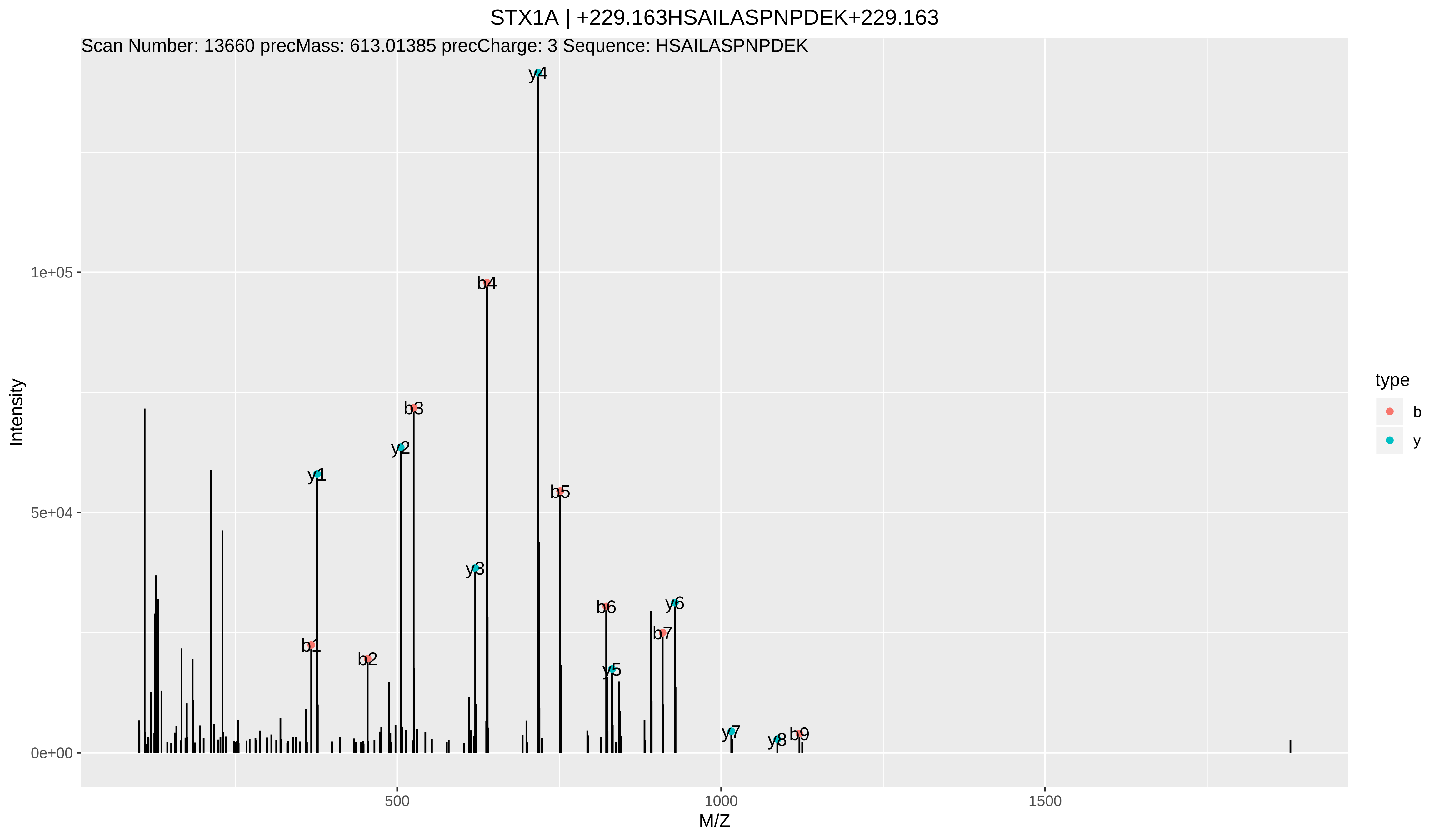
STX1A | +229.163HSAILASPNPDEK+229.163
Scan Number: 13660 precMass: 613.01385 precCharge: 3 Sequence: HSAILASPNPDEK
Intensity
M/Z
0e+00
5e+04
1e+05
500
1000
1500
y4
b4
b3
y2
y1
b5
y3
b6
y6
b7
b1
b2
y5
y7
y8
b9
type
b
y

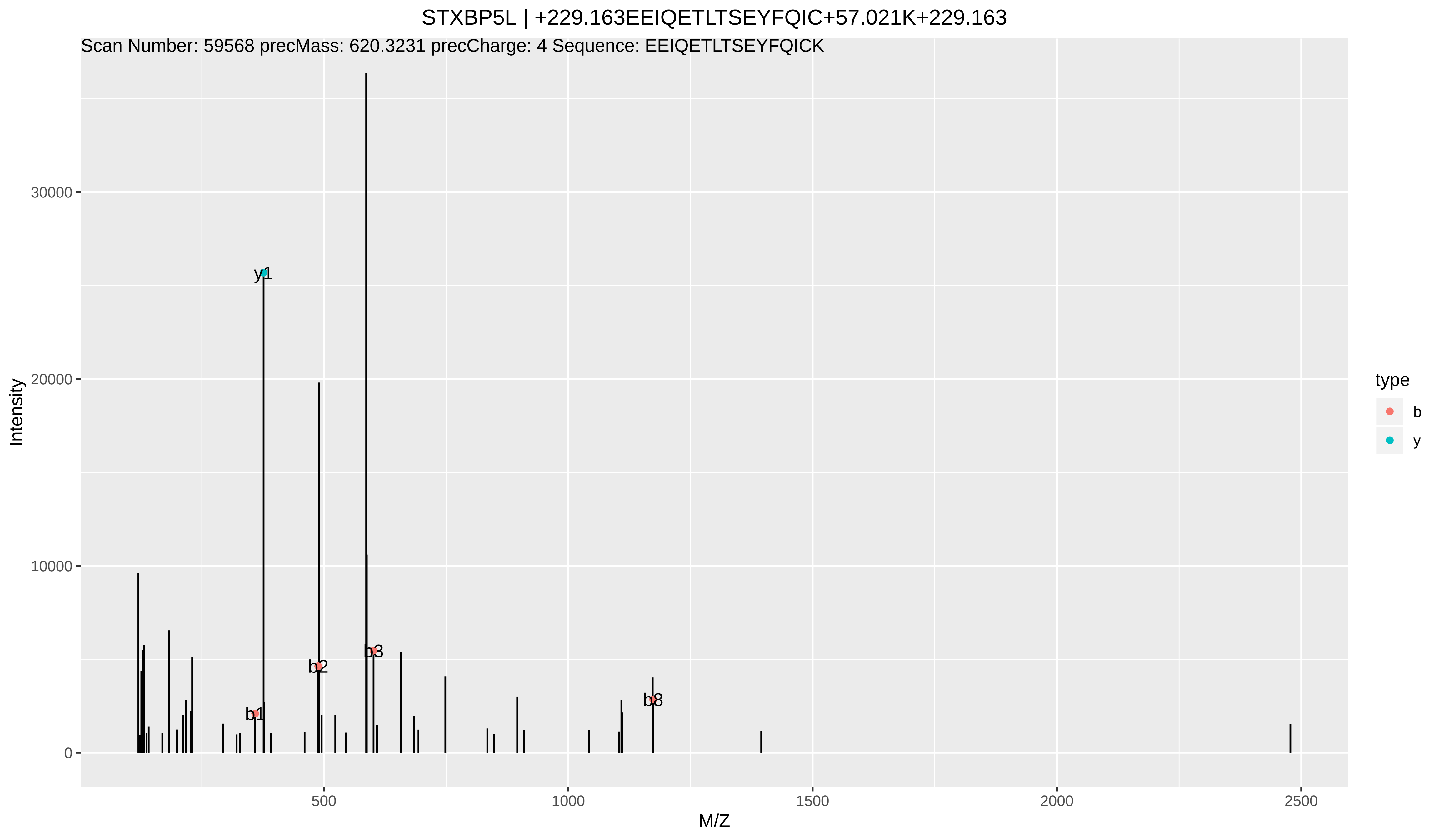

STXBP5L | +229.163EEIQETLTSEYFQIC+57.021K+229.163
Scan Number: 59568 precMass: 620.3231 precCharge: 4 Sequence: EEIQETLTSEYFQICK
Intensity
M/Z
0
10000
20000
30000
500
1000
1500
2000
2500
y1
b1
b2
b3
b8
type
b
y

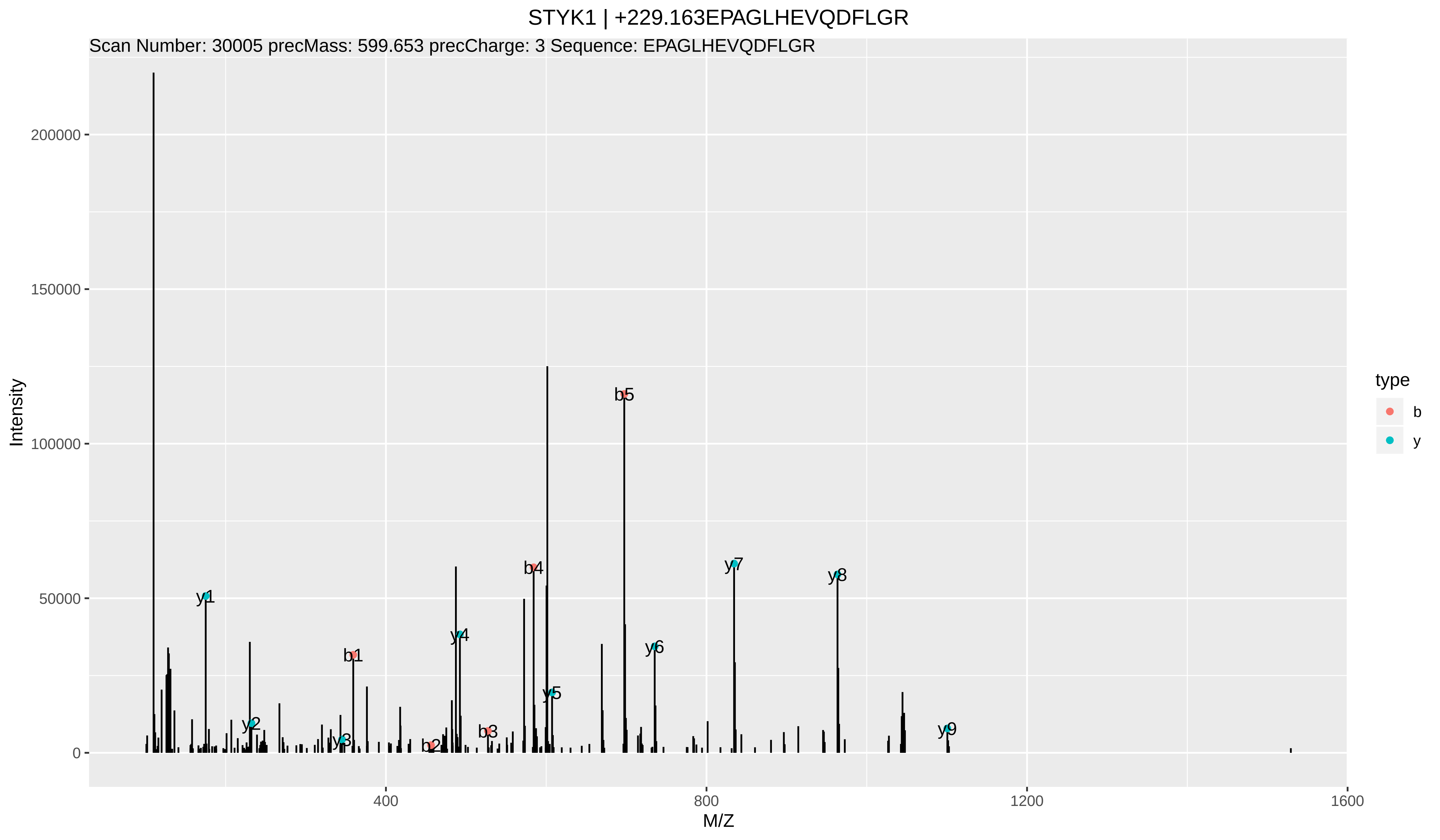
STYK1 | +229.163EPAGLHEVQDFLGR
Scan Number: 30005 precMass: 599.653 precCharge: 3 Sequence: EPAGLHEVQDFLGR
Intensity
200000
150000
100000
50000
0
M/Z
400
800
1200
1600
y1
y2
y3
b1
b2
y4
b3
b4
y5
b5
y6
y7
y8
y9
type
b
y

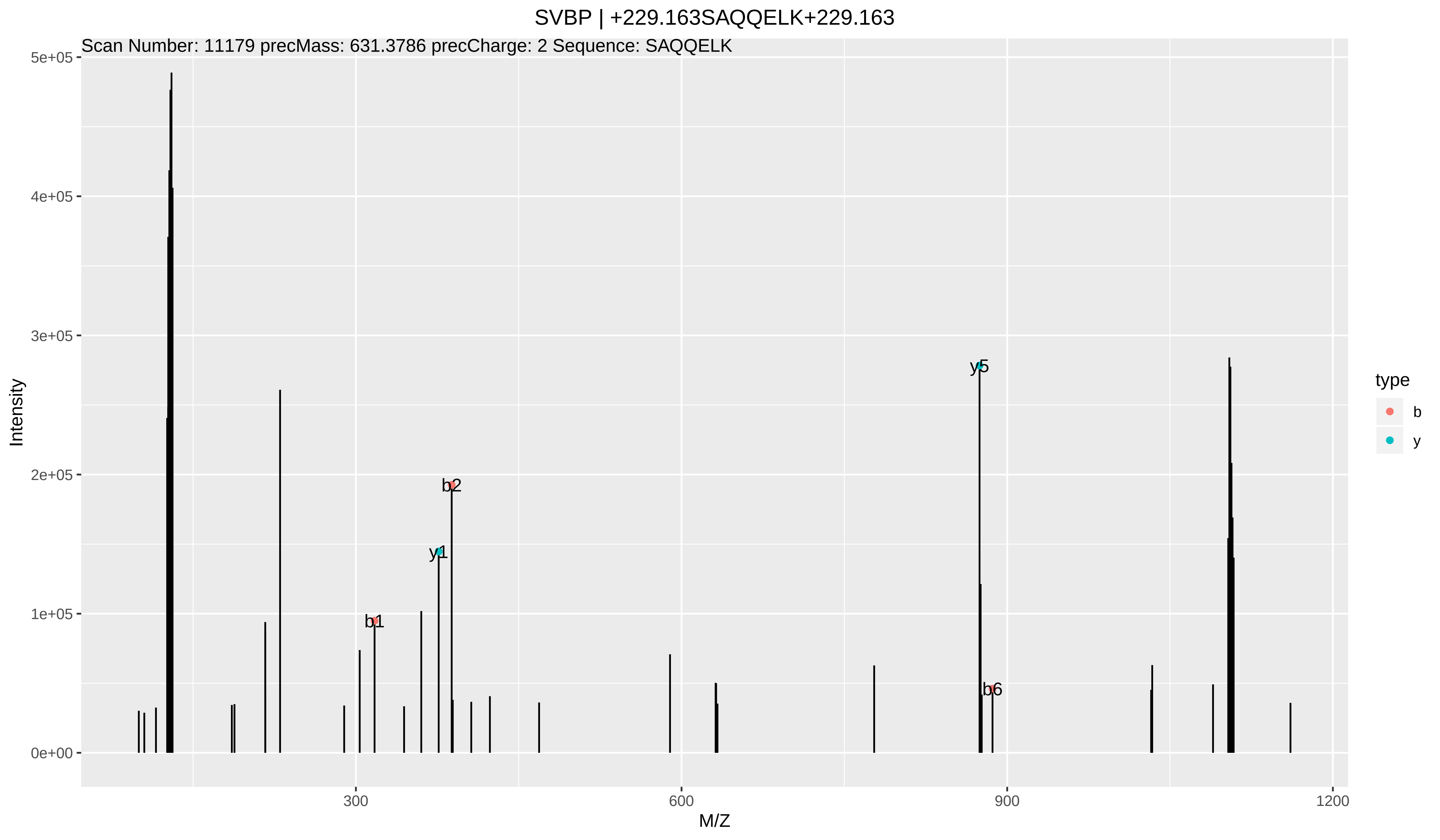
SVBP | +229.163SAQQELK+229.163
Scan Number: 11179 precMass: 631.3786 precCharge: 2 Sequence: SAQQELK
Intensity
5e+05
4e+05
3e+05
2e+05
1e+05
0e+00
M/Z
300
600
900
1200
b1
b2
y1
y5
b6
type
b
y

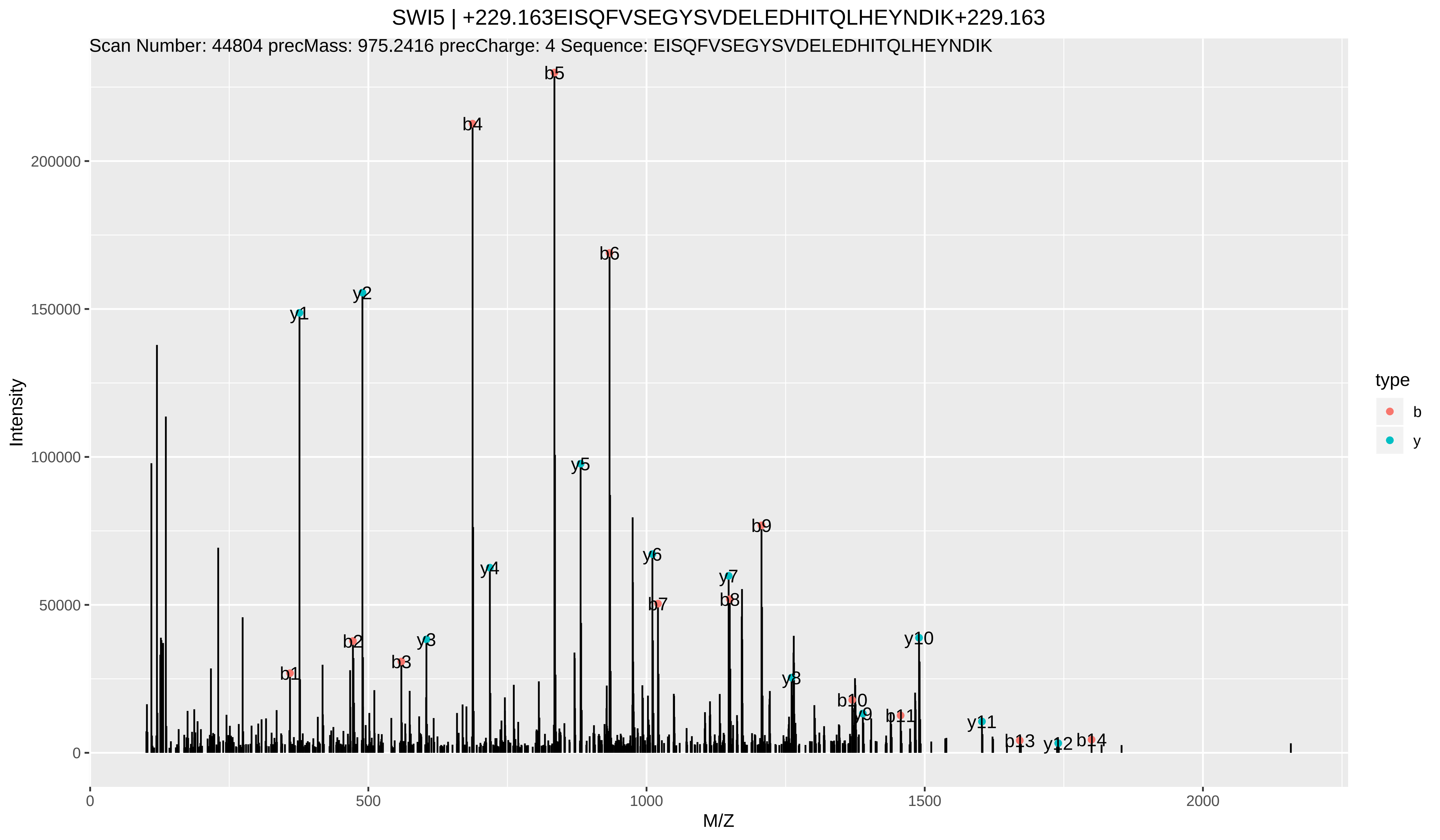

SWI5 | +229.163EISQFVSEGYSVDELEDHITQLHEYNDIK+229.163
Scan Number: 44804 precMass: 975.2416 precCharge: 4 Sequence: EISQFVSEGYSVDELEDHITQLHEYNDIK
Intensity
M/Z
0
50000
100000
150000
200000
500
1000
1500
2000
b1
b2
b3
b4
b5
b6
b7
b8
b9
b10
b11
b13
b14
y1
y2
y3
y4
y5
y6
y7
y8
y9
y10
y11
y12
type
b
y

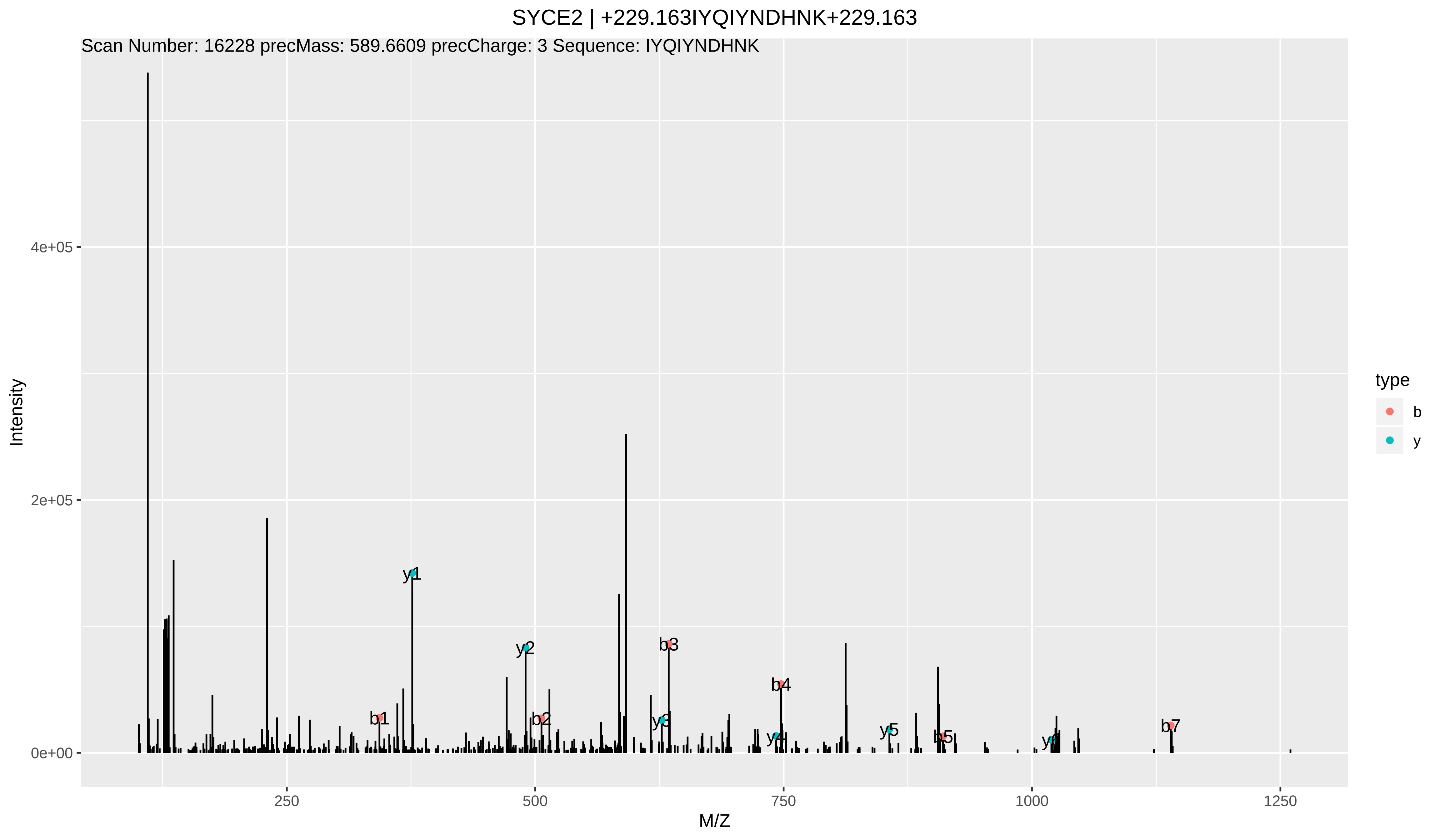
SYCE2 | +229.163IYQIYNDHNK+229.163
Scan Number: 16228 precMass: 589.6609 precCharge: 3 Sequence: IYQIYNDHNK
Intensity
4e+05
2e+05
0e+00
M/Z
250
500
750
1000
1250
y1
y2
b3
b4
b1
b2
y3
y4
y5
b5
y6
b7
type
b
y

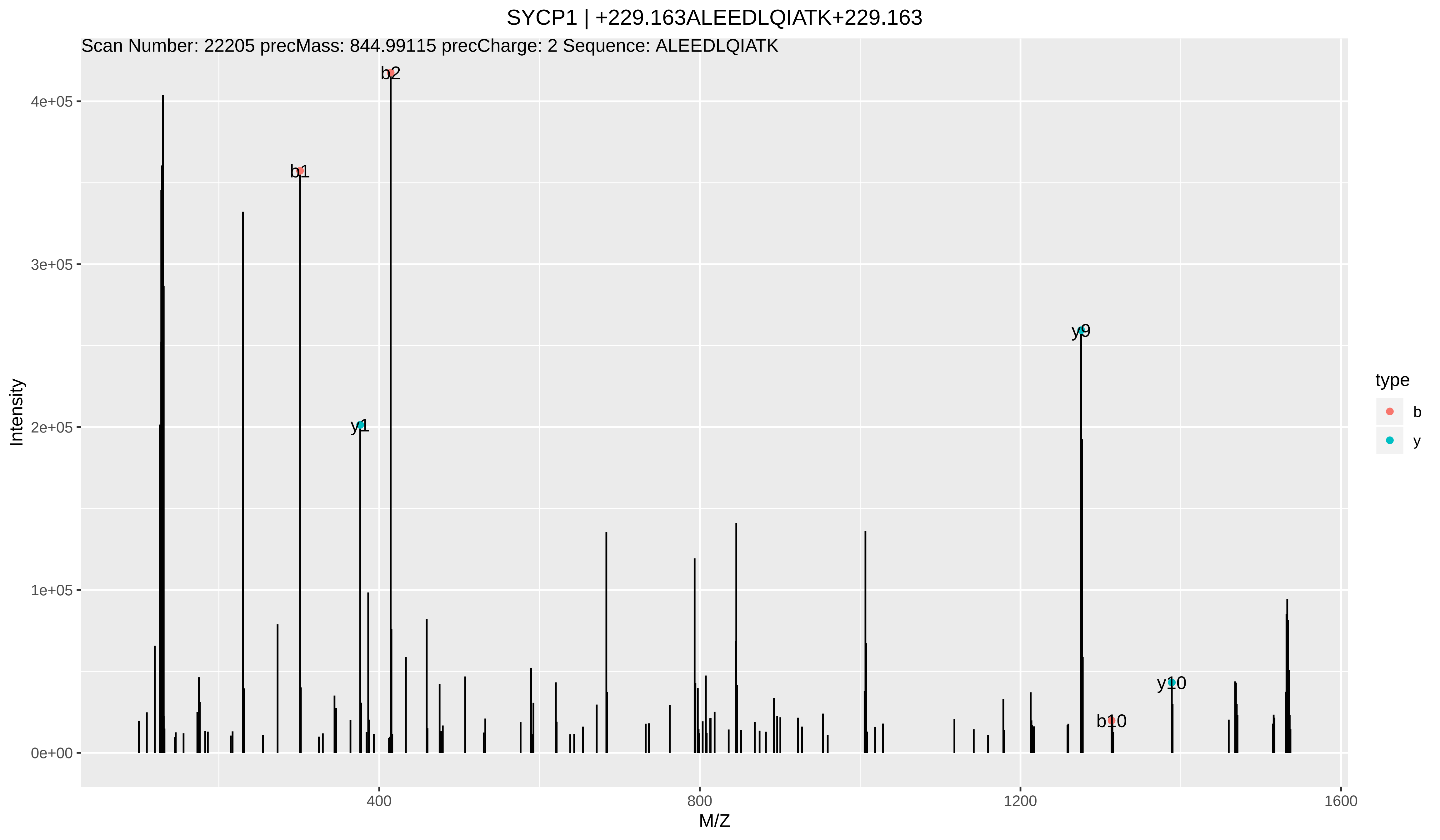

SYCP1 | +229.163ALEEDLQIATK+229.163
Scan Number: 22205 precMass: 844.99115 precCharge: 2 Sequence: ALEEDLQIATK
b2
b1
y1
y9
y10
b10
Intensity
4e+05
3e+05
2e+05
1e+05
0e+00
400
800
1200
1600
M/Z
type
b
y

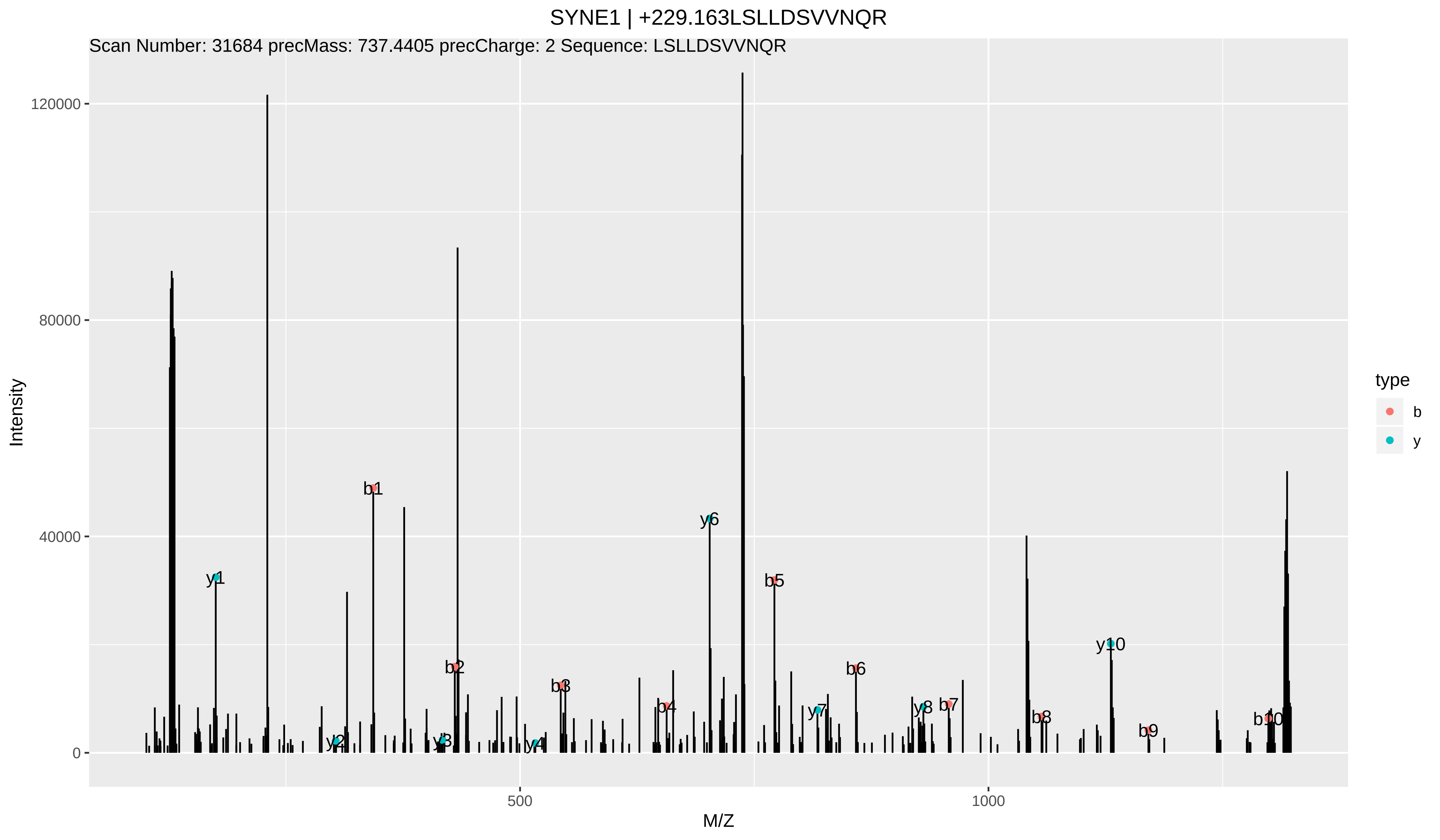
SYNE1 | +229.163LSLLDSVVNQR
Scan Number: 31684 precMass: 737.4405 precCharge: 2 Sequence: LSLLDSVVNQR
Intensity
M/Z
120000
80000
40000
0
500
1000
type
b
y
y1
y2
y3
y4
y6
y7
y8
y10
b1
b2
b3
b4
b5
b6
b7
b8
b9
b10

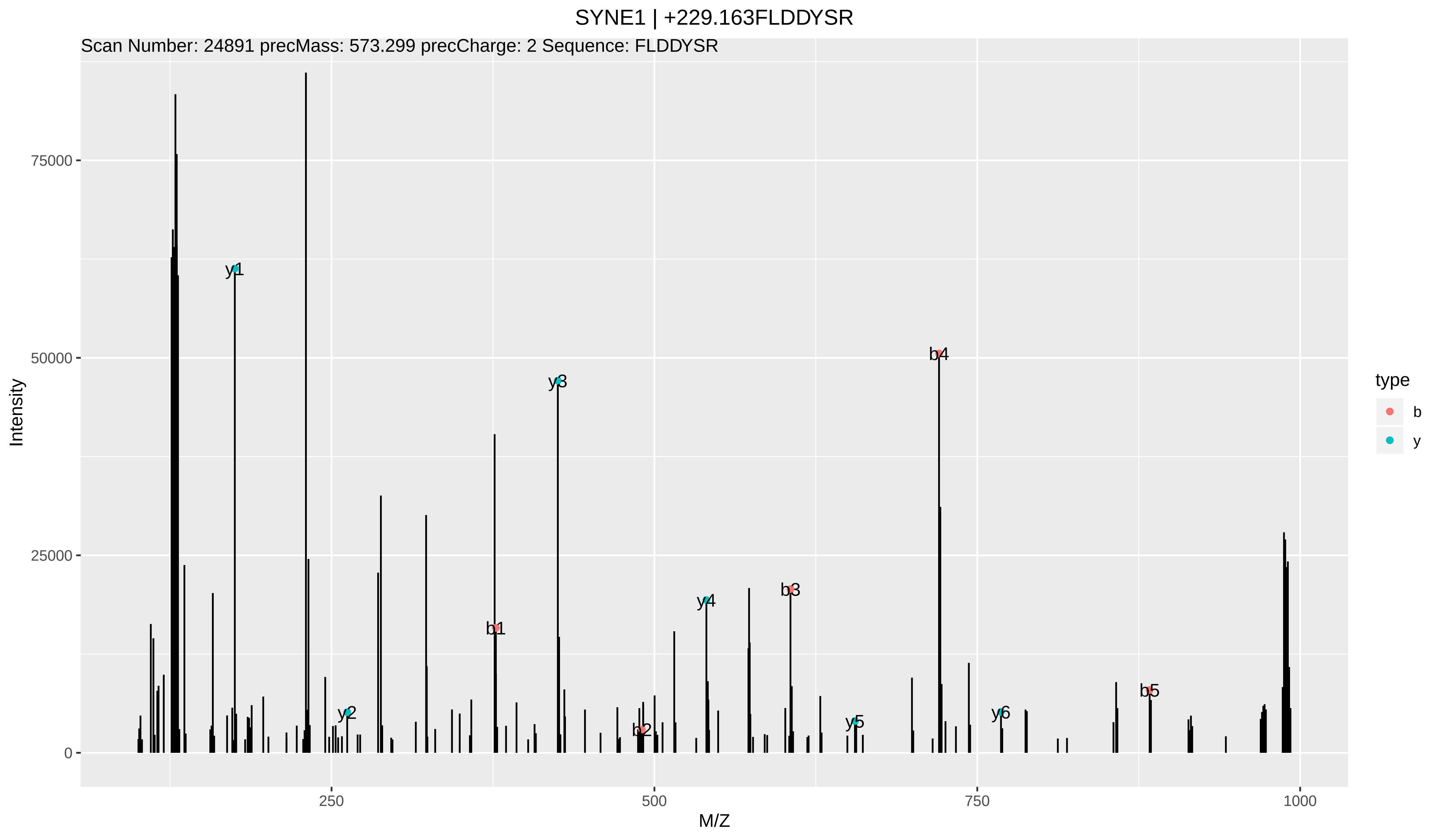

SYNE1 | +229.163FLDDYSR
Scan Number: 24891 precMass: 573.299 precCharge: 2 Sequence: FLDDYSR
Intensity
M/Z
75000
50000
25000
0
250
500
750
1000
y1
y2
y3
y4
y5
y6
b1
b2
b3
b4
b5
type
b
y

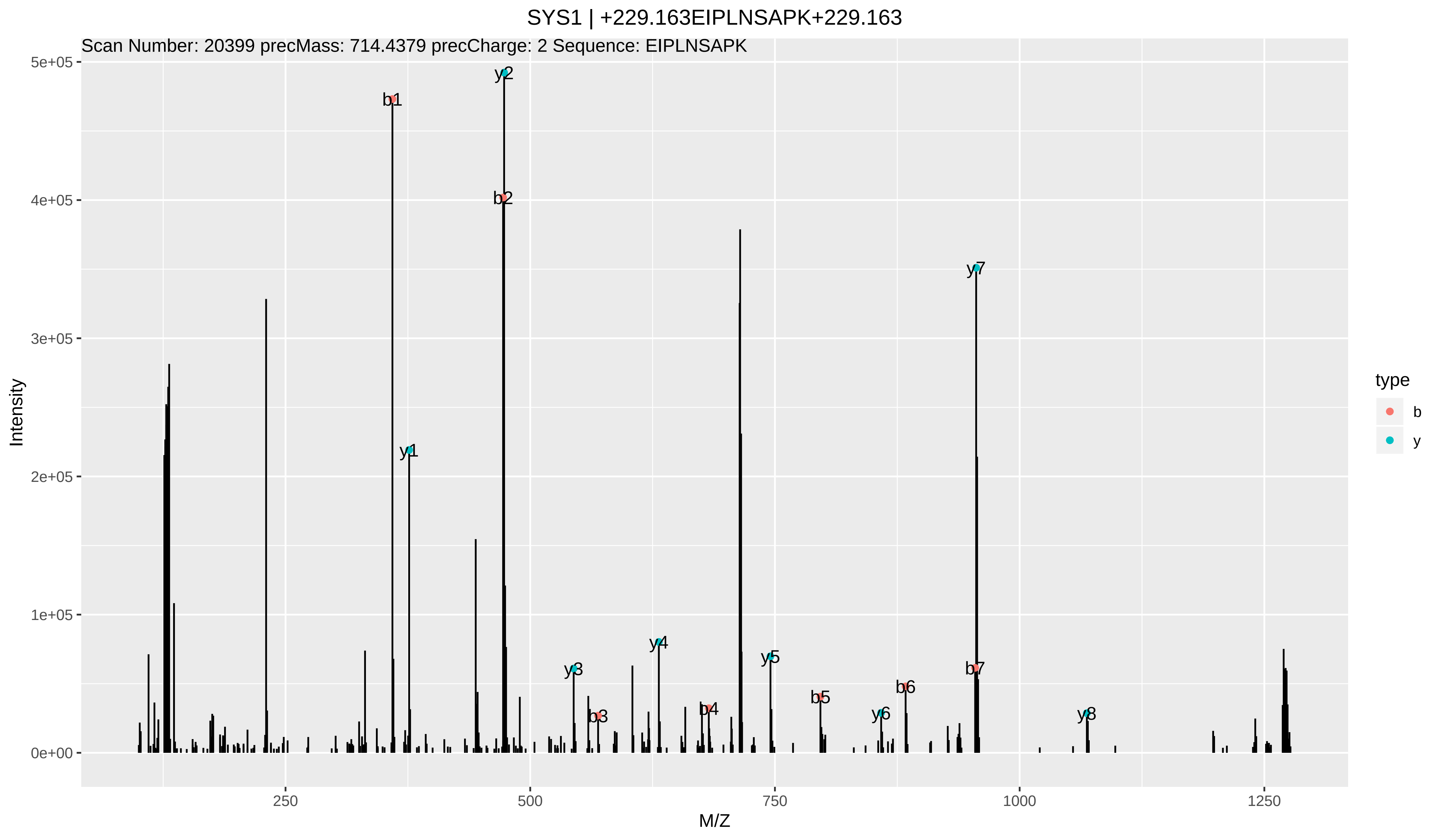

SYS1 | +229.163EIPLNSAPK+229.163
Scan Number: 20399 precMass: 714.4379 precCharge: 2 Sequence: EIPLNSAPK
Intensity
M/Z
type
b
y
b1
b2
b3
b4
b5
b6
b7
y1
y2
y3
y4
y5
y6
y7
y8
0e+00
1e+05
2e+05
3e+05
4e+05
5e+05
250
500
750
1000
1250

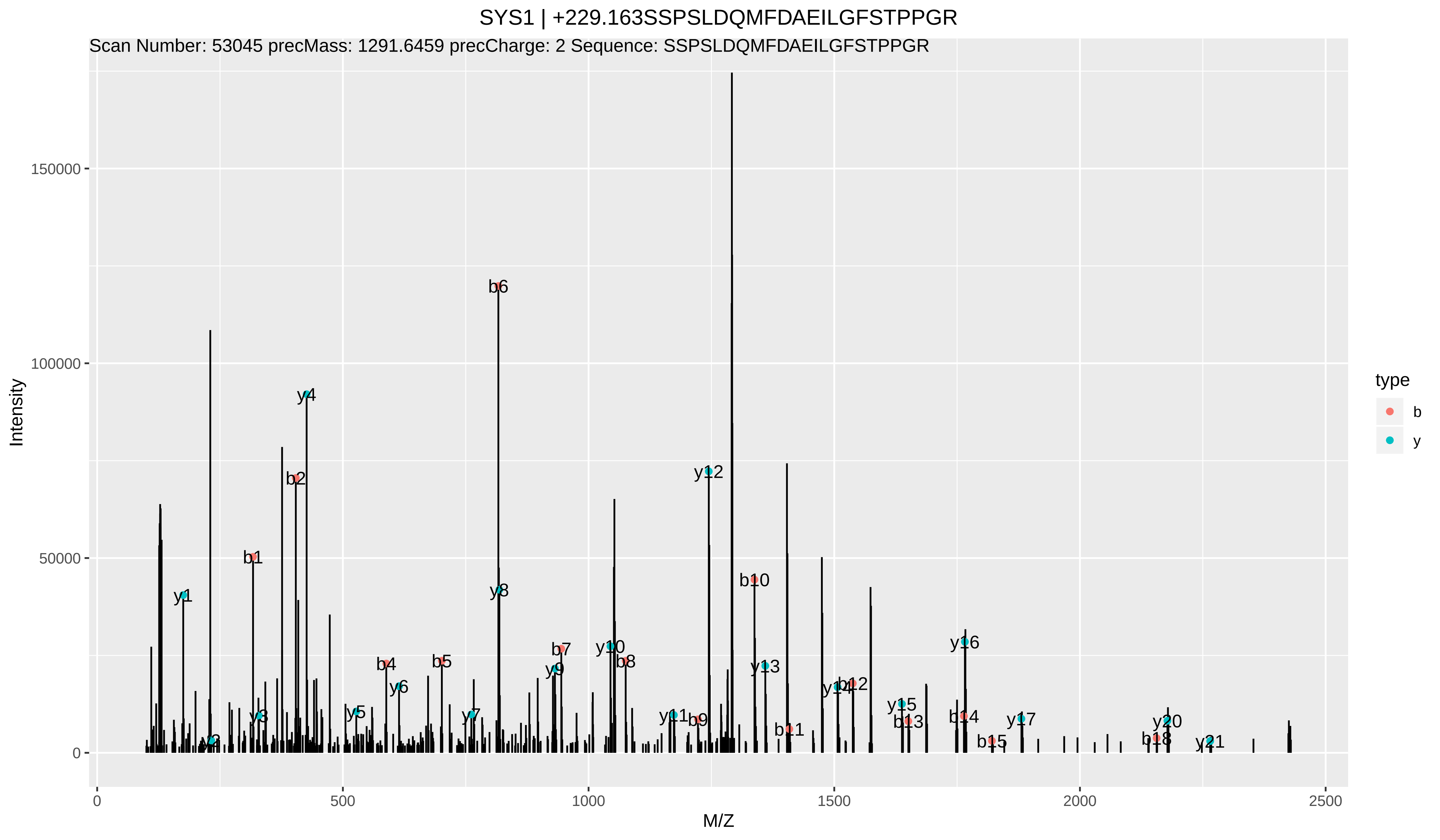

SYS1 | +229.163SSPSLDQMFDAEILGFSTPPGR
Scan Number: 53045 precMass: 1291.6459 precCharge: 2 Sequence: SSPSLDQMFDAEILGFSTPPGR
Intensity
M/Z
type
b
y
b6
y4
b2
y12
b1
y1
y8
b10
y16
b7
y10
b8
b4
b5
y9
y13
y6
y14
b12
y15
y5
y3
y7
y11
b9
b13
b14
y17
y20
b11
b15
b18
y21
y2
150000
100000
50000
0
500
1000
1500
2000
2500

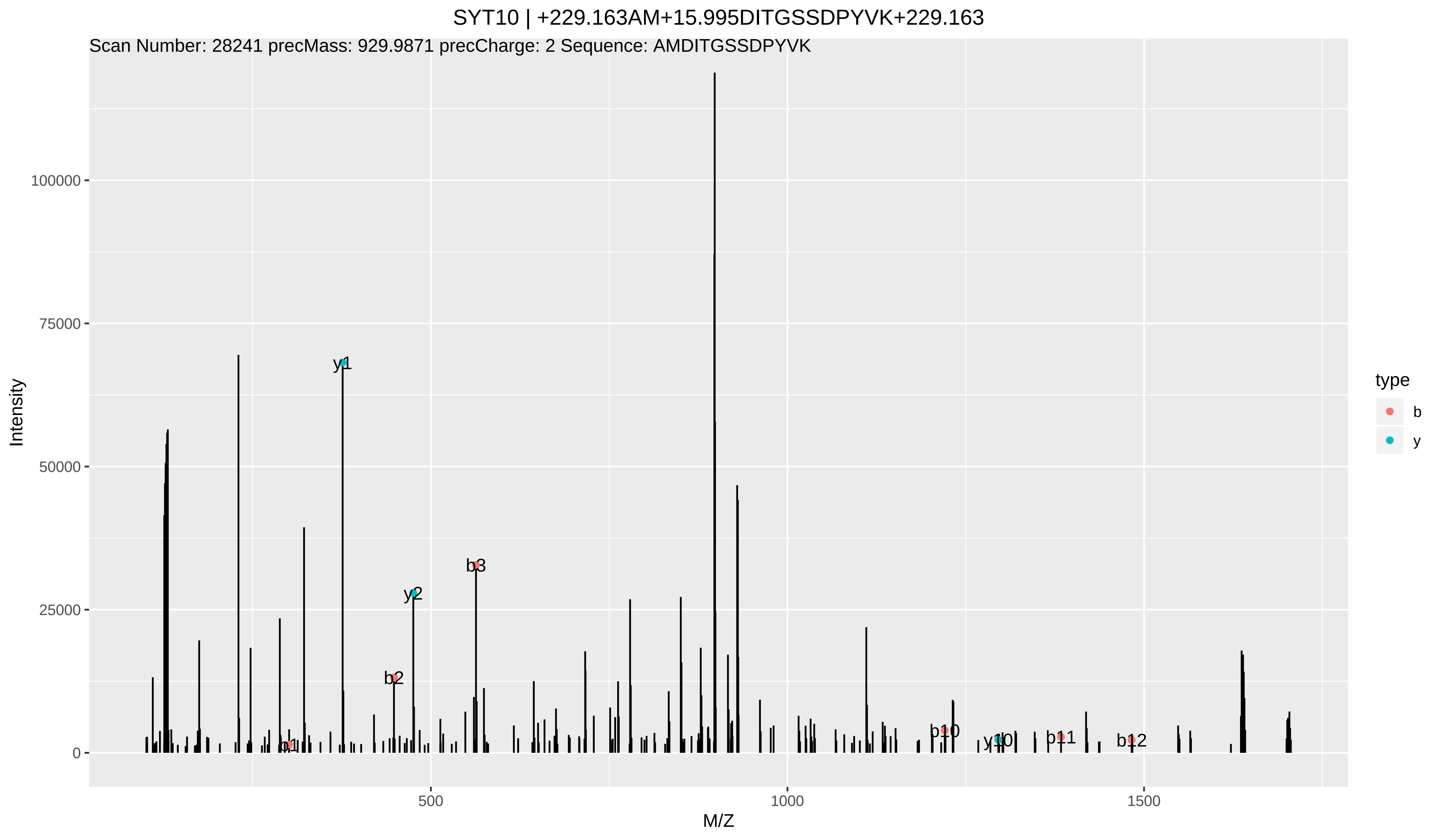

SYT10 | +229.163AM+15.995DITGSSDPYVK+229.163
Scan Number: 28241 precMass: 929.9871 precCharge: 2 Sequence: AMDITGSSDPYVK
Intensity
M/Z
0
25000
50000
75000
100000
500
1000
1500
y1
y2
b2
b3
b10
y10
b11
b12
type
b
y

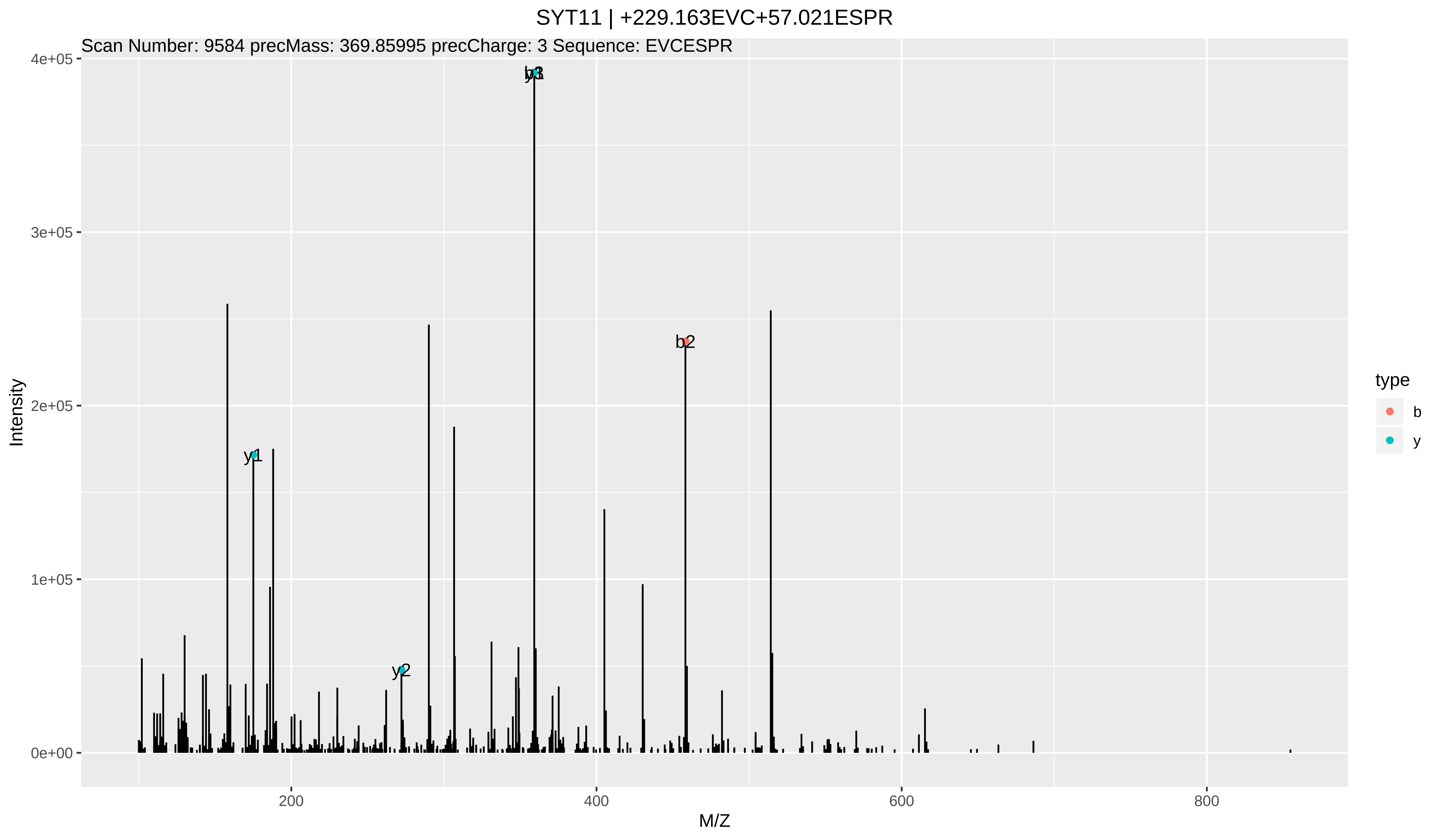
SYT11 | +229.163EVC+57.021ESPR
Scan Number: 9584 precMass: 369.85995 precCharge: 3 Sequence: EVCESPR
Intensity
M/Z
type
b
y
y1
y2
b2
b3
y3
0e+00
1e+05
2e+05
3e+05
4e+05
200
400
600
800

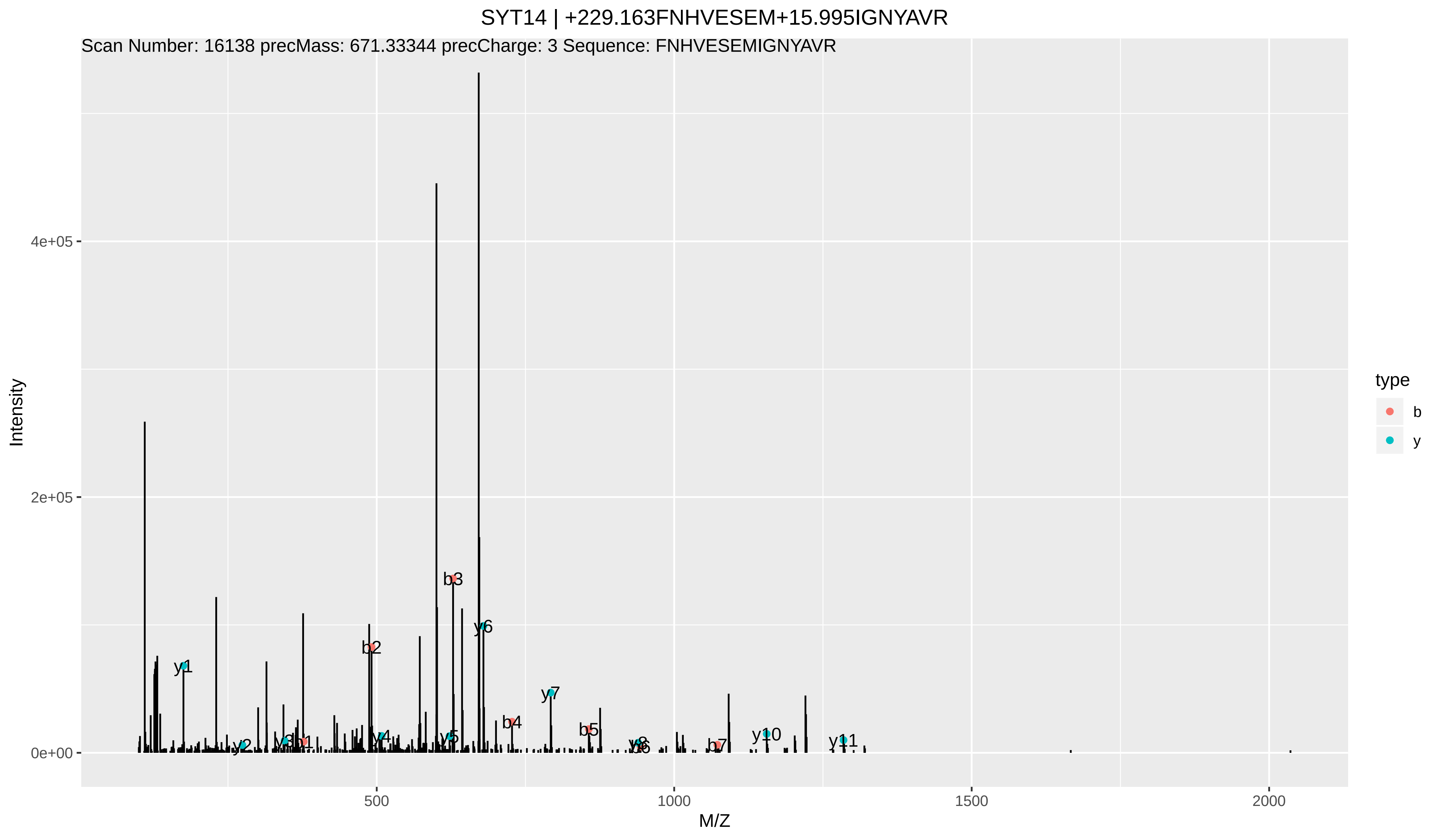

SYT14 | +229.163FNHVESEM+15.995IGNYAVR
Scan Number: 16138 precMass: 671.33344 precCharge: 3 Sequence: FNHVESEMIGNYAVR
Intensity
4e+05
2e+05
0e+00
M/Z
500
1000
1500
2000
type
b
y
b3
y6
b2
y1
y7
b4
b5
y10
y11
y2
y4
y5
b7

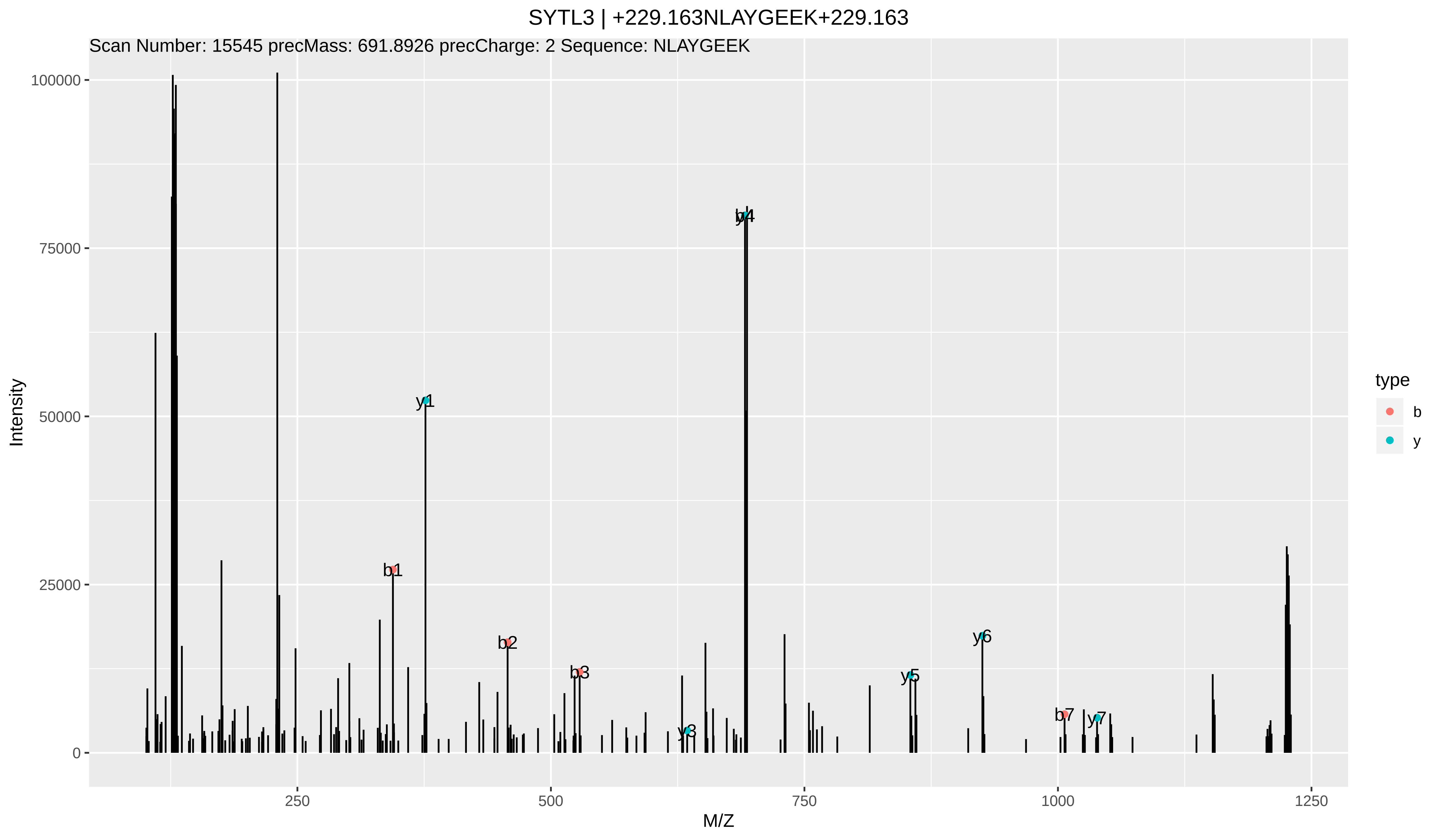
SYTL3 | +229.163NLAYGEEK+229.163
Scan Number: 15545 precMass: 691.8926 precCharge: 2 Sequence: NLAYGEEK
Intensity
M/Z
100000
75000
50000
25000
0
250
500
750
1000
1250
b1
y1
b2
b3
y3
b4
y4
y5
y6
b7
y7
type
b
y

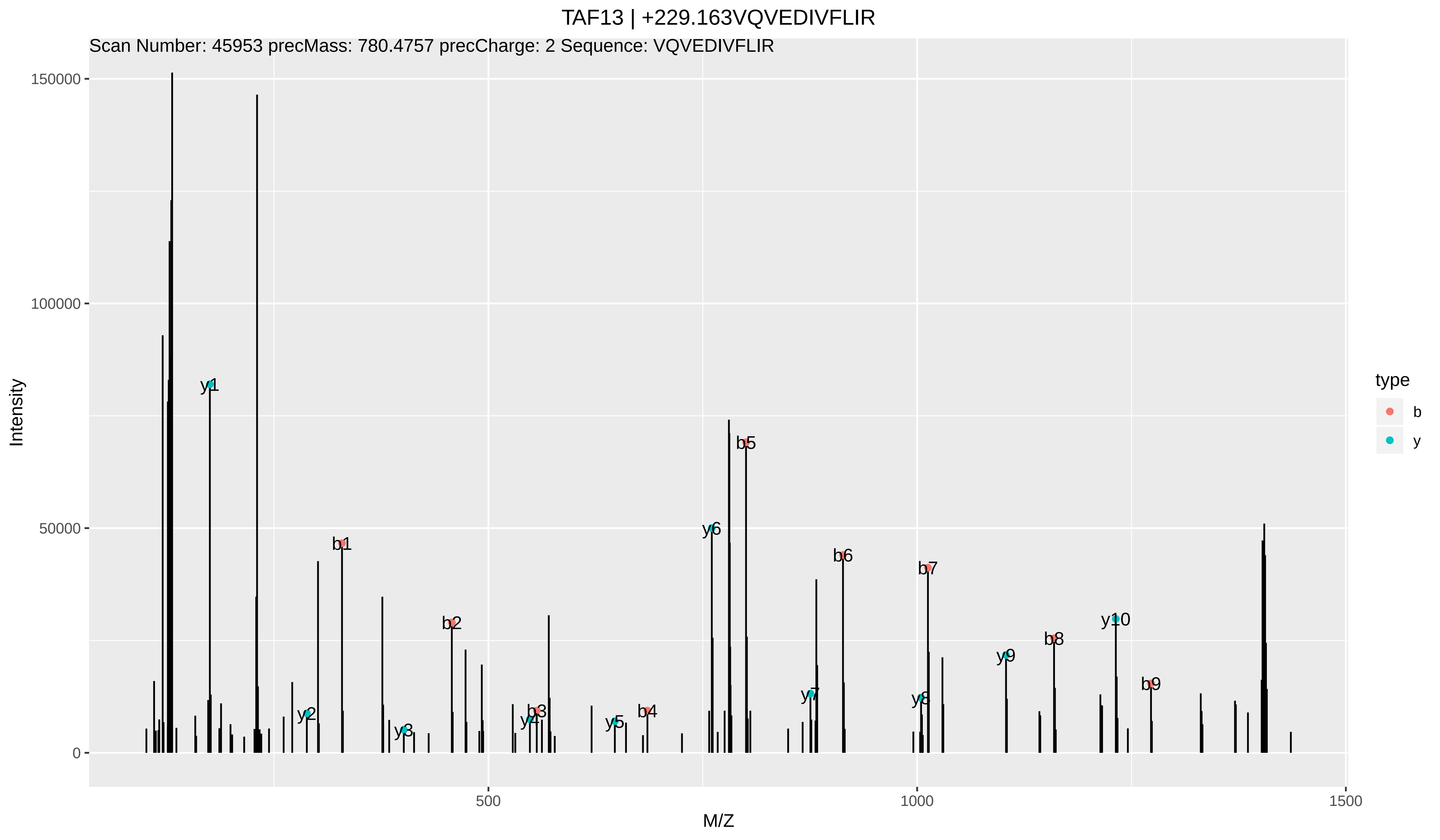
TAF13 | +229.163VQVEDIVFLIR
Scan Number: 45953 precMass: 780.4757 precCharge: 2 Sequence: VQVEDIVFLIR
Intensity
150000
100000
50000
0
M/Z
500
1000
1500
y1
b1
y2
y3
b2
y4
b3
y5
b4
y6
b5
y7
b6
y8
b7
y9
b8
y10
b9
type
b
y

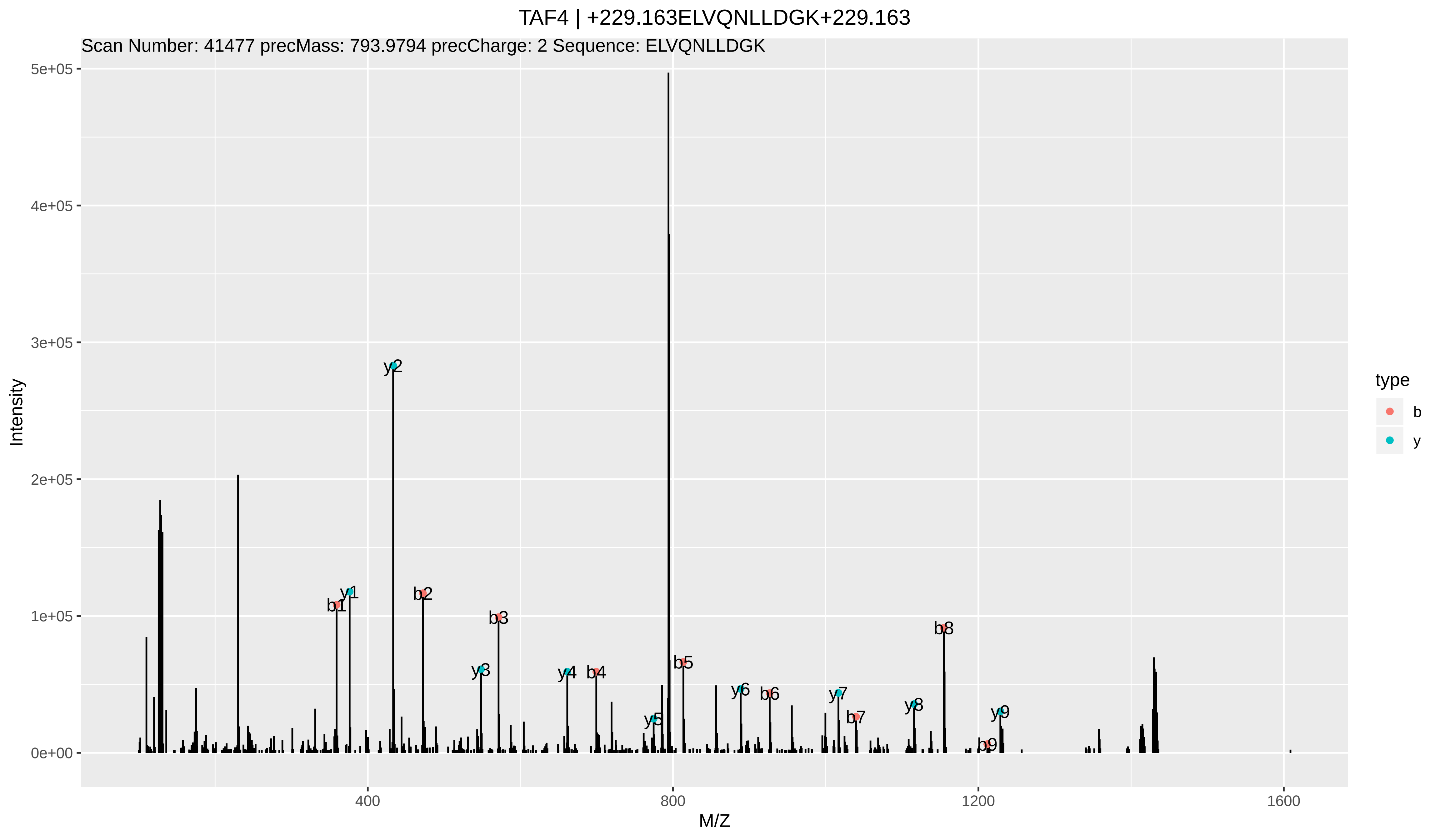

TAF4 | +229.163ELVQNLLDGK+229.163
Scan Number: 41477 precMass: 793.9794 precCharge: 2 Sequence: ELVQNLLDGK
Intensity
5e+05
4e+05
3e+05
2e+05
1e+05
0e+00
M/Z
400
800
1200
1600
y2
y1
b1
b2
b3
y3
y4
b4
b5
y5
y6
b6
y7
b7
y8
b8
y9
b9
type
b
y

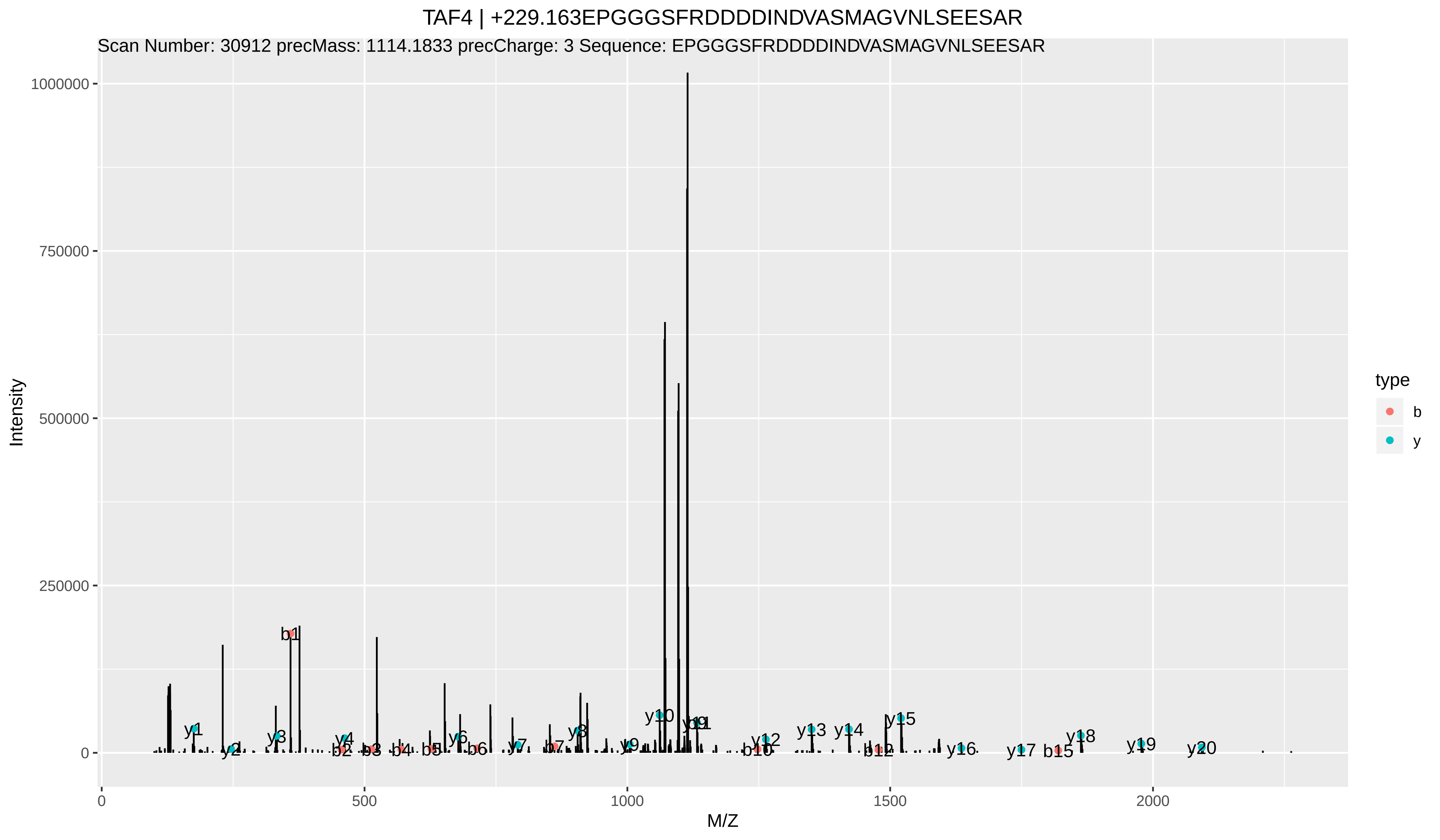
TAF4 | +229.163EPGGGSFRDDDDINDVASMAGVNLSEESAR
Scan Number: 30912 precMass: 1114.1833 precCharge: 3 Sequence: EPGGGSFRDDDDINDVASMAGVNLSEESAR
Intensity
M/Z
type
b
y
0
250000
500000
750000
1000000
0
500
1000
1500
2000
b1
y1
y2
y3
y4
b2
b3
b4
b5
y6
b6
y7
b7
y8
y9
y10
y12
b10
y13
y14
b12
y15
y16
y17
b15
y18
y19
y20

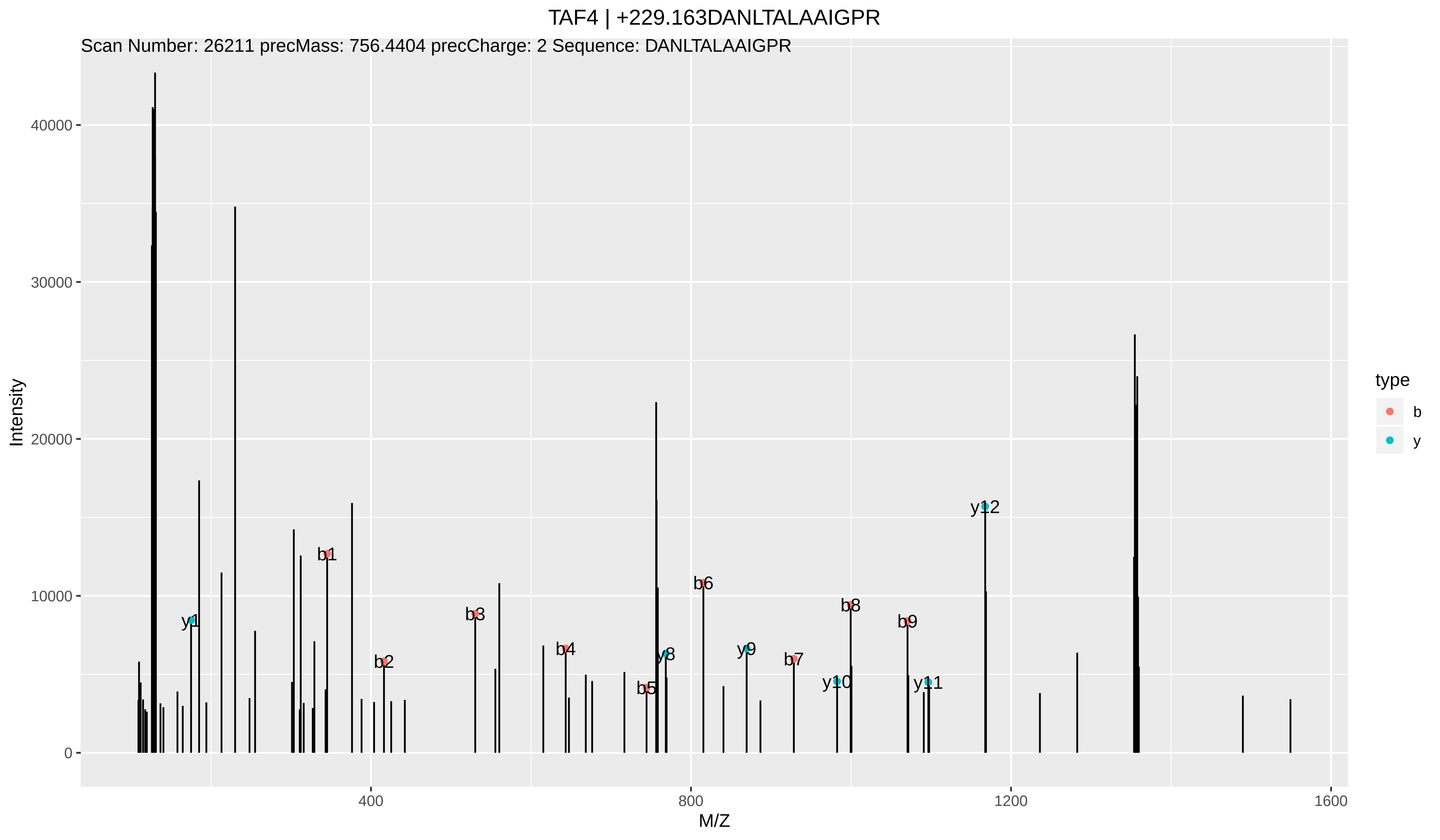

TAF4 | +229.163DANLTALAAIGPR
Scan Number: 26211 precMass: 756.4404 precCharge: 2 Sequence: DANLTALAAIGPR
Intensity
M/Z
0
10000
20000
30000
40000
400
800
1200
1600
y1
b1
b2
b3
b4
b5
y8
b6
y9
b7
y10
b8
b9
y11
y12
type
b
y

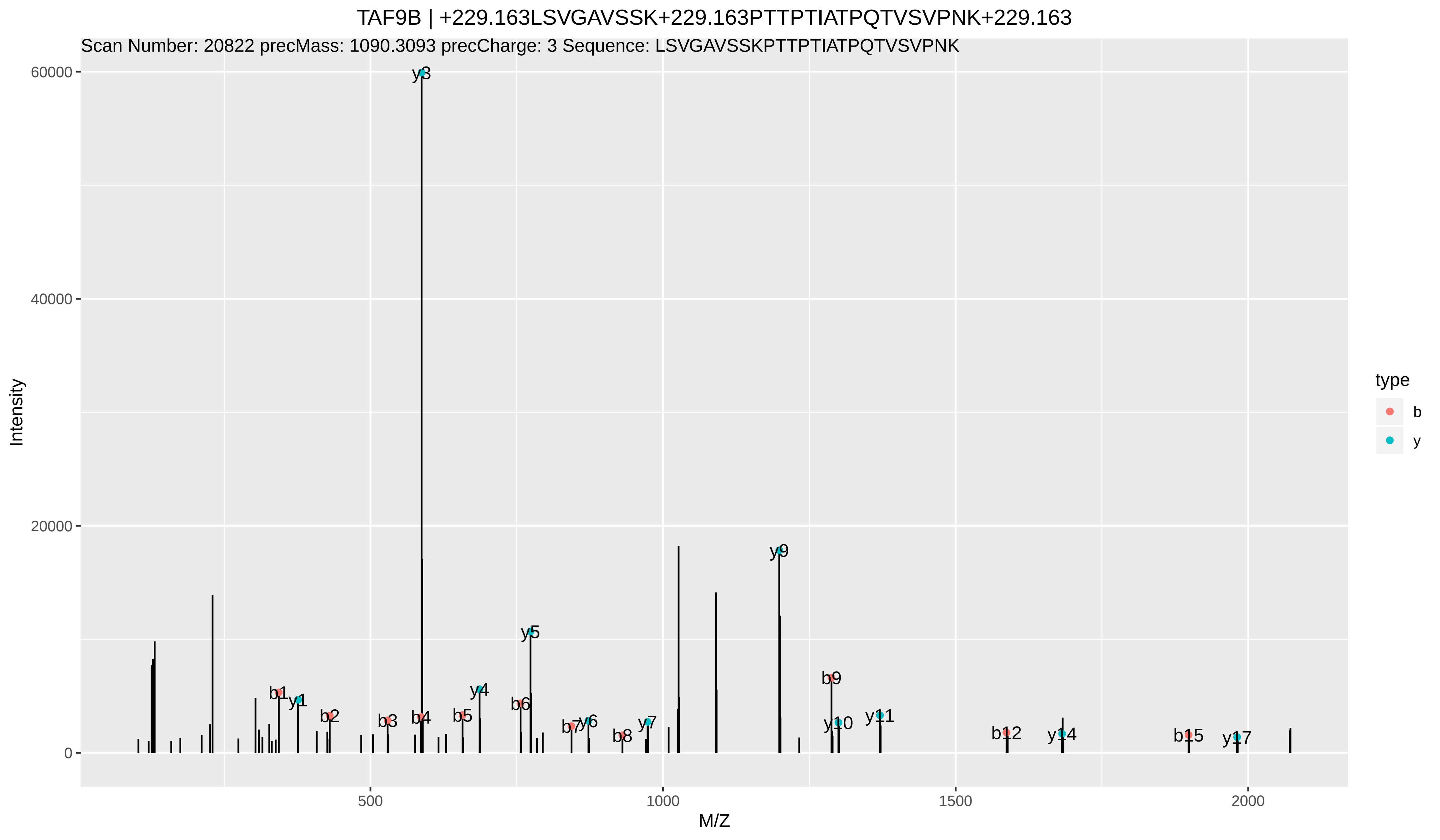

TAF9B | +229.163LSVGAVSSK+229.163PTTPTIATPQTVSVPNK+229.163
Scan Number: 20822 precMass: 1090.3093 precCharge: 3 Sequence: LSVGAVSSKPTTPTIATPQTVSVPNK
Intensity
M/Z
60000
40000
20000
0
500
1000
1500
2000
y3
y9
y5
b9
y4
b1
y1
b6
b2
b3
b4
b5
b7
y6
y7
b8
y10
y11
b12
y14
b15
y17
type
b
y

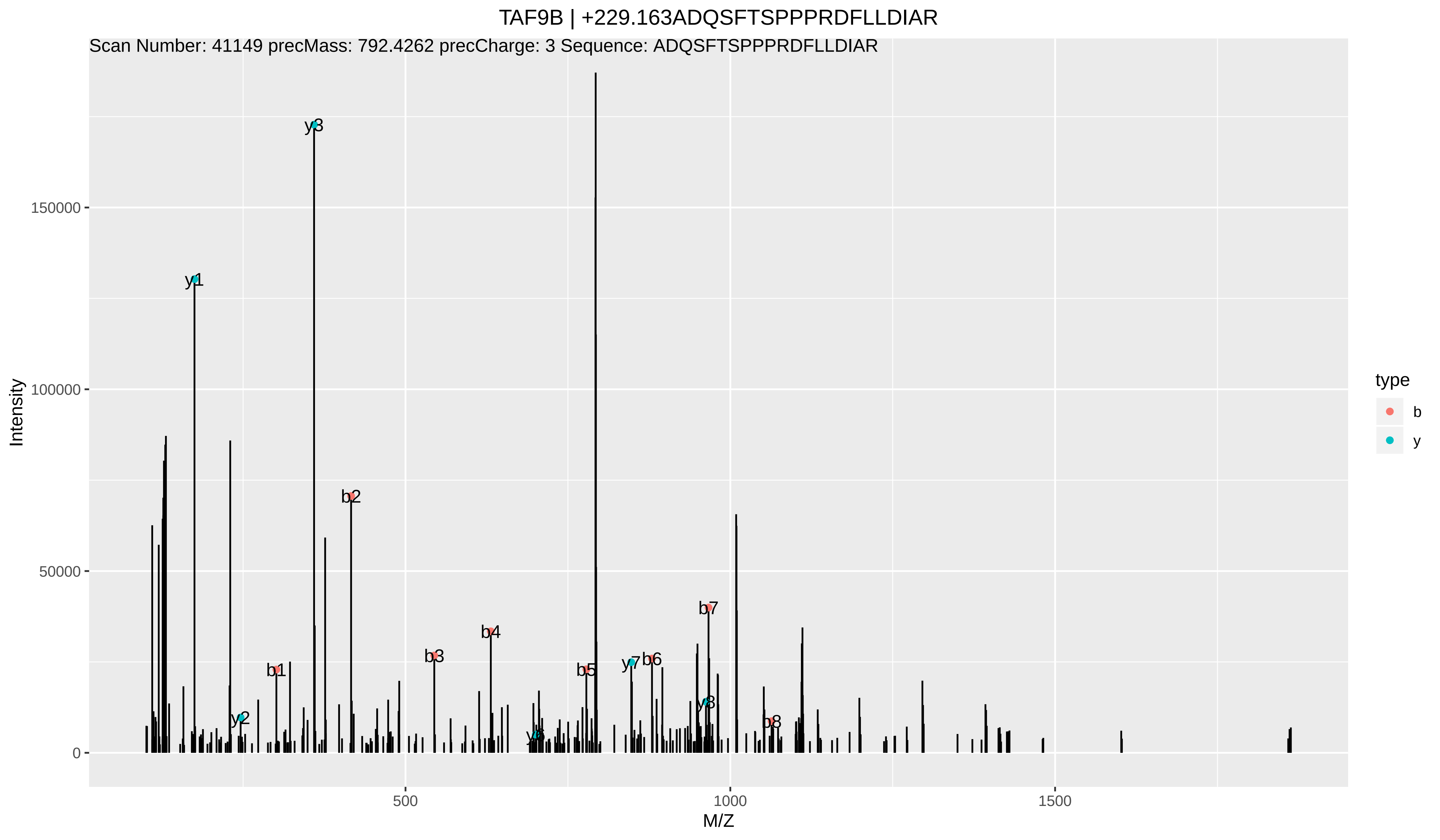
TAF9B | +229.163ADQSFTSPPPRDFLLDIAR
Scan Number: 41149 precMass: 792.4262 precCharge: 3 Sequence: ADQSFTSPPPRDFLLDIAR
Intensity
M/Z
150000
100000
50000
0
500
1000
1500
y1
y2
y3
y6
y7
y8
b1
b2
b3
b4
b5
b6
b7
b8
type
b
y

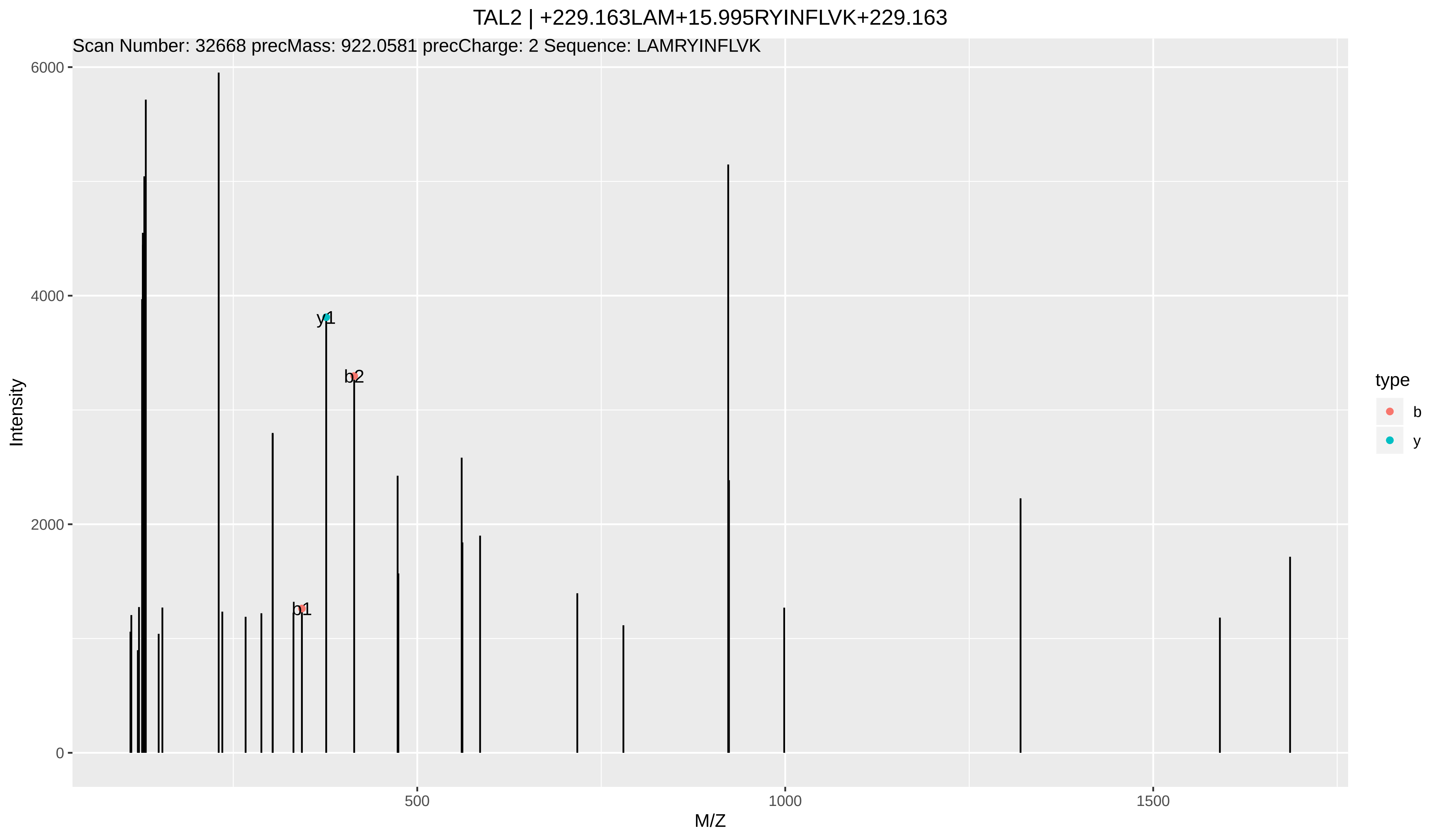
TAL2 | +229.163LAM+15.995RYINFLVK+229.163
Scan Number: 32668 precMass: 922.0581 precCharge: 2 Sequence: LAMRYINFLVK
Intensity
M/Z
y1
b2
b1
type
b
y
0
2000
4000
6000
500
1000
1500

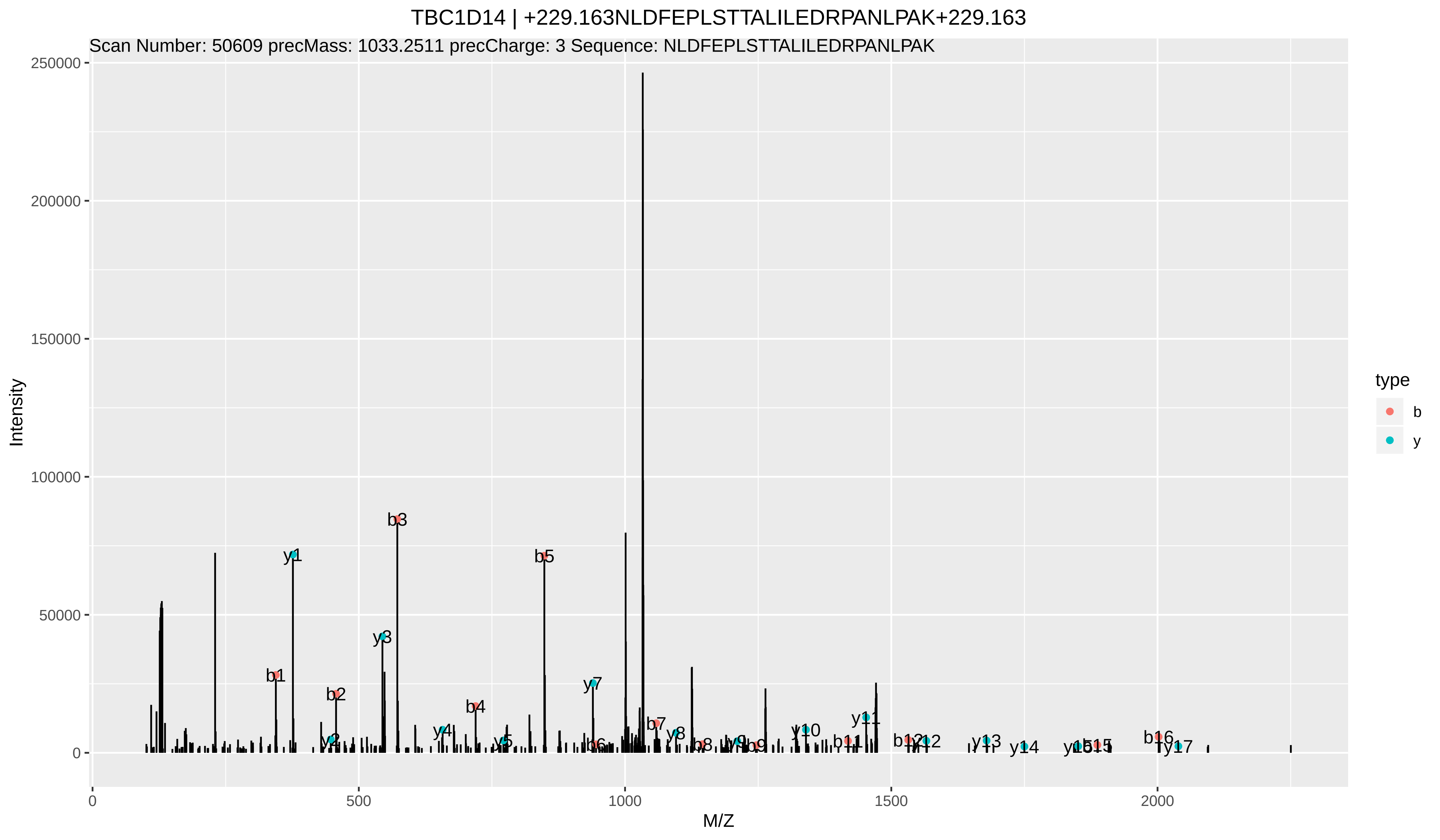
TBC1D14 | +229.163NLDFEPLSTTALILEDRPANLPAK+229.163
Scan Number: 50609 precMass: 1033.2511 precCharge: 3 Sequence: NLDFEPLSTTALILEDRPANLPAK
Intensity
M/Z
250000
200000
150000
100000
50000
0
0
500
1000
1500
2000
type
b
y
b1
b2
b3
b4
b5
b6
b7
b8
b9
b11
b16
y1
y2
y3
y4
y5
y7
y8
y10
y11
y12
y13
y14
y17

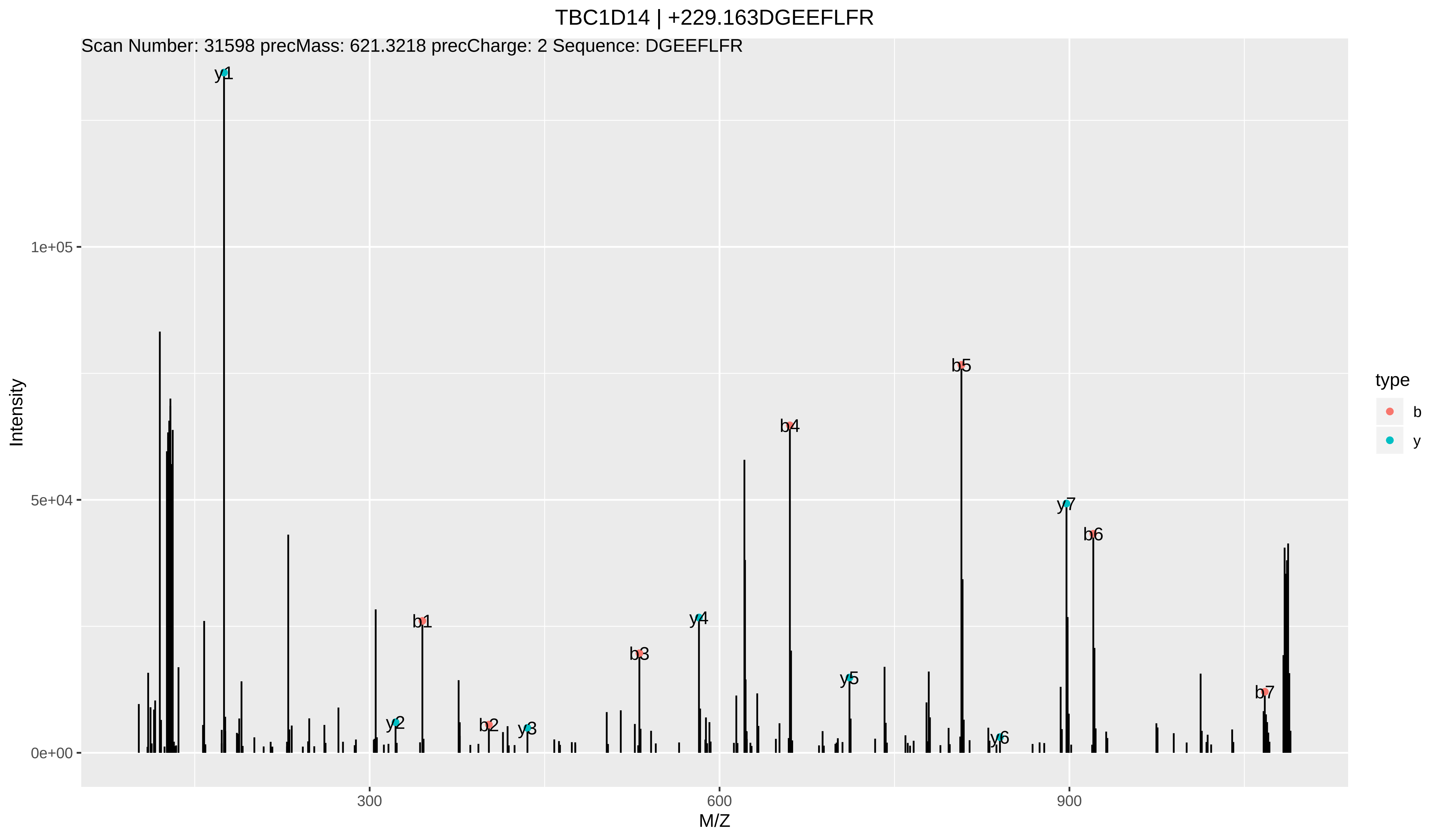
TBC1D14 | +229.163DGEEFLFR
Scan Number: 31598 precMass: 621.3218 precCharge: 2 Sequence: DGEEFLFR
y1
b5
b4
y7
b6
b1
y4
b3
y5
b7
y2
b2
y3
y6
Intensity
1e+05
5e+04
0e+00
300
600
900
M/Z
type
b
y

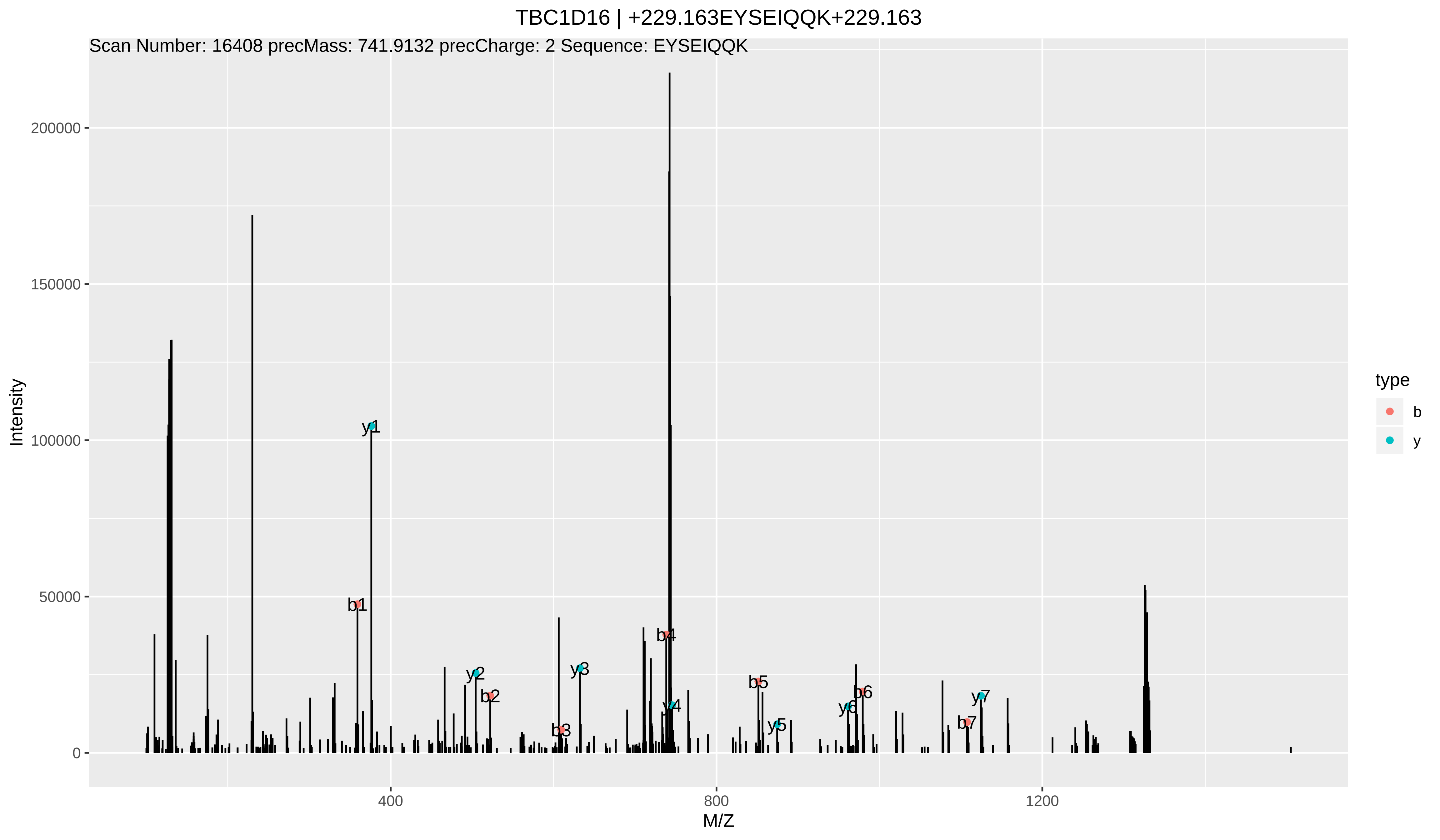

TBC1D16 | +229.163EYSEIQQK+229.163
Scan Number: 16408 precMass: 741.9132 precCharge: 2 Sequence: EYSEIQQK
Intensity
M/Z
200000
150000
100000
50000
0
400
800
1200
y1
b1
b4
y2
b2
y3
b3
b5
y4
y5
y6
b6
y7
b7
type
b
y

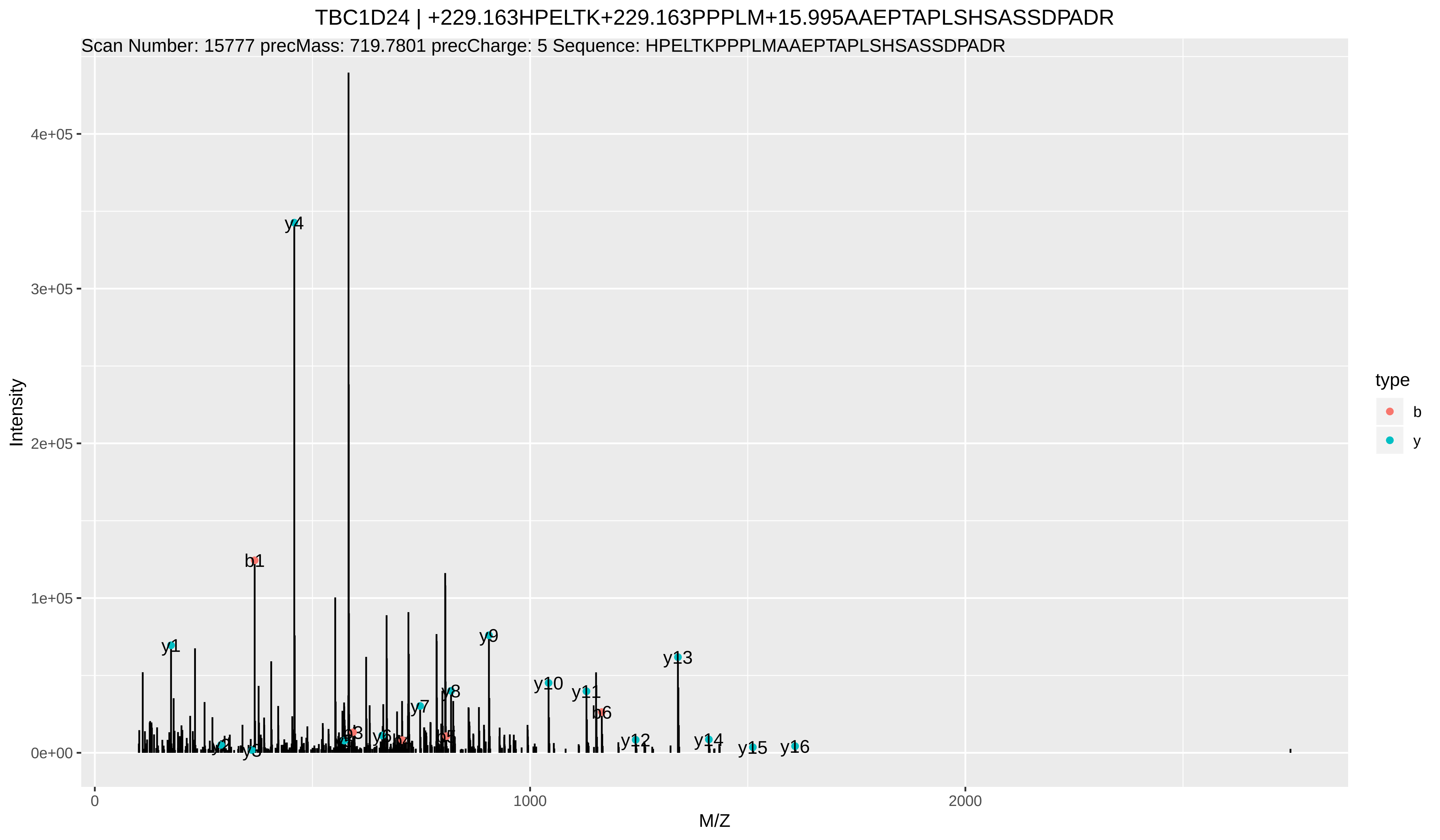
TBC1D24 | +229.163HPELTK+229.163PPPLM+15.995AAEPTAPLSHSASSDPADR
Scan Number: 15777 precMass: 719.7801 precCharge: 5 Sequence: HPELTKPPPLMAAEPTAPLSHSASSDPADR
Intensity
4e+05
3e+05
2e+05
1e+05
0e+00
0
1000
2000
M/Z
y4
b1
y1
y9
y13
y10
y11
b6
y8
y7
y12
y14
y15
y16
type
b
y

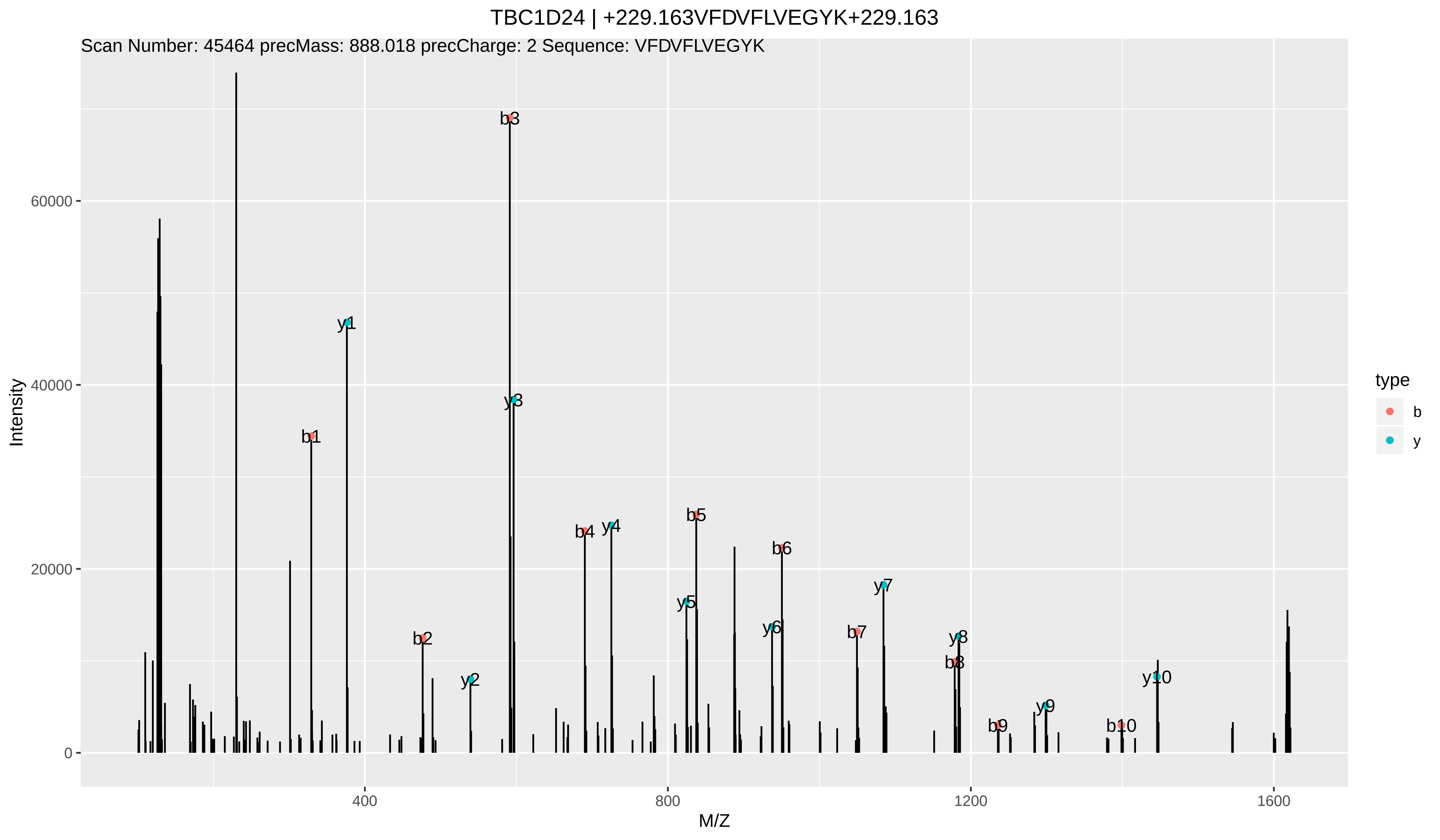

TBC1D24 | +229.163VFDVFLVEGYK+229.163
Scan Number: 45464 precMass: 888.018 precCharge: 2 Sequence: VFDVFLVEGYK
Intensity
M/Z
60000
40000
20000
0
400
800
1200
1600
b1
b2
b3
b4
b5
b6
b7
b8
b9
b10
y1
y2
y3
y4
y5
y6
y7
y8
y9
y10
type
b
y

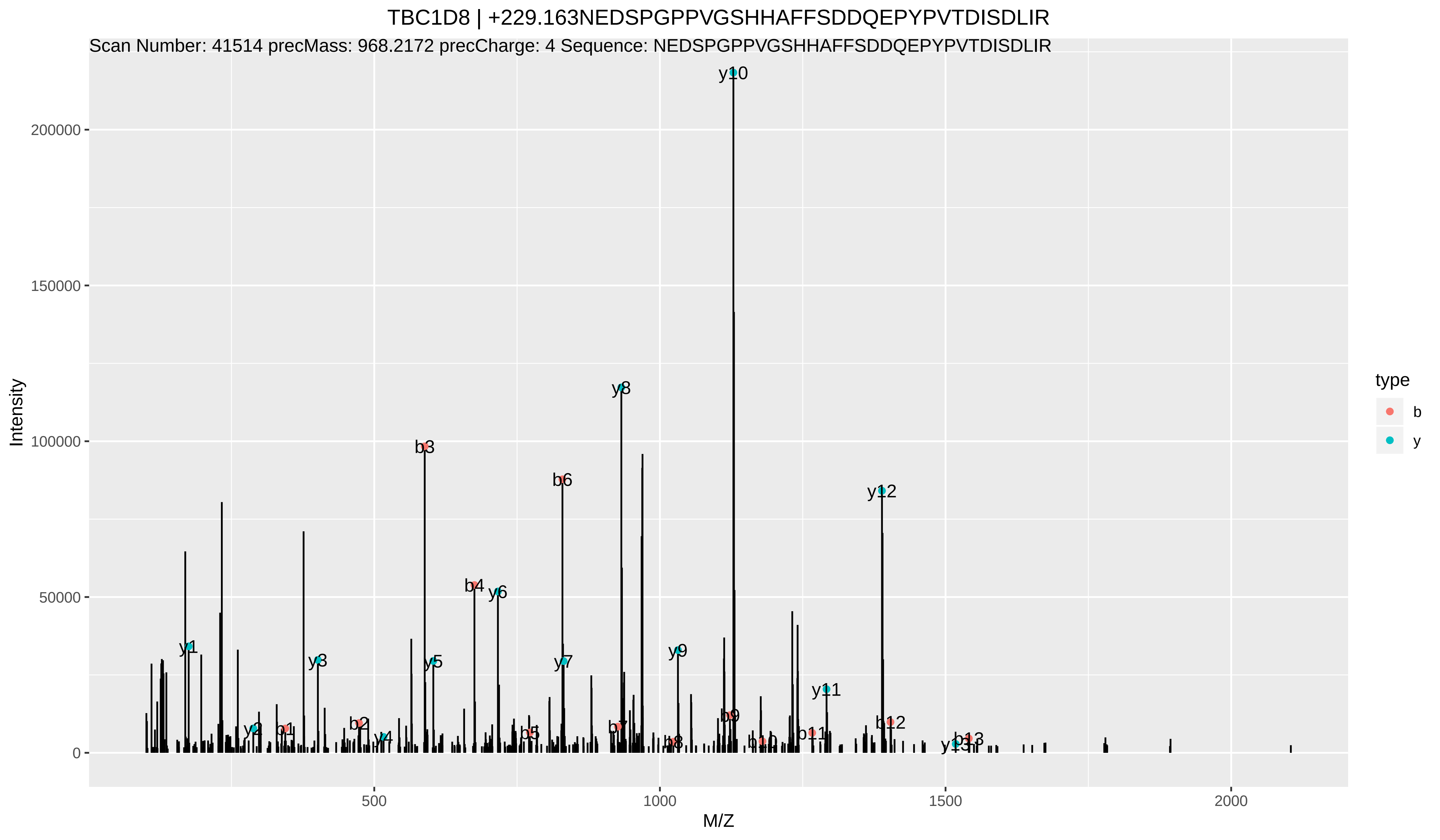

TBC1D8 | +229.163NEDSPGPPVGSHHAFFSDDQEPYPVTDISDLIR
Scan Number: 41514 precMass: 968.2172 precCharge: 4 Sequence: NEDSPGPPVGSHHAFFSDDQEPYPVTDISDLIR
Intensity
M/Z
0
50000
100000
150000
200000
500
1000
1500
2000
y10
y8
b3
b6
y12
b4
y6
y1
y3
y5
y7
y9
y11
b9
b12
y2
b1
b2
y4
b5
b7
b8
b10
b11
b13
y13
type
b
y

TBCCD1 | +229.163SSDTLAGAHVK+229.163

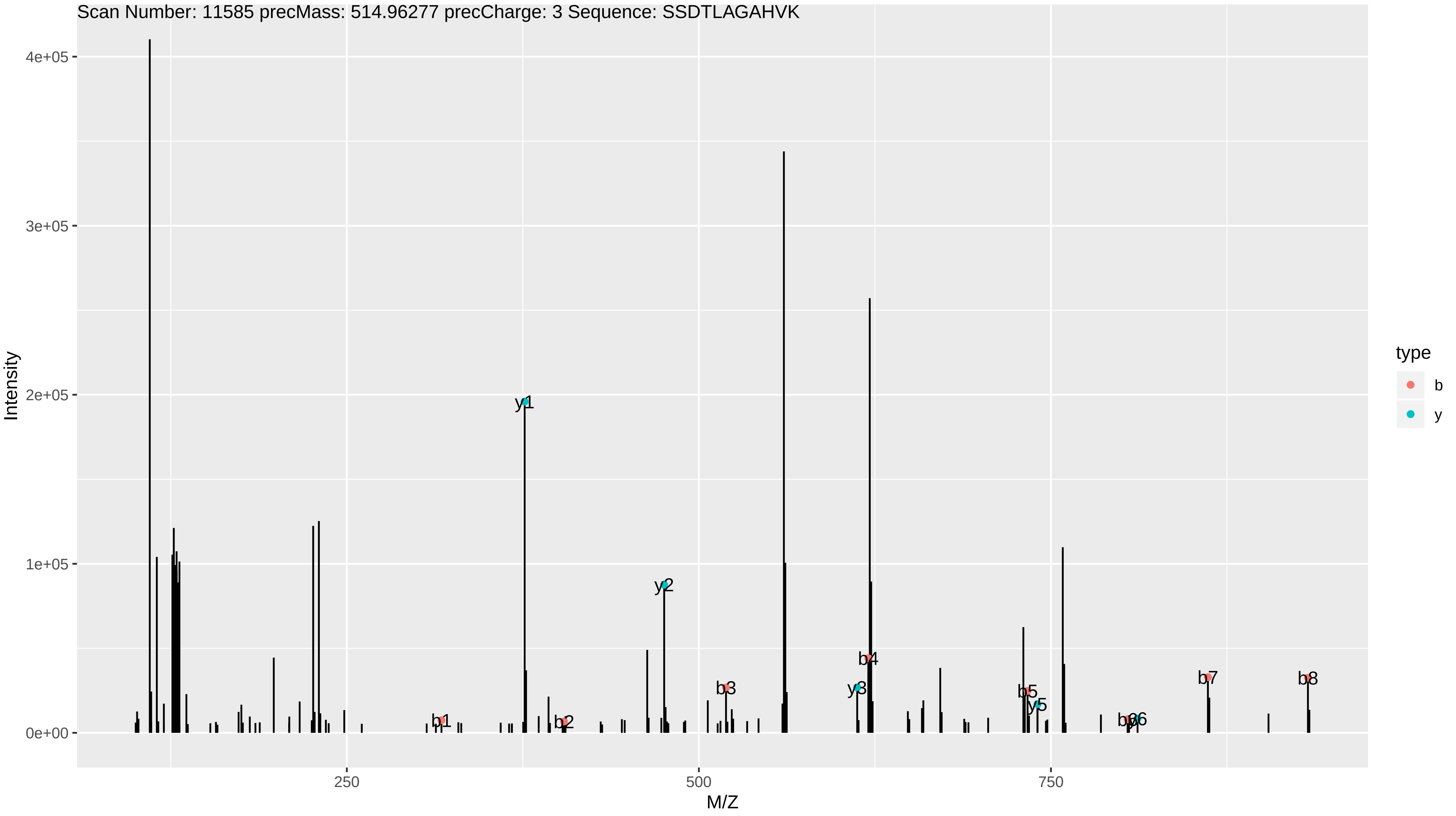

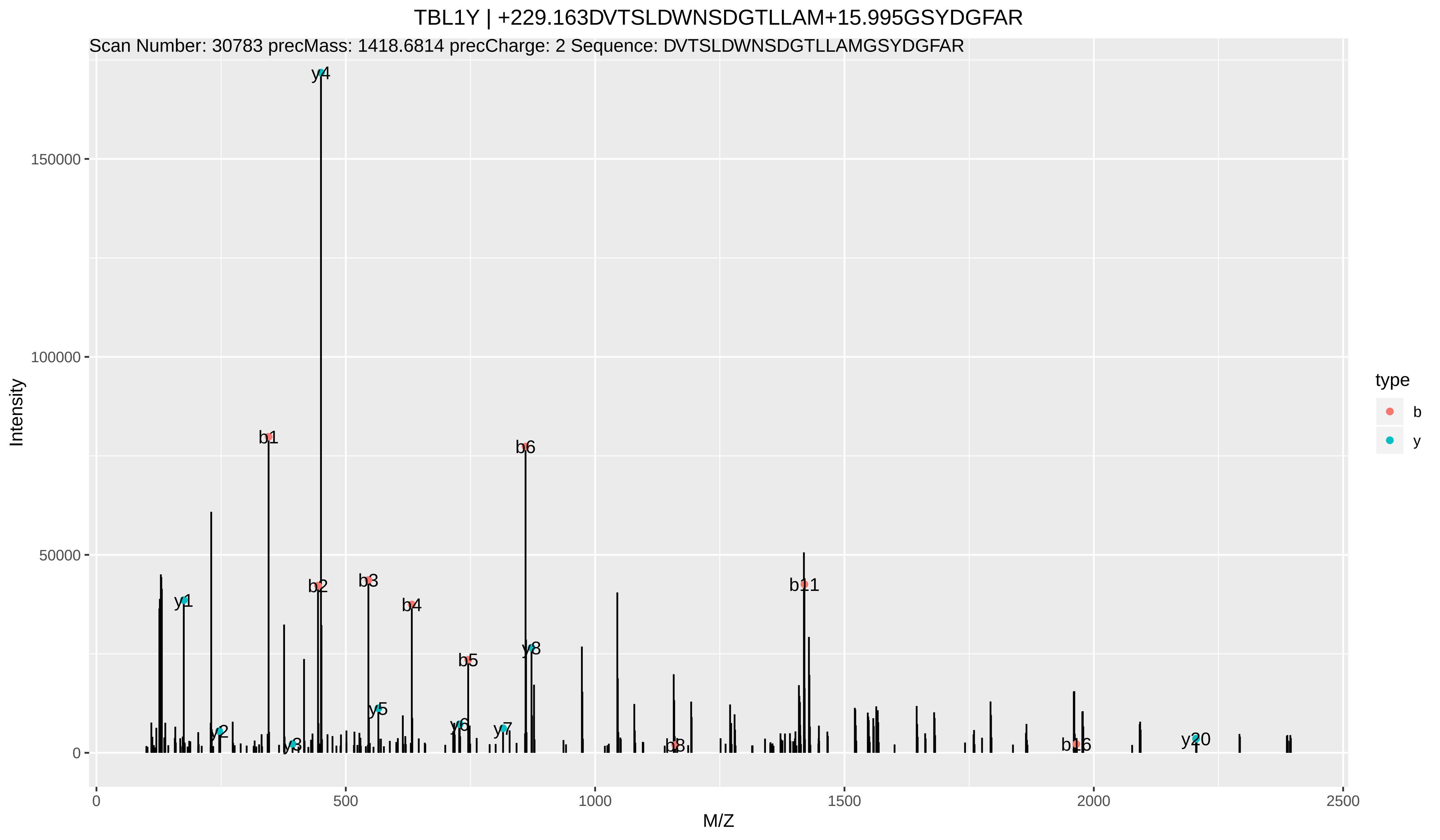
TBL1Y | +229.163DVTSLDWNSDGTLLAM+15.995GSYDGFAR
Scan Number: 30783 precMass: 1418.6814 precCharge: 2 Sequence: DVTSLDWNSDGTLLAMGSYDGFAR
Intensity
M/Z
0
50000
100000
150000
500
1000
1500
2000
2500
y4
b1
b6
b2
b3
b4
b11
y1
y2
y3
y5
y6
y7
y8
b5
b8
b16
y20
type
b
y

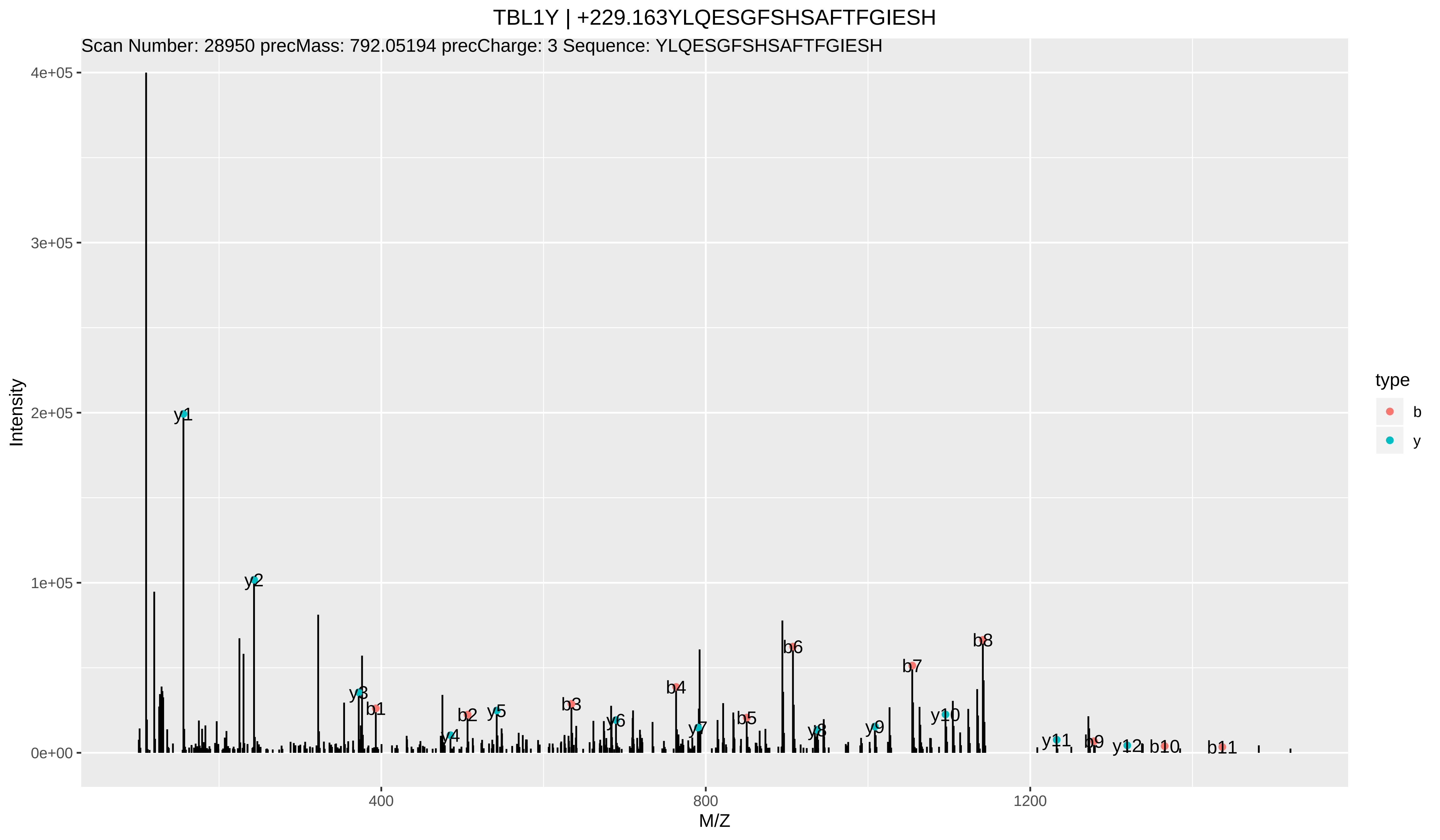
TBL1Y | +229.163YLQESGFSHSAFTFGIESH
Scan Number: 28950 precMass: 792.05194 precCharge: 3 Sequence: YLQESGFSHSAFTFGIESH
Intensity
M/Z
type
b
y
y1
y2
y3
b1
y4
b2
y5
b3
y6
b4
y7
b5
b6
y8
y9
b7
y10
b8
y11
b9
y12
b10
b11
4e+05
3e+05
2e+05
1e+05
0e+00
400
800
1200

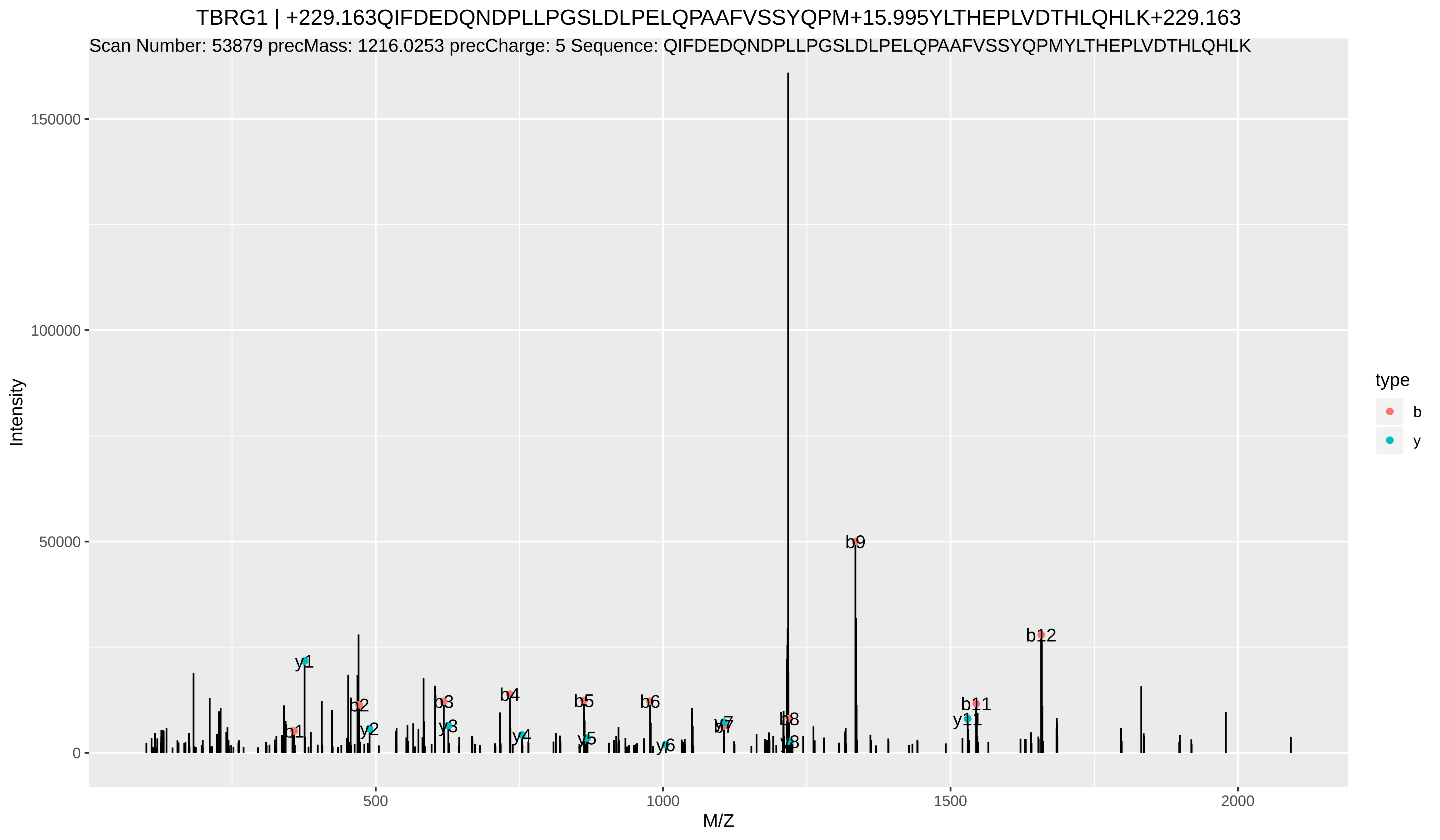
TBRG1 | +229.163QIFDEDQNDPLLPGSLDLPELQPAAFVSSYQPM+15.995YLTHEPLVDTHLQHLK+229.163
Scan Number: 53879 precMass: 1216.0253 precCharge: 5 Sequence: QIFDEDQNDPLLPGSLDLPELQPAAFVSSYQPMYLTHEPLVDTHLQHLK
Intensity
150000
100000
50000
0
M/Z
500
1000
1500
2000
type
b
y
y1
b1
b2
y2
b3
y3
b4
y4
b5
y5
b6
y6
y7
b7
b8
y8
b9
y11
b11
b12

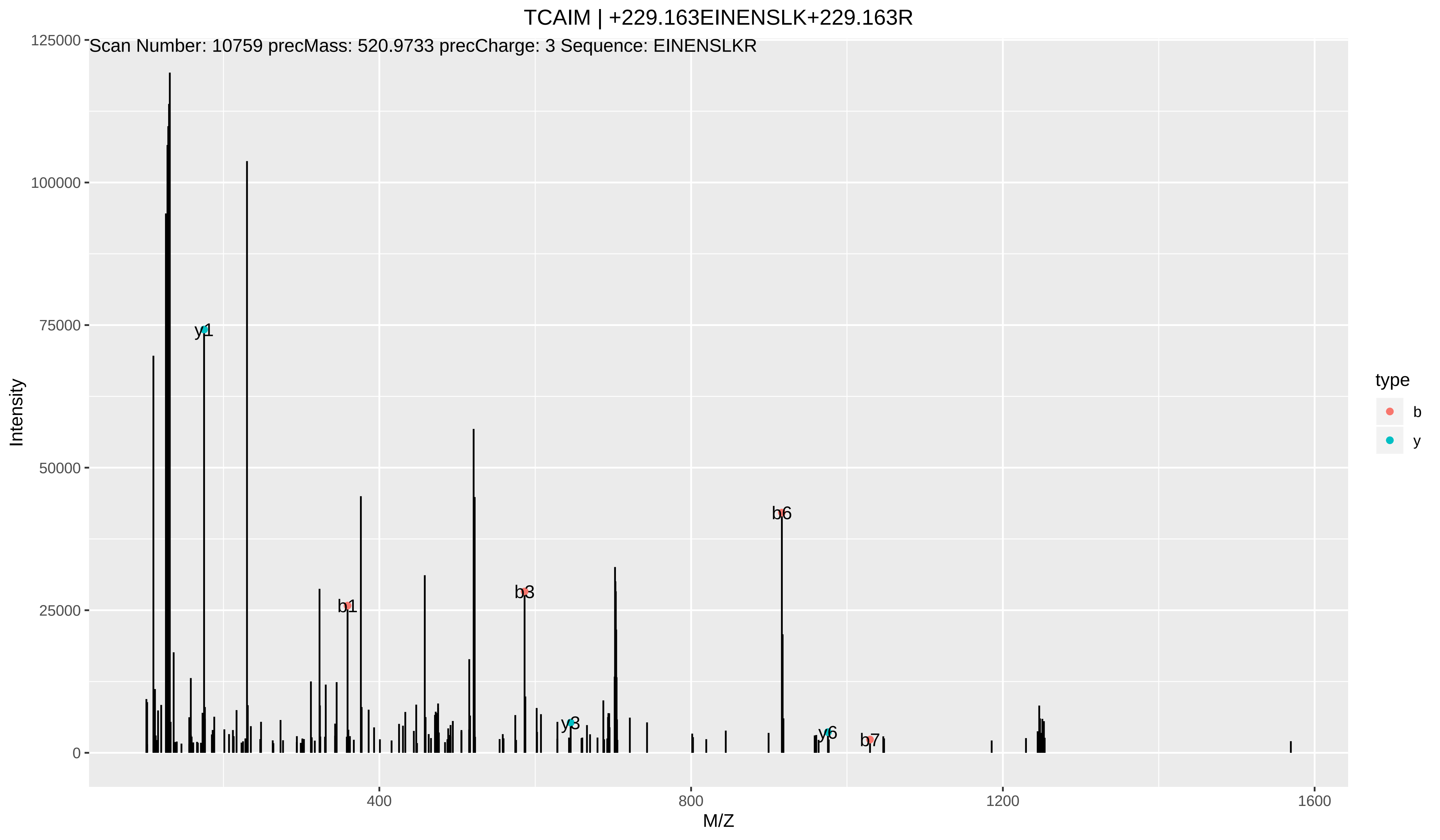

TCAIM | +229.163EINENSLK+229.163R
Scan Number: 10759 precMass: 520.9733 precCharge: 3 Sequence: EINENSLKR
Intensity
M/Z
0
25000
50000
75000
100000
125000
400
800
1200
1600
y1
b1
b3
y3
b6
y6
b7
type
b
y

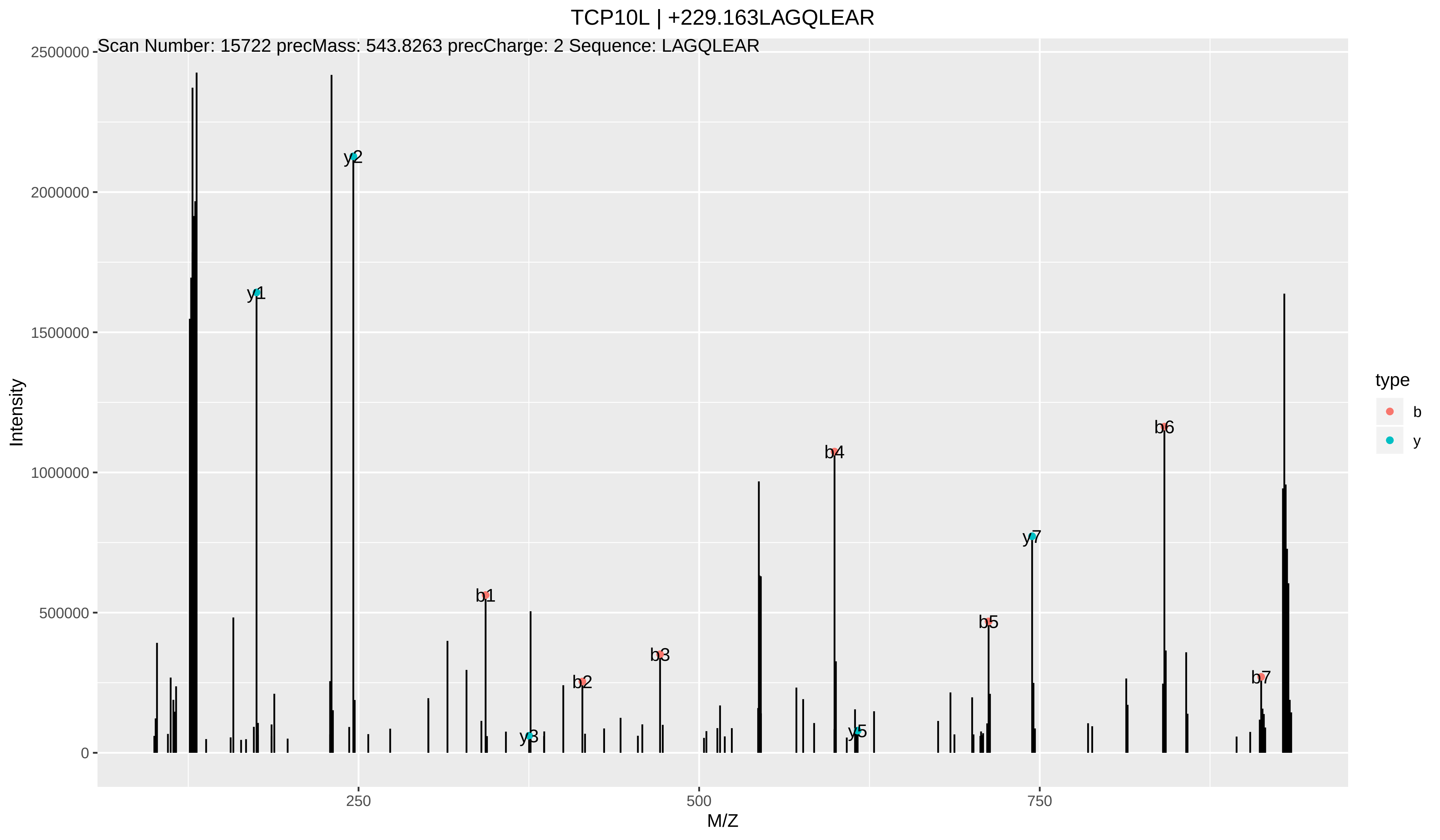
TCP10L | +229.163LAGQLEAR
Scan Number: 15722 precMass: 543.8263 precCharge: 2 Sequence: LAGQLEAR
Intensity
2500000
2000000
1500000
1000000
500000
0
M/Z
250
500
750
y1
y2
y3
y5
y7
b1
b2
b3
b4
b5
b6
b7
type
b
y

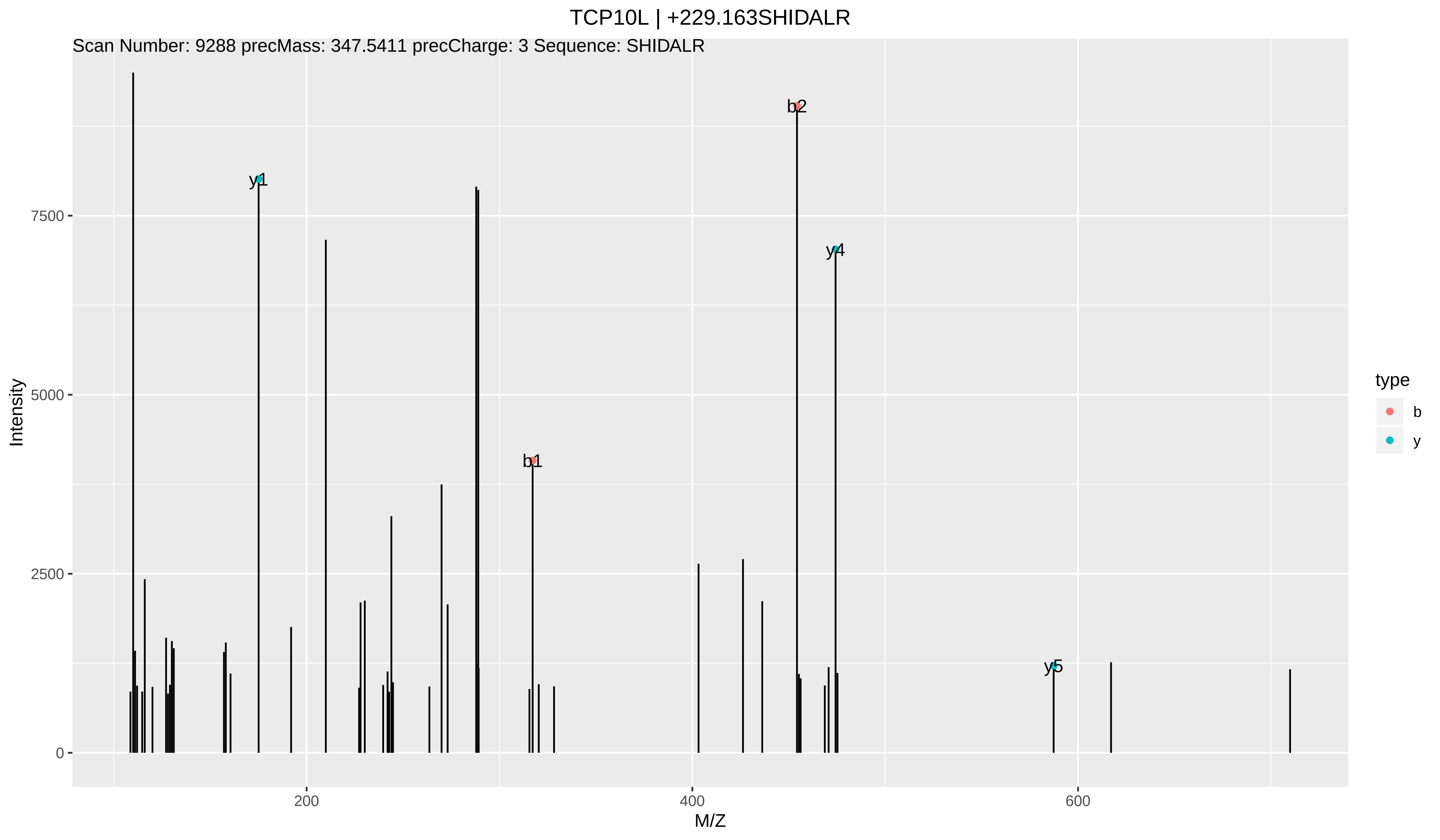

TCP10L | +229.163SHIDALR
Scan Number: 9288 precMass: 347.5411 precCharge: 3 Sequence: SHIDALR
Intensity
0
2500
5000
7500
M/Z
200
400
600
y1
b1
b2
y4
y5
type
b
y

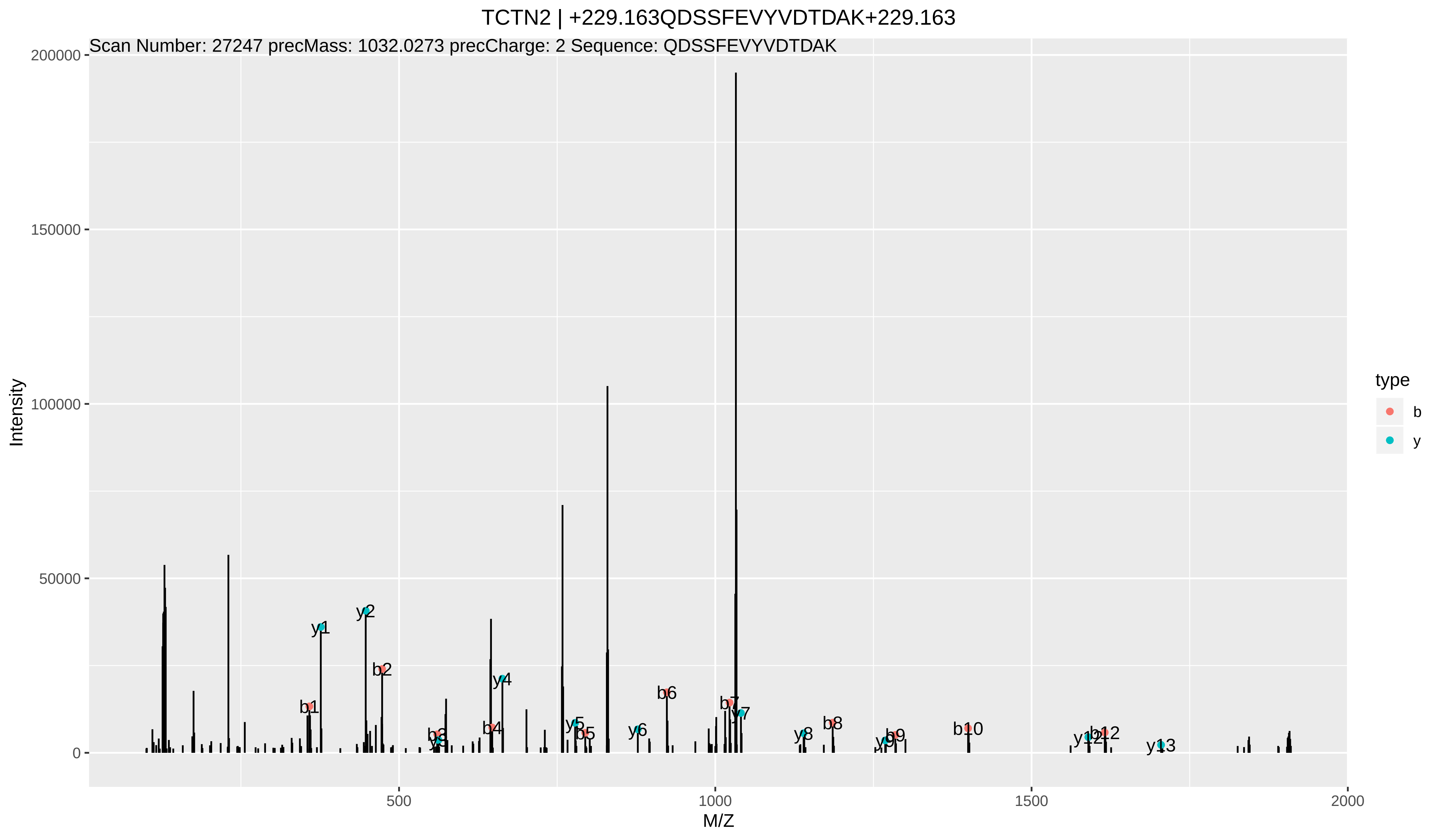
TCTN2 | +229.163QDSSFEVYVDTDAK+229.163
Scan Number: 27247 precMass: 1032.0273 precCharge: 2 Sequence: QDSSFEVYVDTDAK
Intensity
M/Z
type
b
y
0
50000
100000
150000
200000
500
1000
1500
2000
y1
y2
b1
b2
b3
y3
b4
y4
y5
b5
y6
b6
b7
y7
y8
b8
y9
b9
b10
y12
b12
y13

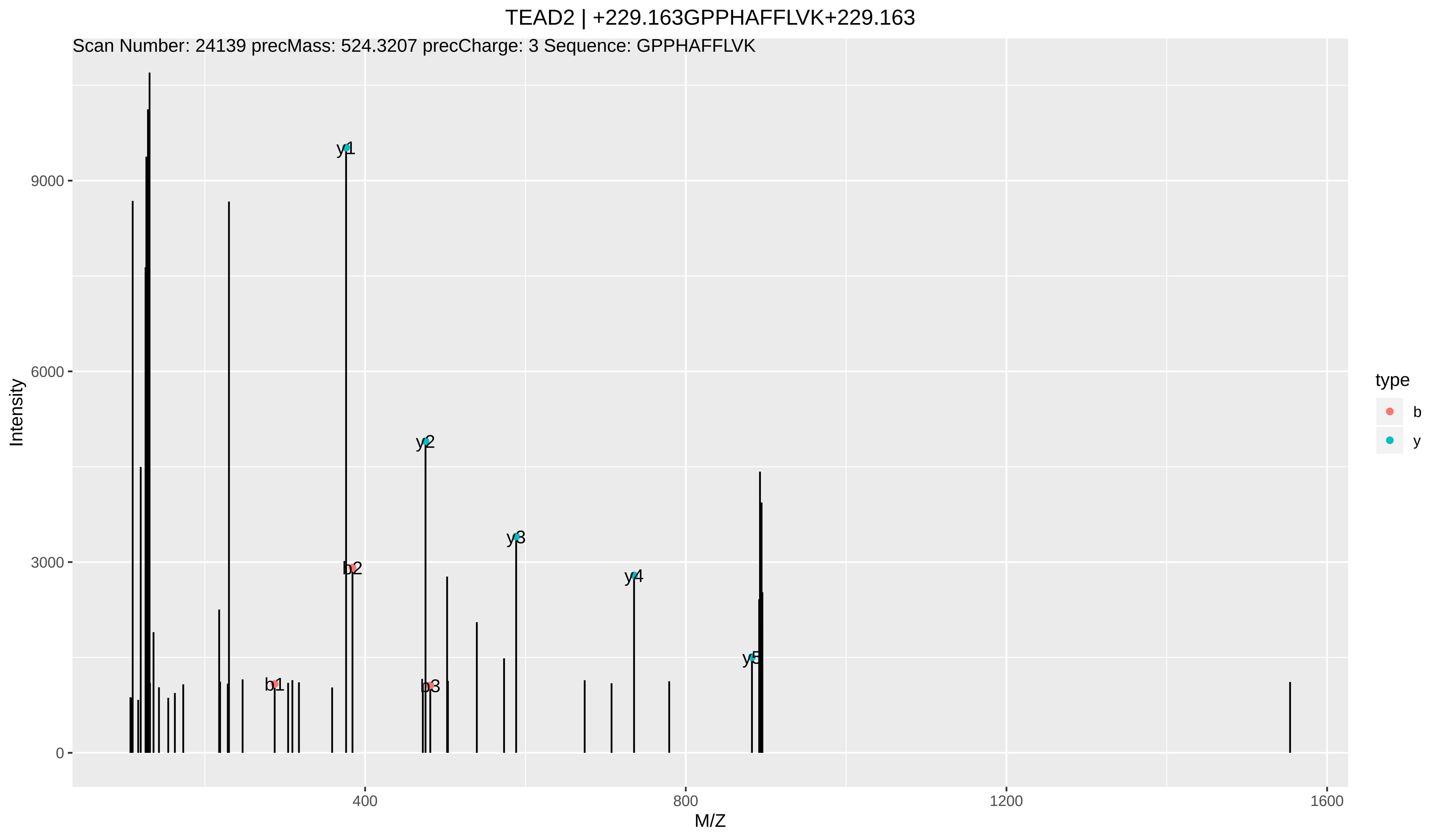
TEAD2 | +229.163GPPHAFFLVK+229.163
Scan Number: 24139 precMass: 524.3207 precCharge: 3 Sequence: GPPHAFFLVK
Intensity
9000
6000
3000
0
400
800
1200
1600
M/Z
y1
y2
y3
y4
y5
b1
b2
b3
type
b
y

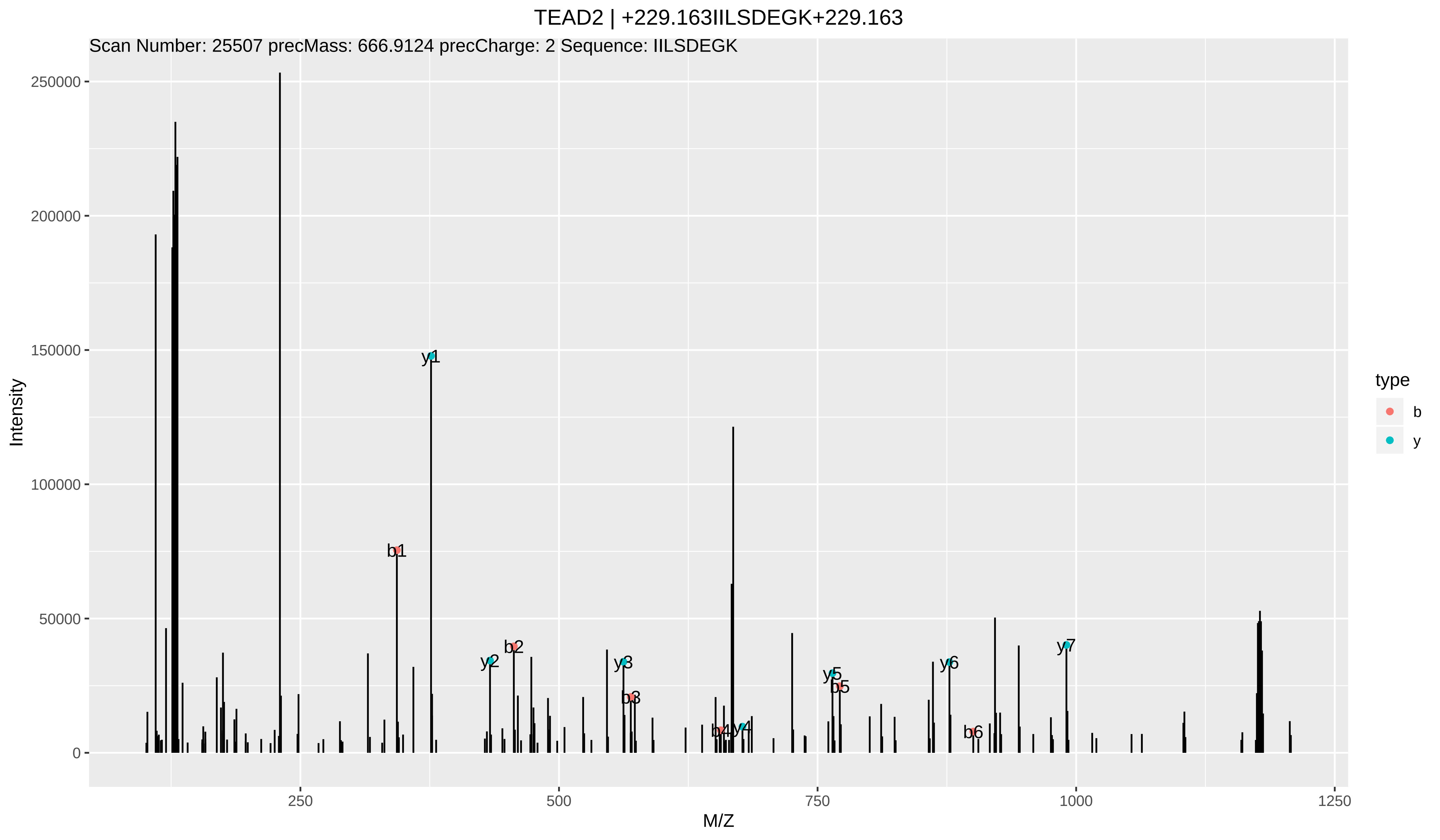
TEAD2 | +229.163IILSDEGK+229.163
Scan Number: 25507 precMass: 666.9124 precCharge: 2 Sequence: IILSDEGK
Intensity
M/Z
type
b
y
y1
b1
y2
b2
y3
b3
b4
y4
y5
b5
y6
b6
y7
250000
200000
150000
100000
50000
0
250
500
750
1000
1250

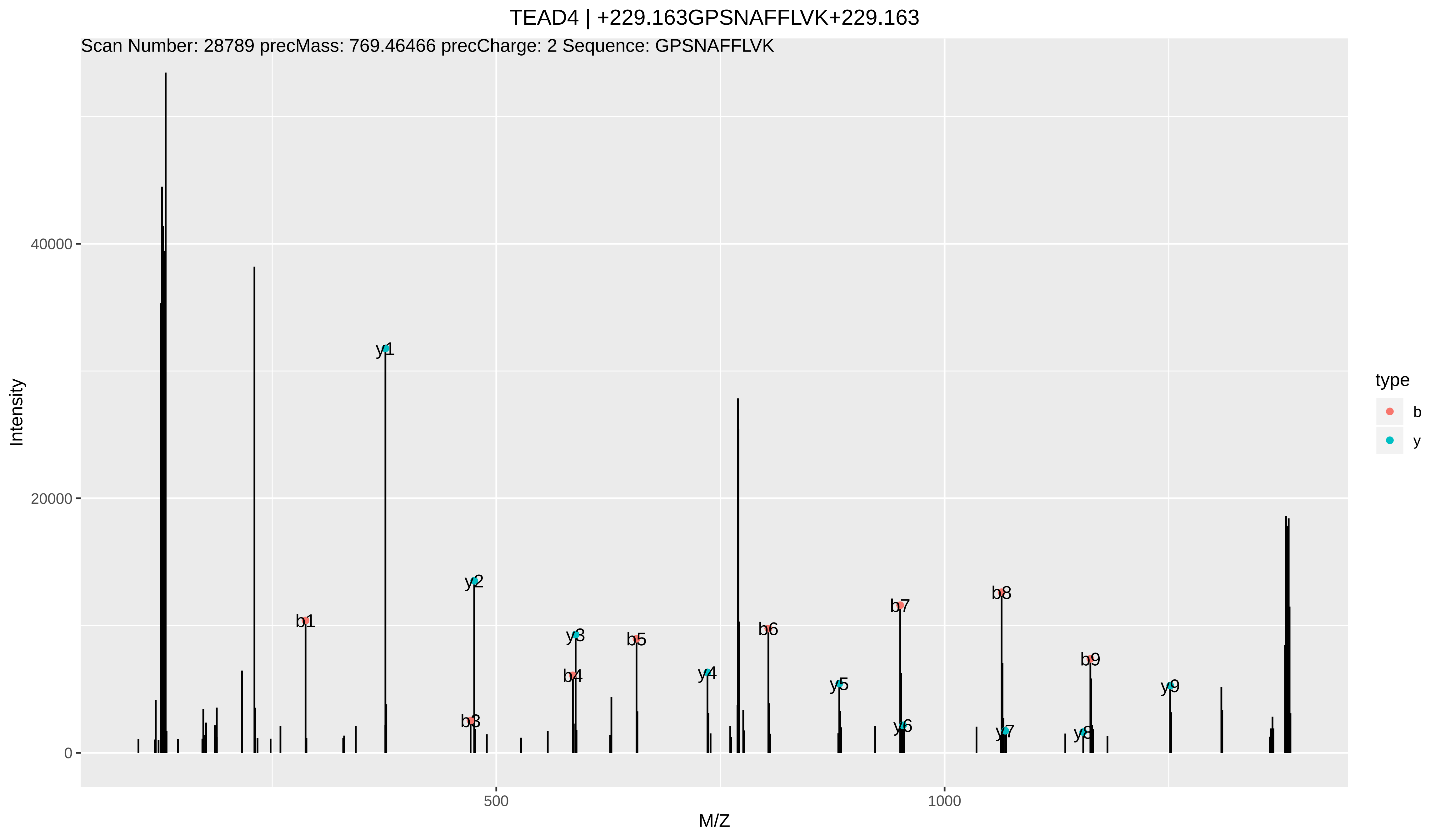
TEAD4 | +229.163GPSNAFFLVK+229.163
Scan Number: 28789 precMass: 769.46466 precCharge: 2 Sequence: GPSNAFFLVK
Intensity
40000
20000
0
M/Z
500
1000
y1
y2
b1
y3
b5
b4
b3
y4
b6
y5
b7
y6
b8
y7
b9
y8
y9
type
b
y

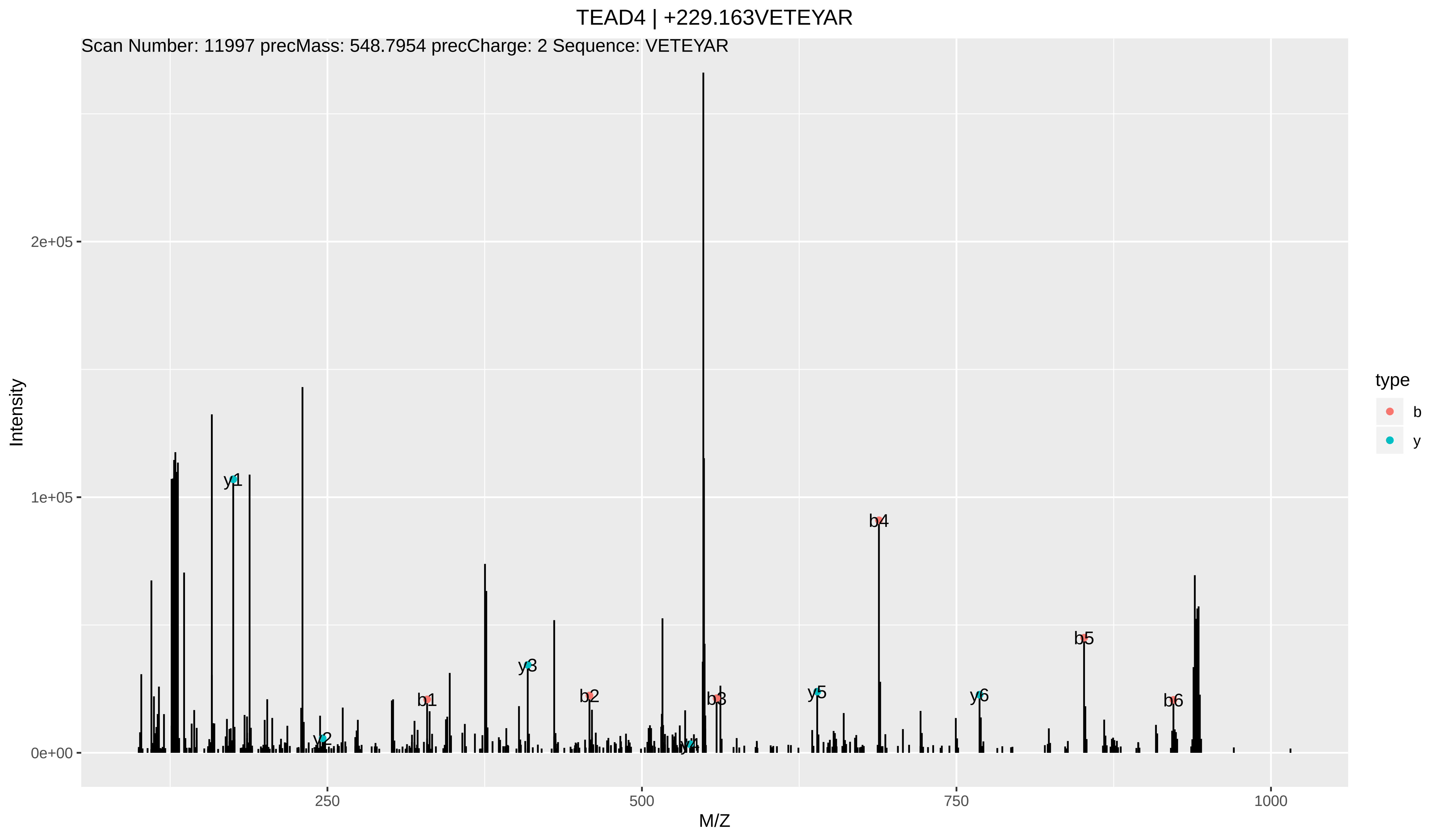
TEAD4 | +229.163VETEYAR
Scan Number: 11997 precMass: 548.7954 precCharge: 2 Sequence: VETEYAR
Intensity
2e+05
1e+05
0e+00
M/Z
250
500
750
1000
y1
y2
b1
y3
b2
y4
b3
y5
b4
y6
b5
b6
type
b
y

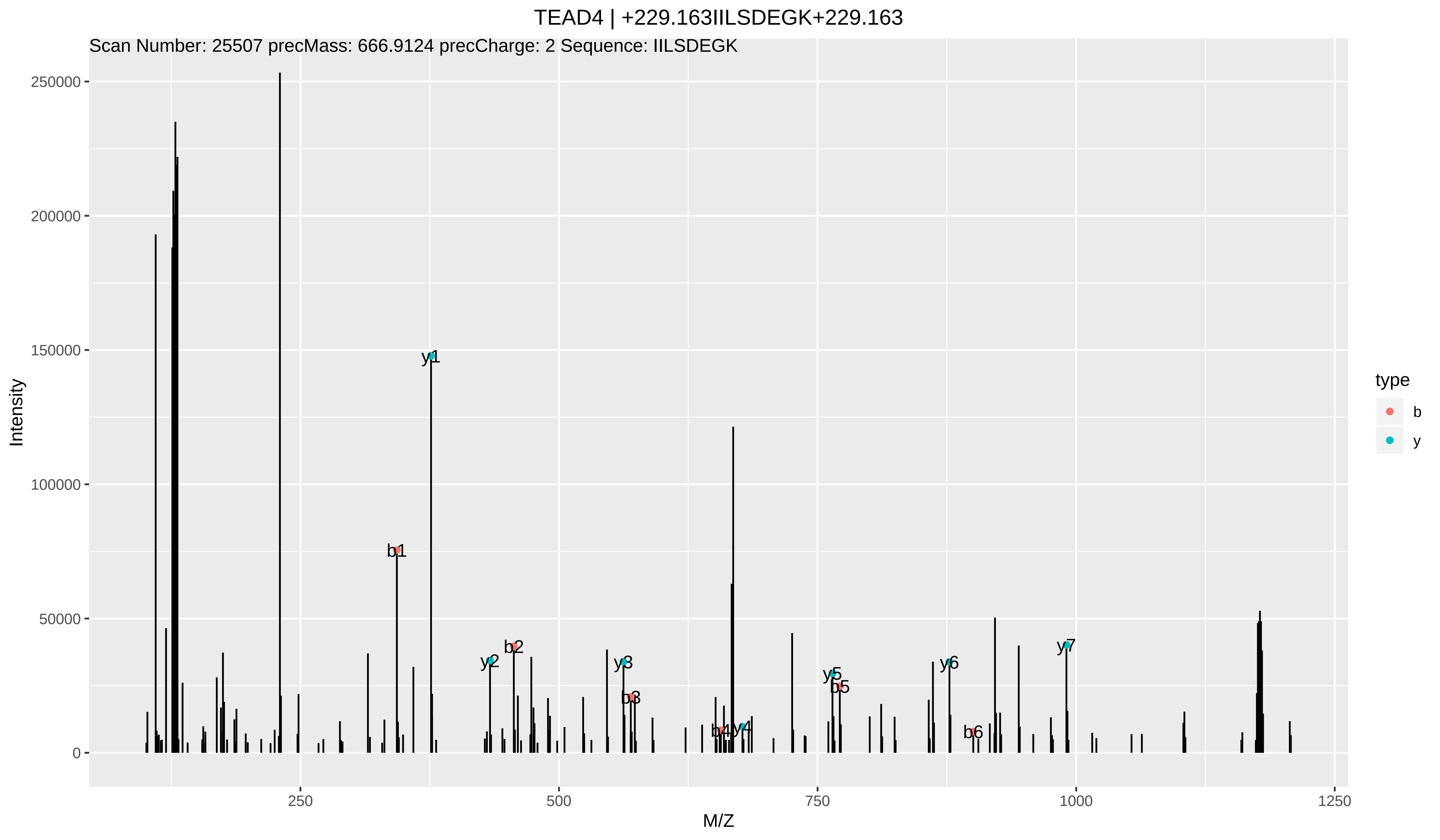

TEAD4 | +229.163IILSDEGK+229.163
Scan Number: 25507 precMass: 666.9124 precCharge: 2 Sequence: IILSDEGK
Intensity
M/Z
type
b
y
y1
b1
y2
b2
y3
b3
b4
y4
y5
b5
y6
b6
y7
250000
200000
150000
100000
50000
0
250
500
750
1000
1250

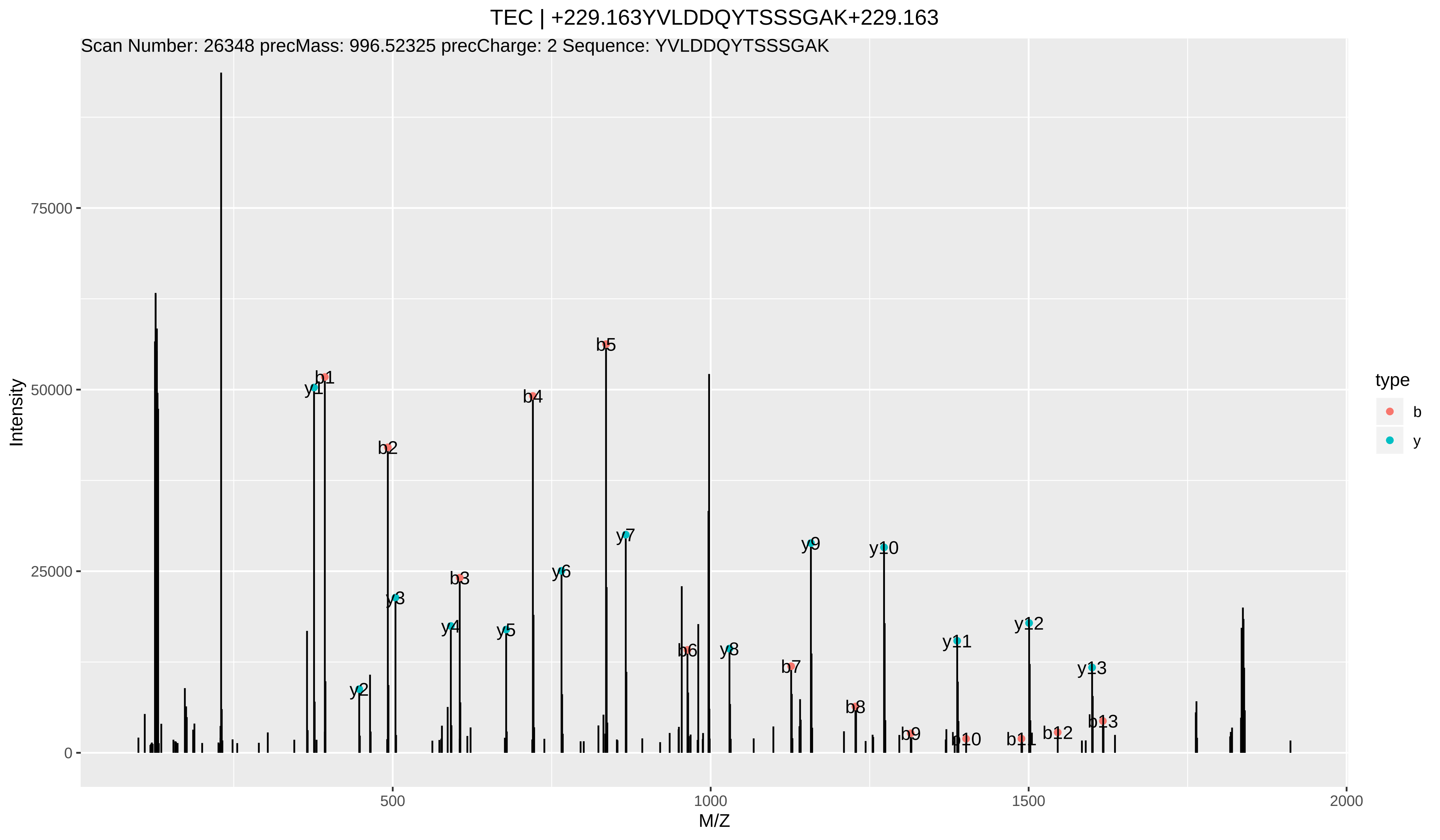

TEC | +229.163YVLDDQYTSSSGAK+229.163
Scan Number: 26348 precMass: 996.52325 precCharge: 2 Sequence: YVLDDQYTSSSGAK
Intensity
M/Z
type
b
y
b1
b2
b3
b4
b5
b6
b7
b8
b9
b10
b11
b12
b13
y1
y2
y3
y4
y5
y6
y7
y8
y9
y10
y11
y12
y13

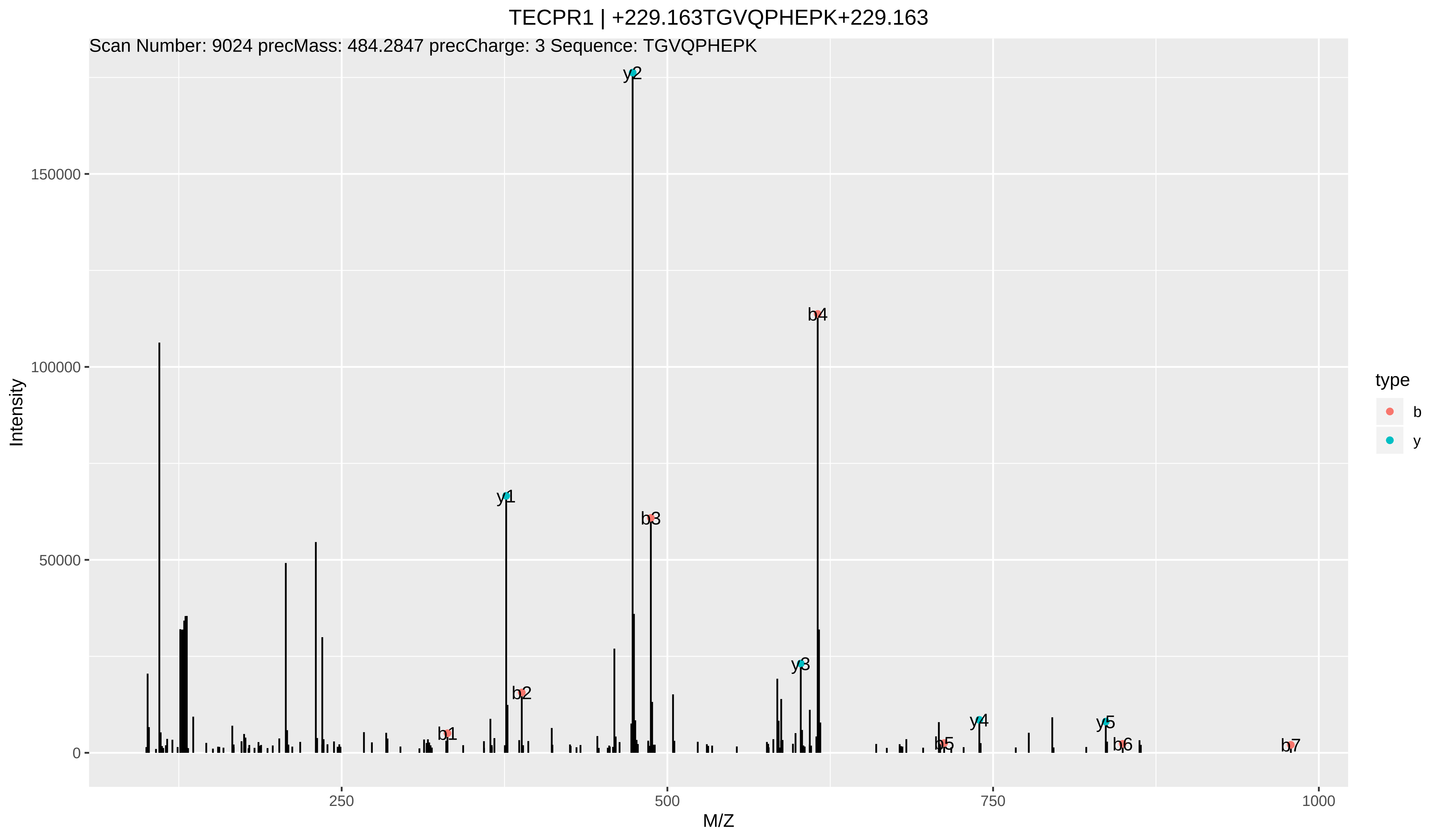
TECPR1 | +229.163TGVQPHEPK+229.163
Scan Number: 9024 precMass: 484.2847 precCharge: 3 Sequence: TGVQPHEPK
y2
b4
y1
b3
y3
b2
b1
y4
y5
b5
b6
b7
150000
100000
50000
0
Intensity
250
500
750
1000
M/Z
type
b
y

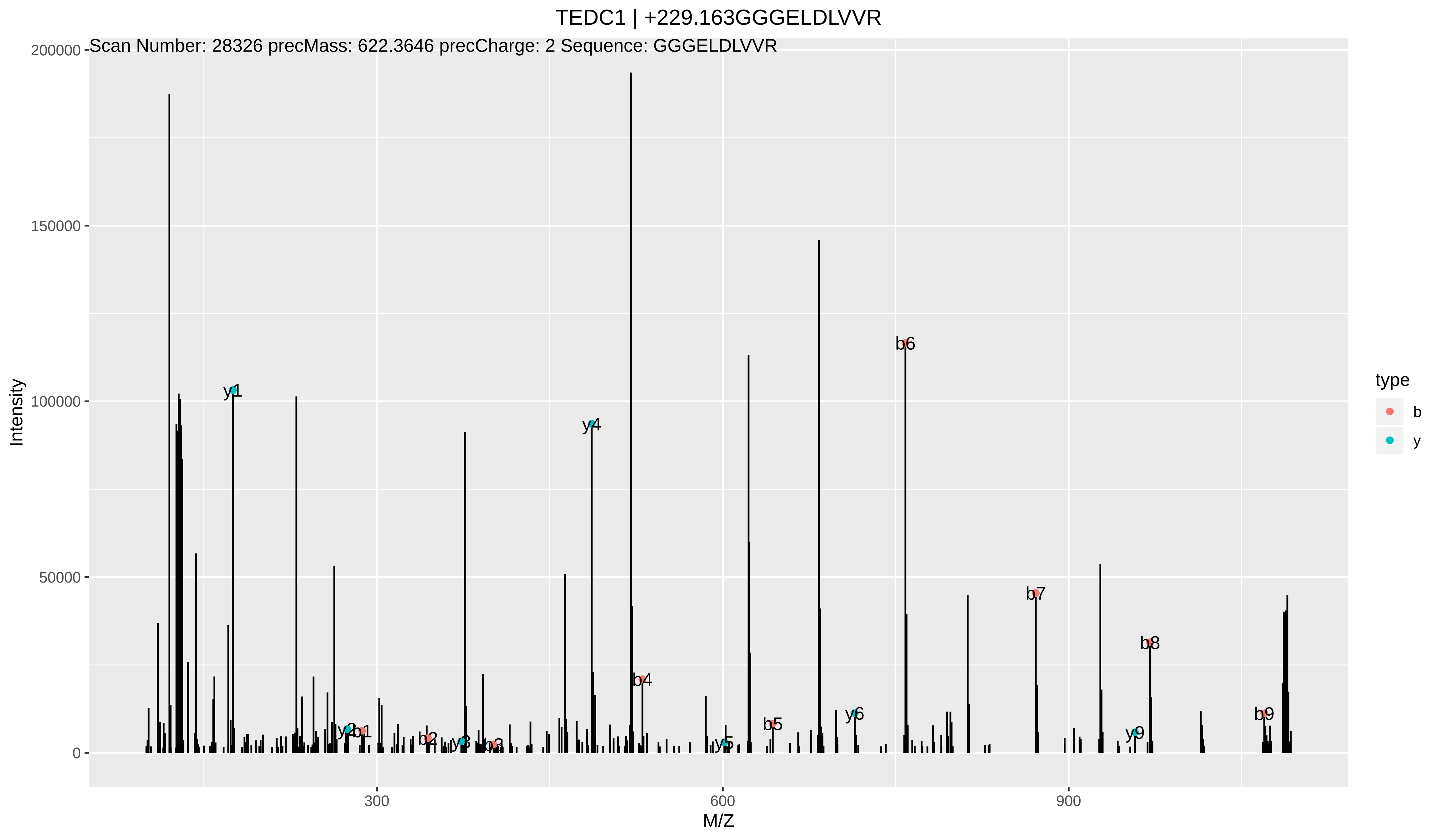

TEDC1 | +229.163GGGELDLVVR
Scan Number: 28326 precMass: 622.3646 precCharge: 2 Sequence: GGGELDLVVR
Intensity
200000
150000
100000
50000
0
300
600
900
M/Z
y1
y2
b1
b2
y3
b3
y4
b4
y5
b5
y6
b6
b7
y9
b8
b9
type
b
y

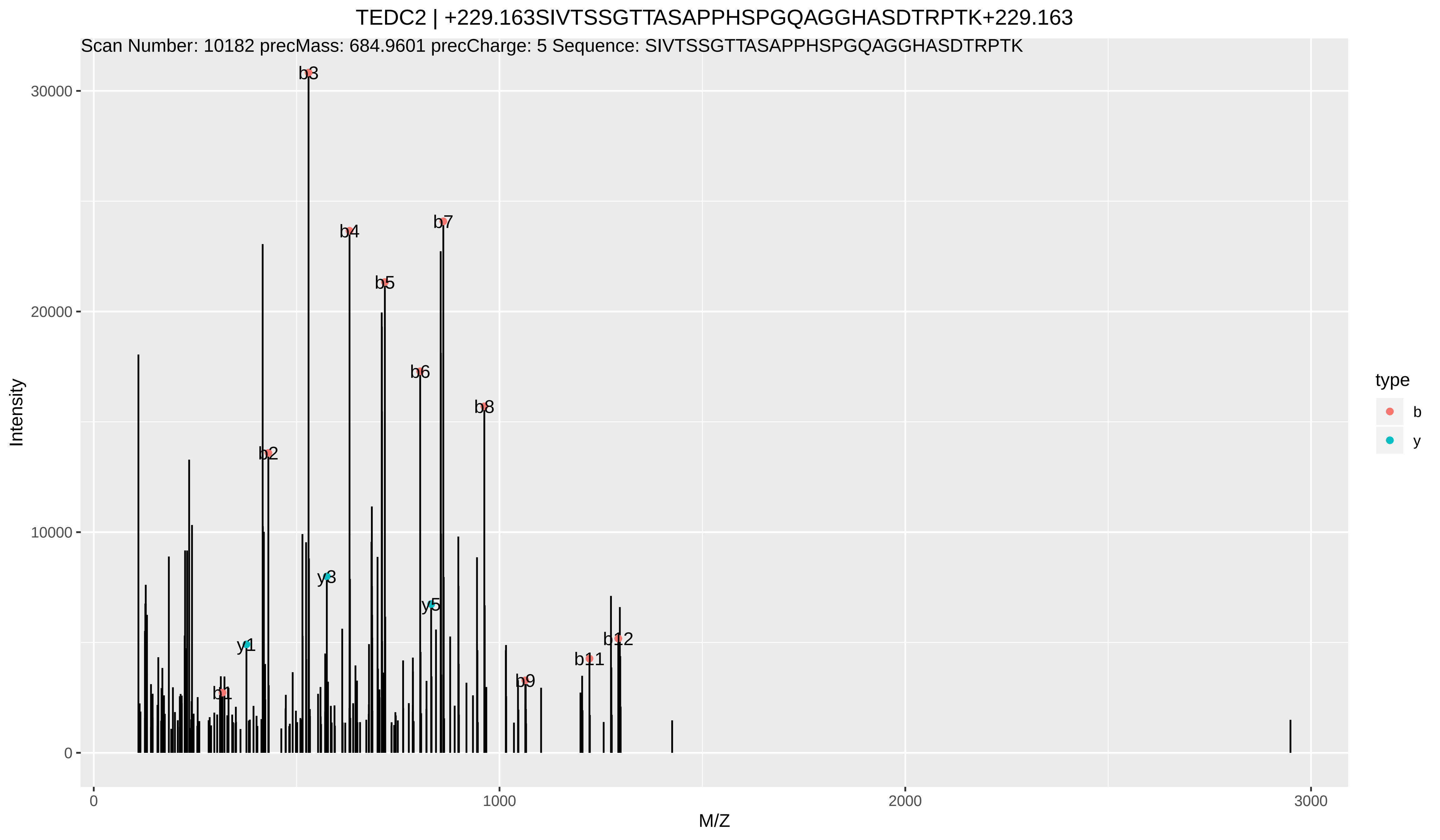
TEDC2 | +229.163SIVTSSGTTASAPPHSPGQAGGHASDTRPTK+229.163
Scan Number: 10182 precMass: 684.9601 precCharge: 5 Sequence: SIVTSSGTTASAPPHSPGQAGGHASDTRPTK
Intensity
M/Z
type
b
y
b1
b2
b3
b4
b5
b6
b7
b8
b9
b11
b12
y1
y3
y5
0
10000
20000
30000
1000
2000
3000

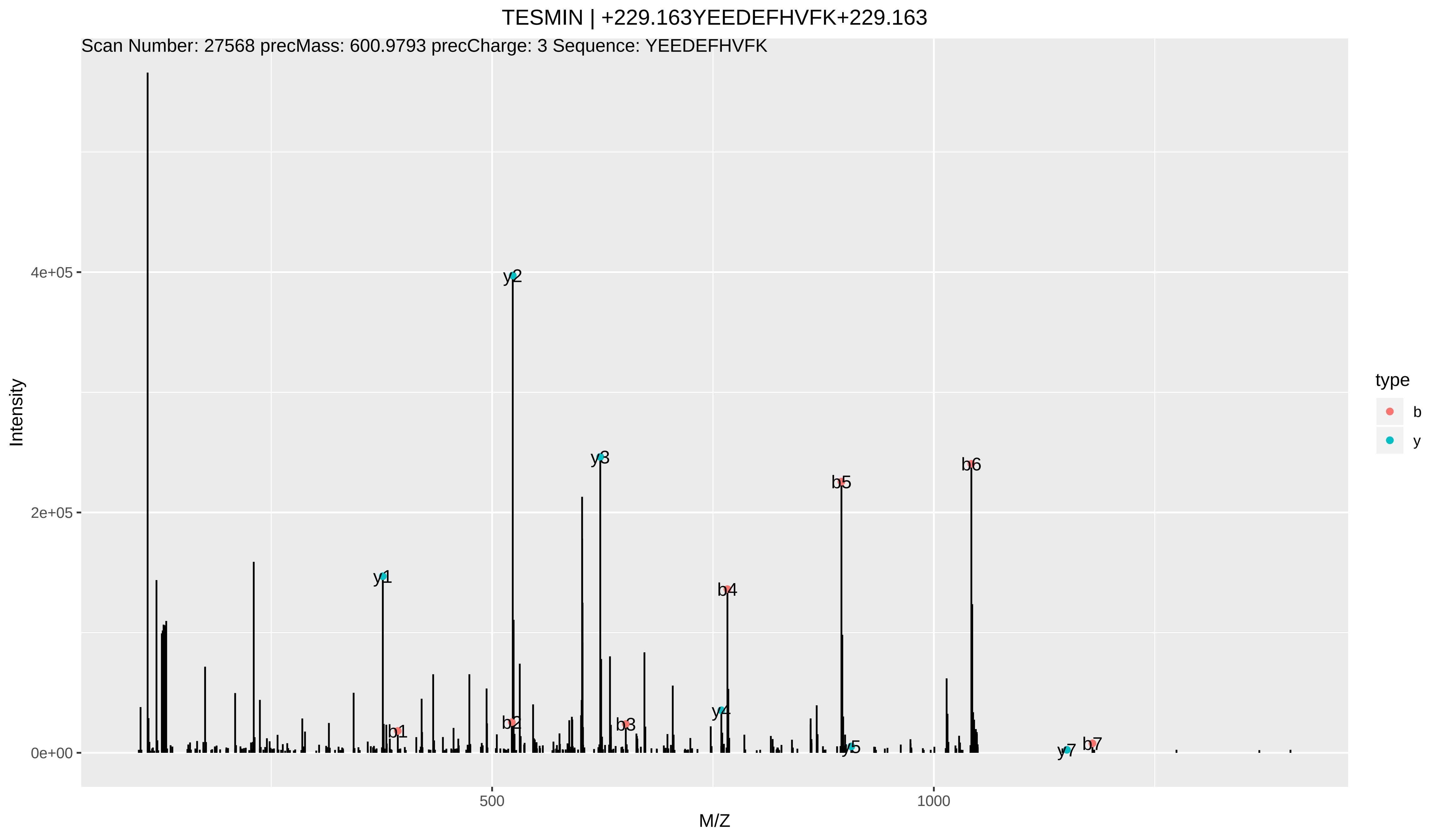
TESMIN | +229.163YEEDEFHVFK+229.163
Scan Number: 27568 precMass: 600.9793 precCharge: 3 Sequence: YEEDEFHVFK
Intensity
4e+05
2e+05
0e+00
M/Z
500
1000
y1
y2
y3
y4
y5
y7
b1
b2
b3
b4
b5
b6
b7
type
b
y

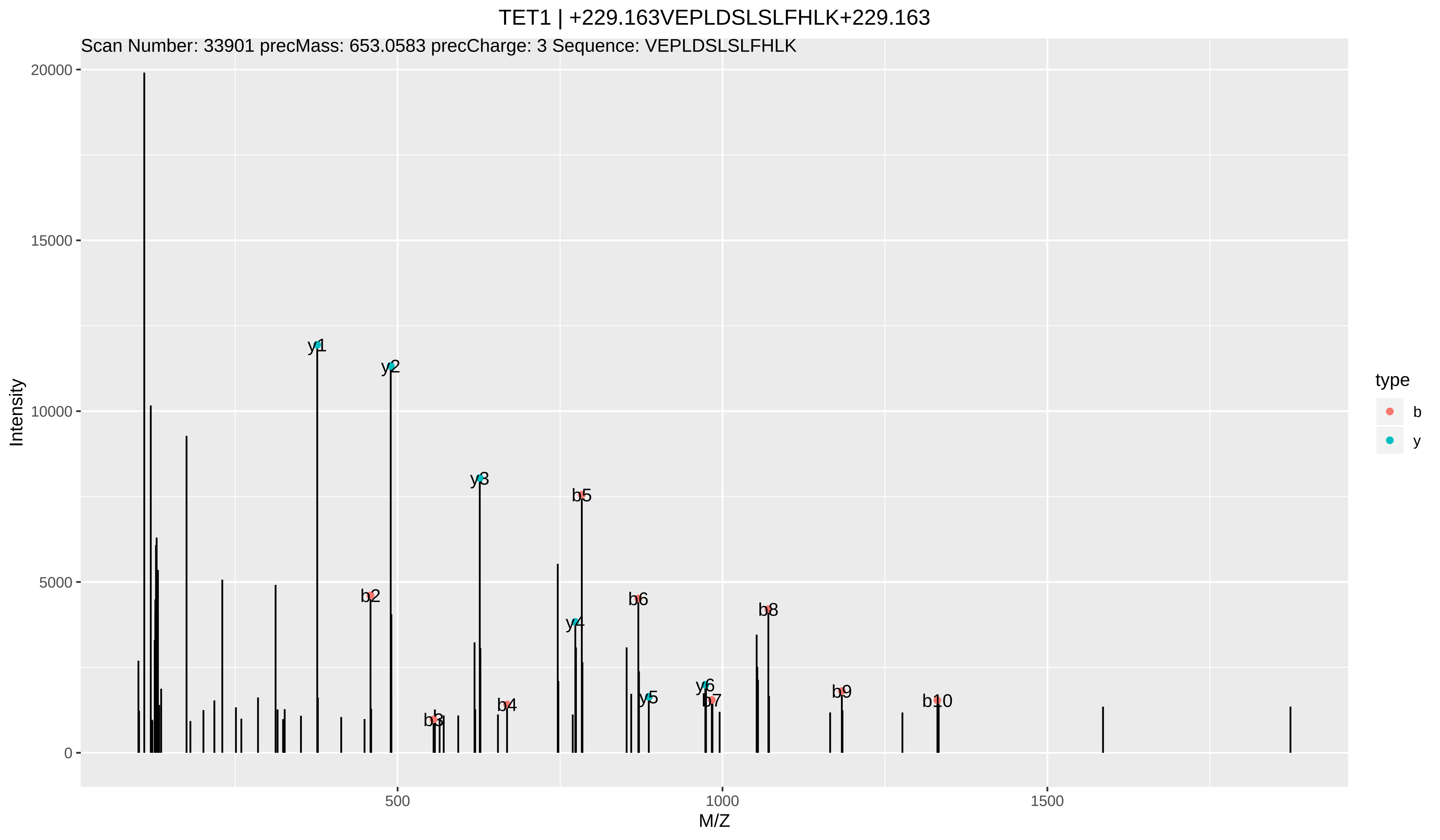

TET1 | +229.163VEPLDSLSLFHLK+229.163
Scan Number: 33901 precMass: 653.0583 precCharge: 3 Sequence: VEPLDSLSLFHLK
Intensity
M/Z
0
5000
10000
15000
20000
500
1000
1500
y1
y2
y3
y4
y5
y6
b2
b3
b4
b5
b6
b7
b8
b9
b10
type
b
y

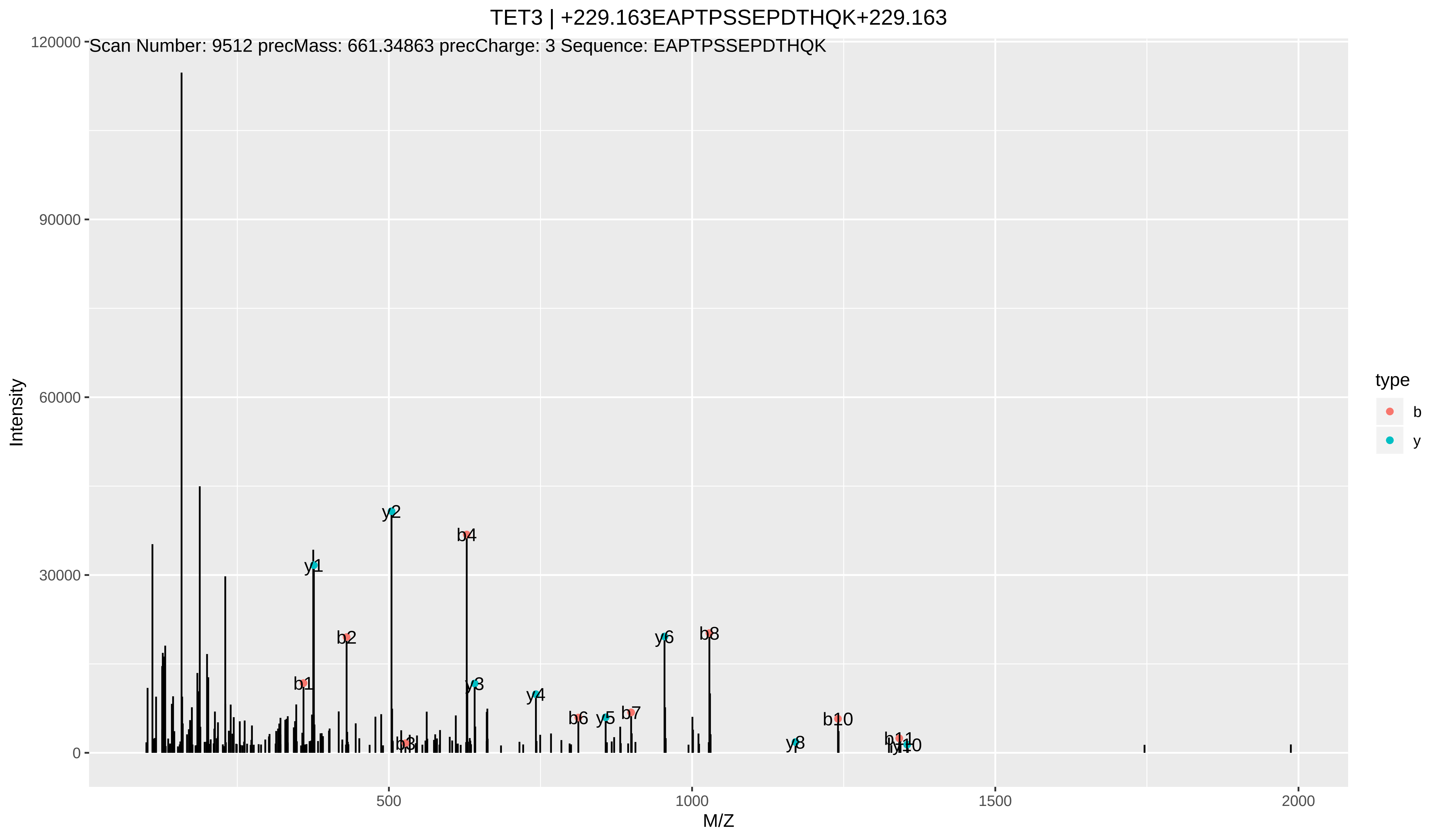

TET3 | +229.163EAPTPSSEPDTHQK+229.163
Scan Number: 9512 precMass: 661.34863 precCharge: 3 Sequence: EAPTPSSEPDTHQK
Intensity
120000
90000
60000
30000
0
M/Z
500
1000
1500
2000
y2
b4
y1
b2
b1
y3
y4
b6
y5
b7
y6
b8
b3
y8
b10
b11
y10
type
b
y

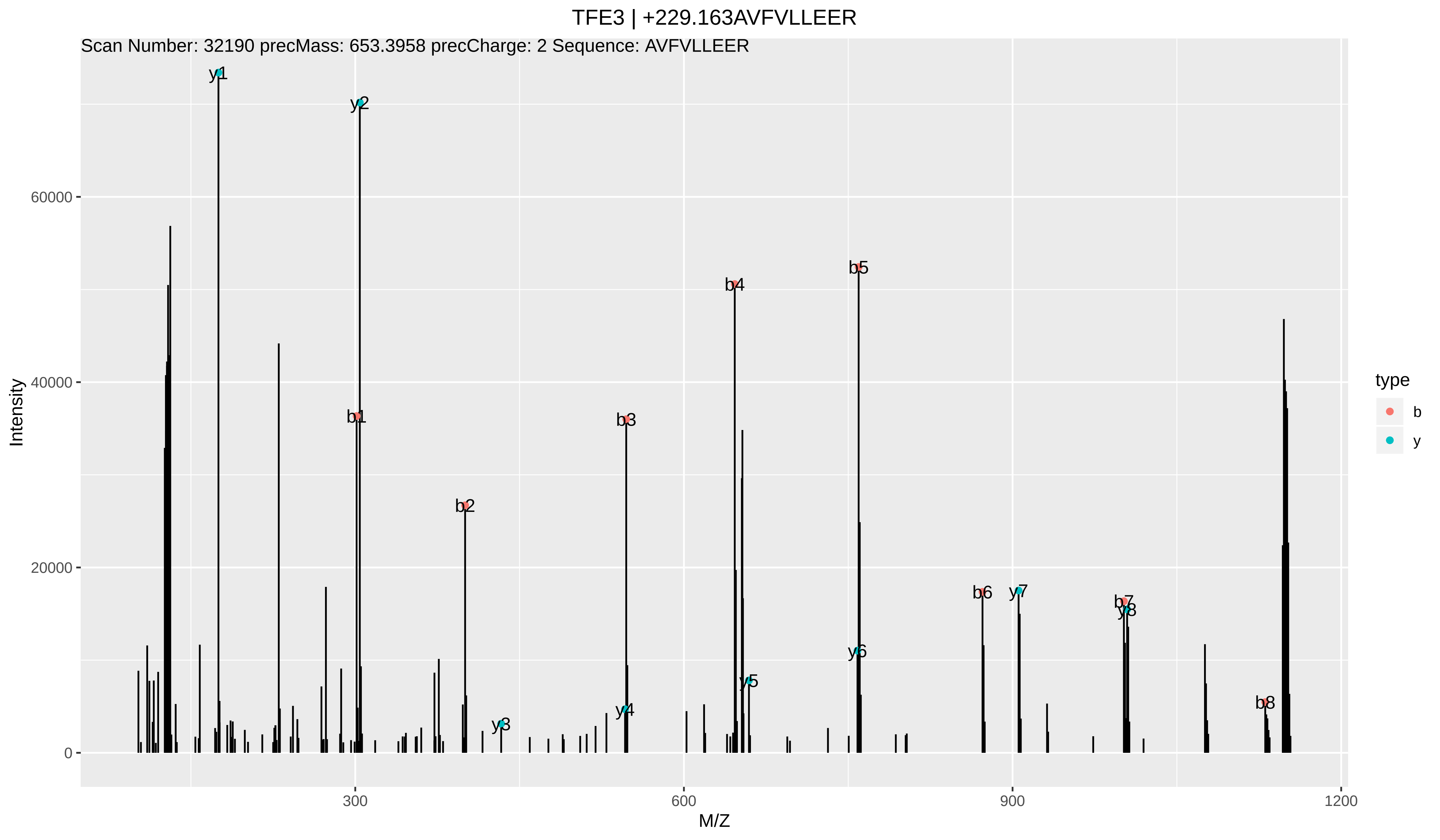

TFE3 | +229.163AVFVLLEER
Scan Number: 32190 precMass: 653.3958 precCharge: 2 Sequence: AVFVLLEER
y1
y2
b1
b2
y3
b3
y4
b4
y5
b5
y6
b6
y7
b7
y8
b8
Intensity
60000
40000
20000
0
300
600
900
1200
M/Z
type
b
y

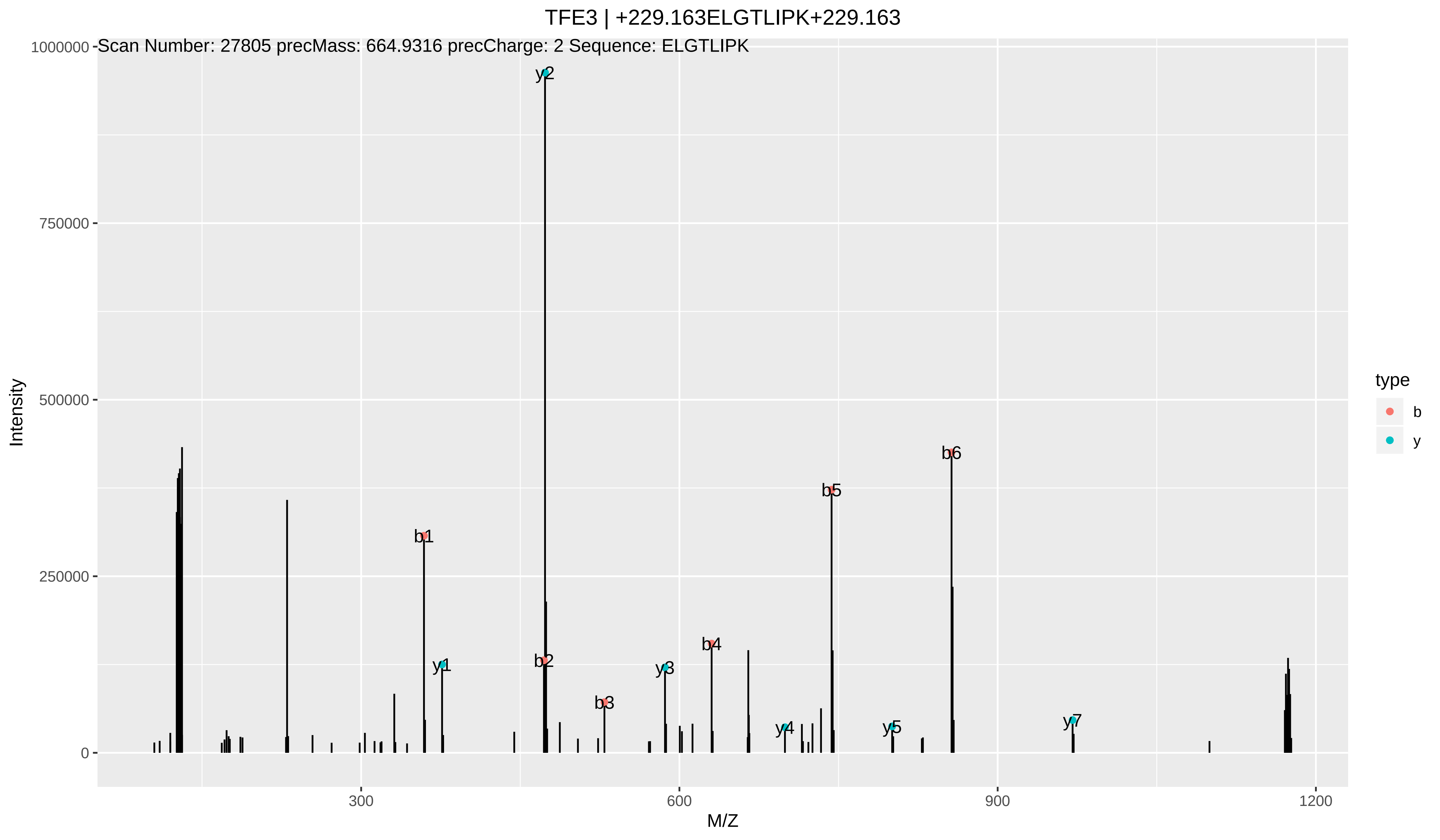
TFE3 | +229.163ELGTLIPK+229.163
Scan Number: 27805 precMass: 664.9316 precCharge: 2 Sequence: ELGTLIPK
Intensity
1000000
750000
500000
250000
0
M/Z
300
600
900
1200
y2
b6
b5
b1
y1
b2
b3
y3
b4
y4
y5
y7
type
b
y

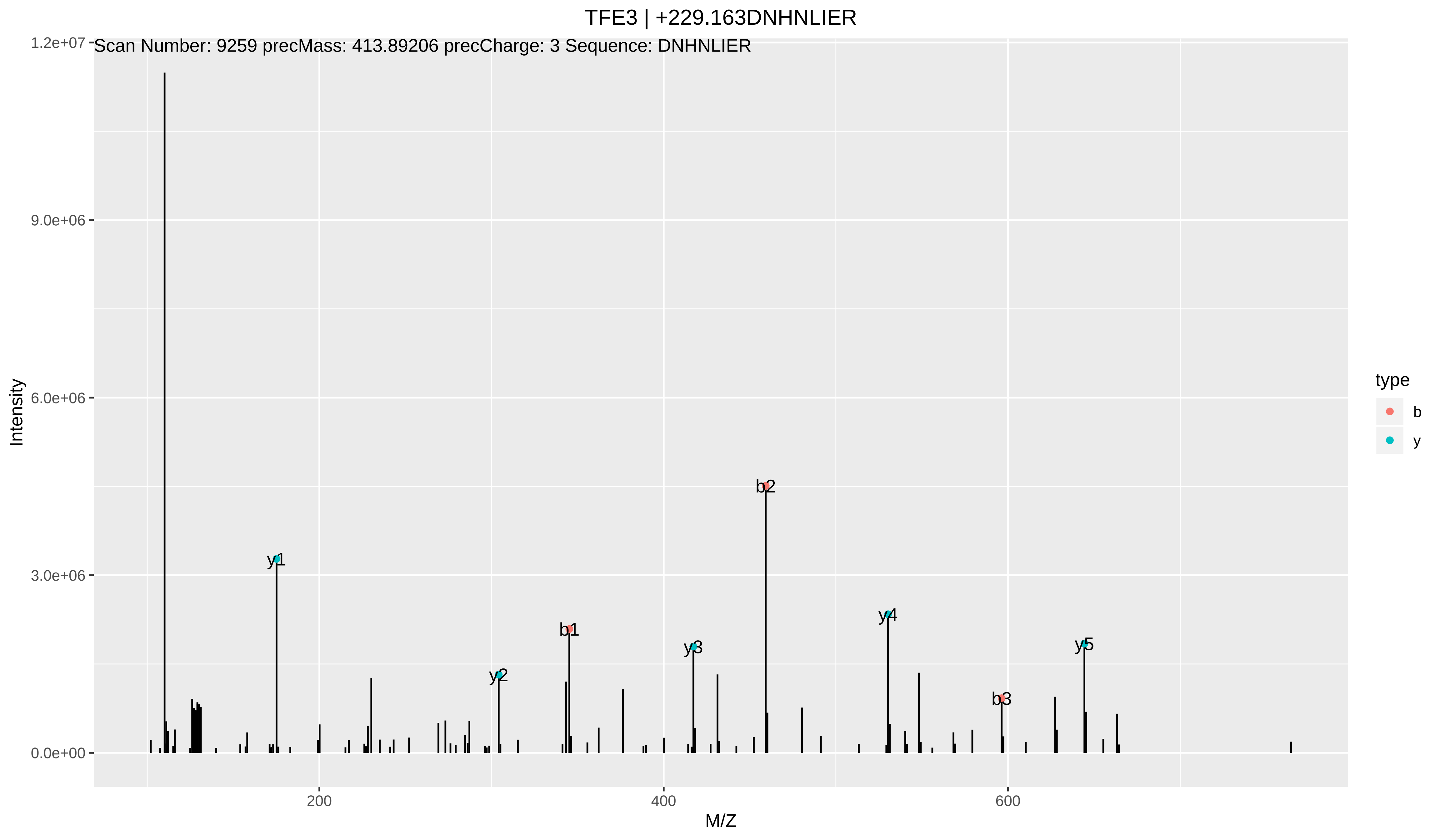
TFE3 | +229.163DNHNLIER
Scan Number: 9259 precMass: 413.89206 precCharge: 3 Sequence: DNHNLIER
Intensity
0.0e+00
3.0e+06
6.0e+06
9.0e+06
1.2e+07
M/Z
200
400
600
y1
y2
b1
y3
b2
y4
b3
y5
type
b
y

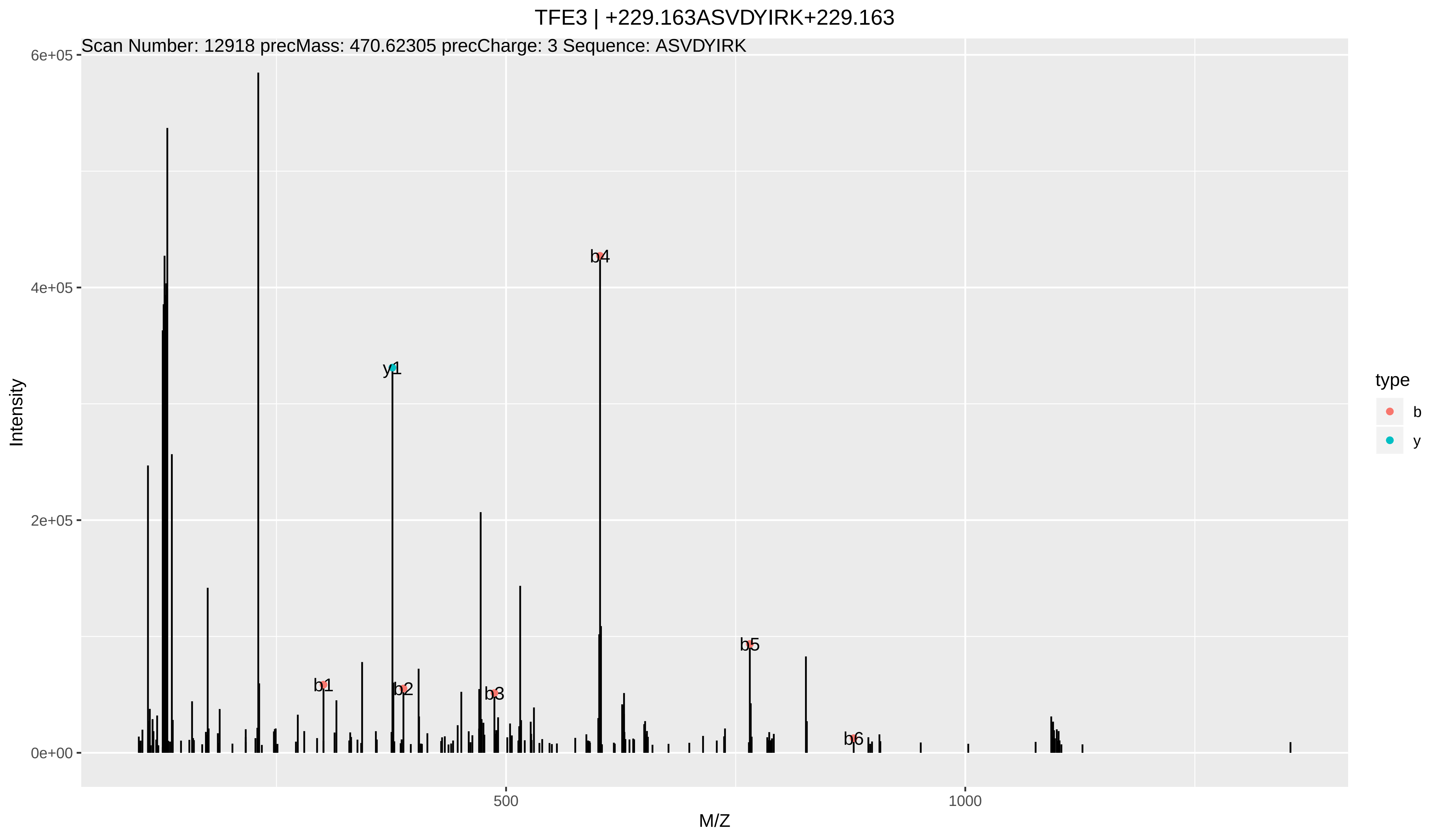
TFE3 | +229.163ASVDYIRK+229.163
Scan Number: 12918 precMass: 470.62305 precCharge: 3 Sequence: ASVDYIRK
Intensity
M/Z
6e+05
4e+05
2e+05
0e+00
500
1000
y1
b1
b2
b3
b4
b5
b6
type
b
y

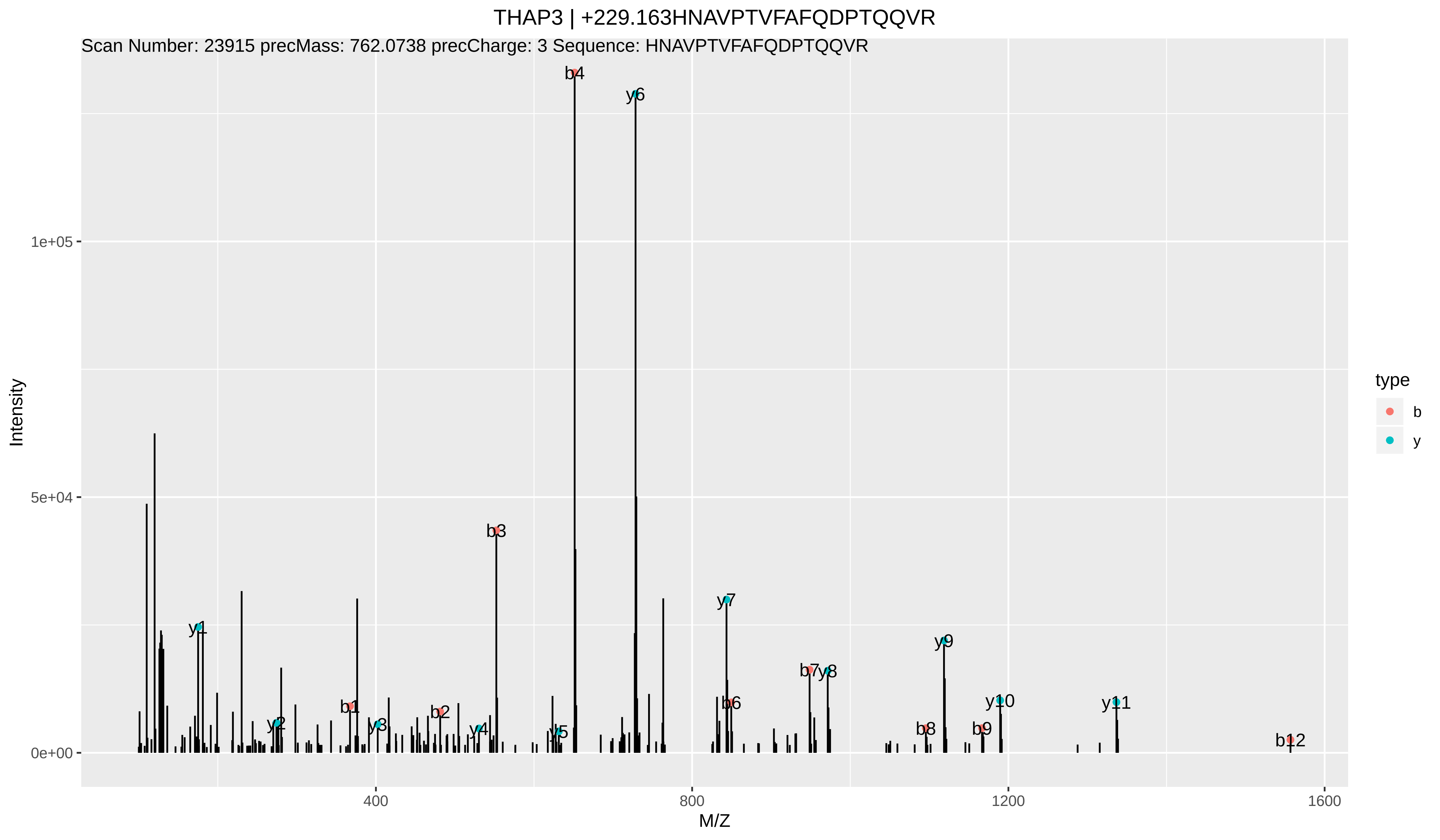
THAP3 | +229.163HNAVPTVFAFQDPTQQVR
Scan Number: 23915 precMass: 762.0738 precCharge: 3 Sequence: HNAVPTVFAFQDPTQQVR
Intensity
M/Z
1e+05
5e+04
0e+00
400
800
1200
1600
type
b
y
b4
y6
b3
y7
y1
y9
b7
y8
b1
b2
y2
y3
y4
y5
b6
y10
y11
b8
b9
b12

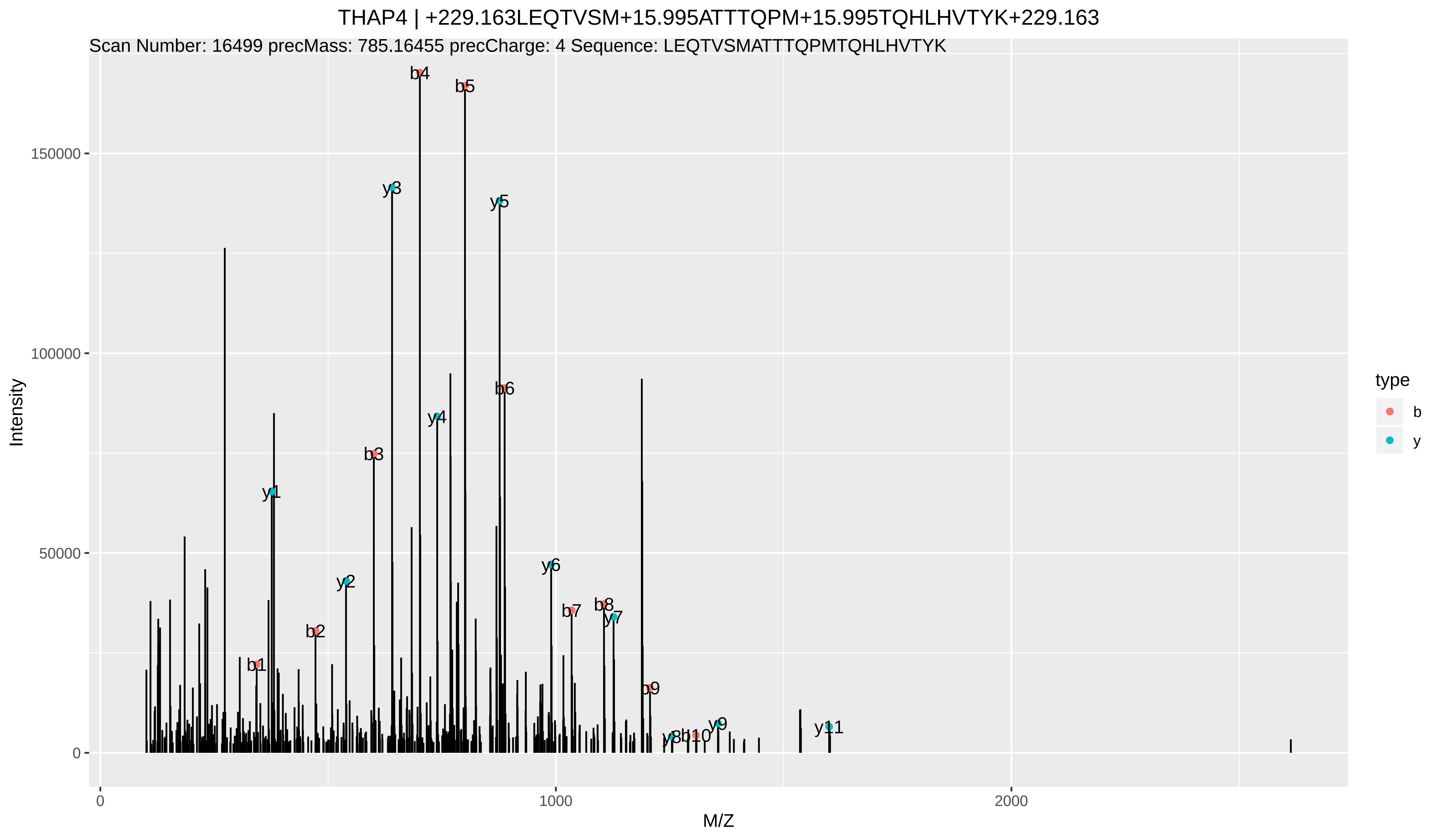
THAP4 | +229.163LEQTVSM+15.995ATTTQPM+15.995TQHLHVTYK+229.163
Scan Number: 16499 precMass: 785.16455 precCharge: 4 Sequence: LEQTVSMATTTQPMTQHLHVTYK
Intensity
M/Z
0
50000
100000
150000
0
1000
2000
b4
b5
y3
y5
b6
y4
b3
y1
y6
y2
b7
b8
y7
b2
b1
b9
y8
b10
y9
y11
type
b
y

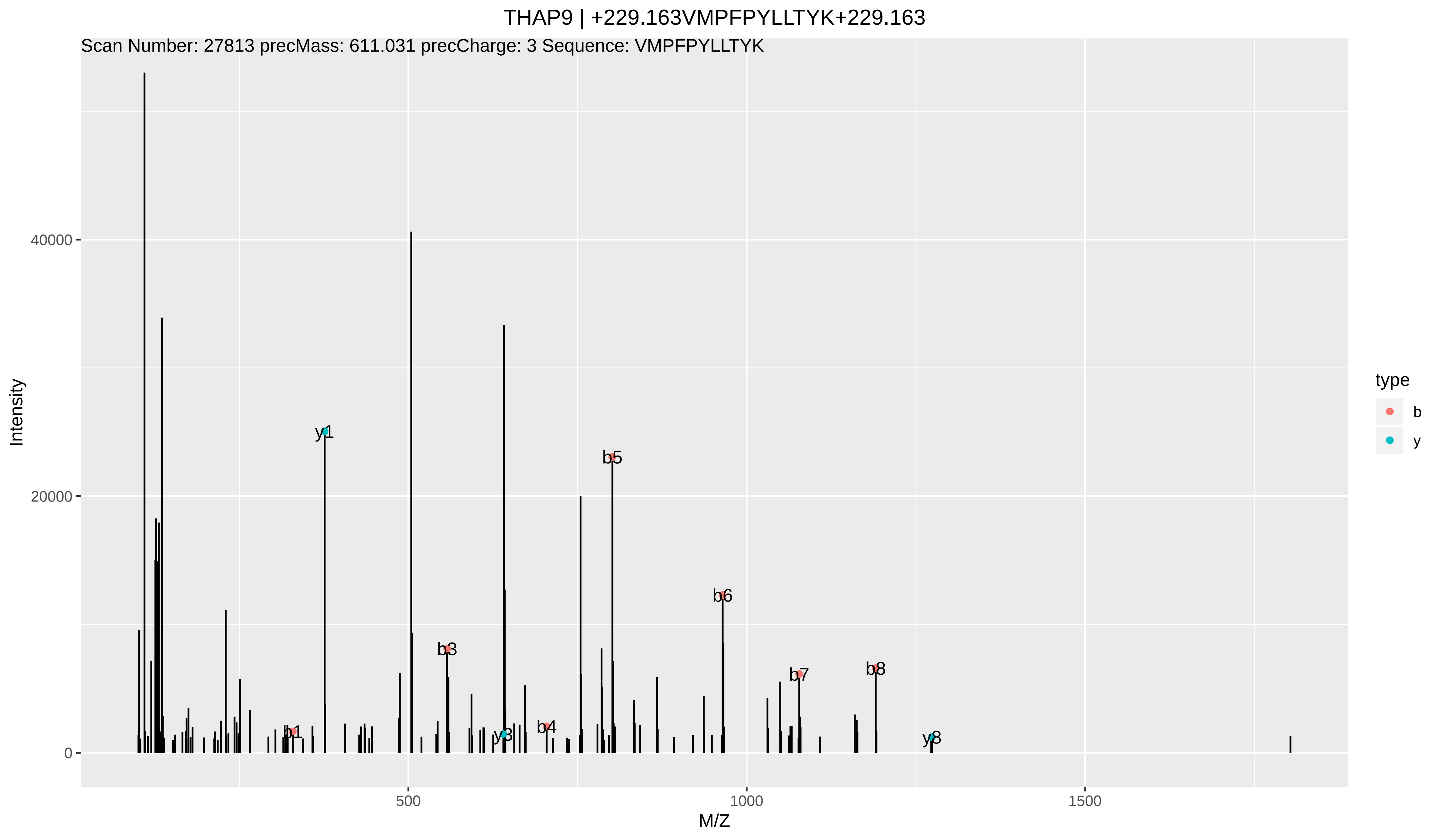
THAP9 | +229.163VMPFPYLLTYK+229.163
Scan Number: 27813 precMass: 611.031 precCharge: 3 Sequence: VMPFPYLLTYK
Intensity
M/Z
0
20000
40000
500
1000
1500
y1
b5
b6
b3
b7
b8
b1
y3
b4
y8
type
b
y

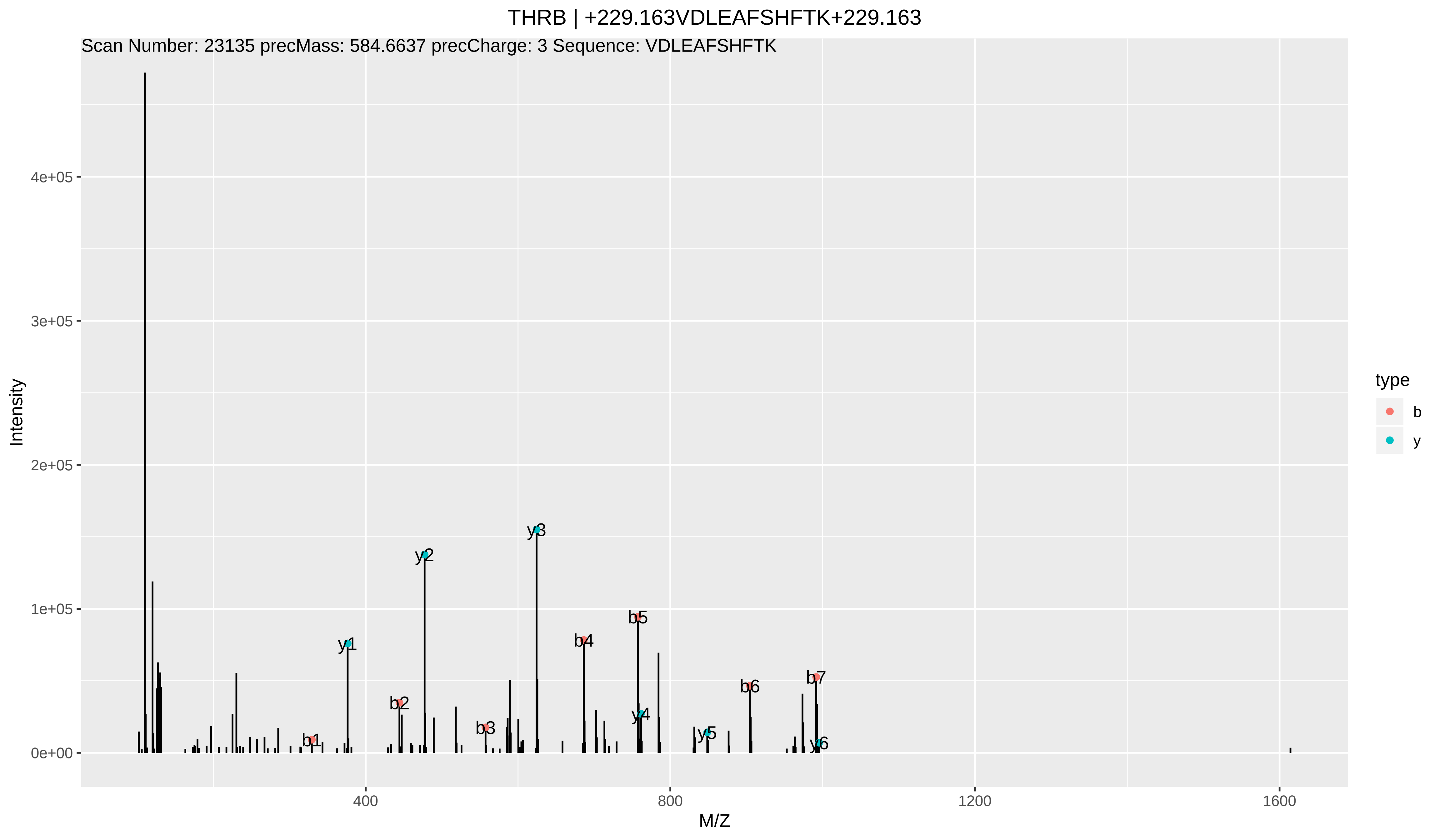
THRB | +229.163VDLEAFSHFTK+229.163
Scan Number: 23135 precMass: 584.6637 precCharge: 3 Sequence: VDLEAFSHFTK
Intensity
M/Z
4e+05
3e+05
2e+05
1e+05
0e+00
400
800
1200
1600
y1
y2
y3
y4
y5
y6
b1
b2
b3
b4
b5
b6
b7
type
b
y

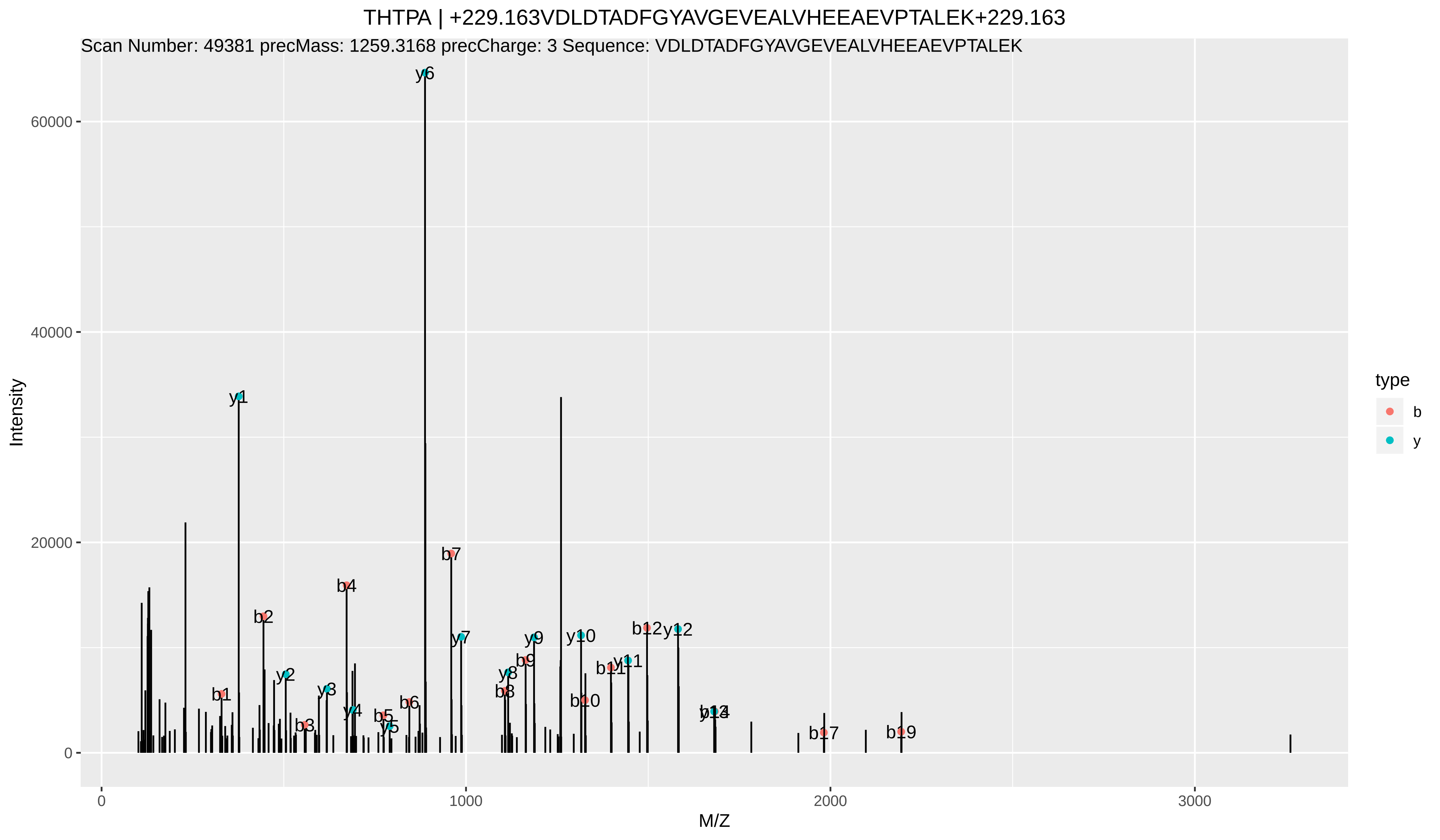
THTPA | +229.163VDLDTADFGYAVGEVEALVHEEAEVPTALEK+229.163
Scan Number: 49381 precMass: 1259.3168 precCharge: 3 Sequence: VDLDTADFGYAVGEVEALVHEEAEVPTALEK
Intensity
M/Z
0
20000
40000
60000
0
1000
2000
3000
type
b
y
y6
y1
b7
b4
b2
b12
y12
y10
y9
y7
b9
y8
b8
y11
b11
b10
y2
y3
b1
b3
y4
b5
y5
b6
b17
b19

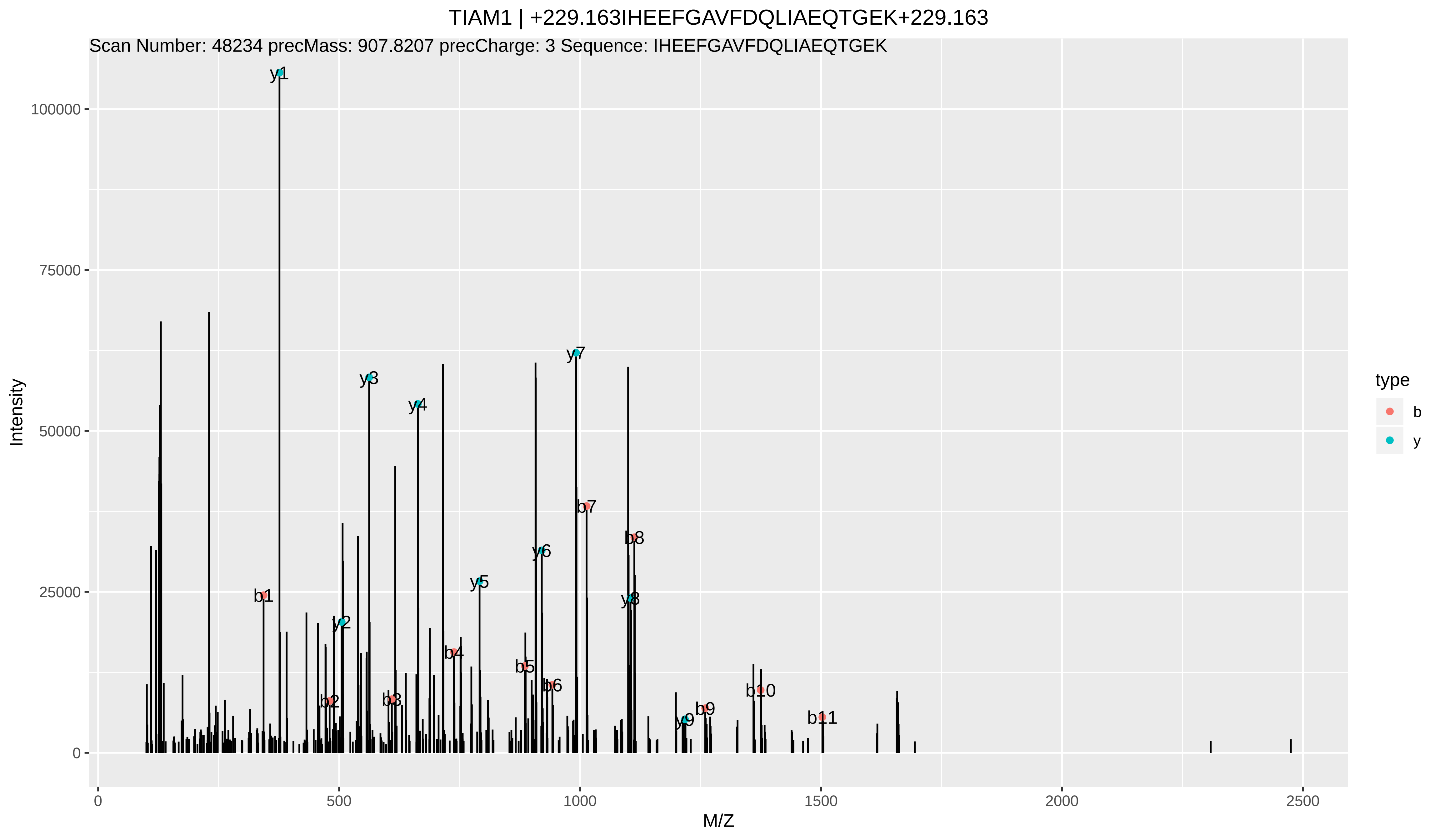

TIAM1 | +229.163IHEEFGAVFDQLIAEQTGEK+229.163
Scan Number: 48234 precMass: 907.8207 precCharge: 3 Sequence: IHEEFGAVFDQLIAEQTGEK
Intensity
M/Z
0
25000
50000
75000
100000
0
500
1000
1500
2000
2500
y1
y2
y3
y4
y5
y6
y7
y8
y9
b1
b2
b3
b4
b5
b6
b7
b8
b9
b10
b11
type
b
y

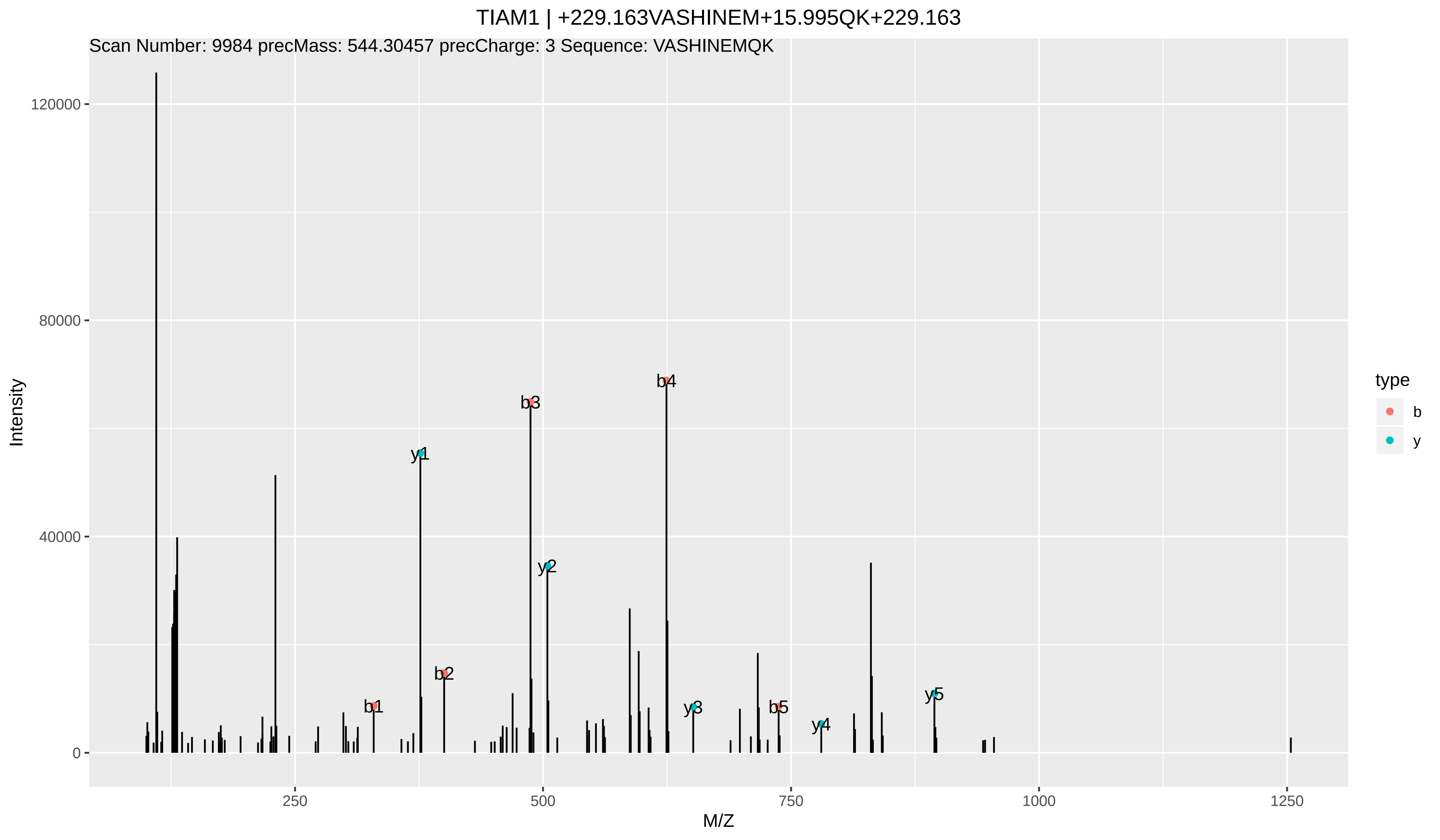
TIAM1 | +229.163VASHINEM+15.995QK+229.163
Scan Number: 9984 precMass: 544.30457 precCharge: 3 Sequence: VASHINEMQK
Intensity
M/Z
120000
80000
40000
0
250
500
750
1000
1250
b1
y1
b2
b3
y2
b4
y3
b5
y4
y5
type
b
y

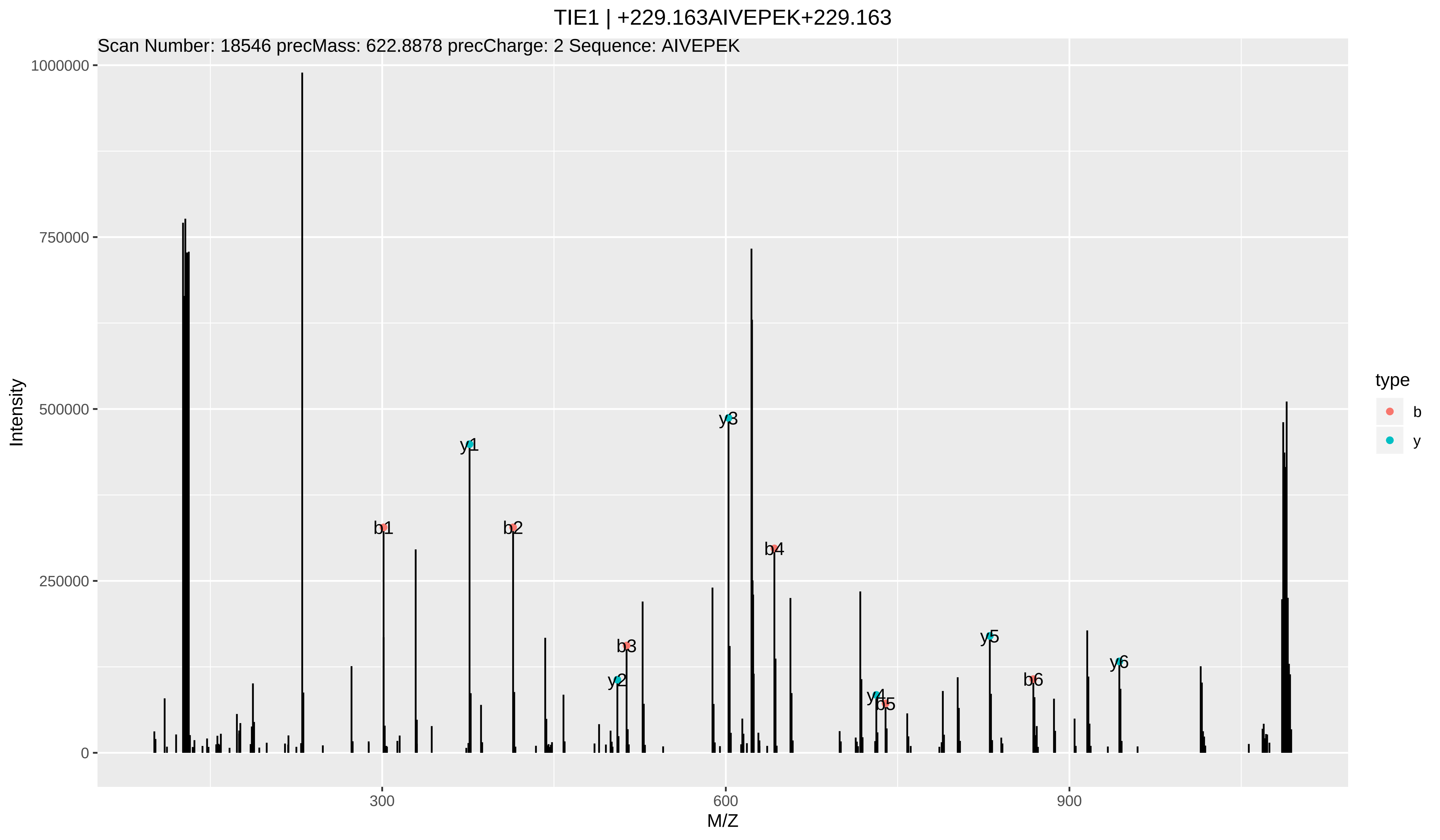

TIE1 | +229.163AIVEPEK+229.163
Scan Number: 18546 precMass: 622.8878 precCharge: 2 Sequence: AIVEPEK
Intensity
1000000
750000
500000
250000
0
M/Z
300
600
900
y1
y2
y3
y4
y5
y6
b1
b2
b3
b4
b5
b6
type
b
y

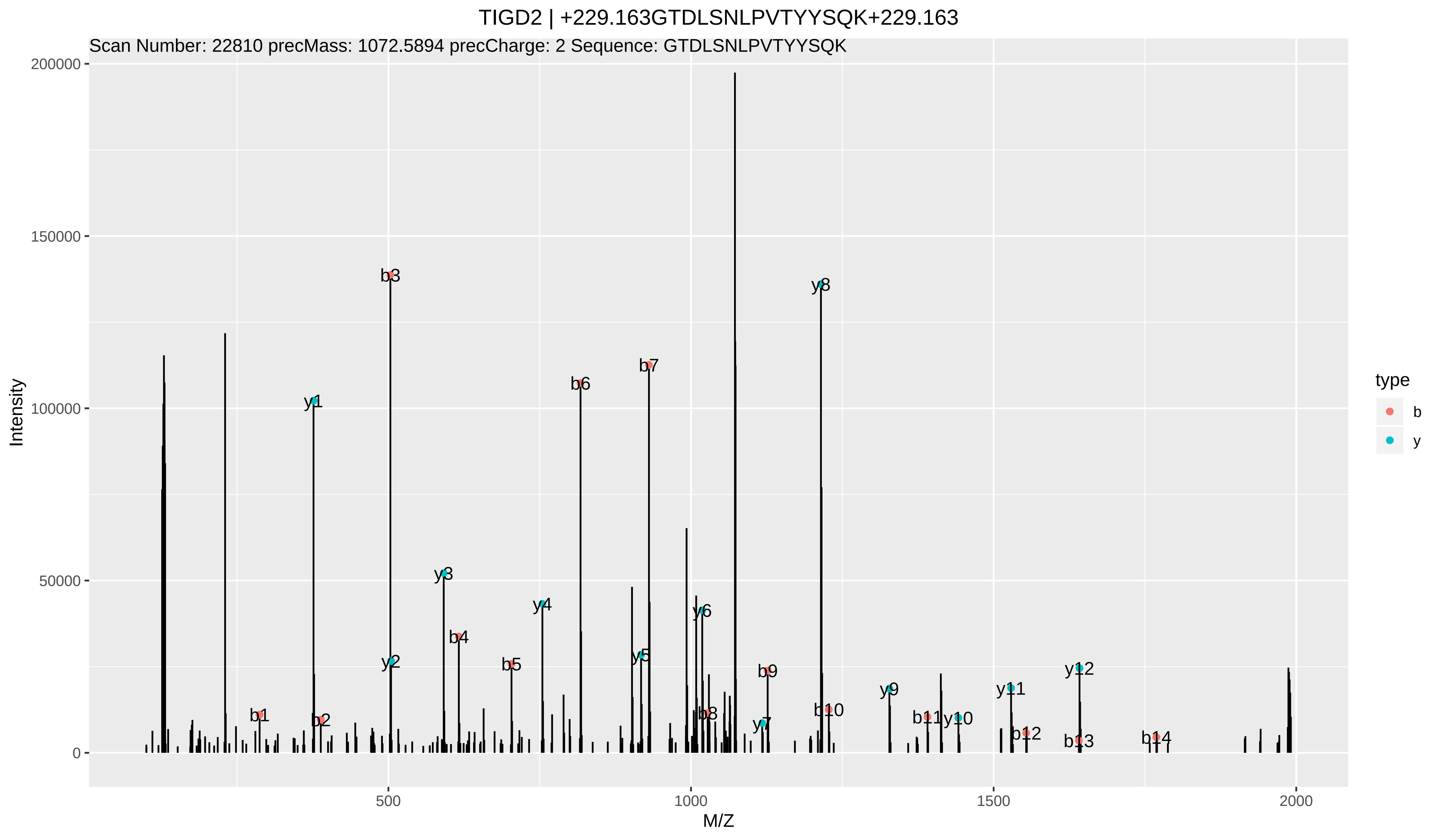

TIGD2 | +229.163GTDLSNLPVTYYSQK+229.163
Scan Number: 22810 precMass: 1072.5894 precCharge: 2 Sequence: GTDLSNLPVTYYSQK
Intensity
M/Z
0
50000
100000
150000
200000
500
1000
1500
2000
b1
b2
b3
b4
b5
b6
b7
b8
b9
b10
b11
b12
b13
b14
y1
y2
y3
y4
y5
y6
y7
y8
y9
y10
y11
y12
type
b
y

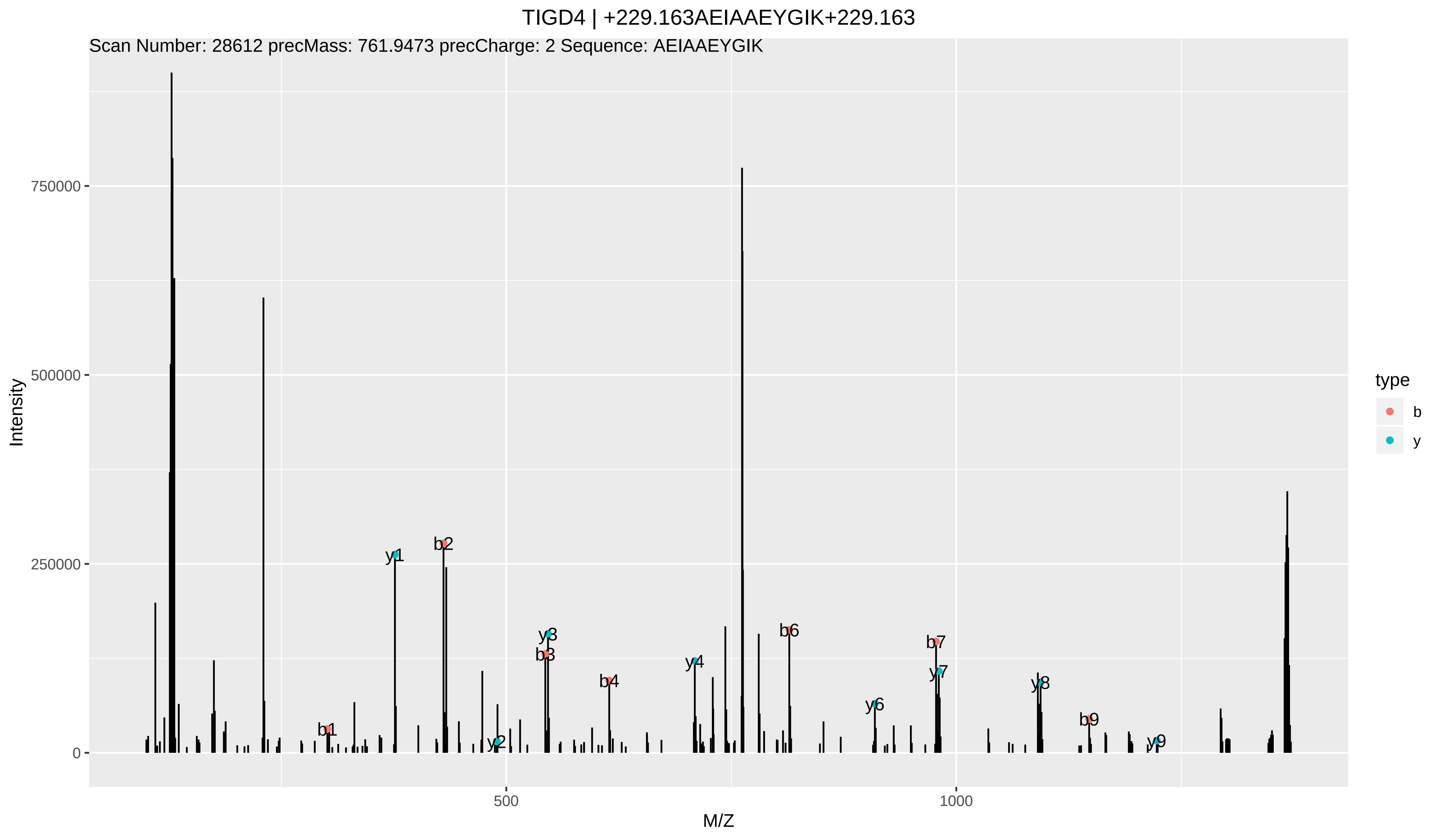

TIGD4 | +229.163AEIAAEYGIK+229.163
Scan Number: 28612 precMass: 761.9473 precCharge: 2 Sequence: AEIAAEYGIK
Intensity
750000
500000
250000
0
M/Z
500
1000
type
b
y
y1
b2
y2
y3
b3
b4
y4
b6
y6
b7
y7
y8
b9
y9
b1

TIMM23 | +229.163TTGGLAGFFGAGGAGYSHADLAGVPLTGM+15.995NPLSPYLNVDPR

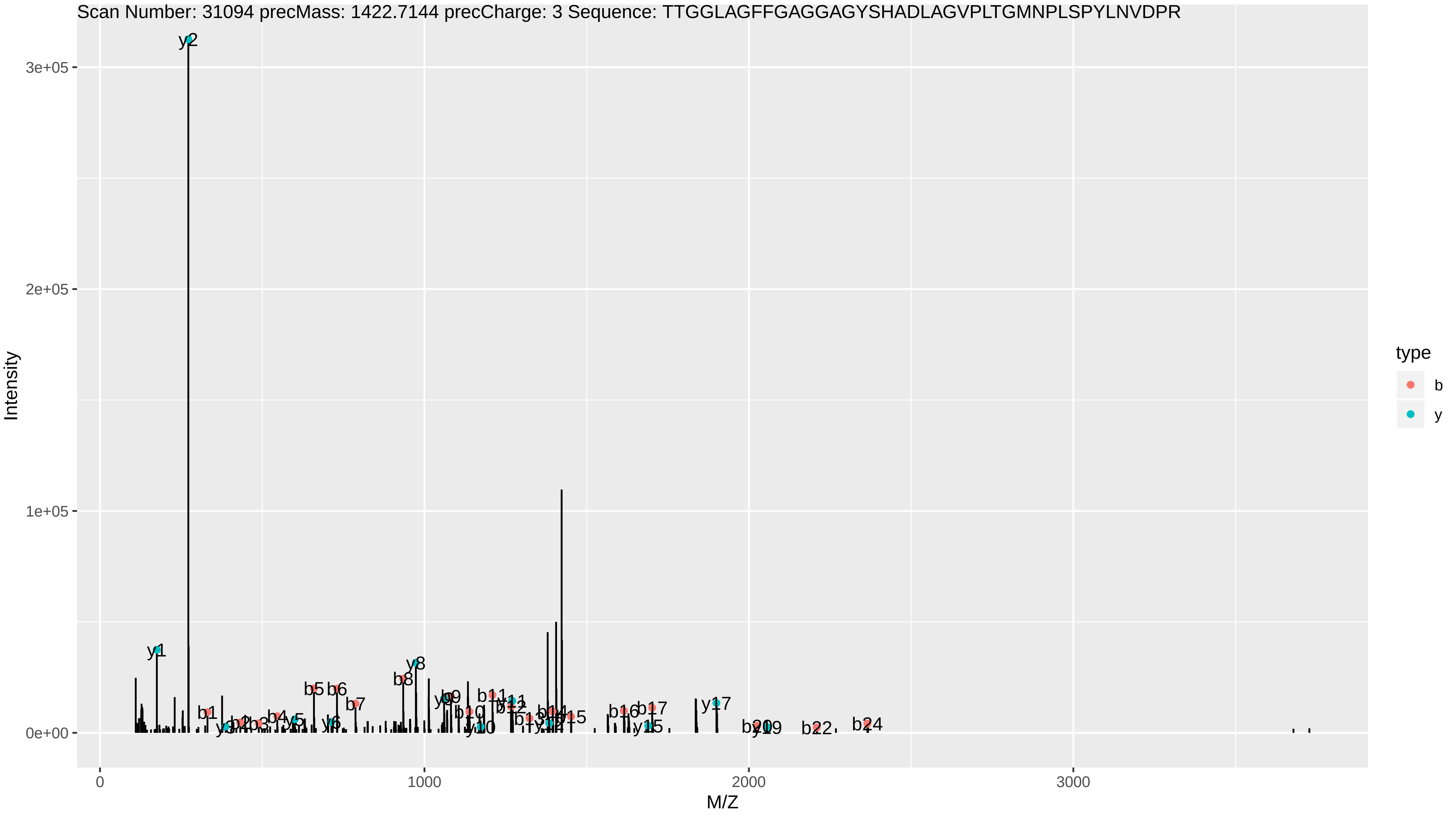

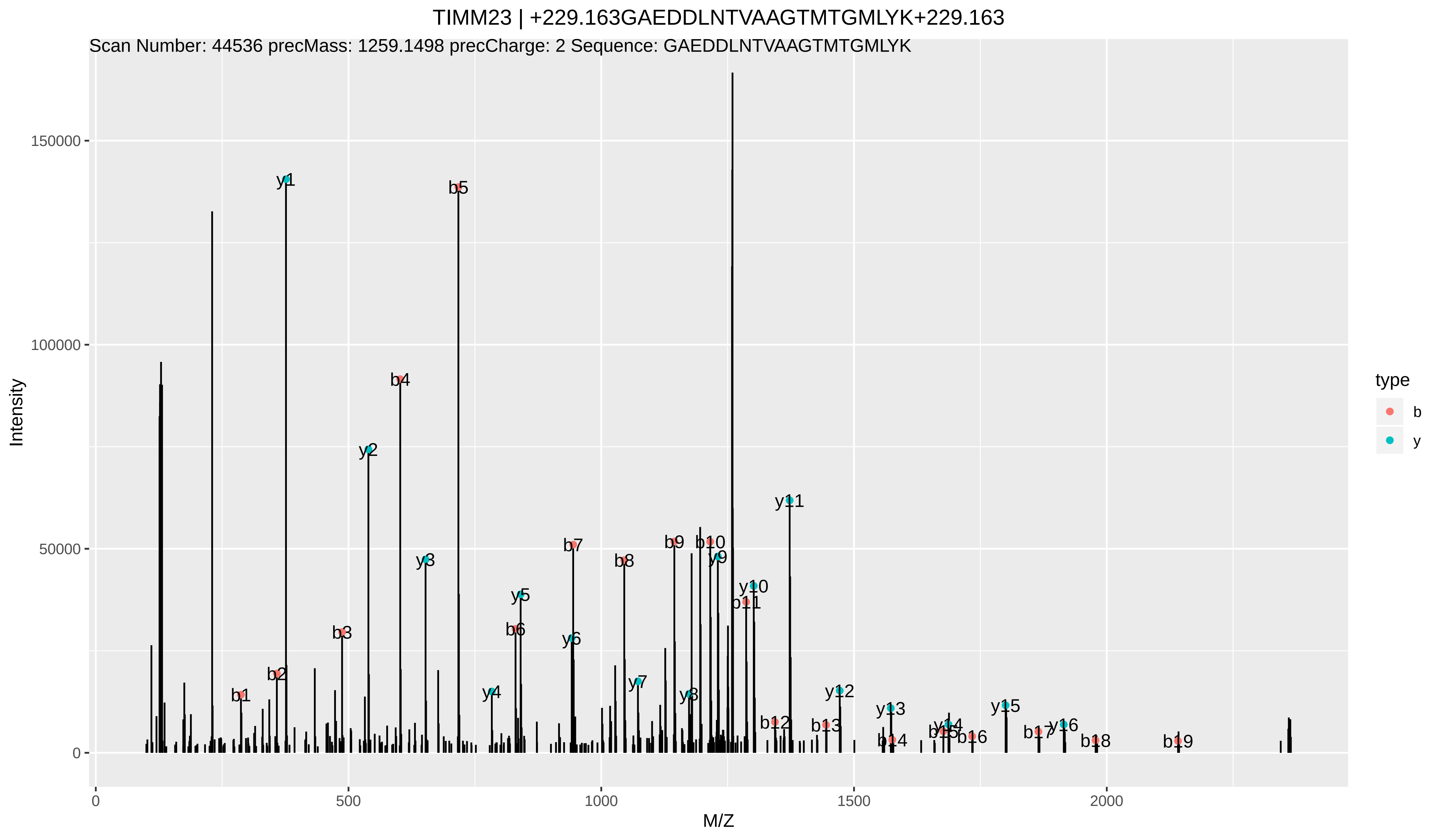
TIMM23 | +229.163GAEDDLNTVAAGTMTGMLYK+229.163
Scan Number: 44536 precMass: 1259.1498 precCharge: 2 Sequence: GAEDDLNTVAAGTMTGMLYK
Intensity
M/Z
type
b
y
0
50000
100000
150000
0
500
1000
1500
2000
y1
b5
b4
y2
y11
b7
b9
b10
y9
b8
y3
y10
y5
b11
b3
b6
y6
b2
y7
b1
y4
y8
y12
y13
y15
b12
b13
y14
y16
b15
b16
b17
b14
b18
b19

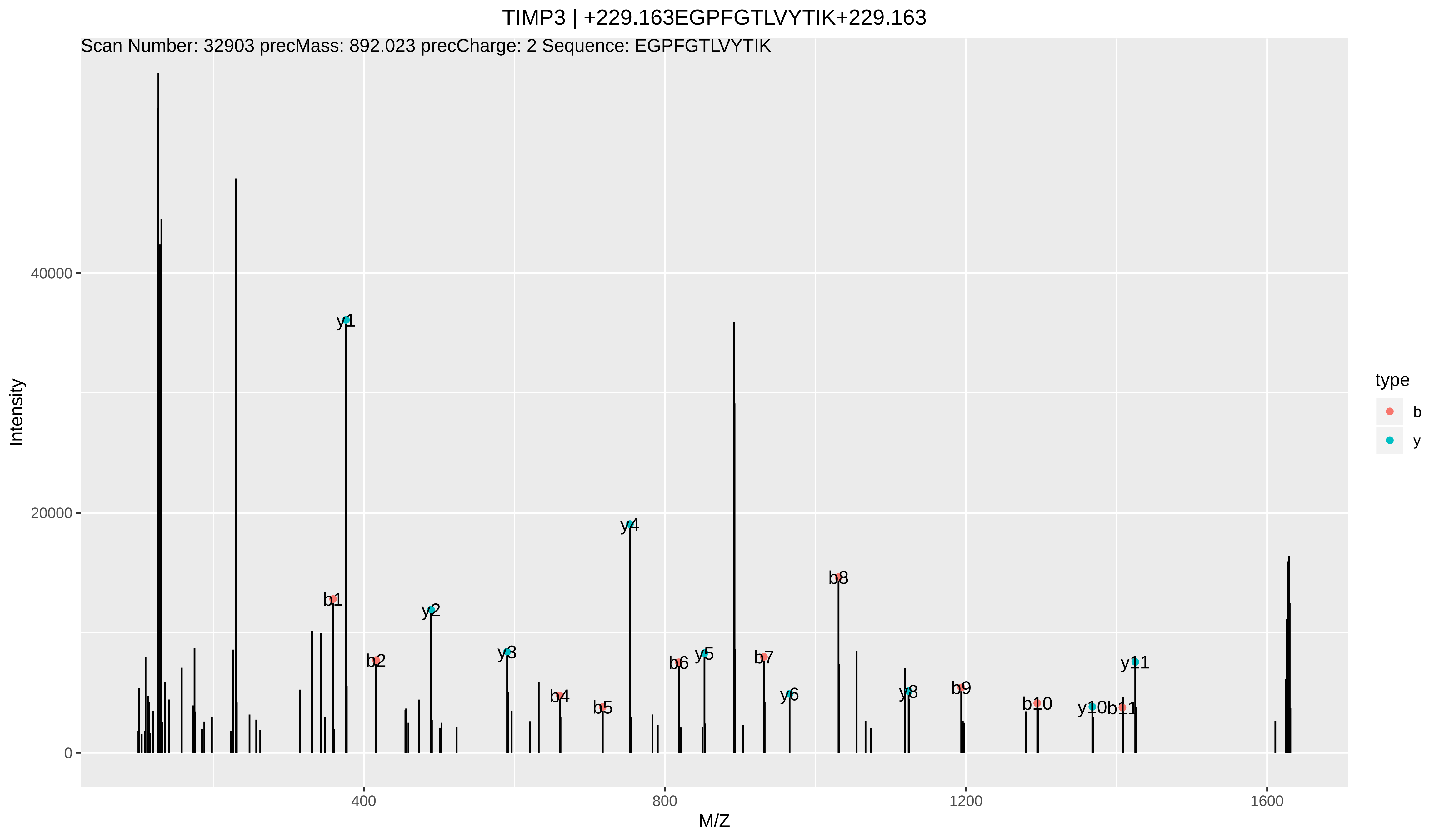

TIMP3 | +229.163EGPFGTLVYTIK+229.163
Scan Number: 32903 precMass: 892.023 precCharge: 2 Sequence: EGPFGTLVYTIK
Intensity
M/Z
0
20000
40000
400
800
1200
1600
y1
b1
y2
b2
y3
b4
b5
y4
b6
y5
b7
y6
b8
y8
b9
b10
y10
b11
y11
type
b
y

TJP1 | +229.163LPHC+57.021ASM+15.995TPDGDPK+229.163

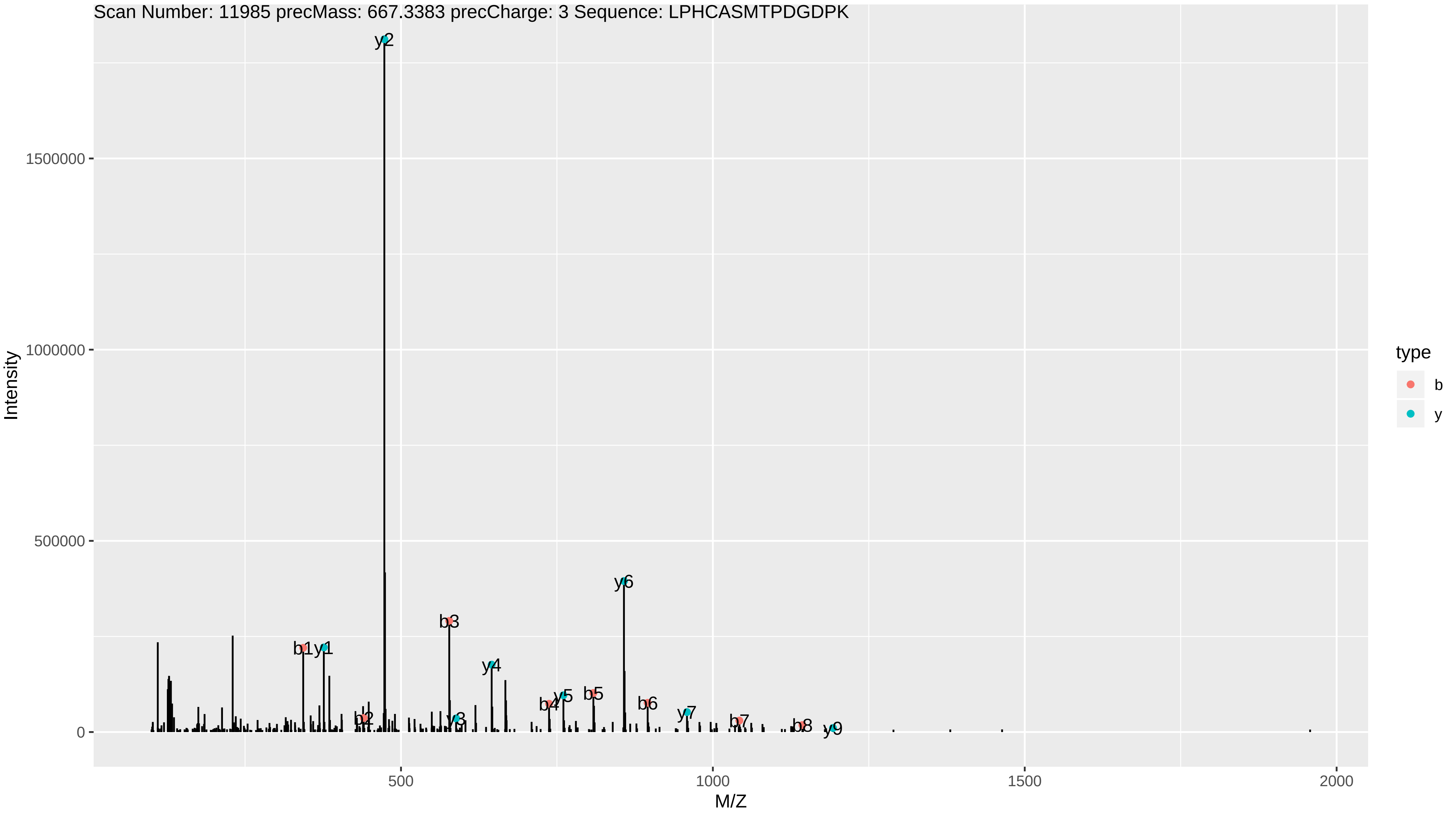

TJP1 | +229.163HTSDYEDTDTEGGAYTDQELDETLNDEVGTPPESAITR

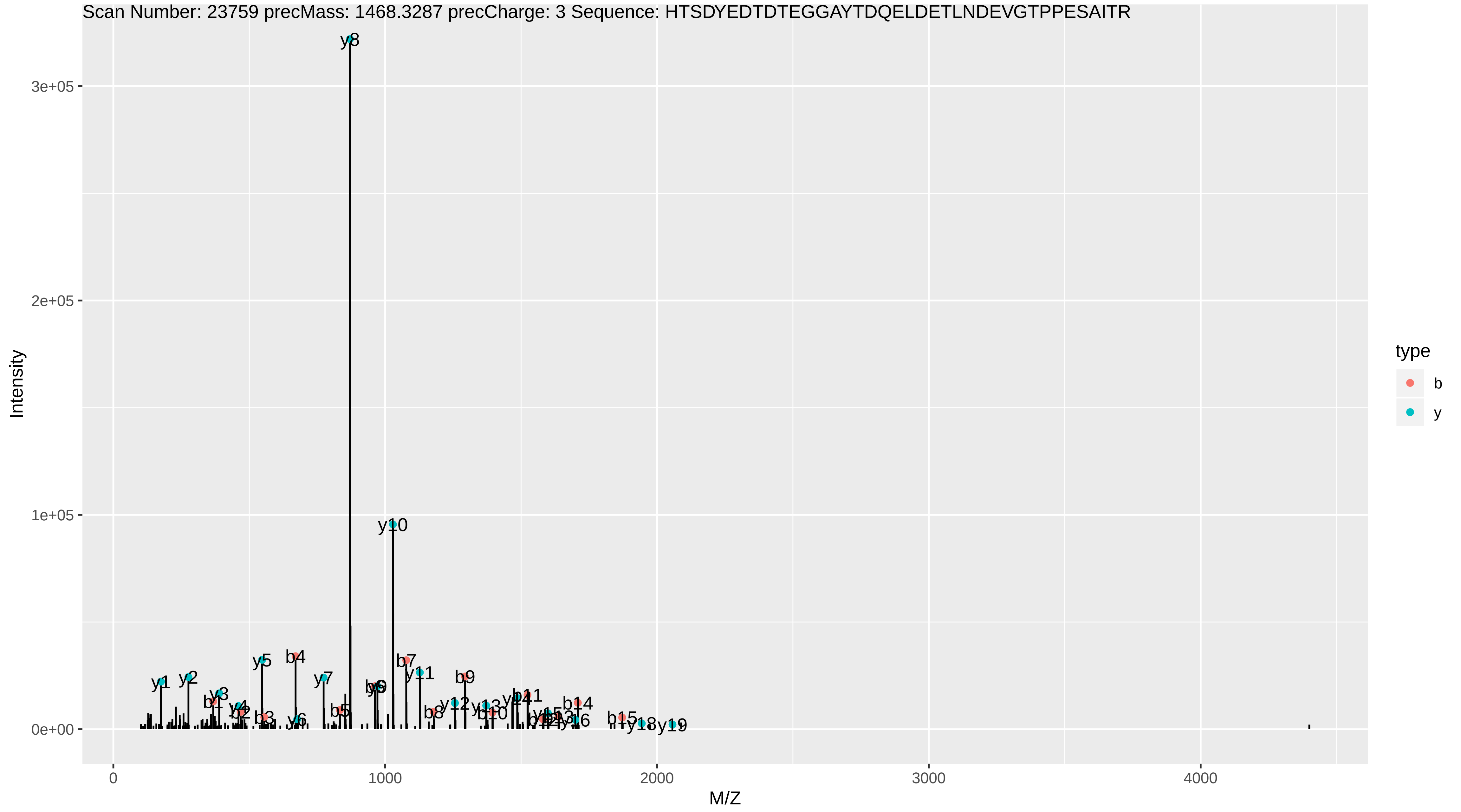

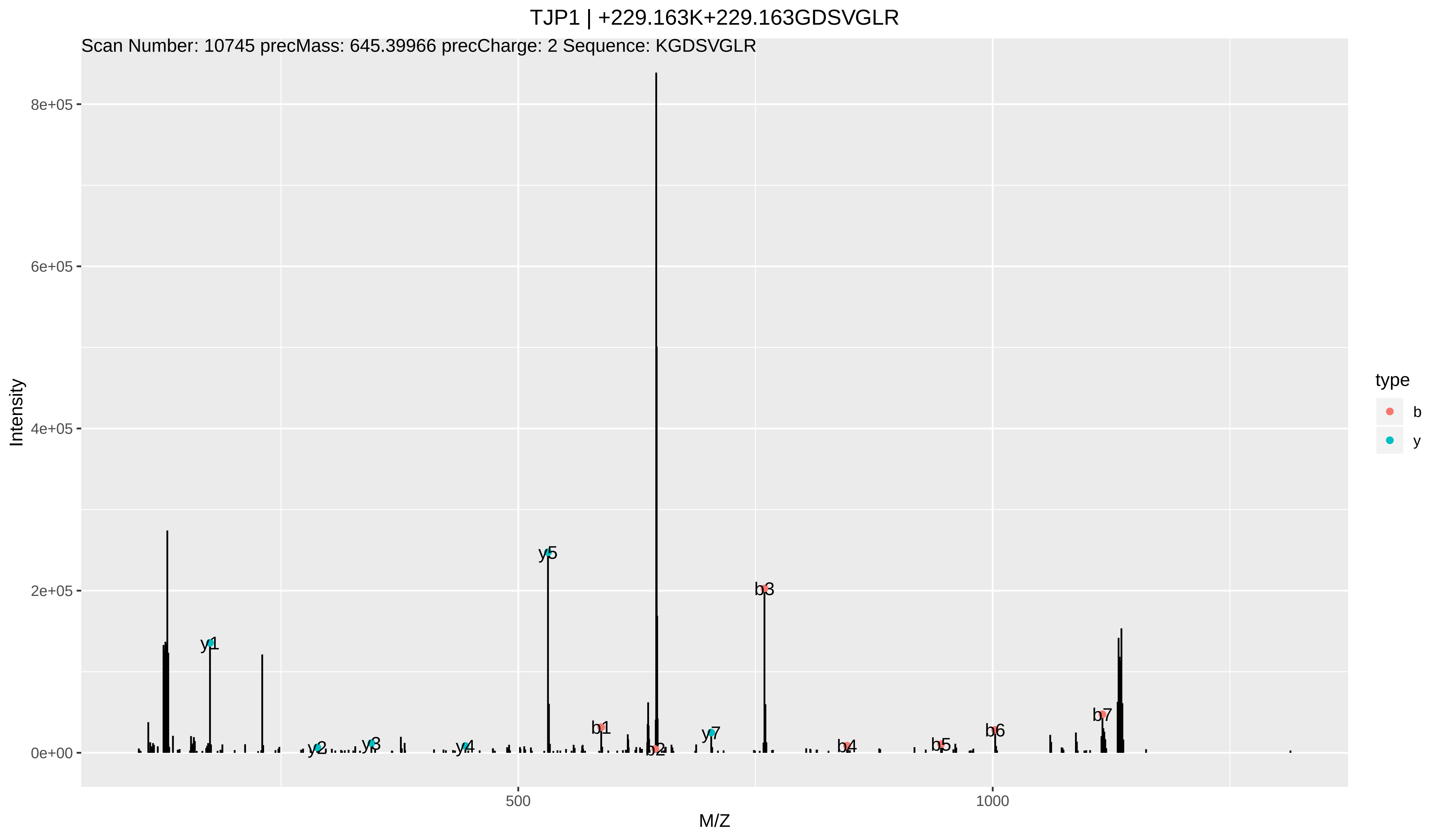

TJP1 | +229.163K+229.163GDSVGLR
Scan Number: 10745 precMass: 645.39966 precCharge: 2 Sequence: KGDSVGLR
Intensity
M/Z
8e+05
6e+05
4e+05
2e+05
0e+00
500
1000
y1
y2
y3
y4
y5
y7
b1
b2
b3
b4
b5
b6
b7
type
b
y

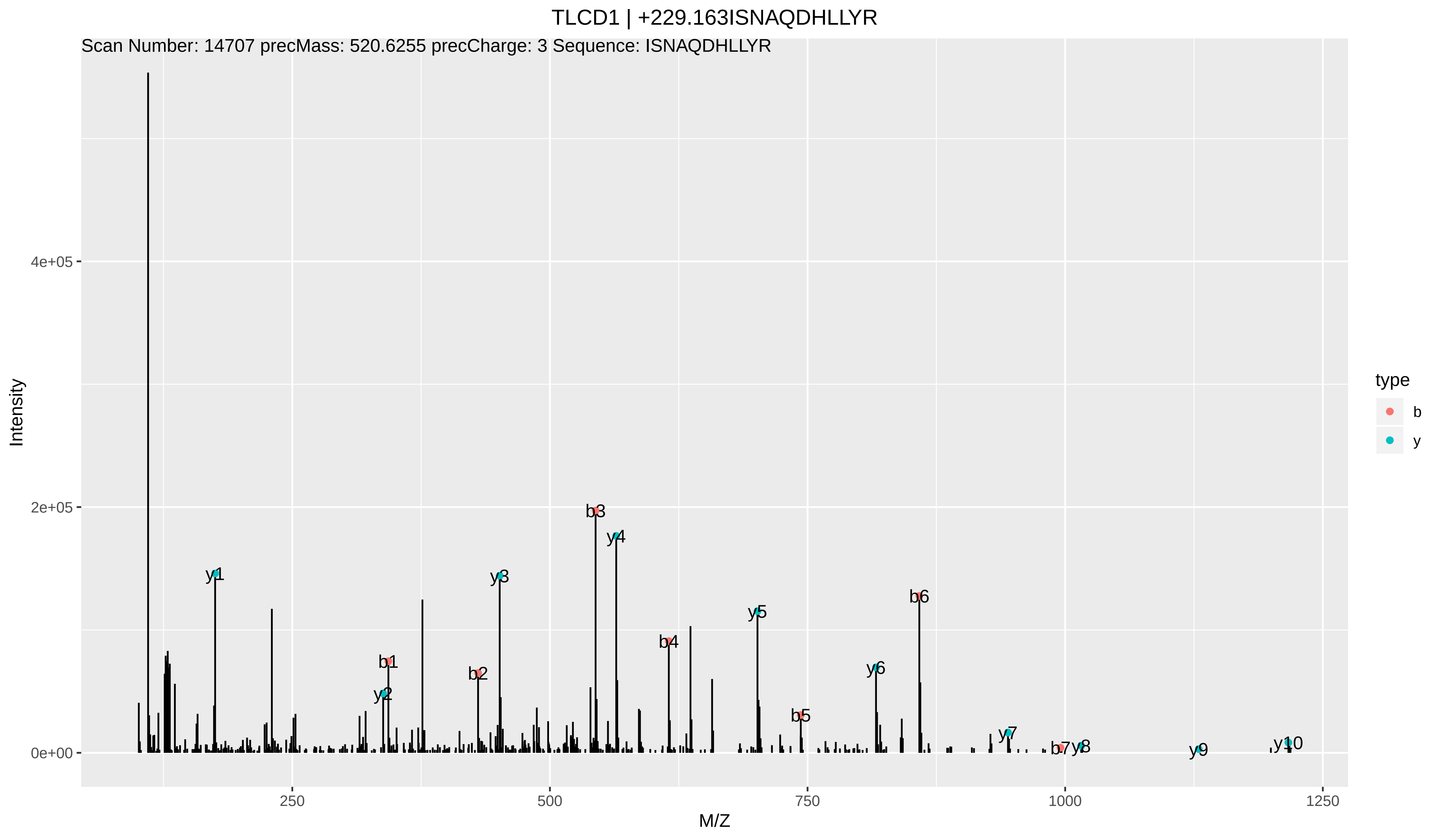
TLCD1 | +229.163ISNAQDHLLYR
Scan Number: 14707 precMass: 520.6255 precCharge: 3 Sequence: ISNAQDHLLYR
Intensity
4e+05
2e+05
0e+00
M/Z
250
500
750
1000
1250
y1
y2
y3
y4
y5
y6
y7
y8
y9
y10
b1
b2
b3
b4
b5
b6
b7
type
b
y

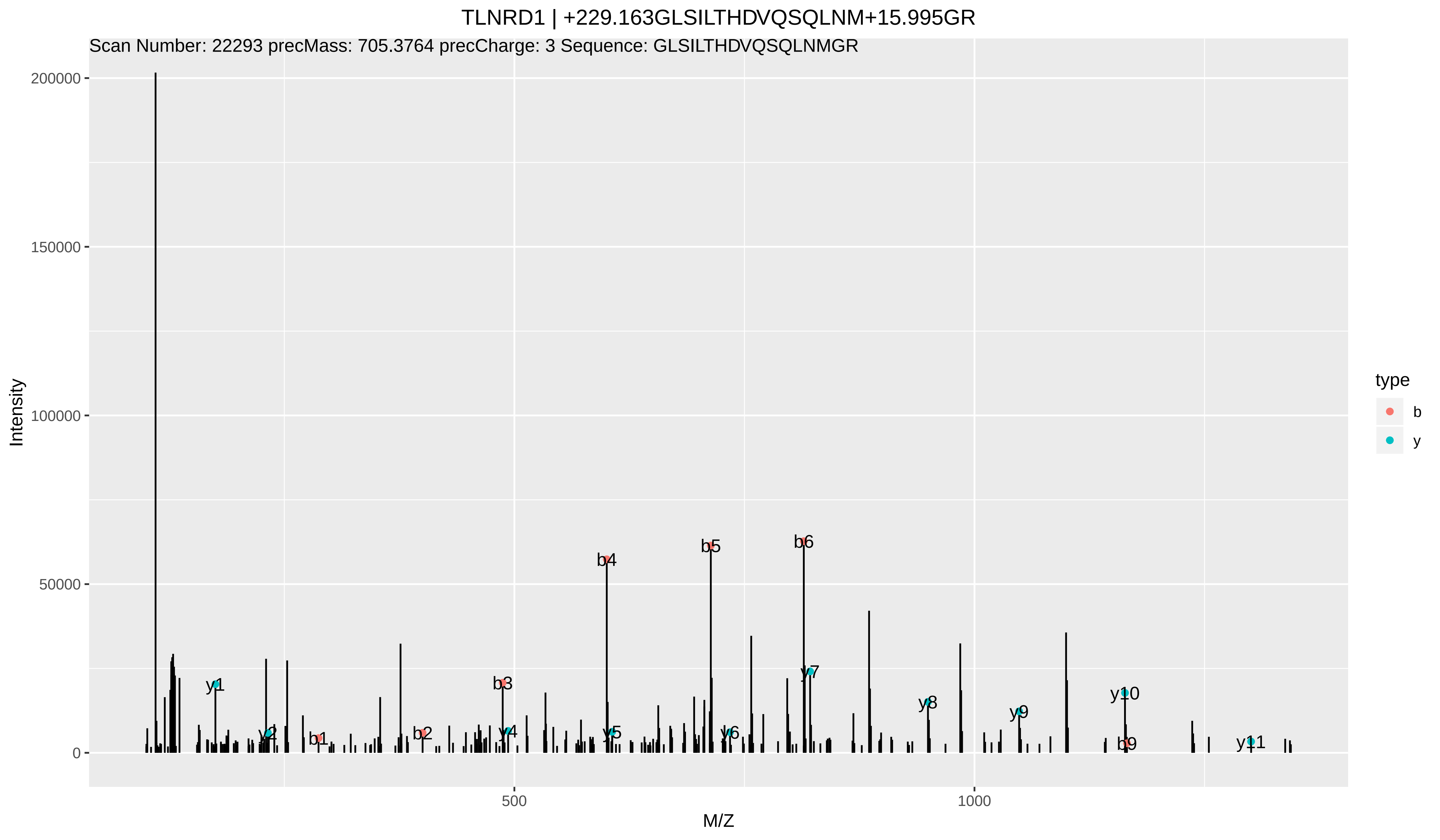

TLNRD1 | +229.163GLSILTHDVQSQLNM+15.995GR
Scan Number: 22293 precMass: 705.3764 precCharge: 3 Sequence: GLSILTHDVQSQLNMGR
Intensity
200000
150000
100000
50000
0
M/Z
500
1000
y1
y2
b1
b2
b3
y4
b4
y5
b5
y6
b6
y7
y8
y9
y10
b9
y11
type
b
y

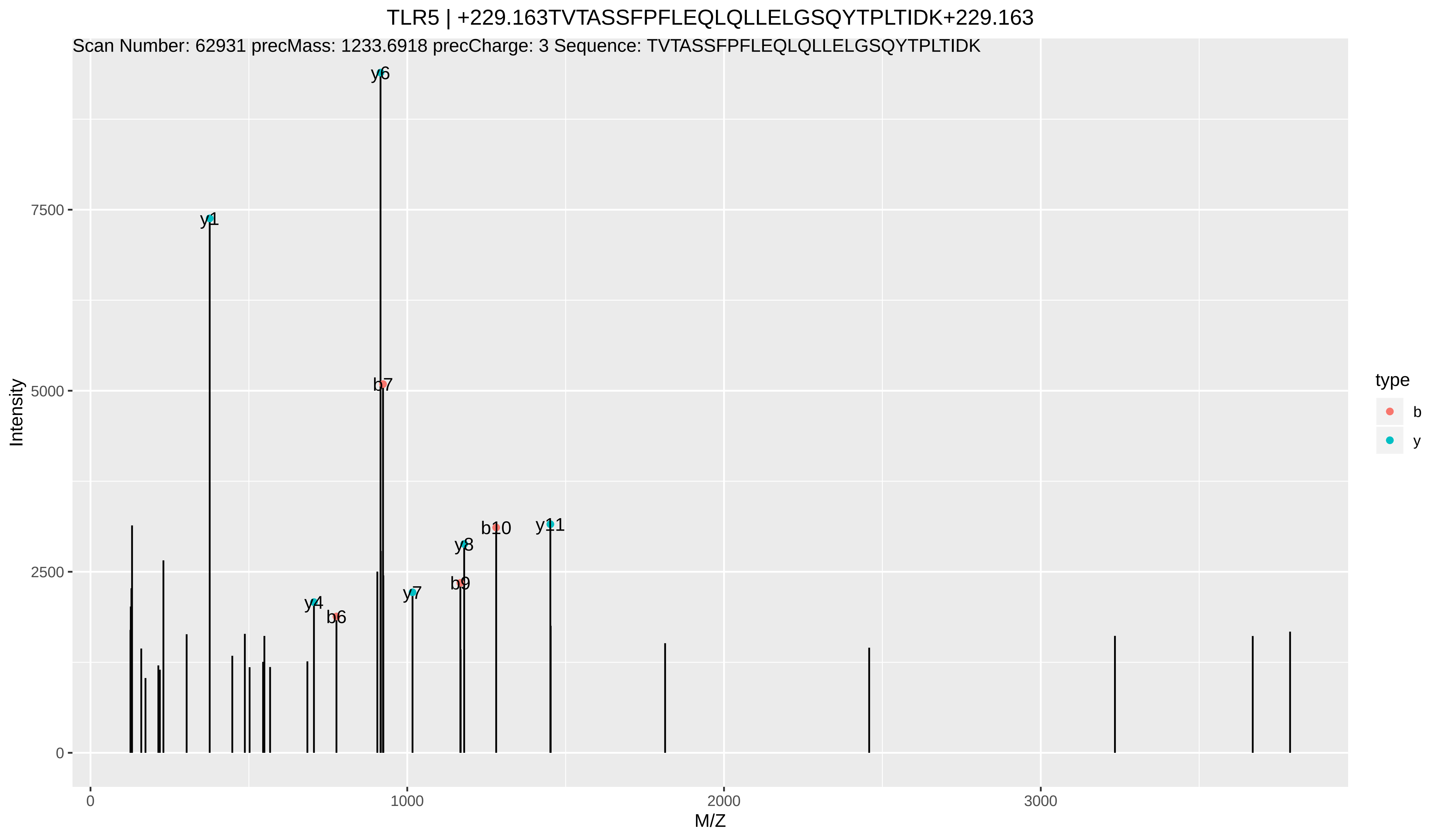
TLR5 | +229.163TVTASSFPFLEQLQLLELGSQYTPLTIDK+229.163
Scan Number: 62931 precMass: 1233.6918 precCharge: 3 Sequence: TVTASSFPFLEQLQLLELGSQYTPLTIDK
y6
y1
b7
y11
b10
y8
b9
y7
y4
b6
Intensity
7500
5000
2500
0
0
1000
2000
3000
M/Z
type
b
y

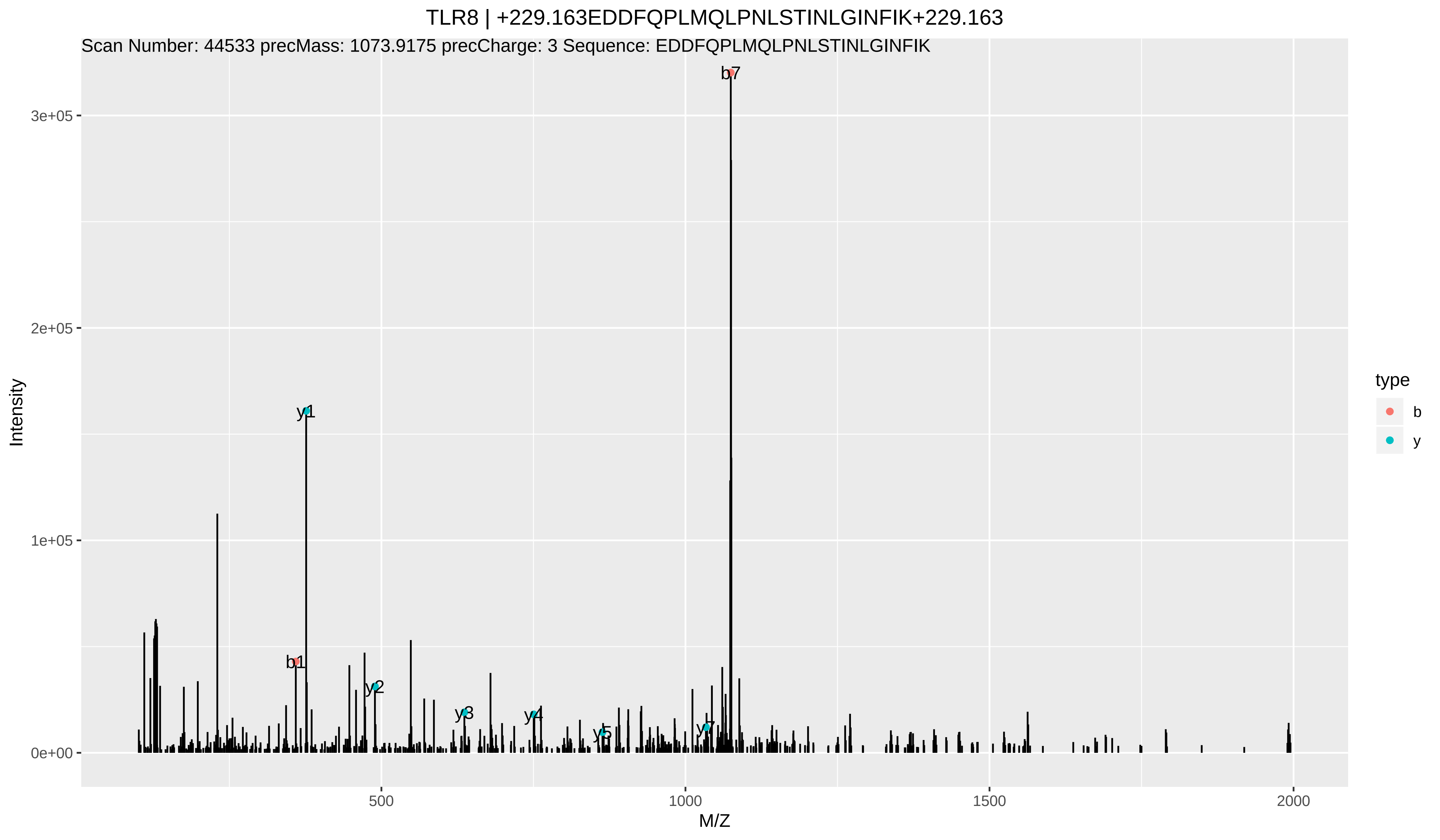

TLR8 | +229.163EDDFQPLMQLPNLSTINLGINFIK+229.163
Scan Number: 44533 precMass: 1073.9175 precCharge: 3 Sequence: EDDFQPLMQLPNLSTINLGINFIK
Intensity
M/Z
3e+05
2e+05
1e+05
0e+00
500
1000
1500
2000
b7
y1
b1
y2
y3
y4
y5
y7
type
b
y

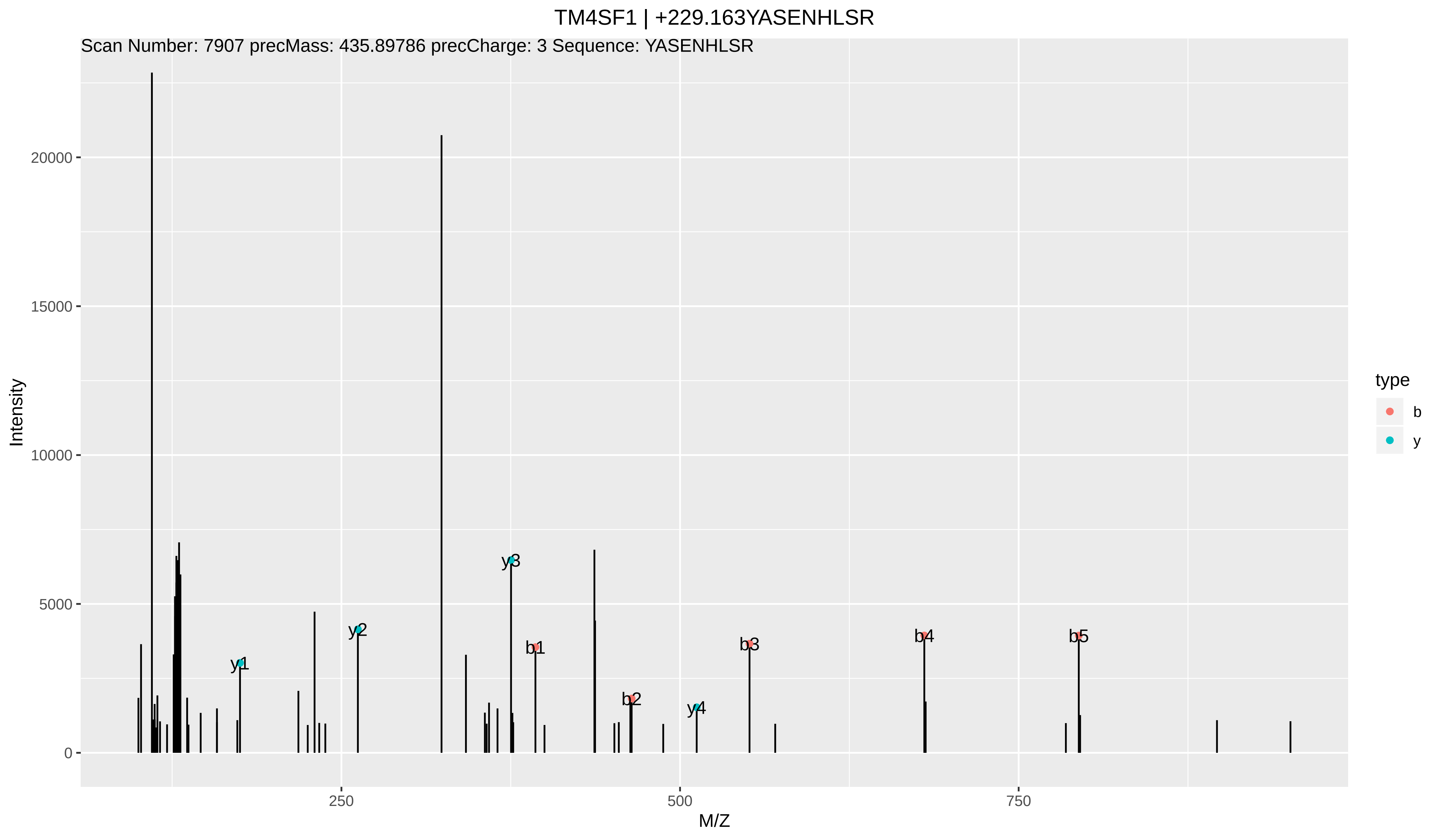
TM4SF1 | +229.163YASENHLSR
Scan Number: 7907 precMass: 435.89786 precCharge: 3 Sequence: YASENHLSR
Intensity
20000
15000
10000
5000
0
250
500
750
M/Z
y1
y2
y3
y4
b1
b2
b3
b4
b5
type
b
y

TM7SF2 | +229.163AQVAPVSALAPGGNSGNPIYDFFLGR

Scan Number: 39409 precMass: 1424.2544 precCharge: 2 Sequence: AQVAPVSALAPGGNSGNPIYDFFLGR

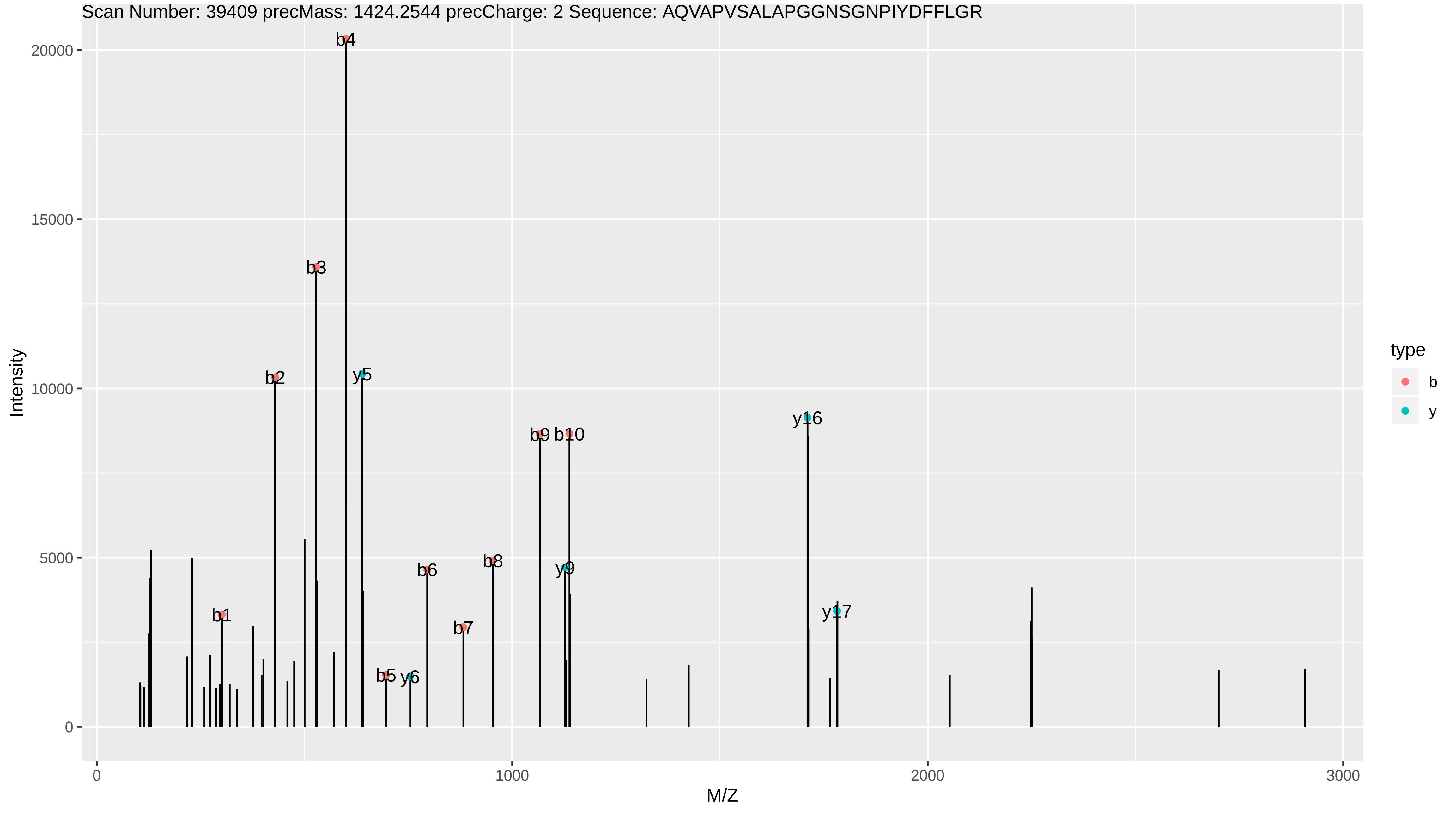

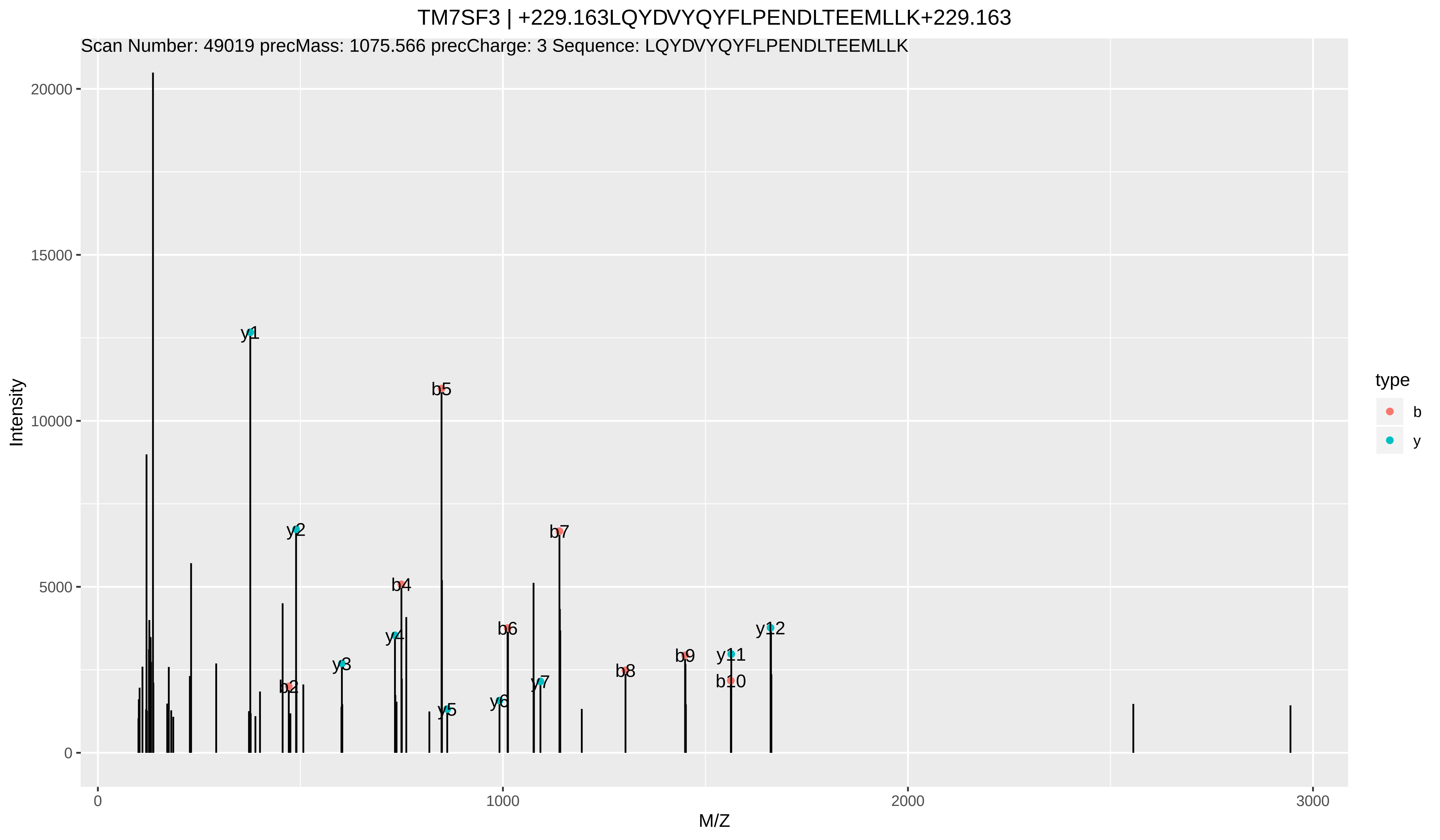

TM7SF3 | +229.163LQYDVYQYFLPENDLTEEMLLK+229.163
Scan Number: 49019 precMass: 1075.566 precCharge: 3 Sequence: LQYDVYQYFLPENDLTEEMLLK
Intensity
M/Z
type
b
y
y1
y2
y3
y4
y5
y6
y7
y11
y12
b2
b4
b5
b6
b7
b8
b9
b10
0
5000
10000
15000
20000
0
1000
2000
3000

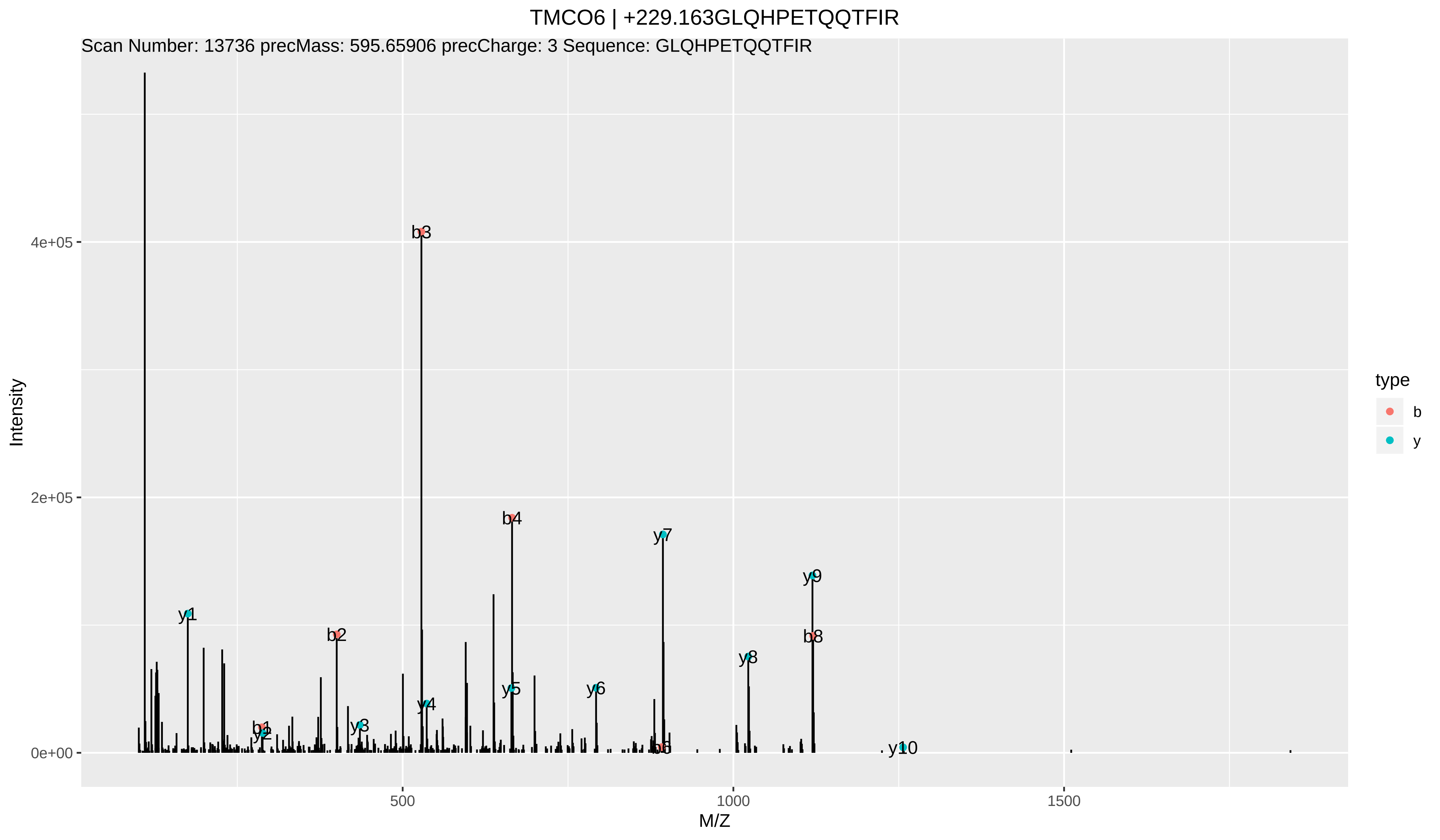

TMCO6 | +229.163GLQHPETQQTFIR
Scan Number: 13736 precMass: 595.65906 precCharge: 3 Sequence: GLQHPETQQTFIR
Intensity
4e+05
2e+05
0e+00
M/Z
500
1000
1500
b3
b4
y7
y9
y1
b2
b8
y8
y5
y6
y4
b1
y2
y3
b6
y10
type
b
y

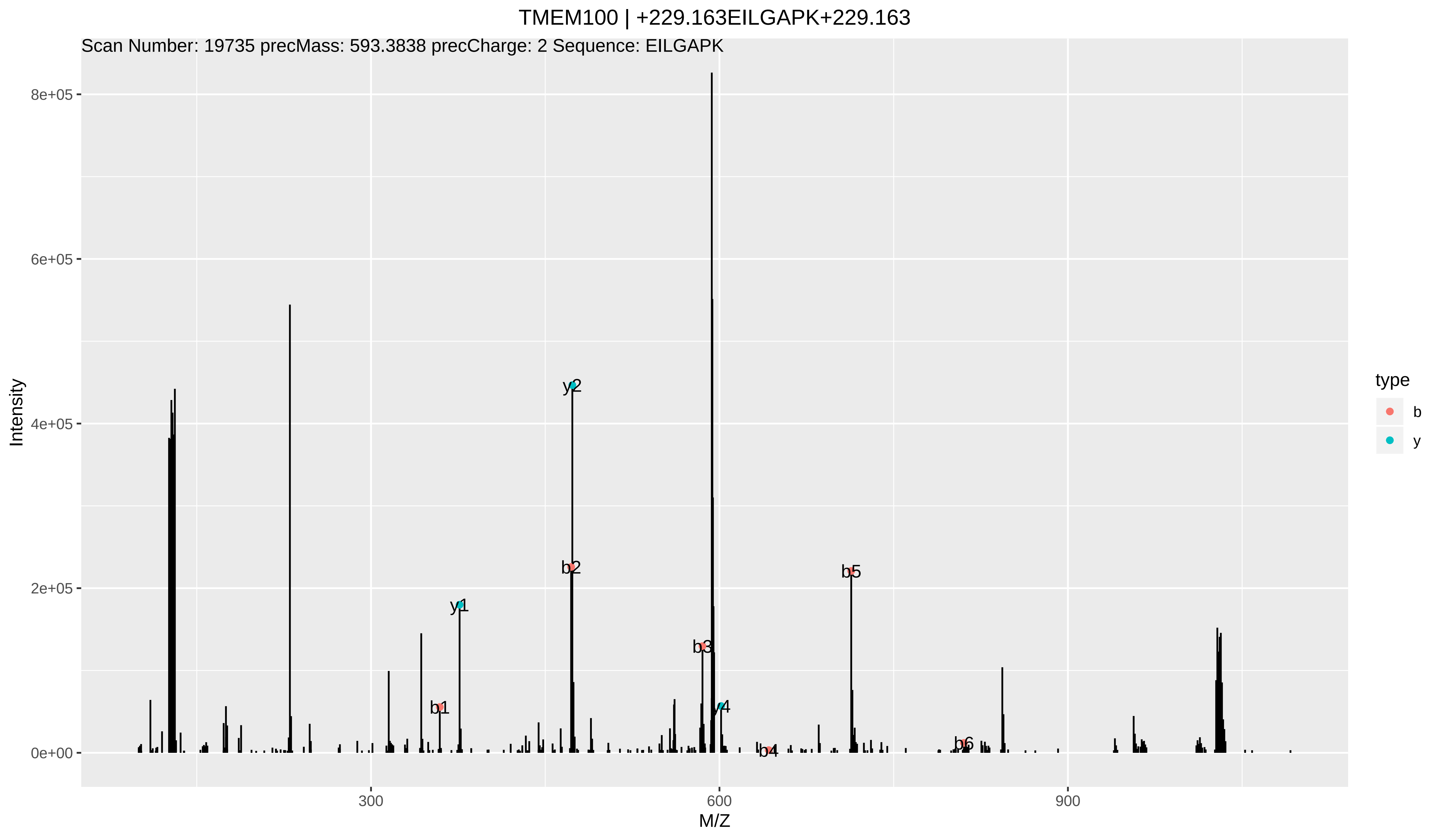

TMEM100 | +229.163EILGAPK+229.163
Scan Number: 19735 precMass: 593.3838 precCharge: 2 Sequence: EILGAPK
Intensity
8e+05
6e+05
4e+05
2e+05
0e+00
M/Z
300
600
900
y2
b2
y1
b3
b1
y4
b4
b5
b6
type
b
y

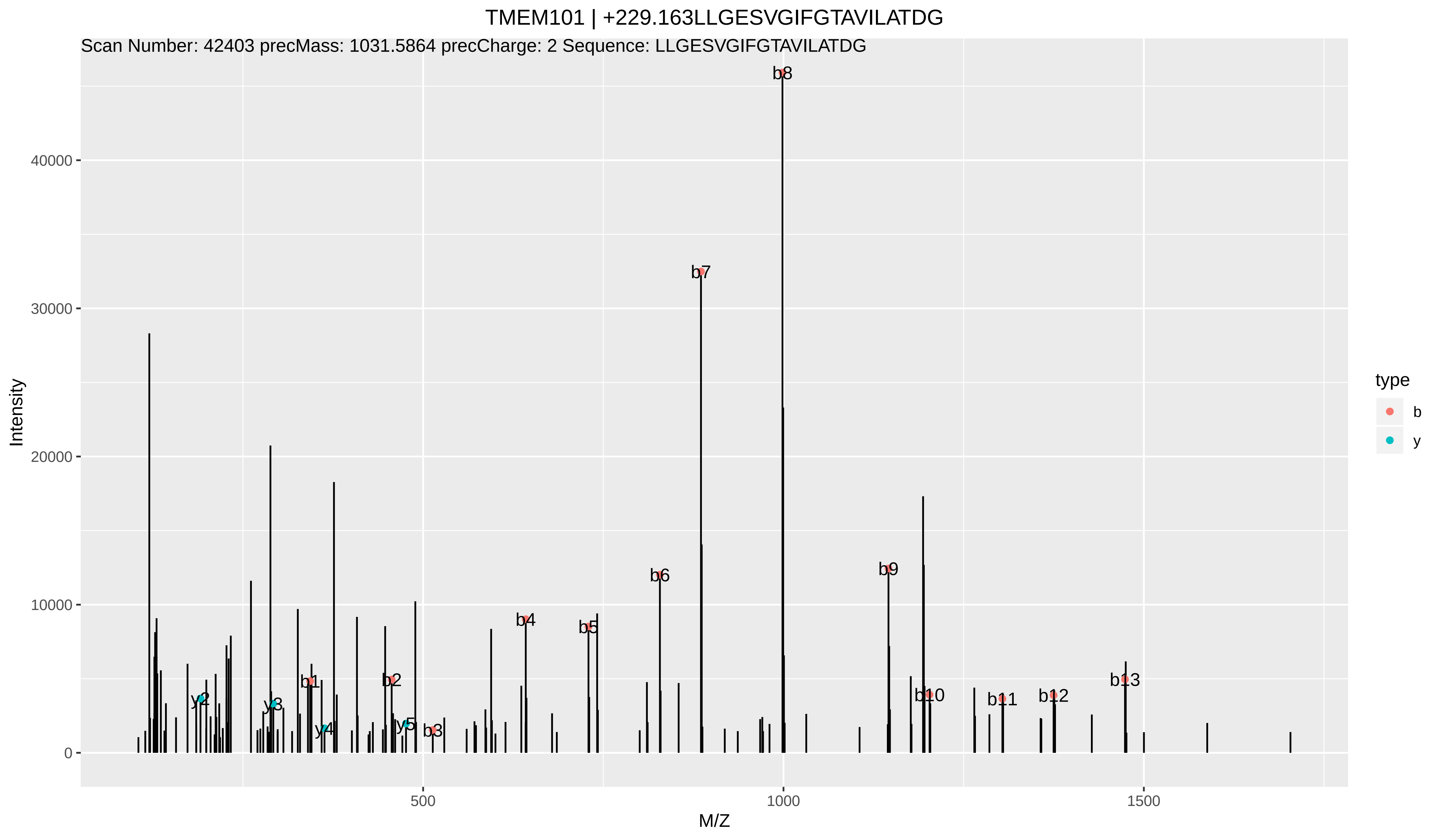

TMEM101 | +229.163LLGESVGIFGTAVILATDG
Scan Number: 42403 precMass: 1031.5864 precCharge: 2 Sequence: LLGESVGIFGTAVILATDG
Intensity
M/Z
type
b
y
b1
b2
b3
b4
b5
b6
b7
b8
b9
b10
b11
b12
b13
y2
y3
y4
y5
0
10000
20000
30000
40000
500
1000
1500

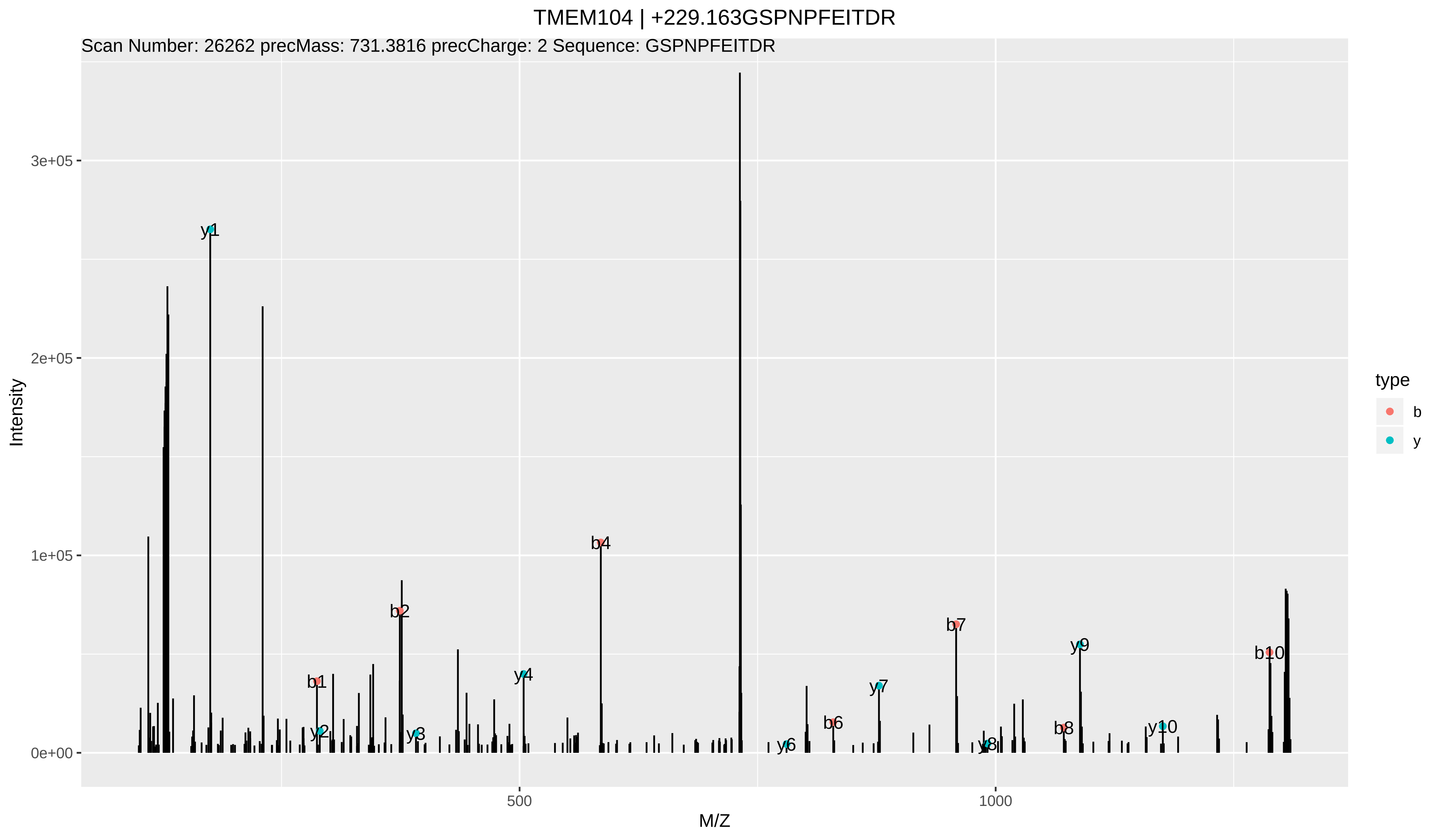
TMEM104 | +229.163GSPNPFEITDR
Scan Number: 26262 precMass: 731.3816 precCharge: 2 Sequence: GSPNPFEITDR
Intensity
3e+05
2e+05
1e+05
0e+00
M/Z
500
1000
type
b
y
y1
y2
y3
y4
y6
y7
y8
y9
y10
b1
b2
b4
b6
b7
b8
b10

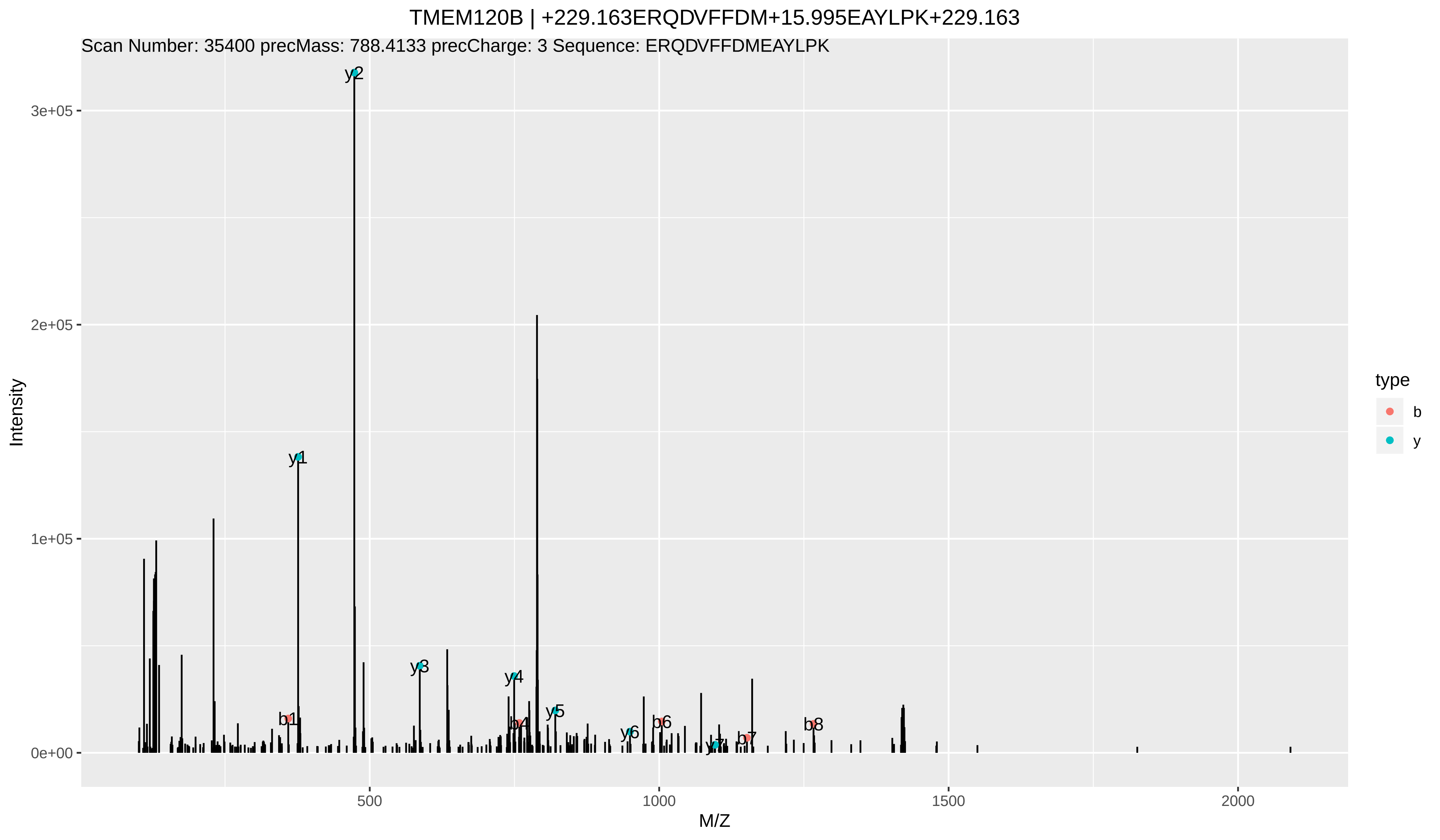
TMEM120B | +229.163ERQDVFFDM+15.995EAYLPK+229.163
Scan Number: 35400 precMass: 788.4133 precCharge: 3 Sequence: ERQDVFFDMEAYLPK
Intensity
M/Z
type
b
y
y2
y1
y3
y4
y5
y6
y7
b1
b4
b6
b7
b8
3e+05
2e+05
1e+05
0e+00
500
1000
1500
2000

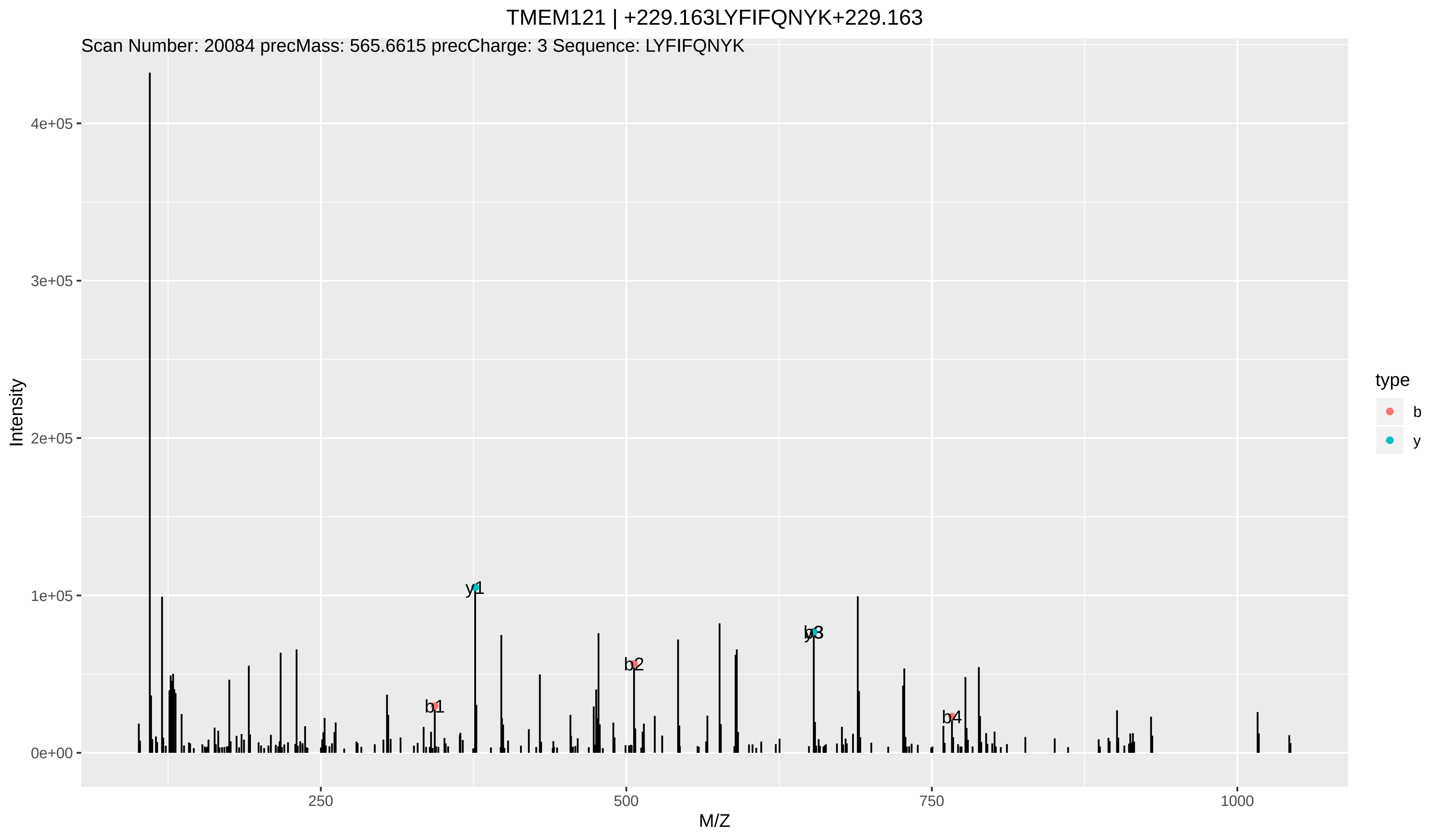
TMEM121 | +229.163LYFIFQNYK+229.163
Scan Number: 20084 precMass: 565.6615 precCharge: 3 Sequence: LYFIFQNYK
Intensity
M/Z
0e+00
1e+05
2e+05
3e+05
4e+05
250
500
750
1000
y1
b1
b2
b3
y3
b4
type
b
y

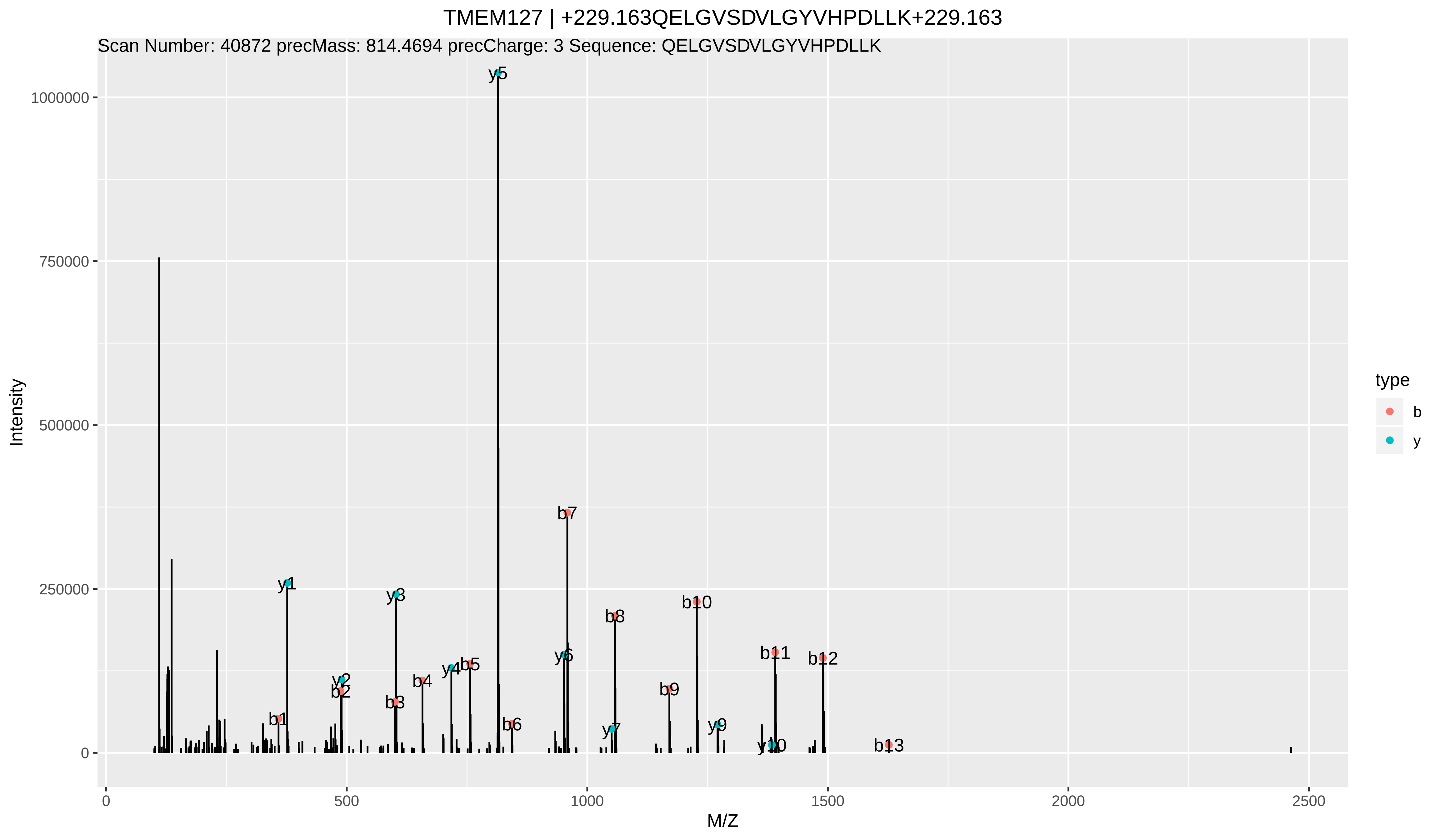

TMEM127 | +229.163QELGVSDVLGYVHPDLLK+229.163
Scan Number: 40872 precMass: 814.4694 precCharge: 3 Sequence: QELGVSDVLGYVHPDLLK
Intensity
1000000
750000
500000
250000
0
M/Z
0
500
1000
1500
2000
2500
type
b
y
y5
b7
y1
y3
b10
b8
y6
b11
b12
y4
b5
y2
b4
b2
b9
b3
b1
b6
y9
y7
y10
b13

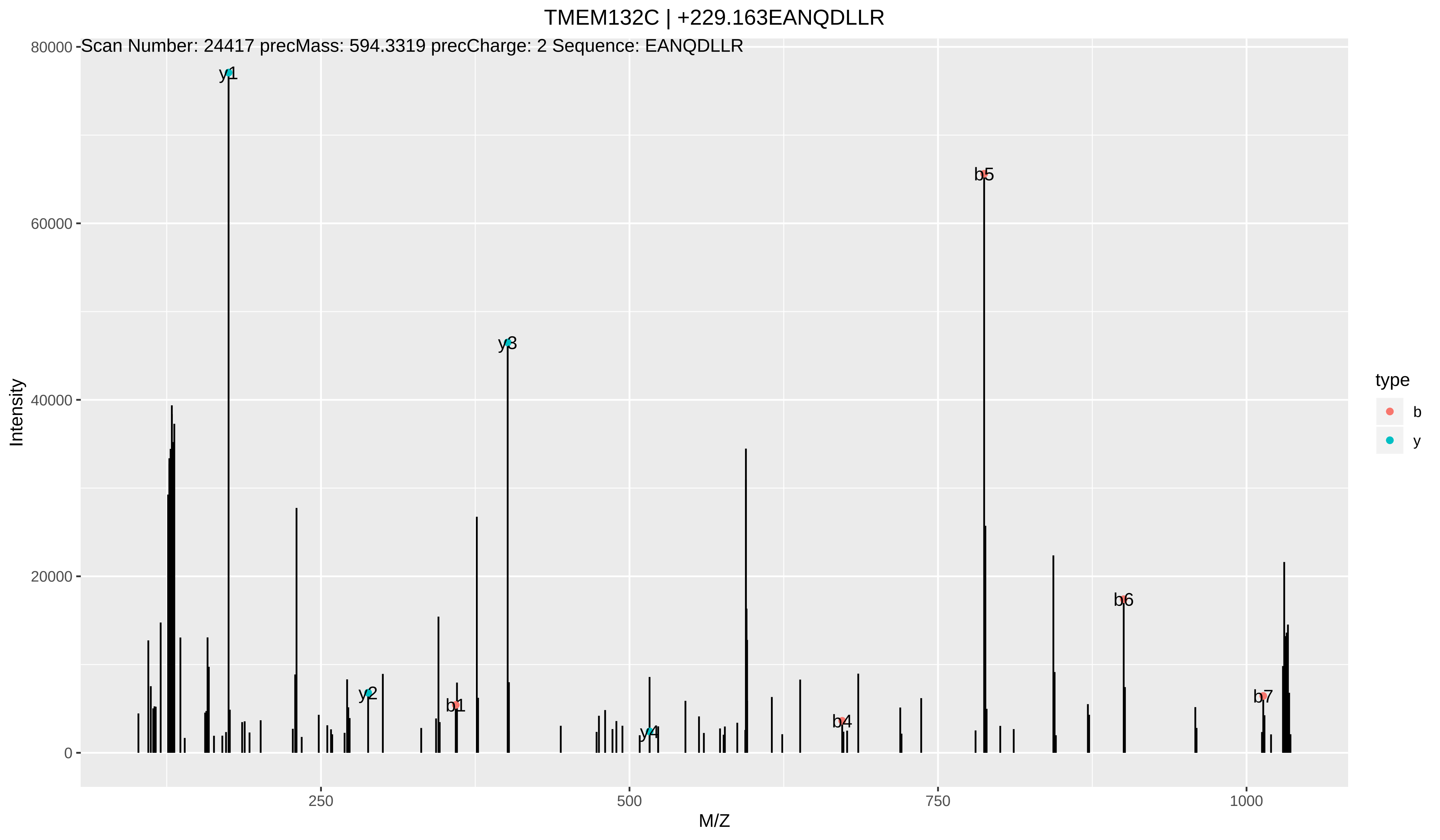

TMEM132C | +229.163EANQDLLR
Scan Number: 24417 precMass: 594.3319 precCharge: 2 Sequence: EANQDLLR
y1
y2
y3
y4
b1
b4
b5
b6
b7
Intensity
M/Z
type
b
y
80000
60000
40000
20000
0
250
500
750
1000

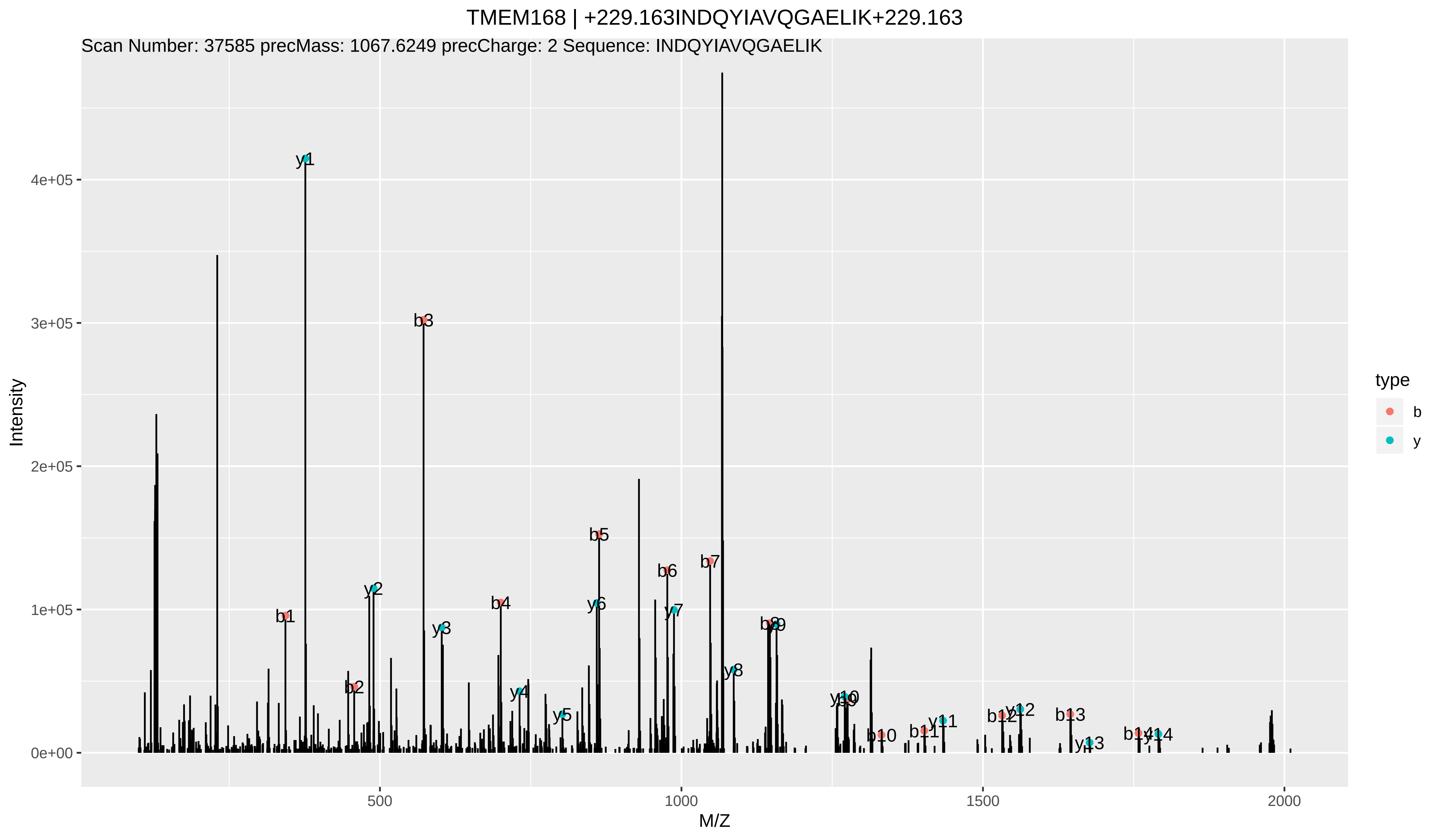
TMEM168 | +229.163INDQYIAVQGAELIK+229.163
Scan Number: 37585 precMass: 1067.6249 precCharge: 2 Sequence: INDQYIAVQGAELIK
Intensity
M/Z
type
b
y
4e+05
3e+05
2e+05
1e+05
0e+00
500
1000
1500
2000

TMEM175 | +229.163EPSAHPVEVFSFDLHEPLSK+229.163

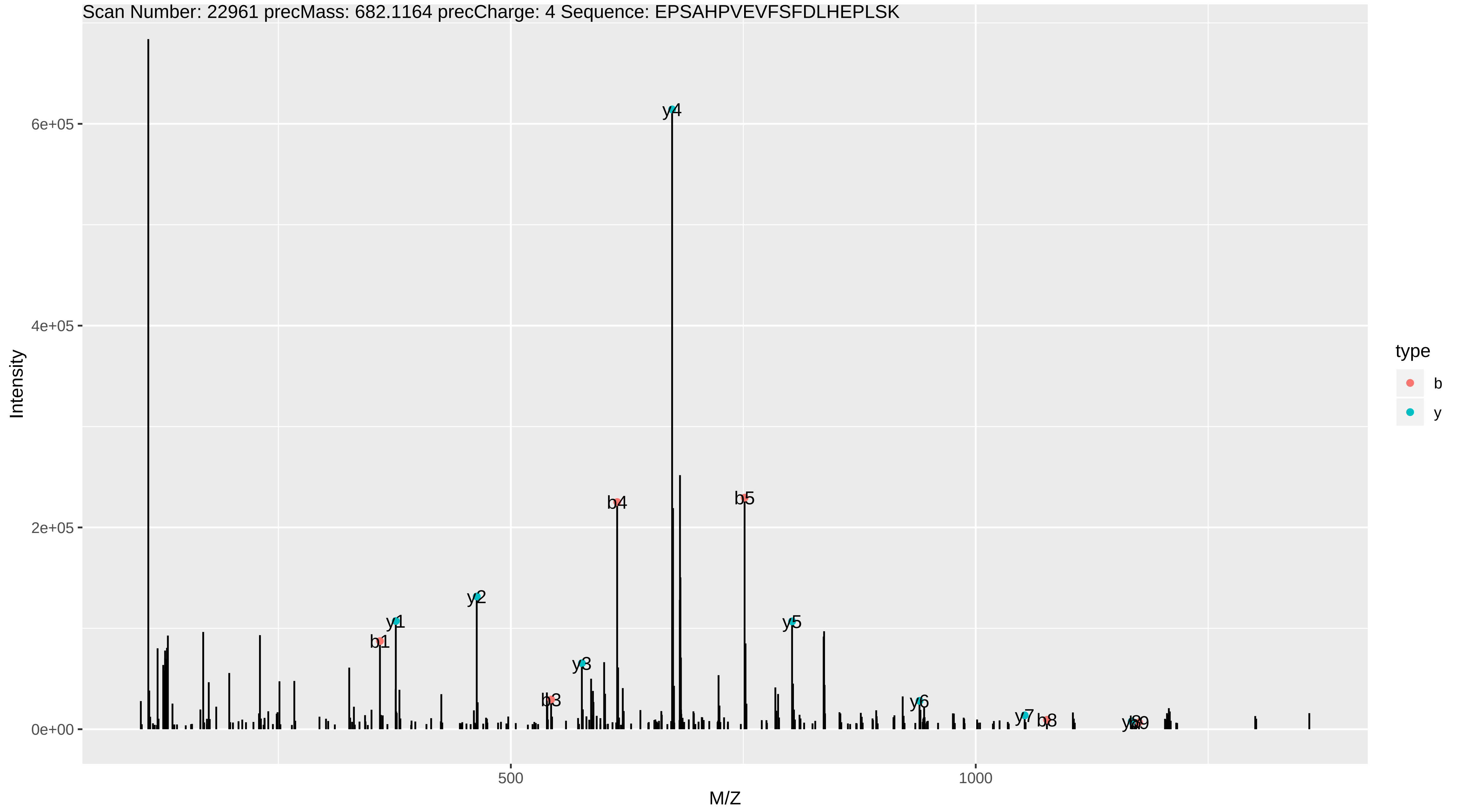

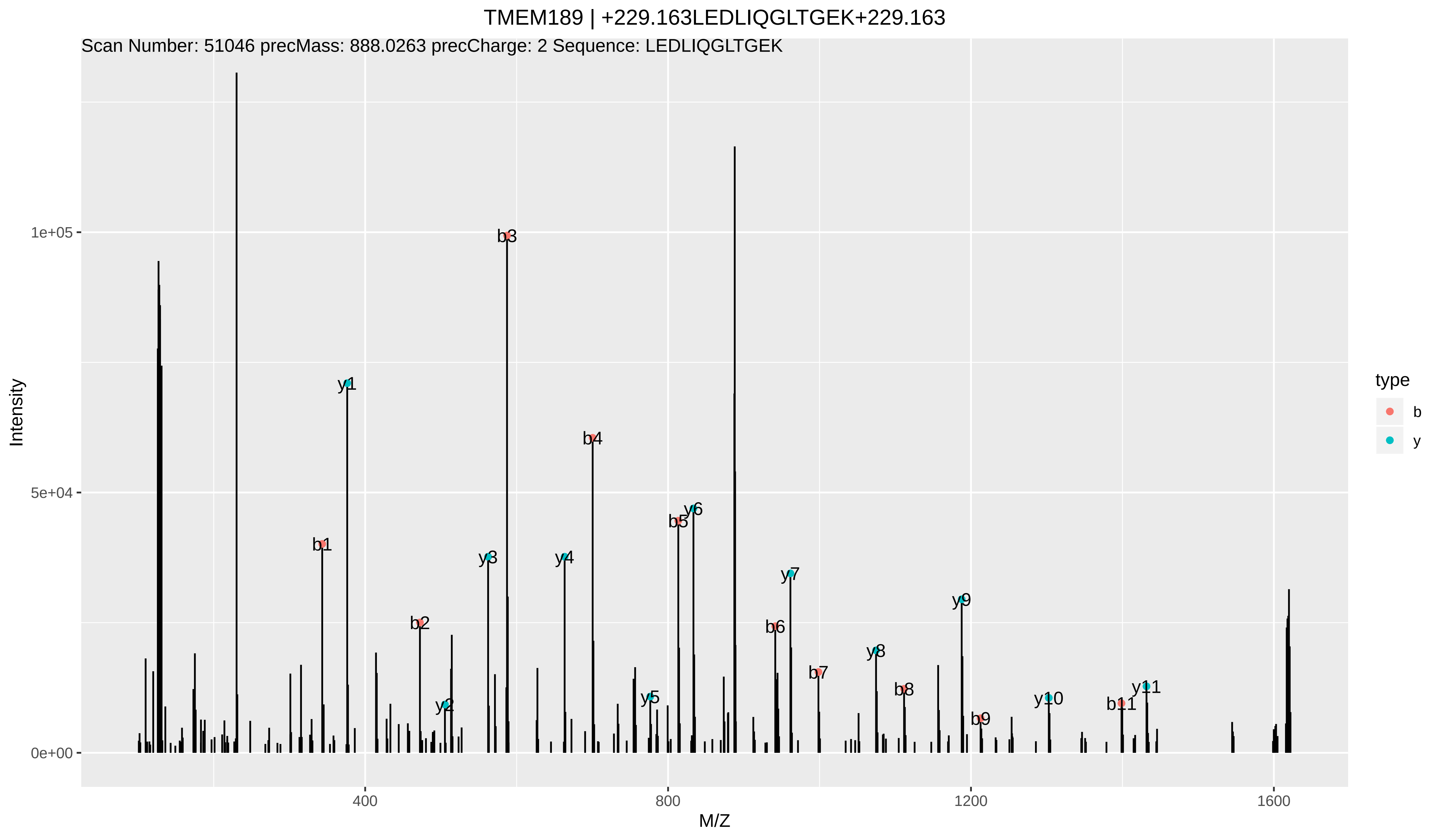

TMEM189 | +229.163LEDLIQGLTGEK+229.163
Scan Number: 51046 precMass: 888.0263 precCharge: 2 Sequence: LEDLIQGLTGEK
Intensity
1e+05
5e+04
0e+00
M/Z
400
800
1200
1600
b3
y1
b4
y6
b5
b1
y3
y4
y7
y9
b2
b6
y8
b7
b8
y5
y2
y10
y11
b11
b9
type
b
y

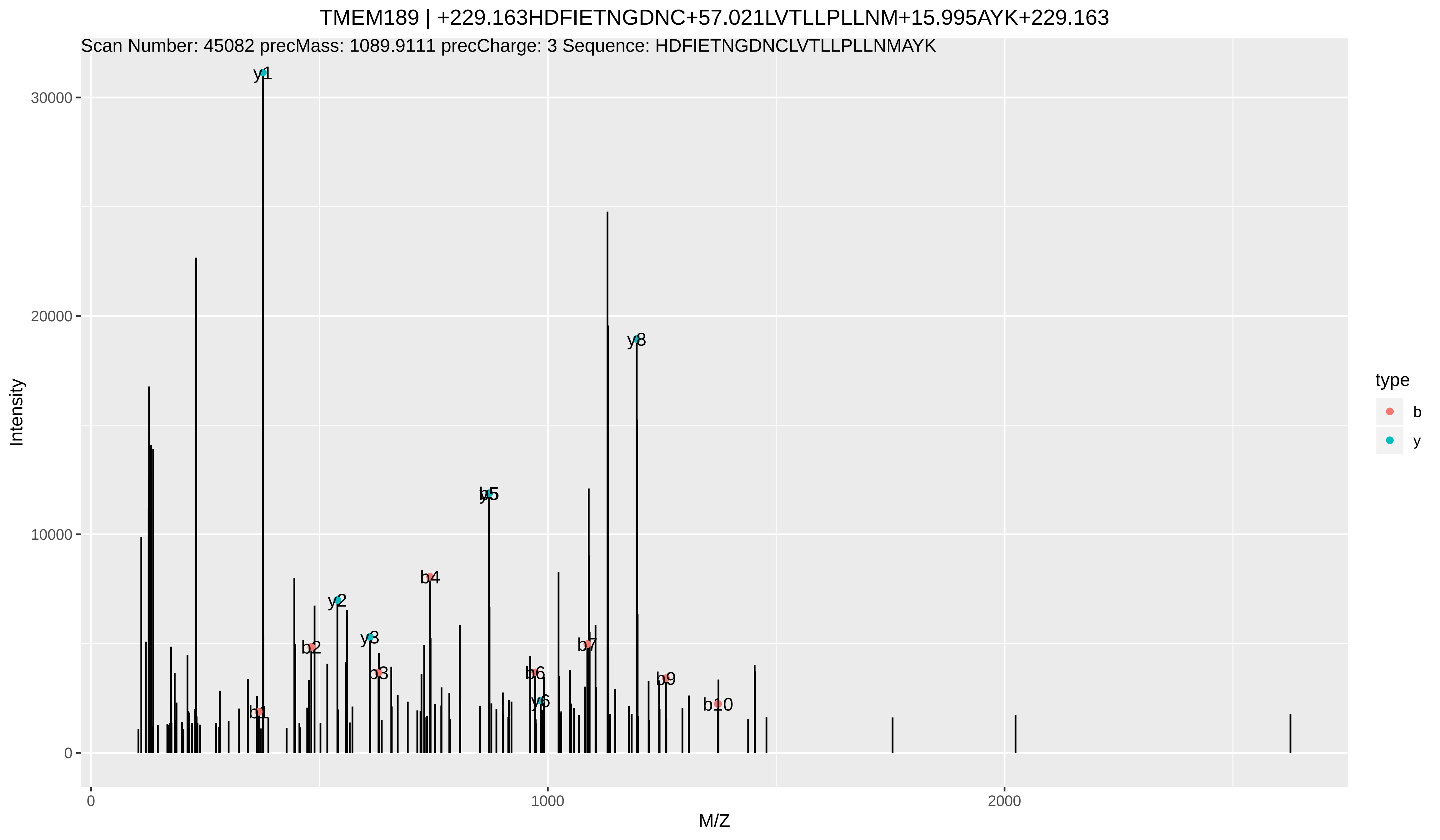
TMEM189 | +229.163HDFIETNGDNC+57.021LVTLLPLLNM+15.995AYK+229.163
Scan Number: 45082 precMass: 1089.9111 precCharge: 3 Sequence: HDFIETNGDNCLVTLLPLLNMAYK
y1
y8
b5
y5
b4
y2
y3
b2
b7
b3
b6
b9
y6
b10
b1
Intensity
M/Z
30000
20000
10000
0
1000
2000
type
b
y

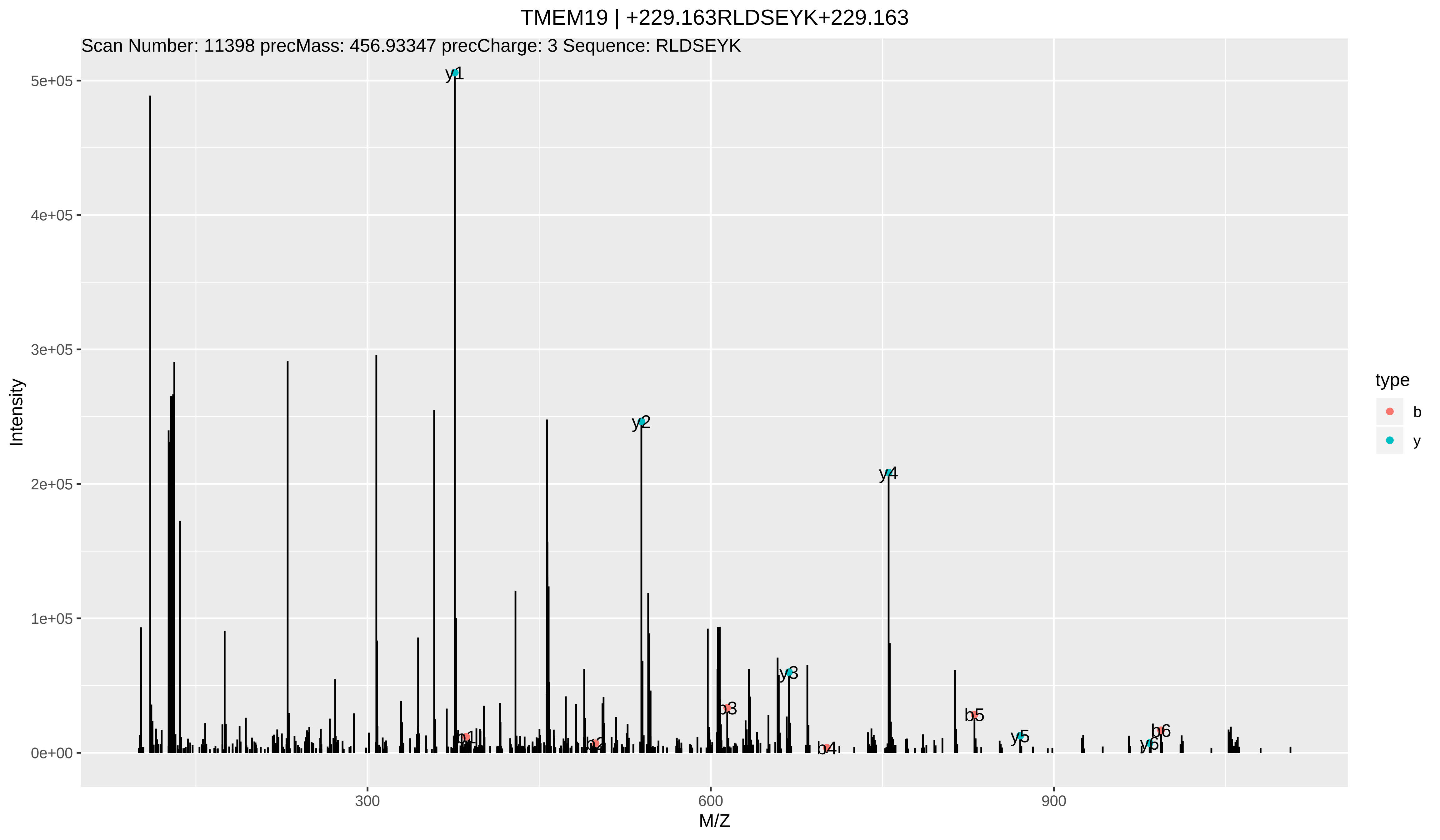
TMEM19 | +229.163RLDSEYK+229.163
Scan Number: 11398 precMass: 456.93347 precCharge: 3 Sequence: RLDSEYK
Intensity
5e+05
4e+05
3e+05
2e+05
1e+05
0e+00
300
600
900
M/Z
y1
y2
y3
y4
y5
y6
b1
b2
b3
b4
b5
b6
type
b
y

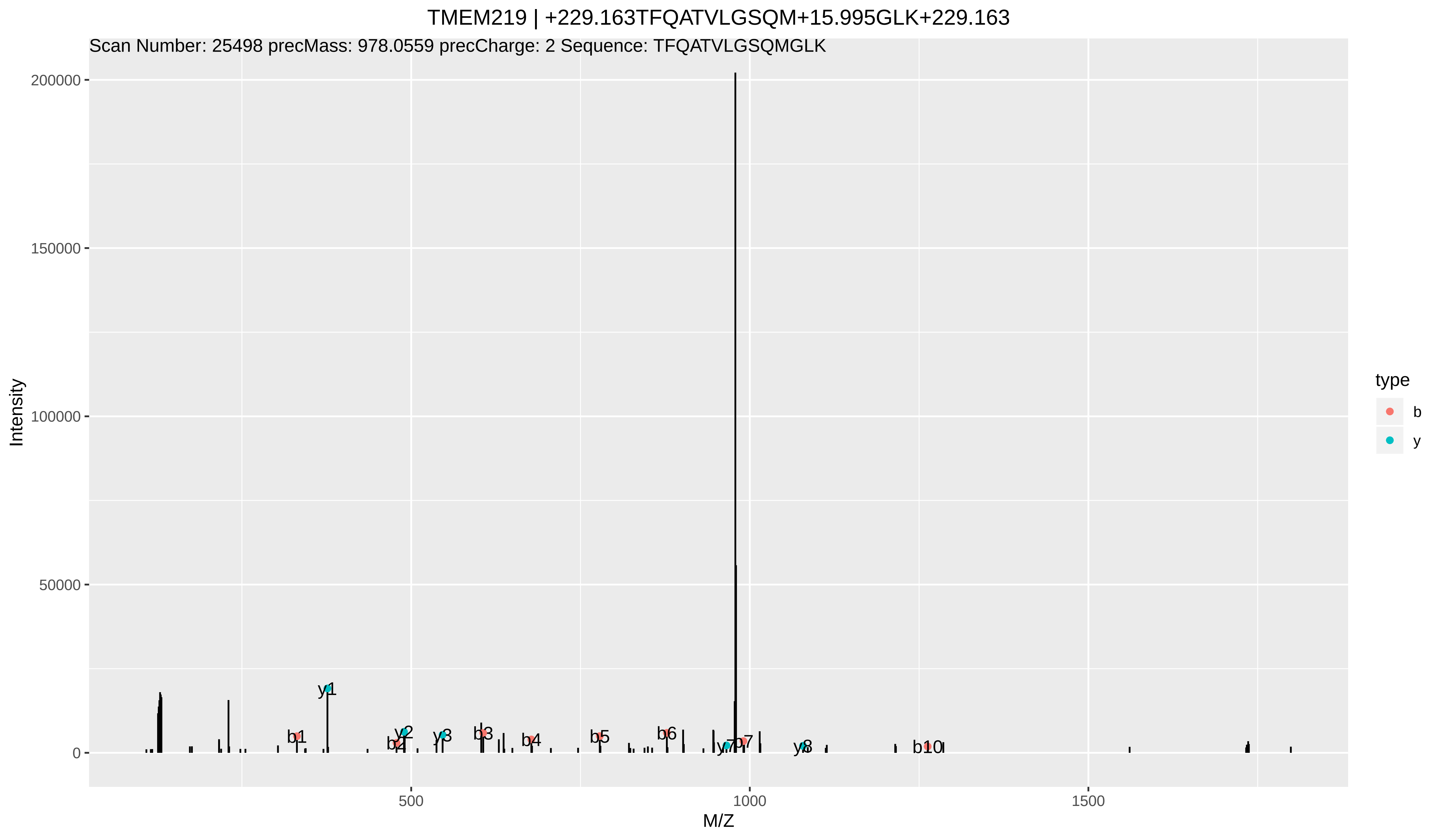

TMEM219 | +229.163TFQATVLGSQM+15.995GLK+229.163
Scan Number: 25498 precMass: 978.0559 precCharge: 2 Sequence: TFQATVLGSQMGLK
Intensity
200000
150000
100000
50000
0
500
1000
1500
M/Z
y1
b1
y2
b2
y3
b3
b4
b5
b6
y7
b7
y8
b10
type
b
y

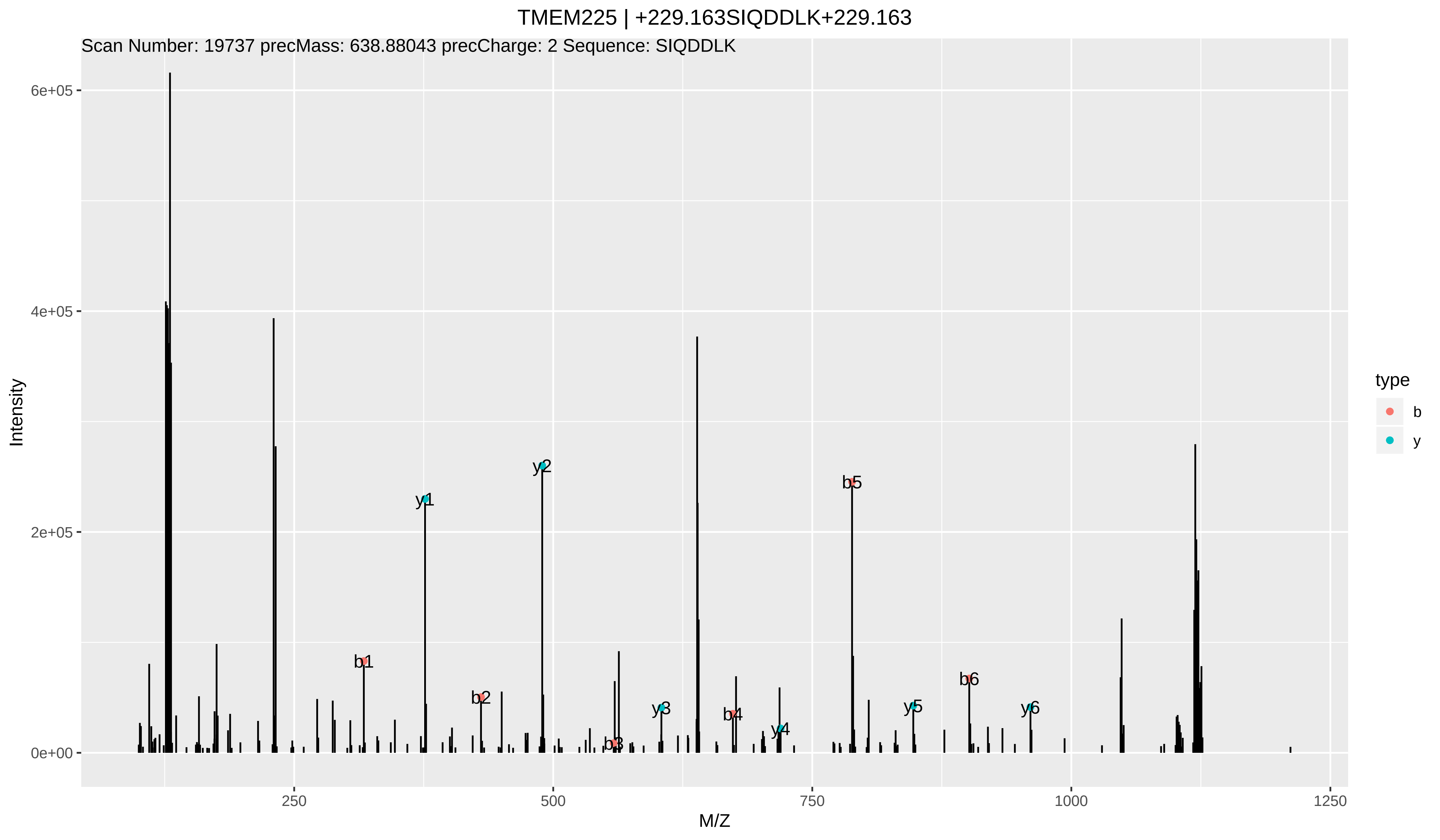
TMEM225 | +229.163SIQDDLK+229.163
Scan Number: 19737 precMass: 638.88043 precCharge: 2 Sequence: SIQDDLK
Intensity
6e+05
4e+05
2e+05
0e+00
M/Z
250
500
750
1000
1250
type
b
y
y1
y2
b5
b1
b2
y3
b4
y4
y5
b6
y6
b3

TMEM238 | +229.163DYGLRPSALAR

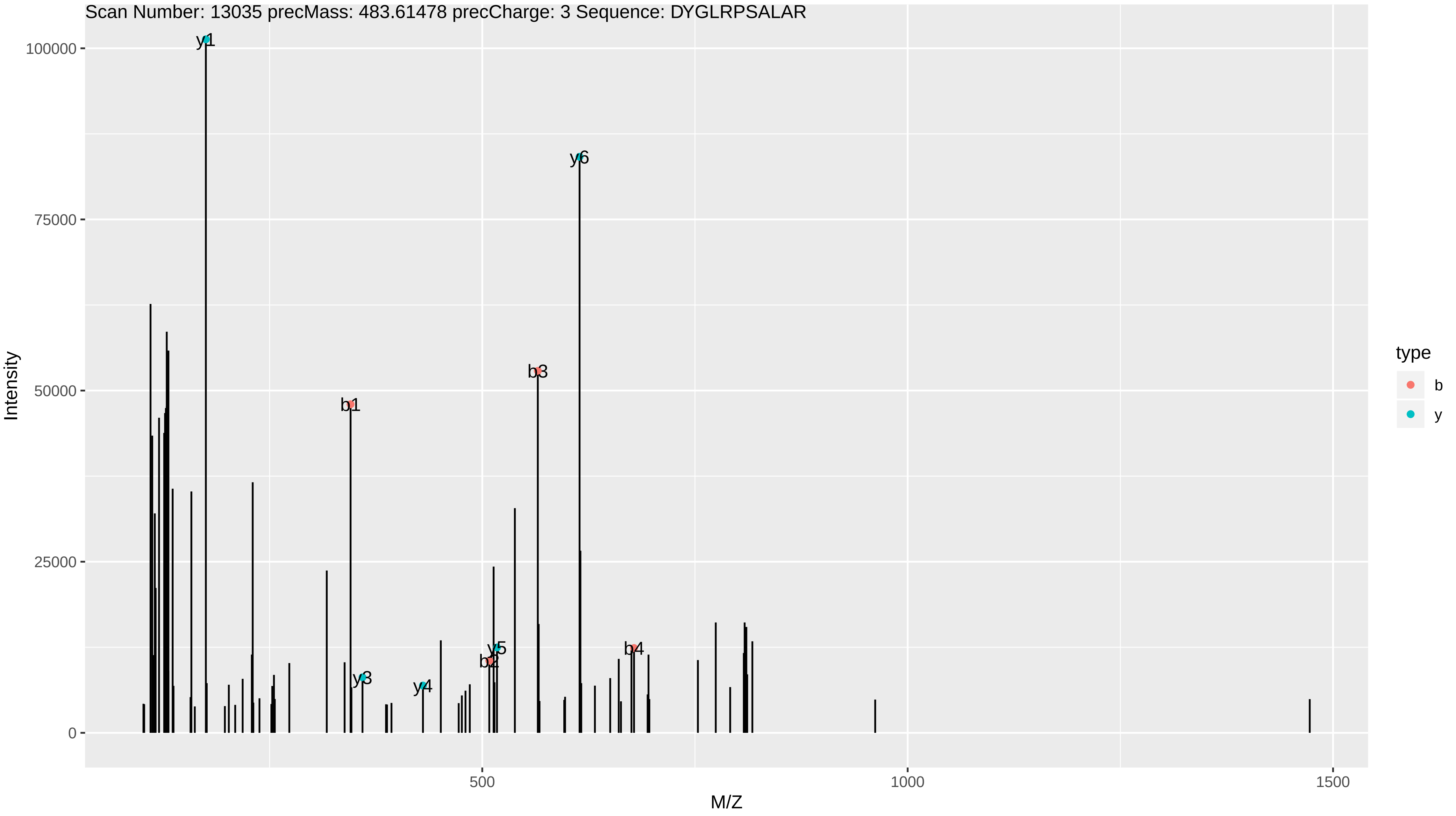

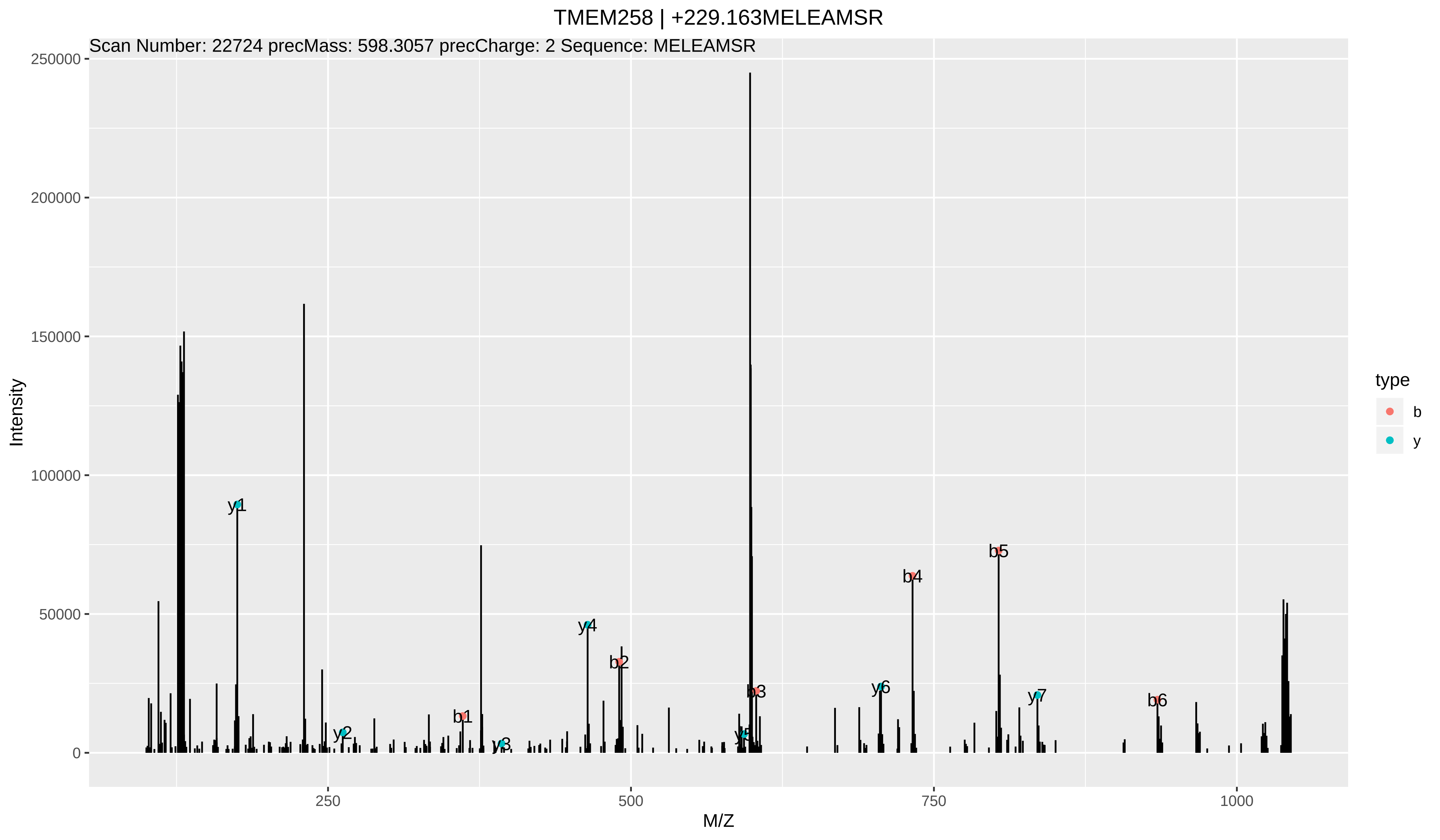
TMEM258 | +229.163MELEAMSR
Scan Number: 22724 precMass: 598.3057 precCharge: 2 Sequence: MELEAMSR
Intensity
M/Z
250000
200000
150000
100000
50000
0
250
500
750
1000
y1
y2
b1
y3
y4
b2
y5
b3
y6
b4
b5
y7
b6
type
b
y

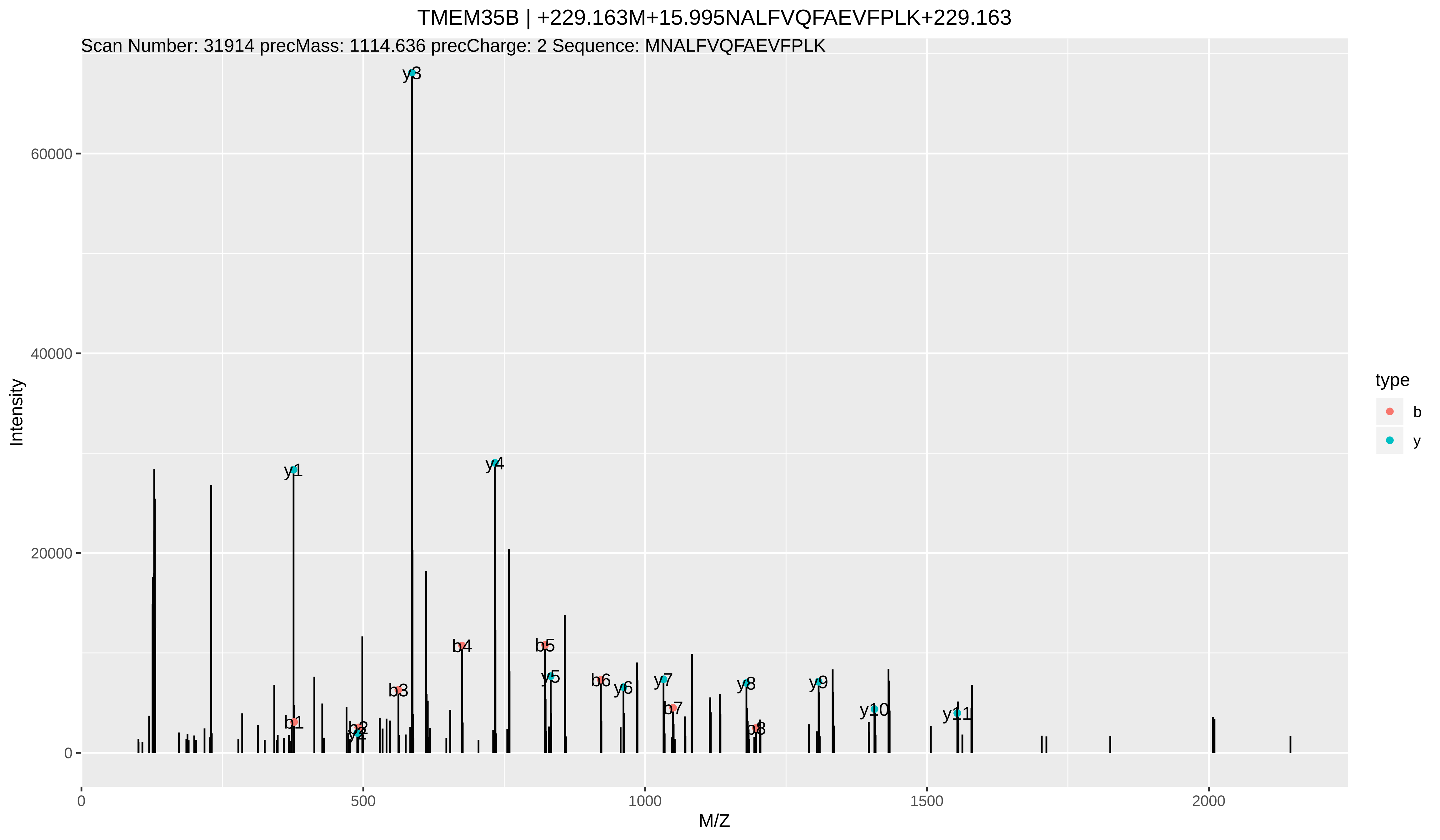
TMEM35B | +229.163M+15.995NALFVQFAEVFPLK+229.163
Scan Number: 31914 precMass: 1114.636 precCharge: 2 Sequence: MNALFVQFAEVFPLK
Intensity
M/Z
type
b
y
0
20000
40000
60000
500
1000
1500
2000
y1
y2
y3
y4
y5
y6
y7
y8
y9
y10
y11
b1
b2
b3
b4
b5
b6
b7
b8

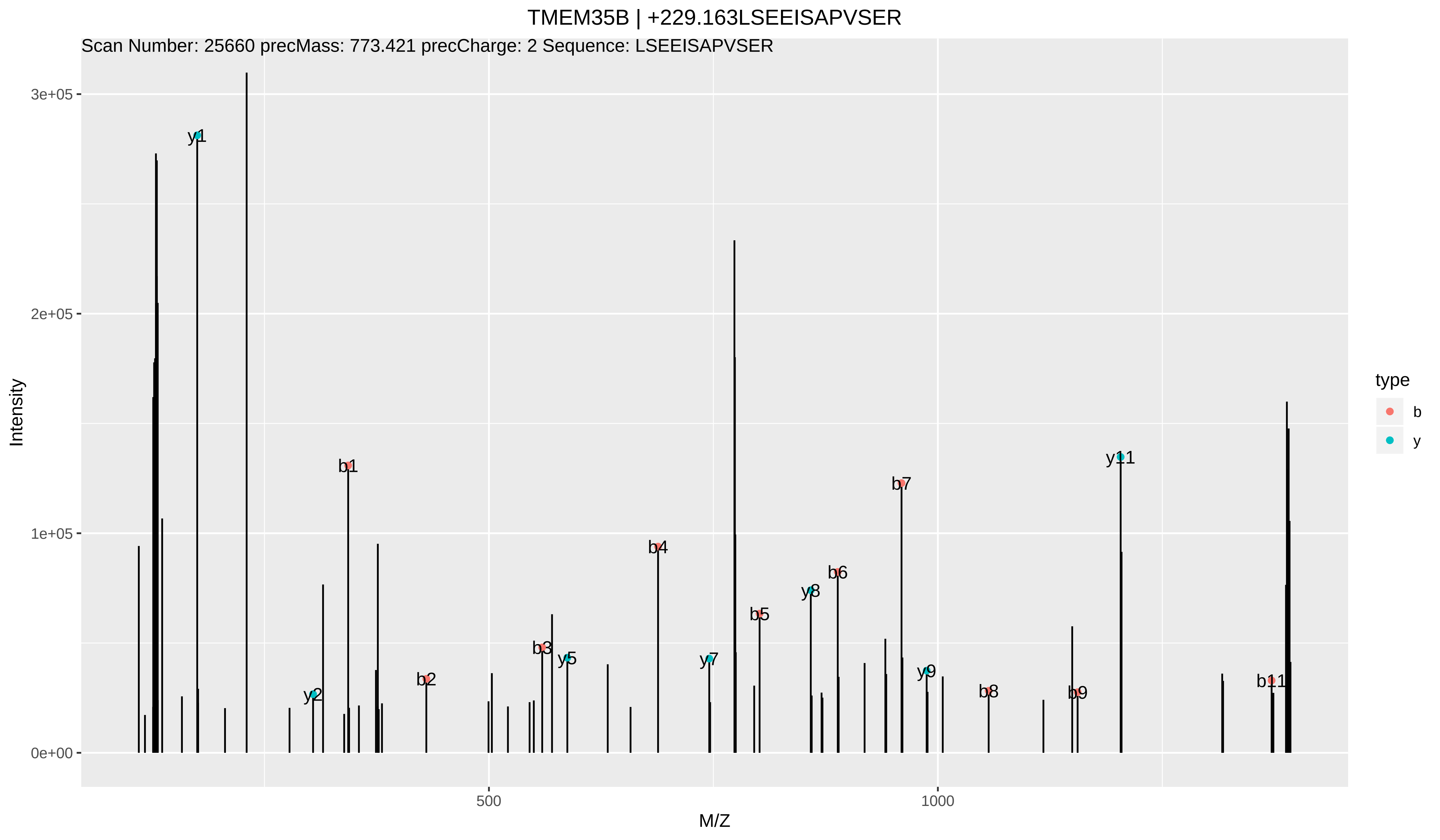
TMEM35B | +229.163LSEEISAPVSER
Scan Number: 25660 precMass: 773.421 precCharge: 2 Sequence: LSEEISAPVSER
Intensity
3e+05
2e+05
1e+05
0e+00
M/Z
500
1000
y1
b1
y2
b2
b3
y5
b4
y7
b5
y8
b6
b7
y9
b8
b9
y11
b11
type
b
y

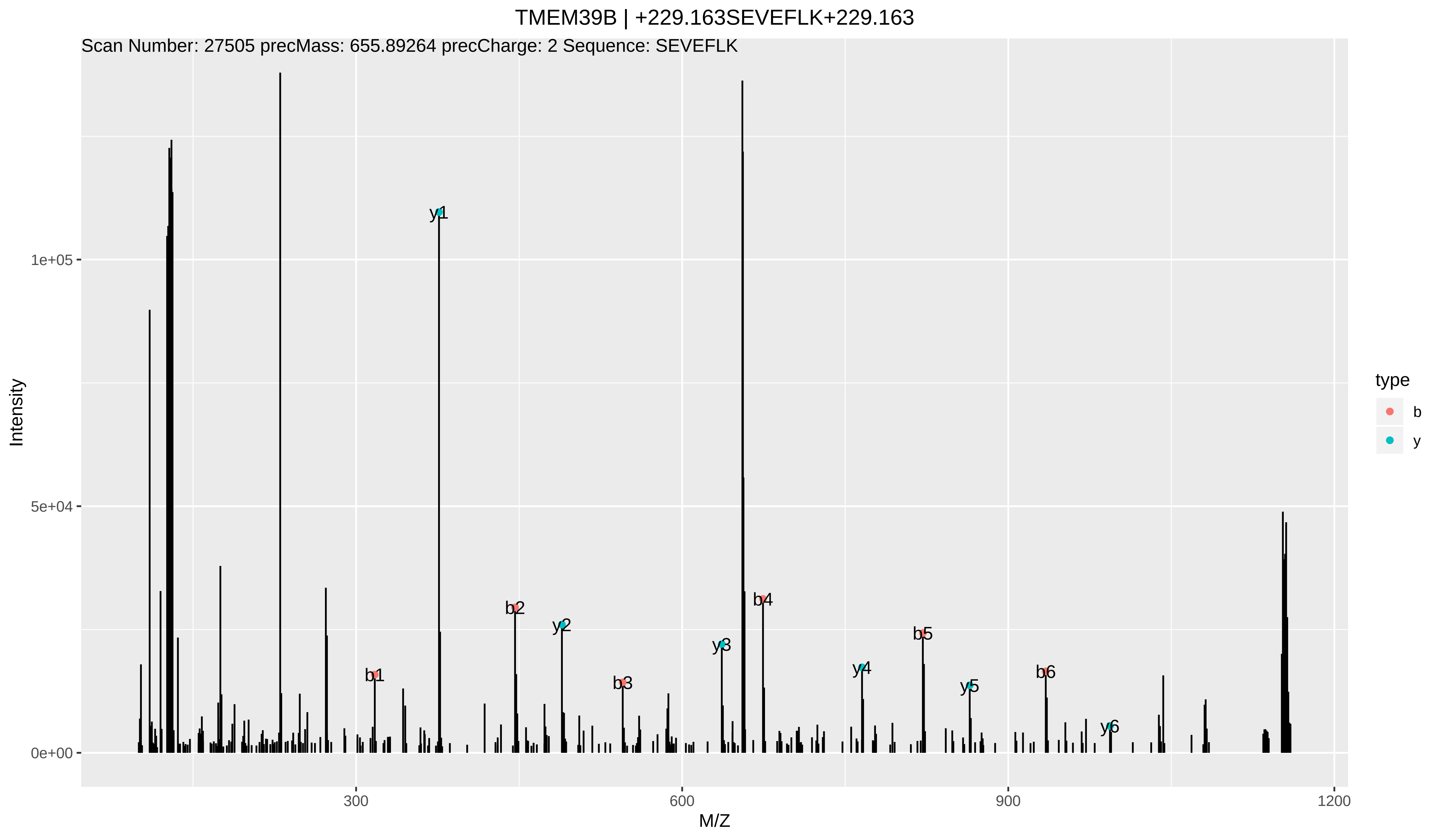

TMEM39B | +229.163SEVEFLK+229.163
Scan Number: 27505 precMass: 655.89264 precCharge: 2 Sequence: SEVEFLK
Intensity
1e+05
5e+04
0e+00
y1
b2
y2
b4
y3
b5
y4
b1
b3
b6
y5
y6
300
600
900
1200
M/Z
type
b
y

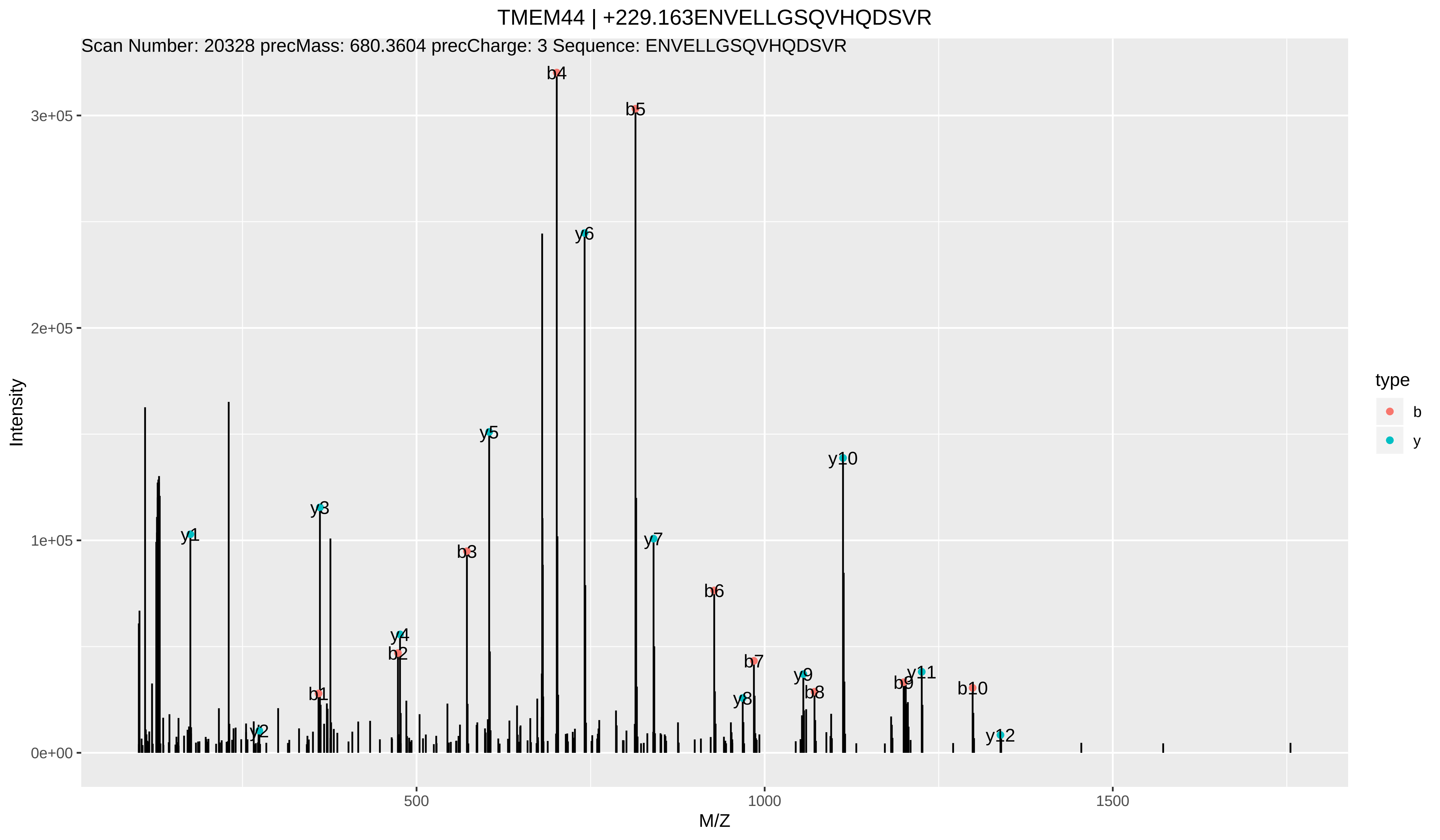

TMEM44 | +229.163ENVELLGSQVHQDSVR
Scan Number: 20328 precMass: 680.3604 precCharge: 3 Sequence: ENVELLGSQVHQDSVR
Intensity
3e+05
2e+05
1e+05
0e+00
M/Z
500
1000
1500
b4
b5
y6
y5
y10
y3
y1
y7
b3
b6
y4
b2
b7
y9
y11
b9
b8
b10
b1
y8
y2
y12
type
b
y

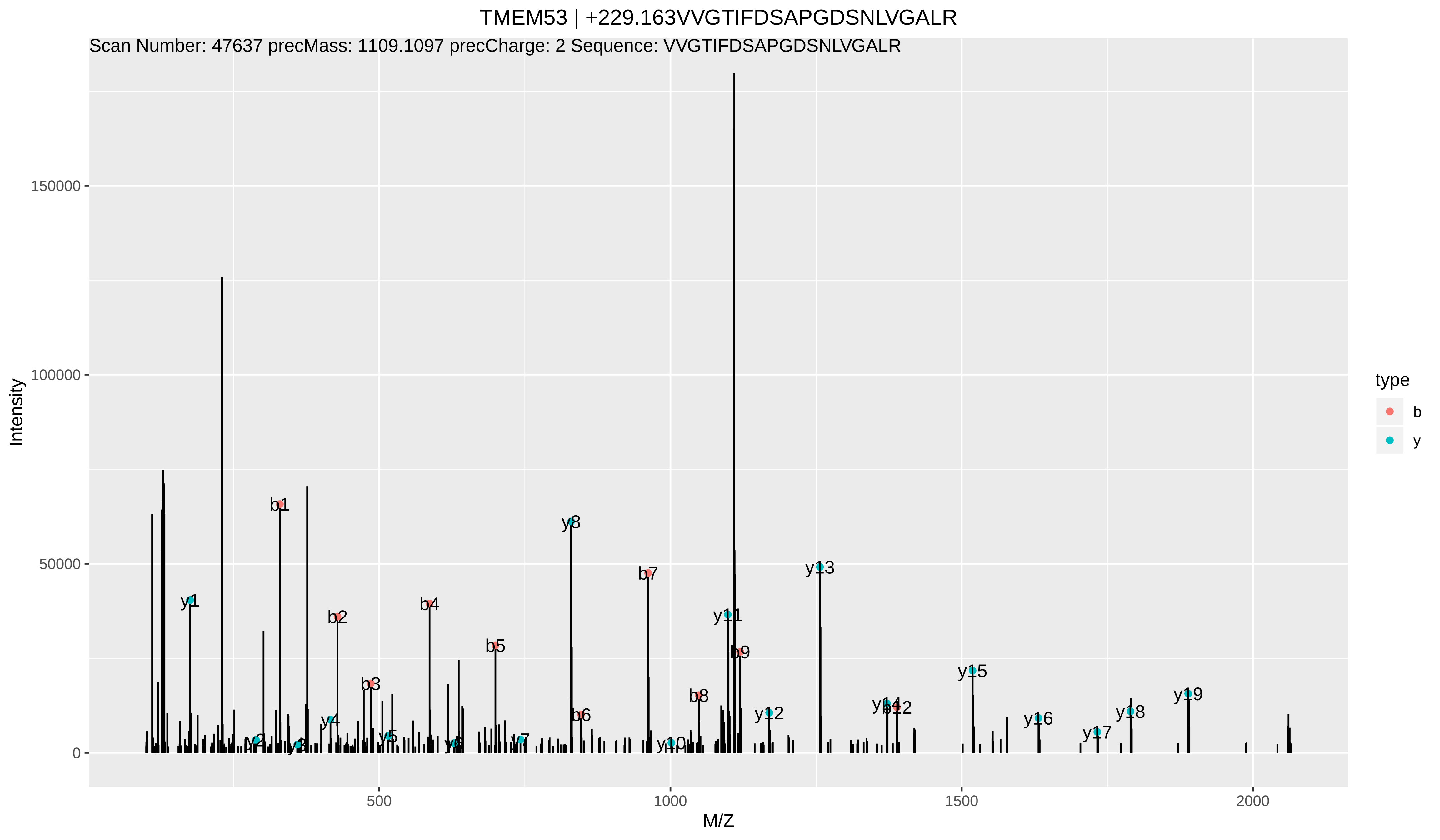
TMEM53 | +229.163VVGTIFDSAPGDSNLVGALR
Scan Number: 47637 precMass: 1109.1097 precCharge: 2 Sequence: VVGTIFDSAPGDSNLVGALR
Intensity
M/Z
type
b
y
0
50000
100000
150000
500
1000
1500
2000
b1
y8
b7
y13
y1
b4
b2
y11
b5
b9
y15
b3
b8
y19
y14
b12
y12
y18
b6
y16
y4
y17
y2
y5
y7
y10

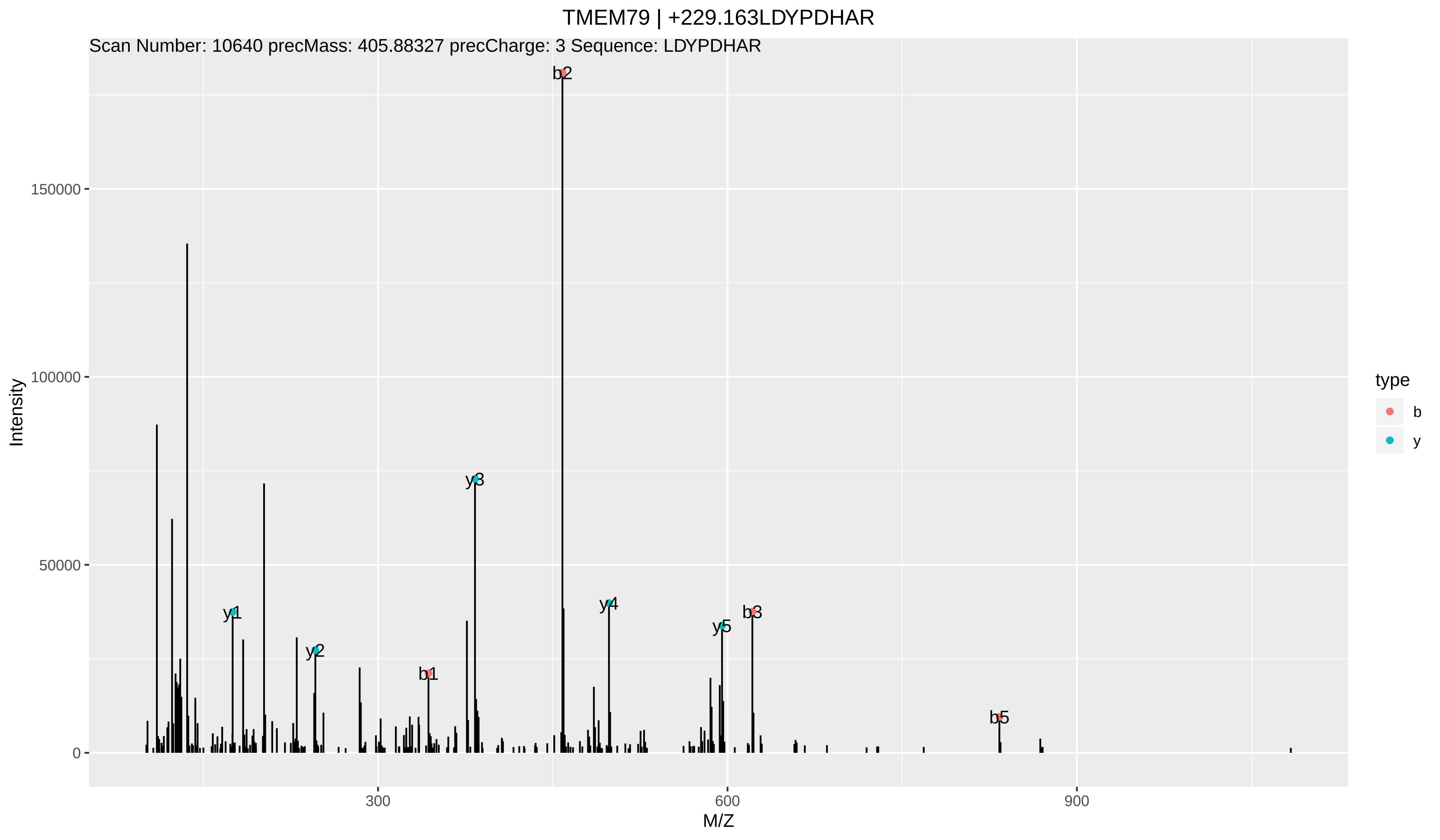
TMEM79 | +229.163LDYPDHAR
Scan Number: 10640 precMass: 405.88327 precCharge: 3 Sequence: LDYPDHAR
b2
y3
y1
y2
b1
y4
y5
b3
b5
Intensity
150000
100000
50000
0
300
600
900
M/Z
type
b
y

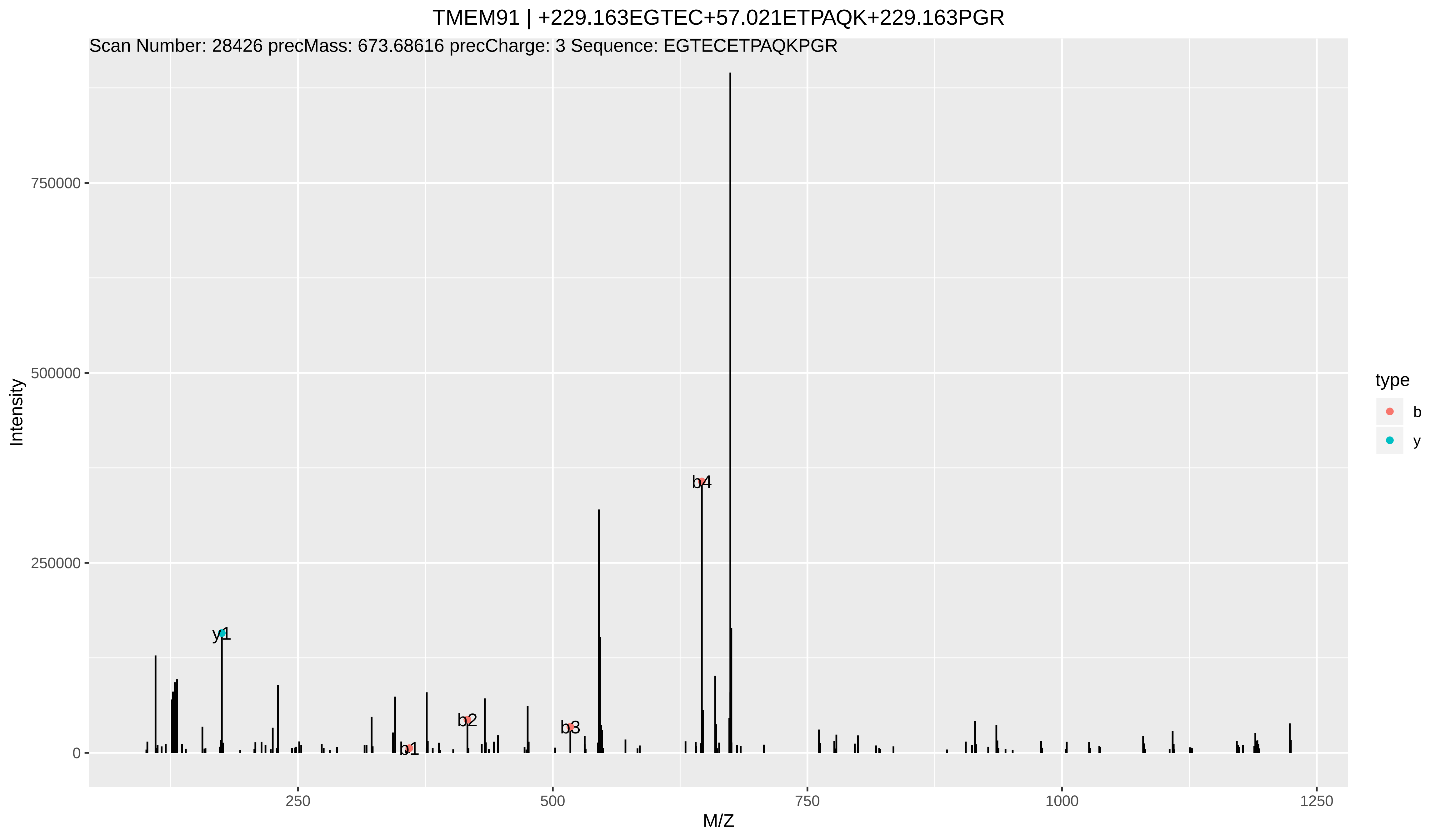

TMEM91 | +229.163EGTEC+57.021ETPAQK+229.163PGR
Scan Number: 28426 precMass: 673.68616 precCharge: 3 Sequence: EGTECETPAQKPGR
Intensity
750000
500000
250000
0
M/Z
250
500
750
1000
1250
y1
b1
b2
b3
b4
type
b
y

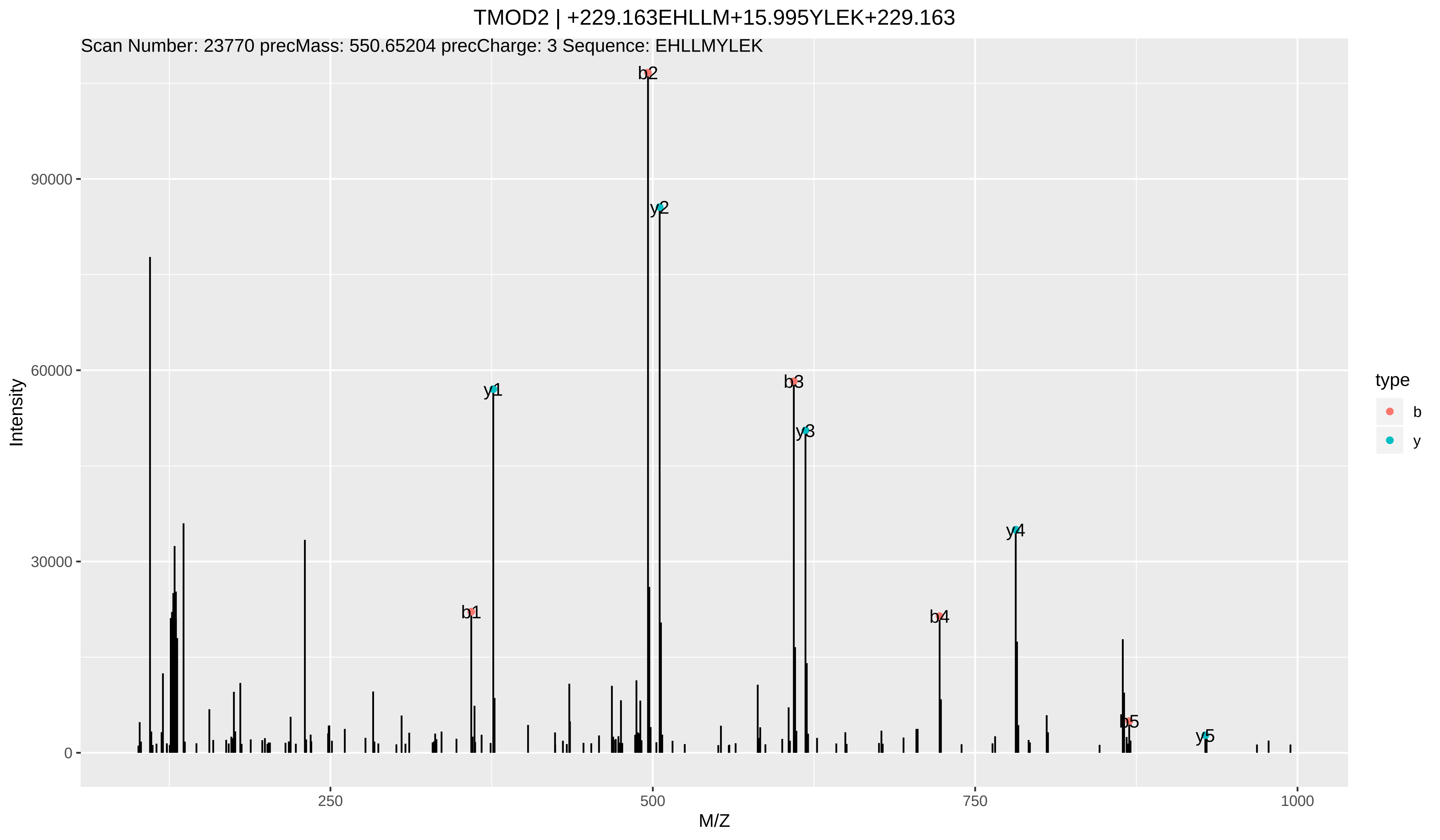
TMOD2 | +229.163EHLLM+15.995YLEK+229.163
Scan Number: 23770 precMass: 550.65204 precCharge: 3 Sequence: EHLLMYLEK
Intensity
M/Z
type
b
y
b1
y1
b2
y2
b3
y3
b4
y4
b5
y5
0
30000
60000
90000
250
500
750
1000

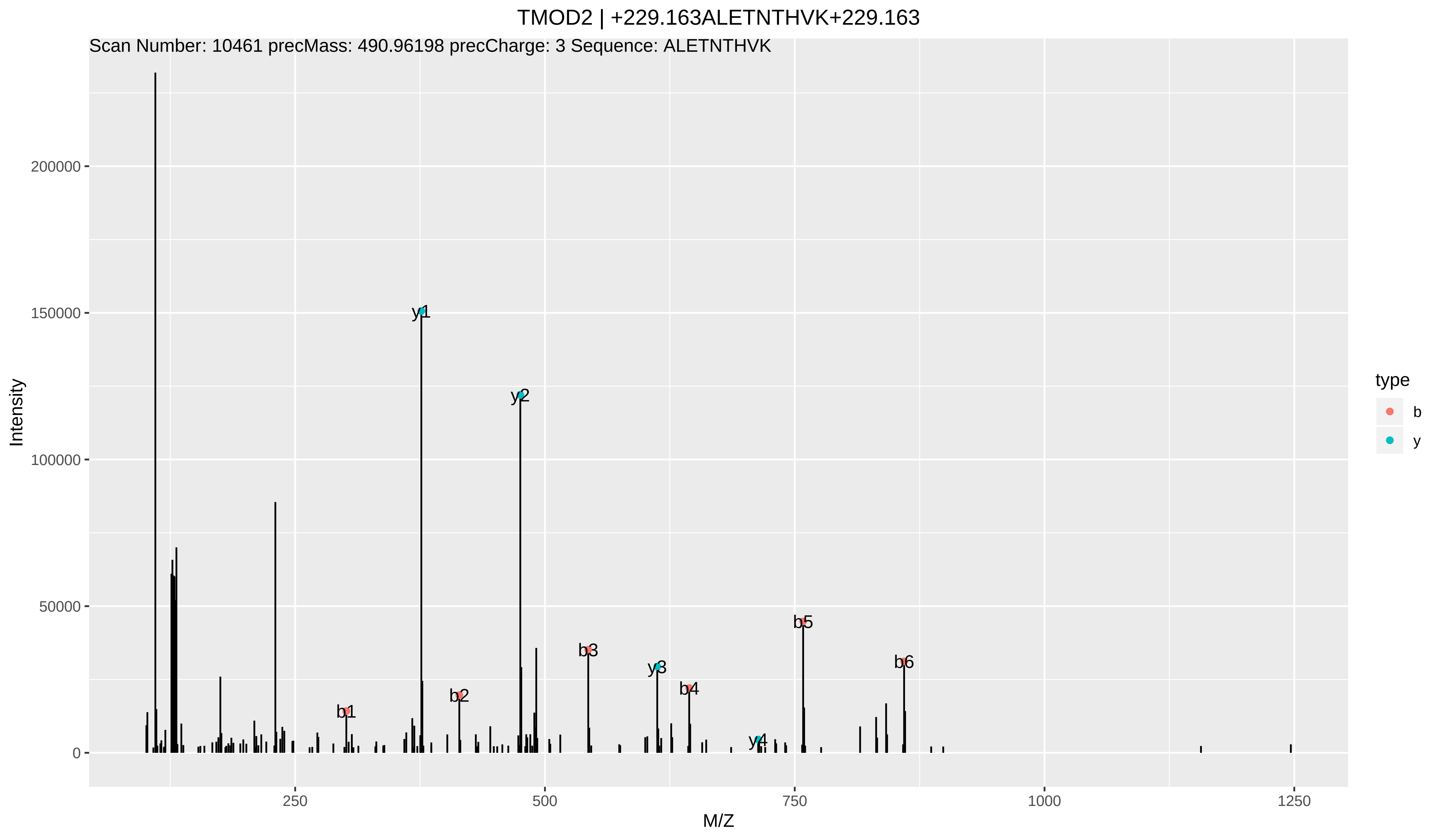
TMOD2 | +229.163ALETNTHVK+229.163
Scan Number: 10461 precMass: 490.96198 precCharge: 3 Sequence: ALETNTHVK
Intensity
M/Z
0
50000
100000
150000
200000
250
500
750
1000
1250
y1
y2
b3
y3
b5
b6
b4
b2
b1
y4
type
b
y

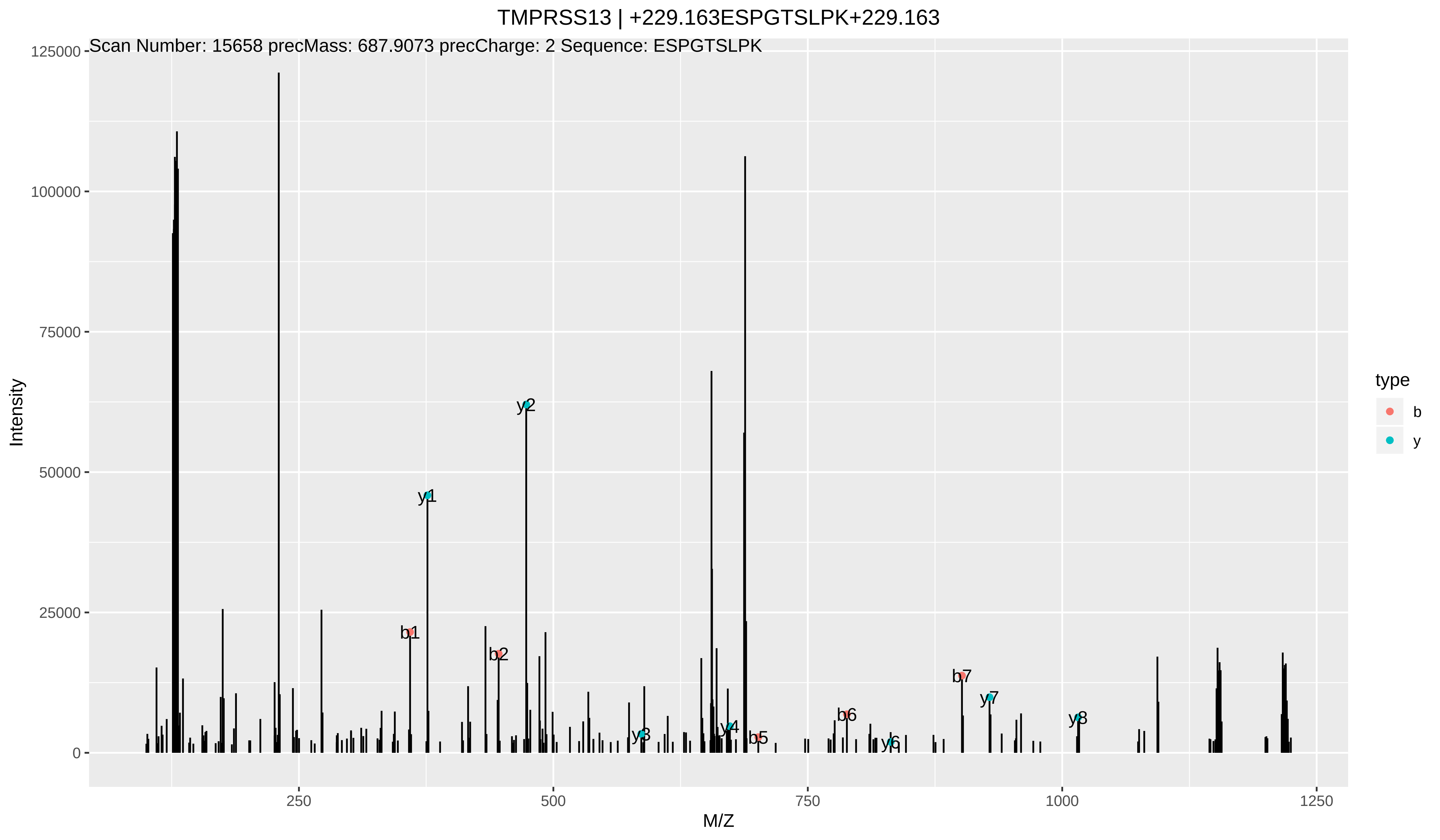

TMPRSS13 | +229.163ESPGTSLPK+229.163
Scan Number: 15658 precMass: 687.9073 precCharge: 2 Sequence: ESPGTSLPK
Intensity
M/Z
125000
100000
75000
50000
25000
0
250
500
750
1000
1250
y1
y2
b1
b2
y3
y4
b5
b6
y6
b7
y7
y8
type
b
y

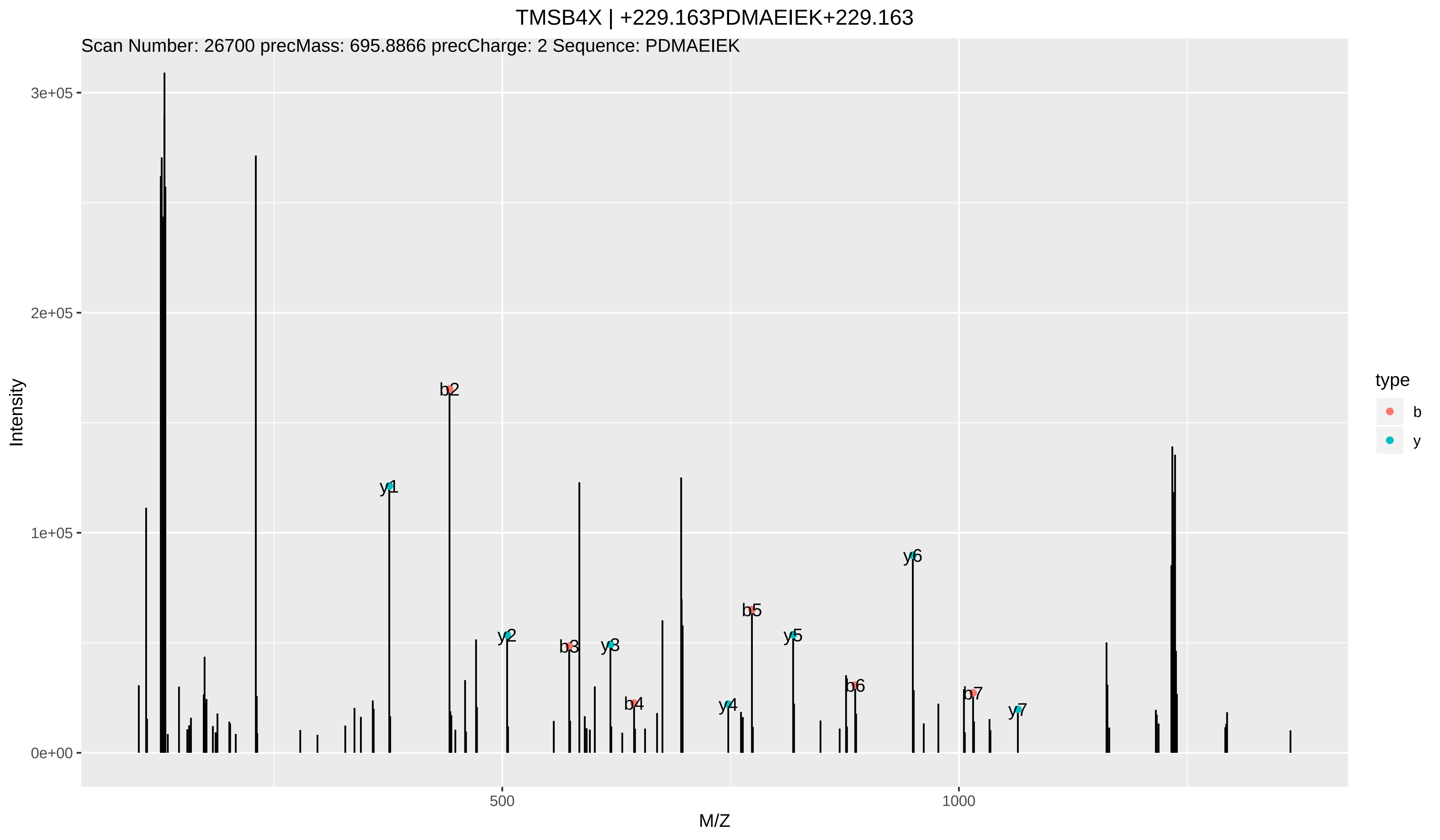
TMSB4X | +229.163PDMAEIEK+229.163
Scan Number: 26700 precMass: 695.8866 precCharge: 2 Sequence: PDMAEIEK
Intensity
3e+05
2e+05
1e+05
0e+00
M/Z
500
1000
b2
y1
y2
b3
y3
b4
y4
b5
y5
b6
y6
b7
y7
type
b
y

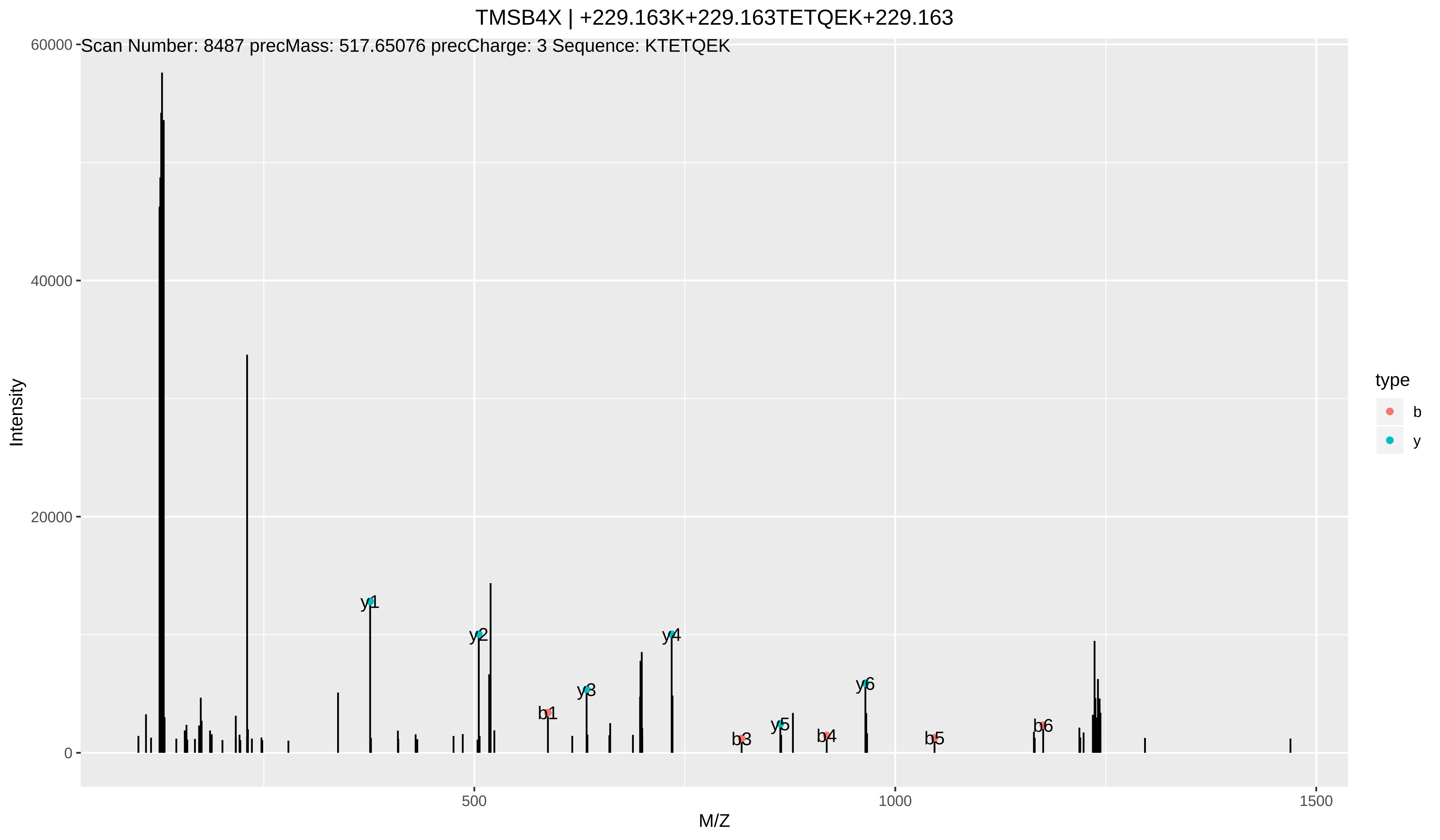

TMSB4X | +229.163K+229.163TETQEK+229.163
Scan Number: 8487 precMass: 517.65076 precCharge: 3 Sequence: KTETQEK
Intensity
M/Z
60000
40000
20000
0
500
1000
1500
y1
y2
b1
y3
y4
b3
y5
b4
y6
b5
b6
type
b
y

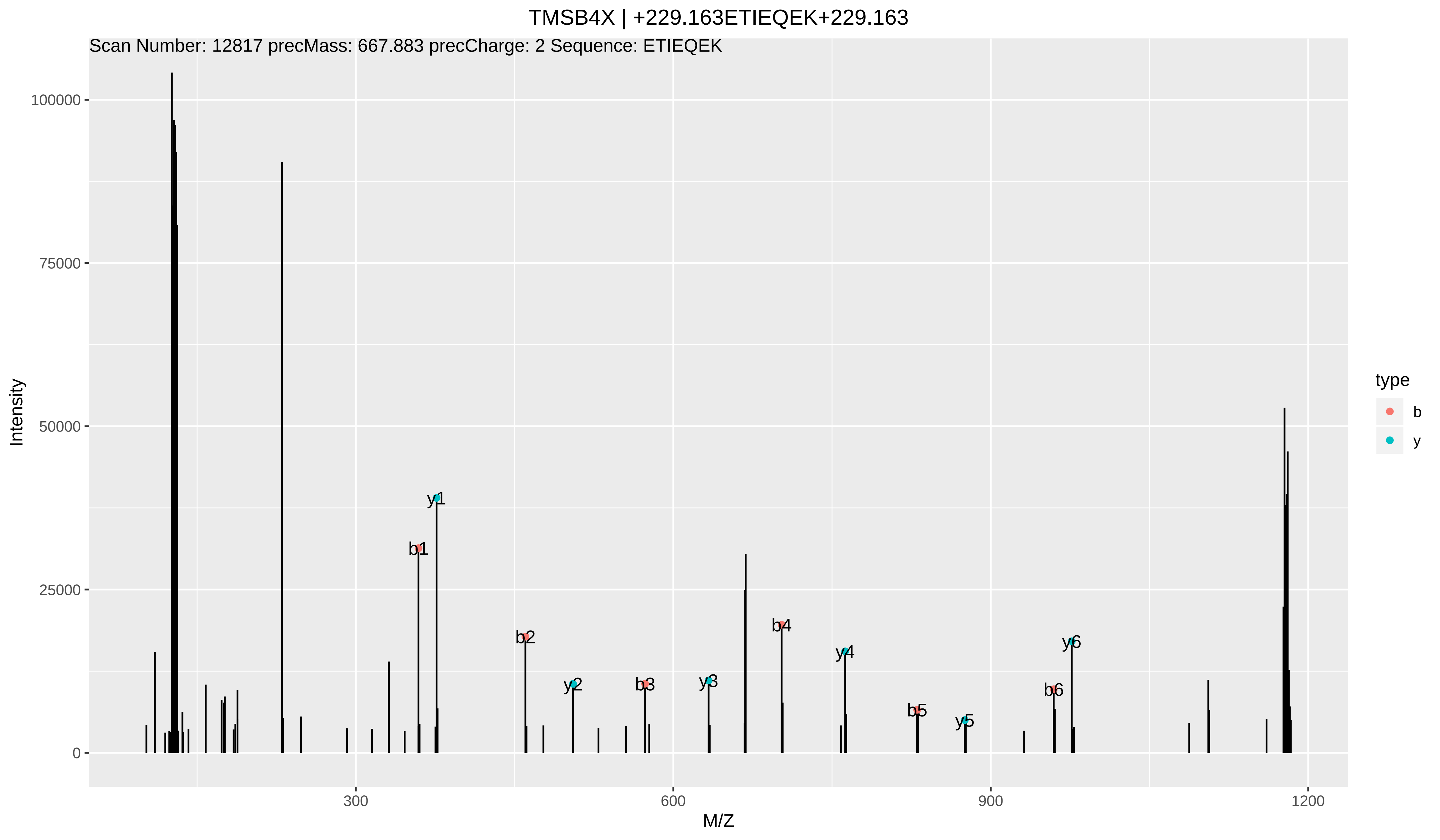
TMSB4X | +229.163ETIEQEK+229.163
Scan Number: 12817 precMass: 667.883 precCharge: 2 Sequence: ETIEQEK
Intensity
100000
75000
50000
25000
0
M/Z
300
600
900
1200
y1
b1
b2
y2
b3
y3
b4
y4
b5
y5
b6
y6
type
b
y

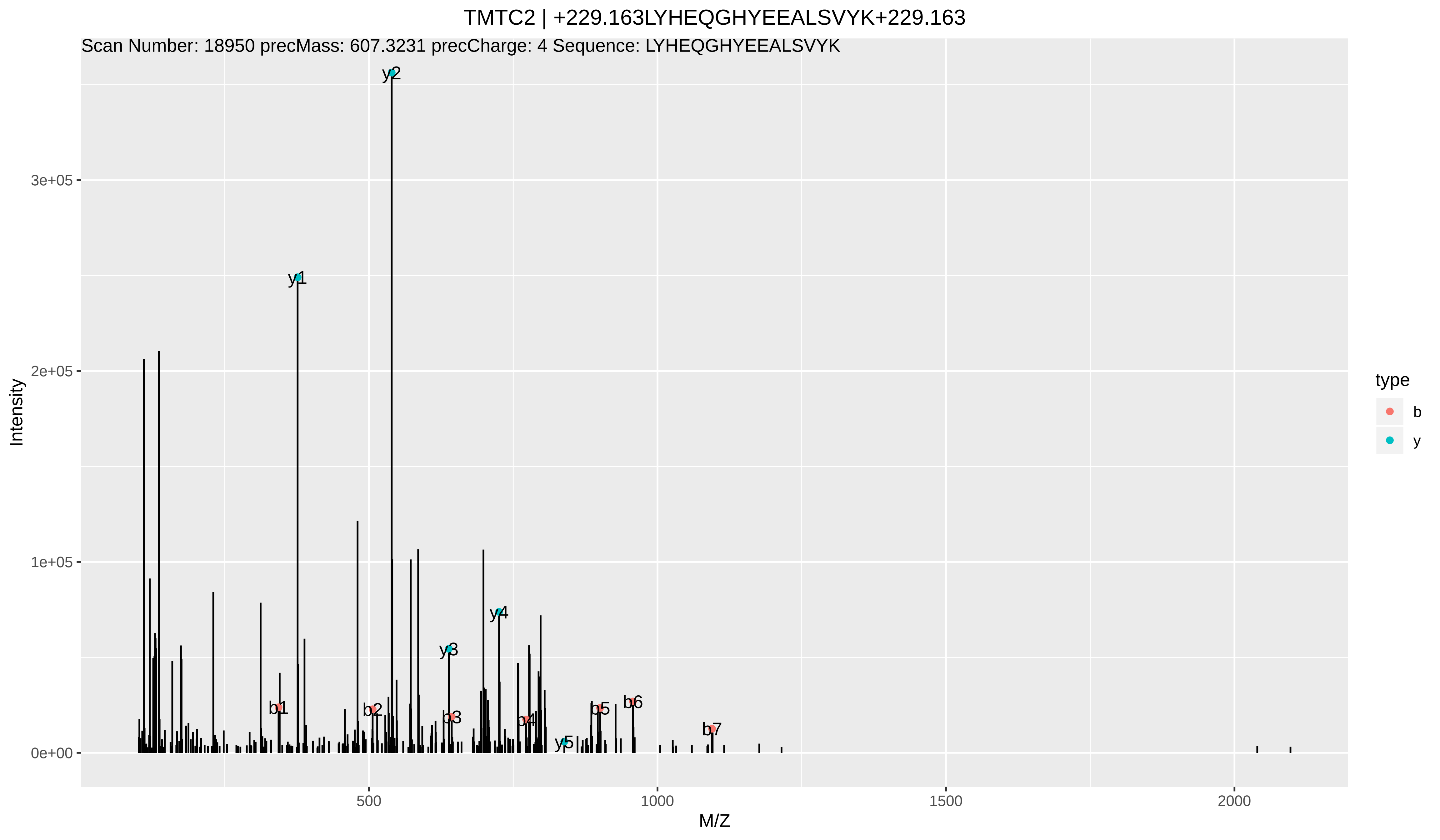

TMTC2 | +229.163LYHEQGHYEEALSVYK+229.163
Scan Number: 18950 precMass: 607.3231 precCharge: 4 Sequence: LYHEQGHYEEALSVYK
y2
y1
y4
y3
y5
b1
b2
b3
b4
b5
b6
b7
Intensity
3e+05
2e+05
1e+05
0e+00
M/Z
500
1000
1500
2000
type
b
y

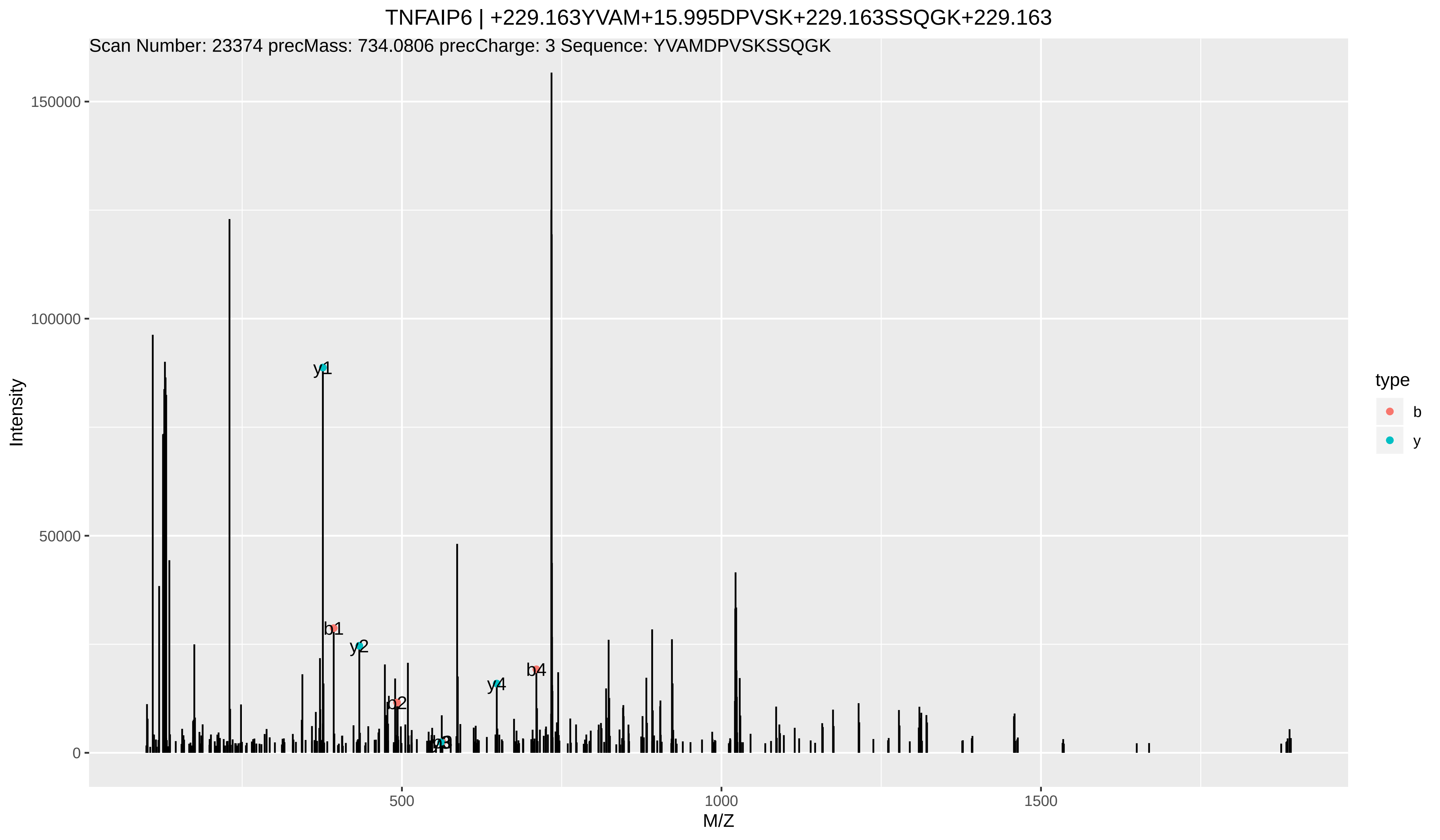

TNFAIP6 | +229.163YVAM+15.995DPVSK+229.163SSQGK+229.163
Scan Number: 23374 precMass: 734.0806 precCharge: 3 Sequence: YVAMDPVSKSSQGK
Intensity
150000
100000
50000
0
M/Z
500
1000
1500
y1
b1
y2
b4
y4
b2
type
b
y

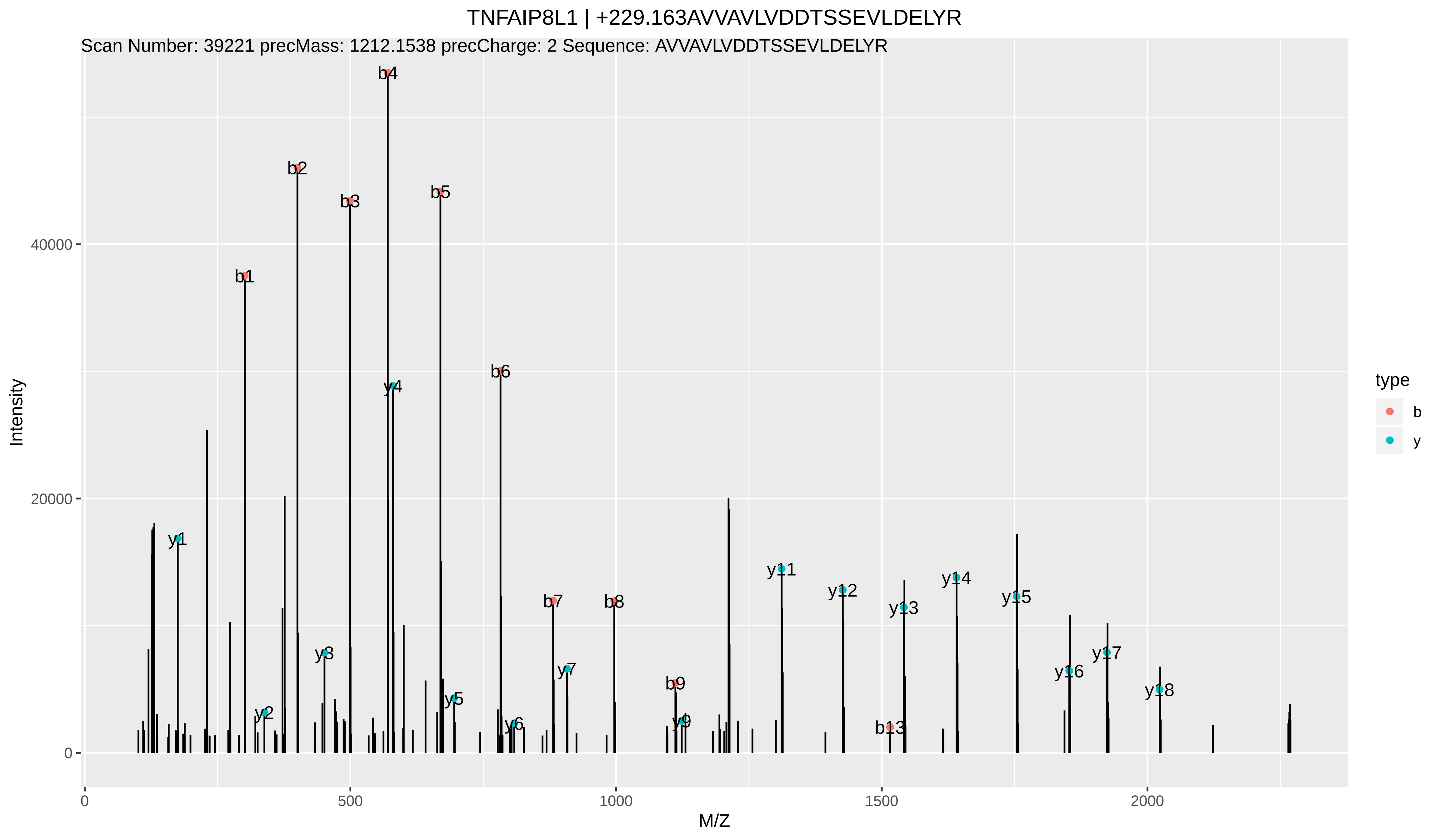
TNFAIP8L1 | +229.163AVVAVLVDDTSSEVLDELYR
Scan Number: 39221 precMass: 1212.1538 precCharge: 2 Sequence: AVVAVLVDDTSSEVLDELYR
Intensity
M/Z
0
20000
40000
500
1000
1500
2000
b1
b2
b3
b4
b5
b6
b7
b8
b9
b13
y1
y2
y3
y4
y5
y6
y7
y9
y11
y12
y13
y14
y15
y16
y17
y18
type
b
y

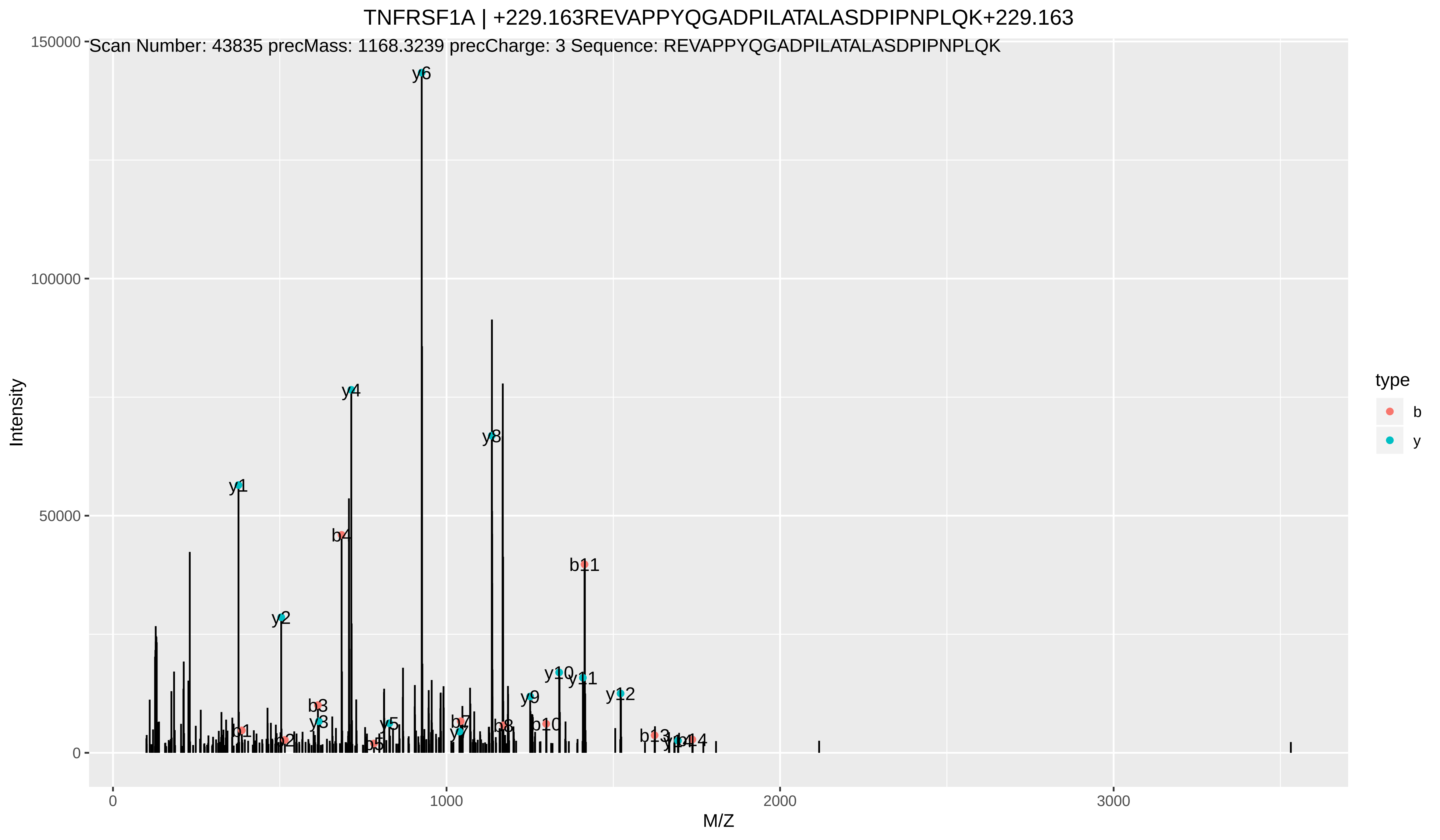
TNFRSF1A | +229.163REVAPPYQGADPILATALASDPIPNPLQK+229.163
Scan Number: 43835 precMass: 1168.3239 precCharge: 3 Sequence: REVAPPYQGADPILATALASDPIPNPLQK
Intensity
150000
100000
50000
0
M/Z
0
1000
2000
3000
type
b
y
y6
y4
y8
y1
b4
b11
y2
y10
y11
y12
y9
b3
y3
y5
b7
y7
b8
b10
b13

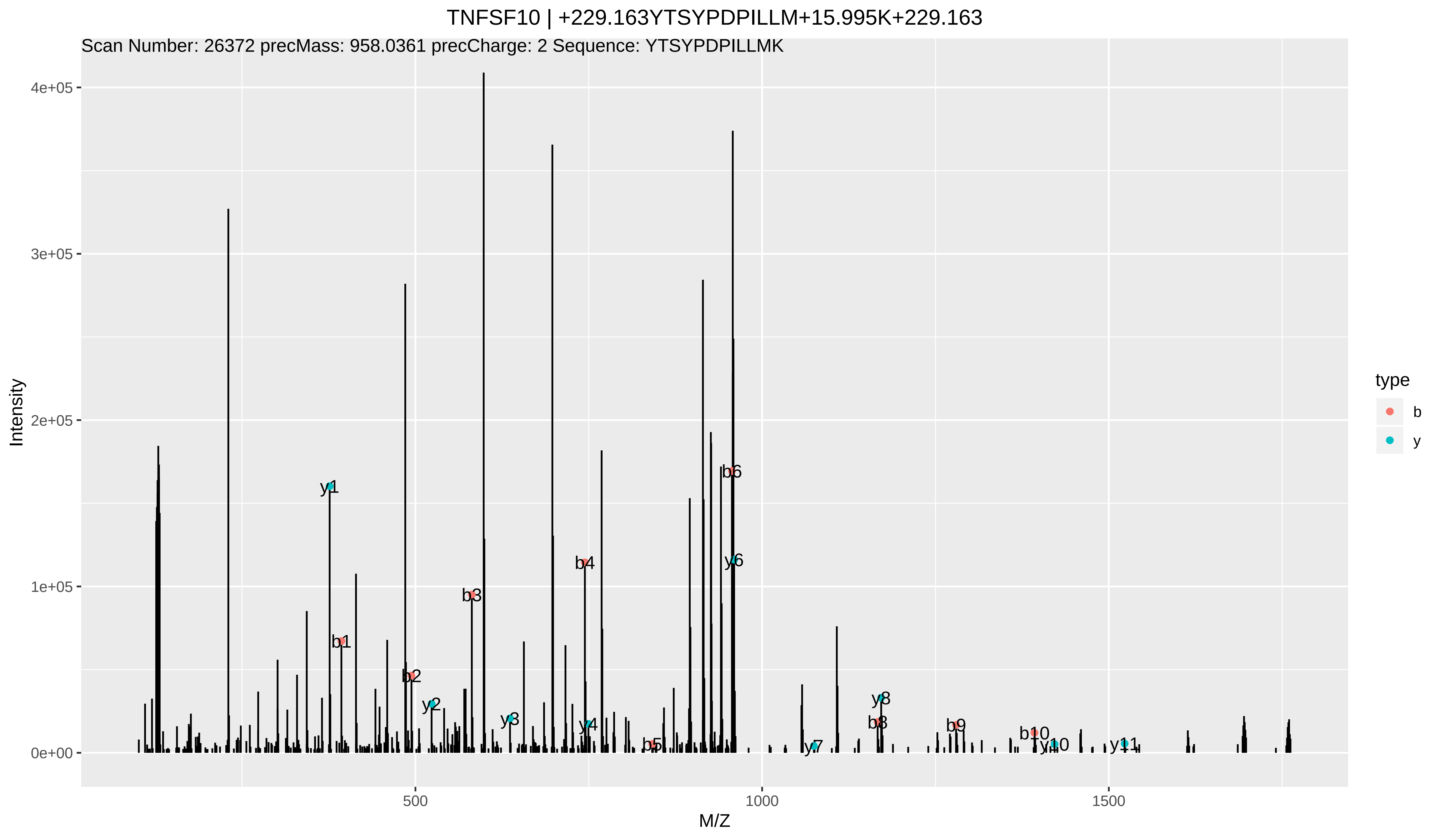
TNFSF10 | +229.163YTSYPDPILLM+15.995K+229.163
Scan Number: 26372 precMass: 958.0361 precCharge: 2 Sequence: YTSYPDPILLMK
Intensity
4e+05
3e+05
2e+05
1e+05
0e+00
M/Z
500
1000
1500
type
b
y
y1
b1
b2
y2
b3
y3
b4
y4
b5
b6
y6
y7
y8
b8
b9
b10
y10
y11

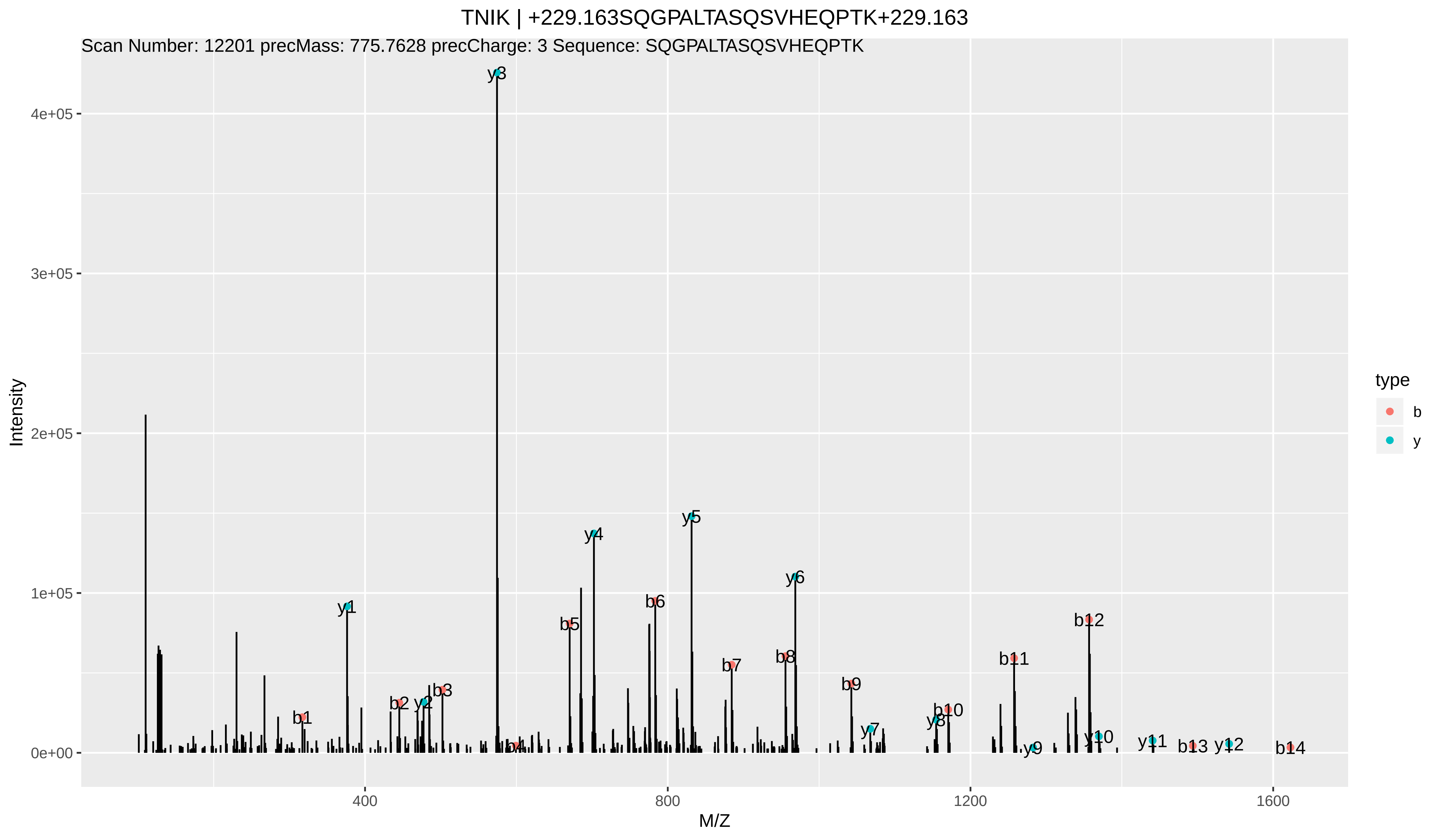

TNIK | +229.163SQGPALTASQSVHEQPTK+229.163
Scan Number: 12201 precMass: 775.7628 precCharge: 3 Sequence: SQGPALTASQSVHEQPTK
Intensity
4e+05
3e+05
2e+05
1e+05
0e+00
M/Z
400
800
1200
1600
y3
y4
y5
y6
y1
b5
b6
b7
b8
b9
b11
b12
b1
b2
y2
b3
b4
y7
y8
b10
y9
y10
y11
b13
y12
b14
type
b
y

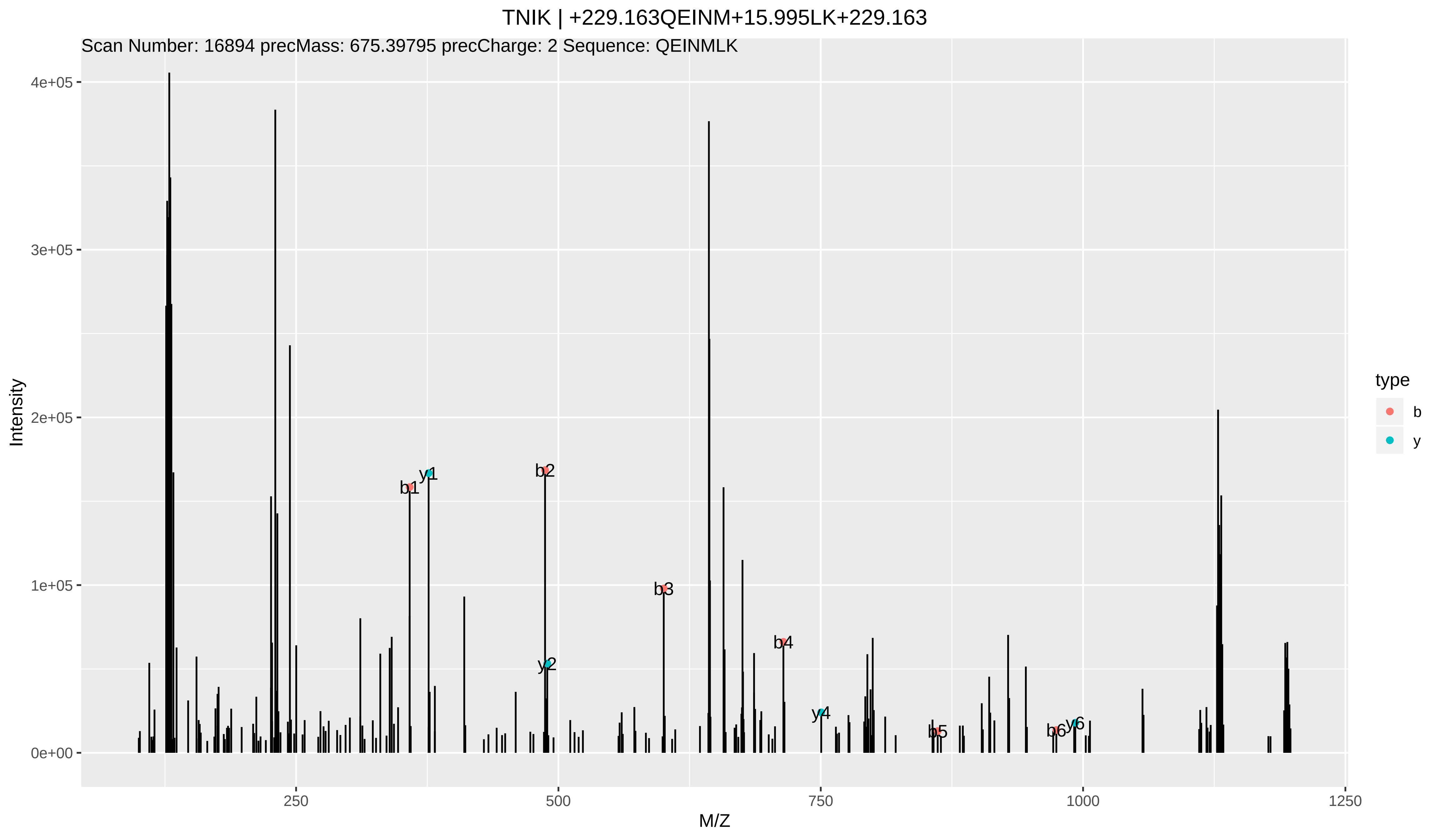
TNIK | +229.163QEINM+15.995LK+229.163
Scan Number: 16894 precMass: 675.39795 precCharge: 2 Sequence: QEINMLK
Intensity
4e+05
3e+05
2e+05
1e+05
0e+00
M/Z
250
500
750
1000
1250
b1
y1
b2
y2
b3
b4
y4
b5
b6
y6
type
b
y

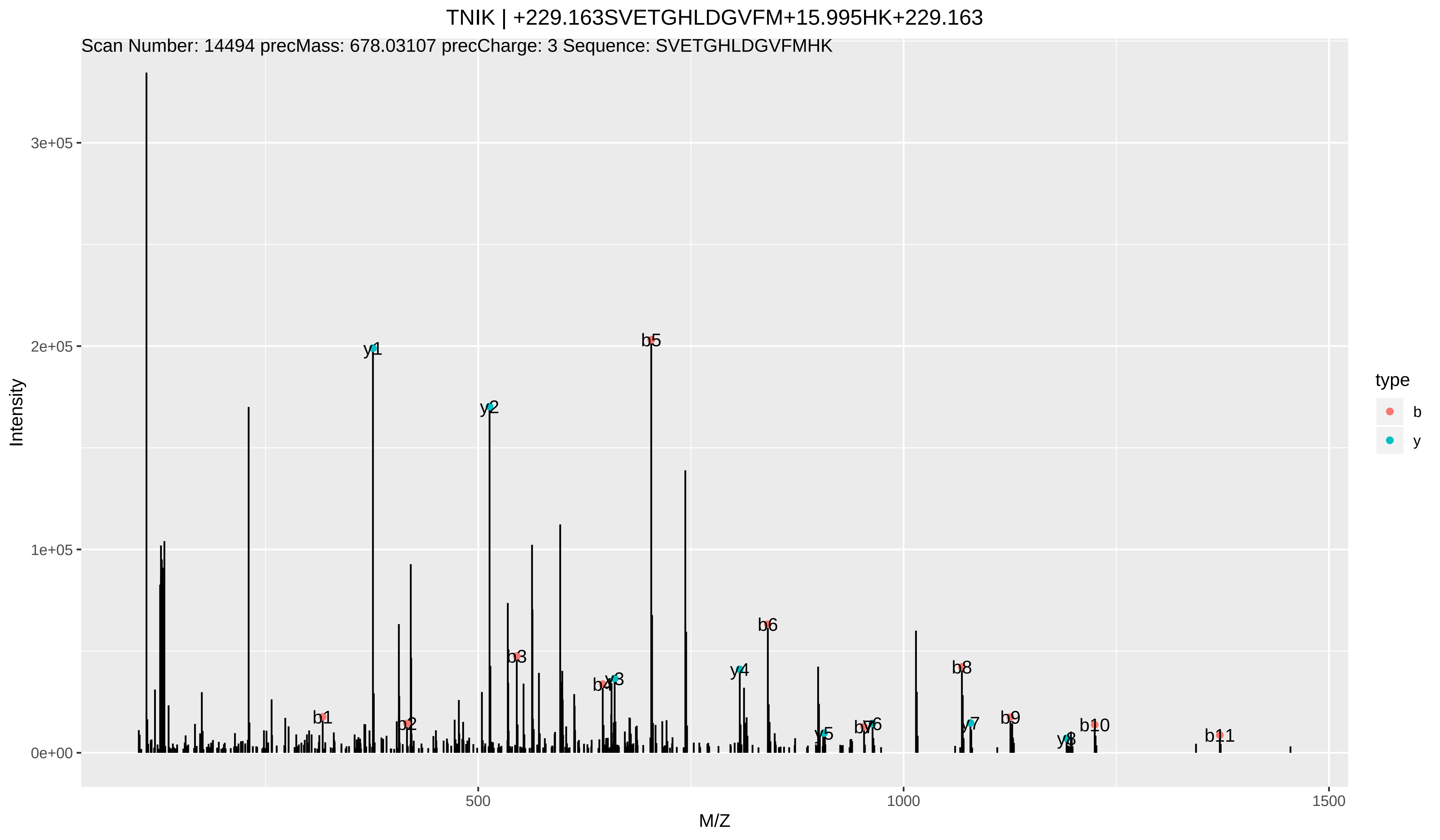

TNIK | +229.163SVETGHLDGVFM+15.995HK+229.163
Scan Number: 14494 precMass: 678.03107 precCharge: 3 Sequence: SVETGHLDGVFMHK
Intensity
M/Z
type
b
y
3e+05
2e+05
1e+05
0e+00
500
1000
1500
y1
y2
b5
b3
b4
y3
y4
b6
b8
b1
b2
y5
b7
y6
y7
b9
y8
b10
b11

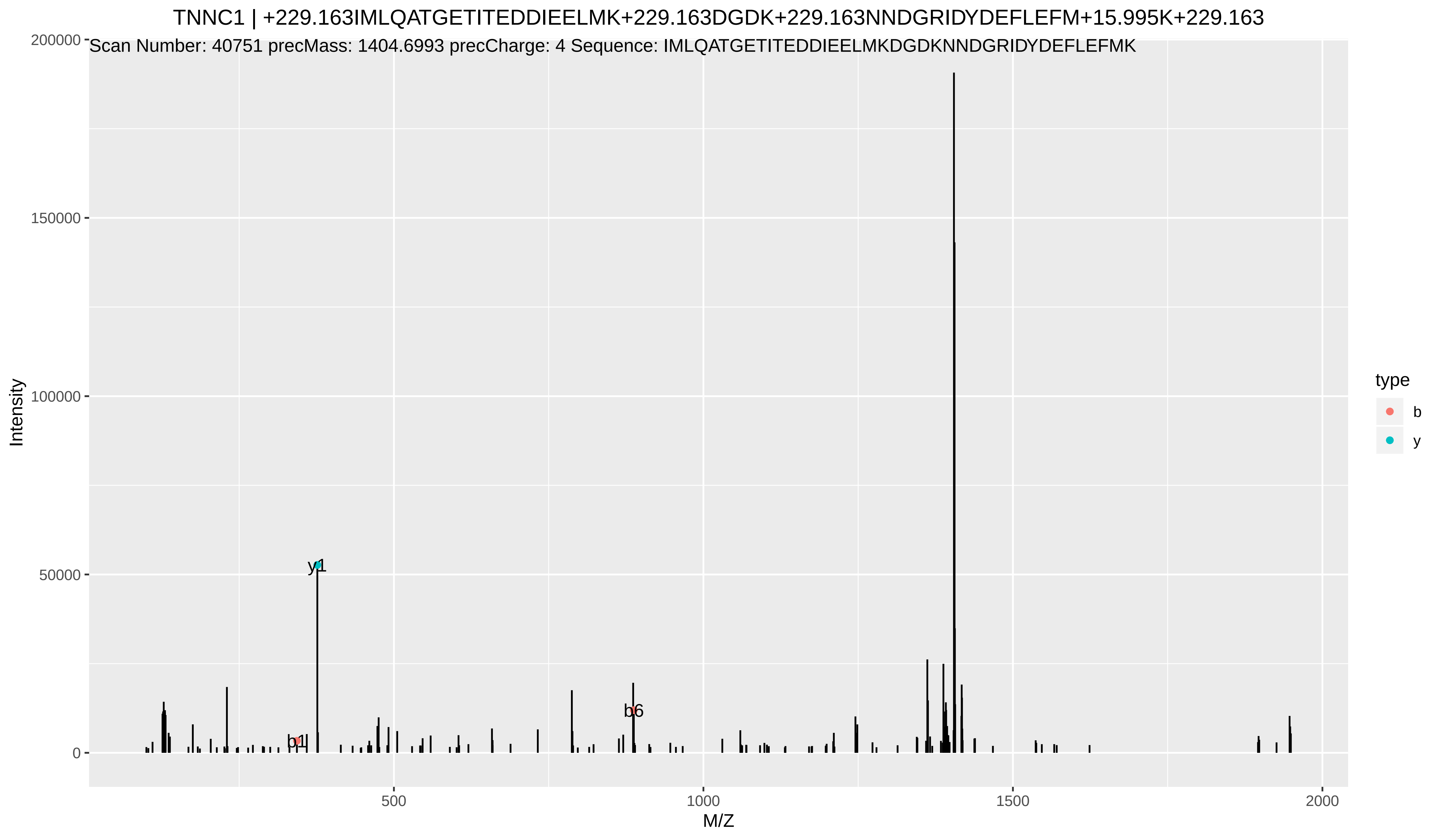
TNNC1 | +229.163IMLQATGETITEDDIEELMK+229.163DGDK+229.163NNDGRIDYDEFLEFM+15.995K+229.163
Scan Number: 40751 precMass: 1404.6993 precCharge: 4 Sequence: IMLQATGETITEDDIEELMKDGDKNNDGRIDYDEFLEFMK
Intensity
200000
150000
100000
50000
0
M/Z
500
1000
1500
2000
y1
b1
b6
type
b
y

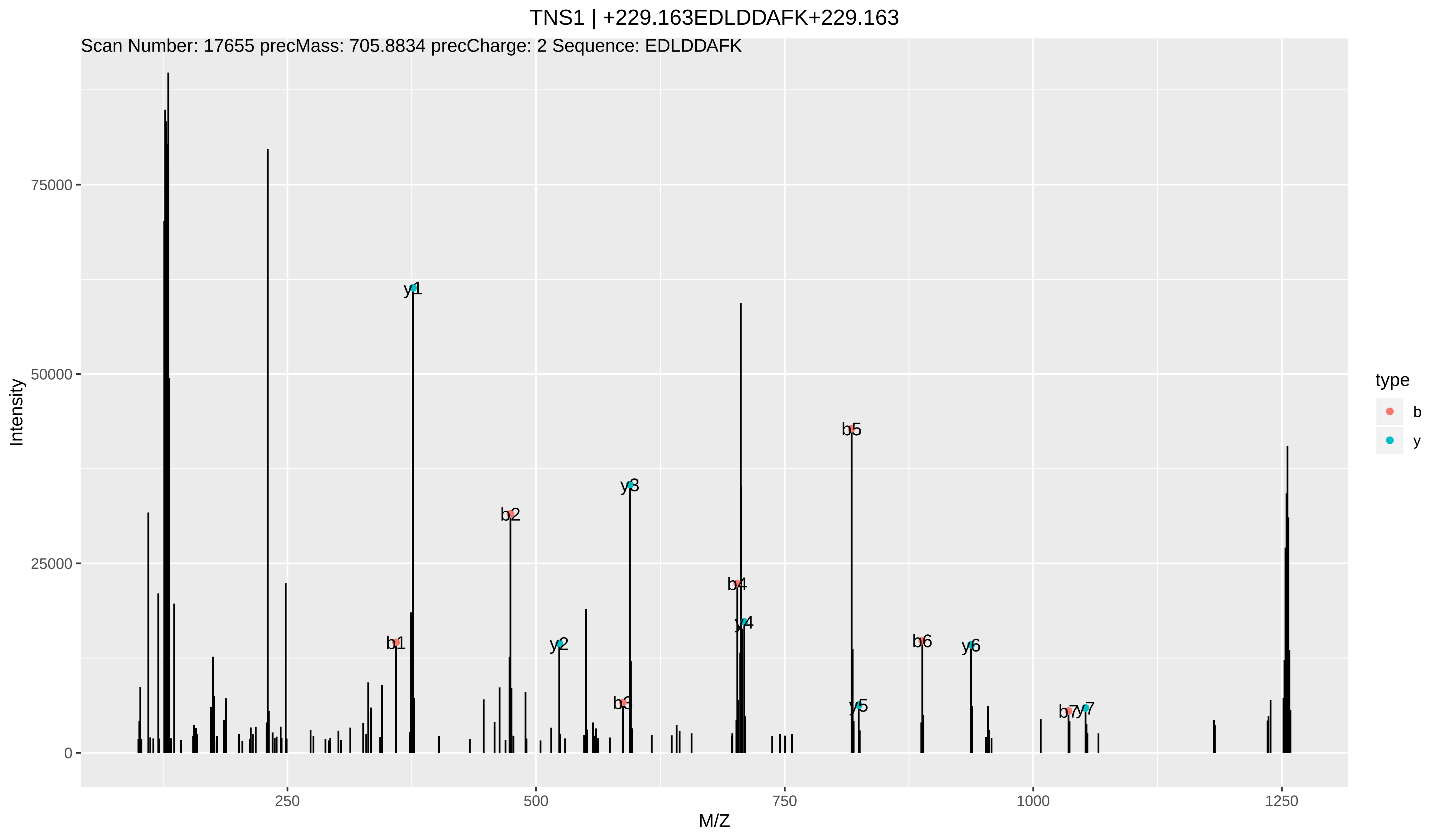

TNS1 | +229.163EDLDDAFK+229.163
Scan Number: 17655 precMass: 705.8834 precCharge: 2 Sequence: EDLDDAFK
Intensity
M/Z
0
25000
50000
75000
250
500
750
1000
1250
y1
b1
b2
y2
b3
y3
b4
y4
b5
y5
b6
y6
b7
y7
type
b
y

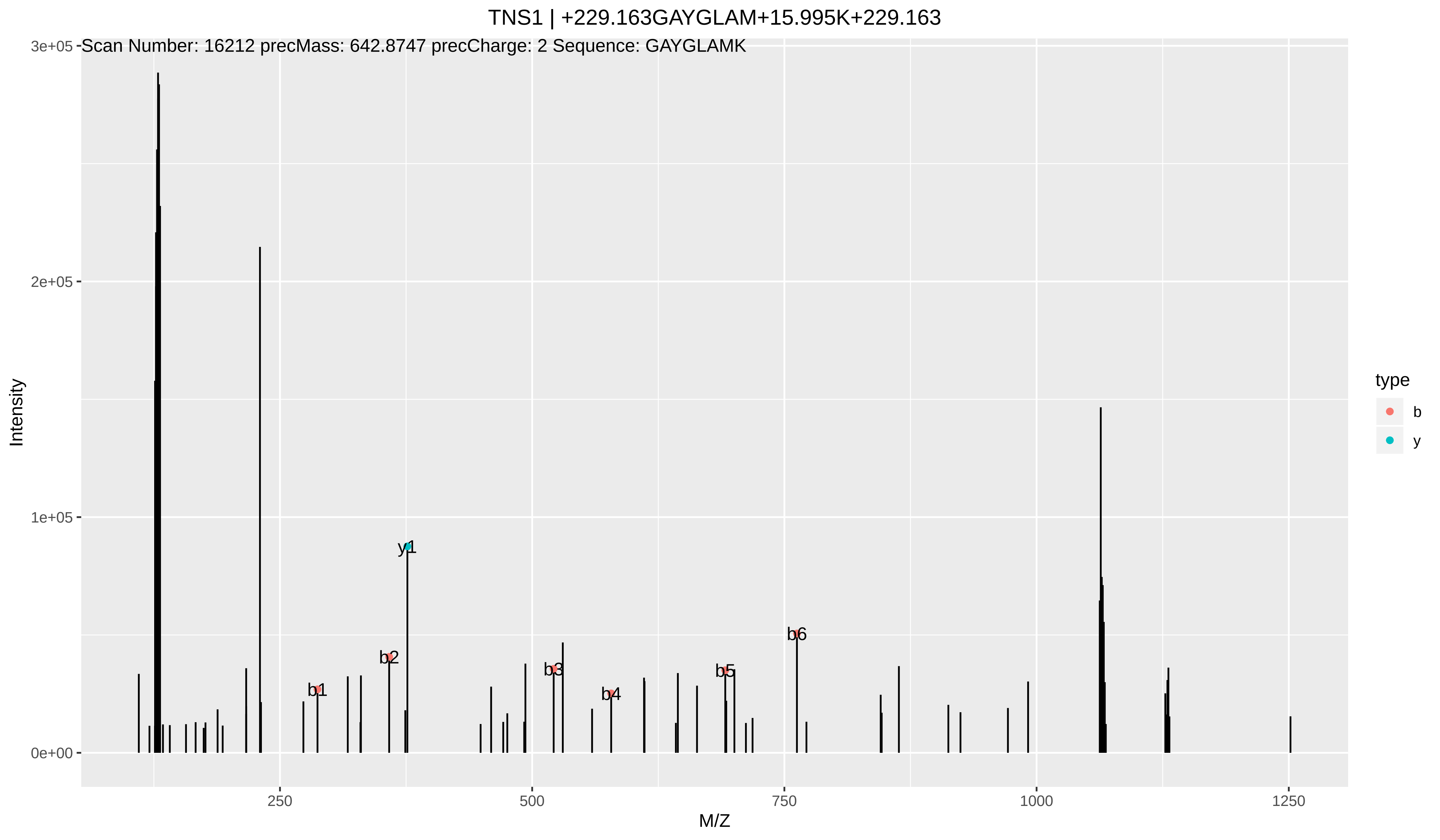

TNS1 | +229.163GAYGLAM+15.995K+229.163
Scan Number: 16212 precMass: 642.8747 precCharge: 2 Sequence: GAYGLAMK
Intensity
M/Z
3e+05
2e+05
1e+05
0e+00
250
500
750
1000
1250
y1
b1
b2
b3
b4
b5
b6
type
b
y

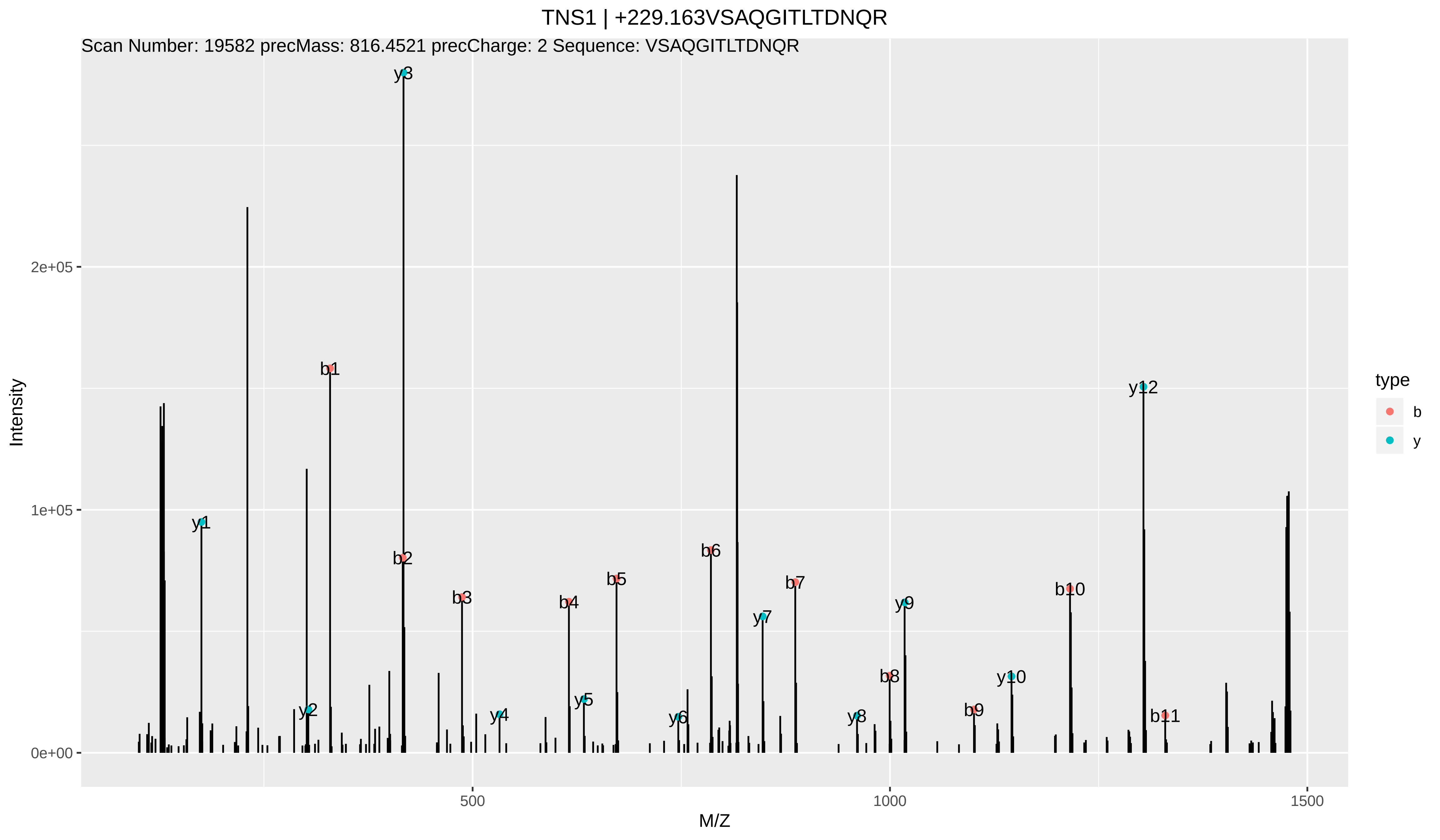

TNS1 | +229.163VSAQGITLTDNQR
Scan Number: 19582 precMass: 816.4521 precCharge: 2 Sequence: VSAQGITLTDNQR
Intensity
M/Z
0e+00
1e+05
2e+05
500
1000
1500
y3
b1
y12
y1
b2
b6
b5
b7
b10
b3
b4
y9
y7
b8
y10
y5
y2
b9
y4
b11
y6
y8
type
b
y

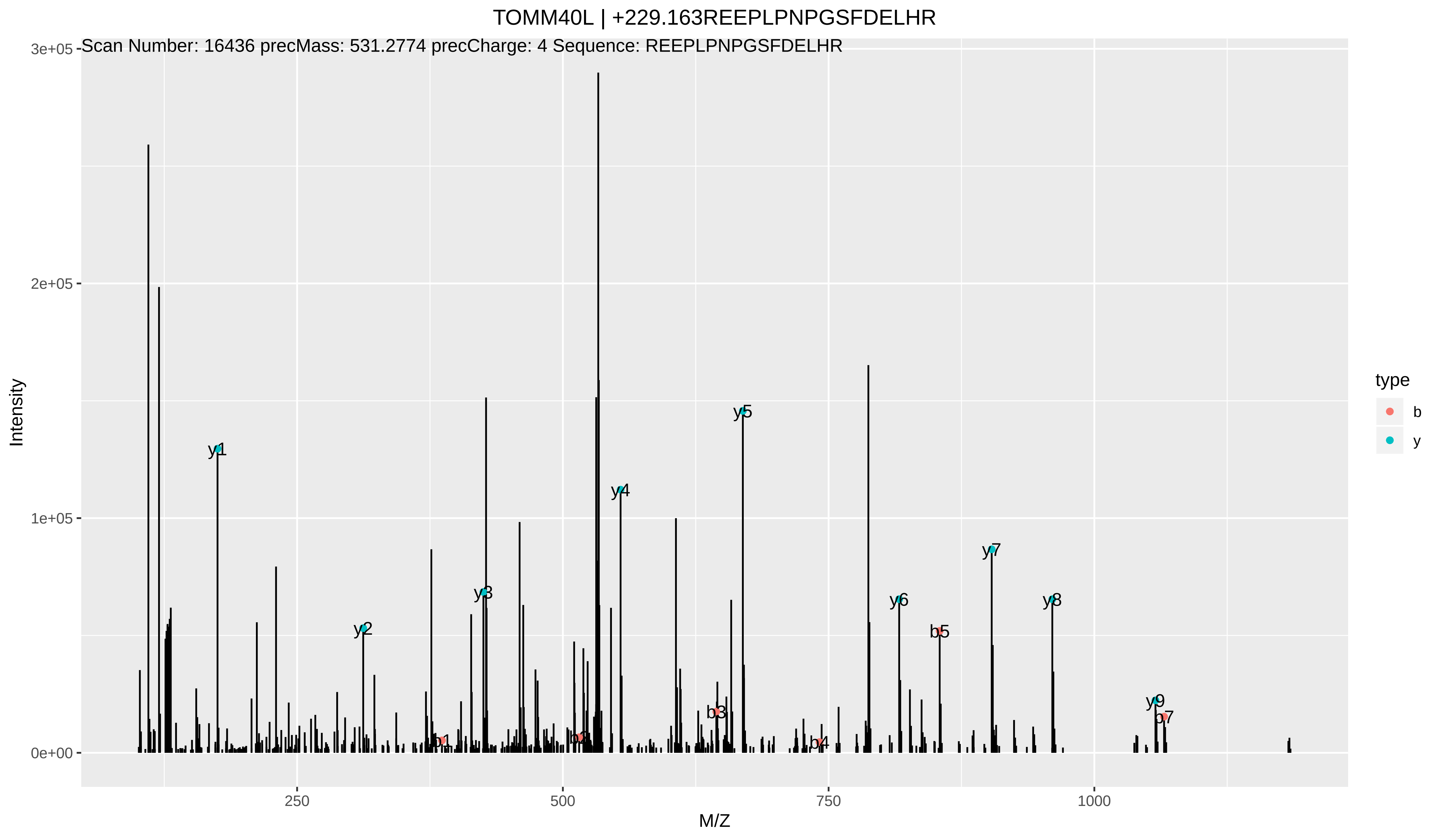

TOMM40L | +229.163REEPLPNPGSFDELHR
Scan Number: 16436 precMass: 531.2774 precCharge: 4 Sequence: REEPLPNPGSFDELHR
Intensity
M/Z
0e+00
1e+05
2e+05
3e+05
250
500
750
1000
y1
y2
y3
y4
y5
y6
y7
y8
y9
b1
b2
b3
b4
b5
b7
type
b
y

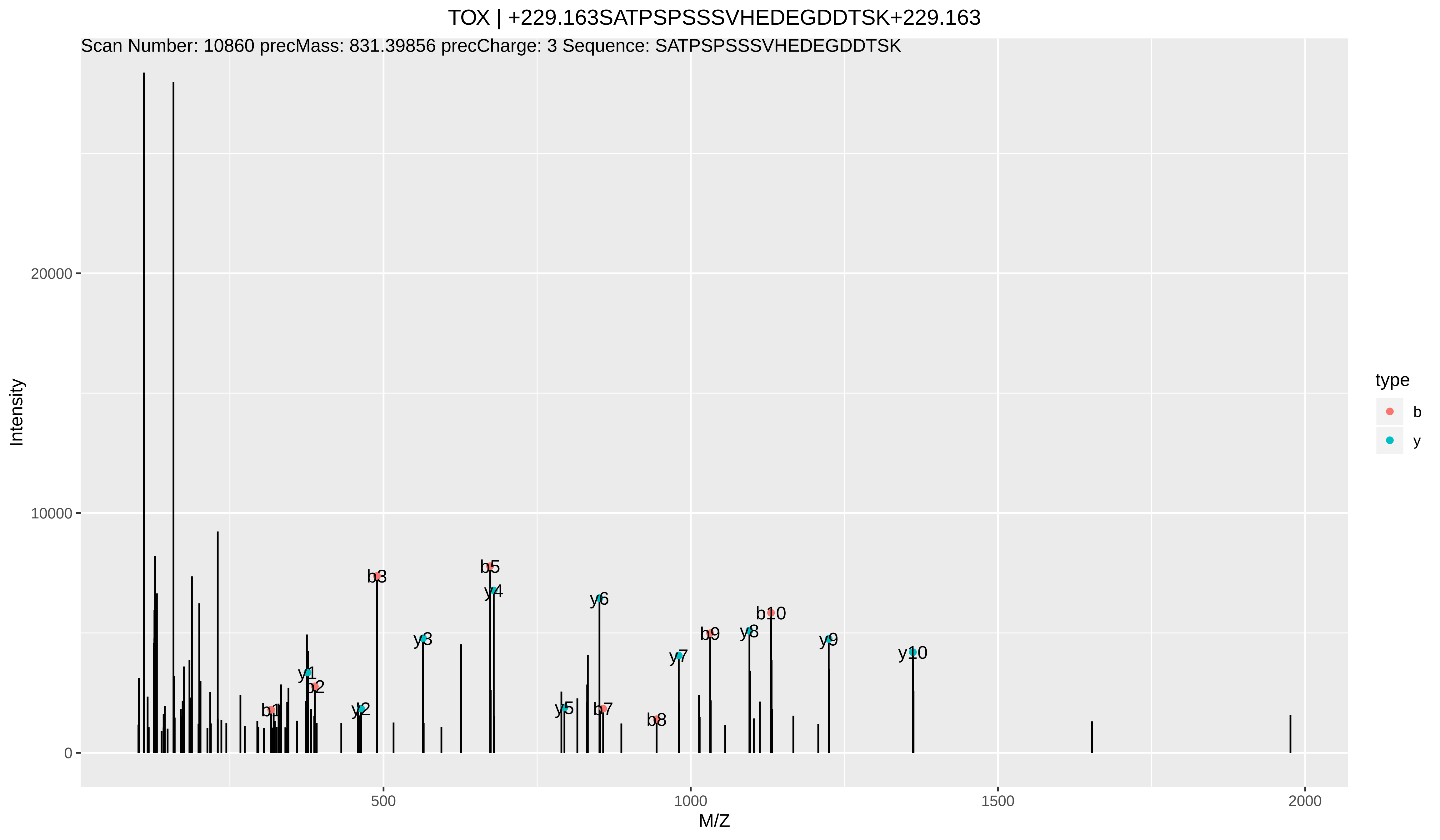

TOX | +229.163SATPSPSSSVHEDEGDDTSK+229.163
Scan Number: 10860 precMass: 831.39856 precCharge: 3 Sequence: SATPSPSSSVHEDEGDDTSK
Intensity
M/Z
20000
10000
0
500
1000
1500
2000
b1
b2
b3
b5
b7
b8
b9
b10
y1
y2
y3
y4
y5
y6
y7
y8
y9
y10
type
b
y

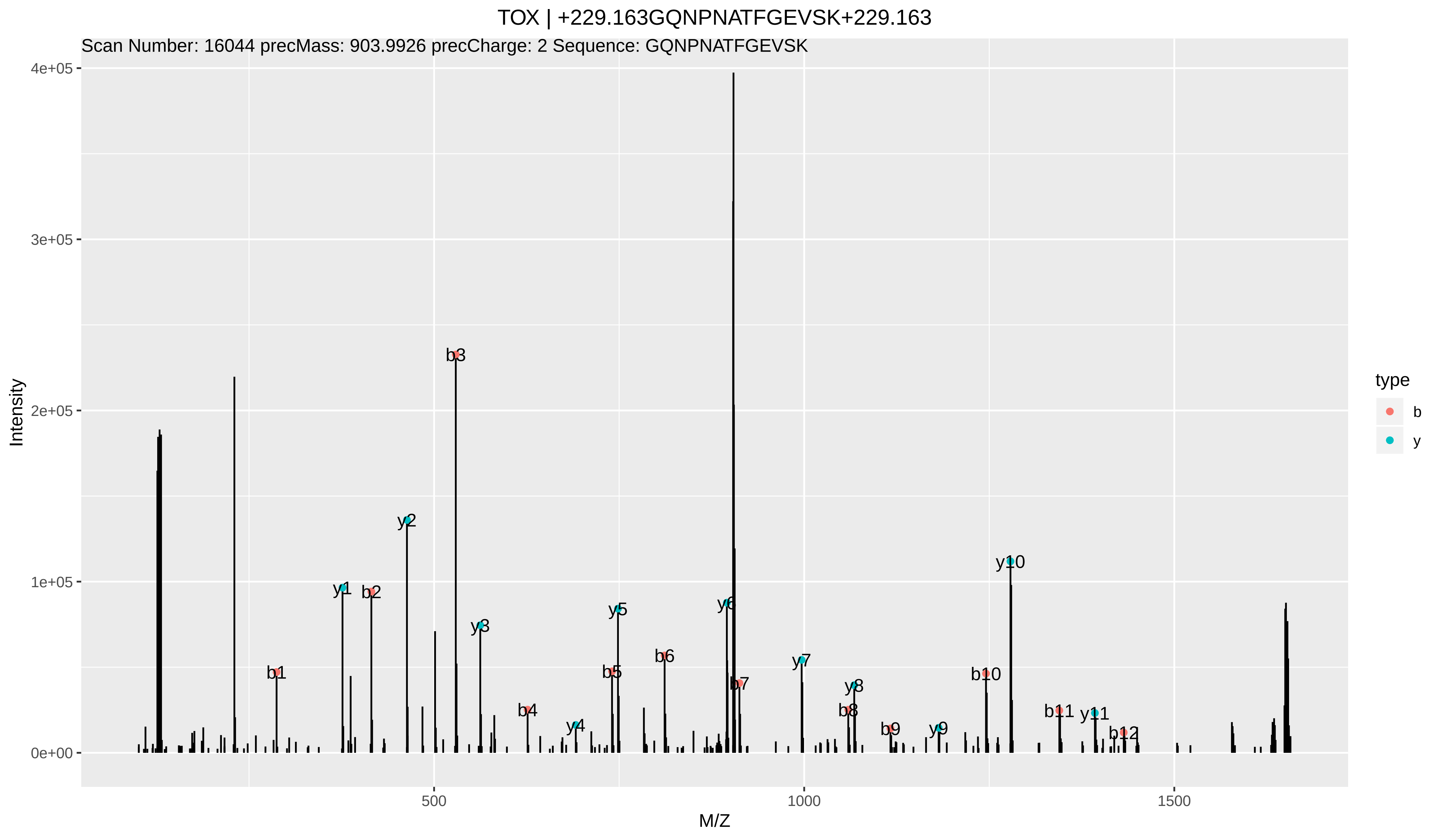

TOX | +229.163GQNPNATFGEVSK+229.163
Scan Number: 16044 precMass: 903.9926 precCharge: 2 Sequence: GQNPNATFGEVSK
Intensity
M/Z
4e+05
3e+05
2e+05
1e+05
0e+00
500
1000
1500
b1
y1
b2
y2
b3
y3
b4
y4
b5
y5
b6
y6
b7
y7
b8
y8
b9
y9
b10
y10
b11
y11
b12
type
b
y

TOX | +229.163DTQAAIK+229.163

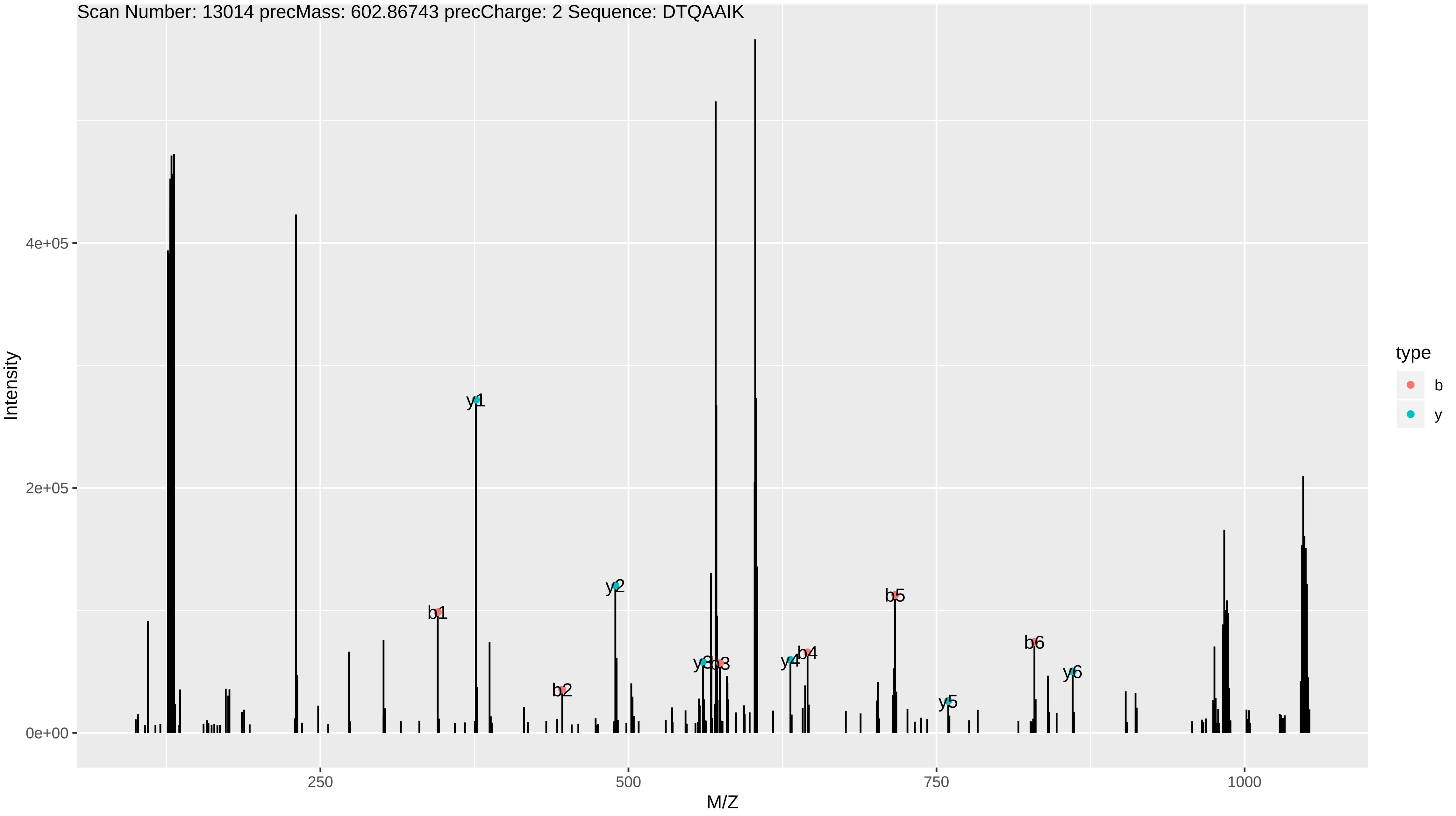

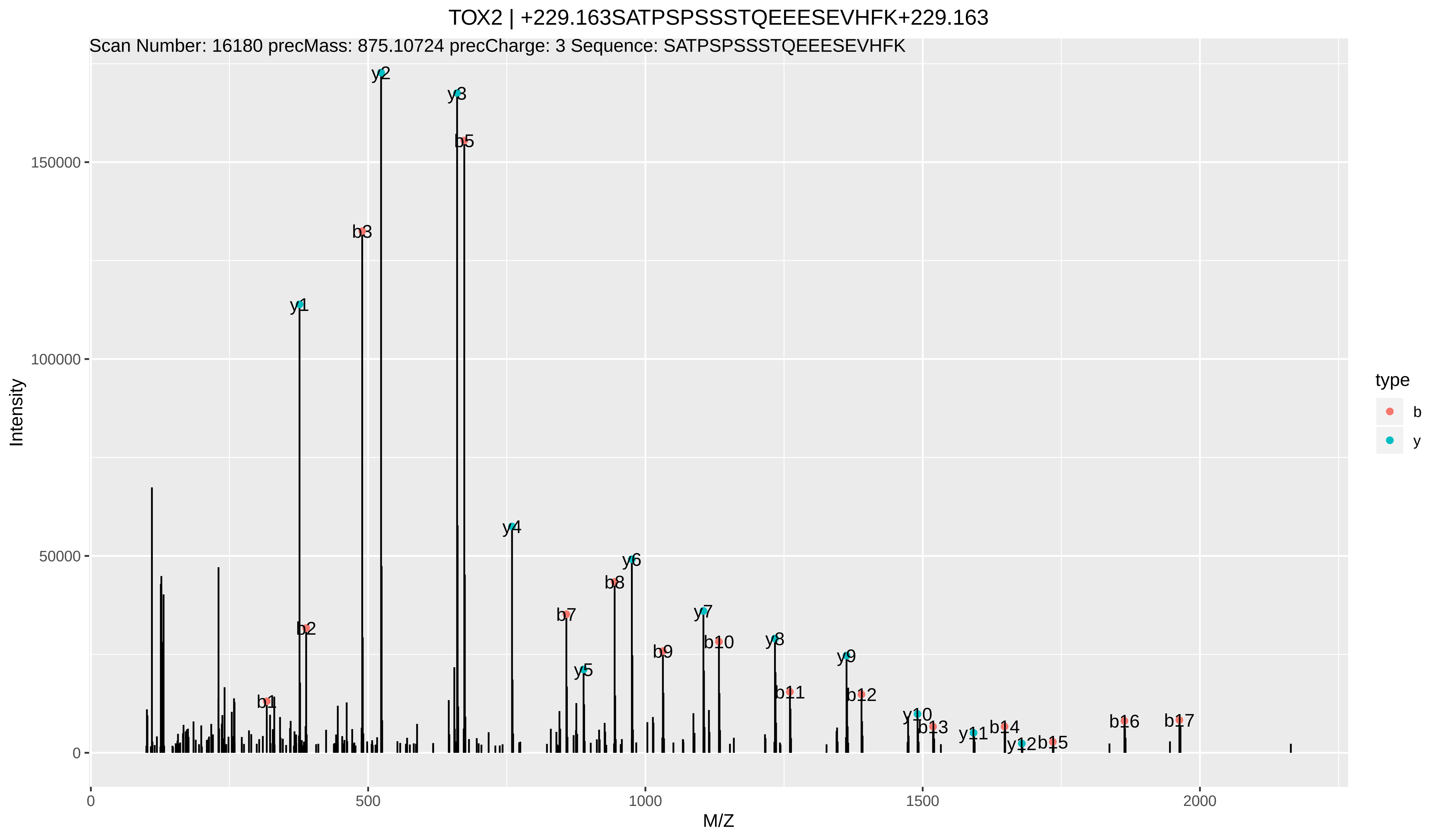
TOX2 | +229.163SATPSPSSSTQEEESEVHFK+229.163
Scan Number: 16180 precMass: 875.10724 precCharge: 3 Sequence: SATPSPSSSTQEEESEVHFK
Intensity
M/Z
type
b
y
y1
y2
y3
y4
y5
y6
y7
y8
y9
y10
y11
y12
b1
b2
b3
b5
b7
b8
b9
b10
b11
b12
b13
b14
b15
b16
b17
0
50000
100000
150000
500
1000
1500
2000

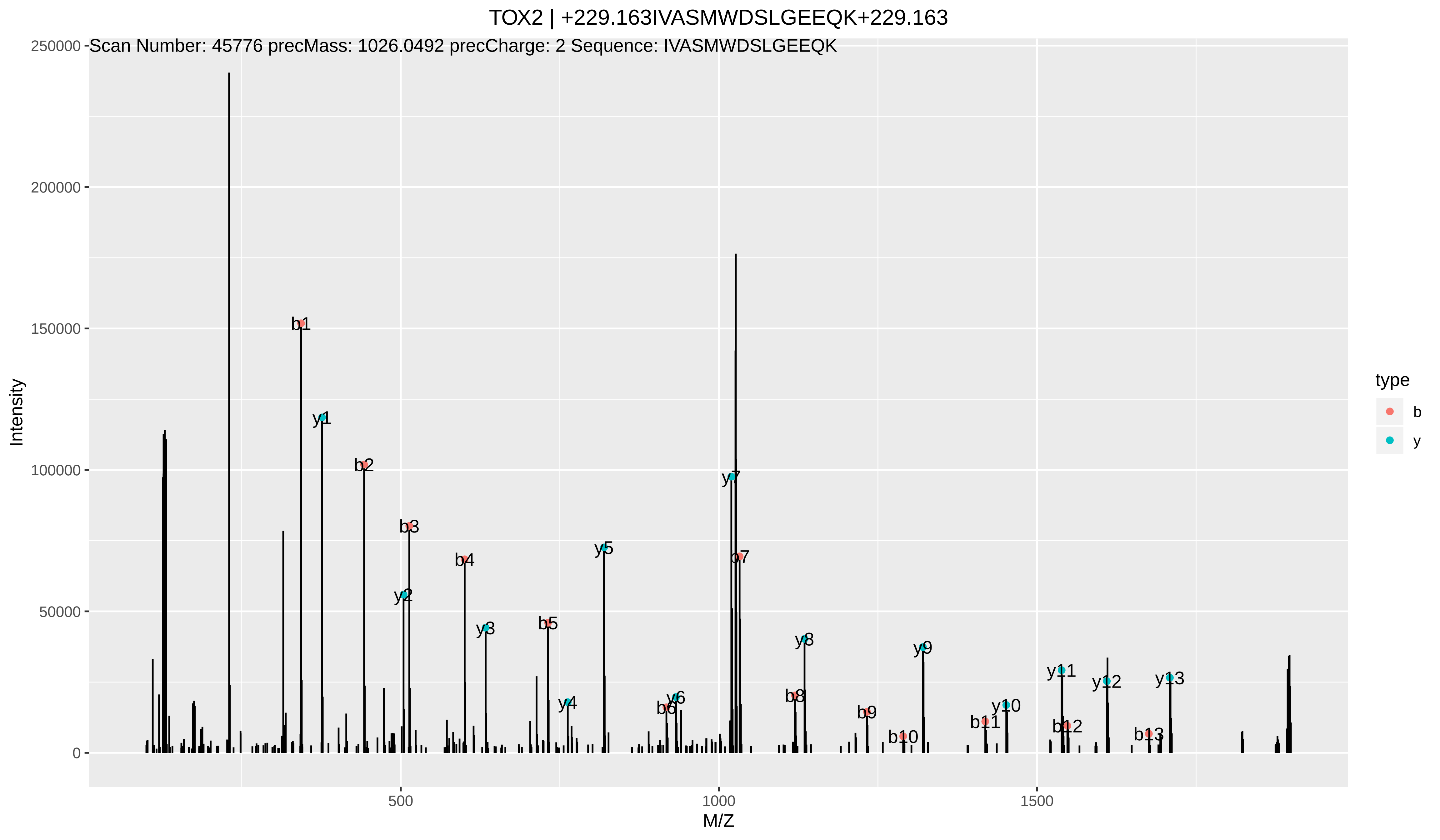
TOX2 | +229.163IVASMWDSLGEEQK+229.163
Scan Number: 45776 precMass: 1026.0492 precCharge: 2 Sequence: IVASMWDSLGEEQK
Intensity
M/Z
0
50000
100000
150000
200000
250000
500
1000
1500
b1
y1
b2
b3
y2
b4
y3
b5
y4
y5
b6
y6
y7
b7
b8
y8
b9
y9
b10
b11
y10
y11
b12
y12
b13
y13
type
b
y

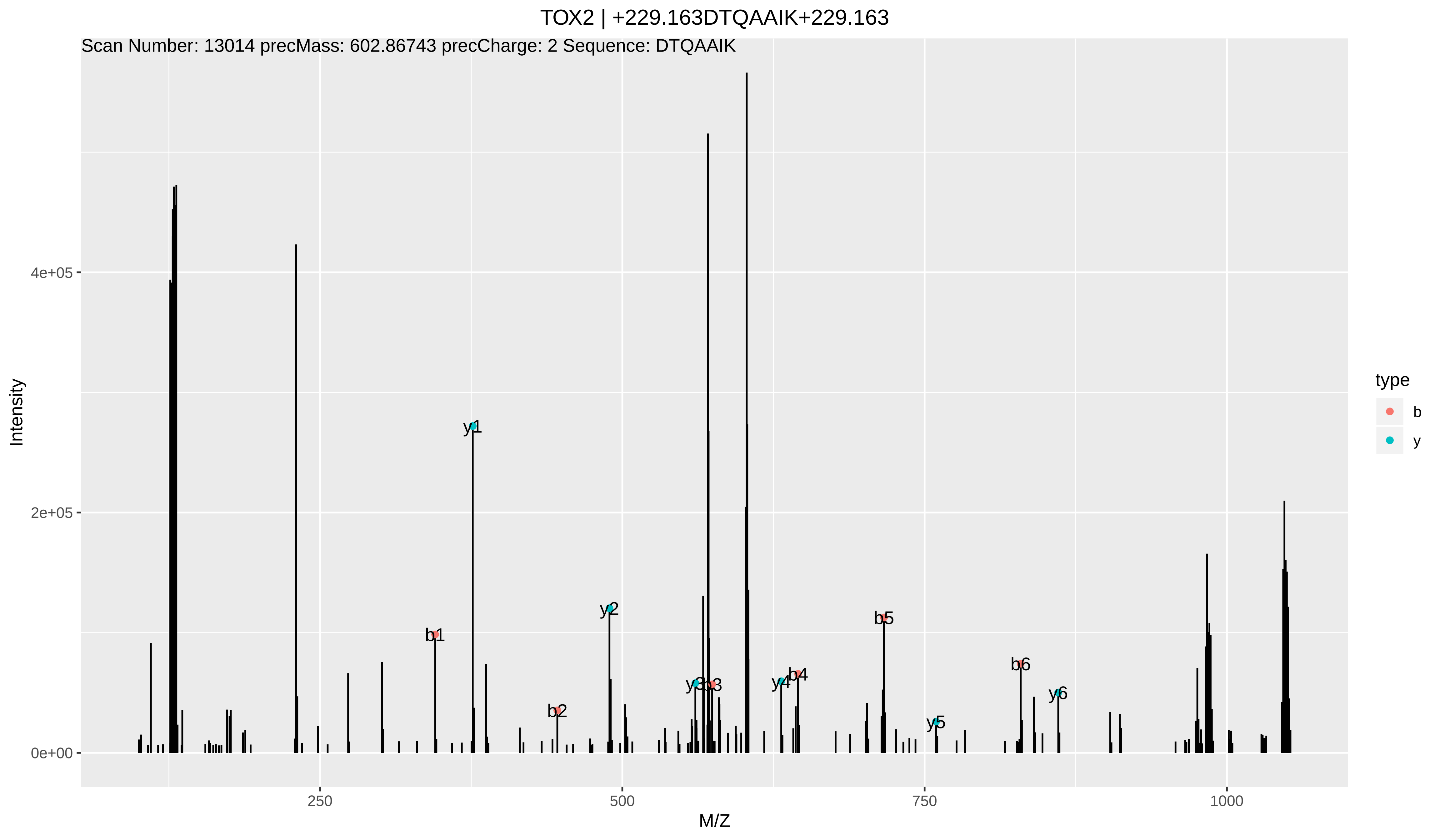

TOX2 | +229.163DTQAAIK+229.163
Scan Number: 13014 precMass: 602.86743 precCharge: 2 Sequence: DTQAAIK
Intensity
4e+05
2e+05
0e+00
M/Z
250
500
750
1000
y1
y2
y3
b3
y4
b4
b1
b2
b5
y5
b6
y6
type
b
y

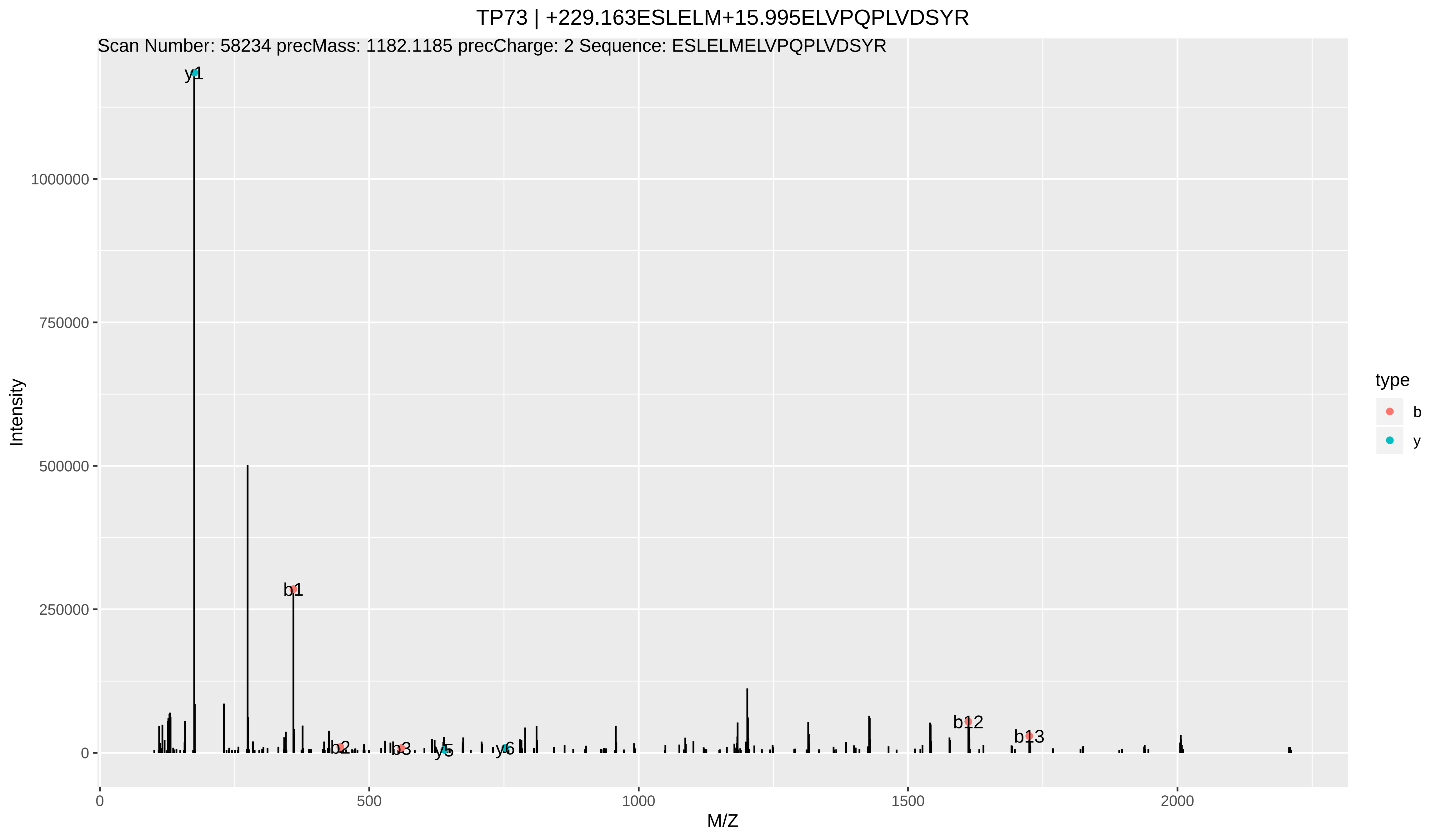

TP73 | +229.163ESLELM+15.995ELVPQPLVDSYR
Scan Number: 58234 precMass: 1182.1185 precCharge: 2 Sequence: ESLELMELVPQPLVDSYR
y1
b1
b2
b3
y5
y6
b12
b13
Intensity
1000000
750000
500000
250000
0
0
500
1000
1500
2000
M/Z
type
b
y

TPGS1 | +229.163LGPDSLALALDR

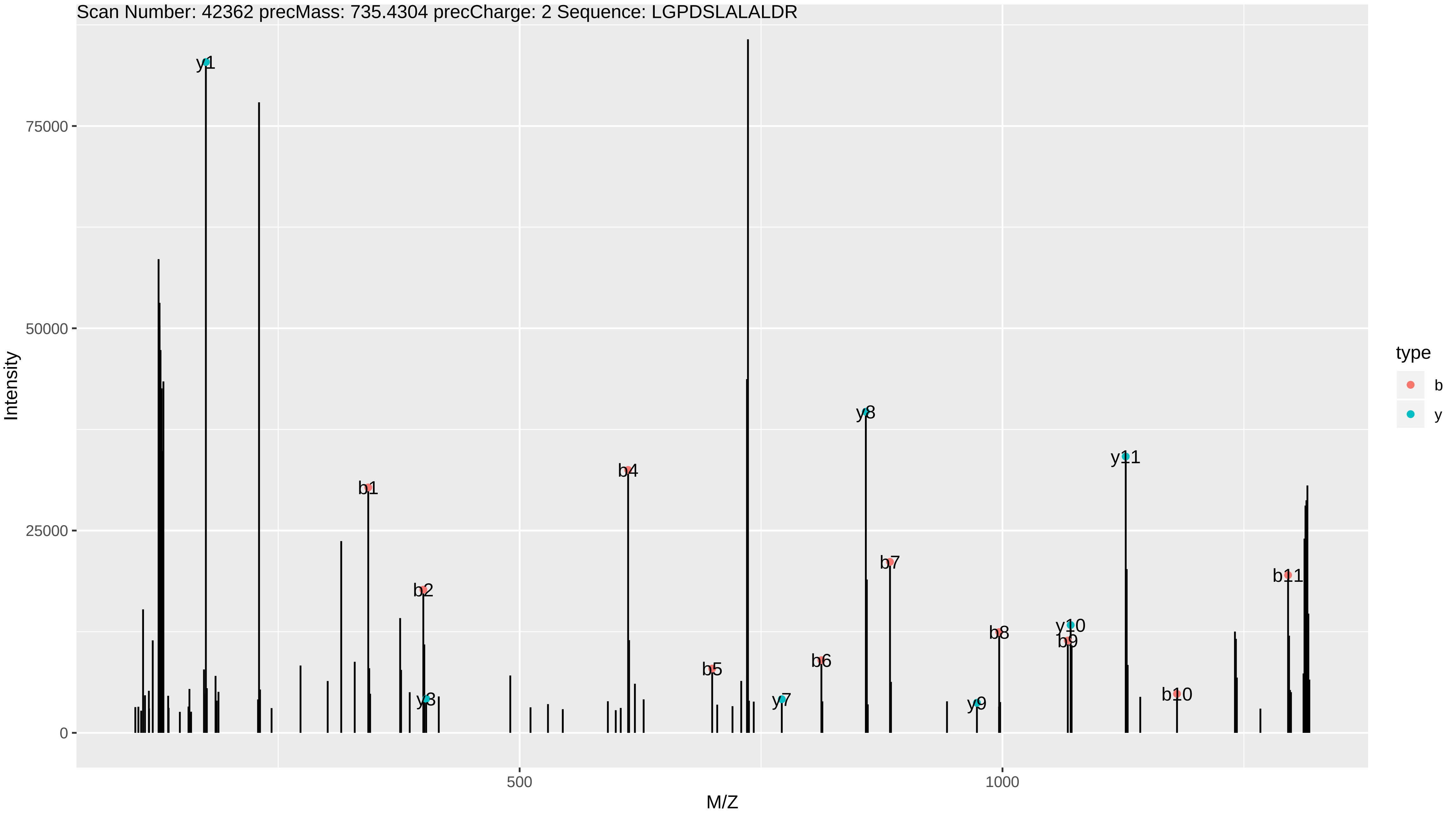

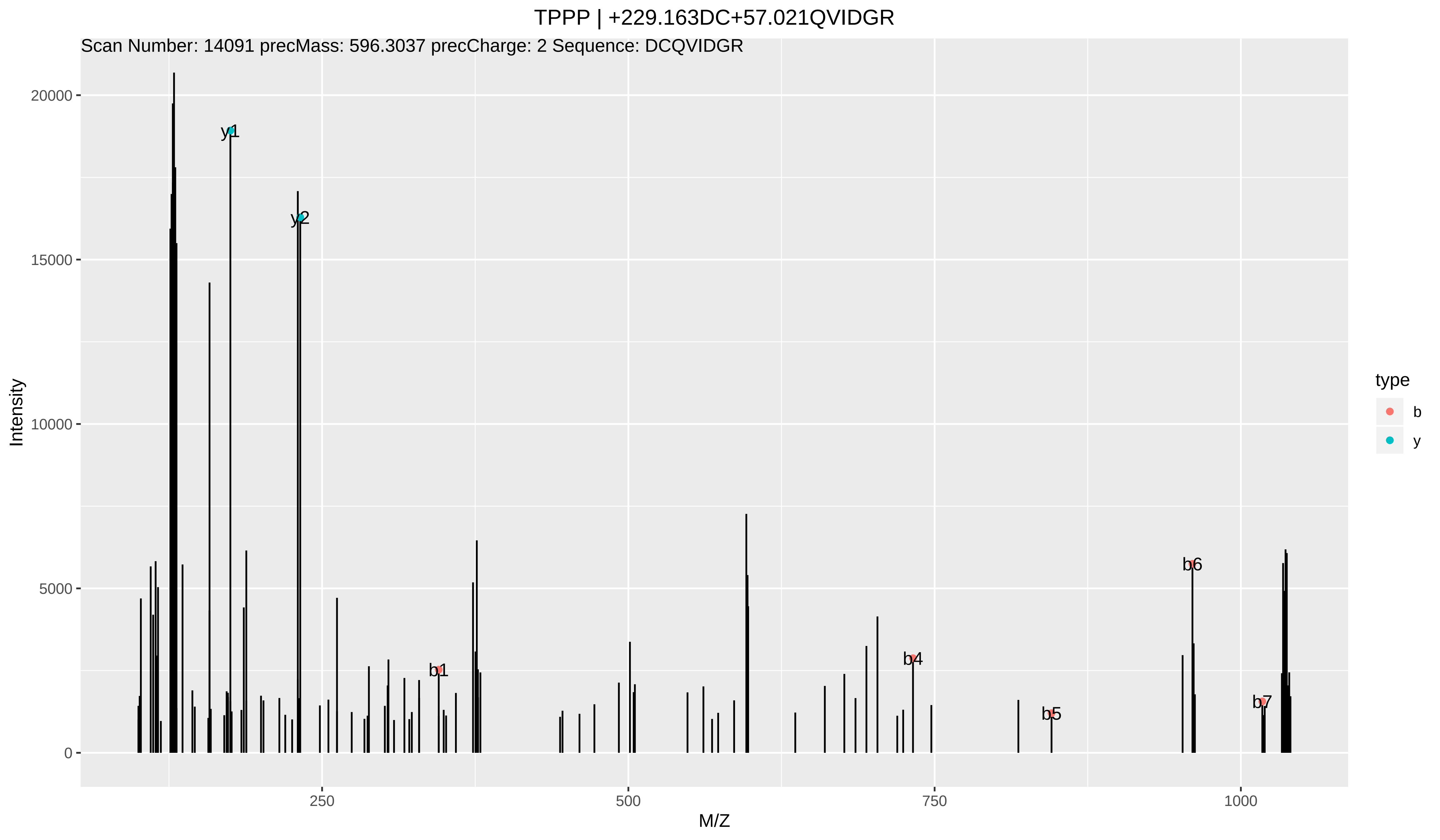

TPPP | +229.163DC+57.021QVIDGR
Scan Number: 14091 precMass: 596.3037 precCharge: 2 Sequence: DCQVIDGR
y1
y2
b1
b4
b5
b6
b7
Intensity
20000
15000
10000
5000
0
250
500
750
1000
M/Z
type
b
y

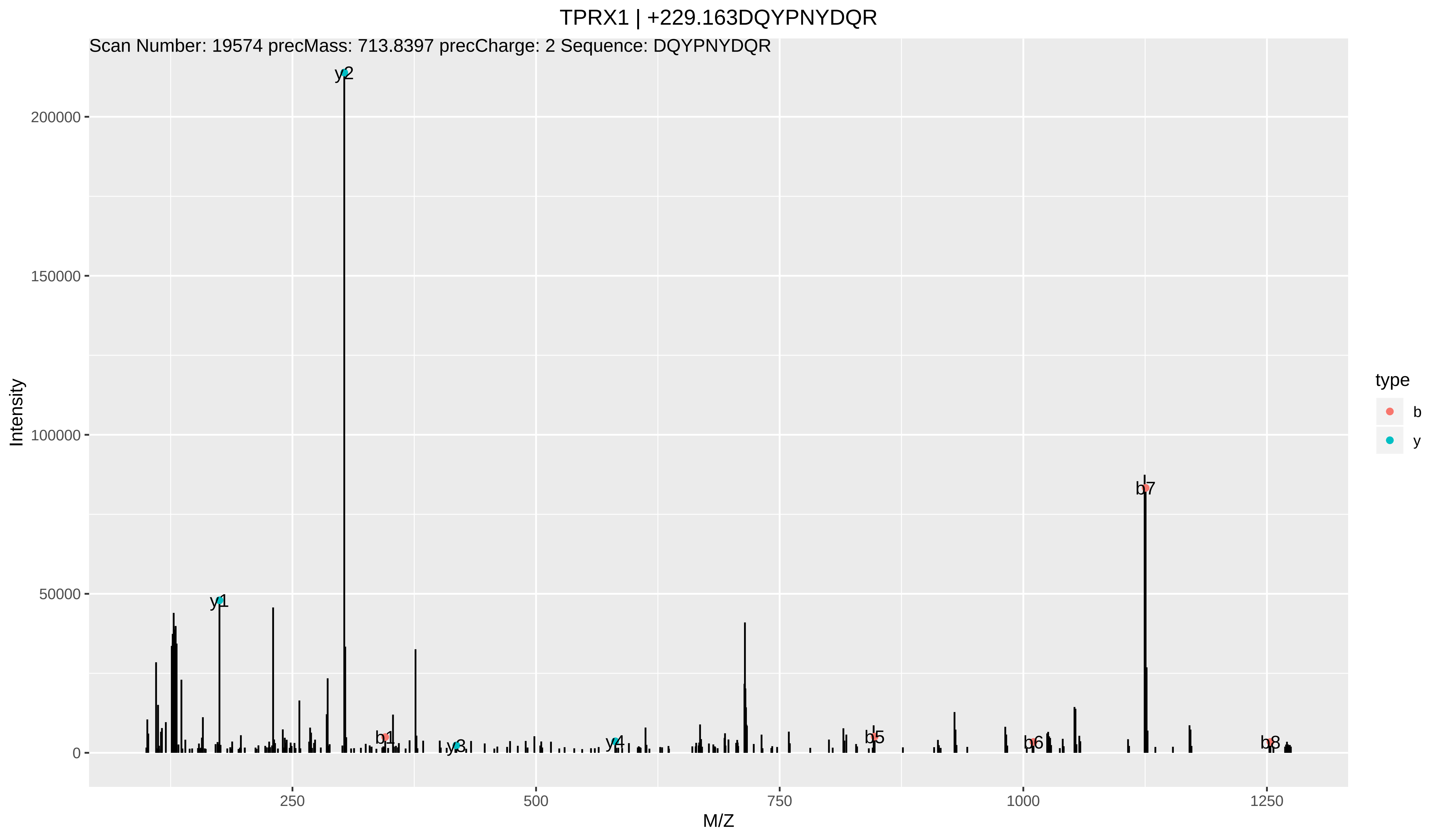

TPRX1 | +229.163DQYPNYDQR
Scan Number: 19574 precMass: 713.8397 precCharge: 2 Sequence: DQYPNYDQR
y2
y1
b1
y3
y4
b5
b6
b7
b8
Intensity
200000
150000
100000
50000
0
250
500
750
1000
1250
M/Z
type
b
y

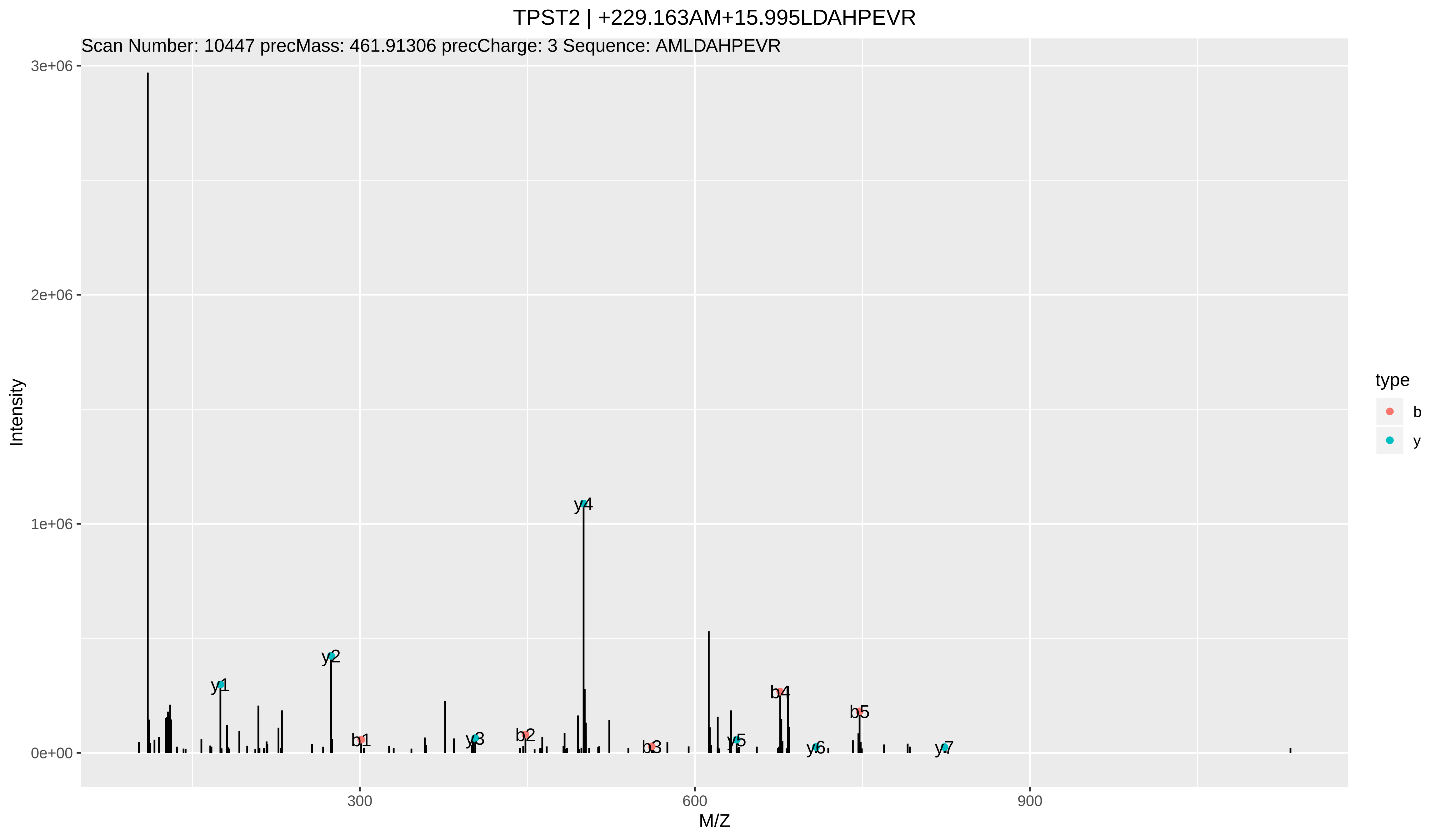
TPST2 | +229.163AM+15.995LDAHPEVR
Scan Number: 10447 precMass: 461.91306 precCharge: 3 Sequence: AMLDAHPEVR
Intensity
M/Z
3e+06
2e+06
1e+06
0e+00
300
600
900
y1
y2
b1
y3
b2
y4
b3
y5
b4
y6
b5
y7
type
b
y

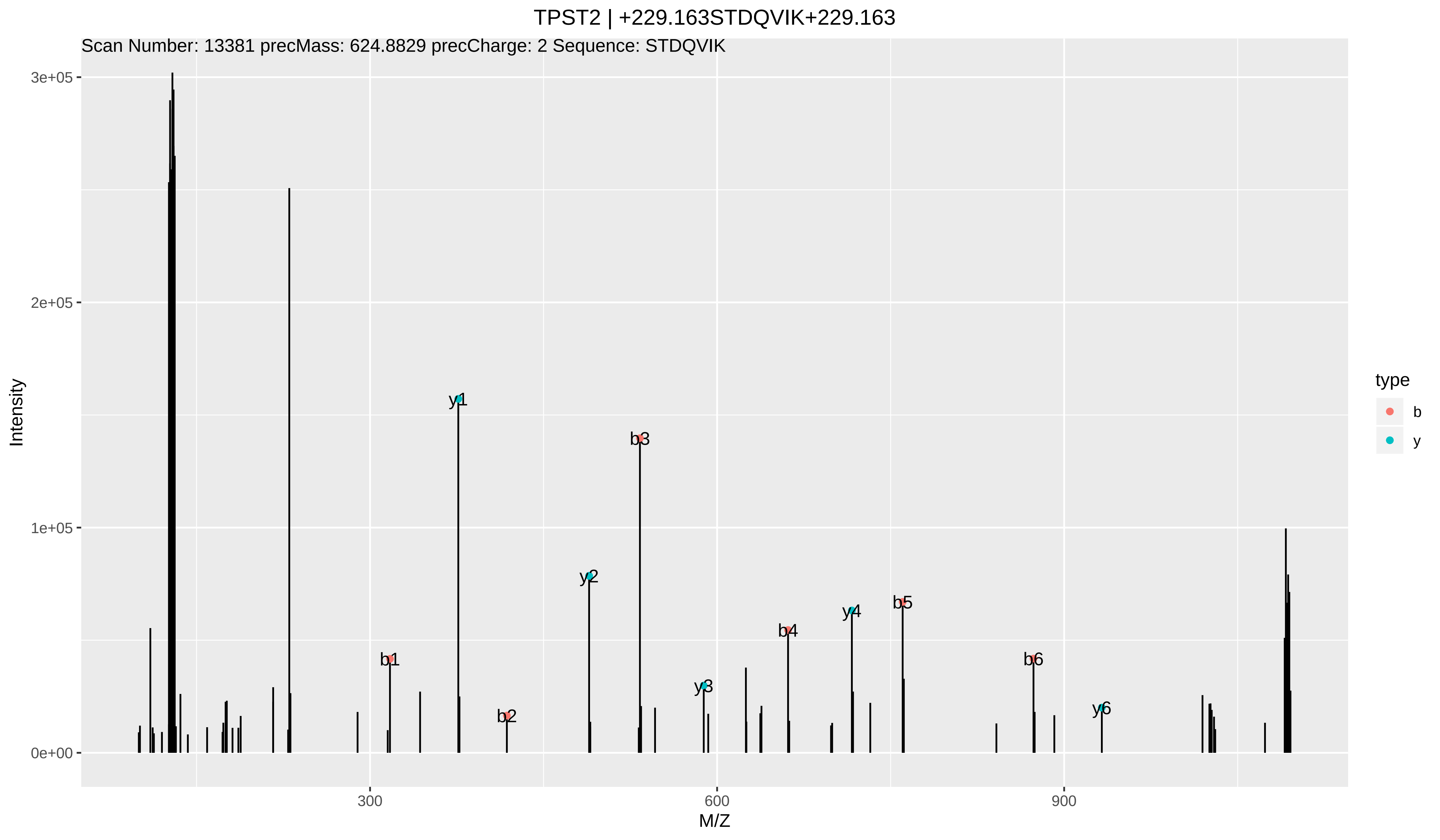
TPST2 | +229.163STDQVIK+229.163
Scan Number: 13381 precMass: 624.8829 precCharge: 2 Sequence: STDQVIK
Intensity
3e+05
2e+05
1e+05
0e+00
M/Z
300
600
900
y1
b3
y2
b1
b2
y3
b4
y4
b5
b6
y6
type
b
y

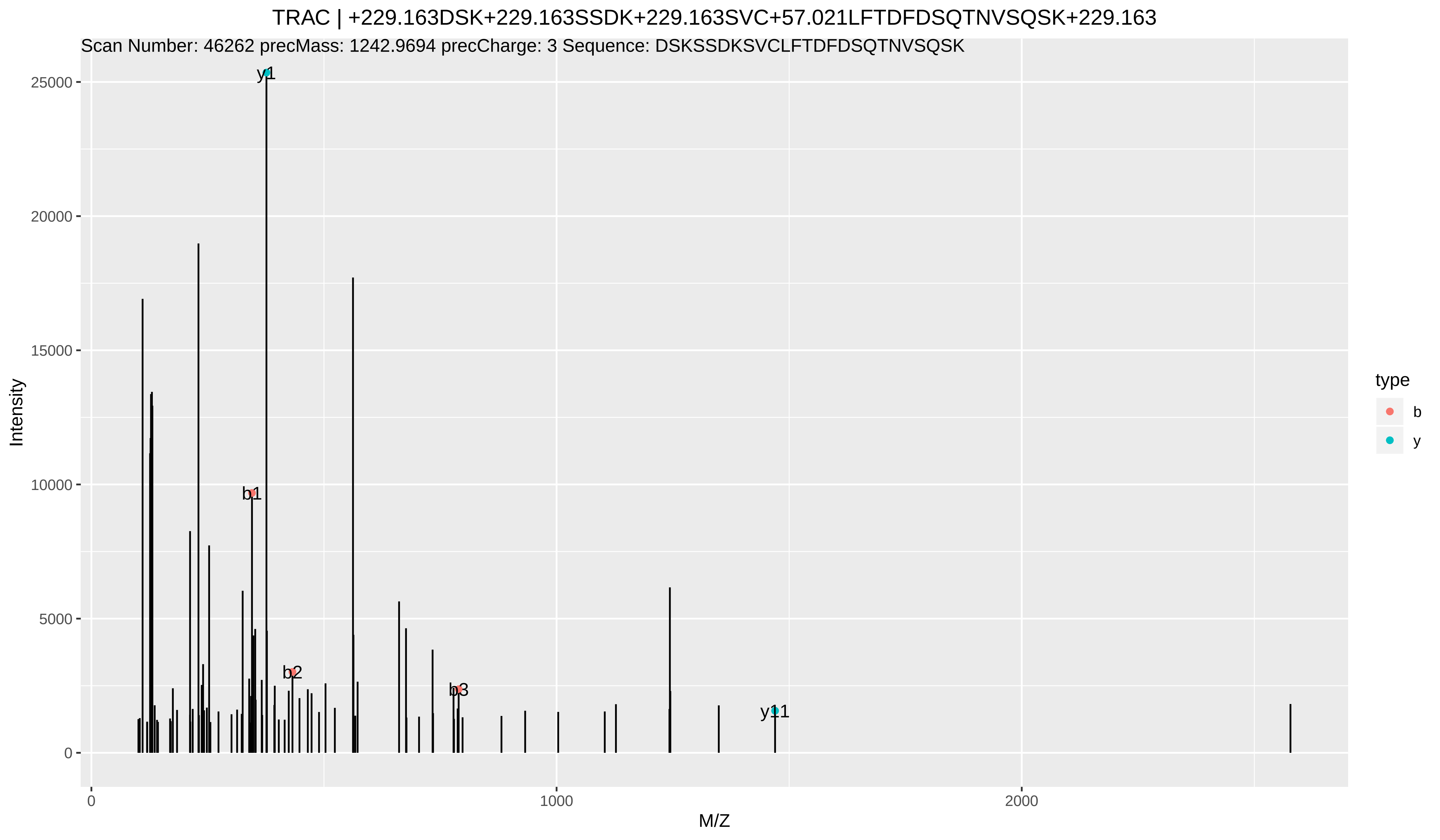

TRAC | +229.163DSK+229.163SSDK+229.163SVC+57.021LFTDFDSQTNVSQSK+229.163
Scan Number: 46262 precMass: 1242.9694 precCharge: 3 Sequence: DSKSSDKSVCLFTDFDSQTNVSQSK
y1
b1
b2
b3
y11
25000
20000
15000
10000
5000
0
Intensity
0
1000
2000
M/Z
type
b
y

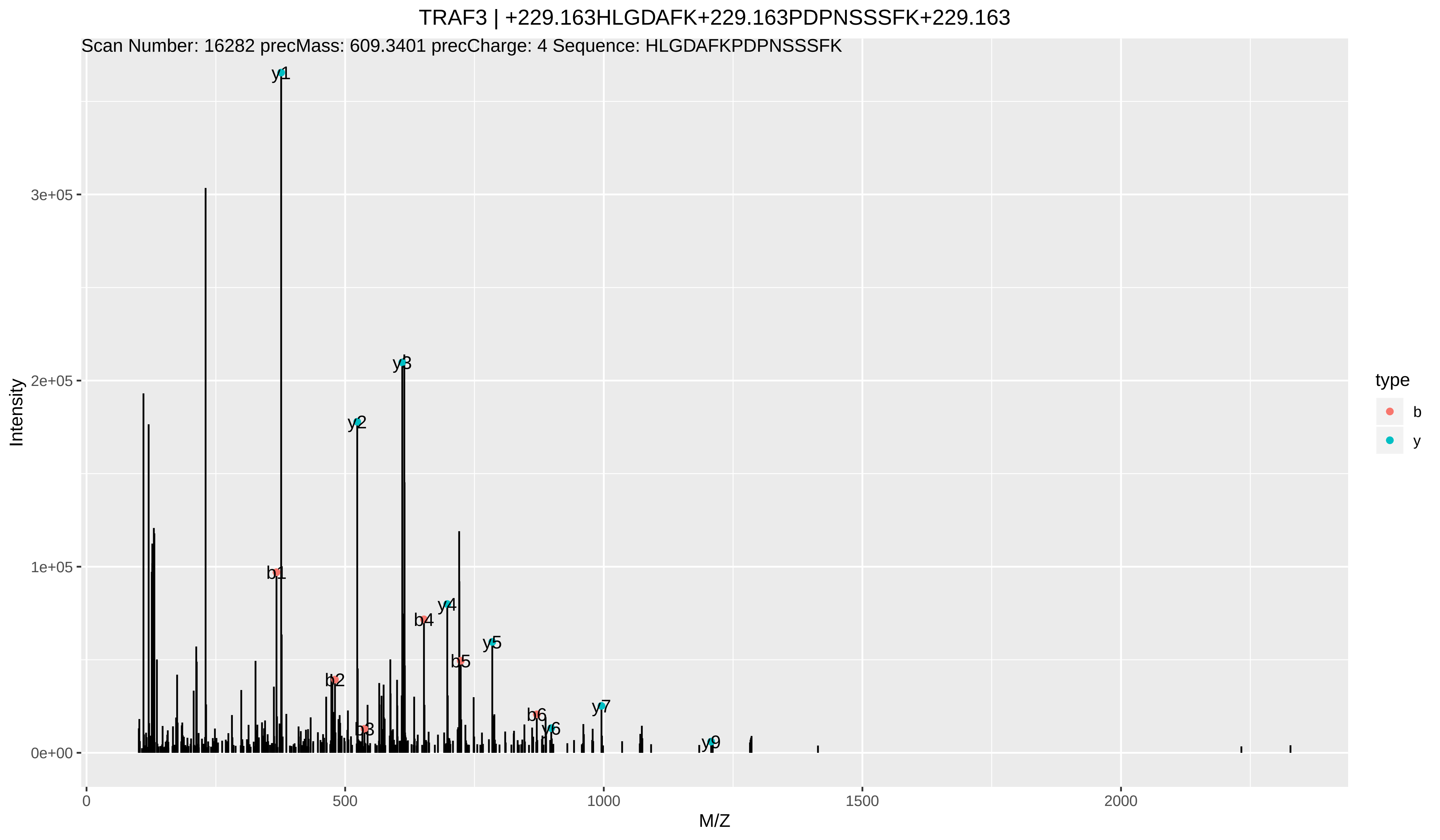

TRAF3 | +229.163HLGDAFK+229.163PDPNSSSFK+229.163
Scan Number: 16282 precMass: 609.3401 precCharge: 4 Sequence: HLGDAFKPDPNSSSFK
Intensity
M/Z
3e+05
2e+05
1e+05
0e+00
0
500
1000
1500
2000
y1
y3
y2
b1
y4
b4
y5
b5
b2
b6
y7
b3
y6
y9
type
b
y

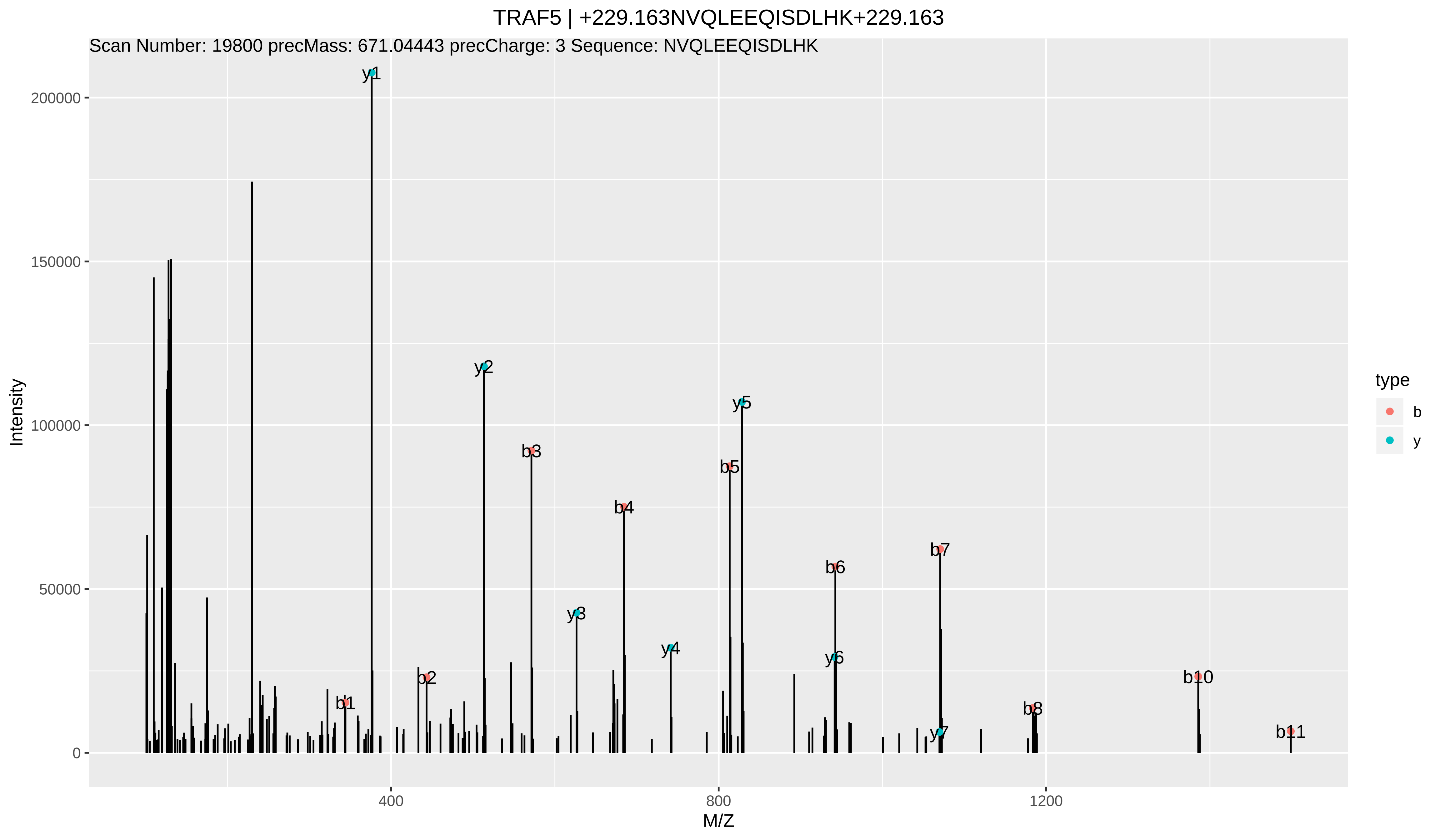

TRAF5 | +229.163NVQLEEQISDLHK+229.163
Scan Number: 19800 precMass: 671.04443 precCharge: 3 Sequence: NVQLEEQISDLHK
Intensity
M/Z
0
50000
100000
150000
200000
400
800
1200
y1
y2
b3
y5
b5
b4
y3
y4
b7
b6
y6
b2
b1
b10
b8
y7
b11
type
b
y

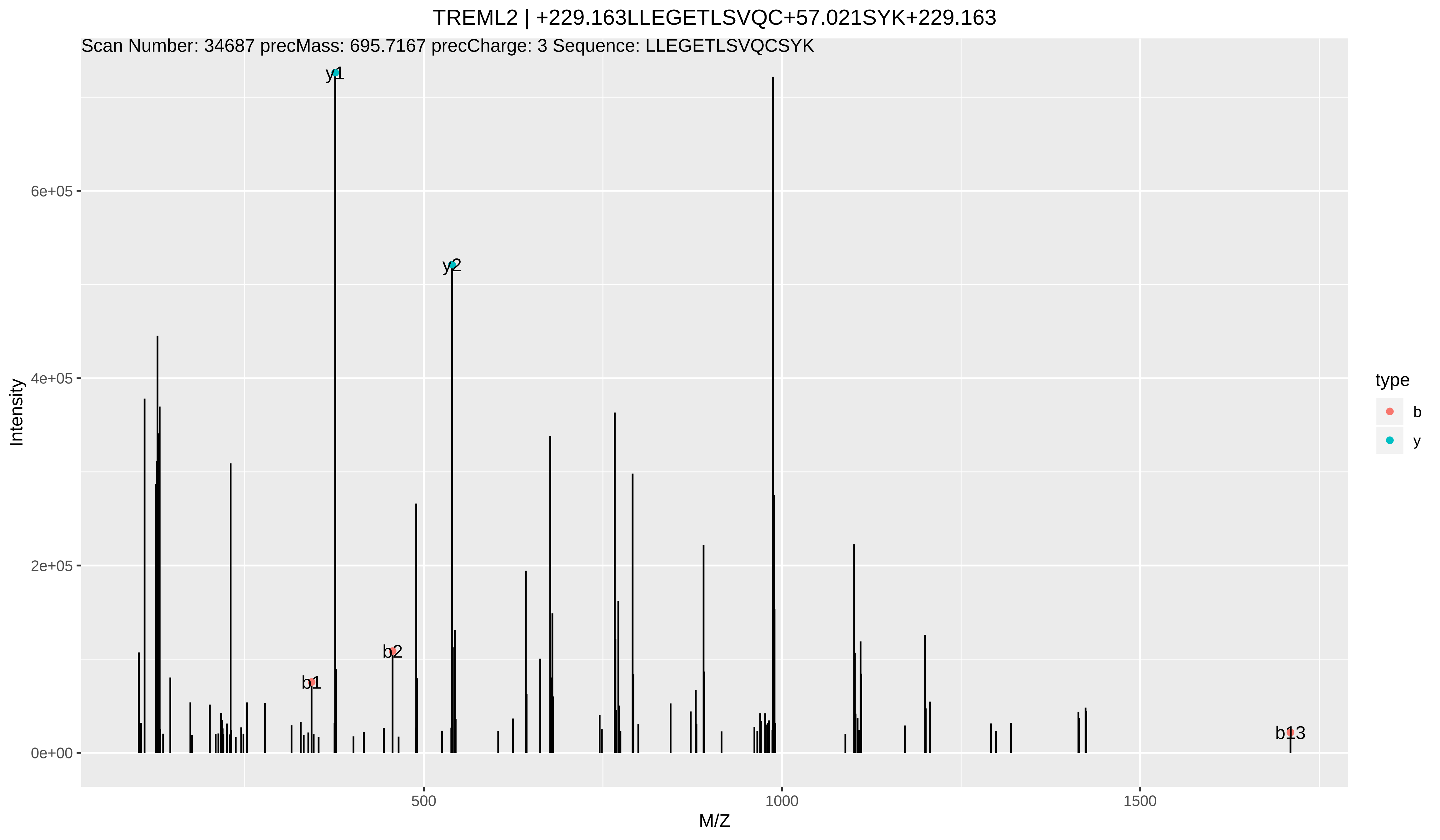

TREML2 | +229.163LLEGETLSVQC+57.021SYK+229.163
Scan Number: 34687 precMass: 695.7167 precCharge: 3 Sequence: LLEGETLSVQCSYK
y1
y2
b1
b2
b13
Intensity
6e+05
4e+05
2e+05
0e+00
500
1000
1500
M/Z
type
b
y

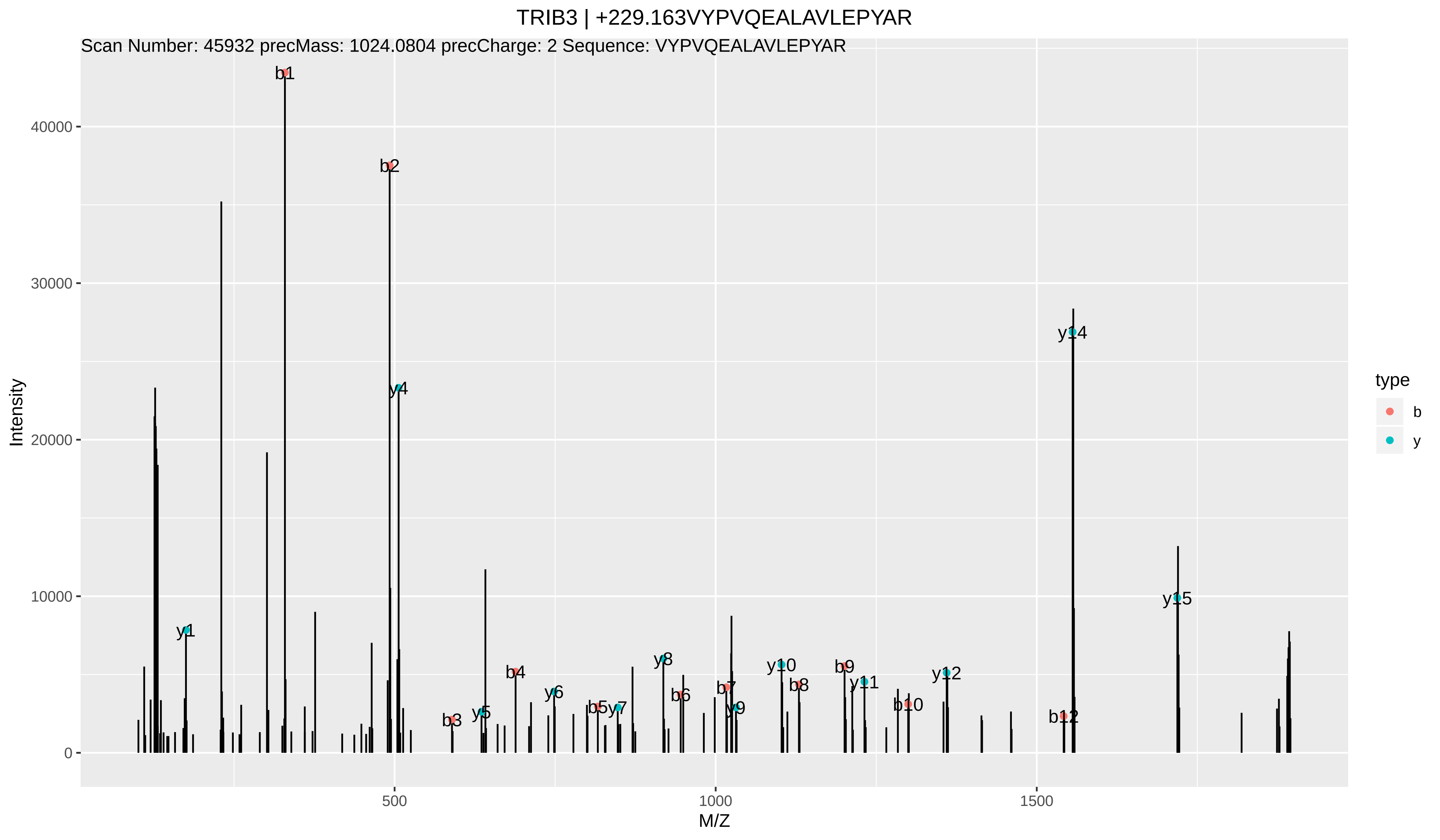
TRIB3 | +229.163VYPVQEALAVLEPYAR
Scan Number: 45932 precMass: 1024.0804 precCharge: 2 Sequence: VYPVQEALAVLEPYAR
Intensity
M/Z
0
10000
20000
30000
40000
500
1000
1500
b1
b2
b3
b4
b5
b6
b7
b8
b9
b10
b12
y1
y4
y5
y6
y7
y8
y9
y10
y11
y12
y14
y15
type
b
y

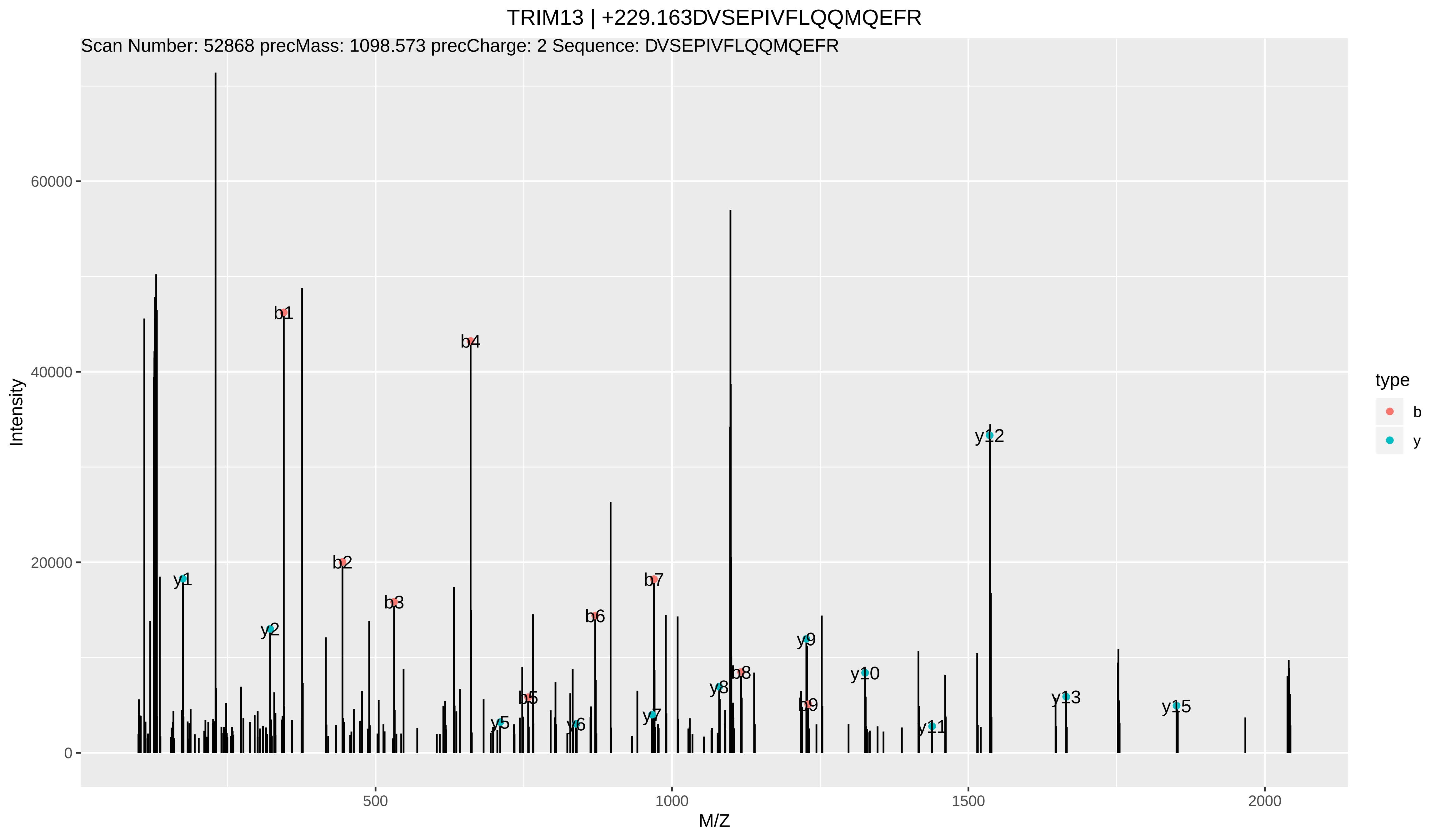

TRIM13 | +229.163DVSEPIVFLQQMQEFR
Scan Number: 52868 precMass: 1098.573 precCharge: 2 Sequence: DVSEPIVFLQQMQEFR
Intensity
M/Z
0
20000
40000
60000
500
1000
1500
2000
y1
y2
b1
b2
b3
b4
y5
b5
y6
b6
b7
y7
y8
b8
y9
b9
y10
y11
y12
y13
y15
type
b
y

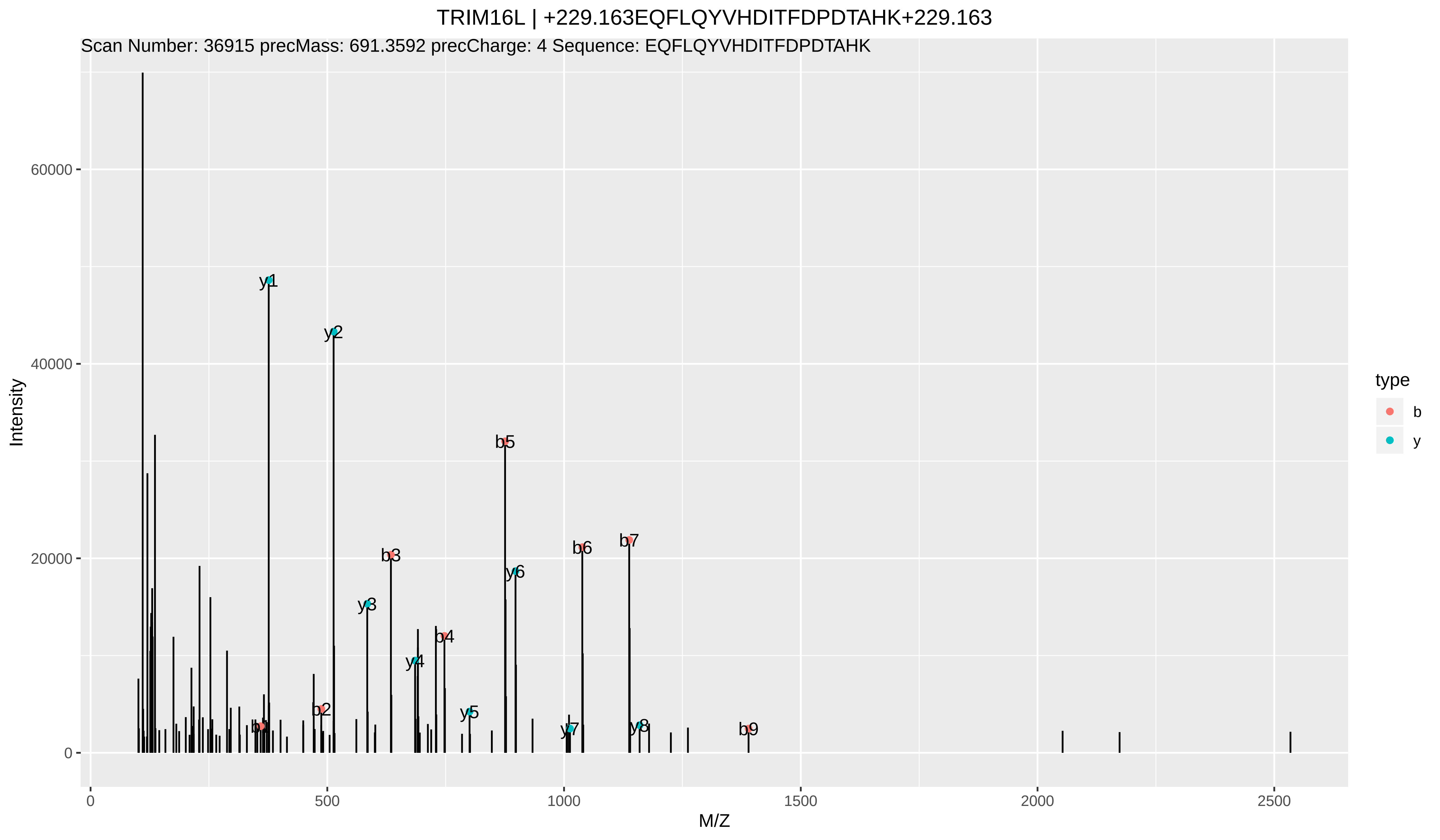

TRIM16L | +229.163EQFLQYVHDITFDPDTAHK+229.163
Scan Number: 36915 precMass: 691.3592 precCharge: 4 Sequence: EQFLQYVHDITFDPDTAHK
Intensity
M/Z
60000
40000
20000
0
0
500
1000
1500
2000
2500
y1
y2
y3
y4
y5
y6
y7
y8
b2
b3
b4
b5
b6
b7
b9
type
b
y

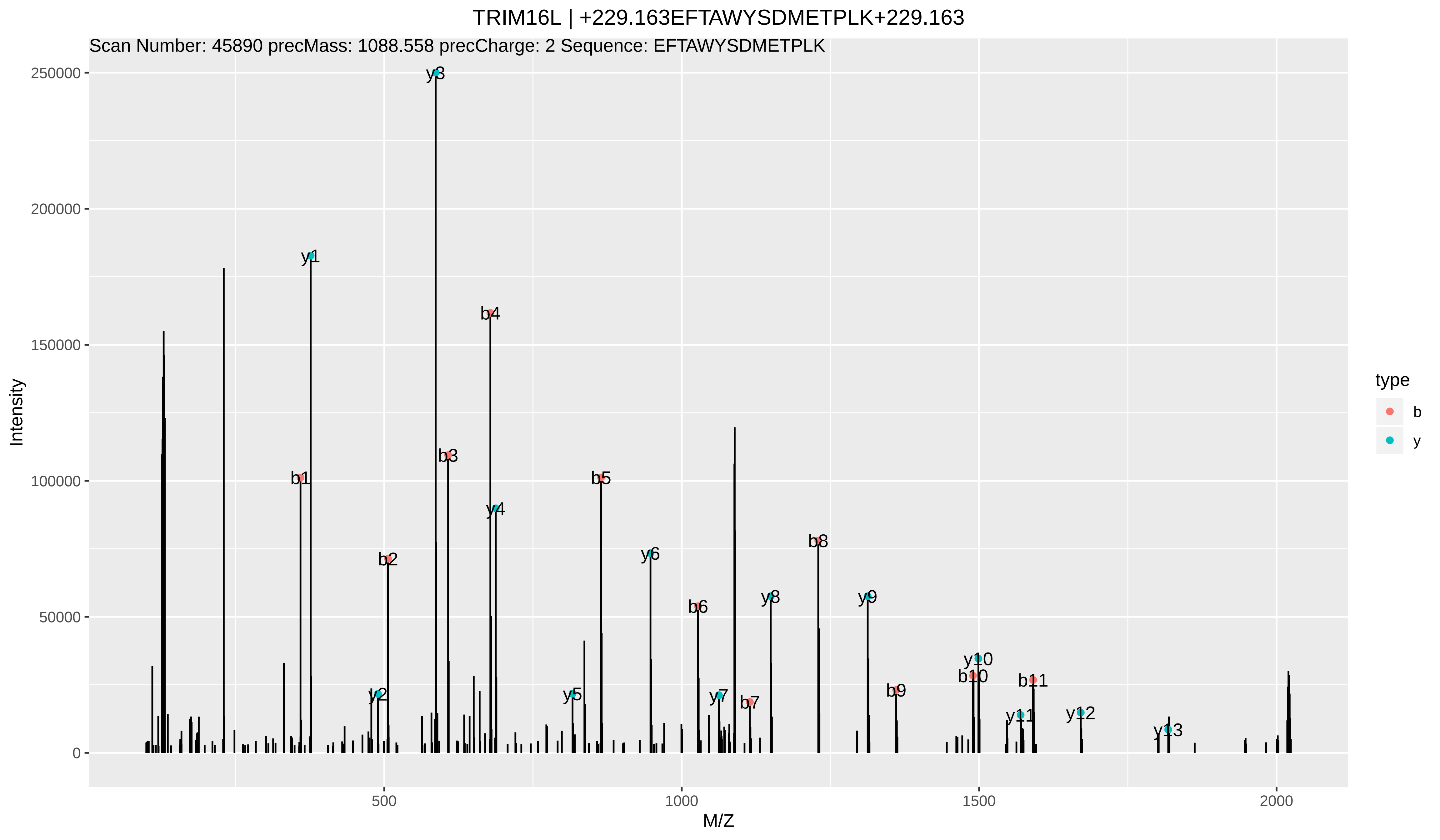
TRIM16L | +229.163EFTAWYSDMETPLK+229.163
Scan Number: 45890 precMass: 1088.558 precCharge: 2 Sequence: EFTAWYSDMETPLK
Intensity
M/Z
0
50000
100000
150000
200000
250000
500
1000
1500
2000
type
b
y
y3
y1
b4
b3
b1
b5
y4
b2
y6
b8
b6
y8
y9
y10
b10
b11
b9
y2
y5
y7
b7
y11
y12
y13

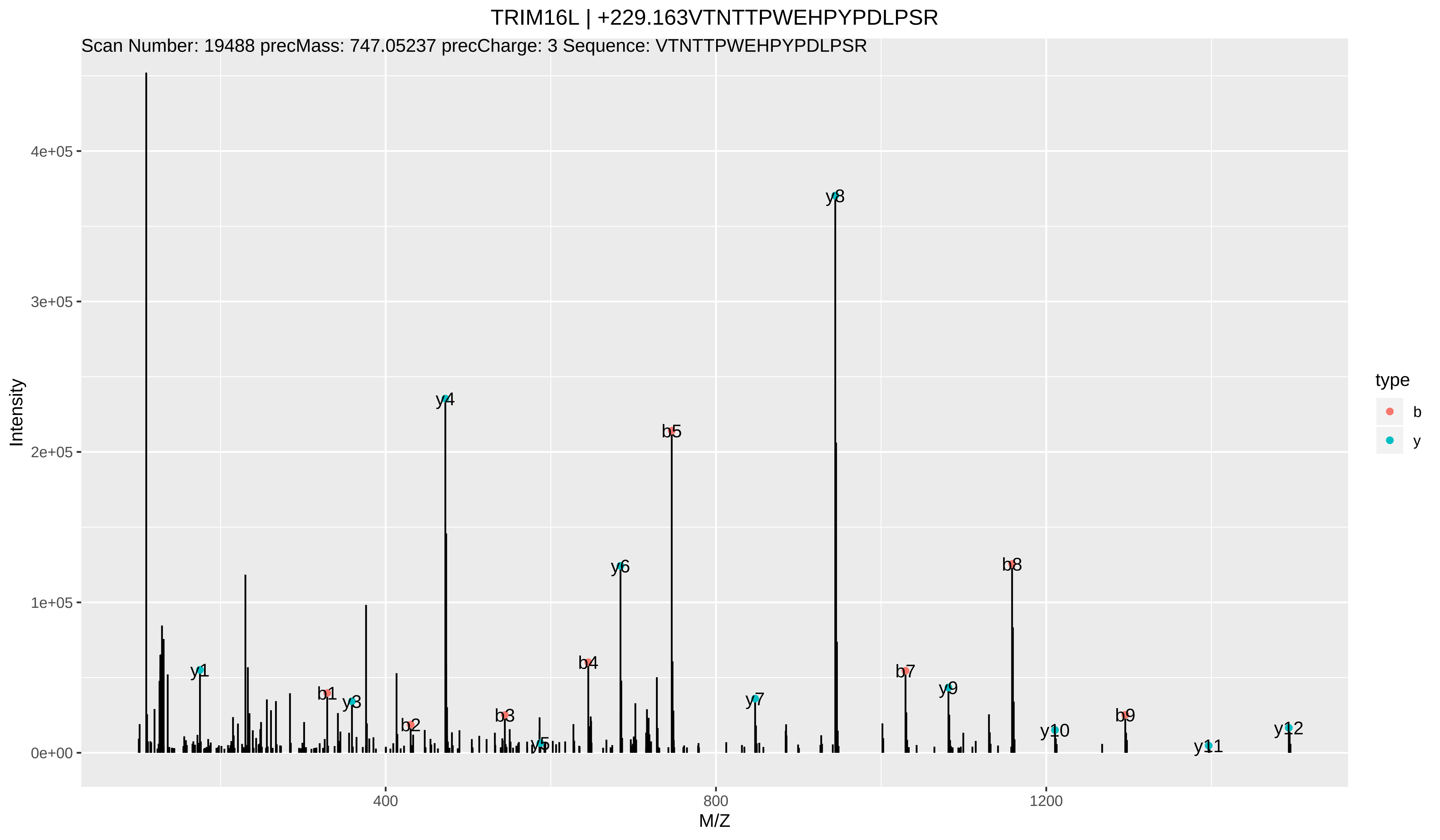
TRIM16L | +229.163VTNTTPWEHPYPDLPSR
Scan Number: 19488 precMass: 747.05237 precCharge: 3 Sequence: VTNTTPWEHPYPDLPSR
Intensity
0e+00
1e+05
2e+05
3e+05
4e+05
M/Z
400
800
1200
y1
b1
y3
b2
y4
b3
y5
b4
y6
b5
y7
y8
b7
y9
b8
y10
b9
y11
y12
type
b
y

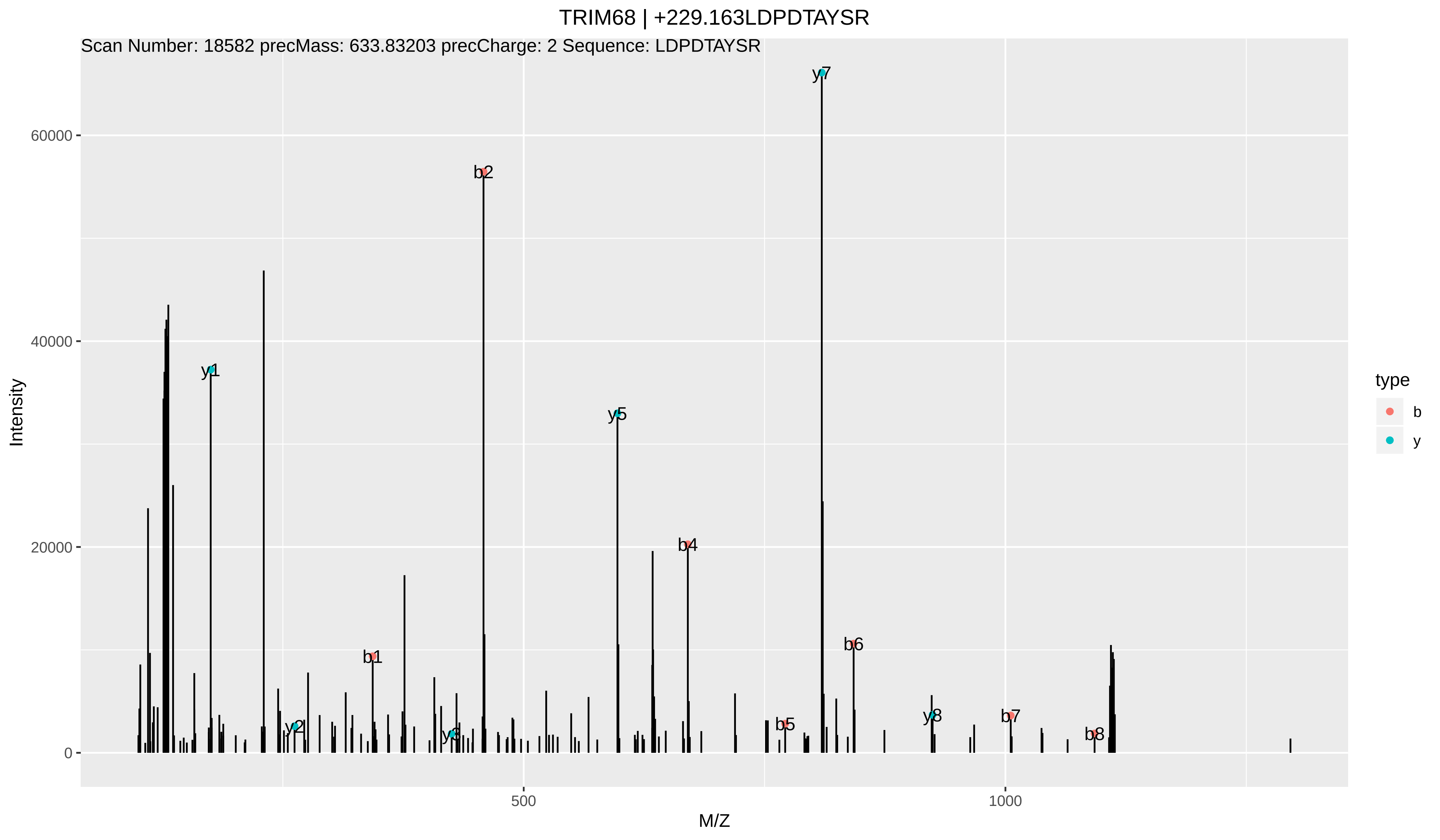

TRIM68 | +229.163LDPDTAYSR
Scan Number: 18582 precMass: 633.83203 precCharge: 2 Sequence: LDPDTAYSR
Intensity
60000
40000
20000
0
500
1000
M/Z
y7
b2
y1
y5
b4
b6
b1
y2
y3
b5
y8
b7
b8
type
b
y

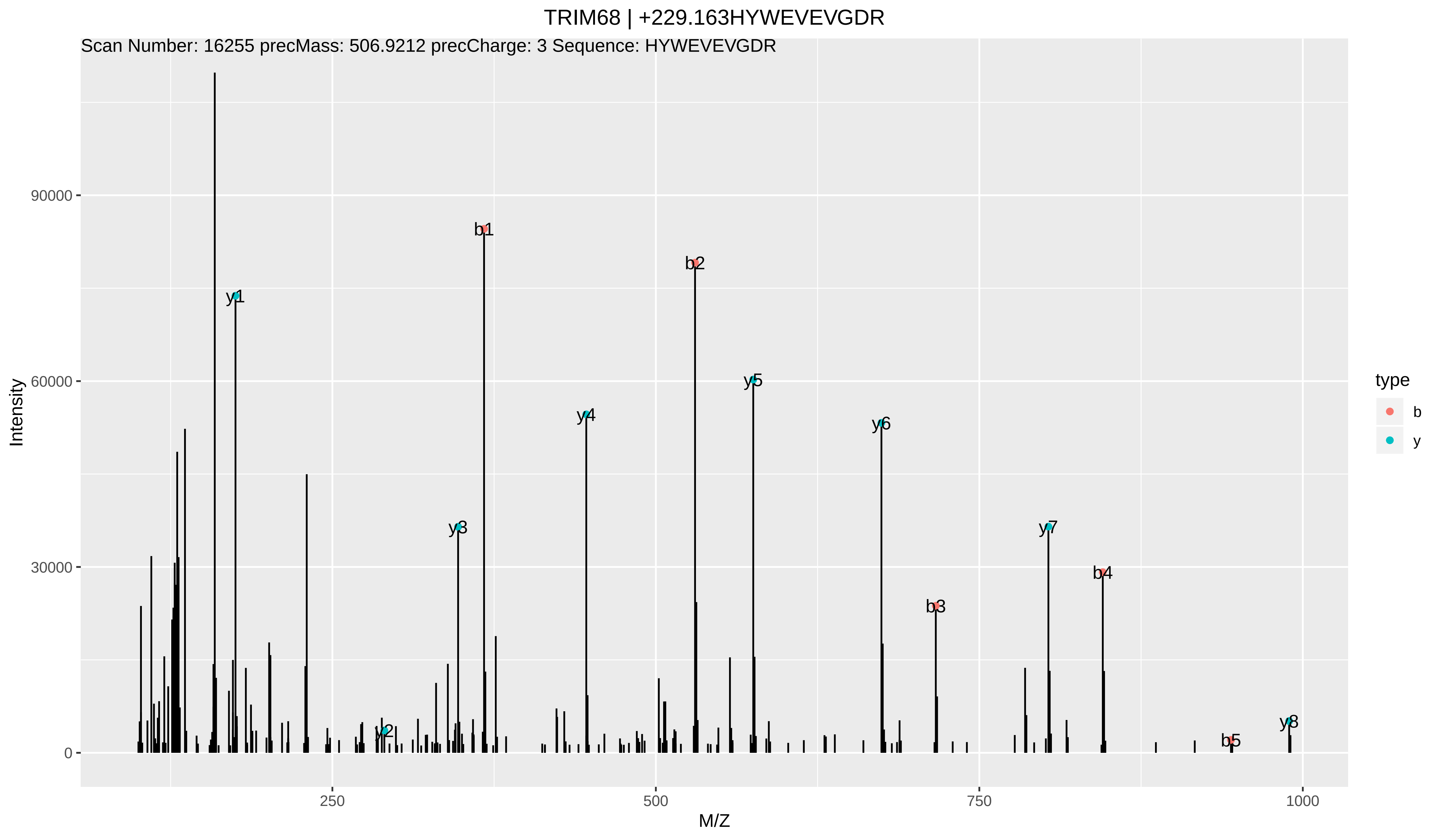

TRIM68 | +229.163HYWEVEVGDR
Scan Number: 16255 precMass: 506.9212 precCharge: 3 Sequence: HYWEVEVGDR
Intensity
M/Z
90000
60000
30000
0
250
500
750
1000
b1
b2
b3
b4
b5
y1
y2
y3
y4
y5
y6
y7
y8
type
b
y

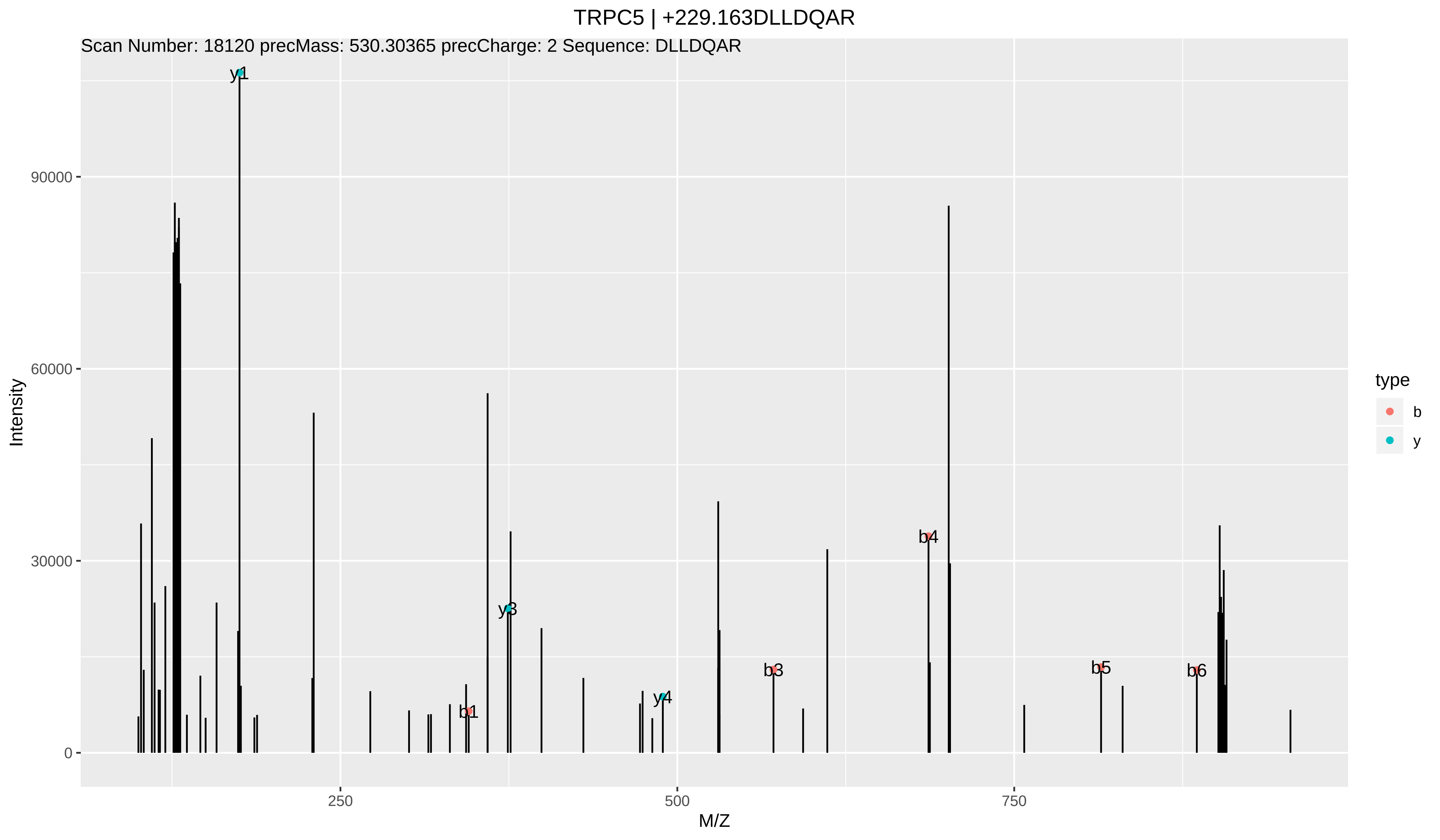

TRPC5 | +229.163DLLDQAR
Scan Number: 18120 precMass: 530.30365 precCharge: 2 Sequence: DLLDQAR
y1
y3
y4
b1
b3
b4
b5
b6
Intensity
90000
60000
30000
0
250
500
750
M/Z
type
b
y

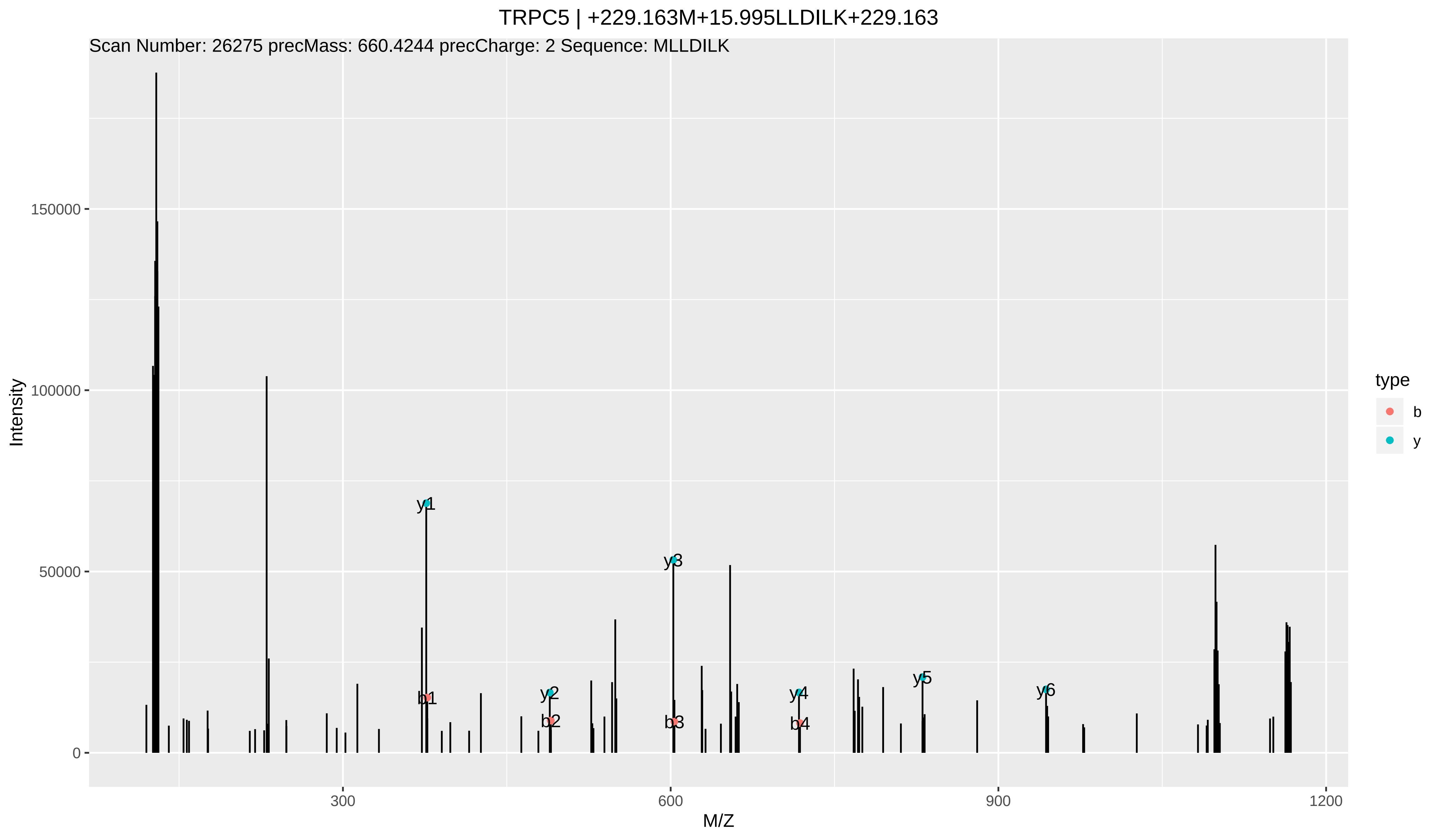
TRPC5 | +229.163M+15.995LLDILK+229.163
Scan Number: 26275 precMass: 660.4244 precCharge: 2 Sequence: MLLLDILK
Intensity
150000
100000
50000
0
300
600
900
1200
M/Z
type
b
y
y1
b1
y2
b2
y3
b3
y4
b4
y5
y6

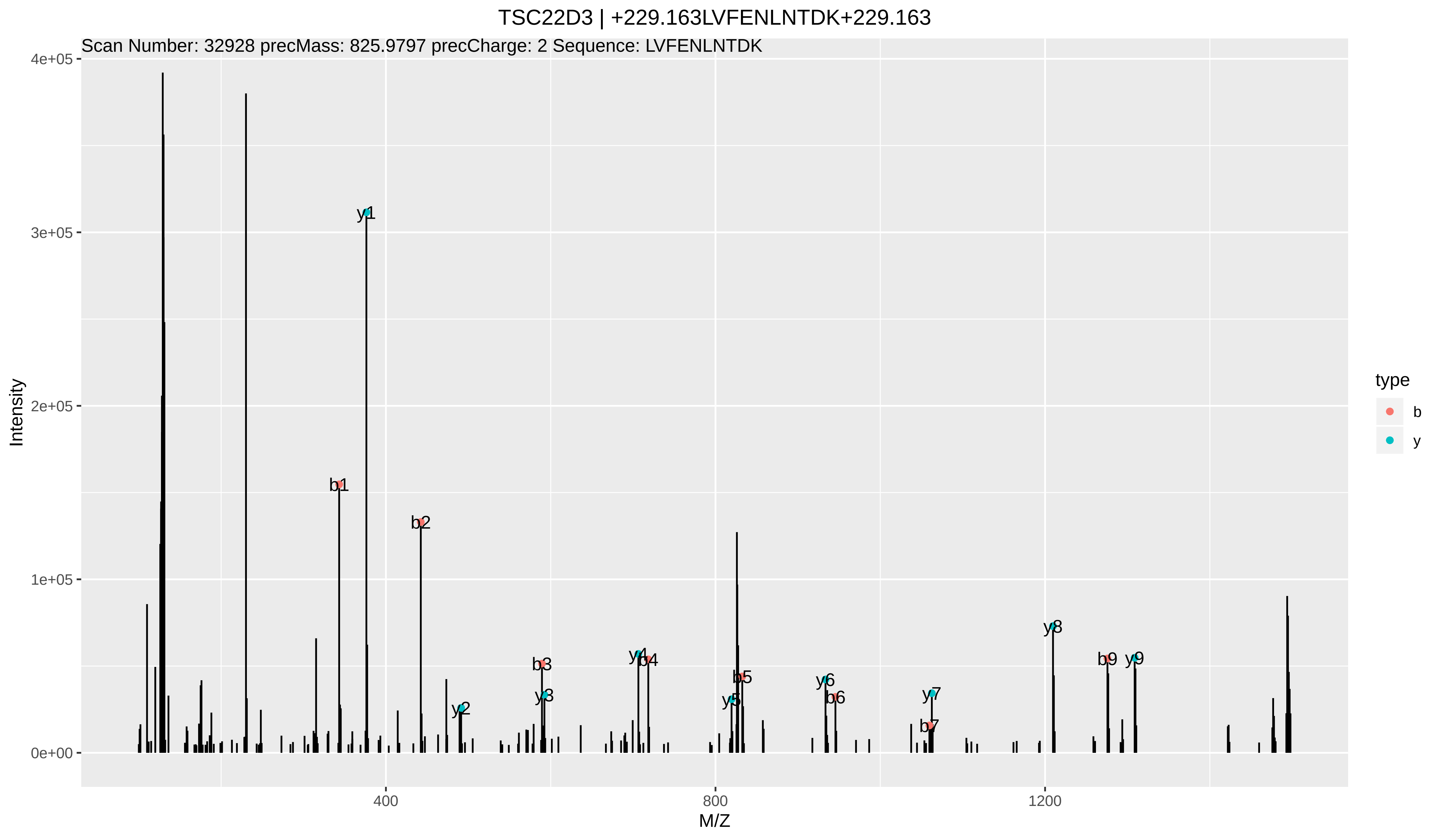
TSC22D3 | +229.163LVFENLNTDK+229.163
Scan Number: 32928 precMass: 825.9797 precCharge: 2 Sequence: LVFENLNTDK
Intensity
4e+05
3e+05
2e+05
1e+05
0e+00
M/Z
400
800
1200
y1
b1
b2
y2
b3
y3
y4
b4
b5
y5
y6
b6
y7
b7
y8
b9
y9
type
b
y

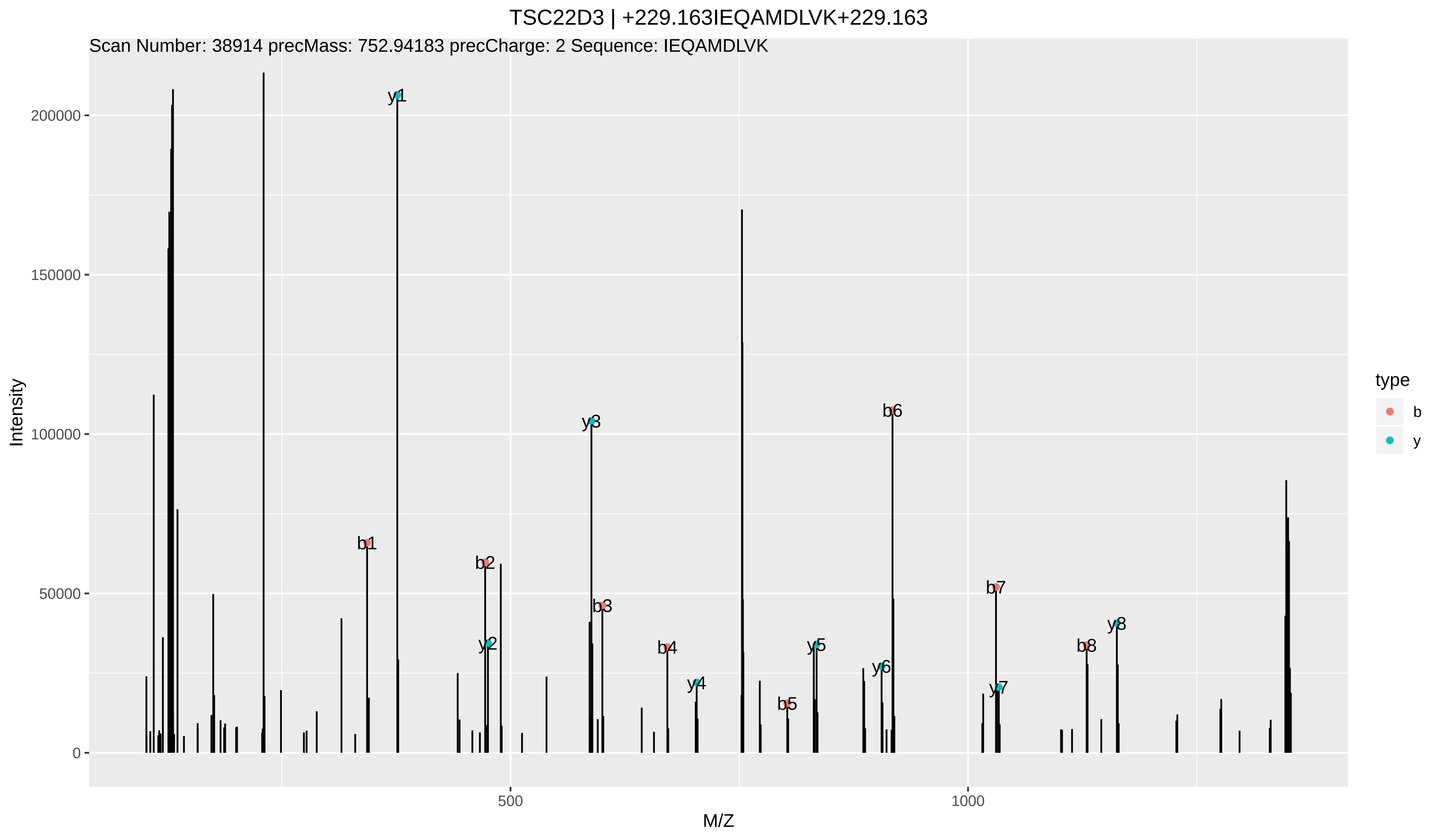

TSC22D3 | +229.163IEQAMDLVK+229.163
Scan Number: 38914 precMass: 752.94183 precCharge: 2 Sequence: IEQAMDLVK
Intensity
M/Z
0
50000
100000
150000
200000
500
1000
y1
b1
b2
y2
y3
b3
b4
y4
b5
y5
y6
b6
b7
y7
b8
y8
type
b
y

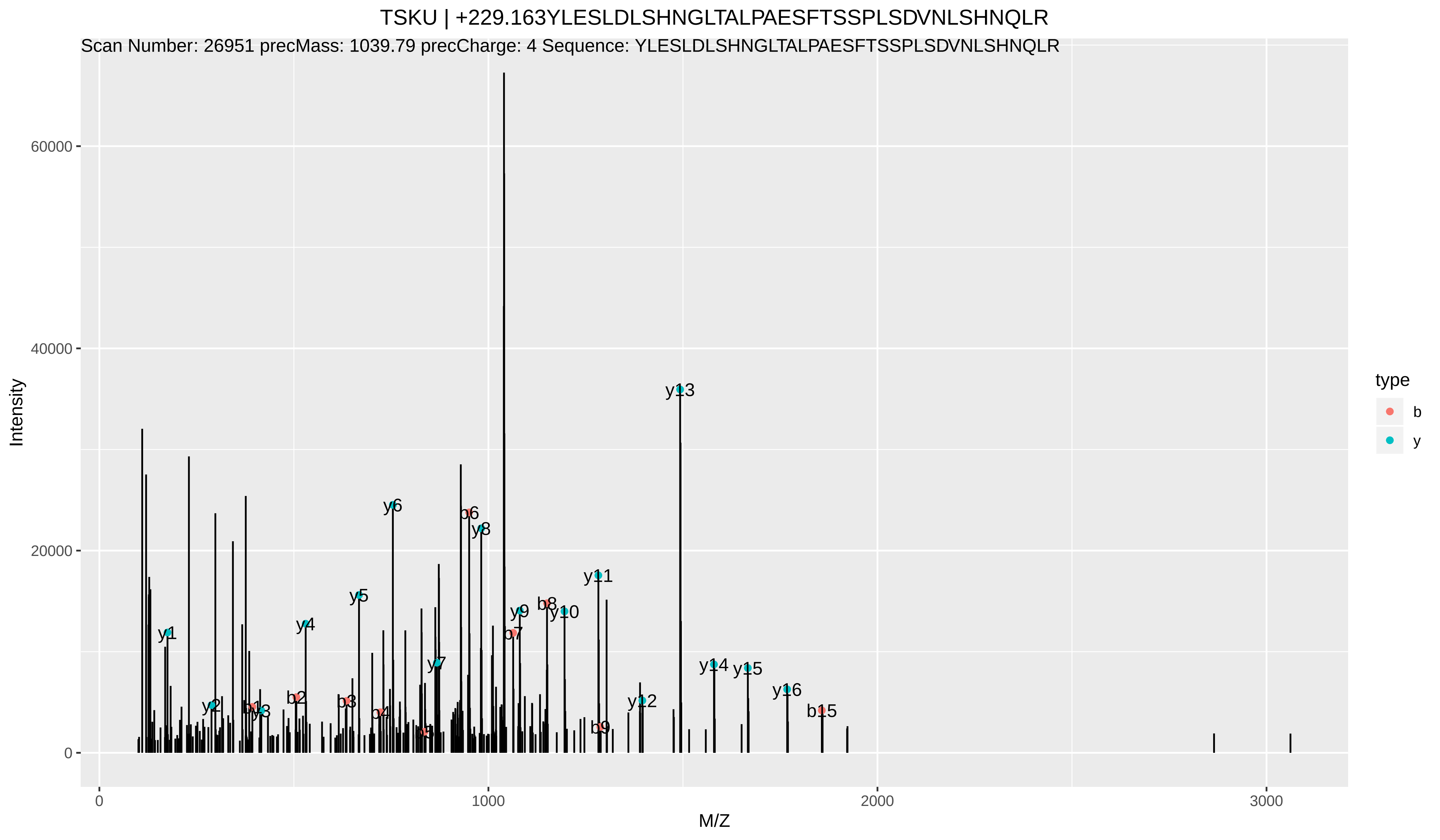

TSKU | +229.163YLESLDLSHNGLTALPAESFTSSPLSDVNLSHNQLR
Scan Number: 26951 precMass: 1039.79 precCharge: 4 Sequence: YLESLDLSHNGLTALPAESFTSSPLSDVNLSHNQLR
Intensity
M/Z
type
b
y

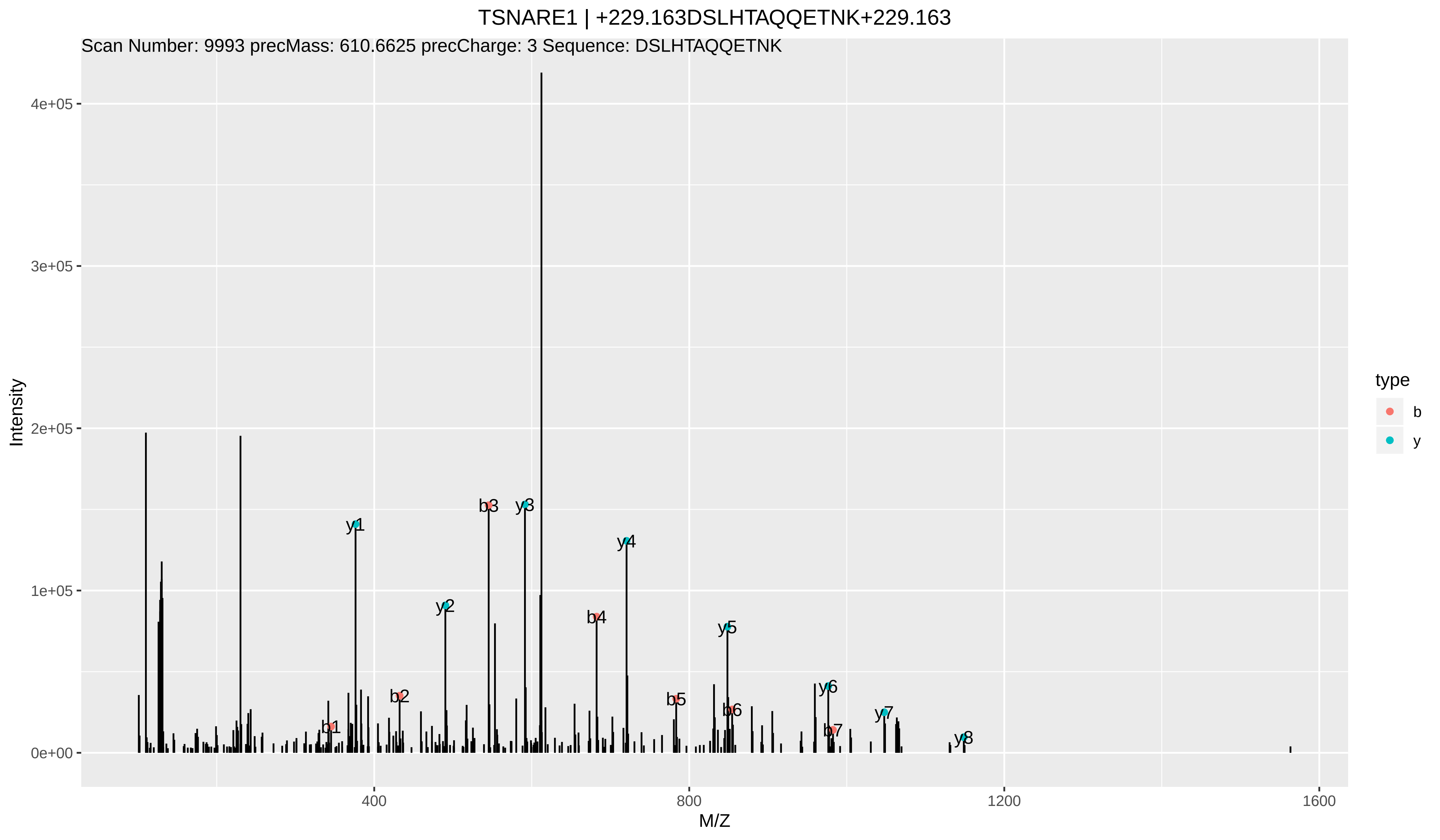
TSNARE1 | +229.163DSLHTAQQETNK+229.163
Scan Number: 9993 precMass: 610.6625 precCharge: 3 Sequence: DSLHTAQQETNK
Intensity
4e+05
3e+05
2e+05
1e+05
0e+00
M/Z
400
800
1200
1600
y1
y2
y3
y4
y5
y6
y7
y8
b1
b2
b3
b4
b5
b6
b7
type
b
y

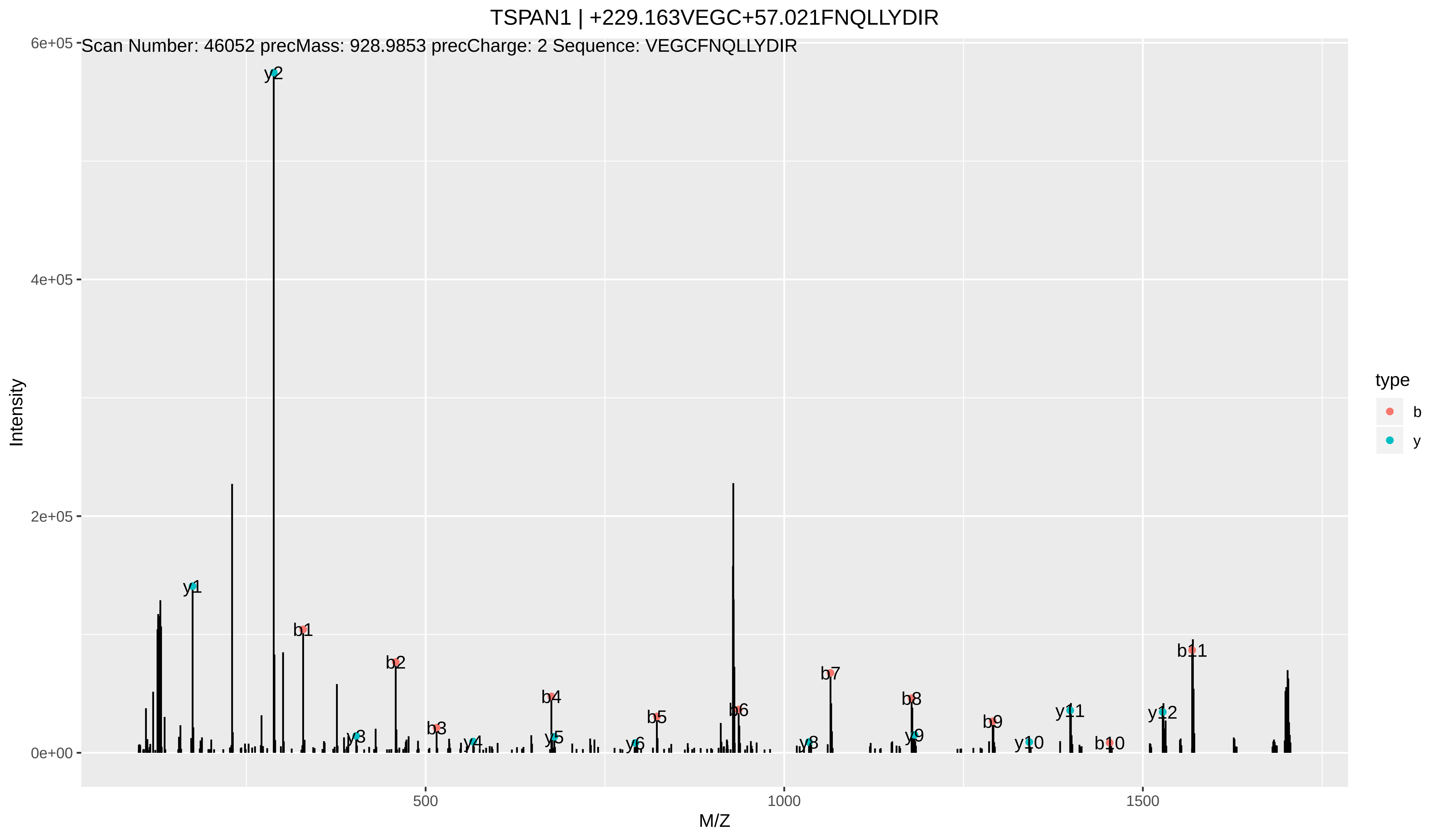
TSPAN1 | +229.163VEGC+57.021FNQLLYDIR
Scan Number: 46052 precMass: 928.9853 precCharge: 2 Sequence: VEGCFNQLLYDIR
Intensity
M/Z
type
b
y
y1
y2
y3
y4
y5
y6
y8
y9
y10
y11
y12
b1
b2
b3
b4
b5
b6
b7
b8
b9
b10
b11
0e+00
2e+05
4e+05
6e+05
500
1000
1500

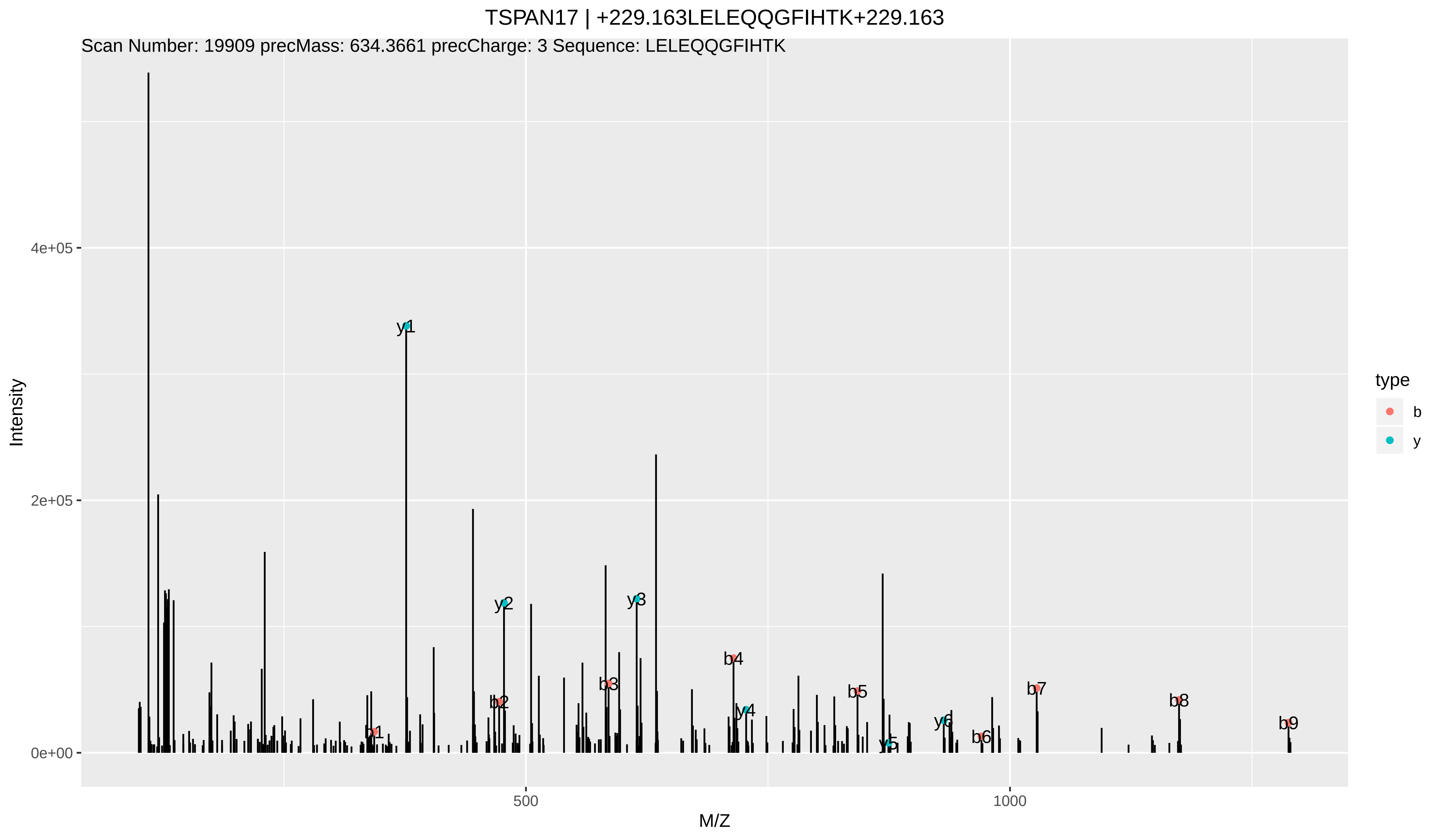
TSPAN17 | +229.163LELEQQGFIHTK+229.163
Scan Number: 19909 precMass: 634.3661 precCharge: 3 Sequence: LELEQQGFIHTK
Intensity
4e+05
2e+05
0e+00
M/Z
500
1000
y1
y2
y3
y4
y5
y6
b1
b2
b3
b4
b5
b6
b7
b8
b9
type
b
y

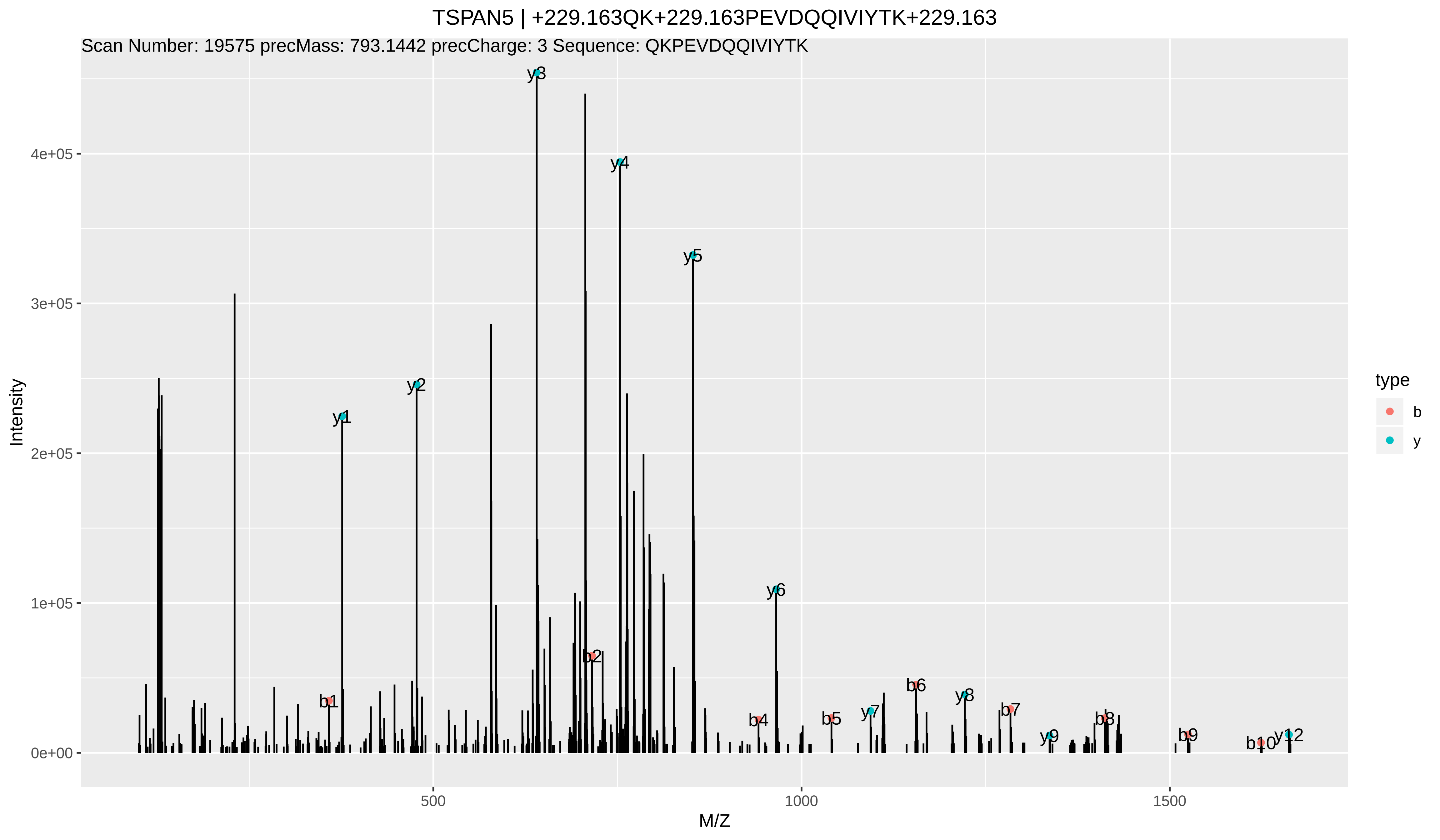
TSPAN5 | +229.163QK+229.163PEVDQQIVIYTK+229.163
Scan Number: 19575 precMass: 793.1442 precCharge: 3 Sequence: QKPEVDQQIVIYTK
Intensity
M/Z
type
b
y
y3
y4
y5
y2
y1
y6
b1
b2
b4
b5
y7
b6
y8
b7
y9
b8
b9
b10
y12
0e+00
1e+05
2e+05
3e+05
4e+05
500
1000
1500

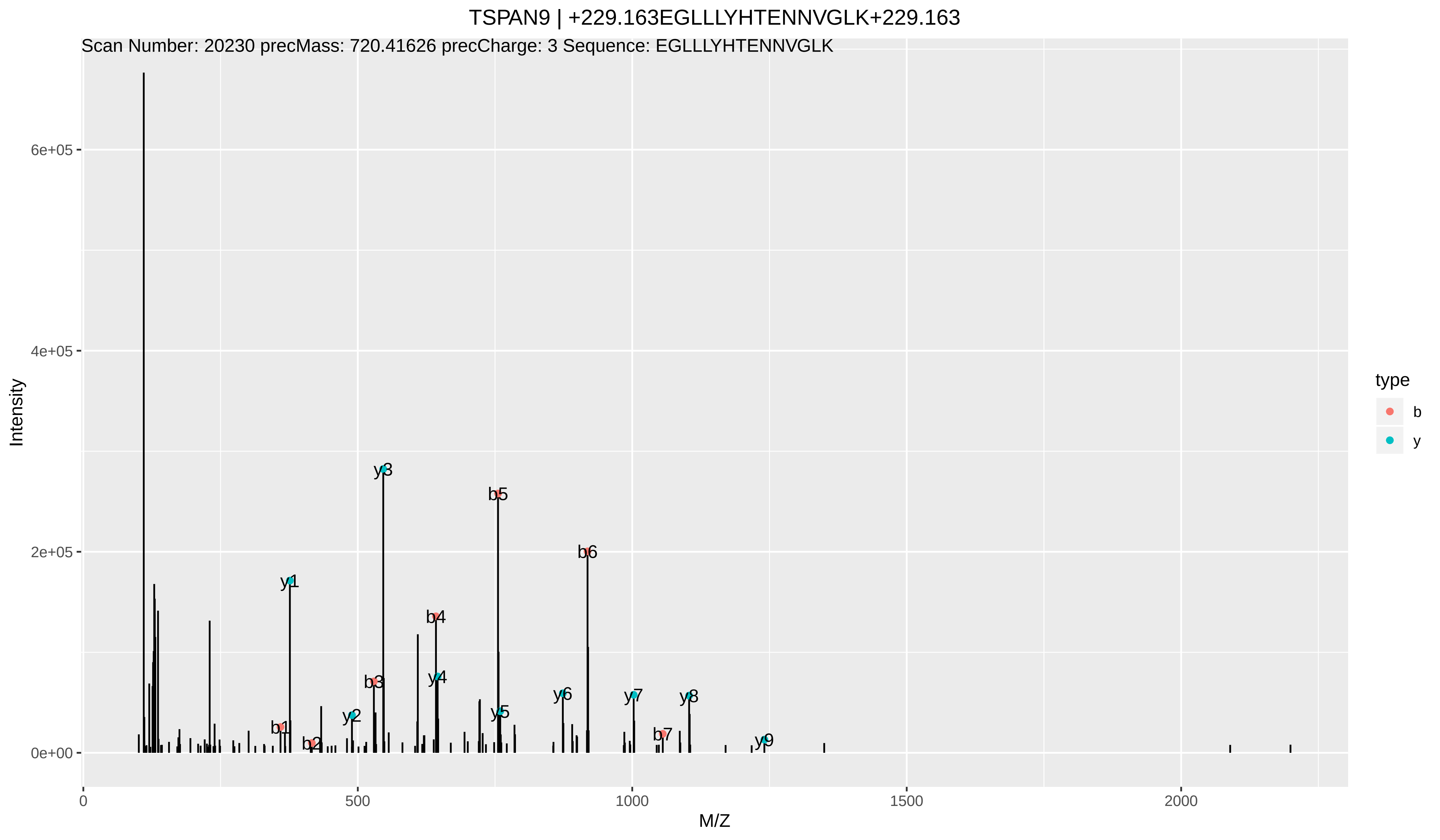

TSPAN9 | +229.163EGLLLYHTENNVGLK+229.163
Scan Number: 20230 precMass: 720.41626 precCharge: 3 Sequence: EGLLLYHTENNVGLK
Intensity
M/Z
0e+00
2e+05
4e+05
6e+05
0
500
1000
1500
2000
type
b
y
y1
y2
y3
y4
y5
y6
y7
y8
y9
b1
b2
b3
b4
b5
b6
b7

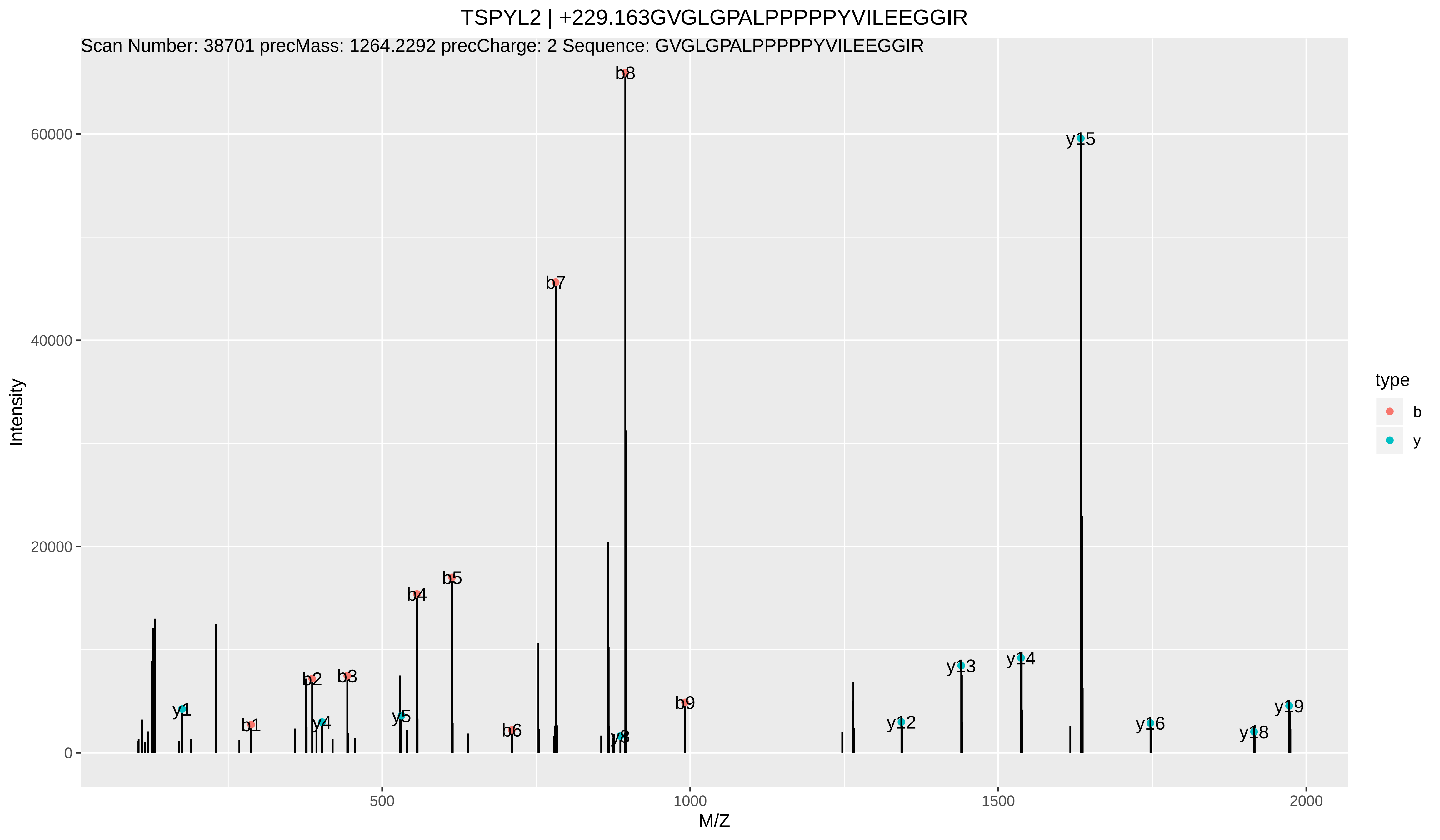
TSPYL2 | +229.163GVGLGPALPPPPPYVILEEGGIR
Scan Number: 38701 precMass: 1264.2292 precCharge: 2 Sequence: GVGLGPALPPPPPYVILEEGGIR
Intensity
60000
40000
20000
0
M/Z
500
1000
1500
2000
b8
y15
b7
b5
b4
b3
b2
y1
b1
y4
y5
b6
y8
b9
y12
y13
y14
y16
y18
y19
type
b
y

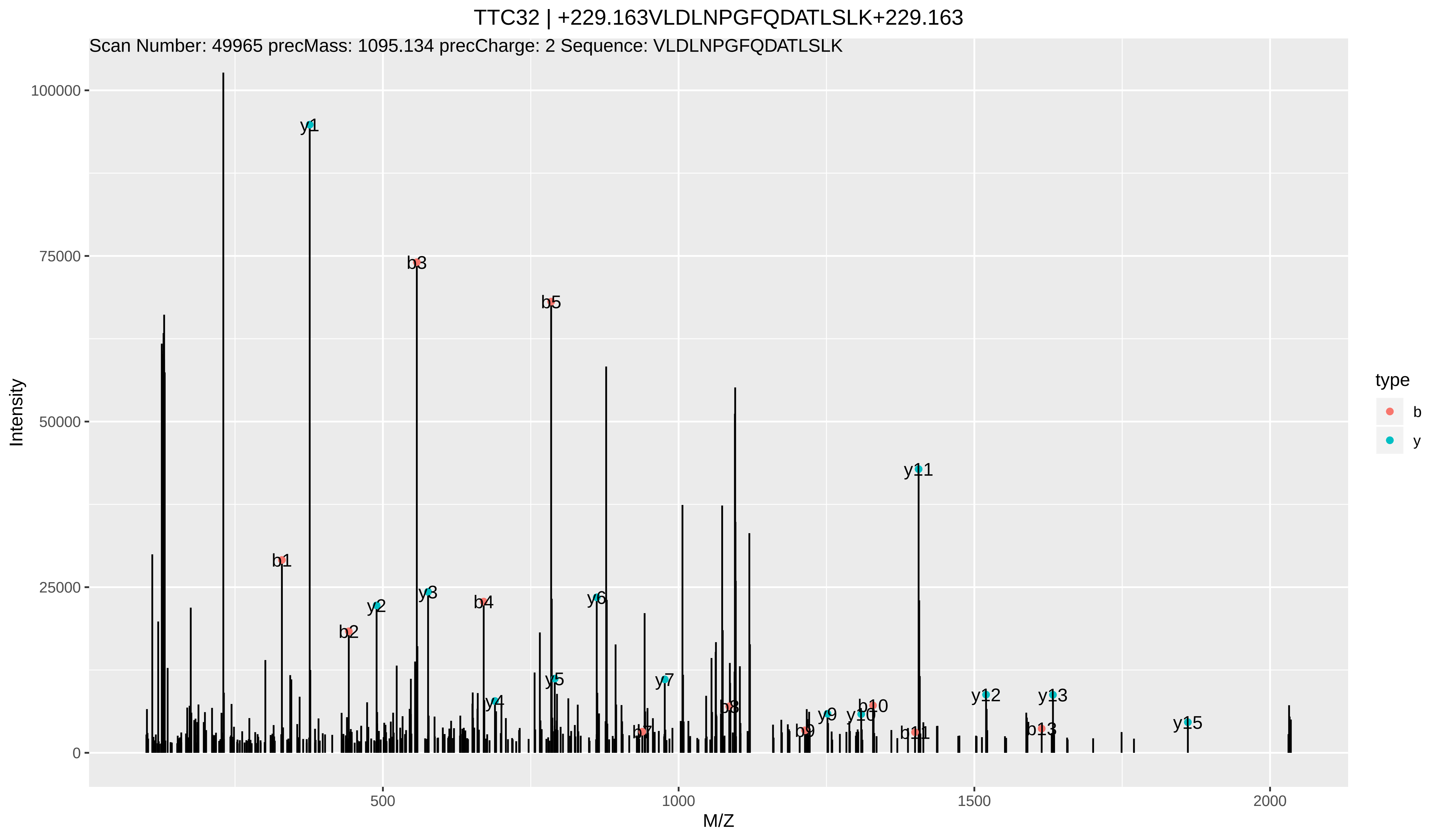
TTC32 | +229.163VLDLNPGFQDATLSLK+229.163
Scan Number: 49965 precMass: 1095.134 precCharge: 2 Sequence: VLDLNPGFQDATLSLK
Intensity
100000
75000
50000
25000
0
500
1000
1500
2000
M/Z
y1
b3
b5
b1
y2
b2
y3
b4
y6
y4
y5
y7
b7
b8
y9
b9
y10
b10
y11
b11
y12
y13
b13
y15
type
b
y

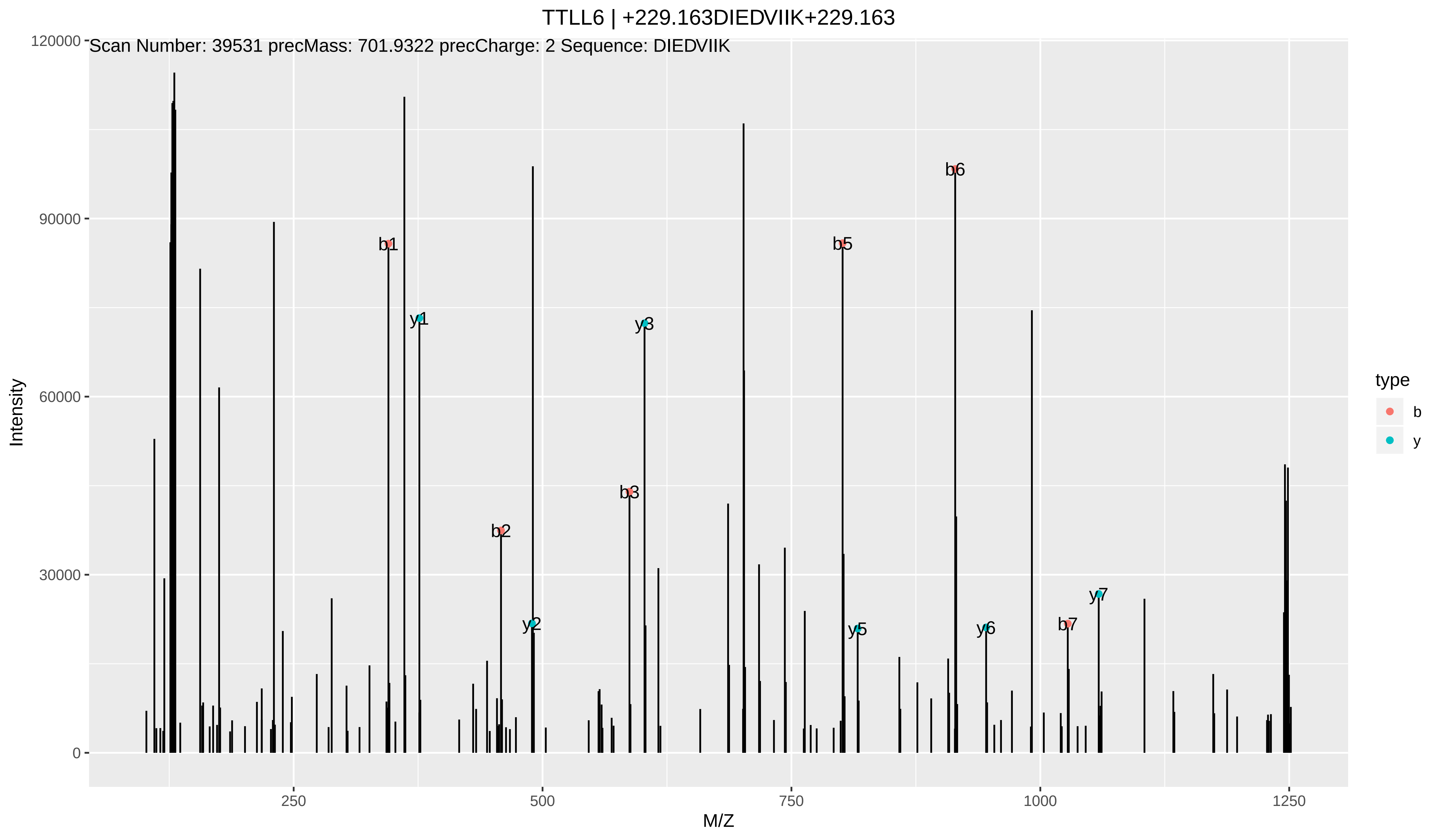

TTLL6 | +229.163DIEDVIIK+229.163
Scan Number: 39531 precMass: 701.9322 precCharge: 2 Sequence: DIEDVIIK
Intensity
M/Z
type
b
y
b1
b2
b3
b5
b6
b7
y1
y2
y3
y5
y6
y7
0
30000
60000
90000
120000
250
500
750
1000
1250

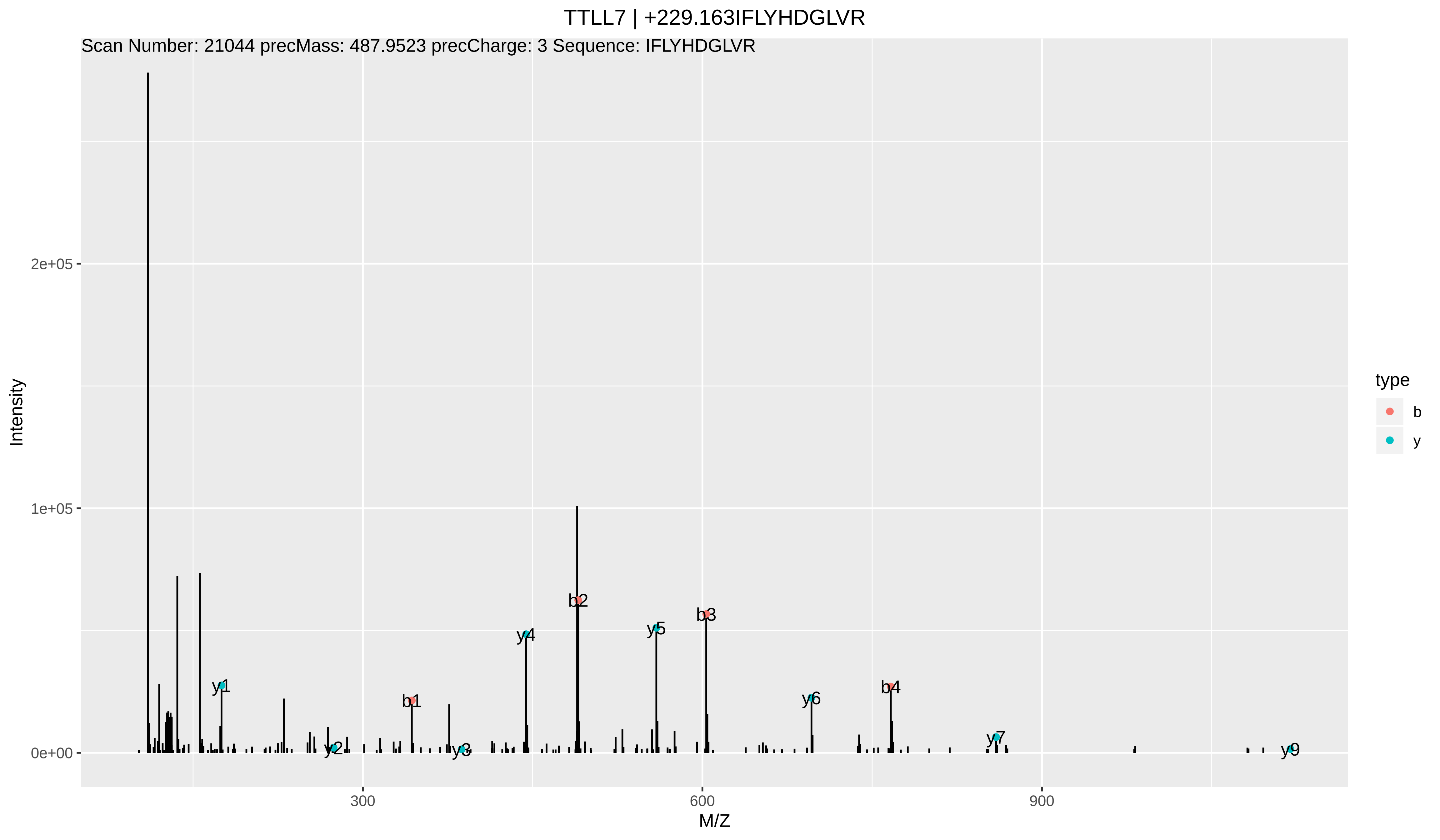
TTLL7 | +229.163IFLYHDGLVR
Scan Number: 21044 precMass: 487.9523 precCharge: 3 Sequence: IFLYHDGLVR
Intensity
2e+05
1e+05
0e+00
300
600
900
M/Z
y1
y2
y3
y4
y5
y6
y7
y9
b1
b2
b3
b4
type
b
y

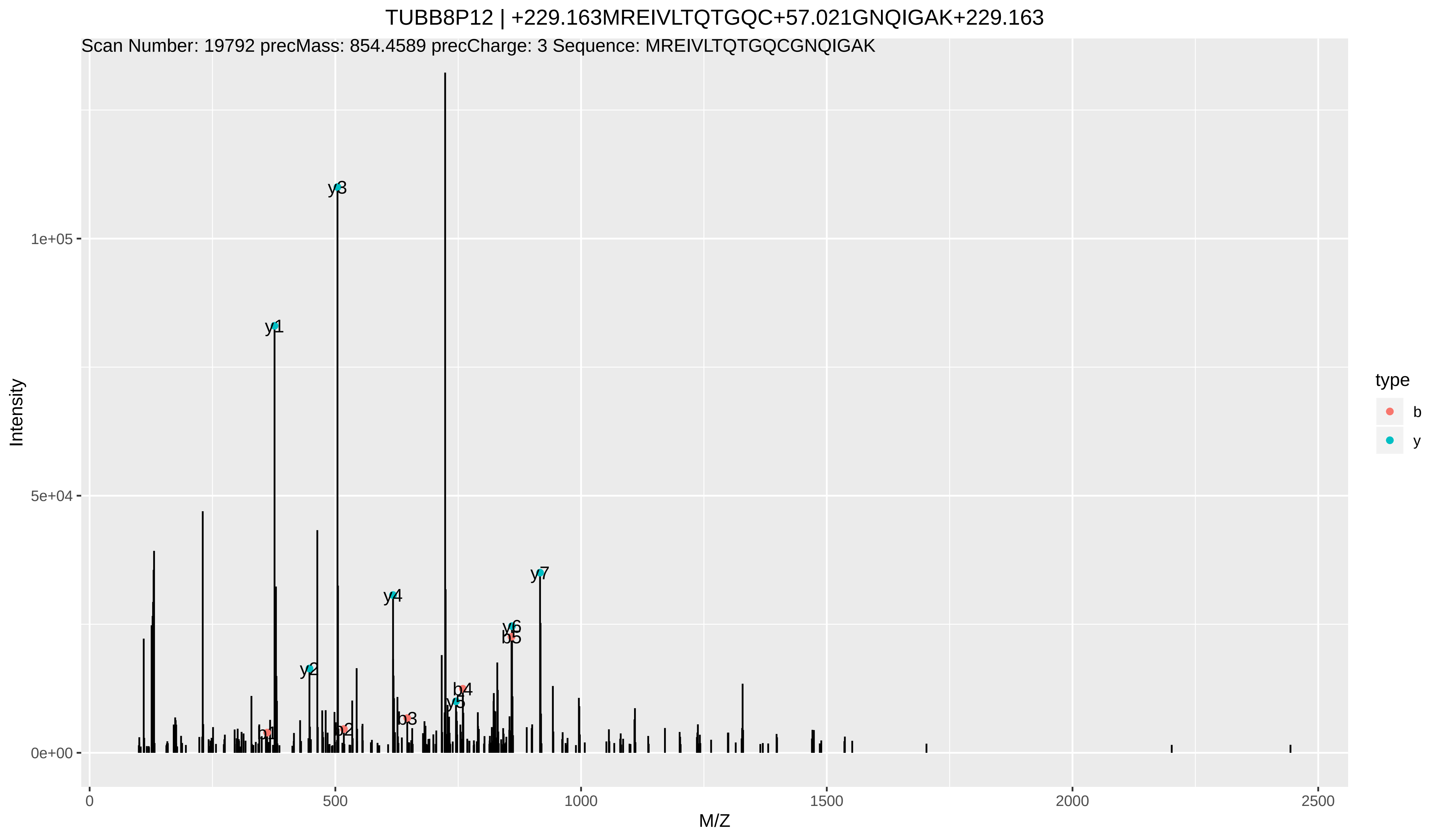

TUBB8P12 | +229.163MREIVLTQTGQC+57.021GNQIGAK+229.163
Scan Number: 19792 precMass: 854.4589 precCharge: 3 Sequence: MREIVLTQTGQCGNQIGAK
Intensity
M/Z
1e+05
5e+04
0e+00
0
500
1000
1500
2000
2500
y1
y2
y3
y4
y5
y6
y7
b1
b2
b3
b4
b5
type
b
y

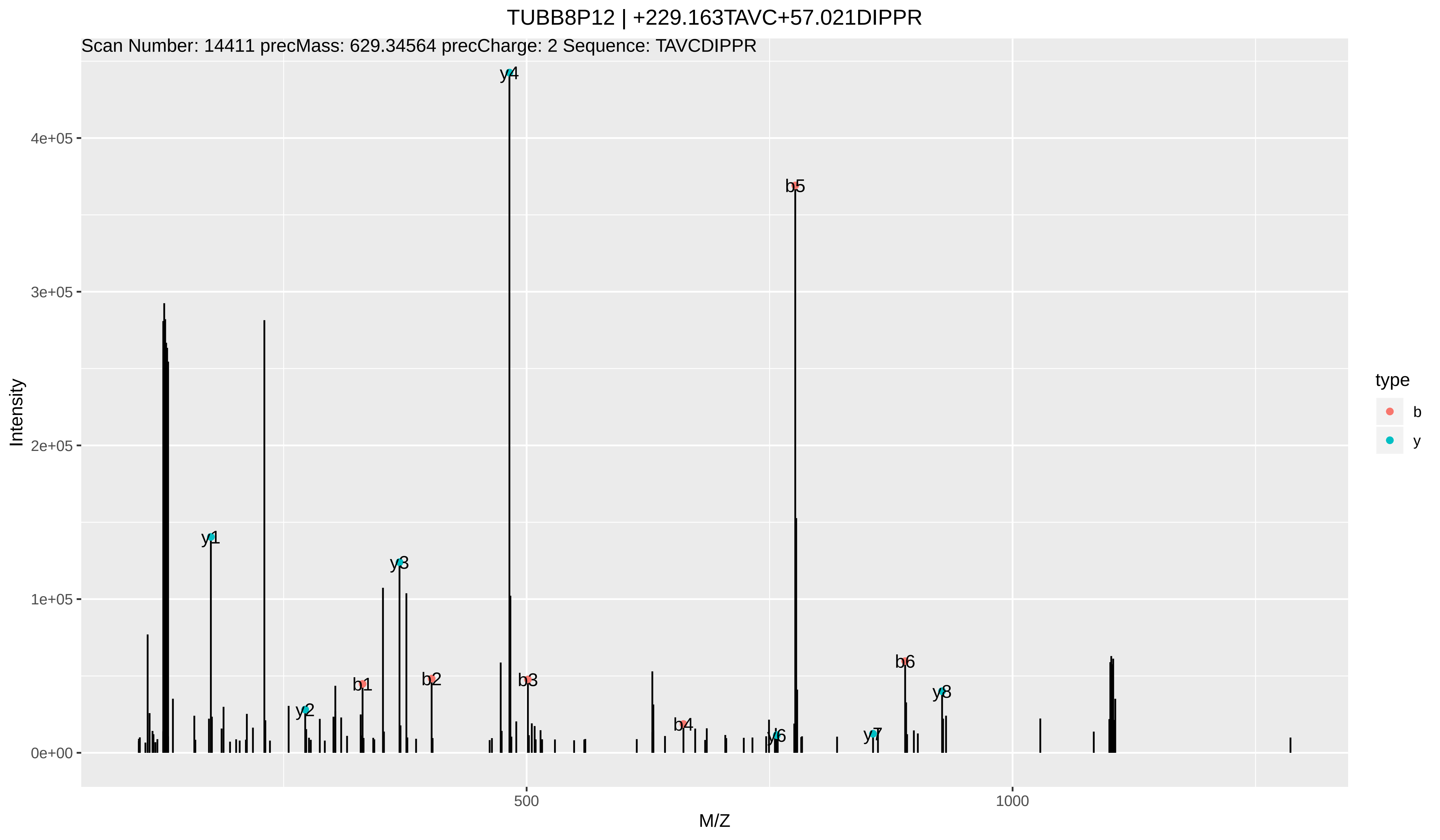
TUBB8P12 | +229.163TAVC+57.021DIPPR
Scan Number: 14411 precMass: 629.34564 precCharge: 2 Sequence: TAVCDIPPR
y4
b5
y1
y3
b1
b2
b3
y2
b4
y6
y7
b6
y8
4e+05
3e+05
2e+05
1e+05
0e+00
Intensity
500
1000
M/Z
type
b
y

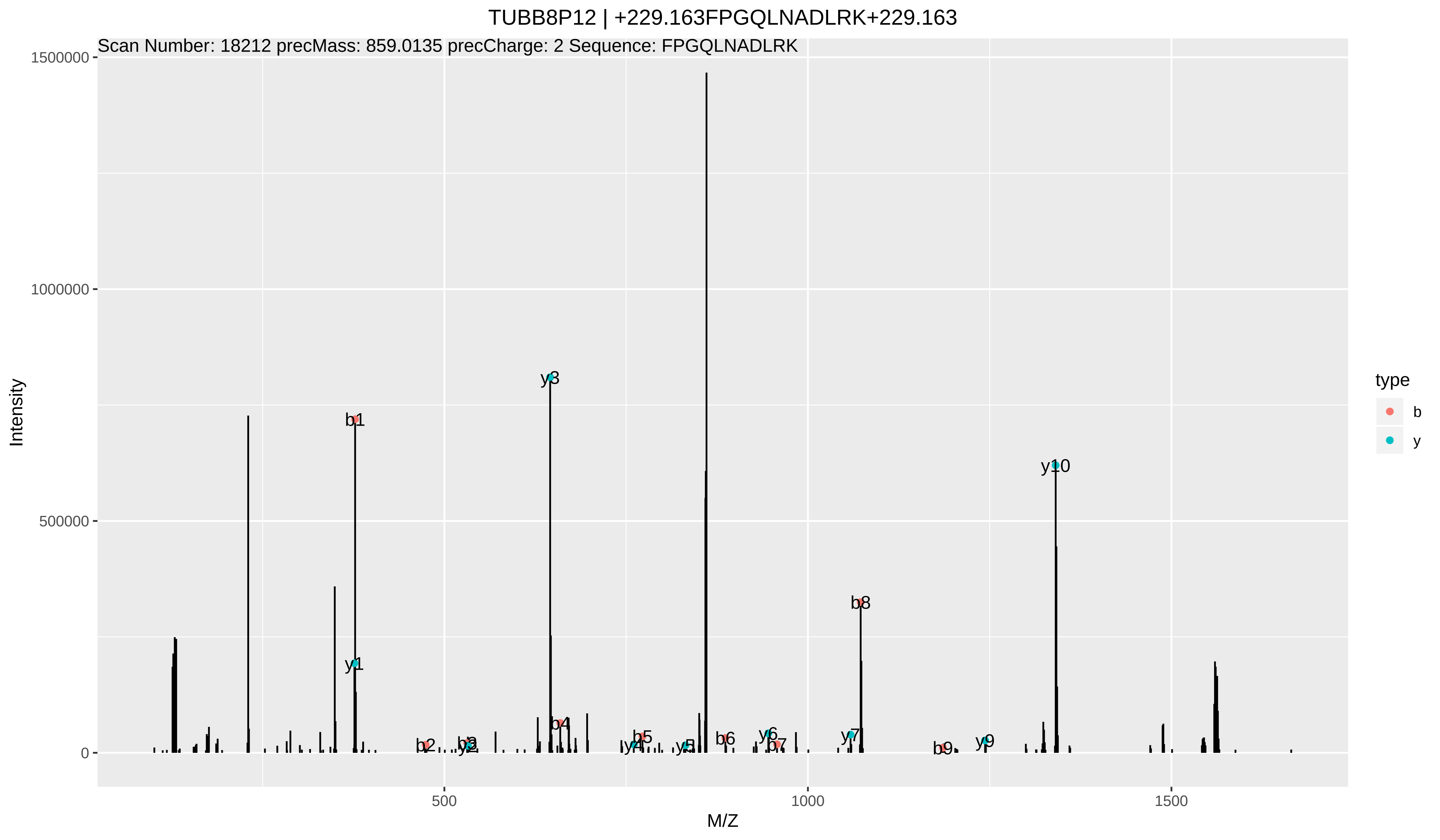
TUBB8P12 | +229.163FPGQLNADLRK+229.163
Scan Number: 18212 precMass: 859.0135 precCharge: 2 Sequence: FPGQLNADLRK
Intensity
M/Z
1500000
1000000
500000
0
500
1000
1500
type
b
y
b1
y1
b2
b3
y2
y3
b4
y4
b5
y5
b6
y6
b7
y7
b8
b9
y9
y10

TUBB8P12 | +229.163NMMAAC+57.021DPR

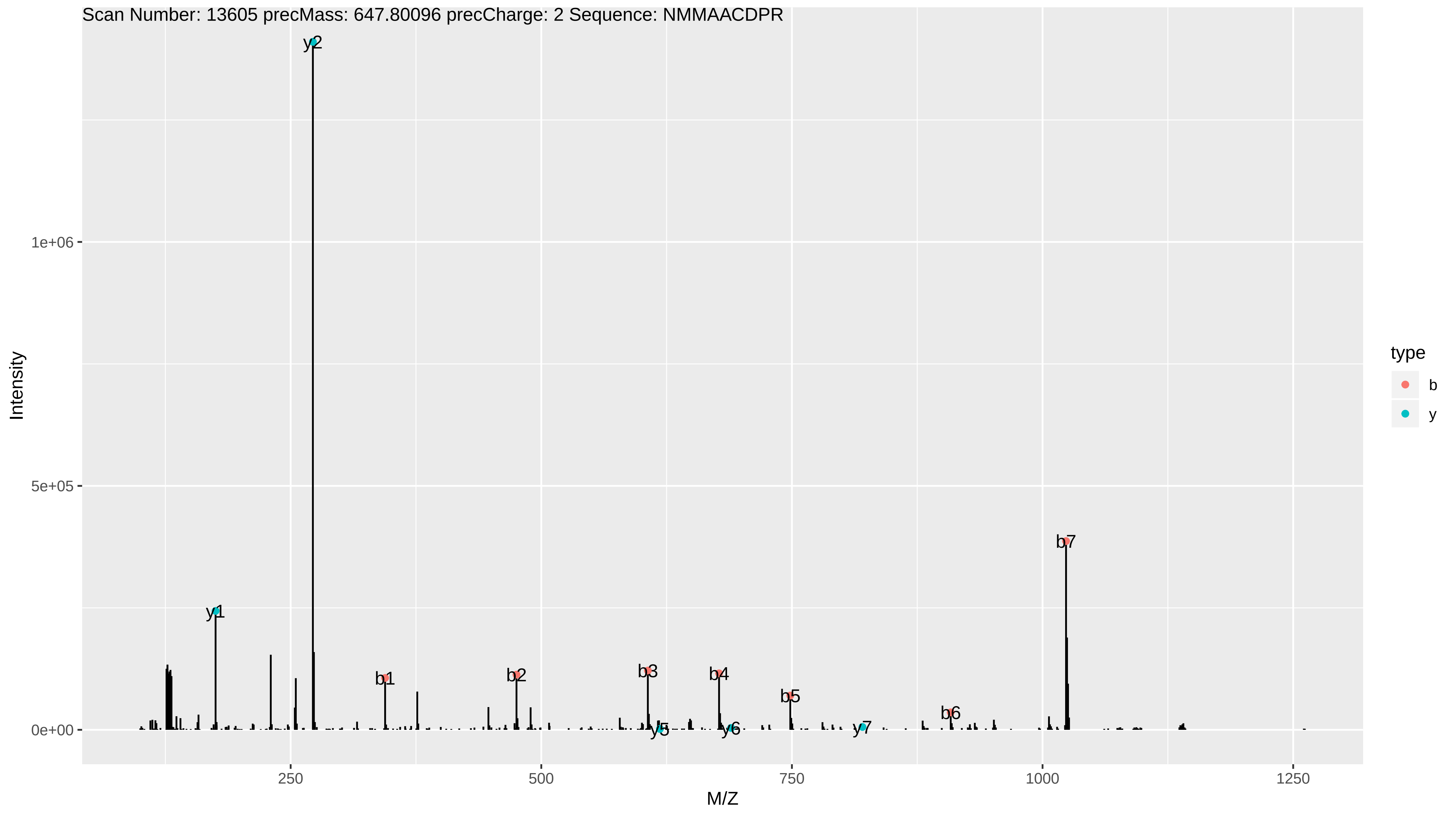

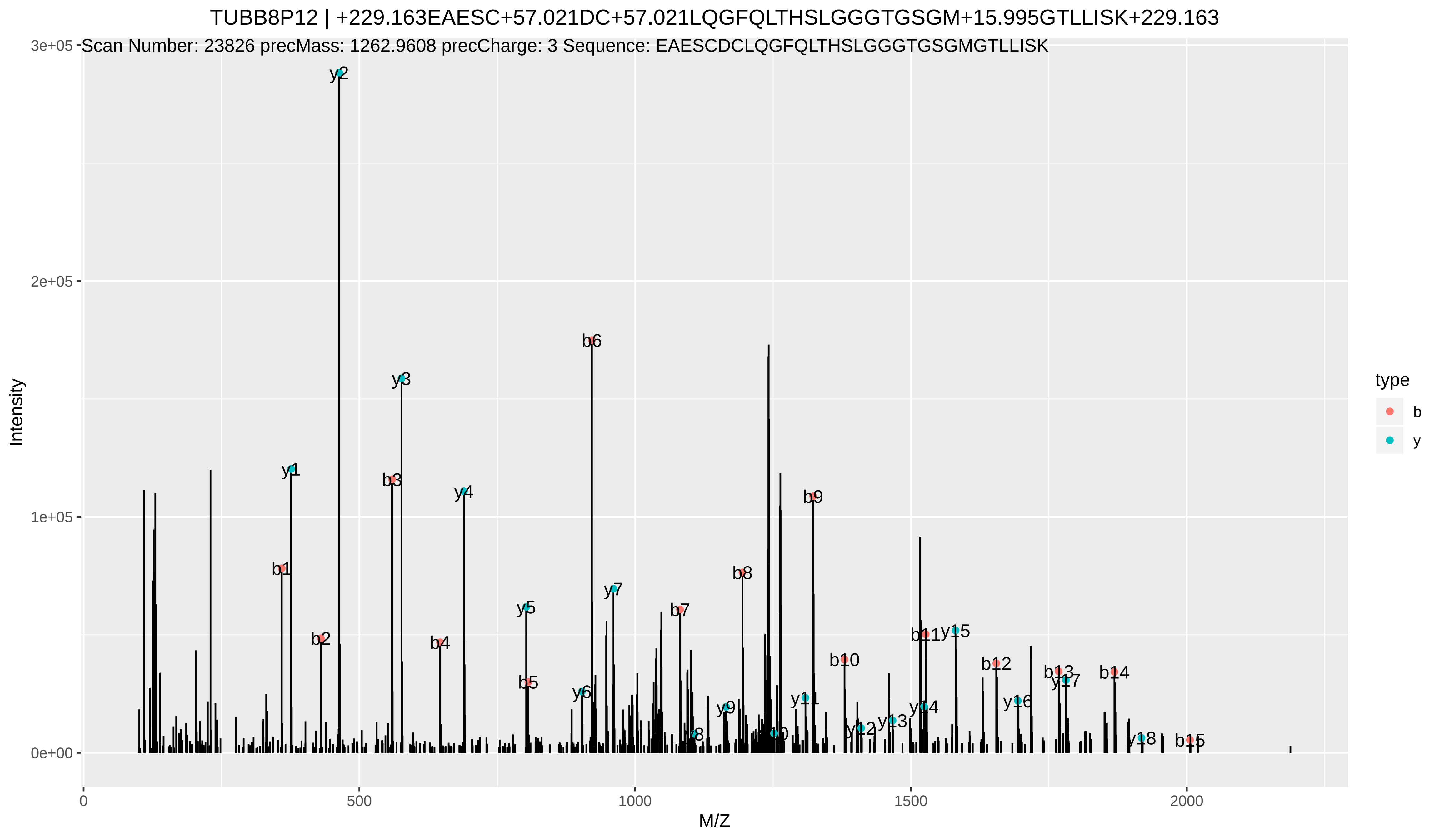
TUBB8P12 | +229.163EAESC+57.021DC+57.021LQGFQLTHSLGGGTGSGM+15.995GTLLISK+229.163
Scan Number: 23826 precMass: 1262.9608 precCharge: 3 Sequence: EAESCDCLQGFQLTHSLGGGTGSGMGTLLISK
Intensity
3e+05
2e+05
1e+05
0e+00
M/Z
0
500
1000
1500
2000
type
b
y
y2
b6
y3
y1
b3
y4
b9
b1
b8
y7
b7
y5
b2
b4
b5
y6
y9
y11
b10
b11
y15
b12
b13
y17
b14
y16
y14
y13
y12
y18
b15

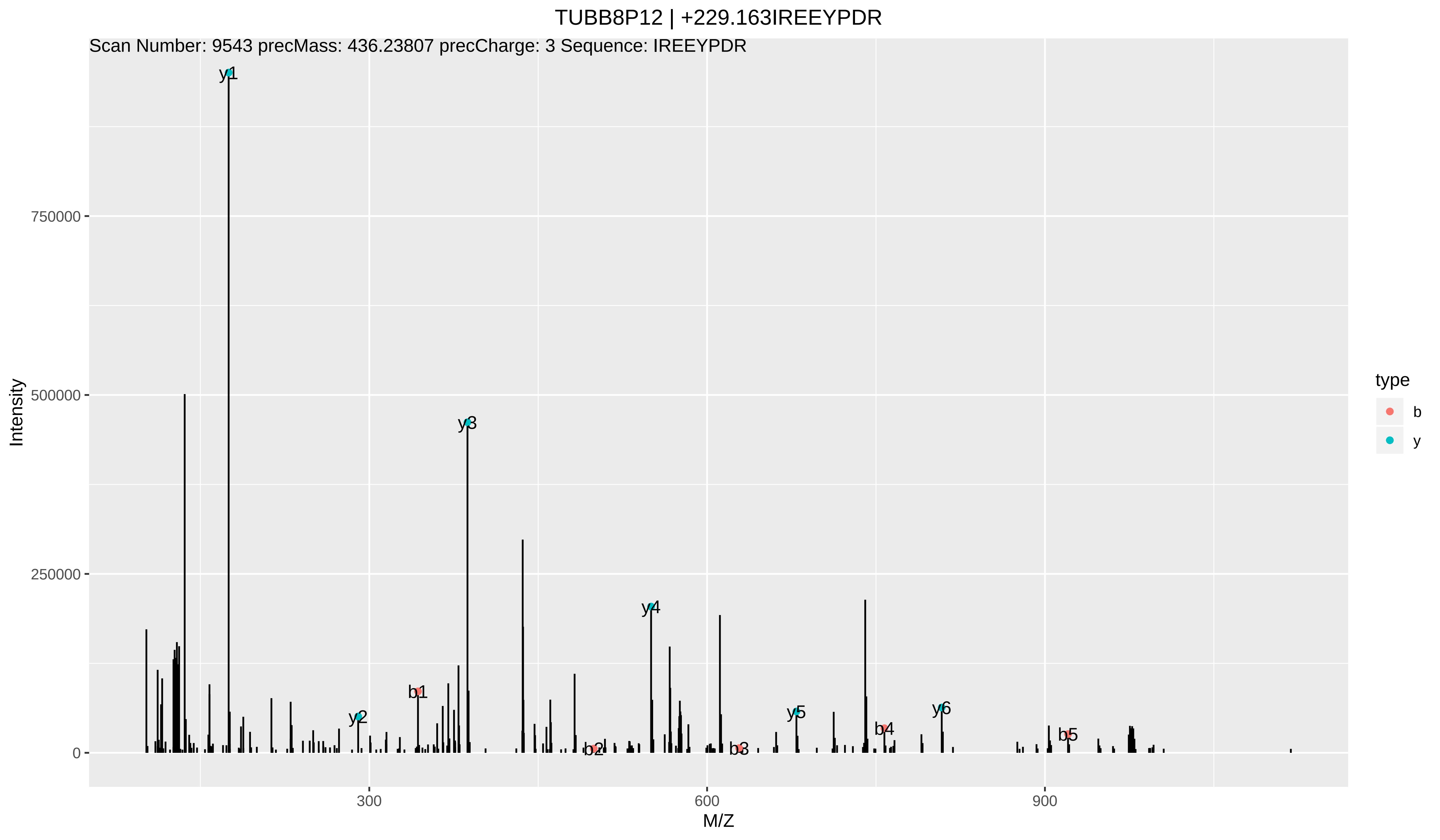
TUBB8P12 | +229.163IREEYPDR
Scan Number: 9543 precMass: 436.23807 precCharge: 3 Sequence: IREEYPDR
y1
y3
y4
y2
b1
b2
b3
y5
y6
b4
b5
Intensity
750000
500000
250000
0
300
600
900
M/Z
type
b
y

TUBB8P12 | +229.163ALTVAELTQQM+15.995FDAK+229.163

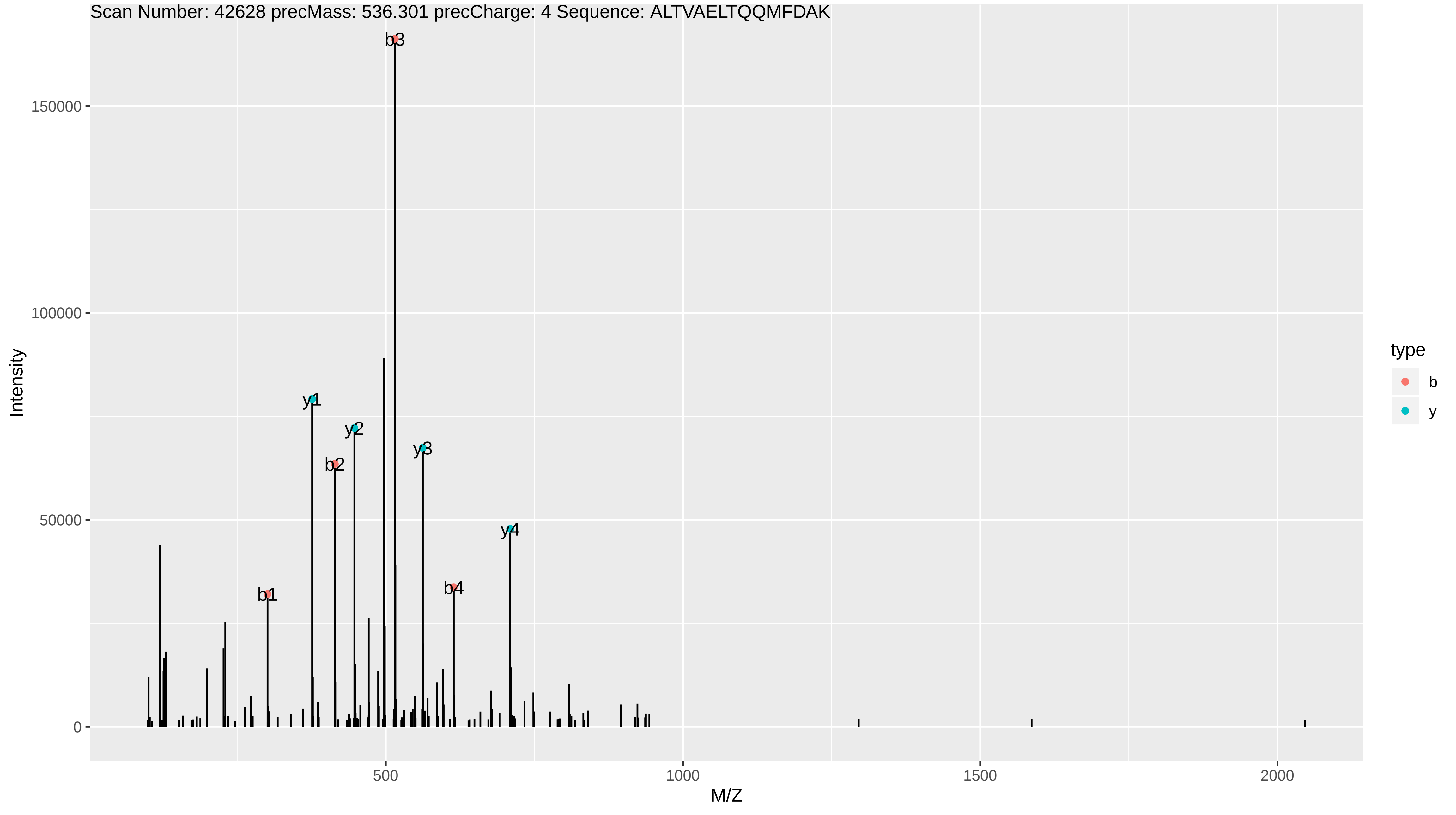

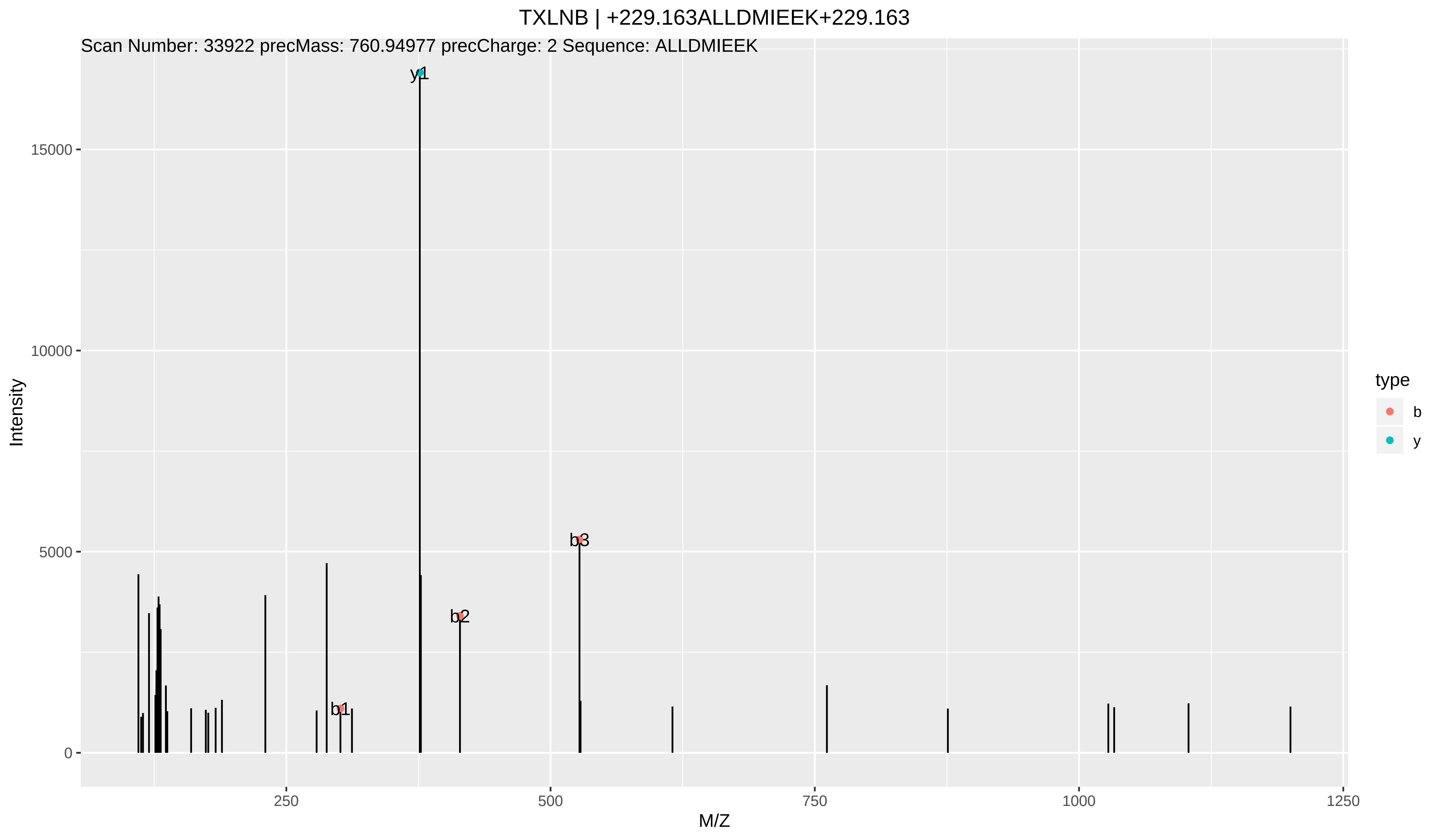

TXLNB | +229.163ALLLDMIEEK+229.163
Scan Number: 33922 precMass: 760.94977 precCharge: 2 Sequence: ALLDMIEEK
y1
b3
b2
b1
15000
10000
5000
0
Intensity
250
500
750
1000
1250
M/Z
type
b
y

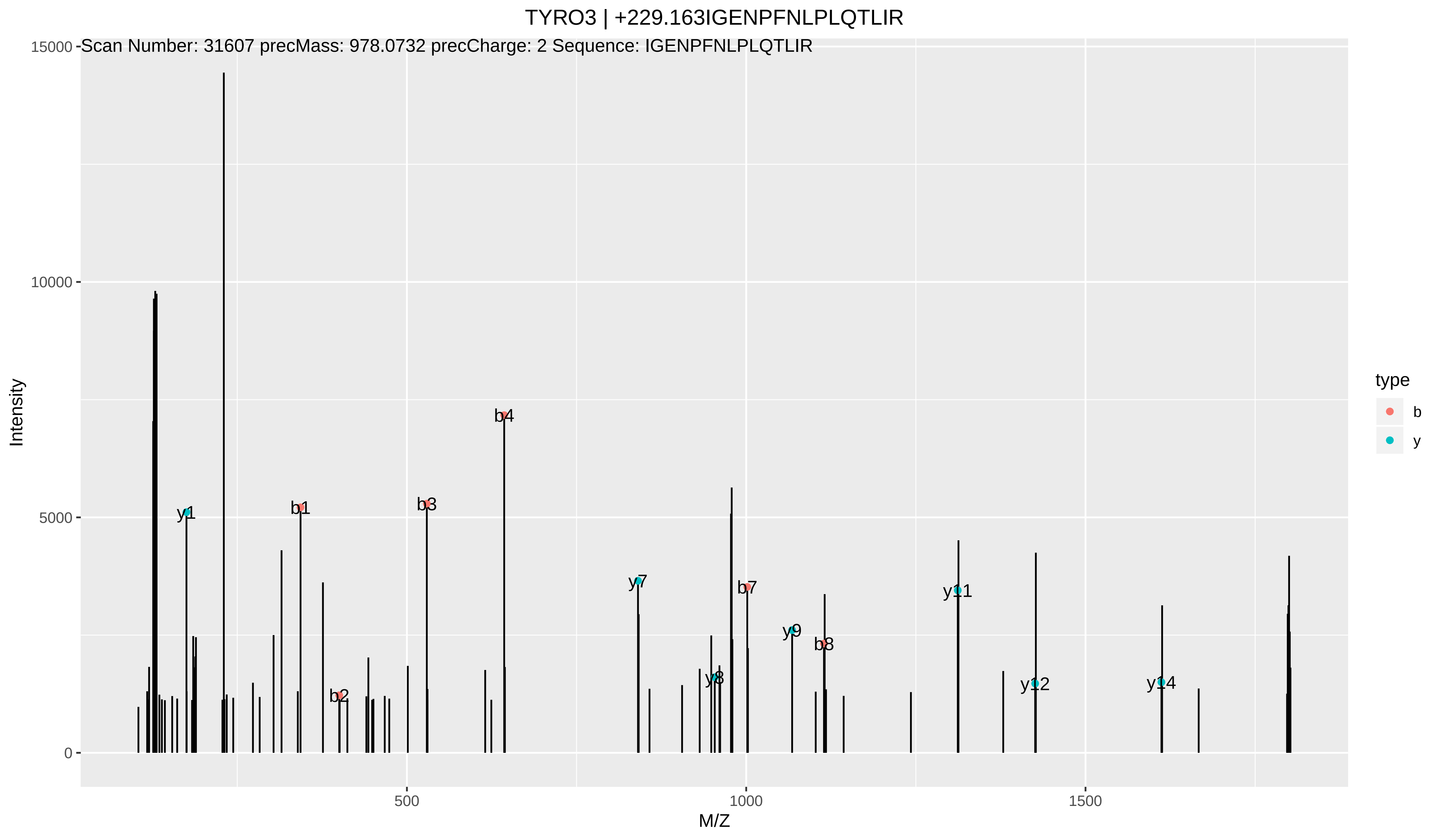
TYRO3 | +229.163IGENPFNLPLQTLIR
Scan Number: 31607 precMass: 978.0732 precCharge: 2 Sequence: IGENPFNLPLQTLIR
Intensity
15000
10000
5000
0
M/Z
500
1000
1500
y1
b1
b2
b3
b4
y7
y8
b7
y9
b8
y11
y12
y14
type
b
y

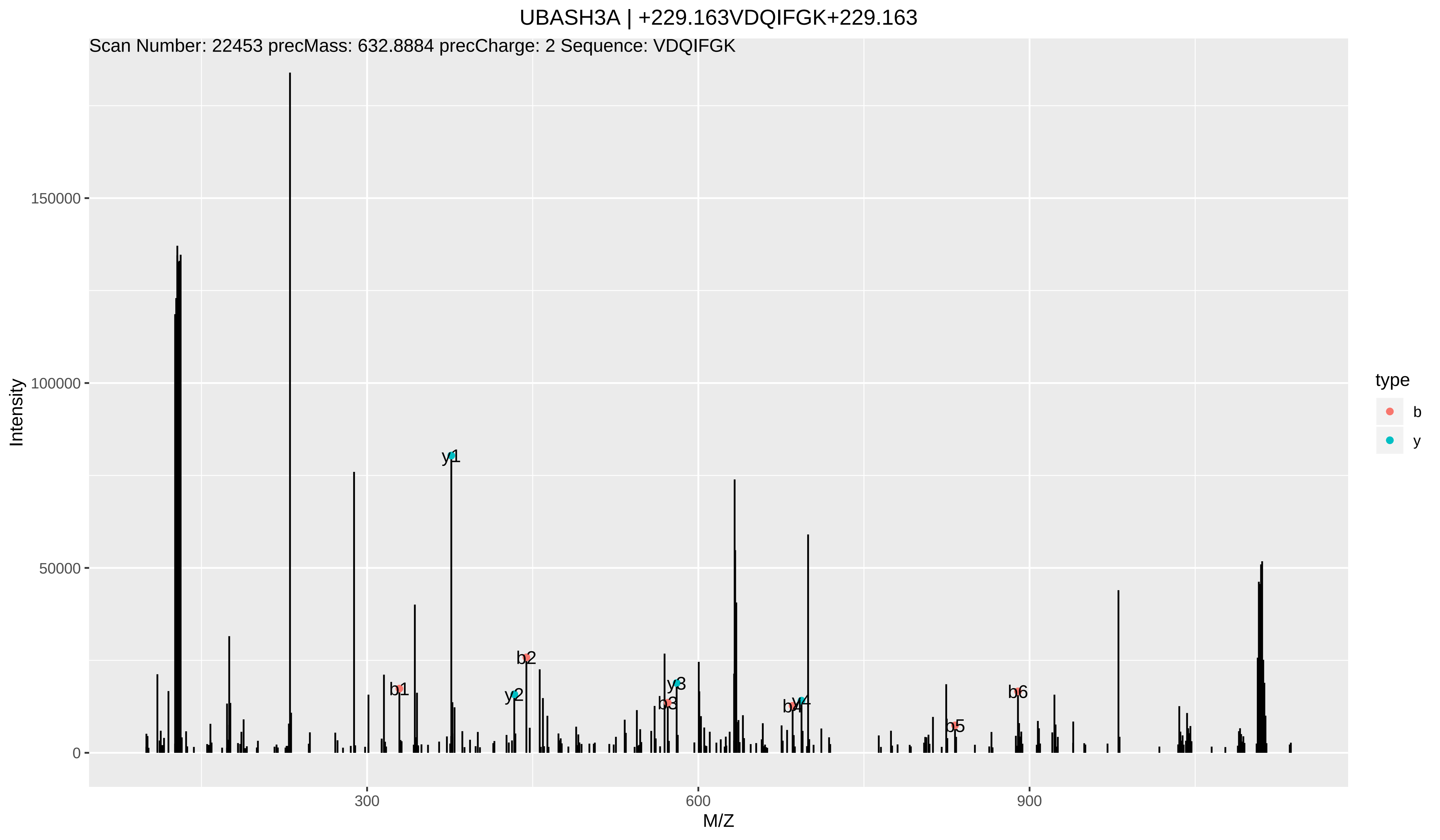

UBASH3A | +229.163VDQIFGK+229.163
Scan Number: 22453 precMass: 632.8884 precCharge: 2 Sequence: VDQIFGK
Intensity
150000
100000
50000
0
M/Z
300
600
900
y1
b1
y2
b2
b3
y3
b4
y4
b5
b6
type
b
y

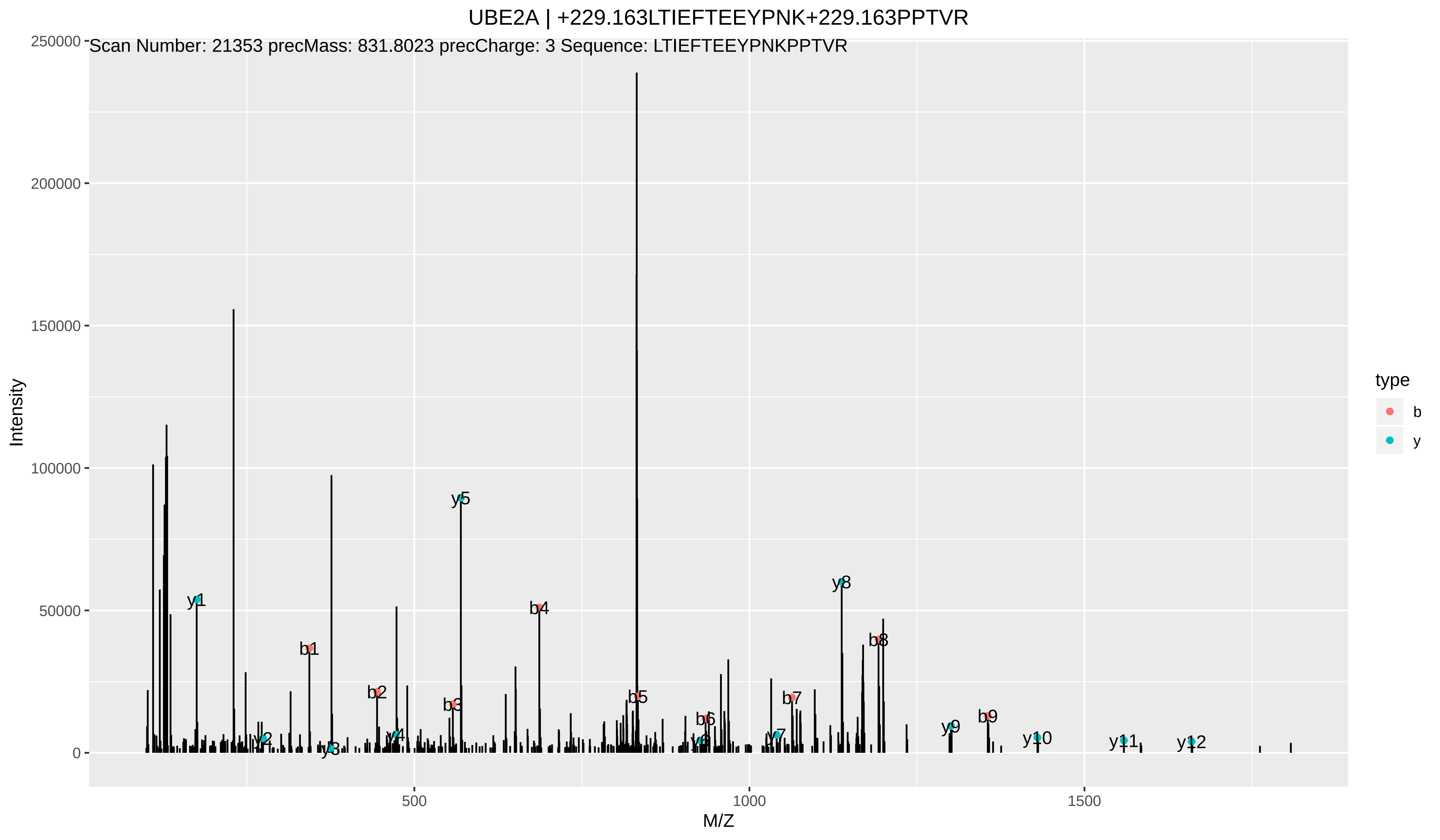

UBE2A | +229.163LTIEFTEEYPNK+229.163PPTVR
Scan Number: 21353 precMass: 831.8023 precCharge: 3 Sequence: LTIEFTEEYPNKPPTVR
Intensity
M/Z
250000
200000
150000
100000
50000
0
500
1000
1500
type
b
y
y1
y2
y3
y4
y5
y6
y7
y8
y9
y10
y11
y12
b1
b2
b3
b4
b5
b6
b7
b8
b9

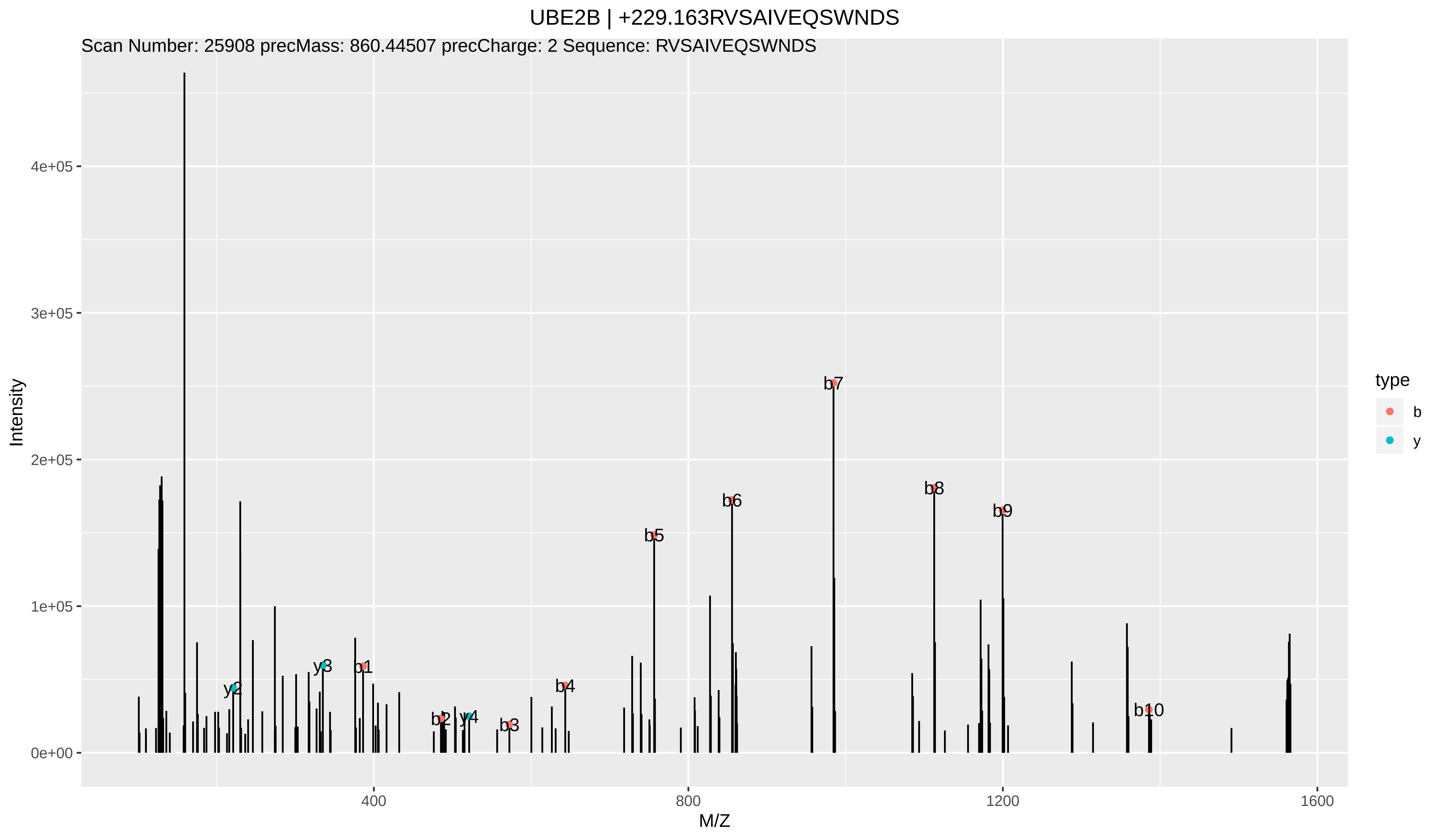

UBE2B | +229.163RVSAIVEQSWNDS
Scan Number: 25908 precMass: 860.44507 precCharge: 2 Sequence: RVSAIVEQSWNDS
Intensity
4e+05
3e+05
2e+05
1e+05
0e+00
M/Z
400
800
1200
1600
b7
b8
b6
b9
b5
y3
b1
y2
b4
b2
y4
b3
b10
type
b
y

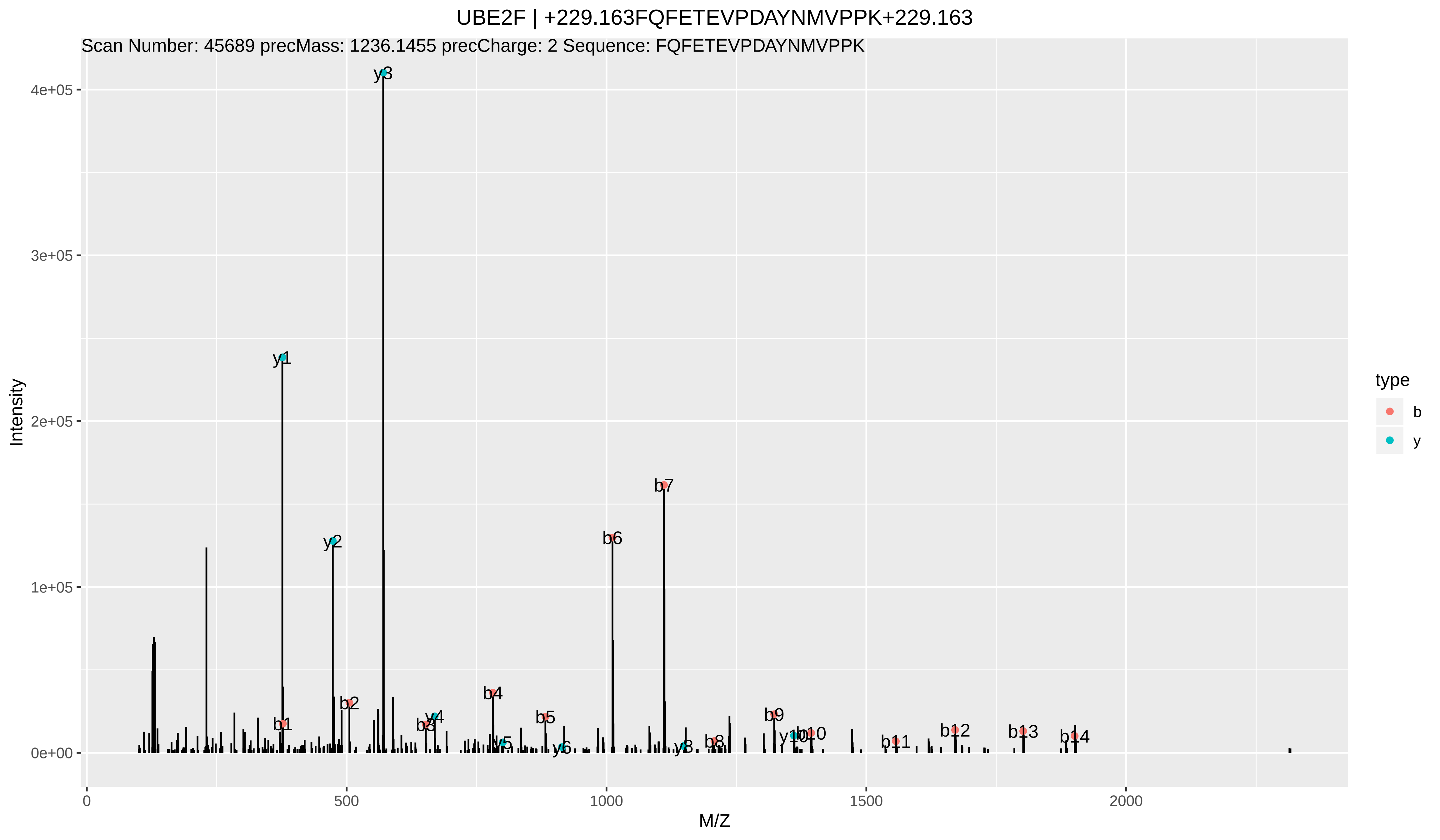

UBE2F | +229.163FQFETEVPDAYNMVPPK+229.163
Scan Number: 45689 precMass: 1236.1455 precCharge: 2 Sequence: FQFETEVPDAYNMVPPK
Intensity
M/Z
type
b
y

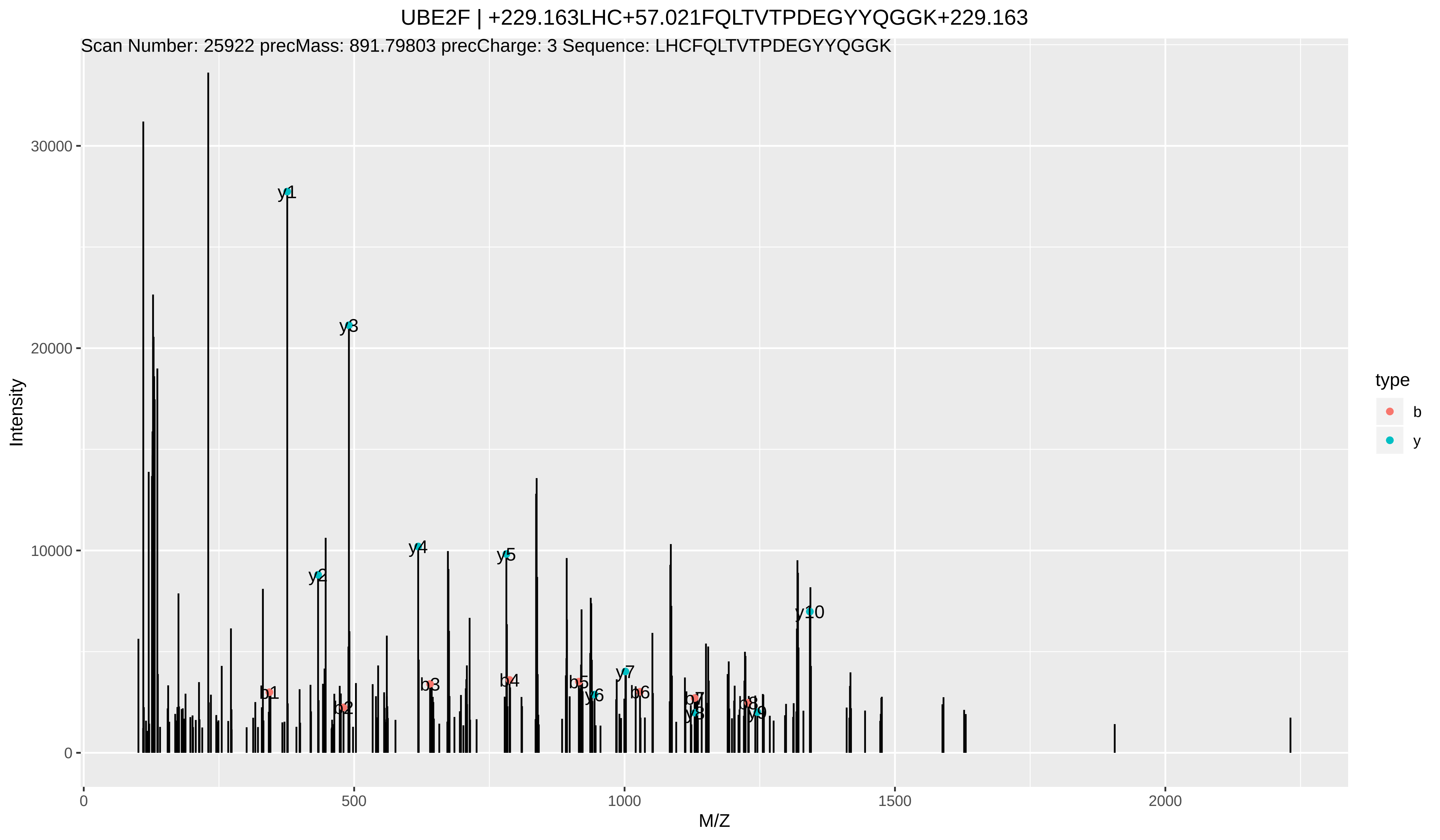

UBE2F | +229.163LHC+57.021FQLTVTPDEGYYQGGK+229.163
Scan Number: 25922 precMass: 891.79803 precCharge: 3 Sequence: LHCFQLTVTPDEGYYQGGK
Intensity
M/Z
0
10000
20000
30000
0
500
1000
1500
2000
y1
y2
y3
y4
y5
y6
y7
y8
y9
y10
b1
b2
b3
b4
b5
b6
b7
b8
type
b
y

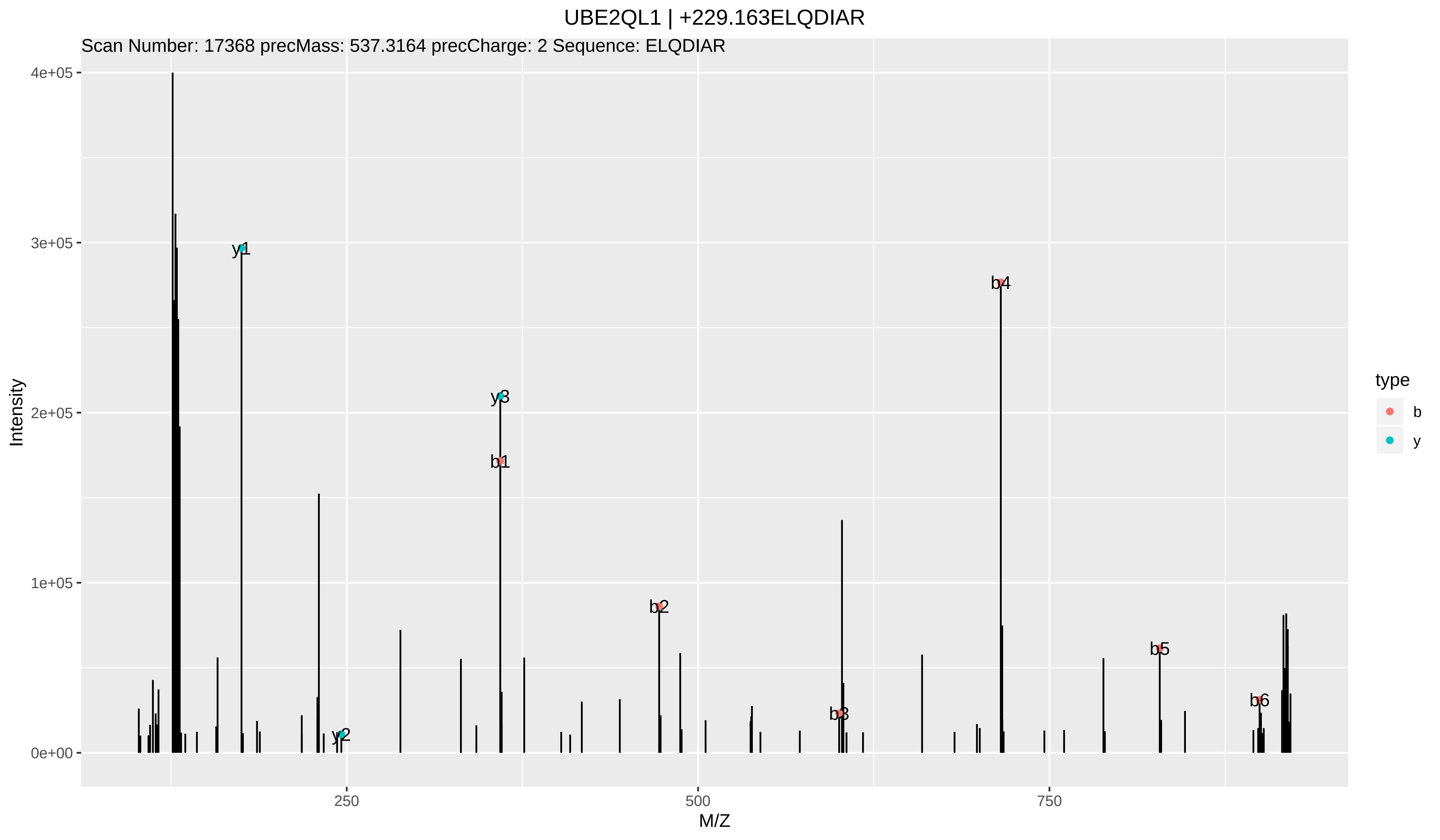
UBE2QL1 | +229.163ELQDIAR
Scan Number: 17368 precMass: 537.3164 precCharge: 2 Sequence: ELQDIAR
Intensity
4e+05
3e+05
2e+05
1e+05
0e+00
y1
y2
y3
b1
b2
b3
b4
b5
b6
250
500
750
M/Z
type
b
y

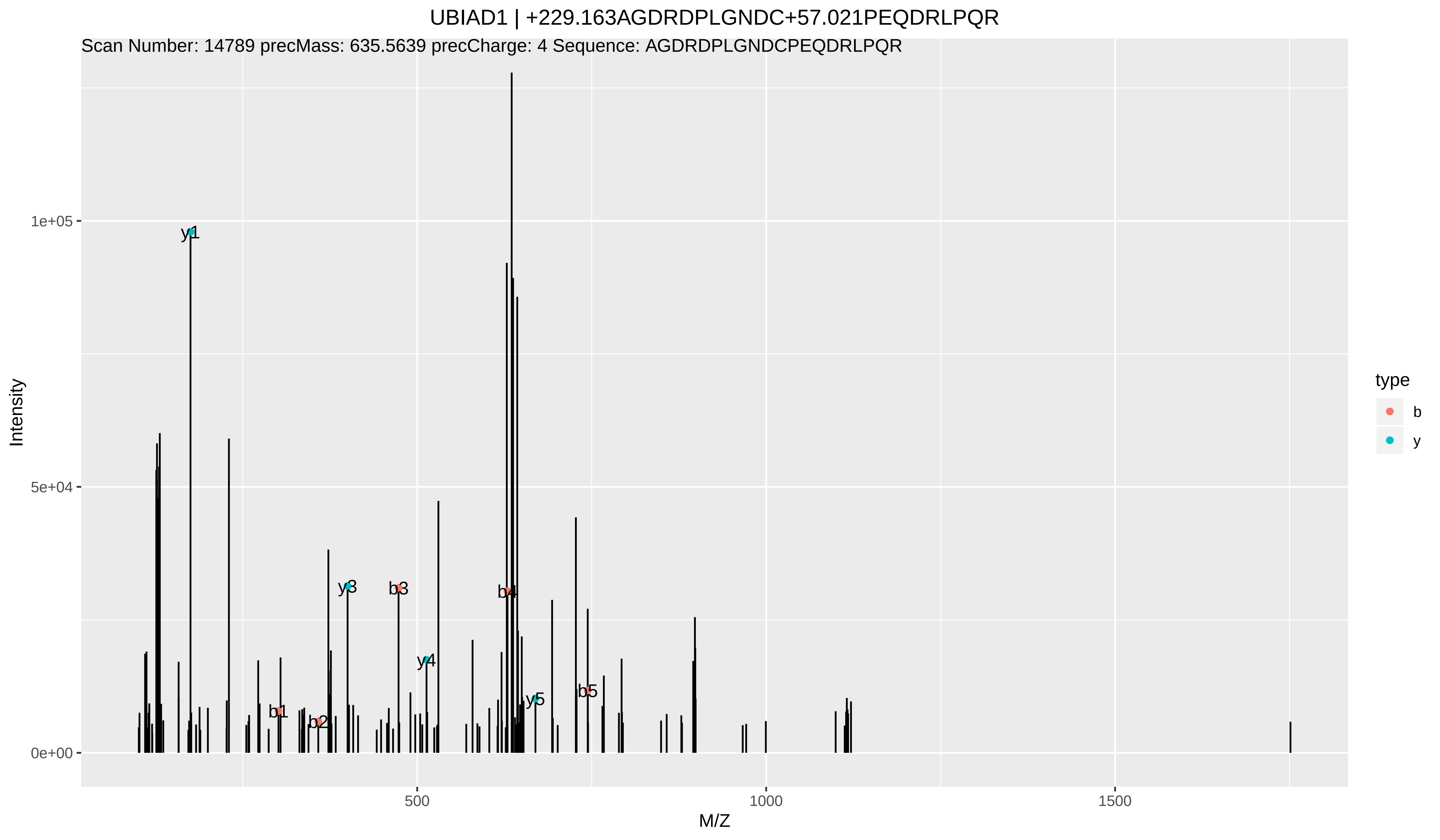
UBIAD1 | +229.163AGDRDPLGNDC+57.021PEQDRLPQR
Scan Number: 14789 precMass: 635.5639 precCharge: 4 Sequence: AGDRDPLGNDCPEQDRLPQR
Intensity
1e+05
5e+04
0e+00
M/Z
500
1000
1500
y1
b1
b2
y3
b3
y4
b4
y5
b5
type
b
y

UBL3 | +229.163EFLFSPNDSASDIAK+229.163

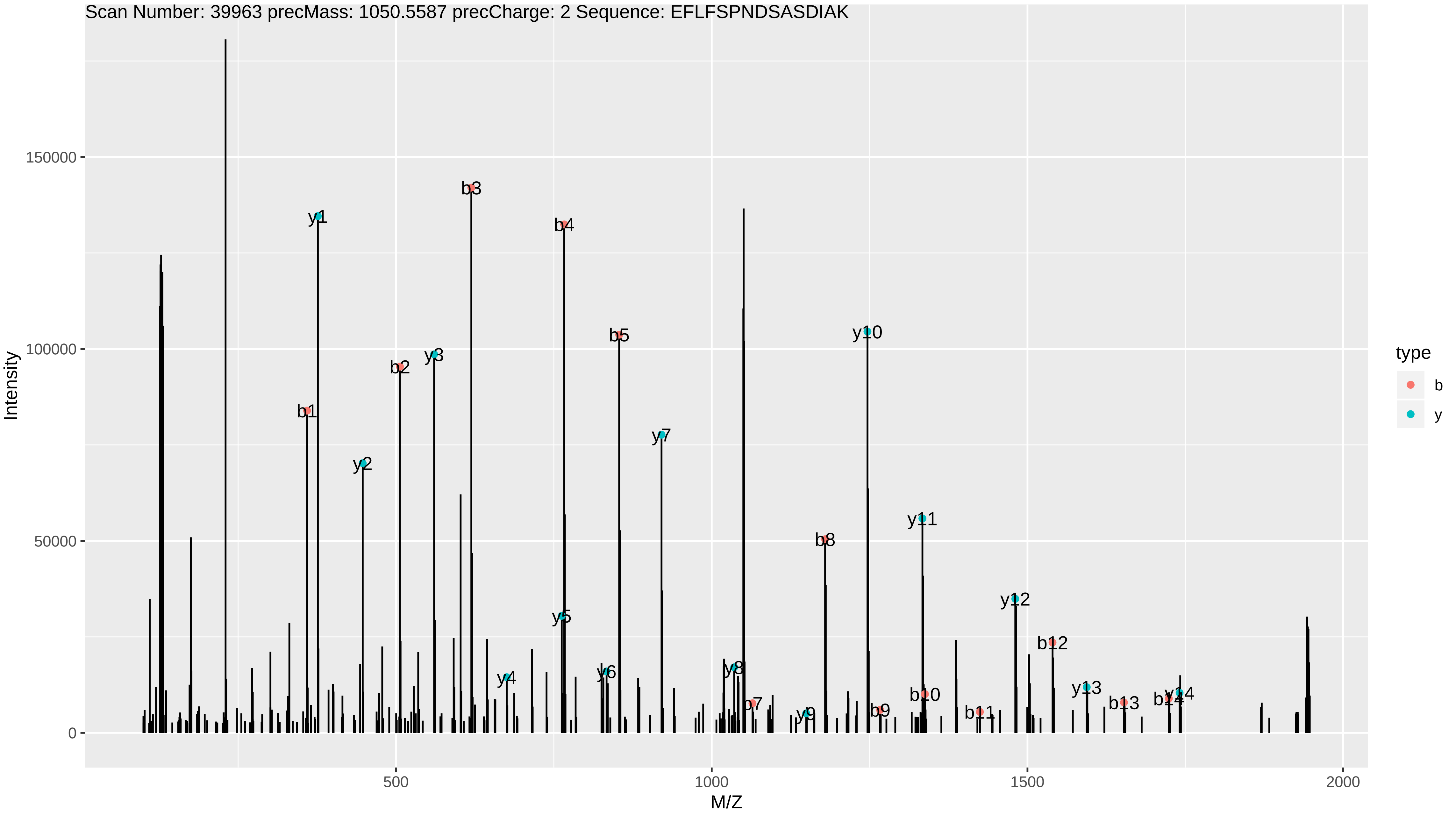

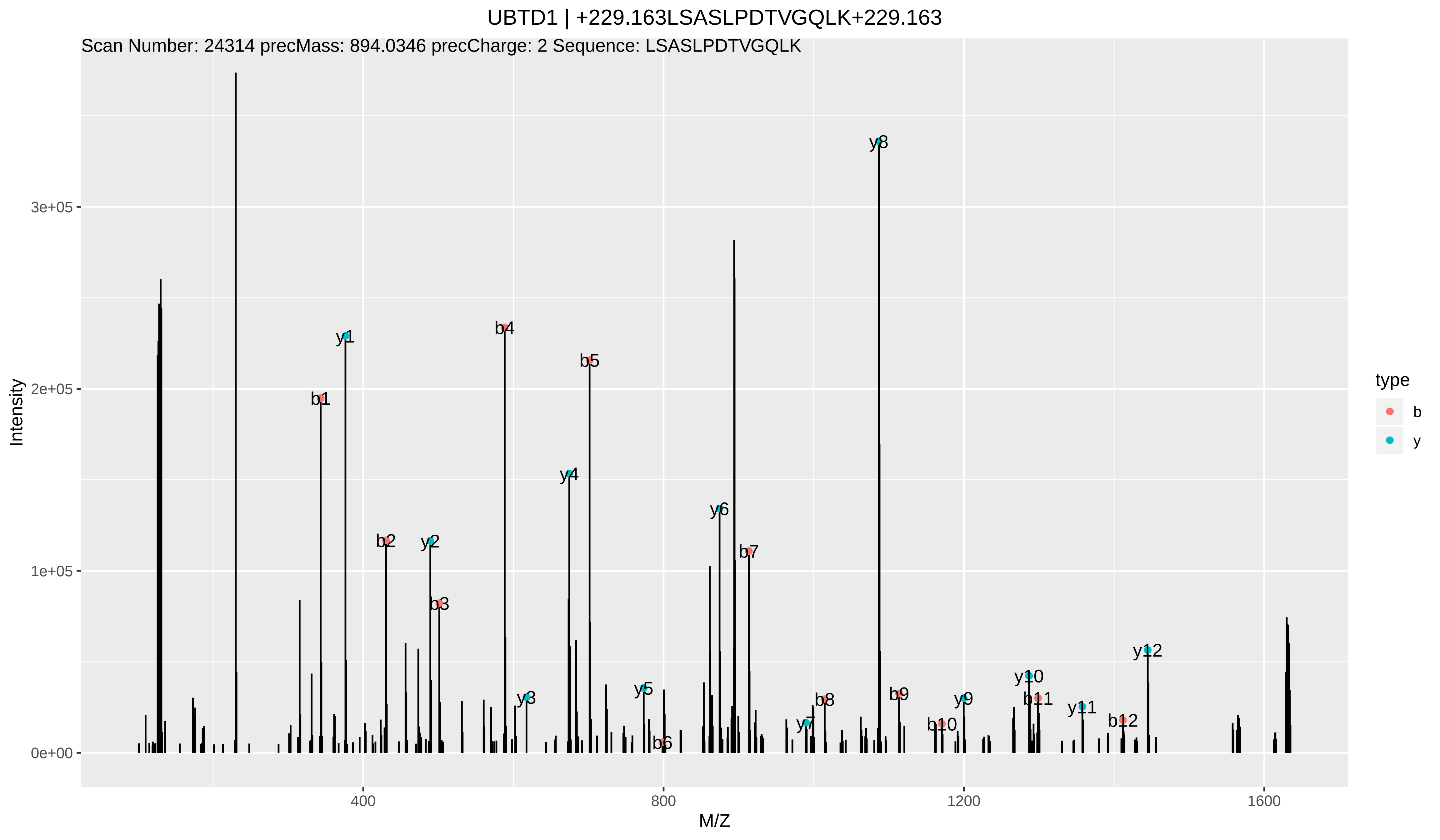

UBTD1 | +229.163LSASLPDTVGQLK+229.163
Scan Number: 24314 precMass: 894.0346 precCharge: 2 Sequence: LSASLPDTVGQLK
Intensity
M/Z
3e+05
2e+05
1e+05
0e+00
400
800
1200
1600
y8
y1
b4
b5
b1
y4
y6
b2
y2
b7
b3
y12
y10
y5
y3
b8
b9
y9
b11
y11
b12
y7
b10
b6
type
b
y

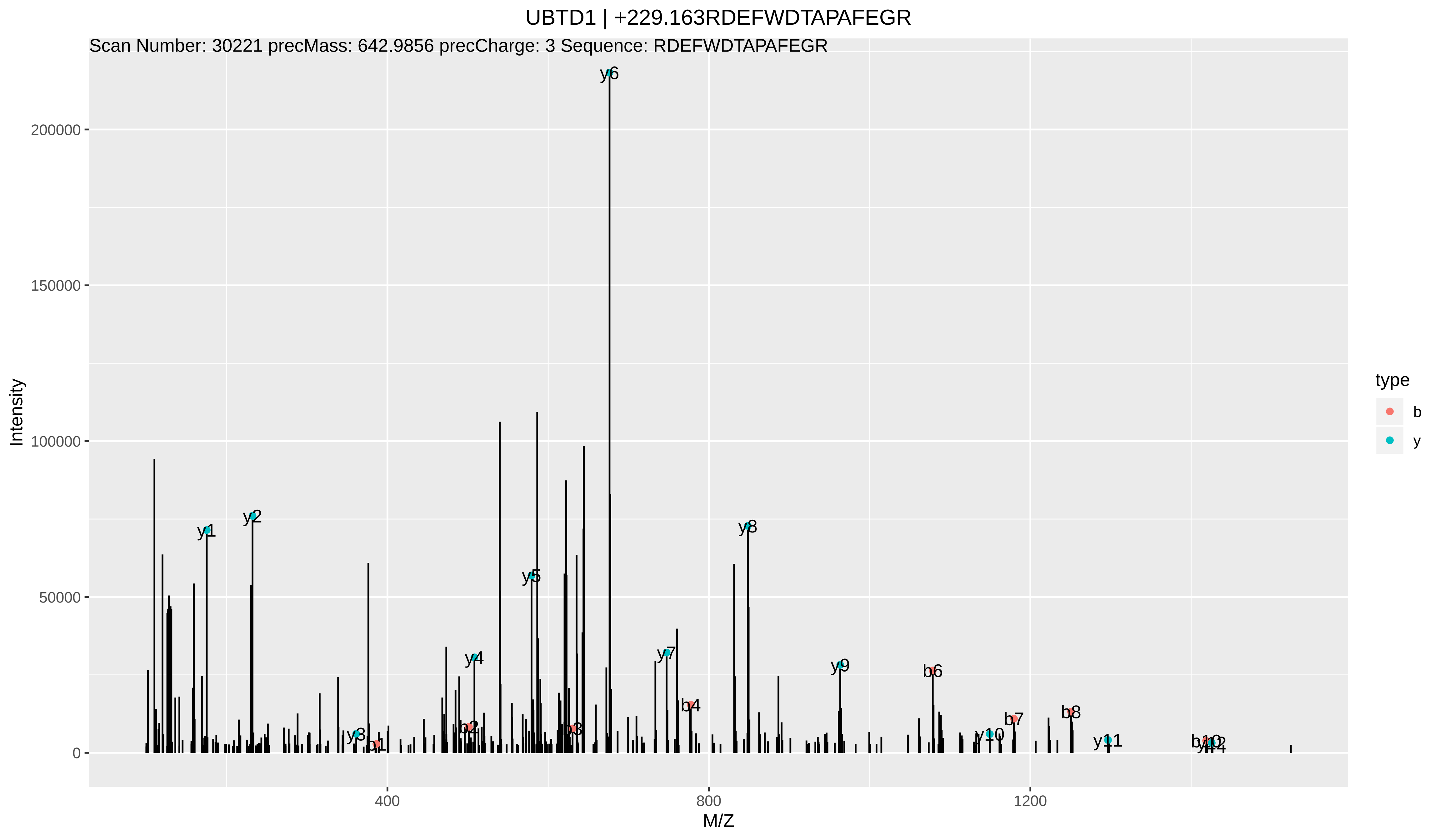
UBTD1 | +229.163RDEFWDTAPAFEGR
Scan Number: 30221 precMass: 642.9856 precCharge: 3 Sequence: RDEFWDTAPAFEGR
Intensity
M/Z
200000
150000
100000
50000
0
400
800
1200
y1
y2
y3
y4
y5
y6
y7
y8
y9
y10
y11
b1
b2
b3
b4
b6
b7
b8
type
b
y

UCK1 | +229.163DLEQILTQYTTFVK+229.163

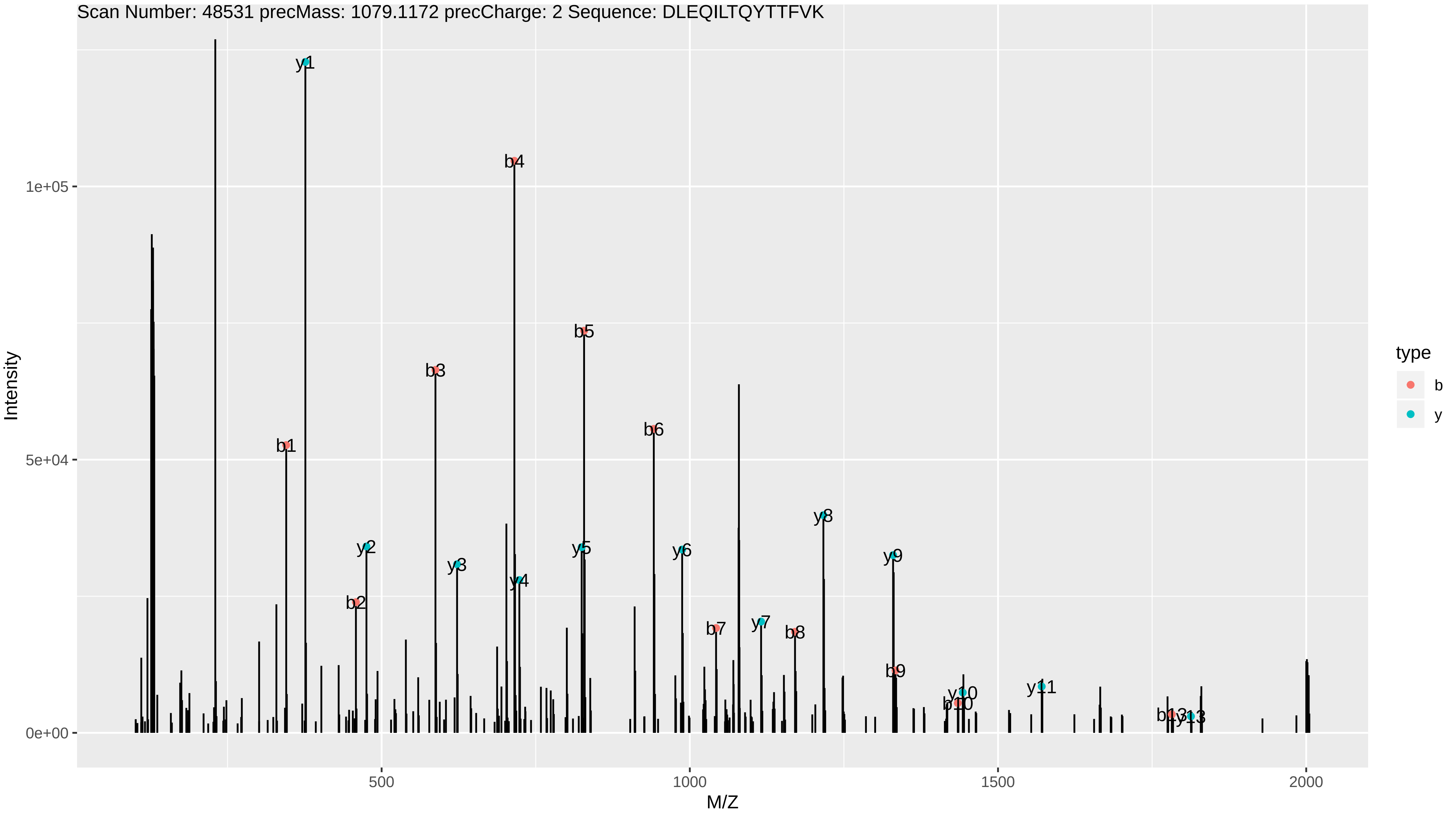

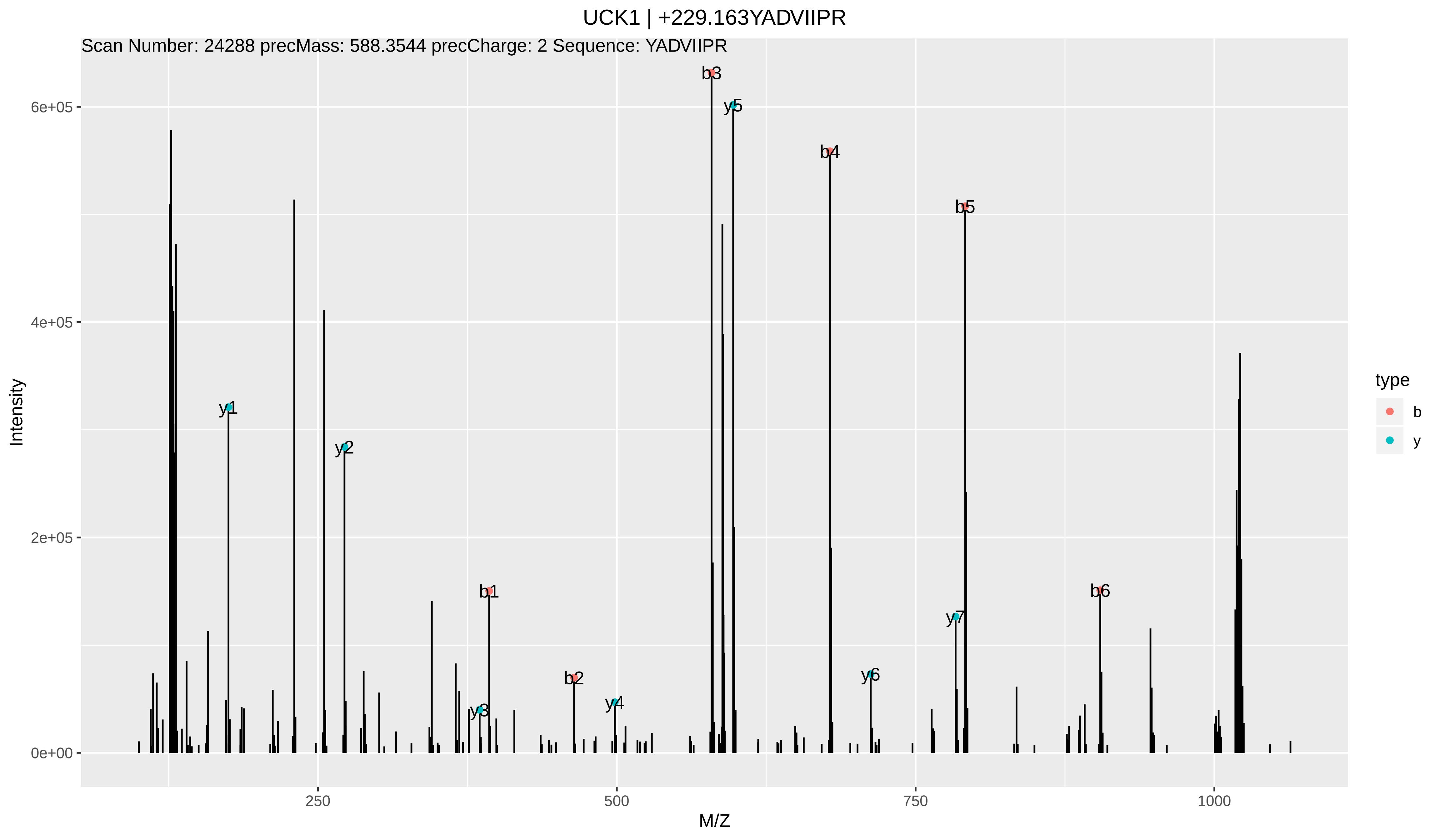

UCK1 | +229.163YADVIIPR
Scan Number: 24288 precMass: 588.3544 precCharge: 2 Sequence: YADVIIPR
Intensity
M/Z
0e+00
2e+05
4e+05
6e+05
250
500
750
1000
b3
y5
b4
b5
y1
y2
b1
y7
b6
b2
y6
y4
y3
type
b
y

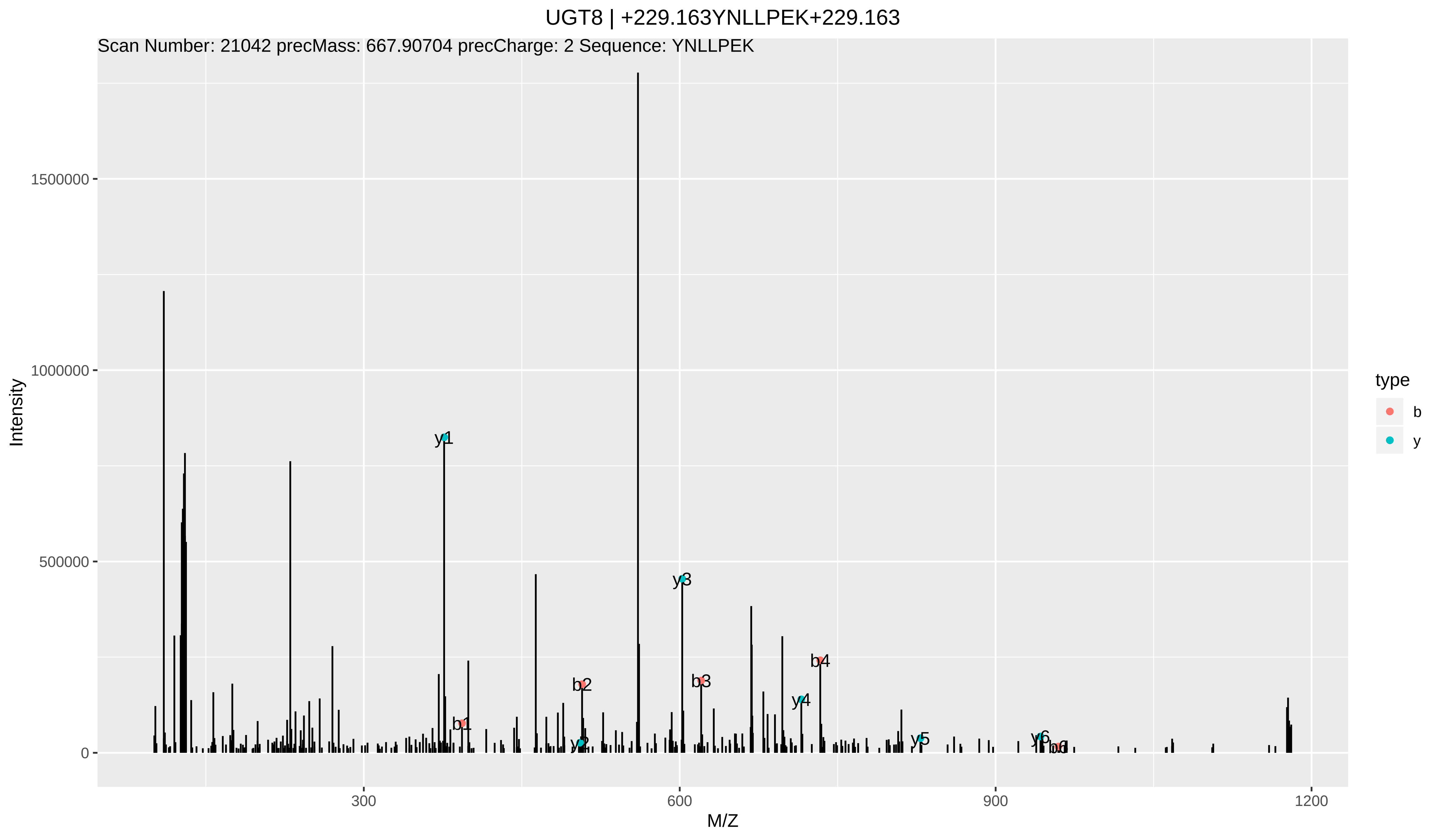
UGT8 | +229.163YNLLPEK+229.163
Scan Number: 21042 precMass: 667.90704 precCharge: 2 Sequence: YNLLPEK
Intensity
1500000
1000000
500000
0
300
600
900
1200
M/Z
y1
b1
b2
y2
y3
b3
y4
b4
y5
y6
b6
type
b
y

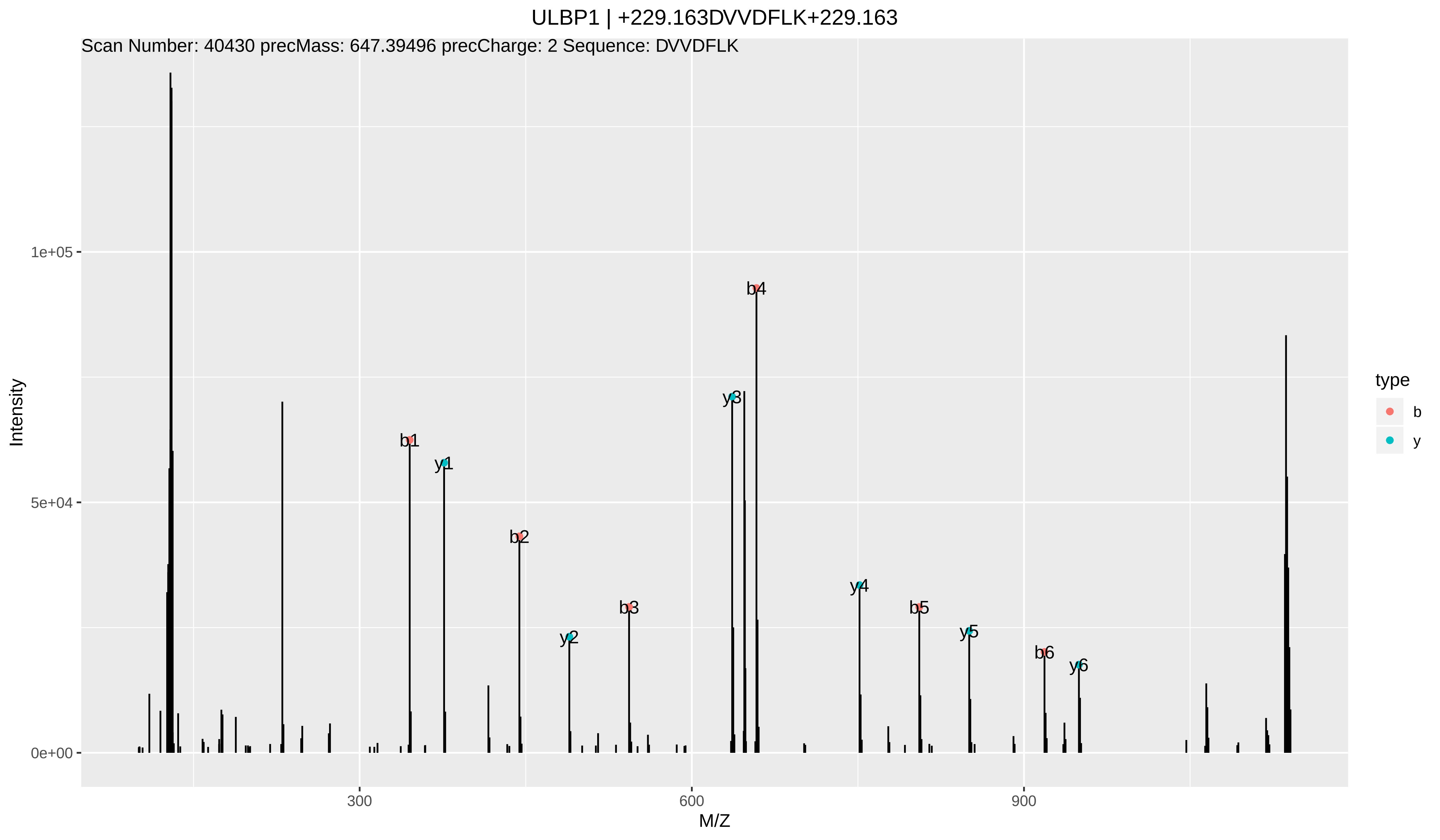
ULBP1 | +229.163DVVDFLK+229.163
Scan Number: 40430 precMass: 647.39496 precCharge: 2 Sequence: DVVDFLK
Intensity
0e+00
5e+04
1e+05
M/Z
300
600
900
b1
y1
b2
y2
b3
y3
b4
y4
b5
y5
b6
y6
type
b
y

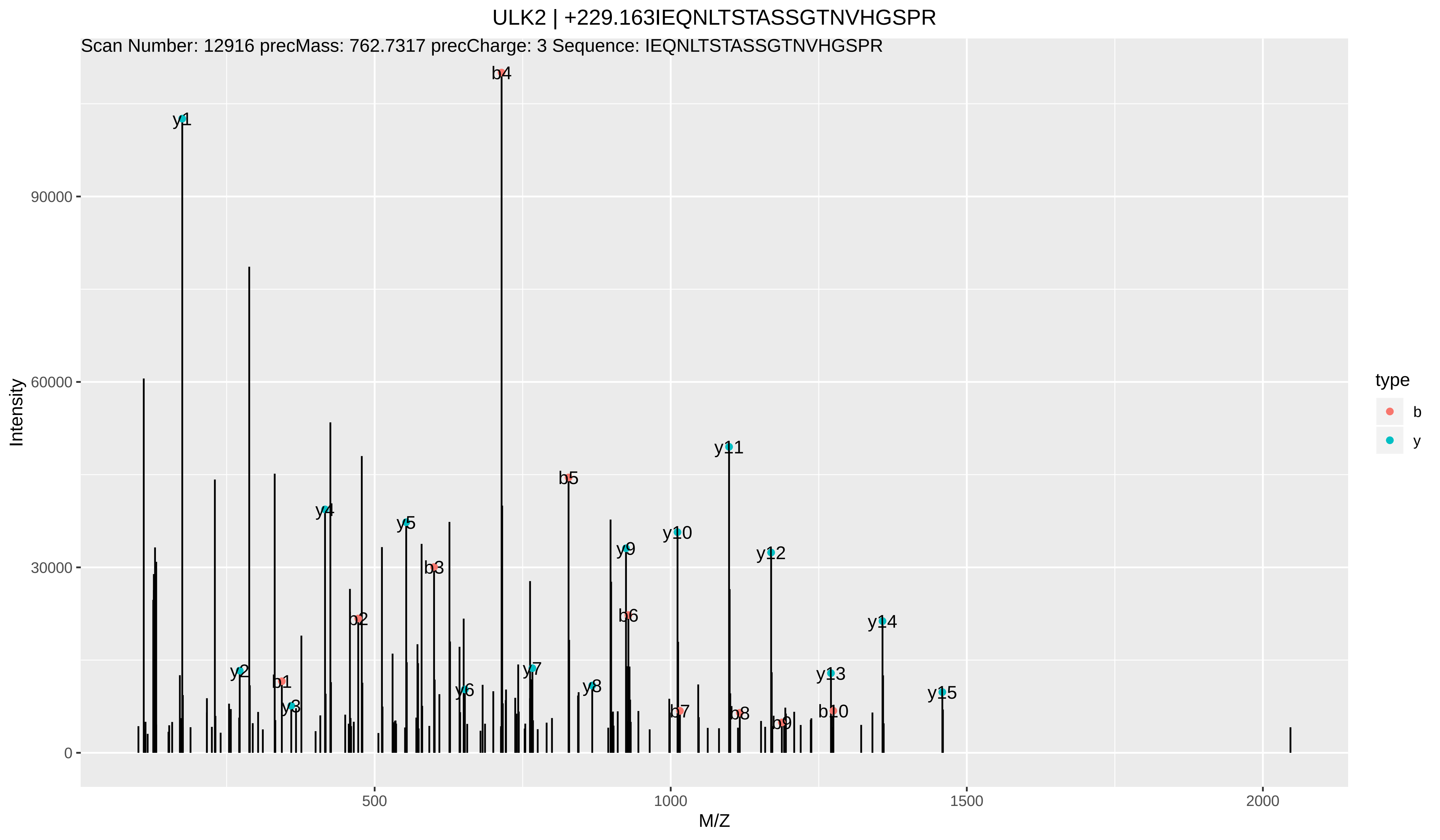

ULK2 | +229.163IEQNLTSTASSGTNVHGSPR
Scan Number: 12916 precMass: 762.7317 precCharge: 3 Sequence: IEQNLTSTASSGTNVHGSPR
Intensity
M/Z
90000
60000
30000
0
500
1000
1500
2000
type
b
y
b4
y1
b5
y11
y4
y5
y10
y9
b3
y12
b2
b6
y14
y2
y7
b1
y13
y8
y3
y6
y15
b7
b8
b10
b9

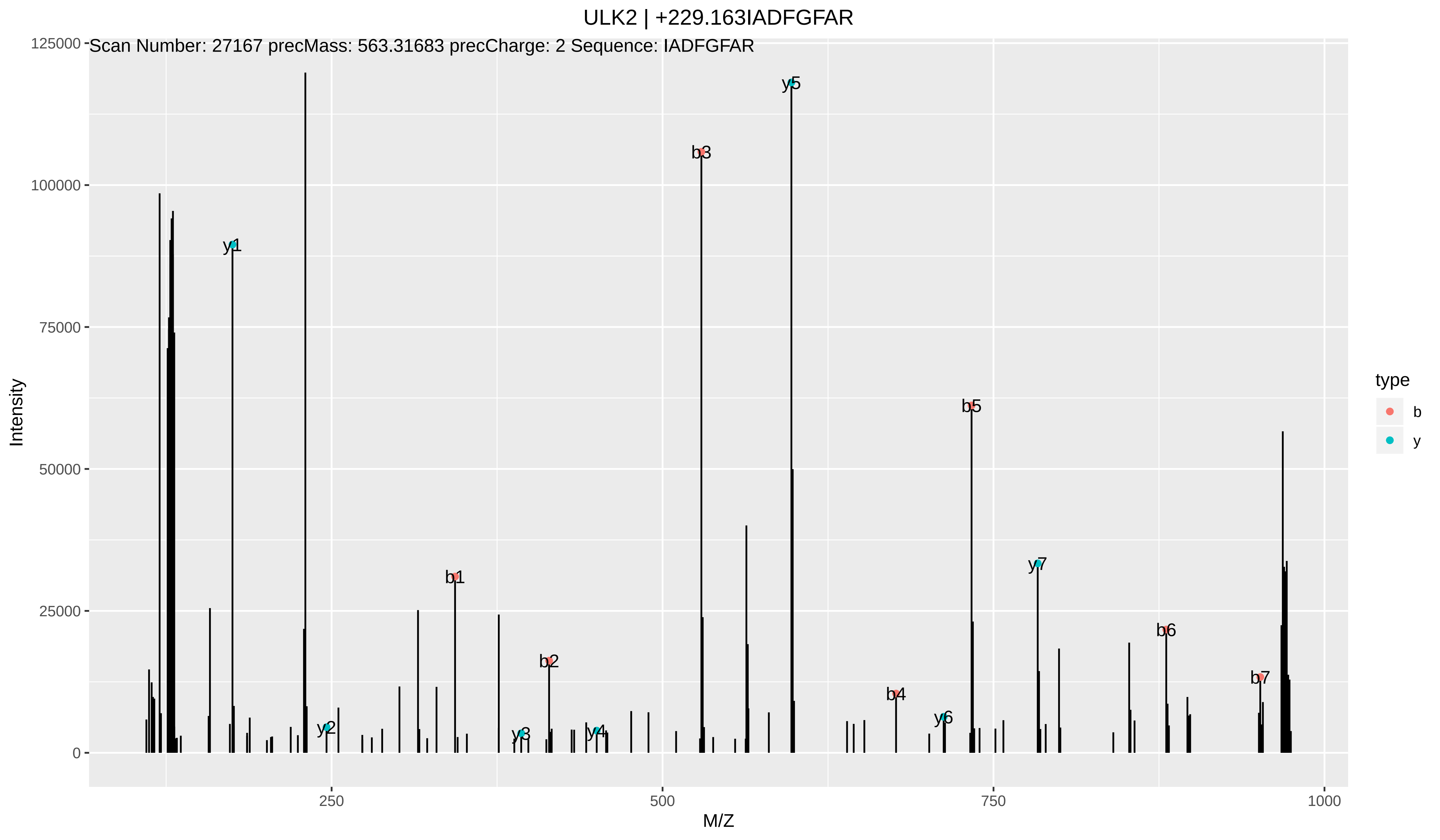

ULK2 | +229.163IADFGFAR
Scan Number: 27167 precMass: 563.31683 precCharge: 2 Sequence: IADFGFAR
Intensity
M/Z
0
25000
50000
75000
100000
125000
250
500
750
1000
y1
y2
y3
y4
y5
y6
y7
b1
b2
b3
b4
b5
b6
b7
type
b
y

UNC5D | +229.163EVFINVTR

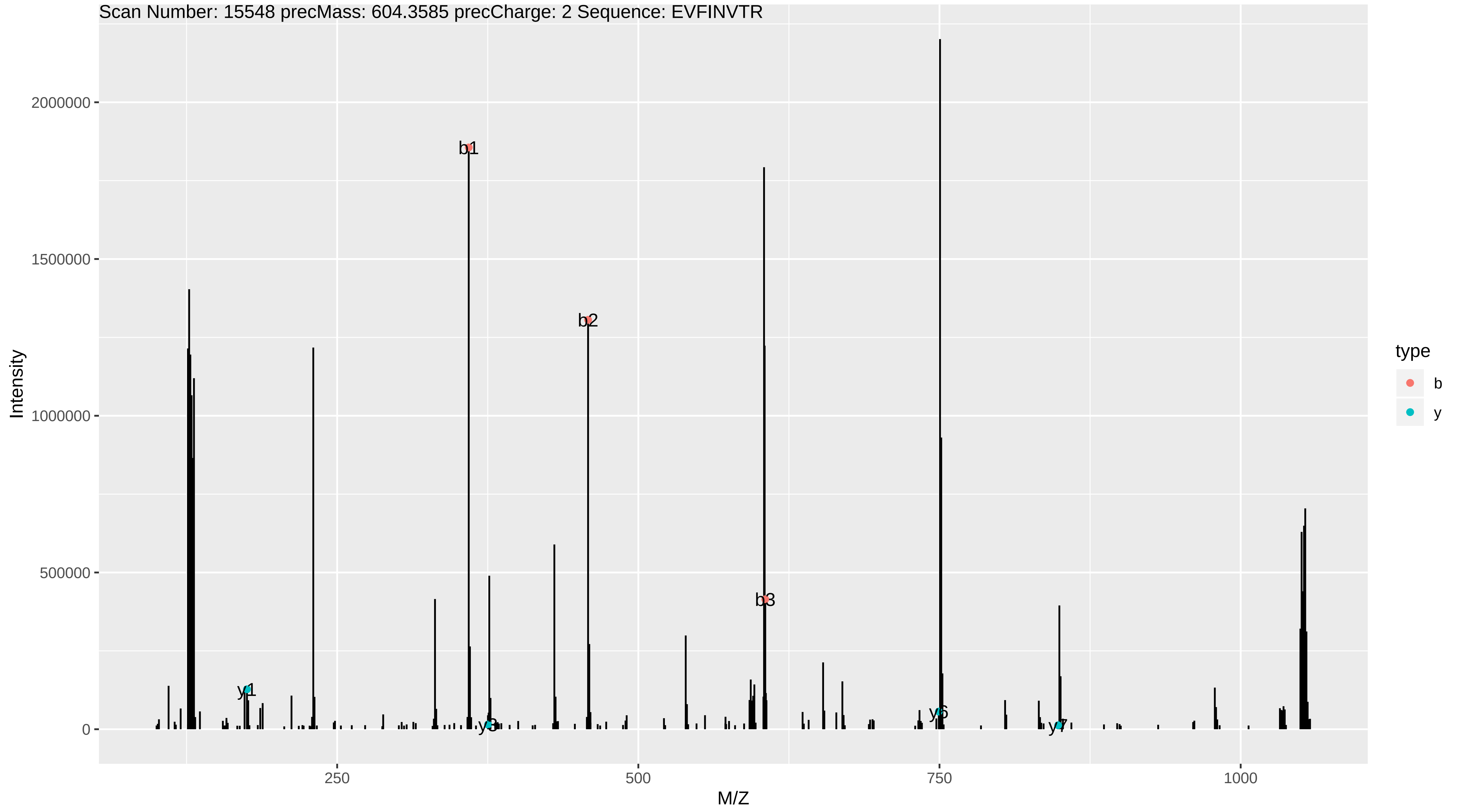

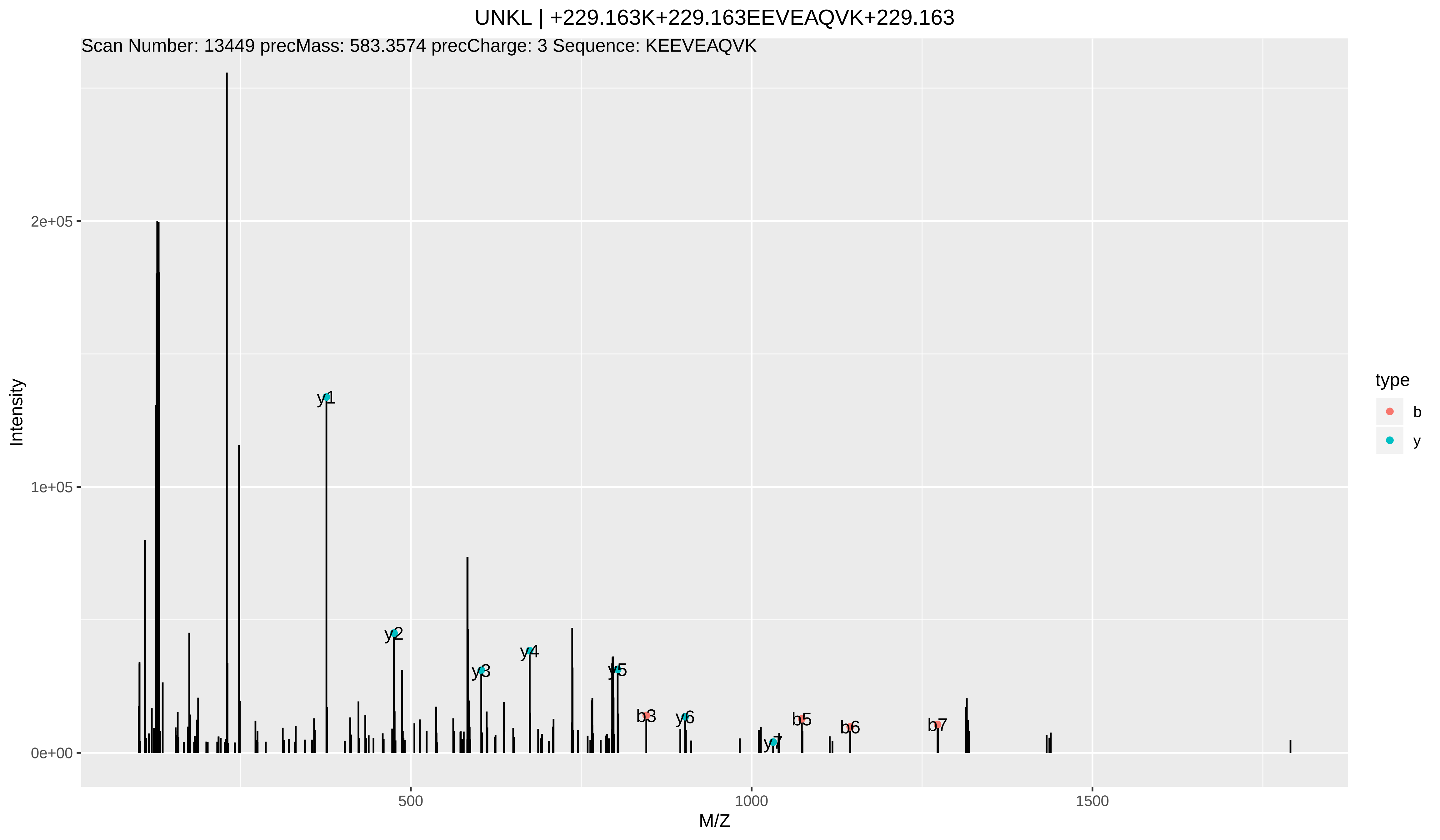

UNKL | +229.163K+229.163EEVEAQVK+229.163
Scan Number: 13449 precMass: 583.3574 precCharge: 3 Sequence: KEEVEAQVK
Intensity
2e+05
1e+05
0e+00
M/Z
500
1000
1500
y1
y2
y3
y4
y5
b3
y6
y7
b5
b6
b7
type
b
y

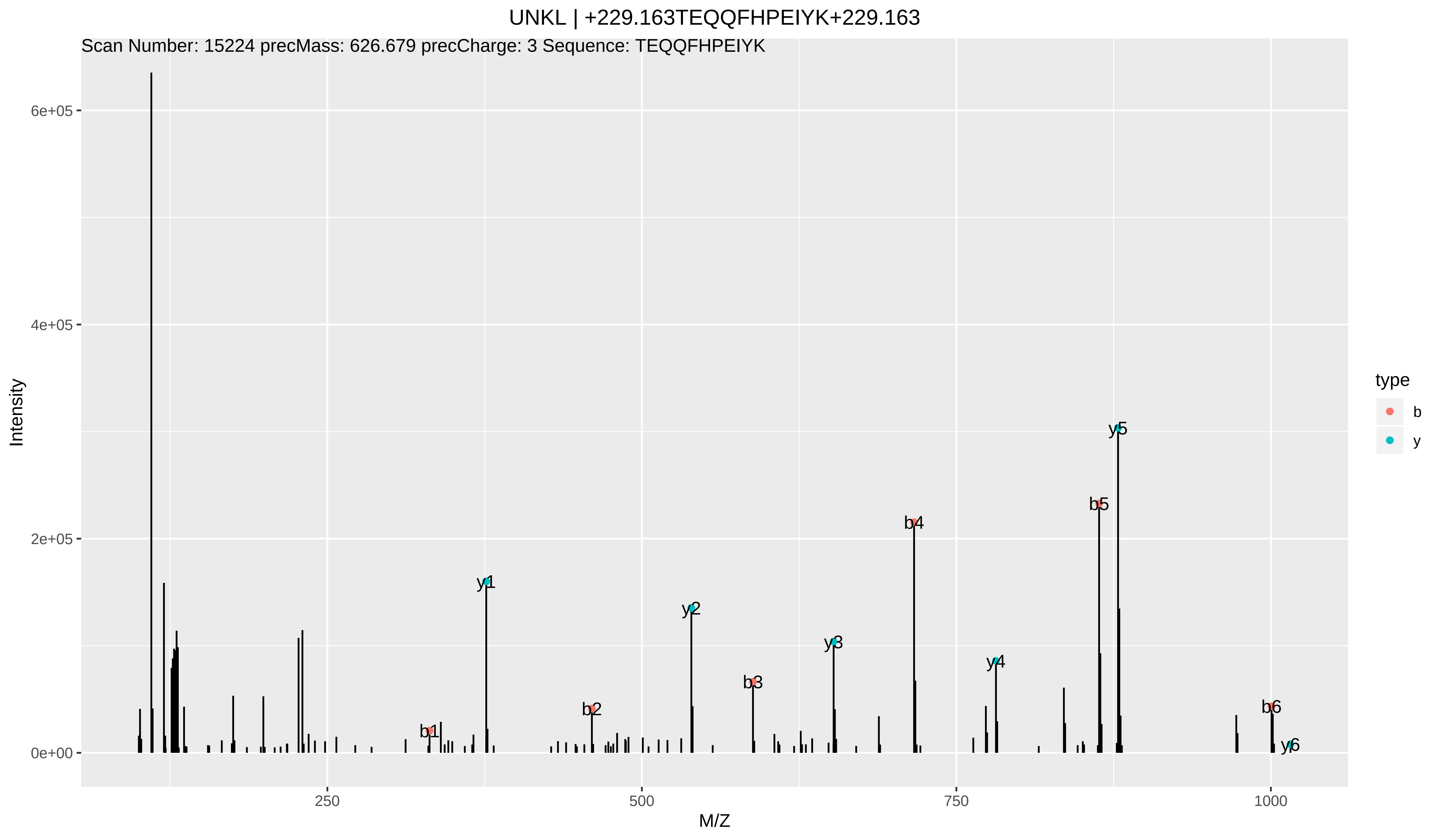
UNKL | +229.163TEQQFHPEIYK+229.163
Scan Number: 15224 precMass: 626.679 precCharge: 3 Sequence: TEQQFHPEIYK
Intensity
6e+05
4e+05
2e+05
0e+00
M/Z
250
500
750
1000
y1
y2
y3
y4
y5
y6
b1
b2
b3
b4
b5
b6
type
b
y

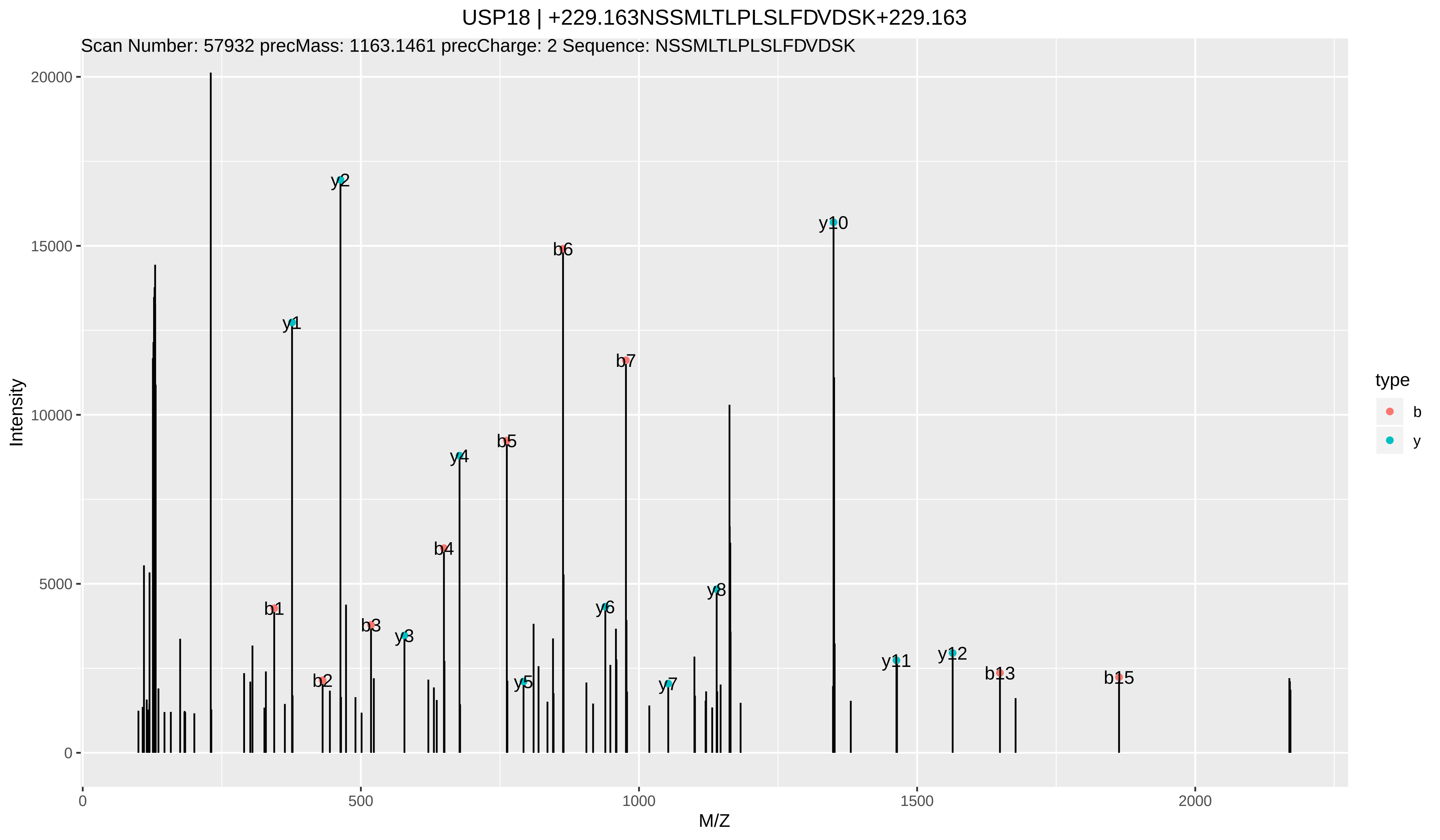

USP18 | +229.163NSSMLTLPLSLFDVDSK+229.163
Scan Number: 57932 precMass: 1163.1461 precCharge: 2 Sequence: NSSMLTLPLSLFDVDSK
Intensity
M/Z
type
b
y
y2
y10
b6
y1
b7
b5
y4
b4
y8
b1
y6
b3
y3
y12
y11
b13
b15
b2
y5
y7
20000
15000
10000
5000
0
0
500
1000
1500
2000

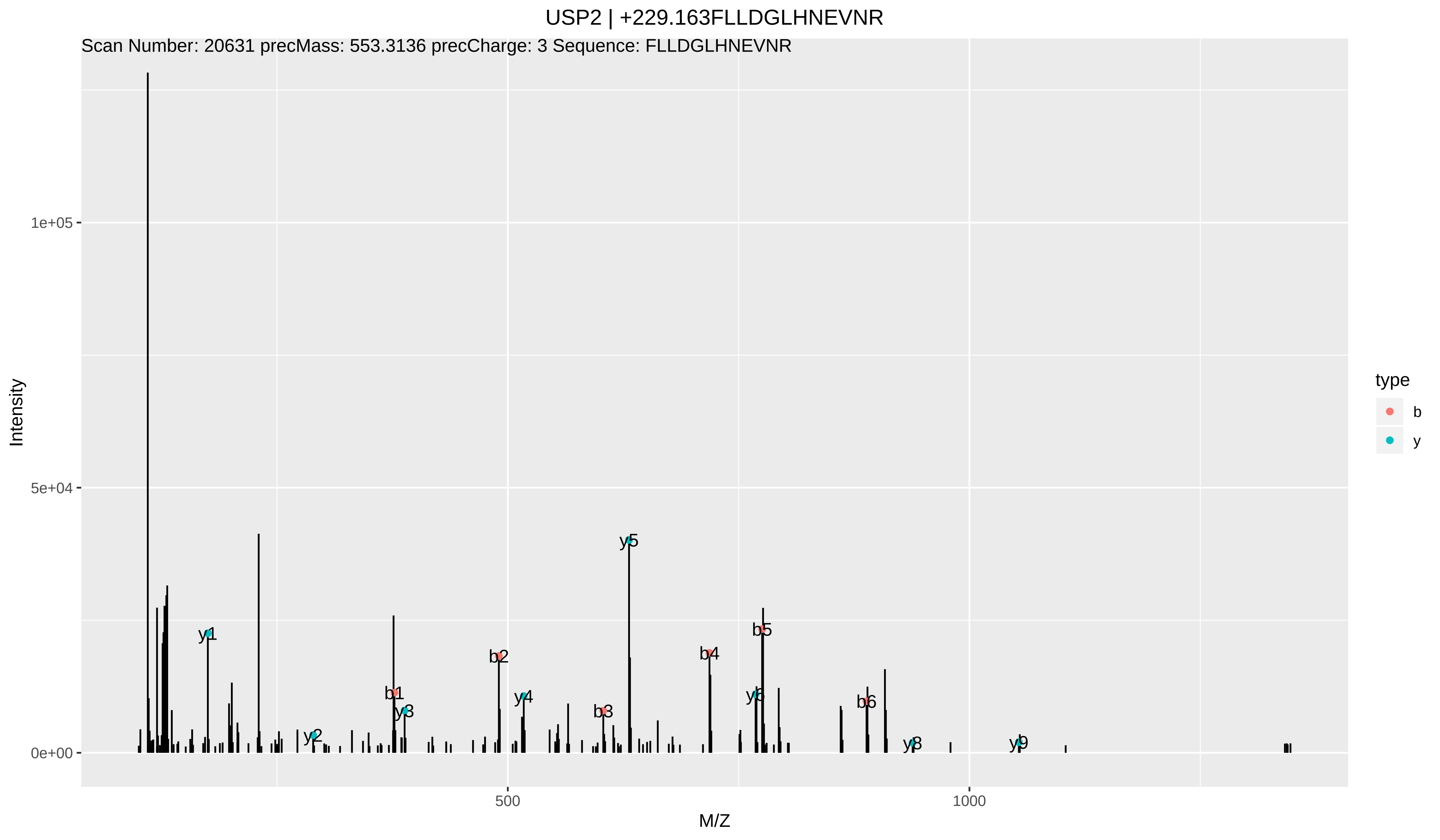

USP2 | +229.163FLLLDGLHNEVNR
Scan Number: 20631 precMass: 553.3136 precCharge: 3 Sequence: FLLLDGLHNEVNR
Intensity
1e+05
5e+04
0e+00
M/Z
500
1000
y1
y2
b1
y3
b2
y4
y5
b3
b4
b5
y6
b6
y8
y9
type
b
y

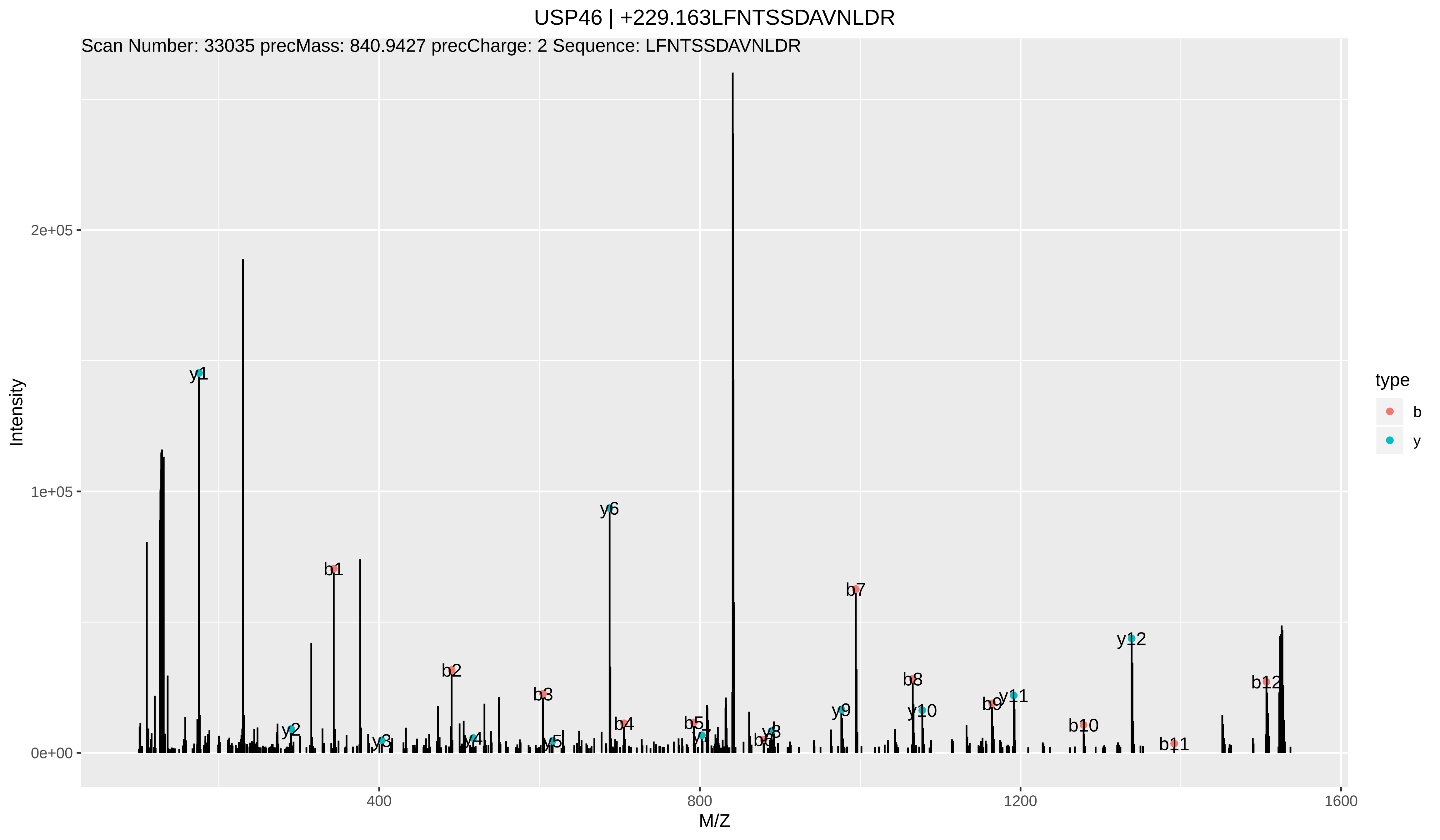

USP46 | +229.163LFNTSSDAVNLDR
Scan Number: 33035 precMass: 840.9427 precCharge: 2 Sequence: LFNTSSDAVNLDR
Intensity
M/Z
0e+00
1e+05
2e+05
400
800
1200
1600
y1
y6
b1
b7
y12
b12
b2
b8
b3
y11
b9
y10
y9
b4
b5
b10
y8
b11
type
b
y

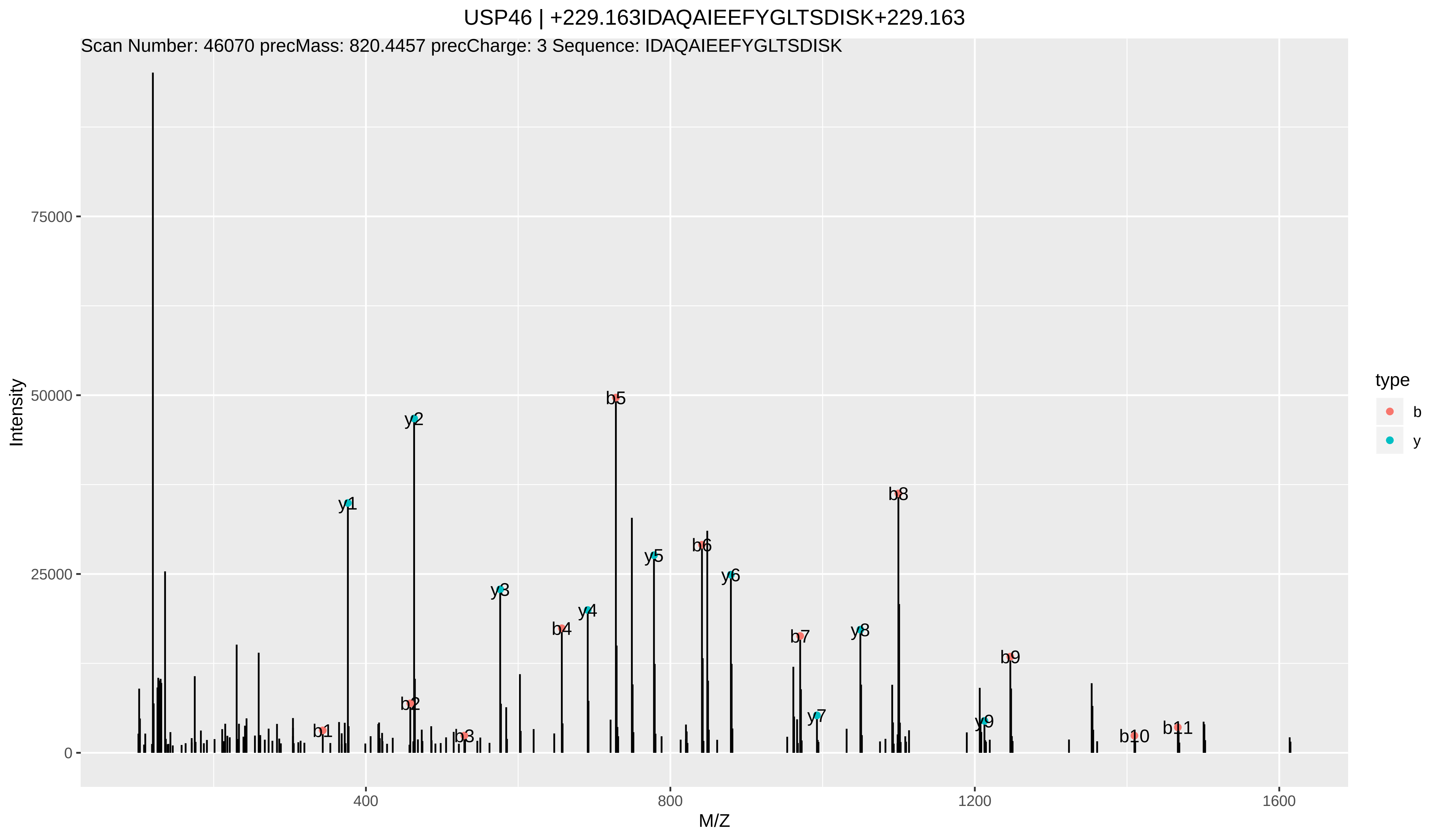
USP46 | +229.163IDAQAIEEFYGLTSDISK+229.163
Scan Number: 46070 precMass: 820.4457 precCharge: 3 Sequence: IDAQAIEEFYGLTSDISK
Intensity
M/Z
0
25000
50000
75000
400
800
1200
1600
y1
y2
y3
y4
y5
y6
y7
y8
y9
b1
b2
b3
b4
b5
b6
b7
b8
b9
b10
b11
type
b
y

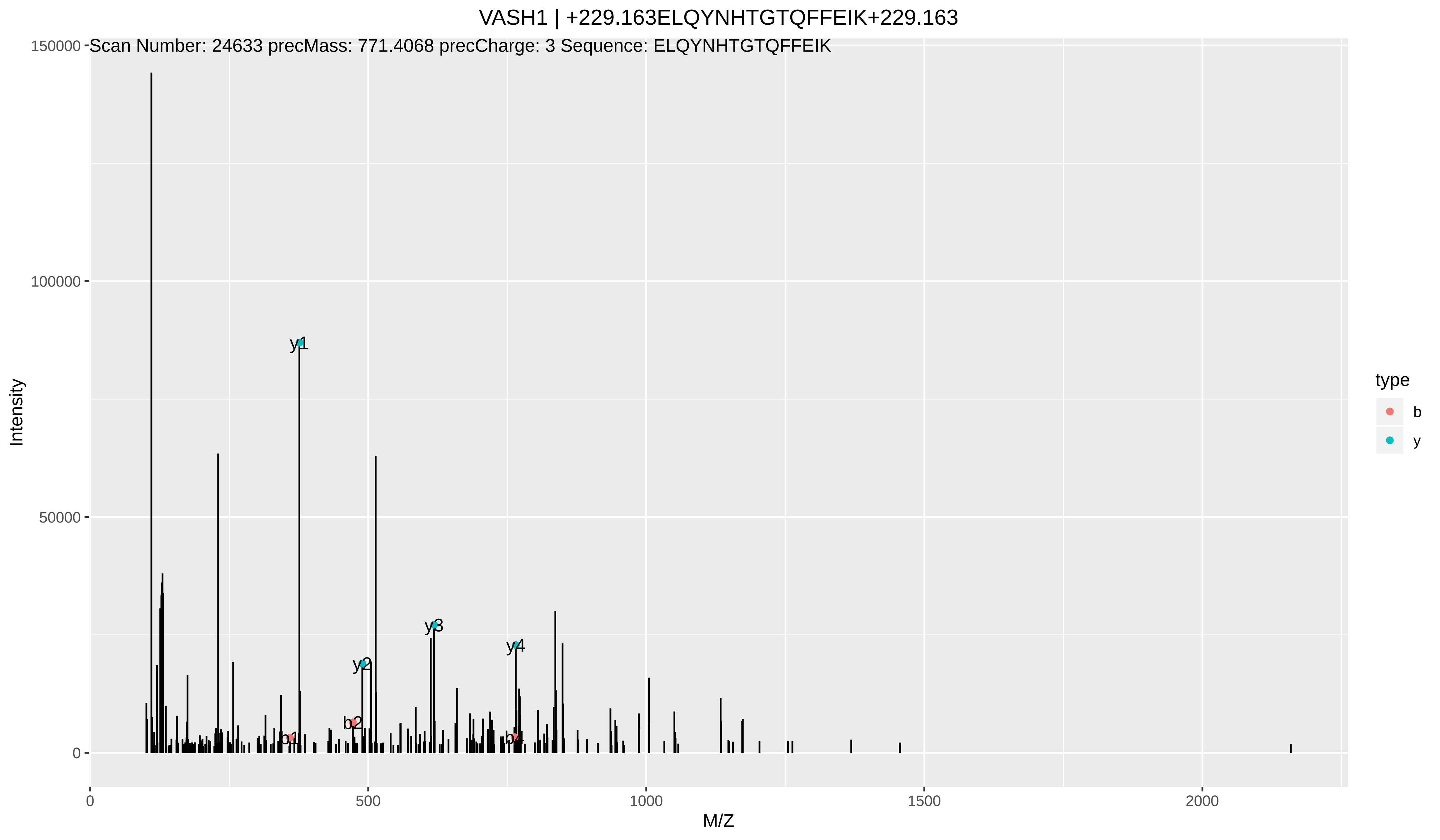

VASH1 | +229.163ELQYNHTGTQFFEIK+229.163
Scan Number: 24633 precMass: 771.4068 precCharge: 3 Sequence: ELQYNHTGTQFFEIK
Intensity
M/Z
150000
100000
50000
0
0
500
1000
1500
2000
y1
y2
y3
y4
b1
b2
b4
type
b
y

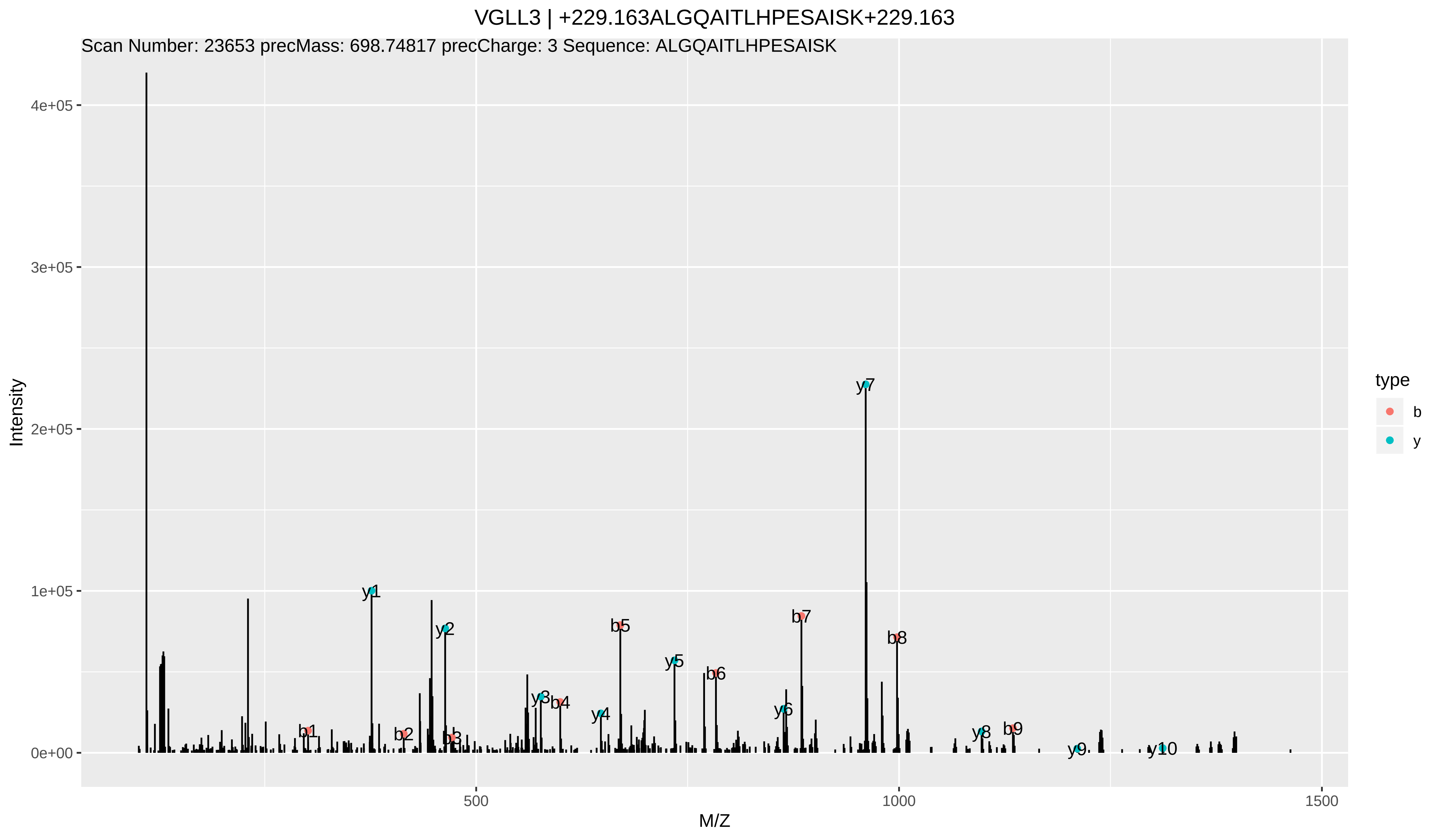
VGLL3 | +229.163ALGQAITLHPESAISK+229.163
Scan Number: 23653 precMass: 698.74817 precCharge: 3 Sequence: ALGQAITLHPESAISK
Intensity
4e+05
3e+05
2e+05
1e+05
0e+00
M/Z
500
1000
1500
type
b
y
y1
y2
y3
y4
y5
y6
y7
y8
y9
y10
b1
b2
b3
b4
b5
b6
b7
b8
b9

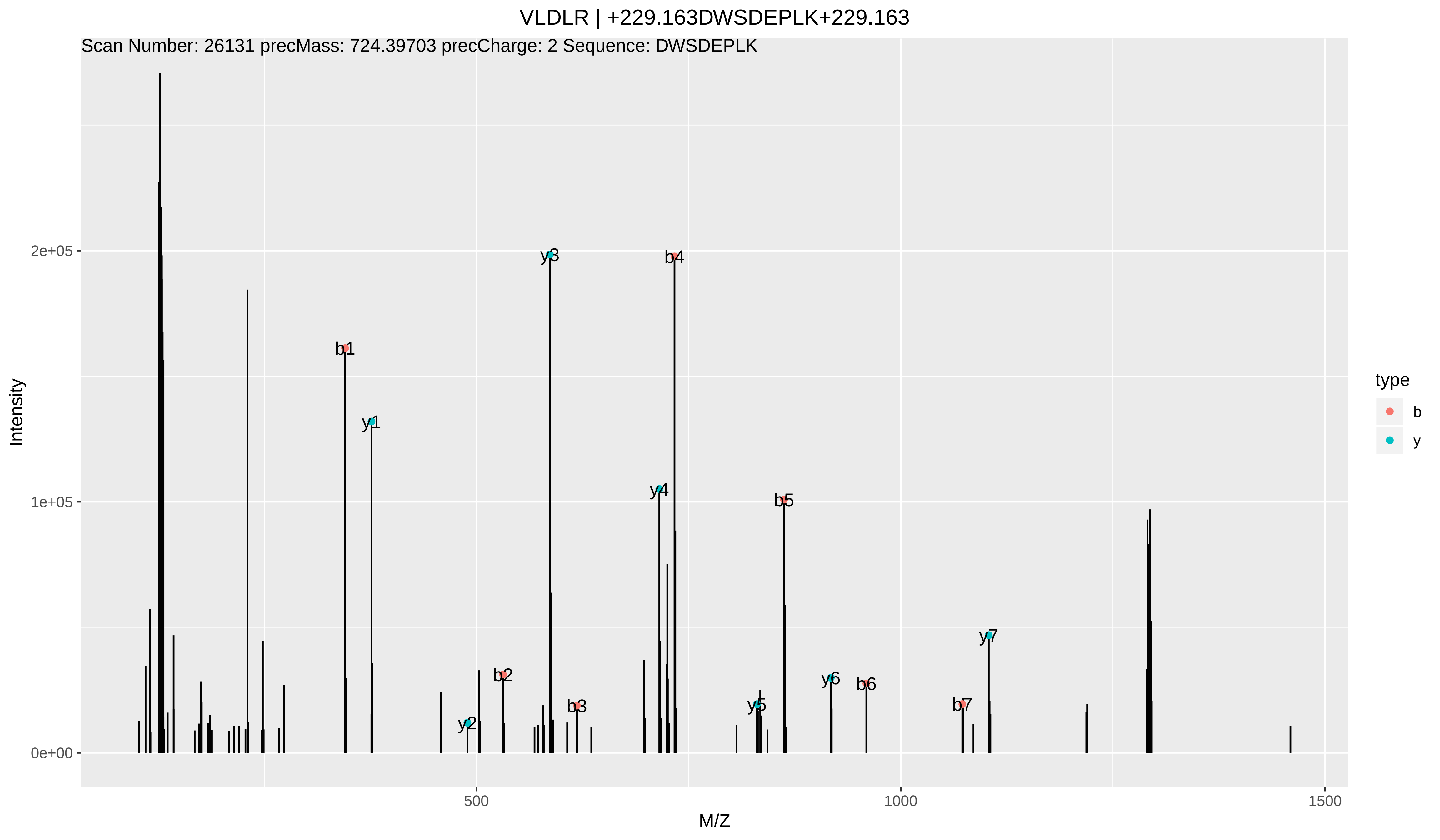

VLDLR | +229.163DWSDEPLK+229.163
Scan Number: 26131 precMass: 724.39703 precCharge: 2 Sequence: DWSDEPLK
Intensity
2e+05
1e+05
0e+00
M/Z
500
1000
1500
b1
y1
y2
b2
y3
b3
y4
b4
y5
b5
y6
b6
b7
y7
type
b
y

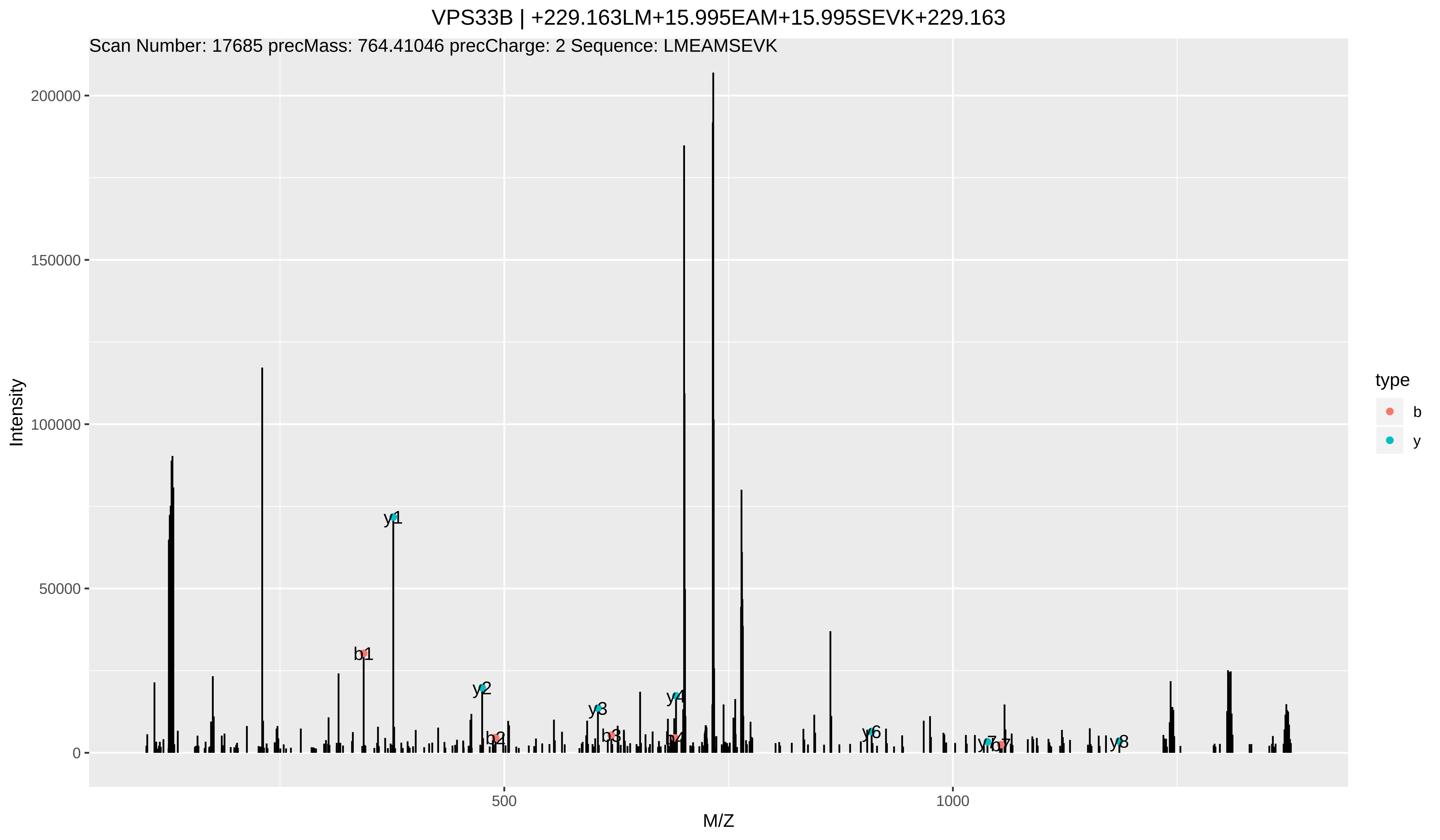

VPS33B | +229.163LM+15.995EAM+15.995SEVK+229.163
Scan Number: 17685 precMass: 764.41046 precCharge: 2 Sequence: LMEAMSEVK
Intensity
M/Z
type
b
y
200000
150000
100000
50000
0
500
1000
y1
b1
y2
b2
y3
b3
y4
b4
y6
y7
b7
y8

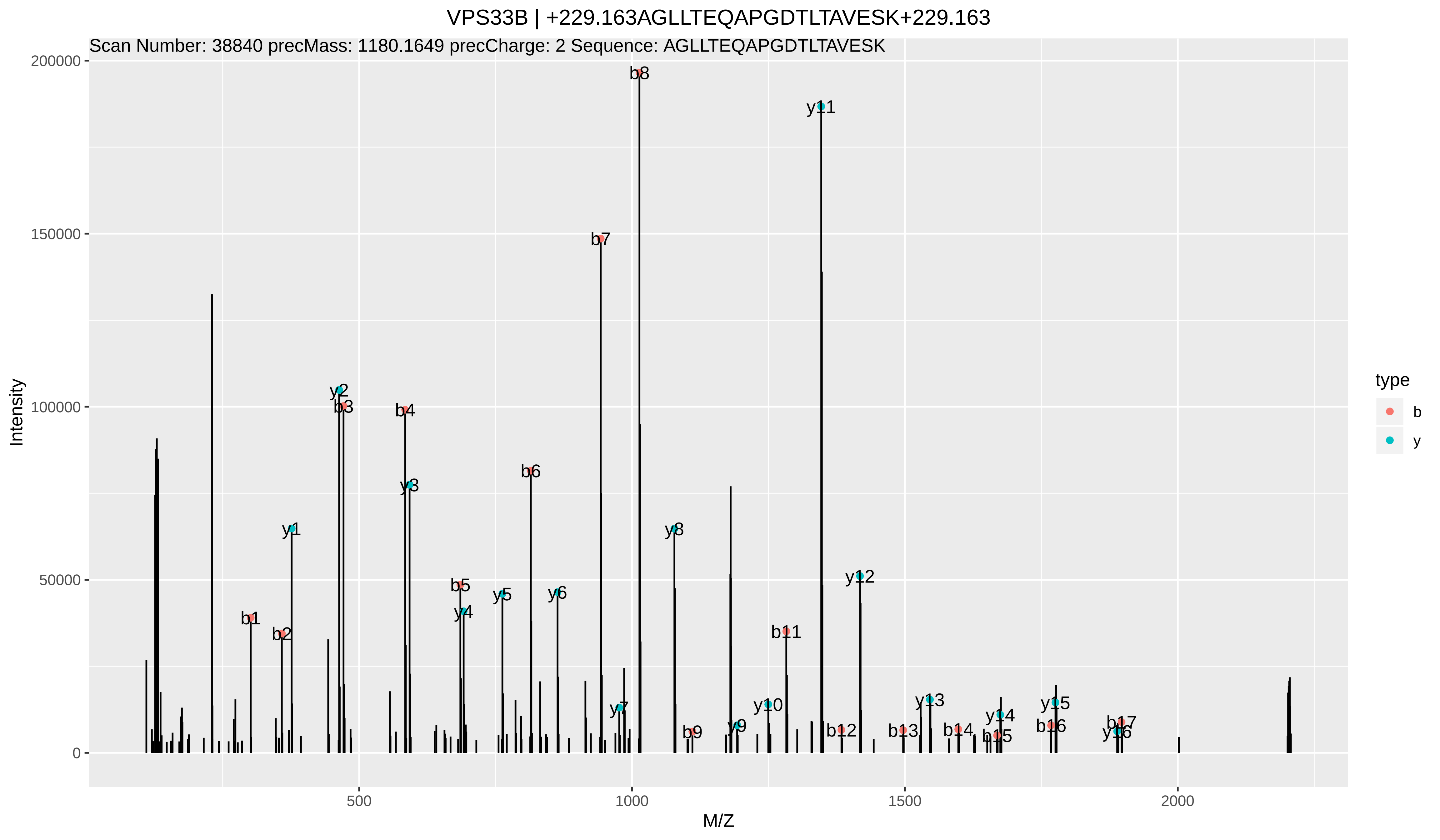

VPS33B | +229.163AGLLTEQAPGDTLTAVESK+229.163
Scan Number: 38840 precMass: 1180.1649 precCharge: 2 Sequence: AGLLTEQAPGDTLTAVESK
Intensity
M/Z
type
b
y
b1
b2
b3
b4
b5
b6
b7
b8
b9
b11
b12
b13
b14
b15
b16
b17
y1
y2
y3
y4
y5
y6
y7
y8
y9
y10
y11
y12
y13
y14
y15
y16
0
50000
100000
150000
200000
500
1000
1500
2000

VPS9D1 | +229.163TAVEEIHNAVDR

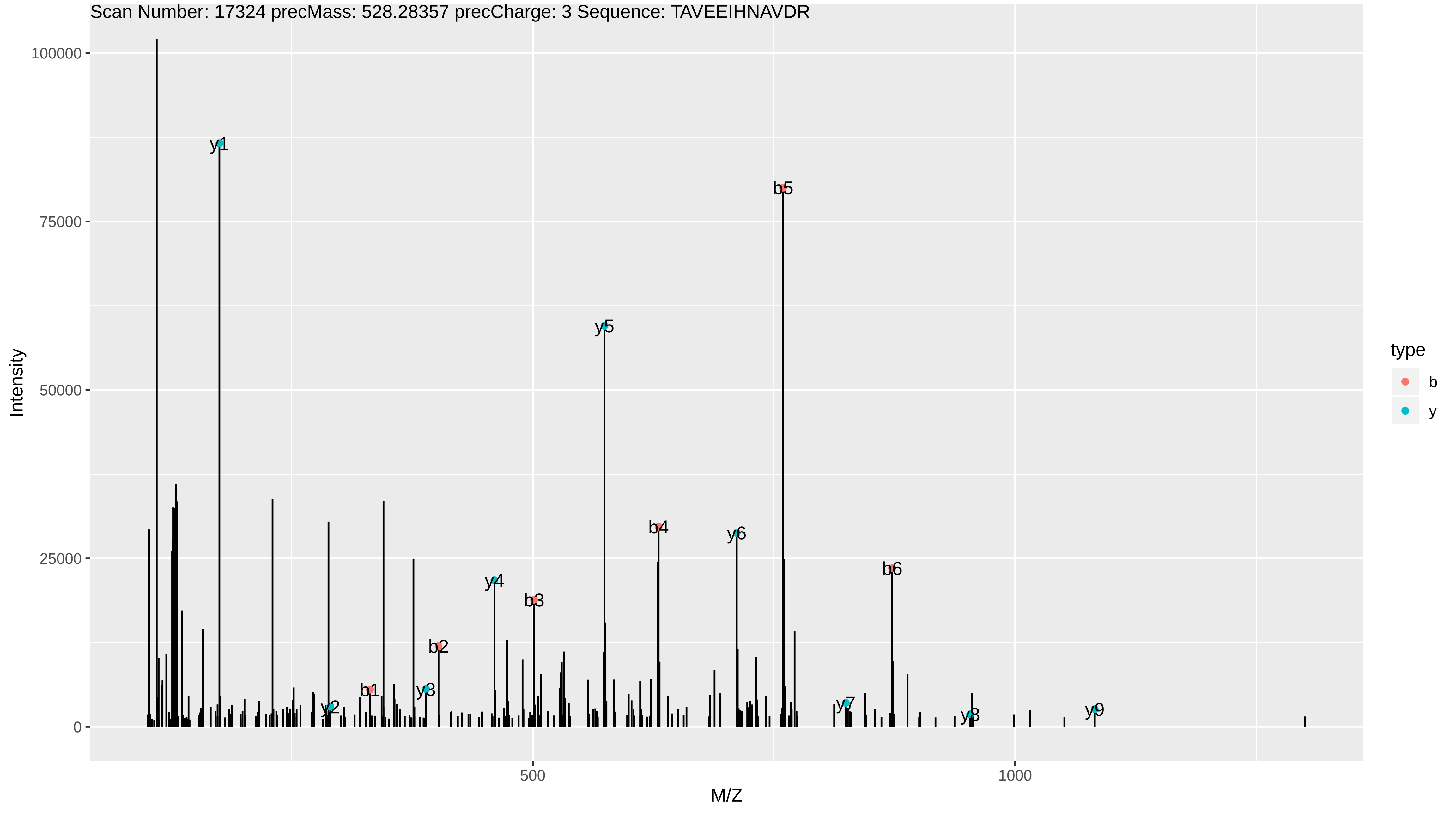

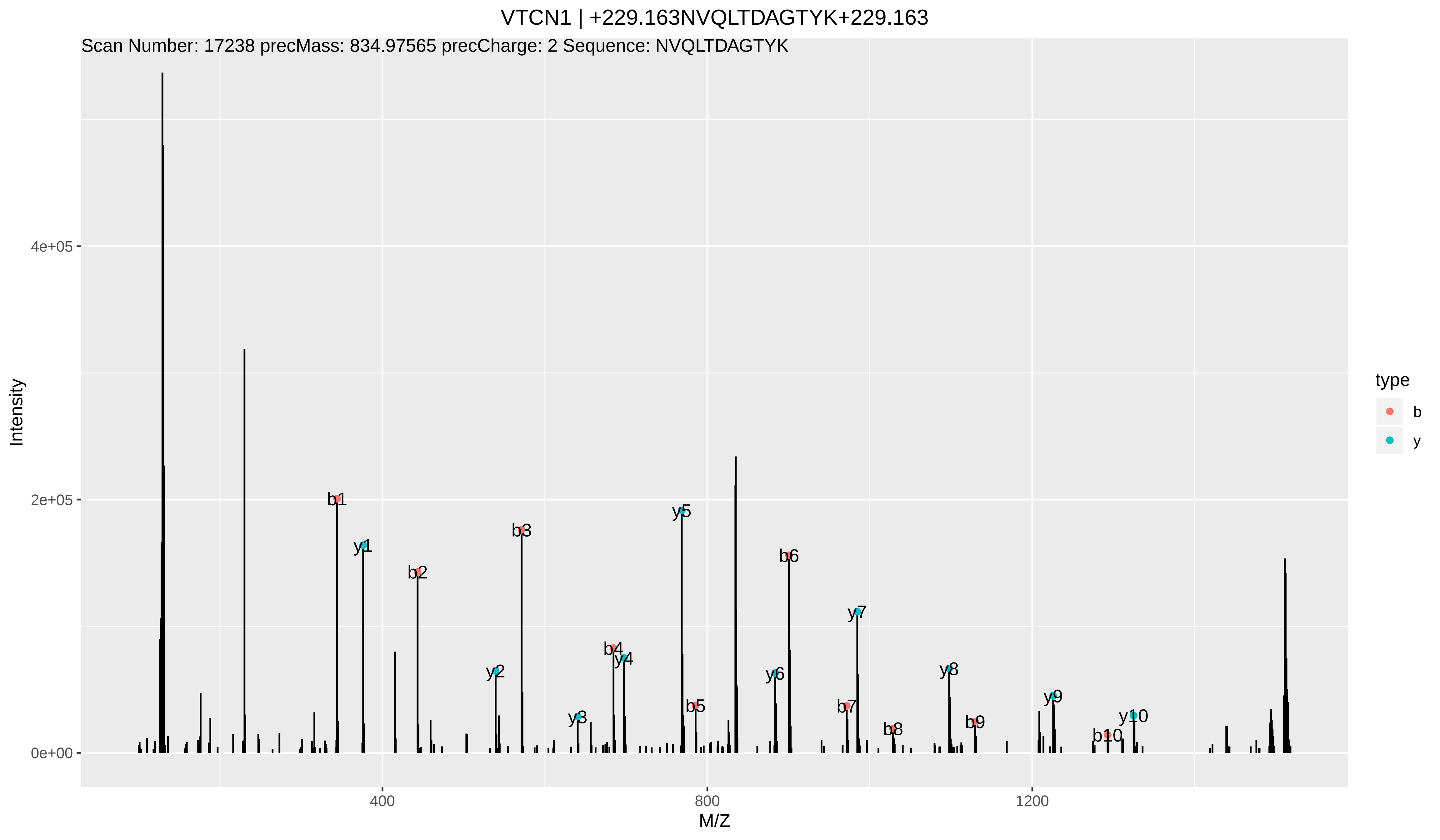

VTCN1 | +229.163NVQLTDAGTYK+229.163
Scan Number: 17238 precMass: 834.97565 precCharge: 2 Sequence: NVQLTDAGTYK
Intensity
0e+00
2e+05
4e+05
M/Z
400
800
1200
b1
y1
b2
y2
b3
y3
b4
y4
y5
b5
y6
b6
b7
y7
b8
y8
b9
y9
b10
y10
type
b
y

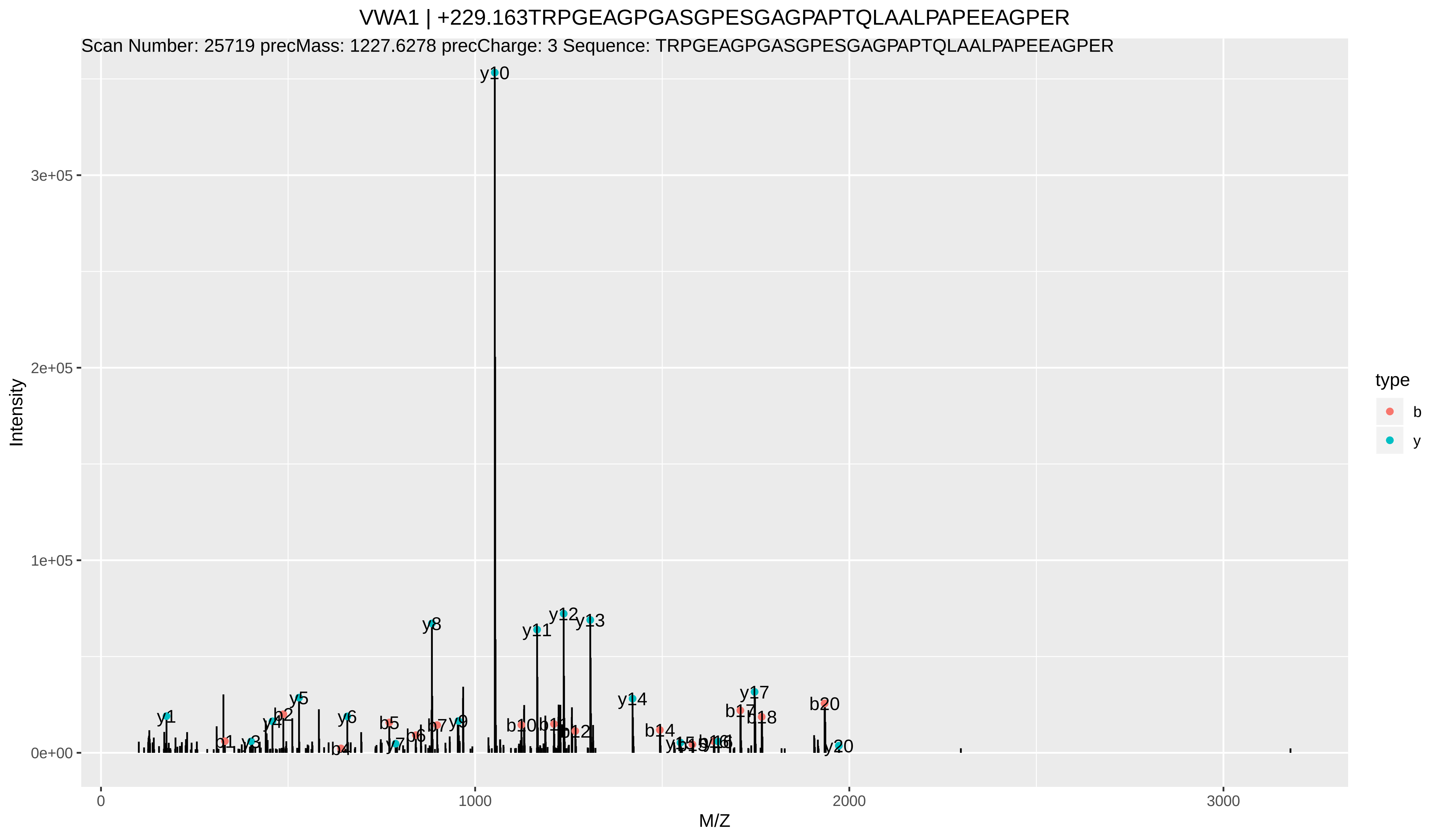
VWA1 | +229.163TRPGEAGPGASGPESGAGPAPTQLAALPAPEEAGPER
Scan Number: 25719 precMass: 1227.6278 precCharge: 3 Sequence: TRPGEAGPGASGPESGAGPAPTQLAALPAPEEAGPER
Intensity
0e+00
1e+05
2e+05
3e+05
M/Z
0
1000
2000
3000
type
b
y
y10
y8
y11
y12
y13
y14
y17
b20
b17
b18
b14
b10
y1
y5
y6
b5
y9
b7
b2
b1
y3
b4
y20

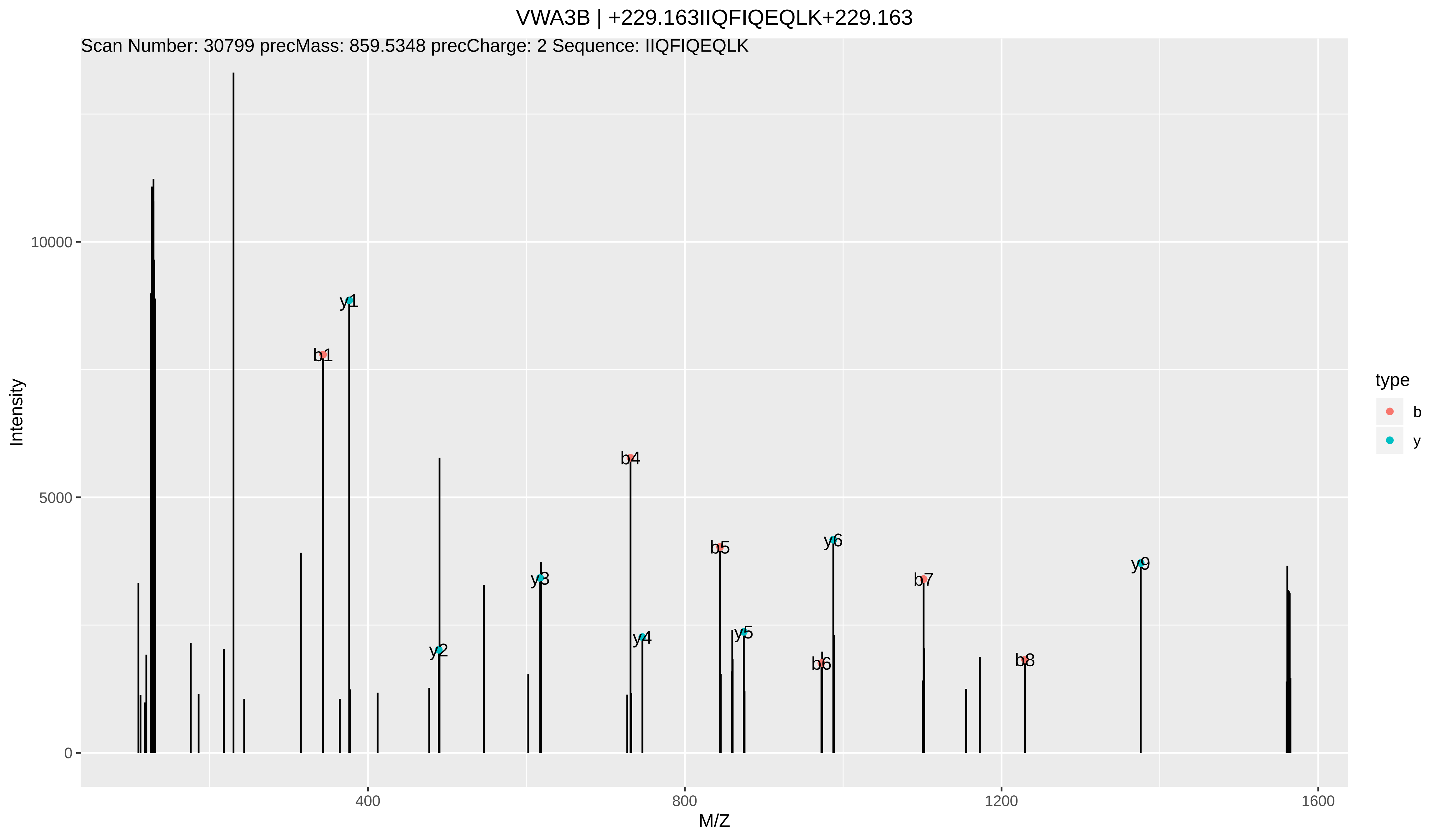
VWA3B | +229.163IIQFIQEQLK+229.163
Scan Number: 30799 precMass: 859.5348 precCharge: 2 Sequence: IIQFIQEQLK
Intensity
M/Z
10000
5000
0
400
800
1200
1600
y1
b1
b4
y2
y3
y4
b5
y5
b6
y6
b7
b8
y9
type
b
y

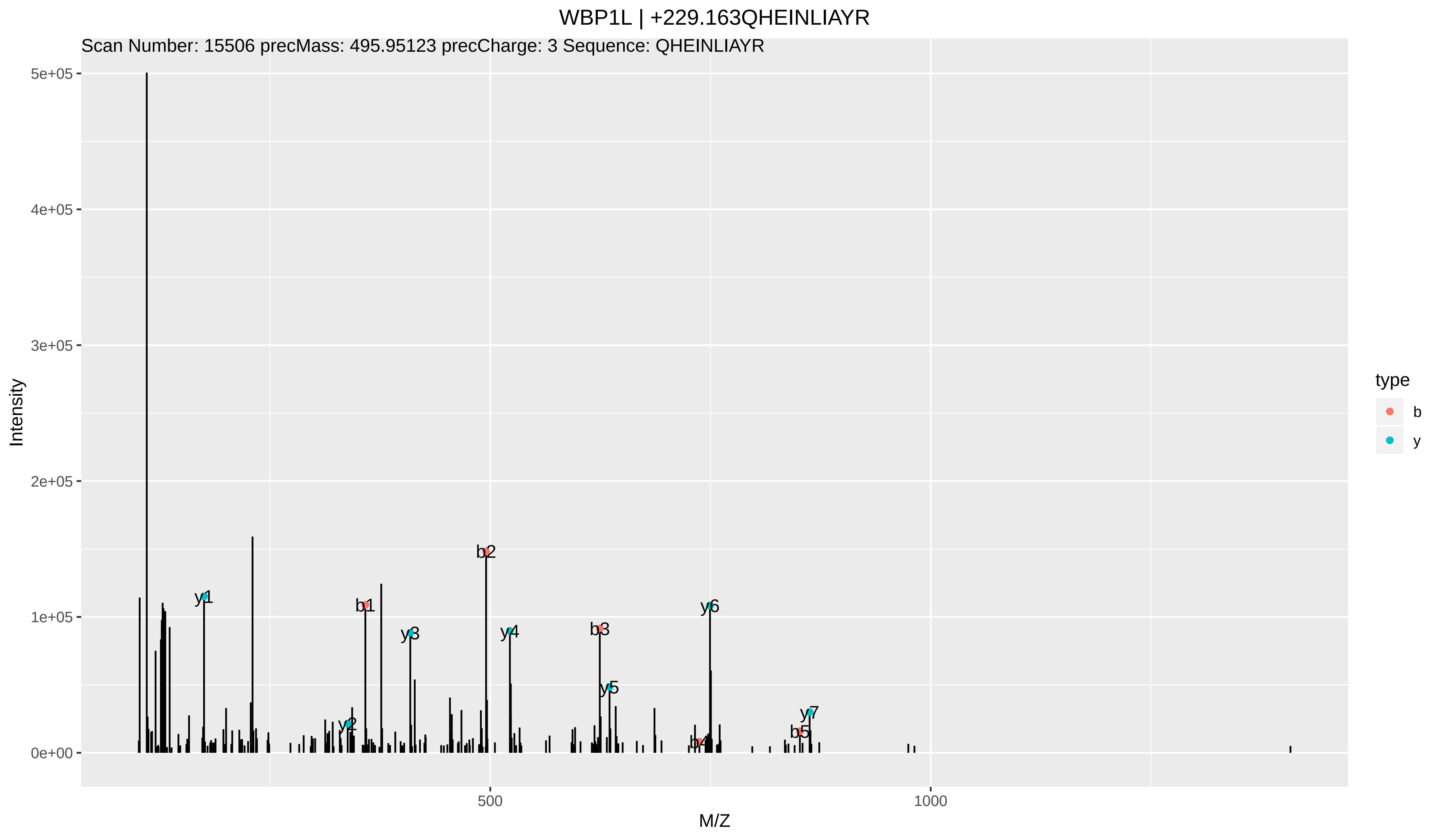

WBP1L | +229.163QHEINLIAYR
Scan Number: 15506 precMass: 495.95123 precCharge: 3 Sequence: QHEINLIAYR
Intensity
M/Z
5e+05
4e+05
3e+05
2e+05
1e+05
0e+00
500
1000
y1
b1
y2
y3
b2
y4
b3
y5
b4
y6
b5
y7
type
b
y

WDR83 | +229.163TYSGHGYEVLDAAGSFDNSSLC+57.021SGGGDK+229.163

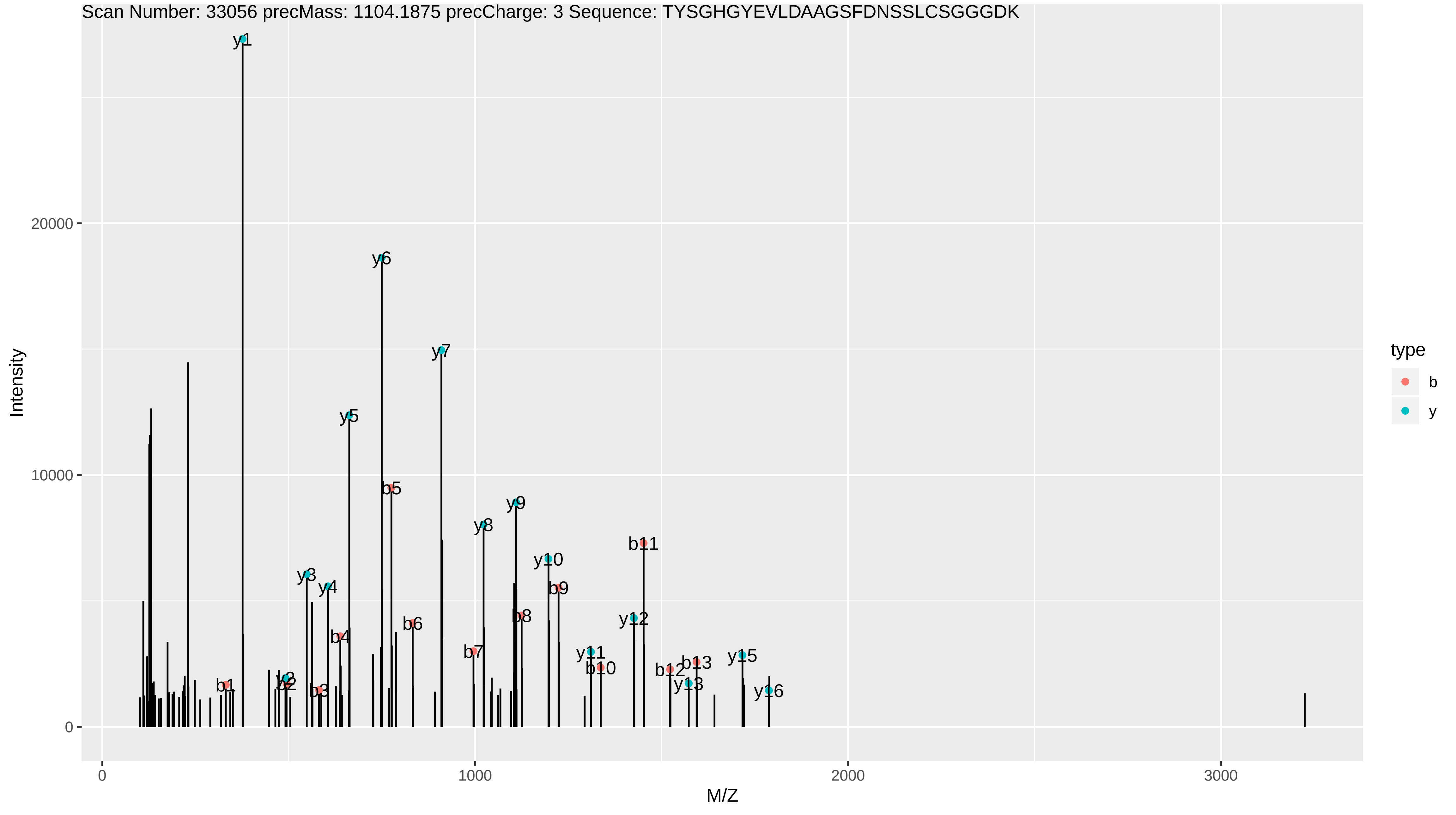

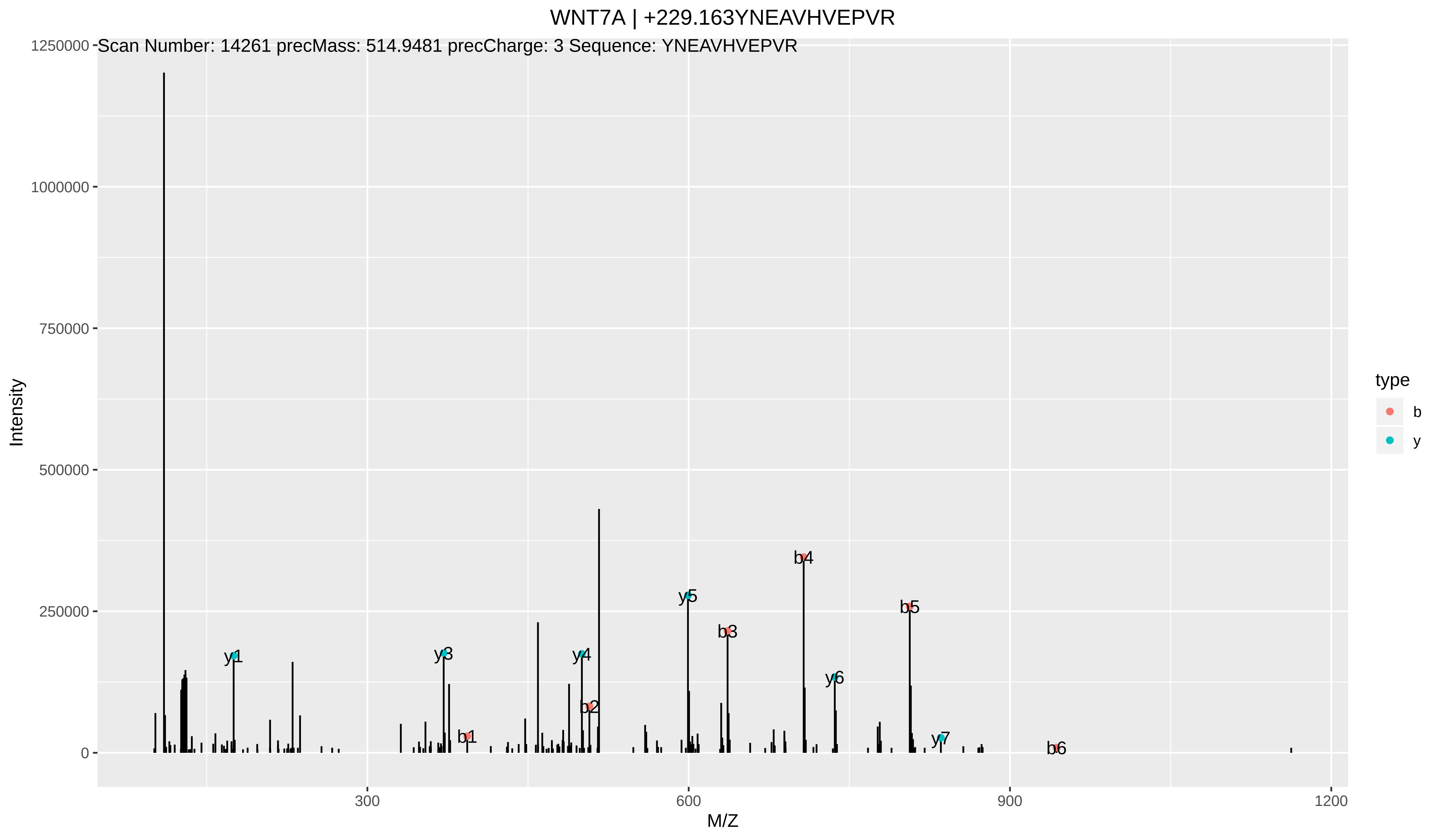

WNT7A | +229.163YNEAVHVEPVR
Scan Number: 14261 precMass: 514.9481 precCharge: 3 Sequence: YNEAVHVEPVR
Intensity
1250000
1000000
750000
500000
250000
0
M/Z
300
600
900
1200
y1
y3
b1
y4
b2
y5
b3
b4
y6
b5
y7
b6
type
b
y

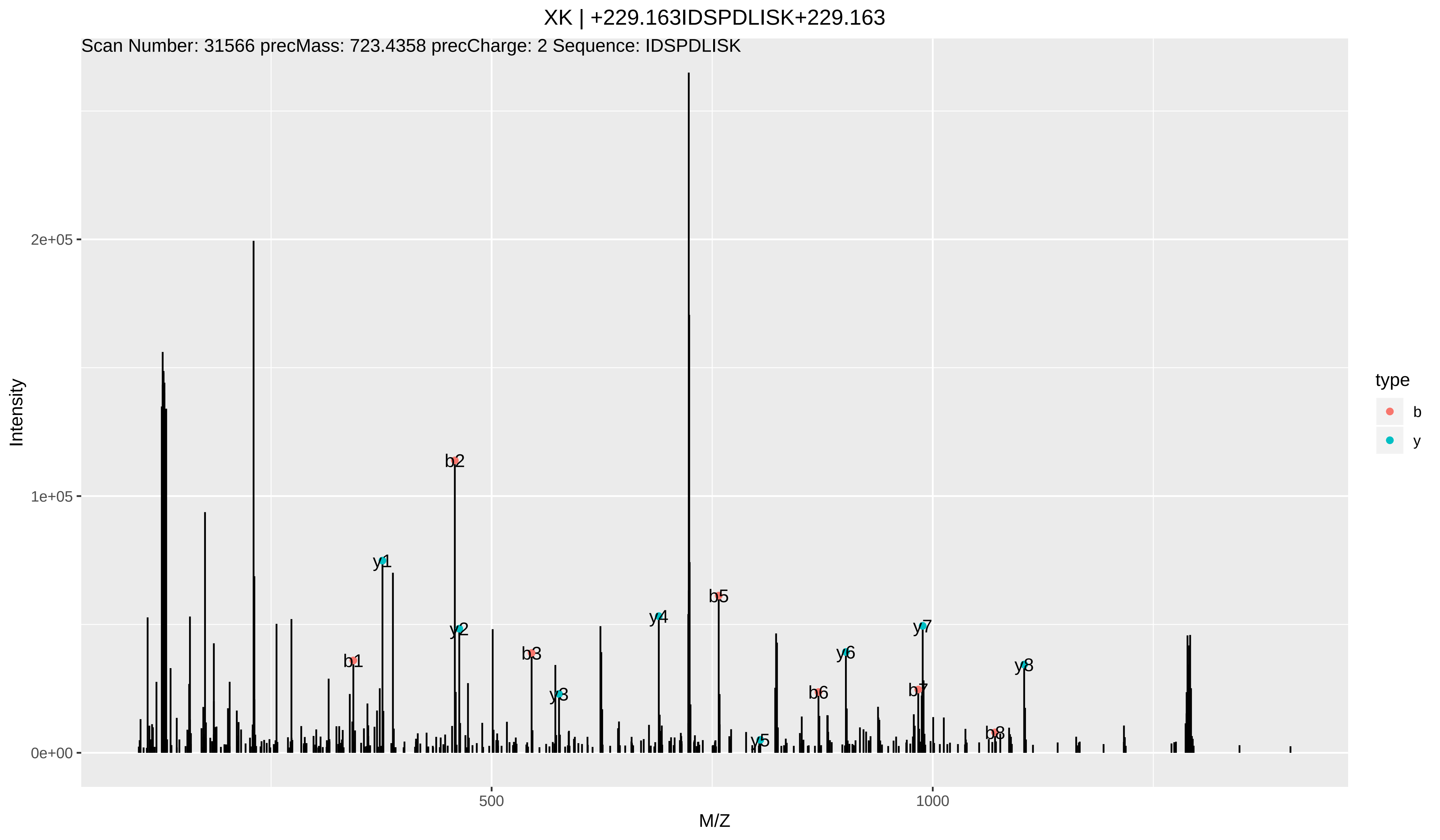
XK | +229.163IDSPDLISK+229.163
Scan Number: 31566 precMass: 723.4358 precCharge: 2 Sequence: IDSPDLISK
Intensity
2e+05
1e+05
0e+00
M/Z
500
1000
type
b
y
b1
b2
b3
b5
b6
b7
b8
y1
y2
y3
y4
y5
y6
y7
y8

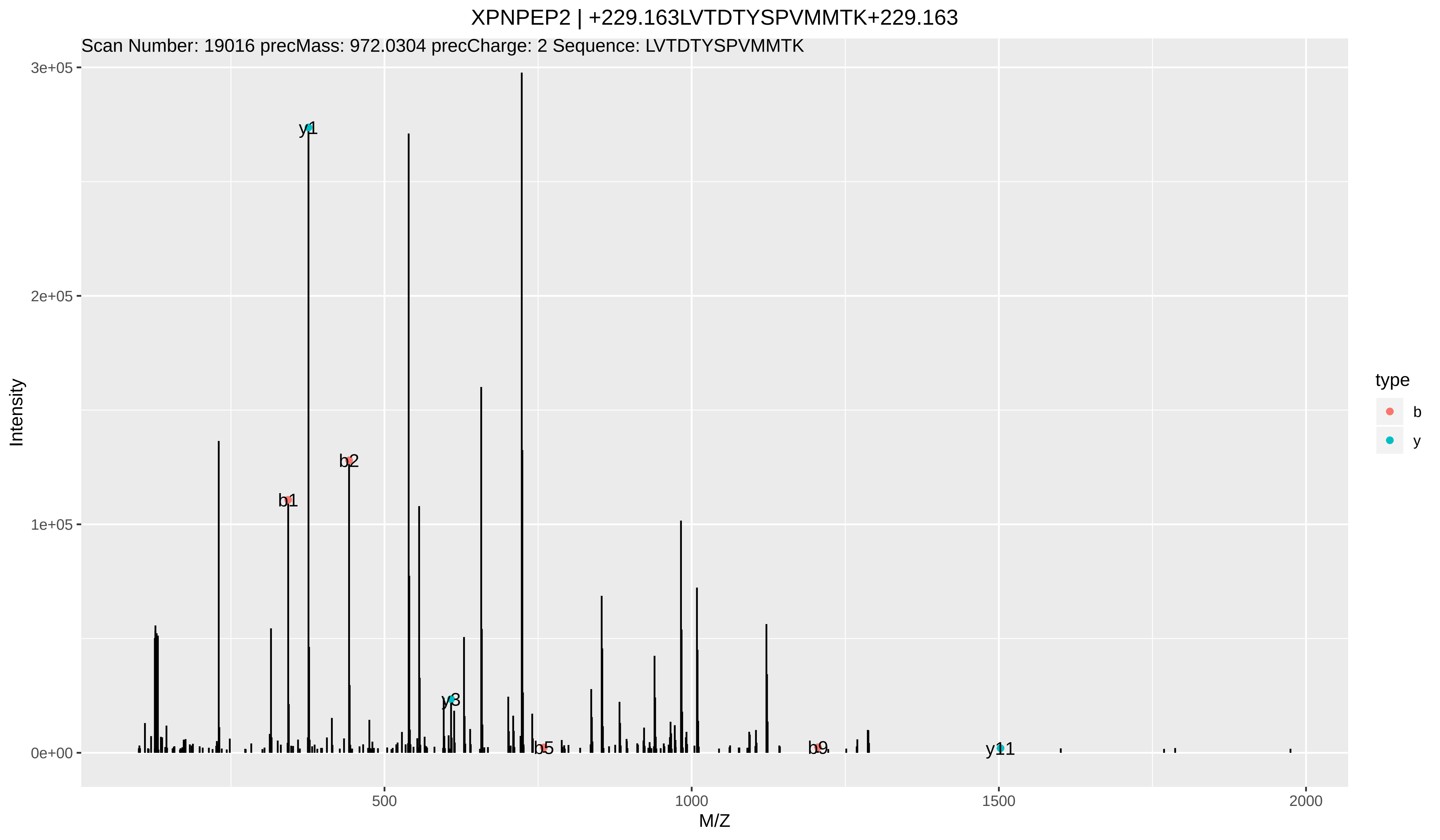

XPNPEP2 | +229.163LVTDTYSPVMMTK+229.163
Scan Number: 19016 precMass: 972.0304 precCharge: 2 Sequence: LVTDTYSPVMMTK
Intensity
M/Z
3e+05
2e+05
1e+05
0e+00
500
1000
1500
2000
y1
b2
b1
y3
b5
b9
y11
type
b
y

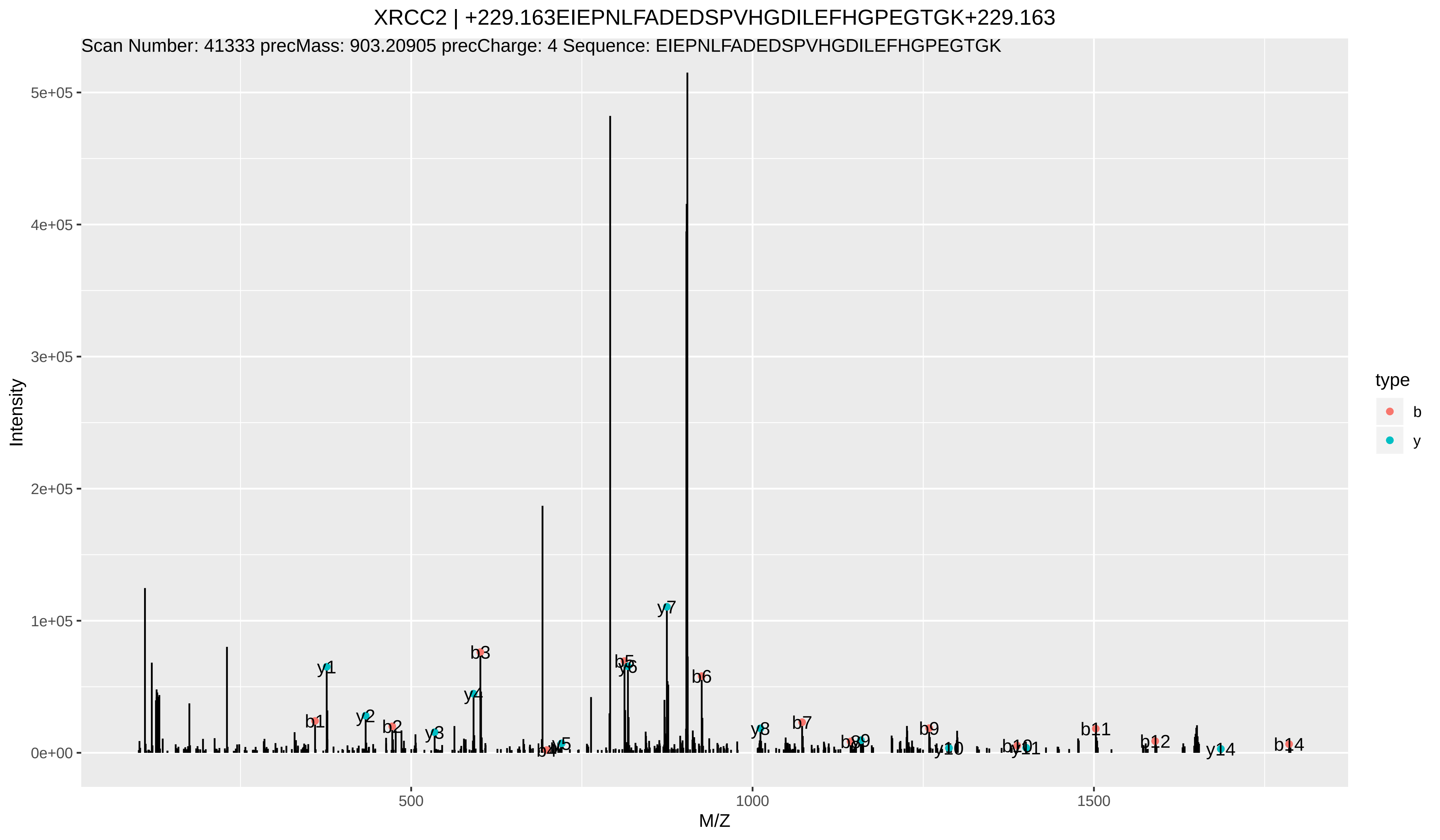
XRCC2 | +229.163EIEPNLFADEDSPVHGDILEFHGPEGTGK+229.163
Scan Number: 41333 precMass: 903.20905 precCharge: 4 Sequence: EIEPNLFADEDSPVHGDILEFHGPEGTGK
Intensity
M/Z
5e+05
4e+05
3e+05
2e+05
1e+05
0e+00
500
1000
1500
type
b
y
y1
b1
y2
b2
y3
y4
b3
b4
y5
b5
y6
y7
b6
y8
b7
b8
y9
b9
y10
b10
y11
b11
b12
y14
b14

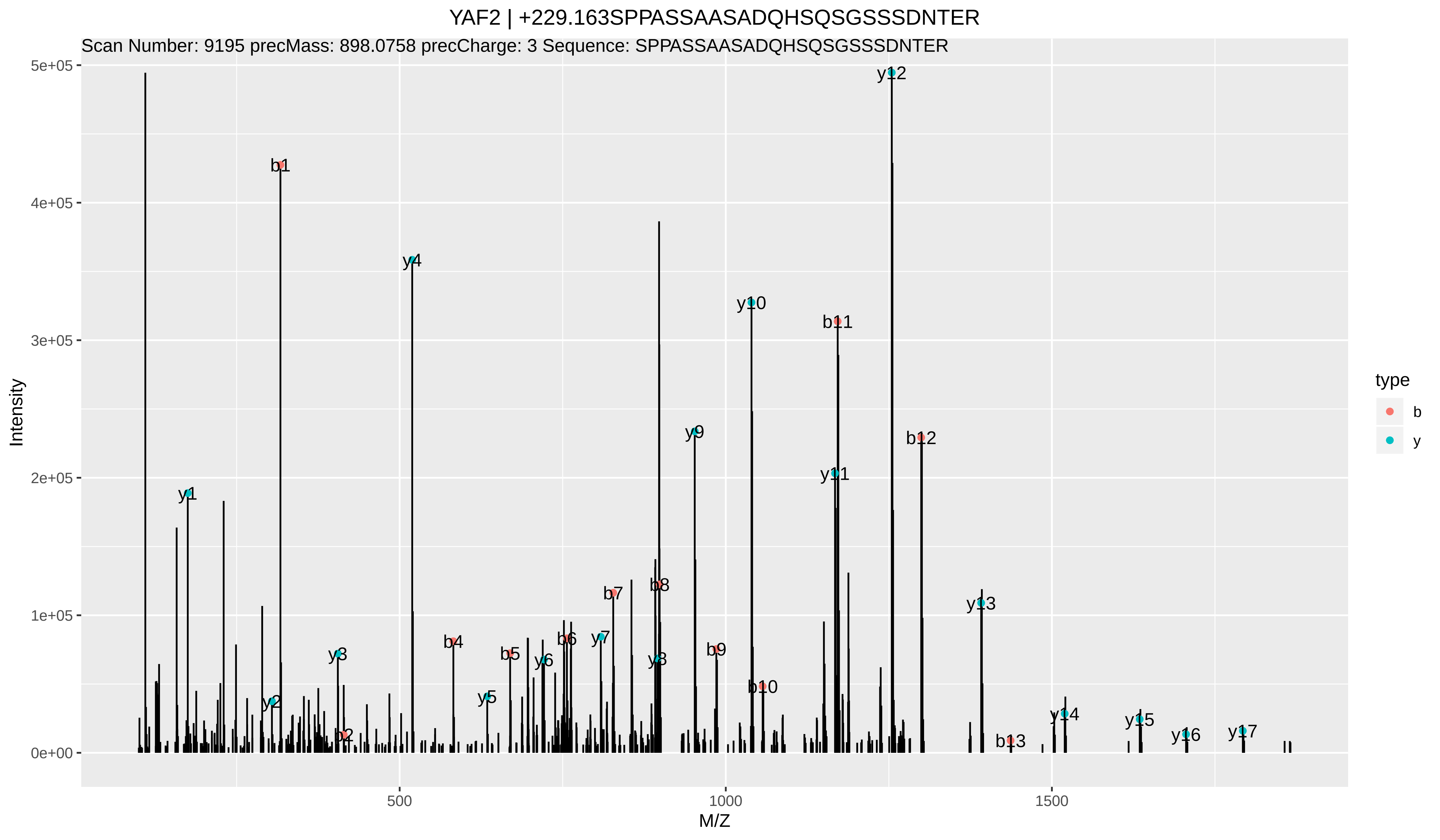
YAF2 | +229.163SPPASSAASADQHSQSGSSSDNTER
Scan Number: 9195 precMass: 898.0758 precCharge: 3 Sequence: SPPASSAASADQHSQSGSSSDNTER
Intensity
M/Z
type
b
y
y12
b1
y4
y10
b11
y9
b12
y11
y1
y13
b8
b7
b6
y7
b4
b5
y6
y3
y8
b9
b10
y5
y2
b2
b13
y14
y15
y16
y17
0e+00
1e+05
2e+05
3e+05
4e+05
5e+05
500
1000
1500

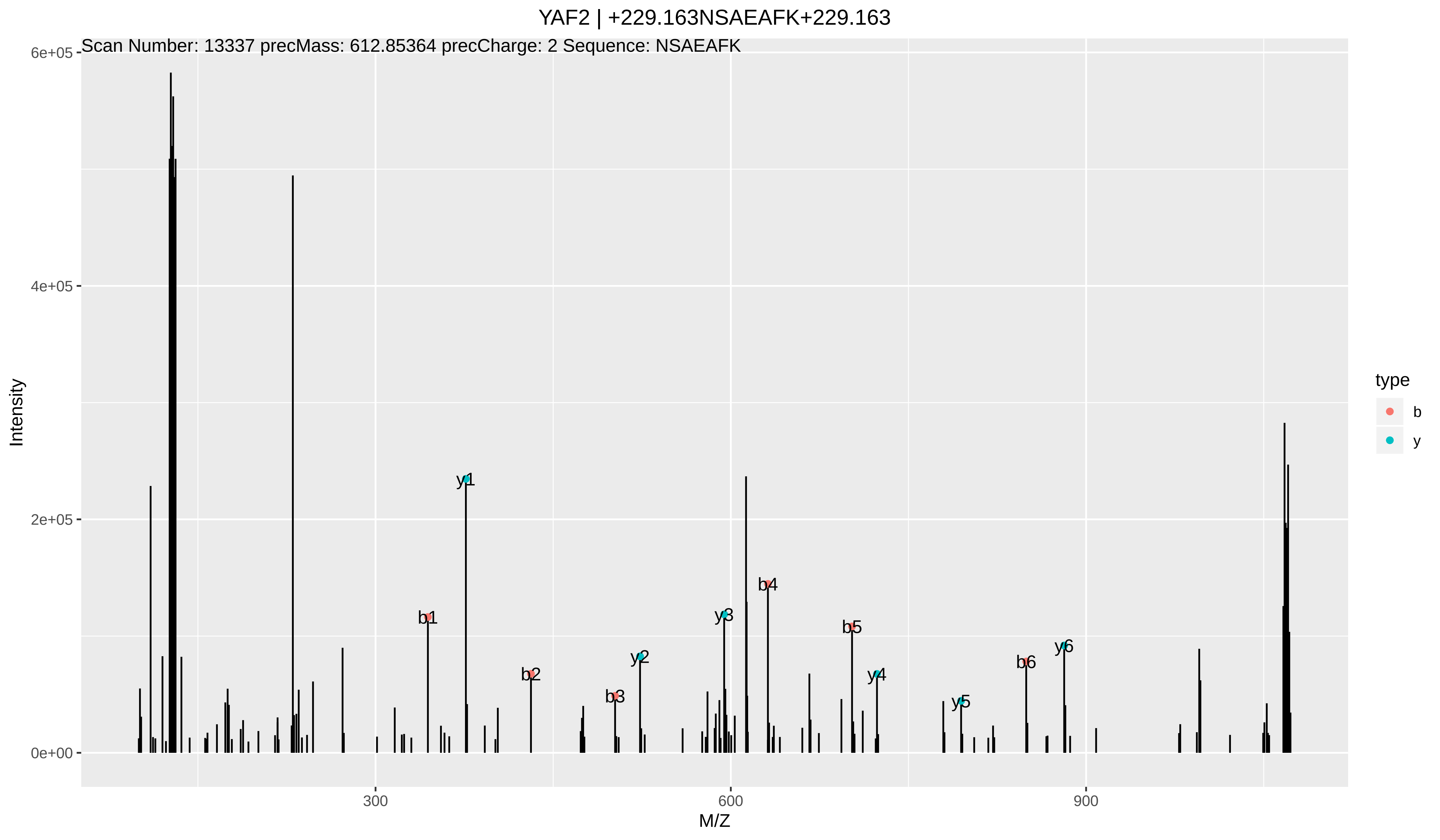

YAF2 | +229.163NSAEAFK+229.163
Scan Number: 13337 precMass: 612.85364 precCharge: 2 Sequence: NSAEAFK
Intensity
M/Z
6e+05
4e+05
2e+05
0e+00
300
600
900
y1
b1
b2
b3
y2
y3
b4
b5
y4
y5
b6
y6
type
b
y

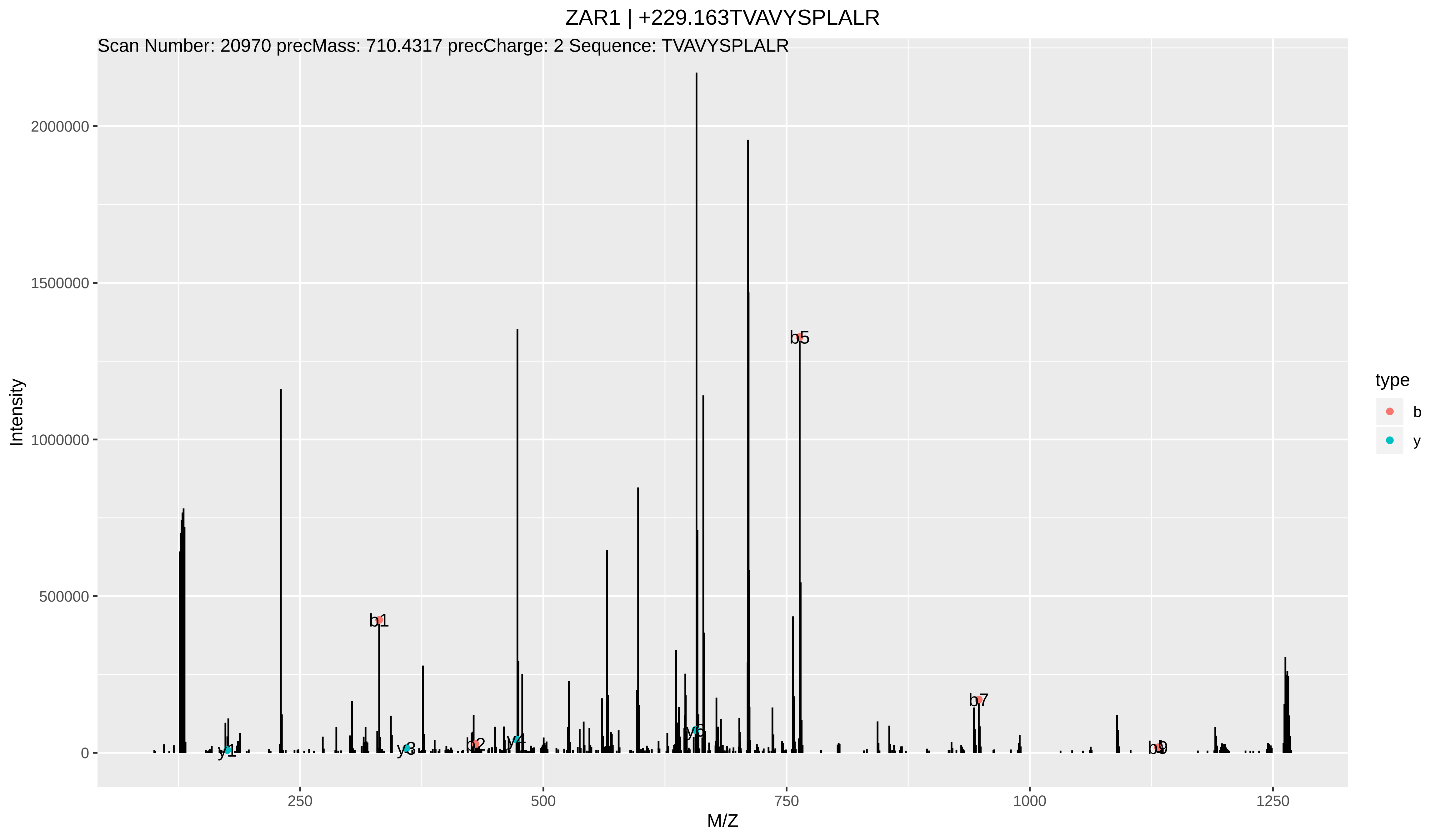

ZAR1 | +229.163TVAVYSPLALR
Scan Number: 20970 precMass: 710.4317 precCharge: 2 Sequence: TVAVYSPLALR
Intensity
M/Z
0
500000
1000000
1500000
2000000
250
500
750
1000
1250
b1
b2
b5
b7
b9
y1
y3
y4
y6
type
b
y

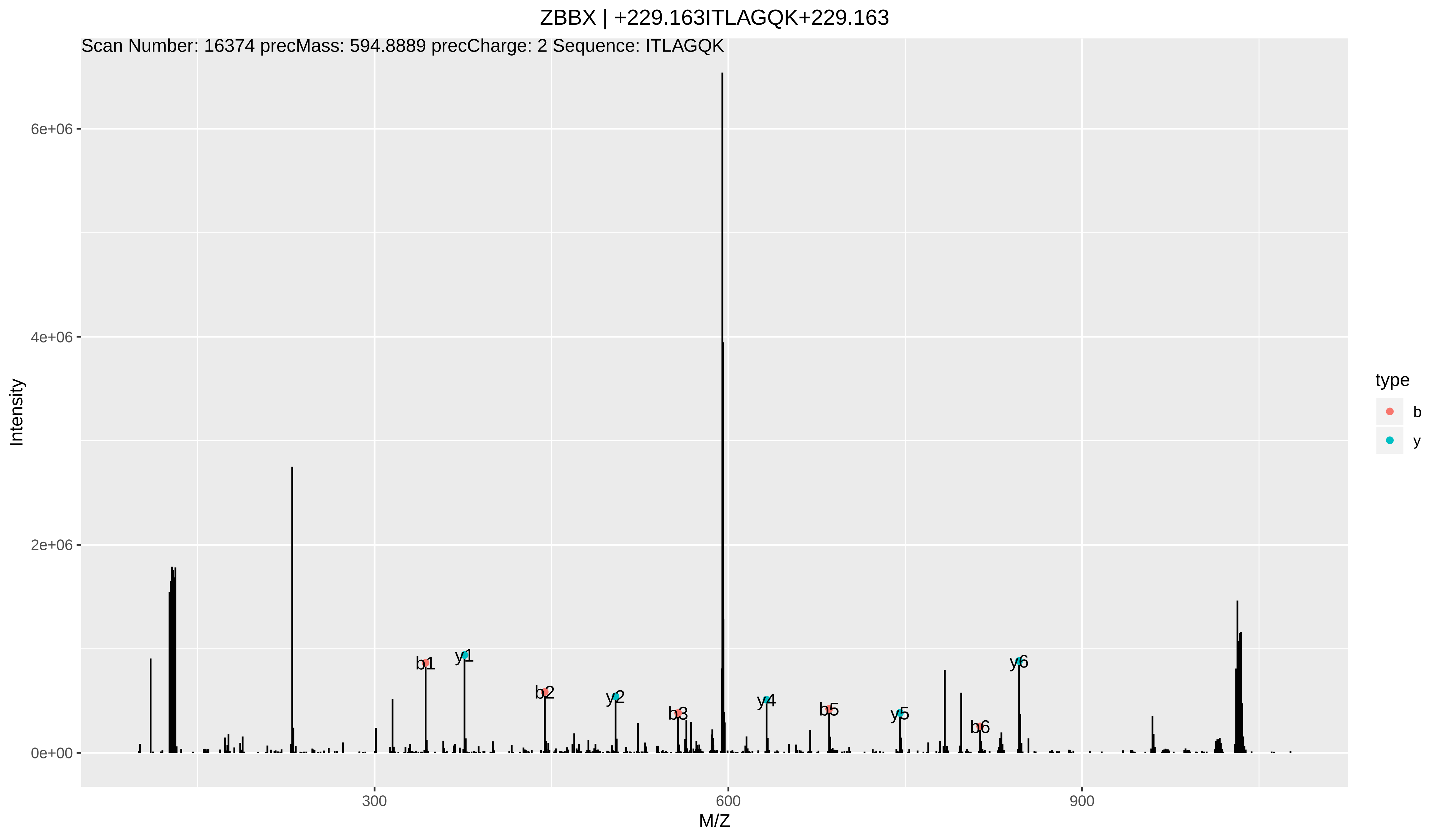
ZBBX | +229.163ITLAGQK+229.163
Scan Number: 16374 precMass: 594.8889 precCharge: 2 Sequence: ITLAGQK
Intensity
6e+06
4e+06
2e+06
0e+00
300
600
900
M/Z
b1
y1
b2
y2
b3
y4
b5
y5
b6
y6
type
b
y

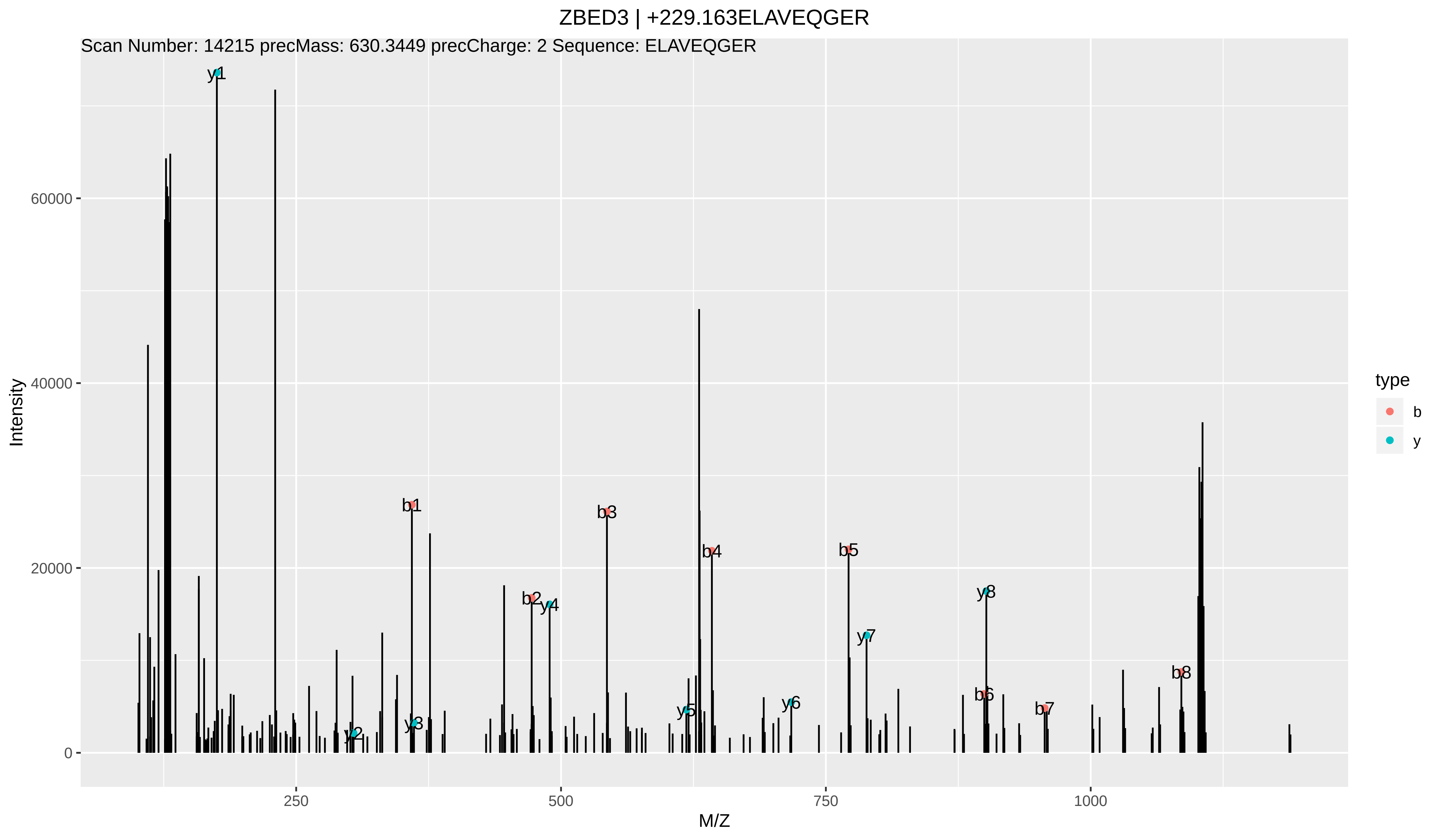

ZBED3 | +229.163ELAVEQGER
Scan Number: 14215 precMass: 630.3449 precCharge: 2 Sequence: ELAVEQGER
Intensity
M/Z
type
b
y
y1
b1
b2
b3
b4
b5
b6
b7
b8
y2
y3
y4
y5
y6
y7
y8
0
20000
40000
60000
250
500
750
1000

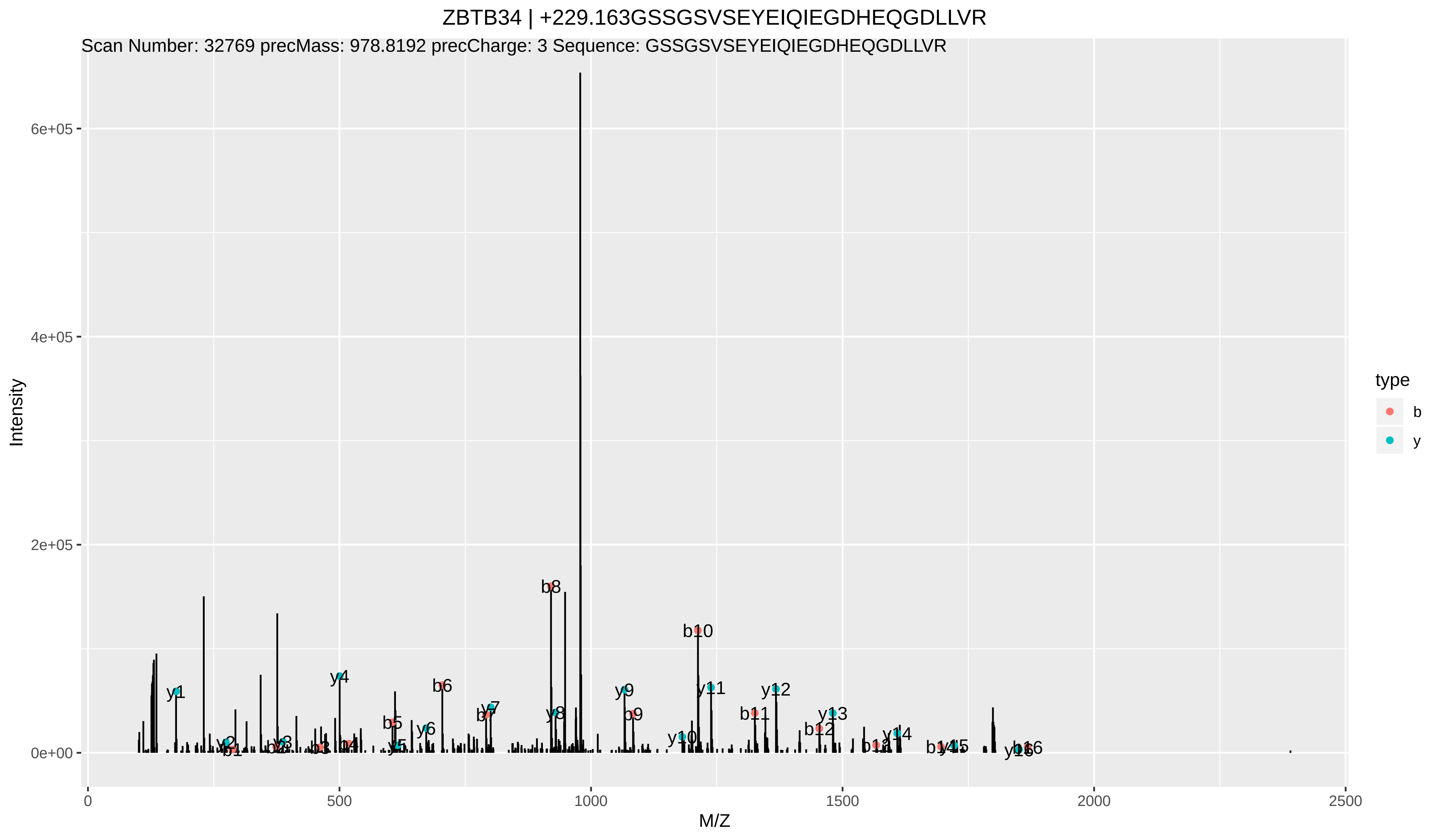
ZBTB34 | +229.163GSSGSVSEYEIQIEGDHEQGDLLVR
Scan Number: 32769 precMass: 978.8192 precCharge: 3 Sequence: GSSGSVSEYEIQIEGDHEQGDLLVR
Intensity
M/Z
type
b
y
6e+05
4e+05
2e+05
0e+00
0
500
1000
1500
2000
2500
y1
y4
b6
y7
b8
y8
y9
b9
b10
y10
y11
y12
b11
b12
y13
y14
b5
y6
y5
b1
y2
y3
b2
b3
b4

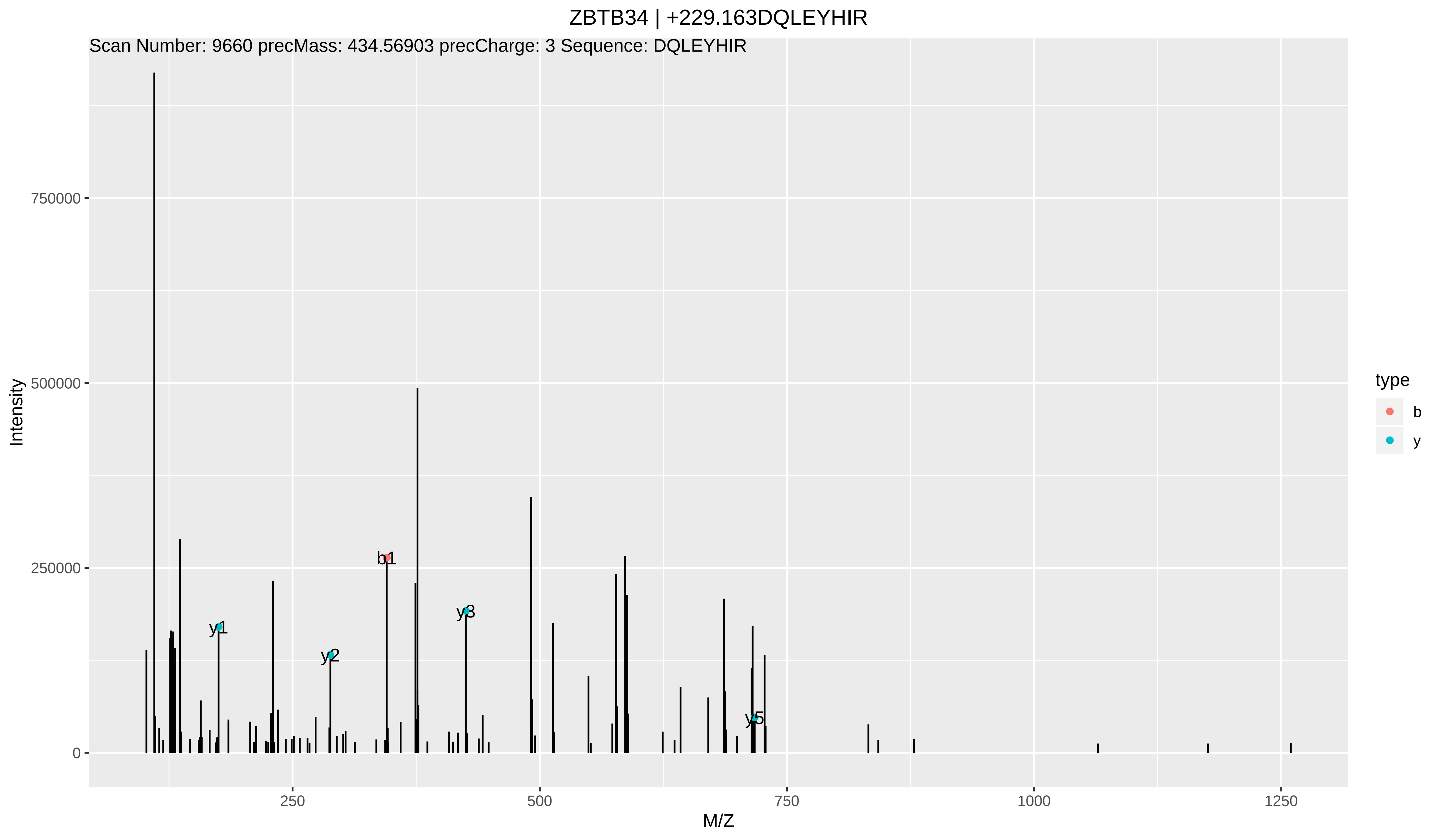
ZBTB34 | +229.163DQLEYHIR
Scan Number: 9660 precMass: 434.56903 precCharge: 3 Sequence: DQLEYHIR
Intensity
750000
500000
250000
0
M/Z
250
500
750
1000
1250
b1
y1
y2
y3
y5
type
b
y

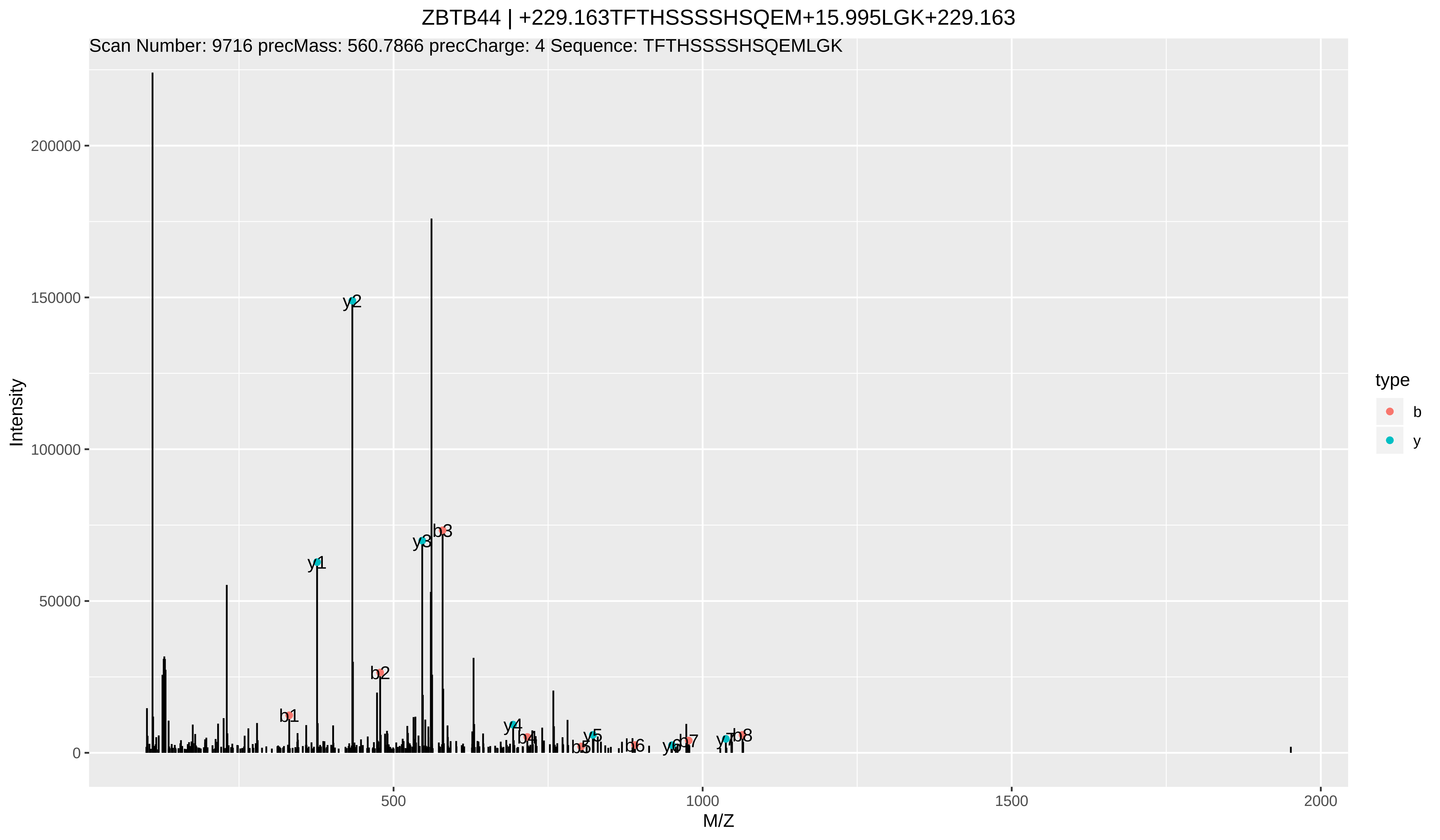

ZBTB44 | +229.163TFTHSSSSHSQEM+15.995LGK+229.163
Scan Number: 9716 precMass: 560.7866 precCharge: 4 Sequence: TFTHSSSSHSQEMLGK
Intensity
M/Z
0
50000
100000
150000
200000
500
1000
1500
2000
type
b
y
y1
y2
y3
y4
y5
y6
y7
b1
b2
b3
b4
b5
b6
b7
b8

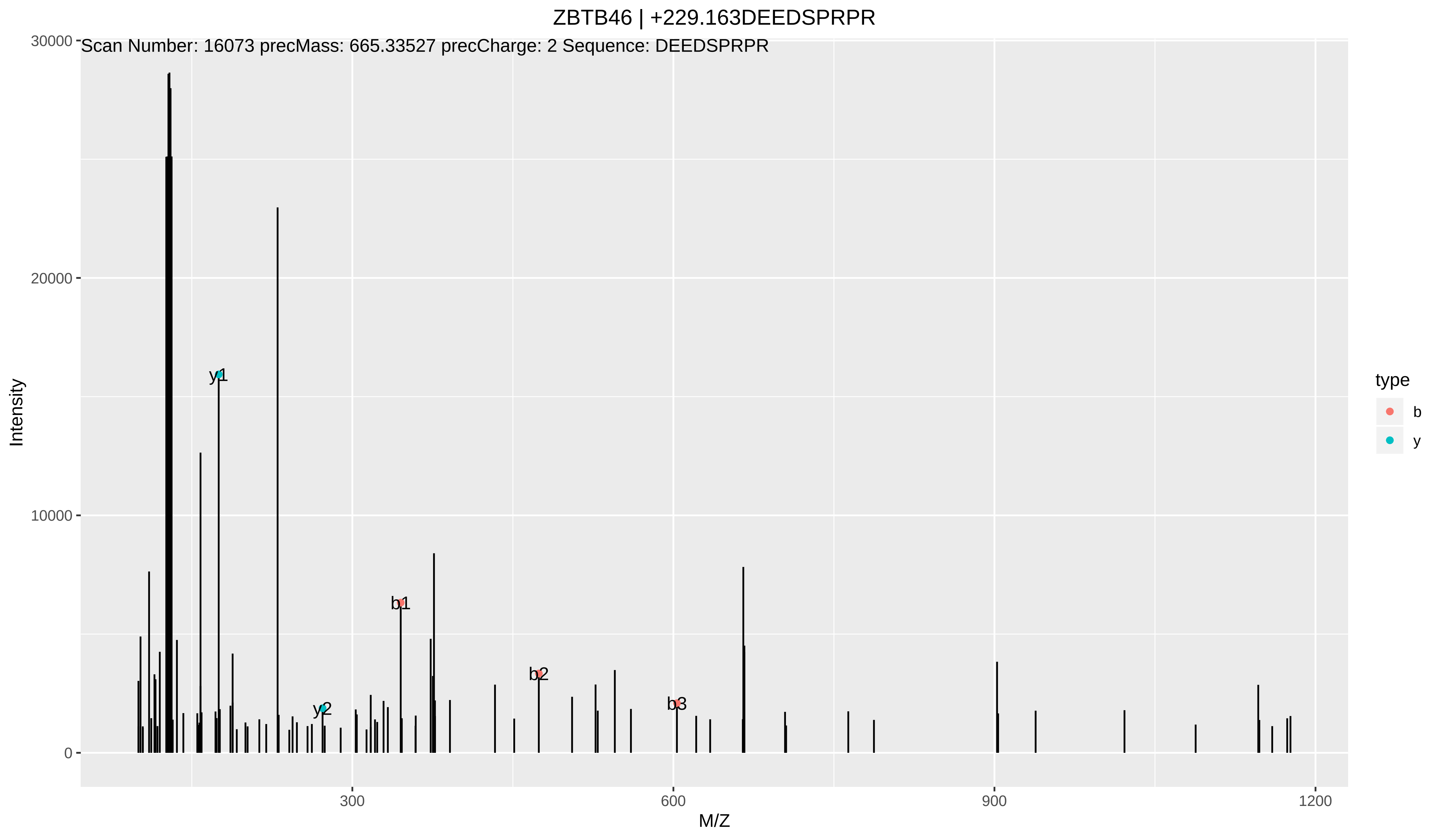
ZBTB46 | +229.163DEEDSPRPR
Scan Number: 16073 precMass: 665.33527 precCharge: 2 Sequence: DEEDSPRPR
Intensity
M/Z
30000
20000
10000
0
300
600
900
1200
y1
y2
b1
b2
b3
type
b
y

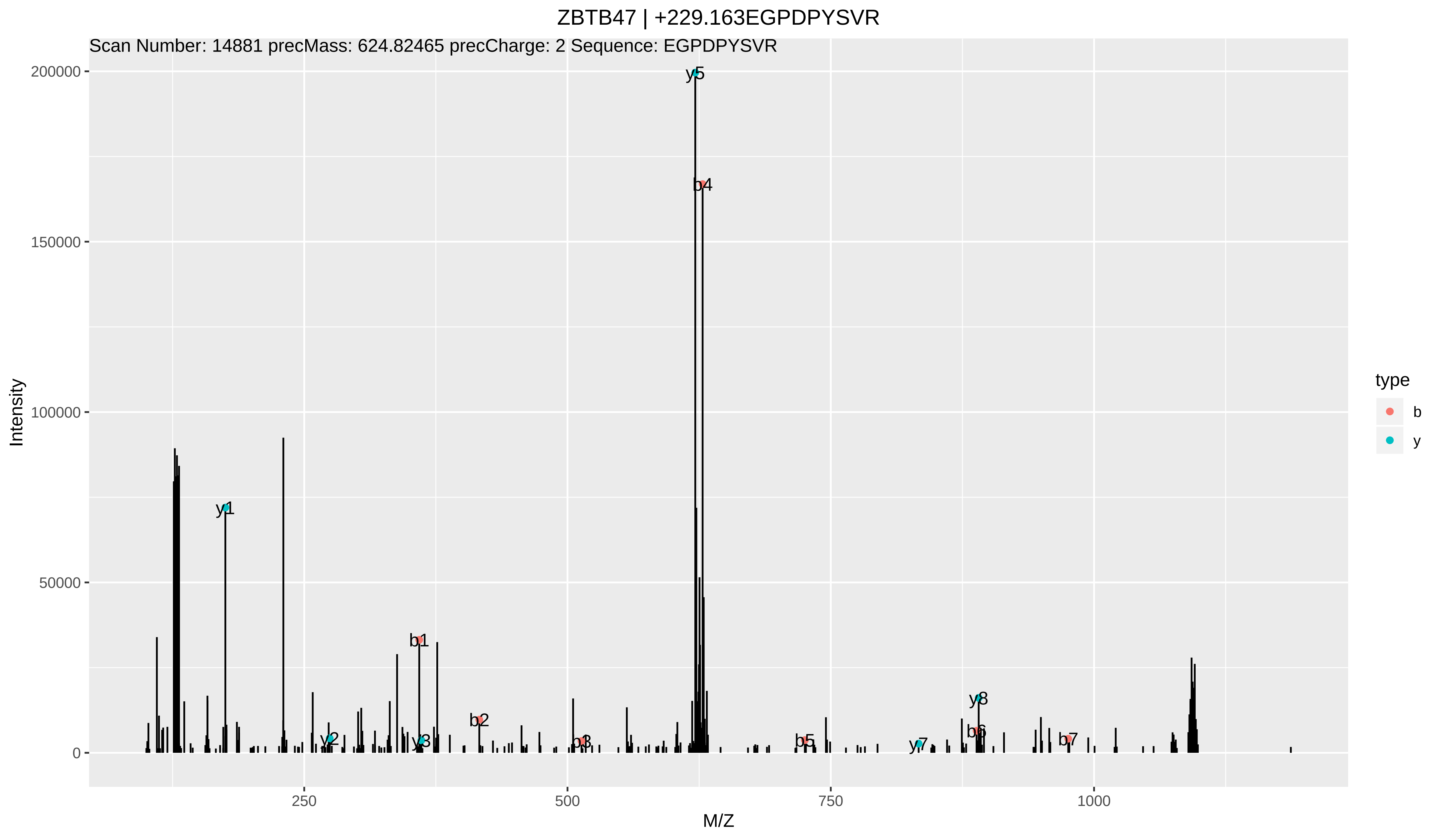

ZBTB47 | +229.163EGPDPYSVR
Scan Number: 14881 precMass: 624.82465 precCharge: 2 Sequence: EGPDPYSVR
Intensity
200000
150000
100000
50000
0
M/Z
250
500
750
1000
y5
b4
y1
b1
b2
y2
y3
b3
b5
y7
y8
b6
b7
type
b
y

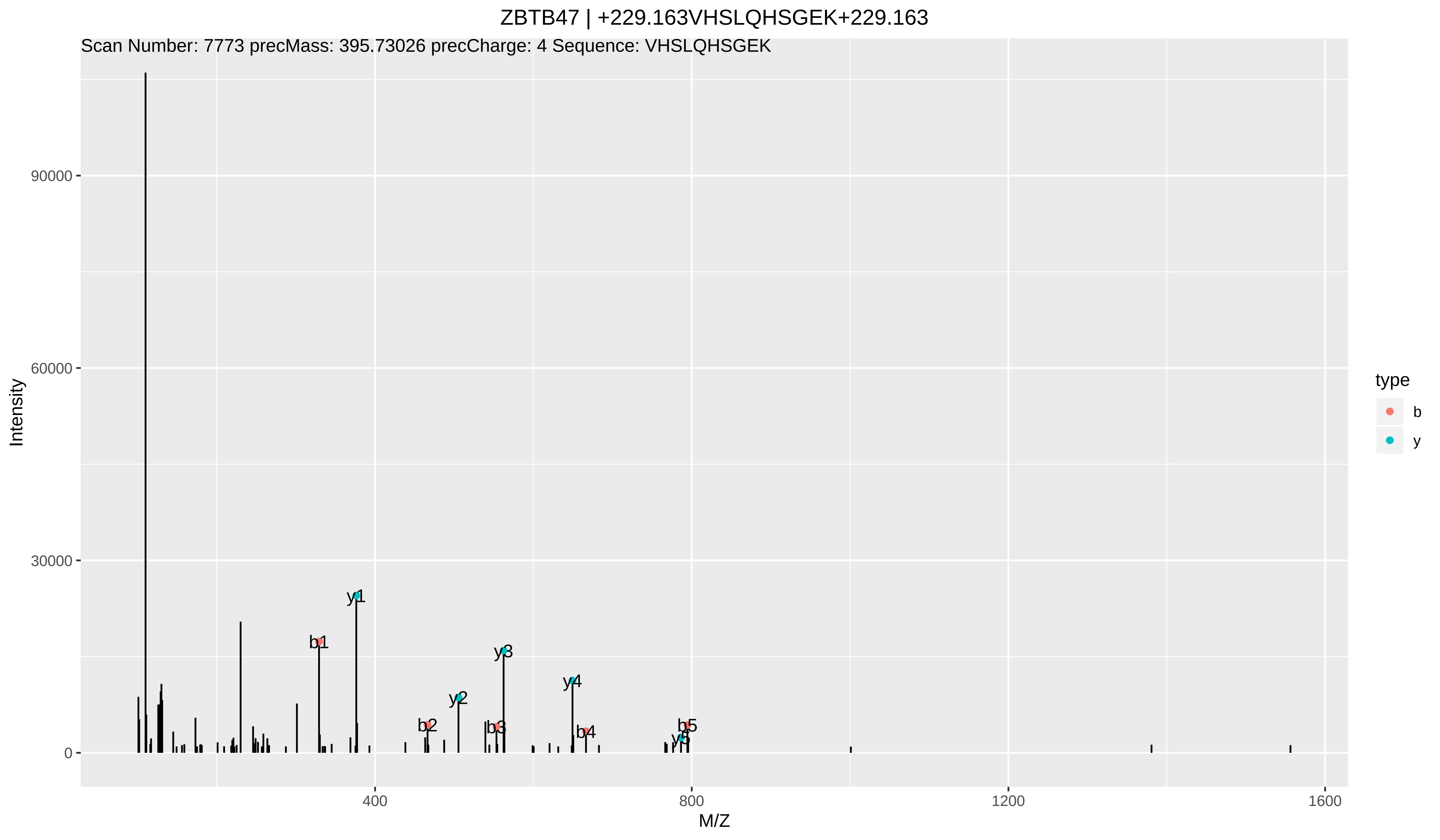

ZBTB47 | +229.163VHSLQHSGEK+229.163
Scan Number: 7773 precMass: 395.73026 precCharge: 4 Sequence: VHSLQHSGEK
Intensity
90000
60000
30000
0
M/Z
400
800
1200
1600
y1
b1
y3
y4
y2
b2
b3
b4
b5
y5
type
b
y

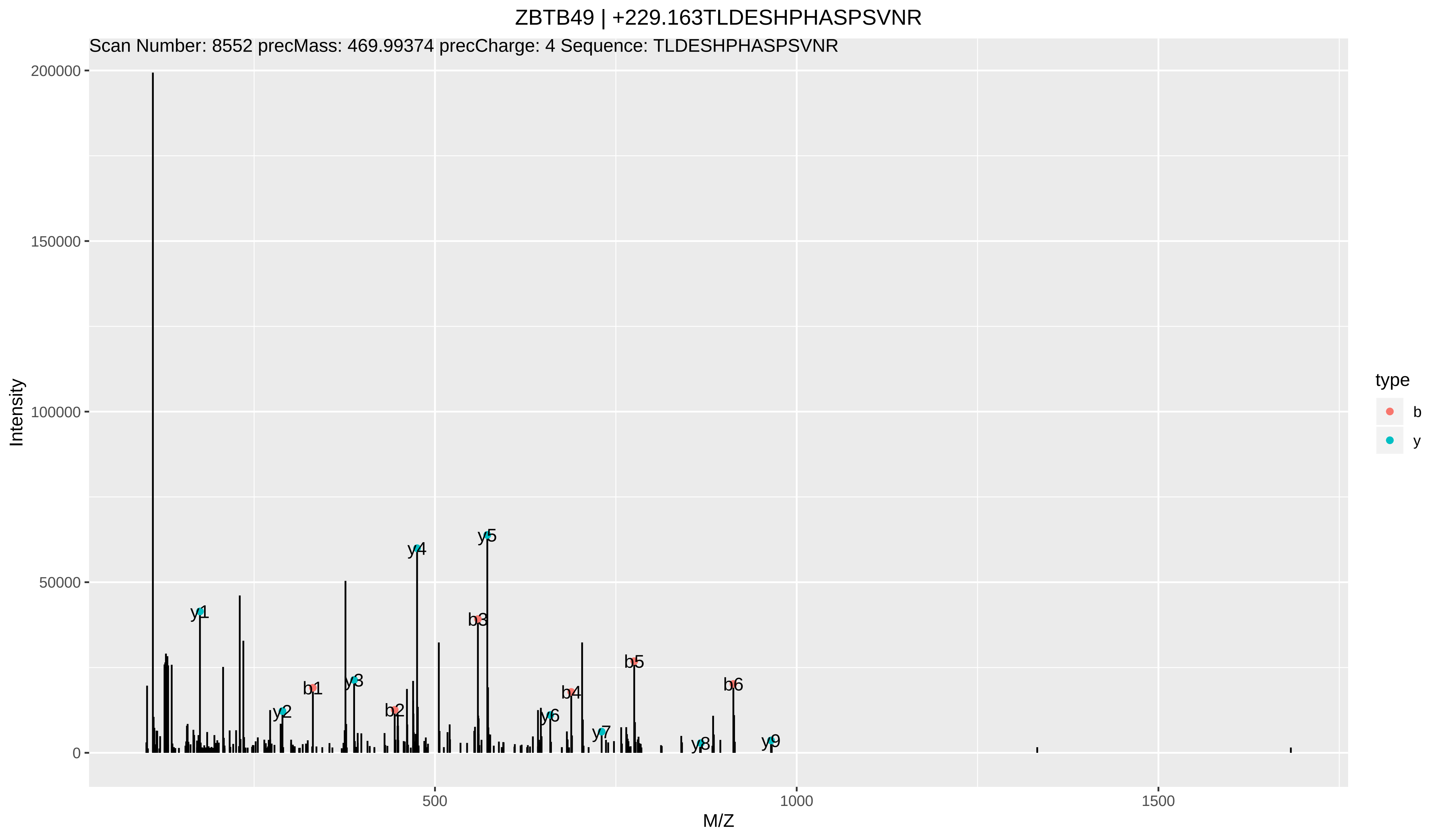

ZBTB49 | +229.163TLDESHPHASPSVNR
Scan Number: 8552 precMass: 469.99374 precCharge: 4 Sequence: TLDESHPHASPSVNR
Intensity
200000
150000
100000
50000
0
M/Z
500
1000
1500
y1
y2
b1
y3
b2
y4
y5
b3
y6
b4
y7
b5
y8
b6
y9
type
b
y

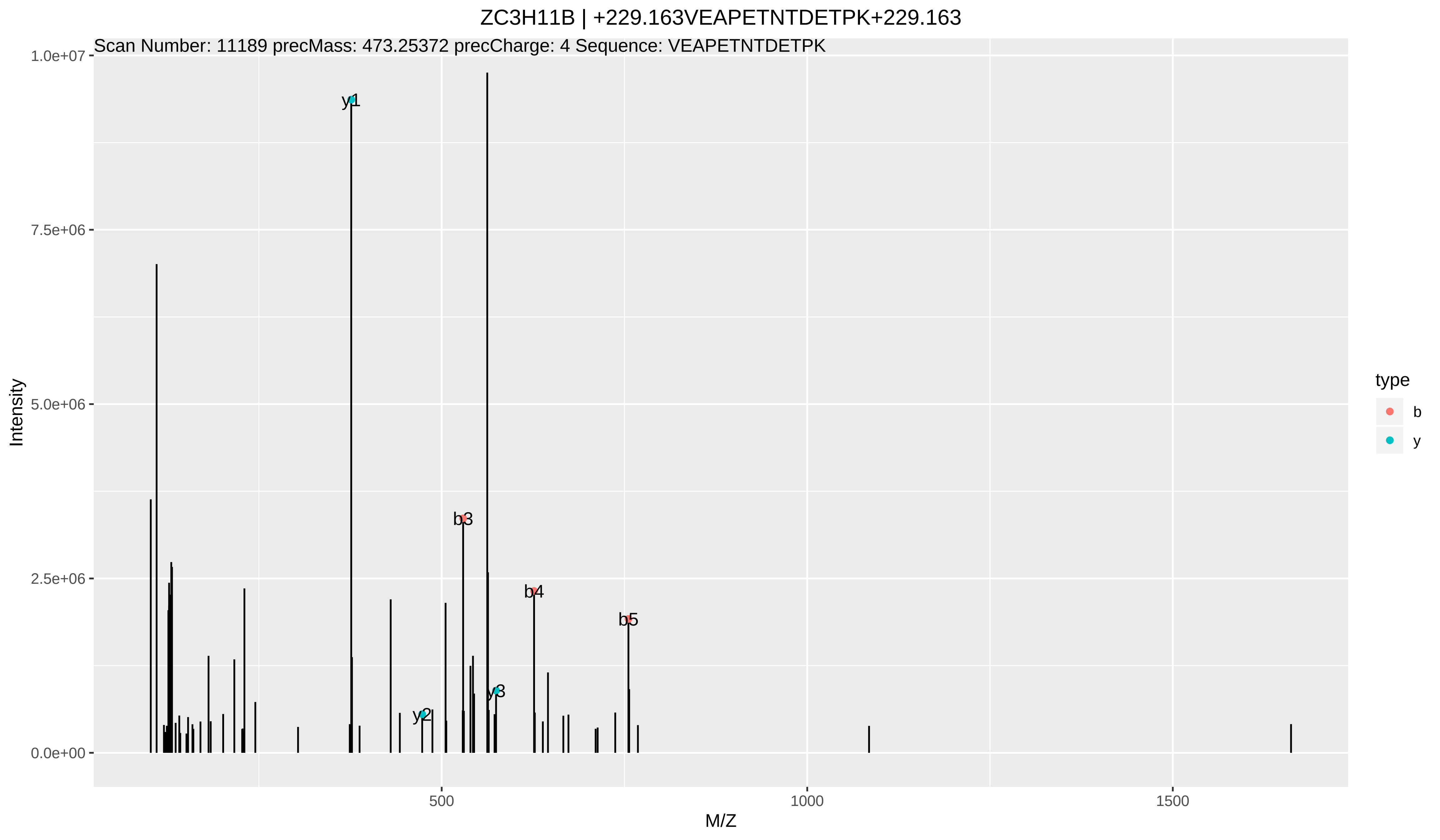

ZC3H11B | +229.163VEAPETNTDETPK+229.163
Scan Number: 11189 precMass: 473.25372 precCharge: 4 Sequence: VEAPETNTDETPK
Intensity
1.0e+07
7.5e+06
5.0e+06
2.5e+06
0.0e+00
y1
b3
b4
b5
y2
y3
500
1000
1500
M/Z
type
b
y

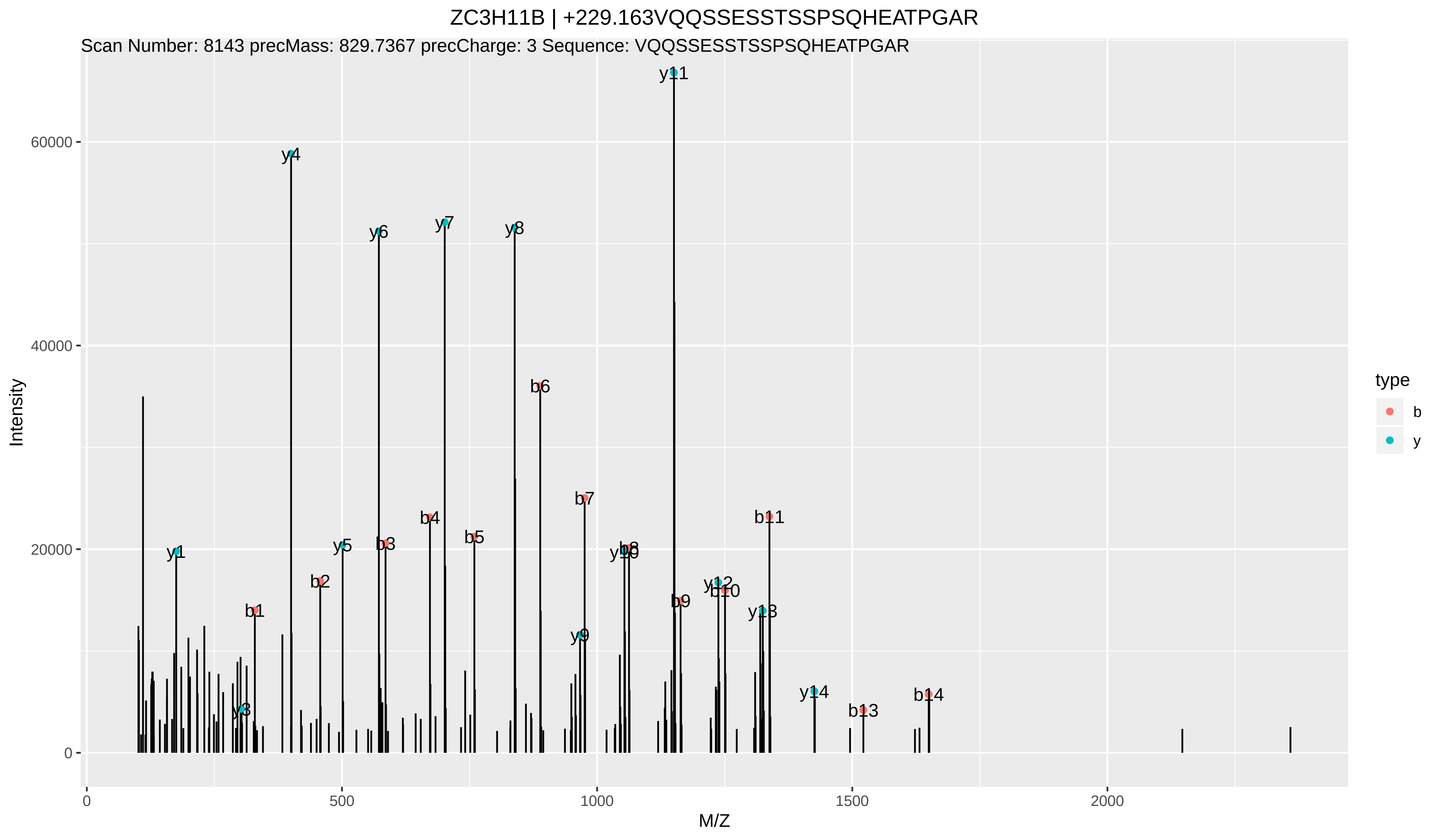

ZC3H11B | +229.163VQQSSESSTSSPSQHEATPGAR
Scan Number: 8143 precMass: 829.7367 precCharge: 3 Sequence: VQQSSESSTSSPSQHEATPGAR
Intensity
M/Z
type
b
y
y11
y4
y6
y7
y8
b6
b7
b4
b11
b5
b3
y5
y1
b8
y10
b2
y12
b10
b9
y13
b1
y9
y14
b14
b13
y3
0
20000
40000
60000
0
500
1000
1500
2000

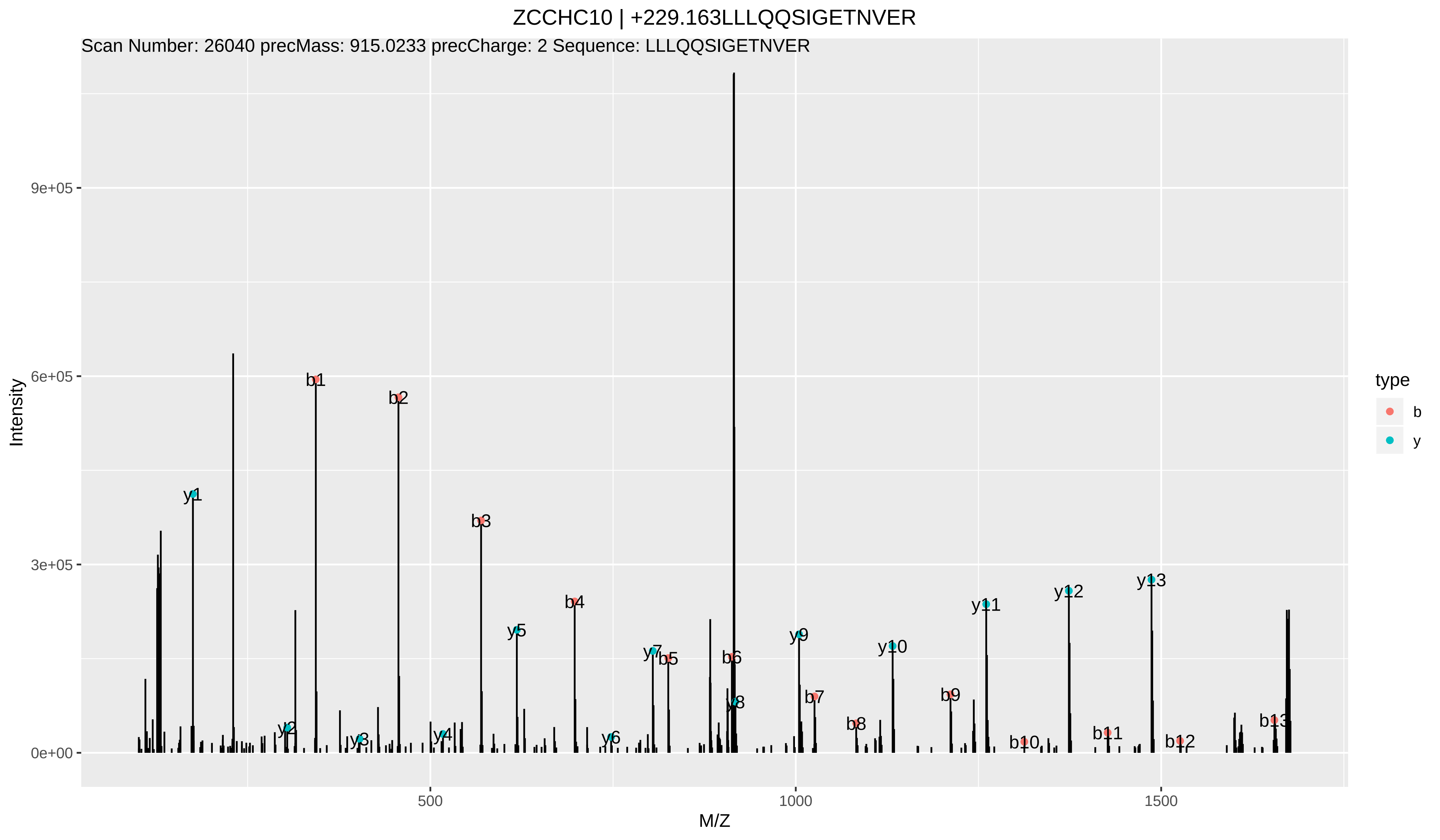
ZCCHC10 | +229.163LLLQQSIGETNVER
Scan Number: 26040 precMass: 915.0233 precCharge: 2 Sequence: LLLQQSIGETNVER
Intensity
9e+05
6e+05
3e+05
0e+00
M/Z
500
1000
1500
type
b
y
y1
y2
y3
y4
y5
y6
y7
y8
y9
y10
y11
y12
y13
b1
b2
b3
b4
b5
b6
b7
b8
b9
b10
b11
b12
b13

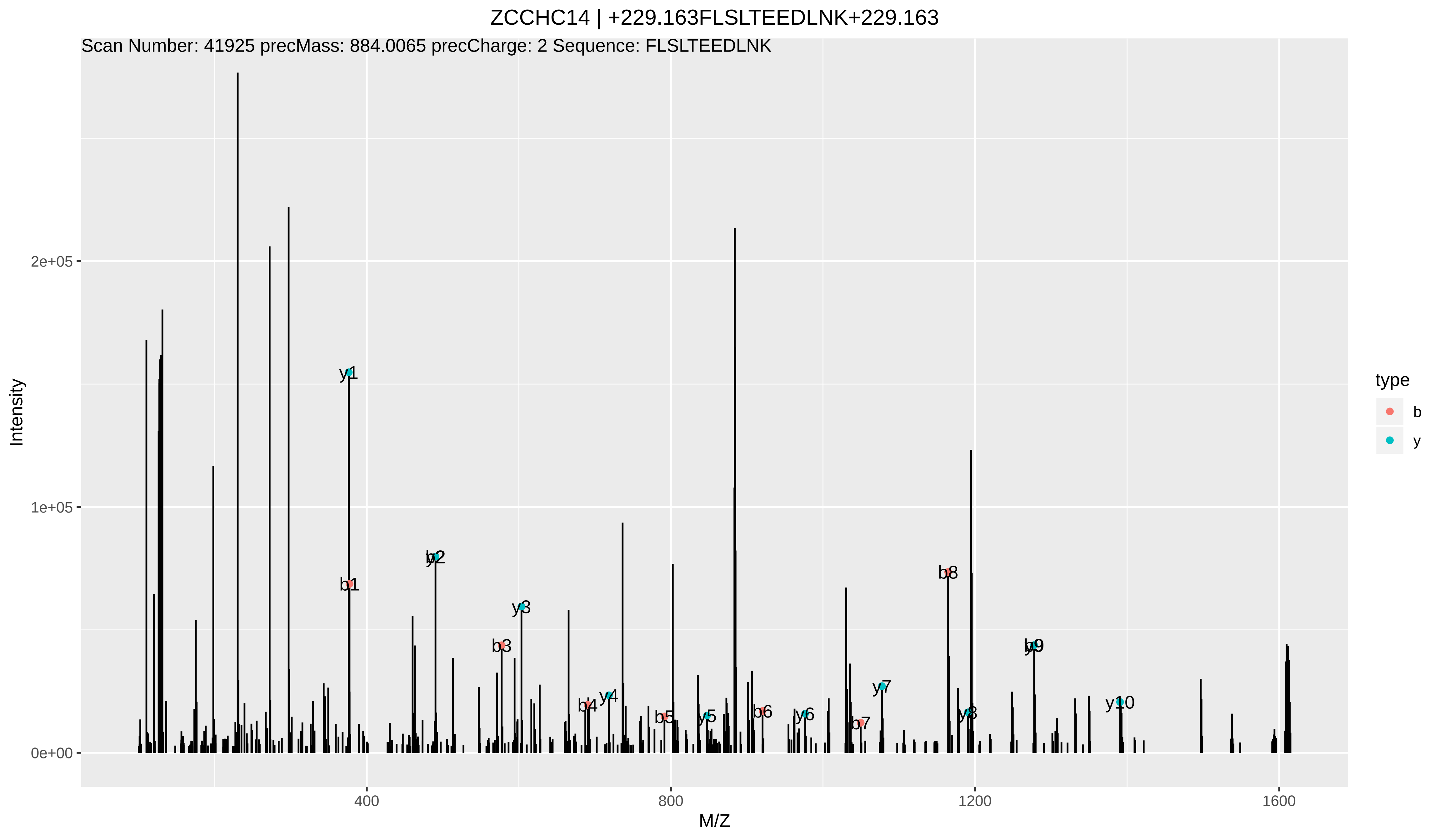

ZCCHC14 | +229.163FLSLTEEDLNK+229.163
Scan Number: 41925 precMass: 884.0065 precCharge: 2 Sequence: FLSLTEEDLNK
Intensity
2e+05
1e+05
0e+00
M/Z
400
800
1200
1600
y1
b1
b2
y2
y3
b3
b4
y4
b5
y5
b6
y6
b7
y7
b8
y8
b9
y9
y10
type
b
y

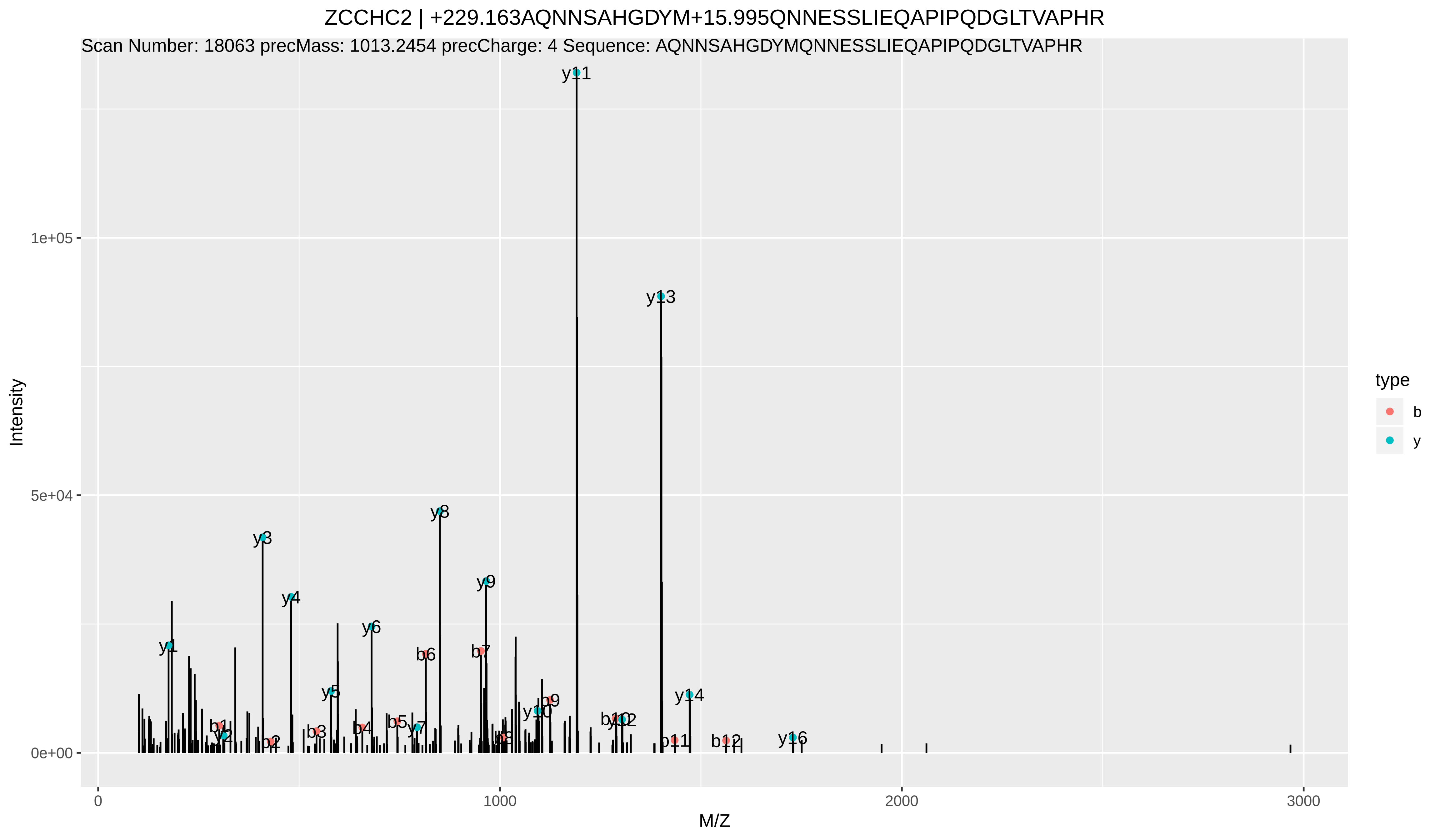

ZCCHC2 | +229.163AQNNSAHGDYM+15.995QNNESSLIEQAPIPQDGLTVAPHR
Scan Number: 18063 precMass: 1013.2454 precCharge: 4 Sequence: AQNNSAHGDYMQNNESSLIEQAPIPQDGLTVAPHR
Intensity
M/Z
0e+00
5e+04
1e+05
0
1000
2000
3000
type
b
y
y11
y13
y8
y3
y9
y4
y6
y1
b6
b7
y5
y14
b9
y10
b1
y2
b2
b3
b4
b5
y7
b10
y12
b11
b12
y16

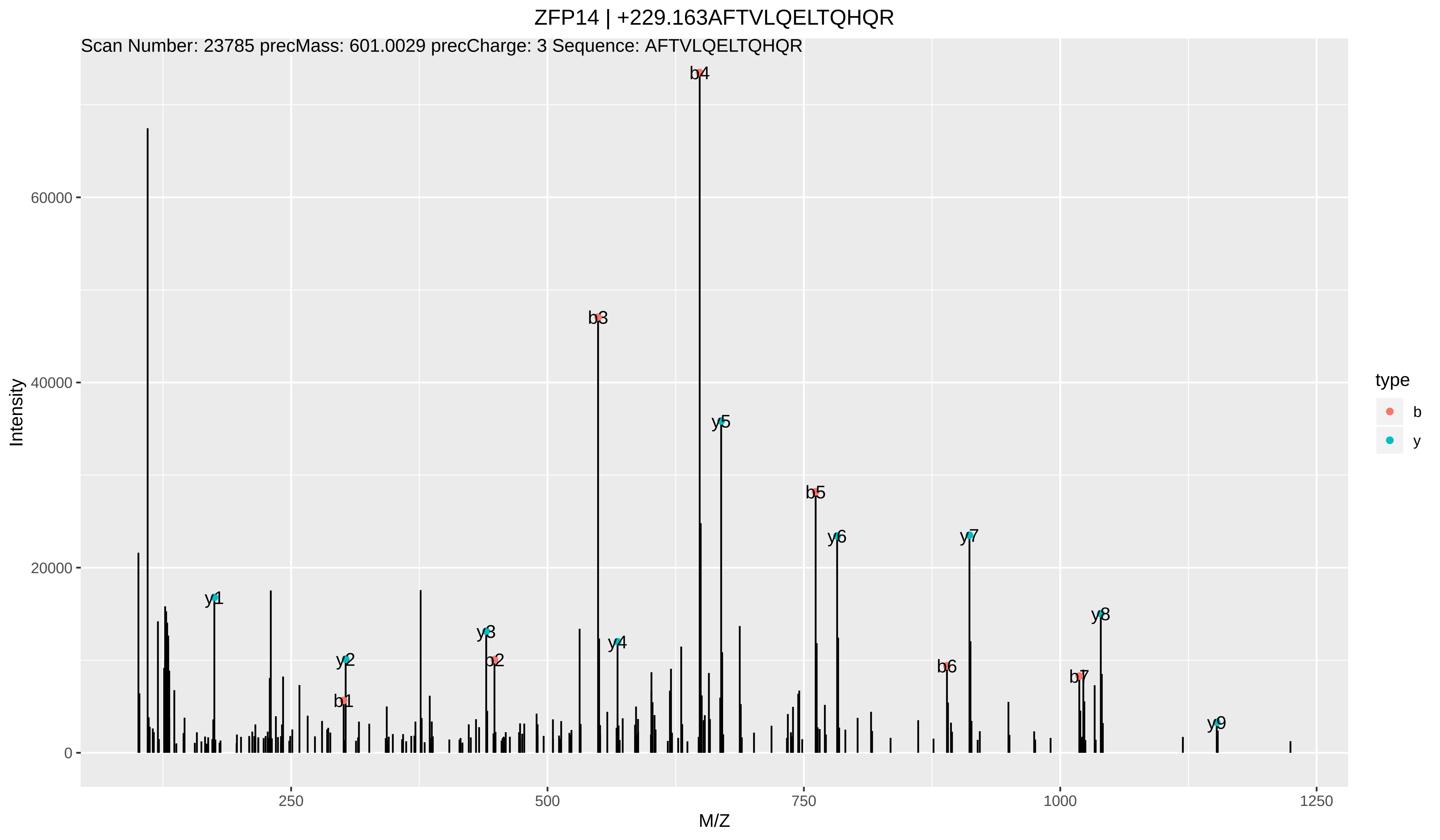

ZFP14 | +229.163AFTVLQELTQHQR
Scan Number: 23785 precMass: 601.0029 precCharge: 3 Sequence: AFTVLQELTQHQR
Intensity
M/Z
0
20000
40000
60000
250
500
750
1000
1250
b4
b3
y5
b5
y6
y7
y1
y8
y3
y4
y2
b2
b6
b7
b1
y9
type
b
y

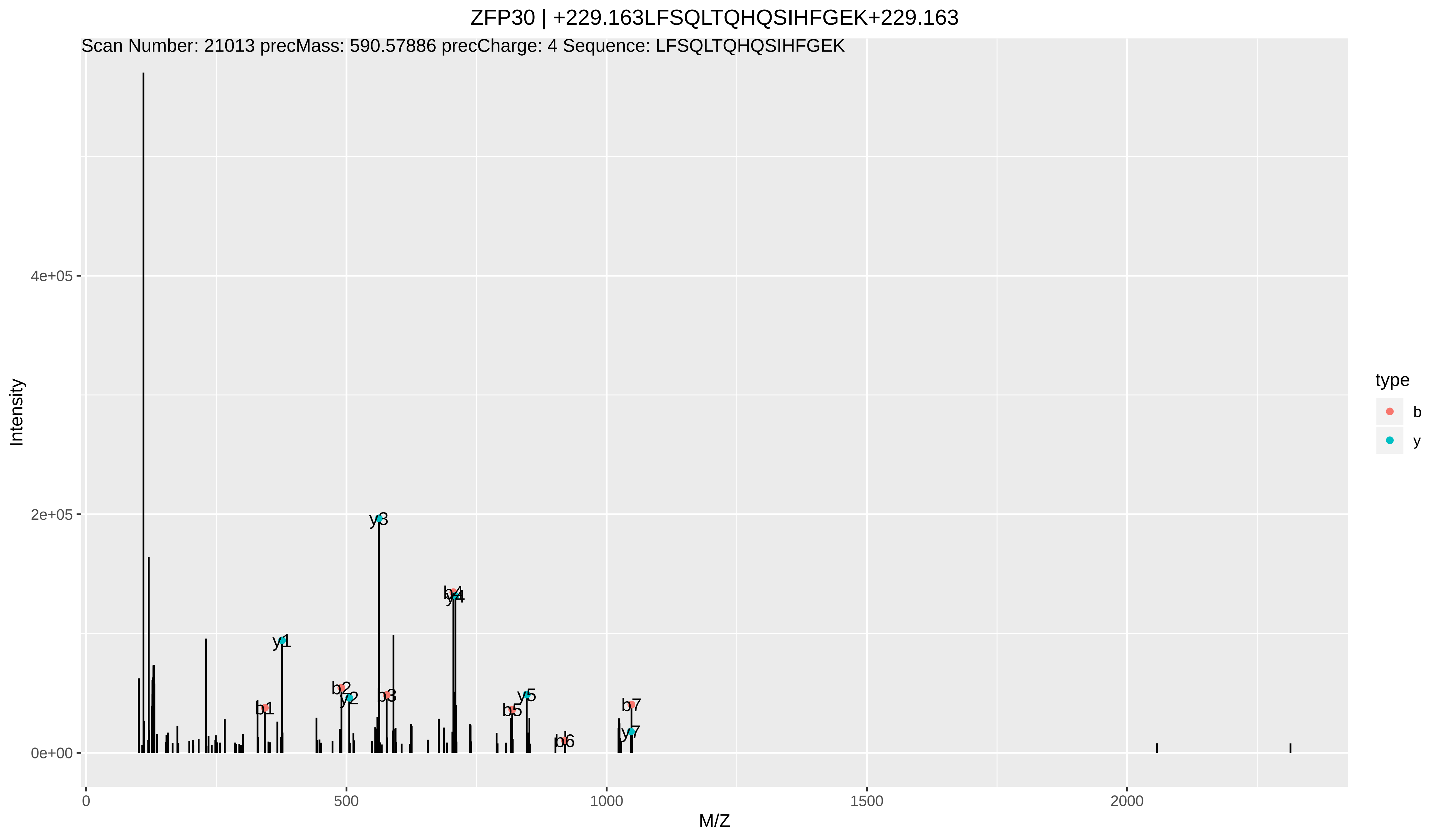

ZFP30 | +229.163LFSQLTQHQSIHFGEK+229.163
Scan Number: 21013 precMass: 590.57886 precCharge: 4 Sequence: LFSQLTQHQSIHFGEK
Intensity
4e+05
2e+05
0e+00
0
500
1000
1500
2000
M/Z
y1
y2
y3
y4
y5
y7
b1
b2
b3
b4
b5
b6
b7
type
b
y

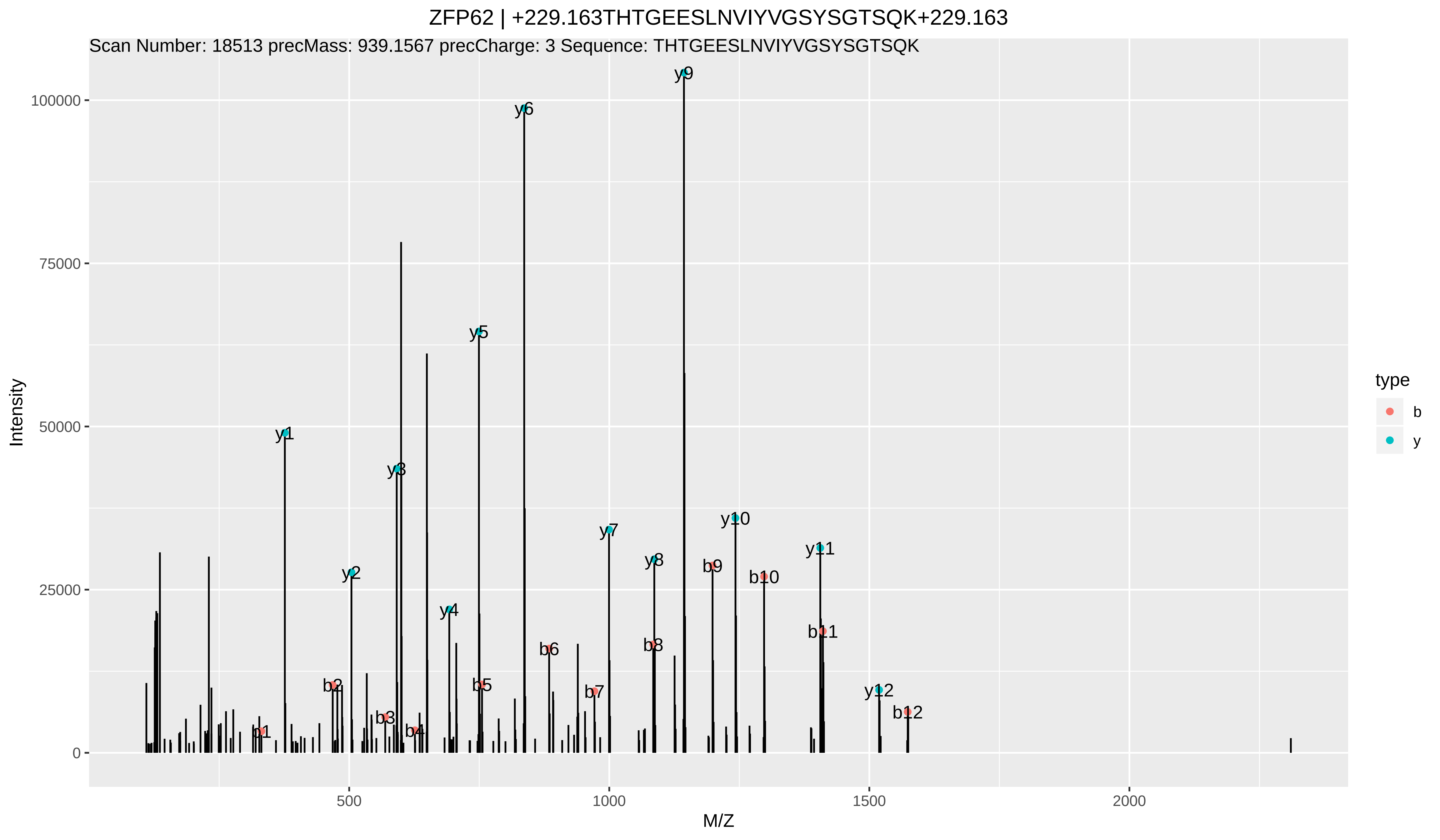
ZFP62 | +229.163THTGEESLNVIYVGSYSGTSQK+229.163
Scan Number: 18513 precMass: 939.1567 precCharge: 3 Sequence: THTGEESLNVIYVGSYSGTSQK
Intensity
M/Z
type
b
y
y1
y2
y3
y4
y5
y6
y7
y8
y9
y10
y11
y12
b1
b2
b3
b4
b5
b6
b7
b8
b9
b10
b11
b12
0
25000
50000
75000
100000
500
1000
1500
2000

ZFP62 | +229.163VIHTGEK+229.163

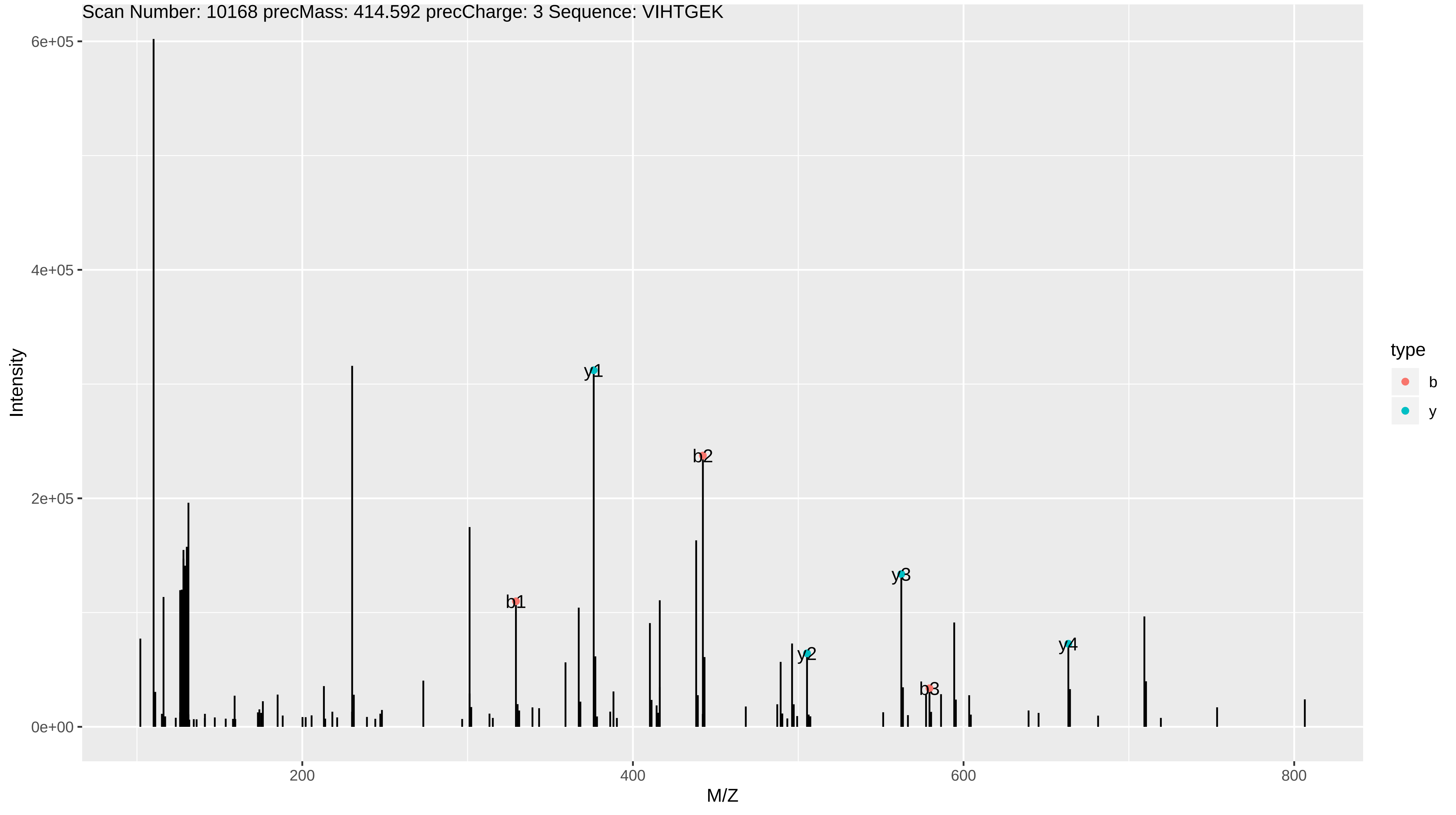

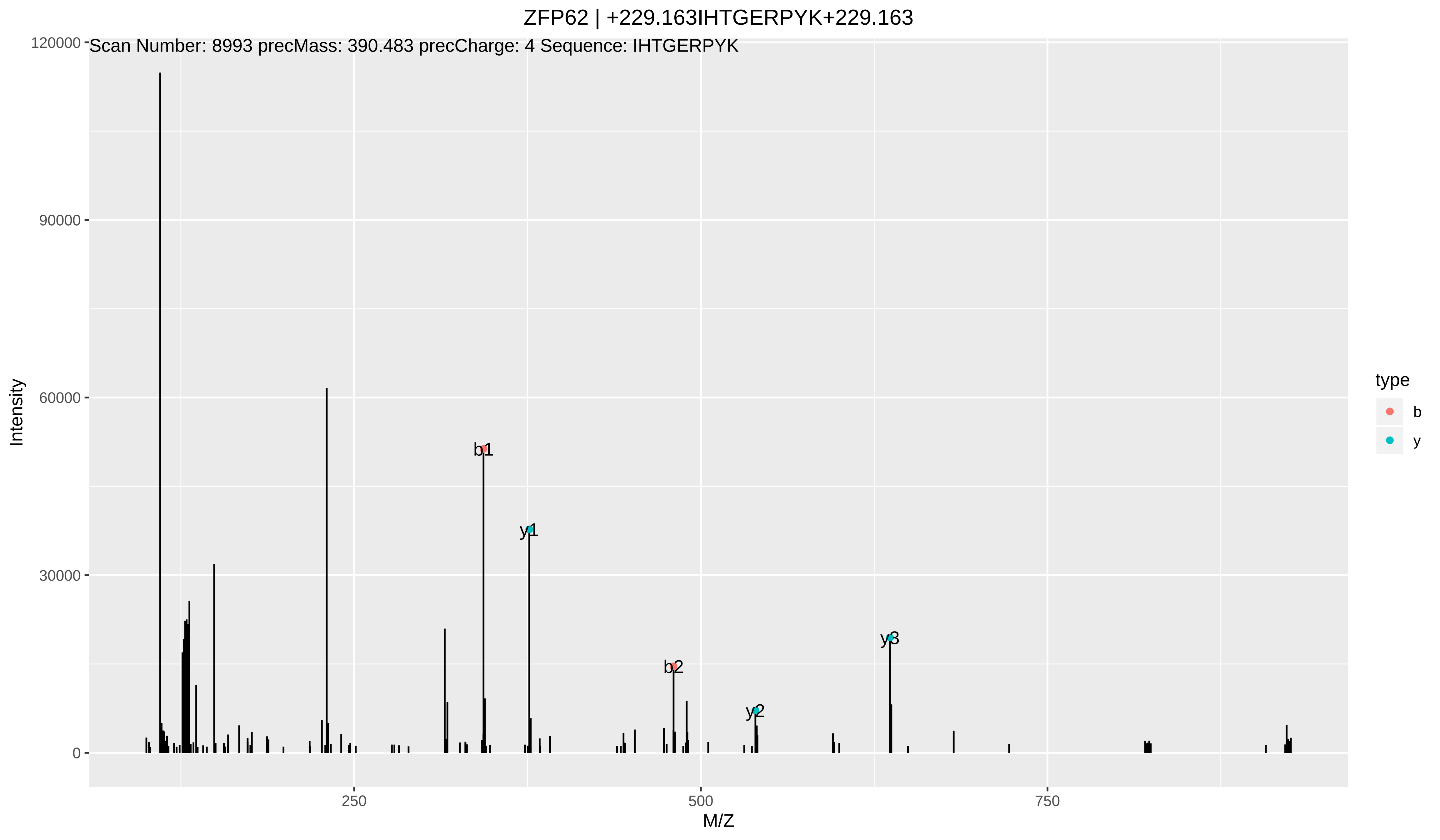

ZFP62 | +229.163IHTGERPYK+229.163
Scan Number: 8993 precMass: 390.483 precCharge: 4 Sequence: IHTGERPYK
120000
90000
60000
30000
0
Intensity
250
500
750
M/Z
b1
y1
b2
y2
y3
type
b
y

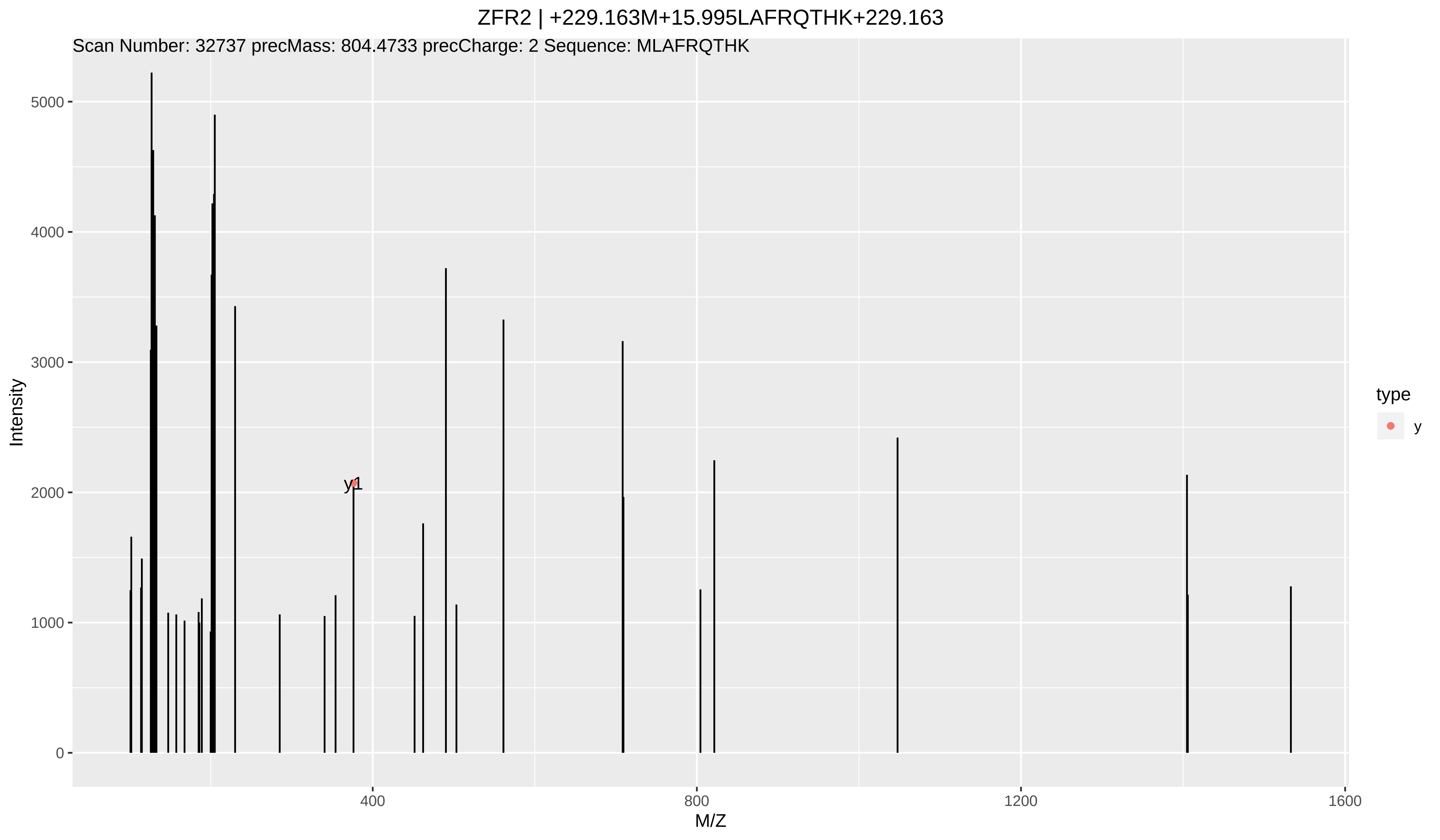

ZFR2 | +229.163M+15.995LAFRQTHK+229.163
Scan Number: 32737 precMass: 804.4733 precCharge: 2 Sequence: MLAFRQTHK
Intensity
5000
4000
3000
2000
1000
0
400
800
1200
1600
M/Z
y1
type
y

ZFX | +229.163EVQQHALIHQESK+229.163

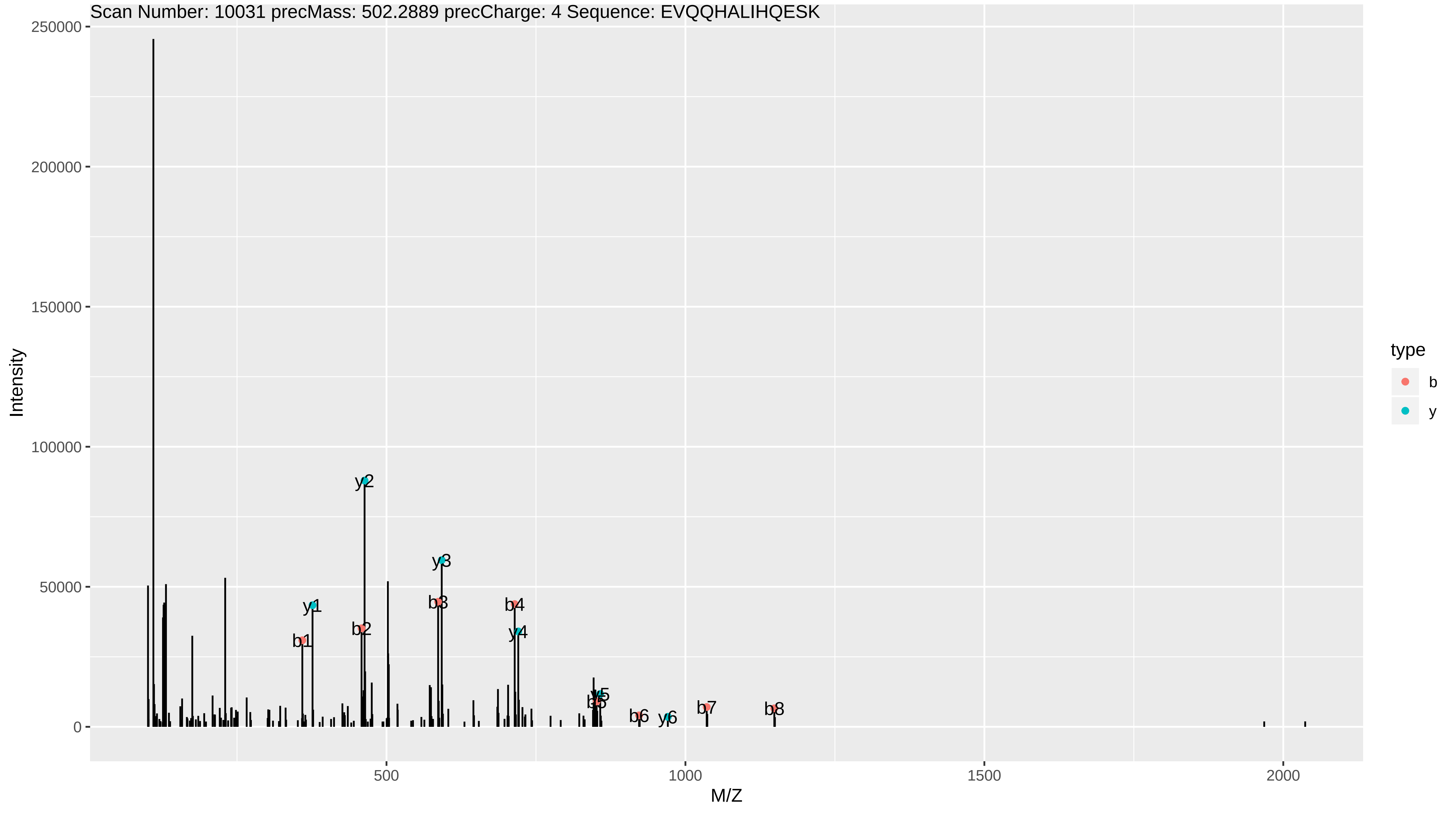

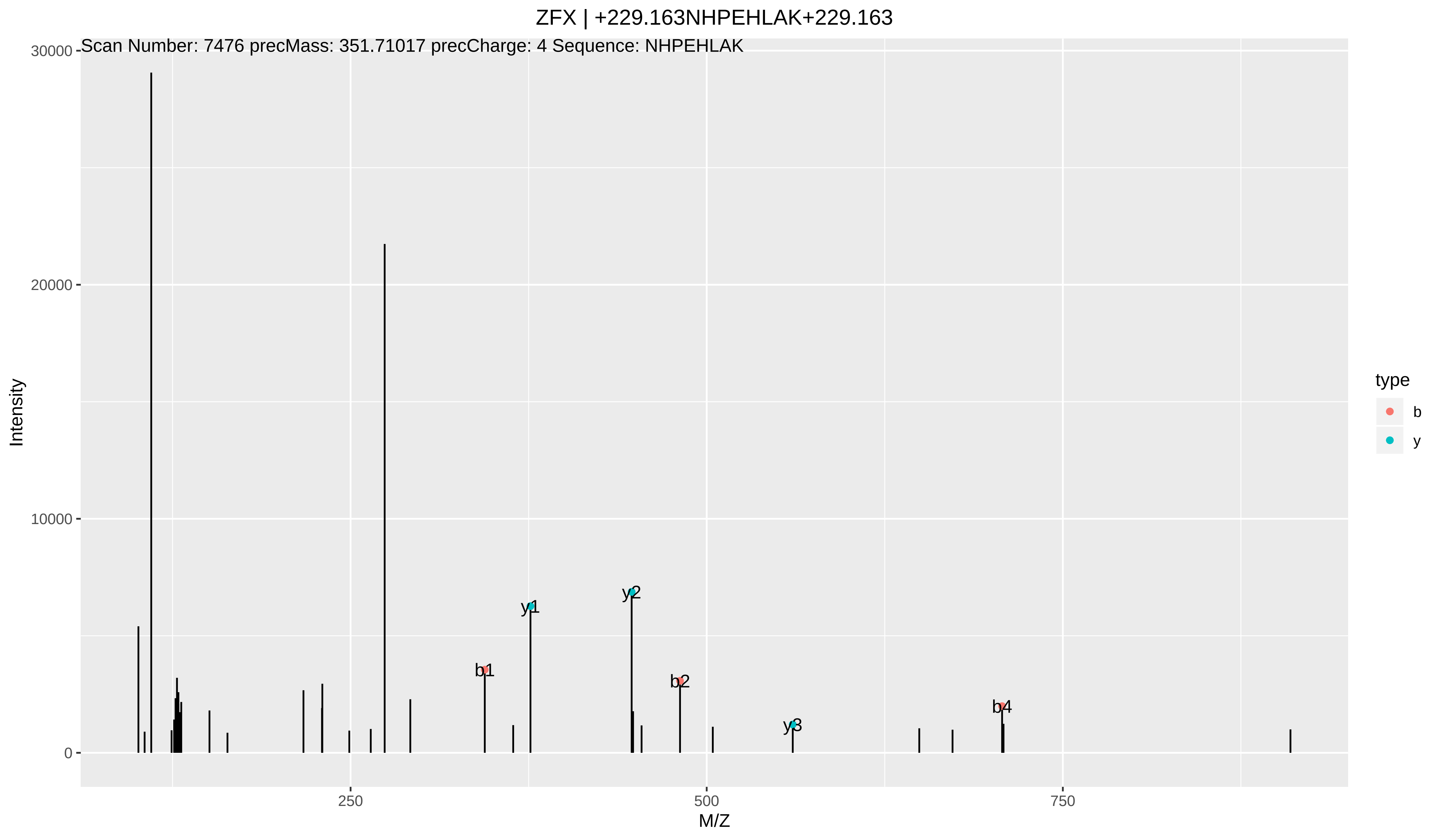
ZFX | +229.163NHPEHLAK+229.163
Scan Number: 7476 precMass: 351.71017 precCharge: 4 Sequence: NHPEHLAK
Intensity
30000
20000
10000
0
250
500
750
M/Z
b1
y1
y2
b2
y3
b4
type
b
y

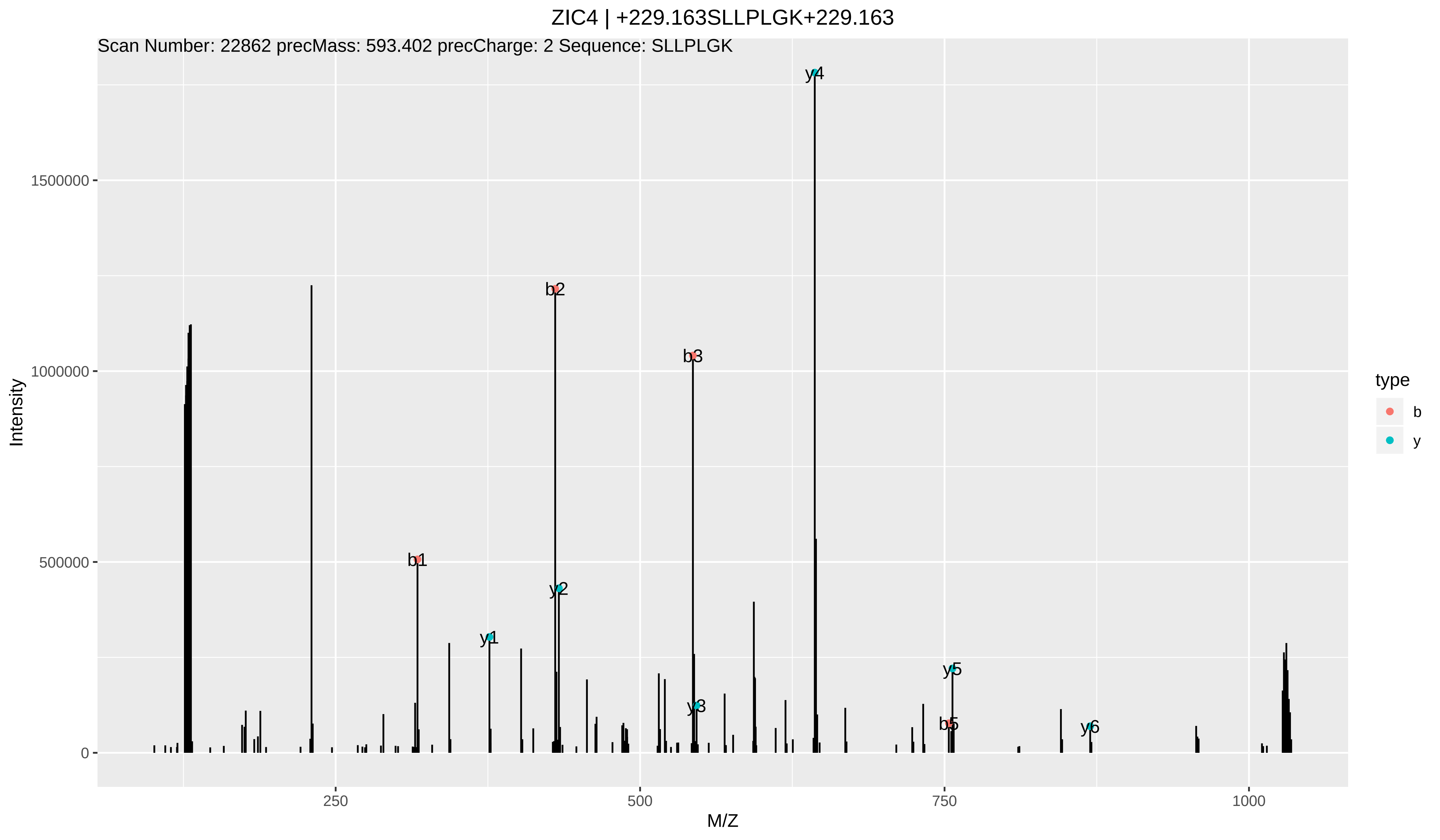

ZIC4 | +229.163SLLPLGK+229.163
Scan Number: 22862 precMass: 593.402 precCharge: 2 Sequence: SLLPLGK
y4
b2
b3
b1
y2
y1
y5
y3
b5
y6
1500000
1000000
500000
0
Intensity
250
500
750
1000
M/Z
type
b
y

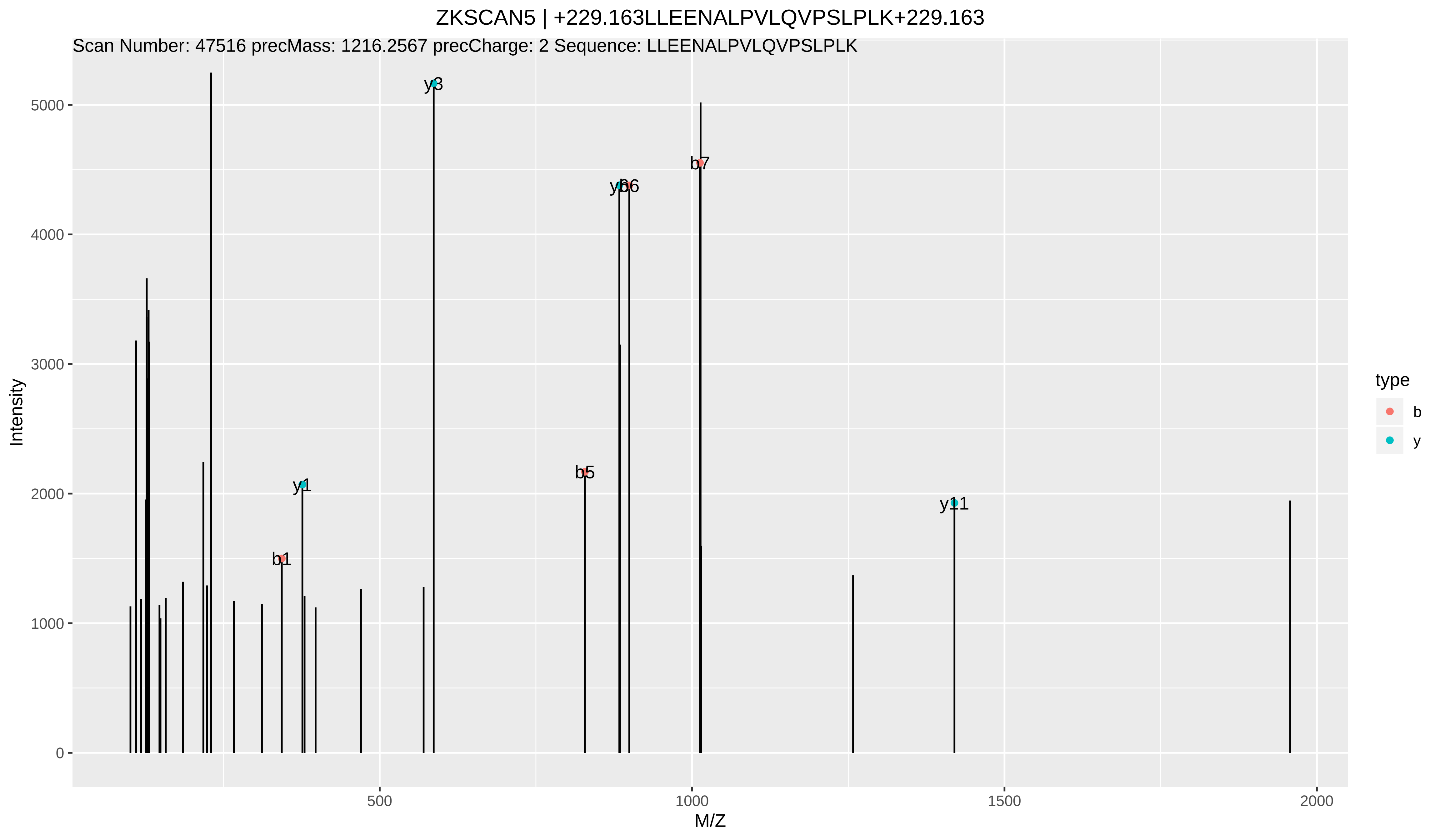

ZKSCAN5 | +229.163LLEENALPVLQVPSLPLK+229.163
Scan Number: 47516 precMass: 1216.2567 precCharge: 2 Sequence: LLEENALPVLQVPSLPLK
Intensity
M/Z
0
1000
2000
3000
4000
5000
500
1000
1500
2000
y3
b7
y6
b6
b5
y1
b1
y11
type
b
y

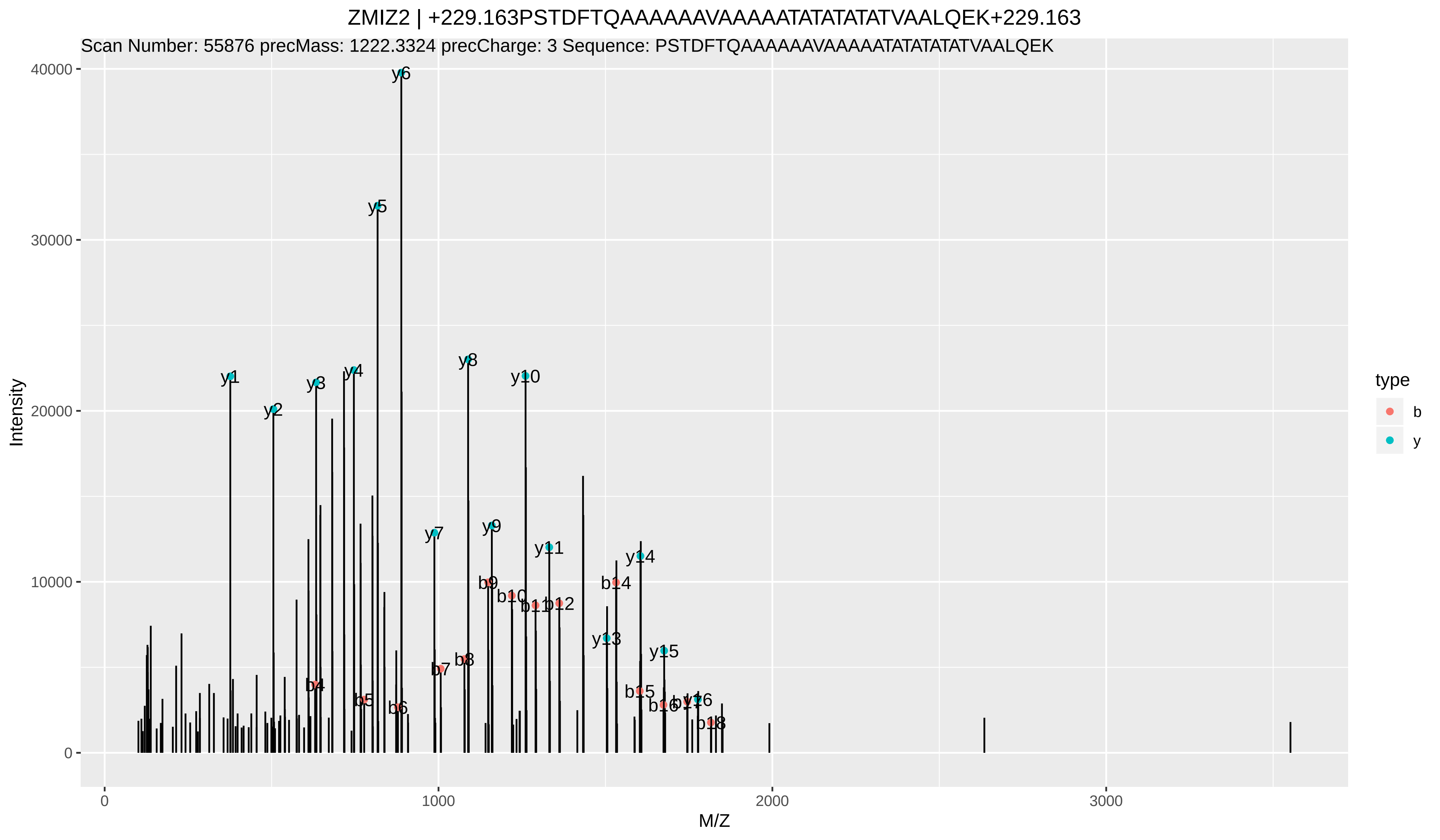
ZMIZ2 | +229.163PSTDFTQAAAAAAVAAAAATATATATATVAALQEK+229.163
Scan Number: 55876 precMass: 1222.3324 precCharge: 3 Sequence: PSTDFTQAAAAAAVAAAAATATATATATVAALQEK
Intensity
M/Z
type
b
y
y1
y2
y3
y4
y5
y6
y7
y8
y9
y10
y11
y13
y14
y15
y16
b4
b5
b6
b7
b8
b9
b10
b11
b12
b14
b15
b16
b17
b18
0
10000
20000
30000
40000
1000
2000
3000

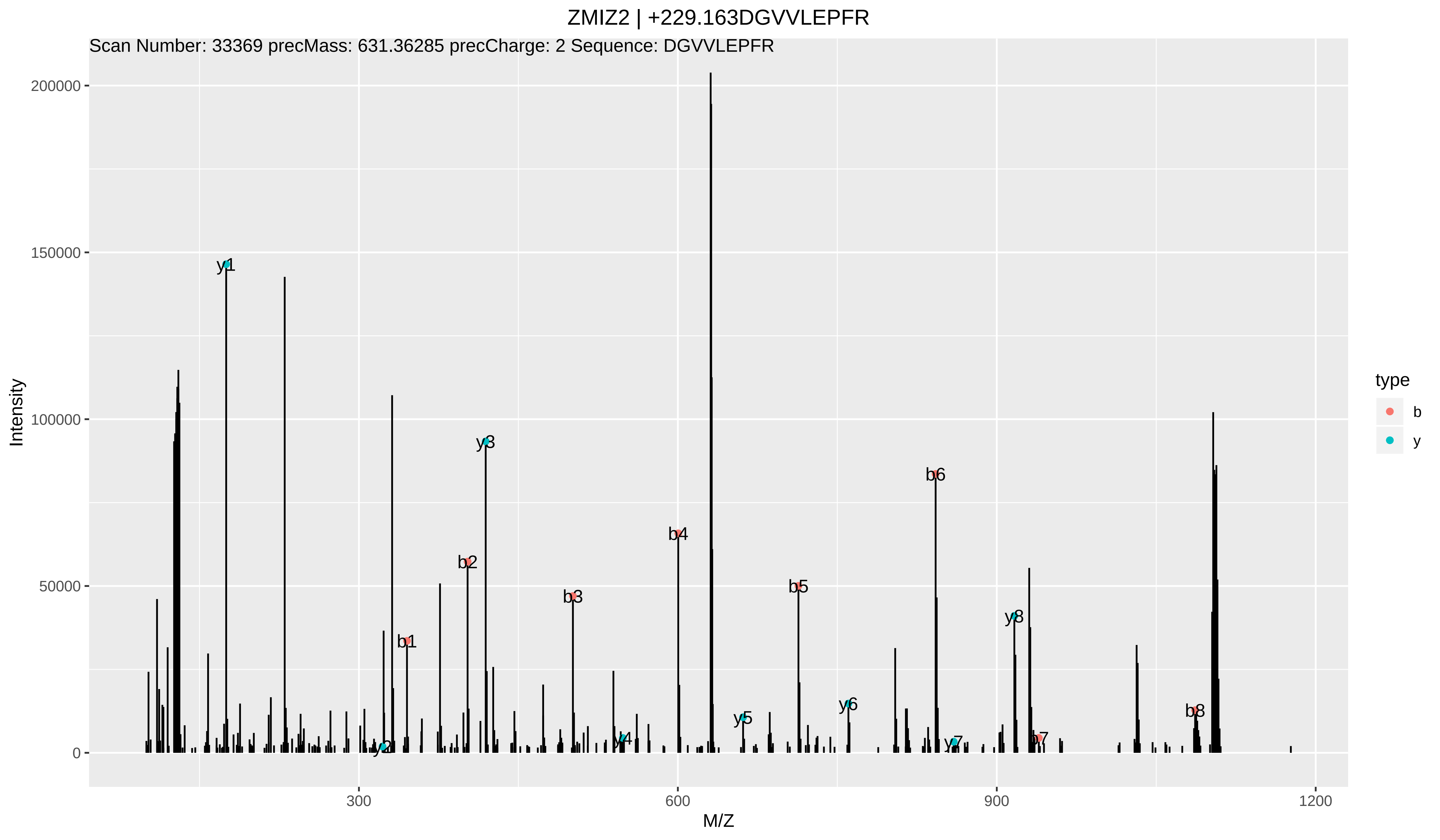
ZMIZ2 | +229.163DGVVLEPFR
Scan Number: 33369 precMass: 631.36285 precCharge: 2 Sequence: DGVVLEPFR
Intensity
M/Z
type
b
y
0
50000
100000
150000
200000
300
600
900
1200
y1
y2
y3
y4
y5
y6
y7
y8
b1
b2
b3
b4
b5
b6
b7
b8

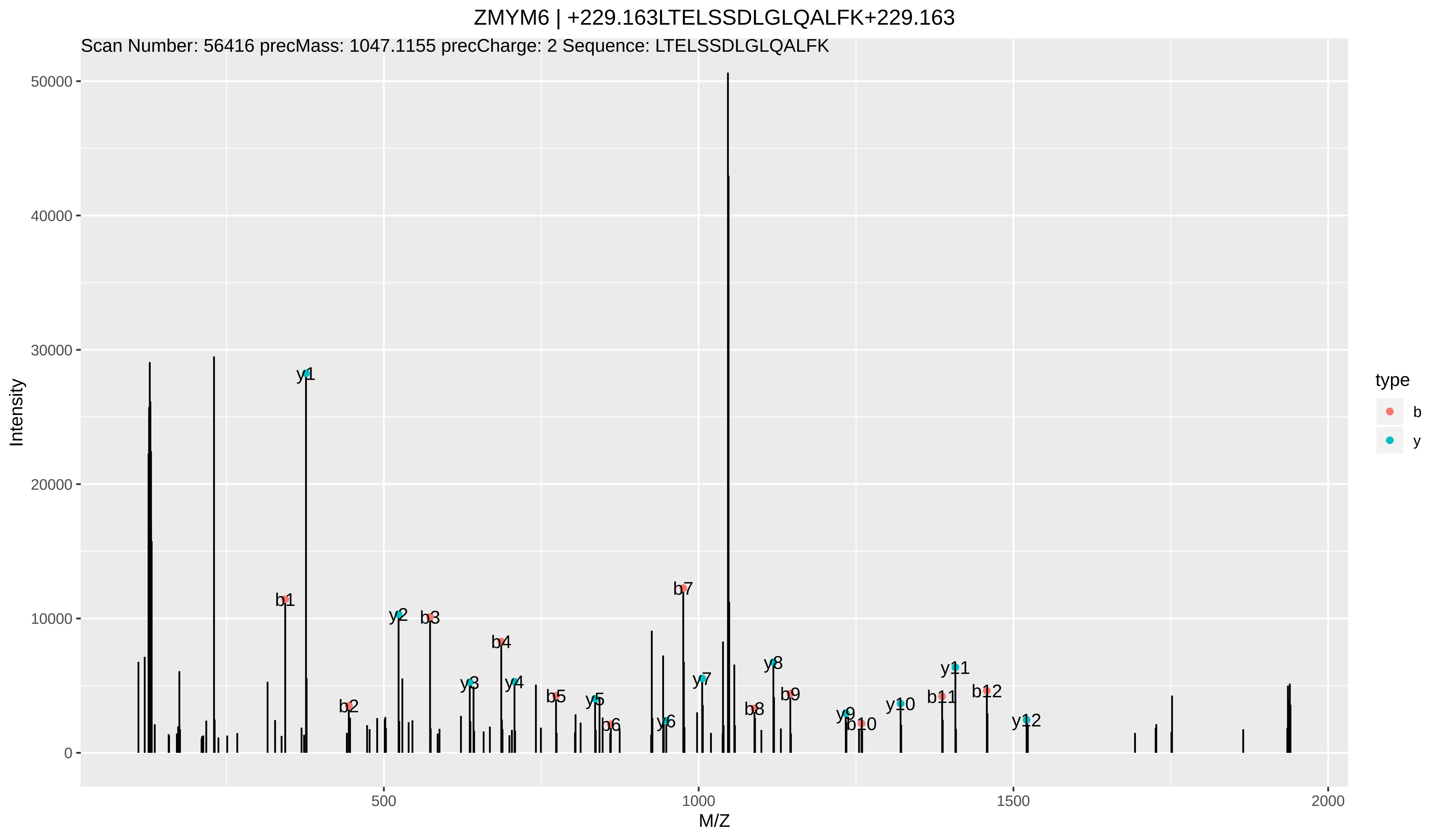

ZMYM6 | +229.163LTELSSDLGLQALFK+229.163
Scan Number: 56416 precMass: 1047.1155 precCharge: 2 Sequence: LTELSSDLGLQALFK
Intensity
0
10000
20000
30000
40000
50000
M/Z
500
1000
1500
2000
y1
b1
y2
b3
b2
y3
b4
y4
b5
y5
b6
b7
y6
y7
b8
y8
b9
y9
b10
y10
b11
y11
b12
y12
type
b
y

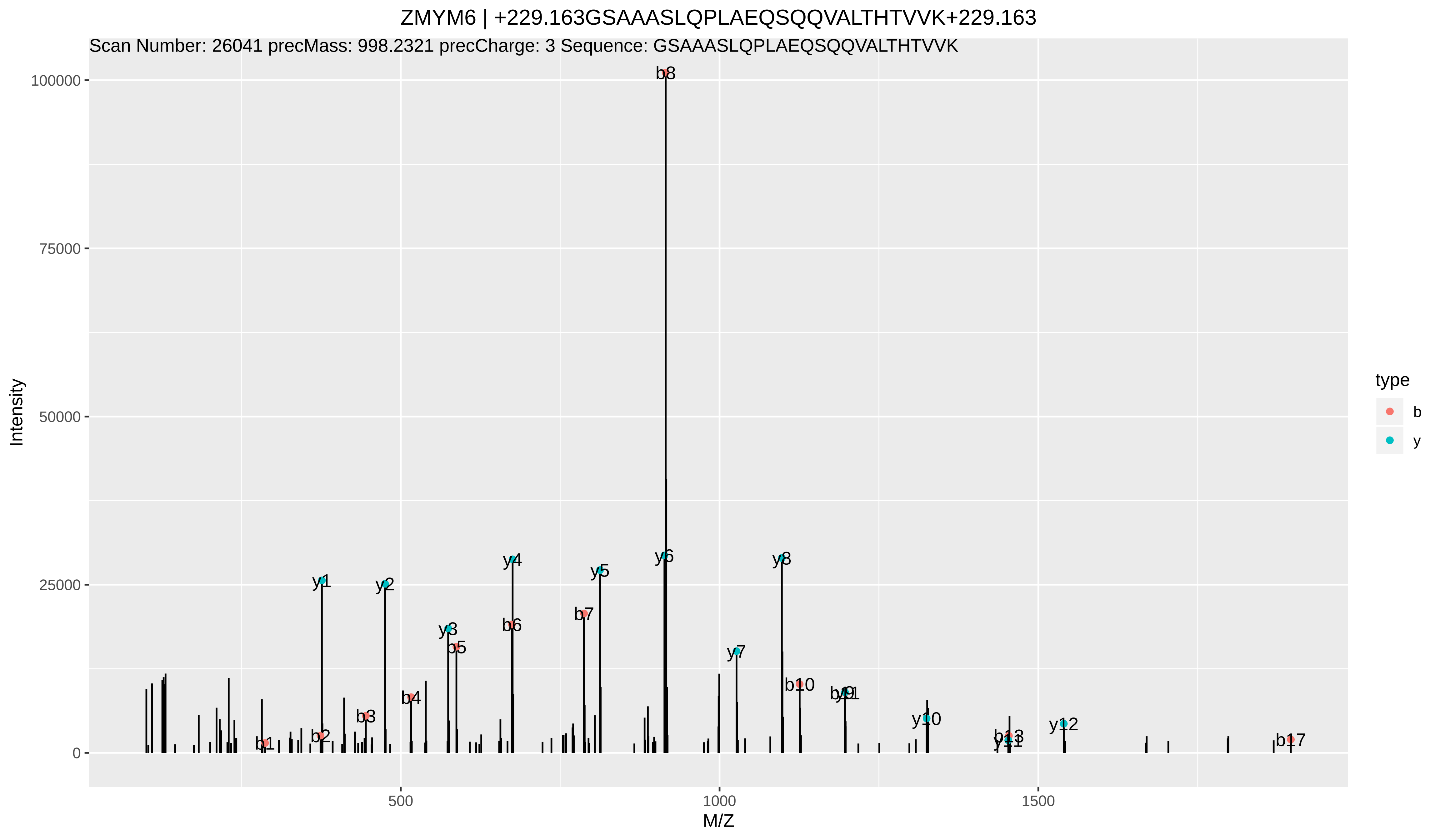

ZMYM6 | +229.163GSAAASLQPLAEQSQQVALTHTVVK+229.163
Scan Number: 26041 precMass: 998.2321 precCharge: 3 Sequence: GSAAASLQPLAEQSQQVALTHTVVK
Intensity
M/Z
type
b
y
b8
y1
y2
y3
y4
y5
y6
y7
y8
y10
y12
b1
b2
b3
b4
b5
b6
b7
b10
b17
0
25000
50000
75000
100000
500
1000
1500

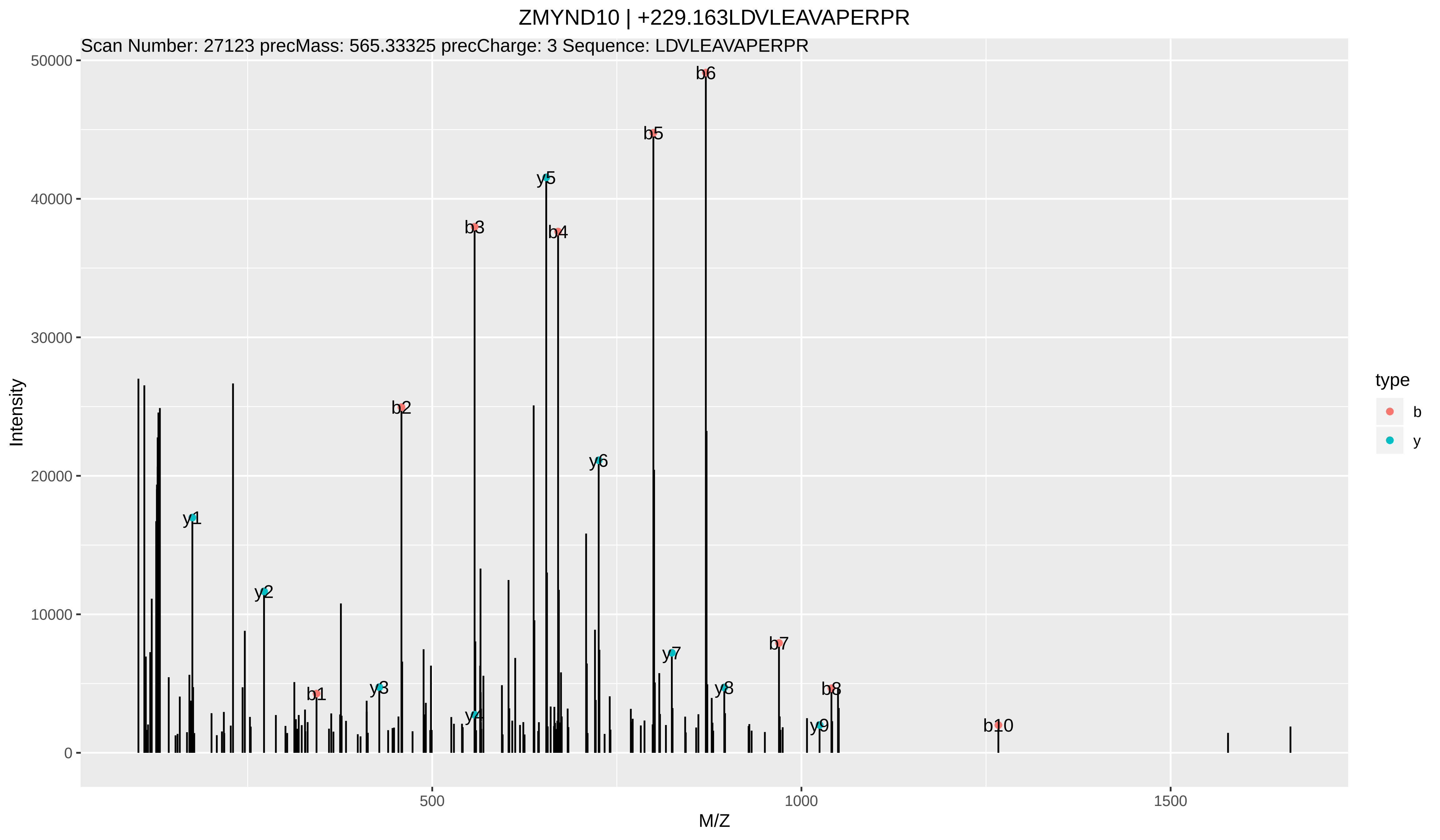
ZMYND10 | +229.163LDVLEAVAPERPR
Scan Number: 27123 precMass: 565.33325 precCharge: 3 Sequence: LDVLEAVAPERPR
Intensity
M/Z
0
10000
20000
30000
40000
50000
500
1000
1500
b6
b5
y5
b3
b4
b2
y6
y1
y2
b1
y3
y4
y7
y8
b7
b8
y9
b10
type
b
y

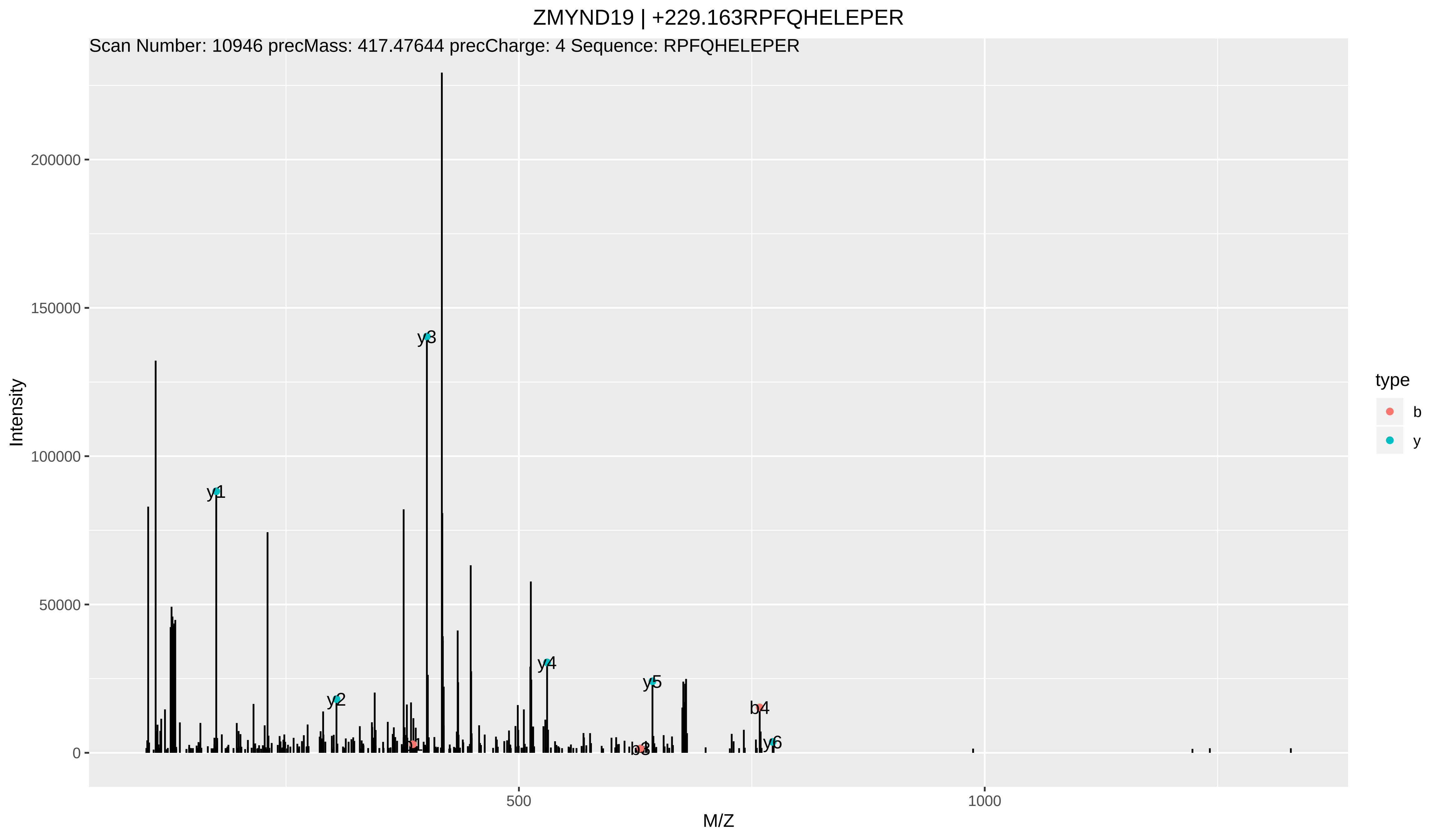
ZMYND19 | +229.163RPFQHELEPER
Scan Number: 10946 precMass: 417.47644 precCharge: 4 Sequence: RPFQHELEPER
Intensity
M/Z
0
50000
100000
150000
200000
500
1000
y1
y2
y3
y4
y5
y6
b1
b3
b4
type
b
y

ZNF107 | +229.163ILHTGENLYK+229.163

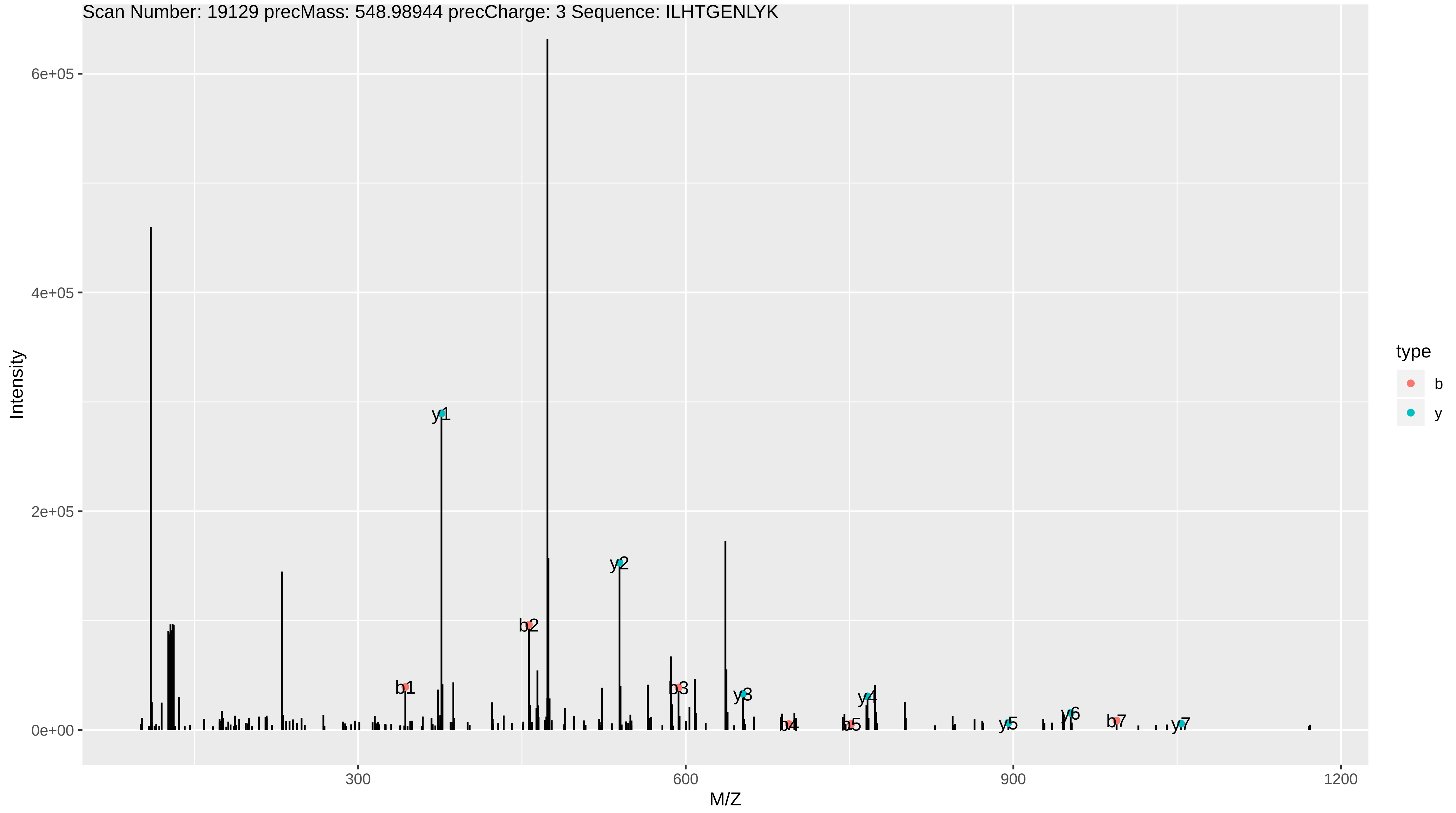

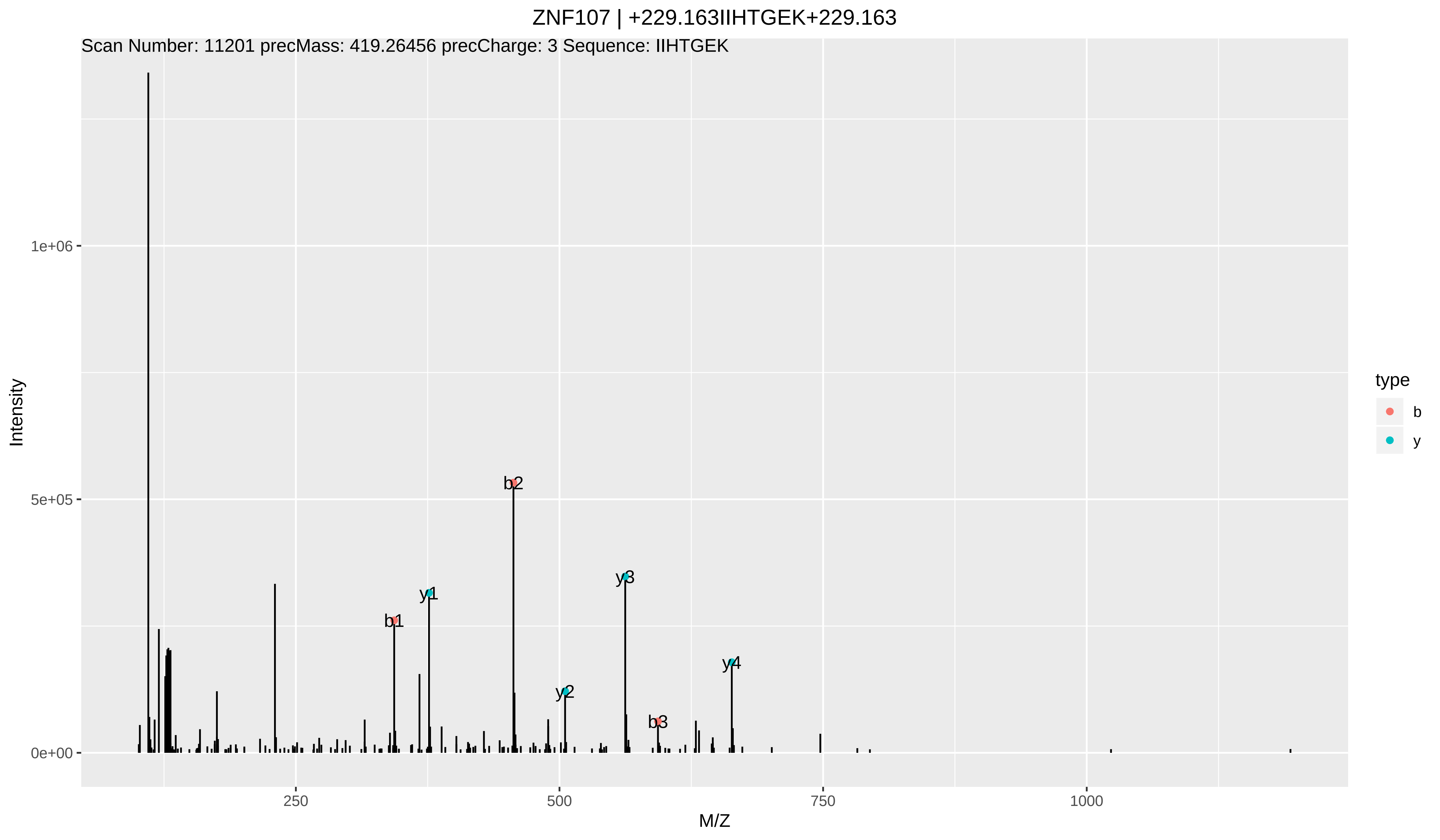
ZNF107 | +229.163IIHTGEK+229.163
Scan Number: 11201 precMass: 419.26456 precCharge: 3 Sequence: IIHTGEK
Intensity
1e+06
5e+05
0e+00
M/Z
250
500
750
1000
b1
y1
b2
y2
y3
b3
y4
type
b
y

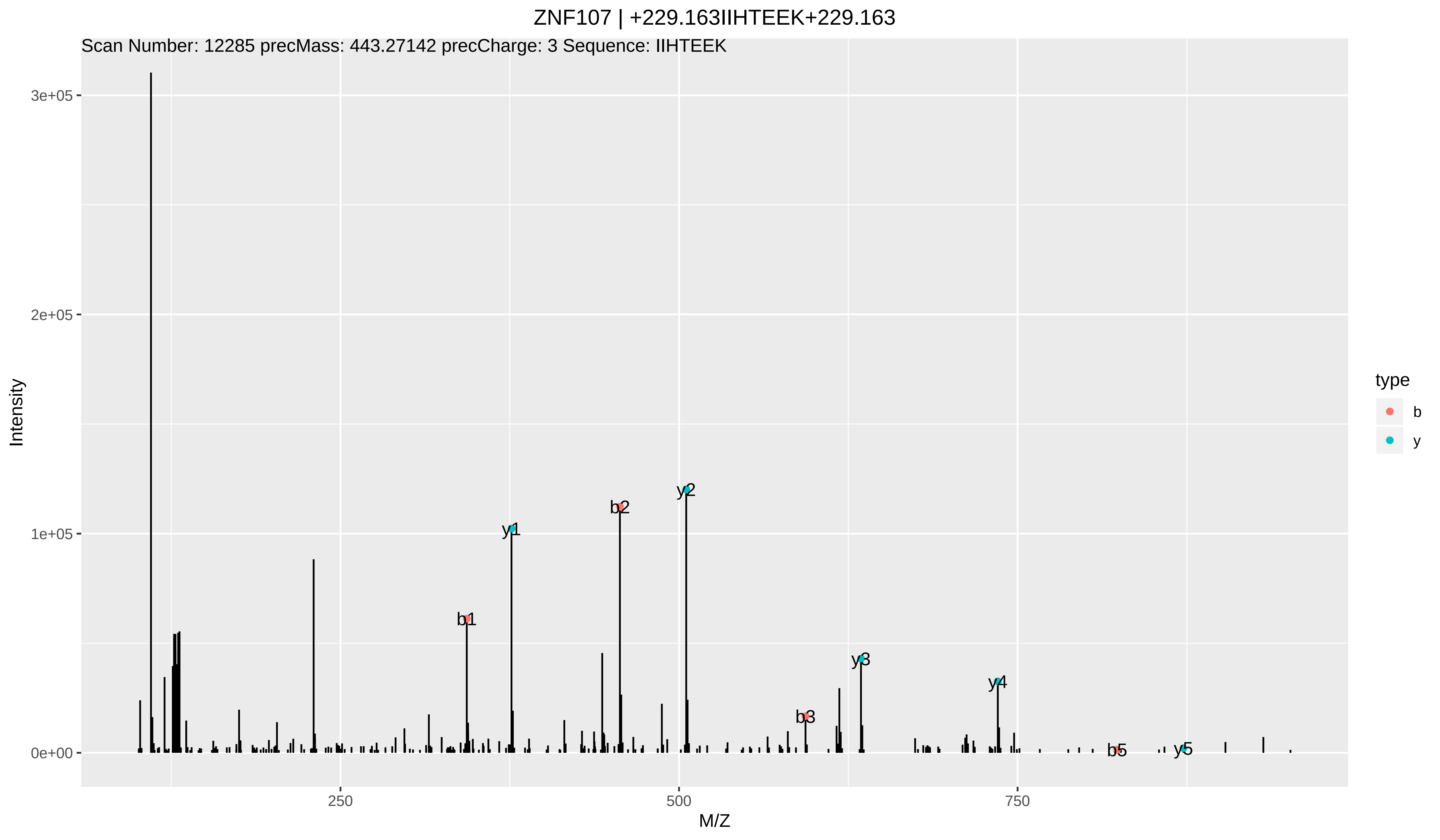
ZNF107 | +229.163IIHTEEK+229.163
Scan Number: 12285 precMass: 443.27142 precCharge: 3 Sequence: IIHTEEK
Intensity
M/Z
3e+05
2e+05
1e+05
0e+00
250
500
750
b1
y1
b2
y2
b3
y3
y4
b5
y5
type
b
y

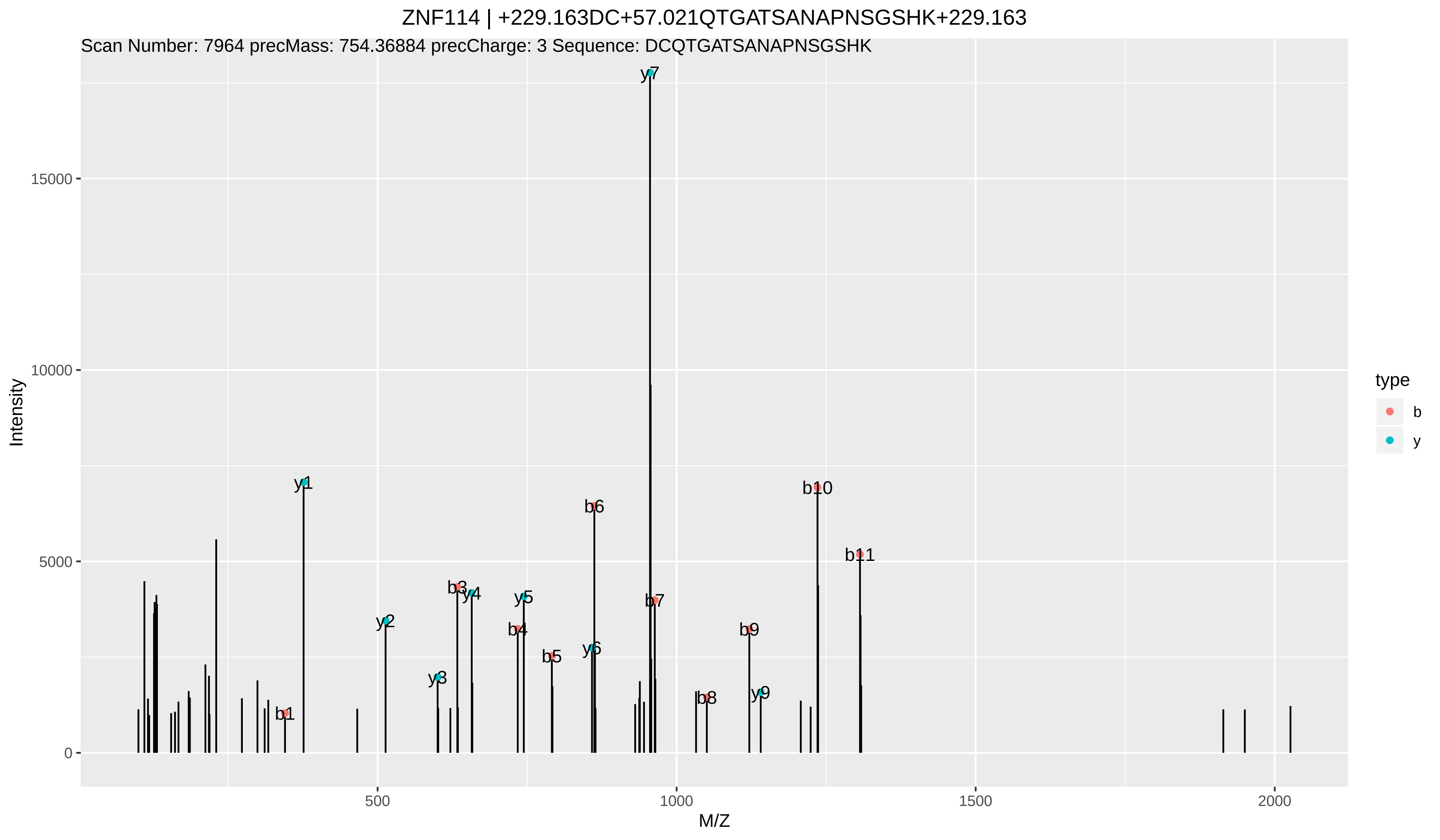

ZNF114 | +229.163DC+57.021QTGATSANAPNSGSHK+229.163
Scan Number: 7964 precMass: 754.36884 precCharge: 3 Sequence: DCQTGATSANAPNSGSHK
Intensity
M/Z
0
5000
10000
15000
500
1000
1500
2000
type
b
y
y7
y1
b10
b6
b11
b3
y4
y5
b7
y2
b4
b9
y6
b5
y3
y9
b8
b1

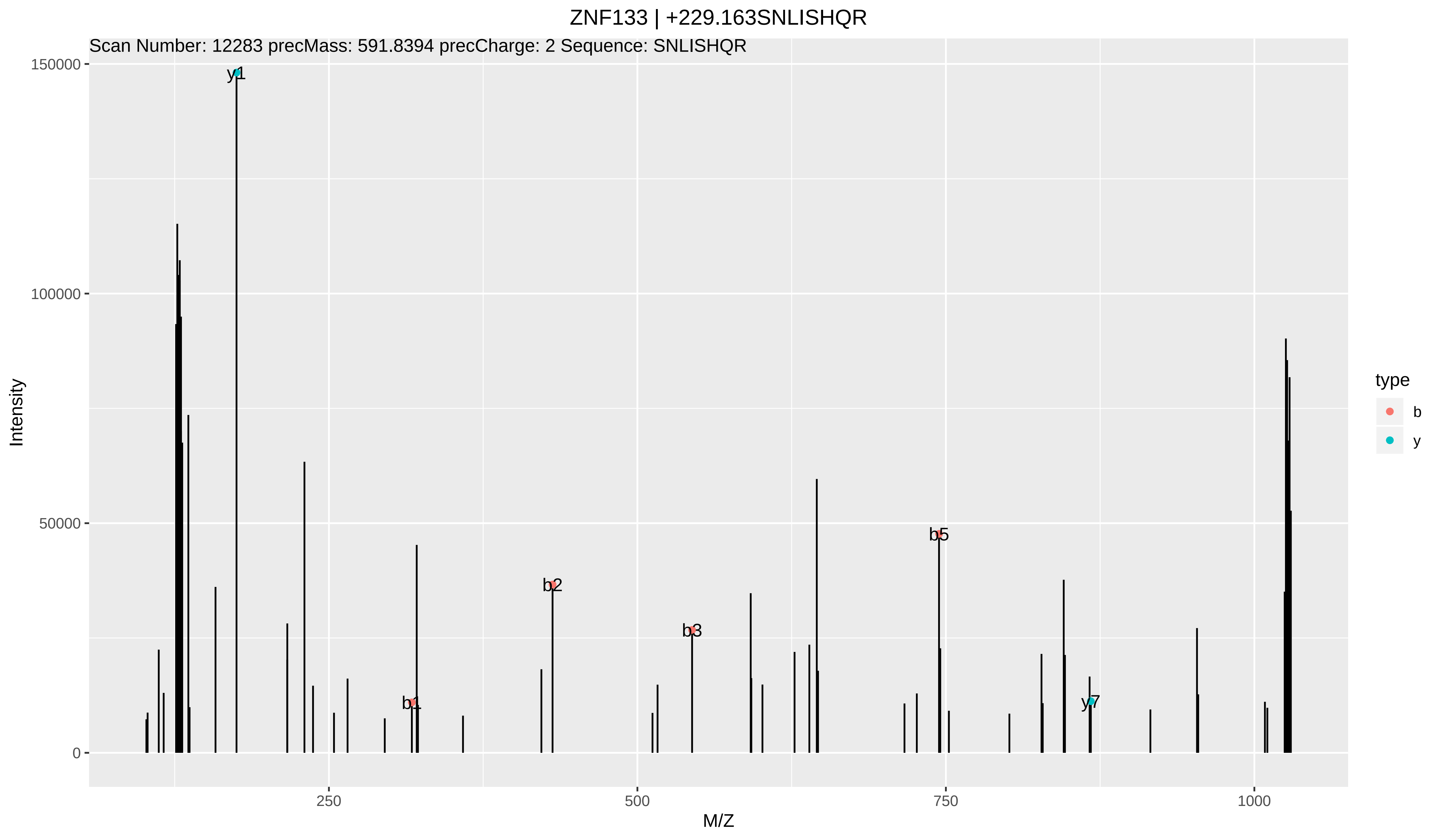
ZNF133 | +229.163SNLISHQR
Scan Number: 12283 precMass: 591.8394 precCharge: 2 Sequence: SNLISHQR
Intensity
M/Z
150000
100000
50000
0
250
500
750
1000
y1
b1
b2
b3
b5
y7
type
b
y

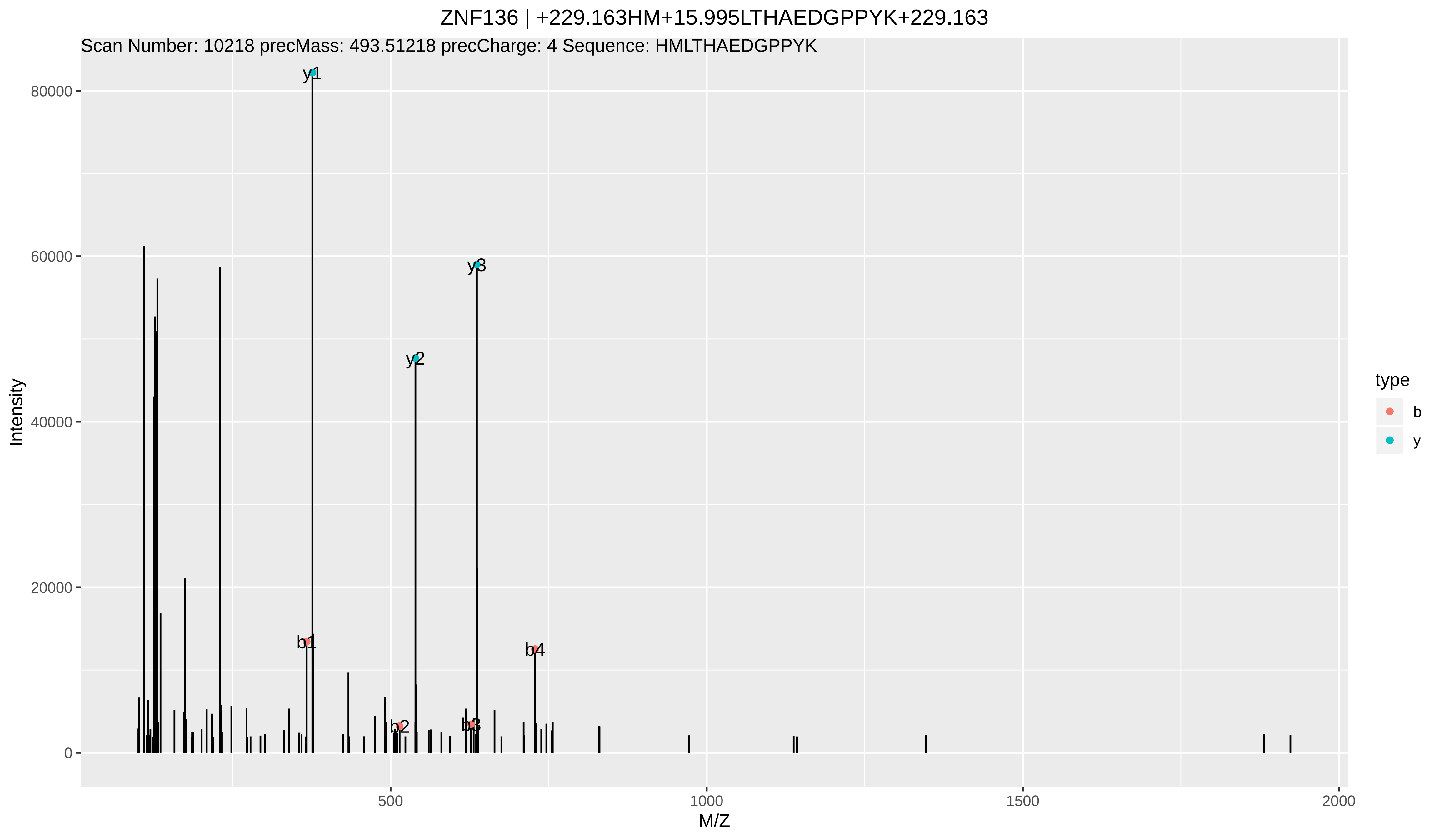
ZNF136 | +229.163HM+15.995LTHAEDGPPYK+229.163
Scan Number: 10218 precMass: 493.51218 precCharge: 4 Sequence: HMLTHAEDGPPYK
y1
y2
y3
b1
b2
b3
b4
Intensity
M/Z
type
b
y
0
20000
40000
60000
80000
500
1000
1500
2000

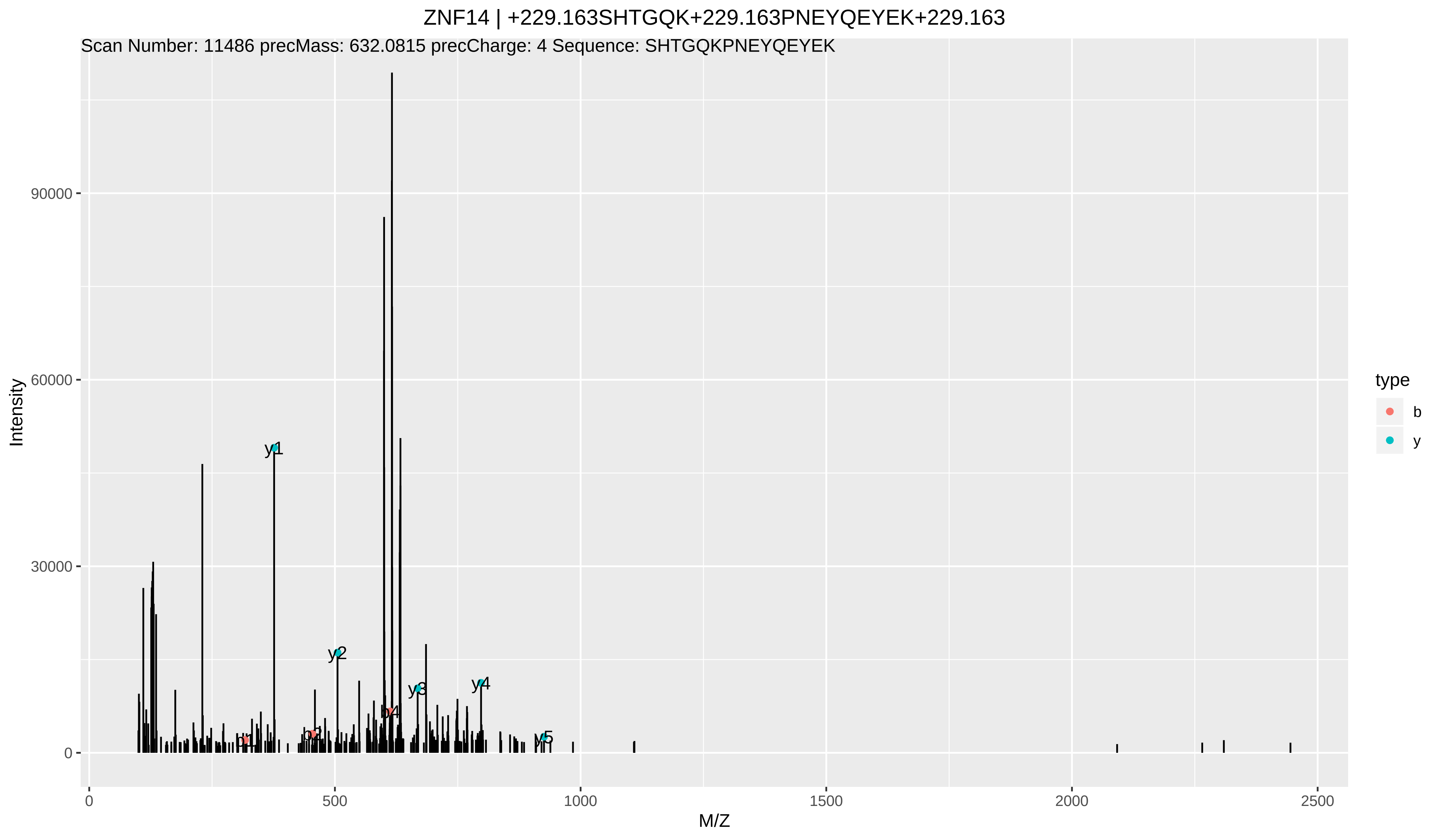

ZNF14 | +229.163SHTGQK+229.163PNEYQEYEK+229.163
Scan Number: 11486 precMass: 632.0815 precCharge: 4 Sequence: SHTGQKPNEYQEYEK
Intensity
M/Z
type
b
y
y1
y2
y3
y4
y5
b1
b2
b4
90000
60000
30000
0
0
500
1000
1500
2000
2500

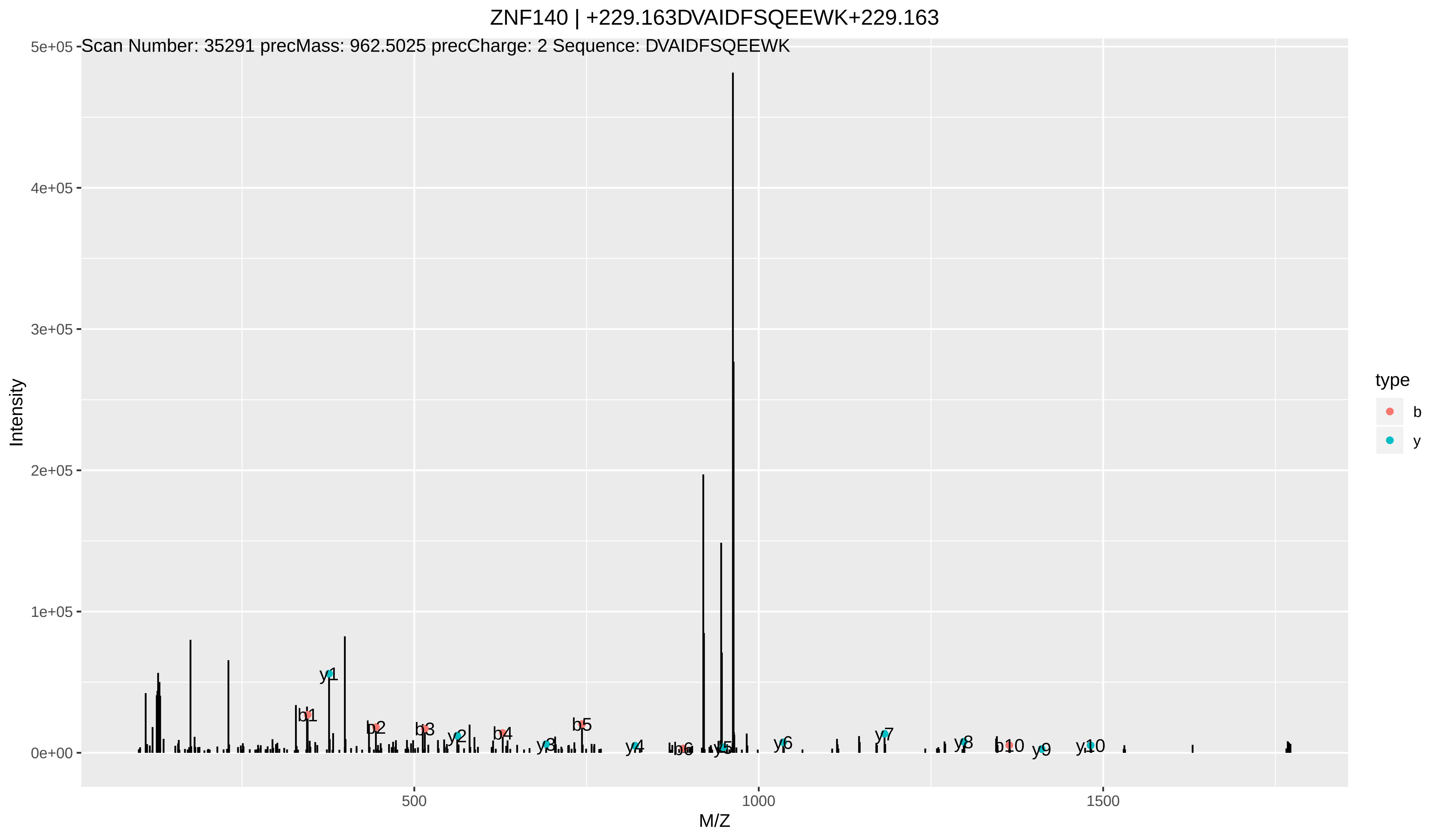

ZNF140 | +229.163DVAIDFSQEEWK+229.163
Scan Number: 35291 precMass: 962.5025 precCharge: 2 Sequence: DVAIDFSQEEWK
Intensity
M/Z
type
b
y
b1
b2
b3
b4
b5
b6
b10
y1
y2
y3
y4
y5
y6
y7
y8
y9
y10

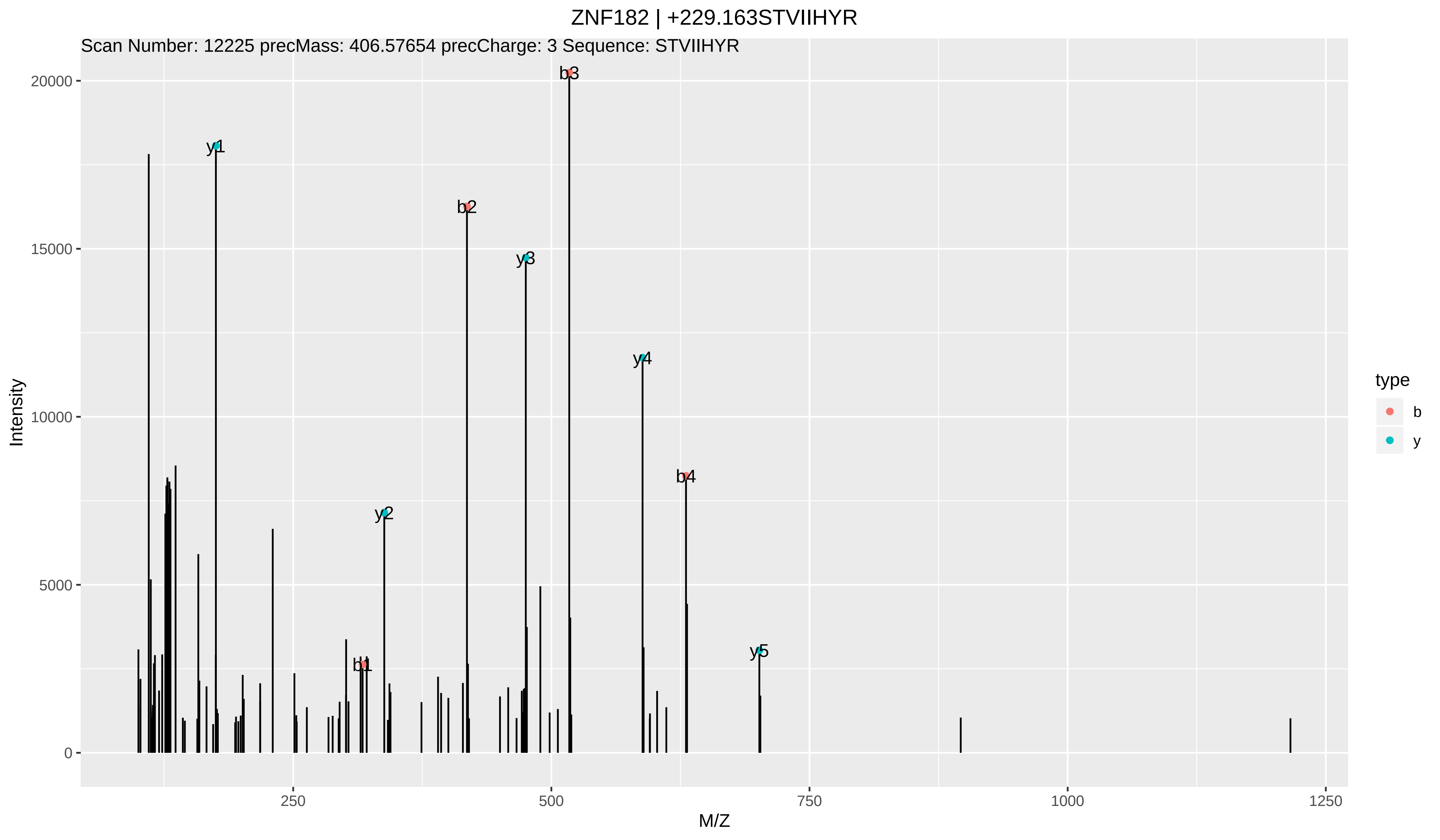

ZNF182 | +229.163STVIIHYR
Scan Number: 12225 precMass: 406.57654 precCharge: 3 Sequence: STVIIHYR
Intensity
M/Z
0
5000
10000
15000
20000
250
500
750
1000
1250
b3
y1
b2
y3
y4
b4
y2
b1
y5
type
b
y

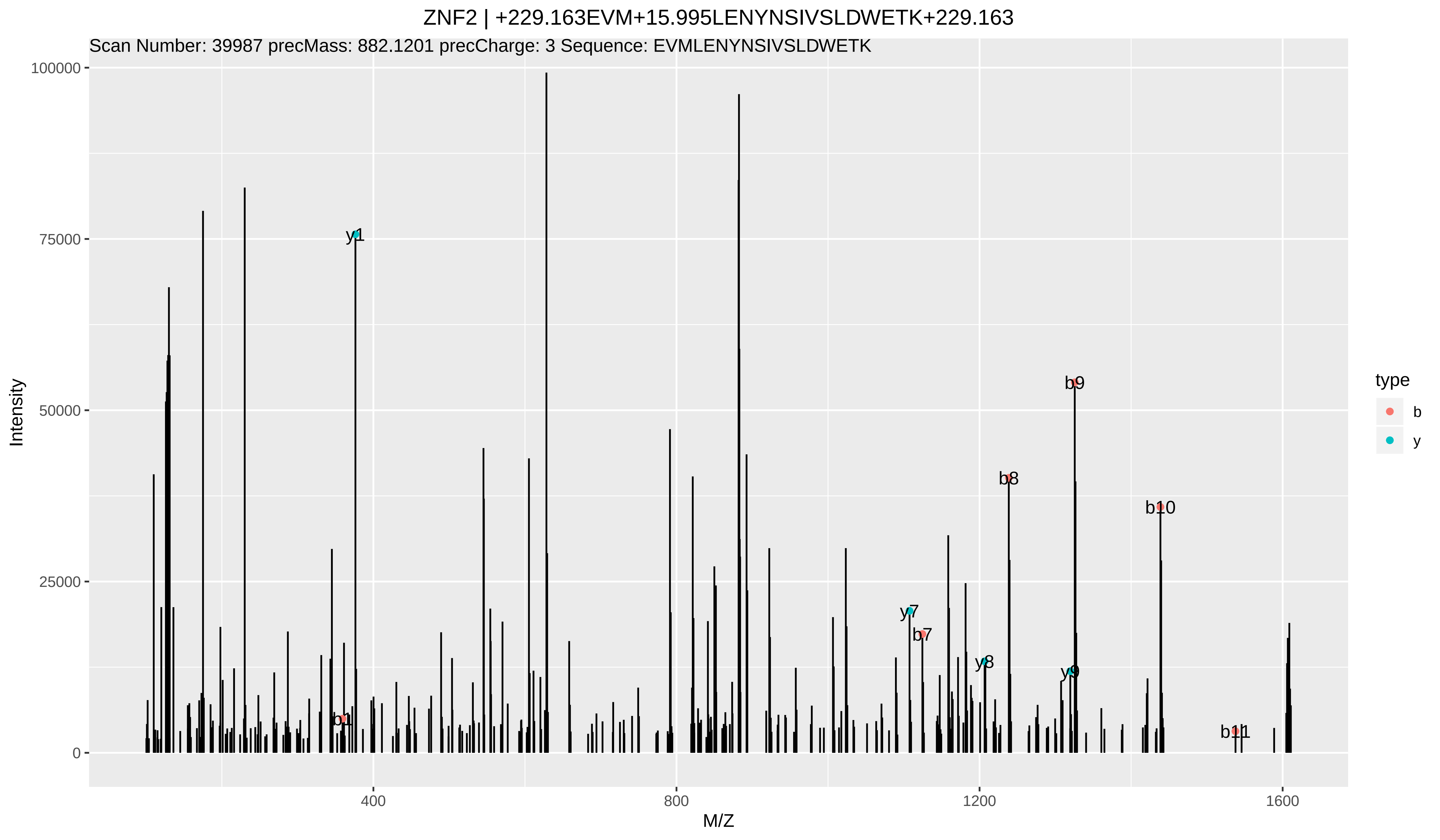

ZNF2 | +229.163EVM+15.995LENYNSIVSLDWETK+229.163
Scan Number: 39987 precMass: 882.1201 precCharge: 3 Sequence: EVMLENYNSIVSLDWETK
Intensity
M/Z
100000
75000
50000
25000
0
400
800
1200
1600
y1
b1
y7
b7
y8
b8
y9
b9
b10
b11
type
b
y

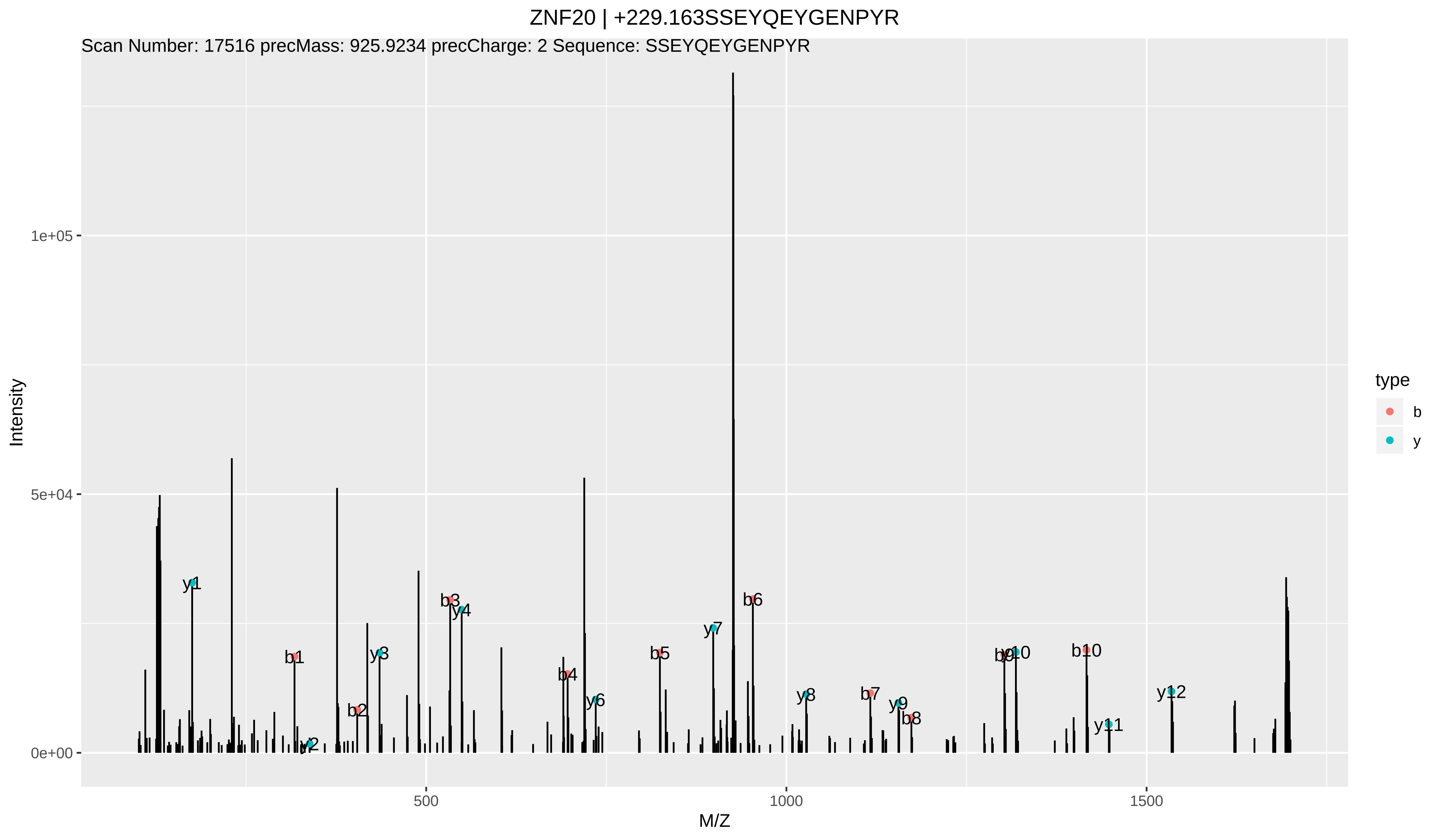

ZNF20 | +229.163SSEYQEYGENPYR
Scan Number: 17516 precMass: 925.9234 precCharge: 2 Sequence: SSEYQEYGENPYR
Intensity
1e+05
5e+04
0e+00
M/Z
500
1000
1500
y1
b1
y2
b2
y3
b3
y4
b4
y6
b5
y7
b6
y8
b7
y9
b8
b9
y10
b10
y11
y12
type
b
y

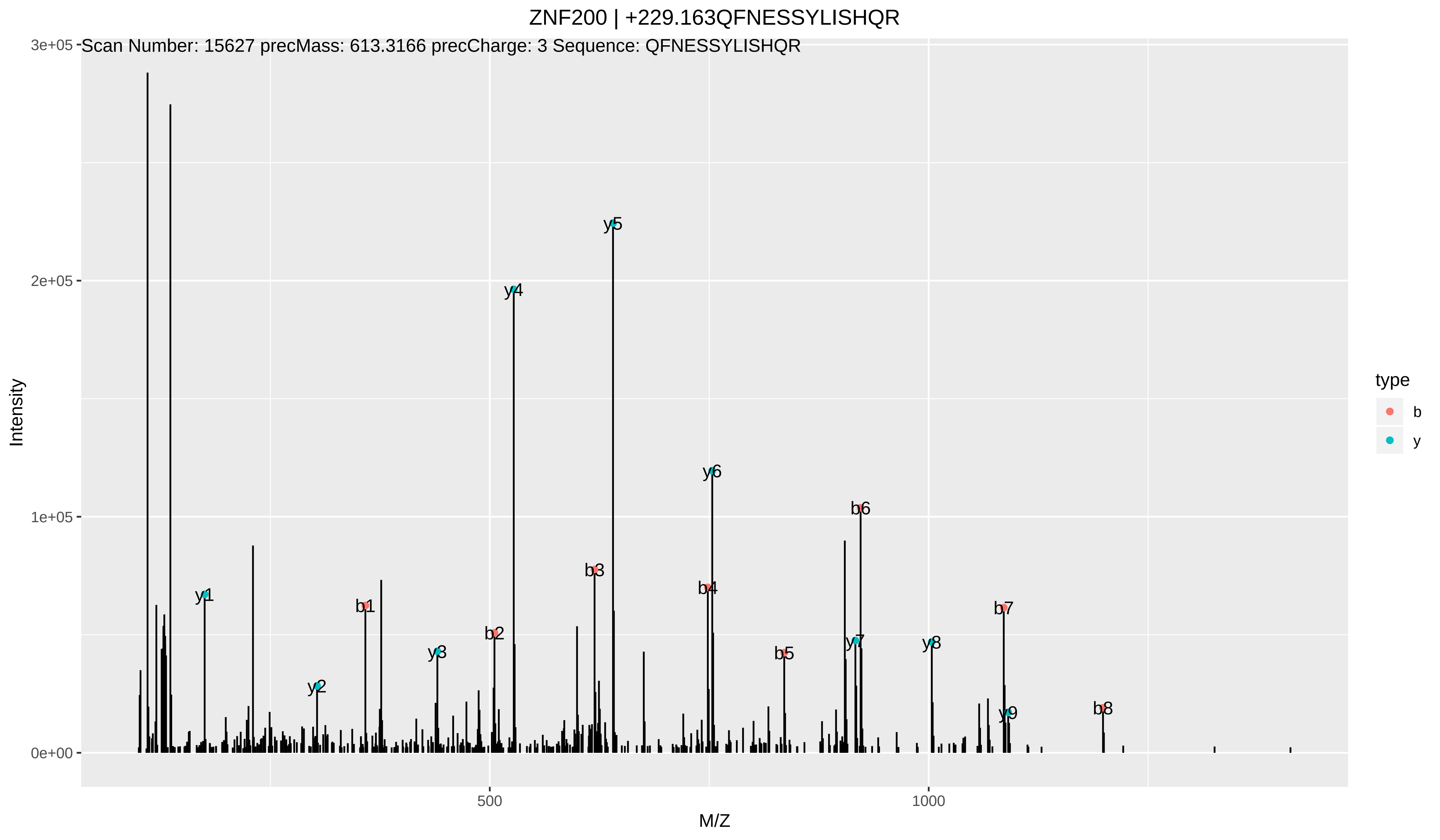
ZNF200 | +229.163QFNESSYLISHQR
Scan Number: 15627 precMass: 613.3166 precCharge: 3 Sequence: QFNESSYLISHQR
Intensity
M/Z
3e+05
2e+05
1e+05
0e+00
500
1000
y1
y2
y3
y4
y5
y6
y7
y8
y9
b1
b2
b3
b4
b5
b6
b7
b8
type
b
y

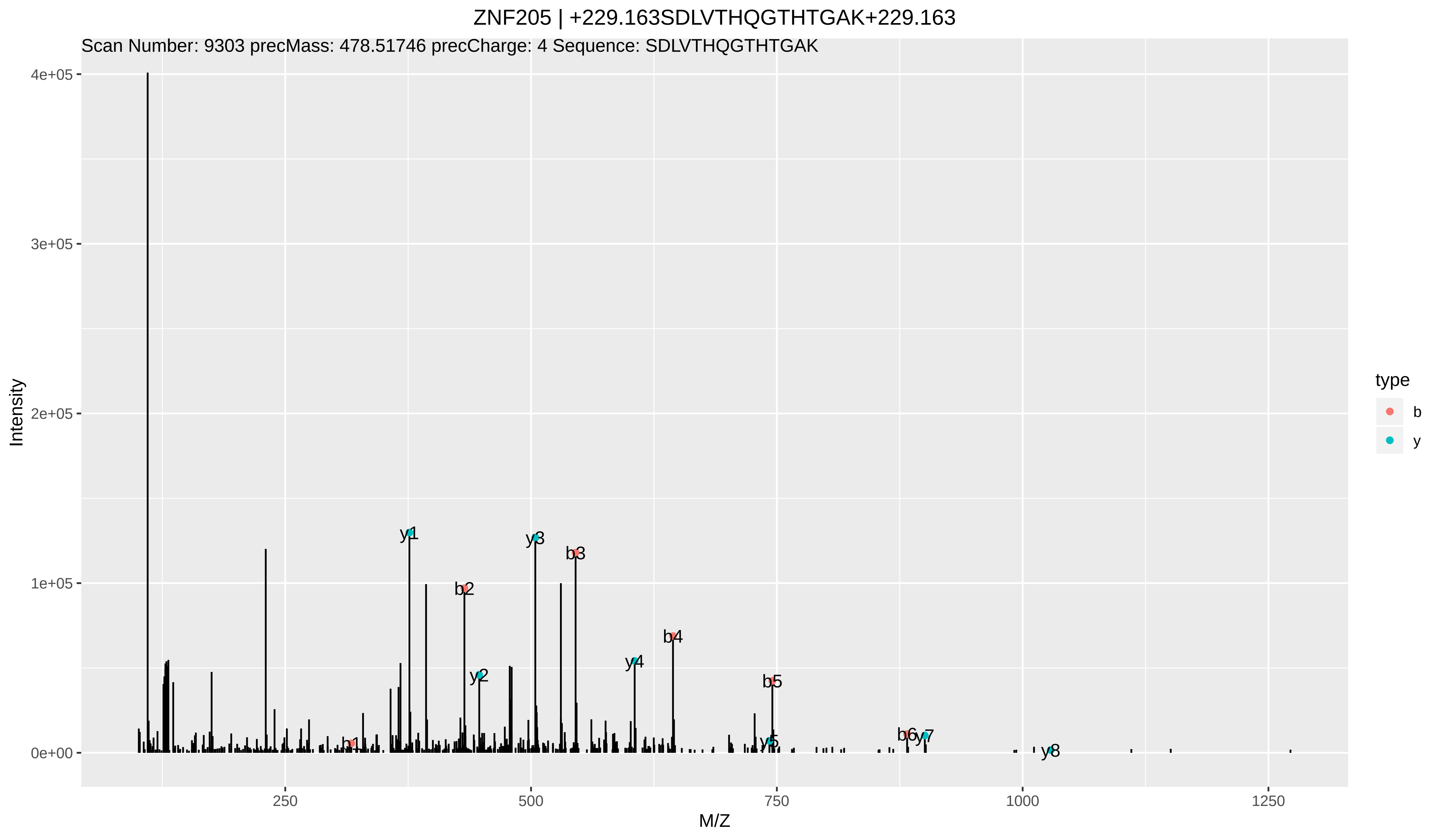
ZNF205 | +229.163SDLVTHQGTHTGAK+229.163
Scan Number: 9303 precMass: 478.51746 precCharge: 4 Sequence: SDLVTHQGTHTGAK
Intensity
4e+05
3e+05
2e+05
1e+05
0e+00
M/Z
250
500
750
1000
1250
y1
y3
b3
b2
b4
y4
y2
b5
y5
b6
y7
y8
b1
type
b
y

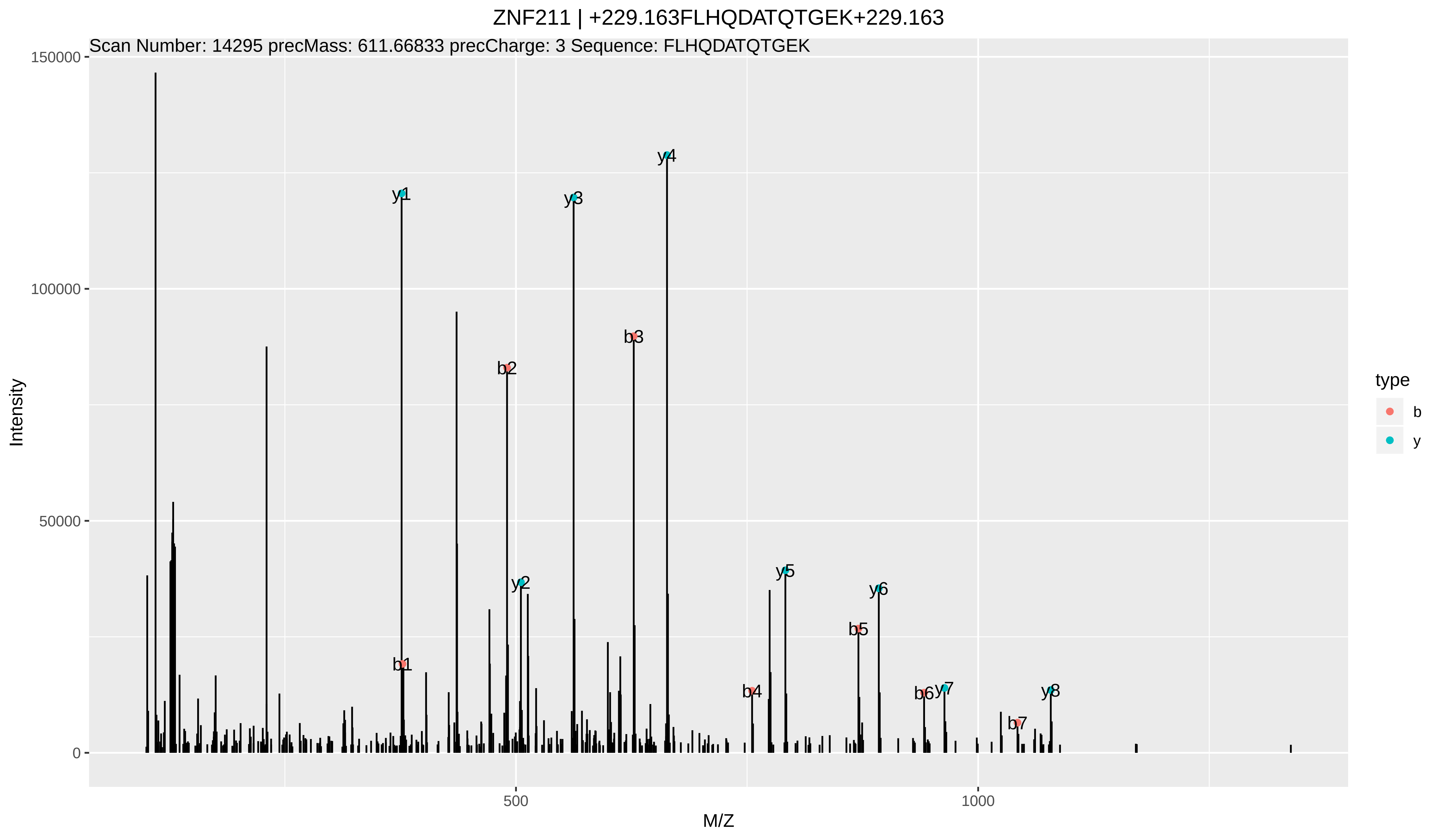

ZNF211 | +229.163FLHQDATQTGEK+229.163
Scan Number: 14295 precMass: 611.66833 precCharge: 3 Sequence: FLHQDATQTGEK
Intensity
150000
100000
50000
0
M/Z
500
1000
y1
y3
y4
b3
b2
y2
b1
y5
y6
b5
b4
b6
y7
y8
b7
type
b
y

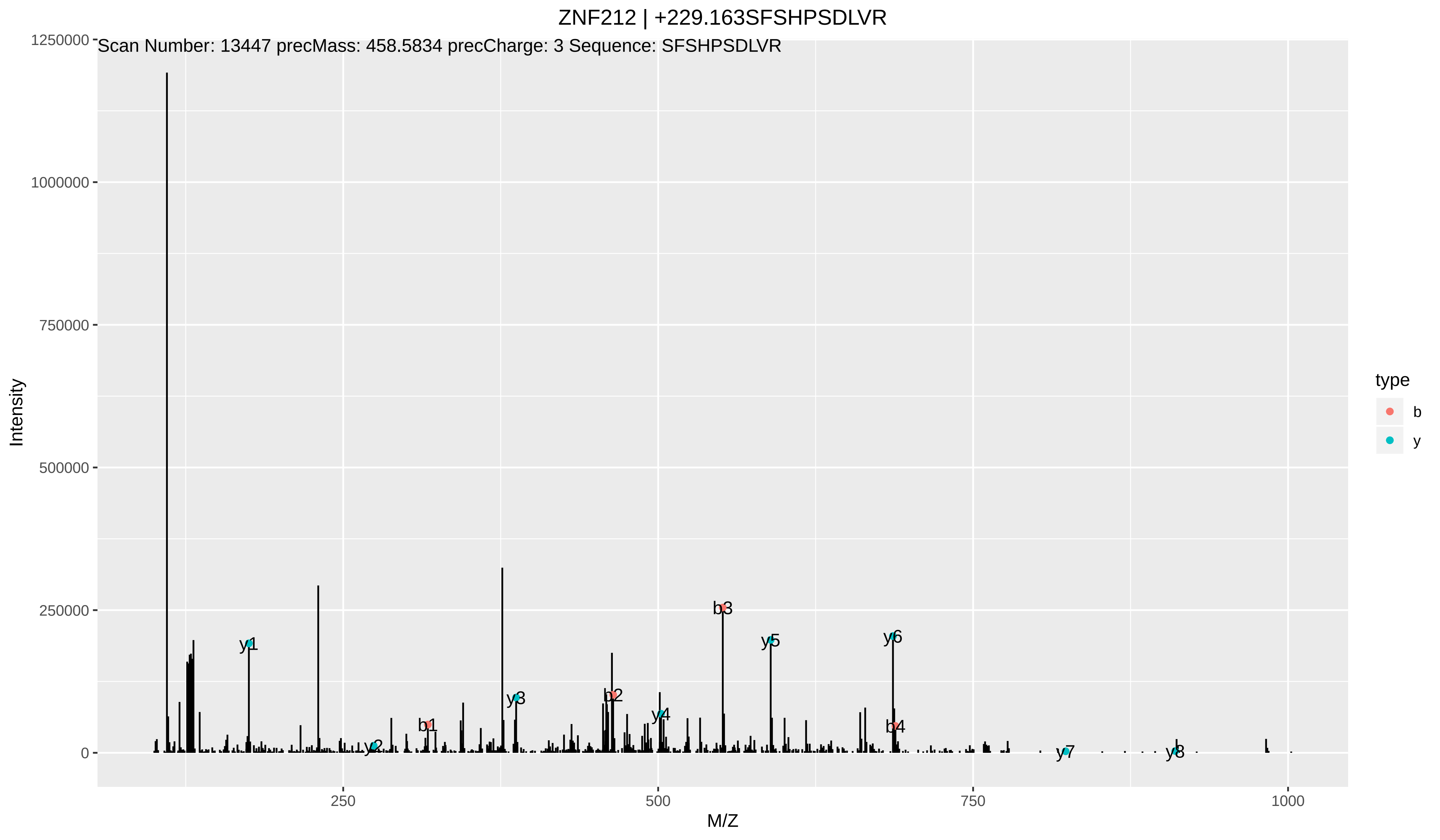
ZNF212 | +229.163SFSHPSDLVR
Scan Number: 13447 precMass: 458.5834 precCharge: 3 Sequence: SFSHPSDLVR
Intensity
1250000
1000000
750000
500000
250000
0
M/Z
250
500
750
1000
y1
y2
b1
y3
b2
y4
b3
y5
y6
b4
y7
y8
type
b
y

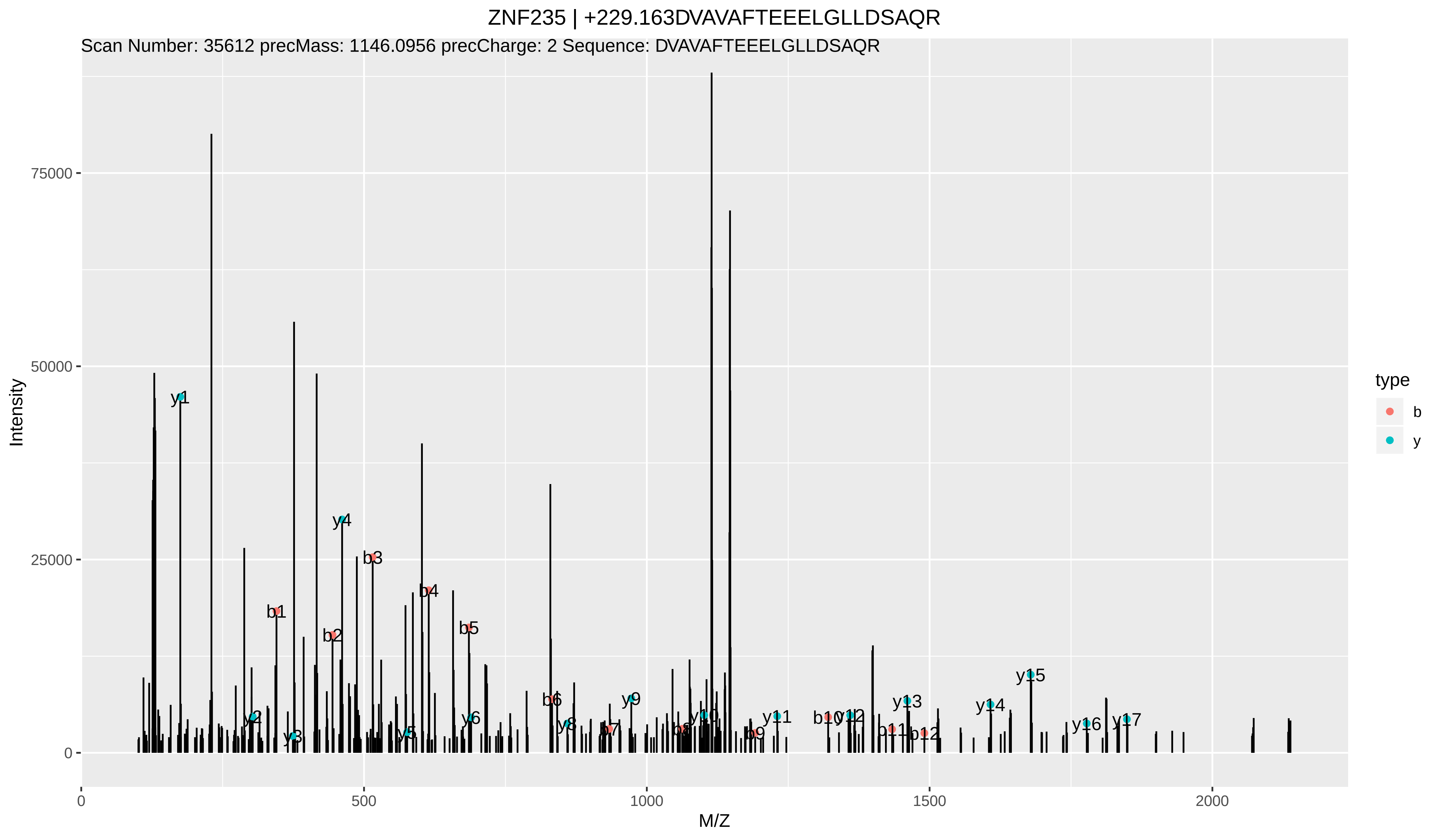
ZNF235 | +229.163DVAVAFTEEELGLLDSAQR
Scan Number: 35612 precMass: 1146.0956 precCharge: 2 Sequence: DVAVAFTEEELGLLDSAQR
Intensity
M/Z
type
b
y
0
25000
50000
75000
500
1000
1500
2000
y1
y2
y3
y4
y5
y6
y8
y9
y10
y11
y12
y13
y14
y15
y16
y17
b1
b2
b3
b4
b5
b6
b7
b8
b9
b10
b11
b12

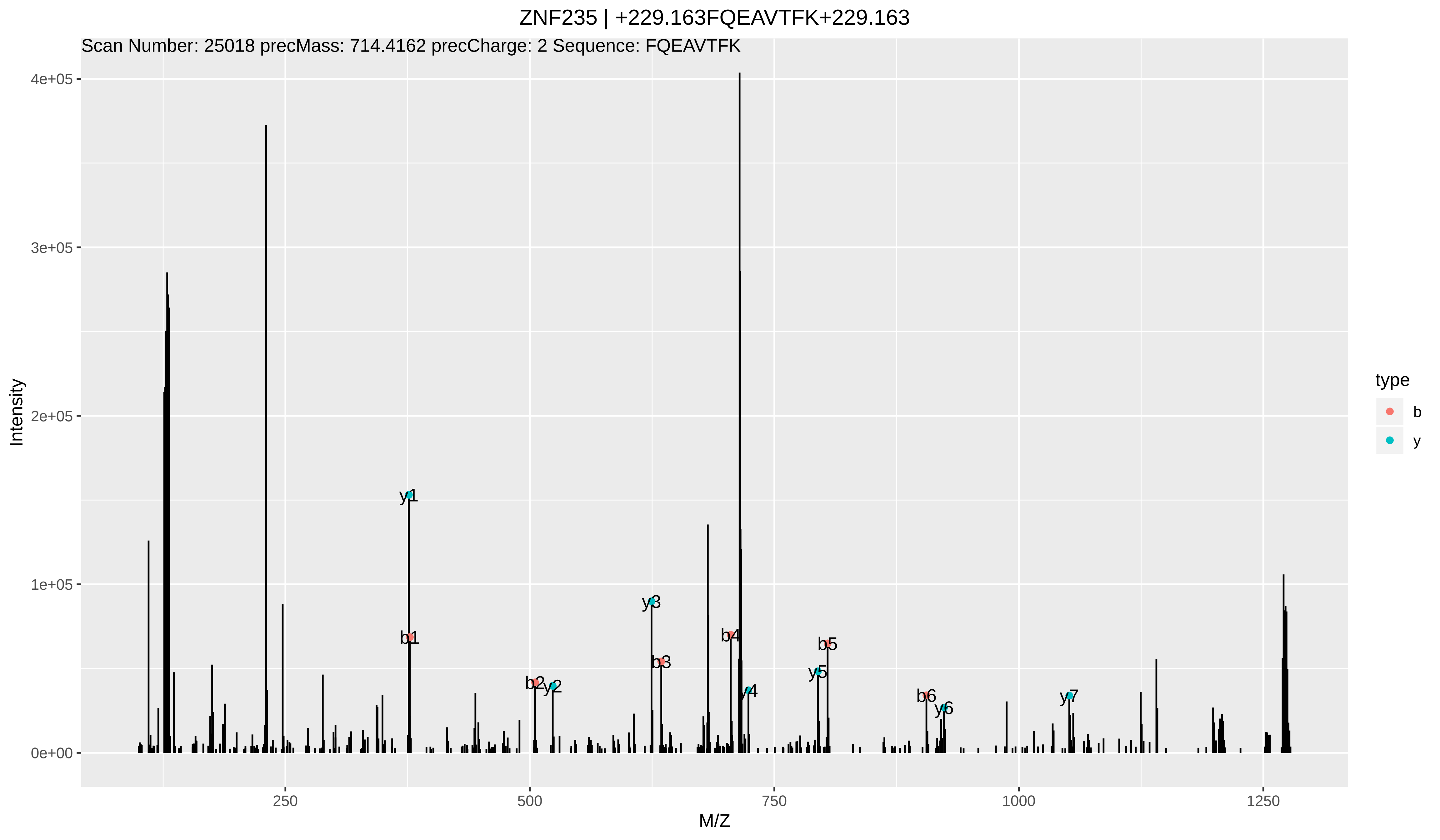

ZNF235 | +229.163FQEAVTFK+229.163
Scan Number: 25018 precMass: 714.4162 precCharge: 2 Sequence: FQEAVTFK
Intensity
4e+05
3e+05
2e+05
1e+05
0e+00
250
500
750
1000
1250
M/Z
y1
b1
b2
y2
y3
b3
b4
y4
y5
b5
b6
y6
y7
type
b
y

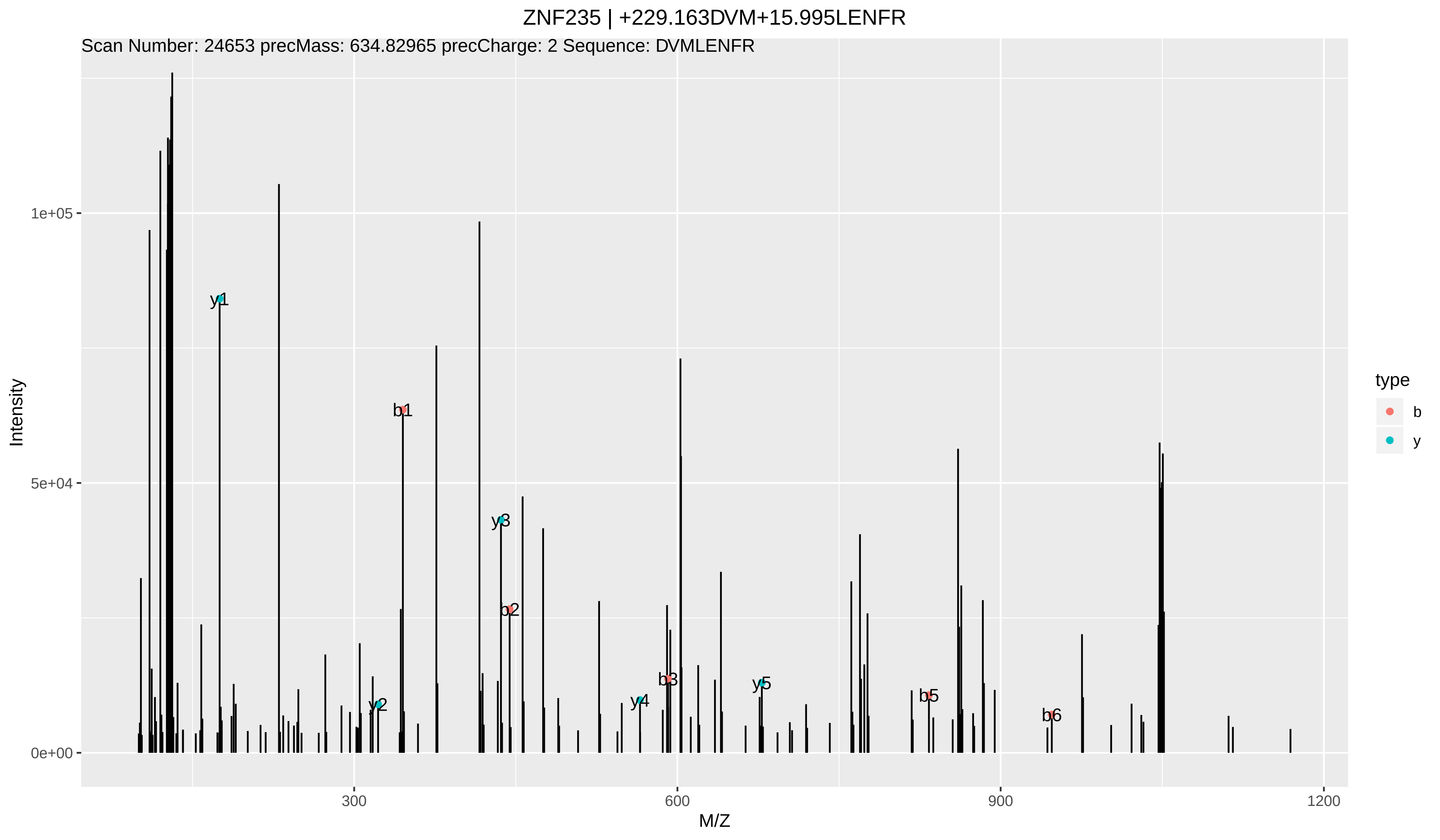
ZNF235 | +229.163DVM+15.995LENFR
Scan Number: 24653 precMass: 634.82965 precCharge: 2 Sequence: DVMLENFR
Intensity
1e+05
5e+04
0e+00
M/Z
300
600
900
1200
y1
b1
y3
b2
y2
y4
b3
y5
b5
b6
type
b
y

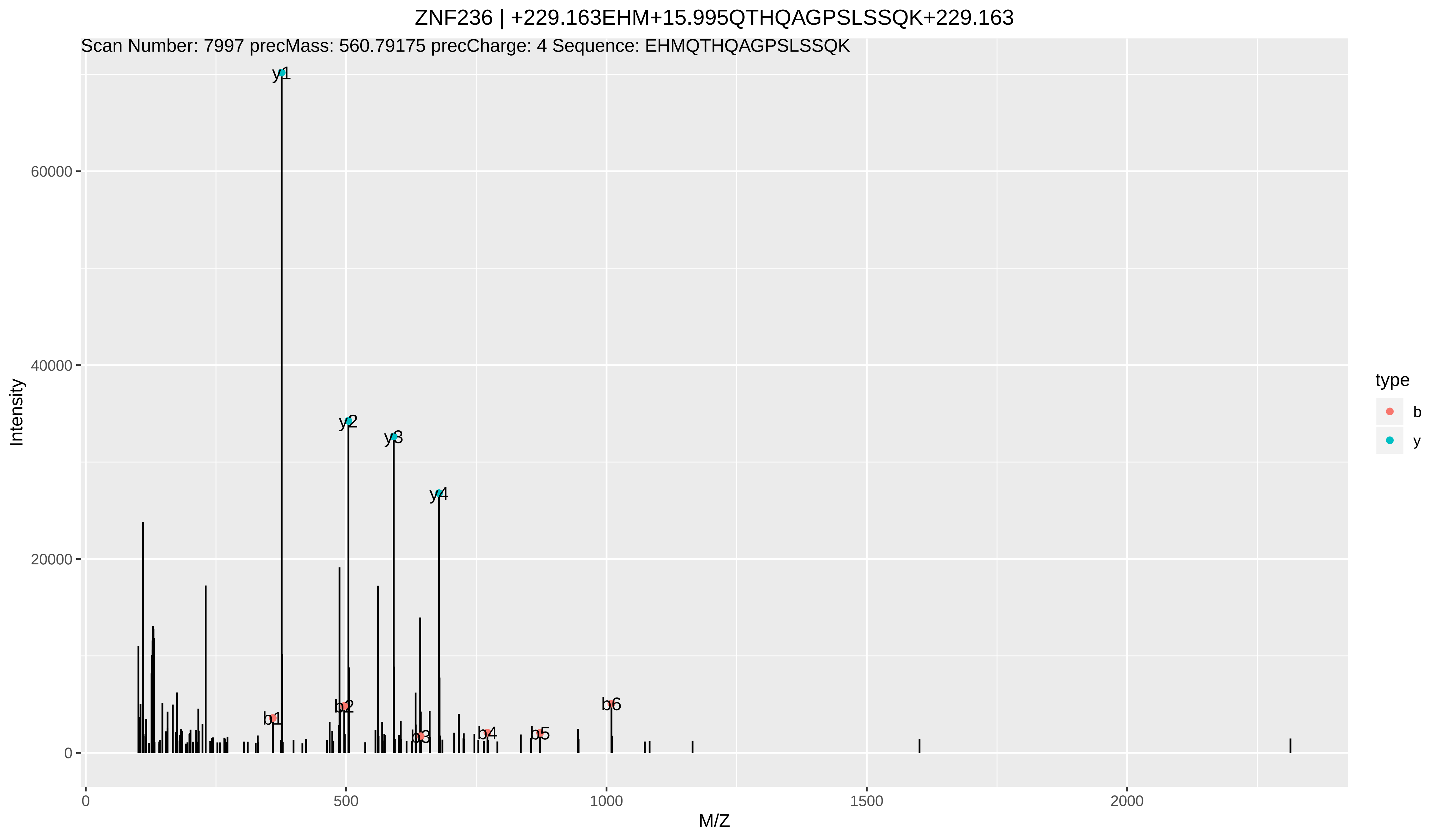
ZNF236 | +229.163EHM+15.995QTHQAGPSLSSQK+229.163
Scan Number: 7997 precMass: 560.79175 precCharge: 4 Sequence: EHMQTHQAGPSLSSQK
Intensity
M/Z
0
20000
40000
60000
0
500
1000
1500
2000
y1
y2
y3
y4
b1
b2
b3
b4
b5
b6
type
b
y

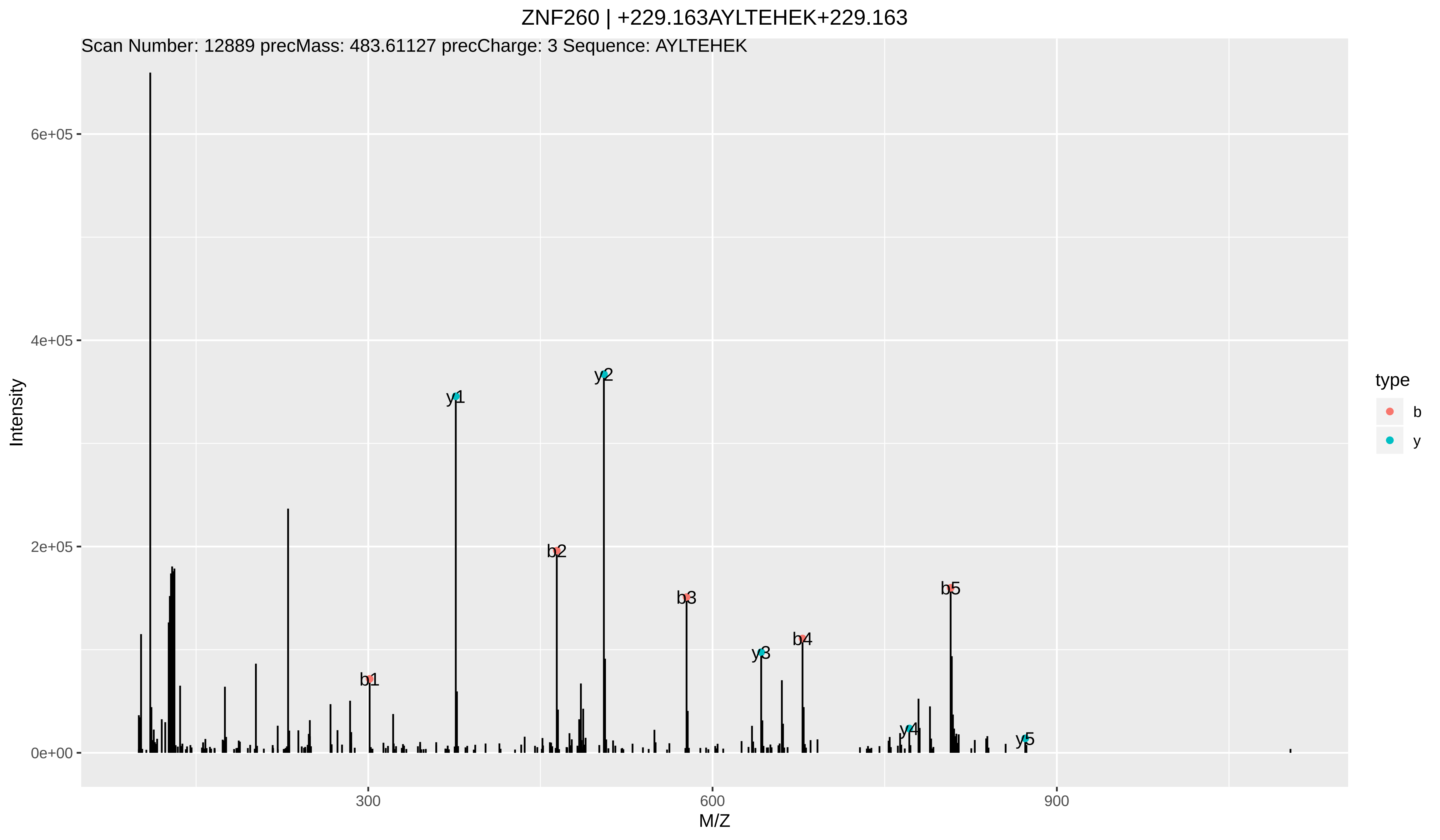
ZNF260 | +229.163AYLTEHEK+229.163
Scan Number: 12889 precMass: 483.61127 precCharge: 3 Sequence: AYLTEHEK
Intensity
M/Z
6e+05
4e+05
2e+05
0e+00
300
600
900
y1
y2
b2
b3
b5
y3
b4
b1
y4
y5
type
b
y

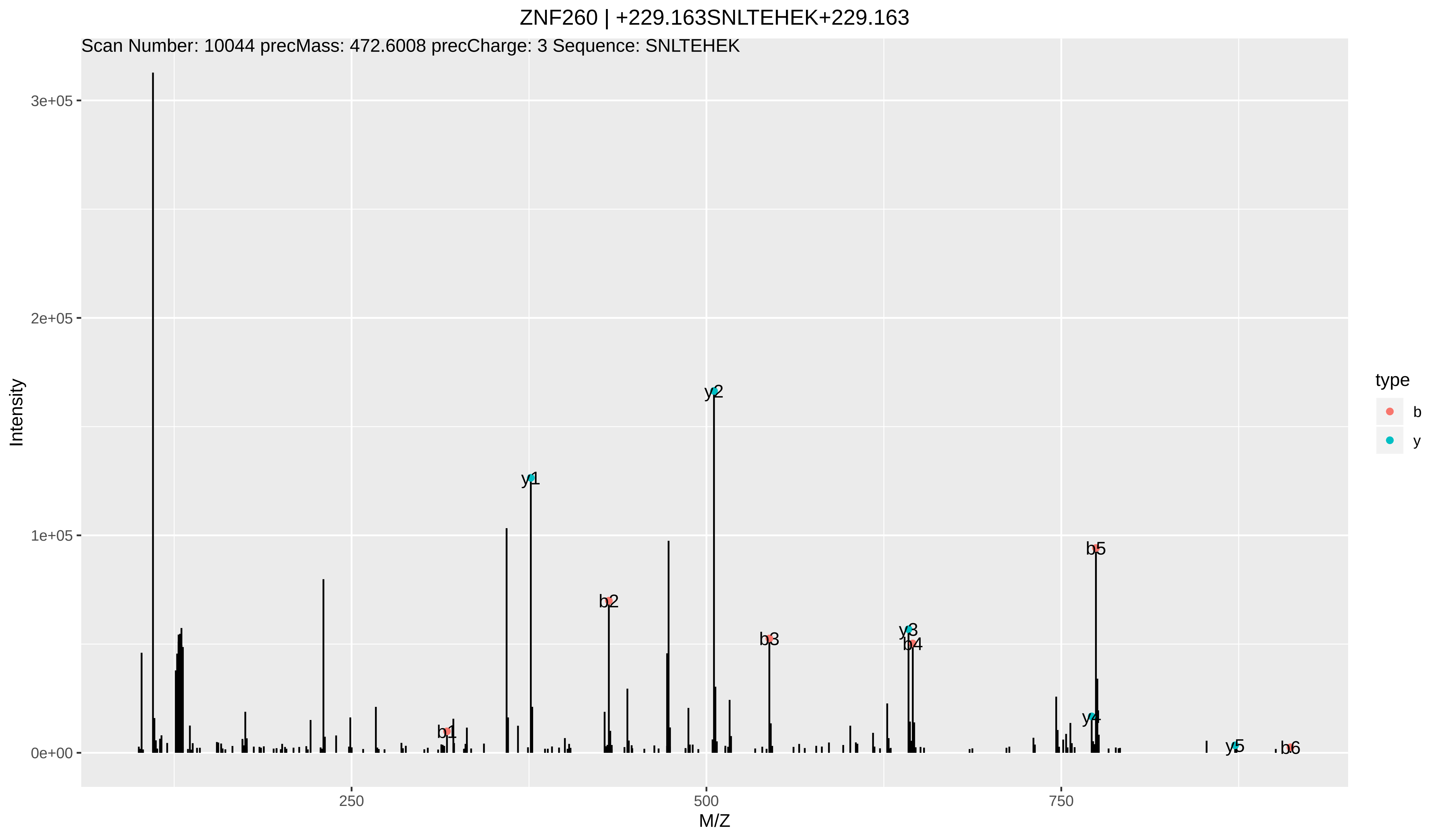

ZNF260 | +229.163SNLTEHEK+229.163
Scan Number: 10044 precMass: 472.6008 precCharge: 3 Sequence: SNLTEHEK
Intensity
3e+05
2e+05
1e+05
0e+00
M/Z
250
500
750
y1
y2
y3
y4
y5
b1
b2
b3
b4
b5
b6
type
b
y

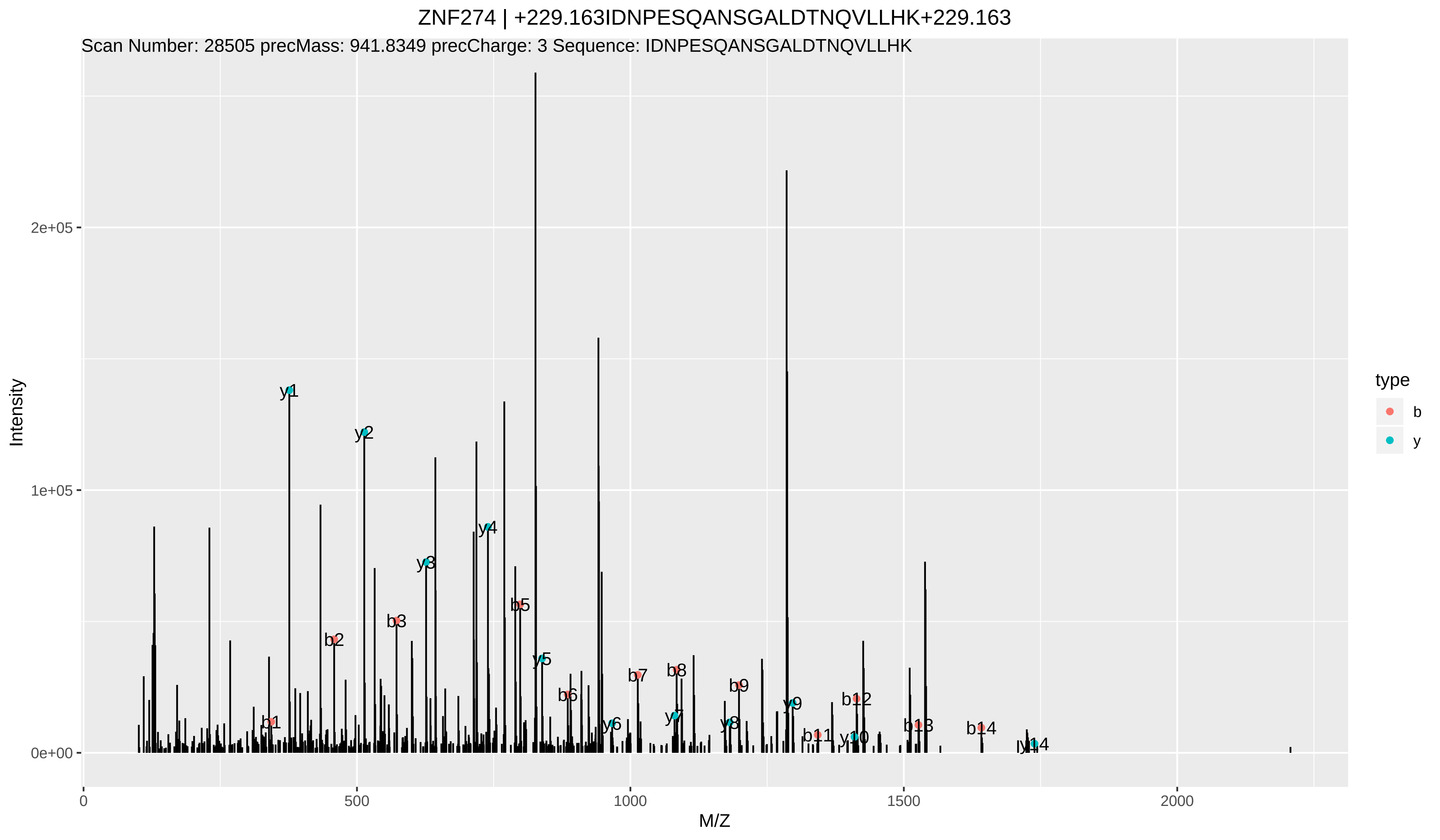
ZNF274 | +229.163IDNPESQANSGALDTNQVLLHK+229.163
Scan Number: 28505 precMass: 941.8349 precCharge: 3 Sequence: IDNPESQANSGALDTNQVLLHK
Intensity
M/Z
0e+00
1e+05
2e+05
0
500
1000
1500
2000
y1
y2
y3
y4
y5
y6
y7
y8
y9
y10
y14
b1
b2
b3
b5
b6
b7
b8
b9
b11
b12
b13
b14
type
b
y

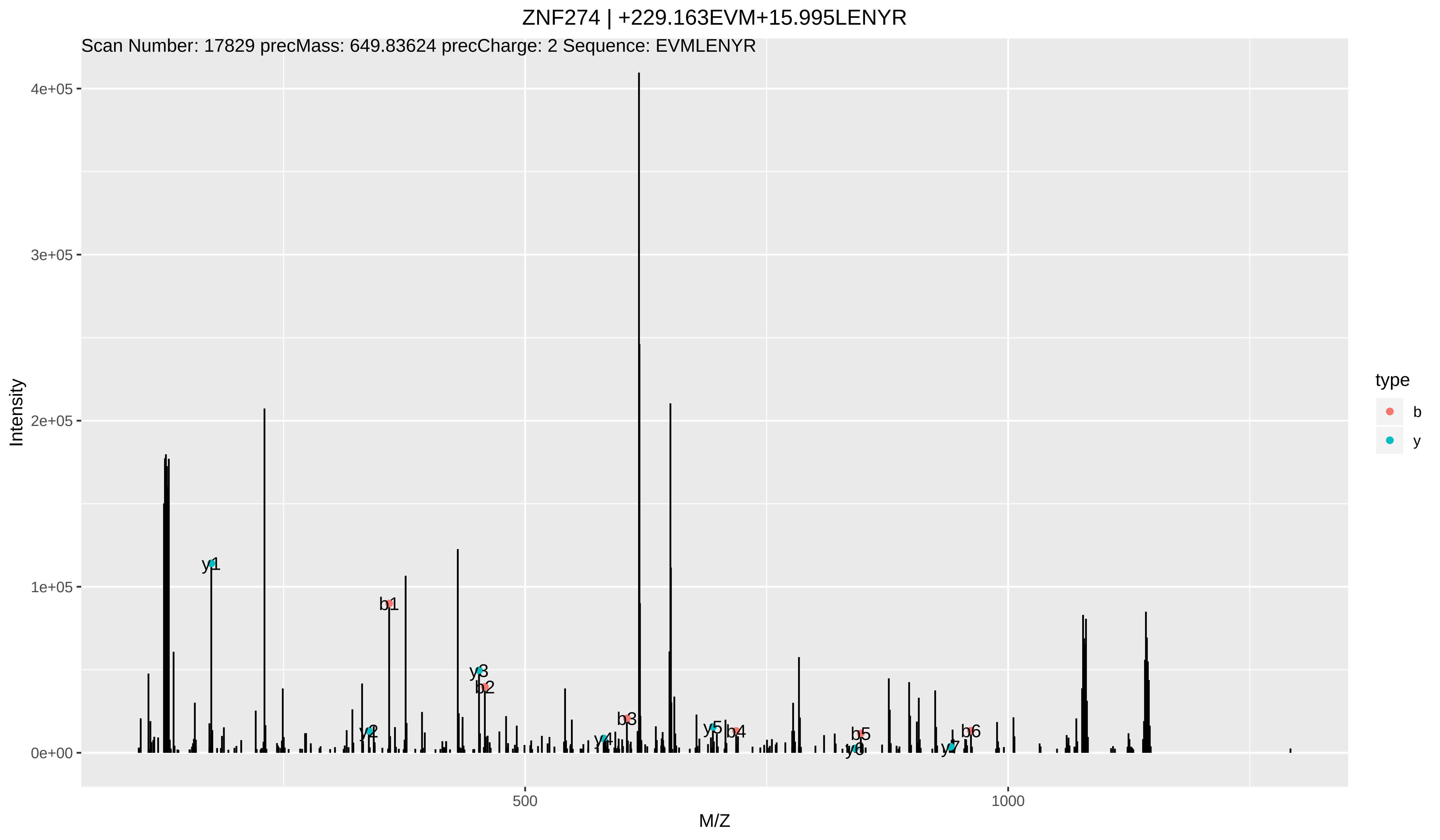
ZNF274 | +229.163EVM+15.995LENYR
Scan Number: 17829 precMass: 649.83624 precCharge: 2 Sequence: EVMLENYR
Intensity
4e+05
3e+05
2e+05
1e+05
0e+00
500
1000
M/Z
y1
b1
y2
y3
b2
y4
b3
y5
b4
y6
b5
y7
b6
type
b
y

ZNF276 | +229.163AHNLNVHM+15.995SM+15.995VHPLTQTQDK+229.163

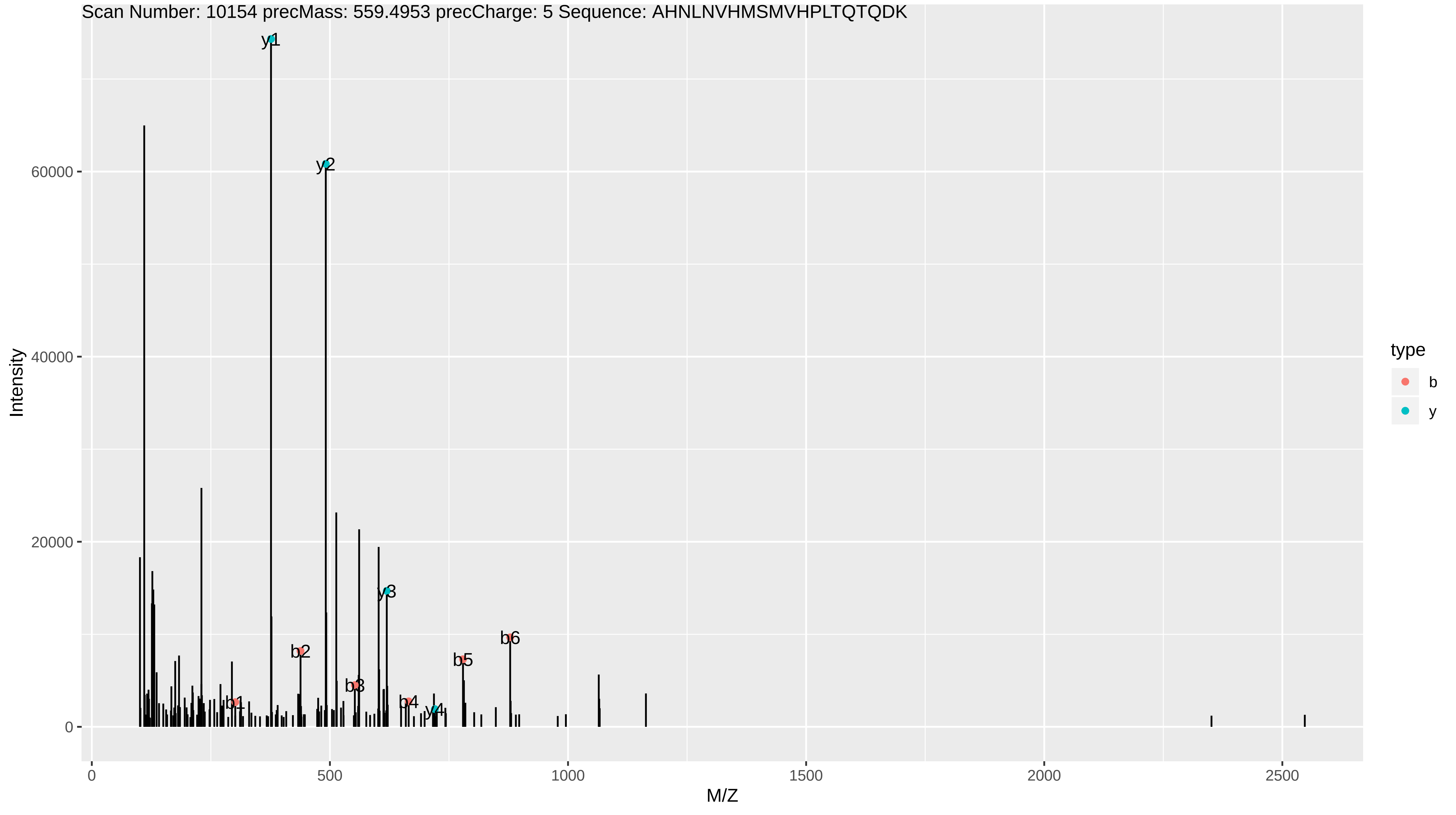

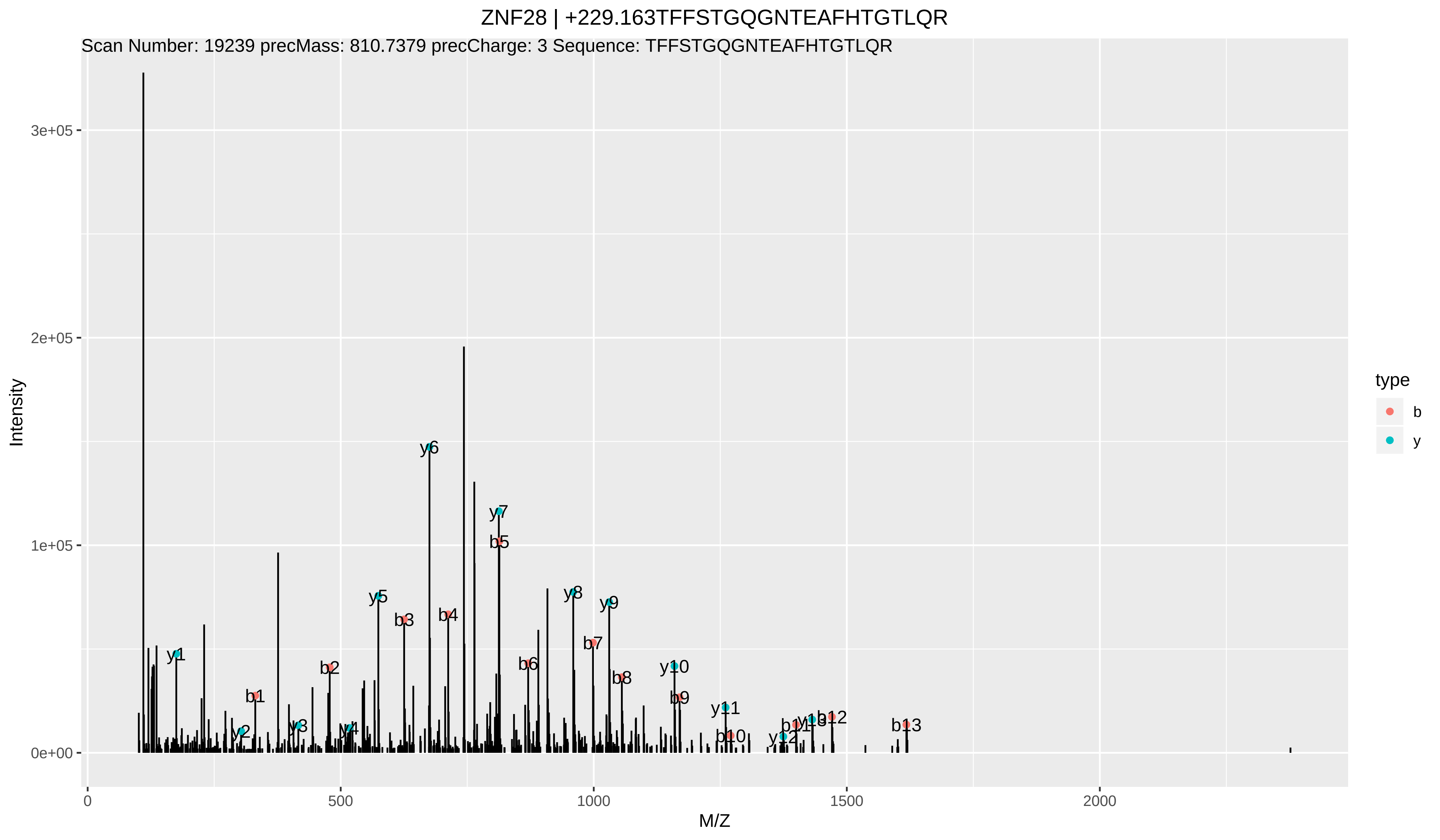

ZNF28 | +229.163TFFSTGQGNTEAFHTGTLQR
Scan Number: 19239 precMass: 810.7379 precCharge: 3 Sequence: TFFSTGQGNTEAFHTGTLQR
Intensity
M/Z
0e+00
1e+05
2e+05
3e+05
0
500
1000
1500
2000
type
b
y
y1
y2
y3
y4
y5
y6
y7
y8
y9
y10
y11
y12
b1
b2
b3
b4
b5
b6
b7
b8
b9
b10
b11
b12
b13

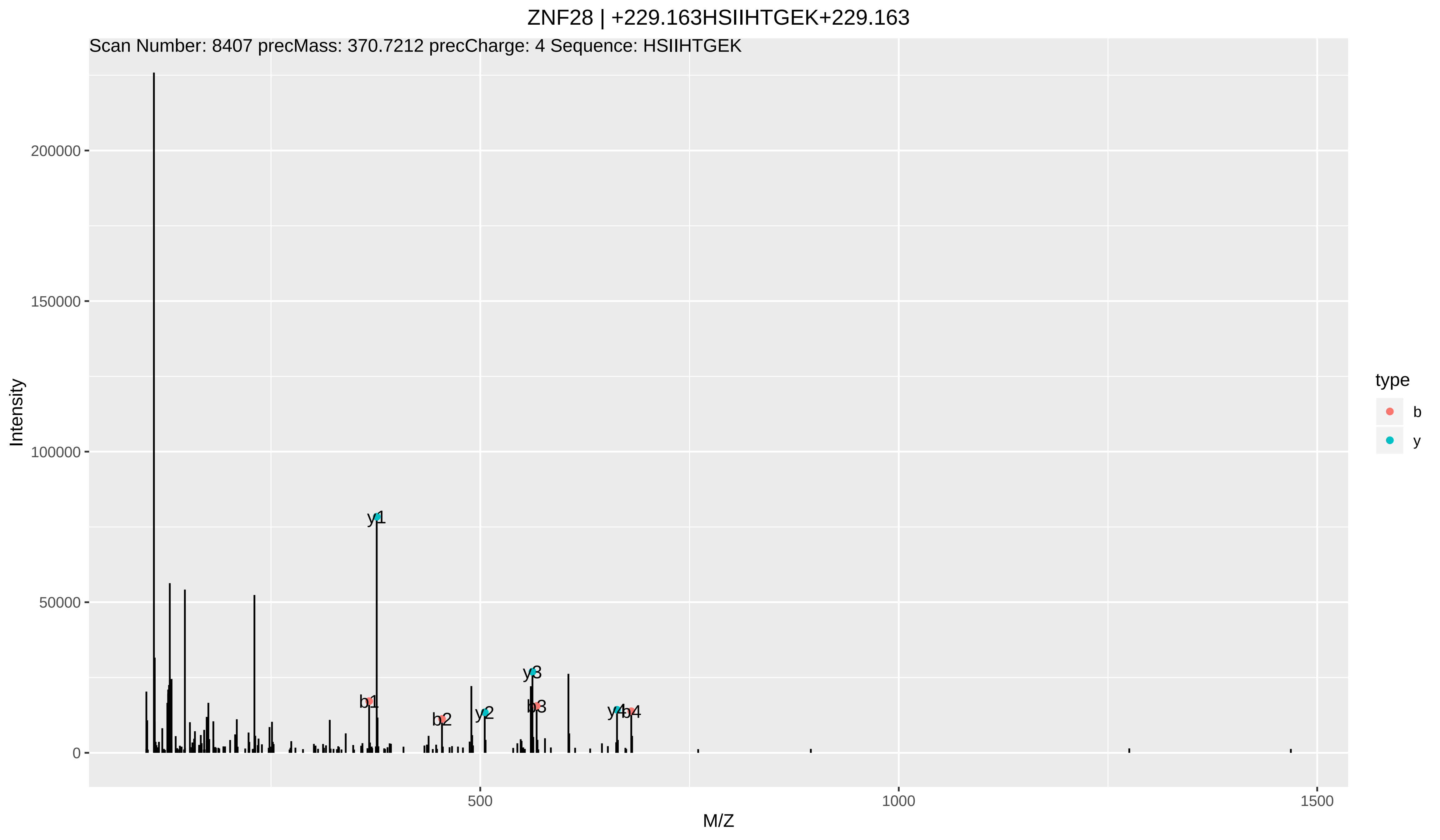

ZNF28 | +229.163HSIIHTGEK+229.163
Scan Number: 8407 precMass: 370.7212 precCharge: 4 Sequence: HSIIHTGEK
Intensity
200000
150000
100000
50000
0
M/Z
500
1000
1500
y1
b1
b2
y2
y3
b3
y4
b4
type
b
y

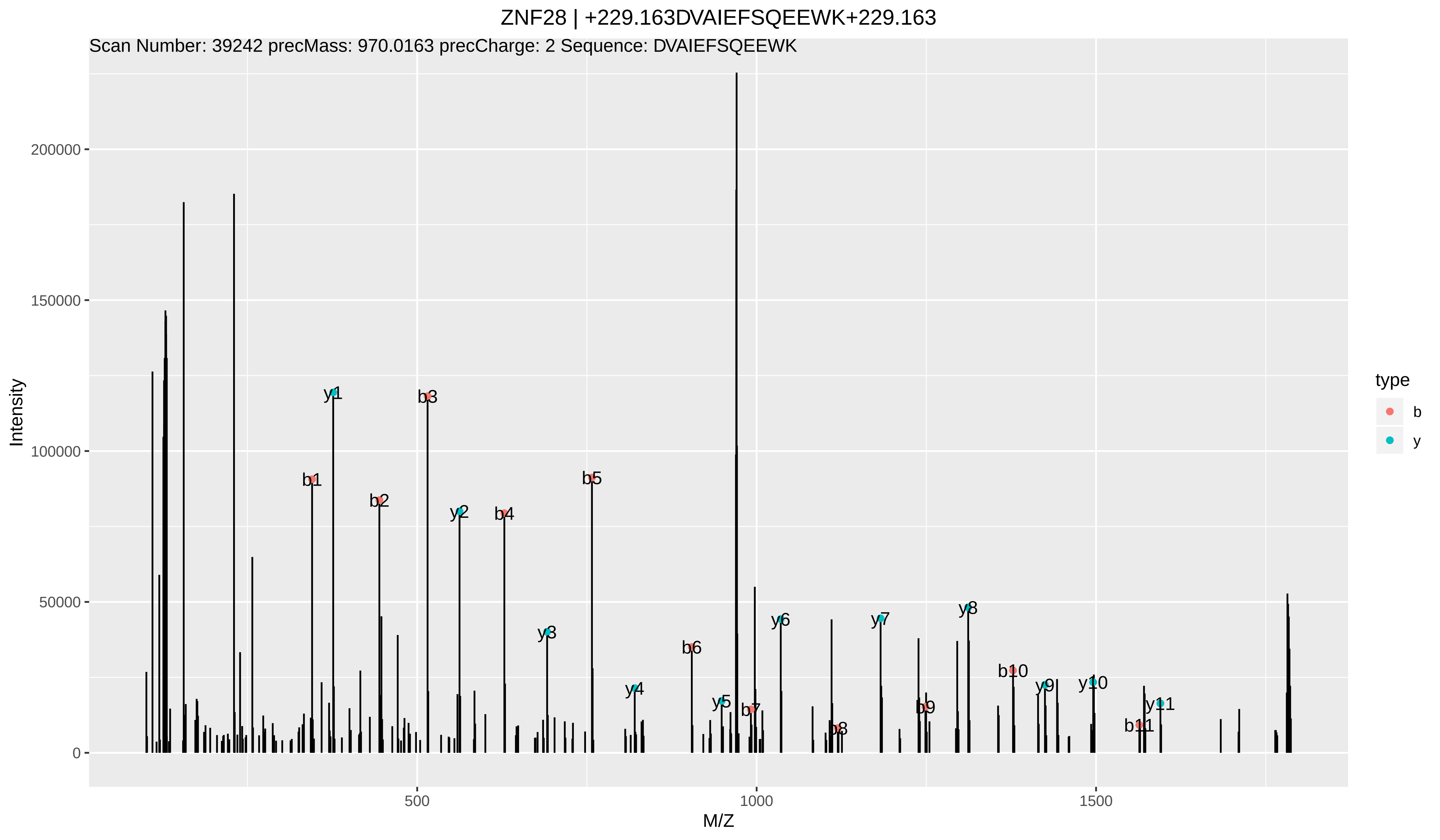

ZNF28 | +229.163DVAIEFSQEEWK+229.163
Scan Number: 39242 precMass: 970.0163 precCharge: 2 Sequence: DVAIEFSQEEWK
Intensity
M/Z
type
b
y
y1
b1
b2
b3
y2
b4
b5
y3
y4
b6
y5
b7
y6
b8
y7
b9
y8
b10
y9
y10
b11
y11

ZNF282 | +229.163TAVEFGNHM+15.995ESK+229.163

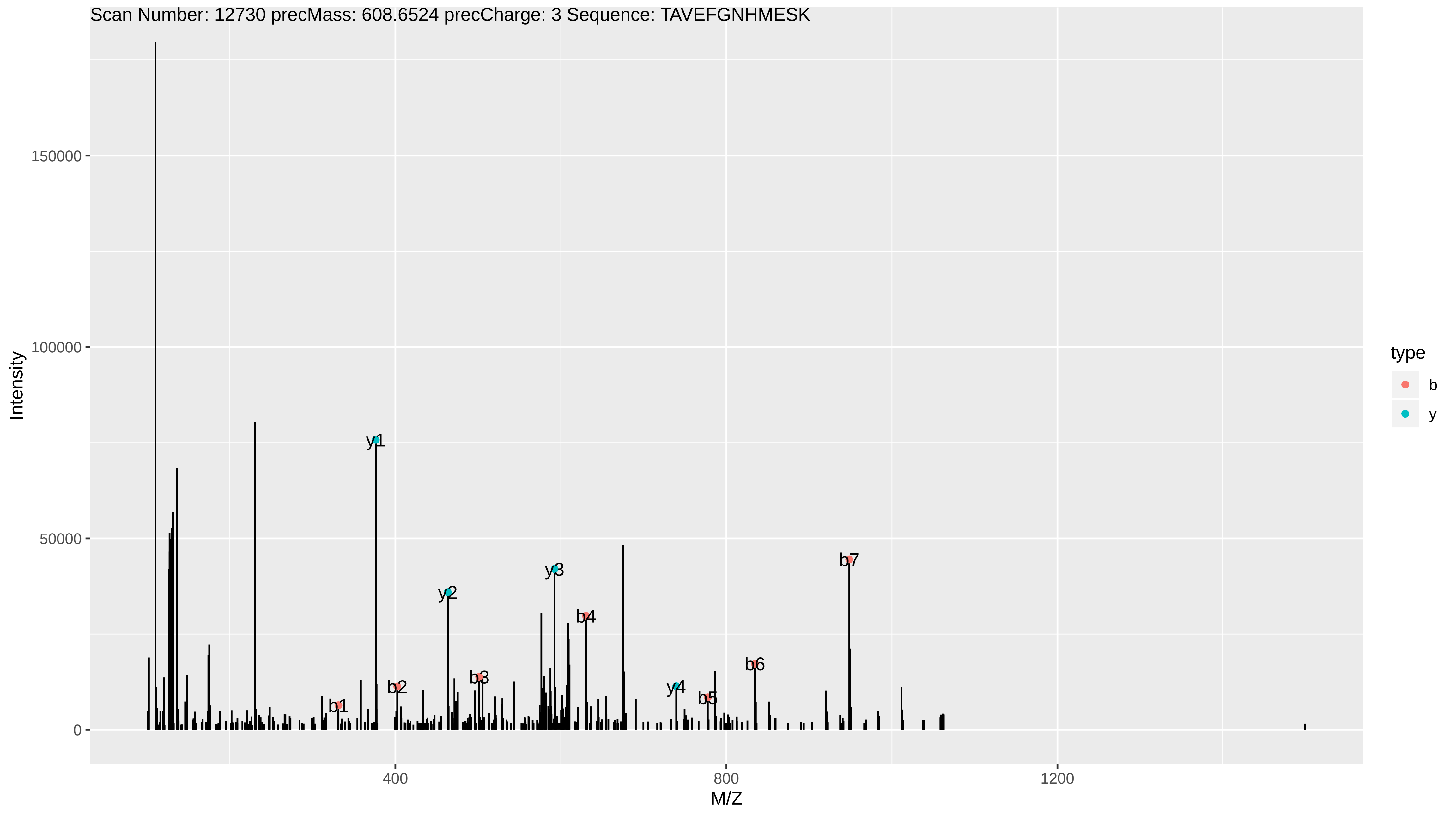

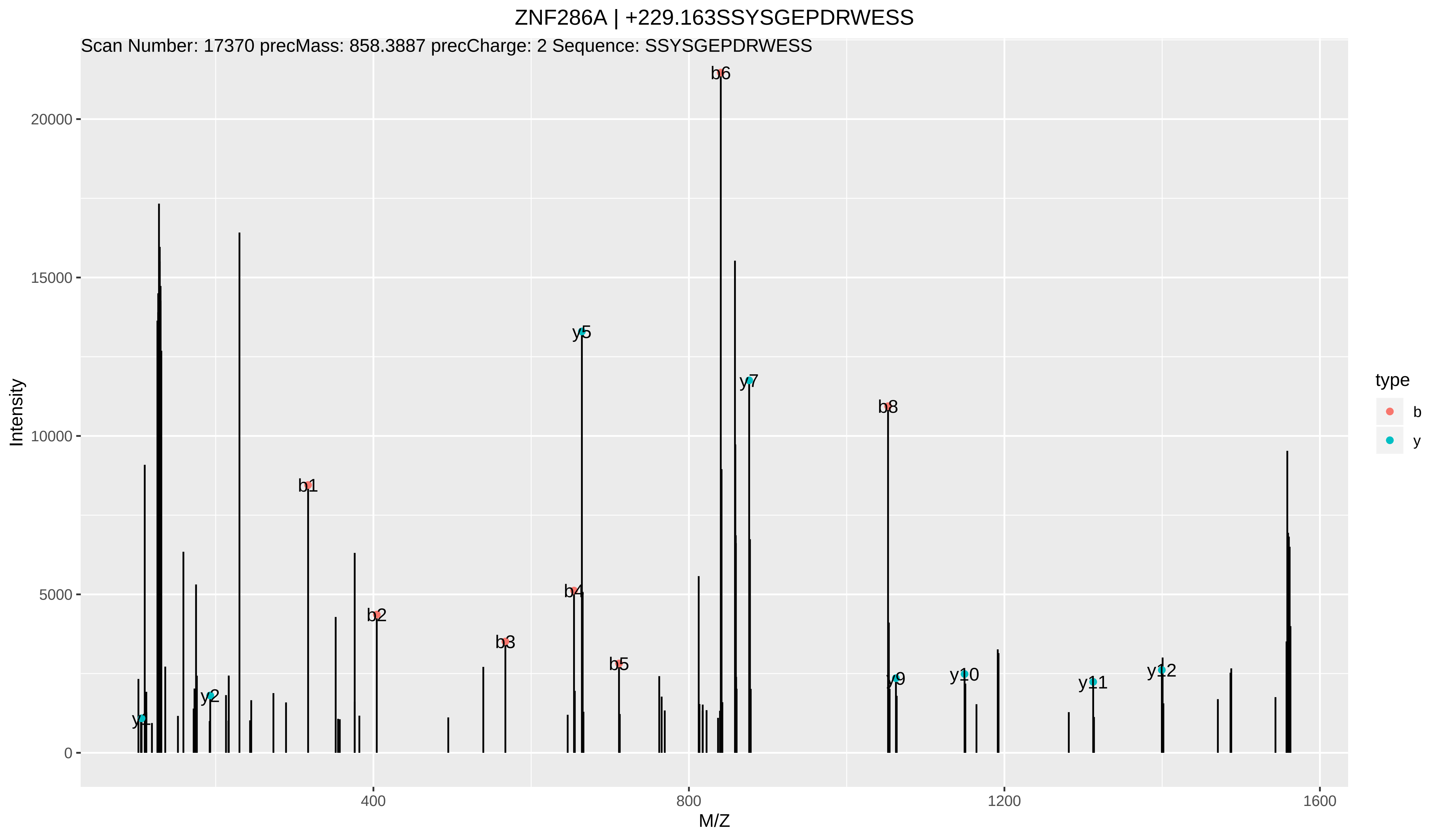

ZNF286A | +229.163SSYSGEPDRWESS
Scan Number: 17370 precMass: 858.3887 precCharge: 2 Sequence: SSYSGEPDRWESS
Intensity
M/Z
0
5000
10000
15000
20000
400
800
1200
1600
b6
y5
y7
b8
b1
b4
b2
b3
b5
y1
y2
y9
y10
y11
y12
type
b
y

ZNF286A | +229.163TFTESSSLATHQR

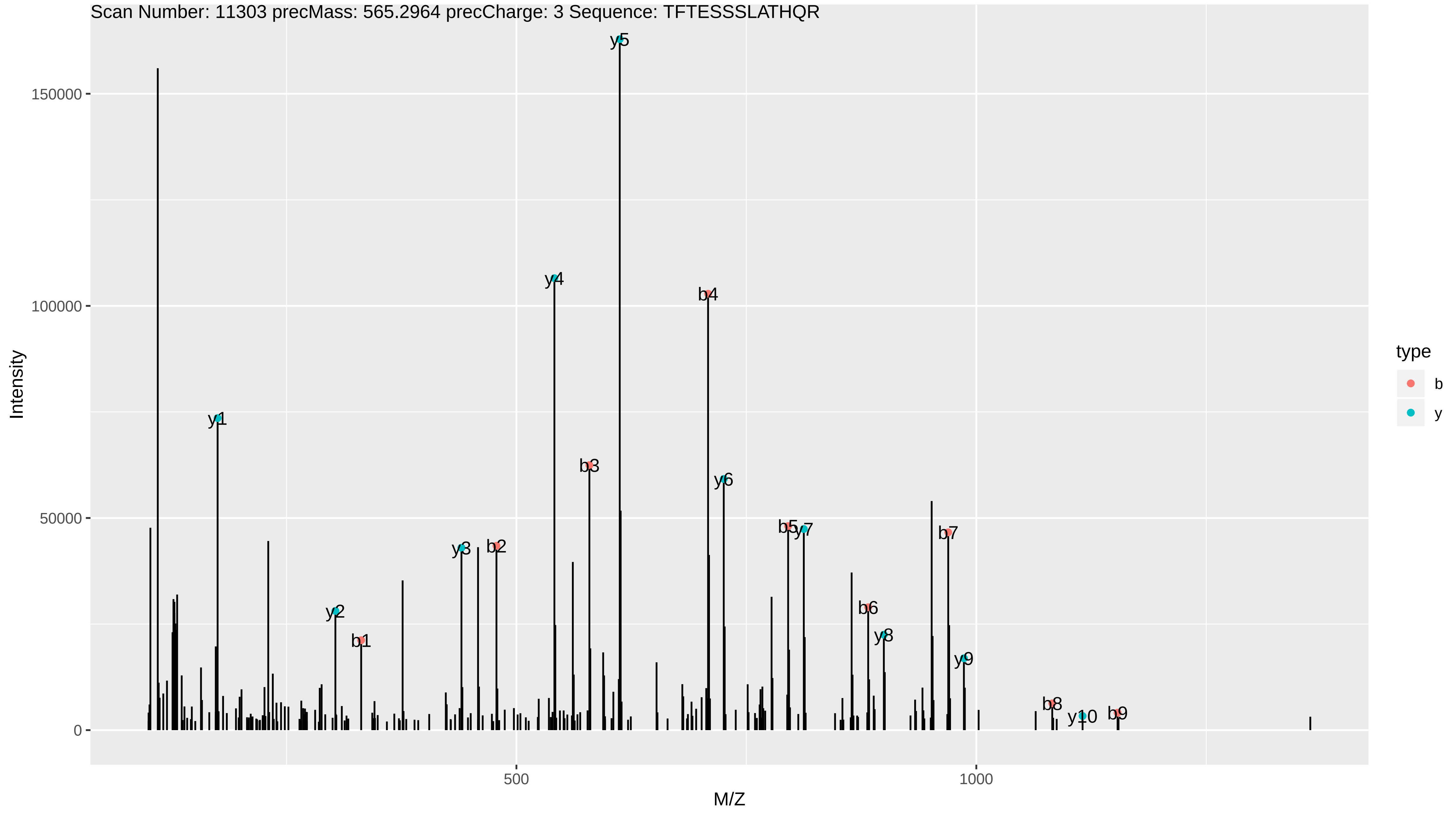

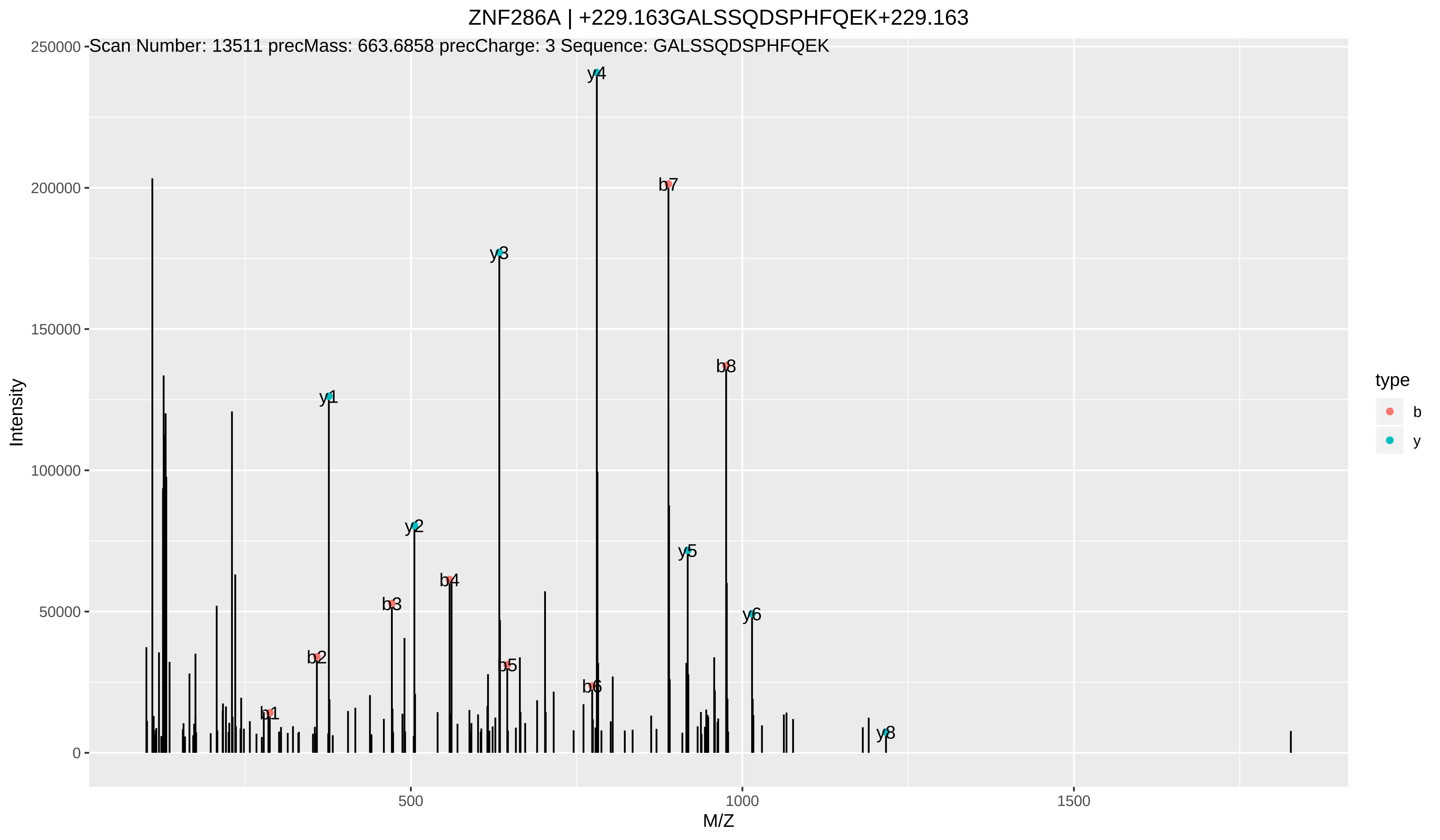

ZNF286A | +229.163GALSSQDSPHFQEK+229.163
Scan Number: 13511 precMass: 663.6858 precCharge: 3 Sequence: GALSSQDSPHFQEK
Intensity
M/Z
0
50000
100000
150000
200000
250000
500
1000
1500
y1
y2
y3
y4
y5
y6
y8
b1
b2
b3
b4
b5
b6
b7
b8
type
b
y

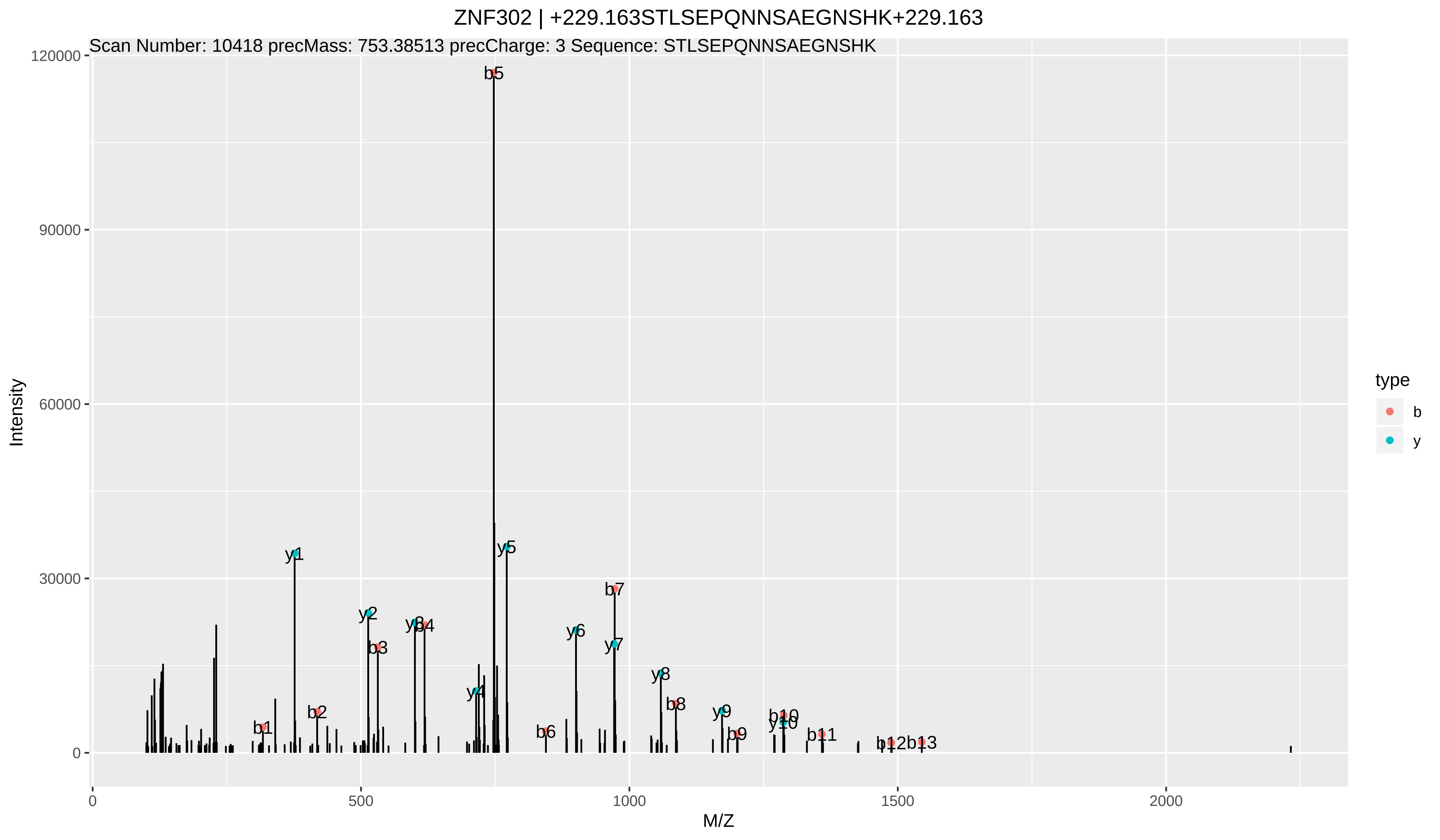

ZNF302 | +229.163STLSEPQNNSAEGNSHK+229.163
Scan Number: 10418 precMass: 753.38513 precCharge: 3 Sequence: STLSEPQNNSAEGNSHK
Intensity
M/Z
type
b
y
0
30000
60000
90000
120000
0
500
1000
1500
2000
b1
b2
b3
b4
b5
b6
b7
b8
b9
b10
b11
b12
b13
y1
y2
y3
y4
y5
y6
y7
y8
y9
y10

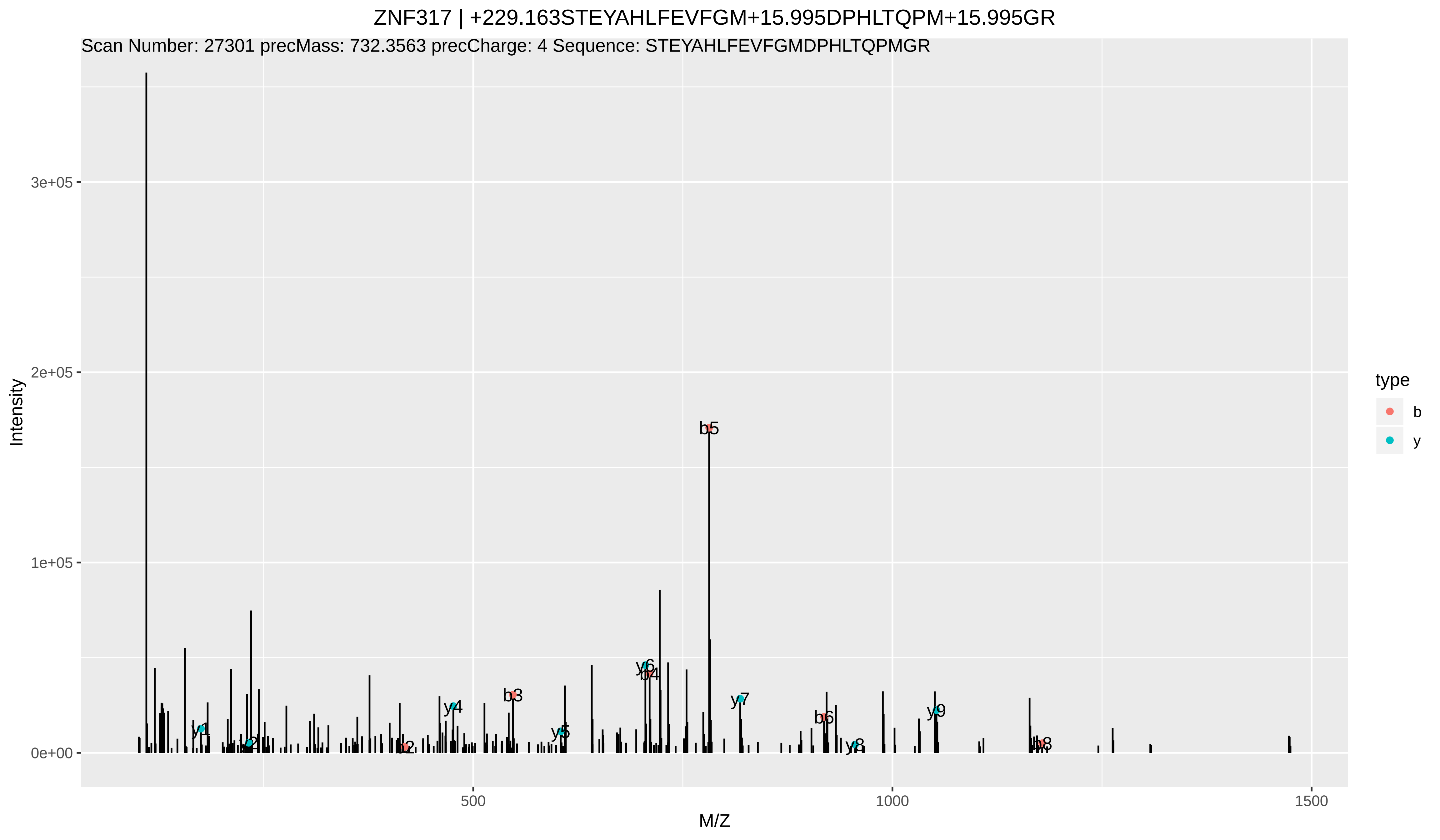

ZNF317 | +229.163STEYAHLFEVFGM+15.995DPHLTQPM+15.995GR
Scan Number: 27301 precMass: 732.3563 precCharge: 4 Sequence: STEYAHLFEVFGMDPHLTQPMGR
Intensity
3e+05
2e+05
1e+05
0e+00
M/Z
500
1000
1500
type
b
y
b5
y6
b4
b3
y7
y4
b6
y9
y1
y5
y2
b2
y8
b8

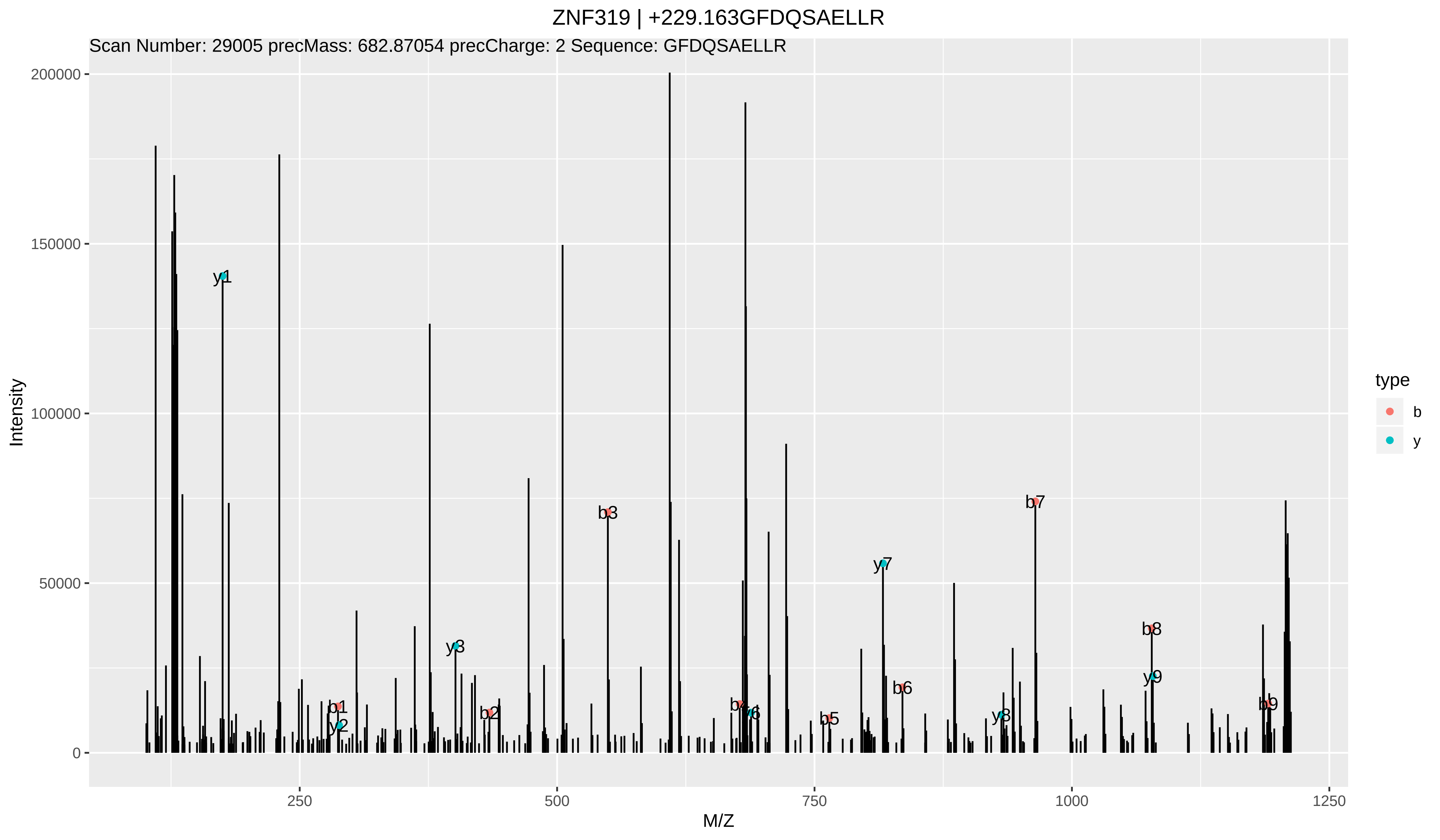

ZNF319 | +229.163GFDQSAELLR
Scan Number: 29005 precMass: 682.87054 precCharge: 2 Sequence: GFDQSAELLR
Intensity
M/Z
type
b
y
y1
y2
y3
y6
y7
y8
y9
b1
b2
b3
b4
b5
b6
b7
b8
b9
0
50000
100000
150000
200000
250
500
750
1000
1250

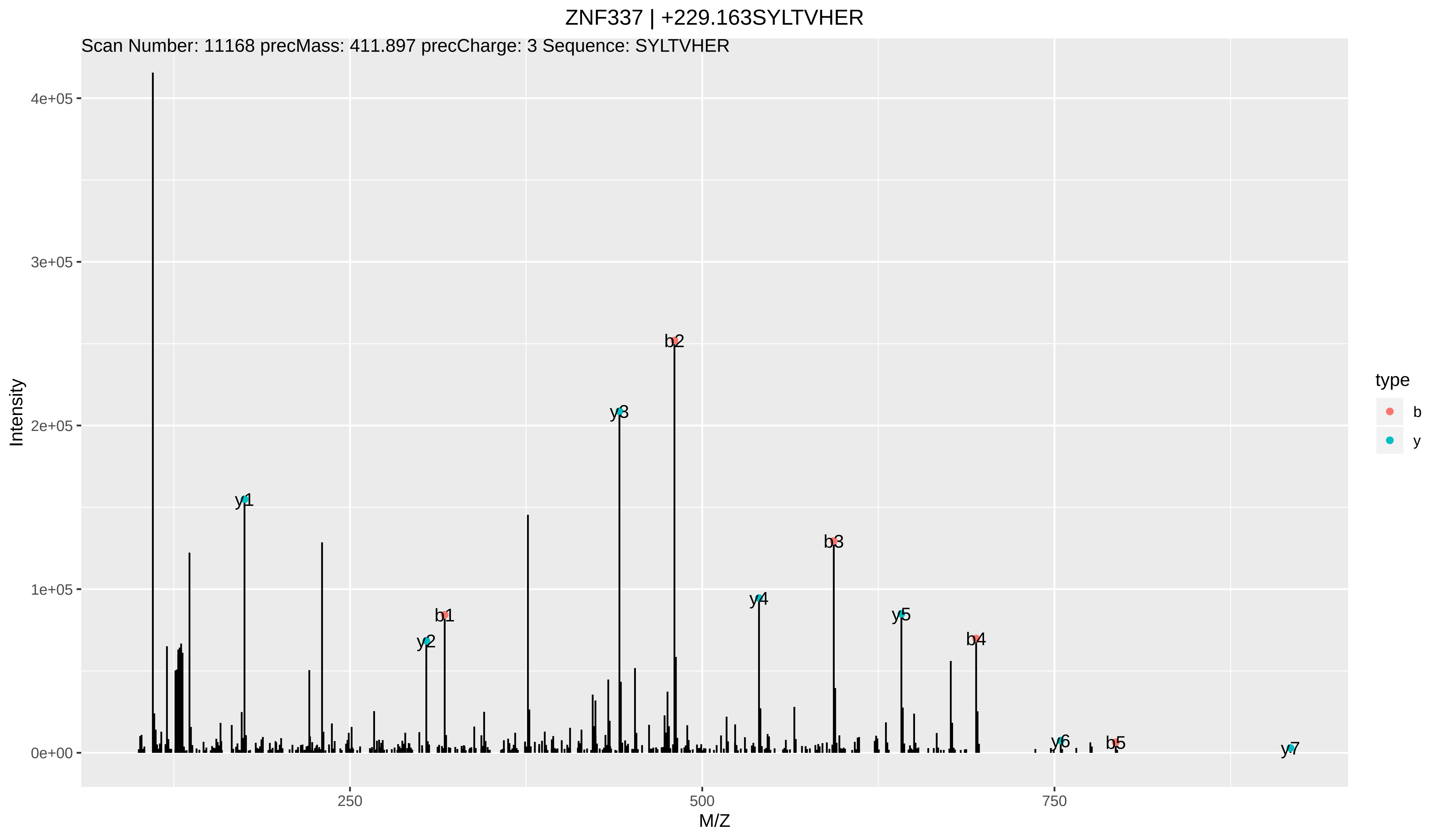
ZNF337 | +229.163SYLTVHER
Scan Number: 11168 precMass: 411.897 precCharge: 3 Sequence: SYLTVHER
Intensity
M/Z
4e+05
3e+05
2e+05
1e+05
0e+00
250
500
750
y1
y2
b1
y3
b2
y4
b3
y5
b4
y6
b5
y7
type
b
y

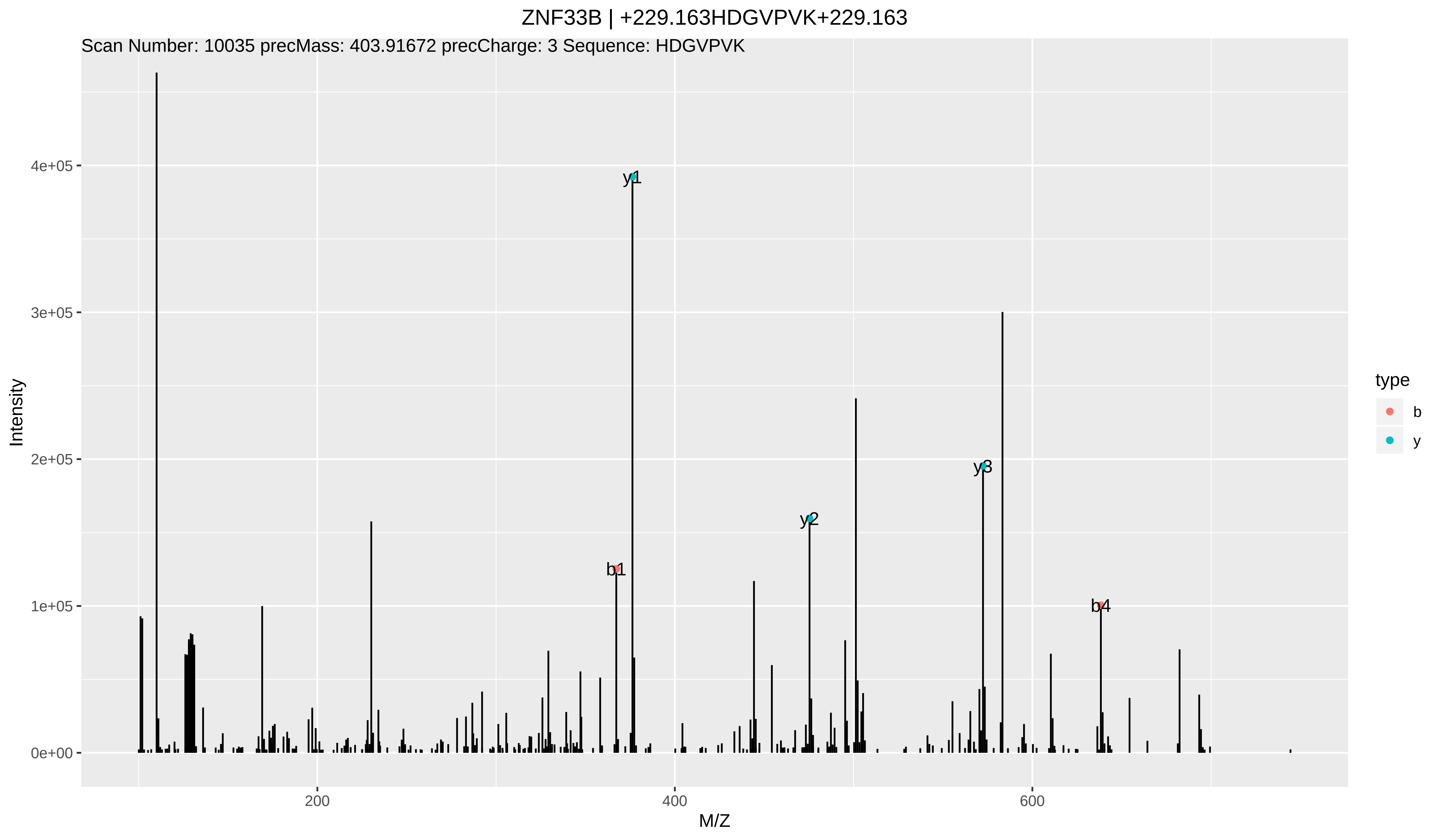

ZNF33B | +229.163HDGVPVK+229.163
Scan Number: 10035 precMass: 403.91672 precCharge: 3 Sequence: HDGVPVK
Intensity
4e+05
3e+05
2e+05
1e+05
0e+00
M/Z
200
400
600
y1
y2
y3
b1
b4
type
b
y

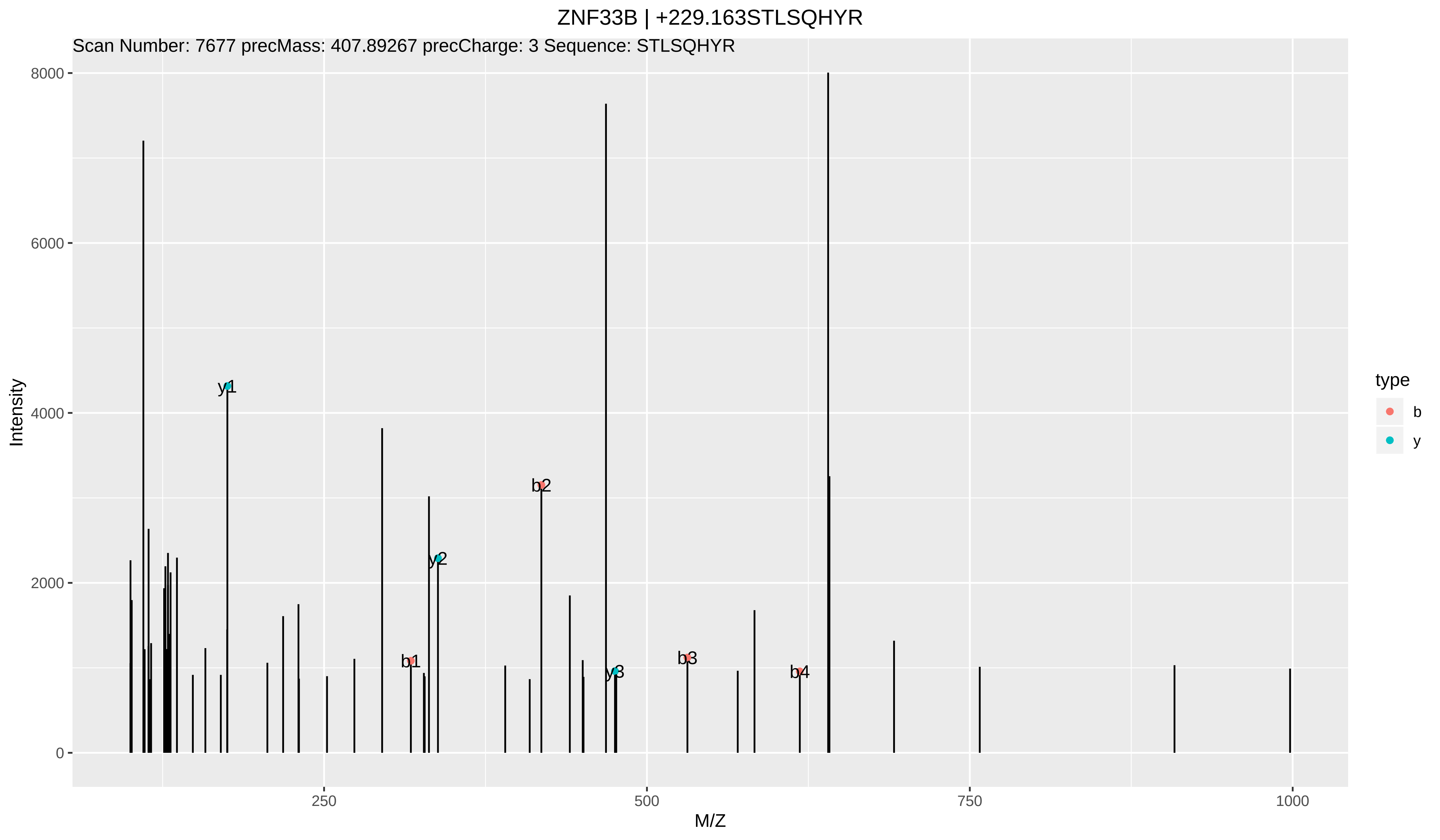

ZNF33B | +229.163STLSQHYR
Scan Number: 7677 precMass: 407.89267 precCharge: 3 Sequence: STLSQHYR
Intensity
M/Z
0
2000
4000
6000
8000
250
500
750
1000
y1
y2
y3
b1
b2
b3
b4
type
b
y

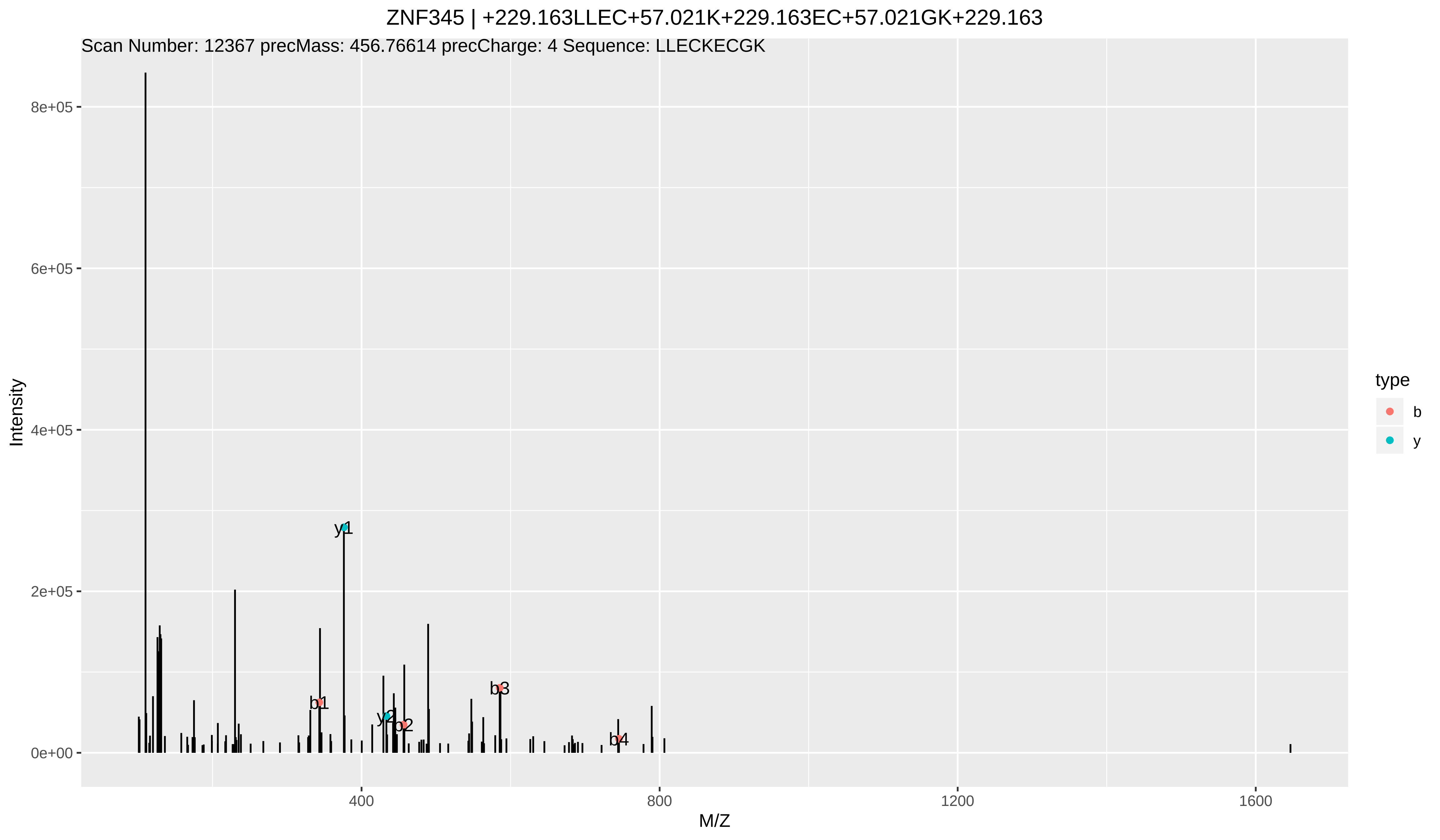

ZNF345 | +229.163LLEC+57.021K+229.163EC+57.021GK+229.163
Scan Number: 12367 precMass: 456.76614 precCharge: 4 Sequence: LLECKECGK
Intensity
8e+05
6e+05
4e+05
2e+05
0e+00
M/Z
400
800
1200
1600
y1
b1
y2
b2
b3
b4
type
b
y

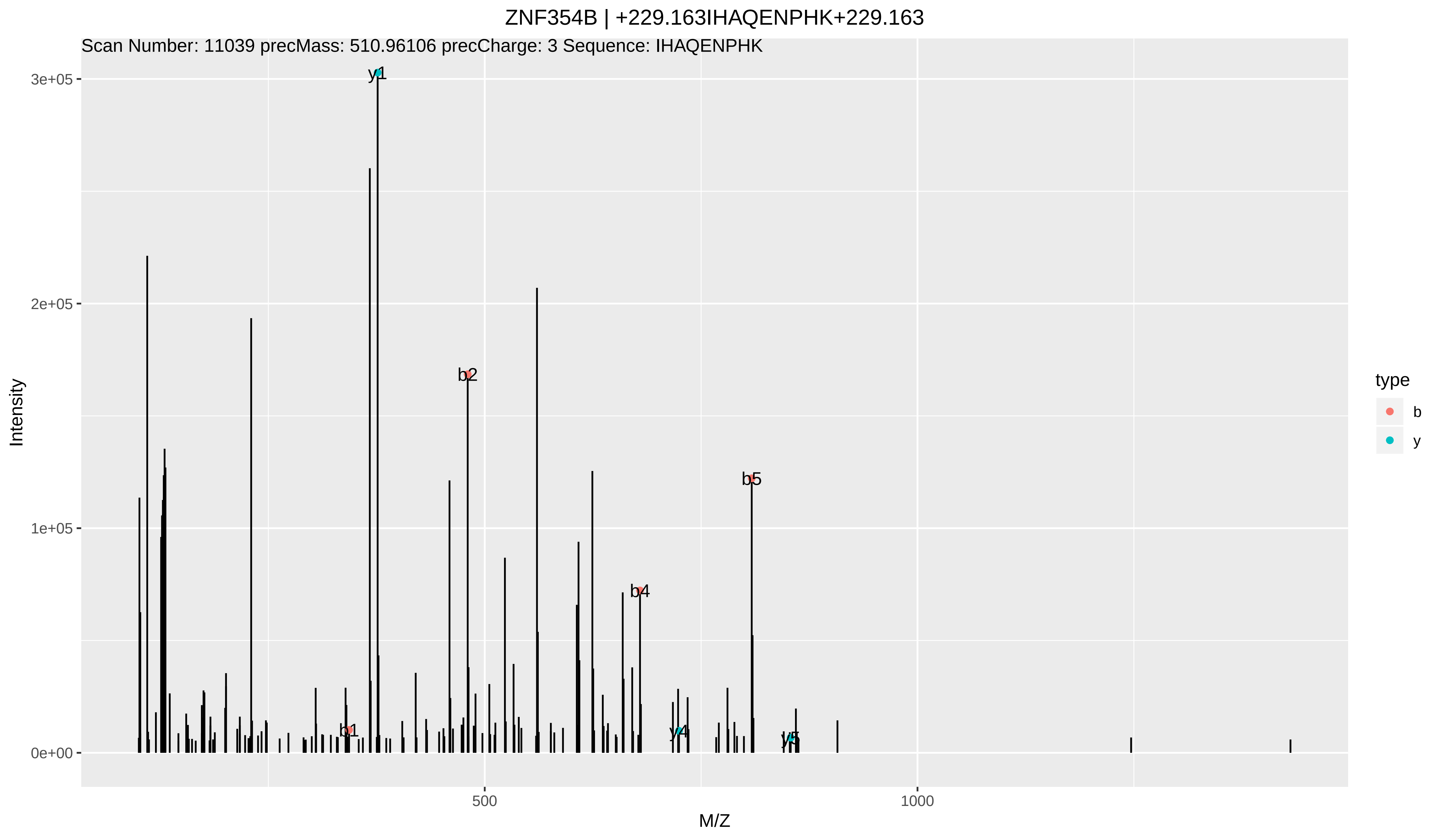
ZNF354B | +229.163IHAQENPHK+229.163
Scan Number: 11039 precMass: 510.96106 precCharge: 3 Sequence: IHAQENPHK
Intensity
M/Z
3e+05
2e+05
1e+05
0e+00
500
1000
y1
b2
b5
b4
b1
y4
y5
type
b
y

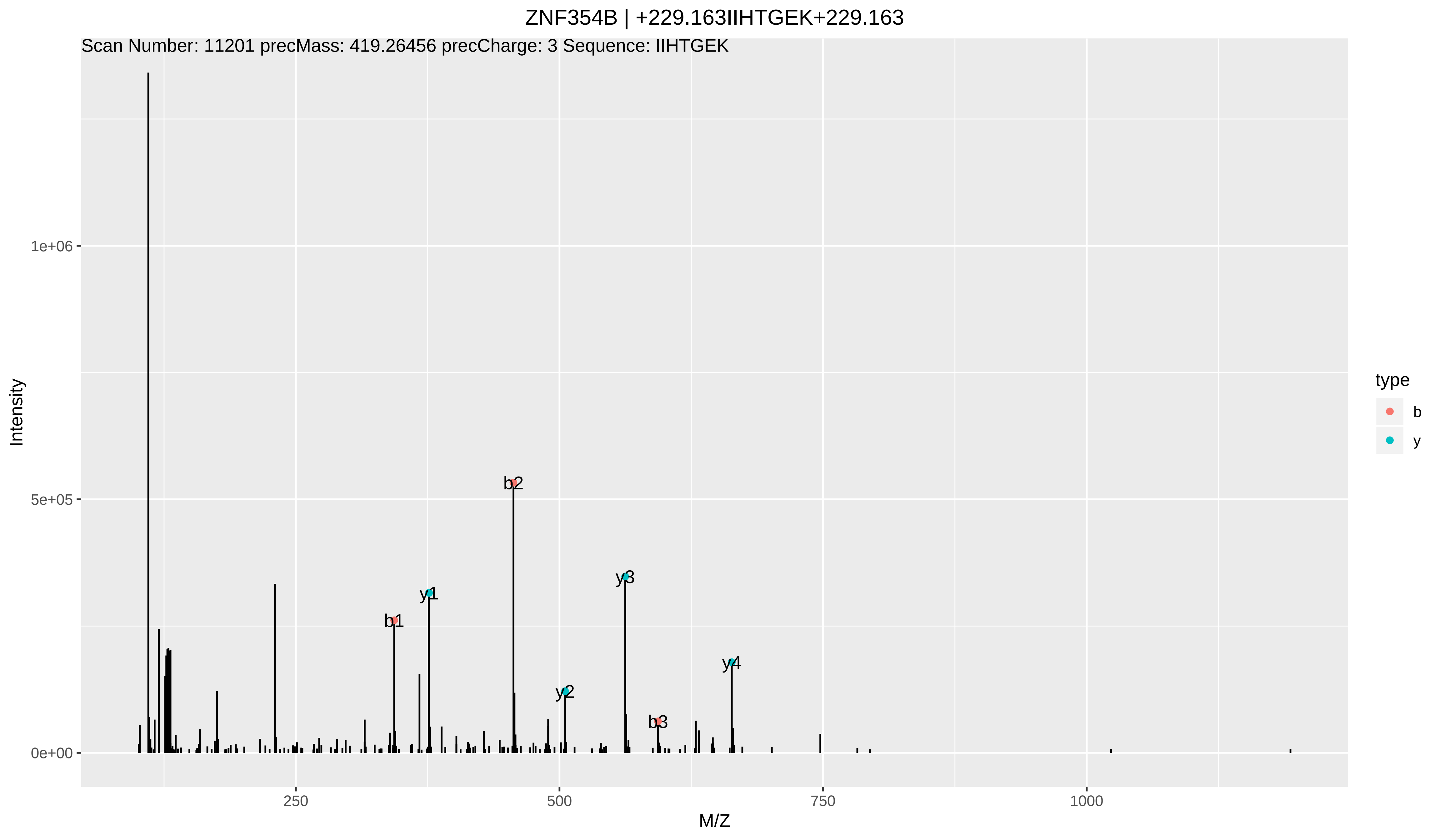

ZNF354B | +229.163IIHTGEK+229.163
Scan Number: 11201 precMass: 419.26456 precCharge: 3 Sequence: IIHTGEK
Intensity
1e+06
5e+05
0e+00
b1
y1
b2
y2
y3
b3
y4
250
500
750
1000
M/Z
type
b
y

ZNF362 | +229.163LIASSPTLISGITSPPLLDSIK+229.163

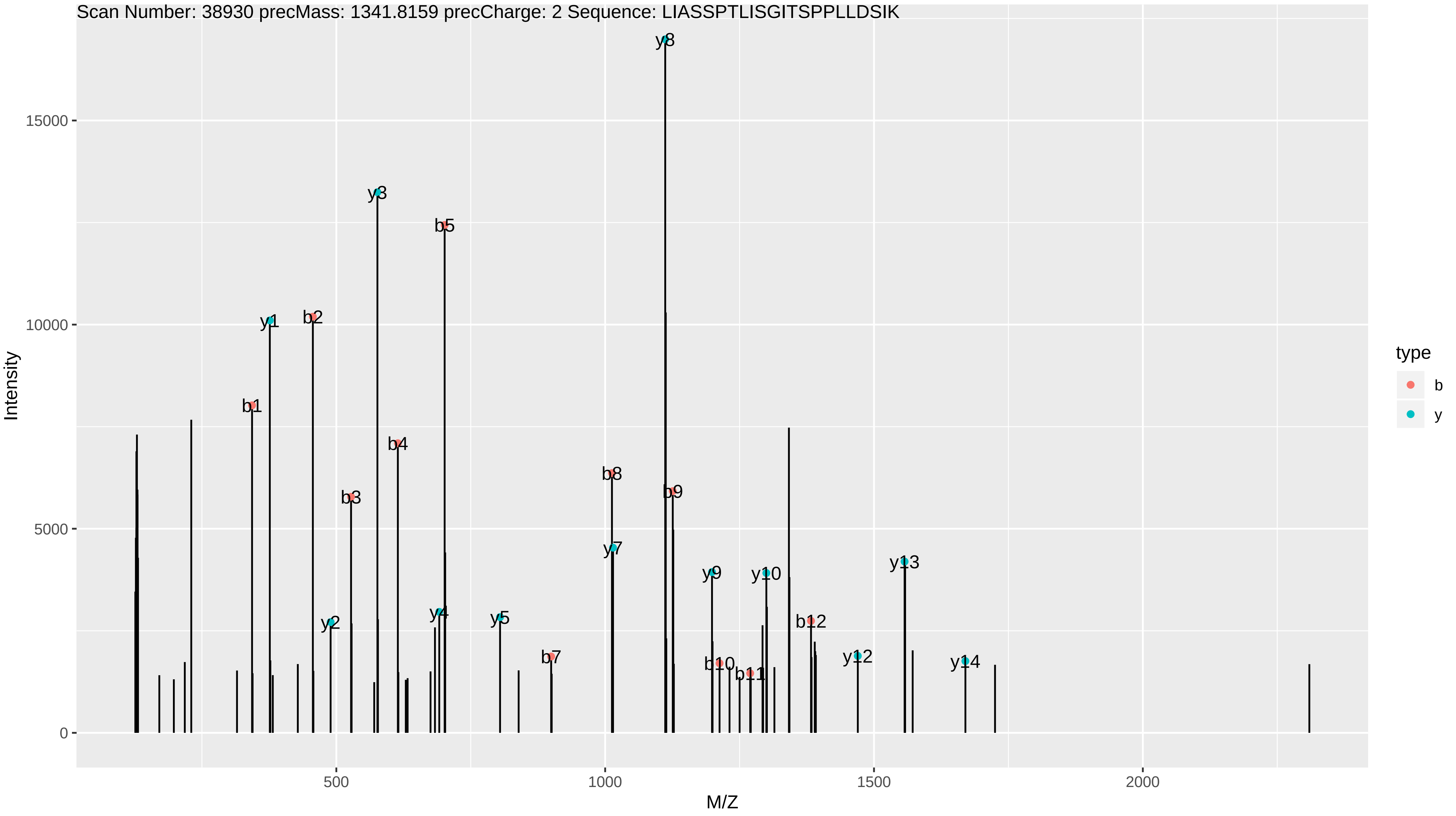

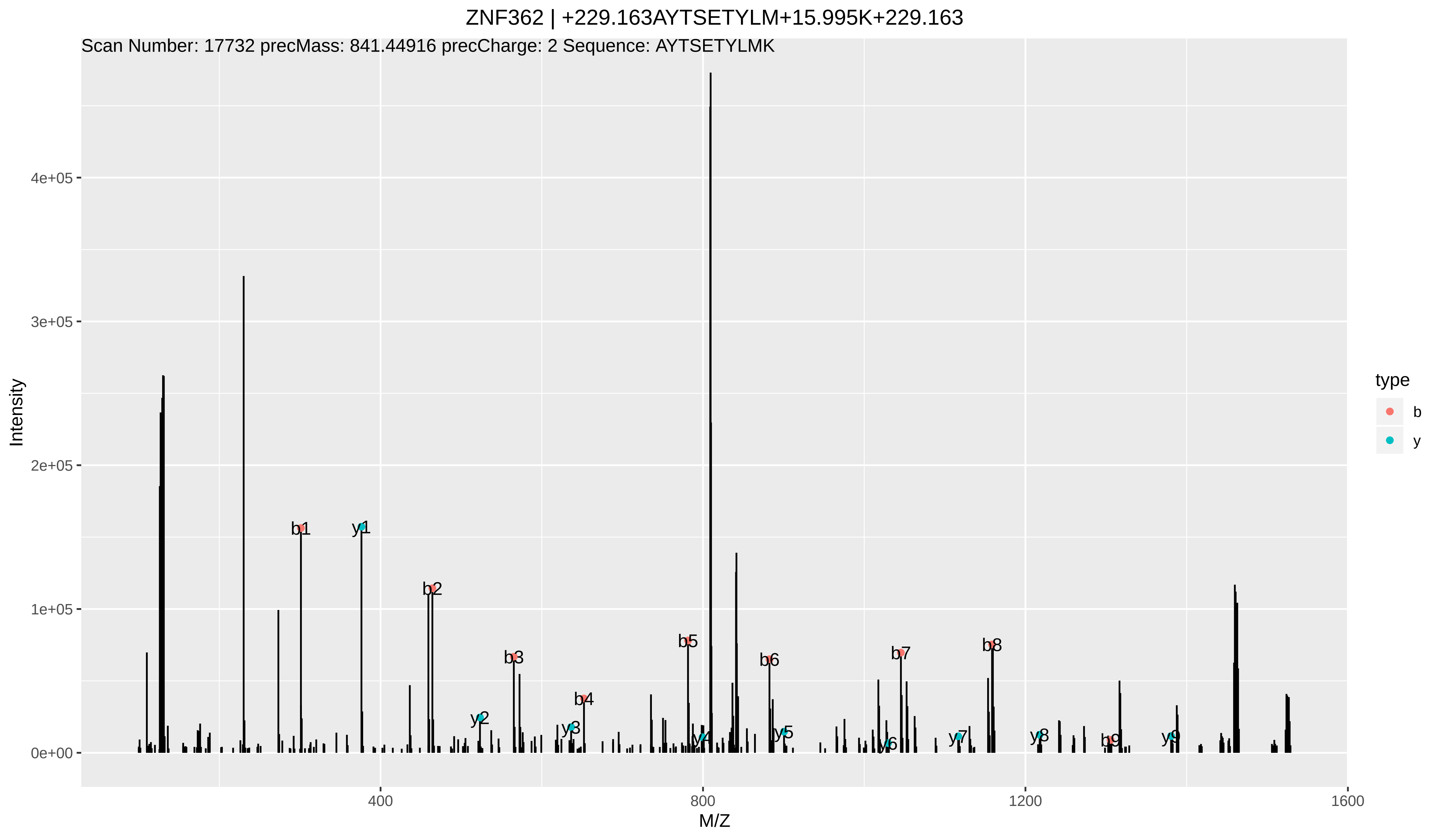
ZNF362 | +229.163AYTSETYLM+15.995K+229.163
Scan Number: 17732 precMass: 841.44916 precCharge: 2 Sequence: AYTSETYLMK
Intensity
M/Z
type
b
y
0e+00
1e+05
2e+05
3e+05
4e+05
400
800
1200
1600
b1
y1
b2
b3
y2
b4
y3
b5
y4
b6
y5
b7
y6
b8
y7
y8
b9
y9

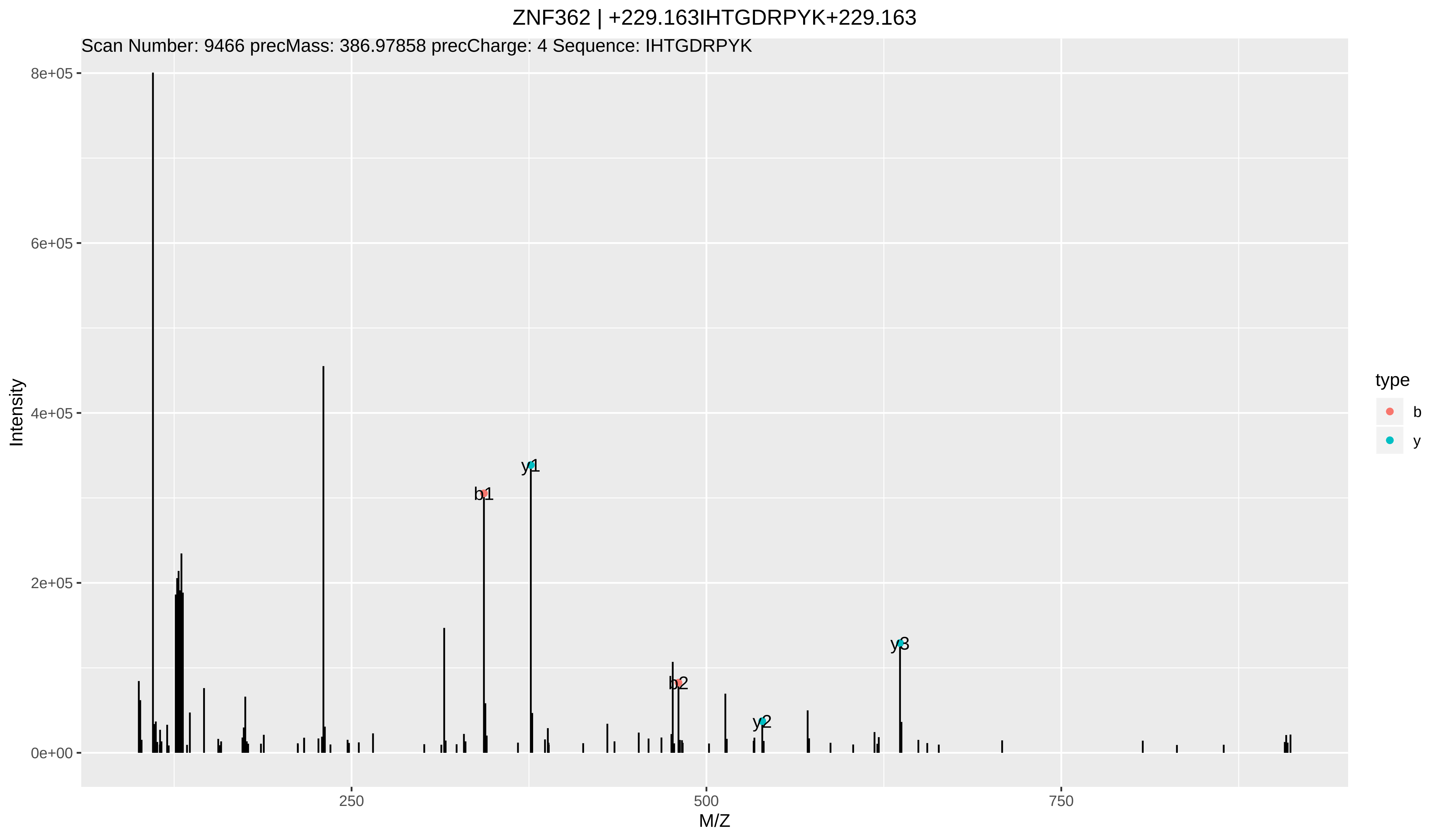
ZNF362 | +229.163IHTGDRPYK+229.163
Scan Number: 9466 precMass: 386.97858 precCharge: 4 Sequence: IHTGDRPYK
Intensity
8e+05
6e+05
4e+05
2e+05
0e+00
M/Z
250
500
750
y1
b1
b2
y2
y3
type
b
y

ZNF394 | +229.163QLHEERPYK+229.163

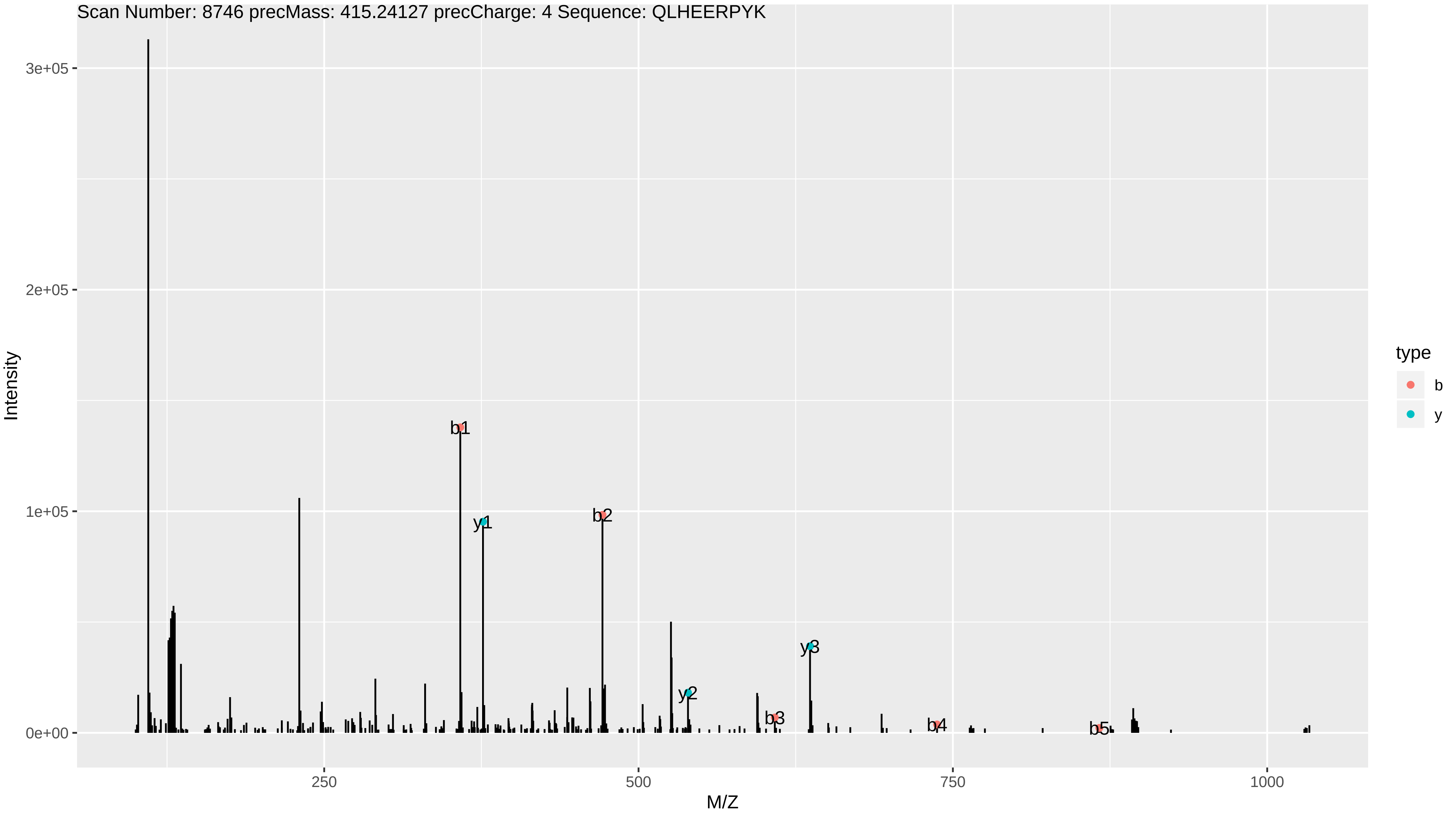

ZNF425 | +229.163VSITAYDPDK+229.163

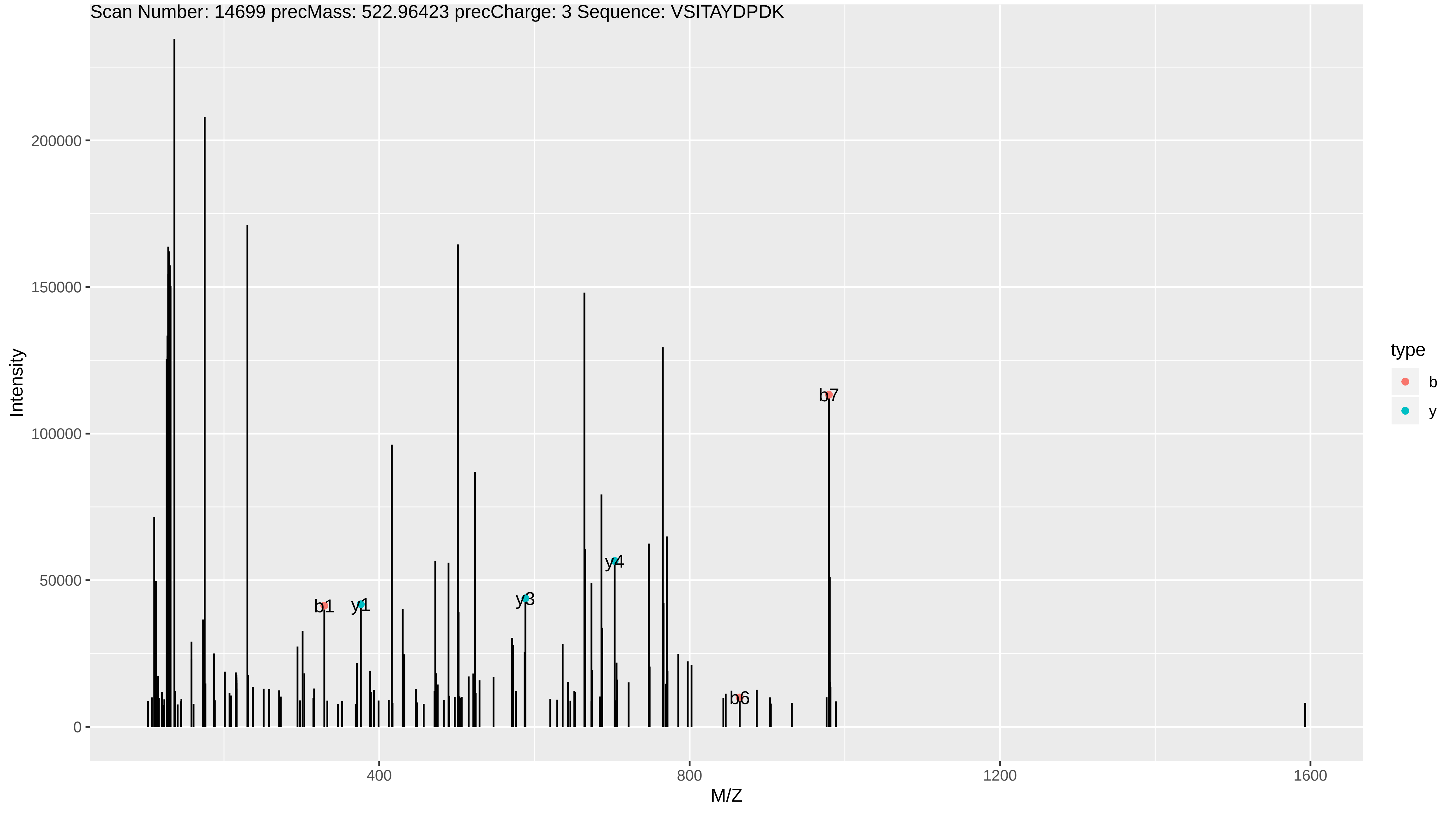

ZNF440 | +229.163YVNNLQSHER

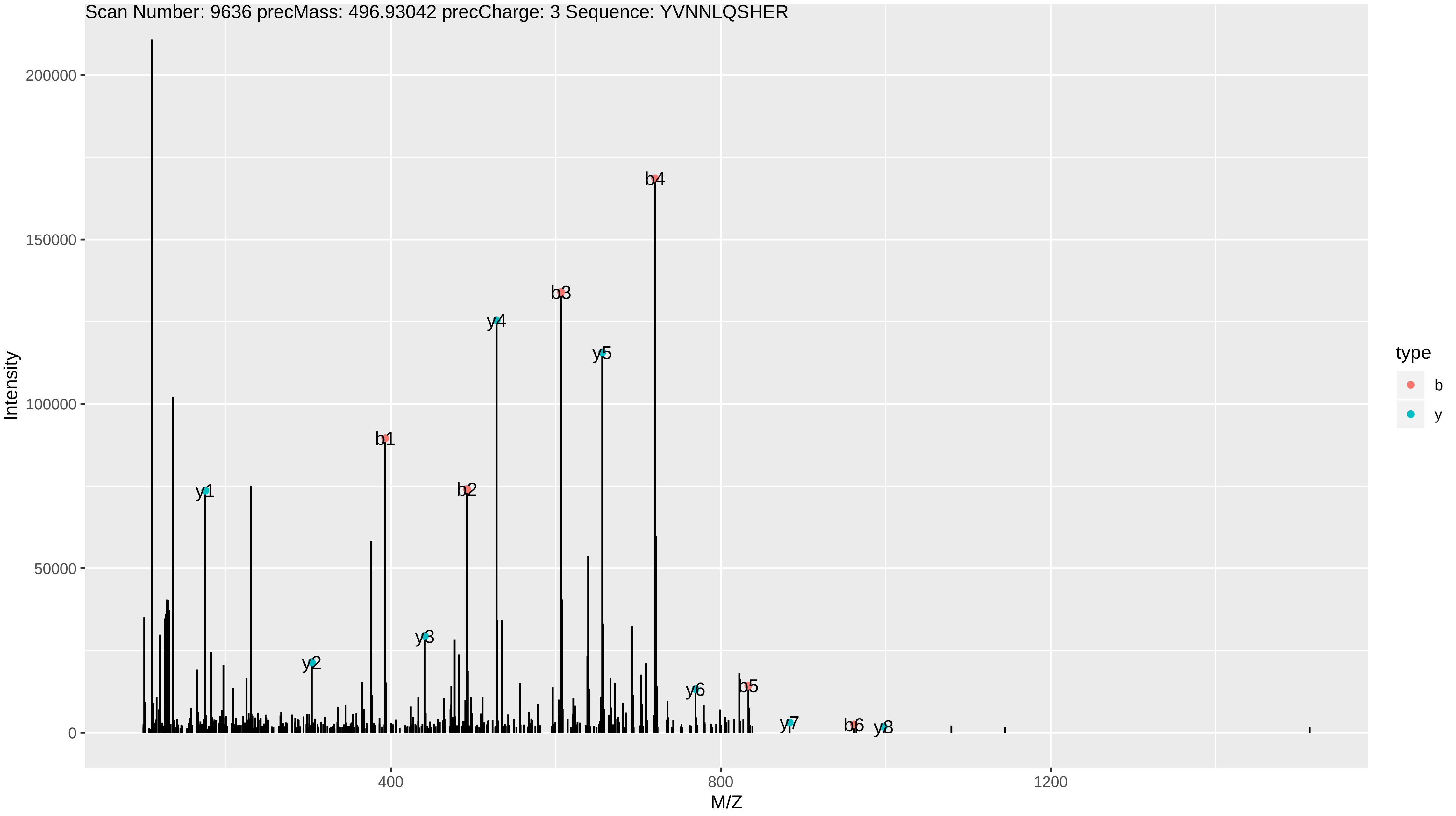

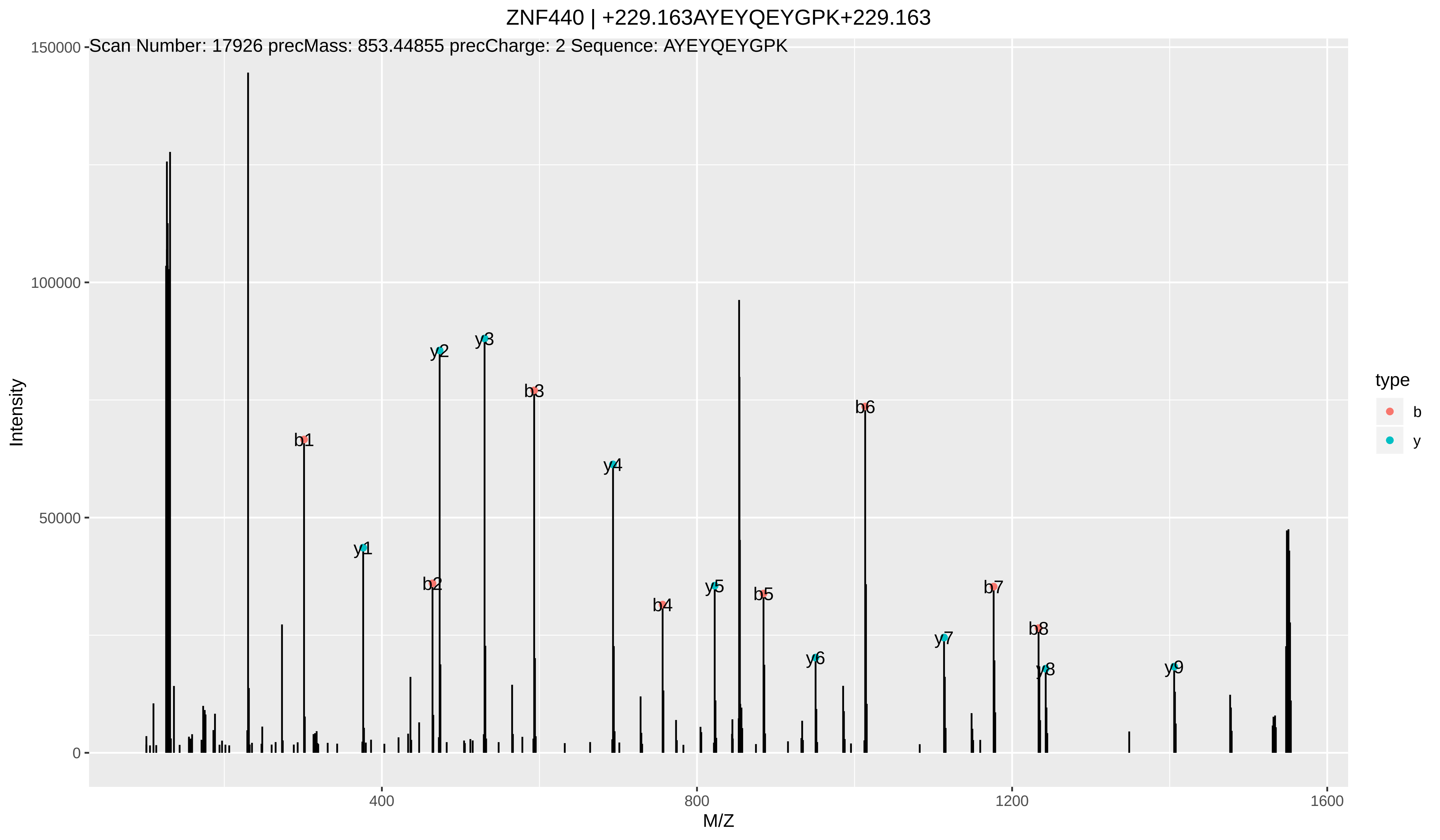
ZNF440 | +229.163AYEYQEYGPK+229.163
Scan Number: 17926 precMass: 853.44855 precCharge: 2 Sequence: AYEYQEYGPK
Intensity
M/Z
150000
100000
50000
0
400
800
1200
1600
b1
y1
b2
y2
y3
b3
y4
b4
y5
b5
y6
b6
y7
b7
b8
y8
y9
type
b
y

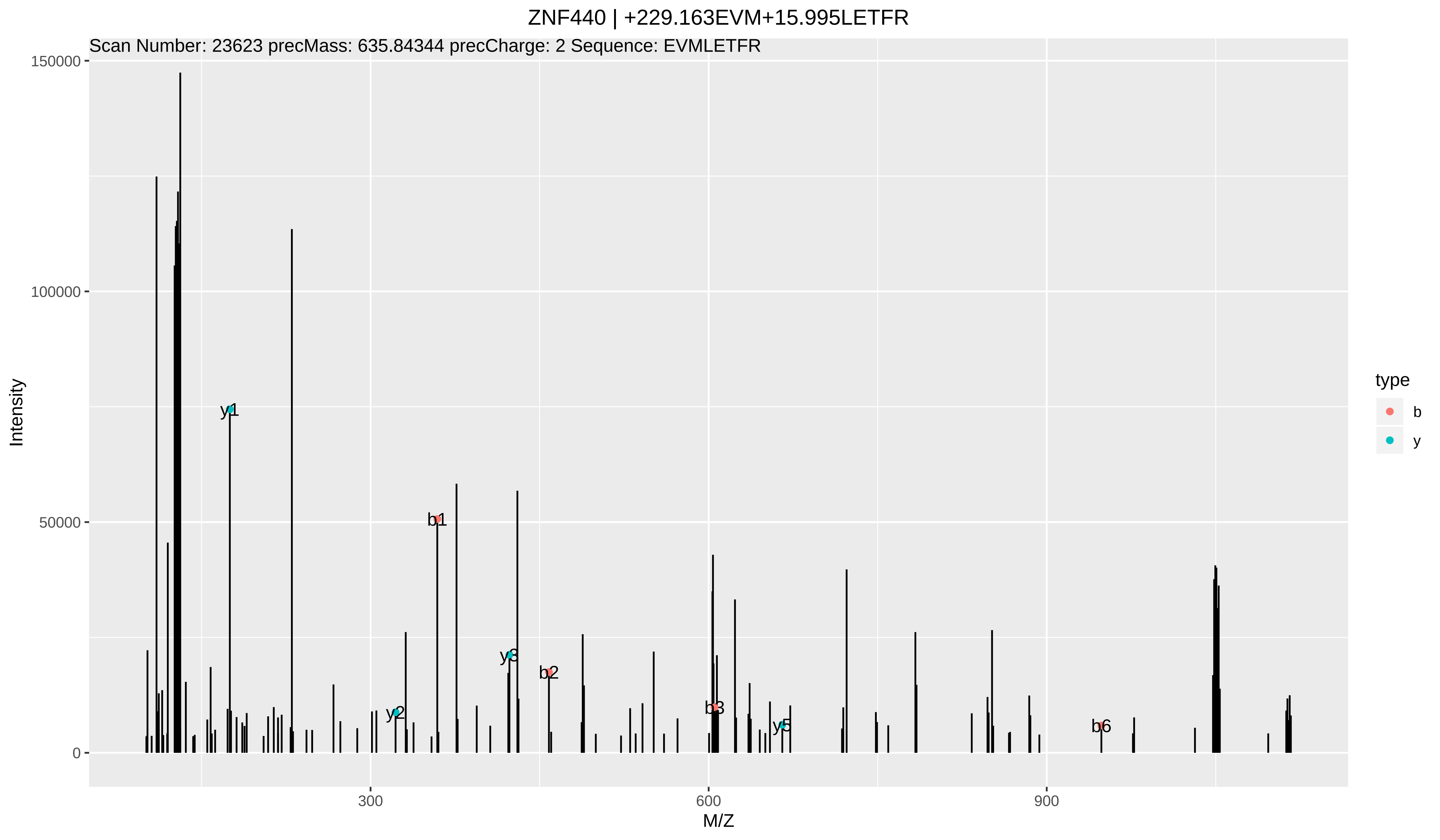

ZNF440 | +229.163EVM+15.995LETFR
Scan Number: 23623 precMass: 635.84344 precCharge: 2 Sequence: EVMLETFR
Intensity
M/Z
150000
100000
50000
0
300
600
900
y1
b1
y3
b2
y2
b3
y5
b6
type
b
y

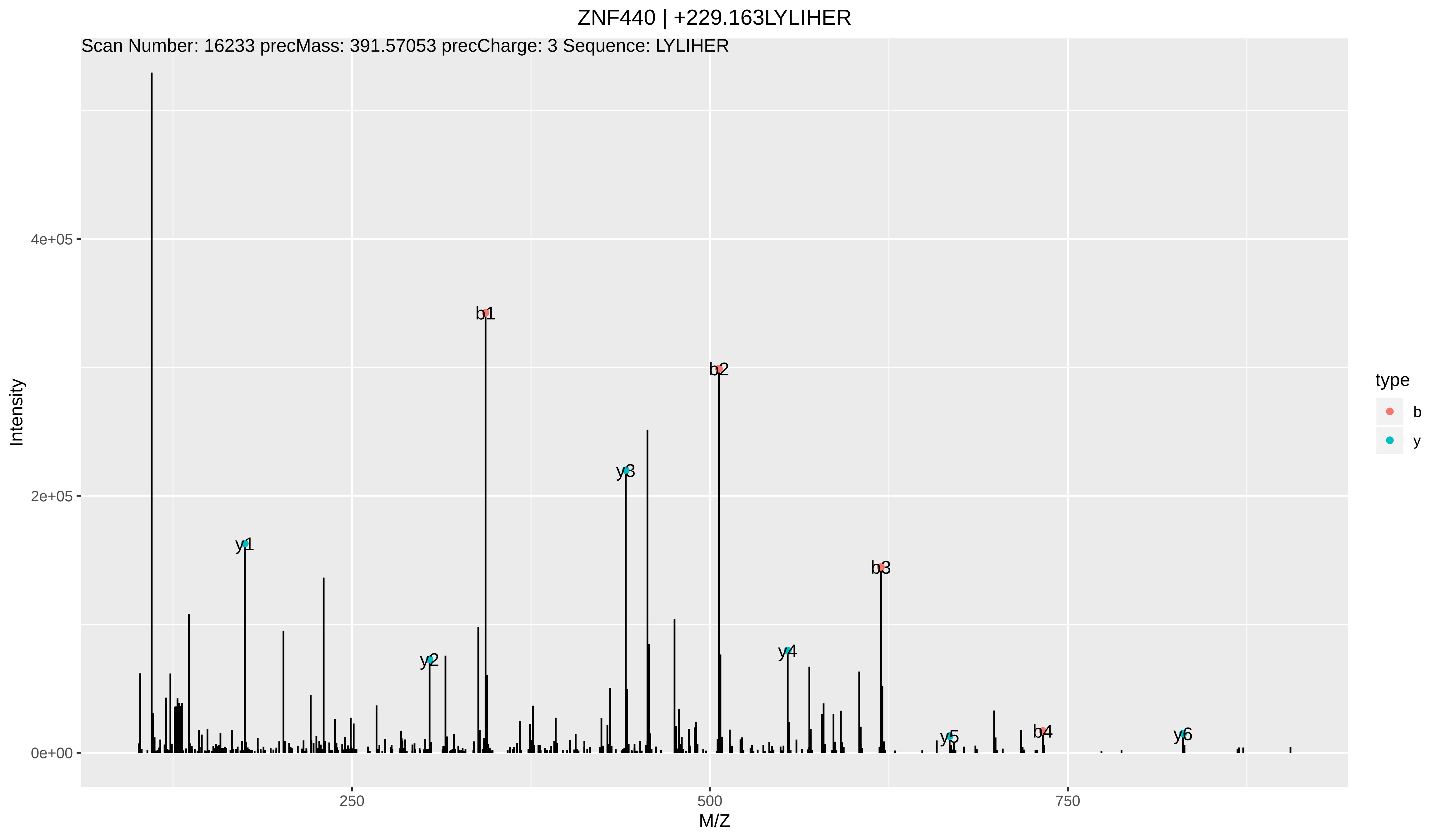

ZNF440 | +229.163LYLIHER
Scan Number: 16233 precMass: 391.57053 precCharge: 3 Sequence: LYLIHER
Intensity
4e+05
2e+05
0e+00
M/Z
250
500
750
b1
b2
b3
b4
y1
y2
y3
y4
y5
y6
type
b
y

ZNF449 | +229.163EAAGPHEAFNK+229.163

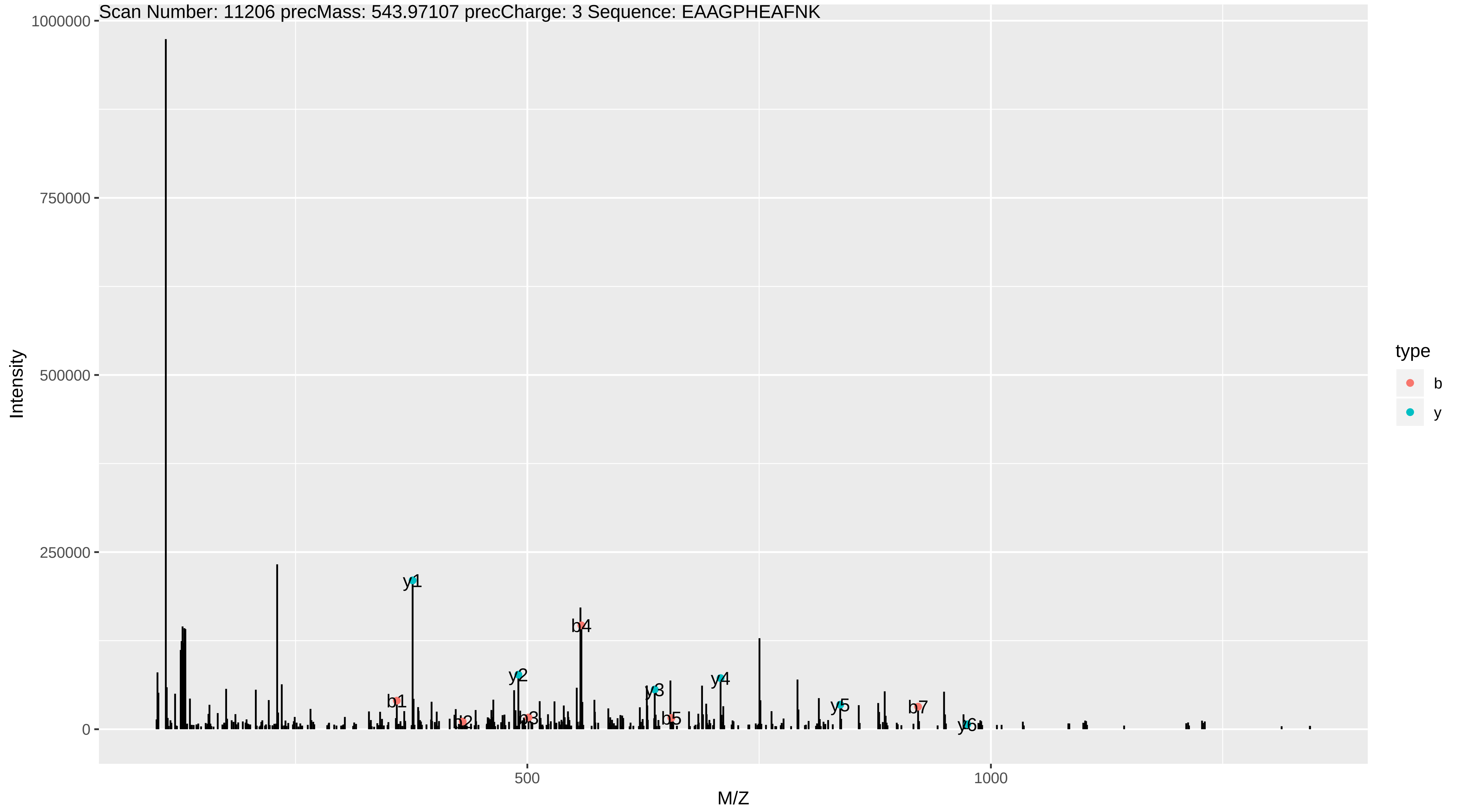

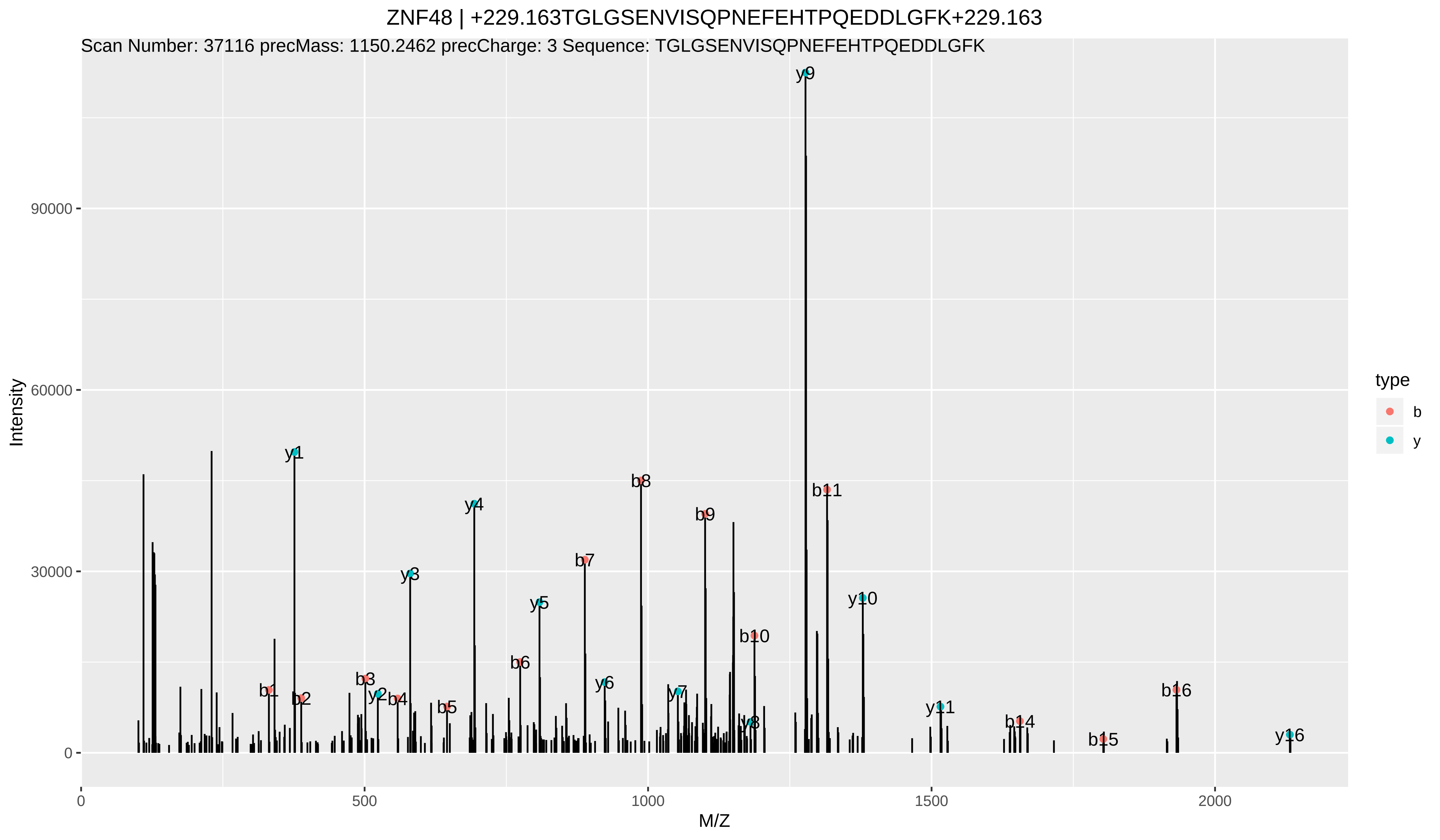
ZNF48 | +229.163TGLGSENVISQPNEFEHTPQEDDLGFK+229.163
Scan Number: 37116 precMass: 1150.2462 precCharge: 3 Sequence: TGLGSENVISQPNEFEHTPQEDDLGFK
Intensity
M/Z
type
b
y
y9
y1
b8
b11
y4
b9
b7
y3
y10
y5
b10
b6
b3
y6
b1
y2
b2
b4
y7
b5
y11
b16
b14
y8
b15
y16
90000
60000
30000
0
0
500
1000
1500
2000

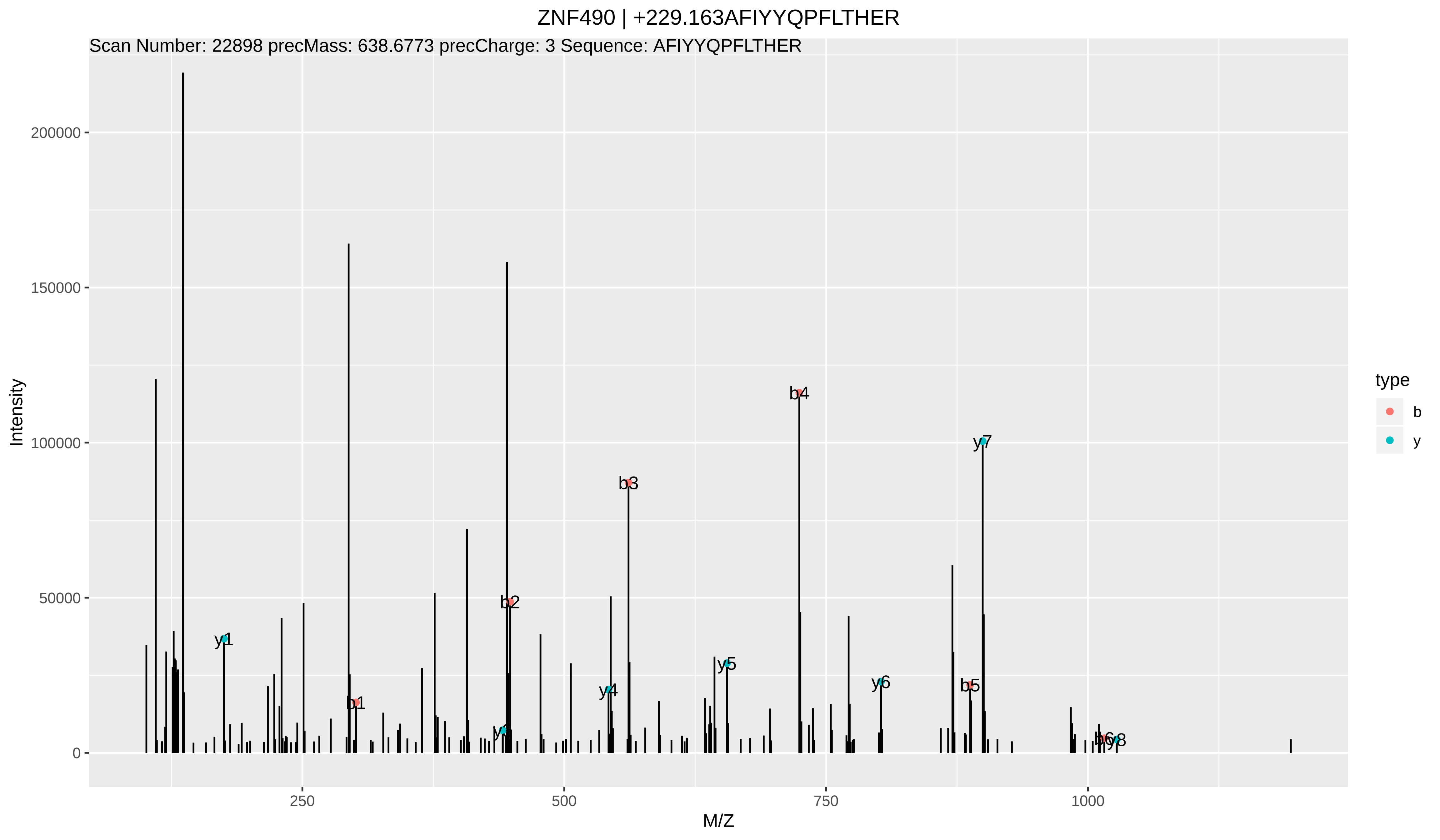

ZNF490 | +229.163AFIYYQPFLTHER
Scan Number: 22898 precMass: 638.6773 precCharge: 3 Sequence: AFIYYQPFLTHER
Intensity
200000
150000
100000
50000
0
M/Z
250
500
750
1000
y1
b1
y3
b2
y4
b3
y5
b4
y6
b5
y7
b6
y8
type
b
y

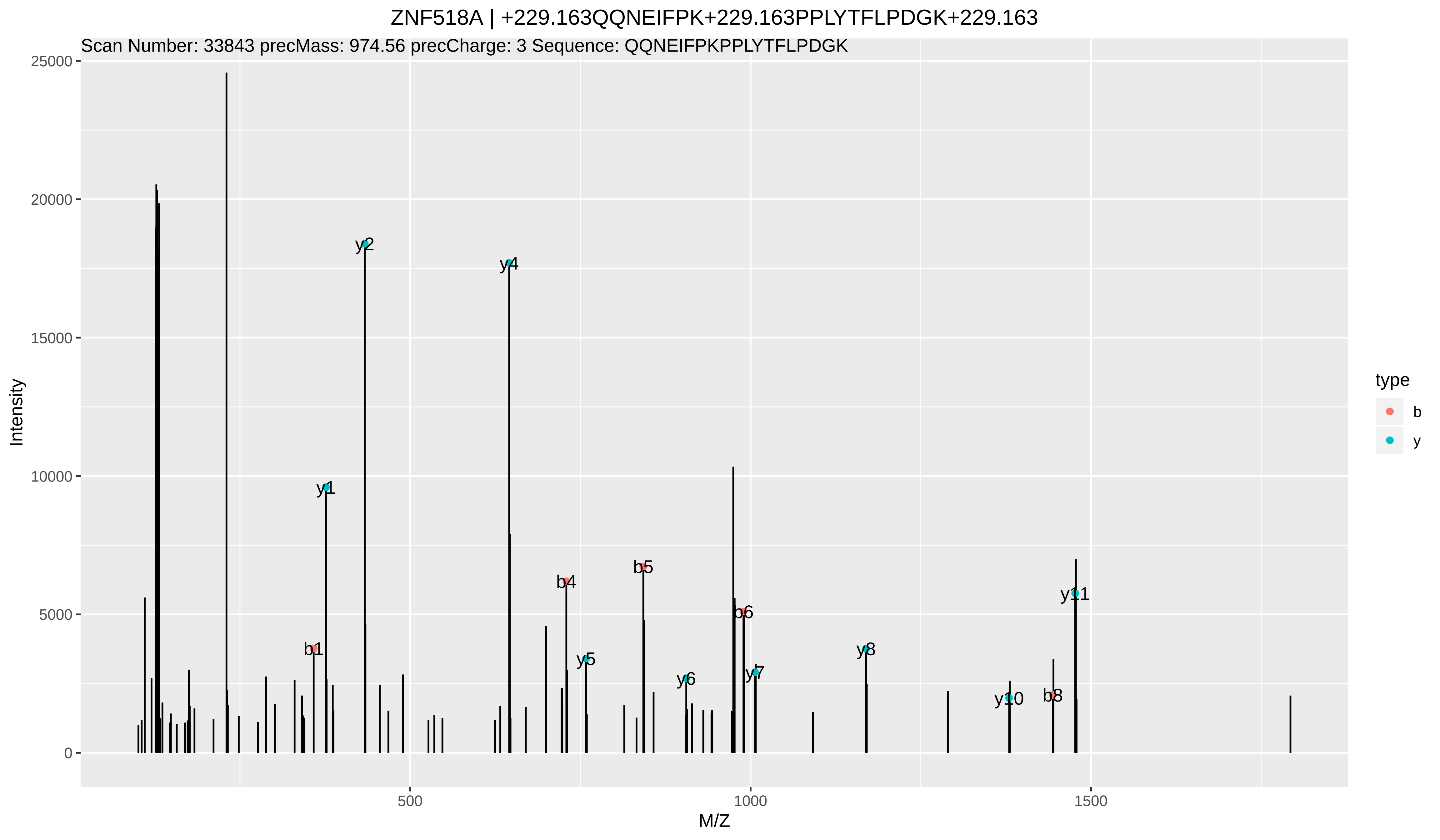

ZNF518A | +229.163QQNEIFPK+229.163PPLYTFLPDGK+229.163
Scan Number: 33843 precMass: 974.56 precCharge: 3 Sequence: QQNEIFPKPPLYTFLPDGK
Intensity
M/Z
25000
20000
15000
10000
5000
0
500
1000
1500
y2
y4
y1
b4
b5
b6
y11
b1
y5
y6
y7
y8
y10
b8
type
b
y

ZNF524 | +229.163AFPYLSDLER

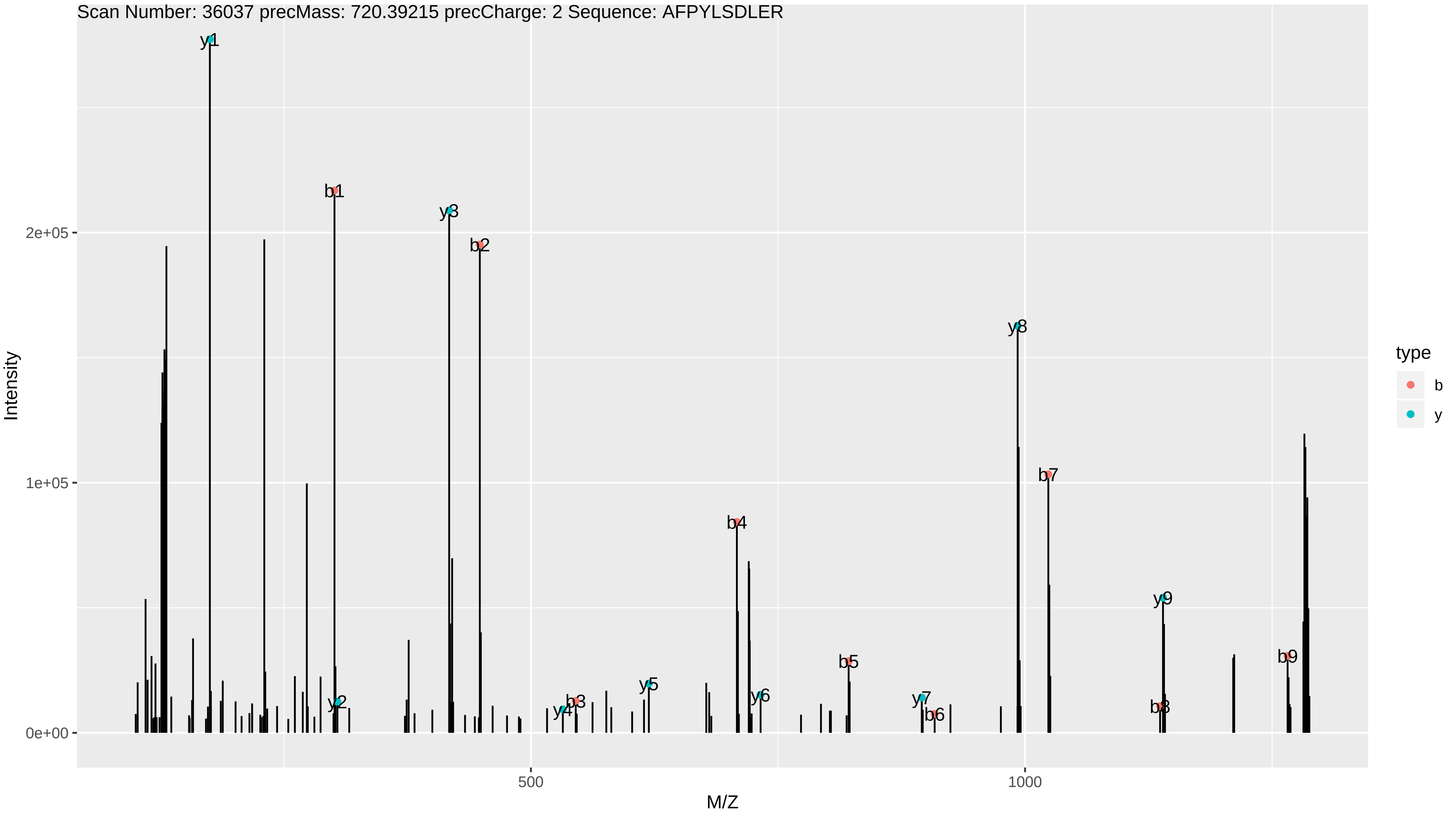

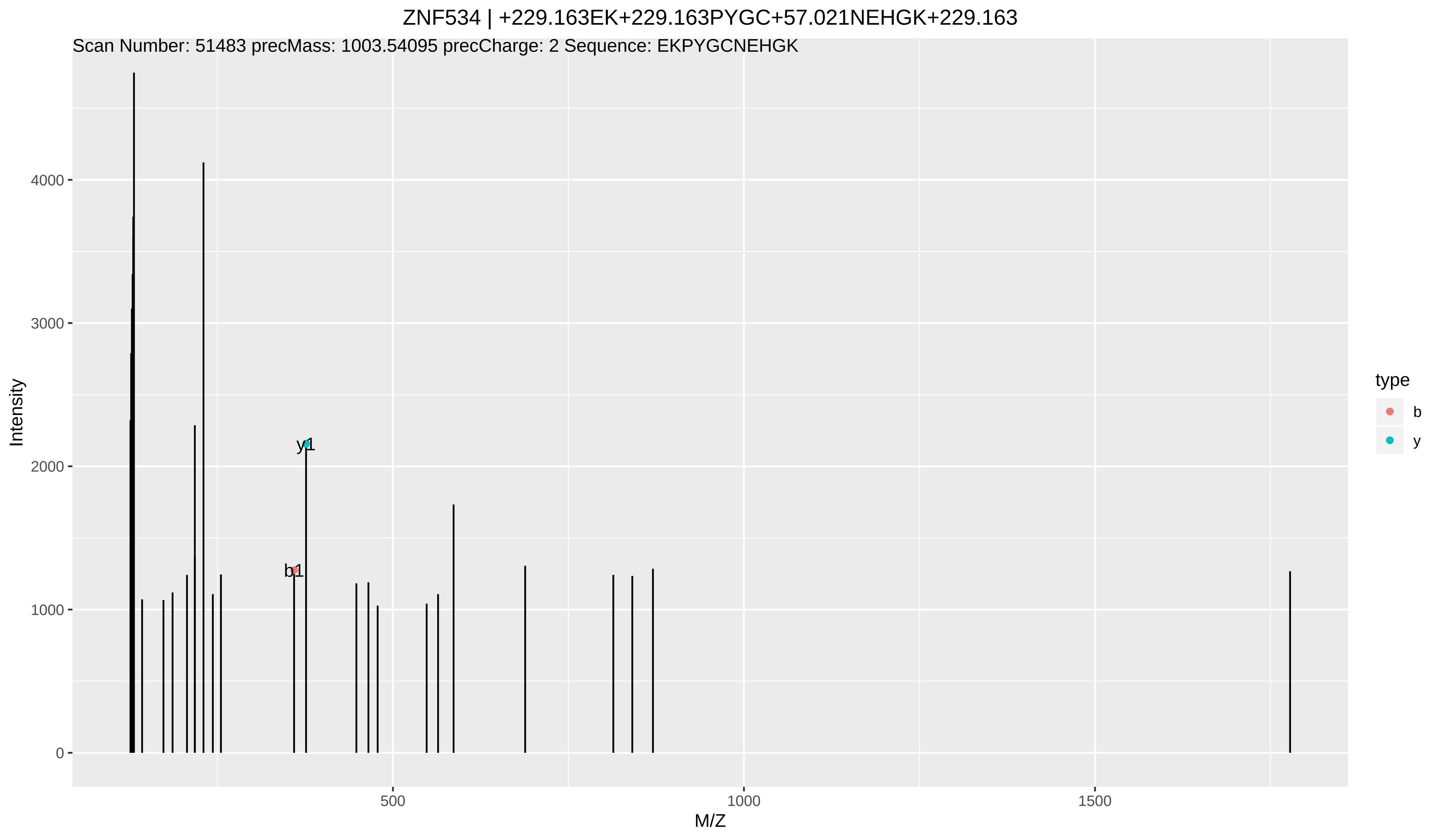
ZNF534 | +229.163EK+229.163PYGC+57.021NEHGK+229.163
Scan Number: 51483 precMass: 1003.54095 precCharge: 2 Sequence: EKPYGCNEHGK
Intensity
M/Z
0
1000
2000
3000
4000
500
1000
1500
y1
b1
type
b
y

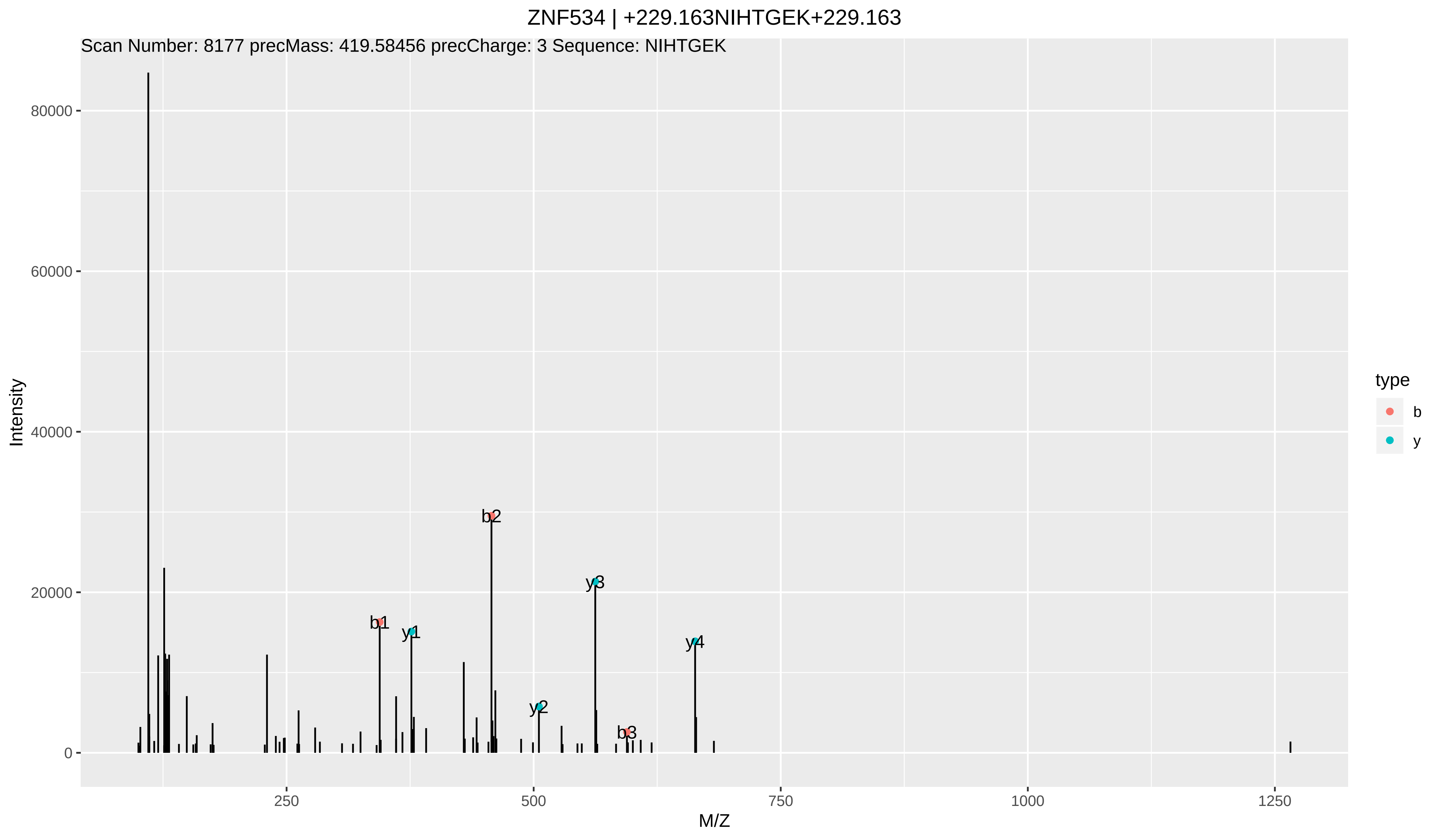
ZNF534 | +229.163NIHTGEK+229.163
Scan Number: 8177 precMass: 419.58456 precCharge: 3 Sequence: NIHTGEK
Intensity
0
20000
40000
60000
80000
M/Z
250
500
750
1000
1250
b1
y1
b2
y2
y3
b3
y4
type
b
y

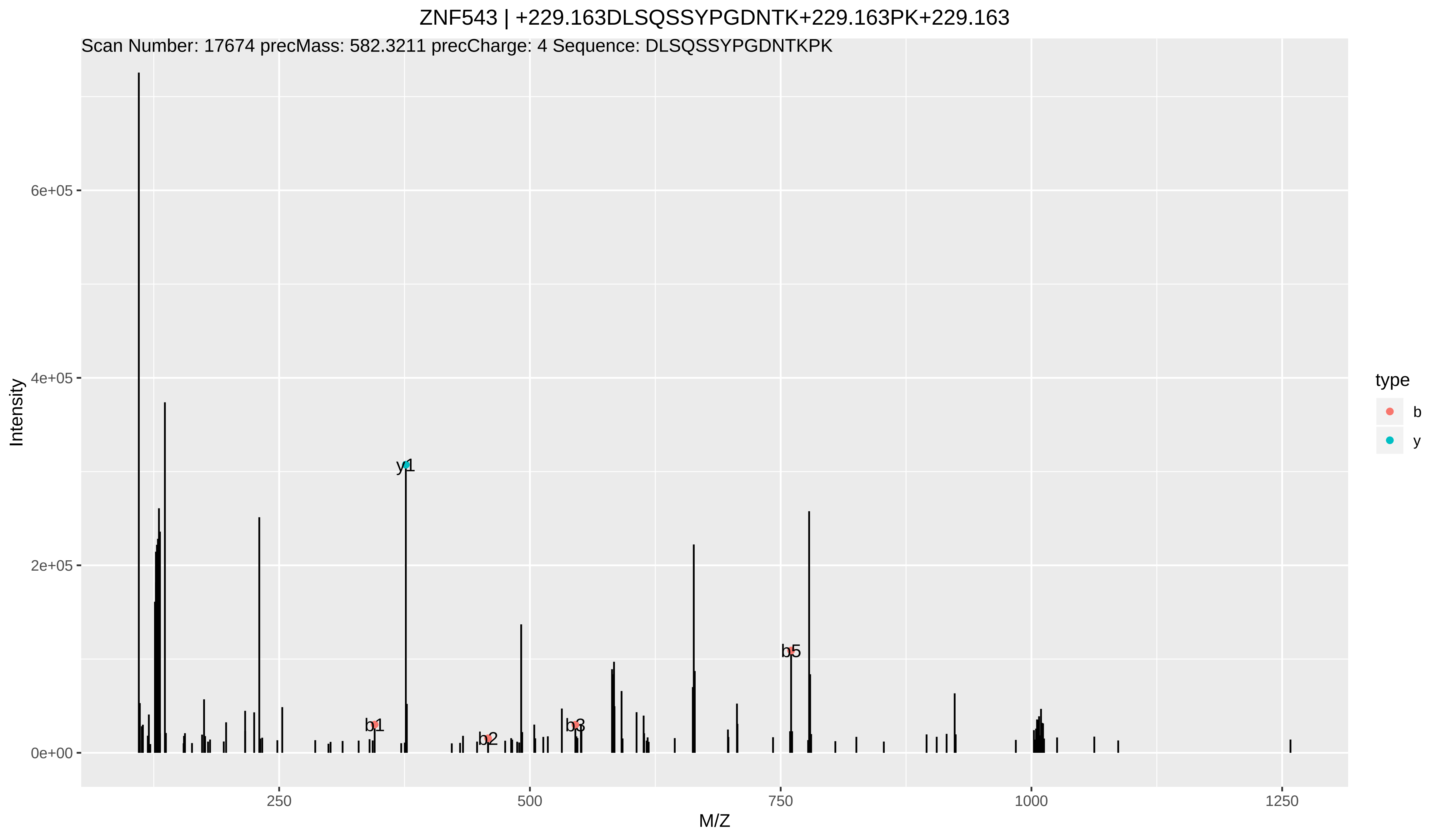
ZNF543 | +229.163DLSQSSYPGDNTK+229.163PK+229.163
Scan Number: 17674 precMass: 582.3211 precCharge: 4 Sequence: DLSQSSYPGDNTKPK
Intensity
6e+05
4e+05
2e+05
0e+00
M/Z
250
500
750
1000
1250
y1
b1
b2
b3
b5
type
b
y

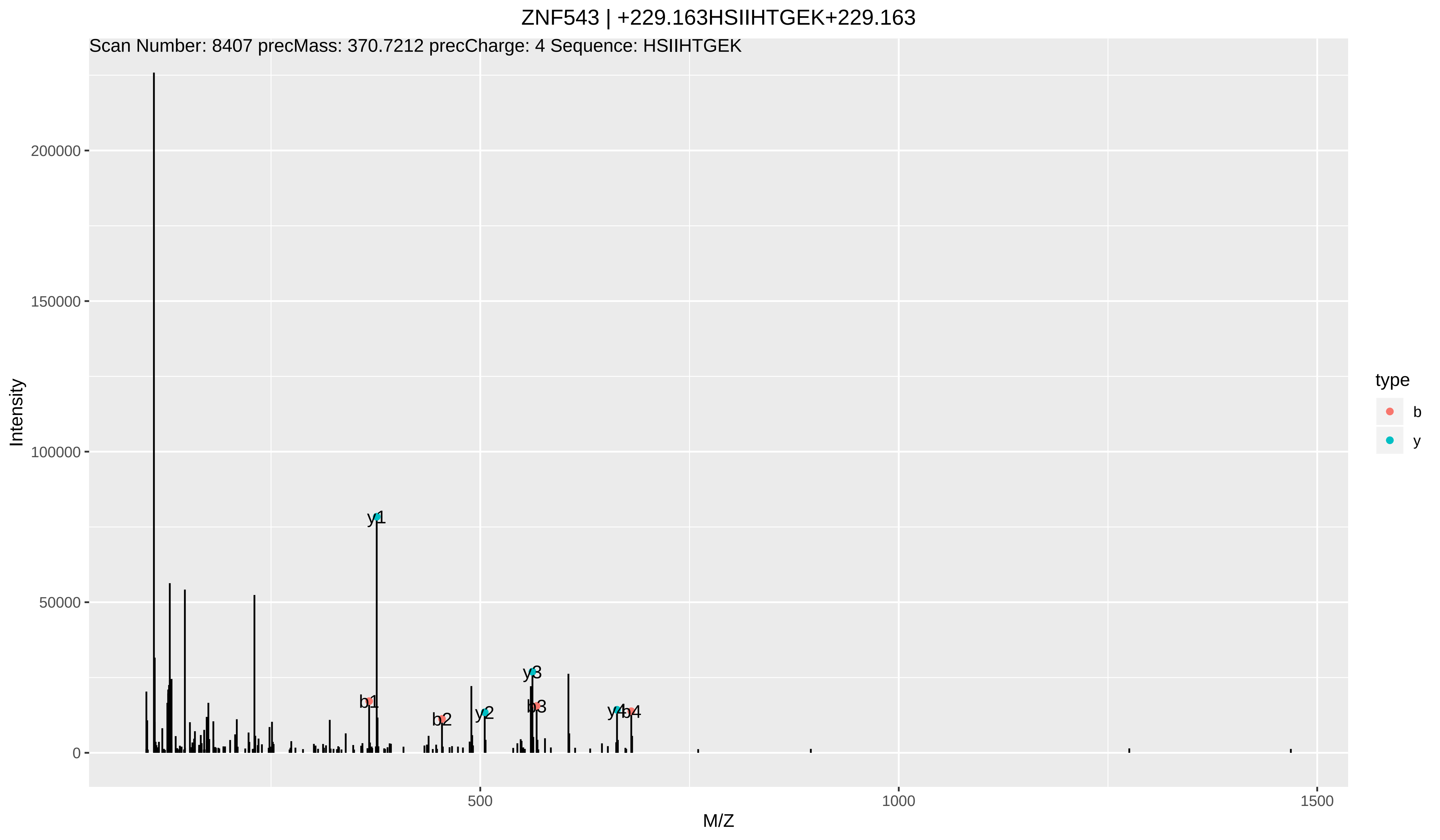

ZNF543 | +229.163HSIIHTGEK+229.163
Scan Number: 8407 precMass: 370.7212 precCharge: 4 Sequence: HSIIHTGEK
Intensity
M/Z
y1
b1
b2
y2
y3
b3
y4
b4
type
b
y

ZNF543 | +229.163HFSIHTGEK+229.163

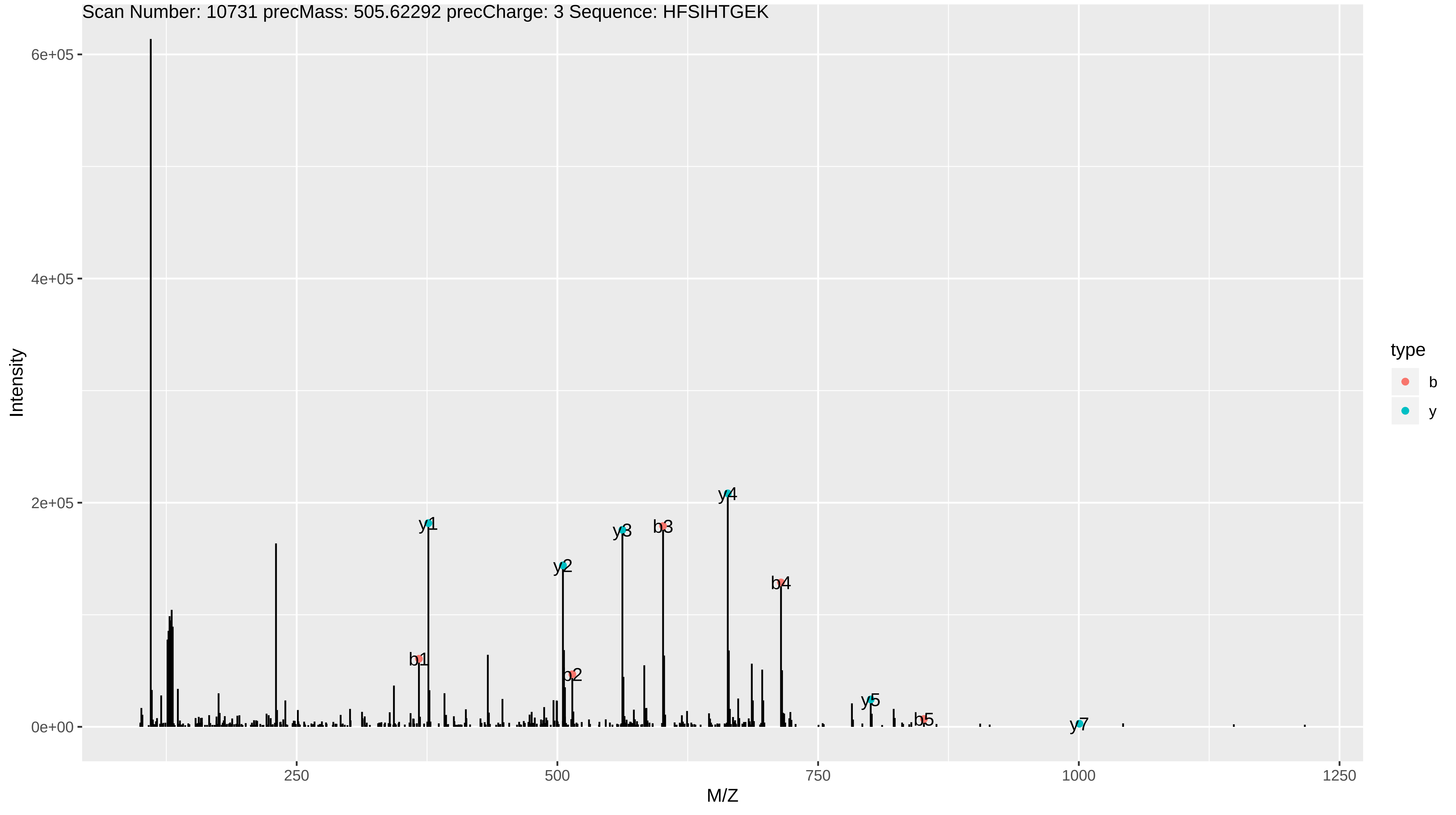

ZNF551 | +229.163NTLVQHQQIHTGQK+229.163

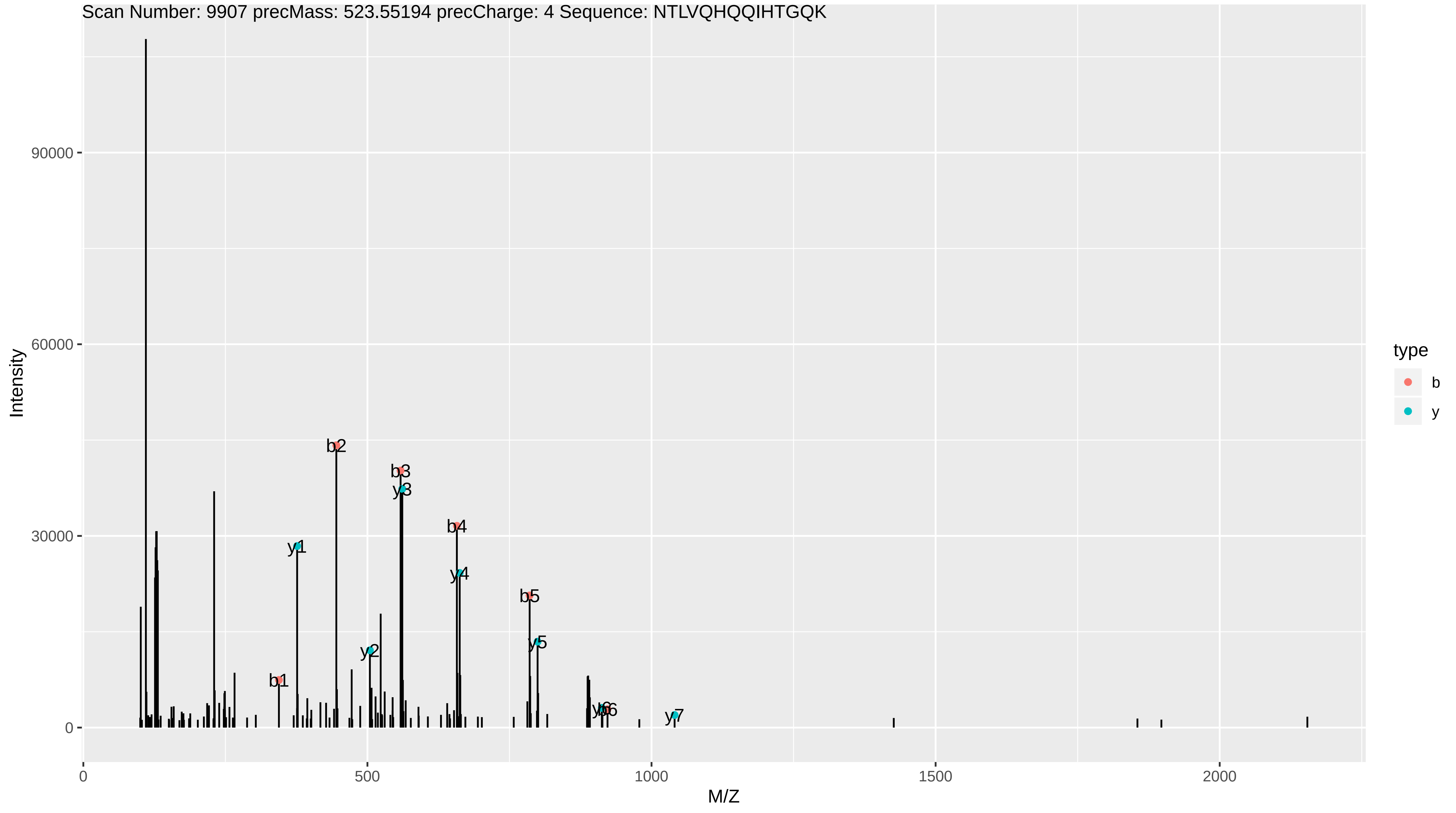

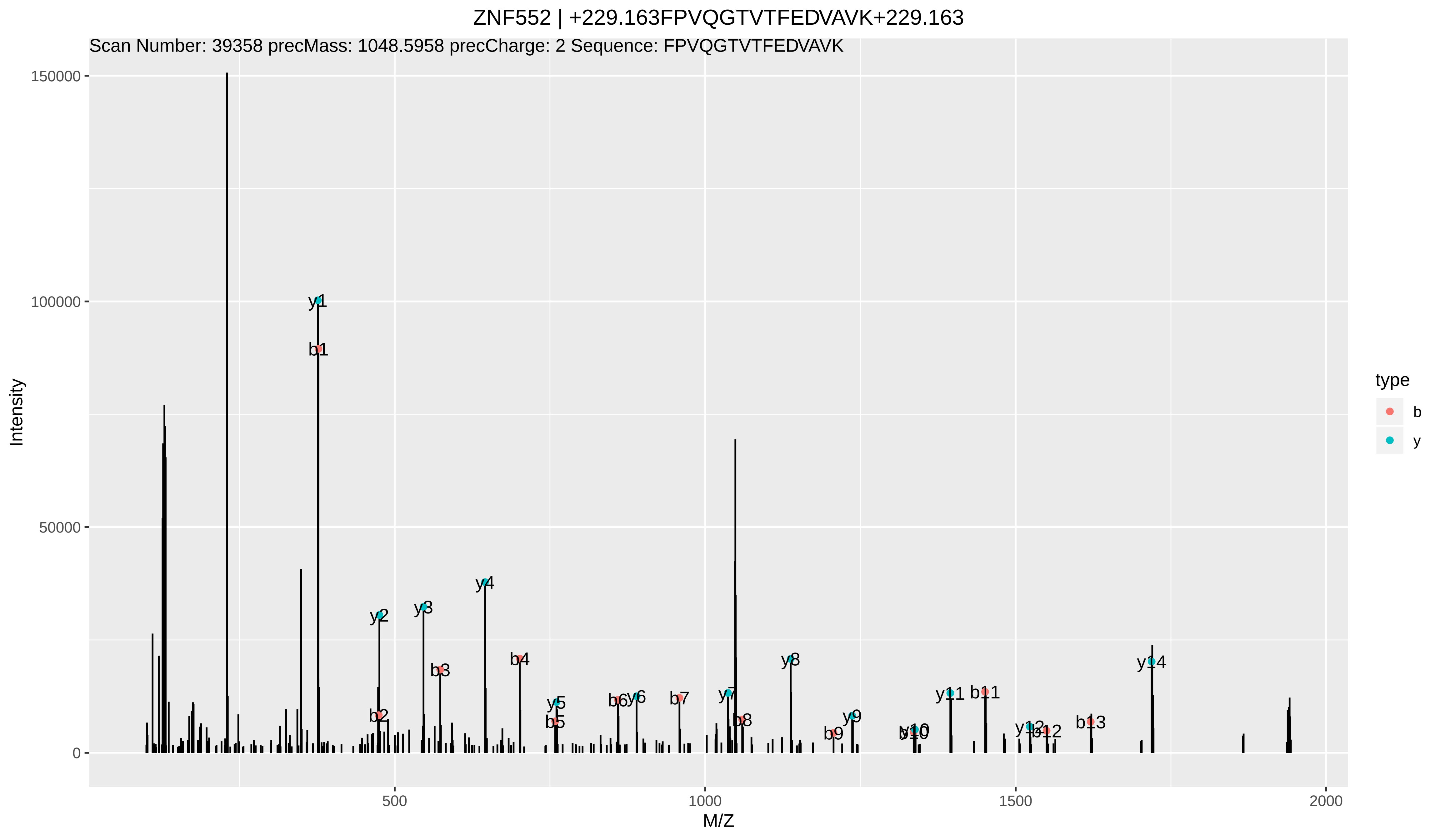

ZNF552 | +229.163FPVQGTVTFEDVAVK+229.163
Scan Number: 39358 precMass: 1048.5958 precCharge: 2 Sequence: FPVQGTVTFEDVAVK
Intensity
150000
100000
50000
0
500
1000
1500
2000
M/Z
y1
b1
y2
b2
y3
b3
y4
b4
y5
b5
b6
y6
b7
y7
b8
y8
b9
y9
y10
b10
y11
b11
y12
b12
b13
y14
type
b
y

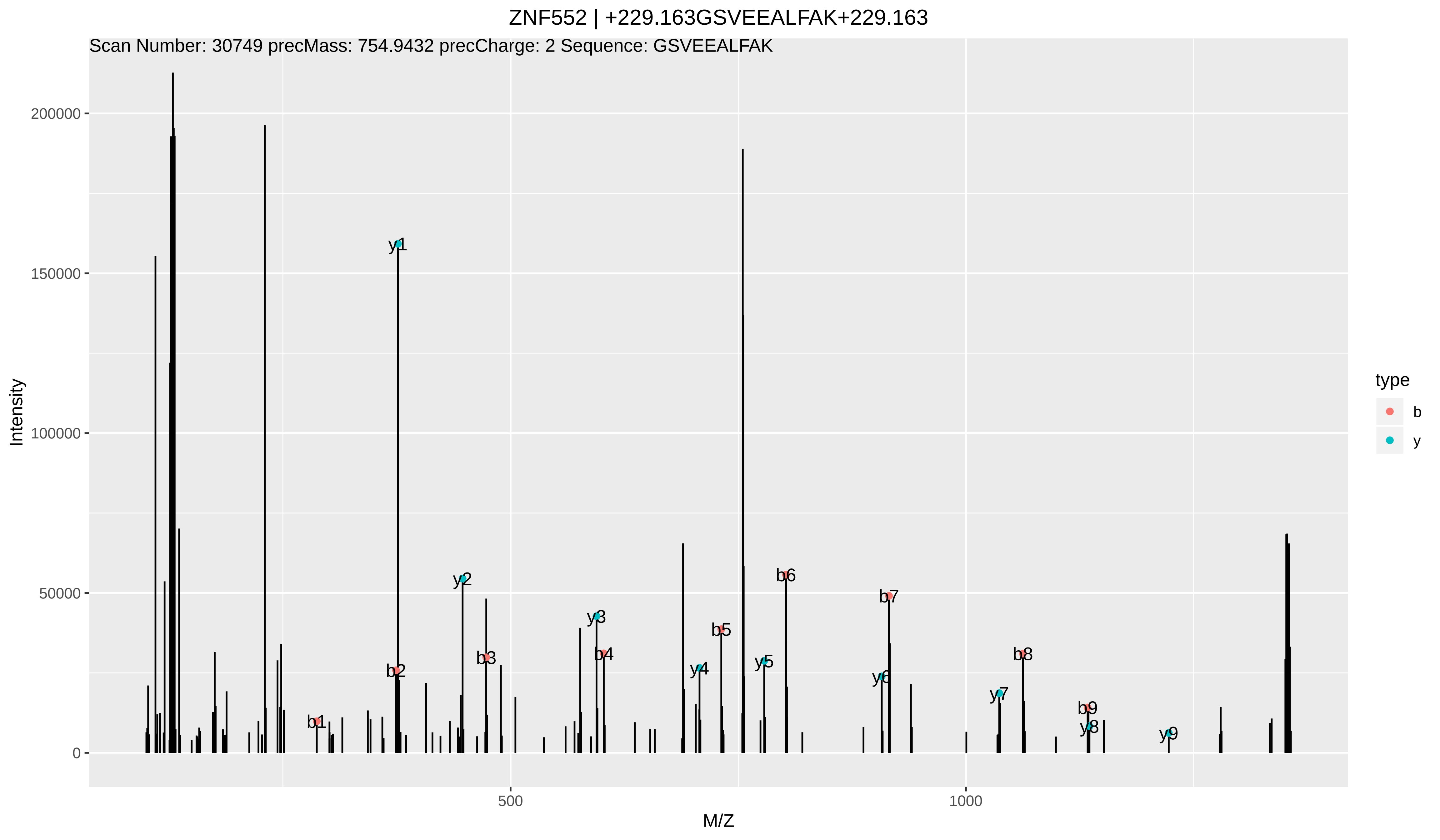

ZNF552 | +229.163GSVEEALFAK+229.163
Scan Number: 30749 precMass: 754.9432 precCharge: 2 Sequence: GSVEEALFAK
Intensity
200000
150000
100000
50000
0
500
1000
M/Z
y1
y2
b2
b1
b3
y3
b4
y4
b5
y5
b6
y6
b7
y7
b8
b9
y8
y9
type
b
y

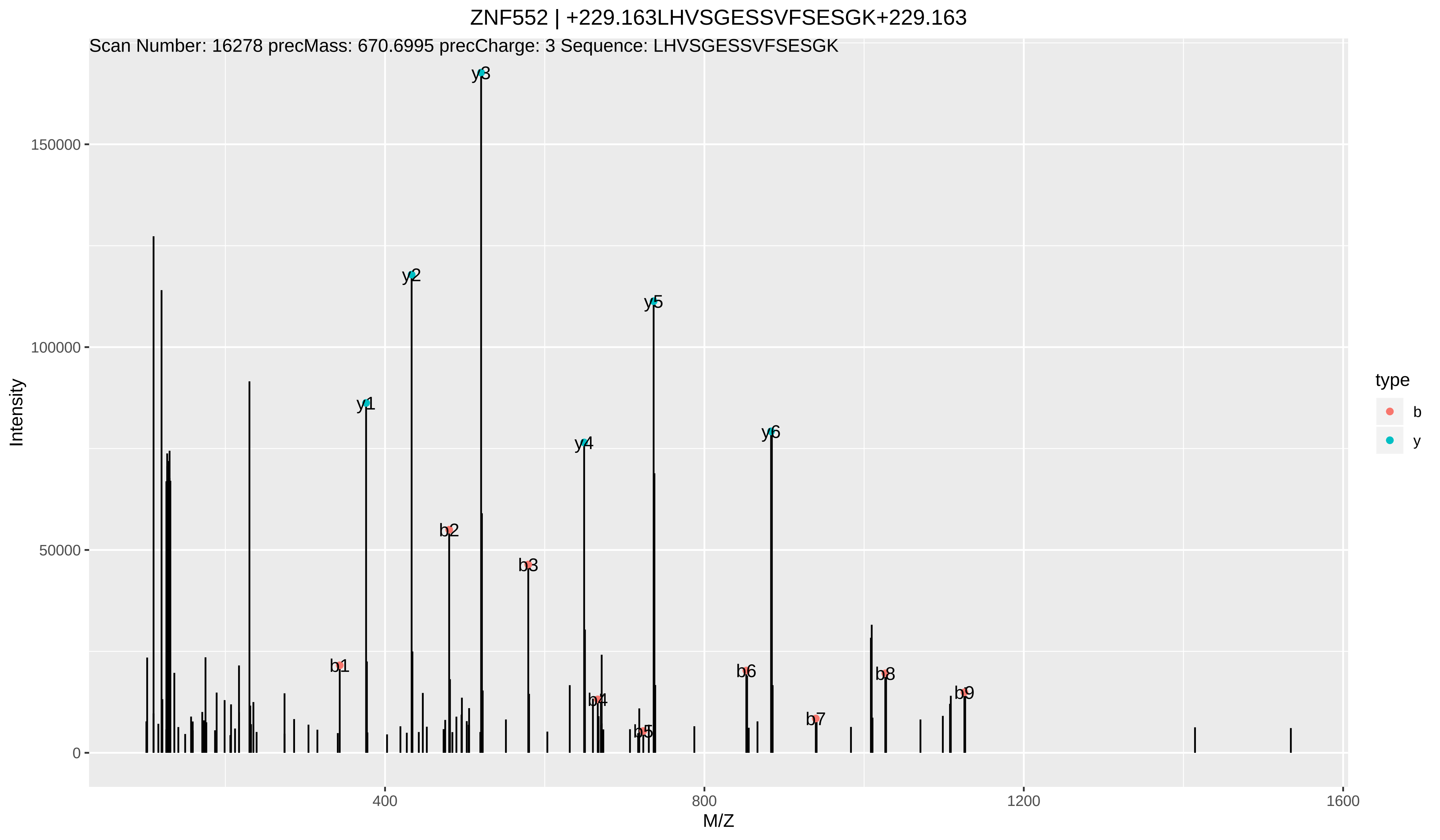

ZNF552 | +229.163LHVSGESSVFSESGK+229.163
Scan Number: 16278 precMass: 670.6995 precCharge: 3 Sequence: LHVSGESSVFSESGK
Intensity
M/Z
type
b
y
y1
y2
y3
y4
y5
y6
b1
b2
b3
b4
b5
b6
b7
b8
b9
0
50000
100000
150000
400
800
1200
1600

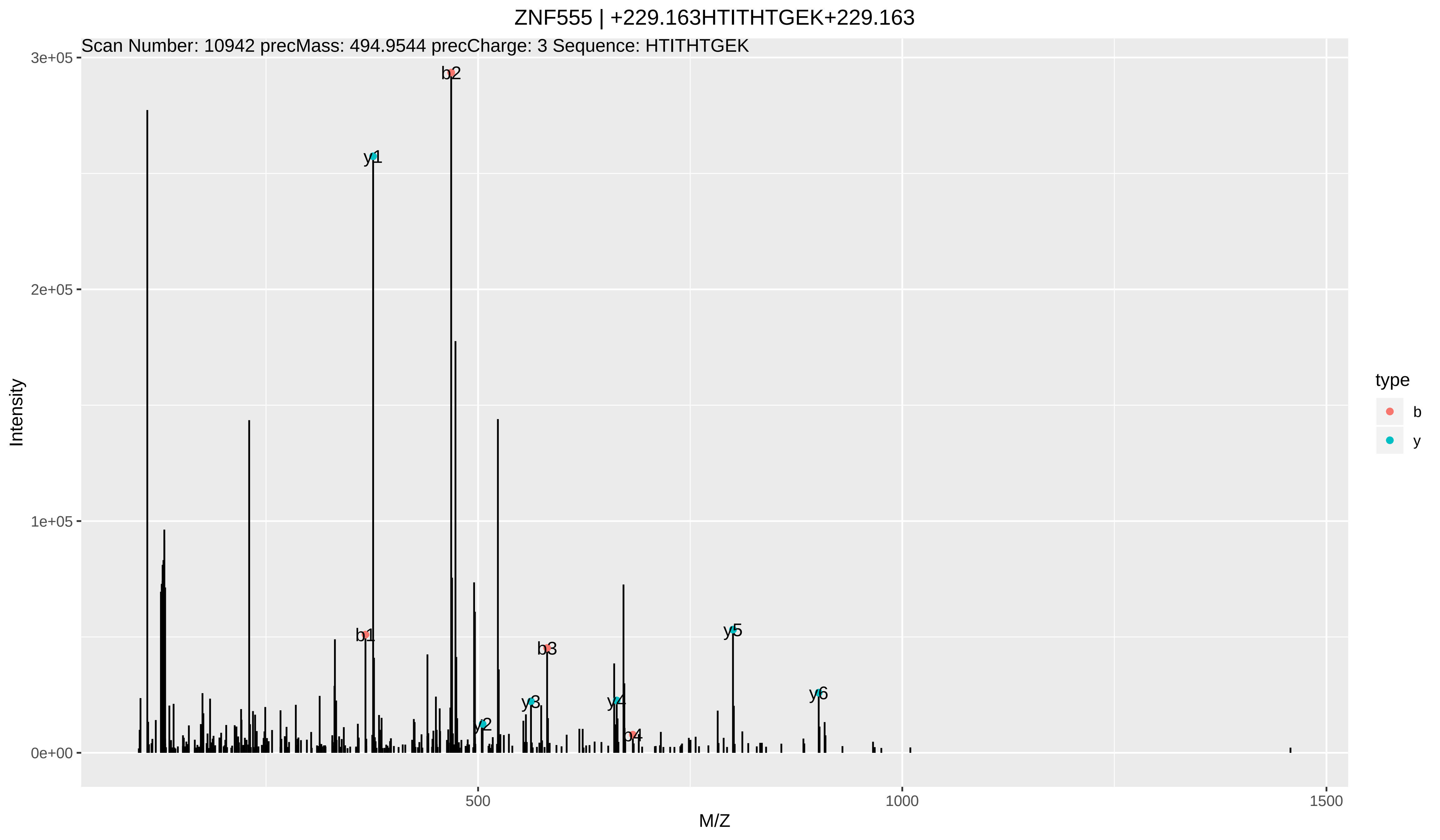

ZNF555 | +229.163HTITHTGEK+229.163
Scan Number: 10942 precMass: 494.9544 precCharge: 3 Sequence: HTITHTGEK
Intensity
M/Z
3e+05
2e+05
1e+05
0e+00
500
1000
1500
b2
y1
b1
b3
y5
y3
y4
y2
b4
y6
type
b
y

ZNF561 | +229.163SFTNFSQLYAPVK+229.163

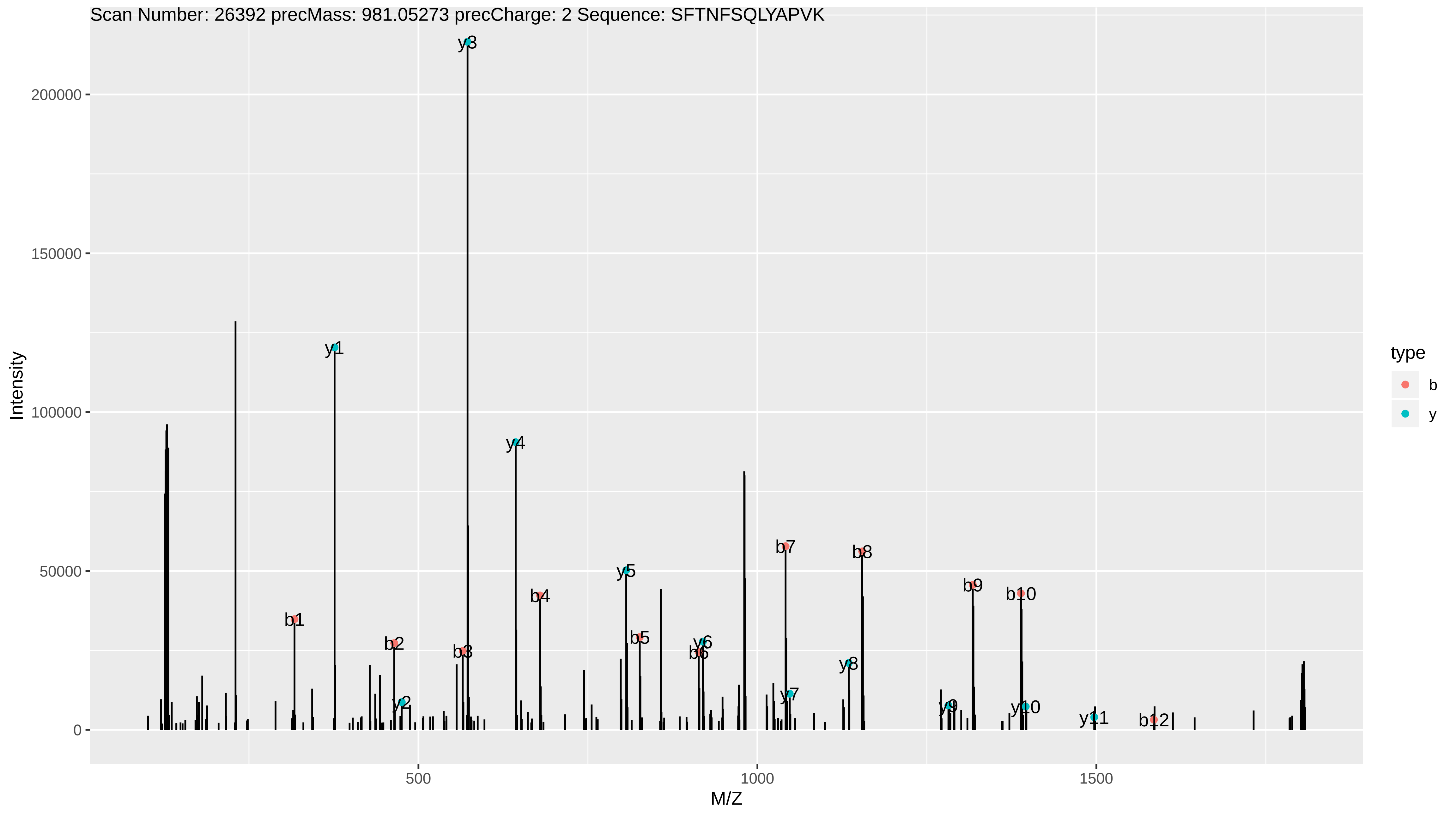

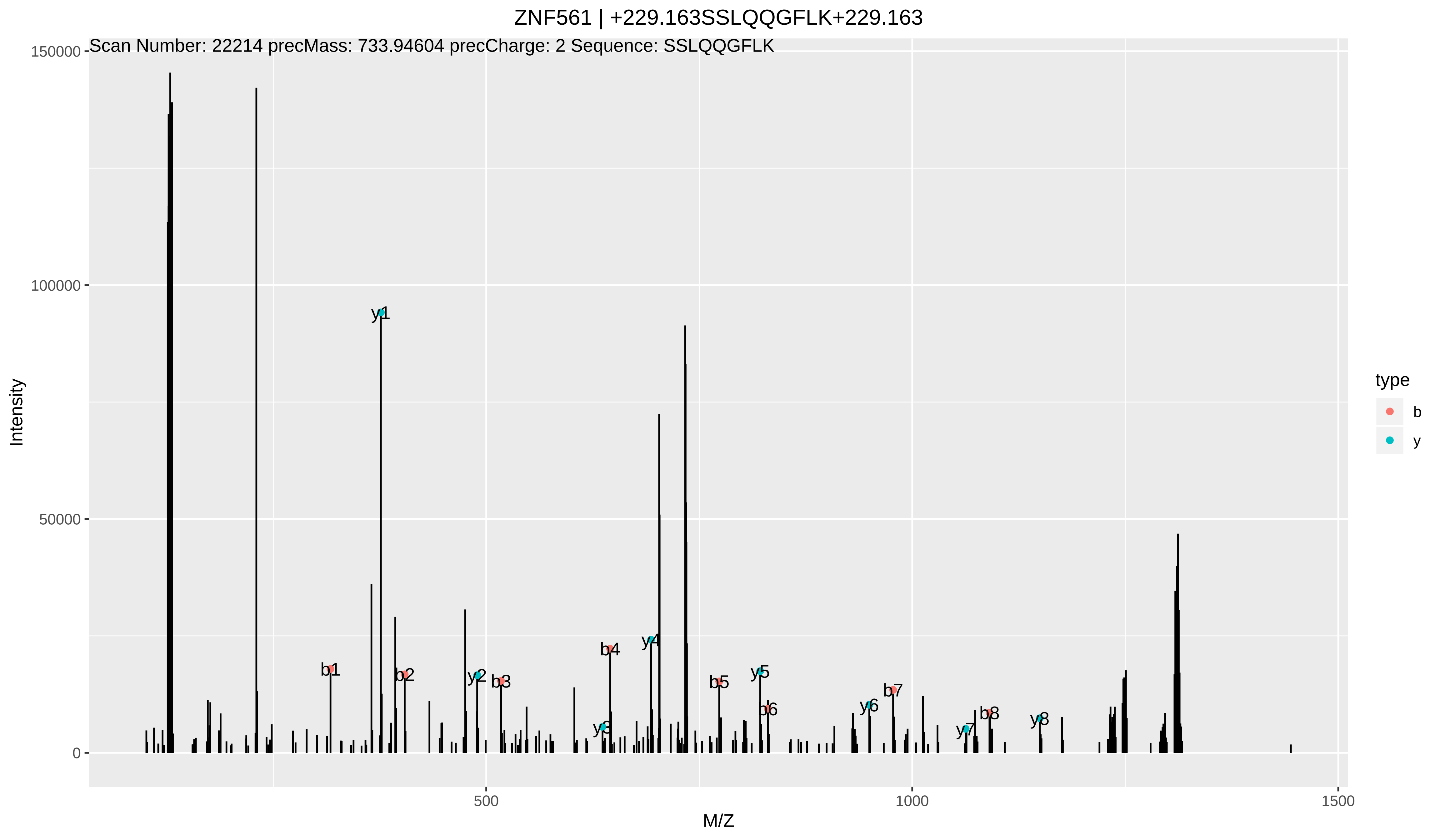
ZNF561 | +229.163SSLQQGFLK+229.163
Scan Number: 22214 precMass: 733.94604 precCharge: 2 Sequence: SSLQQGFLK
Intensity
M/Z
0
50000
100000
150000
500
1000
1500
y1
b1
b2
y2
b3
b4
y4
y3
b5
y5
b6
y6
b7
y7
b8
y8
type
b
y

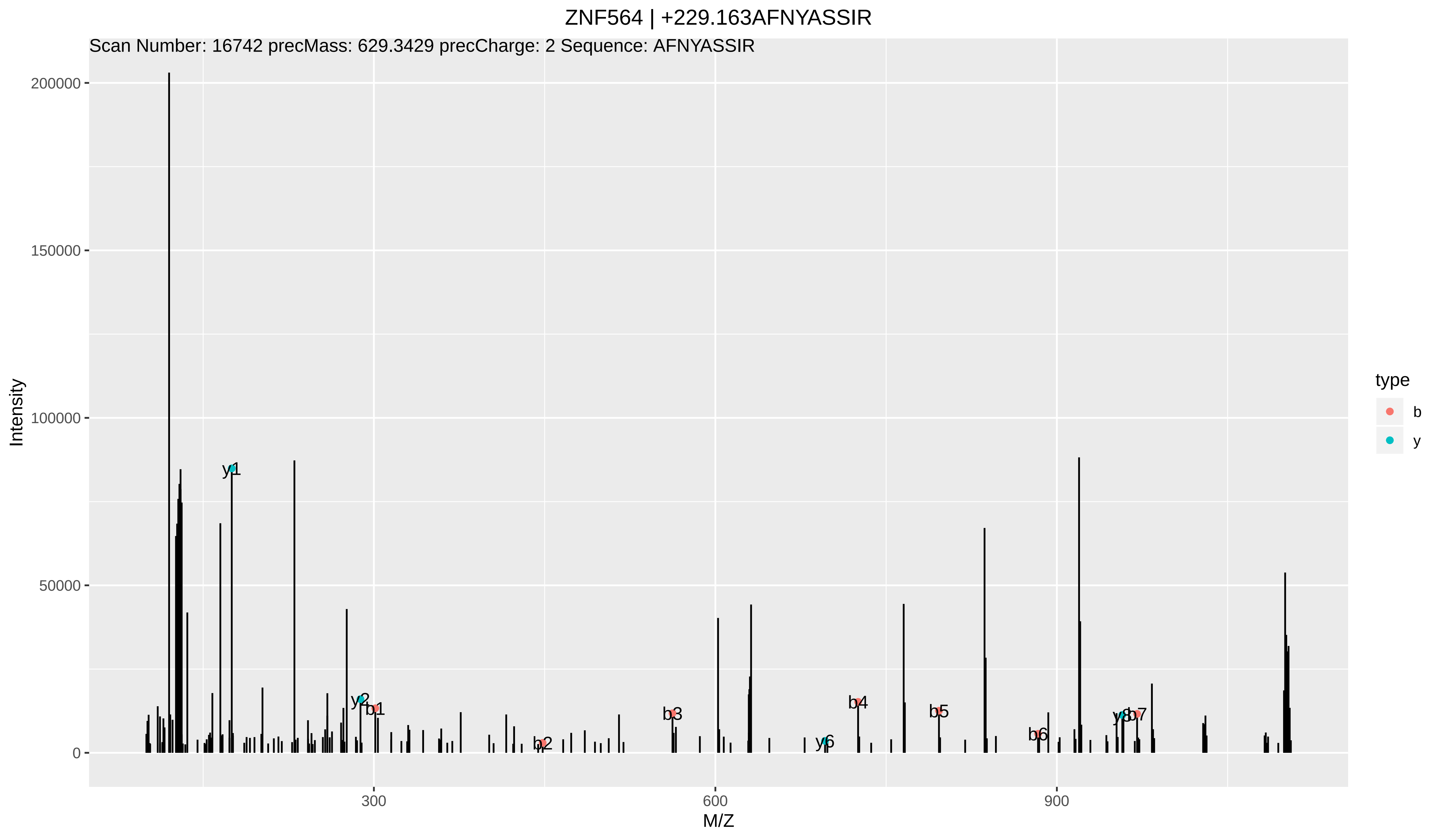
ZNF564 | +229.163AFNYASSIR
Scan Number: 16742 precMass: 629.3429 precCharge: 2 Sequence: AFNYASSIR
Intensity
200000
150000
100000
50000
0
M/Z
300
600
900
y1
y2
b1
b2
b3
y6
b4
b5
b6
y8
b7
type
b
y

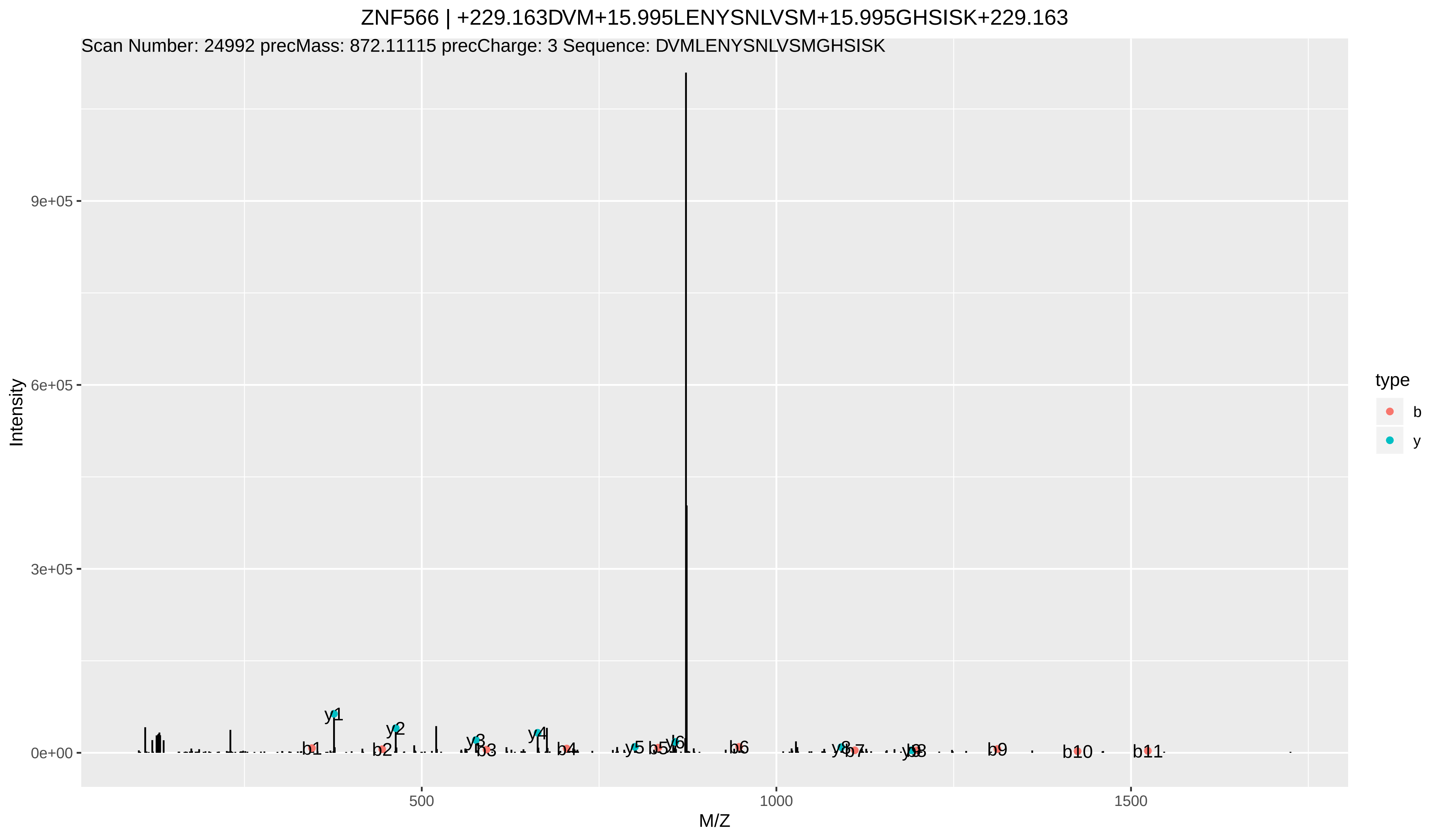

ZNF566 | +229.163DVM+15.995LENYSNLVSM+15.995GHSISK+229.163
Scan Number: 24992 precMass: 872.11115 precCharge: 3 Sequence: DVMLENYSNLVSMGHSISK
Intensity
M/Z
type
b
y

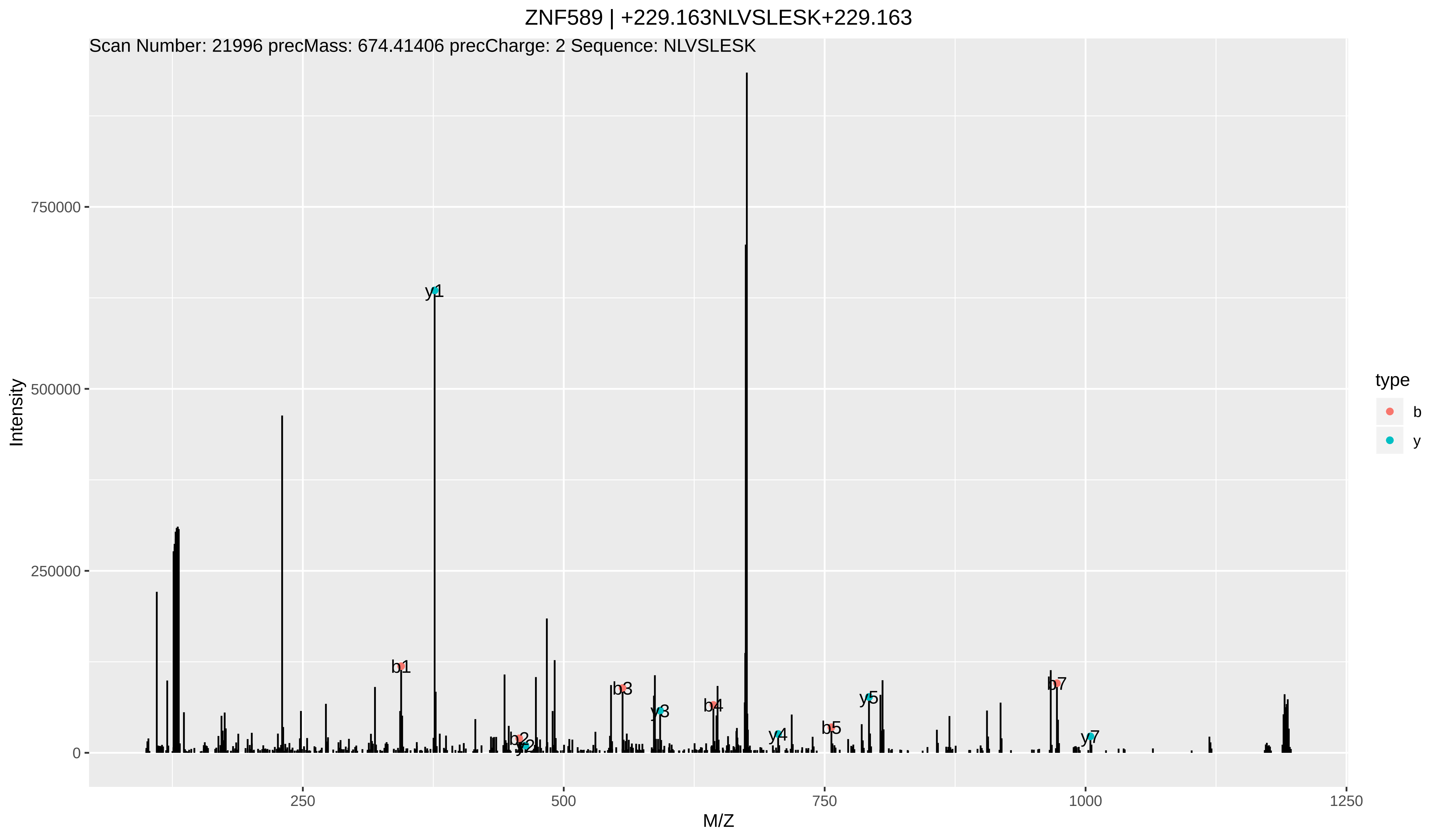
ZNF589 | +229.163NLVSLESK+229.163
Scan Number: 21996 precMass: 674.41406 precCharge: 2 Sequence: NLVSLESK
Intensity
750000
500000
250000
0
M/Z
250
500
750
1000
1250
y1
b1
b2
y2
b3
y3
b4
y4
b5
y5
b7
y7
type
b
y

ZNF616 | +229.163EVPVPHENNLTGK+229.163

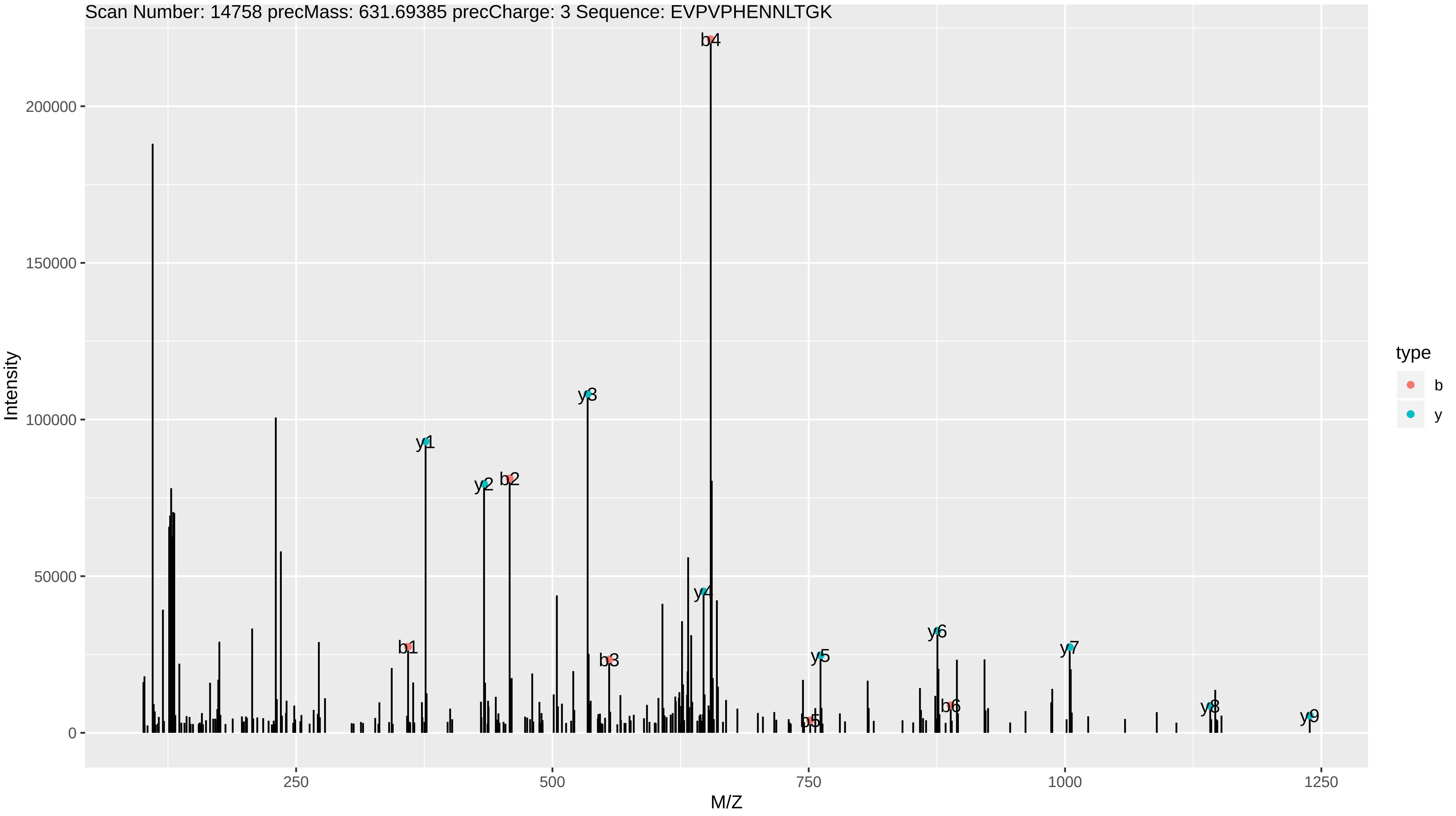

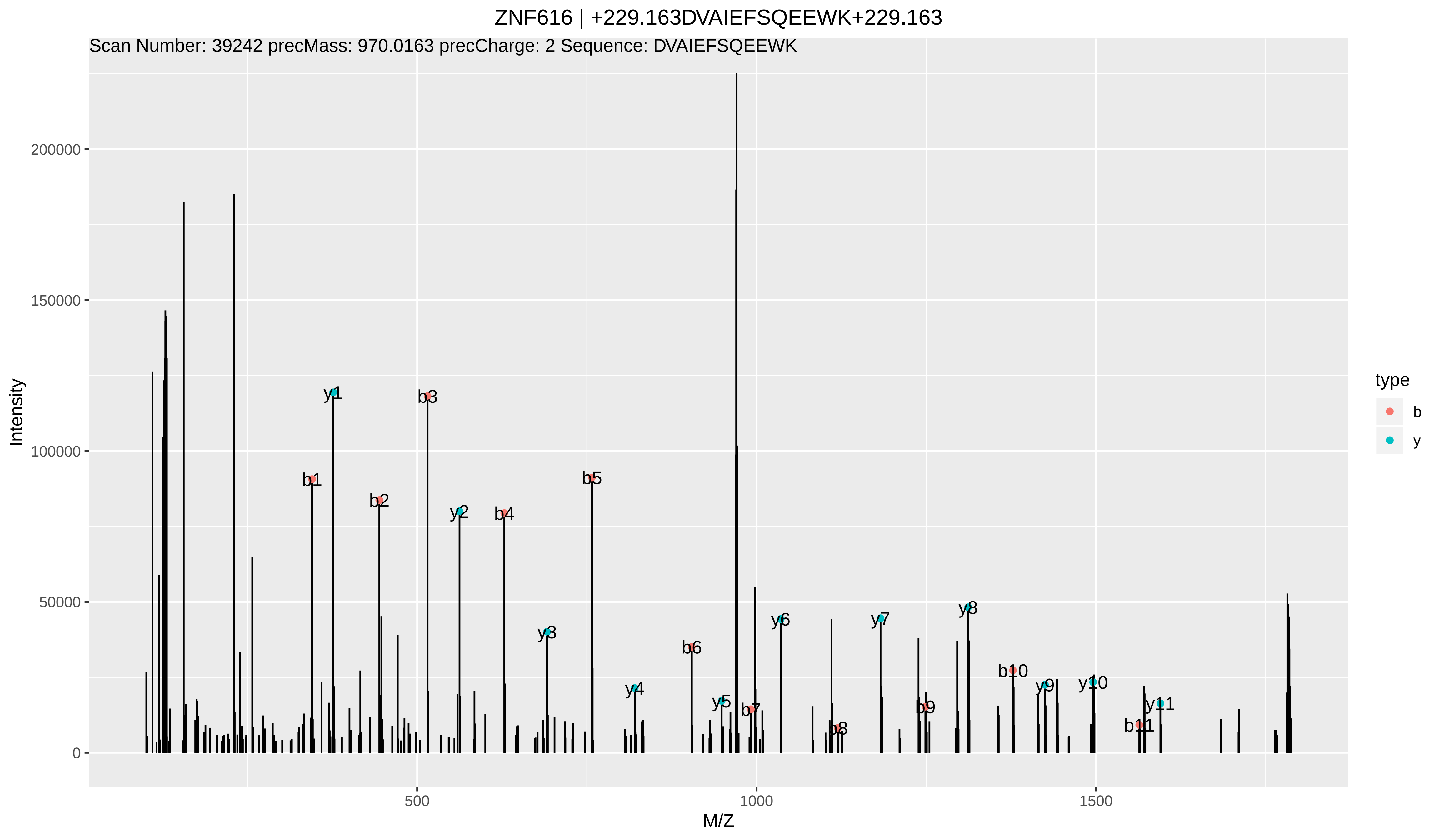
ZNF616 | +229.163DVAIEFSQEEWK+229.163
Scan Number: 39242 precMass: 970.0163 precCharge: 2 Sequence: DVAIEFSQEEWK
Intensity
M/Z
type
b
y
y1
b3
b1
b5
b2
y2
b4
y3
b6
y4
y5
b7
y6
y7
b8
y8
b9
b10
y9
y10
b11
y11
0
50000
100000
150000
200000
500
1000
1500

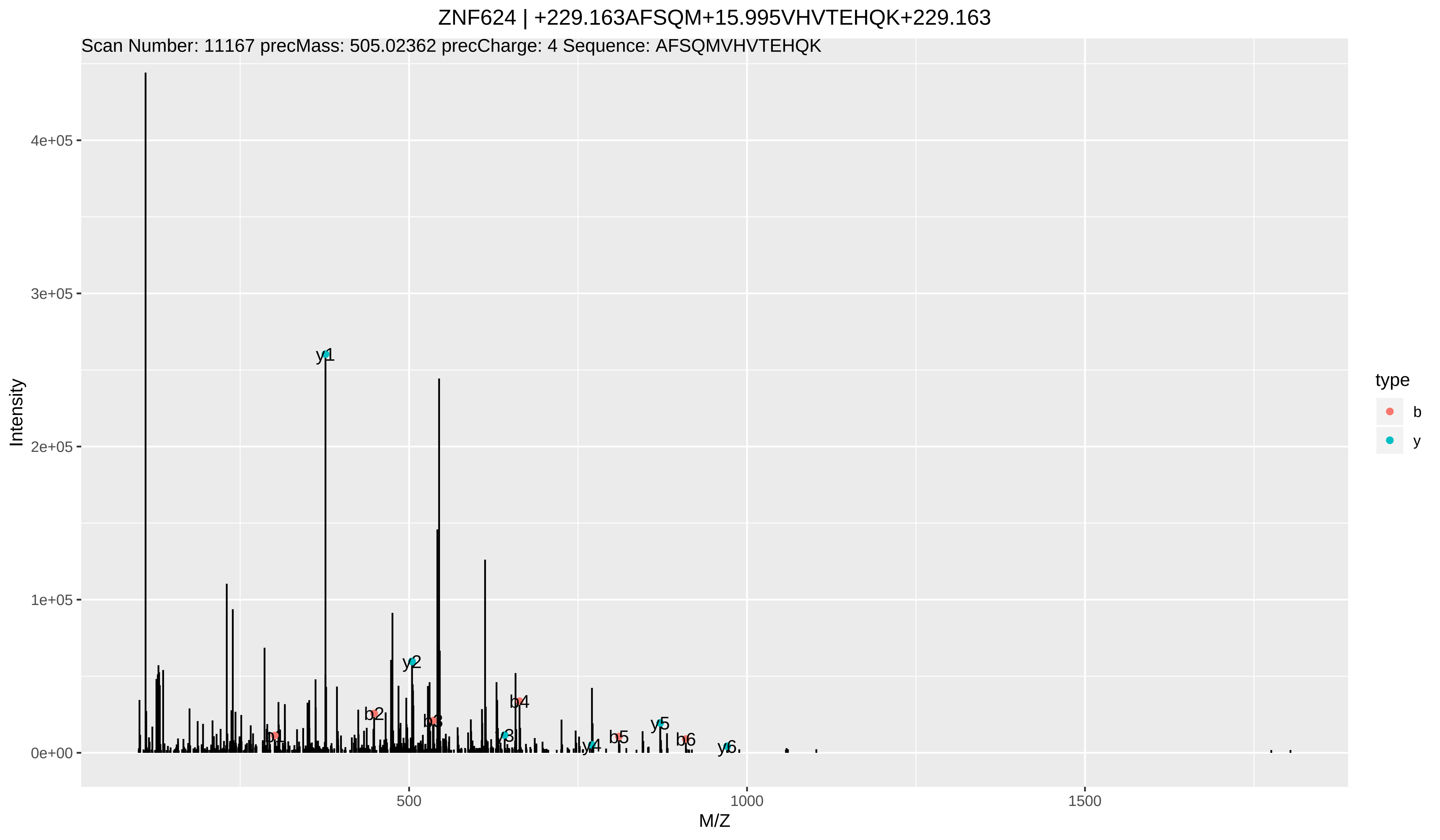

ZNF624 | +229.163AFSQM+15.995VHVTEHQK+229.163
Scan Number: 11167 precMass: 505.02362 precCharge: 4 Sequence: AFSQMVHVTEHQK
Intensity
4e+05
3e+05
2e+05
1e+05
0e+00
M/Z
500
1000
1500
y1
y2
y3
y4
y5
y6
b1
b2
b3
b4
b5
b6
type
b
y

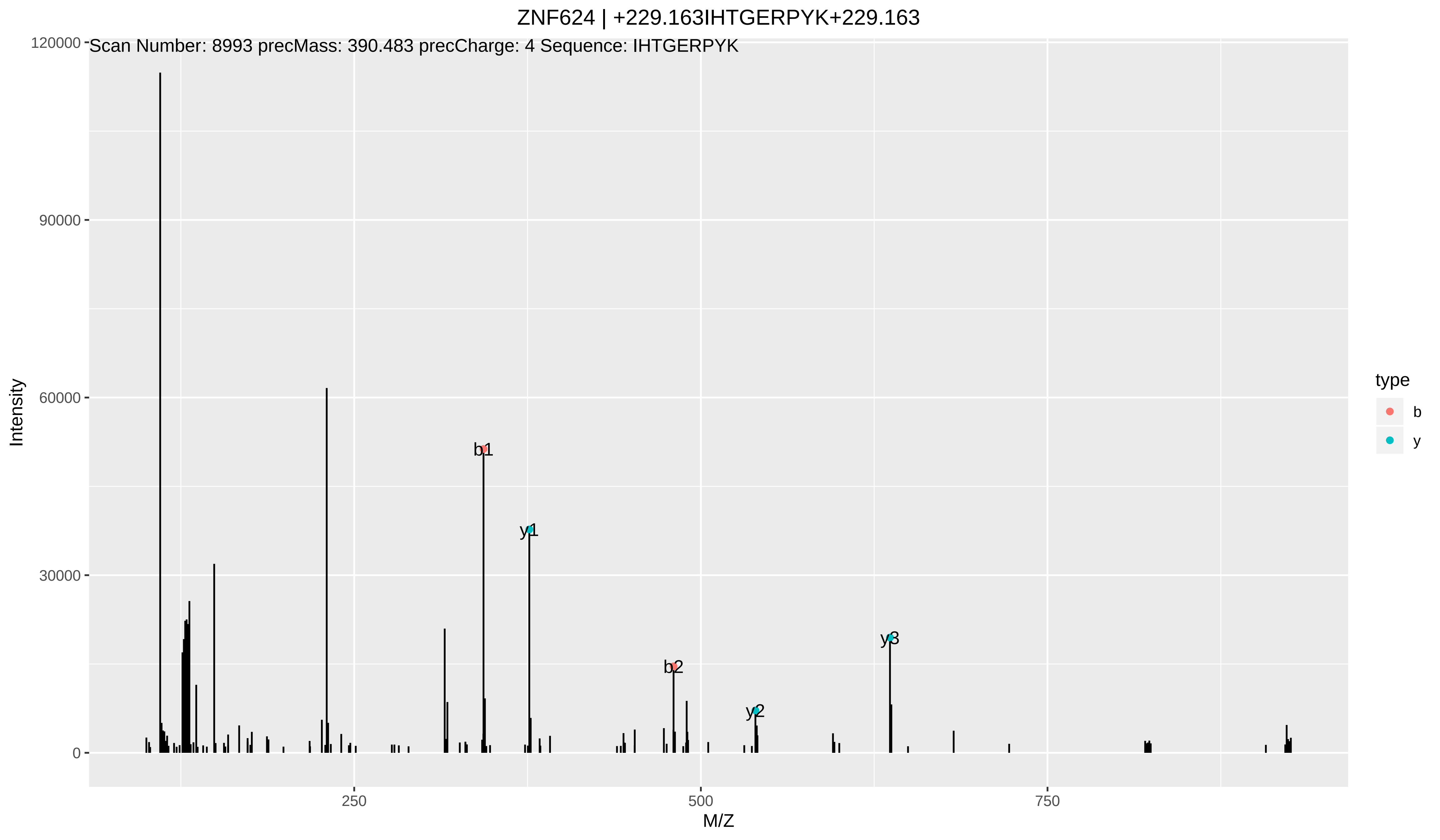
ZNF624 | +229.163IHTGERPYK+229.163
Scan Number: 8993 precMass: 390.483 precCharge: 4 Sequence: IHTGERPYK
Intensity
120000
90000
60000
30000
0
M/Z
250
500
750
b1
y1
b2
y2
y3
type
b
y

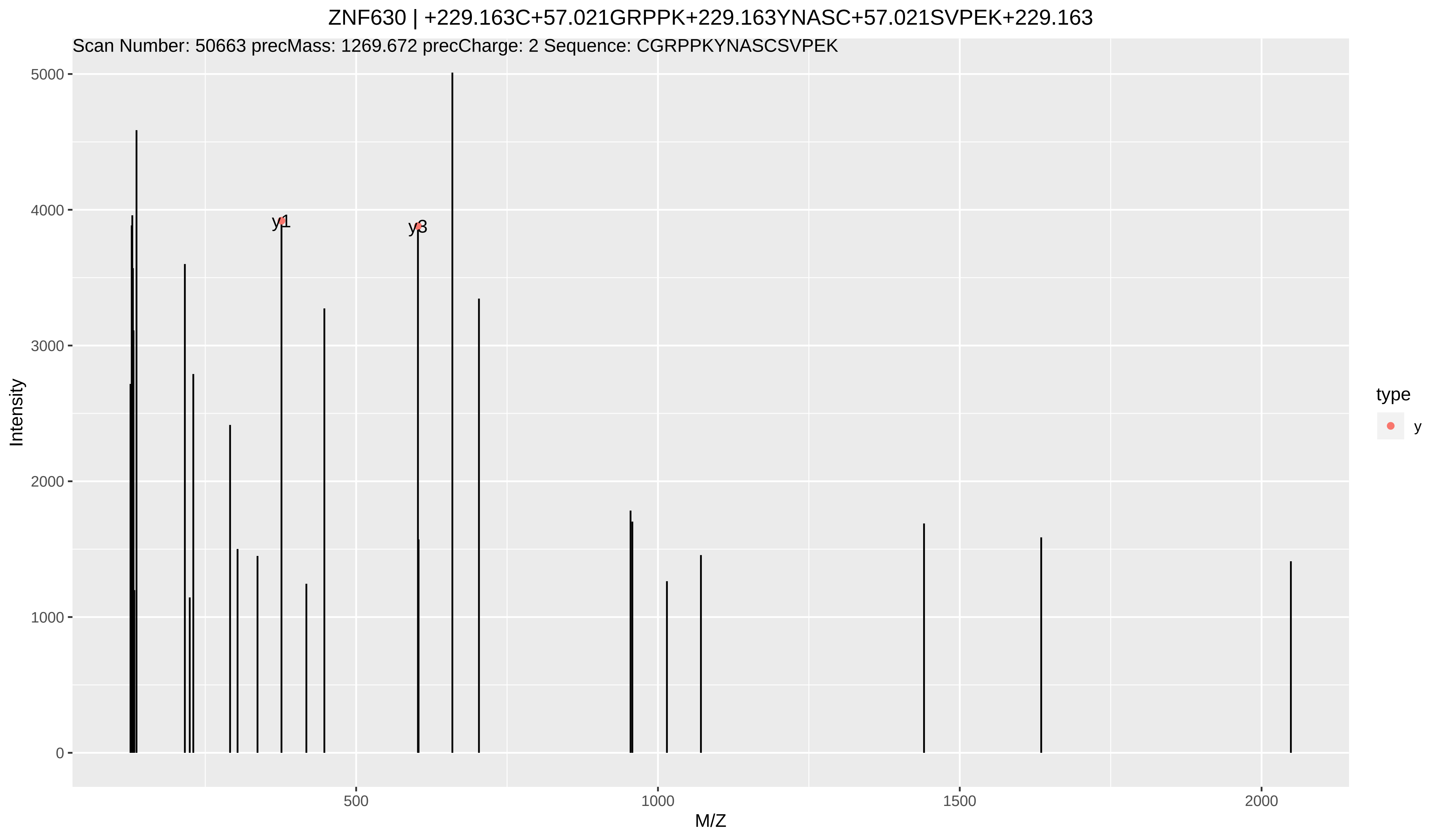

ZNF630 | +229.163C+57.021GRPPK+229.163YNASC+57.021SVPEK+229.163
Scan Number: 50663 precMass: 1269.672 precCharge: 2 Sequence: CGRPPKYNASCSVPEK
Intensity
M/Z
0
1000
2000
3000
4000
5000
500
1000
1500
2000
y1
y3
type
y

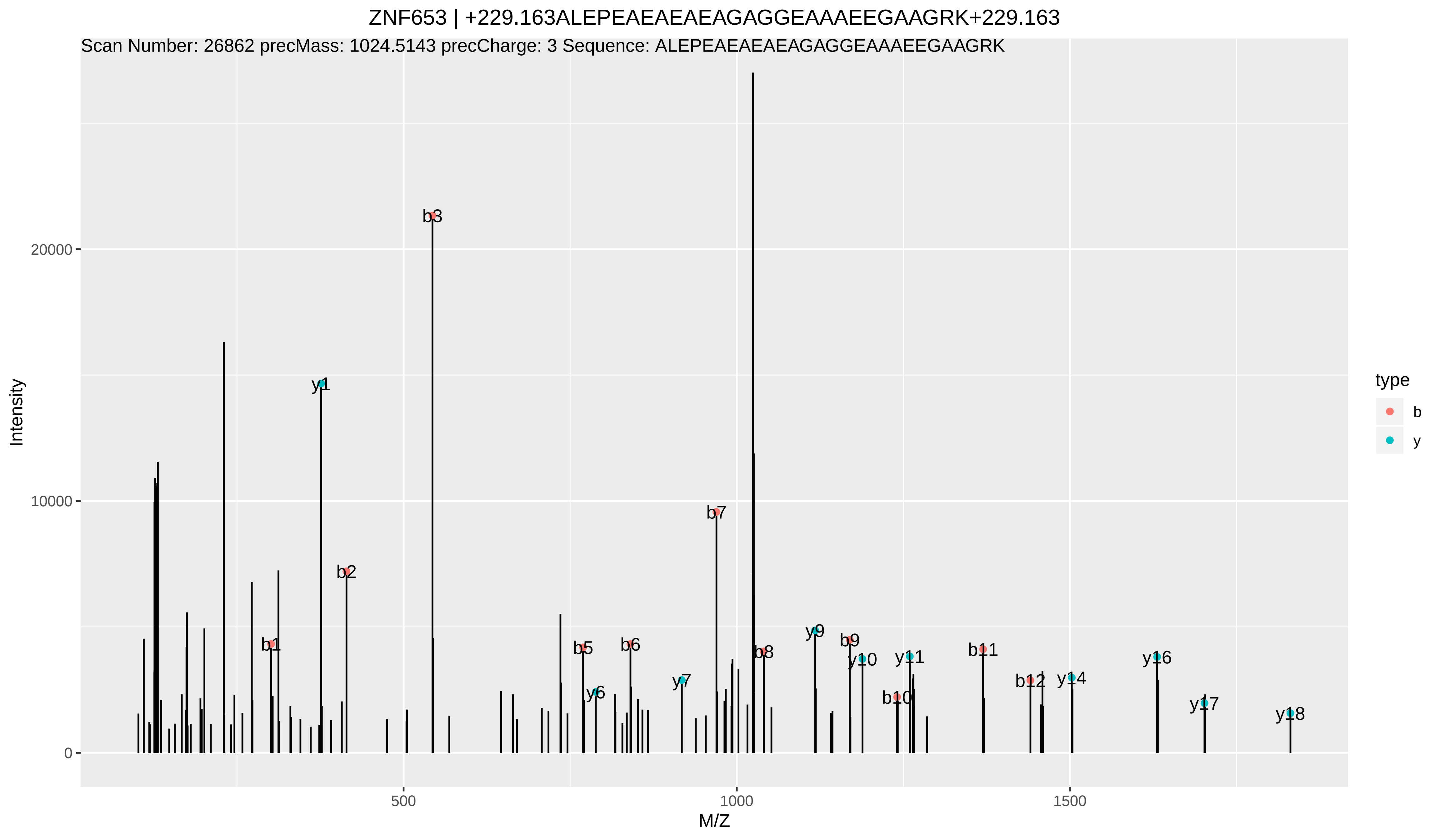
ZNF653 | +229.163ALEPEAEAEAEAGAGGEAAAEEGAAGRK+229.163
Scan Number: 26862 precMass: 1024.5143 precCharge: 3 Sequence: ALEPEAEAEAEAGAGGEAAAEEGAAGRK
Intensity
M/Z
0
10000
20000
500
1000
1500
b3
y1
b2
b1
b7
b5
b6
y6
y7
b8
y9
b9
y10
b10
y11
b11
b12
y14
y16
y17
y18
type
b
y

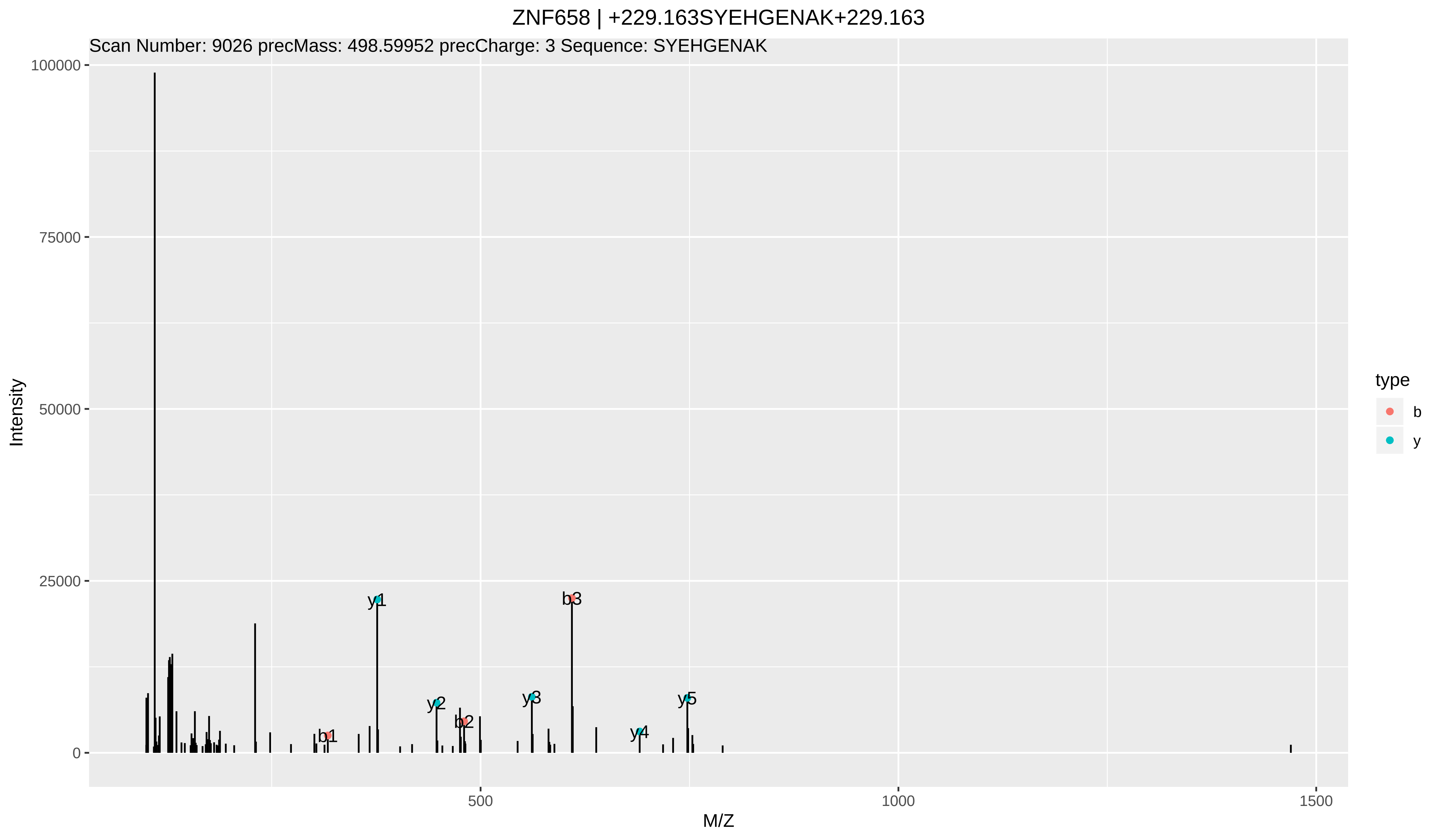

ZNF658 | +229.163SYEHGENAK+229.163
Scan Number: 9026 precMass: 498.59952 precCharge: 3 Sequence: SYEHGENAK
100000
75000
50000
25000
0
Intensity
500
1000
1500
M/Z
y1
b3
y2
b2
y3
y5
b1
y4
type
b
y

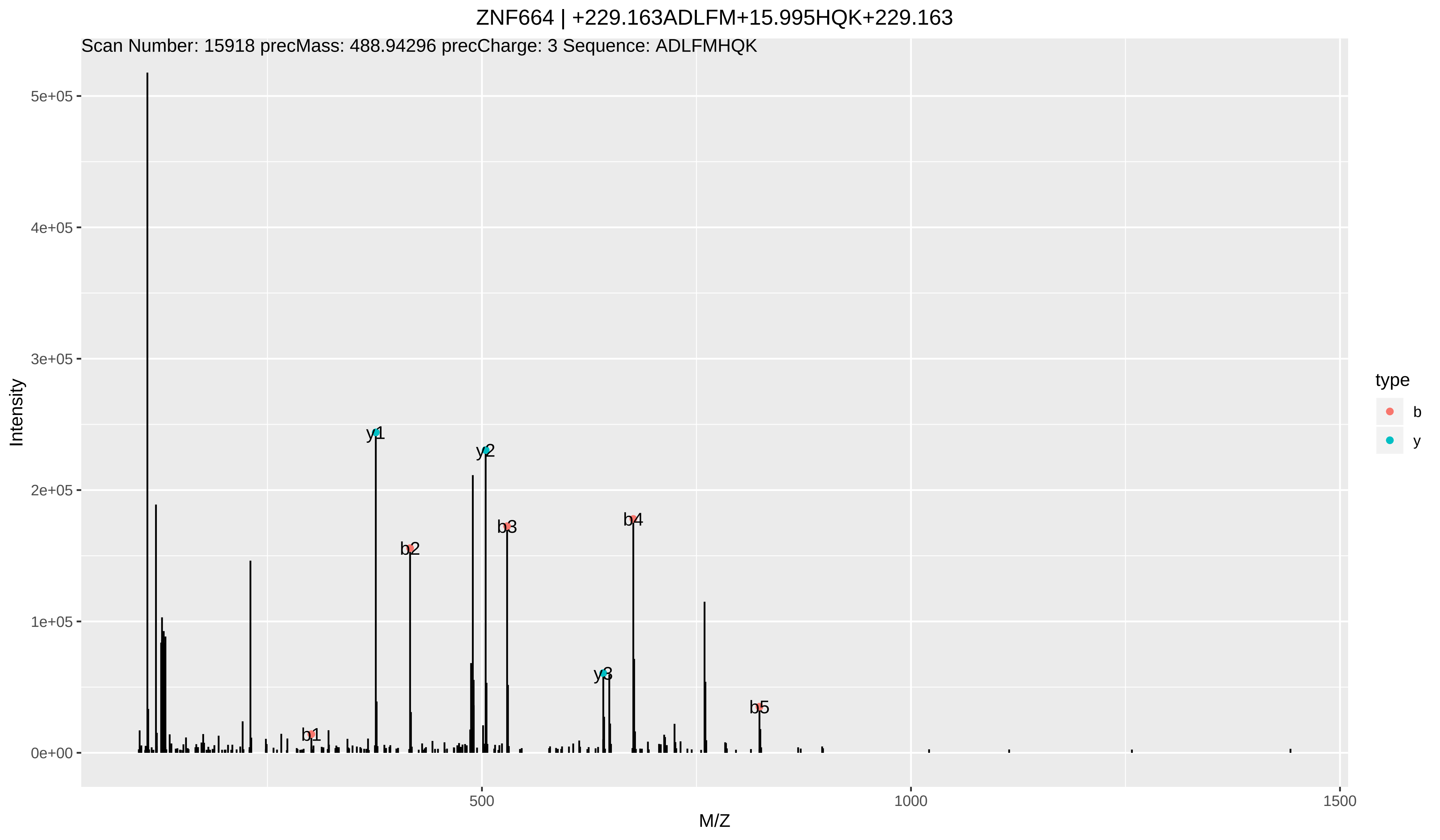

ZNF664 | +229.163ADLFM+15.995HQK+229.163
Scan Number: 15918 precMass: 488.94296 precCharge: 3 Sequence: ADLFMHQK
Intensity
5e+05
4e+05
3e+05
2e+05
1e+05
0e+00
M/Z
500
1000
1500
y1
y2
y3
b1
b2
b3
b4
b5
type
b
y

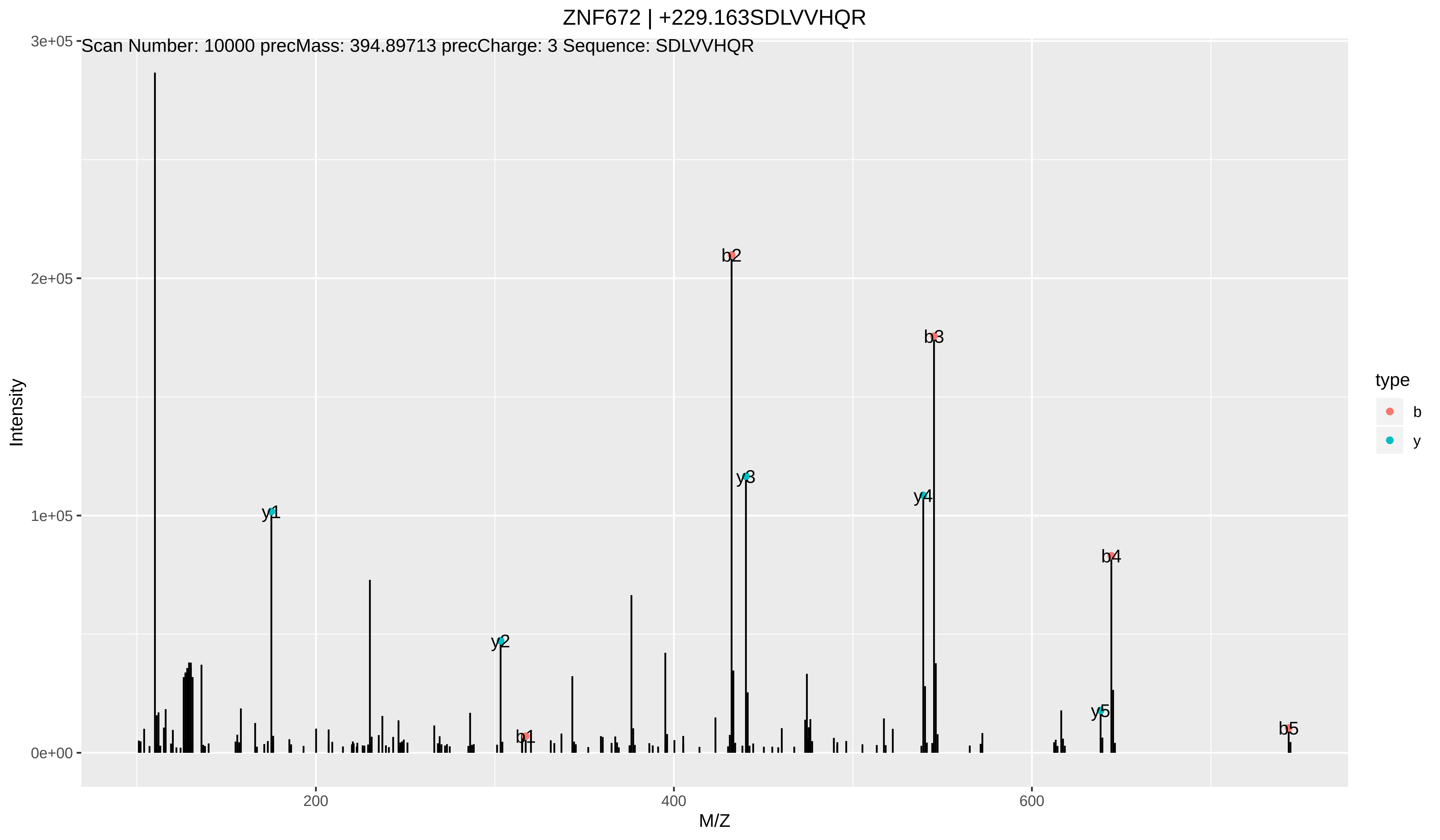

ZNF672 | +229.163SDLVVHQR
Scan Number: 10000 precMass: 394.89713 precCharge: 3 Sequence: SDLVVHQR
Intensity
3e+05
2e+05
1e+05
0e+00
M/Z
200
400
600
y1
y2
b1
b2
y3
b3
y4
b4
y5
b5
type
b
y

ZNF675 | +229.163NIHTGEQPYK+229.163

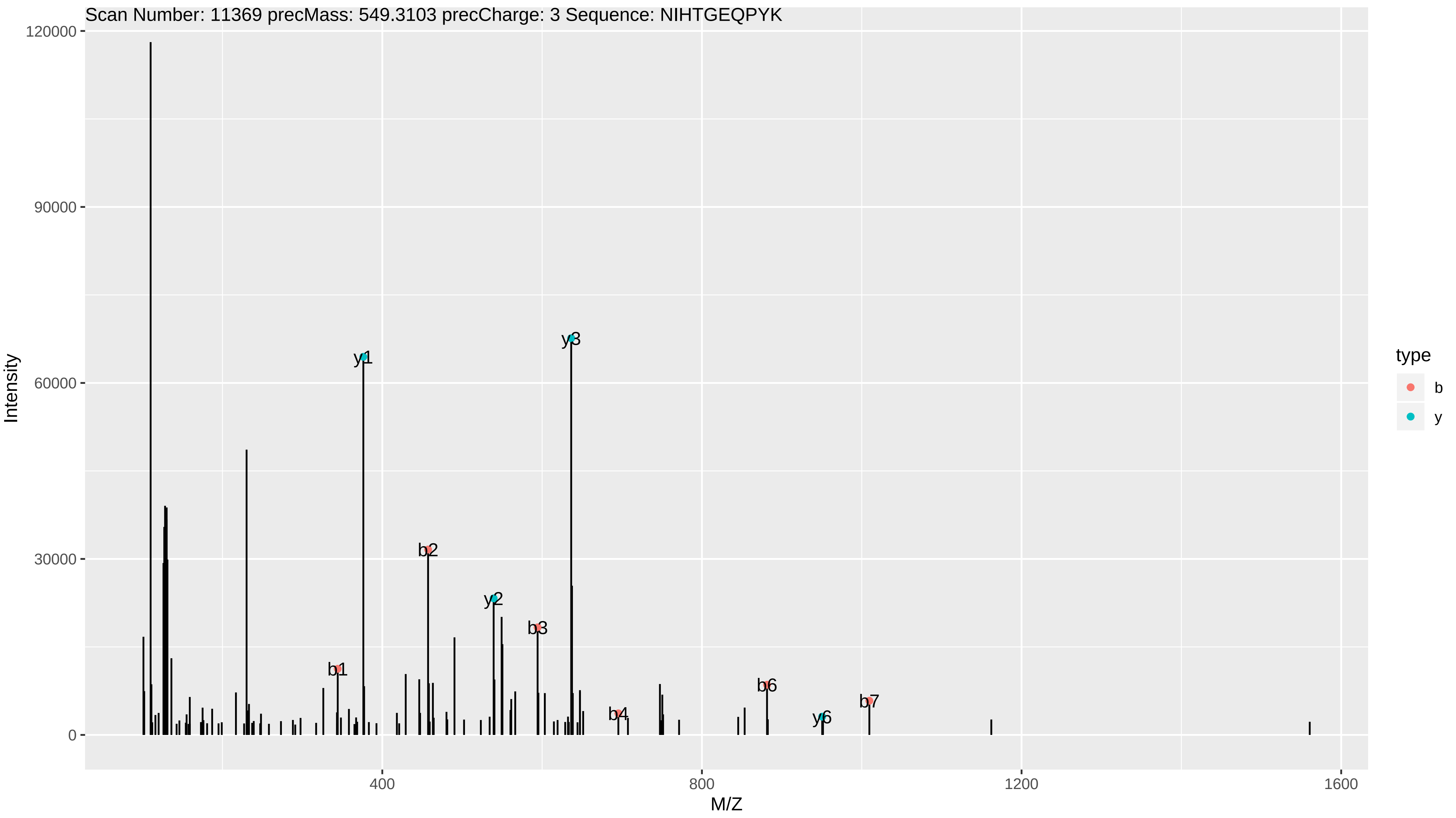

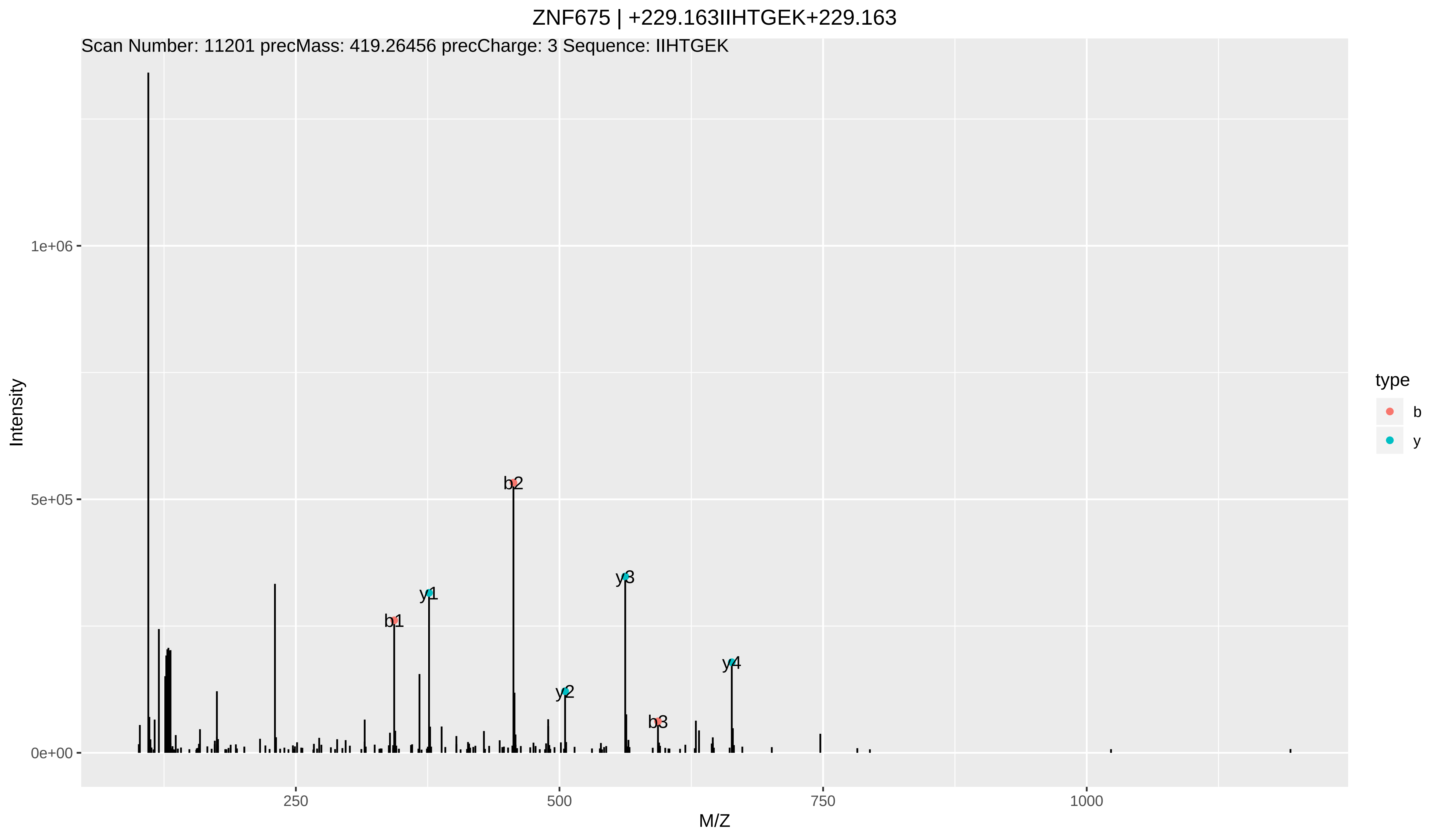

ZNF675 | +229.163IIHTGEK+229.163
Scan Number: 11201 precMass: 419.26456 precCharge: 3 Sequence: IIHTGEK
Intensity
1e+06
5e+05
0e+00
M/Z
250
500
750
1000
b1
b2
b3
y1
y2
y3
y4
type
b
y

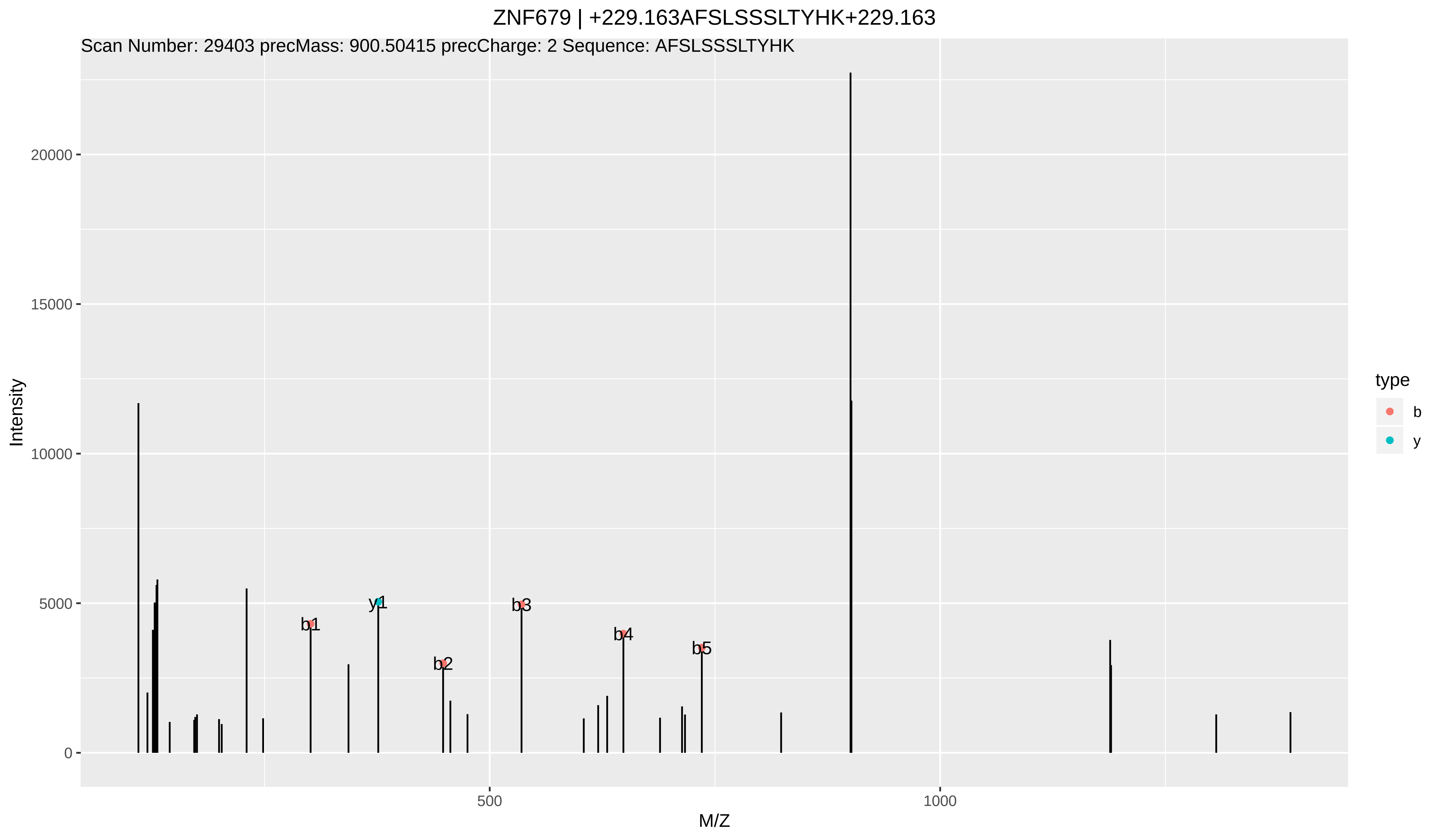

ZNF679 | +229.163AFSLSSSLTYHK+229.163
Scan Number: 29403 precMass: 900.50415 precCharge: 2 Sequence: AFSLSSSLTYHK
Intensity
M/Z
0
5000
10000
15000
20000
500
1000
b1
y1
b2
b3
b4
b5
type
b
y

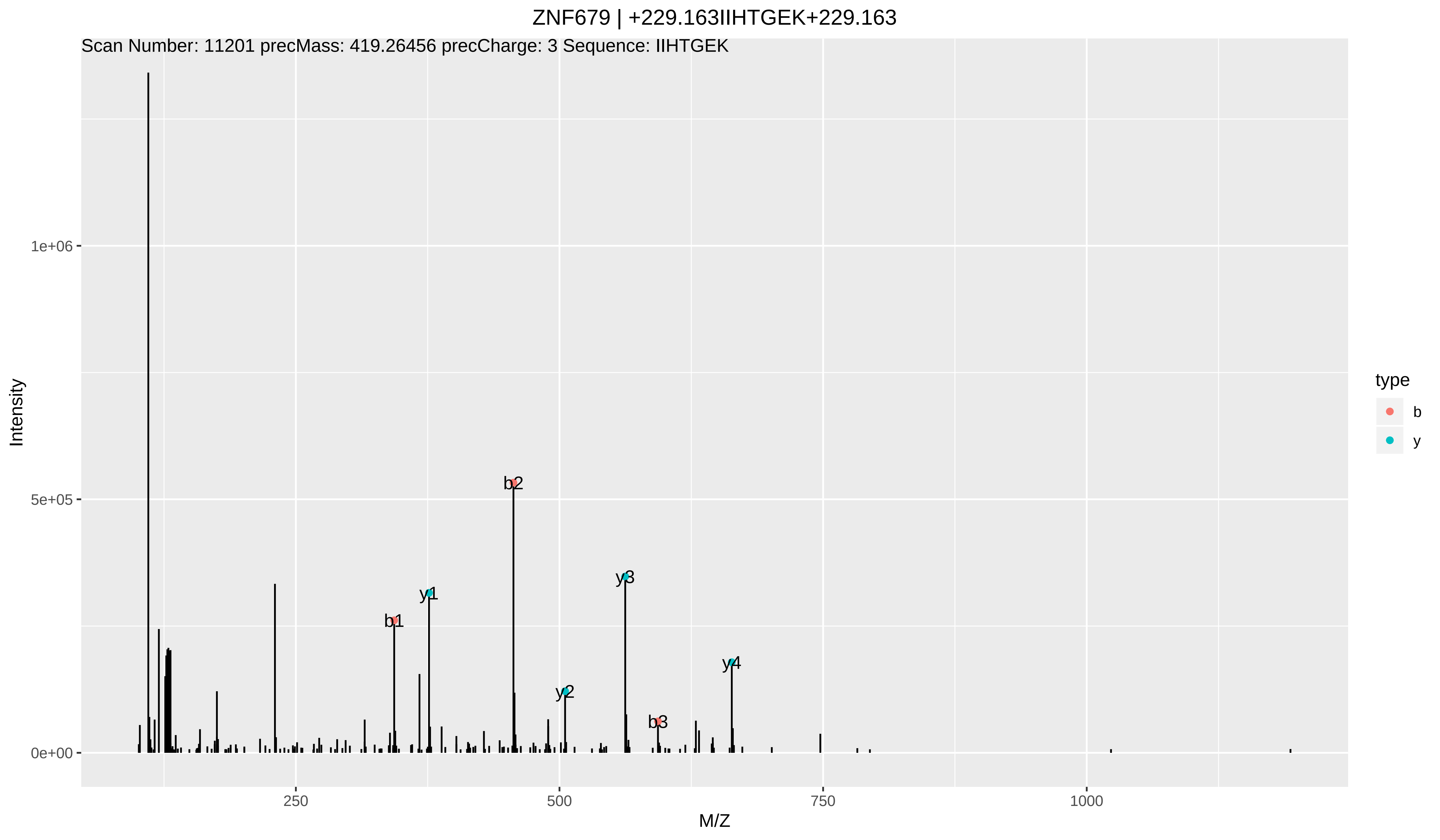

ZNF679 | +229.163IIHTGEK+229.163
Scan Number: 11201 precMass: 419.26456 precCharge: 3 Sequence: IIHTGEK
Intensity
1e+06
5e+05
0e+00
M/Z
250
500
750
1000
b1
y1
b2
y2
y3
b3
y4
type
b
y

ZNF691 | +229.163EHGQESLSDELQETHPK+229.163

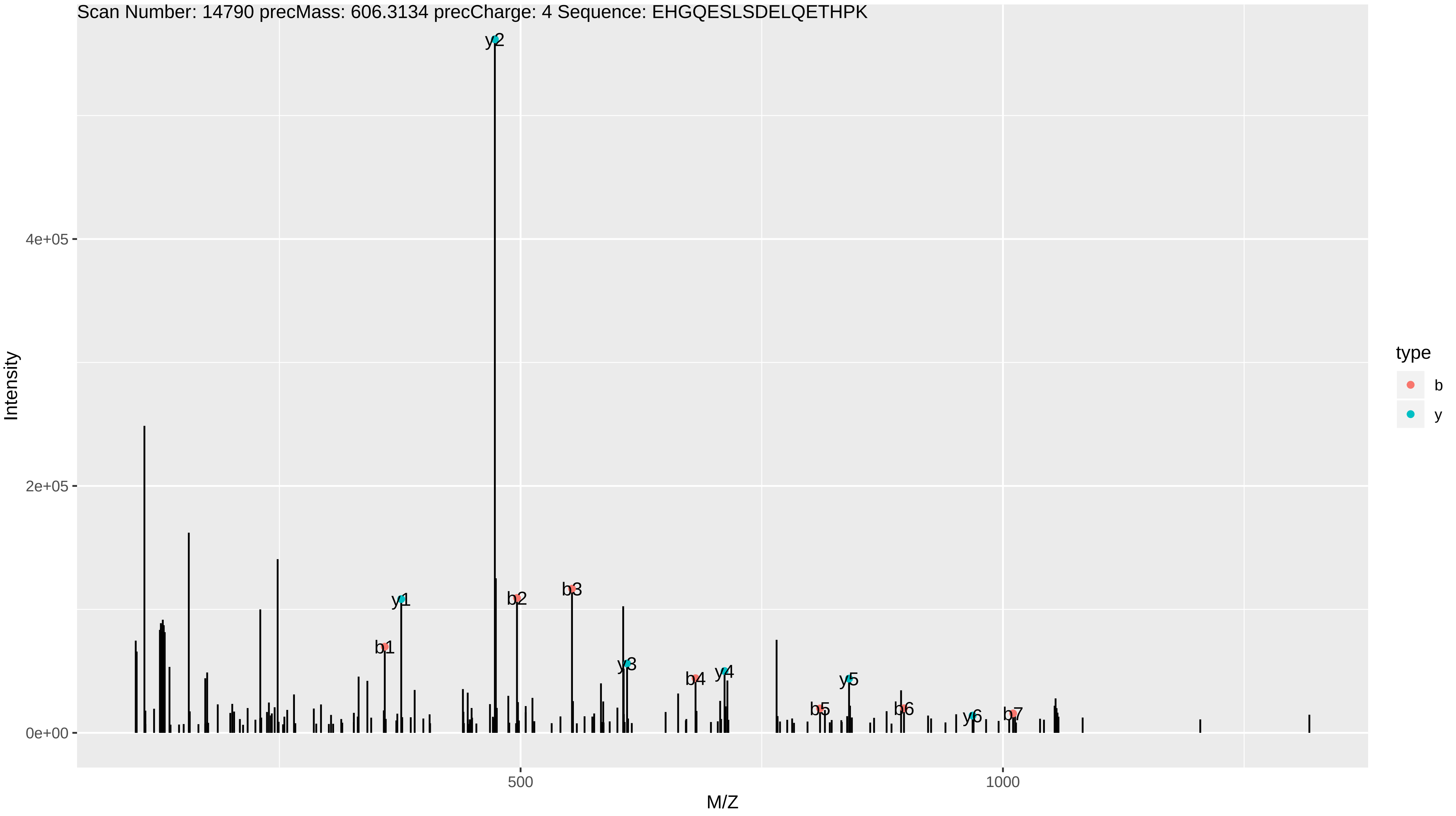

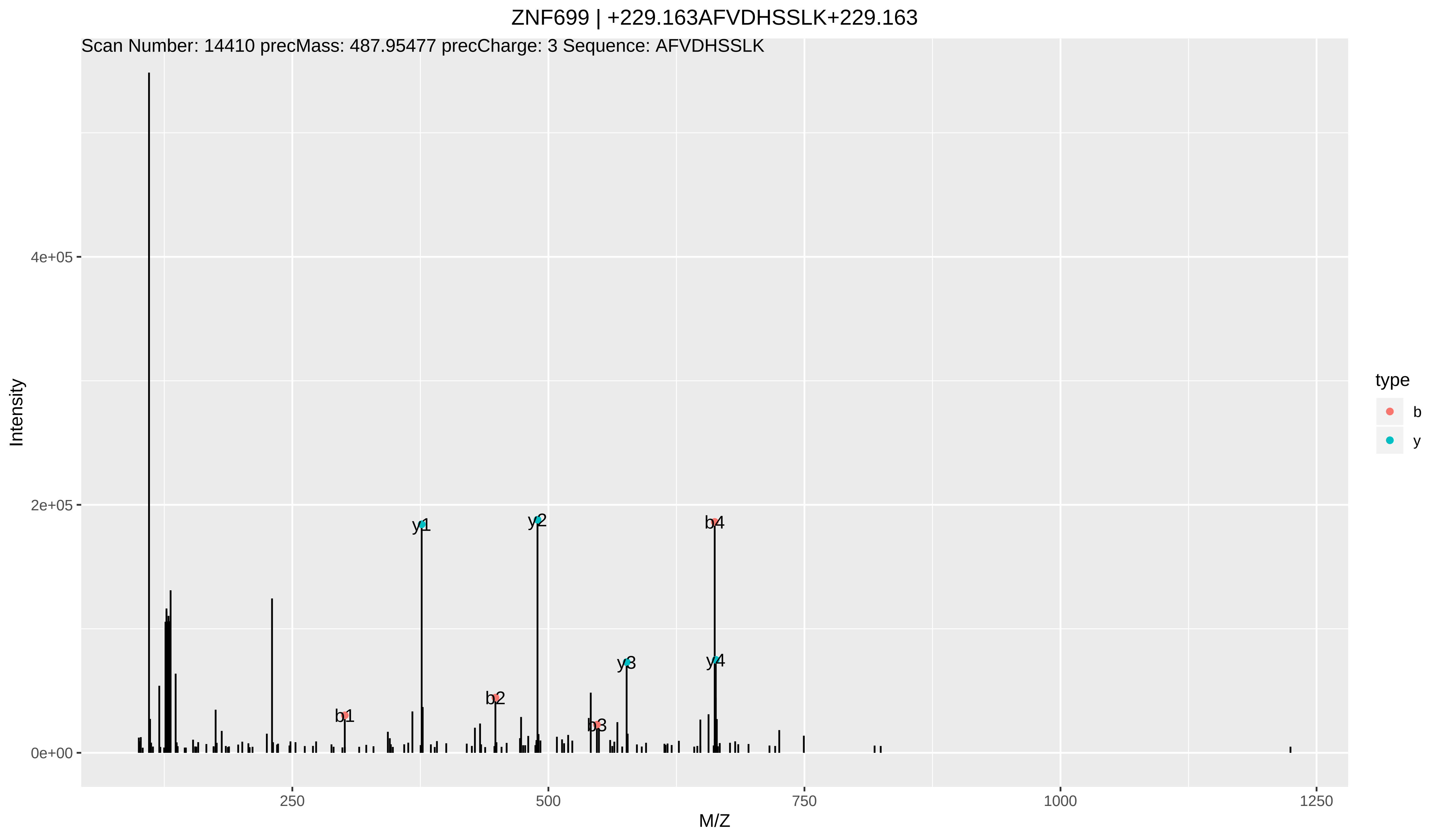
ZNF699 | +229.163AFVDHSSLK+229.163
Scan Number: 14410 precMass: 487.95477 precCharge: 3 Sequence: AFVDHSSLK
Intensity
4e+05
2e+05
0e+00
M/Z
250
500
750
1000
1250
y1
y2
b4
y3
y4
b2
b1
b3
type
b
y

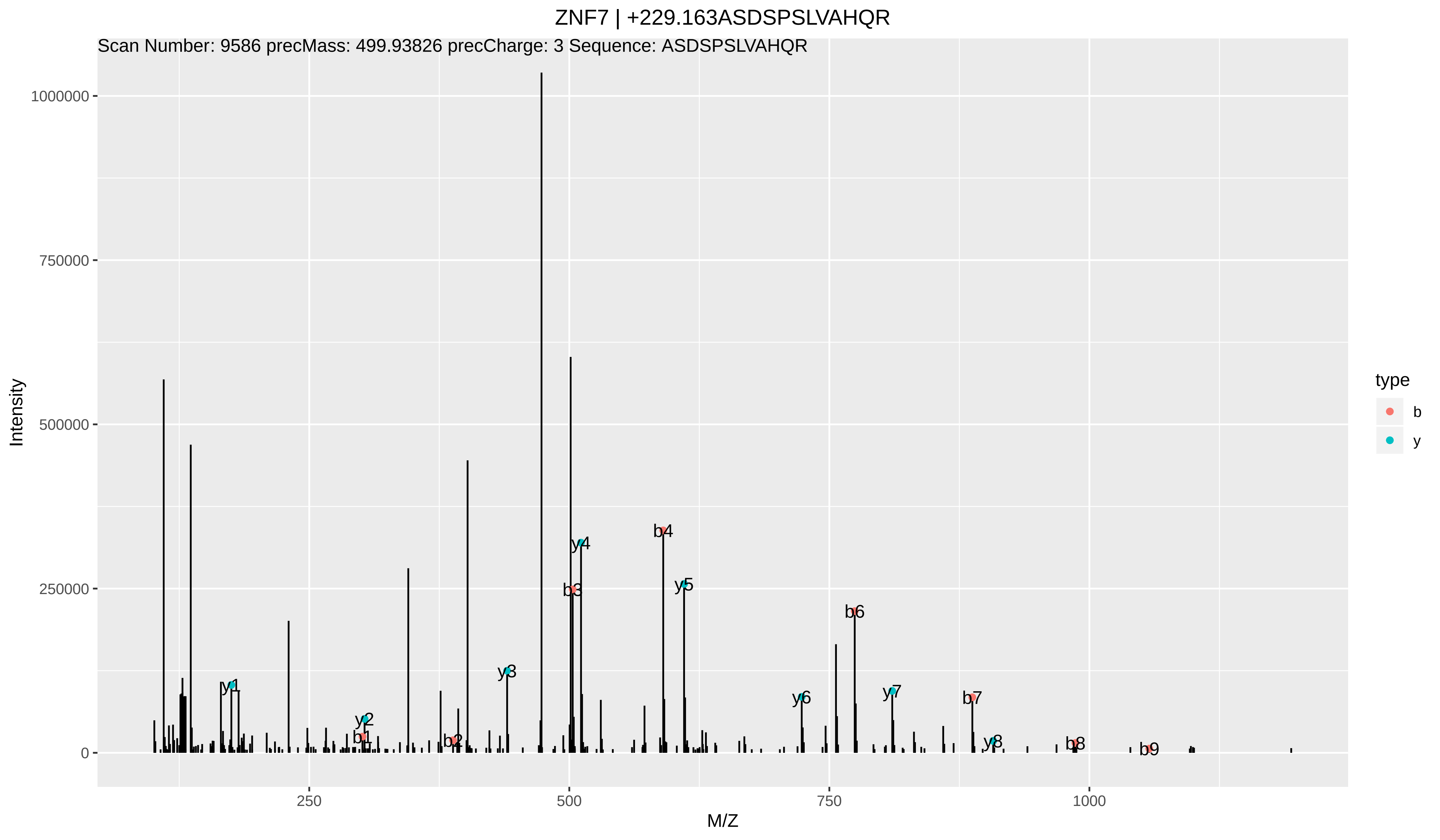
ZNF7 | +229.163ASDSPSLVAHQR
Scan Number: 9586 precMass: 499.93826 precCharge: 3 Sequence: ASDSPSLVAHQR
Intensity
M/Z
type
b
y
y1
y2
b1
b2
y3
b3
y4
b4
y5
y6
b6
y7
b7
y8
b8
b9
0
250000
500000
750000
1000000
250
500
750
1000

ZNF703 | +229.163SEPPSALVGAGHVAPVSPYK+229.163

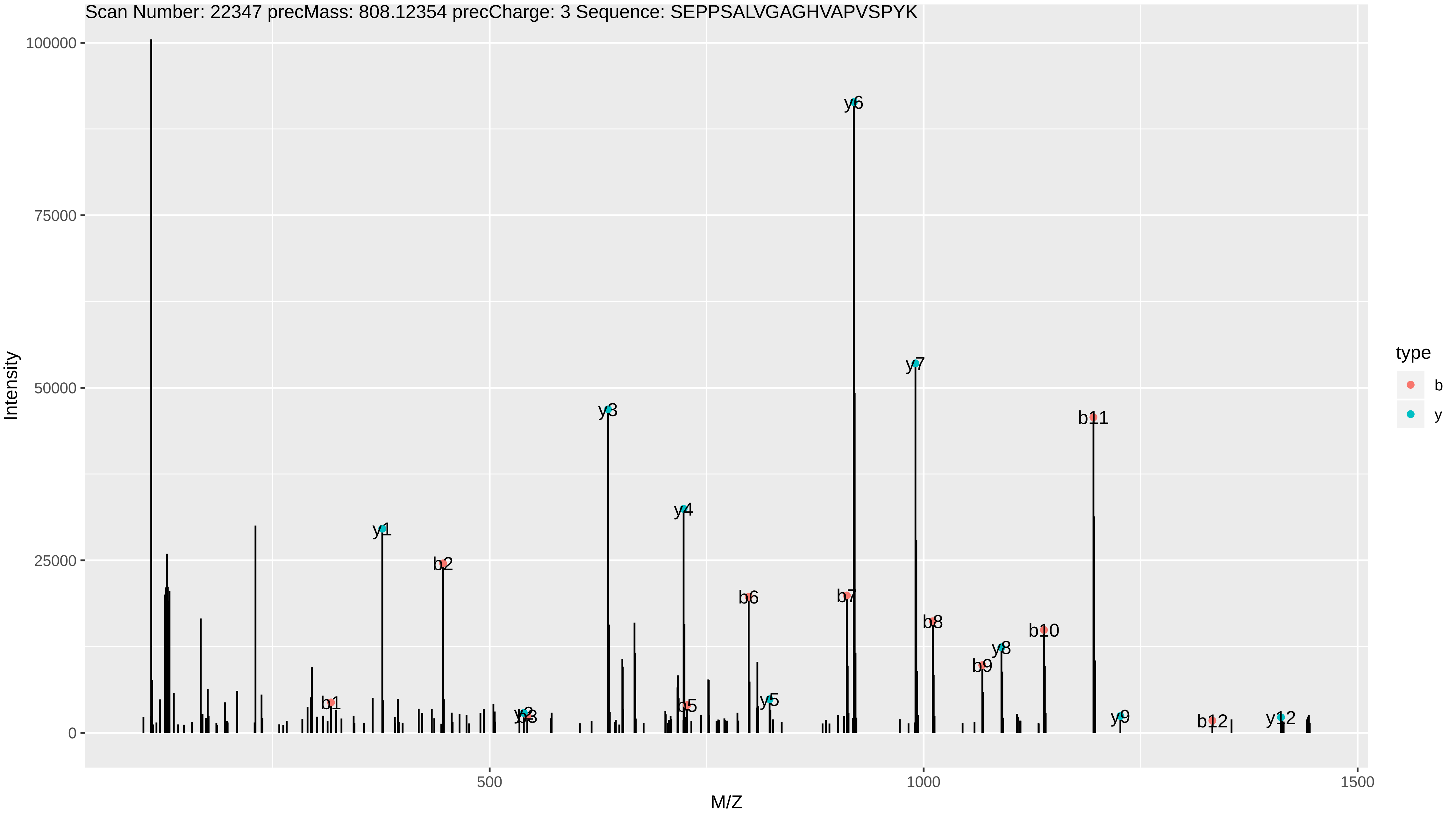

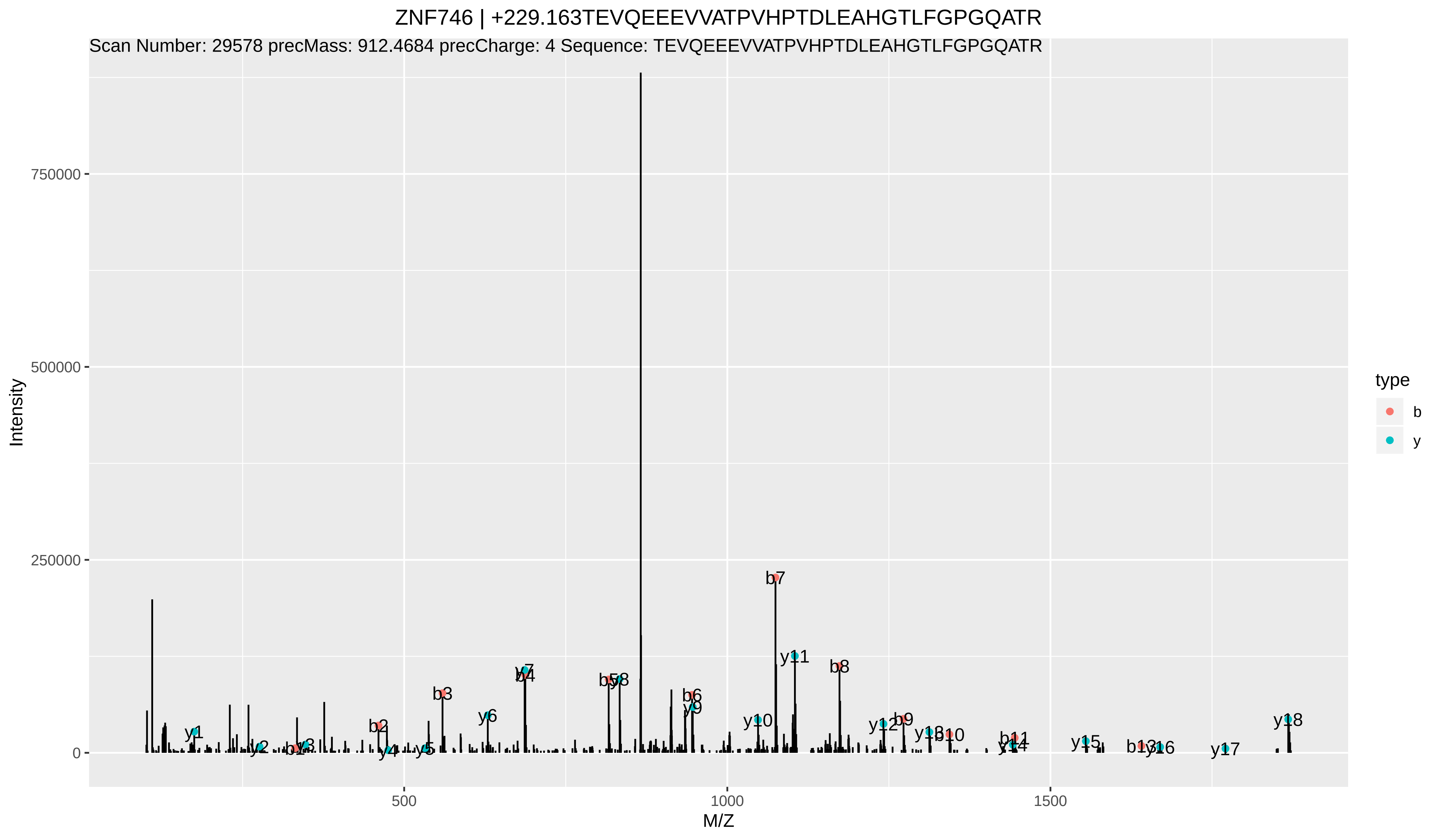
ZNF746 | +229.163TEVQEEEVVATPVHPTDLEAHGTLFGPGQATR
Scan Number: 29578 precMass: 912.4684 precCharge: 4 Sequence: TEVQEEEVVATPVHPTDLEAHGTLFGPGQATR
Intensity
750000
500000
250000
0
M/Z
500
1000
1500
type
b
y
y1
y2
y3
y4
y5
y6
y7
y8
y9
y10
y11
y12
y14
y15
y16
y17
y18
b1
b2
b3
b4
b5
b6
b7
b8
b9
b10
b11

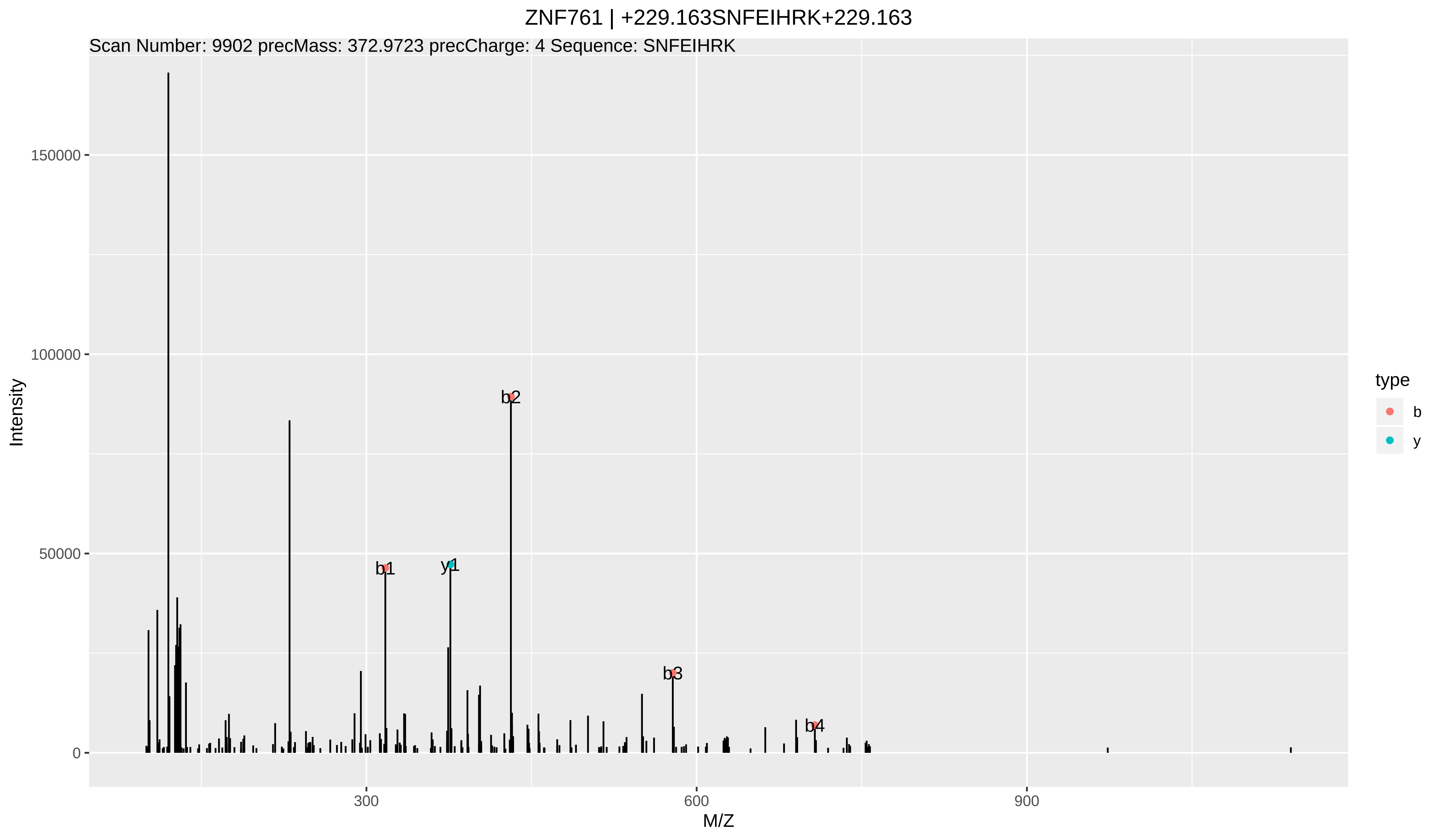
ZNF761 | +229.163SNFEIHRK+229.163
Scan Number: 9902 precMass: 372.9723 precCharge: 4 Sequence: SNFEIHRK
Intensity
150000
100000
50000
0
300
600
900
M/Z
b1
y1
b2
b3
b4
type
b
y

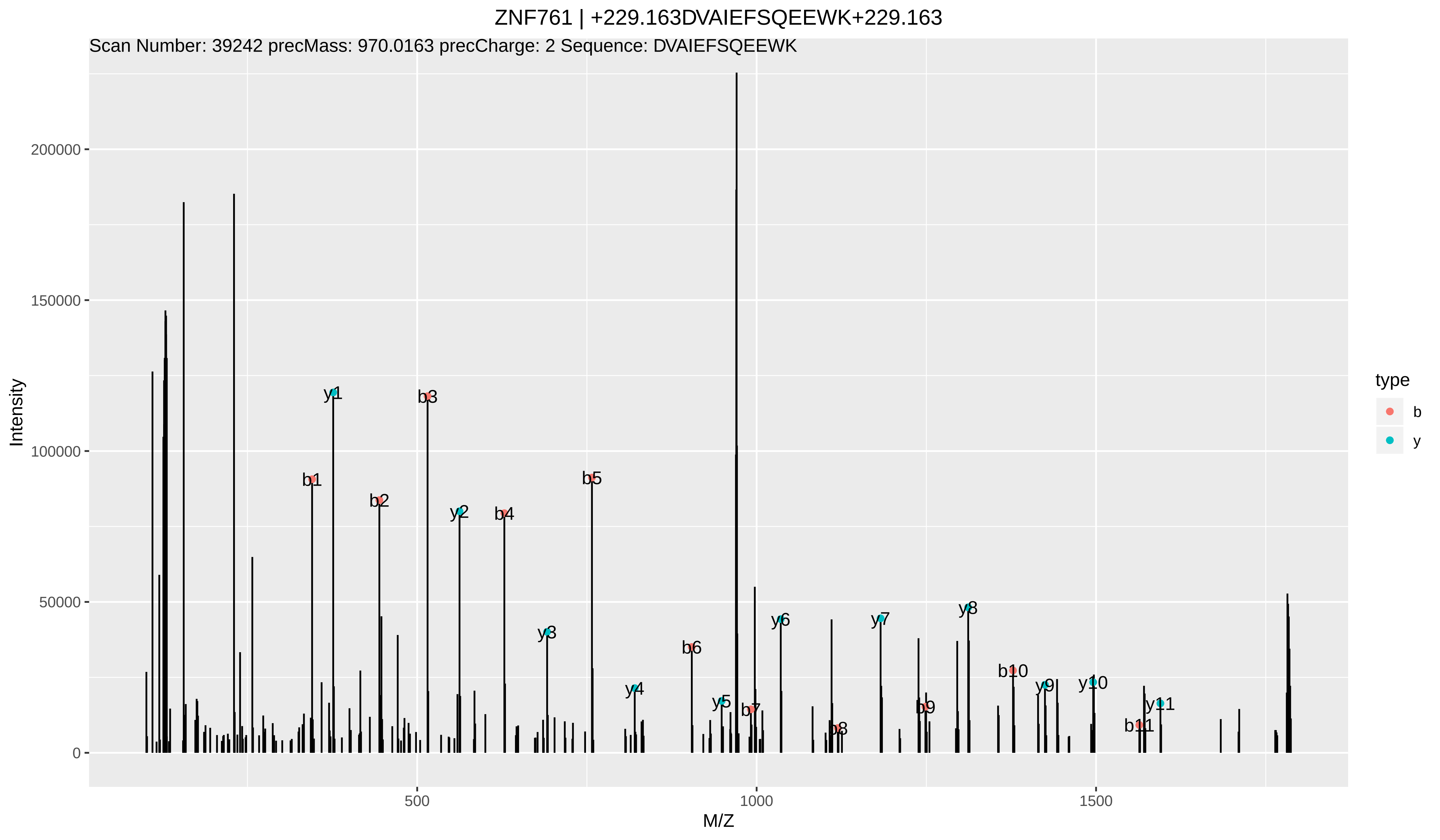
ZNF761 | +229.163DVAIEFSQEEWK+229.163
Scan Number: 39242 precMass: 970.0163 precCharge: 2 Sequence: DVAIEFSQEEWK
Intensity
M/Z
type
b
y
y1
b1
b2
b3
y2
b4
y3
b5
y4
b6
y5
b7
y6
b8
y7
b9
y8
b10
y9
y10
b11
y11

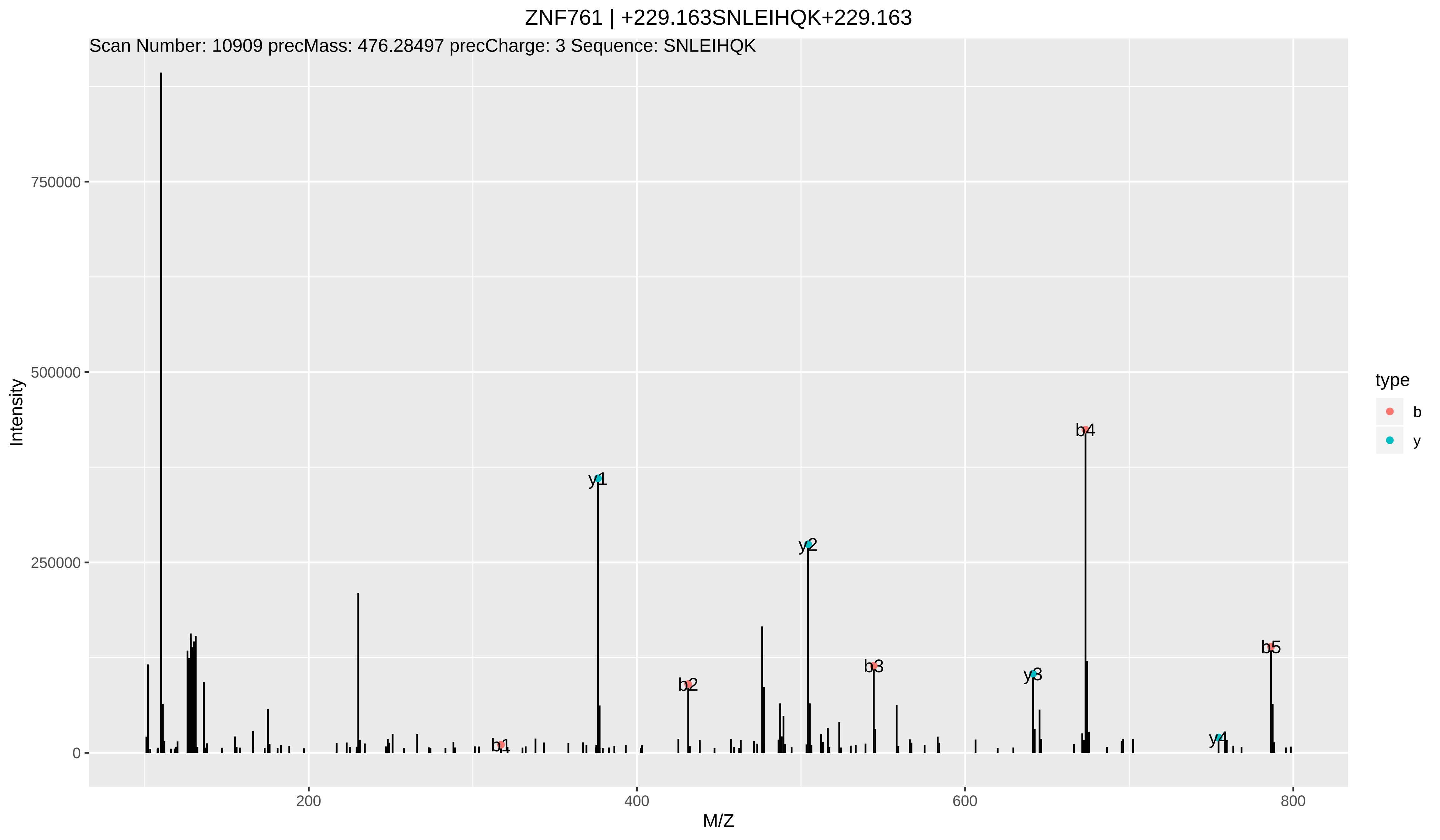

ZNF761 | +229.163SNLEIHQK+229.163
Scan Number: 10909 precMass: 476.28497 precCharge: 3 Sequence: SNLEIHQK
Intensity
750000
500000
250000
0
M/Z
200
400
600
800
y1
y2
b4
b2
b3
y3
b5
b1
y4
type
b
y

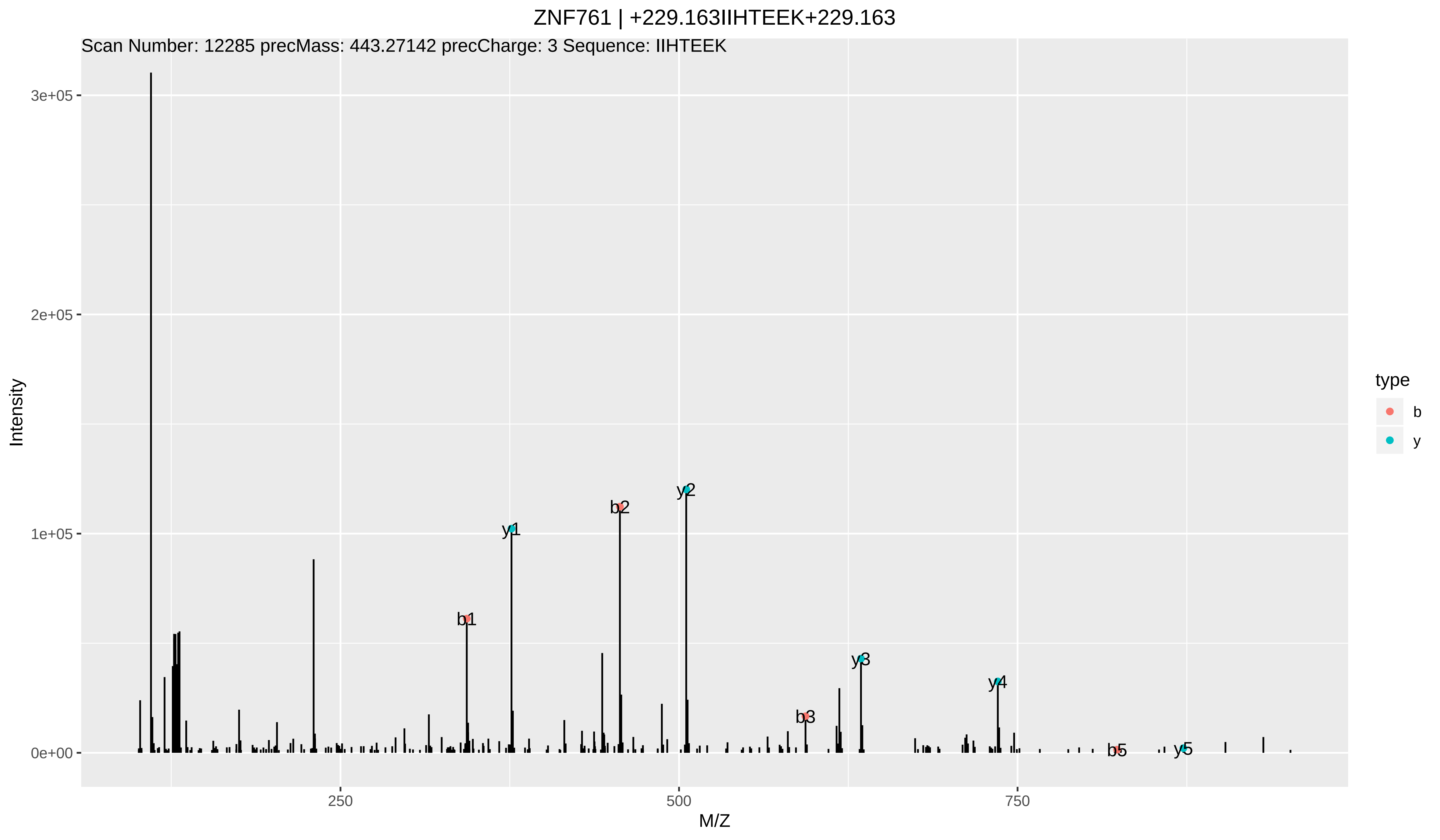

ZNF761 | +229.163IIHTEEK+229.163
Scan Number: 12285 precMass: 443.27142 precCharge: 3 Sequence: IIHTEEK
Intensity
3e+05
2e+05
1e+05
0e+00
M/Z
250
500
750
b1
y1
b2
y2
b3
y3
y4
b5
y5
type
b
y

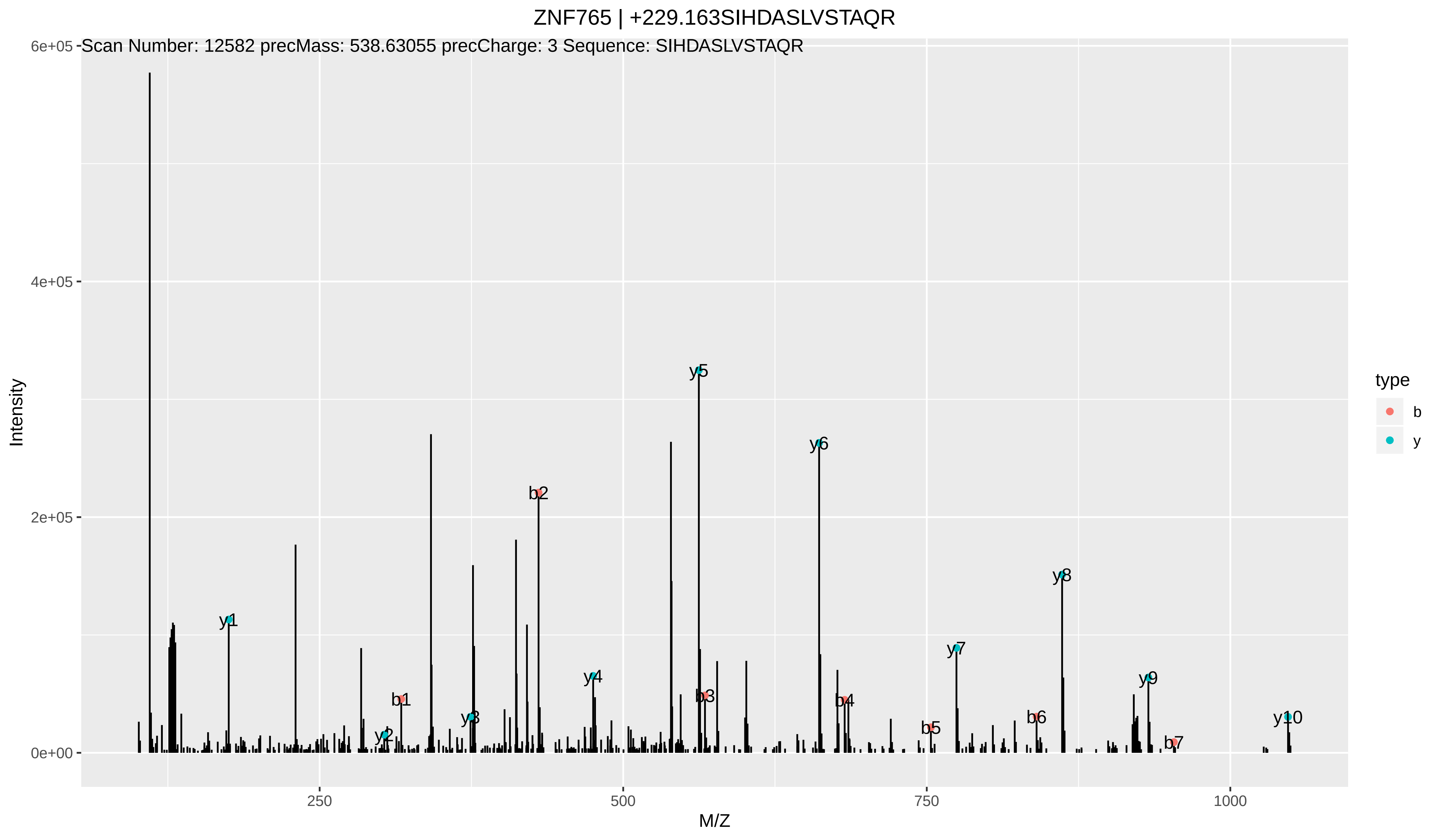
ZNF765 | +229.163SIHDASLVSTAQR
Scan Number: 12582 precMass: 538.63055 precCharge: 3 Sequence: SIHDASLVSTAQR
Intensity
M/Z
6e+05
4e+05
2e+05
0e+00
250
500
750
1000
y1
y2
y3
y4
y5
y6
y7
y8
y9
y10
b1
b2
b3
b4
b5
b6
b7
type
b
y

ZNF765 | +229.163DVAIEFSQEEWK+229.163

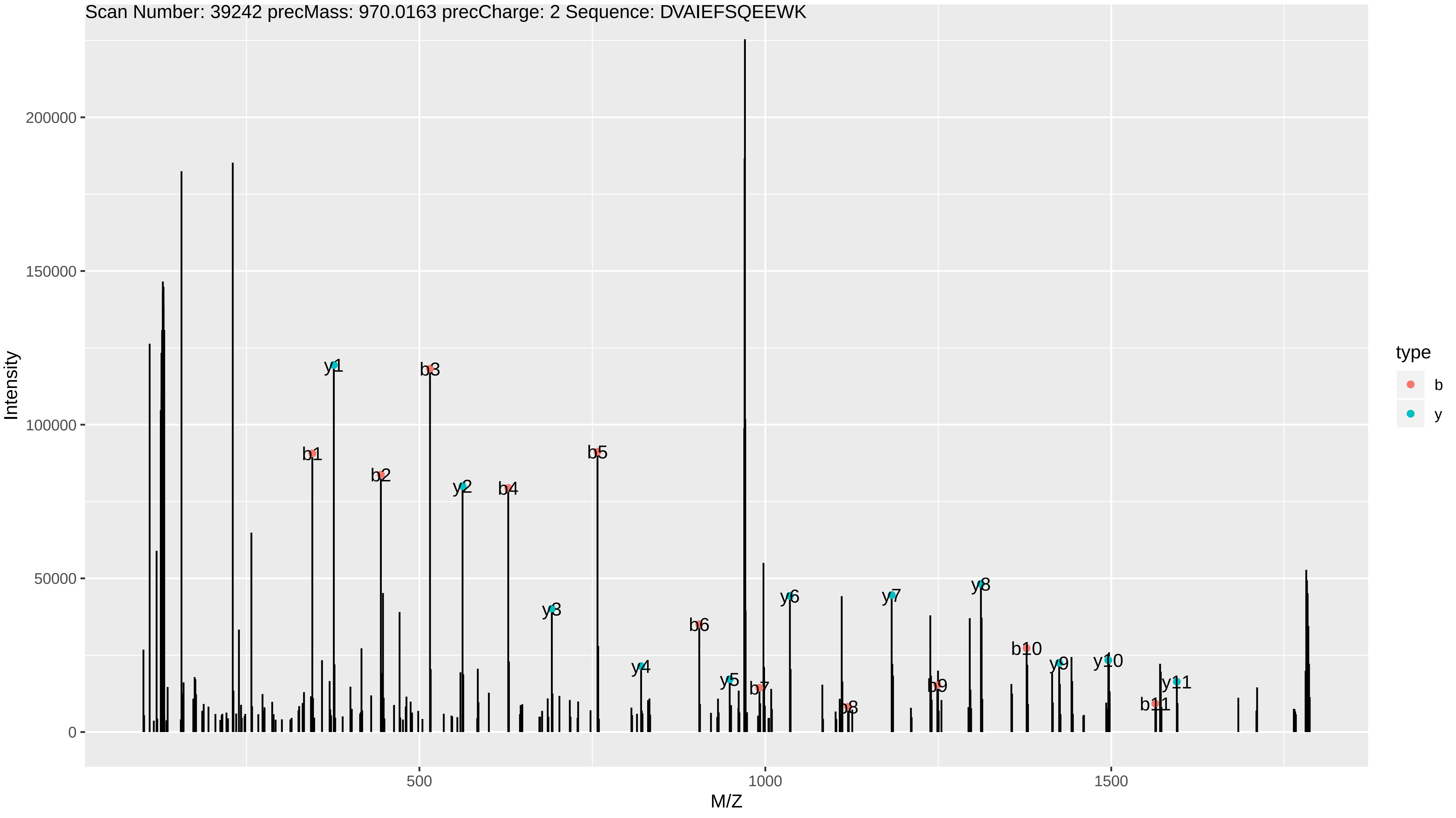

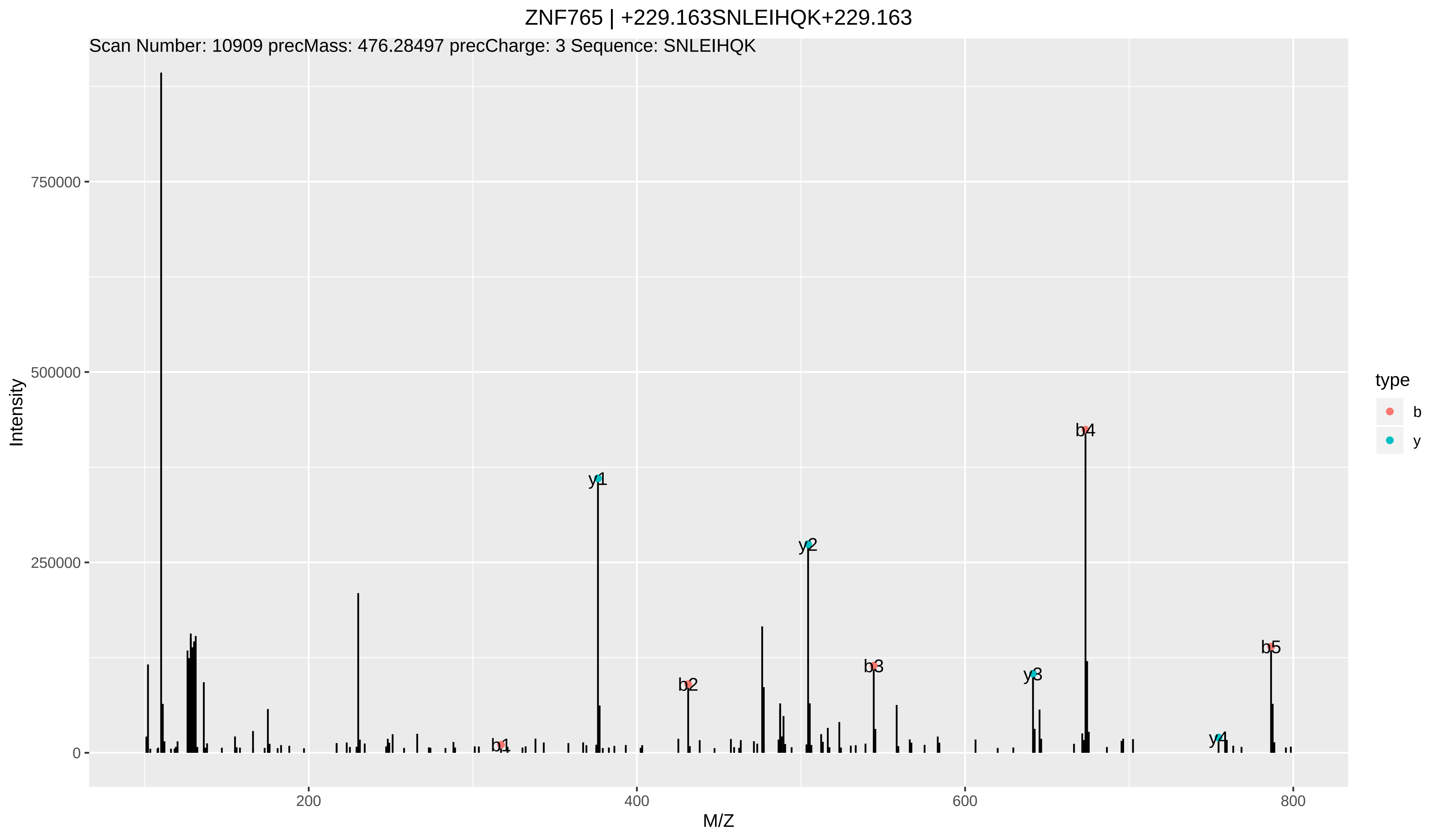

ZNF765 | +229.163SNLEIHQK+229.163
Scan Number: 10909 precMass: 476.28497 precCharge: 3 Sequence: SNLEIHQK
Intensity
750000
500000
250000
0
M/Z
200
400
600
800
y1
y2
b4
b5
b3
y3
b2
b1
y4
type
b
y

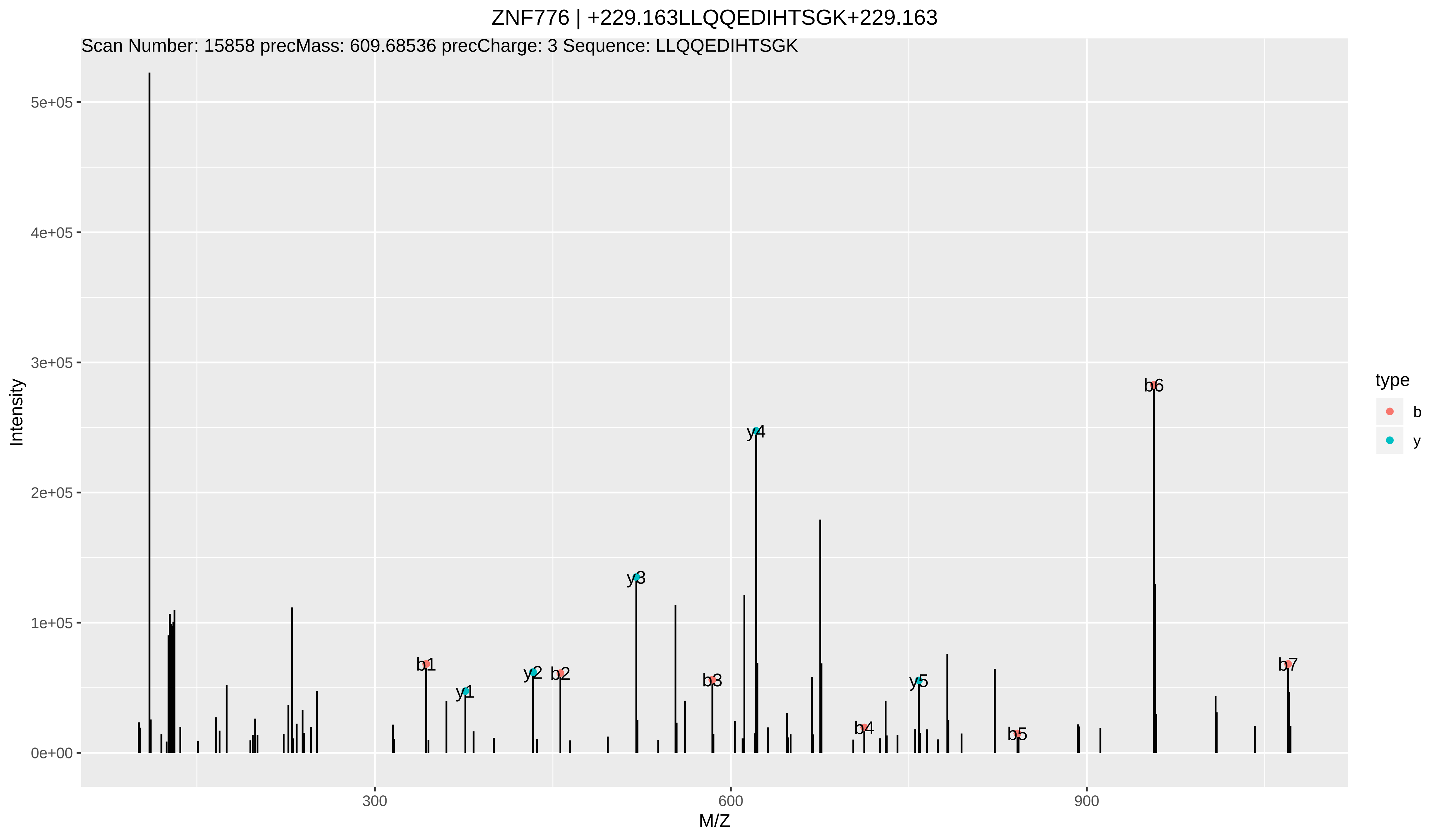
ZNF776 | +229.163LLQQEDIHTSGK+229.163
Scan Number: 15858 precMass: 609.68536 precCharge: 3 Sequence: LLQQEDIHTSGK
Intensity
5e+05
4e+05
3e+05
2e+05
1e+05
0e+00
M/Z
300
600
900
b1
y1
y2
b2
y3
b3
y4
b4
y5
b5
b6
b7
type
b
y

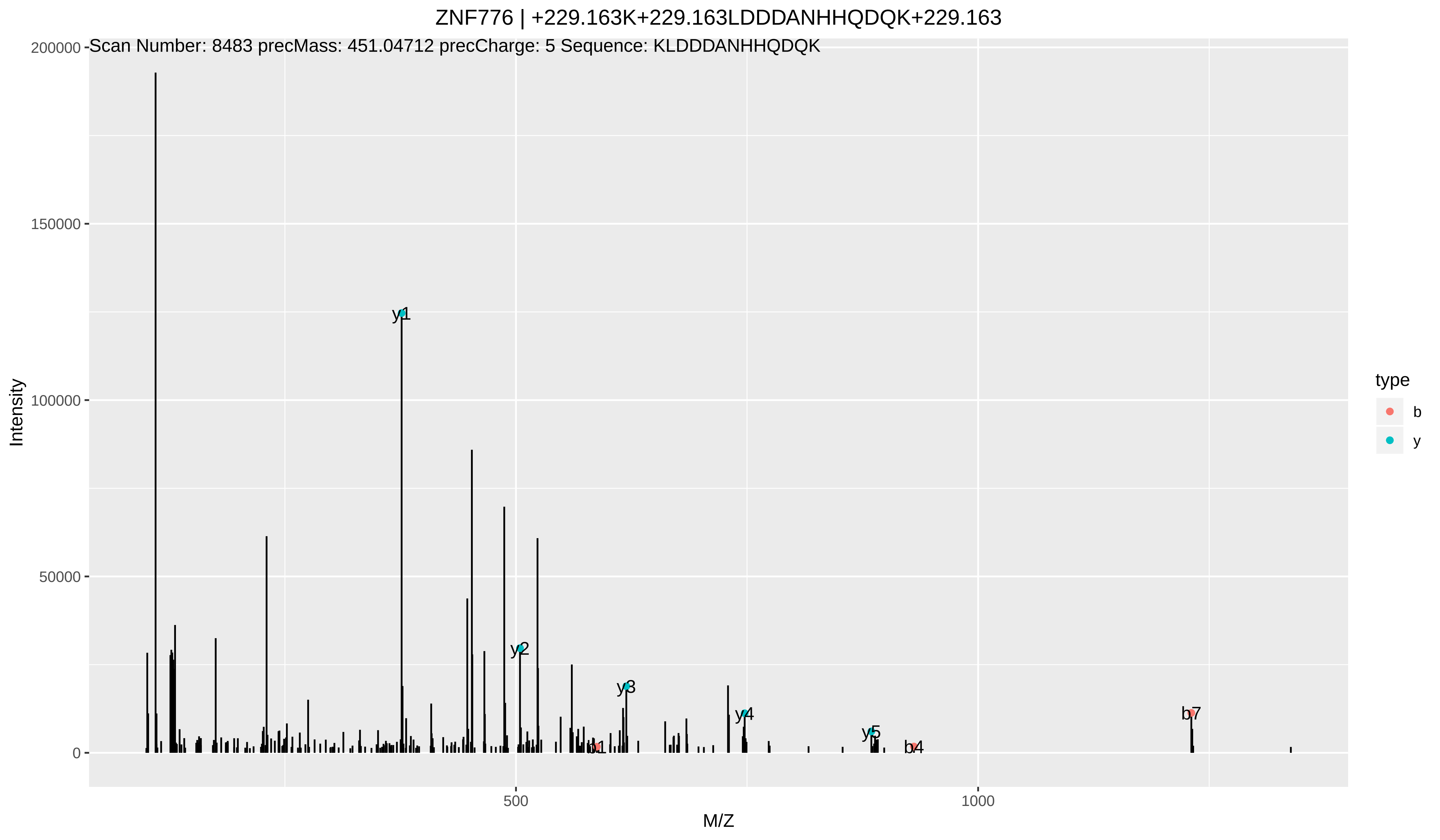
ZNF776 | +229.163K+229.163LDDDANHHQDQK+229.163
Scan Number: 8483 precMass: 451.04712 precCharge: 5 Sequence: KLDDDANHHQDQK
Intensity
200000
150000
100000
50000
0
M/Z
500
1000
y1
y2
y3
y4
y5
b1
b4
b7
type
b
y

ZNF777 | +229.163TEEQDEEEEEEEEDELPQHLQSLGQLSGR

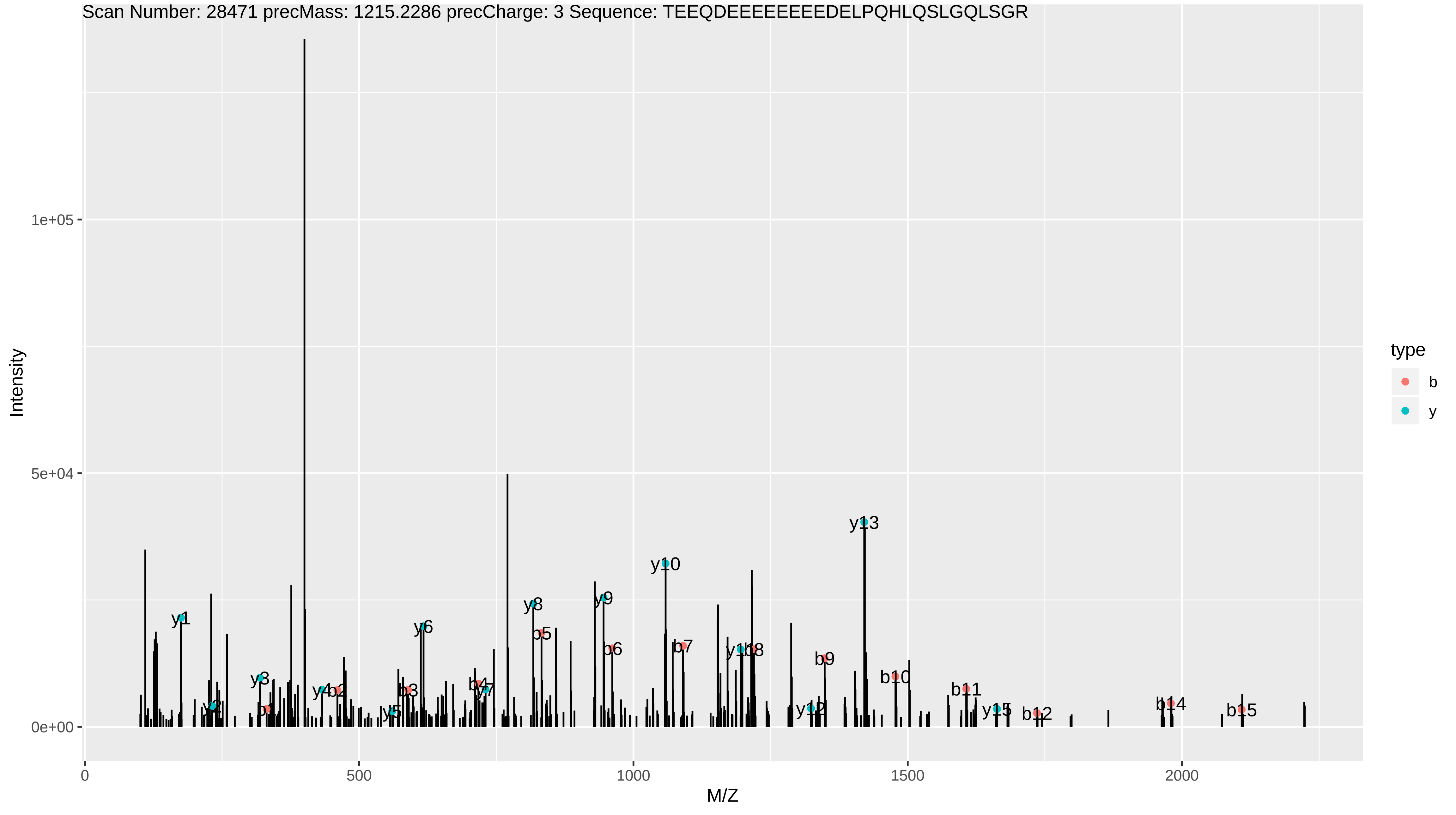

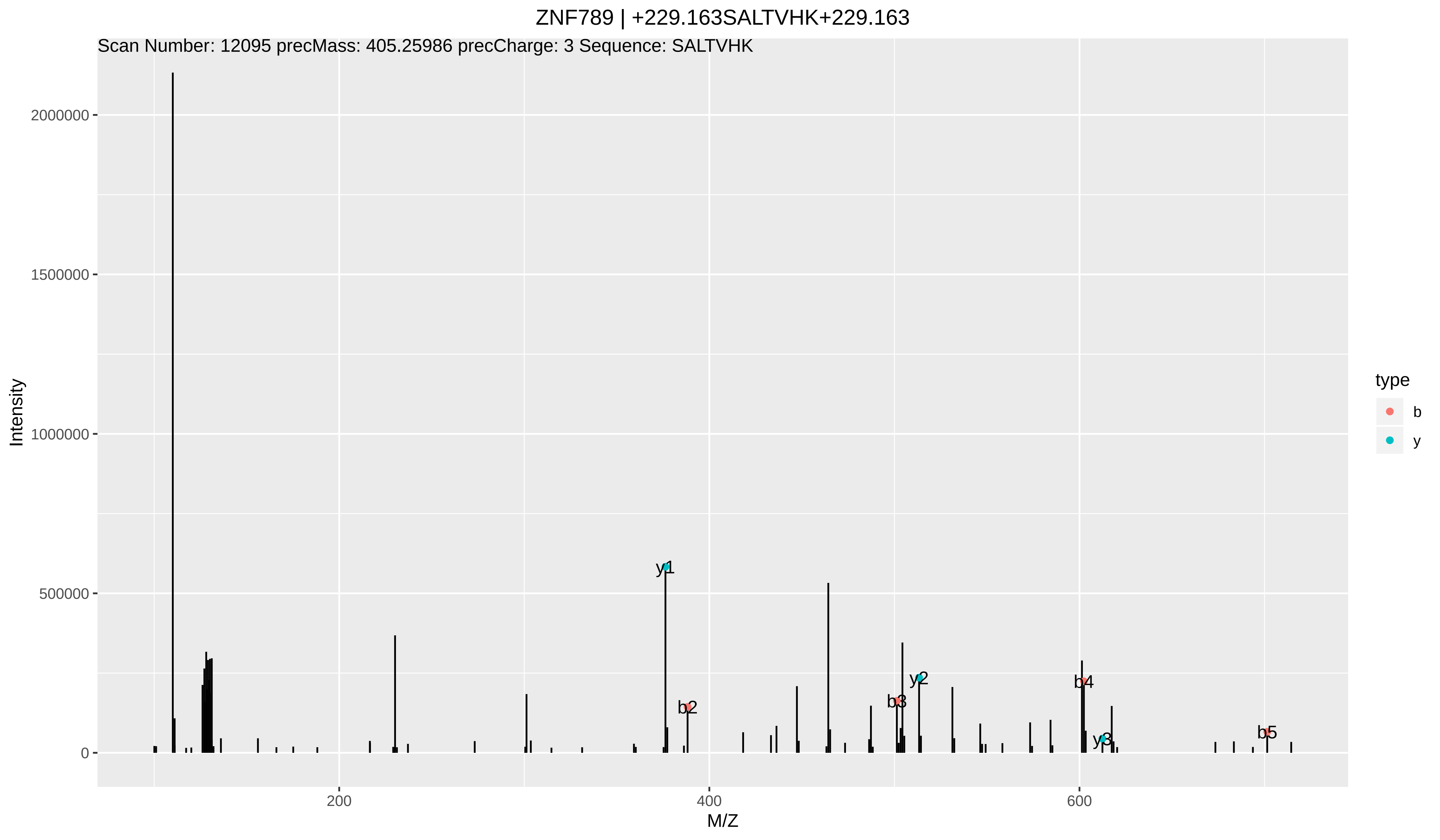
ZNF789 | +229.163SALTVHK+229.163
Scan Number: 12095 precMass: 405.25986 precCharge: 3 Sequence: SALTVHK
Intensity
2000000
1500000
1000000
500000
0
200
400
600
M/Z
y1
b2
b3
y2
b4
y3
b5
type
b
y

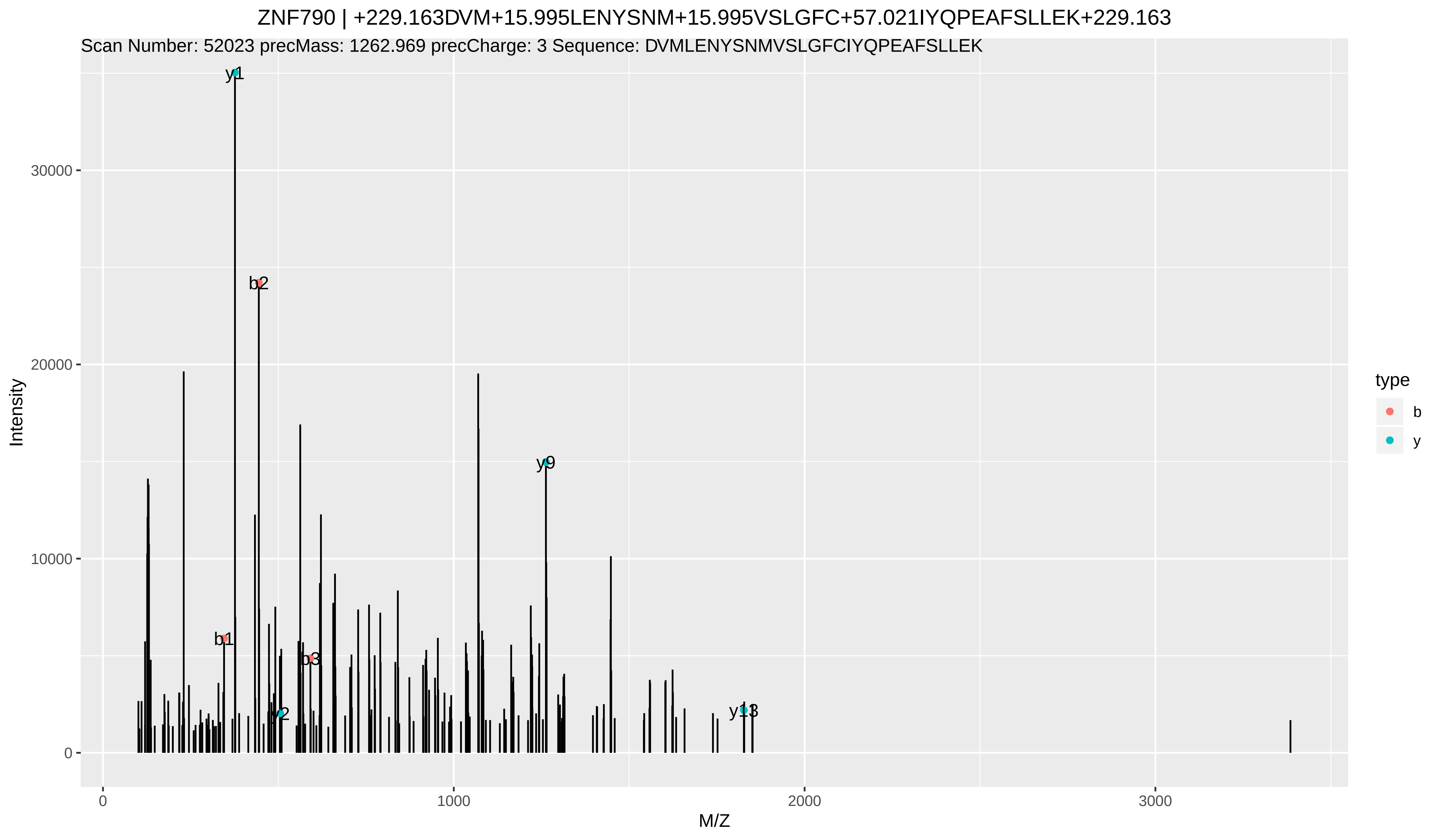

ZNF790 | +229.163DVM+15.995LENYSNM+15.995VSLGFC+57.021IYQPEAFSLLEK+229.163
Scan Number: 52023 precMass: 1262.969 precCharge: 3 Sequence: DVMLENYSNMVSLGFCIYQPEAFSLLEK
y1
b2
y9
b1
b3
y2
y13
Intensity
M/Z
0
10000
20000
30000
1000
2000
3000
type
b
y

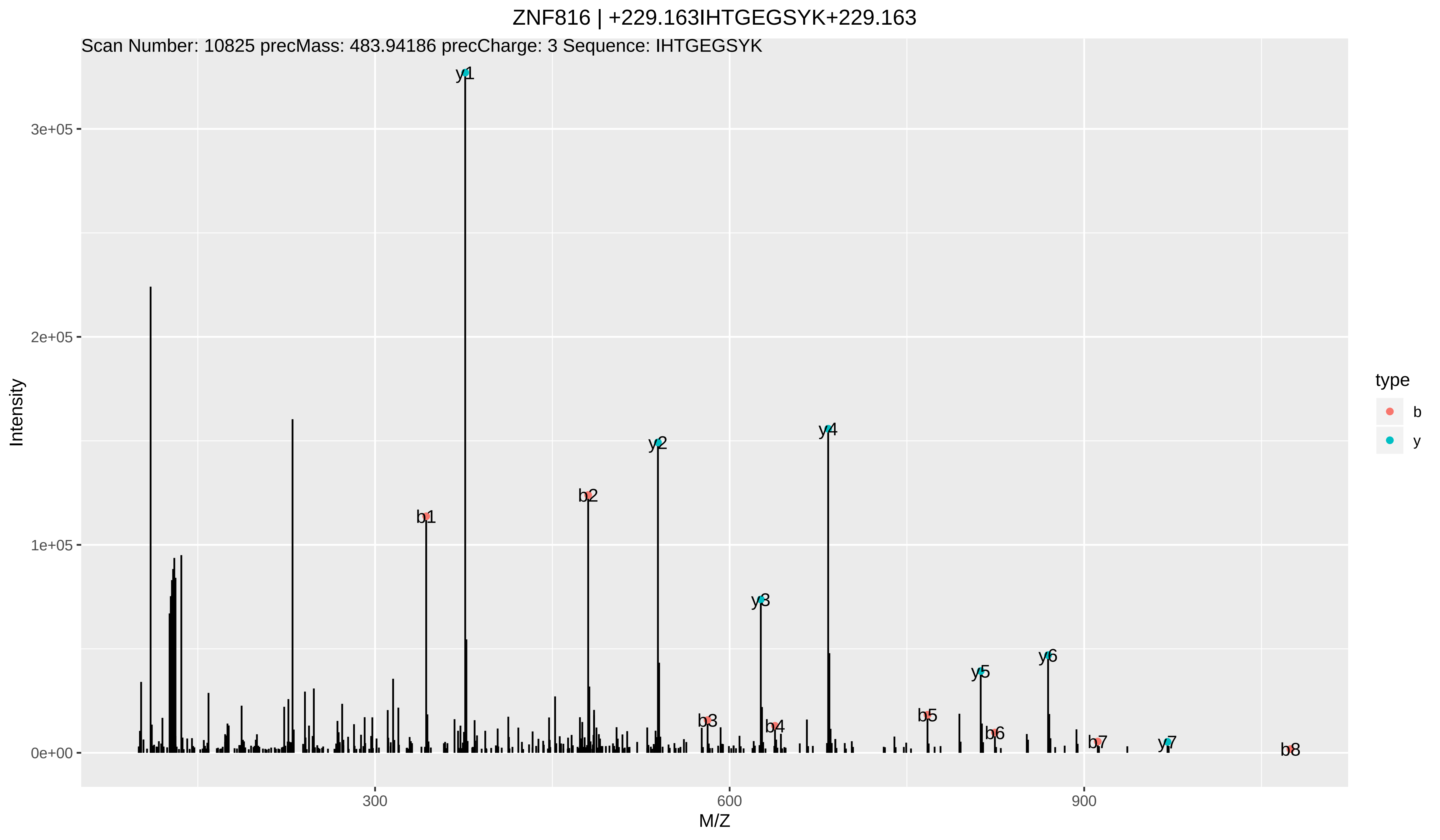

ZNF816 | +229.163IHTGEGSYK+229.163
Scan Number: 10825 precMass: 483.94186 precCharge: 3 Sequence: IHTGEGSYK
Intensity
3e+05
2e+05
1e+05
0e+00
M/Z
300
600
900
y1
b1
b2
y2
b3
y3
b4
y4
b5
y5
b6
y6
b7
y7
b8
type
b
y

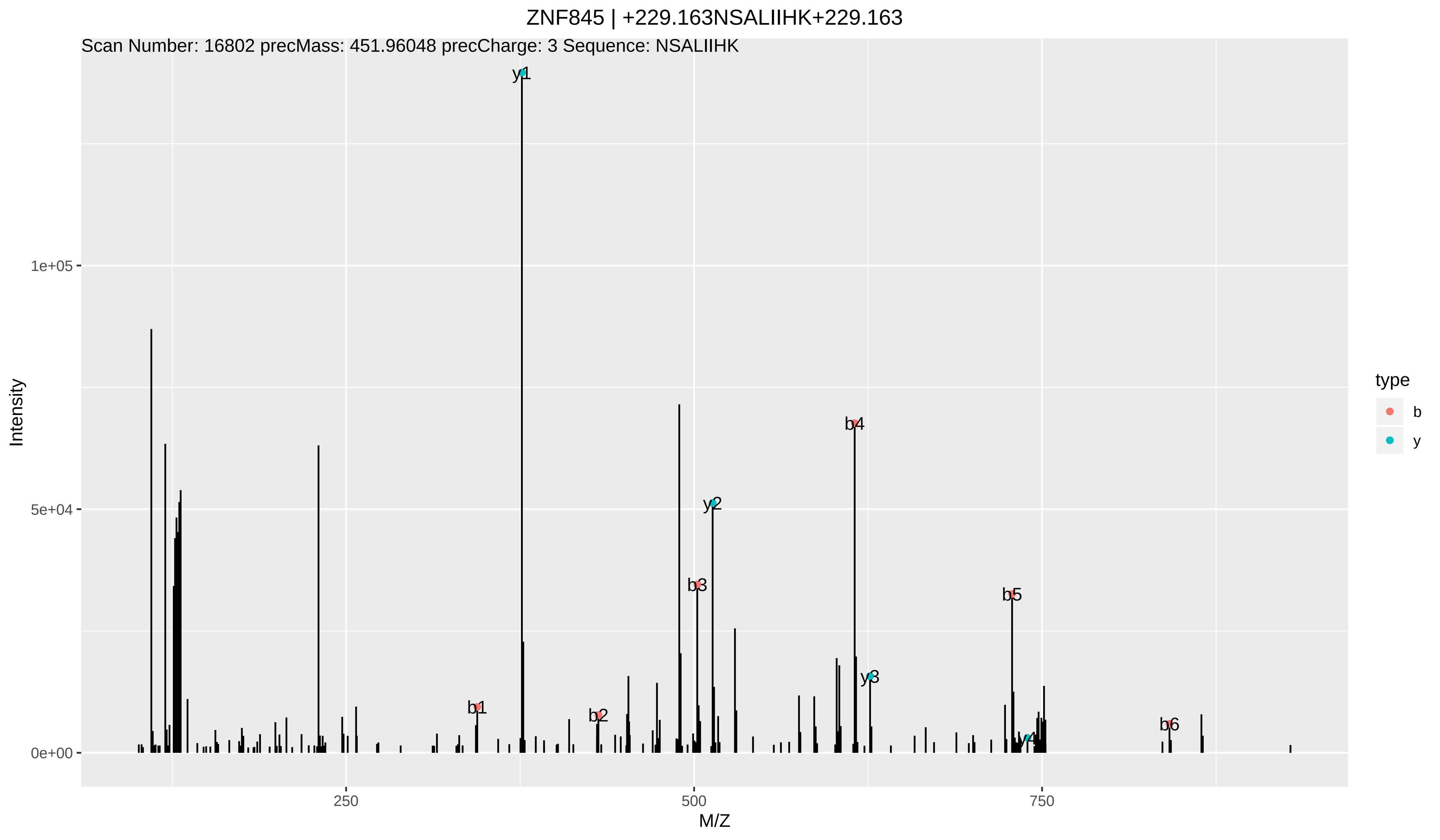
ZNF845 | +229.163NSALIIHK+229.163
Scan Number: 16802 precMass: 451.96048 precCharge: 3 Sequence: NSALIIHK
y1
b4
y2
b3
b5
y3
b1
b2
b6
y4
Intensity
1e+05
5e+04
0e+00
250
500
750
M/Z
type
b
y

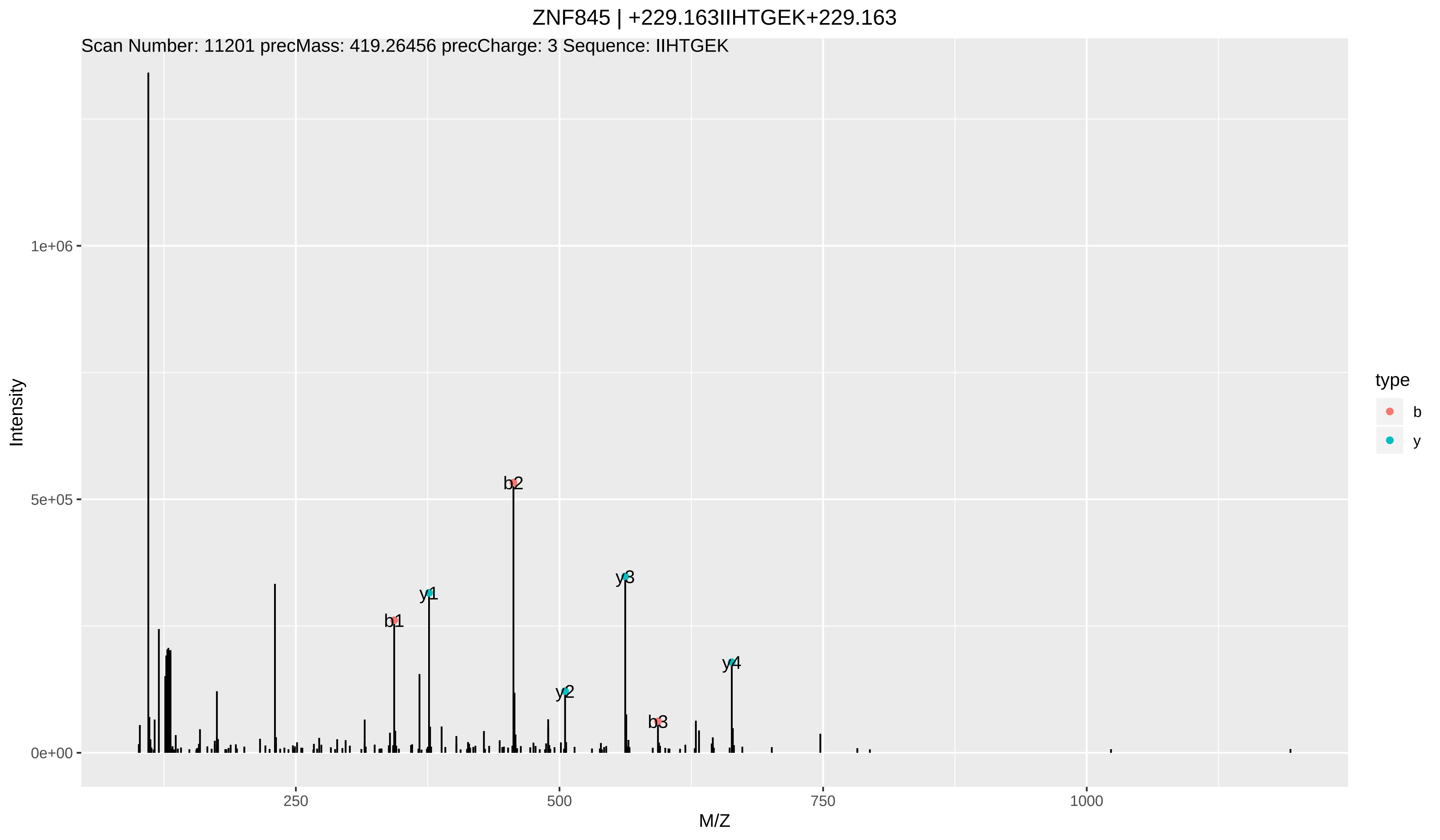
ZNF845 | +229.163IIHTGEK+229.163
Scan Number: 11201 precMass: 419.26456 precCharge: 3 Sequence: IIHTGEK
Intensity
1e+06
5e+05
0e+00
M/Z
250
500
750
1000
b1
b2
b3
y1
y2
y3
y4
type
b
y

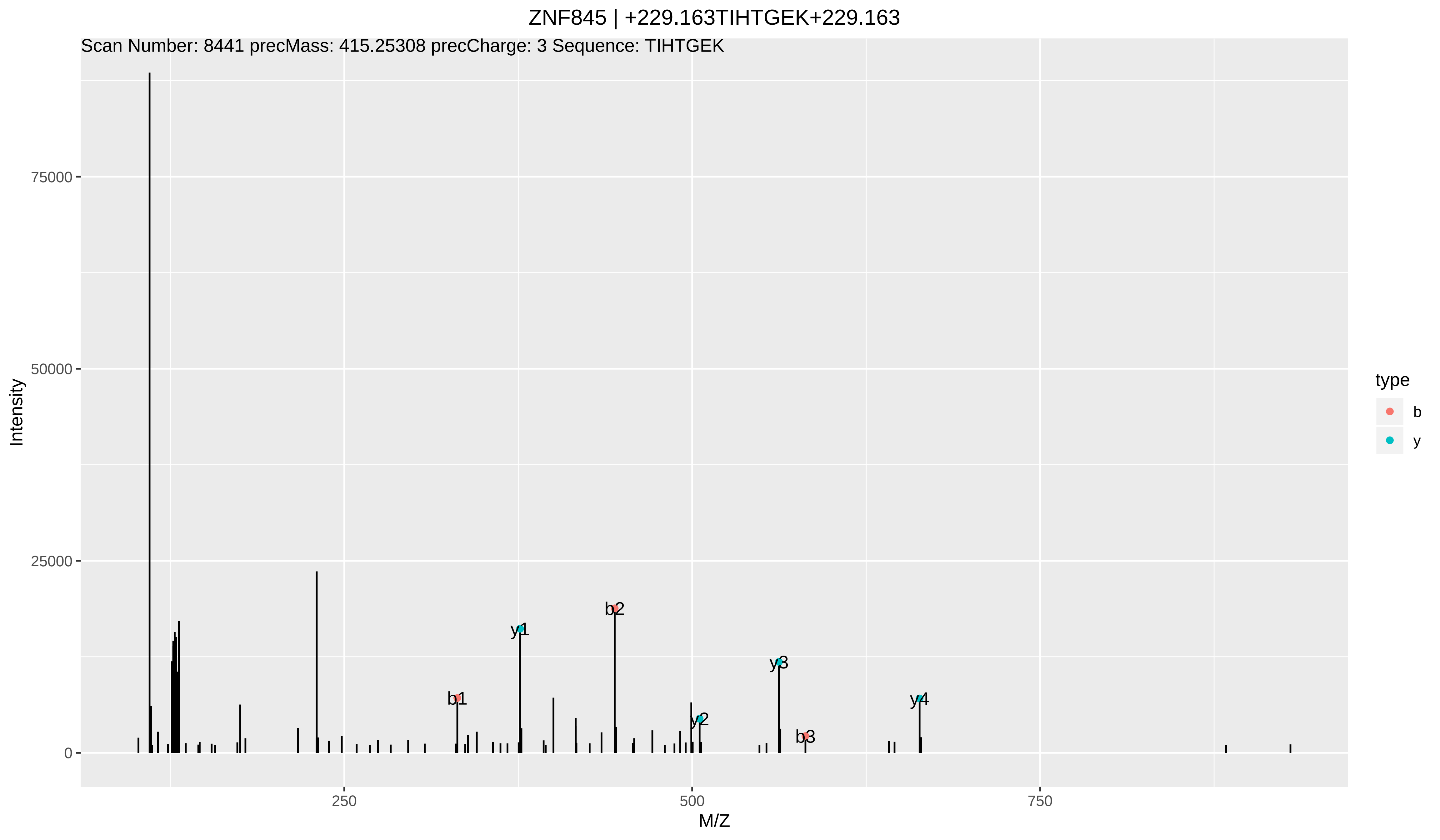
ZNF845 | +229.163TIHTGEK+229.163
Scan Number: 8441 precMass: 415.25308 precCharge: 3 Sequence: TIHTGEK
Intensity
75000
50000
25000
0
M/Z
250
500
750
b1
y1
b2
y2
y3
b3
y4
type
b
y

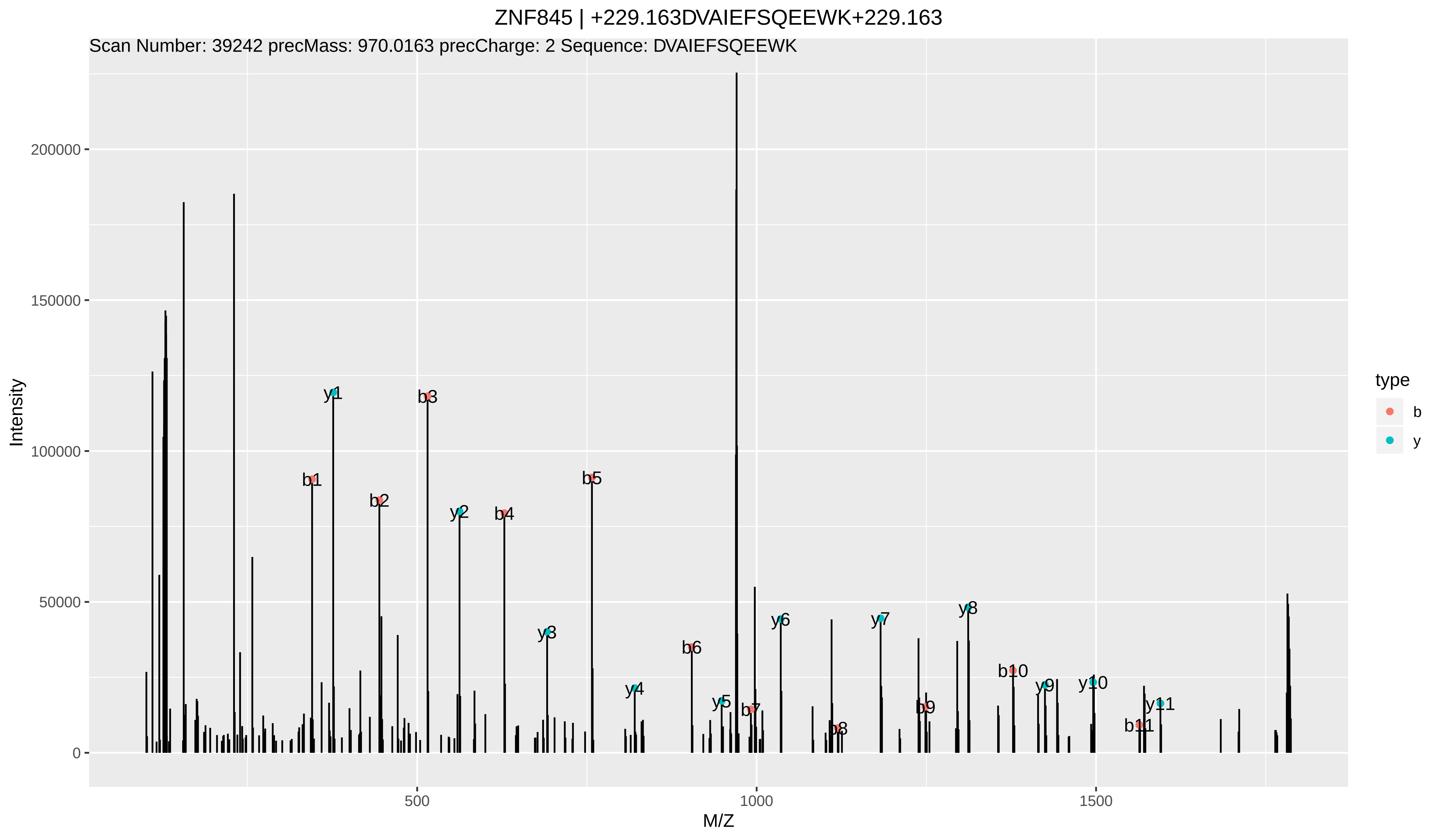

ZNF845 | +229.163DVAIEFSQEEWK+229.163
Scan Number: 39242 precMass: 970.0163 precCharge: 2 Sequence: DVAIEFSQEEWK
Intensity
M/Z
type
b
y
b1
y1
b2
b3
y2
b4
b5
y3
y4
b6
y5
b7
y6
b8
y7
b9
y8
b10
y9
y10
b11
y11

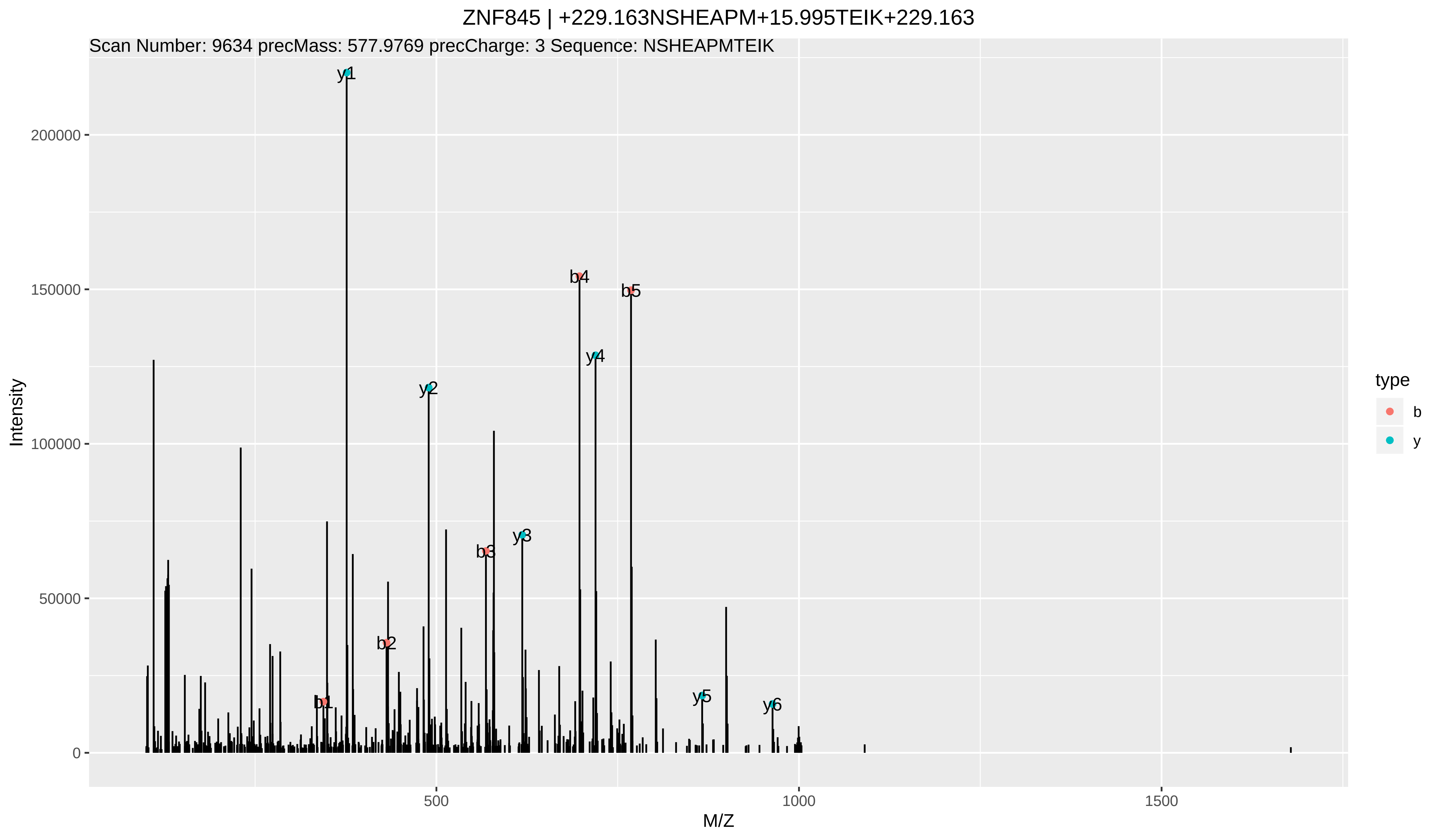

ZNF845 | +229.163NSHEAPM+15.995TEIK+229.163
Scan Number: 9634 precMass: 577.9769 precCharge: 3 Sequence: NSHEAPMTEIK
Intensity
200000
150000
100000
50000
0
500
1000
1500
M/Z
y1
y2
y3
y4
y5
y6
b1
b2
b3
b4
b5
type
b
y

ZNF92 | +229.163AFNQFSILNK+229.163

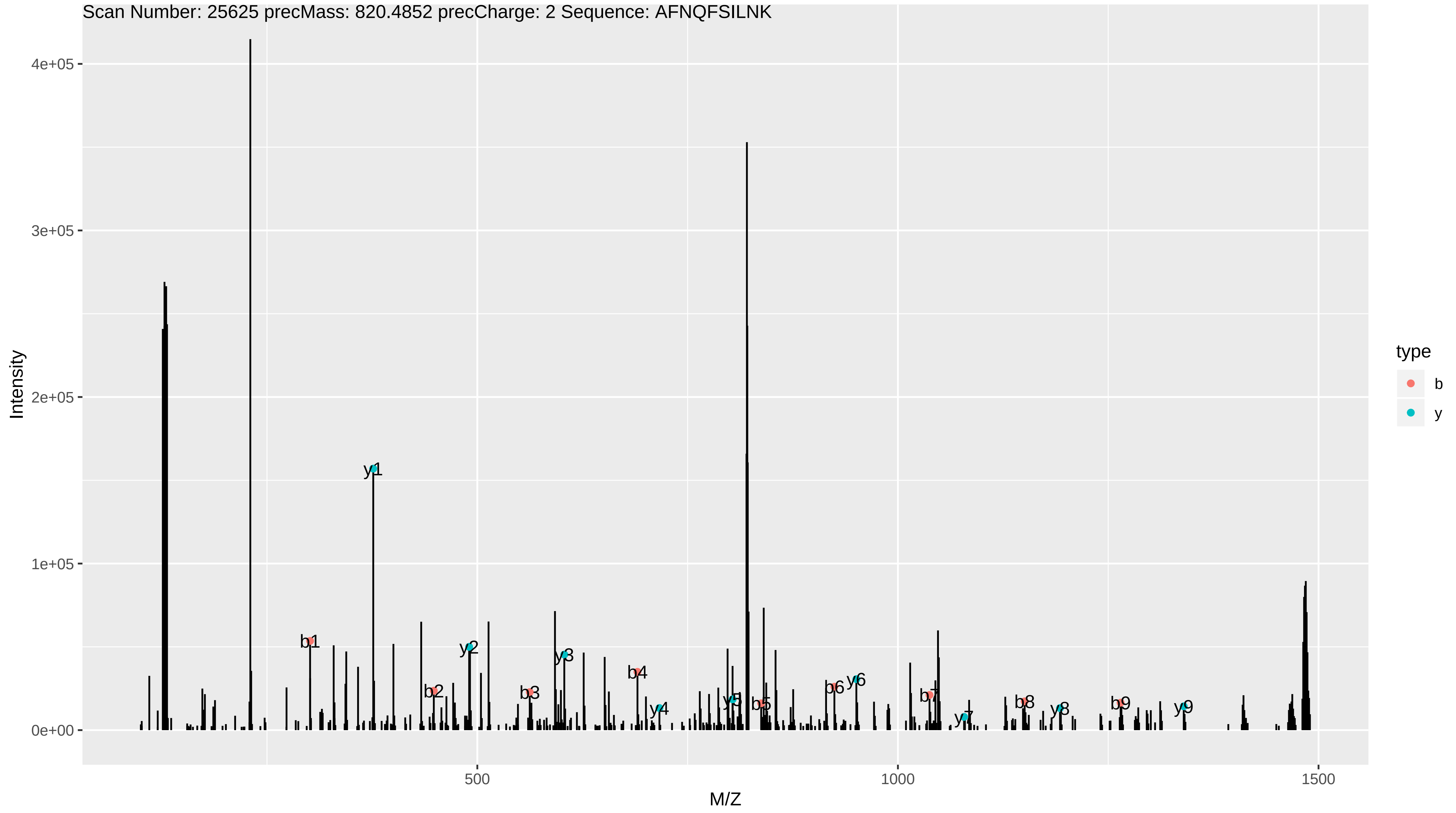

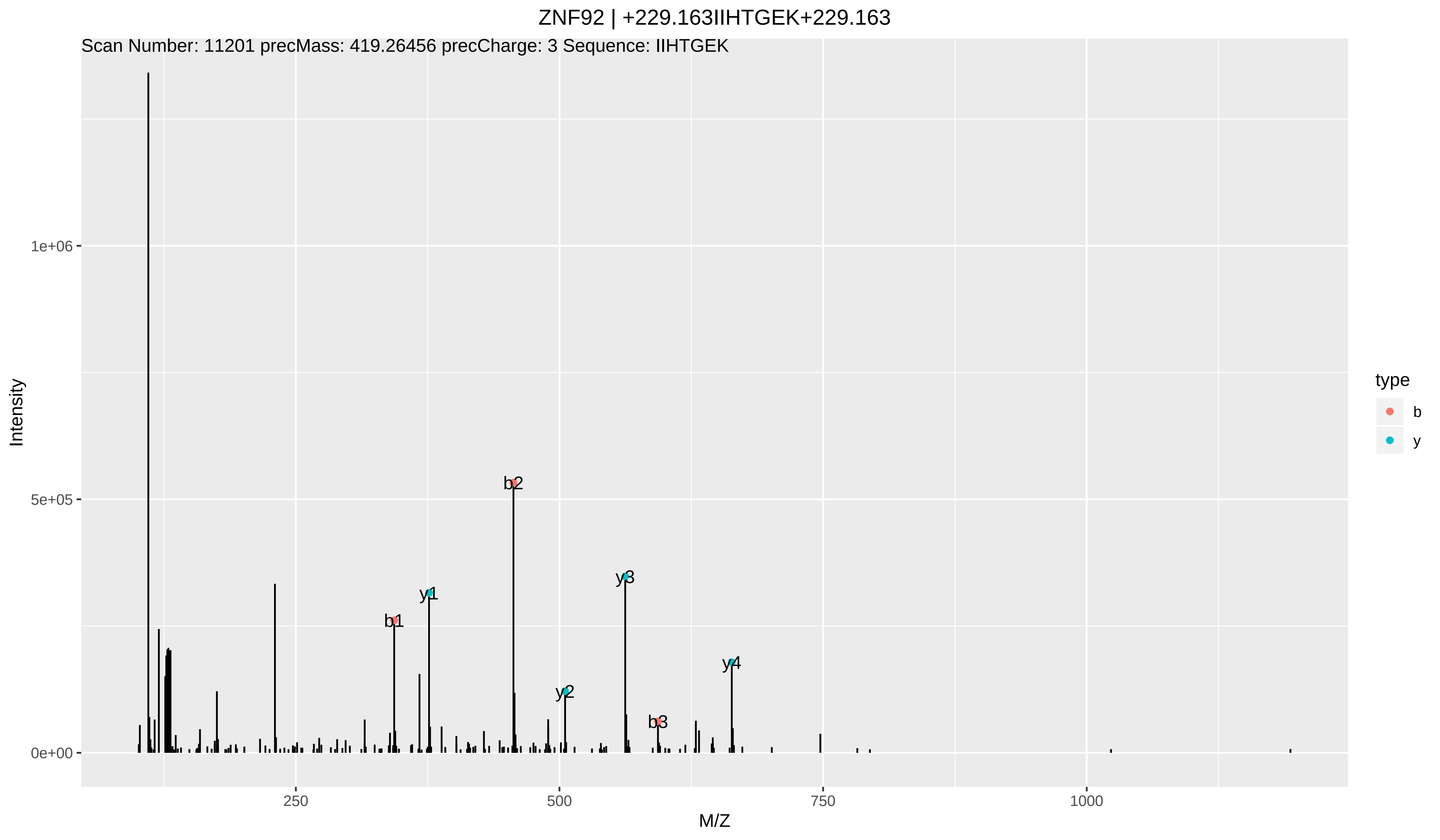

ZNF92 | +229.163IIHTGEK+229.163
Scan Number: 11201 precMass: 419.26456 precCharge: 3 Sequence: IIHTGEK
Intensity
1e+06
5e+05
0e+00
M/Z
250
500
750
1000
b1
y1
b2
y2
y3
b3
y4
type
b
y

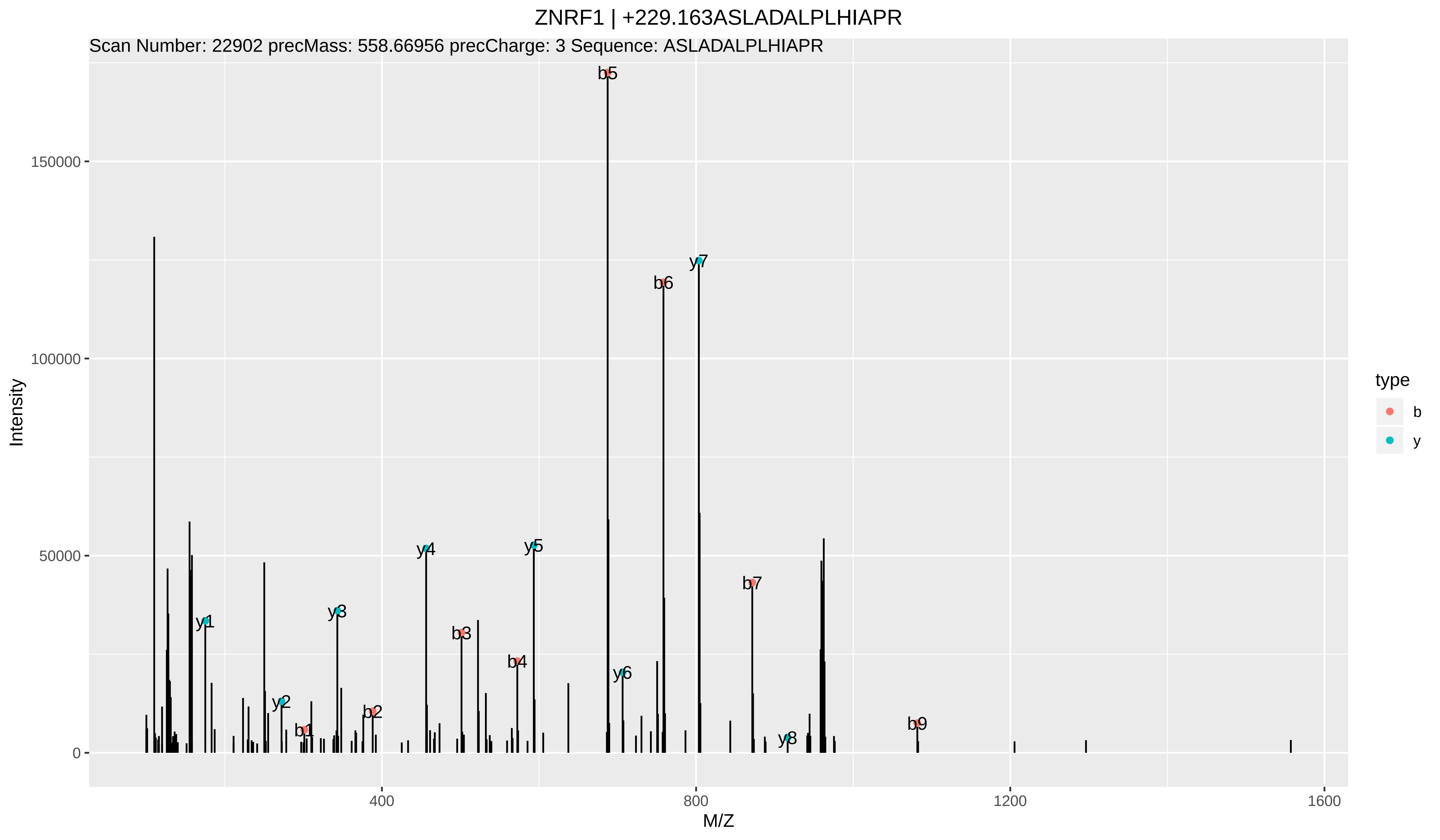

ZNRF1 | +229.163ASLADALPLHIAPR
Scan Number: 22902 precMass: 558.66956 precCharge: 3 Sequence: ASLADALPLHIAPR
Intensity
150000
100000
50000
0
M/Z
400
800
1200
1600
b5
y7
b6
y4
y5
b7
y1
y3
b3
b4
y6
y2
b2
b1
b9
y8
type
b
y

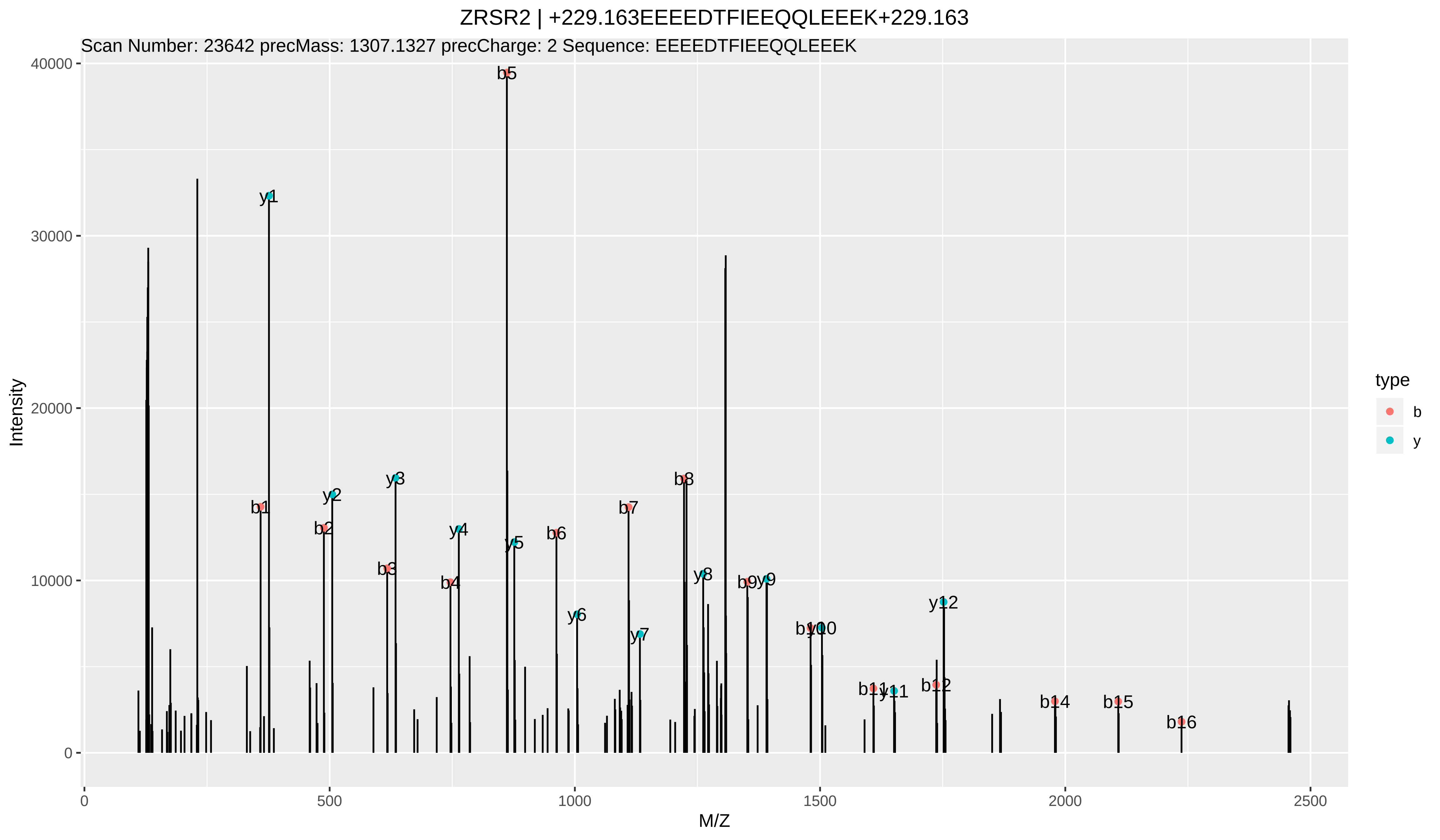
ZRSR2 | +229.163EEEEDTFIEEQQLEEEK+229.163
Scan Number: 23642 precMass: 1307.1327 precCharge: 2 Sequence: EEEEDTFIEEQQLEEEK
Intensity
M/Z
type
b
y
b5
y1
b1
b2
y2
b3
y3
b4
y4
y5
b6
y6
b7
y7
b8
y8
b9
y9
b10
y10
b11
y11
b12
y12
b14
b15
b16
0
10000
20000
30000
40000
500
1000
1500
2000
2500

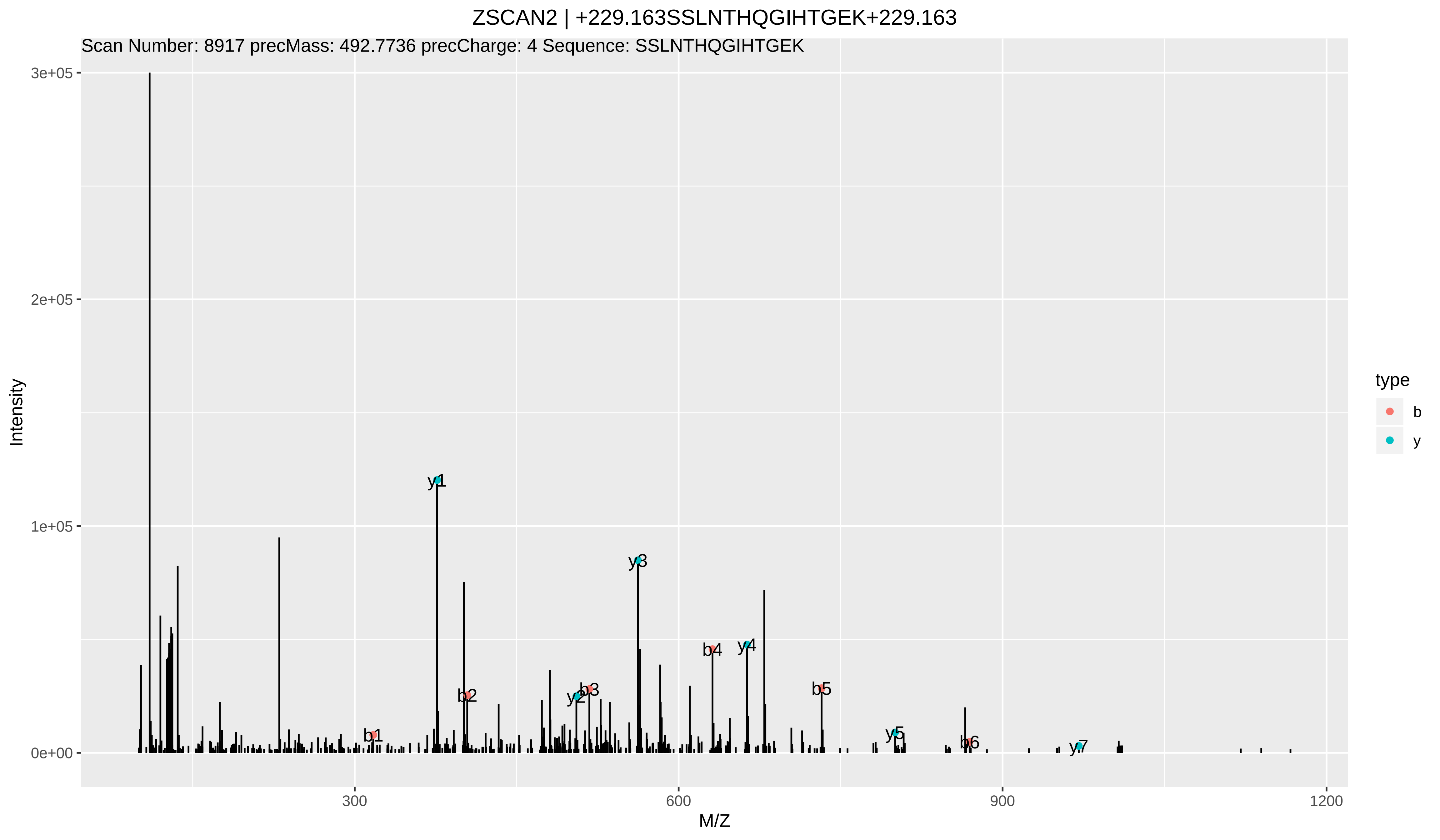
ZSCAN2 | +229.163SSLNTHQGIHTGEK+229.163
Scan Number: 8917 precMass: 492.7736 precCharge: 4 Sequence: SSLNTHQGIHTGEK
Intensity
3e+05
2e+05
1e+05
0e+00
300
600
900
1200
M/Z
y1
y2
y3
y4
y5
y7
b1
b2
b3
b4
b5
b6
type
b
y

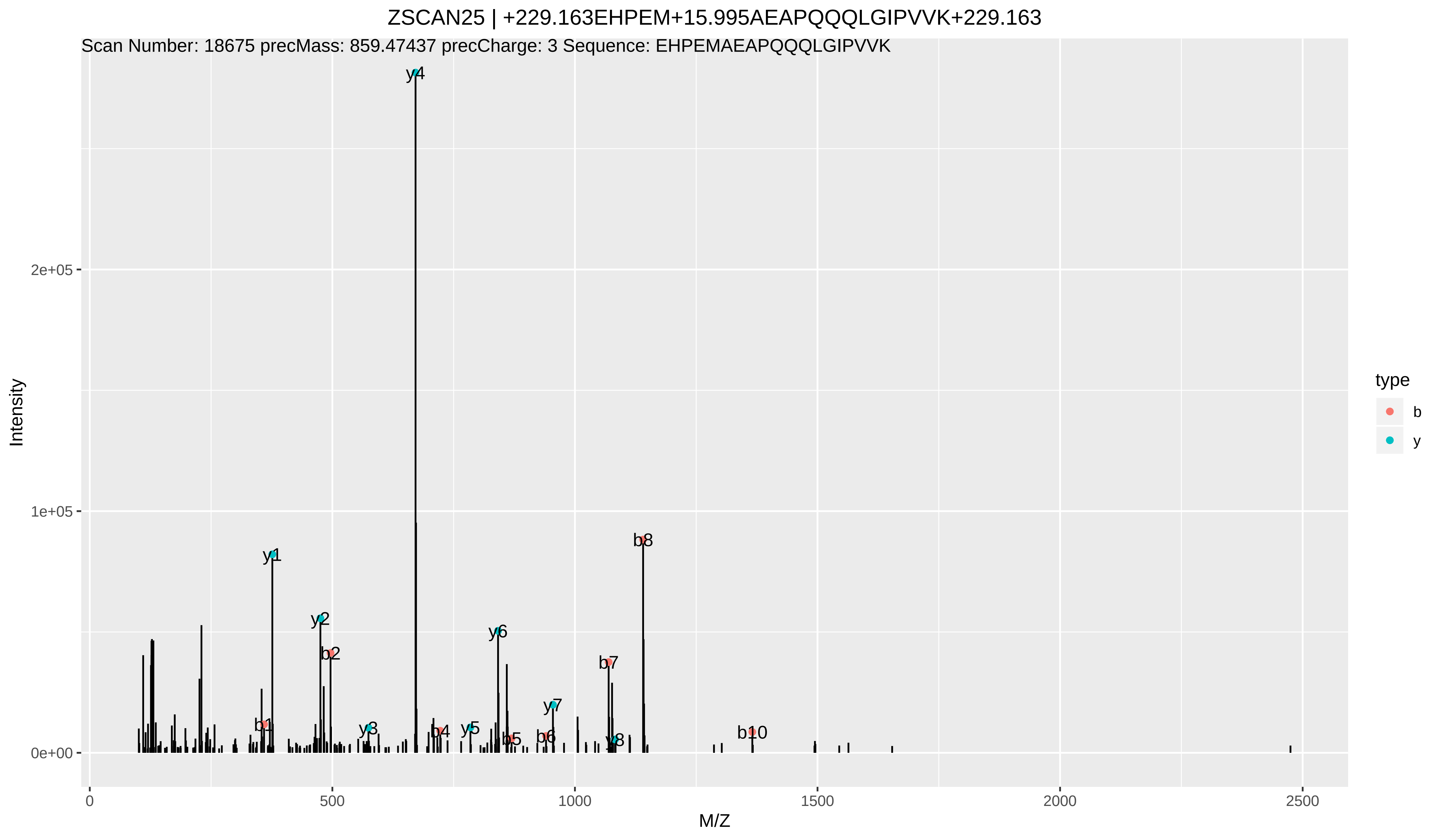
ZSCAN25 | +229.163EHPEM+15.995AEAPQQQLGIPVVK+229.163
Scan Number: 18675 precMass: 859.47437 precCharge: 3 Sequence: EHPEMAEAPQQQLGIPVVK
Intensity
M/Z
0e+00
1e+05
2e+05
0
500
1000
1500
2000
2500
y4
y1
y2
b2
y6
b8
b7
y7
b1
y3
b4
y5
b5
b6
y8
b10
type
b
y

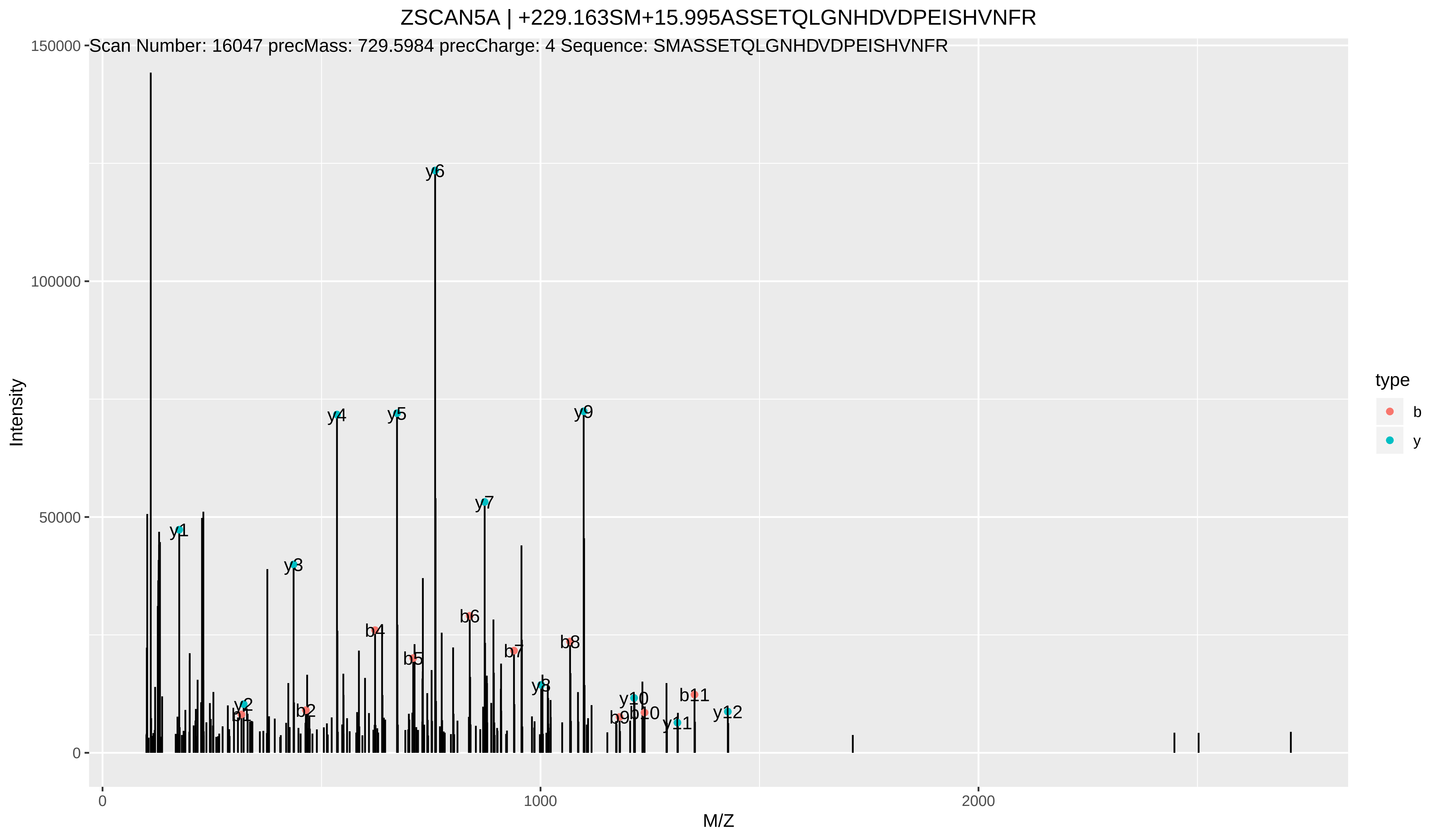

ZSCAN5A | +229.163SM+15.995ASSETQLGNHDVDPEISHVNFR
Scan Number: 16047 precMass: 729.5984 precCharge: 4 Sequence: SMASSETQLGNHDVDPEISHVNFR
Intensity
M/Z
150000
100000
50000
0
0
1000
2000
y1
y2
b1
y3
b2
y4
b4
y5
b5
y6
b6
y7
b7
y8
b8
y9
y10
b9
b10
y11
b11
y12
type
b
y

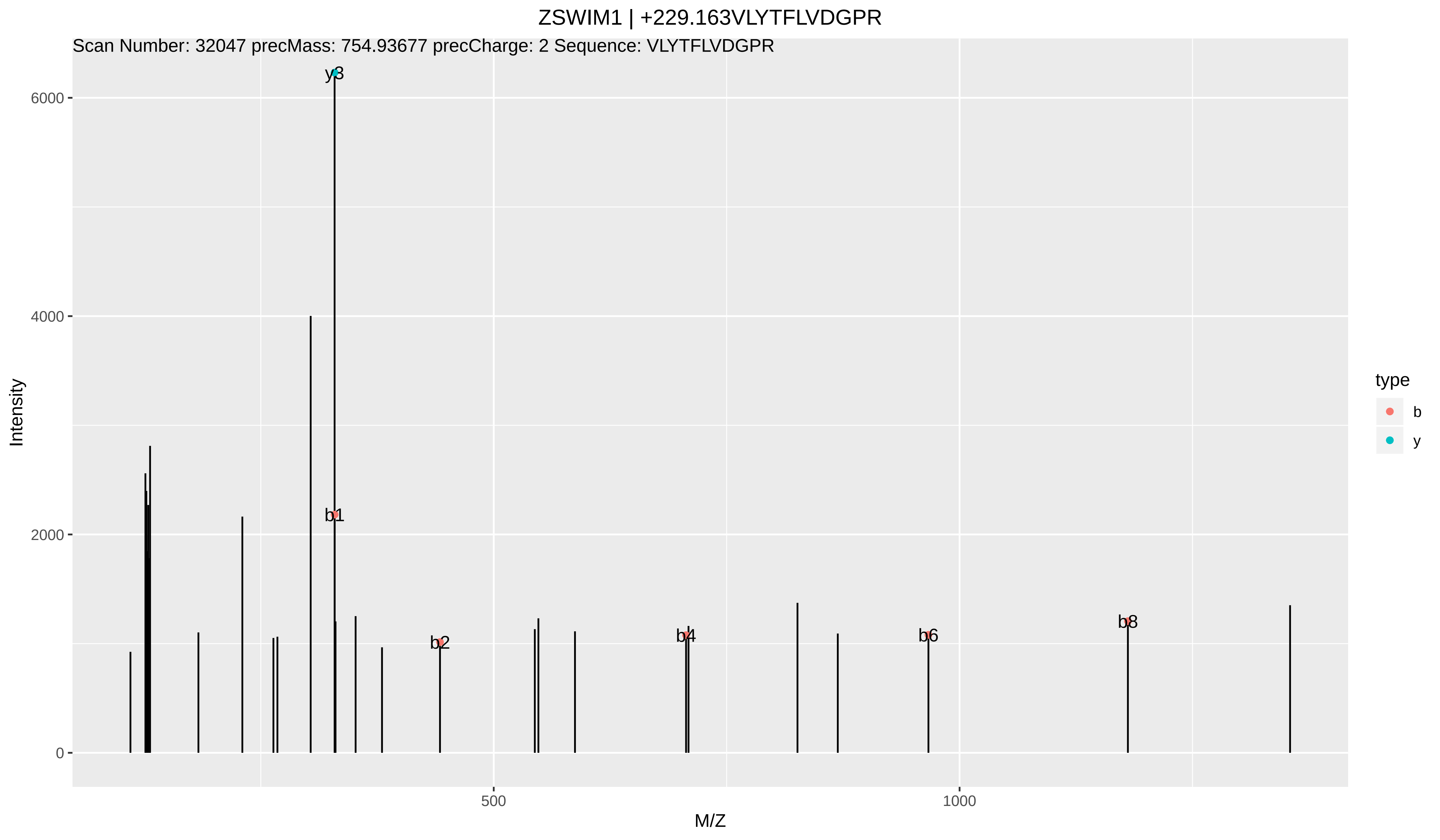

ZSWIM1 | +229.163VLYTFLVDGPR
Scan Number: 32047 precMass: 754.93677 precCharge: 2 Sequence: VLYTFLVDGPR
y3
b1
b2
b4
b6
b8
Intensity
6000
4000
2000
0
500
1000
M/Z
type
b
y

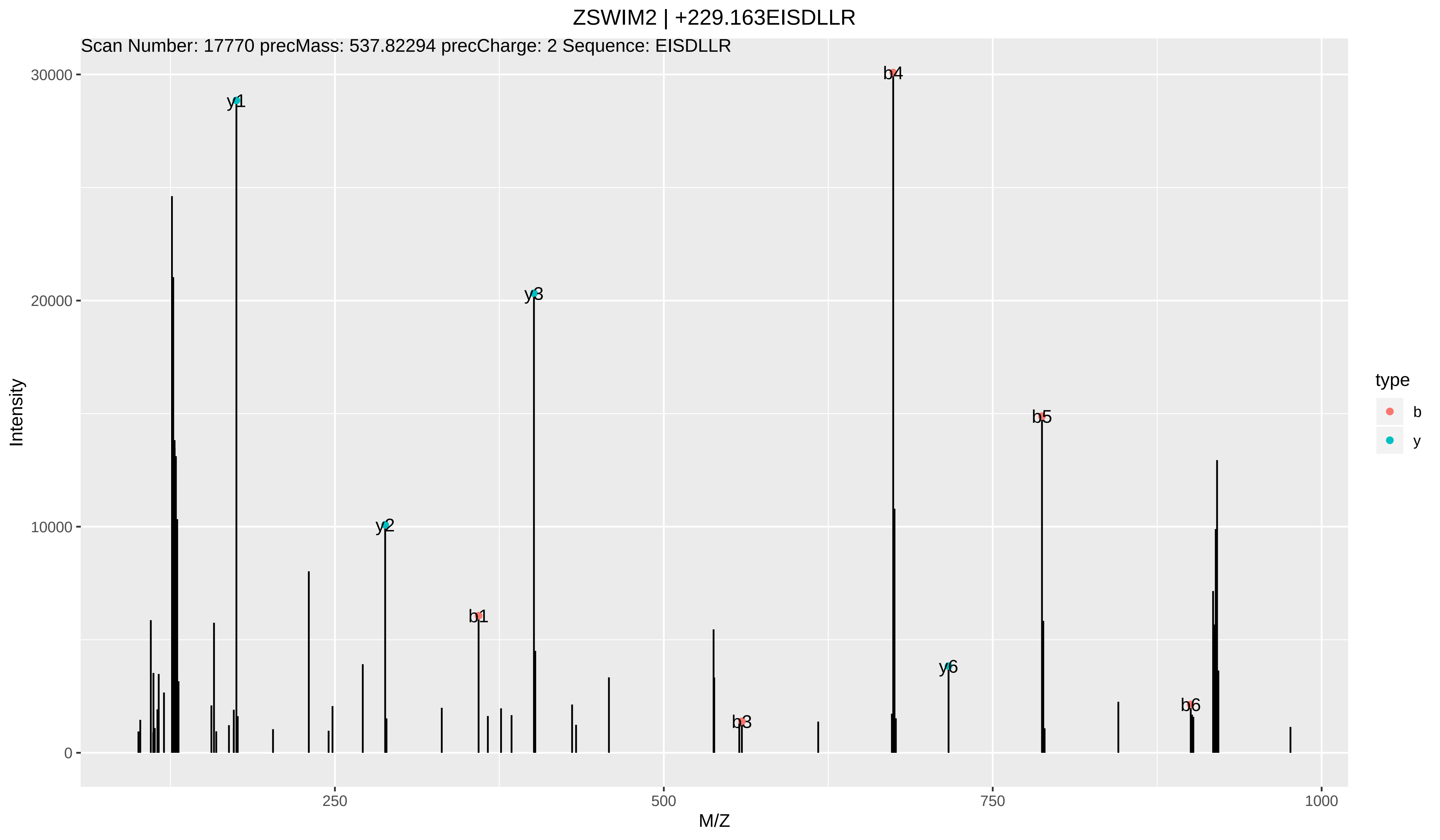
ZSWIM2 | +229.163EISDLLR
Scan Number: 17770 precMass: 537.82294 precCharge: 2 Sequence: EISDLLR
Intensity
M/Z
30000
20000
10000
0
250
500
750
1000
y1
y2
y3
y6
b1
b3
b4
b5
b6
type
b
y

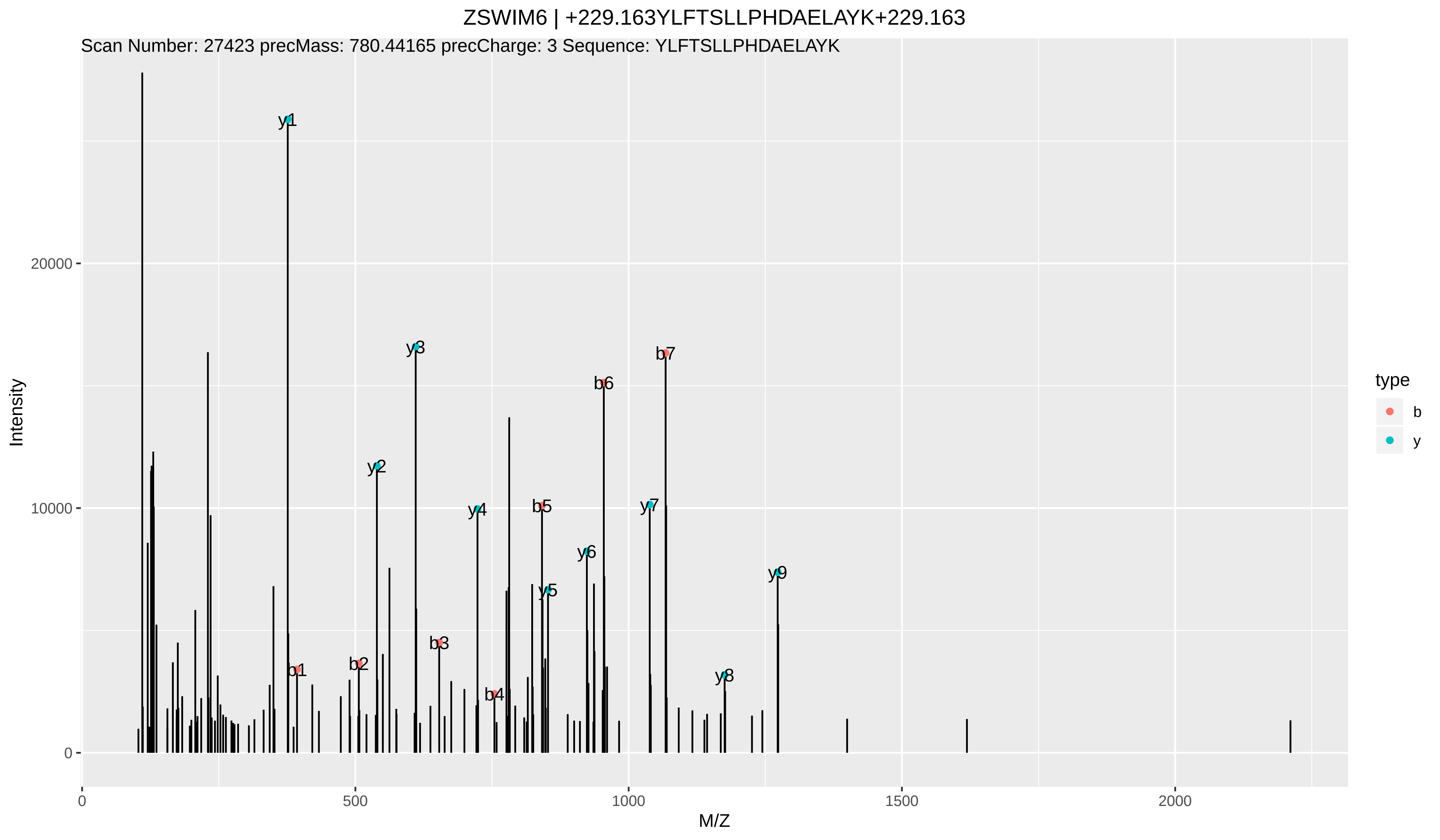
ZSWIM6 | +229.163YLFTSLLPHDAELAYK+229.163
Scan Number: 27423 precMass: 780.44165 precCharge: 3 Sequence: YLFTSLLPHDAELAYK
Intensity
M/Z
0
10000
20000
0
500
1000
1500
2000
y1
y2
y3
y4
y5
y6
y7
y8
y9
b1
b2
b3
b4
b5
b6
b7
type
b
y

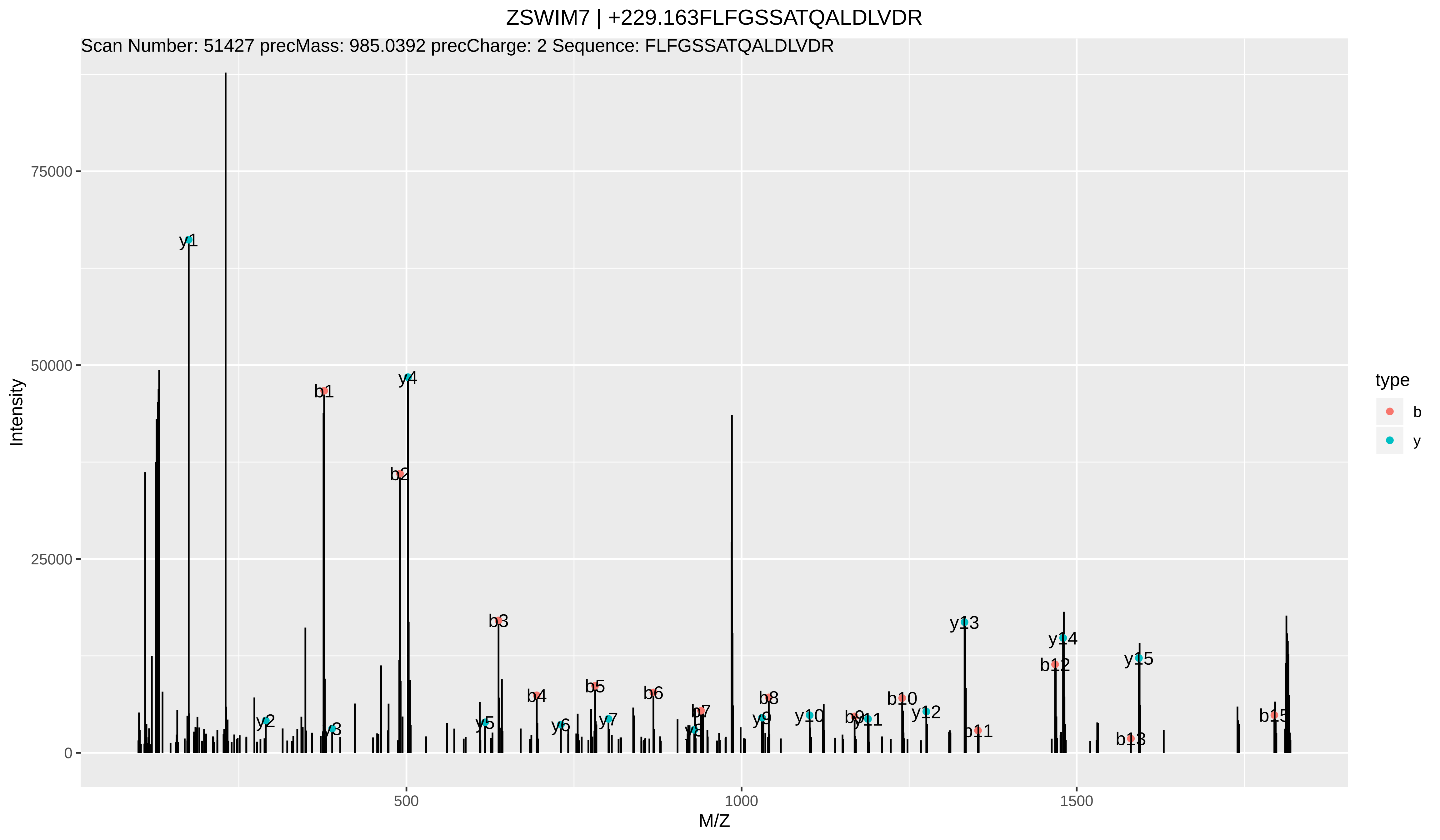

ZSWIM7 | +229.163FLFGSSATQALDLVDR
Scan Number: 51427 precMass: 985.0392 precCharge: 2 Sequence: FLFGSSATQALDLVDR
Intensity
M/Z
0
25000
50000
75000
500
1000
1500
type
b
y
y1
y2
y3
y4
y5
y6
y7
y8
y9
y10
y11
y12
y13
y14
y15
b1
b2
b3
b4
b5
b6
b7
b8
b9
b10
b11
b12
b13
b15
