# Supplementary material for: Immediate Adaptation Analysis Implicates BCL6 as an EGFR-TKI Combination Therapy Target in NSCLC
Source: Mol Cell Proteomics. 2020 Mar 30;19(6):928–43. doi: 10.1074/mcp.RA120.002036 (PMC7261823; doi:10.1074/mcp.RA120.002036)
Supplement: Annotated spectra [file PSM.annotated.spectra_siRNA_BCL6_set1.pdf › PSM.annotated.spectra_siRNA_BCL6_set1.pdf]

A2ML1 | +229.163FIFIDPK+229.163

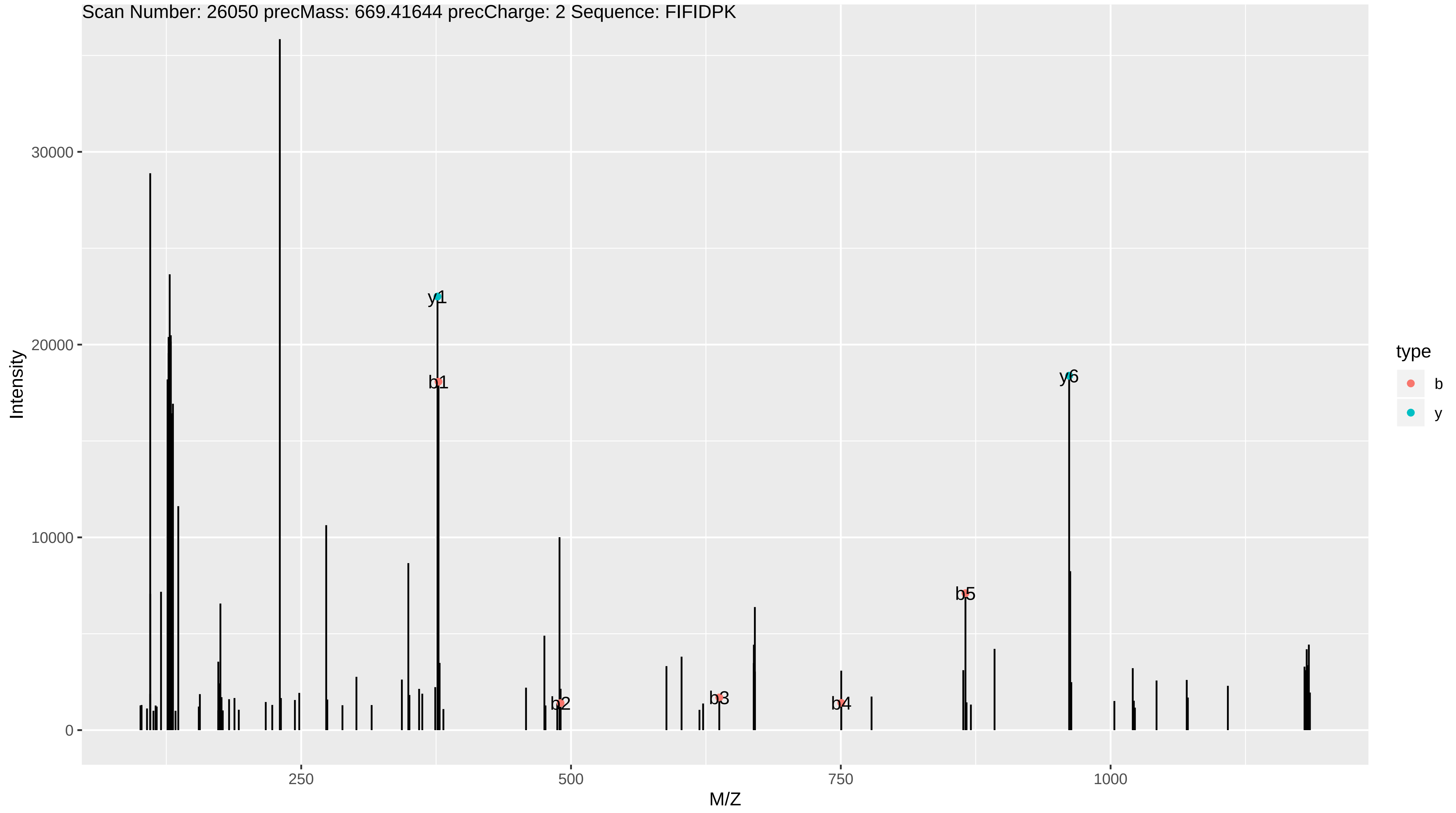

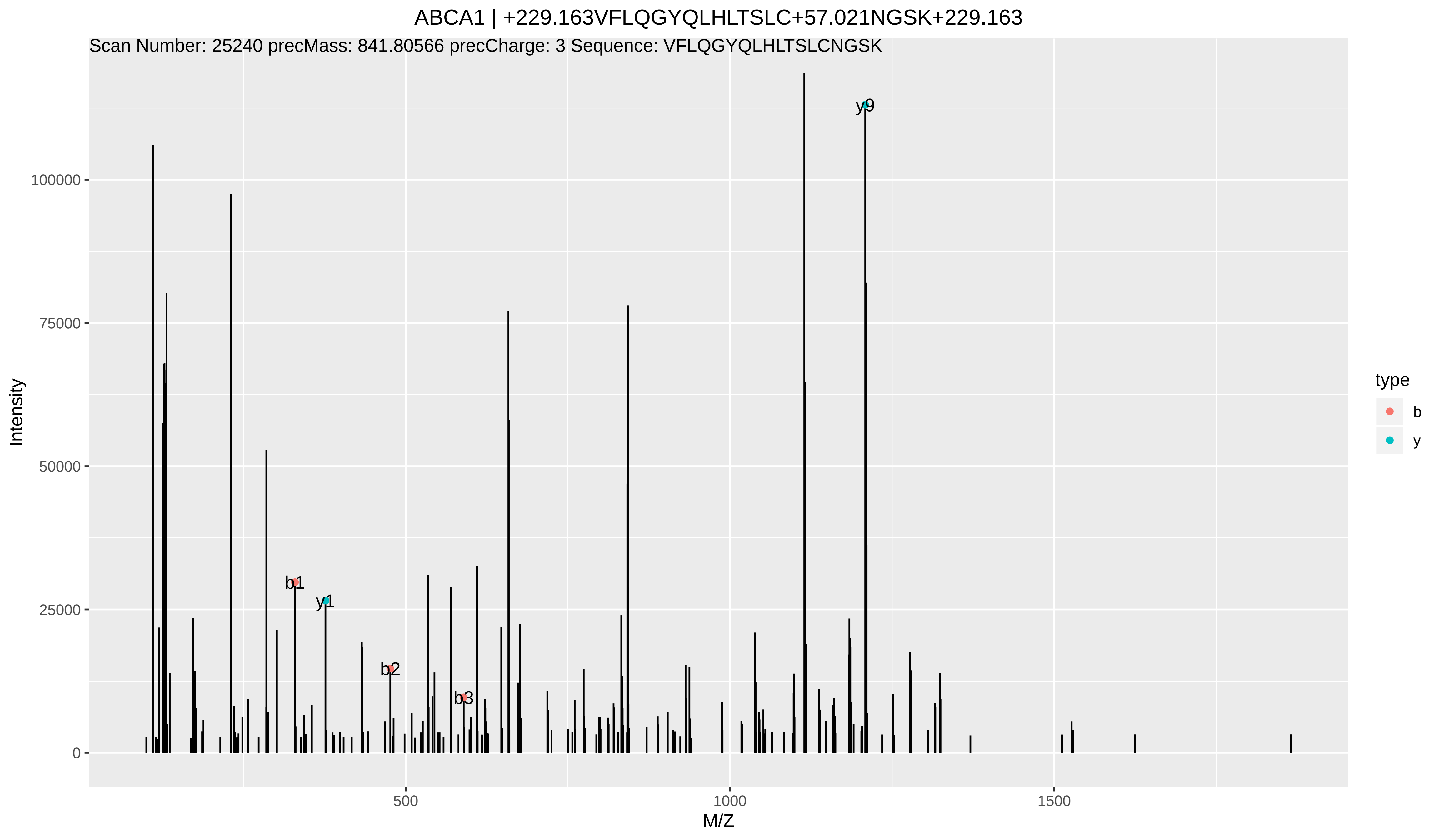

ABCA1 | +229.163VFLQGYQLHLTSLC+57.021NGSK+229.163
Scan Number: 25240 precMass: 841.80566 precCharge: 3 Sequence: VFLQGYQLHLTSLCNGSK
Intensity
M/Z
100000
75000
50000
25000
0
500
1000
1500
y9
b1
y1
b2
b3
type
b
y

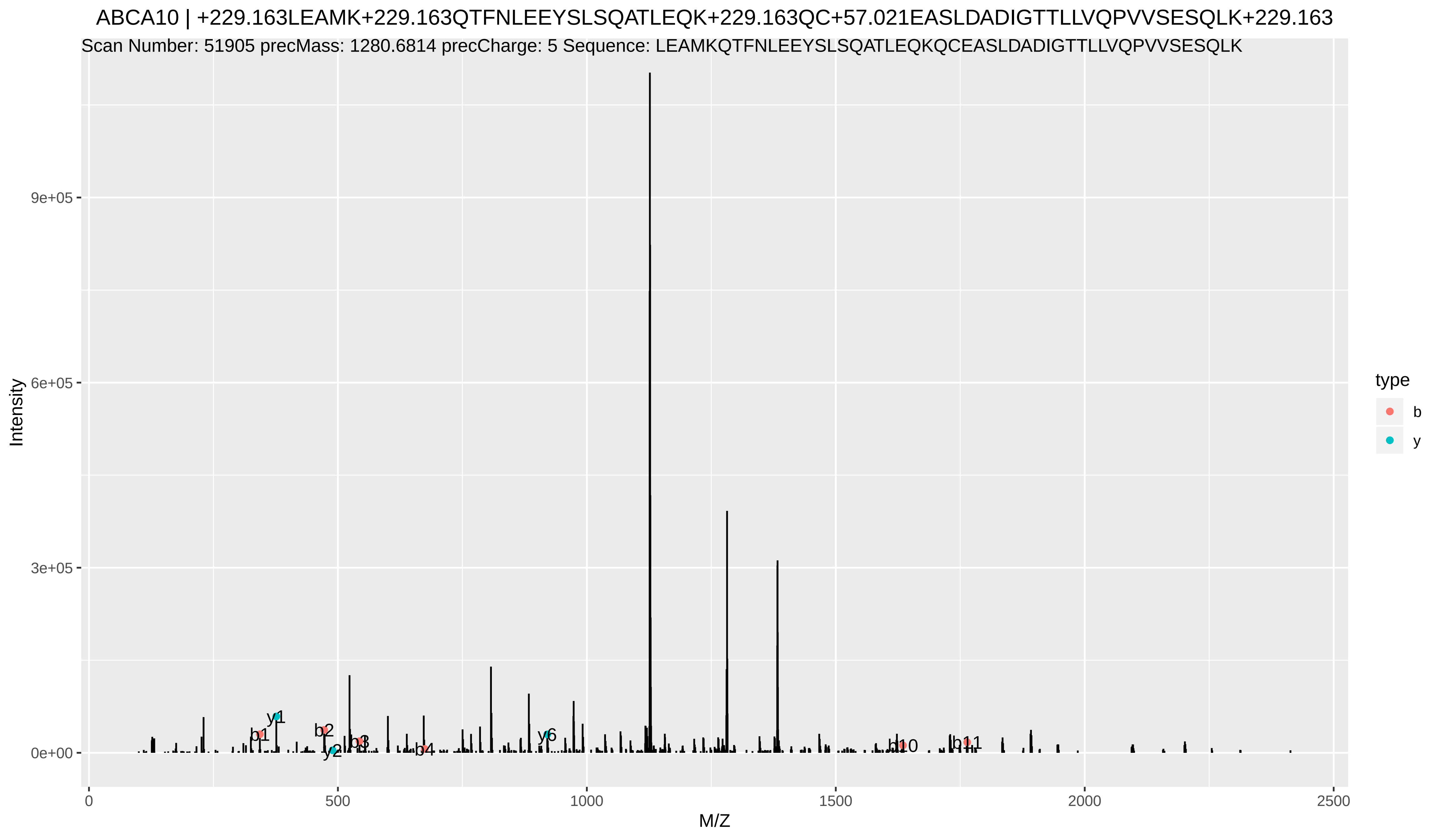
ABCA10 | +229.163LEAMK+229.163QTFNLEEYSLSQATLEQK+229.163QC+57.021EASLDADIGTTLLVQPVVSESQLK+229.163
Scan Number: 51905 precMass: 1280.6814 precCharge: 5 Sequence: LEAMKQTFNLEEYSLSQATLEQKQCEASLDADIGTTLLVQPVVSESQLK
Intensity
9e+05
6e+05
3e+05
0e+00
M/Z
0
500
1000
1500
2000
2500
y1
b1
b2
y2
b3
b4
y6
b10
b11
type
b
y

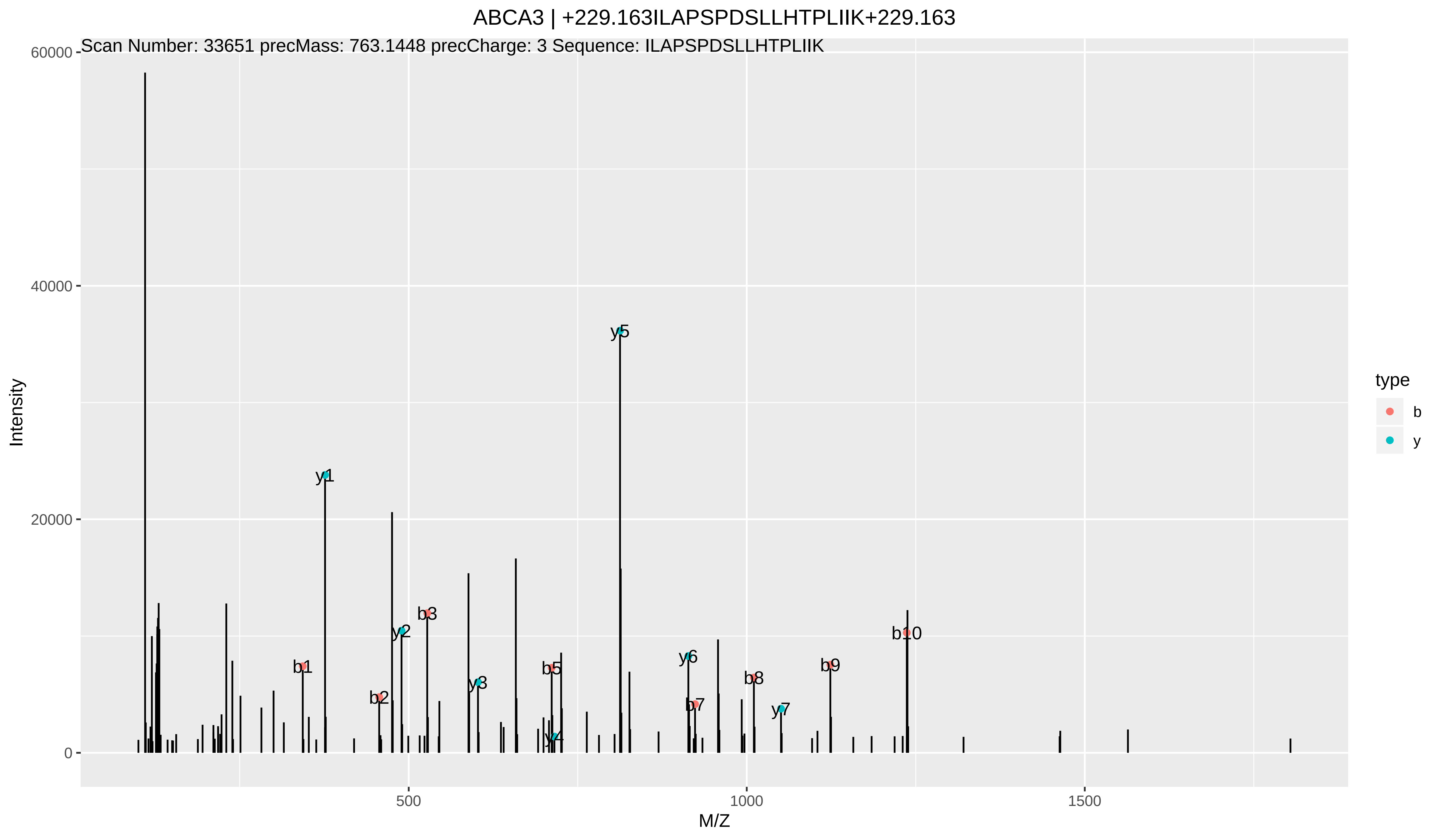

ABCA3 | +229.163ILAPSPDSLLHTPLIIK+229.163
Scan Number: 33651 precMass: 763.1448 precCharge: 3 Sequence: ILAPSPDSLLHTPLIIK
Intensity
60000
40000
20000
0
M/Z
500
1000
1500
y1
y2
y3
y4
y5
y6
y7
b1
b2
b3
b5
b7
b8
b9
b10
type
b
y

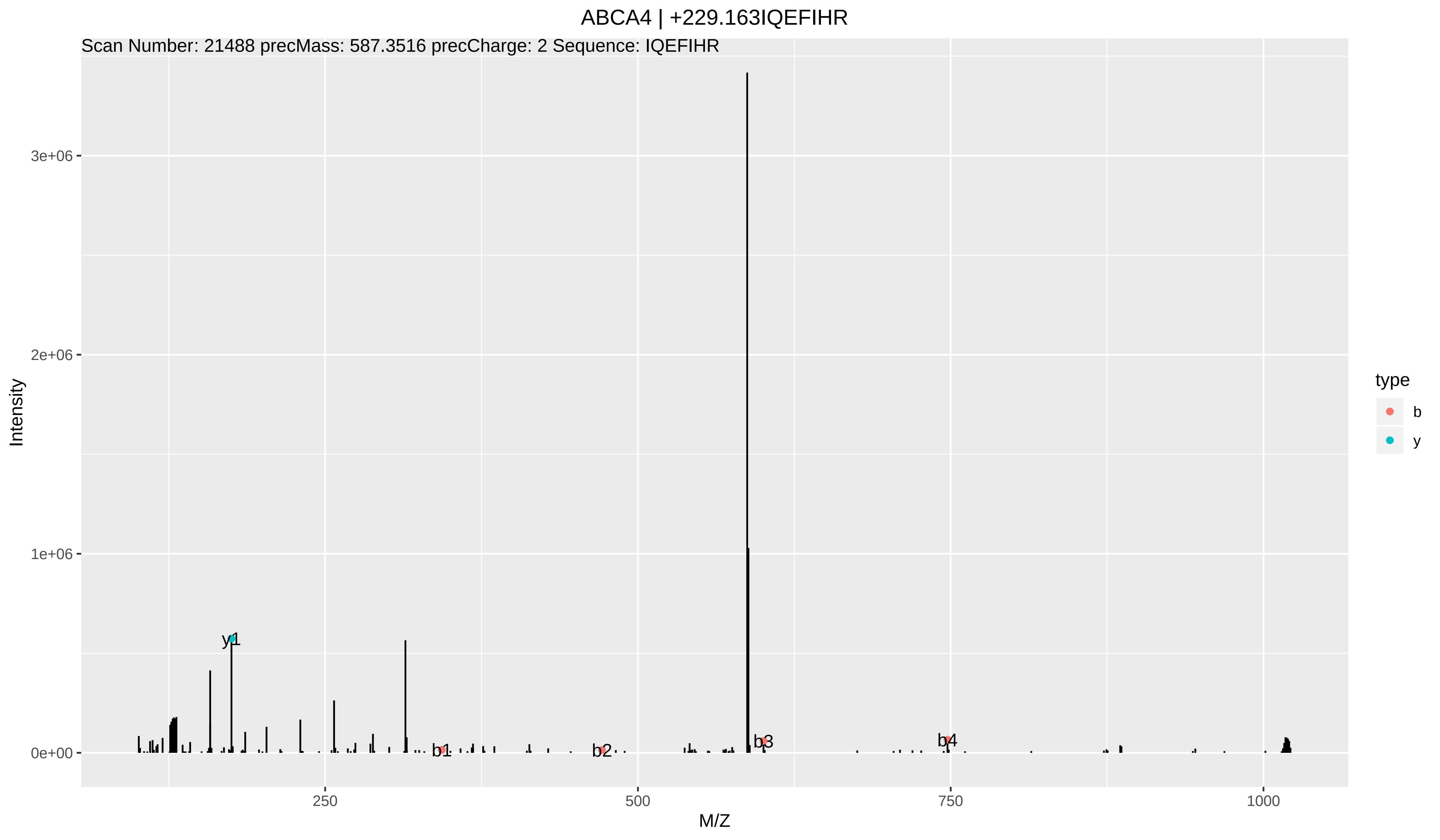
ABCA4 | +229.163IQEFIHR
Scan Number: 21488 precMass: 587.3516 precCharge: 2 Sequence: IQEFIHR
Intensity
3e+06
2e+06
1e+06
0e+00
M/Z
250
500
750
1000
y1
b1
b2
b3
b4
type
b
y

ABCC9 | +229.163SSLLLAILGEM+15.995QTLEGK+229.163

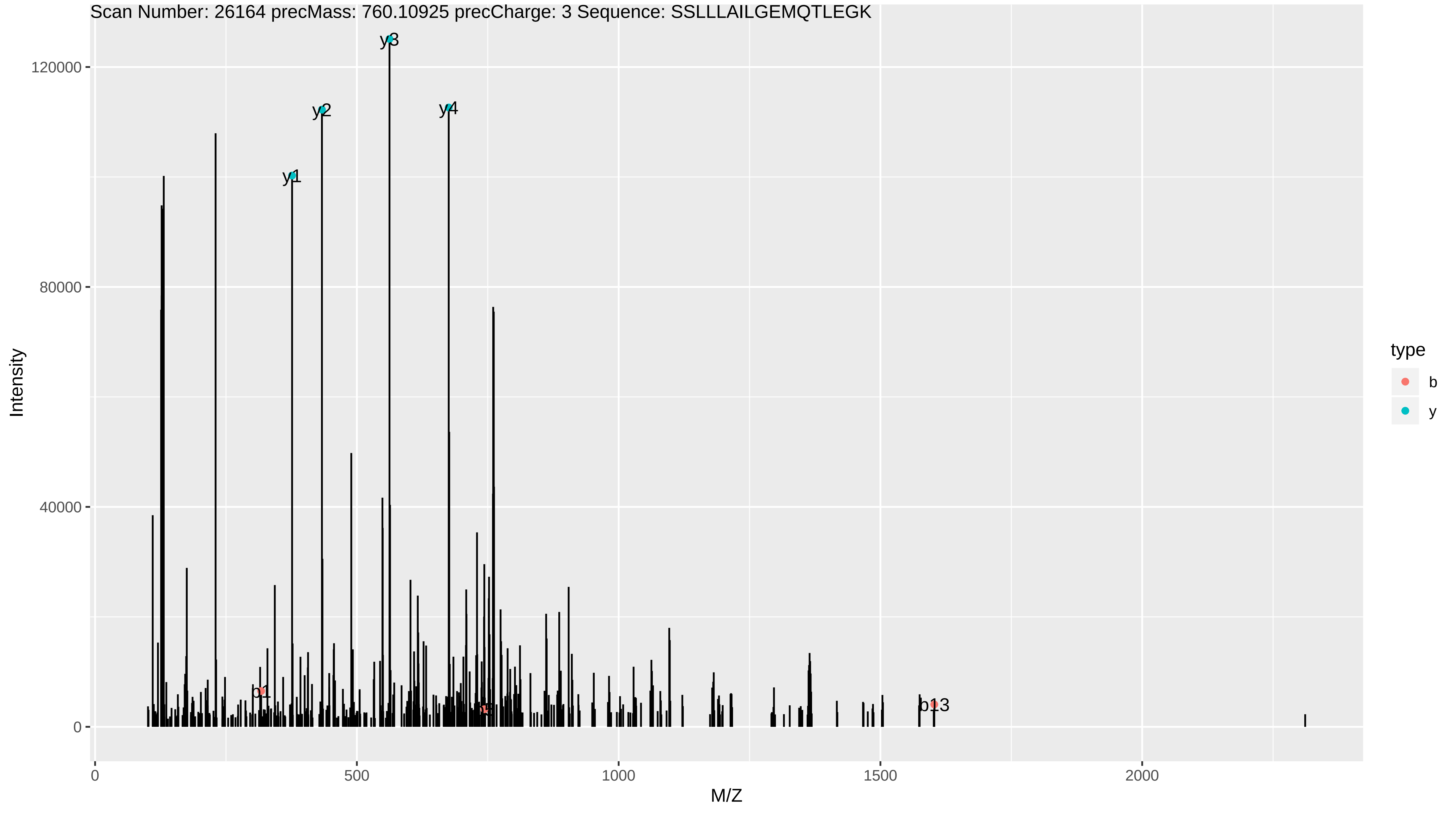

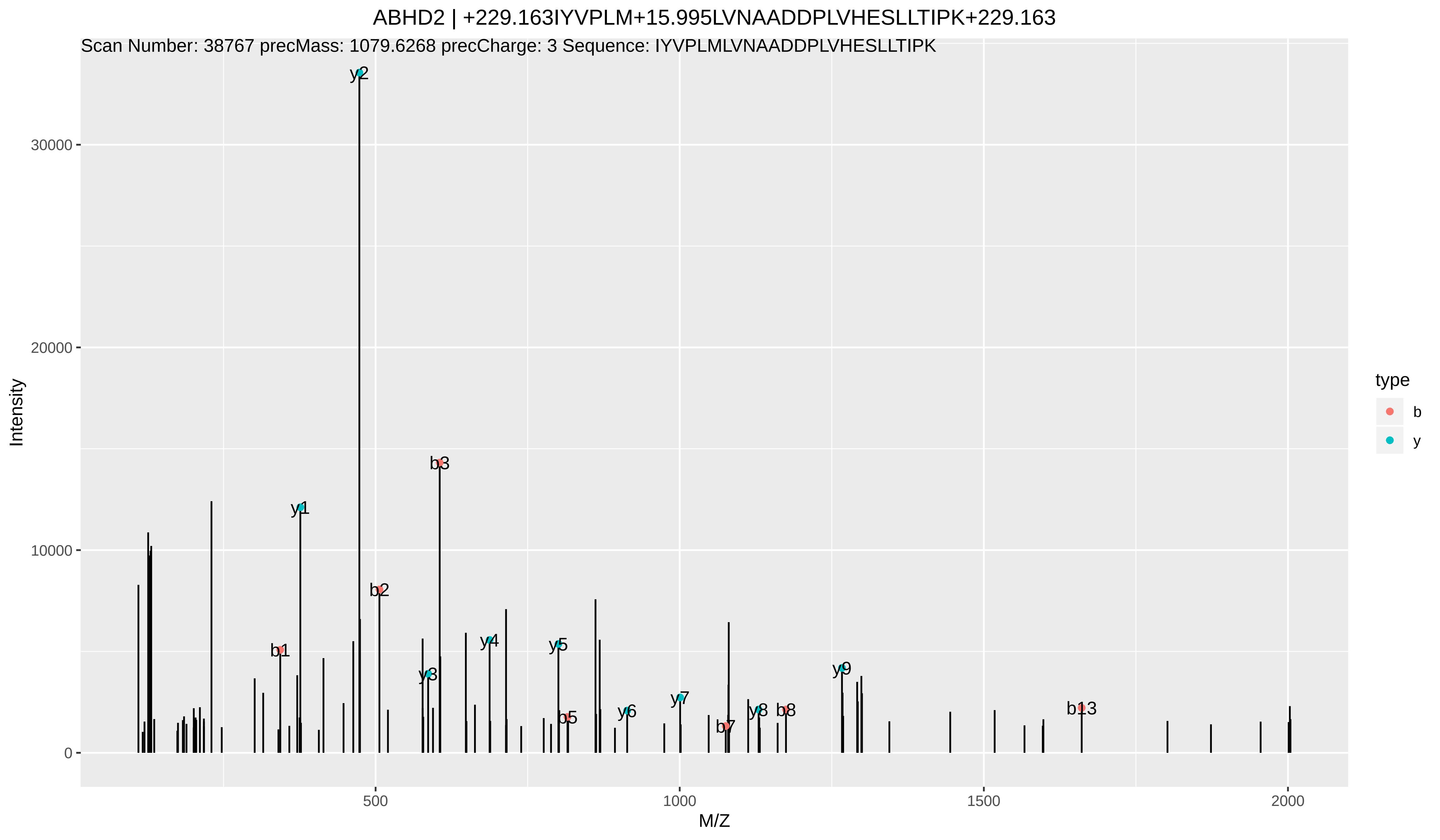
ABHD2 | +229.163IYVPLM+15.995LVNAADDPLVHESLLTIPK+229.163
Scan Number: 38767 precMass: 1079.6268 precCharge: 3 Sequence: IYVPLMLVNAADDPLVHESLLTIPK
Intensity
30000
20000
10000
0
500
1000
1500
2000
M/Z
type
b
y
y2
b3
y1
b2
b1
y4
y5
y3
y9
b13
y7
b5
y6
y8
b8
b7

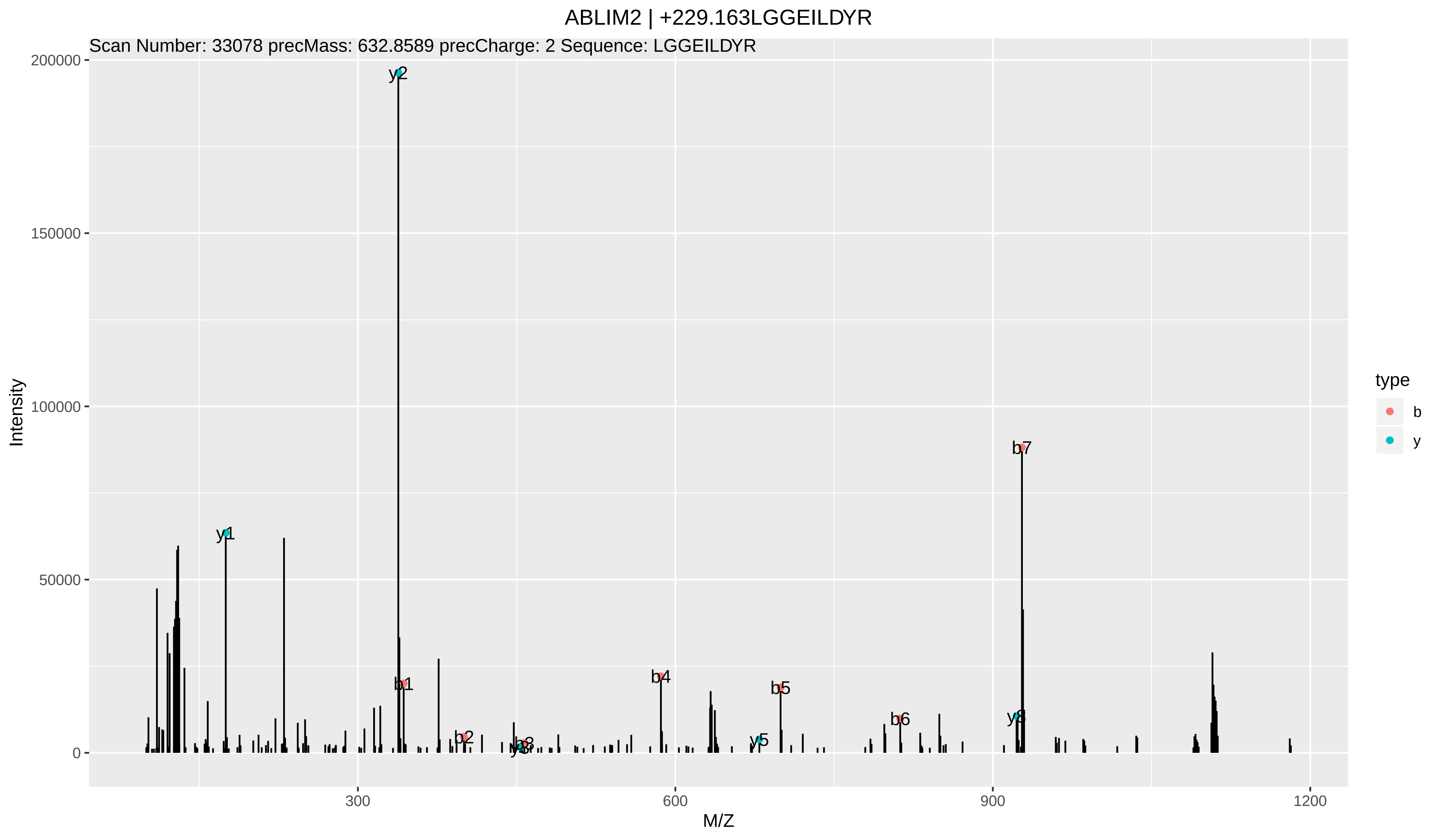

ABLIM2 | +229.163LGGEILDYR
Scan Number: 33078 precMass: 632.8589 precCharge: 2 Sequence: LGGEILDYR
Intensity
M/Z
200000
150000
100000
50000
0
300
600
900
1200
y2
y1
b1
b2
y3
b3
b4
y5
b5
b6
y8
b7
type
b
y

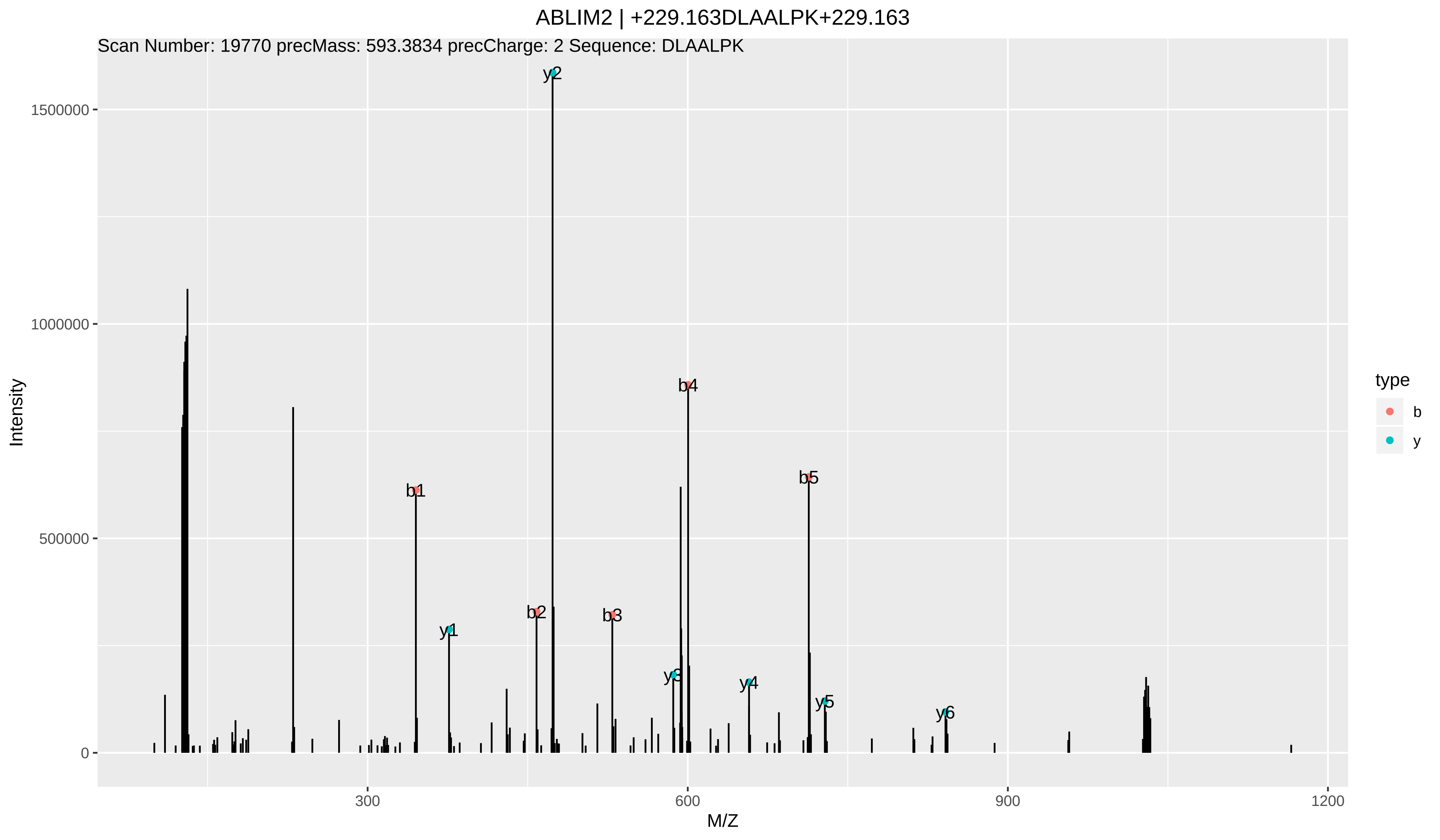

ABLIM2 | +229.163DLAALPK+229.163
Scan Number: 19770 precMass: 593.3834 precCharge: 2 Sequence: DLAALPK
Intensity
1500000
1000000
500000
0
300
600
900
1200
M/Z
y2
b4
b5
b1
b2
b3
y1
y3
y4
y5
y6
type
b
y

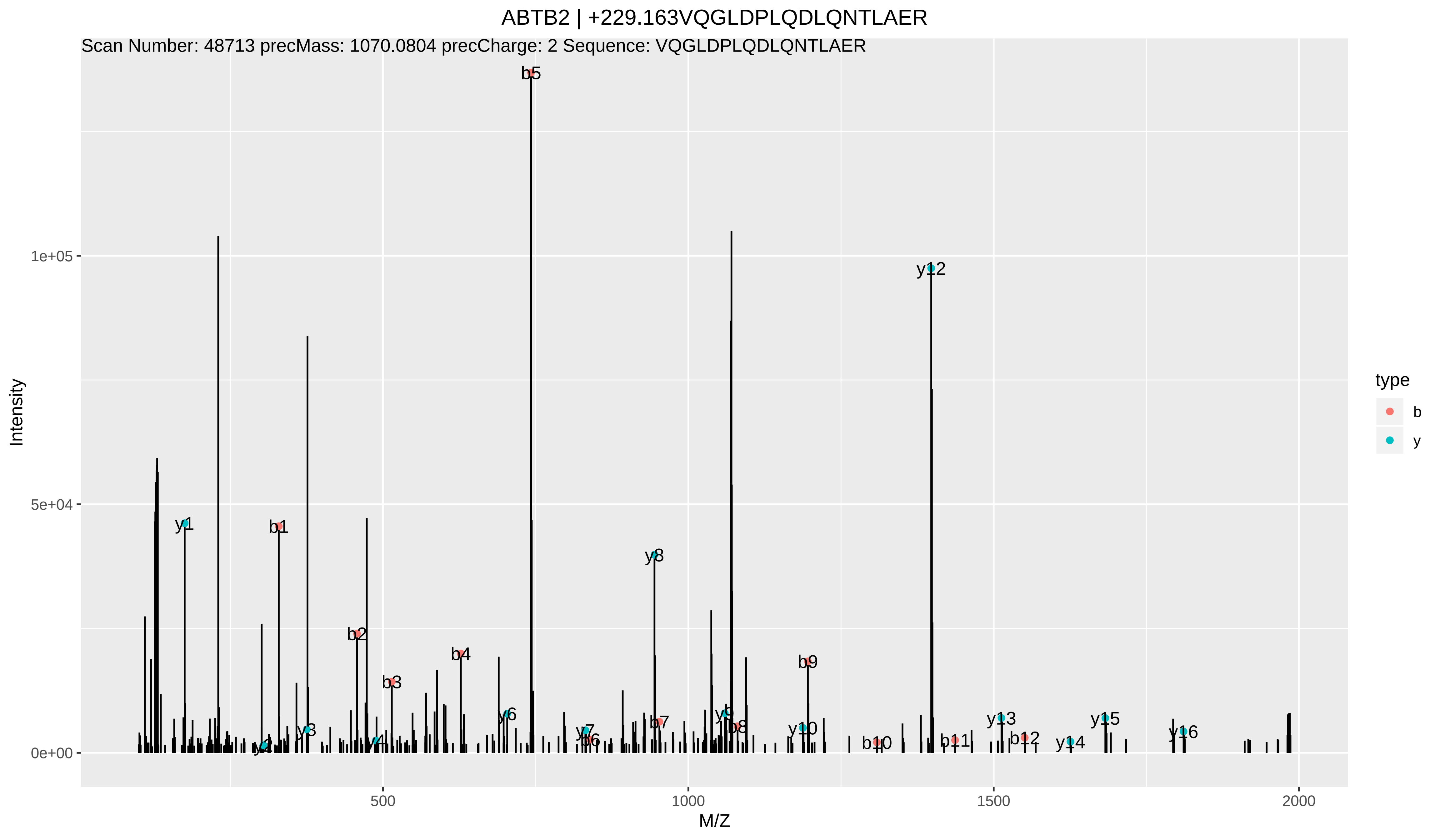

ABTB2 | +229.163VQGLDPLQDLQNTLAER
Scan Number: 48713 precMass: 1070.0804 precCharge: 2 Sequence: VQGLDPLQDLQNTLAER
Intensity
M/Z
0e+00
5e+04
1e+05
500
1000
1500
2000
type
b
y
b5
y12
y1
b1
y8
b2
b4
b3
b9
y6
y3
y9
b7
b8
y7
b6
y13
y15
y16
y10
b12
b11
y14
b10
y2
y4

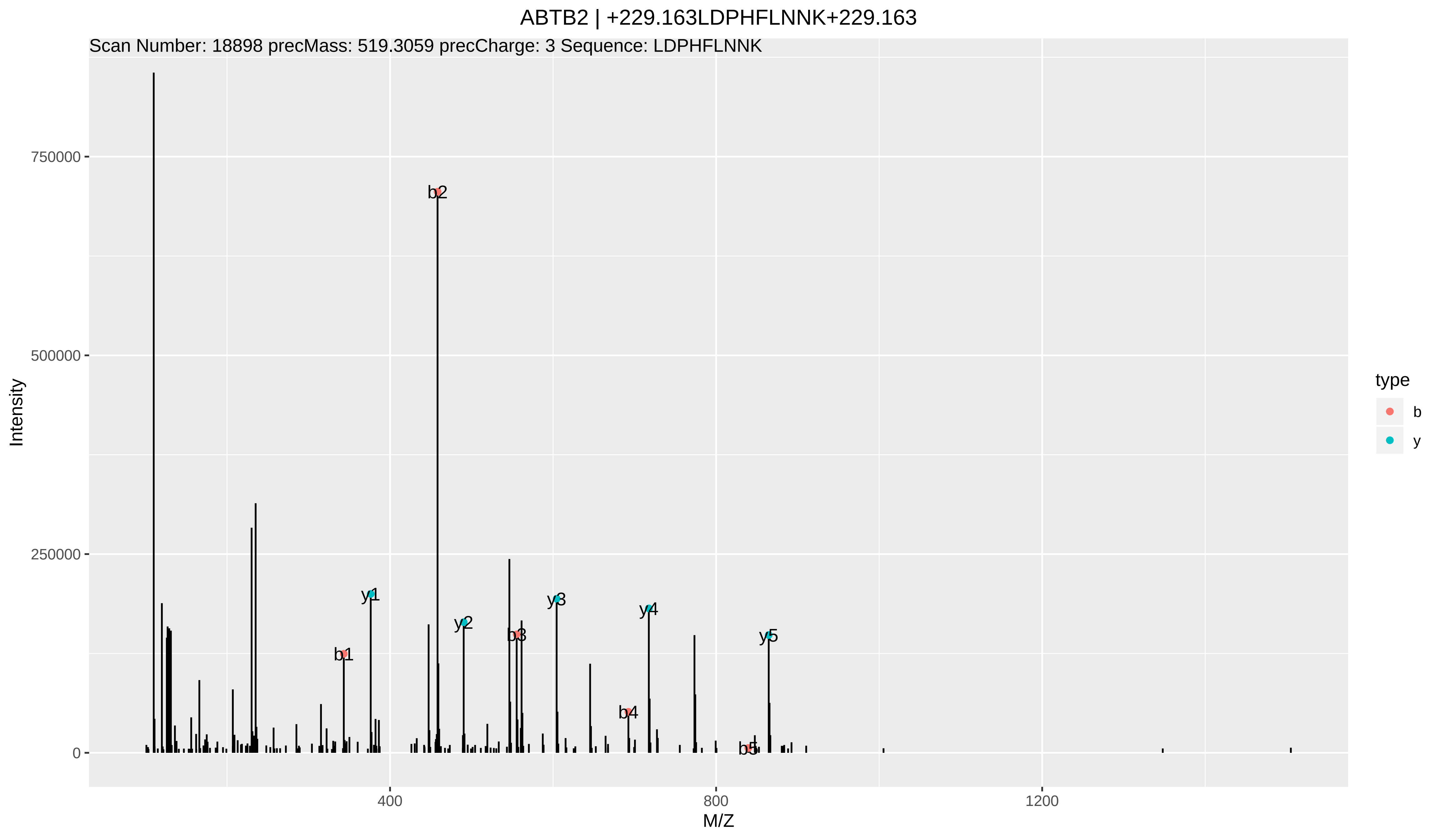

ABTB2 | +229.163LDPHFLNNK+229.163
Scan Number: 18898 precMass: 519.3059 precCharge: 3 Sequence: LDPHFLNNK
Intensity
750000
500000
250000
0
M/Z
400
800
1200
b2
y1
y2
b3
y3
y4
y5
b1
b4
b5
type
b
y

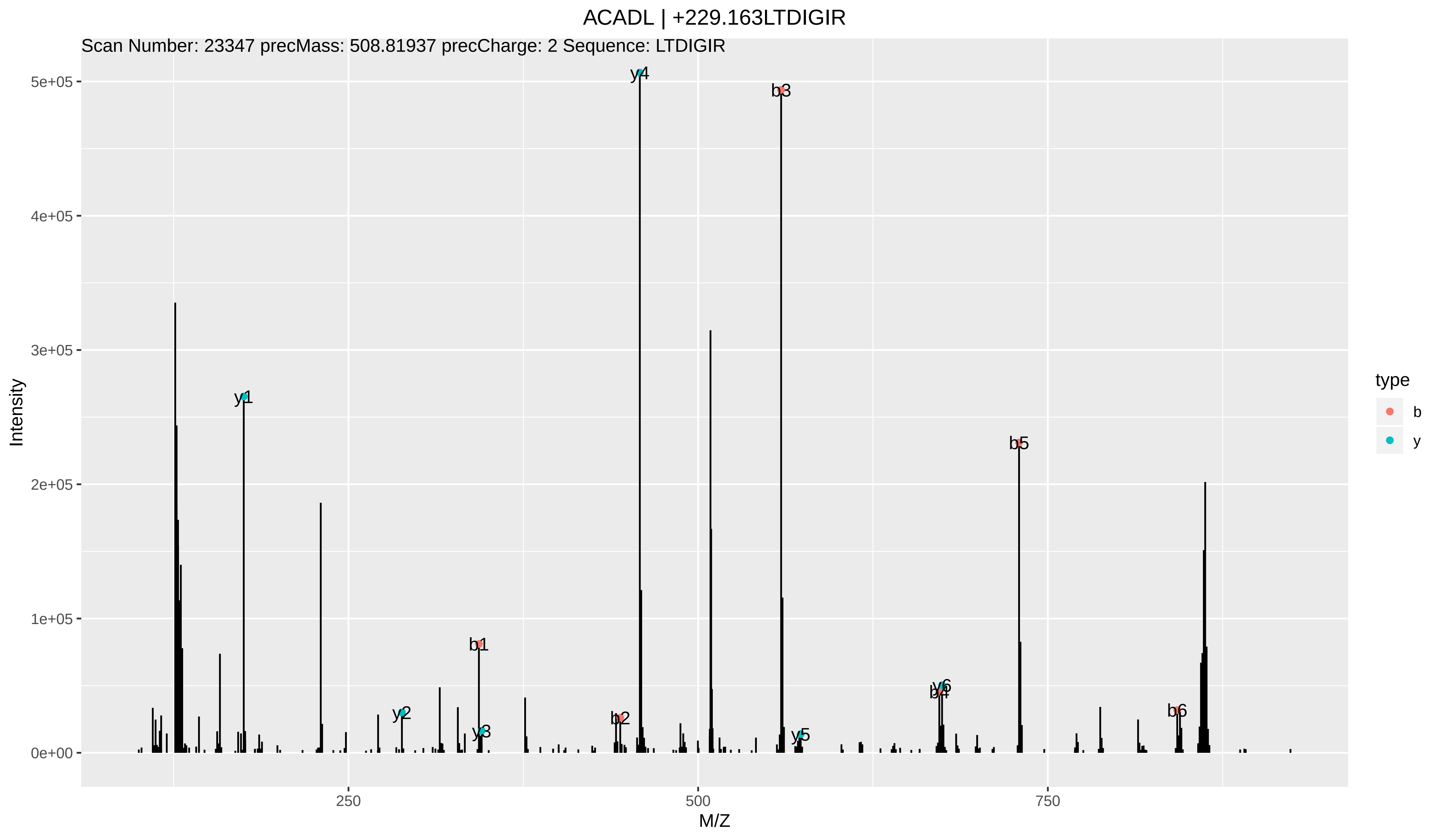

ACADL | +229.163LTDIGIR
Scan Number: 23347 precMass: 508.81937 precCharge: 2 Sequence: LTDIGIR
Intensity
M/Z
0e+00
1e+05
2e+05
3e+05
4e+05
5e+05
250
500
750
y1
y2
y3
y4
y5
y6
b1
b2
b3
b4
b5
b6
type
b
y

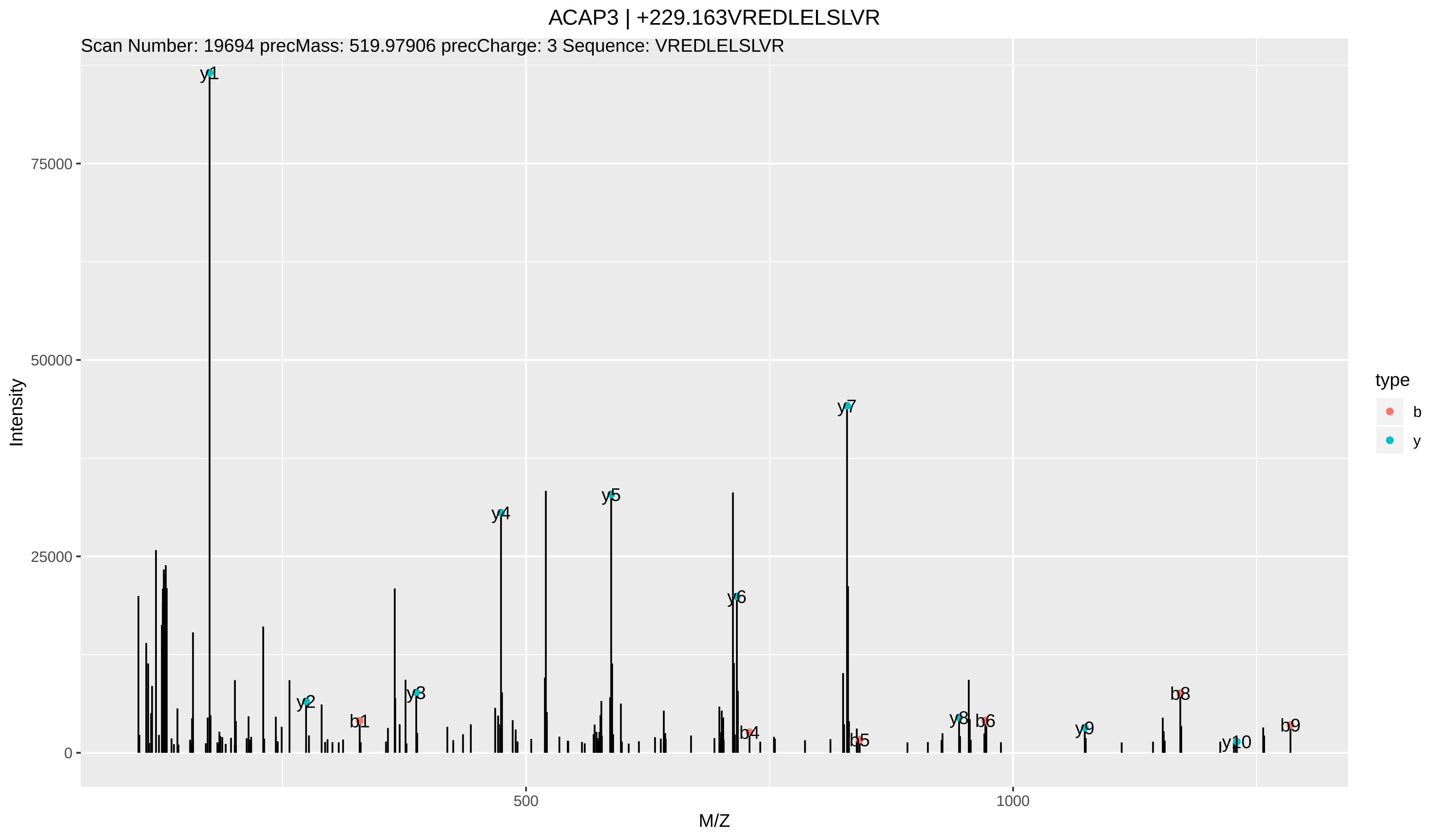
ACAP3 | +229.163VREDLELSLVR
Scan Number: 19694 precMass: 519.97906 precCharge: 3 Sequence: VREDLELSLVR
Intensity
75000
50000
25000
0
M/Z
500
1000
type
b
y
y1
y2
y3
y4
y5
y6
y7
y8
y9
y10
b1
b4
b5
b6
b8
b9

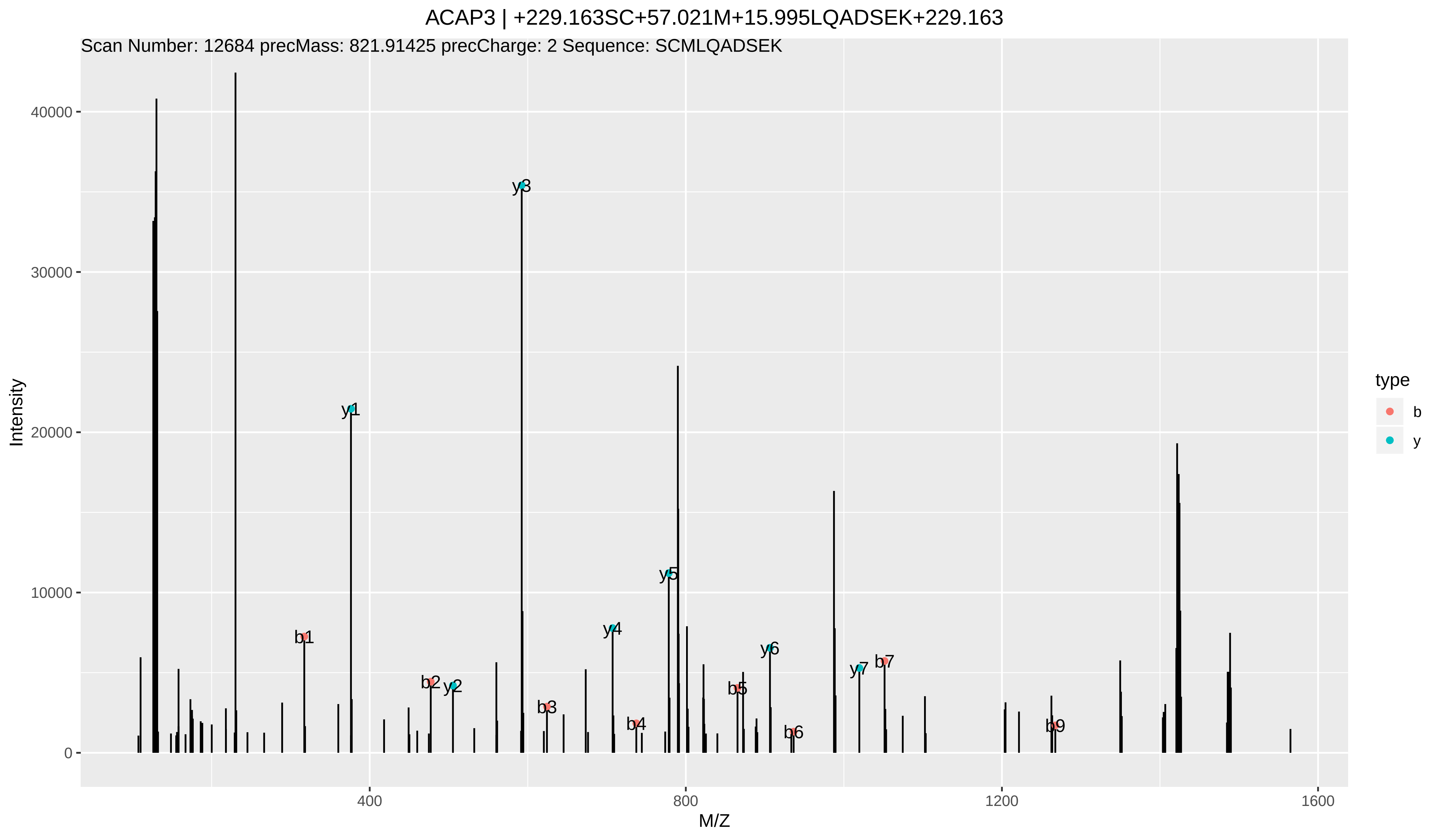

ACAP3 | +229.163SC+57.021M+15.995LQADSEK+229.163
Scan Number: 12684 precMass: 821.91425 precCharge: 2 Sequence: SCMLQADSEK
Intensity
M/Z
0
10000
20000
30000
40000
400
800
1200
1600
y1
y2
y3
y4
y5
y6
y7
b1
b2
b3
b4
b5
b6
b7
b9
type
b
y

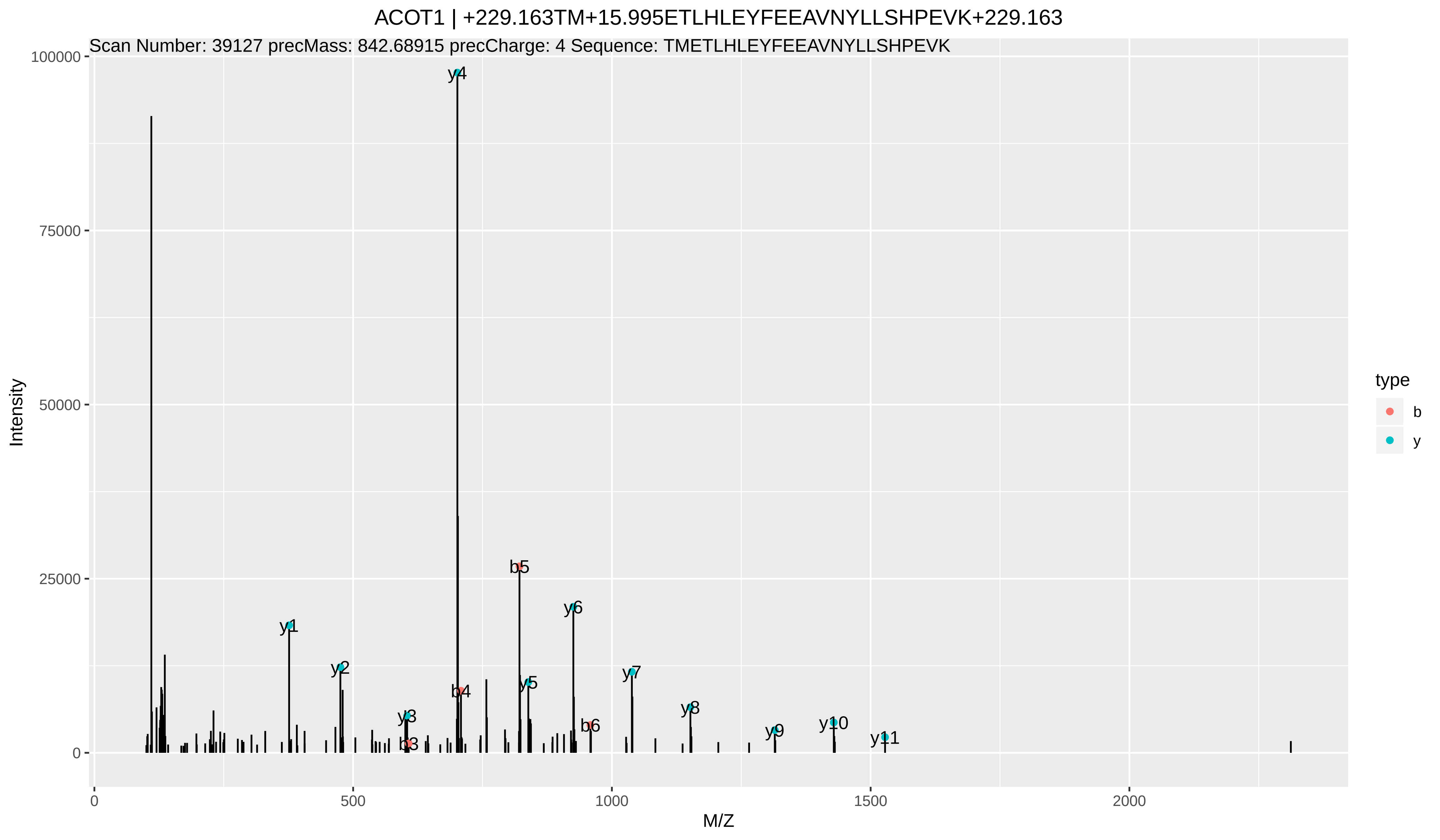
ACOT1 | +229.163TM+15.995ETLHLEYFEEAVNYLLSHPEVK+229.163
Scan Number: 39127 precMass: 842.68915 precCharge: 4 Sequence: TMETLHLEYFEEAVNYLLSHPEVK
Intensity
100000
75000
50000
25000
0
M/Z
0
500
1000
1500
2000
y1
y2
y3
y4
y5
y6
y7
y8
y9
y10
y11
b3
b4
b5
b6
type
b
y

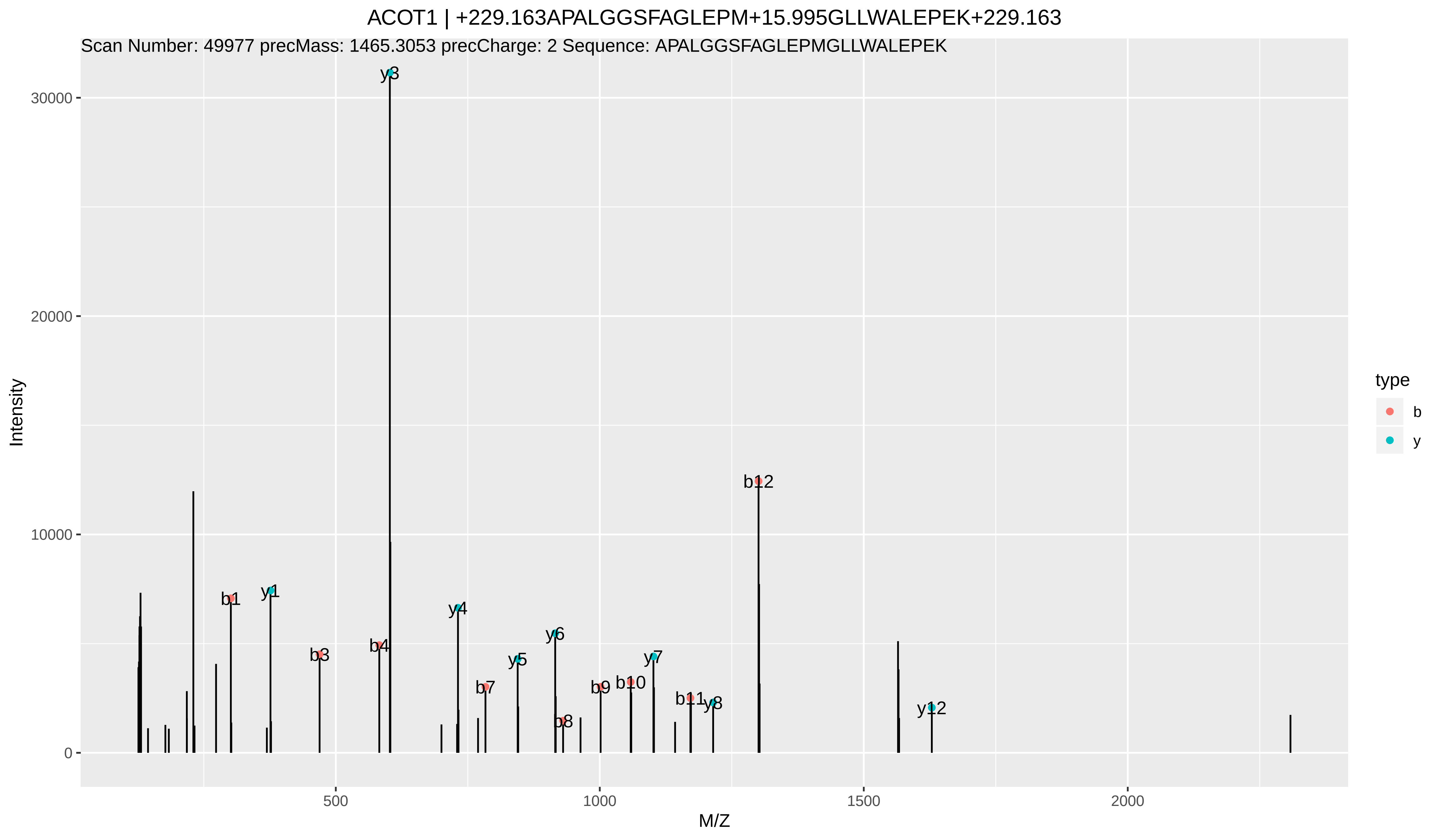
ACOT1 | +229.163APALGGSFAGLEPM+15.995GLLWALEPEK+229.163
Scan Number: 49977 precMass: 1465.3053 precCharge: 2 Sequence: APALGGSFAGLEPMGLLWALEPEK
Intensity
M/Z
0
10000
20000
30000
500
1000
1500
2000
y3
b12
b1
y1
y4
b3
b4
y5
y6
y7
b7
b8
b9
b10
b11
y8
y12
type
b
y

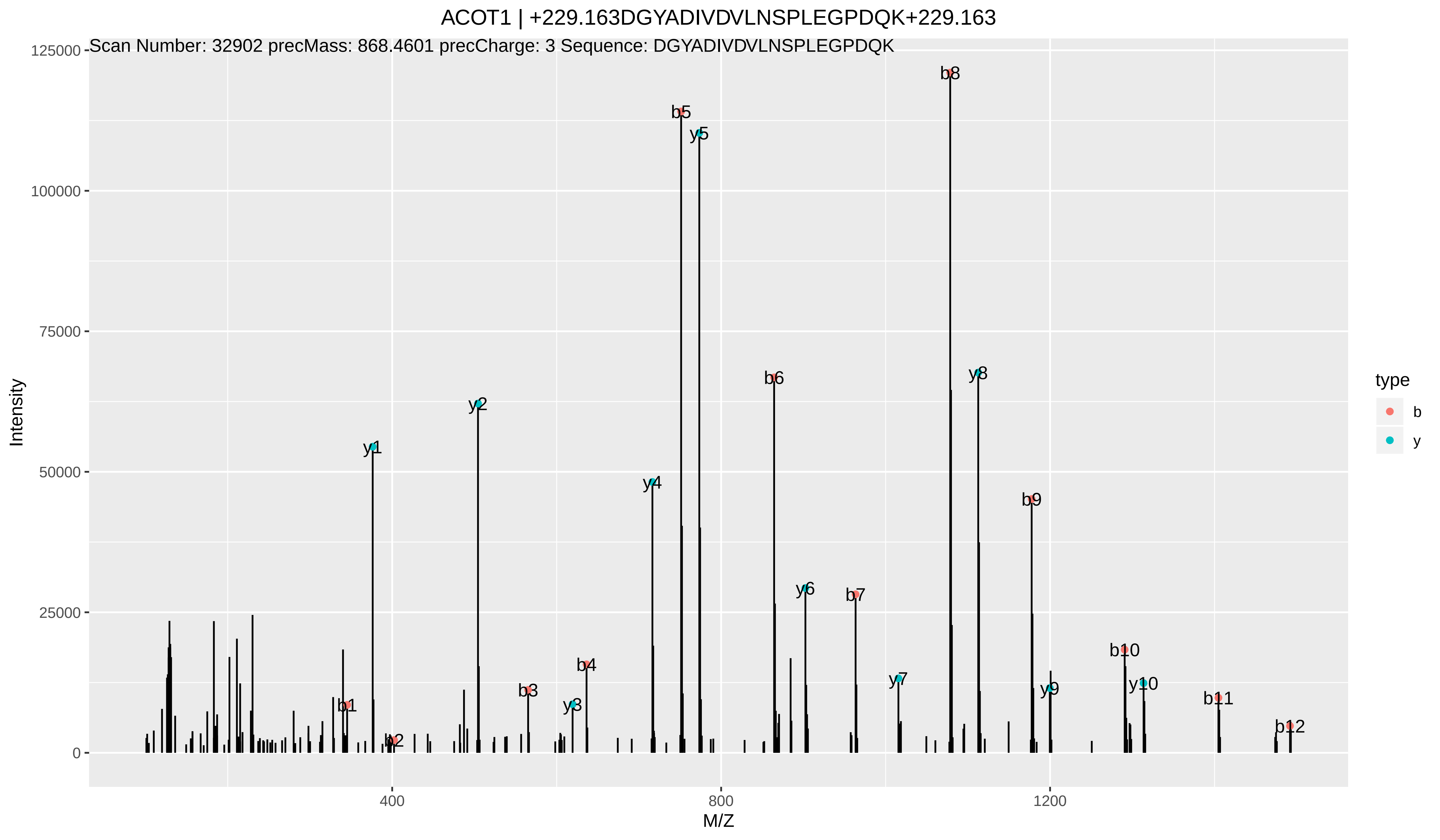
ACOT1 | +229.163DGYADIVDVLNSPLEGPDQK+229.163
Scan Number: 32902 precMass: 868.4601 precCharge: 3 Sequence: DGYADIVDVLNSPLEGPDQK
Intensity
125000
100000
75000
50000
25000
0
400
800
1200
M/Z
b8
b5
y5
b6
y8
y2
y1
y4
b9
y6
b7
b10
y10
y7
b11
y9
b4
b3
y3
b1
b2
b12
type
b
y

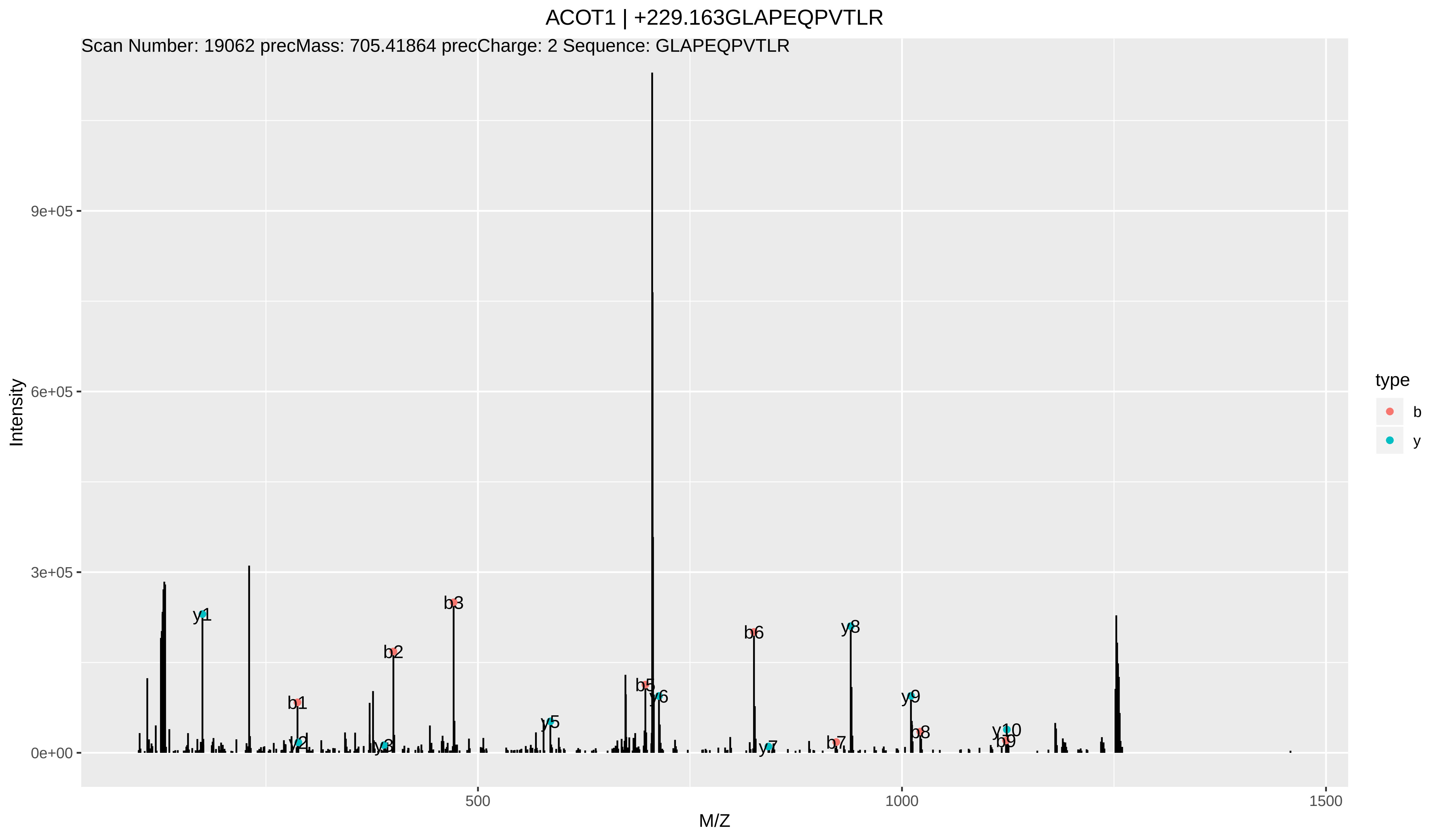

ACOT1 | +229.163GLAPEQPVTLR
Scan Number: 19062 precMass: 705.41864 precCharge: 2 Sequence: GLAPEQPVTLR
Intensity
9e+05
6e+05
3e+05
0e+00
M/Z
500
1000
1500
y1
b1
y2
y3
b2
b3
y5
b5
y6
b6
y7
b7
y8
y9
b8
y10
b9
type
b
y

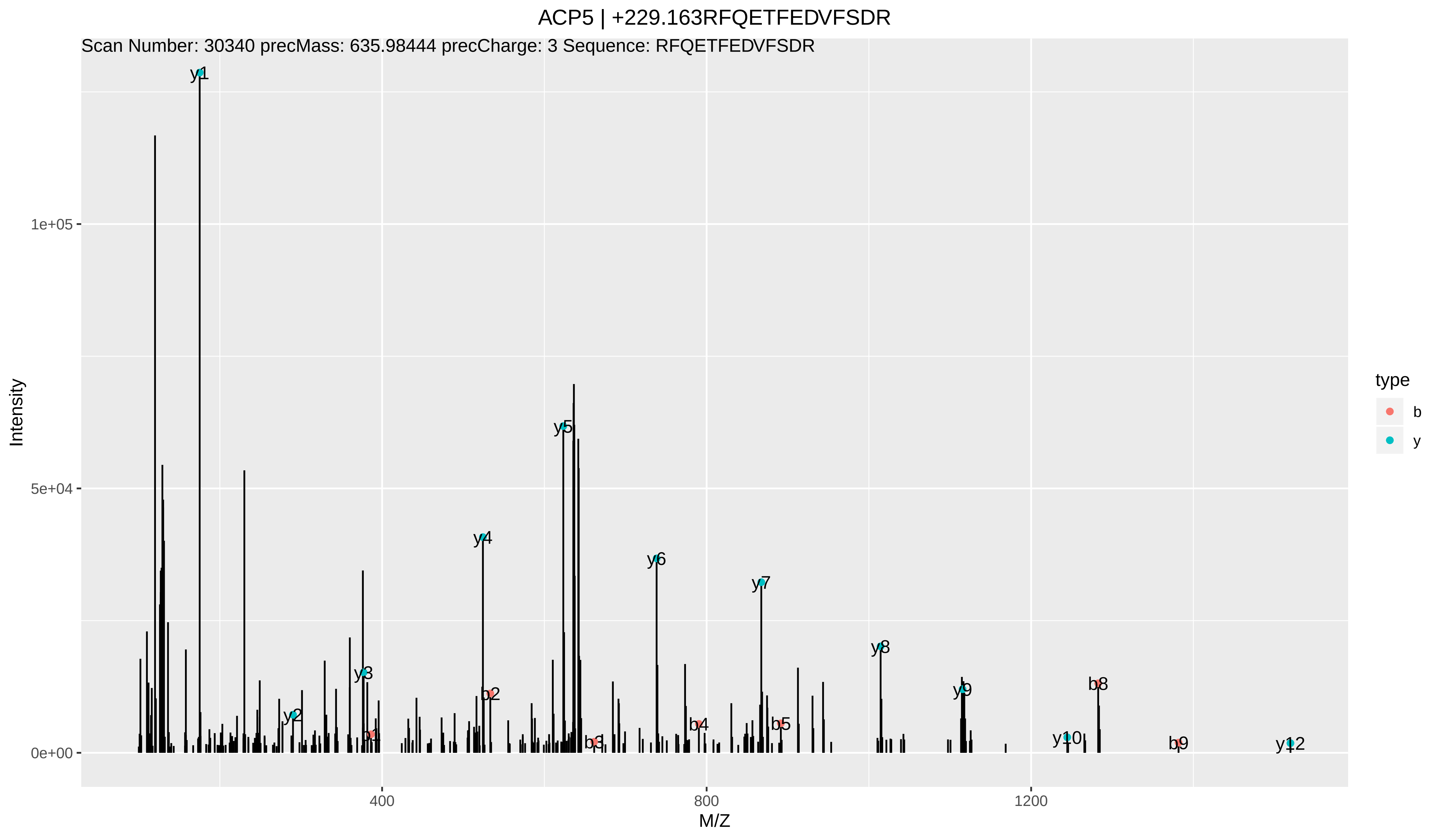
ACP5 | +229.163RFQETFEDVFSDR
Scan Number: 30340 precMass: 635.98444 precCharge: 3 Sequence: RFQETFEDVFSDR
Intensity
M/Z
1e+05
5e+04
0e+00
400
800
1200
type
b
y
y1
y2
y3
y4
y5
y6
y7
y8
y9
y10
y12
b2
b3
b4
b5
b8
b9

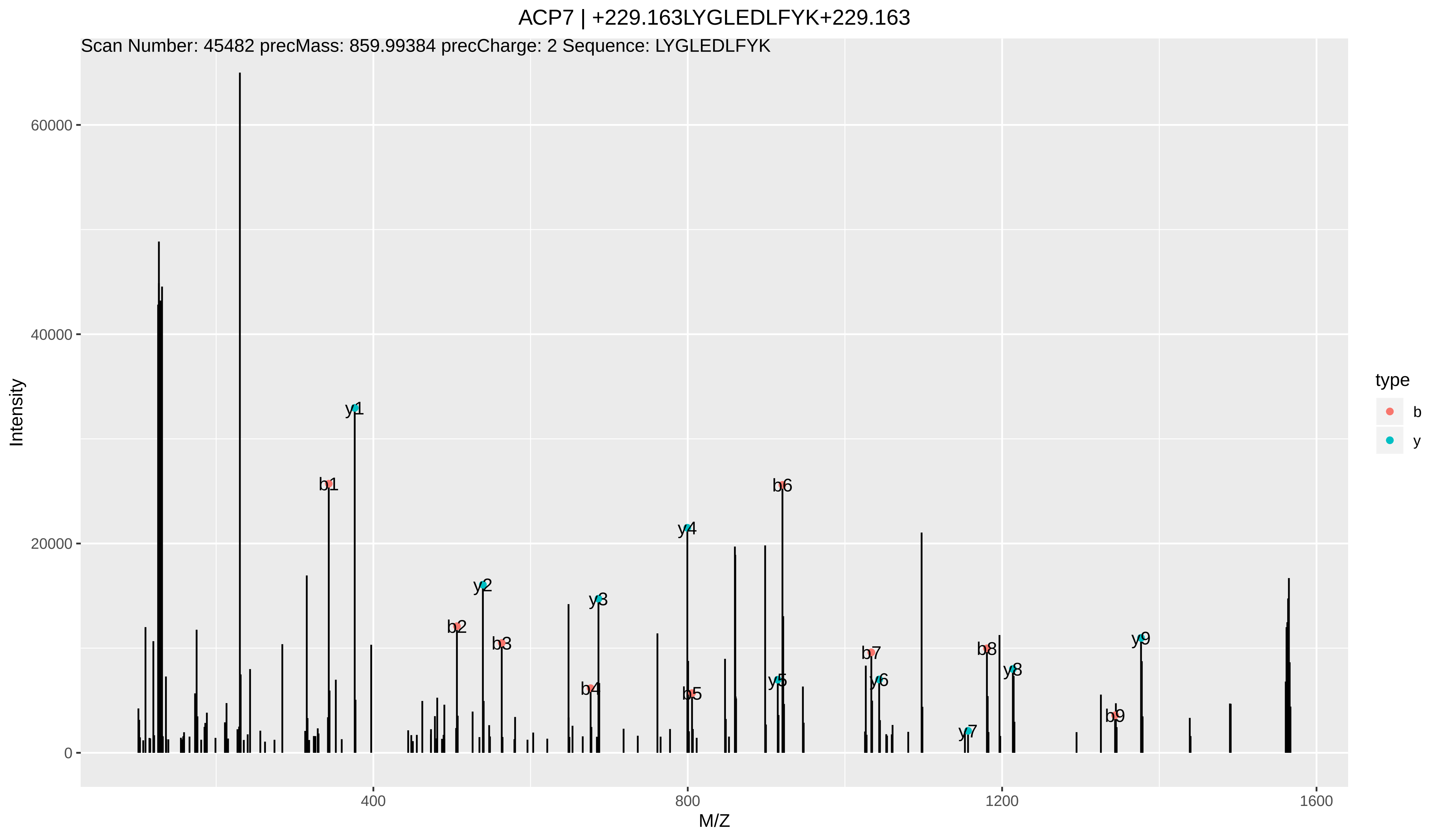

ACP7 | +229.163LYGLEDLFYK+229.163
Scan Number: 45482 precMass: 859.99384 precCharge: 2 Sequence: LYGLEDLFYK
Intensity
60000
40000
20000
0
400
800
1200
1600
M/Z
y1
b1
b6
y4
y2
y3
b2
b3
b4
b5
y5
y6
b7
b8
y8
y9
y7
b9
type
b
y

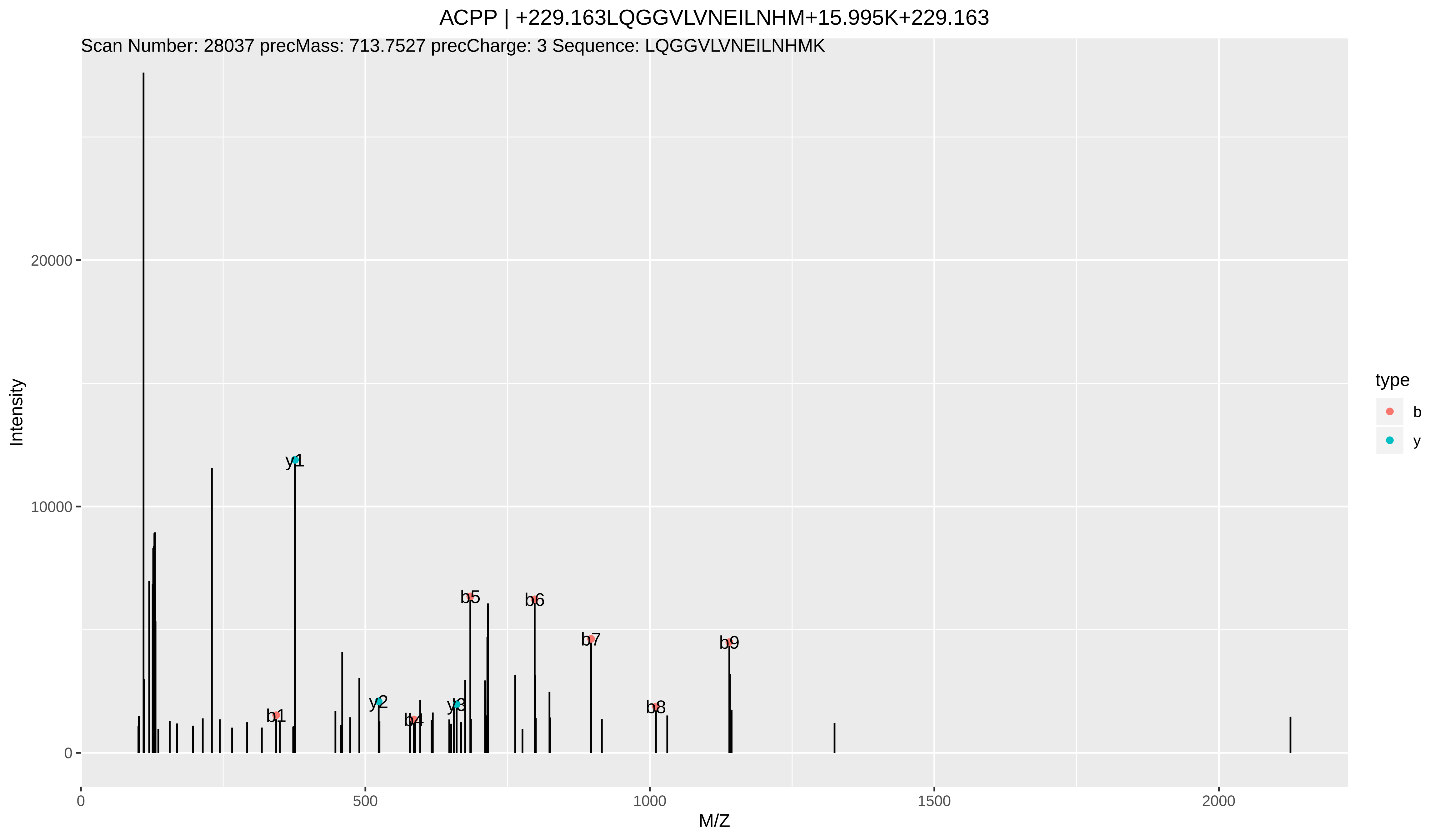

ACPP | +229.163LQGGVLVNEILNHM+15.995K+229.163
Scan Number: 28037 precMass: 713.7527 precCharge: 3 Sequence: LQGGVLVNEILNHMK
Intensity
20000
10000
0
M/Z
0
500
1000
1500
2000
y1
b1
y2
b4
y3
b5
b6
b7
b8
b9
type
b
y

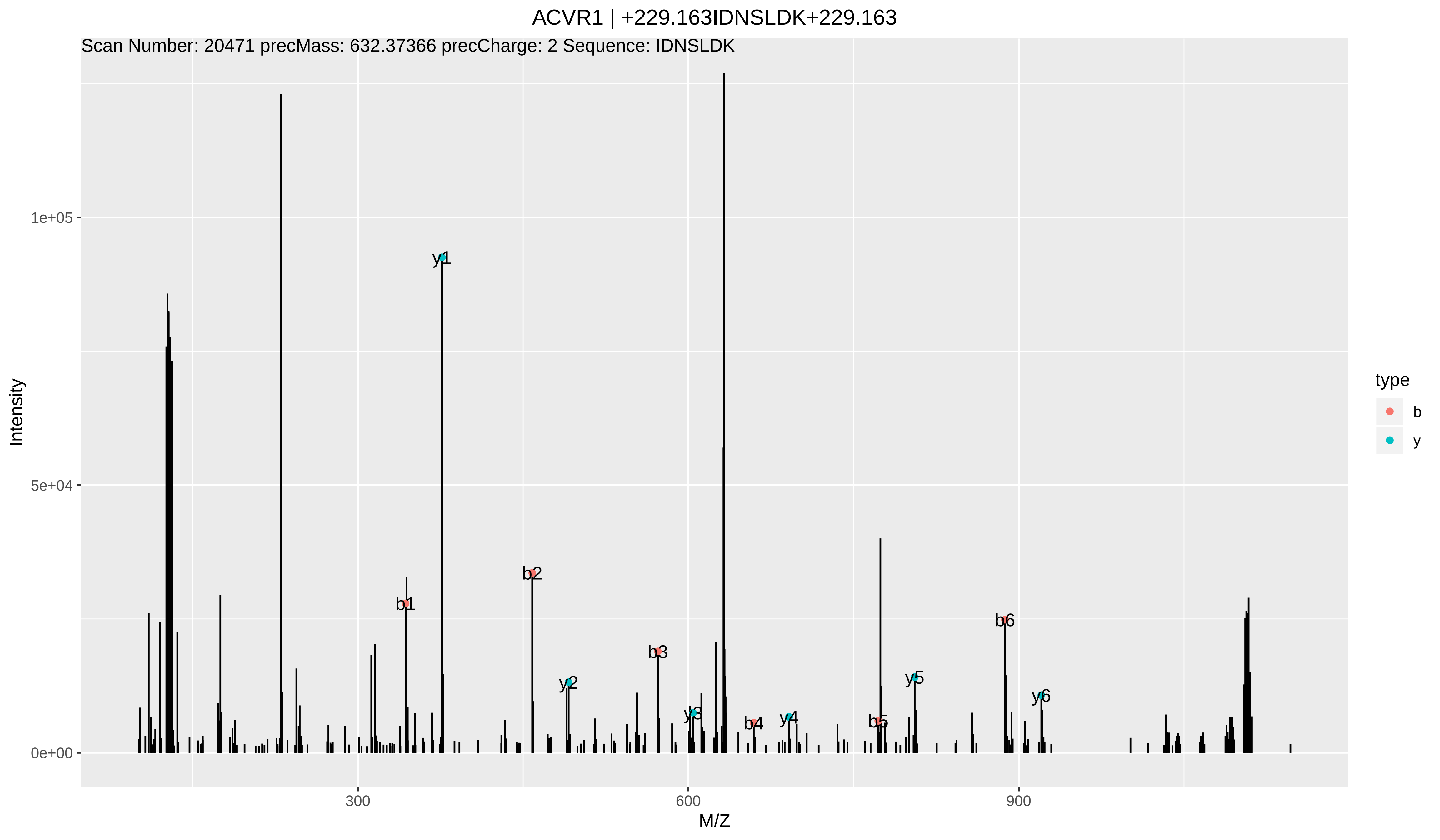
ACVR1 | +229.163IDNSLDK+229.163
Scan Number: 20471 precMass: 632.37366 precCharge: 2 Sequence: IDNSLDK
Intensity
1e+05
5e+04
0e+00
M/Z
300
600
900
y1
b2
b1
b6
b3
y2
y5
y6
y3
y4
b4
b5
type
b
y

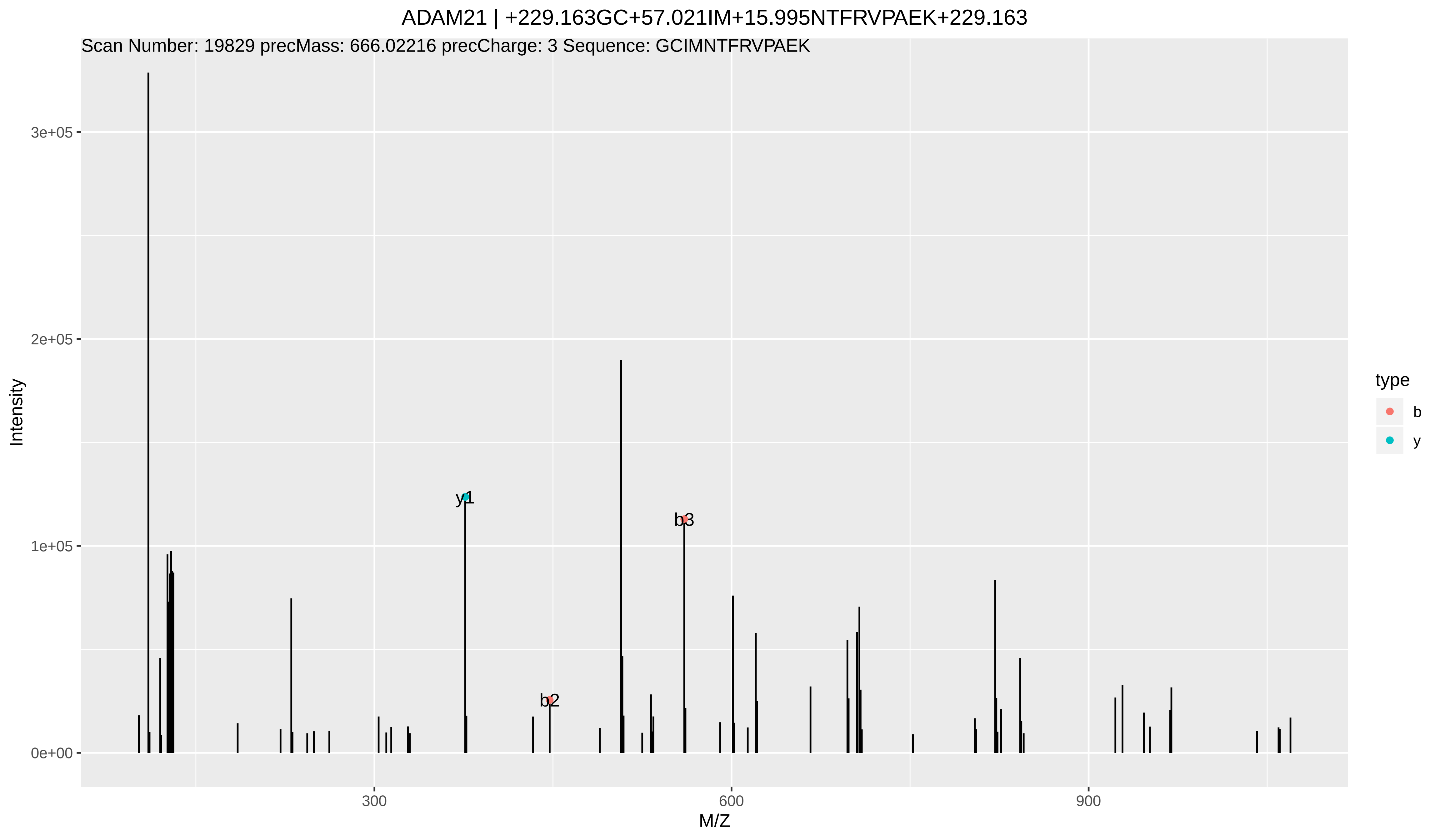
ADAM21 | +229.163GC+57.021IM+15.995NTFRVPAEK+229.163
Scan Number: 19829 precMass: 666.02216 precCharge: 3 Sequence: GCIMNTFRVPAEK
Intensity
3e+05
2e+05
1e+05
0e+00
M/Z
300
600
900
y1
b2
b3
type
b
y

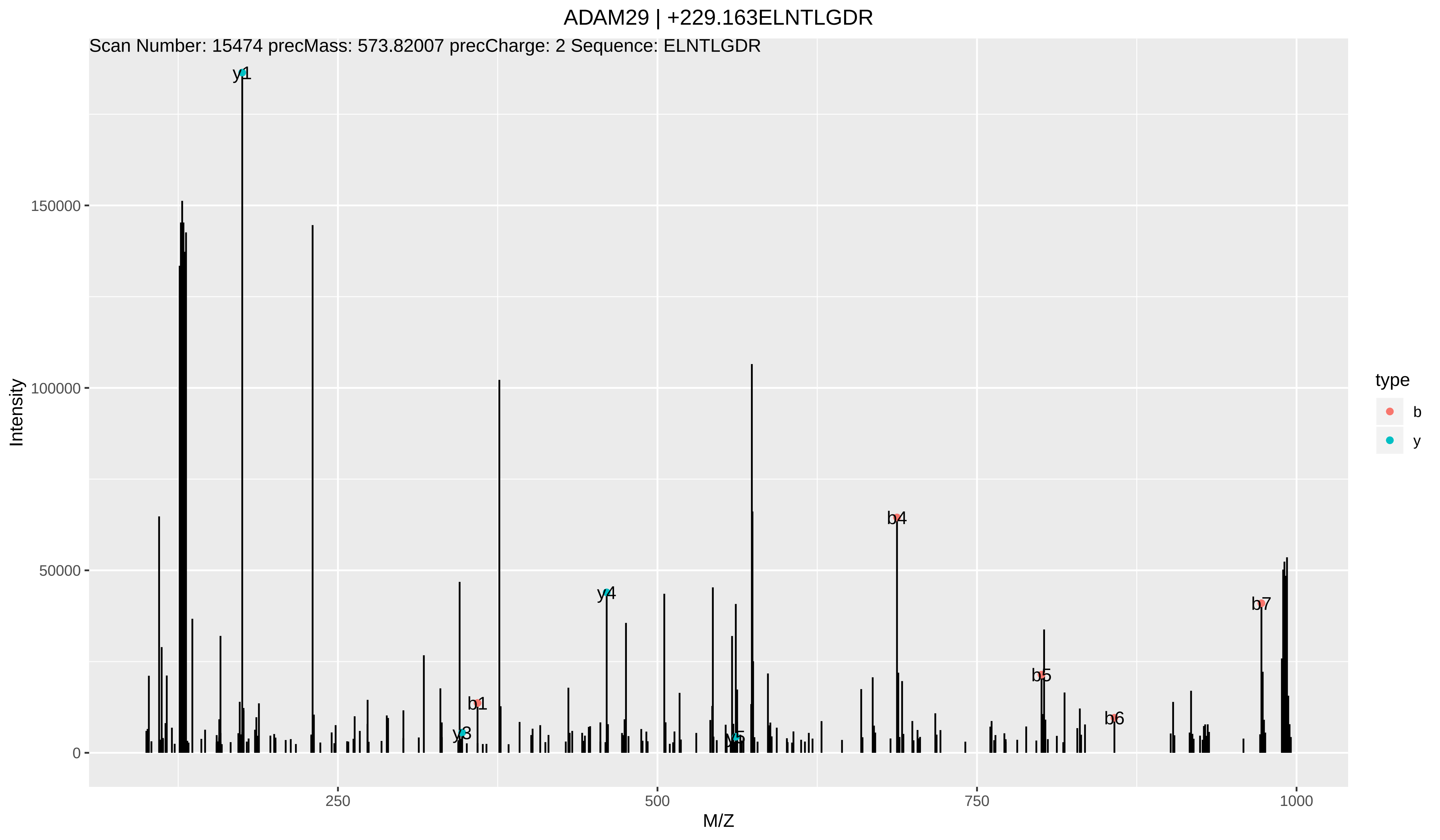
ADAM29 | +229.163ELNTLGDR
Scan Number: 15474 precMass: 573.82007 precCharge: 2 Sequence: ELNTLGDR
y1
y3
b1
y4
y5
b4
b5
b6
b7
Intensity
150000
100000
50000
0
250
500
750
1000
M/Z
type
b
y

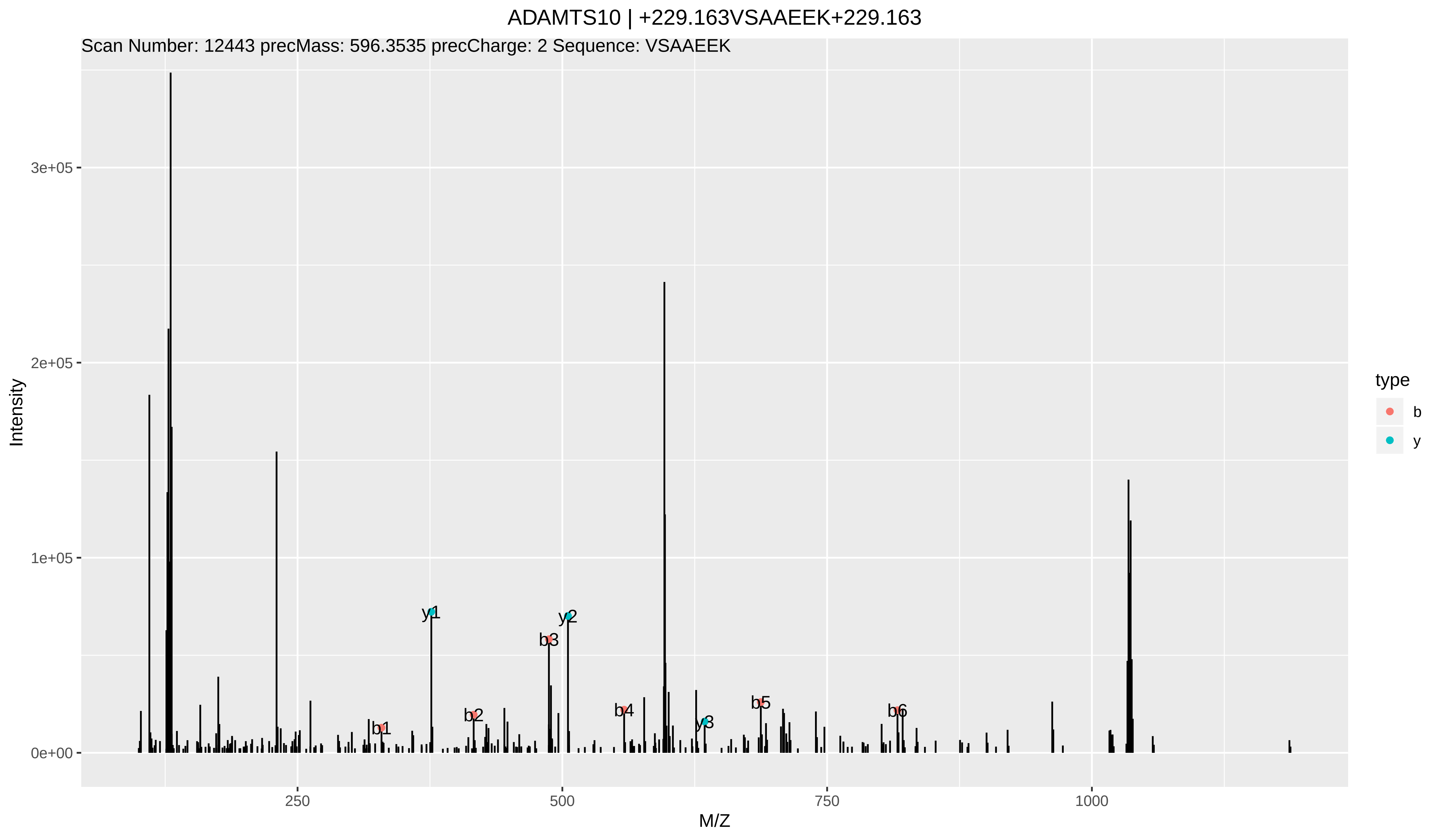

ADAMTS10 | +229.163VSAAEEK+229.163
Scan Number: 12443 precMass: 596.3535 precCharge: 2 Sequence: VSAAEEK
Intensity
3e+05
2e+05
1e+05
0e+00
y1
y2
b3
b1
b2
b4
y3
b5
b6
250
500
750
1000
M/Z
type
b
y

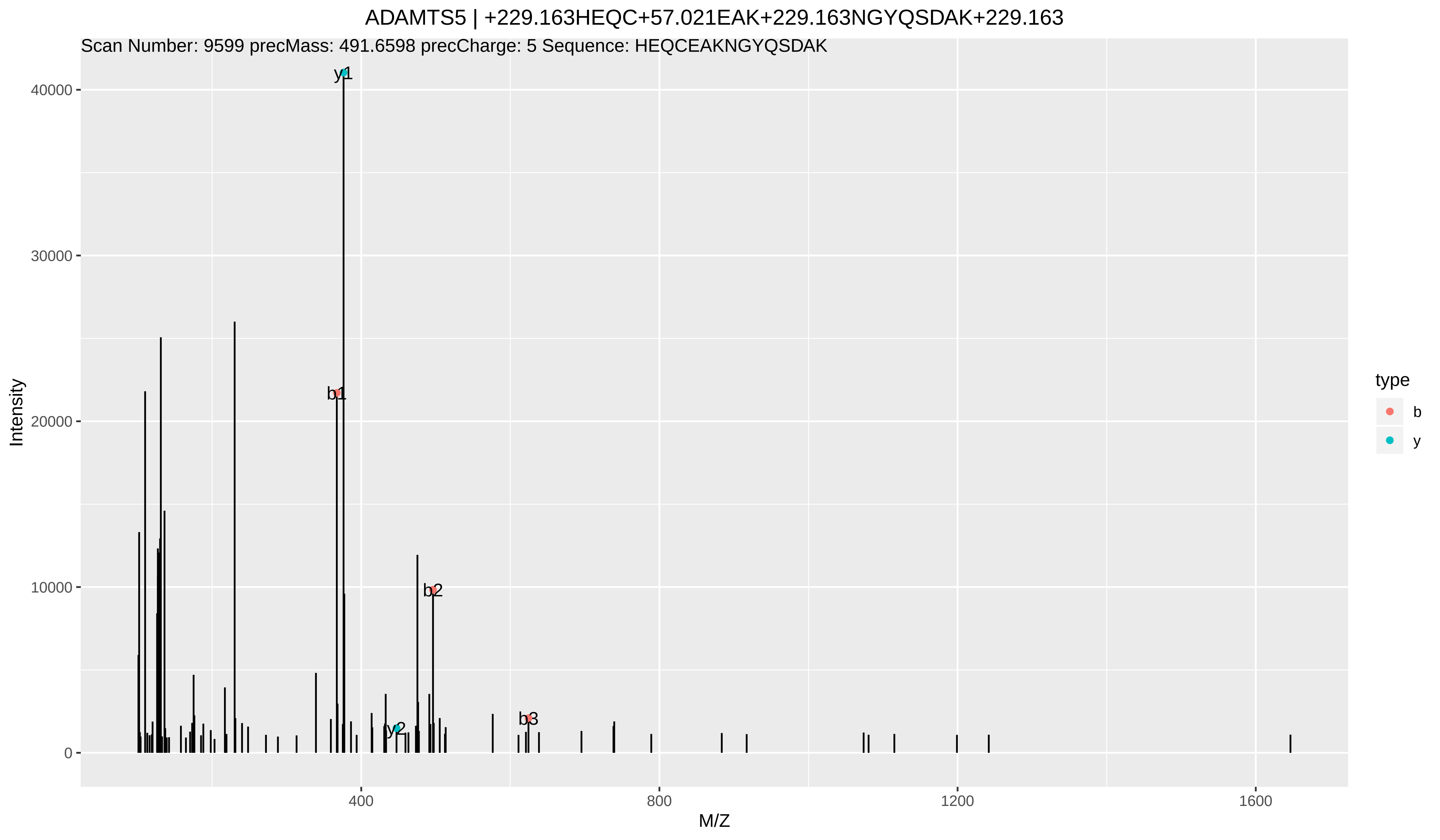

ADAMTS5 | +229.163HEQC+57.021EAK+229.163NGYQSDAK+229.163
Scan Number: 9599 precMass: 491.6598 precCharge: 5 Sequence: HEQCEAKNGYQSDAK
y1
b1
b2
y2
b3
Intensity
40000
30000
20000
10000
0
400
800
1200
1600
M/Z
type
b
y

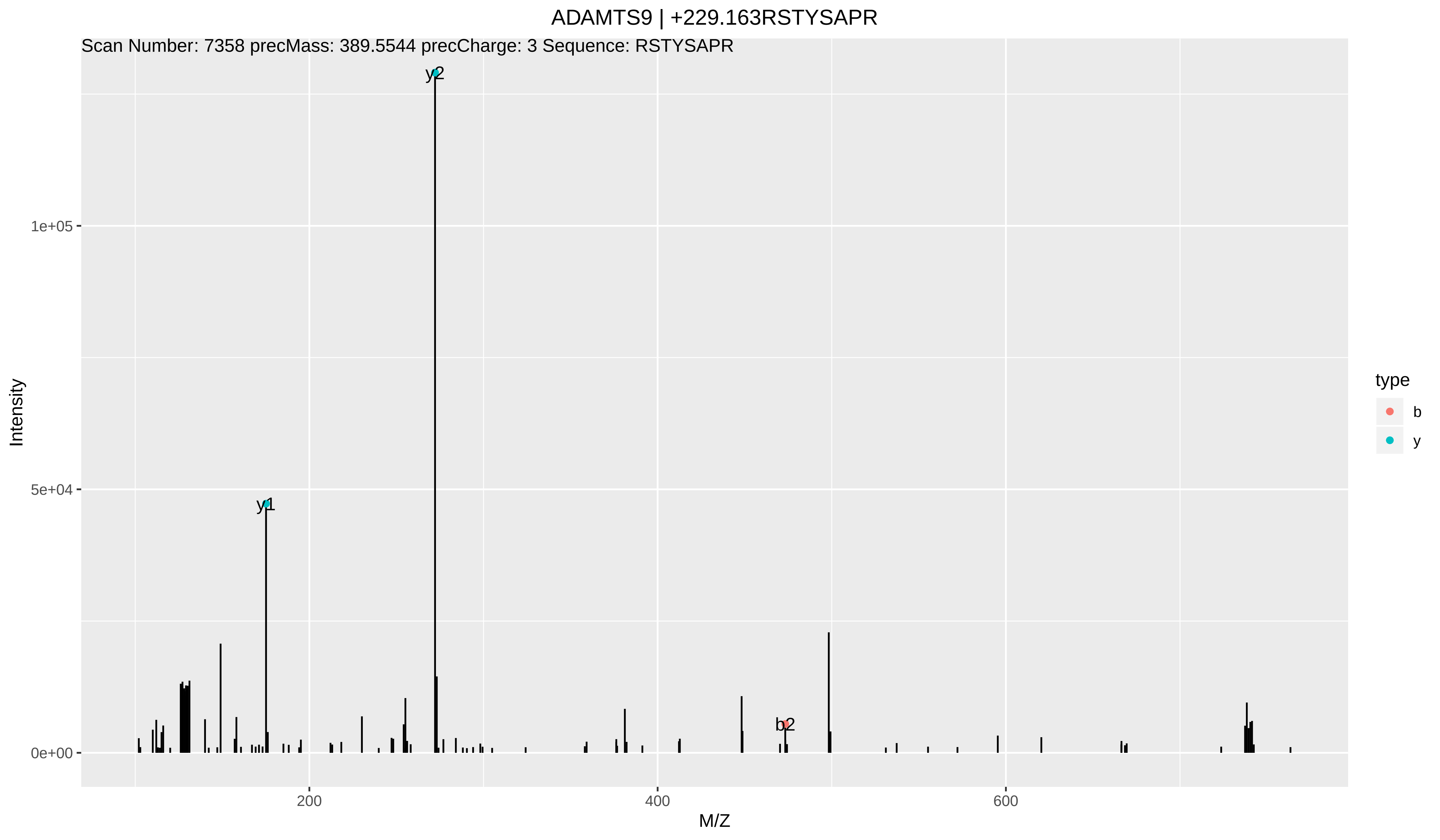
ADAMTS9 | +229.163RSTYSAPR
Scan Number: 7358 precMass: 389.5544 precCharge: 3 Sequence: RSTYSAPR
y2
y1
b2
Intensity
1e+05
5e+04
0e+00
200
400
600
M/Z
type
b
y

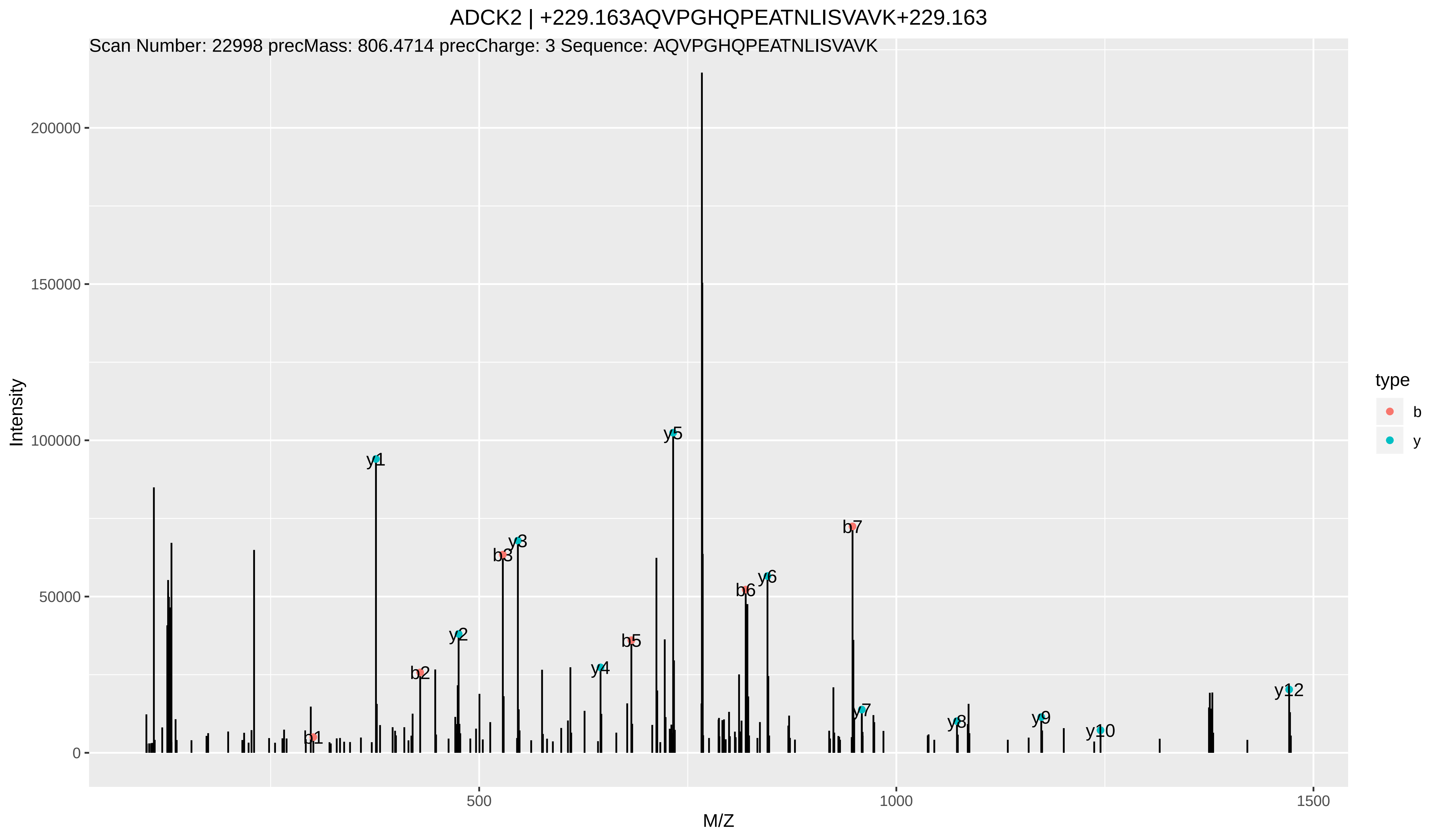

ADCK2 | +229.163AQVPGHQPEATNLISVAVK+229.163
Scan Number: 22998 precMass: 806.4714 precCharge: 3 Sequence: AQVPGHQPEATNLISVAVK
Intensity
200000
150000
100000
50000
0
M/Z
500
1000
1500
y1
y2
y3
y4
y5
y6
y7
y8
y9
y10
y12
b1
b2
b3
b5
b6
b7
type
b
y

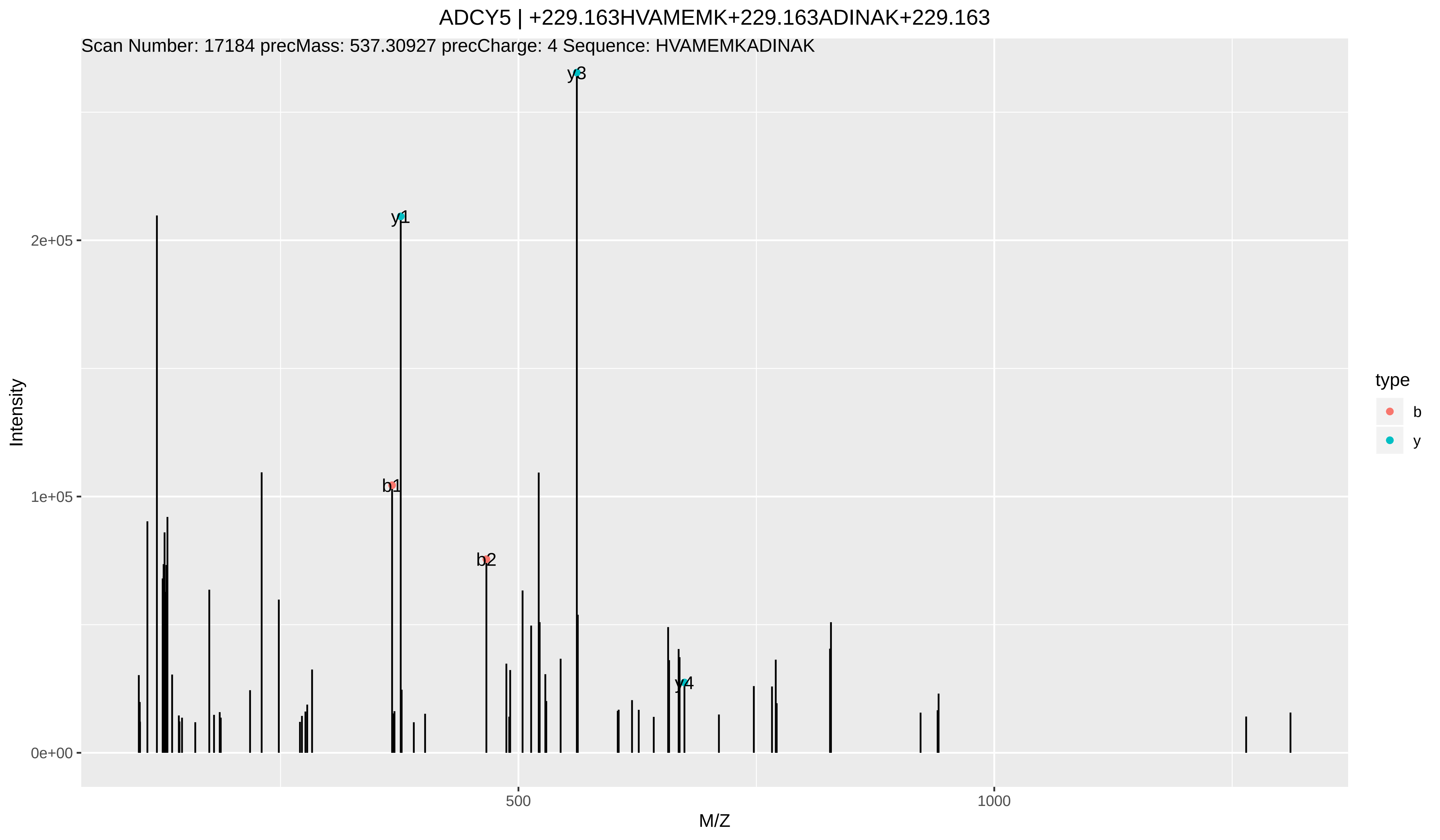
ADCY5 | +229.163HVAMEMK+229.163ADINAK+229.163
Scan Number: 17184 precMass: 537.30927 precCharge: 4 Sequence: HVAMEMKADINAK
y3
y1
b1
b2
y4
Intensity
2e+05
1e+05
0e+00
500
1000
M/Z
type
b
y

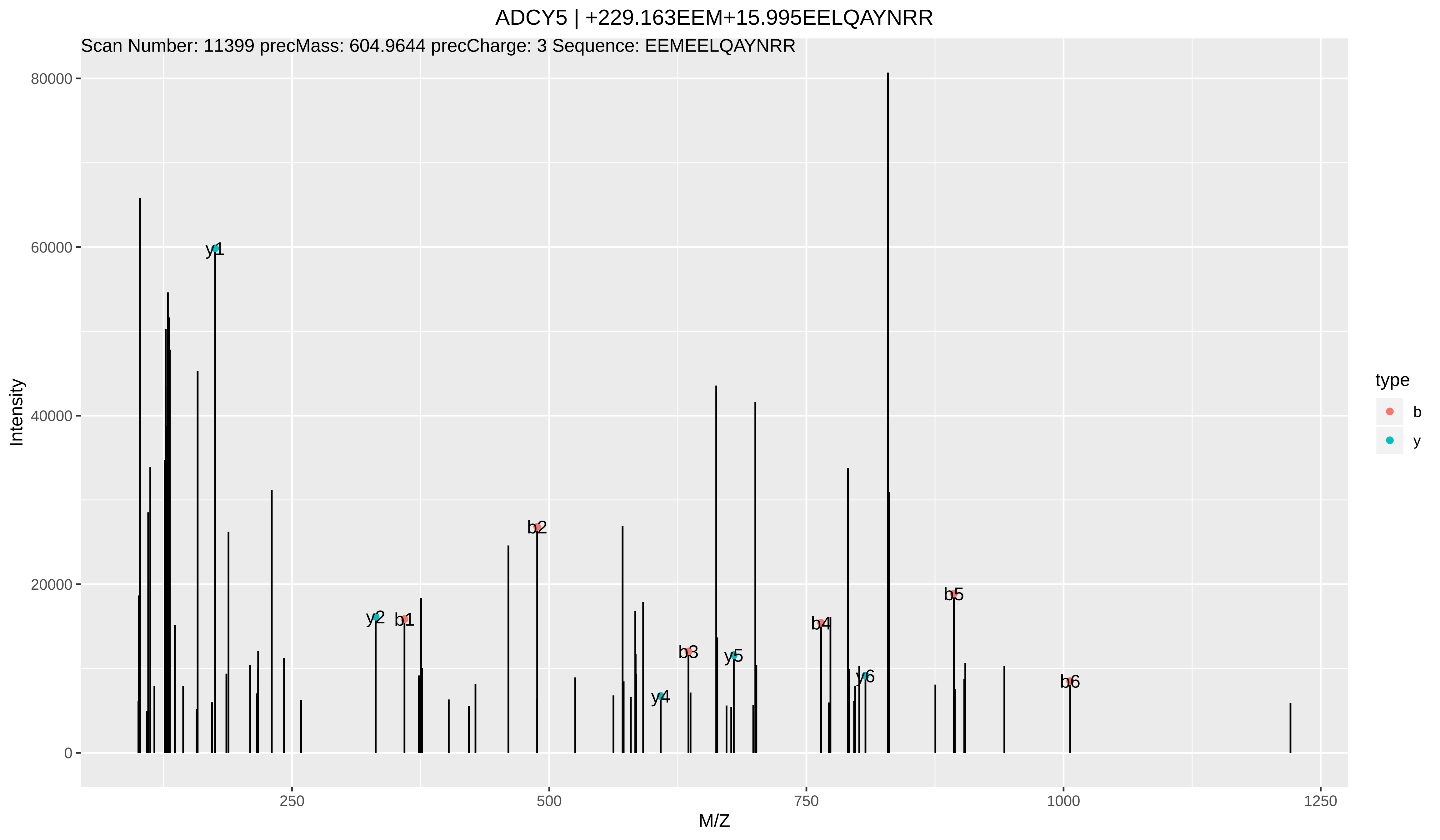
ADCY5 | +229.163EEM+15.995EELQAYNRR
Scan Number: 11399 precMass: 604.9644 precCharge: 3 Sequence: EEMEELQAYNRR
Intensity
M/Z
type
b
y
y1
y2
b1
b2
y4
b3
y5
b4
y6
b5
b6
0
20000
40000
60000
80000
250
500
750
1000
1250

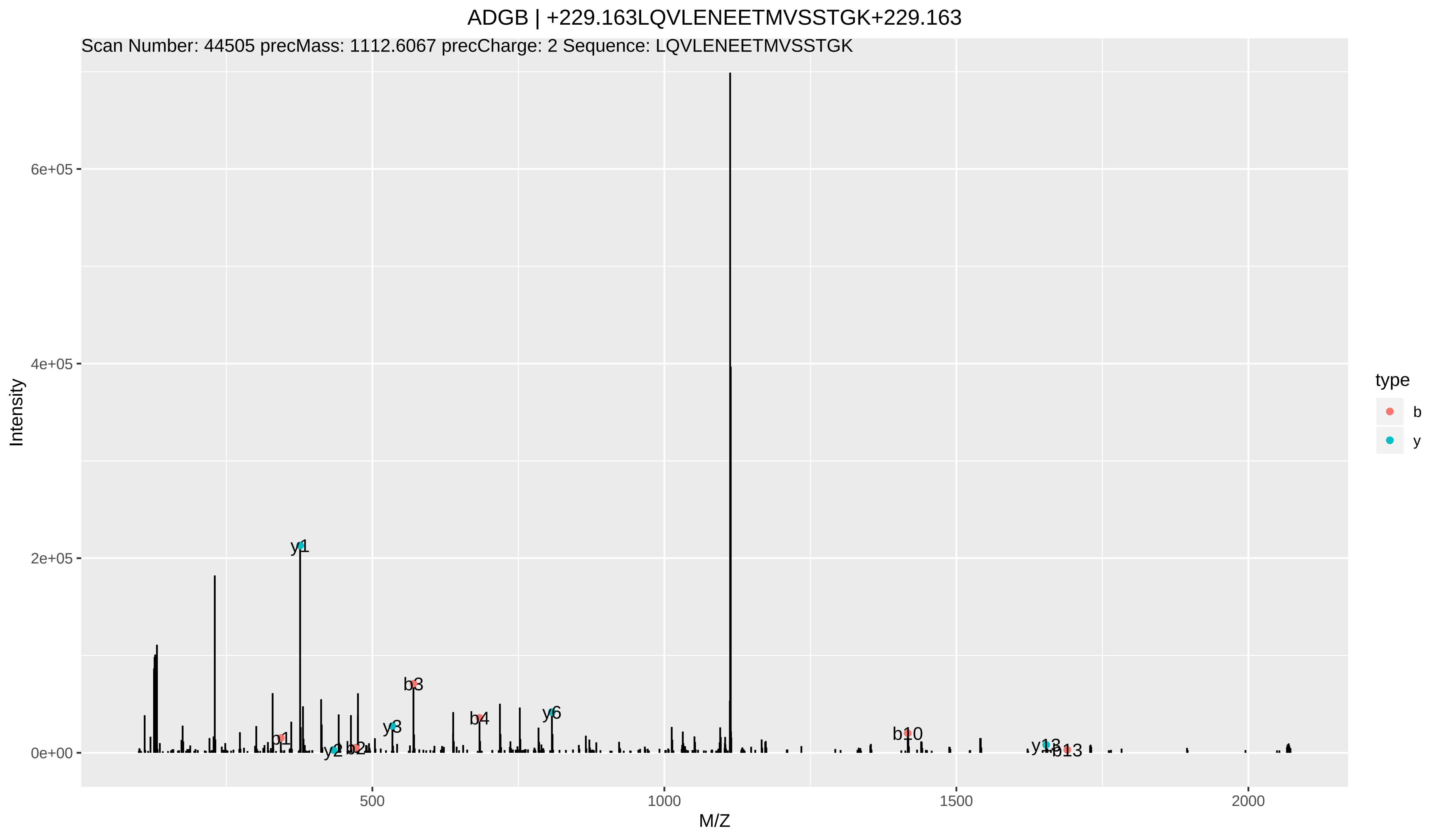

ADGB | +229.163LQVLENEETMVSSTGK+229.163
Scan Number: 44505 precMass: 1112.6067 precCharge: 2 Sequence: LQVLENEETMVSSTGK
Intensity
6e+05
4e+05
2e+05
0e+00
M/Z
500
1000
1500
2000
y1
b1
y2
b2
y3
b3
b4
y6
b10
y13
b13
type
b
y

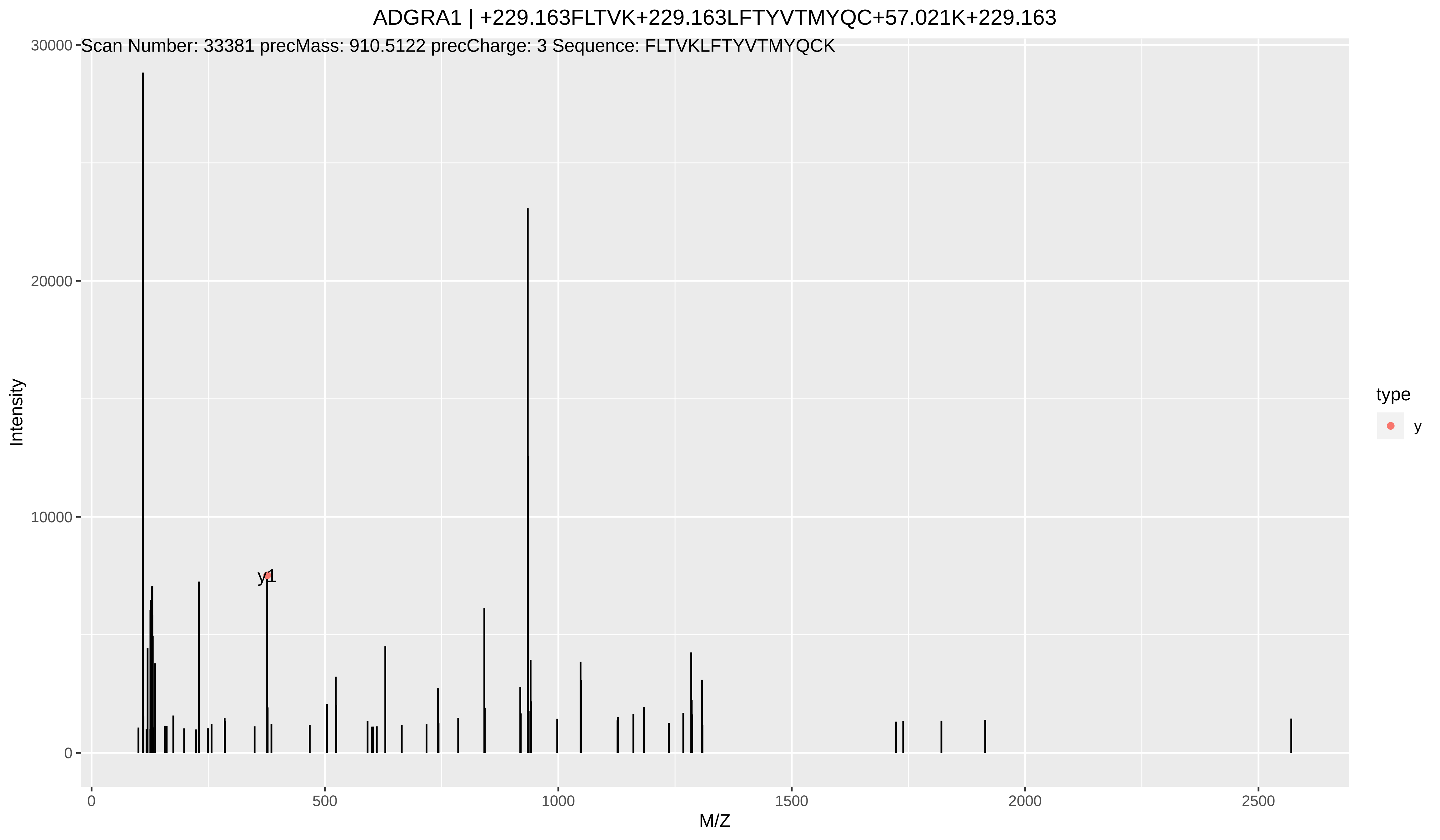
ADGRA1 | +229.163FLTVK+229.163LFTYVTMYQC+57.021K+229.163
Scan Number: 33381 precMass: 910.5122 precCharge: 3 Sequence: FLTVKLFTYVTMYQCK
30000
20000
10000
0
Intensity
y1
0
500
1000
1500
2000
2500
M/Z
type
y

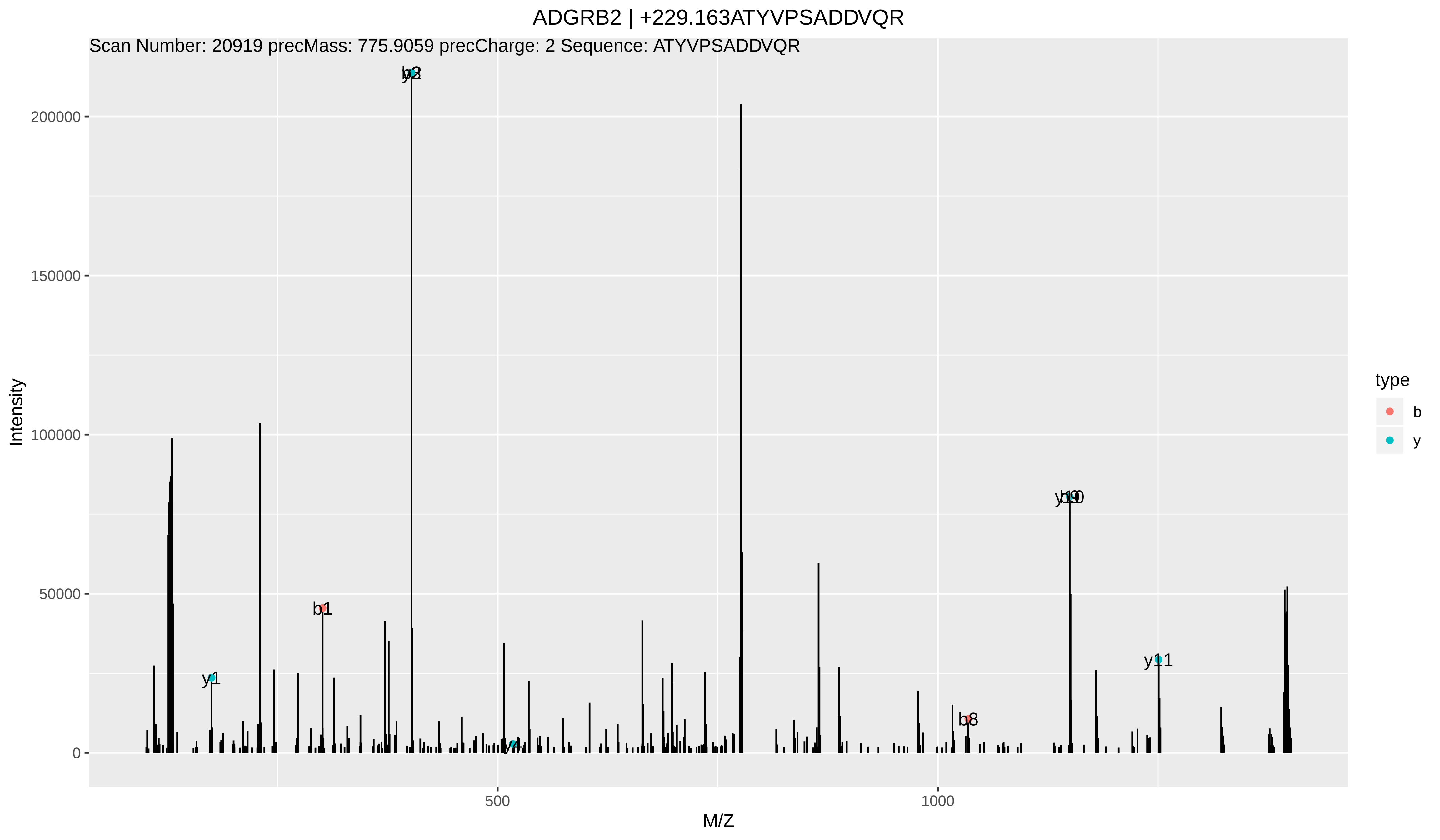
ADGRB2 | +229.163ATYVPSADDVQR
Scan Number: 20919 precMass: 775.9059 precCharge: 2 Sequence: ATYVPSADDVQR
Intensity
200000
150000
100000
50000
0
500
1000
M/Z
y1
b1
b2
y3
y4
b8
y9
b10
y11
type
b
y

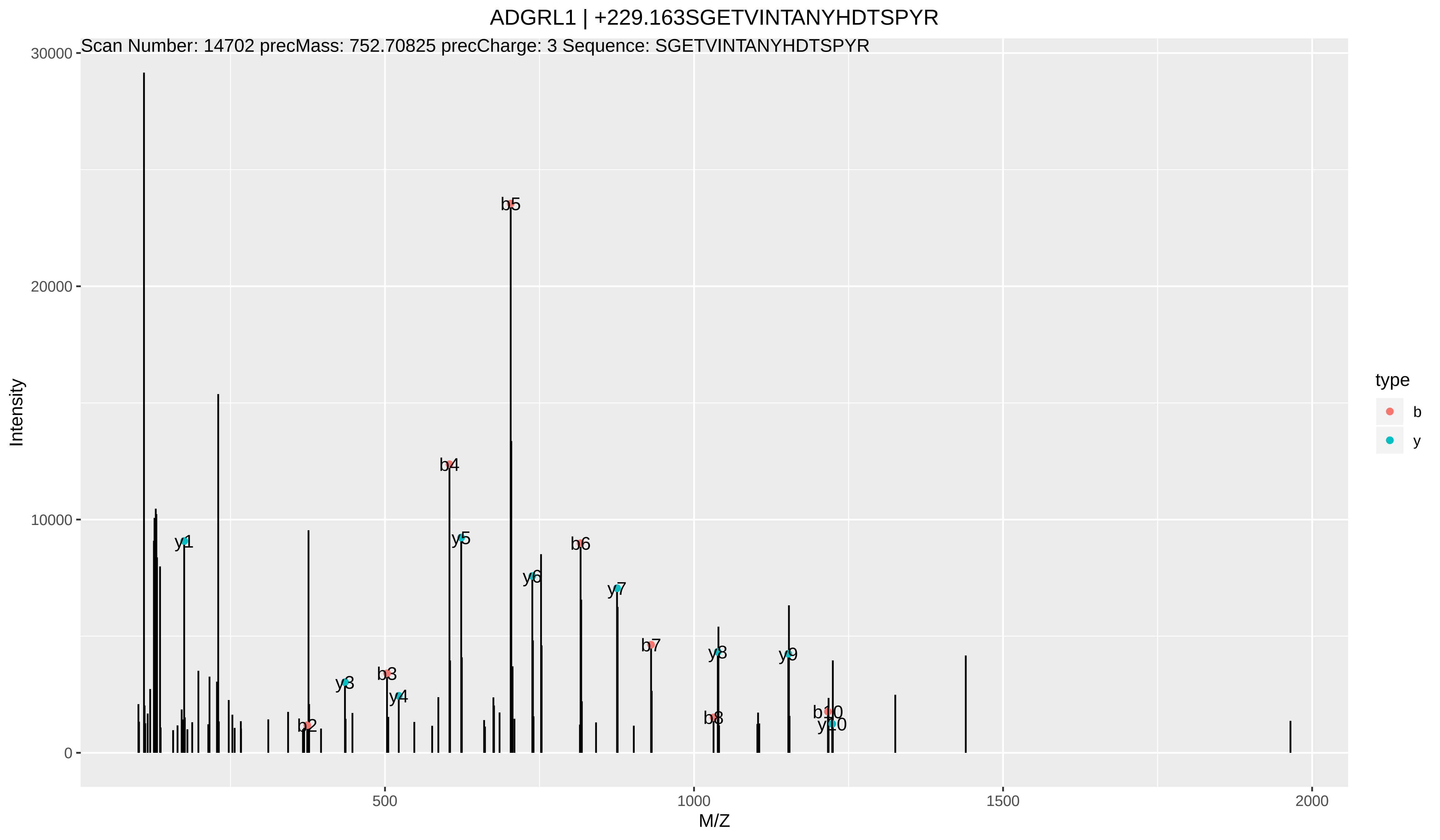

ADGRL1 | +229.163SGETVINTANYHDTSPYR
Scan Number: 14702 precMass: 752.70825 precCharge: 3 Sequence: SGETVINTANYHDTSPYR
Intensity
M/Z
0
10000
20000
30000
500
1000
1500
2000
y1
b2
y3
b3
y4
b4
y5
b5
y6
b6
y7
b7
y8
b8
y9
b10
y10
type
b
y

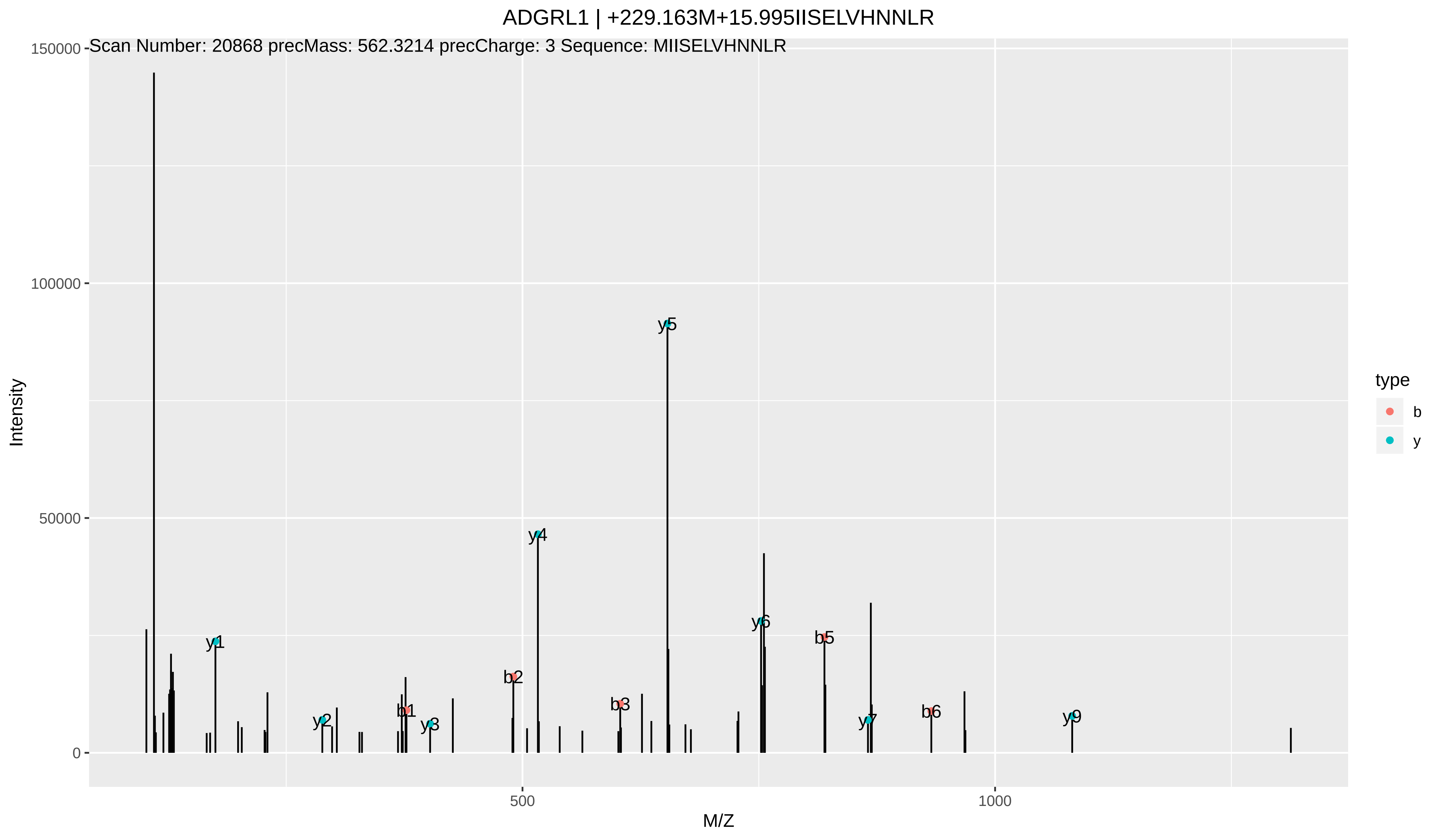

ADGRL1 | +229.163M+15.995IISELVHNNLR
Scan Number: 20868 precMass: 562.3214 precCharge: 3 Sequence: MIISELVHNNLR
150000
100000
50000
0
500
1000
M/Z
Intensity
type
b
y
y1
y2
b1
y3
b2
y4
b3
y5
y6
b5
y7
b6
y9

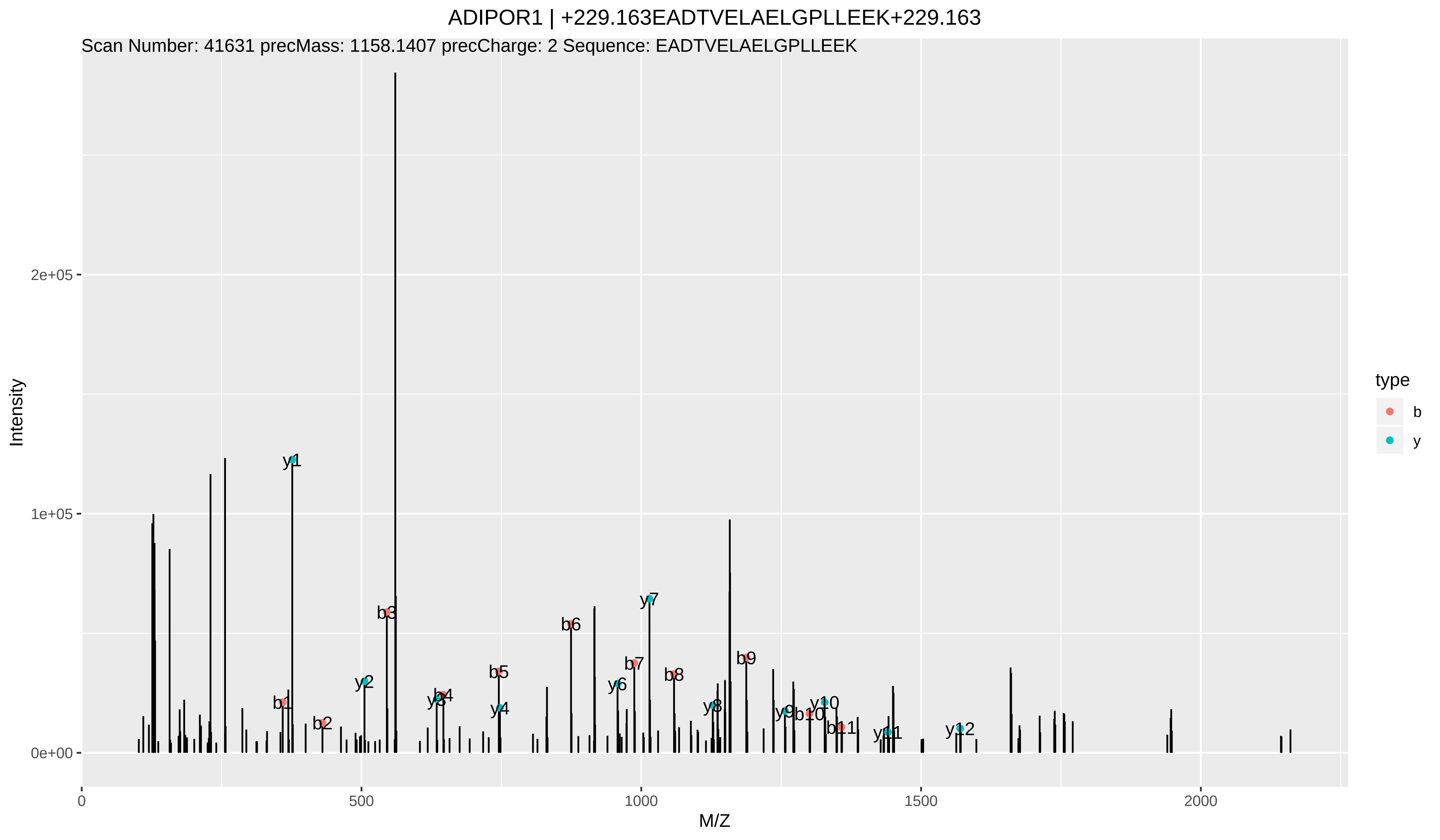
ADIPOR1 | +229.163EADTVELAELGPLLEEK+229.163
Scan Number: 41631 precMass: 1158.1407 precCharge: 2 Sequence: EADTVELAELGPLLEEK
Intensity
2e+05
1e+05
0e+00
M/Z
0
500
1000
1500
2000
type
b
y
y1
y2
y3
y4
y6
y7
y8
y9
y10
y11
y12
b1
b2
b3
b4
b5
b6
b7
b8
b9
b10
b11

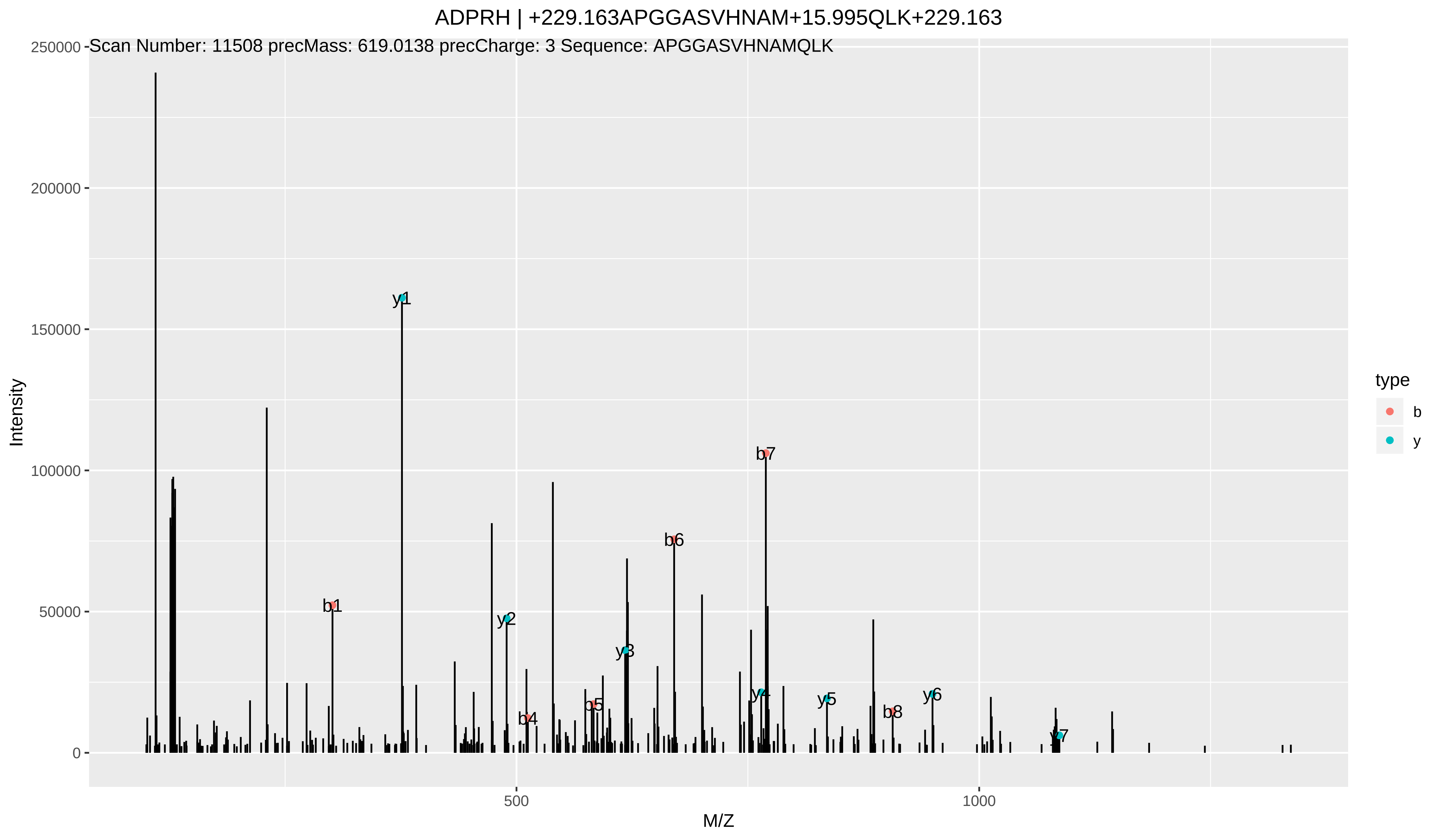
ADPRH | +229.163APGGASVHNAM+15.995QLK+229.163
Scan Number: 11508 precMass: 619.0138 precCharge: 3 Sequence: APGGASVHNAMQLK
Intensity
250000
200000
150000
100000
50000
0
M/Z
500
1000
y1
b1
y2
b4
b5
y3
b6
y4
b7
y5
b8
y6
y7
type
b
y

ADPRM | +229.163FLEDQIVHHPETM+15.995PSEDYYAYHFVPFPK+229.163

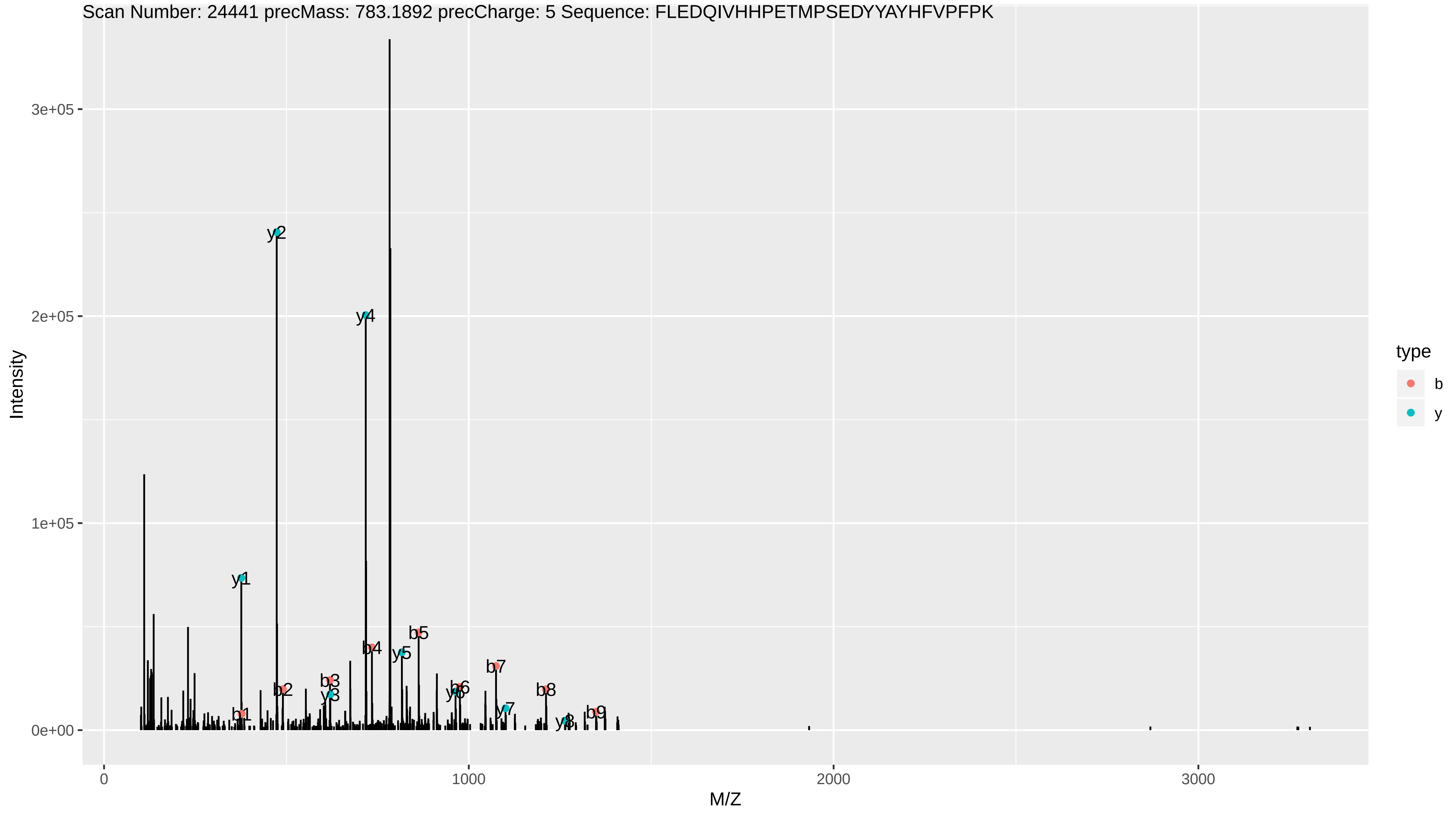

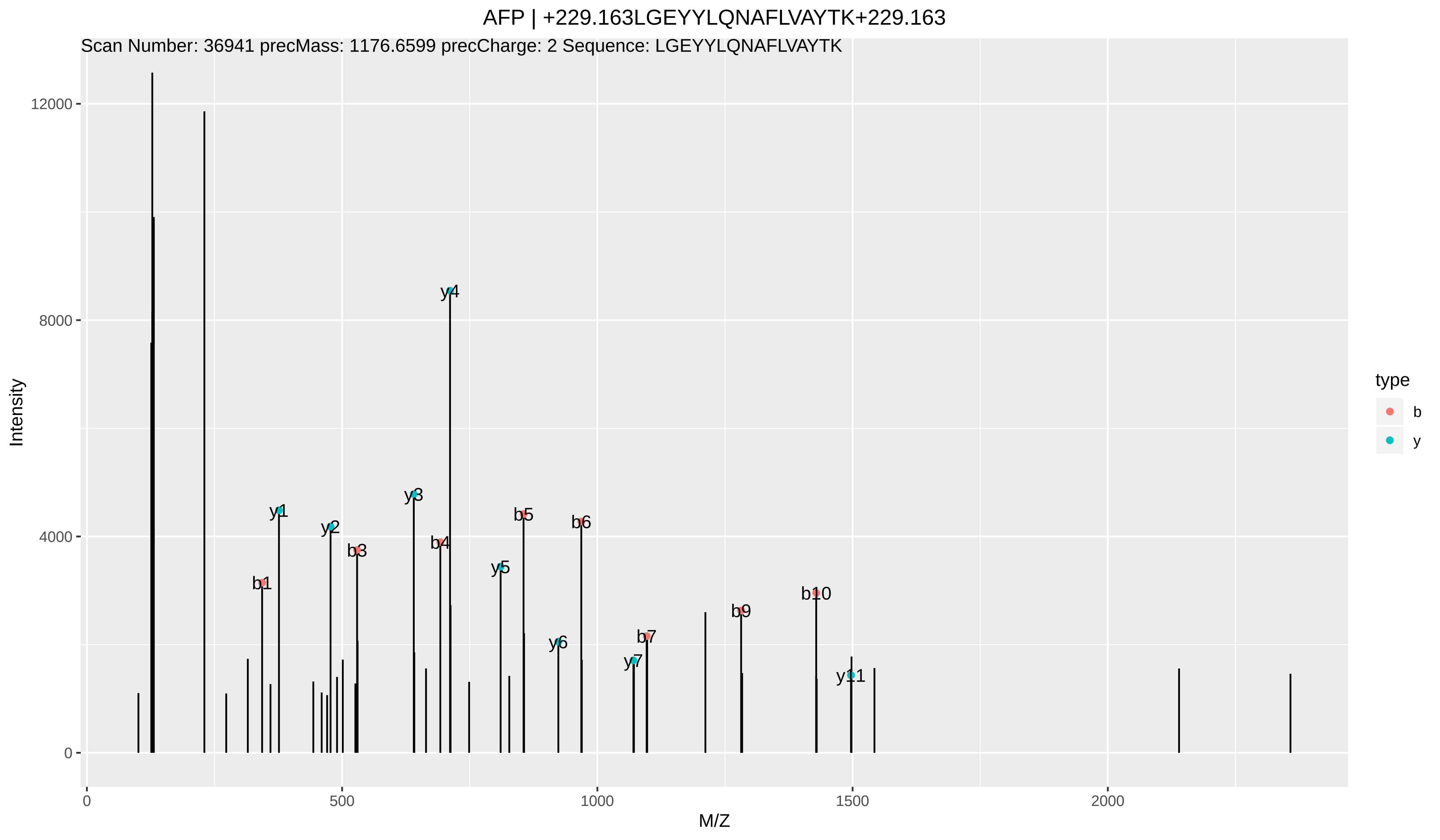

AFP | +229.163LGEYYLQNAFLVAYTK+229.163
Scan Number: 36941 precMass: 1176.6599 precCharge: 2 Sequence: LGEYYLQNAFLVAYTK
Intensity
12000
8000
4000
0
M/Z
0
500
1000
1500
2000
y4
y3
y1
y2
b3
b4
b5
b6
y5
b1
b10
b9
b7
y6
y7
y11
type
b
y

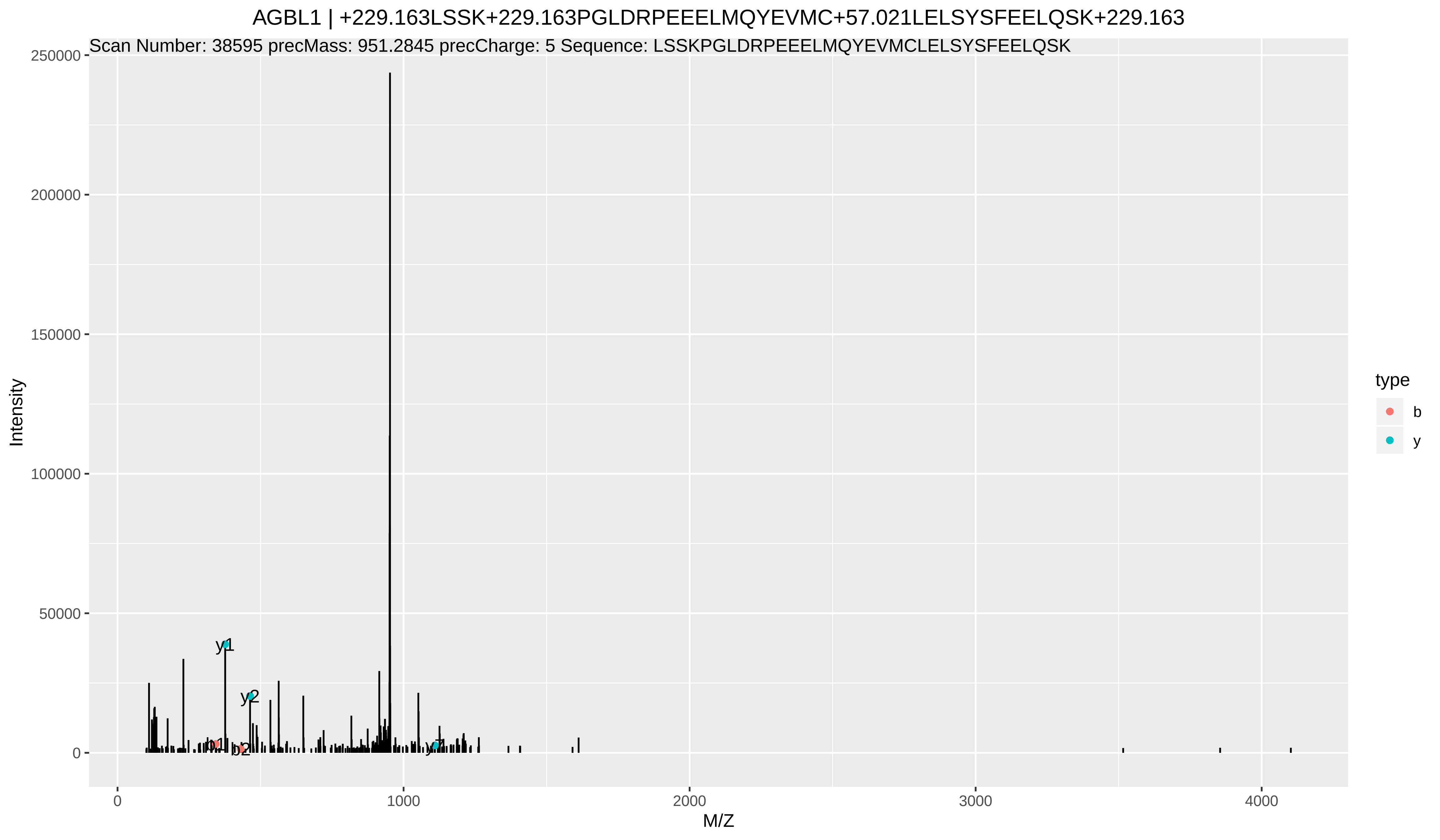
AGBL1 | +229.163LSSK+229.163PGLDRPEEELMQYEVMC+57.021LELSYSFEELQSK+229.163
Scan Number: 38595 precMass: 951.2845 precCharge: 5 Sequence: LSSKPGLDRPEEELMQYEVMCLELSYSFEELQSK
Intensity
250000
200000
150000
100000
50000
0
y1
y2
b1
b2
y7
0
1000
2000
3000
4000
M/Z
type
b
y

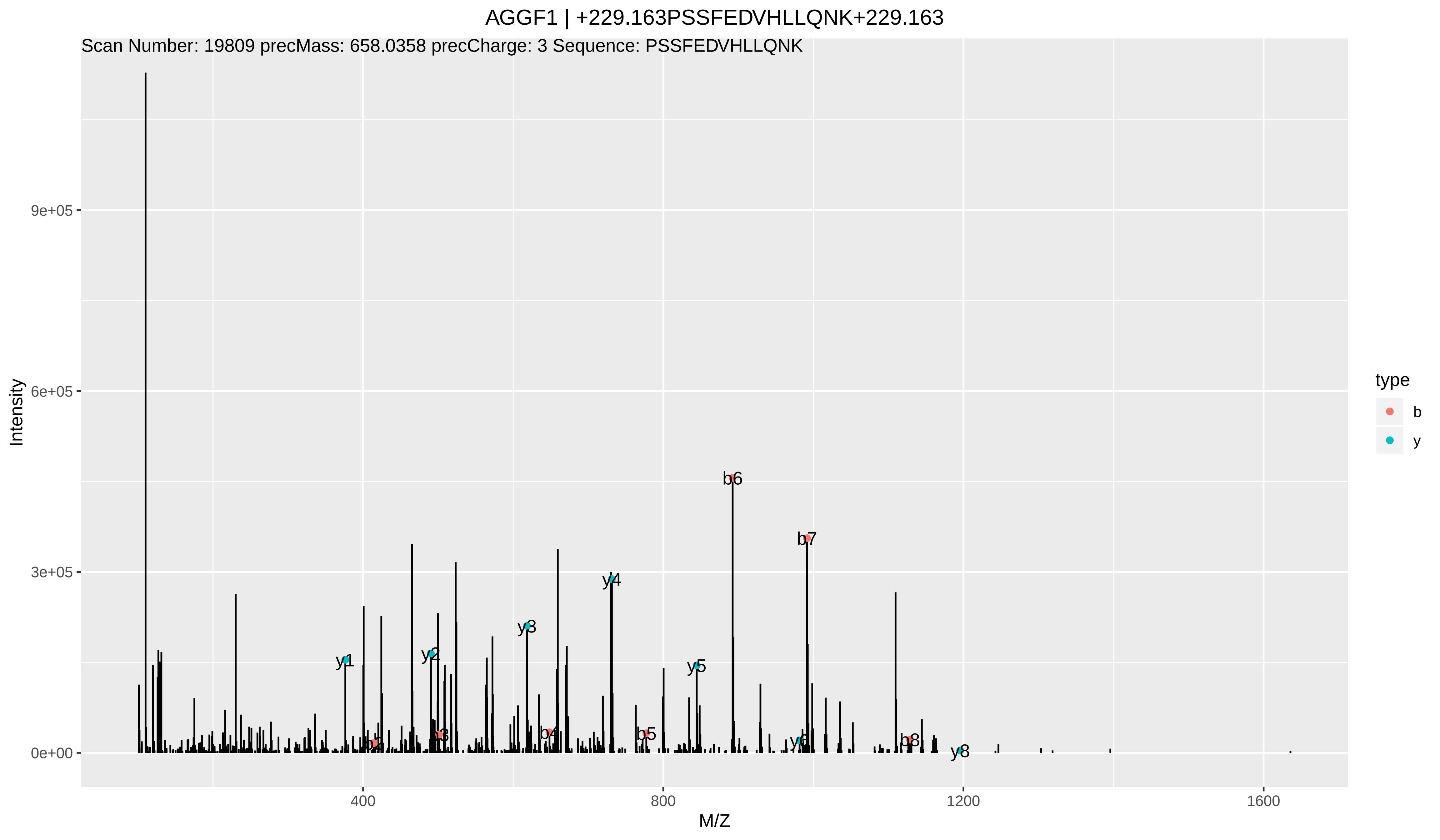

AGGF1 | +229.163PSSFEDVHLLQNK+229.163
Scan Number: 19809 precMass: 658.0358 precCharge: 3 Sequence: PSSFEDVHLLQNK
Intensity
9e+05
6e+05
3e+05
0e+00
400
800
1200
1600
M/Z
y1
y2
y3
y4
y5
y6
y8
b2
b3
b4
b5
b6
b7
b8
type
b
y

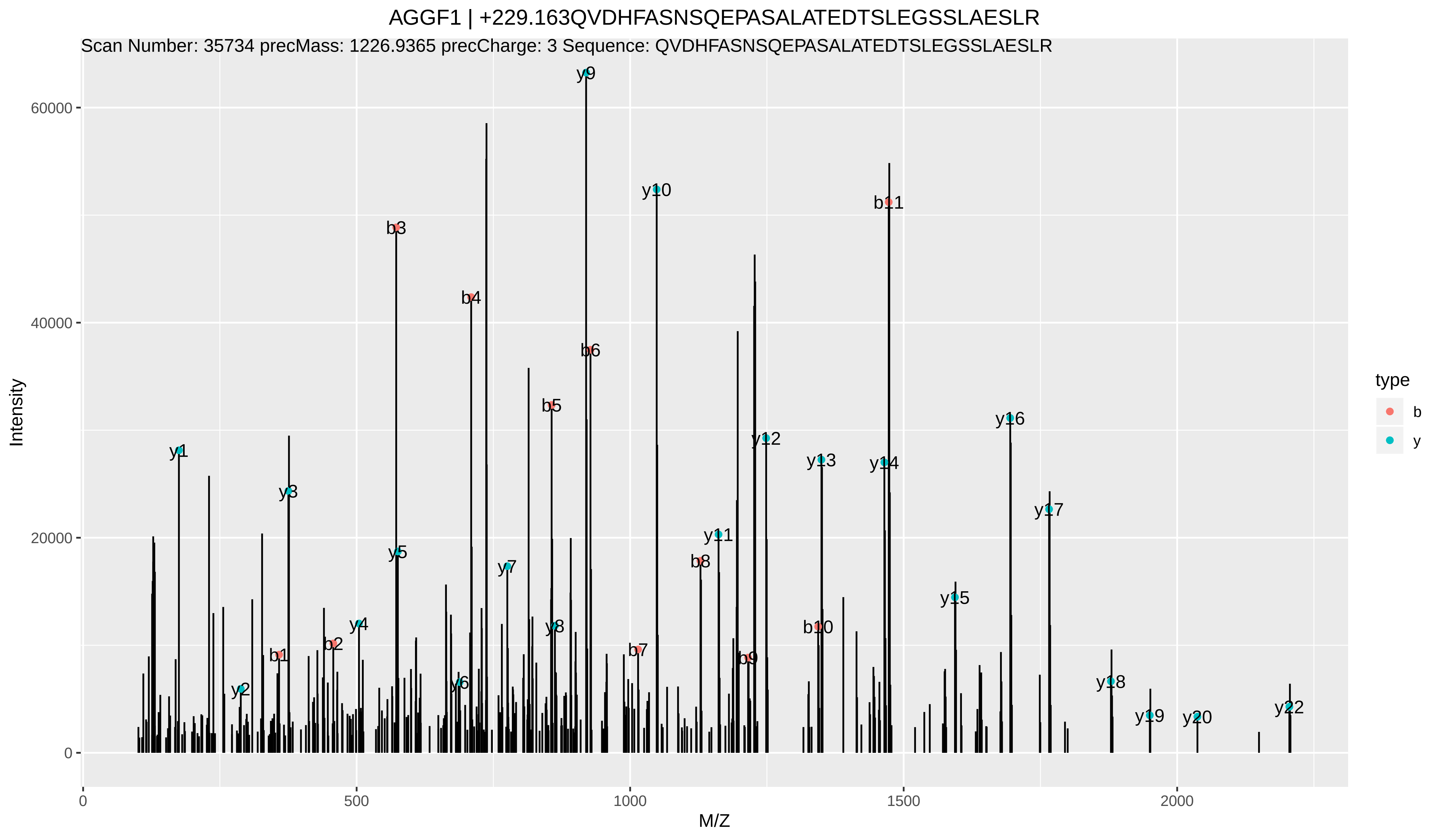

AGGF1 | +229.163QVDHFASNSQEPASALATEDTSLEGSSLAESLR
Scan Number: 35734 precMass: 1226.9365 precCharge: 3 Sequence: QVDHFASNSQEPASALATEDTSLEGSSLAESLR
Intensity
M/Z
type
b
y
y9
y10
b11
b3
b4
b6
b5
y16
y12
y13
y14
y1
y3
y17
y11
y5
b8
y7
y15
y4
b10
y8
b2
b1
b7
b9
y6
y18
y2
y22
y19
y20

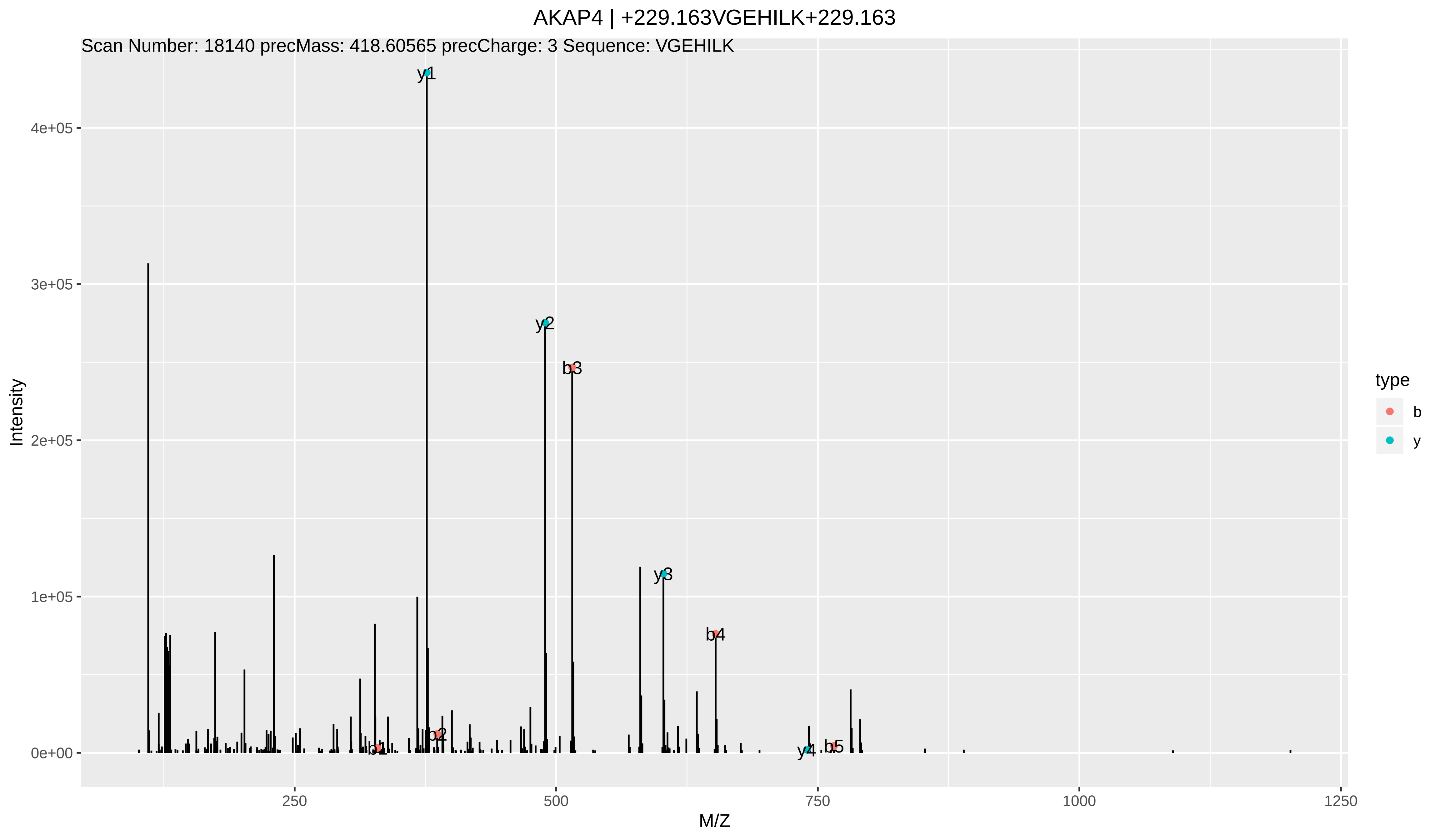

AKAP4 | +229.163VGEHILK+229.163
Scan Number: 18140 precMass: 418.60565 precCharge: 3 Sequence: VGEHILK
y1
y2
b3
y3
b4
y4
b5
b1
b2
Intensity
4e+05
3e+05
2e+05
1e+05
0e+00
250
500
750
1000
1250
M/Z
type
b
y

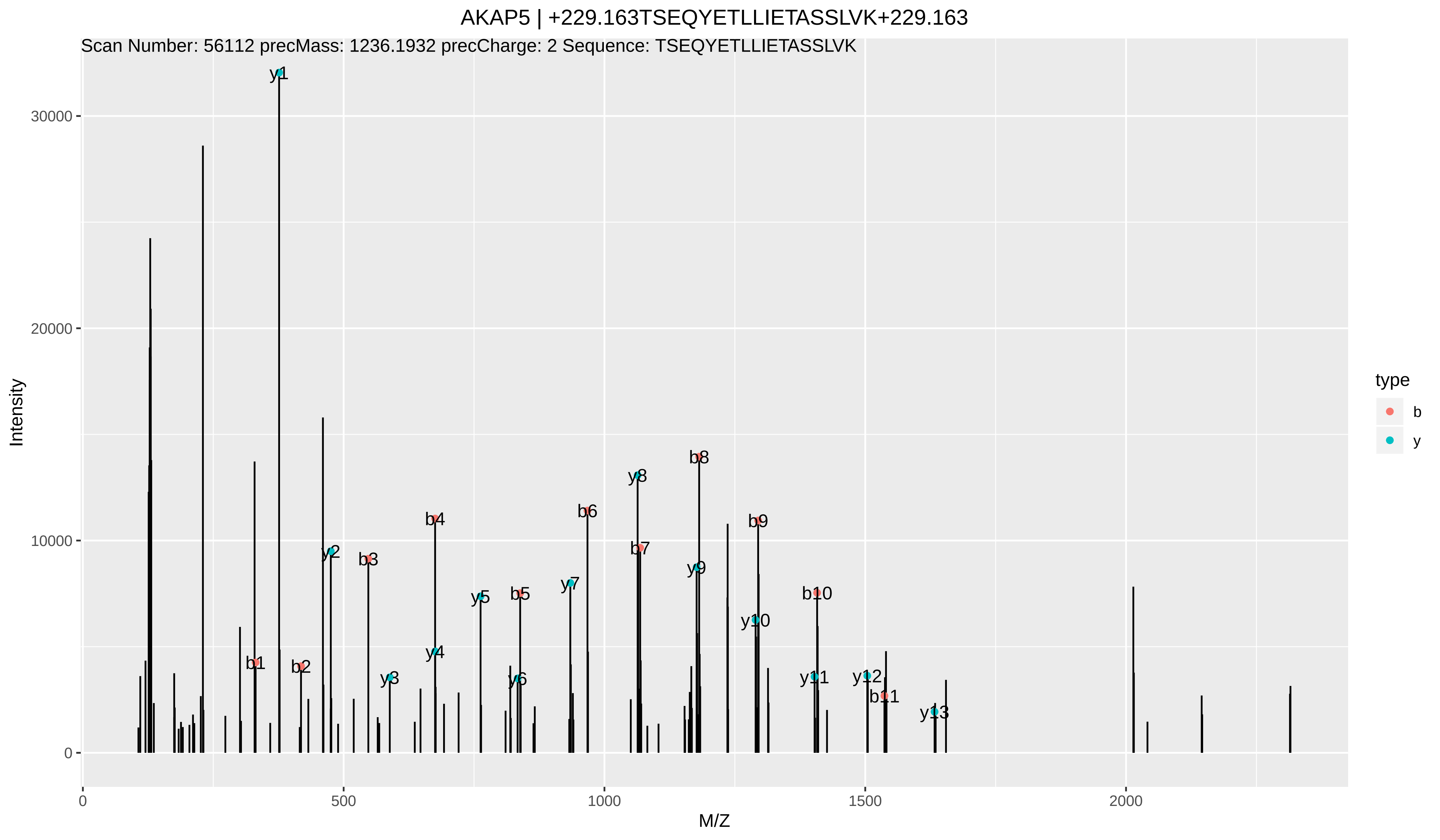
AKAP5 | +229.163TSEQYETLLIETASSLVK+229.163
Scan Number: 56112 precMass: 1236.1932 precCharge: 2 Sequence: TSEQYETLLIETASSLVK
Intensity
M/Z
type
b
y
y1
y2
y3
y4
y5
y6
y7
y8
y9
y10
y11
y12
y13
b1
b2
b3
b4
b5
b6
b7
b8
b9
b10
b11
0
10000
20000
30000
500
1000
1500
2000

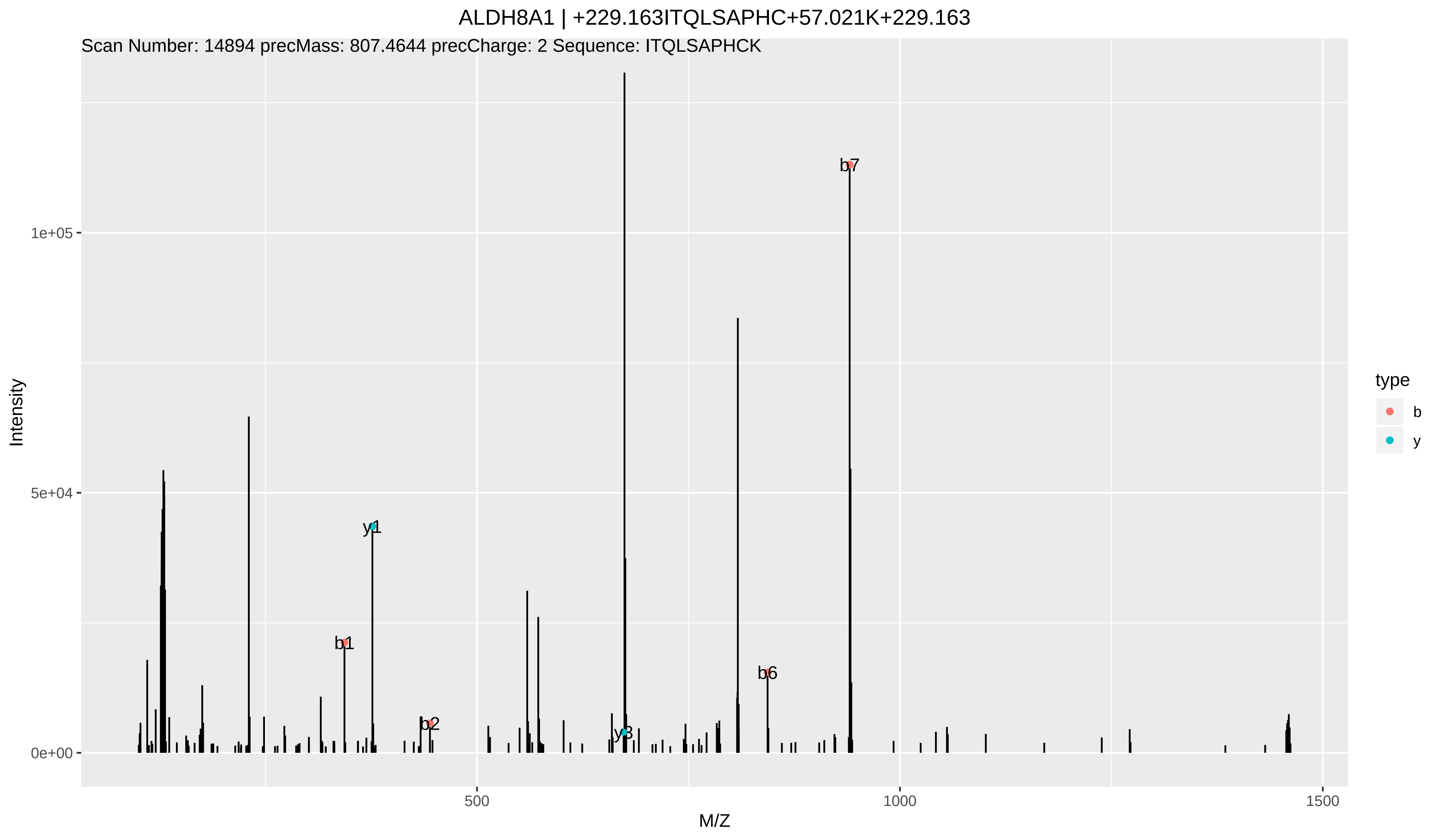

ALDH8A1 | +229.163ITQLSAPHC+57.021K+229.163
Scan Number: 14894 precMass: 807.4644 precCharge: 2 Sequence: ITQLSAPHCK
Intensity
M/Z
1e+05
5e+04
0e+00
500
1000
1500
b7
y1
b1
b6
b2
y3
type
b
y

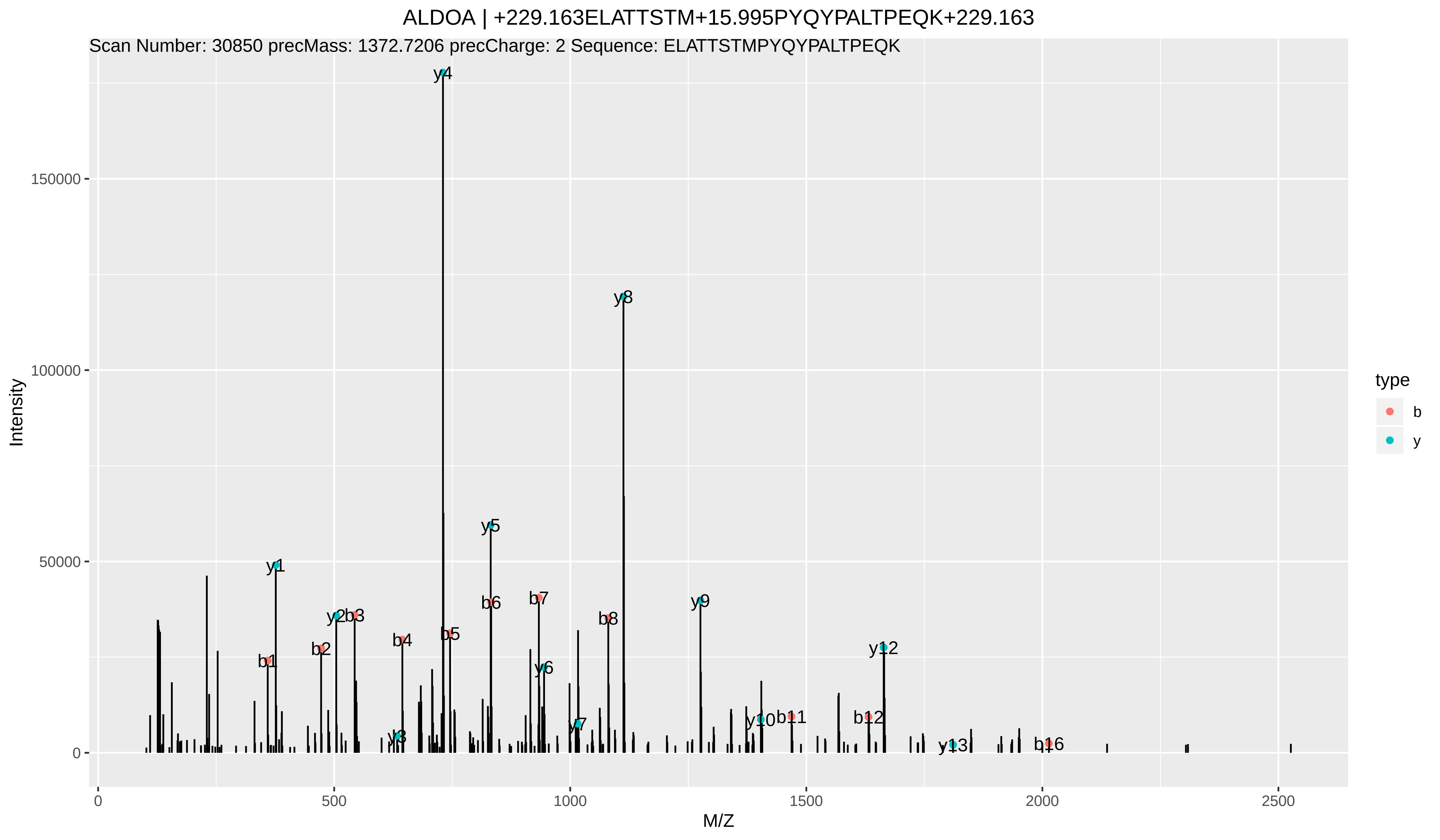

ALDOA | +229.163ELATTSTM+15.995PYQYPALTPEQK+229.163
Scan Number: 30850 precMass: 1372.7206 precCharge: 2 Sequence: ELATTSTMPYQYPALTPEQK
y4
y8
y5
y1
y2
b3
b6
b7
y9
b8
b5
b4
b2
b1
y6
y12
y10
b11
b12
y7
y3
y13
b16
Intensity
150000
100000
50000
0
0
500
1000
1500
2000
2500
M/Z
type
b
y

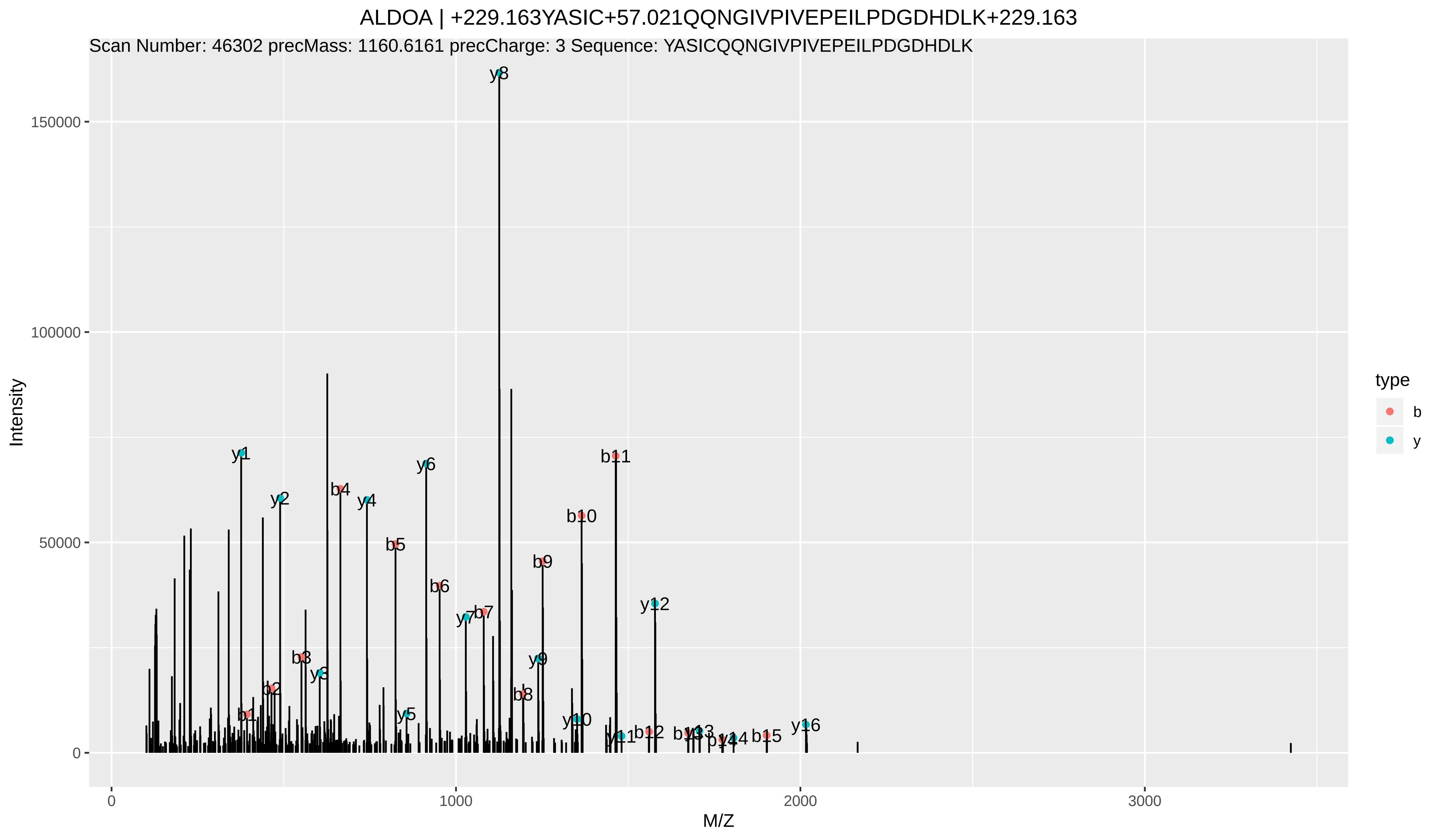

ALDOA | +229.163YASIC+57.021QQNGIVPIVEPEILPDGDHDLK+229.163
Scan Number: 46302 precMass: 1160.6161 precCharge: 3 Sequence: YASICQQNGIVPIVEPEILPDGDHDLK
Intensity
M/Z
type
b
y
y8
y1
y2
b4
y4
y6
b11
b10
b5
b9
b6
y12
y7
b7
b3
y3
y9
b2
b8
b1
y5
y10
y11
b12
b13
y13
b14
y14
b15
y16
150000
100000
50000
0
1000
2000
3000

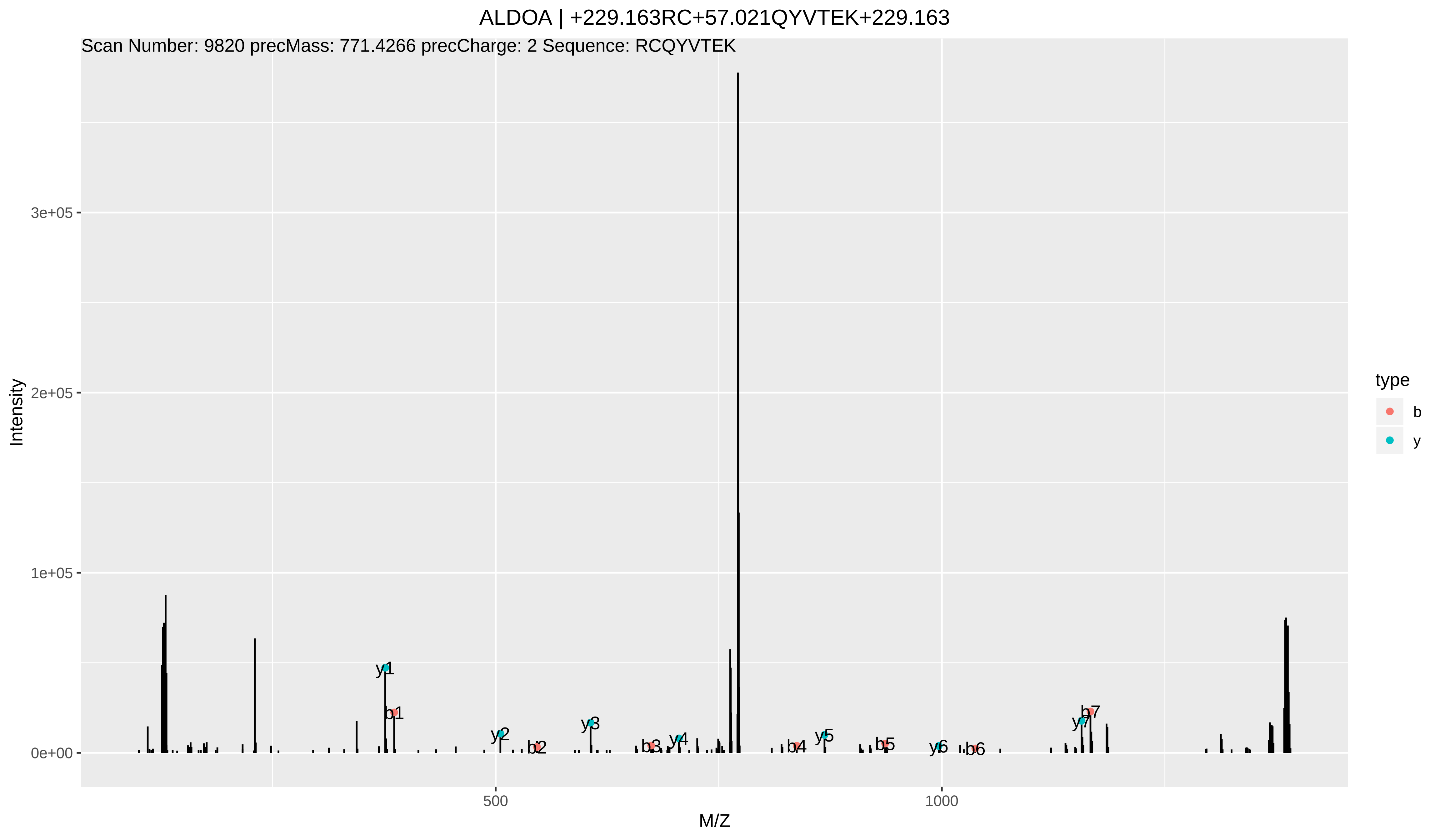

ALDOA | +229.163RC+57.021QYVTEK+229.163
Scan Number: 9820 precMass: 771.4266 precCharge: 2 Sequence: RCQYVTEK
Intensity
3e+05
2e+05
1e+05
0e+00
M/Z
500
1000
y1
b1
y2
b2
y3
b3
y4
b4
y5
b5
y6
b6
y7
b7
type
b
y

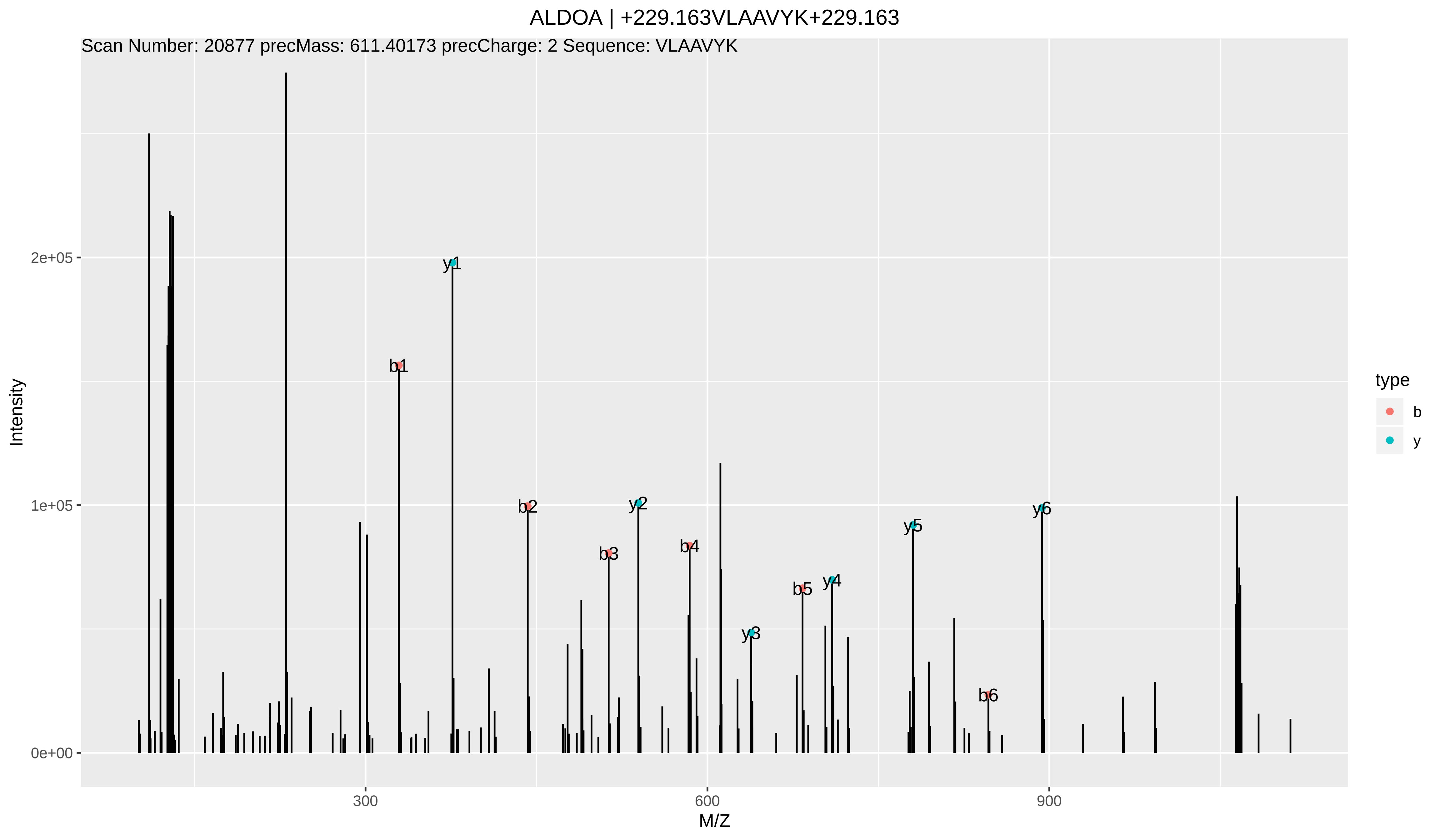

ALDOA | +229.163VLAAVYK+229.163
Scan Number: 20877 precMass: 611.40173 precCharge: 2 Sequence: VLAAVYK
Intensity
M/Z
0e+00
1e+05
2e+05
300
600
900
y1
b1
b2
y2
b3
b4
y3
b5
y4
y5
y6
b6
type
b
y

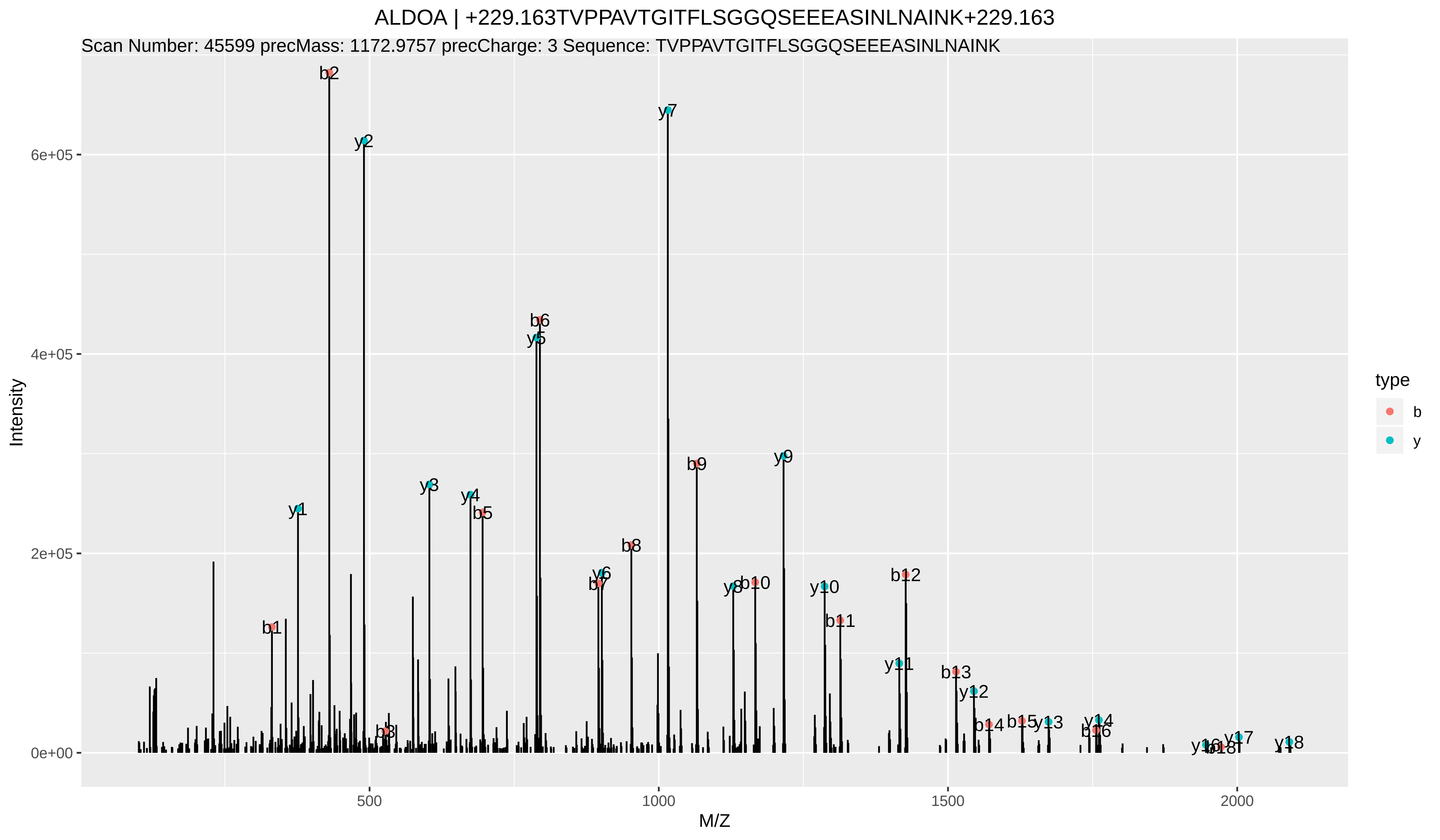
ALDOA | +229.163TVPPAVTGITFLSGGQSEEEASINLNAINK+229.163
Scan Number: 45599 precMass: 1172.9757 precCharge: 3 Sequence: TVPPAVTGITFLSGGQSEEEASINLNAINK
Intensity
M/Z
type
b
y
0e+00
2e+05
4e+05
6e+05
500
1000
1500
2000
b1
b2
b3
b5
b6
b7
b8
b9
b10
b11
b12
b13
b14
b15
b16
b18
y1
y2
y3
y4
y5
y6
y7
y8
y9
y10
y11
y12
y13
y14
y16
y17
y18

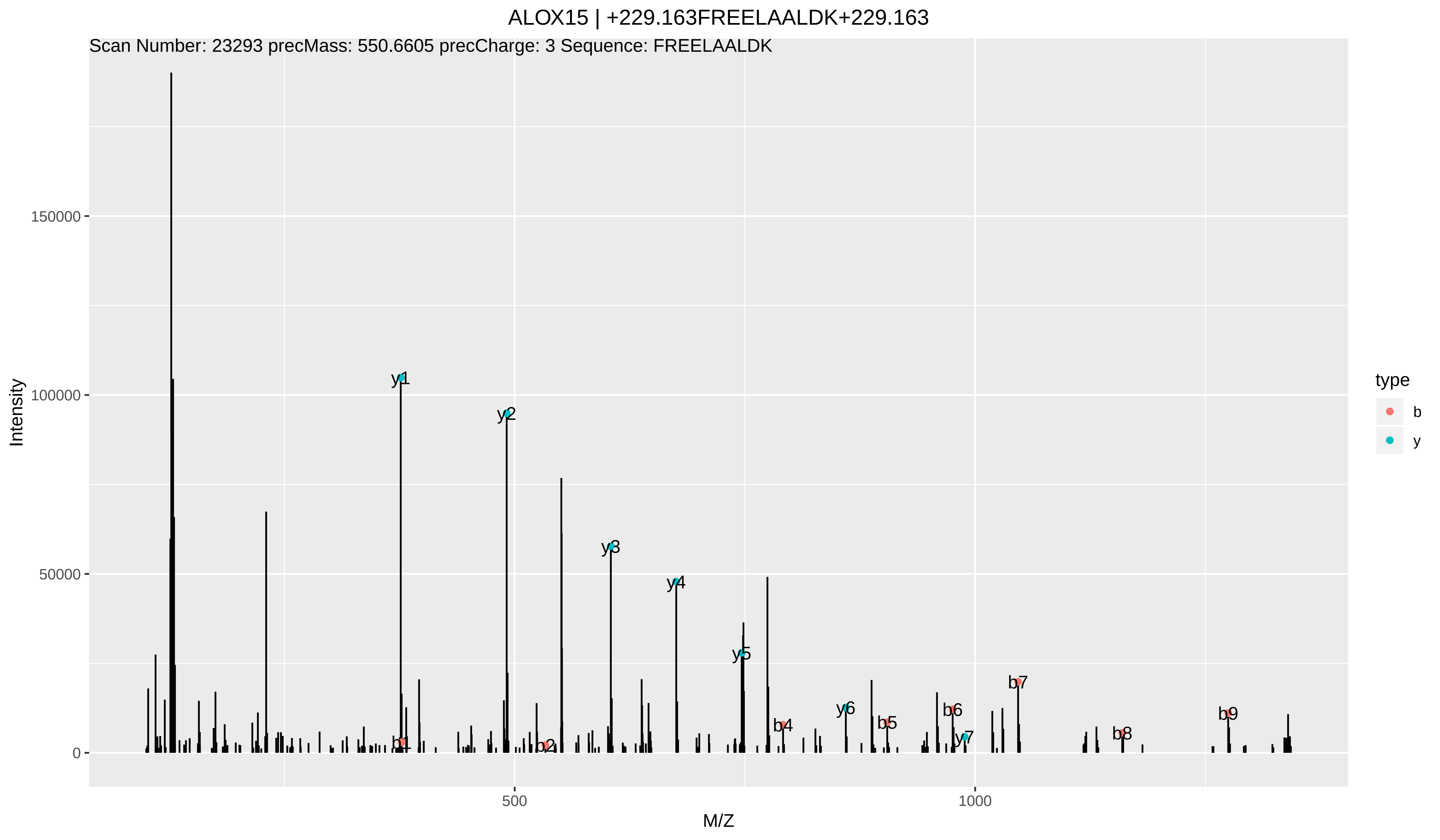

ALOX15 | +229.163FREELAALDK+229.163
Scan Number: 23293 precMass: 550.6605 precCharge: 3 Sequence: FREELAALDK
Intensity
150000
100000
50000
0
M/Z
500
1000
y1
y2
y3
y4
y5
y6
y7
b1
b2
b4
b5
b6
b7
b8
b9
type
b
y

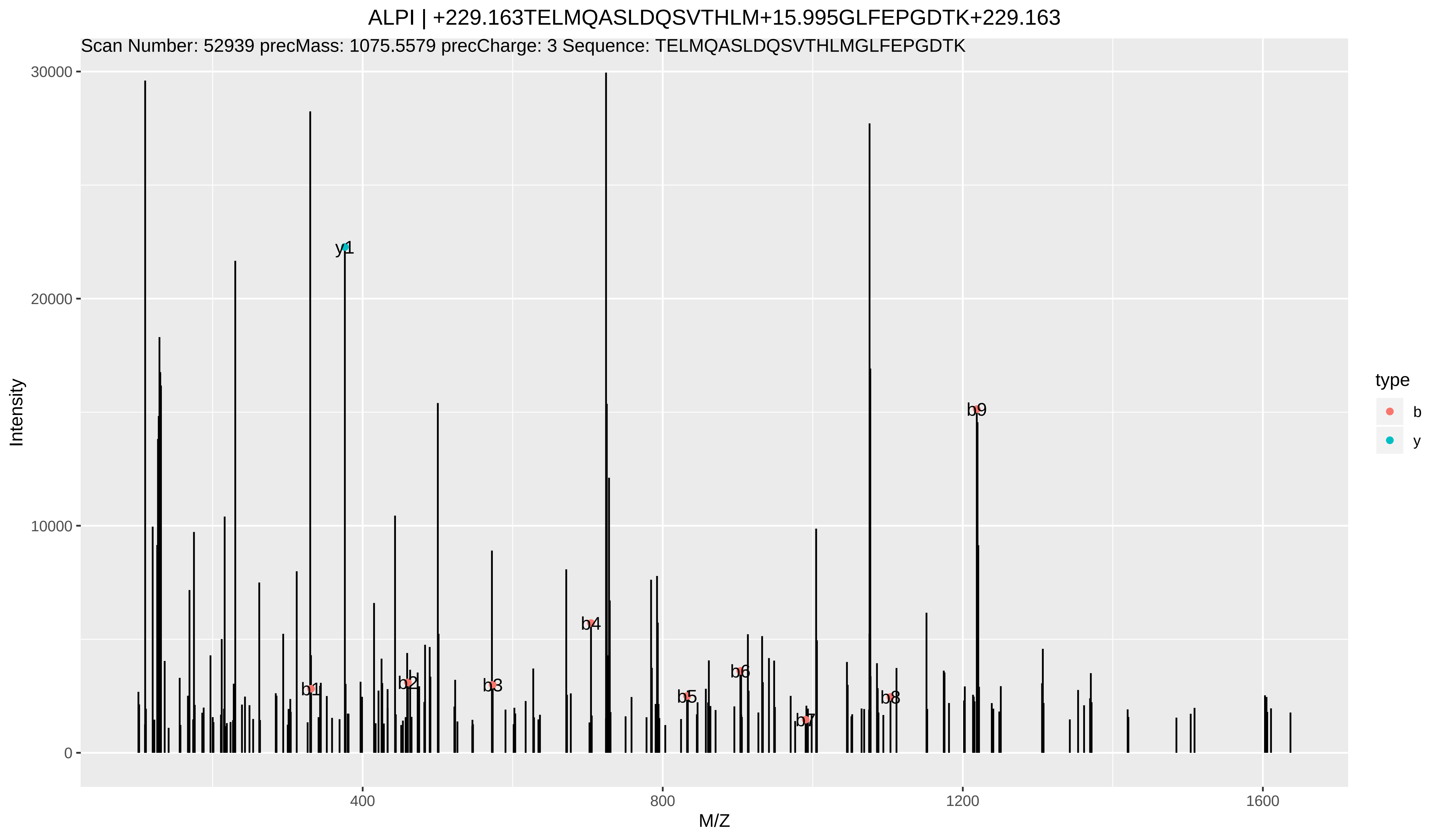

ALPI | +229.163TELMQASLDQSVTHLM+15.995GLFEPGDTK+229.163
Scan Number: 52939 precMass: 1075.5579 precCharge: 3 Sequence: TELMQASLDQSVTHLMGLFEPGDTK
Intensity
M/Z
30000
20000
10000
0
400
800
1200
1600
y1
b1
b2
b3
b4
b5
b6
b7
b8
b9
type
b
y

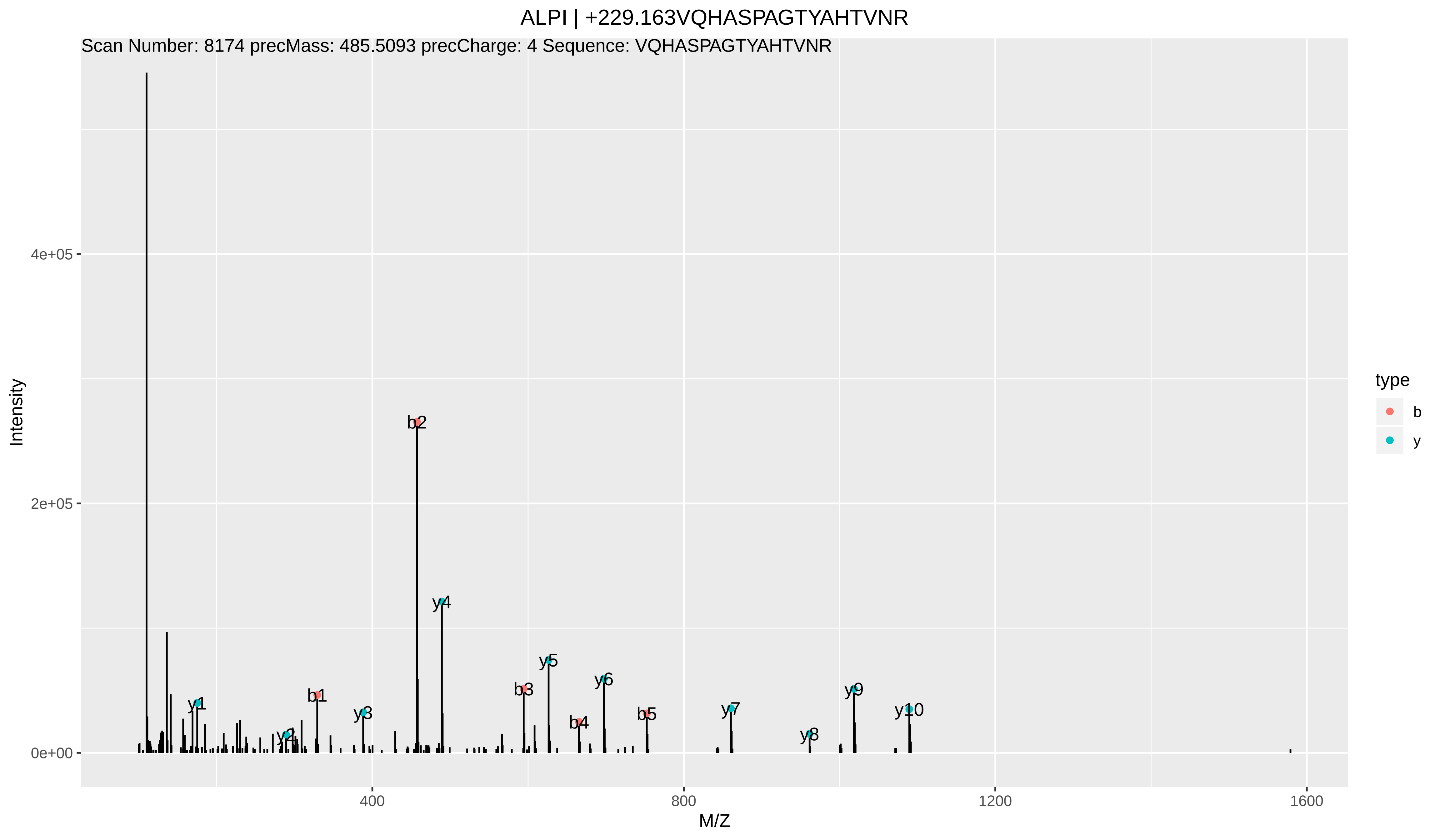
ALPI | +229.163VQHASPAGTYAHTVNR
Scan Number: 8174 precMass: 485.5093 precCharge: 4 Sequence: VQHASPAGTYAHTVNR
Intensity
4e+05
2e+05
0e+00
M/Z
400
800
1200
1600
b2
y4
y5
y6
b3
y9
b1
y1
y3
b5
y7
y10
b4
y2
y8
type
b
y

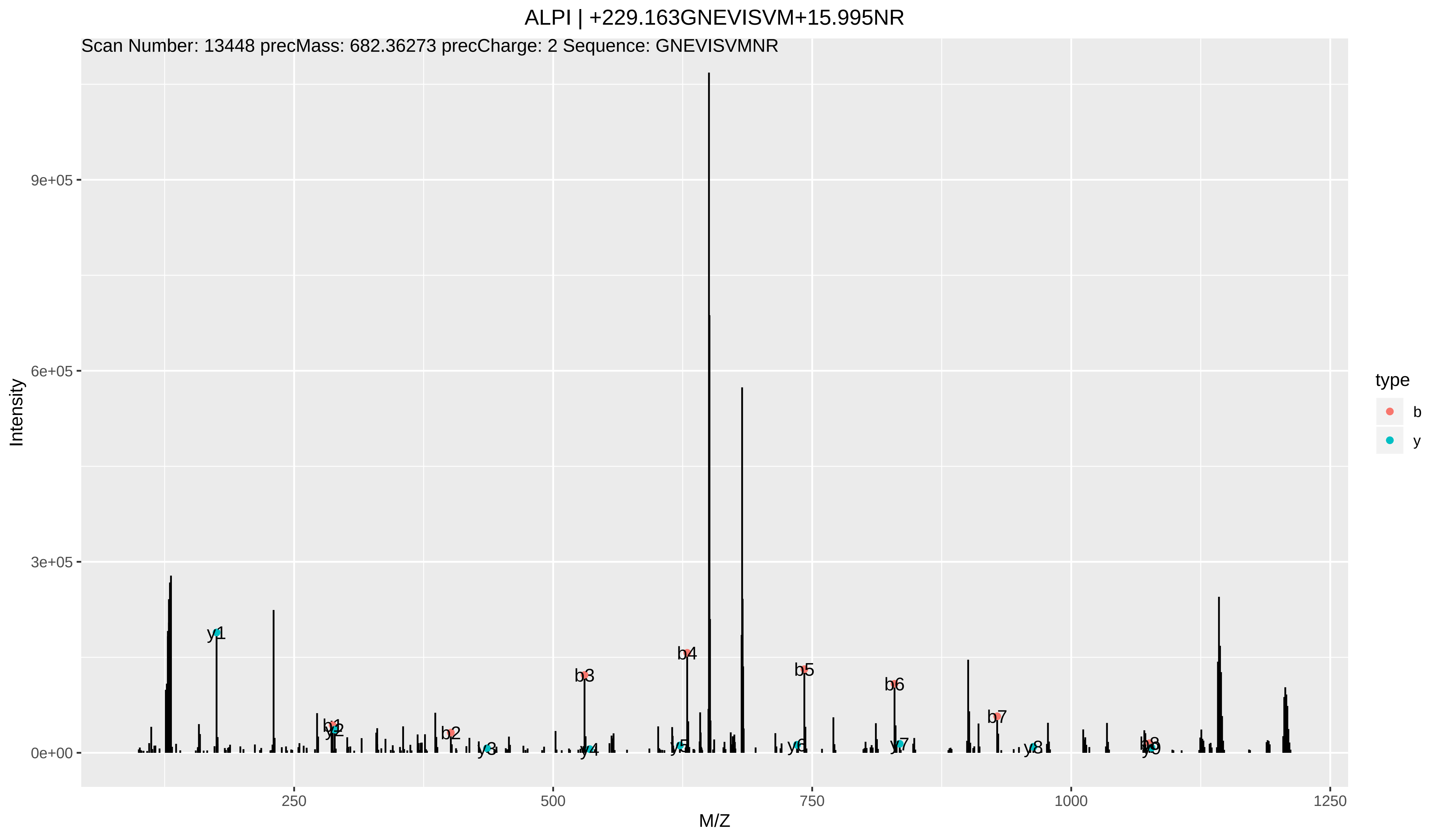

ALPI | +229.163GNEVISVM+15.995NR
Scan Number: 13448 precMass: 682.36273 precCharge: 2 Sequence: GNEVISVMNR
Intensity
9e+05
6e+05
3e+05
0e+00
M/Z
250
500
750
1000
1250
y1
b1
y2
b2
y3
b3
y4
b4
y5
b5
y6
b6
y7
b7
y8
b8
y9
type
b
y

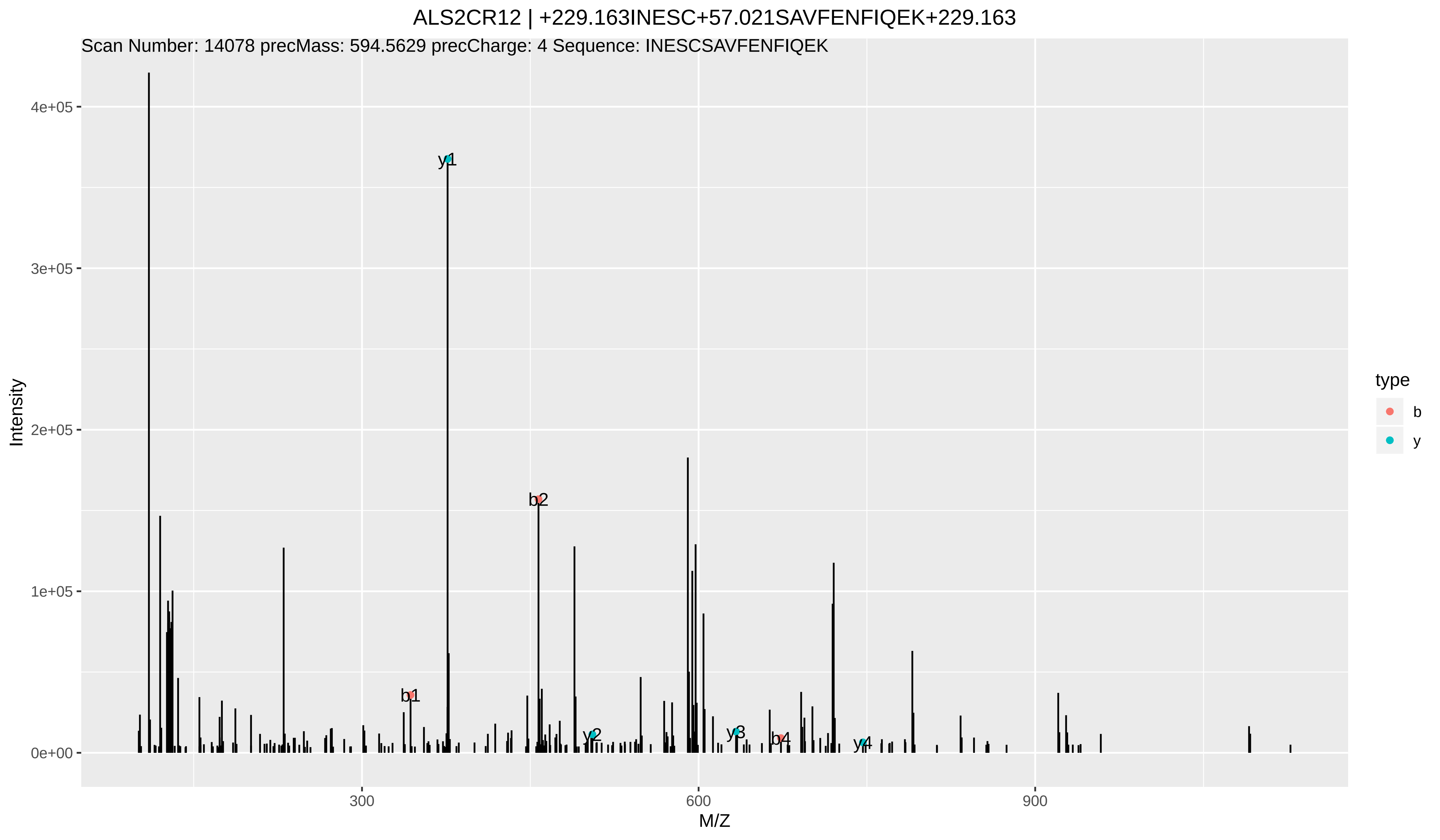

ALS2CR12 | +229.163INESC+57.021SAVFENFIQEK+229.163
Scan Number: 14078 precMass: 594.5629 precCharge: 4 Sequence: INESCSAVFENFIQEK
Intensity
M/Z
4e+05
3e+05
2e+05
1e+05
0e+00
300
600
900
y1
b2
b1
y2
y3
b4
y4
type
b
y

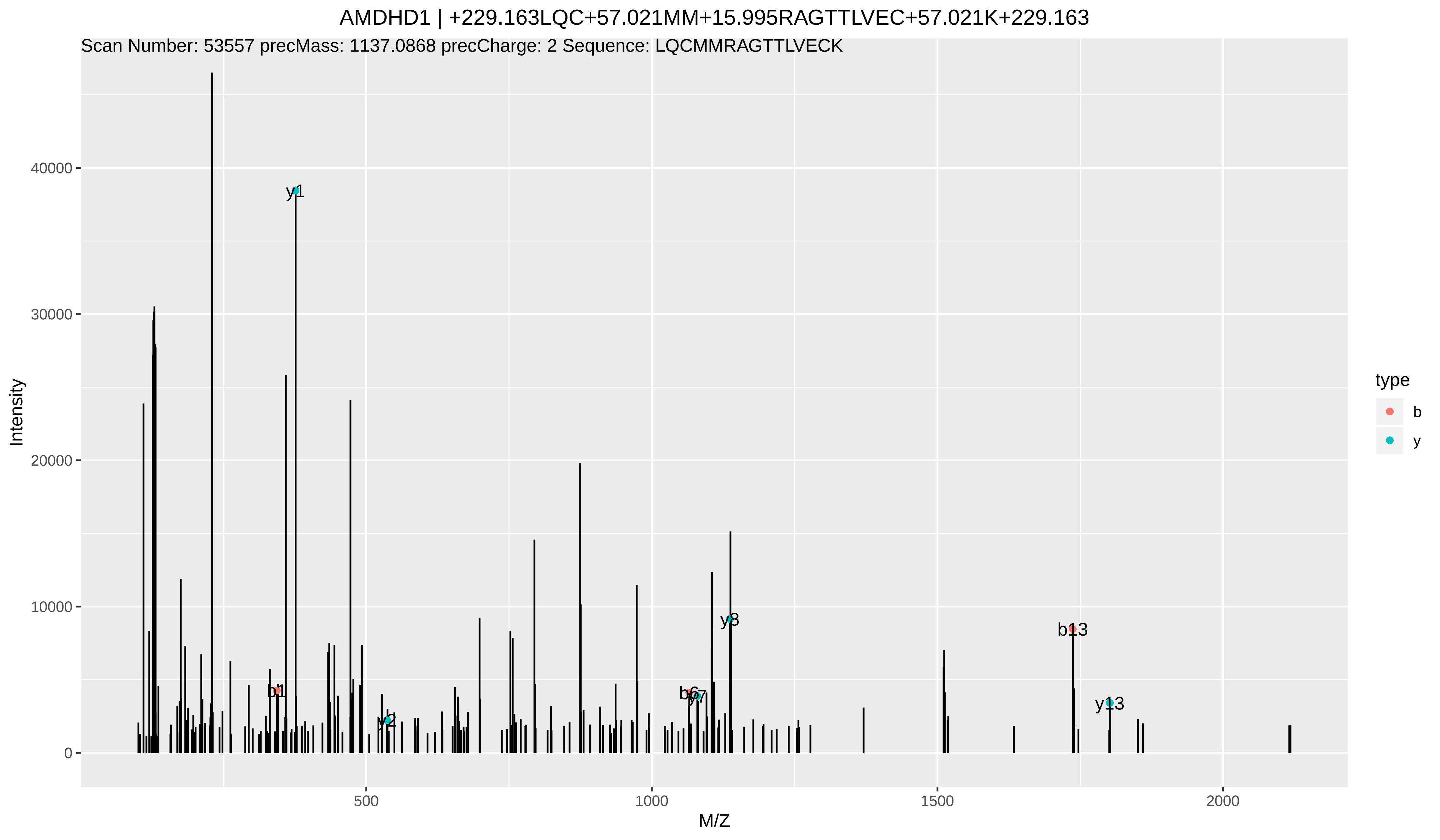

AMDHD1 | +229.163LQC+57.021MM+15.995RAGTTLVEC+57.021K+229.163
Scan Number: 53557 precMass: 1137.0868 precCharge: 2 Sequence: LQCMMRAGTTLVECK
Intensity
M/Z
type
b
y
y1
b1
y2
b6
y7
y8
b13
y13
0
10000
20000
30000
40000
500
1000
1500
2000

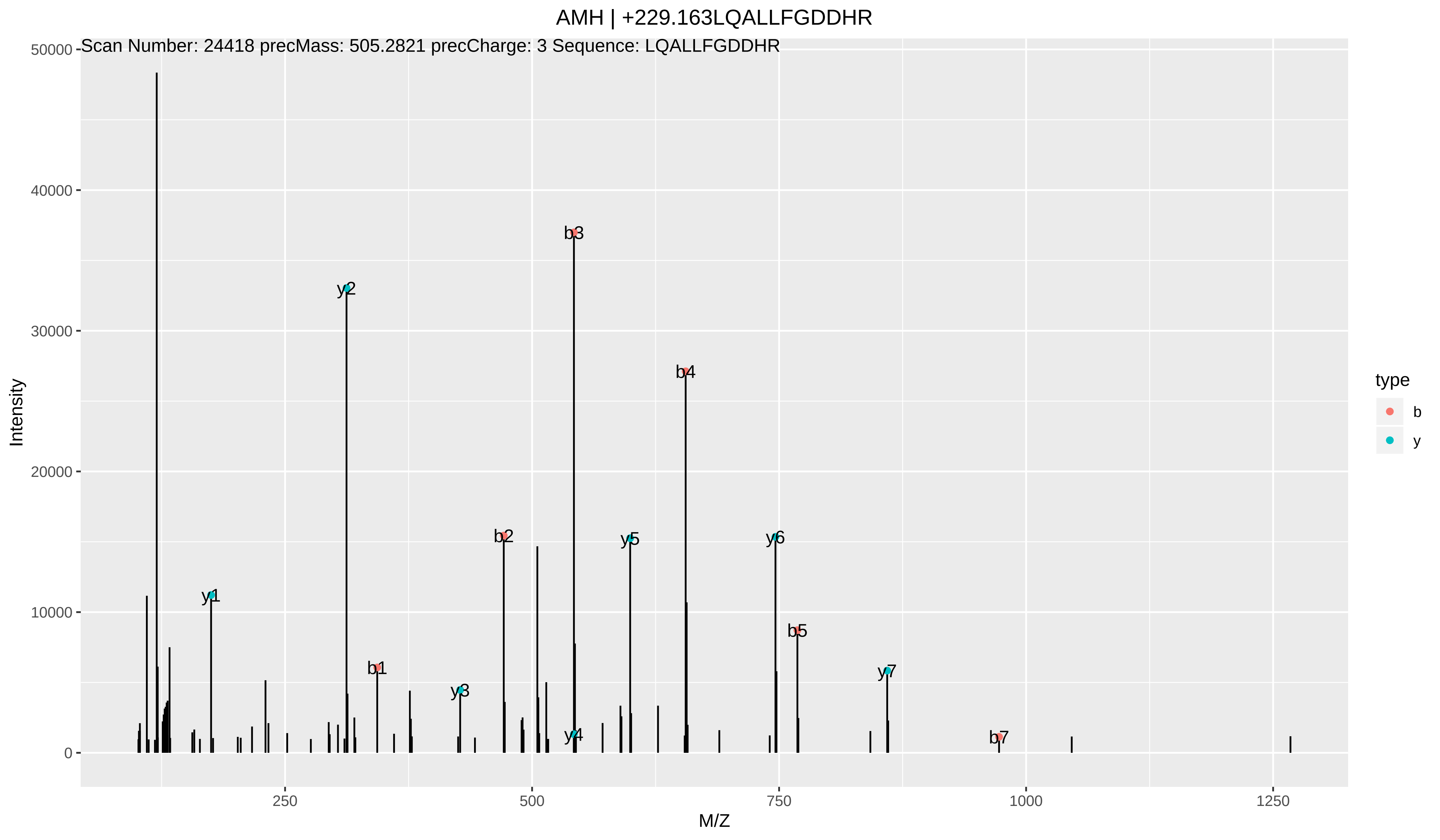

AMH | +229.163LQALLFGDDHR
Scan Number: 24418 precMass: 505.2821 precCharge: 3 Sequence: LQALLFGDDHR
Intensity
M/Z
0
10000
20000
30000
40000
50000
250
500
750
1000
1250
y1
y2
y3
y4
y5
y6
y7
b1
b2
b3
b4
b5
b7
type
b
y

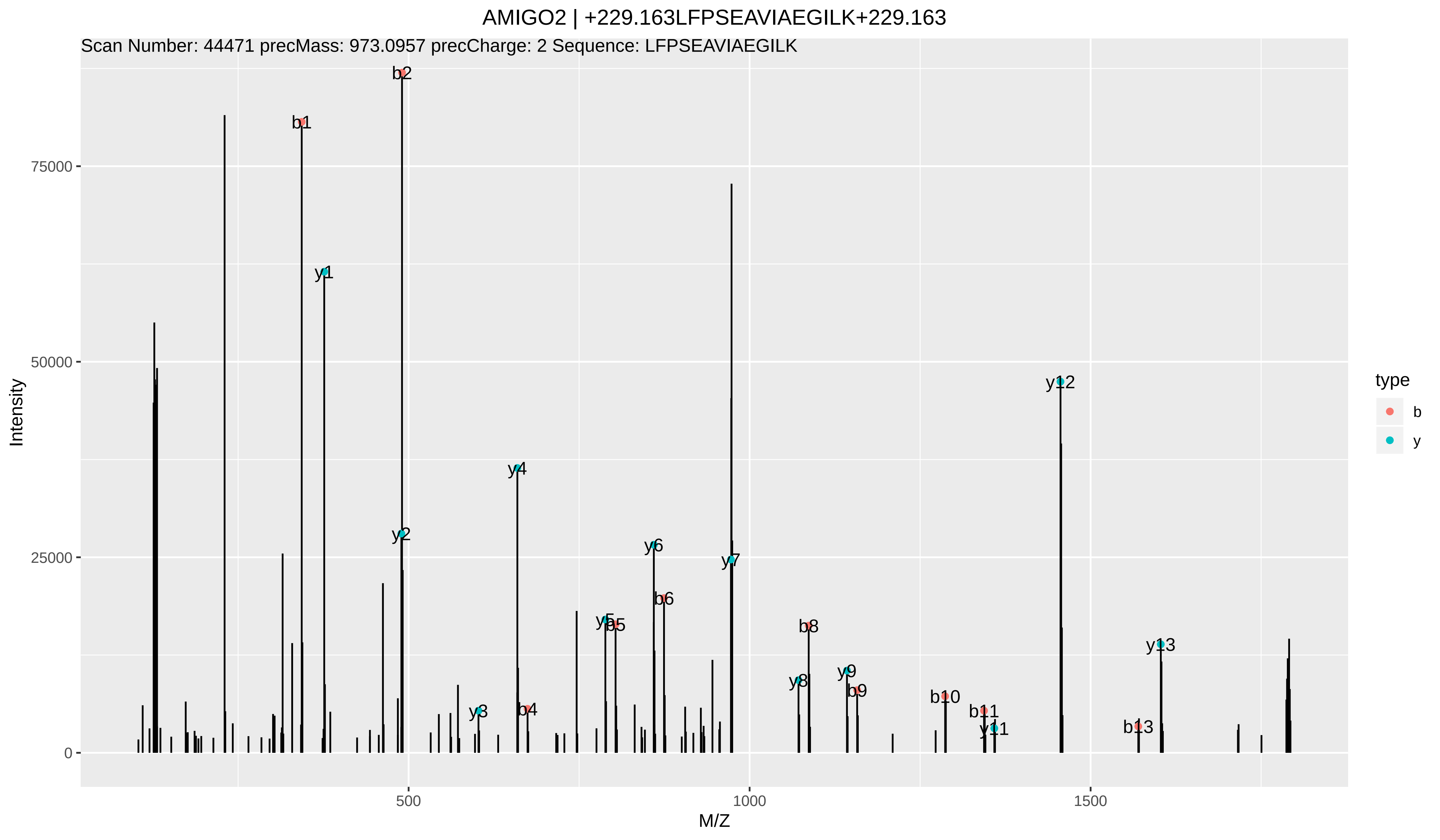

AMIGO2 | +229.163LFPSEAVIAEGILK+229.163
Scan Number: 44471 precMass: 973.0957 precCharge: 2 Sequence: LFPSEAVIAEGILK
Intensity
75000
50000
25000
0
M/Z
500
1000
1500
type
b
y
b1
b2
b4
b5
b6
b8
b9
b10
b11
b13
y1
y2
y3
y4
y5
y6
y7
y8
y9
y11
y12
y13

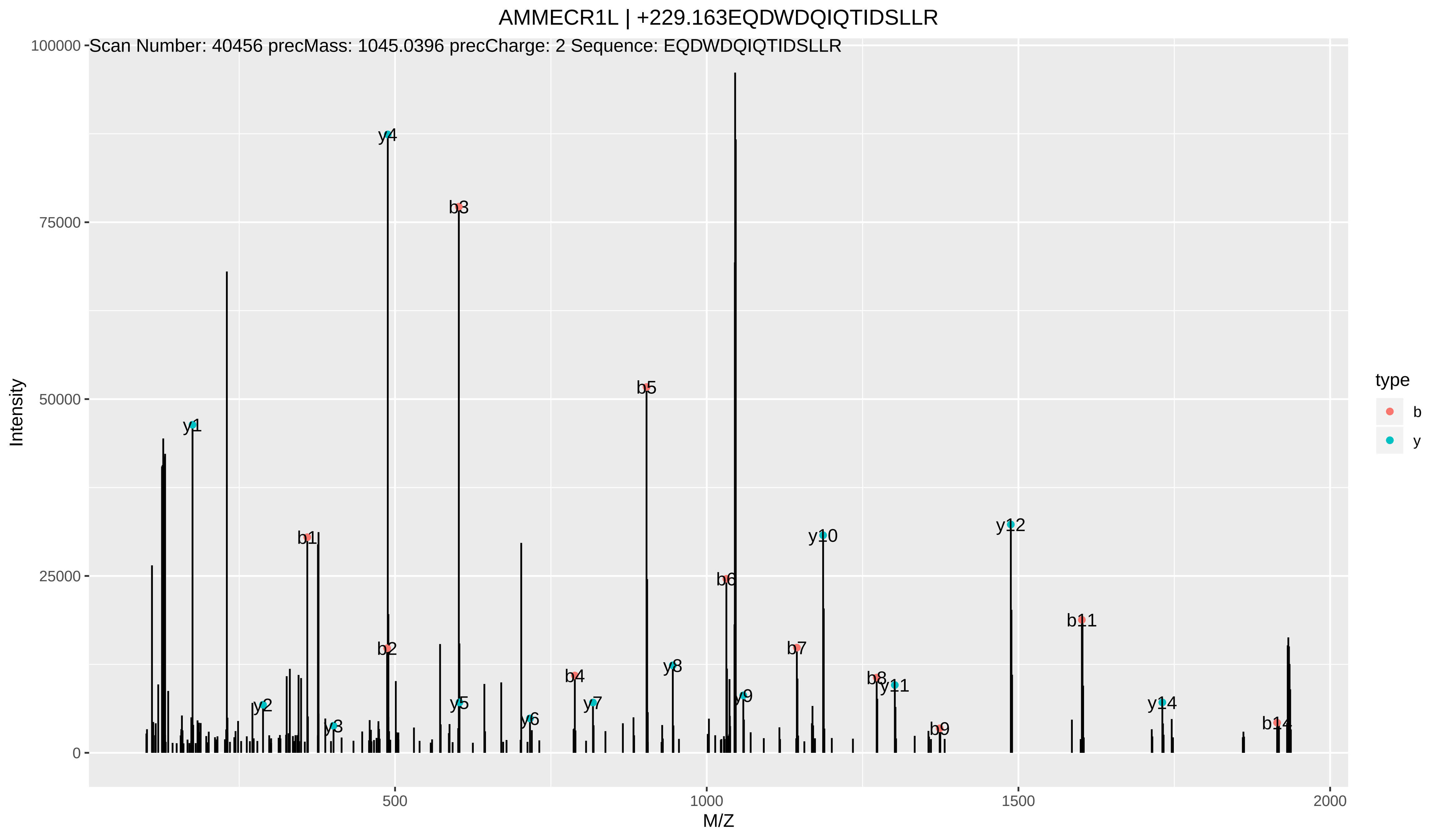

AMMECR1L | +229.163EQDWDQIQTIDSLLR
Scan Number: 40456 precMass: 1045.0396 precCharge: 2 Sequence: EQDWDQIQTIDSLLR
Intensity
M/Z
type
b
y
y1
y2
y3
y4
y5
y6
y7
y8
y9
y10
y11
y12
y14
b1
b2
b3
b4
b5
b6
b7
b8
b9
b11
b14

AMMECR1L | +229.163TATYLPEVAK+229.163

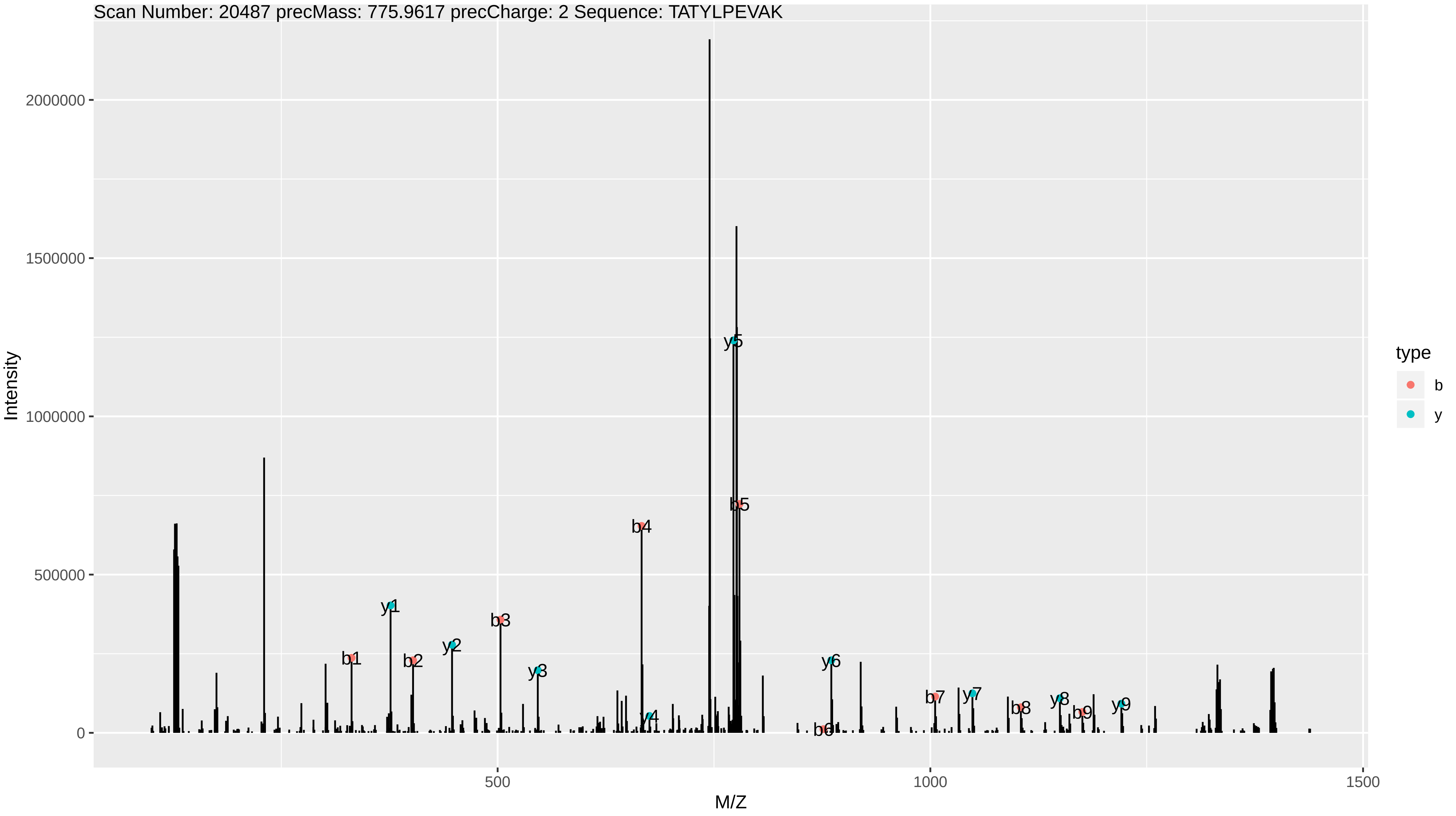

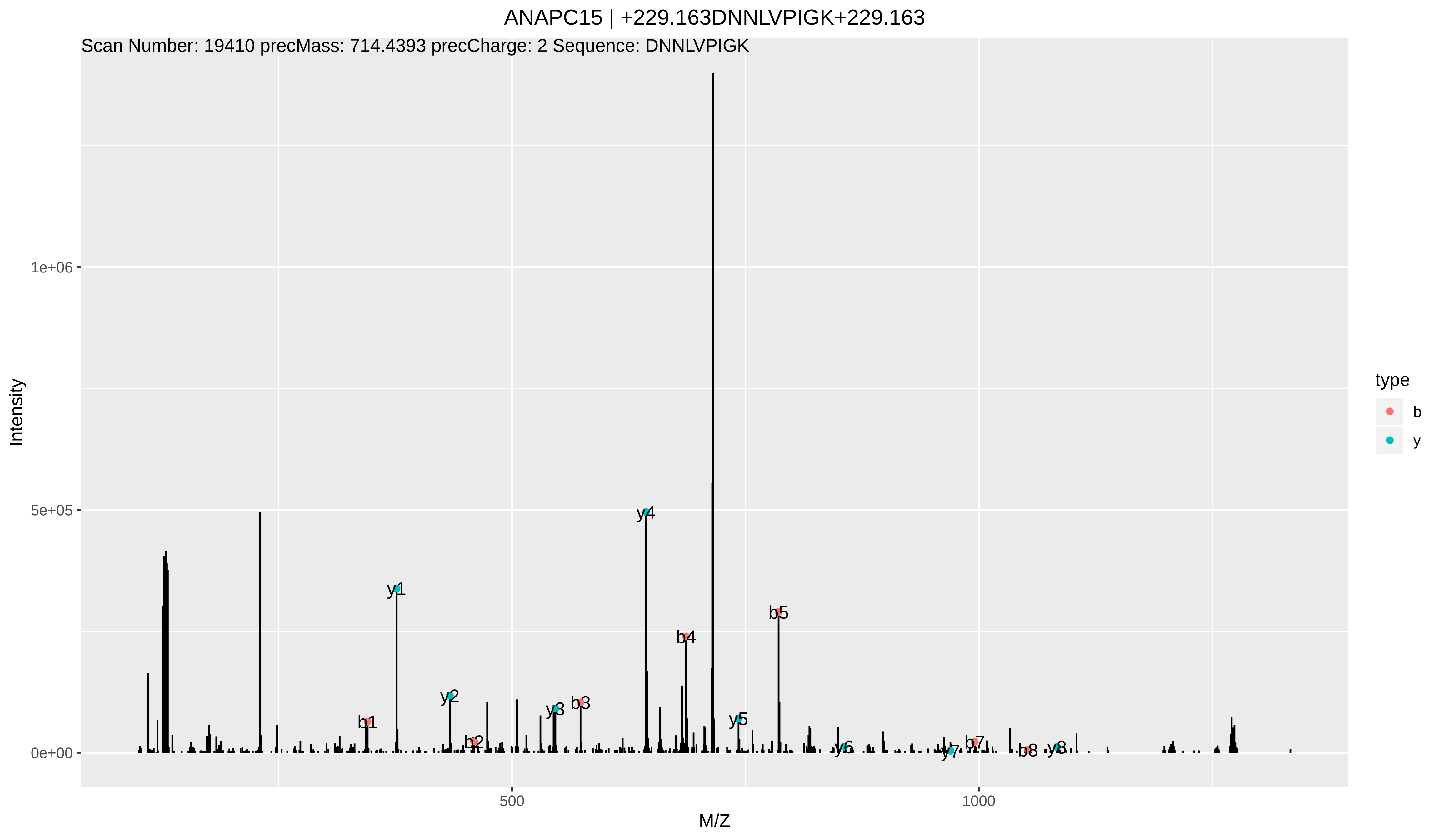
ANAPC15 | +229.163DNNLVPIGK+229.163
Scan Number: 19410 precMass: 714.4393 precCharge: 2 Sequence: DNNLVPIGK
Intensity
M/Z
1e+06
5e+05
0e+00
500
1000
y1
y2
y3
y4
y5
y6
y7
y8
b1
b2
b3
b4
b5
b7
b8
type
b
y

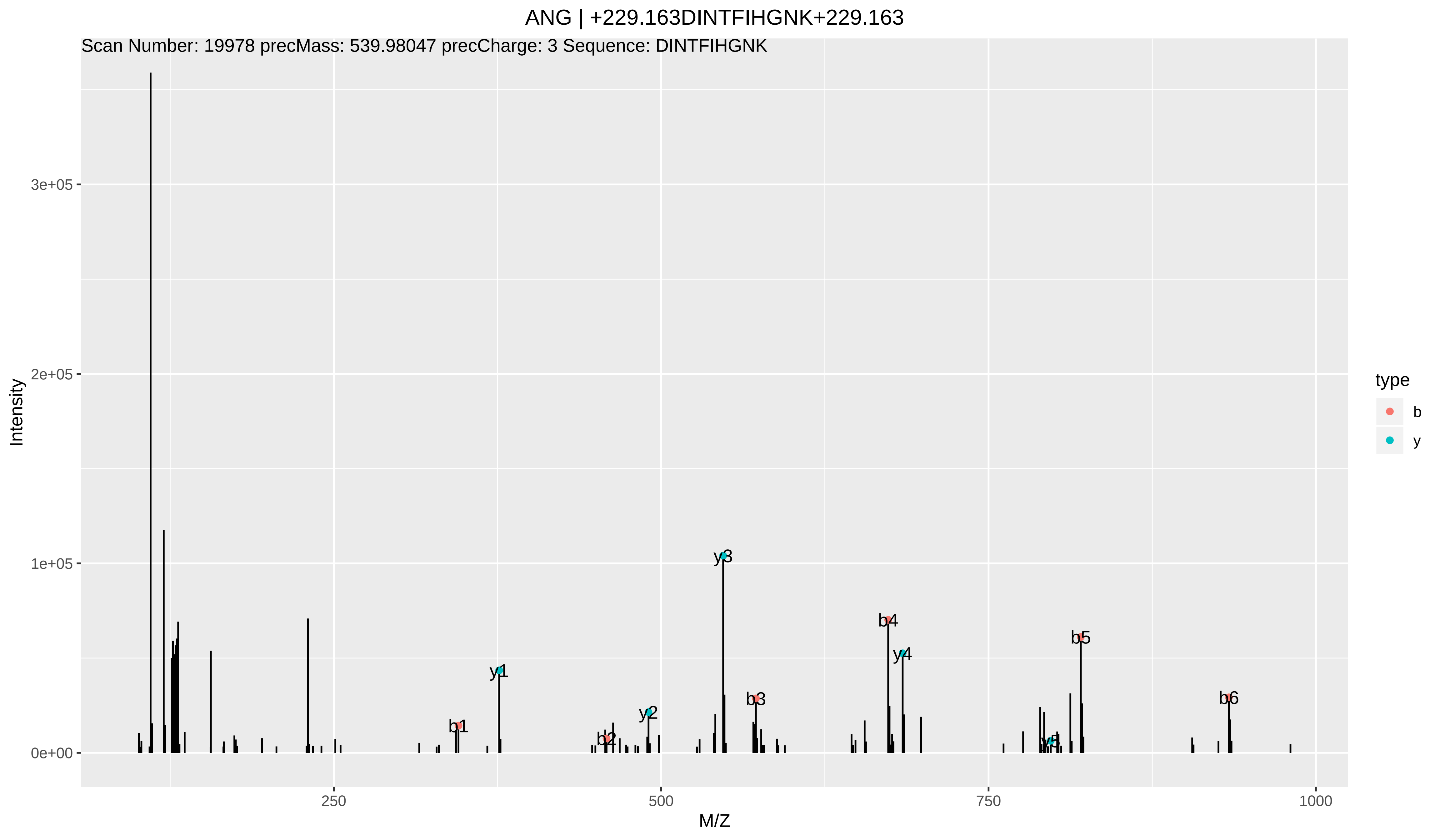
ANG | +229.163DINTFIHGNK+229.163
Scan Number: 19978 precMass: 539.98047 precCharge: 3 Sequence: DINTFIHGNK
Intensity
3e+05
2e+05
1e+05
0e+00
M/Z
250
500
750
1000
y1
y2
y3
y4
b1
b2
b3
b4
b5
b6
type
b
y

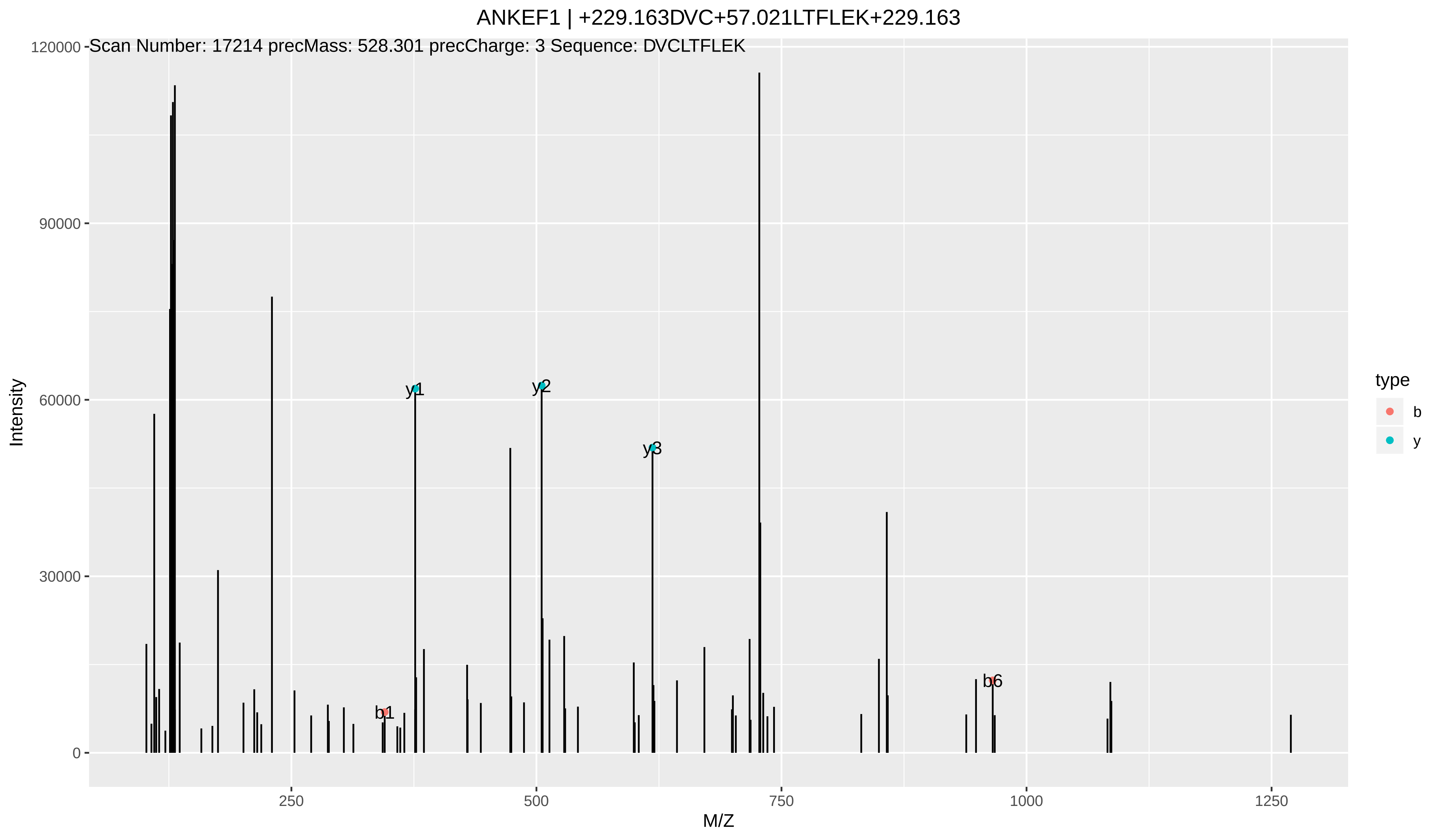
ANKEF1 | +229.163DVC+57.021LTFLEK+229.163
Scan Number: 17214 precMass: 528.301 precCharge: 3 Sequence: DVCLTFLEK
Intensity
M/Z
120000
90000
60000
30000
0
250
500
750
1000
1250
y1
y2
y3
b1
b6
type
b
y

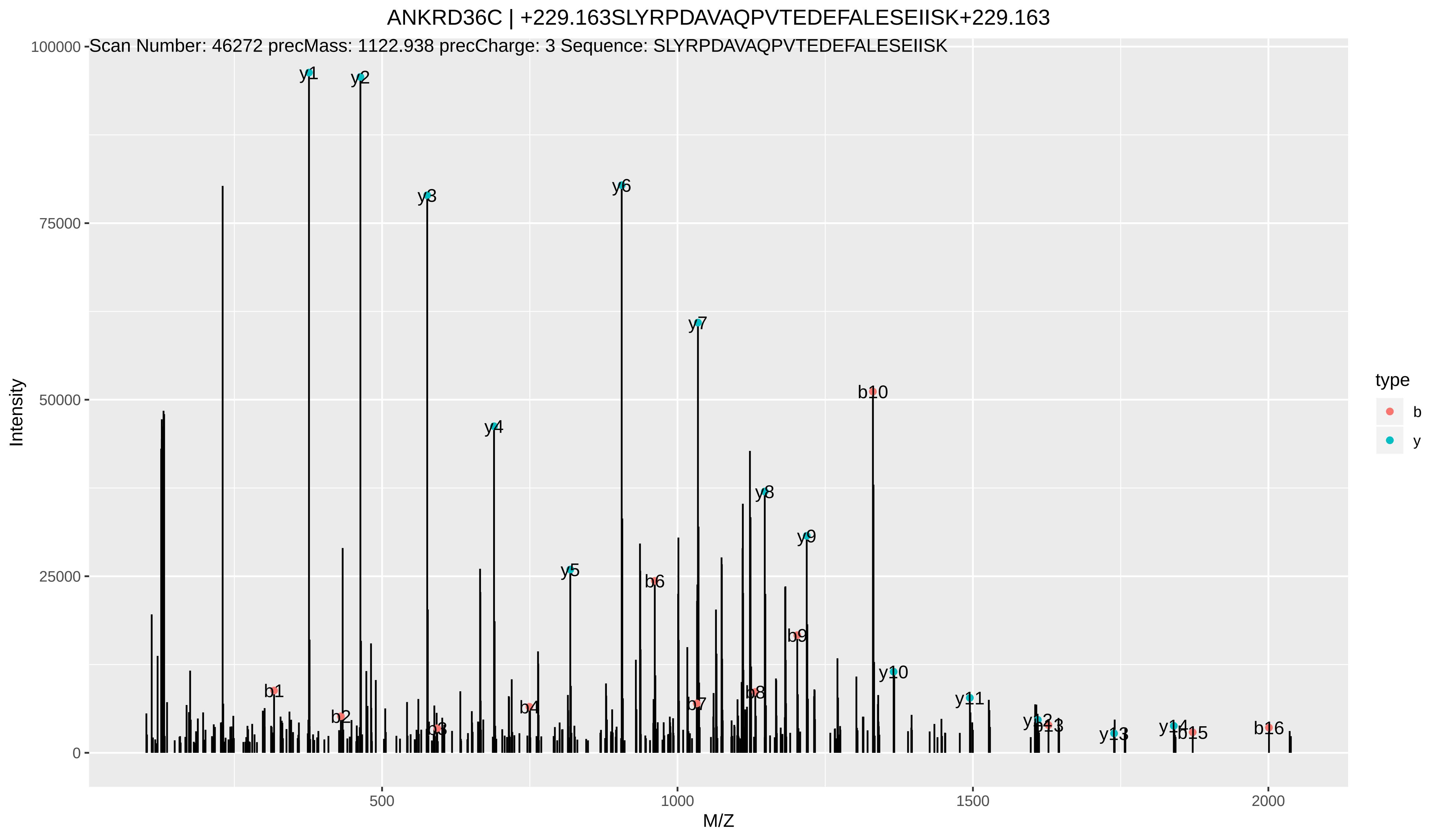

ANKRD36C | +229.163SLYRPDAVAQPVTEDEFALESEIISK+229.163
Scan Number: 46272 precMass: 1122.938 precCharge: 3 Sequence: SLYRPDAVAQPVTEDEFALESEIISK
Intensity
M/Z
type
b
y
y1
y2
y3
y4
y5
y6
y7
y8
y9
y10
y11
y12
y13
y14
b1
b2
b3
b4
b6
b7
b8
b9
b10
b13
b15
b16
0
25000
50000
75000
100000
500
1000
1500
2000

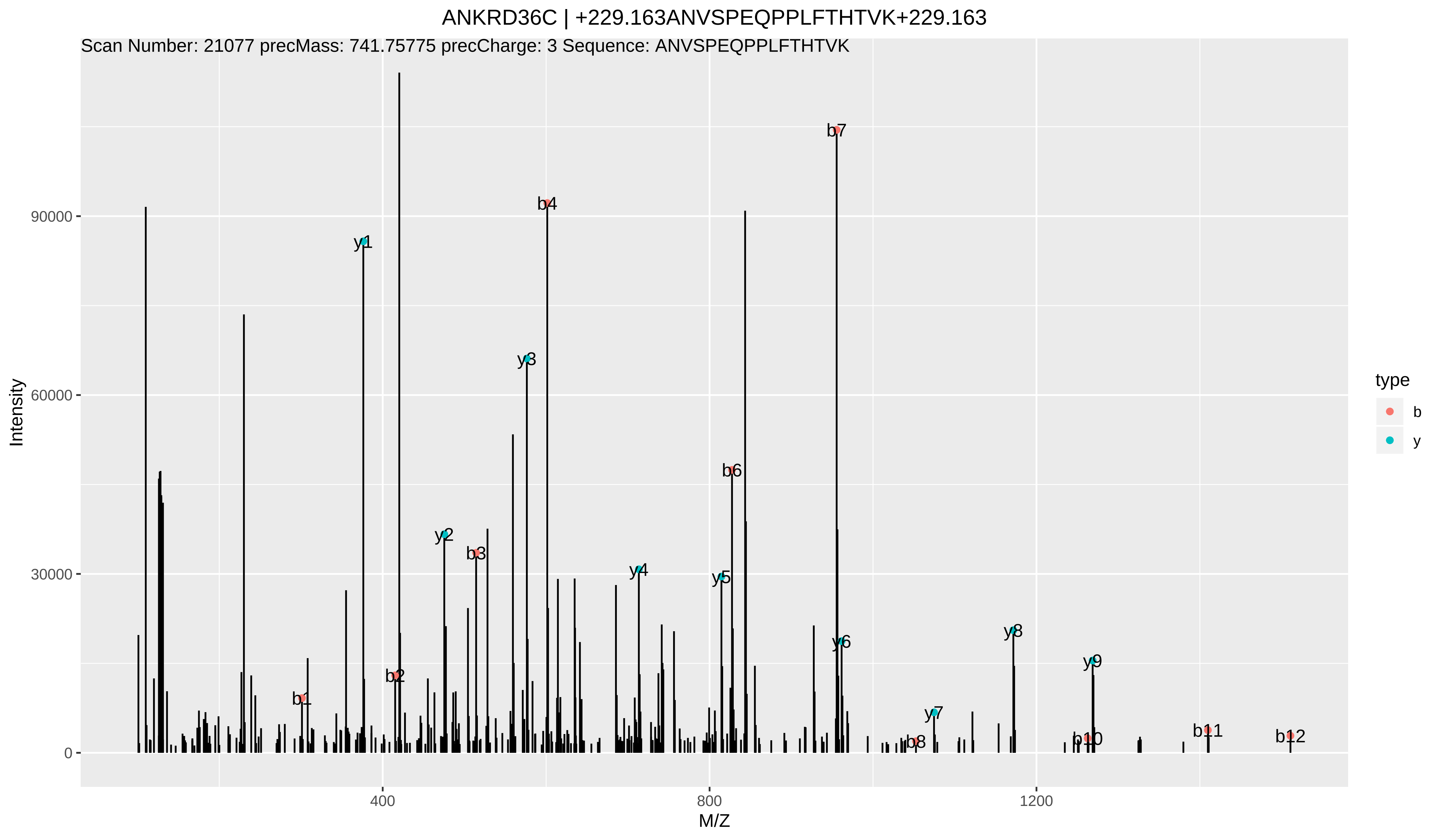
ANKRD36C | +229.163ANVSPEQPPLFTHTVK+229.163
Scan Number: 21077 precMass: 741.75775 precCharge: 3 Sequence: ANVSPEQPPLFTHTVK
Intensity
M/Z
type
b
y
0
30000
60000
90000
400
800
1200
b1
b2
b3
b4
b6
b7
b8
b10
b11
b12
y1
y2
y3
y4
y5
y6
y7
y8
y9

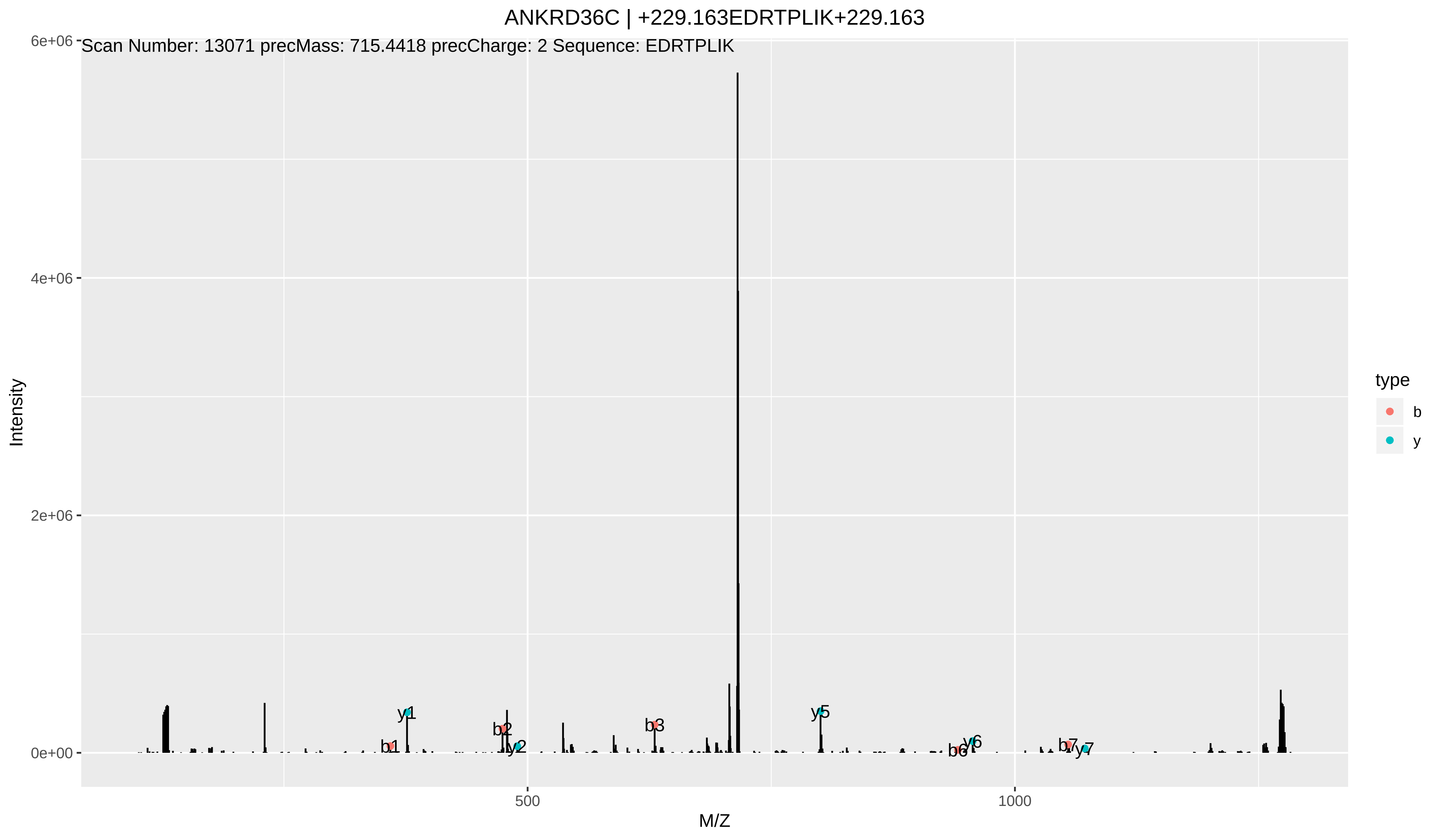
ANKRD36C | +229.163EDRTPLIK+229.163
Scan Number: 13071 precMass: 715.4418 precCharge: 2 Sequence: EDRTPLIK
Intensity
M/Z
type
b
y
6e+06
4e+06
2e+06
0e+00
500
1000
y1
b1
b2
y2
b3
y5
b6
y6
b7
y7

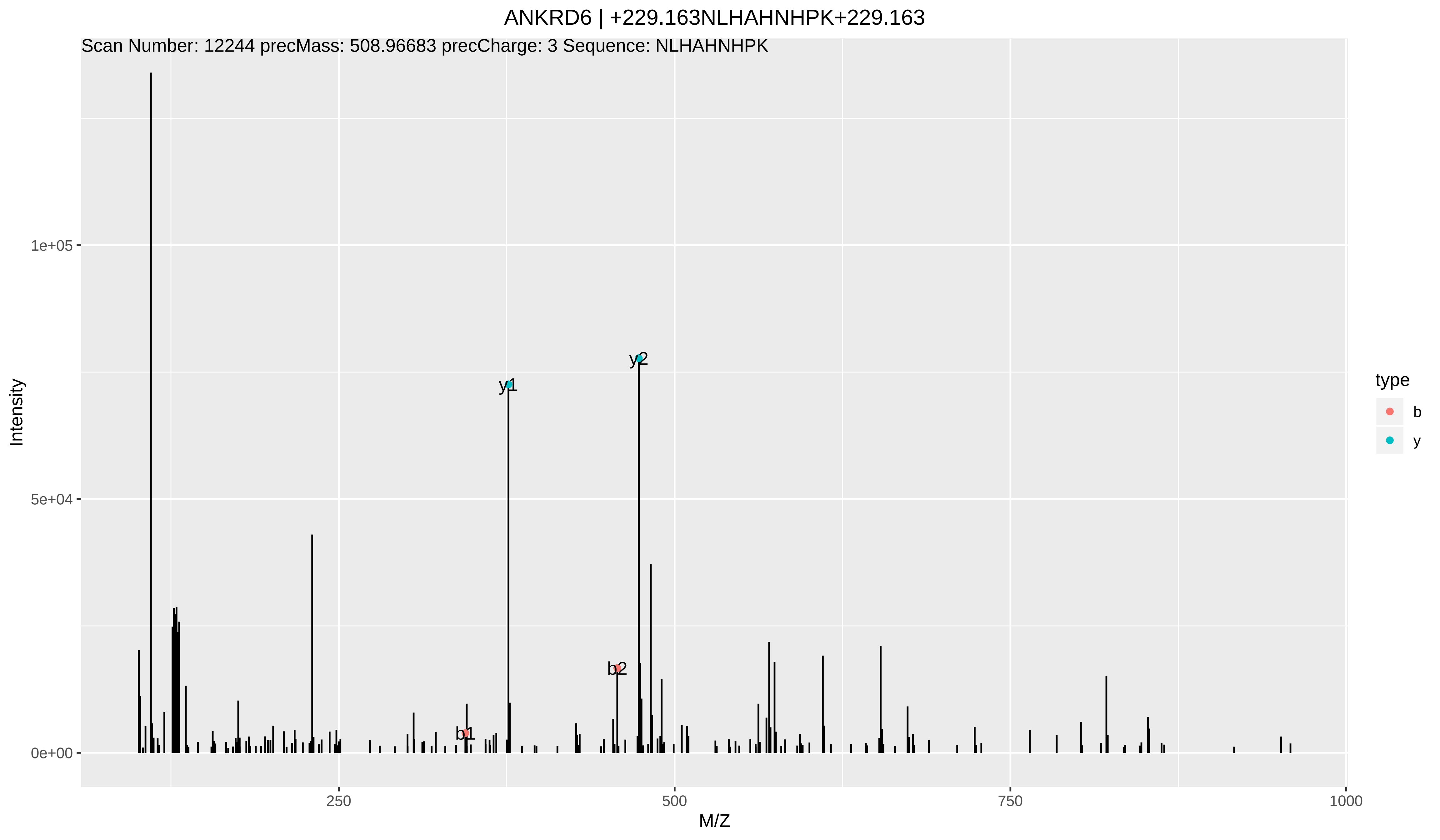

ANKRD6 | +229.163NLHAHNHPK+229.163
Scan Number: 12244 precMass: 508.96683 precCharge: 3 Sequence: NLHAHNHPK
Intensity
1e+05
5e+04
0e+00
M/Z
250
500
750
1000
y1
y2
b1
b2
type
b
y

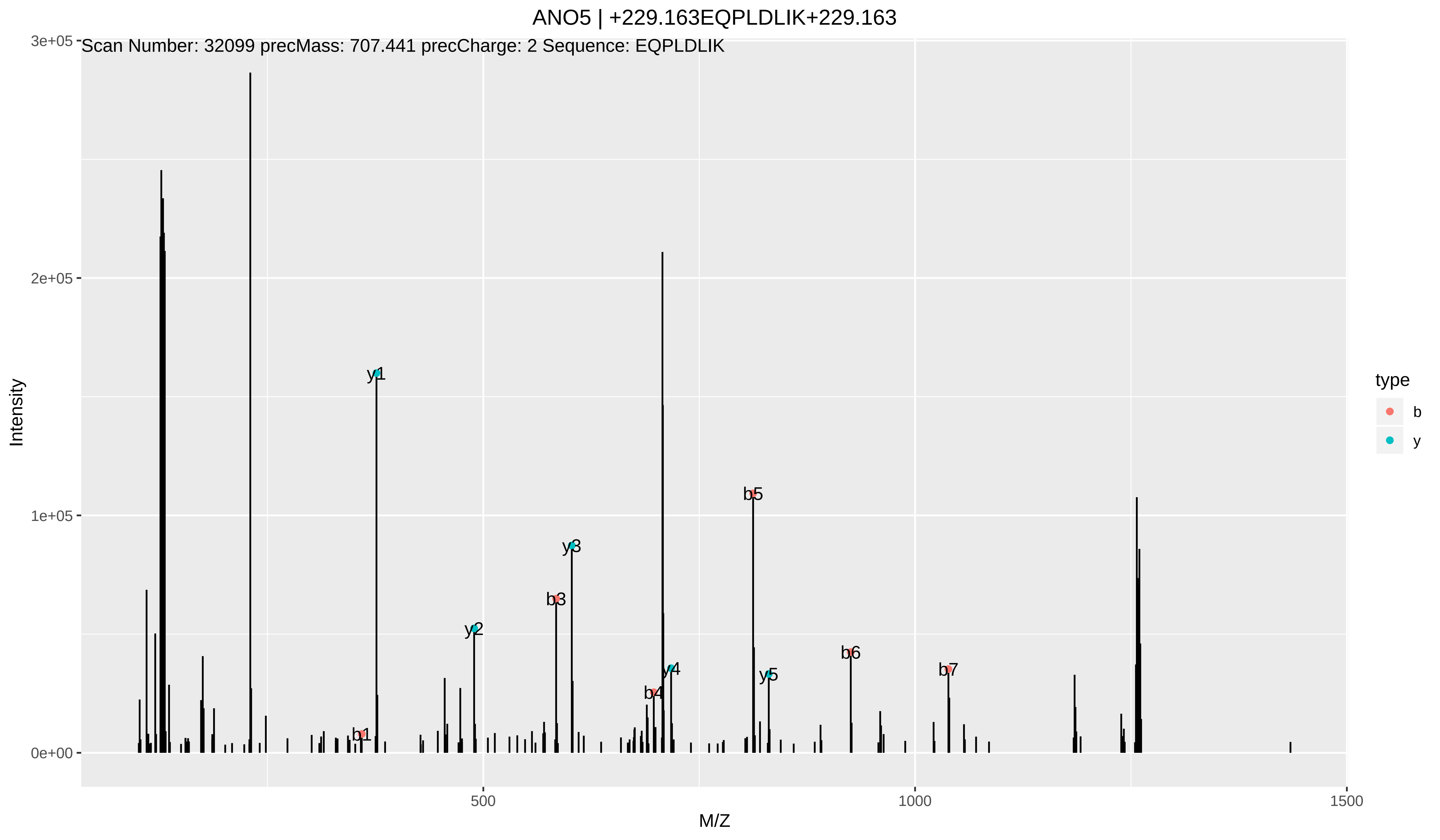

ANO5 | +229.163EQPLDLIK+229.163
Scan Number: 32099 precMass: 707.441 precCharge: 2 Sequence: EQPLDLIK
Intensity
M/Z
0e+00
1e+05
2e+05
3e+05
500
1000
1500
y1
y2
y3
y4
y5
b1
b3
b4
b5
b6
b7
type
b
y

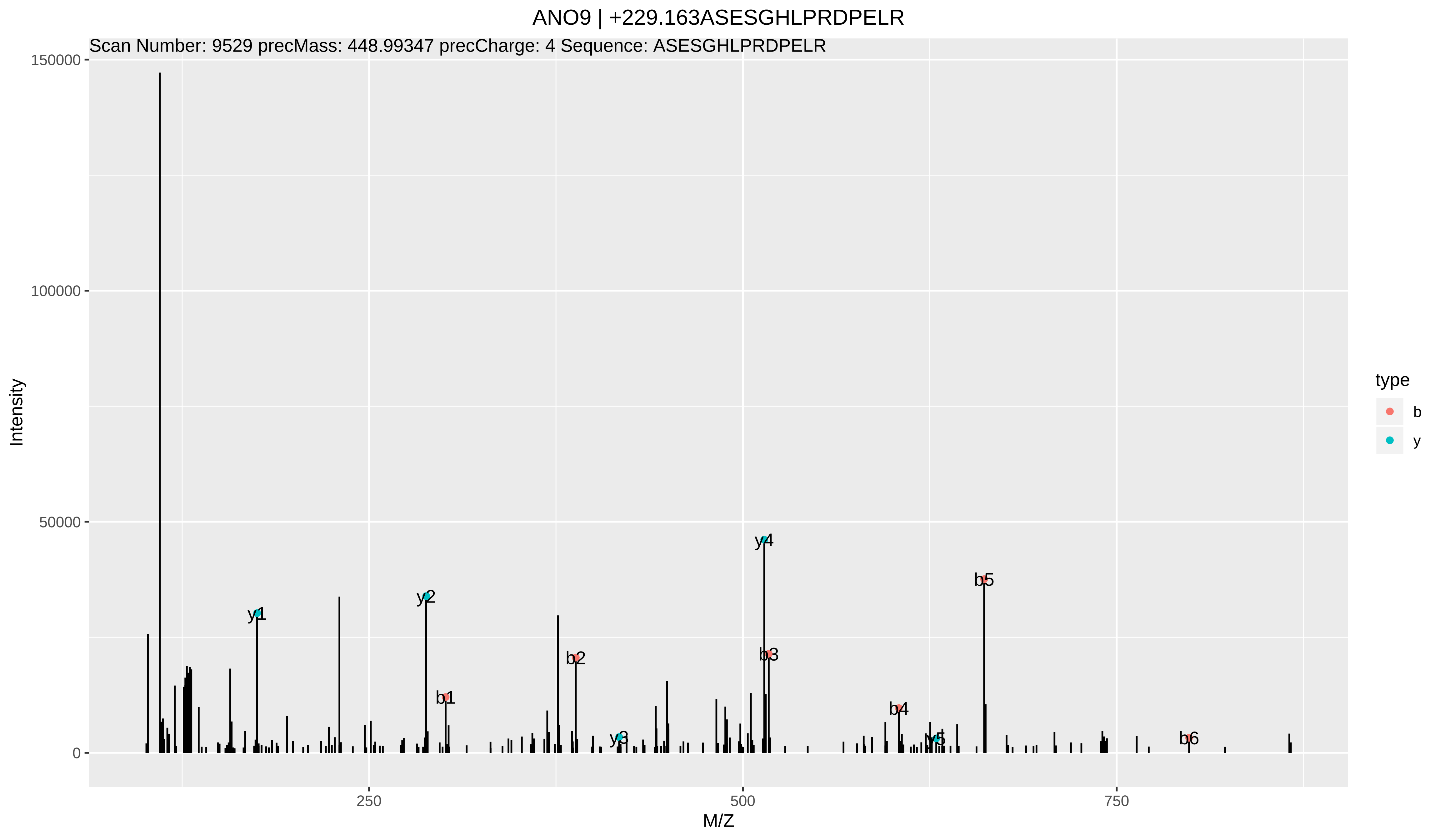

ANO9 | +229.163ASESGHLPRDPELR
Scan Number: 9529 precMass: 448.99347 precCharge: 4 Sequence: ASESGHLPRDPELR
150000
100000
50000
0
Intensity
250
500
750
M/Z
y1
y2
b1
b2
y3
y4
b3
b4
y5
b5
b6
type
b
y

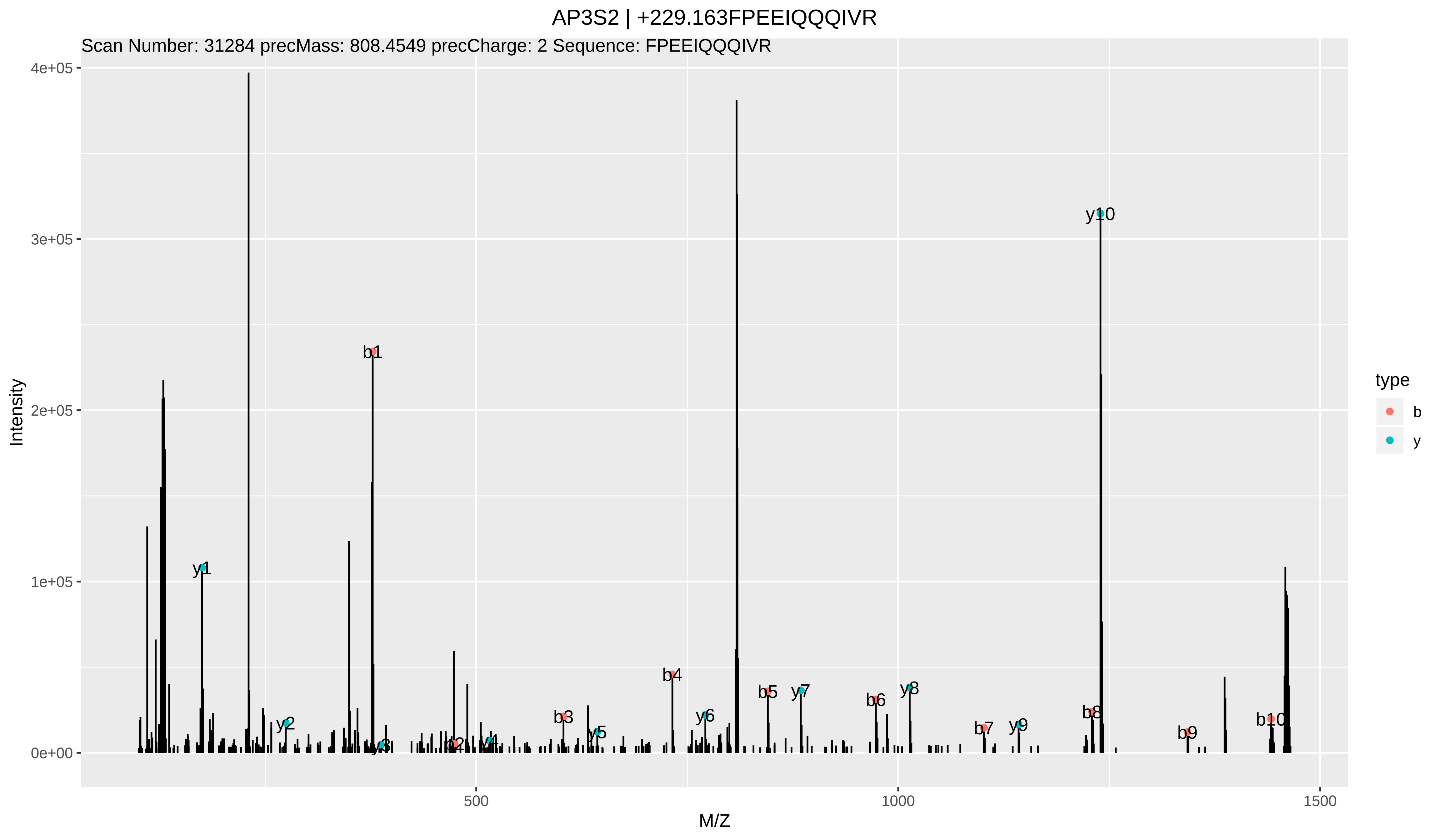

AP3S2 | +229.163FPEEIQQQIVR
Scan Number: 31284 precMass: 808.4549 precCharge: 2 Sequence: FPEEIQQQIVR
Intensity
M/Z
4e+05
3e+05
2e+05
1e+05
0e+00
500
1000
1500
type
b
y
y1
y2
y3
y4
y5
y6
y7
y8
y9
y10
b1
b2
b3
b4
b5
b6
b7
b8
b9
b10

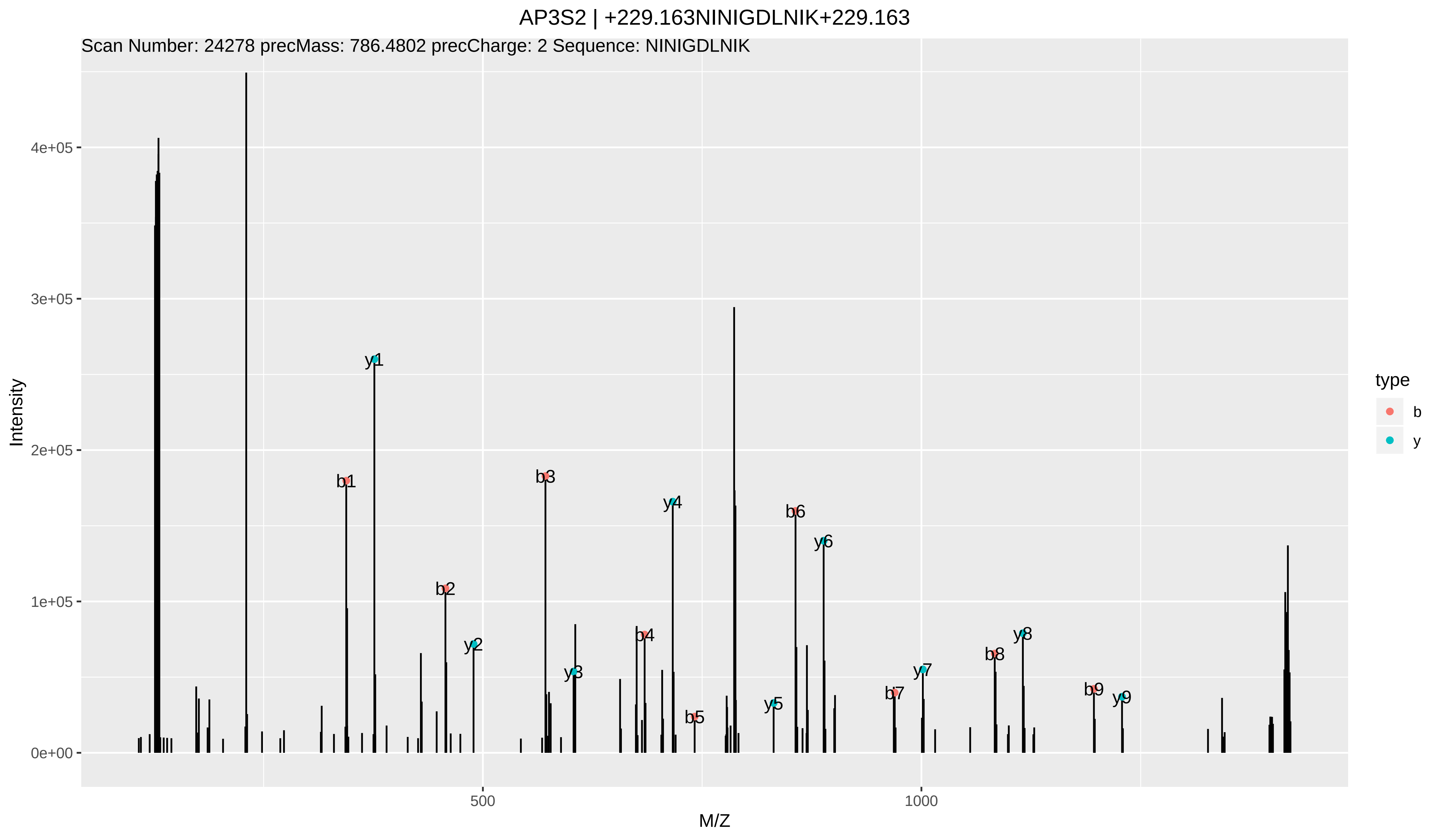

AP3S2 | +229.163NINIGDLNIK+229.163
Scan Number: 24278 precMass: 786.4802 precCharge: 2 Sequence: NINIGDLNIK
Intensity
M/Z
0e+00
1e+05
2e+05
3e+05
4e+05
500
1000
type
b
y
b1
y1
b2
y2
b3
y3
b4
y4
b5
y5
b6
y6
b7
y7
b8
y8
b9
y9

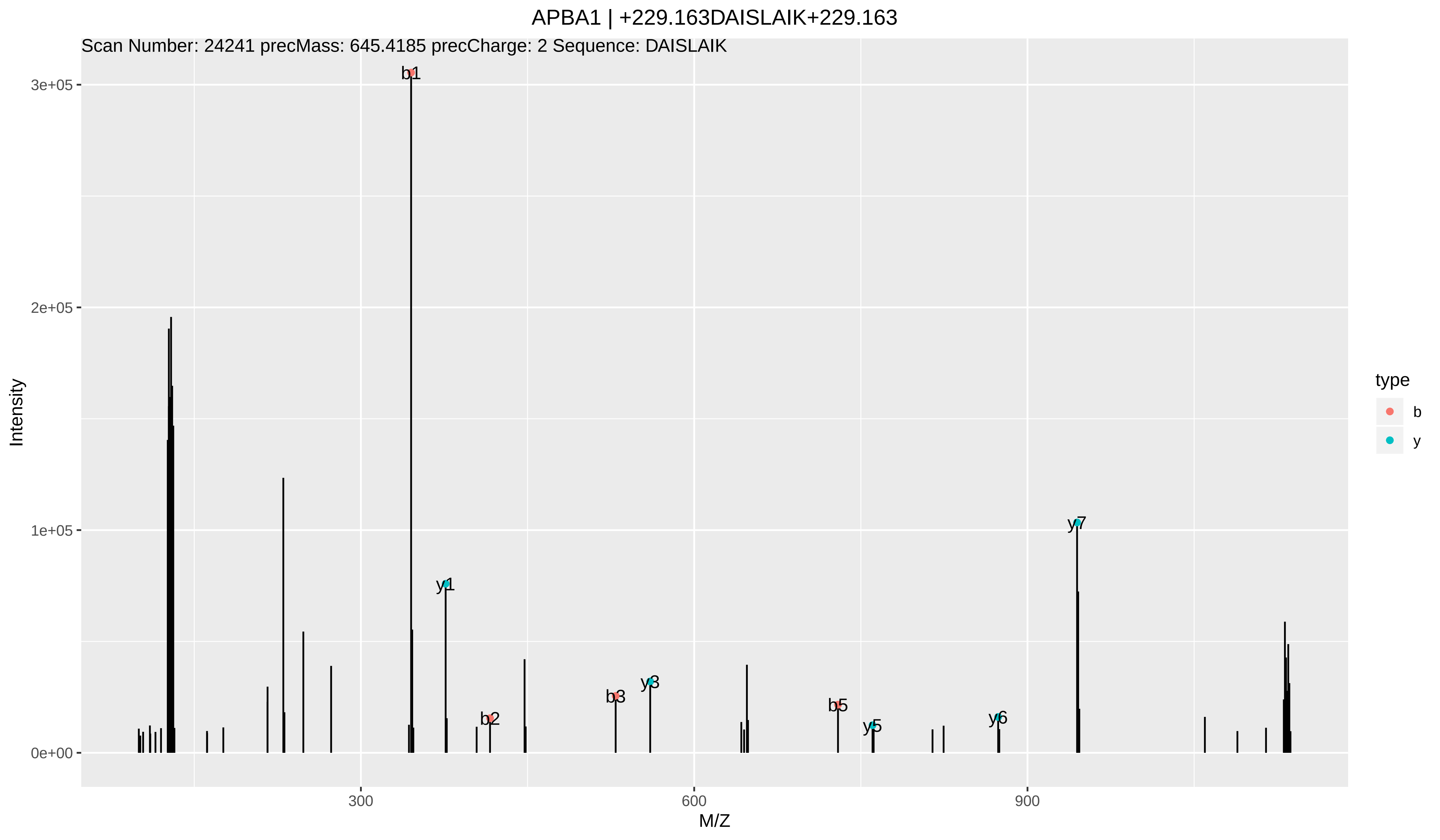

APBA1 | +229.163DAISLAIK+229.163
Scan Number: 24241 precMass: 645.4185 precCharge: 2 Sequence: DAISLAIK
Intensity
3e+05
2e+05
1e+05
0e+00
M/Z
300
600
900
b1
y1
b2
b3
y3
b5
y5
y6
y7
type
b
y

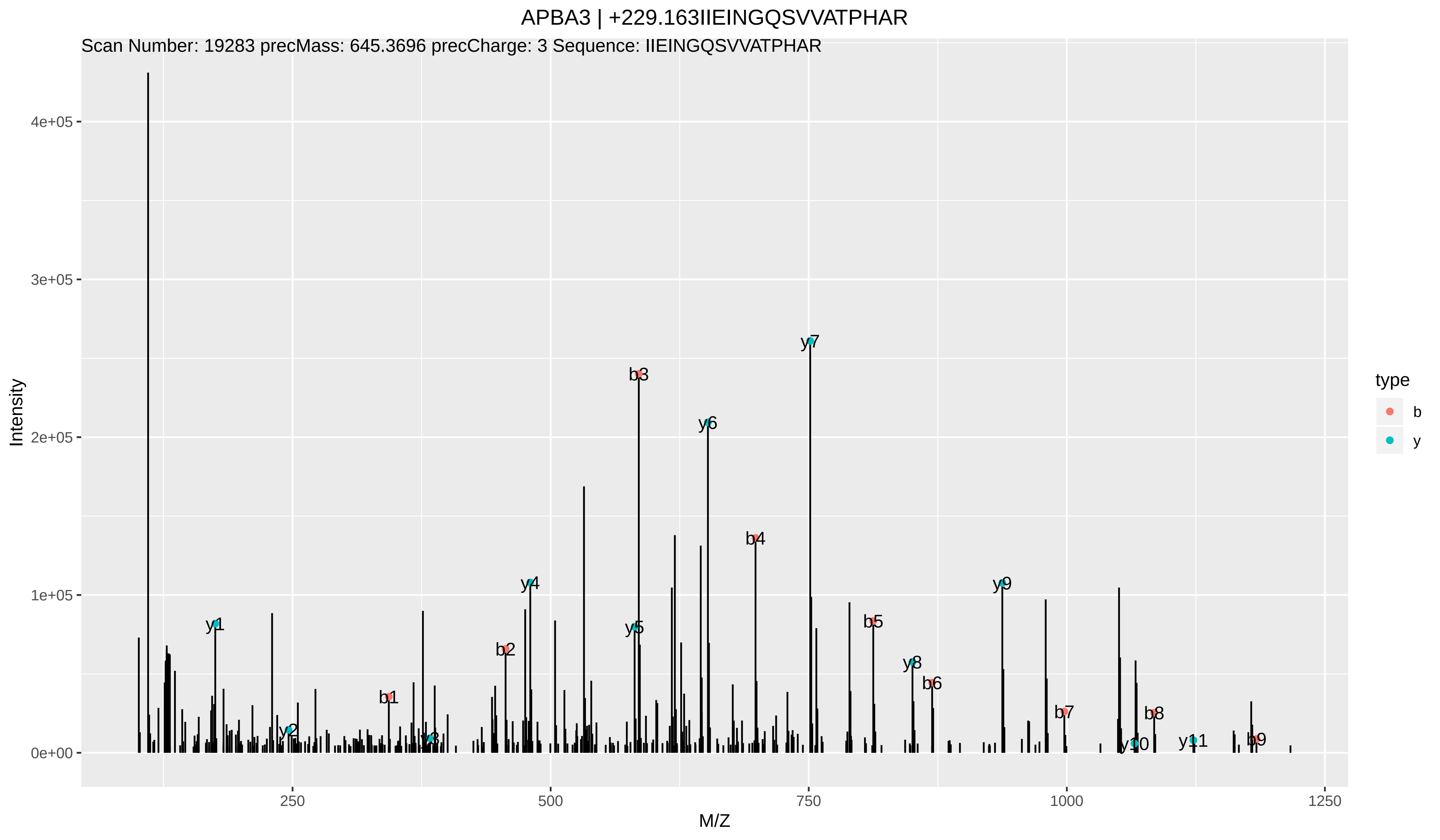
APBA3 | +229.163IIEINGQSVVATPHAR
Scan Number: 19283 precMass: 645.3696 precCharge: 3 Sequence: IIEINGQSVVATPHAR
Intensity
M/Z
4e+05
3e+05
2e+05
1e+05
0e+00
250
500
750
1000
1250
y1
y2
b1
y3
b2
y4
y5
b3
y6
b4
y7
b5
y8
b6
y9
b7
y10
b8
y11
b9
type
b
y

APOBEC3G | +229.163DM+15.995ATFLAEDPK+229.163

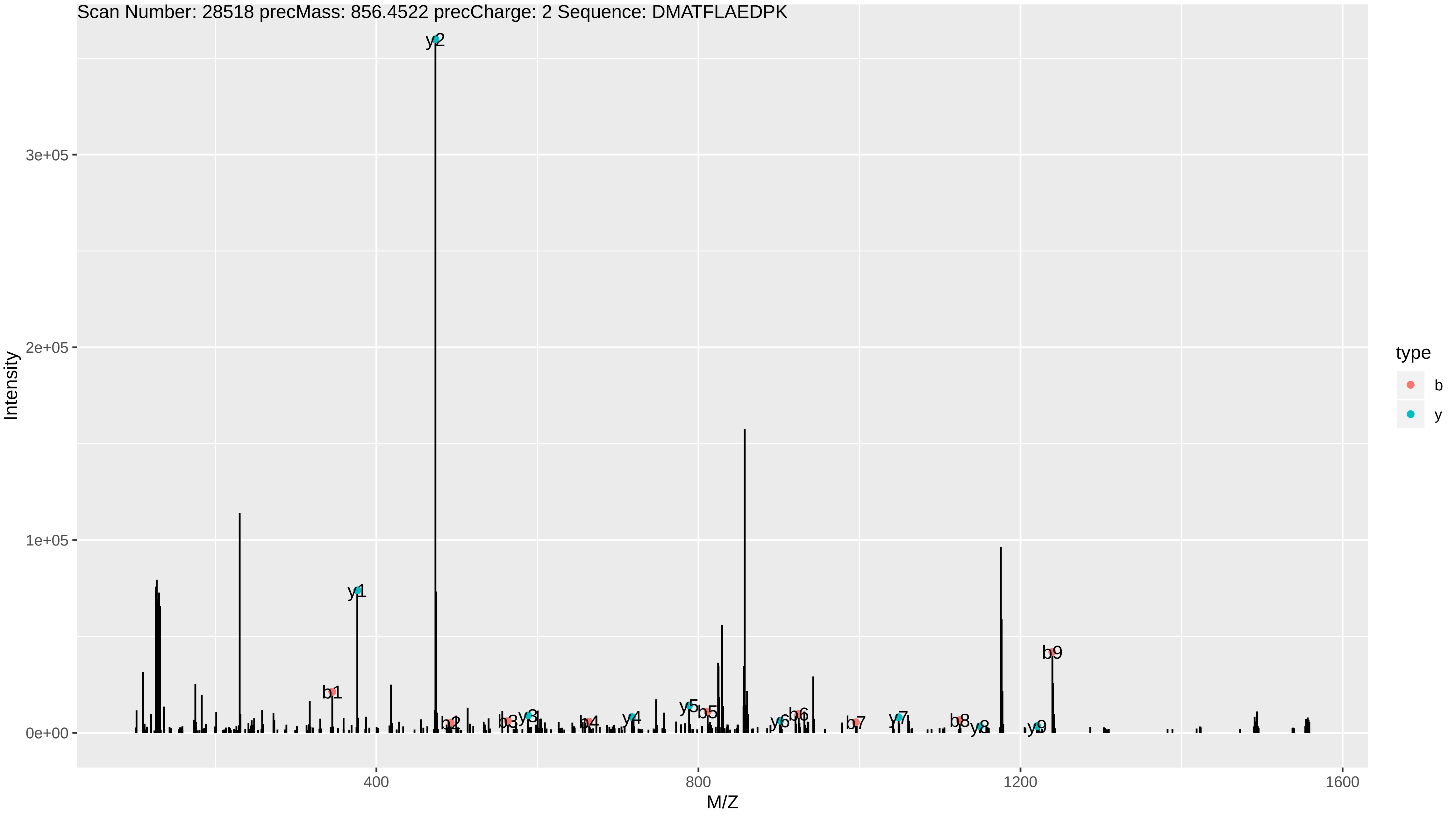

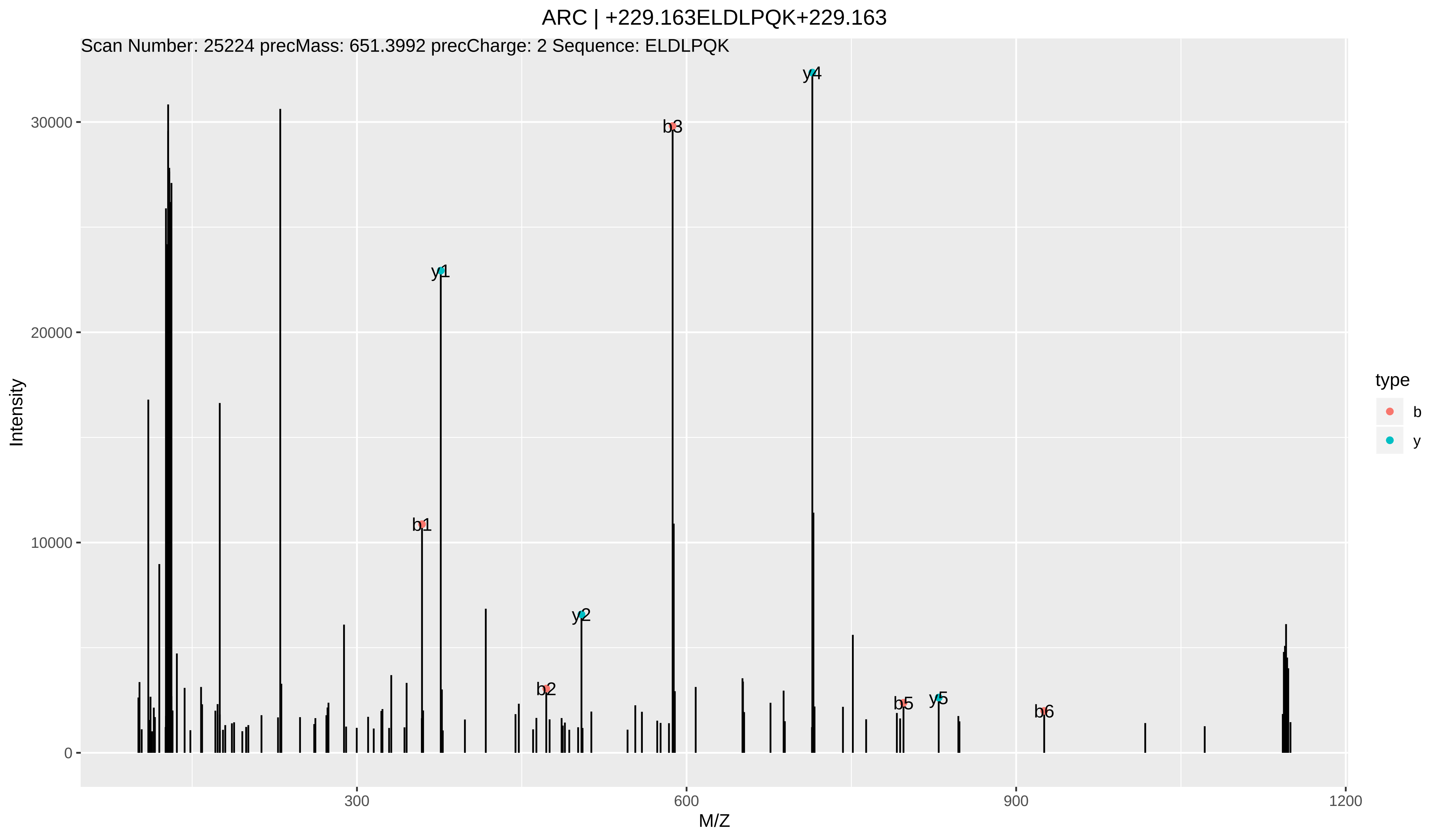
ARC | +229.163ELDLPQK+229.163
Scan Number: 25224 precMass: 651.3992 precCharge: 2 Sequence: ELDLPQK
Intensity
M/Z
type
b
y
y4
b3
y1
b1
y2
b2
b5
y5
b6
30000
20000
10000
0
300
600
900
1200

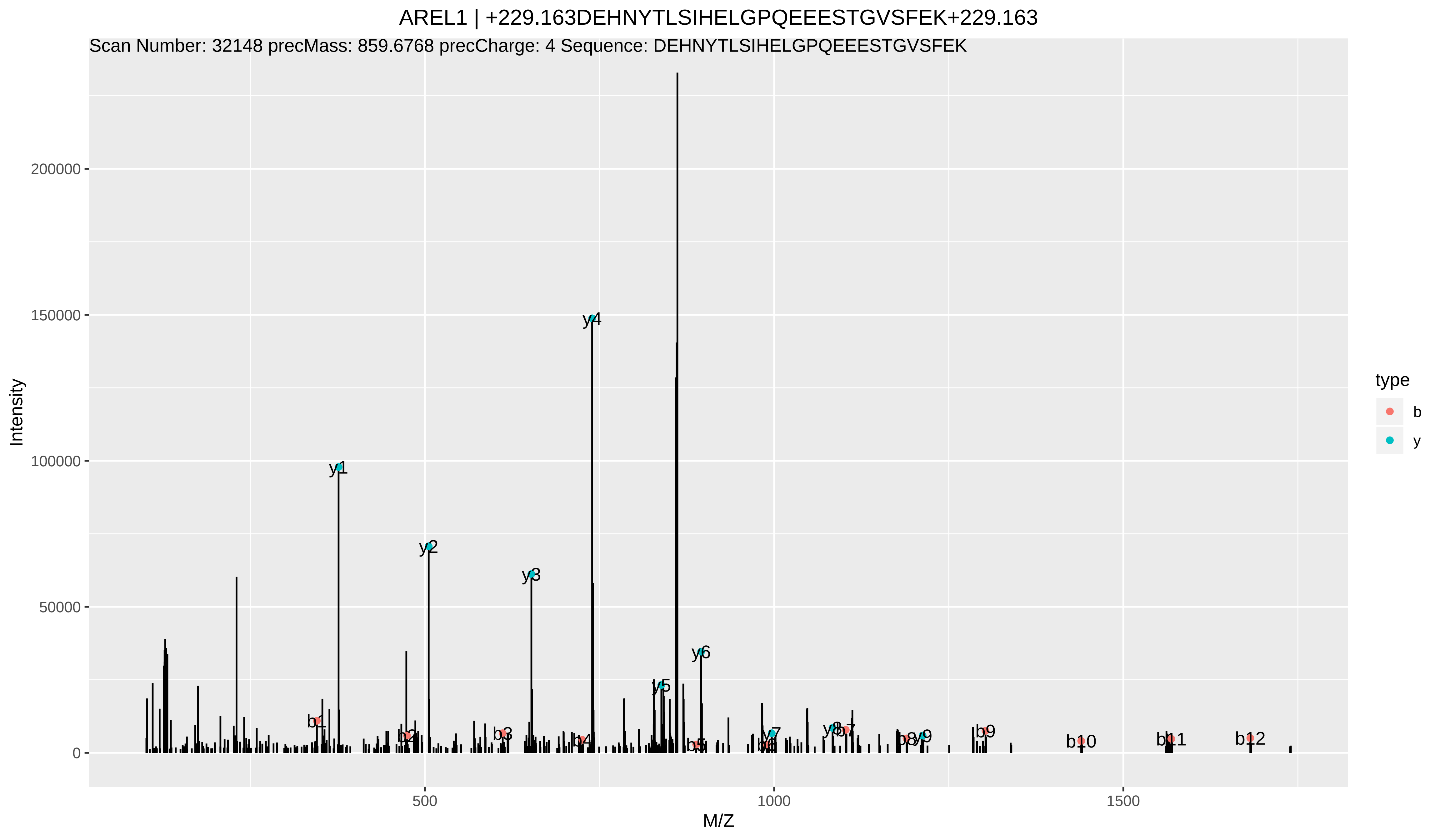

AREL1 | +229.163DEHNYTLSIHELGPQEEESTGVSFEK+229.163
Scan Number: 32148 precMass: 859.6768 precCharge: 4 Sequence: DEHNYTLSIHELGPQEEESTGVSFEK
Intensity
M/Z
0
50000
100000
150000
200000
500
1000
1500
y1
y2
y3
y4
y5
y6
y7
y8
y9
b1
b2
b3
b4
b5
b6
b7
b8
b9
b10
b11
b12
type
b
y

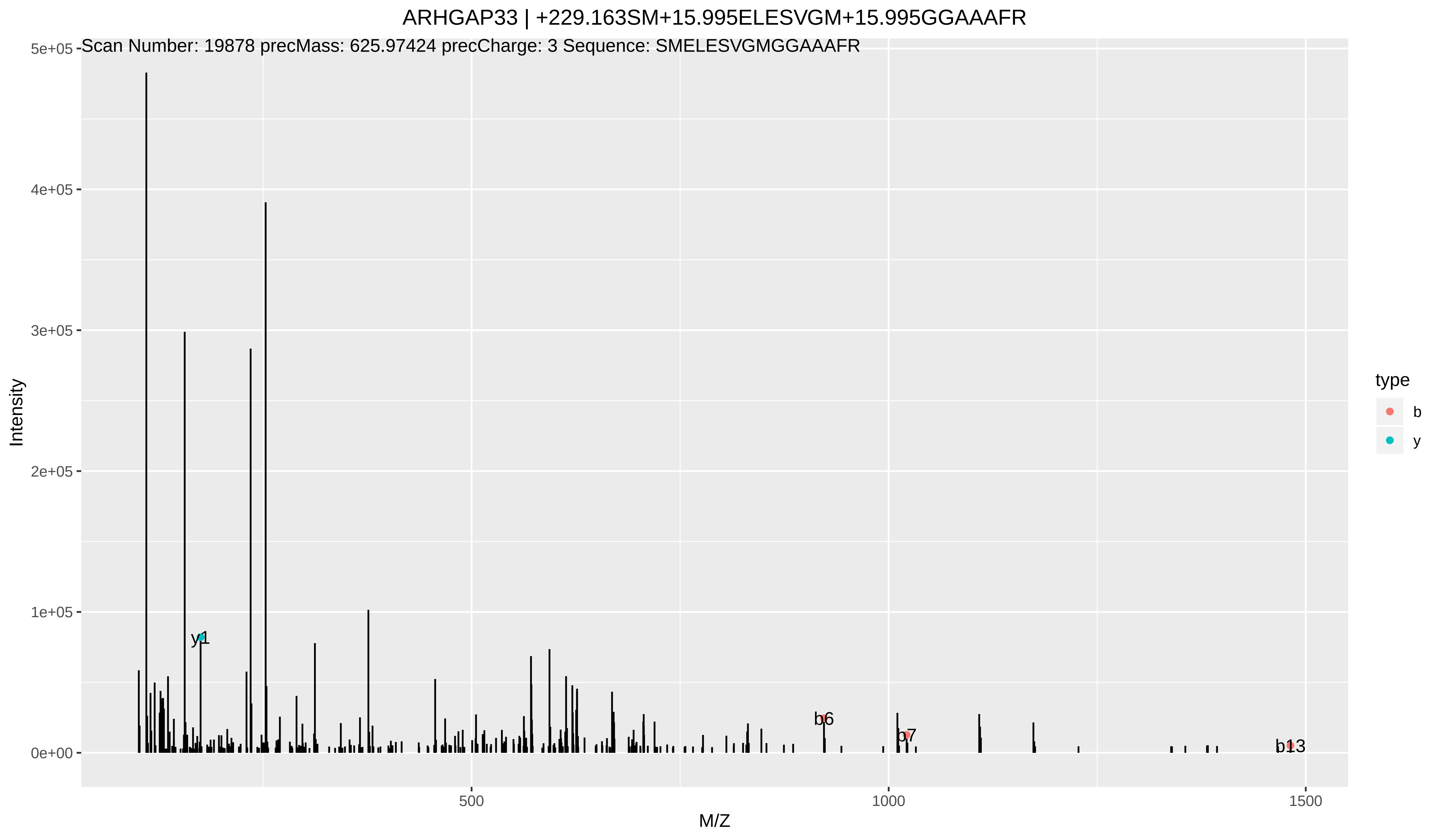

ARHGAP33 | +229.163SM+15.995ELESVGM+15.995GGAAAFR
Scan Number: 19878 precMass: 625.97424 precCharge: 3 Sequence: SMELESVGMGGAAAFR
Intensity
5e+05
4e+05
3e+05
2e+05
1e+05
0e+00
M/Z
500
1000
1500
y1
b6
b7
b13
type
b
y

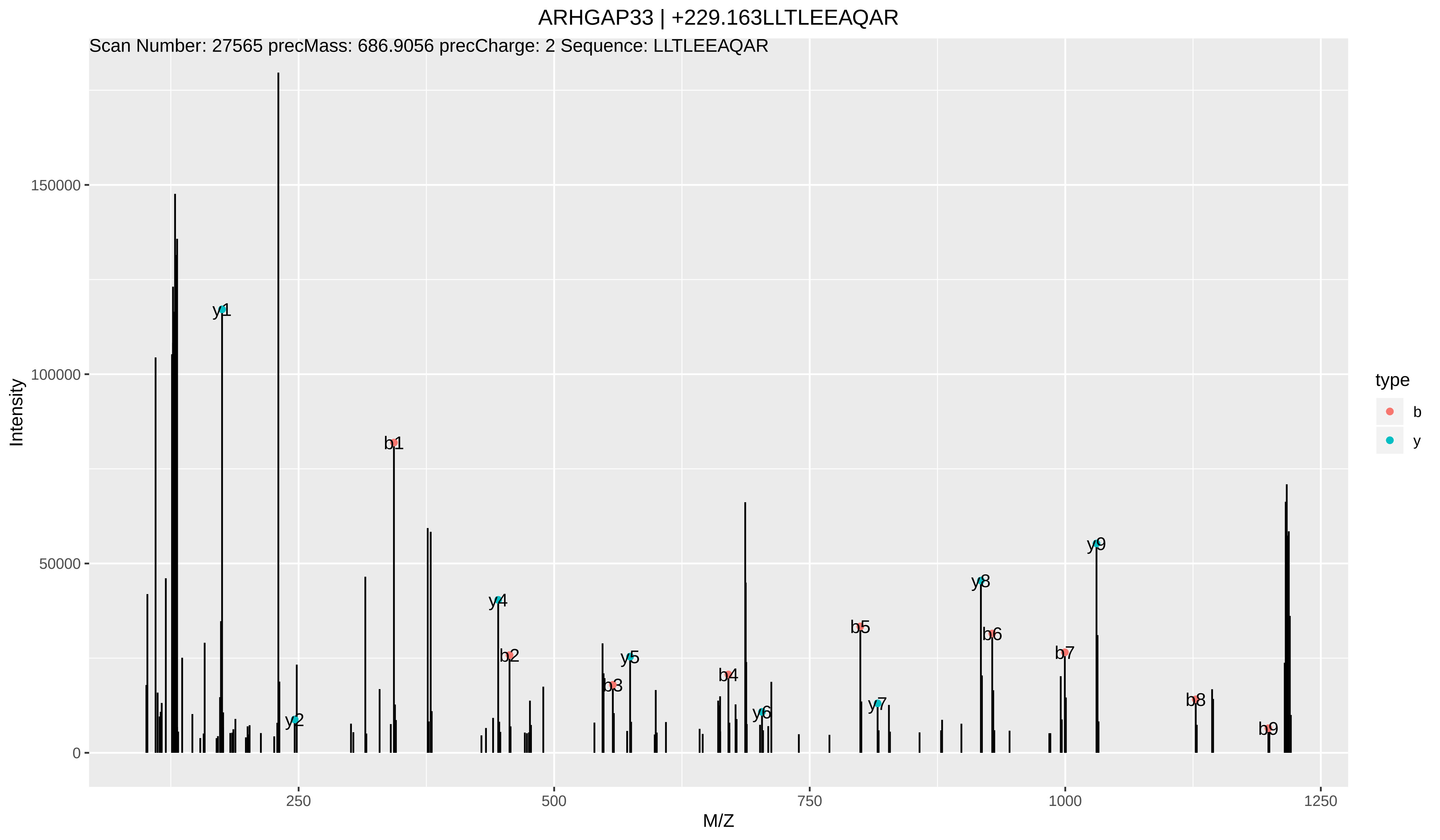

ARHGAP33 | +229.163LLTLEEAQAR
Scan Number: 27565 precMass: 686.9056 precCharge: 2 Sequence: LLTLEEAQAR
Intensity
M/Z
150000
100000
50000
0
250
500
750
1000
1250
y1
y2
y4
y5
y6
y7
y8
y9
b1
b2
b3
b4
b5
b6
b7
b8
b9
type
b
y

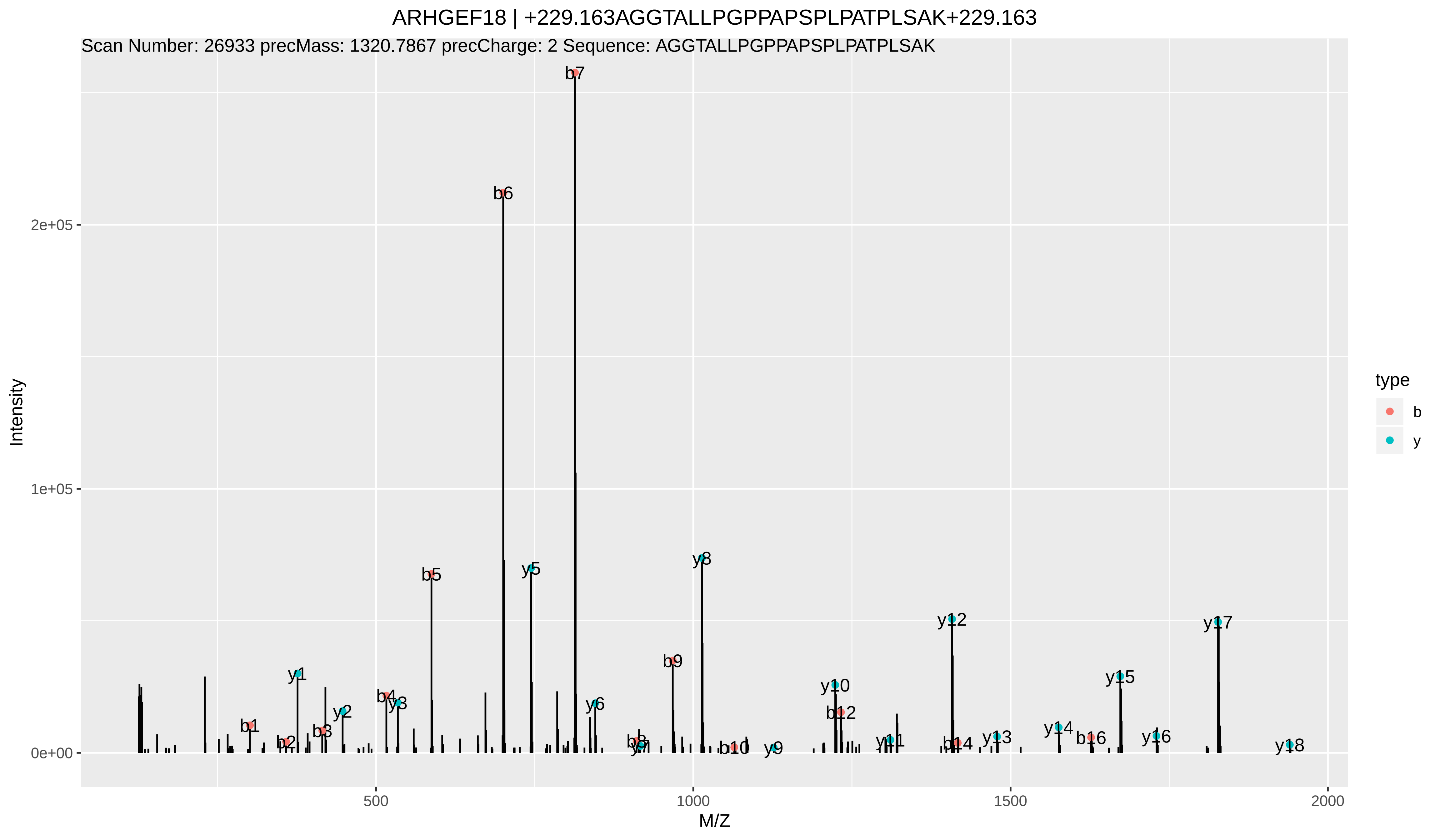

ARHGEF18 | +229.163AGGTALLPGPPAPSPLPATPLSAK+229.163
Scan Number: 26933 precMass: 1320.7867 precCharge: 2 Sequence: AGGTALLPGPPAPSPLPATPLSAK
Intensity
M/Z
type
b
y
b7
b6
y8
y5
b5
y12
y17
b9
y15
y10
y1
b4
y3
y6
y2
b12
b1
y14
b3
b16
y13
y16
b2
y11
b14
y18
b10
y9
0e+00
1e+05
2e+05
500
1000
1500
2000

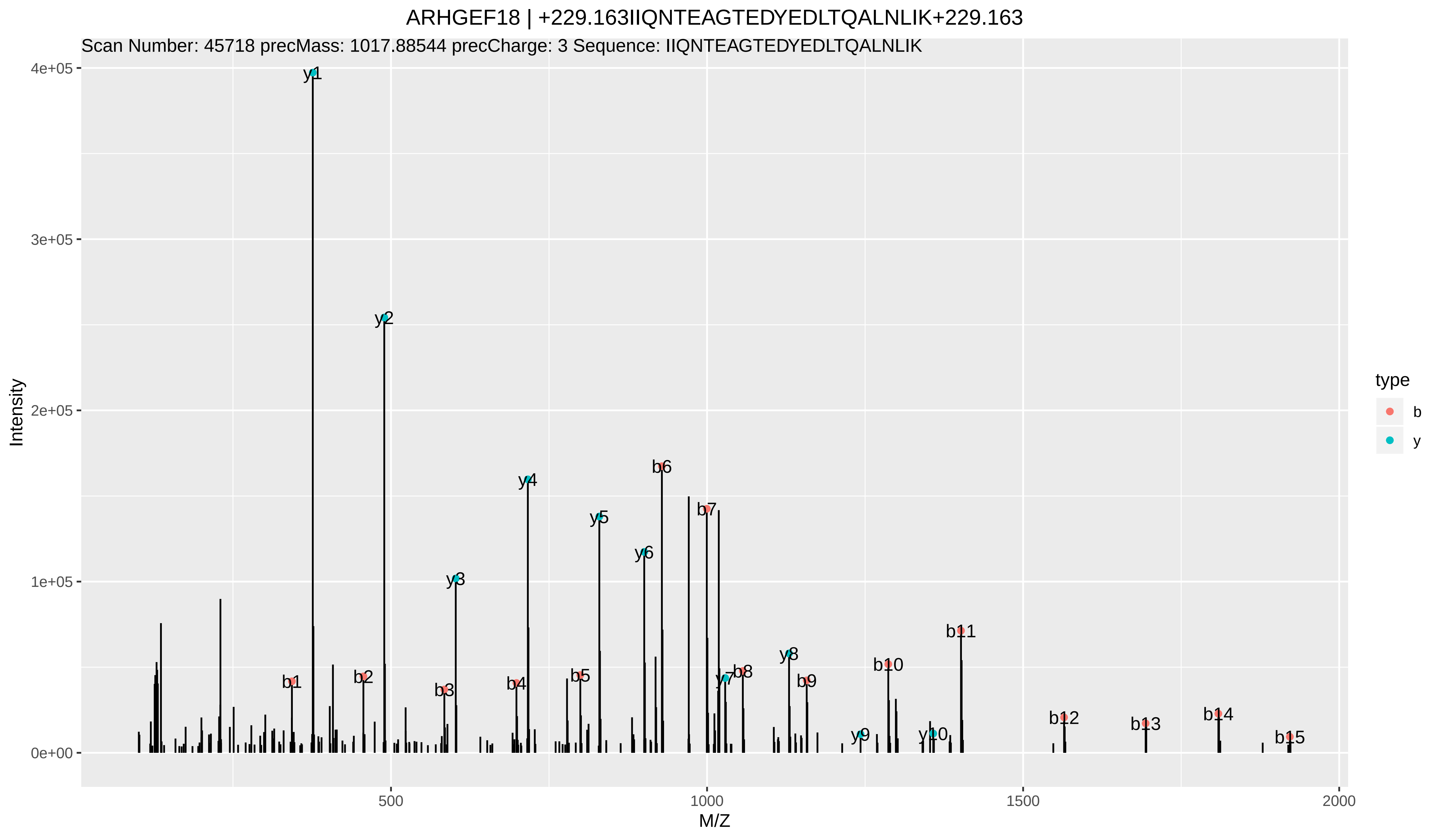
ARHGEF18 | +229.163IIQNTEAGTEDYEDLTQALNLIK+229.163
Scan Number: 45718 precMass: 1017.88544 precCharge: 3 Sequence: IIQNTEAGTEDYEDLTQALNLIK
Intensity
4e+05
3e+05
2e+05
1e+05
0e+00
M/Z
500
1000
1500
2000
y1
y2
y3
y4
y5
y6
y7
y8
y9
y10
b1
b2
b3
b4
b5
b6
b7
b8
b9
b10
b11
b12
b13
b14
b15
type
b
y

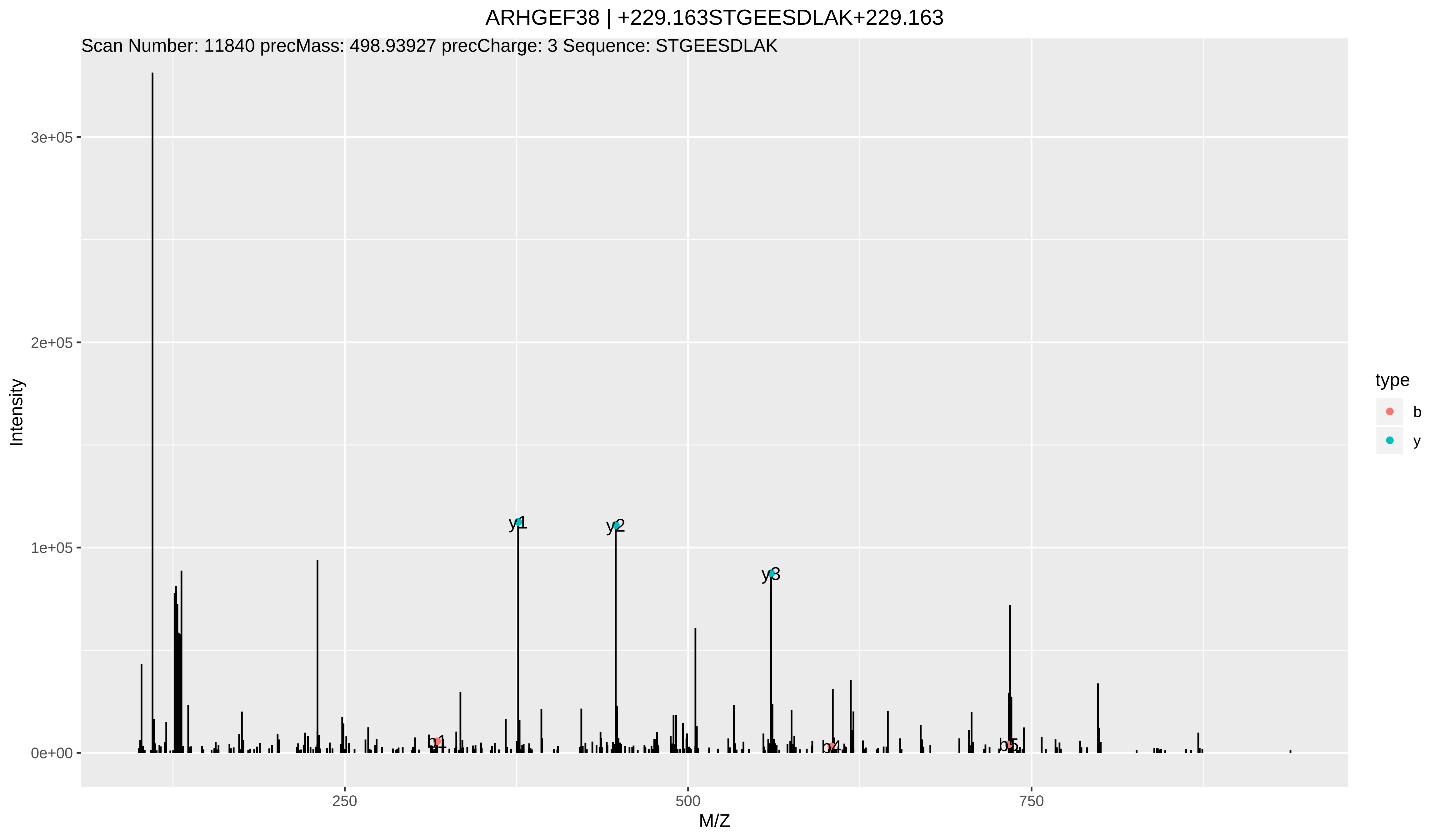
ARHGEF38 | +229.163STGEESDLAK+229.163
Scan Number: 11840 precMass: 498.93927 precCharge: 3 Sequence: STGEESDLAK
Intensity
3e+05
2e+05
1e+05
0e+00
M/Z
250
500
750
y1
y2
y3
b1
b4
b5
type
b
y

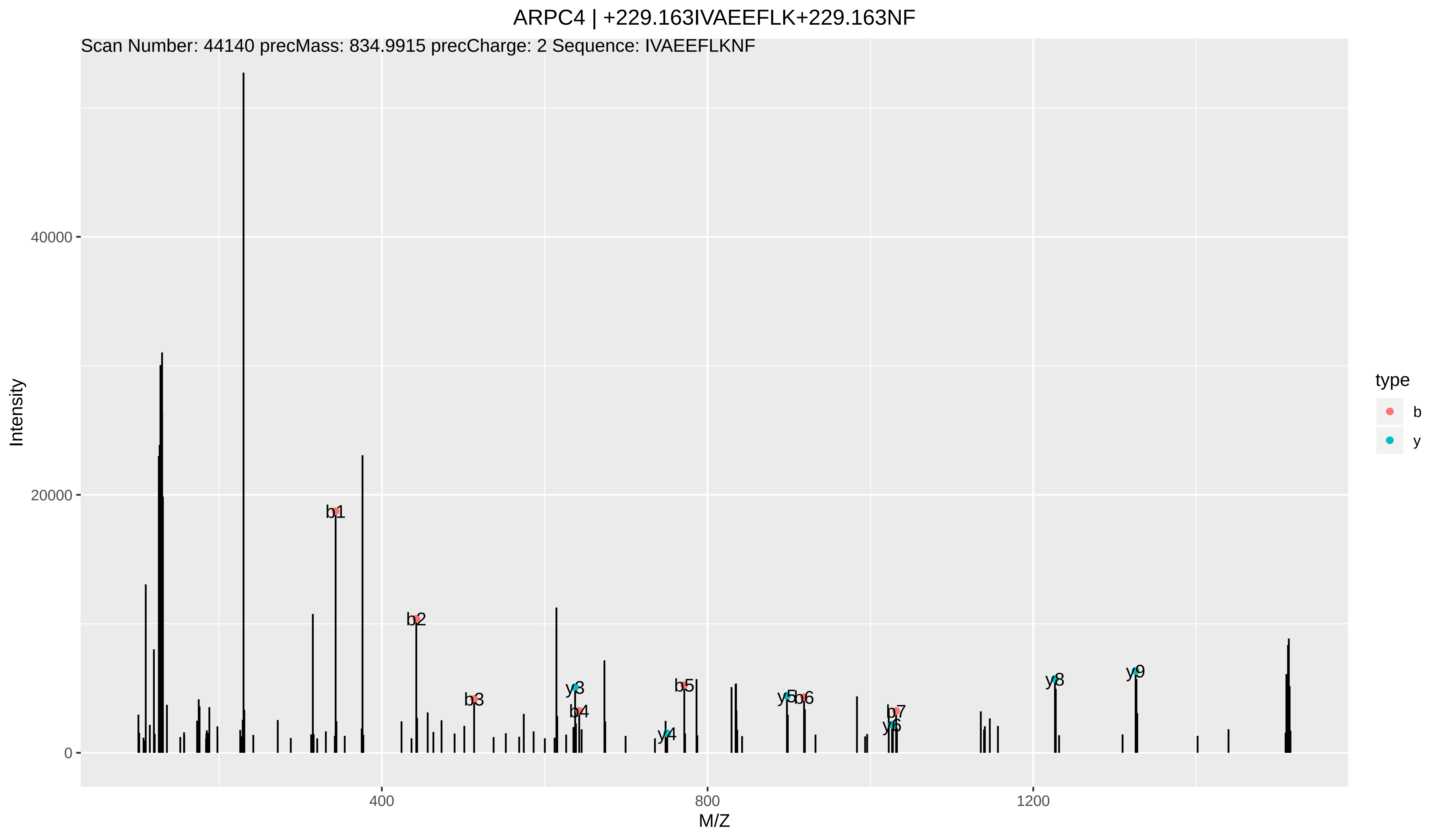
ARPC4 | +229.163IVAEEFLK+229.163NF
Scan Number: 44140 precMass: 834.9915 precCharge: 2 Sequence: IVAEEFLKNF
Intensity
M/Z
40000
20000
0
400
800
1200
b1
b2
b3
b4
b5
b6
b7
y3
y4
y5
y6
y8
y9
type
b
y

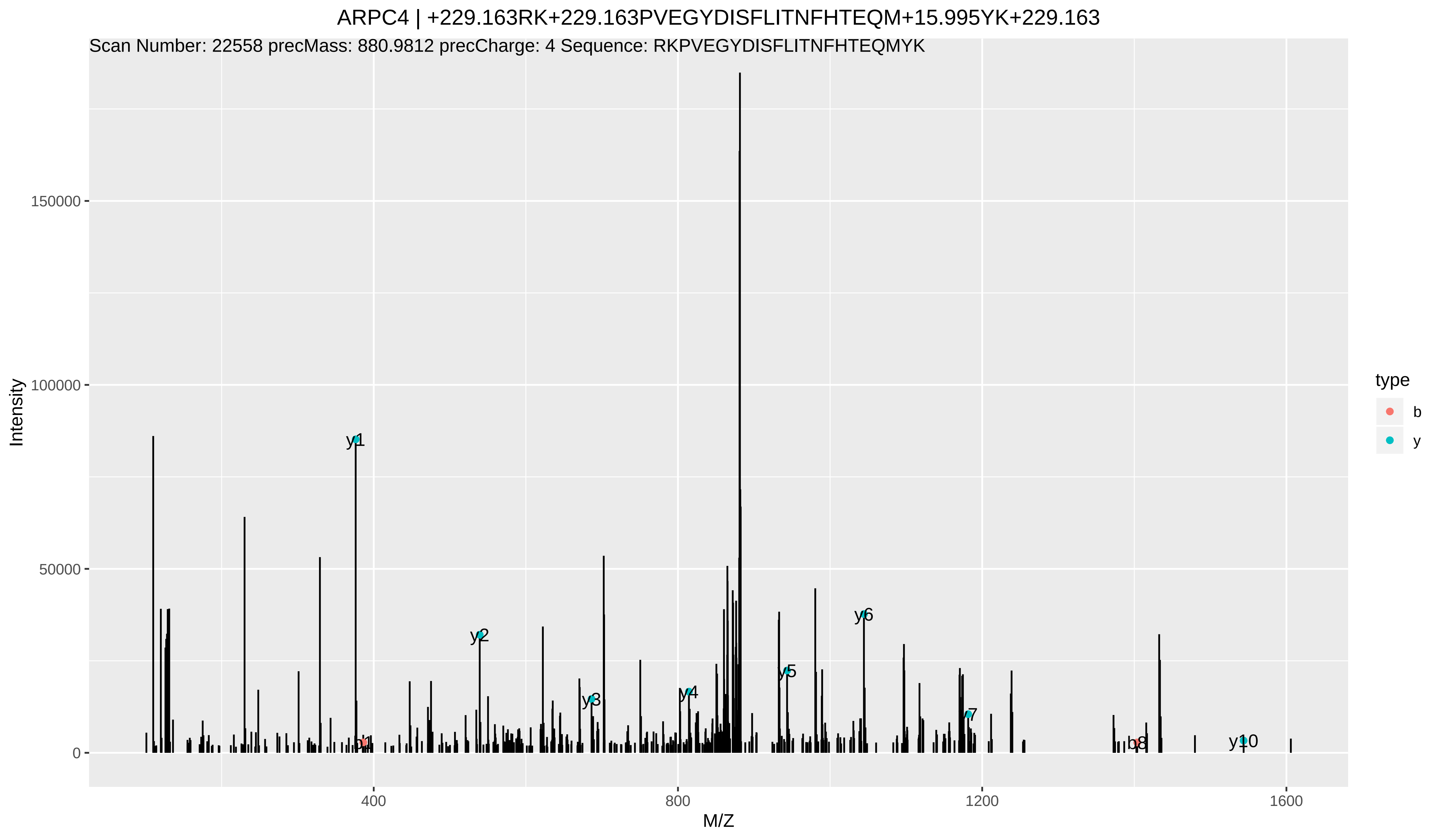
ARPC4 | +229.163RK+229.163PVEGYDISFLITNFHTEQM+15.995YK+229.163
Scan Number: 22558 precMass: 880.9812 precCharge: 4 Sequence: RKPVEGYDISFLITNFHTEQMYK
Intensity
150000
100000
50000
0
M/Z
400
800
1200
1600
y1
y2
y3
y4
y5
y6
y7
y10
b1
b8
type
b
y

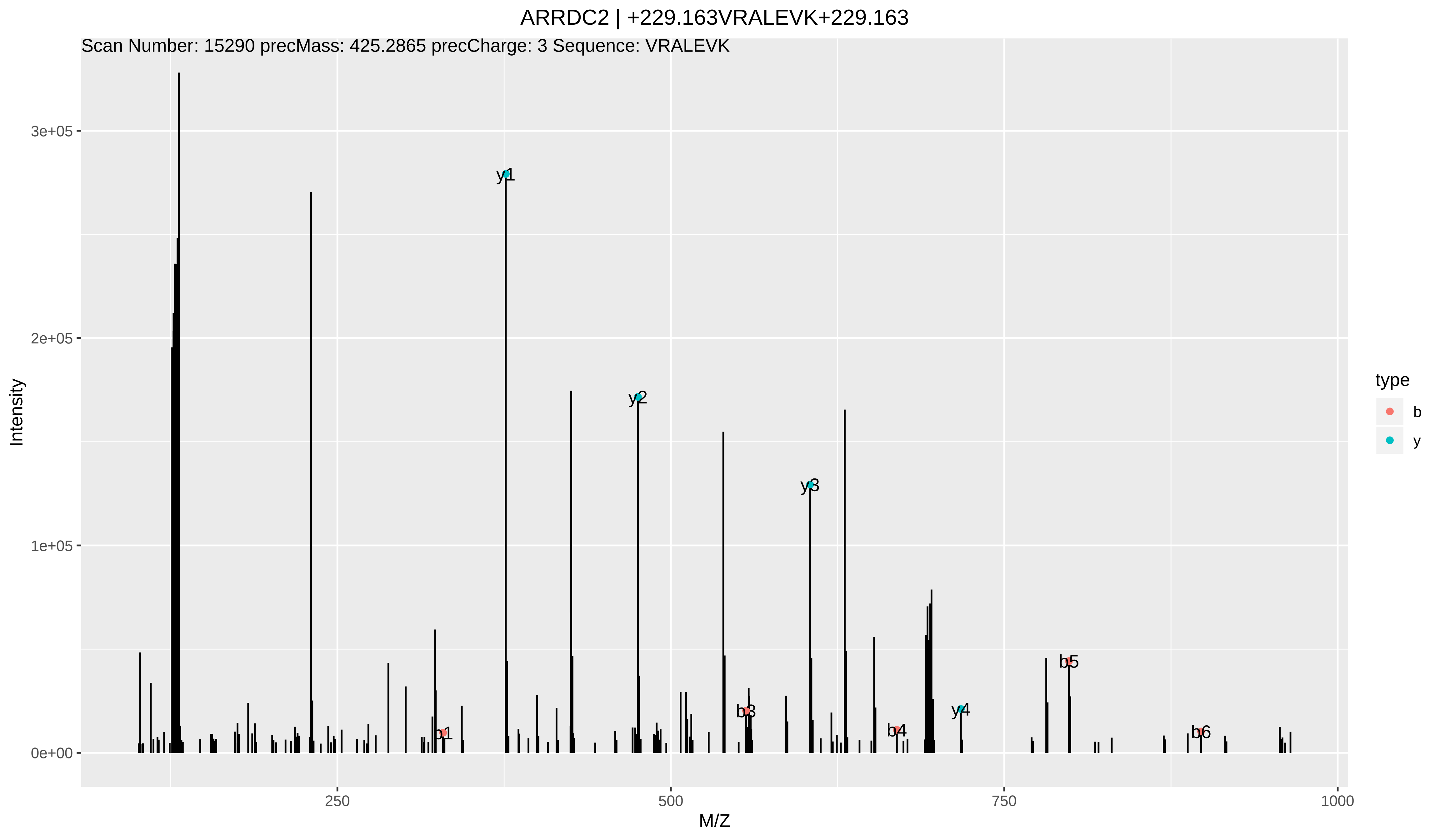

ARRDC2 | +229.163VRALEVK+229.163
Scan Number: 15290 precMass: 425.2865 precCharge: 3 Sequence: VRALEVK
Intensity
3e+05
2e+05
1e+05
0e+00
M/Z
250
500
750
1000
y1
y2
y3
y4
b1
b3
b4
b5
b6
type
b
y

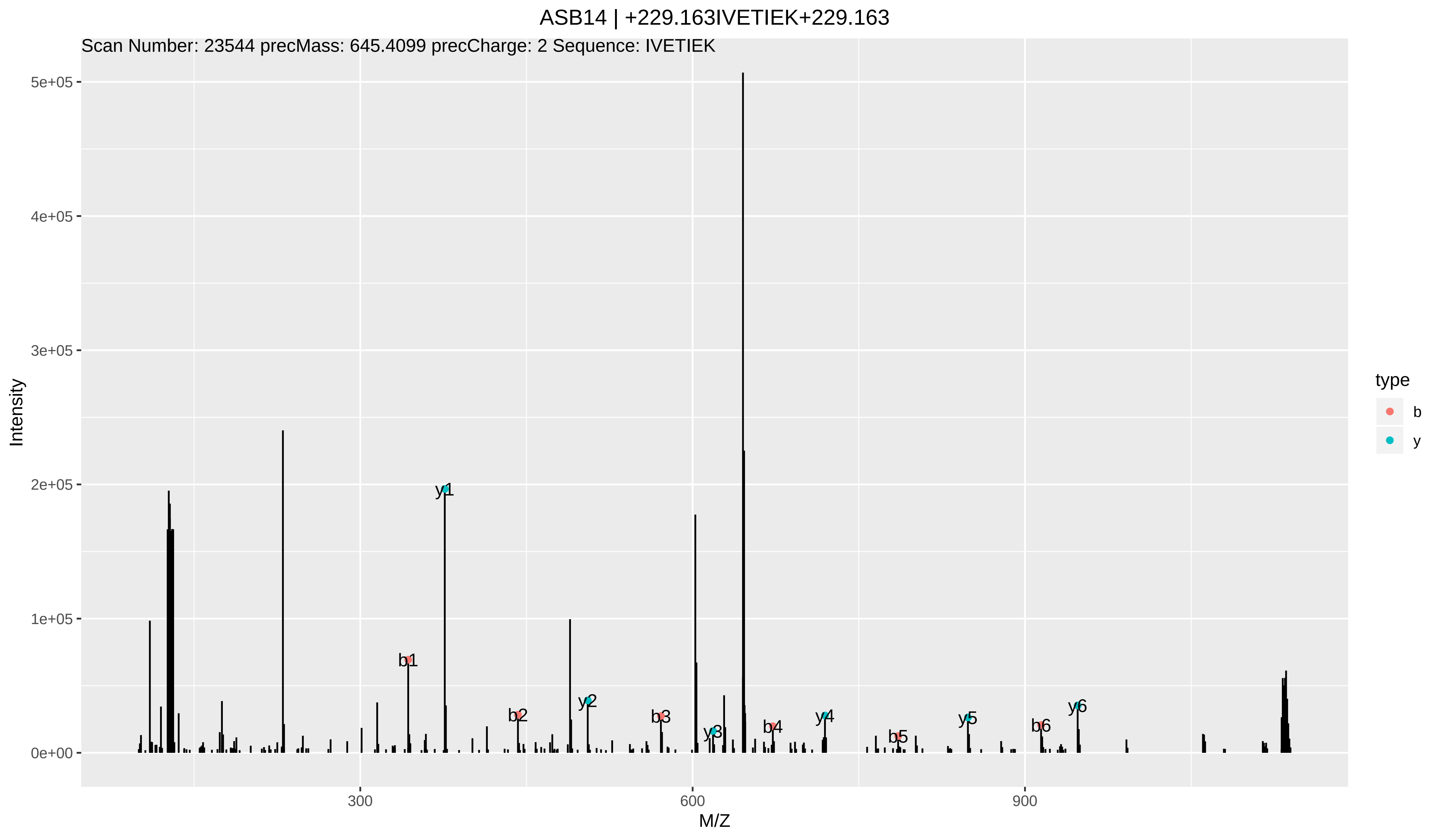
ASB14 | +229.163IVETIEK+229.163
Scan Number: 23544 precMass: 645.4099 precCharge: 2 Sequence: IVETIEK
Intensity
M/Z
5e+05
4e+05
3e+05
2e+05
1e+05
0e+00
300
600
900
y1
b1
b2
y2
b3
y3
b4
y4
b5
y5
b6
y6
type
b
y

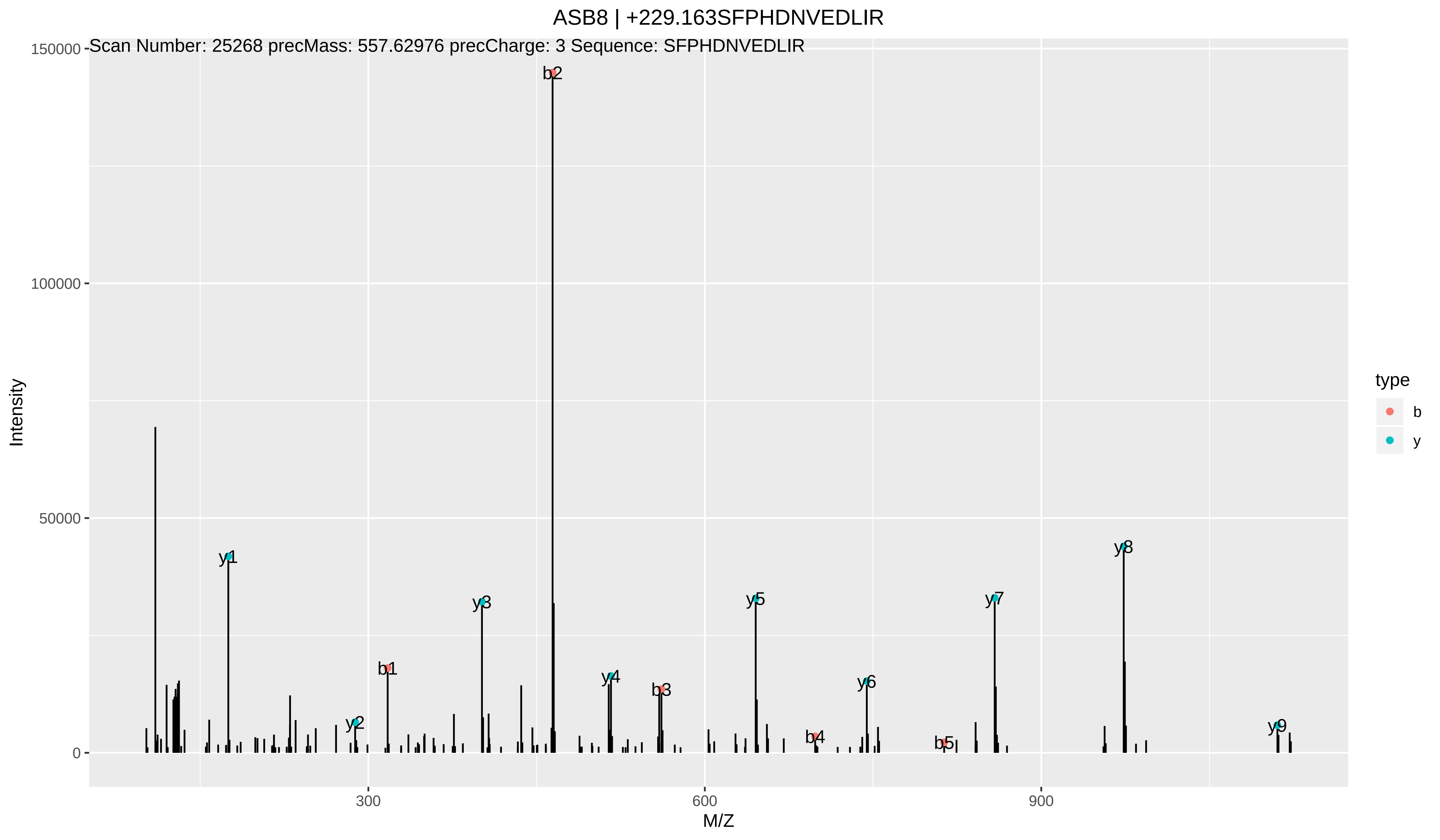
ASB8 | +229.163SFPHDNVEDLIR
Scan Number: 25268 precMass: 557.62976 precCharge: 3 Sequence: SFPHDNVEDLIR
Intensity
150000
100000
50000
0
300
600
900
M/Z
b2
y1
y2
b1
y3
y4
b3
y5
b4
y6
b5
y7
y8
y9
type
b
y

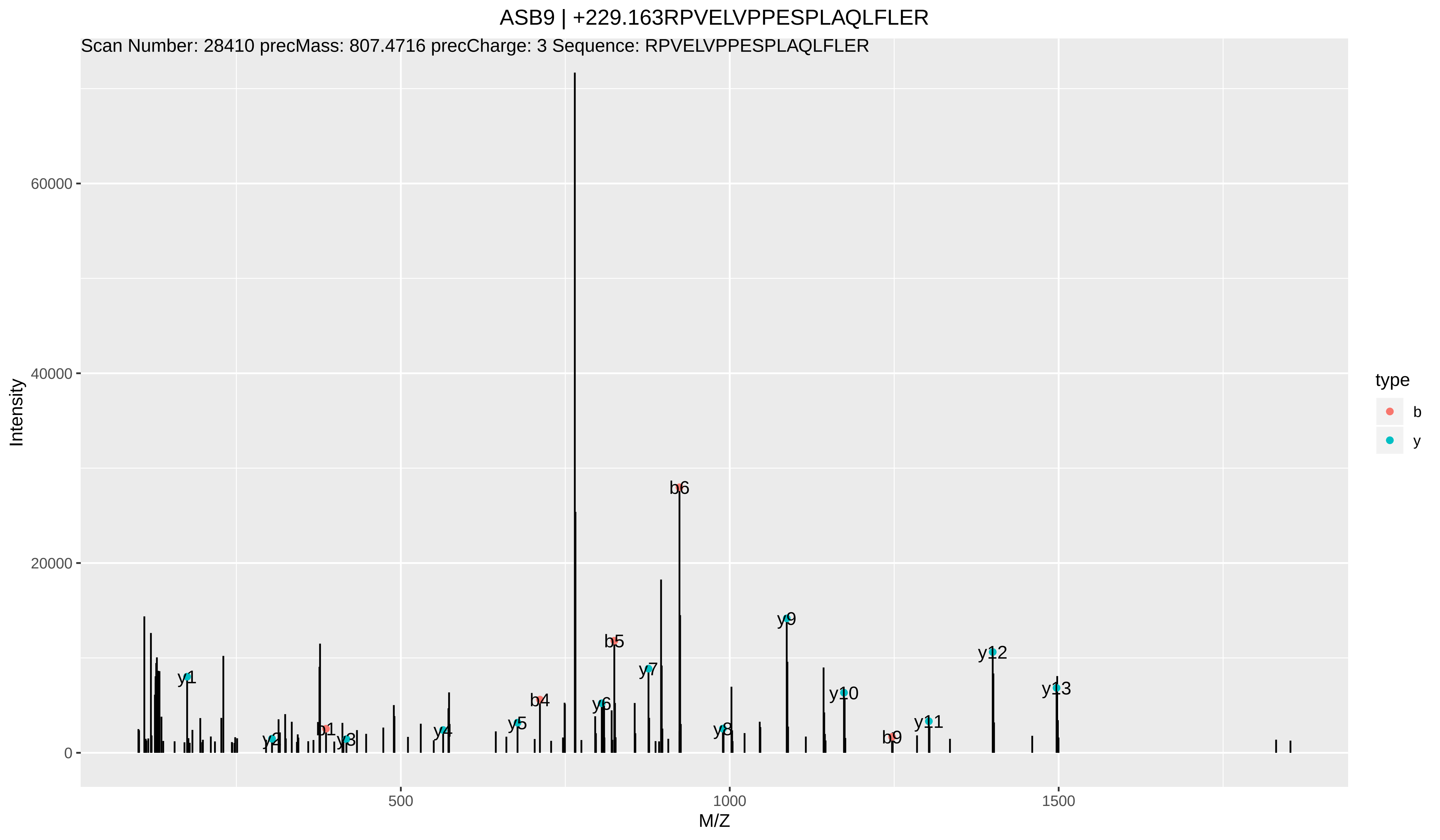

ASB9 | +229.163RPVELVPPESPLAQLFLER
Scan Number: 28410 precMass: 807.4716 precCharge: 3 Sequence: RPVELVPPESPLAQLFLER
Intensity
60000
40000
20000
0
500
1000
1500
M/Z
y1
y2
b1
y3
y4
y5
b4
y6
b5
y7
b6
y8
y9
y10
b9
y11
y12
y13
type
b
y

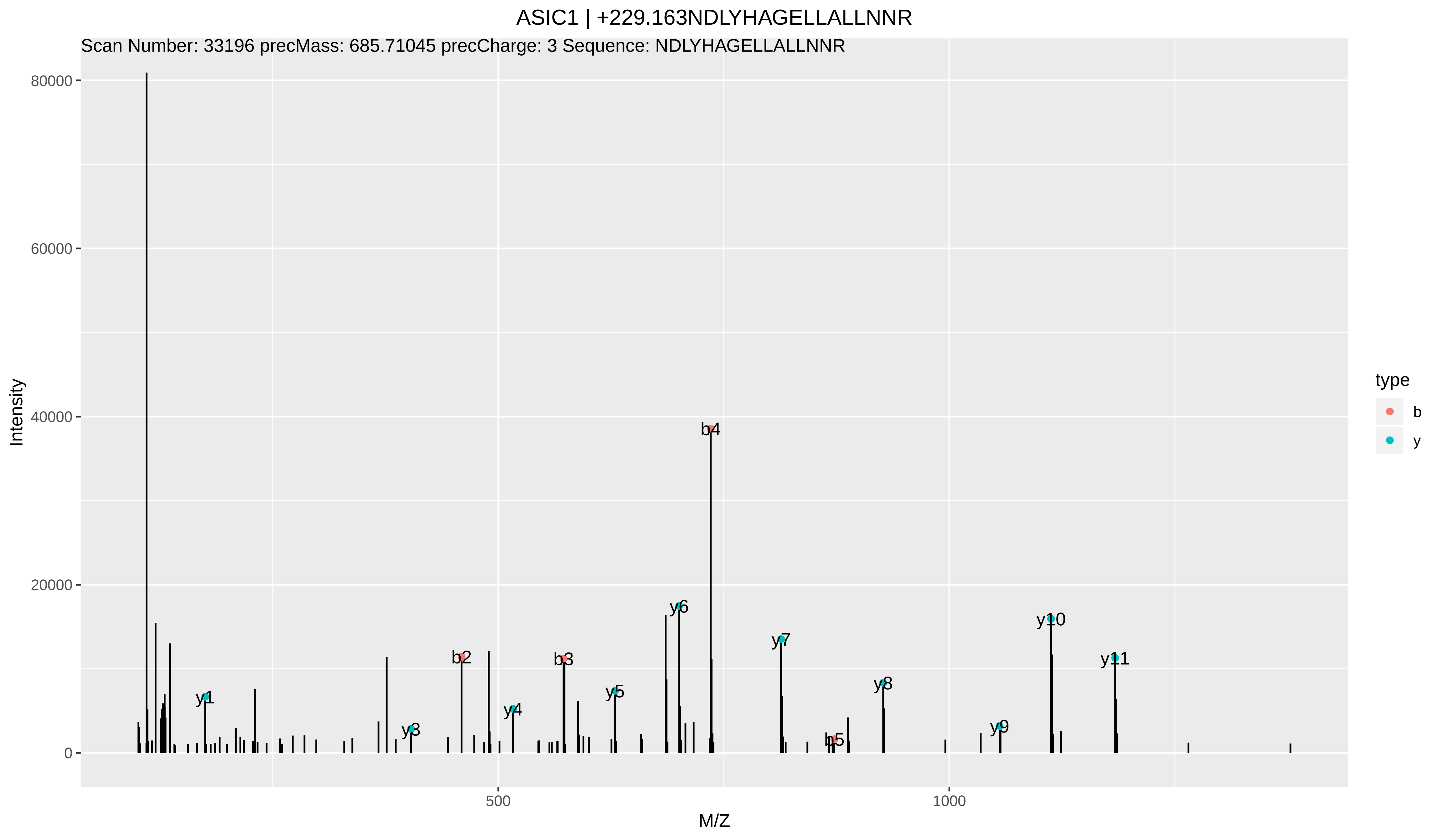

ASIC1 | +229.163NDLYHAGELLALLNNR
Scan Number: 33196 precMass: 685.71045 precCharge: 3 Sequence: NDLYHAGELLALLNNR
Intensity
M/Z
0
20000
40000
60000
80000
500
1000
y1
y3
b2
y4
b3
y5
y6
b4
y7
b5
y8
y9
y10
y11
type
b
y

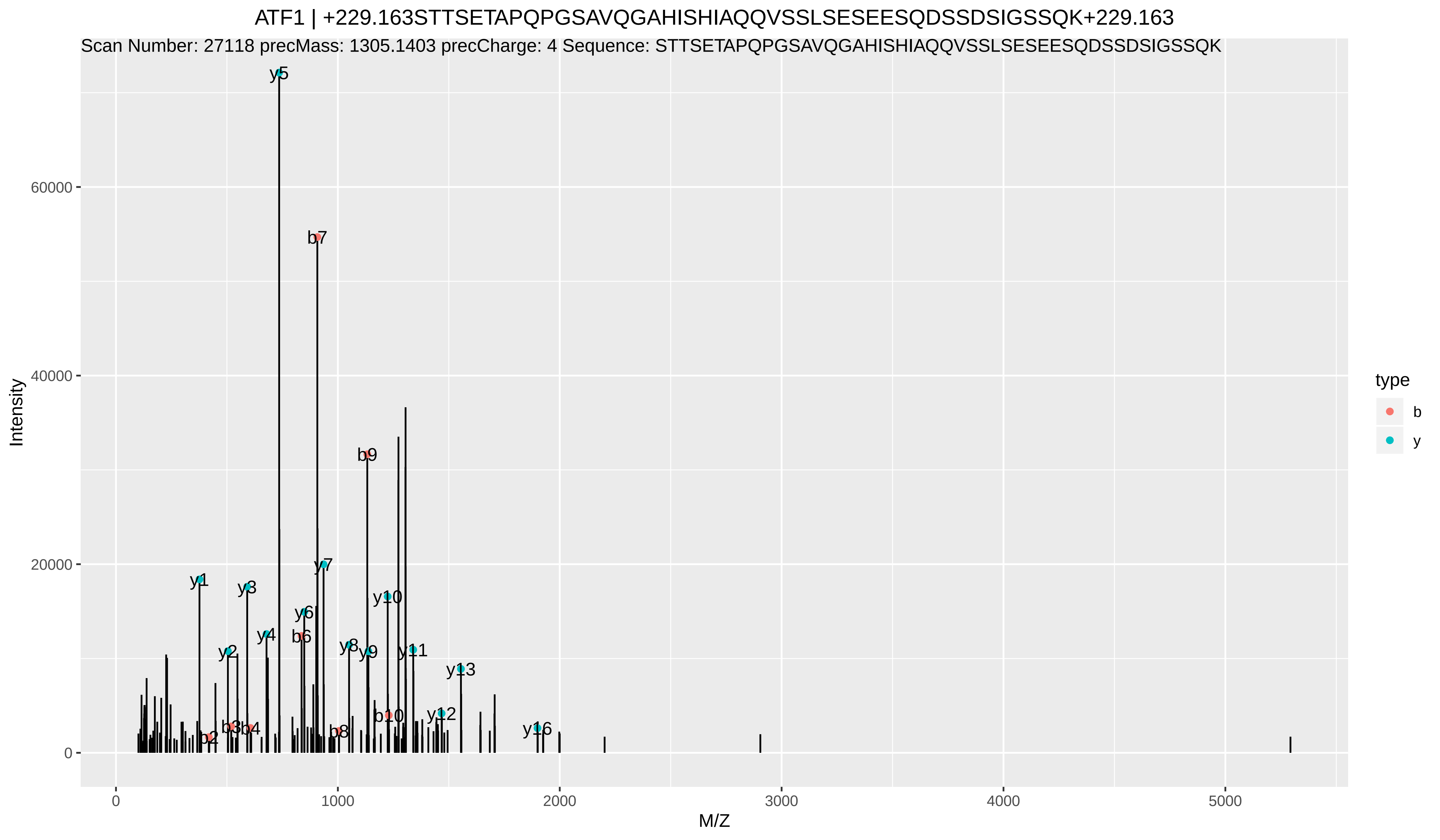

ATF1 | +229.163STTSETAPQPGSAVQGAHISHIAQQVSSLSESEESQDSSDSIGSSQK+229.163
Scan Number: 27118 precMass: 1305.1403 precCharge: 4 Sequence: STTSETAPQPGSAVQGAHISHIAQQVSSLSESEESQDSSDSIGSSQK
Intensity
M/Z
type
b
y
y5
b7
b9
y1
y3
y7
y10
y6
y4
b6
y2
y8
y9
y11
y13
b10
y12
y16
b2
b3
b4
b8

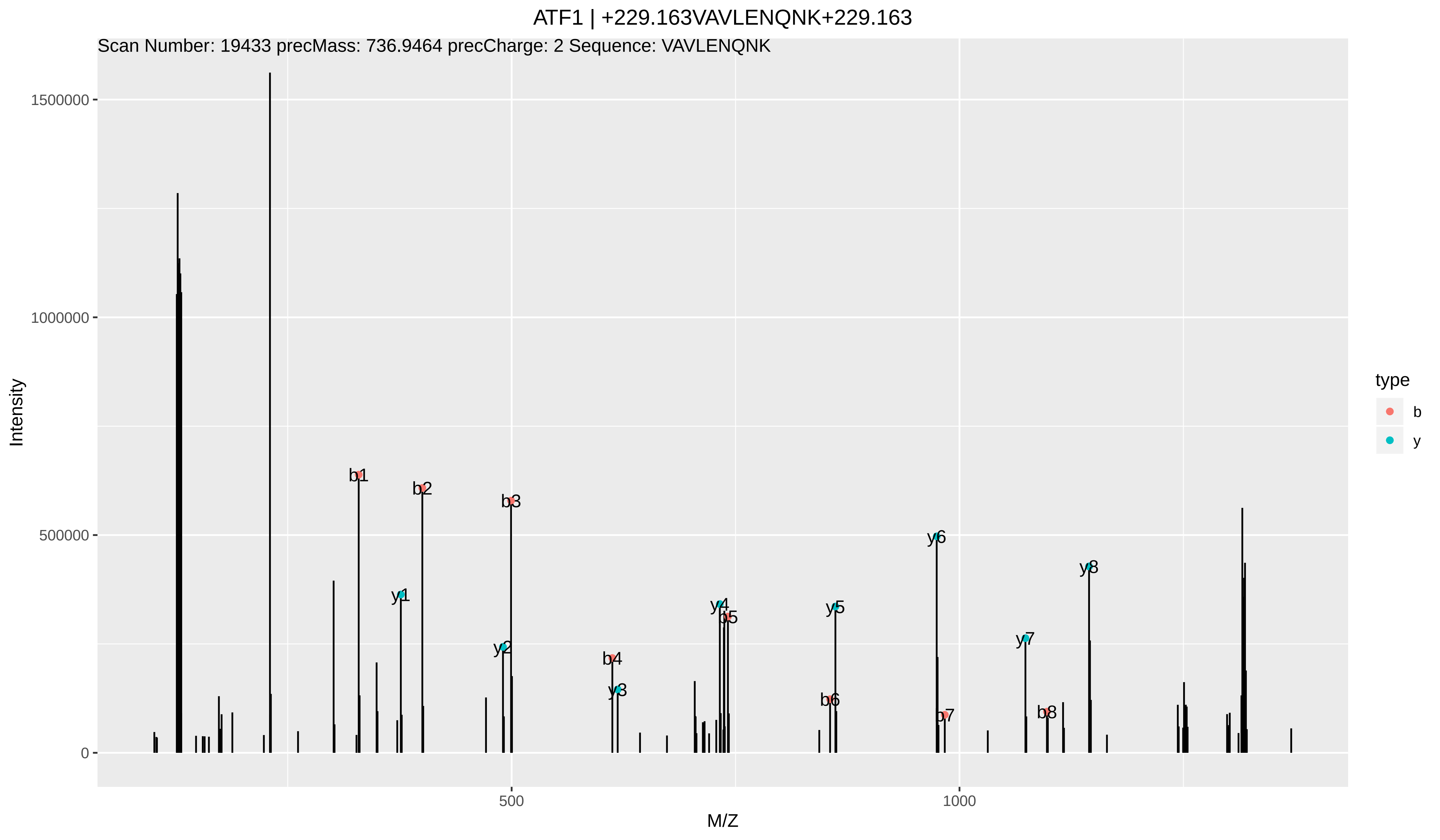
ATF1 | +229.163VAVLENQNK+229.163
Scan Number: 19433 precMass: 736.9464 precCharge: 2 Sequence: VAVLENQNK
Intensity
M/Z
0
500000
1000000
1500000
500
1000
b1
b2
b3
b4
b5
b6
b7
b8
y1
y2
y3
y4
y5
y6
y7
y8
type
b
y

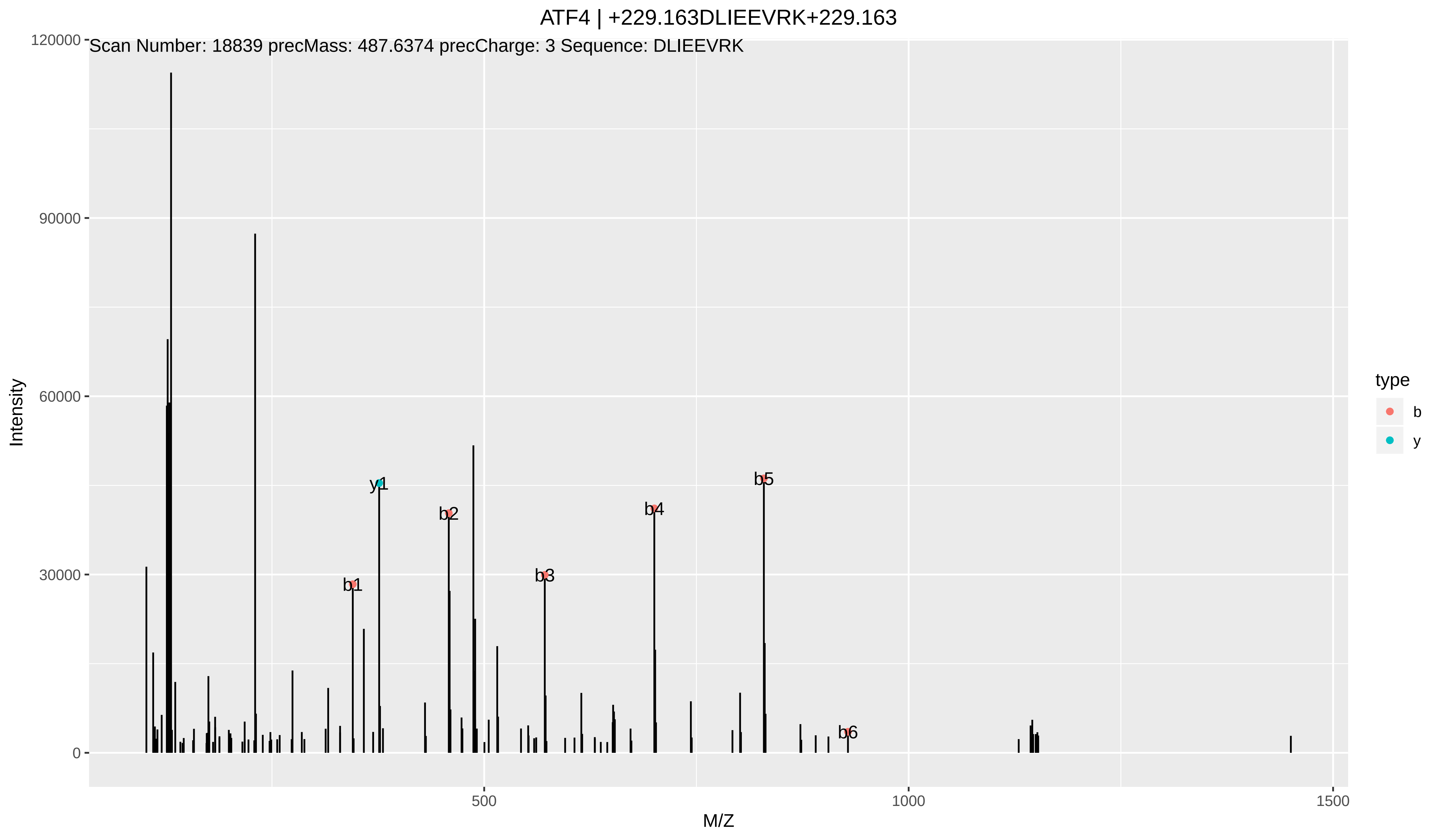

ATF4 | +229.163DLIEEVRK+229.163
Scan Number: 18839 precMass: 487.6374 precCharge: 3 Sequence: DLIEEVRK
Intensity
120000
90000
60000
30000
0
M/Z
500
1000
1500
y1
b1
b2
b3
b4
b5
b6
type
b
y

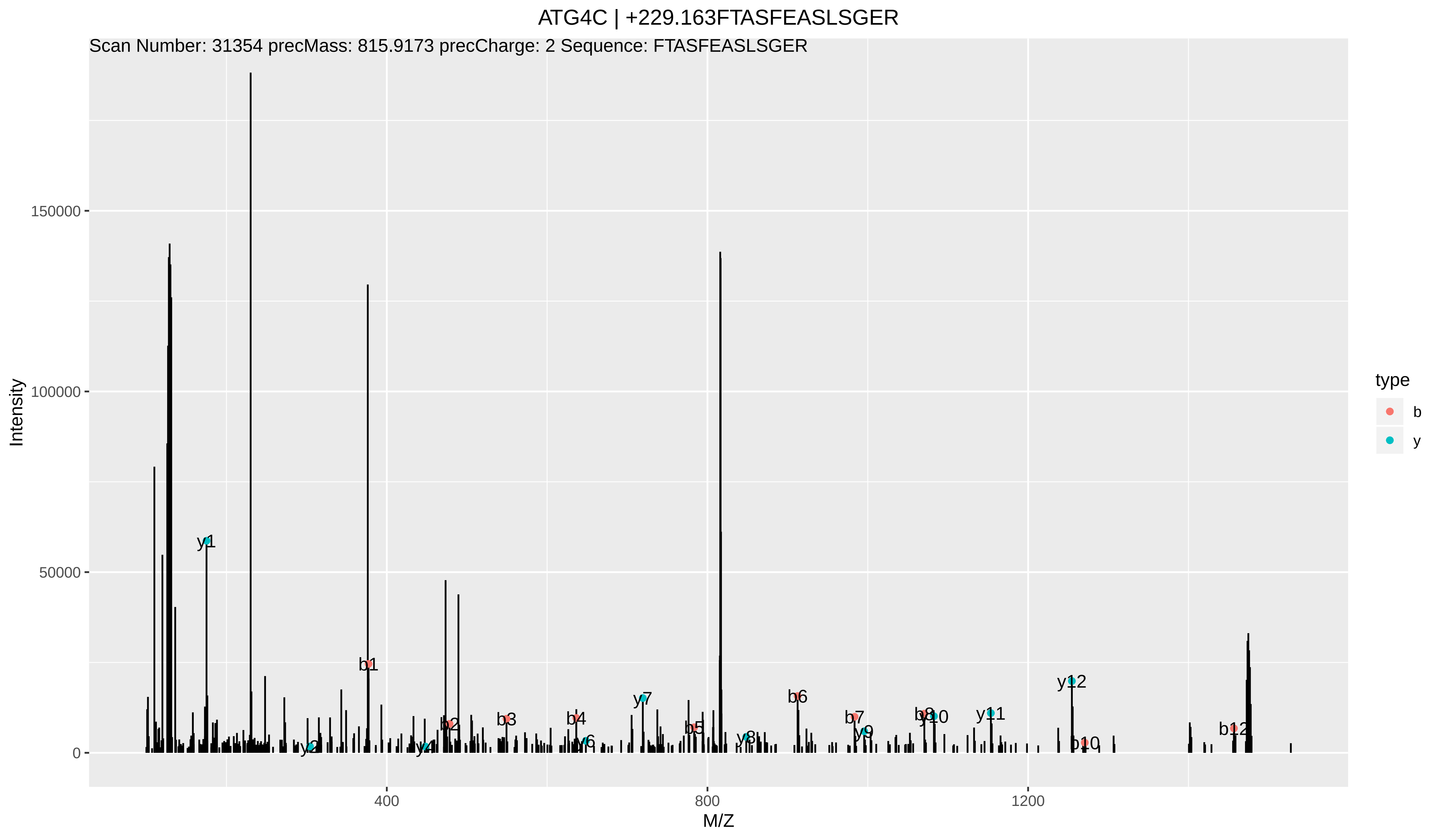
ATG4C | +229.163FTASFEASLSGER
Scan Number: 31354 precMass: 815.9173 precCharge: 2 Sequence: FTASFEASLSGER
Intensity
150000
100000
50000
0
400
800
1200
M/Z
y1
b1
y2
y4
b2
b3
b4
y6
y7
b5
y8
b6
b7
y9
b8
y10
y11
y12
b10
b12
type
b
y

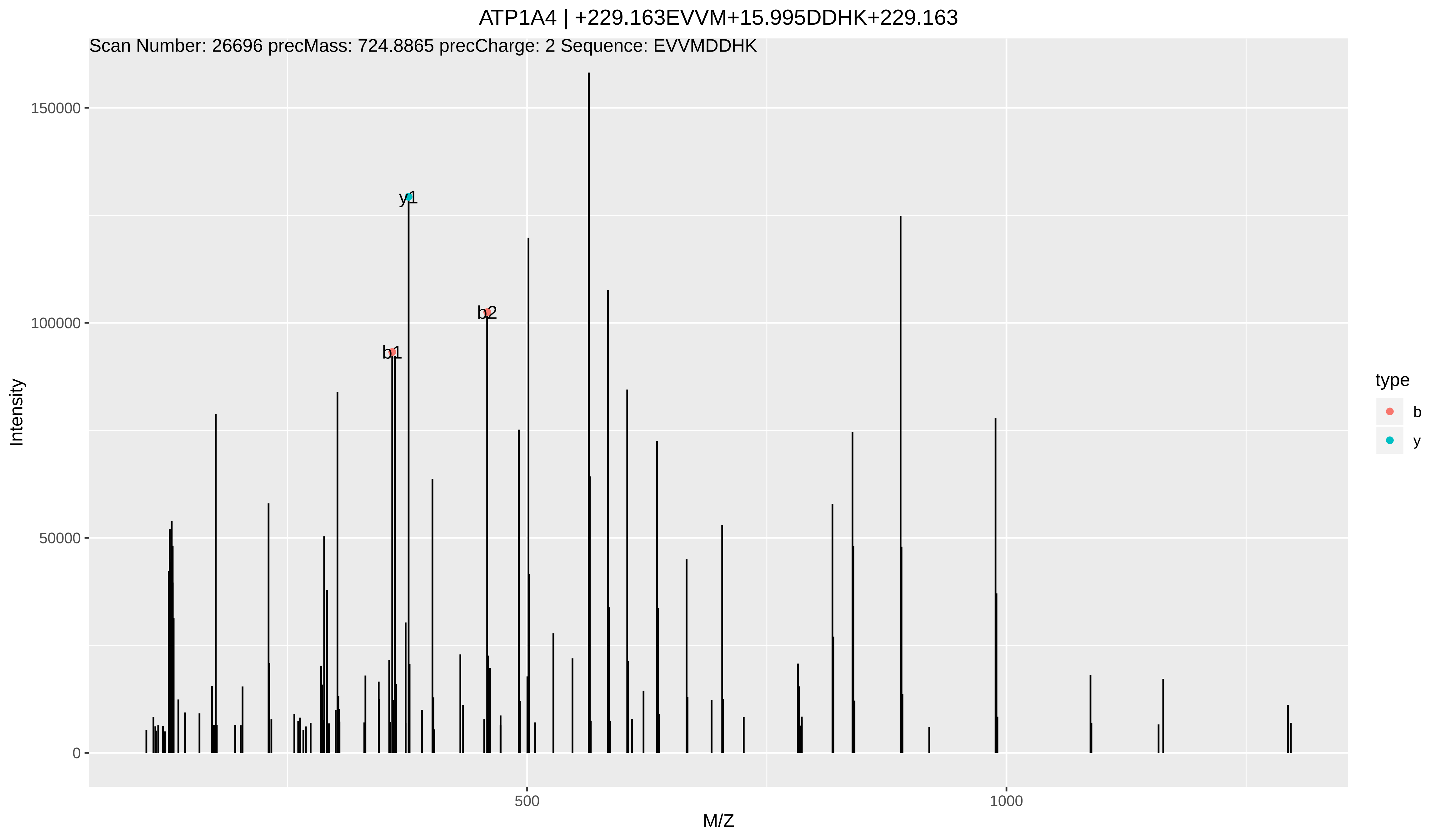

ATP1A4 | +229.163EVVM+15.995DDHK+229.163
Scan Number: 26696 precMass: 724.8865 precCharge: 2 Sequence: EVVMDDHK
y1
b2
b1
150000
100000
50000
0
Intensity
500
1000
M/Z
type
b
y

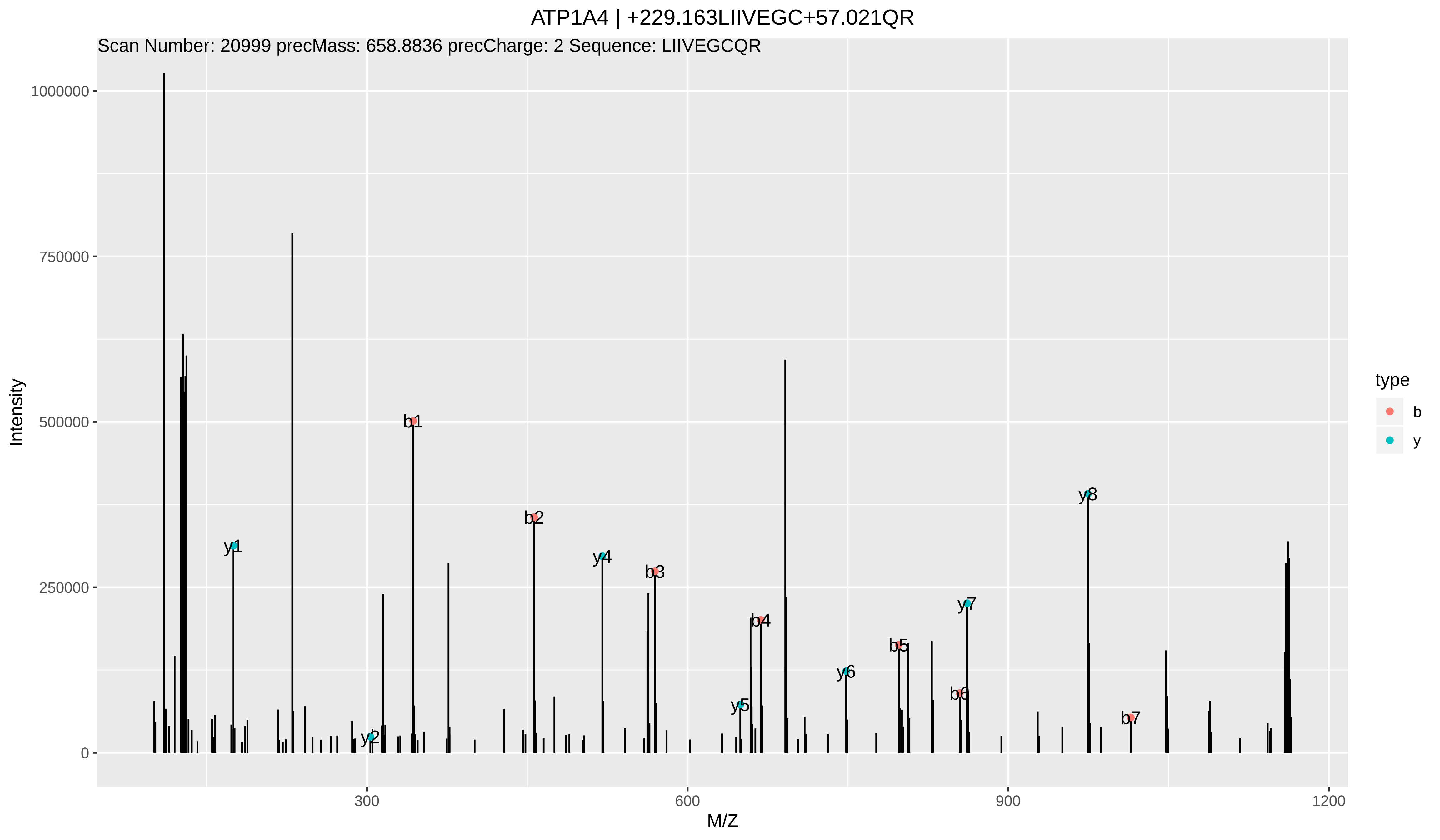

ATP1A4 | +229.163LIIVEGC+57.021QR
Scan Number: 20999 precMass: 658.8836 precCharge: 2 Sequence: LIIVEGCQR
Intensity
1000000
750000
500000
250000
0
300
600
900
1200
M/Z
y1
y2
b1
b2
y4
b3
y5
b4
y6
b5
b6
y7
y8
b7
type
b
y

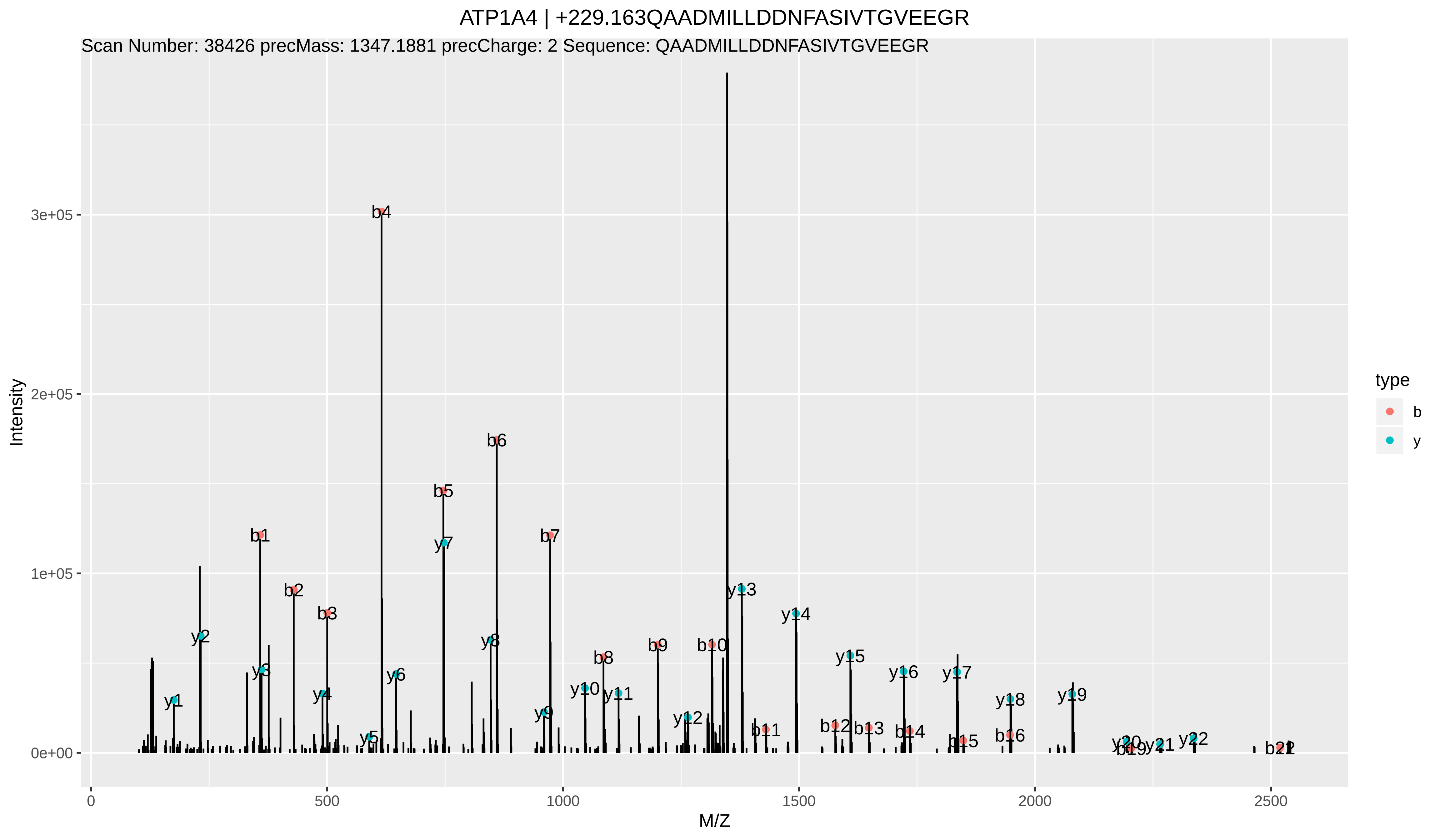

ATP1A4 | +229.163QAADMILLDDNFASIVTGVEEGR
Scan Number: 38426 precMass: 1347.1881 precCharge: 2 Sequence: QAADMILLDDNFASIVTGVEEGR
Intensity
M/Z
type
b
y

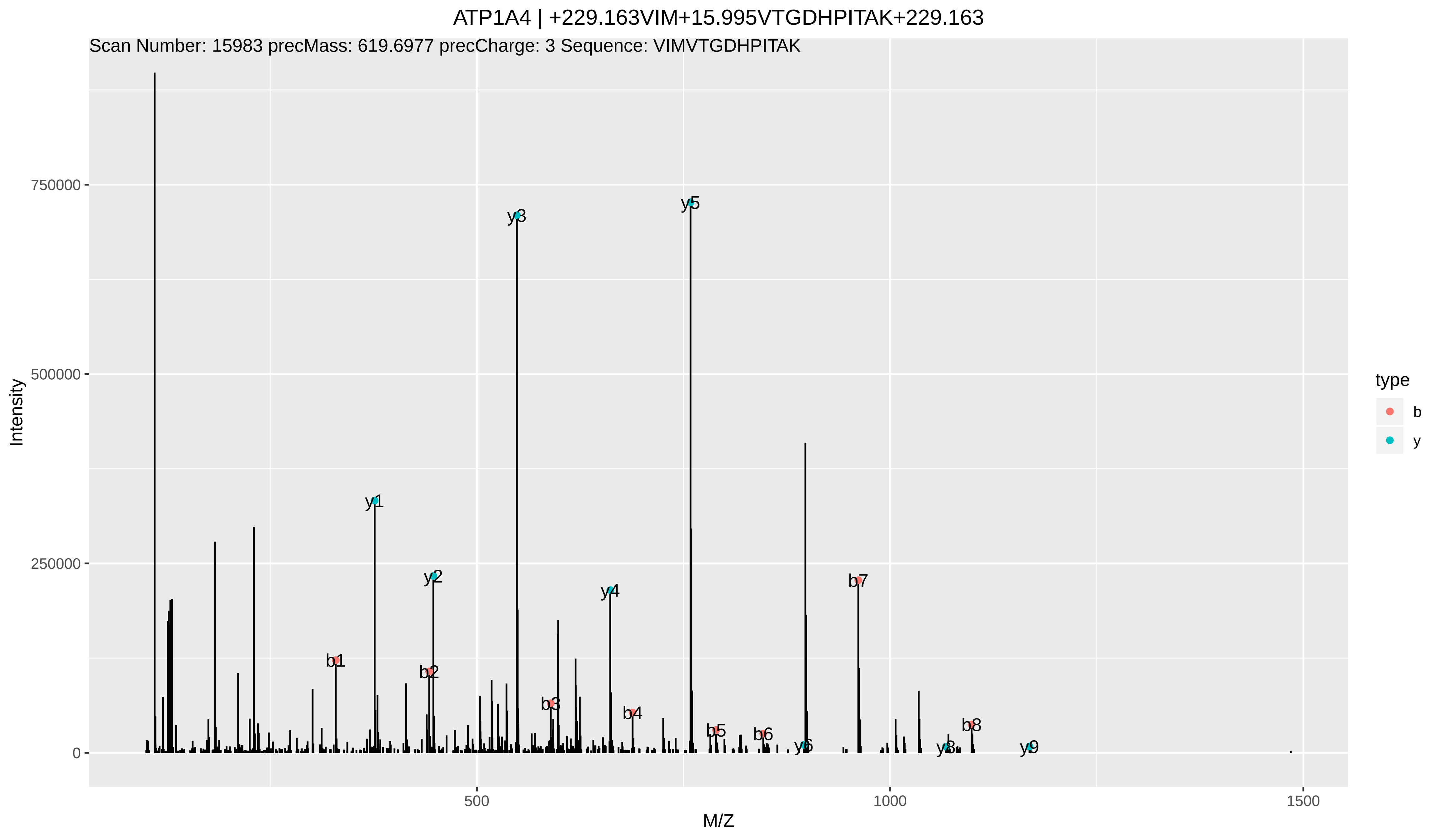

ATP1A4 | +229.163VIM+15.995VTGDHPITAK+229.163
Scan Number: 15983 precMass: 619.6977 precCharge: 3 Sequence: VIMVTGDHPITAK
Intensity
750000
500000
250000
0
M/Z
500
1000
1500
type
b
y
y1
y2
y3
y4
y5
y6
y8
y9
b1
b2
b3
b4
b5
b6
b7
b8

ATP2C2 | +229.163EAANMILVDDDFSAIMNAVEEGK+229.163

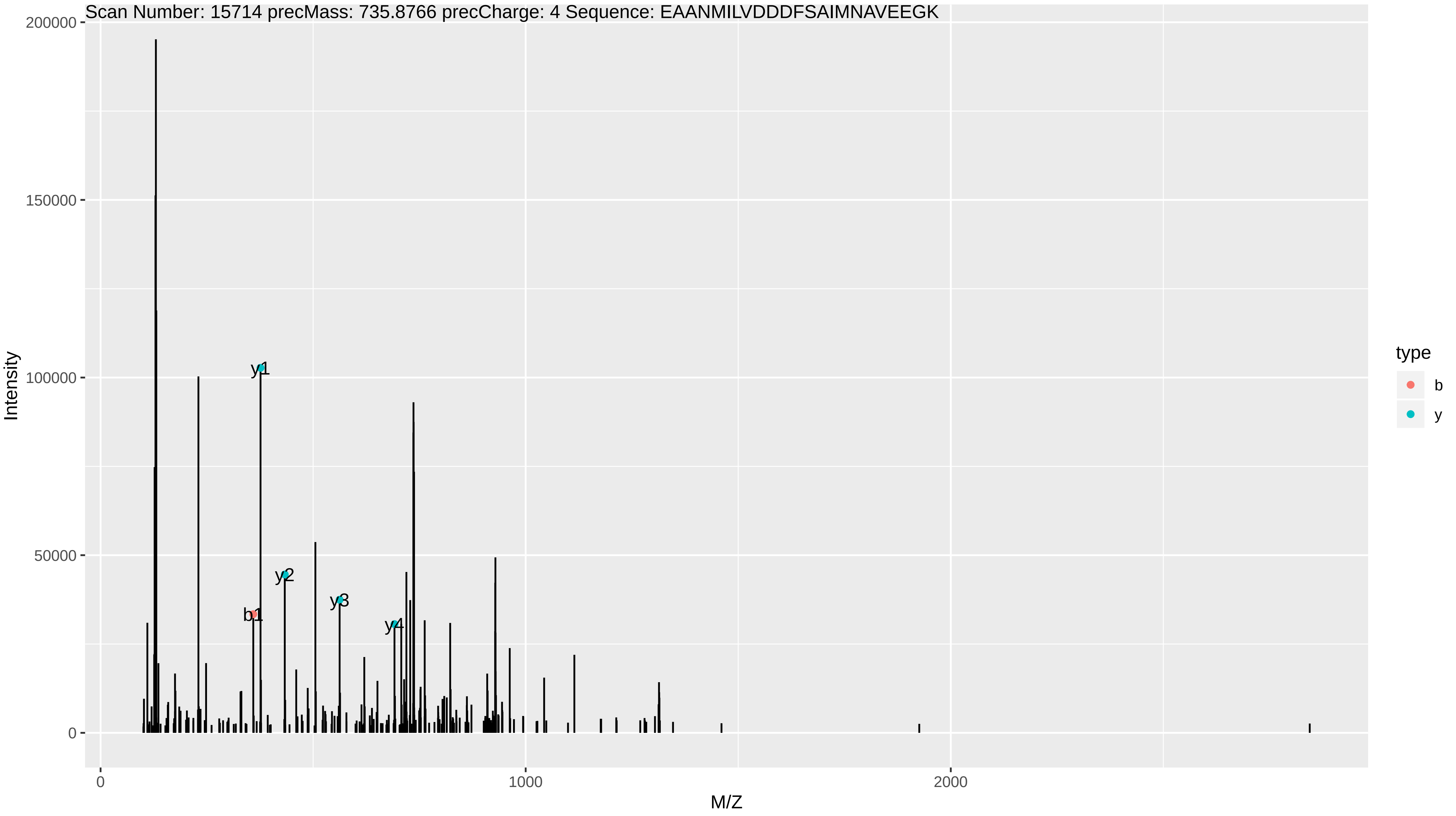

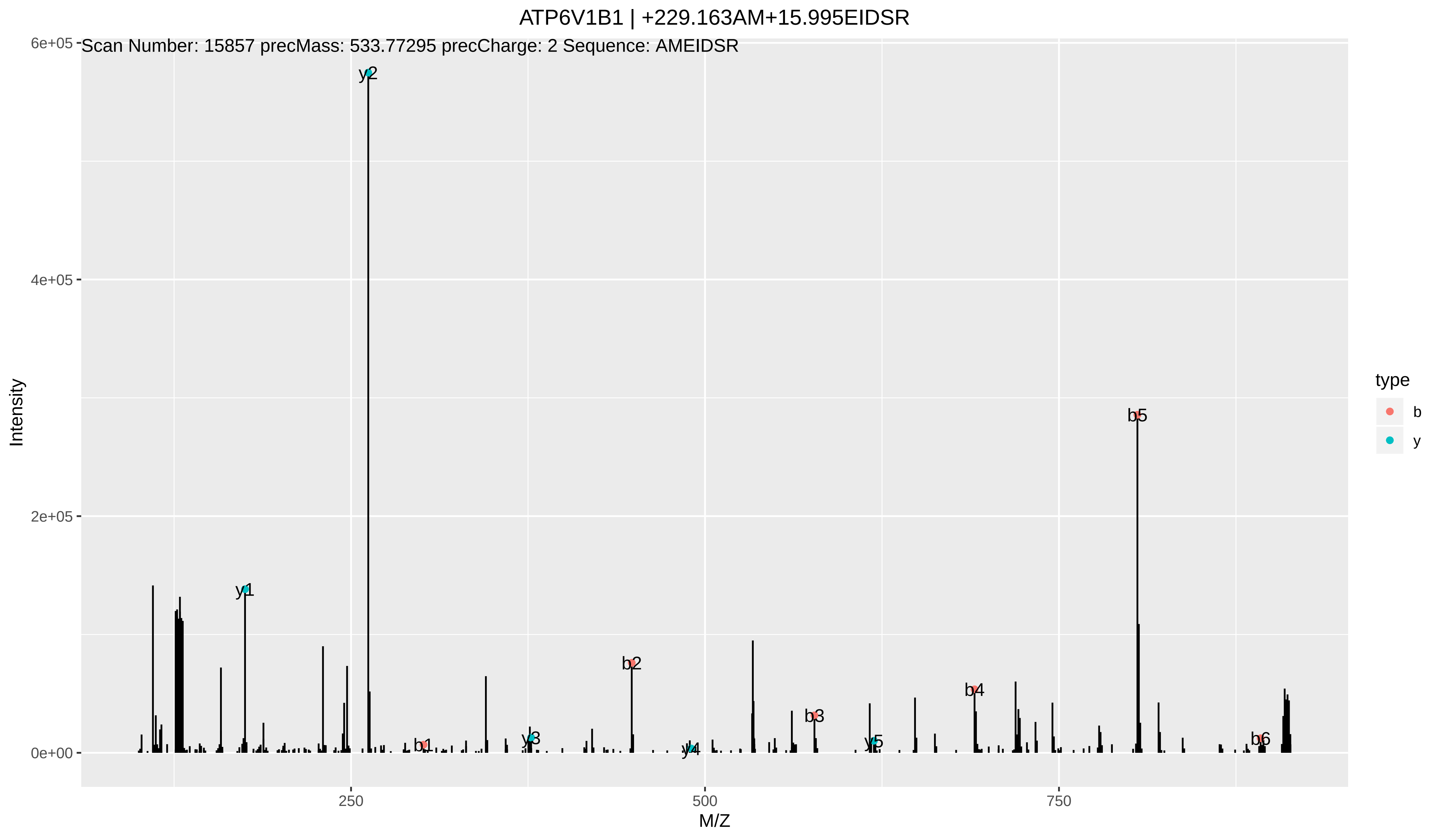
ATP6V1B1 | +229.163AM+15.995EIDSR
Scan Number: 15857 precMass: 533.77295 precCharge: 2 Sequence: AMEIDSR
Intensity
6e+05
4e+05
2e+05
0e+00
M/Z
250
500
750
y1
y2
y3
y4
y5
b1
b2
b3
b4
b5
b6
type
b
y

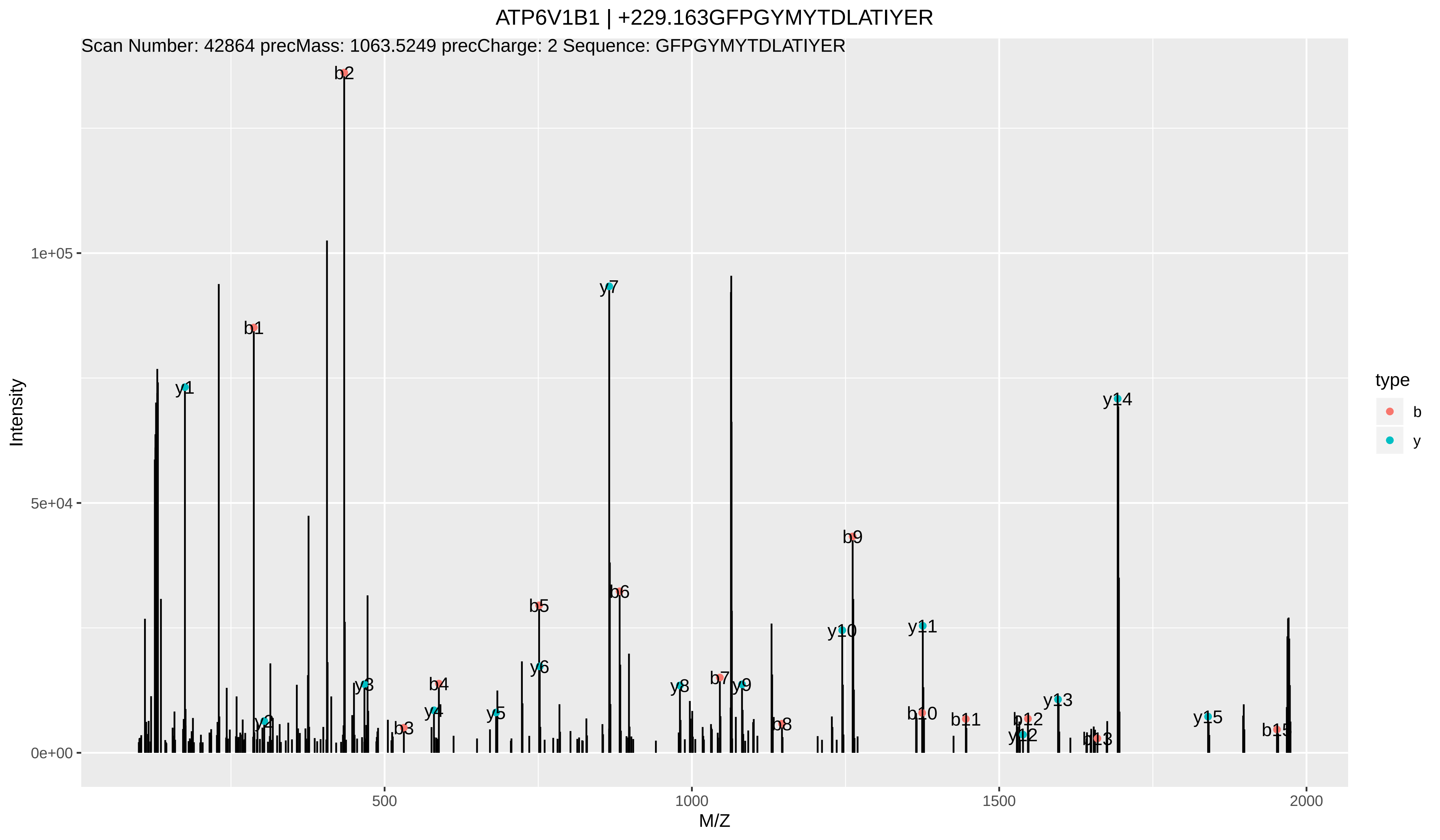

ATP6V1B1 | +229.163GFPGYMYTDLATIYER
Scan Number: 42864 precMass: 1063.5249 precCharge: 2 Sequence: GFPGYMYTDLATIYER
Intensity
M/Z
0e+00
5e+04
1e+05
500
1000
1500
2000
b2
b1
y1
y7
b6
b5
y6
y3
y2
b3
b4
y4
y5
y8
b7
y9
b8
b9
y10
y11
b10
b11
b12
y12
y13
b13
y14
y15
b15
type
b
y

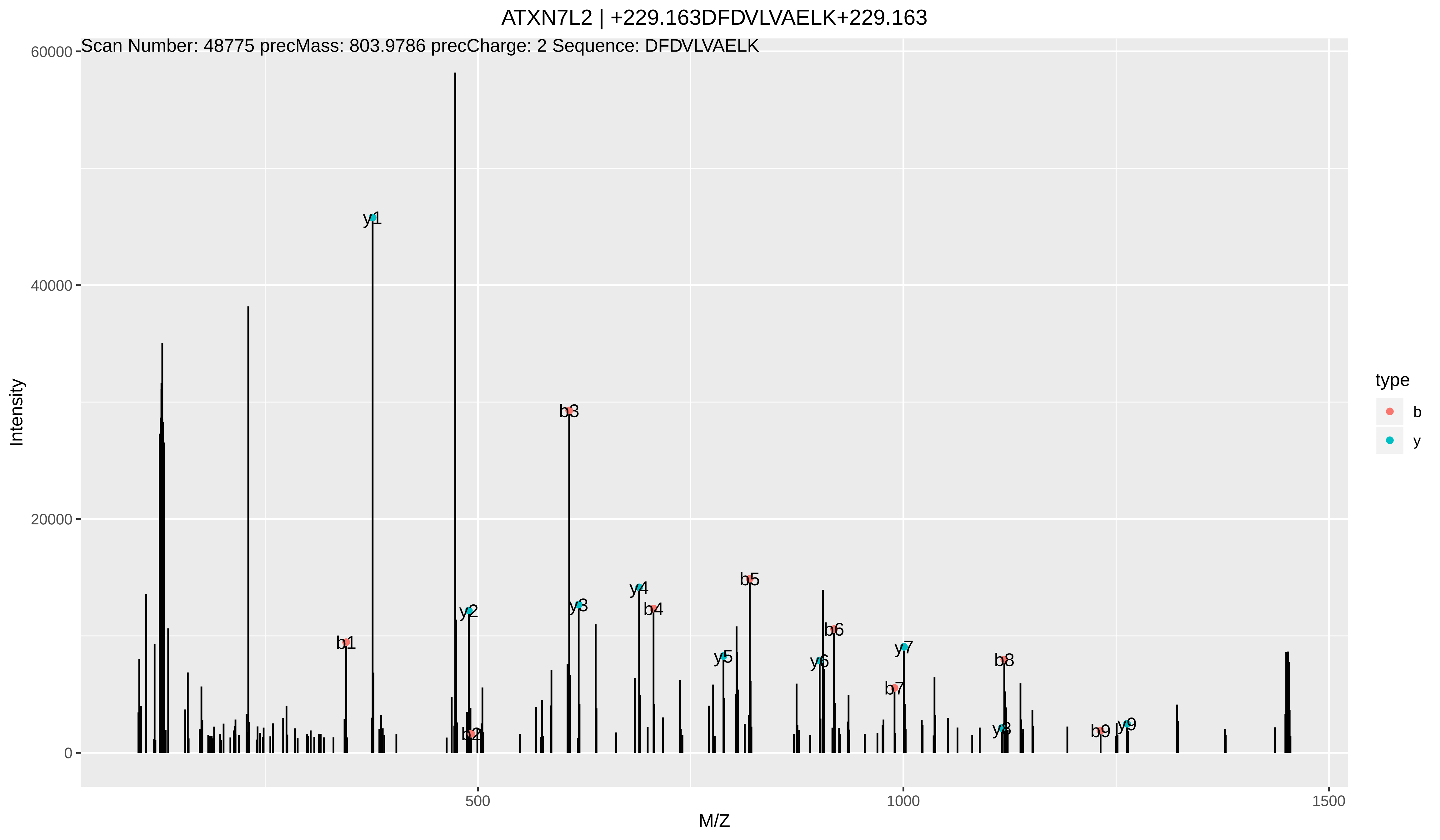
ATXN7L2 | +229.163DFDVLVAELK+229.163
Scan Number: 48775 precMass: 803.9786 precCharge: 2 Sequence: DFDVLVAELK
60000
40000
20000
0
Intensity
500
1000
1500
M/Z
y1
b3
b1
y2
b2
y3
y4
b4
y5
b5
y6
b6
y7
b7
b8
y8
b9
y9
type
b
y

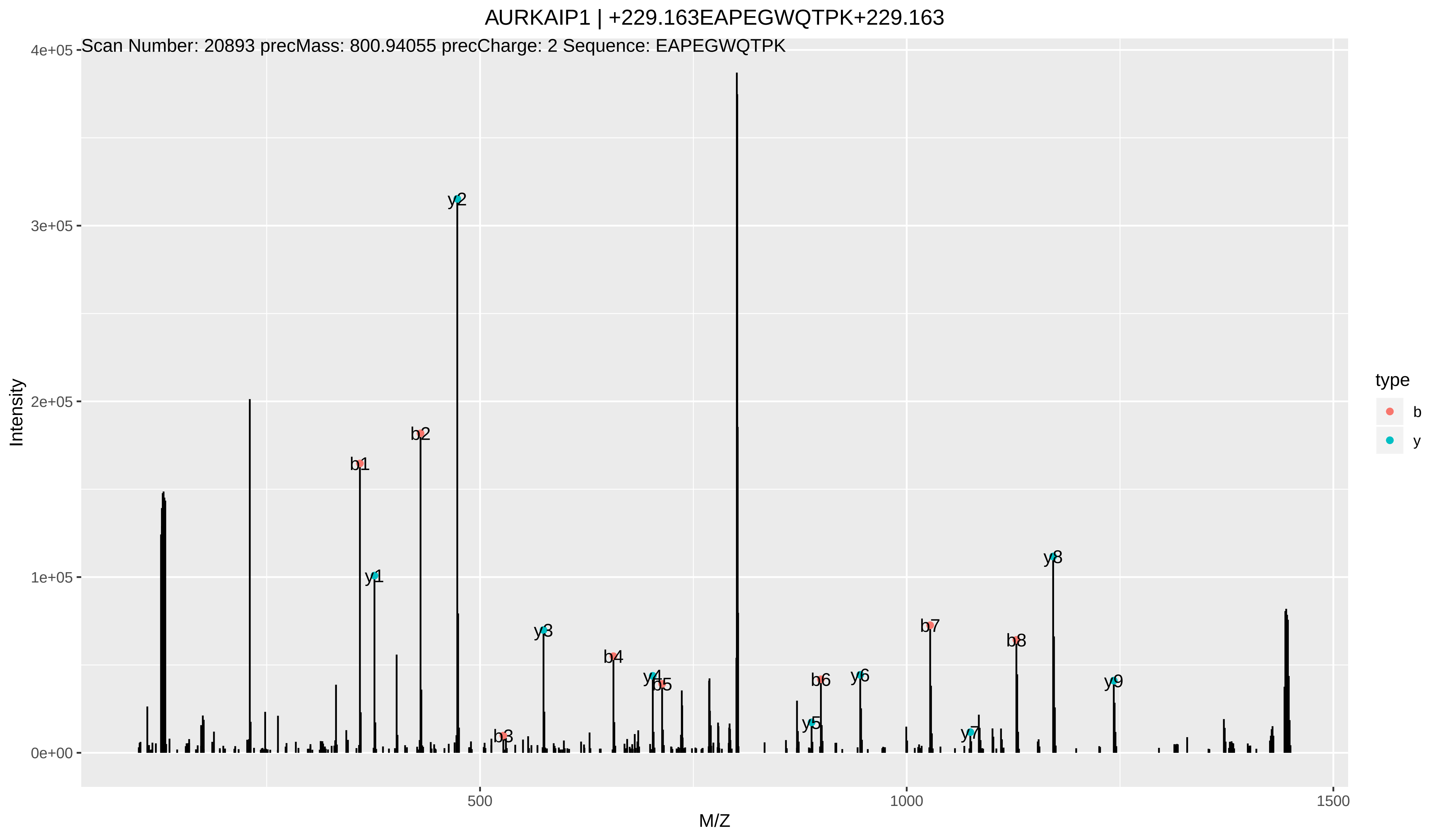
AURKAIP1 | +229.163EAPEGWQTPK+229.163
Scan Number: 20893 precMass: 800.94055 precCharge: 2 Sequence: EAPEGWQTPK
Intensity
M/Z
0e+00
1e+05
2e+05
3e+05
4e+05
500
1000
1500
type
b
y
y2
b2
b1
y1
y3
b3
b4
y4
b5
y5
b6
y6
b7
y7
b8
y8
y9

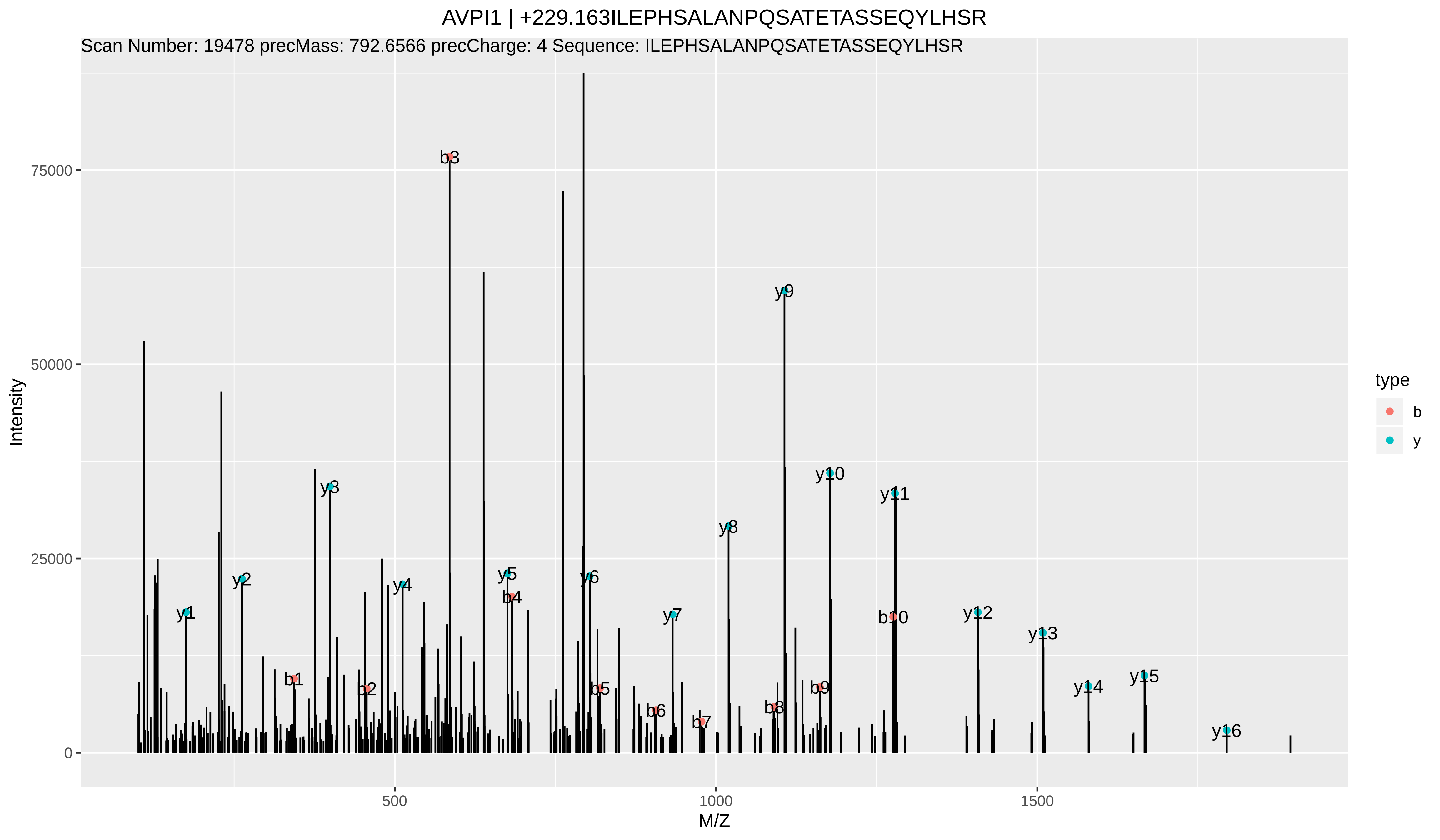
AVPI1 | +229.163ILEPHSALANPQSATETASSEQYLHSR
Scan Number: 19478 precMass: 792.6566 precCharge: 4 Sequence: ILEPHSALANPQSATETASSEQYLHSR
Intensity
M/Z
type
b
y
75000
50000
25000
0
500
1000
1500
b1
b2
b3
b4
b5
b6
b7
b8
b9
b10
y1
y2
y3
y4
y5
y6
y7
y8
y9
y10
y11
y12
y13
y14
y15
y16

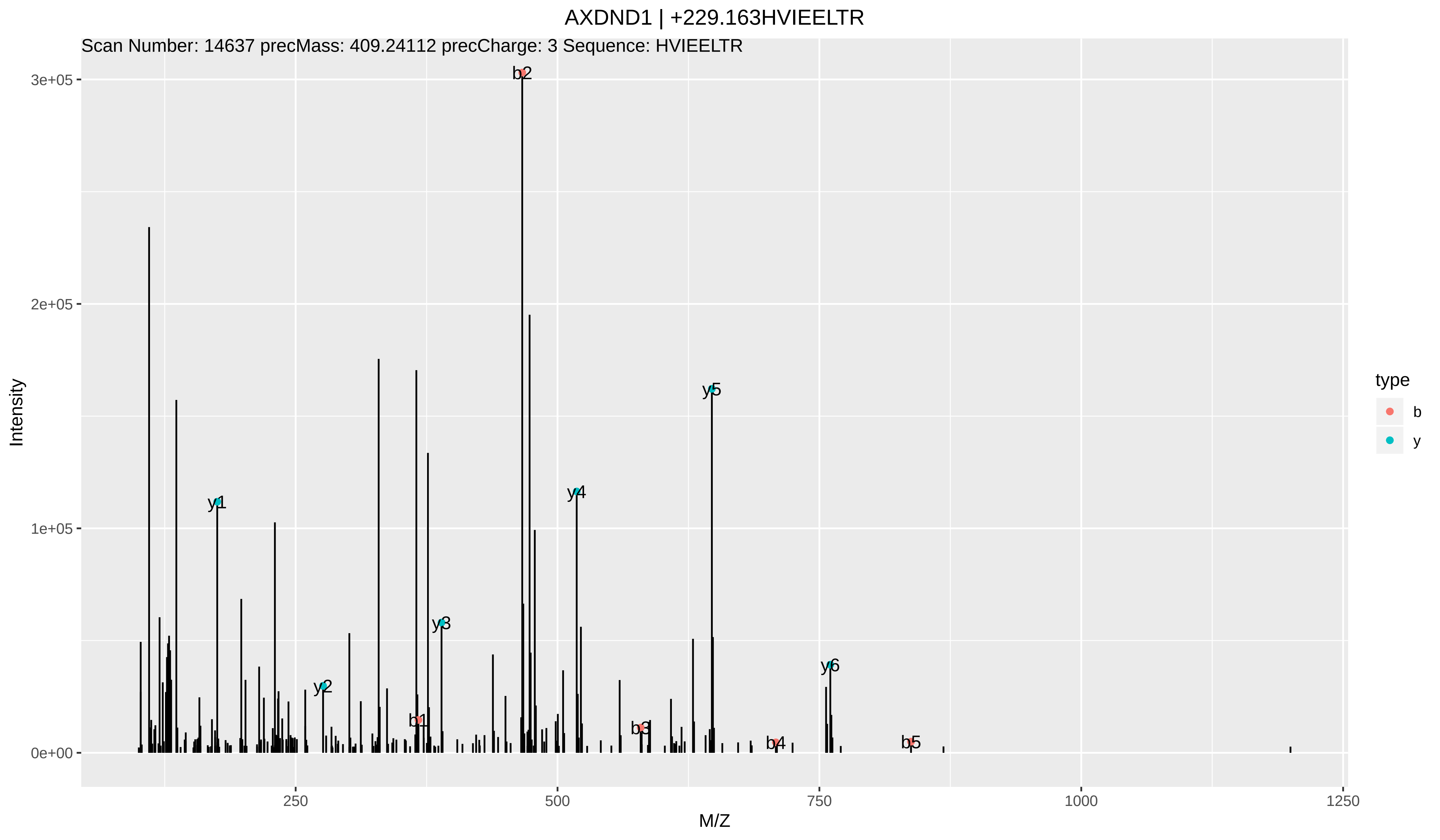

AXDND1 | +229.163HVIEELTR
Scan Number: 14637 precMass: 409.24112 precCharge: 3 Sequence: HVIEELTR
Intensity
M/Z
3e+05
2e+05
1e+05
0e+00
250
500
750
1000
1250
b2
y1
y2
y3
y4
y5
y6
b1
b3
b4
b5
type
b
y

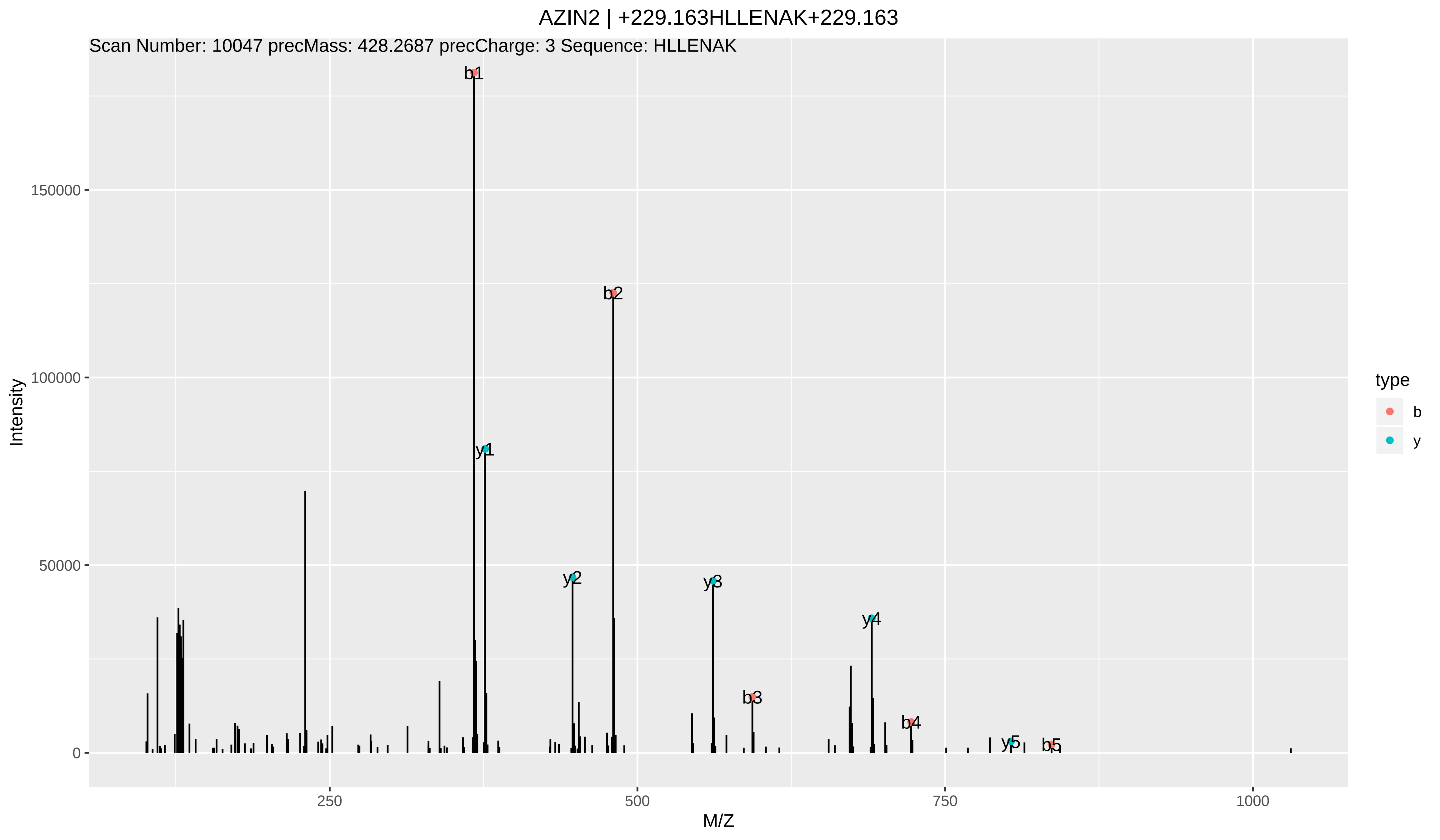

AZIN2 | +229.163HLLENAK+229.163
Scan Number: 10047 precMass: 428.2687 precCharge: 3 Sequence: HLLENAK
b1
b2
y1
y2
y3
b3
y4
b4
y5
b5
Intensity
150000
100000
50000
0
250
500
750
1000
M/Z
type
b
y

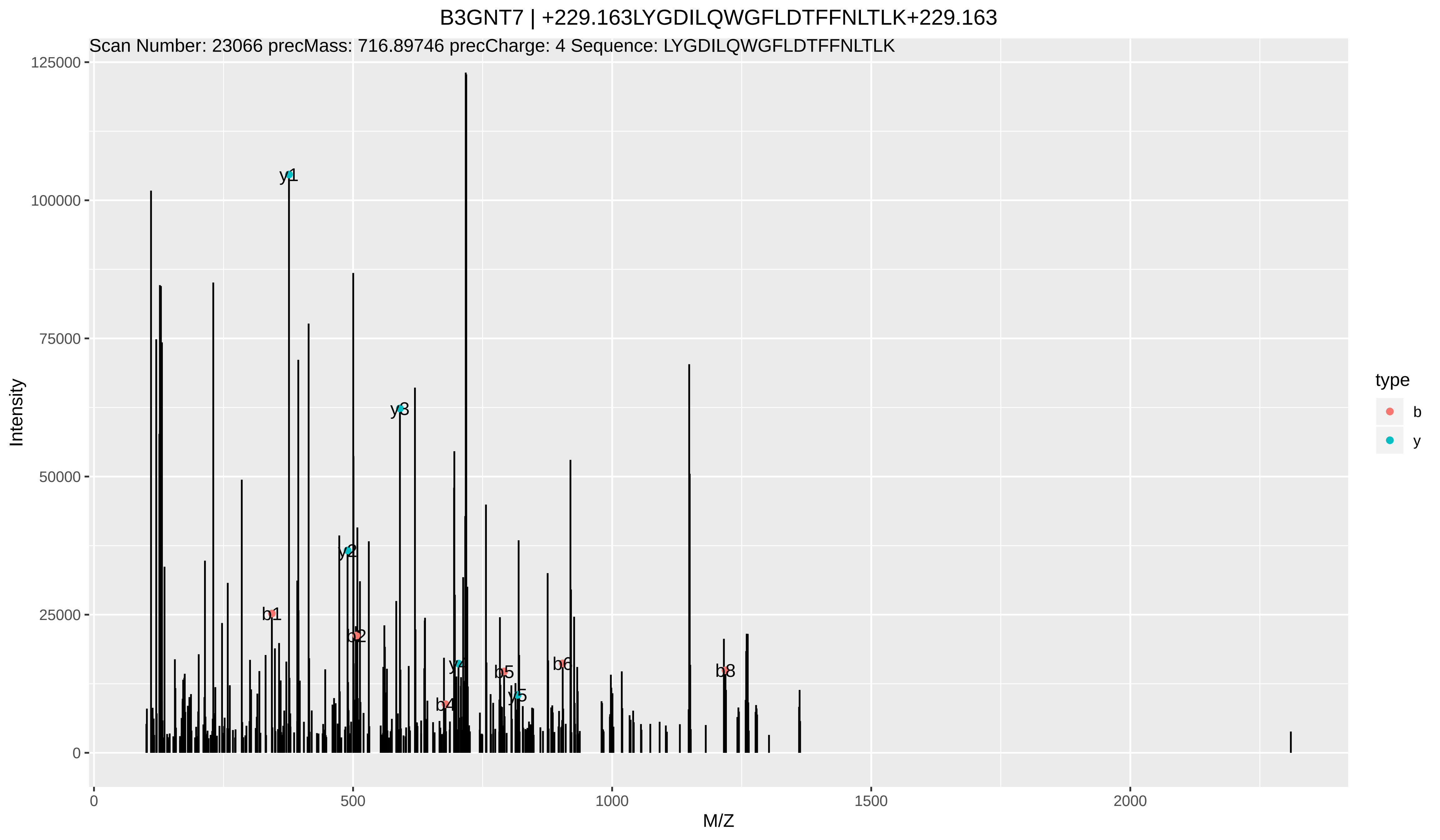
B3GNT7 | +229.163LYGDILQWGFLDTFFNLTLK+229.163
Scan Number: 23066 precMass: 716.89746 precCharge: 4 Sequence: LYGDILQWGFLDTFFNLTLK
Intensity
M/Z
type
b
y
y1
y2
y3
y4
y5
b1
b2
b4
b5
b6
b8
0
25000
50000
75000
100000
125000
0
500
1000
1500
2000

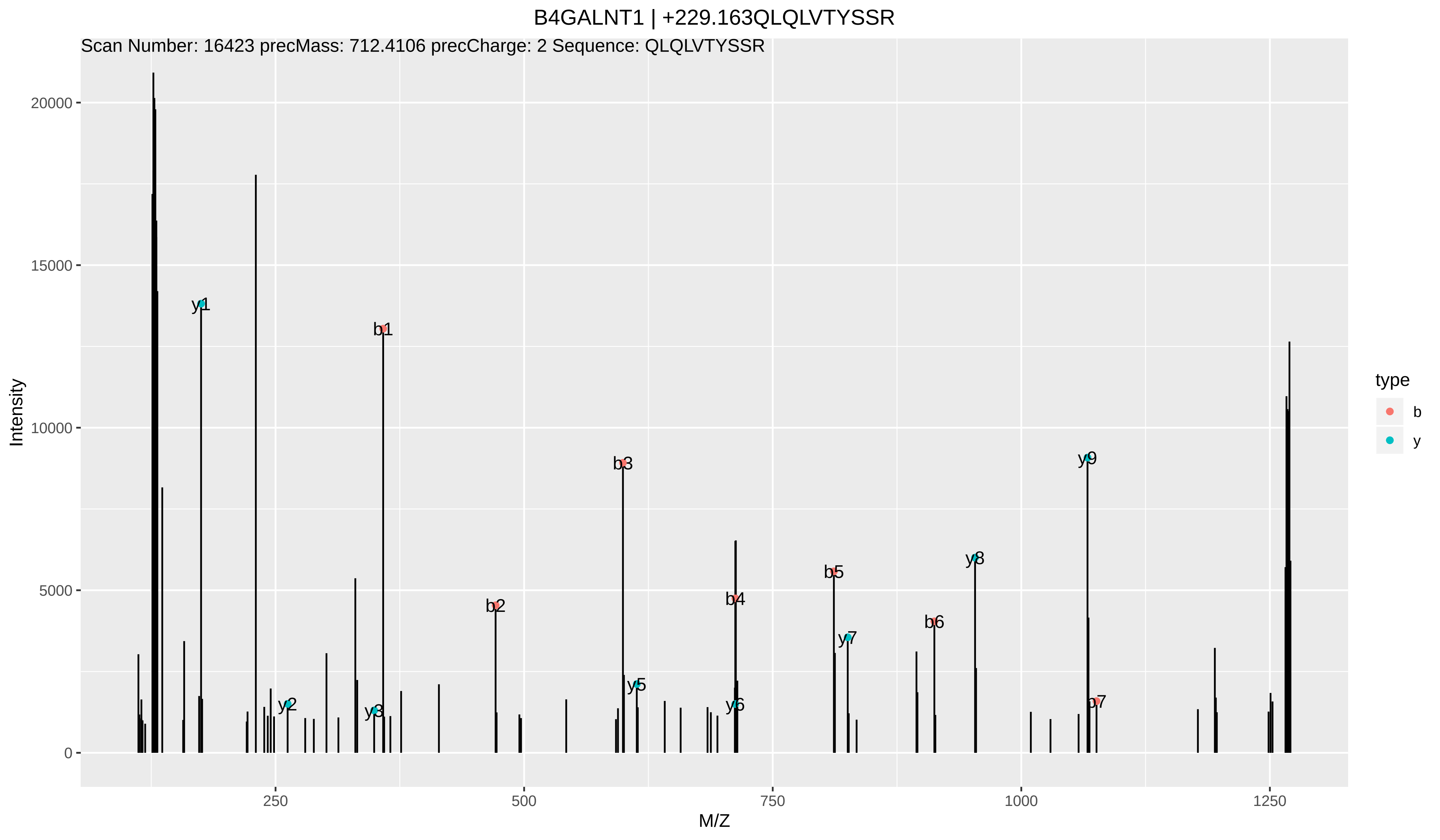
B4GALNT1 | +229.163QLQLVTYSSR
Scan Number: 16423 precMass: 712.4106 precCharge: 2 Sequence: QLQLVTYSSR
Intensity
M/Z
y1
b1
b2
b3
b4
b5
b6
b7
y2
y3
y5
y6
y7
y8
y9
type
b
y
0
5000
10000
15000
20000
250
500
750
1000
1250

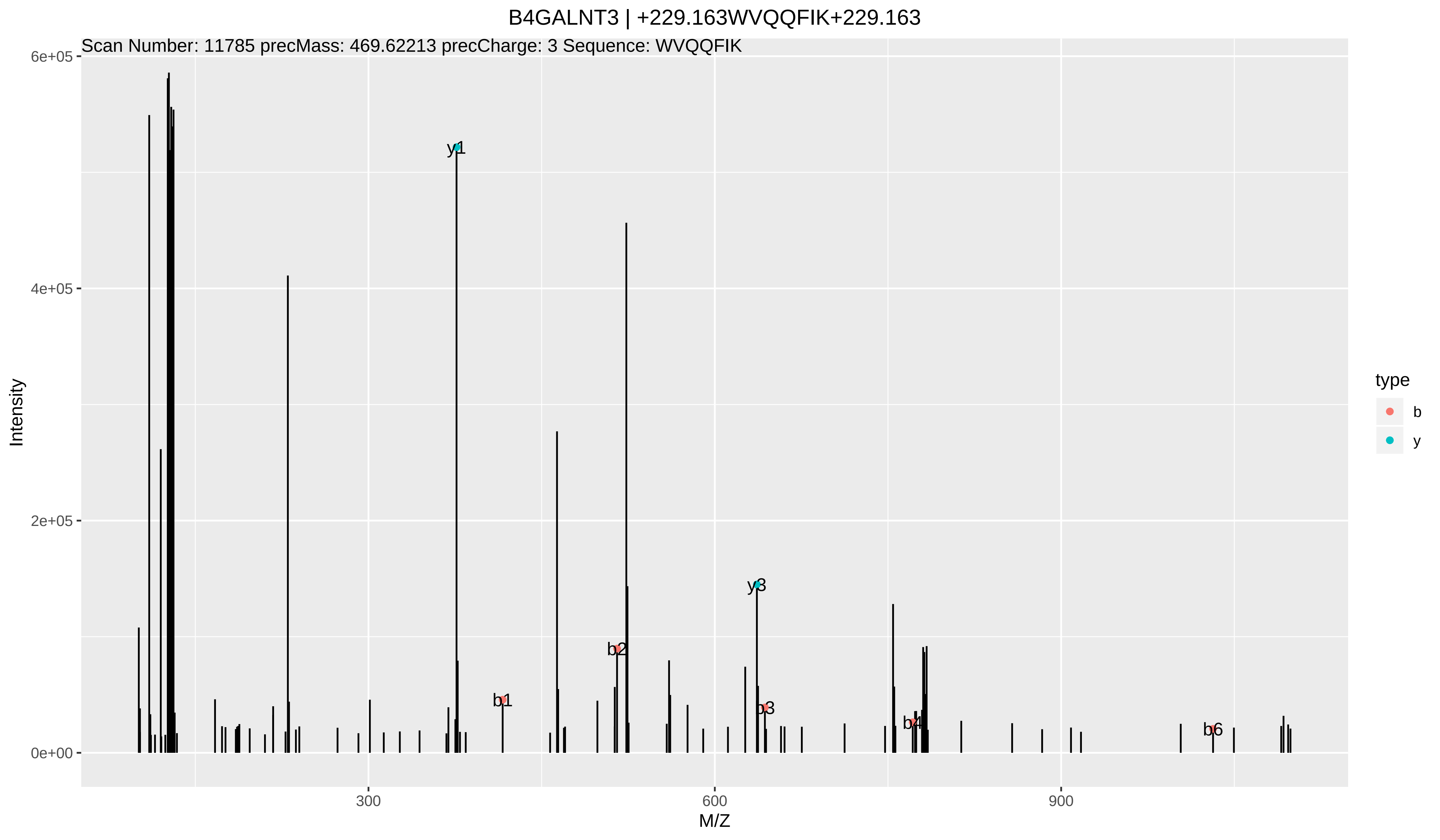

B4GALNT3 | +229.163WVQQFIK+229.163
Scan Number: 11785 precMass: 469.62213 precCharge: 3 Sequence: WVQQFIK
Intensity
6e+05
4e+05
2e+05
0e+00
M/Z
300
600
900
y1
y3
b1
b2
b3
b4
b6
type
b
y

B9D1 | +229.163GYGAVHVPFSPGR

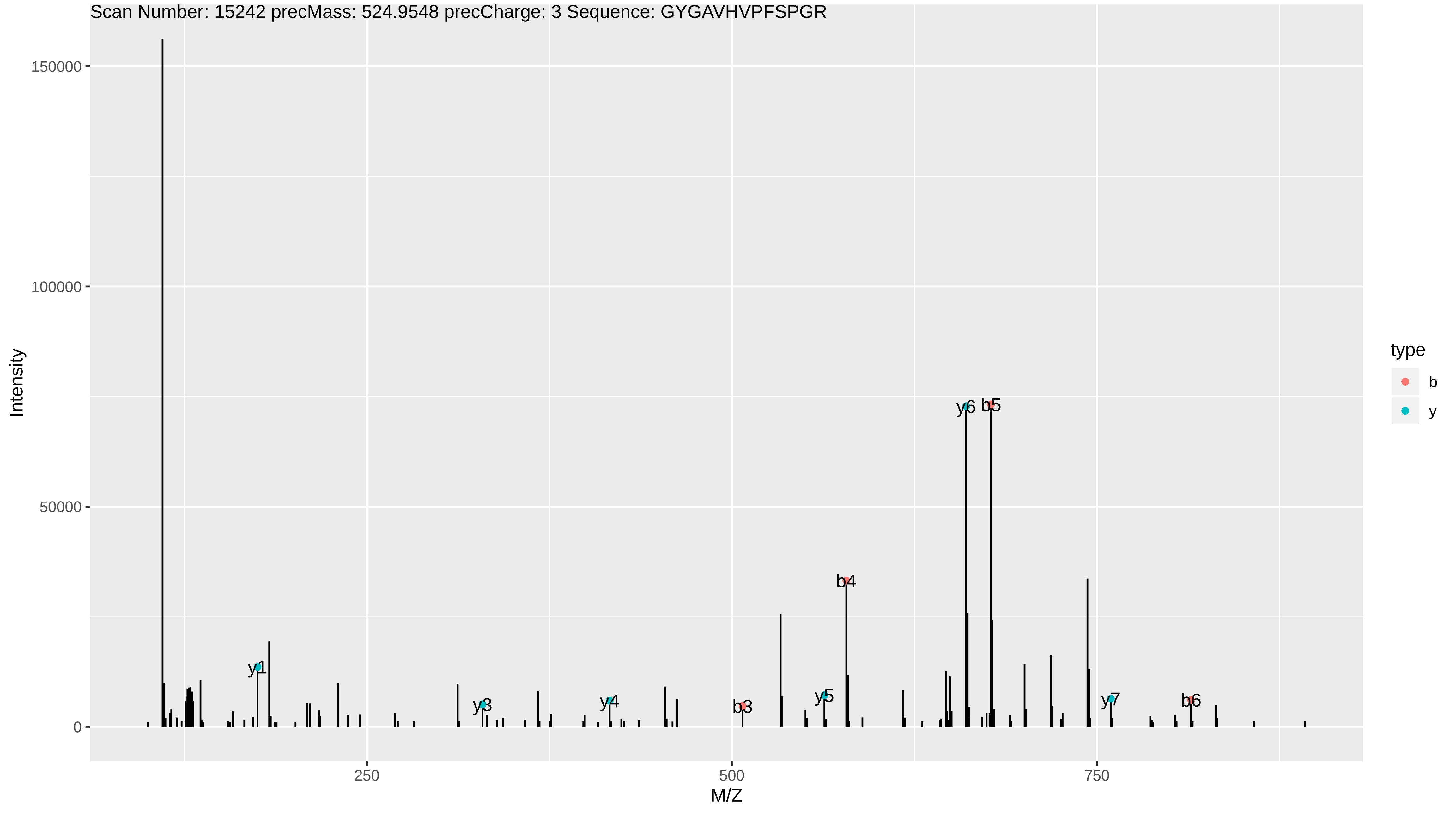

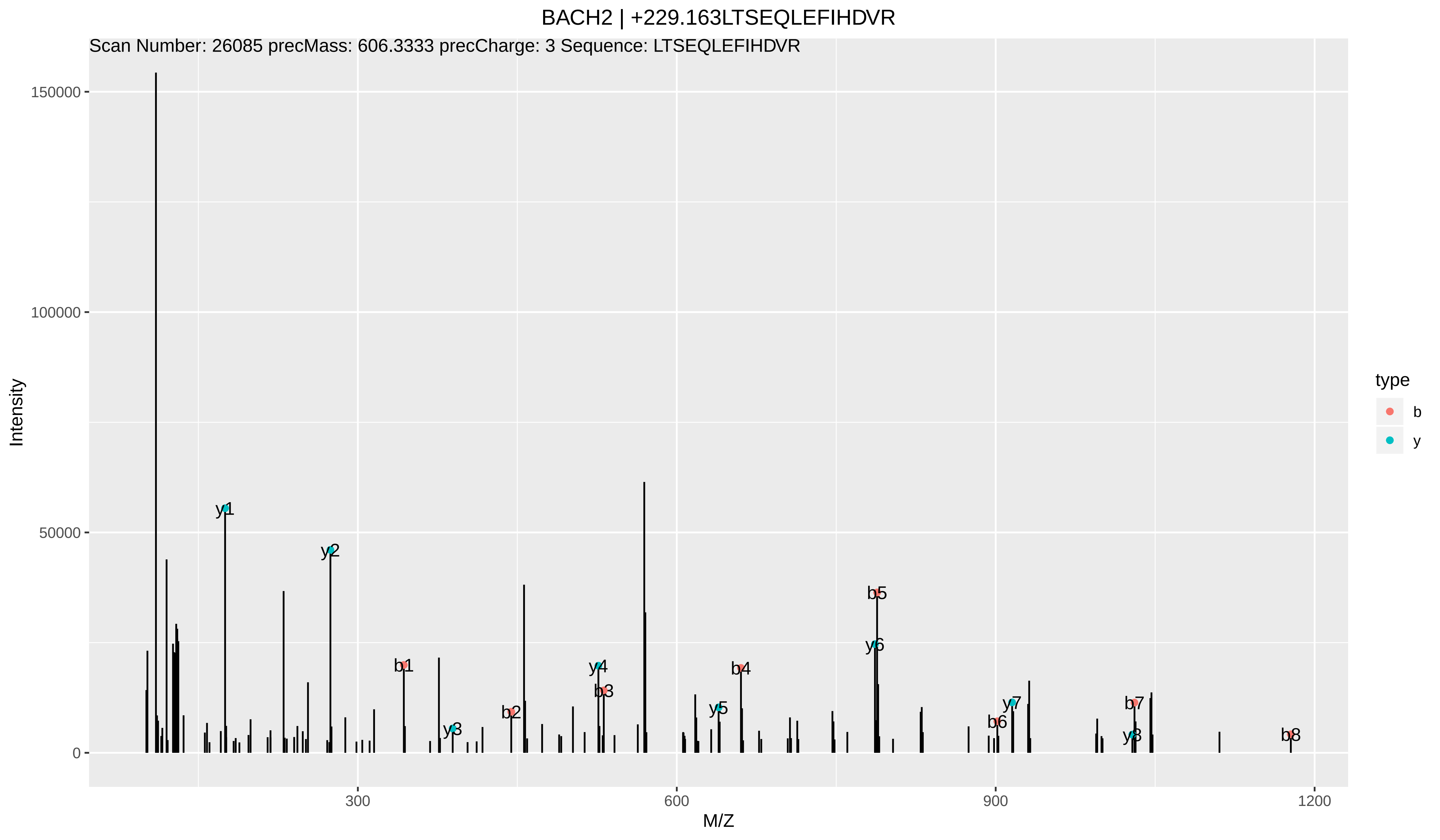
BACH2 | +229.163LTSEQLEFIHDVR
Scan Number: 26085 precMass: 606.3333 precCharge: 3 Sequence: LTSEQLEFIHDVR
Intensity
150000
100000
50000
0
M/Z
300
600
900
1200
y1
y2
b1
y3
b2
y4
b3
y5
b4
b5
y6
b6
y7
b7
y8
b8
type
b
y

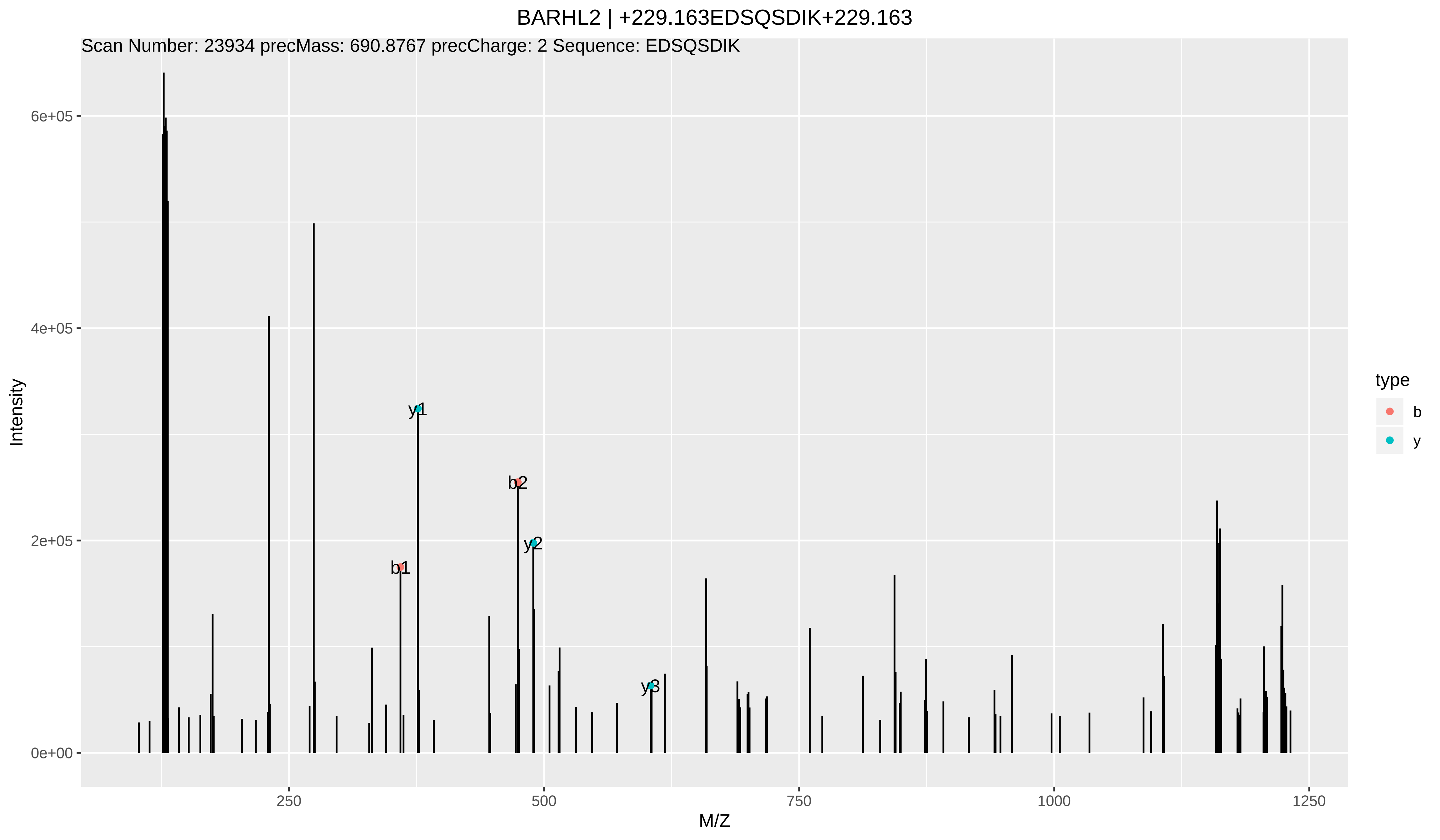

BARHL2 | +229.163EDSQSDIK+229.163
Scan Number: 23934 precMass: 690.8767 precCharge: 2 Sequence: EDSQSDIK
Intensity
M/Z
6e+05
4e+05
2e+05
0e+00
250
500
750
1000
1250
y1
b2
y2
b1
y3
type
b
y

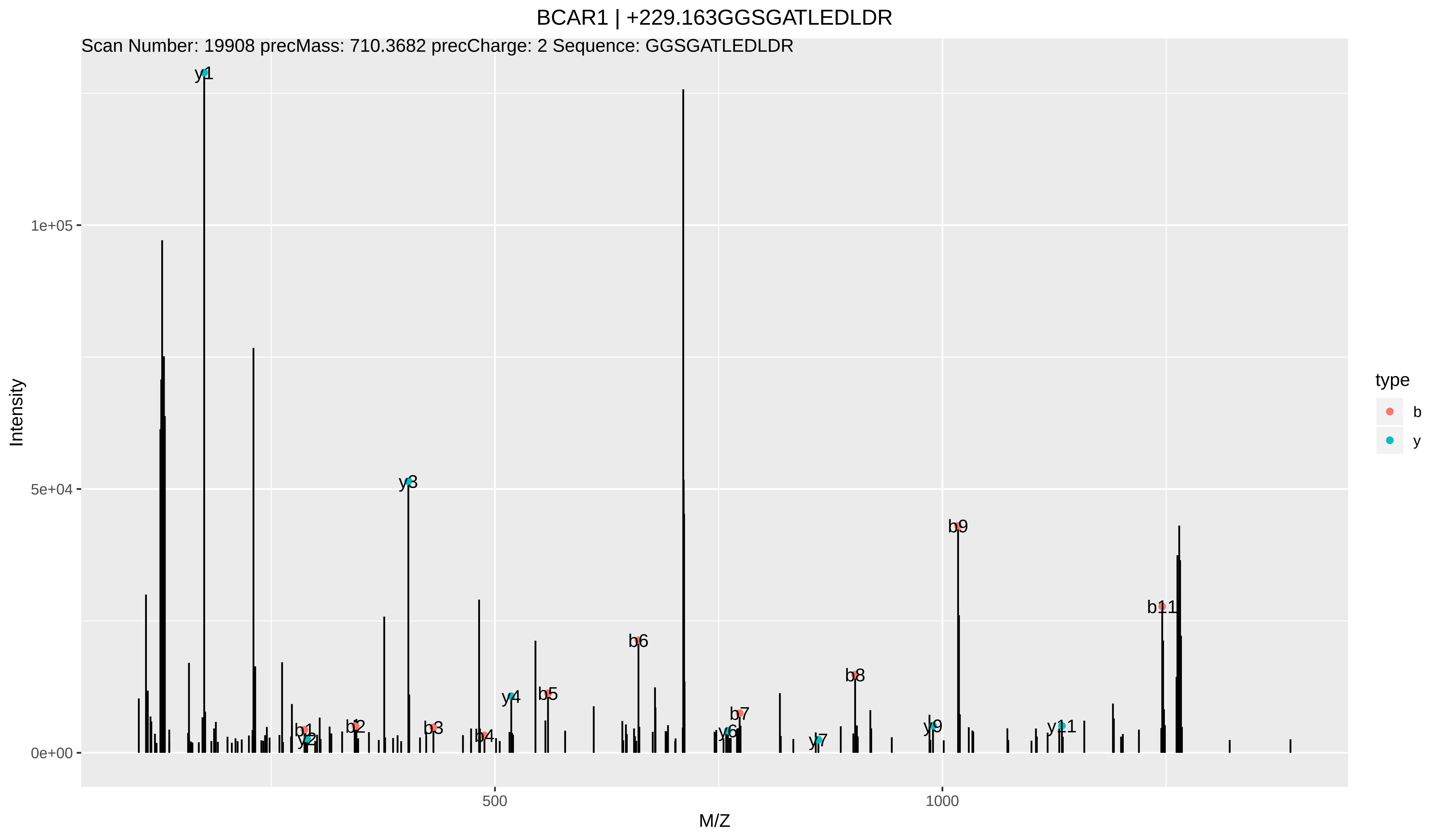

BCAR1 | +229.163GGSGATLEDLDR
Scan Number: 19908 precMass: 710.3682 precCharge: 2 Sequence: GGSGATLEDLDR
Intensity
0e+00
5e+04
1e+05
M/Z
500
1000
y1
y2
y3
y4
y6
y7
y9
y11
b1
b2
b3
b4
b5
b6
b7
b8
b9
b11
type
b
y

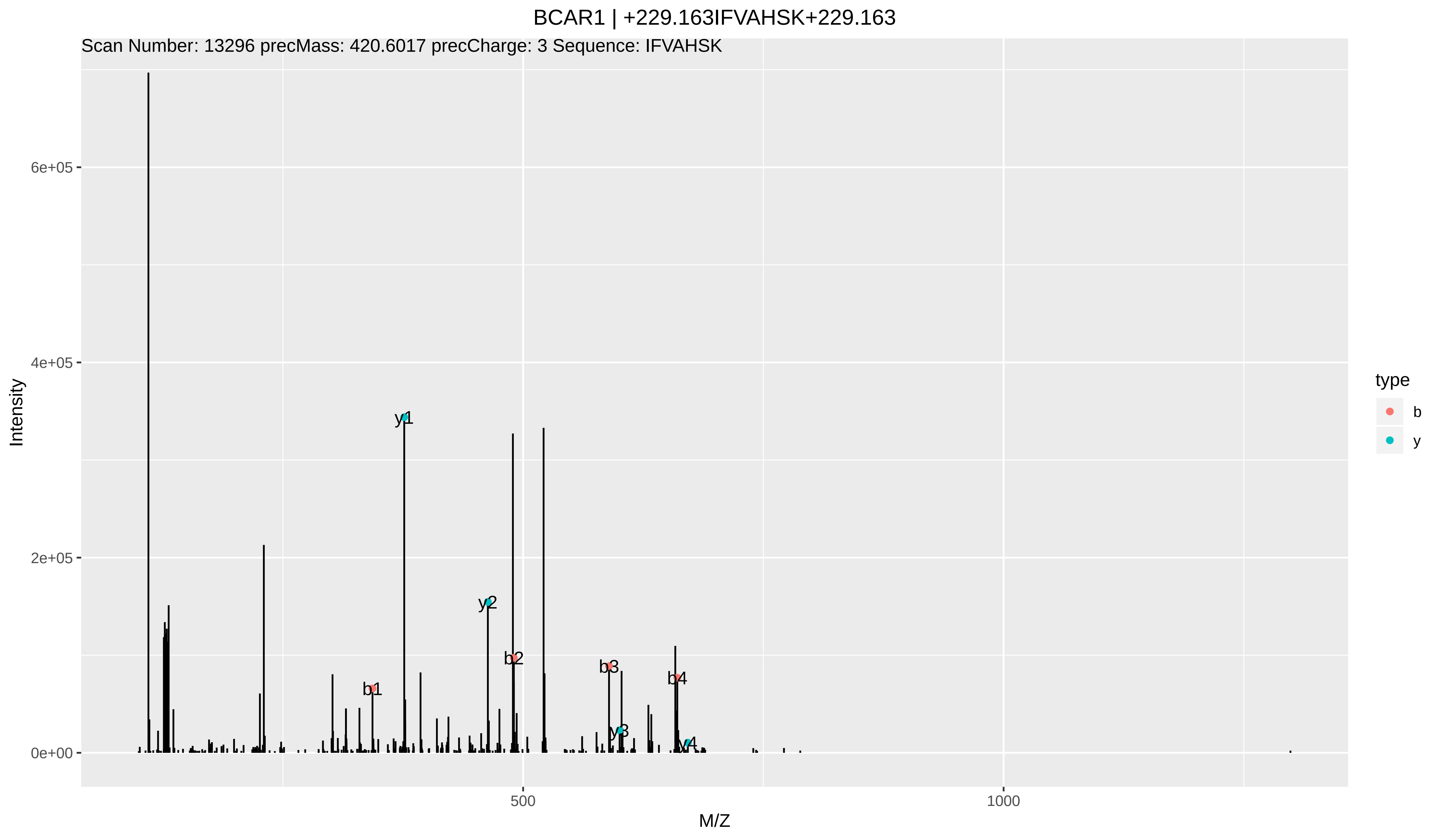
BCAR1 | +229.163IFVAHSK+229.163
Scan Number: 13296 precMass: 420.6017 precCharge: 3 Sequence: IFVAHSK
Intensity
6e+05
4e+05
2e+05
0e+00
M/Z
500
1000
y1
y2
b2
b3
b1
b4
y3
y4
type
b
y

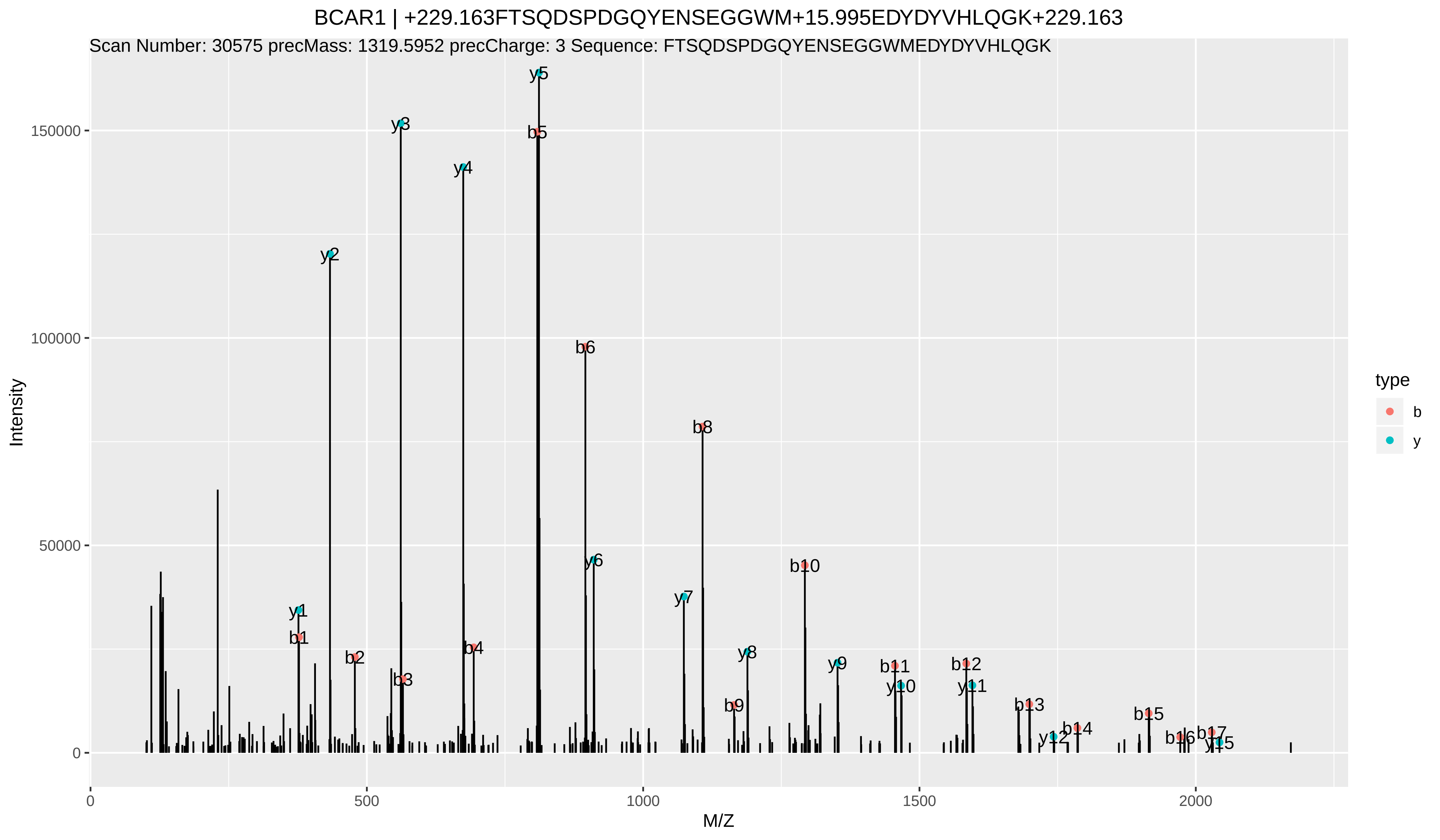

BCAR1 | +229.163FTSQDSPDGQYENSEGGWM+15.995EDYDYVHLQGK+229.163
Scan Number: 30575 precMass: 1319.5952 precCharge: 3 Sequence: FTSQDSPDGQYENSEGGWMEDYDYVHLQGK
Intensity
M/Z
type
b
y
y5
y3
b5
y4
y2
b6
b8
y6
b10
y7
y1
b1
b2
b3
b4
y8
b9
y9
b11
y10
b12
y11
b13
b14
y12
b15
b16
b17
y15
0
50000
100000
150000
500
1000
1500
2000

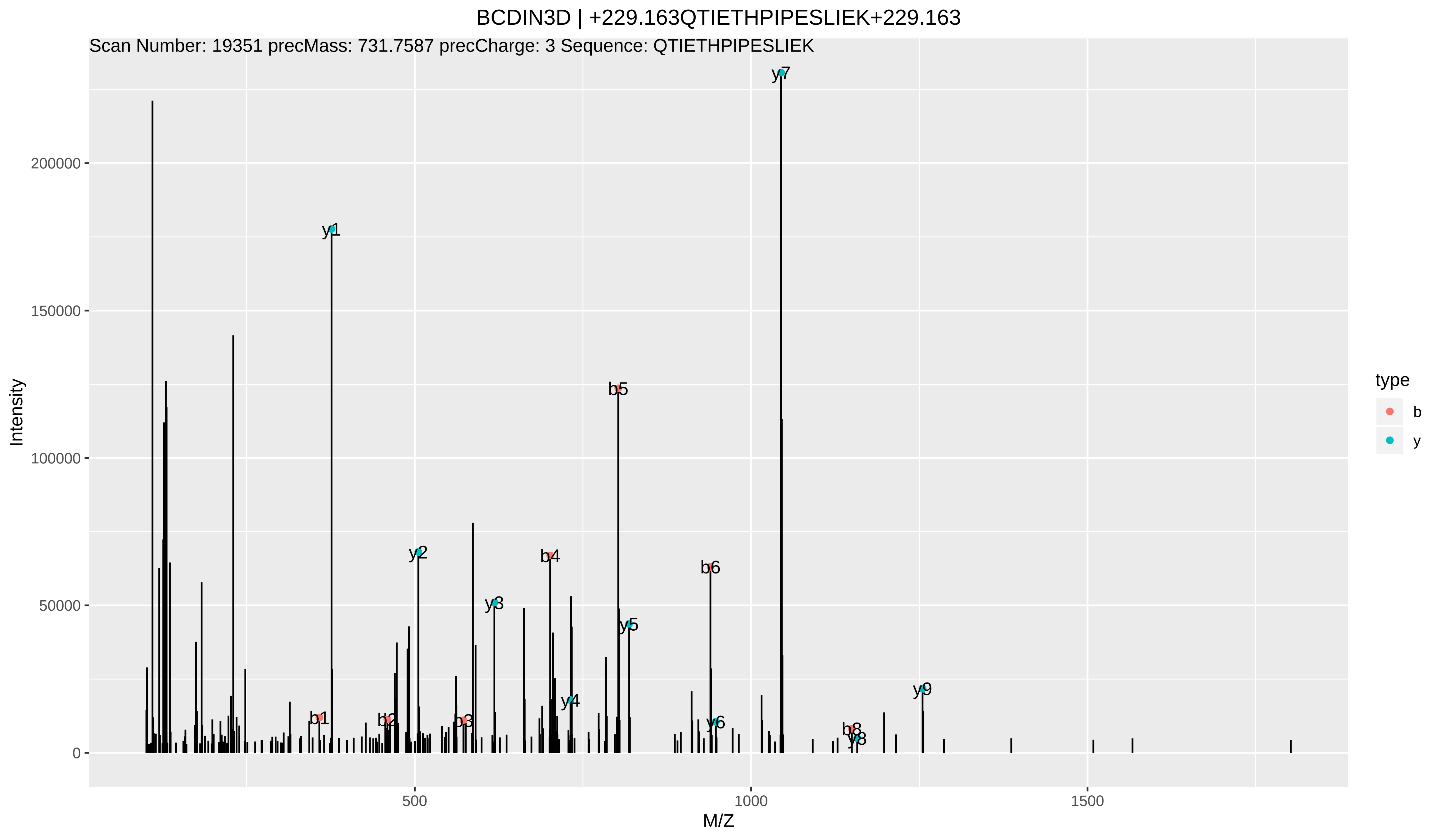
BCDIN3D | +229.163QTIETHPIPESLIEK+229.163
Scan Number: 19351 precMass: 731.7587 precCharge: 3 Sequence: QTIETHPIPESLIEK
Intensity
M/Z
type
b
y
y7
y1
b5
y2
b4
b6
y3
y5
y9
y4
b1
b2
b3
y6
b8
y8
0
50000
100000
150000
200000
500
1000
1500

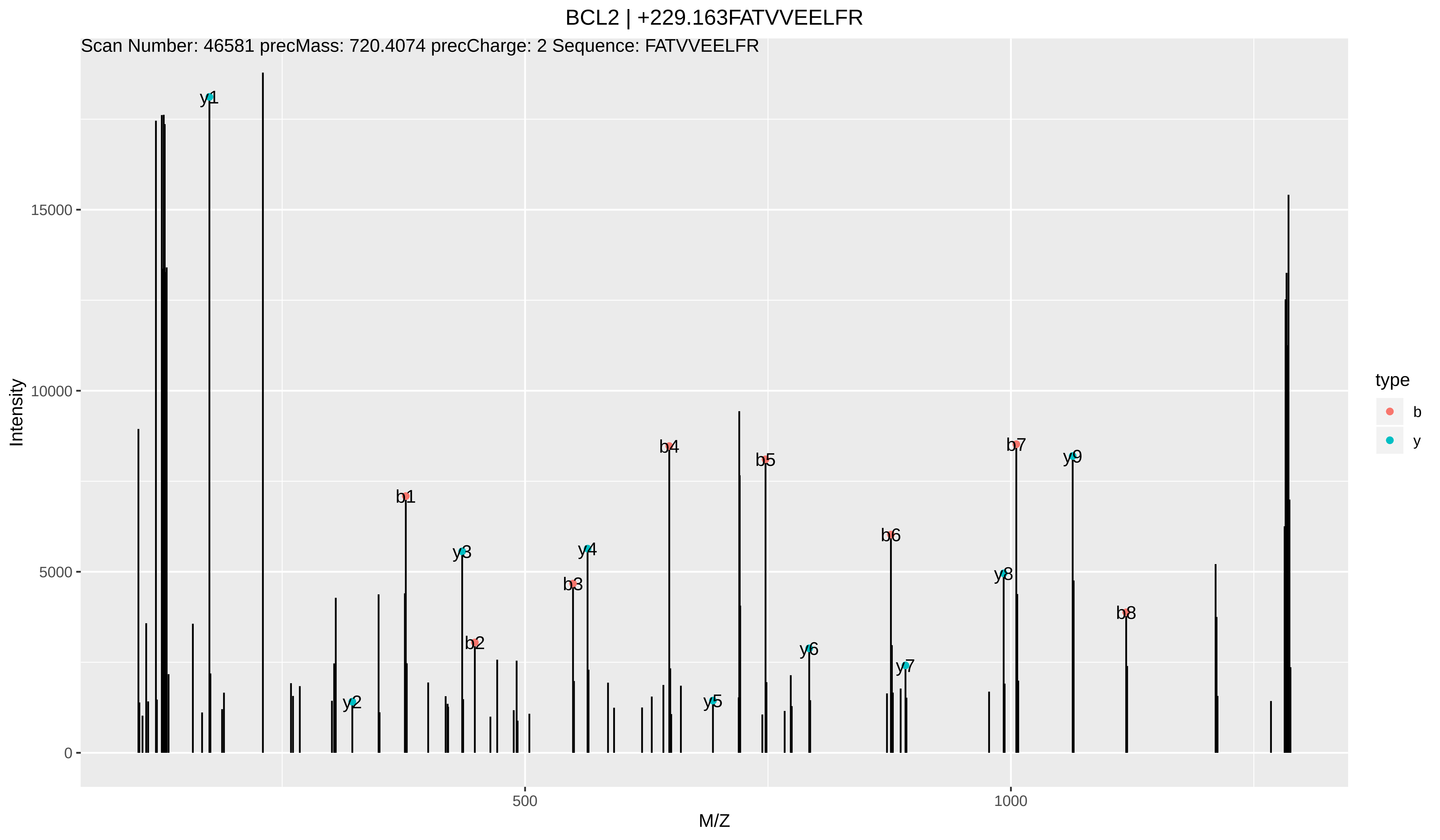
BCL2 | +229.163FATVVEELFR
Scan Number: 46581 precMass: 720.4074 precCharge: 2 Sequence: FATVVEELFR
Intensity
15000
10000
5000
0
M/Z
500
1000
y1
b1
y3
b2
y2
b3
y4
b4
y5
b5
y6
b6
y7
y8
b7
y9
b8
type
b
y

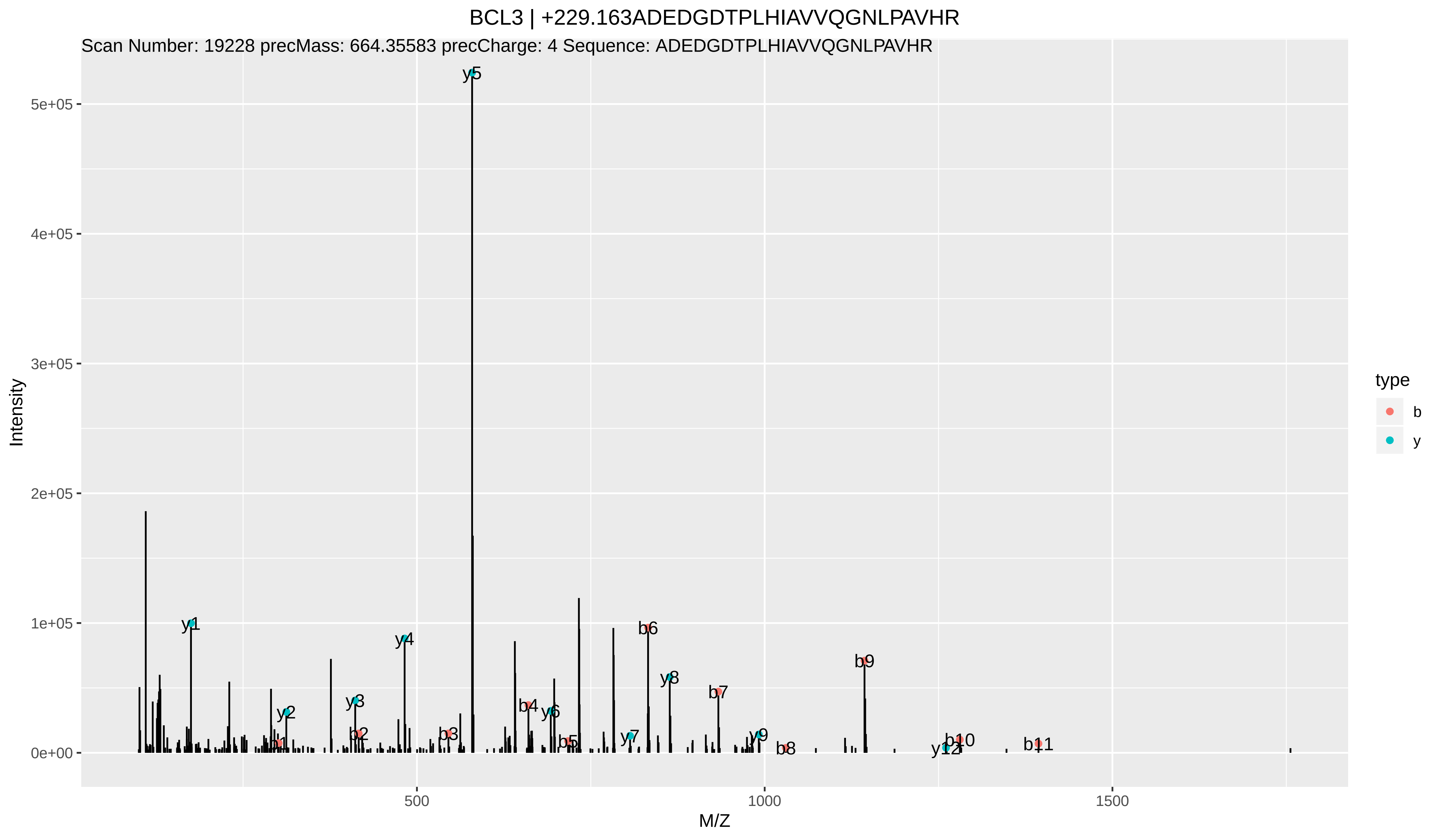
BCL3 | +229.163ADEDGDTPLHIAVVQGNLPAVHR
Scan Number: 19228 precMass: 664.35583 precCharge: 4 Sequence: ADEDGDTPLHIAVVQGNLPAVHR
Intensity
5e+05
4e+05
3e+05
2e+05
1e+05
0e+00
M/Z
500
1000
1500
y5
y1
y4
b6
b9
y8
b7
b4
y6
y3
y2
b2
b3
b1
b5
y7
y9
b8
y12
b10
b11
type
b
y

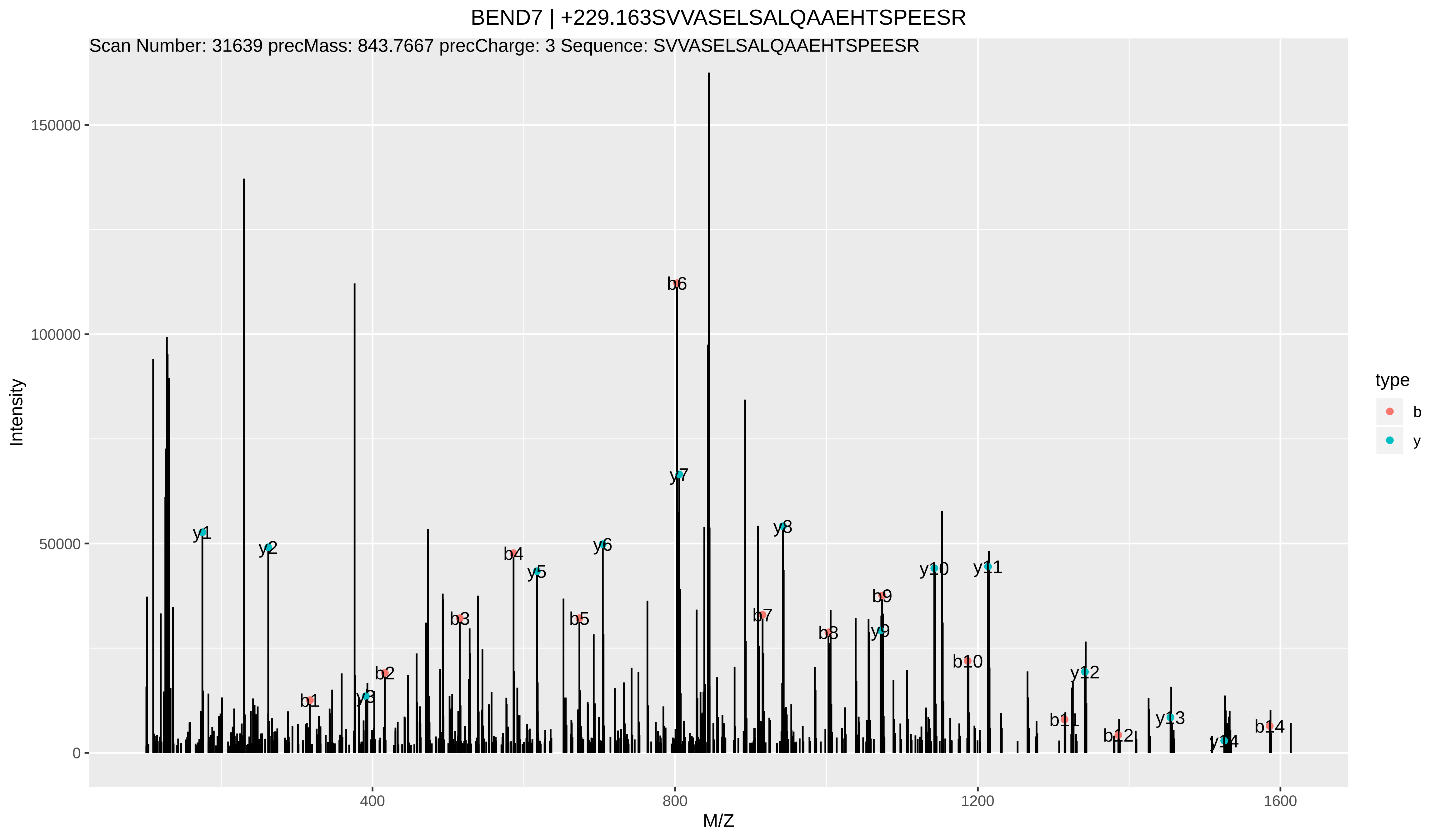

BEND7 | +229.163SVVASELSALQAAEHTSPEESR
Scan Number: 31639 precMass: 843.7667 precCharge: 3 Sequence: SVVASELSALQAAEHTSPEESR
Intensity
M/Z
0
50000
100000
150000
400
800
1200
1600
type
b
y
y1
y2
b1
y3
b2
b3
b4
y5
b5
y6
b6
y7
b7
y8
b8
b9
y9
y10
b10
y11
b11
y12
b12
y13
y14
b14

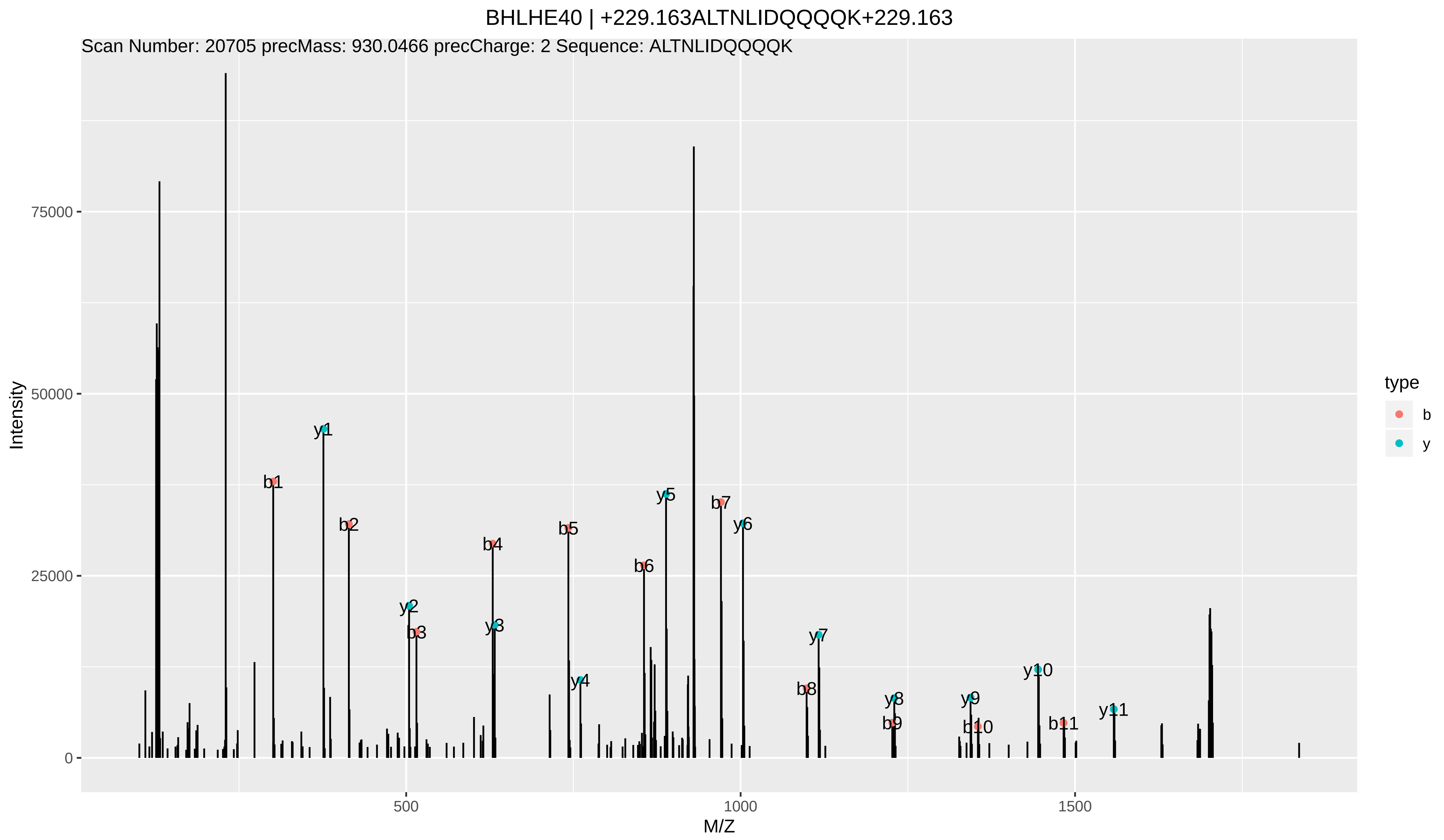
BHLHE40 | +229.163ALTNLIDQQQQK+229.163
Scan Number: 20705 precMass: 930.0466 precCharge: 2 Sequence: ALTNLIDQQQQK
Intensity
75000
50000
25000
0
M/Z
500
1000
1500
type
b
y
y1
b1
b2
y2
b3
b4
y3
b5
y4
b6
y5
b7
y6
b8
y7
y8
b9
y9
b10
y10
b11
y11

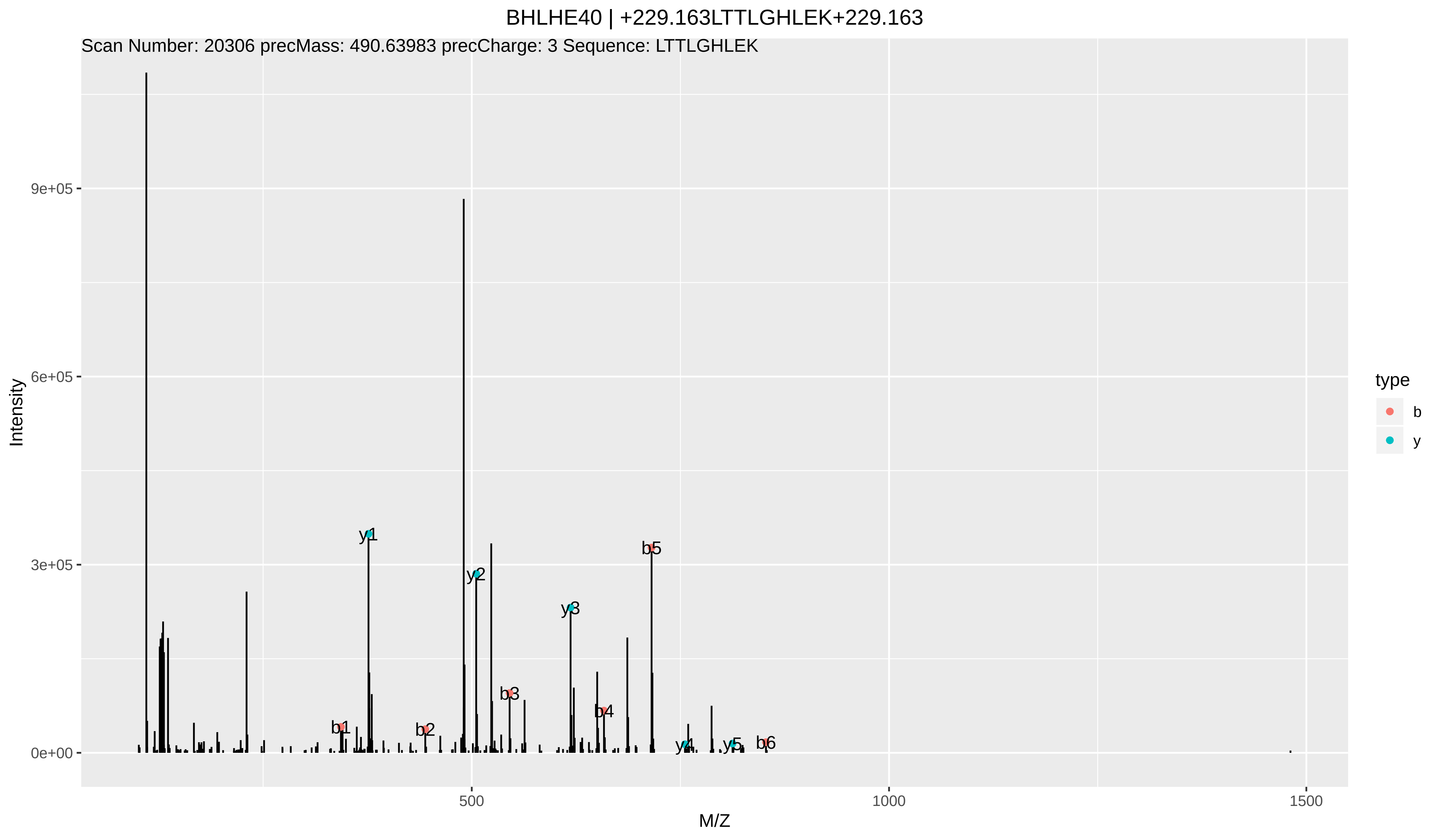
BHLHE40 | +229.163LTTLGHLEK+229.163
Scan Number: 20306 precMass: 490.63983 precCharge: 3 Sequence: LTTLGHLEK
Intensity
9e+05
6e+05
3e+05
0e+00
M/Z
500
1000
1500
y1
b5
y2
y3
b3
b4
b1
b2
y4
y5
b6
type
b
y

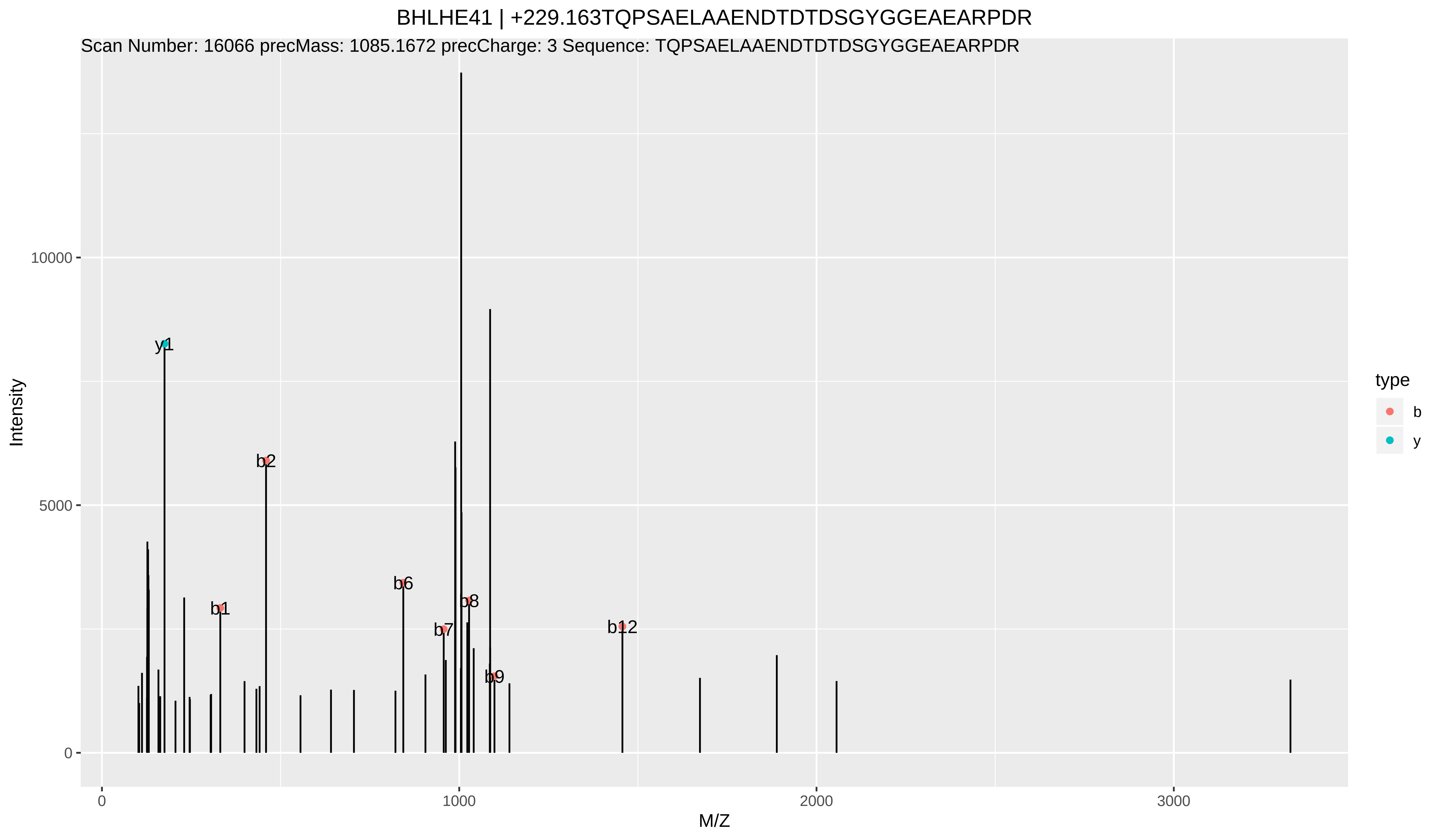
BHLHE41 | +229.163TQPSAELAAENDTDTDSGYGGEAEARPDR
Scan Number: 16066 precMass: 1085.1672 precCharge: 3 Sequence: TQPSAELAAENDTDTDSGYGGEAEARPDR
Intensity
M/Z
type
b
y
y1
b1
b2
b6
b7
b8
b9
b12
0
5000
10000
0
1000
2000
3000

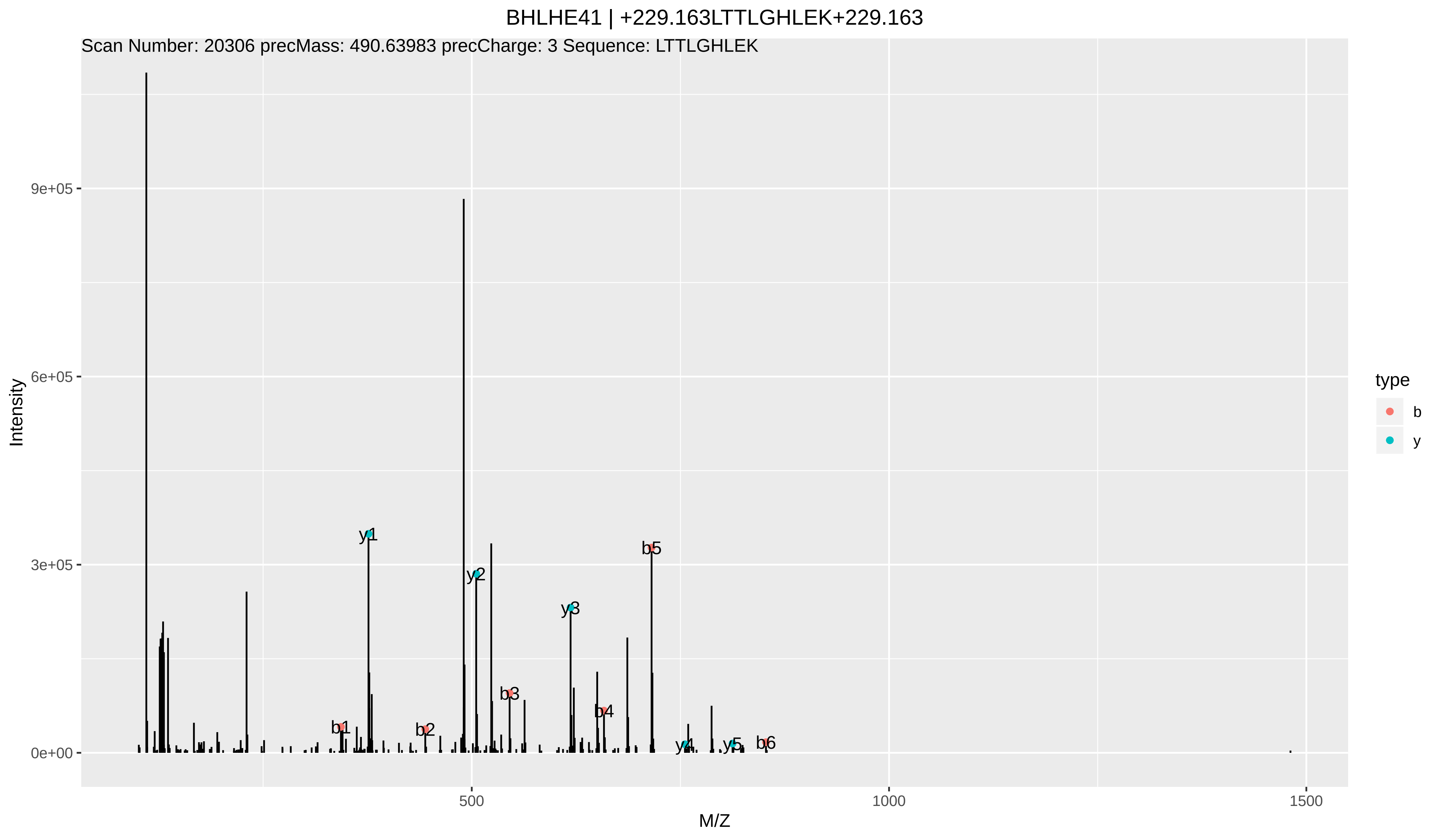
BHLHE41 | +229.163LTTLGHLEK+229.163
Scan Number: 20306 precMass: 490.63983 precCharge: 3 Sequence: LTTLGHLEK
Intensity
9e+05
6e+05
3e+05
0e+00
M/Z
500
1000
1500
y1
b5
y2
y3
b3
b4
b1
b2
y4
y5
b6
type
b
y

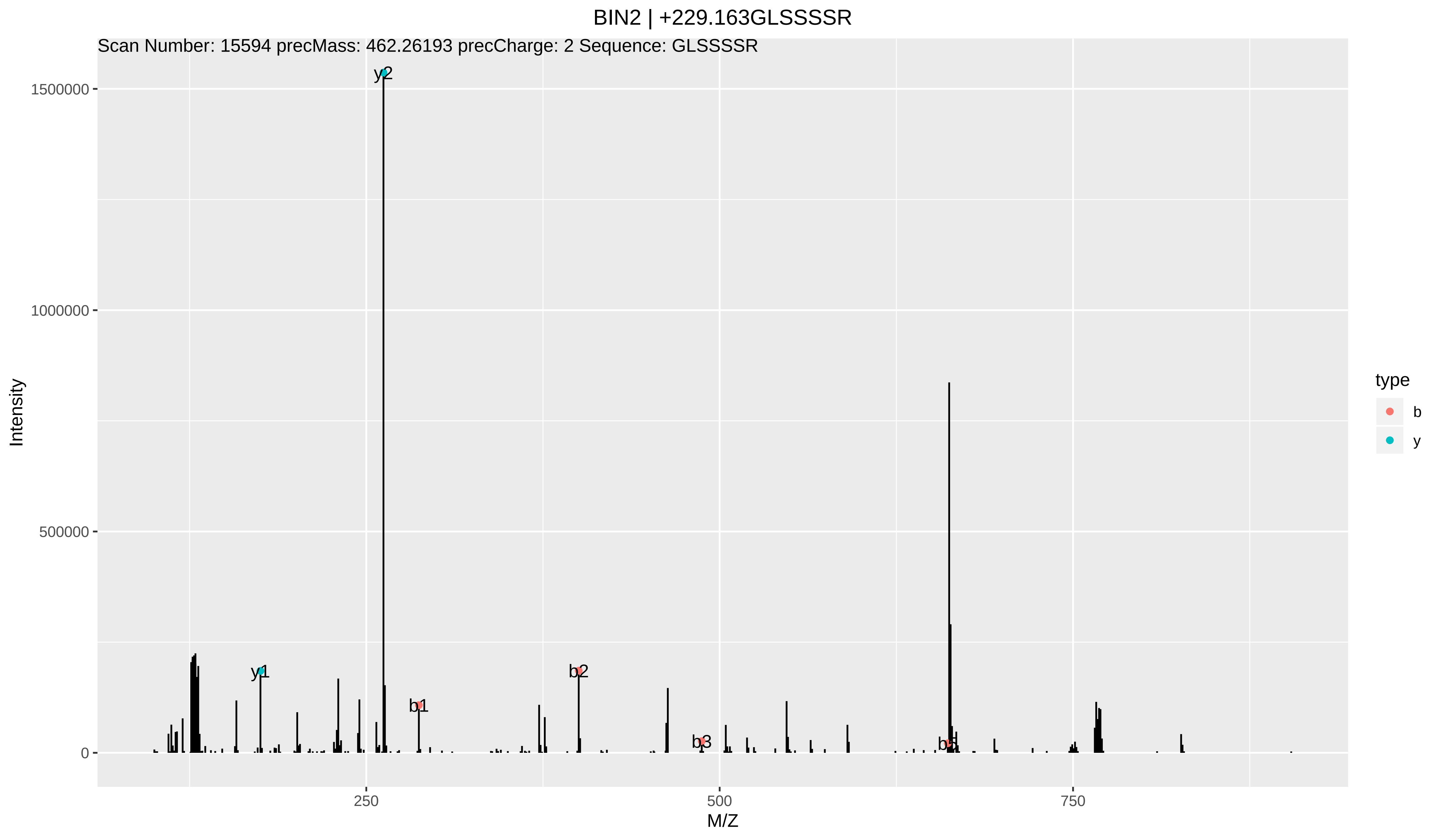
BIN2 | +229.163GLSSSSR
Scan Number: 15594 precMass: 462.26193 precCharge: 2 Sequence: GLSSSSR
Intensity
1500000
1000000
500000
0
250
500
750
M/Z
y2
y1
b1
b2
b3
b5
type
b
y

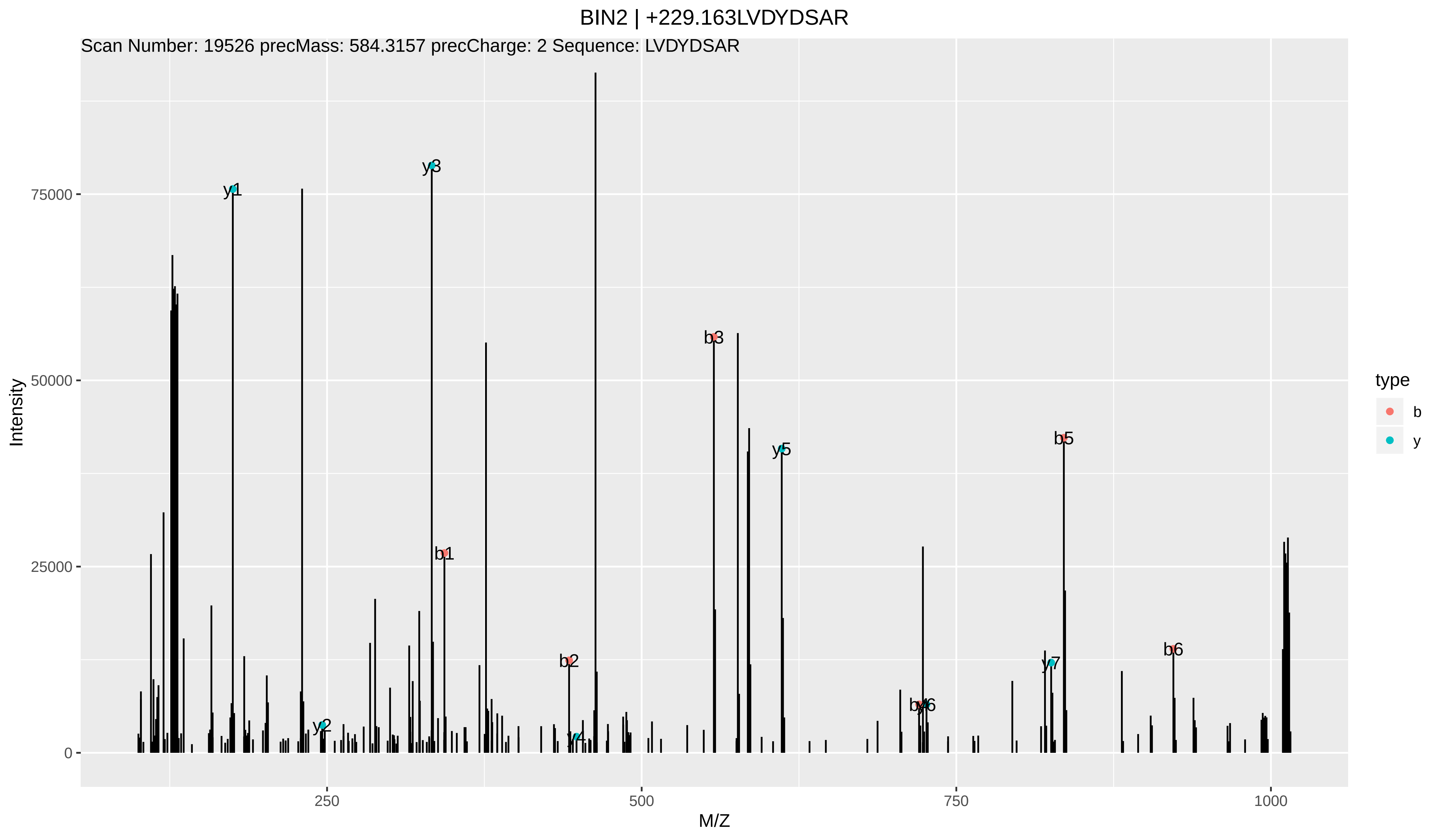
BIN2 | +229.163LVDYDSAR
Scan Number: 19526 precMass: 584.3157 precCharge: 2 Sequence: LVDYDSAR
Intensity
M/Z
75000
50000
25000
0
250
500
750
1000
y1
y3
b1
b2
y2
y4
b3
y5
b4
y6
y7
b5
b6
type
b
y

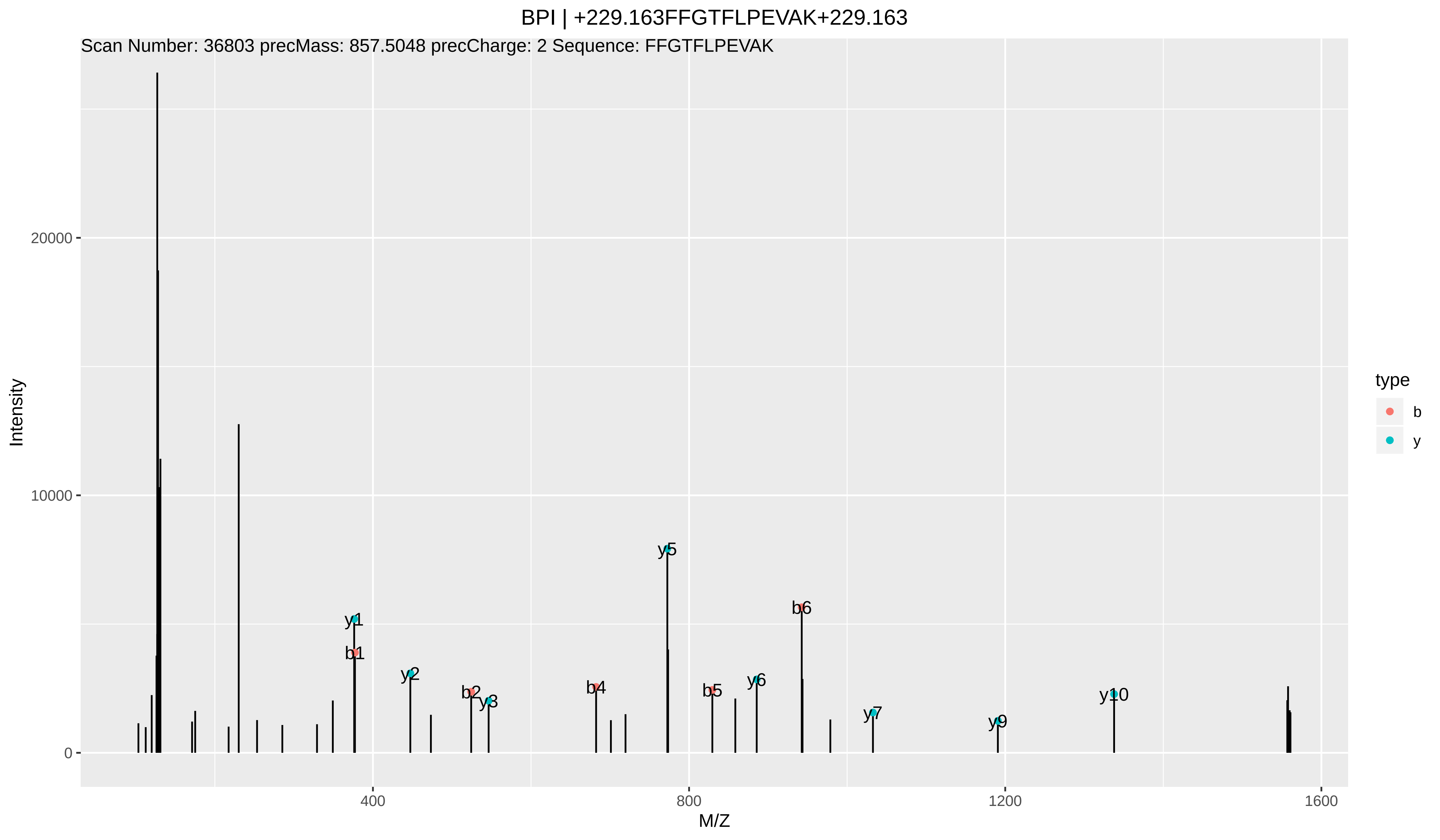

BPI | +229.163FFGTFLPEVAK+229.163
Scan Number: 36803 precMass: 857.5048 precCharge: 2 Sequence: FFGTFLPEVAK
Intensity
20000
10000
0
400
800
1200
1600
M/Z
y1
b1
y2
b2
y3
b4
y5
b5
y6
b6
y7
y9
y10
type
b
y

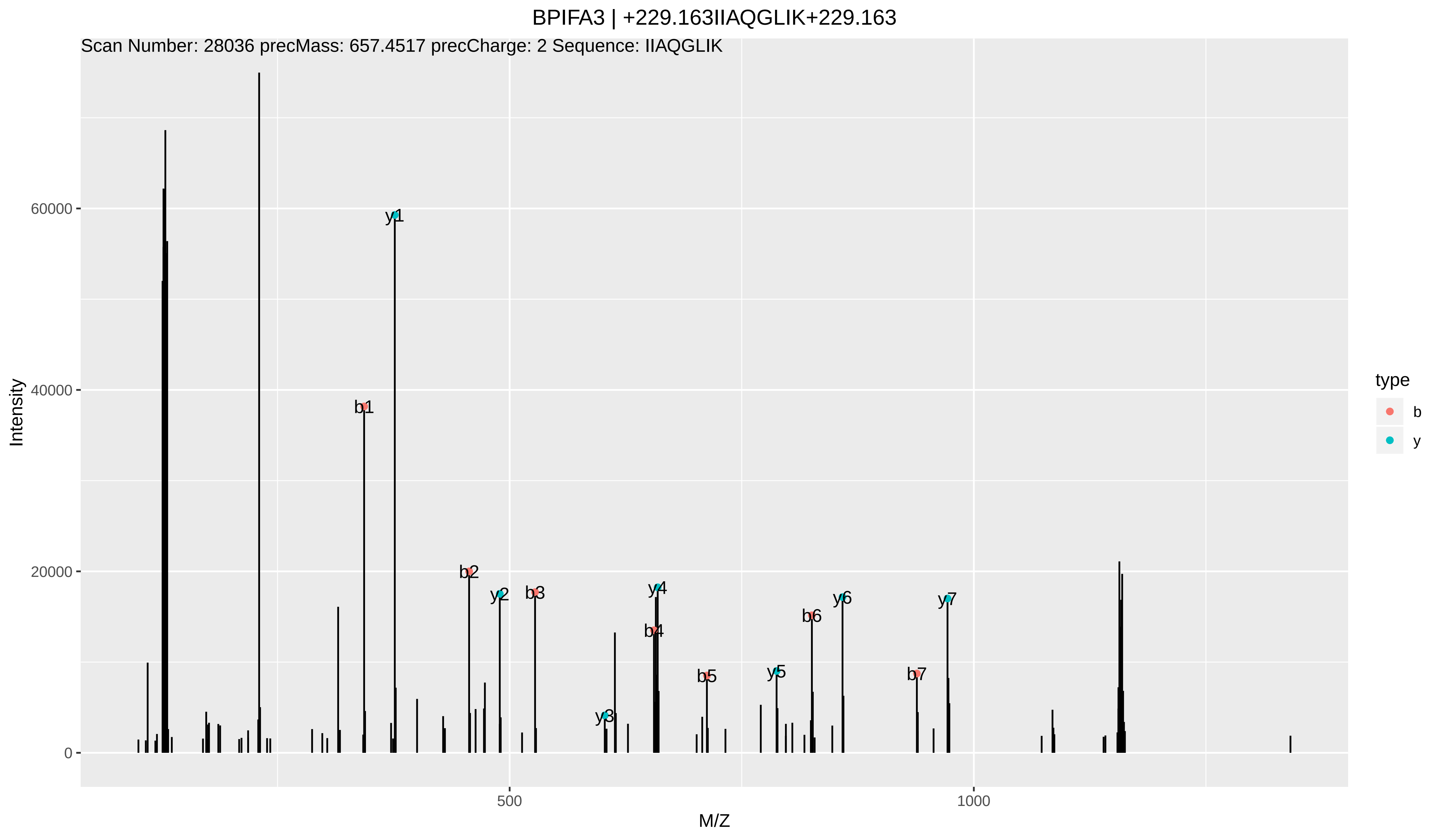

BPIFA3 | +229.163IIAQGLIK+229.163
Scan Number: 28036 precMass: 657.4517 precCharge: 2 Sequence: IIAQGLIK
Intensity
M/Z
60000
40000
20000
0
500
1000
y1
b1
b2
y2
b3
y3
y4
b4
b5
y5
b6
y6
b7
y7
type
b
y

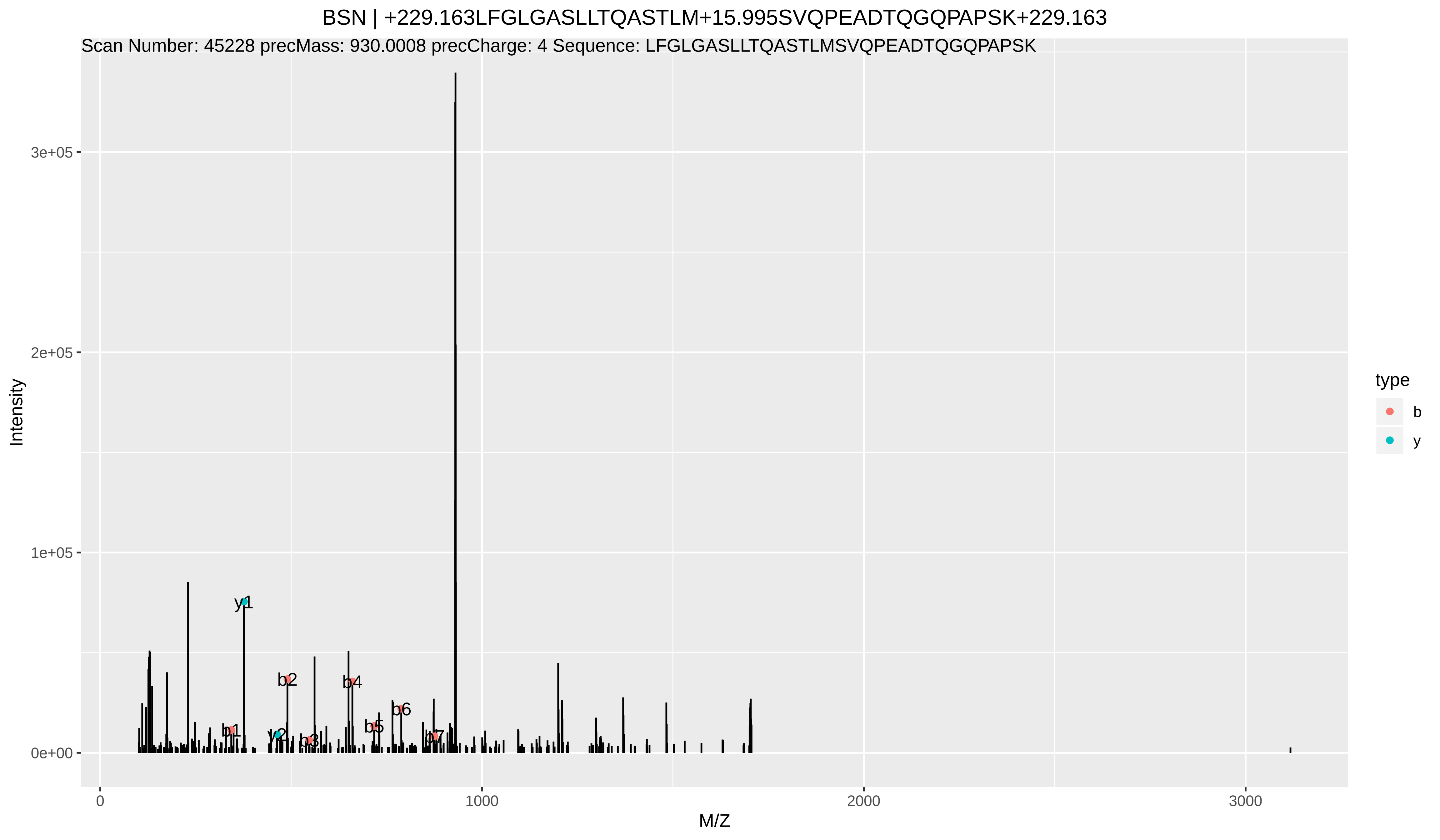

BSN | +229.163LFGLGASLLTQASTLM+15.995SVQPEADTQGQPAPSK+229.163
Scan Number: 45228 precMass: 930.0008 precCharge: 4 Sequence: LFGLGASLLTQASTLMSVQPEADTQGQPAPSK
Intensity
3e+05
2e+05
1e+05
0e+00
M/Z
0
1000
2000
3000
y1
b1
y2
b2
b3
b4
b5
b6
b7
type
b
y

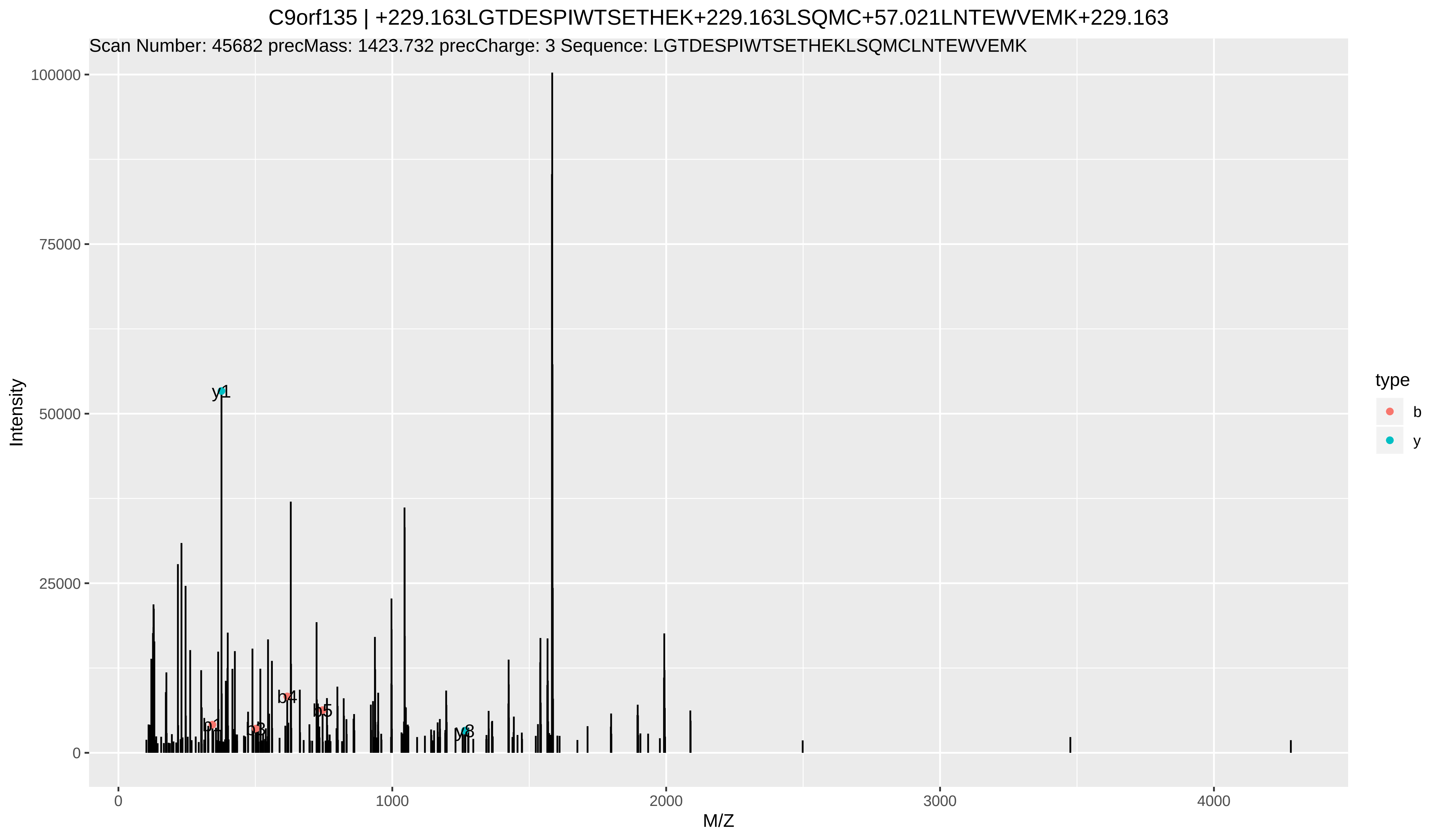
C9orf135 | +229.163LGTDESPIWTSETHEK+229.163LSQMC+57.021LNTEWVEMK+229.163
Scan Number: 45682 precMass: 1423.732 precCharge: 3 Sequence: LGTDESPIWTSETHEKLSQMCLNTEWVEMK
Intensity
100000
75000
50000
25000
0
0
1000
2000
3000
4000
M/Z
y1
b4
b5
y8
type
b
y

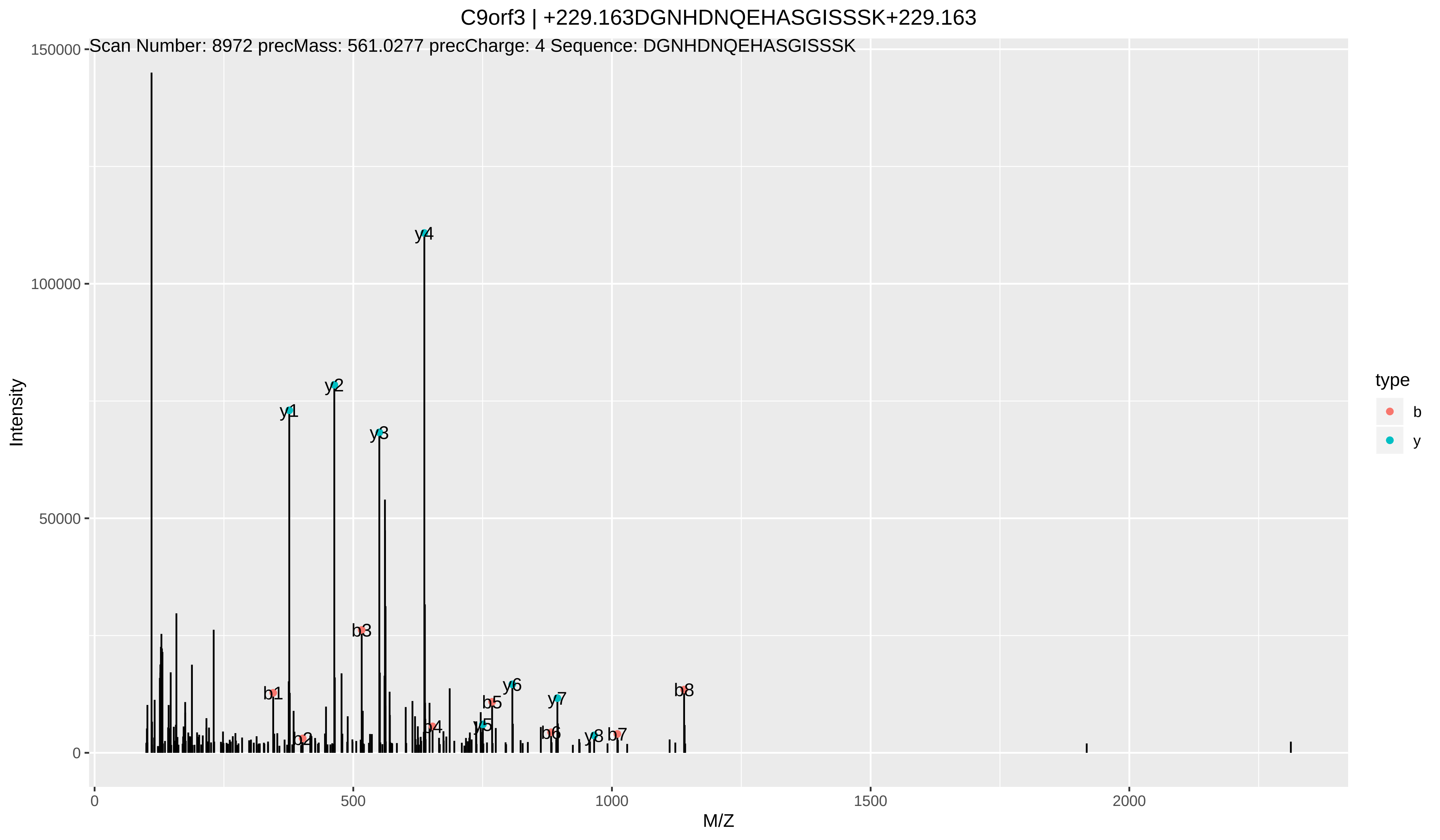

C9orf3 | +229.163DGNHDNQEHASGISSSK+229.163
Scan Number: 8972 precMass: 561.0277 precCharge: 4 Sequence: DGNHDNQEHASGISSSK
Intensity
150000
100000
50000
0
M/Z
0
500
1000
1500
2000
y4
y2
y1
y3
b3
b1
b2
b4
y5
b5
y6
b6
y7
y8
b7
b8
type
b
y

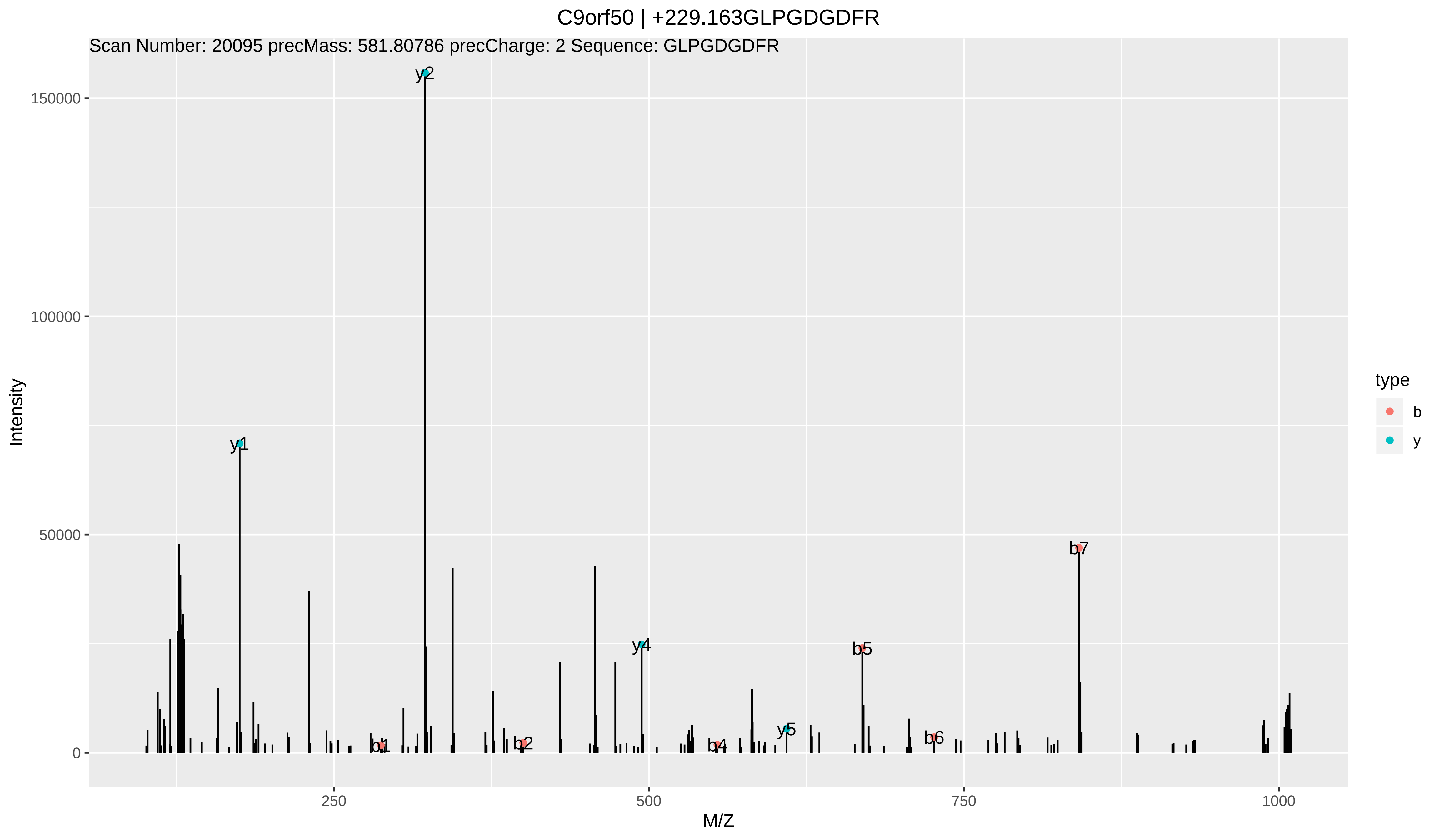
C9orf50 | +229.163GLPGDGDFR
Scan Number: 20095 precMass: 581.80786 precCharge: 2 Sequence: GLPGDGDFR
Intensity
M/Z
type
b
y
y1
y2
y4
y5
b1
b2
b4
b5
b6
b7
150000
100000
50000
0
250
500
750
1000

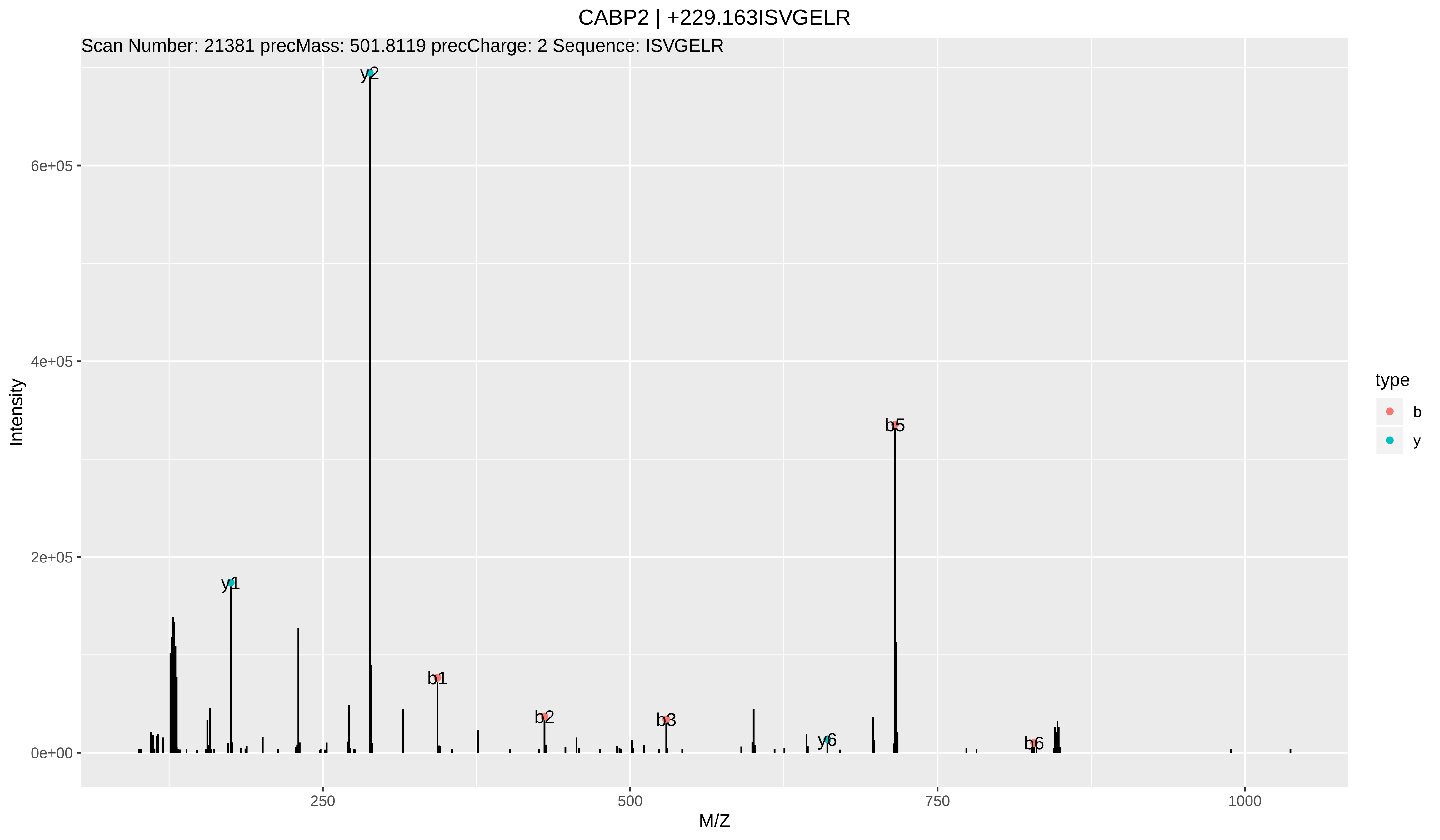
CABP2 | +229.163ISVGELR
Scan Number: 21381 precMass: 501.8119 precCharge: 2 Sequence: ISVGELR
Intensity
6e+05
4e+05
2e+05
0e+00
M/Z
250
500
750
1000
y2
y1
b1
b2
b3
y6
b5
b6
type
b
y

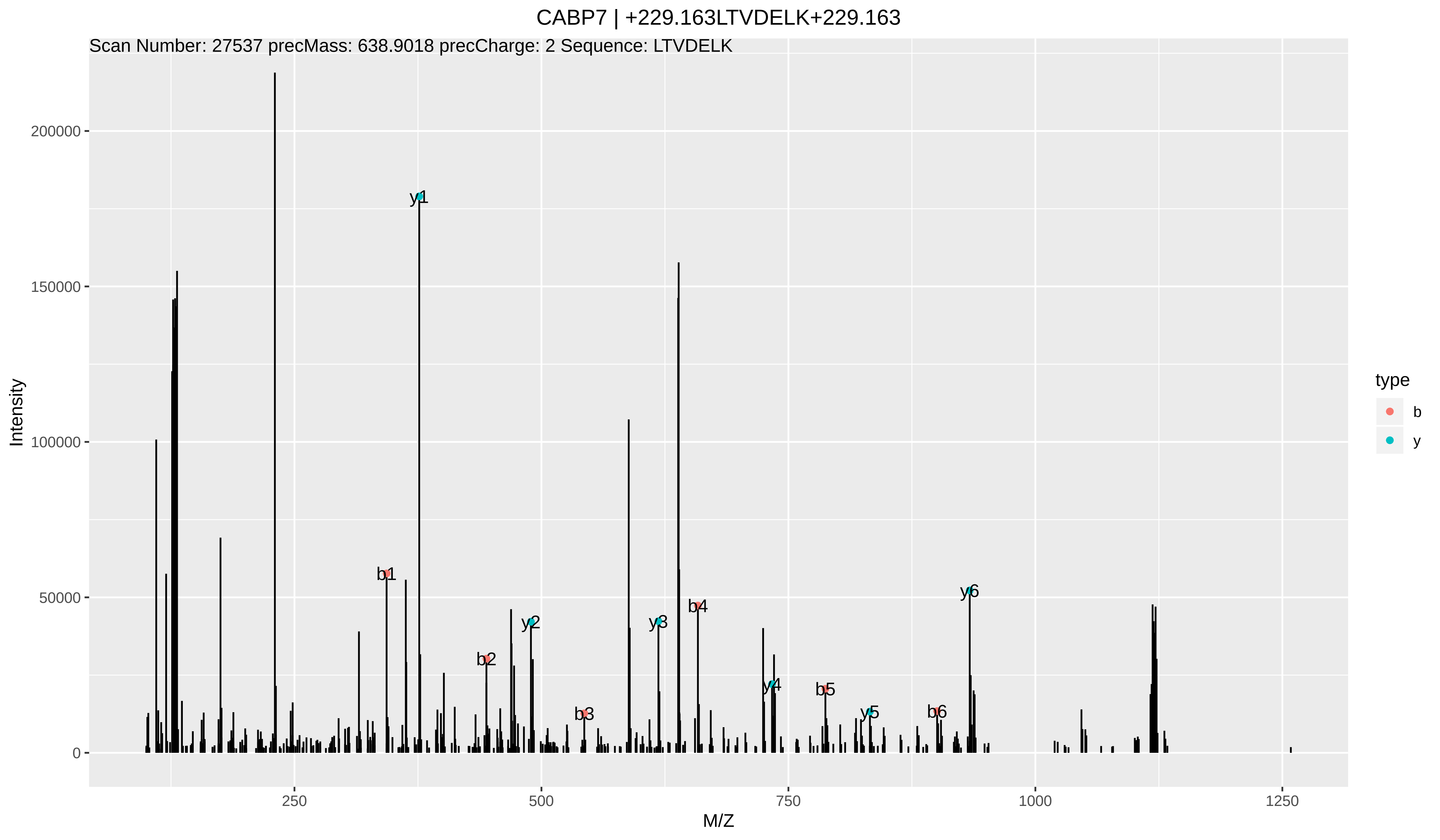
CABP7 | +229.163LTVDELK+229.163
Scan Number: 27537 precMass: 638.9018 precCharge: 2 Sequence: LTVDELK
Intensity
M/Z
0
50000
100000
150000
200000
250
500
750
1000
1250
y1
b1
b2
y2
b3
y3
b4
y4
b5
y5
b6
y6
type
b
y

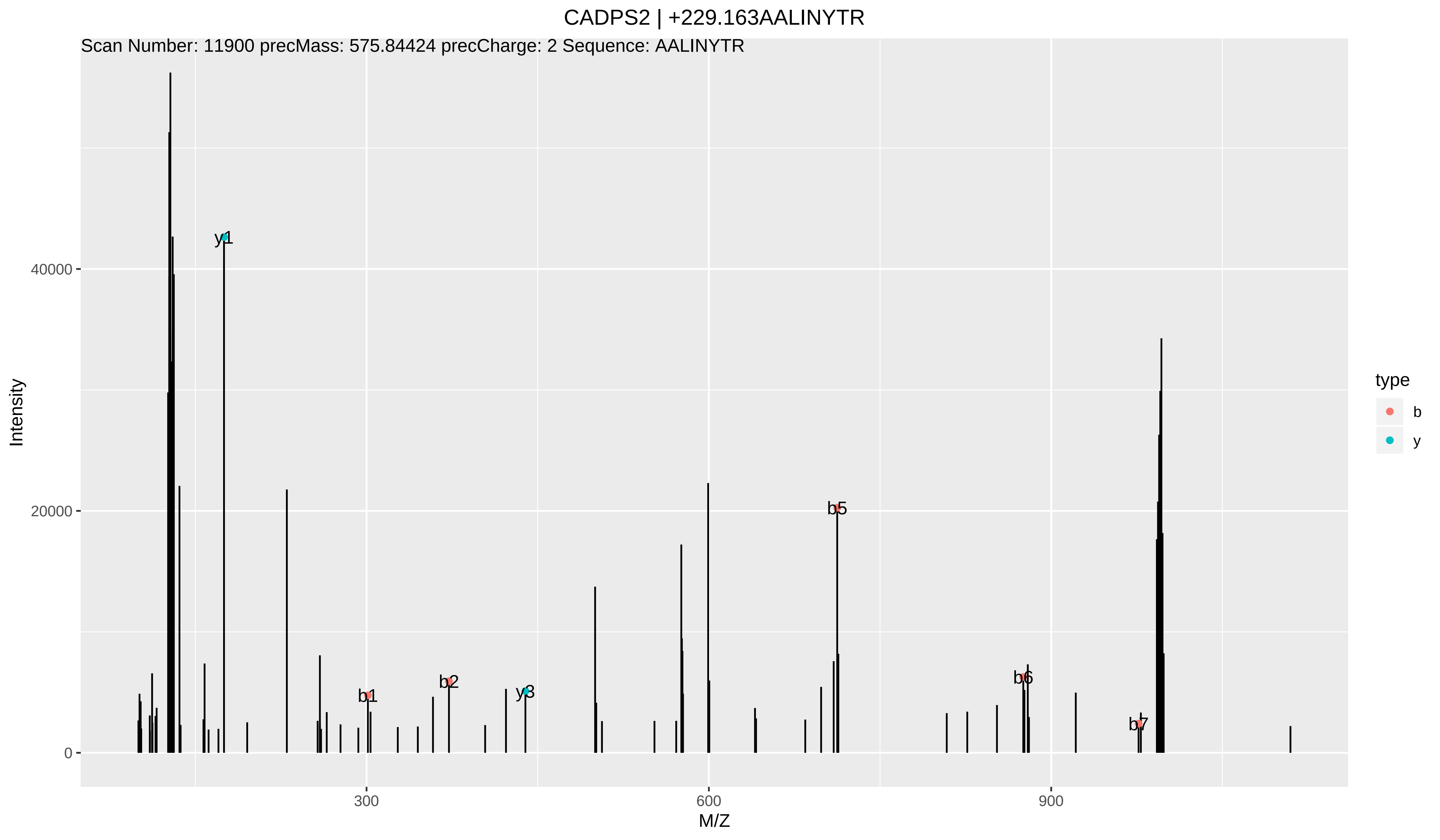

CADPS2 | +229.163AALINYTR
Scan Number: 11900 precMass: 575.84424 precCharge: 2 Sequence: AALINYTR
Intensity
40000
20000
0
300
600
900
M/Z
y1
b1
b2
y3
b5
b6
b7
type
b
y

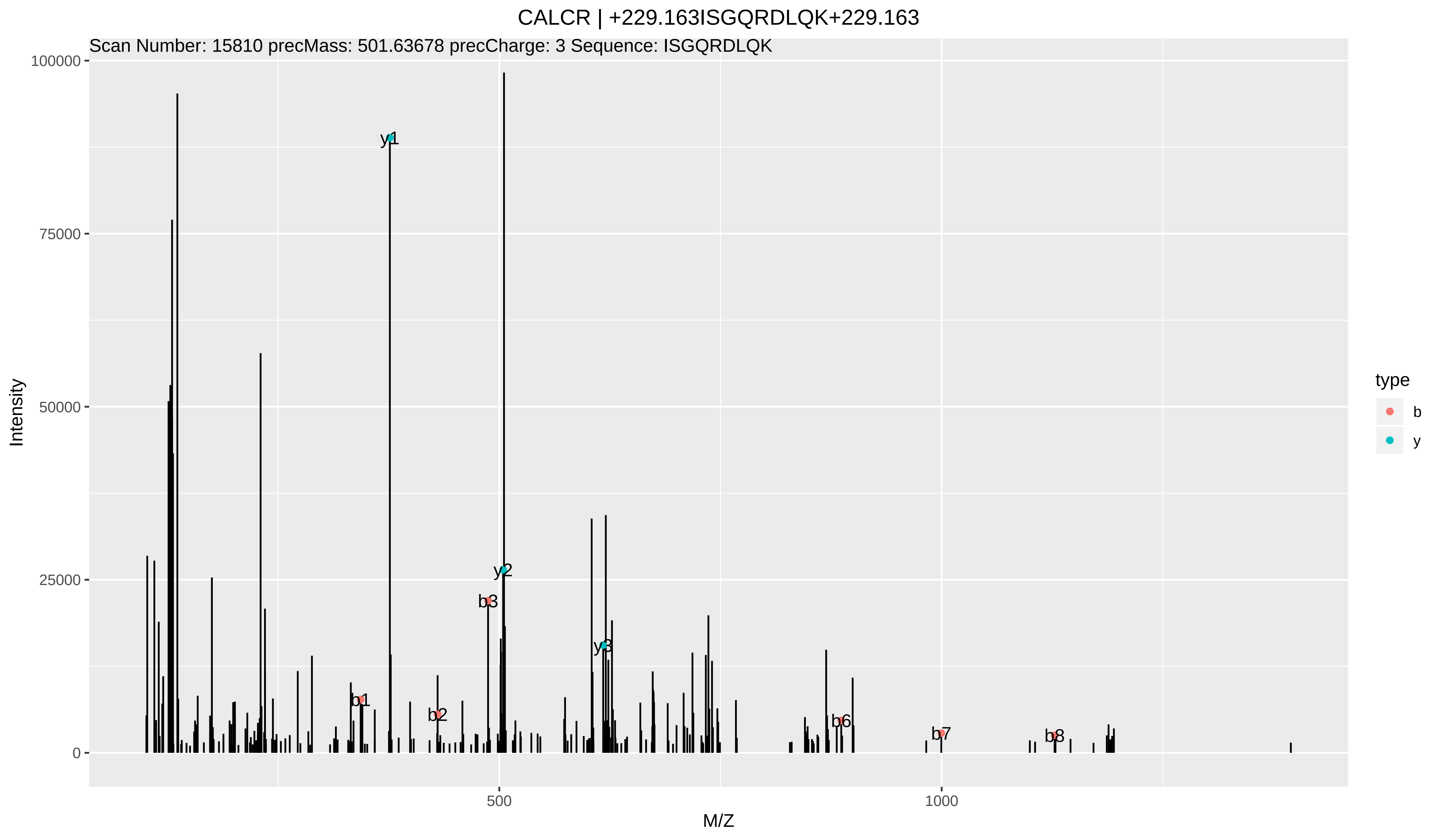

CALCR | +229.163ISGQRDLQK+229.163
Scan Number: 15810 precMass: 501.63678 precCharge: 3 Sequence: ISGQRDLQK
Intensity
100000
75000
50000
25000
0
M/Z
500
1000
y1
y2
y3
b1
b2
b3
b6
b7
b8
type
b
y

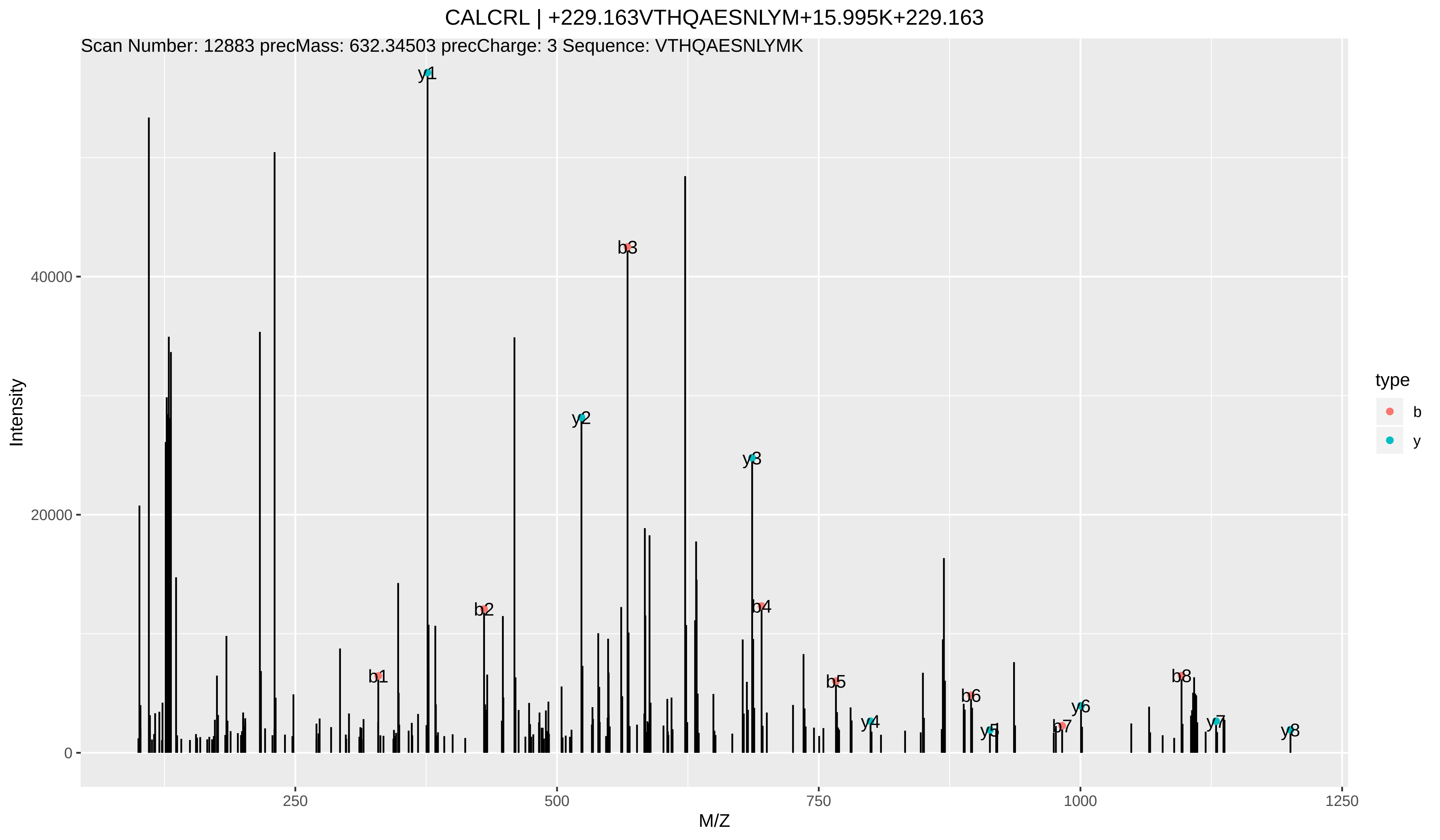

CALCRL | +229.163VTHQAESNLYM+15.995K+229.163
Scan Number: 12883 precMass: 632.34503 precCharge: 3 Sequence: VTHQAESNLYMK
y1
b3
y2
y3
b2
b4
b1
b5
b6
y4
y5
b7
y6
b8
y7
y8
Intensity
40000
20000
0
250
500
750
1000
1250
M/Z
type
b
y

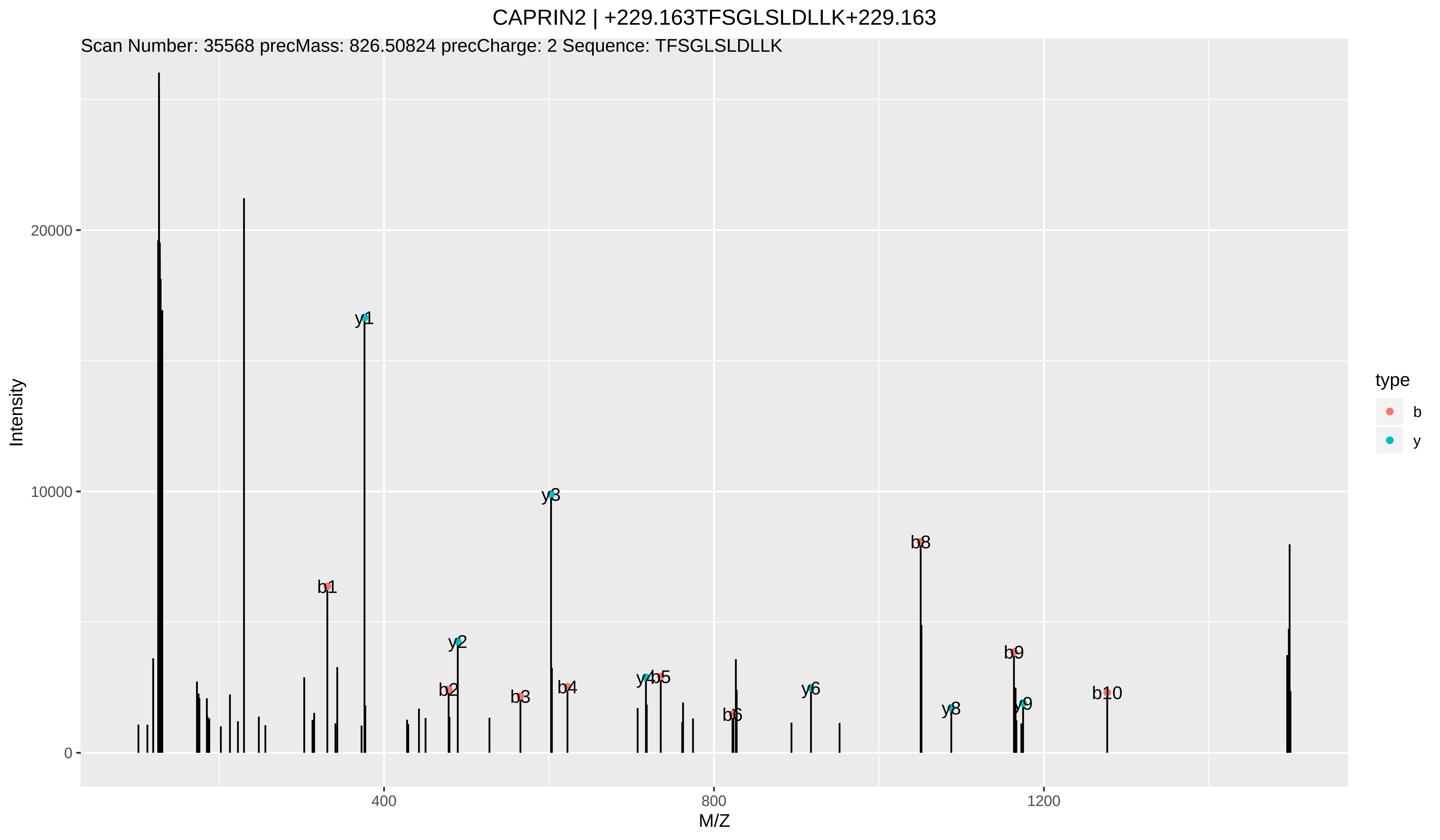
CAPRIN2 | +229.163TFSGLSLDLLK+229.163
Scan Number: 35568 precMass: 826.50824 precCharge: 2 Sequence: TFSGLSLDLLK
Intensity
M/Z
0
10000
20000
400
800
1200
y1
y2
y3
y4
y6
y8
y9
b1
b2
b3
b4
b5
b6
b8
b9
b10
type
b
y

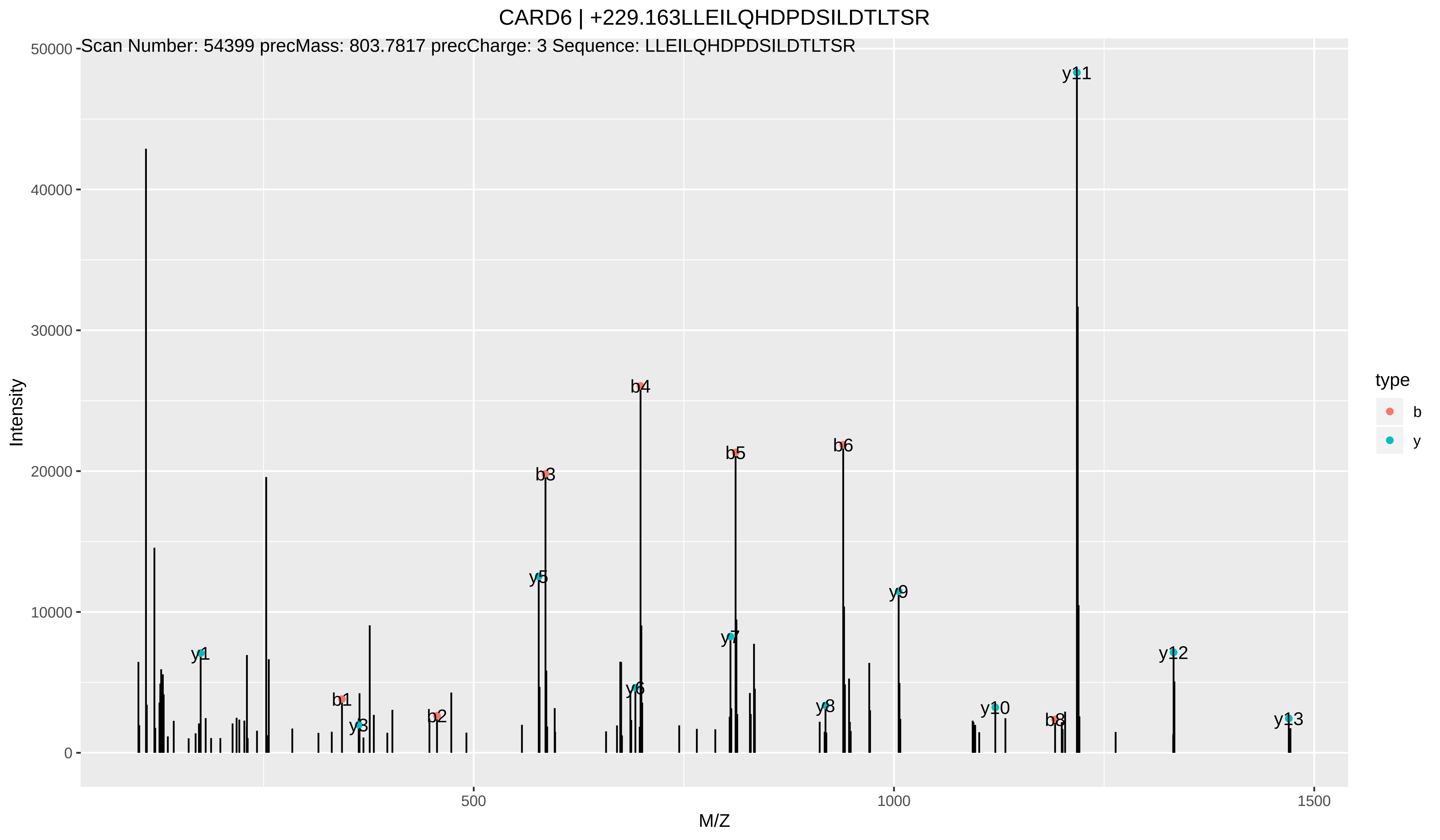
CARD6 | +229.163LLEILQHDPDSILDTLTSR
Scan Number: 54399 precMass: 803.7817 precCharge: 3 Sequence: LLEILQHDPDSILDTLTSR
Intensity
0
10000
20000
30000
40000
50000
M/Z
500
1000
1500
y11
b4
b6
b5
b3
y5
y9
y7
y1
y12
y6
b1
y8
y10
b2
y3
b8
y13
type
b
y

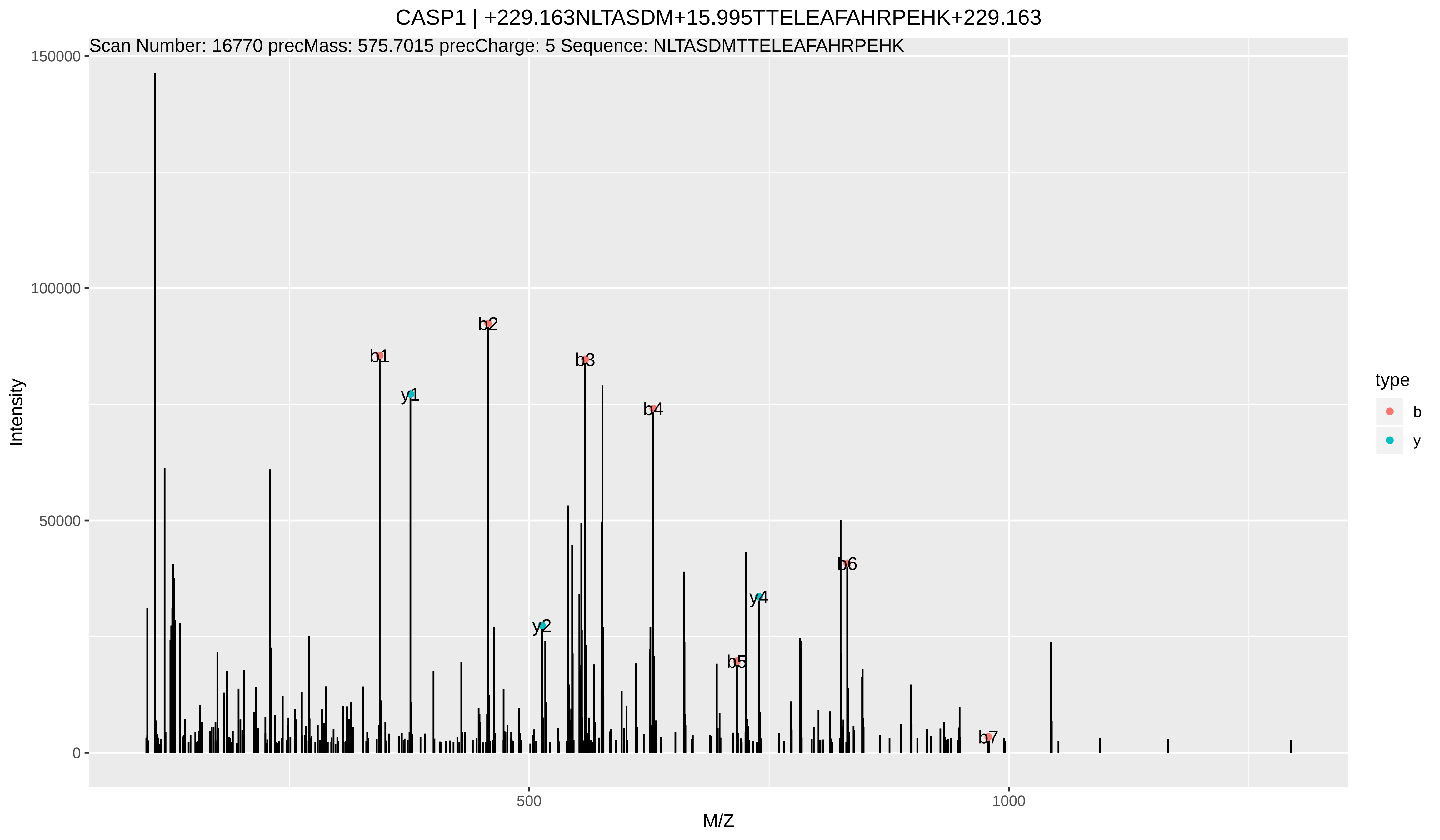
CASP1 | +229.163NLTASDM+15.995TTELEAFAHRPEHK+229.163
Scan Number: 16770 precMass: 575.7015 precCharge: 5 Sequence: NLTASDMTTELEAFAHRPEHK
Intensity
150000
100000
50000
0
500
1000
M/Z
b1
y1
b2
b3
b4
y2
b5
y4
b6
b7
type
b
y

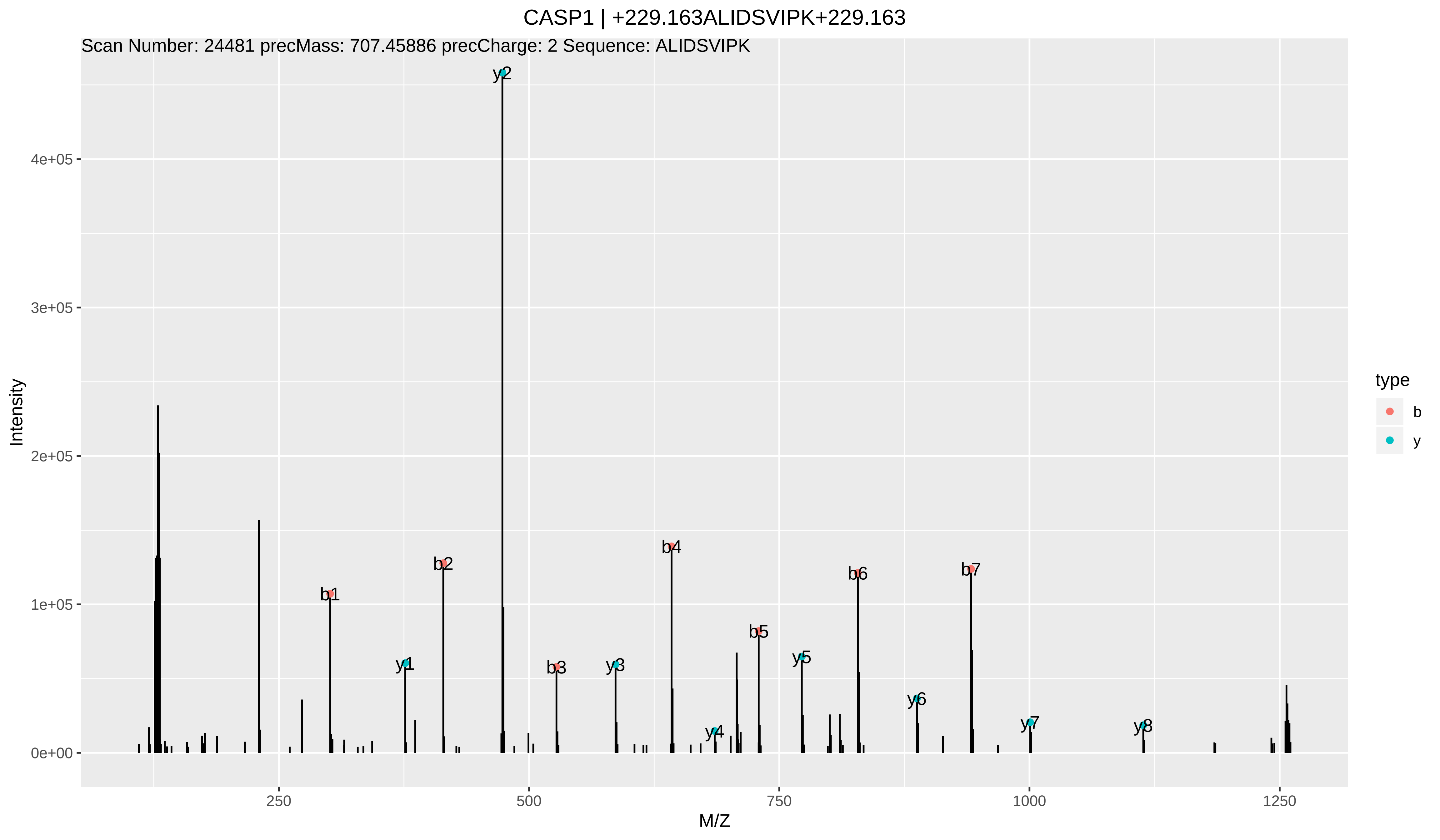

CASP1 | +229.163ALIDSVIPK+229.163
Scan Number: 24481 precMass: 707.45886 precCharge: 2 Sequence: ALIDSVIPK
Intensity
4e+05
3e+05
2e+05
1e+05
0e+00
M/Z
250
500
750
1000
1250
y2
b1
b2
b3
b4
b5
b6
b7
y1
y3
y4
y5
y6
y7
y8
type
b
y

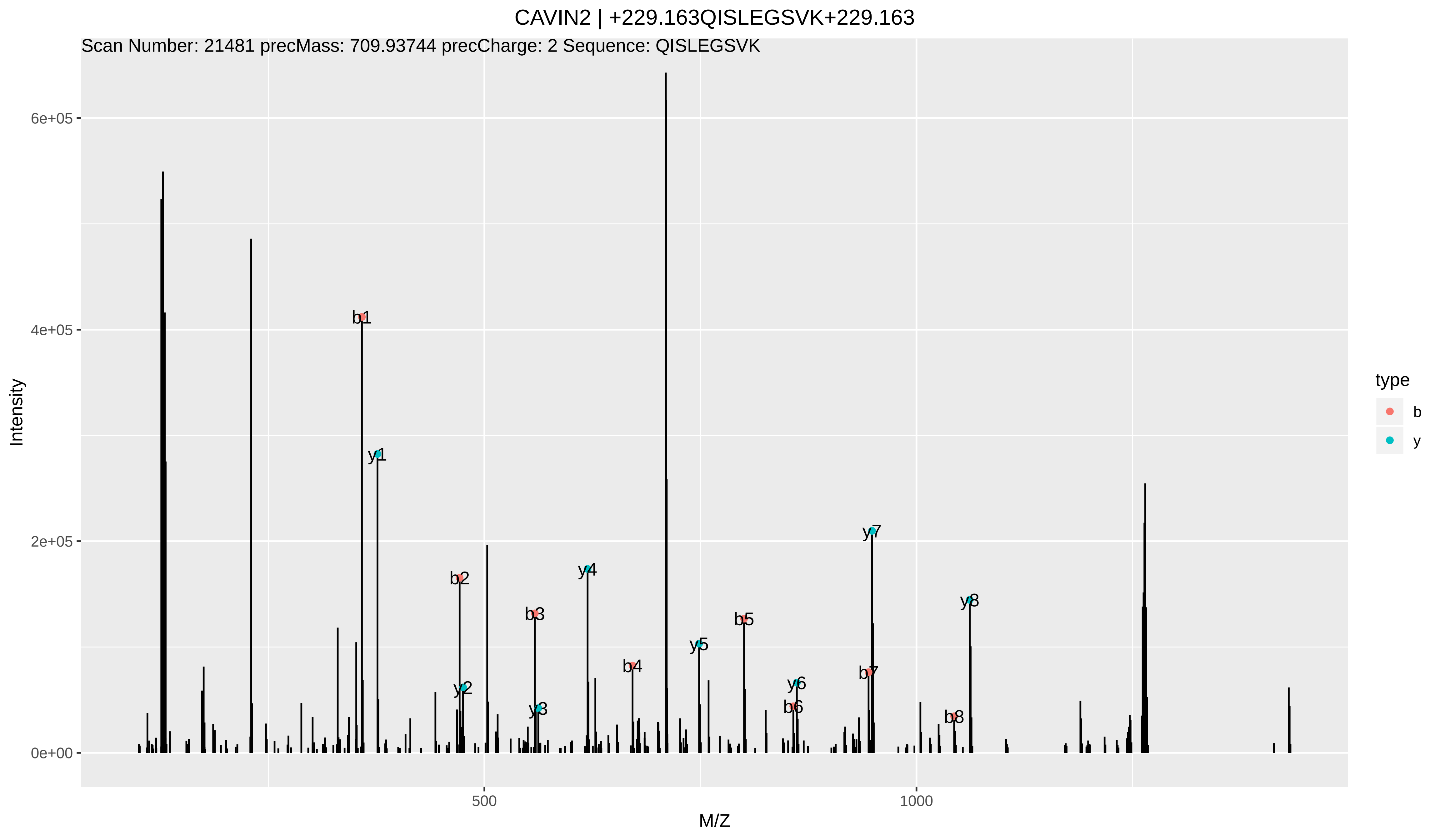
CAVIN2 | +229.163QISLEGSVK+229.163
Scan Number: 21481 precMass: 709.93744 precCharge: 2 Sequence: QISLEGSVK
Intensity
6e+05
4e+05
2e+05
0e+00
M/Z
500
1000
b1
y1
b2
y2
b3
y3
y4
b4
y5
b5
y6
b6
y7
b7
y8
b8
type
b
y

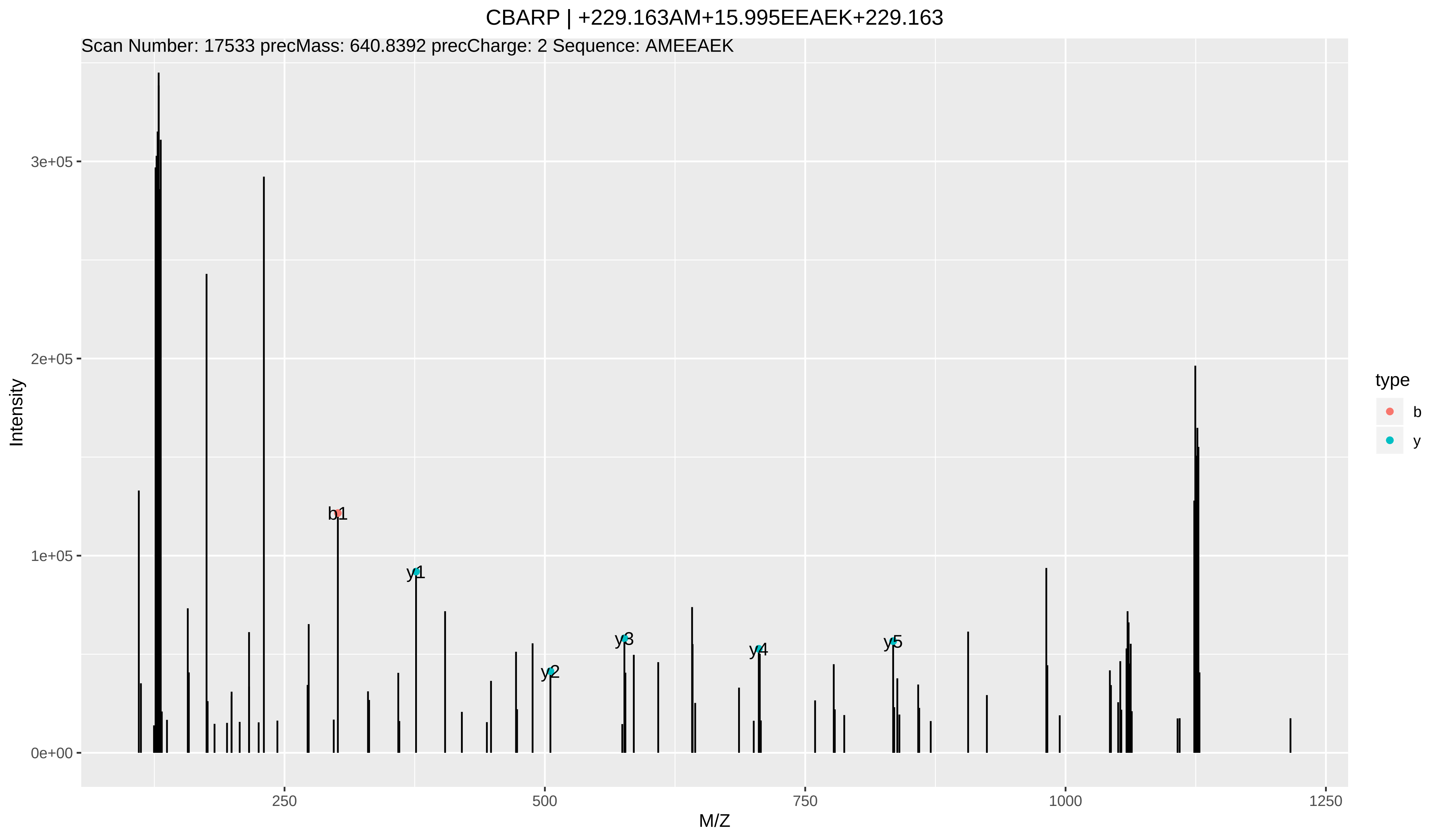
CBARP | +229.163AM+15.995EEAEK+229.163
Scan Number: 17533 precMass: 640.8392 precCharge: 2 Sequence: AMEEAEK
Intensity
3e+05
2e+05
1e+05
0e+00
M/Z
250
500
750
1000
1250
b1
y1
y2
y3
y4
y5
type
b
y

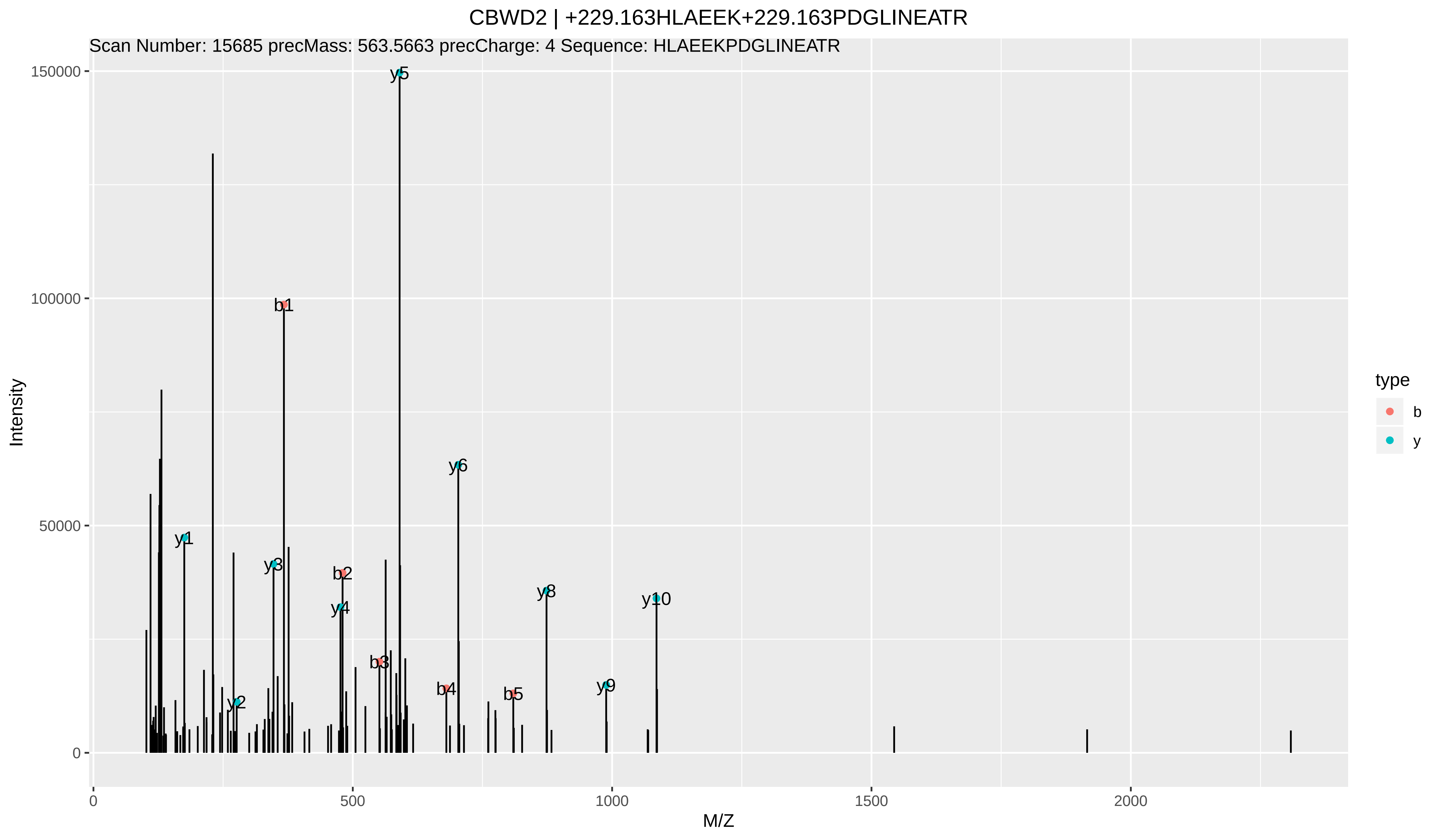
CBWD2 | +229.163HLAEEK+229.163PDGLINEATR
Scan Number: 15685 precMass: 563.5663 precCharge: 4 Sequence: HLAEEKPDGLINEATR
Intensity
150000
100000
50000
0
M/Z
0
500
1000
1500
2000
y5
b1
y6
y1
y3
b2
y4
y8
y10
b3
y2
b4
b5
y9
type
b
y

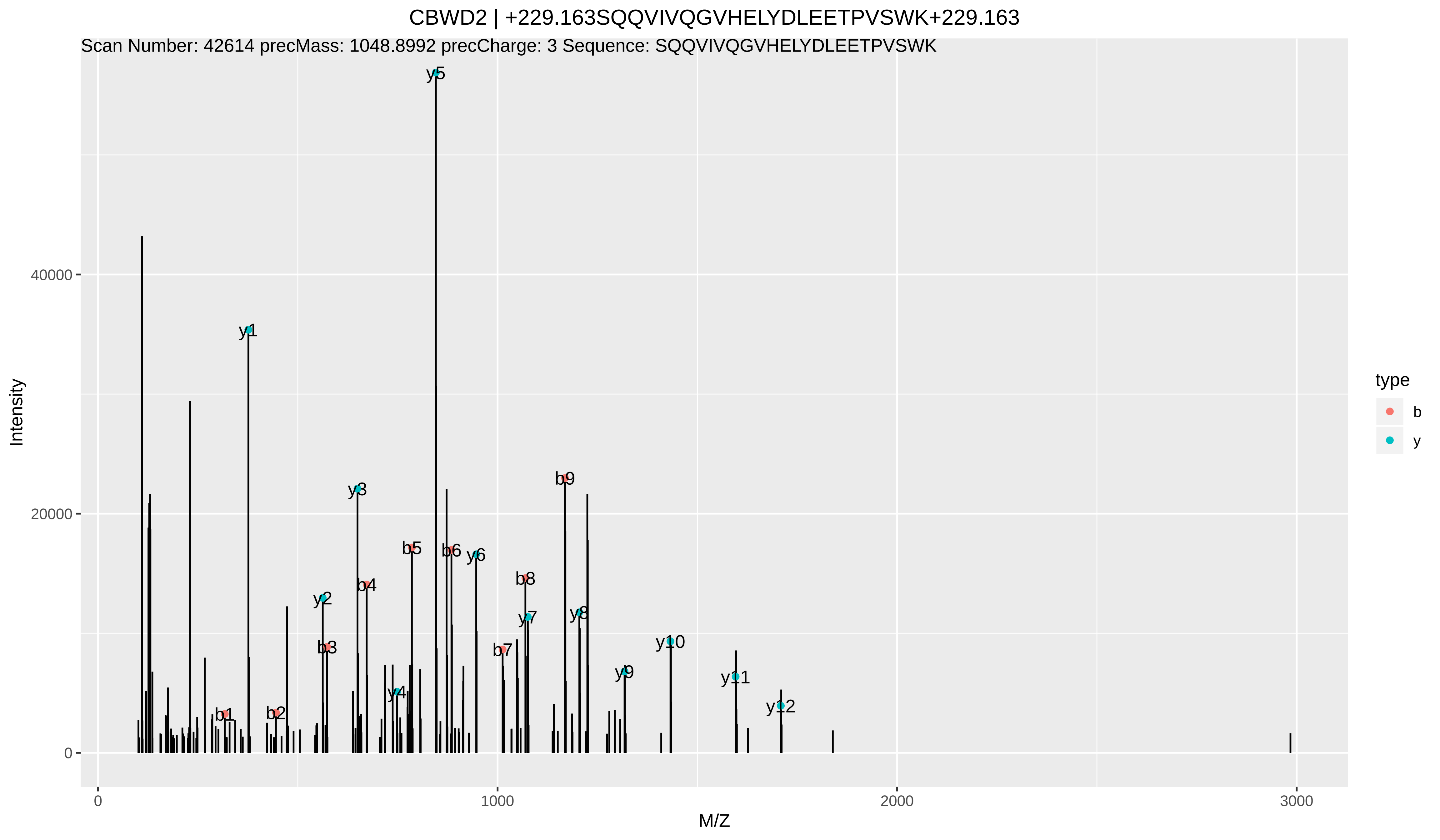
CBWD2 | +229.163SQQVIVQGVHELYDLEETPVSWK+229.163
Scan Number: 42614 precMass: 1048.8992 precCharge: 3 Sequence: SQQVIVQGVHELYDLEETPVSWK
Intensity
M/Z
type
b
y
y5
y1
y3
b9
b5
b6
y6
b4
y2
b8
y7
y8
b3
b7
y10
y9
y11
y4
y12
b1
b2
40000
20000
0
0
1000
2000
3000

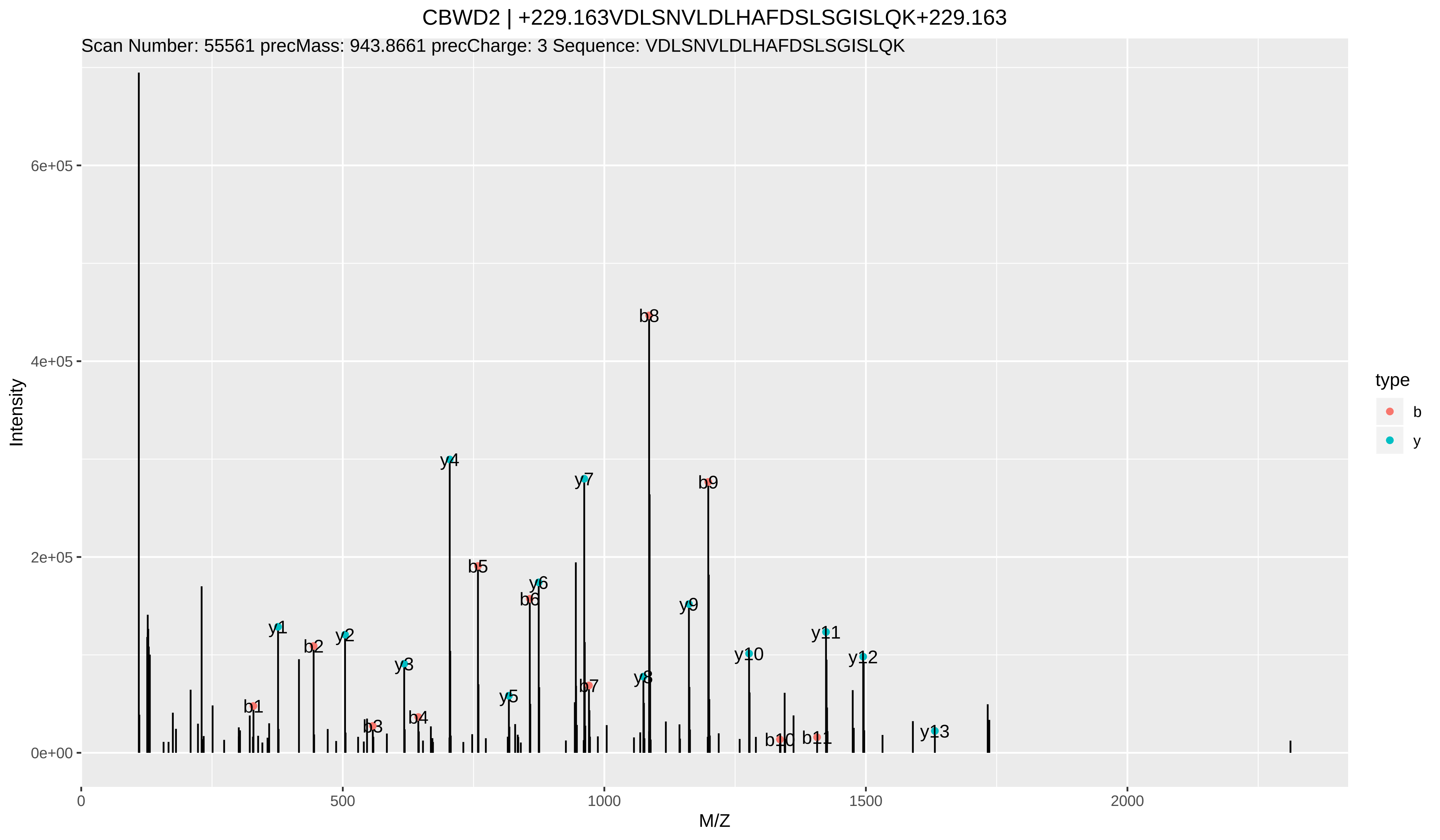
CBWD2 | +229.163VDLSNVLDLHAFDSLSGISLQK+229.163
Scan Number: 55561 precMass: 943.8661 precCharge: 3 Sequence: VDLSNVLDLHAFDSLSGISLQK
Intensity
6e+05
4e+05
2e+05
0e+00
M/Z
0
500
1000
1500
2000
type
b
y
b1
b2
b3
b4
b5
b6
b7
b8
b9
b10
b11
y1
y2
y3
y4
y5
y6
y7
y8
y9
y10
y11
y12
y13

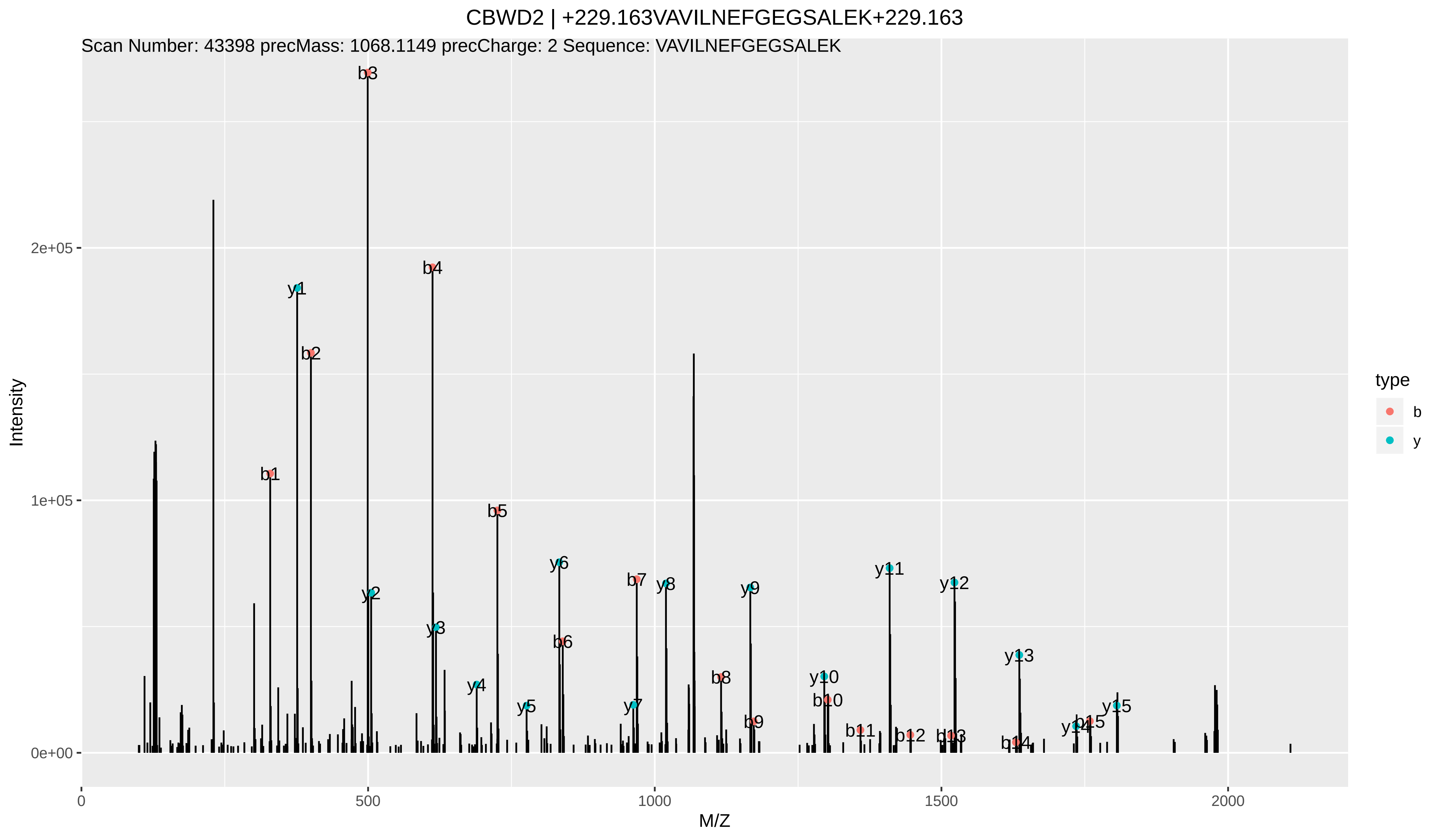

CBWD2 | +229.163VAVILNEFGEGSALEK+229.163
Scan Number: 43398 precMass: 1068.1149 precCharge: 2 Sequence: VAVILNEFGEGSALEK
Intensity
2e+05
1e+05
0e+00
M/Z
0
500
1000
1500
2000
type
b
y
b1
b2
b3
b4
b5
b6
b7
b8
b9
b10
b11
b12
b13
b14
b15
y1
y2
y3
y4
y5
y6
y7
y8
y9
y10
y11
y12
y13
y14
y15

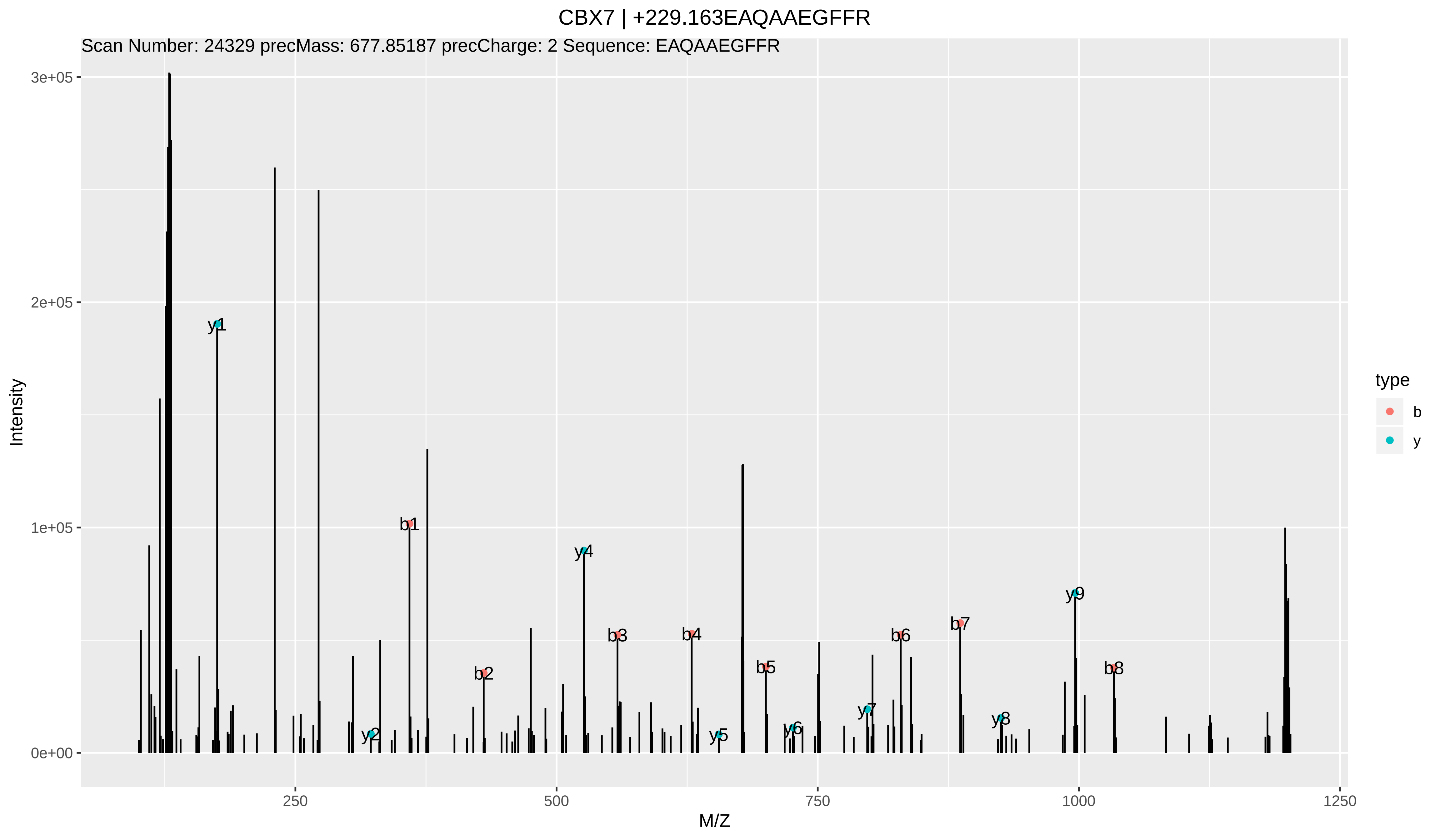

CBX7 | +229.163EAQAAEGFFR
Scan Number: 24329 precMass: 677.85187 precCharge: 2 Sequence: EAQAAEGFFR
Intensity
M/Z
0e+00
1e+05
2e+05
3e+05
250
500
750
1000
1250
y1
y2
b1
b2
y4
b3
b4
y5
b5
y6
y7
b6
b7
y8
y9
b8
type
b
y

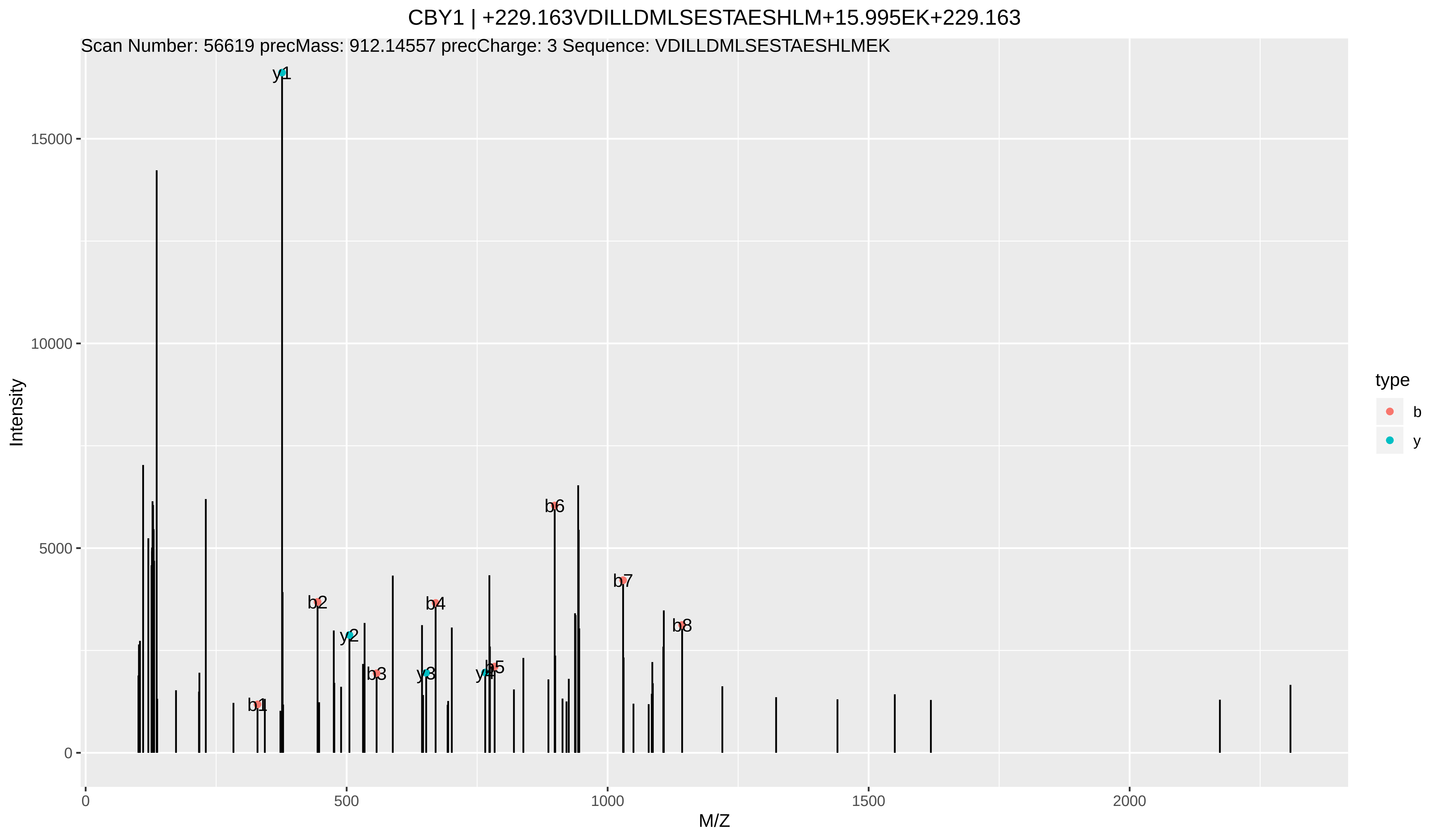
CBY1 | +229.163VDILLDMLSESTAESHLM+15.995EK+229.163
Scan Number: 56619 precMass: 912.14557 precCharge: 3 Sequence: VDILLDMLSESTAESHLMEK
y1
b1
b2
y2
b3
y3
b4
y4
b5
b6
b7
b8
Intensity
15000
10000
5000
0
M/Z
0
500
1000
1500
2000
type
b
y

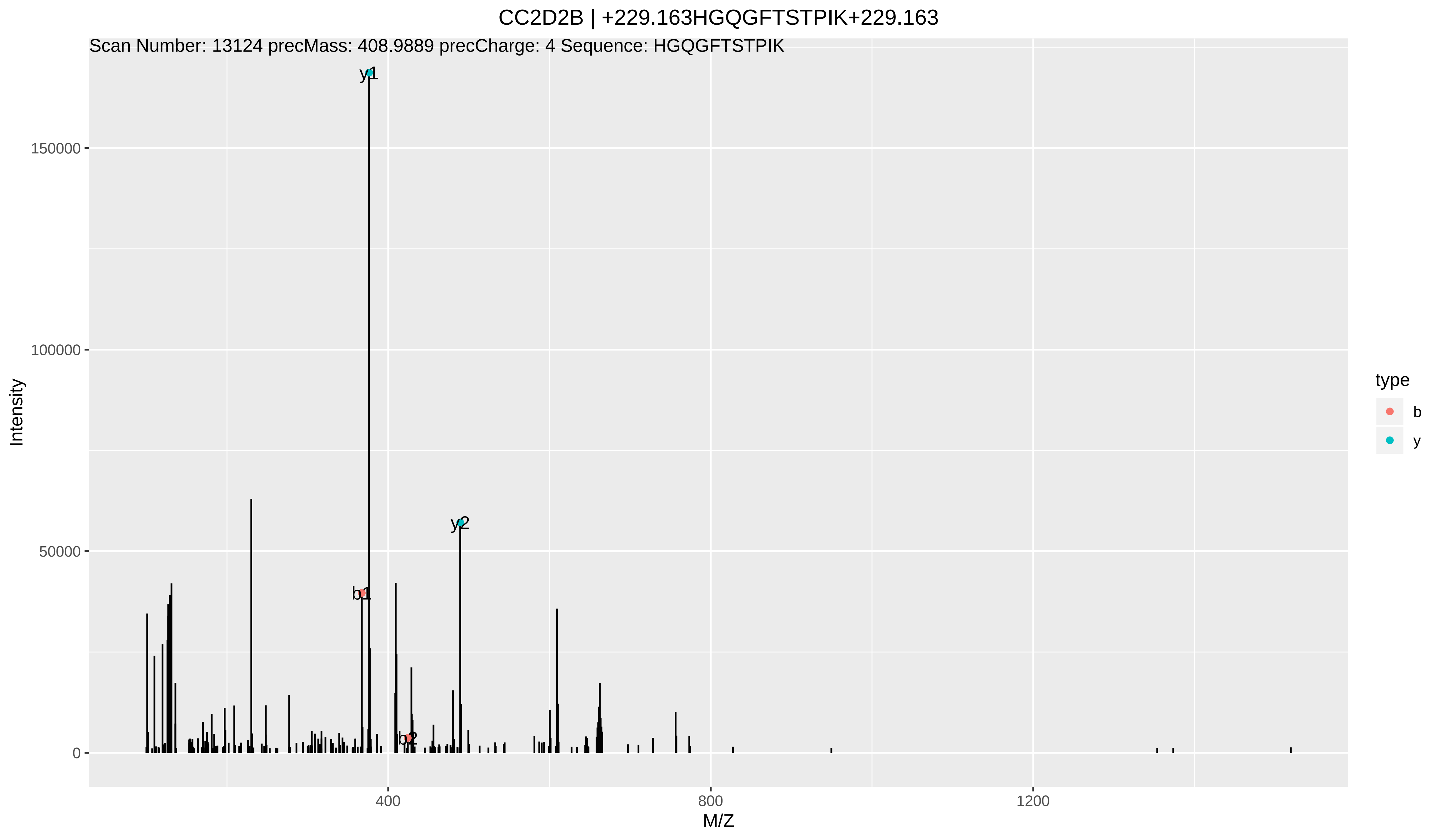

CC2D2B | +229.163HGQGFTSTPIK+229.163
Scan Number: 13124 precMass: 408.9889 precCharge: 4 Sequence: HGQGFTSTPIK
y1
y2
b1
b2
Intensity
150000
100000
50000
0
400
800
1200
M/Z
type
b
y

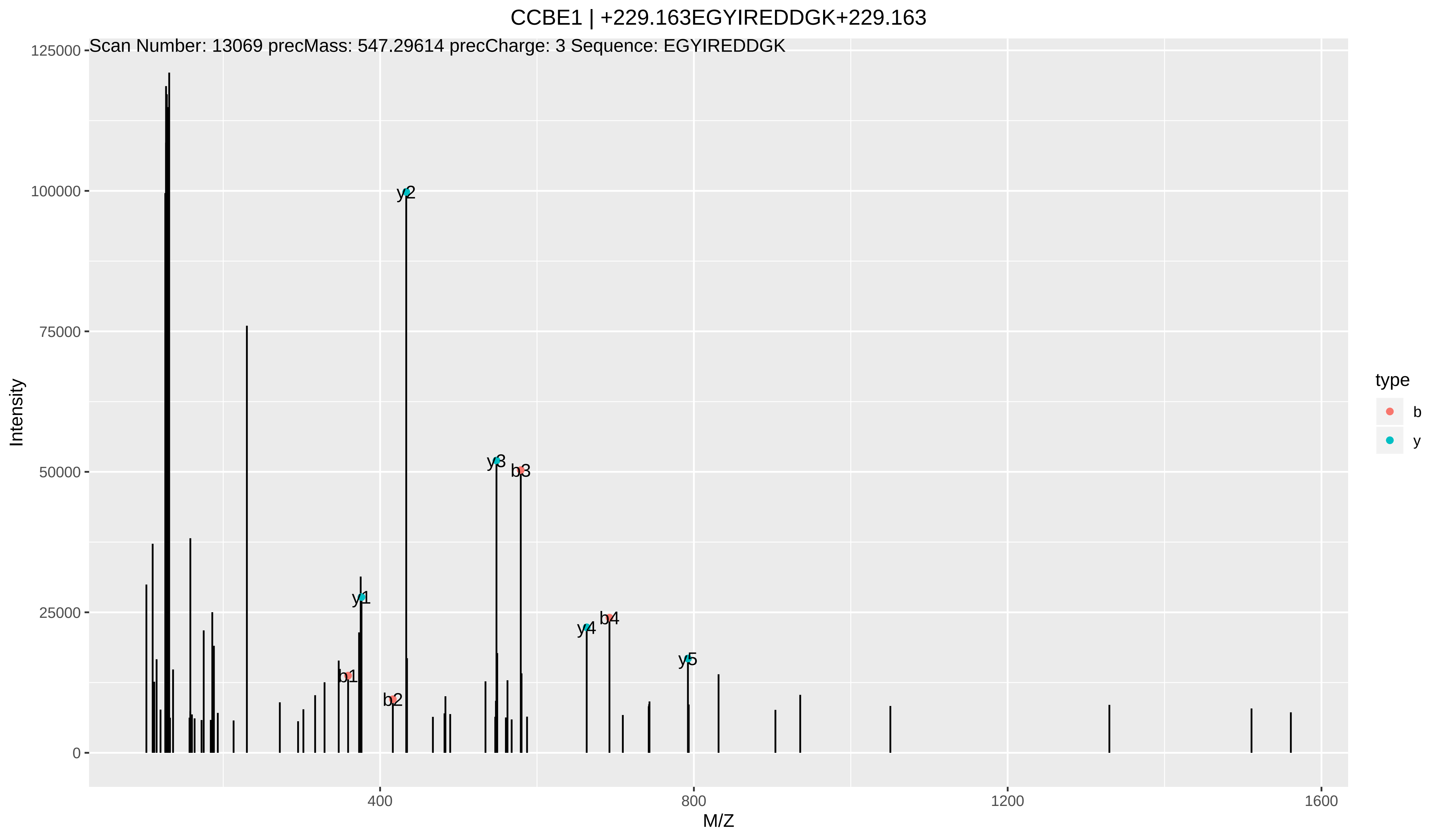
CCBE1 | +229.163EGYIREDDGK+229.163
Scan Number: 13069 precMass: 547.29614 precCharge: 3 Sequence: EGYIREDDGK
Intensity
M/Z
0
25000
50000
75000
100000
125000
400
800
1200
1600
y2
y3
b3
y1
b1
b2
y4
b4
y5
type
b
y

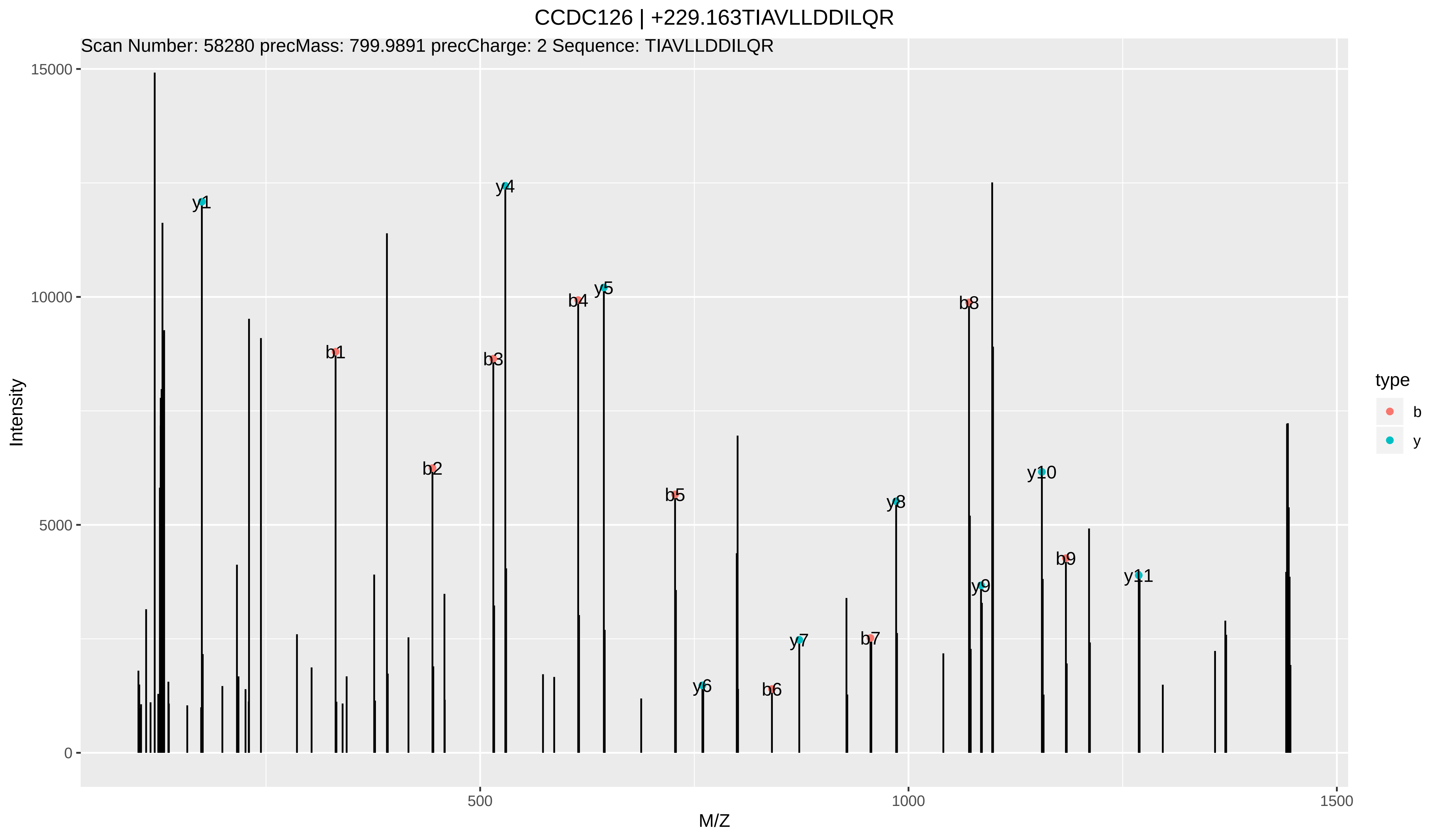
CCDC126 | +229.163TIAVLLDDILQR
Scan Number: 58280 precMass: 799.9891 precCharge: 2 Sequence: TIAVLLDDILQR
Intensity
15000
10000
5000
0
M/Z
500
1000
1500
y1
y4
y5
y6
y7
y8
y9
y10
y11
b1
b2
b3
b4
b5
b6
b7
b8
b9
type
b
y

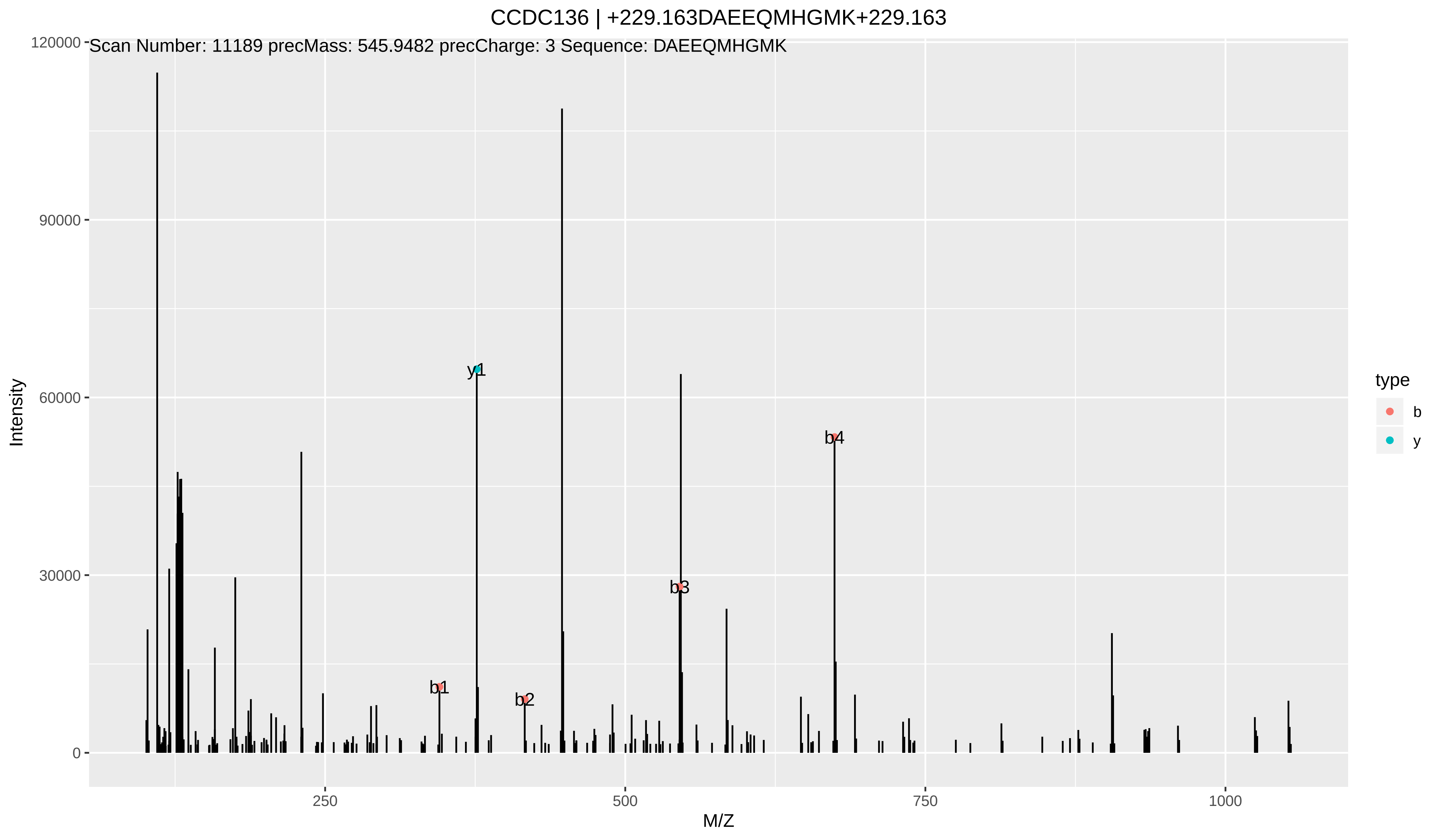
CCDC136 | +229.163DAEEQMHGMK+229.163
Scan Number: 11189 precMass: 545.9482 precCharge: 3 Sequence: DAEEQMHGMK
Intensity
M/Z
120000
90000
60000
30000
0
250
500
750
1000
y1
b1
b2
b3
b4
type
b
y

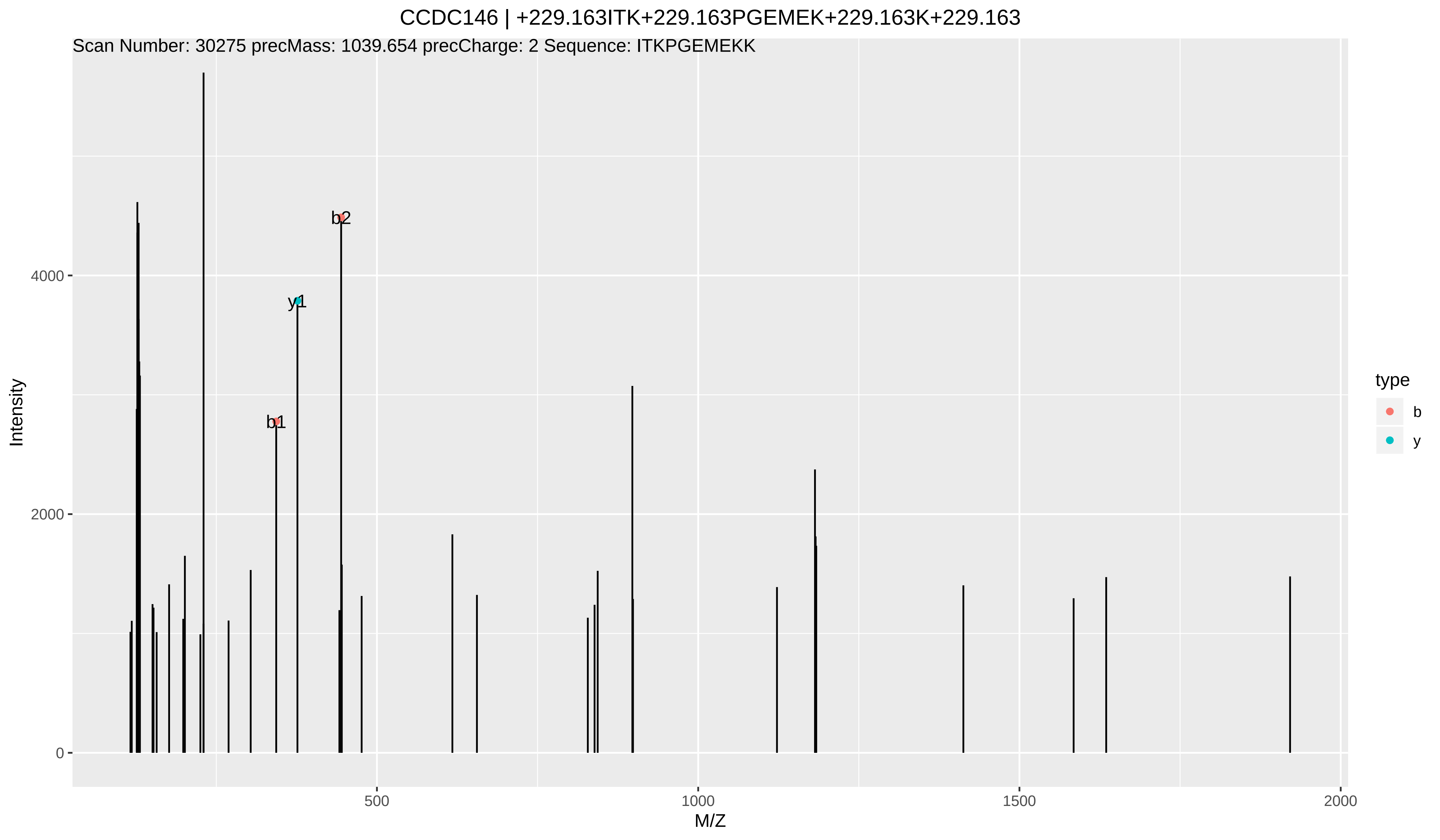

CCDC146 | +229.163ITK+229.163PGEMEK+229.163K+229.163
Scan Number: 30275 precMass: 1039.654 precCharge: 2 Sequence: ITKPGEMEKK
Intensity
M/Z
b1
y1
b2
0
2000
4000
500
1000
1500
2000
type
b
y

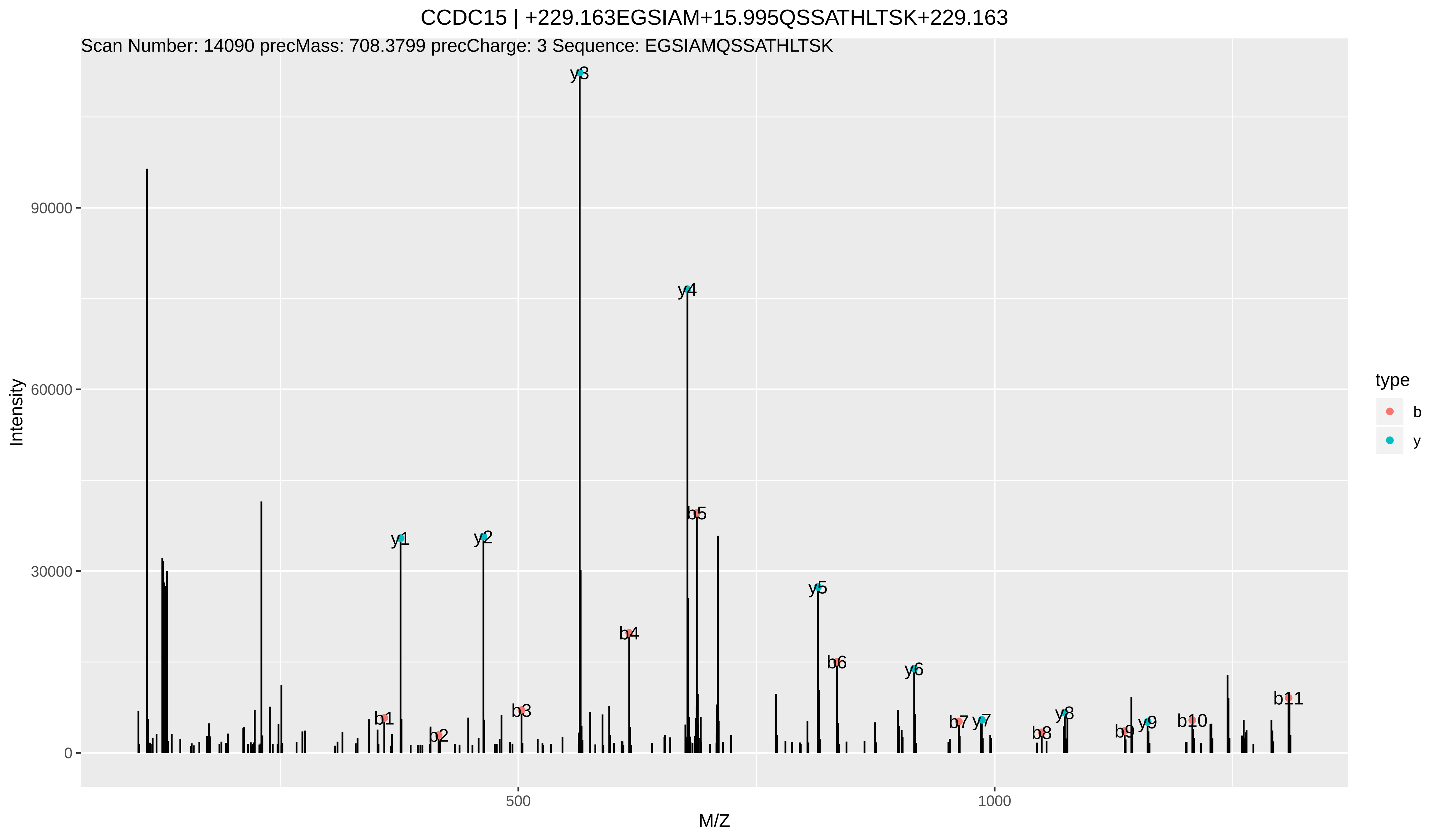

CCDC15 | +229.163EGSIAM+15.995QSSATHLTSK+229.163
Scan Number: 14090 precMass: 708.3799 precCharge: 3 Sequence: EGSIAMQSSATHLTSK
Intensity
M/Z
90000
60000
30000
0
500
1000
y3
y4
b5
y1
y2
y5
b4
b6
y6
b11
b1
b2
b3
b7
y7
b8
y8
b9
y9
b10
type
b
y

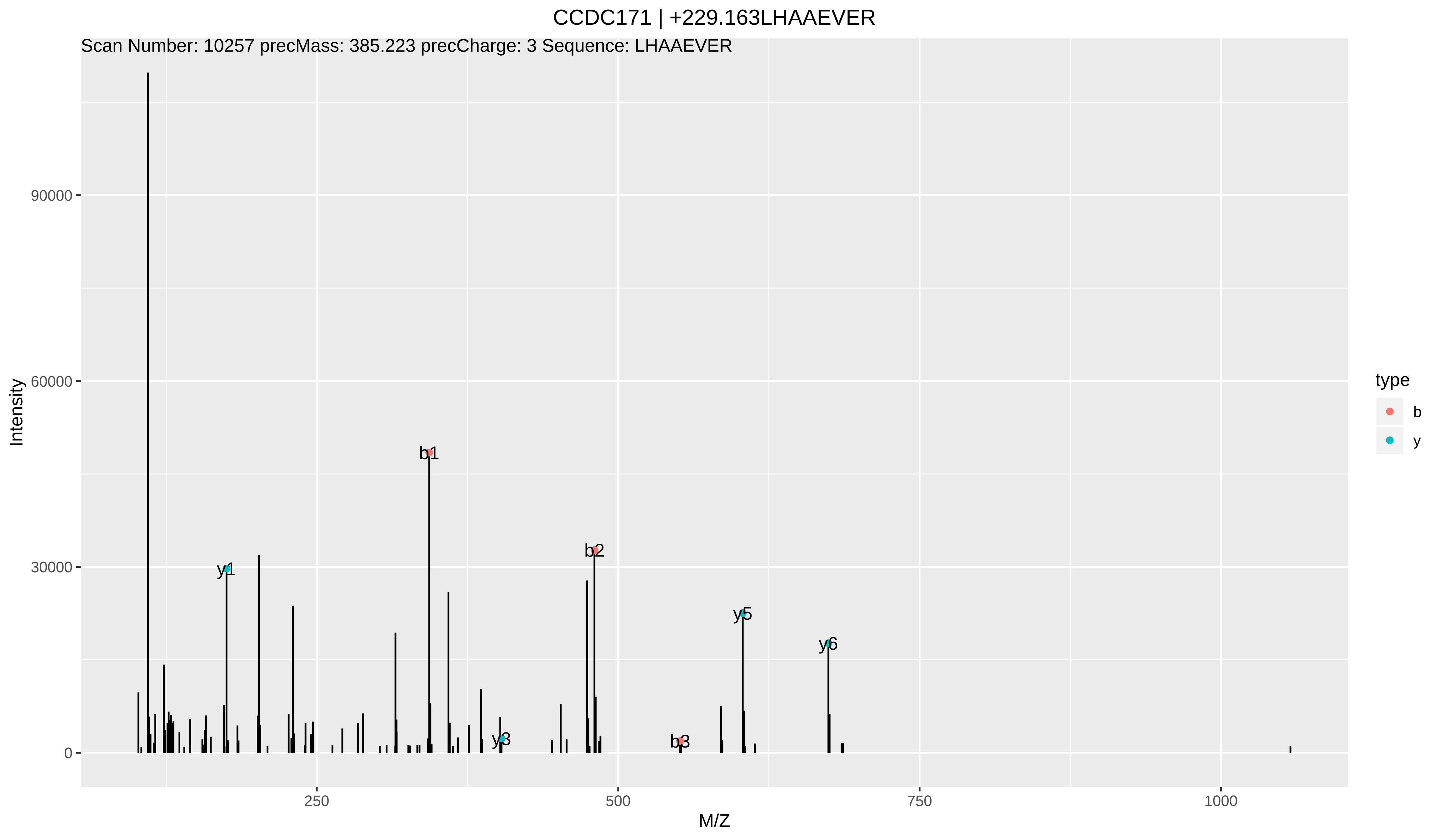
CCDC171 | +229.163LHAAEVER
Scan Number: 10257 precMass: 385.223 precCharge: 3 Sequence: LHAAEVER
Intensity
M/Z
90000
60000
30000
0
250
500
750
1000
y1
b1
b2
y3
b3
y5
y6
type
b
y

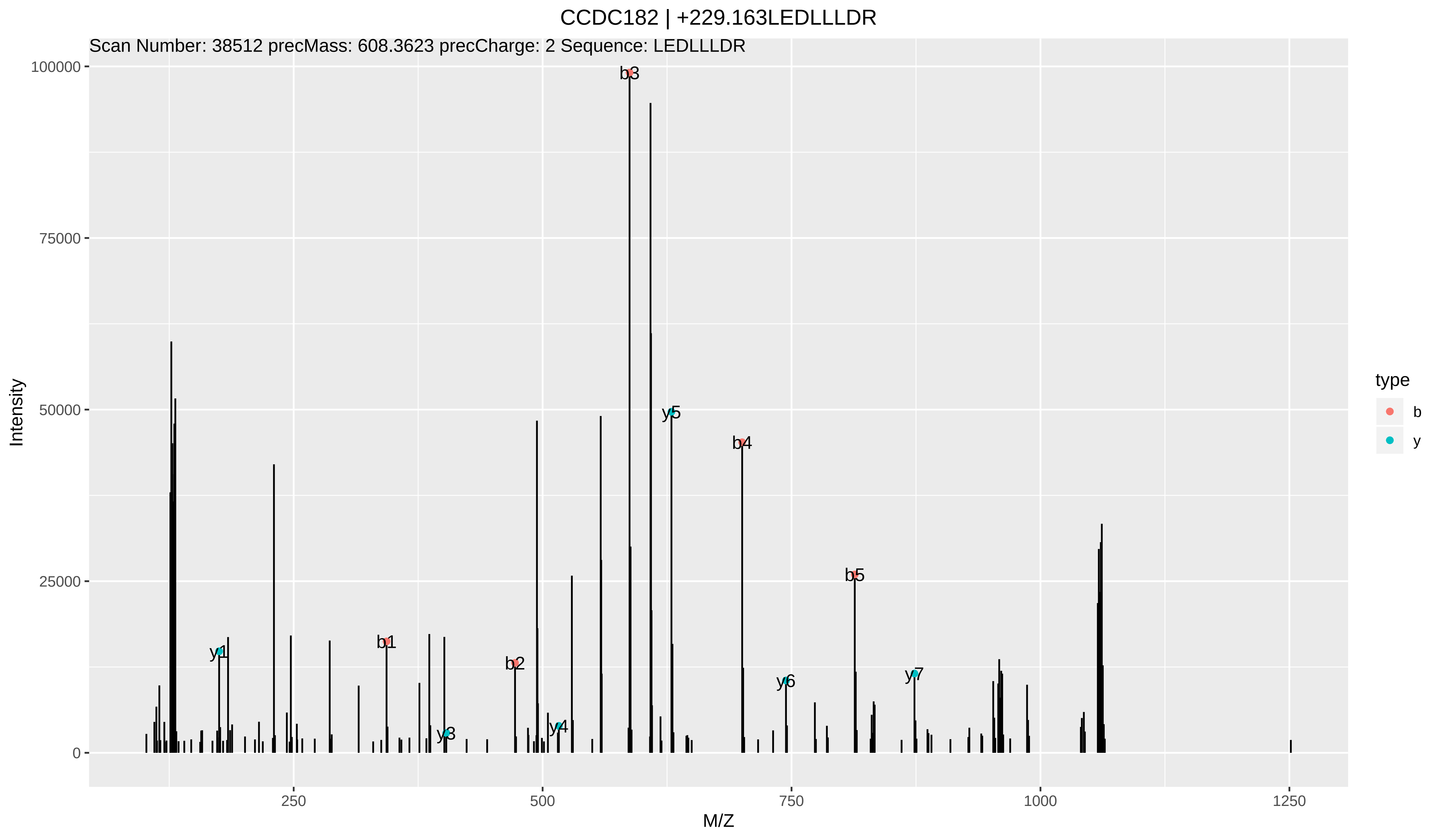
CCDC182 | +229.163LEDLLLDR
Scan Number: 38512 precMass: 608.3623 precCharge: 2 Sequence: LEDLLLDR
Intensity
100000
75000
50000
25000
0
M/Z
250
500
750
1000
1250
b3
y5
b4
b5
y1
b1
b2
y3
y4
y6
y7
type
b
y

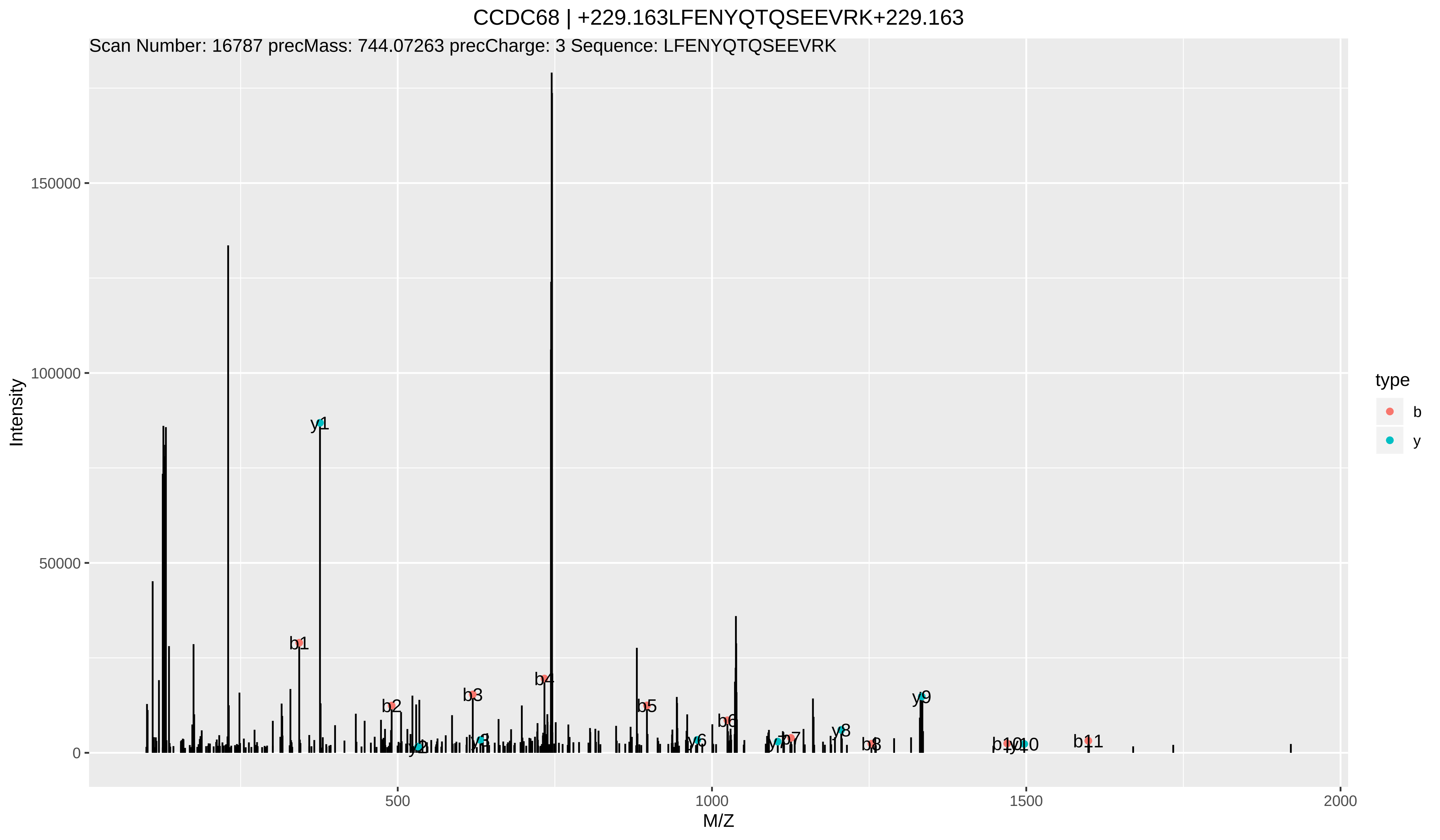

CCDC68 | +229.163LFENYQTQSEEVRK+229.163
Scan Number: 16787 precMass: 744.07263 precCharge: 3 Sequence: LFENYQTQSEEVRK
Intensity
M/Z
type
b
y
0
50000
100000
150000
500
1000
1500
2000
y1
b1
b2
y2
b3
y3
b4
b5
y6
b6
y7
b7
y8
b8
y9
b10
y10
b11

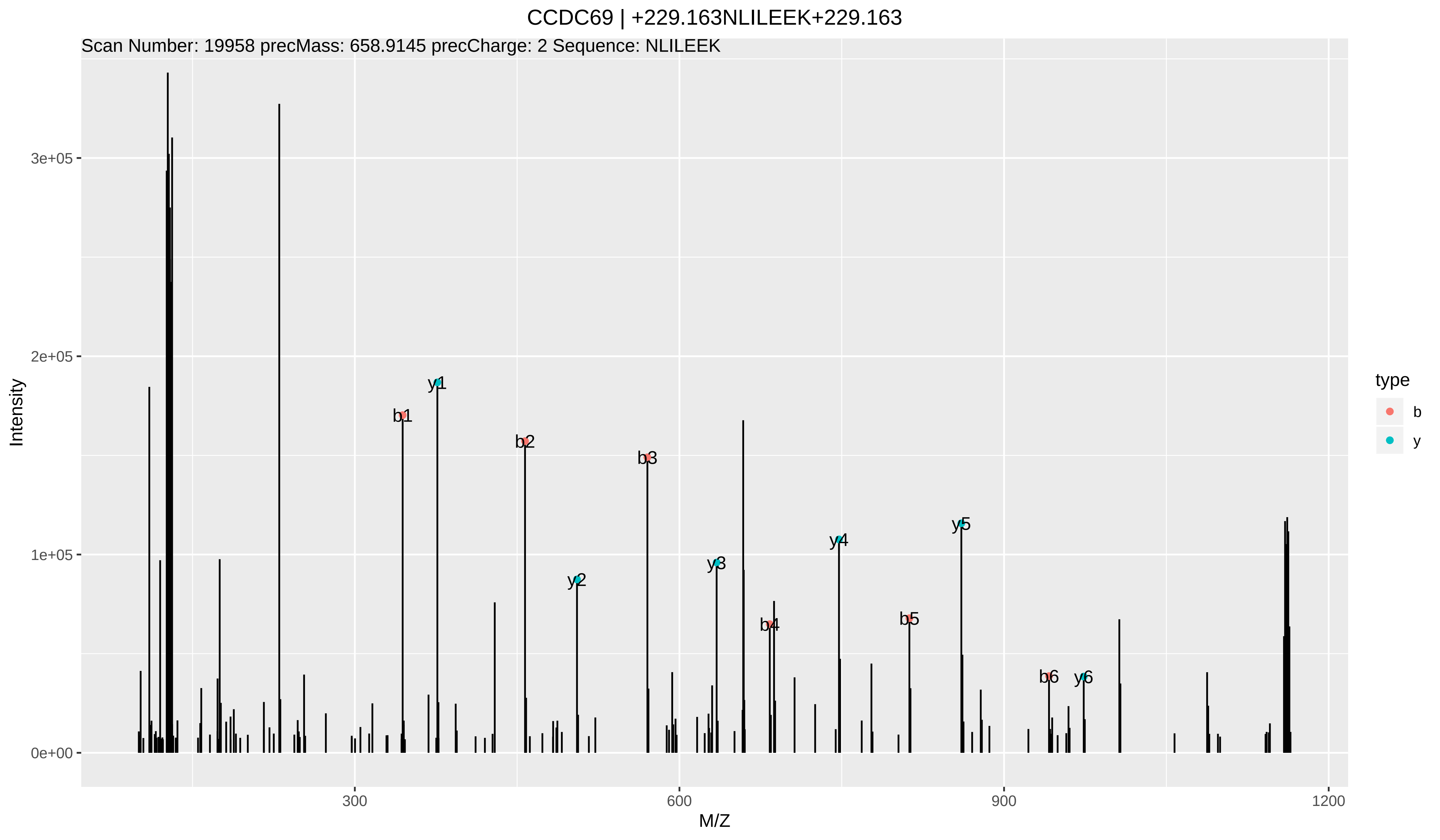

CCDC69 | +229.163NLILEEK+229.163
Scan Number: 19958 precMass: 658.9145 precCharge: 2 Sequence: NLILEEK
Intensity
M/Z
0e+00
1e+05
2e+05
3e+05
300
600
900
1200
y1
b1
b2
y2
b3
y3
b4
y4
b5
y5
b6
y6
type
b
y

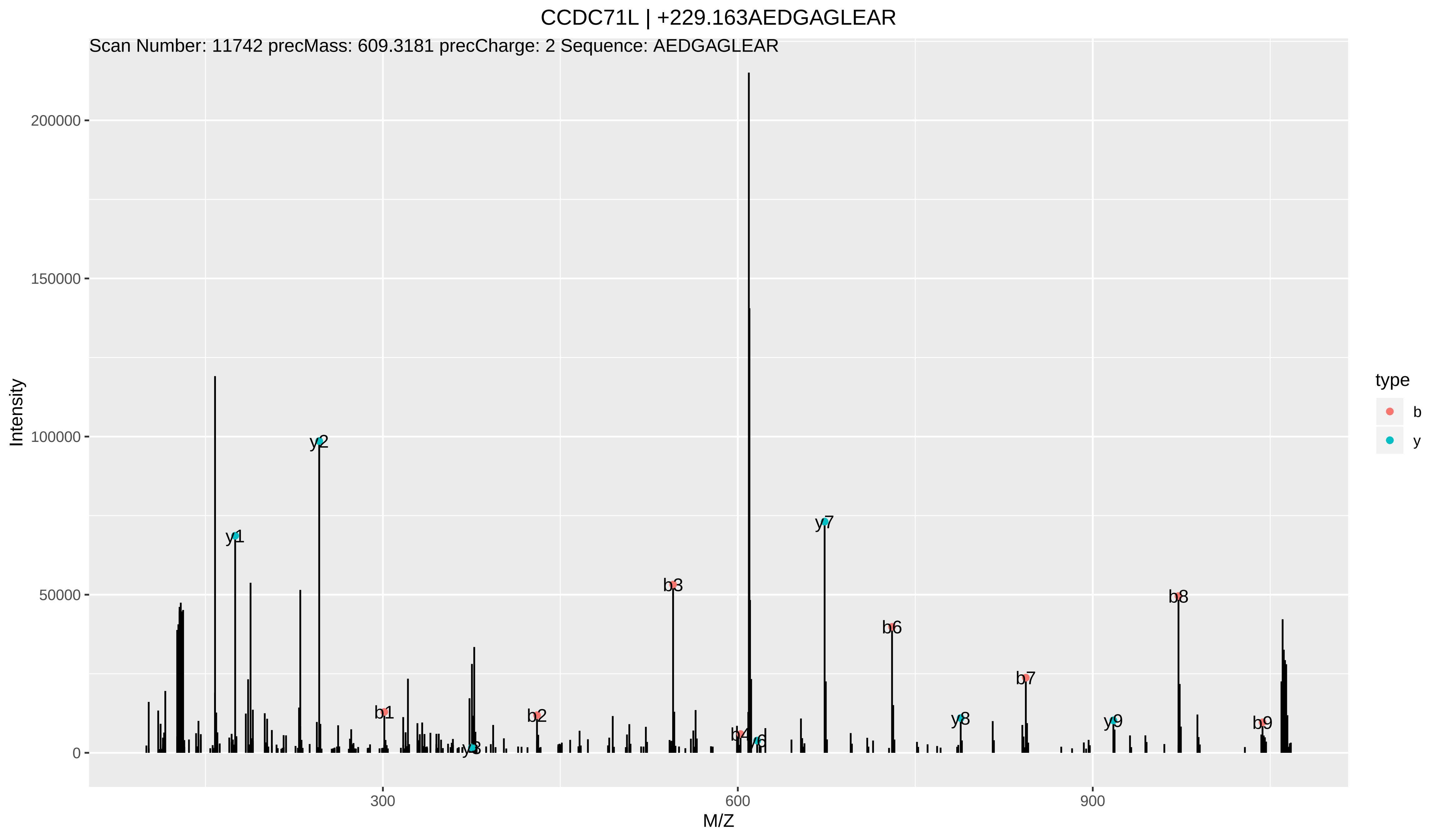
CCDC71L | +229.163AEDGAGLEAR
Scan Number: 11742 precMass: 609.3181 precCharge: 2 Sequence: AEDGAGLEAR
Intensity
0
50000
100000
150000
200000
M/Z
300
600
900
type
b
y
y1
y2
y3
y6
y7
y8
y9
b1
b2
b3
b4
b6
b7
b8
b9

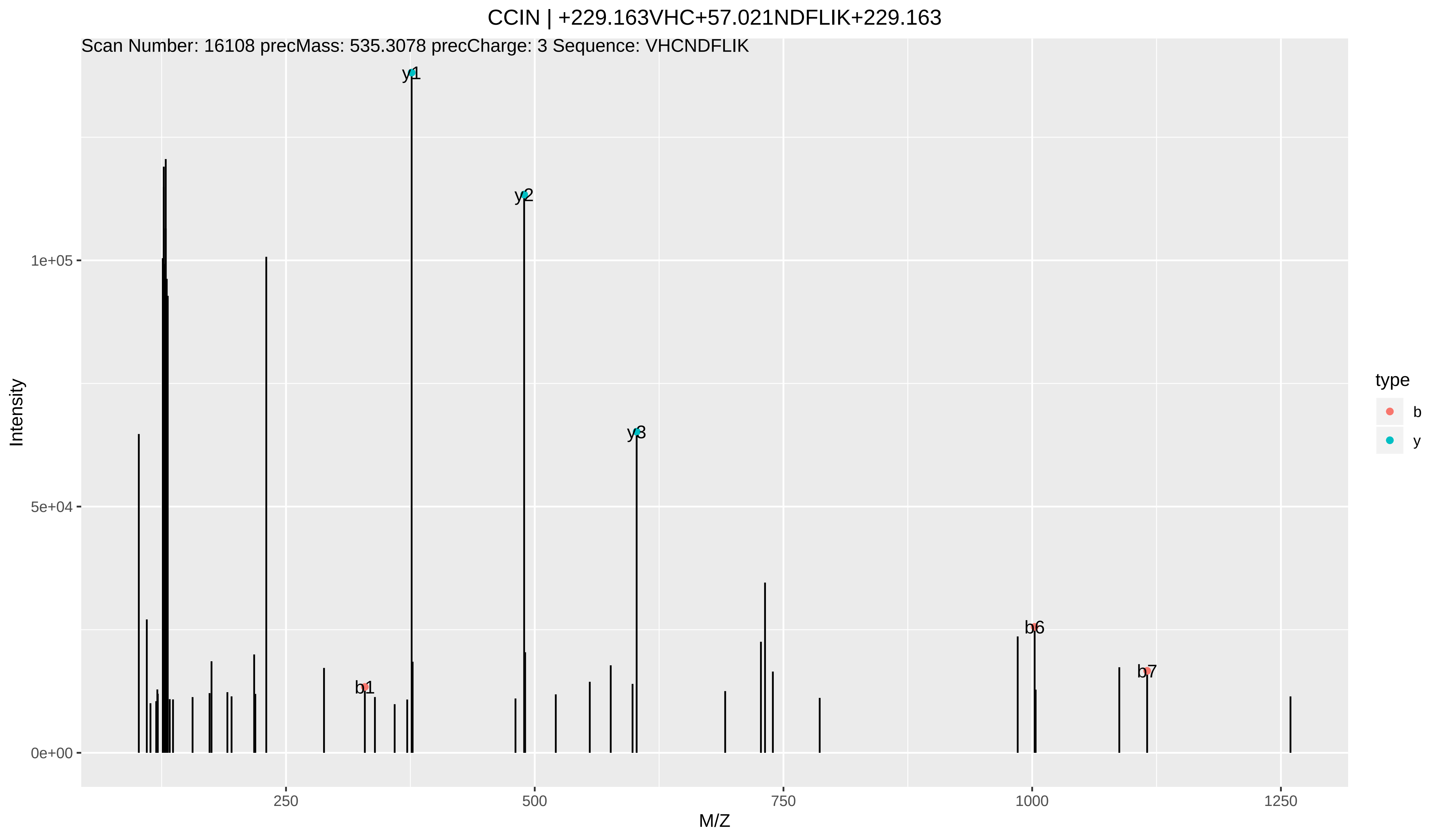

CCIN | +229.163VHC+57.021NDFLIK+229.163
Scan Number: 16108 precMass: 535.3078 precCharge: 3 Sequence: VHCNDFLIK
y1
y2
y3
b1
b6
b7
Intensity
1e+05
5e+04
0e+00
250
500
750
1000
1250
M/Z
type
b
y

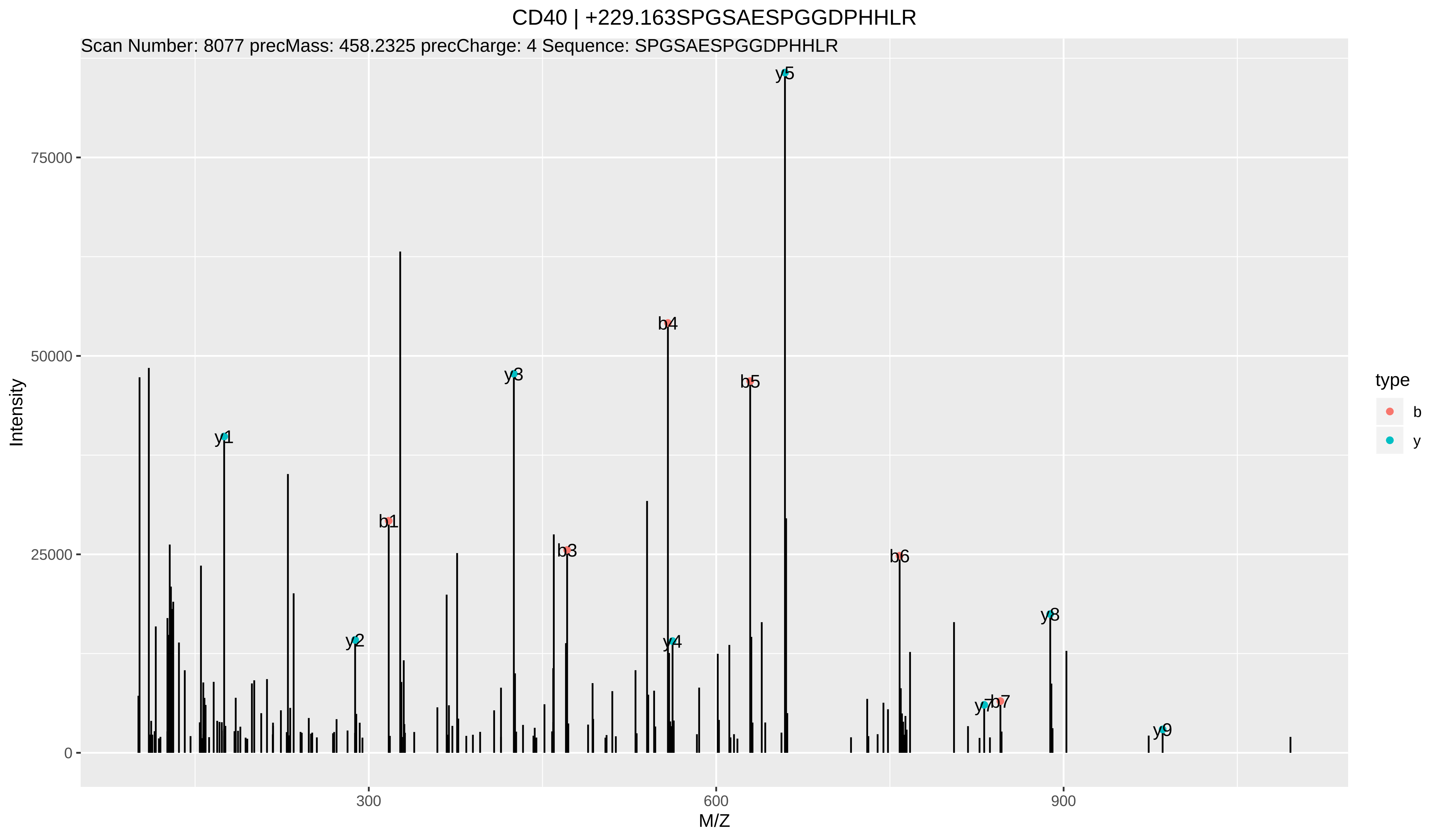

CD40 | +229.163SPGSAESPGGDPHHLR
Scan Number: 8077 precMass: 458.2325 precCharge: 4 Sequence: SPGSAESPGGDPHHLR
Intensity
M/Z
0
25000
50000
75000
300
600
900
type
b
y
y1
y2
b1
y3
b3
b4
y4
b5
y5
b6
y7
b7
y8
y9

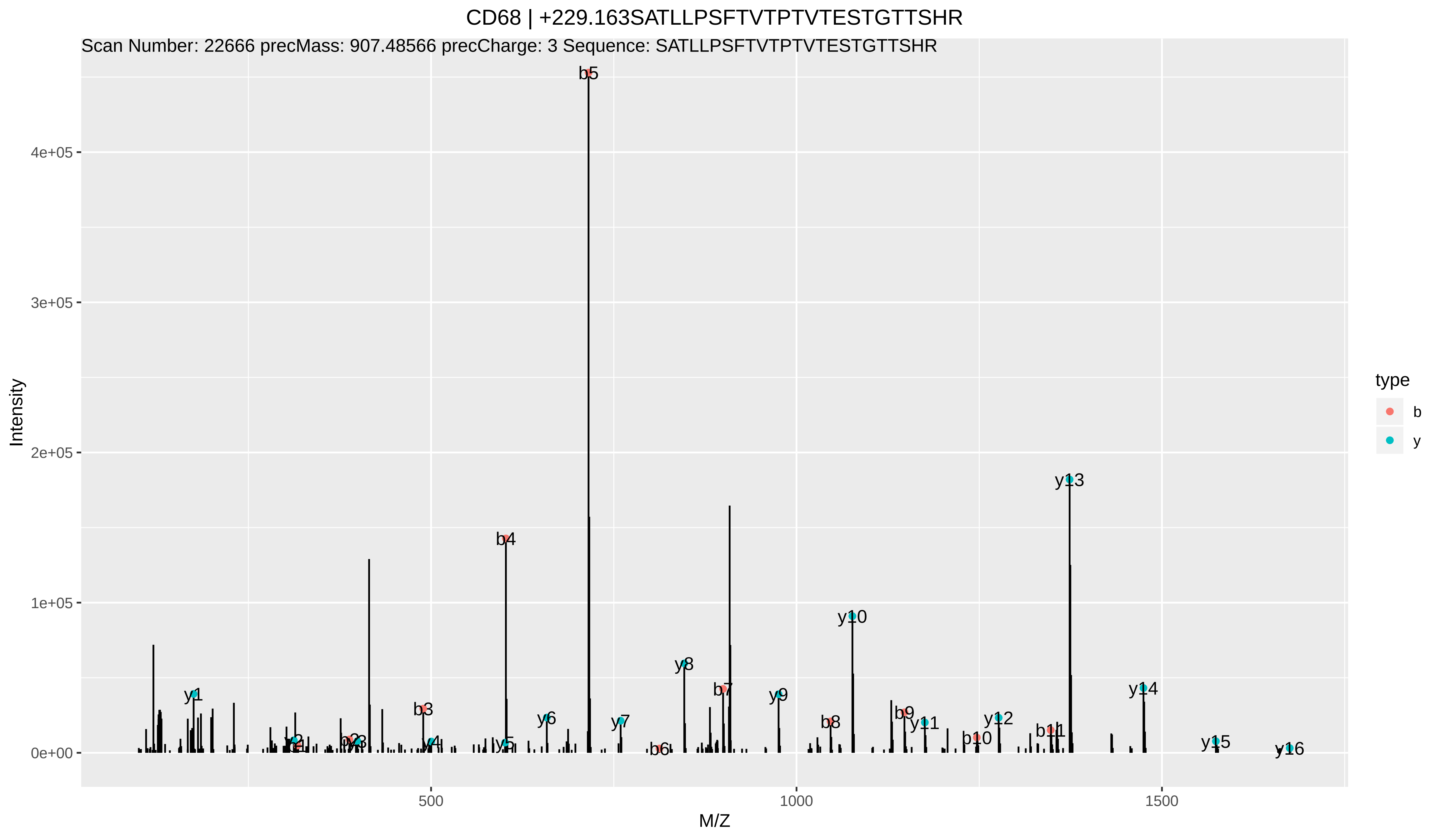
CD68 | +229.163SATLLPSFTVTPTVTESTGTTSHR
Scan Number: 22666 precMass: 907.48566 precCharge: 3 Sequence: SATLLPSFTVTPTVTESTGTTSHR
Intensity
4e+05
3e+05
2e+05
1e+05
0e+00
M/Z
500
1000
1500
type
b
y
b3
b4
b5
b6
b7
b8
b9
b10
b11
y1
y4
y5
y6
y7
y8
y9
y10
y11
y12
y13
y14
y15
y16

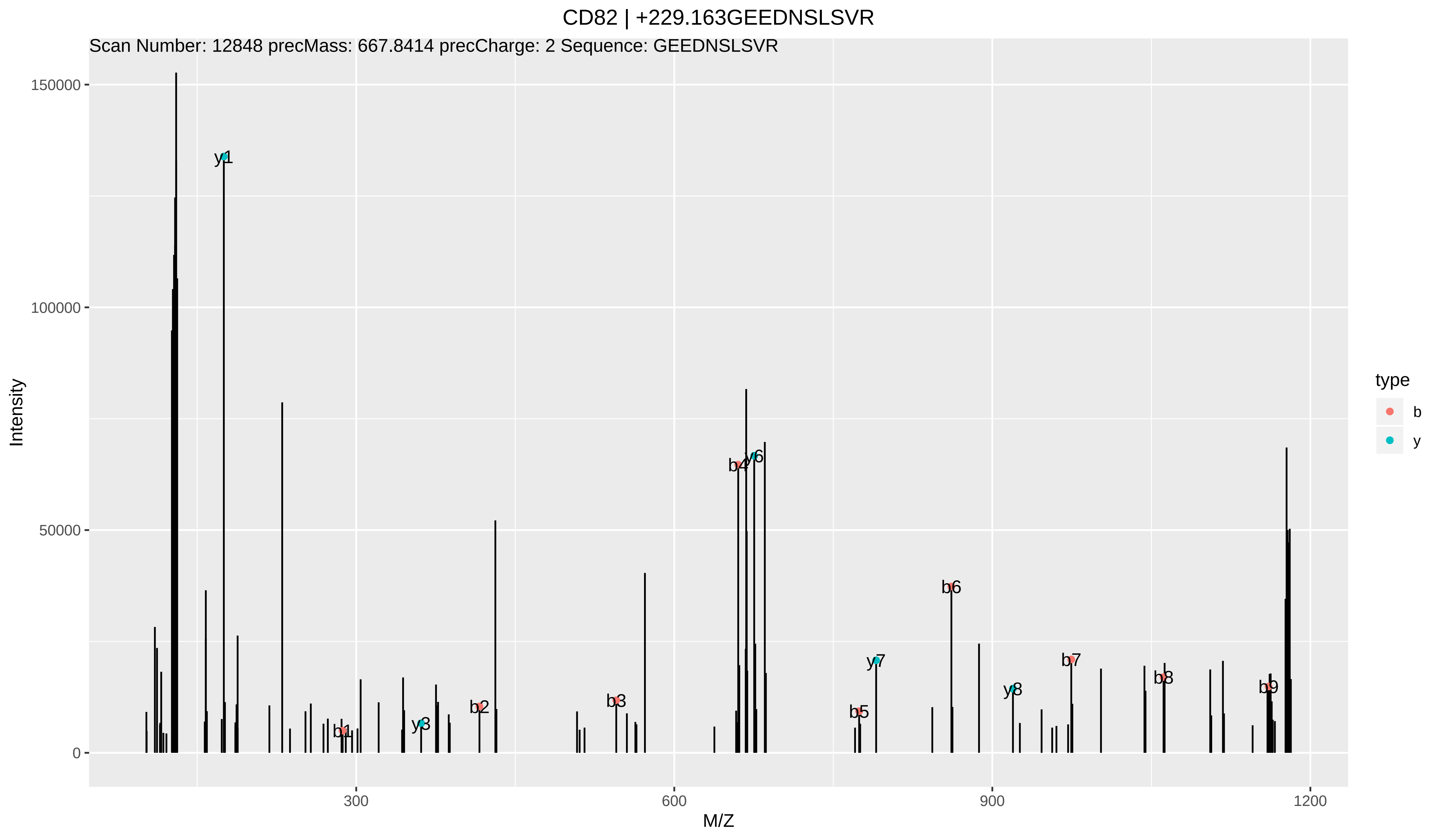
CD82 | +229.163GEEDNSLSVR
Scan Number: 12848 precMass: 667.8414 precCharge: 2 Sequence: GEEDNSLSVR
Intensity
M/Z
type
b
y
y1
b1
y3
b2
b3
b4
y6
b5
y7
b6
y8
b7
b8
b9
0
50000
100000
150000
300
600
900
1200

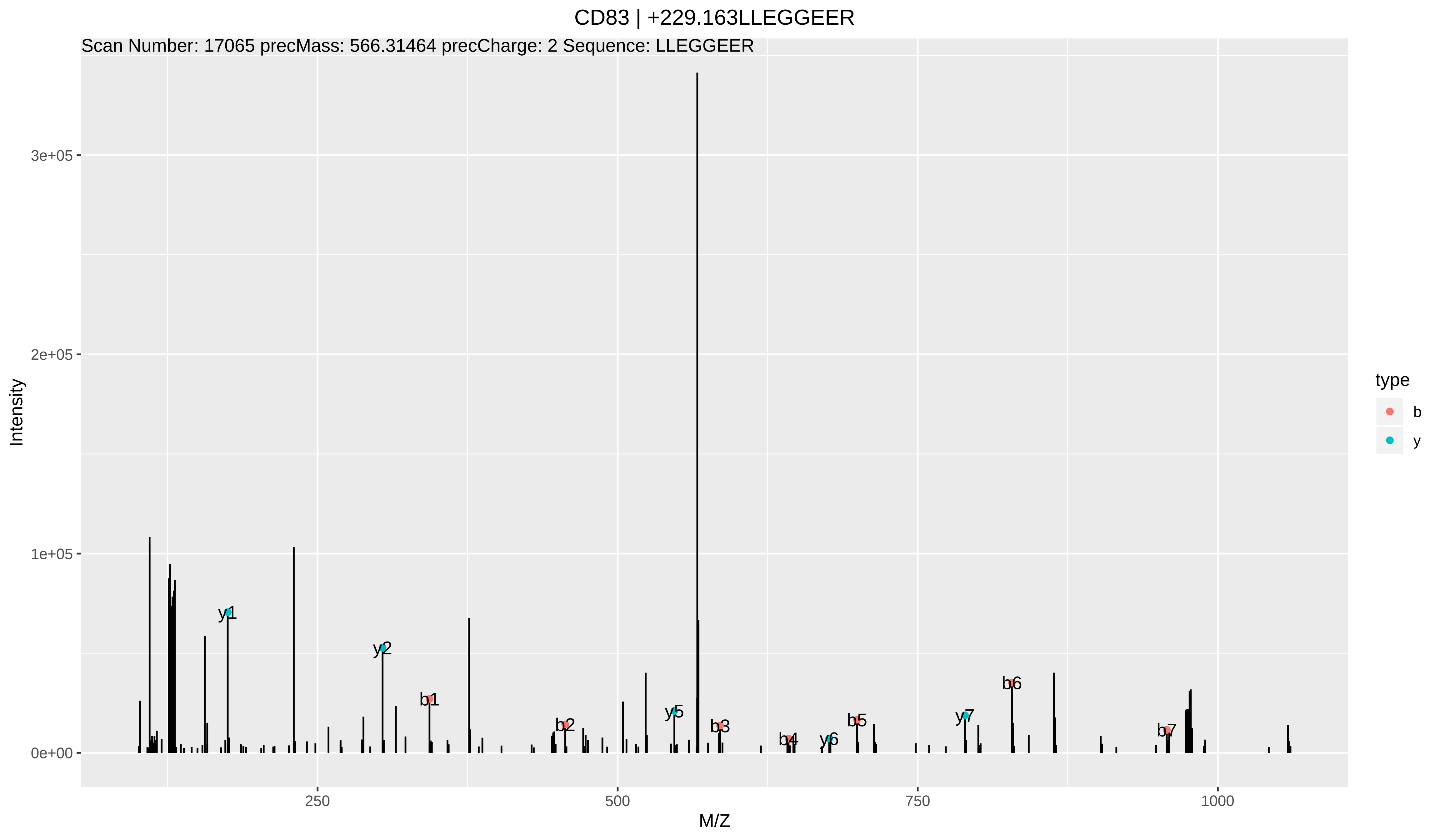
CD83 | +229.163LLEGGEER
Scan Number: 17065 precMass: 566.31464 precCharge: 2 Sequence: LLEGGEER
Intensity
3e+05
2e+05
1e+05
0e+00
M/Z
250
500
750
1000
y1
y2
b1
b2
y5
b3
b4
y6
b5
y7
b6
b7
type
b
y

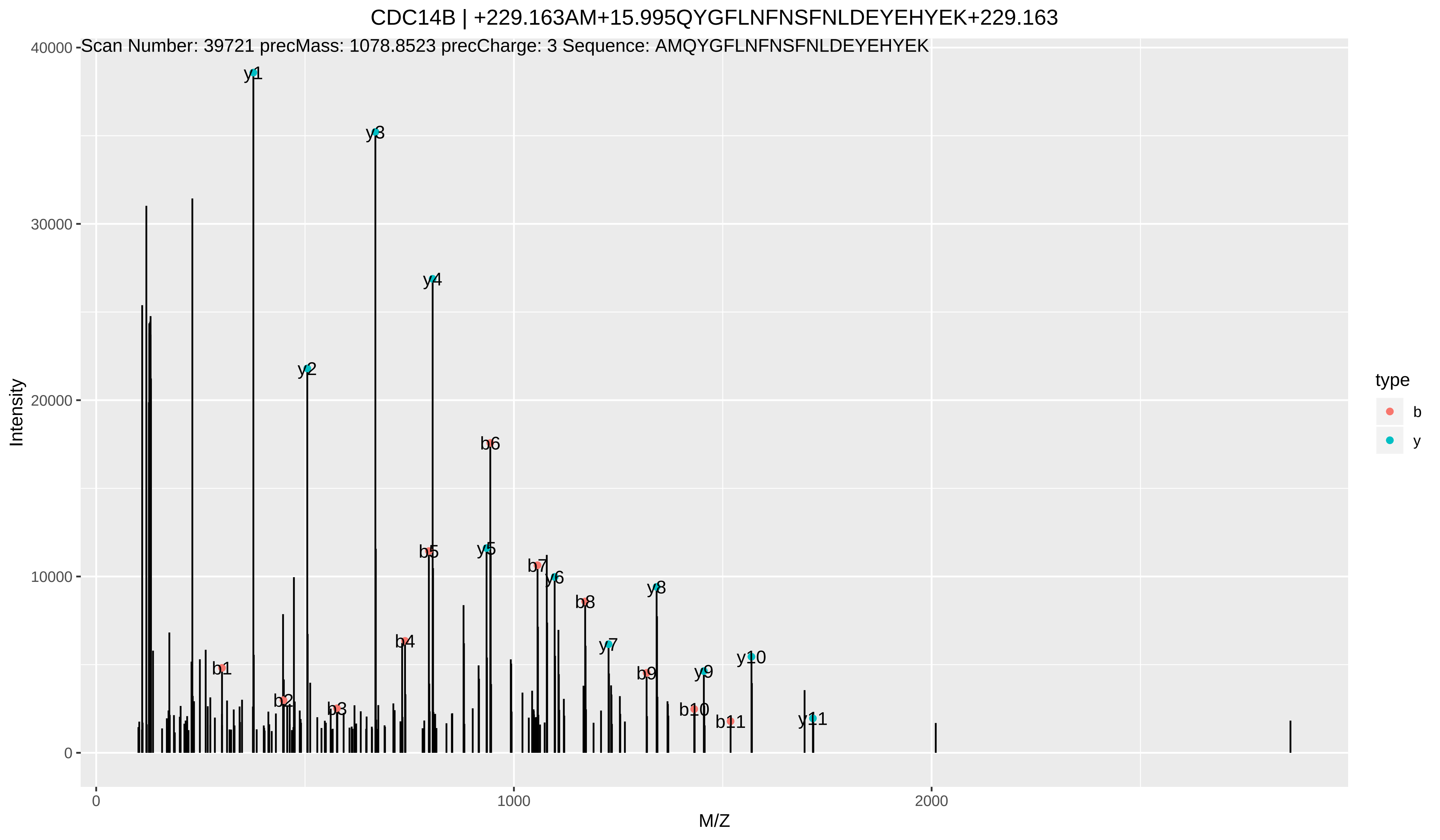

CDC14B | +229.163AM+15.995QYGFLNFNSFNLDEYEHYEK+229.163
Scan Number: 39721 precMass: 1078.8523 precCharge: 3 Sequence: AMQYGFLNFNSFNLDEYEHYEK
Intensity
M/Z
0
10000
20000
30000
40000
0
1000
2000
y1
y2
y3
y4
y5
y6
y7
y8
y9
y10
y11
b1
b2
b3
b4
b5
b6
b7
b8
b9
b10
b11
type
b
y

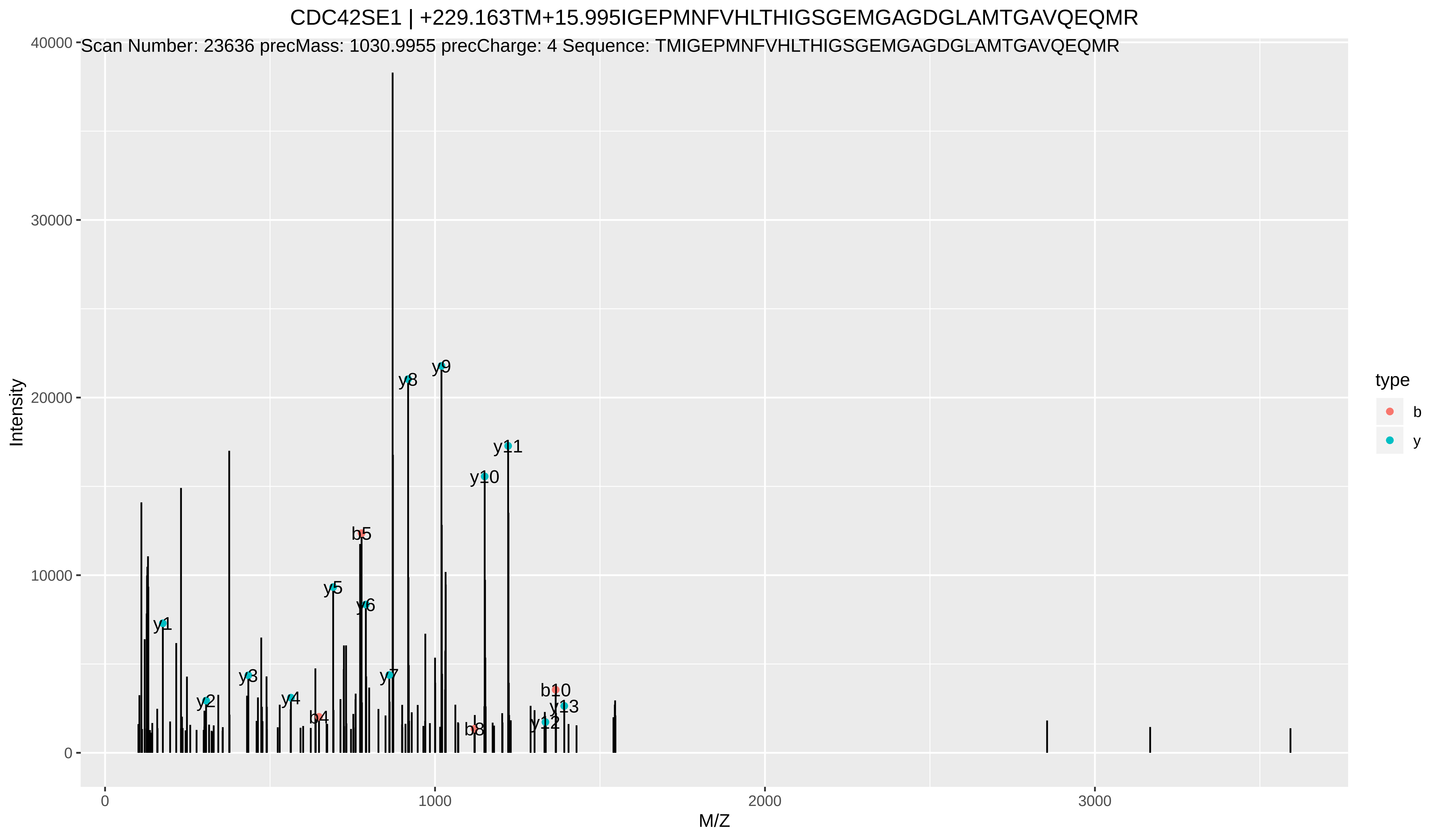

CDC42SE1 | +229.163TM+15.995IGEPMNFVHLTHIGSGEMGAGDGLAMTGAVQEQMR
Scan Number: 23636 precMass: 1030.9955 precCharge: 4 Sequence: TMIGEPMNFVHLTHIGSGEMGAGDGLAMTGAVQEQMR
Intensity
M/Z
0
10000
20000
30000
40000
0
1000
2000
3000
y1
y2
y3
y4
b4
y5
b5
y6
y7
y8
y9
b8
y10
y11
y12
b10
y13
type
b
y

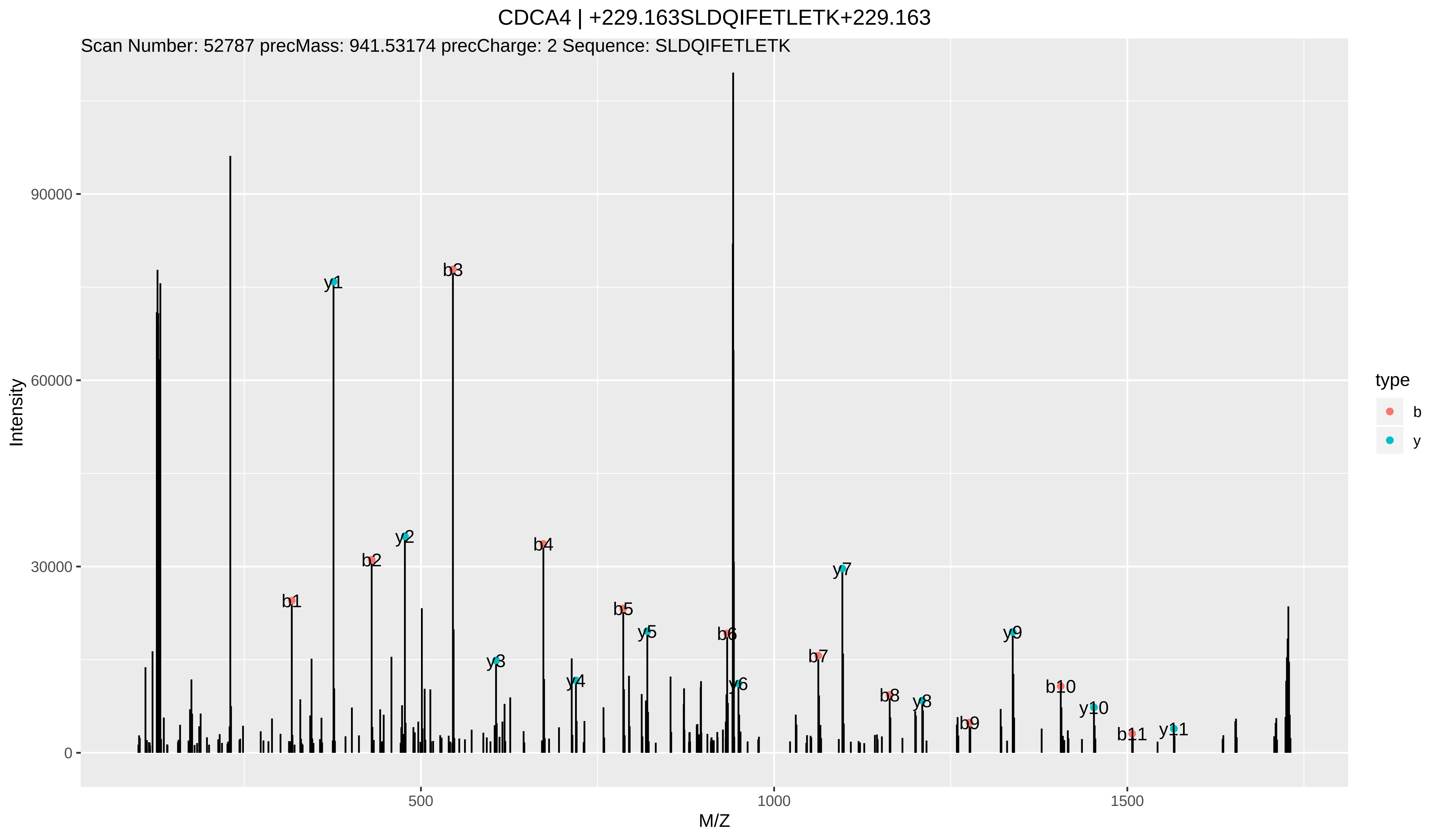
CDCA4 | +229.163SLDQIFETLETK+229.163
Scan Number: 52787 precMass: 941.53174 precCharge: 2 Sequence: SLDQIFETLETK
Intensity
M/Z
0
30000
60000
90000
500
1000
1500
type
b
y
b1
b2
b3
b4
b5
b6
b7
b8
b9
b10
b11
y1
y2
y3
y4
y5
y6
y7
y8
y9
y10
y11

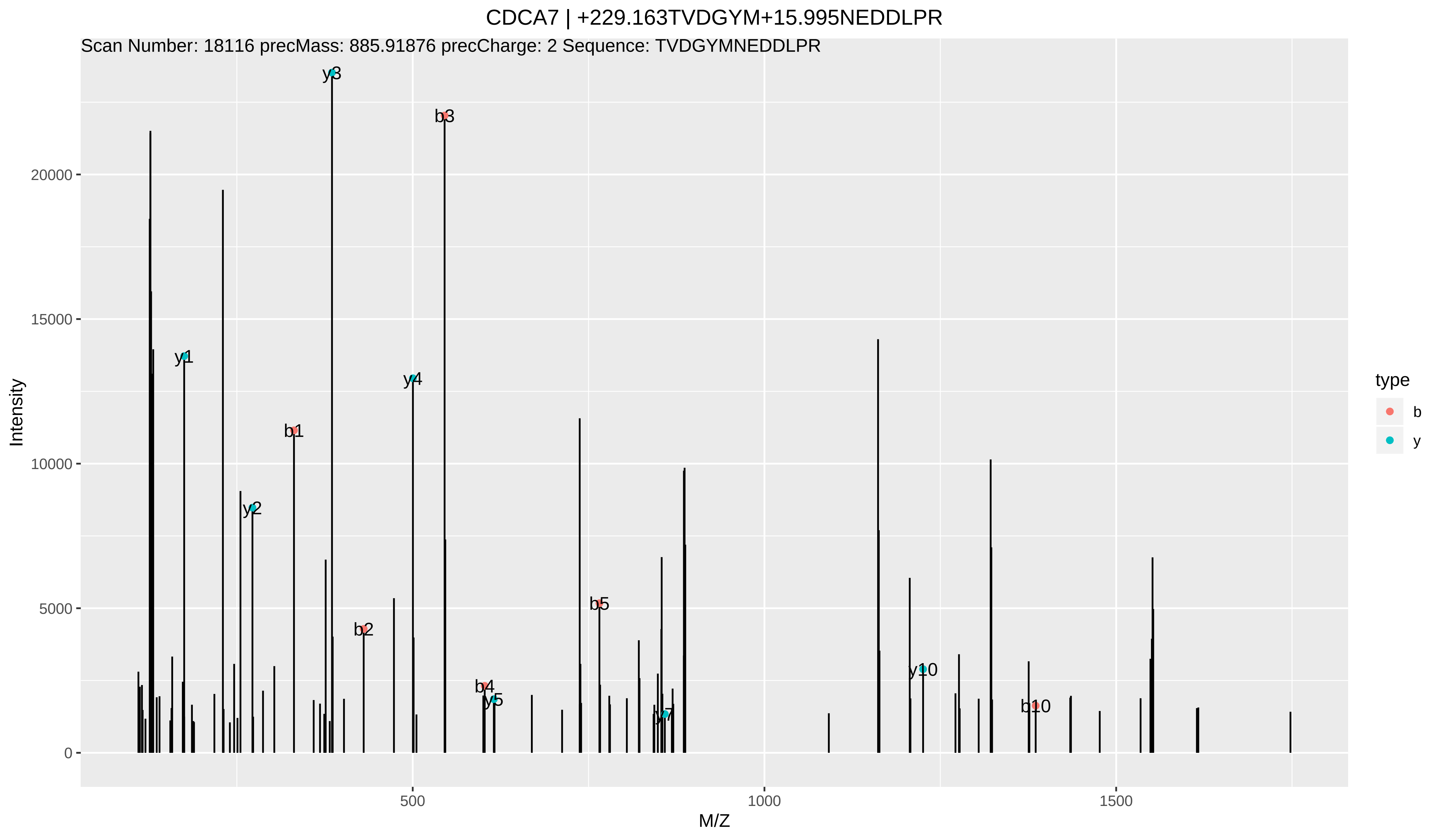

CDCA7 | +229.163TVDGYM+15.995NEDDLPR
Scan Number: 18116 precMass: 885.91876 precCharge: 2 Sequence: TVDGYMNEDDLPR
Intensity
M/Z
y3
b3
y1
y4
b1
y2
b2
b5
b4
y5
y7
y10
b10
0
5000
10000
15000
20000
500
1000
1500
type
b
y

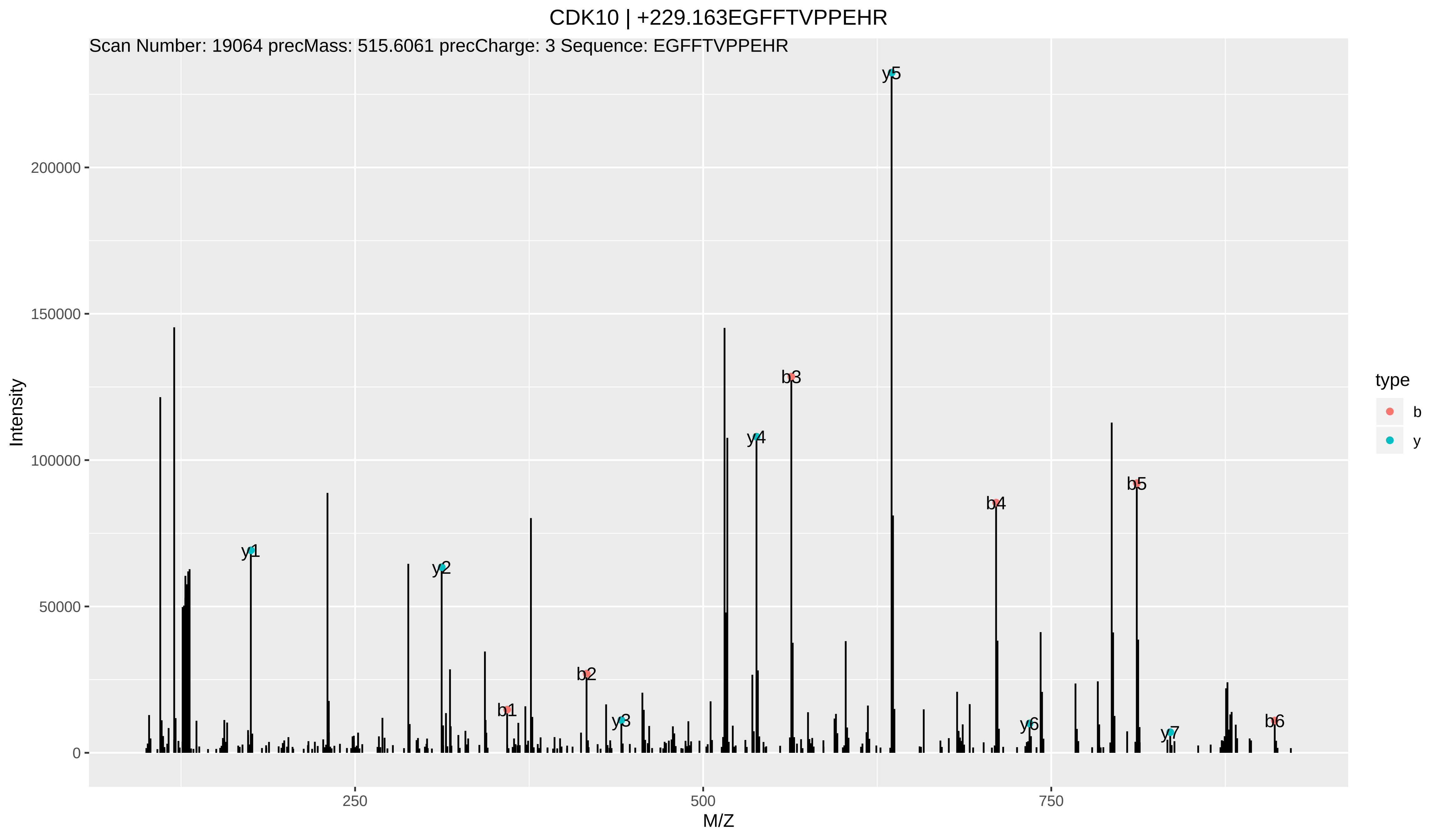
CDK10 | +229.163EGFFTVPPEHR
Scan Number: 19064 precMass: 515.6061 precCharge: 3 Sequence: EGFFTVPPEHR
Intensity
0
50000
100000
150000
200000
M/Z
250
500
750
y1
y2
y3
y4
y5
y6
y7
b1
b2
b3
b4
b5
b6
type
b
y

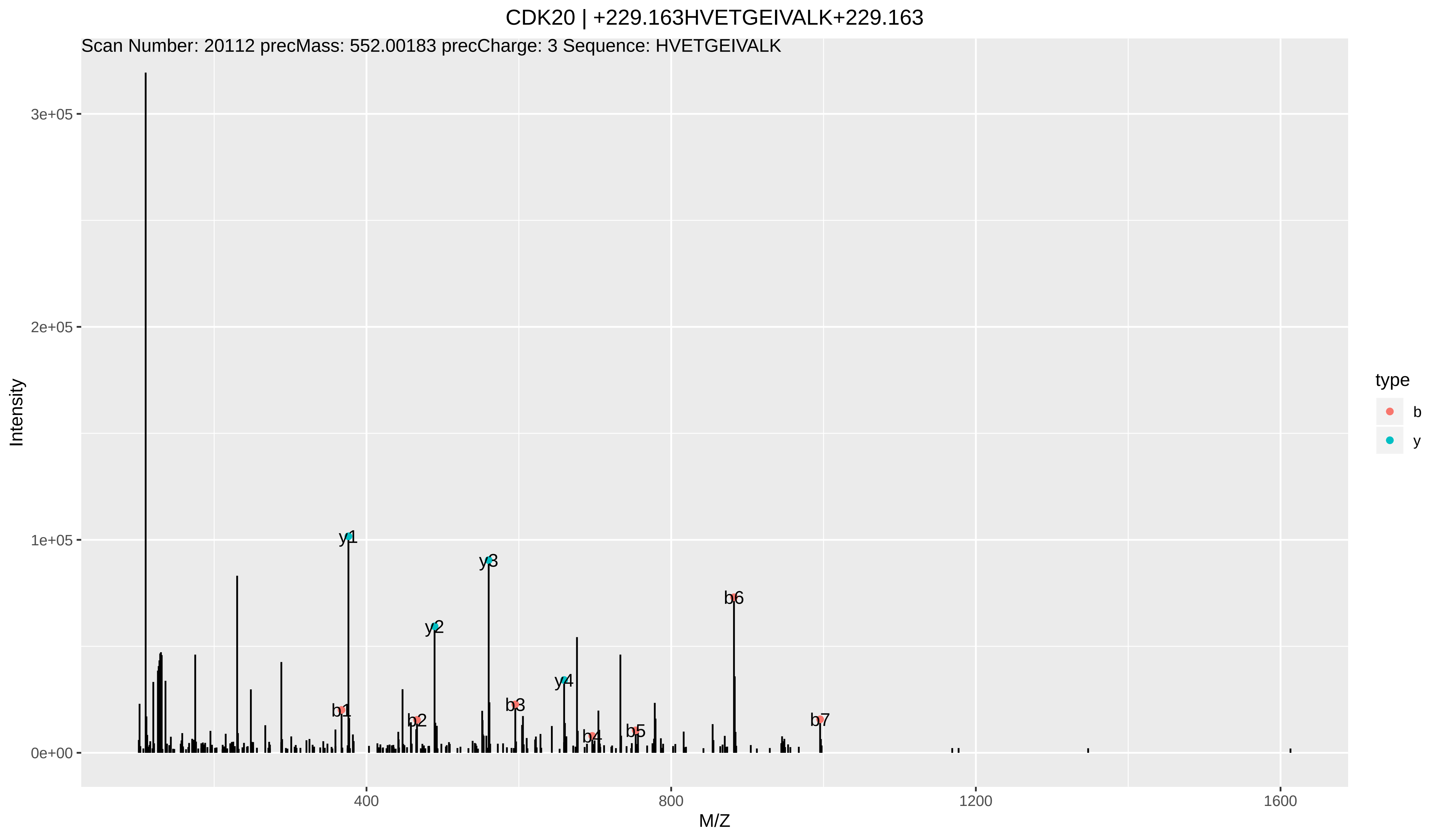
CDK20 | +229.163HVETGEIVALK+229.163
Scan Number: 20112 precMass: 552.00183 precCharge: 3 Sequence: HVETGEIVALK
Intensity
M/Z
type
b
y
y1
y2
y3
y4
b1
b2
b3
b4
b5
b6
b7
0e+00
1e+05
2e+05
3e+05
400
800
1200
1600

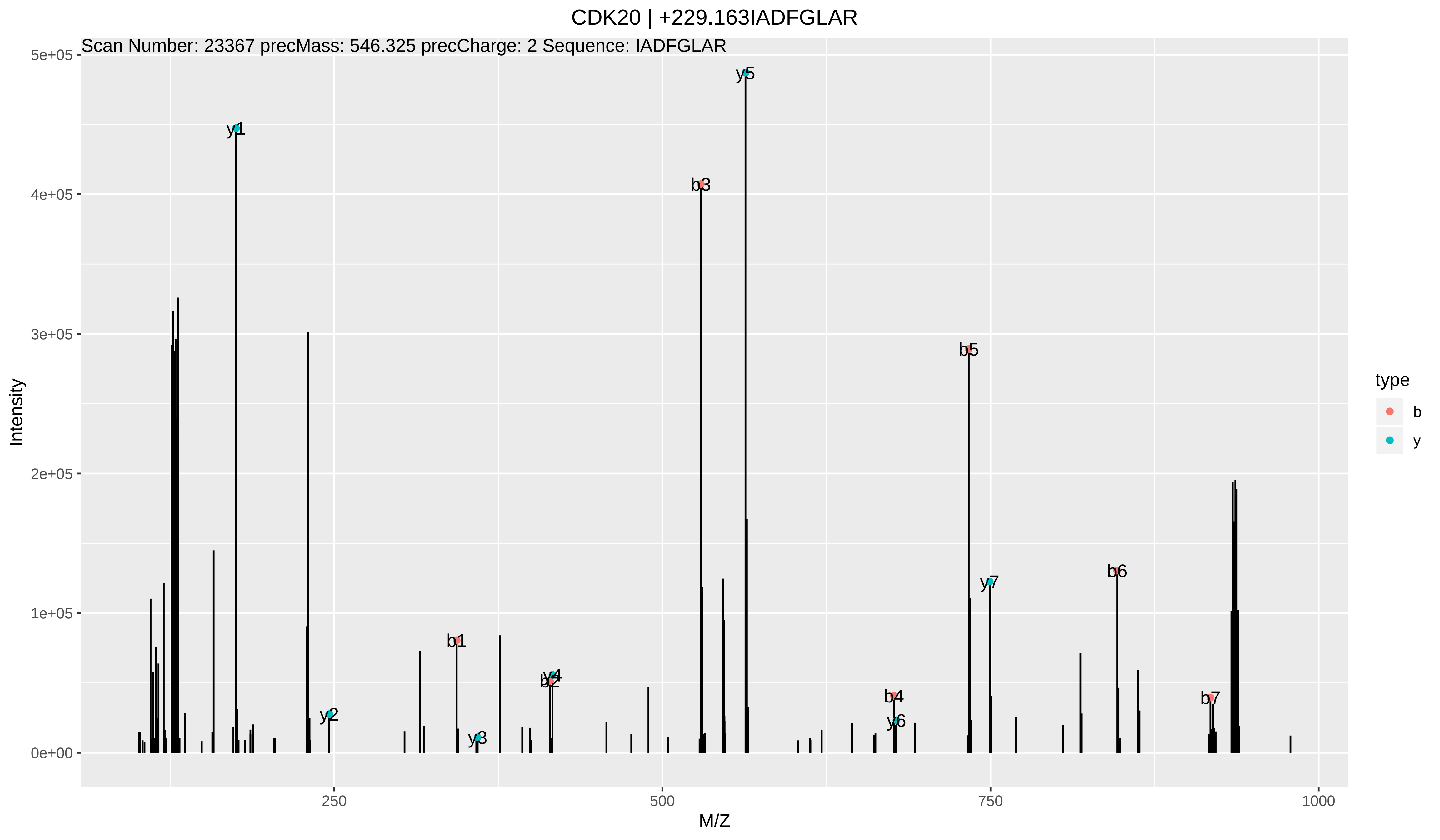
CDK20 | +229.163IADFGLAR
Scan Number: 23367 precMass: 546.325 precCharge: 2 Sequence: IADFGLAR
Intensity
M/Z
type
b
y
y1
y5
b3
b5
y7
b6
b1
y4
b2
y2
y3
b4
y6
b7
5e+05
4e+05
3e+05
2e+05
1e+05
0e+00
250
500
750
1000

CDK2AP1 | +229.163YAELLAIIEELGK+229.163

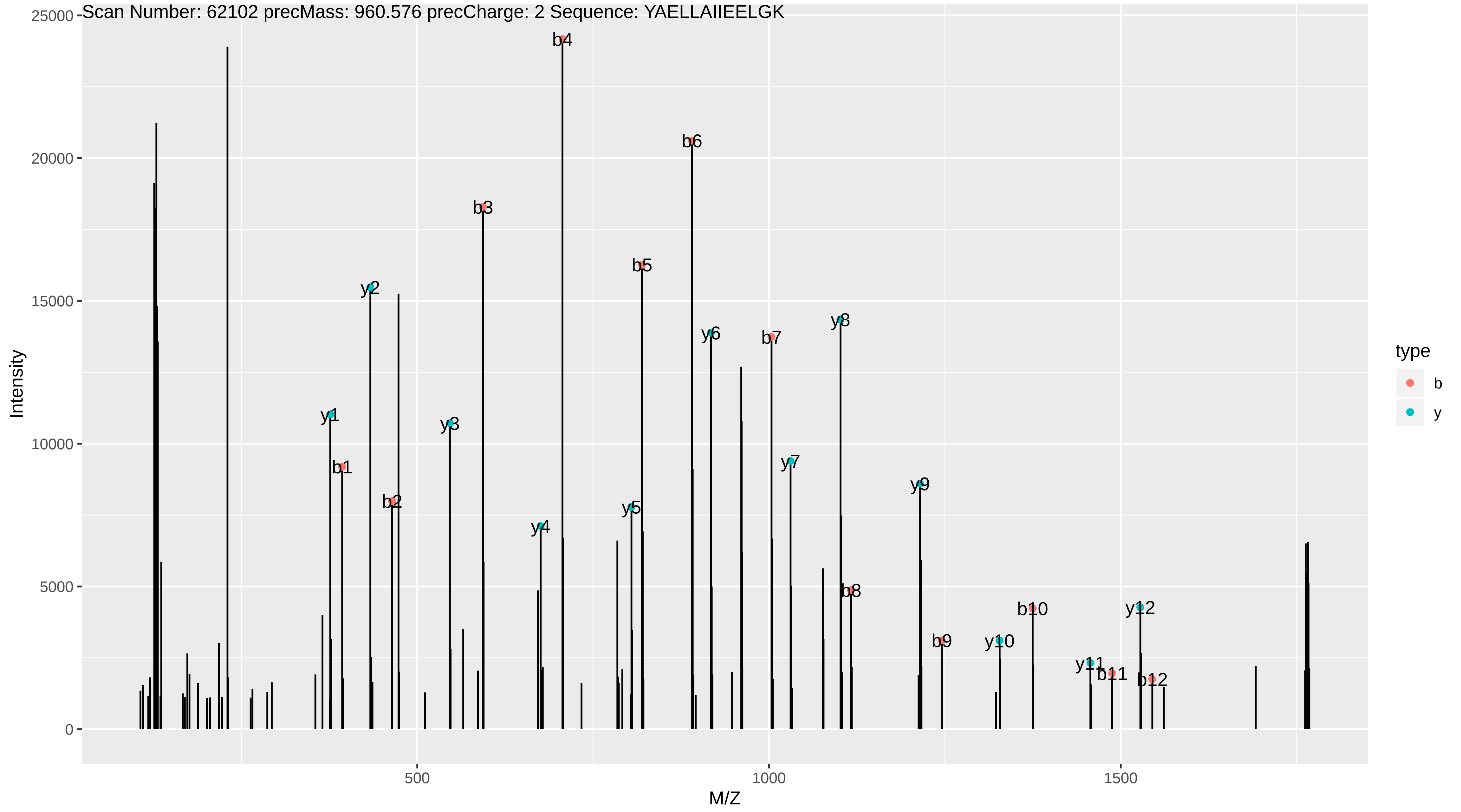

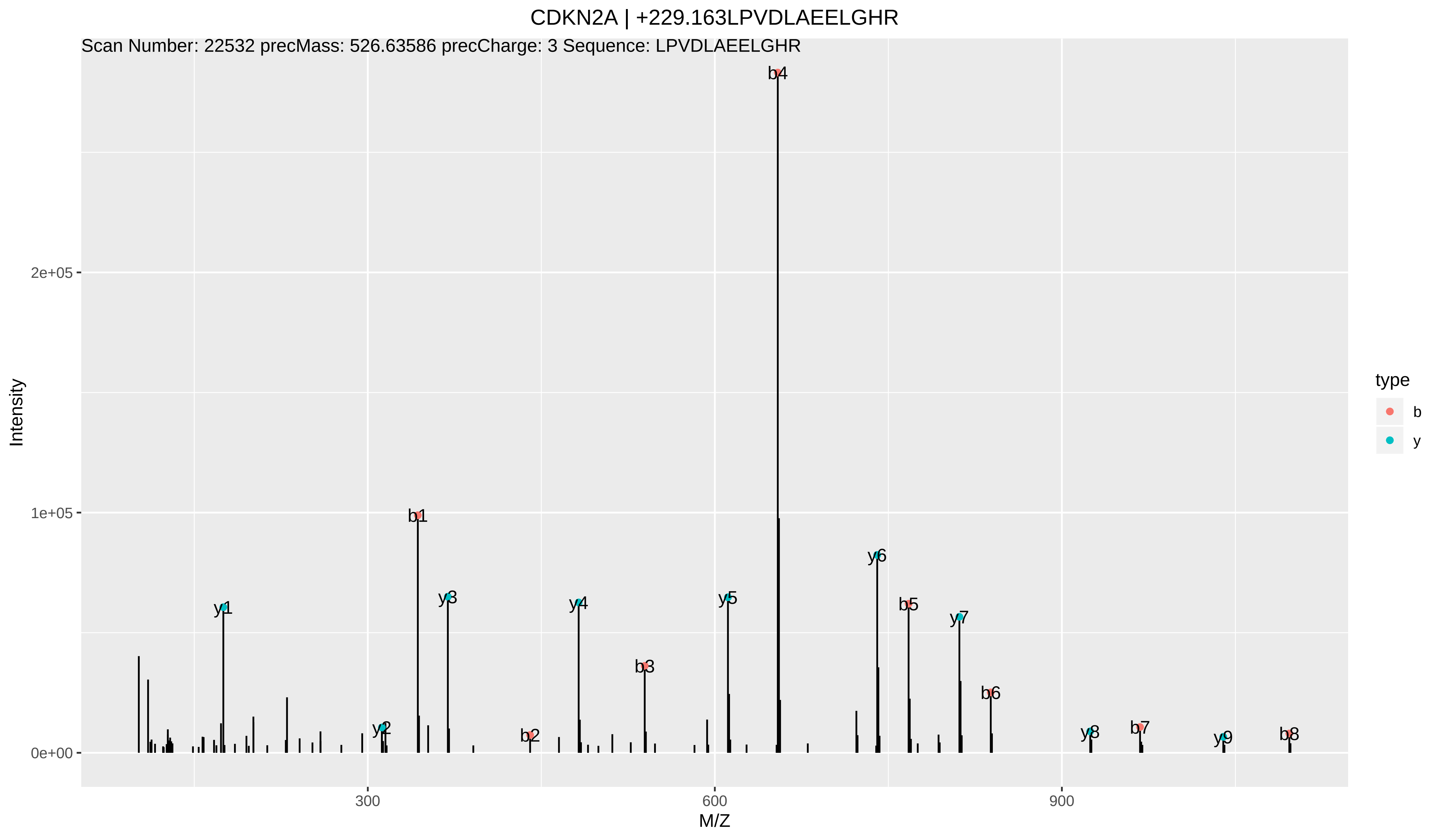
CDKN2A | +229.163LPVDLAEELGHR
Scan Number: 22532 precMass: 526.63586 precCharge: 3 Sequence: LPVDLAEELGHR
Intensity
M/Z
0e+00
1e+05
2e+05
300
600
900
type
b
y
b1
b2
b3
b4
b5
b6
b7
b8
y1
y2
y3
y4
y5
y6
y7
y8
y9

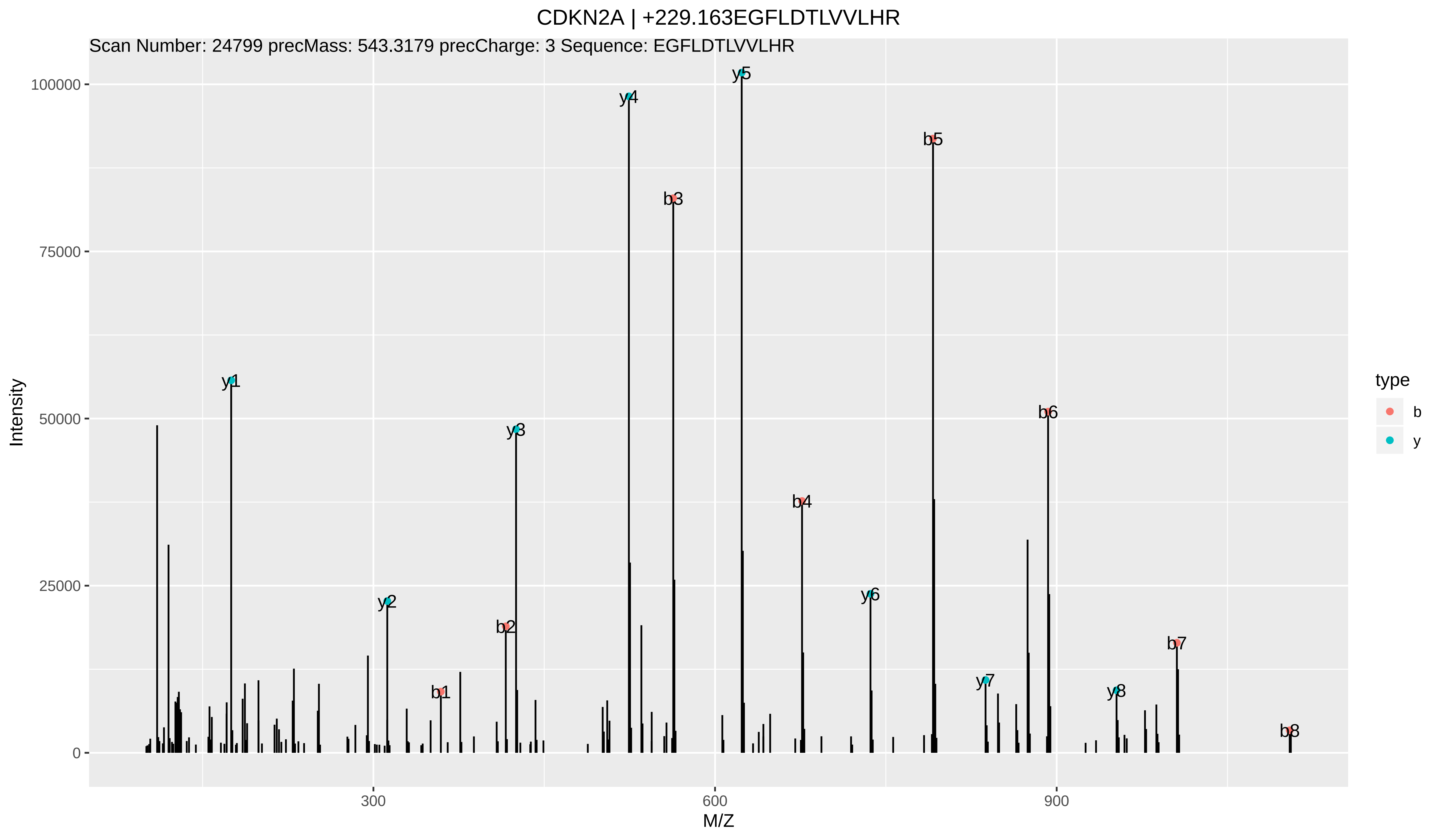

CDKN2A | +229.163EGFLDTLVVLHR
Scan Number: 24799 precMass: 543.3179 precCharge: 3 Sequence: EGFLDTLVVLHR
Intensity
M/Z
0
25000
50000
75000
100000
300
600
900
y1
y2
y3
y4
y5
y6
y7
y8
b1
b2
b3
b4
b5
b6
b7
b8
type
b
y

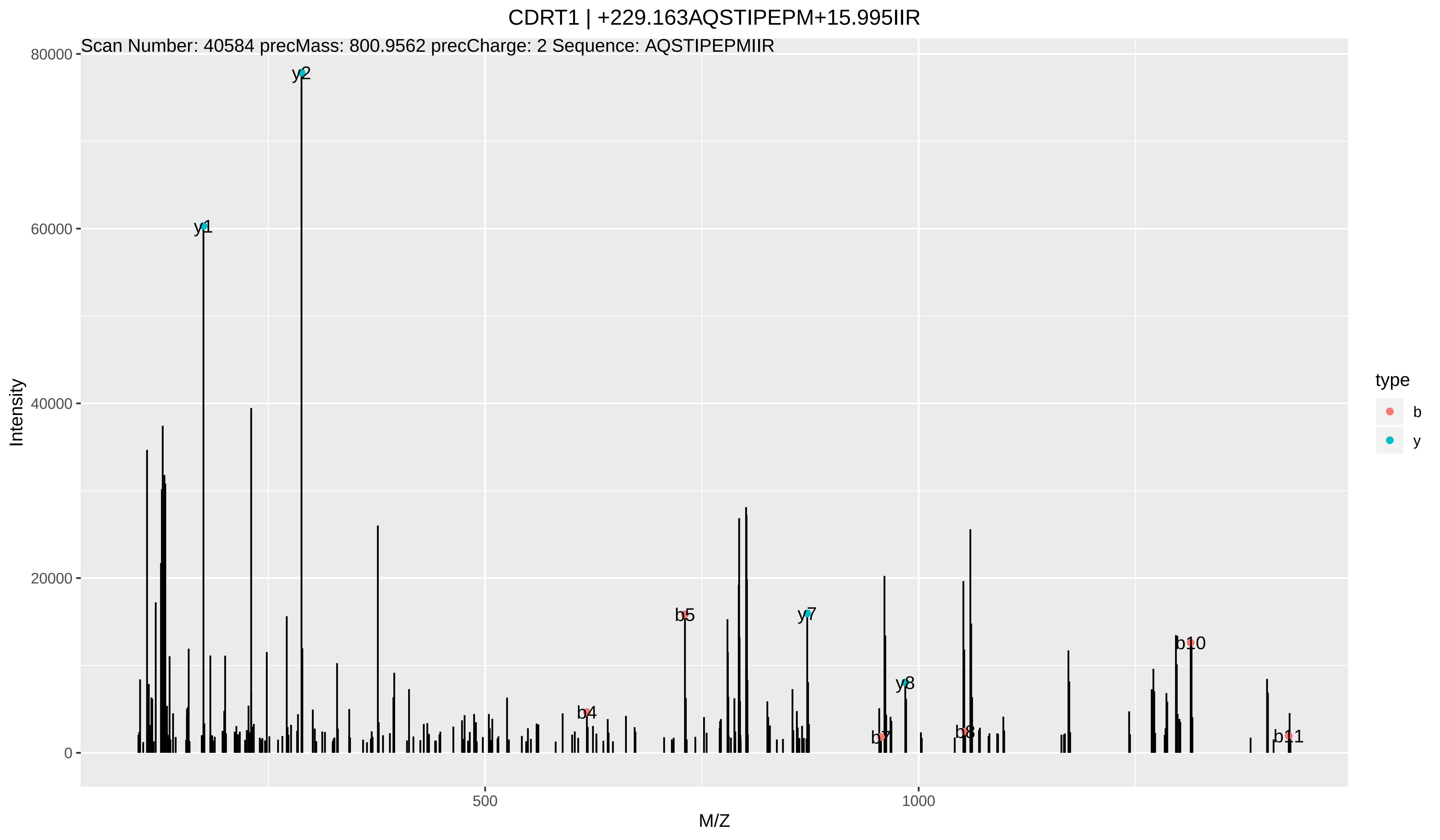

CDRT1 | +229.163AQSTIPEPM+15.995IIR
Scan Number: 40584 precMass: 800.9562 precCharge: 2 Sequence: AQSTIPEPMIIR
Intensity
M/Z
y1
y2
b4
b5
y7
b7
y8
b8
b10
b11
type
b
y
0
20000
40000
60000
80000
500
1000

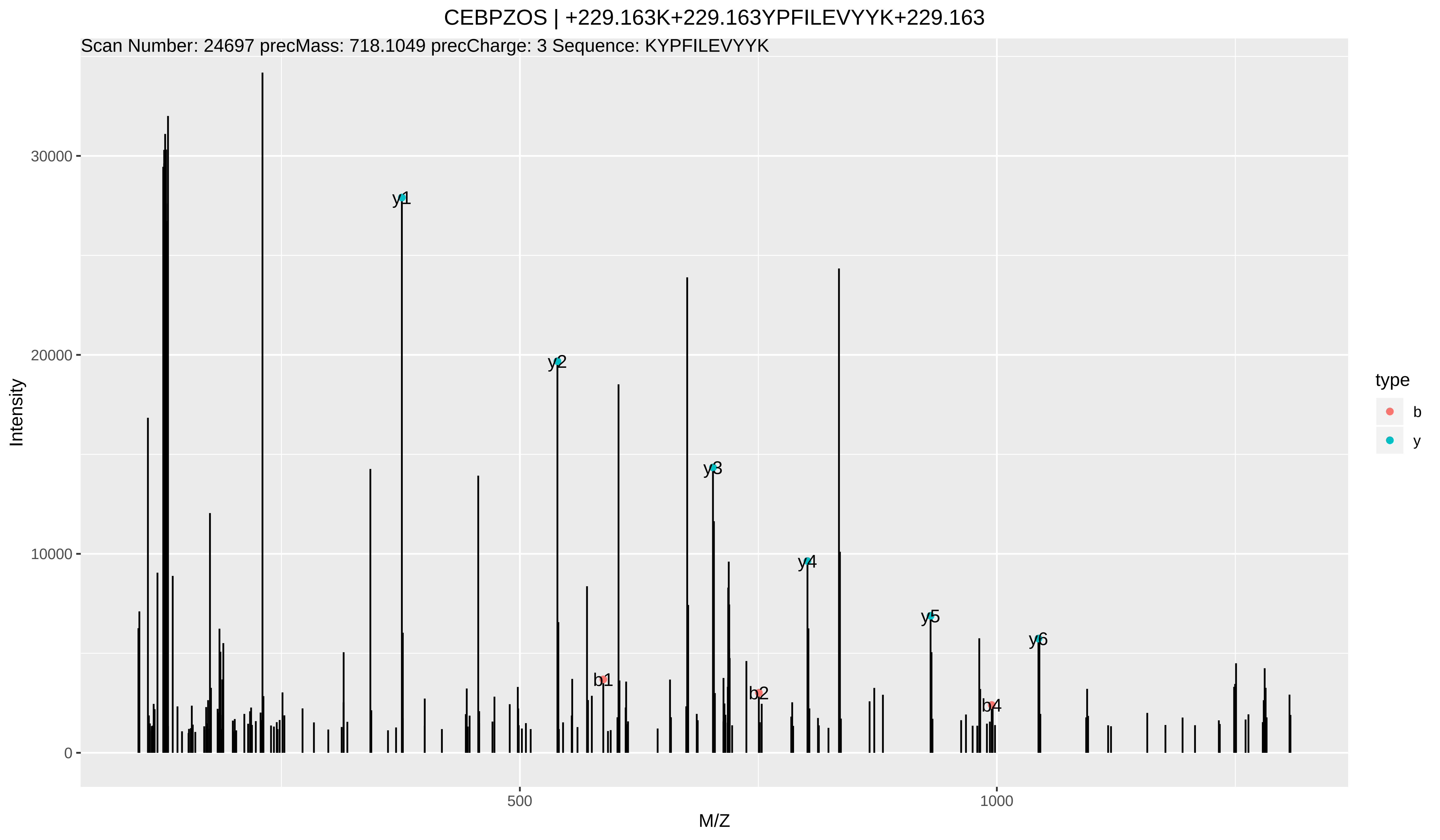
CEBPZOS | +229.163K+229.163YPFILEVYYK+229.163
Scan Number: 24697 precMass: 718.1049 precCharge: 3 Sequence: KYPFILEVYYK
Intensity
M/Z
type
b
y
y1
y2
y3
y4
y5
y6
b1
b2
b4
30000
20000
10000
0
500
1000

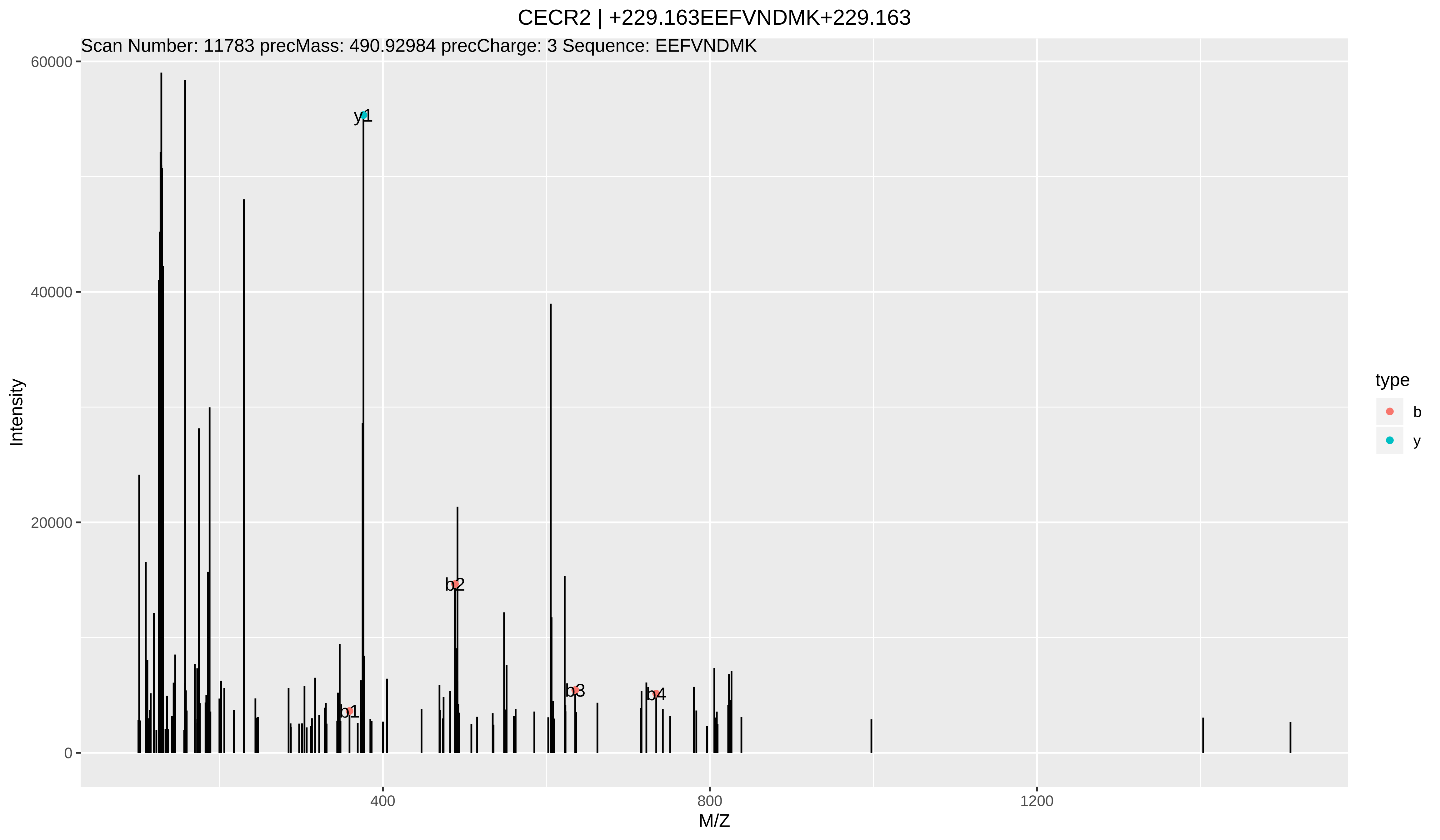

CECR2 | +229.163EEFVNDMK+229.163
Scan Number: 11783 precMass: 490.92984 precCharge: 3 Sequence: EEFVNDMK
Intensity
M/Z
60000
40000
20000
0
400
800
1200
y1
b1
b2
b3
b4
type
b
y

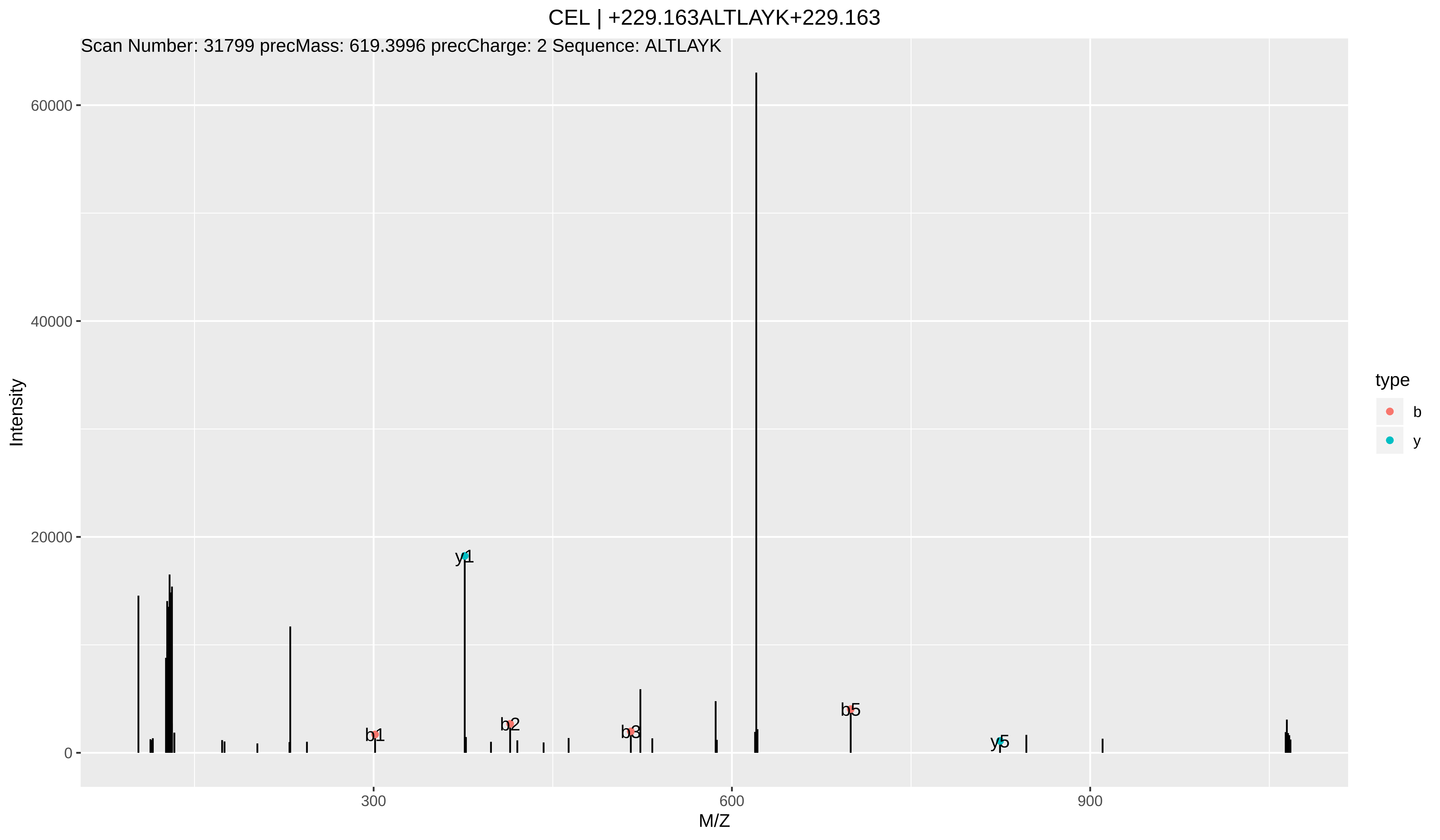

CEL | +229.163ALTLAYK+229.163
Scan Number: 31799 precMass: 619.3996 precCharge: 2 Sequence: ALTLAYK
Intensity
60000
40000
20000
0
y1
b1
b2
b3
b5
y5
300
600
900
M/Z
type
b
y

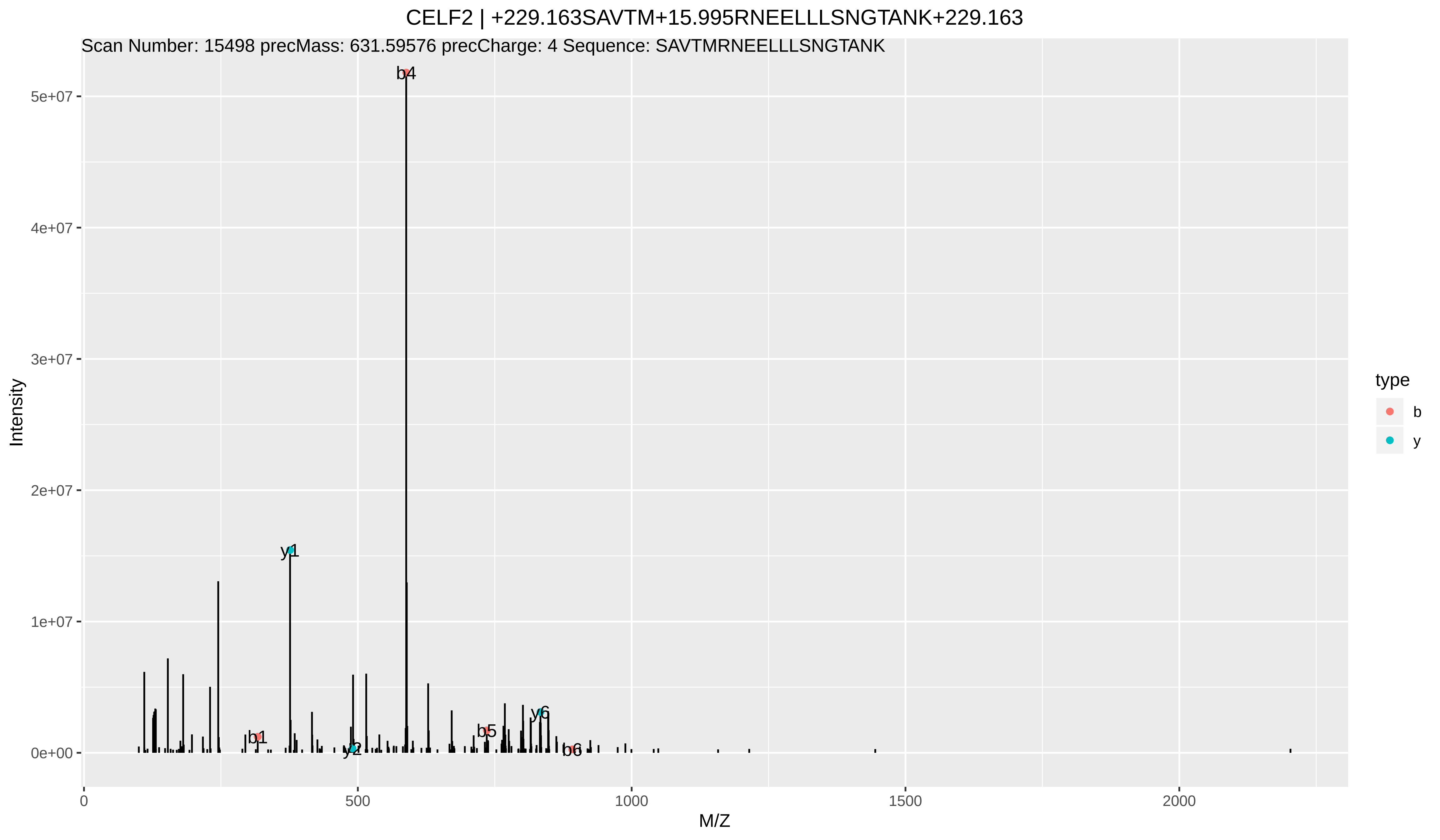
CELF2 | +229.163SAVTM+15.995RNEELLLSNGTANK+229.163
Scan Number: 15498 precMass: 631.59576 precCharge: 4 Sequence: SAVTMRNEELLLSNGTANK
Intensity
M/Z
0e+00
1e+07
2e+07
3e+07
4e+07
5e+07
0
500
1000
1500
2000
b4
y1
b1
y2
b5
y6
b6
type
b
y

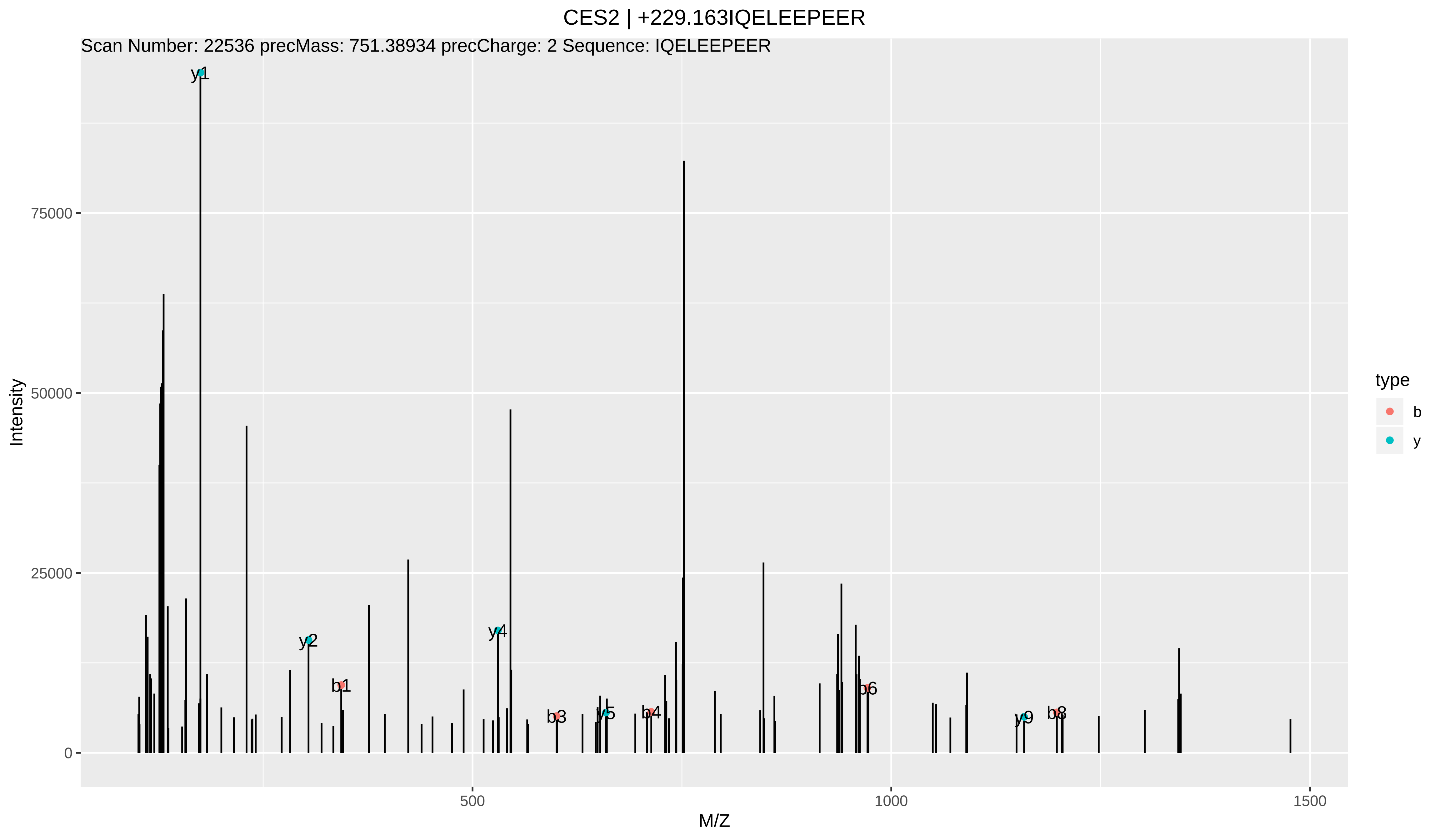

CES2 | +229.163IQELEEPEER
Scan Number: 22536 precMass: 751.38934 precCharge: 2 Sequence: IQELEEPEER
y1
y2
b1
y4
b3
y5
b4
b6
y9
b8
Intensity
0
25000
50000
75000
M/Z
500
1000
1500
type
b
y

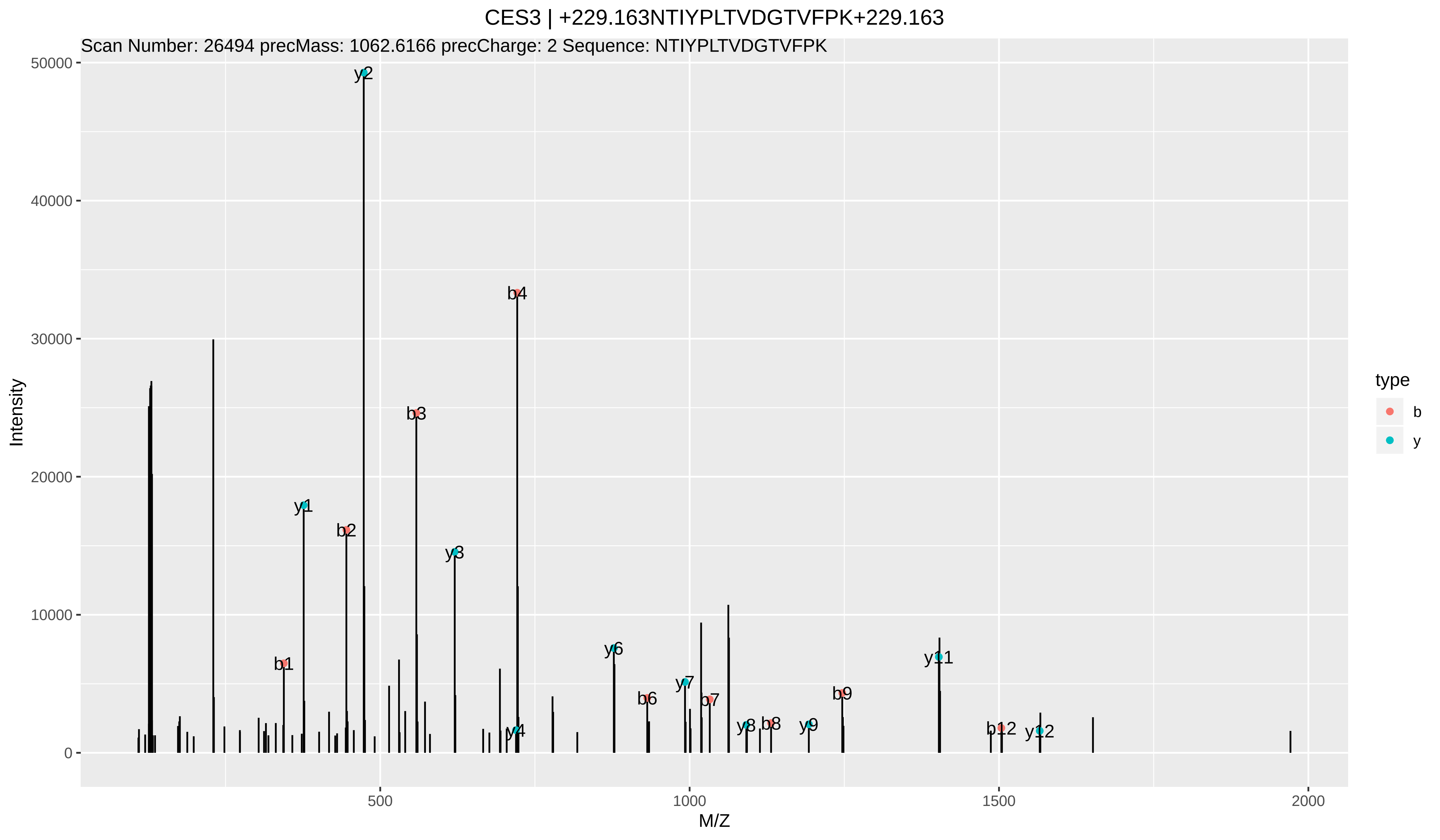

CES3 | +229.163NTIYPLTVDGTVFPK+229.163
Scan Number: 26494 precMass: 1062.6166 precCharge: 2 Sequence: NTIYPLTVDGTVFPK
Intensity
M/Z
0
10000
20000
30000
40000
50000
500
1000
1500
2000
y2
b4
b3
y1
b2
y3
b1
y6
y11
b6
y7
b7
b9
y4
y8
b8
y9
b12
y12
type
b
y

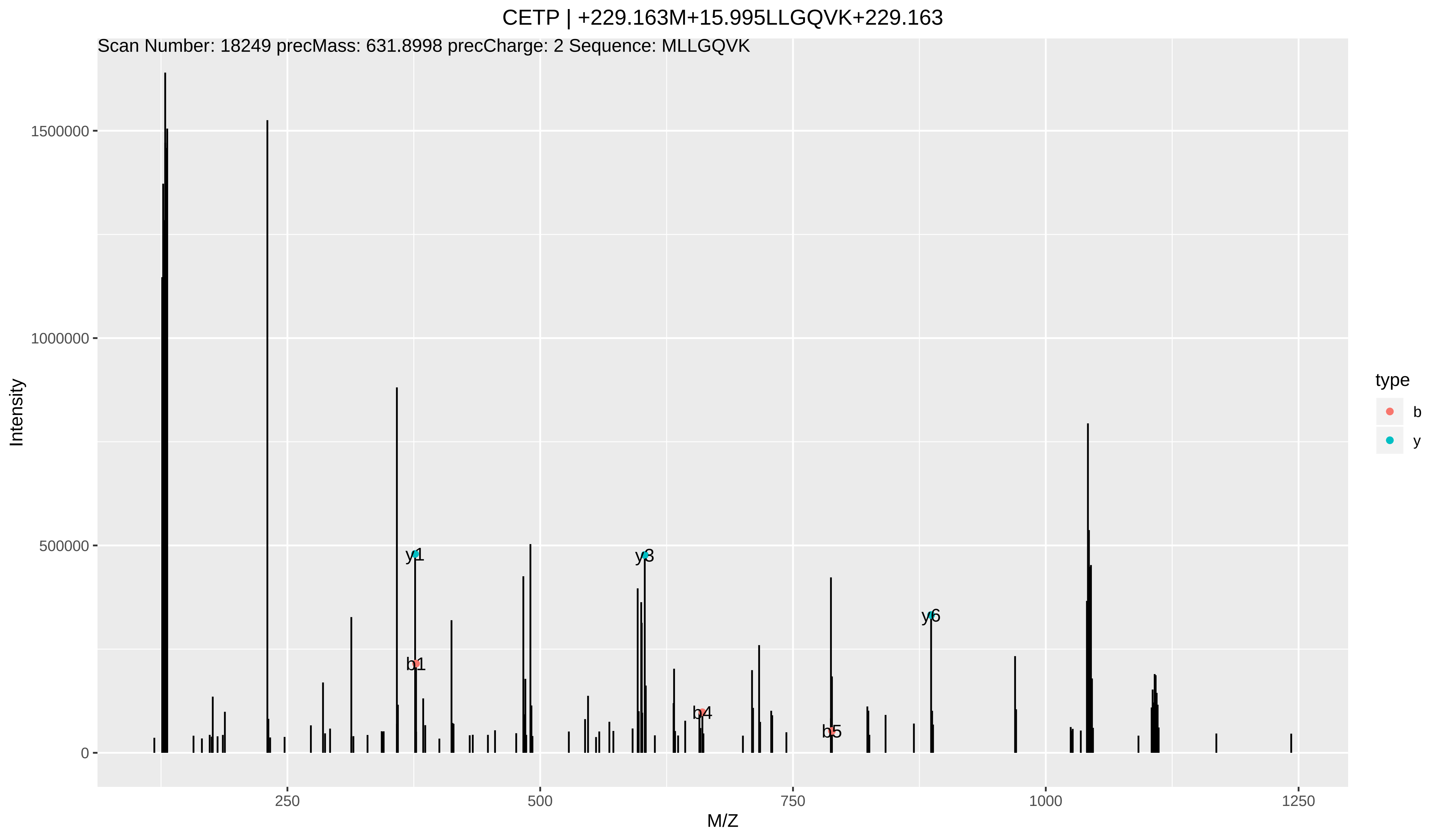
CETP | +229.163M+15.995LLGQVK+229.163
Scan Number: 18249 precMass: 631.8998 precCharge: 2 Sequence: MLLGQVK
Intensity
M/Z
1500000
1000000
500000
0
250
500
750
1000
1250
y1
b1
y3
b4
b5
y6
type
b
y

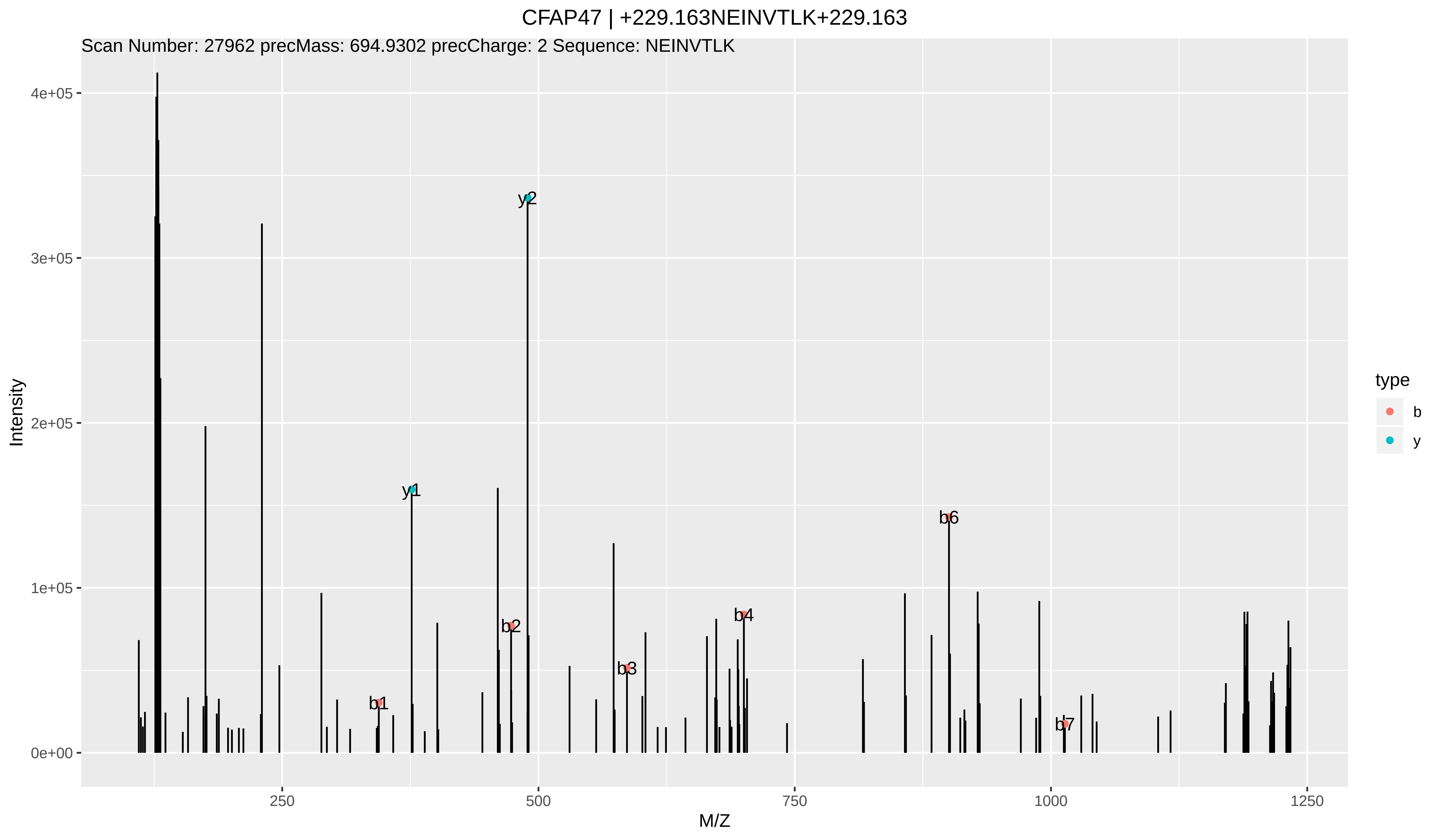
CFAP47 | +229.163NEINVTLK+229.163
Scan Number: 27962 precMass: 694.9302 precCharge: 2 Sequence: NEINVTLK
Intensity
4e+05
3e+05
2e+05
1e+05
0e+00
y2
y1
b6
b4
b2
b3
b1
b7
250
500
750
1000
1250
M/Z
type
b
y

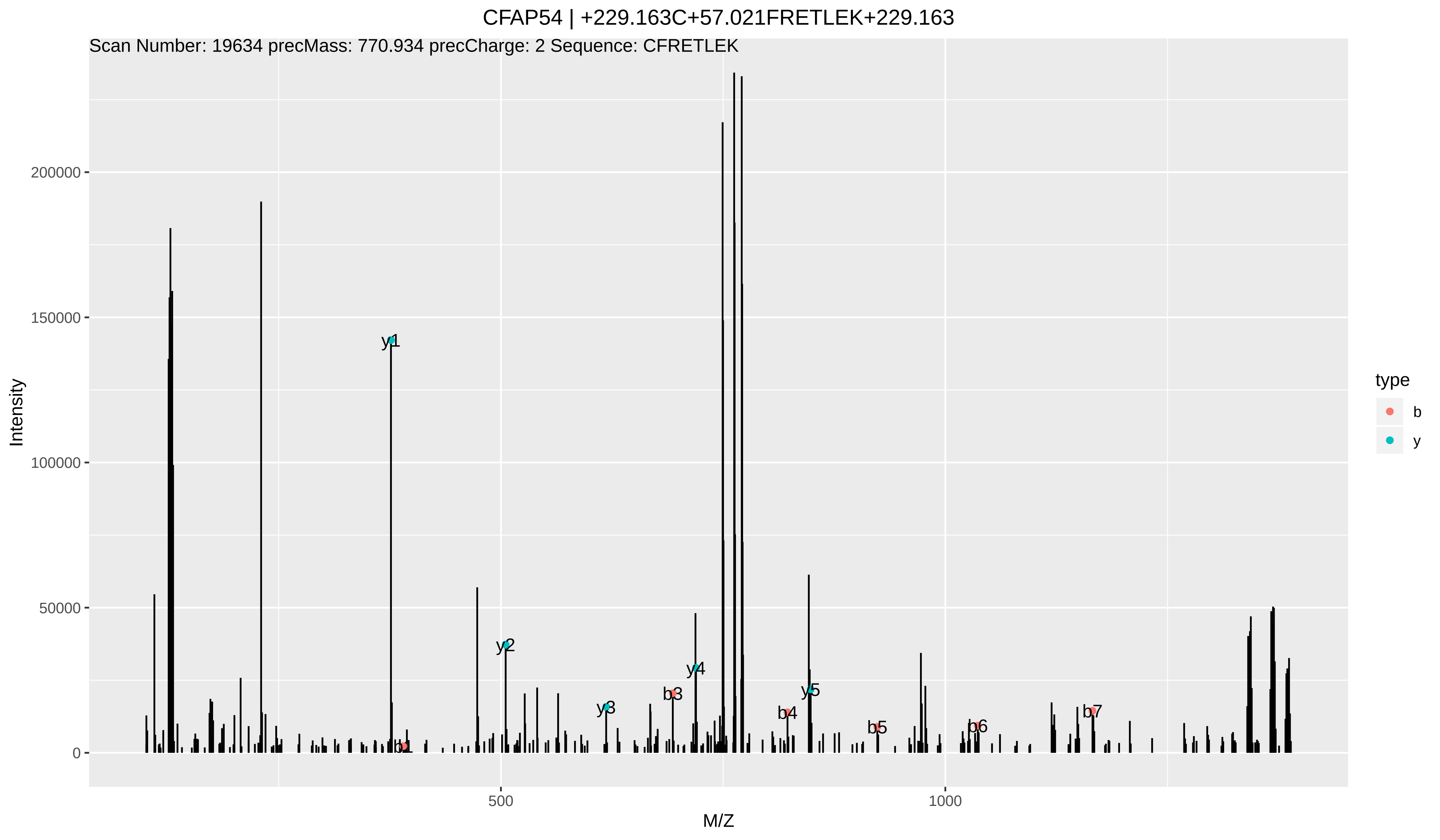

CFAP54 | +229.163C+57.021FRETLEK+229.163
Scan Number: 19634 precMass: 770.934 precCharge: 2 Sequence: CFRETLEK
Intensity
M/Z
type
b
y
y1
y2
y3
y4
y5
b1
b3
b4
b5
b6
b7
0
50000
100000
150000
200000
500
1000

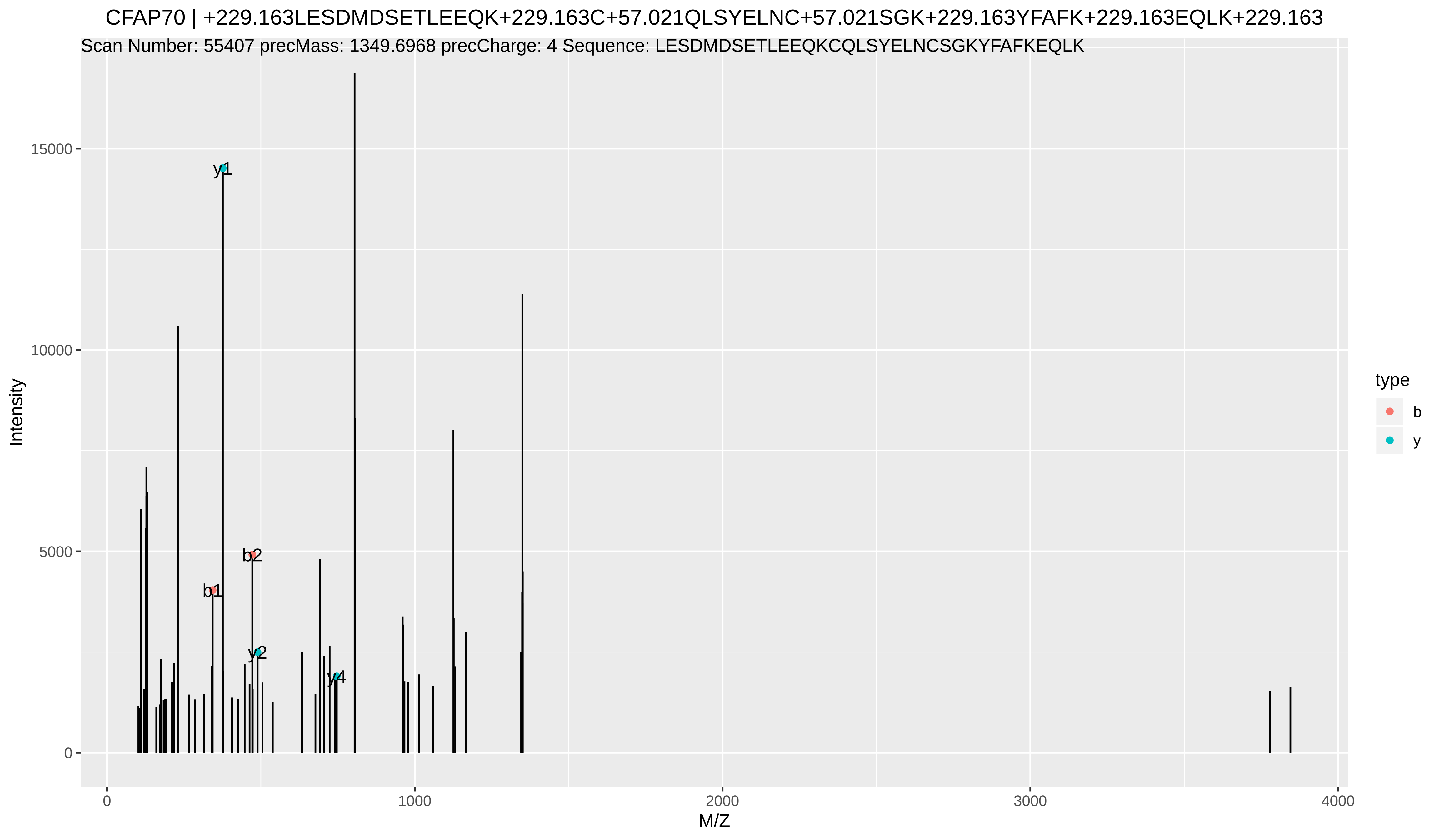

CFAP70 | +229.163LESDMDSETLEEQK+229.163C+57.021QLSYELNC+57.021SGK+229.163YFAFK+229.163EQLK+229.163
Scan Number: 55407 precMass: 1349.6968 precCharge: 4 Sequence: LESDMDSETLEEQKCQLSYELNCSGKYFAFKEQLK
Intensity
M/Z
0
5000
10000
15000
0
1000
2000
3000
4000
y1
b1
b2
y2
y4
type
b
y

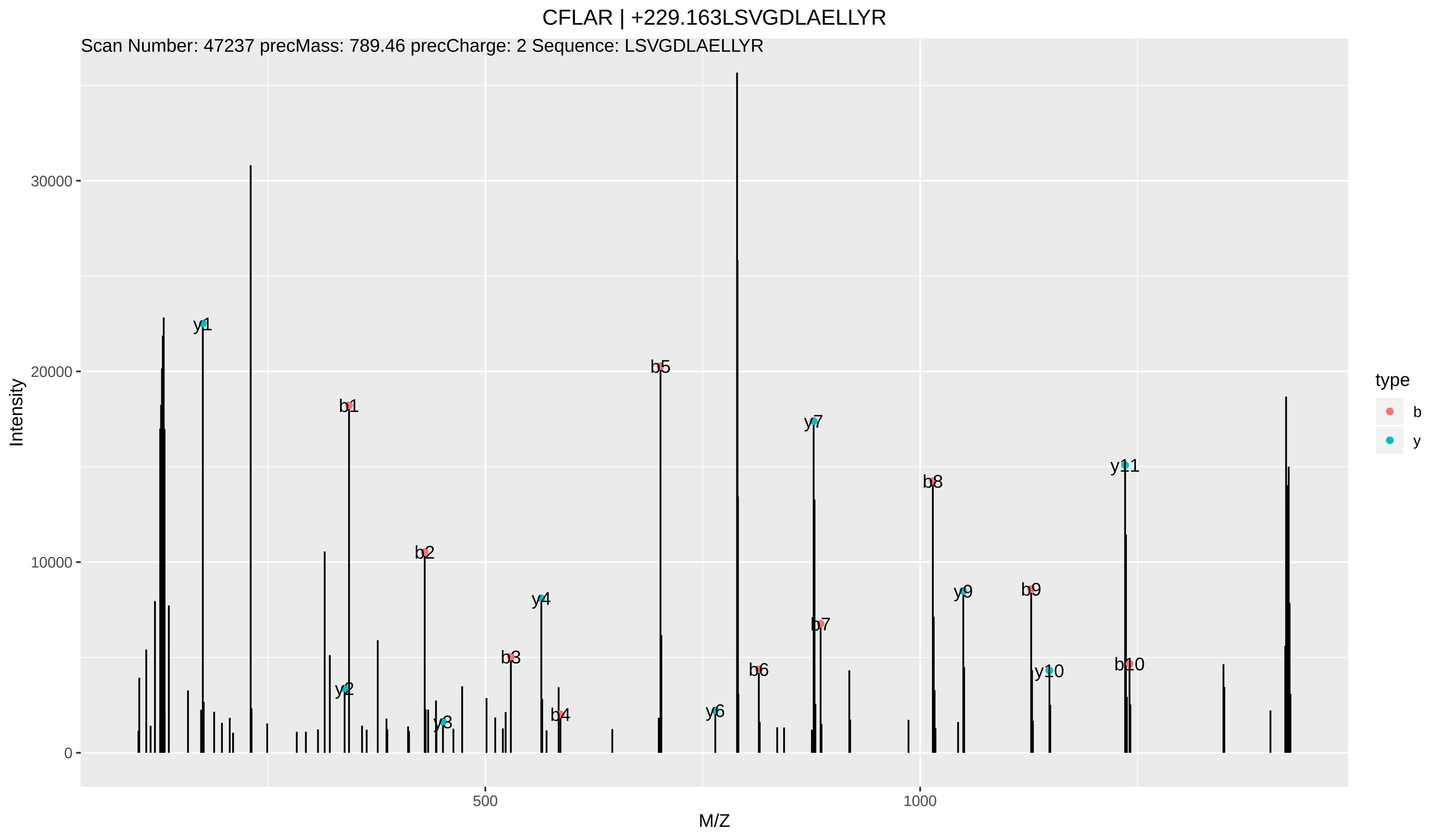
CFLAR | +229.163LSVGDLAELLYR
Scan Number: 47237 precMass: 789.46 precCharge: 2 Sequence: LSVGDLAELLYR
Intensity
M/Z
30000
20000
10000
0
500
1000
y1
b1
b2
y2
y3
b3
y4
b4
b5
y6
b6
y7
b7
b8
y9
b9
y10
y11
b10
type
b
y

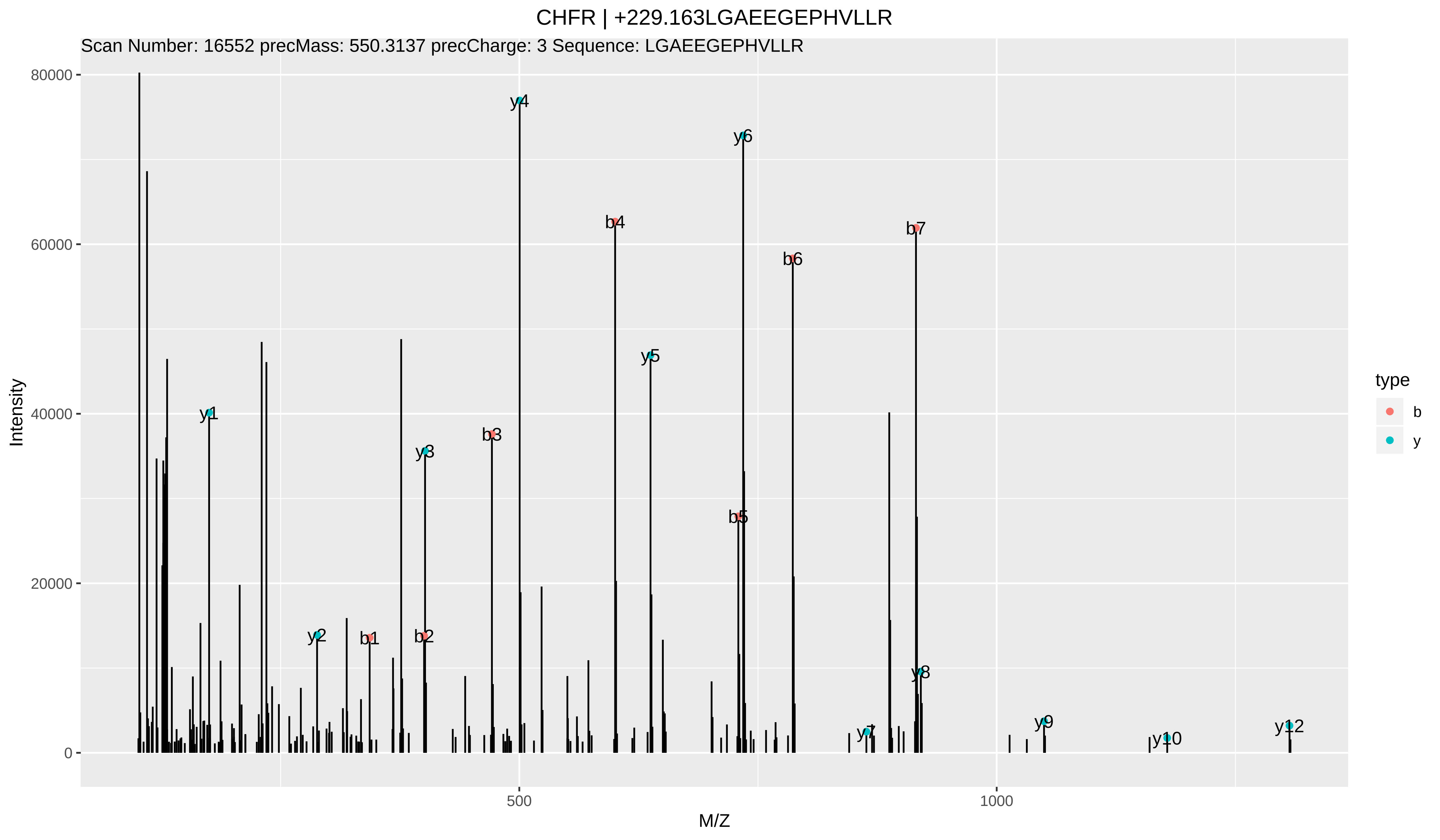
CHFR | +229.163LGAEEGEPHVLLR
Scan Number: 16552 precMass: 550.3137 precCharge: 3 Sequence: LGAEEGEPHVLLR
Intensity
80000
60000
40000
20000
0
M/Z
500
1000
type
b
y
y1
y2
y3
y4
y5
y6
y7
y8
y9
y10
y12
b1
b2
b3
b4
b5
b6
b7

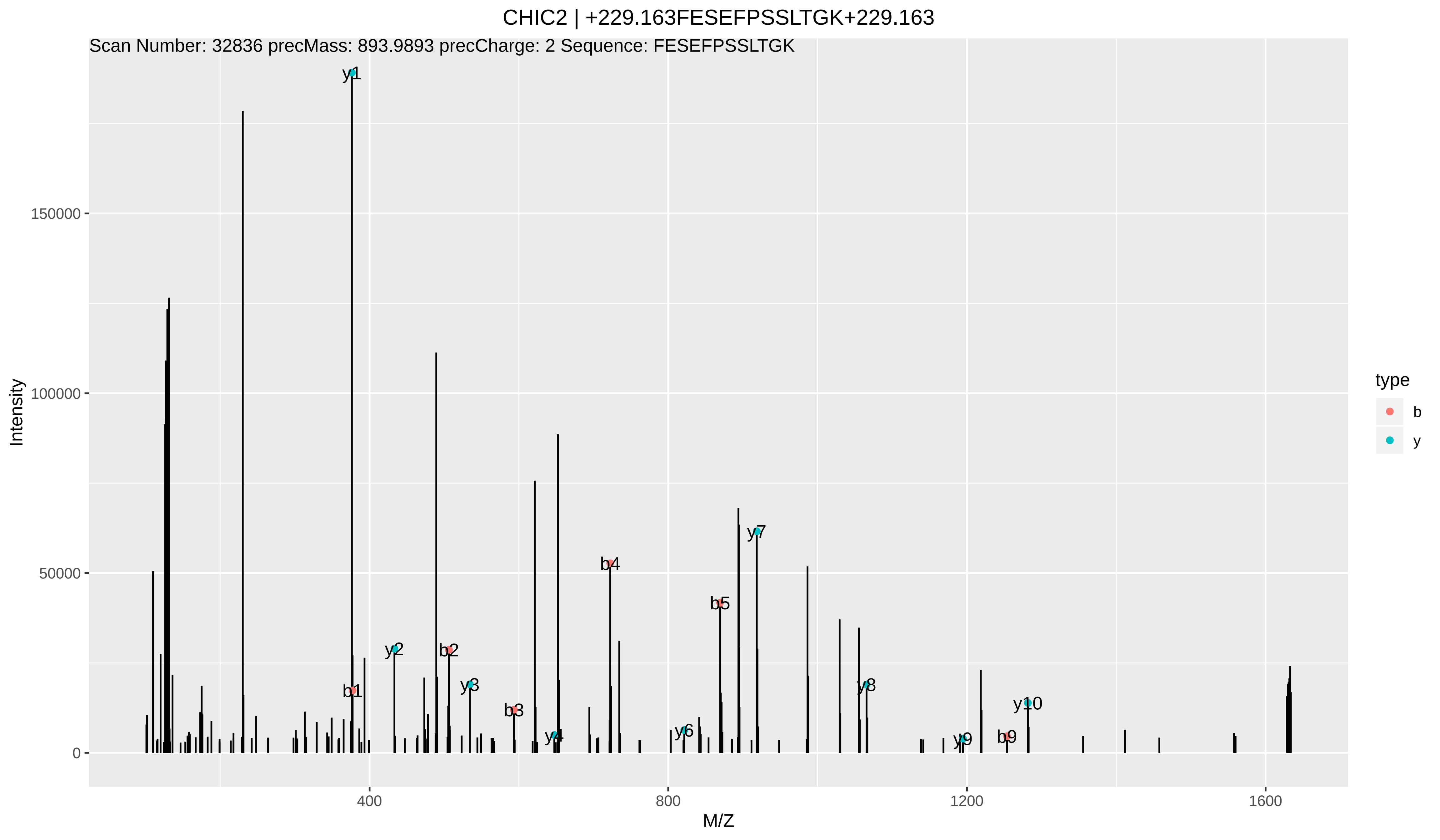
CHIC2 | +229.163FESEFPSSLTGK+229.163
Scan Number: 32836 precMass: 893.9893 precCharge: 2 Sequence: FESEFPSSLTGK
y1
y2
b2
y3
b1
b3
y4
b4
b5
y7
y6
y8
y9
b9
y10
Intensity
150000
100000
50000
0
400
800
1200
1600
M/Z
type
b
y

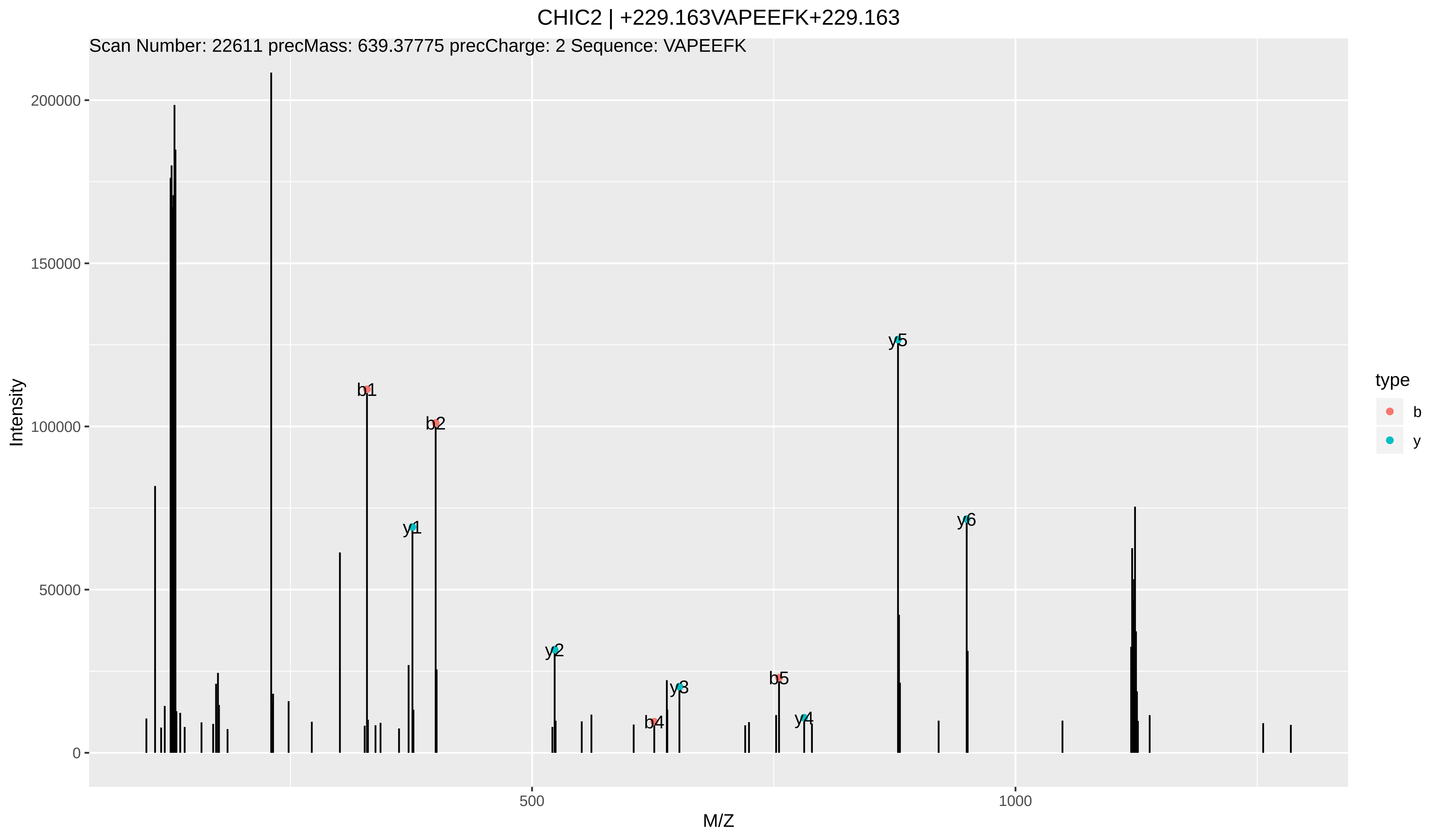

CHIC2 | +229.163VAPEEFK+229.163
Scan Number: 22611 precMass: 639.37775 precCharge: 2 Sequence: VAPEEFK
Intensity
200000
150000
100000
50000
0
M/Z
500
1000
b1
b2
y1
y2
b4
y3
b5
y4
y5
y6
type
b
y

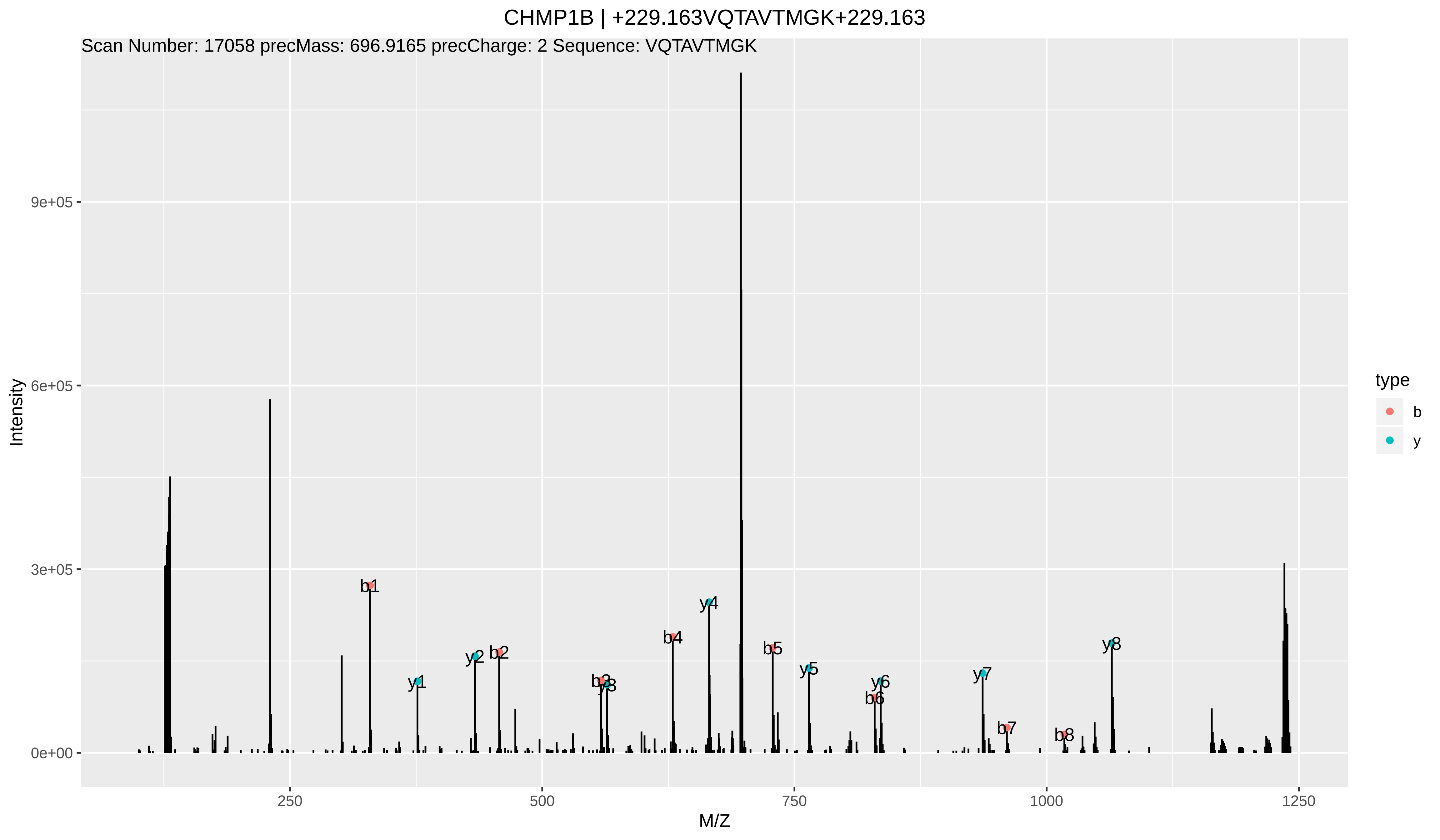
CHMP1B | +229.163VQTAVTMGK+229.163
Scan Number: 17058 precMass: 696.9165 precCharge: 2 Sequence: VQTAVTMGK
Intensity
M/Z
9e+05
6e+05
3e+05
0e+00
250
500
750
1000
1250
b1
y1
y2
b2
b3
y3
b4
y4
b5
y5
b6
y6
y7
b7
b8
y8
type
b
y

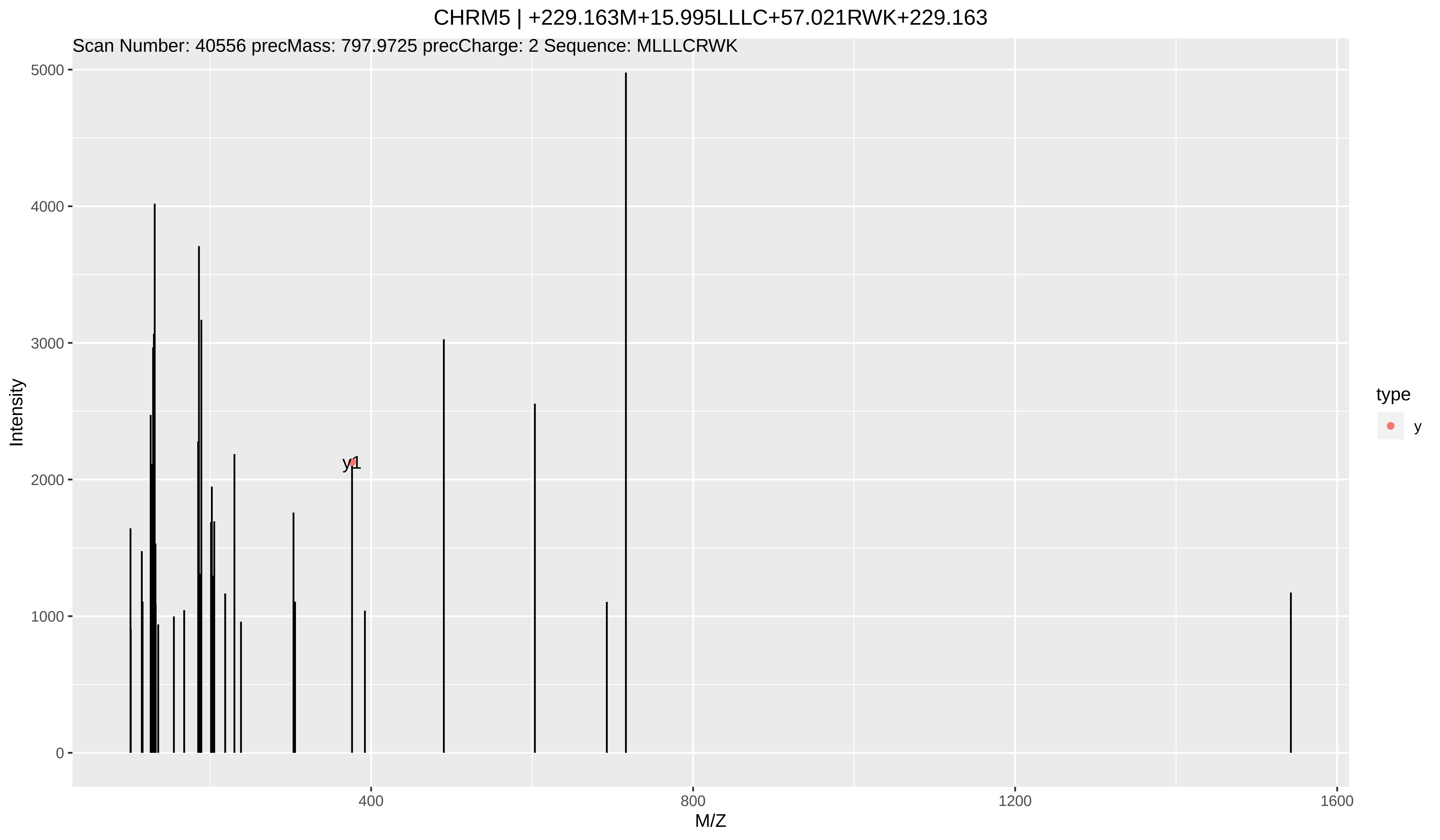
CHRM5 | +229.163M+15.995LLLC+57.021RWK+229.163
Scan Number: 40556 precMass: 797.9725 precCharge: 2 Sequence: MLLLCRWK
Intensity
M/Z
y1
type
y
0
1000
2000
3000
4000
5000
400
800
1200
1600

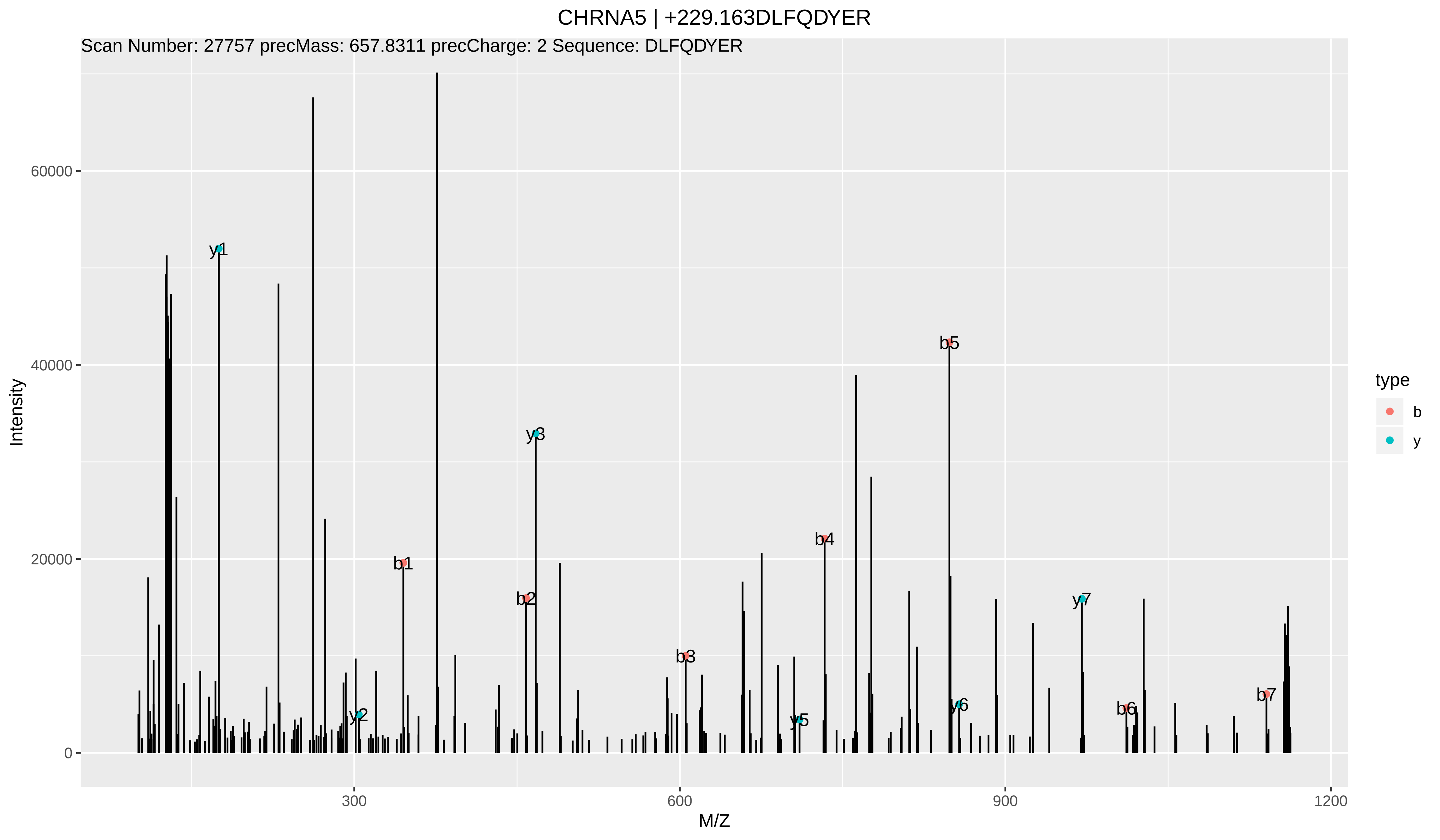

CHRNA5 | +229.163DLFQDYER
Scan Number: 27757 precMass: 657.8311 precCharge: 2 Sequence: DLFQDYER
Intensity
60000
40000
20000
0
300
600
900
1200
M/Z
y1
y2
y3
y5
y6
y7
b1
b2
b3
b4
b5
b6
b7
type
b
y

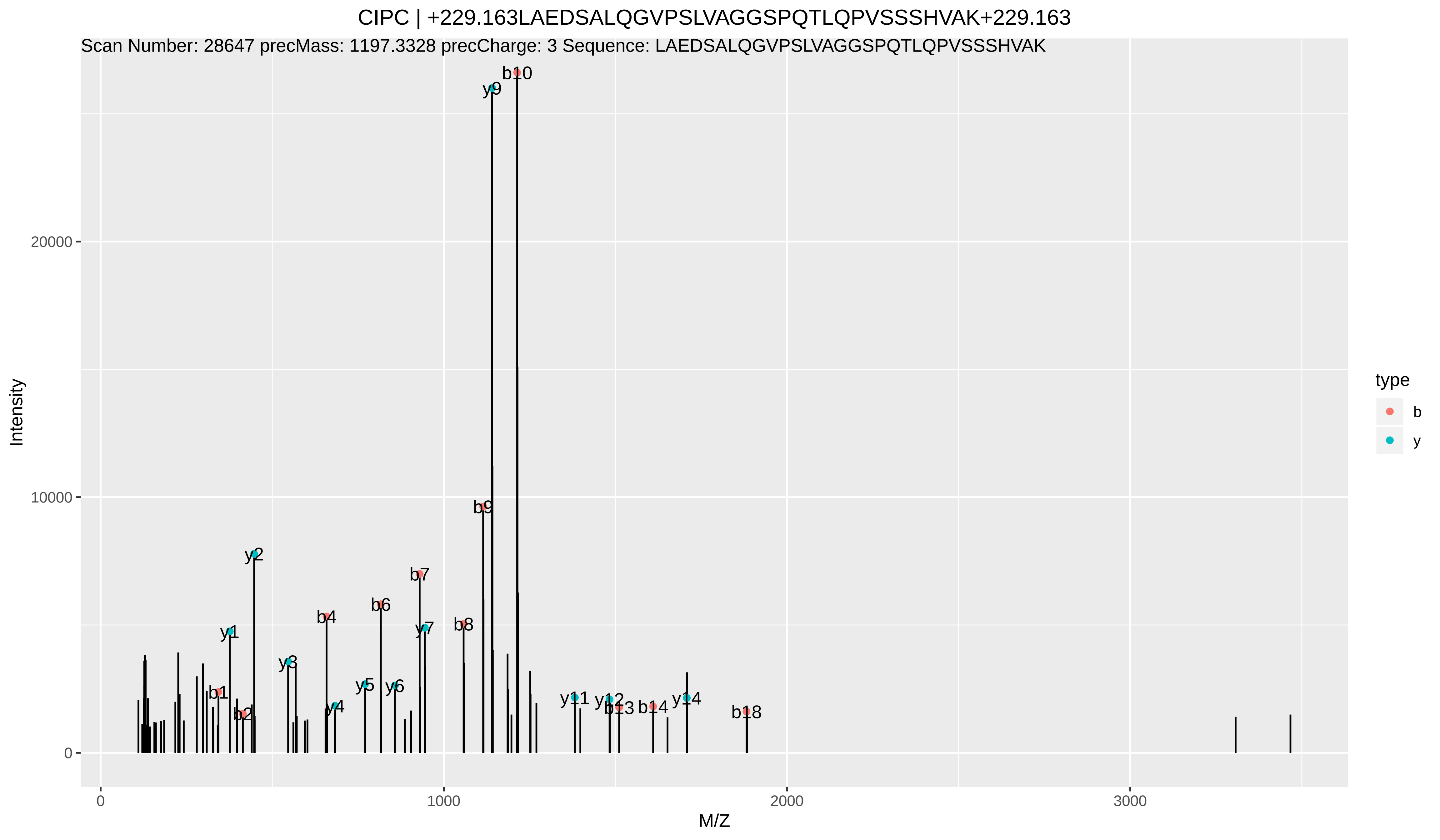

CIPC | +229.163LAEDSALQGVPSLVAGGSPQTLQPVSSSHVAK+229.163
Scan Number: 28647 precMass: 1197.3328 precCharge: 3 Sequence: LAEDSALQGVPSLVAGGSPQTLQPVSSSHVAK
Intensity
M/Z
0
10000
20000
0
1000
2000
3000
b10
y9
b9
y2
b7
b6
b4
y7
b8
y1
y3
y5
y6
b1
b2
y4
y11
y12
b13
b14
y14
b18
type
b
y

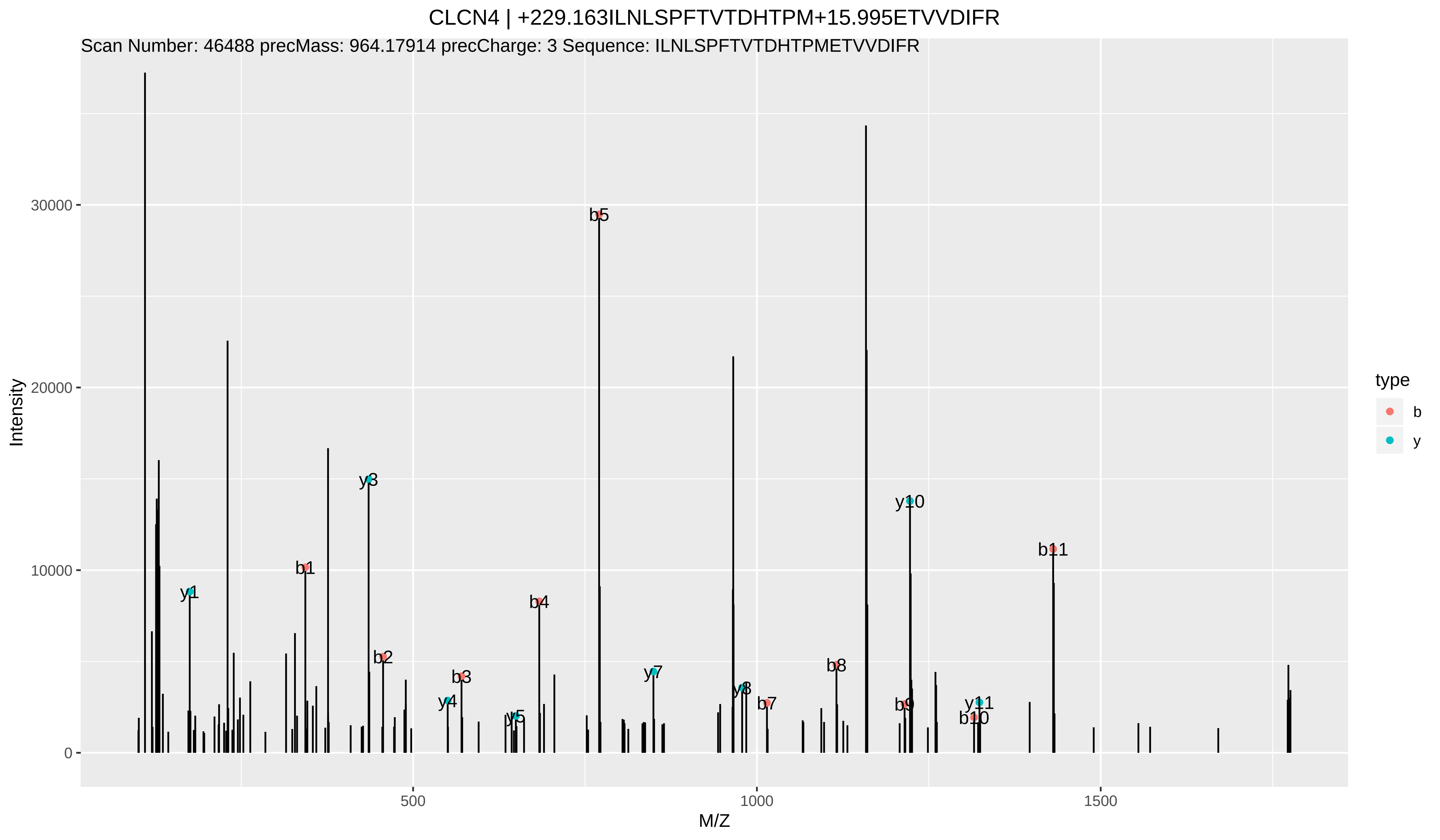

CLCN4 | +229.163ILNLSPFTVTDHTPM+15.995ETVVDIFR
Scan Number: 46488 precMass: 964.17914 precCharge: 3 Sequence: ILNLSPFTVTDHTPMETVVDIFR
Intensity
30000
20000
10000
0
500
1000
1500
M/Z
type
b
y
b5
y3
y10
b11
b1
y1
b4
b2
b8
y7
b3
y8
b7
b9
y11
b10
y4
y5

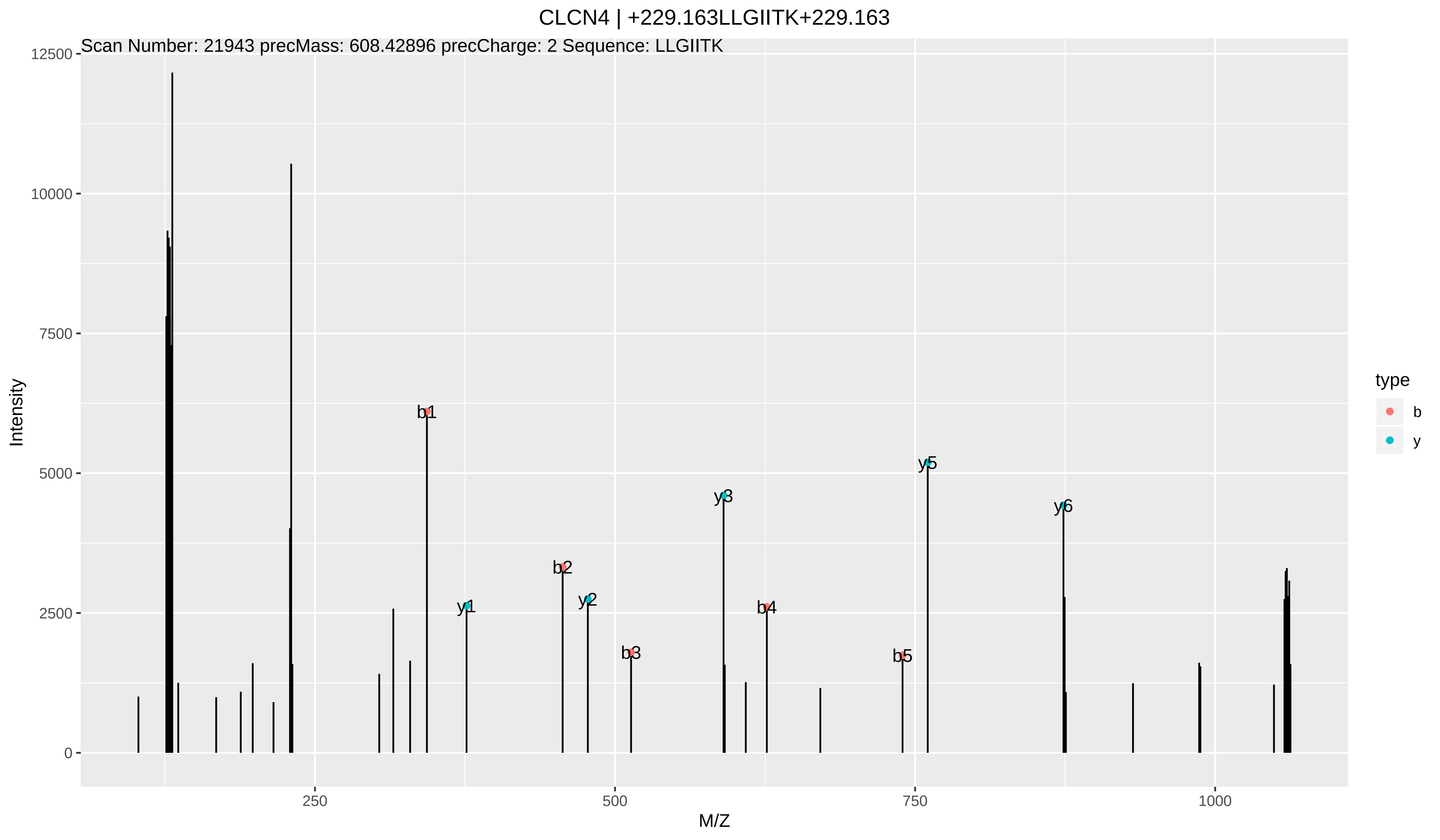

CLCN4 | +229.163LLGIITK+229.163
Scan Number: 21943 precMass: 608.42896 precCharge: 2 Sequence: LLGIITK
Intensity
M/Z
type
b
y
b1
y1
b2
y2
b3
y3
b4
b5
y5
y6

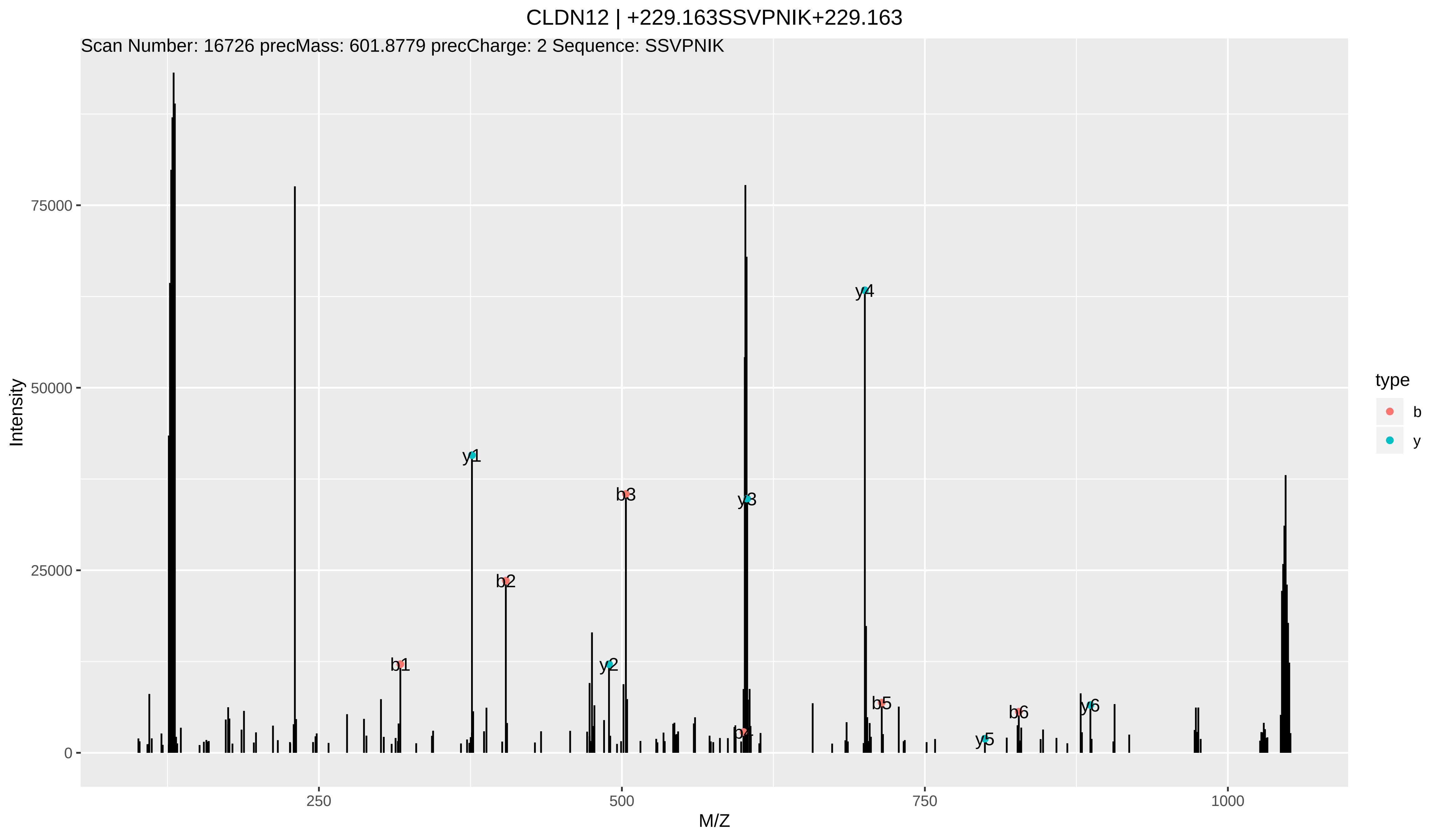
CLDN12 | +229.163SSVPNIK+229.163
Scan Number: 16726 precMass: 601.8779 precCharge: 2 Sequence: SSVPNIK
Intensity
M/Z
75000
50000
25000
0
250
500
750
1000
y1
b3
y3
y4
b2
b1
y2
b5
b6
y6
y5
type
b
y

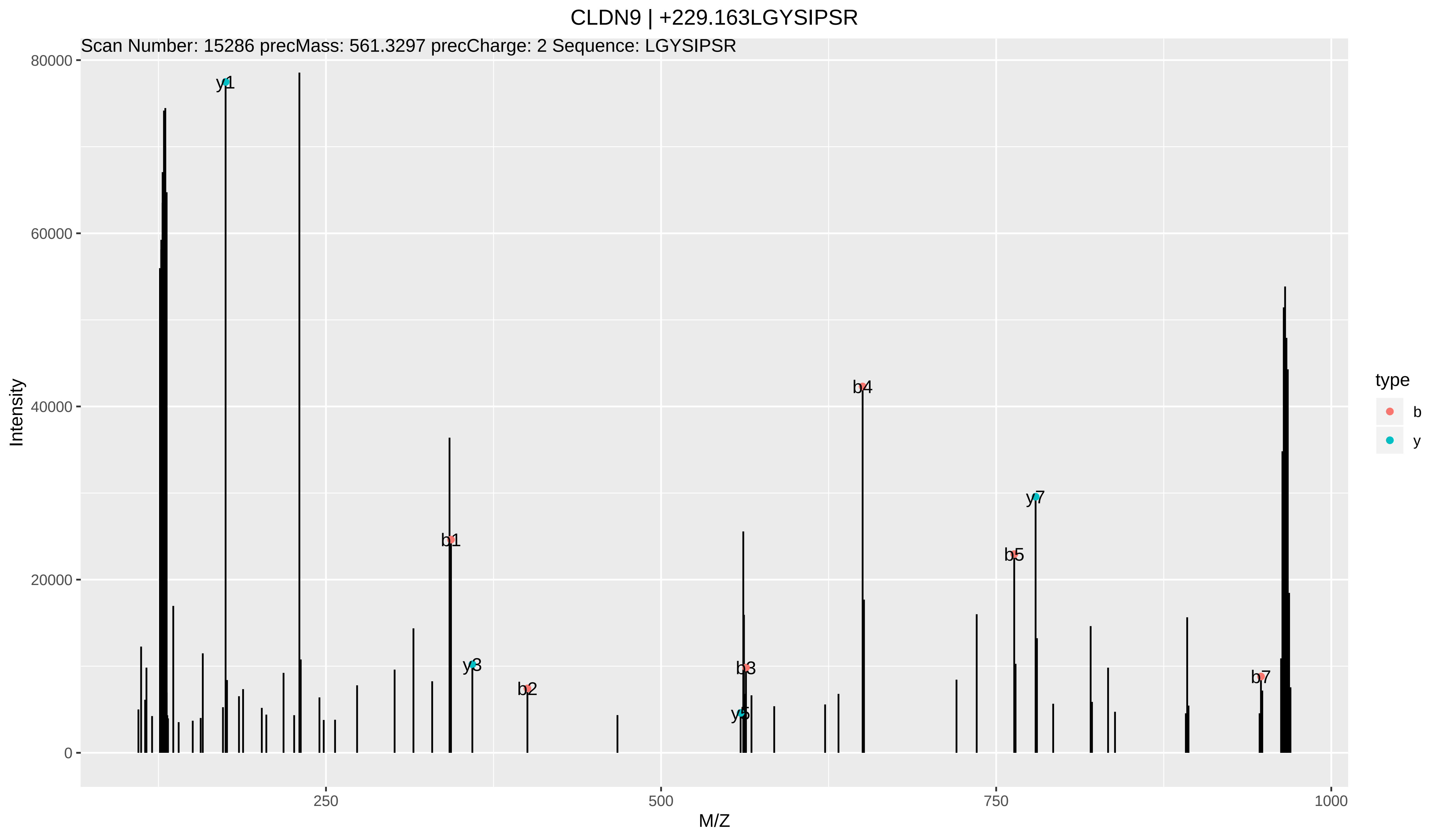

CLDN9 | +229.163LGYSIPSR
Scan Number: 15286 precMass: 561.3297 precCharge: 2 Sequence: LGYSIPSR
Intensity
M/Z
y1
b1
y3
b2
b3
y5
b4
b5
y7
b7
type
b
y
0
20000
40000
60000
80000
250
500
750
1000

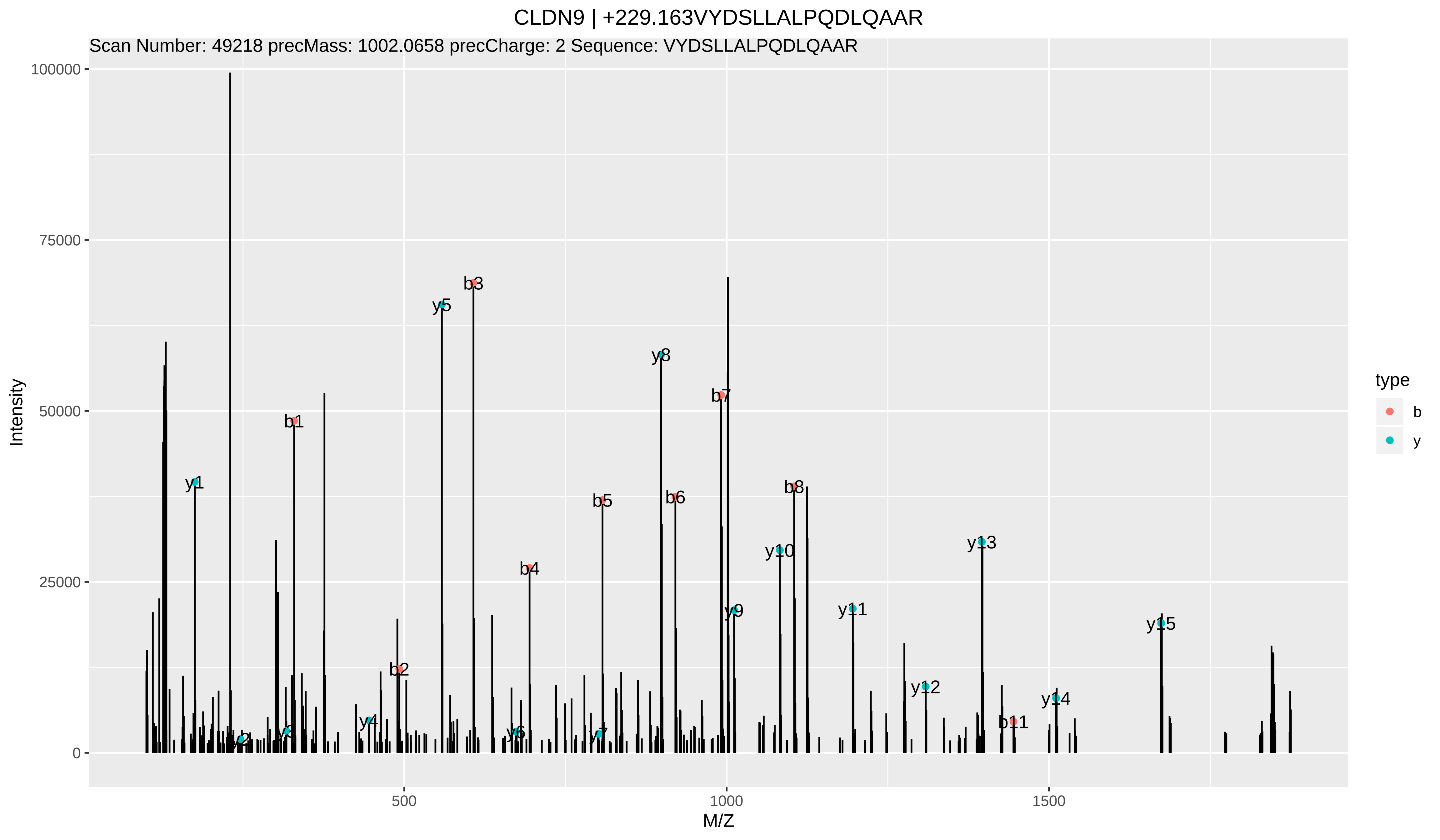

CLDN9 | +229.163VYDSLLALPQDLQAAR
Scan Number: 49218 precMass: 1002.0658 precCharge: 2 Sequence: VYDSLLALPQDLQAAR
Intensity
M/Z
type
b
y

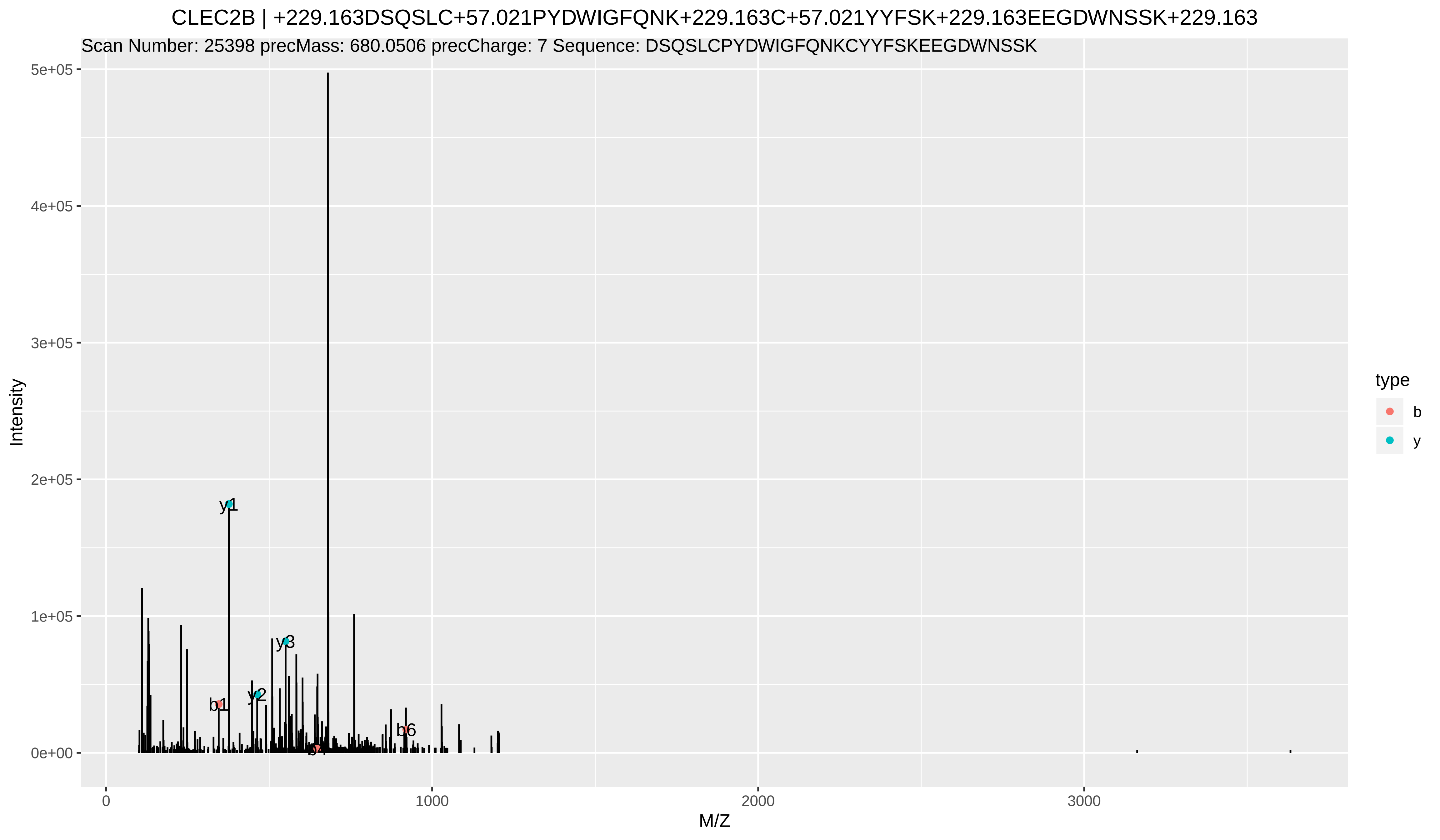
CLEC2B | +229.163DSQSLC+57.021PYDWIGFQNK+229.163C+57.021YYFSK+229.163EEGDWNSSK+229.163
Scan Number: 25398 precMass: 680.0506 precCharge: 7 Sequence: DSQSLCPYDWIGFQNKCYYFSKEEGDWNSSK
Intensity
5e+05
4e+05
3e+05
2e+05
1e+05
0e+00
y1
y3
y2
b1
b6
0
1000
2000
3000
M/Z
type
b
y

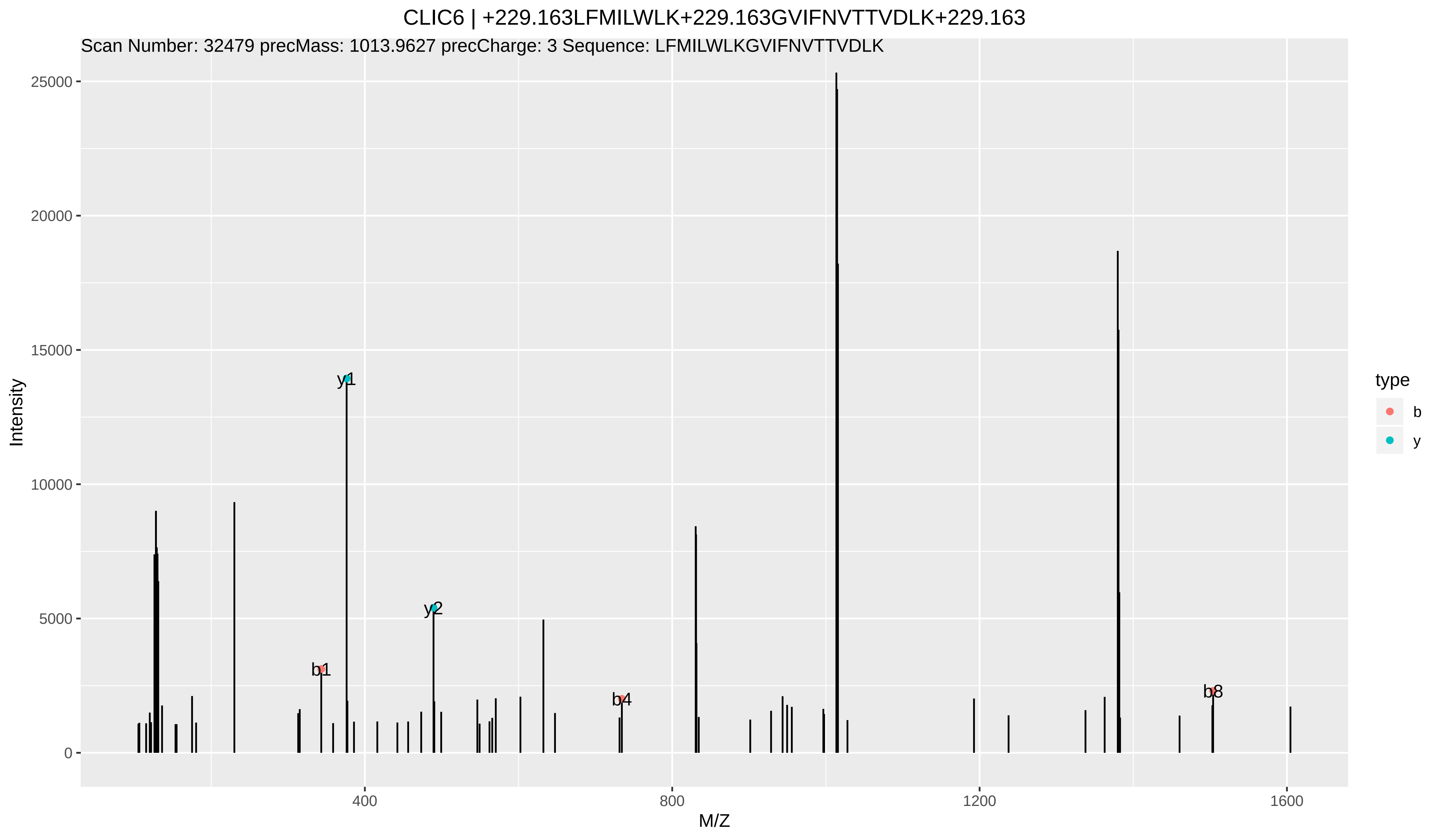
CLIC6 | +229.163LFMILWLK+229.163GVIFNVTTVDLK+229.163
Scan Number: 32479 precMass: 1013.9627 precCharge: 3 Sequence: LFMILWLKGVIFNVTTVDLK
Intensity
M/Z
type
b
y
y1
y2
b1
b4
b8
25000
20000
15000
10000
5000
0
400
800
1200
1600

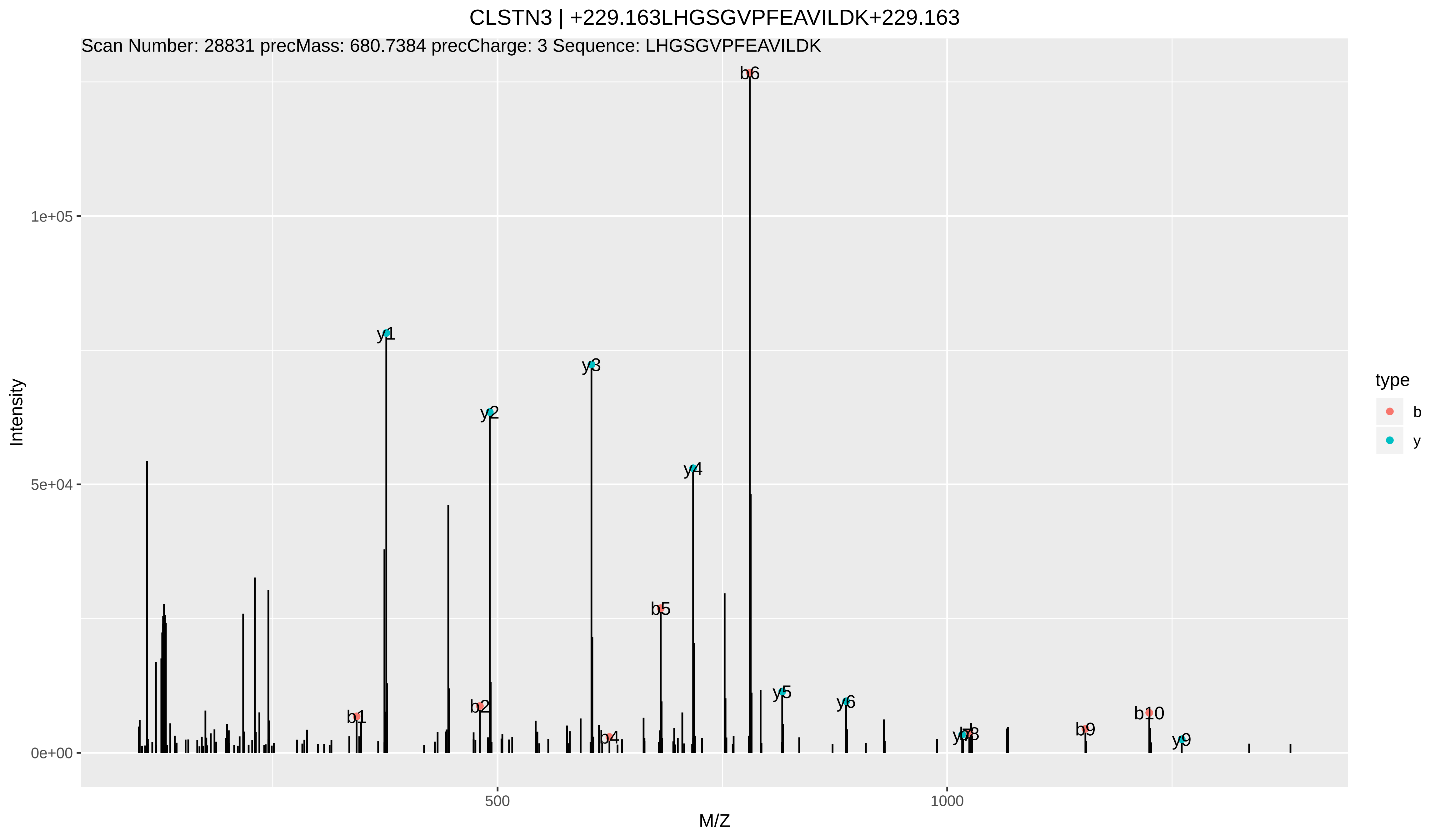

CLSTN3 | +229.163LHGSGVPFEAVILDK+229.163
Scan Number: 28831 precMass: 680.7384 precCharge: 3 Sequence: LHGSGVPFEAVILDK
Intensity
1e+05
5e+04
0e+00
500
1000
M/Z
type
b
y
b6
y1
y3
y2
y4
b5
y5
y6
b2
b1
b4
b10
b9
y9

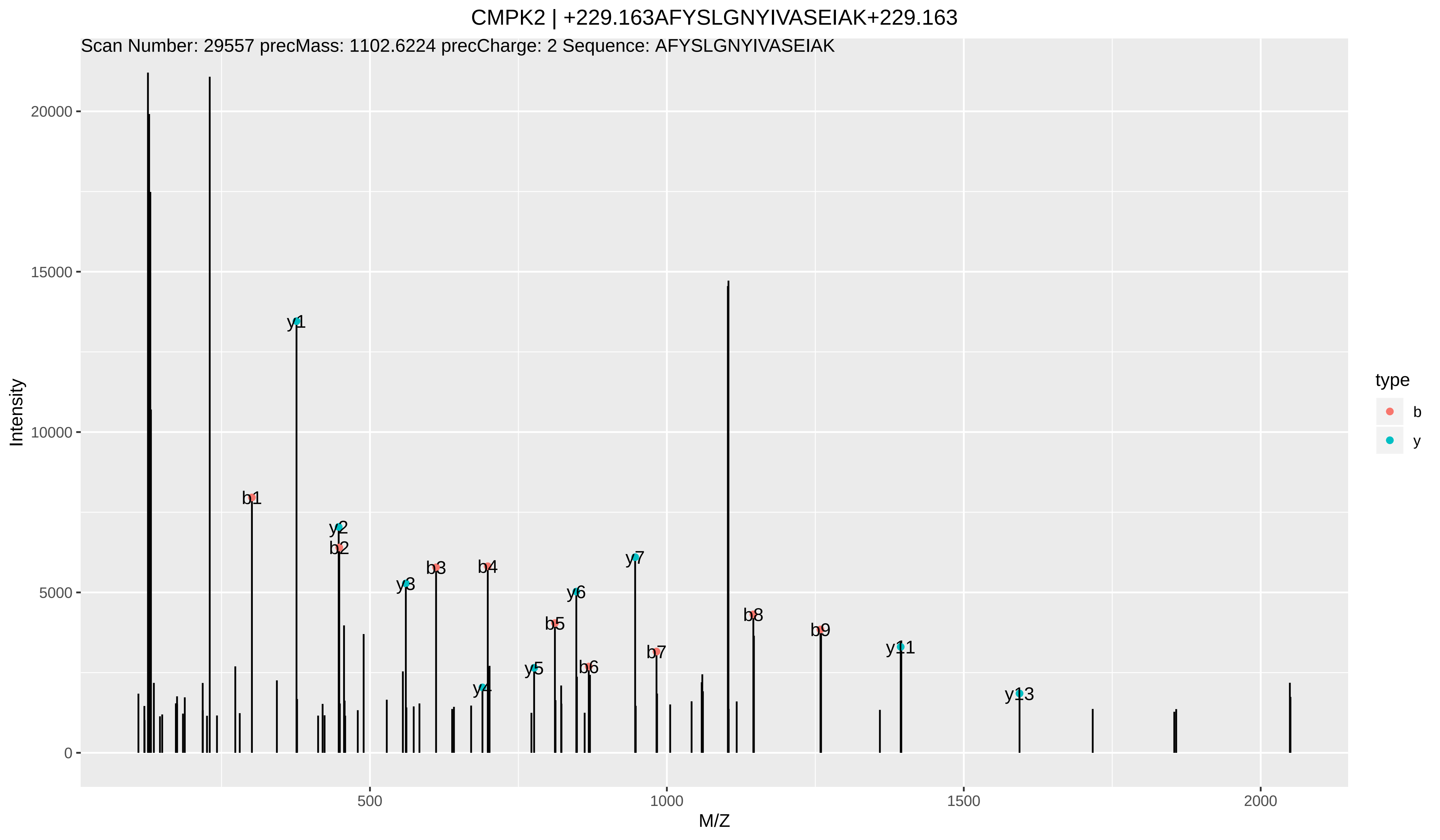

CMPK2 | +229.163AFYSLGNYIVASEIAK+229.163
Scan Number: 29557 precMass: 1102.6224 precCharge: 2 Sequence: AFYSLGNYIVASEIAK
Intensity
M/Z
0
5000
10000
15000
20000
500
1000
1500
2000
y1
b1
y2
b2
y3
b3
b4
y4
y5
b5
y6
b6
y7
b7
b8
b9
y11
y13
type
b
y

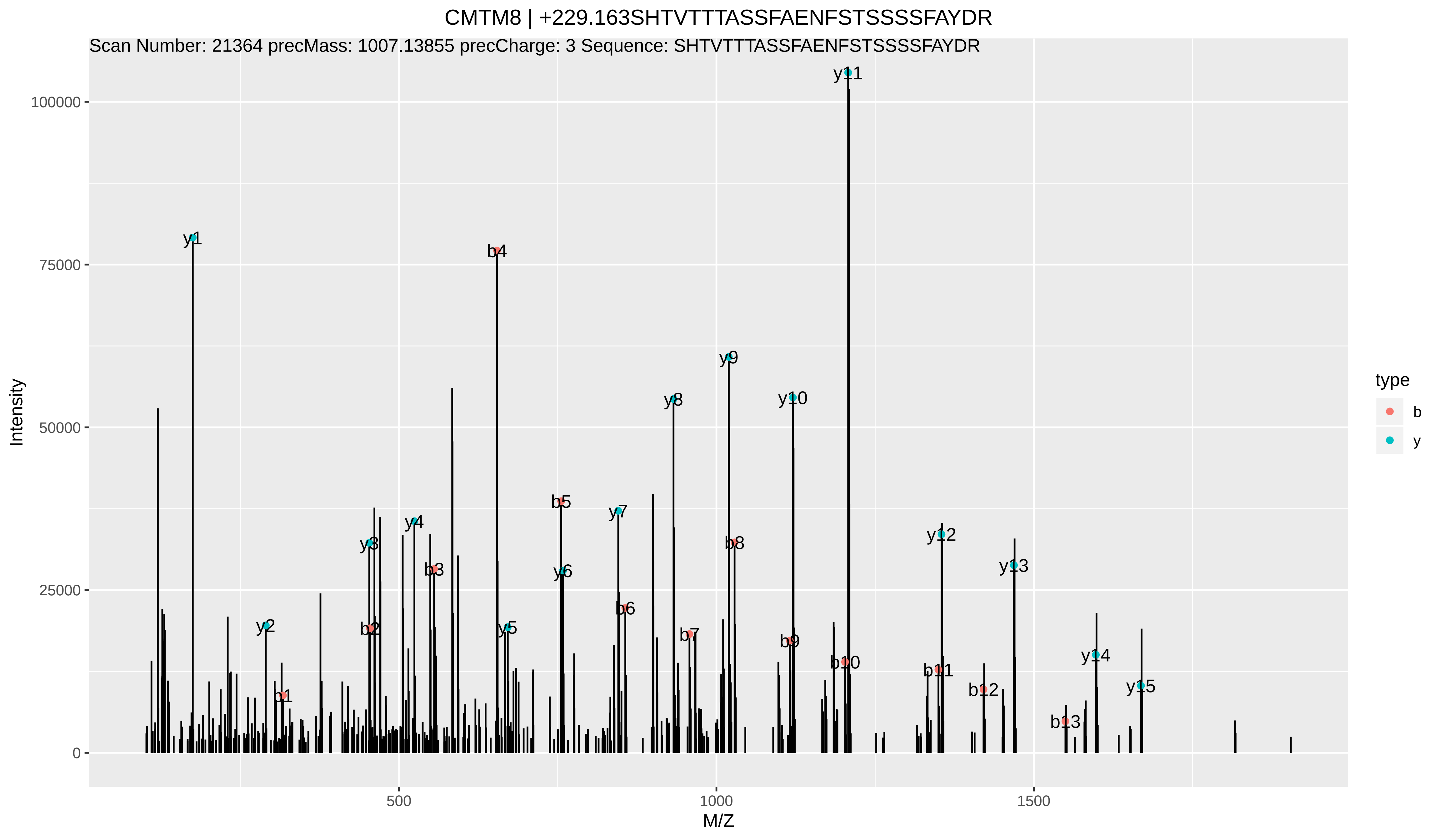

CMTM8 | +229.163SHTVTTTASSFAENFSTSSSSFAYDR
Scan Number: 21364 precMass: 1007.13855 precCharge: 3 Sequence: SHTVTTTASSFAENFSTSSSSFAYDR
Intensity
M/Z
type
b
y
0
25000
50000
75000
100000
500
1000
1500
y1
y2
y3
y4
y5
y6
y7
y8
y9
y10
y11
y12
y13
y14
y15
b1
b2
b3
b4
b5
b6
b7
b8
b9
b10
b11
b12
b13

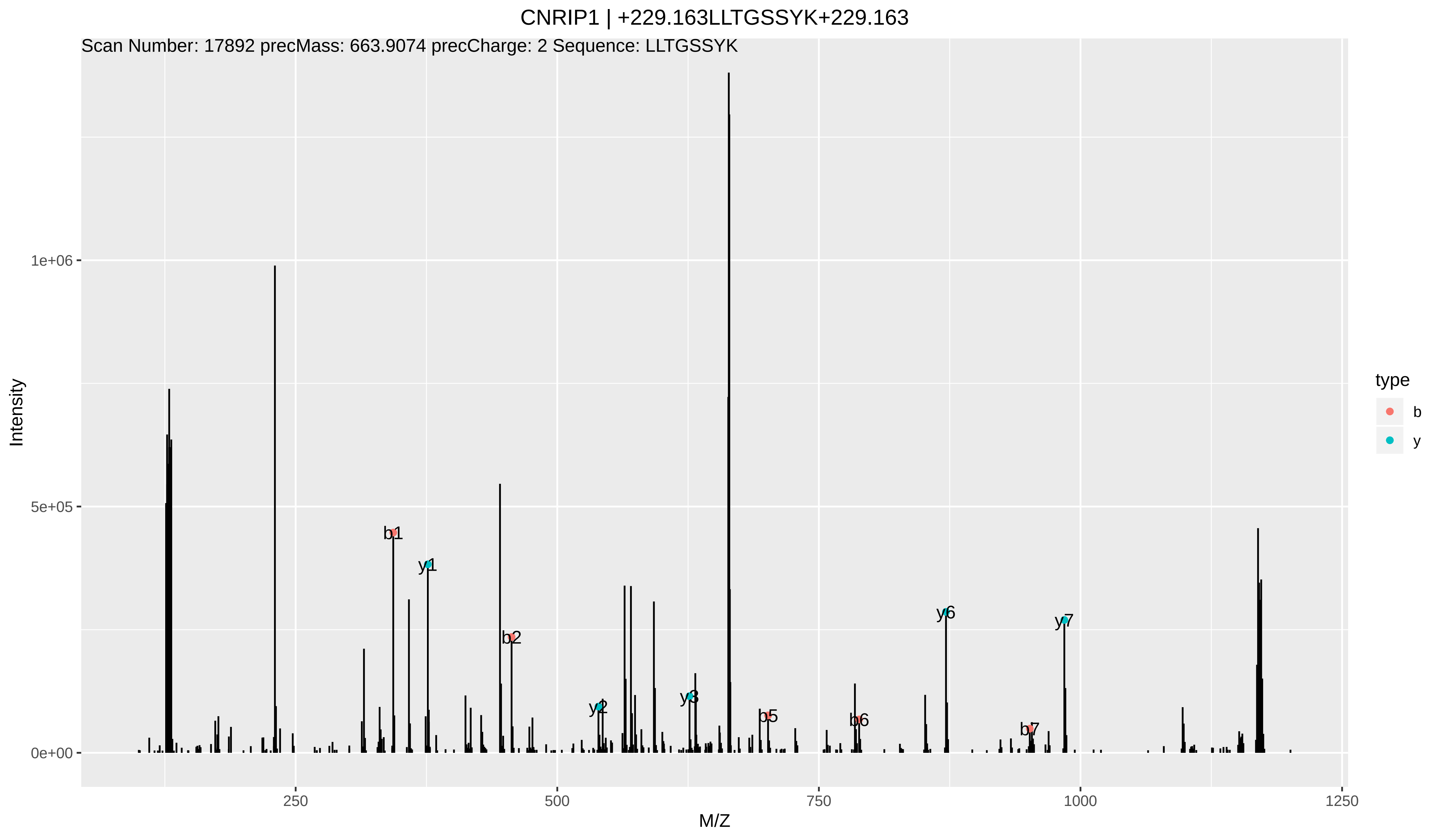
CNRIP1 | +229.163LLTGSSYK+229.163
Scan Number: 17892 precMass: 663.9074 precCharge: 2 Sequence: LLTGSSYK
Intensity
1e+06
5e+05
0e+00
250
500
750
1000
1250
M/Z
b1
y1
b2
y2
y3
b5
b6
y6
y7
b7
type
b
y

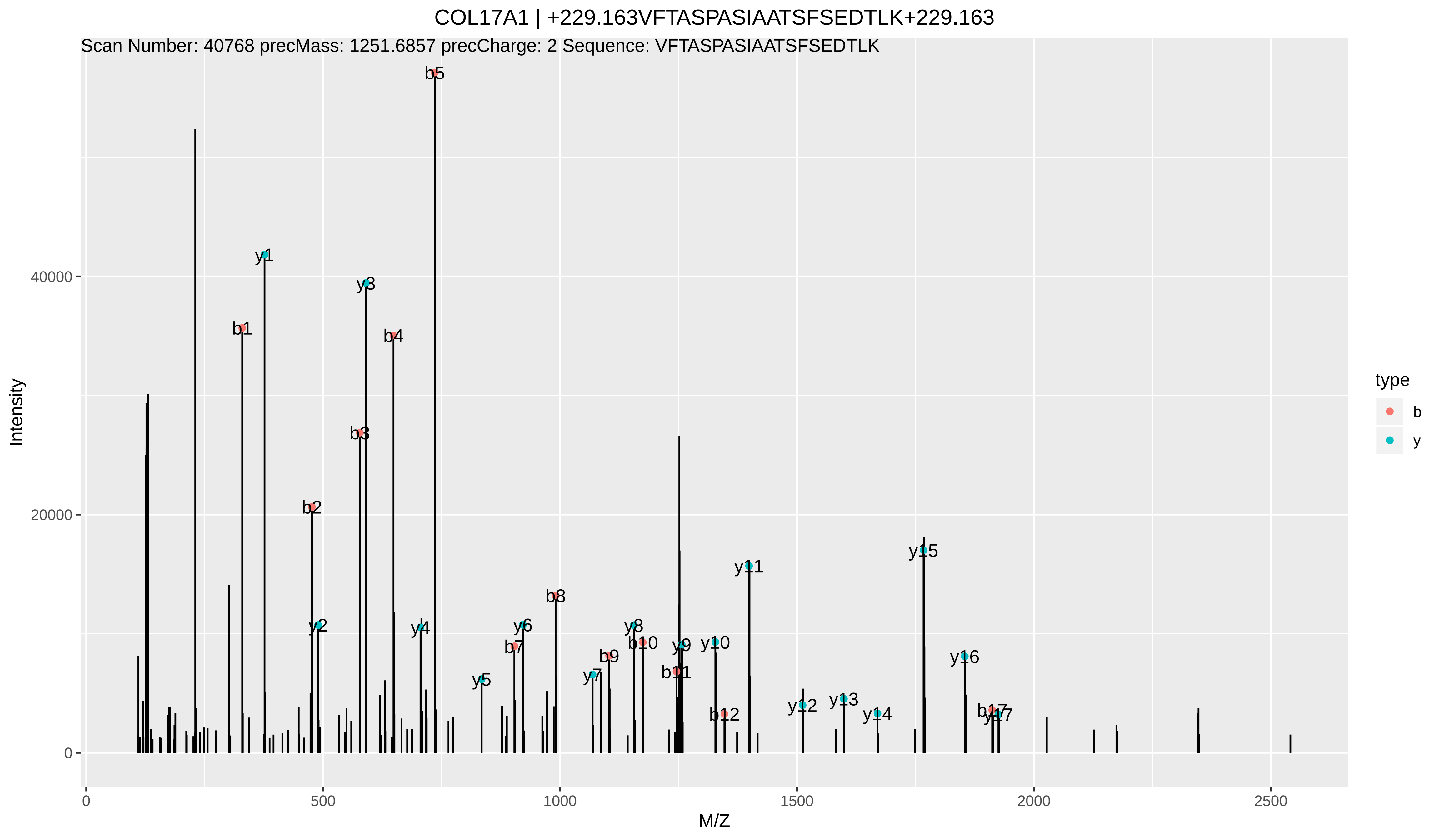
COL17A1 | +229.163VFTASPASIAATSFSEDTLK+229.163
Scan Number: 40768 precMass: 1251.6857 precCharge: 2 Sequence: VFTASPASIAATSFSEDTLK
Intensity
M/Z
type
b
y
b5
y1
y3
b1
b4
b3
b2
y2
y4
b8
y6
y8
b10
y9
y10
b7
b9
y11
y15
y16
b11
y5
y7
b12
y12
y13
y14
b17
y17
0
20000
40000
500
1000
1500
2000
2500

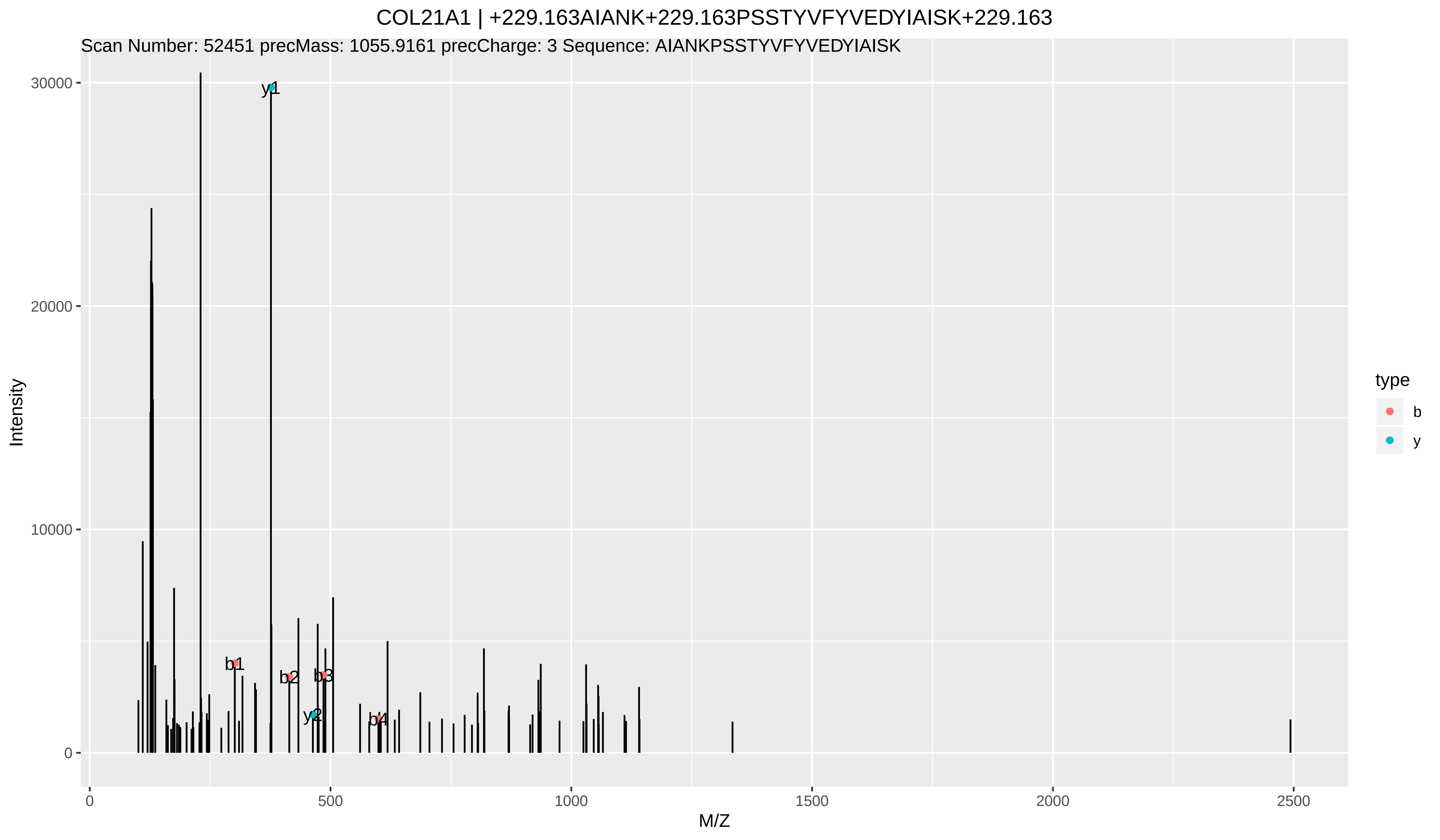
COL21A1 | +229.163AIANK+229.163PSSTYVFYVEDYIAISK+229.163
Scan Number: 52451 precMass: 1055.9161 precCharge: 3 Sequence: AIANKPSSTYVFYVEDYIAISK
Intensity
M/Z
type
b
y
y1
y2
b1
b2
b3
b4
0
10000
20000
30000
0
500
1000
1500
2000
2500

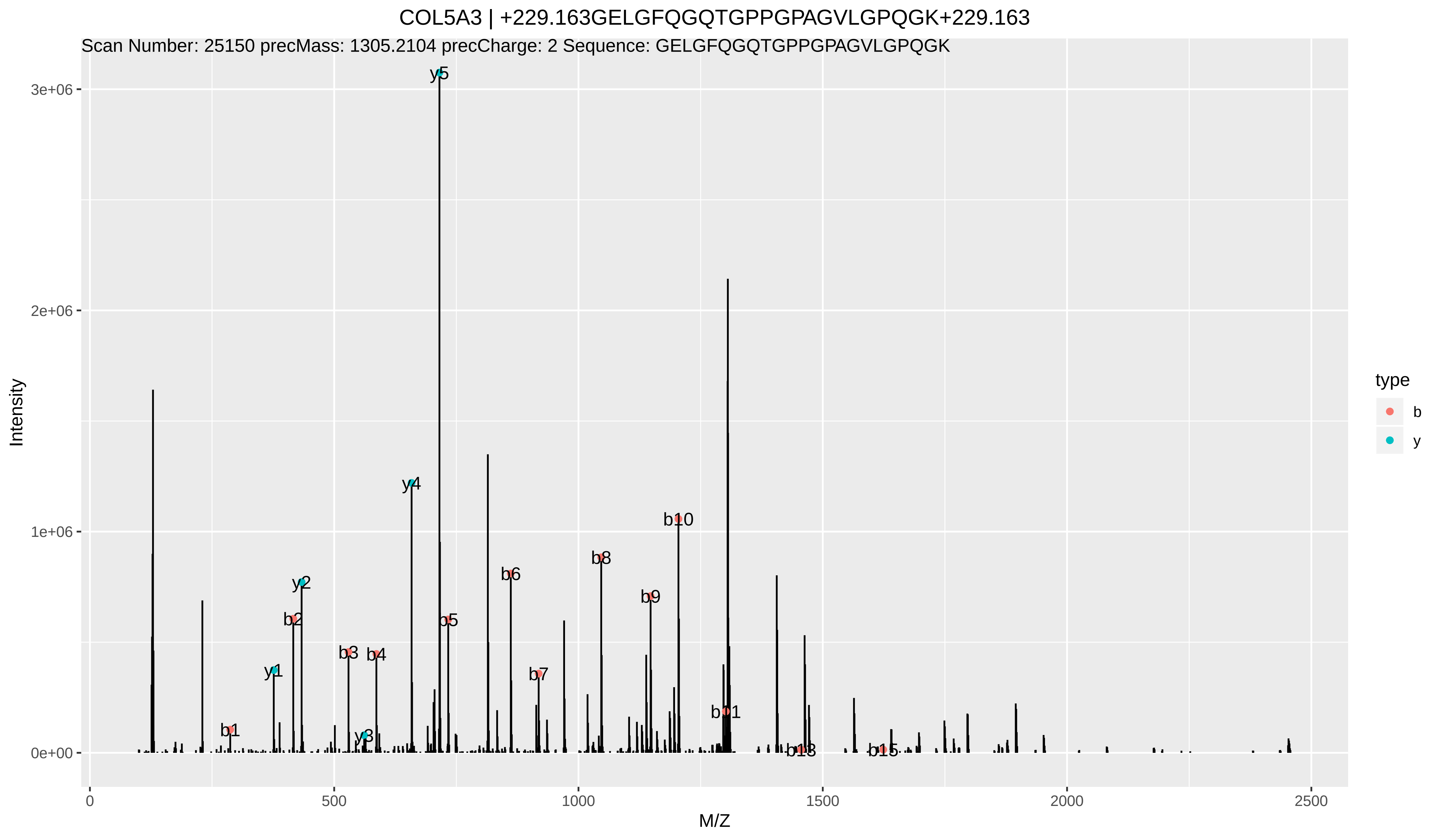

COL5A3 | +229.163GELGFQGQTGPPGPAGVLGPQGK+229.163
Scan Number: 25150 precMass: 1305.2104 precCharge: 2 Sequence: GELGFQGQTGPPGPAGVLGPQGK
Intensity
M/Z
type
b
y
y5
y4
y2
y1
y3
b1
b2
b3
b4
b5
b6
b7
b8
b9
b10
b11
b13
b15

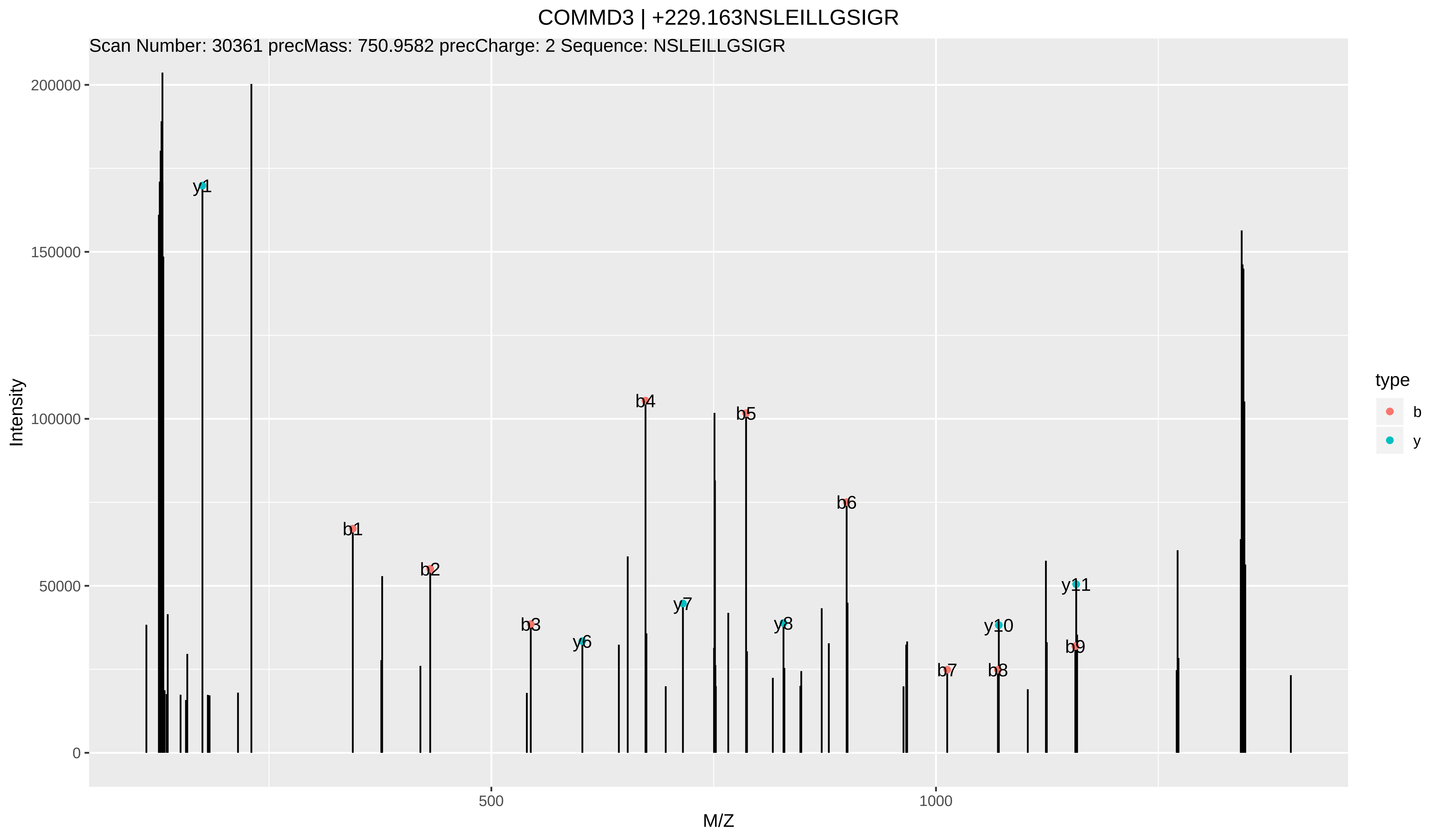

COMMD3 | +229.163NSLEILLGSIGR
Scan Number: 30361 precMass: 750.9582 precCharge: 2 Sequence: NSLEILLGSIGR
Intensity
200000
150000
100000
50000
0
500
1000
M/Z
y1
b1
b2
b3
y6
b4
y7
b5
y8
b6
b7
b8
y10
y11
b9
type
b
y

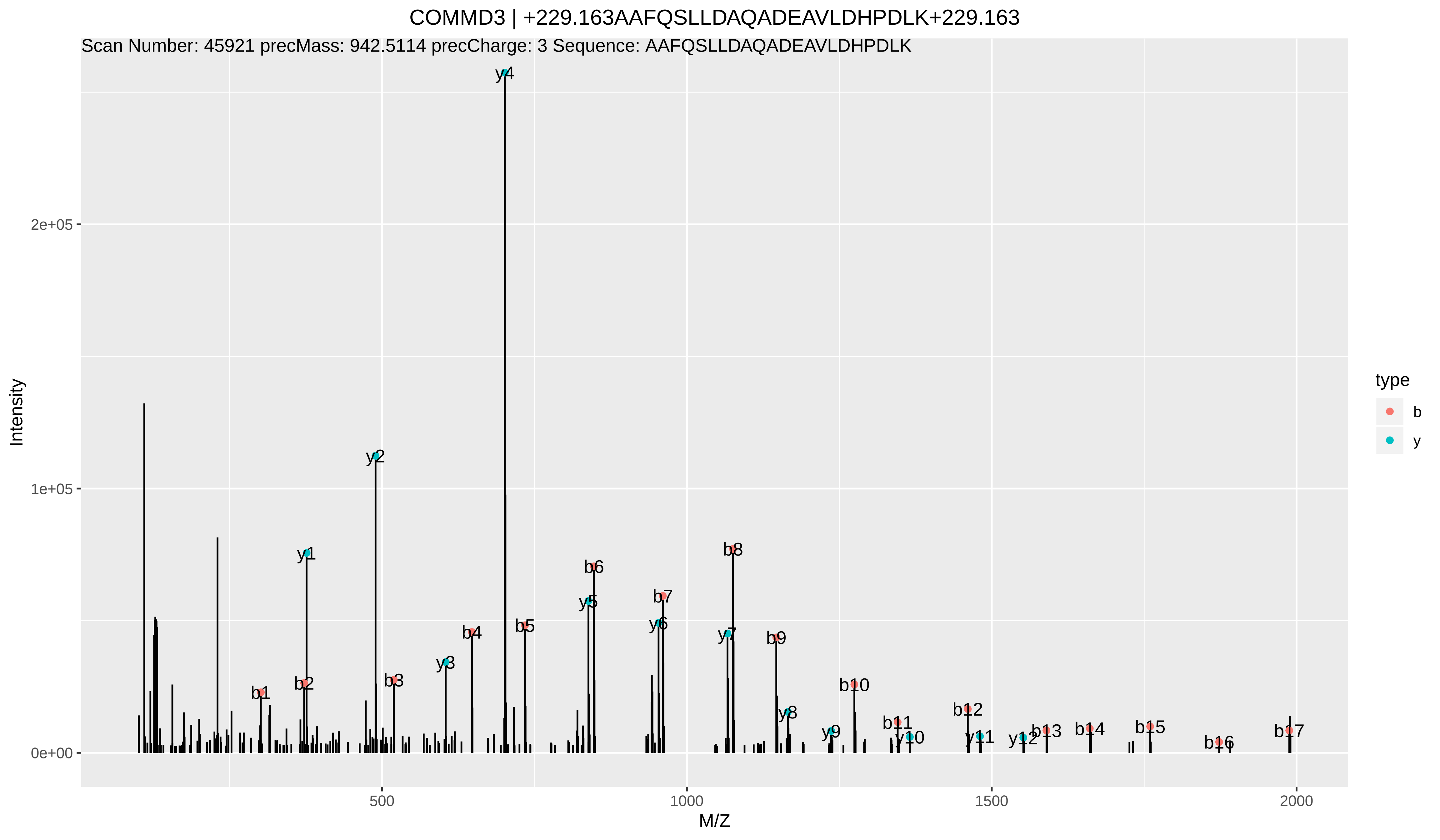
COMMD3 | +229.163AAFQSLLDAQADEAVLDHPDLK+229.163
Scan Number: 45921 precMass: 942.5114 precCharge: 3 Sequence: AAFQSLLDAQADEAVLDHPDLK
Intensity
M/Z
0e+00
1e+05
2e+05
500
1000
1500
2000
type
b
y
y1
y2
y3
y4
y5
y6
y7
y8
y9
y10
y11
y12
b1
b2
b3
b4
b5
b6
b7
b8
b9
b10
b11
b12
b13
b14
b15
b16
b17

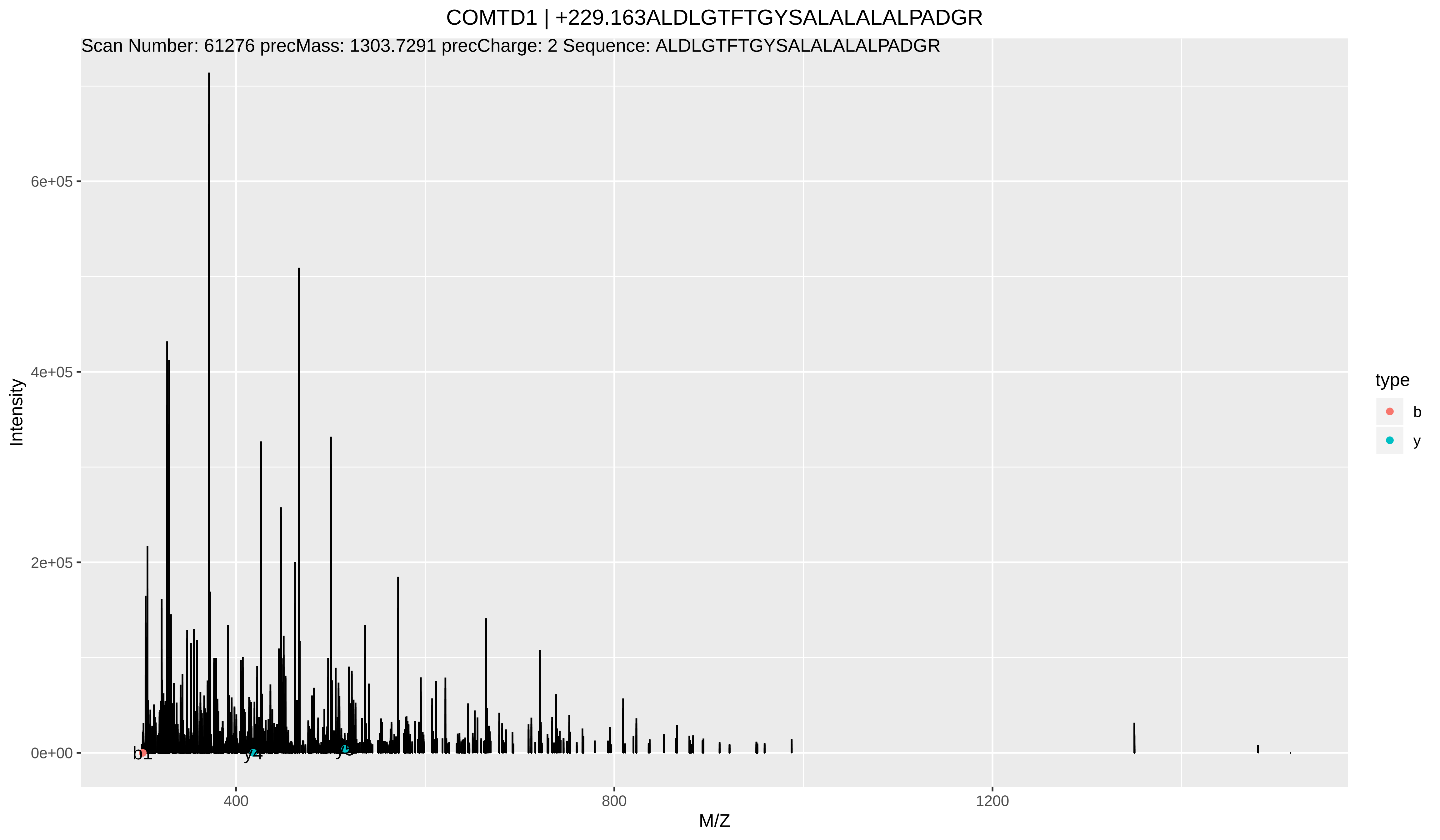
COMTD1 | +229.163ALDLGTFTGYSALALALALPADGR
Scan Number: 61276 precMass: 1303.7291 precCharge: 2 Sequence: ALDLGTFTGYSALALALALPADGR
Intensity
6e+05
4e+05
2e+05
0e+00
400
800
1200
M/Z
b1
y4
y5
type
b
y

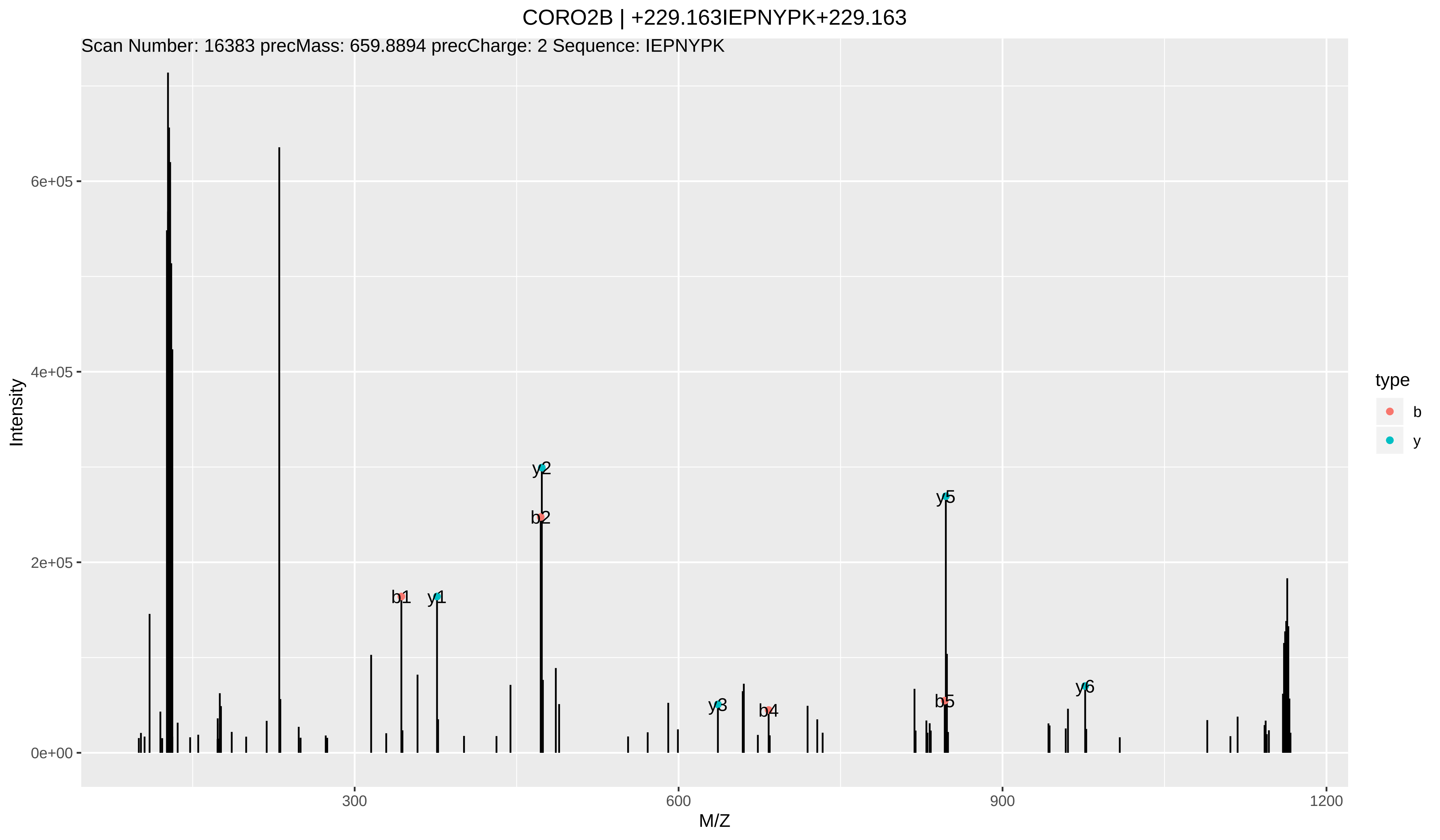
CORO2B | +229.163IEPNYPK+229.163
Scan Number: 16383 precMass: 659.8894 precCharge: 2 Sequence: IEPNYPK
Intensity
M/Z
0e+00
2e+05
4e+05
6e+05
300
600
900
1200
b1
y1
y2
b2
y3
b4
y5
b5
y6
type
b
y

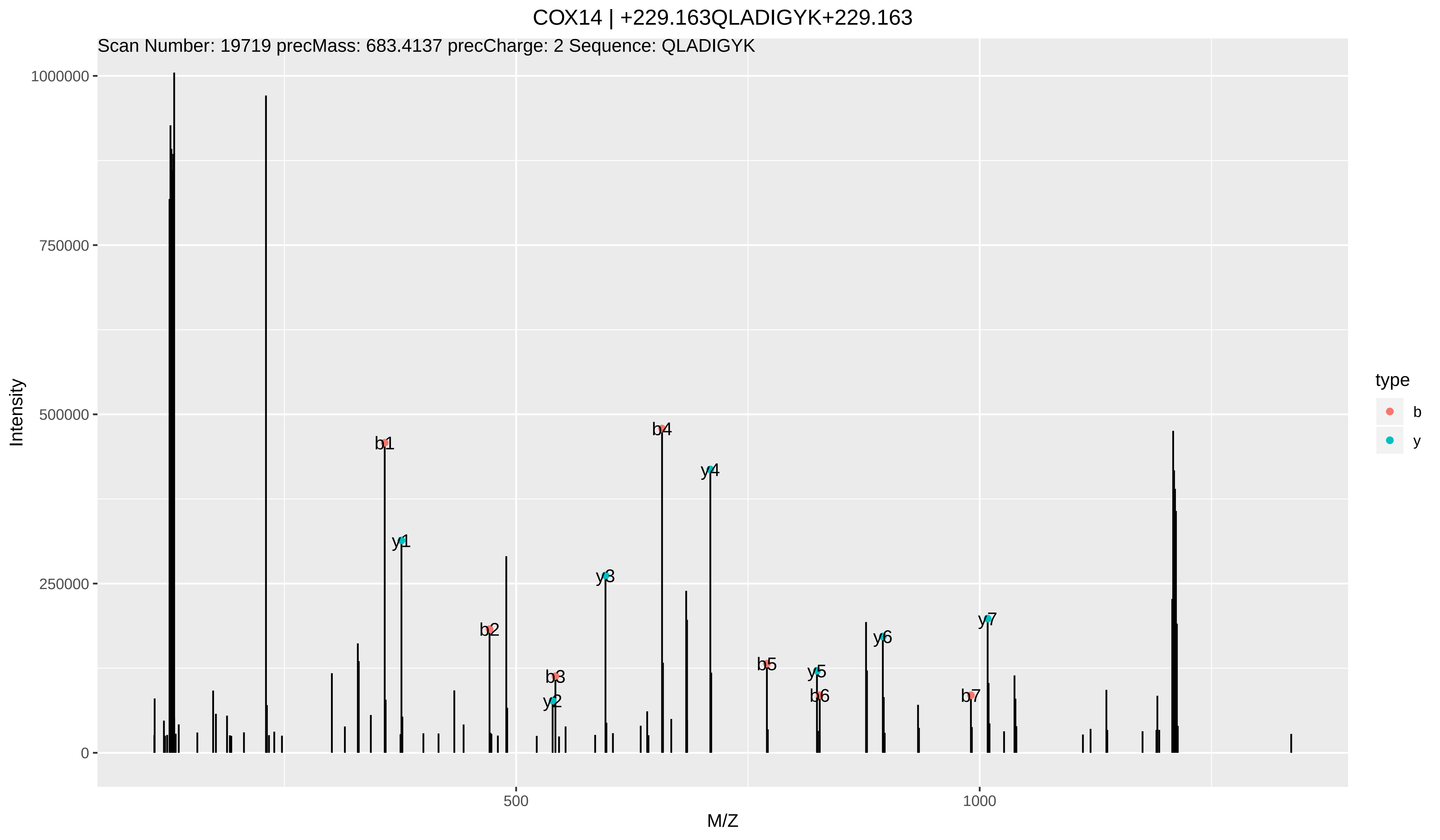
COX14 | +229.163QLADIGYK+229.163
Scan Number: 19719 precMass: 683.4137 precCharge: 2 Sequence: QLADIGYK
Intensity
1000000
750000
500000
250000
0
M/Z
500
1000
b1
y1
b2
b3
y2
y3
b4
y4
b5
y5
b6
y6
b7
y7
type
b
y

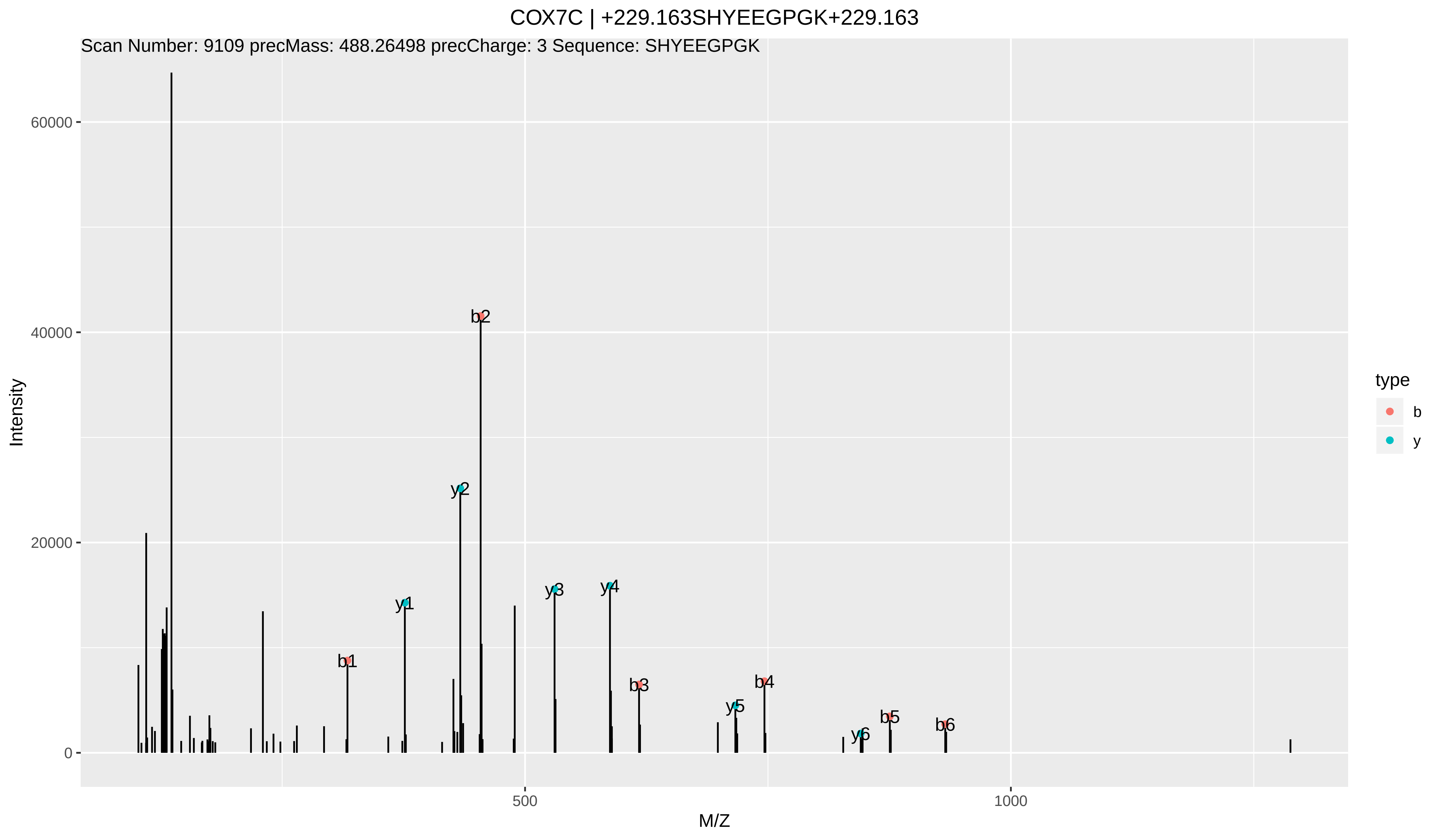
COX7C | +229.163SHYEEGPGK+229.163
Scan Number: 9109 precMass: 488.26498 precCharge: 3 Sequence: SHYEEGPGK
Intensity
M/Z
60000
40000
20000
0
500
1000
b2
y2
y1
y3
y4
b1
b3
b4
y5
b5
b6
y6
type
b
y

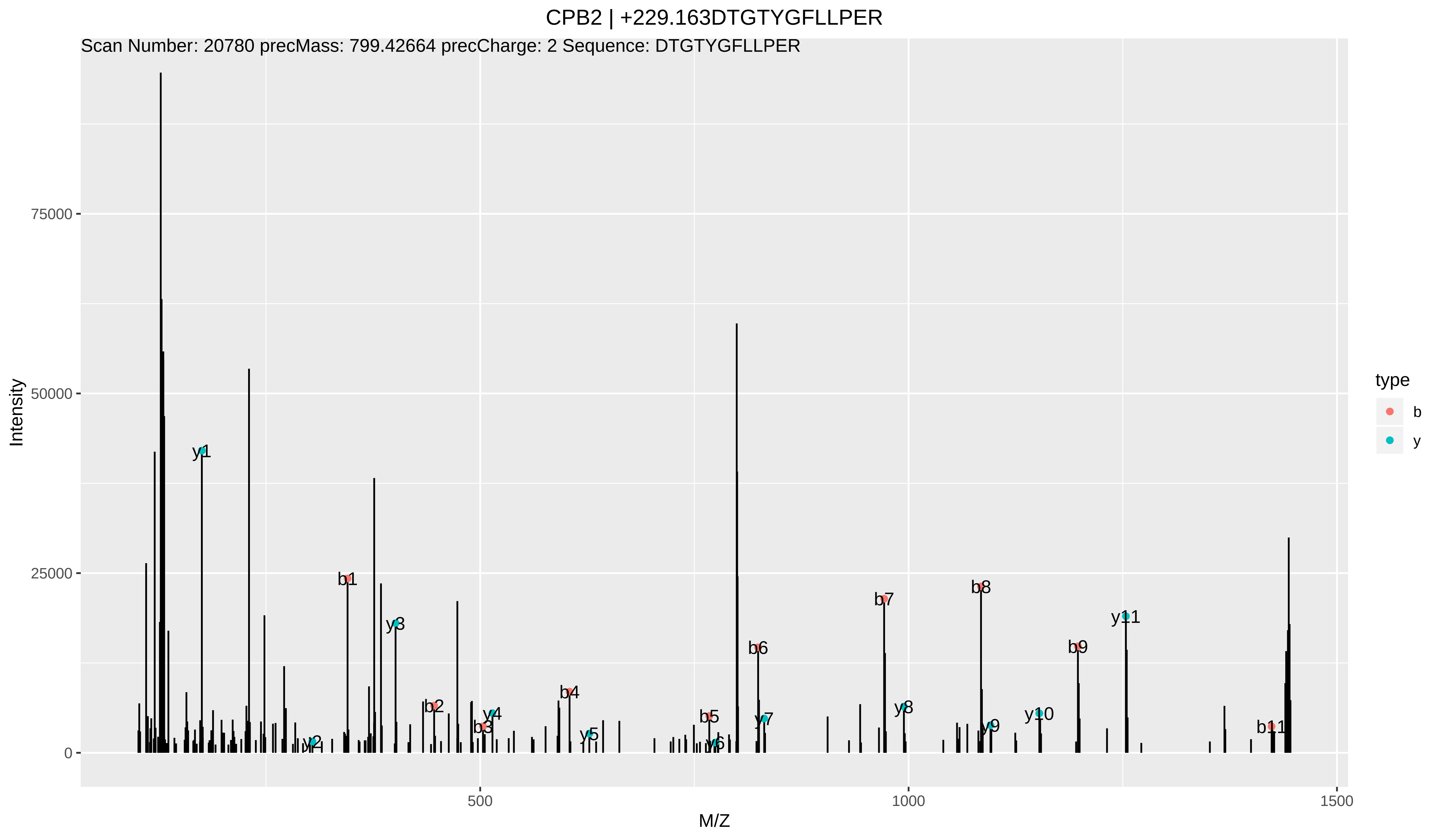
CPB2 | +229.163DTGTYGFLLPER
Scan Number: 20780 precMass: 799.42664 precCharge: 2 Sequence: DTGTYGFLLPER
Intensity
M/Z
type
b
y
y1
y2
b1
y3
b2
b3
y4
b4
y5
b5
y6
b6
y7
b7
y8
b8
y9
y10
b9
y11
b11
0
25000
50000
75000
500
1000
1500

CPEB3 | +229.163SSLFPFEDAFLDDSHGDQALSSGLSSPTR

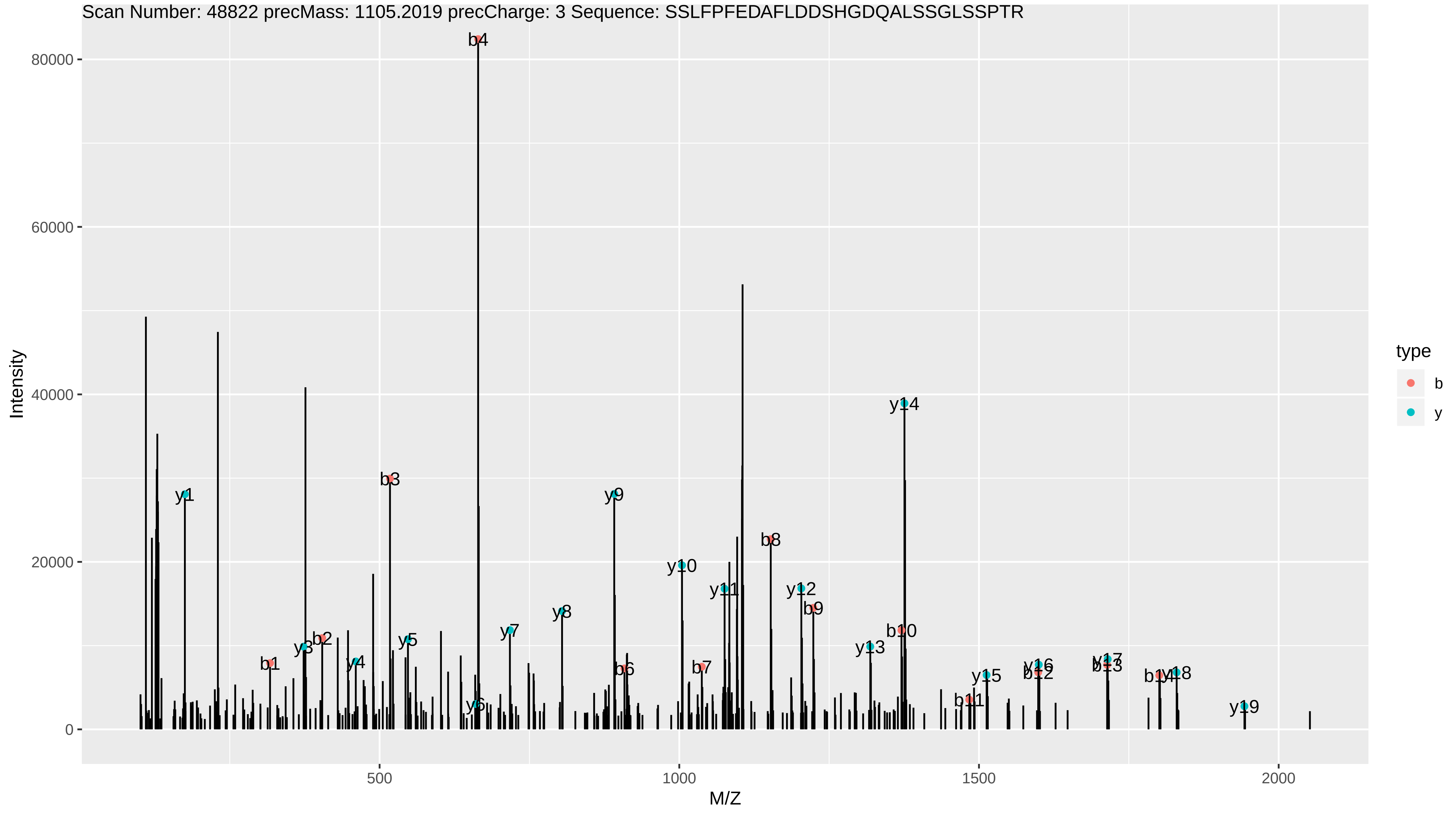

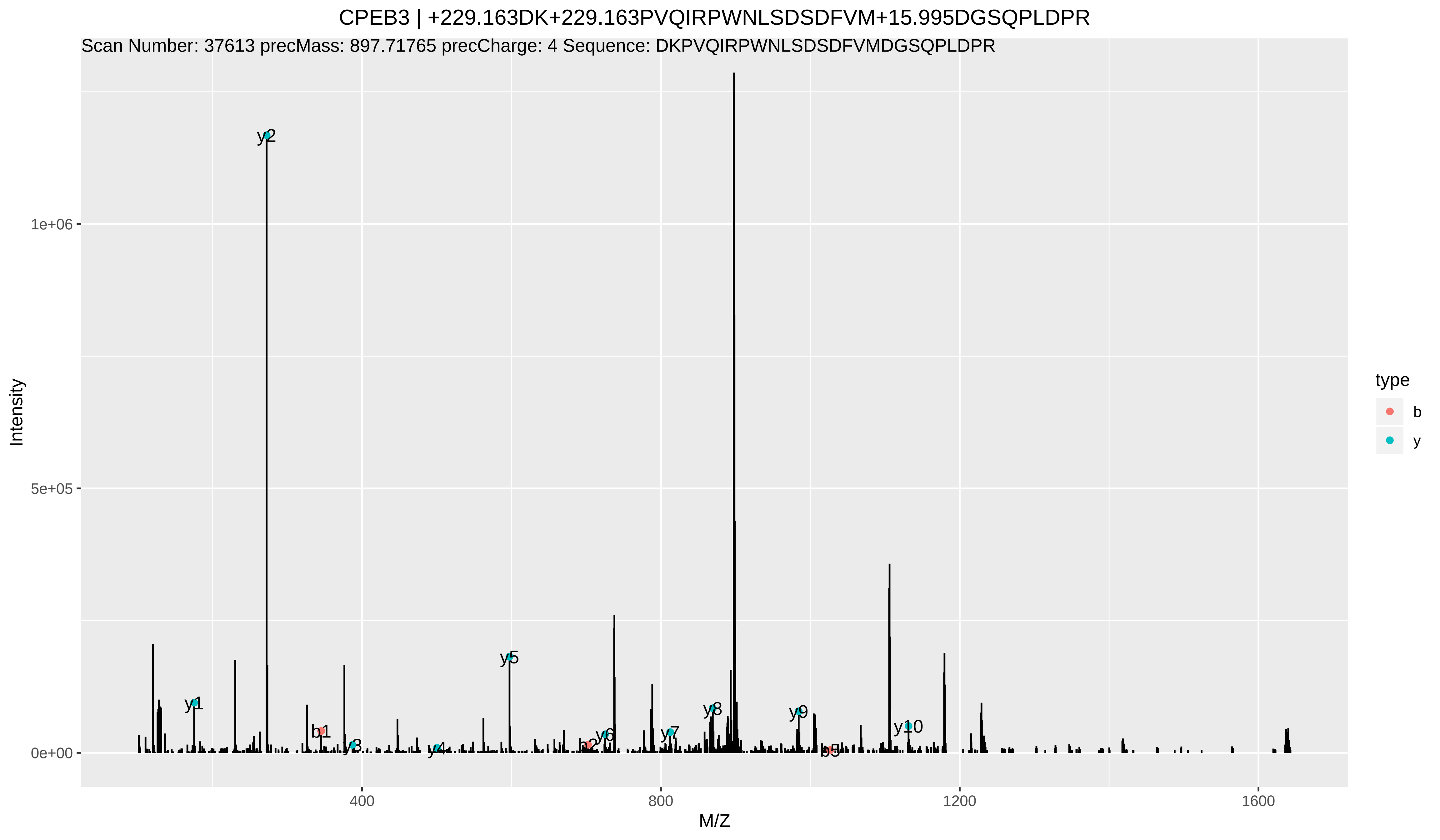
CPEB3 | +229.163DK+229.163PVQIRPWNLSDSDFVM+15.995DGSQPLDPR
Scan Number: 37613 precMass: 897.71765 precCharge: 4 Sequence: DKPVQIRPWNLSDSDFVMDGSQPLDPR
Intensity
1e+06
5e+05
0e+00
M/Z
400
800
1200
1600
y1
y2
y3
y4
y5
y6
y7
y8
y9
y10
b1
b2
b5
type
b
y

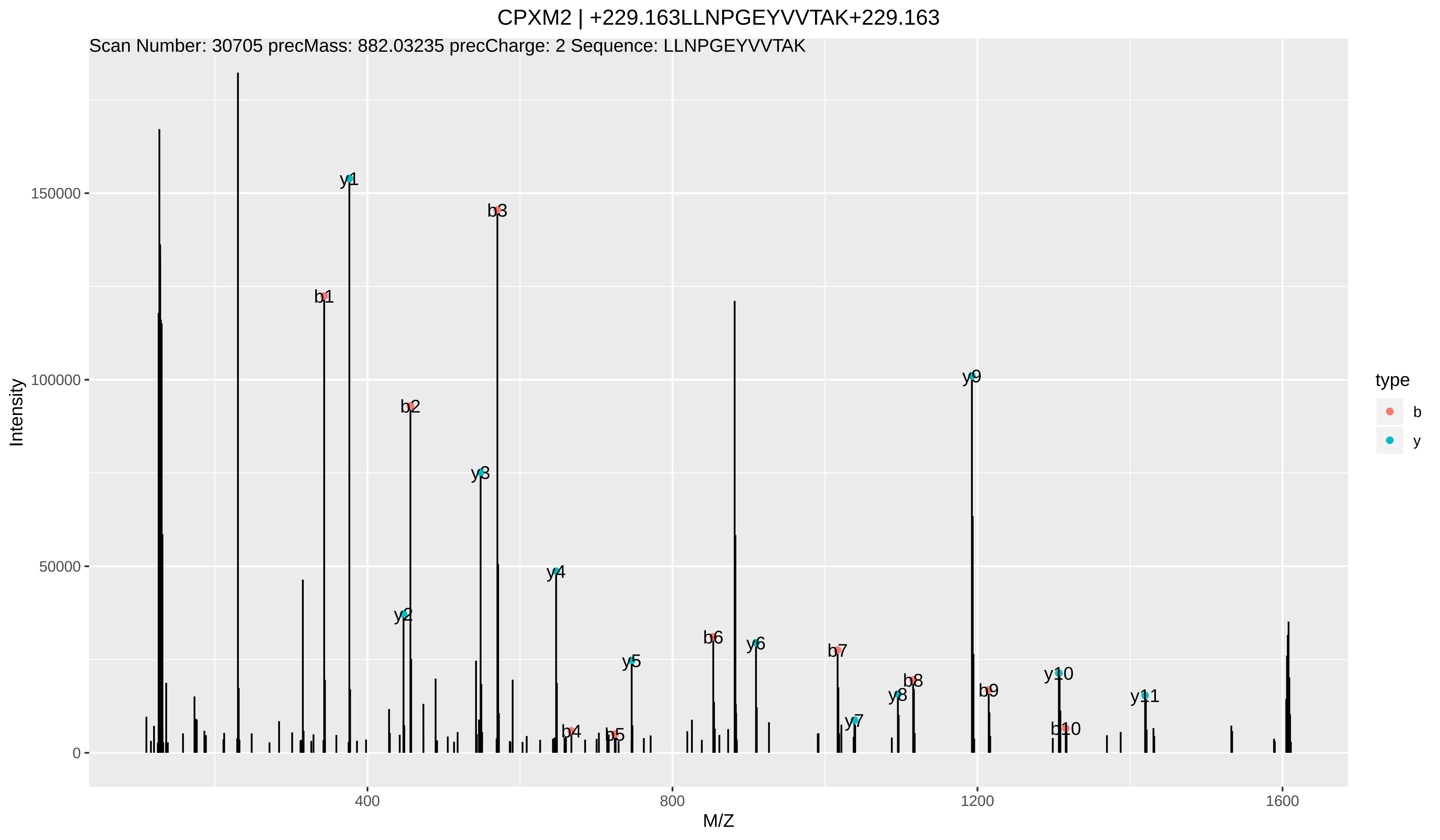
CPXM2 | +229.163LLNPGEYVVTAK+229.163
Scan Number: 30705 precMass: 882.03235 precCharge: 2 Sequence: LLNPGEYVVTAK
Intensity
M/Z
type
b
y
y1
b3
b1
y9
b2
y3
y4
y2
b6
y6
b7
y5
b8
y10
b9
y8
y11
y7
b10
b4
b5
0
50000
100000
150000
400
800
1200
1600

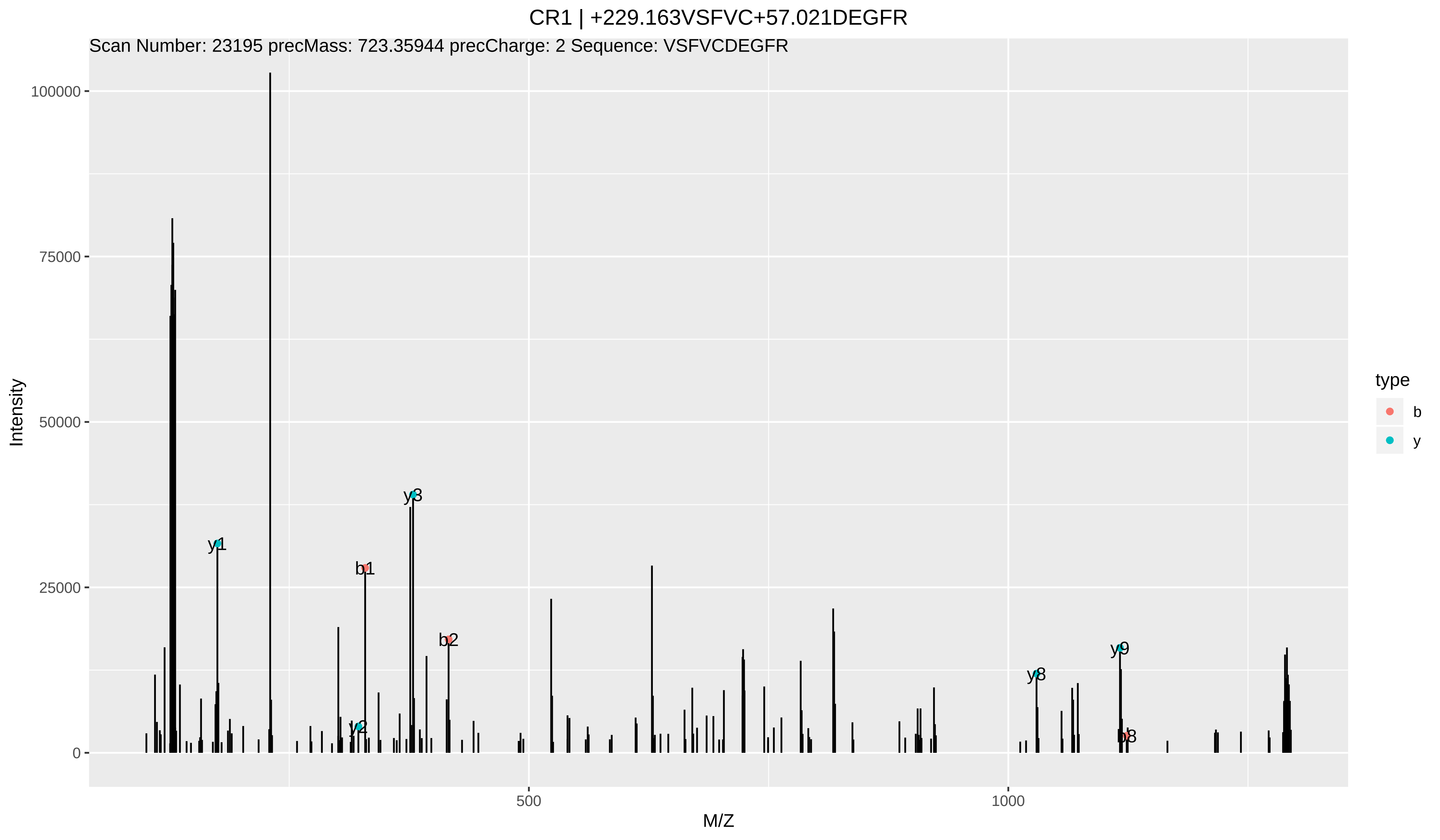

CR1 | +229.163VSFVC+57.021DEGFR
Scan Number: 23195 precMass: 723.35944 precCharge: 2 Sequence: VSFVCDEGFR
Intensity
100000
75000
50000
25000
0
M/Z
500
1000
y1
b1
y2
y3
b2
y8
y9
b8
type
b
y

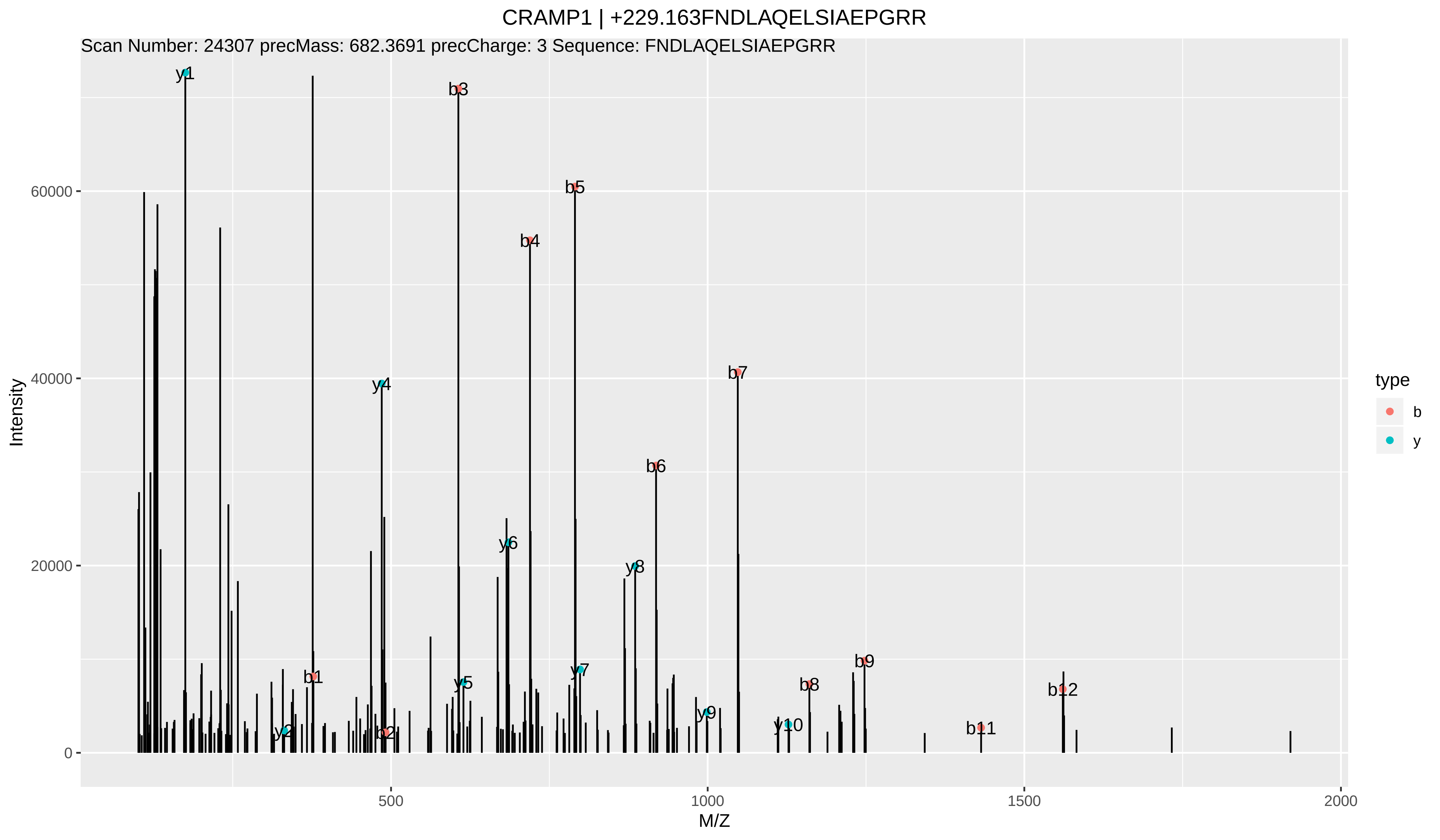
CRAMP1 | +229.163FNDLAQELSIAEPGRR
Scan Number: 24307 precMass: 682.3691 precCharge: 3 Sequence: FNDLAQELSIAEPGRR
y1
b3
b5
b4
y4
b7
b6
y6
y8
b9
y7
b1
y5
b8
b12
y9
y10
b11
y2
b2
Intensity
0
20000
40000
60000
M/Z
500
1000
1500
2000
type
b
y

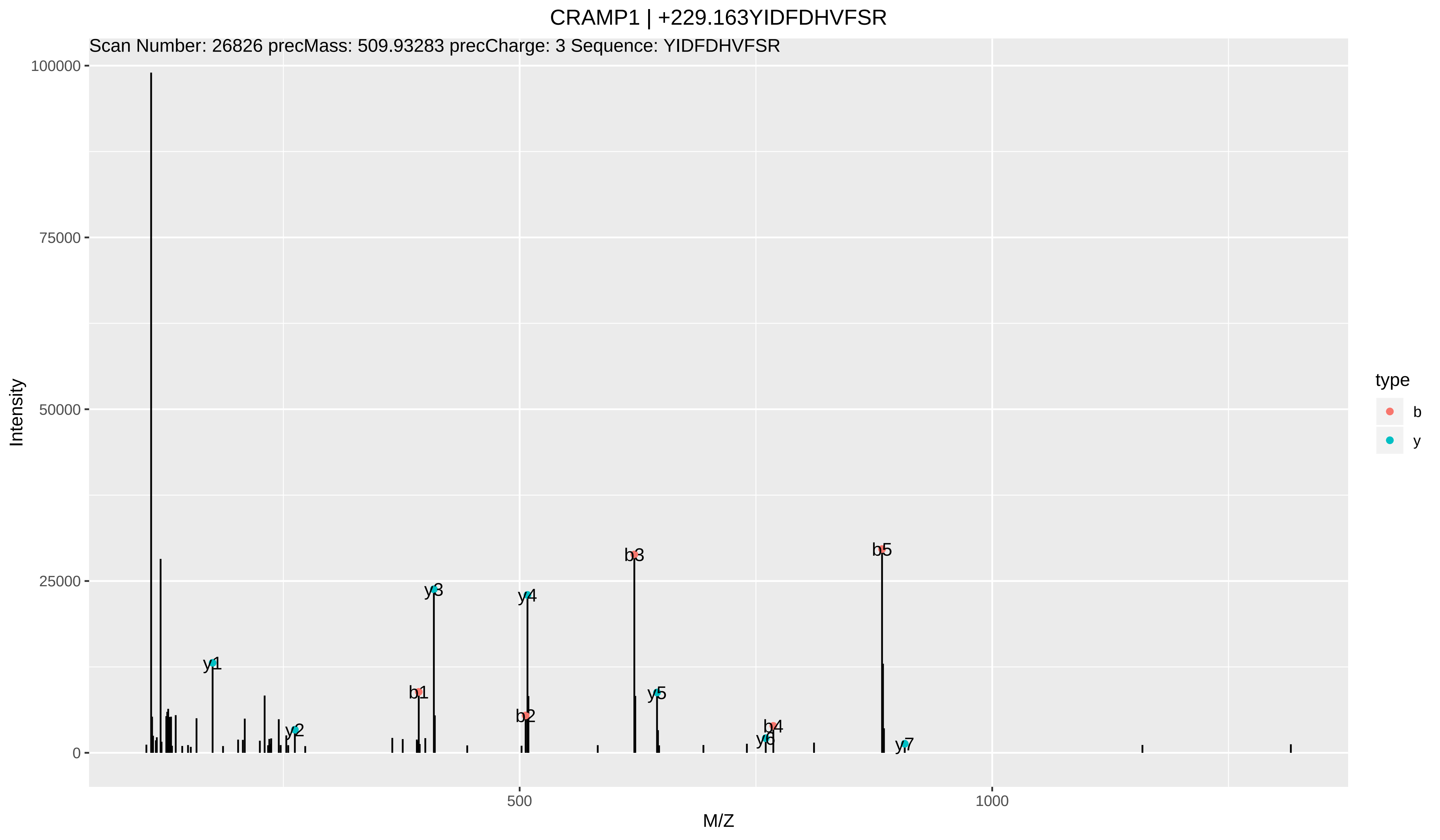

CRAMP1 | +229.163YIDFDHVFSR
Scan Number: 26826 precMass: 509.93283 precCharge: 3 Sequence: YIDFDHVFSR
100000
75000
50000
25000
0
Intensity
500
1000
M/Z
y1
y2
b1
y3
b2
y4
b3
y5
y6
b4
b5
y7
type
b
y

CRAT | +229.163EPVGILTSNHR

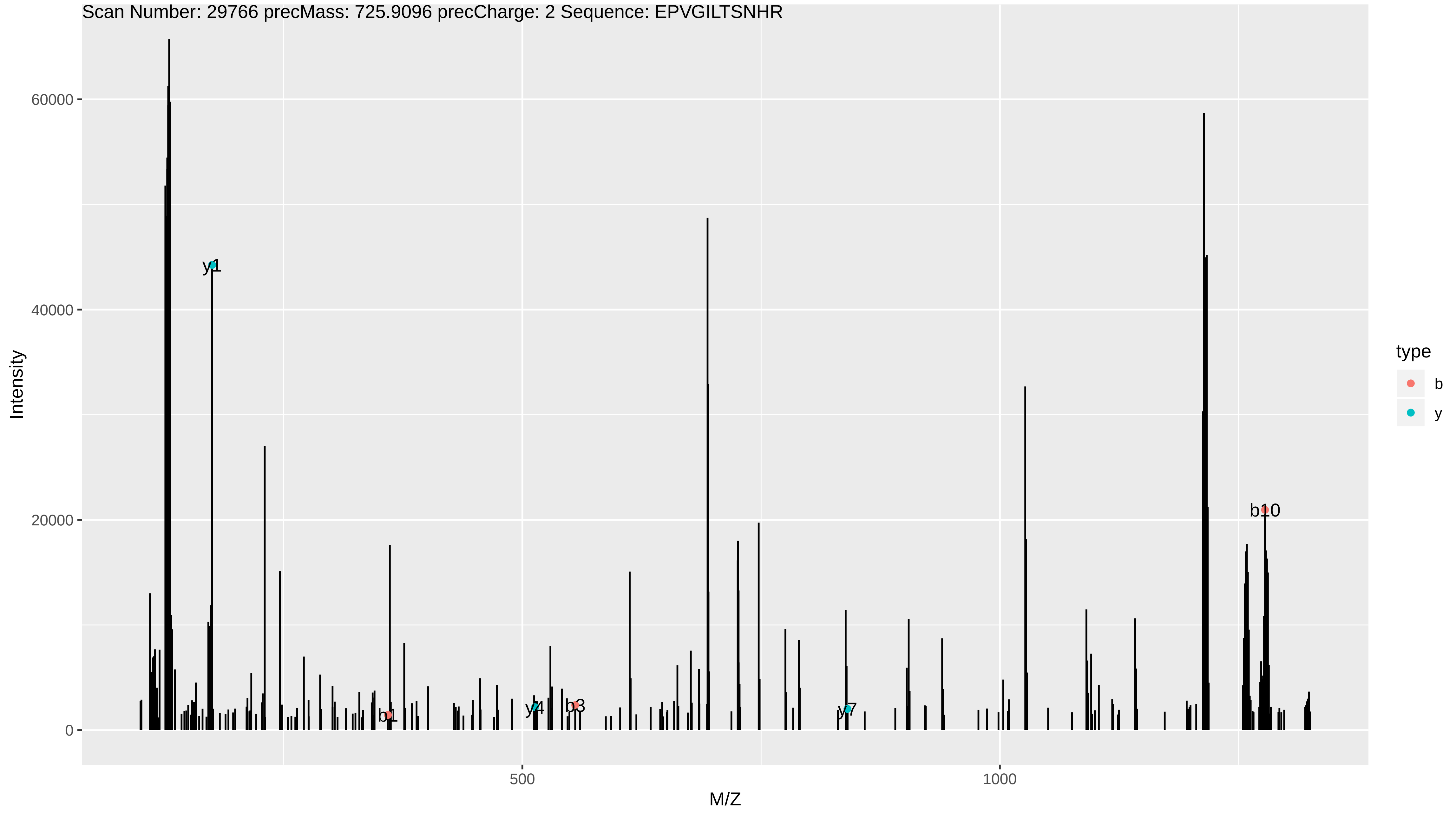

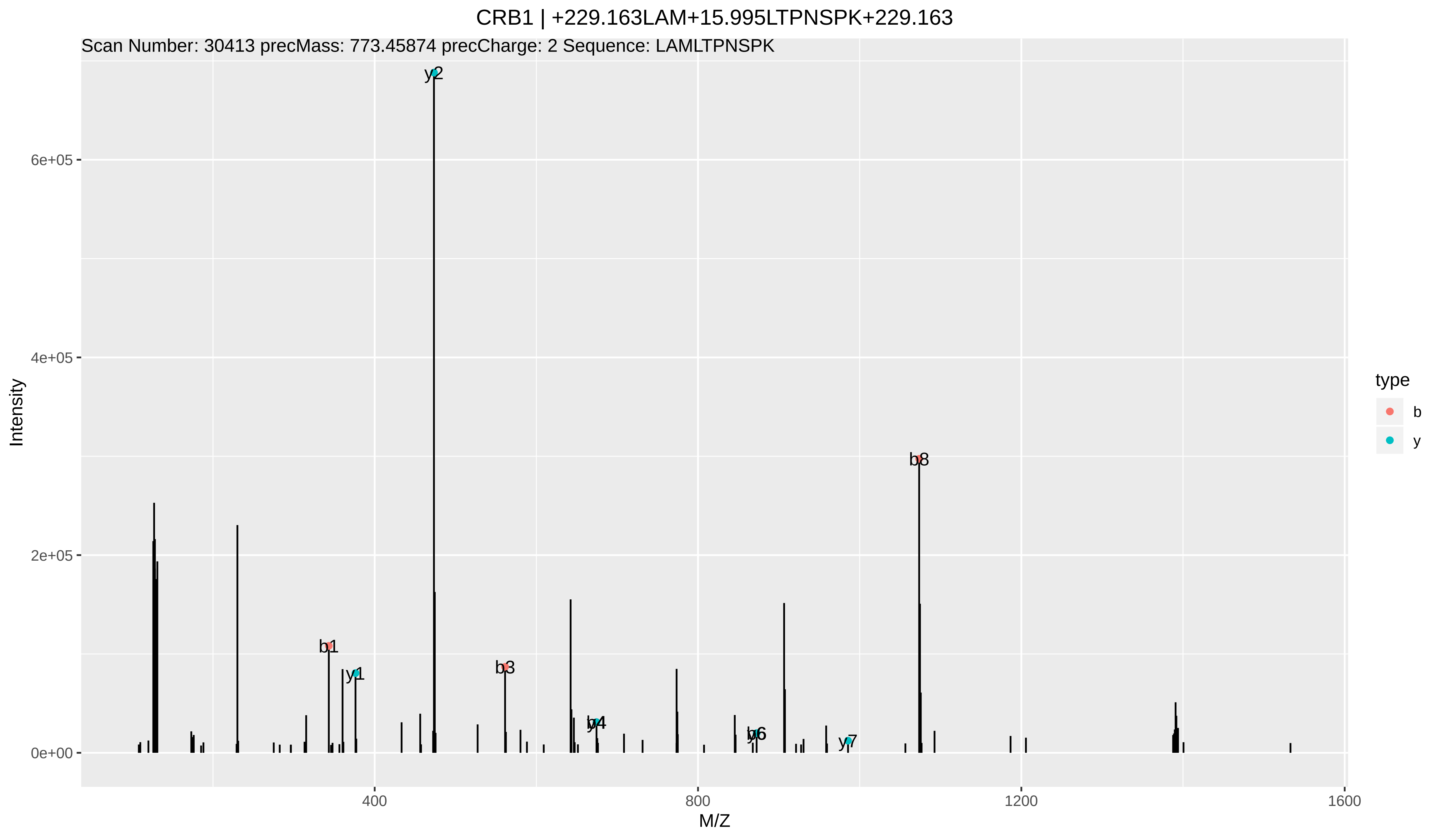

CRB1 | +229.163LAM+15.995LTPNSPK+229.163
Scan Number: 30413 precMass: 773.45874 precCharge: 2 Sequence: LAMLTPNSPK
Intensity
M/Z
type
b
y
y2
b8
b1
y1
b3
y4
y6
y7

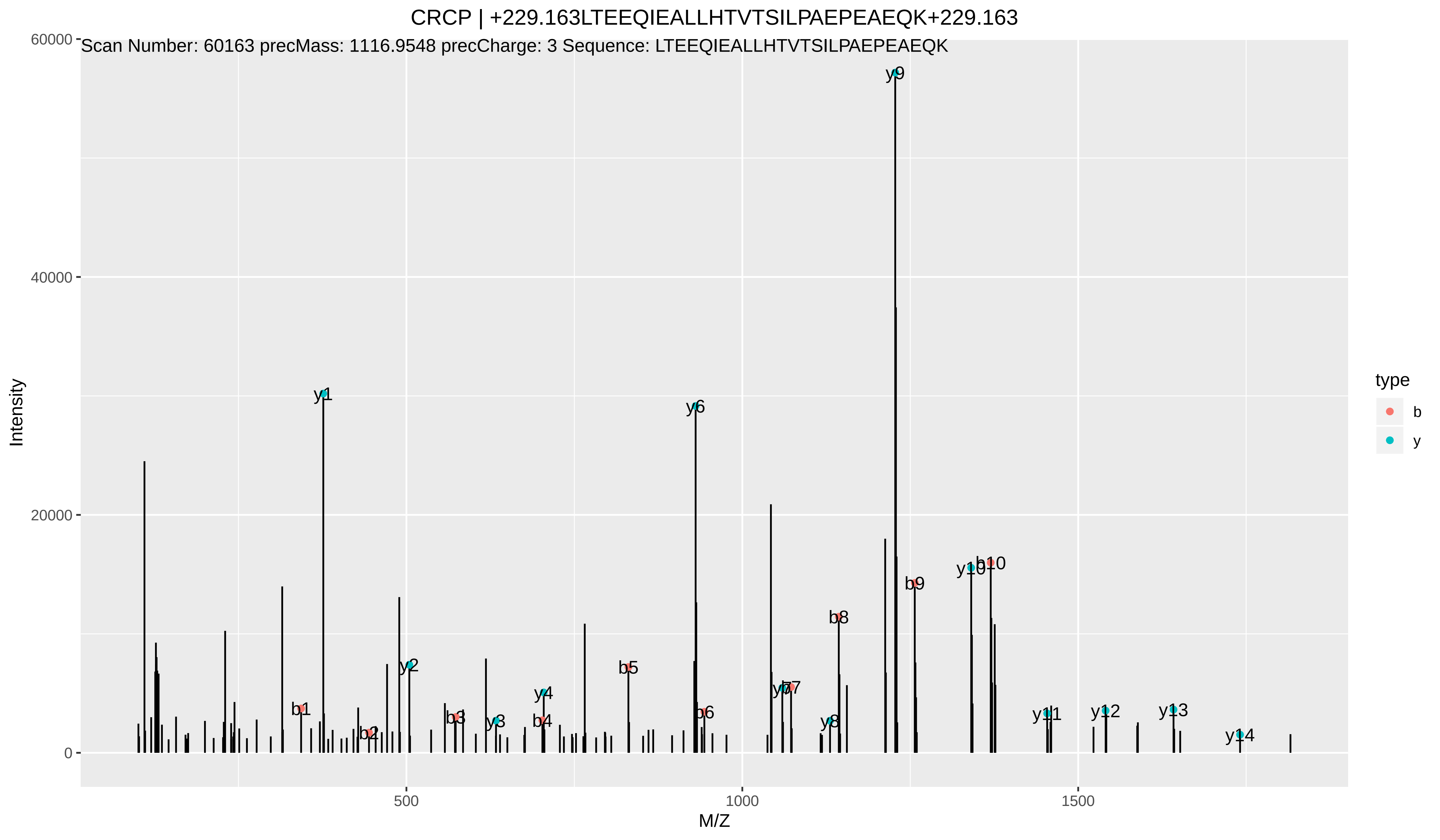

CRCP | +229.163LTEEQIEALLHTVTSILPAEPEAEQK+229.163
Scan Number: 60163 precMass: 1116.9548 precCharge: 3 Sequence: LTEEQIEALLHTVTSILPAEPEAEQK
Intensity
M/Z
type
b
y
0
20000
40000
60000
500
1000
1500
y1
y2
y3
y4
y6
y7
y8
y9
y10
y11
y12
y13
y14
b1
b2
b3
b4
b5
b6
b7
b8
b9
b10

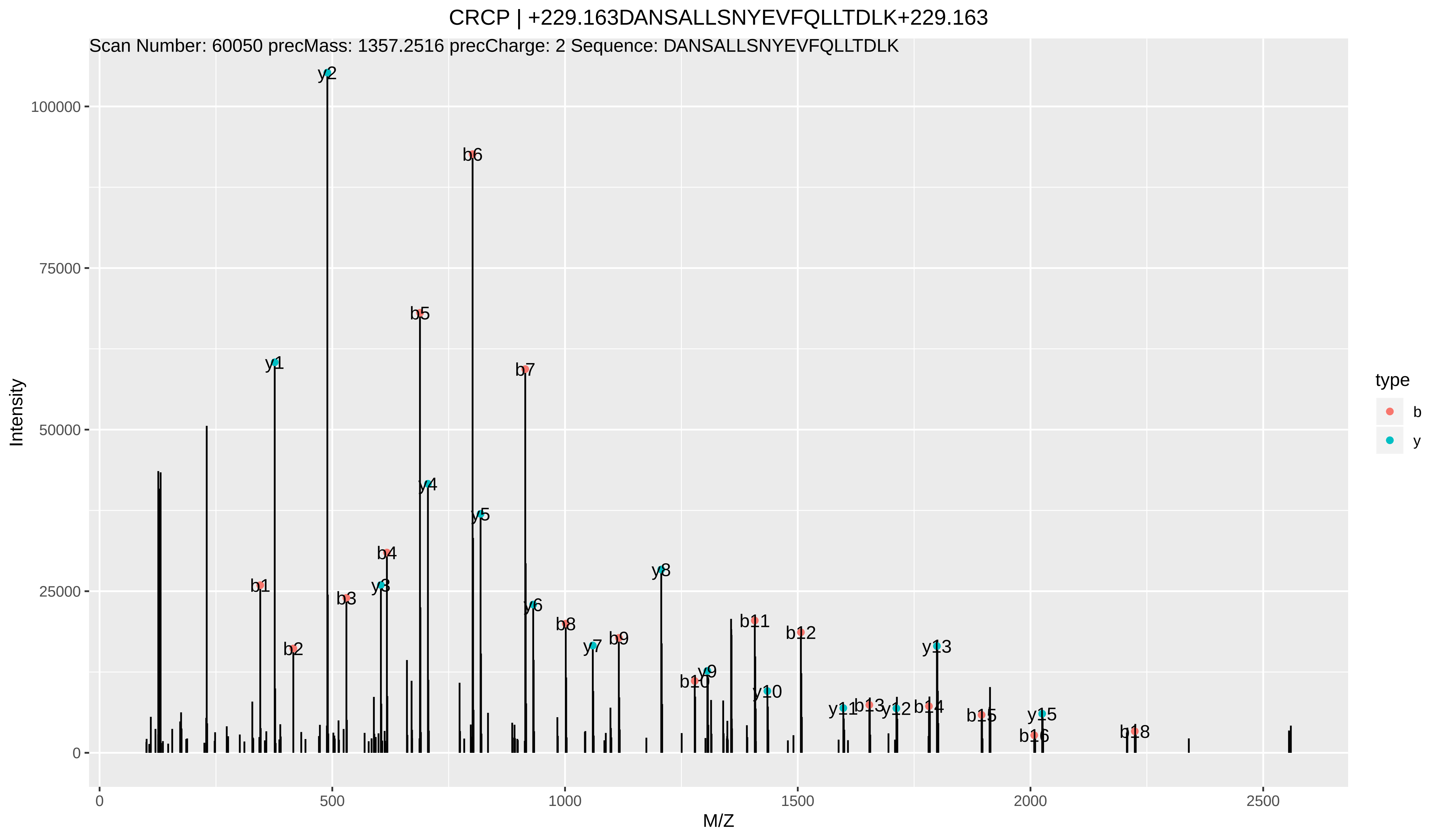
CRCP | +229.163DANSALLSNYEVFQLLTDLK+229.163
Scan Number: 60050 precMass: 1357.2516 precCharge: 2 Sequence: DANSALLSNYEVFQLLTDLK
Intensity
100000
75000
50000
25000
0
M/Z
0
500
1000
1500
2000
2500
y2
b6
b5
y1
b7
y4
y5
b4
b1
y3
b3
y8
y6
b2
b8
y7
b9
b11
b12
y13
b10
y9
y10
y11
b13
y12
b14
b15
y15
b16
b18
type
b
y

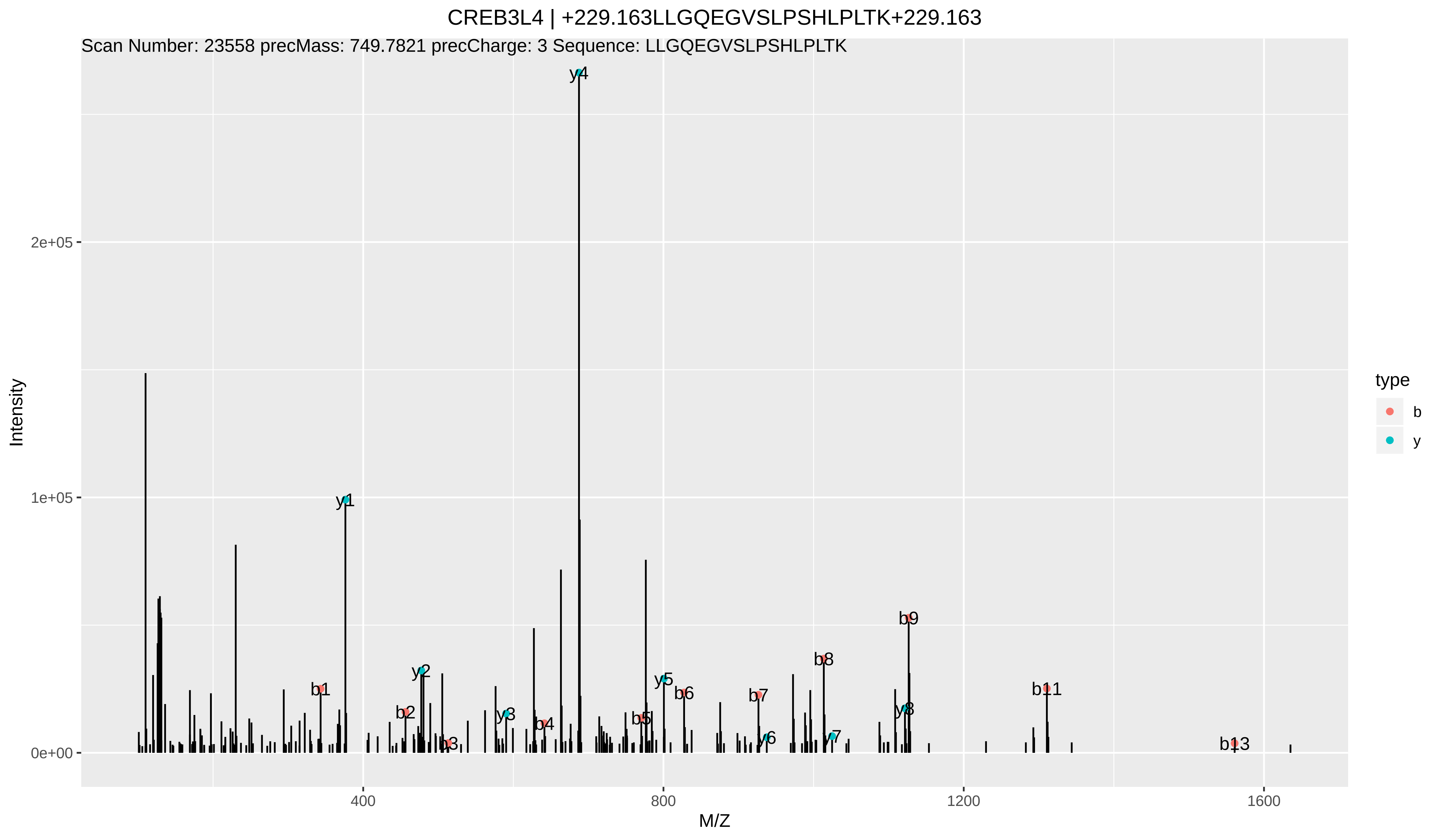

CREB3L4 | +229.163LLGQEGVSLPSHLPLTK+229.163
Scan Number: 23558 precMass: 749.7821 precCharge: 3 Sequence: LLGQEGVSLPSHLPLTK
Intensity
M/Z
0e+00
1e+05
2e+05
400
800
1200
1600
y4
y1
b9
b8
b11
y2
b1
y5
b6
b7
b2
y8
y3
b5
b4
y6
y7
b3
b13
type
b
y

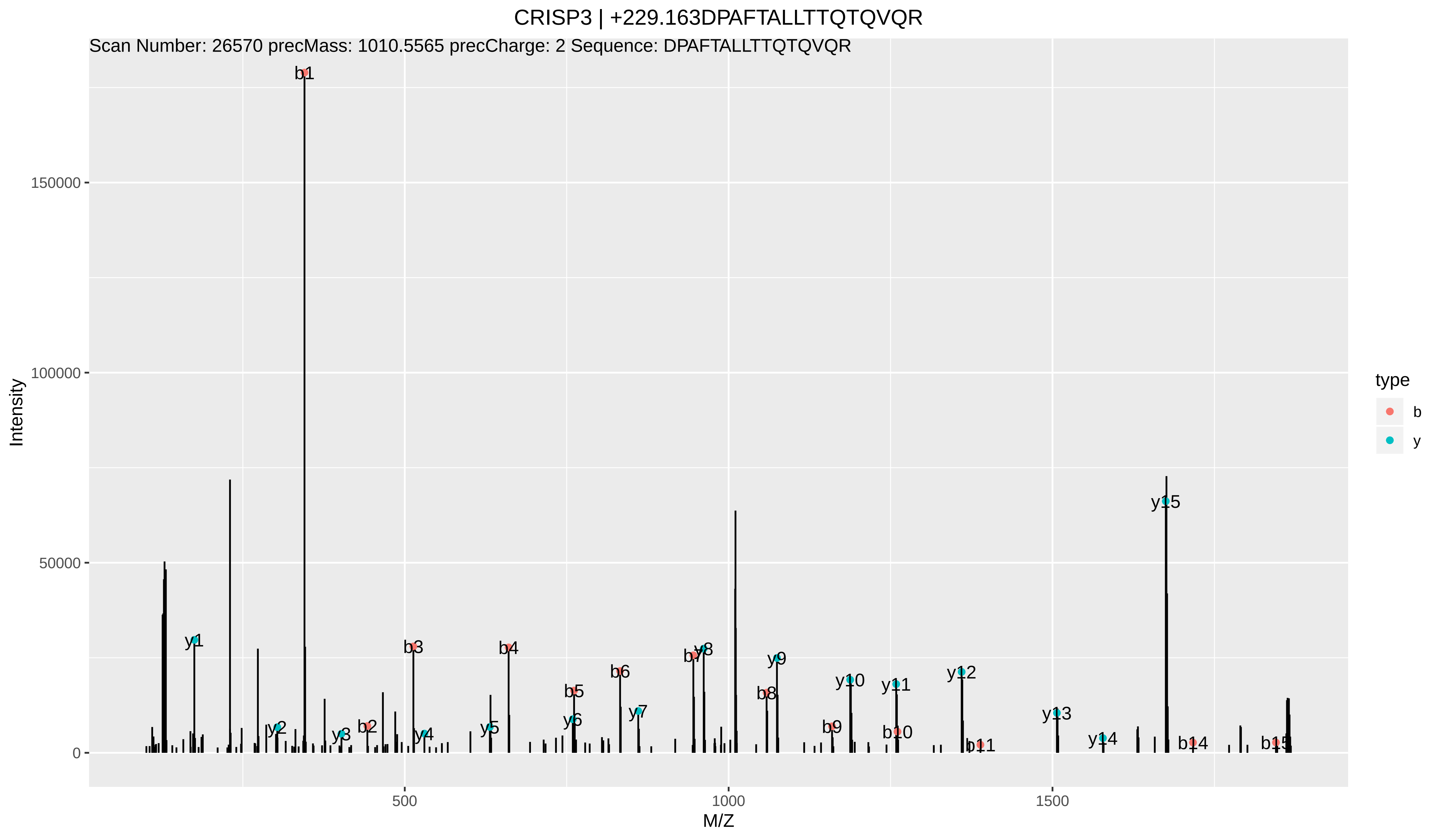
CRISP3 | +229.163DPAFTALLTTQTQVQR
Scan Number: 26570 precMass: 1010.5565 precCharge: 2 Sequence: DPAFTALLTTQTQVQR
Intensity
M/Z
0
50000
100000
150000
500
1000
1500
type
b
y
b1
y1
y2
y3
b2
b3
y4
y5
b4
b5
y6
b6
y7
b7
y8
y9
b8
b9
y10
y11
b10
y12
b11
y13
y14
y15
b14
b15

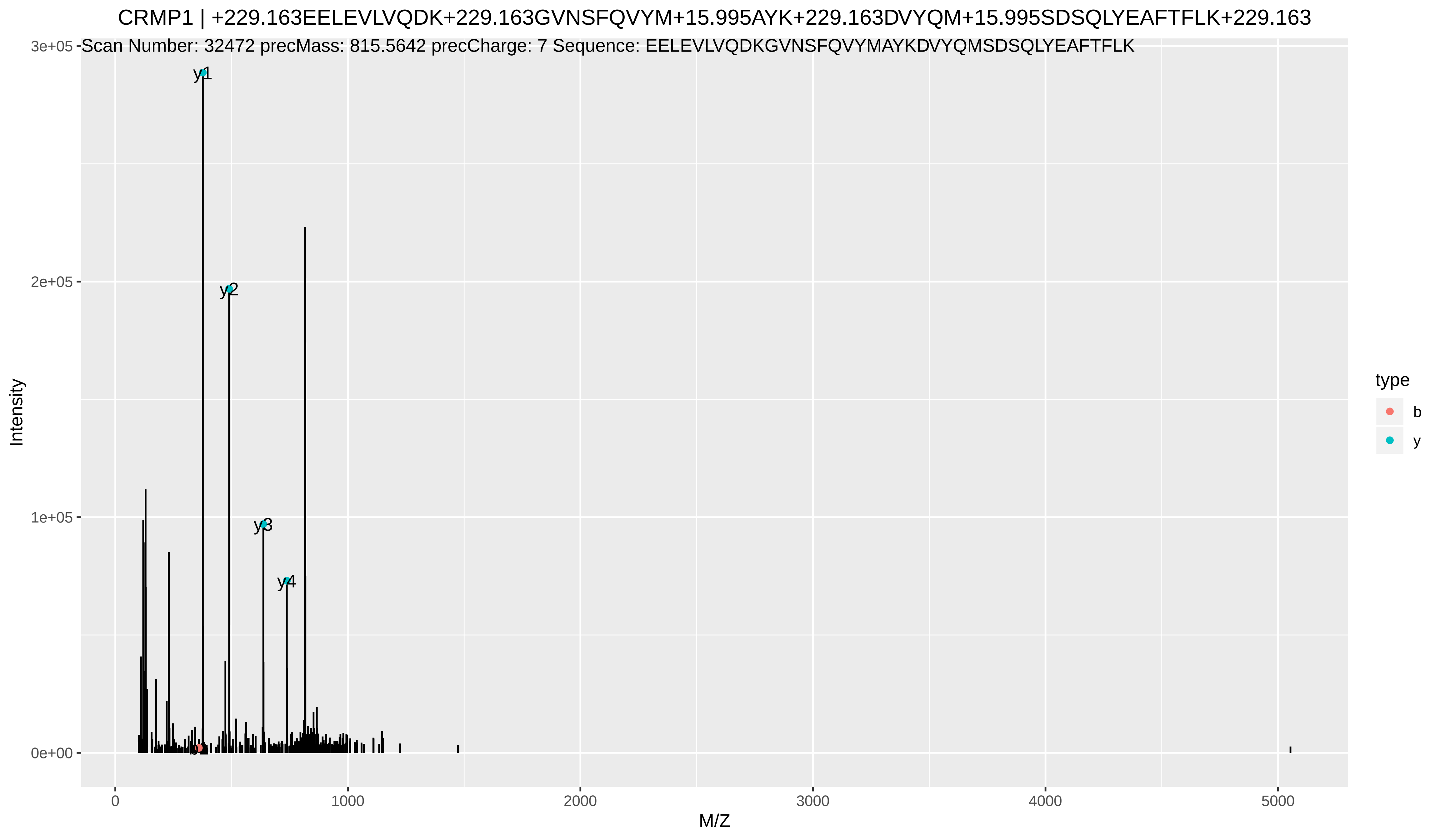
CRMP1 | +229.163EELEVLVQDK+229.163GVNSFQVYM+15.995AYK+229.163DVYQM+15.995SDSQLYEAFTFLK+229.163
Scan Number: 32472 precMass: 815.5642 precCharge: 7 Sequence: EELEVLVQDKGVNSFQVYMAYKDVYQMSDSQLYEAFTFLK
Intensity
3e+05
2e+05
1e+05
0e+00
y1
y2
y3
y4
b1
M/Z
0
1000
2000
3000
4000
5000
type
b
y

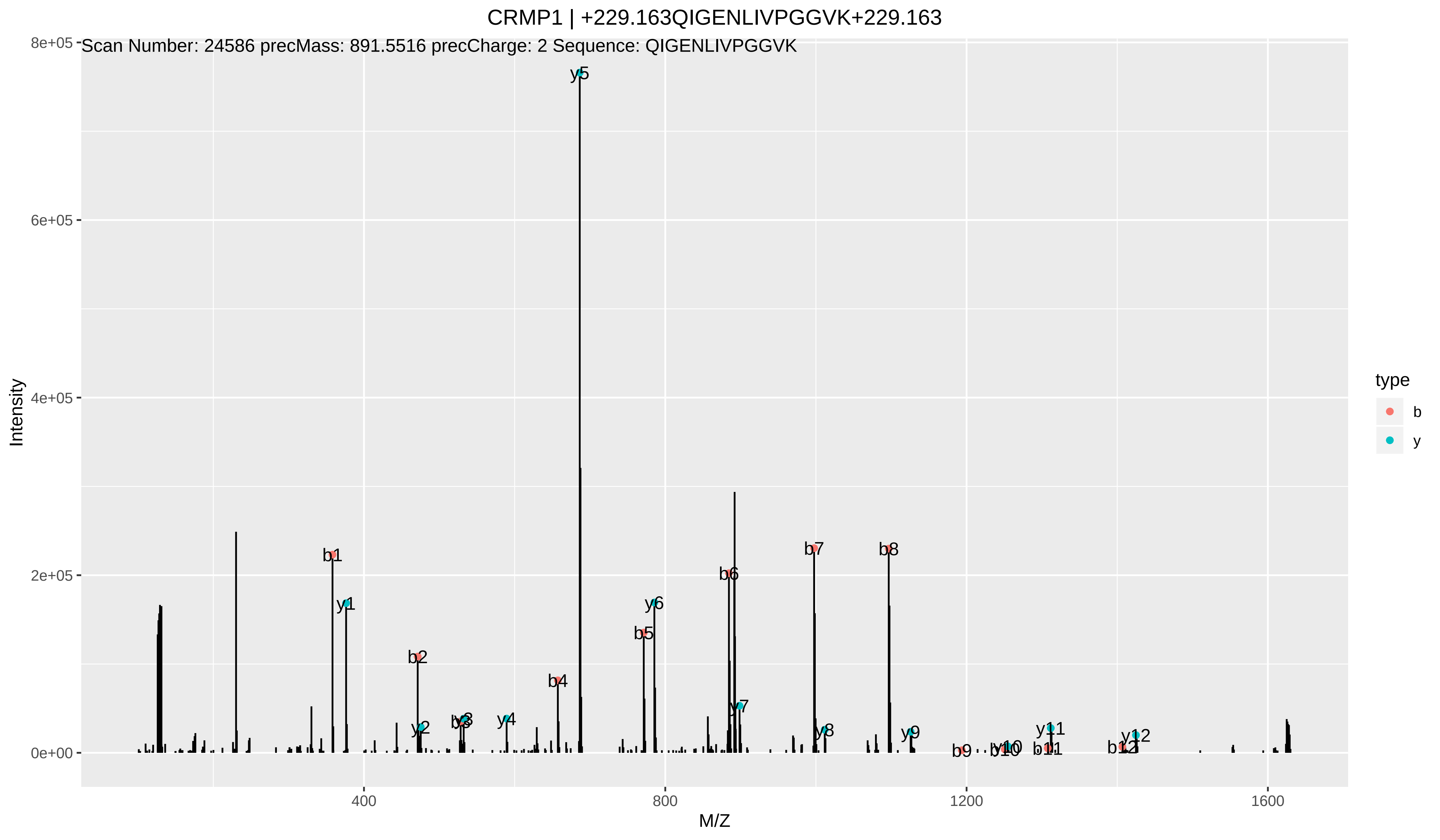

CRMP1 | +229.163QIGENLIVPGGVK+229.163
Scan Number: 24586 precMass: 891.5516 precCharge: 2 Sequence: QIGENLIVPGGVK
Intensity
M/Z
type
b
y
y5
b1
y1
b2
y2
b3
y3
y4
b4
b5
y6
b6
y7
b7
b8
y8
y9
b9
y10
b10
y11
b11
y12
b12
8e+05
6e+05
4e+05
2e+05
0e+00
400
800
1200
1600

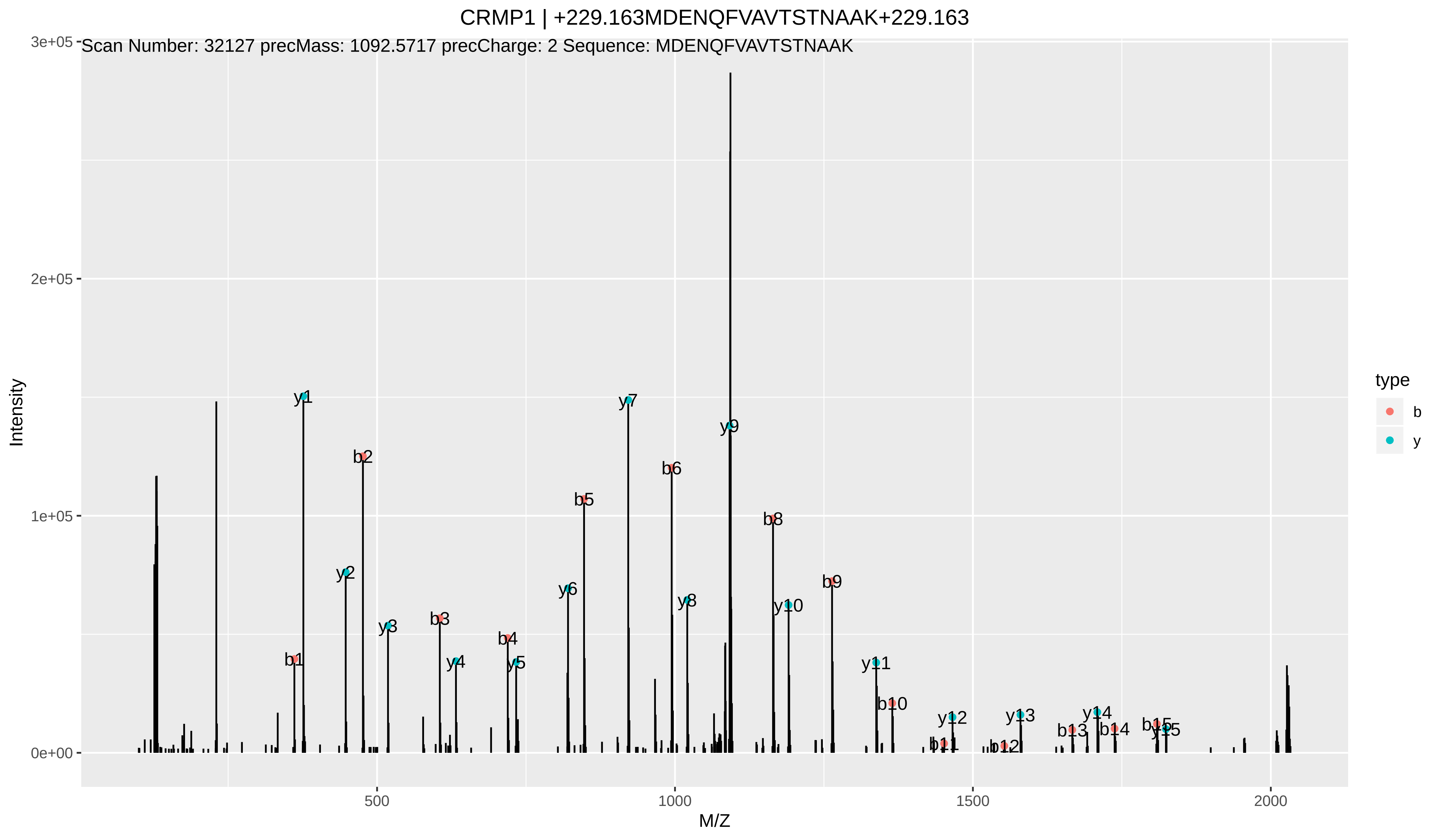
CRMP1 | +229.163MDENQFVAVTSTNAAK+229.163
Scan Number: 32127 precMass: 1092.5717 precCharge: 2 Sequence: MDENQFVAVTSTNAAK
Intensity
M/Z
type
b
y
3e+05
2e+05
1e+05
0e+00
500
1000
1500
2000
y1
y2
y3
y4
y5
y6
y7
y8
y9
y10
y11
y12
y13
y14
y15
b1
b2
b3
b4
b5
b6
b8
b9
b10
b11
b12
b13
b14
b15

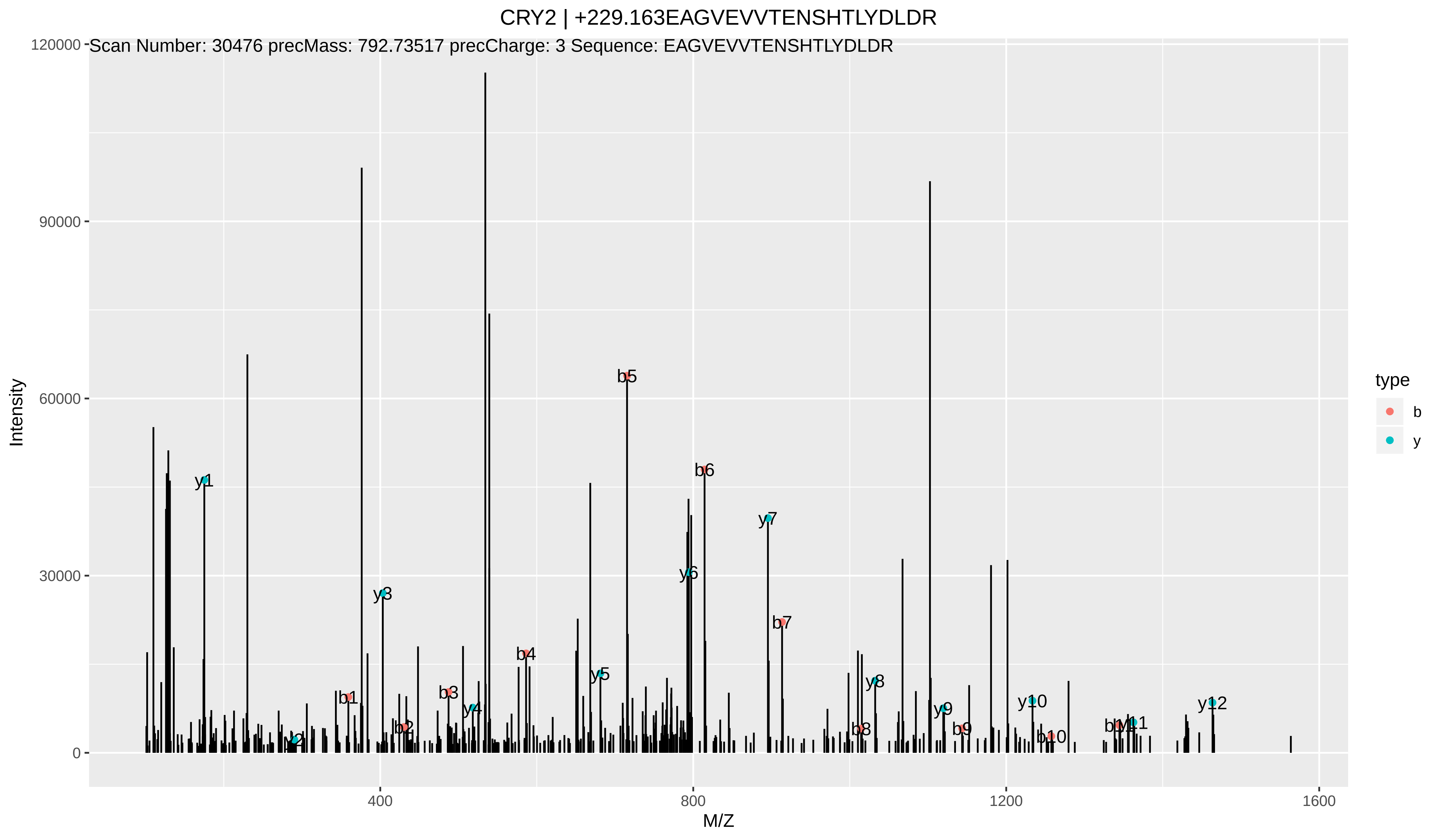
CRY2 | +229.163EAGVEVVTENSHTLYDLDR
Scan Number: 30476 precMass: 792.73517 precCharge: 3 Sequence: EAGVEVVTENSHTLYDLDR
Intensity
120000
90000
60000
30000
0
M/Z
400
800
1200
1600
type
b
y
y1
y2
y3
y4
y5
y6
y7
y8
y9
y10
y11
y12
b1
b2
b3
b4
b5
b6
b7
b8
b9
b10
b11

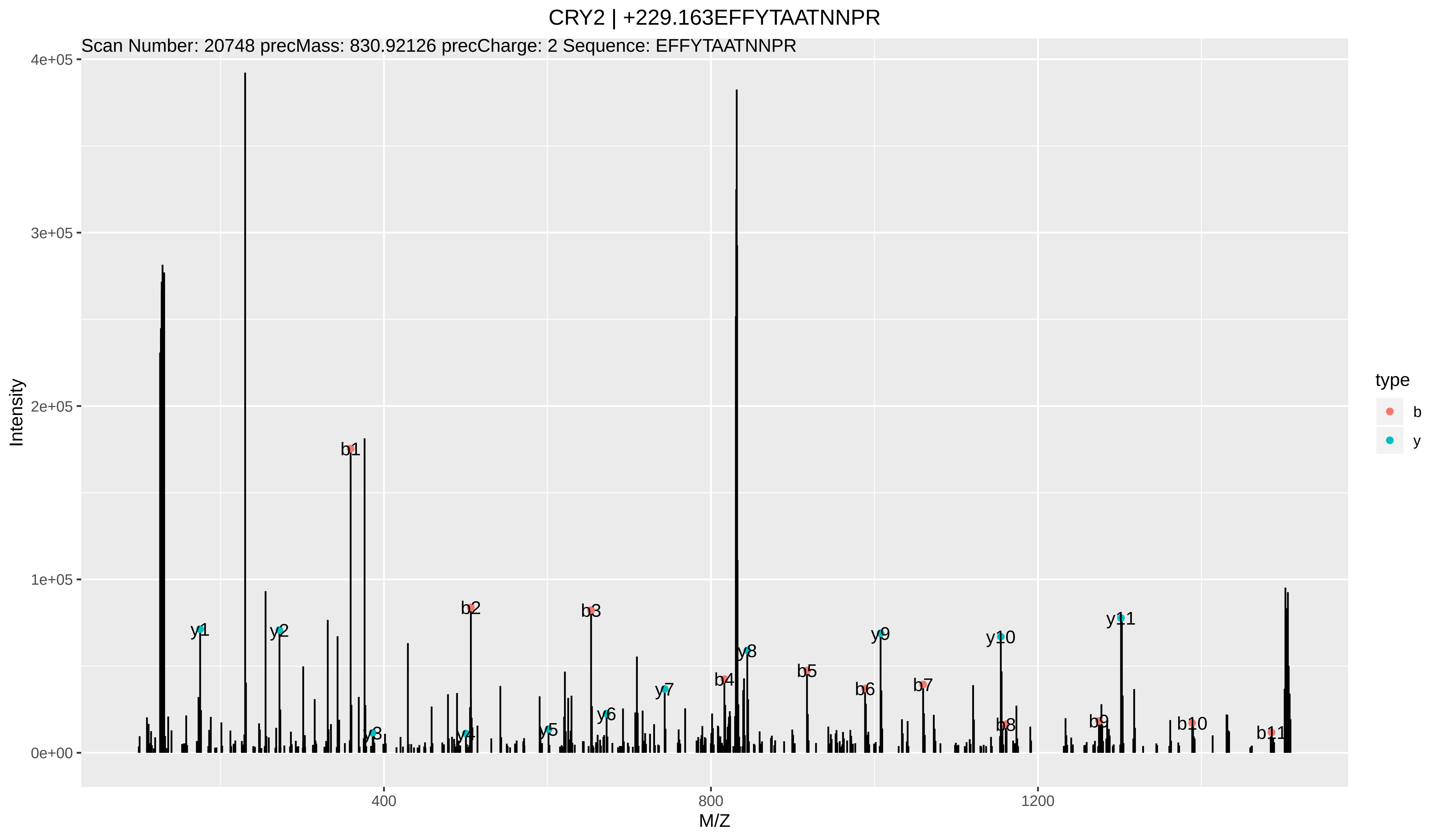

CRY2 | +229.163EFFYTAATNNPR
Scan Number: 20748 precMass: 830.92126 precCharge: 2 Sequence: EFFYTAATNNPR
Intensity
4e+05
3e+05
2e+05
1e+05
0e+00
M/Z
400
800
1200
b1
b2
b3
b4
b5
b6
b7
b8
b9
b10
b11
y1
y2
y3
y4
y5
y6
y7
y8
y9
y10
y11
type
b
y

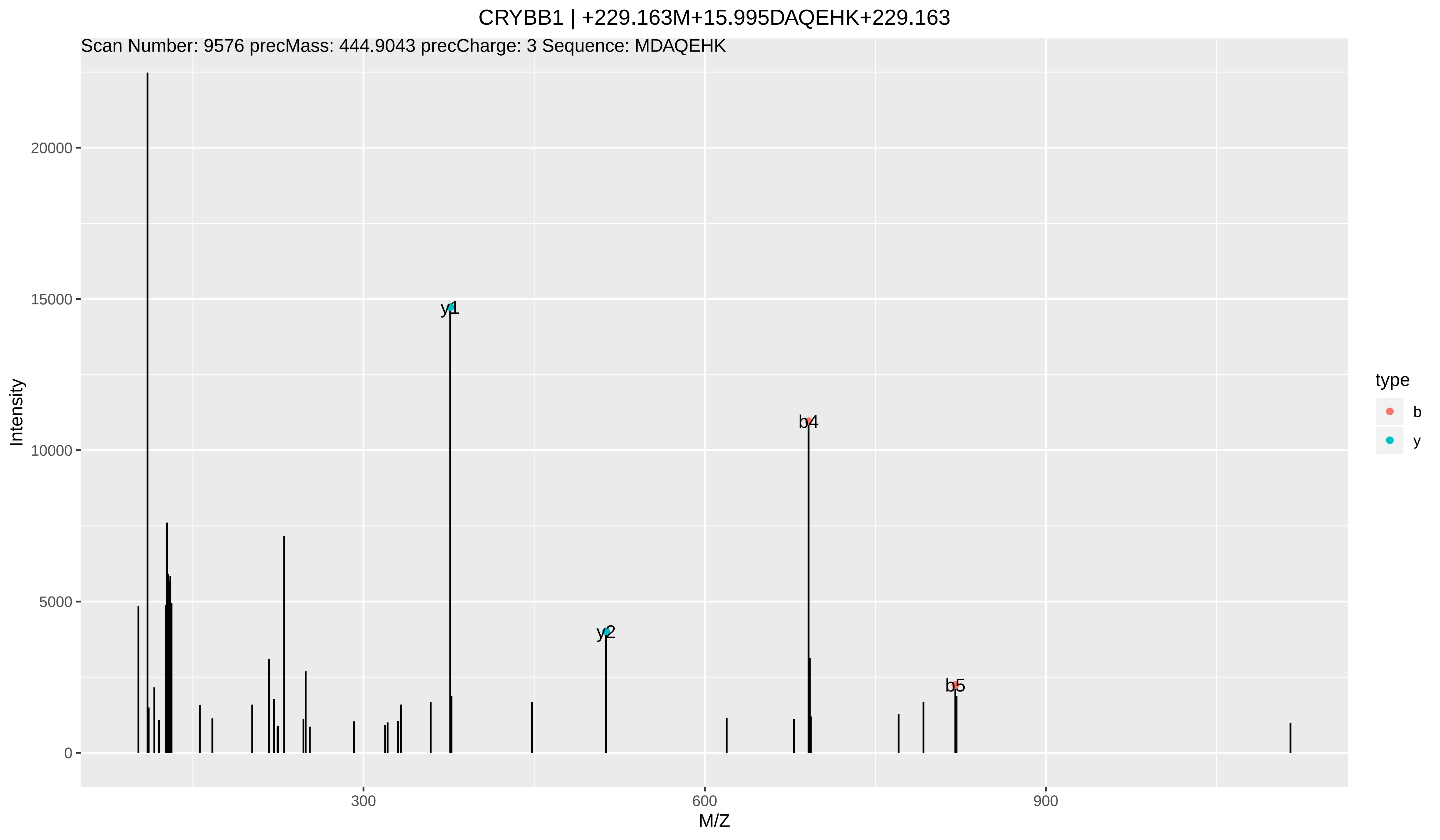

CRYBB1 | +229.163M+15.995DAQEHK+229.163
Scan Number: 9576 precMass: 444.9043 precCharge: 3 Sequence: MDAQEHK
Intensity
M/Z
0
5000
10000
15000
20000
300
600
900
y1
y2
b4
b5
type
b
y

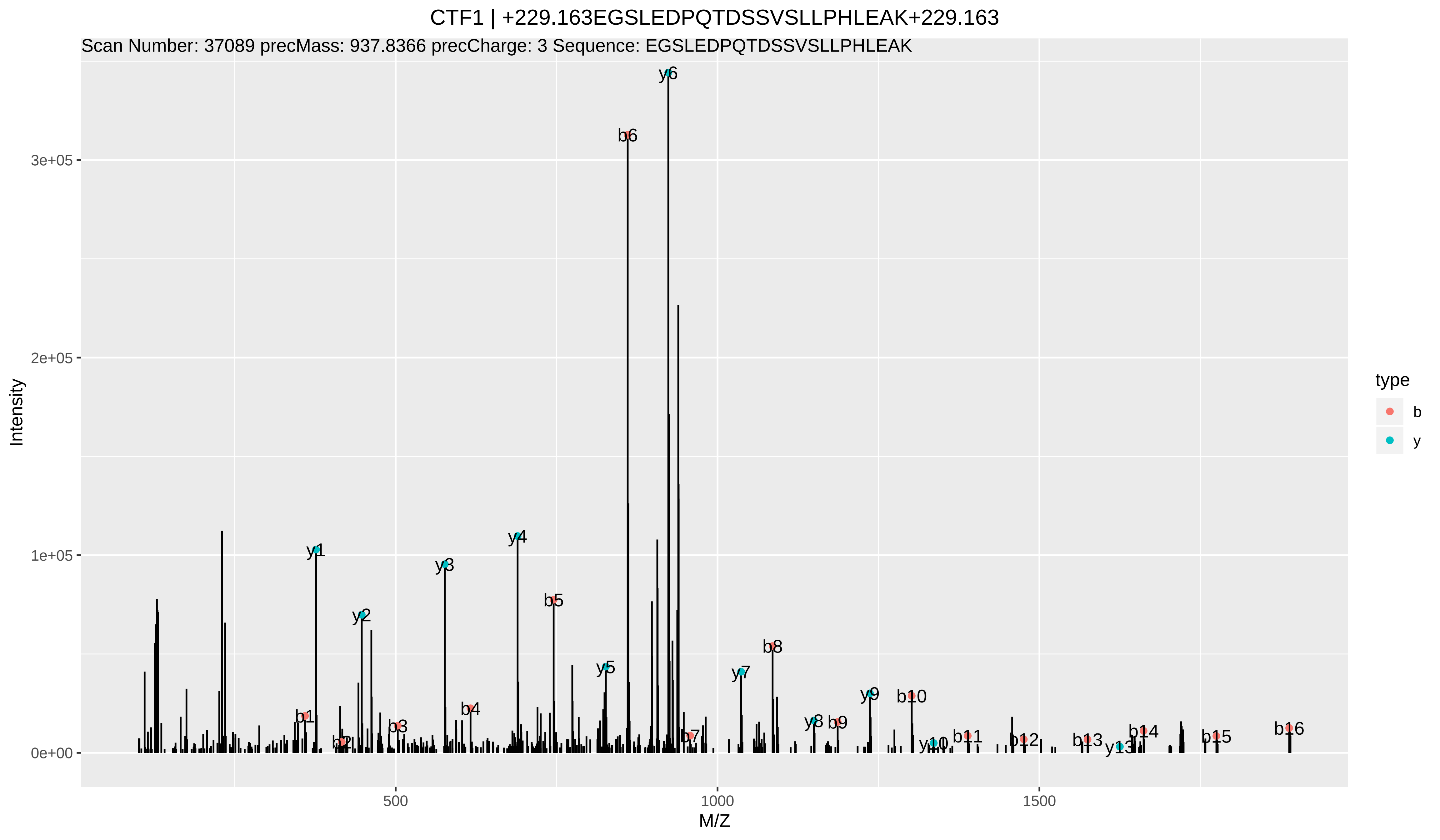
CTF1 | +229.163EGSLEDPQTDSSVSLLPHLEAK+229.163
Scan Number: 37089 precMass: 937.8366 precCharge: 3 Sequence: EGSLEDPQTDSSVSLLPHLEAK
Intensity
M/Z
0e+00
1e+05
2e+05
3e+05
500
1000
1500
type
b
y
y6
b6
y4
y1
y3
b5
y2
b8
y5
y7
y9
b10
b1
b4
b3
b2
b7
y8
b9
b11
b12
b13
y10
y13
b14
b15
b16

CTNNA2 | +229.163AIMAQLPQEEK+229.163

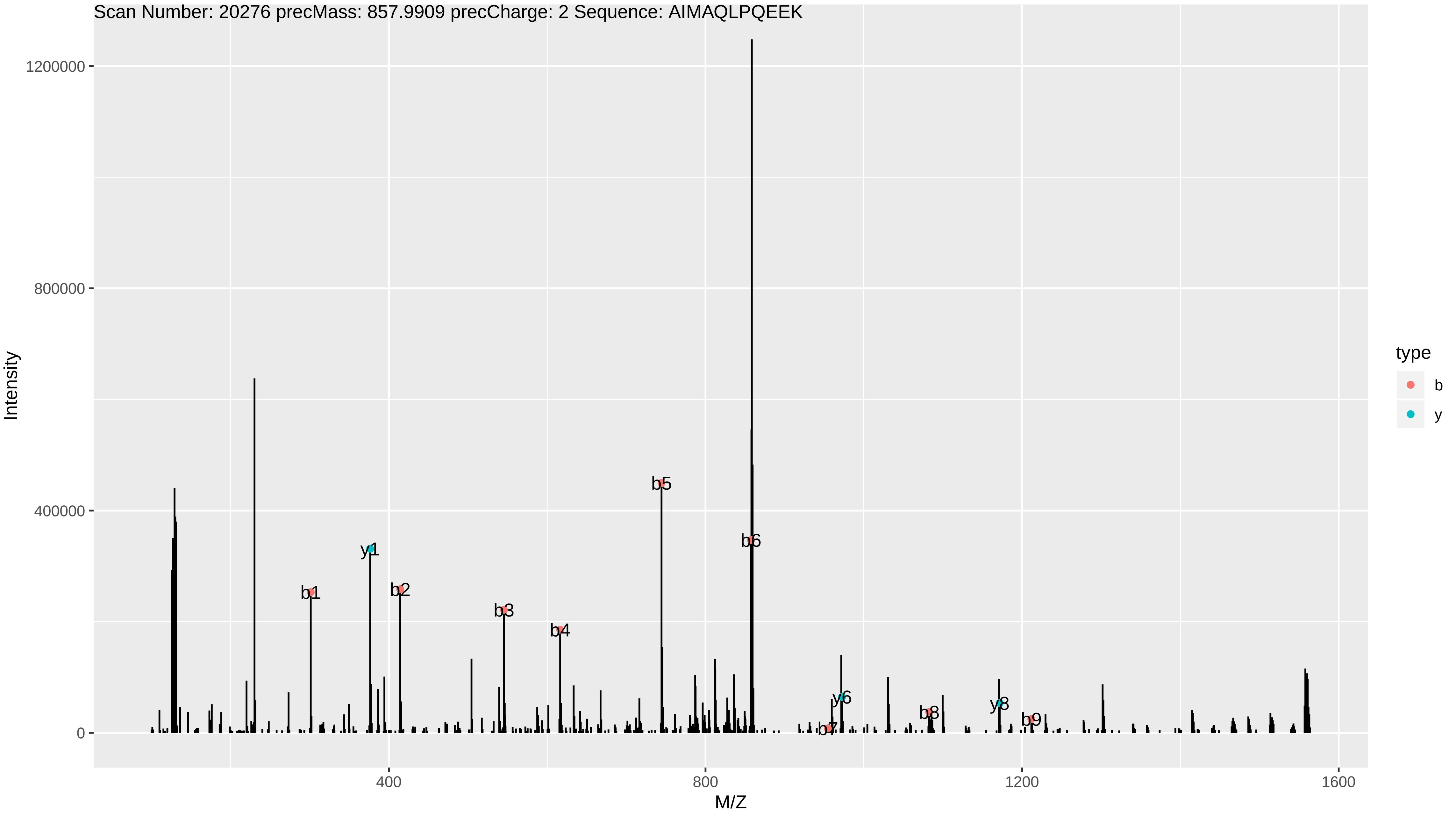

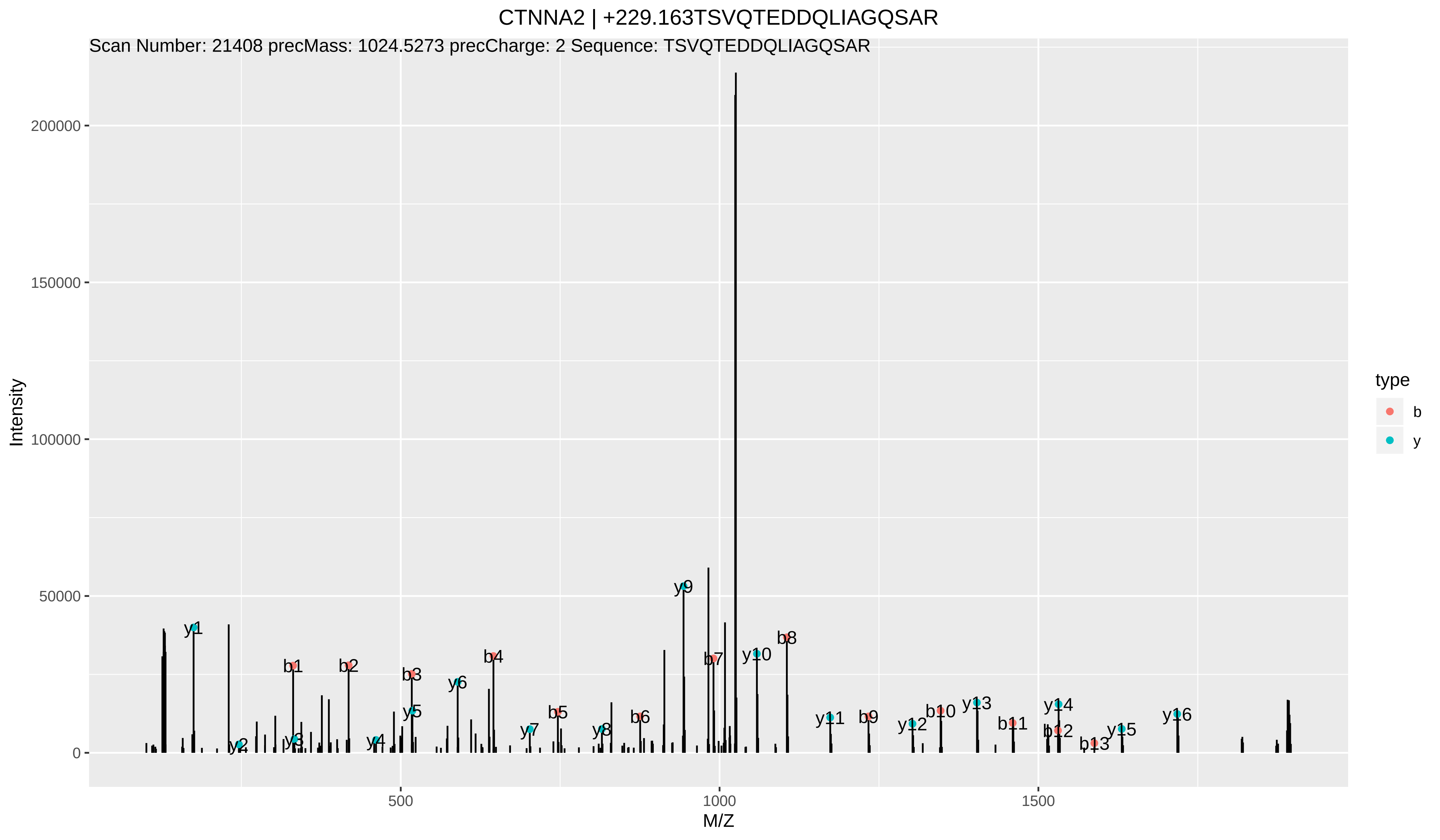
CTNNA2 | +229.163TSVQTEDDQLIAGQSAR
Scan Number: 21408 precMass: 1024.5273 precCharge: 2 Sequence: TSVQTEDDQLIAGQSAR
Intensity
M/Z
type
b
y
y1
y2
y3
y4
y5
y6
y7
y8
y9
y10
y11
y12
y13
y14
y15
y16
b1
b2
b3
b4
b5
b6
b7
b8
b9
b10
b11
b12
b13
0
50000
100000
150000
200000
500
1000
1500

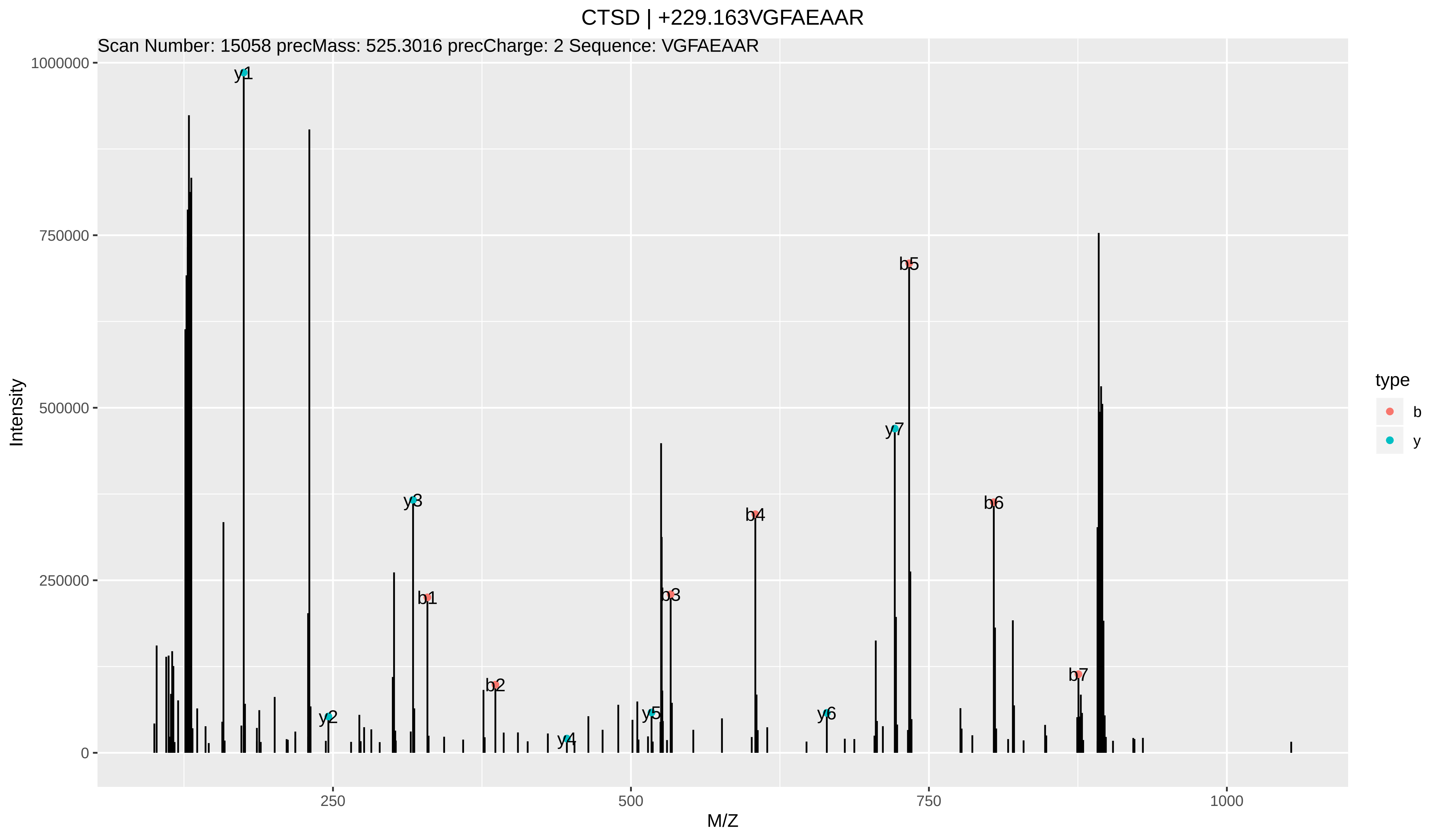

CTSD | +229.163VGFAEAAR
Scan Number: 15058 precMass: 525.3016 precCharge: 2 Sequence: VGFAEAAR
Intensity
1000000
750000
500000
250000
0
M/Z
250
500
750
1000
y1
y2
y3
y4
y5
y6
y7
b1
b2
b3
b4
b5
b6
b7
type
b
y

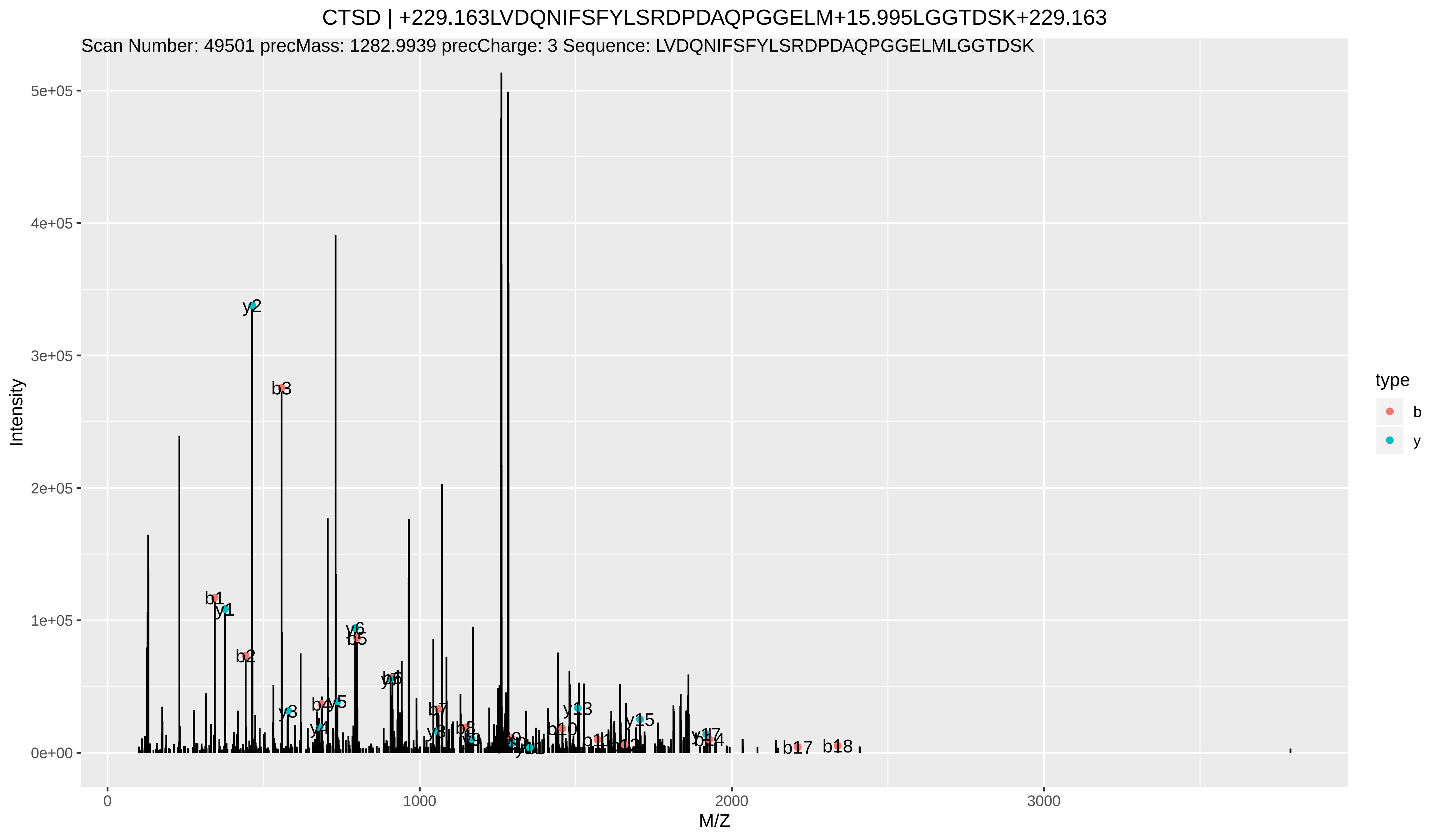
CTSD | +229.163LVDQNIFSFYLSRDPDAQPGGELM+15.995LGGTDSK+229.163
Scan Number: 49501 precMass: 1282.9939 precCharge: 3 Sequence: LVDQNIFSFYLSRDPDAQPGGELMLGGTDSK
Intensity
M/Z
type
b
y
y2
b3
b1
y1
b2
y6
b5
y3
b4
y5
y4
b7
y8
b8
b10
y13
y15
b17
b18
0e+00
1e+05
2e+05
3e+05
4e+05
5e+05
0
1000
2000
3000

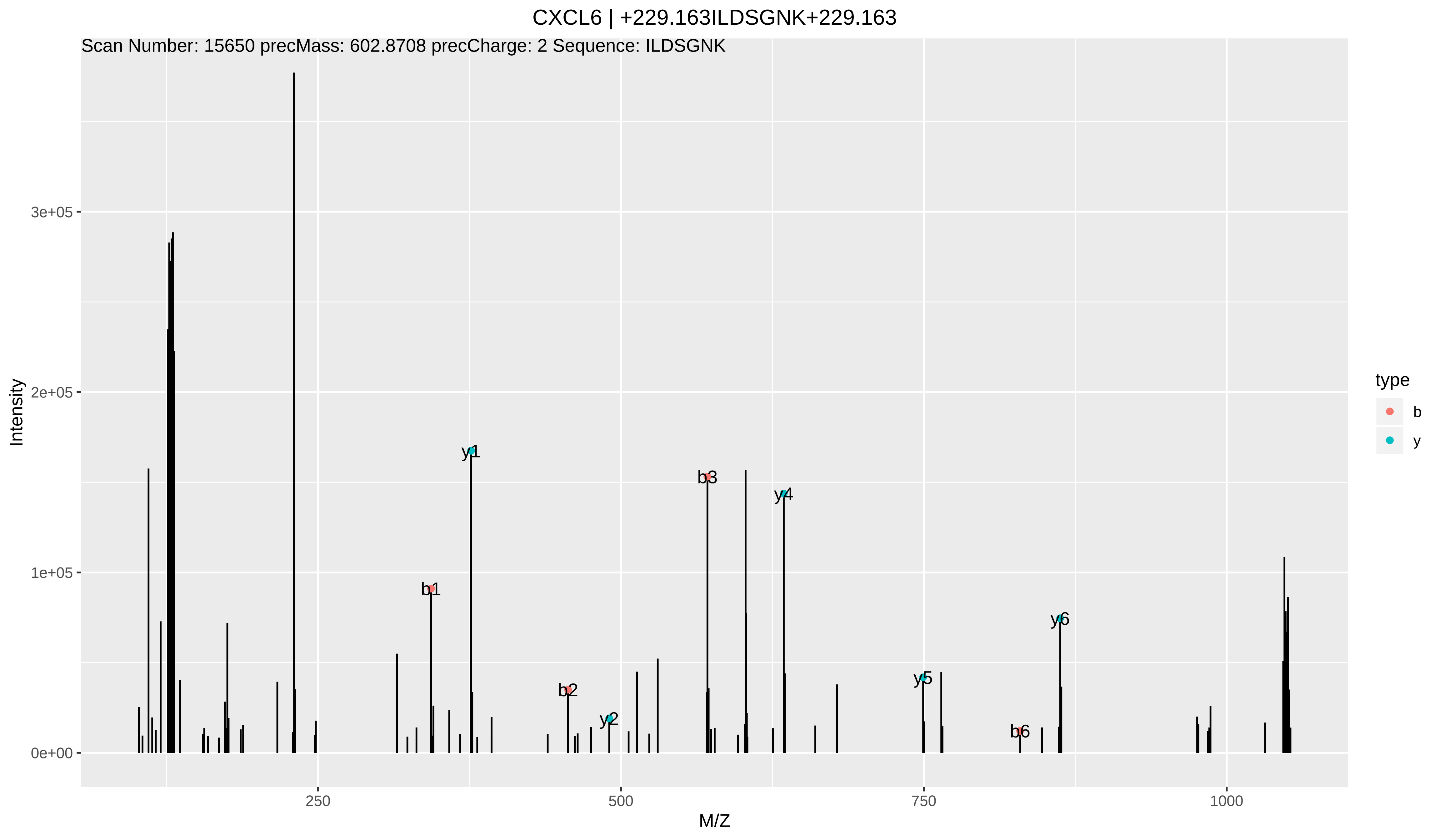
CXCL6 | +229.163ILDSGNK+229.163
Scan Number: 15650 precMass: 602.8708 precCharge: 2 Sequence: ILDSGNK
Intensity
3e+05
2e+05
1e+05
0e+00
M/Z
250
500
750
1000
y1
b1
b2
y2
b3
y4
y5
b6
y6
type
b
y

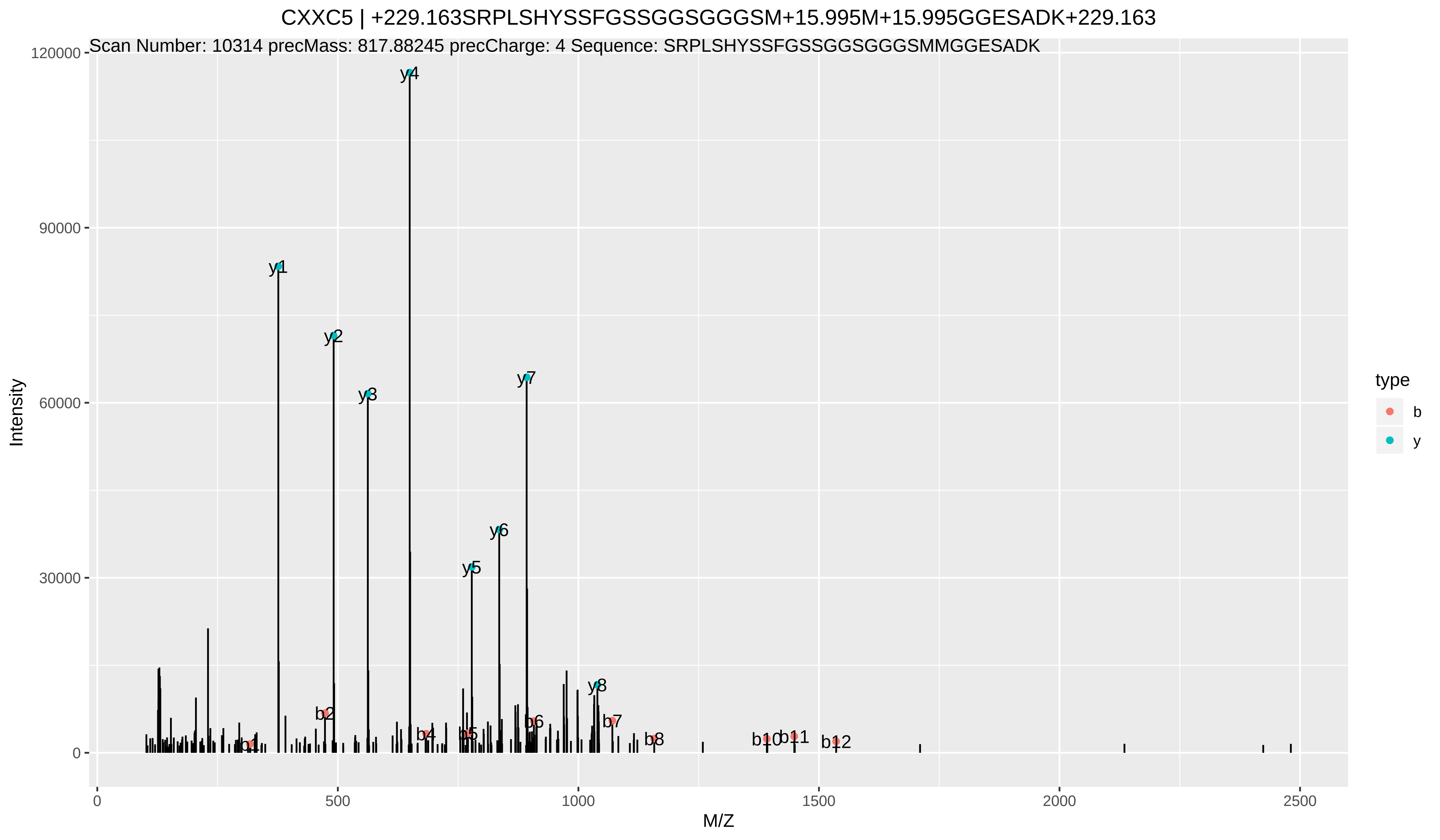

CXXC5 | +229.163SRPLSHYSSFGSSGGSGGGSM+15.995M+15.995GGESADK+229.163
Scan Number: 10314 precMass: 817.88245 precCharge: 4 Sequence: SRPLSHYSSFGSSGGSGGGSMMGGESADK
Intensity
M/Z
type
b
y
y1
y2
y3
y4
y5
y6
y7
y8
b1
b2
b4
b5
b6
b7
b8
b10
b11
b12

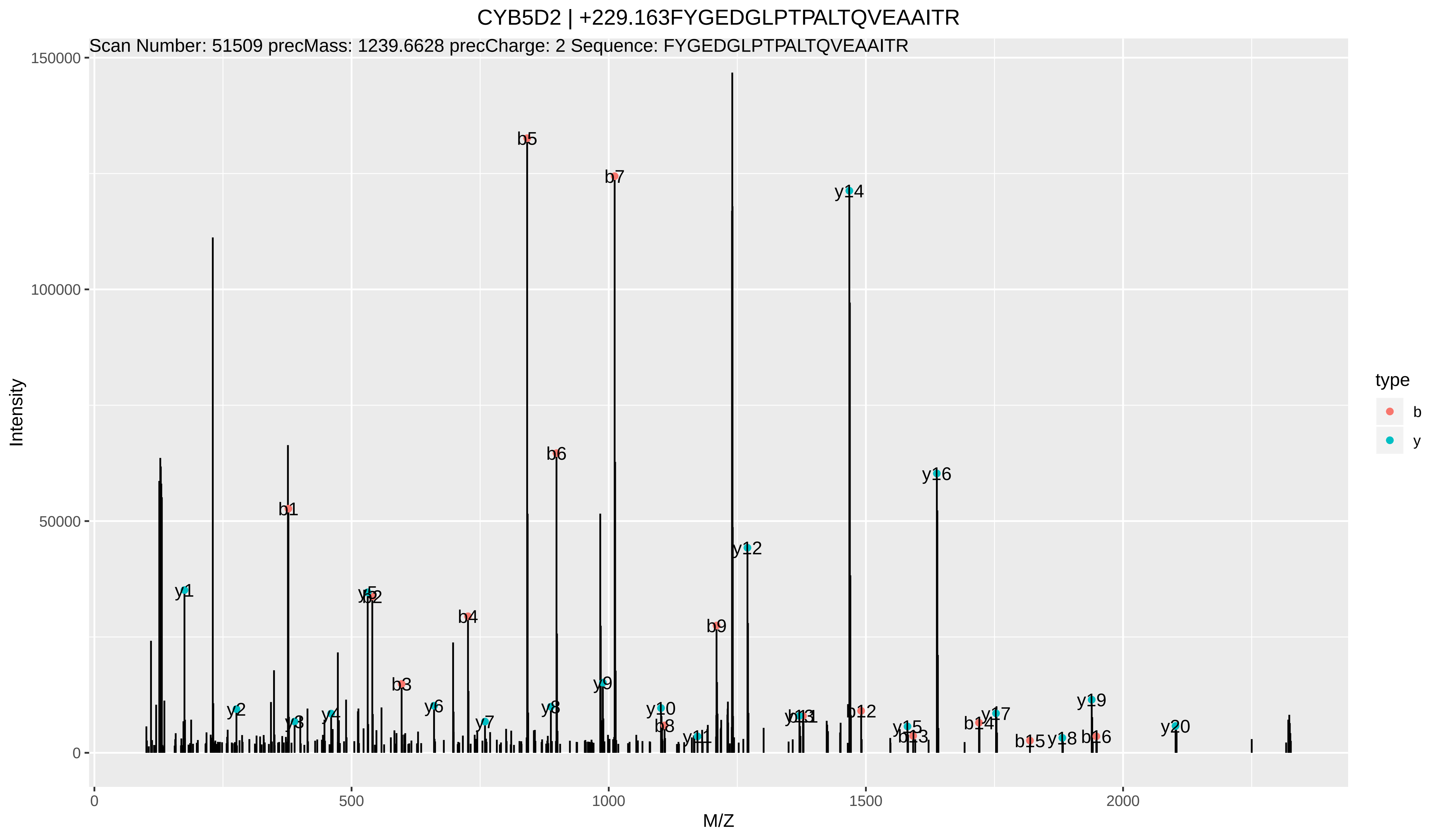

CYB5D2 | +229.163FYGEDGLPTPALTQVEAAITR
Scan Number: 51509 precMass: 1239.6628 precCharge: 2 Sequence: FYGEDGLPTPALTQVEAAITR
Intensity
M/Z
type
b
y

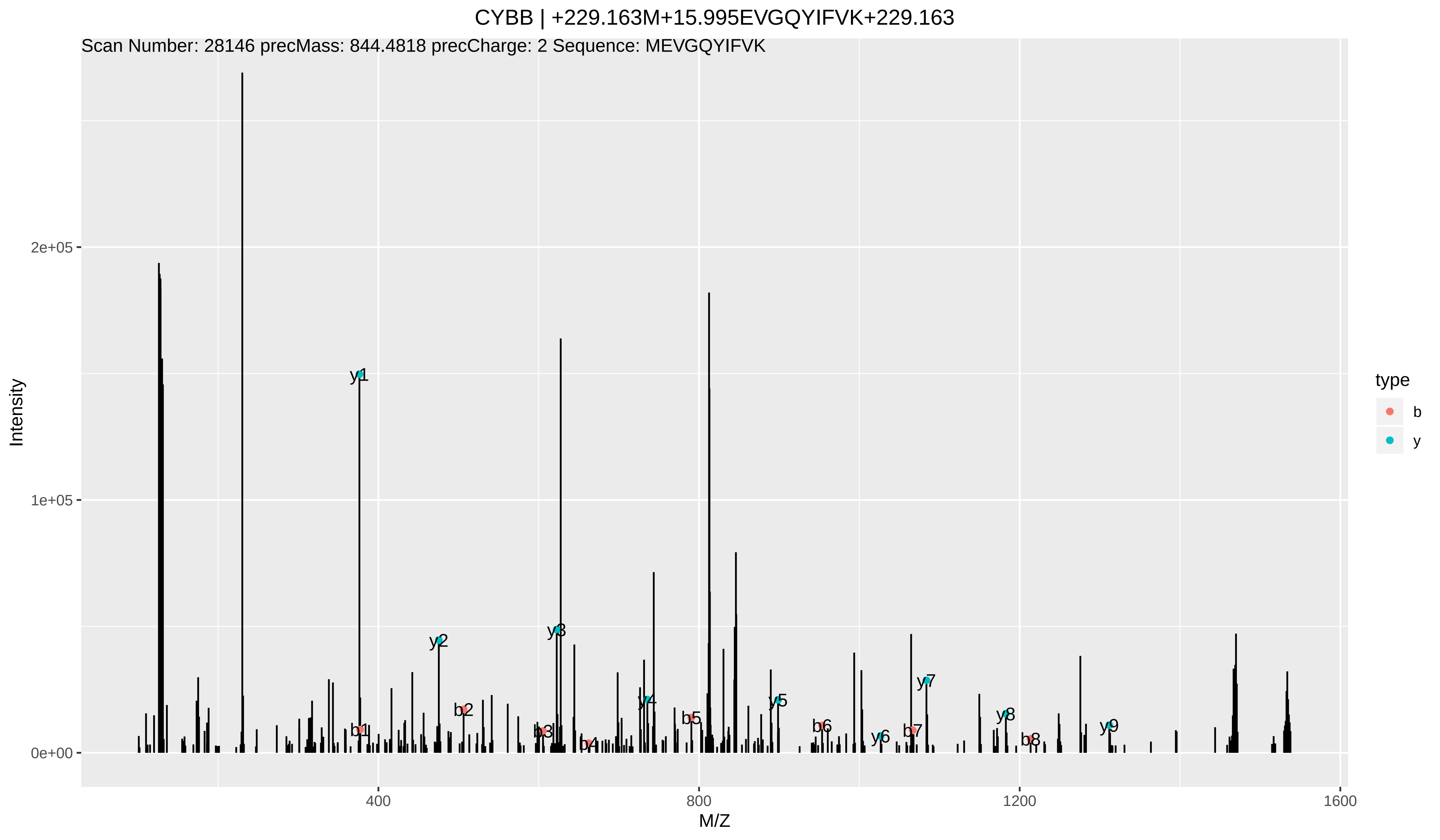

CYBB | +229.163M+15.995EVGQYIFVK+229.163
Scan Number: 28146 precMass: 844.4818 precCharge: 2 Sequence: MEVGQYIFVK
Intensity
2e+05
1e+05
0e+00
M/Z
400
800
1200
1600
y1
y2
y3
y4
y5
y6
y7
y8
y9
b1
b2
b3
b4
b5
b6
b7
b8
type
b
y

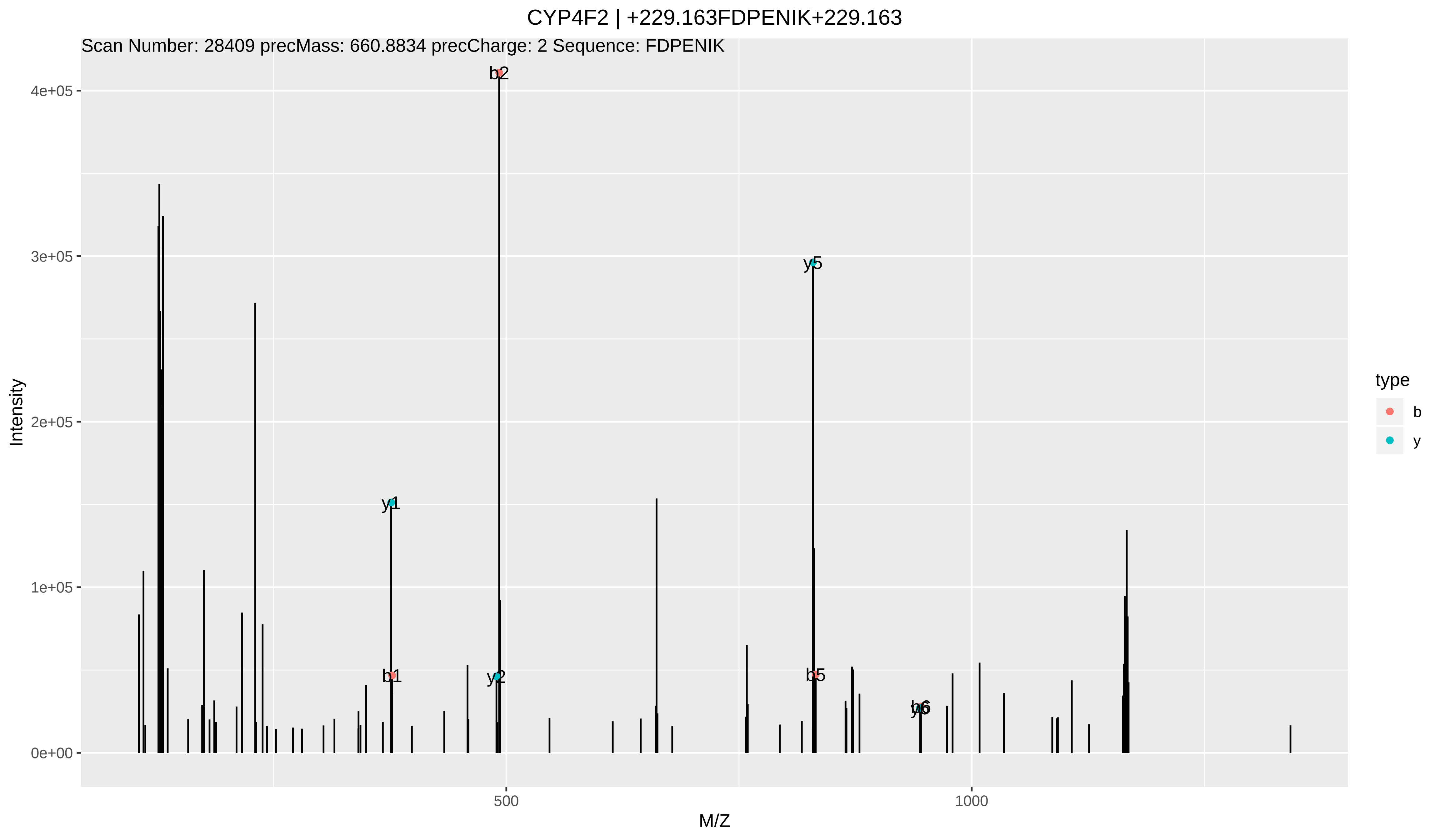
CYP4F2 | +229.163FDPENIK+229.163
Scan Number: 28409 precMass: 660.8834 precCharge: 2 Sequence: FDPENIK
Intensity
4e+05
3e+05
2e+05
1e+05
0e+00
M/Z
500
1000
b2
y5
y1
b1
y2
b5
y6
b6
type
b
y

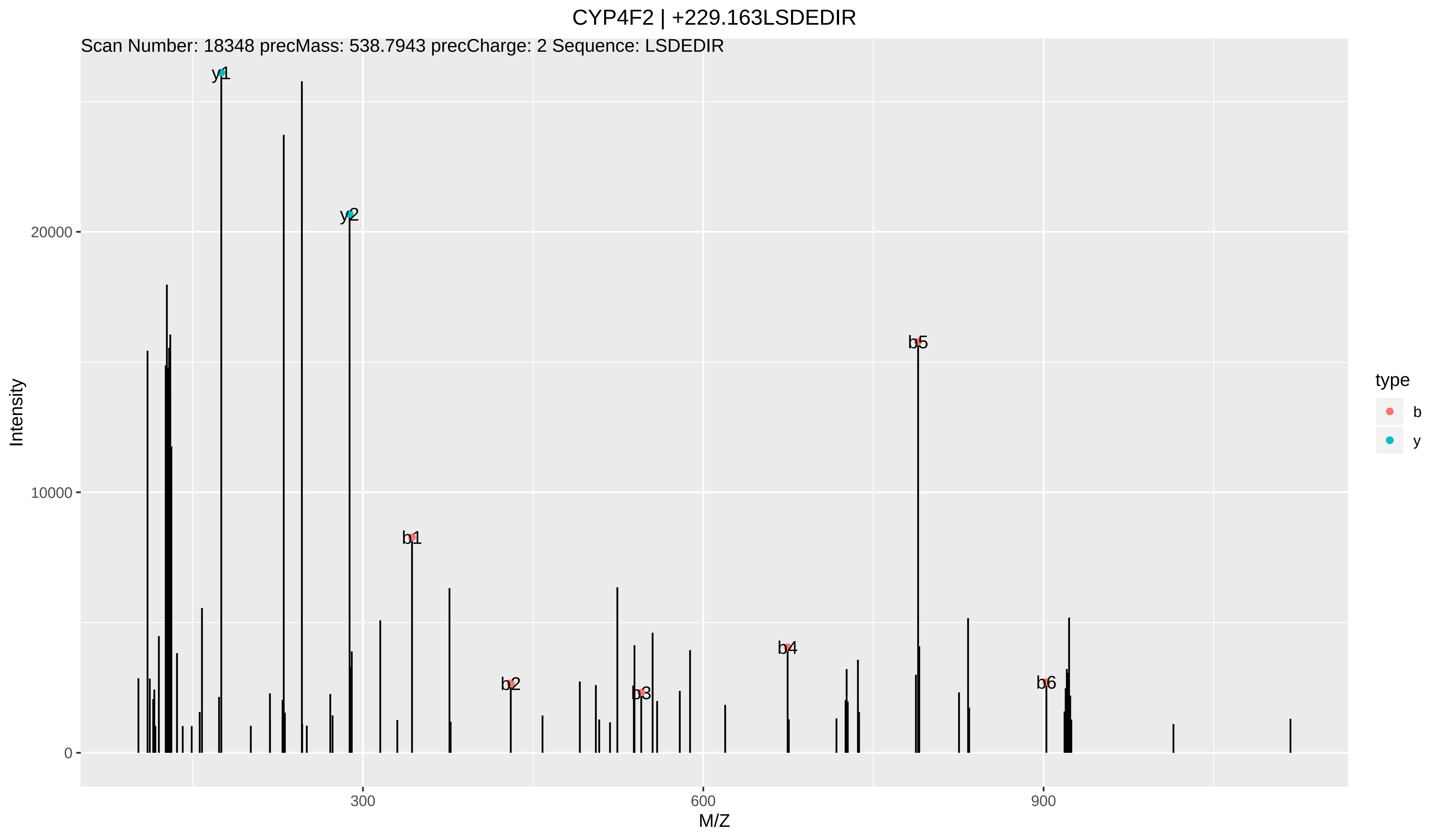

CYP4F2 | +229.163LSDEDIR
Scan Number: 18348 precMass: 538.7943 precCharge: 2 Sequence: LSDEDIR
Intensity
M/Z
0
10000
20000
300
600
900
y1
y2
b1
b2
b3
b4
b5
b6
type
b
y

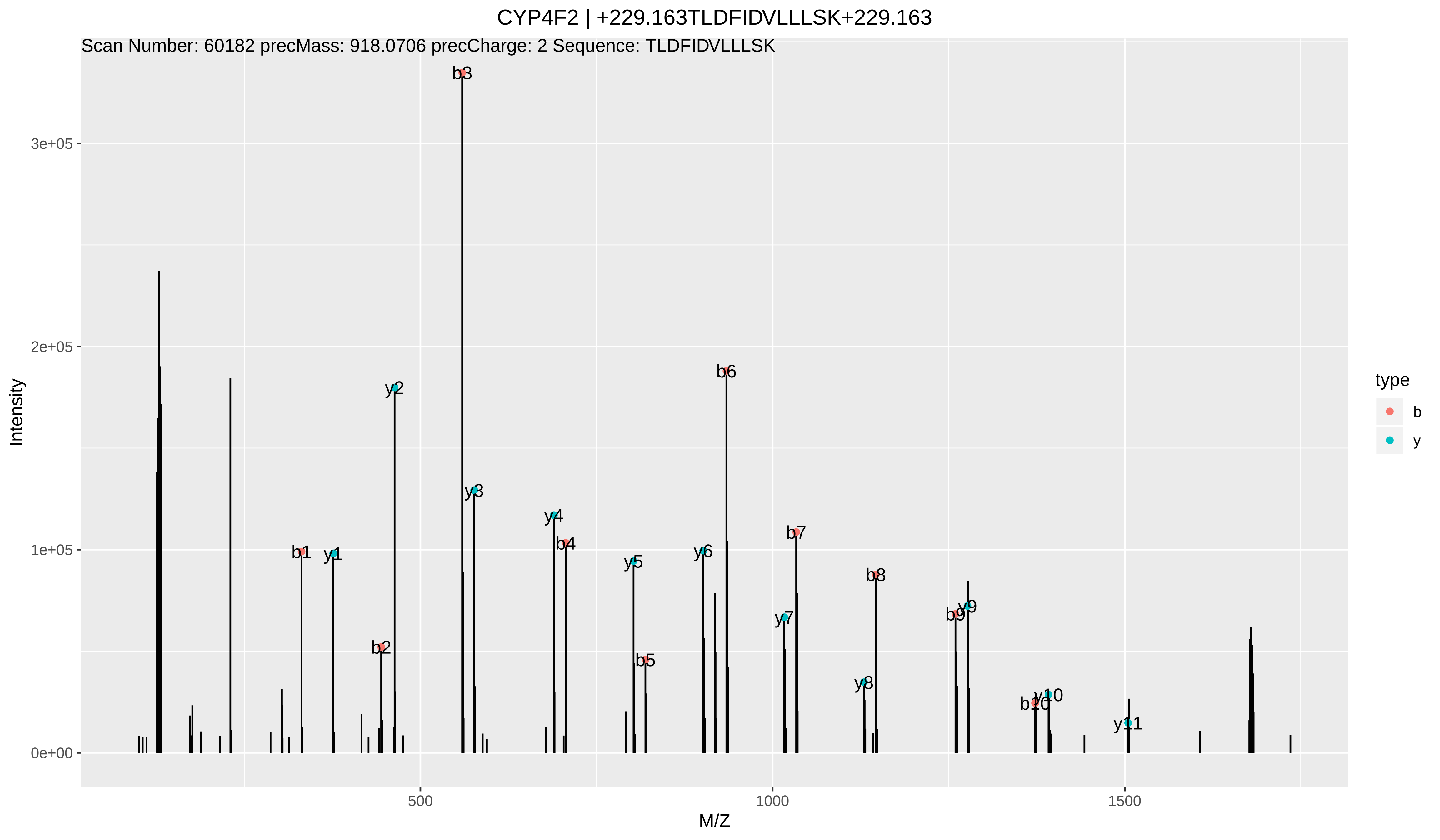
CYP4F2 | +229.163TLDFIDVLLLSK+229.163
Scan Number: 60182 precMass: 918.0706 precCharge: 2 Sequence: TLDFIDVLLLSK
Intensity
M/Z
type
b
y
0e+00
1e+05
2e+05
3e+05
500
1000
1500
b1
b2
b3
b4
b5
b6
b7
b8
b9
b10
y1
y2
y3
y4
y5
y6
y7
y8
y9
y10
y11

CYP4F2 | +229.163QEVQELLK+229.163

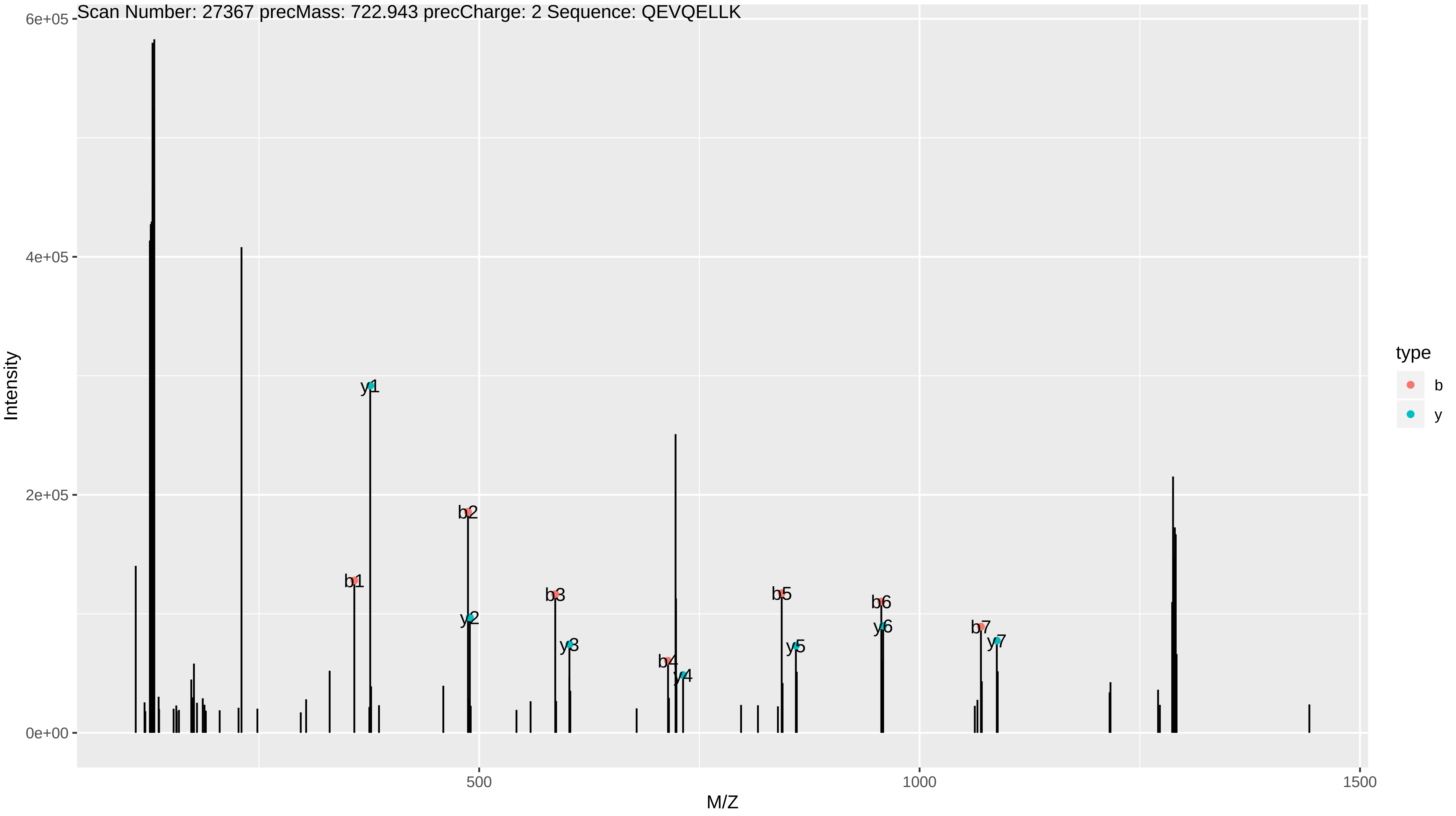

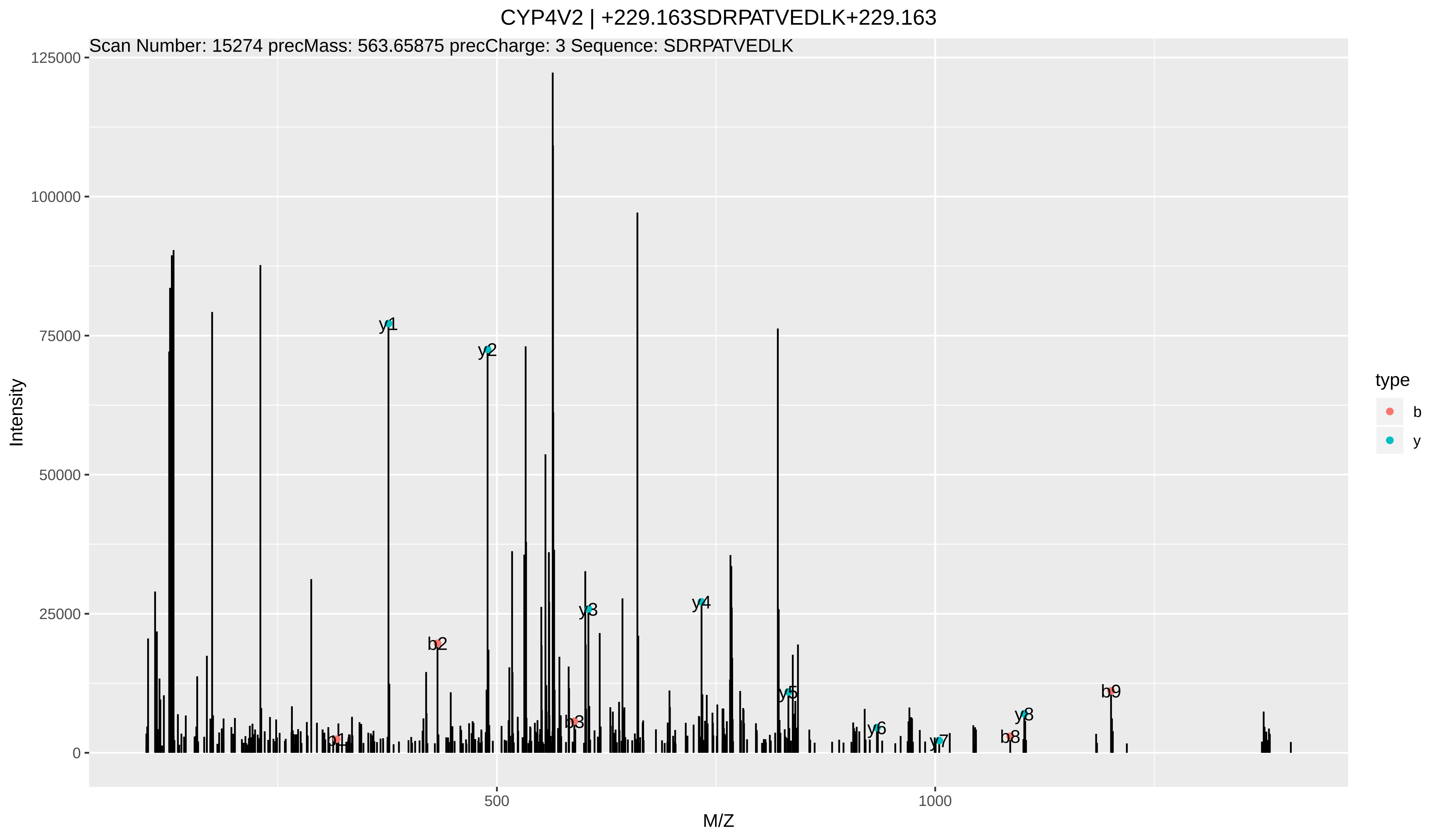

CYP4V2 | +229.163SDRPATVEDLK+229.163
Scan Number: 15274 precMass: 563.65875 precCharge: 3 Sequence: SDRPATVEDLK
Intensity
125000
100000
75000
50000
25000
0
M/Z
500
1000
y1
y2
b2
y3
b3
y4
y5
y6
y7
b8
y8
b9
b1
type
b
y

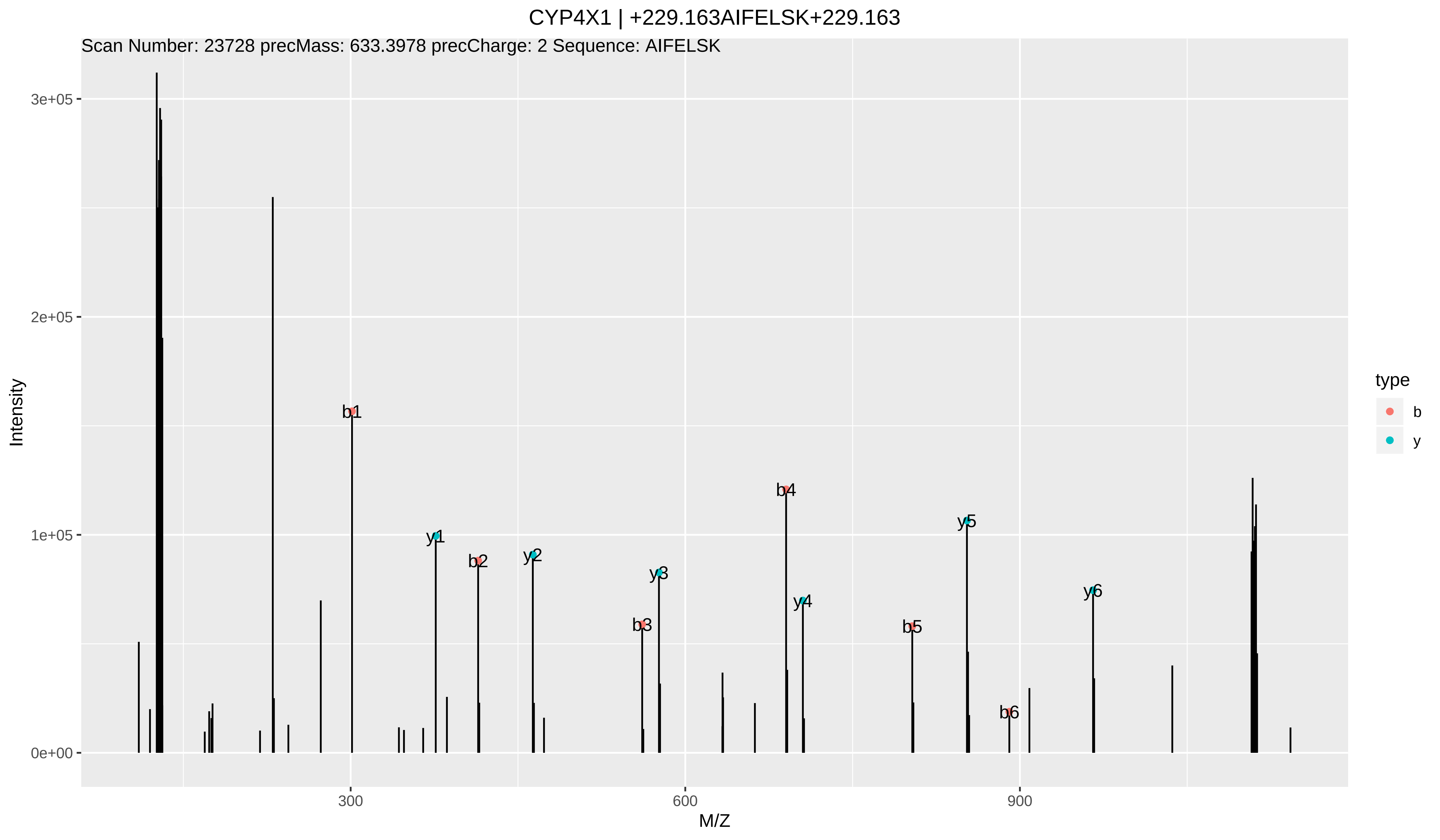
CYP4X1 | +229.163AIFELSK+229.163
Scan Number: 23728 precMass: 633.3978 precCharge: 2 Sequence: AIFELSK
Intensity
3e+05
2e+05
1e+05
0e+00
M/Z
300
600
900
b1
y1
b2
y2
b3
y3
b4
y4
b5
y5
b6
y6
type
b
y

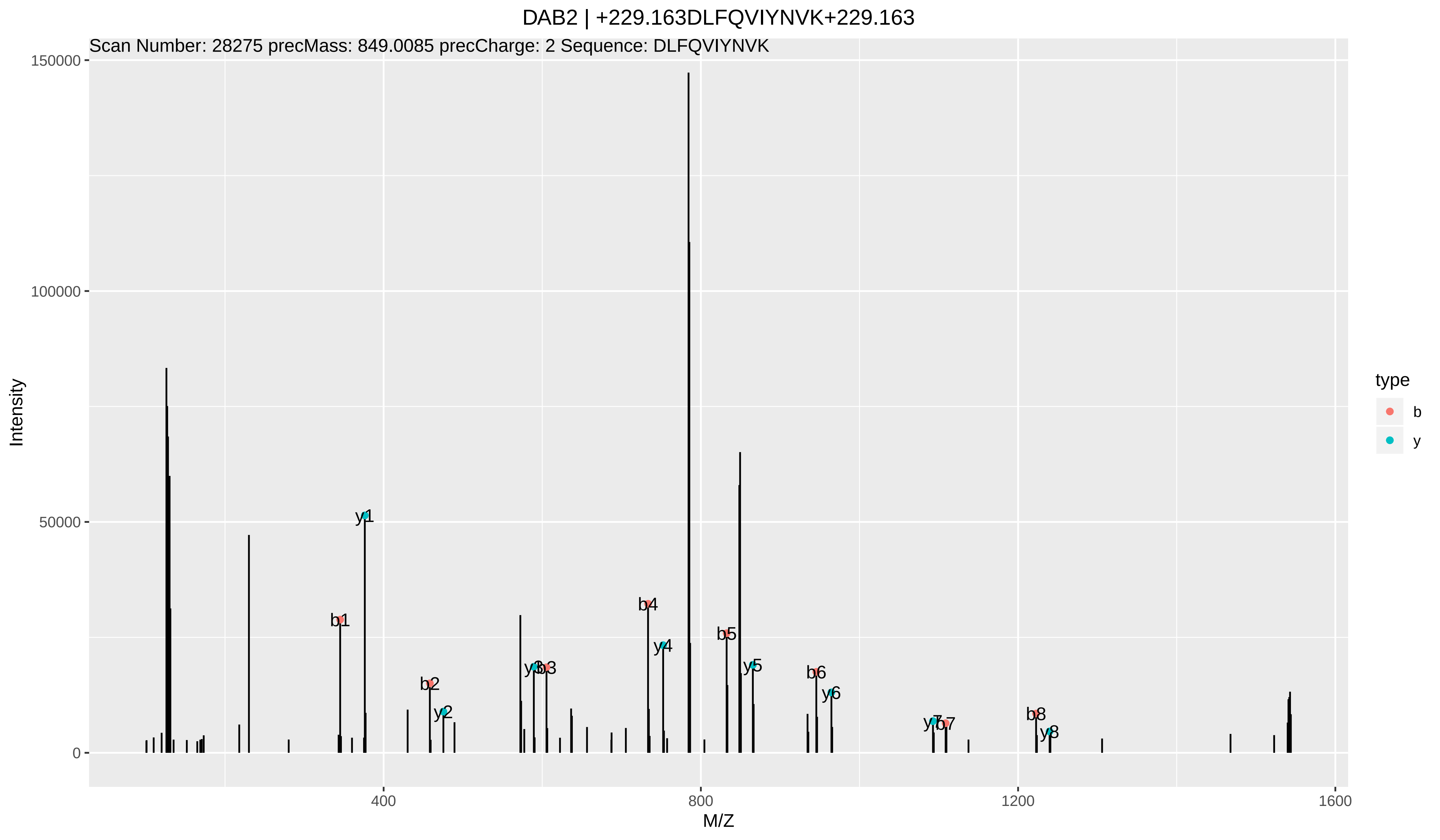

DAB2 | +229.163DLFQVIYNVK+229.163
Scan Number: 28275 precMass: 849.0085 precCharge: 2 Sequence: DLFQVIYNVK
Intensity
150000
100000
50000
0
400
800
1200
1600
M/Z
b1
y1
b2
y2
y3
b3
b4
y4
b5
y5
b6
y6
y7
b7
b8
y8
type
b
y

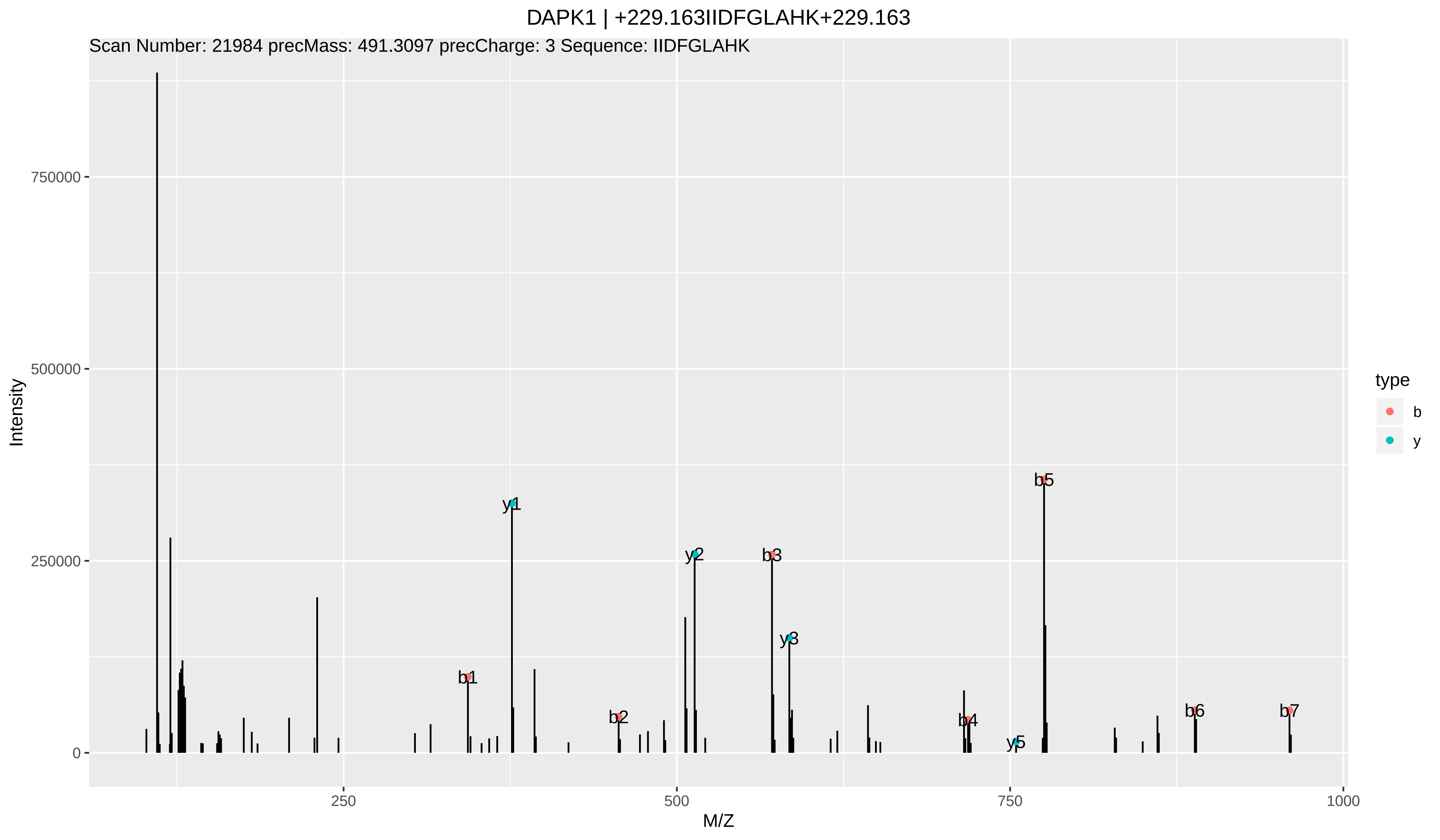
DAPK1 | +229.163IIDFGLAHK+229.163
Scan Number: 21984 precMass: 491.3097 precCharge: 3 Sequence: IIDFGLAHK
Intensity
750000
500000
250000
0
250
500
750
1000
M/Z
y1
b1
b2
y2
b3
y3
b4
y5
b5
b6
b7
type
b
y

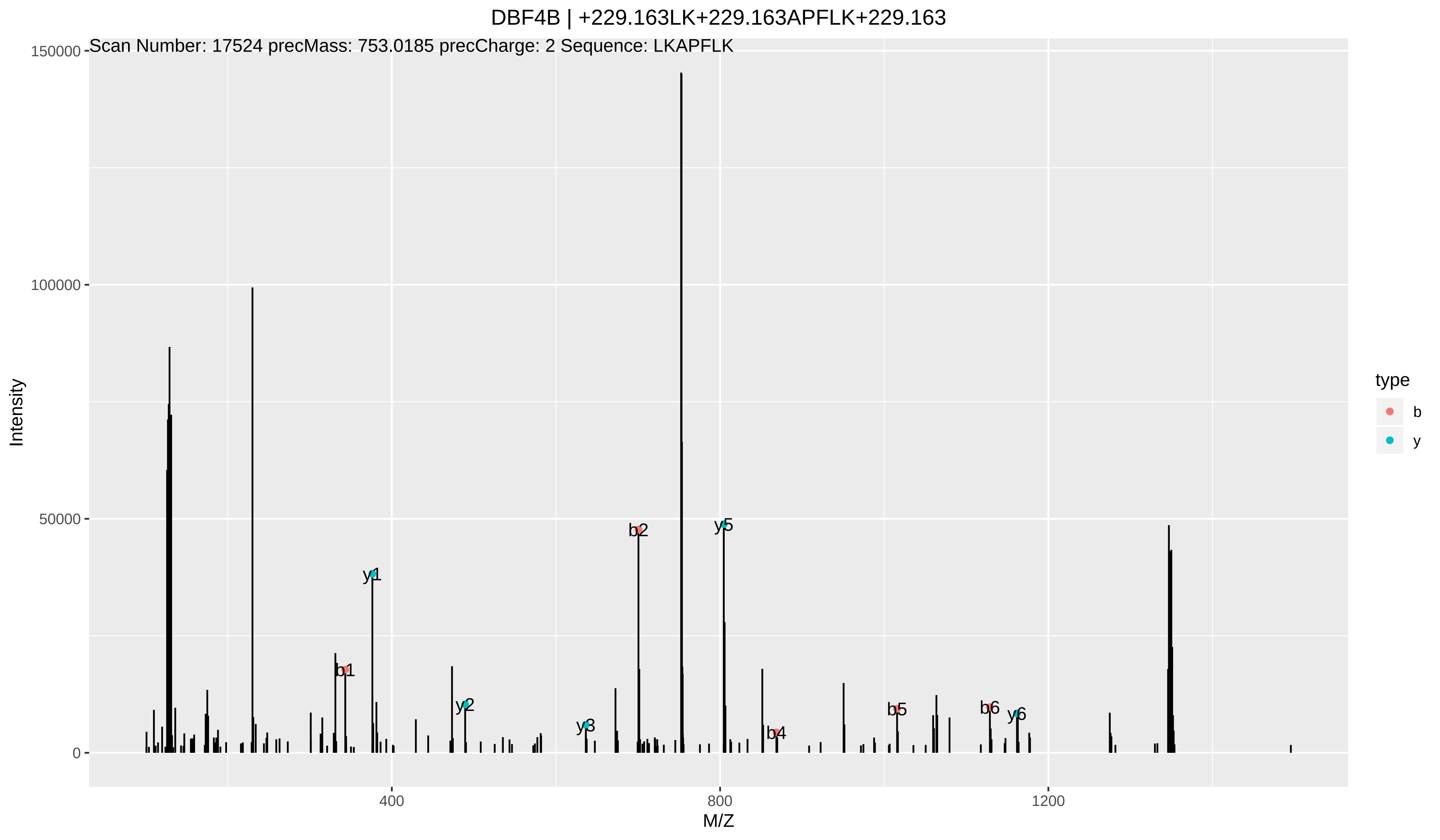

DBF4B | +229.163LK+229.163APFLK+229.163
Scan Number: 17524 precMass: 753.0185 precCharge: 2 Sequence: LKAPFLK
150000
100000
50000
0
Intensity
400
800
1200
M/Z
y1
b1
y2
y3
b2
y5
b4
b5
b6
y6
type
b
y

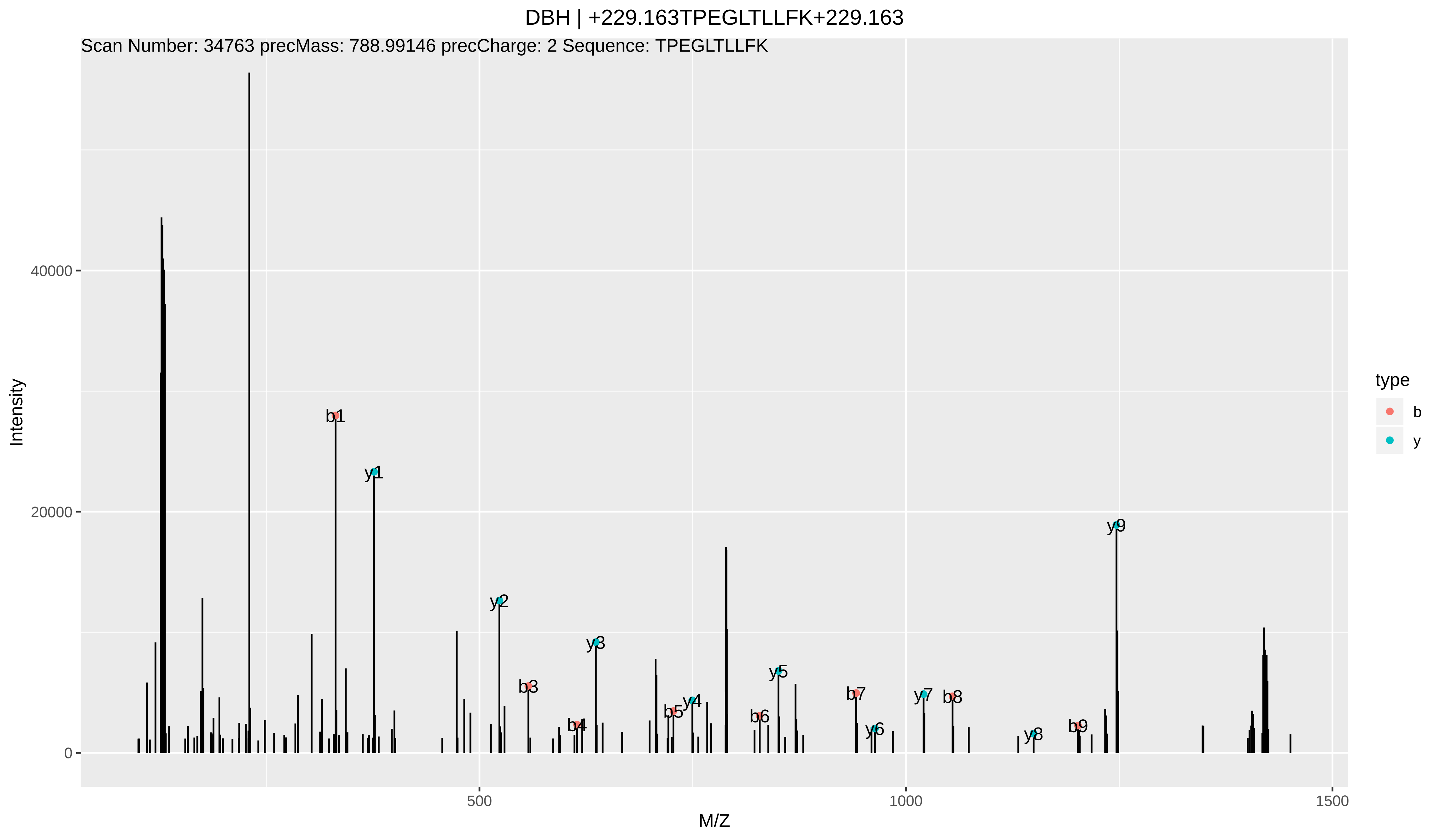
DBH | +229.163TPEGLTLLFK+229.163
Scan Number: 34763 precMass: 788.99146 precCharge: 2 Sequence: TPEGLTLLFK
Intensity
M/Z
0
20000
40000
500
1000
1500
b1
y1
y2
b3
y3
b4
b5
y4
y5
b6
b7
y6
y7
b8
y8
b9
y9
type
b
y

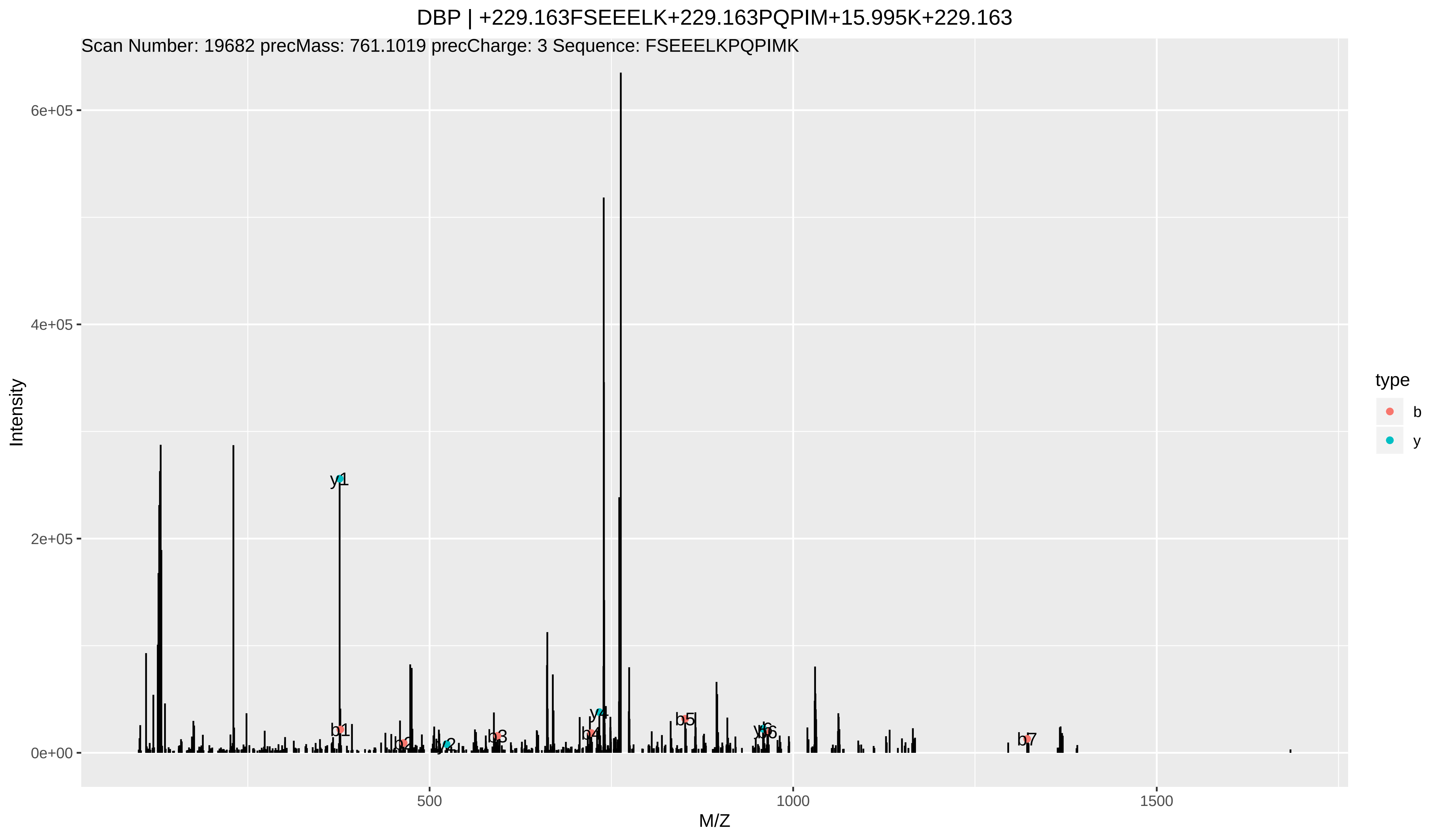
DBP | +229.163FSEEELK+229.163PQPIM+15.995K+229.163
Scan Number: 19682 precMass: 761.1019 precCharge: 3 Sequence: FSEEELKPQPIMK
Intensity
6e+05
4e+05
2e+05
0e+00
M/Z
500
1000
1500
y1
b1
b2
y2
b3
y4
b4
b5
y6
b6
b7
type
b
y

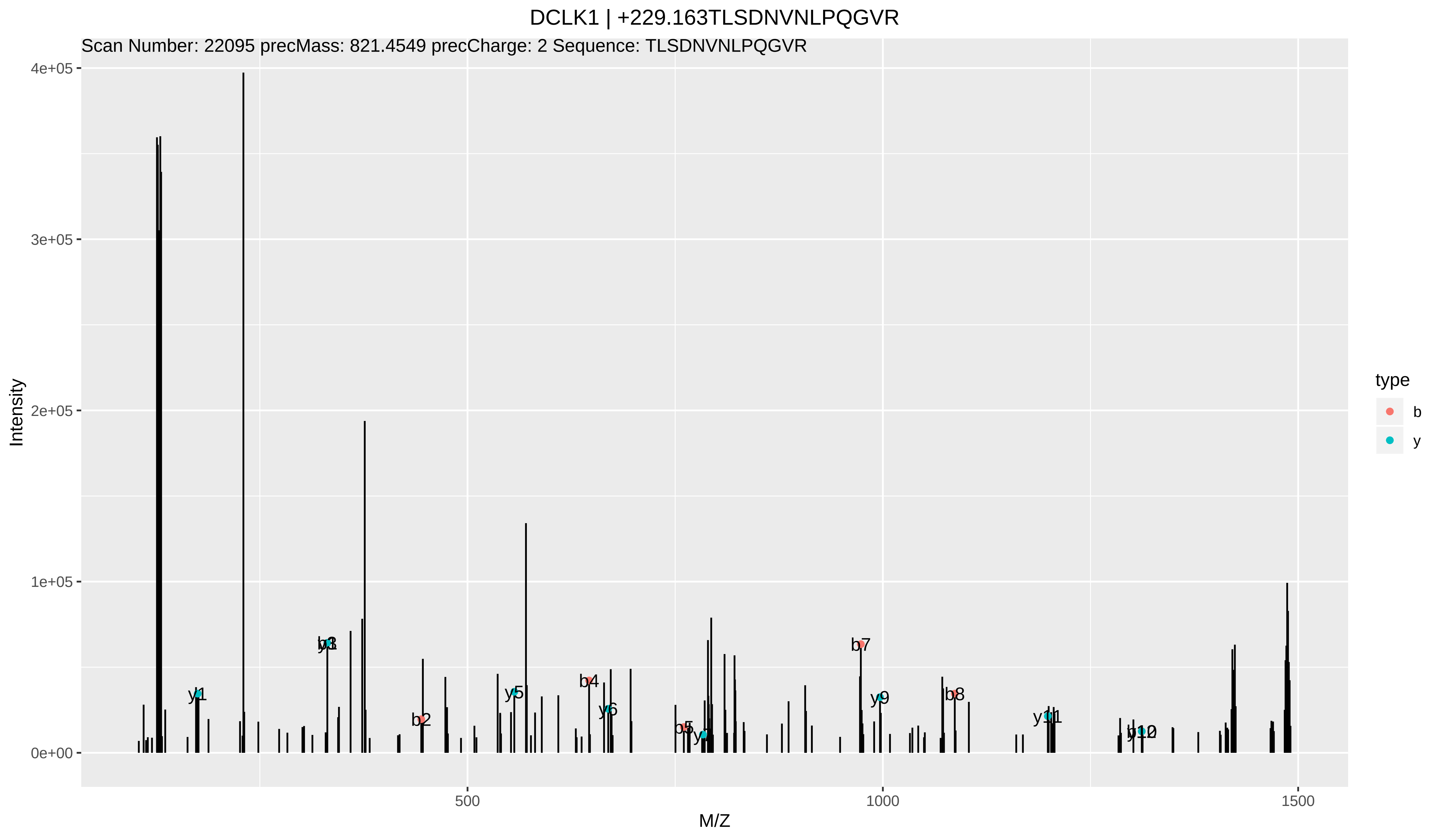
DCLK1 | +229.163TLSDNVNLPQGVR
Scan Number: 22095 precMass: 821.4549 precCharge: 2 Sequence: TLSDNVNLPQGVR
Intensity
4e+05
3e+05
2e+05
1e+05
0e+00
M/Z
500
1000
1500
type
b
y
y1
b2
b4
y5
y6
b7
y9
b8
y11

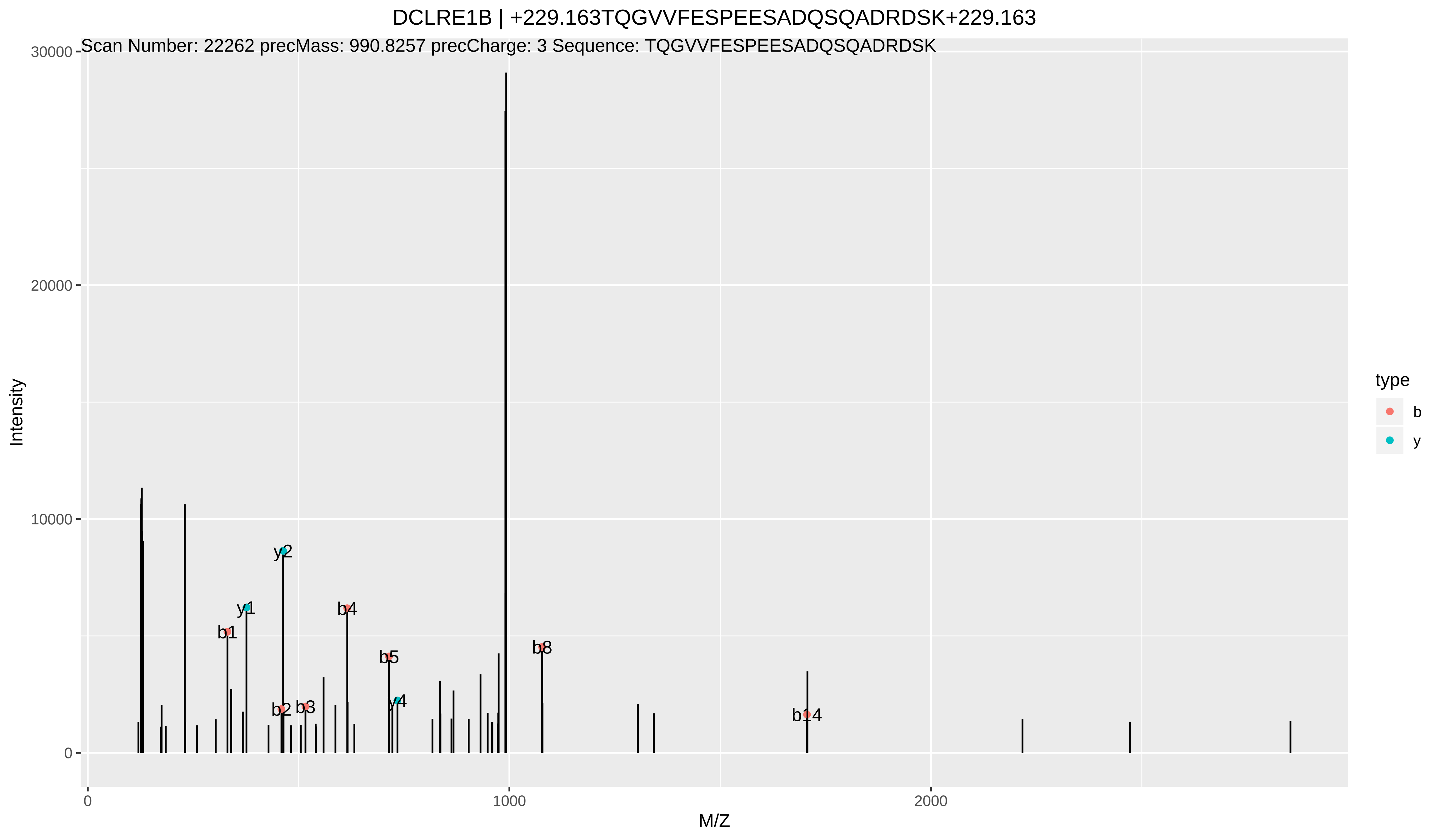

DCLRE1B | +229.163TQGVVFESPEESADQSQADRDSK+229.163
Scan Number: 22262 precMass: 990.8257 precCharge: 3 Sequence: TQGVVFESPEESADQSQADRDSK
Intensity
M/Z
0
10000
20000
30000
0
1000
2000
y2
y1
b1
b4
b5
b8
b2
b3
y4
b14
type
b
y

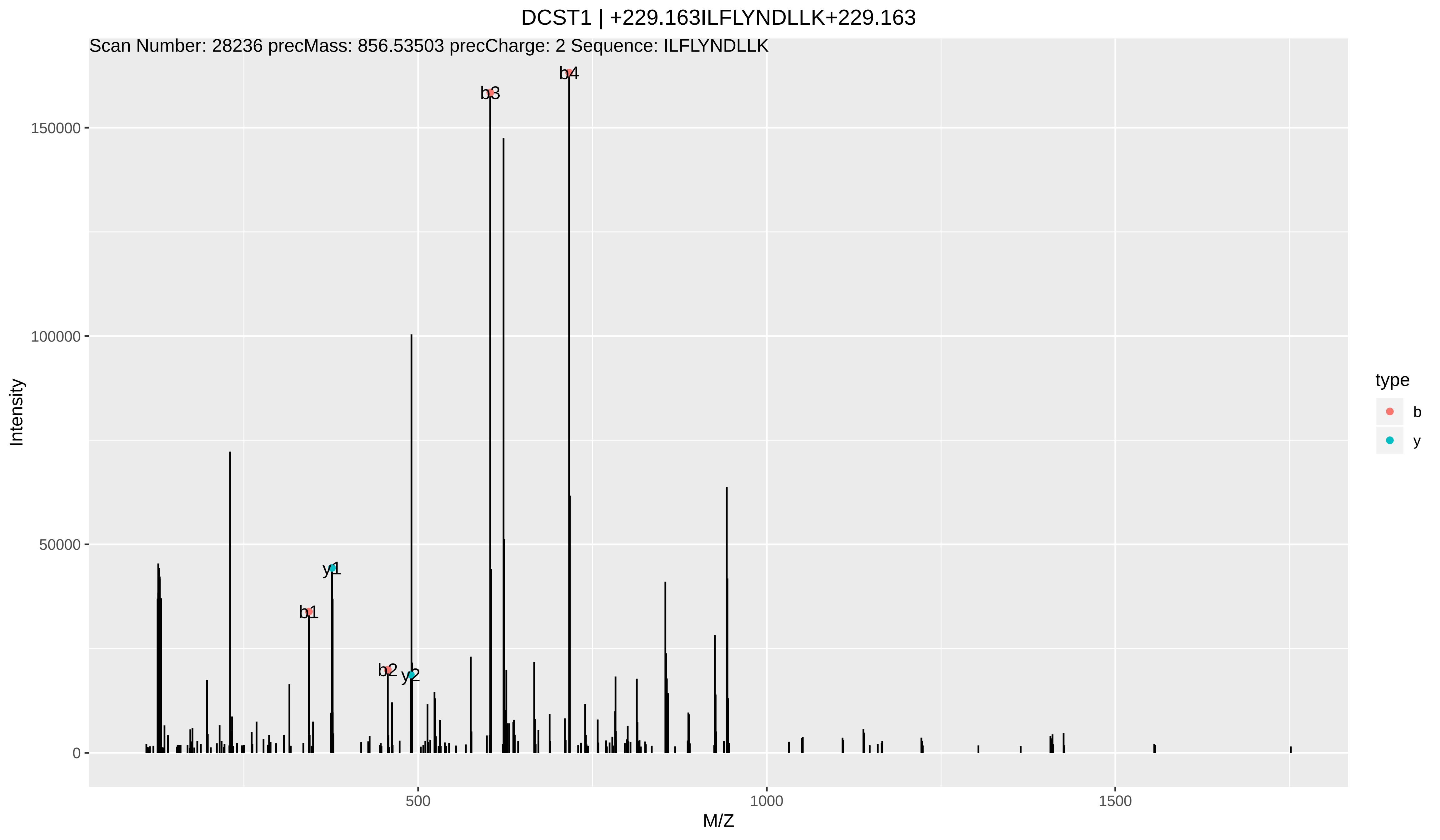
DCST1 | +229.163ILFLYNDLLK+229.163
Scan Number: 28236 precMass: 856.53503 precCharge: 2 Sequence: ILFLYNDLLK
b4
b3
y1
b1
b2
y2
Intensity
150000
100000
50000
0
500
1000
1500
M/Z
type
b
y

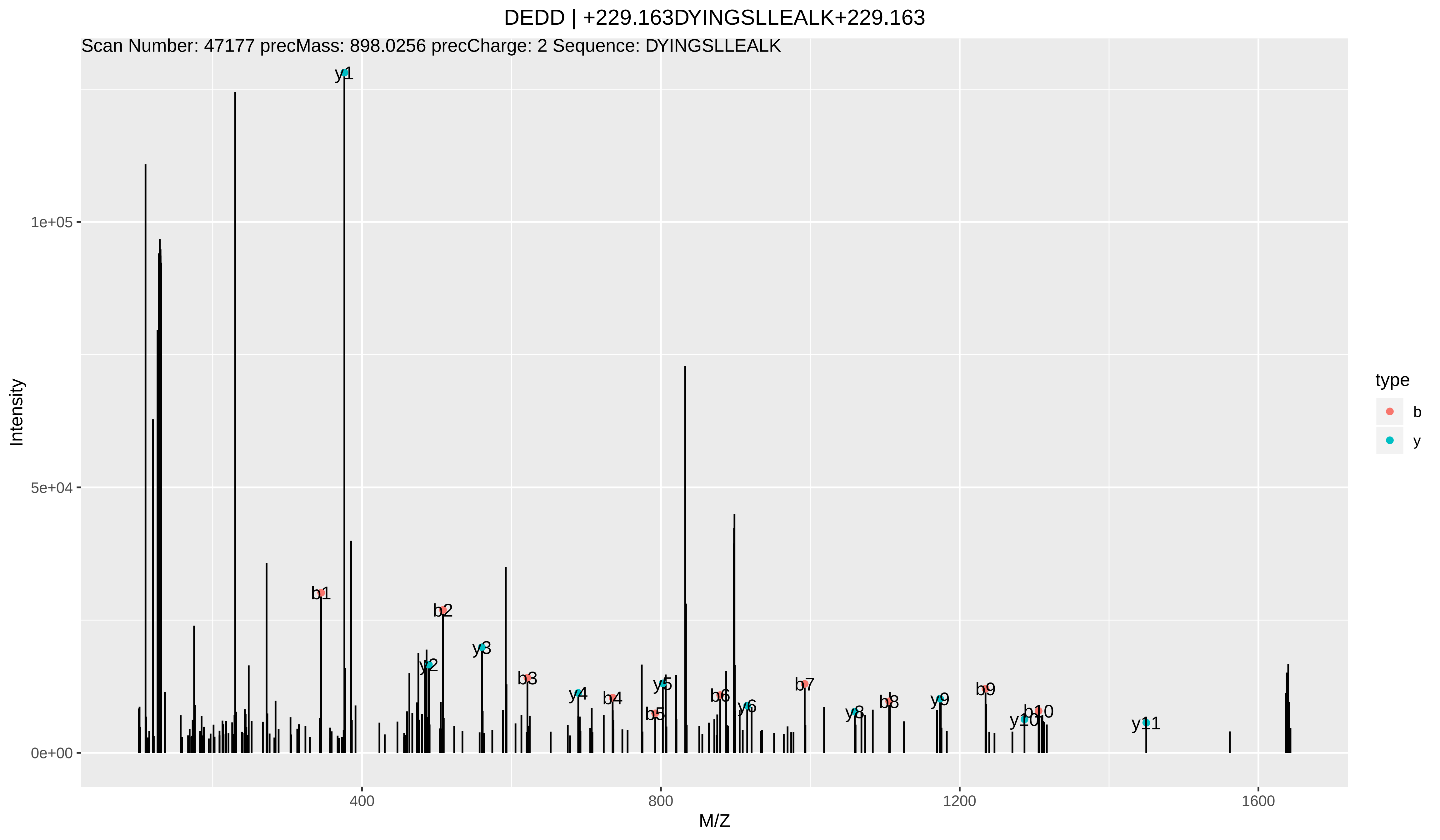
DEDD | +229.163DYINGSLLEALK+229.163
Scan Number: 47177 precMass: 898.0256 precCharge: 2 Sequence: DYINGSLLEALK
y1
b1
b2
y2
y3
b3
y4
b4
b5
y5
b6
y6
b7
y8
b8
y9
b9
y10
b10
y11
Intensity
1e+05
5e+04
0e+00
400
800
1200
1600
M/Z
type
b
y

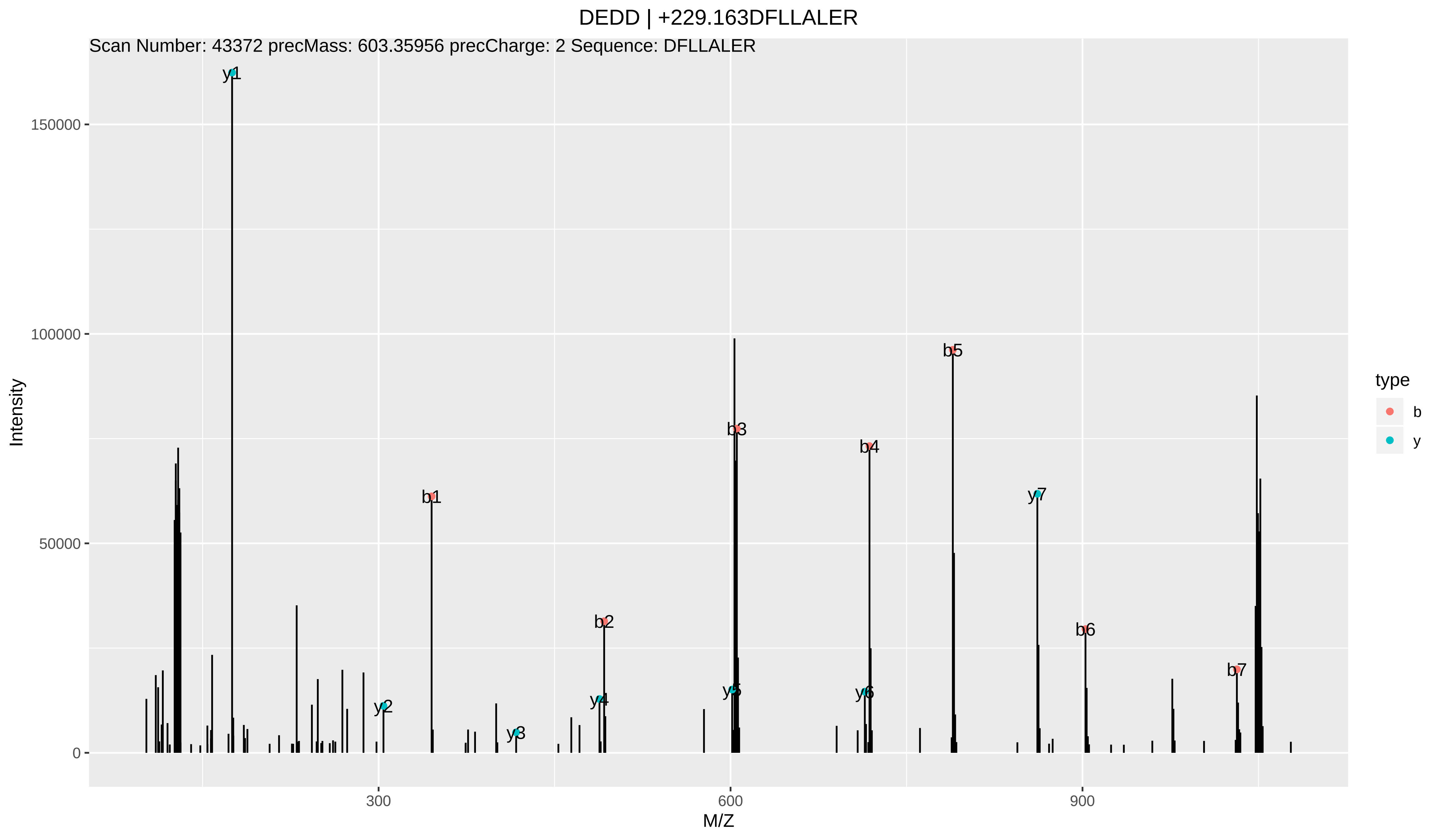
DEDD | +229.163DFLLALER
Scan Number: 43372 precMass: 603.35956 precCharge: 2 Sequence: DFLLALER
y1
b1
y2
y3
b2
y4
b3
y5
b4
y6
b5
y7
b6
b7
150000
100000
50000
0
Intensity
300
600
900
M/Z
type
b
y

DENND5B | +229.163FIETQMFATFIDNK+229.163

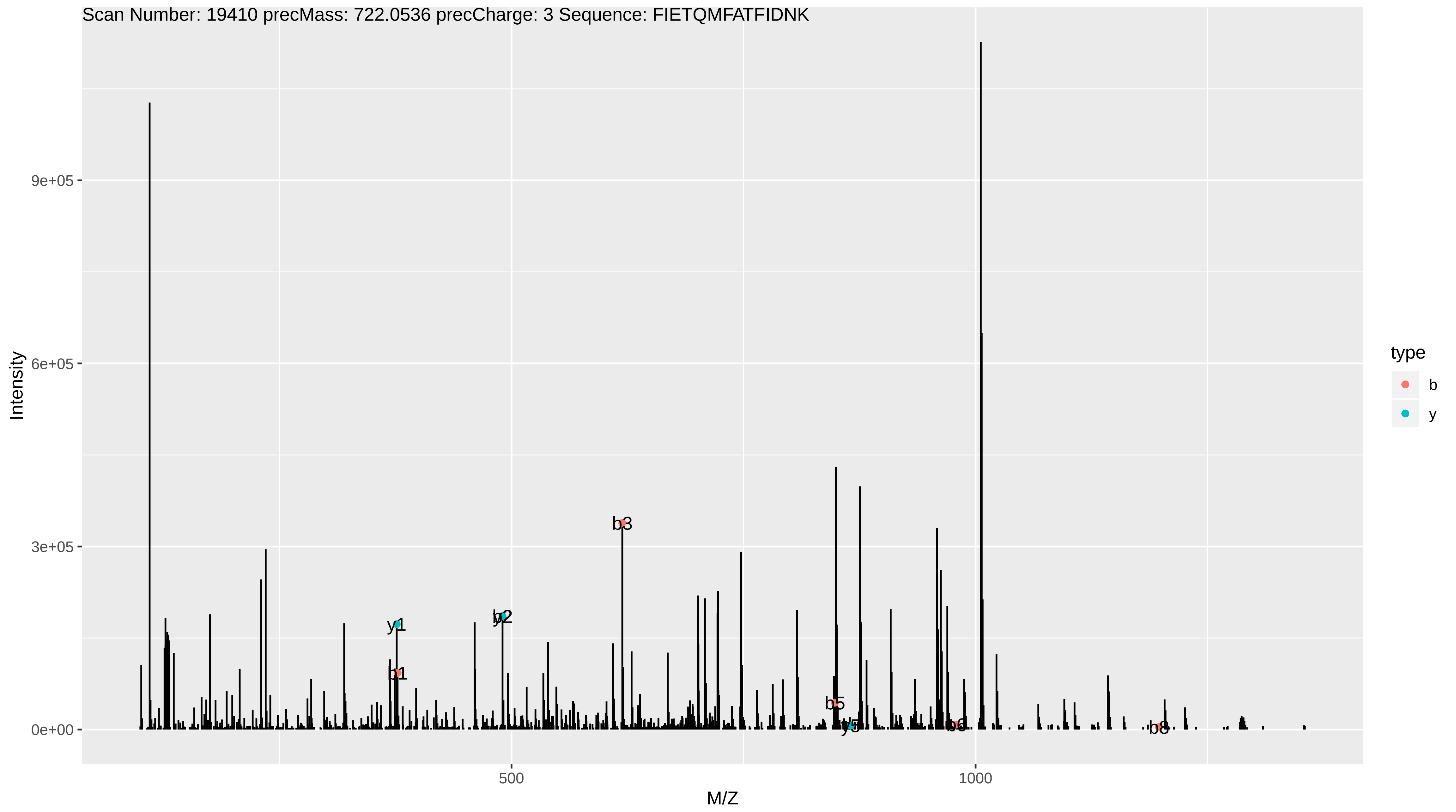

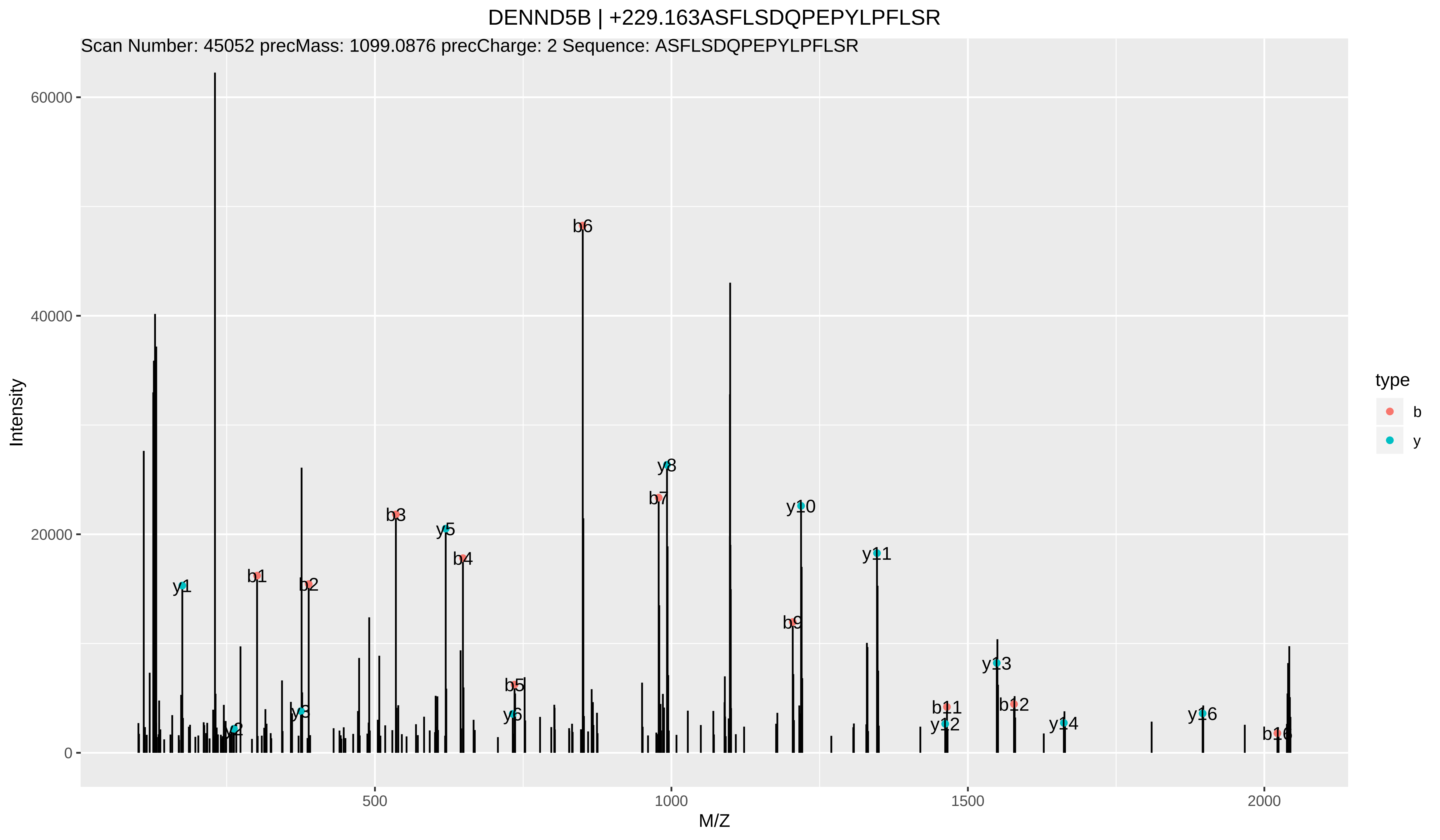
DENND5B | +229.163ASFLSDQPEPYLPFLSR
Scan Number: 45052 precMass: 1099.0876 precCharge: 2 Sequence: ASFLSDQPEPYLPFLSR
Intensity
60000
40000
20000
0
500
1000
1500
2000
M/Z
type
b
y
y1
b1
b2
y2
y3
b3
y5
b4
b5
y6
b6
b7
y8
b9
y10
y11
b11
y12
y13
b12
y14
y16
b16

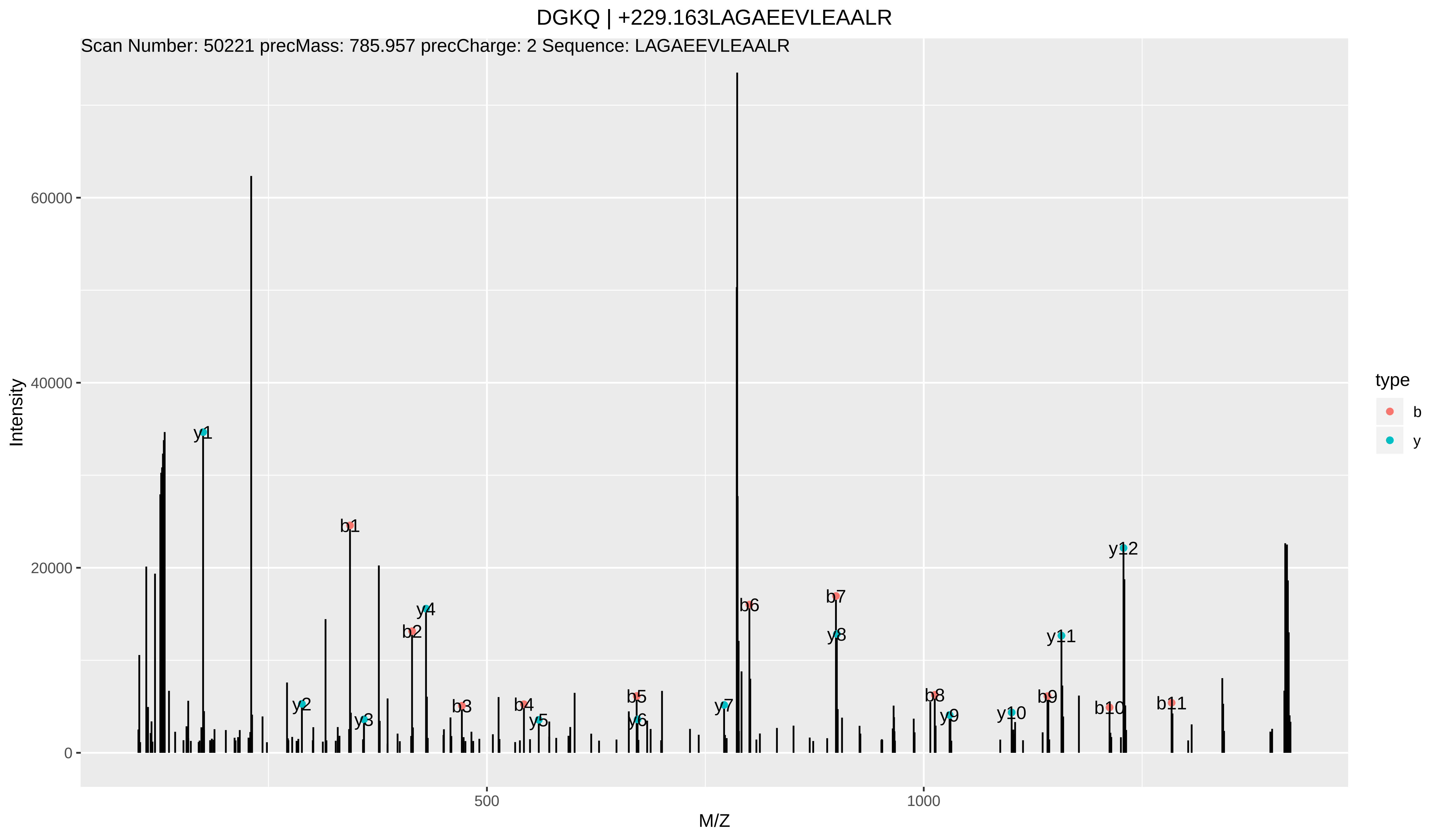

DGKQ | +229.163LAGAEEVLEAALR
Scan Number: 50221 precMass: 785.957 precCharge: 2 Sequence: LAGAEEVLEAALR
Intensity
60000
40000
20000
0
500
1000
M/Z
type
b
y
y1
b1
y2
y3
b2
y4
b3
b4
y5
b5
y6
y7
b6
b7
y8
b8
y9
y10
b9
y11
b10
y12
b11

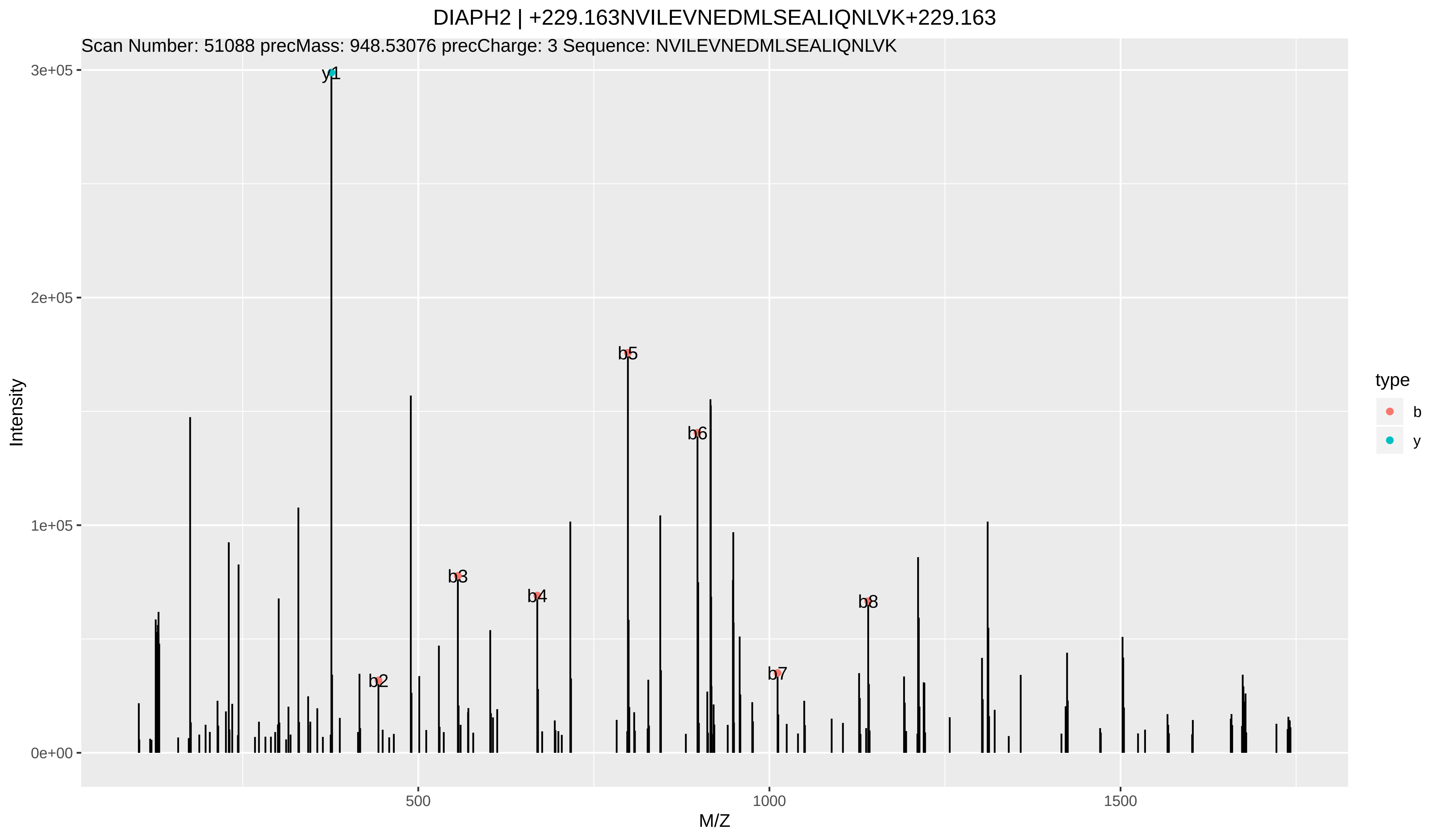
DIAPH2 | +229.163NVILEVNEDMLSEALIQNLVK+229.163
Scan Number: 51088 precMass: 948.53076 precCharge: 3 Sequence: NVILEVNEDMLSEALIQNLVK
Intensity
M/Z
3e+05
2e+05
1e+05
0e+00
500
1000
1500
y1
b2
b3
b4
b5
b6
b7
b8
type
b
y

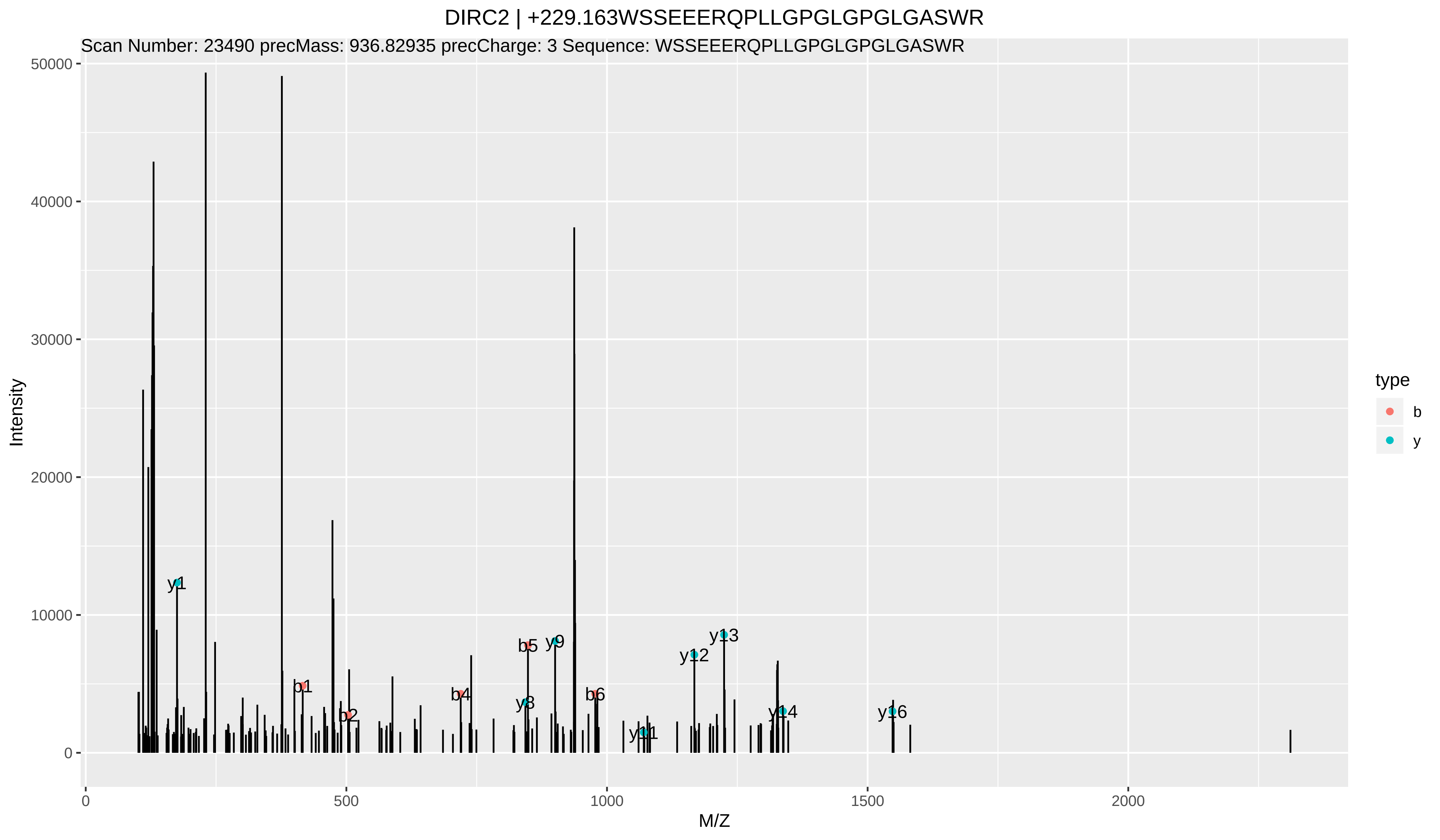
DIRC2 | +229.163WSSEEERQPLLGPGLGPGLGASWR
Scan Number: 23490 precMass: 936.82935 precCharge: 3 Sequence: WSSEEERQPLLGPGLGPGLGASWR
Intensity
M/Z
0
10000
20000
30000
40000
50000
500
1000
1500
2000
y1
b1
b2
b4
b5
y8
y9
b6
y11
y12
y13
y14
y16
type
b
y

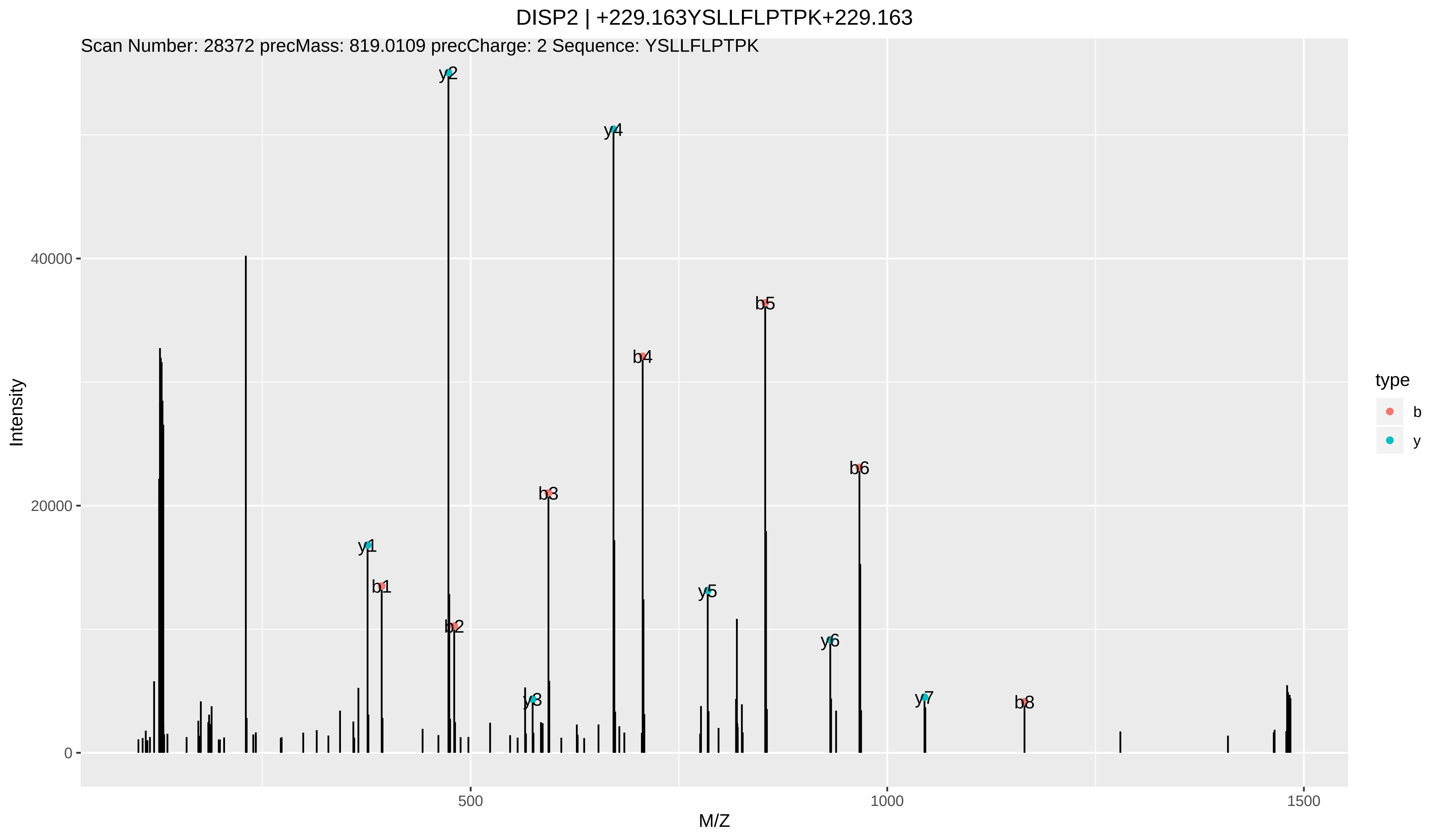
DISP2 | +229.163YSLLFLPTPK+229.163
Scan Number: 28372 precMass: 819.0109 precCharge: 2 Sequence: YSLLFLPTPK
y2
y4
b5
b4
b6
b3
y1
b1
y5
b2
y6
y3
y7
b8
40000
20000
0
500
1000
1500
M/Z
Intensity
type
b
y

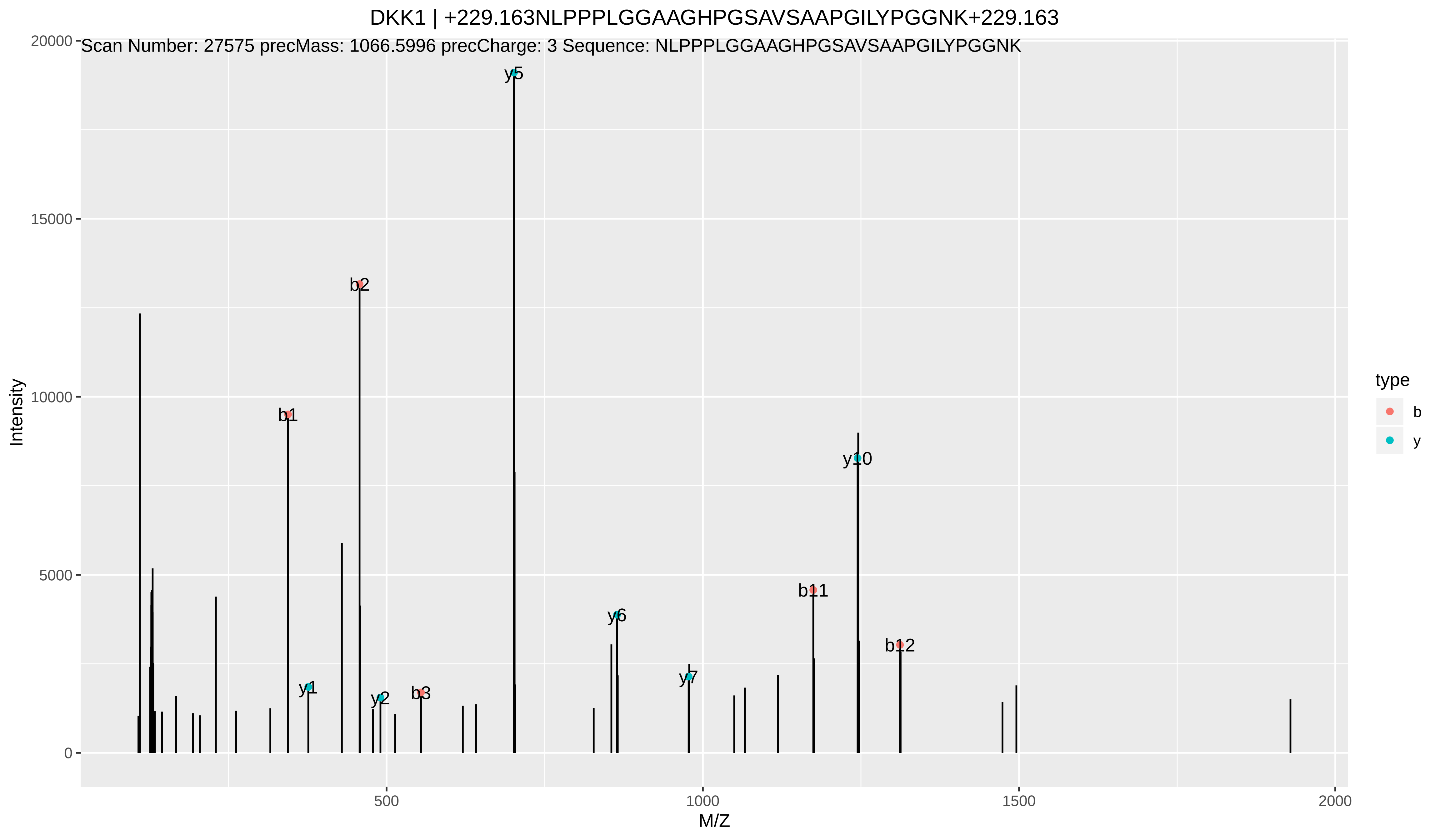

DKK1 | +229.163NLPPPLGGAAGHPGSAVSAAPGILYPGGNK+229.163
Scan Number: 27575 precMass: 1066.5996 precCharge: 3 Sequence: NLPPPLGGAAGHPGSAVSAAPGILYPGGNK
Intensity
M/Z
y5
b2
b1
y10
b11
y6
b12
y7
y1
y2
b3
type
b
y

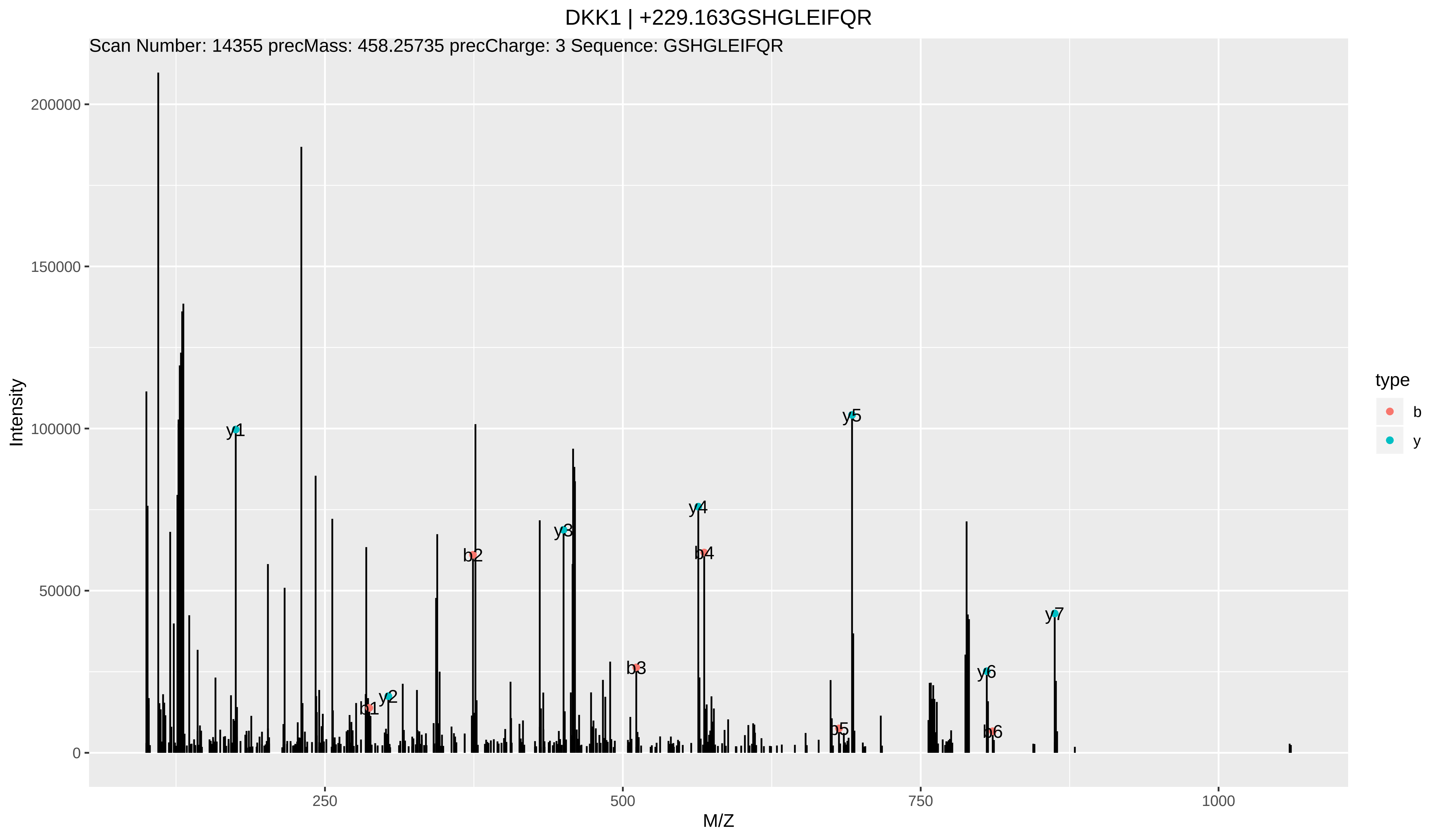
DKK1 | +229.163GSHGLEIFQR
Scan Number: 14355 precMass: 458.25735 precCharge: 3 Sequence: GSHGLEIFQR
Intensity
M/Z
type
b
y
y1
y2
y3
y4
y5
y6
y7
b1
b2
b3
b4
b5
b6
0
50000
100000
150000
200000
250
500
750
1000

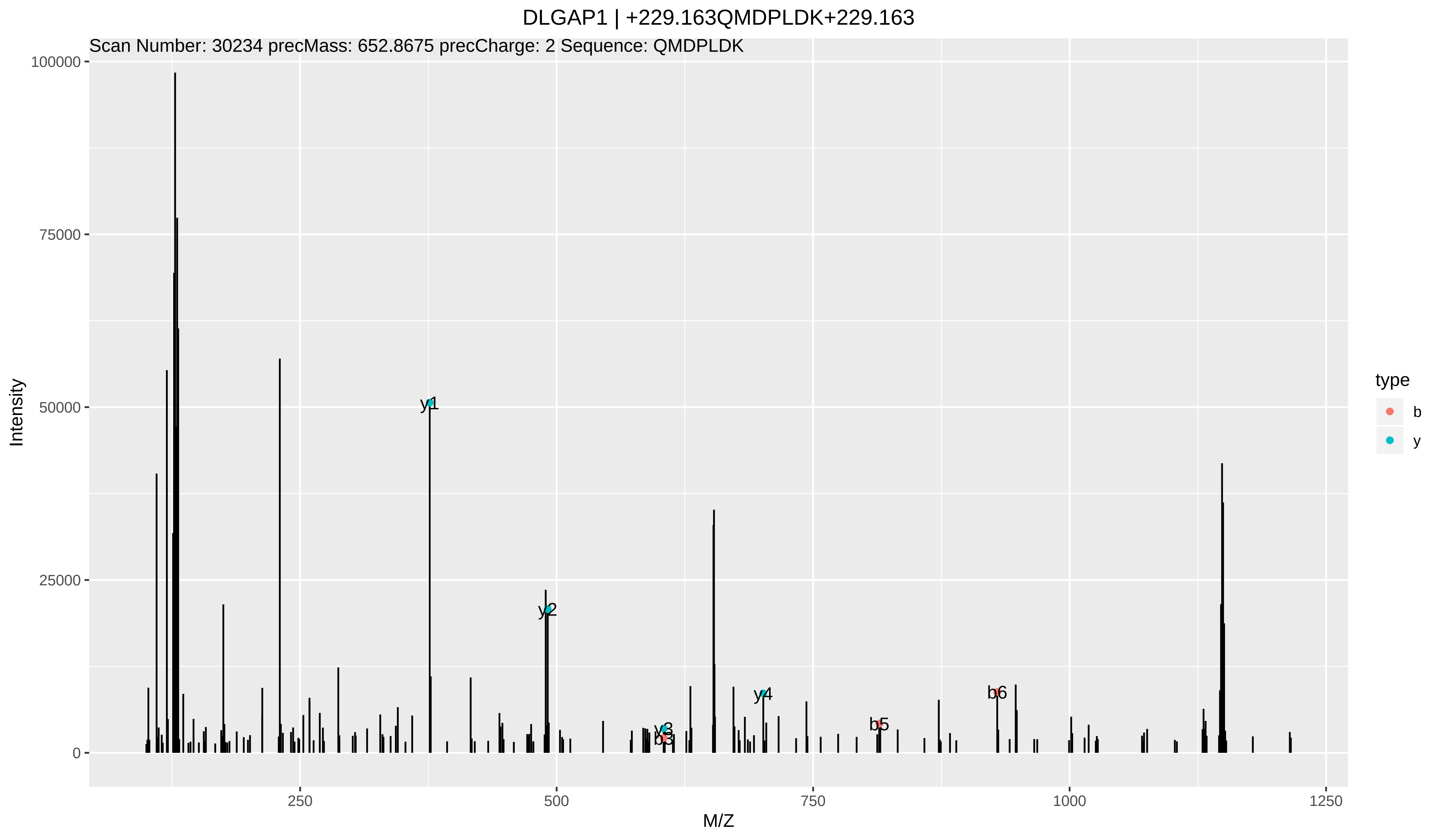

DLGAP1 | +229.163QMDPLDK+229.163
Scan Number: 30234 precMass: 652.8675 precCharge: 2 Sequence: QMDPLDK
Intensity
100000
75000
50000
25000
0
250
500
750
1000
1250
M/Z
y1
y2
y3
b3
y4
b5
b6
type
b
y

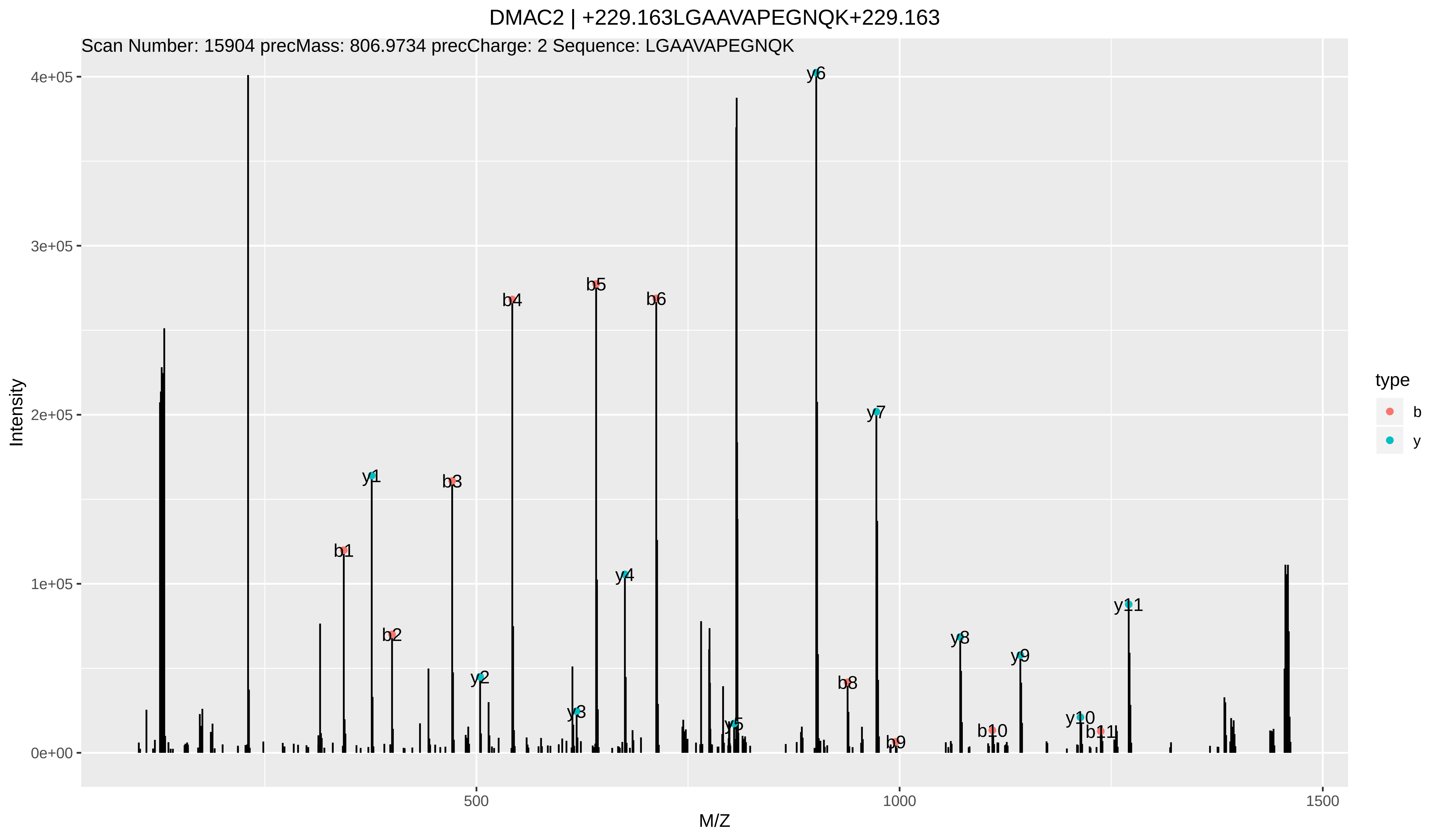

DMAC2 | +229.163LGAAVAPEGNQK+229.163
Scan Number: 15904 precMass: 806.9734 precCharge: 2 Sequence: LGAAVAPEGNQK
Intensity
4e+05
3e+05
2e+05
1e+05
0e+00
M/Z
500
1000
1500
y6
b4
b5
b6
y7
y1
b3
b1
y4
y11
b2
y8
y9
y2
b8
y3
y5
y10
b10
b11
b9
type
b
y

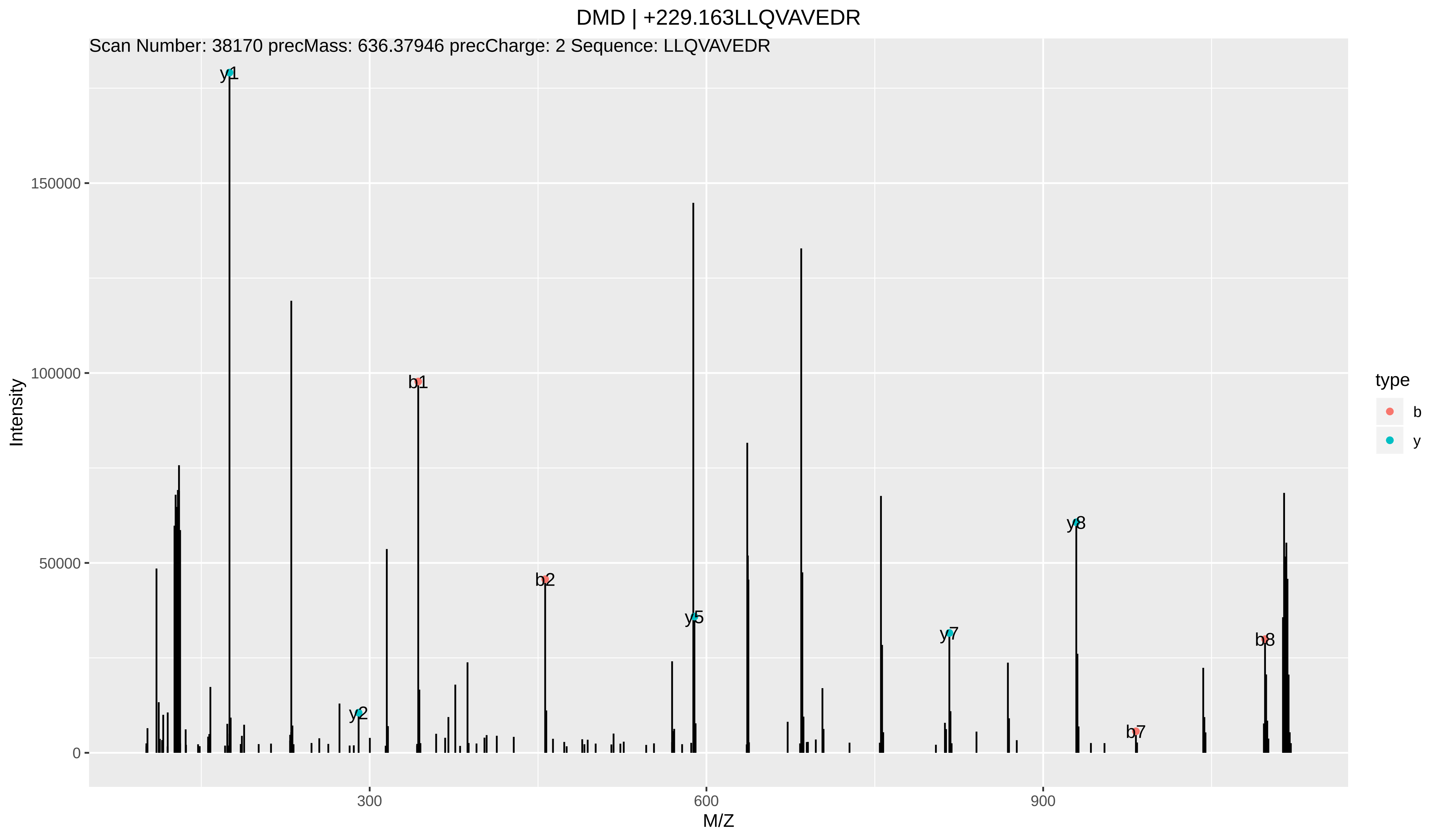

DMD | +229.163LLQVAVEDR
Scan Number: 38170 precMass: 636.37946 precCharge: 2 Sequence: LLQVAVEDR
y1
b1
y2
b2
y5
y7
y8
b7
b8
Intensity
150000
100000
50000
0
300
600
900
M/Z
type
b
y

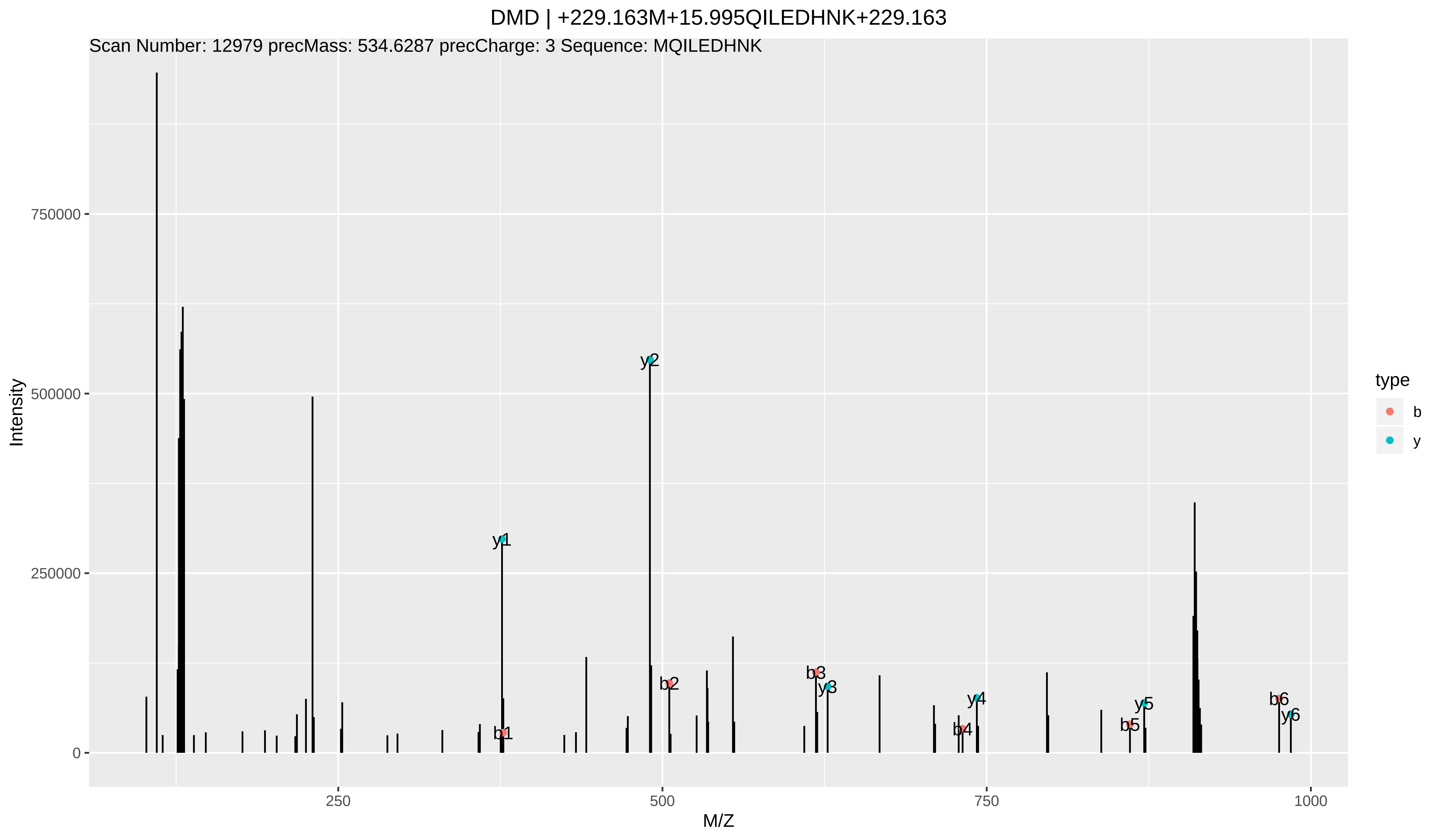
DMD | +229.163M+15.995QILEDHNK+229.163
Scan Number: 12979 precMass: 534.6287 precCharge: 3 Sequence: MQILEDHNK
Intensity
750000
500000
250000
0
M/Z
250
500
750
1000
y1
b1
y2
b2
b3
y3
b4
y4
b5
y5
b6
y6
type
b
y

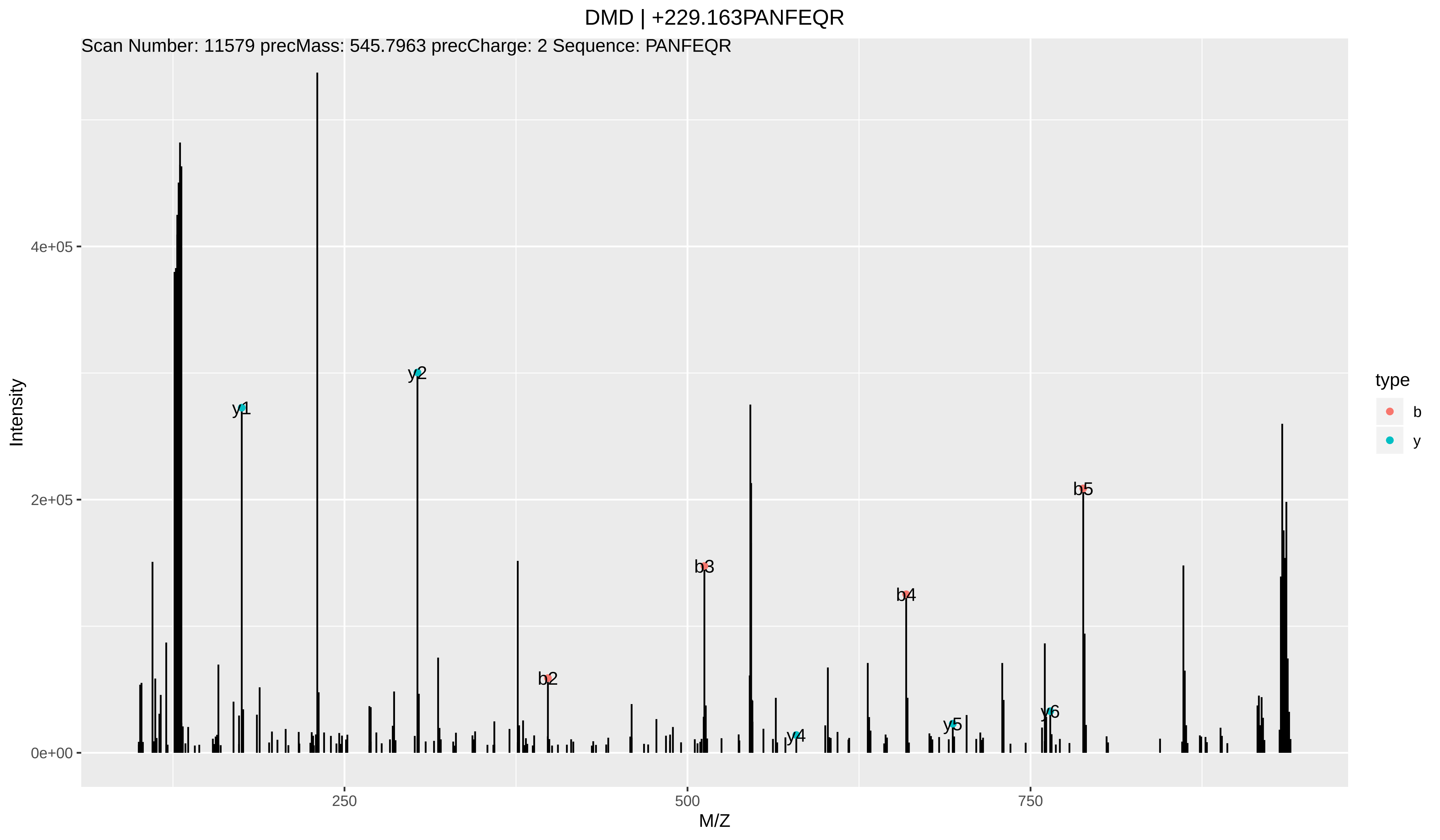

DMD | +229.163PANFEQR
Scan Number: 11579 precMass: 545.7963 precCharge: 2 Sequence: PANFEQR
Intensity
M/Z
0e+00
2e+05
4e+05
250
500
750
y1
y2
b2
b3
y4
b4
y5
y6
b5
type
b
y

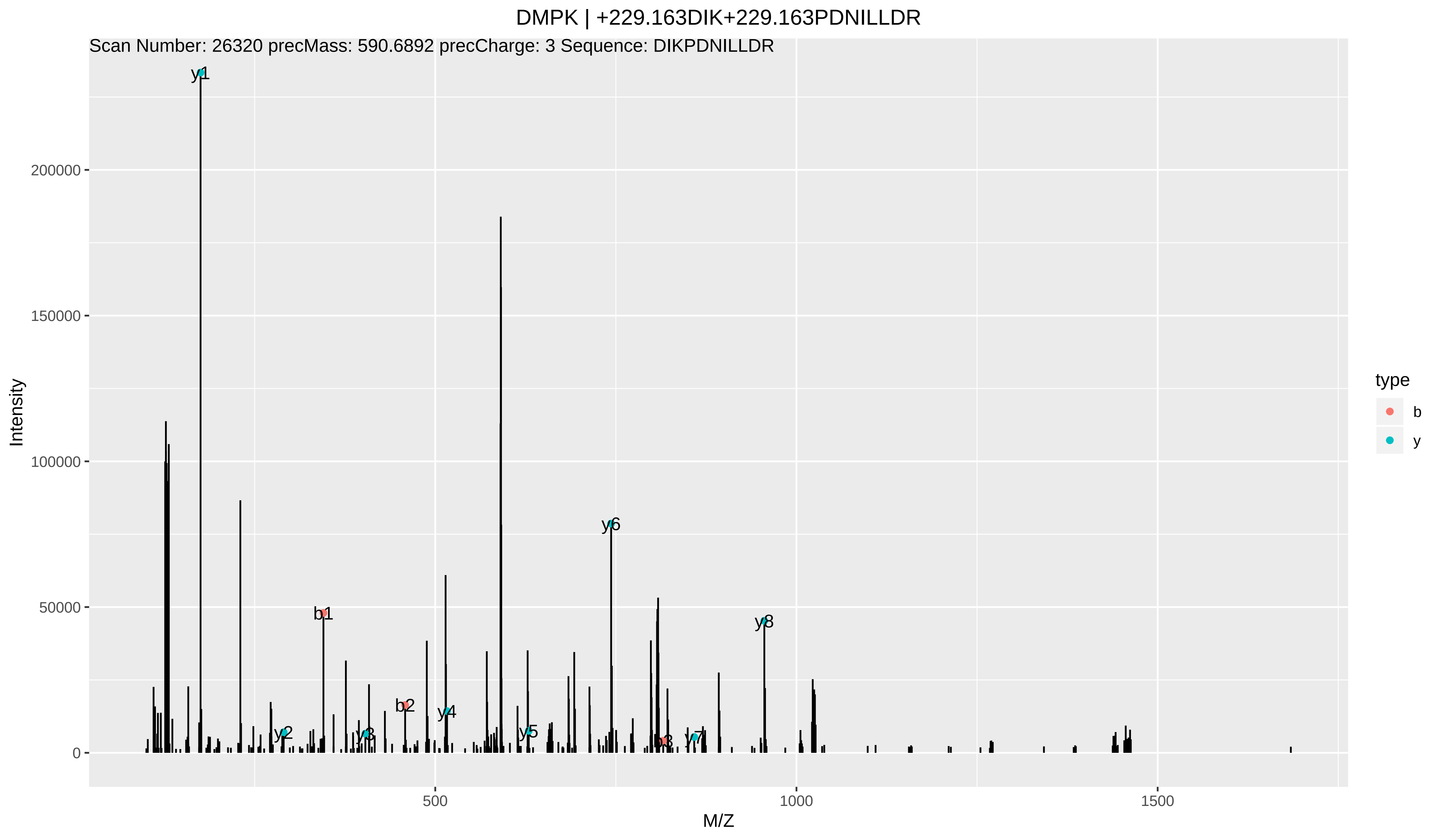
DMPK | +229.163DIK+229.163PDNILLDR
Scan Number: 26320 precMass: 590.6892 precCharge: 3 Sequence: DIKPDNILLDR
y1
y2
b1
y3
b2
y4
y5
y6
b3
y7
y8
Intensity
200000
150000
100000
50000
0
500
1000
1500
M/Z
type
b
y

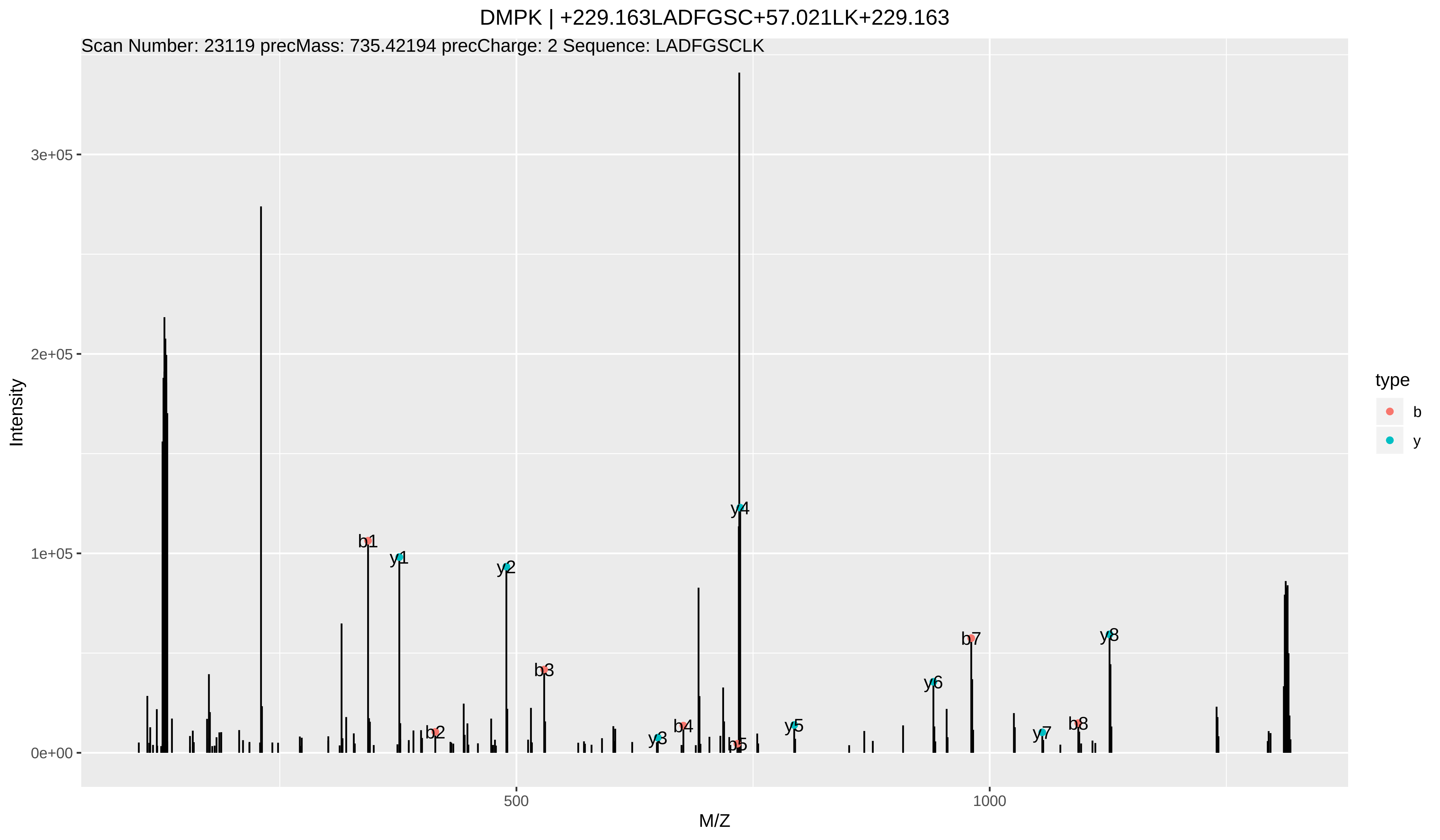
DMPK | +229.163LADFGSC+57.021LK+229.163
Scan Number: 23119 precMass: 735.42194 precCharge: 2 Sequence: LADFGSCLK
Intensity
3e+05
2e+05
1e+05
0e+00
M/Z
500
1000
b1
y1
y2
b2
b3
y3
b4
b5
y4
y5
y6
b7
y7
b8
y8
type
b
y

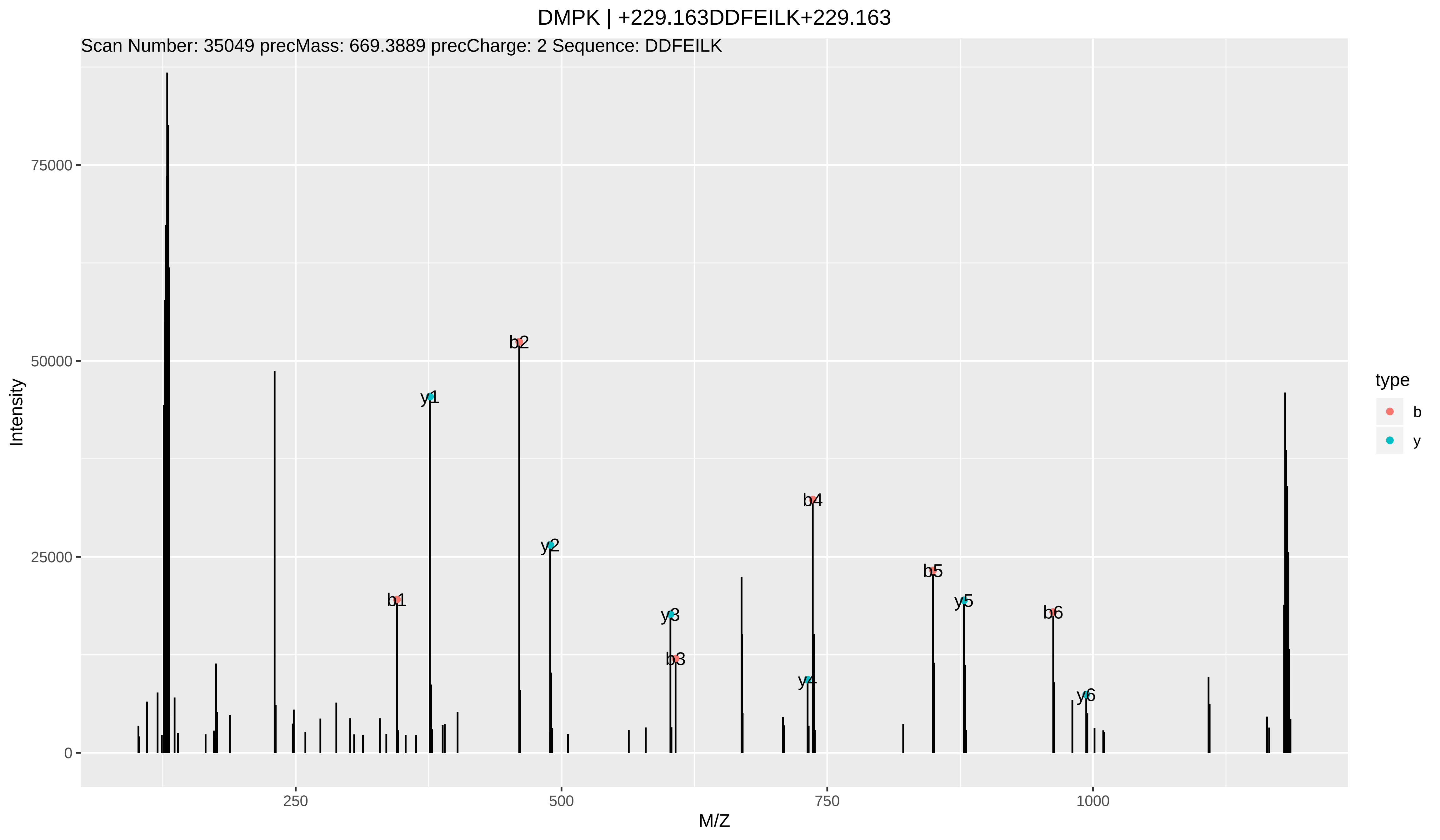

DMPK | +229.163DDFEILK+229.163
Scan Number: 35049 precMass: 669.3889 precCharge: 2 Sequence: DDFEILK
Intensity
M/Z
75000
50000
25000
0
250
500
750
1000
b1
b2
b3
b4
b5
b6
y1
y2
y3
y4
y5
y6
type
b
y

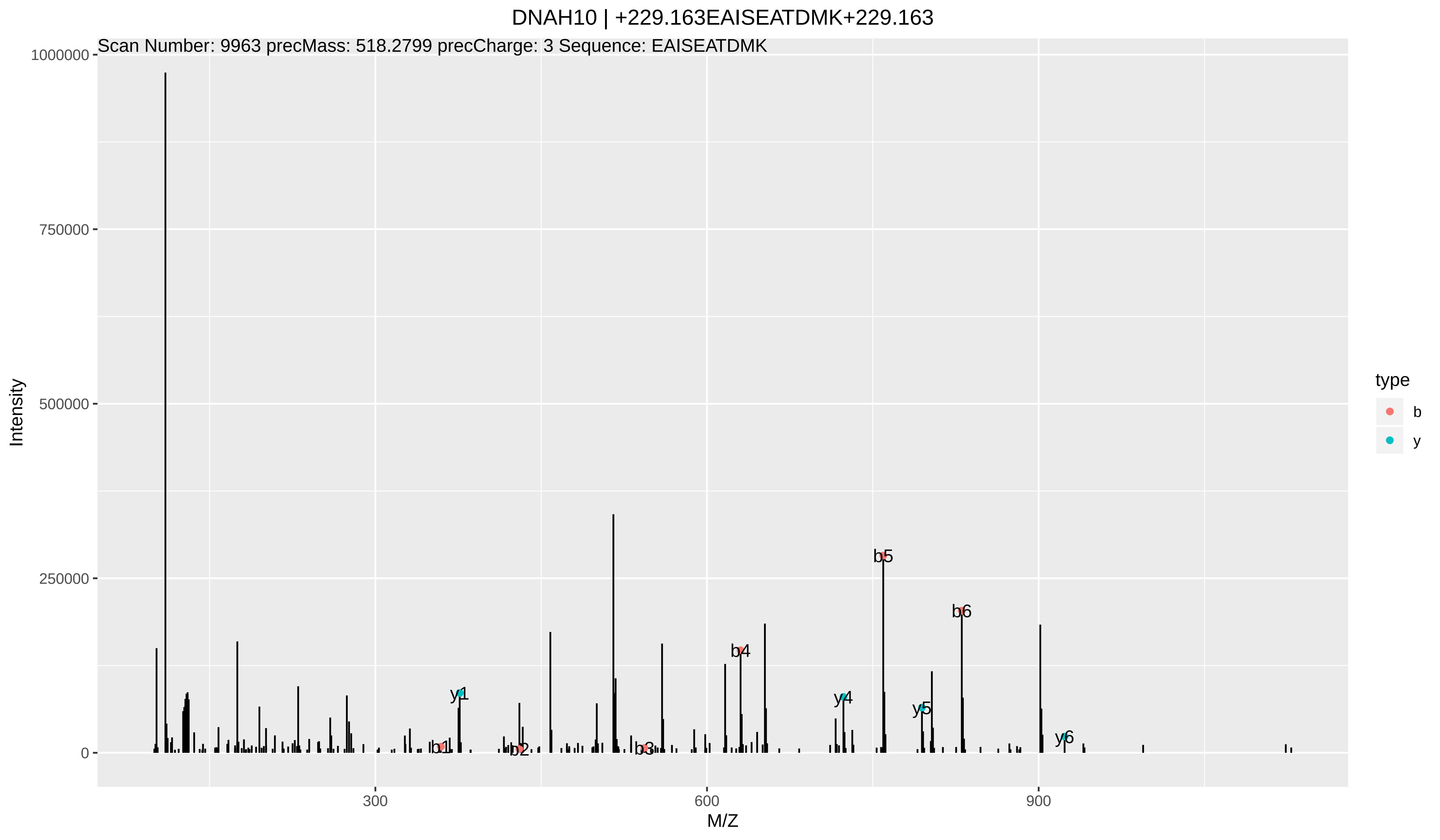
DNAH10 | +229.163EAISEATDMK+229.163
Scan Number: 9963 precMass: 518.2799 precCharge: 3 Sequence: EAISEATDMK
Intensity
1000000
750000
500000
250000
0
M/Z
300
600
900
b1
b2
b3
b4
b5
b6
y1
y4
y5
y6
type
b
y

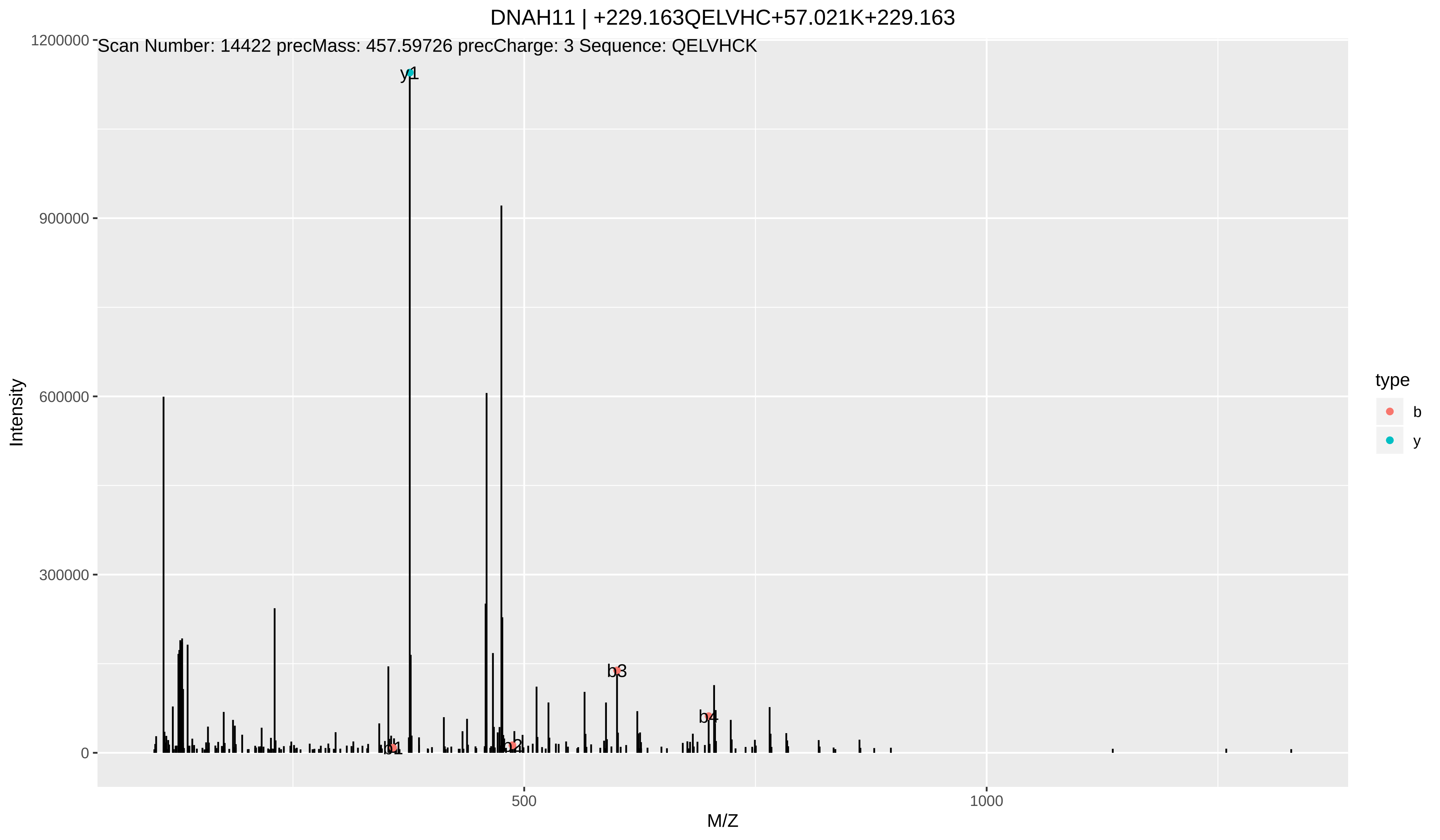

DNAH11 | +229.163QELVHC+57.021K+229.163
Scan Number: 14422 precMass: 457.59726 precCharge: 3 Sequence: QELVHCK
Intensity
1200000
900000
600000
300000
0
M/Z
500
1000
y1
b1
b2
b3
b4
type
b
y

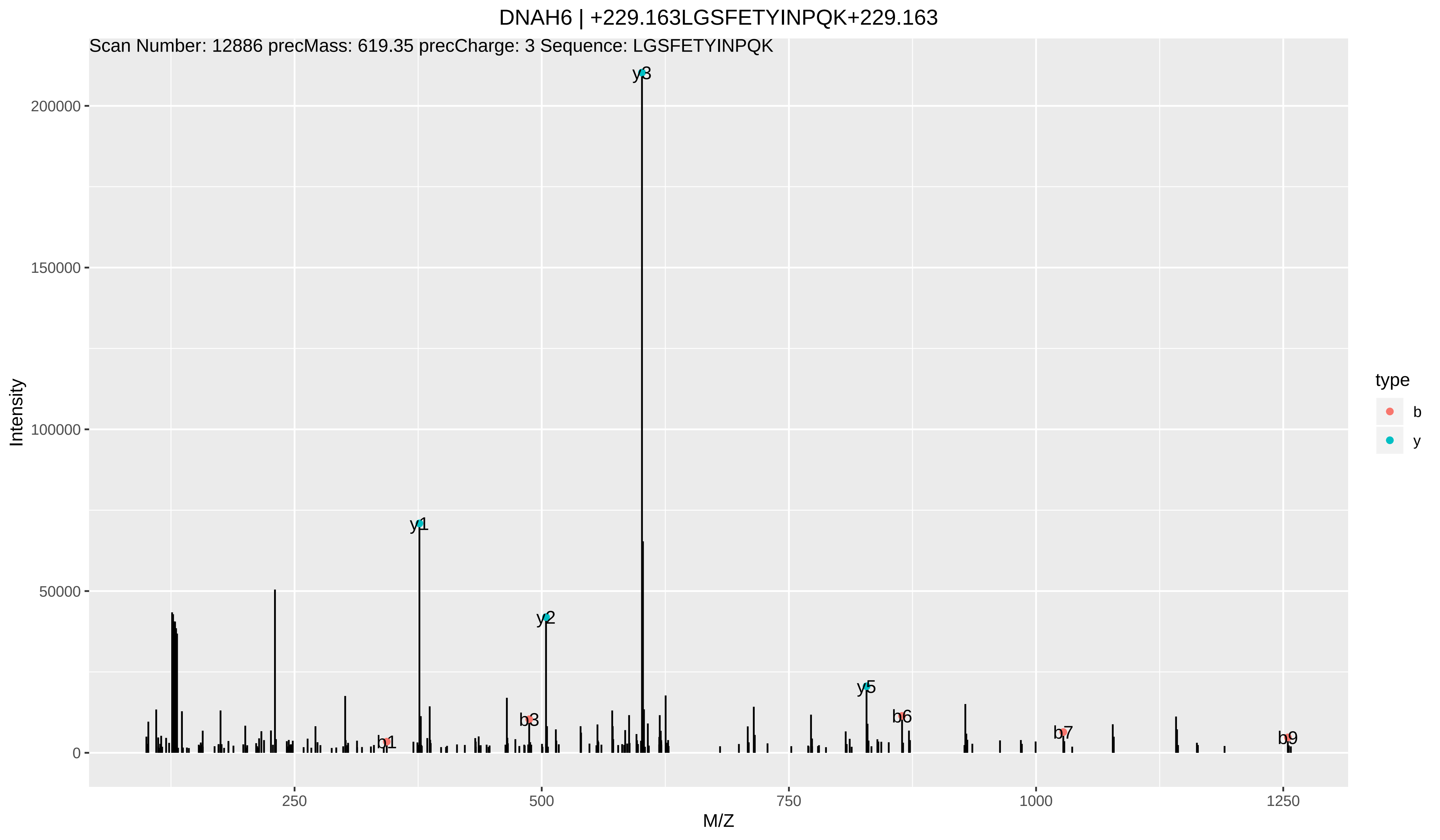

DNAH6 | +229.163LGSFETYINPQK+229.163
Scan Number: 12886 precMass: 619.35 precCharge: 3 Sequence: LGSFETYINPQK
Intensity
0
50000
100000
150000
200000
M/Z
250
500
750
1000
1250
y3
y1
y2
y5
b1
b3
b6
b7
b9
type
b
y

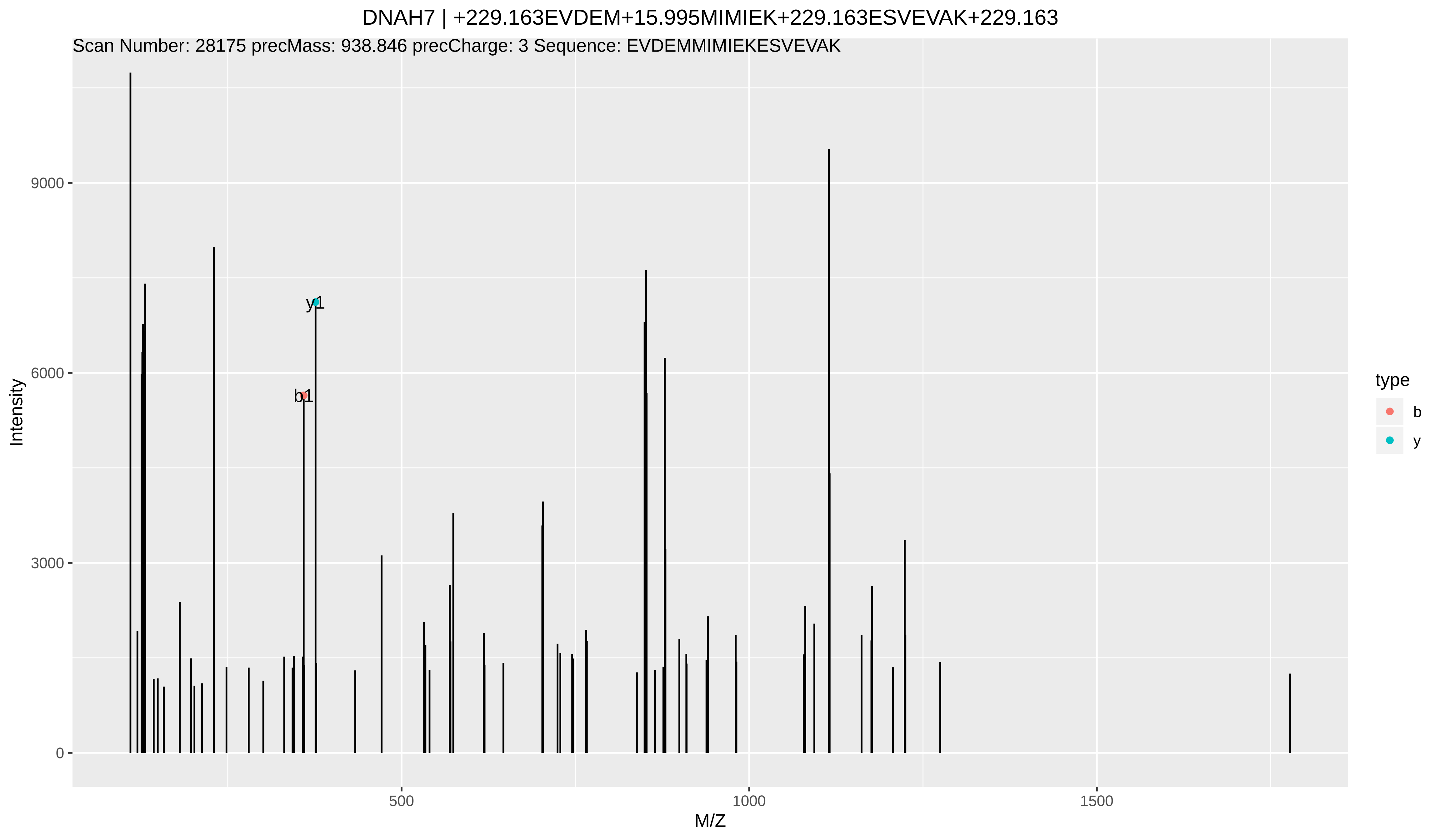

DNAH7 | +229.163EVDEM+15.995MIMIEK+229.163ESVEVAK+229.163
Scan Number: 28175 precMass: 938.846 precCharge: 3 Sequence: EVDEMMIMIEKESVEVAK
y1
b1
Intensity
M/Z
type
b
y

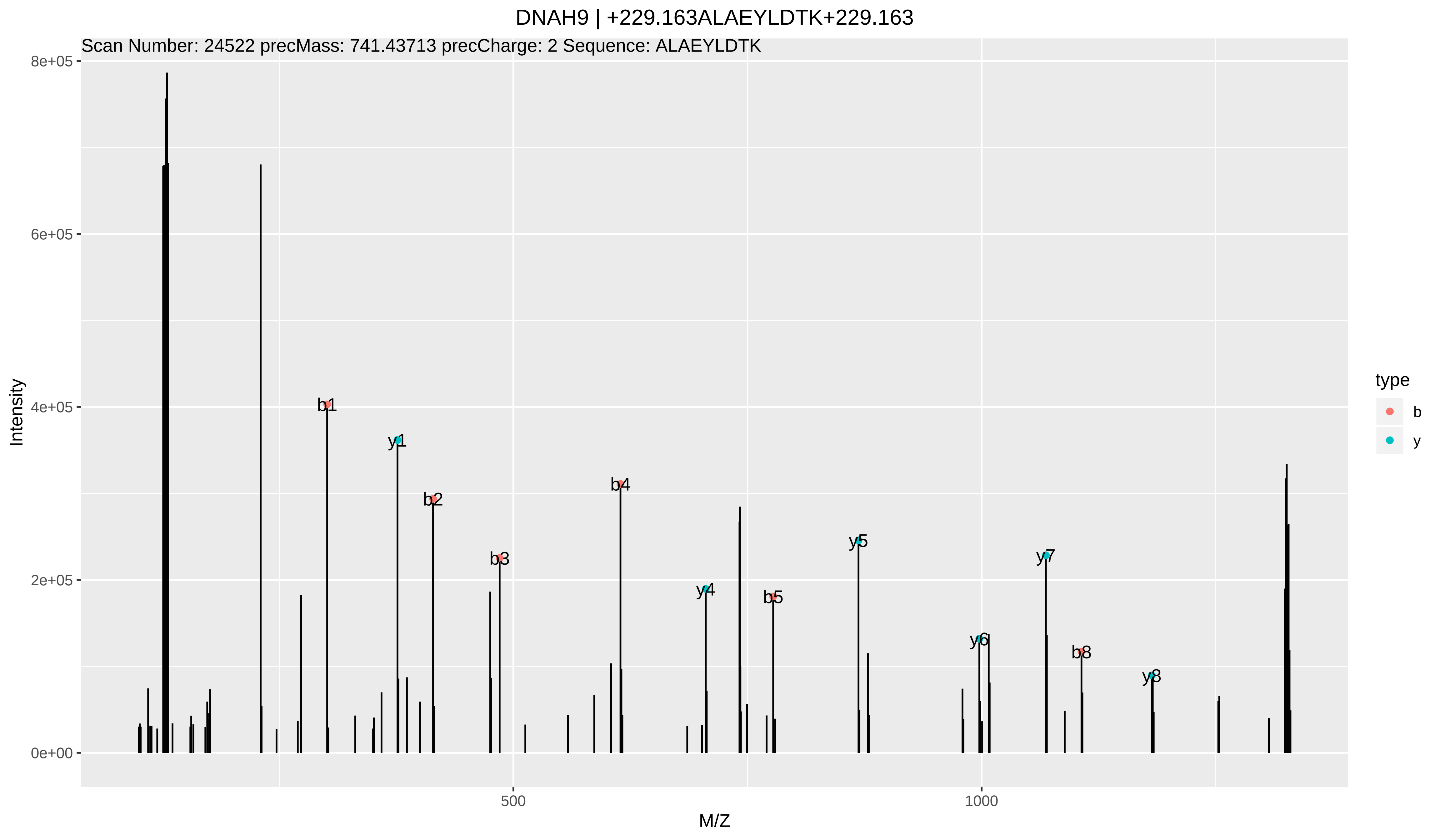
DNAH9 | +229.163ALAEYLDTK+229.163
Scan Number: 24522 precMass: 741.43713 precCharge: 2 Sequence: ALAEYLDTK
Intensity
M/Z
8e+05
6e+05
4e+05
2e+05
0e+00
500
1000
b1
y1
b2
b3
b4
y4
b5
y5
y6
y7
b8
y8
type
b
y

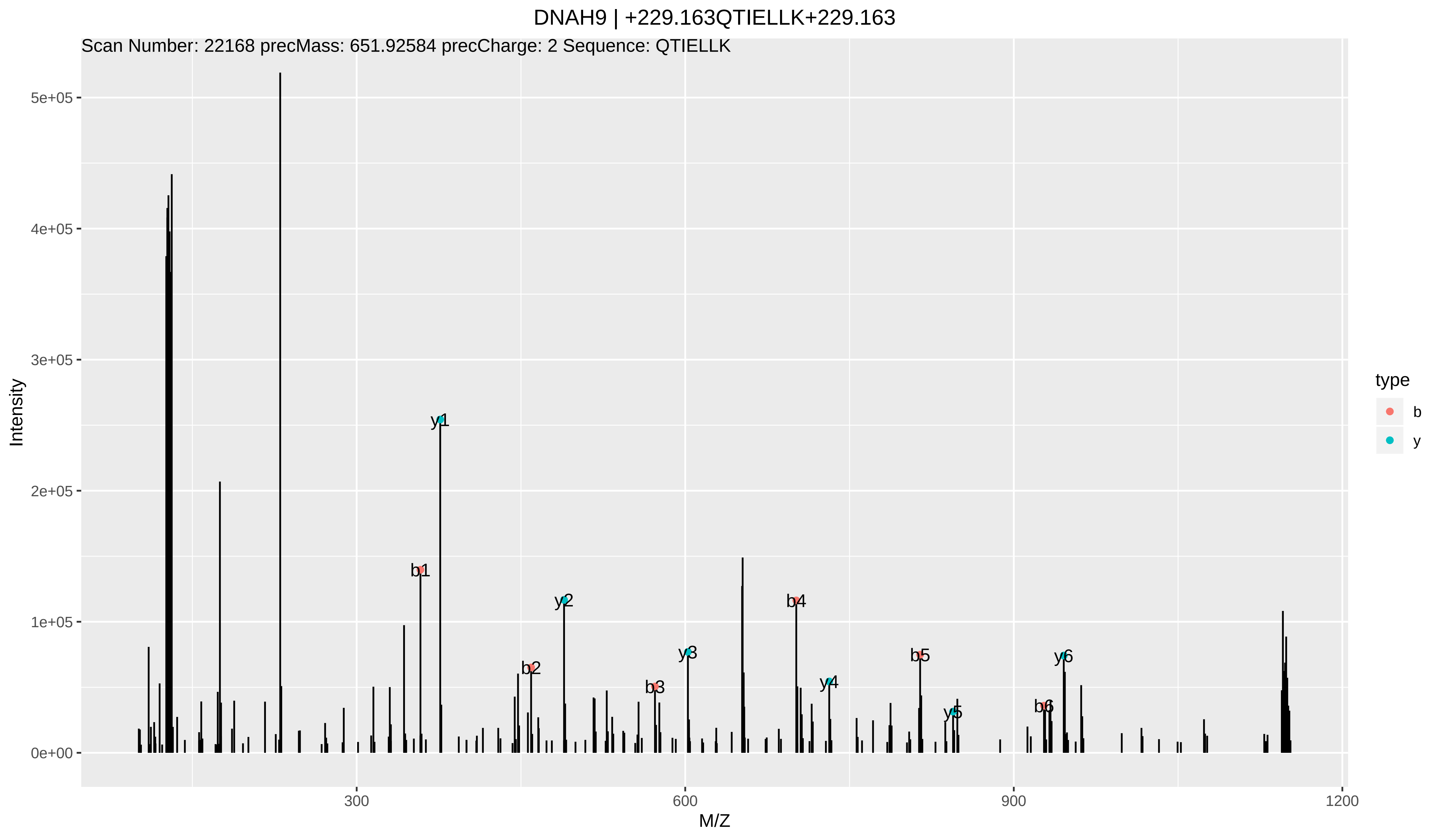
DNAH9 | +229.163QTIELLK+229.163
Scan Number: 22168 precMass: 651.92584 precCharge: 2 Sequence: QTIELLK
Intensity
M/Z
0e+00
1e+05
2e+05
3e+05
4e+05
5e+05
300
600
900
1200
y1
b1
y2
b2
b3
y3
b4
y4
b5
y5
b6
y6
type
b
y

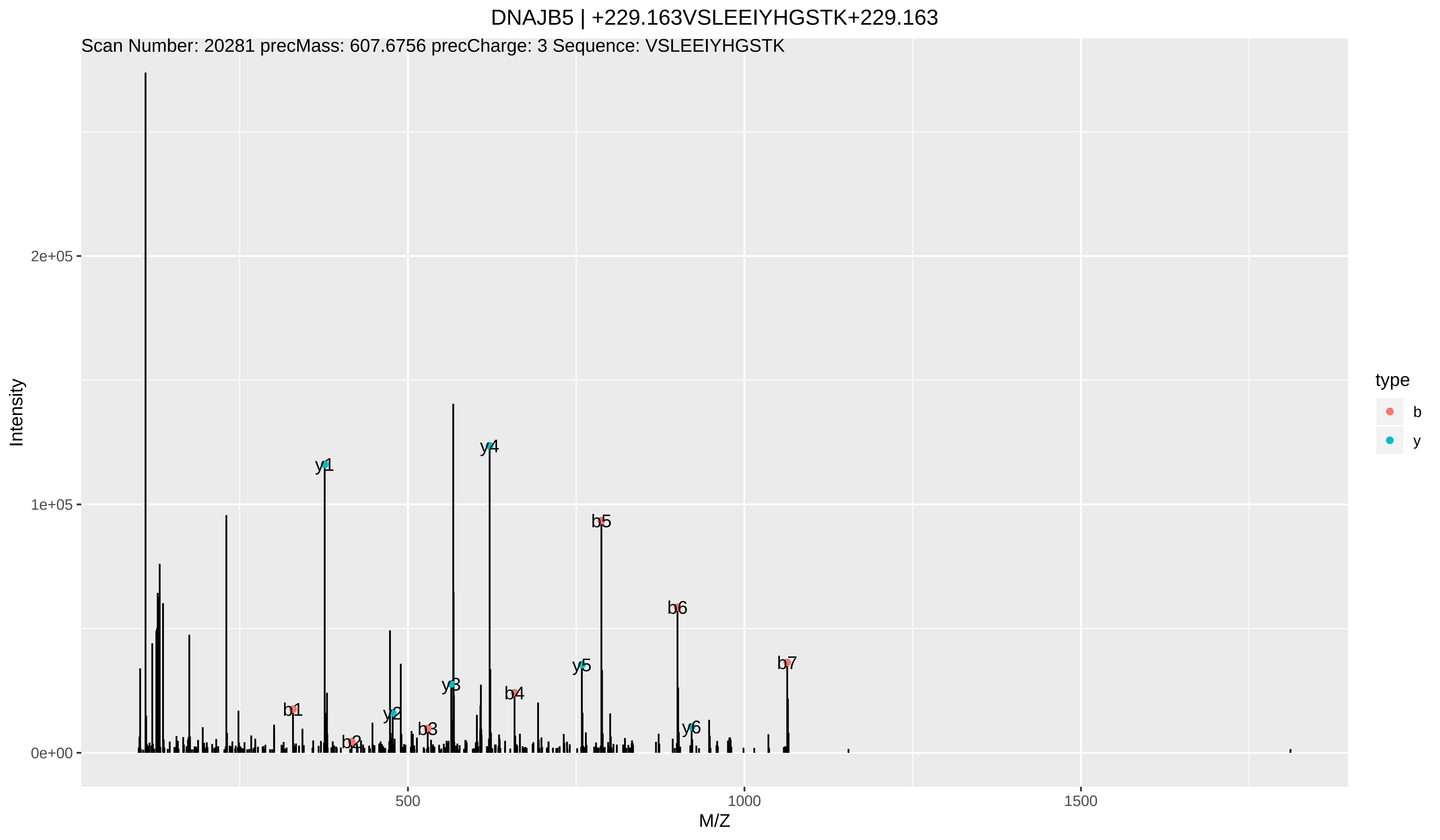
DNAJB5 | +229.163VSLEEIYHGSTK+229.163
Scan Number: 20281 precMass: 607.6756 precCharge: 3 Sequence: VSLEEIYHGSTK
Intensity
2e+05
1e+05
0e+00
M/Z
500
1000
1500
y1
y4
b5
b6
y5
b7
y3
b4
b1
y2
b3
y6
b2
type
b
y

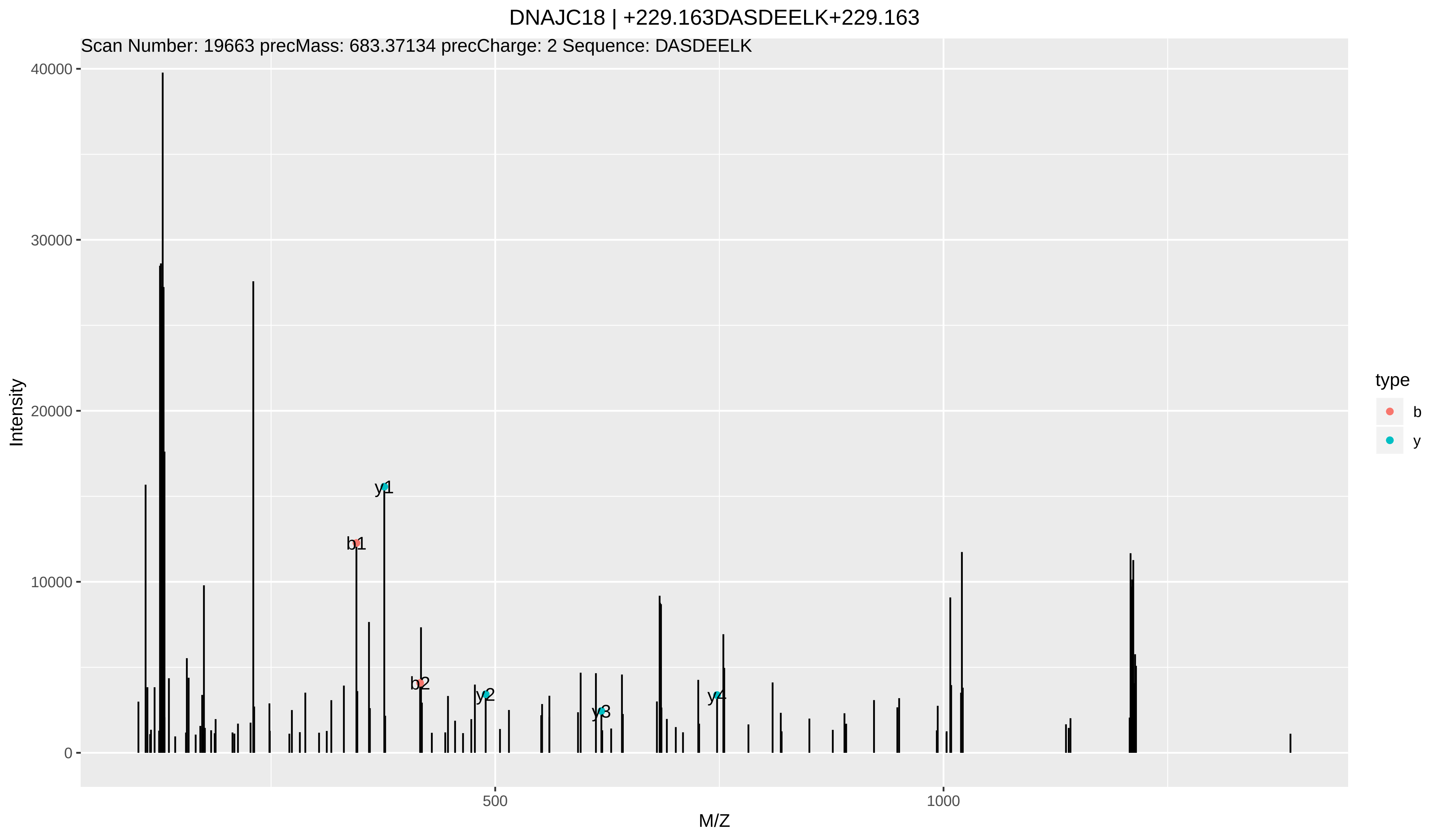

DNAJC18 | +229.163DASDEELK+229.163
Scan Number: 19663 precMass: 683.37134 precCharge: 2 Sequence: DASDEELK
Intensity
40000
30000
20000
10000
0
500
1000
M/Z
y1
b1
b2
y2
y3
y4
type
b
y

DNAL4 | +229.163FSNNNESAAK+229.163

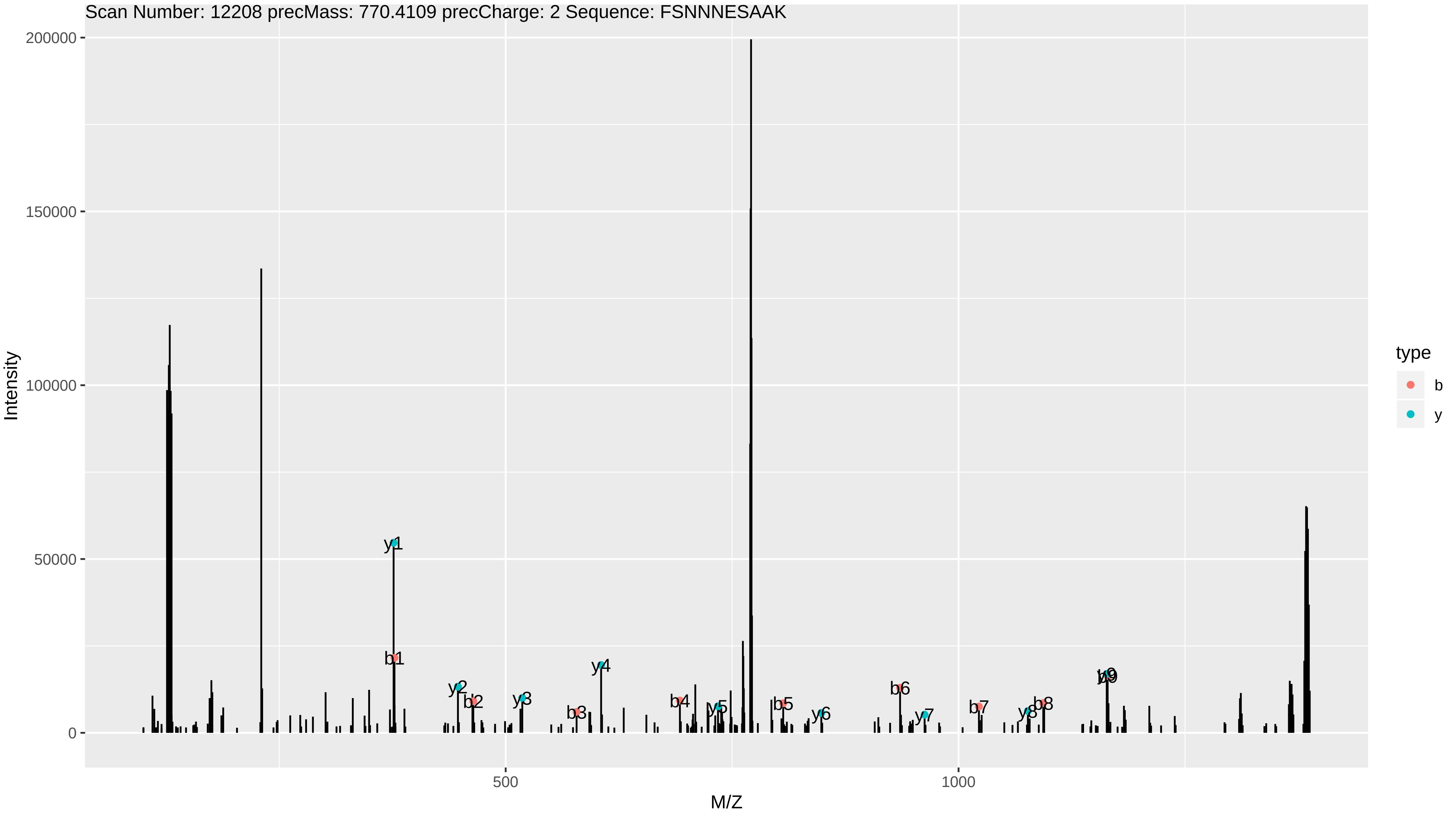

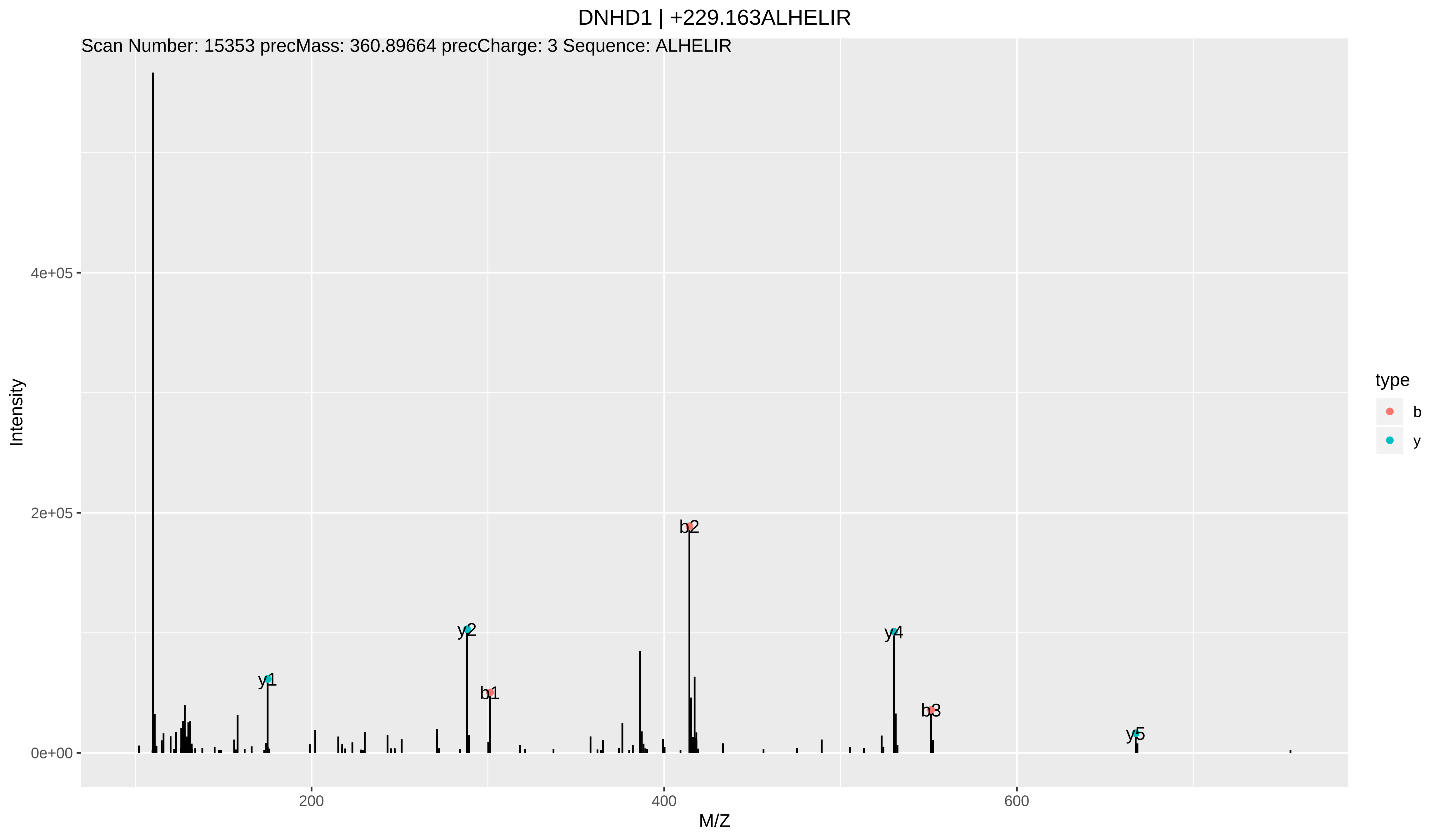
DNHD1 | +229.163ALHELIR
Scan Number: 15353 precMass: 360.89664 precCharge: 3 Sequence: ALHELIR
Intensity
4e+05
2e+05
0e+00
200
400
600
M/Z
y1
y2
b1
b2
y4
b3
y5
type
b
y

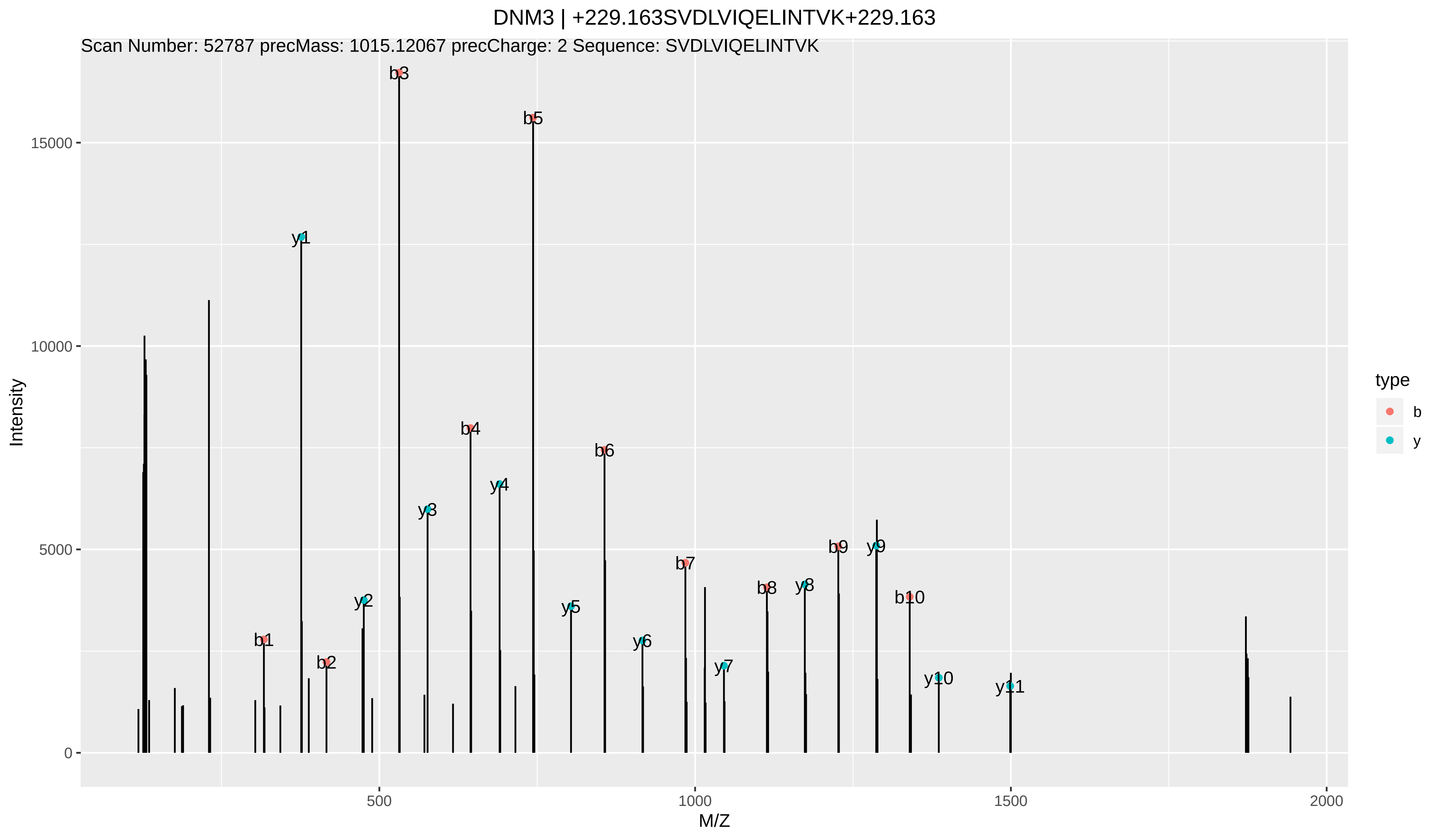
DNM3 | +229.163SVDLVIQELINTVK+229.163
Scan Number: 52787 precMass: 1015.12067 precCharge: 2 Sequence: SVDLVIQELINTVK
Intensity
M/Z
type
b
y
b1
b2
b3
b4
b5
b6
b7
b8
b9
b10
y1
y2
y3
y4
y5
y6
y7
y8
y9
y10
y11
0
5000
10000
15000
500
1000
1500
2000

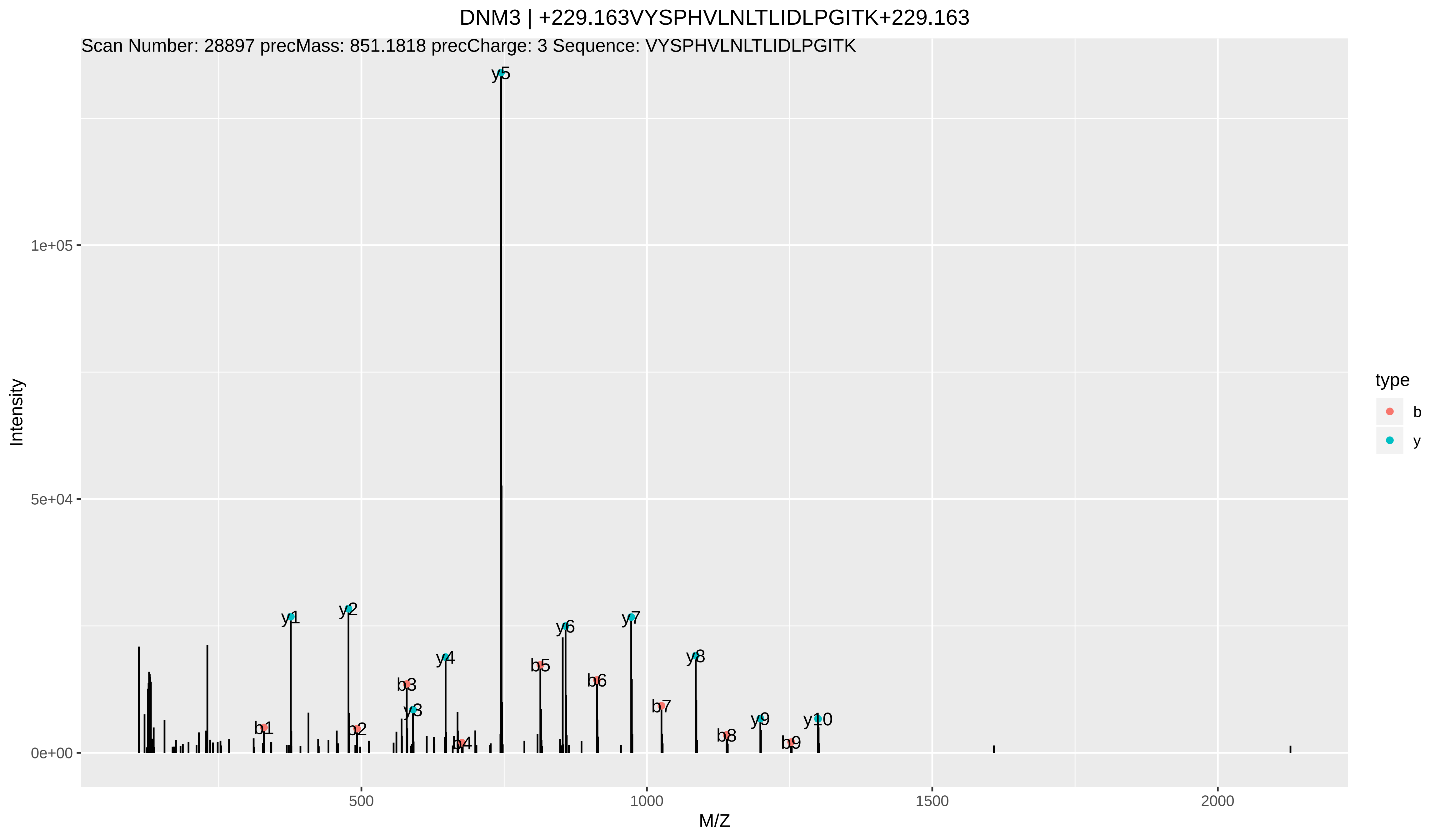

DNM3 | +229.163VYSPHVLNLTLIDLPGITK+229.163
Scan Number: 28897 precMass: 851.1818 precCharge: 3 Sequence: VYSPHVLNLTLIDLPGITK
y5
y1
y2
y6
y7
y8
y4
b5
b6
b3
y3
b7
b1
b2
y9
y10
b8
b4
b9
1e+05
5e+04
0e+00
500
1000
1500
2000
Intensity
M/Z
type
b
y

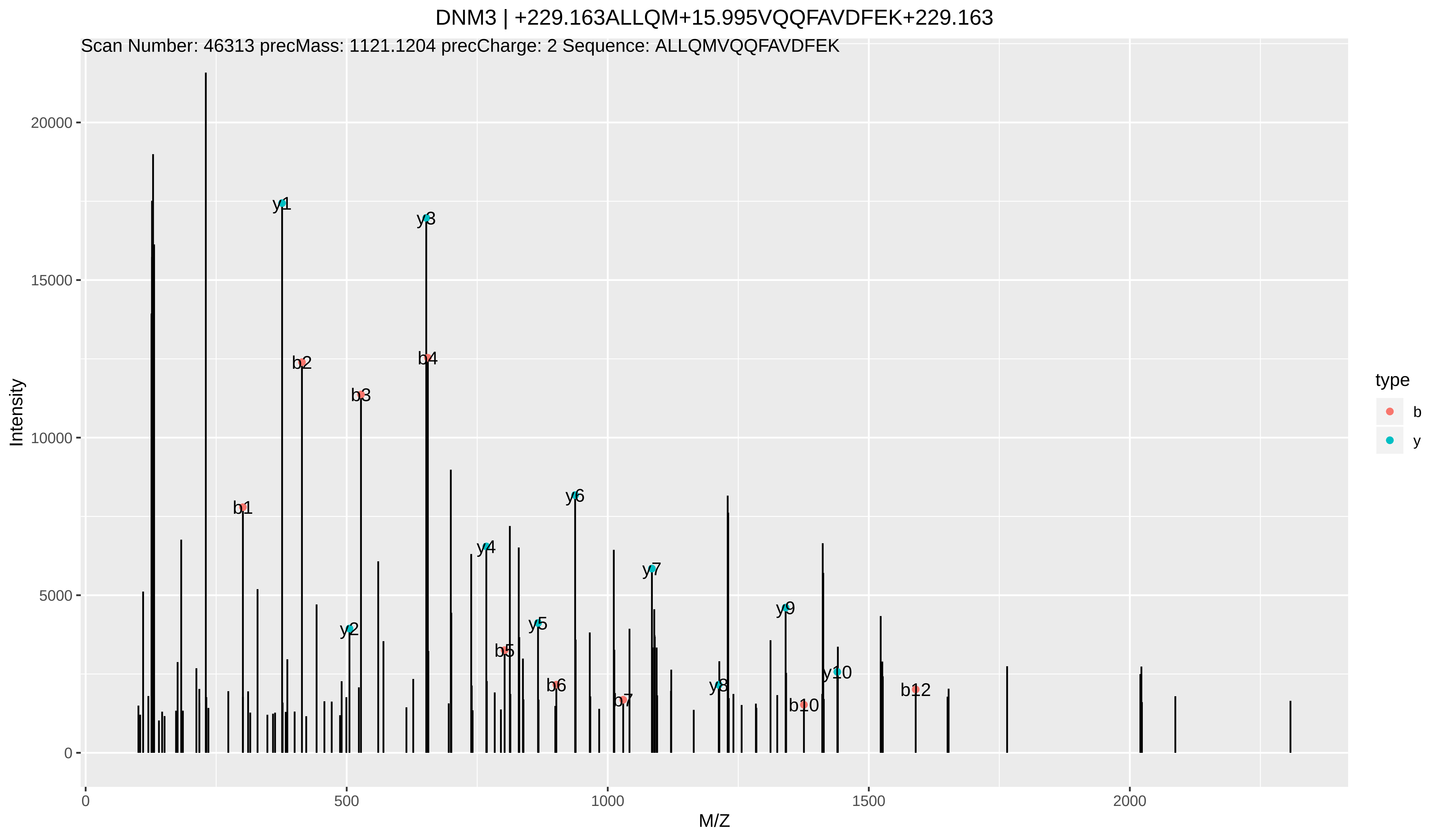
DNM3 | +229.163ALLQM+15.995VQQFAVDFEK+229.163
Scan Number: 46313 precMass: 1121.1204 precCharge: 2 Sequence: ALLQMVQQFAVDFEK
Intensity
M/Z
type
b
y
y1
y2
y3
y4
y5
y6
y7
y8
y9
y10
b1
b2
b3
b4
b5
b6
b7
b10
b12
0
5000
10000
15000
20000
500
1000
1500
2000

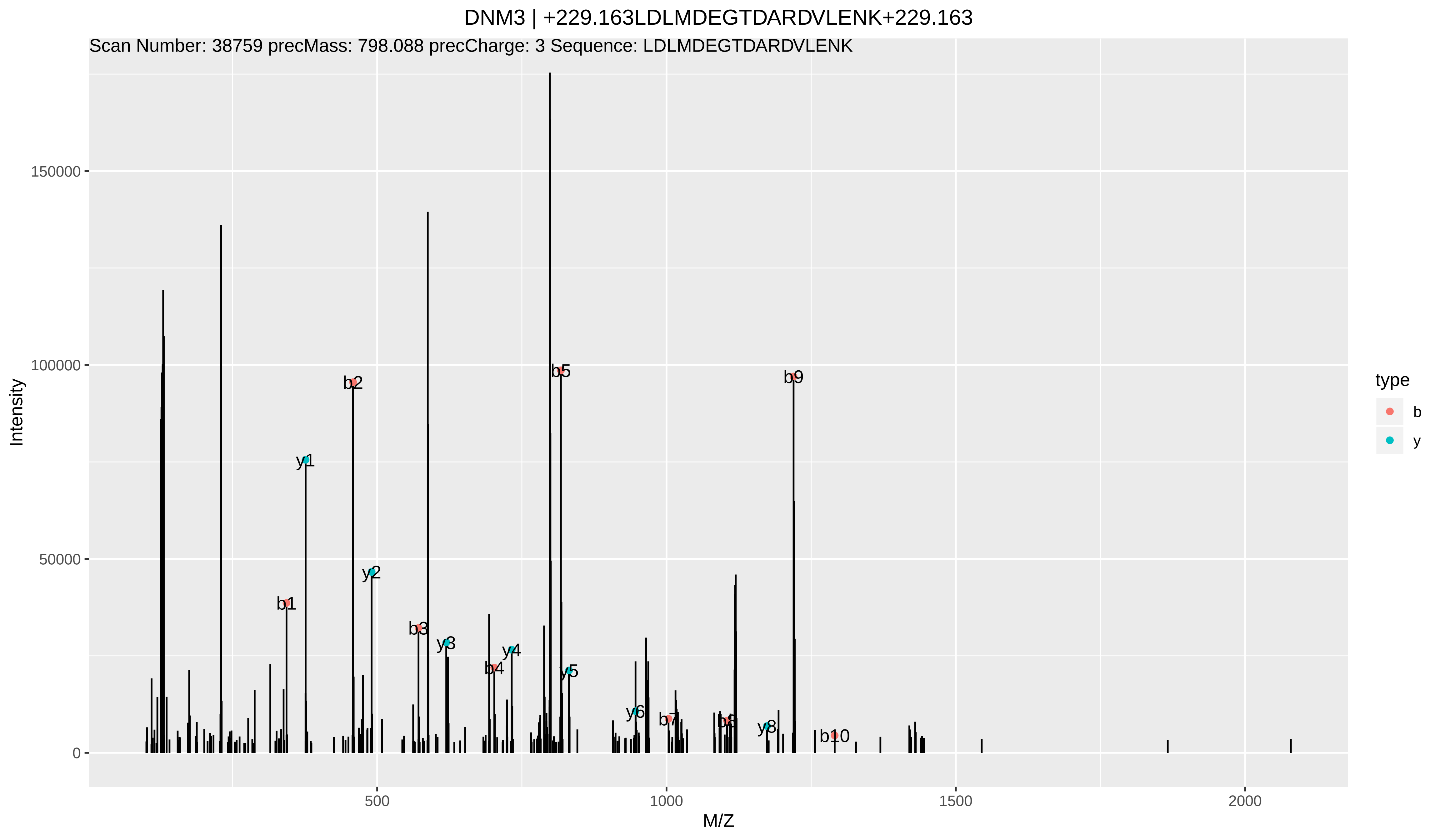
DNM3 | +229.163LDLMDEGTDARDVLENK+229.163
Scan Number: 38759 precMass: 798.088 precCharge: 3 Sequence: LDLMDEGTDARDVLENK
Intensity
150000
100000
50000
0
M/Z
500
1000
1500
2000
b1
b2
b3
b4
b5
b7
b8
b9
b10
y1
y2
y3
y4
y5
y6
y8
type
b
y

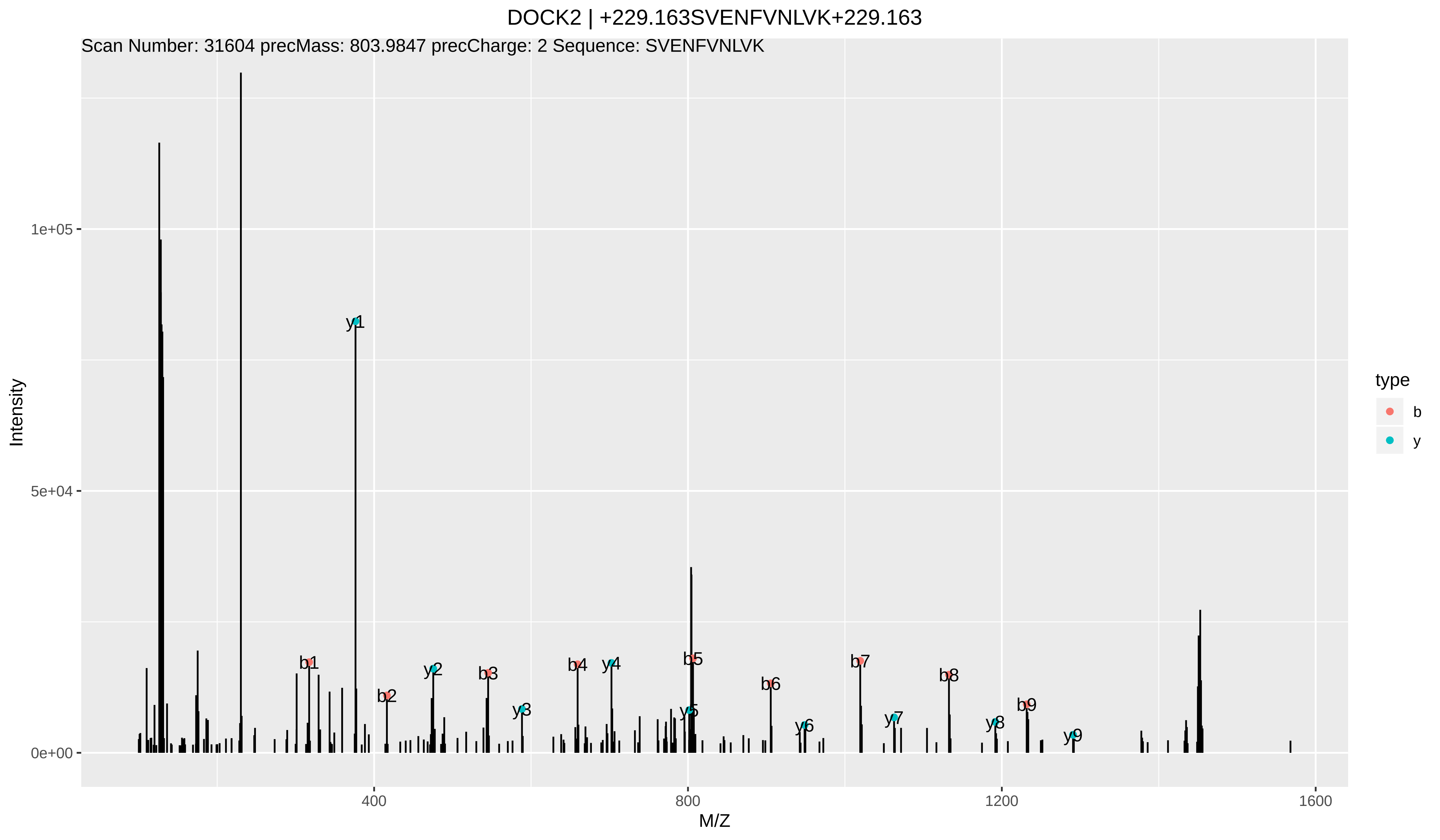
DOCK2 | +229.163SVENFVNLVK+229.163
Scan Number: 31604 precMass: 803.9847 precCharge: 2 Sequence: SVENFVNLVK
Intensity
1e+05
5e+04
0e+00
M/Z
400
800
1200
1600
y1
b1
b2
y2
b3
y3
b4
y4
b5
y5
b6
y6
b7
y7
b8
y8
b9
y9
type
b
y

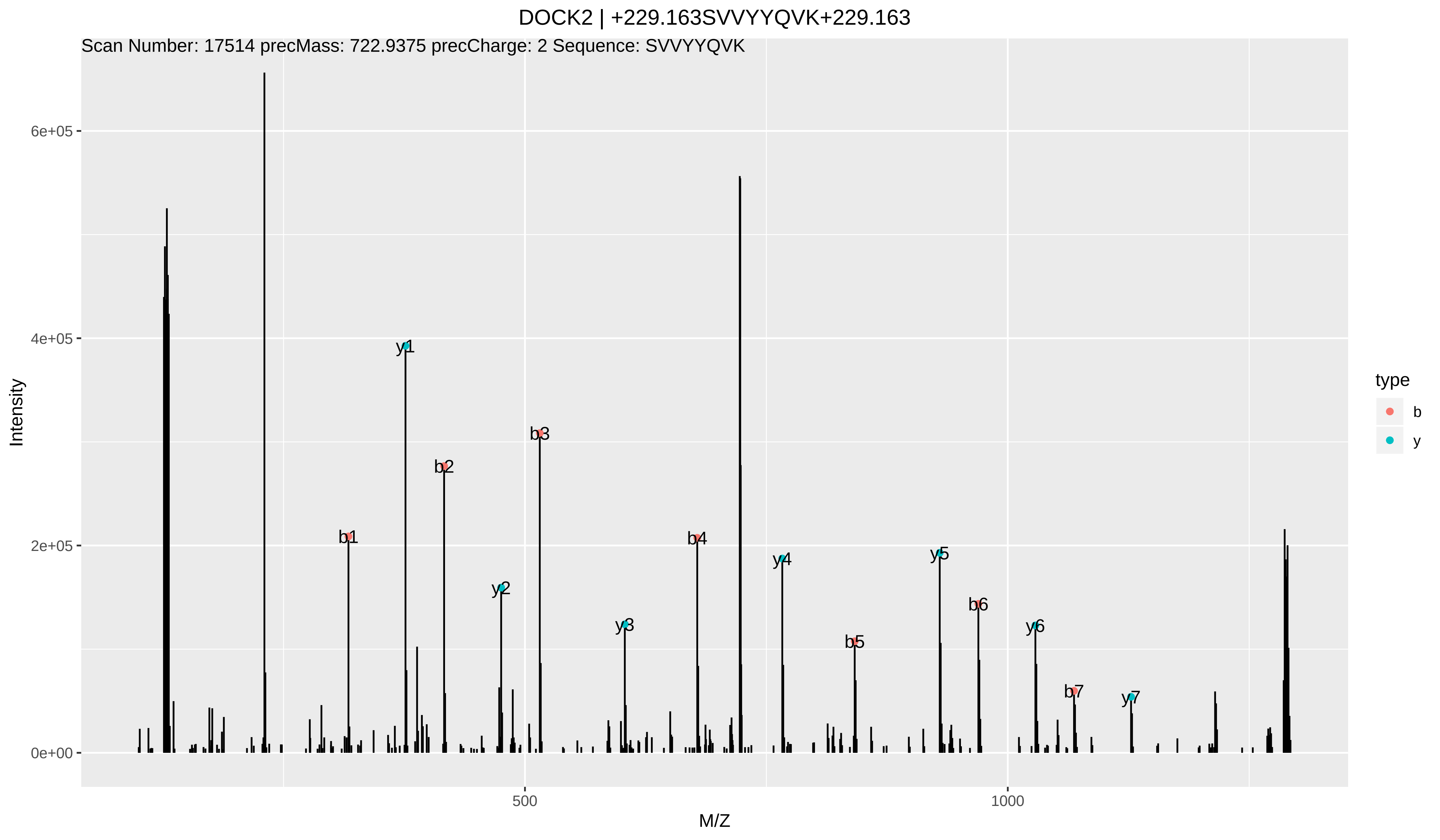
DOCK2 | +229.163SVVYYQVK+229.163
Scan Number: 17514 precMass: 722.9375 precCharge: 2 Sequence: SVVYYQVK
Intensity
M/Z
6e+05
4e+05
2e+05
0e+00
500
1000
y1
b1
b2
b3
y2
y3
b4
y4
b5
y5
b6
y6
b7
y7
type
b
y

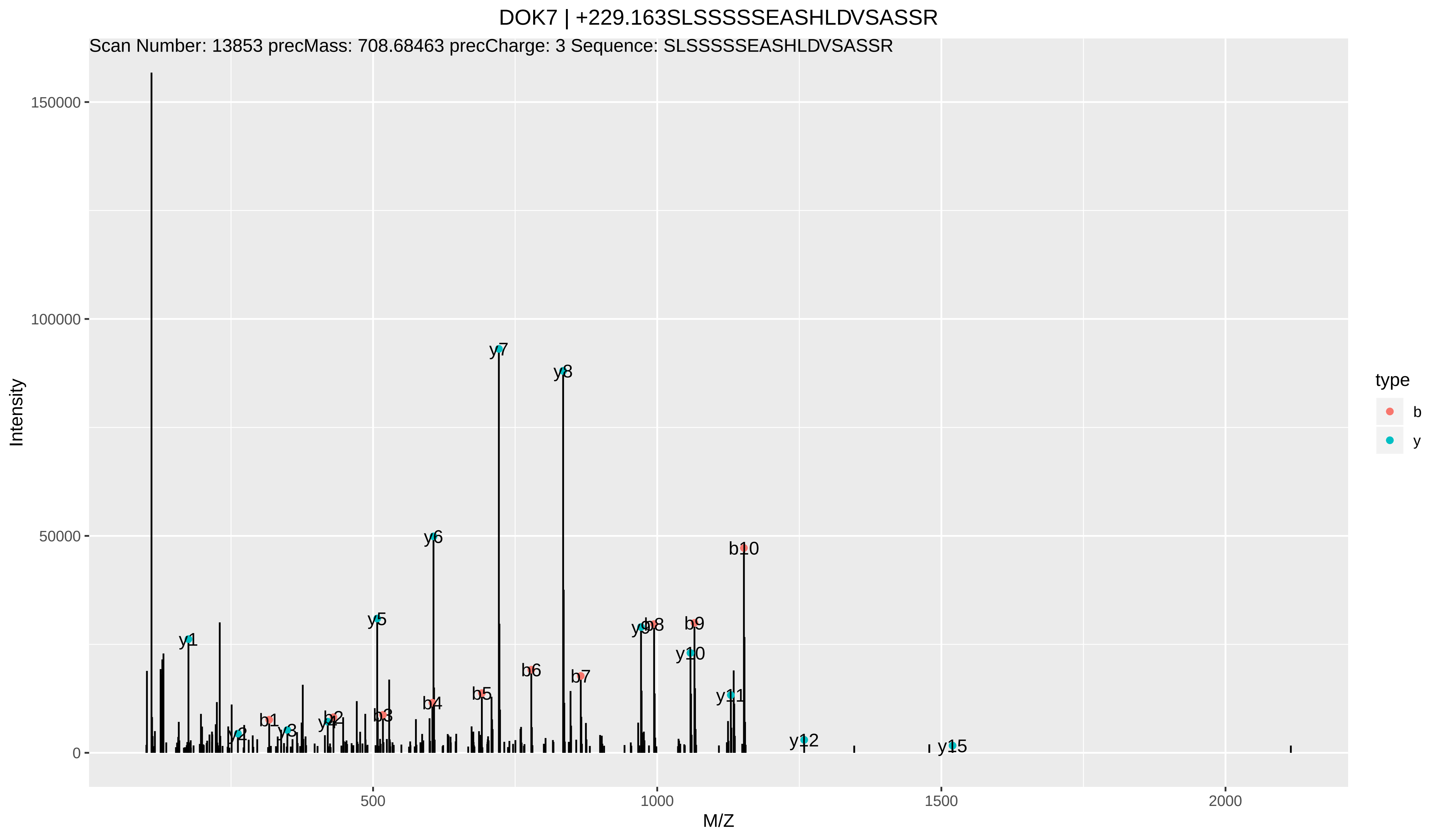

DOK7 | +229.163SLSSSSSEASHLDVSASSR
Scan Number: 13853 precMass: 708.68463 precCharge: 3 Sequence: SLSSSSSEASHLDVSASSR
Intensity
M/Z
150000
100000
50000
0
500
1000
1500
2000
type
b
y
y1
y2
y3
y4
y5
y6
y7
y8
y9
y10
y11
y12
y15
b1
b2
b3
b4
b5
b6
b7
b8
b9
b10

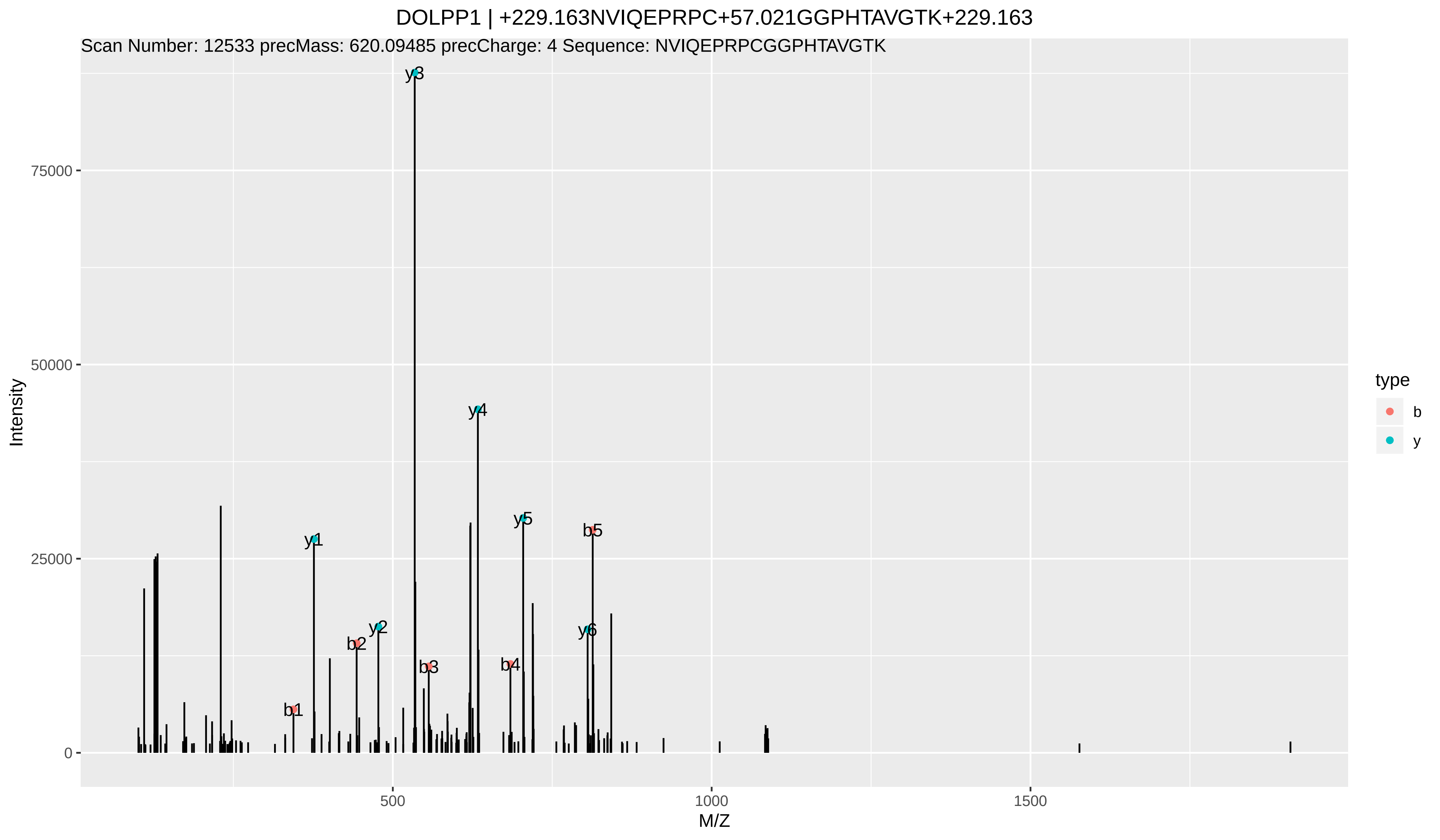
DOLPP1 | +229.163NVIQEPRPC+57.021GGPHTAVGTK+229.163
Scan Number: 12533 precMass: 620.09485 precCharge: 4 Sequence: NVIQEPRPCGGPHTAVGTK
y3
y4
y5
b5
y1
y2
b2
y6
b3
b4
b1
75000
50000
25000
0
Intensity
500
1000
1500
M/Z
type
b
y

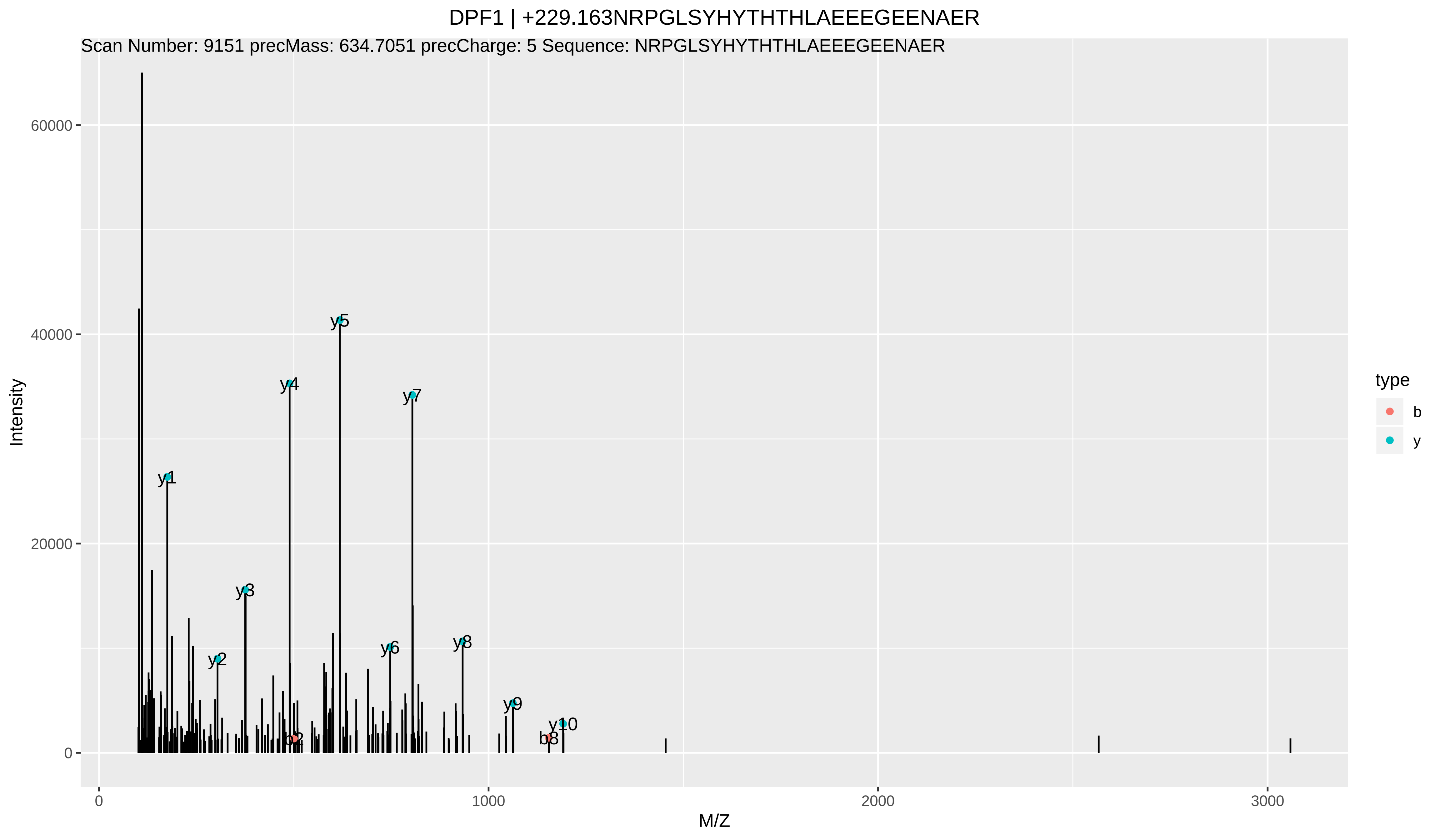
DPF1 | +229.163NRPGLSYHYTHTHLAEEEGEENAER
Scan Number: 9151 precMass: 634.7051 precCharge: 5 Sequence: NRPGLSYHYTHTHLAEEEGEENAER
Intensity
60000
40000
20000
0
0
1000
2000
3000
M/Z
y1
y2
y3
y4
y5
y6
y7
y8
y9
y10
b2
b8
type
b
y

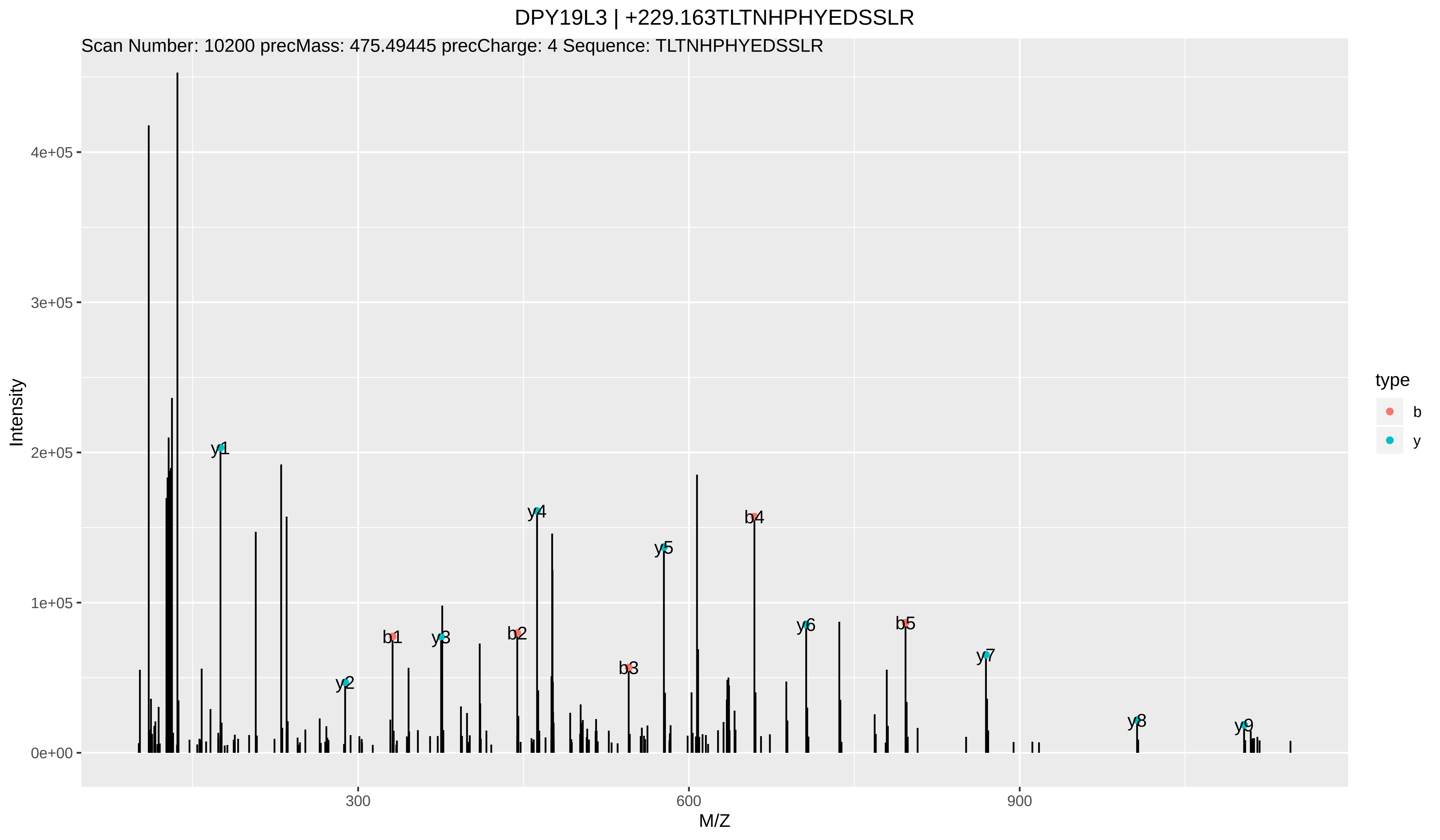
DPY19L3 | +229.163TLTNHPHYEDSSLR
Scan Number: 10200 precMass: 475.49445 precCharge: 4 Sequence: TLTNHPHYEDSSLR
Intensity
4e+05
3e+05
2e+05
1e+05
0e+00
M/Z
300
600
900
y1
y2
b1
y3
b2
y4
b3
y5
b4
y6
b5
y7
y8
y9
type
b
y

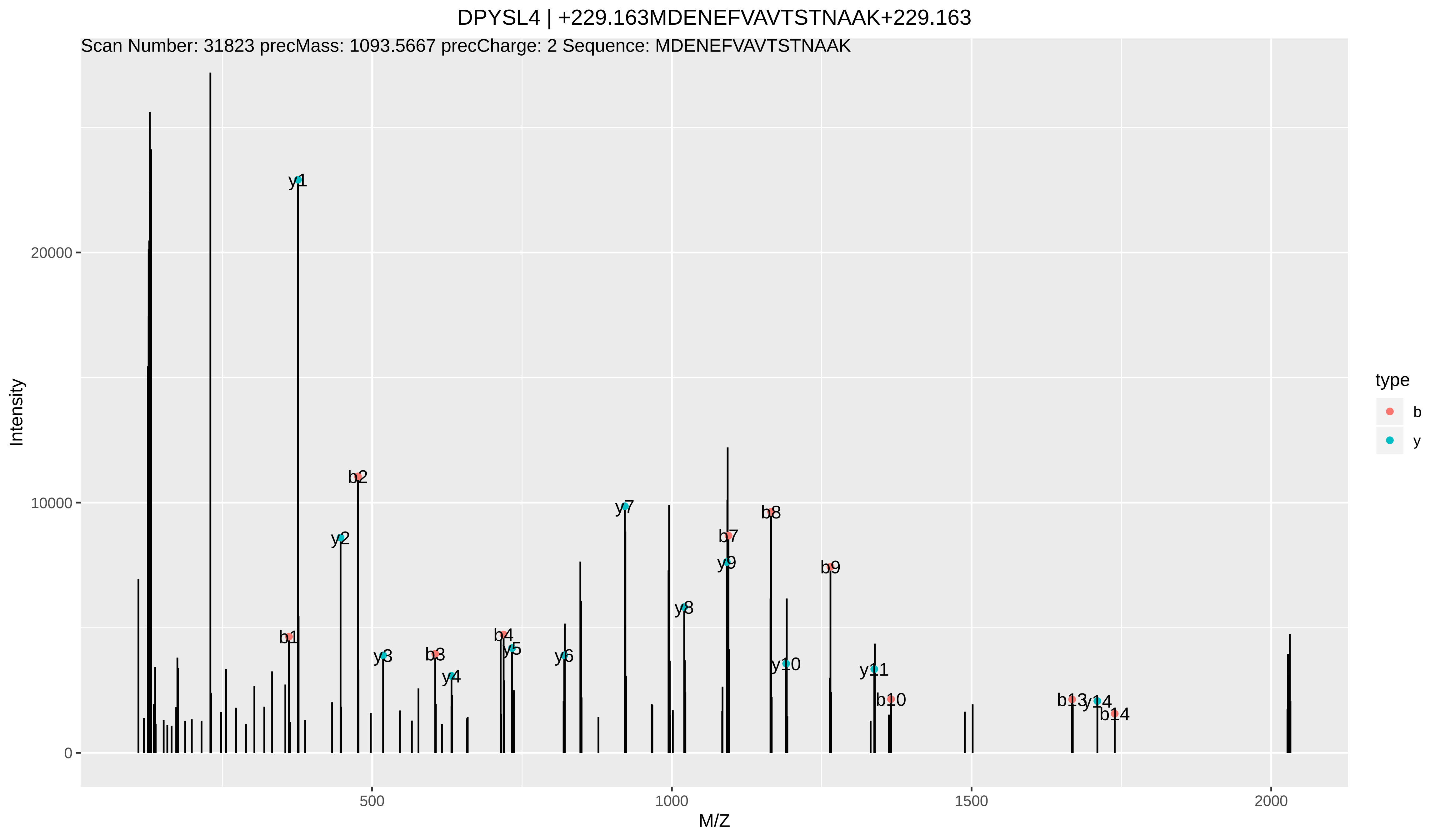
DPYSL4 | +229.163MDENEFVAVTSTNAAK+229.163
Scan Number: 31823 precMass: 1093.5667 precCharge: 2 Sequence: MDENEFVAVTSTNAAK
Intensity
M/Z
type
b
y
y1
b2
y2
y7
b8
b7
y9
b9
y8
b1
b4
y5
y3
b3
y6
y10
y11
y4
b10
b13
y14
b14
0
10000
20000
500
1000
1500
2000

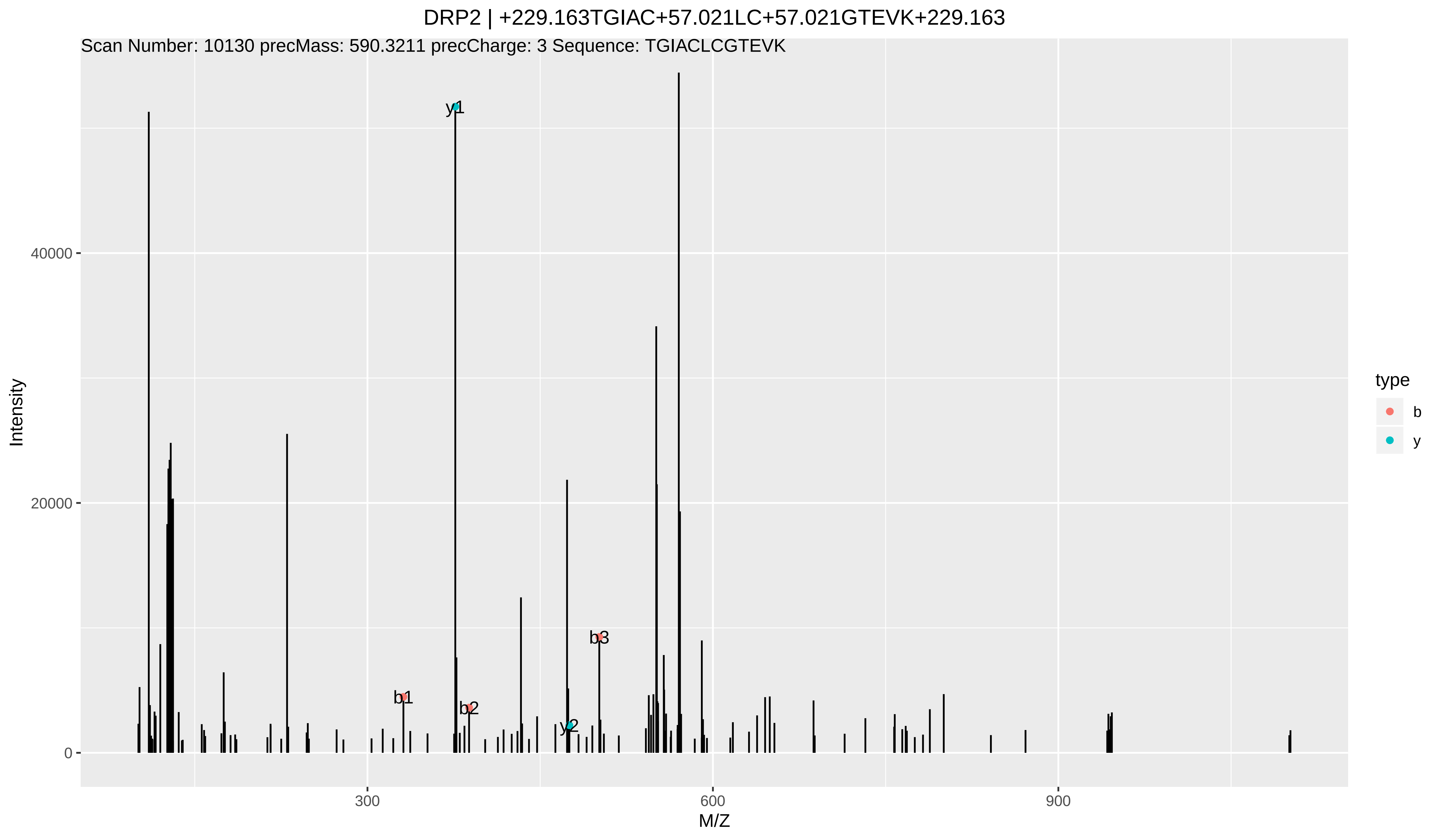

DRP2 | +229.163TGIAC+57.021LC+57.021GTEVK+229.163
Scan Number: 10130 precMass: 590.3211 precCharge: 3 Sequence: TGIACLCGTEVK
y1
b1
b2
y2
b3
Intensity
40000
20000
0
300
600
900
M/Z
type
b
y

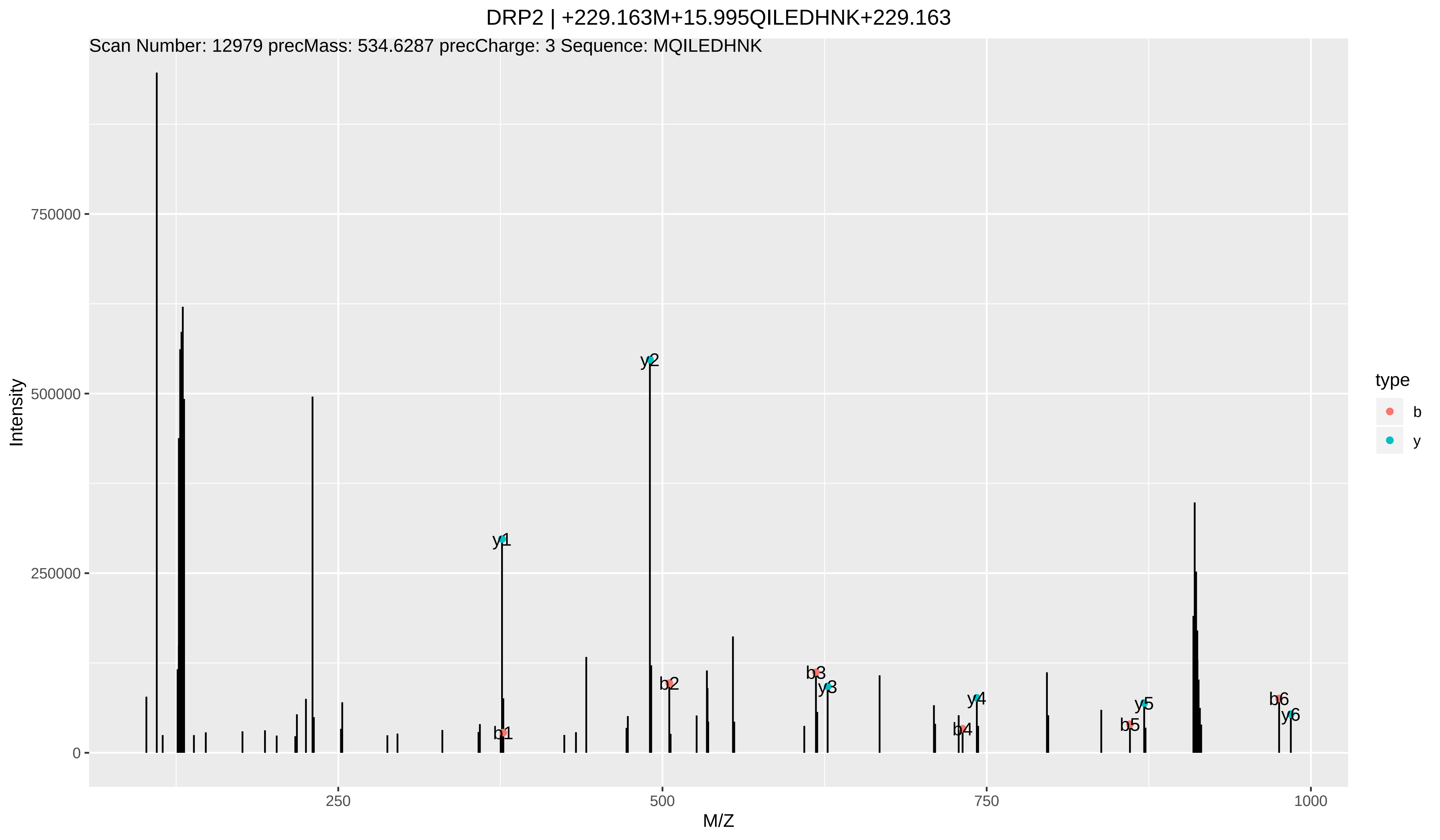

DRP2 | +229.163M+15.995QILEDHNK+229.163
Scan Number: 12979 precMass: 534.6287 precCharge: 3 Sequence: MQILEDHNK
Intensity
750000
500000
250000
0
250
500
750
1000
M/Z
y1
b1
y2
b2
b3
y3
y4
b4
y5
b5
b6
y6
type
b
y

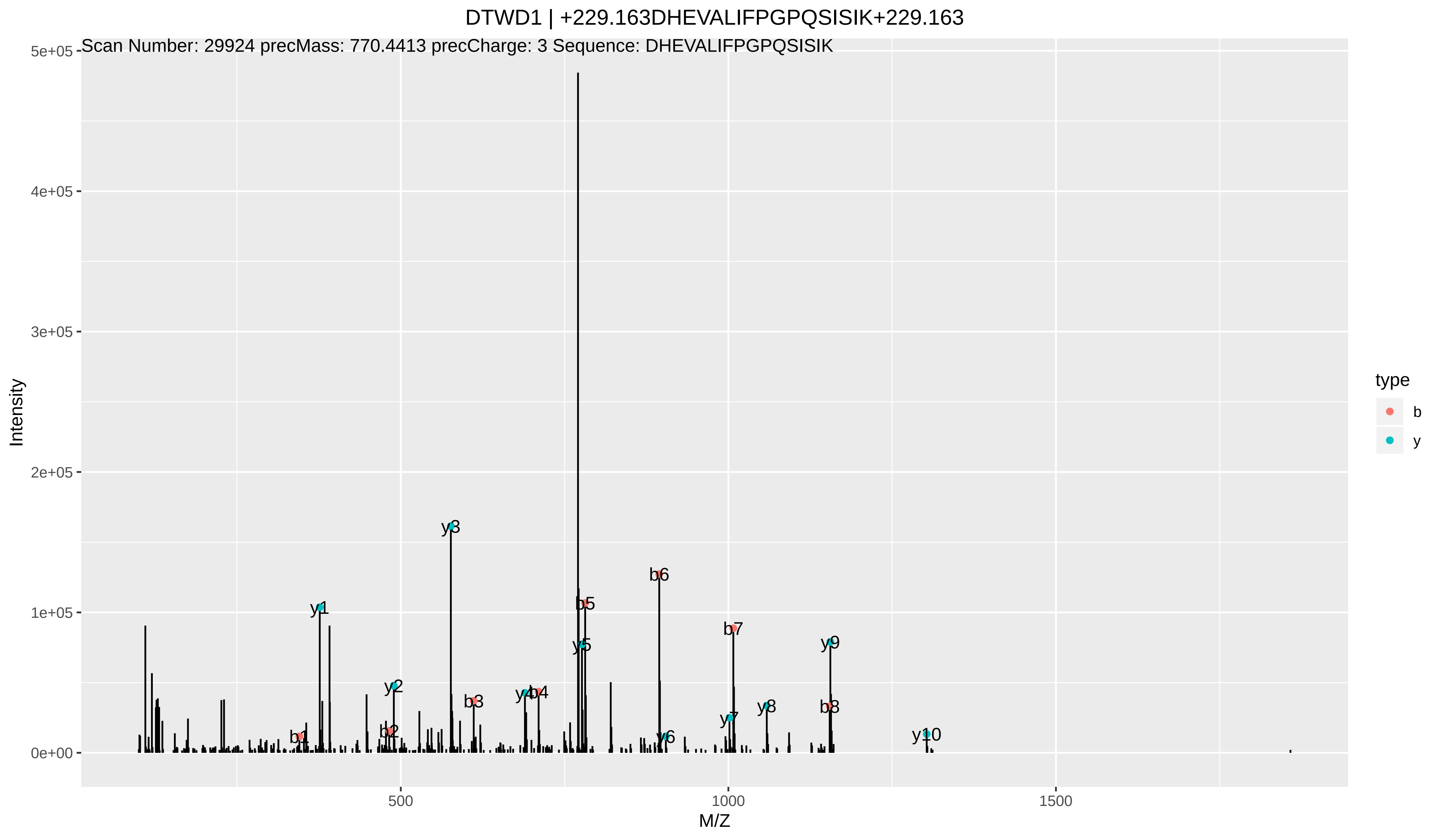

DTWD1 | +229.163DHEVALIFPGPQSISIK+229.163
Scan Number: 29924 precMass: 770.4413 precCharge: 3 Sequence: DHEVALIFPGPQSISIK
Intensity
M/Z
type
b
y
0e+00
1e+05
2e+05
3e+05
4e+05
5e+05
500
1000
1500
y1
y2
y3
y4
y5
y6
y7
y8
y9
y10
b1
b2
b3
b4
b5
b6
b7
b8

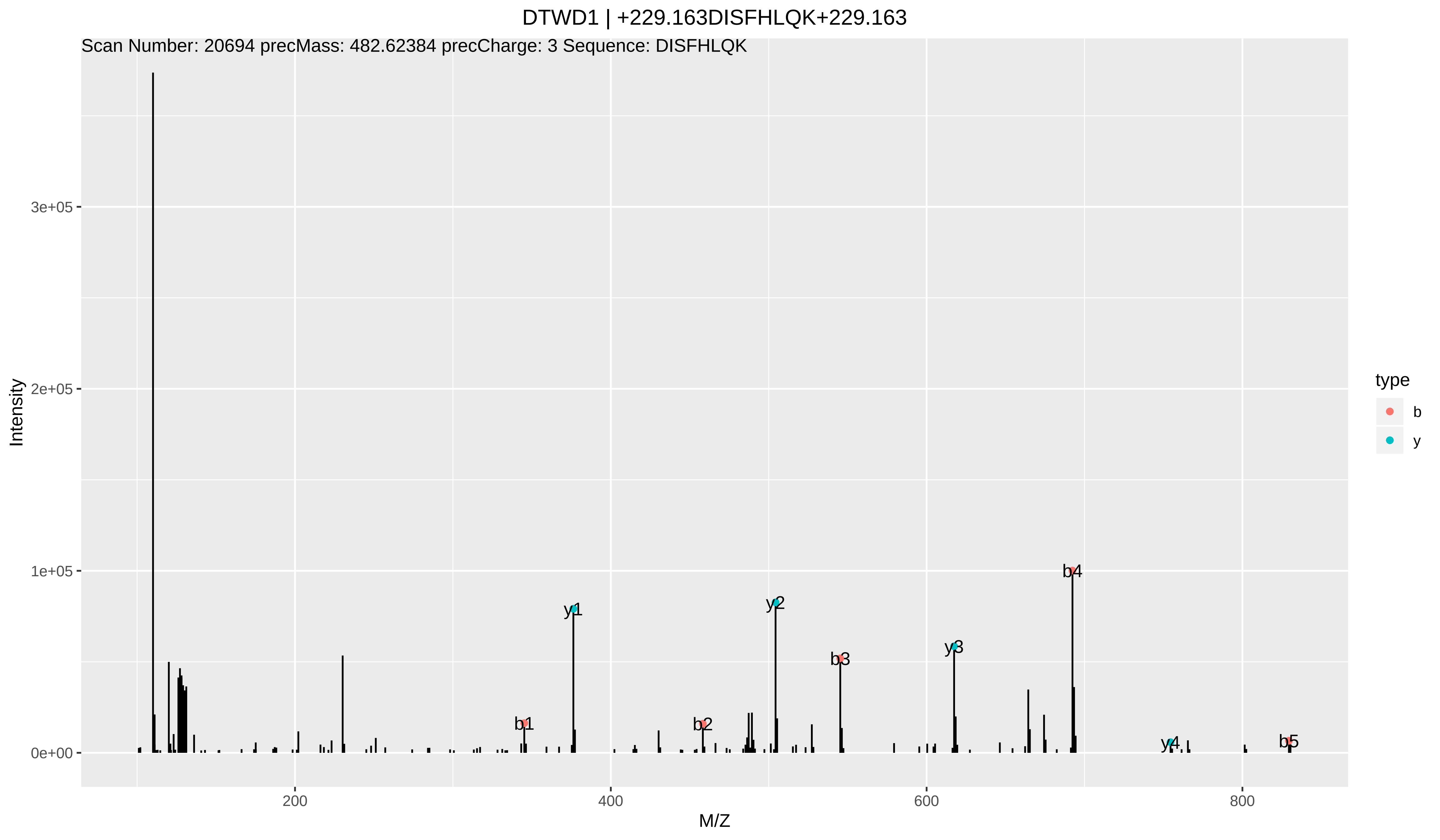
DTWD1 | +229.163DISFHLQK+229.163
Scan Number: 20694 precMass: 482.62384 precCharge: 3 Sequence: DISFHLQK
Intensity
3e+05
2e+05
1e+05
0e+00
M/Z
200
400
600
800
y1
y2
y3
y4
b1
b2
b3
b4
b5
type
b
y

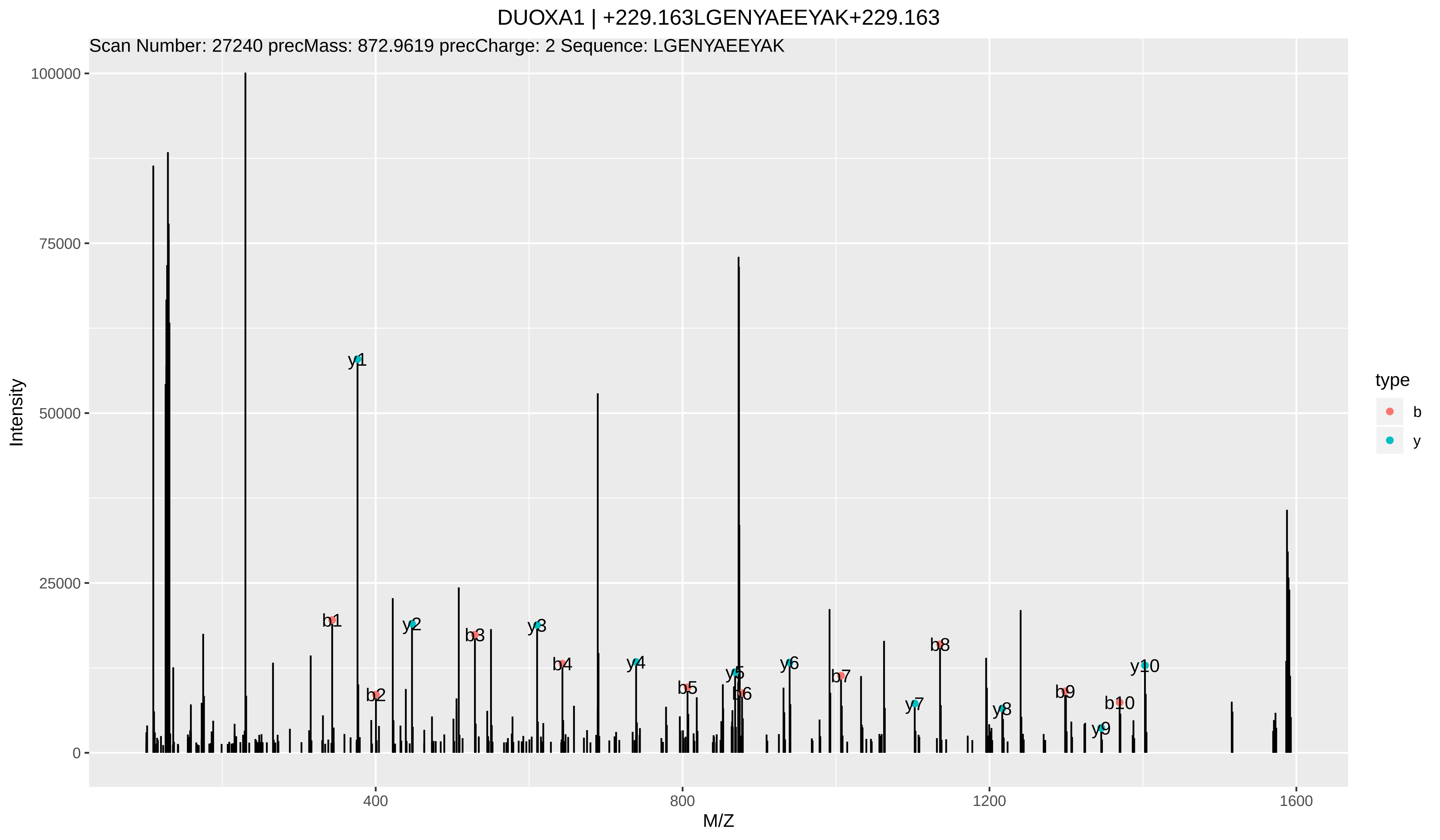

DUOXA1 | +229.163LGENYAEEYAK+229.163
Scan Number: 27240 precMass: 872.9619 precCharge: 2 Sequence: LGENYAEEYAK
Intensity
100000
75000
50000
25000
0
400
800
1200
1600
M/Z
y1
b1
y2
b2
b3
y3
b4
y4
b5
y5
b6
y6
b7
y7
b8
y8
b9
y9
b10
y10
type
b
y

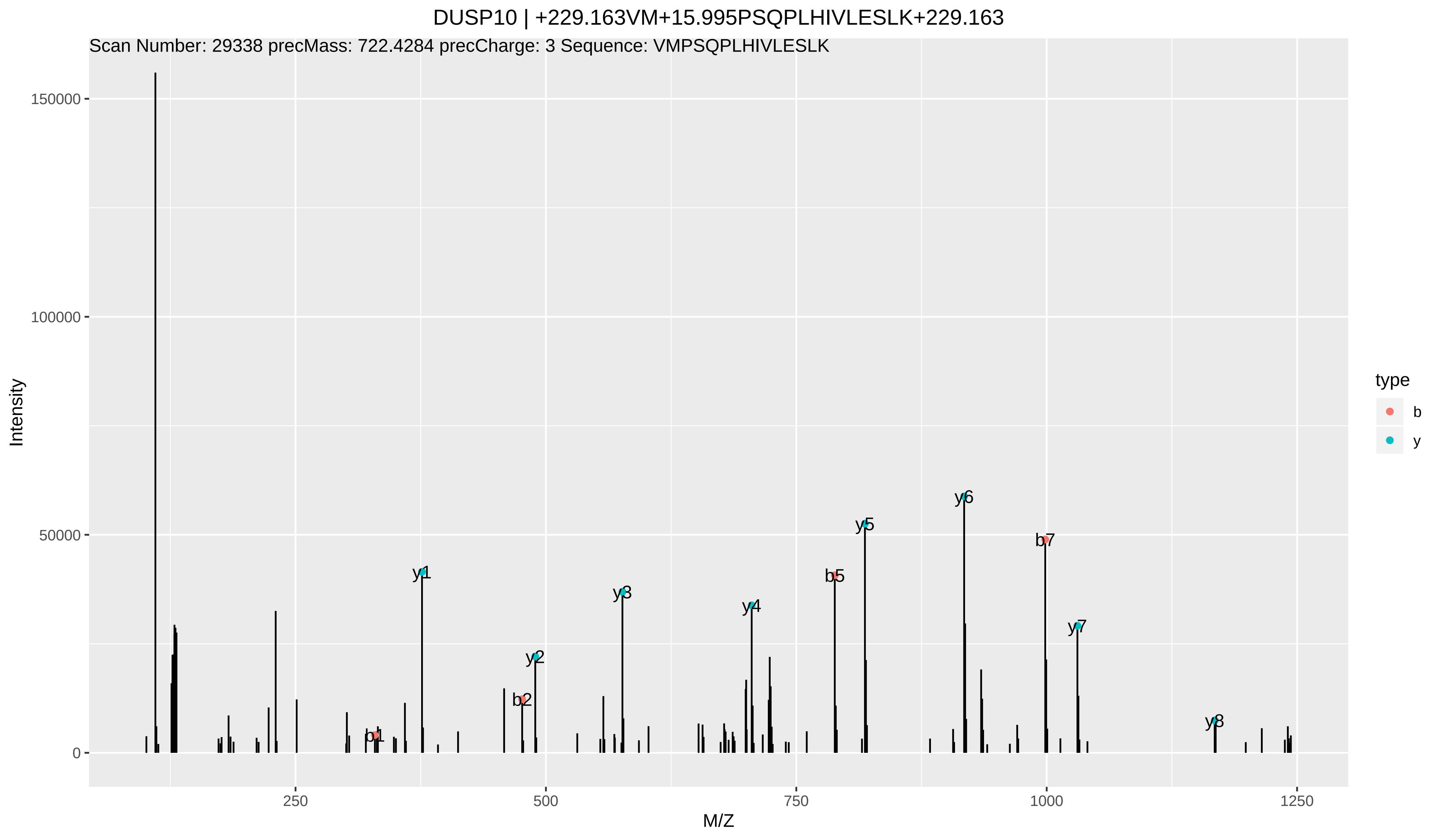

DUSP10 | +229.163VM+15.995PSQPLHIVLESLK+229.163
Scan Number: 29338 precMass: 722.4284 precCharge: 3 Sequence: VMPSQPLHIVLESLK
Intensity
150000
100000
50000
0
250
500
750
1000
1250
M/Z
y1
y2
y3
y4
y5
y6
y7
y8
b1
b2
b5
b7
type
b
y

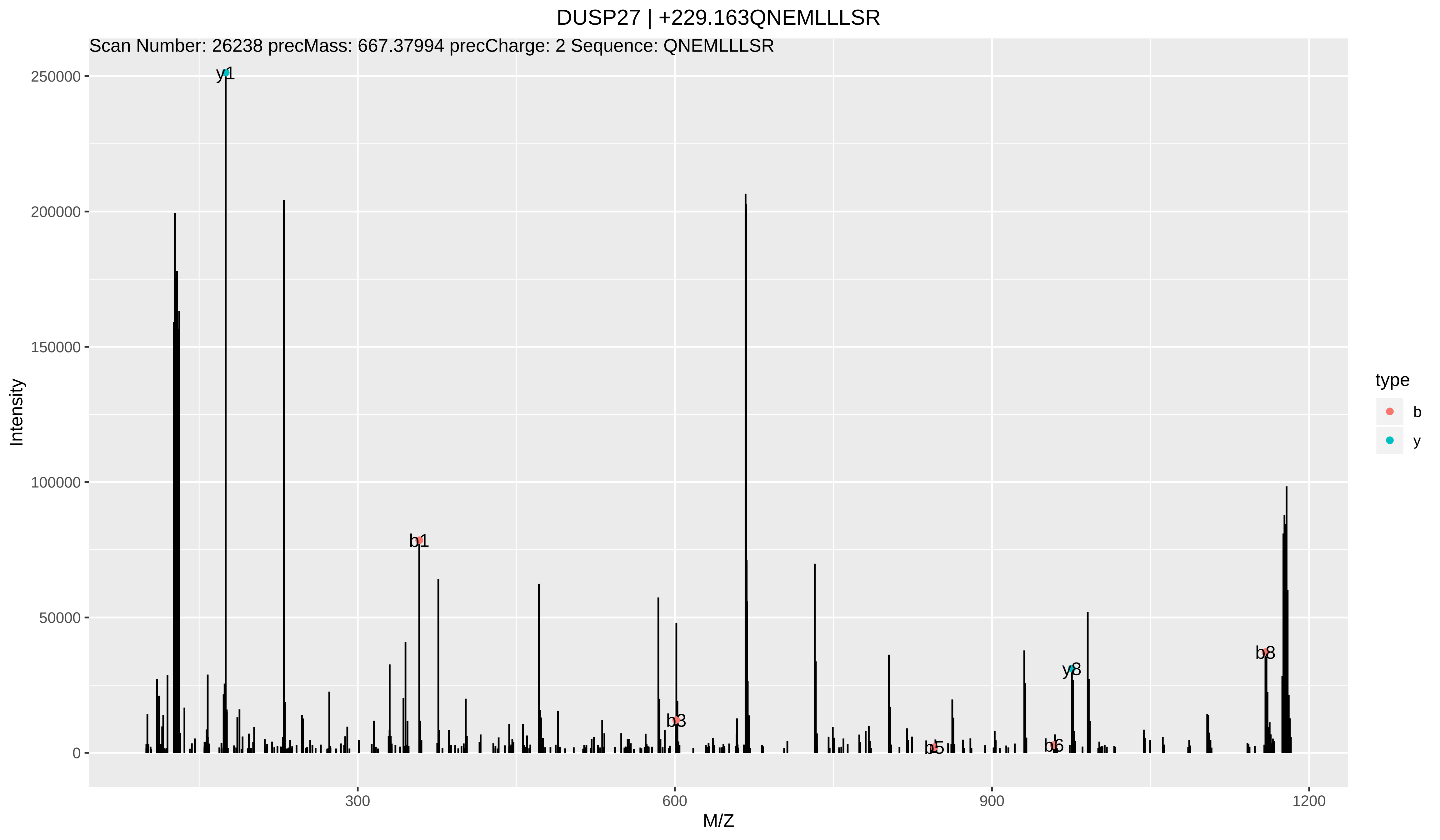

DUSP27 | +229.163QNEMLLLSR
Scan Number: 26238 precMass: 667.37994 precCharge: 2 Sequence: QNEMLLLSR
Intensity
250000
200000
150000
100000
50000
0
M/Z
300
600
900
1200
y1
b1
b3
b5
b6
y8
b8
type
b
y

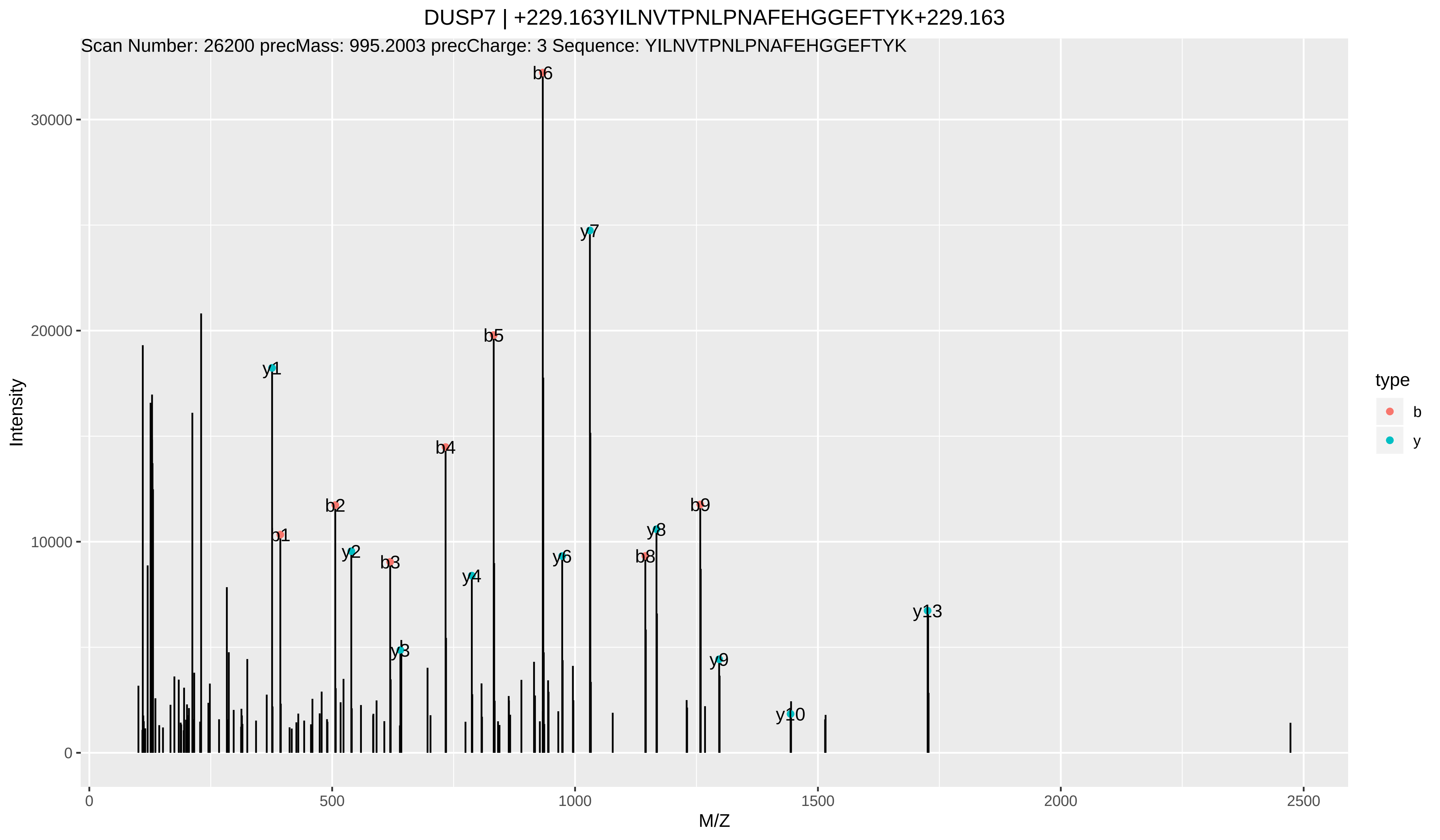

DUSP7 | +229.163YILNVTPNLPNAFEHGGEFTYK+229.163
Scan Number: 26200 precMass: 995.2003 precCharge: 3 Sequence: YILNVTPNLPNAFEHGGEFTYK
Intensity
M/Z
type
b
y
b6
y7
b5
y1
b4
b2
y8
b9
b1
y2
b3
y6
b8
y4
y13
y3
y9
y10
30000
20000
10000
0
0
500
1000
1500
2000
2500

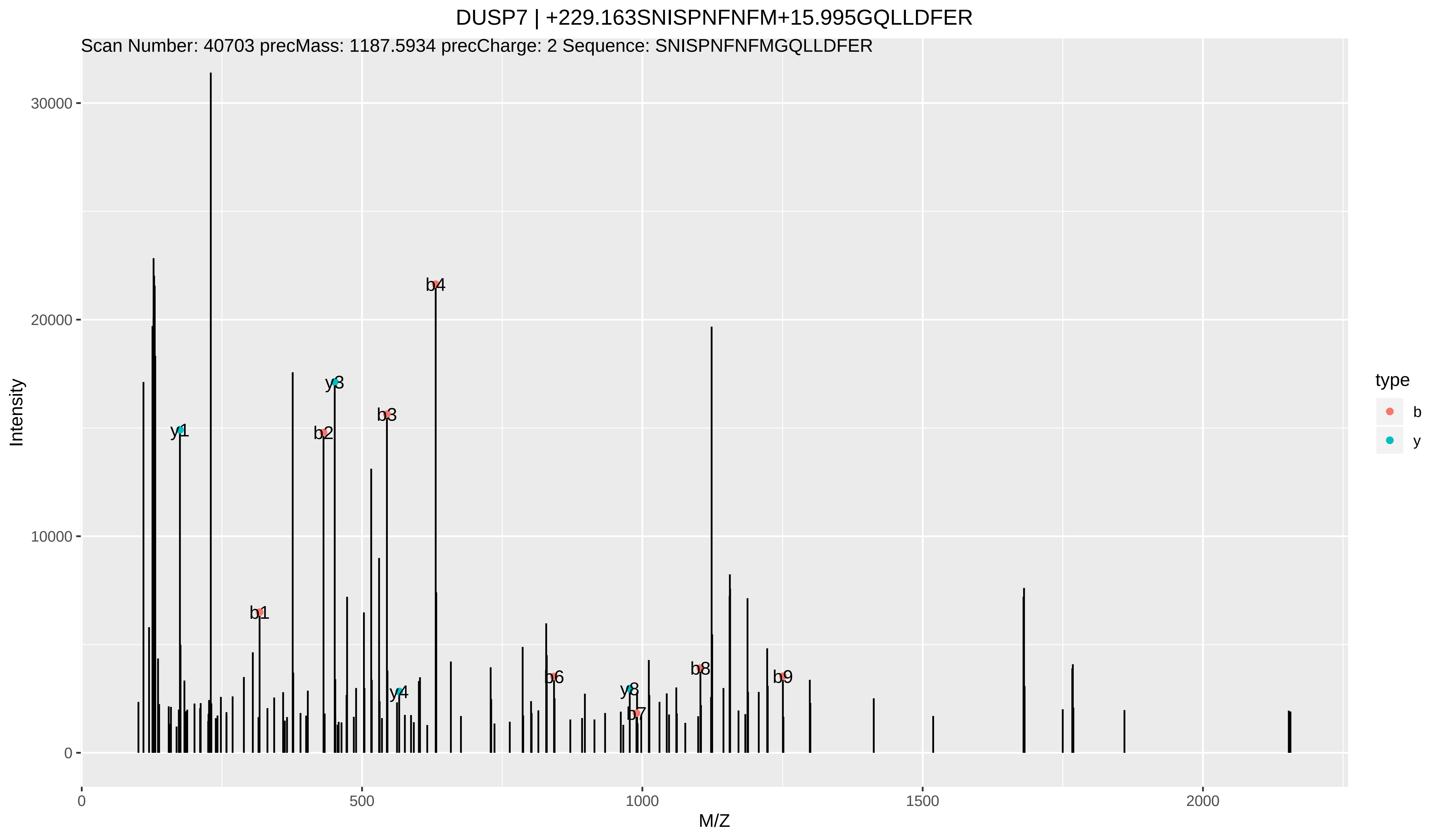

DUSP7 | +229.163SNISPNFNFM+15.995GQLLDFER
Scan Number: 40703 precMass: 1187.5934 precCharge: 2 Sequence: SNISPNFNFMGQLLDFER
Intensity
M/Z
type
b
y
y1
b1
b2
y3
b3
y4
b4
b6
y8
b7
b8
b9
0
10000
20000
30000
0
500
1000
1500
2000

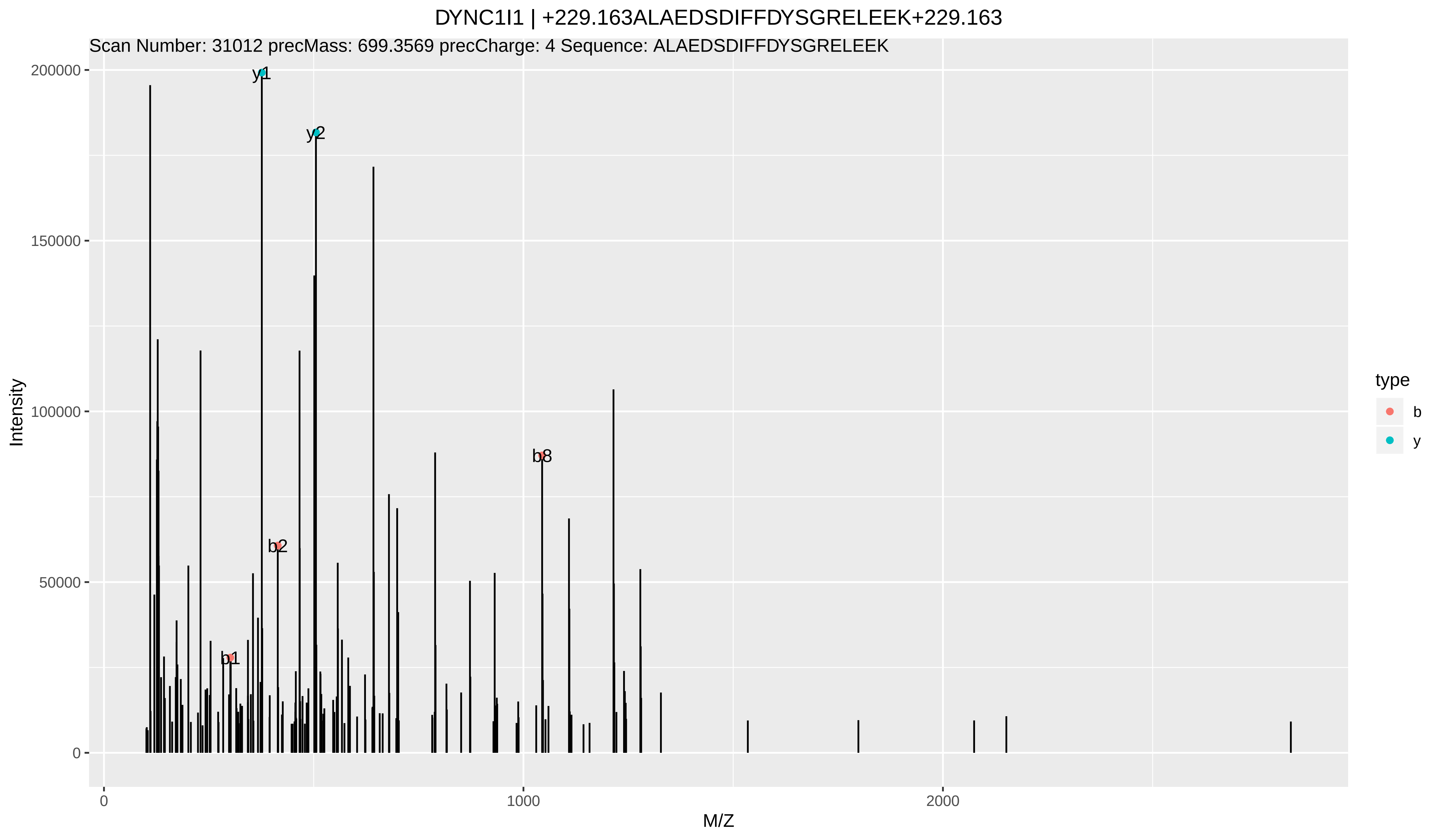
DYNC1I1 | +229.163ALAEDSDIFFDYSGRELEEK+229.163
Scan Number: 31012 precMass: 699.3569 precCharge: 4 Sequence: ALAEDSDIFFDYSGRELEEK
Intensity
200000
150000
100000
50000
0
M/Z
0
1000
2000
y1
y2
b8
b2
b1
type
b
y

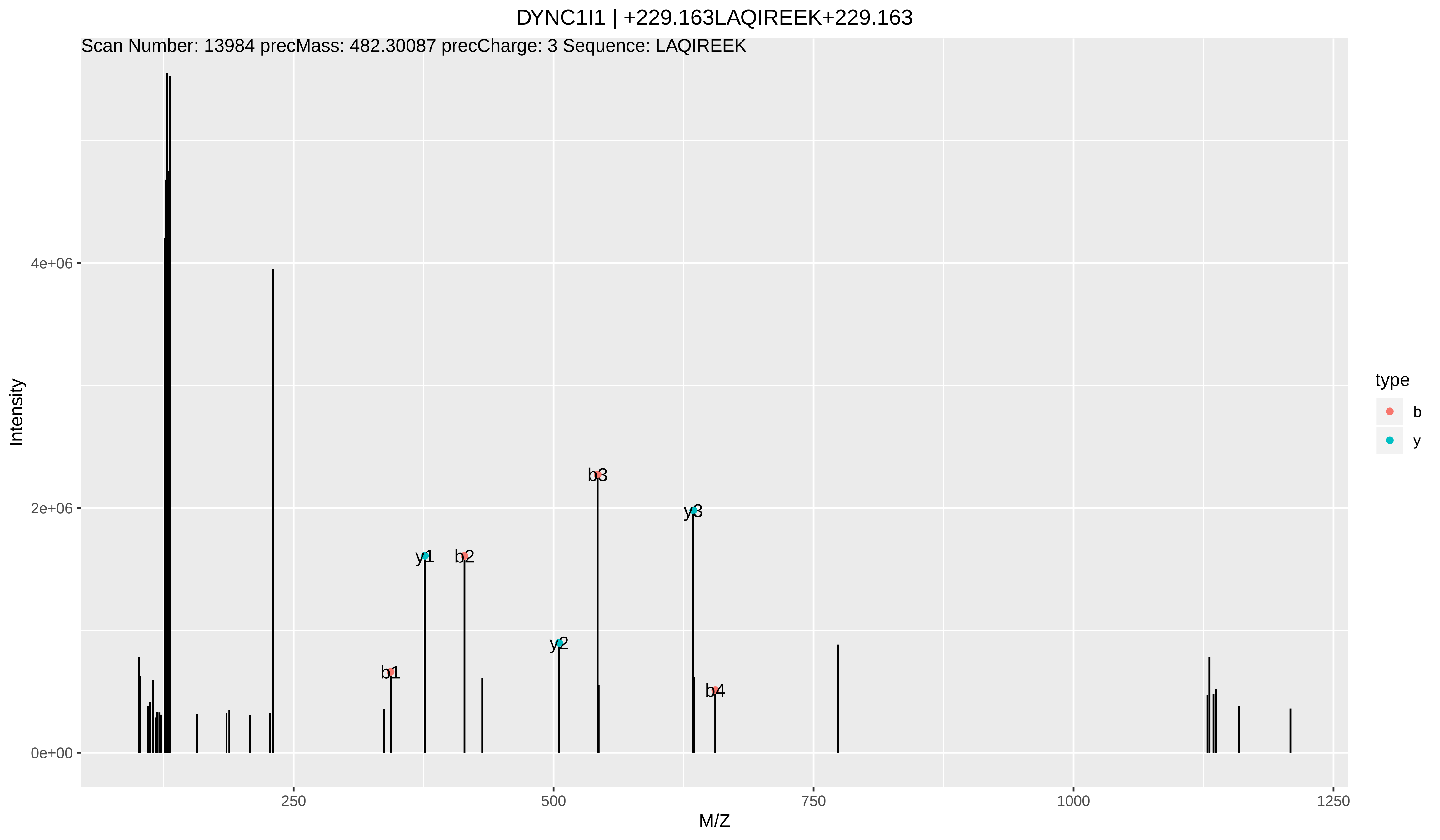

DYNC1I1 | +229.163LAQIREEK+229.163
Scan Number: 13984 precMass: 482.30087 precCharge: 3 Sequence: LAQIREEK
Intensity
4e+06
2e+06
0e+00
M/Z
250
500
750
1000
1250
b1
y1
b2
y2
b3
y3
b4
type
b
y

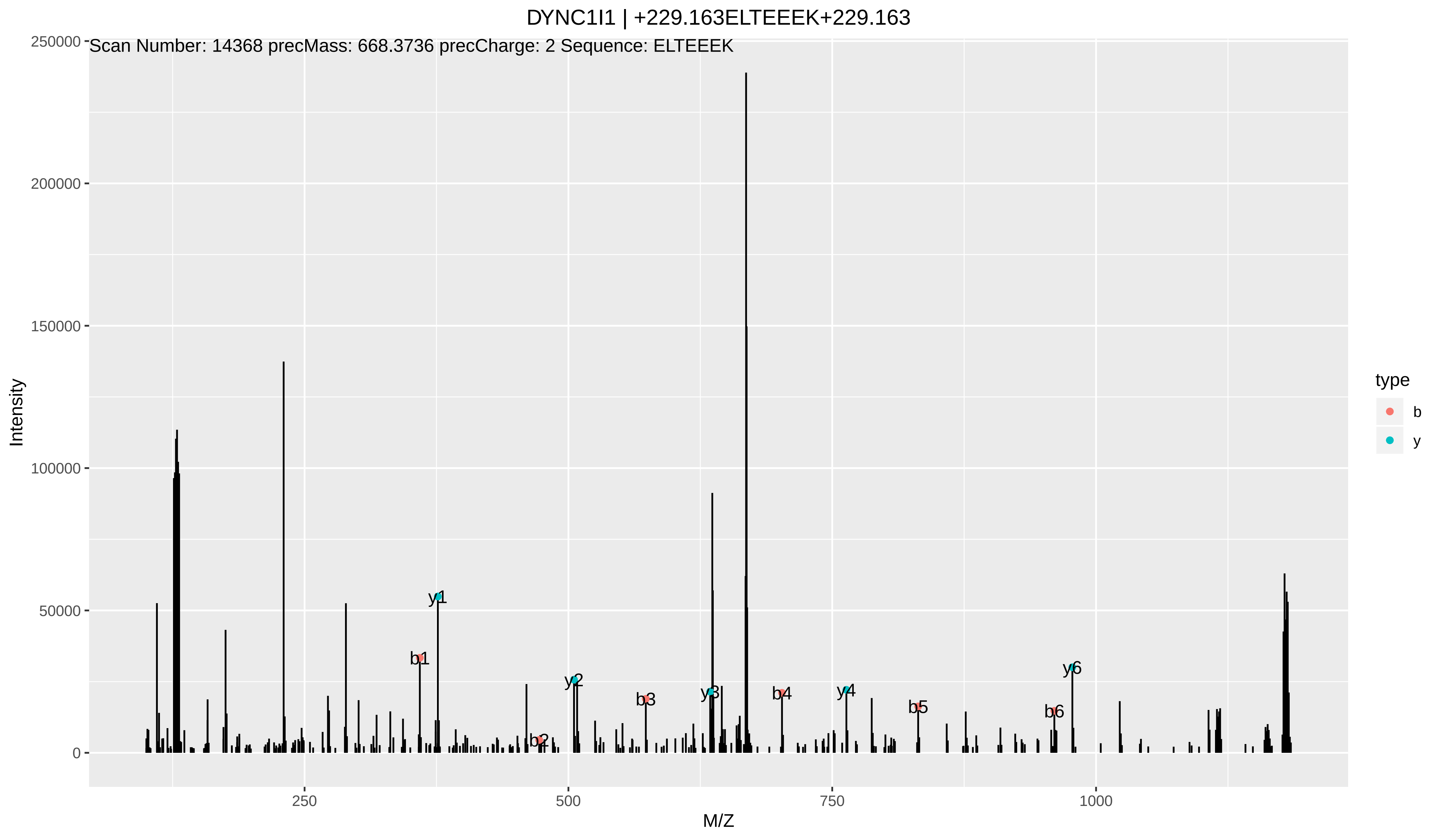

DYNC1I1 | +229.163ELTEEEK+229.163
Scan Number: 14368 precMass: 668.3736 precCharge: 2 Sequence: ELTEEEK
Intensity
M/Z
type
b
y
y1
b1
y2
b2
b3
y3
b4
y4
b5
b6
y6
250000
200000
150000
100000
50000
0
250
500
750
1000

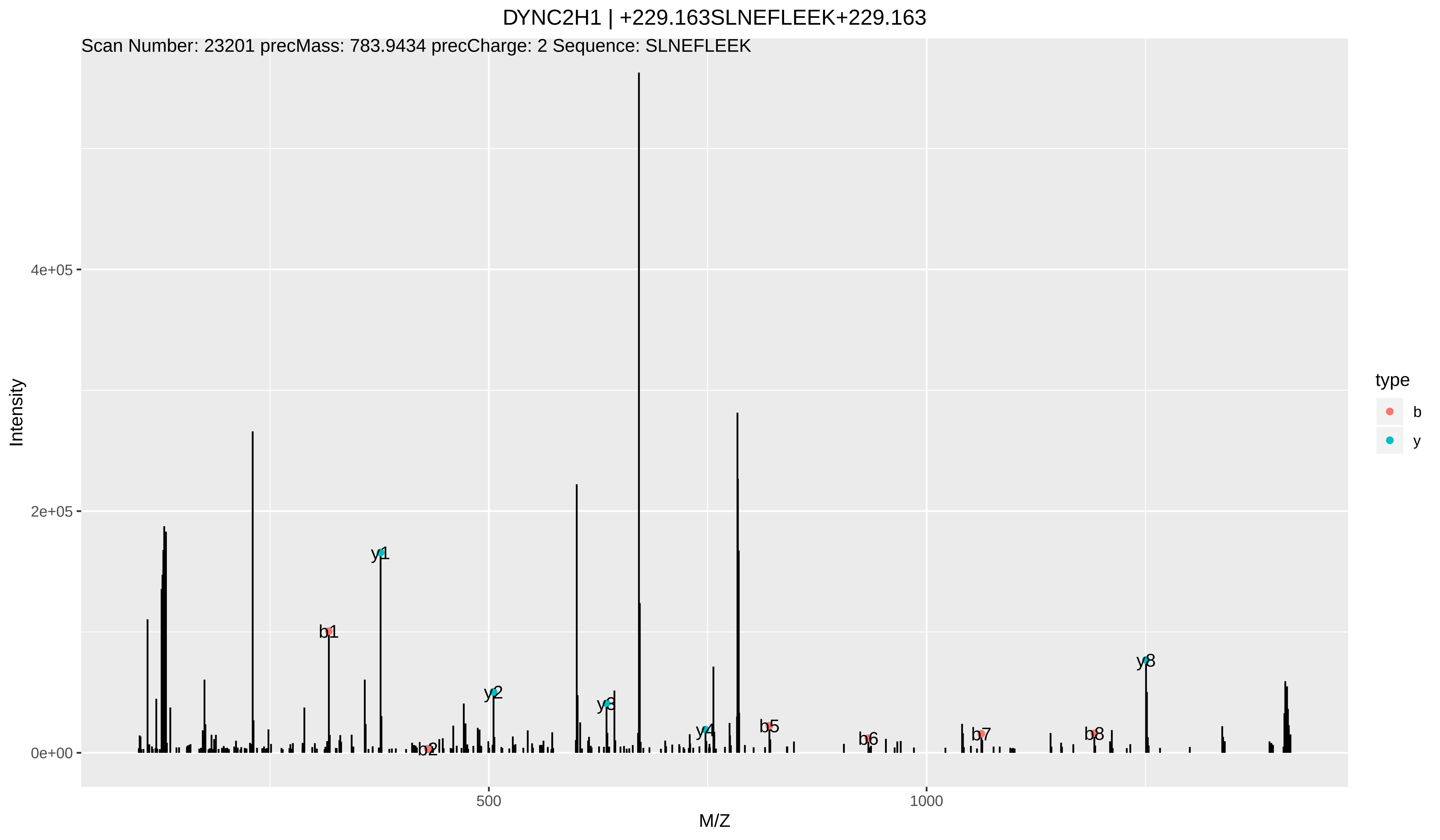

DYNC2H1 | +229.163SLNEFLEEK+229.163
Scan Number: 23201 precMass: 783.9434 precCharge: 2 Sequence: SLNEFLEEK
Intensity
4e+05
2e+05
0e+00
M/Z
500
1000
y1
b1
y2
b2
y3
y4
b5
b6
b7
b8
y8
type
b
y

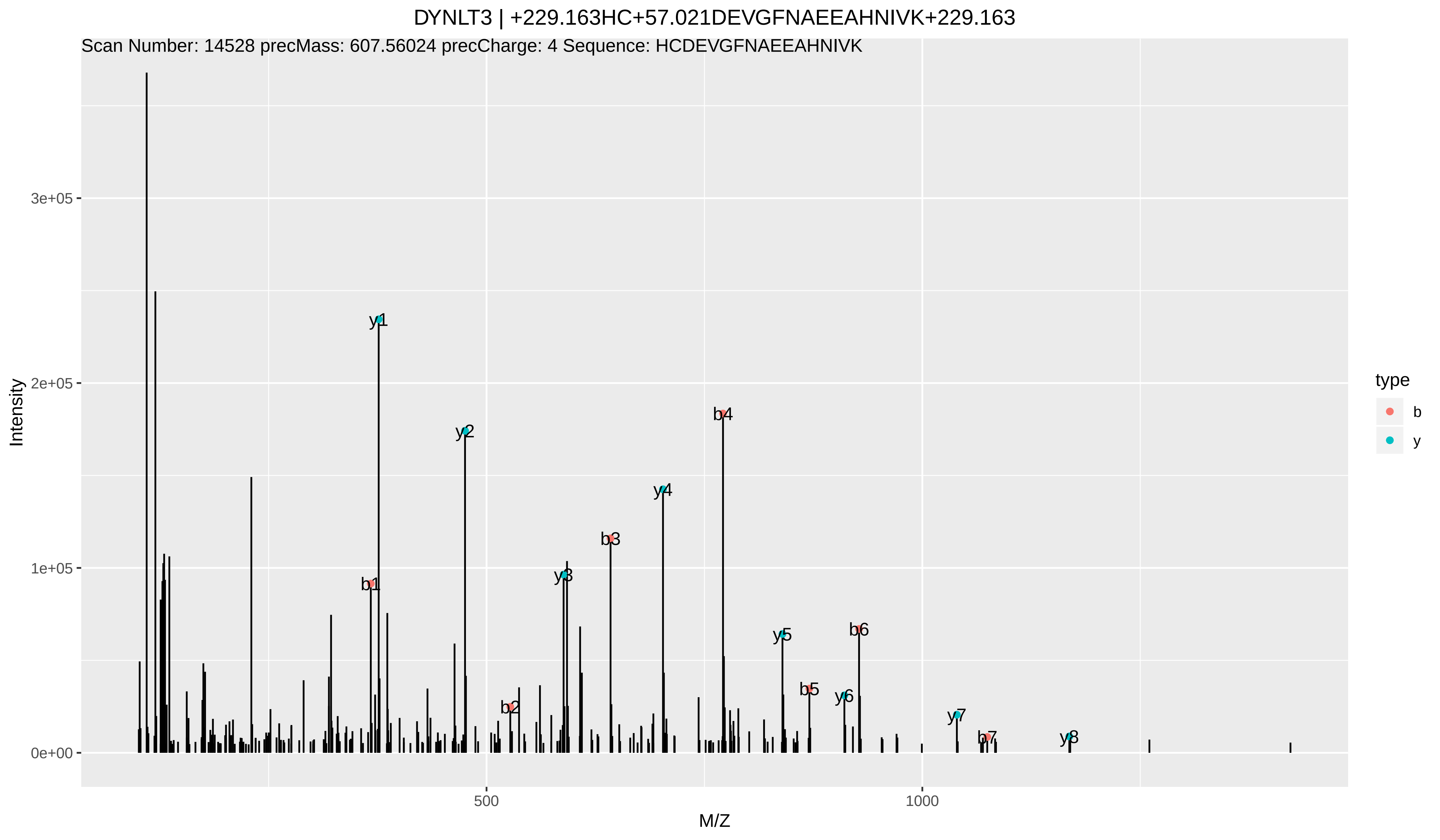
DYNLT3 | +229.163HC+57.021DEVGFNAEEAHNIVK+229.163
Scan Number: 14528 precMass: 607.56024 precCharge: 4 Sequence: HCDEVGFNAEEAHNIVK
Intensity
3e+05
2e+05
1e+05
0e+00
M/Z
500
1000
y1
y2
b4
y4
b3
b1
y3
y5
b6
b5
y6
b2
y7
b7
y8
type
b
y

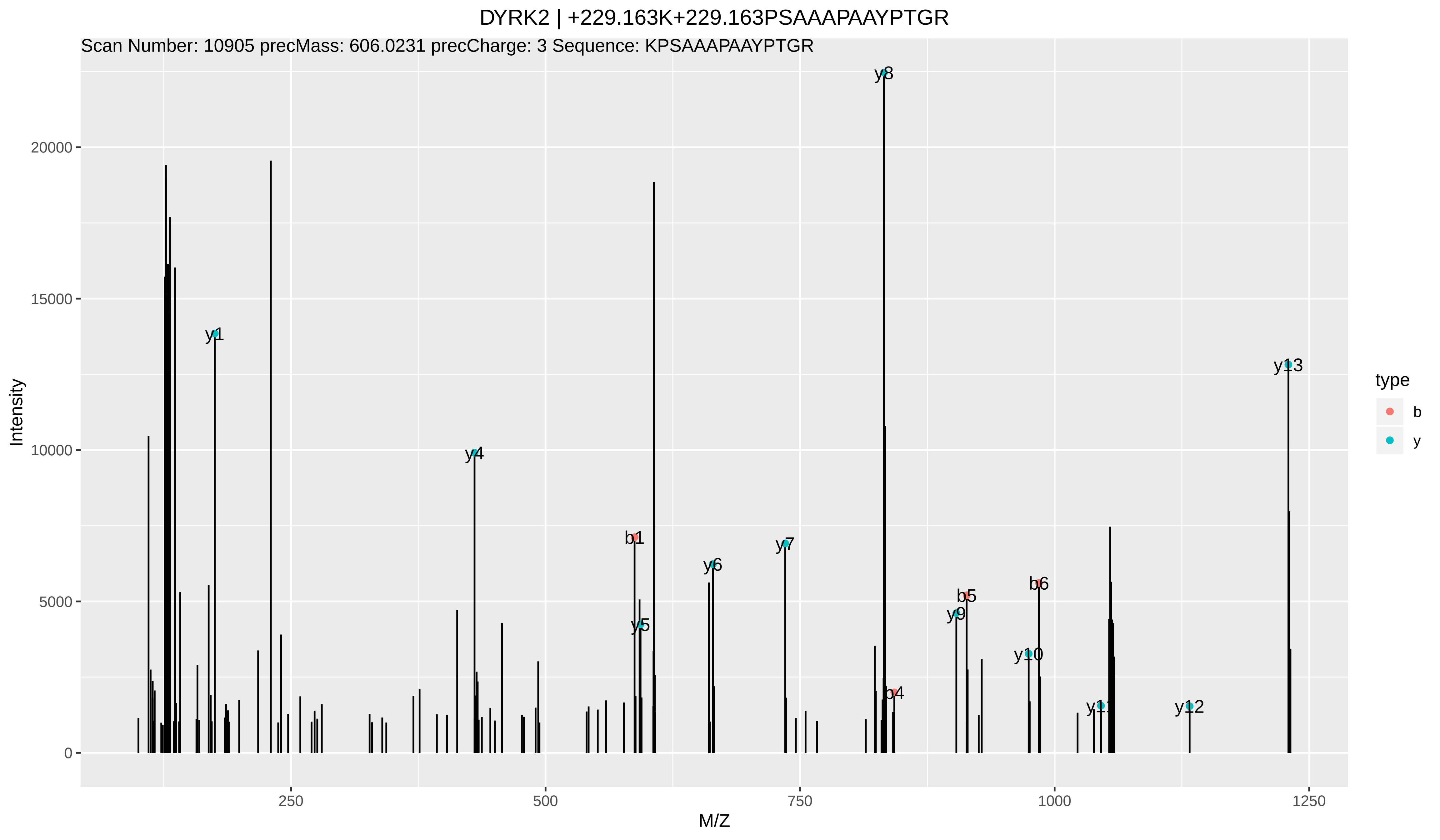
DYRK2 | +229.163K+229.163PSAAAPAAYPTGR
Scan Number: 10905 precMass: 606.0231 precCharge: 3 Sequence: KPSAAAPAAYPTGR
Intensity
M/Z
0
5000
10000
15000
20000
250
500
750
1000
1250
y1
y4
b1
y5
y6
y7
y8
b4
y9
b5
b6
y10
y12
y13
type
b
y

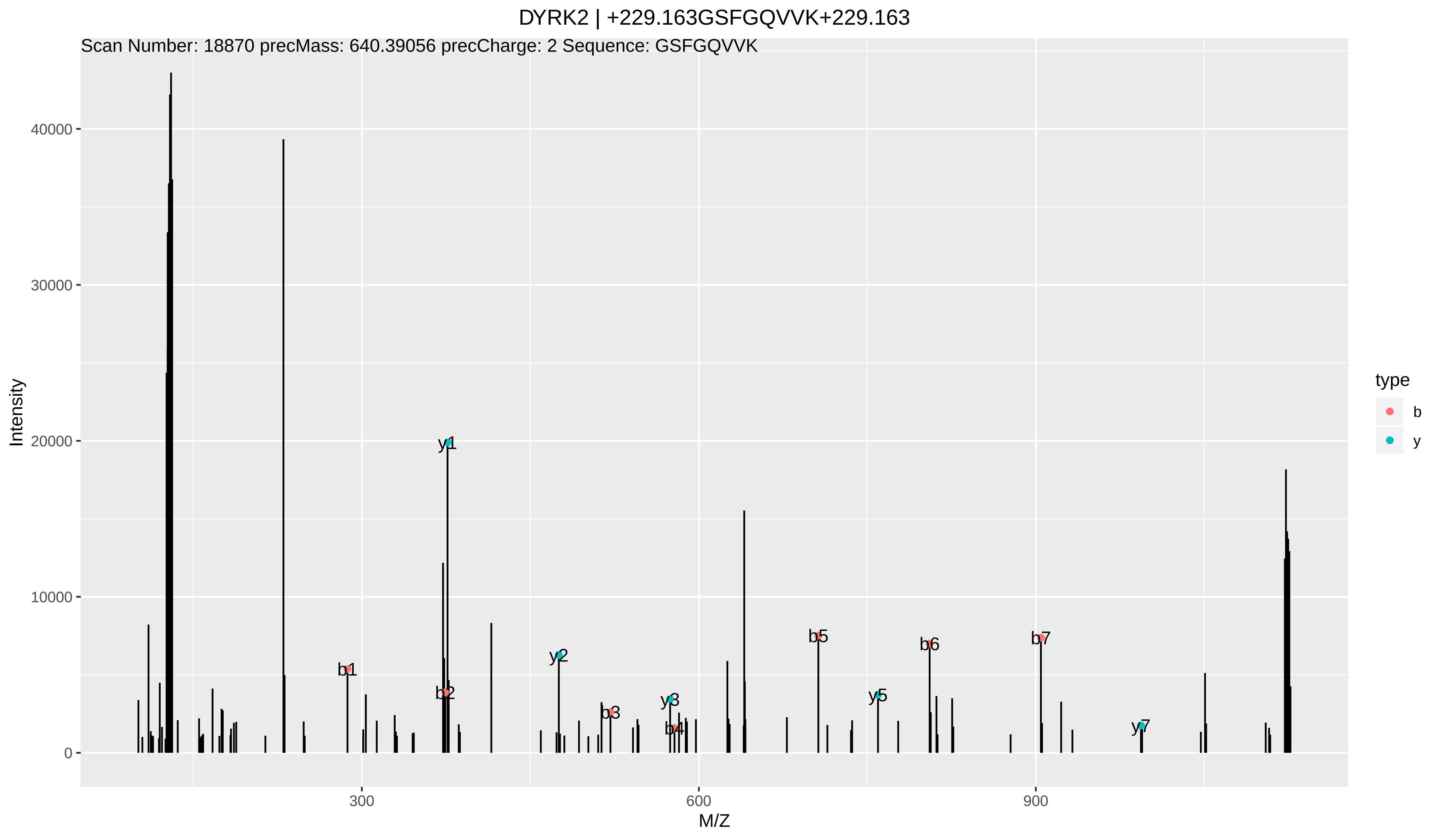
DYRK2 | +229.163GSFGQVVK+229.163
Scan Number: 18870 precMass: 640.39056 precCharge: 2 Sequence: GSFGQVVK
Intensity
0
10000
20000
30000
40000
M/Z
300
600
900
y1
y2
y3
y5
y7
b1
b2
b3
b4
b5
b6
b7
type
b
y

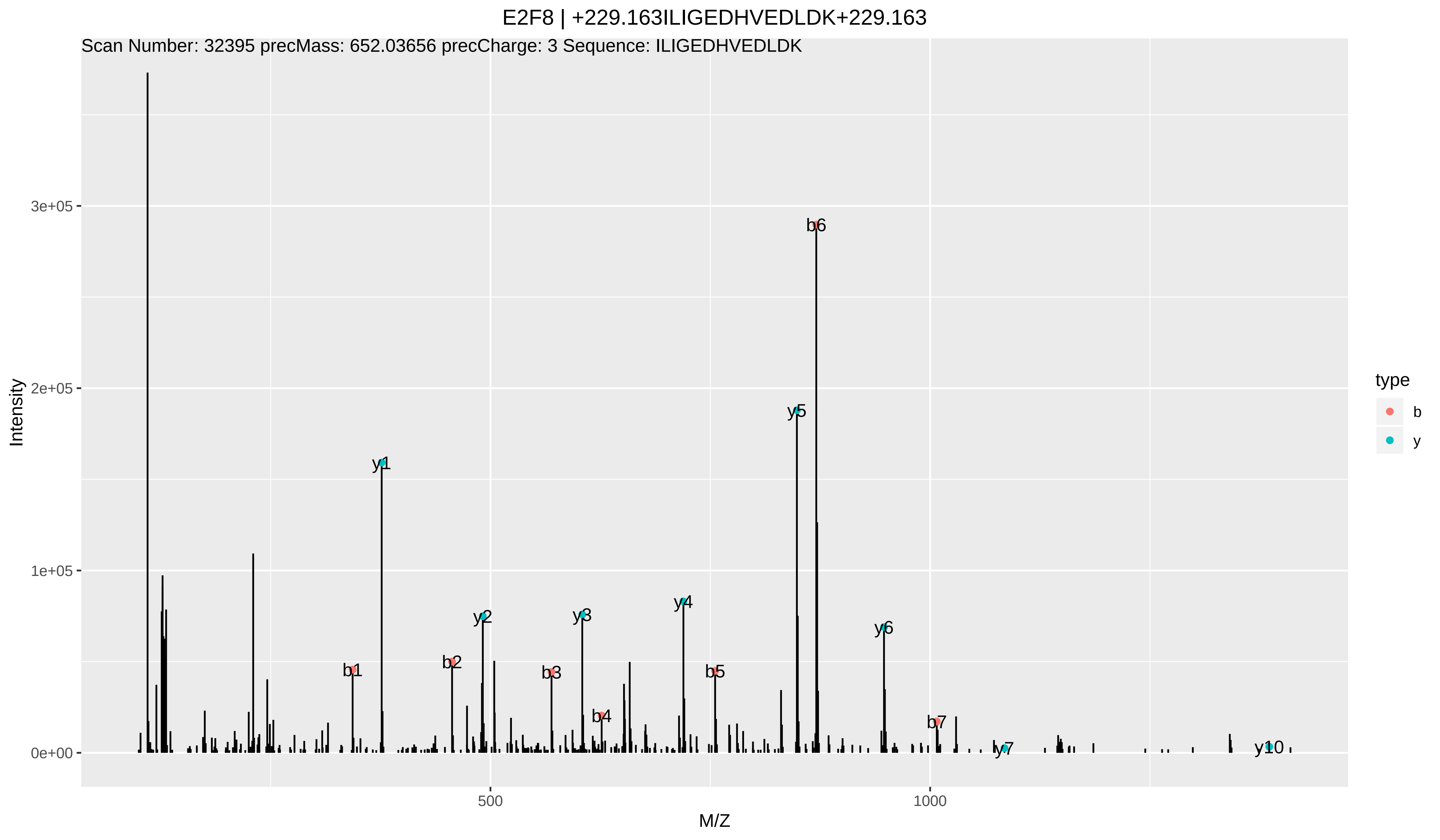

E2F8 | +229.163ILIGEDHVEDLDK+229.163
Scan Number: 32395 precMass: 652.03656 precCharge: 3 Sequence: ILIGEDHVEDLDK
Intensity
3e+05
2e+05
1e+05
0e+00
M/Z
500
1000
b6
y5
y1
y4
y2
y3
y6
b1
b2
b3
b5
b4
b7
y7
y10
type
b
y

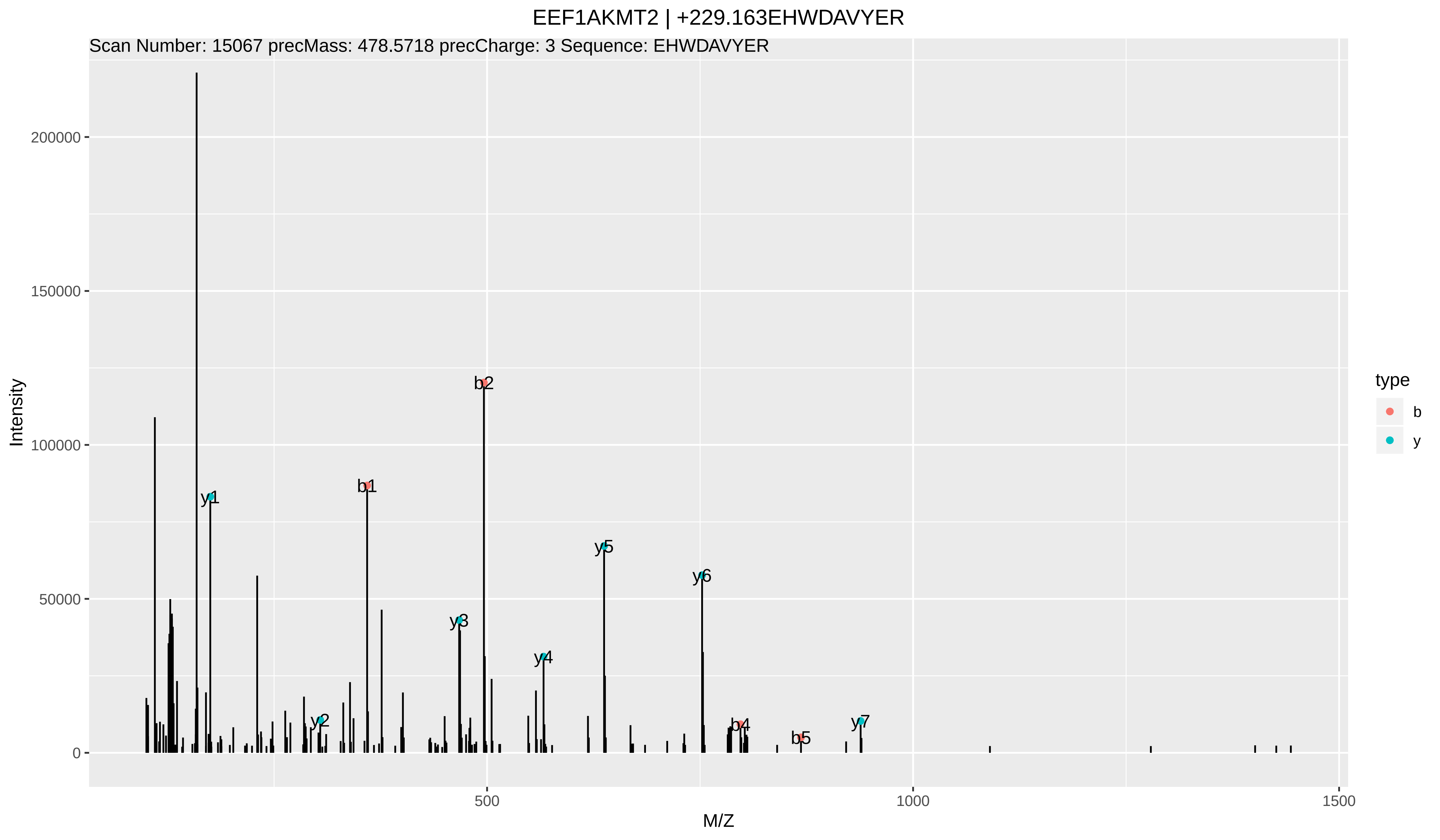

EEF1AKMT2 | +229.163EHWDAVYER
Scan Number: 15067 precMass: 478.5718 precCharge: 3 Sequence: EHWDAVYER
Intensity
M/Z
0
50000
100000
150000
200000
500
1000
1500
y1
y2
y3
y4
y5
y6
y7
b1
b2
b4
b5
type
b
y

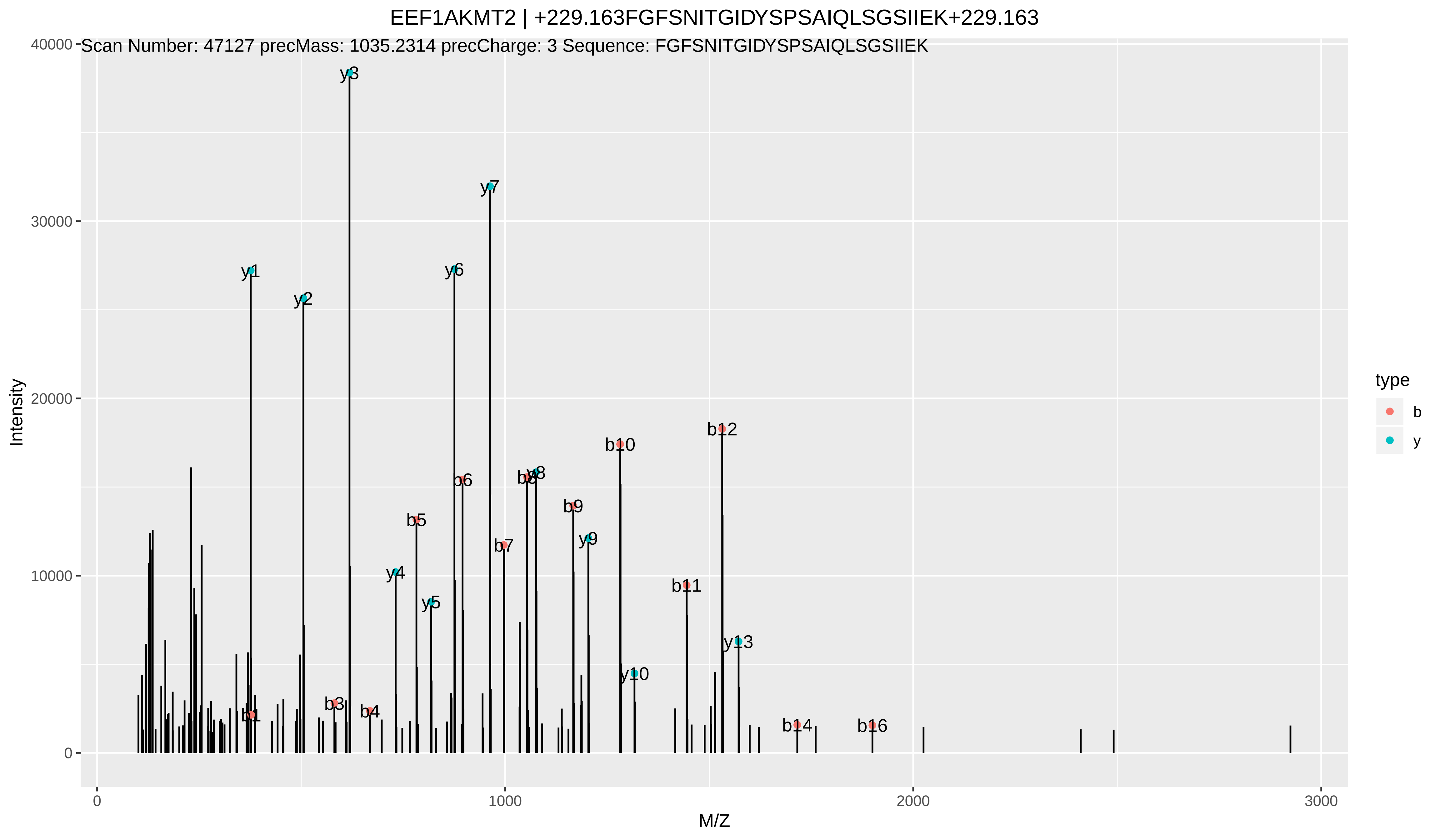
EEF1AKMT2 | +229.163FGFSNITGIDYSPSAIQLSGSIIEK+229.163
Scan Number: 47127 precMass: 1035.2314 precCharge: 3 Sequence: FGFSNITGIDYSPSAIQLSGSIIEK
Intensity
M/Z
0
10000
20000
30000
40000
1000
2000
3000
y1
y2
y3
y4
y5
y6
y7
y8
y9
y10
y13
b1
b3
b4
b5
b6
b7
b8
b9
b10
b11
b12
b14
b16
type
b
y

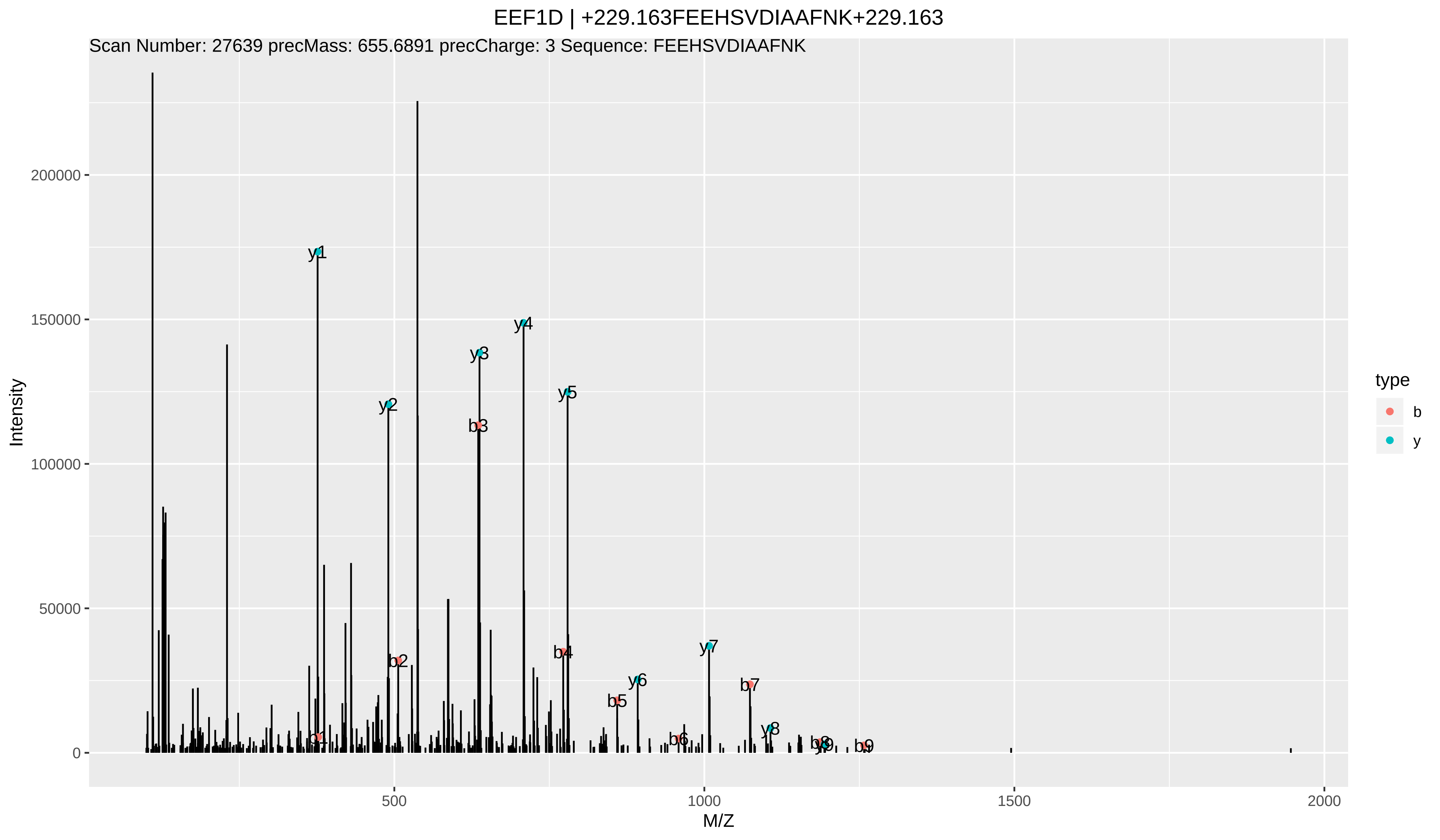
EEF1D | +229.163FEEHSVDIAAFNK+229.163
Scan Number: 27639 precMass: 655.6891 precCharge: 3 Sequence: FEEHSVDIAAFNK
Intensity
M/Z
0
50000
100000
150000
200000
500
1000
1500
2000
y1
y2
y3
y4
y5
y6
y7
y8
b1
b2
b3
b4
b5
b6
b7
b9
type
b
y

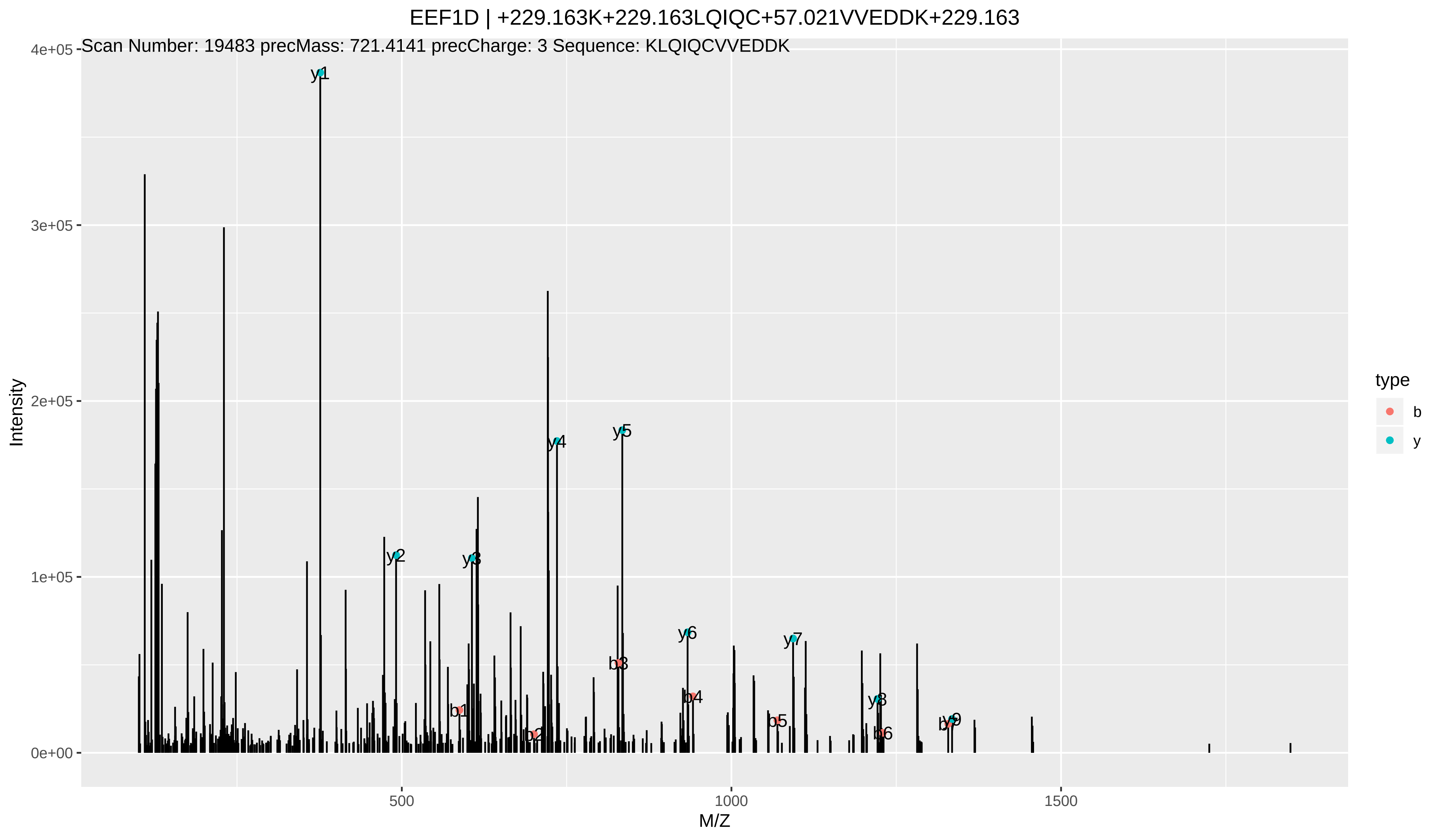

EEF1D | +229.163K+229.163LQIQC+57.021VVEDDK+229.163
Scan Number: 19483 precMass: 721.4141 precCharge: 3 Sequence: KLQIQCVVEDDK
Intensity
M/Z
4e+05
3e+05
2e+05
1e+05
0e+00
500
1000
1500
y1
y2
y3
y4
y5
y6
y7
y8
y9
b1
b2
b3
b4
b5
b6
b7
type
b
y

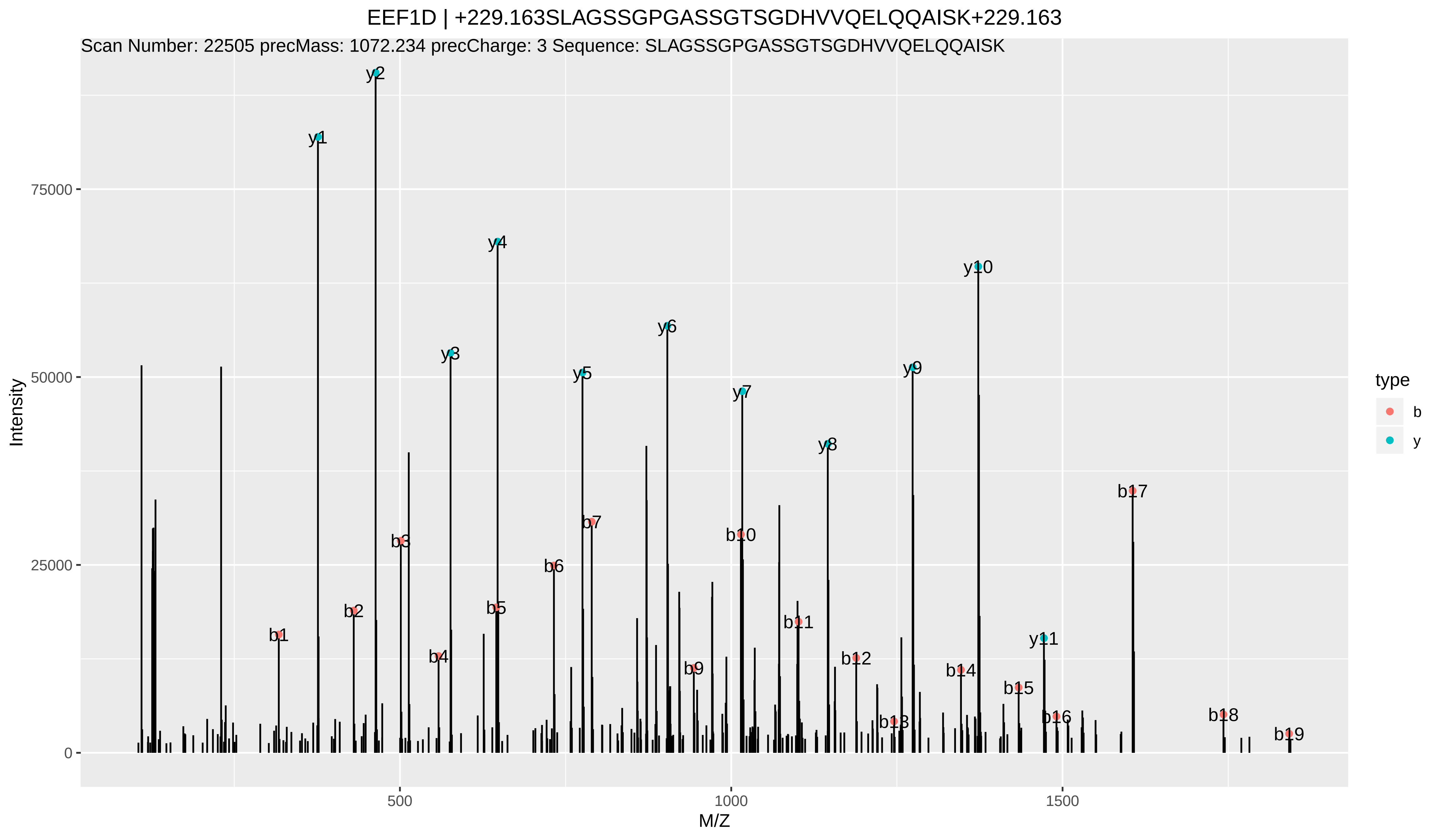
EEF1D | +229.163SLAGSSGPGASSGTSGDHVVQELQQAISK+229.163
Scan Number: 22505 precMass: 1072.234 precCharge: 3 Sequence: SLAGSSGPGASSGTSGDHVVQELQQAISK
Intensity
M/Z
type
b
y
0
25000
50000
75000
500
1000
1500
y1
y2
y3
y4
y5
y6
y7
y8
y9
y10
y11
b1
b2
b3
b4
b5
b6
b7
b9
b10
b11
b12
b13
b14
b15
b16
b17
b18
b19

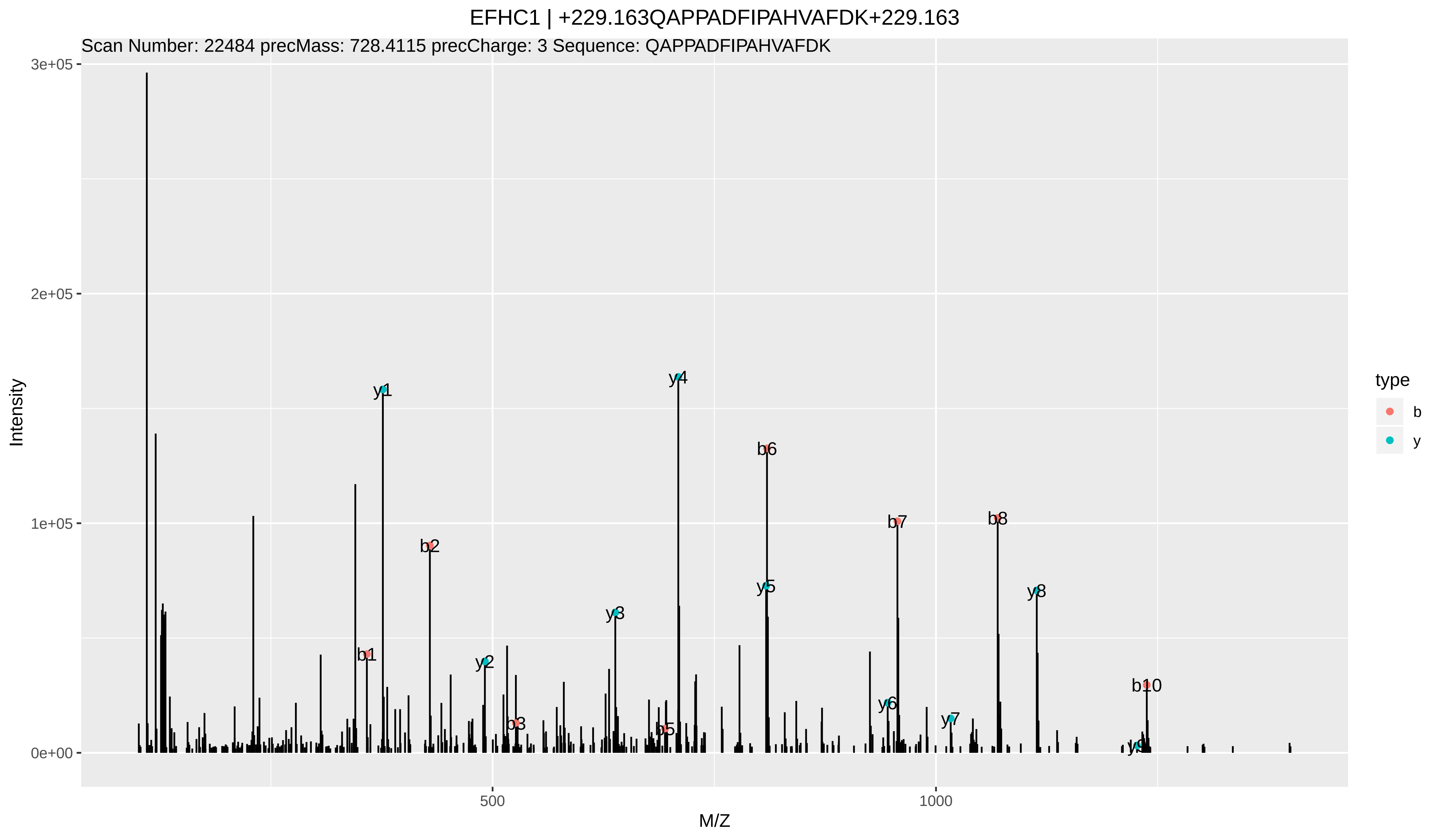

EFHC1 | +229.163QAPPADFIPAHVAFDK+229.163
Scan Number: 22484 precMass: 728.4115 precCharge: 3 Sequence: QAPPADFIPAHVAFDK
Intensity
3e+05
2e+05
1e+05
0e+00
M/Z
500
1000
y1
y2
y3
y4
y5
y6
y7
y8
b1
b2
b3
b5
b6
b7
b8
b10
type
b
y

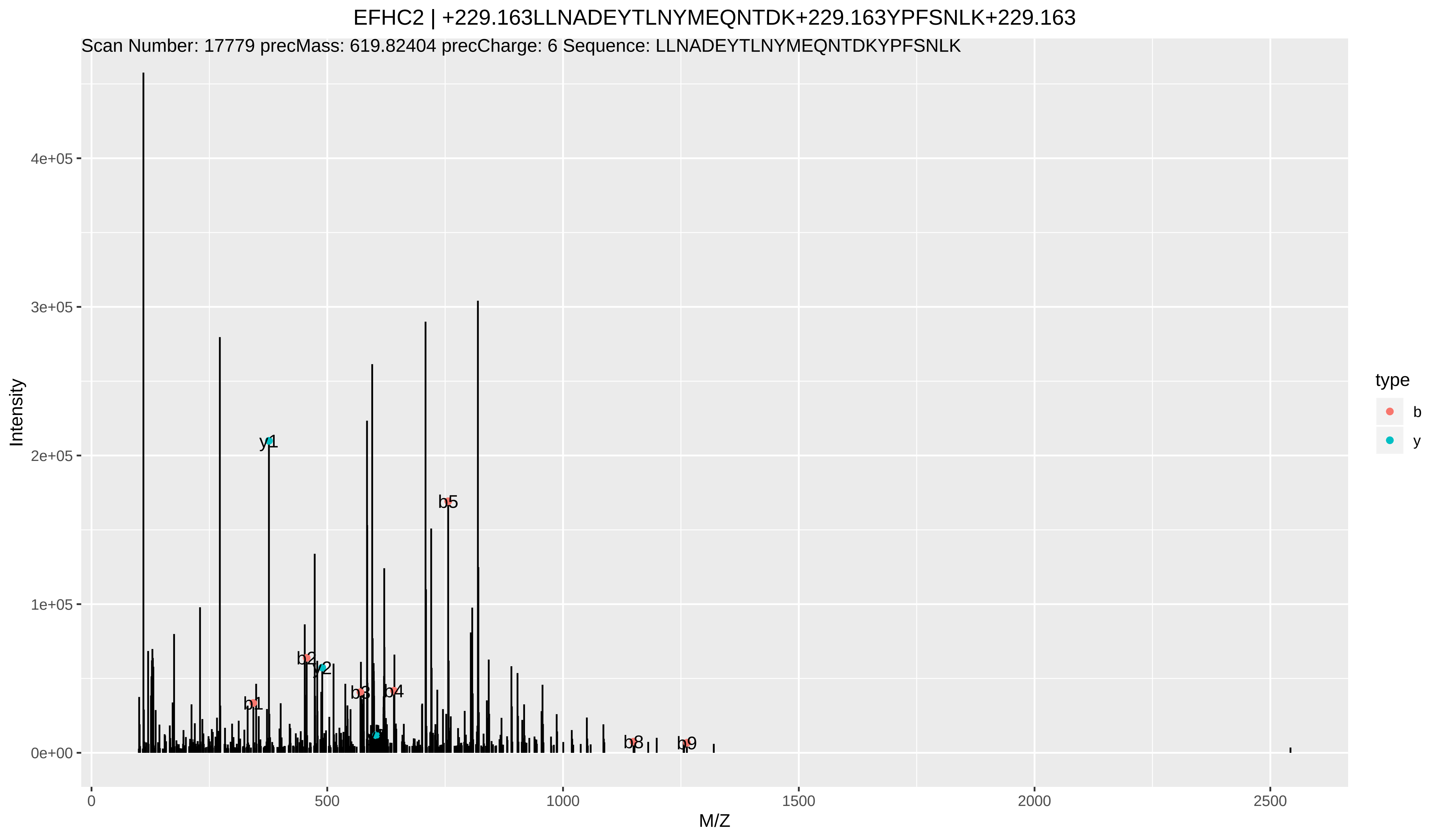

EFHC2 | +229.163LLNADEYTLNYMEQNTDK+229.163YPFSNLK+229.163
Scan Number: 17779 precMass: 619.82404 precCharge: 6 Sequence: LLNADEYTLNYMEQNTDKYPFSNLK
Intensity
4e+05
3e+05
2e+05
1e+05
0e+00
0
500
1000
1500
2000
2500
M/Z
y1
b5
b2
y2
b3
b4
b1
b8
b9
type
b
y

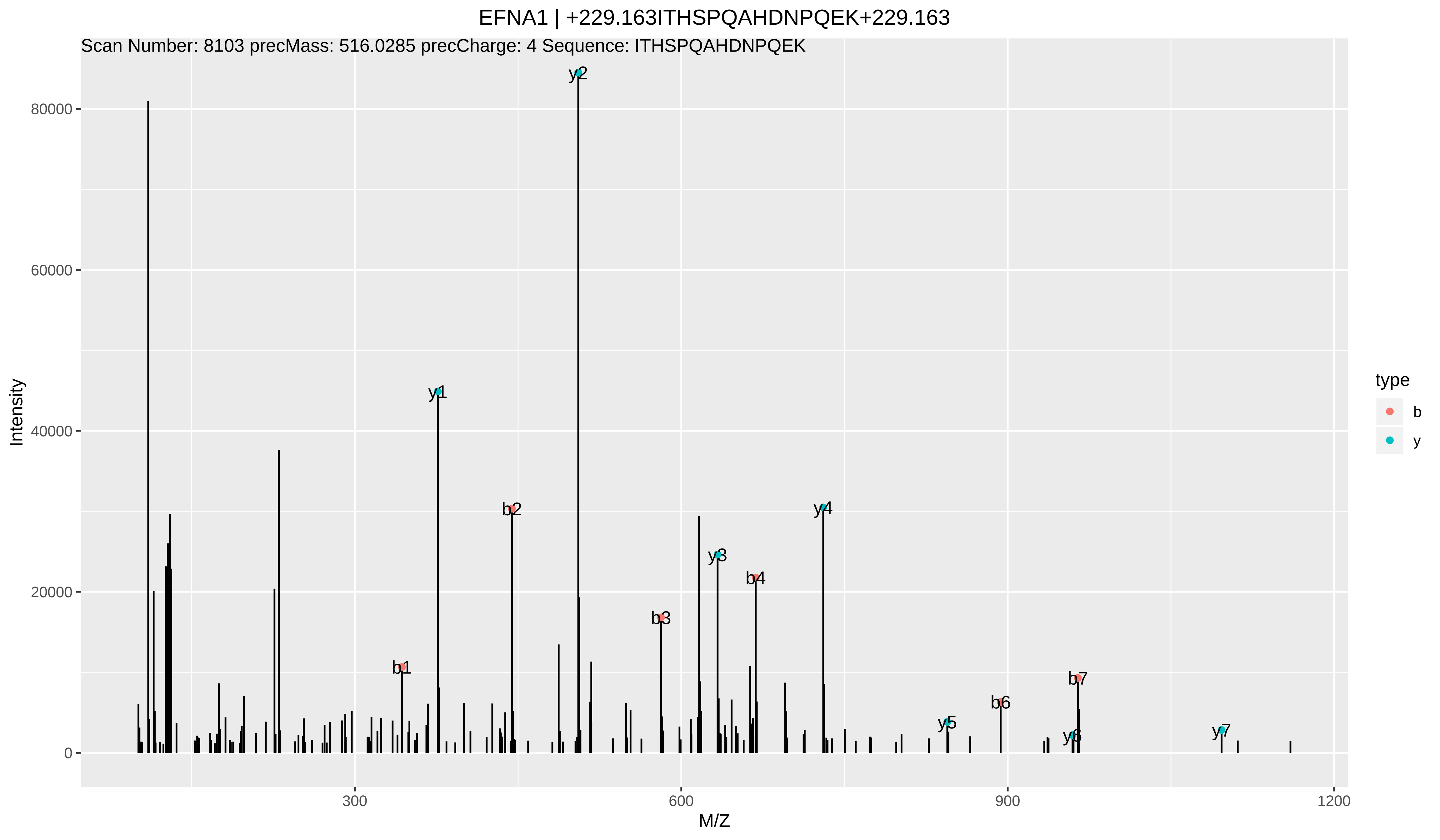

EFNA1 | +229.163ITHSPQAHDNPQEK+229.163
Scan Number: 8103 precMass: 516.0285 precCharge: 4 Sequence: ITHSPQAHDNPQEK
Intensity
M/Z
0
20000
40000
60000
80000
300
600
900
1200
y1
y2
y3
y4
y5
y6
y7
b1
b2
b3
b4
b6
b7
type
b
y

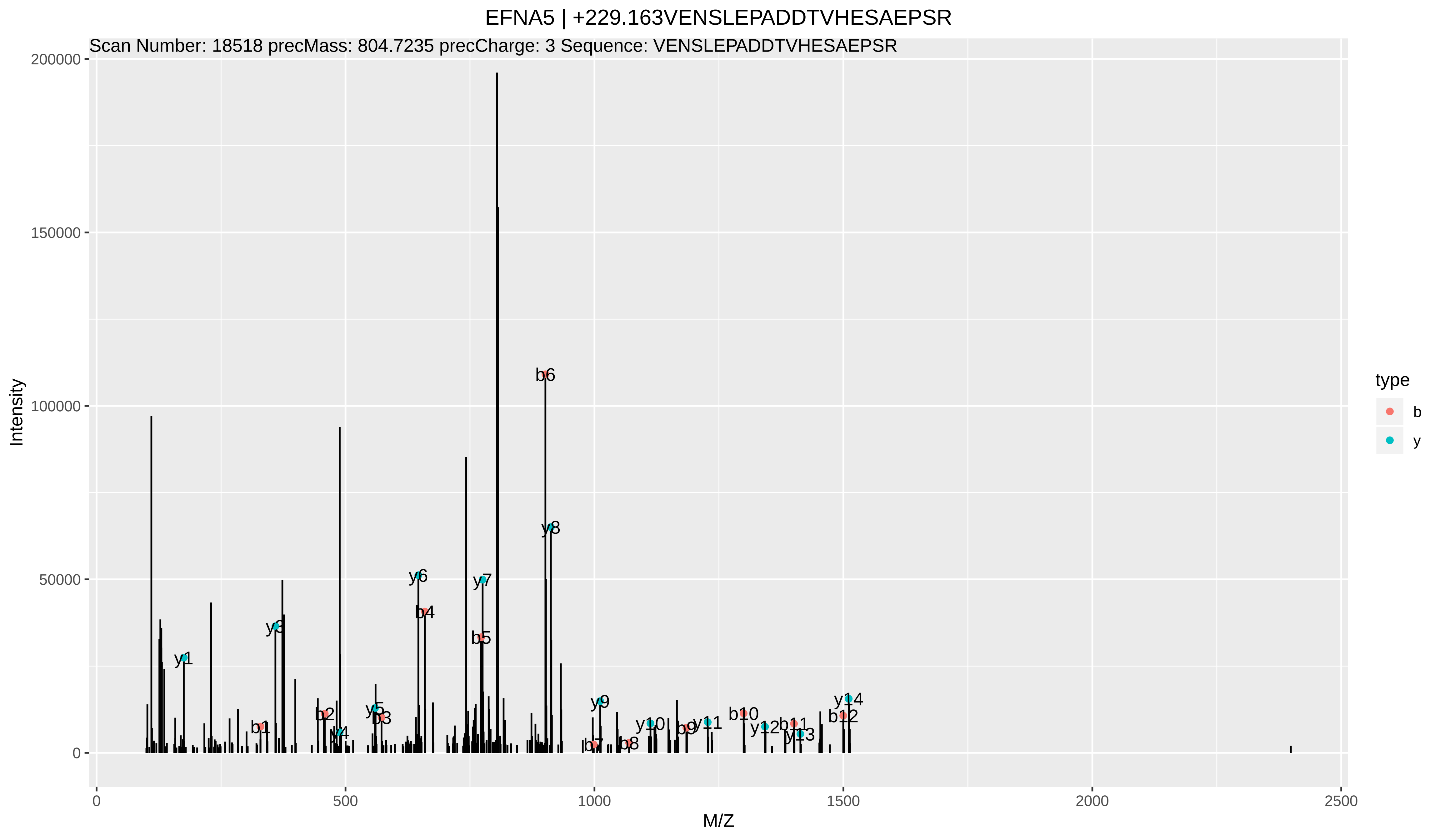
EFNA5 | +229.163VENSLEPADDTVHESAEPSR
Scan Number: 18518 precMass: 804.7235 precCharge: 3 Sequence: VENSLEPADDTVHESAEPSR
Intensity
M/Z
type
b
y
200000
150000
100000
50000
0
0
500
1000
1500
2000
2500
y1
y3
y4
y5
y6
y7
y8
y9
y10
y11
y12
y13
y14
b1
b2
b3
b4
b5
b6
b7
b8
b9
b10
b11
b12

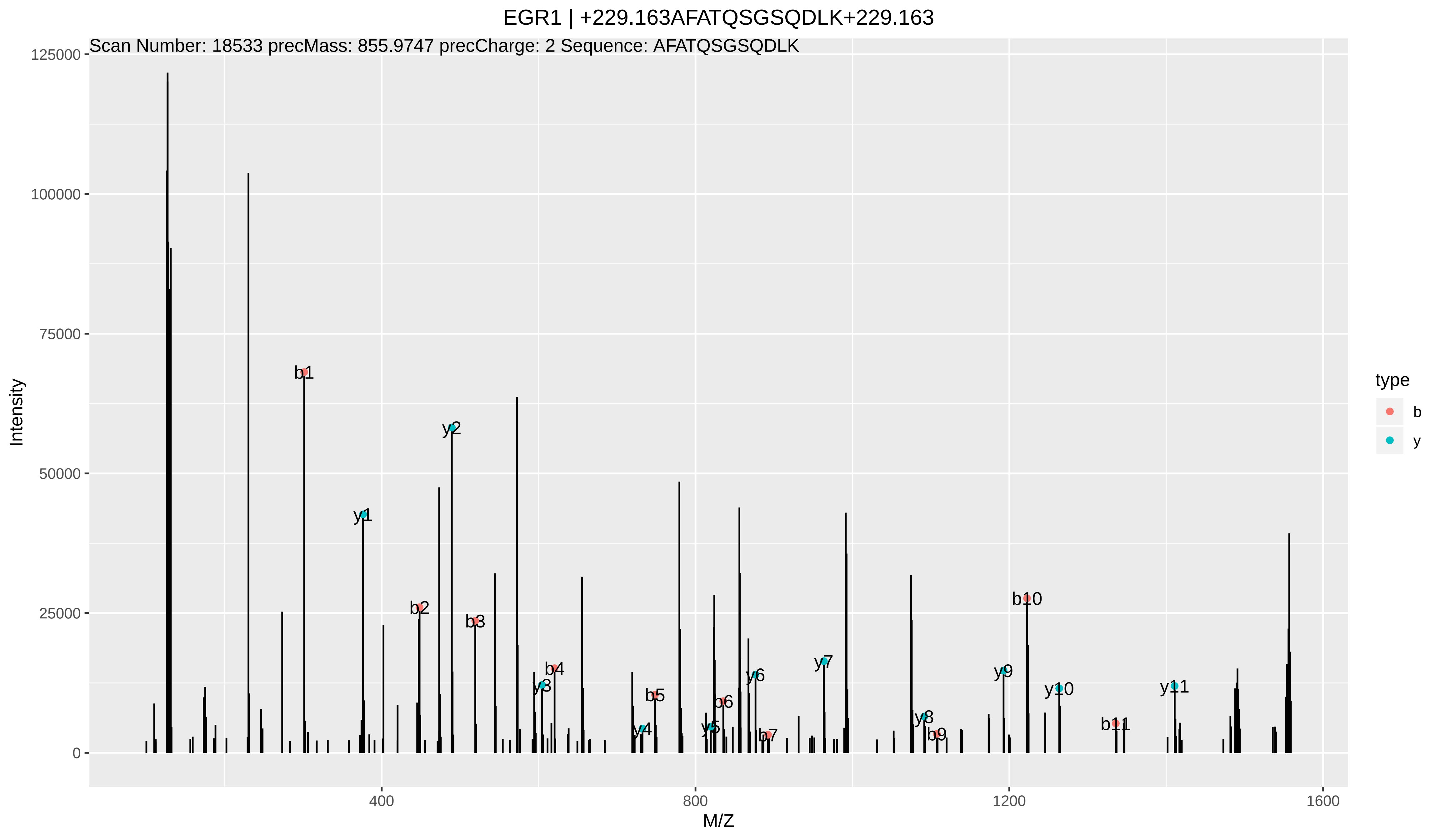

EGR1 | +229.163AFATQSGSQDLK+229.163
Scan Number: 18533 precMass: 855.9747 precCharge: 2 Sequence: AFATQSGSQDLK
Intensity
M/Z
type
b
y
b1
y1
b2
y2
b3
b4
y3
b5
y4
y5
b6
y6
b7
y7
y8
b9
y9
b10
y10
b11
y11
0
25000
50000
75000
100000
125000
400
800
1200
1600

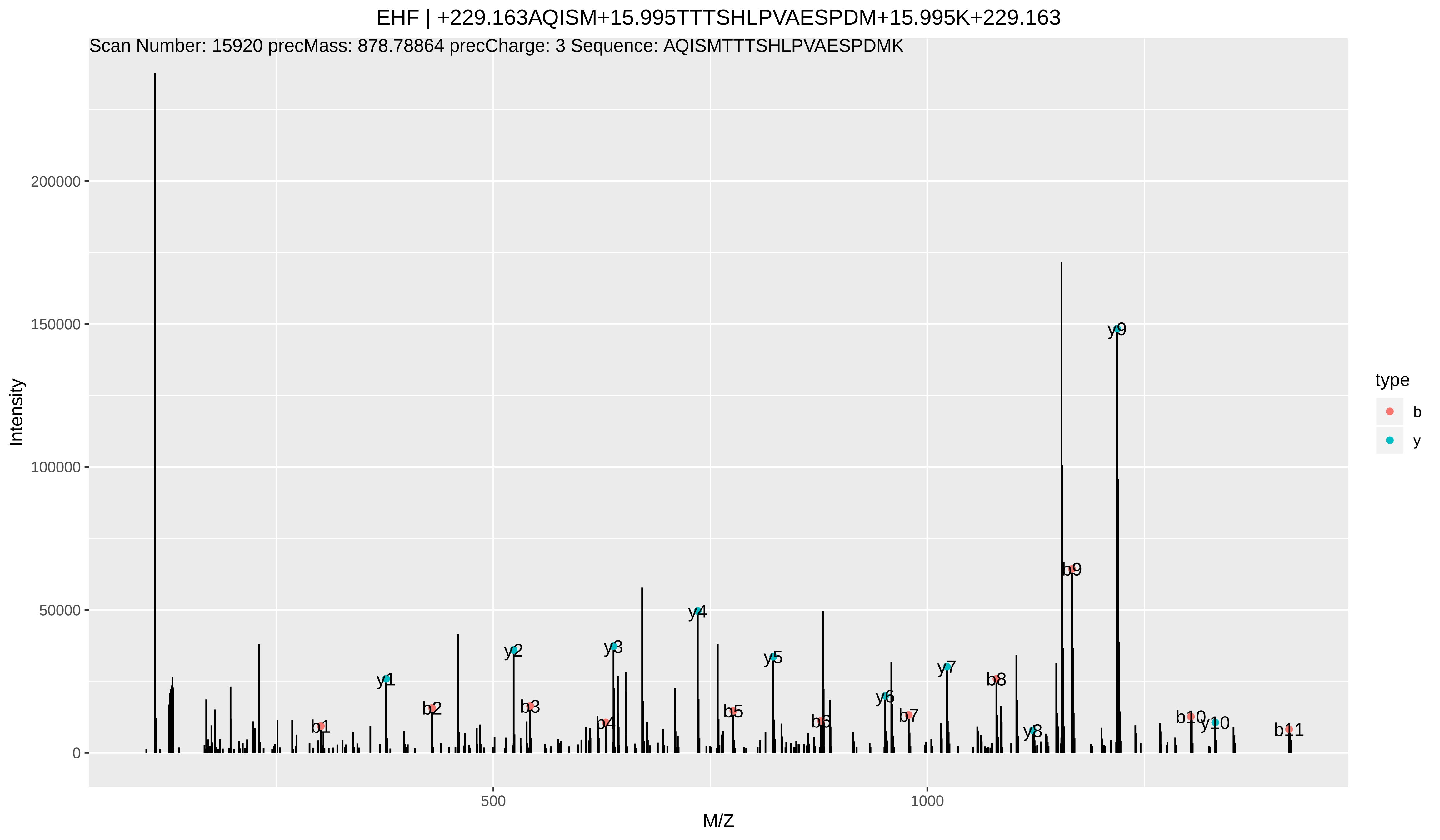
EHF | +229.163AQISM+15.995TTTSHLPVAESPDM+15.995K+229.163
Scan Number: 15920 precMass: 878.78864 precCharge: 3 Sequence: AQISMTTTSHLPVAESPDMK
Intensity
200000
150000
100000
50000
0
500
1000
M/Z
type
b
y
y1
y2
y3
y4
y5
y6
y7
y8
y9
y10
b1
b2
b3
b4
b5
b6
b7
b8
b9
b10
b11

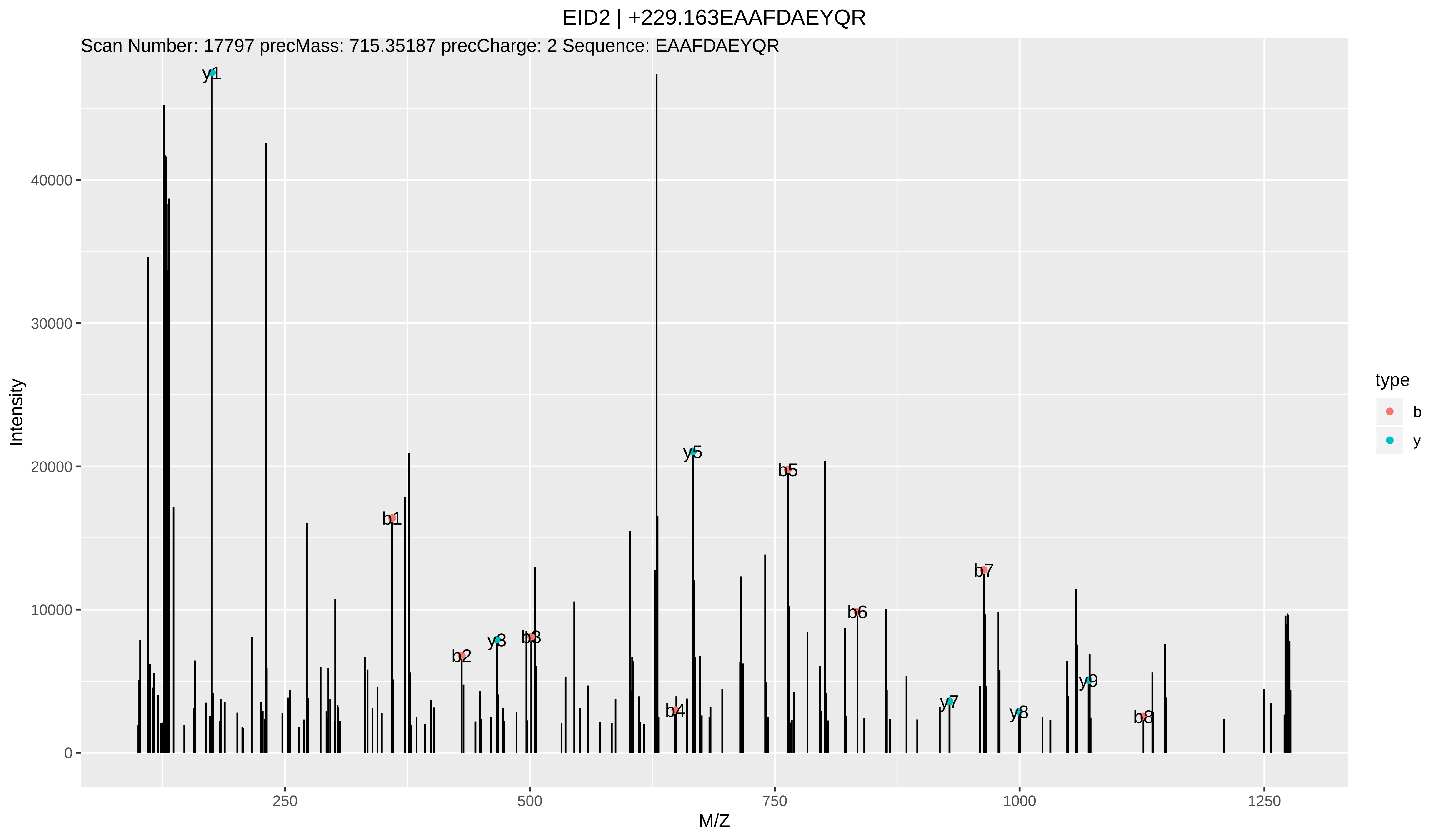

EID2 | +229.163EAAFDAEYQR
Scan Number: 17797 precMass: 715.35187 precCharge: 2 Sequence: EAAFDAEYQR
y1
b1
b2
y3
b3
b4
y5
b5
b6
y7
b7
y8
y9
b8
Intensity
40000
30000
20000
10000
0
M/Z
250
500
750
1000
1250
type
b
y

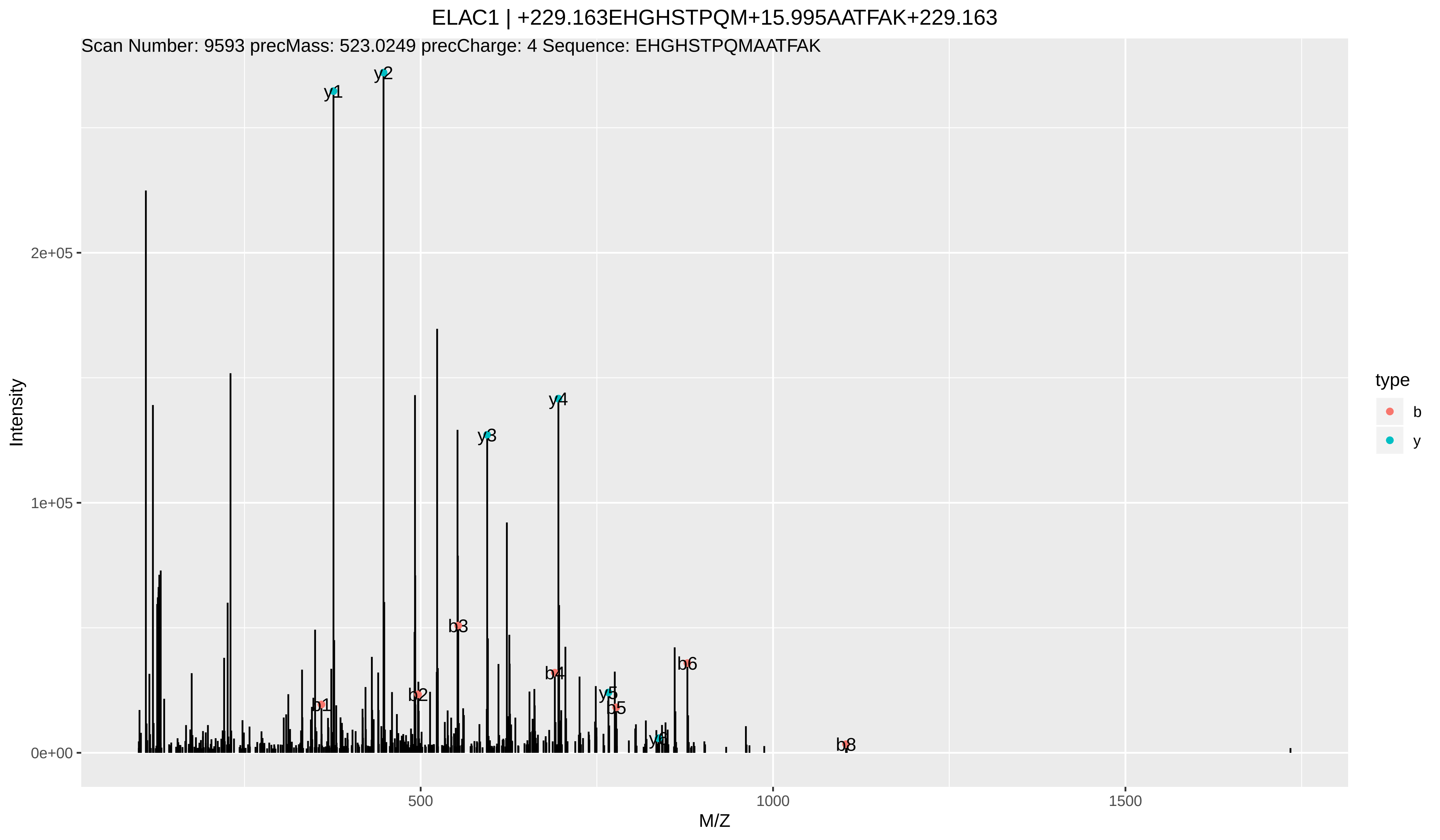

ELAC1 | +229.163EHGHSTPQM+15.995AATFAK+229.163
Scan Number: 9593 precMass: 523.0249 precCharge: 4 Sequence: EHGHSTPQMAATFAK
Intensity
2e+05
1e+05
0e+00
500
1000
1500
M/Z
y1
y2
y3
y4
y5
y6
b1
b2
b3
b4
b5
b6
b8
type
b
y

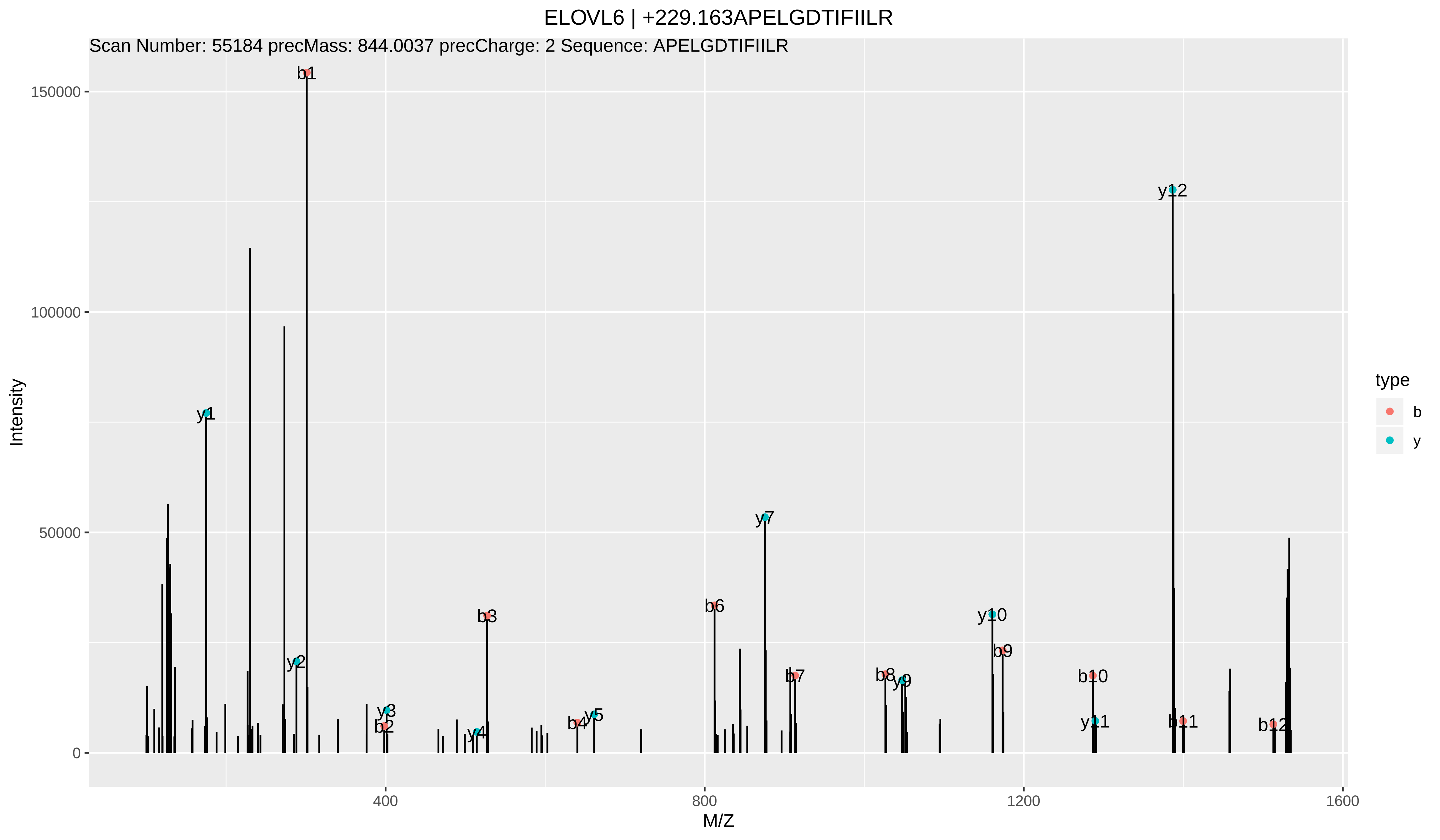
ELOVL6 | +229.163APELGDTIFIILR
Scan Number: 55184 precMass: 844.0037 precCharge: 2 Sequence: APELGDTIFIILR
Intensity
M/Z
type
b
y
b1
y12
y1
y7
b6
b3
y10
b9
y2
b7
b8
y9
b10
y3
b2
y4
b4
y5
y11
b11
b12
150000
100000
50000
0
400
800
1200
1600

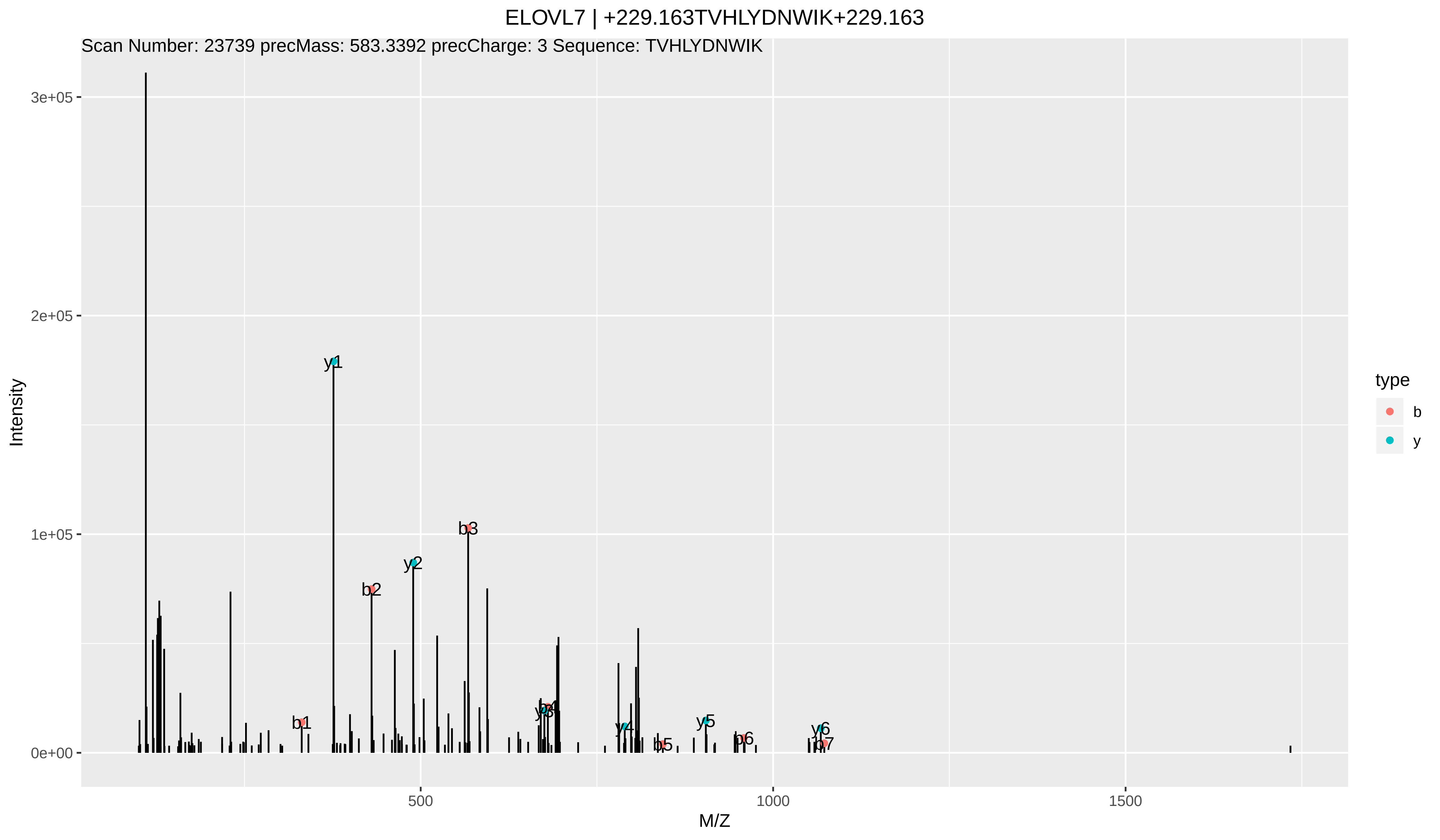
ELOVL7 | +229.163TVHLYDNWIK+229.163
Scan Number: 23739 precMass: 583.3392 precCharge: 3 Sequence: TVHLYDNWIK
Intensity
M/Z
type
b
y

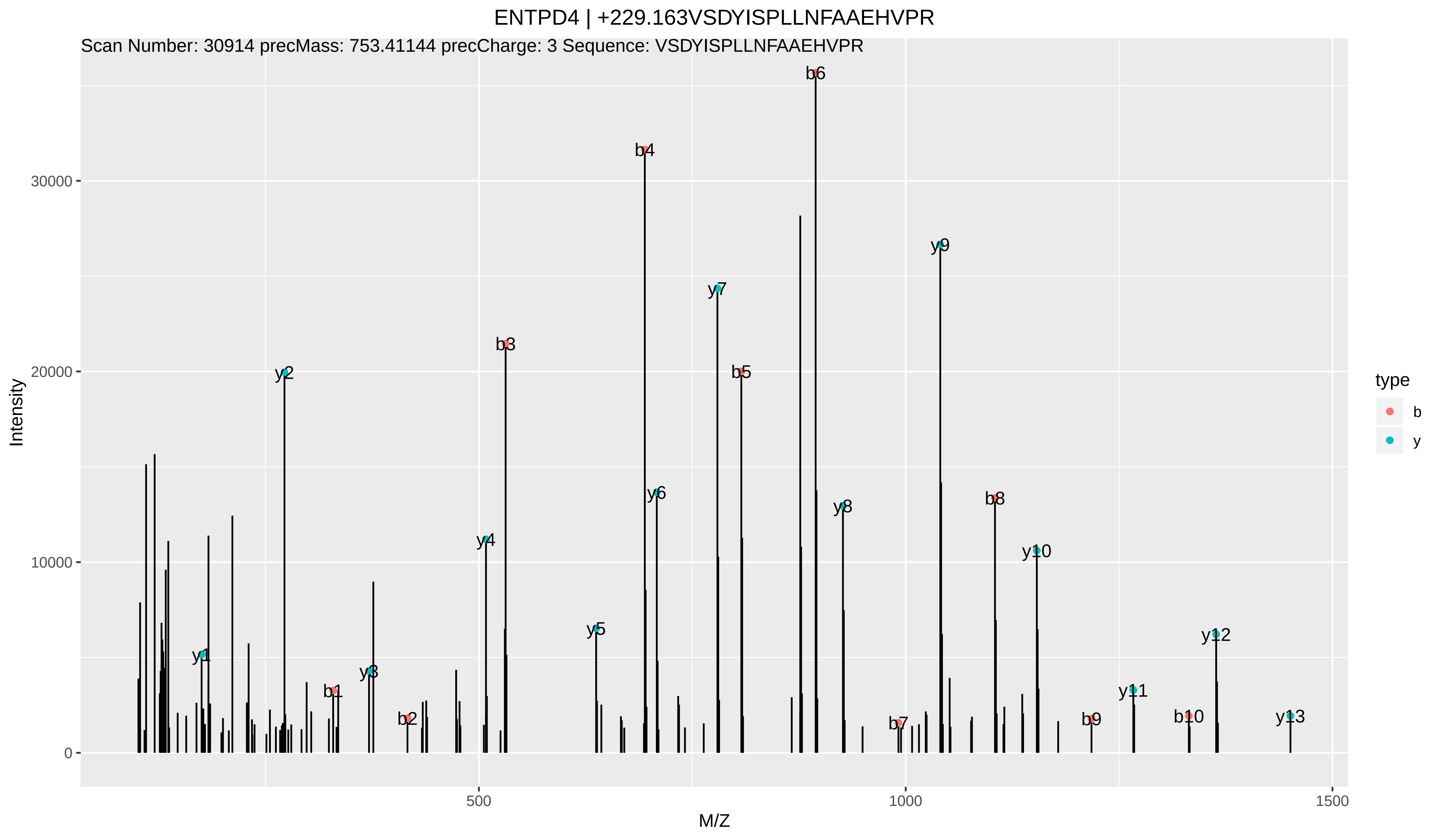

ENTPD4 | +229.163VSDYISPLLNFAAEHVPR
Scan Number: 30914 precMass: 753.41144 precCharge: 3 Sequence: VSDYISPLLNFAAEHVPR
Intensity
M/Z
0
10000
20000
30000
500
1000
1500
type
b
y
y1
y2
b1
y3
b2
y4
b3
y5
b4
y6
y7
b5
b6
y8
b7
y9
b8
y10
b9
y11
b10
y12
y13

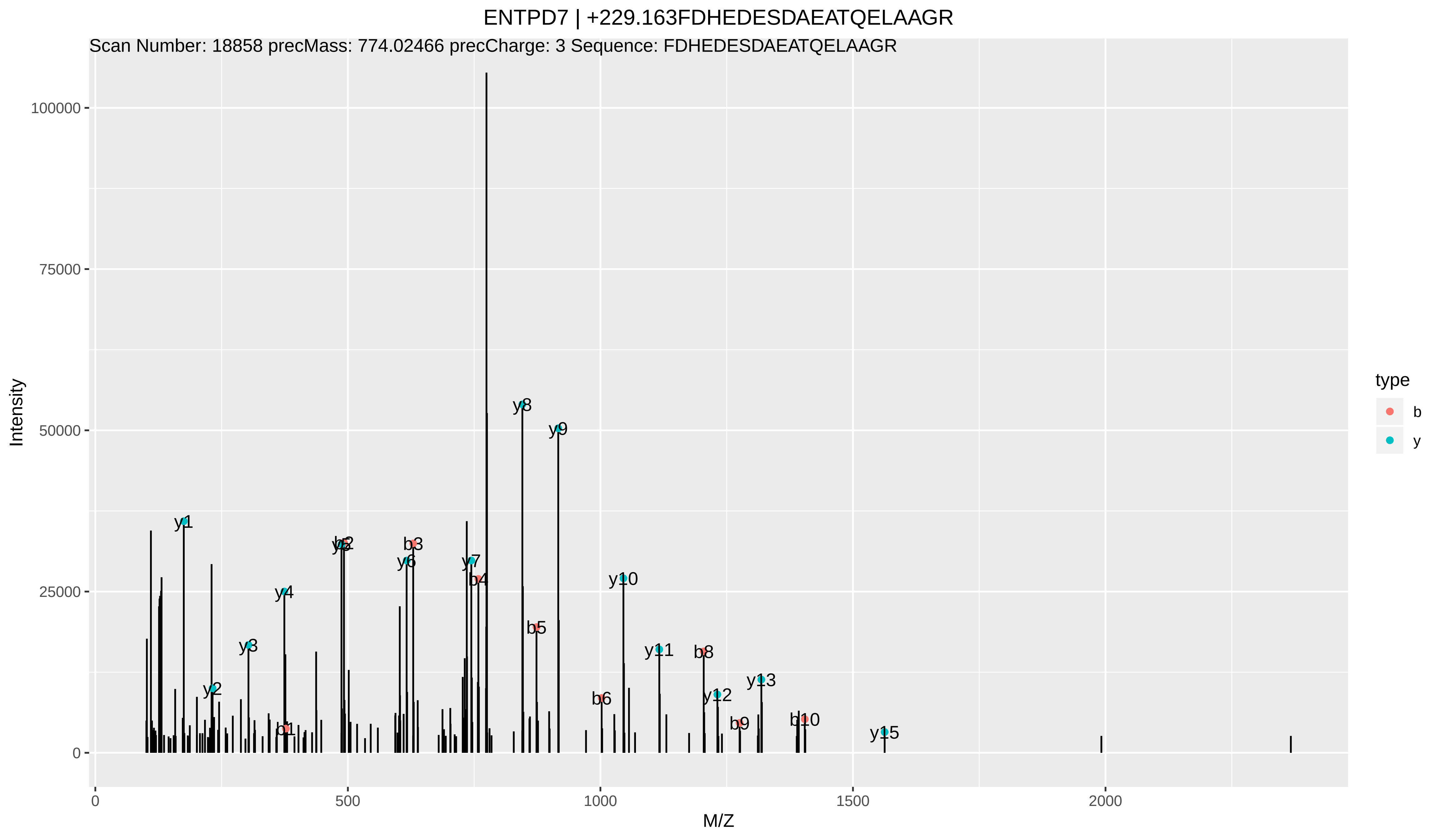

ENTPD7 | +229.163FDHEDESDAEATQELAAGR
Scan Number: 18858 precMass: 774.02466 precCharge: 3 Sequence: FDHEDESDAEATQELAAGR
Intensity
M/Z
type
b
y
y1
y2
y3
y4
y6
y7
y8
y9
y10
y11
y12
y13
y15
b1
b3
b4
b5
b6
b8
b9
b10
0
25000
50000
75000
100000
500
1000
1500
2000

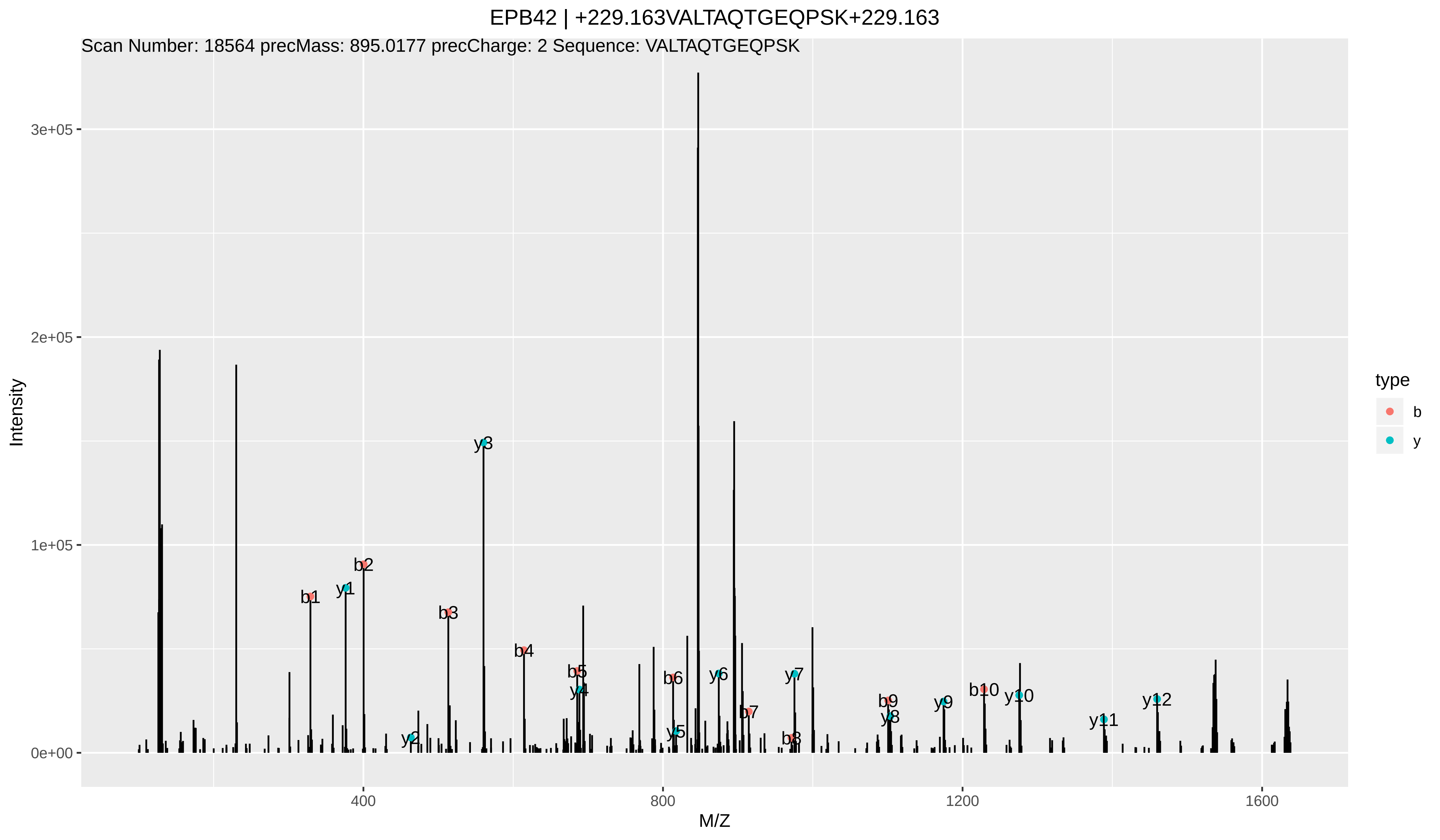
EPB42 | +229.163VALTAQTGEQPSK+229.163
Scan Number: 18564 precMass: 895.0177 precCharge: 2 Sequence: VALTAQTGEQPSK
Intensity
M/Z
3e+05
2e+05
1e+05
0e+00
400
800
1200
1600
b1
y1
b2
b3
y3
y2
b4
b5
y4
b6
y5
y6
b7
y7
b8
b9
y8
y9
b10
y10
y11
y12
type
b
y

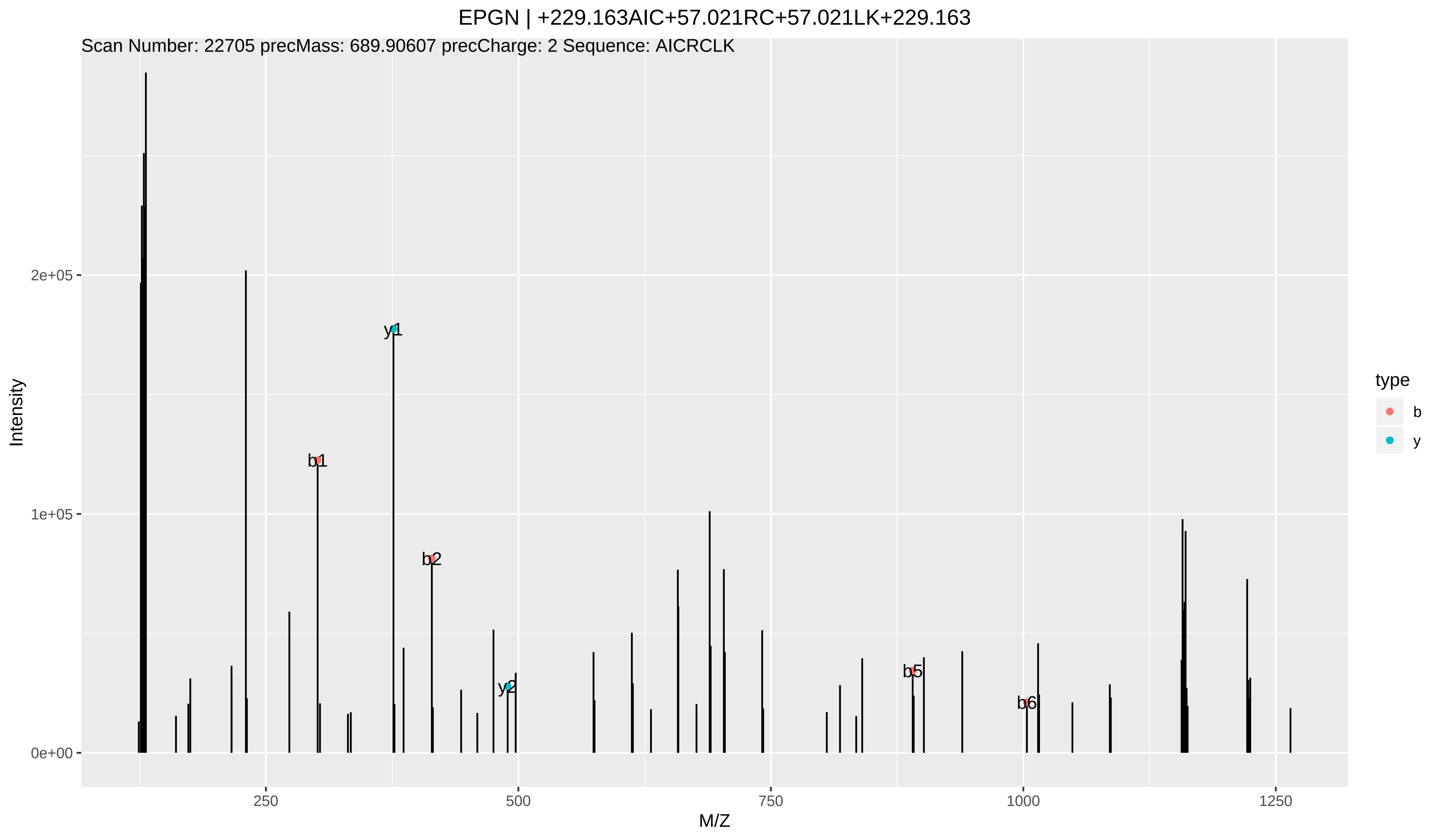
EPGN | +229.163AIC+57.021RC+57.021LK+229.163
Scan Number: 22705 precMass: 689.90607 precCharge: 2 Sequence: AICRCLK
Intensity
M/Z
2e+05
1e+05
0e+00
250
500
750
1000
1250
y1
b1
b2
y2
b5
b6
type
b
y

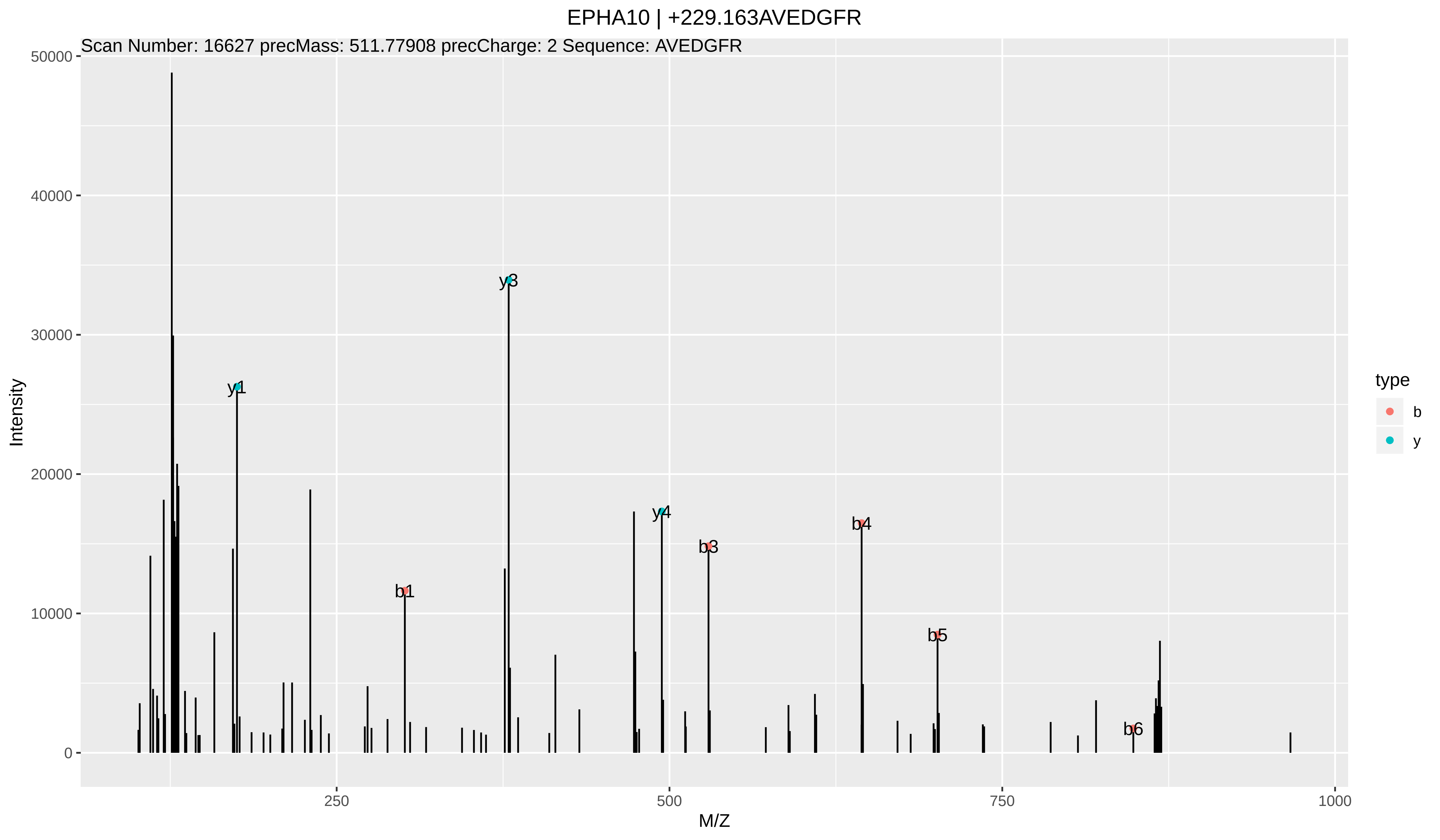
EPHA10 | +229.163AVEDGFR
Scan Number: 16627 precMass: 511.77908 precCharge: 2 Sequence: AVEDGFR
Intensity
50000
40000
30000
20000
10000
0
y1
y3
y4
b1
b3
b4
b5
b6
250
500
750
1000
M/Z
type
b
y

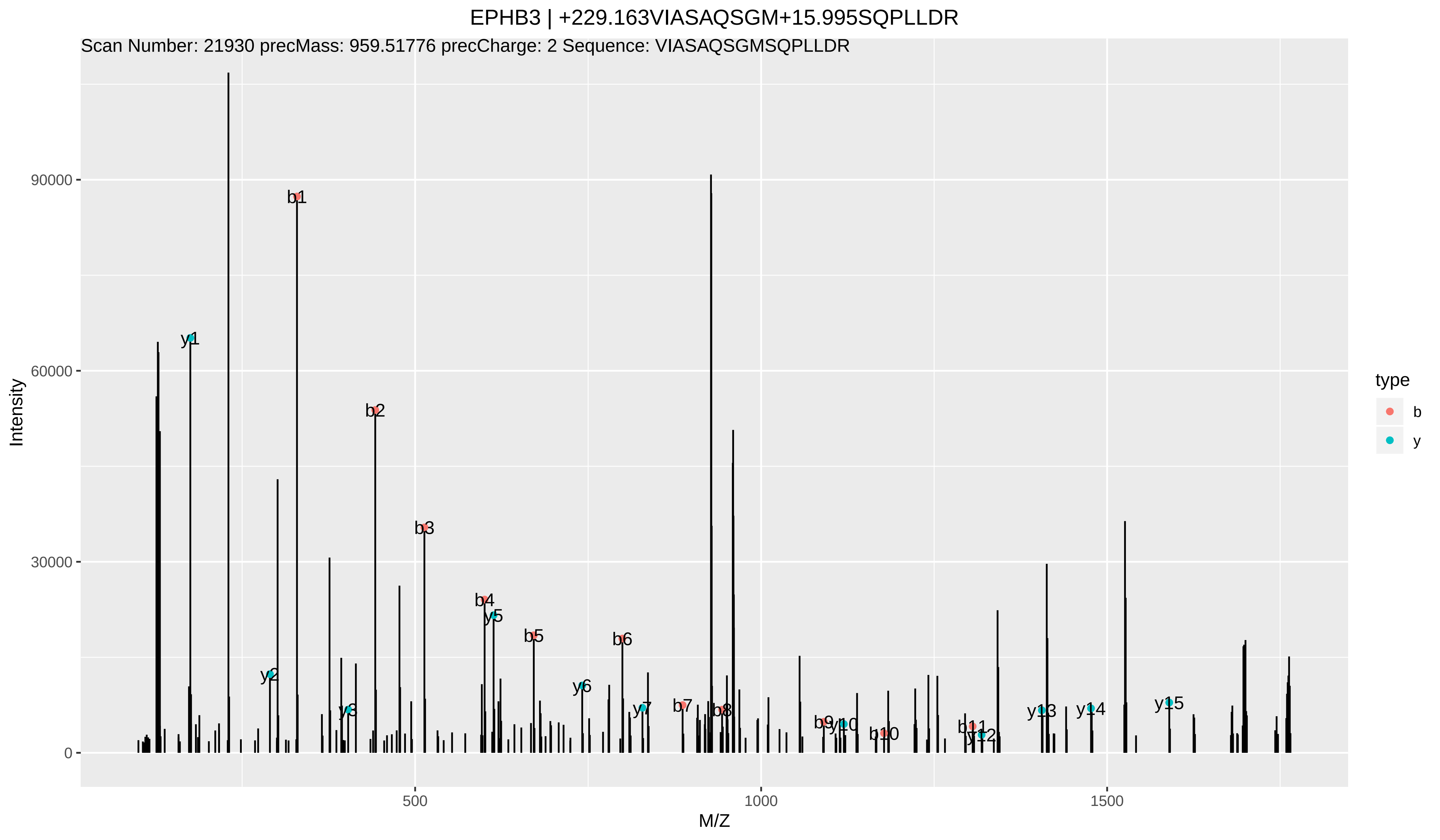

EPHB3 | +229.163VIASAQSGM+15.995SQPLLDR
Scan Number: 21930 precMass: 959.51776 precCharge: 2 Sequence: VIASAQSGMSQPLLDR
Intensity
90000
60000
30000
0
M/Z
500
1000
1500
type
b
y
y1
b1
b2
b3
b4
y5
b5
b6
y2
y3
y6
y7
b7
b8
b9
y10
b10
b11
y12
y13
y14
y15

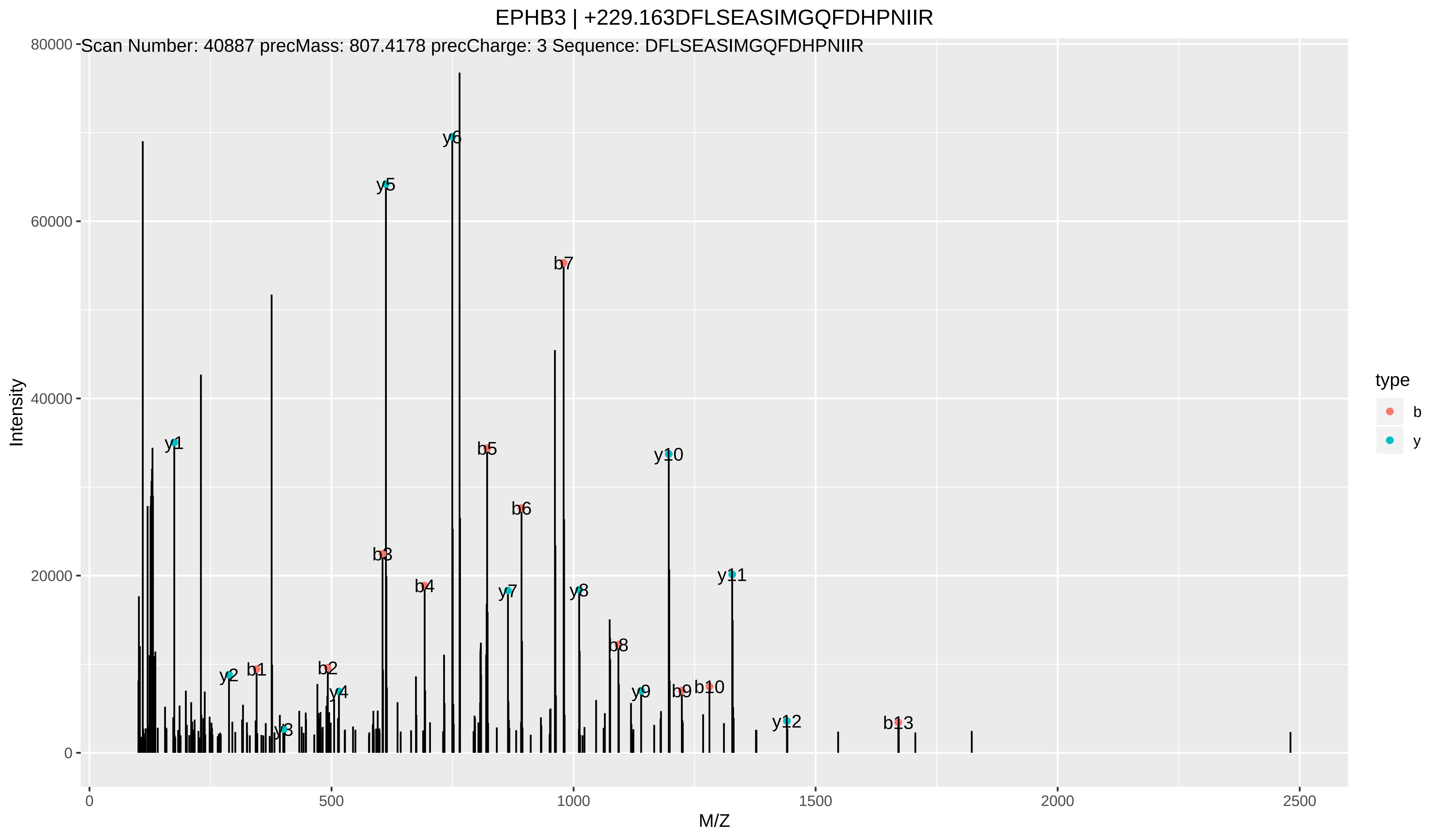
EPHB3 | +229.163DFLSEASIMGQFDHPNIIR
Scan Number: 40887 precMass: 807.4178 precCharge: 3 Sequence: DFLSEASIMGQFDHPNIIR
Intensity
M/Z
0
20000
40000
60000
80000
0
500
1000
1500
2000
2500
y1
y2
b1
y3
b2
y4
b3
y5
b4
y6
b5
y7
b6
b7
y8
b8
y9
y10
b9
b10
y11
y12
b13
type
b
y

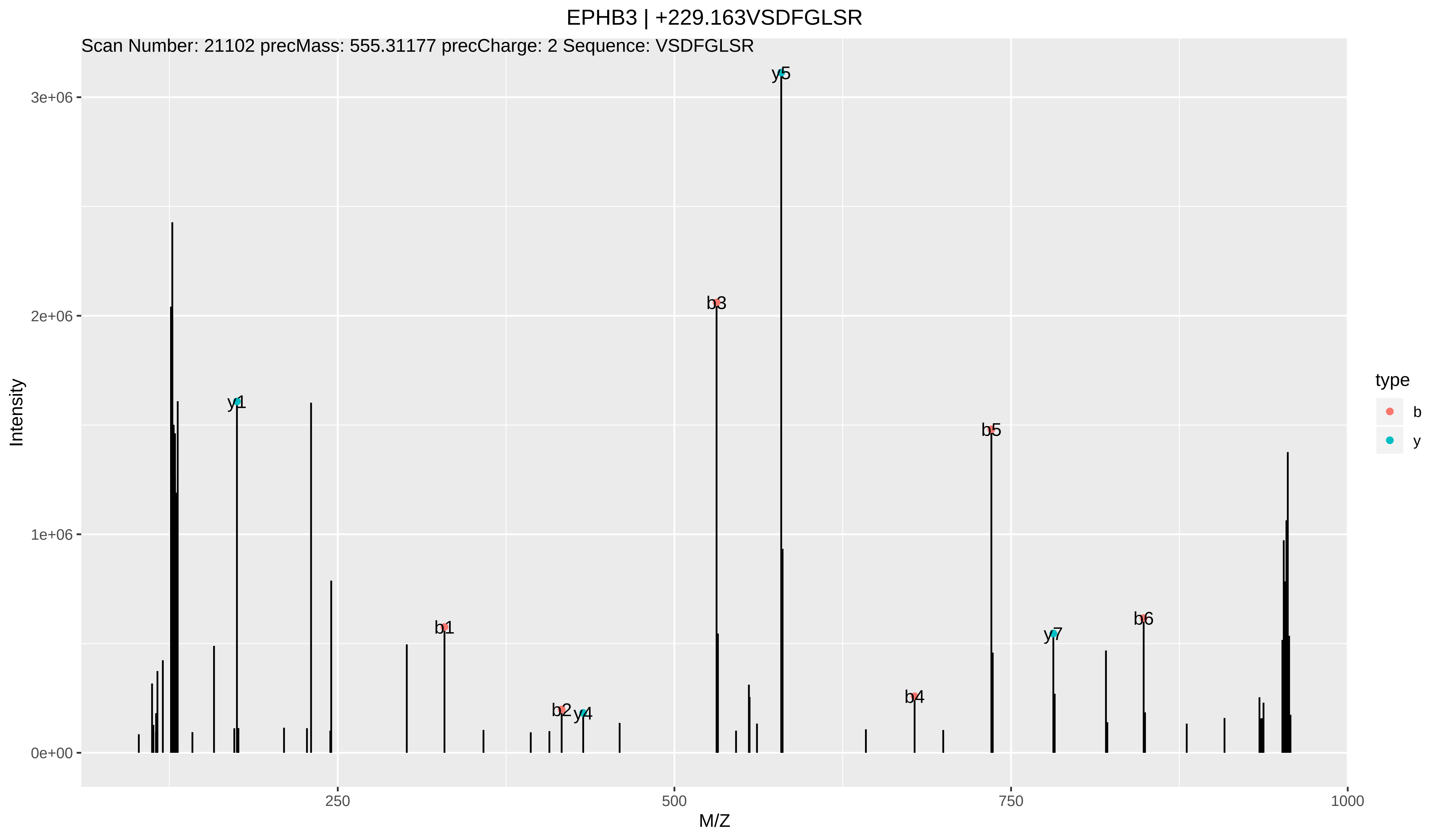
EPHB3 | +229.163VSDFGLSR
Scan Number: 21102 precMass: 555.31177 precCharge: 2 Sequence: VSDFGLSR
Intensity
3e+06
2e+06
1e+06
0e+00
M/Z
250
500
750
1000
y1
b1
b2
y4
b3
y5
b4
b5
y7
b6
type
b
y

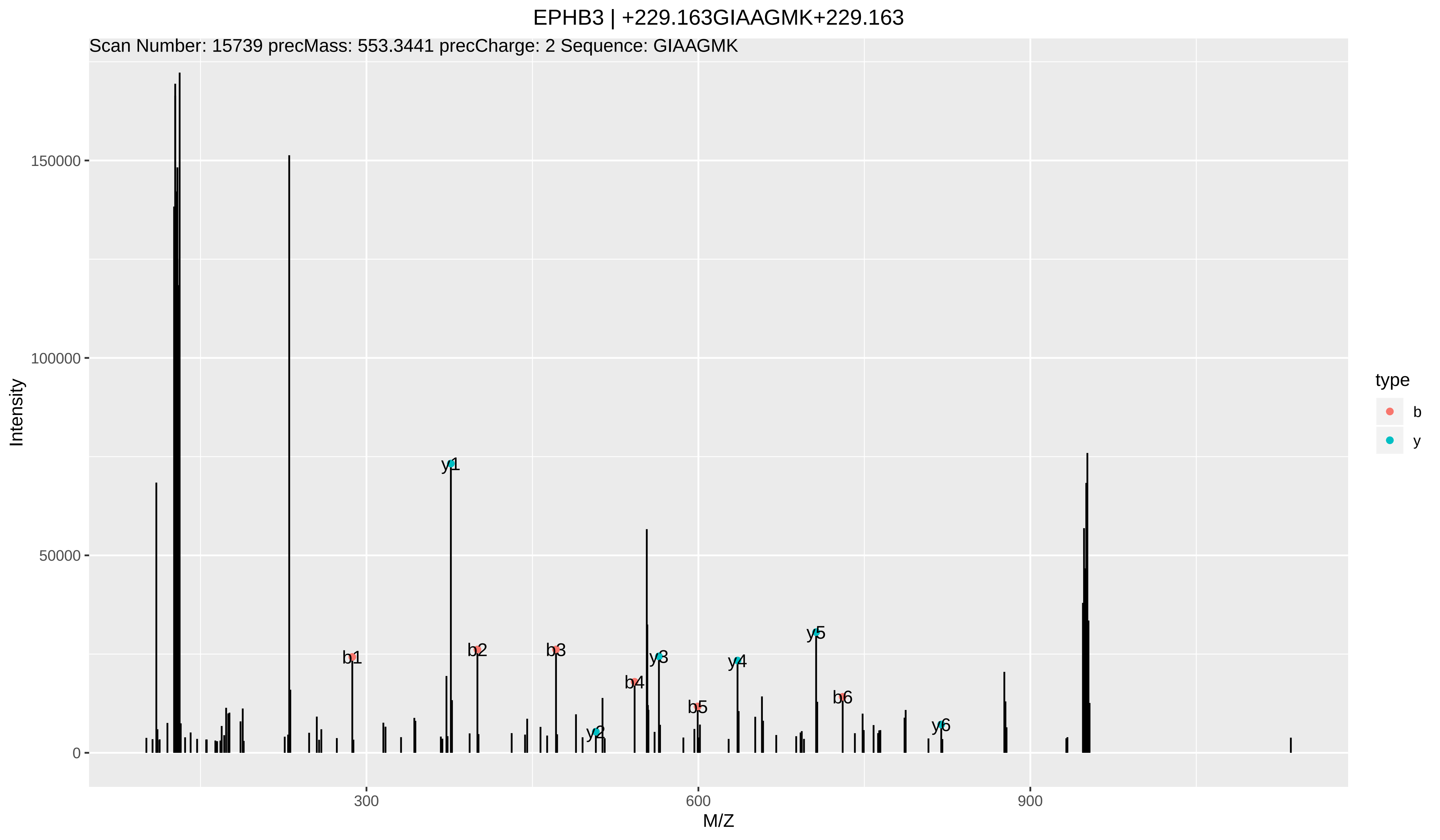
EPHB3 | +229.163GIAAGMK+229.163
Scan Number: 15739 precMass: 553.3441 precCharge: 2 Sequence: GIAAGMK
Intensity
150000
100000
50000
0
300
600
900
M/Z
y1
b1
b2
b3
y3
y4
y5
b4
b5
b6
y2
y6
type
b
y

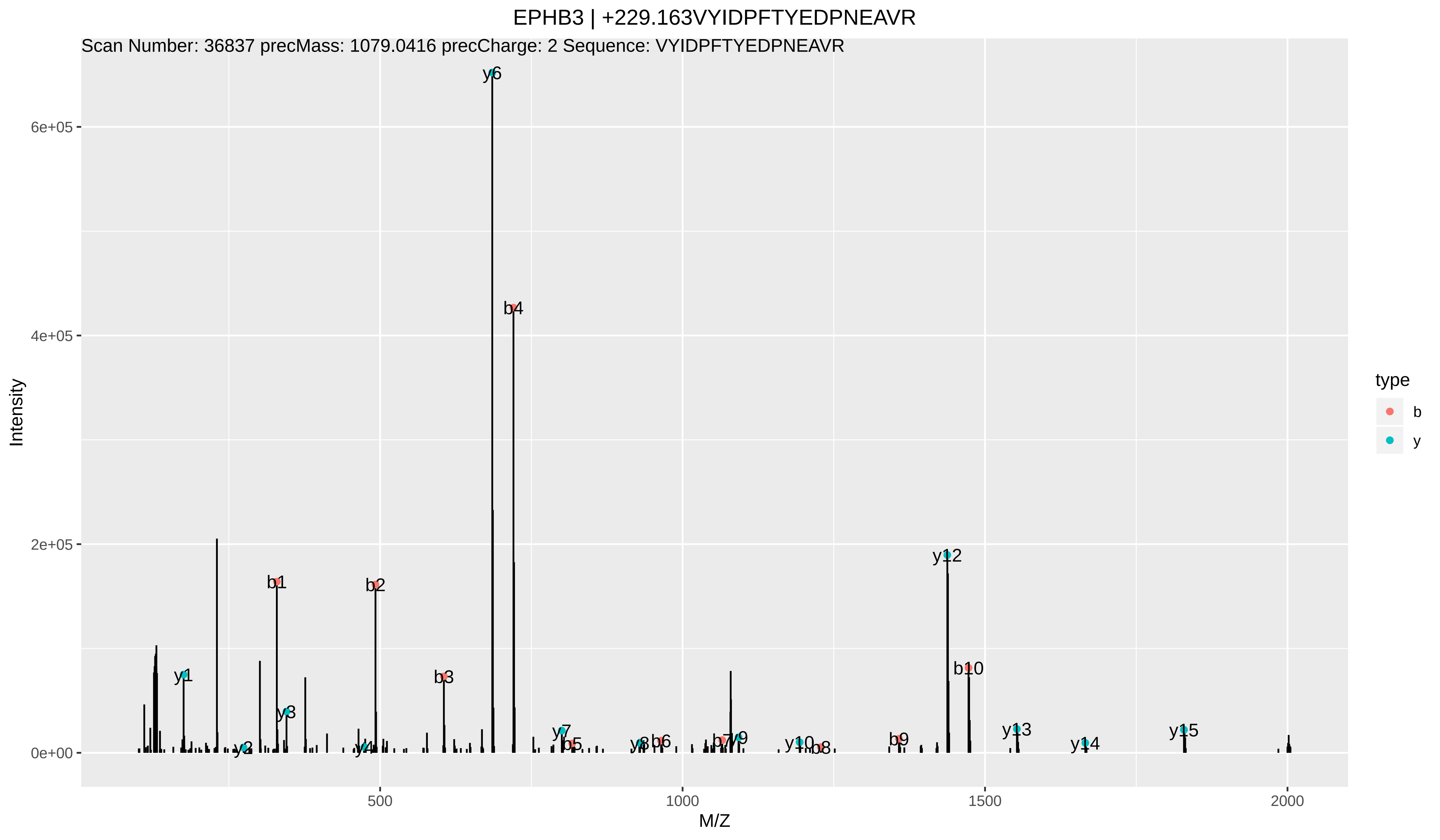

EPHB3 | +229.163VYIDPFTYEDPNEAVR
Scan Number: 36837 precMass: 1079.0416 precCharge: 2 Sequence: VYIDPFTYEDPNEAVR
Intensity
M/Z
type
b
y
y6
b4
y12
b1
b2
b10
y1
b3
y3
y7
y13
y15
b5
y8
b6
b7
y9
y10
b8
b9
y14
y2
y4
6e+05
4e+05
2e+05
0e+00
500
1000
1500
2000

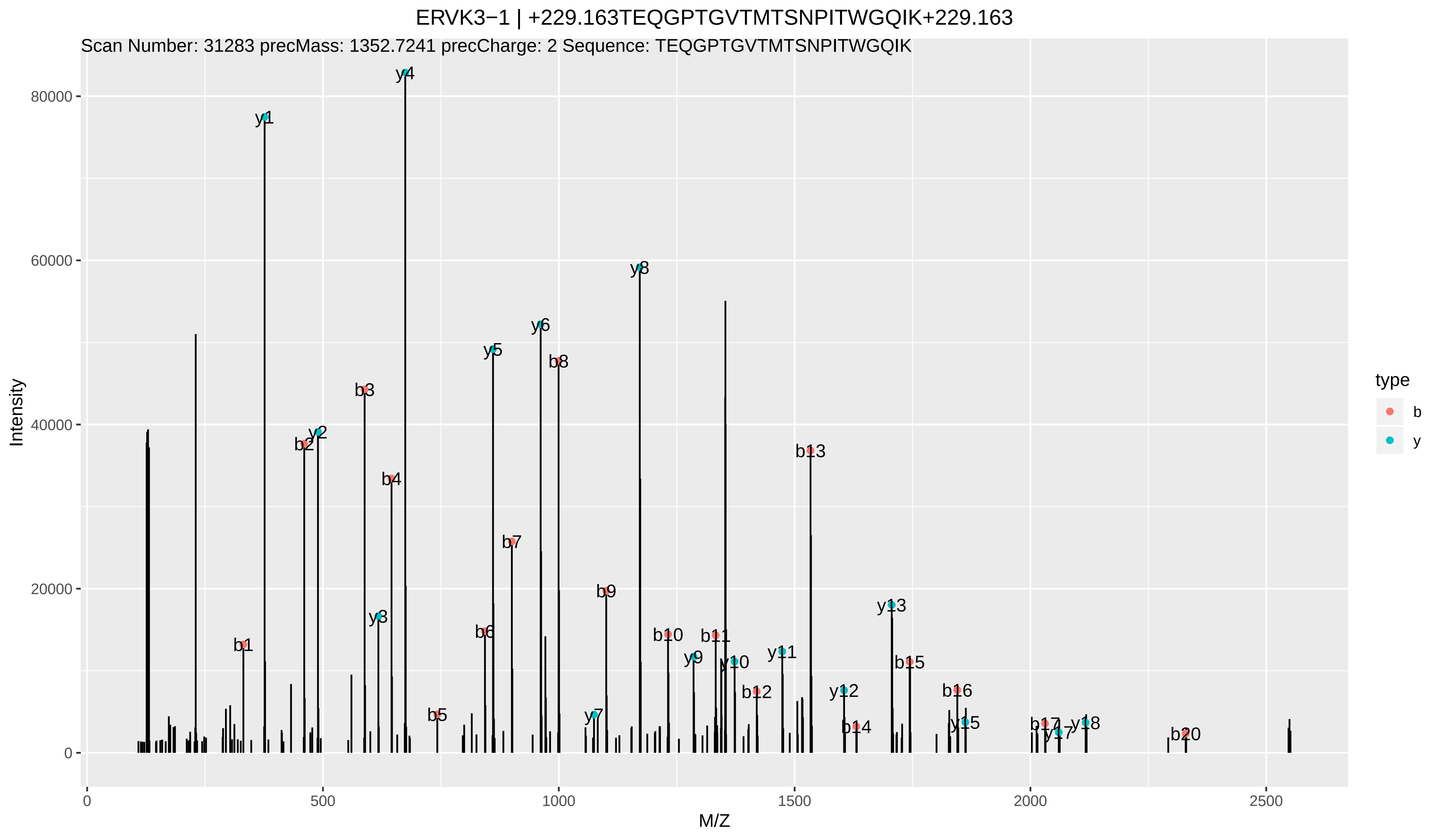

ERVK3−1 | +229.163TEQGPTGVTMTSNPITWGQIK+229.163
Scan Number: 31283 precMass: 1352.7241 precCharge: 2 Sequence: TEQGPTGVTMTSNPITWGQIK
Intensity
M/Z
type
b
y
0
20000
40000
60000
80000
0
500
1000
1500
2000
2500
y1
y2
y3
y4
y5
y6
y7
y8
y9
y10
y11
y12
y13
y15
y17
y18
b1
b2
b3
b4
b5
b6
b7
b8
b9
b10
b11
b12
b13
b14
b15
b16
b17
b20

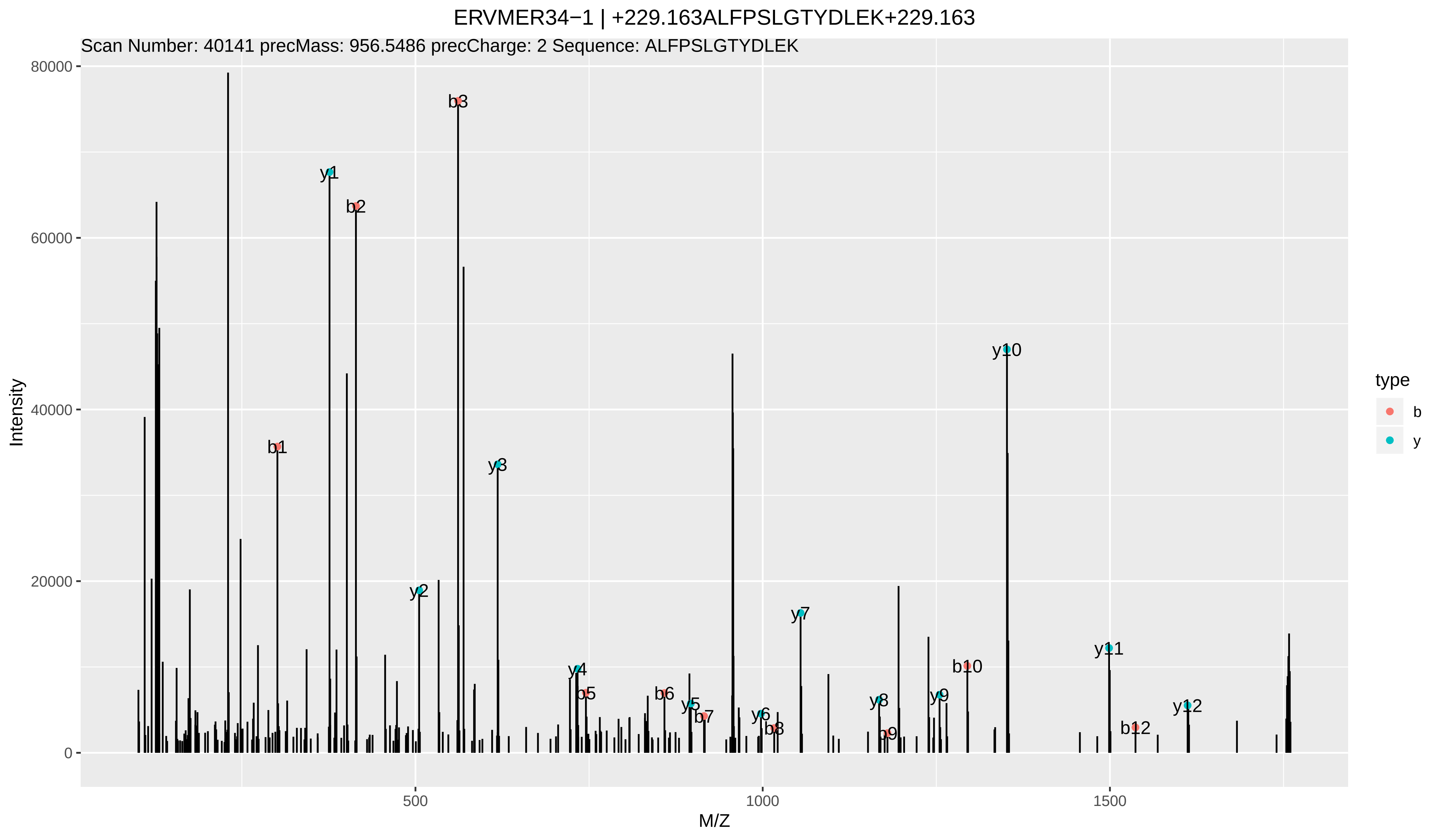

ERVMER34−1 | +229.163ALFPSLGTYDLEK+229.163
Scan Number: 40141 precMass: 956.5486 precCharge: 2 Sequence: ALFPSLGTYDLEK
Intensity
M/Z
0
20000
40000
60000
80000
500
1000
1500
b3
y1
b2
b1
y3
y2
y10
y4
b5
b6
y5
b7
y6
b8
y7
b9
y8
y9
b10
y11
b12
y12
type
b
y

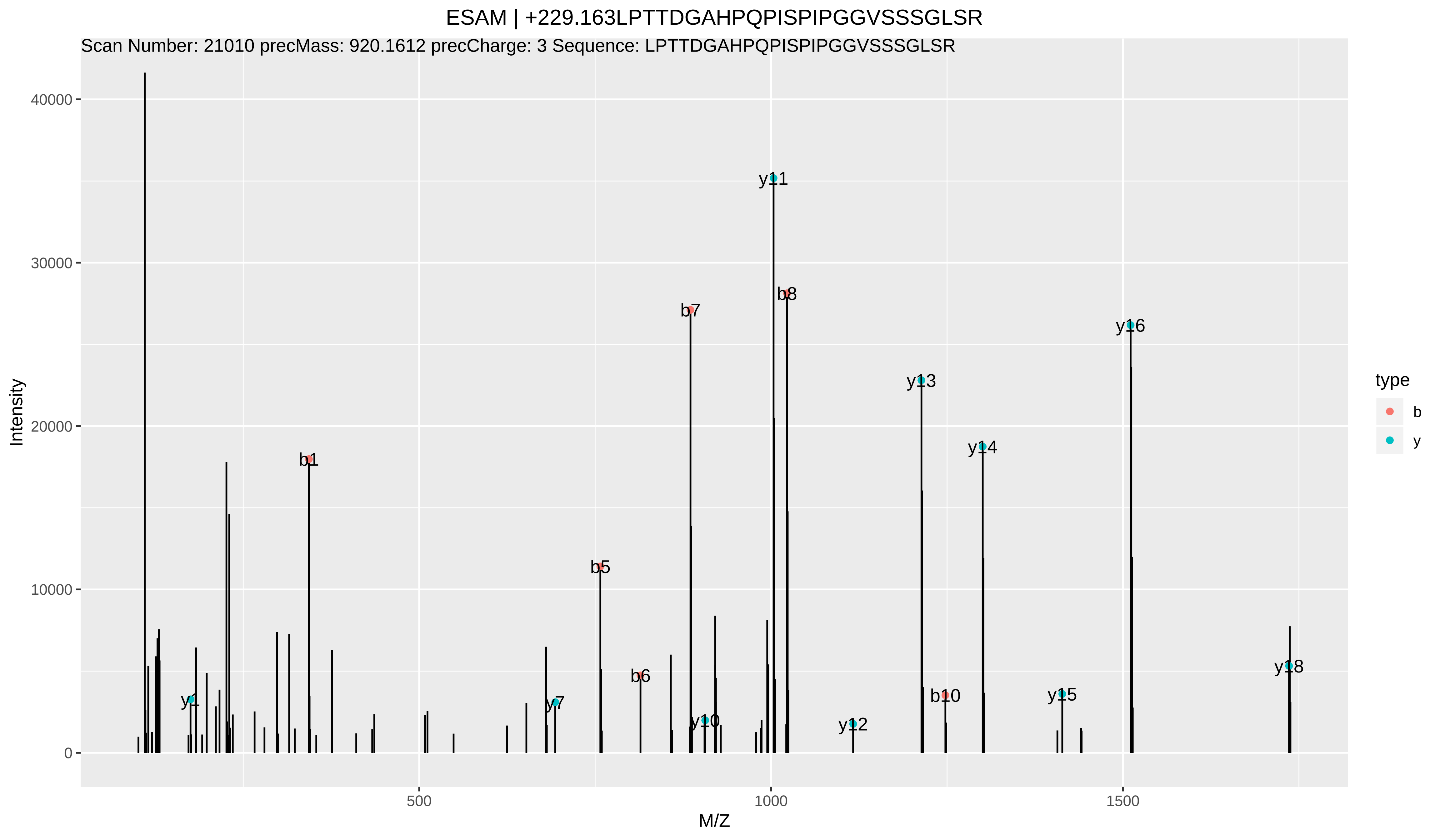

ESAM | +229.163LPTTDGAHPQPISPIPGGVSSSGLSR
Scan Number: 21010 precMass: 920.1612 precCharge: 3 Sequence: LPTTDGAHPQPISPIPGGVSSSGLSR
Intensity
M/Z
0
10000
20000
30000
40000
500
1000
1500
type
b
y
y1
b1
y7
b5
b6
b7
y10
y11
b8
y12
y13
b10
y14
y15
y16
y18

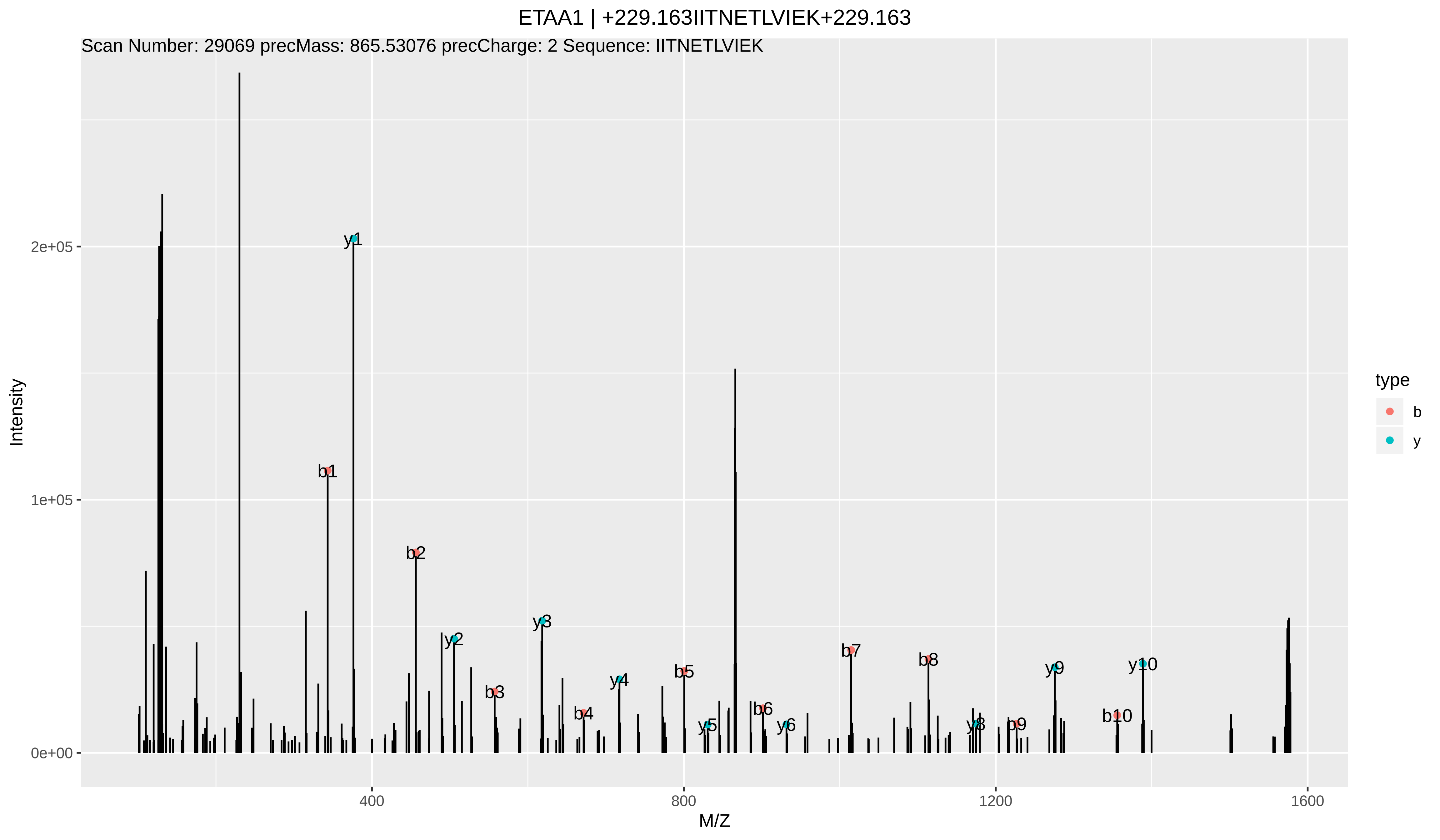

ETAA1 | +229.163IITNETLVIEK+229.163
Scan Number: 29069 precMass: 865.53076 precCharge: 2 Sequence: IITNETLVIEK
Intensity
M/Z
0e+00
1e+05
2e+05
400
800
1200
1600
y1
b1
b2
y2
y3
b3
b4
y4
b5
y5
b6
y6
b7
b8
y8
b9
y9
b10
y10
type
b
y

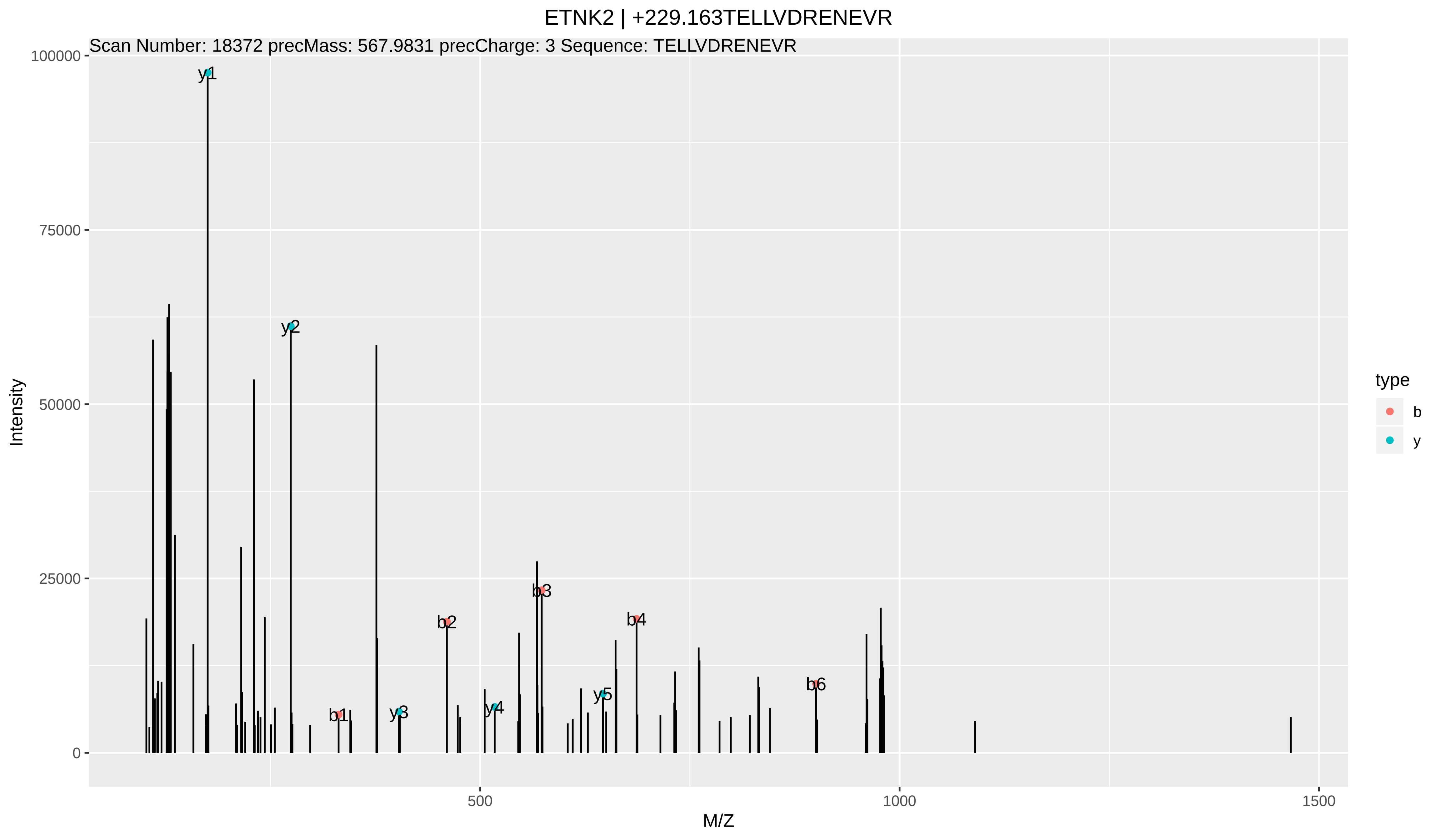

ETNK2 | +229.163TELLVDRENEVR
Scan Number: 18372 precMass: 567.9831 precCharge: 3 Sequence: TELLVDRENEVR
Intensity
100000
75000
50000
25000
0
M/Z
500
1000
1500
y1
y2
y3
y4
y5
b1
b2
b3
b4
b6
type
b
y

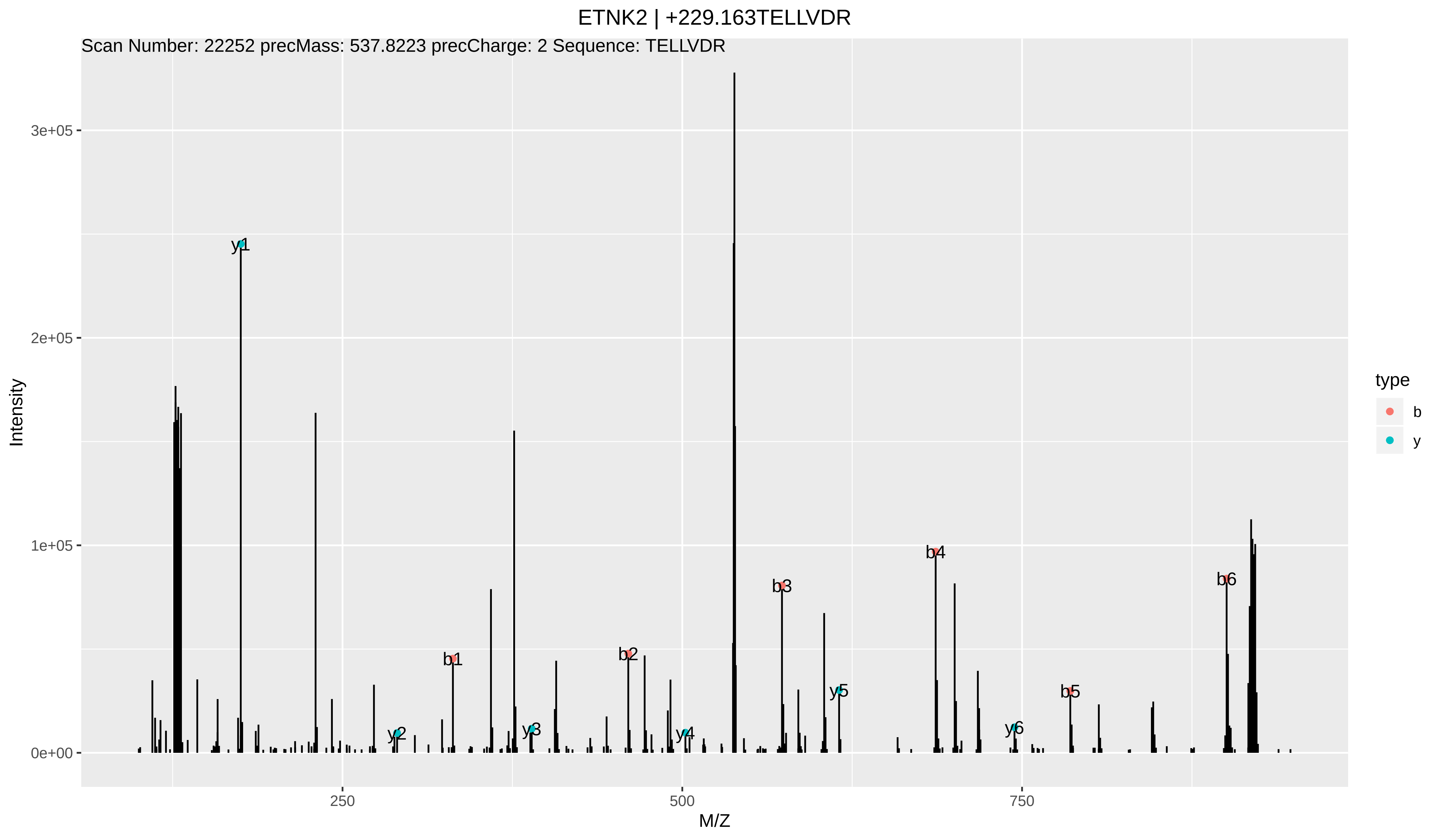

ETNK2 | +229.163TELLVDR
Scan Number: 22252 precMass: 537.8223 precCharge: 2 Sequence: TELLVDR
Intensity
M/Z
0e+00
1e+05
2e+05
3e+05
250
500
750
y1
y2
y3
y4
y5
y6
b1
b2
b3
b4
b5
b6
type
b
y

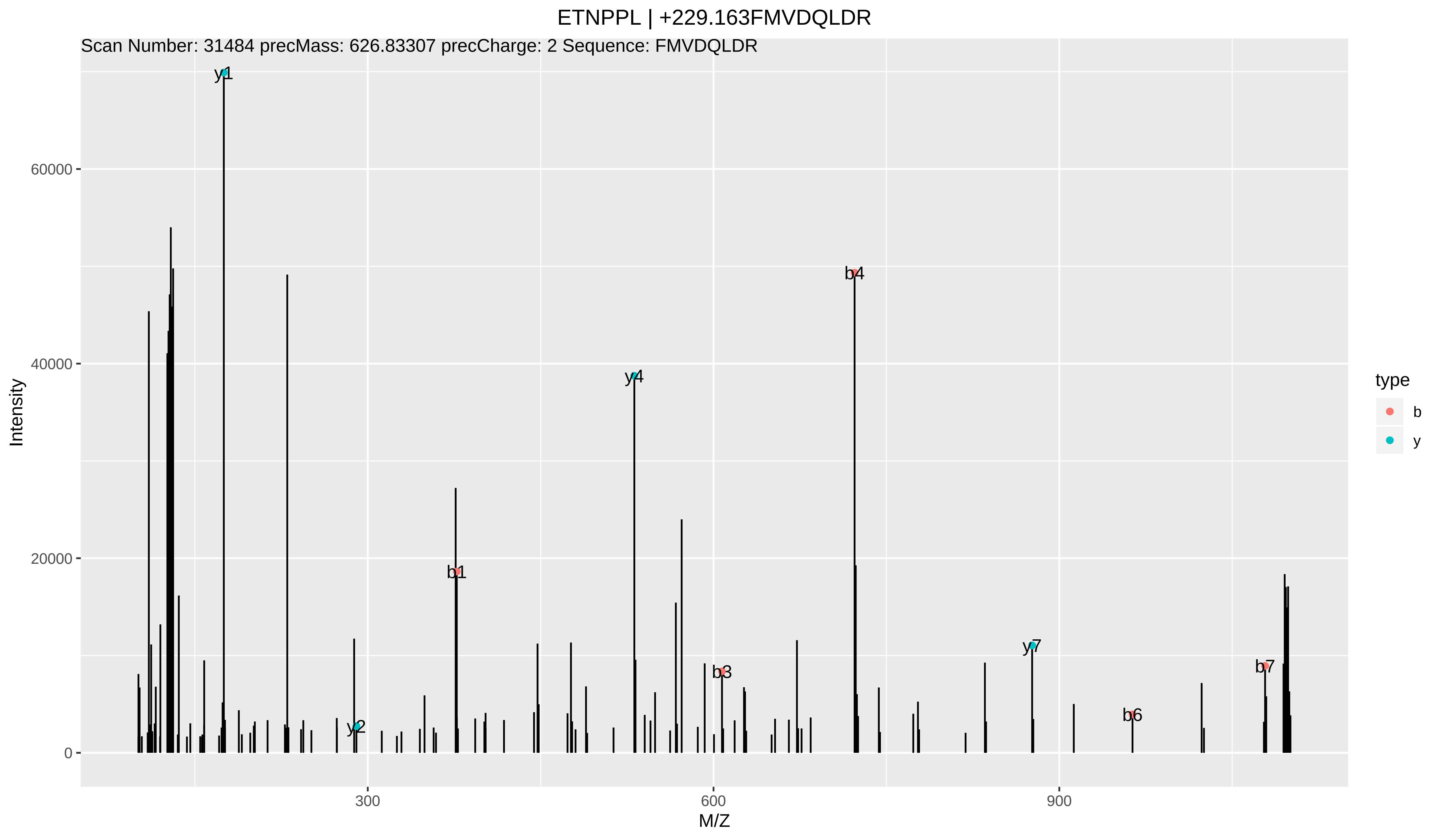
ETNPPL | +229.163FMVDQLDR
Scan Number: 31484 precMass: 626.83307 precCharge: 2 Sequence: FMVDQLDR
Intensity
M/Z
y1
y2
y4
y7
b1
b3
b4
b6
b7
60000
40000
20000
0
300
600
900
type
b
y

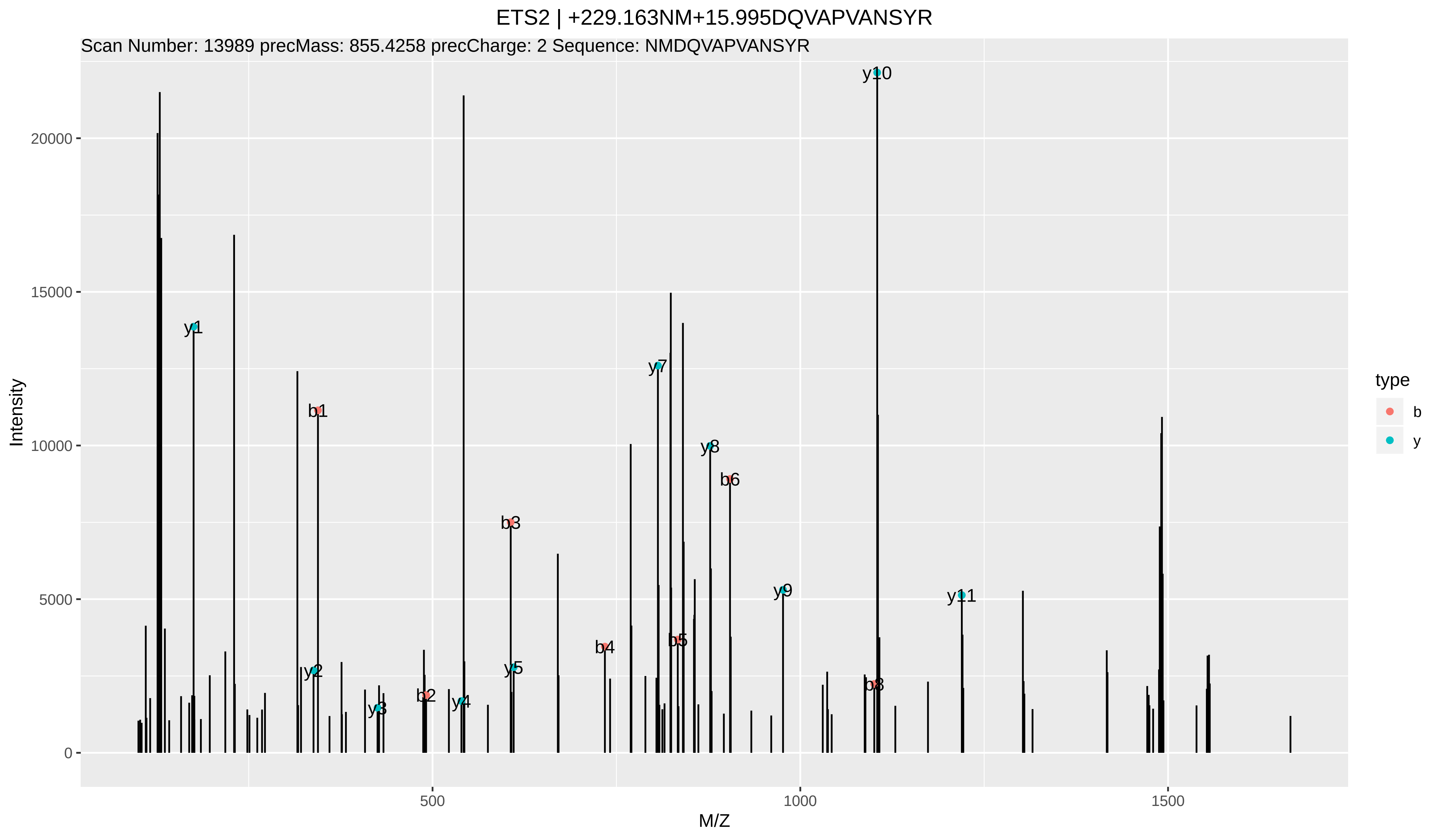
ETS2 | +229.163NM+15.995DQVAPVANSYR
Scan Number: 13989 precMass: 855.4258 precCharge: 2 Sequence: NMDQVAPVANSYR
Intensity
M/Z
0
5000
10000
15000
20000
500
1000
1500
type
b
y
y1
y2
y3
y4
y5
y7
y8
y9
y10
y11
b1
b2
b3
b4
b5
b6
b8

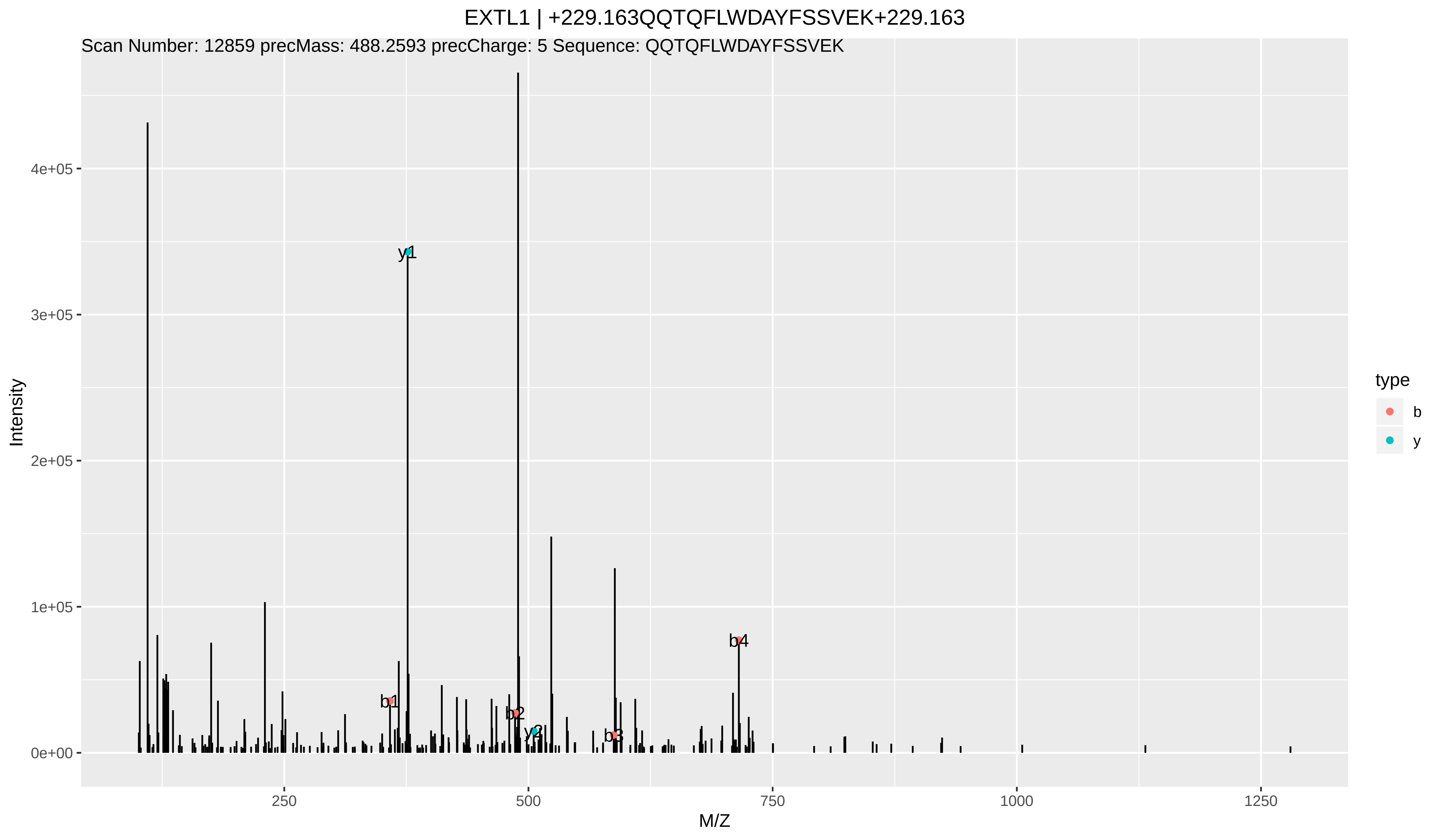

EXTL1 | +229.163QQTQFLWDAYFSSVEK+229.163
Scan Number: 12859 precMass: 488.2593 precCharge: 5 Sequence: QQTQFLWDAYFSSVEK
Intensity
M/Z
type
b
y
y1
b1
b2
y2
b3
b4
4e+05
3e+05
2e+05
1e+05
0e+00
250
500
750
1000
1250

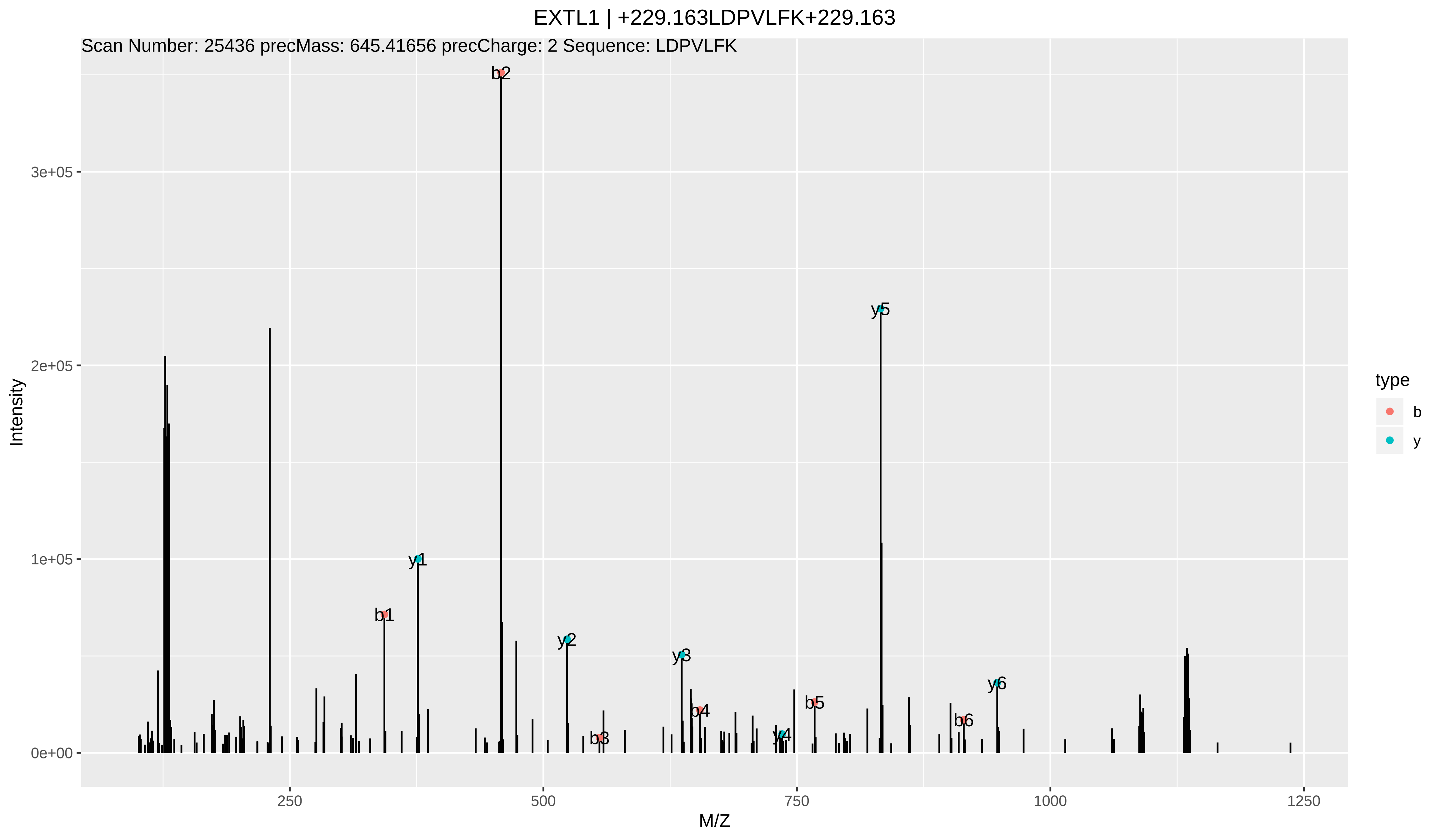
EXTL1 | +229.163LDPVLFK+229.163
Scan Number: 25436 precMass: 645.41656 precCharge: 2 Sequence: LDPVLFK
Intensity
M/Z
type
b
y
b1
b2
b3
b4
b5
b6
y1
y2
y3
y4
y5
y6
0e+00
1e+05
2e+05
3e+05
250
500
750
1000
1250

EZH1 | +229.163NEFISEYC+57.021GELISQDEADRR

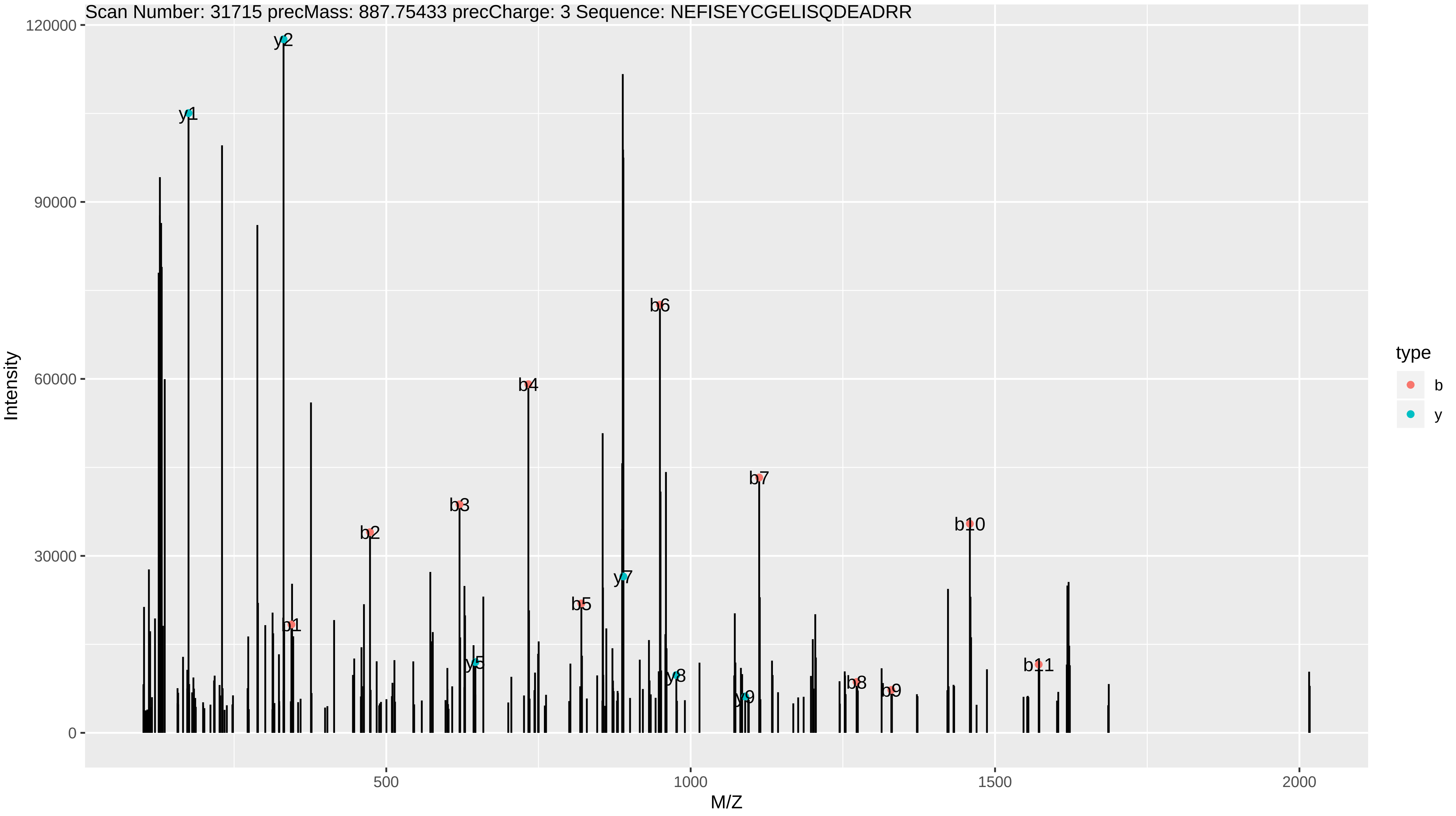

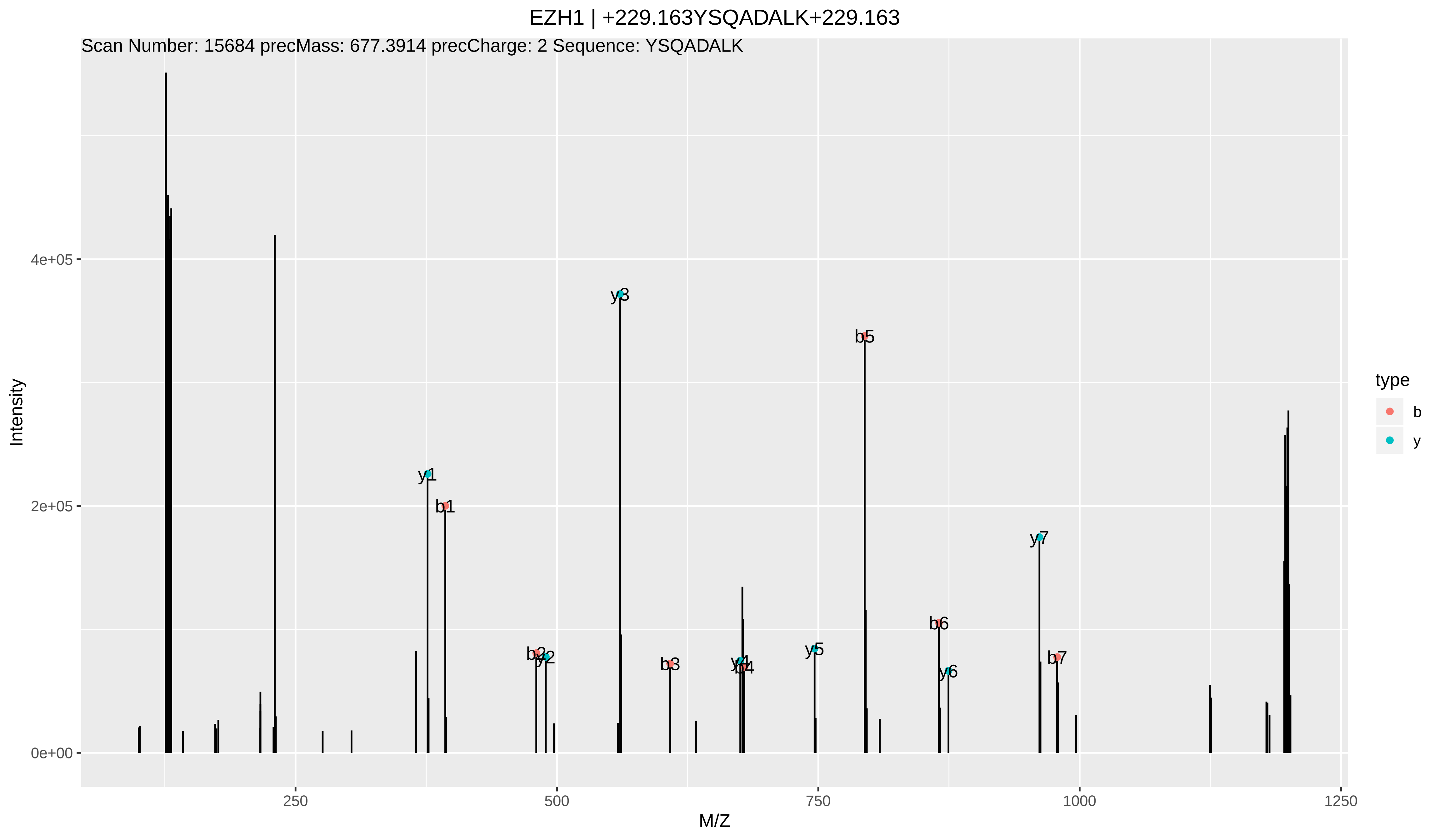
EZH1 | +229.163YSQADALK+229.163
Scan Number: 15684 precMass: 677.3914 precCharge: 2 Sequence: YSQADALK
Intensity
M/Z
0e+00
2e+05
4e+05
250
500
750
1000
1250
y1
b1
b2
y2
y3
b3
y4
b4
y5
b5
b6
y6
y7
b7
type
b
y

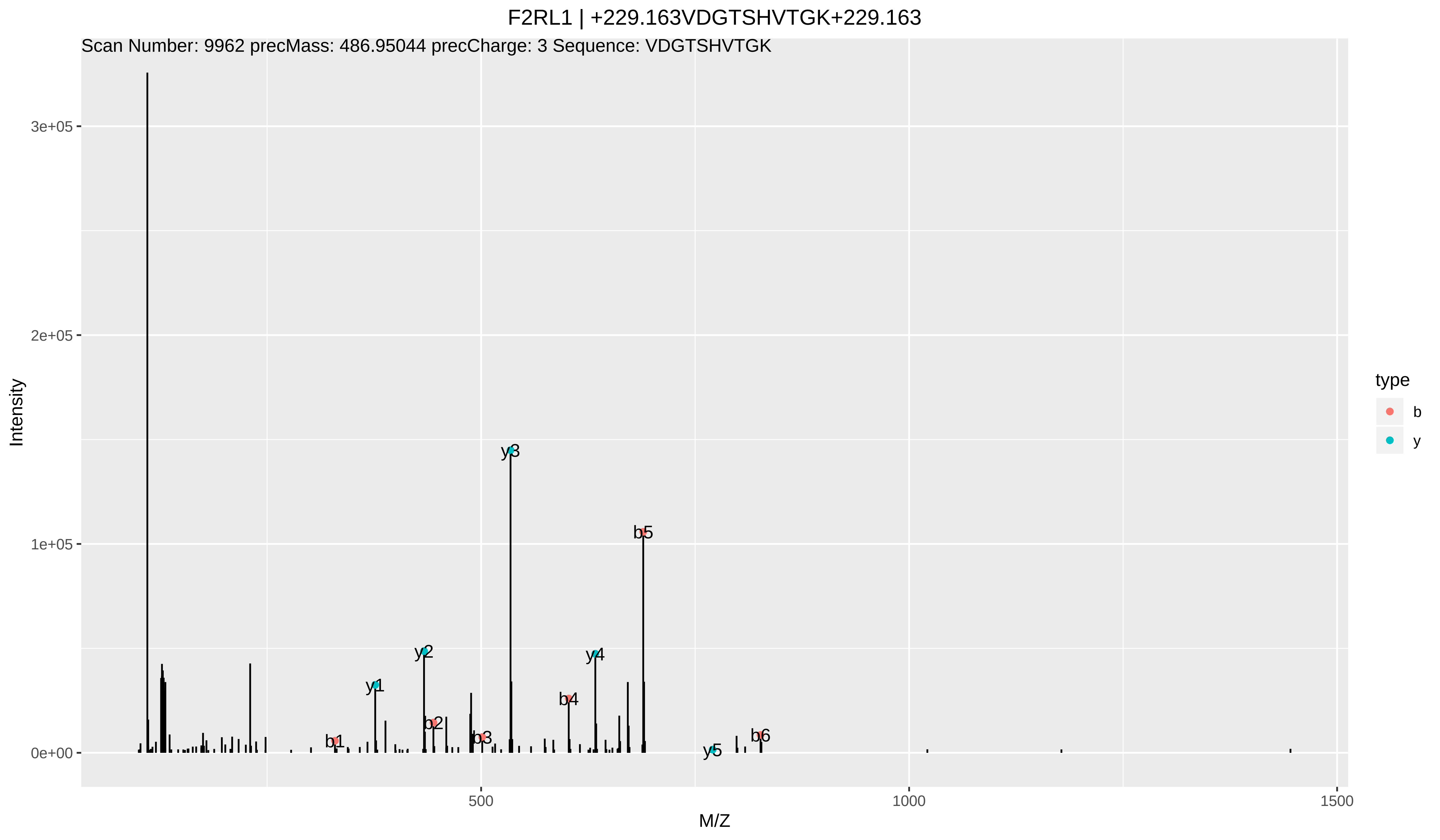
F2RL1 | +229.163VDGTSHVTGK+229.163
Scan Number: 9962 precMass: 486.95044 precCharge: 3 Sequence: VDGTSHVTGK
Intensity
3e+05
2e+05
1e+05
0e+00
M/Z
500
1000
1500
y3
b5
y2
y4
y1
b4
b2
b3
b1
b6
y5
type
b
y

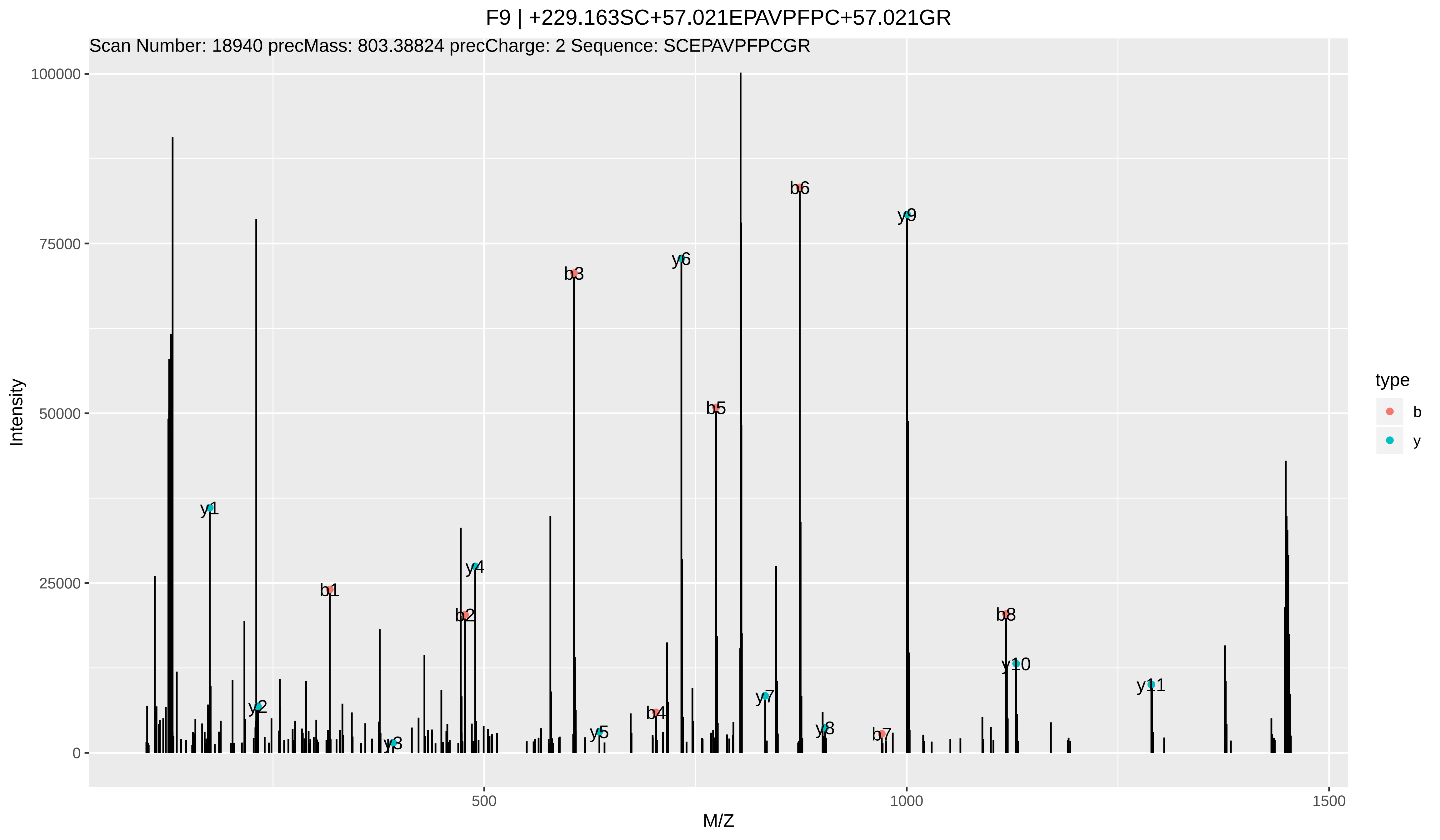

F9 | +229.163SC+57.021EPAVPFPC+57.021GR
Scan Number: 18940 precMass: 803.38824 precCharge: 2 Sequence: SCEPAVPFPCGR
Intensity
100000
75000
50000
25000
0
M/Z
500
1000
1500
y1
y2
y3
y4
y5
y6
y7
y8
y9
y10
y11
b1
b2
b3
b4
b5
b6
b7
b8
type
b
y

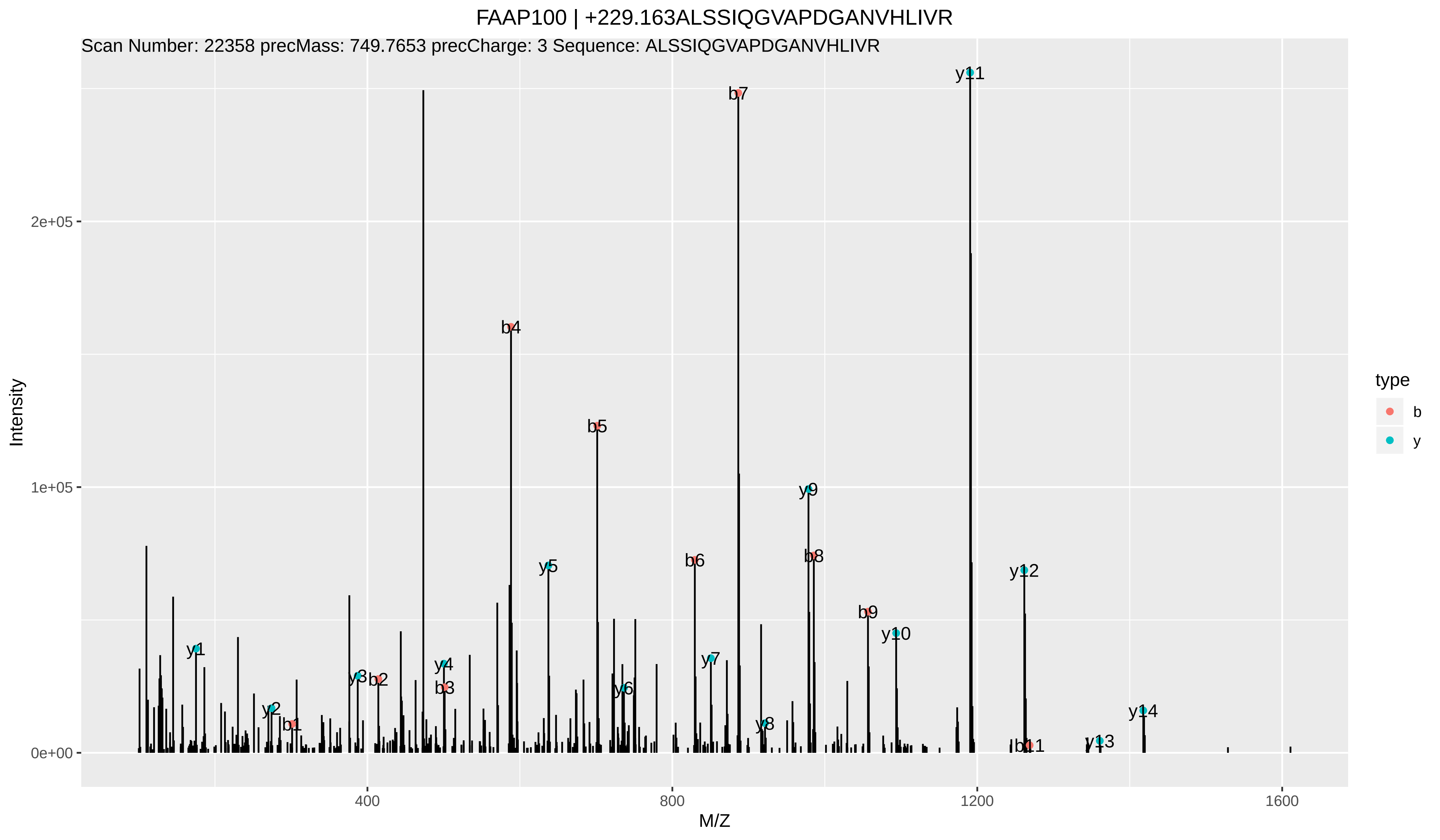

FAAP100 | +229.163ALSSIQGVAPDGANVHLIVR
Scan Number: 22358 precMass: 749.7653 precCharge: 3 Sequence: ALSSIQGVAPDGANVHLIVR
Intensity
M/Z
2e+05
1e+05
0e+00
400
800
1200
1600
y11
b7
b4
b5
y9
b8
b6
y5
y12
b9
y10
y1
y7
y4
y3
b2
b3
y6
y2
b1
y8
y14
y13
b11
type
b
y

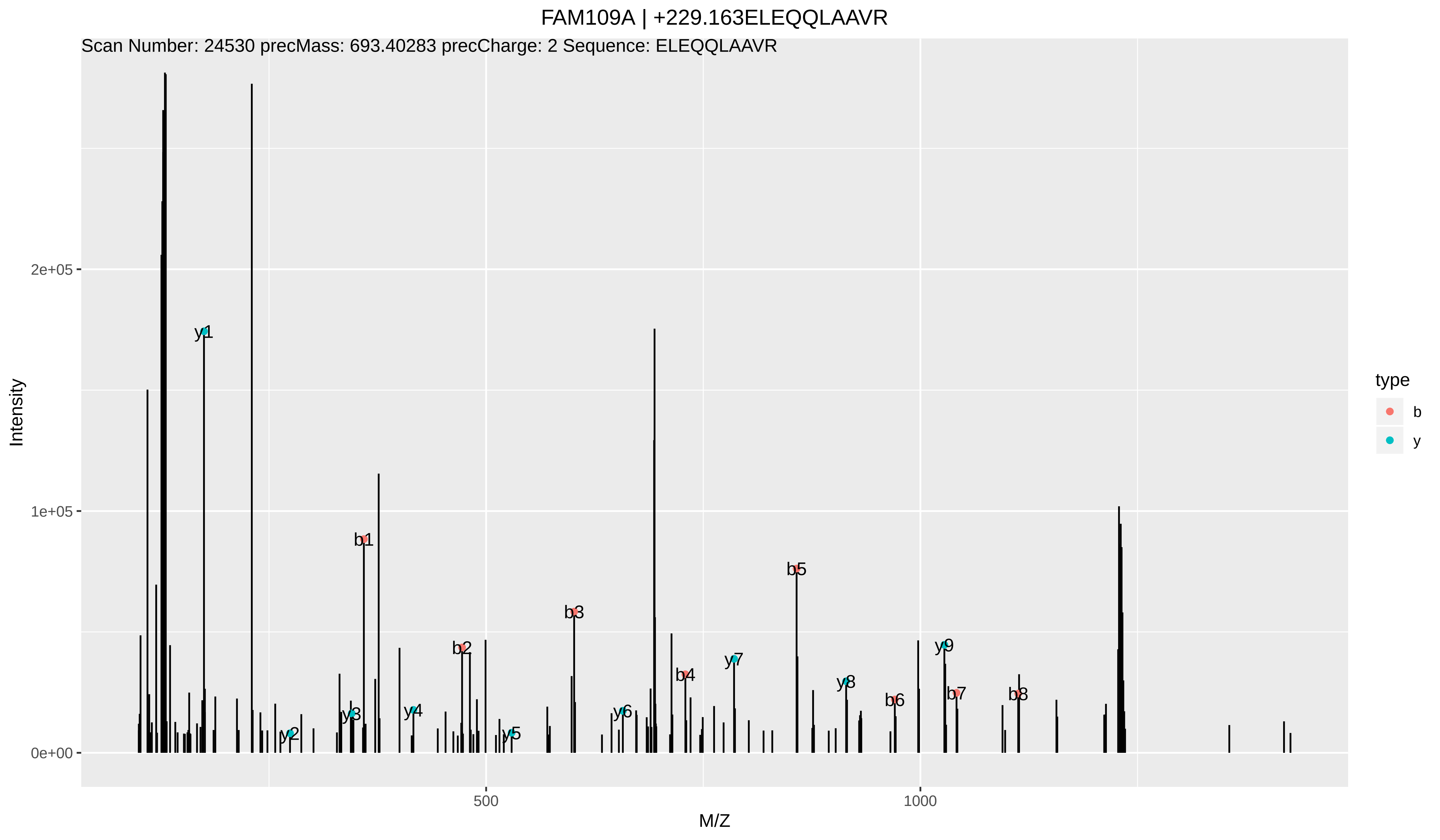

FAM109A | +229.163ELEQQLAAVR
Scan Number: 24530 precMass: 693.40283 precCharge: 2 Sequence: ELEQQLAAVR
Intensity
M/Z
2e+05
1e+05
0e+00
500
1000
y1
b1
b5
b3
b2
y9
y7
b4
y8
b6
b7
b8
y3
y4
y6
y2
y5
type
b
y

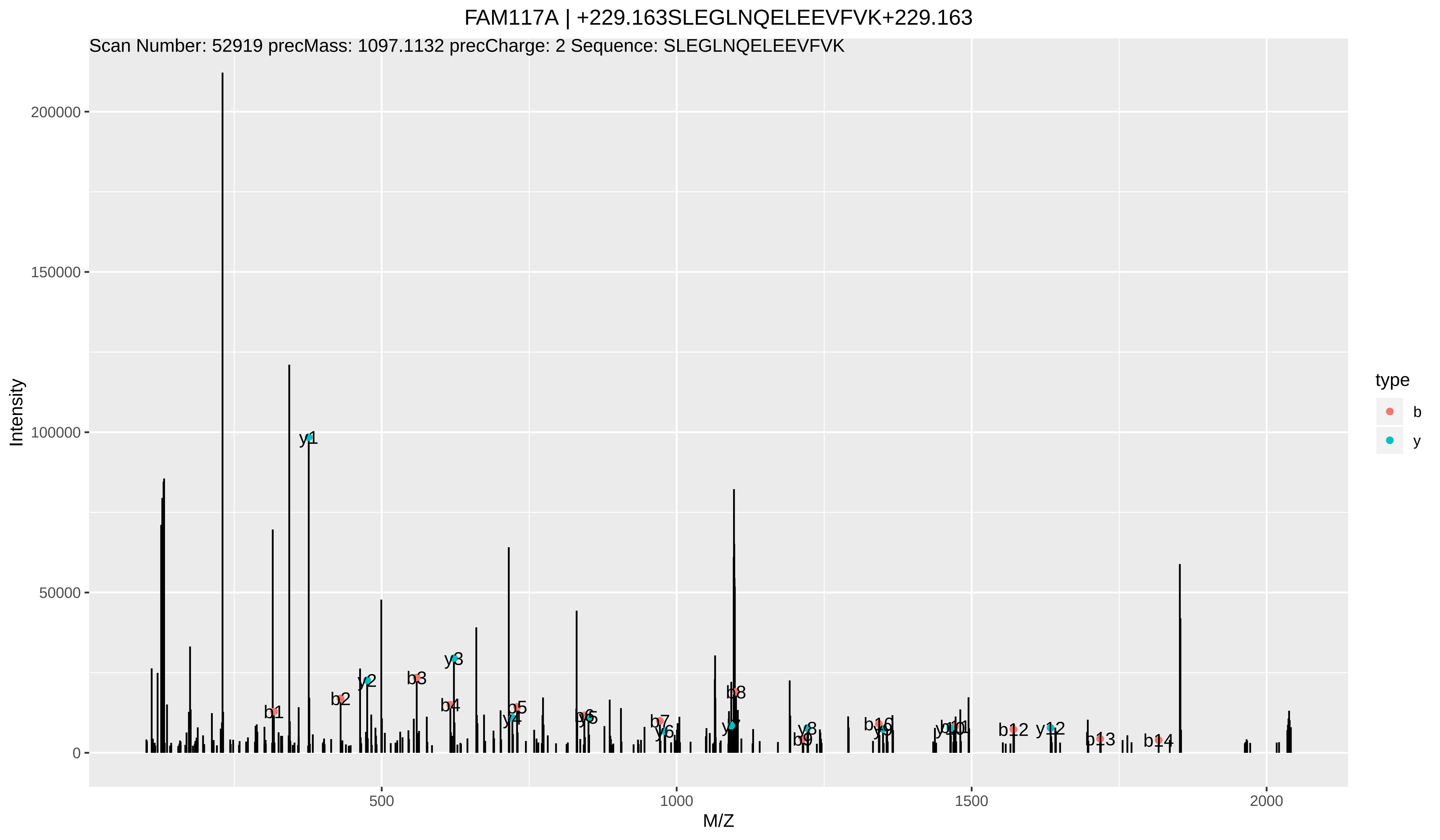

FAM117A | +229.163SLEGLNQELEEVFVK+229.163
Scan Number: 52919 precMass: 1097.1132 precCharge: 2 Sequence: SLEGLNQELEEVFVK
Intensity
200000
150000
100000
50000
0
500
1000
1500
2000
M/Z
type
b
y

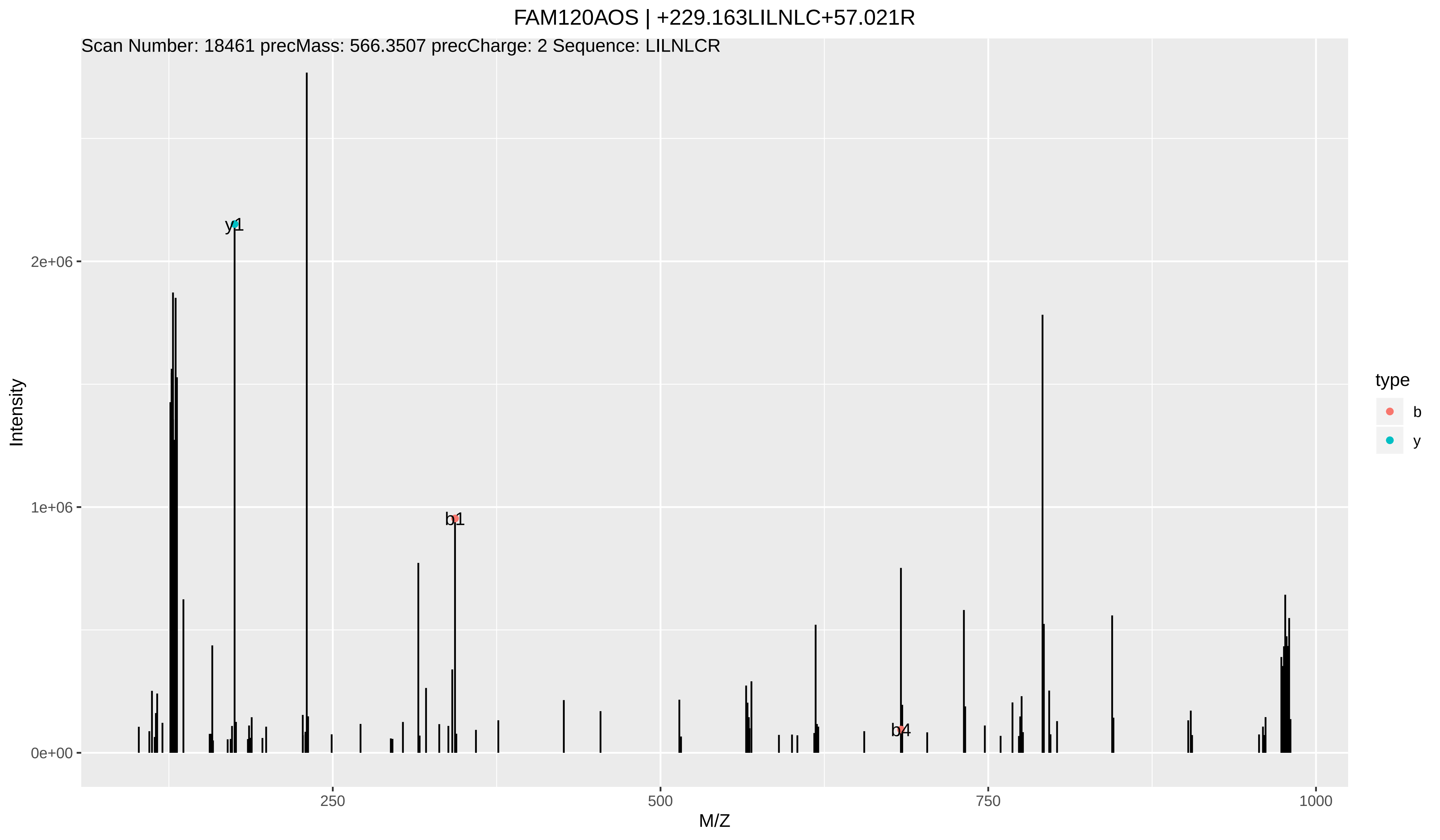

FAM120AOS | +229.163LILNLC+57.021R
Scan Number: 18461 precMass: 566.3507 precCharge: 2 Sequence: LILNLCR
y1
b1
b4
Intensity
2e+06
1e+06
0e+00
250
500
750
1000
M/Z
type
b
y

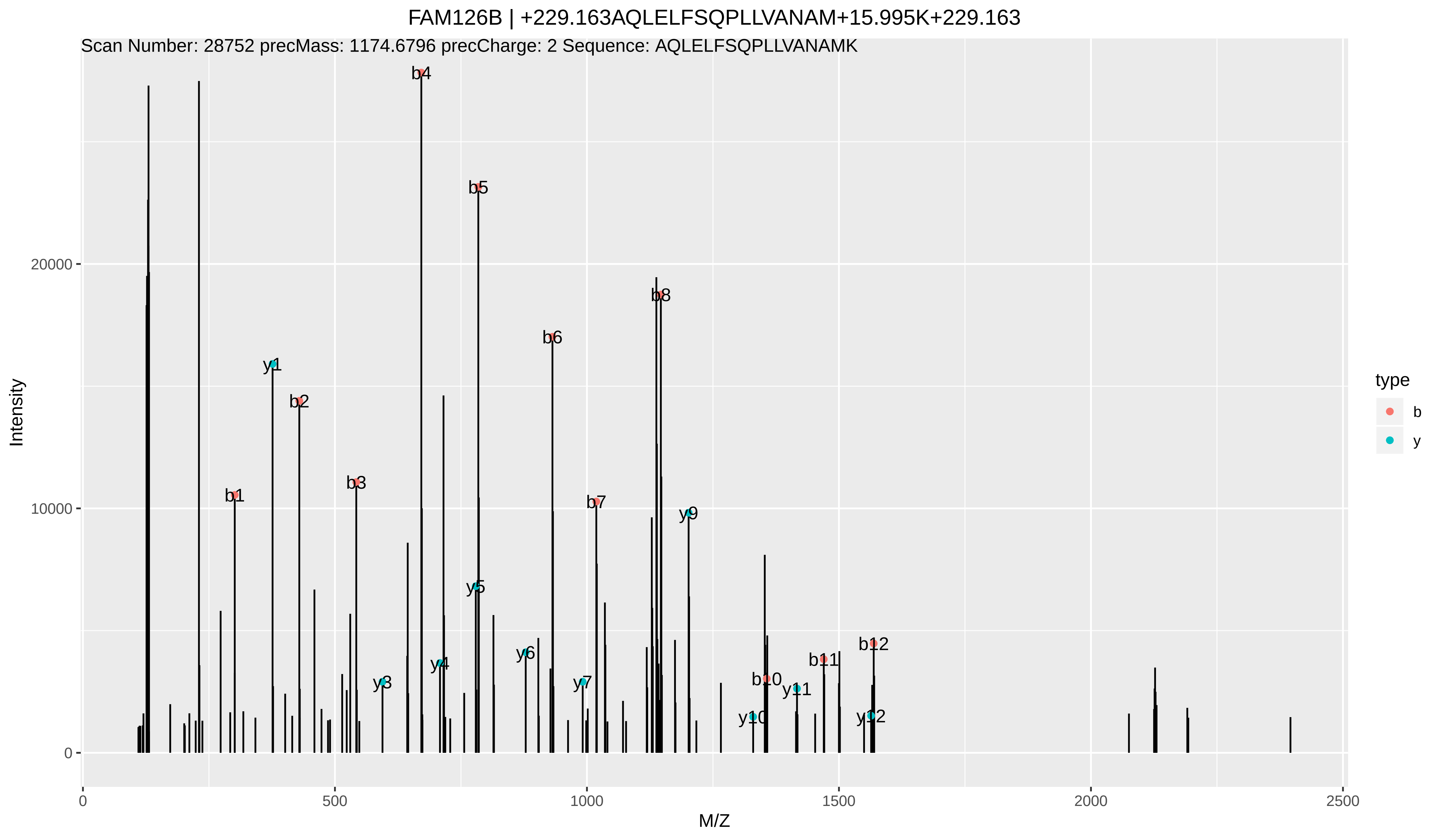
FAM126B | +229.163AQLELFSQPLLVANAM+15.995K+229.163
Scan Number: 28752 precMass: 1174.6796 precCharge: 2 Sequence: AQLELFSQPLLVANAMK
Intensity
M/Z
0
10000
20000
500
1000
1500
2000
2500
b4
b5
b8
b6
y1
b2
b1
b3
b7
y9
y5
b12
b11
b10
y11
y10
y12
y3
y4
y6
y7
type
b
y

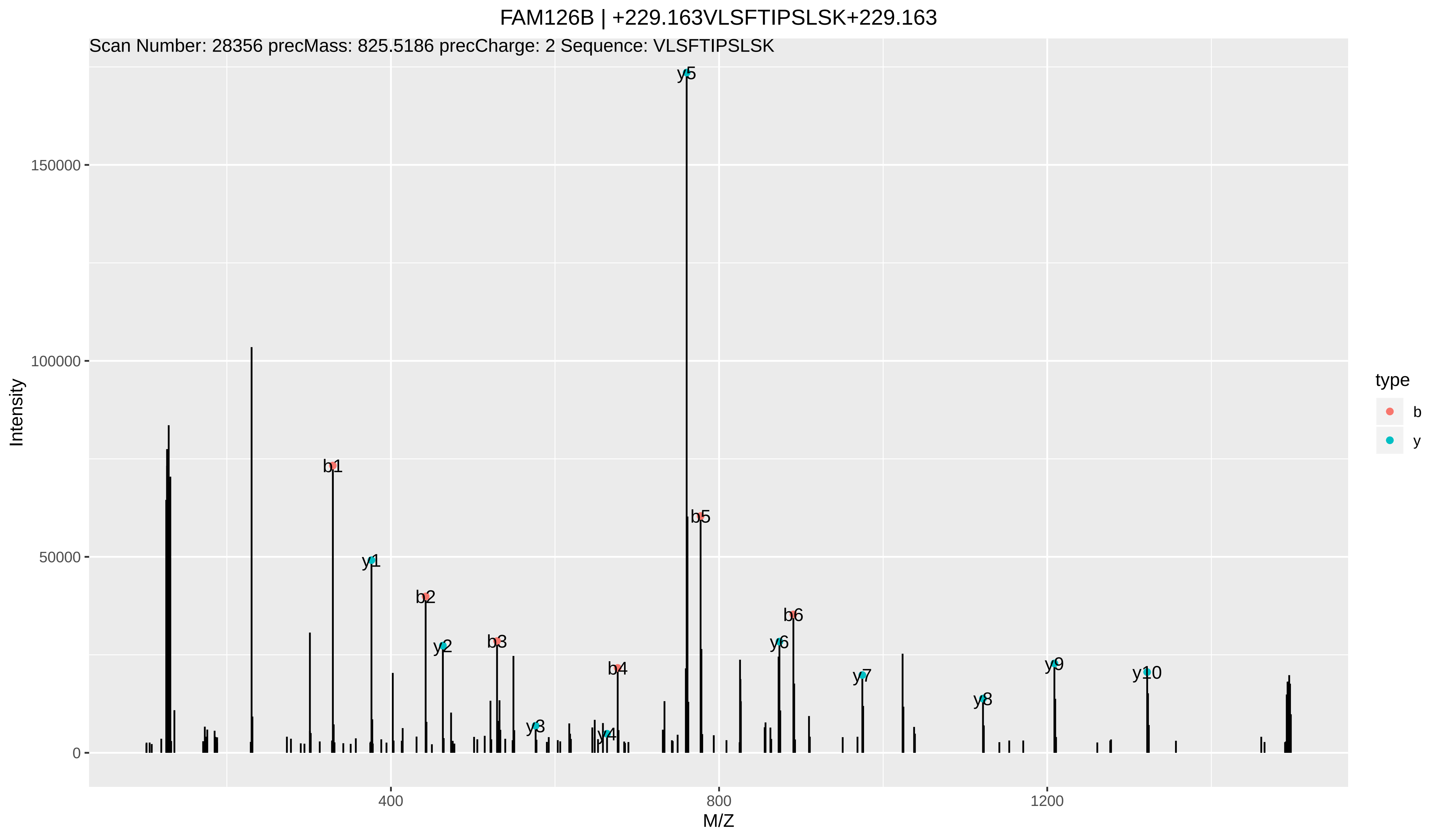
FAM126B | +229.163VLSFTIPSLSK+229.163
Scan Number: 28356 precMass: 825.5186 precCharge: 2 Sequence: VLSFTIPSLSK
Intensity
M/Z
0
50000
100000
150000
400
800
1200
y5
b1
y1
b2
y2
b3
y3
b4
y4
b5
y6
b6
y7
y8
y9
y10
type
b
y

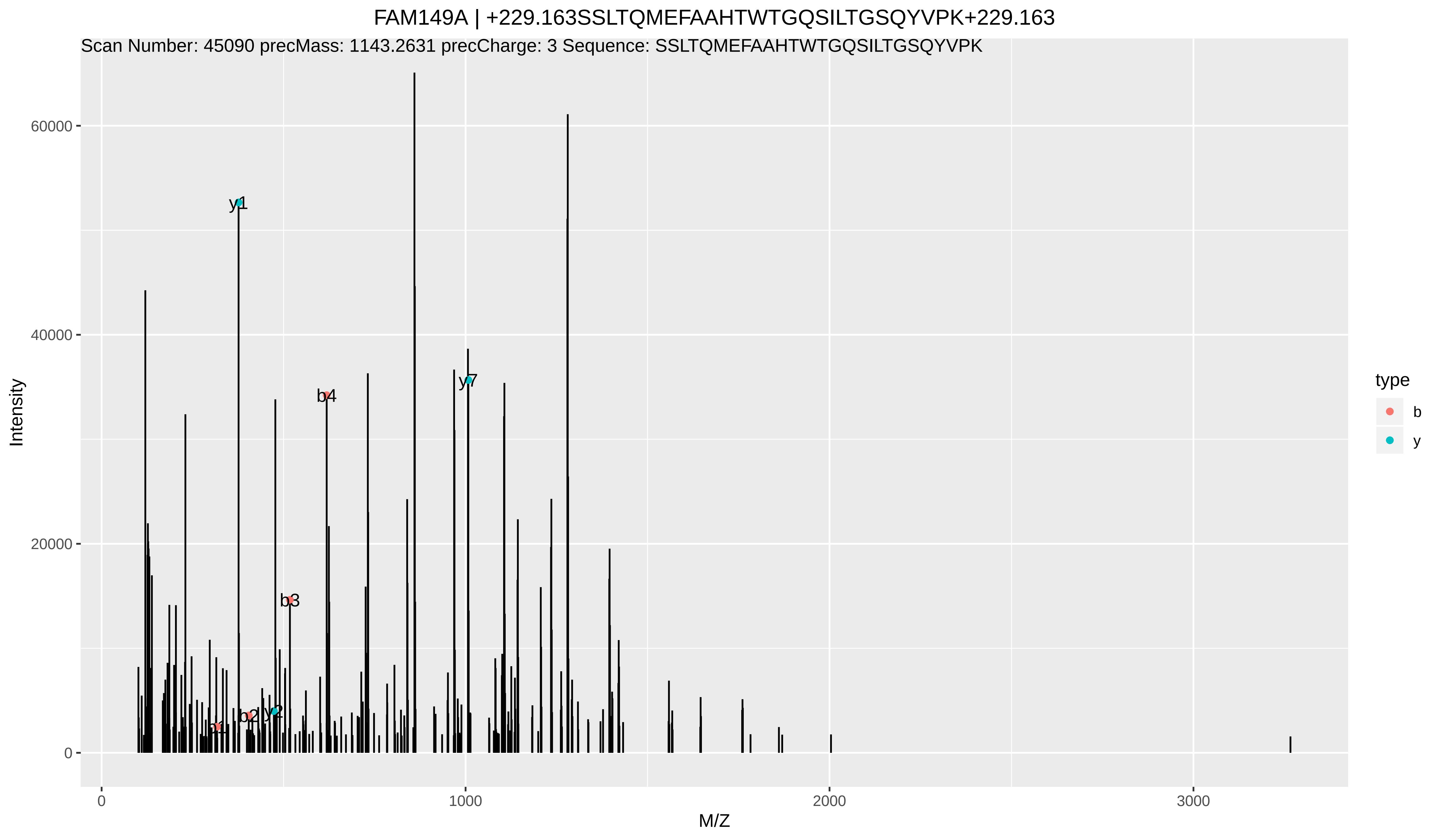

FAM149A | +229.163SSLTQMEFAAHTWTGQSILTGSQYVPK+229.163
Scan Number: 45090 precMass: 1143.2631 precCharge: 3 Sequence: SSLTQMEFAAHTWTGQSILTGSQYVPK
Intensity
M/Z
0
20000
40000
60000
1000
2000
3000
y1
y2
y7
b1
b2
b3
b4
type
b
y

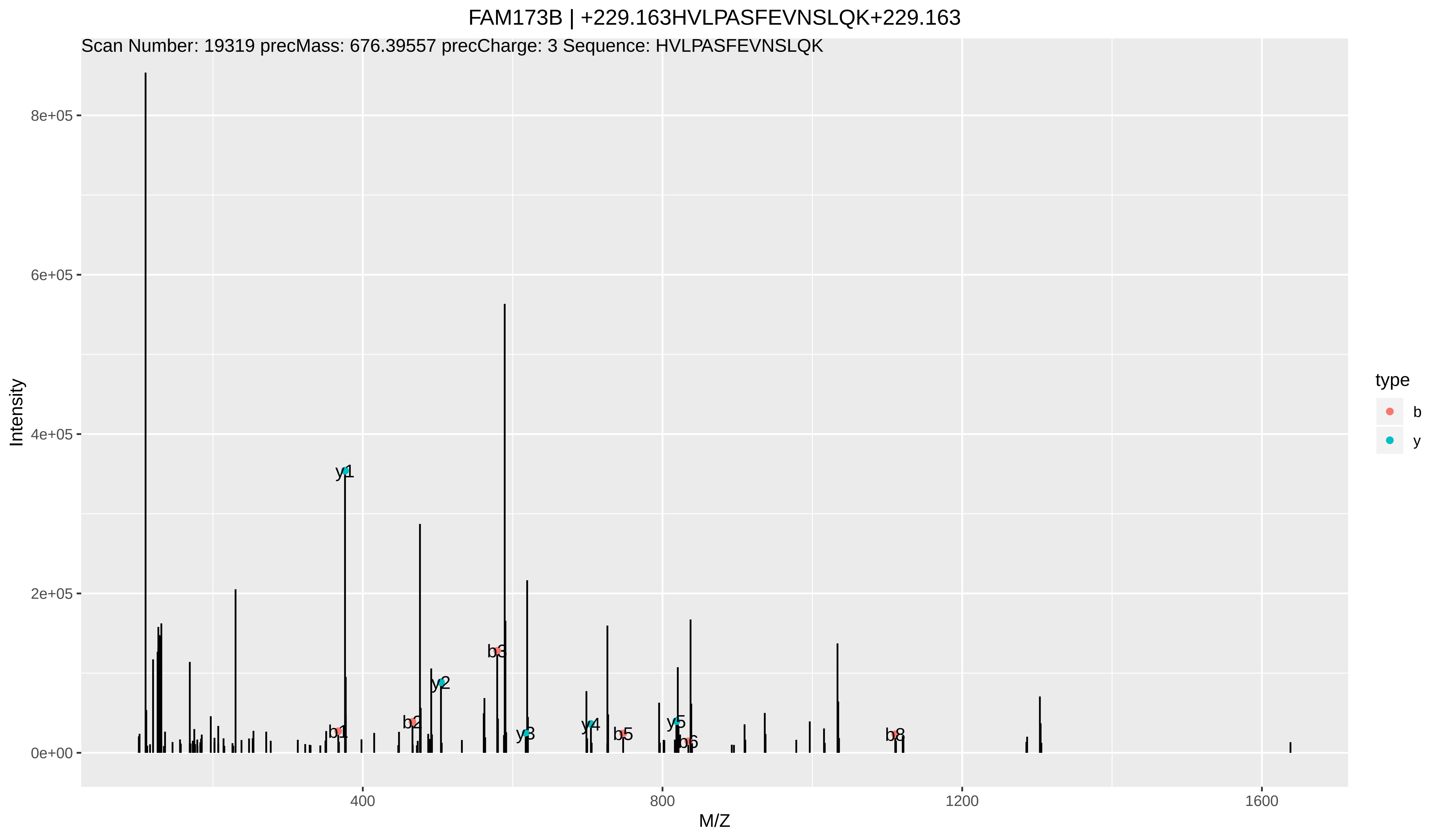
FAM173B | +229.163HVLPASFEVNSLQK+229.163
Scan Number: 19319 precMass: 676.39557 precCharge: 3 Sequence: HVLPASFEVNSLQK
Intensity
8e+05
6e+05
4e+05
2e+05
0e+00
M/Z
400
800
1200
1600
y1
b1
b2
y2
b3
y3
y4
b5
y5
b6
b8
type
b
y

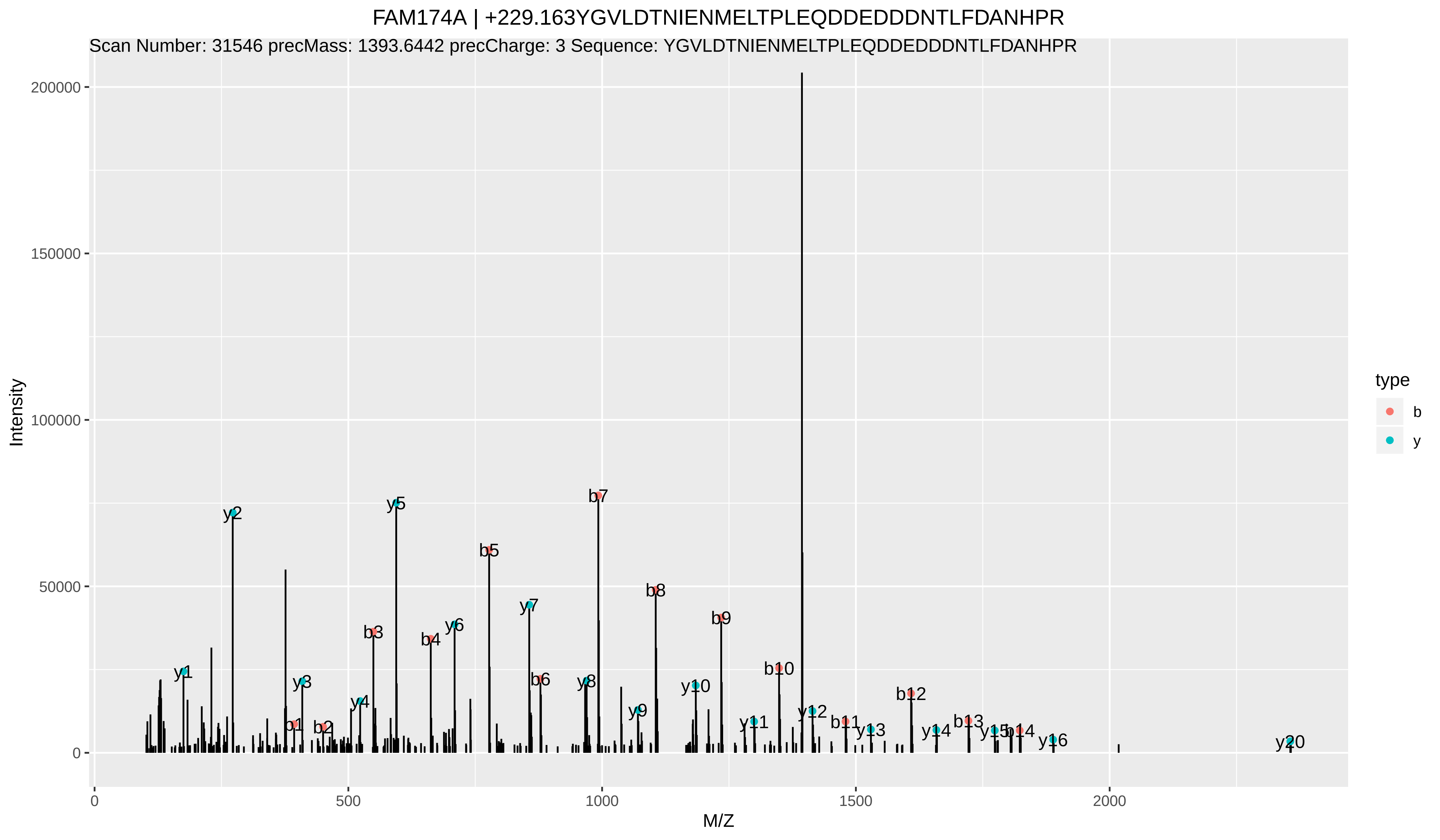

FAM174A | +229.163YGVLDTNIENMELTPLEQDDEDDDNTLFDANHPR
Scan Number: 31546 precMass: 1393.6442 precCharge: 3 Sequence: YGVLDTNIENMELTPLEQDDEDDDNTLFDANHPR
Intensity
M/Z
type
b
y
0
50000
100000
150000
200000
0
500
1000
1500
2000
y1
y2
y3
y4
y5
y6
y7
y8
y9
y10
y11
y12
y13
y14
y15
y16
y20
b1
b2
b3
b4
b5
b6
b7
b8
b9
b10
b11
b12
b13
b14

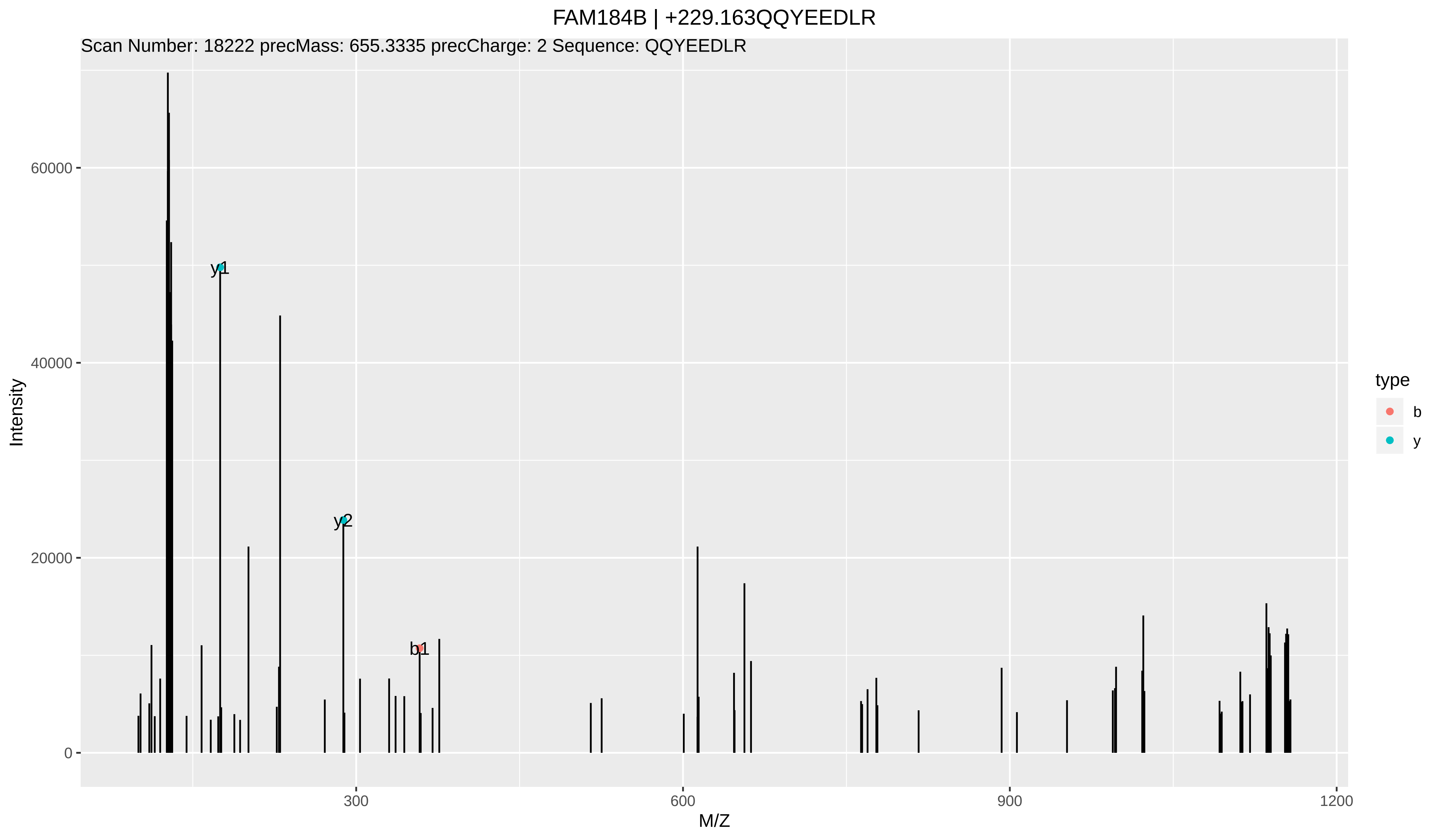
FAM184B | +229.163QQYEEDLR
Scan Number: 18222 precMass: 655.3335 precCharge: 2 Sequence: QQYEEDLR
Intensity
M/Z
y1
y2
b1
0
20000
40000
60000
300
600
900
1200
type
b
y

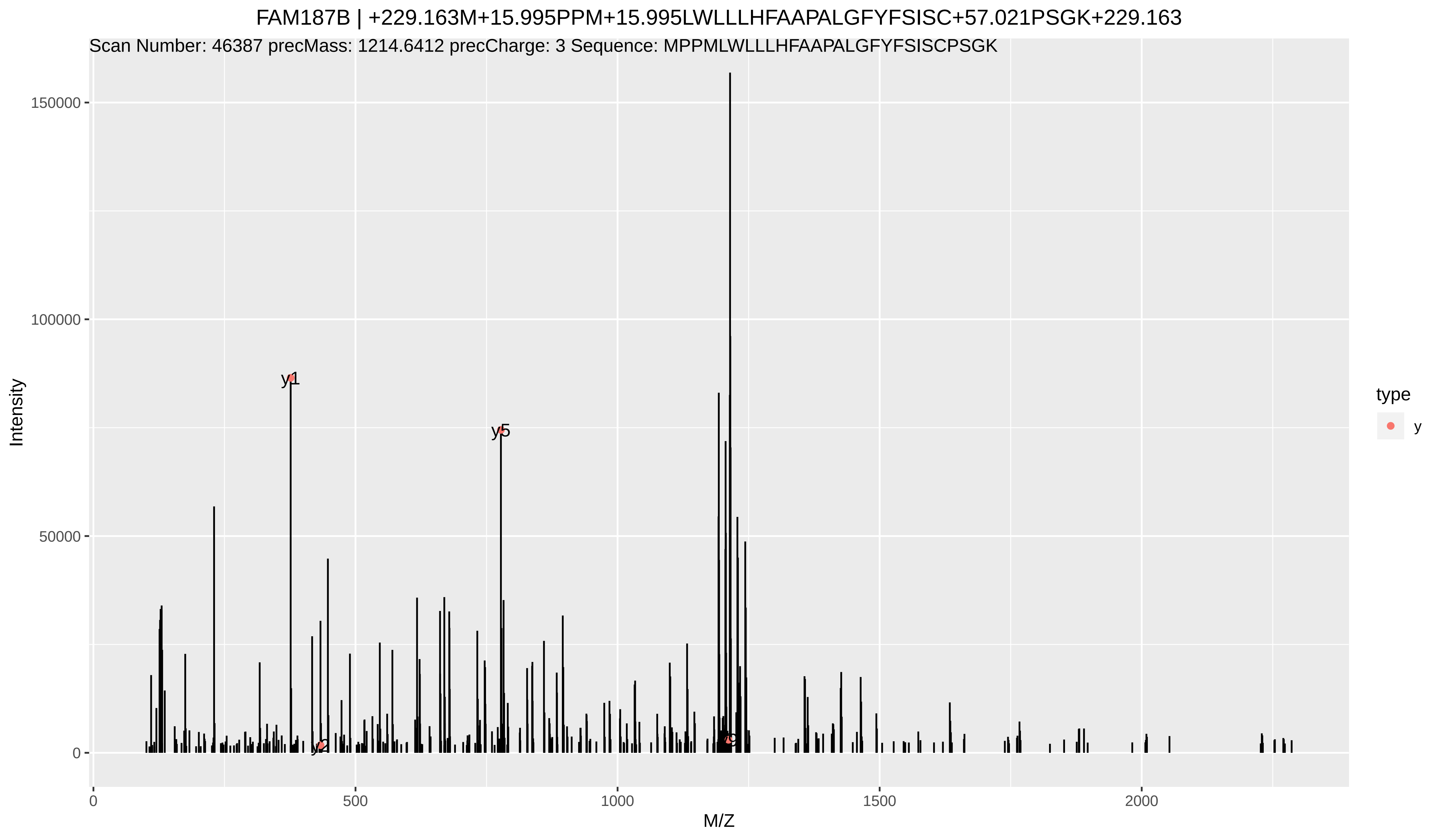
FAM187B | +229.163M+15.995PPM+15.995LWLLLHFAAPALGFYFSISC+57.021PSGK+229.163
Scan Number: 46387 precMass: 1214.6412 precCharge: 3 Sequence: MPPMLWLLLHFAAPALGFYFSISCPSGK
Intensity
150000
100000
50000
0
M/Z
0
500
1000
1500
2000
y1
y5
y2
type
y

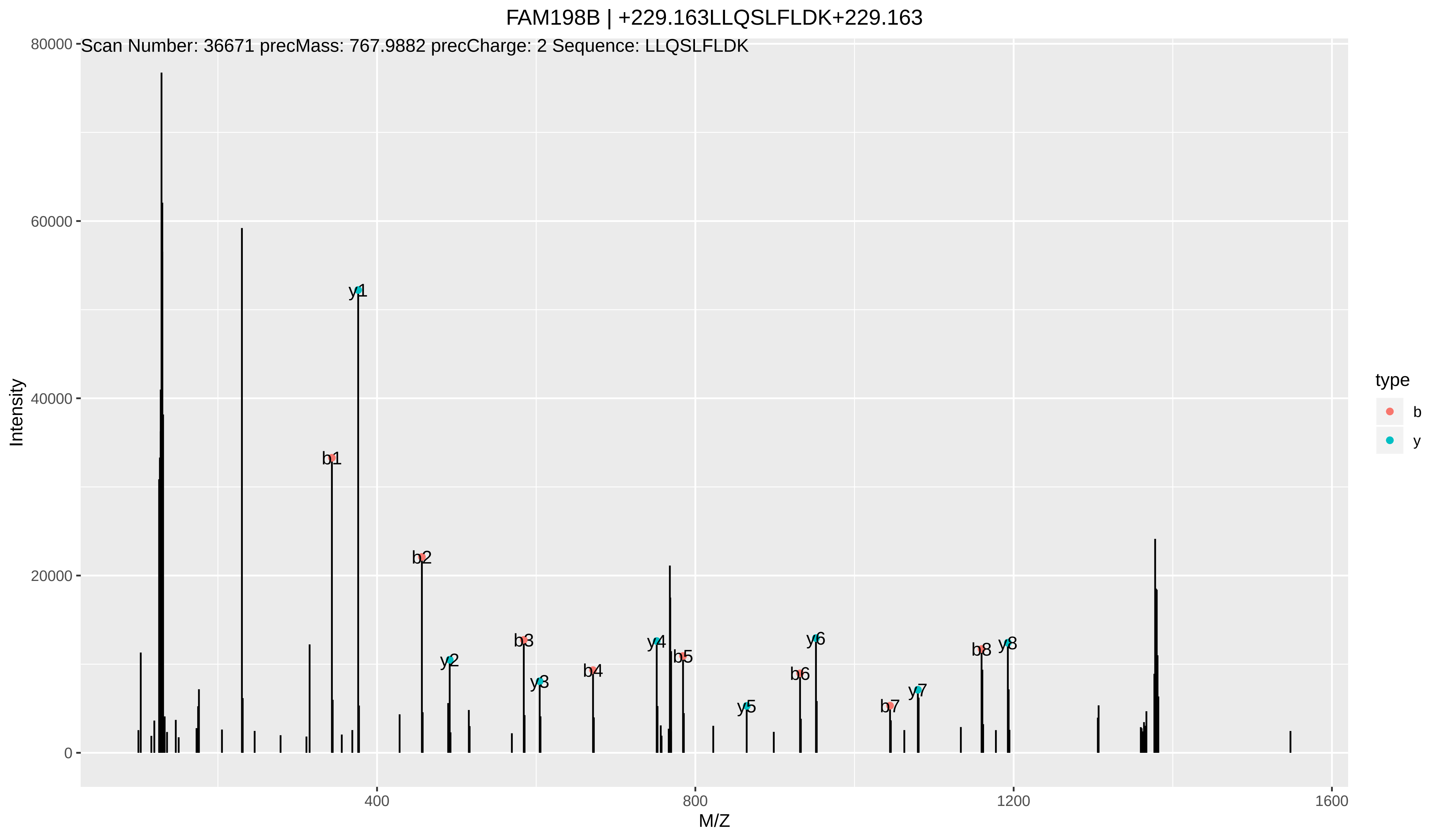

FAM198B | +229.163LLQSLFLDK+229.163
Scan Number: 36671 precMass: 767.9882 precCharge: 2 Sequence: LLQSLFLDK
Intensity
M/Z
type
b
y
y1
b1
b2
y2
b3
y3
b4
y4
b5
y5
b6
y6
b7
y7
b8
y8

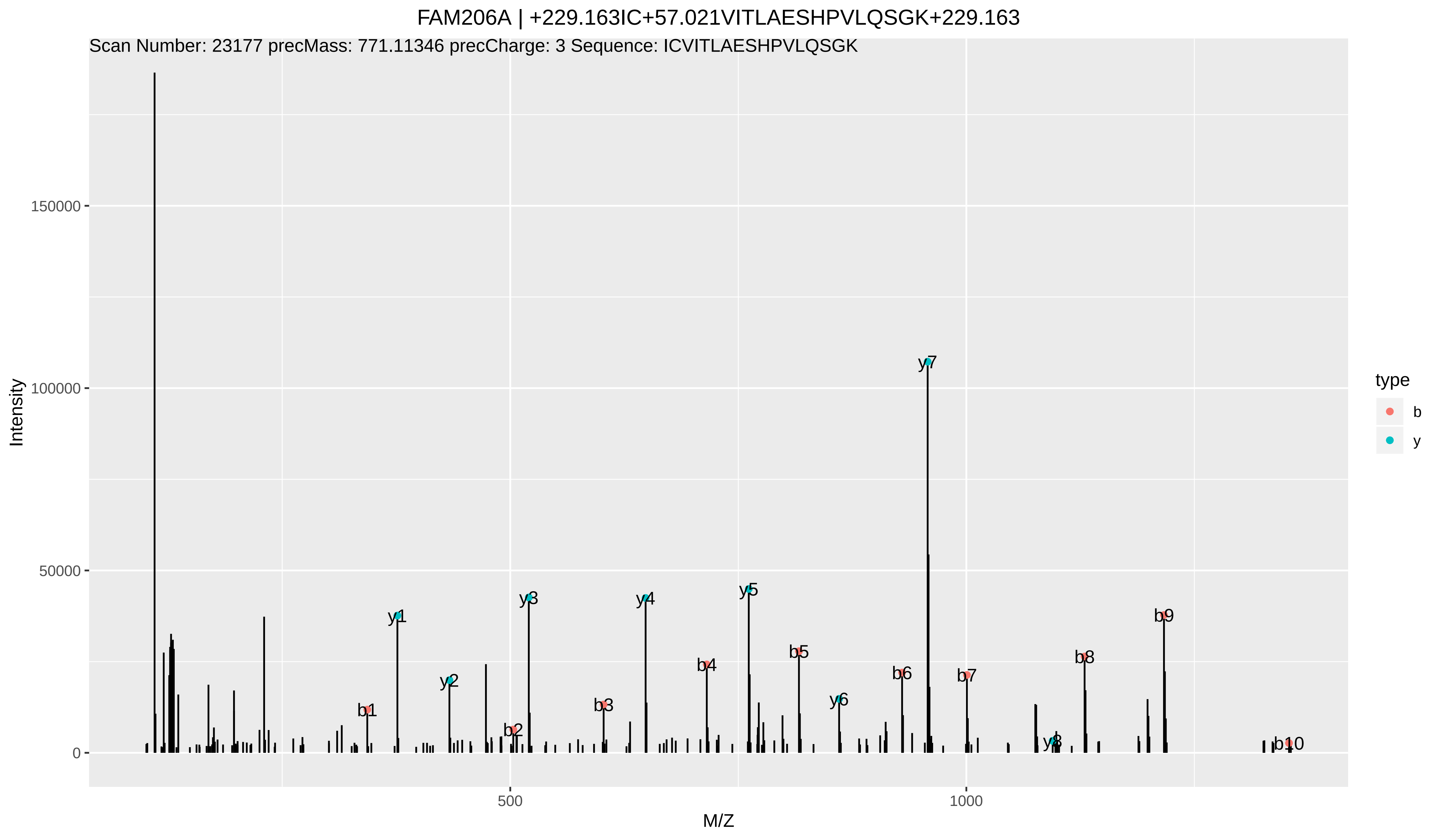
FAM206A | +229.163IC+57.021VITLAESHPVLQSGK+229.163
Scan Number: 23177 precMass: 771.11346 precCharge: 3 Sequence: ICVITLAESHPVLQSGK
Intensity
150000
100000
50000
0
500
1000
M/Z
type
b
y
y1
y2
y3
y4
y5
y6
y7
y8
b1
b2
b3
b4
b5
b6
b7
b8
b9
b10

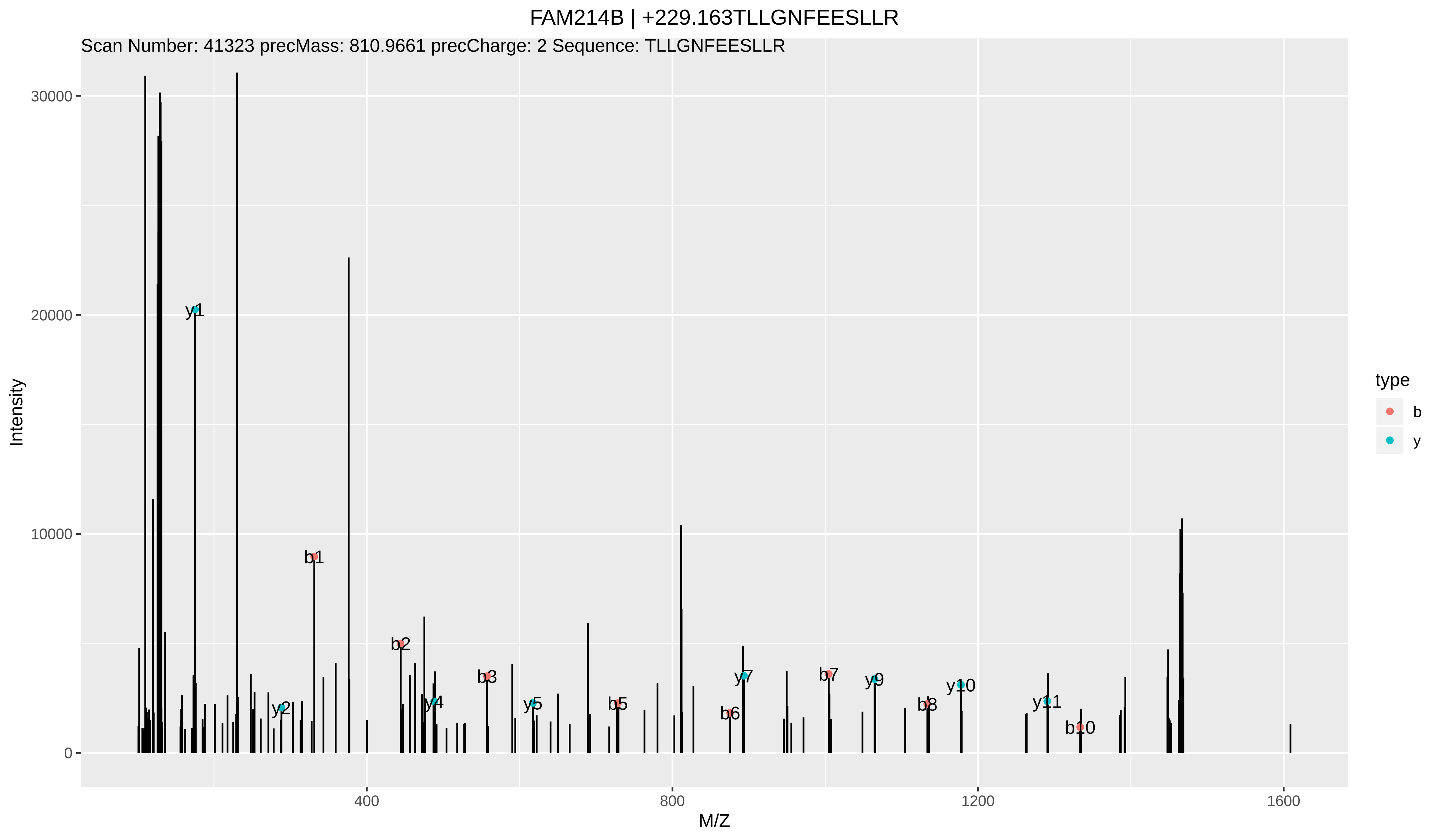

FAM214B | +229.163TLLGNFEESLLR
Scan Number: 41323 precMass: 810.9661 precCharge: 2 Sequence: TLLGNFEESLLR
Intensity
M/Z
30000
20000
10000
0
400
800
1200
1600
y1
b1
b2
b3
y2
y4
y5
b5
b6
y7
b7
y9
b8
y10
y11
b10
type
b
y

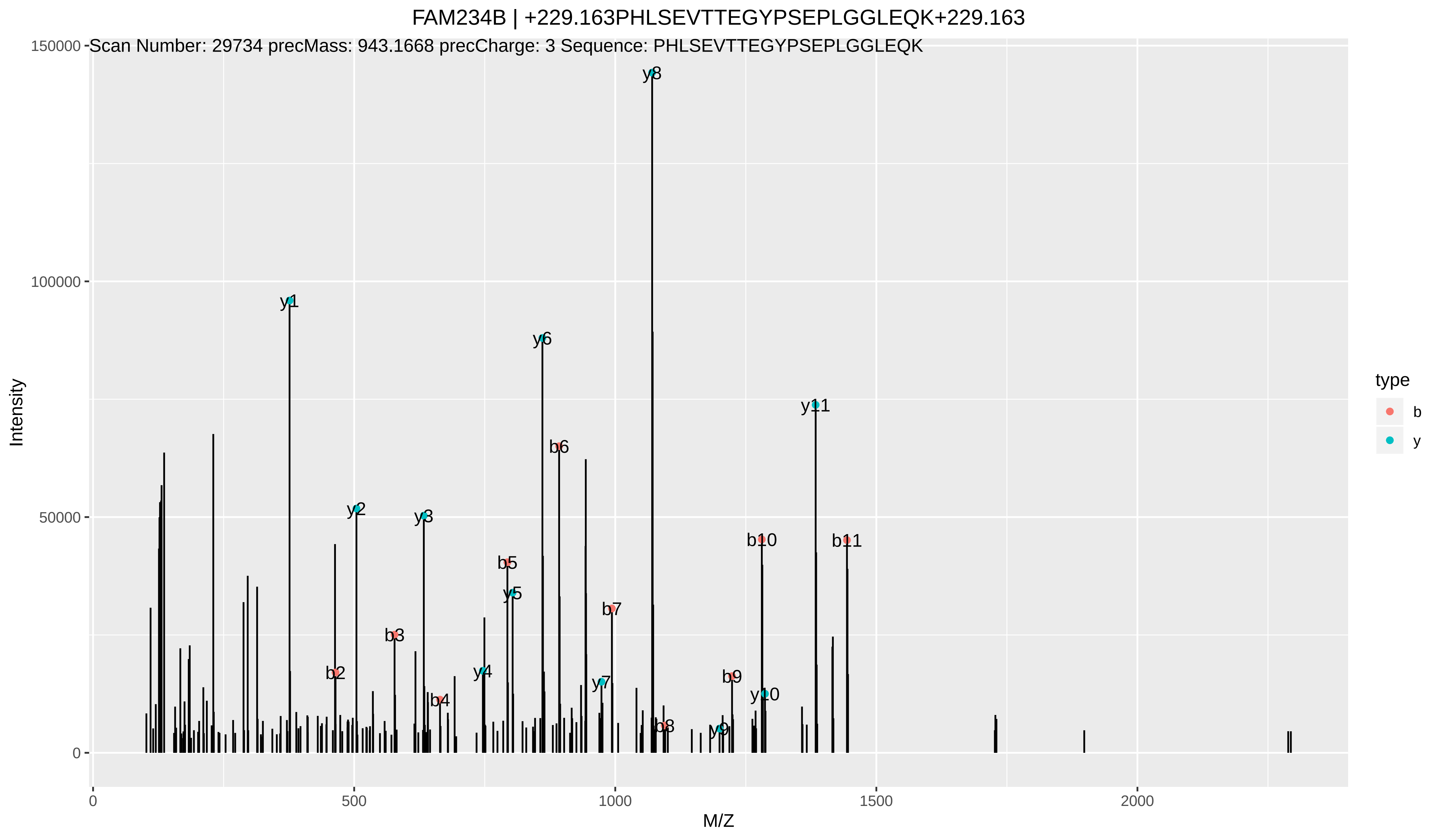
FAM234B | +229.163PHLSEVTTEGYPSEPLGGLEQK+229.163
Scan Number: 29734 precMass: 943.1668 precCharge: 3 Sequence: PHLSEVTTEGYPSEPLGGLEQK
Intensity
M/Z
type
b
y
y8
y1
y6
y11
b6
y2
y3
b10
b11
b5
y5
b7
b3
b2
y4
y7
b9
y10
b4
b8
y9
150000
100000
50000
0
0
500
1000
1500
2000

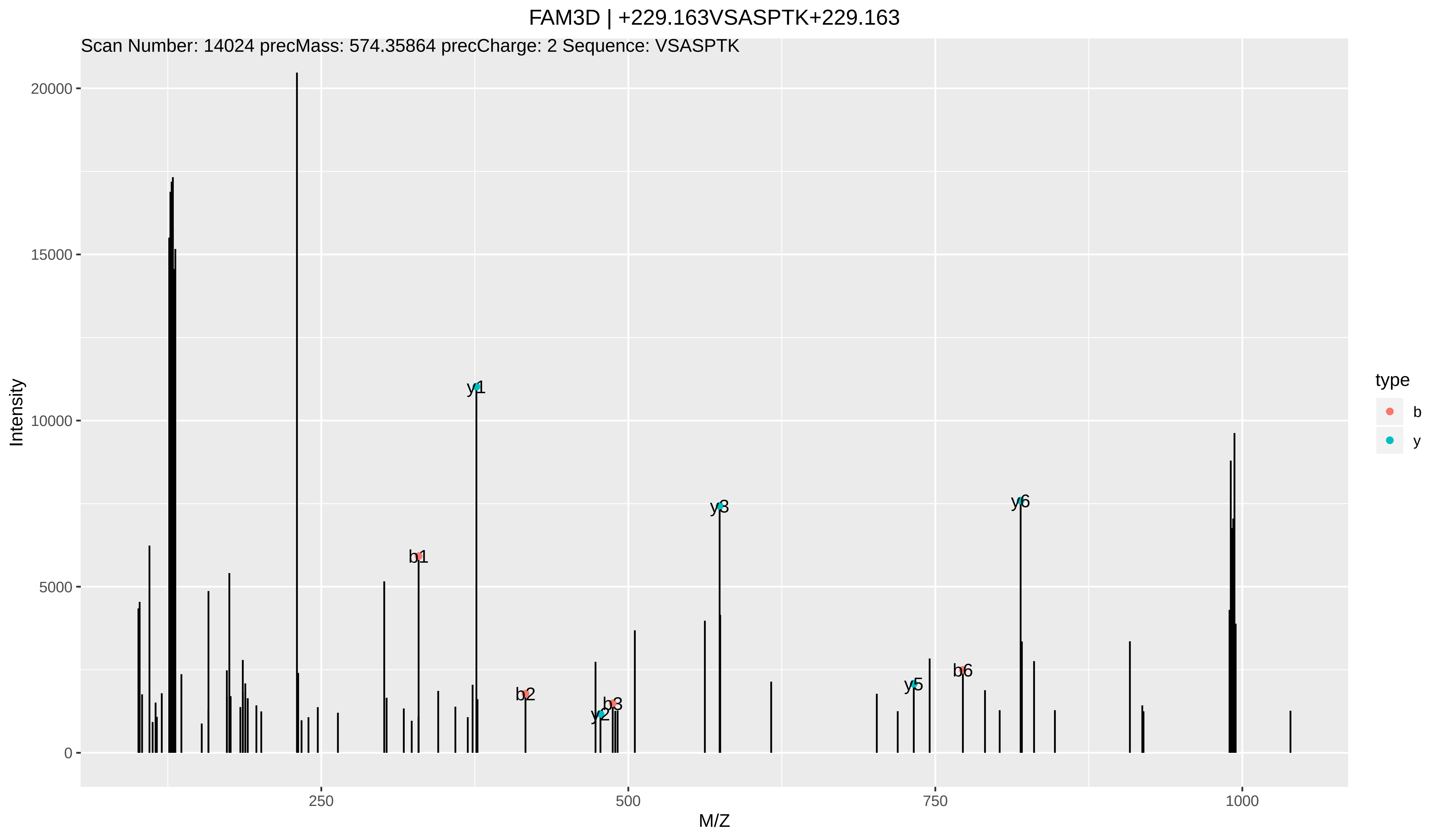
FAM3D | +229.163VSASPTK+229.163
Scan Number: 14024 precMass: 574.35864 precCharge: 2 Sequence: VSASPTK
Intensity
20000
15000
10000
5000
0
250
500
750
1000
M/Z
y1
b1
b2
y2
b3
y3
y5
b6
y6
type
b
y

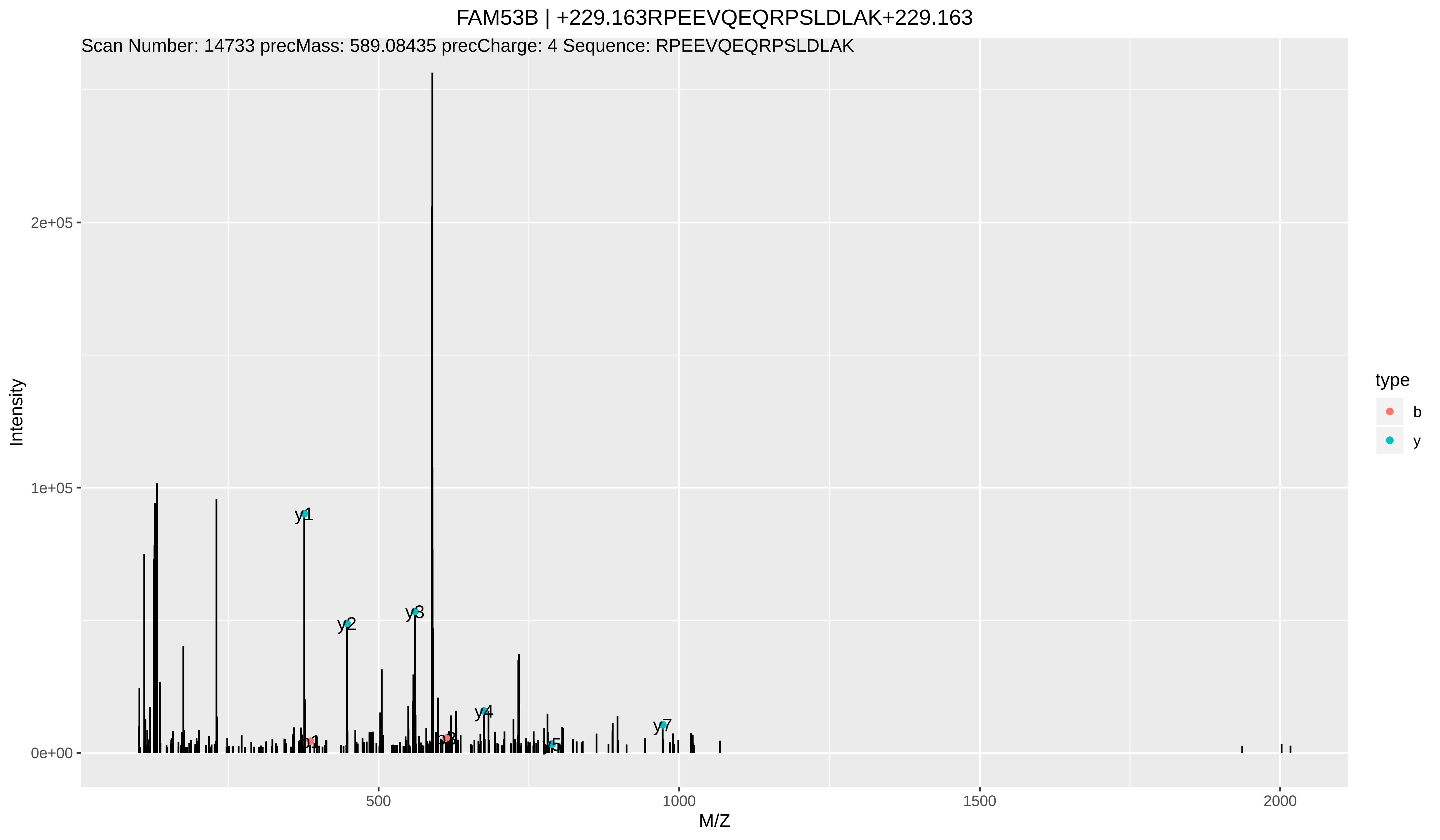
FAM53B | +229.163RPEEVQEQRPSLDLAK+229.163
Scan Number: 14733 precMass: 589.08435 precCharge: 4 Sequence: RPEEVQEQRPSLDLAK
Intensity
M/Z
2e+05
1e+05
0e+00
500
1000
1500
2000
y1
y2
y3
y4
y7
type
b
y

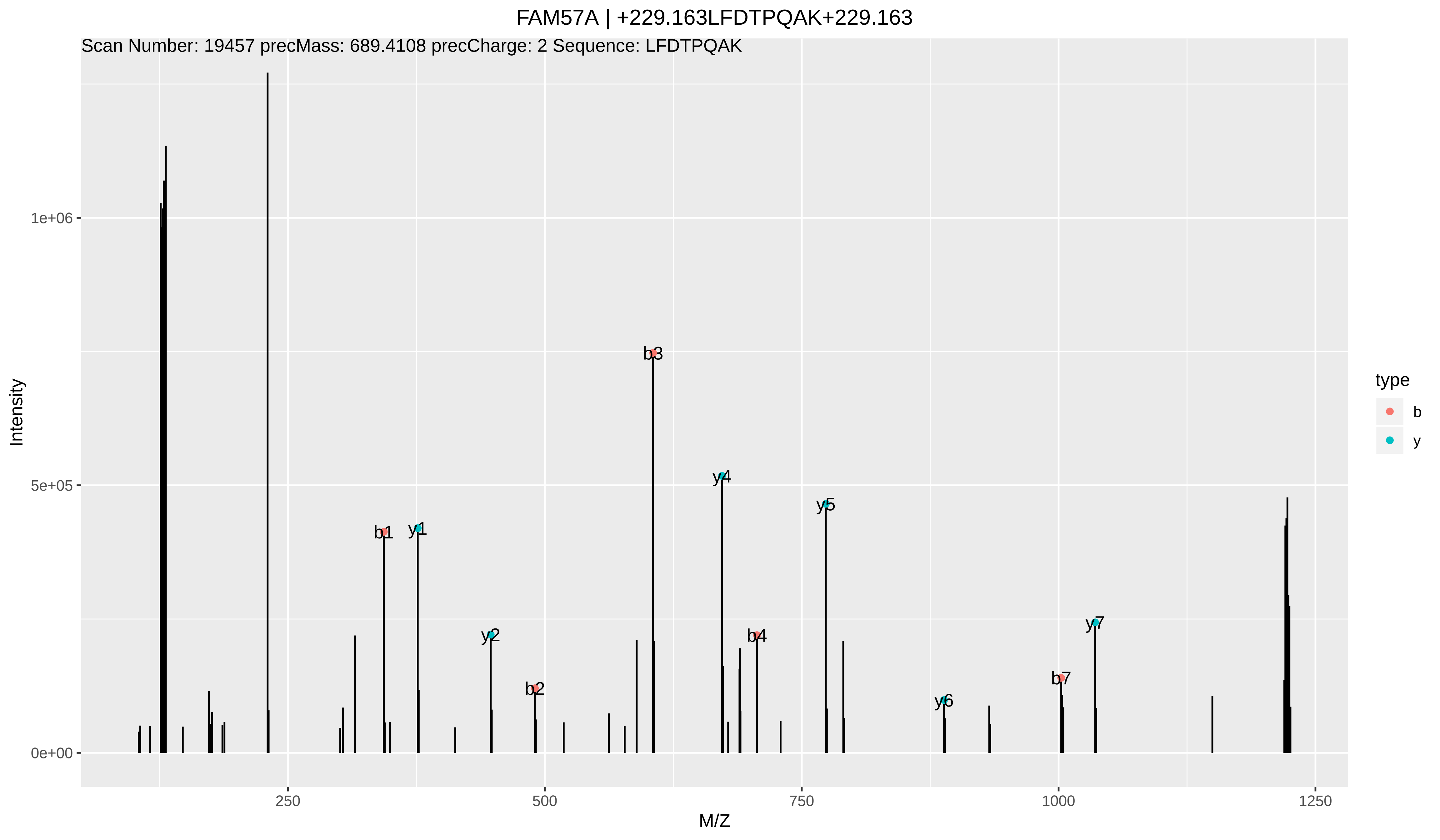

FAM57A | +229.163LFDTPQAK+229.163
Scan Number: 19457 precMass: 689.4108 precCharge: 2 Sequence: LFDTPQAK
Intensity
M/Z
0e+00
5e+05
1e+06
250
500
750
1000
1250
b1
y1
y2
b2
b3
y4
b4
y5
y6
b7
y7
type
b
y

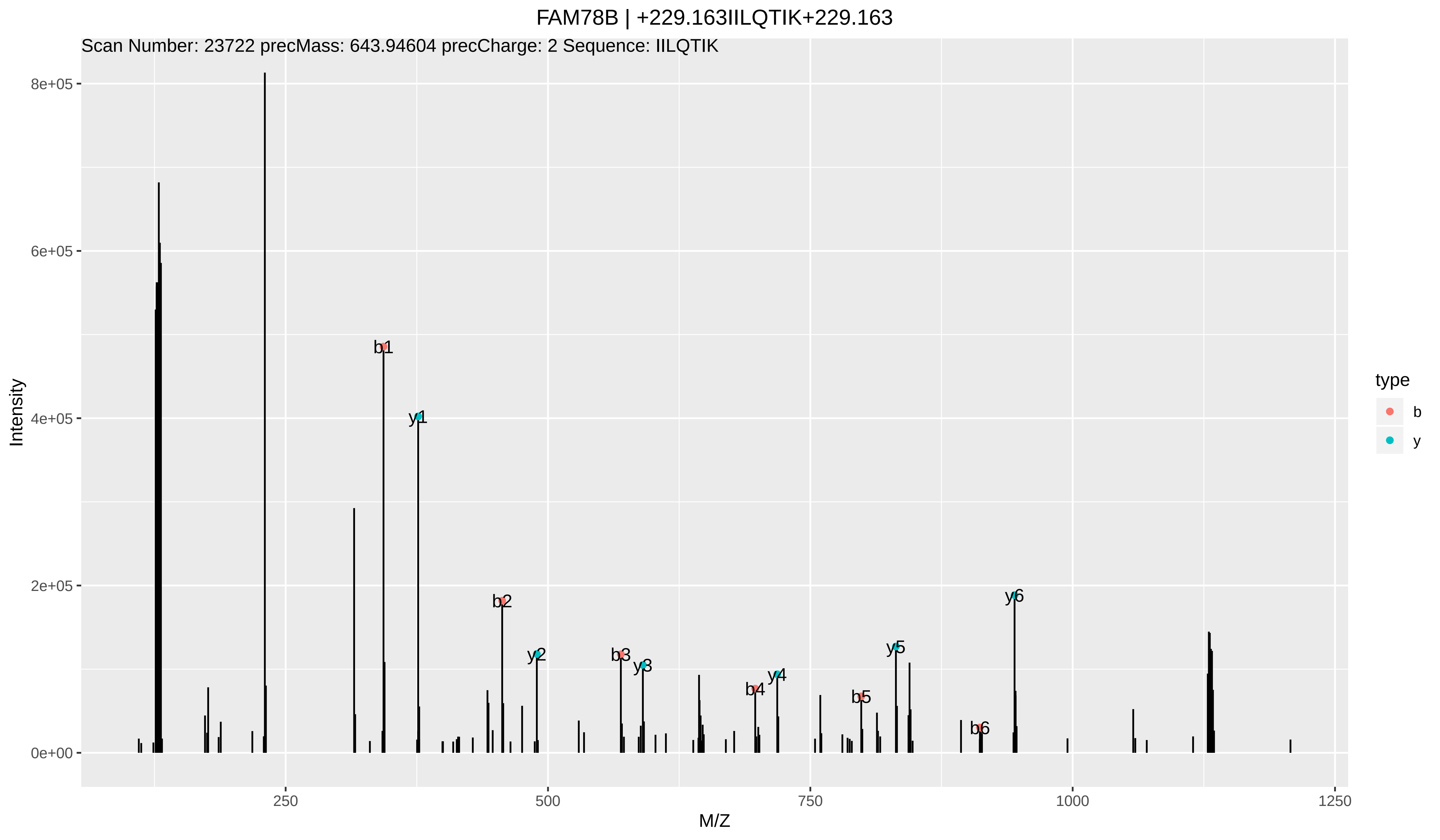

FAM78B | +229.163IILQTIK+229.163
Scan Number: 23722 precMass: 643.94604 precCharge: 2 Sequence: IILQTIK
Intensity
M/Z
8e+05
6e+05
4e+05
2e+05
0e+00
250
500
750
1000
1250
b1
y1
b2
y2
b3
y3
b4
y4
b5
y5
b6
y6
type
b
y

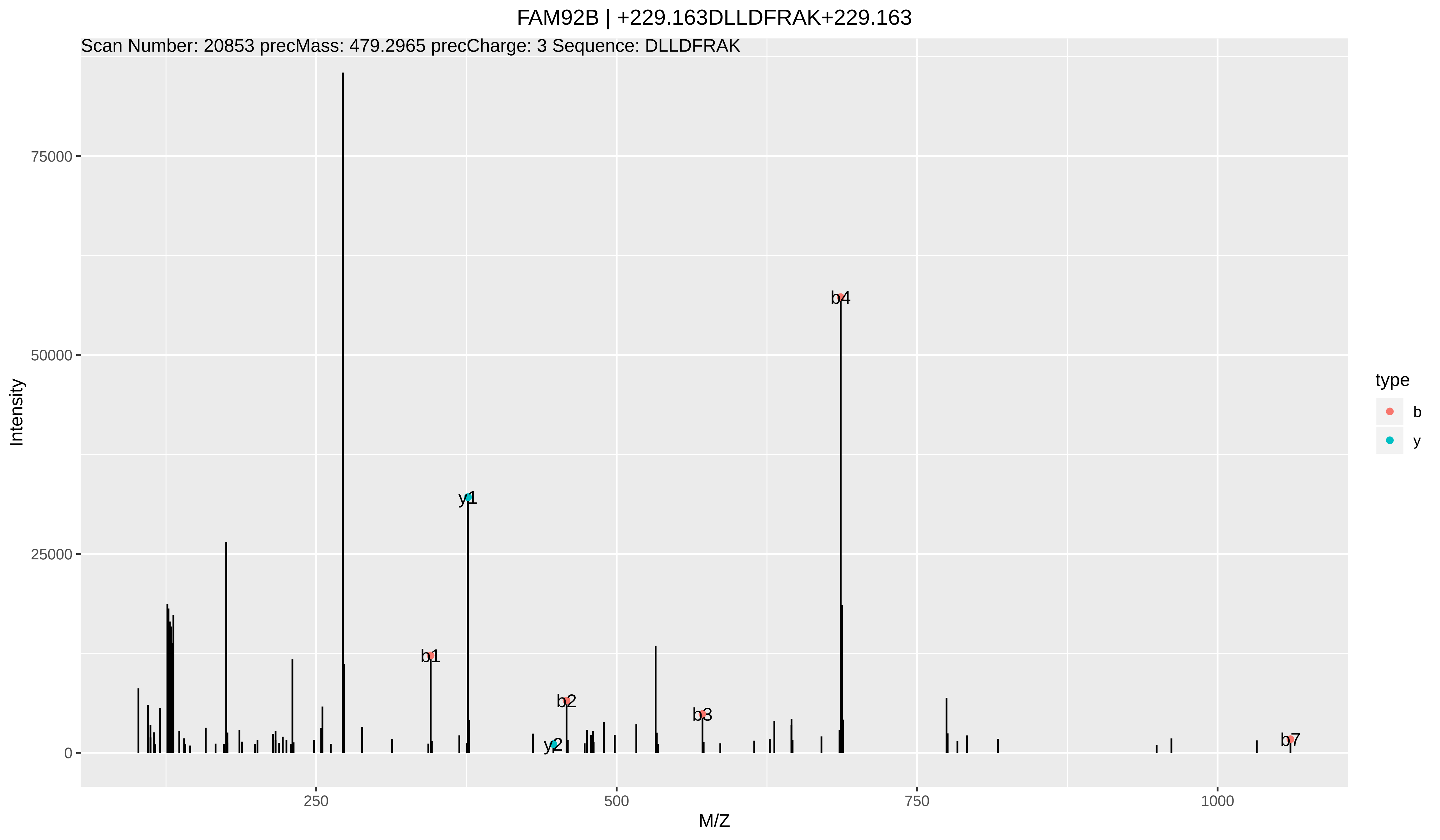

FAM92B | +229.163DLLDFRAK+229.163
Scan Number: 20853 precMass: 479.2965 precCharge: 3 Sequence: DLLDFRAK
Intensity
75000
50000
25000
0
250
500
750
1000
M/Z
b1
y1
y2
b2
b3
b4
b7
type
b
y

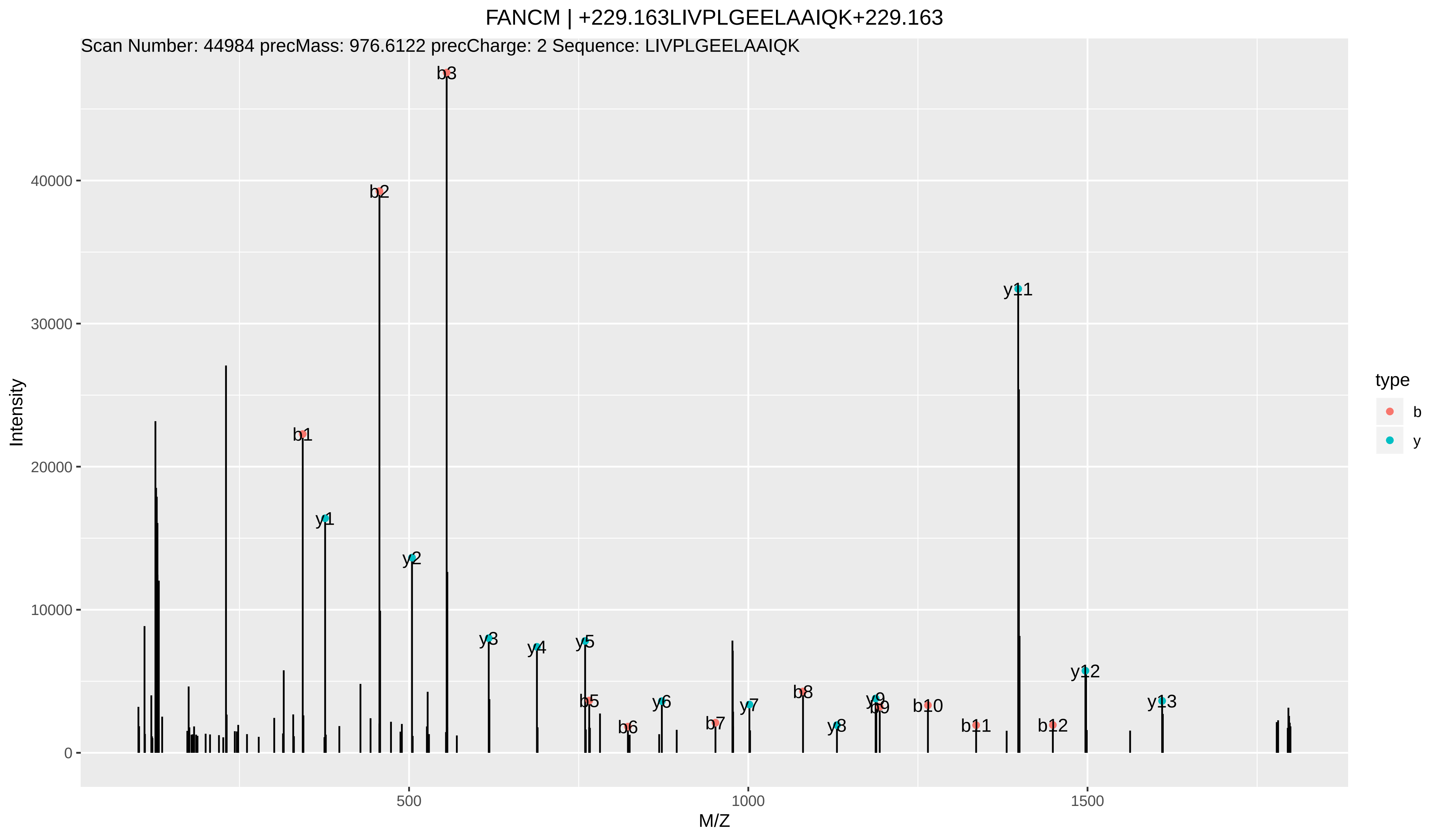

FANCM | +229.163LIVPLGEELAAIQK+229.163
Scan Number: 44984 precMass: 976.6122 precCharge: 2 Sequence: LIVPLGEELAAIQK
b3
b2
y11
b1
y1
y2
y3
y4
y5
b5
y6
b6
b7
y7
b8
y8
y9
b9
b10
b11
b12
y12
y13
Intensity
40000
30000
20000
10000
0
500
1000
1500
M/Z
type
b
y

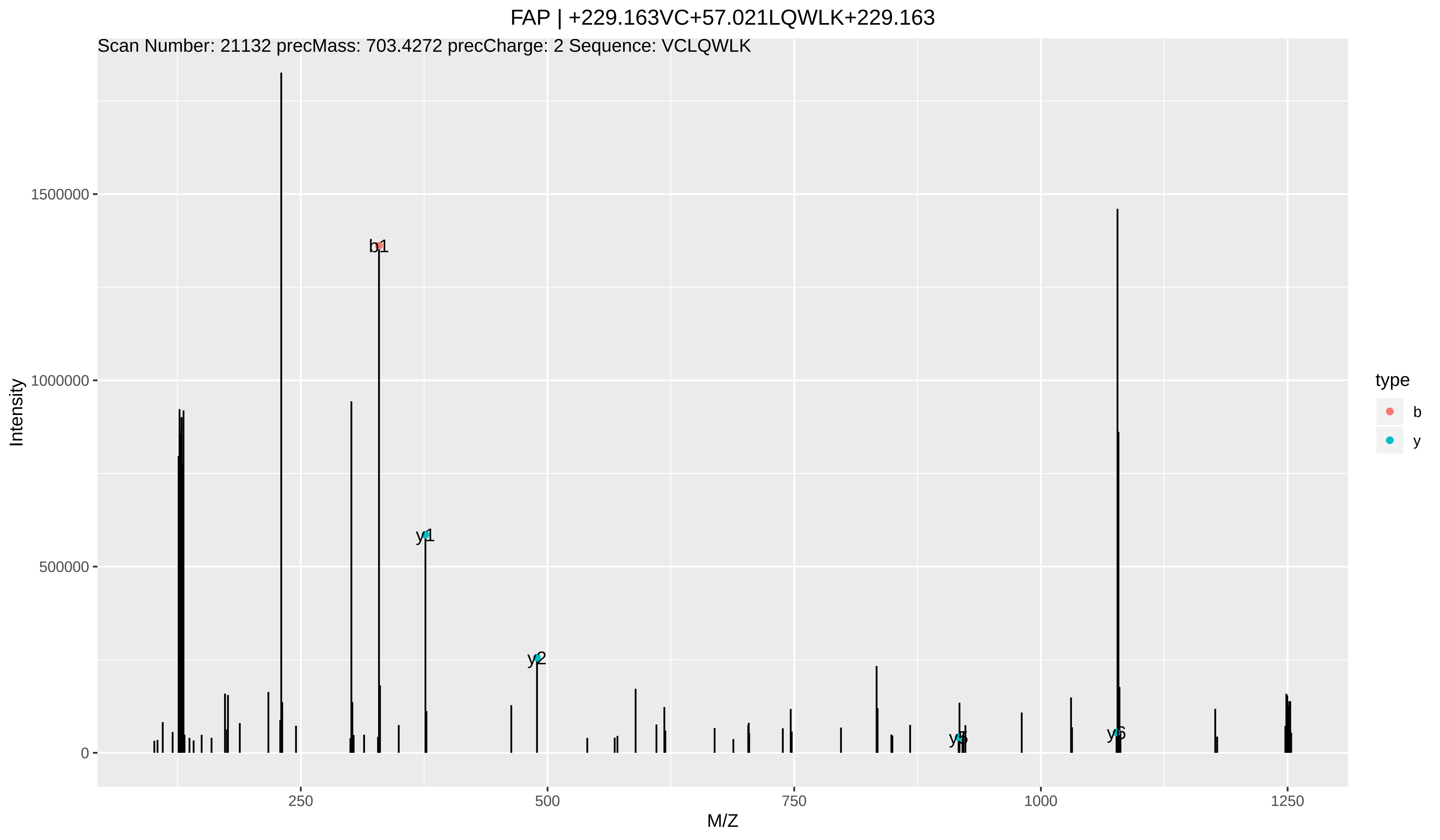
FAP | +229.163VC+57.021LQWLK+229.163
Scan Number: 21132 precMass: 703.4272 precCharge: 2 Sequence: VCLQWLK
Intensity
1500000
1000000
500000
0
250
500
750
1000
1250
M/Z
b1
y1
y2
y5
y6
type
b
y

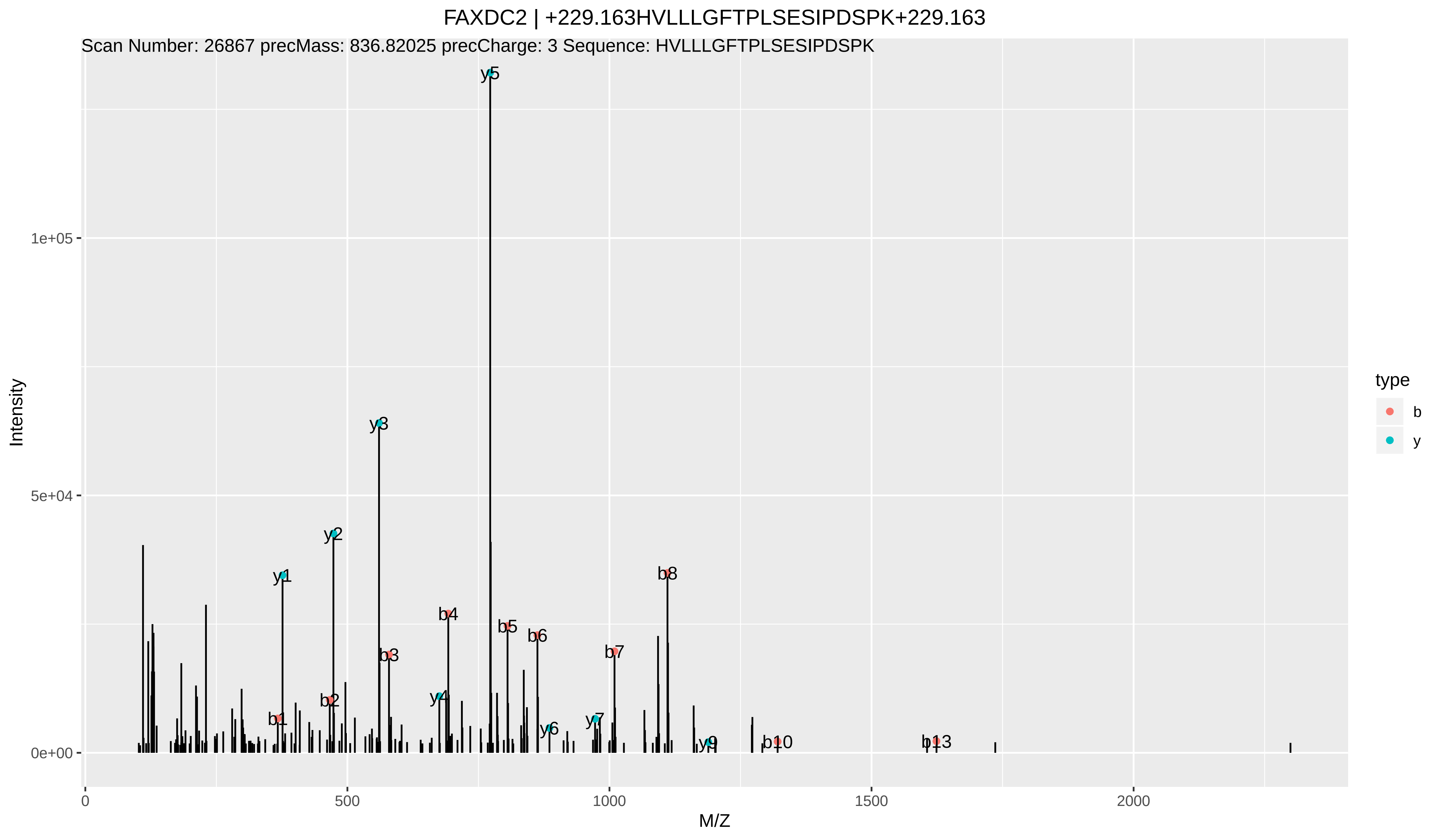

FAXDC2 | +229.163HVLLLGFTPLSESIPDSPK+229.163
Scan Number: 26867 precMass: 836.82025 precCharge: 3 Sequence: HVLLLGFTPLSESIPDSPK
y5
y3
y2
y1
b8
b4
b5
b6
b7
b3
y4
b2
b1
y7
y6
y9
b10
b13
1e+05
5e+04
0e+00
Intensity
0
500
1000
1500
2000
M/Z
type
b
y

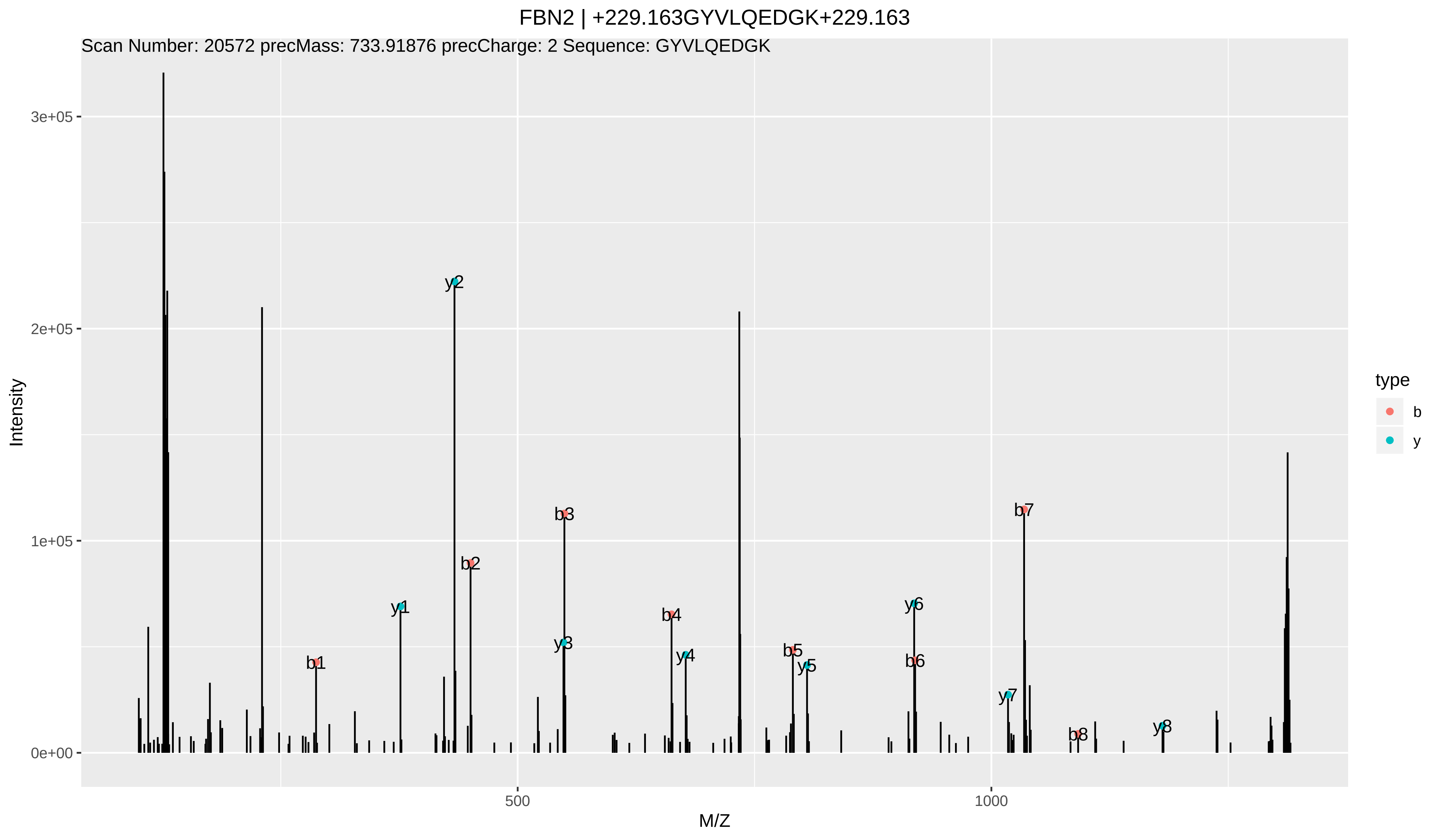

FBN2 | +229.163GYVLQEDGK+229.163
Scan Number: 20572 precMass: 733.91876 precCharge: 2 Sequence: GYVLQEDGK
Intensity
3e+05
2e+05
1e+05
0e+00
M/Z
500
1000
y2
b3
b2
y1
y3
b4
y4
b5
y5
y6
b6
b7
y7
b8
y8
b1
type
b
y

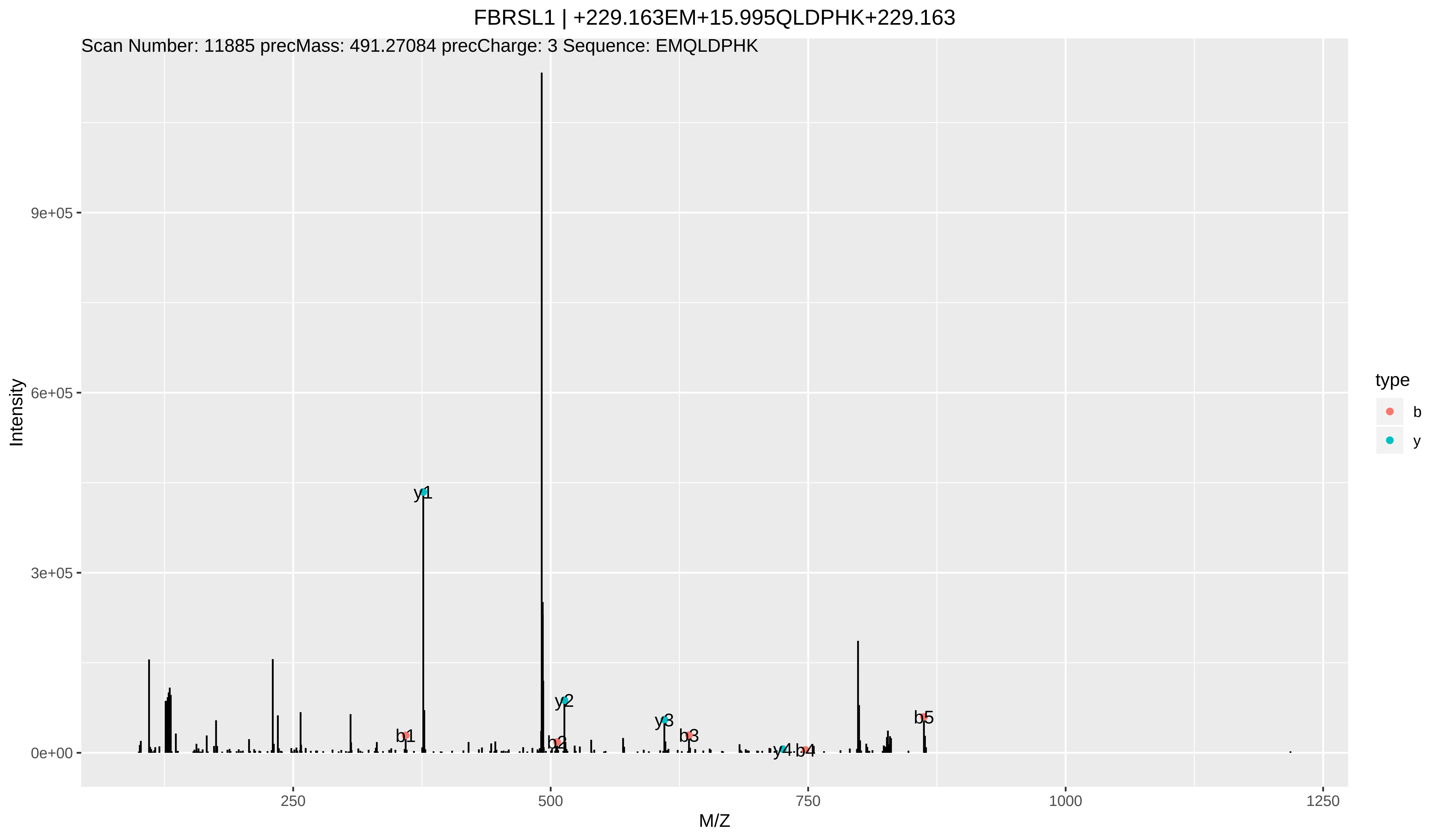
FBRSL1 | +229.163EM+15.995QLDPHK+229.163
Scan Number: 11885 precMass: 491.27084 precCharge: 3 Sequence: EMQLDPHK
Intensity
9e+05
6e+05
3e+05
0e+00
M/Z
250
500
750
1000
1250
y1
y2
y3
y4
b1
b2
b3
b4
b5
type
b
y

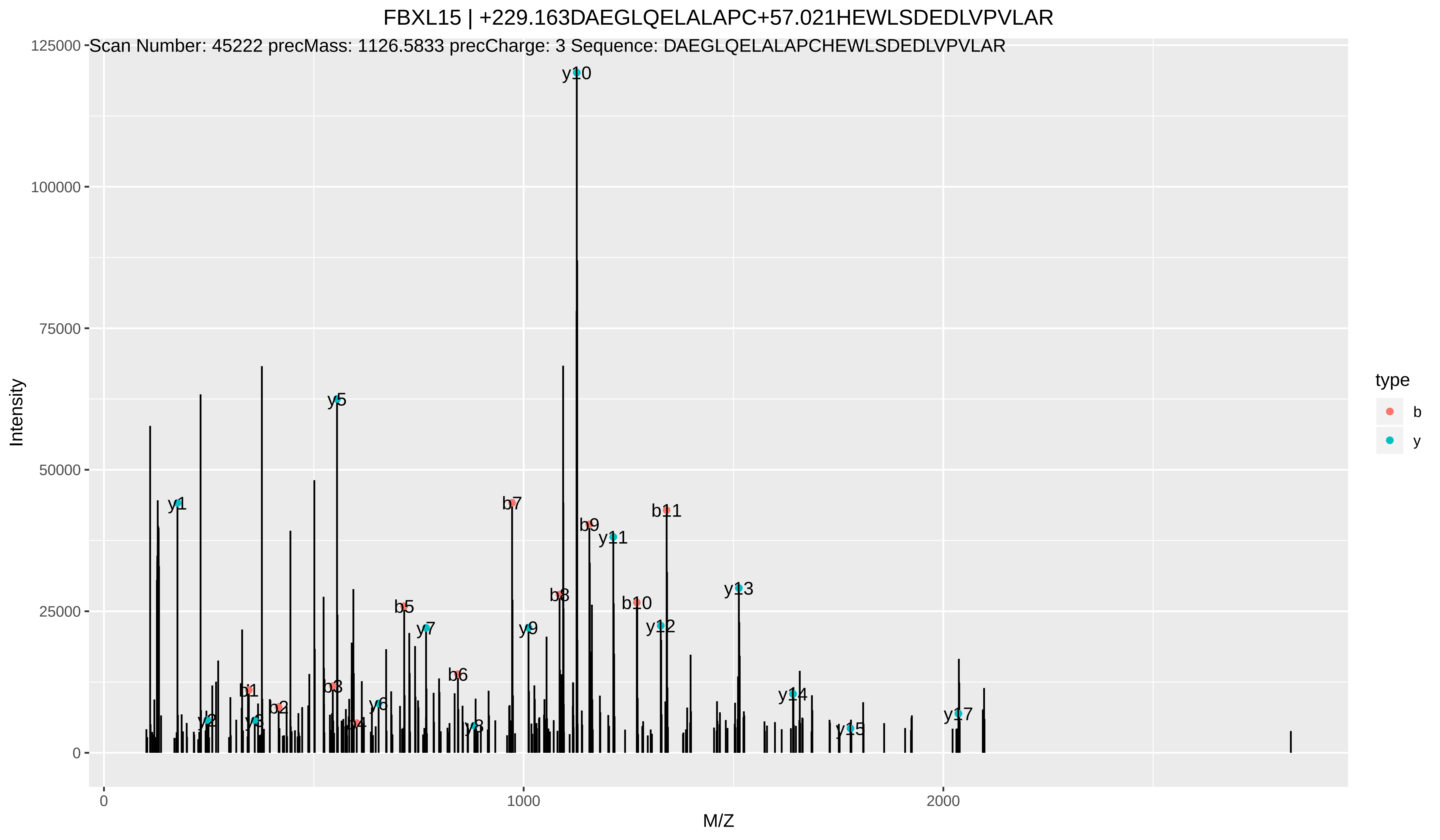
FBXL15 | +229.163DAEGLQELALAPC+57.021HEWLSDEDLVPVLAR
Scan Number: 45222 precMass: 1126.5833 precCharge: 3 Sequence: DAEGLQELALAPCHEWLSDEDLVPVLAR
Intensity
M/Z
type
b
y
y1
y2
y3
y5
y6
y7
y8
y9
y10
y11
y12
y13
y14
y15
y17
b1
b2
b3
b4
b5
b6
b7
b8
b9
b10
b11
0
25000
50000
75000
100000
125000
0
1000
2000

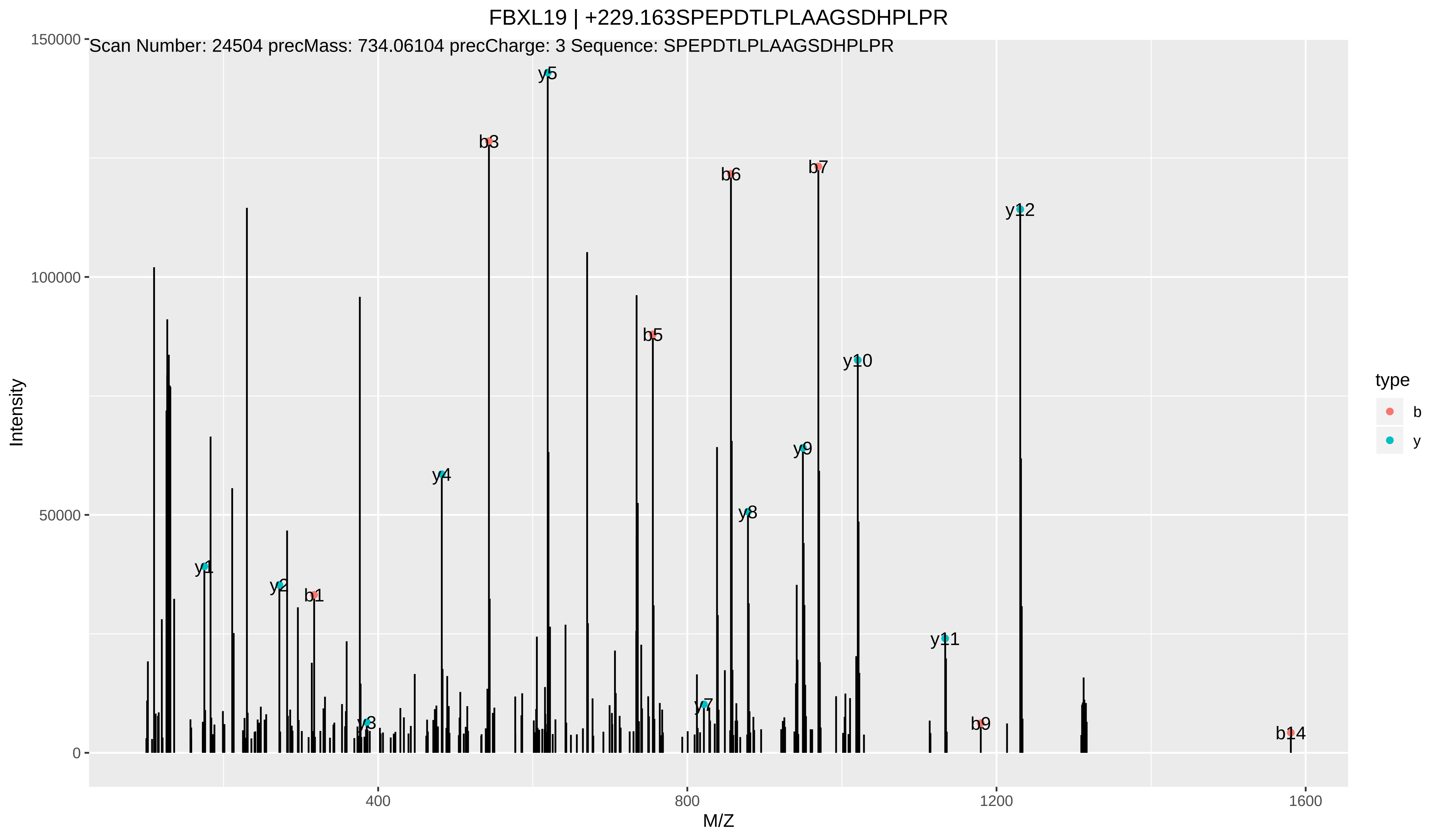
FBXL19 | +229.163SPEPDTLPLAAGSDHPLPR
Scan Number: 24504 precMass: 734.06104 precCharge: 3 Sequence: SPEPDTLPLAAGSDHPLPR
Intensity
M/Z
150000
100000
50000
0
400
800
1200
1600
y5
b3
b6
b7
y12
b5
y10
y9
y4
y8
y1
y2
b1
y11
y7
y3
b9
b14
type
b
y

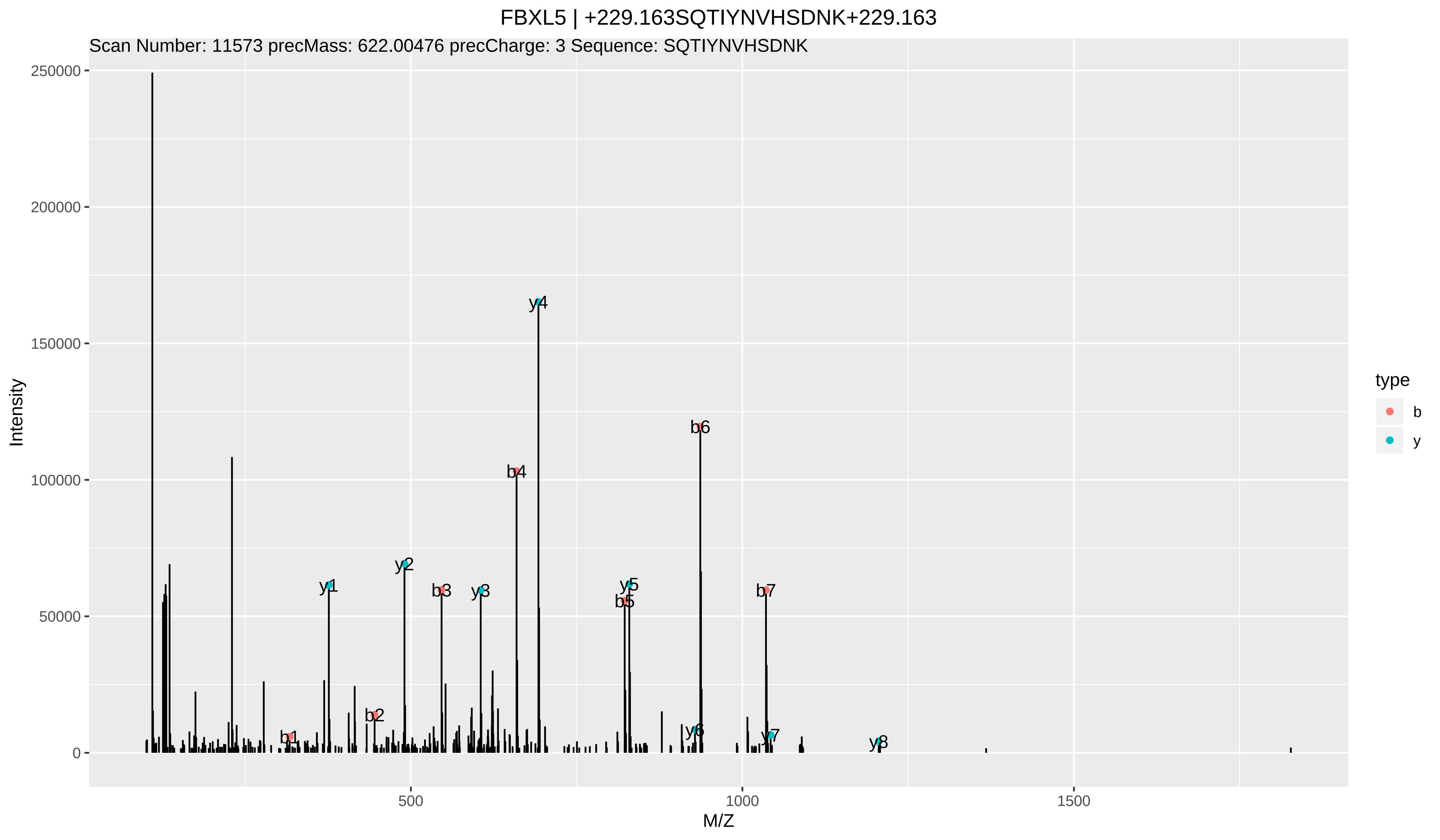

FBXL5 | +229.163SQTIYNVHSDNK+229.163
Scan Number: 11573 precMass: 622.00476 precCharge: 3 Sequence: SQTIYNVHSDNK
Intensity
M/Z
250000
200000
150000
100000
50000
0
500
1000
1500
y4
b6
b4
y2
y1
b3
y3
y5
b5
b7
b2
b1
y6
y7
y8
type
b
y

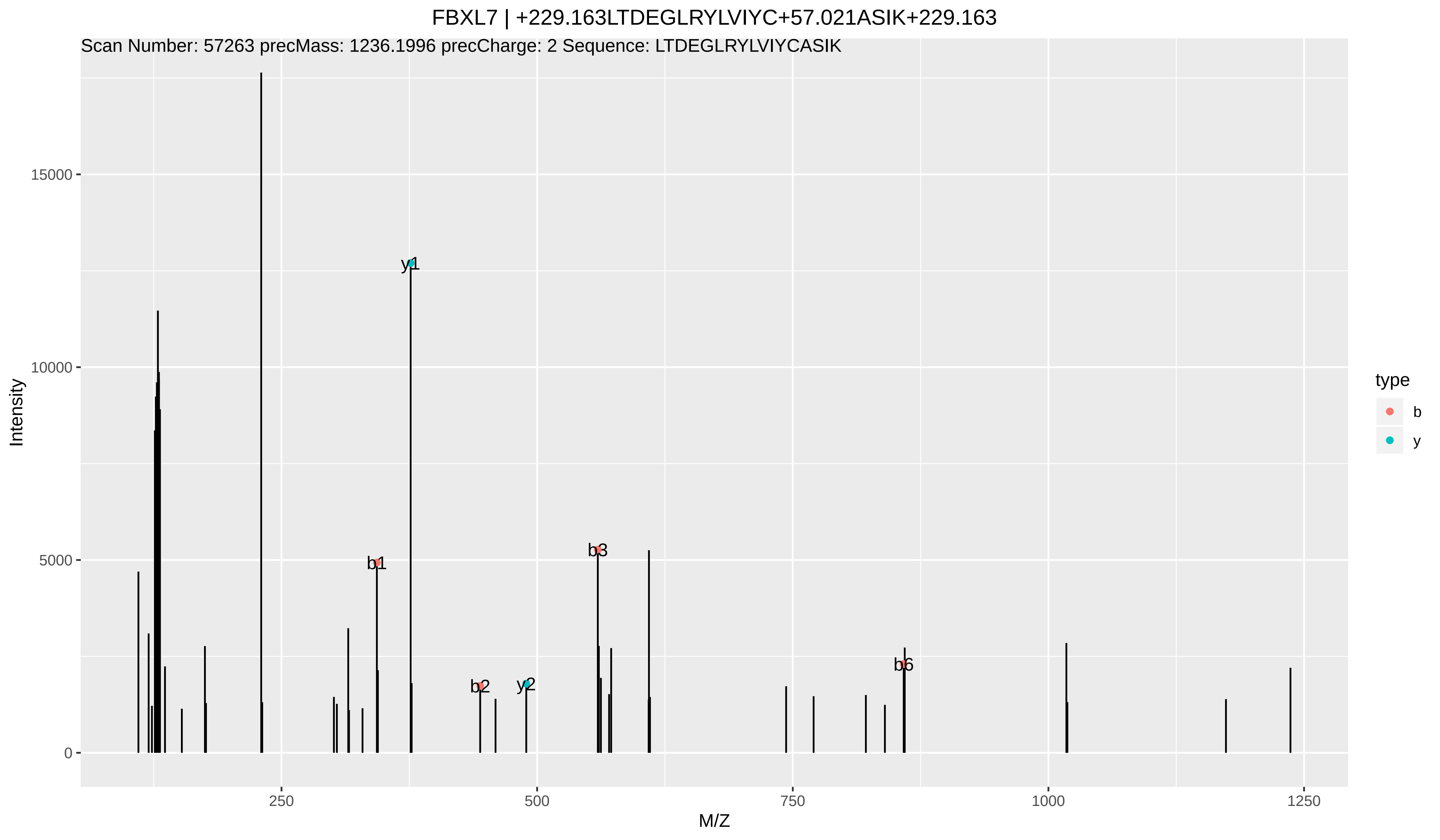
FBXL7 | +229.163LTDEGLRYLVIYC+57.021ASIK+229.163
Scan Number: 57263 precMass: 1236.1996 precCharge: 2 Sequence: LTDEGLRYLVIYCASIK
Intensity
15000
10000
5000
0
250
500
750
1000
1250
M/Z
y1
b1
b2
y2
b3
b6
type
b
y

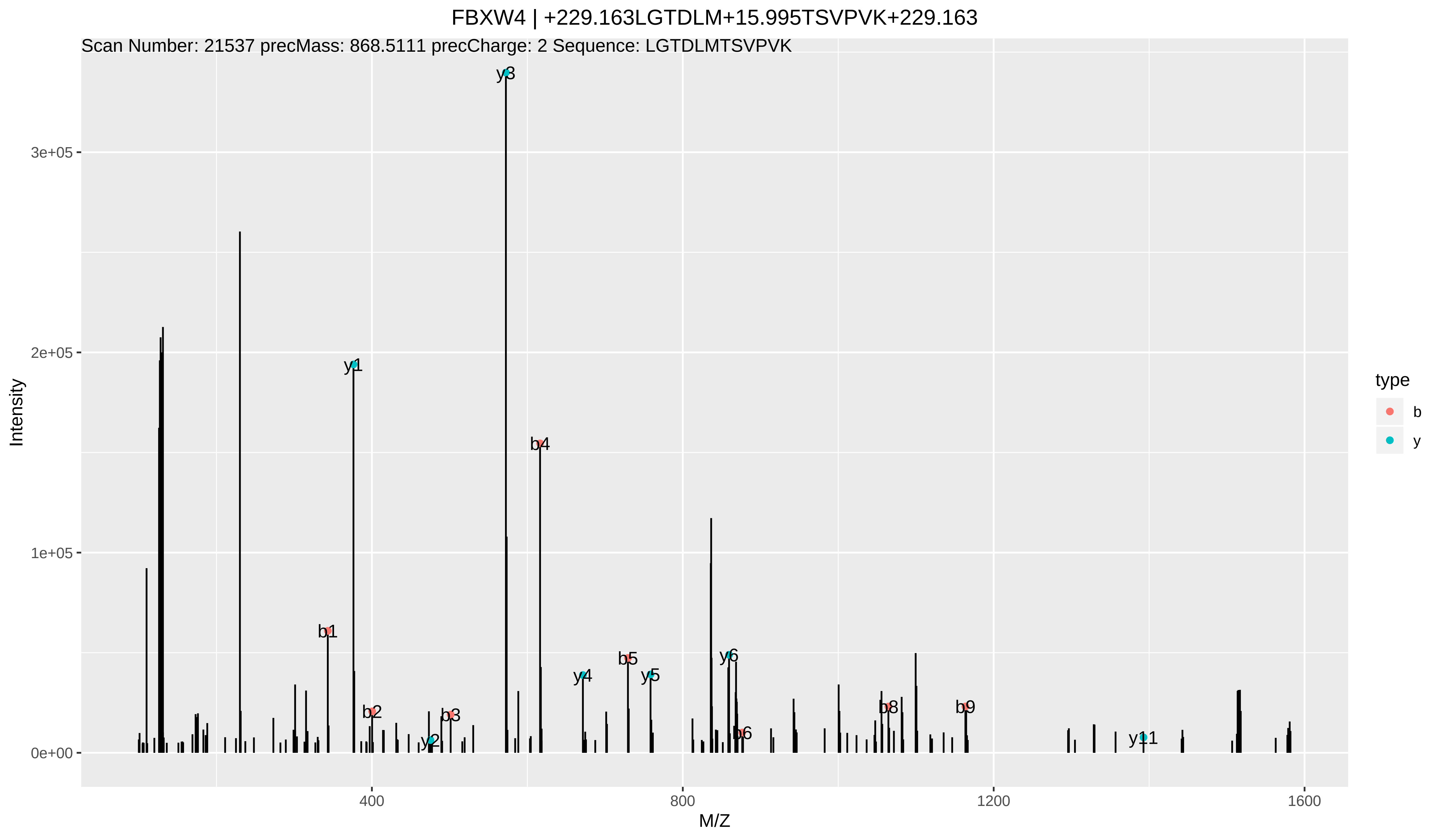

FBXW4 | +229.163LGTDLM+15.995TSVPVK+229.163
Scan Number: 21537 precMass: 868.5111 precCharge: 2 Sequence: LGTDLMTSVPVK
y3
y1
b4
b1
y6
b5
y4
y5
b2
b3
y2
b6
b8
b9
y11
Intensity
3e+05
2e+05
1e+05
0e+00
M/Z
400
800
1200
1600
type
b
y

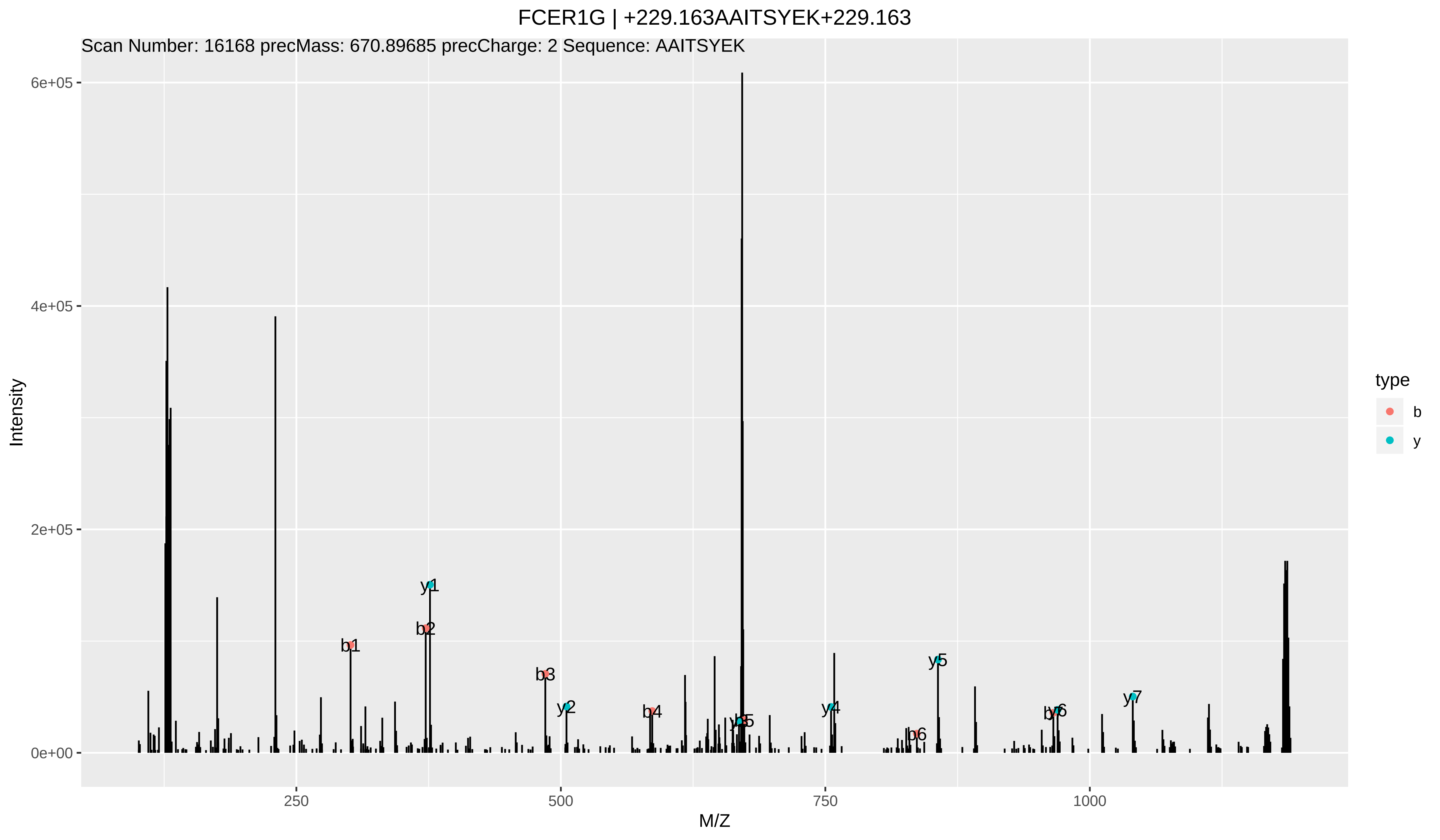

FCER1G | +229.163AAITSYEK+229.163
Scan Number: 16168 precMass: 670.89685 precCharge: 2 Sequence: AAITSYEK
Intensity
6e+05
4e+05
2e+05
0e+00
M/Z
250
500
750
1000
y1
b2
b1
b3
y2
b4
y3
b5
y4
y5
b6
b7
y6
y7
type
b
y

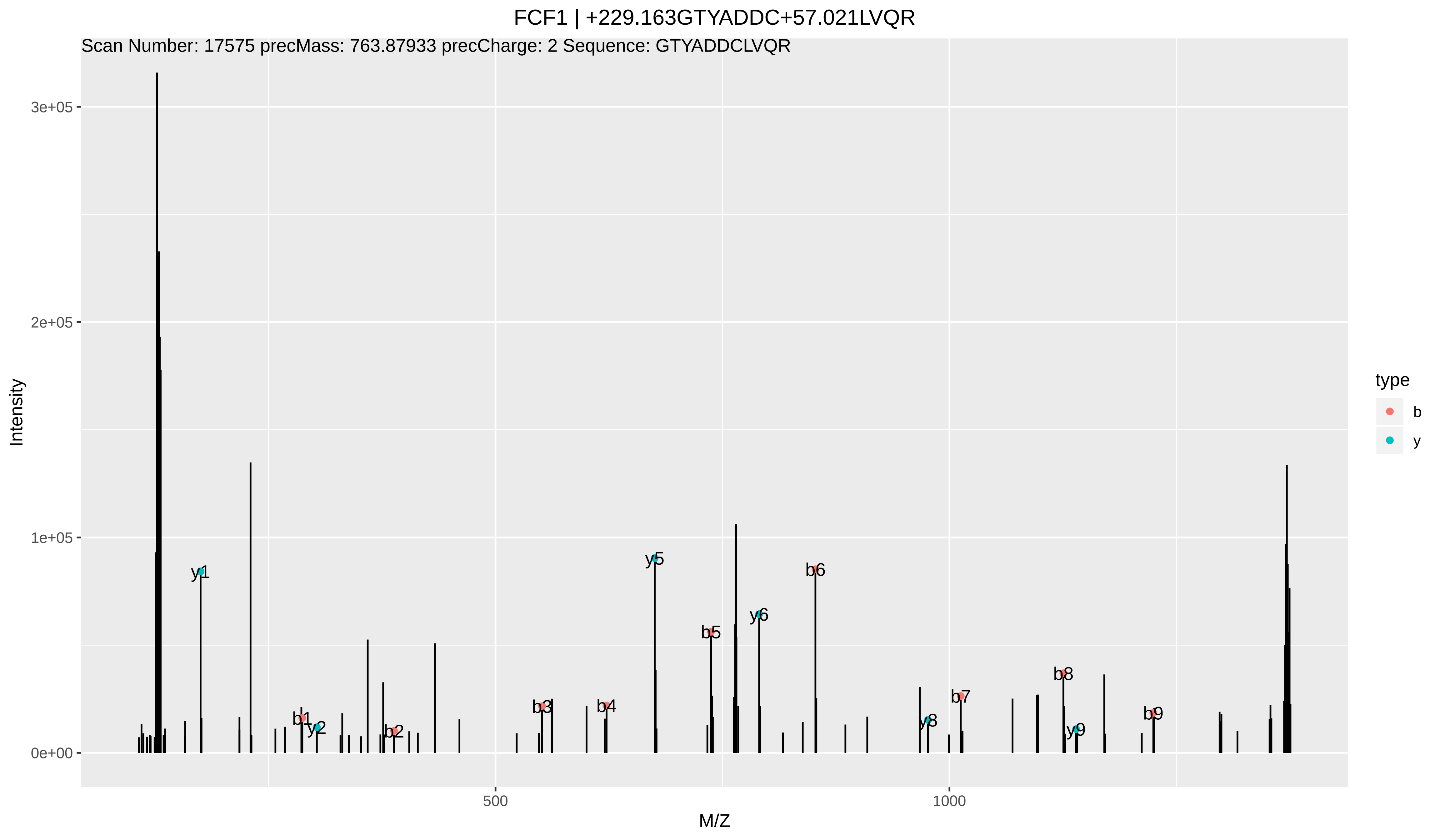

FCF1 | +229.163GTYADDC+57.021LVQR
Scan Number: 17575 precMass: 763.87933 precCharge: 2 Sequence: GTYADDCLVQR
Intensity
3e+05
2e+05
1e+05
0e+00
500
1000
M/Z
y1
b1
y2
b2
b3
b4
y5
b5
y6
b6
y8
b7
b8
y9
b9
type
b
y

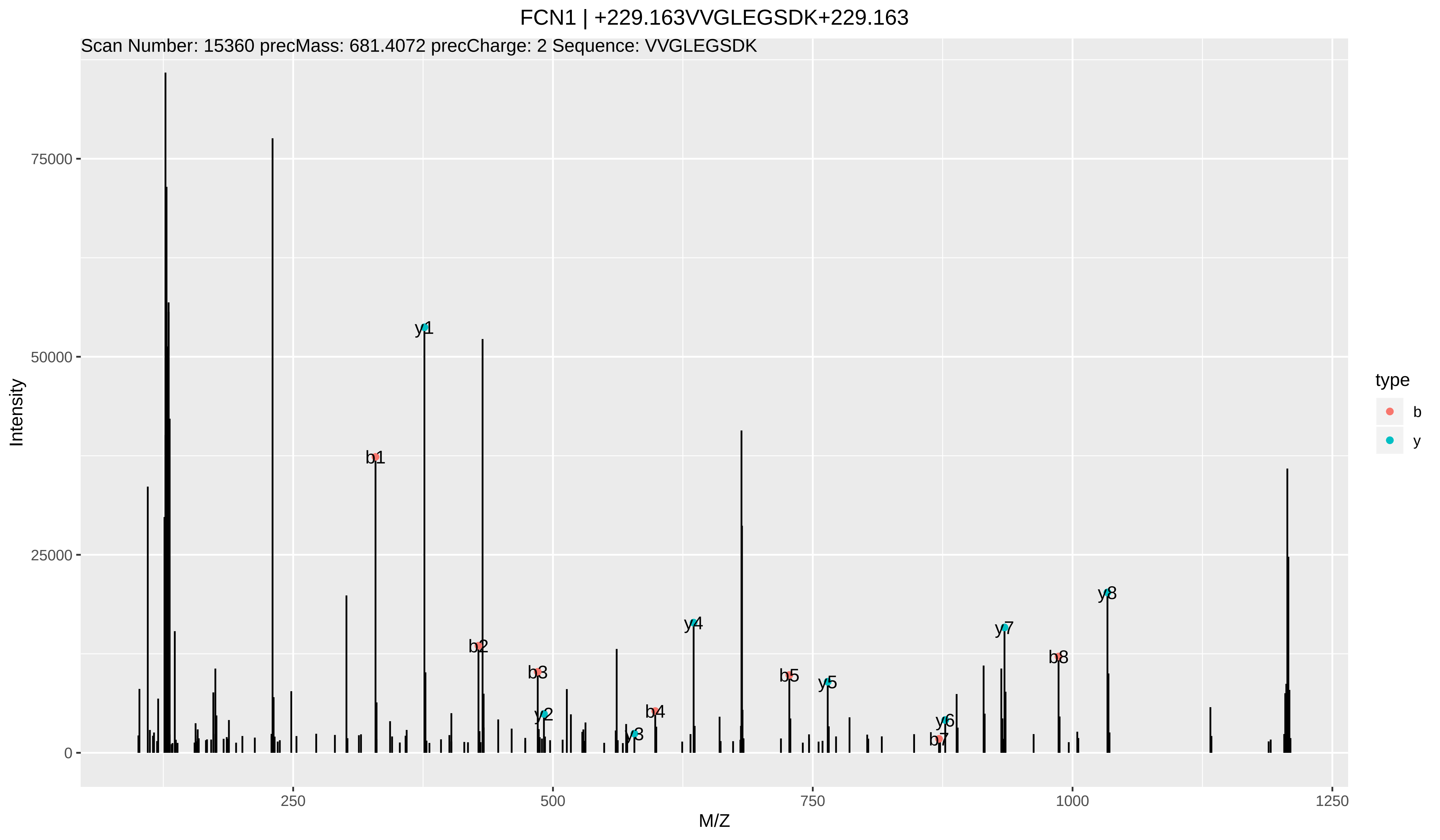
FCN1 | +229.163VVGLEGSDK+229.163
Scan Number: 15360 precMass: 681.4072 precCharge: 2 Sequence: VVGLEGSDK
Intensity
M/Z
0
25000
50000
75000
250
500
750
1000
1250
y1
b1
b2
b3
y2
y3
b4
y4
b5
y5
y6
b7
y7
b8
y8
type
b
y

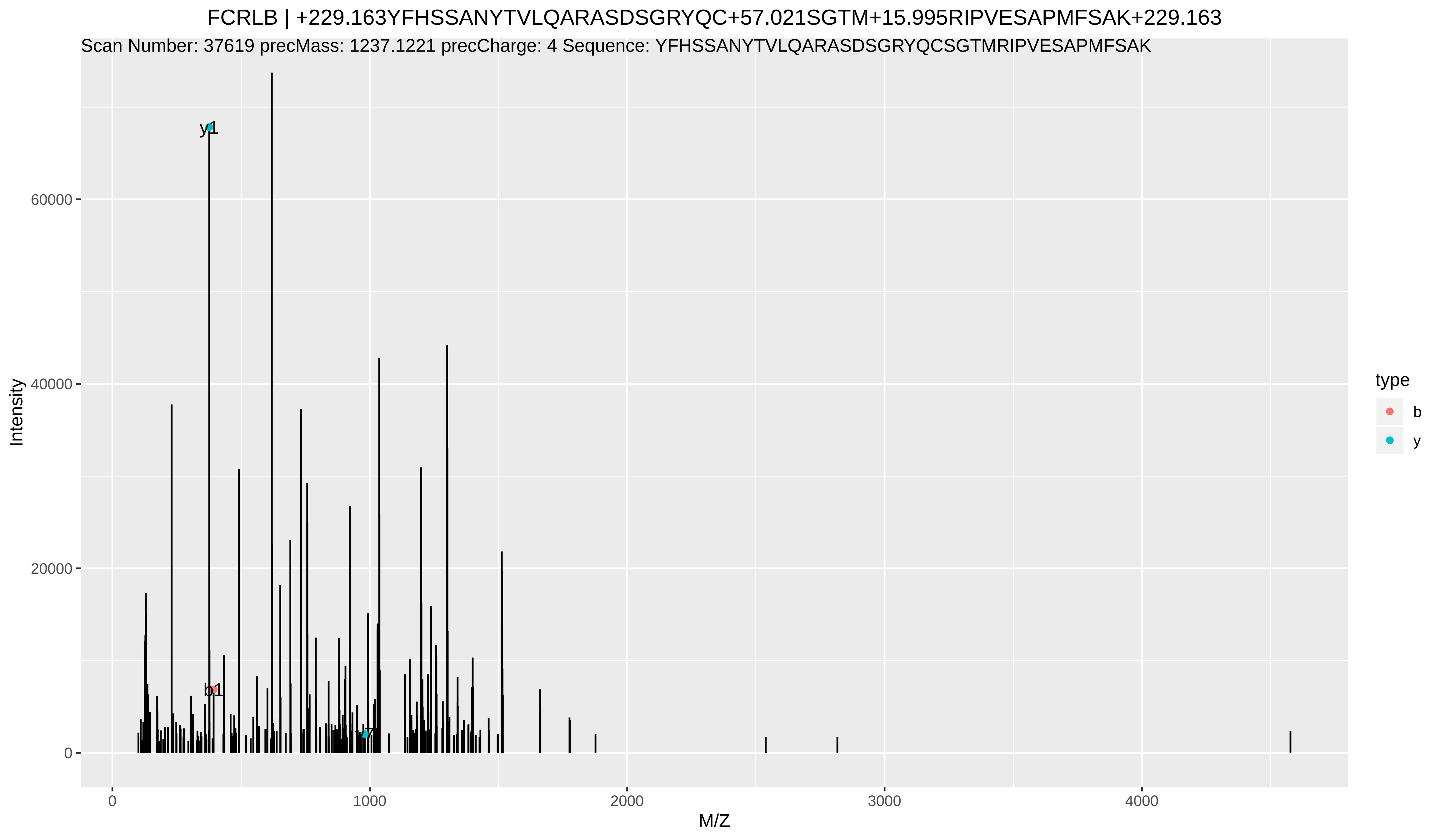

FCRLB | +229.163YFHSSANYTVLQARASDSGRYQC+57.021SGTM+15.995RIPVESAPMFSAK+229.163
Scan Number: 37619 precMass: 1237.1221 precCharge: 4 Sequence: YFHSSANYTVLQARASDSGRYQCSGTMRIPVESAPMFSAK
y1
b1
y7
Intensity
60000
40000
20000
0
M/Z
0
1000
2000
3000
4000
type
b
y

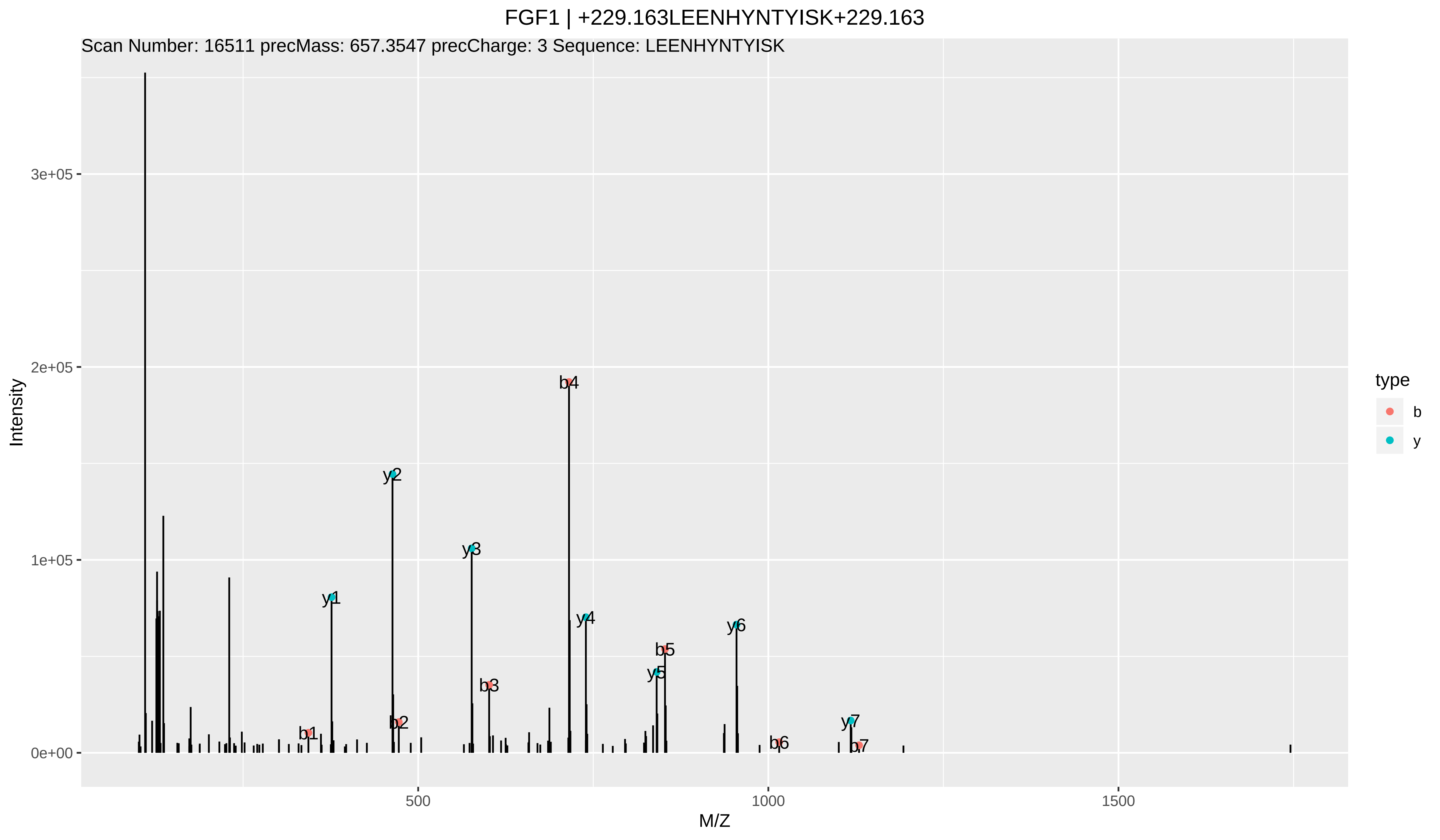

FGF1 | +229.163LEENHYNTYISK+229.163
Scan Number: 16511 precMass: 657.3547 precCharge: 3 Sequence: LEENHYNTYISK
Intensity
3e+05
2e+05
1e+05
0e+00
M/Z
500
1000
1500
type
b
y
b1
b2
b3
b4
b5
b6
b7
y1
y2
y3
y4
y5
y6
y7

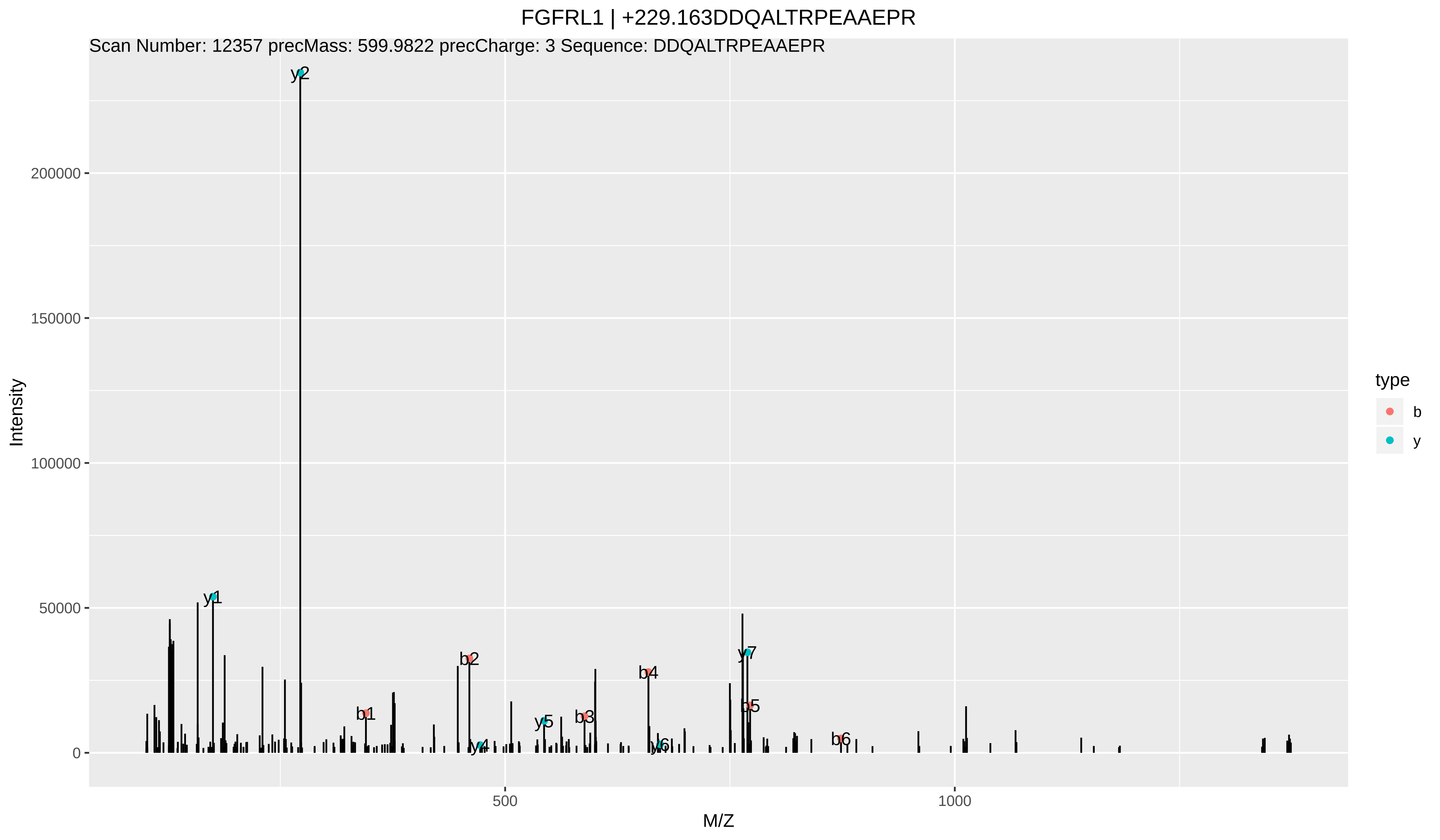
FGFRL1 | +229.163DDQALTRPEAAEPR
Scan Number: 12357 precMass: 599.9822 precCharge: 3 Sequence: DDQALTRPEAAEPR
Intensity
M/Z
type
b
y
y1
y2
y4
y5
y6
y7
b1
b2
b3
b4
b5
b6
0
50000
100000
150000
200000
500
1000

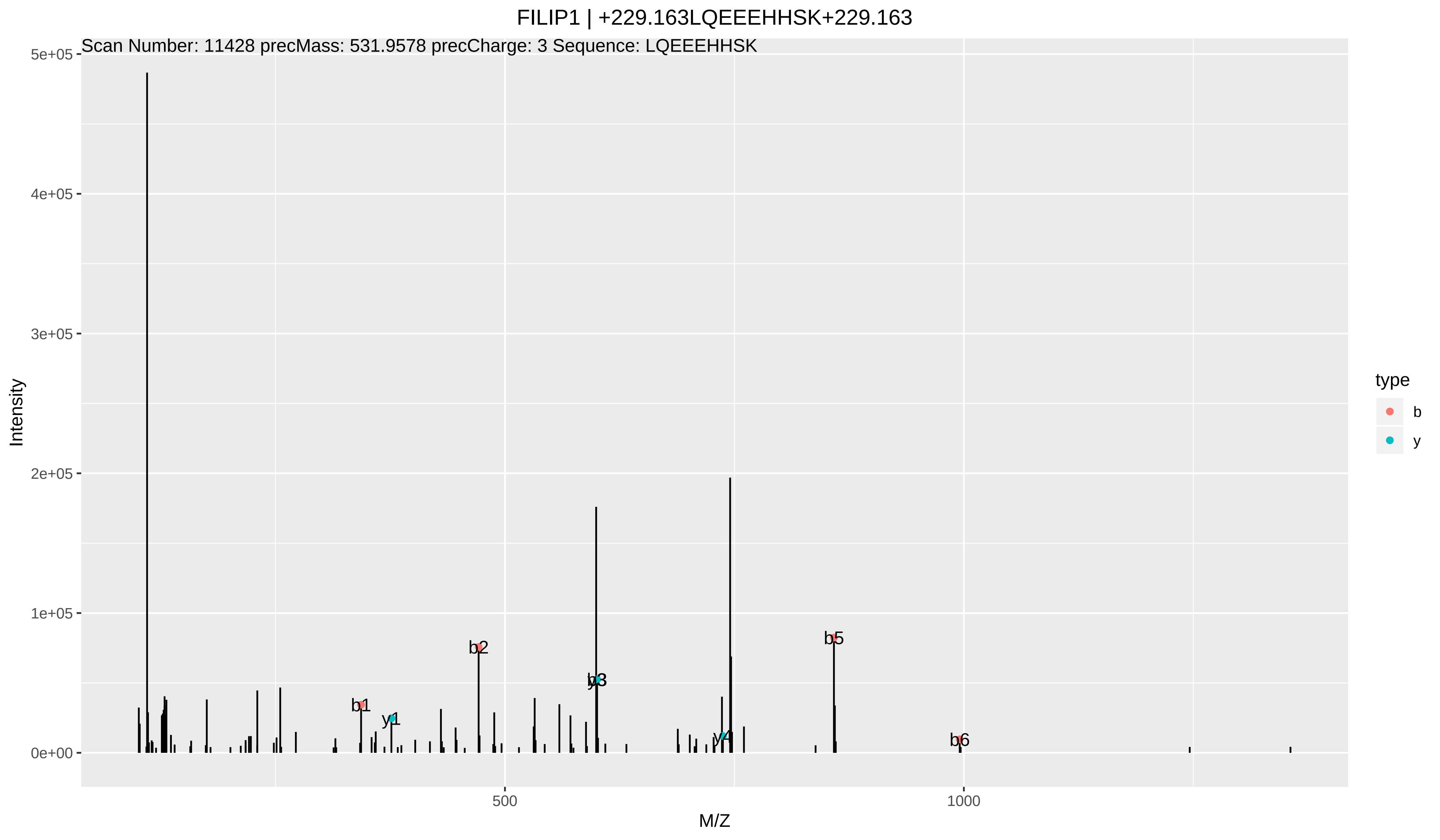

FILIP1 | +229.163LQEEEHHSK+229.163
Scan Number: 11428 precMass: 531.9578 precCharge: 3 Sequence: LQEEEHHSK
Intensity
5e+05
4e+05
3e+05
2e+05
1e+05
0e+00
M/Z
500
1000
b1
y1
b2
b3
y3
y4
b5
b6
type
b
y

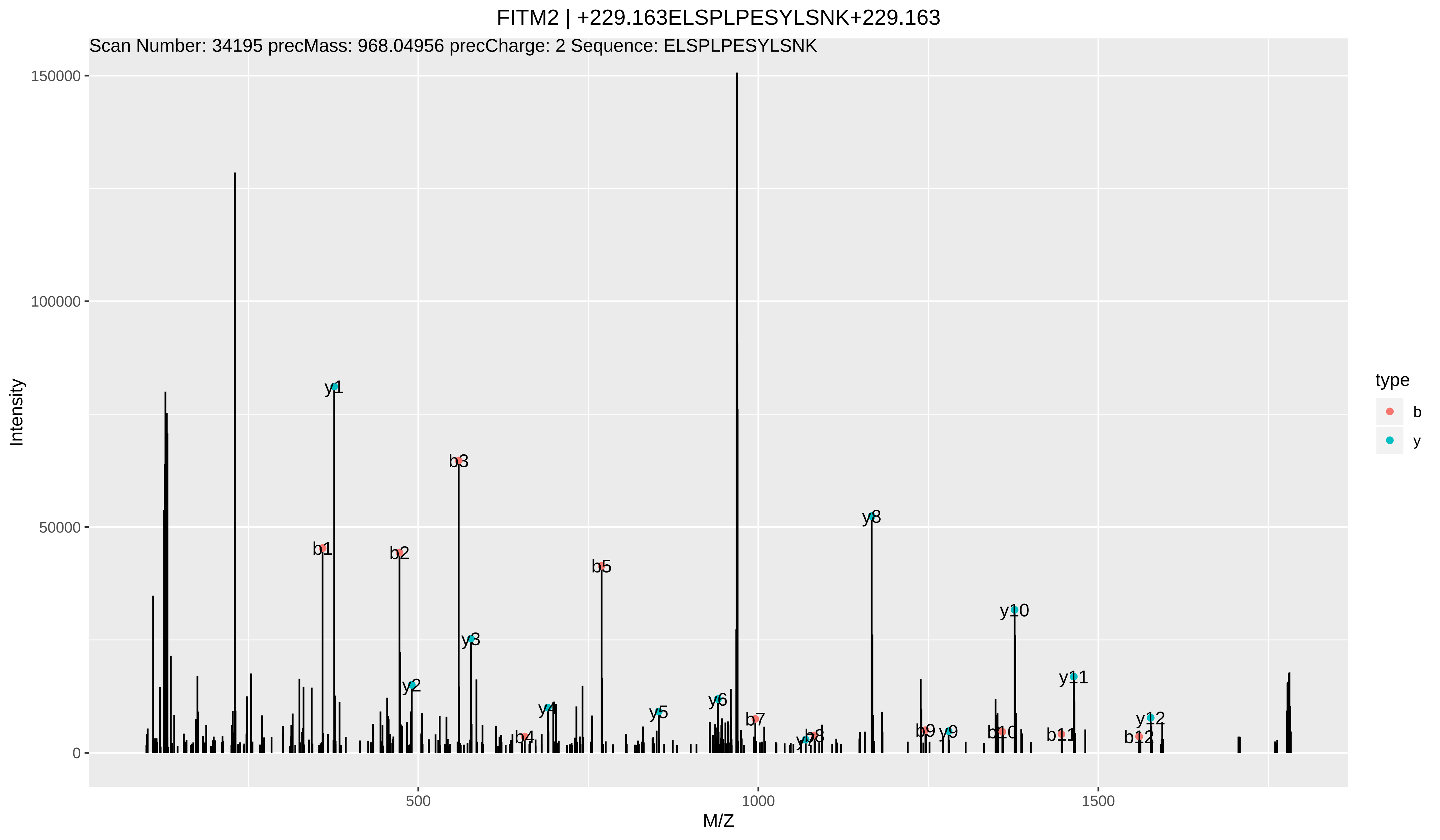
FITM2 | +229.163ELSPLPESYLSNK+229.163
Scan Number: 34195 precMass: 968.04956 precCharge: 2 Sequence: ELSPLPESYLSNK
Intensity
150000
100000
50000
0
M/Z
500
1000
1500
y1
b1
b2
y2
b3
y3
b4
y4
b5
y5
y6
b7
y7
b8
y8
b9
y9
b10
y10
b11
y11
b12
y12
type
b
y

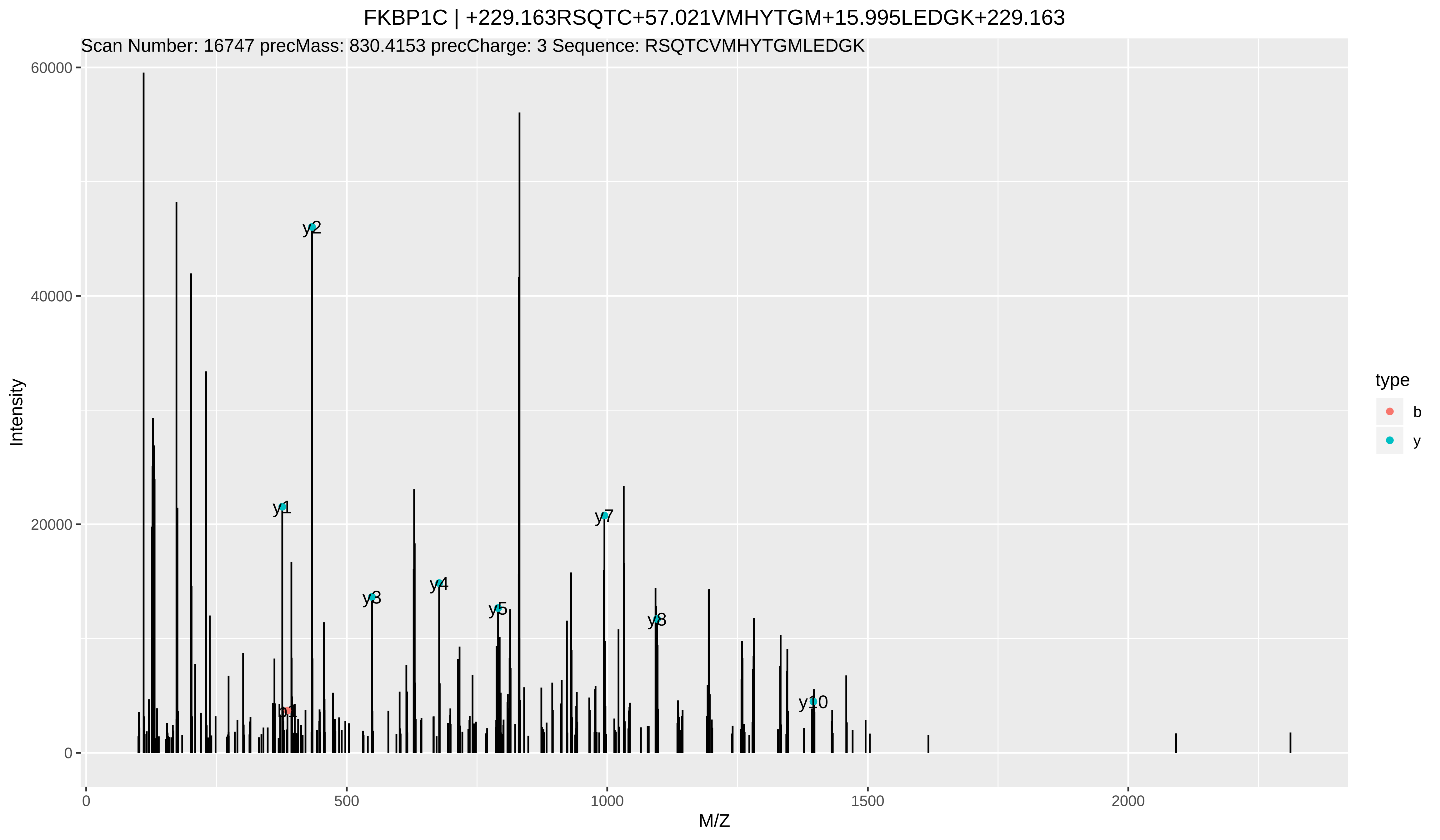
FKBP1C | +229.163RSQTC+57.021VMHYTGM+15.995LEDGK+229.163
Scan Number: 16747 precMass: 830.4153 precCharge: 3 Sequence: RSQTCVMHYTGMLEDGK
Intensity
M/Z
type
b
y
y1
y2
y3
y4
y5
y7
y8
y10
b1
0
20000
40000
60000
0
500
1000
1500
2000

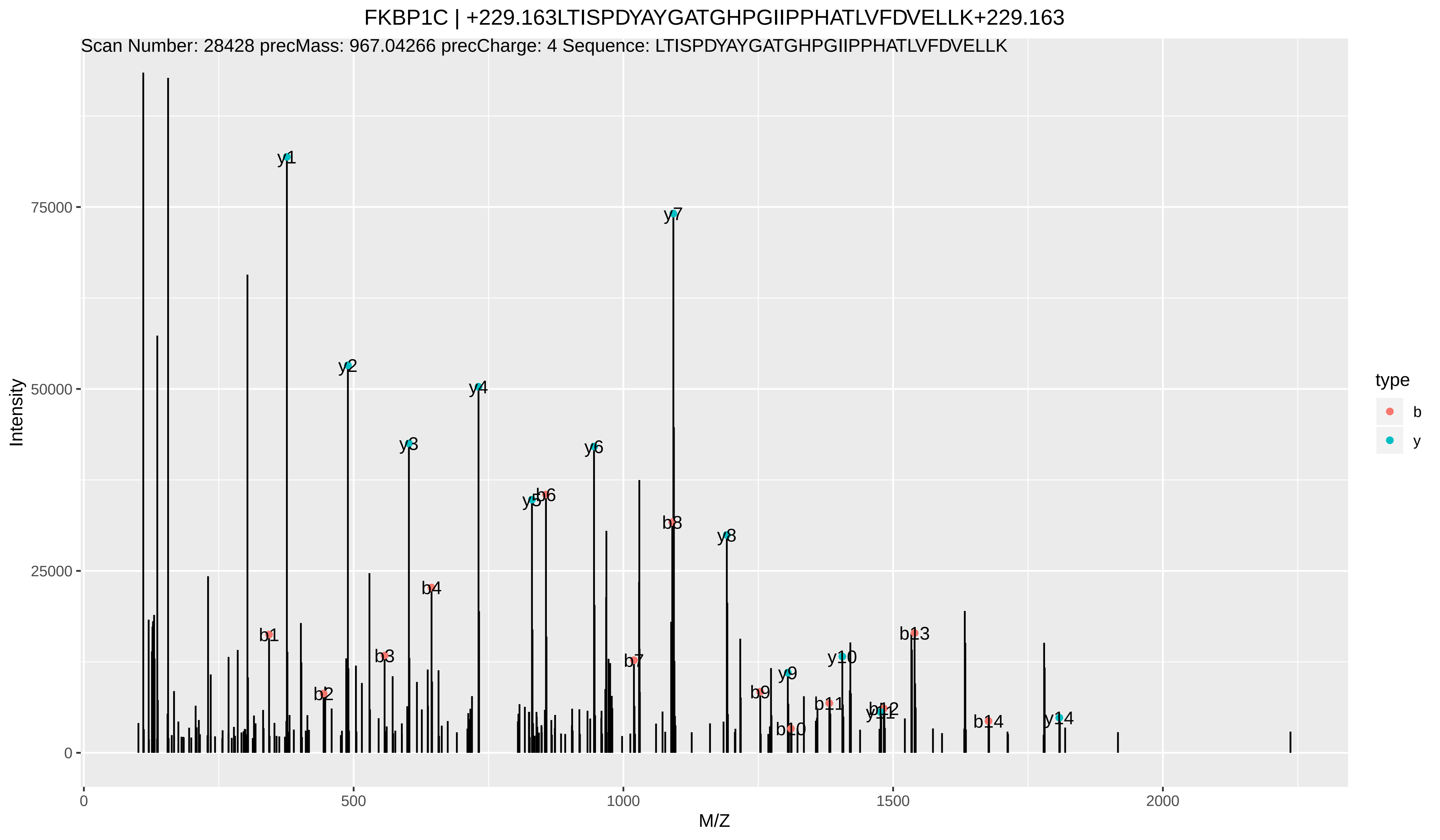

FKBP1C | +229.163LTISPDYAYGATGHPGIIPPHATLVFDVELLK+229.163
Scan Number: 28428 precMass: 967.04266 precCharge: 4 Sequence: LTISPDYAYGATGHPGIIPPHATLVFDVELLK
Intensity
M/Z
type
b
y
y1
y2
y3
y4
y5
y6
y7
y8
y9
y10
y11
y14
b1
b2
b3
b4
b6
b7
b8
b9
b10
b11
b12
b13
b14
0
25000
50000
75000
500
1000
1500
2000

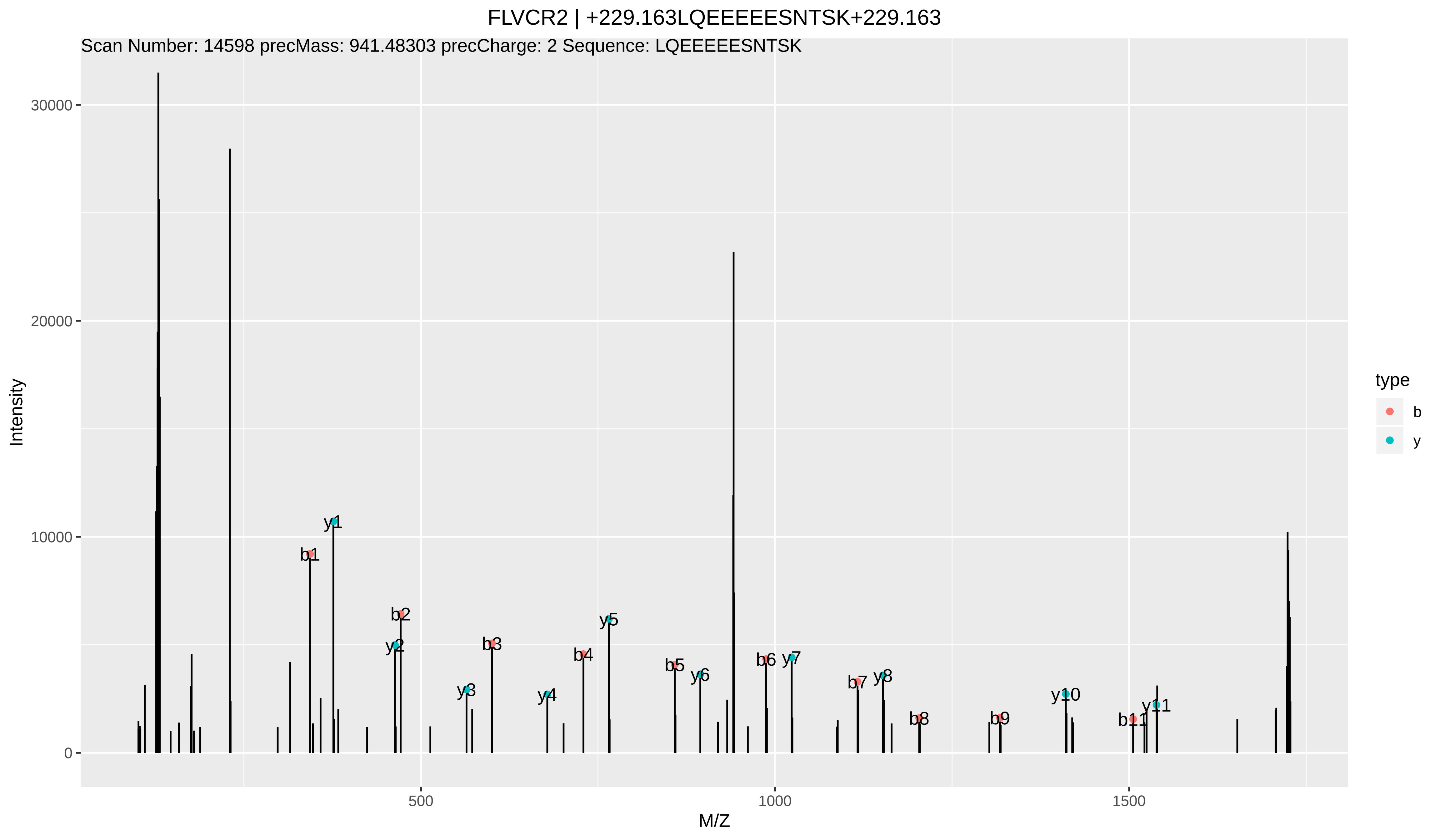
FLVCR2 | +229.163LQEEEEEESNTSK+229.163
Scan Number: 14598 precMass: 941.48303 precCharge: 2 Sequence: LQEEEEEESNTSK
Intensity
M/Z
30000
20000
10000
0
500
1000
1500
type
b
y
y1
b1
b2
y2
y3
b3
y4
b4
y5
b5
y6
b6
y7
b7
y8
b8
b9
y10
b11
y11

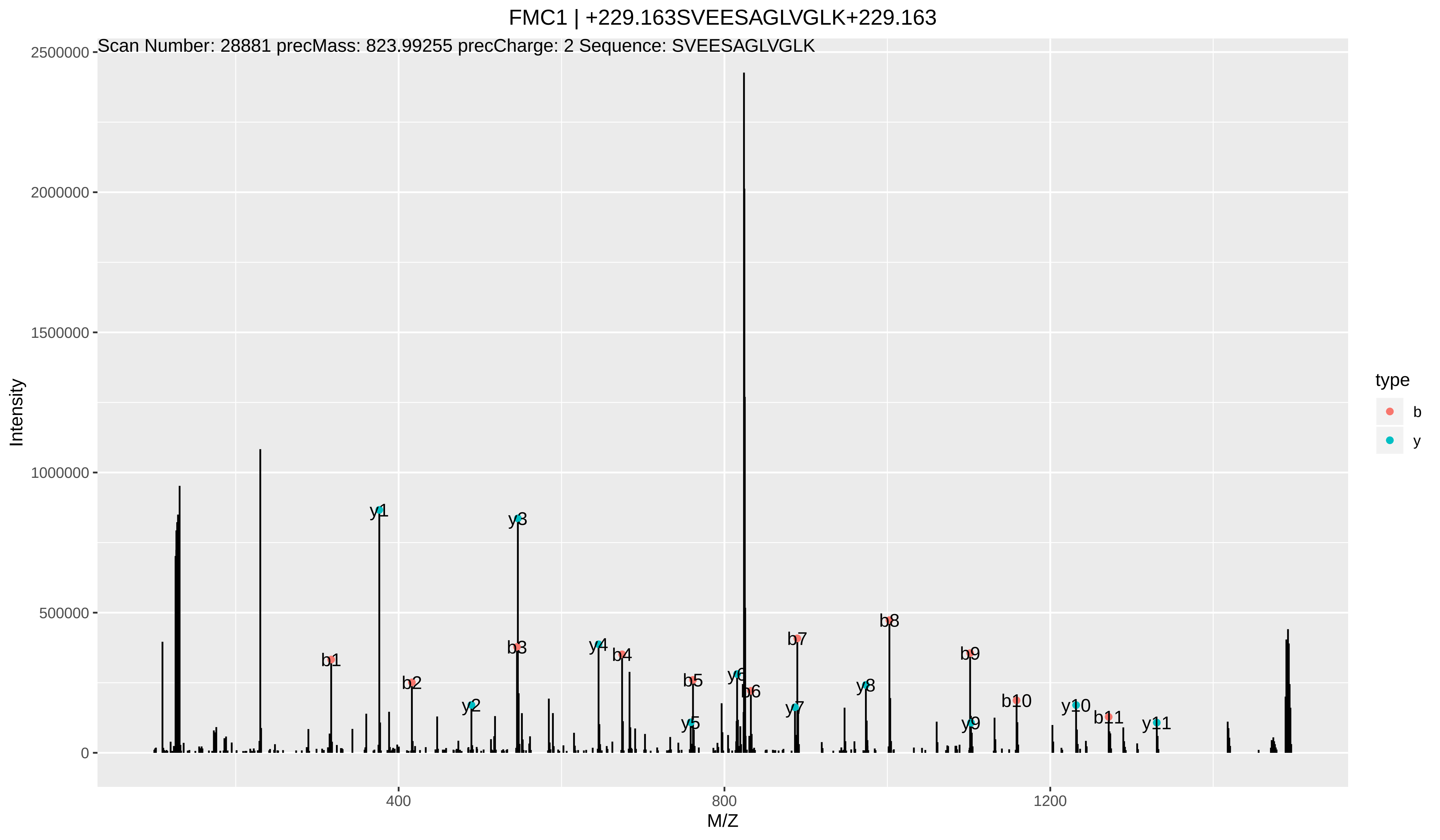

FMC1 | +229.163SVEESAGLVGLK+229.163
Scan Number: 28881 precMass: 823.99255 precCharge: 2 Sequence: SVEESAGLVGLK
Intensity
2500000
2000000
1500000
1000000
500000
0
M/Z
400
800
1200
y1
y3
b1
b2
y2
b3
y4
b4
b5
y5
y6
b6
b7
y7
y8
b8
b9
y9
b10
y10
b11
y11
type
b
y

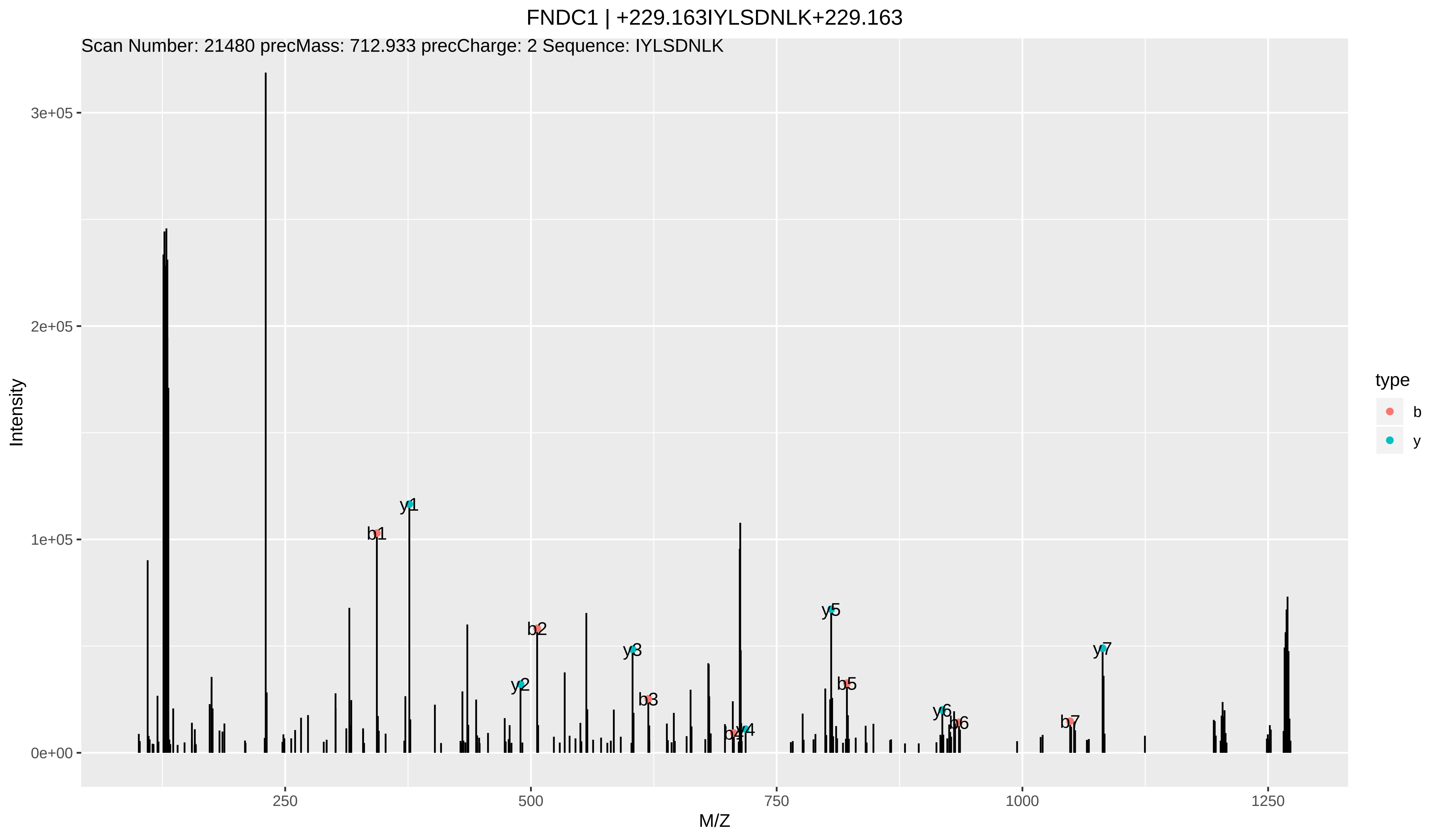

FNDC1 | +229.163IYLSDNLK+229.163
Scan Number: 21480 precMass: 712.933 precCharge: 2 Sequence: IYLSDNLK
Intensity
3e+05
2e+05
1e+05
0e+00
y1
b1
b2
y2
y3
b3
b4
y4
y5
b5
y6
b6
b7
y7
250
500
750
1000
1250
M/Z
type
b
y

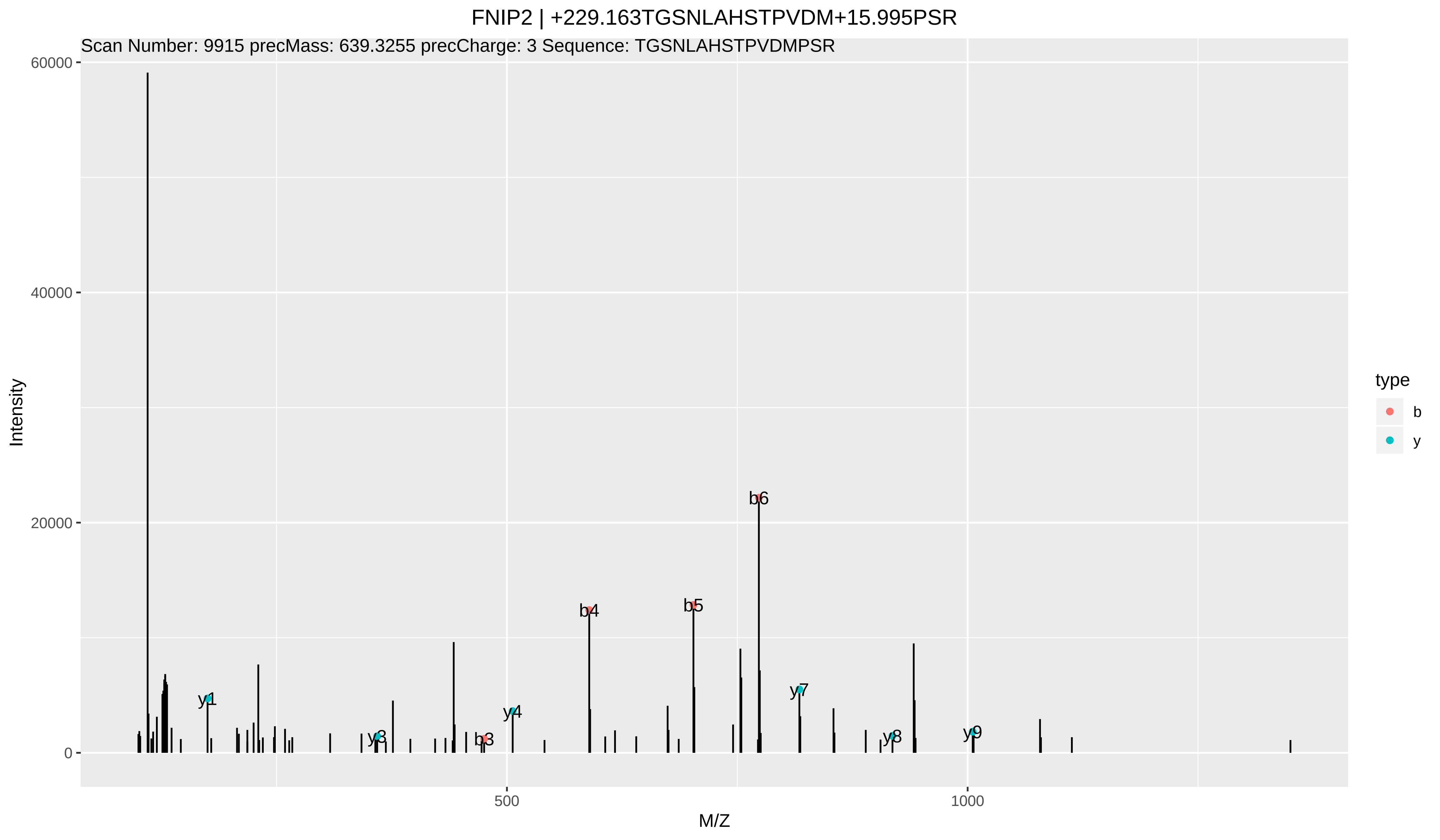
FNIP2 | +229.163TGSNLAHSTPVDM+15.995PSR
Scan Number: 9915 precMass: 639.3255 precCharge: 3 Sequence: TGSNLAHSTPVDMPSR
Intensity
60000
40000
20000
0
500
1000
M/Z
y1
y3
b3
y4
b4
b5
b6
y7
y8
y9
type
b
y

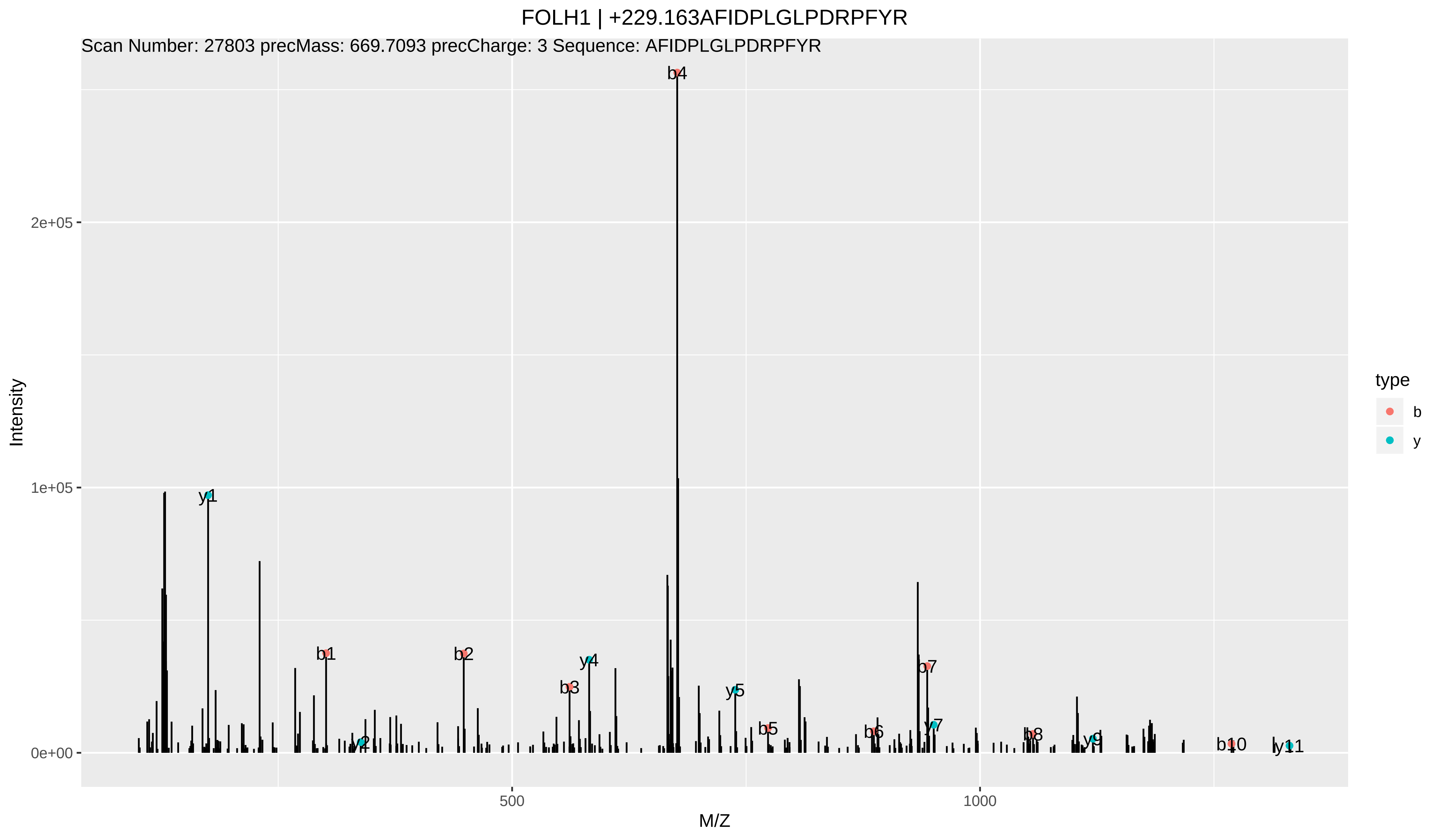

FOLH1 | +229.163AFIDPLGLPDRPFYR
Scan Number: 27803 precMass: 669.7093 precCharge: 3 Sequence: AFIDPLGLPDRPFYR
Intensity
2e+05
1e+05
0e+00
500
1000
M/Z
b4
y1
b1
b2
y4
b3
y5
b5
b6
b7
y7
b8
y9
b10
y11
y2
type
b
y

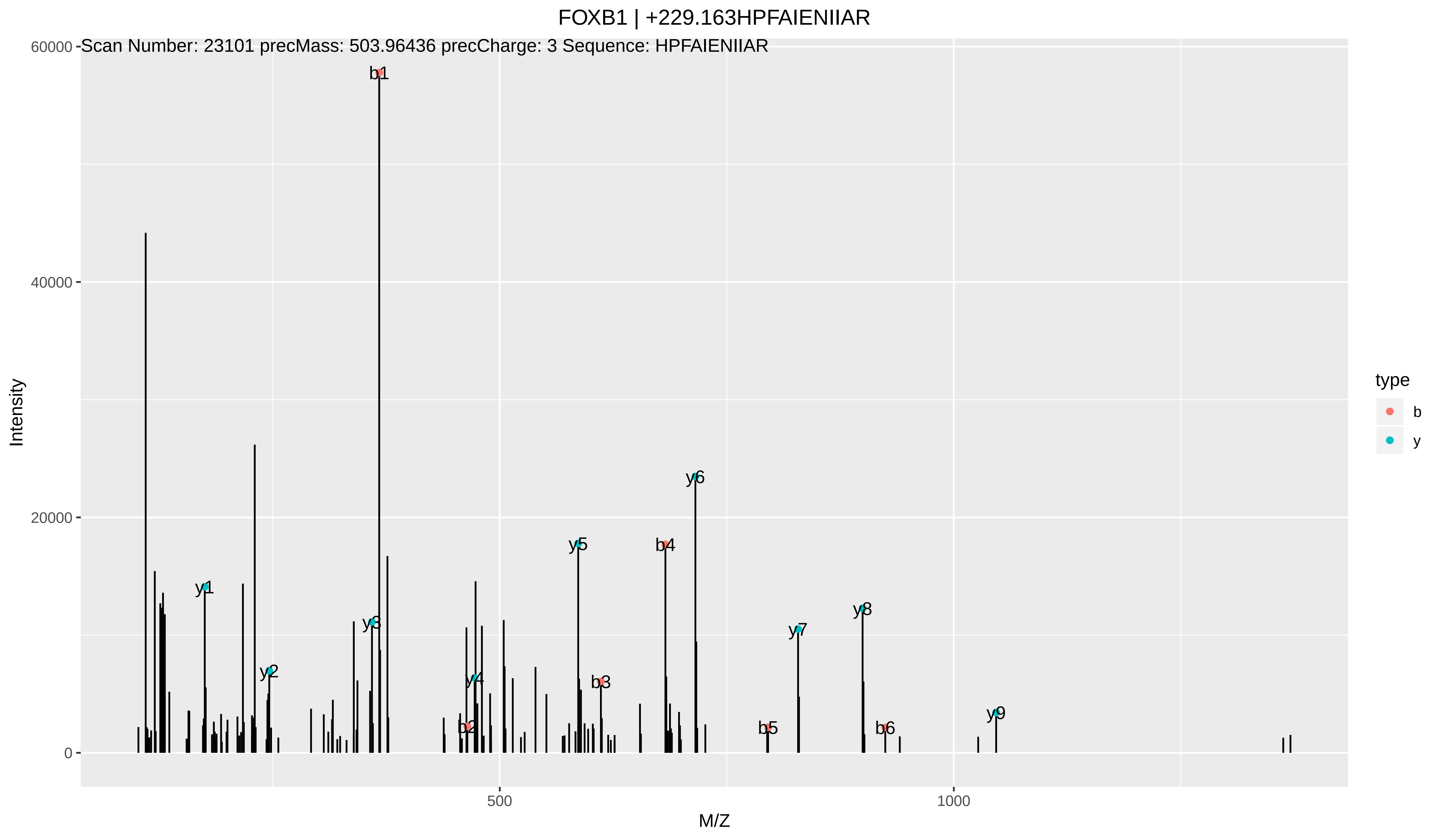
FOXB1 | +229.163HPFAIENIIAR
Scan Number: 23101 precMass: 503.96436 precCharge: 3 Sequence: HPFAIENIIAR
Intensity
M/Z
type
b
y
b1
y1
y2
y3
b2
y4
y5
b3
b4
y6
b5
y7
y8
b6
y9
0
20000
40000
60000
500
1000

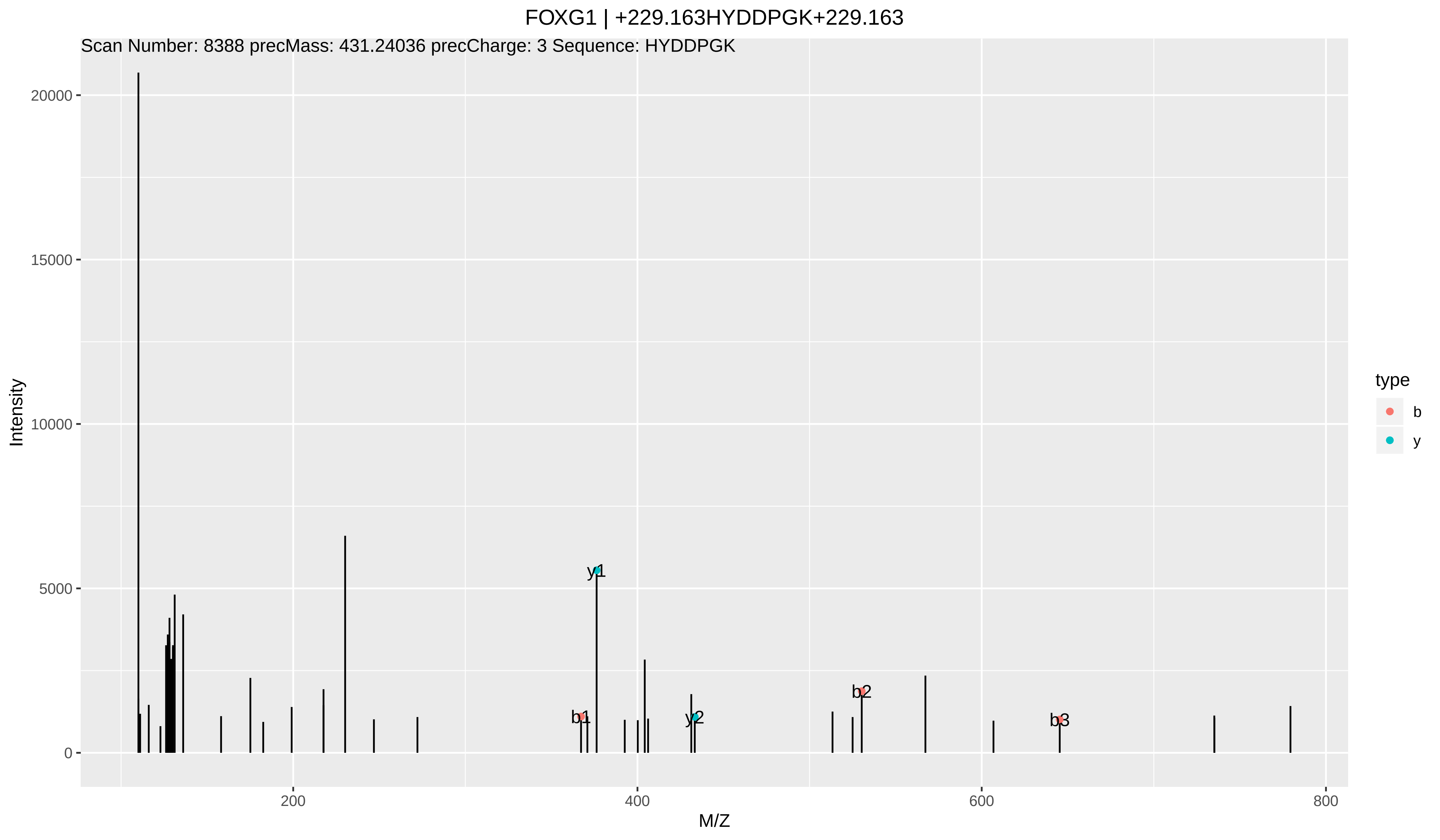
FOXG1 | +229.163HYDDPGK+229.163
Scan Number: 8388 precMass: 431.24036 precCharge: 3 Sequence: HYDDPGK
Intensity
20000
15000
10000
5000
0
200
400
600
800
M/Z
y1
b1
y2
b2
b3
type
b
y

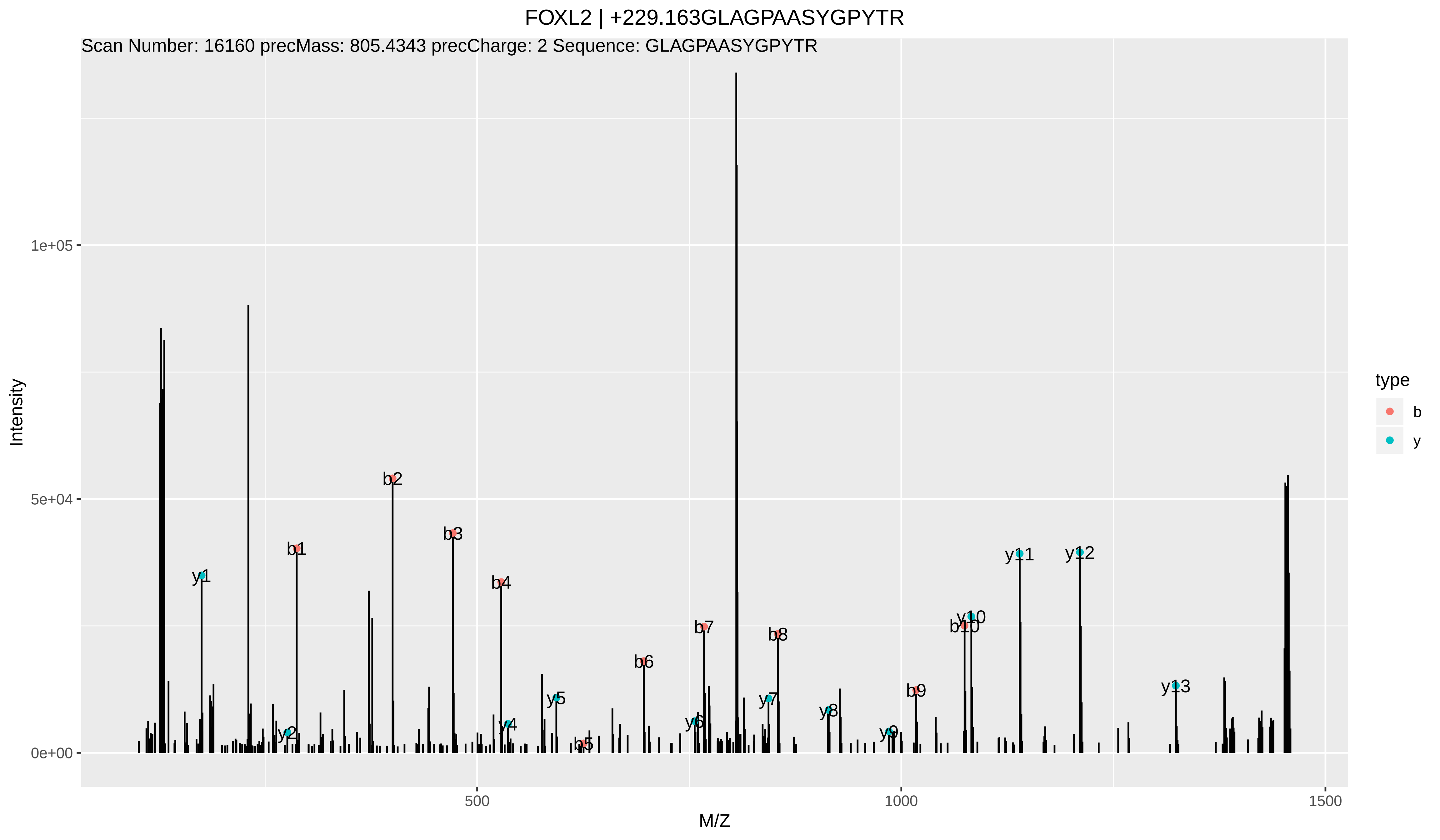
FOXL2 | +229.163GLAGPAASYGPYTR
Scan Number: 16160 precMass: 805.4343 precCharge: 2 Sequence: GLAGPAASYGPYTR
Intensity
1e+05
5e+04
0e+00
M/Z
500
1000
1500
y1
b1
y2
b2
b3
b4
y4
y5
b5
b6
y6
b7
y7
b8
y8
y9
b9
b10
y10
y11
y12
y13
type
b
y

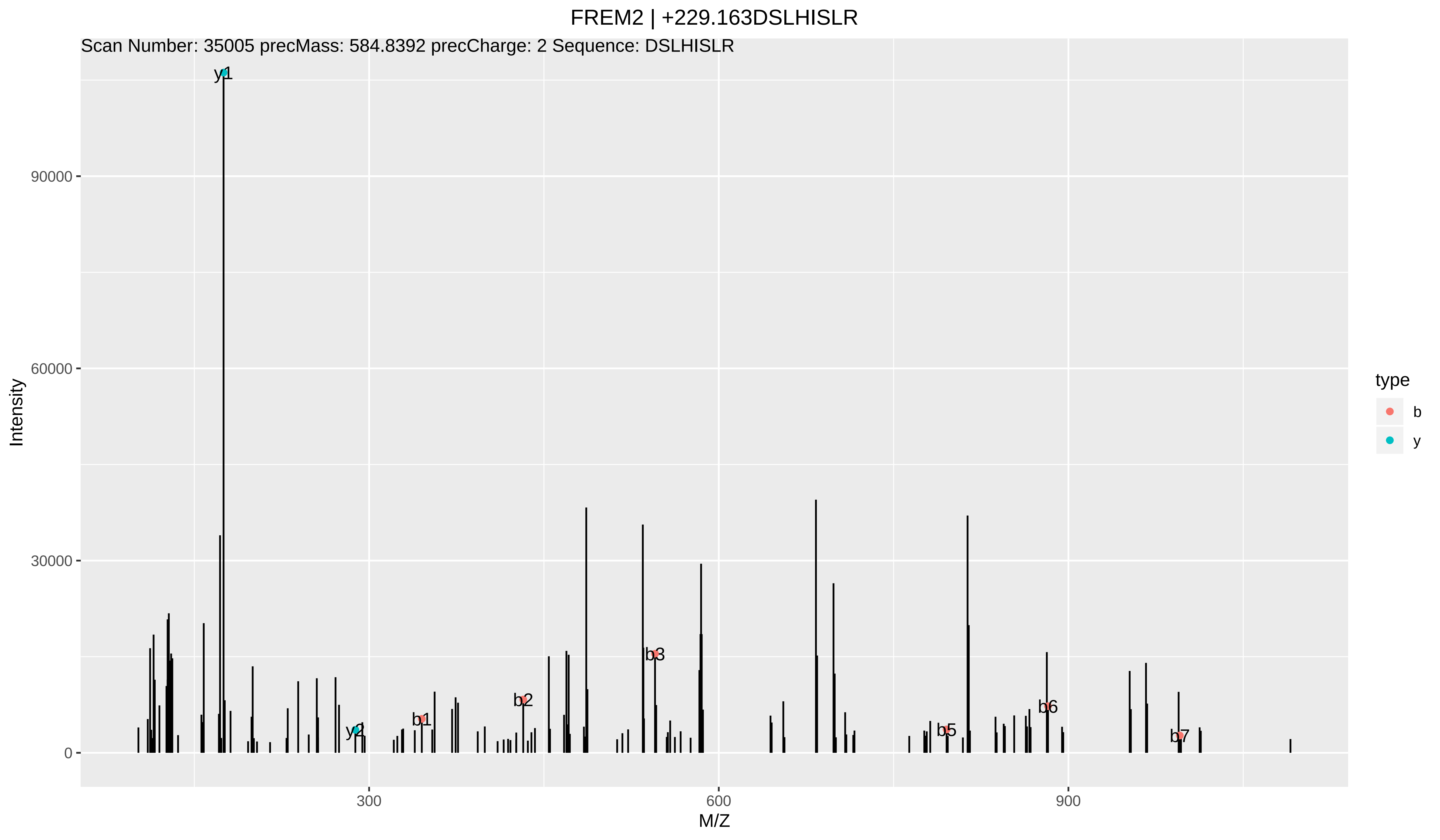
FREM2 | +229.163DSLHISLR
Scan Number: 35005 precMass: 584.8392 precCharge: 2 Sequence: DSLHISLR
y1
y2
b1
b2
b3
b5
b6
b7
Intensity
90000
60000
30000
0
300
600
900
M/Z
type
b
y

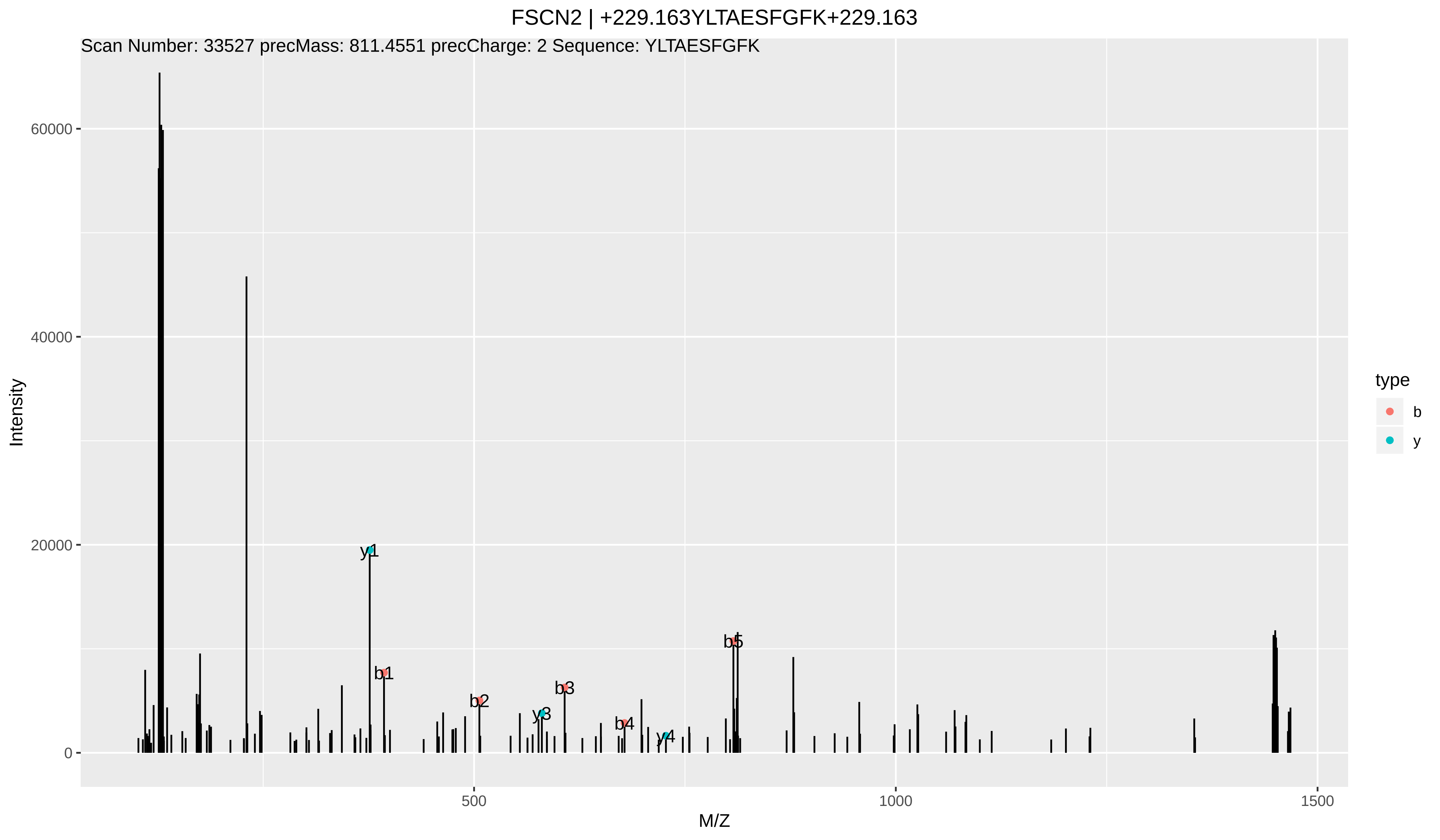

FSCN2 | +229.163YLTAESFGFK+229.163
Scan Number: 33527 precMass: 811.4551 precCharge: 2 Sequence: YLTAESFGFK
Intensity
60000
40000
20000
0
M/Z
500
1000
1500
y1
b1
b2
y3
b3
b4
y4
b5
type
b
y

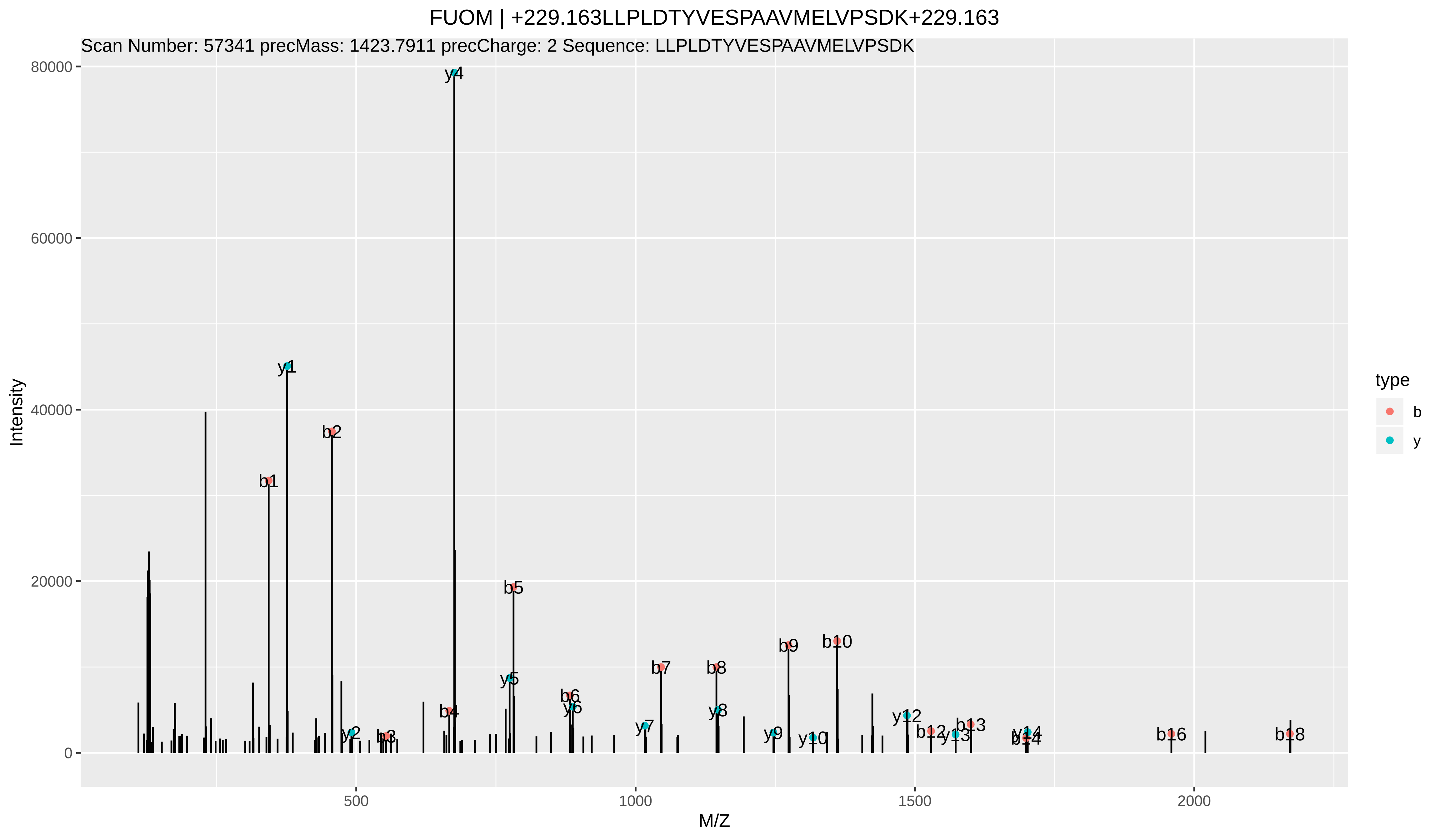

FUOM | +229.163LLPLDTYVESPAAVMELVPSDK+229.163
Scan Number: 57341 precMass: 1423.7911 precCharge: 2 Sequence: LLPLDTYVESPAAVMELVPSDK
Intensity
M/Z
type
b
y
y4
y1
b2
b1
b5
b10
b9
b7
b8
y5
b6
y6
y8
b4
y12
b13
b12
y13
y14
b14
b16
b18
y7
y9
y10
y2
b3
0
20000
40000
60000
80000
500
1000
1500
2000

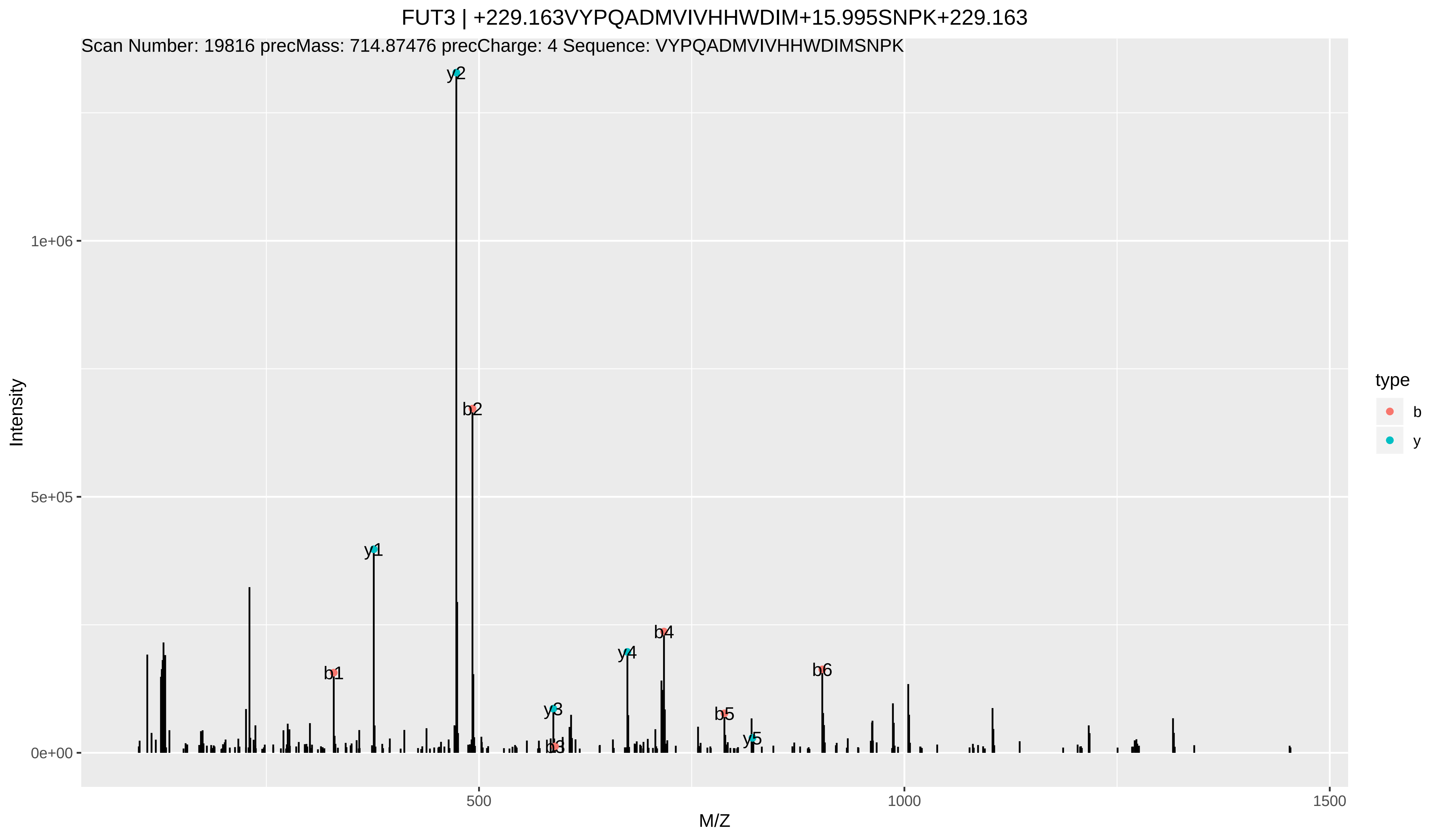

FUT3 | +229.163VYPQADMVIVHHWDIM+15.995SNPK+229.163
Scan Number: 19816 precMass: 714.87476 precCharge: 4 Sequence: VYPQADMVIVHHWDIMSNPK
Intensity
M/Z
0e+00
5e+05
1e+06
500
1000
1500
y2
b2
y1
b1
y3
b3
y4
b4
b5
y5
b6
type
b
y

FUT3 | +229.163FYLAFENSLHPDYITEK+229.163

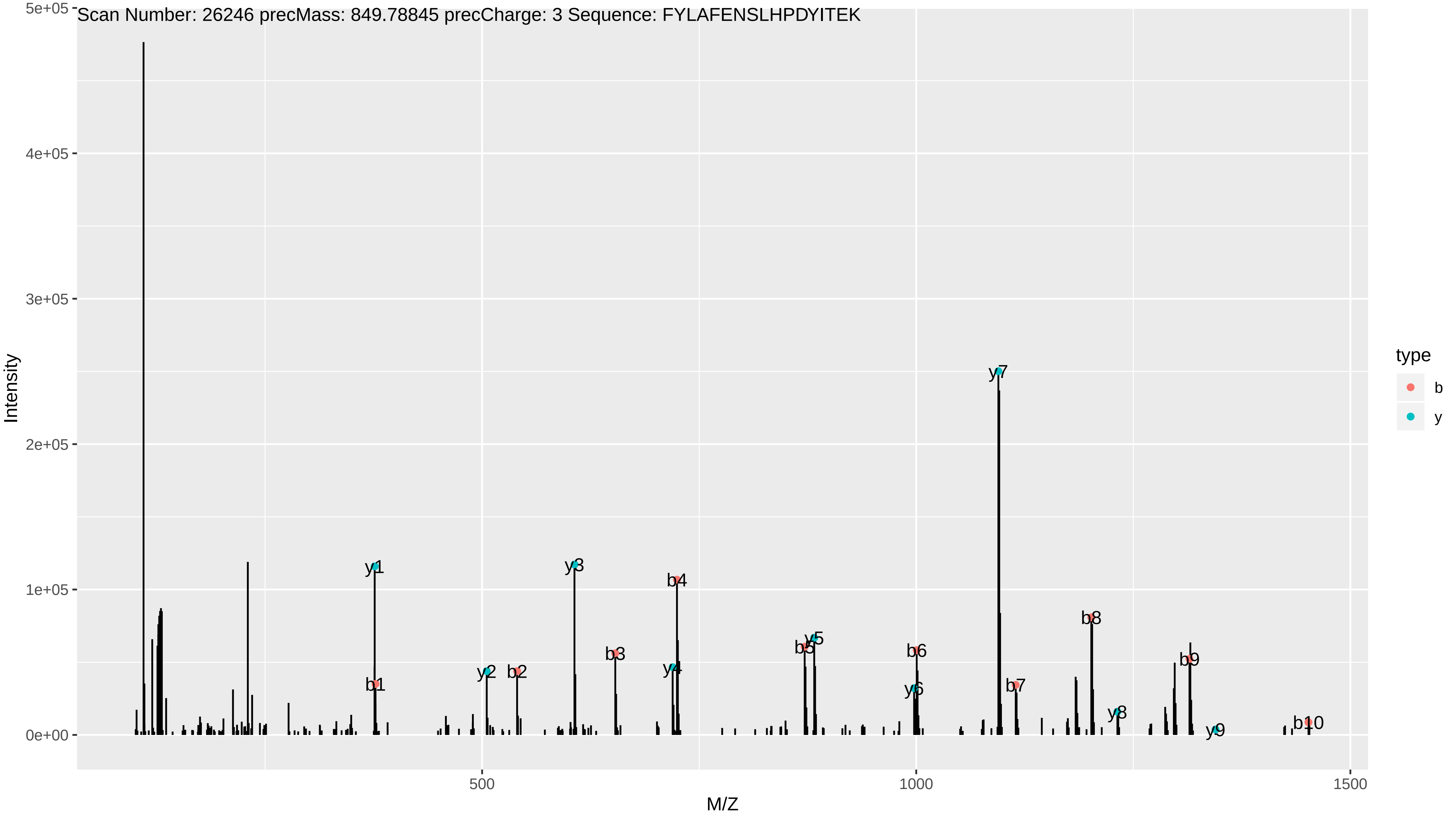

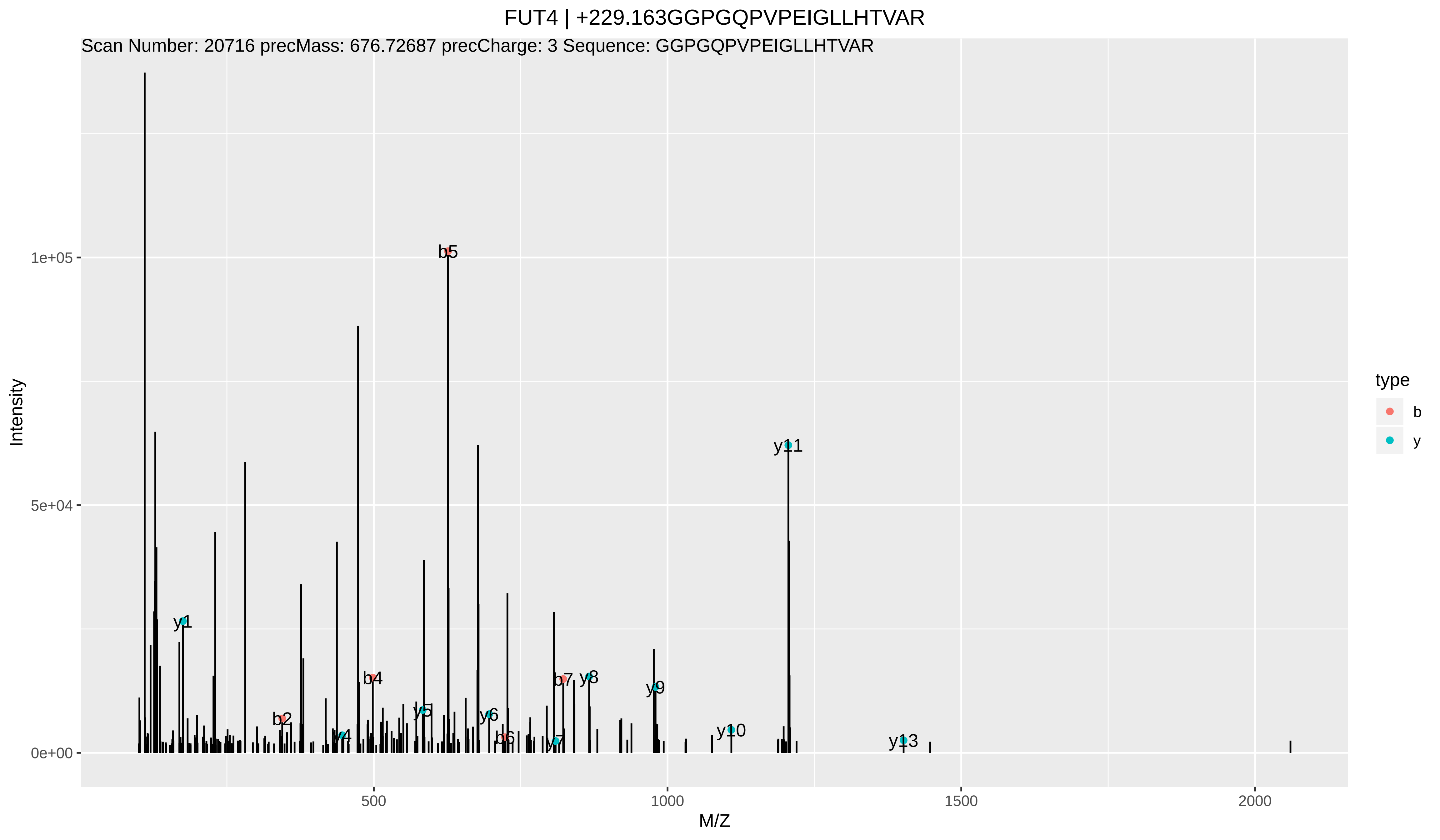
FUT4 | +229.163GGPGQPVPEIGLLHTVAR
Scan Number: 20716 precMass: 676.72687 precCharge: 3 Sequence: GGPGQPVPEIGLLHTVAR
Intensity
M/Z
1e+05
5e+04
0e+00
500
1000
1500
2000
b5
y11
y1
b4
b7
y8
y9
b2
y5
y6
y4
b6
y7
y10
y13
type
b
y

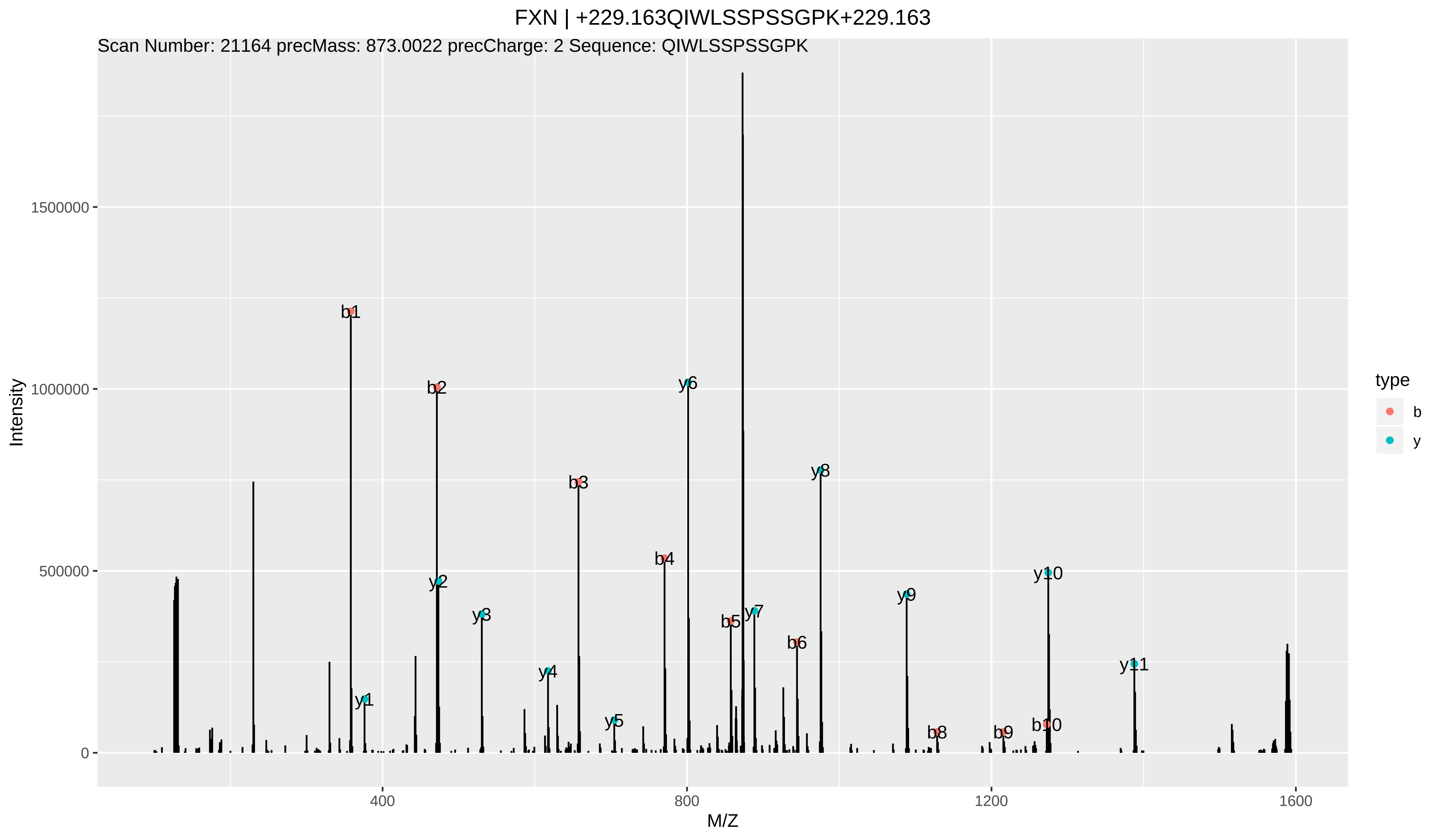
FXN | +229.163QIWLSSPSSGPK+229.163
Scan Number: 21164 precMass: 873.0022 precCharge: 2 Sequence: QIWLSSPSSGPK
Intensity
1500000
1000000
500000
0
M/Z
400
800
1200
1600
b1
b2
y2
y1
y3
b3
y4
y5
b4
y6
b5
y7
b6
y8
y9
b8
b9
b10
y10
y11
type
b
y

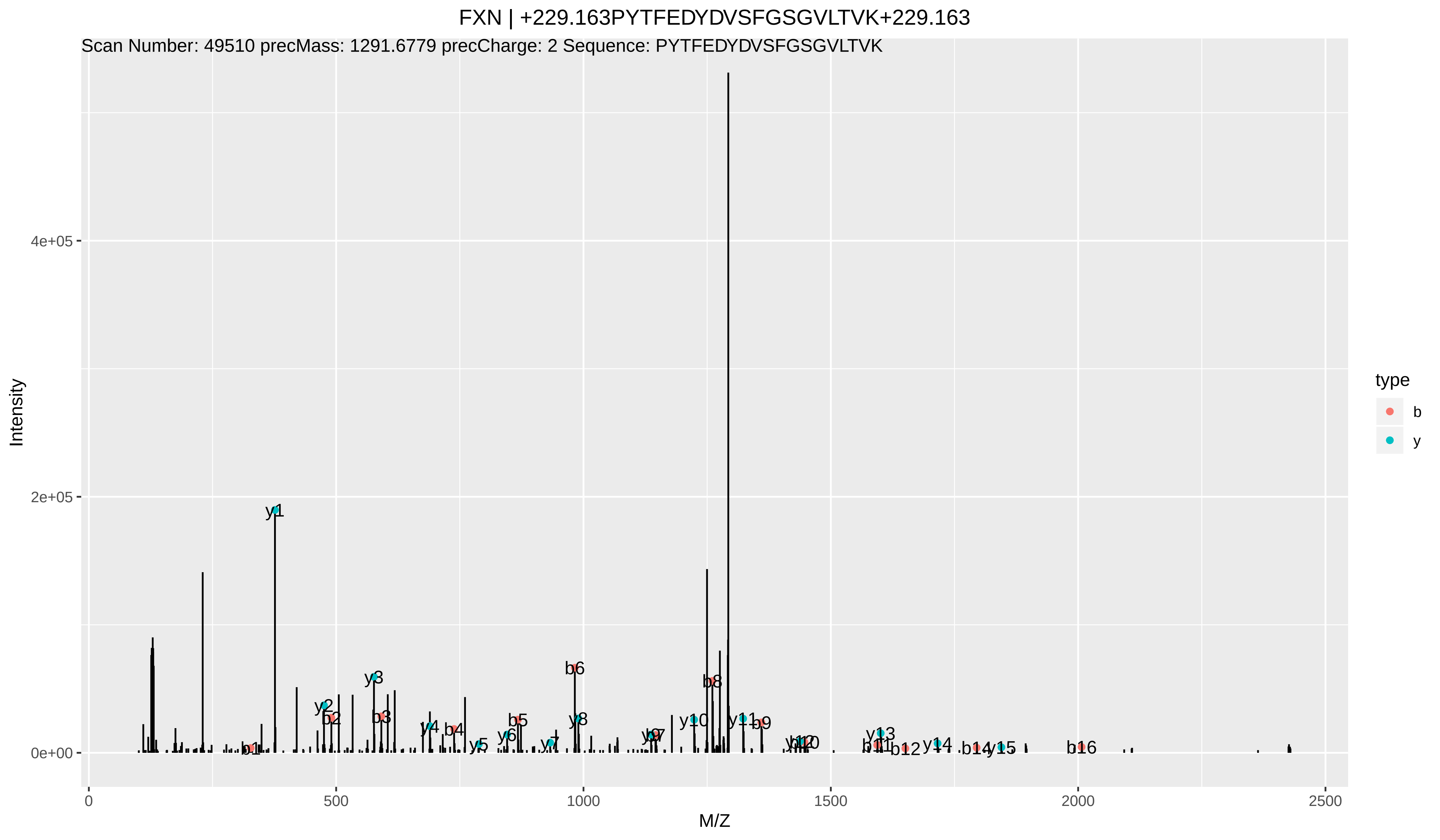
FXN | +229.163PYTFEDYDVSFGSGVLTVK+229.163
Scan Number: 49510 precMass: 1291.6779 precCharge: 2 Sequence: PYTFEDYDVSFGSGVLTVK
Intensity
4e+05
2e+05
0e+00
M/Z
0
500
1000
1500
2000
2500
type
b
y
y1
y2
b2
y3
b3
y4
b4
y5
b5
y6
b6
y7
y8
y10
b8
y11
b9
y13
y14
b12
b14
y15
b16

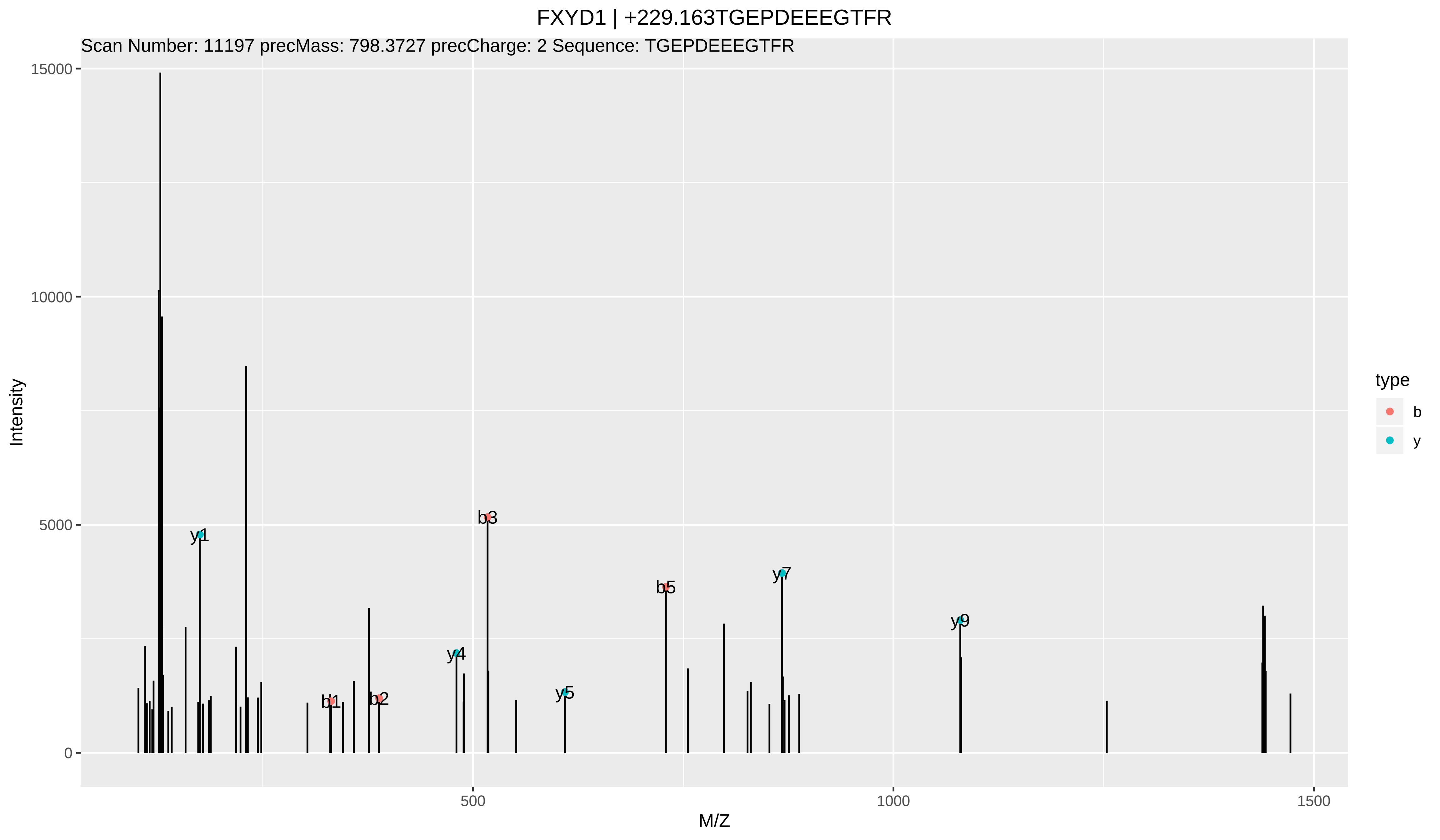

FXYD1 | +229.163TGEPDEEEGTFR
Scan Number: 11197 precMass: 798.3727 precCharge: 2 Sequence: TGEPDEEEGTFR
Intensity
M/Z
15000
10000
5000
0
500
1000
1500
y1
b1
b2
y4
b3
y5
b5
y7
y9
type
b
y

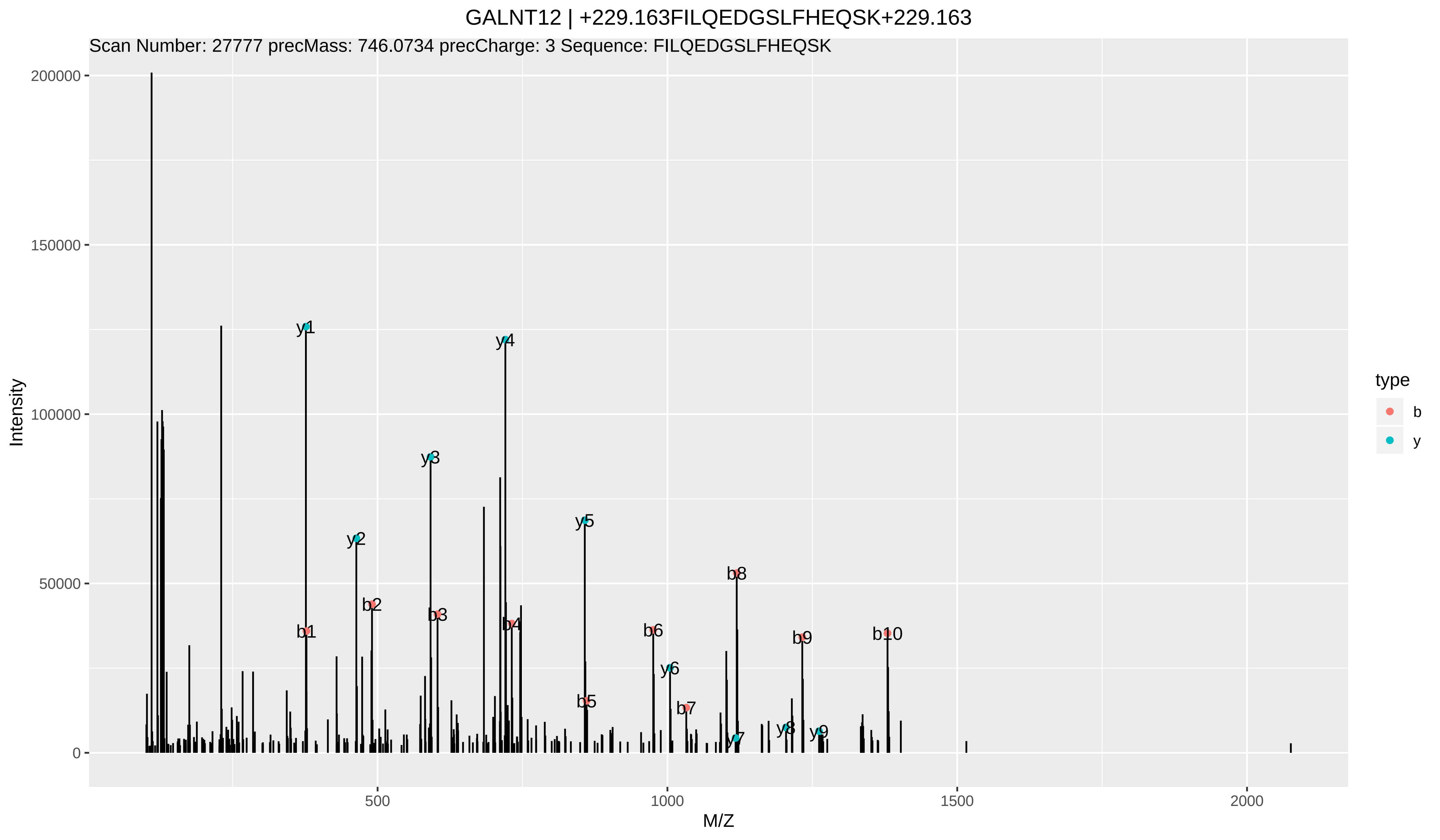
GALNT12 | +229.163FILQEDGSLFHEQSK+229.163
Scan Number: 27777 precMass: 746.0734 precCharge: 3 Sequence: FILQEDGSLFHEQSK
Intensity
M/Z
type
b
y
y1
y2
y3
y4
y5
y6
y7
y8
y9
b1
b2
b3
b4
b5
b6
b7
b8
b9
b10
0
50000
100000
150000
200000
500
1000
1500
2000

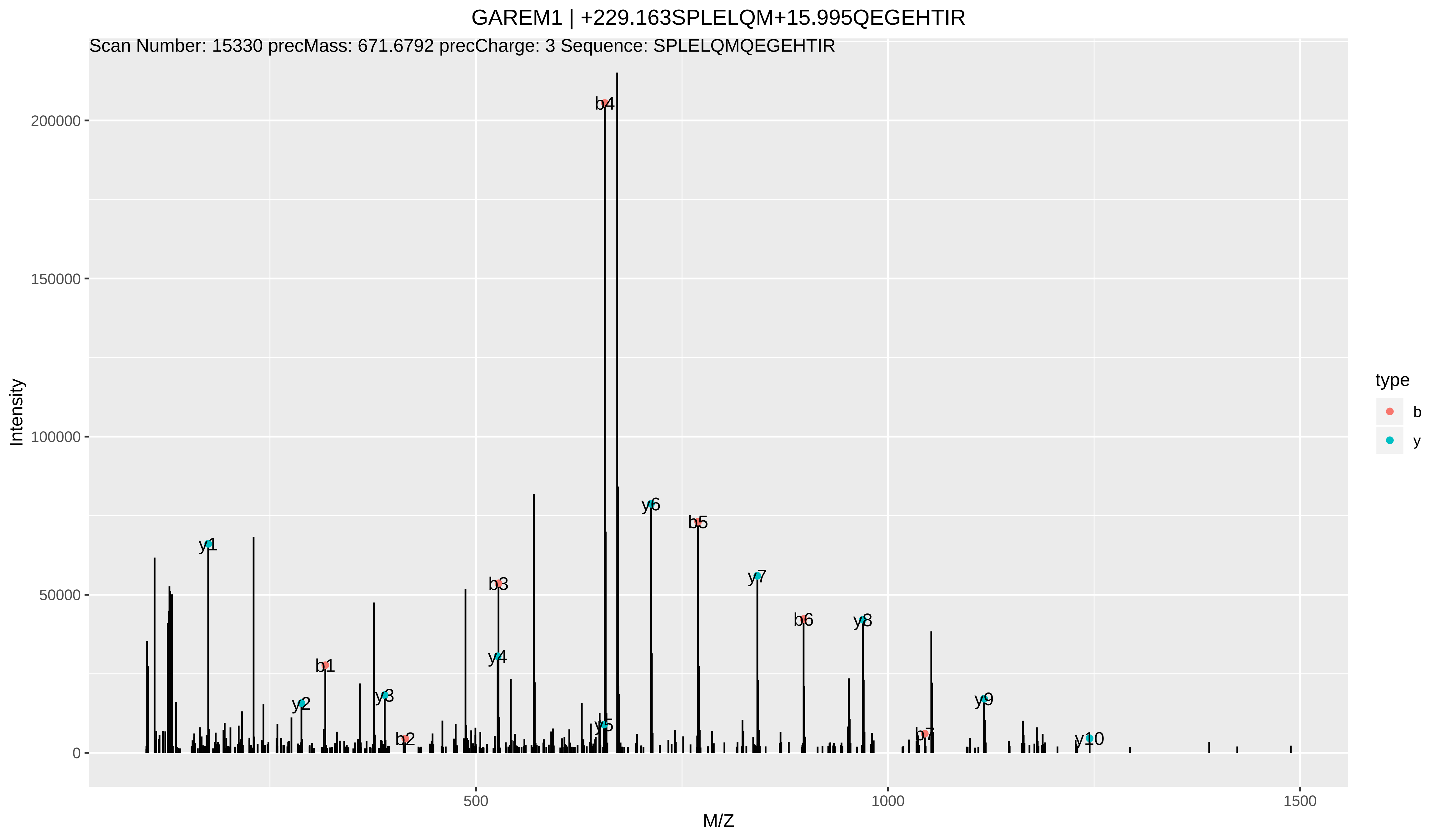
GAREM1 | +229.163SPLELQM+15.995QEGEHTIR
Scan Number: 15330 precMass: 671.6792 precCharge: 3 Sequence: SPLELQMQEGEHTIR
Intensity
M/Z
type
b
y
200000
150000
100000
50000
0
500
1000
1500
b4
y6
b5
y1
y7
b3
b6
y8
y4
b1
y2
y3
y9
y5
b7
b2
y10

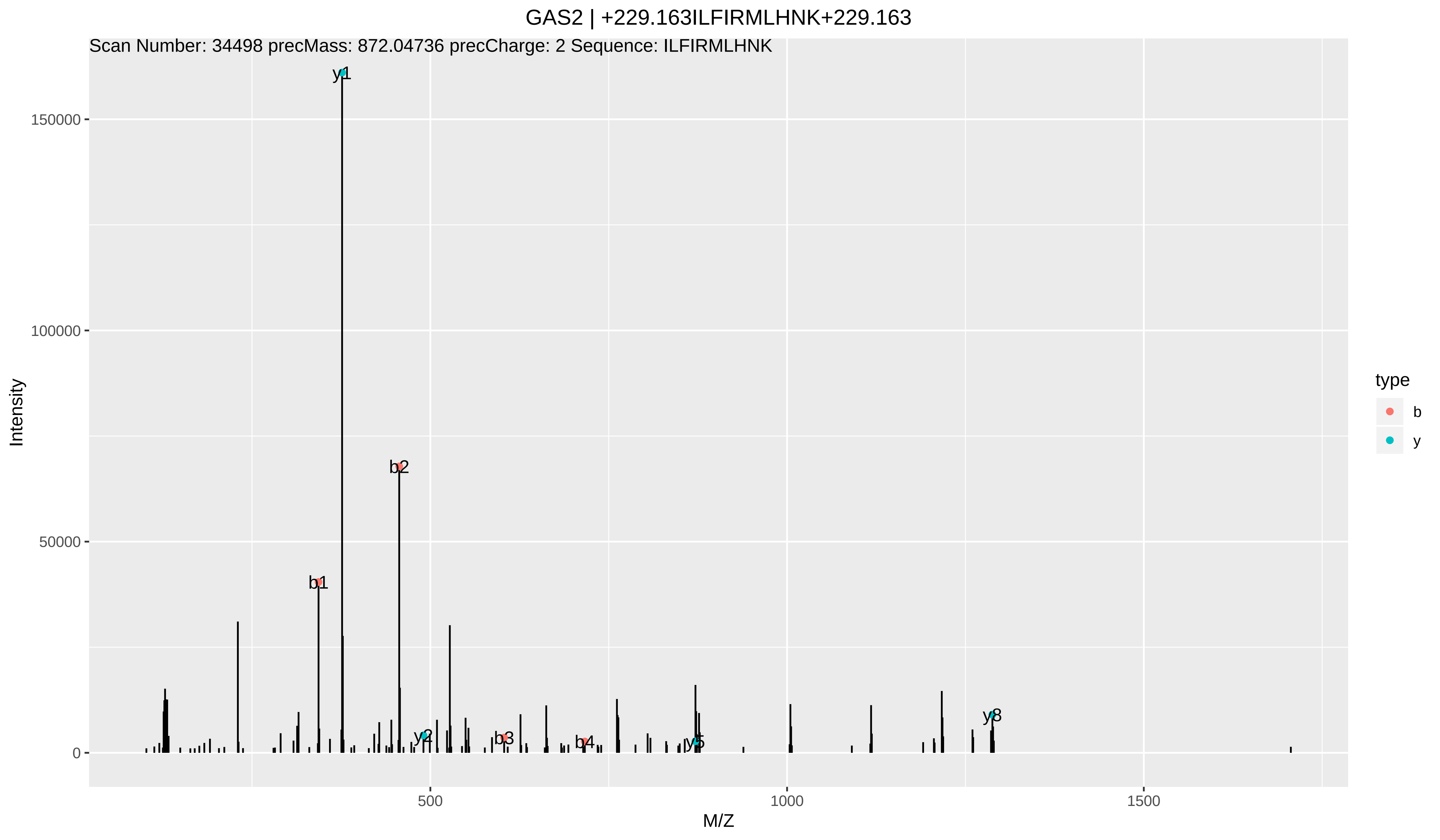
GAS2 | +229.163ILFIRMLHNK+229.163
Scan Number: 34498 precMass: 872.04736 precCharge: 2 Sequence: ILFIRMLHNK
Intensity
M/Z
type
b
y
y1
b2
b1
y2
b3
b4
y5
y8
150000
100000
50000
0
500
1000
1500

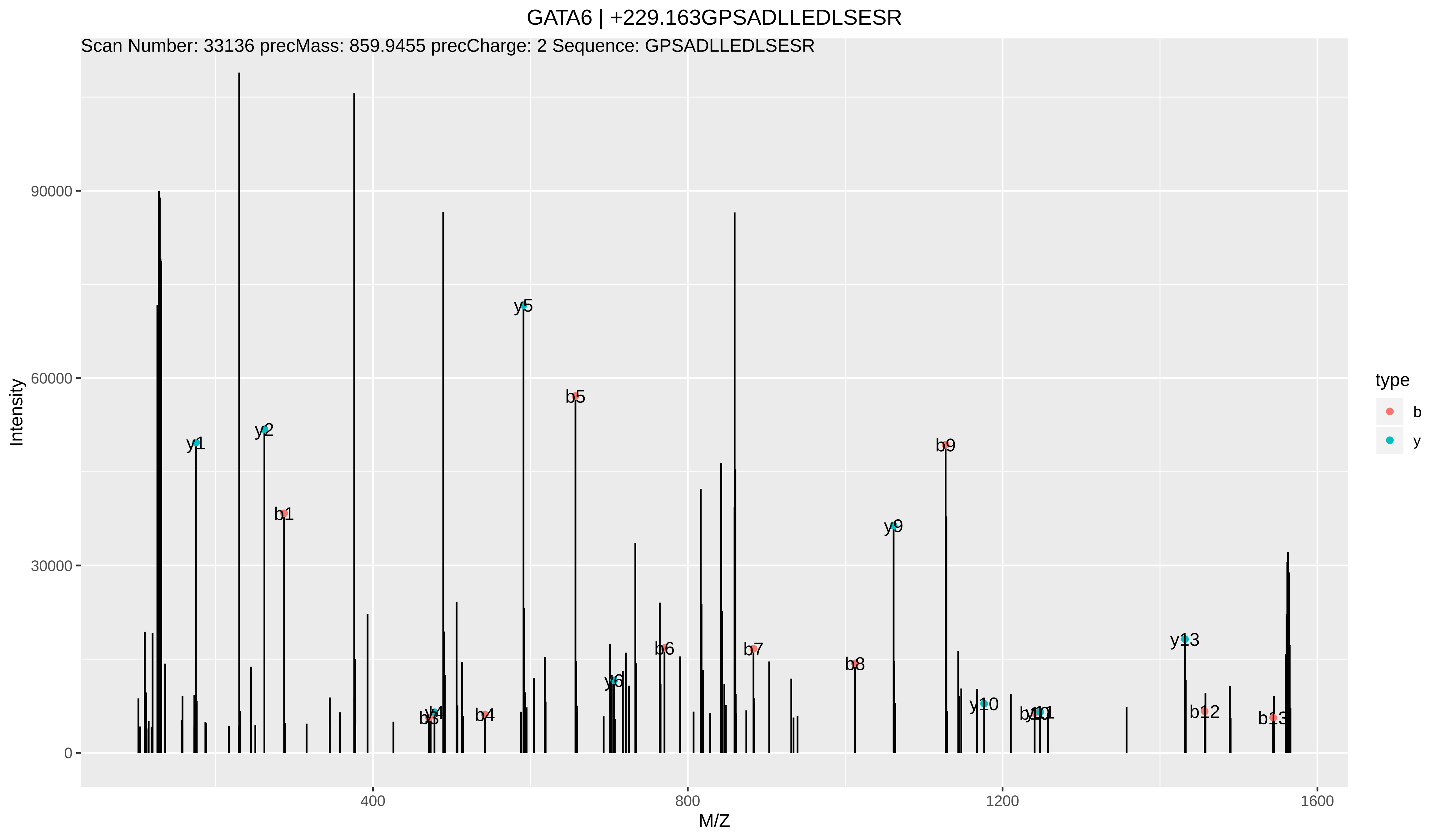

GATA6 | +229.163GPSADLLEDLSESR
Scan Number: 33136 precMass: 859.9455 precCharge: 2 Sequence: GPSADLLEDLSESR
Intensity
90000
60000
30000
0
400
800
1200
1600
M/Z
type
b
y
y1
y2
b1
y5
b5
b9
y9
b6
b7
b8
y6
b4
y13
y10
b12
b13

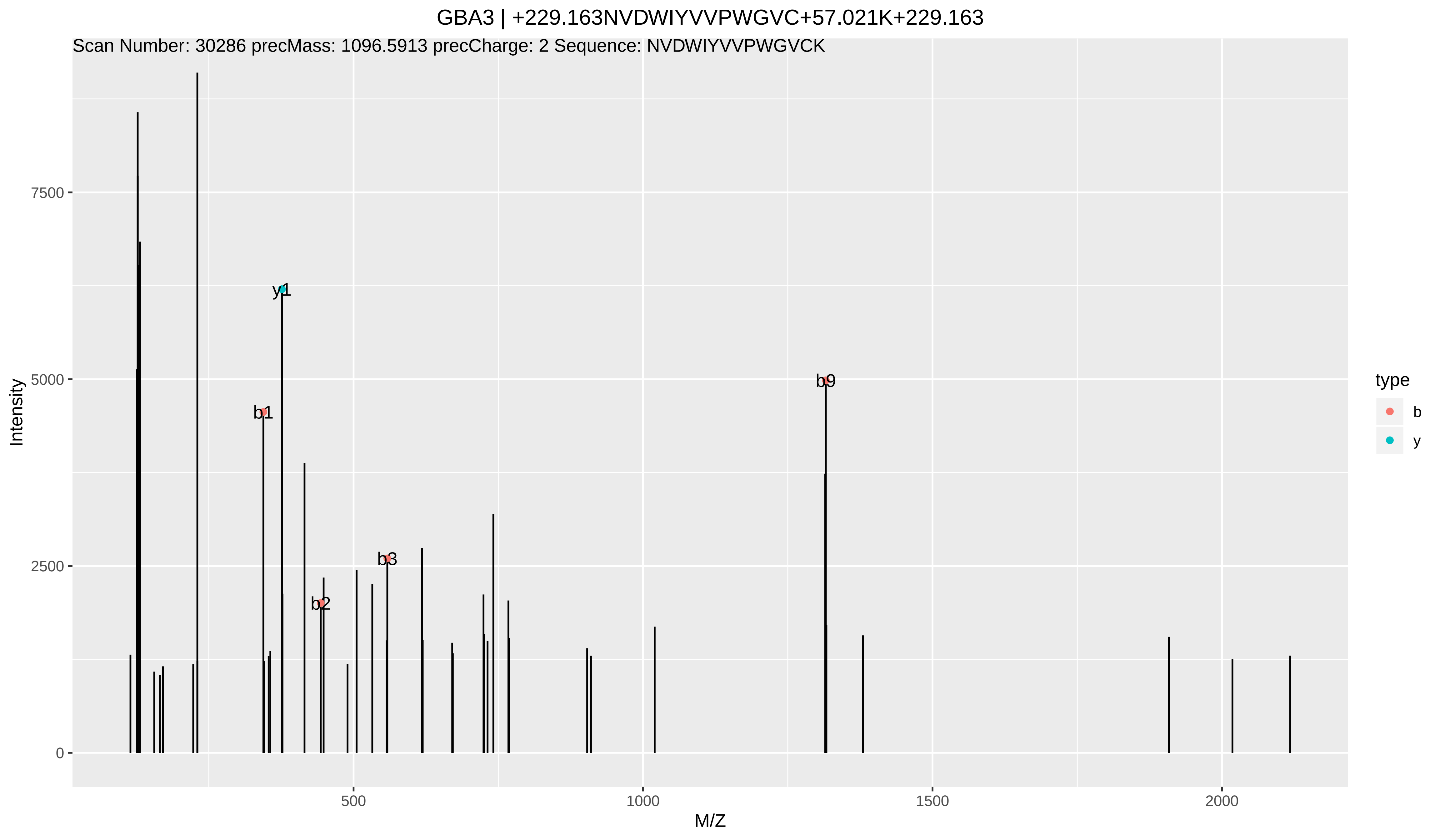
GBA3 | +229.163NVDWIYVVPWGVC+57.021K+229.163
Scan Number: 30286 precMass: 1096.5913 precCharge: 2 Sequence: NVDWIYVVPWGVCK
Intensity
7500
5000
2500
0
M/Z
500
1000
1500
2000
y1
b1
b2
b3
b9
type
b
y

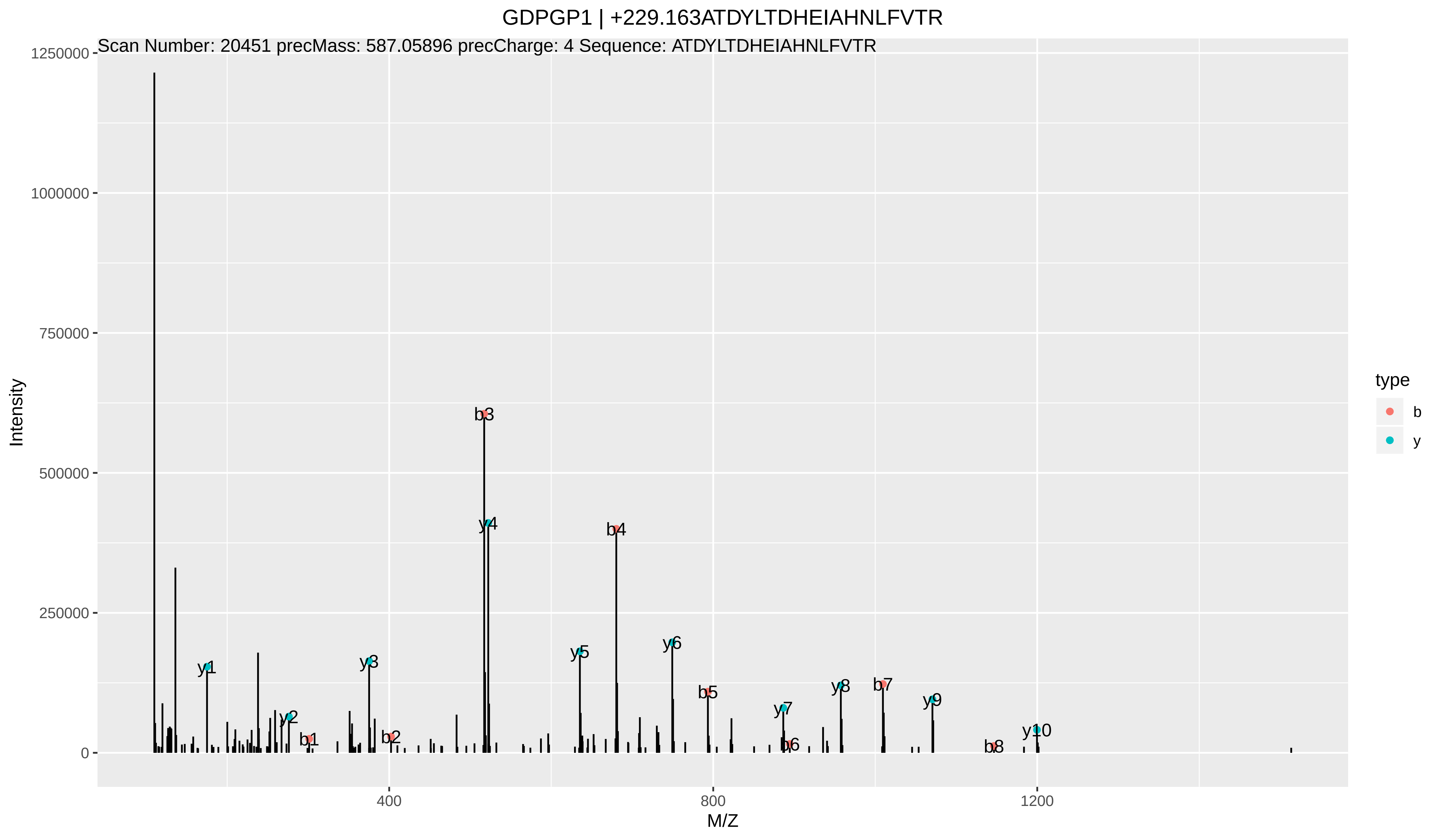
GDPGP1 | +229.163ATDYLTDHEIAHNLFVTR
Scan Number: 20451 precMass: 587.05896 precCharge: 4 Sequence: ATDYLTDHEIAHNLFVTR
Intensity
1250000
1000000
750000
500000
250000
0
M/Z
400
800
1200
y1
y2
b1
y3
b2
b3
y4
y5
b4
y6
b5
y7
b6
y8
b7
y9
b8
y10
type
b
y

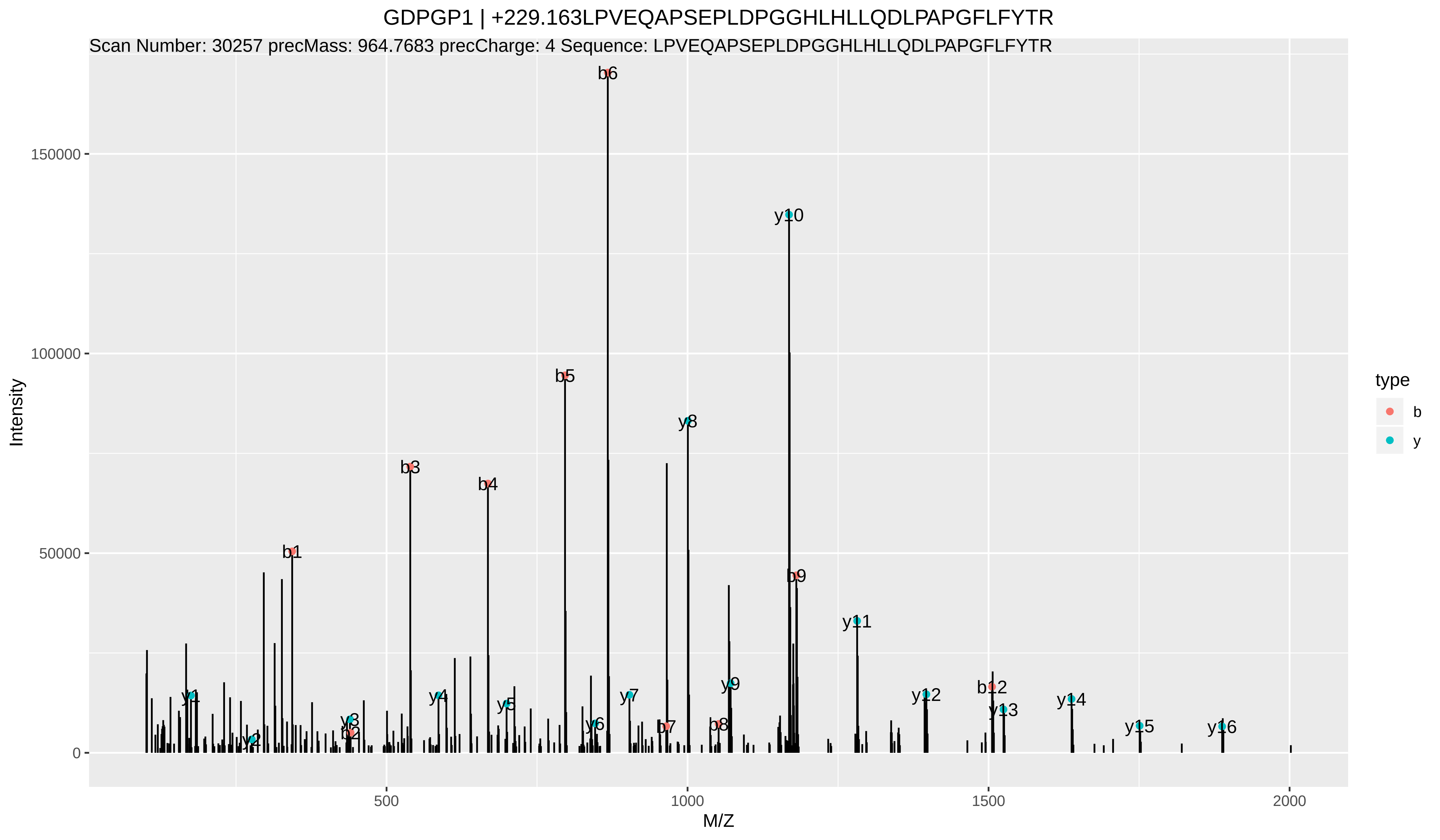
GDPGP1 | +229.163LPVEQAPSEPLDPGGHLHLLQDLPAPGFLFYTR
Scan Number: 30257 precMass: 964.7683 precCharge: 4 Sequence: LPVEQAPSEPLDPGGHLHLLQDLPAPGFLFYTR
Intensity
M/Z
0
50000
100000
150000
500
1000
1500
2000
type
b
y
b1
b2
b3
b4
b5
b6
b7
b8
b9
b12
y1
y2
y3
y4
y5
y6
y7
y8
y9
y10
y11
y12
y13
y14
y15
y16

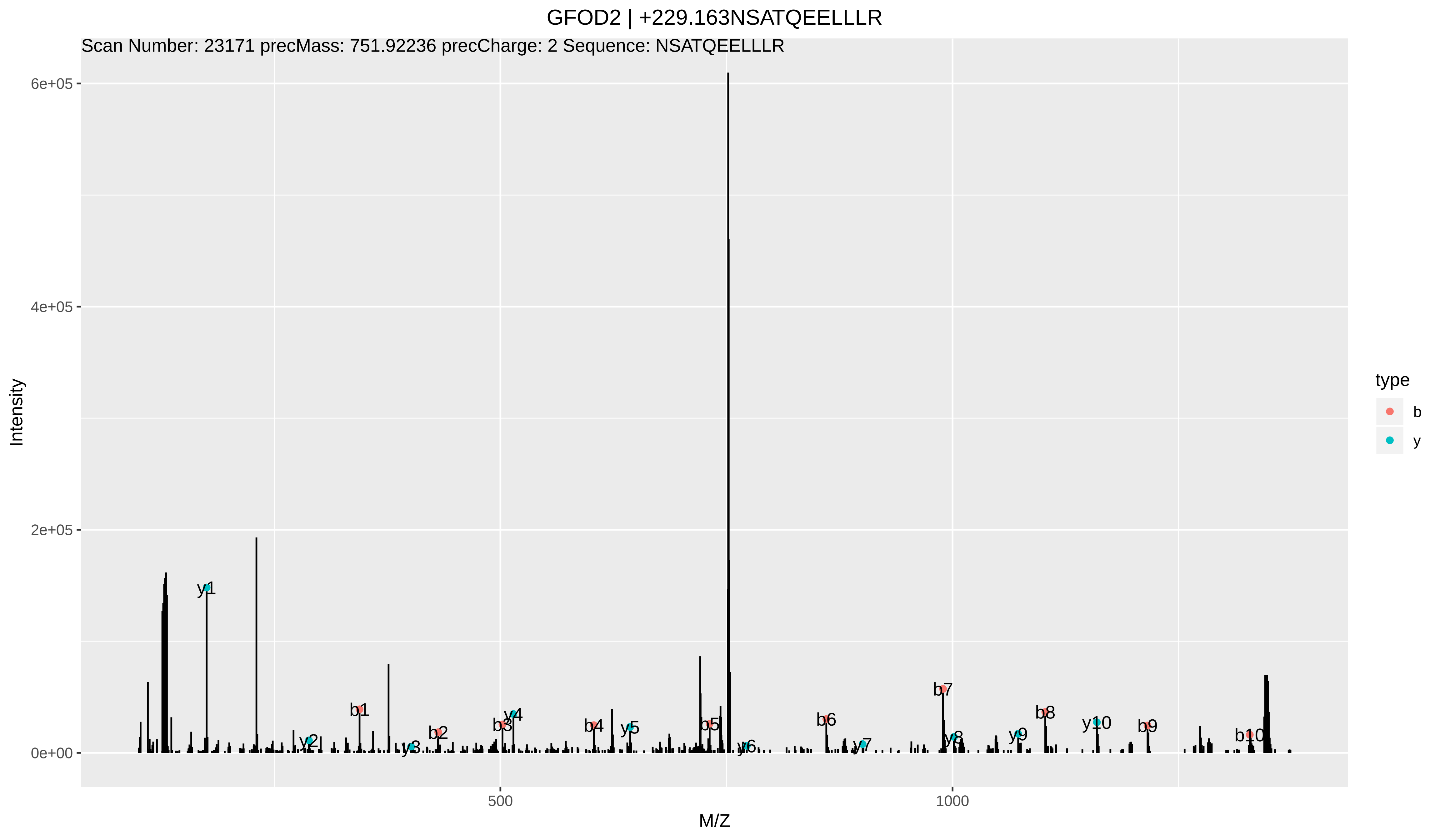

GFOD2 | +229.163NSATQEELLLR
Scan Number: 23171 precMass: 751.92236 precCharge: 2 Sequence: NSATQEELLLR
Intensity
6e+05
4e+05
2e+05
0e+00
M/Z
500
1000
y1
y2
y3
y4
y5
y6
y7
y8
y9
y10
b1
b2
b3
b4
b5
b6
b7
b8
b9
b10
type
b
y

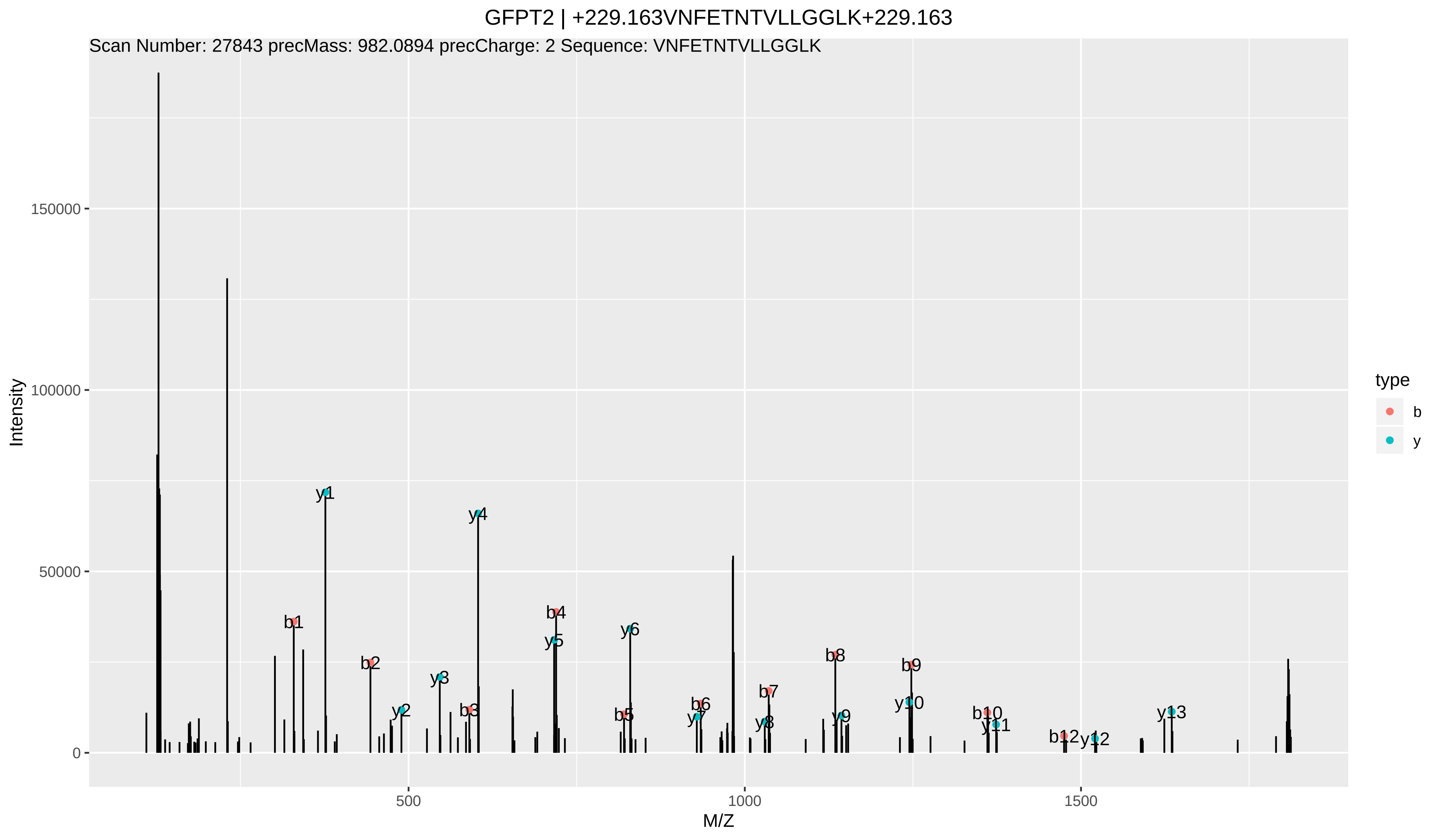
GFPT2 | +229.163VNFETNTVLLGGLK+229.163
Scan Number: 27843 precMass: 982.0894 precCharge: 2 Sequence: VNFETNTVLLGGLK
Intensity
150000
100000
50000
0
M/Z
500
1000
1500
type
b
y
y1
y4
b1
b2
y3
y2
b3
b4
y5
y6
b5
b6
y7
b7
y8
b8
y9
b9
y10
b10
y11
b12
y12
y13

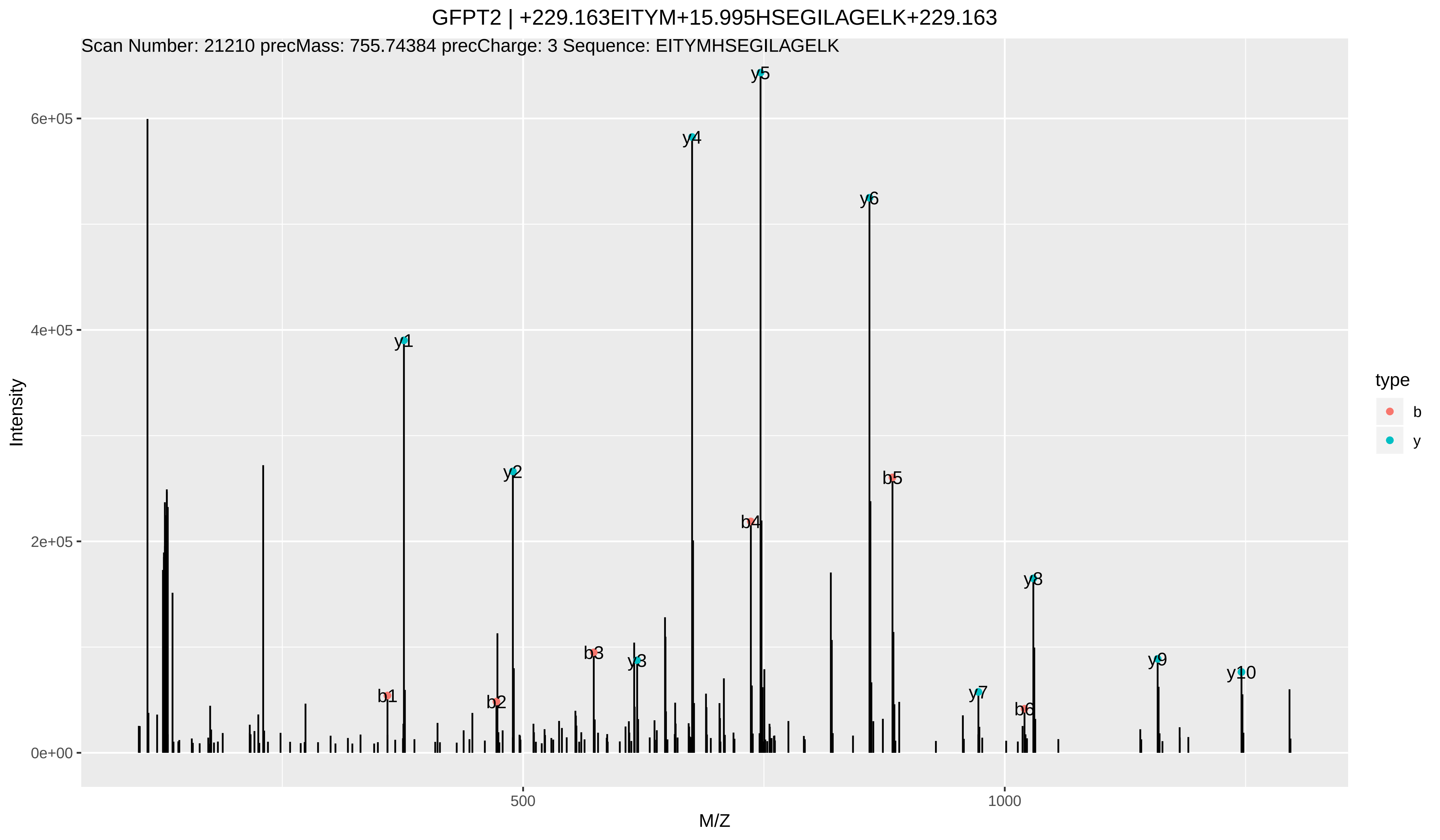

GFPT2 | +229.163EITYM+15.995HSEGILAGELK+229.163
Scan Number: 21210 precMass: 755.74384 precCharge: 3 Sequence: EITYMHSEGILAGELK
Intensity
6e+05
4e+05
2e+05
0e+00
M/Z
500
1000
y1
y2
y3
y4
y5
y6
y7
y8
y9
y10
b1
b2
b3
b4
b5
b6
type
b
y

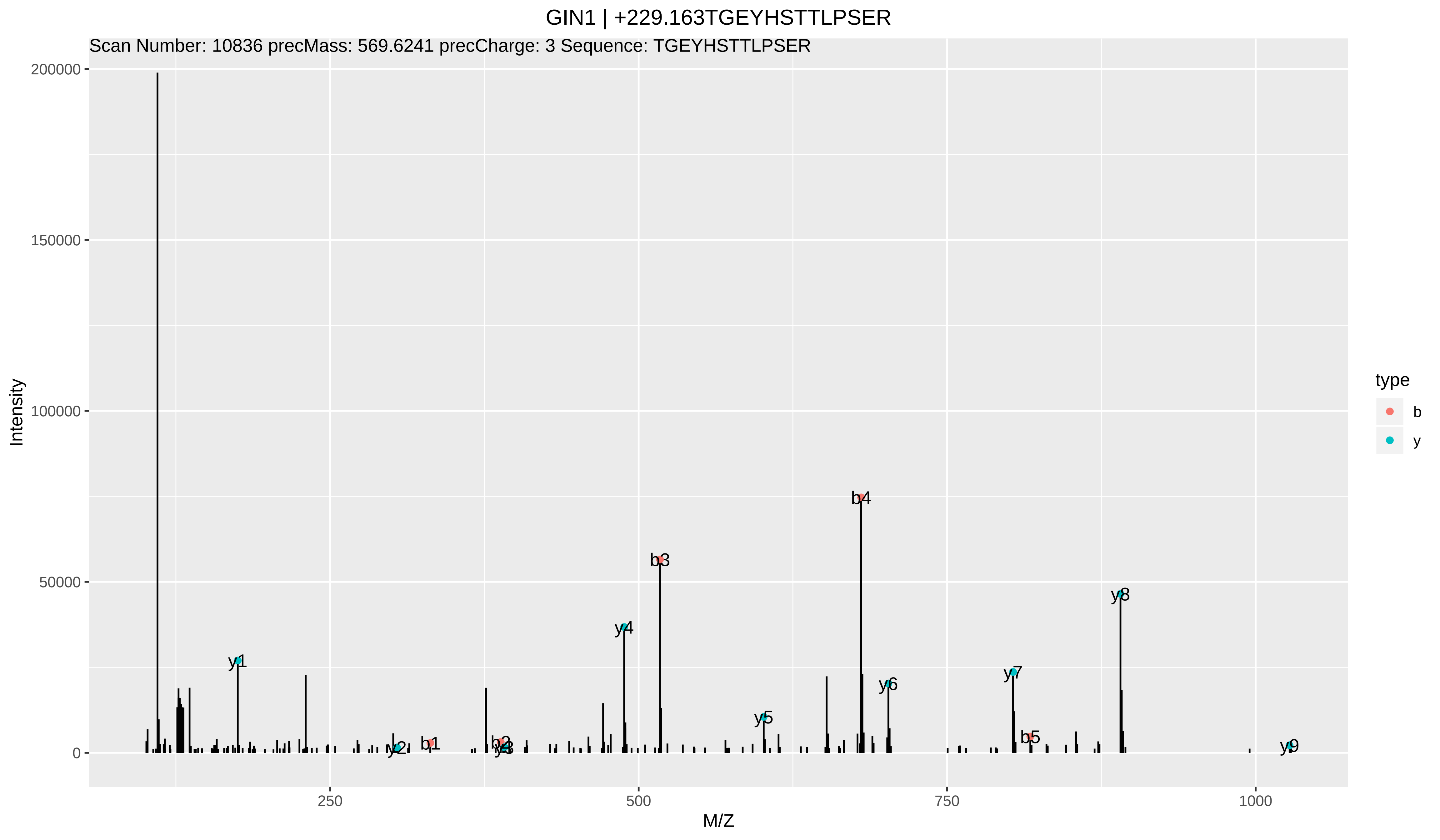
GIN1 | +229.163TGEYHSTTLPSER
Scan Number: 10836 precMass: 569.6241 precCharge: 3 Sequence: TGEYHSTTLPSER
Intensity
200000
150000
100000
50000
0
M/Z
250
500
750
1000
y1
y2
b1
b2
y3
y4
b3
y5
b4
y6
y7
b5
y8
y9
type
b
y

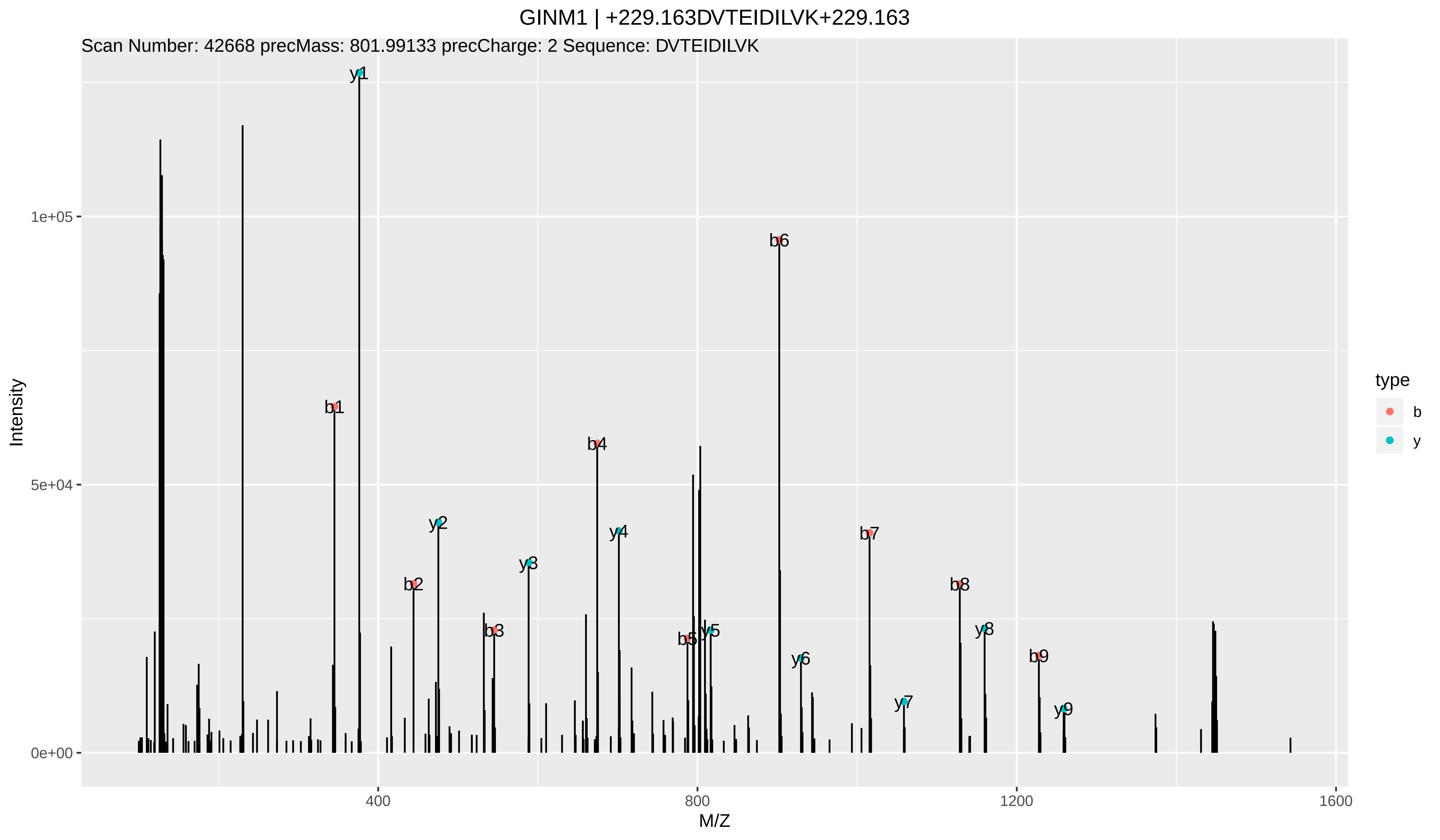
GINM1 | +229.163DVTEIDILVK+229.163
Scan Number: 42668 precMass: 801.99133 precCharge: 2 Sequence: DVTEIDILVK
Intensity
1e+05
5e+04
0e+00
M/Z
400
800
1200
1600
type
b
y
y1
b1
b4
b6
y2
y4
b7
y3
b2
b8
b3
b5
y5
y8
b9
y6
y7
y9

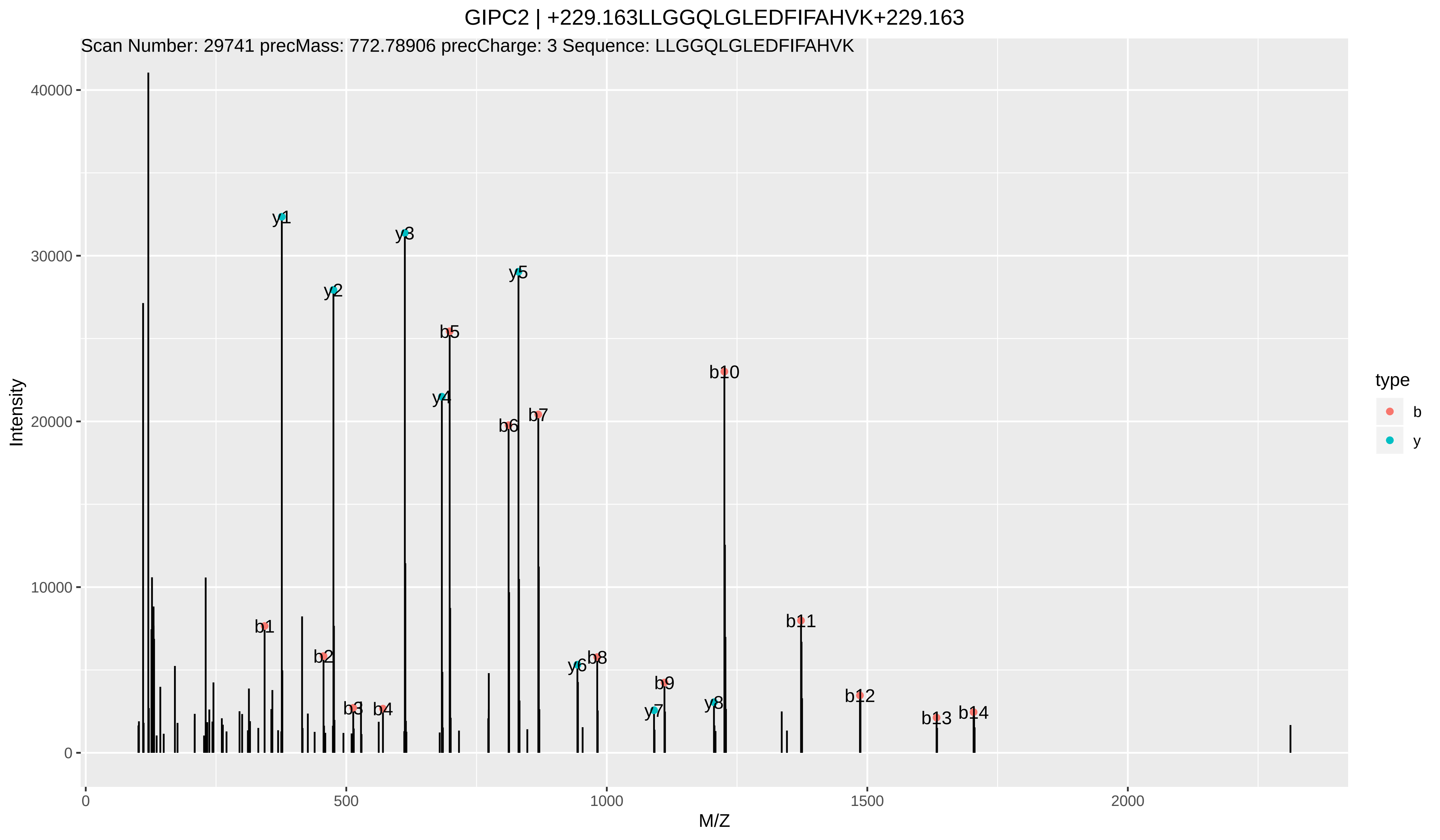

GIPC2 | +229.163LLGGQLGLEDFIFAHVK+229.163
Scan Number: 29741 precMass: 772.78906 precCharge: 3 Sequence: LLGGQLGLEDFIFAHVK
Intensity
40000
30000
20000
10000
0
0
500
1000
1500
2000
M/Z
y1
y2
y3
y4
y5
y6
y7
y8
b1
b2
b3
b4
b5
b6
b7
b8
b9
b10
b11
b12
b13
b14
type
b
y

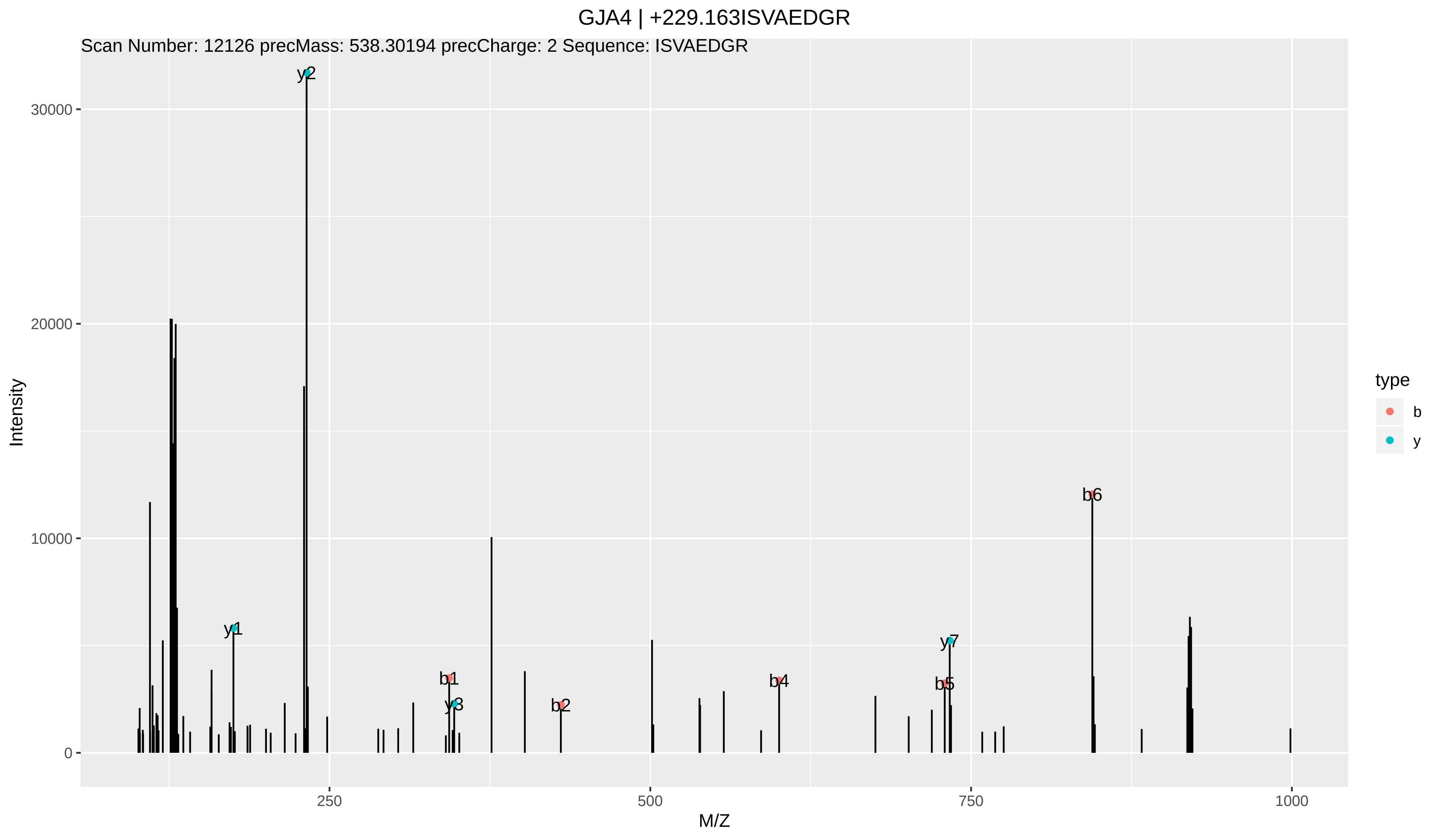
GJA4 | +229.163ISVAEDGR
Scan Number: 12126 precMass: 538.30194 precCharge: 2 Sequence: ISVAEDGR
y2
y1
b1
y3
b2
b4
y7
b5
b6
30000
20000
10000
0
250
500
750
1000
Intensity
M/Z
type
b
y

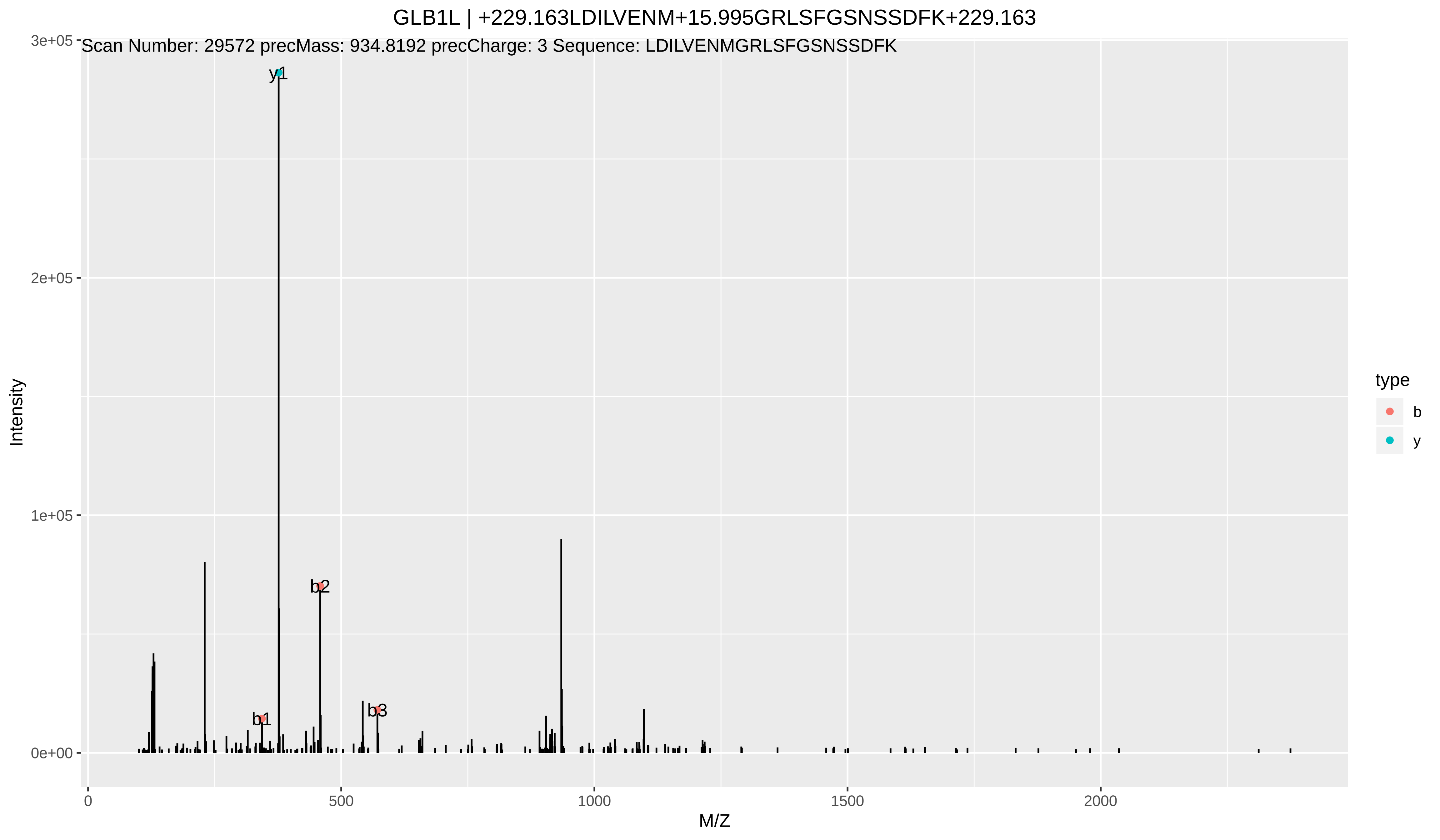
GLB1L | +229.163LDILVENM+15.995GRLSFGSNSSDFK+229.163
Scan Number: 29572 precMass: 934.8192 precCharge: 3 Sequence: LDILVENMGRLSFGSNSSDFK
y1
b2
b1
b3
Intensity
M/Z
type
b
y

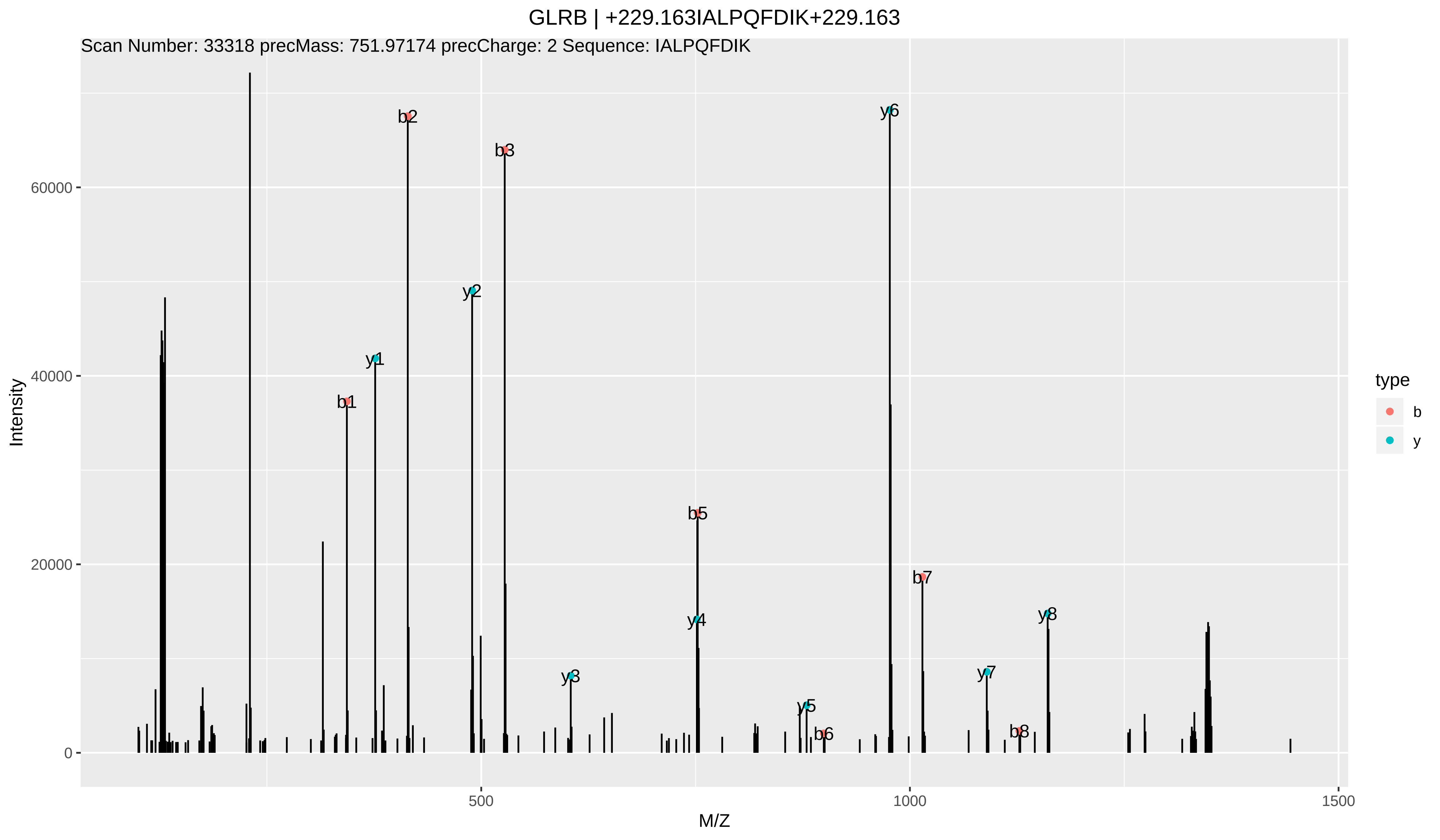

GLRB | +229.163IALPQFDIK+229.163
Scan Number: 33318 precMass: 751.97174 precCharge: 2 Sequence: IALPQFDIK
Intensity
M/Z
0
20000
40000
60000
500
1000
1500
b1
b2
b3
b5
b6
b7
b8
y1
y2
y3
y4
y5
y6
y7
y8
type
b
y

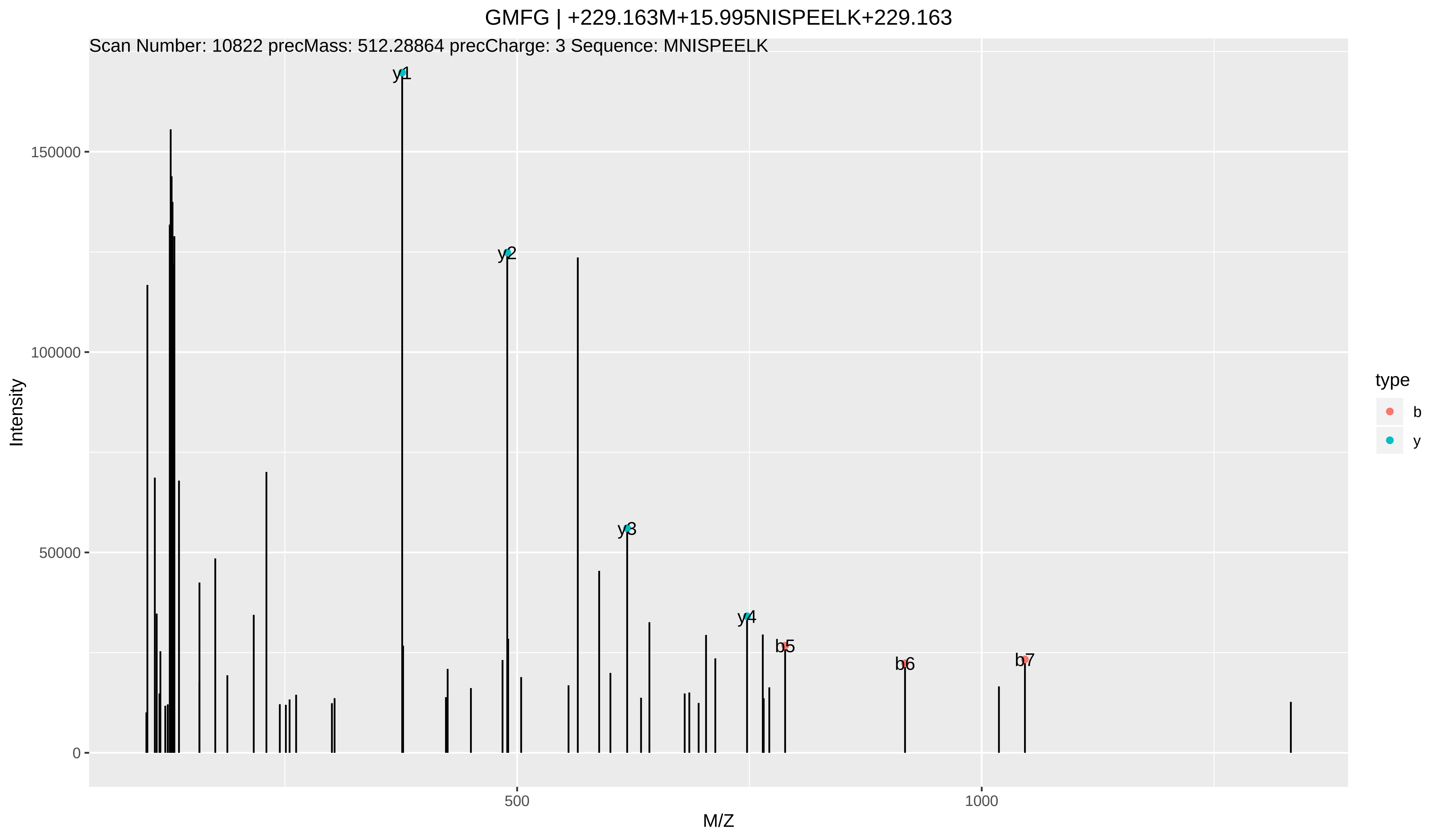
GMFG | +229.163M+15.995NISPEELK+229.163
Scan Number: 10822 precMass: 512.28864 precCharge: 3 Sequence: MNISPEELK
y1
y2
y3
y4
b5
b6
b7
150000
100000
50000
0
Intensity
500
1000
M/Z
type
b
y

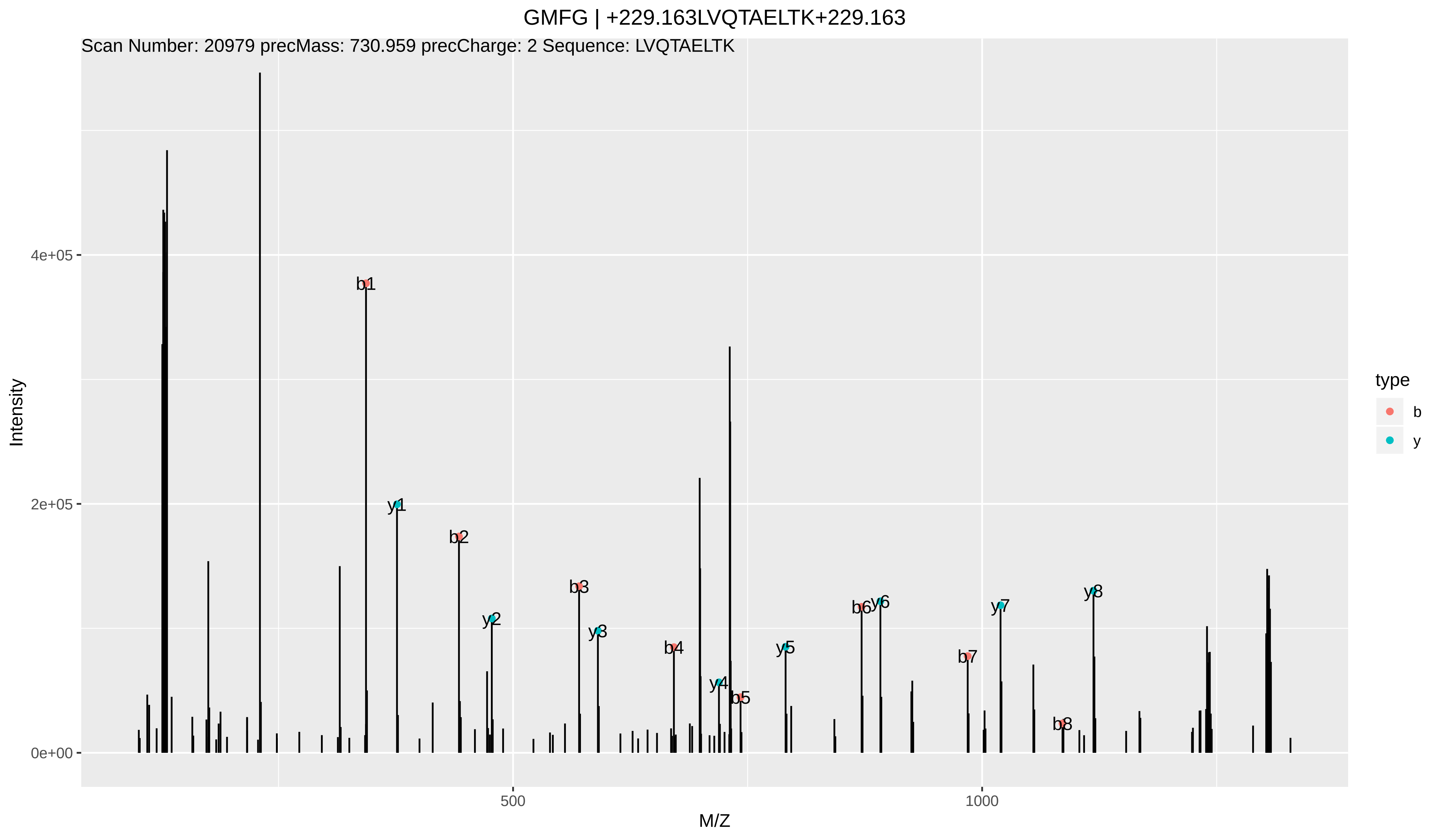

GMFG | +229.163LVQTAELTK+229.163
Scan Number: 20979 precMass: 730.959 precCharge: 2 Sequence: LVQTAELTK
Intensity
4e+05
2e+05
0e+00
M/Z
500
1000
b1
y1
b2
y2
b3
y3
b4
y4
b5
y5
b6
y6
b7
y7
b8
y8
type
b
y

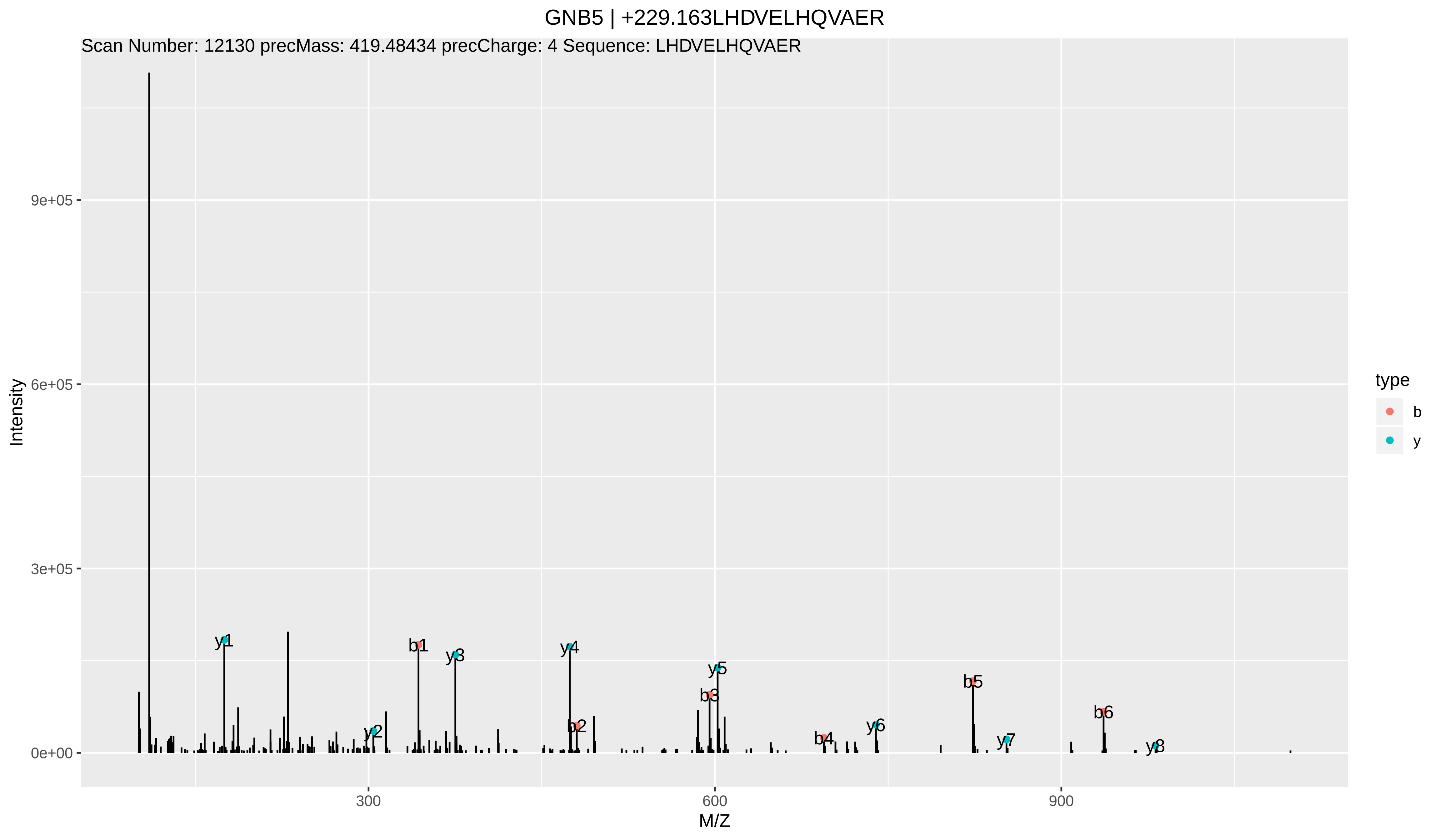
GNB5 | +229.163LHDVELHQVAER
Scan Number: 12130 precMass: 419.48434 precCharge: 4 Sequence: LHDVELHQVAER
Intensity
M/Z
9e+05
6e+05
3e+05
0e+00
300
600
900
type
b
y
y1
y2
y3
y4
y5
y6
y7
y8
b1
b2
b3
b4
b5
b6

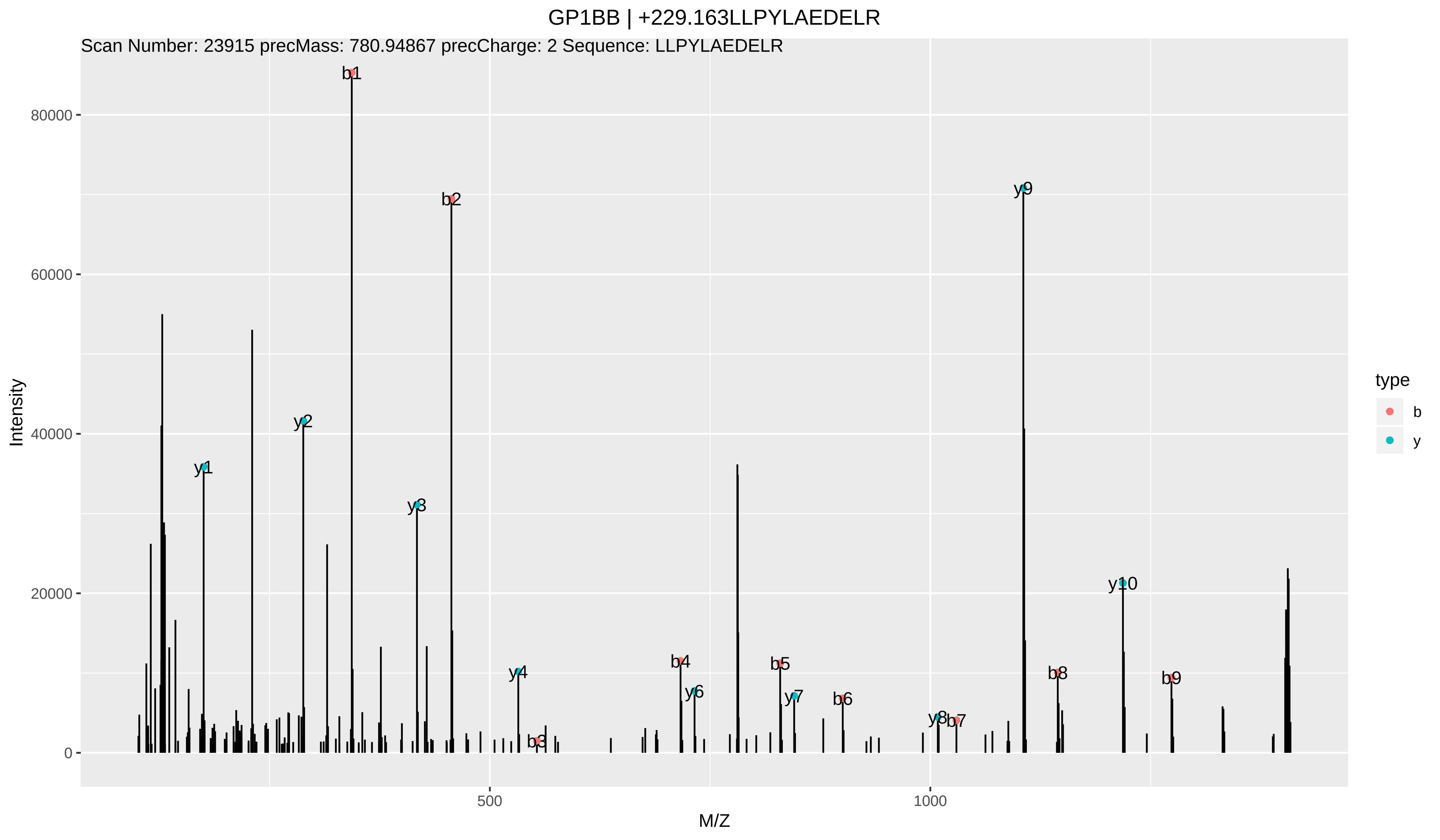
GP1BB | +229.163LLPYLAEDELR
Scan Number: 23915 precMass: 780.94867 precCharge: 2 Sequence: LLPYLAEDELR
Intensity
80000
60000
40000
20000
0
M/Z
500
1000
b1
b2
y9
y2
y1
y3
y10
y4
b3
b4
y6
b5
y7
b6
y8
b7
b8
b9
type
b
y

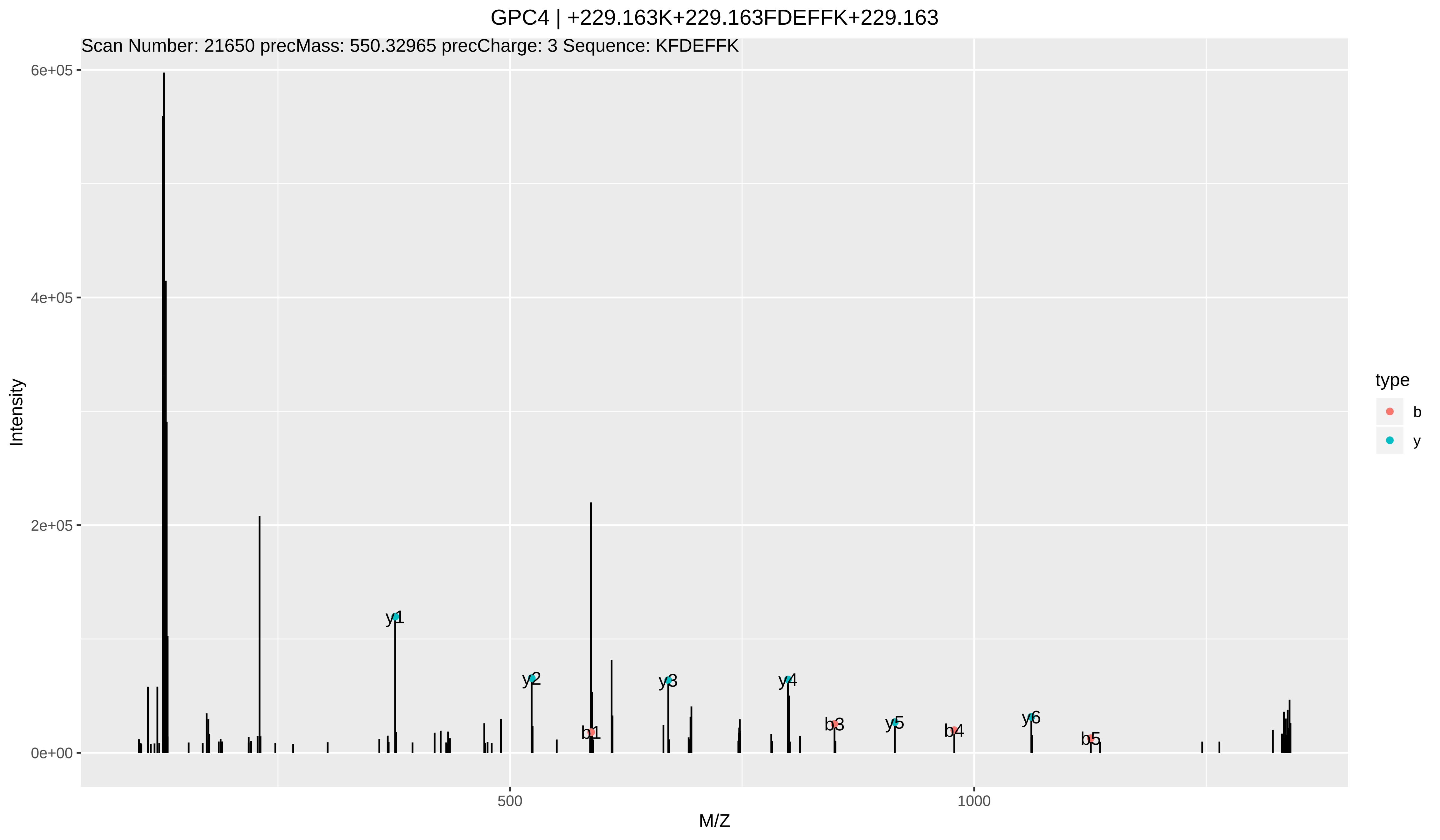

GPC4 | +229.163K+229.163FDEFFK+229.163
Scan Number: 21650 precMass: 550.32965 precCharge: 3 Sequence: KFDEFFK
Intensity
6e+05
4e+05
2e+05
0e+00
M/Z
500
1000
y1
y2
y3
y4
y5
y6
b1
b3
b4
b5
type
b
y

GPR149 | +229.163LSHEESQK+229.163

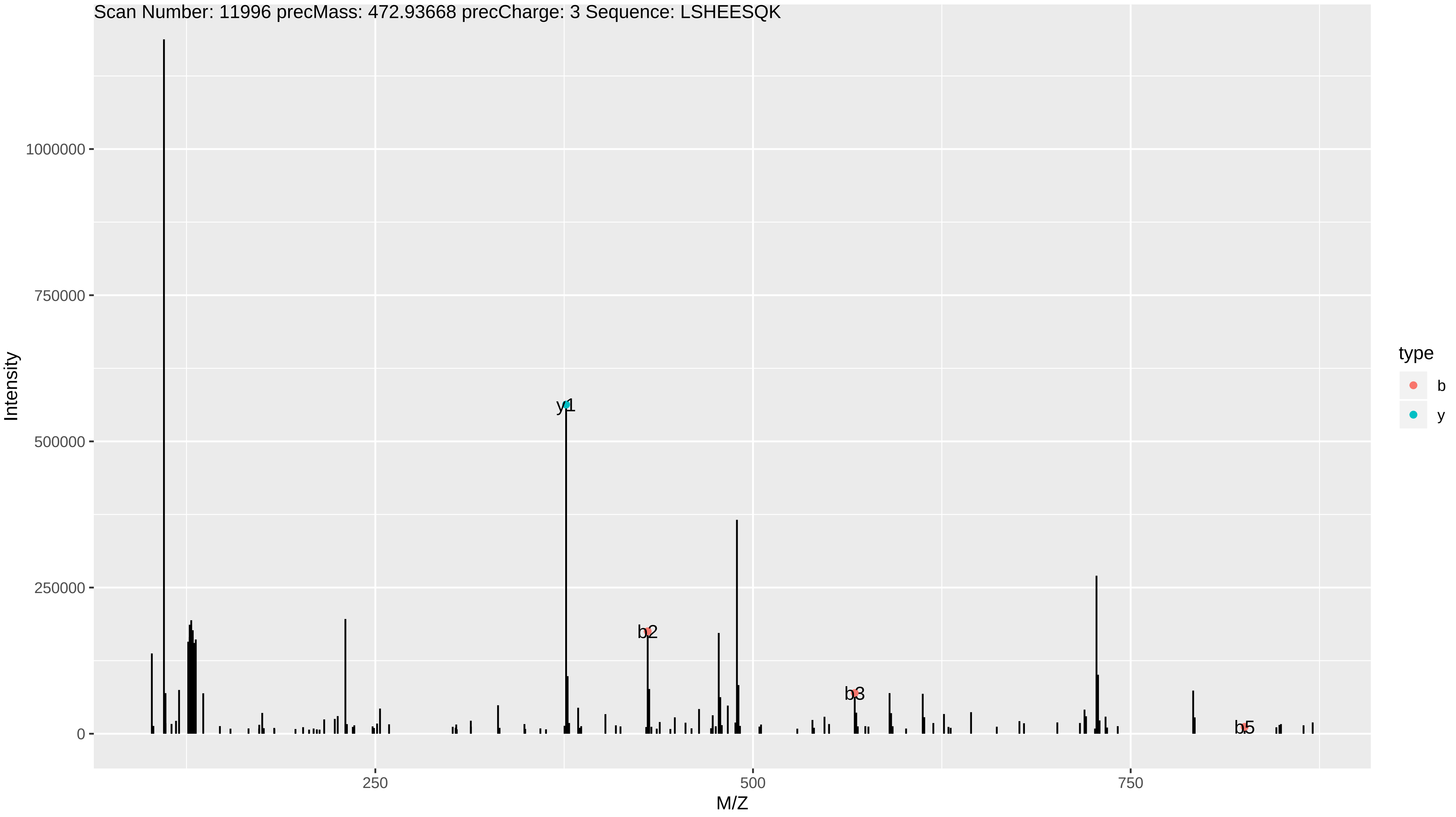

GPR161 | +229.163LQLQSIEEGDVLAAEQR

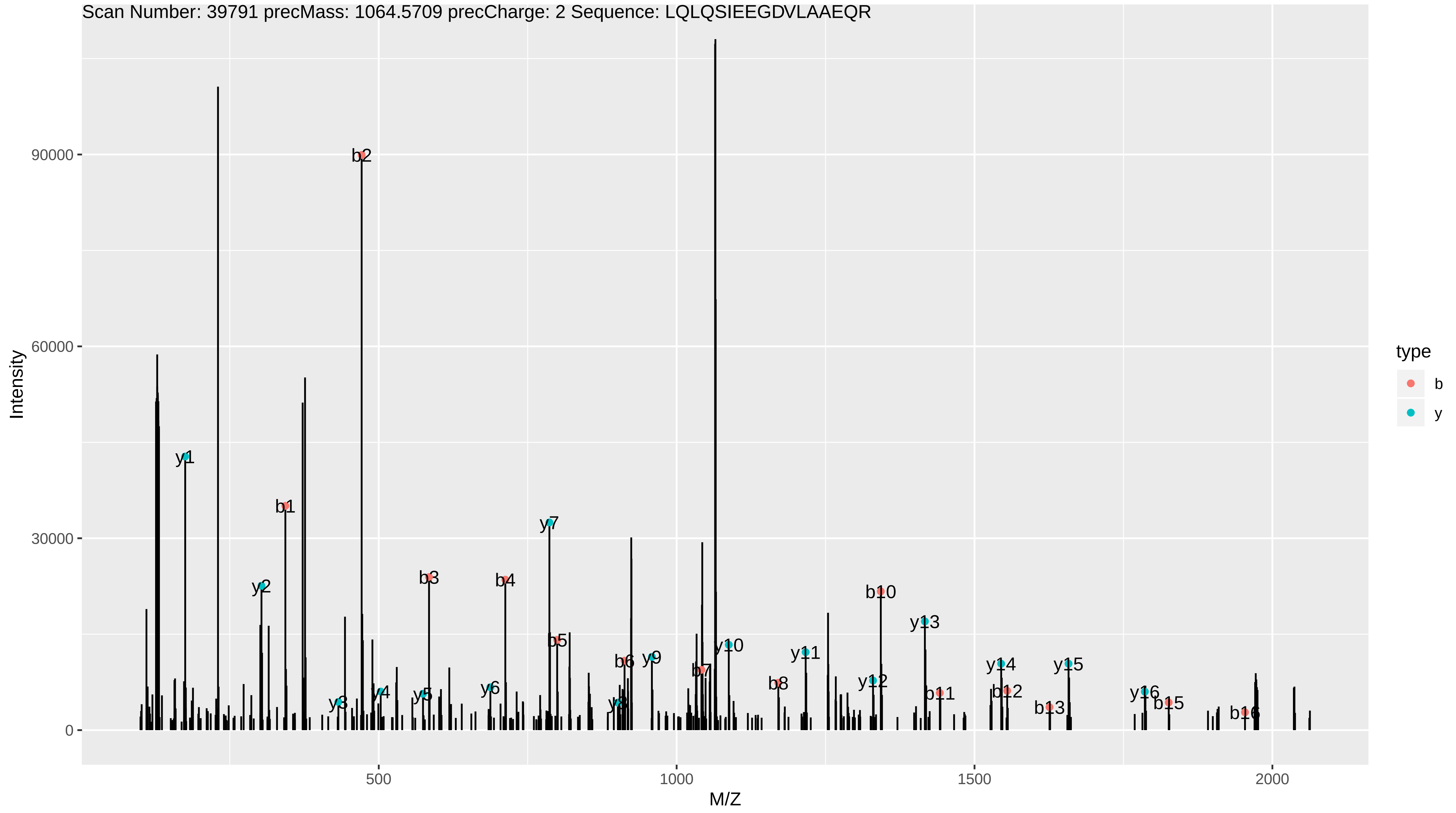

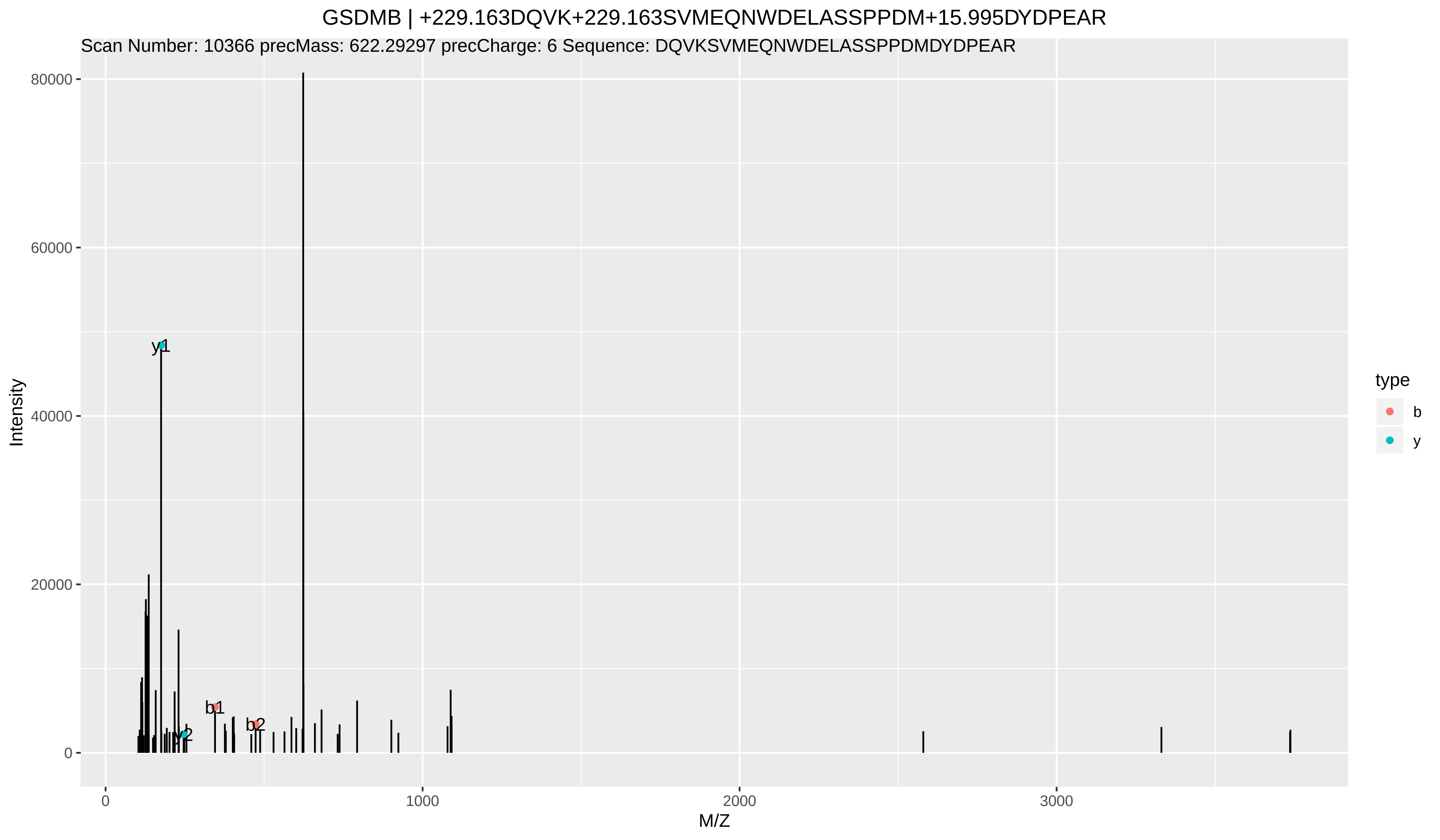
GSDMB | +229.163DQVK+229.163SVMEQNWDELASSPPDM+15.995DYDPEAR
Scan Number: 10366 precMass: 622.29297 precCharge: 6 Sequence: DQVKSVMEQNWDELASSPPDMDYDPEAR
Intensity
80000
60000
40000
20000
0
y1
y2
b1
b2
0
1000
2000
3000
M/Z
type
b
y

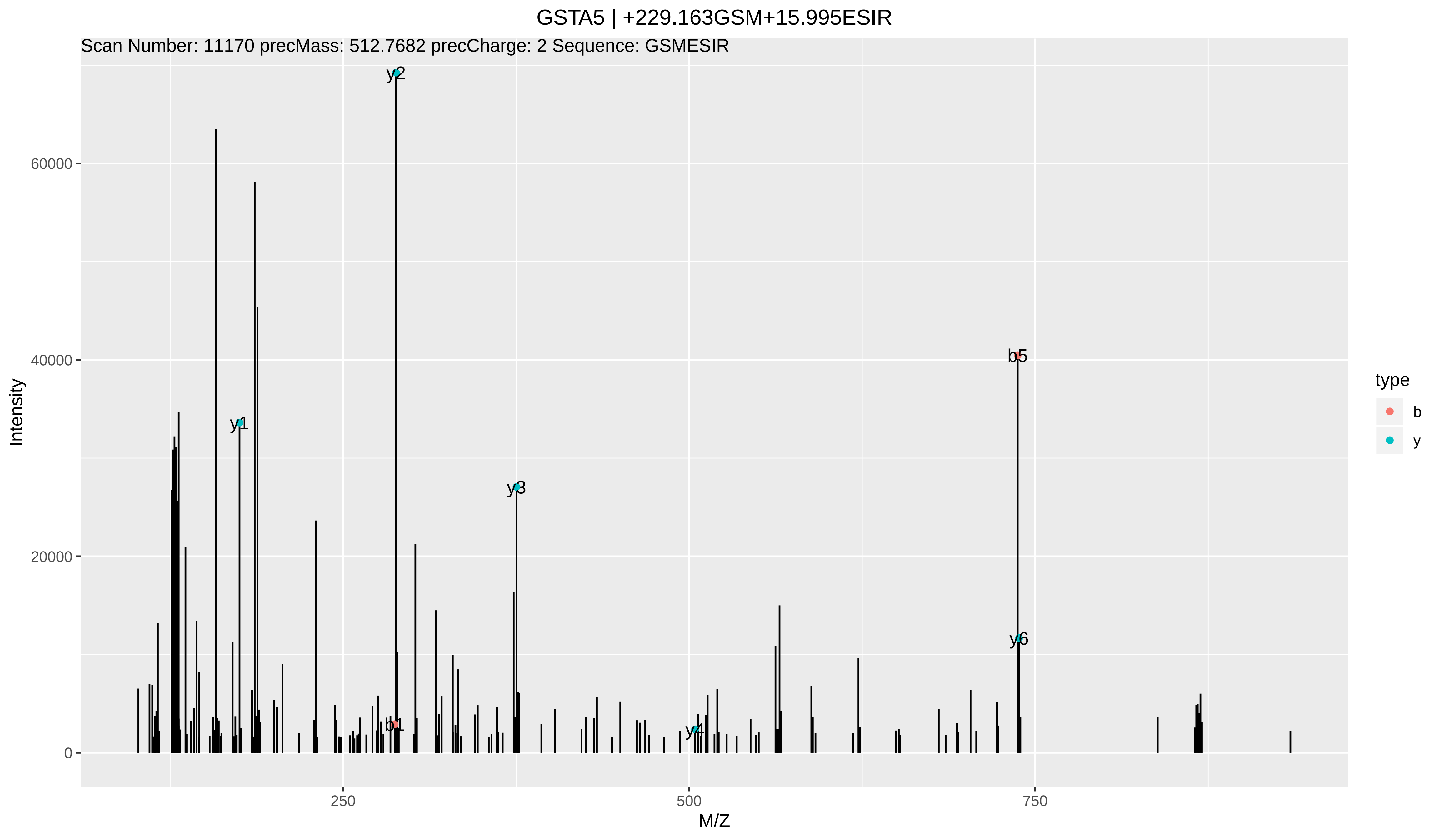
GSTA5 | +229.163GSM+15.995ESIR
Scan Number: 11170 precMass: 512.7682 precCharge: 2 Sequence: GSMESIR
y2
y1
y3
b5
y6
b1
y4
Intensity
60000
40000
20000
0
250
500
750
M/Z
type
b
y

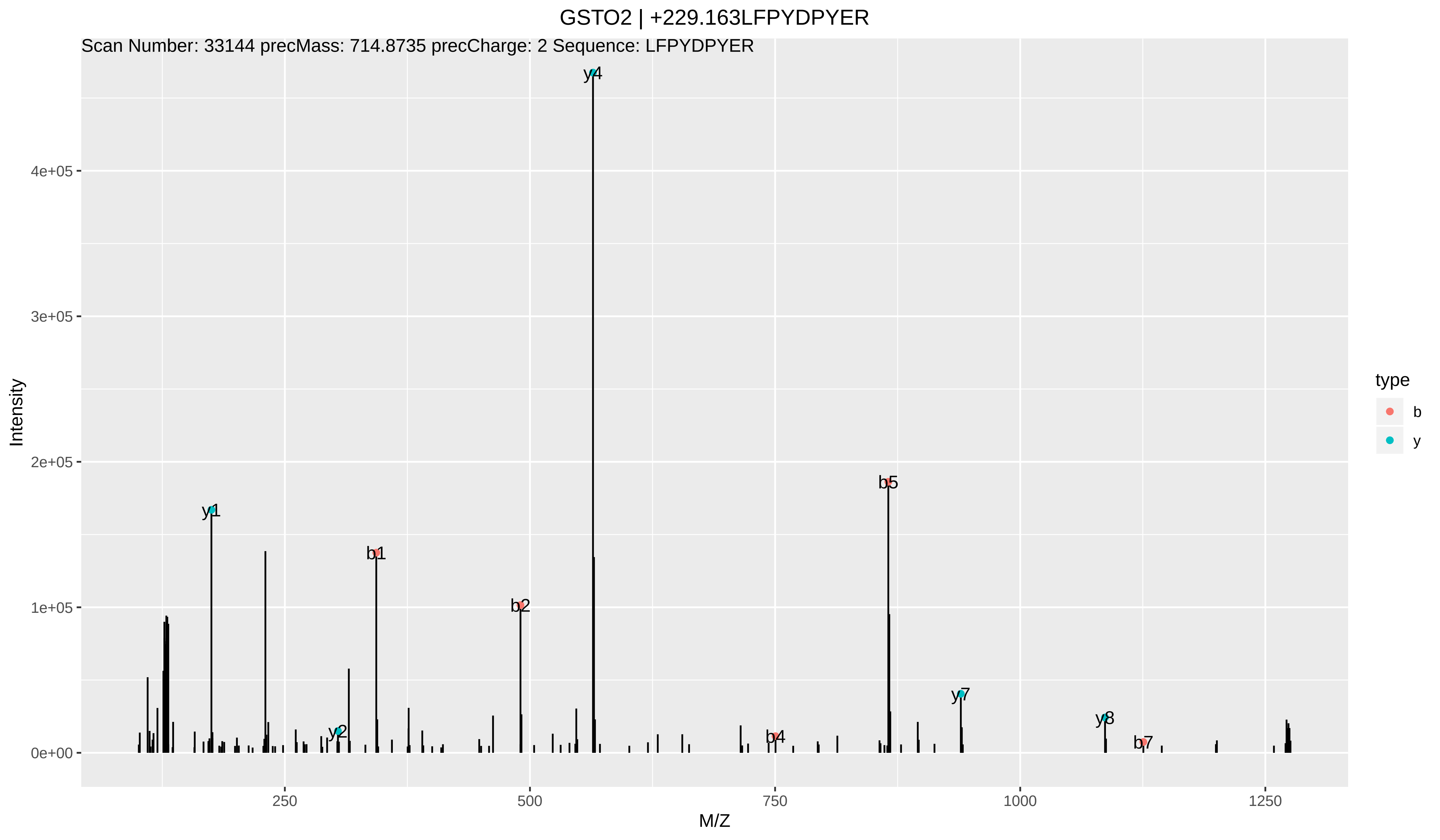

GSTO2 | +229.163LFPYDPYER
Scan Number: 33144 precMass: 714.8735 precCharge: 2 Sequence: LFPYDPYER
Intensity
4e+05
3e+05
2e+05
1e+05
0e+00
M/Z
250
500
750
1000
1250
y4
b5
y1
b1
b2
y7
y8
y2
b4
b7
type
b
y

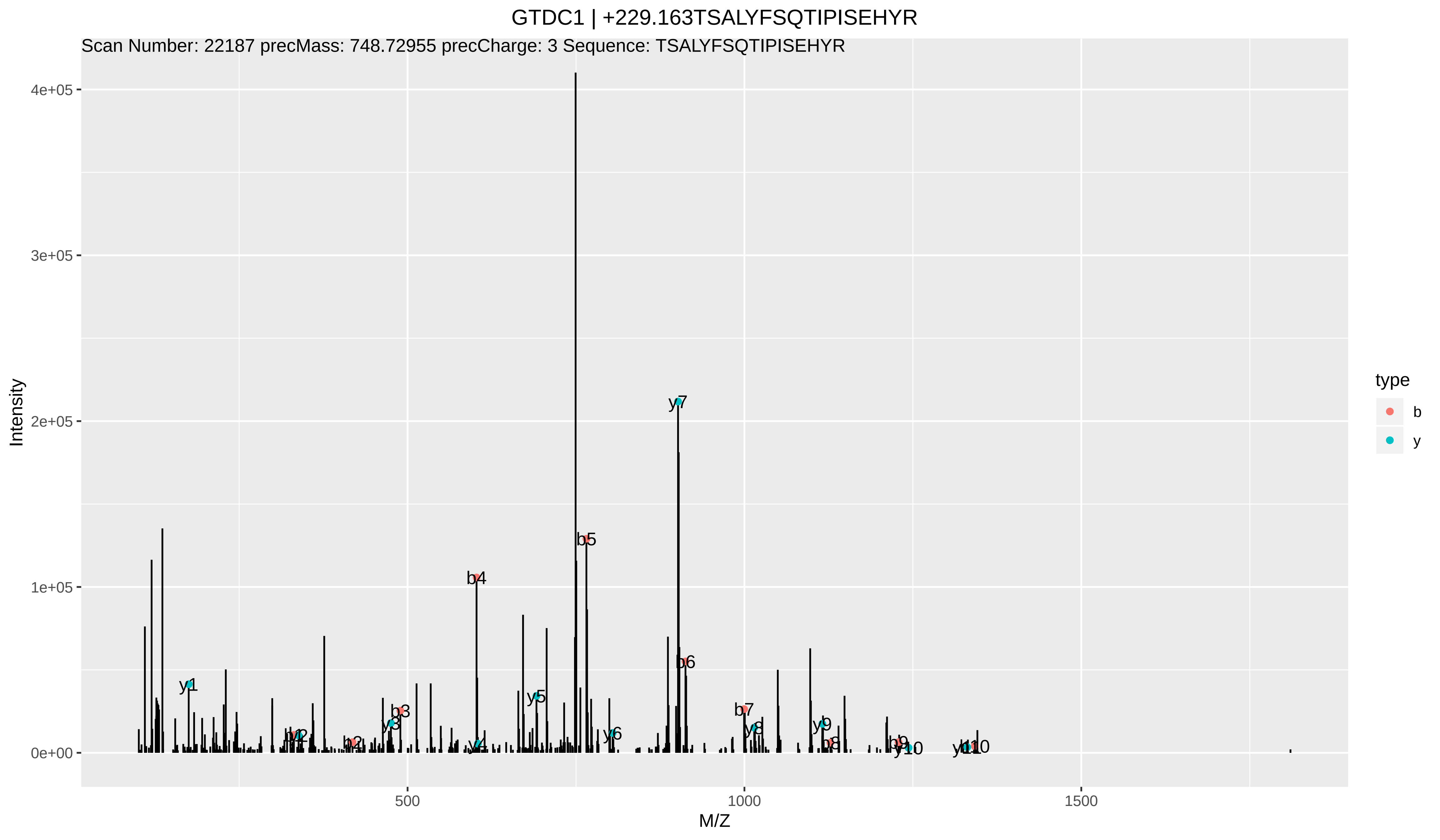
GTDC1 | +229.163TSALYFSQTIPISEHYR
Scan Number: 22187 precMass: 748.72955 precCharge: 3 Sequence: TSALYFSQTIPISEHYR
Intensity
4e+05
3e+05
2e+05
1e+05
0e+00
M/Z
500
1000
1500
type
b
y
y1
b1
y2
b2
y3
b3
b4
y4
y5
b5
y6
b6
y7
b7
y8
b8
y9
b9
y10
y11
b10

GTF3C6 | +229.163K+229.163EGEENIGGVEWLQIK+229.163

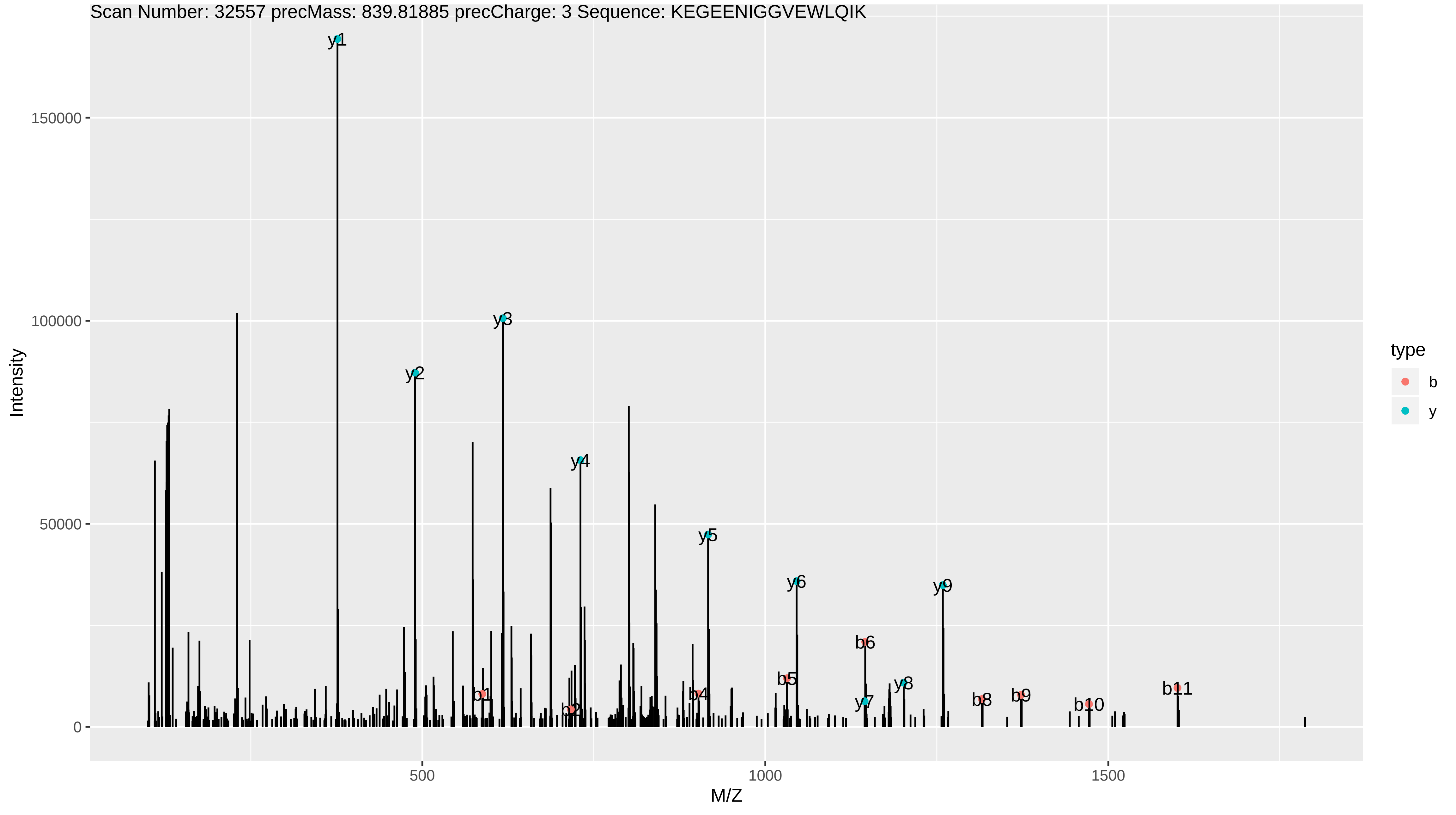

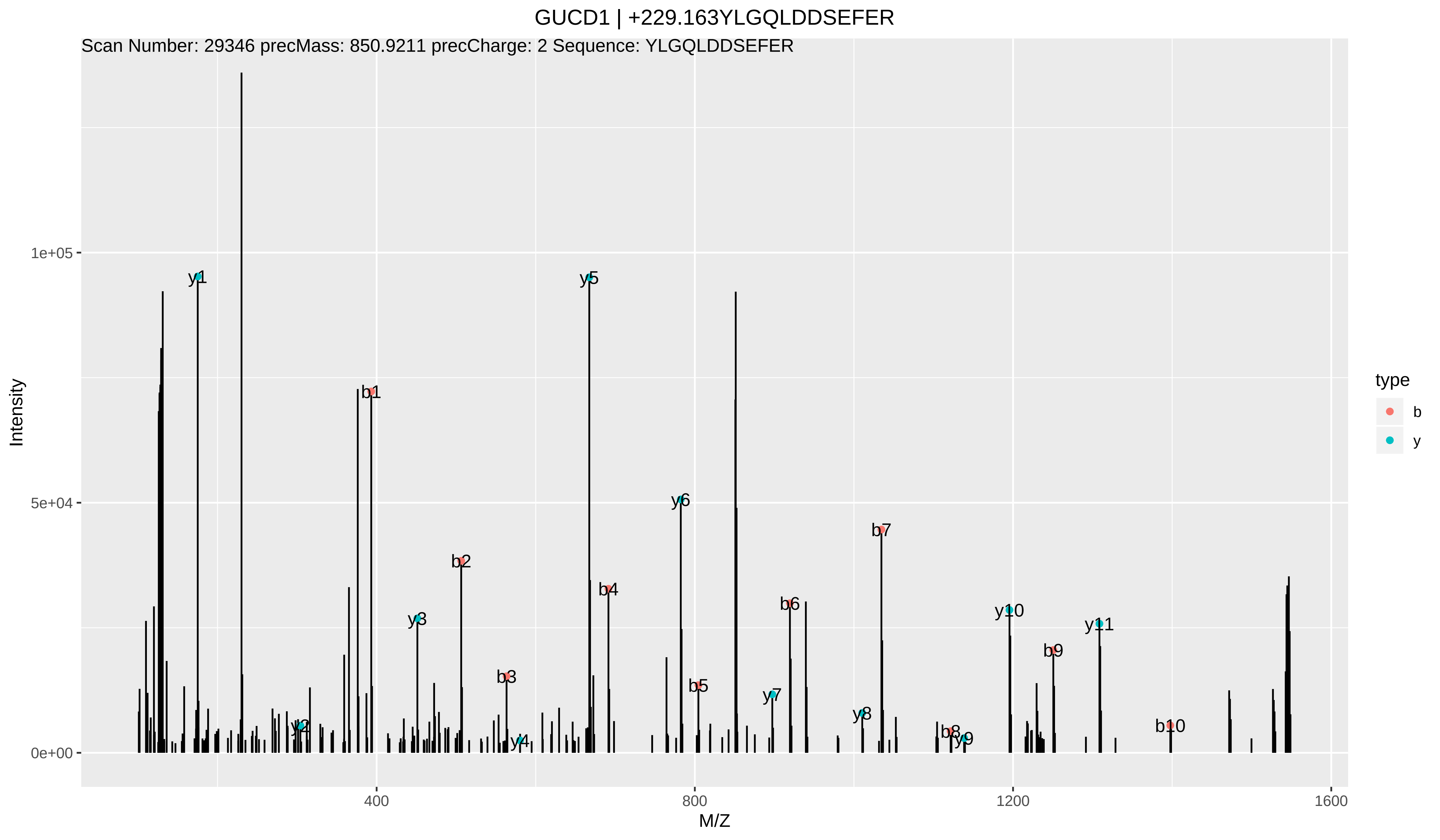
GUCD1 | +229.163YLGQLDDSEFER
Scan Number: 29346 precMass: 850.9211 precCharge: 2 Sequence: YLGQLDDSEFER
Intensity
M/Z
1e+05
5e+04
0e+00
400
800
1200
1600
y1
b1
y5
y6
b7
b2
b4
b6
y3
y10
y11
b9
b3
b5
y7
y8
y2
y4
b8
y9
b10
type
b
y

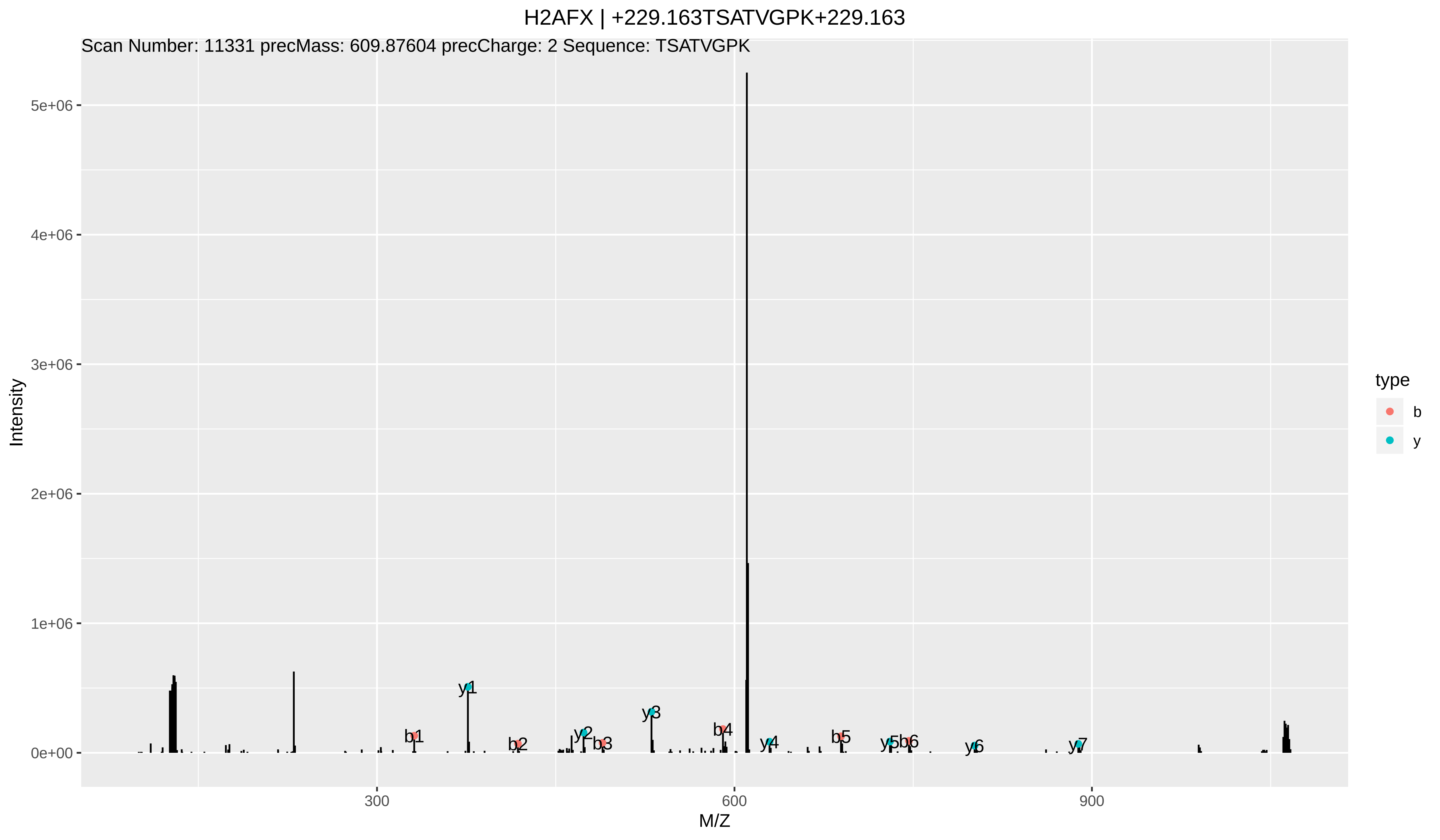
H2AFX | +229.163TSATVGPK+229.163
Scan Number: 11331 precMass: 609.87604 precCharge: 2 Sequence: TSATVGPK
Intensity
5e+06
4e+06
3e+06
2e+06
1e+06
0e+00
M/Z
300
600
900
y1
b1
b2
y2
b3
y3
b4
y4
b5
y5
b6
y6
y7
type
b
y

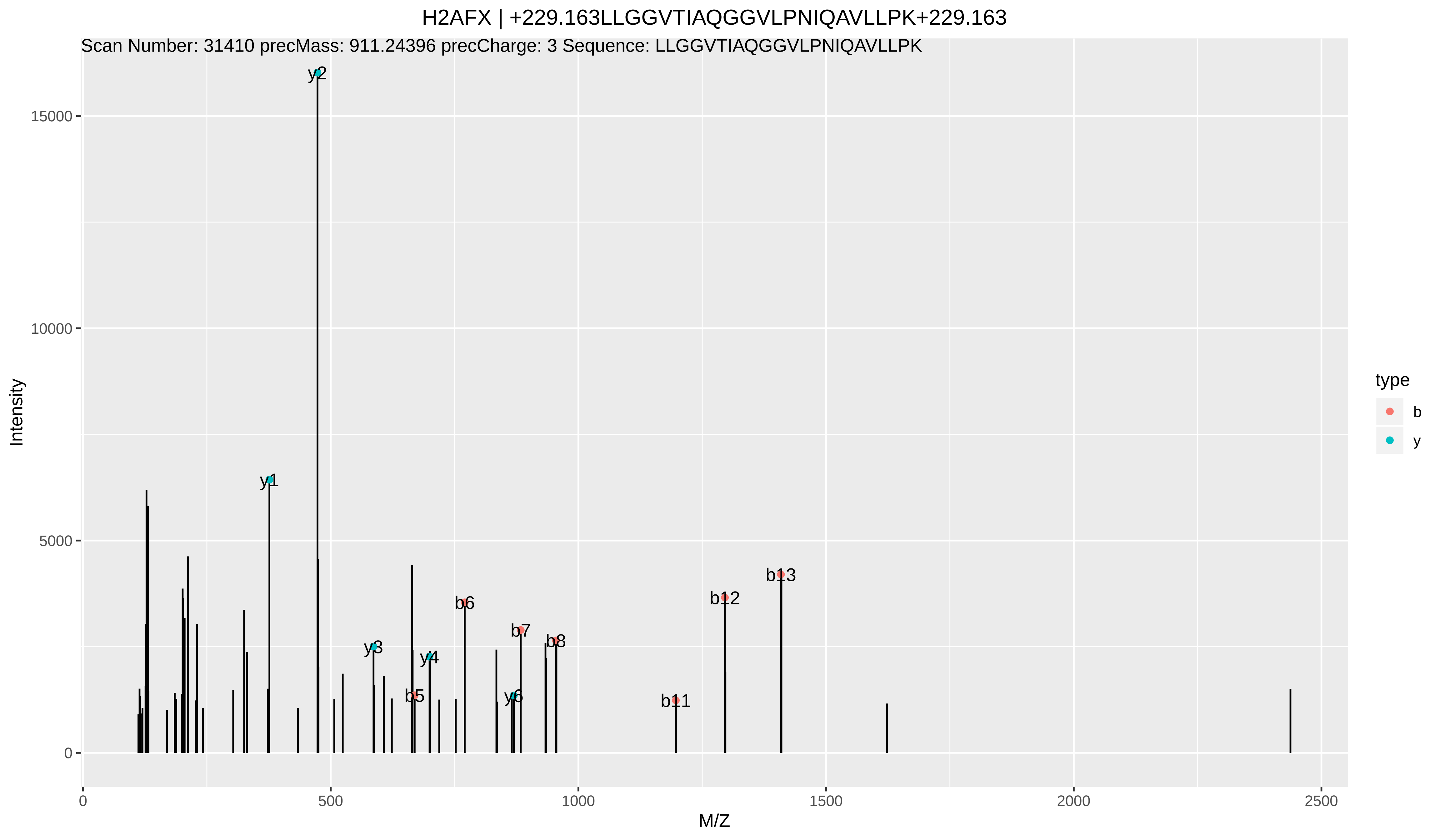

H2AFX | +229.163LLGGVTIAQGGVLPNIQAVLLPK+229.163
Scan Number: 31410 precMass: 911.24396 precCharge: 3 Sequence: LLGGVTIAQGGVLPNIQAVLLPK
Intensity
M/Z
type
b
y
y1
y2
y3
y4
y6
b5
b6
b7
b8
b11
b12
b13
0
5000
10000
15000
500
1000
1500
2000
2500

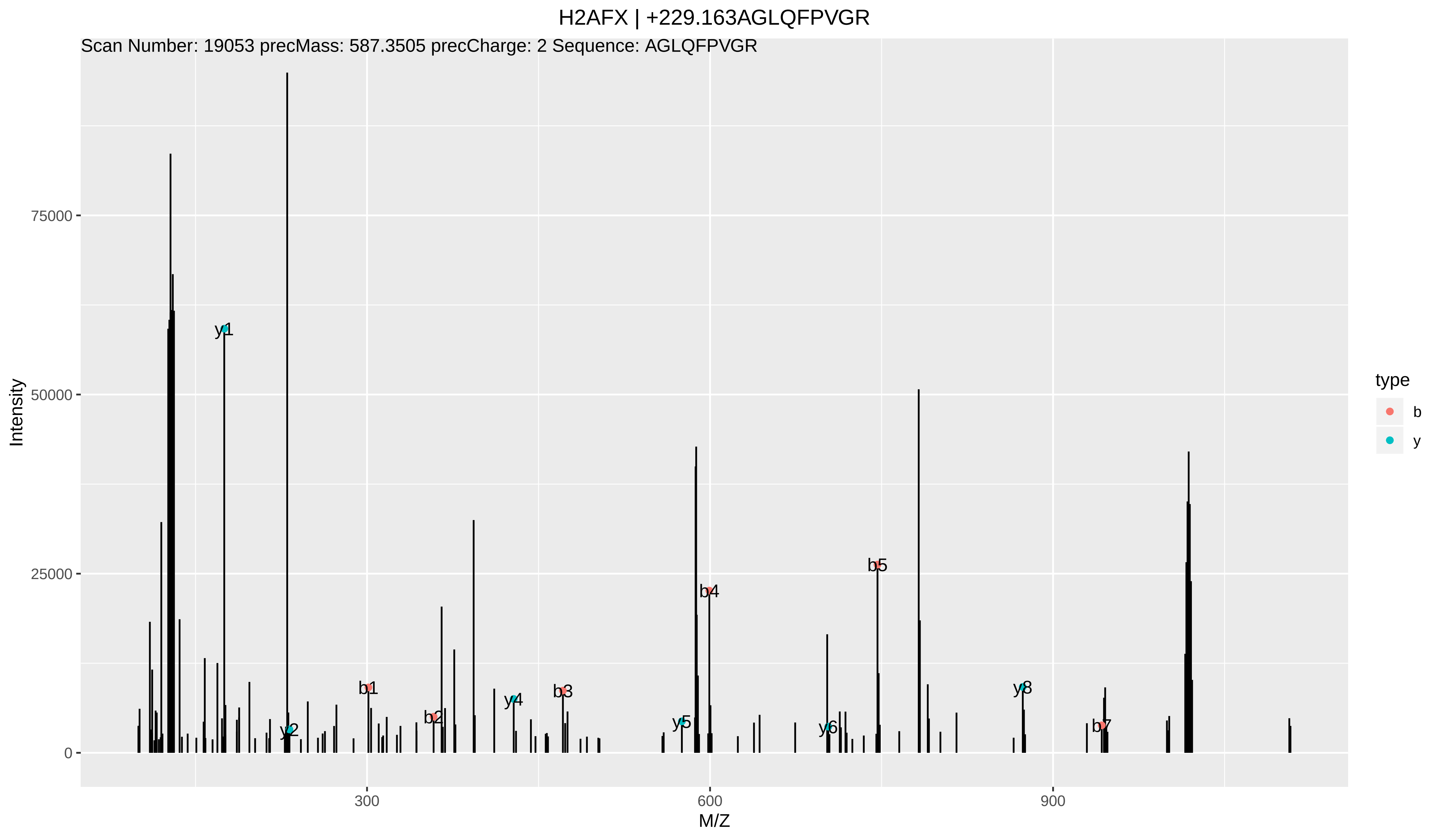
H2AFX | +229.163AGLQFPVGR
Scan Number: 19053 precMass: 587.3505 precCharge: 2 Sequence: AGLQFPVGR
Intensity
75000
50000
25000
0
M/Z
300
600
900
y1
y2
b1
b2
y4
b3
y5
b4
y6
b5
y8
b7
type
b
y

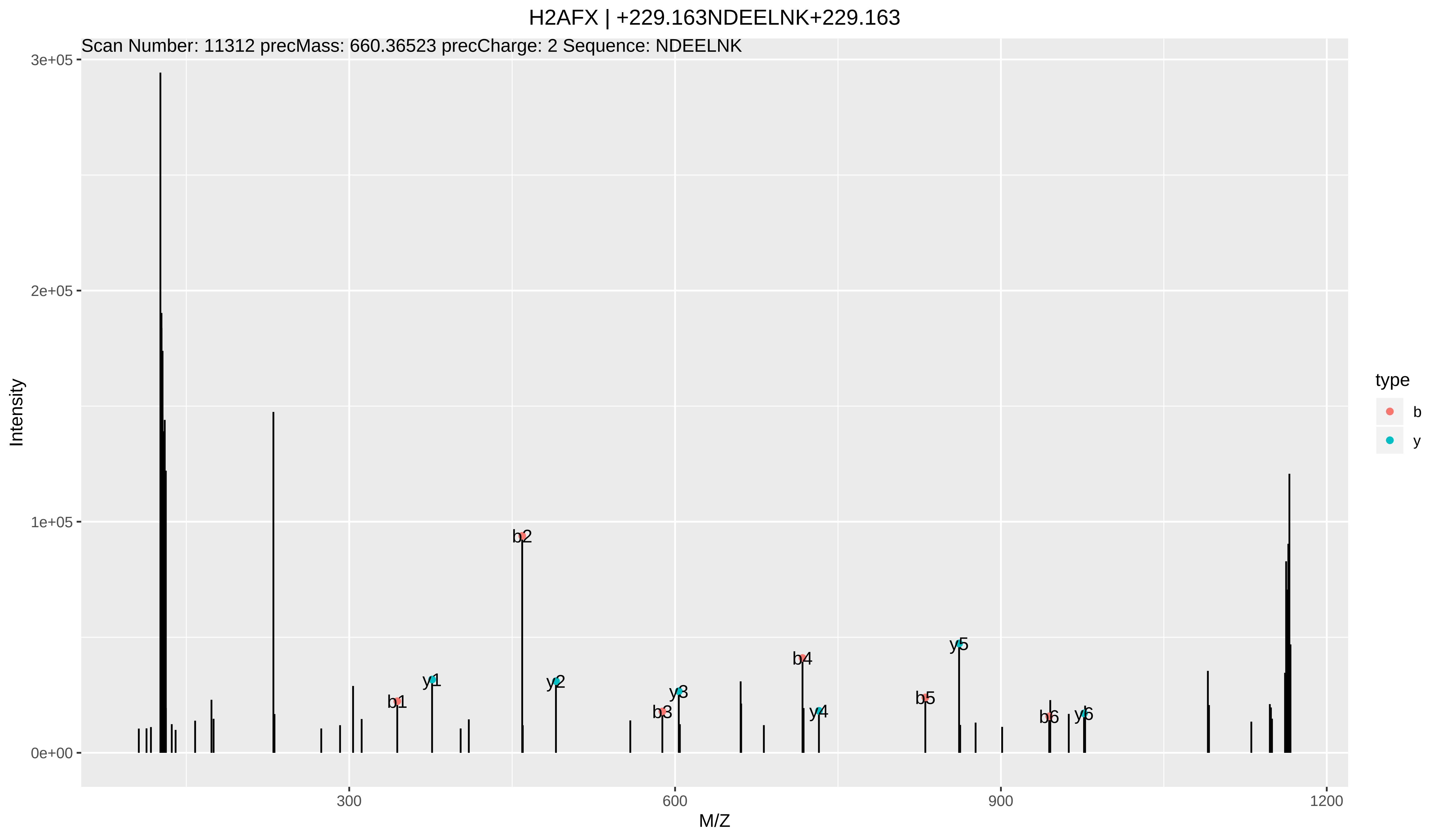
H2AFX | +229.163NDEELNK+229.163
Scan Number: 11312 precMass: 660.36523 precCharge: 2 Sequence: NDEELNK
Intensity
M/Z
3e+05
2e+05
1e+05
0e+00
300
600
900
1200
b1
y1
b2
y2
b3
y3
b4
y4
b5
y5
b6
y6
type
b
y

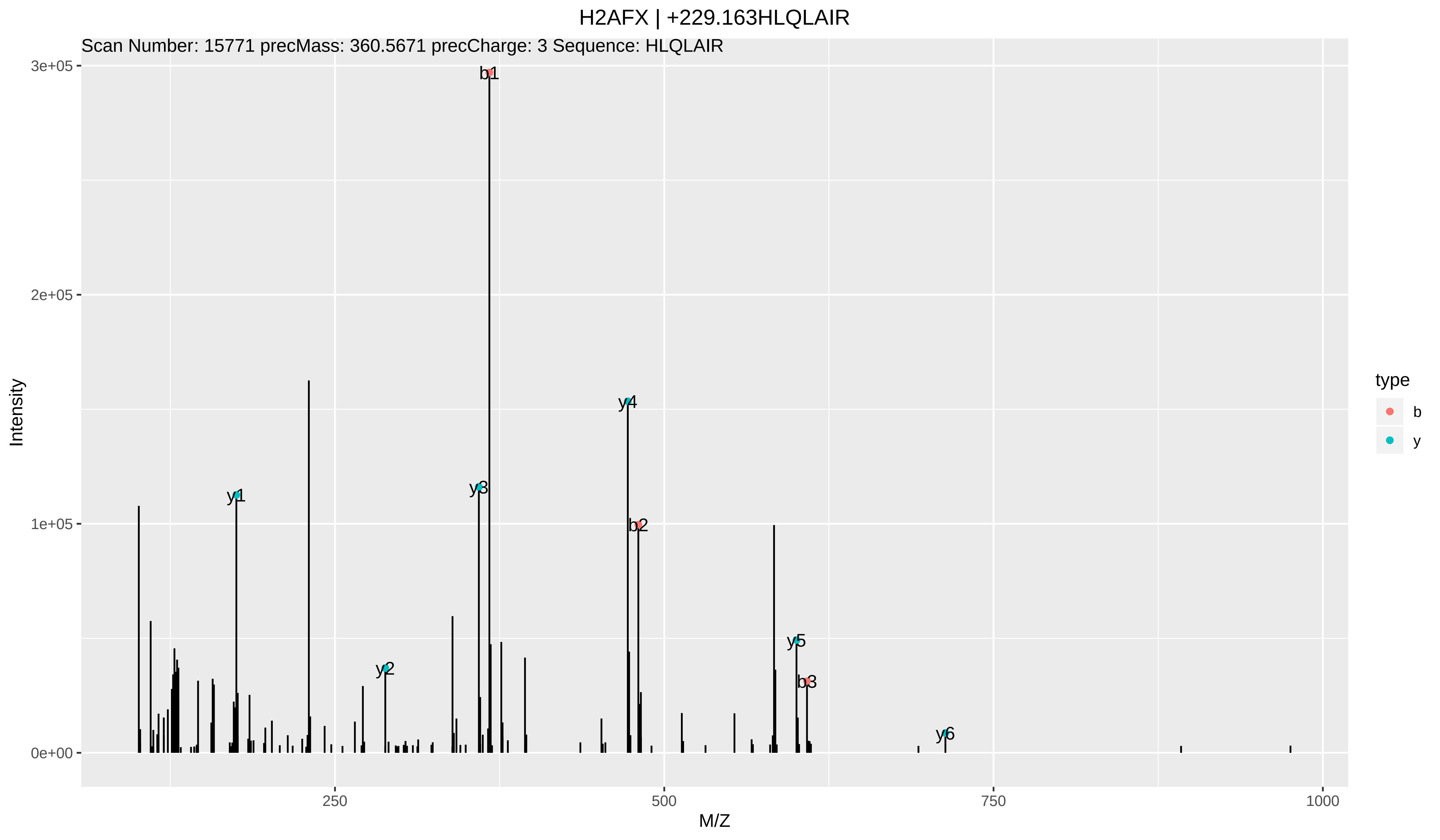
H2AFX | +229.163HLQLAIR
Scan Number: 15771 precMass: 360.5671 precCharge: 3 Sequence: HLQLAIR
Intensity
3e+05
2e+05
1e+05
0e+00
b1
y4
y3
y1
b2
y5
b3
y2
y6
250
500
750
1000
M/Z
type
b
y

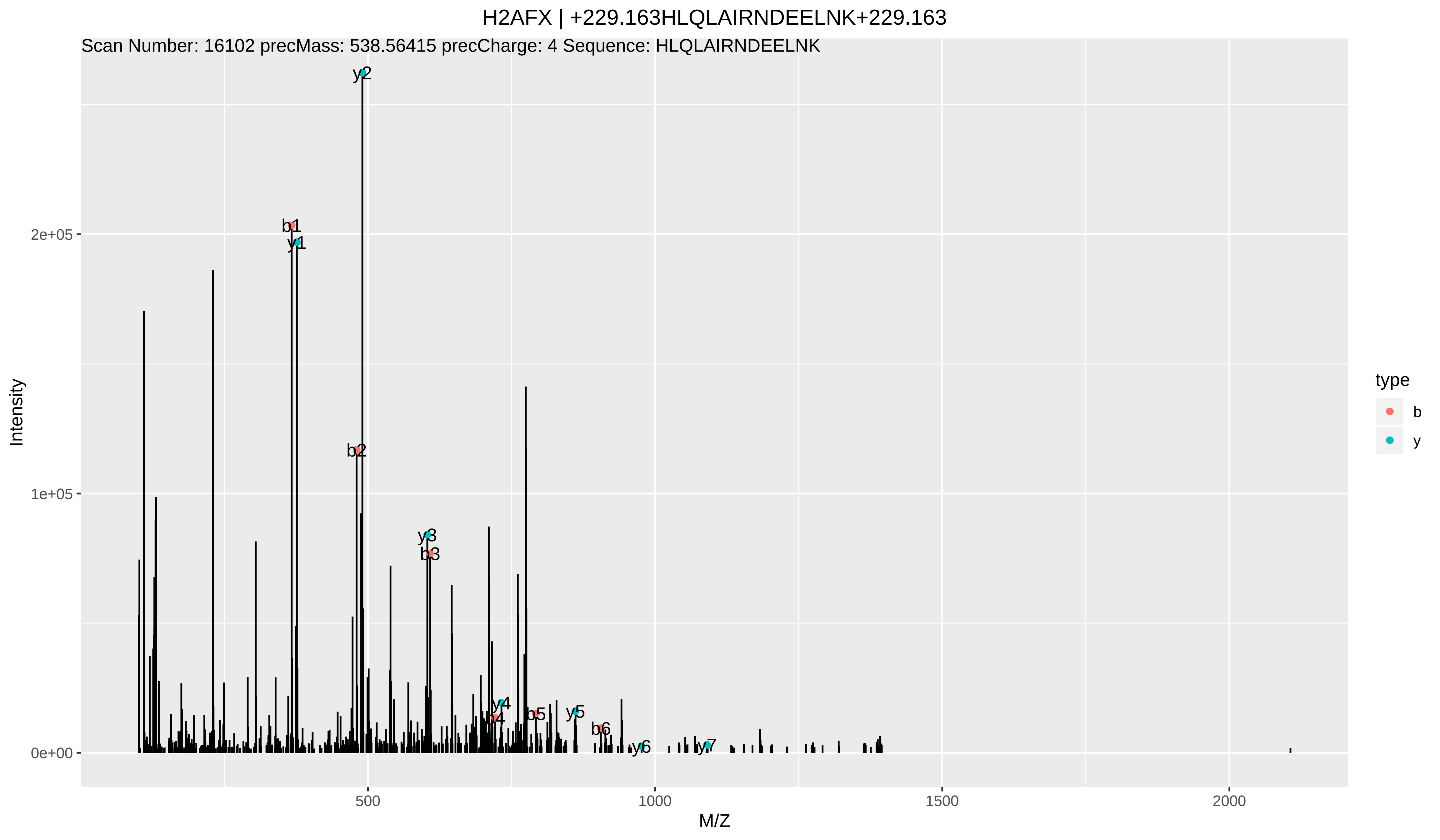

H2AFX | +229.163HLQLAIRNDEELNK+229.163
Scan Number: 16102 precMass: 538.56415 precCharge: 4 Sequence: HLQLAIRNDEELNK
Intensity
M/Z
0e+00
1e+05
2e+05
500
1000
1500
2000
type
b
y
y2
b1
y1
b2
y3
b3
y4
b4
b5
y5
b6
y6
y7

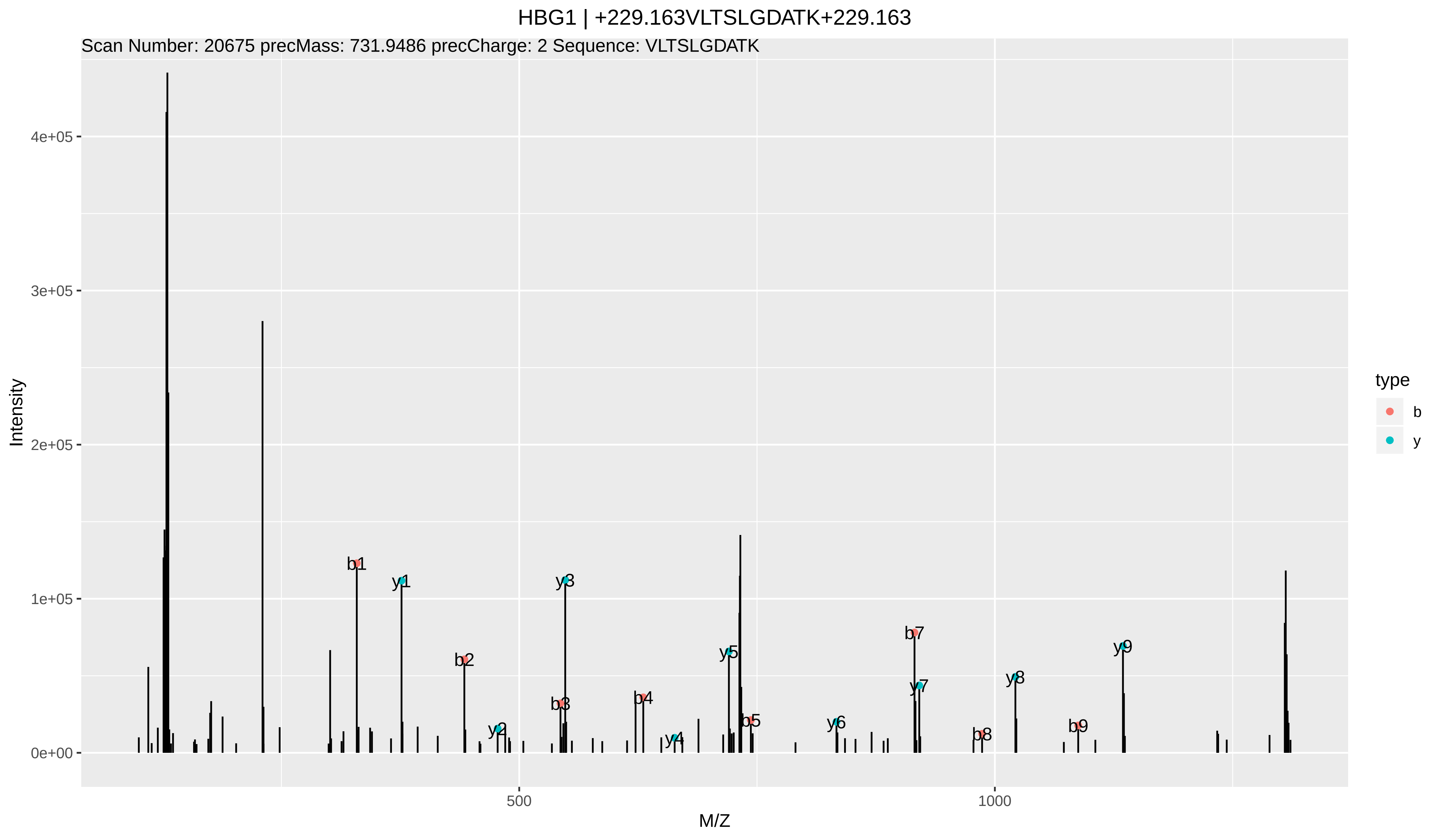

HBG1 | +229.163VLTSLGDATK+229.163
Scan Number: 20675 precMass: 731.9486 precCharge: 2 Sequence: VLTSLGDATK
Intensity
4e+05
3e+05
2e+05
1e+05
0e+00
M/Z
500
1000
b1
y1
y3
b2
y2
b3
b4
y4
y5
b5
y6
b7
y7
b8
y8
b9
y9
type
b
y

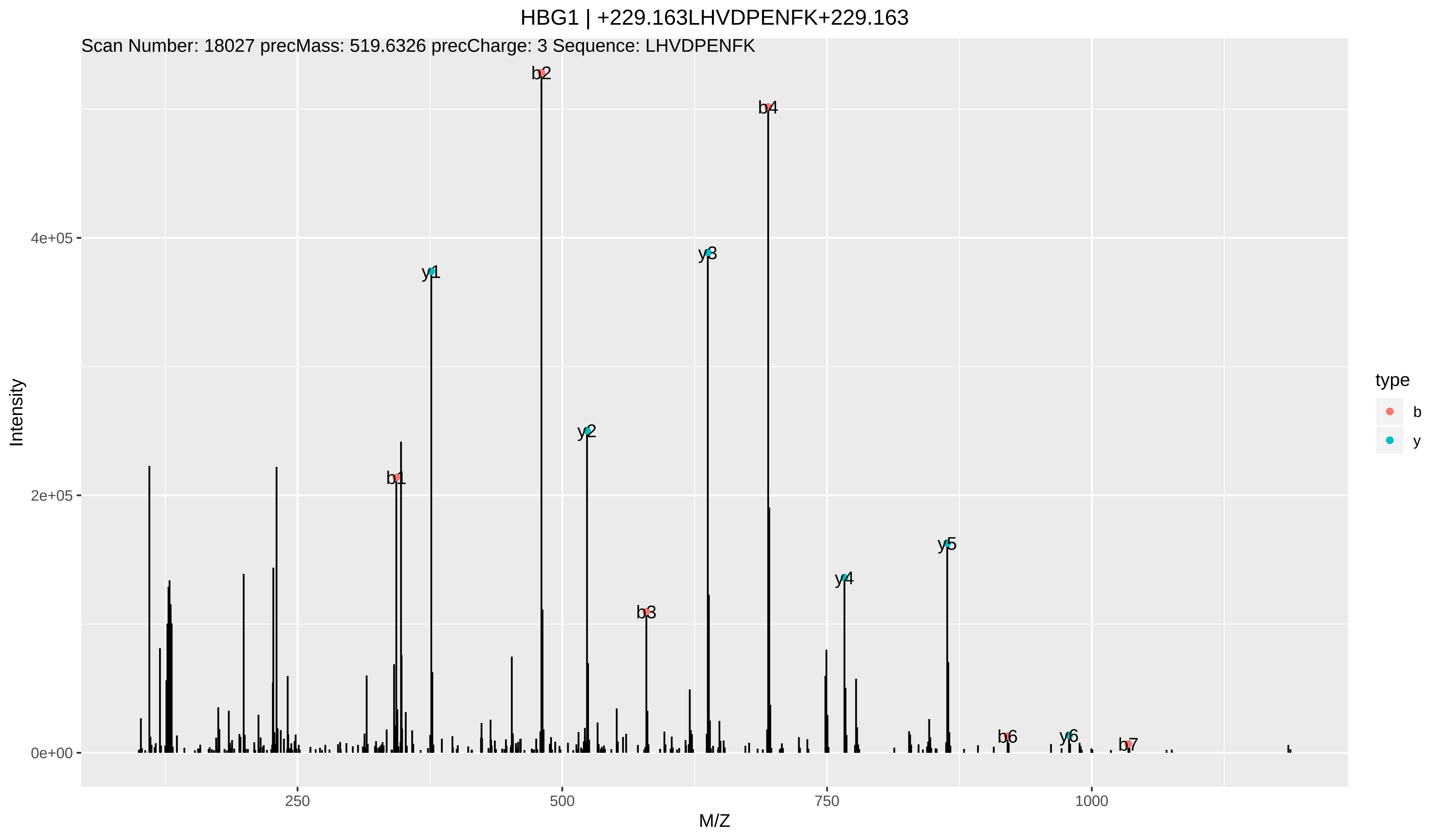

HBG1 | +229.163LHVDPENFK+229.163
Scan Number: 18027 precMass: 519.6326 precCharge: 3 Sequence: LHVDPENFK
Intensity
M/Z
4e+05
2e+05
0e+00
250
500
750
1000
b2
b4
y3
y1
y2
b1
y5
y4
b3
b6
y6
b7
type
b
y

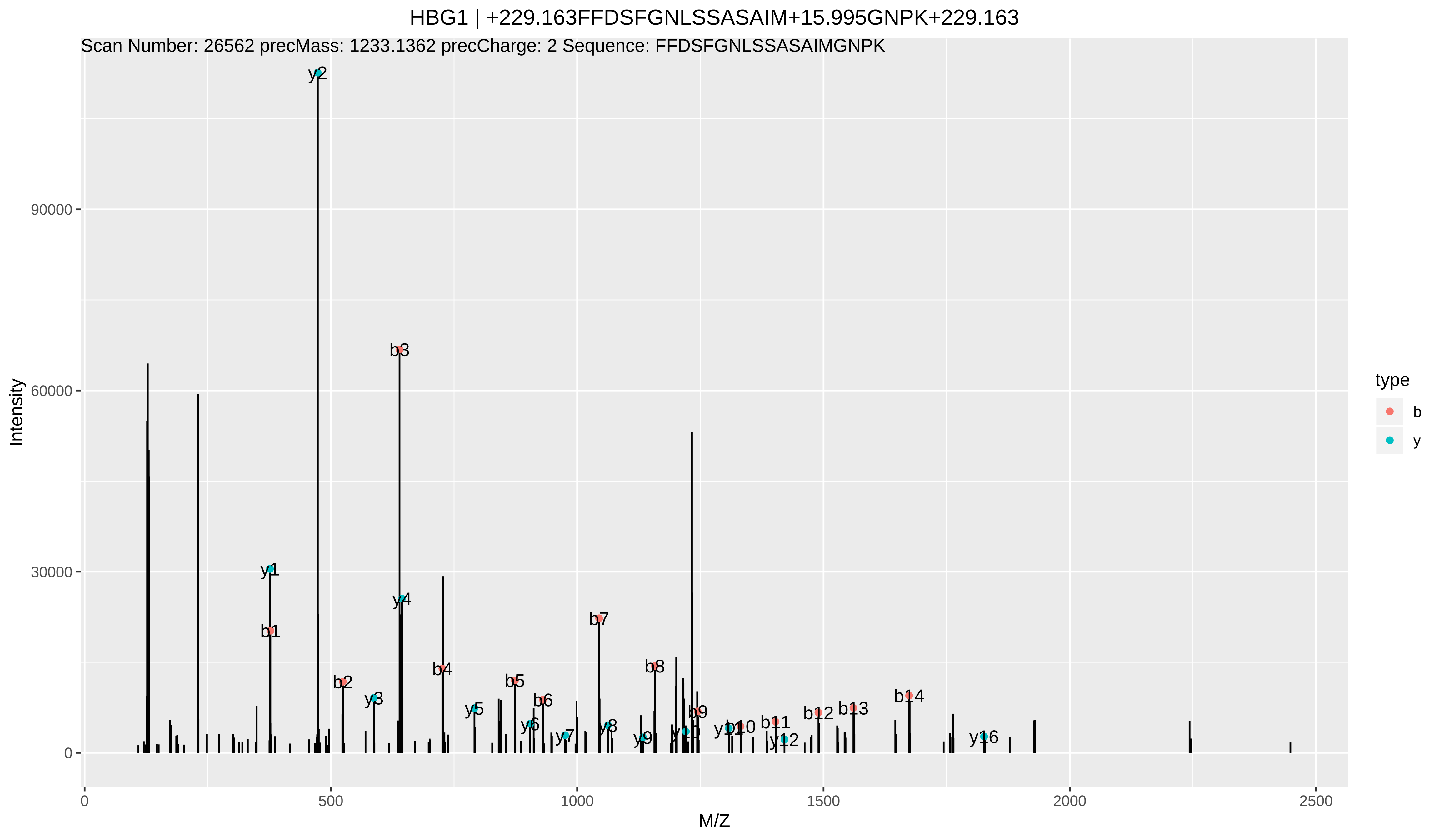

HBG1 | +229.163FFDSFGNLSSASAIM+15.995GNPK+229.163
Scan Number: 26562 precMass: 1233.1362 precCharge: 2 Sequence: FFDSFGNLSSASAIMGNPK
Intensity
M/Z
type
b
y
y2
b3
y1
b1
y4
b7
b2
y3
b4
y5
b5
y6
b6
y7
b8
y8
y9
y10
b9
y11
b10
b11
y12
b12
b13
b14
y16
0
30000
60000
90000
500
1000
1500
2000
2500

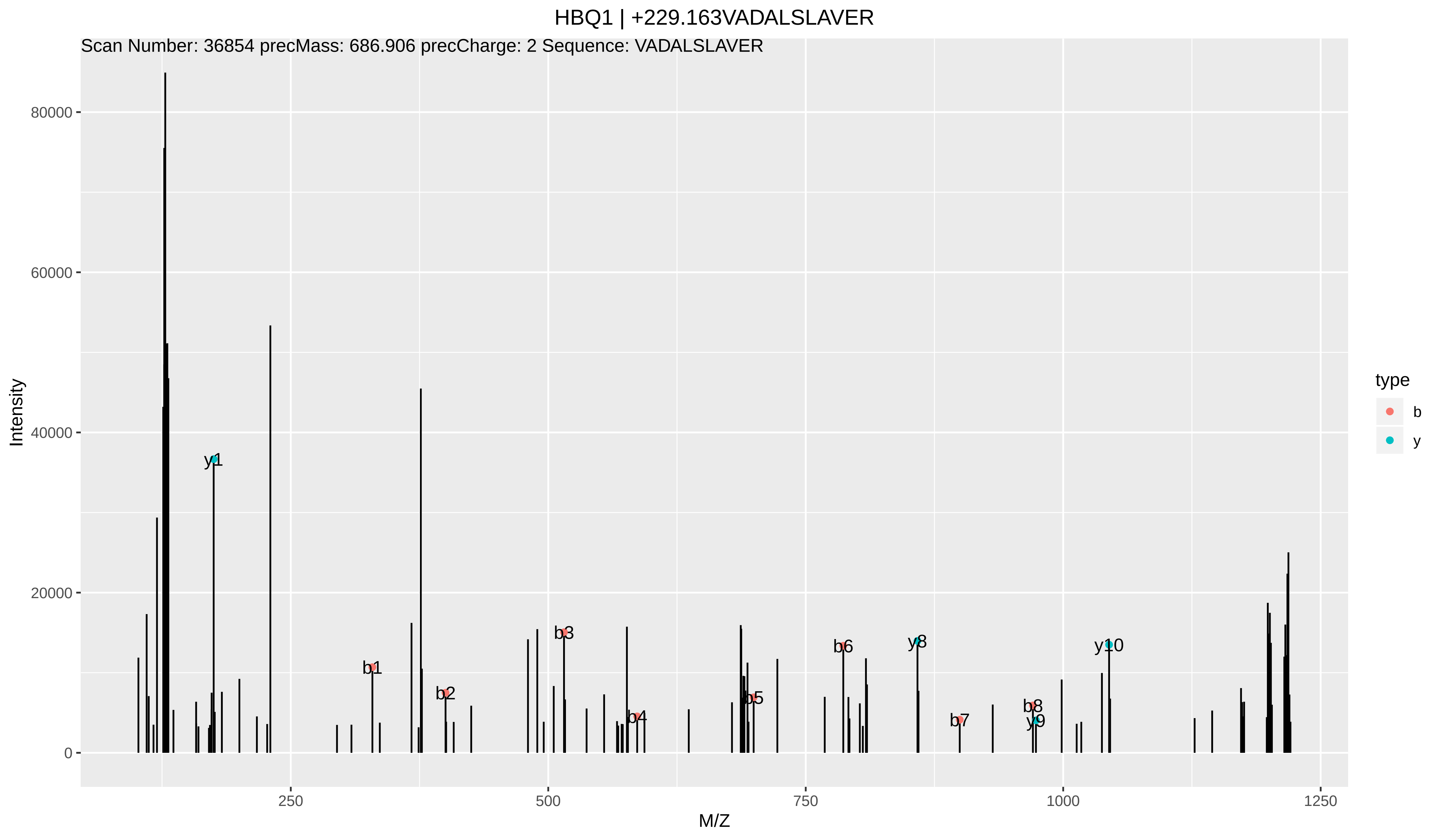
HBQ1 | +229.163VADALSLAVER
Scan Number: 36854 precMass: 686.906 precCharge: 2 Sequence: VADALSLAVER
Intensity
M/Z
type
b
y
y1
b1
b2
b3
b4
b5
b6
y8
b7
b8
y9
y10
0
20000
40000
60000
80000
250
500
750
1000
1250

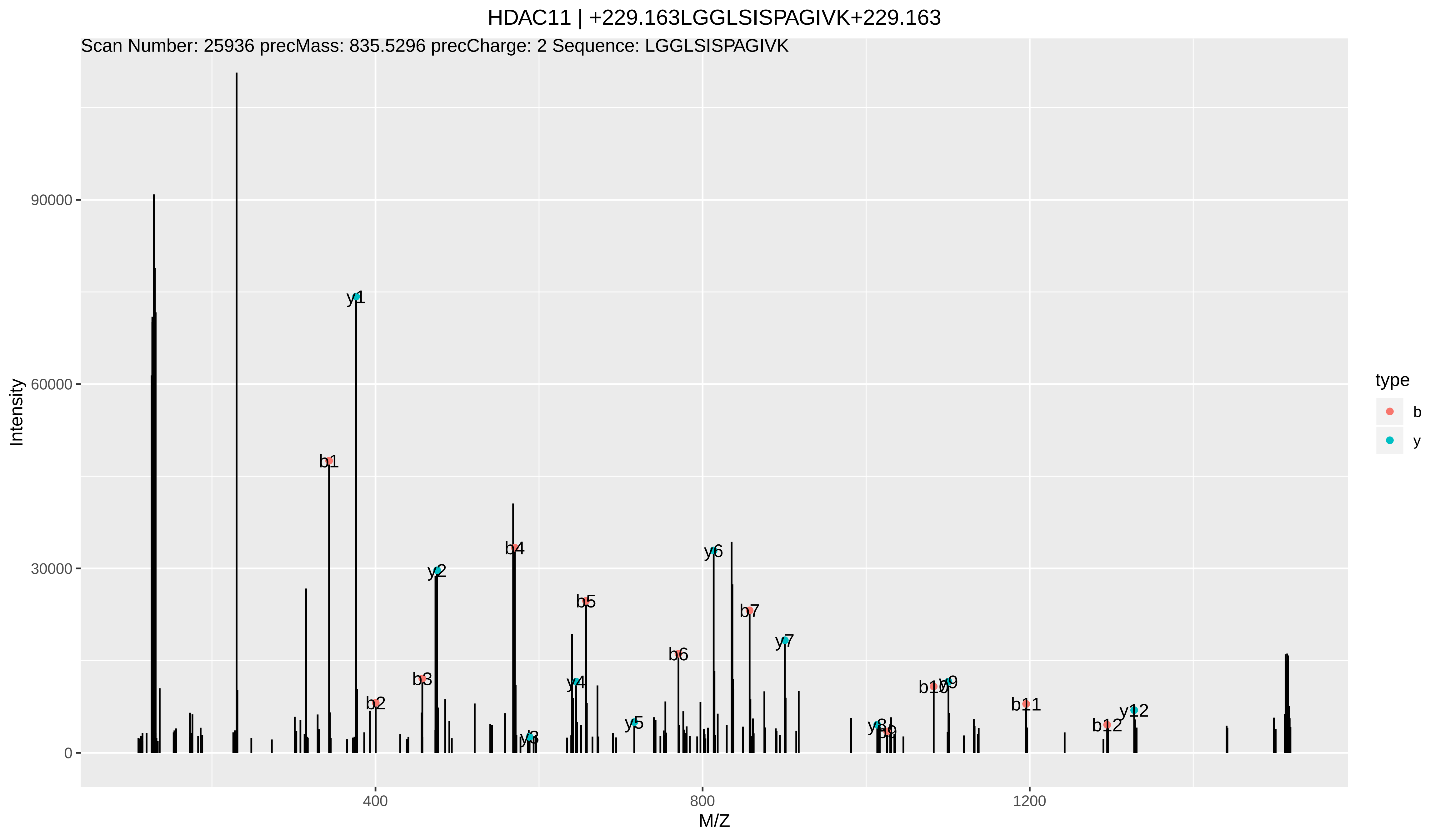
HDAC11 | +229.163LGGLSISPAGIVK+229.163
Scan Number: 25936 precMass: 835.5296 precCharge: 2 Sequence: LGGLSISPAGIVK
Intensity
90000
60000
30000
0
400
800
1200
M/Z
type
b
y
y1
b1
b2
b3
y2
b4
y3
y4
b5
y5
b6
y6
b7
y7
y8
b9
b10
y9
b11
b12
y12

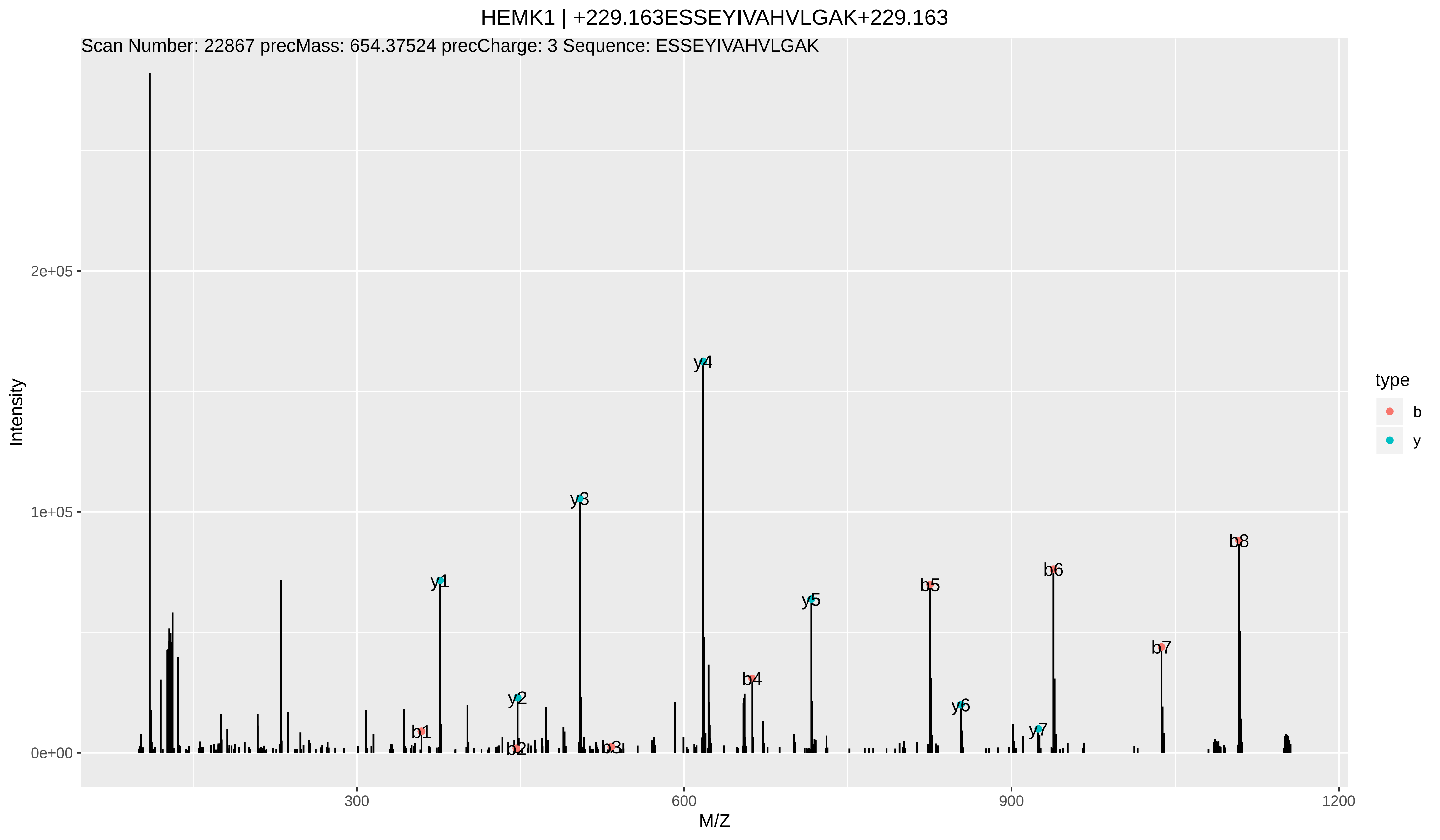
HEMK1 | +229.163ESSEYIVAHVLGAK+229.163
Scan Number: 22867 precMass: 654.37524 precCharge: 3 Sequence: ESSEYIVAHVLGAK
Intensity
2e+05
1e+05
0e+00
300
600
900
1200
M/Z
y1
y2
y3
y4
y5
y6
y7
b1
b2
b3
b4
b5
b6
b7
b8
type
b
y

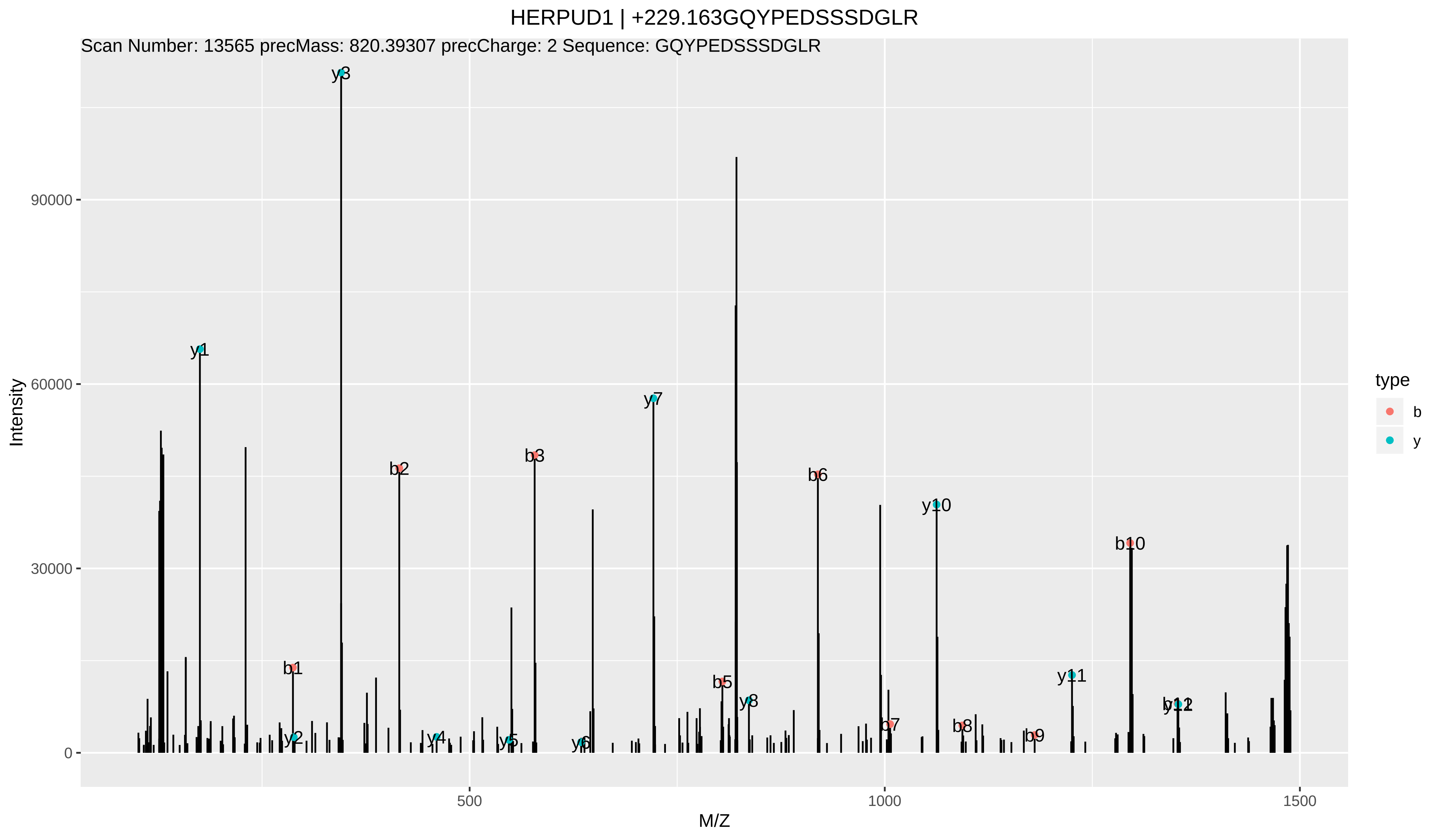

HERPUD1 | +229.163GQYPEDSSSDGLR
Scan Number: 13565 precMass: 820.39307 precCharge: 2 Sequence: GQYPEDSSSDGLR
Intensity
M/Z
type
b
y
y3
y1
y7
b2
b3
b6
y10
b10
b1
b5
y8
y11
y2
y4
y5
y6
b7
b8
b9
90000
60000
30000
0
500
1000
1500

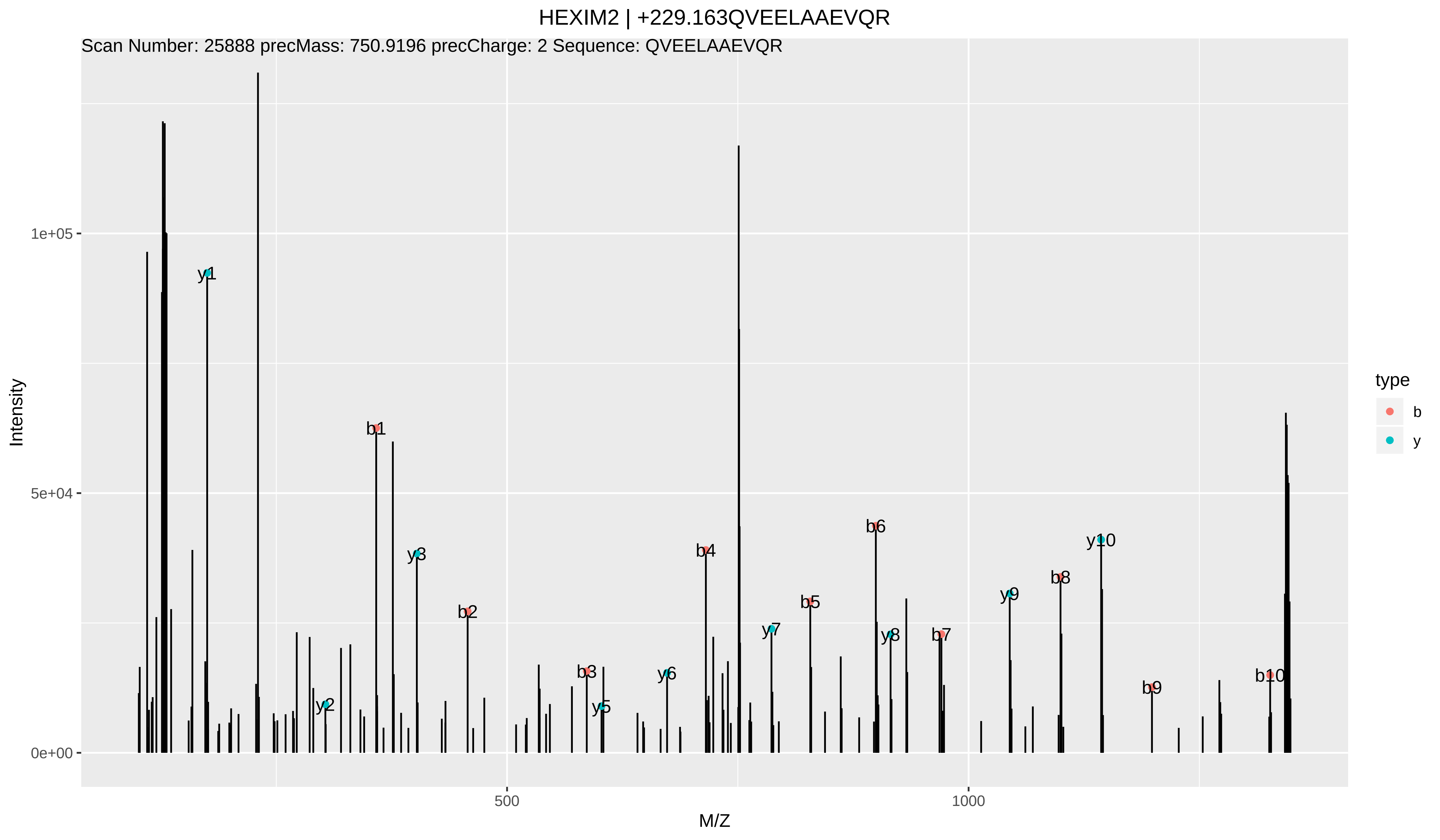
HEXIM2 | +229.163QVEELAAEVQR
Scan Number: 25888 precMass: 750.9196 precCharge: 2 Sequence: QVEELAAEVQR
Intensity
1e+05
5e+04
0e+00
M/Z
500
1000
y1
b1
y3
b2
y2
b3
y5
y6
b4
y7
b5
b6
y8
b7
y9
b8
y10
b9
b10
type
b
y

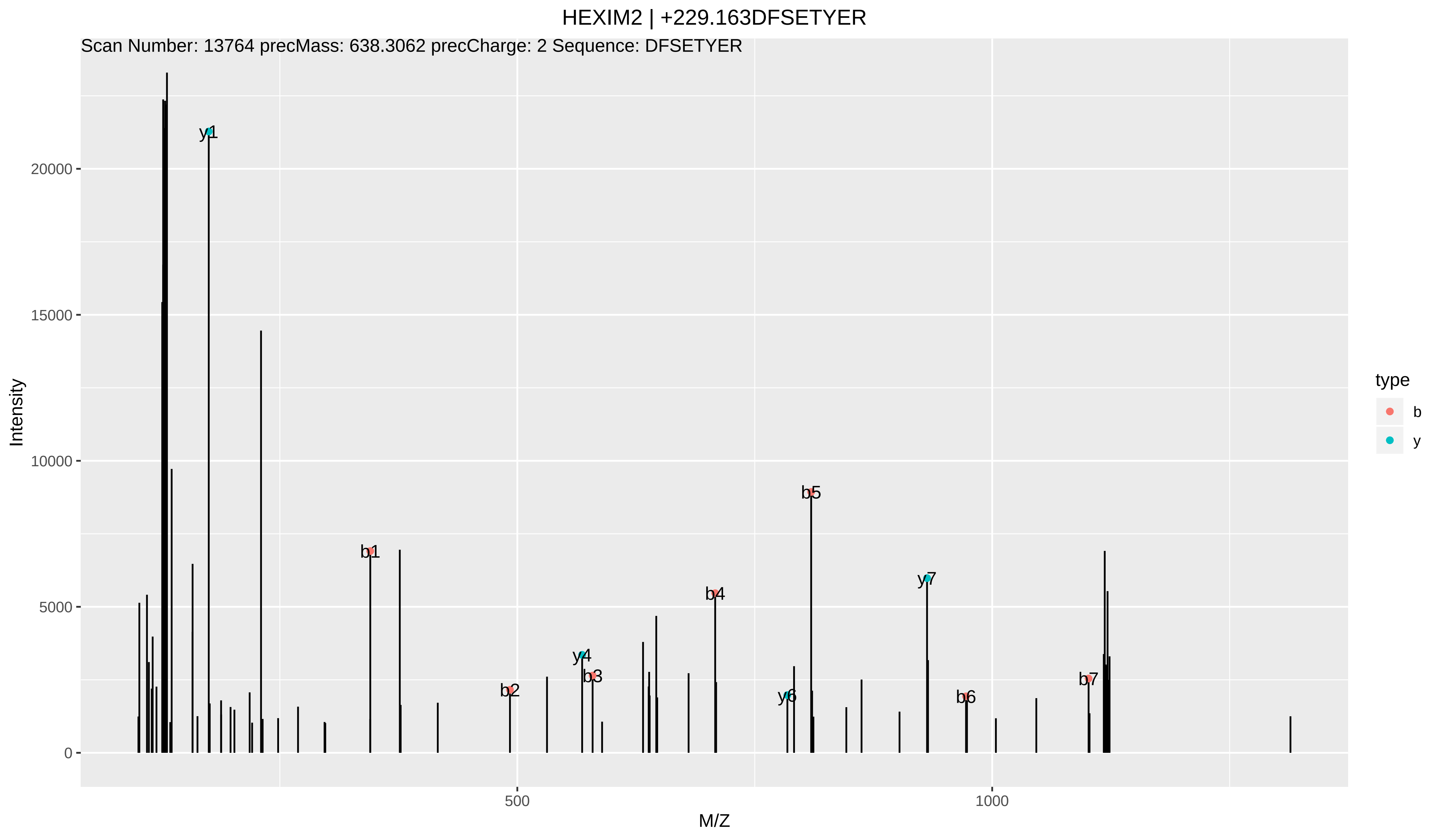
HEXIM2 | +229.163DFSETYER
Scan Number: 13764 precMass: 638.3062 precCharge: 2 Sequence: DFSETYER
Intensity
M/Z
0
5000
10000
15000
20000
500
1000
y1
b1
b2
y4
b3
b4
y6
b5
y7
b6
b7
type
b
y

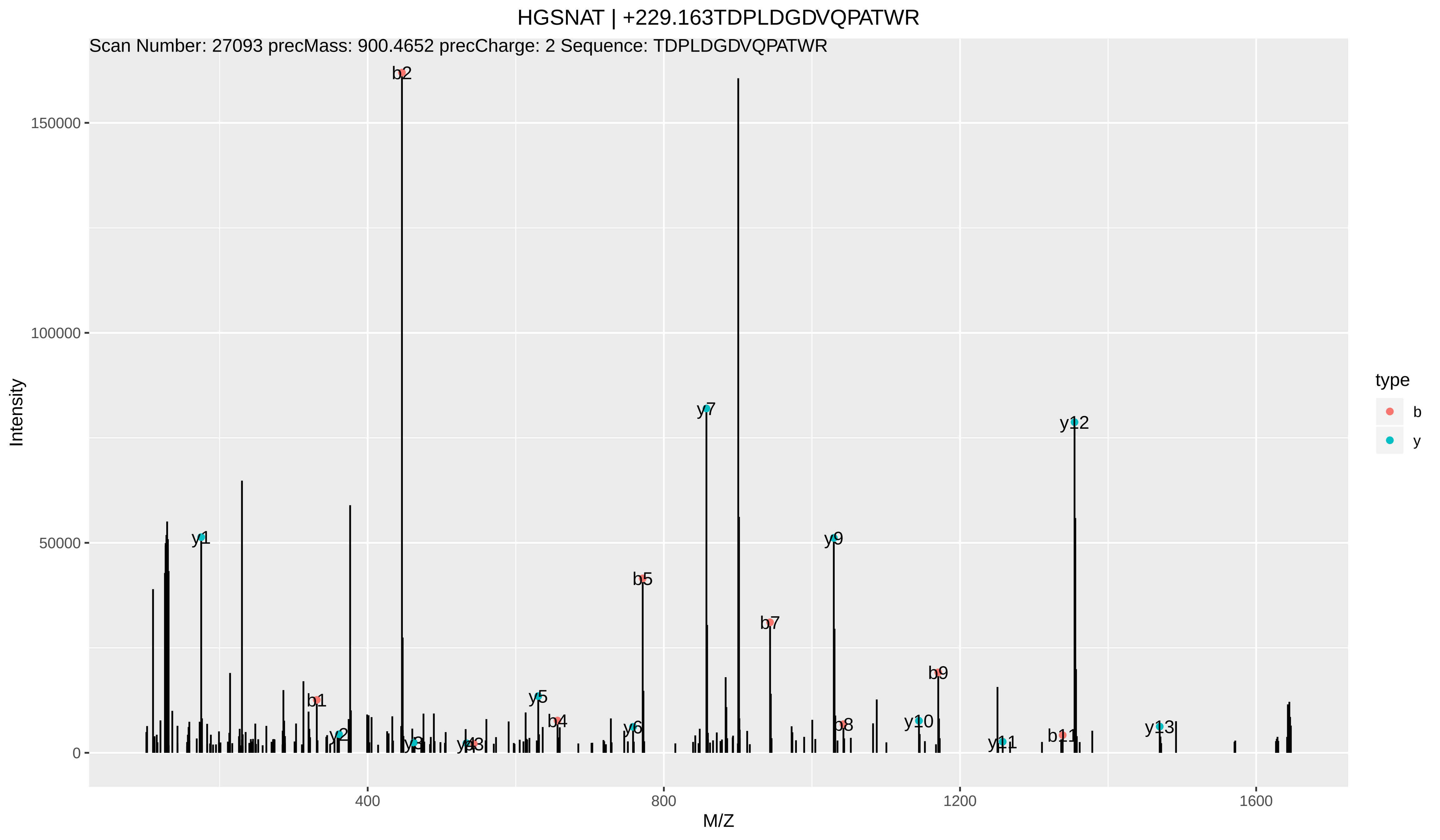
HGSNAT | +229.163TDPLDGDVQPATWR
Scan Number: 27093 precMass: 900.4652 precCharge: 2 Sequence: TDPLDGDVQPATWR
Intensity
M/Z
type
b
y
150000
100000
50000
0
400
800
1200
1600
b2
y7
y12
y1
y9
b5
b7
b9
b1
y5
b4
y10
y6
b8
y13
y2
b11
y3
y4
y11

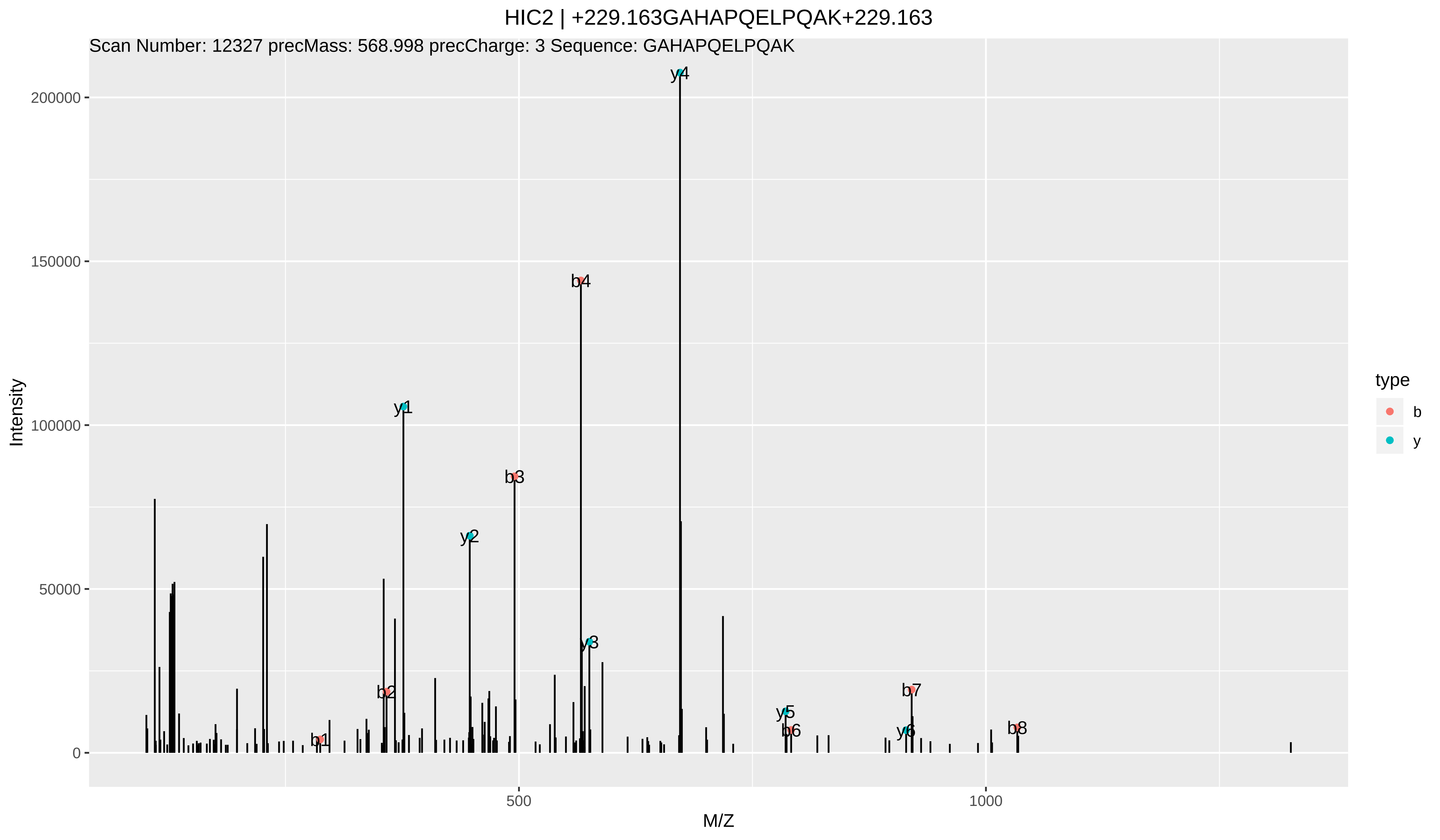
HIC2 | +229.163GAHAPQELPQAK+229.163
Scan Number: 12327 precMass: 568.998 precCharge: 3 Sequence: GAHAPQELPQAK
Intensity
M/Z
200000
150000
100000
50000
0
500
1000
y4
b4
y1
b3
y2
y3
b2
b1
y5
b6
b7
y6
b8
type
b
y

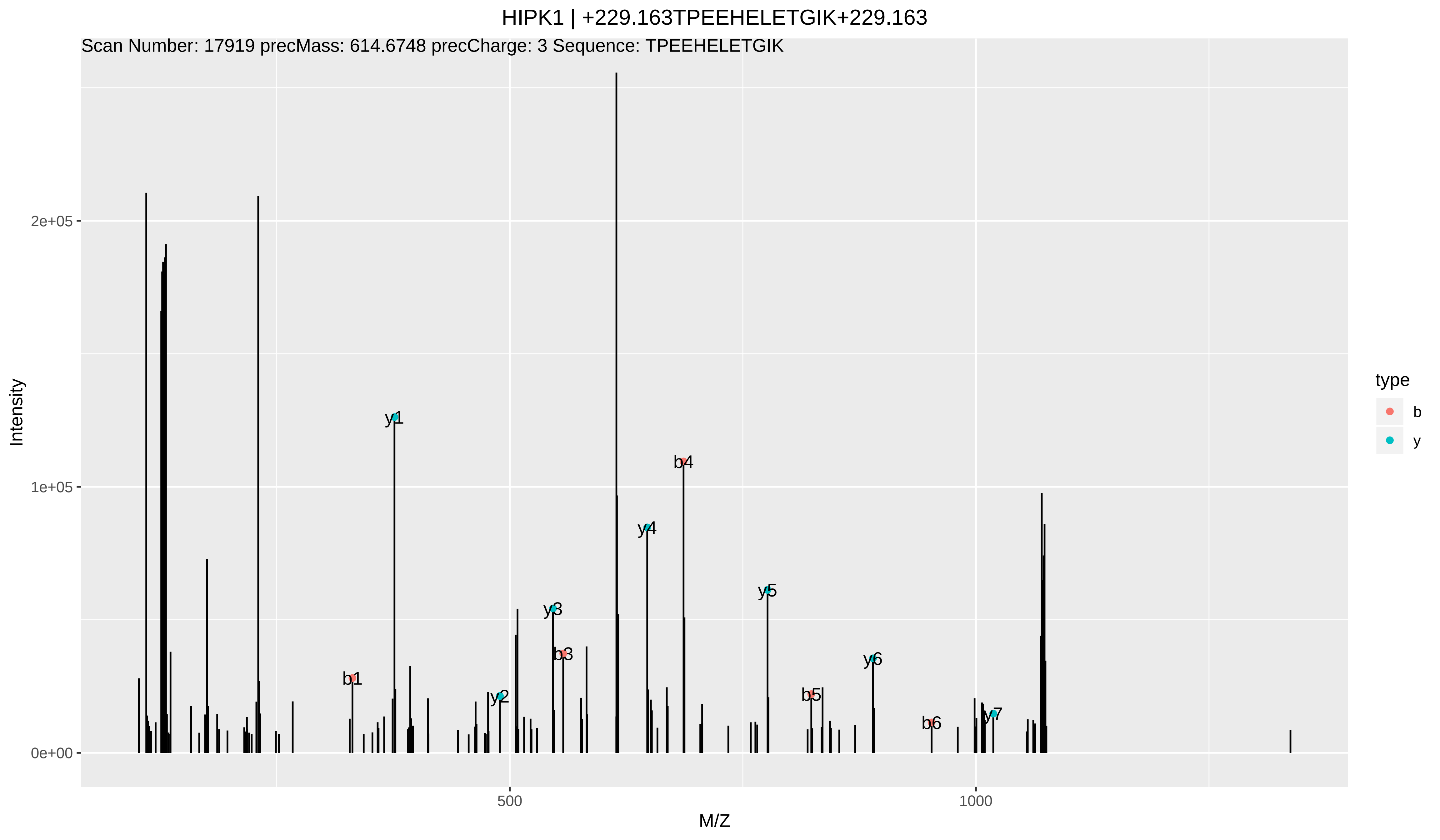
HIPK1 | +229.163TPEEHELETGIK+229.163
Scan Number: 17919 precMass: 614.6748 precCharge: 3 Sequence: TPEEHELETGIK
Intensity
2e+05
1e+05
0e+00
500
1000
M/Z
type
b
y
y1
b4
y4
y5
y3
b3
y6
b1
y2
b5
y7
b6

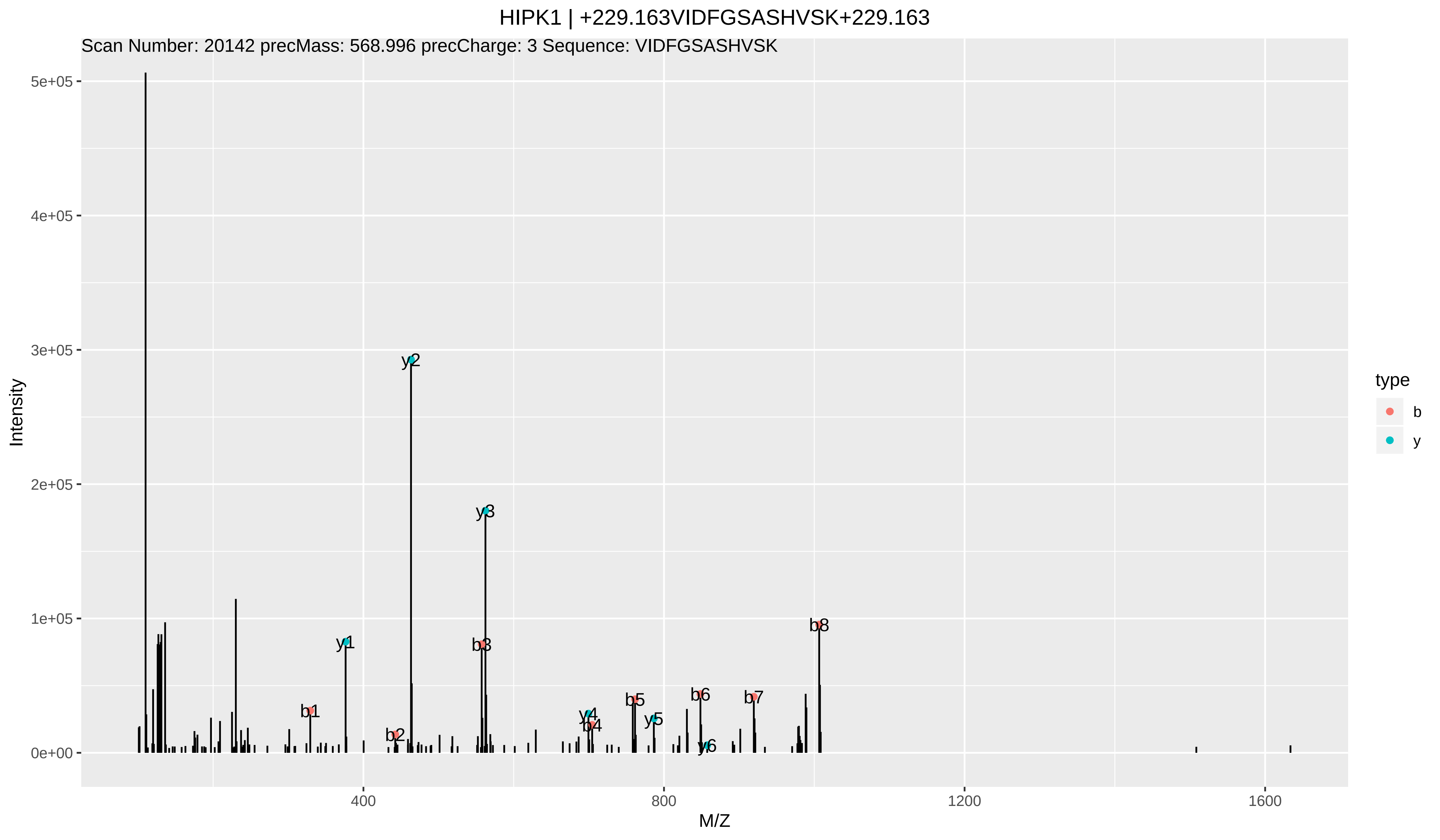

HIPK1 | +229.163VIDFGSASHVSK+229.163
Scan Number: 20142 precMass: 568.996 precCharge: 3 Sequence: VIDFGSASHVSK
Intensity
M/Z
type
b
y
y1
y2
y3
y4
y5
y6
b1
b2
b3
b4
b5
b6
b7
b8
5e+05
4e+05
3e+05
2e+05
1e+05
0e+00
400
800
1200
1600

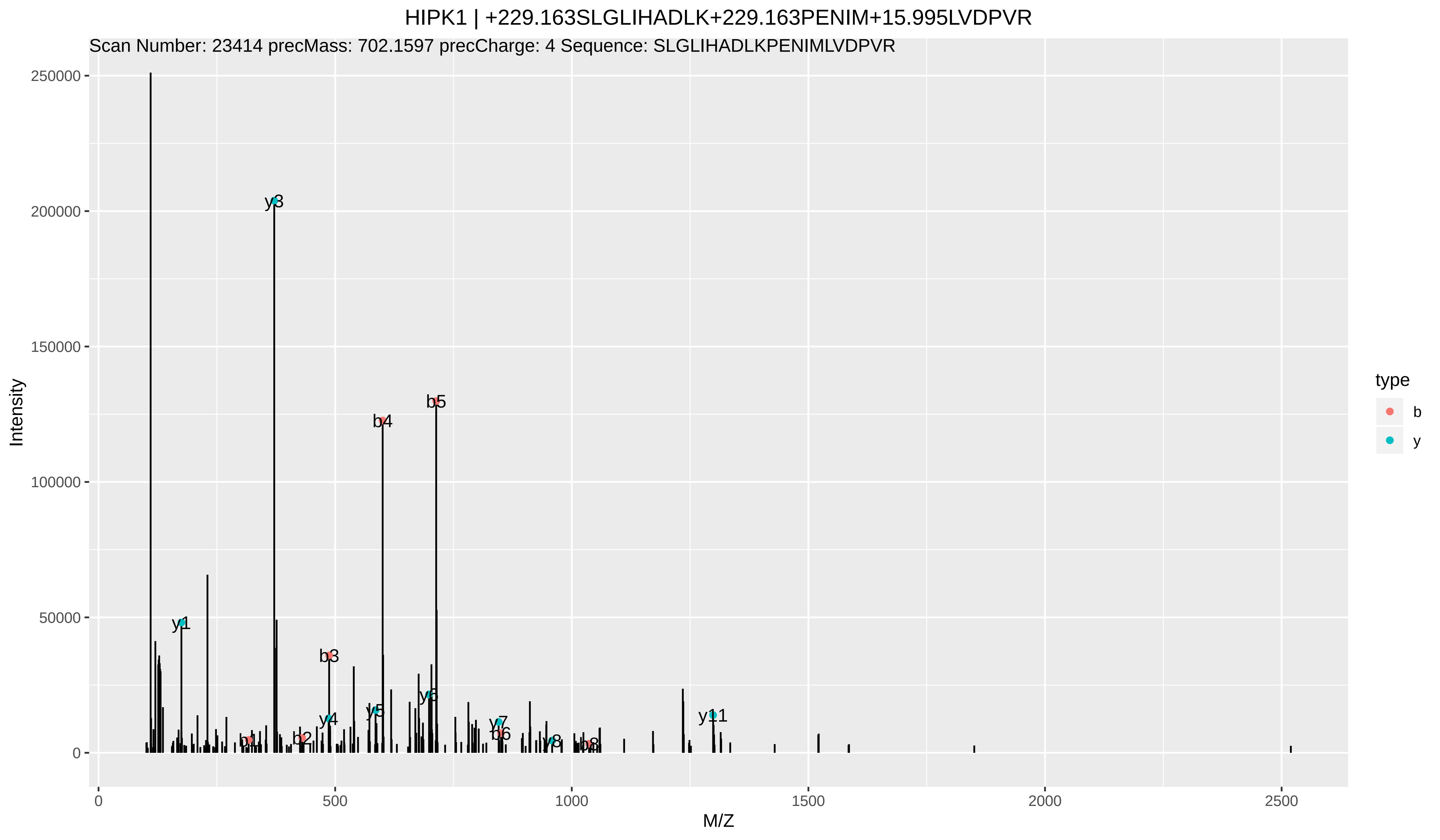

HIPK1 | +229.163SLGLIHADLK+229.163PENIM+15.995LVDPVR
Scan Number: 23414 precMass: 702.1597 precCharge: 4 Sequence: SLGLIHADLKPENIMLVDPVR
Intensity
M/Z
type
b
y
y3
b5
b4
y1
b3
y6
y5
y4
y11
y7
b6
b1
b2
y8
b8

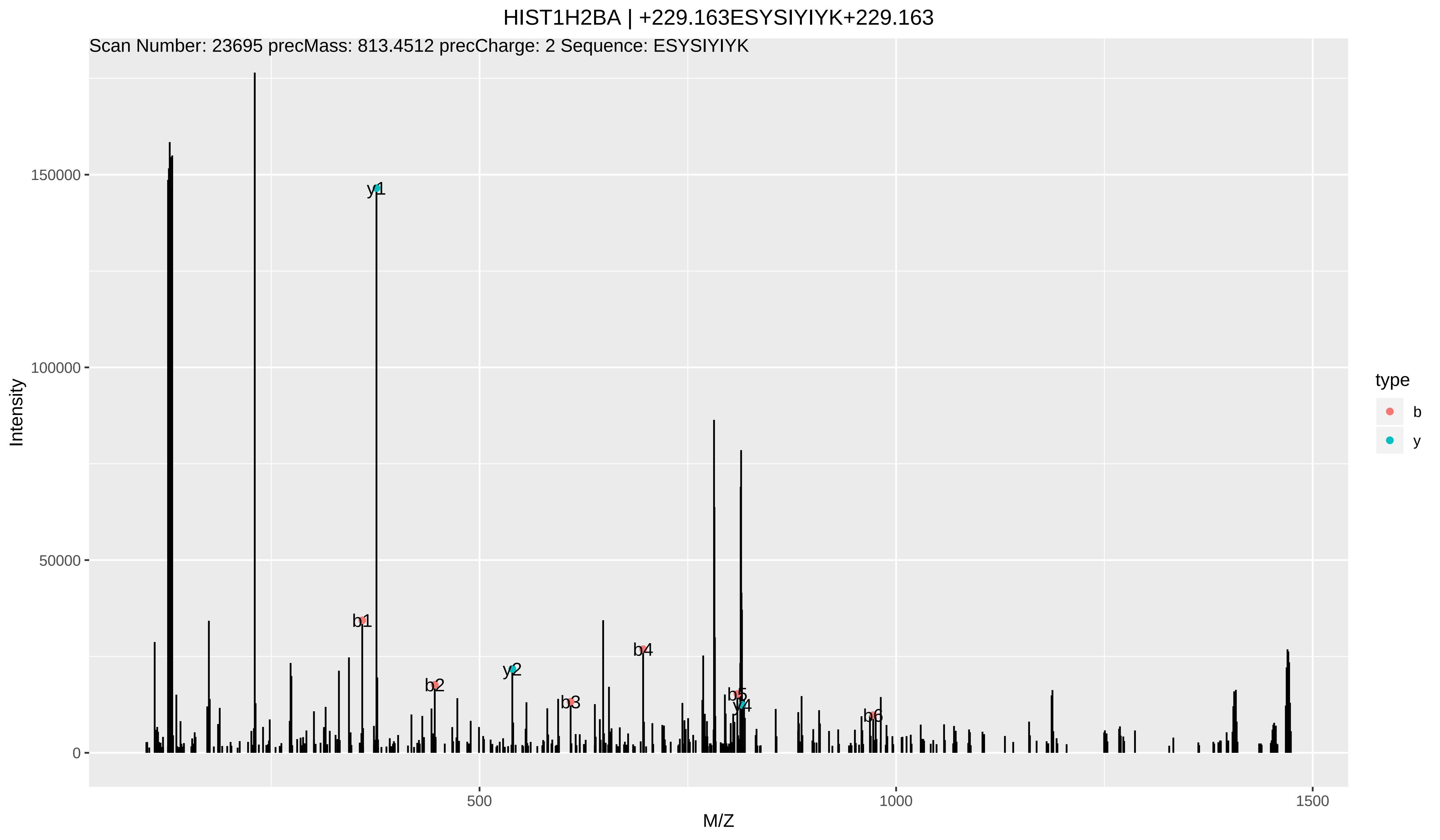
HIST1H2BA | +229.163ESYSIYIYK+229.163
Scan Number: 23695 precMass: 813.4512 precCharge: 2 Sequence: ESYSIYIYK
Intensity
M/Z
y1
b1
b2
y2
b3
b4
b5
y4
b6
0
50000
100000
150000
500
1000
1500
type
b
y

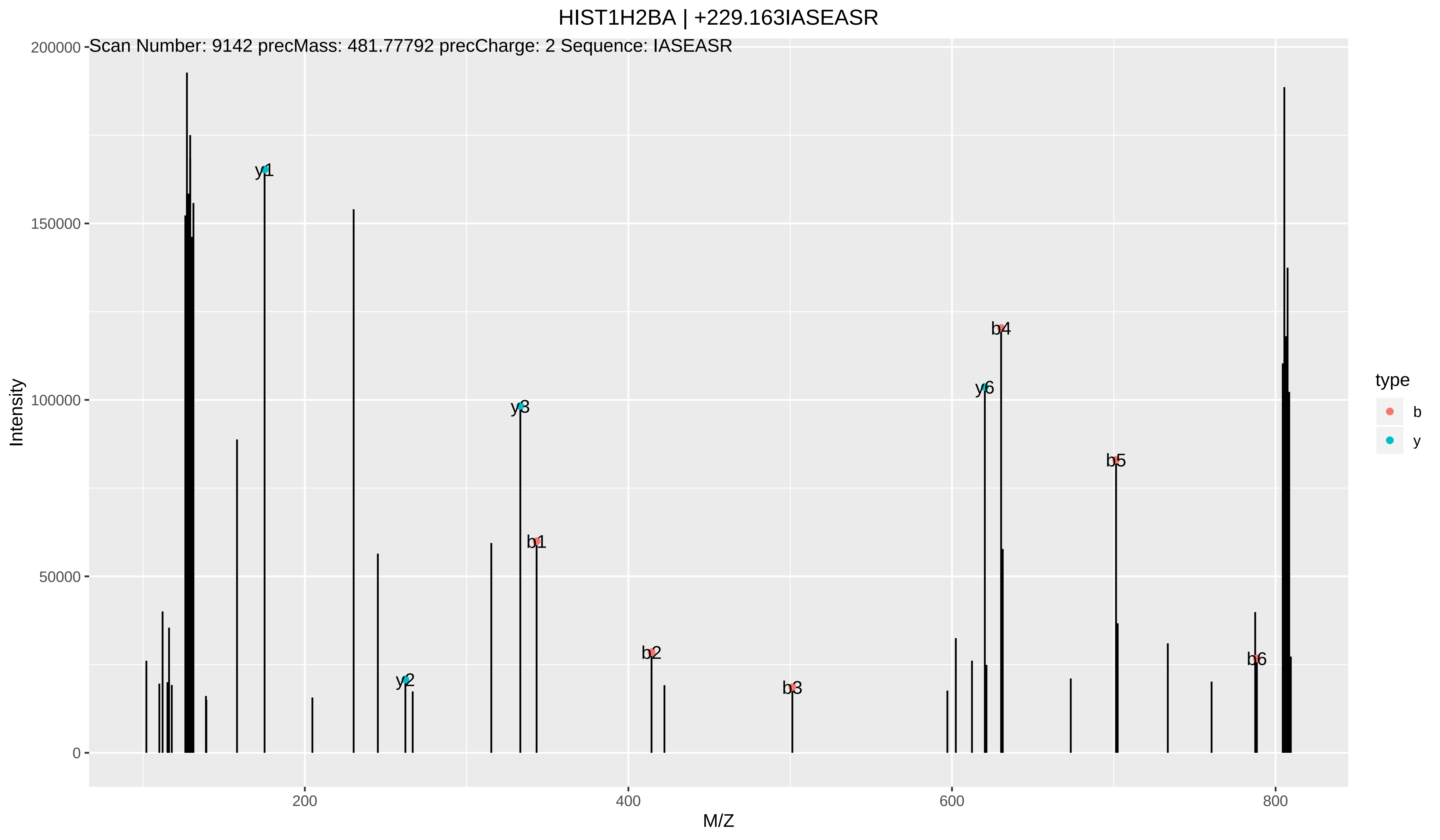

HIST1H2BA | +229.163IASEASR
Scan Number: 9142 precMass: 481.77792 precCharge: 2 Sequence: IASEASR
Intensity
200000
150000
100000
50000
0
M/Z
200
400
600
800
y1
y2
y3
y6
b1
b2
b3
b4
b5
b6
type
b
y

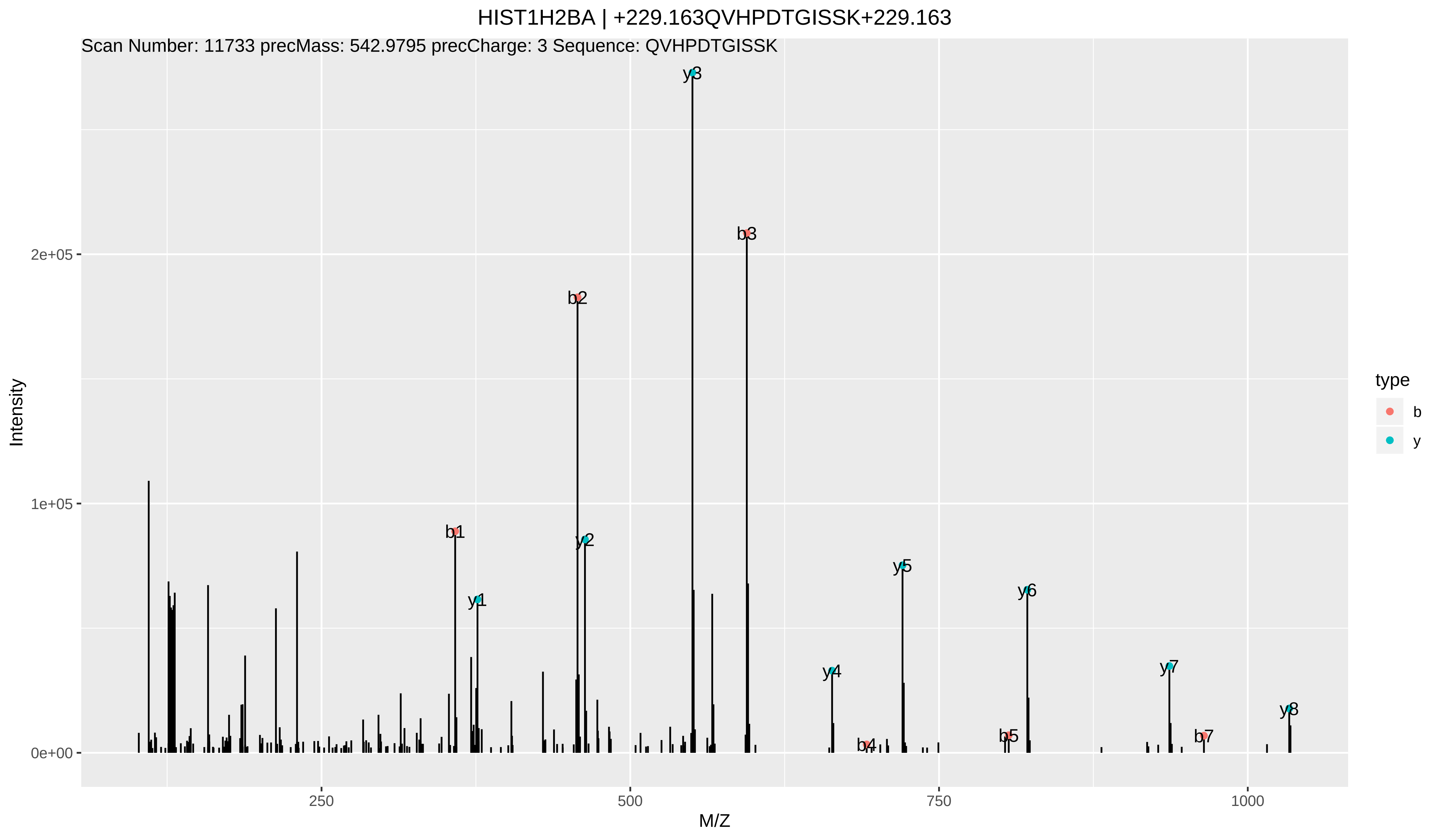

HIST1H2BA | +229.163QVHPDTGISSK+229.163
Scan Number: 11733 precMass: 542.9795 precCharge: 3 Sequence: QVHPDTGISSK
Intensity
M/Z
0e+00
1e+05
2e+05
250
500
750
1000
y3
b3
b2
b1
y2
y1
y5
y6
y4
y7
y8
b4
b5
b7
type
b
y

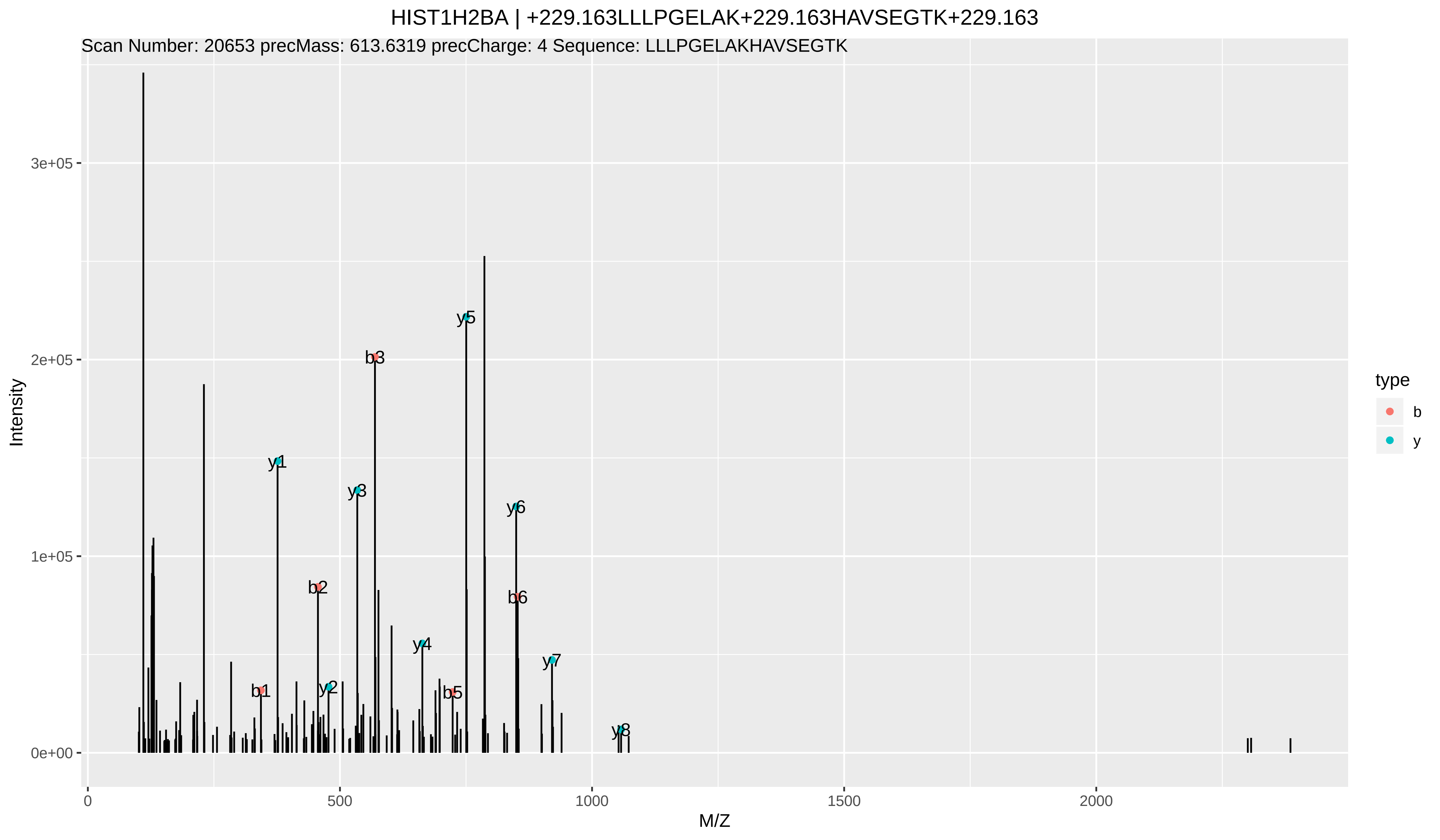
HIST1H2BA | +229.163LLLPGELAK+229.163HAVSEGTK+229.163
Scan Number: 20653 precMass: 613.6319 precCharge: 4 Sequence: LLLPGELAKHAVSEGTK
Intensity
3e+05
2e+05
1e+05
0e+00
M/Z
0
500
1000
1500
2000
y5
b3
y1
y3
y6
b2
b6
y4
y7
b1
y2
b5
y8
type
b
y

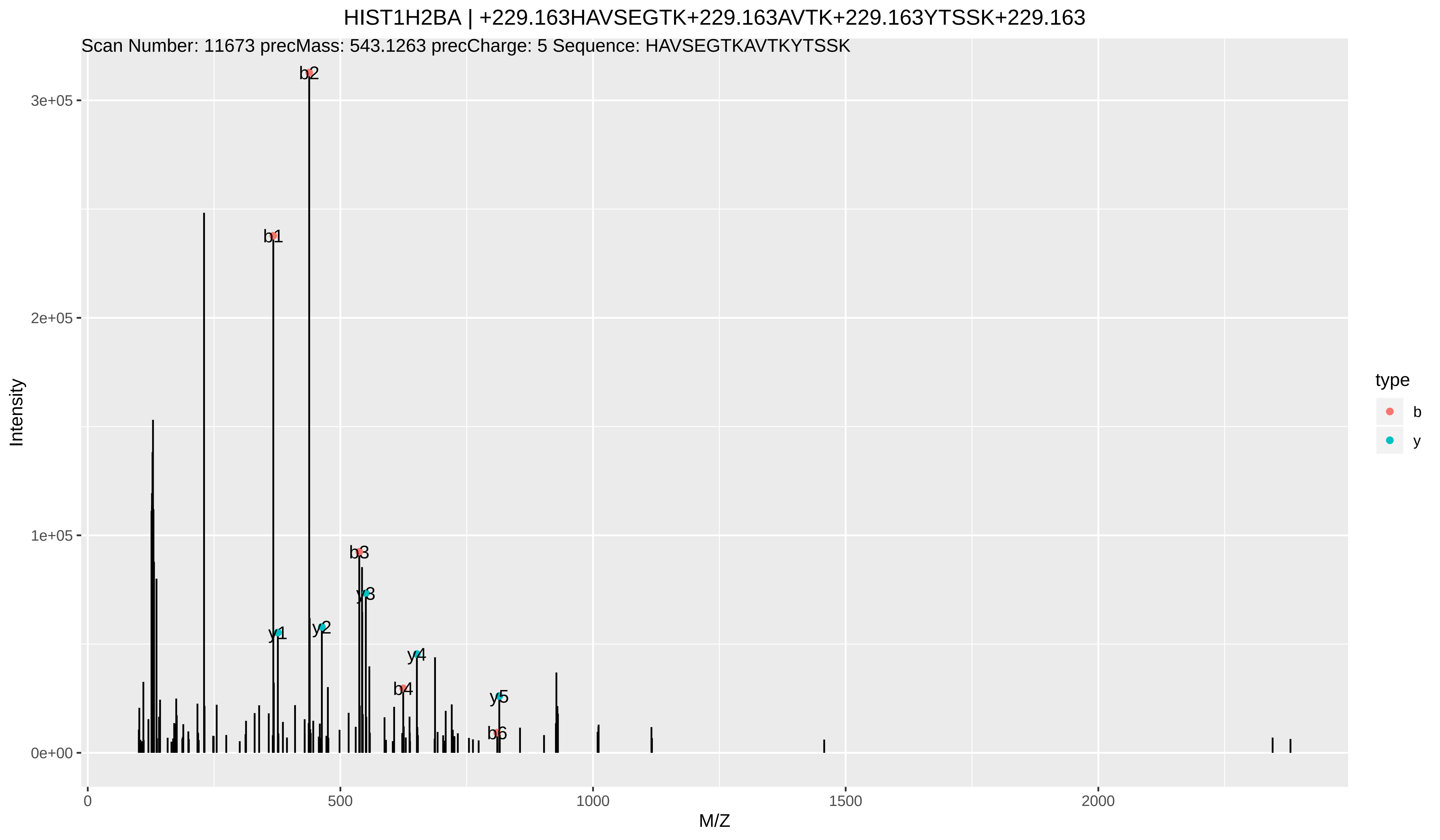
HIST1H2BA | +229.163HAVSEGTK+229.163AVTK+229.163YTSSK+229.163
Scan Number: 11673 precMass: 543.1263 precCharge: 5 Sequence: HAVSEGTKAVTKYTSSK
Intensity
M/Z
3e+05
2e+05
1e+05
0e+00
0
500
1000
1500
2000
b1
b2
b3
b4
b6
y1
y2
y3
y4
y5
type
b
y

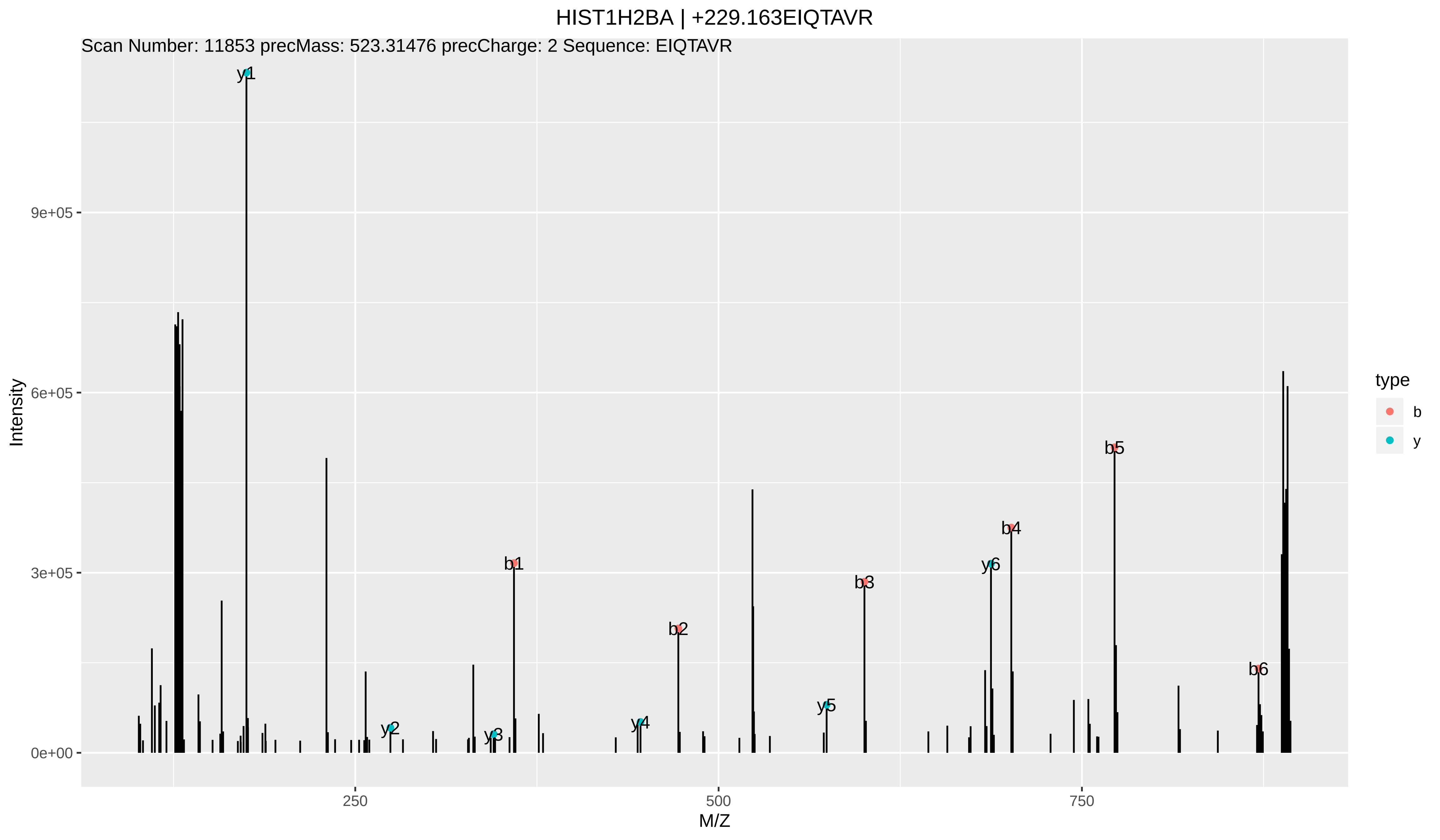
HIST1H2BA | +229.163EIQTAVR
Scan Number: 11853 precMass: 523.31476 precCharge: 2 Sequence: EIQTAVR
y1
b5
b4
b1
y6
b3
b2
b6
y5
y4
y2
y3
9e+05
6e+05
3e+05
0e+00
Intensity
250
500
750
M/Z
type
b
y

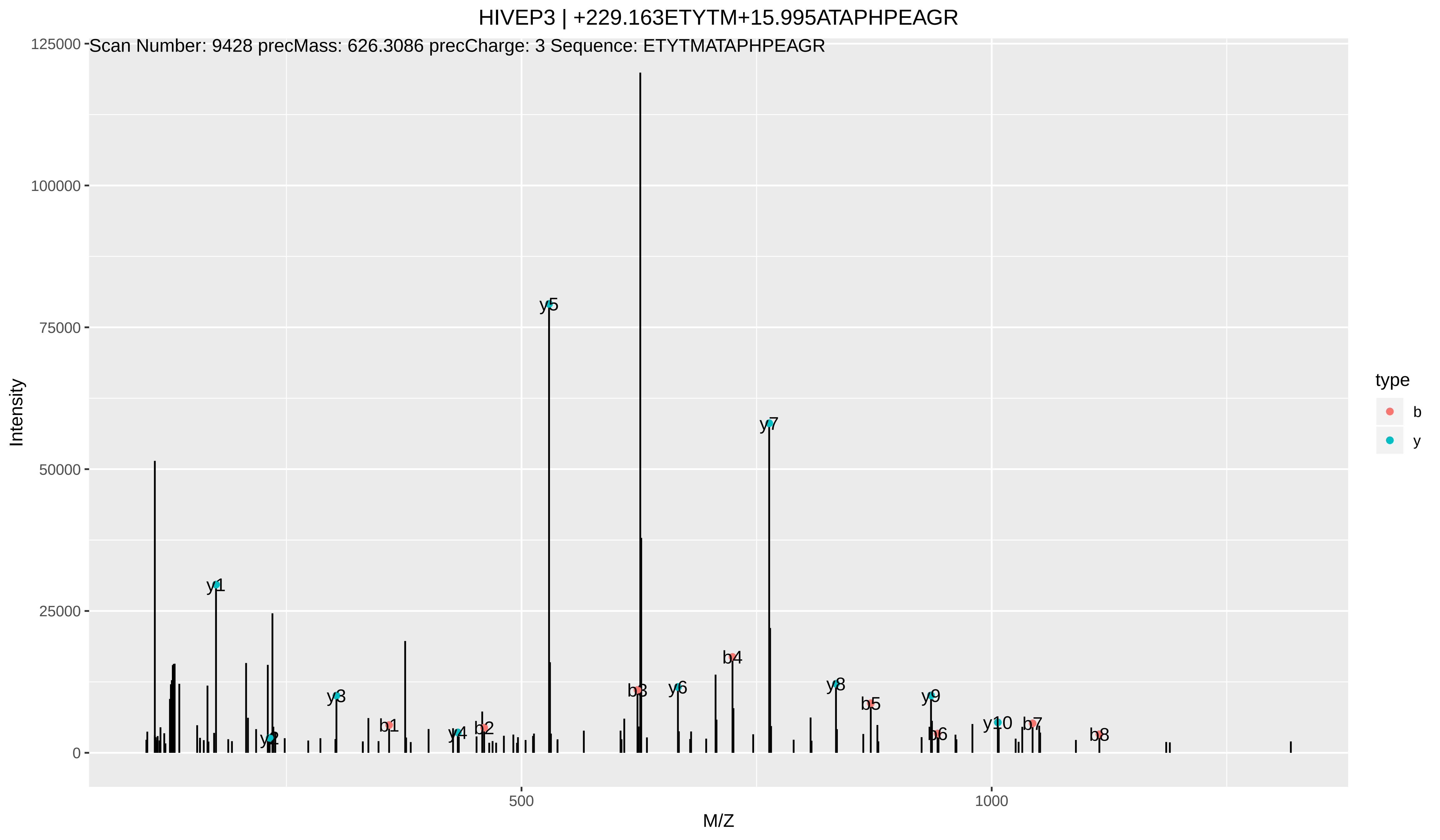
HIVEP3 | +229.163ETYTM+15.995ATAPHPEAGR
Scan Number: 9428 precMass: 626.3086 precCharge: 3 Sequence: ETYTMATAPHPEAGR
Intensity
125000
100000
75000
50000
25000
0
M/Z
500
1000
y1
y2
y3
b1
y4
b2
y5
b3
y6
b4
y7
y8
b5
y9
b6
y10
b7
b8
type
b
y

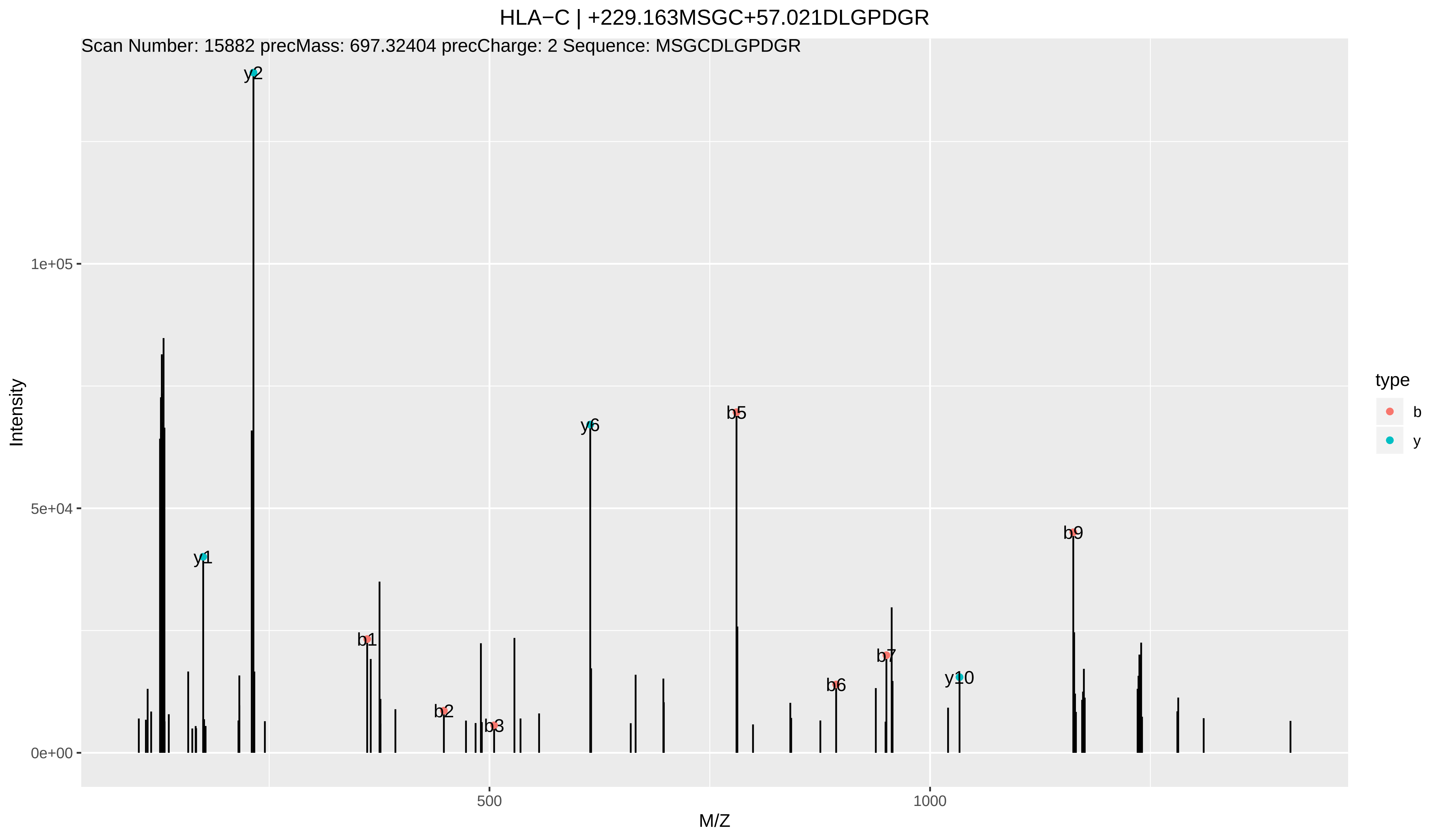
HLA−C | +229.163MSGC+57.021DLGPDGR
Scan Number: 15882 precMass: 697.32404 precCharge: 2 Sequence: MSGCDLGPDGR
y2
y1
b1
b2
b3
y6
b5
b6
b7
y10
b9
Intensity
1e+05
5e+04
0e+00
M/Z
500
1000
type
b
y

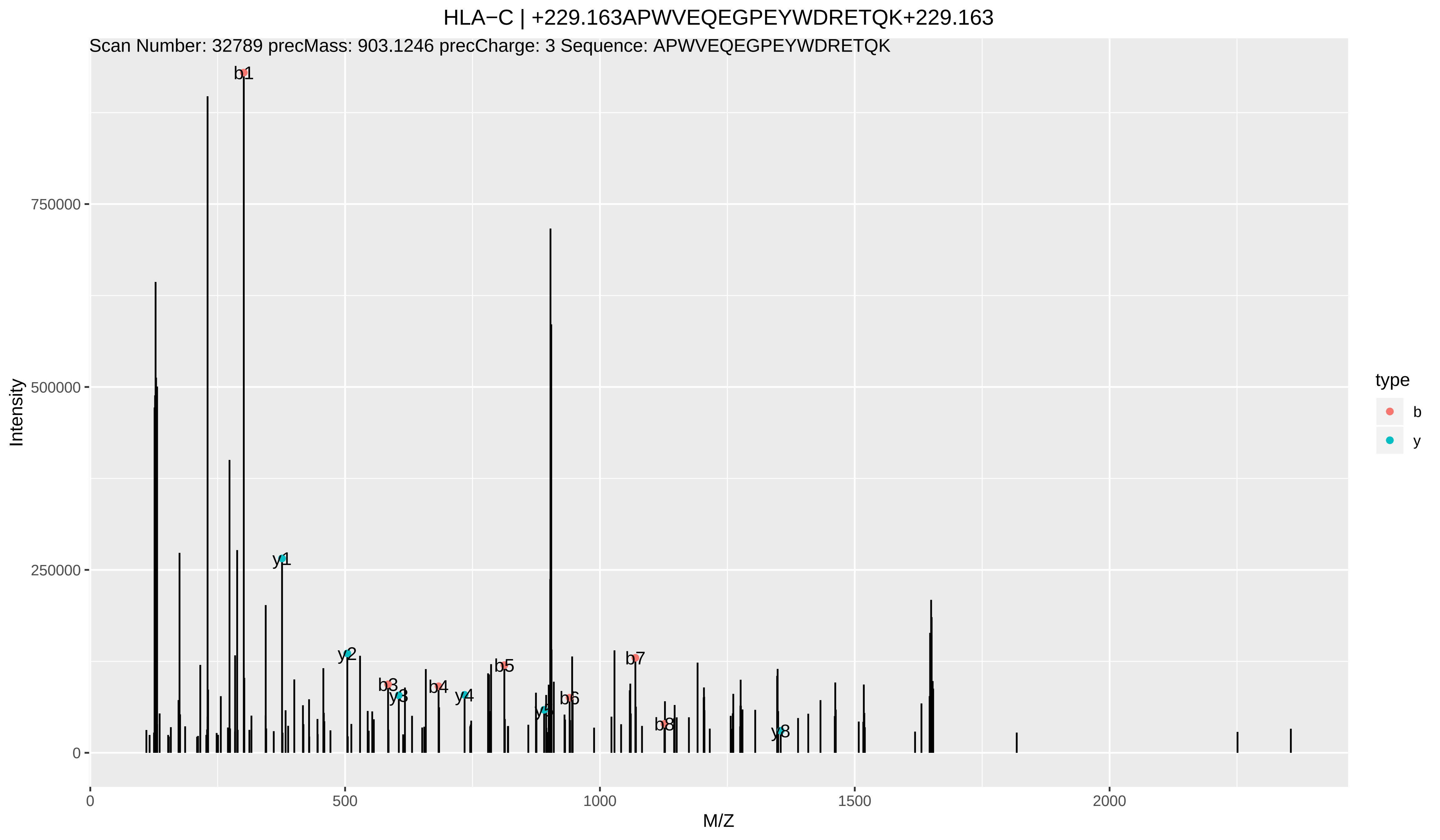
HLA−C | +229.163APWVEQEGPEYWDRETQK+229.163
Scan Number: 32789 precMass: 903.1246 precCharge: 3 Sequence: APWVEQEGPEYWDRETQK
Intensity
M/Z
type
b
y
b1
y1
y2
b3
y3
b4
y4
b5
b6
b7
b8
y8
0
250000
500000
750000
0
500
1000
1500
2000

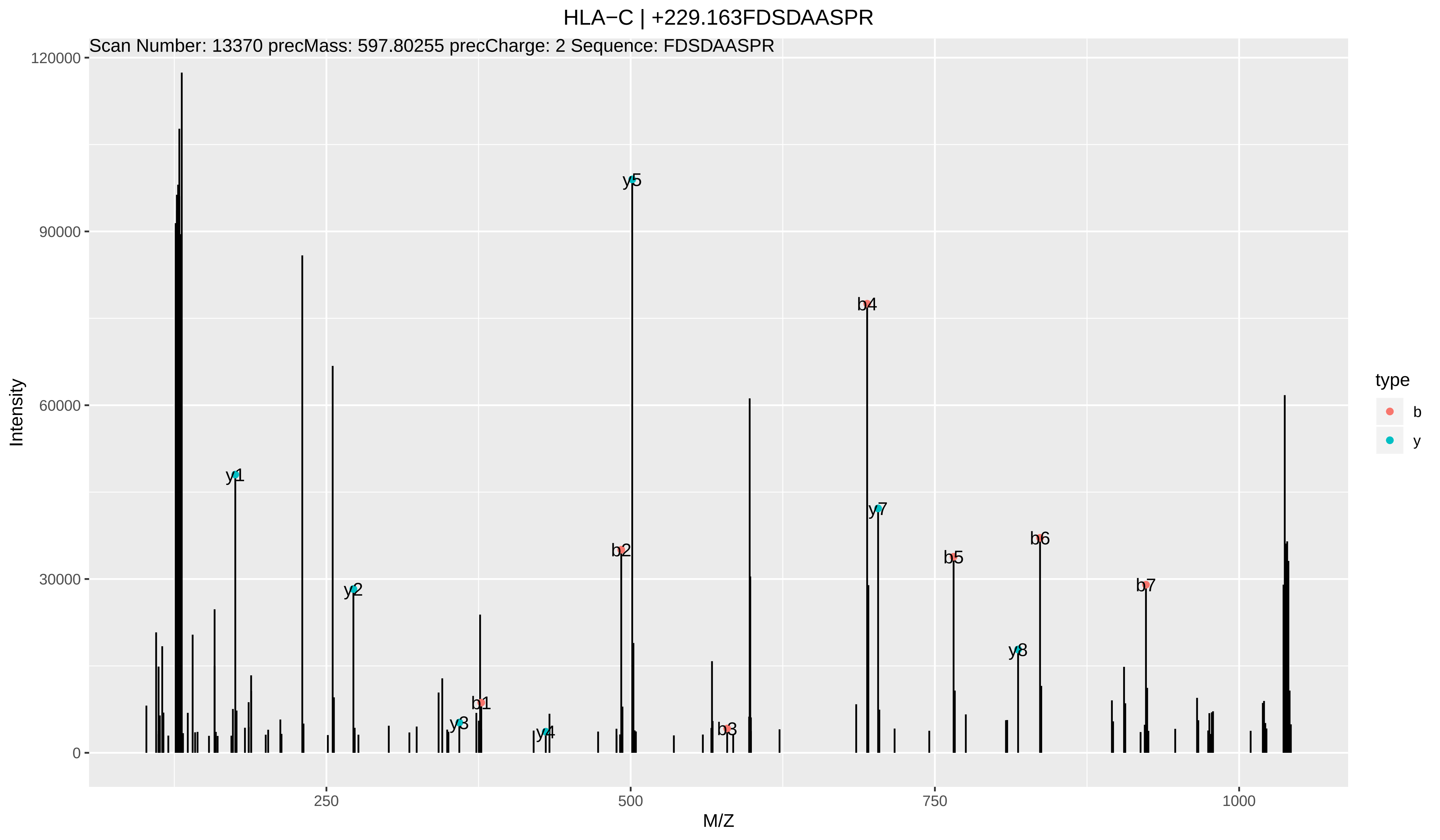

HLA−C | +229.163FDSDAASPR
Scan Number: 13370 precMass: 597.80255 precCharge: 2 Sequence: FDSDAASPR
Intensity
M/Z
0
30000
60000
90000
120000
250
500
750
1000
y1
y2
y3
b1
y4
b2
y5
b3
b4
y7
b5
y8
b6
b7
type
b
y

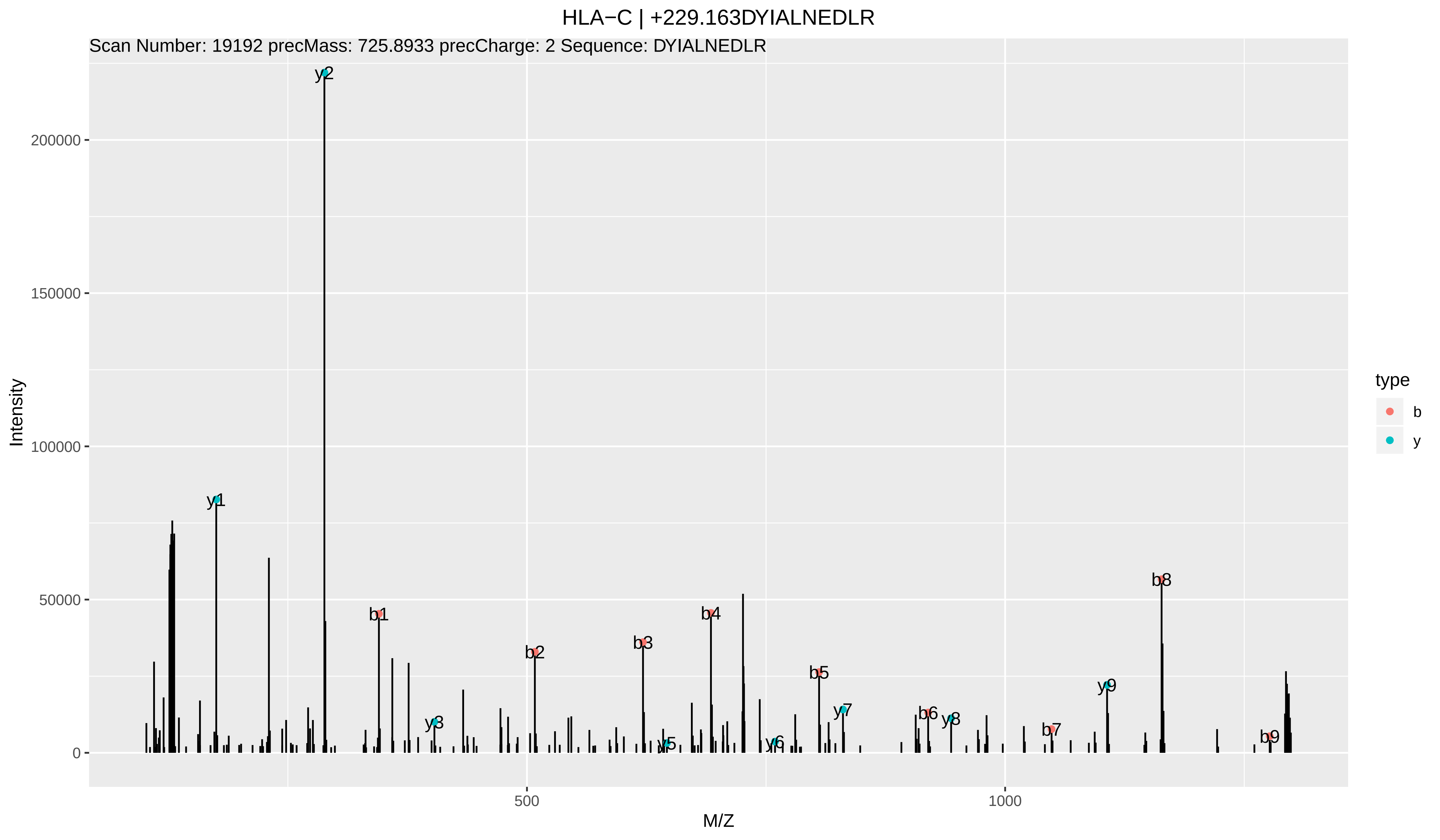
HLA−C | +229.163DYIALNEDLR
Scan Number: 19192 precMass: 725.8933 precCharge: 2 Sequence: DYIALNEDLR
Intensity
M/Z
0
50000
100000
150000
200000
500
1000
y1
y2
y3
y5
y6
y7
y8
y9
b1
b2
b3
b4
b5
b6
b7
b8
b9
type
b
y

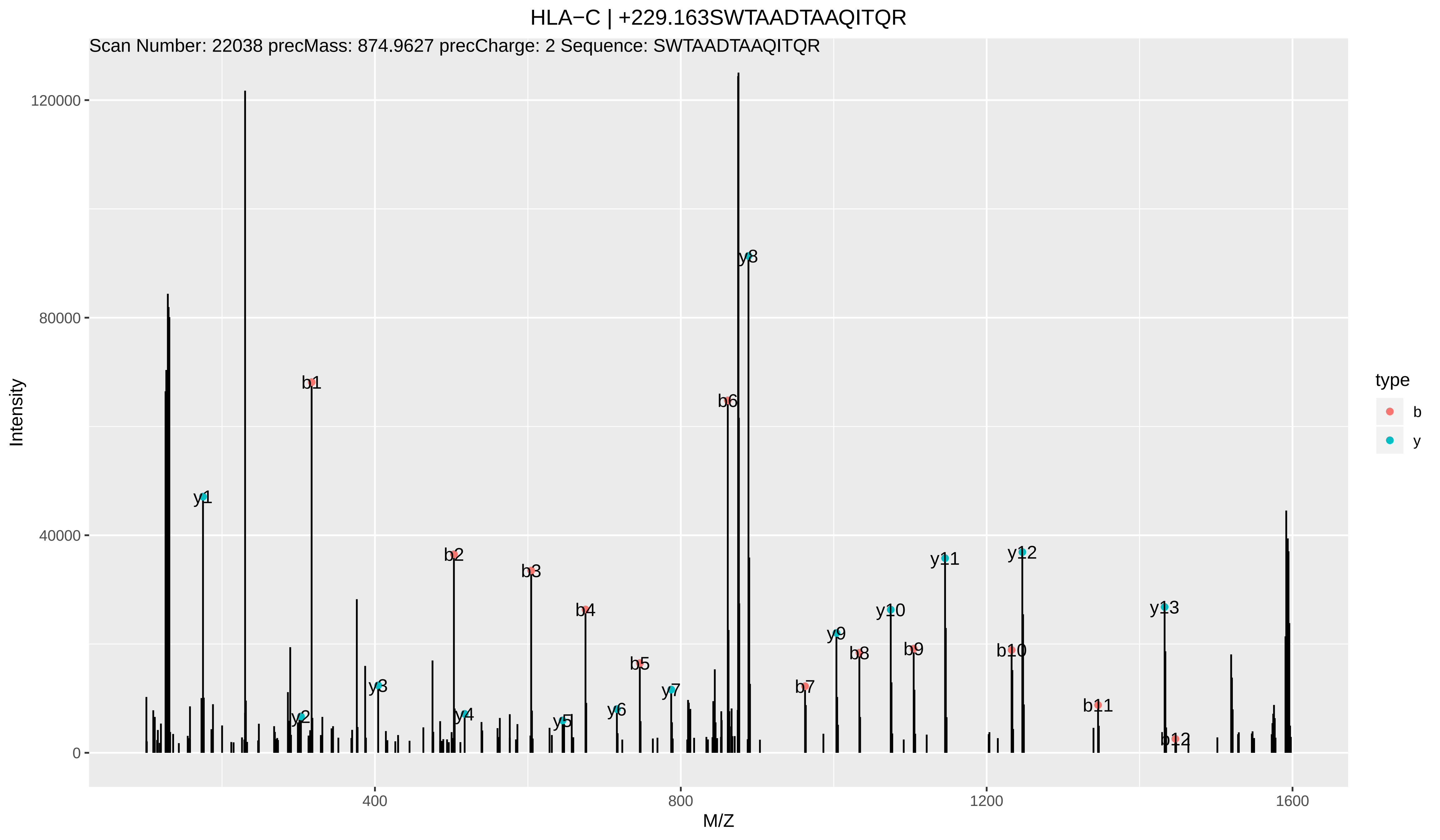
HLA−C | +229.163SWTAADTAAQITQR
Scan Number: 22038 precMass: 874.9627 precCharge: 2 Sequence: SWTAADTAAQITQR
Intensity
M/Z
0
40000
80000
120000
400
800
1200
1600
y1
b1
y2
b2
y3
b3
y4
b4
y5
b5
y6
b6
y7
b7
y8
b8
y9
b9
y10
b10
y11
b11
y12
b12
y13
type
b
y

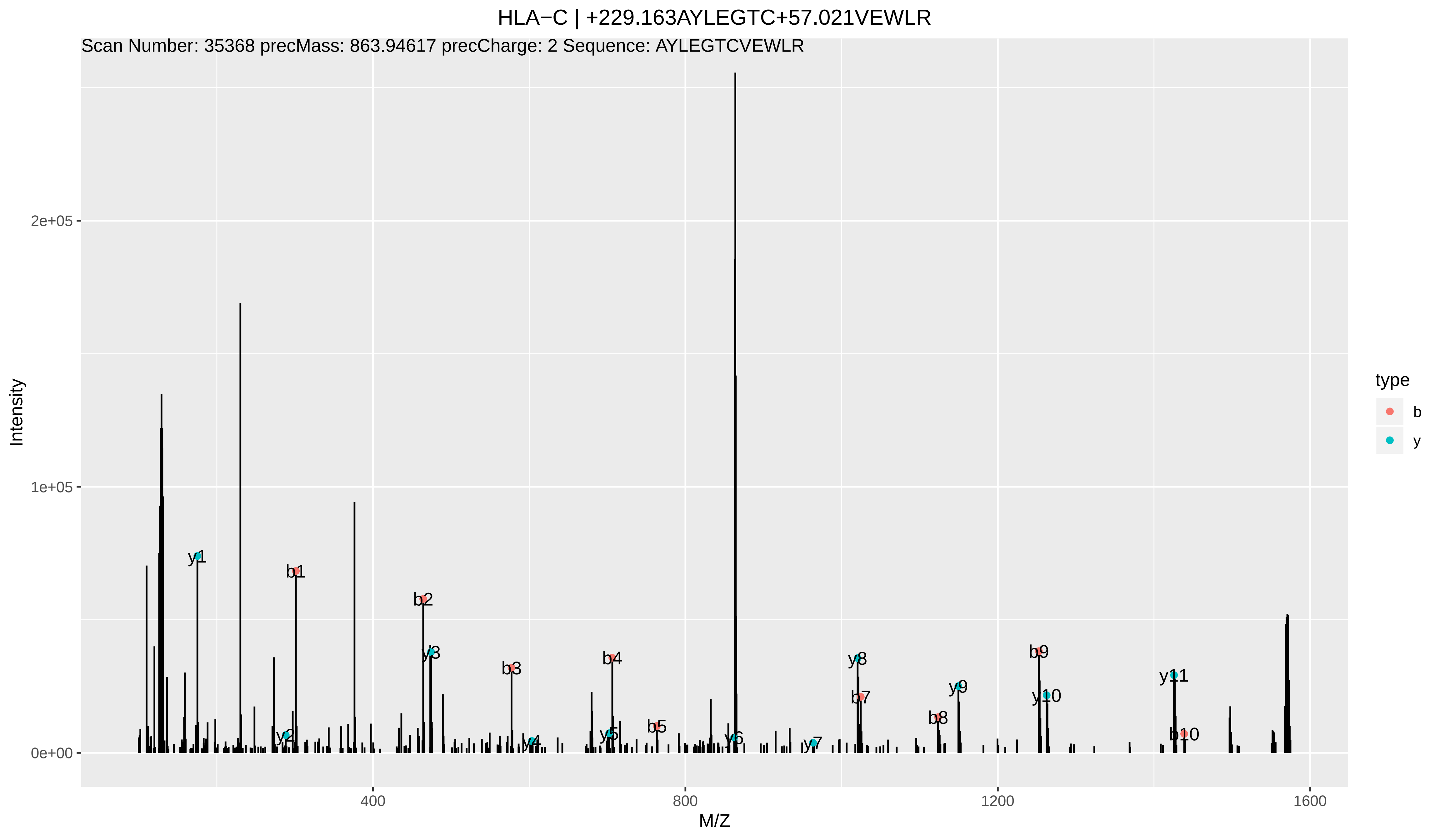
HLA−C | +229.163AYLEGTC+57.021VEWLR
Scan Number: 35368 precMass: 863.94617 precCharge: 2 Sequence: AYLEGTCVEWLR
Intensity
2e+05
1e+05
0e+00
M/Z
400
800
1200
1600
type
b
y
y1
b1
b2
y3
b3
b4
y2
y4
y5
b5
y6
y7
y8
b7
b8
y9
b9
y10
y11
b10

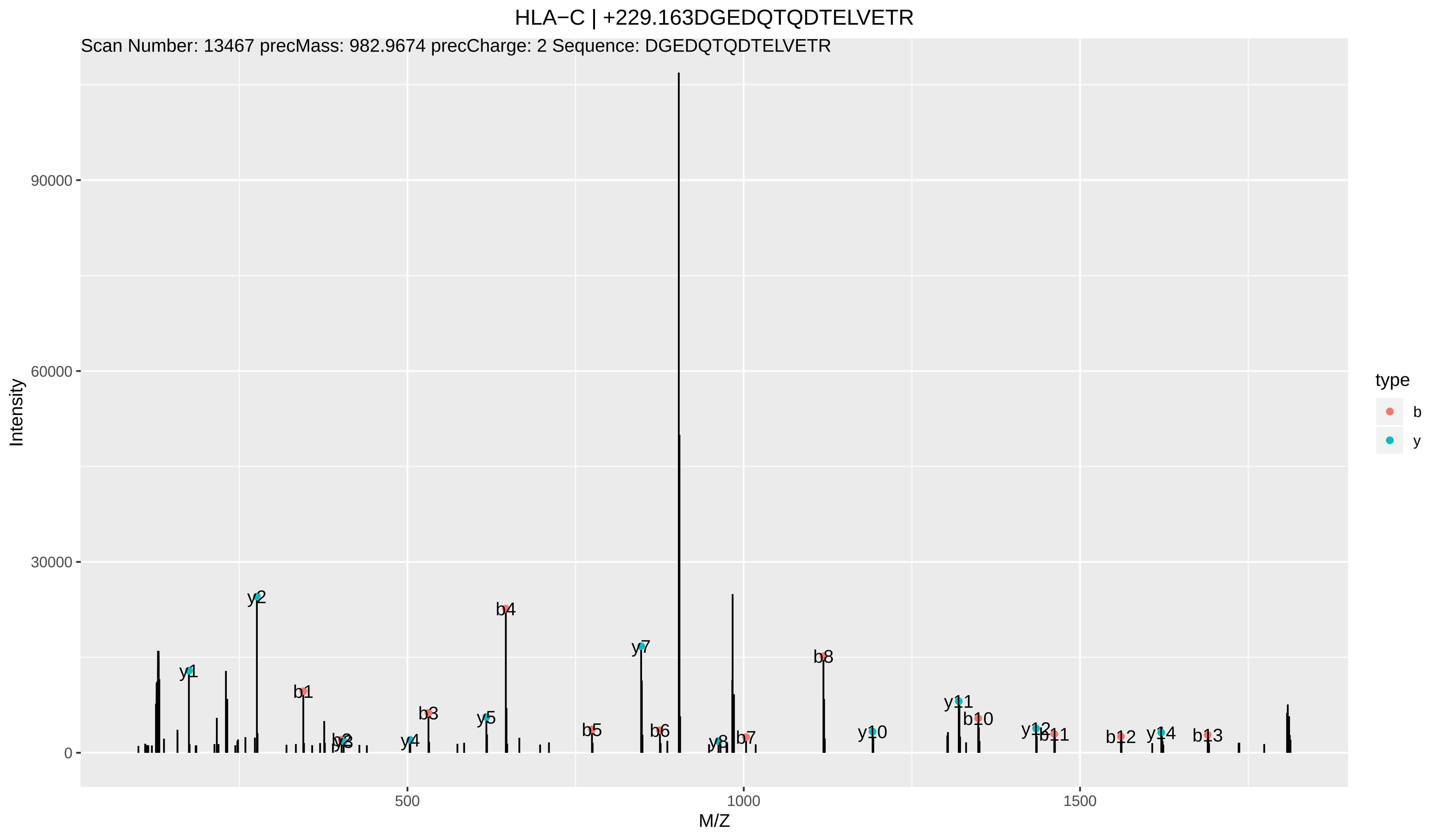

HLA−C | +229.163DGEDQTQDTELVETR
Scan Number: 13467 precMass: 982.9674 precCharge: 2 Sequence: DGEDQTQDTELVETR
Intensity
M/Z
0
30000
60000
90000
500
1000
1500
y1
y2
b1
b2
y3
y4
b3
y5
b4
b5
y7
b6
y8
b7
b8
y10
y11
b10
y12
b11
b12
y14
b13
type
b
y

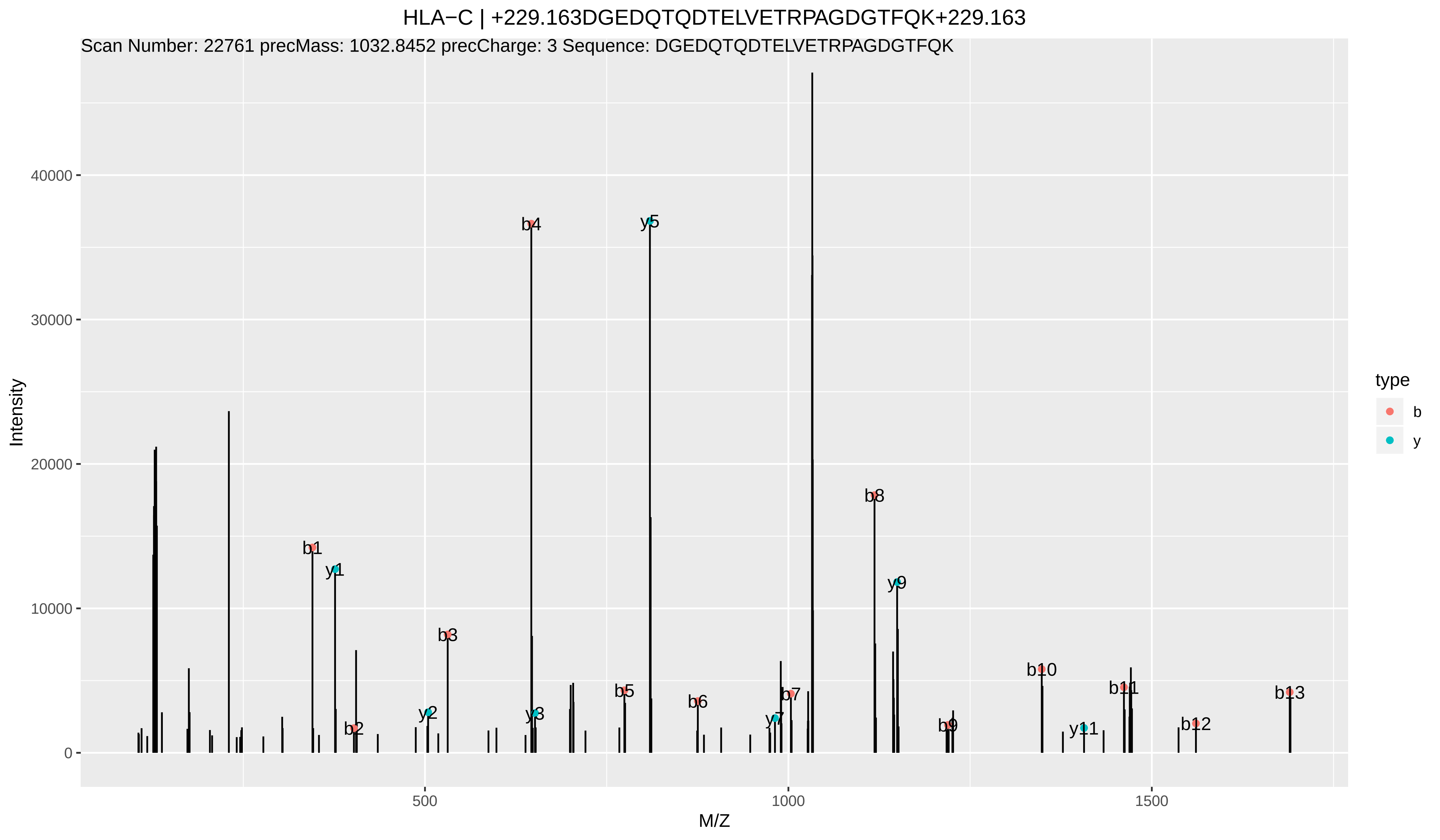
HLA−C | +229.163DGEDQTQDTELVETRPAGDGTFQK+229.163
Scan Number: 22761 precMass: 1032.8452 precCharge: 3 Sequence: DGEDQTQDTELVETRPAGDGTFQK
Intensity
M/Z
0
10000
20000
30000
40000
500
1000
1500
b1
y1
b2
y2
b3
b4
y3
b5
y5
b6
y7
b7
b8
y9
b9
b10
y11
b11
b12
b13
type
b
y

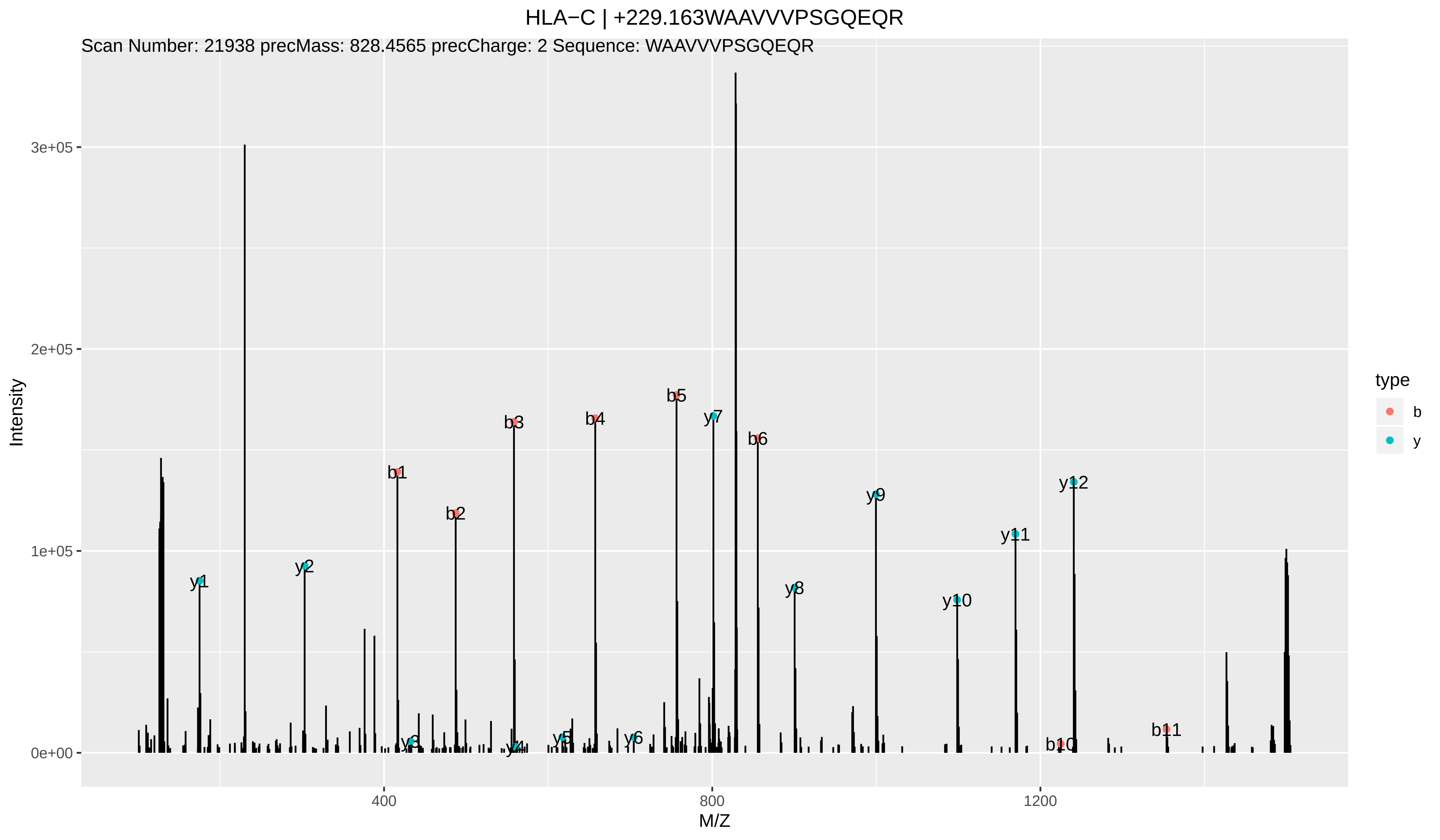

HLA−C | +229.163WAAVVVPSGQEQR
Scan Number: 21938 precMass: 828.4565 precCharge: 2 Sequence: WAAVVVPSGQEQR
Intensity
M/Z
3e+05
2e+05
1e+05
0e+00
400
800
1200
y1
y2
b1
b2
y3
b3
y4
y5
b4
y6
b5
y7
b6
y8
y9
y10
y11
y12
b10
b11
type
b
y

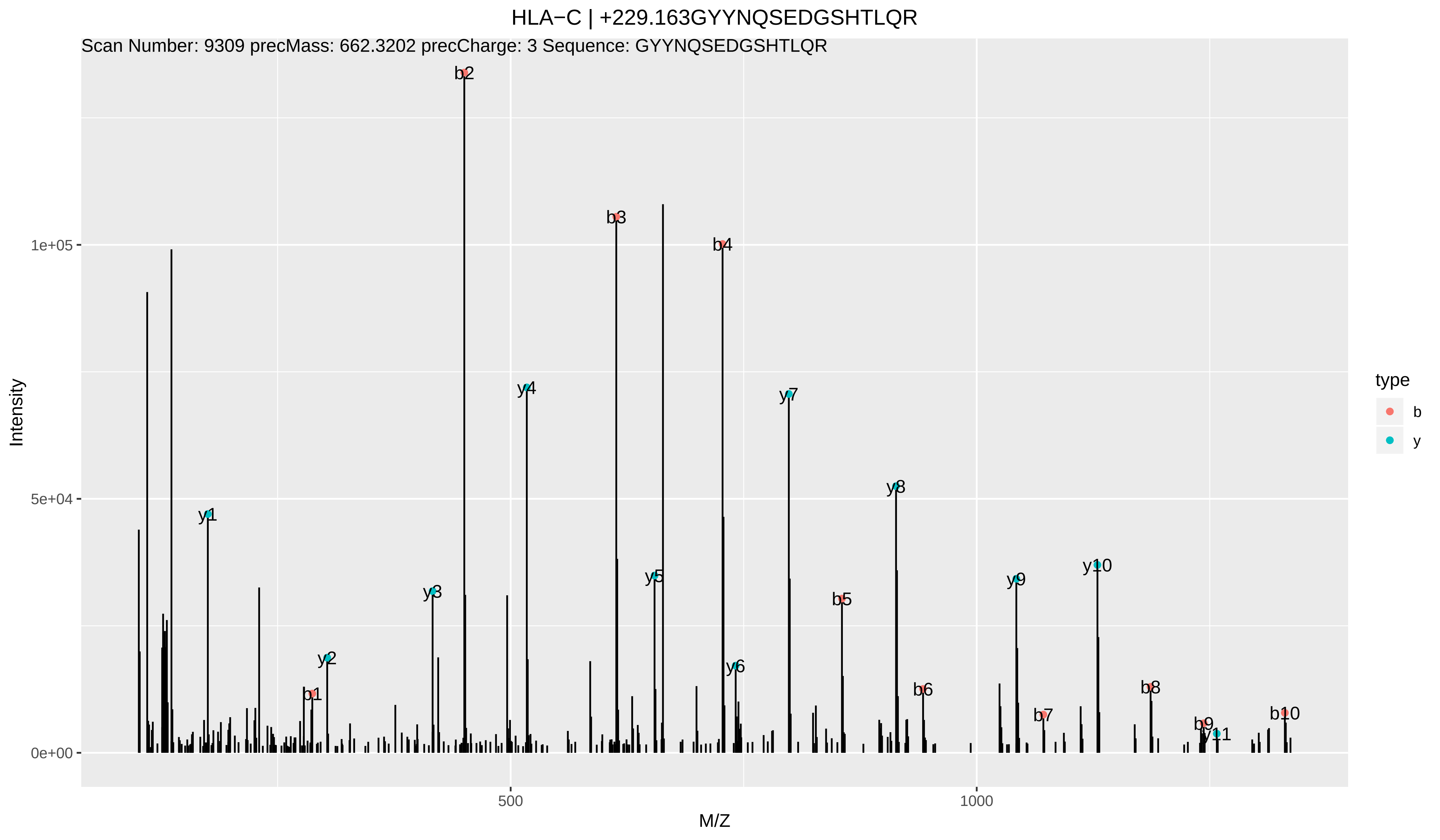

HLA−C | +229.163GYYNQSEDGSHTLQR
Scan Number: 9309 precMass: 662.3202 precCharge: 3 Sequence: GYYNQSEDGSHTLQR
Intensity
M/Z
0e+00
5e+04
1e+05
500
1000
b1
b2
b3
b4
b5
b6
b7
b8
b9
b10
y1
y2
y3
y4
y5
y6
y7
y8
y9
y10
y11
type
b
y

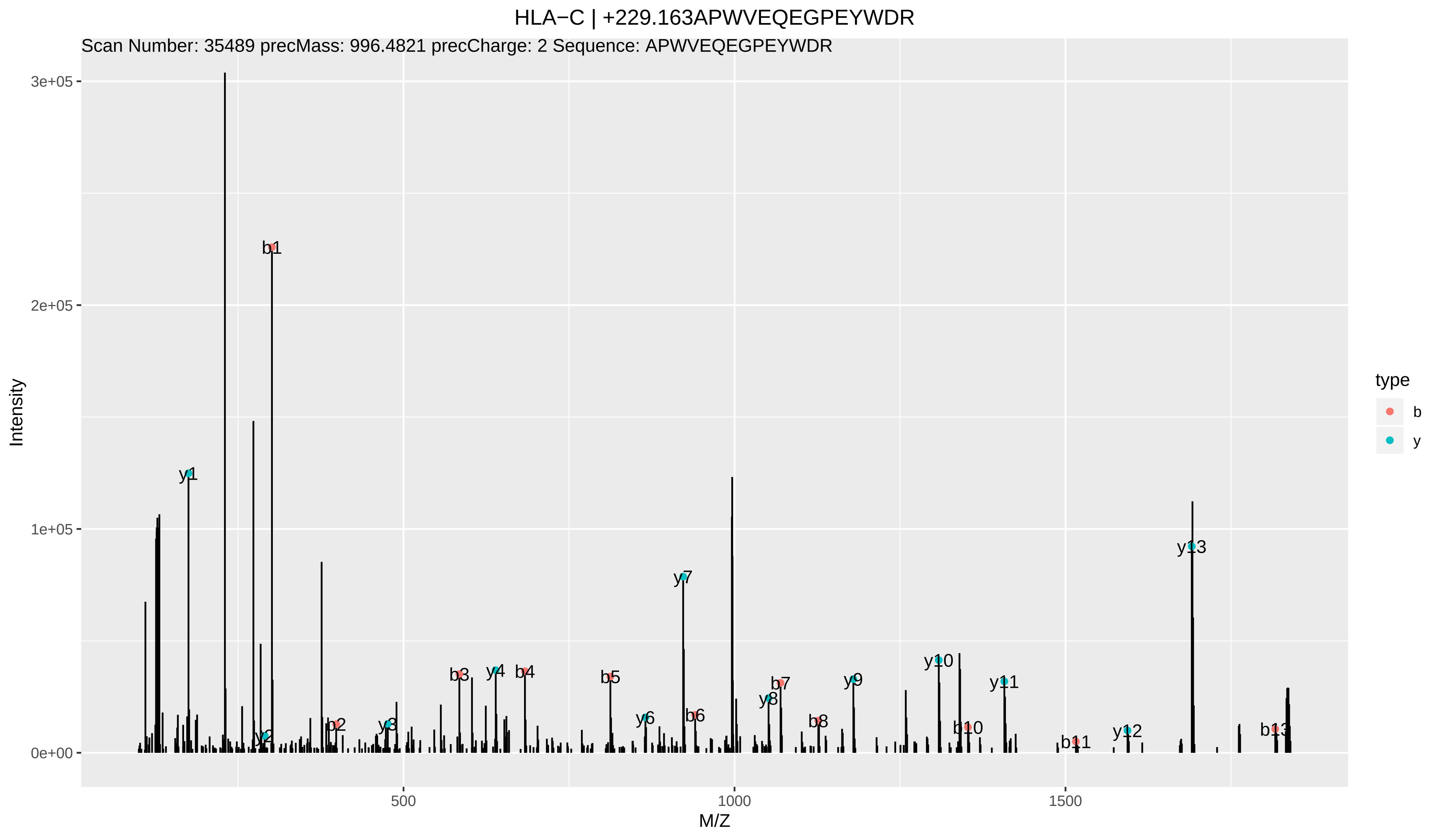

HLA−C | +229.163APWVEQEGPEYWDR
Scan Number: 35489 precMass: 996.4821 precCharge: 2 Sequence: APWVEQEGPEYWDR
Intensity
3e+05
2e+05
1e+05
0e+00
M/Z
500
1000
1500
type
b
y
y1
b1
y2
b2
y3
b3
y4
b4
b5
y6
y7
b6
y8
b7
b8
y9
y10
b10
y11
b11
y12
y13
b13

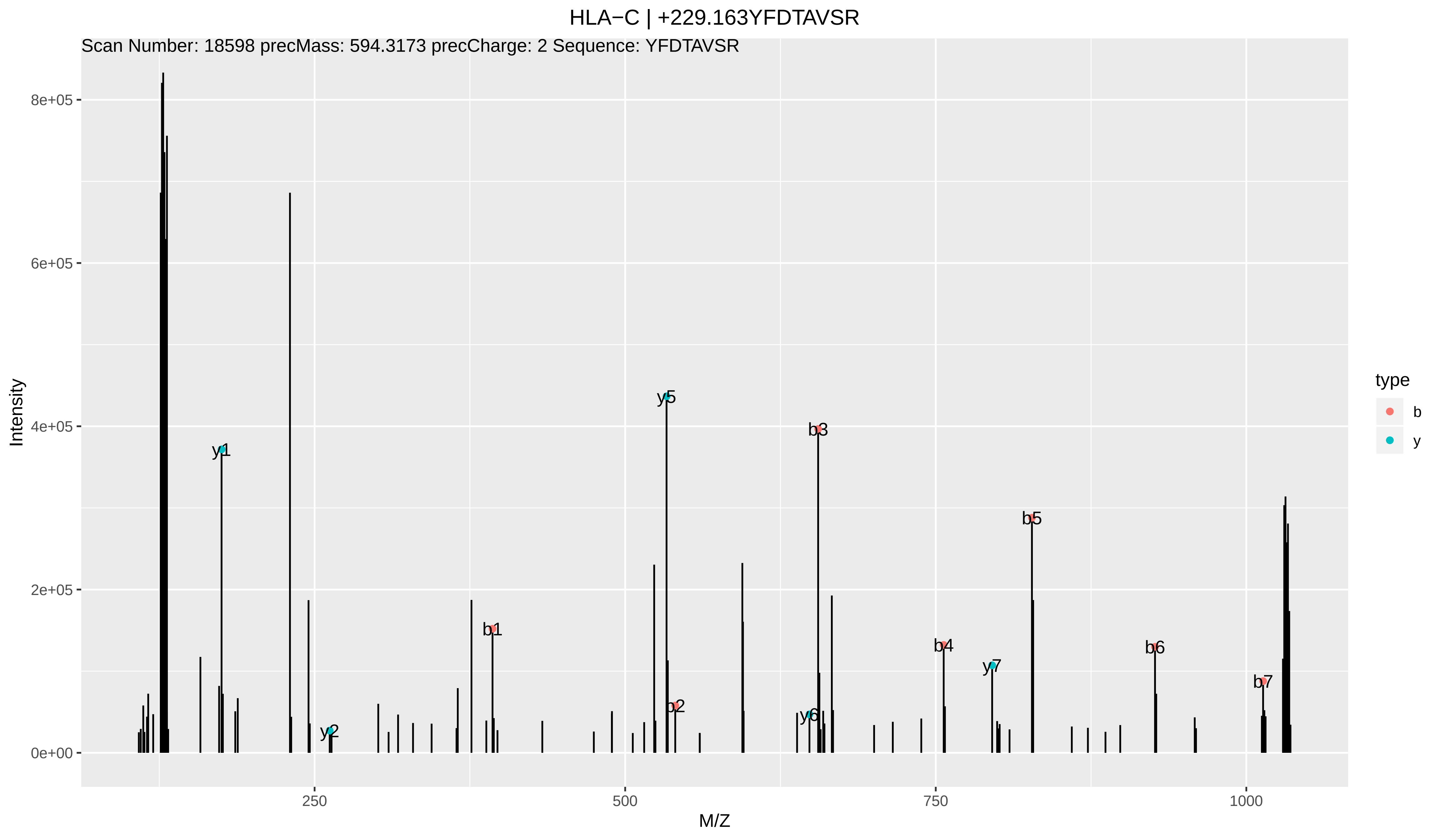

HLA−C | +229.163YFDTAVSR
Scan Number: 18598 precMass: 594.3173 precCharge: 2 Sequence: YFDTAVSR
Intensity
M/Z
8e+05
6e+05
4e+05
2e+05
0e+00
250
500
750
1000
y1
y2
b1
y5
b2
b3
y6
b4
y7
b5
b6
b7
type
b
y

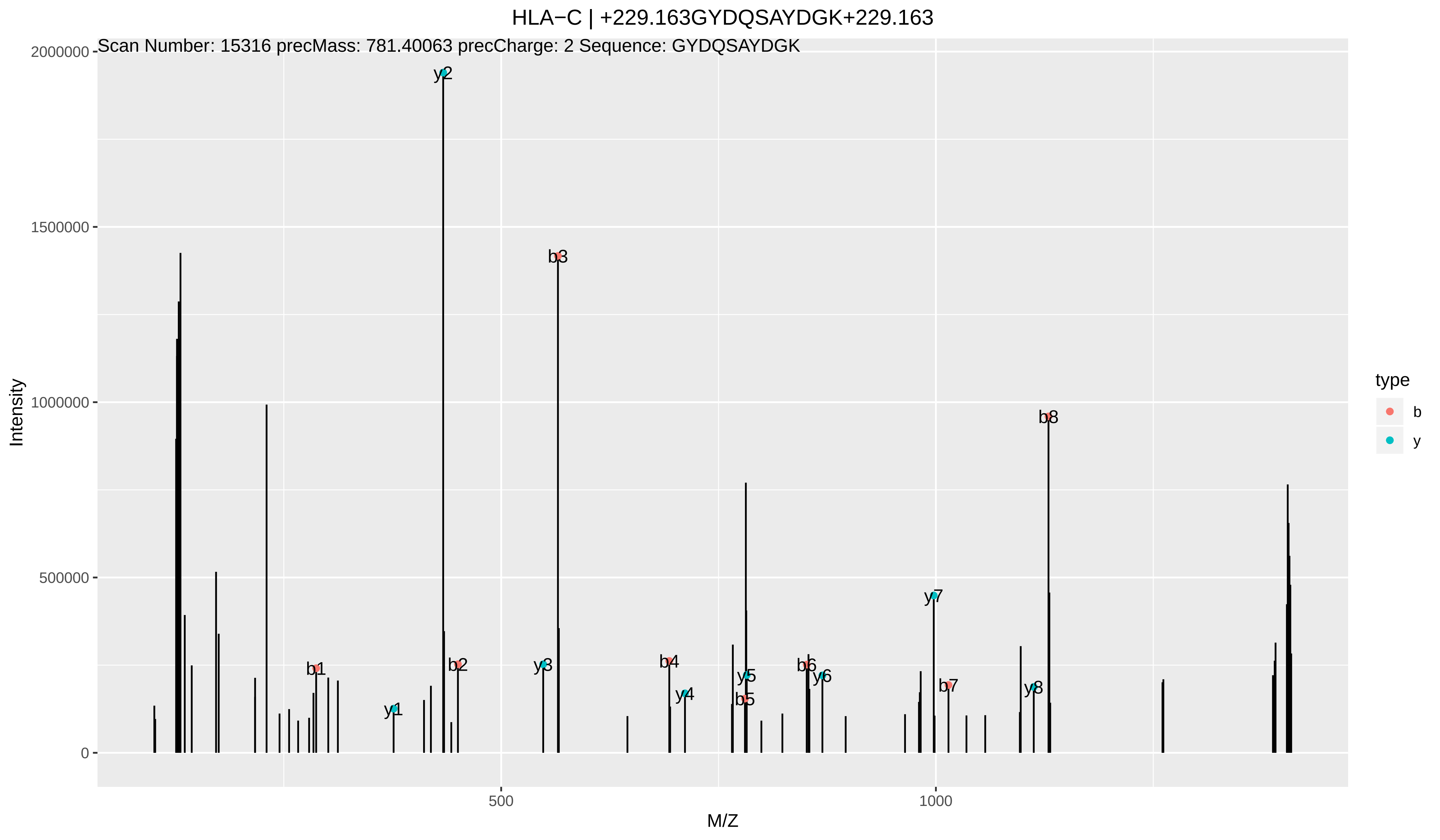

HLA−C | +229.163GYDQSAYDGK+229.163
Scan Number: 15316 precMass: 781.40063 precCharge: 2 Sequence: GYDQSAYDGK
Intensity
2000000
1500000
1000000
500000
0
M/Z
500
1000
y2
b3
b8
y7
b1
y1
b2
y3
b4
y4
y5
b5
b6
y6
b7
y8
type
b
y

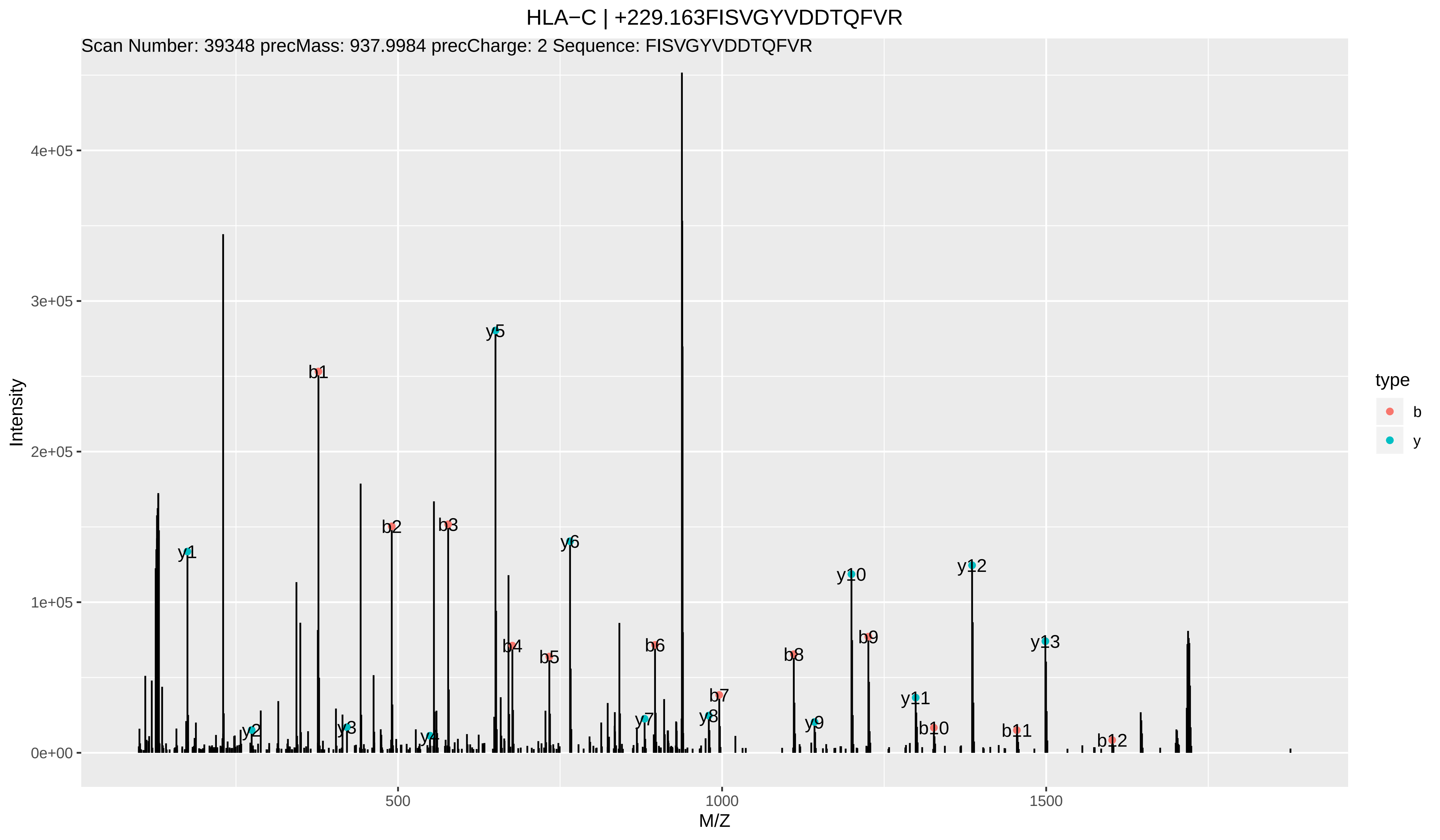
HLA−C | +229.163FISVGYVDDTQFVR
Scan Number: 39348 precMass: 937.9984 precCharge: 2 Sequence: FISVGYVDDTQFVR
Intensity
4e+05
3e+05
2e+05
1e+05
0e+00
M/Z
500
1000
1500
y1
y2
y3
y4
y5
y6
y7
y8
y9
y10
y11
y12
y13
b1
b2
b3
b4
b5
b6
b7
b8
b9
b10
b11
b12
type
b
y

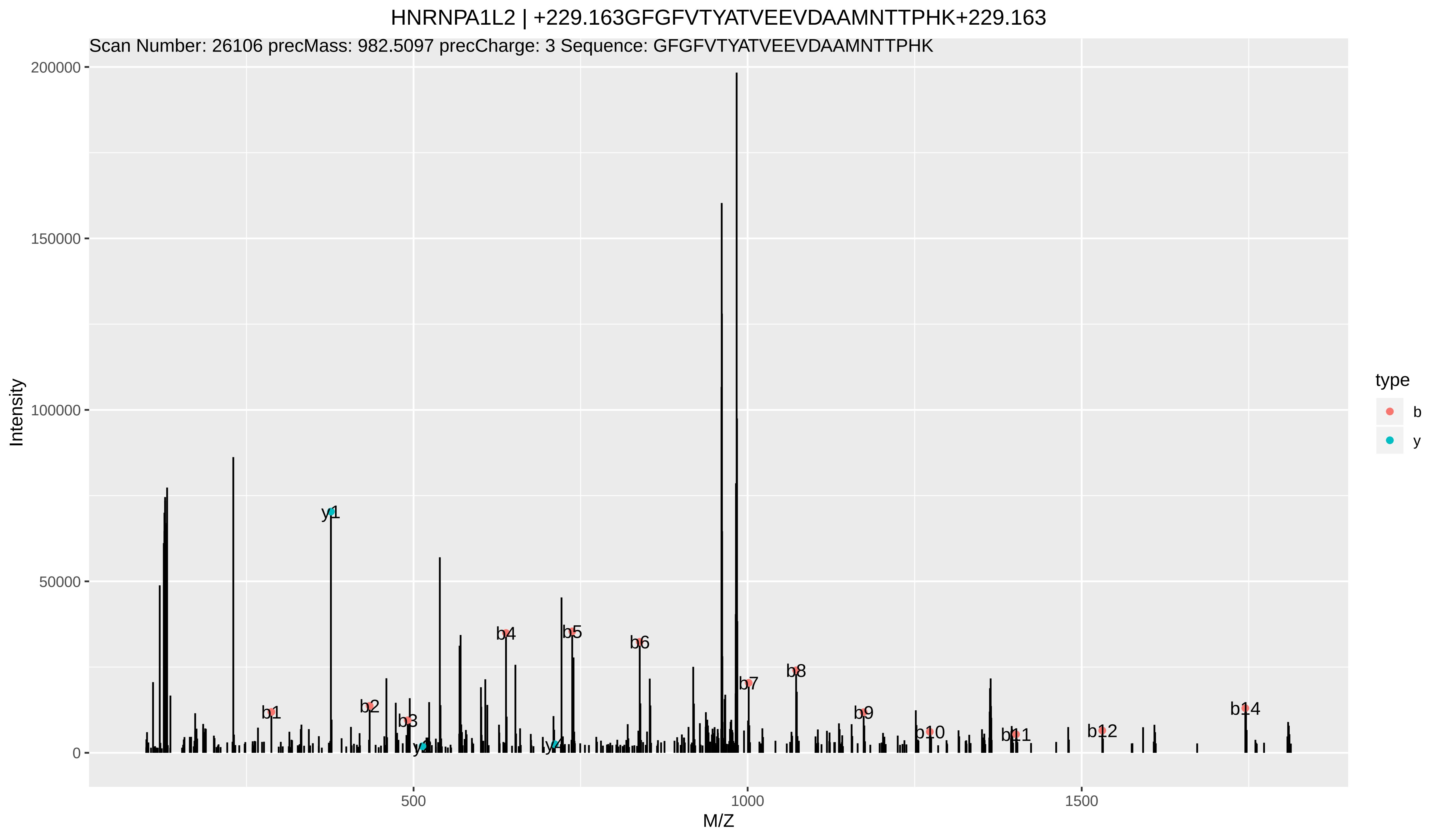

HNRNPA1L2 | +229.163GFGFVTYATVEEVDAAMNTTPHK+229.163
Scan Number: 26106 precMass: 982.5097 precCharge: 3 Sequence: GFGFVTYATVEEVDAAMNTTPHK
Intensity
200000
150000
100000
50000
0
M/Z
500
1000
1500
type
b
y
y1
b1
b2
b3
b4
b5
b6
b7
b8
b9
b10
b11
b12
b14

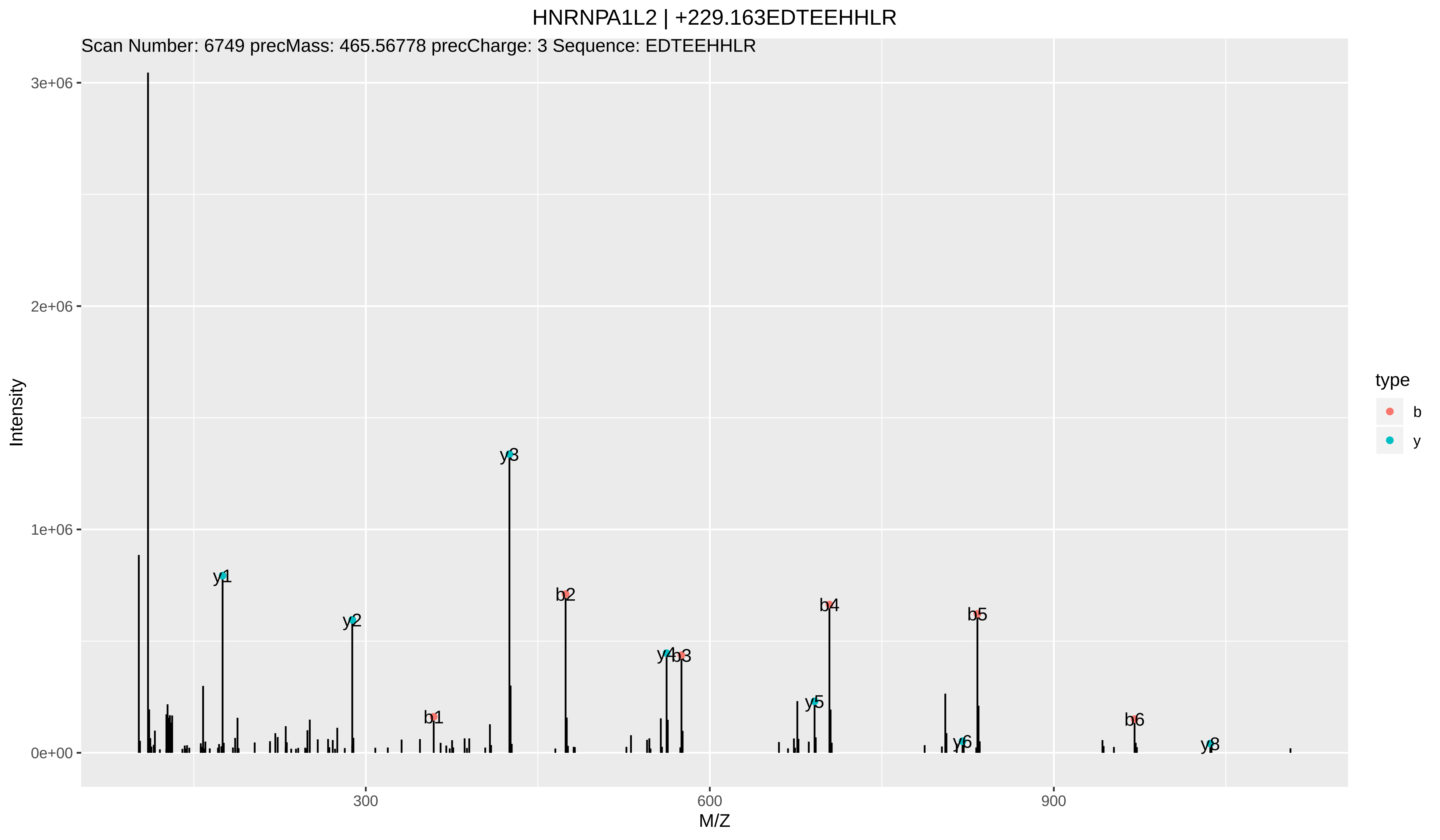

HNRNPA1L2 | +229.163EDTEEHHLR
Scan Number: 6749 precMass: 465.56778 precCharge: 3 Sequence: EDTEEHHLR
Intensity
3e+06
2e+06
1e+06
0e+00
M/Z
300
600
900
y1
y2
b1
y3
b2
y4
b3
y5
b4
y6
b5
b6
y8
type
b
y

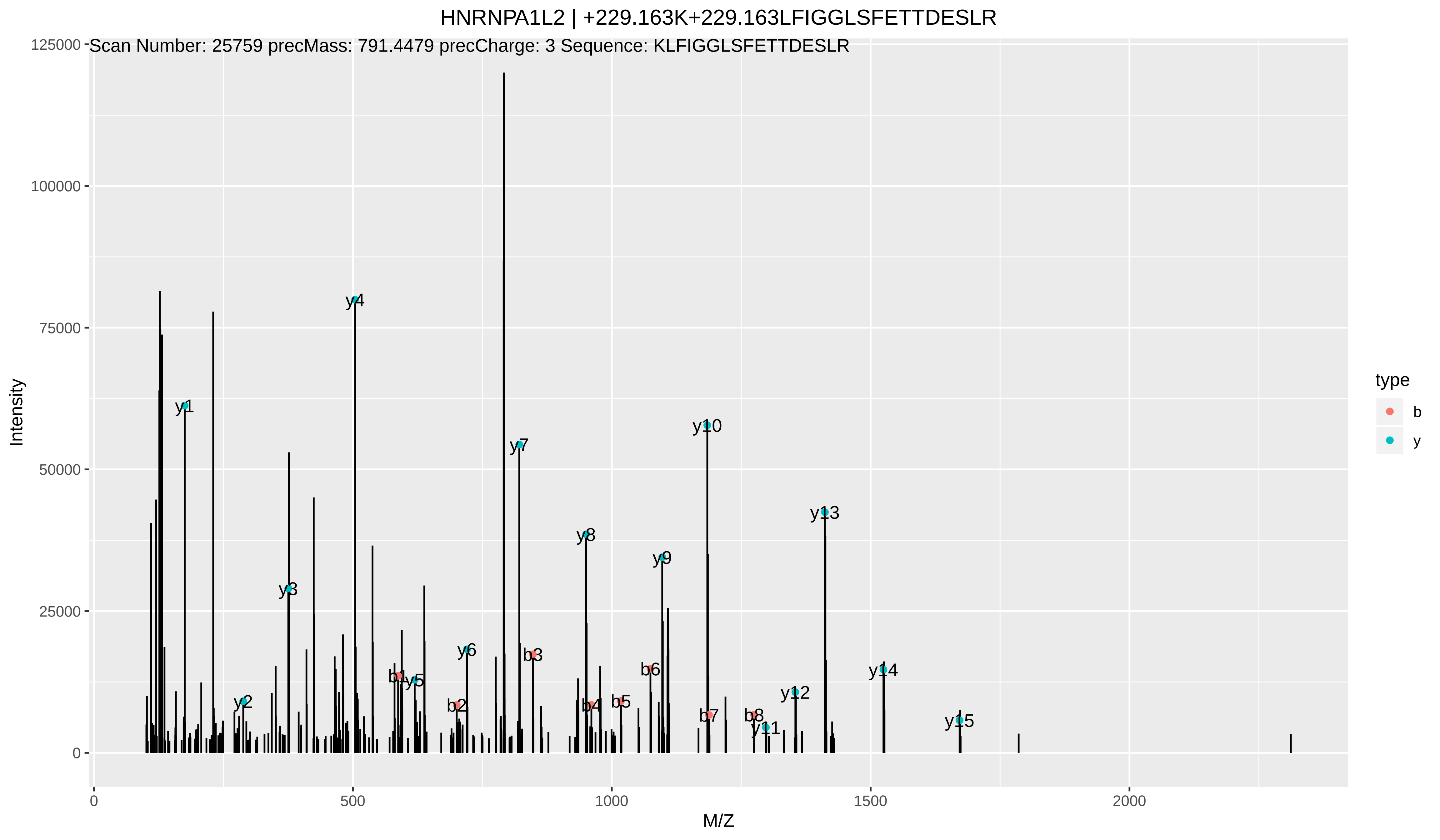
HNRNPA1L2 | +229.163K+229.163LFIGGLSFETTDESLR
Scan Number: 25759 precMass: 791.4479 precCharge: 3 Sequence: KLFIGGLSFETTDESLR
Intensity
M/Z
type
b
y
y1
y2
y3
y4
y5
y6
y7
y8
y9
y10
y11
y12
y13
y14
y15
b1
b2
b3
b4
b5
b6
b7
b8
0
25000
50000
75000
100000
125000
500
1000
1500
2000

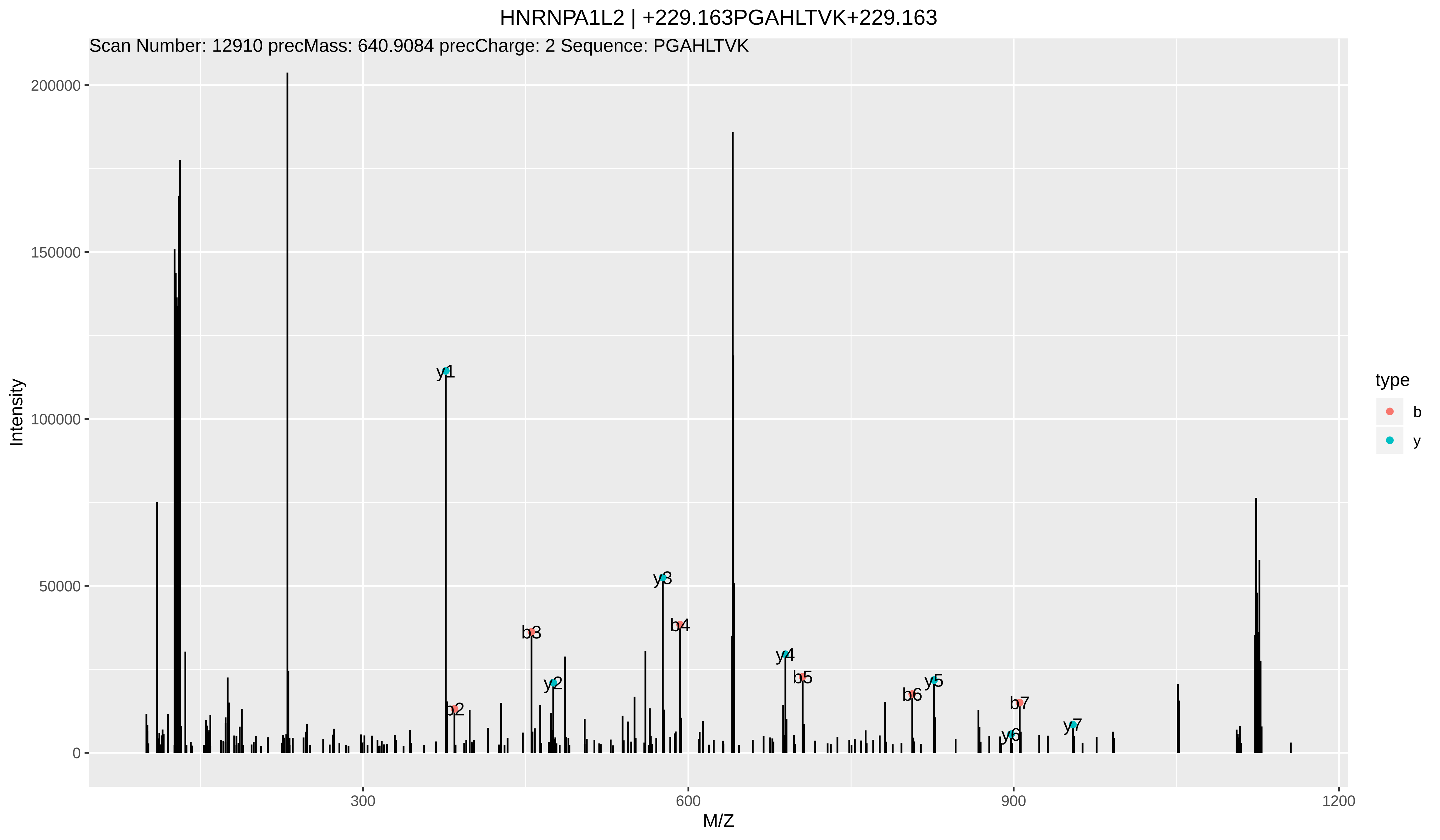
HNRNPA1L2 | +229.163PGAHLTVK+229.163
Scan Number: 12910 precMass: 640.9084 precCharge: 2 Sequence: PGAHLTVK
Intensity
M/Z
200000
150000
100000
50000
0
300
600
900
1200
y1
y2
y3
y4
y5
y6
y7
b2
b3
b4
b5
b6
b7
type
b
y

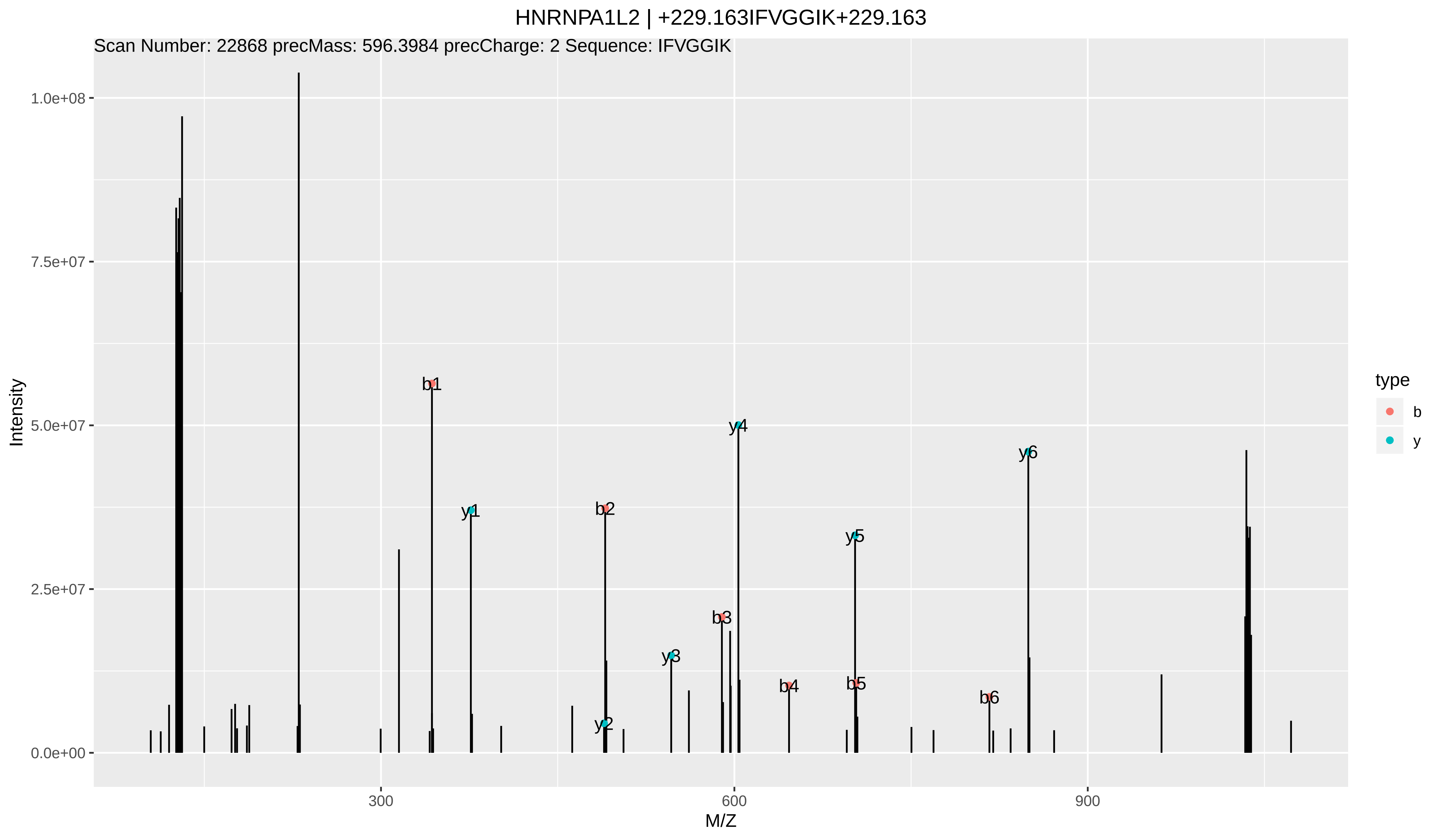

HNRNPA1L2 | +229.163IFVGGIK+229.163
Scan Number: 22868 precMass: 596.3984 precCharge: 2 Sequence: IFVGGIK
Intensity
1.0e+08
7.5e+07
5.0e+07
2.5e+07
0.0e+00
M/Z
300
600
900
b1
y1
b2
y2
y3
b3
y4
b4
y5
b5
b6
y6
type
b
y

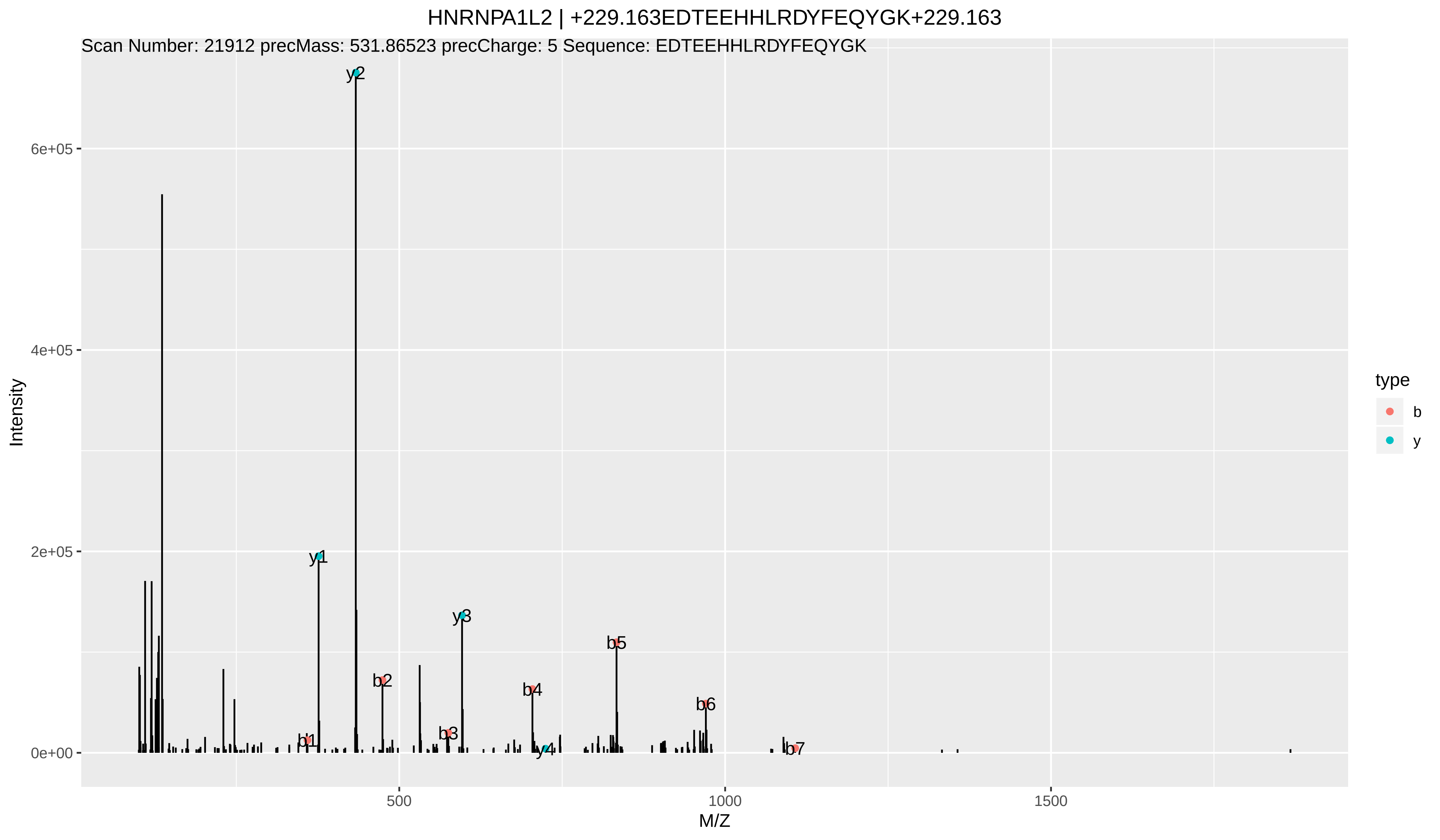

HNRNPA1L2 | +229.163EDTEEHHLRDYFEQYGK+229.163
Scan Number: 21912 precMass: 531.86523 precCharge: 5 Sequence: EDTEEHHLRDYFEQYGK
Intensity
6e+05
4e+05
2e+05
0e+00
M/Z
500
1000
1500
y2
y1
y3
y4
b1
b2
b3
b4
b5
b6
b7
type
b
y

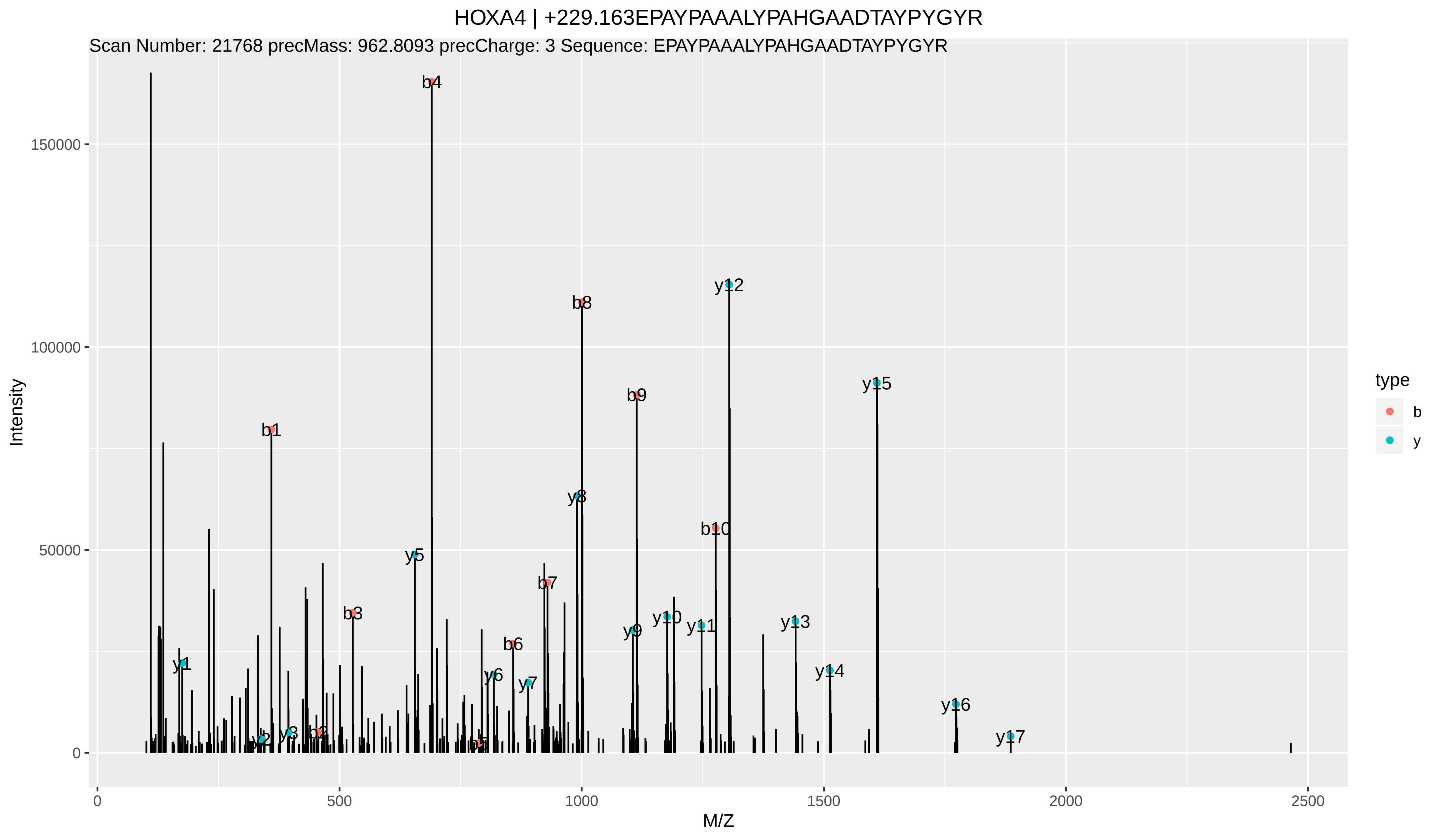
HOXA4 | +229.163EPAYPAAALYPAHGAADTAYPYGYR
Scan Number: 21768 precMass: 962.8093 precCharge: 3 Sequence: EPAYPAAALYPAHGAADTAYPYGYR
Intensity
M/Z
type
b
y
b4
b1
b8
b9
b10
b3
b7
b6
y12
y15
y8
y5
y10
y11
y9
y13
y14
y1
y6
y7
y16
y17
y2
y3
150000
100000
50000
0
0
500
1000
1500
2000
2500

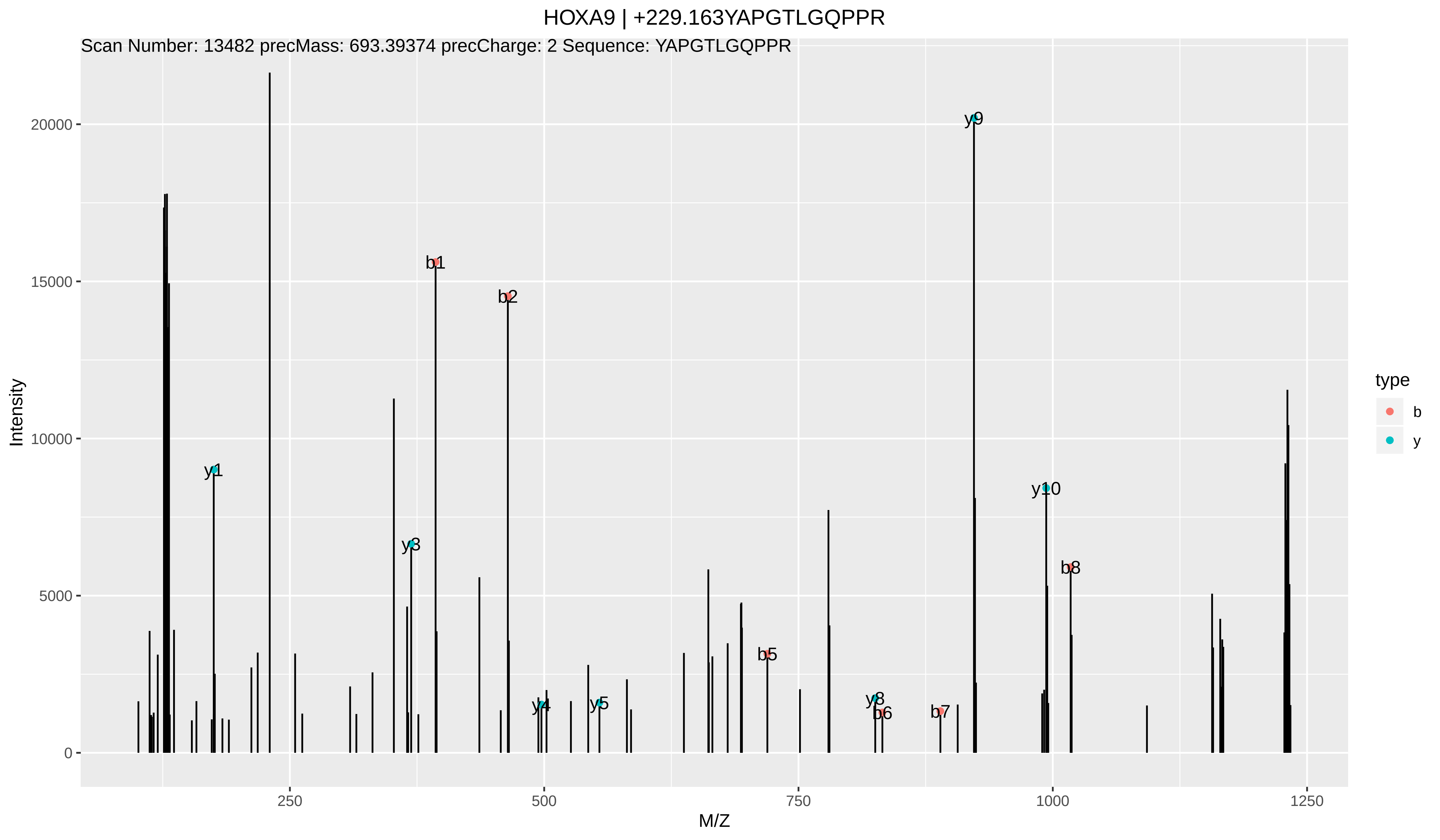

HOXA9 | +229.163YAPGTLGQPPR
Scan Number: 13482 precMass: 693.39374 precCharge: 2 Sequence: YAPGTLGQPPR
Intensity
M/Z
0
5000
10000
15000
20000
250
500
750
1000
1250
y1
y3
b1
b2
y4
y5
b5
y8
b6
b7
y9
y10
b8
type
b
y

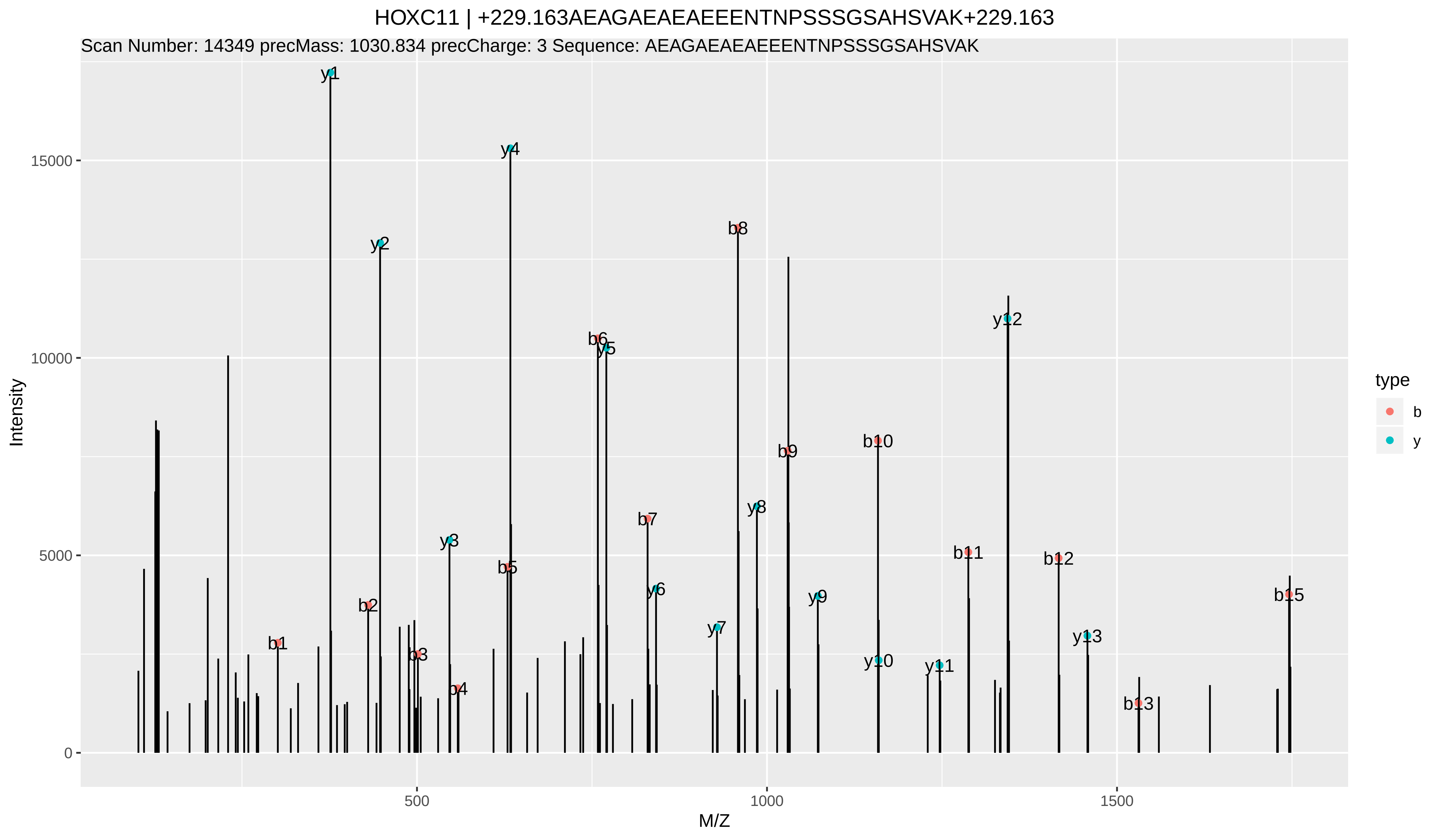

HOXC11 | +229.163AEAGAEAEAEEENTNPSSSGSAHSVAK+229.163
Scan Number: 14349 precMass: 1030.834 precCharge: 3 Sequence: AEAGAEAEAEEENTNPSSSGSAHSVAK
Intensity
M/Z
0
5000
10000
15000
500
1000
1500
y1
y4
y2
b8
y12
b6
y5
b10
b9
y8
b7
y3
b11
b12
b5
y6
b2
y9
b15
y7
y13
b1
b3
y10
y11
b4
b13
type
b
y

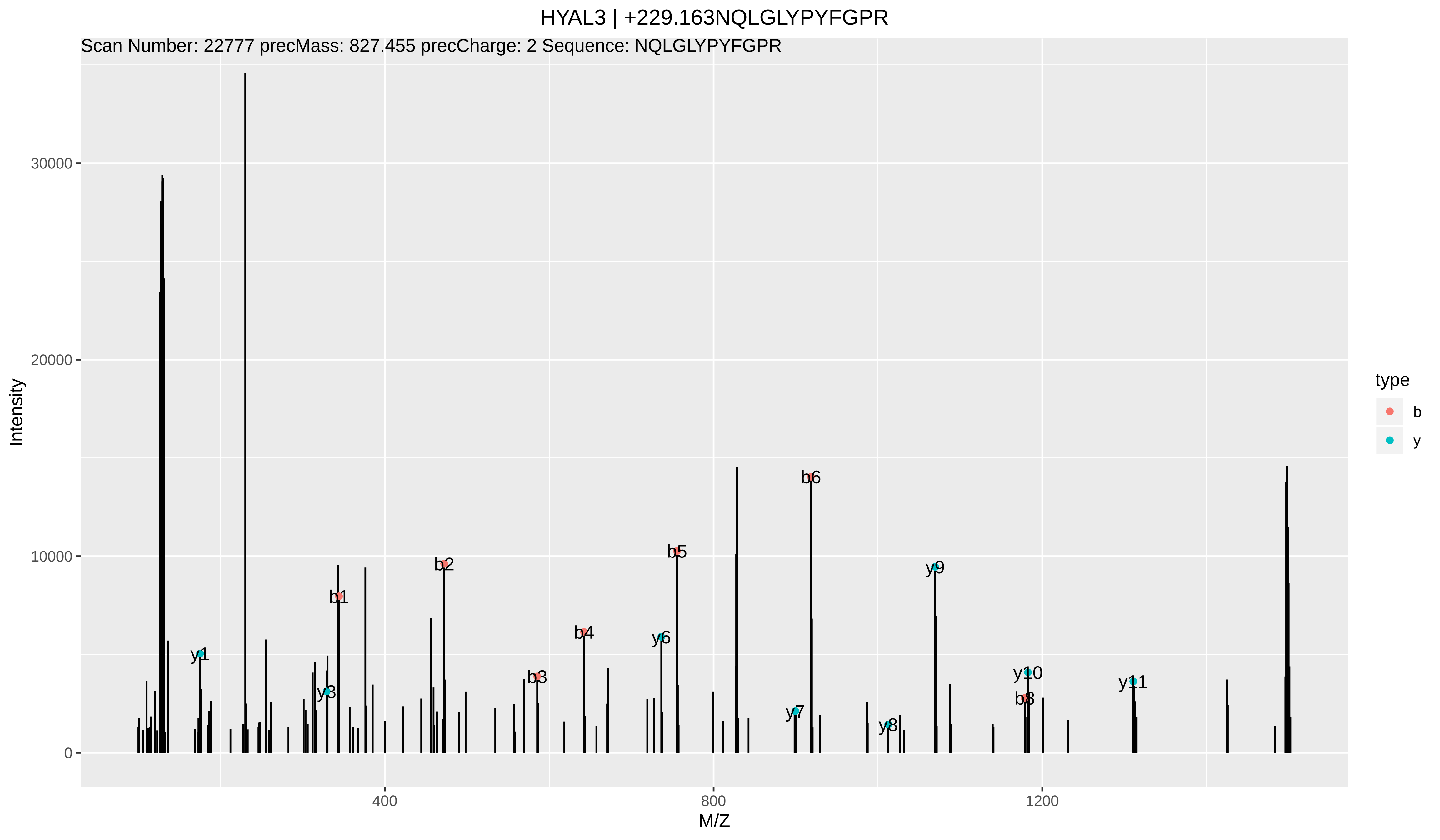
HYAL3 | +229.163NQLGLYPYFGPR
Scan Number: 22777 precMass: 827.455 precCharge: 2 Sequence: NQLGLYPYFGPR
Intensity
M/Z
0
10000
20000
30000
400
800
1200
type
b
y
y1
y3
b1
b2
b3
b4
y6
b5
y7
b6
y8
y9
y10
b8
y11

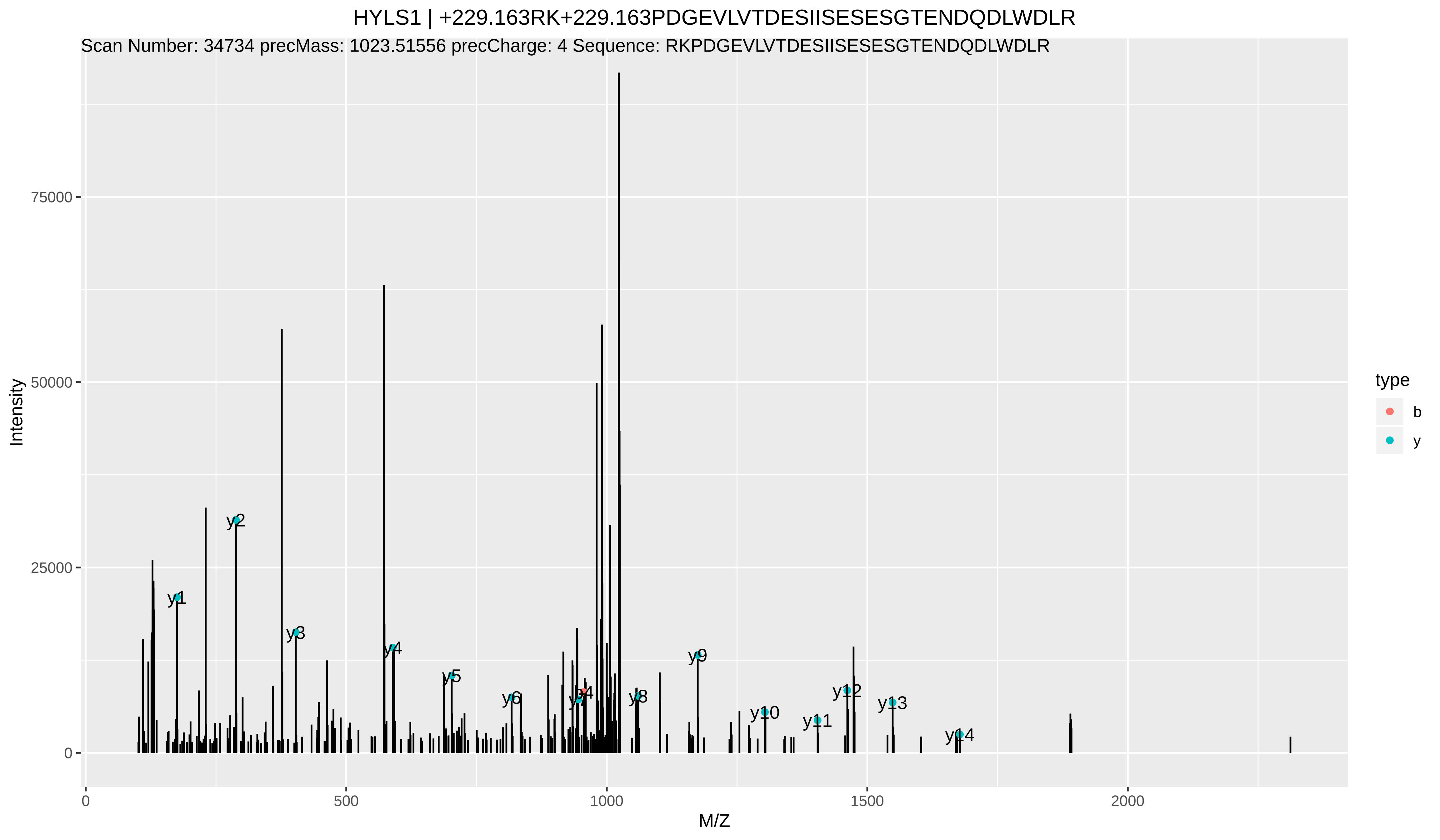
HYLS1 | +229.163RK+229.163PDGEVLVTDESIISESESGTENDQDLWDLR
Scan Number: 34734 precMass: 1023.51556 precCharge: 4 Sequence: RKPDGEVLVTDESIISESESGTENDQDLWDLR
Intensity
75000
50000
25000
0
M/Z
0
500
1000
1500
2000
y1
y2
y3
y4
y5
y6
y7
b4
y8
y9
y10
y11
y12
y13
y14
type
b
y

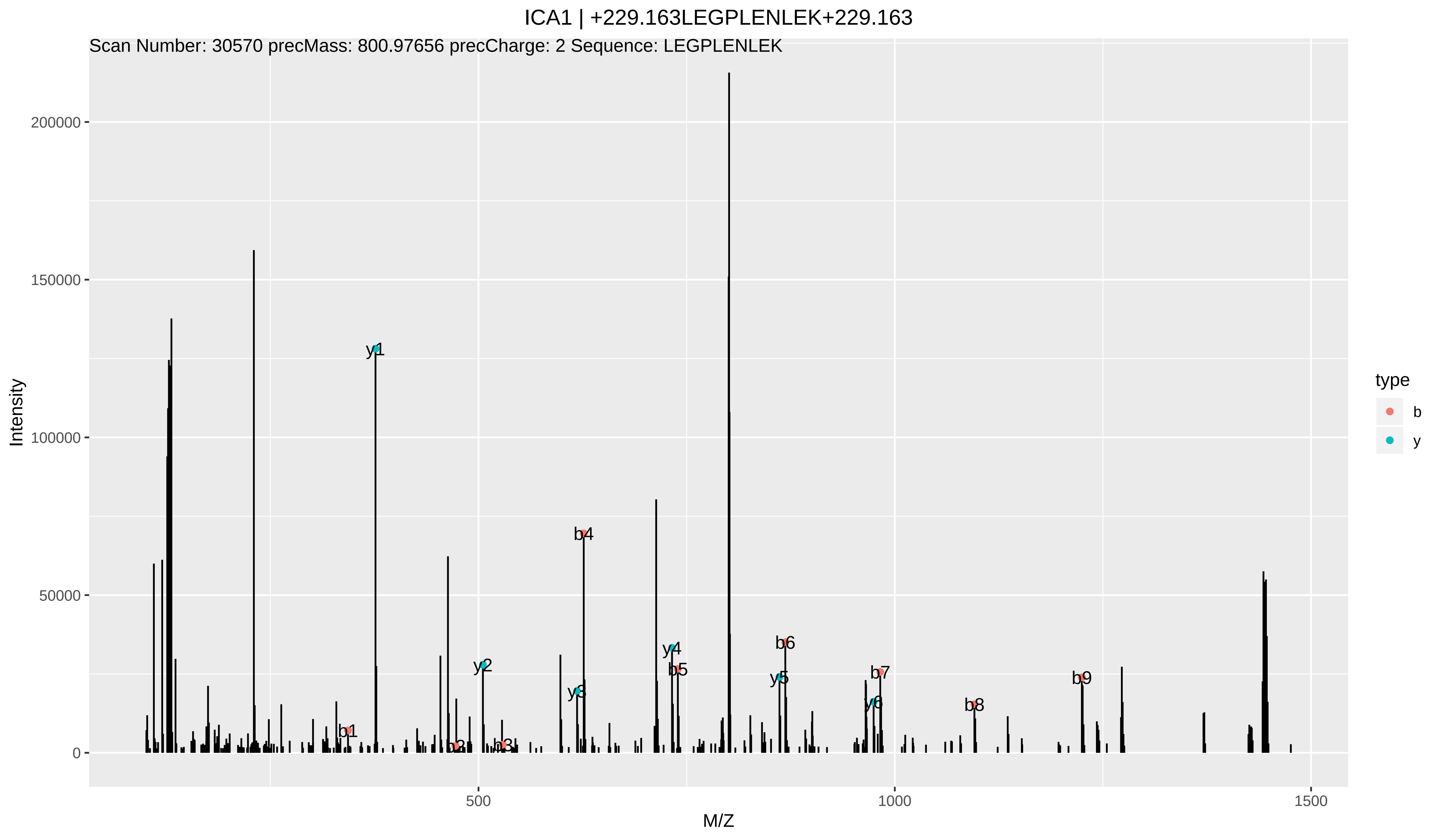
ICA1 | +229.163LEGPLENLEK+229.163
Scan Number: 30570 precMass: 800.97656 precCharge: 2 Sequence: LEGPLENLEK
Intensity
200000
150000
100000
50000
0
500
1000
1500
M/Z
type
b
y
y1
b1
b2
b3
y2
y3
b4
y4
b5
y5
b6
b7
y6
b8
b9

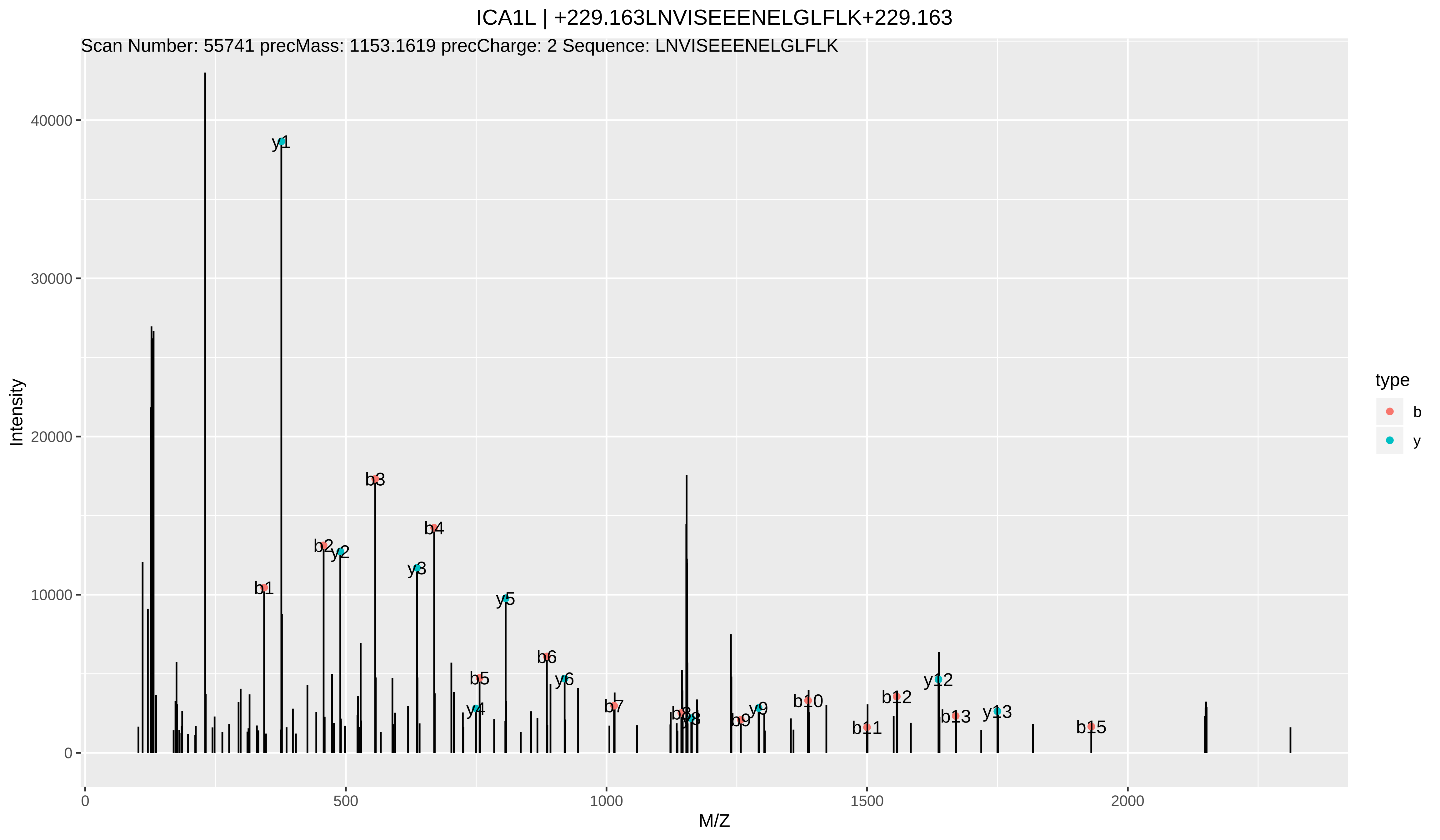
ICA1L | +229.163LNVISEEENELGLFLK+229.163
Scan Number: 55741 precMass: 1153.1619 precCharge: 2 Sequence: LNVISEEENELGLFLK
Intensity
M/Z
type
b
y
y1
b1
b2
y2
b3
y3
b4
y5
b5
y4
b6
y6
b7
b8
y8
b9
y9
b10
b11
b12
y12
b13
y13
b15
0
10000
20000
30000
40000
0
500
1000
1500
2000

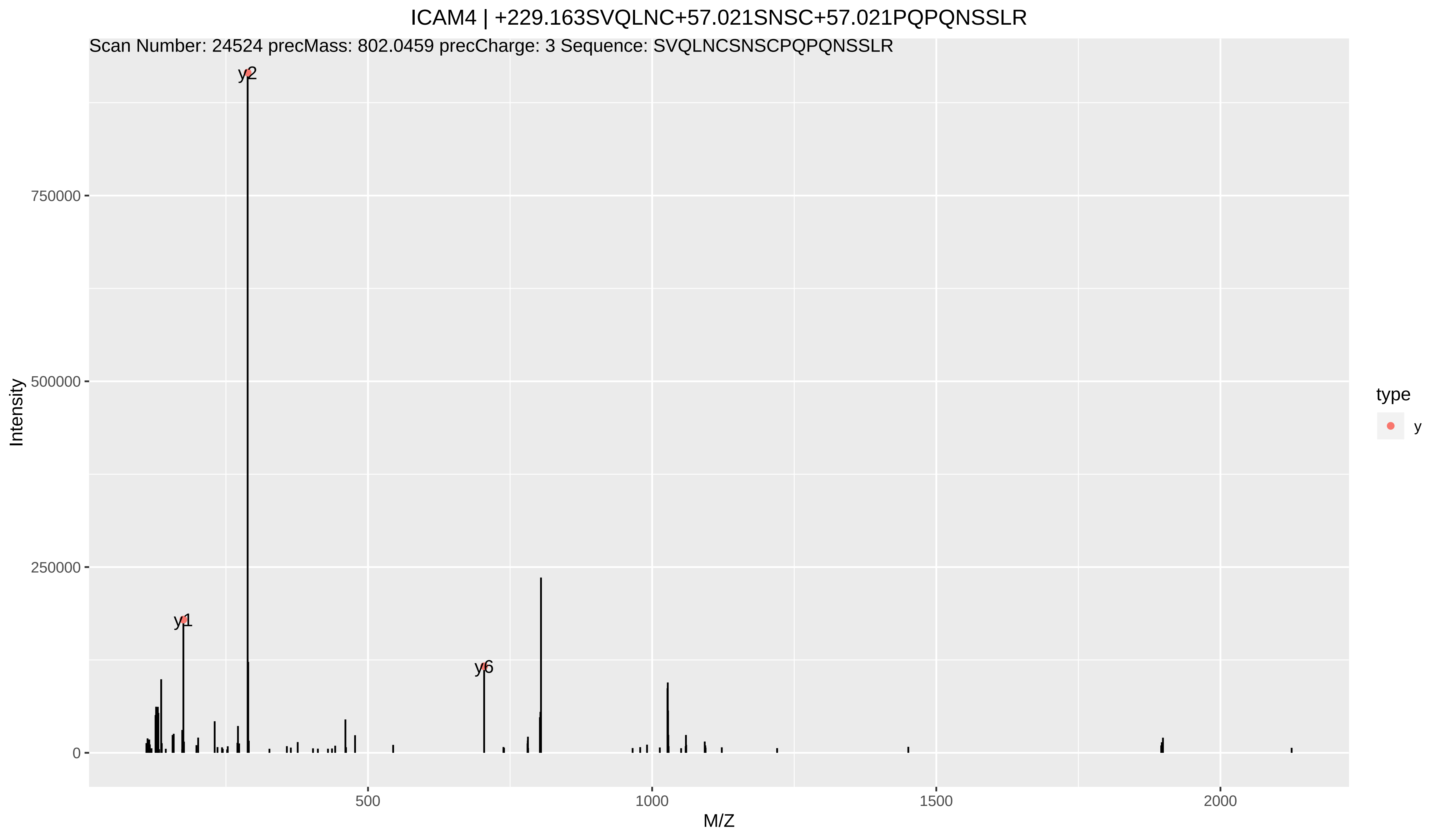

ICAM4 | +229.163SVQLNC+57.021SNSC+57.021PQPQNSSLR
Scan Number: 24524 precMass: 802.0459 precCharge: 3 Sequence: SVQLNCSNSCPQPQNSSLR
y2
y1
y6
Intensity
750000
500000
250000
0
500
1000
1500
2000
M/Z
type
y

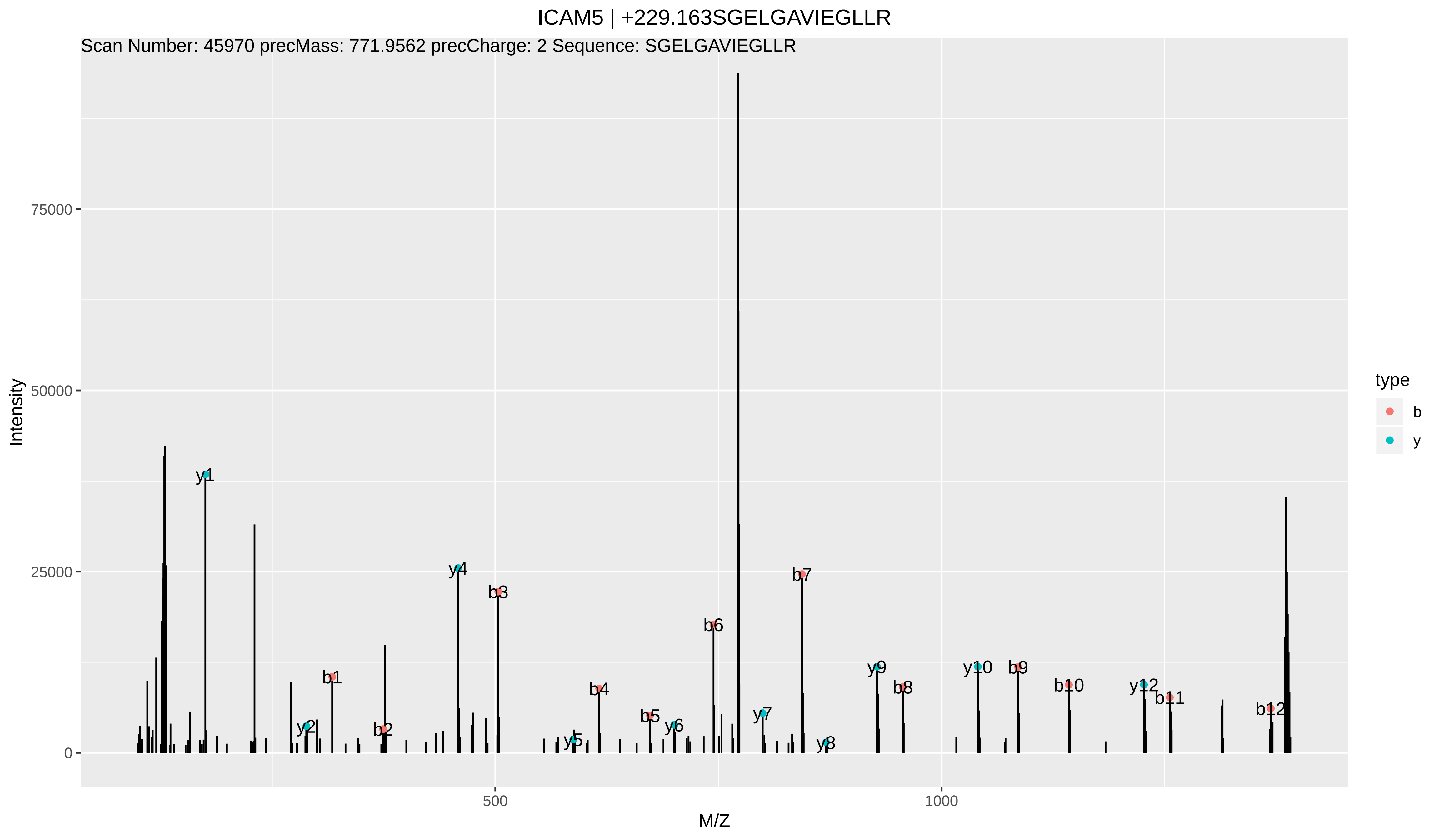

ICAM5 | +229.163SGELGAVIEGLLR
Scan Number: 45970 precMass: 771.9562 precCharge: 2 Sequence: SGELGAVIEGLLR
Intensity
75000
50000
25000
0
500
1000
M/Z
y1
y2
b1
b2
y4
b3
y5
b4
b5
y6
b6
y7
b7
y8
y9
b8
y10
b9
b10
y12
b11
b12
type
b
y

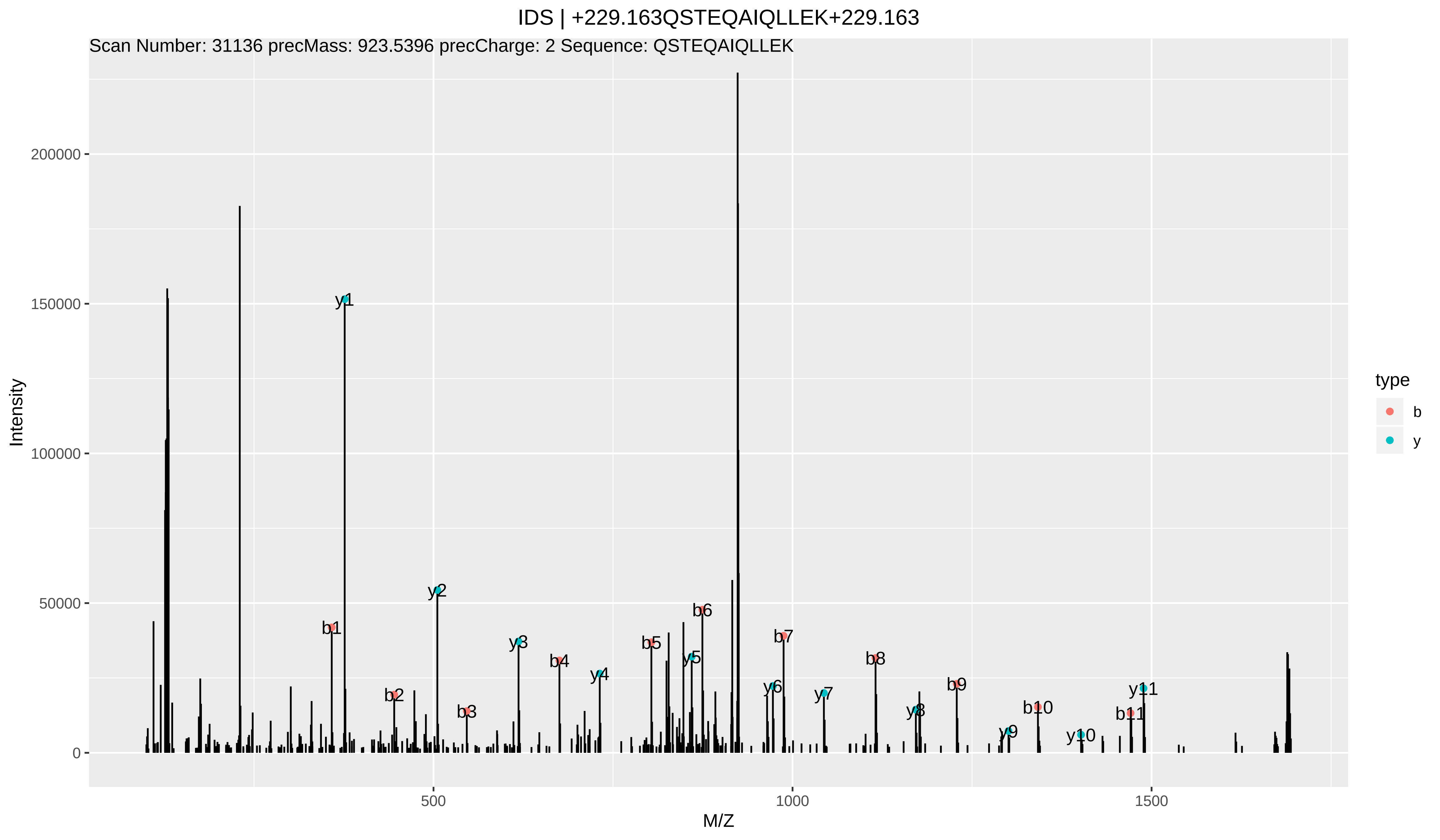
IDS | +229.163QSTEQAIQLLEK+229.163
Scan Number: 31136 precMass: 923.5396 precCharge: 2 Sequence: QSTEQAIQLLEK
Intensity
200000
150000
100000
50000
0
500
1000
1500
M/Z
y1
y2
y3
y4
y5
y6
y7
y8
y9
y10
y11
b1
b2
b3
b4
b5
b6
b7
b8
b9
b10
b11
type
b
y

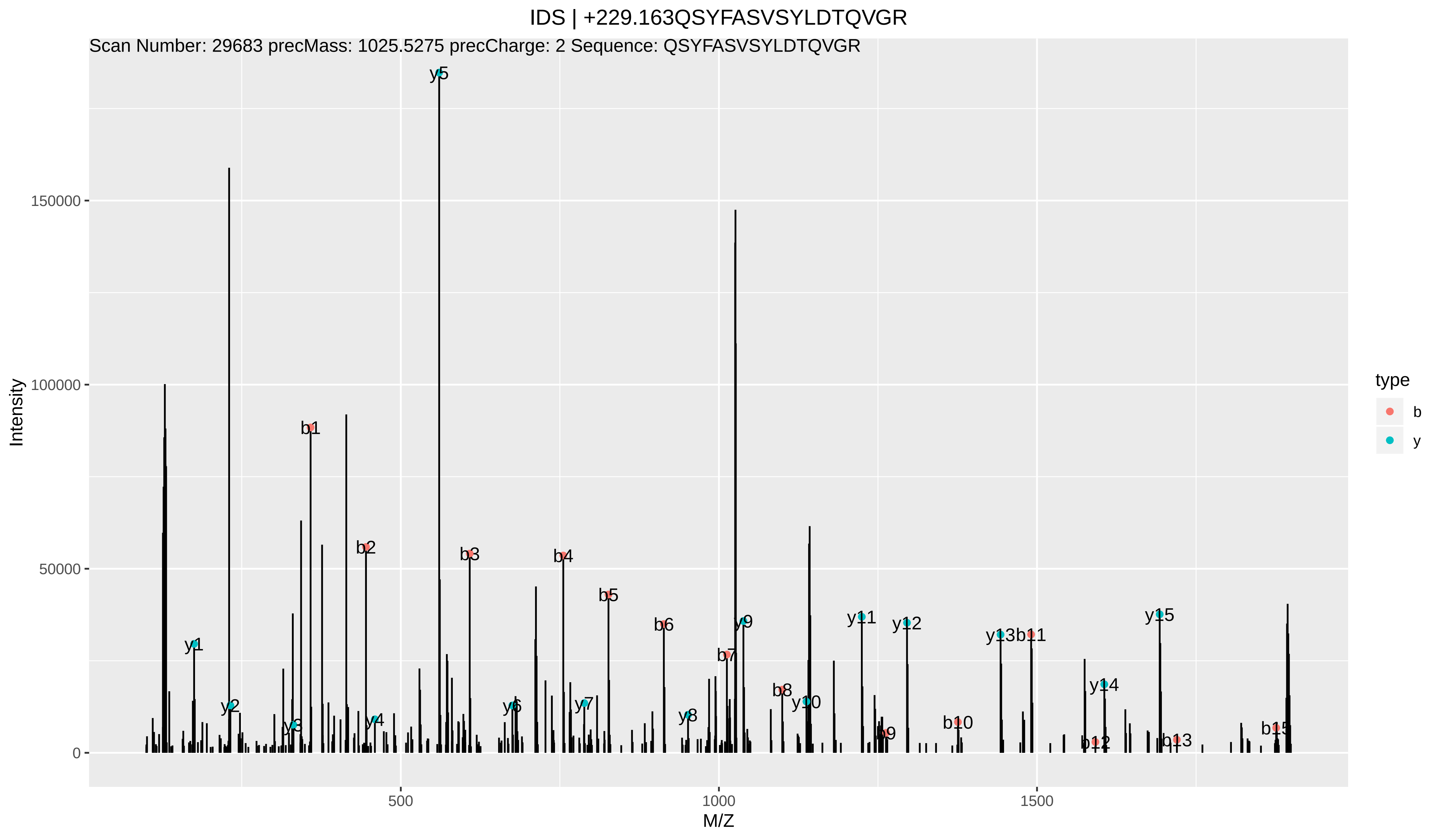
IDS | +229.163QSYFASVSYLDTQVGR
Scan Number: 29683 precMass: 1025.5275 precCharge: 2 Sequence: QSYFASVSYLDTQVGR
Intensity
M/Z
type
b
y
0
50000
100000
150000
500
1000
1500
y1
y2
y3
y4
y5
y6
y7
y8
y9
y10
y11
y12
y13
y14
y15
b1
b2
b3
b4
b5
b6
b7
b8
b10
b11
b12
b13
b15

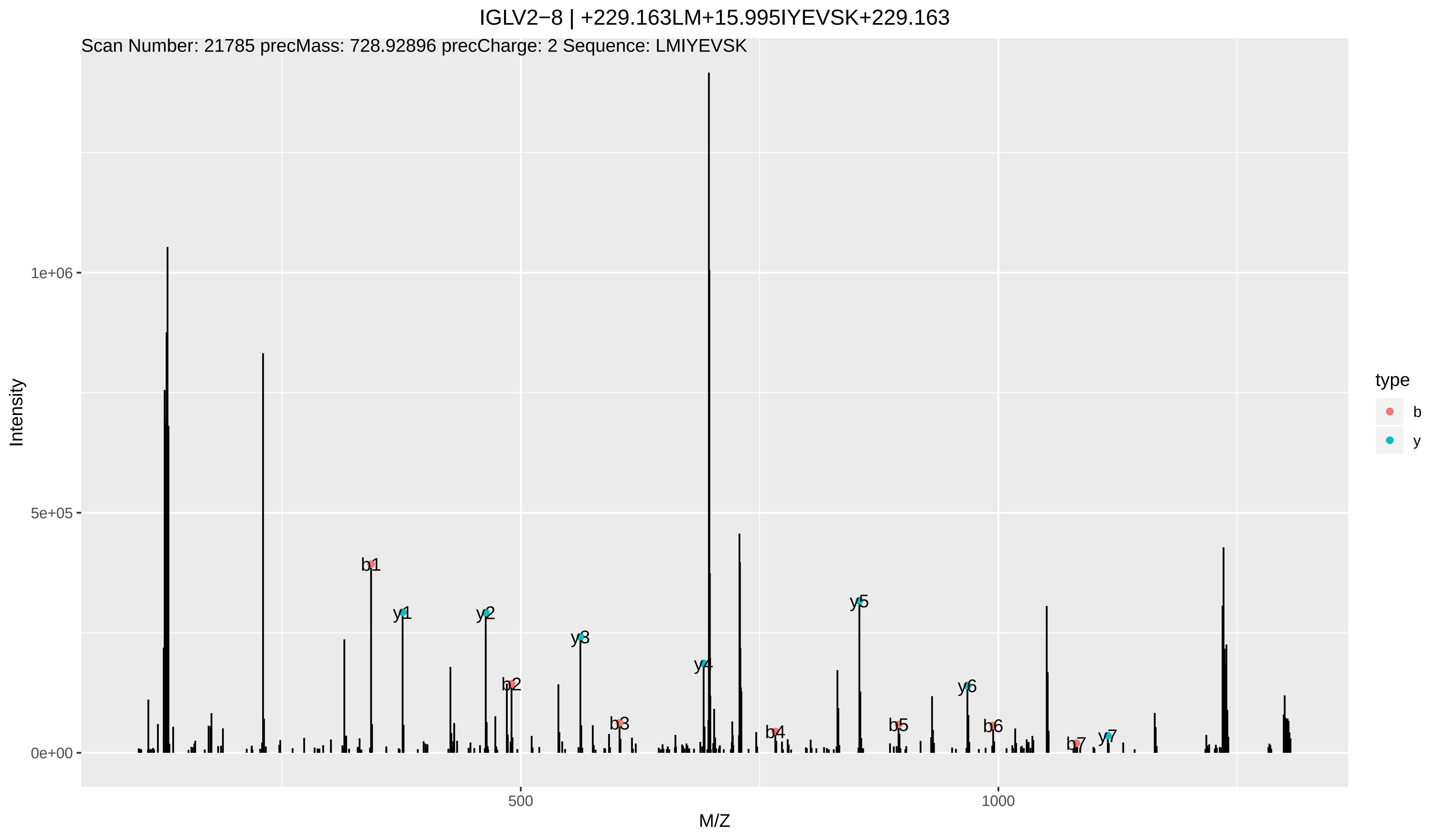

IGLV2−8 | +229.163LM+15.995IYEVSK+229.163
Scan Number: 21785 precMass: 728.92896 precCharge: 2 Sequence: LMIYEVSK
Intensity
1e+06
5e+05
0e+00
M/Z
500
1000
b1
y1
y2
b2
y3
b3
y4
b4
y5
b5
y6
b6
b7
y7
type
b
y

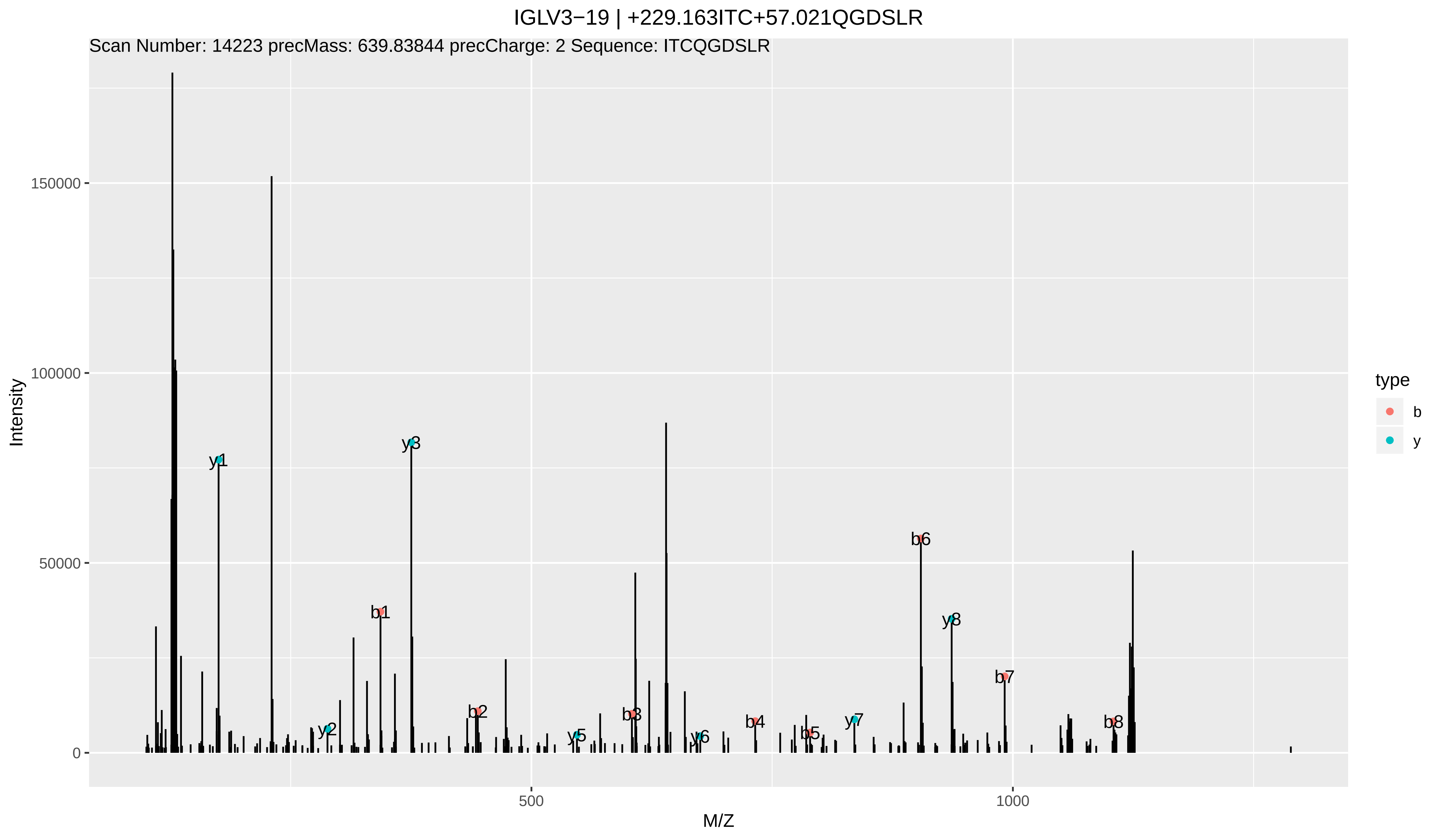

IGLV3−19 | +229.163ITC+57.021QGDSLR
Scan Number: 14223 precMass: 639.83844 precCharge: 2 Sequence: ITCQGDSLR
Intensity
150000
100000
50000
0
M/Z
500
1000
y1
y2
y3
y5
y6
y7
y8
b1
b2
b3
b4
b5
b6
b7
b8
type
b
y

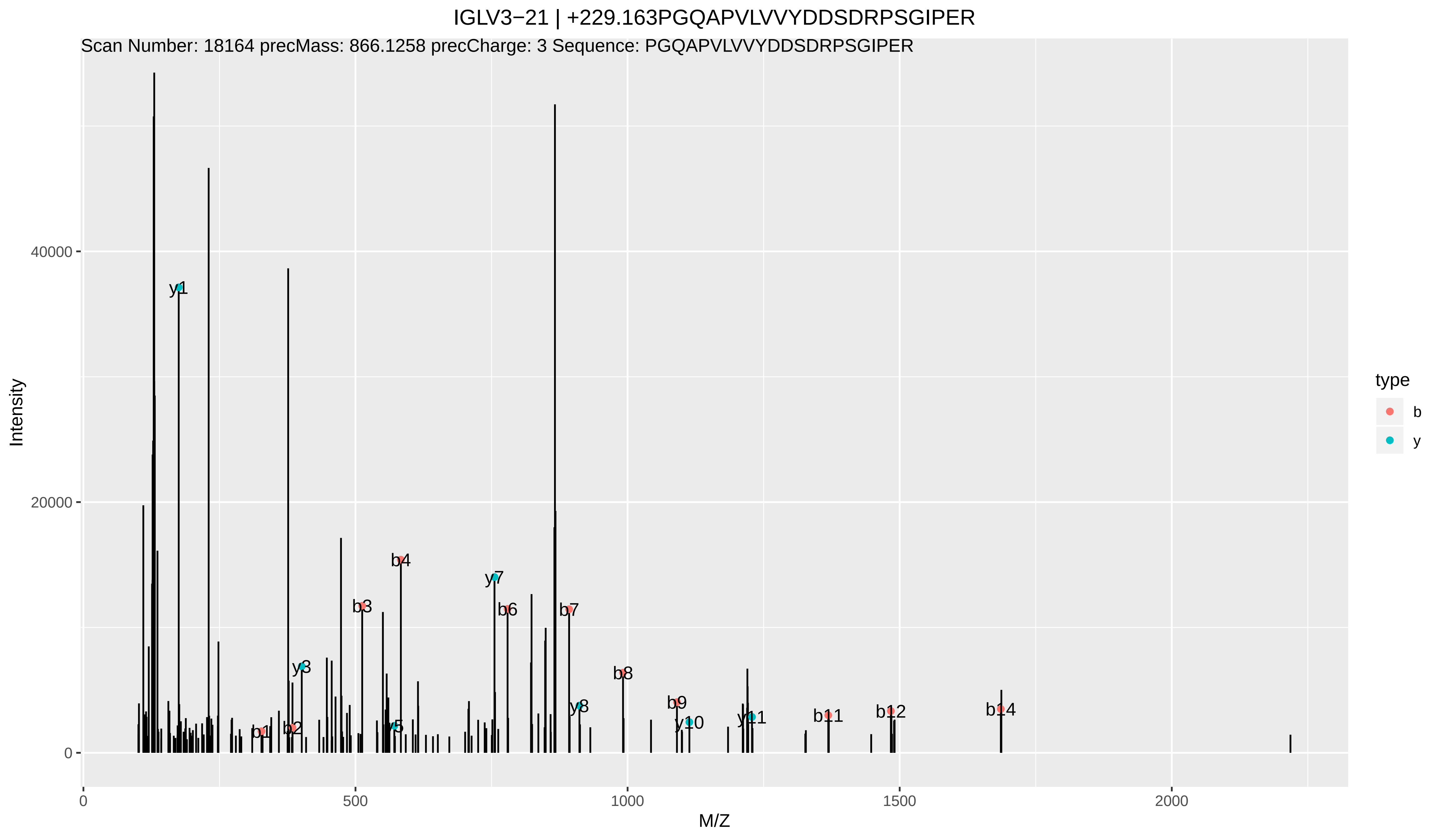

IGLV3−21 | +229.163PGQAPVLVVYDDSDRPSGIPER
Scan Number: 18164 precMass: 866.1258 precCharge: 3 Sequence: PGQAPVLVVYDDSDRPSGIPER
Intensity
40000
20000
0
M/Z
0
500
1000
1500
2000
y1
y3
y5
y7
y8
y10
y11
b1
b2
b3
b4
b6
b7
b8
b9
b11
b12
b14
type
b
y

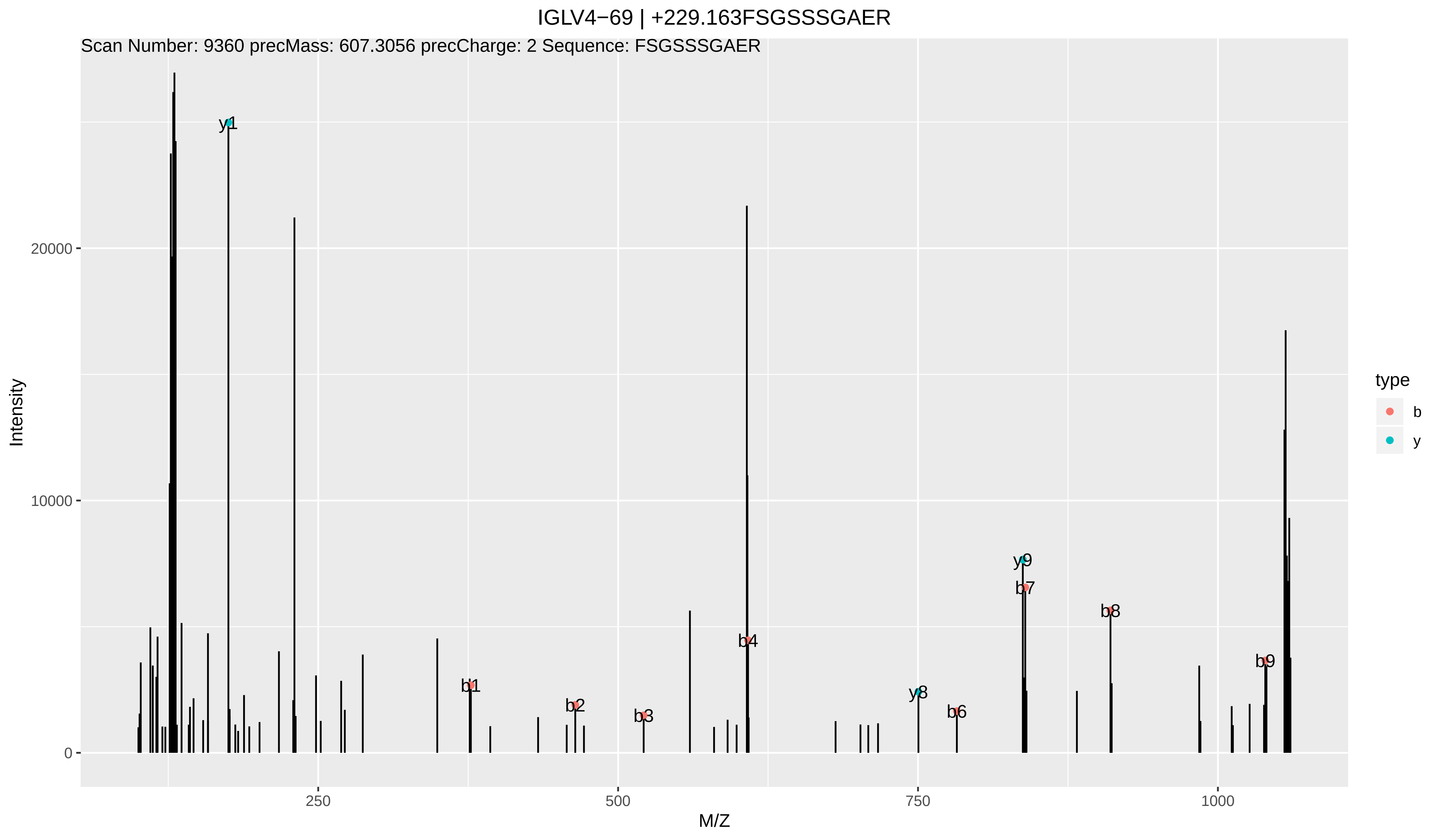
IGLV4−69 | +229.163FSGSSSGAER
Scan Number: 9360 precMass: 607.3056 precCharge: 2 Sequence: FSGSSSGAER
Intensity
M/Z
20000
10000
0
250
500
750
1000
y1
b1
b2
b3
b4
y8
b6
y9
b7
b8
b9
type
b
y

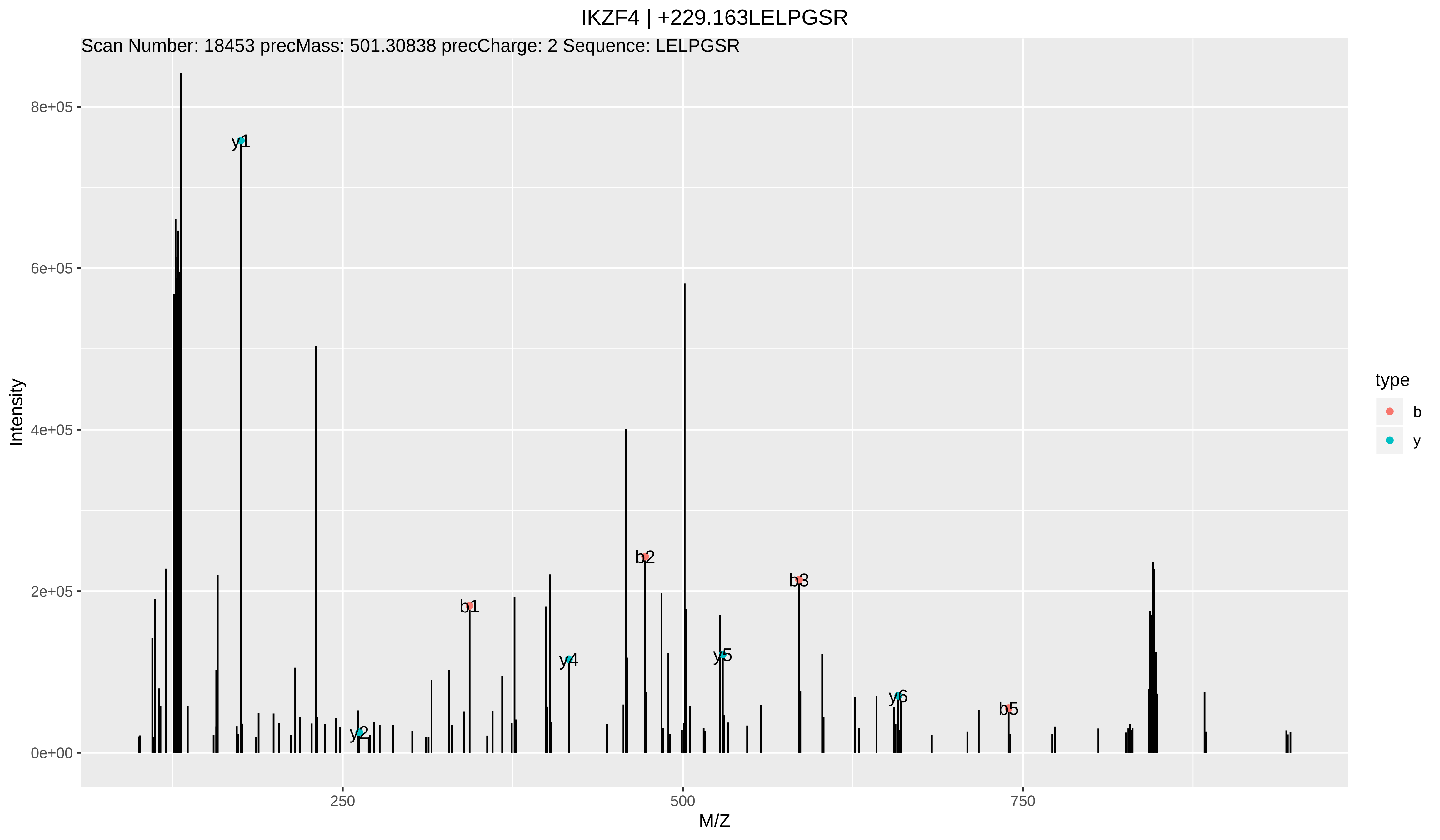
IKZF4 | +229.163LELPGSR
Scan Number: 18453 precMass: 501.30838 precCharge: 2 Sequence: LELPGSR
Intensity
8e+05
6e+05
4e+05
2e+05
0e+00
M/Z
250
500
750
y1
y2
b1
y4
b2
y5
b3
y6
b5
type
b
y

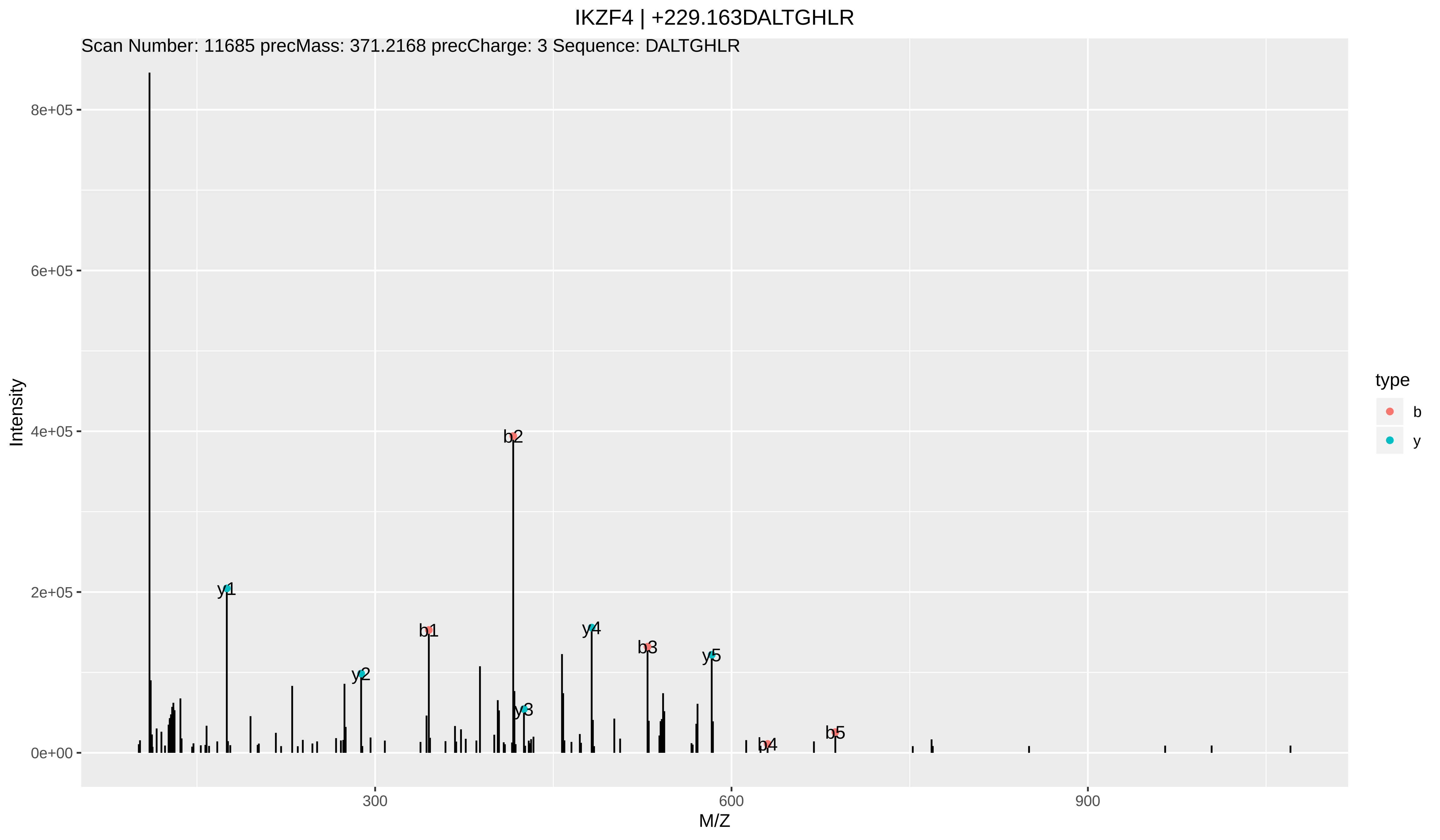

IKZF4 | +229.163DALTGHLR
Scan Number: 11685 precMass: 371.2168 precCharge: 3 Sequence: DALTGHLR
Intensity
8e+05
6e+05
4e+05
2e+05
0e+00
M/Z
300
600
900
y1
y2
b1
b2
y3
y4
b3
y5
b4
b5
type
b
y

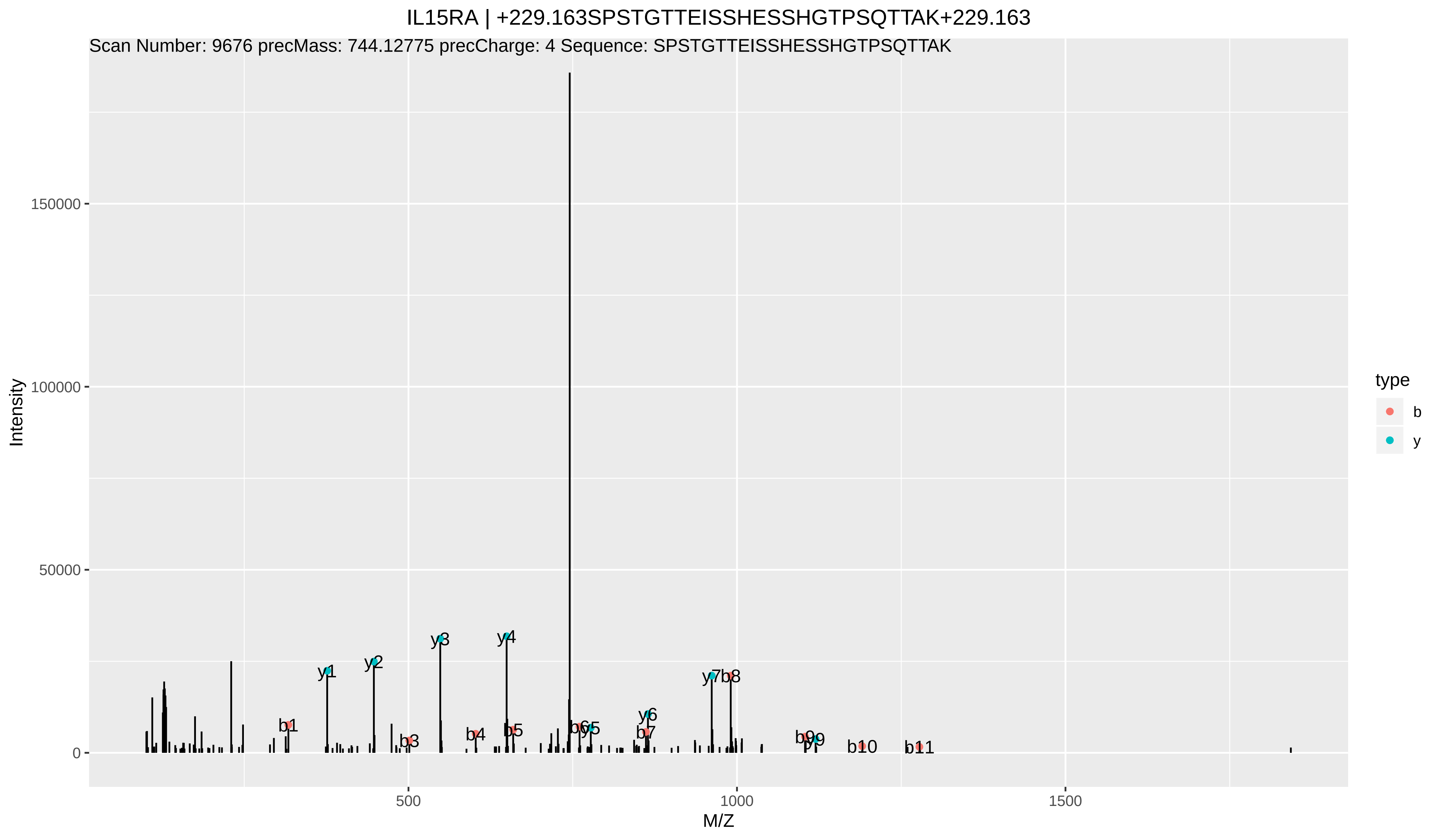
IL15RA | +229.163SPSTGTTEISSHESSHGTPSQTTAK+229.163
Scan Number: 9676 precMass: 744.12775 precCharge: 4 Sequence: SPSTGTTEISSHESSHGTPSQTTAK
Intensity
M/Z
150000
100000
50000
0
500
1000
1500
type
b
y
y1
y2
y3
y4
b1
b3
b4
b5
b6
y5
y6
b7
y7
b8
b9
y9
b10
b11

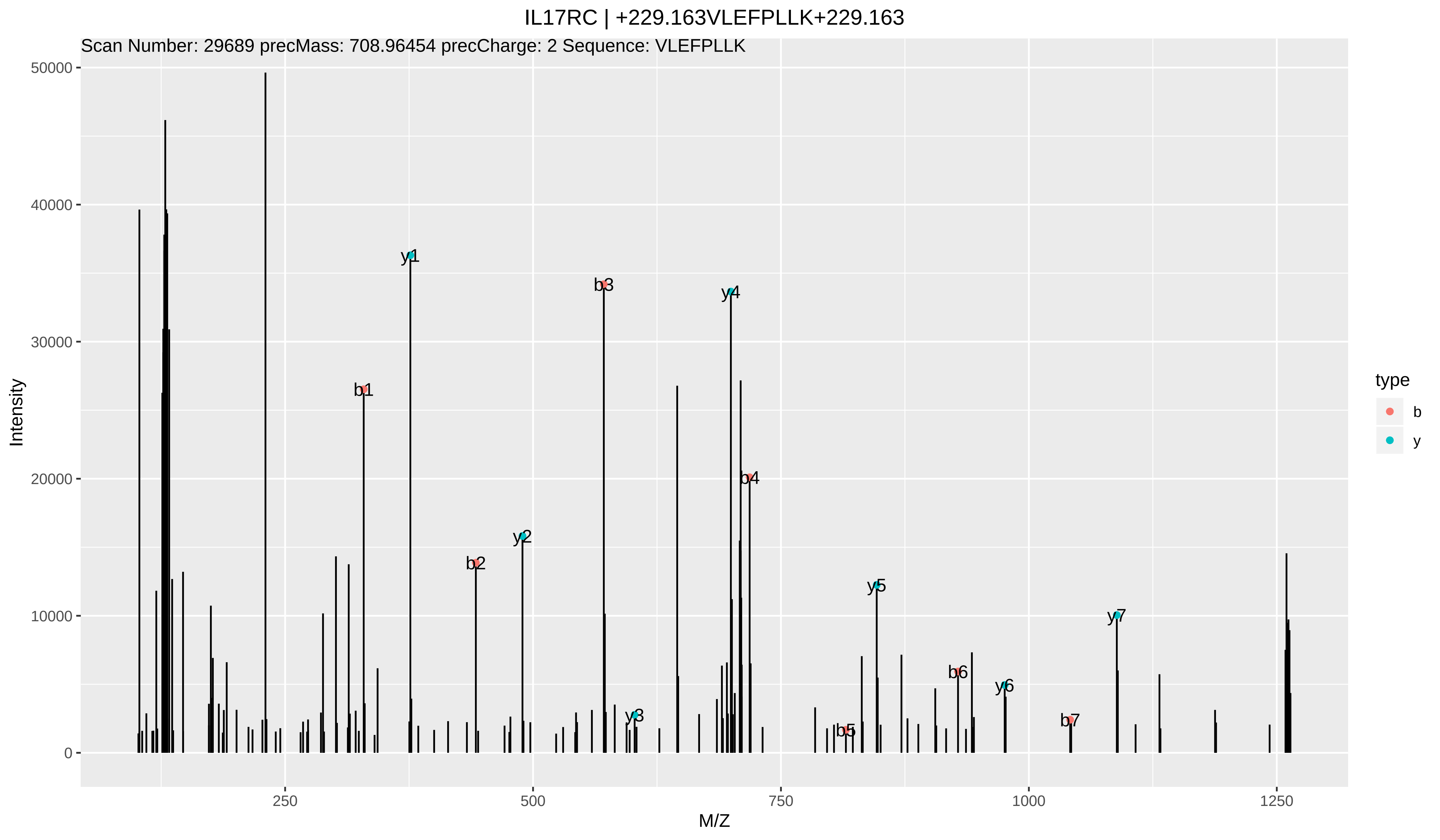

IL17RC | +229.163VLEFPLLK+229.163
Scan Number: 29689 precMass: 708.96454 precCharge: 2 Sequence: VLEFPLLK
Intensity
M/Z
0
10000
20000
30000
40000
50000
250
500
750
1000
1250
y1
b1
b3
y4
b4
y2
b2
y5
y3
b5
b6
y6
b7
y7
type
b
y

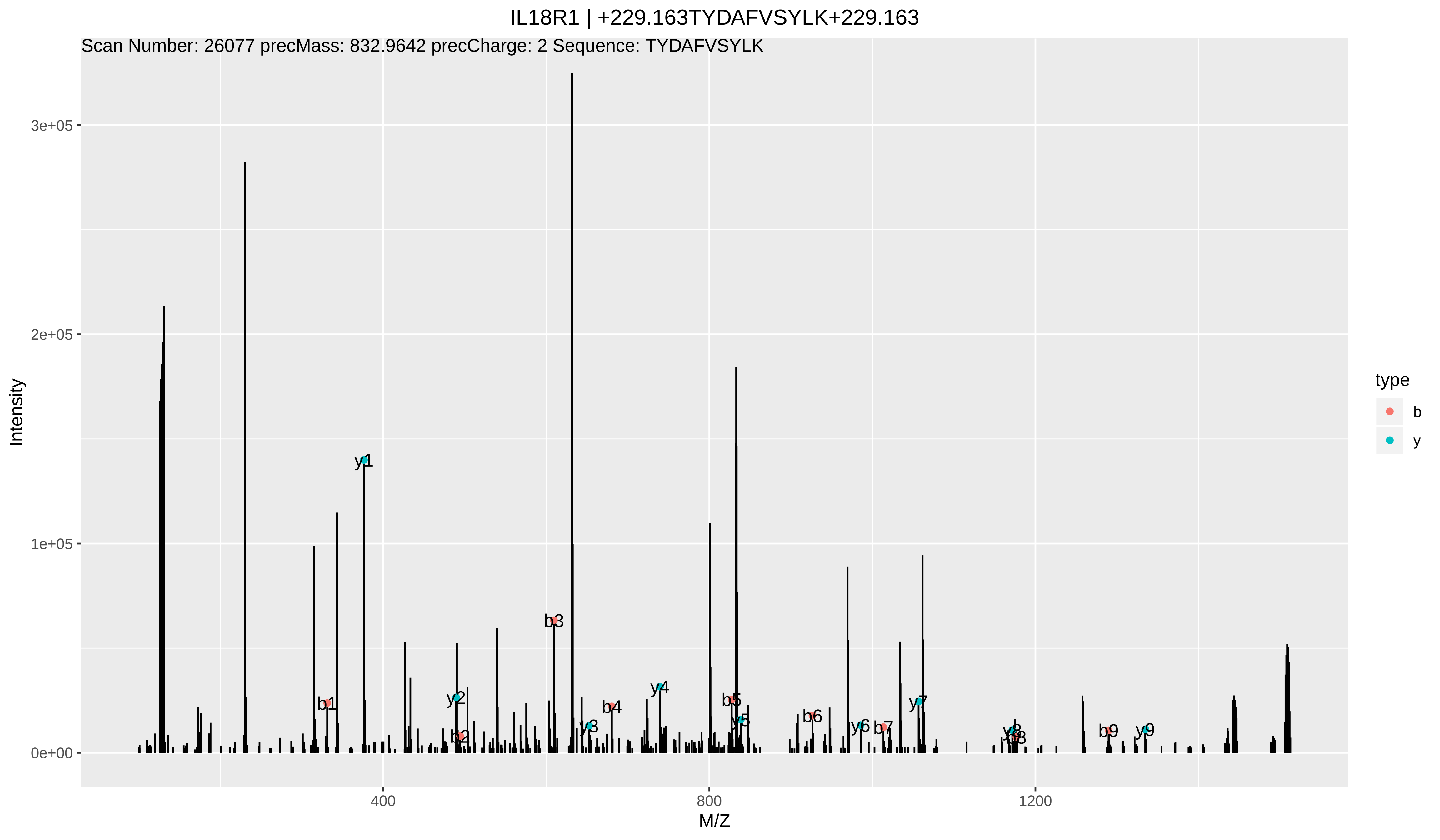

IL18R1 | +229.163TYDAFVSYLK+229.163
Scan Number: 26077 precMass: 832.9642 precCharge: 2 Sequence: TYDAFVSYLK
Intensity
3e+05
2e+05
1e+05
0e+00
M/Z
400
800
1200
type
b
y
y1
y2
y3
y4
y5
y6
y7
y8
y9
b1
b2
b3
b4
b5
b6
b7
b8
b9

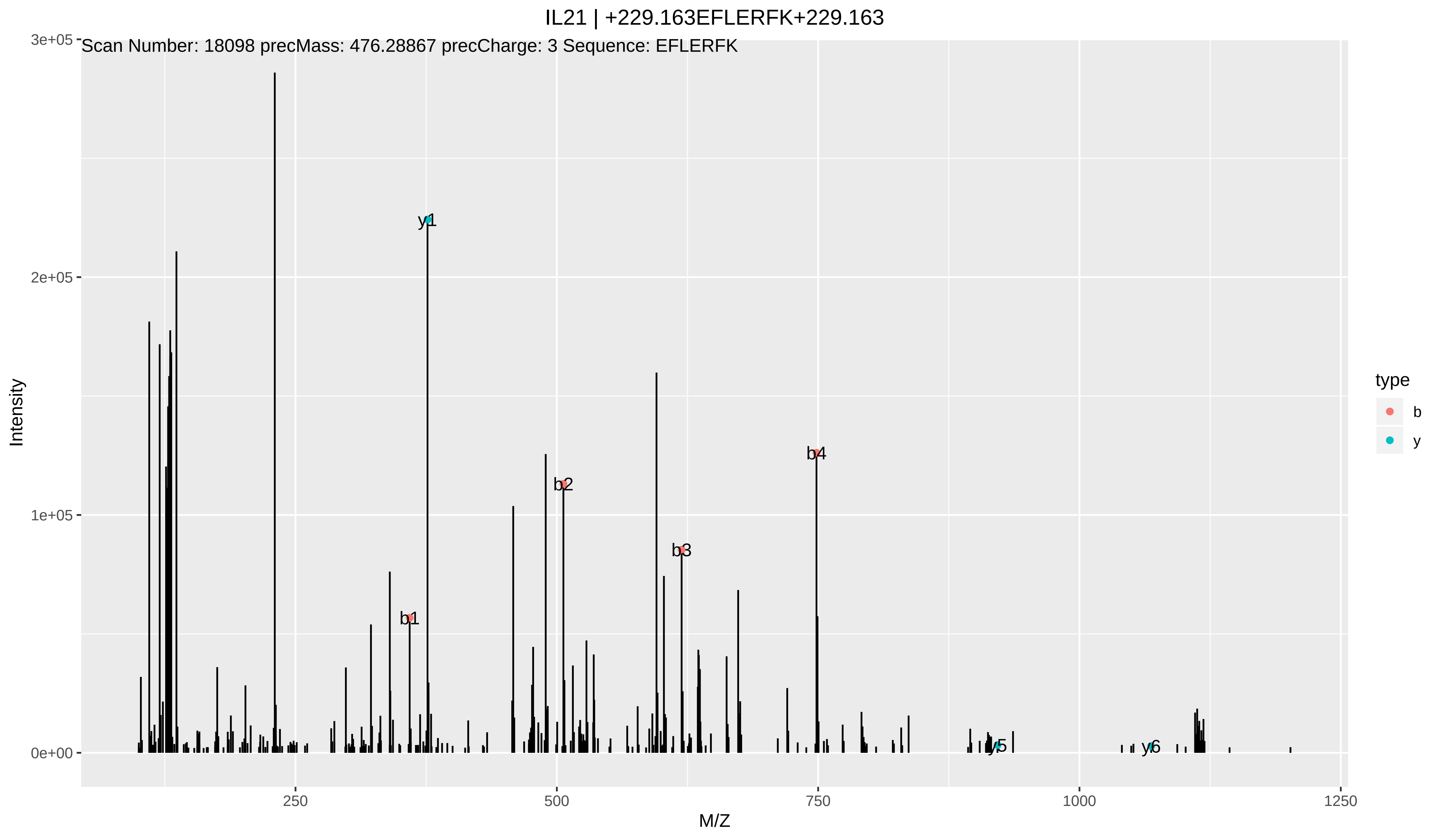

IL21 | +229.163EFLERFK+229.163
Scan Number: 18098 precMass: 476.28867 precCharge: 3 Sequence: EFLERFK
Intensity
3e+05
2e+05
1e+05
0e+00
M/Z
250
500
750
1000
1250
y1
b1
b2
b3
b4
y5
y6
type
b
y

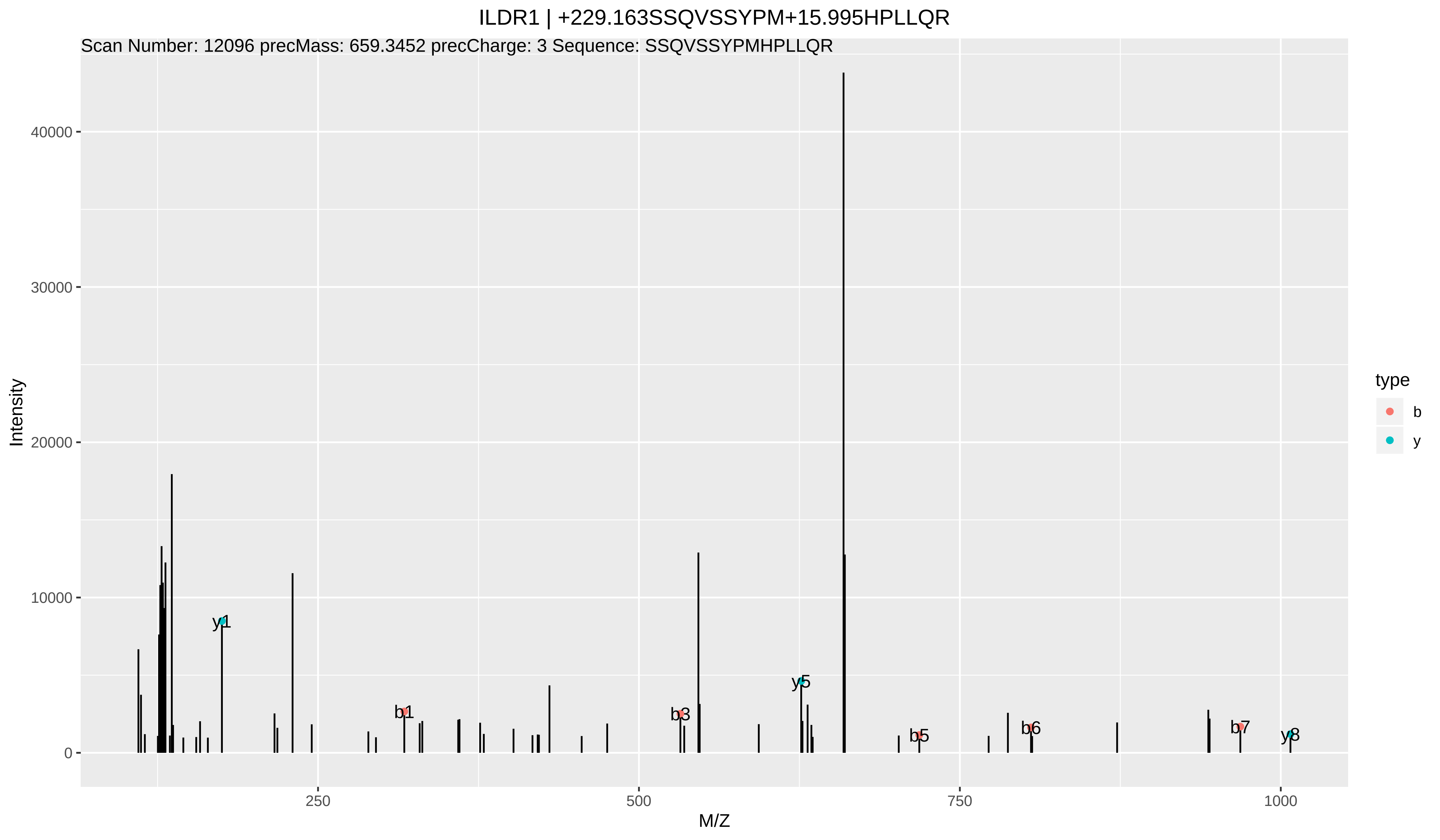
ILDR1 | +229.163SSQVSSYPM+15.995HPLLQR
Scan Number: 12096 precMass: 659.3452 precCharge: 3 Sequence: SSQVSSYPMHPLLQR
Intensity
40000
30000
20000
10000
0
M/Z
250
500
750
1000
y1
b1
b3
y5
b5
b6
b7
y8
type
b
y

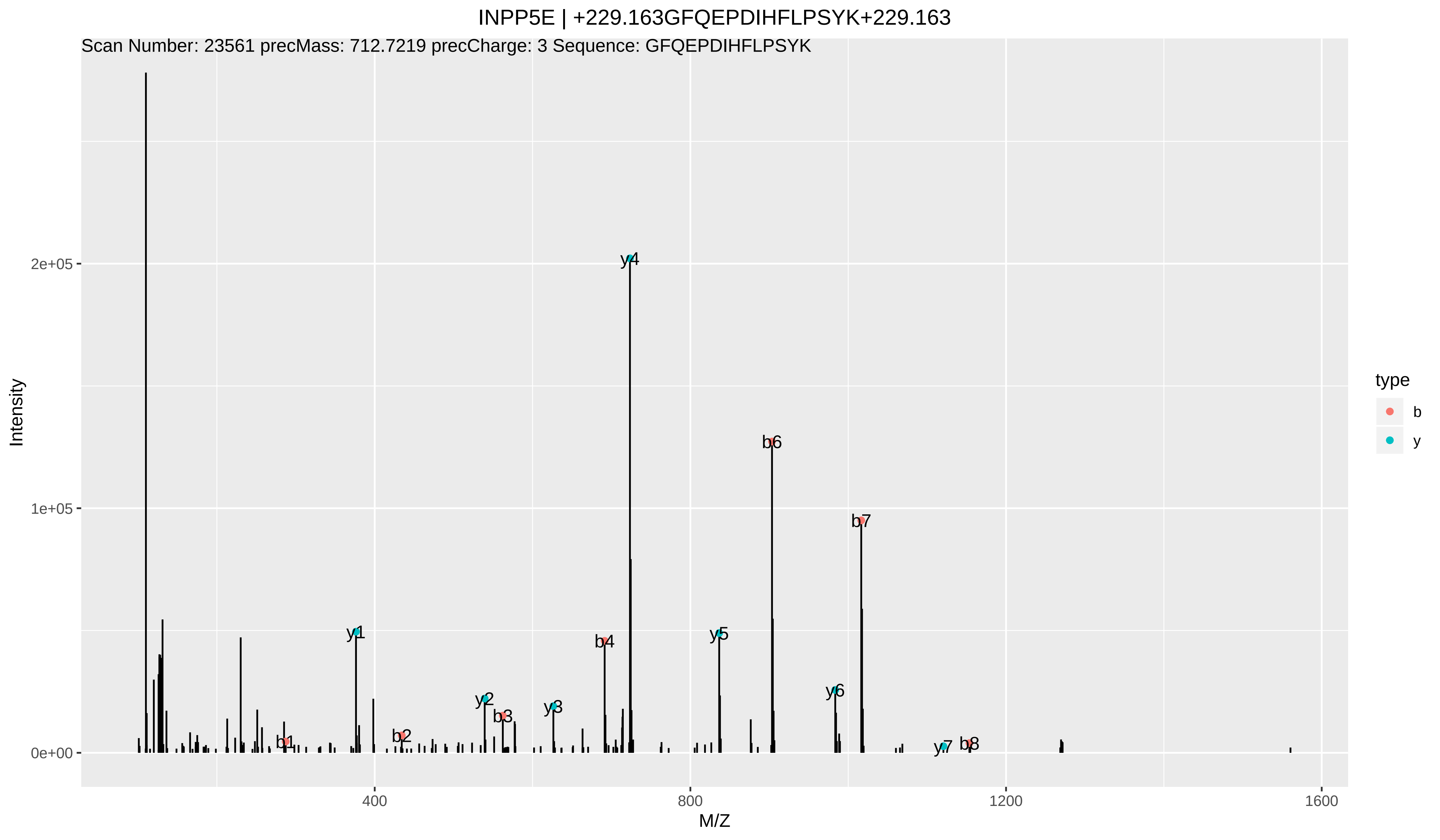
INPP5E | +229.163GFQEPDIHFLPSYK+229.163
Scan Number: 23561 precMass: 712.7219 precCharge: 3 Sequence: GFQEPDIHFLPSYK
Intensity
M/Z
2e+05
1e+05
0e+00
400
800
1200
1600
y4
b6
b7
y1
b4
y5
y6
y2
b3
y3
b2
b1
y7
b8
type
b
y

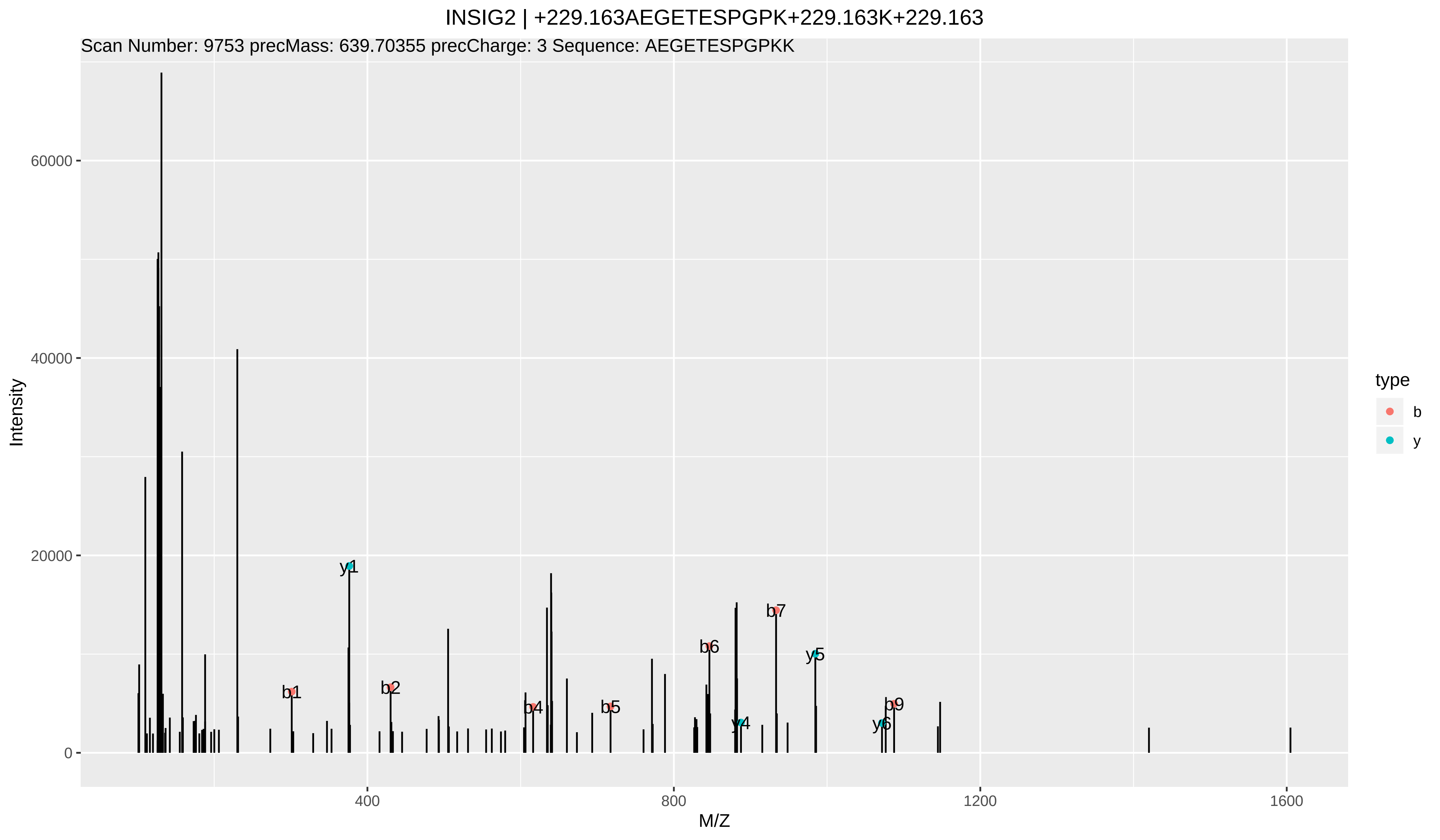
INSIG2 | +229.163AEGETESPGPK+229.163K+229.163
Scan Number: 9753 precMass: 639.70355 precCharge: 3 Sequence: AEGETESPGPKK
Intensity
60000
40000
20000
0
400
800
1200
1600
M/Z
b1
y1
b2
b4
b5
b6
b7
y4
y5
y6
b9
type
b
y

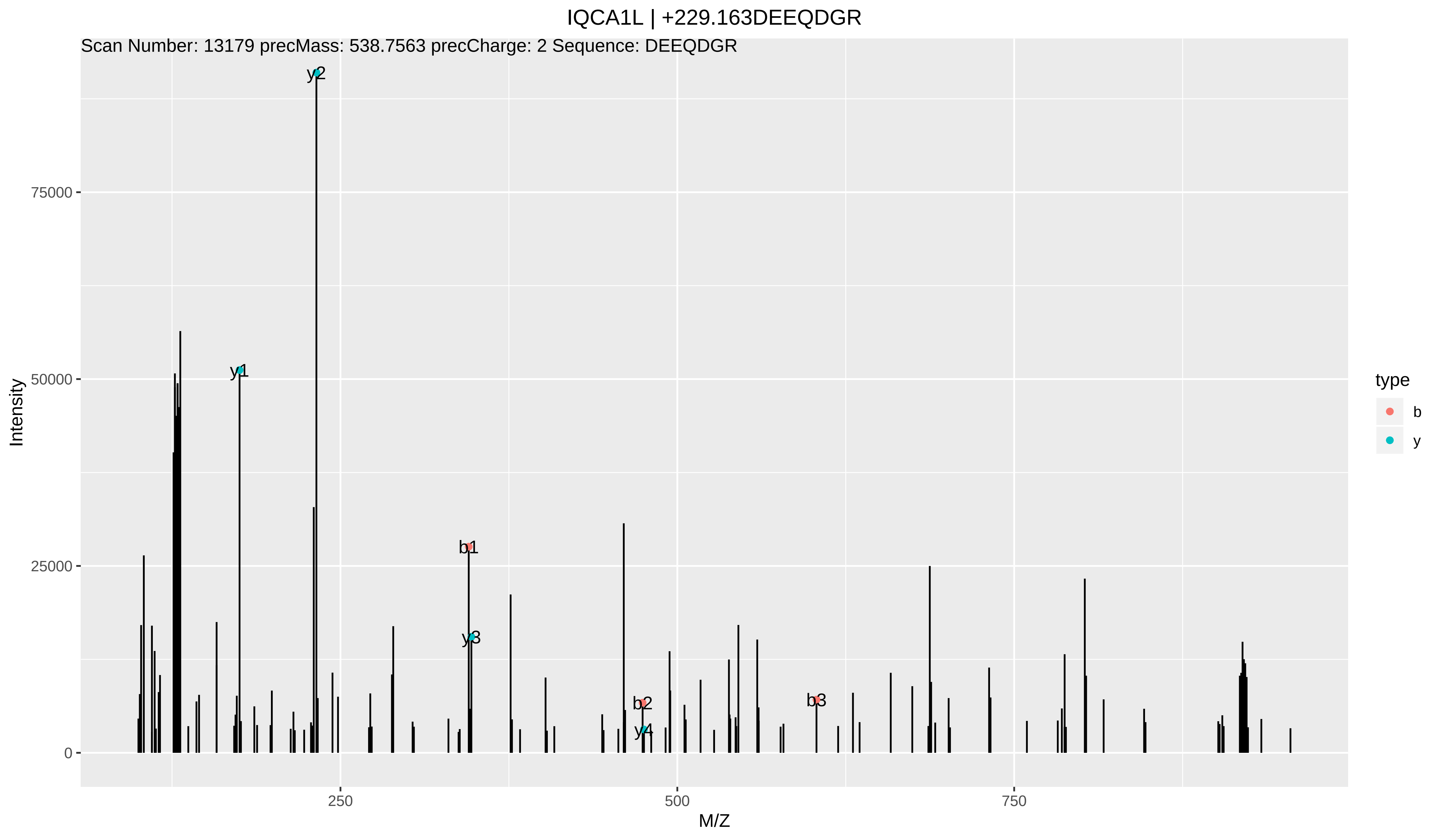
IQCA1L | +229.163DEEQDGR
Scan Number: 13179 precMass: 538.7563 precCharge: 2 Sequence: DEEQDGR
y2
y1
b1
y3
b2
y4
b3
Intensity
75000
50000
25000
0
250
500
750
M/Z
type
b
y

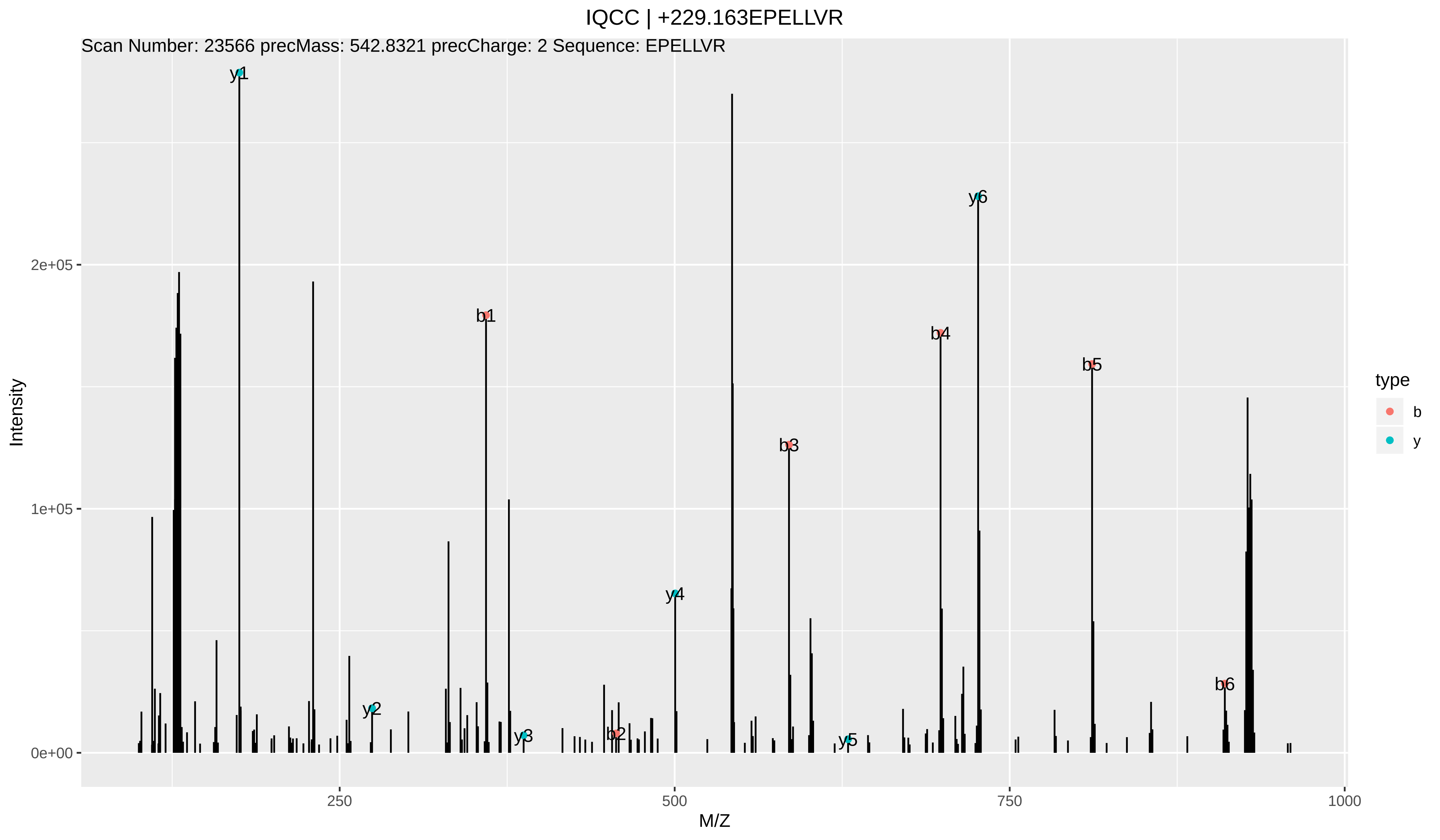

IQCC | +229.163EPELLVR
Scan Number: 23566 precMass: 542.8321 precCharge: 2 Sequence: EPELLVR
Intensity
M/Z
type
b
y
2e+05
1e+05
0e+00
250
500
750
1000
y1
b1
b4
y6
b5
b3
y4
y2
y3
b2
y5
b6

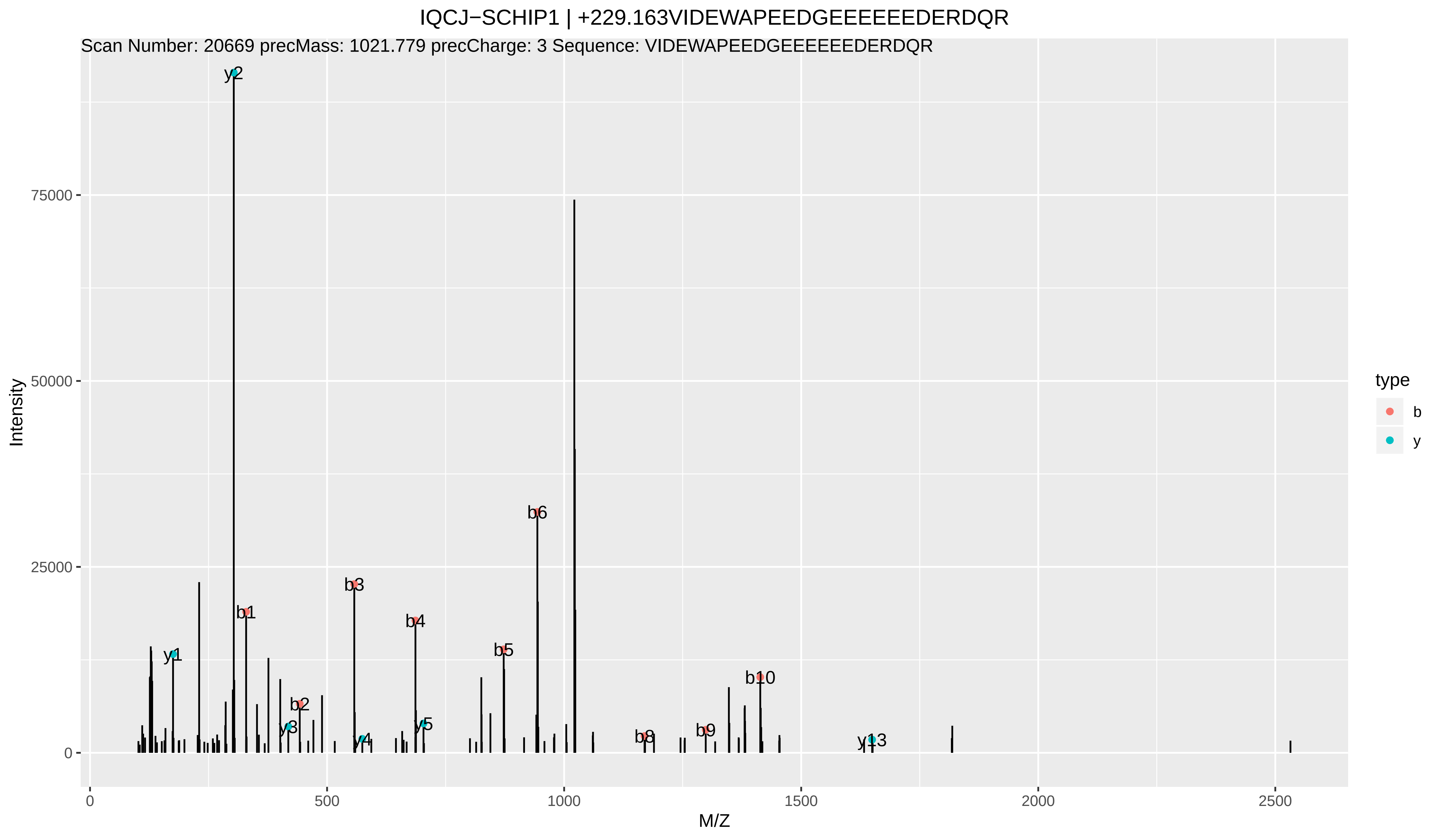

IQCJ−SCHIP1 | +229.163VIDEWAPEEDGEEEEEEDERDQR
Scan Number: 20669 precMass: 1021.779 precCharge: 3 Sequence: VIDEWAPEEDGEEEEEEDERDQR
Intensity
M/Z
0
25000
50000
75000
0
500
1000
1500
2000
2500
y1
y2
y3
y4
y5
y13
b1
b2
b3
b4
b5
b6
b8
b9
b10
type
b
y

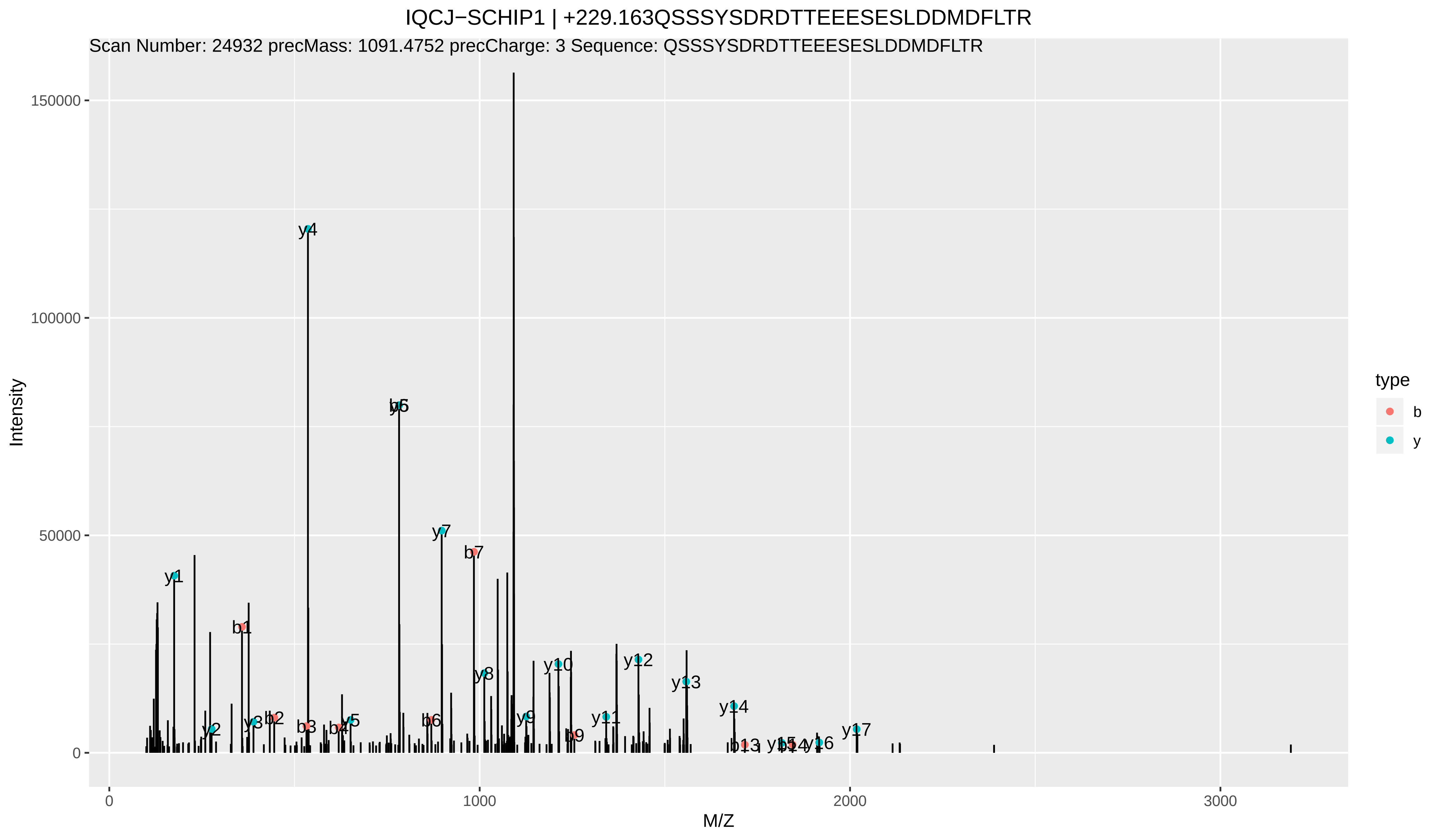
IQCJ−SCHIP1 | +229.163QSSSYSDRDTTEEESESLDDMDFLTR
Scan Number: 24932 precMass: 1091.4752 precCharge: 3 Sequence: QSSSYSDRDTTEEESESLDDMDFLTR
Intensity
M/Z
type
b
y
0
50000
100000
150000
1000
2000
3000
y1
y2
y3
y4
y5
y6
y7
y8
y9
y10
y11
y12
y13
y14
y16
y17
b1
b2
b3
b4
b5
b6
b7
b9
b13

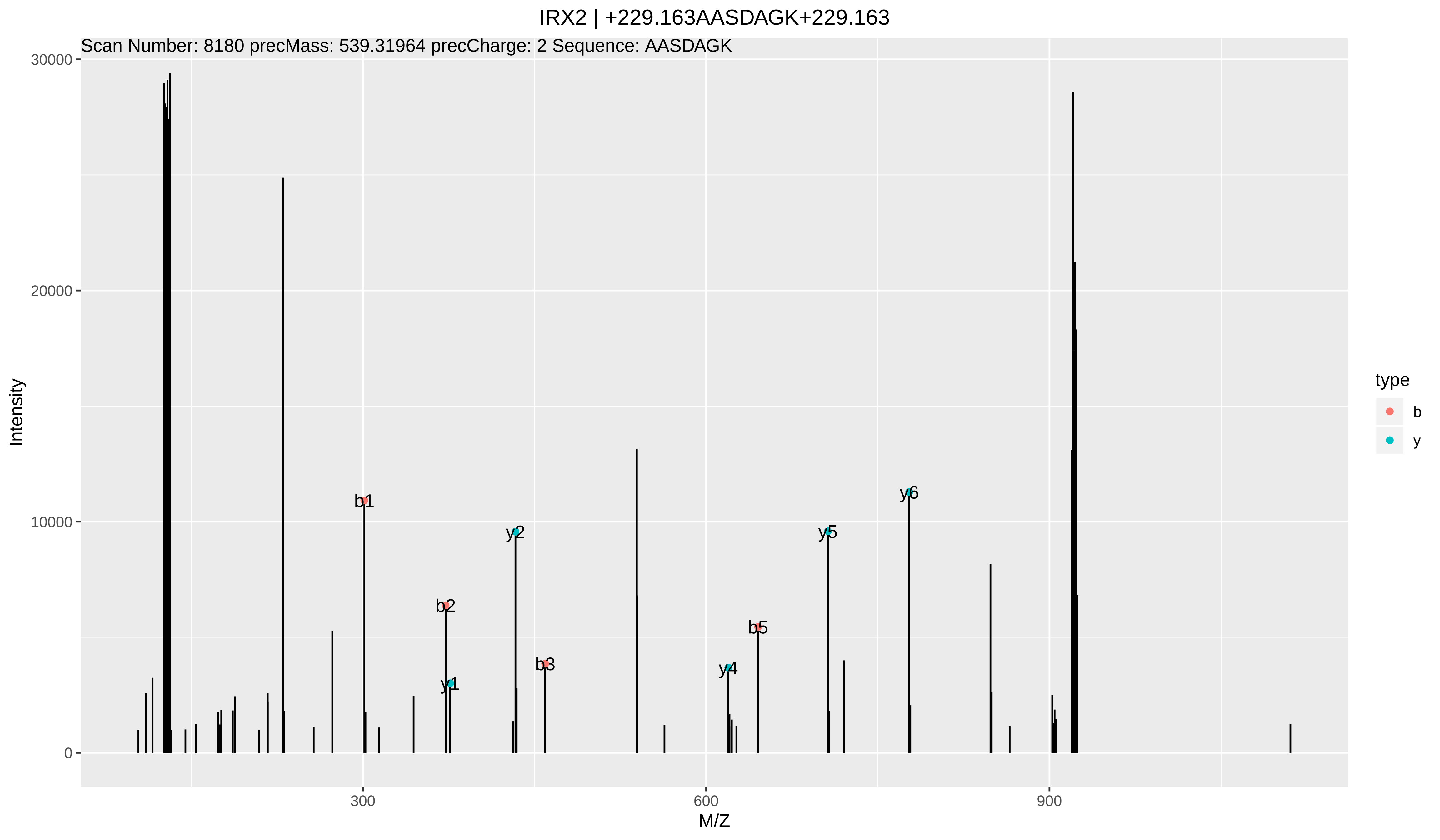
IRX2 | +229.163AASDAGK+229.163
Scan Number: 8180 precMass: 539.31964 precCharge: 2 Sequence: AASDAGK
30000
20000
10000
0
Intensity
300
600
900
M/Z
b1
b2
y1
y2
b3
y4
b5
y5
y6
type
b
y

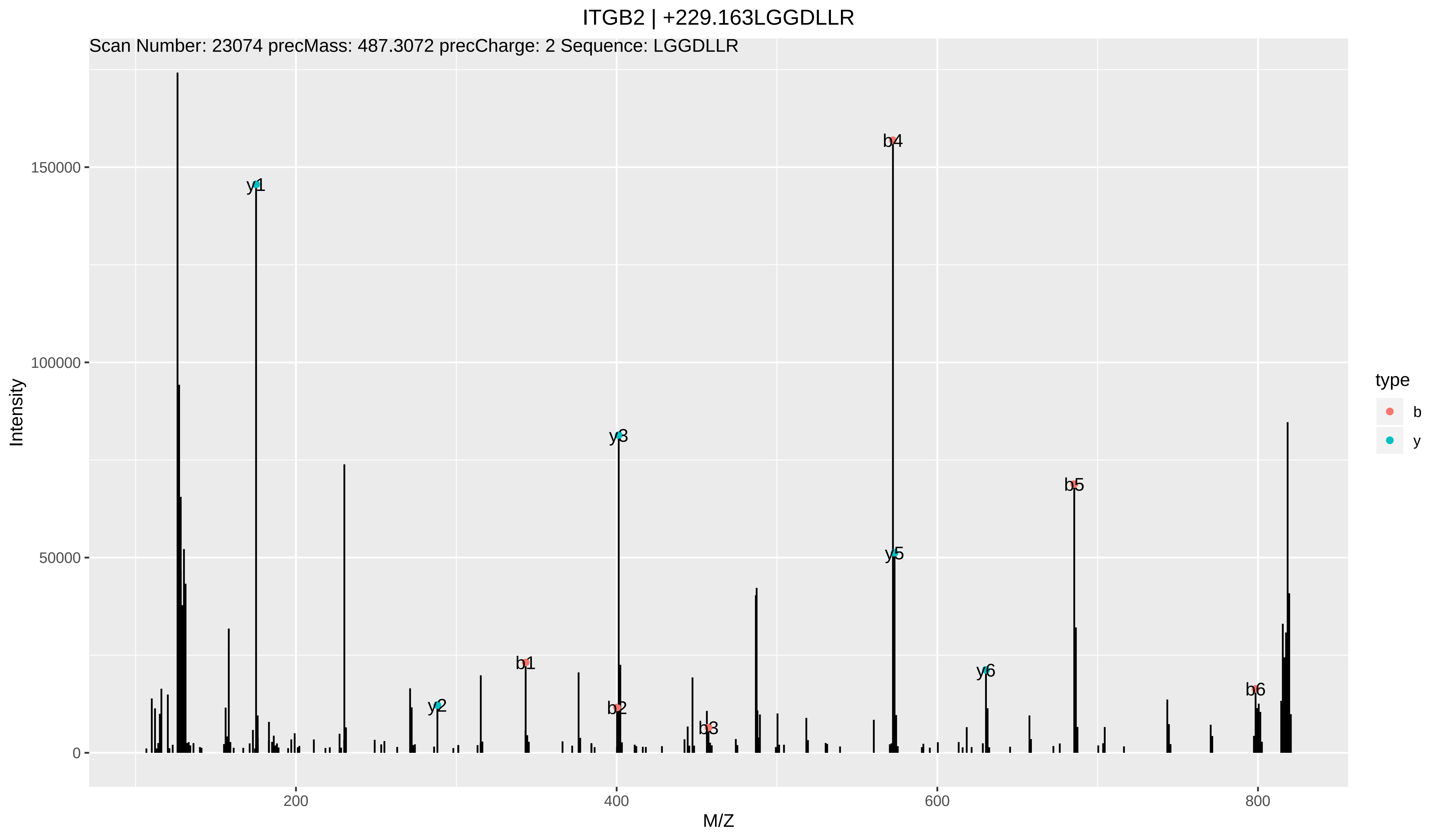
ITGB2 | +229.163LGGDLLR
Scan Number: 23074 precMass: 487.3072 precCharge: 2 Sequence: LGGDLLR
Intensity
M/Z
150000
100000
50000
0
200
400
600
800
y1
y2
y3
y5
y6
b1
b2
b3
b4
b5
b6
type
b
y

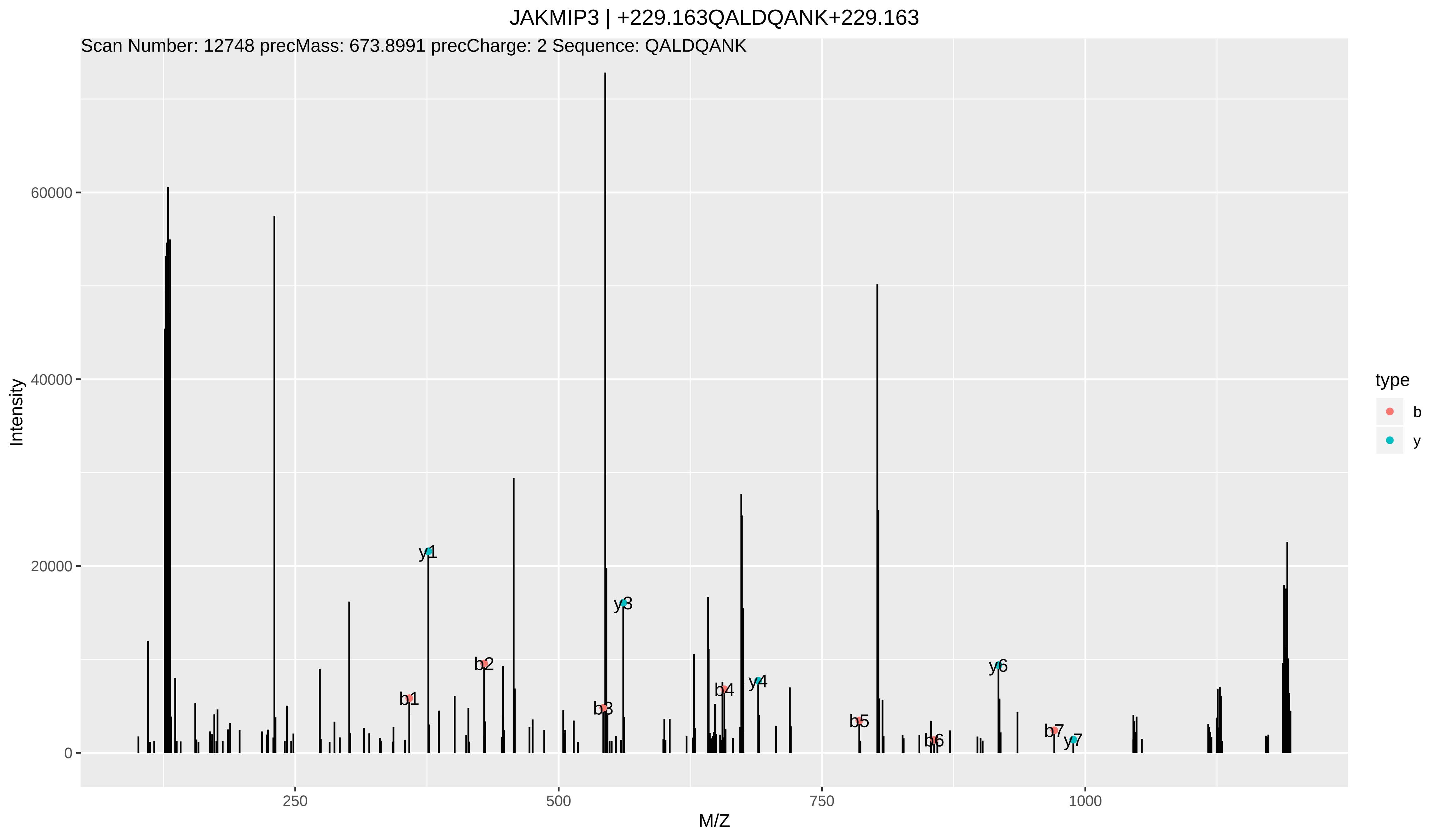

JAKMIP3 | +229.163QALDQANK+229.163
Scan Number: 12748 precMass: 673.8991 precCharge: 2 Sequence: QALDQANK
Intensity
60000
40000
20000
0
M/Z
250
500
750
1000
y1
b1
b2
b3
y3
b4
y4
b5
b6
y6
b7
y7
type
b
y

JOSD1 | +229.163SESLELPQAAPPQIYHEK+229.163

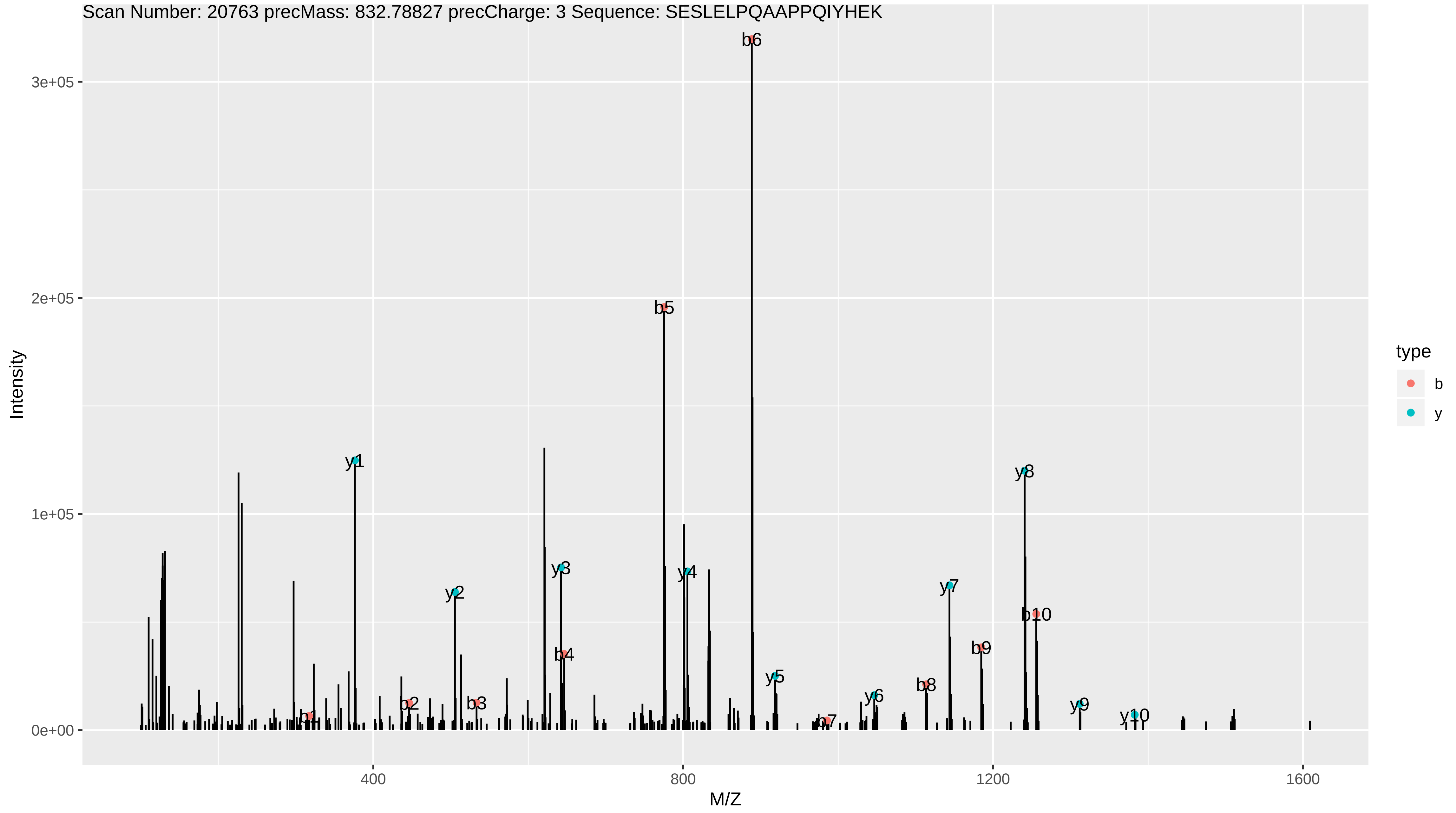

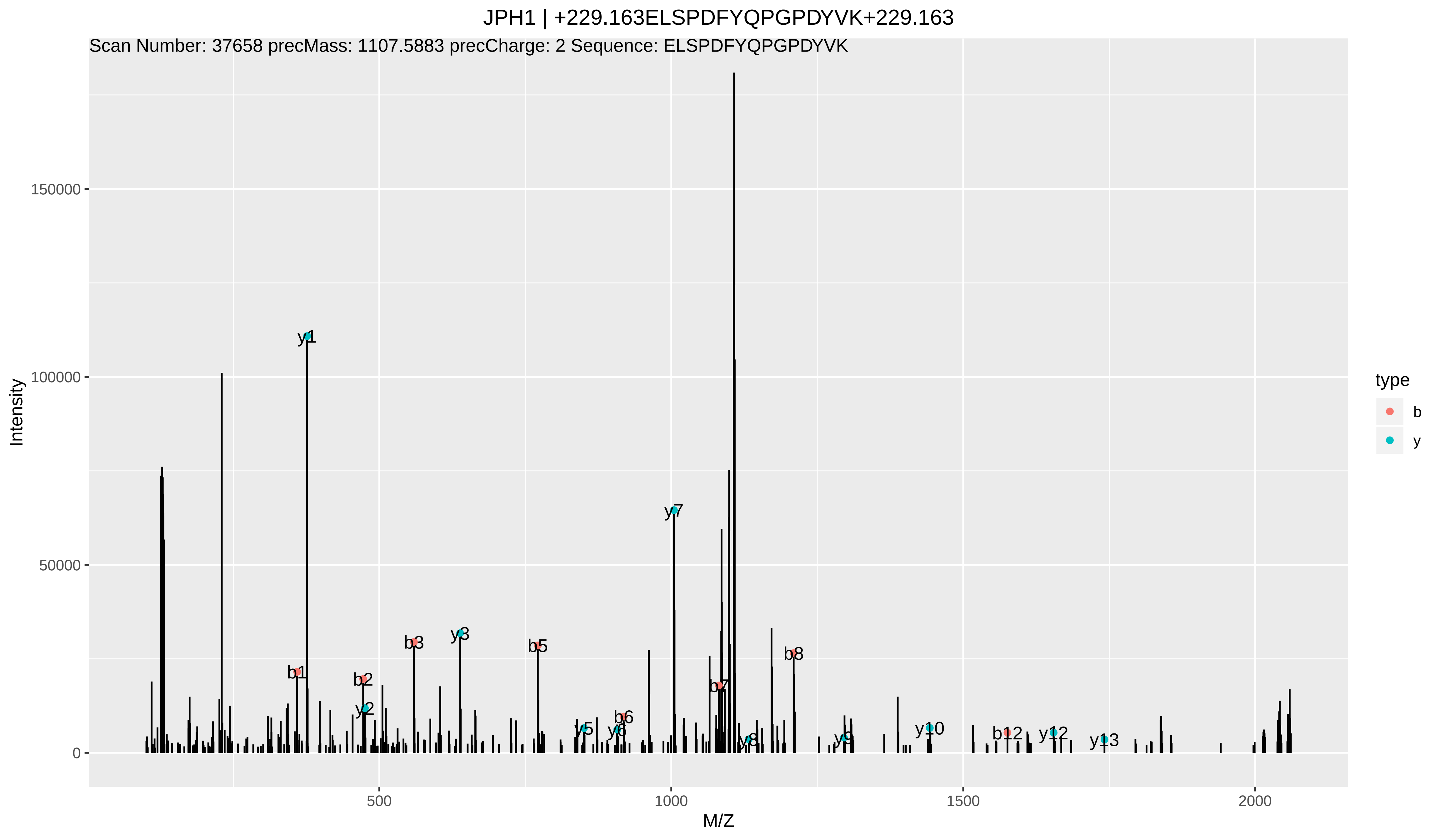
JPH1 | +229.163ELSPDFYQPGPDYVK+229.163
Scan Number: 37658 precMass: 1107.5883 precCharge: 2 Sequence: ELSPDFYQPGPDYVK
Intensity
150000
100000
50000
0
500
1000
1500
2000
M/Z
y1
b1
b2
y2
b3
y3
b5
y5
y6
b6
y7
b7
y8
b8
y9
y10
b12
y12
y13
type
b
y

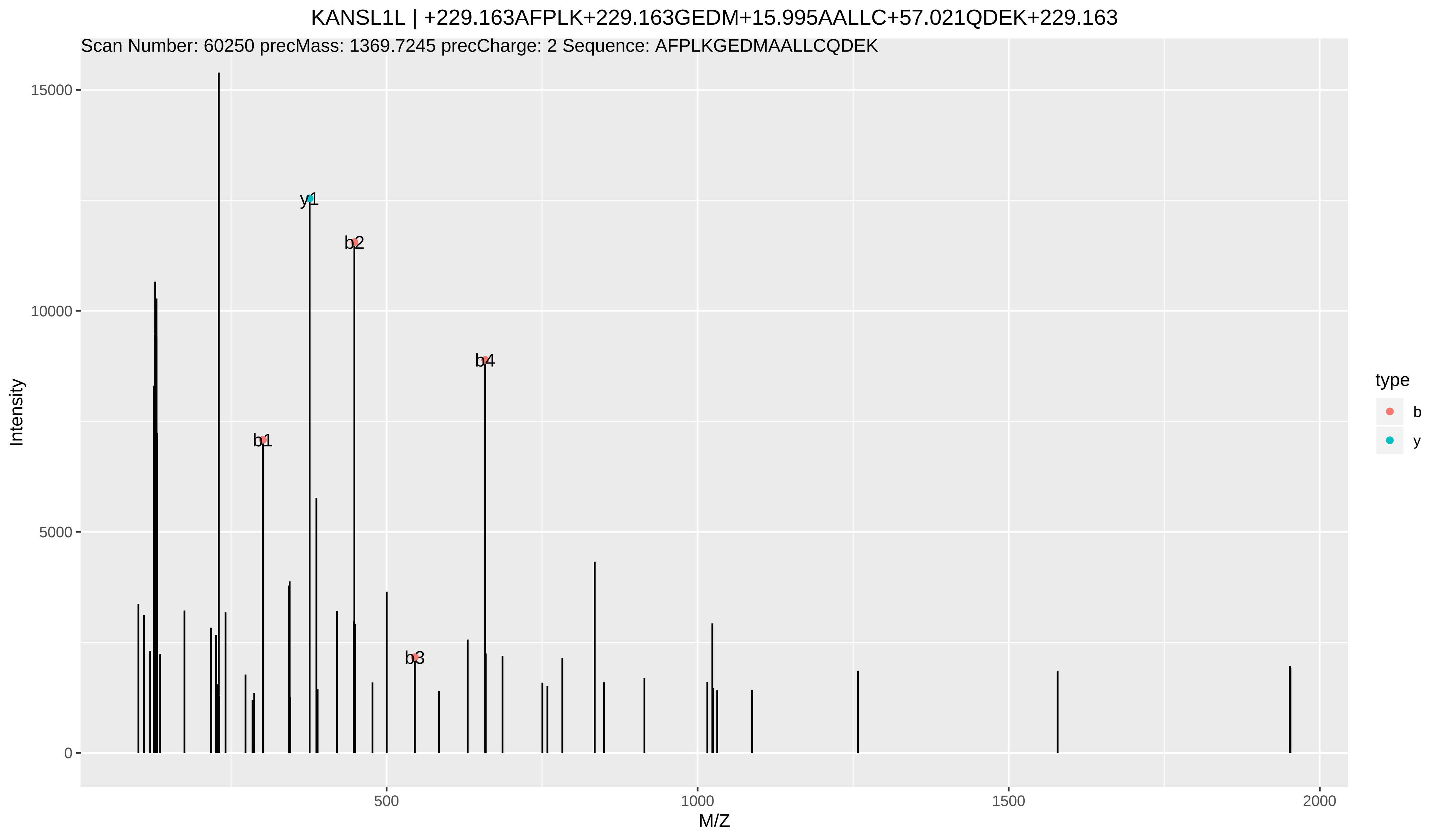
KANSL1L | +229.163AFPLK+229.163GEDM+15.995AALLC+57.021QDEK+229.163
Scan Number: 60250 precMass: 1369.7245 precCharge: 2 Sequence: AFPLKGEDMAALLCQDEK
Intensity
M/Z
15000
10000
5000
0
500
1000
1500
2000
y1
b1
b2
b3
b4
type
b
y

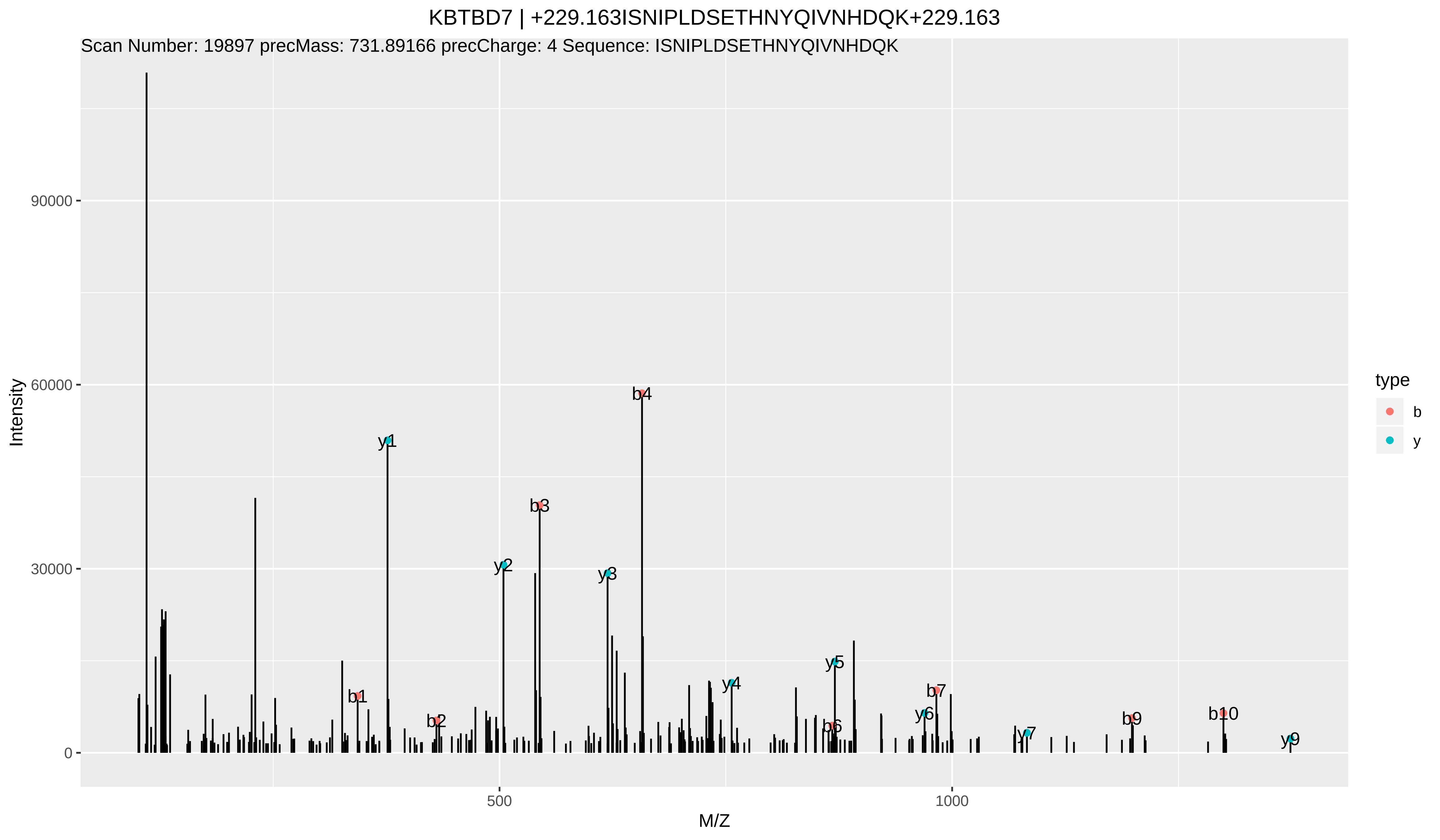

KBTBD7 | +229.163ISNIPLDSETHNYQIVNHDQK+229.163
Scan Number: 19897 precMass: 731.89166 precCharge: 4 Sequence: ISNIPLDSETHNYQIVNHDQK
Intensity
90000
60000
30000
0
500
1000
M/Z
y1
y2
y3
y4
y5
y6
y7
y9
b1
b2
b3
b4
b6
b7
b9
b10
type
b
y

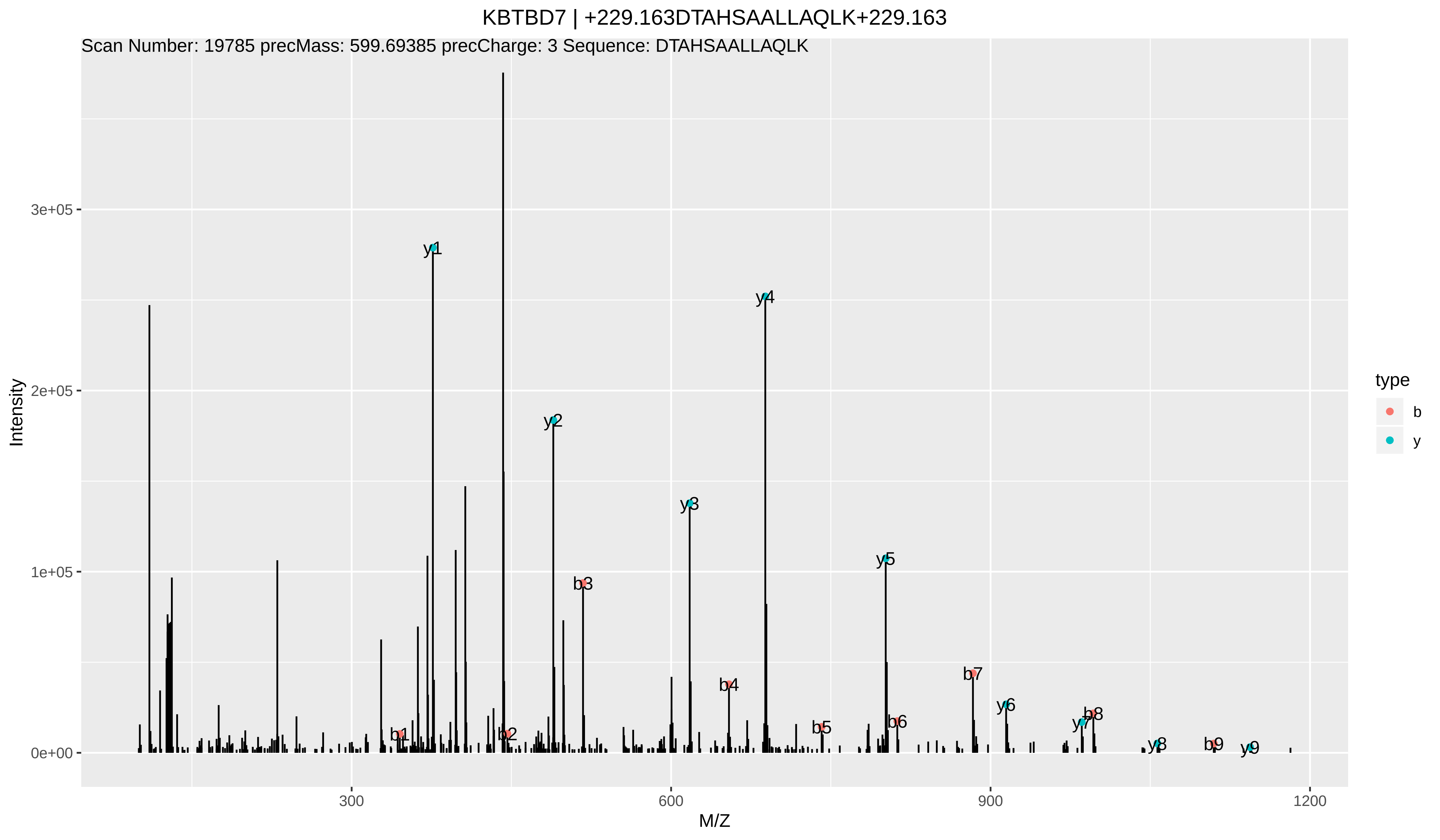
KBTBD7 | +229.163DTAHSAALLAQLK+229.163
Scan Number: 19785 precMass: 599.69385 precCharge: 3 Sequence: DTAHSAALLAQLK
Intensity
M/Z
3e+05
2e+05
1e+05
0e+00
300
600
900
1200
y1
y2
y3
y4
y5
y6
y7
y8
y9
b1
b2
b3
b4
b5
b6
b7
b8
b9
type
b
y

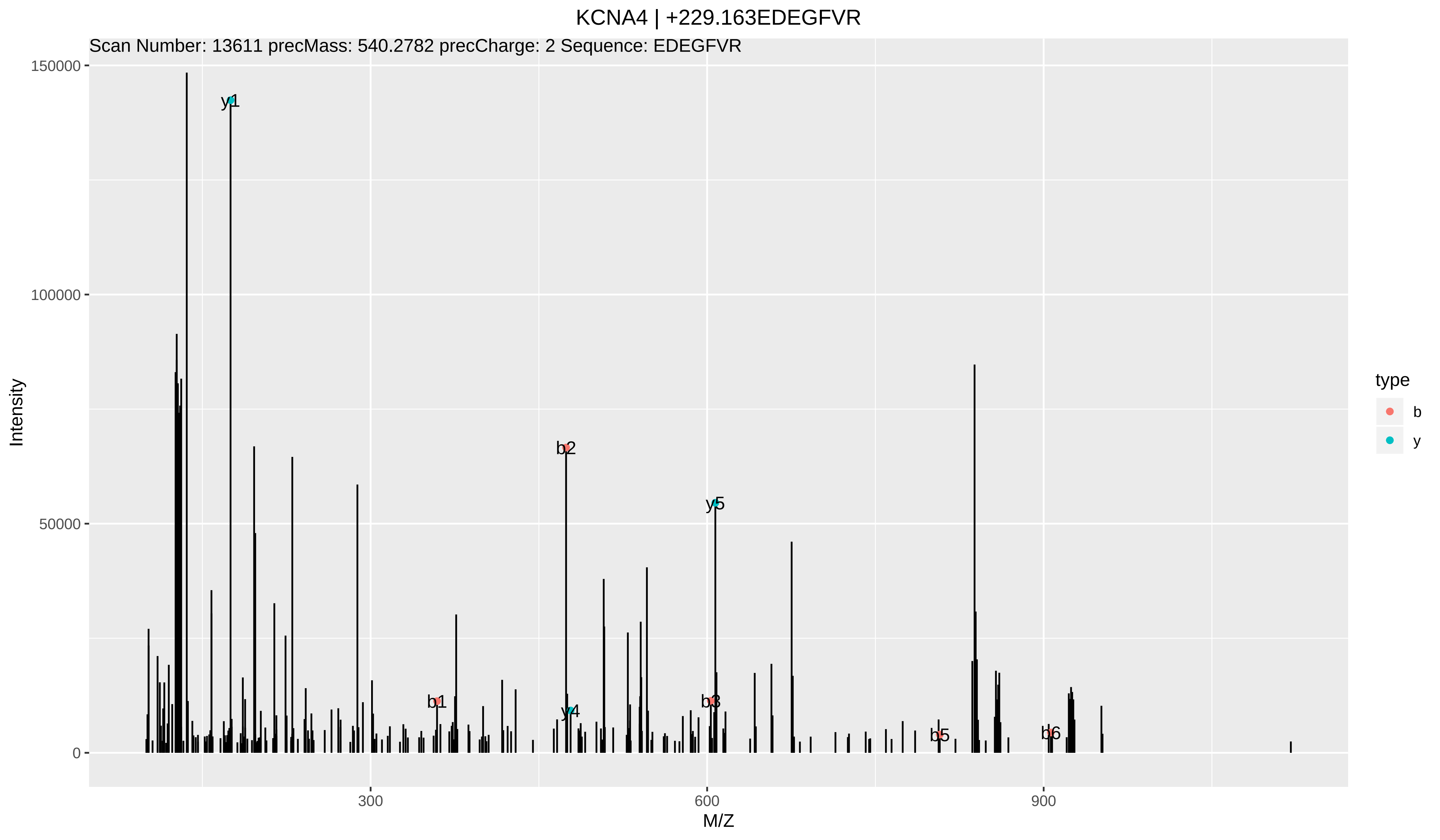
KCNA4 | +229.163EDEGFVR
Scan Number: 13611 precMass: 540.2782 precCharge: 2 Sequence: EDEGFVR
Intensity
150000
100000
50000
0
M/Z
300
600
900
y1
b2
y5
b1
y4
b3
b5
b6
type
b
y

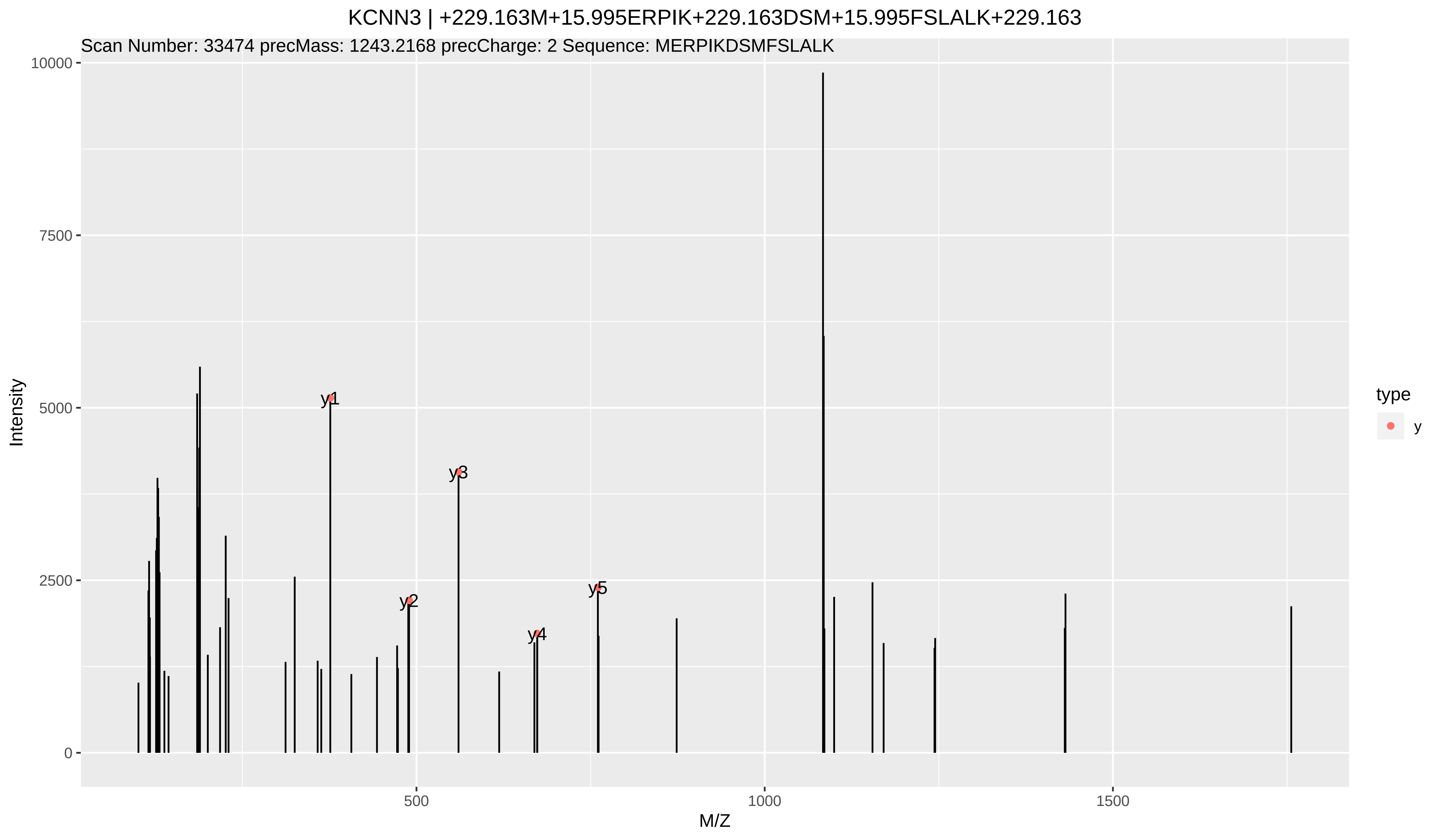

KCNN3 | +229.163M+15.995ERPIK+229.163DSM+15.995FSLALK+229.163
Scan Number: 33474 precMass: 1243.2168 precCharge: 2 Sequence: MERPIKDSMFSLALK
Intensity
M/Z
type
y
y1
y2
y3
y4
y5
0
2500
5000
7500
10000
500
1000
1500

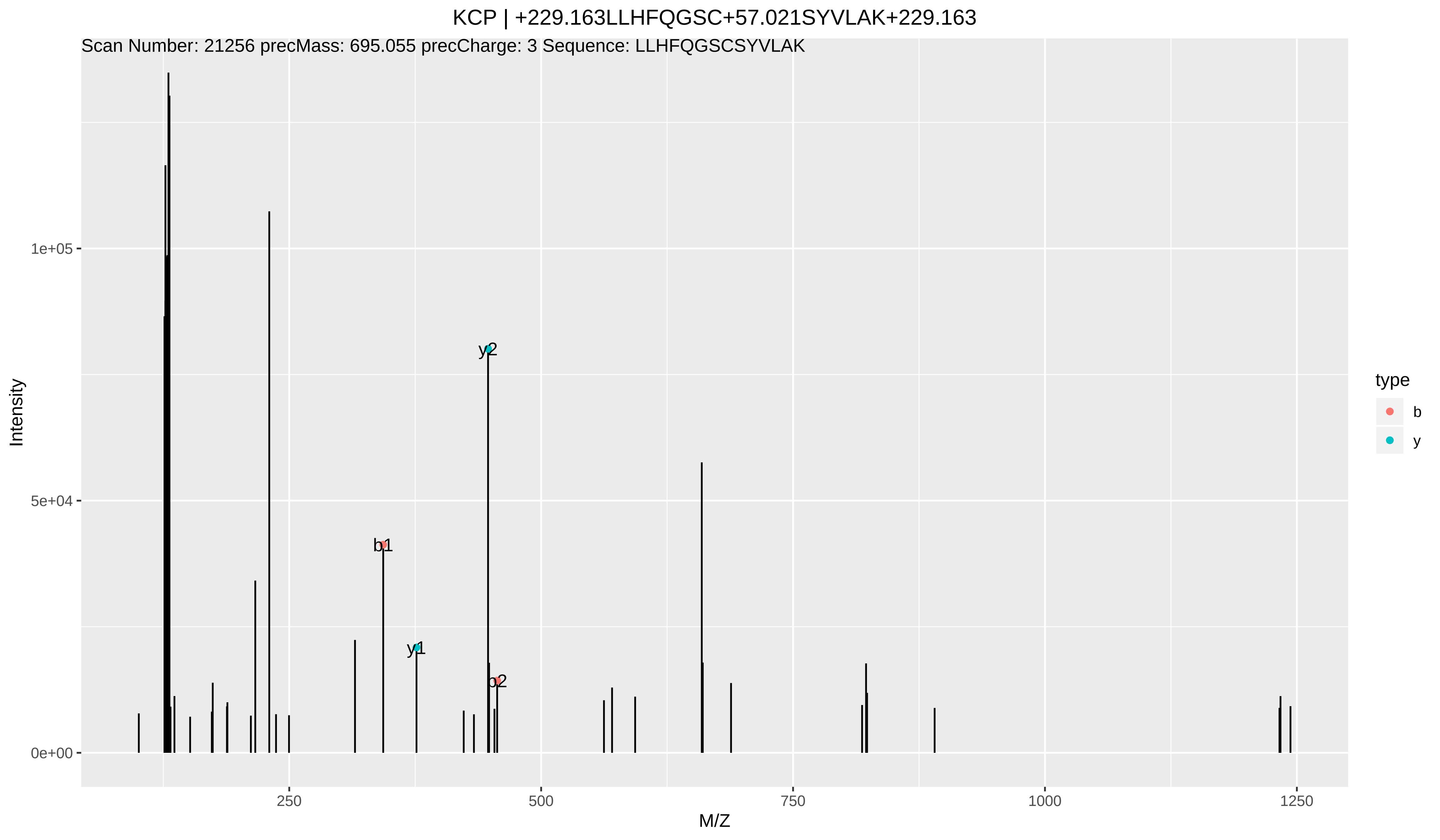
KCP | +229.163LLHFQGSC+57.021SYVLAK+229.163
Scan Number: 21256 precMass: 695.055 precCharge: 3 Sequence: LLHFQGSCSYVLAK
y2
b1
y1
b2
Intensity
1e+05
5e+04
0e+00
250
500
750
1000
1250
M/Z
type
b
y

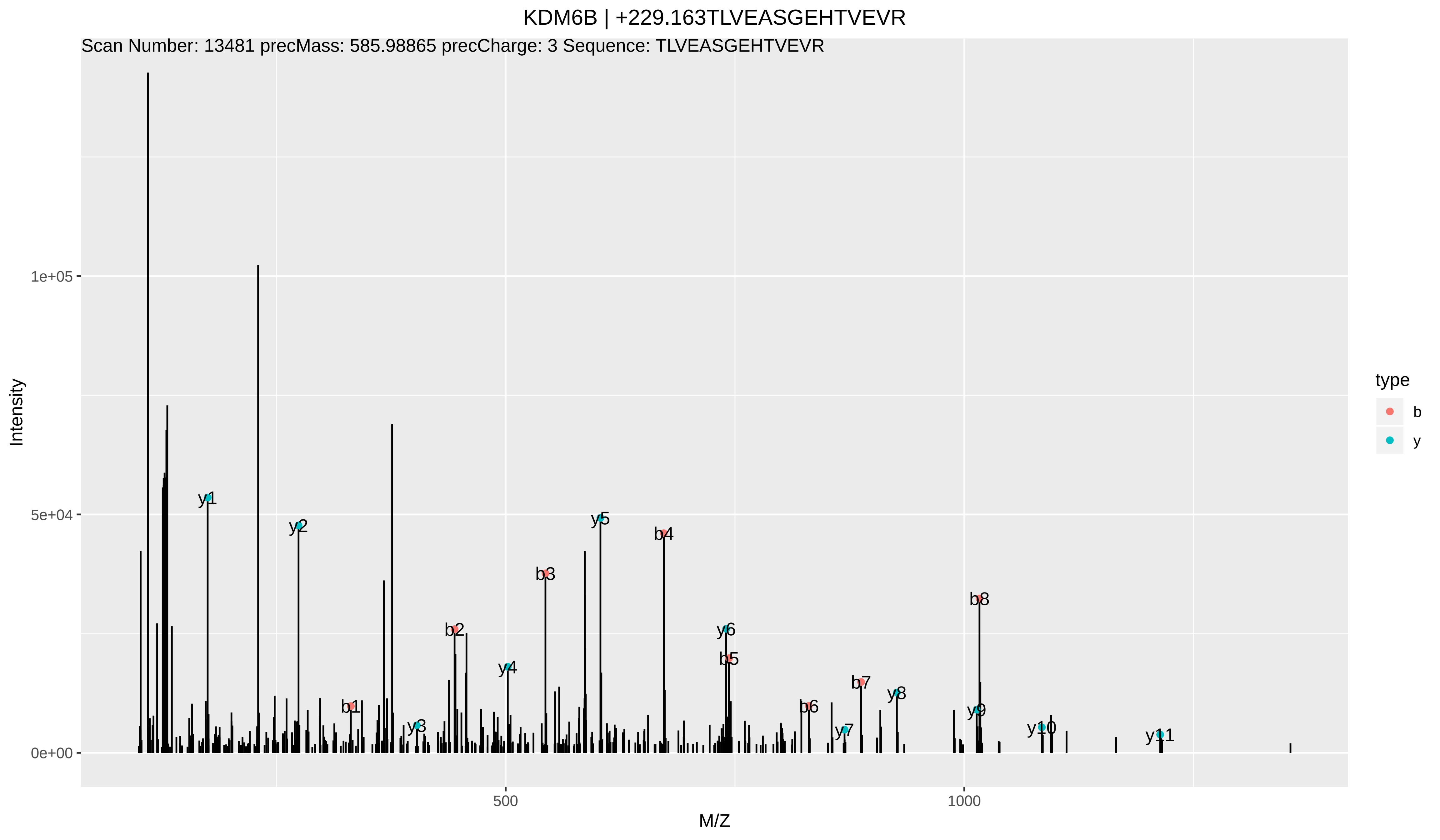

KDM6B | +229.163TLVEASGEHTVEVR
Scan Number: 13481 precMass: 585.98865 precCharge: 3 Sequence: TLVEASGEHTVEVR
Intensity
1e+05
5e+04
0e+00
M/Z
500
1000
y1
y2
b1
y3
b2
y4
b3
y5
b4
y6
b5
b6
y7
b7
y8
b8
y9
y10
y11
type
b
y

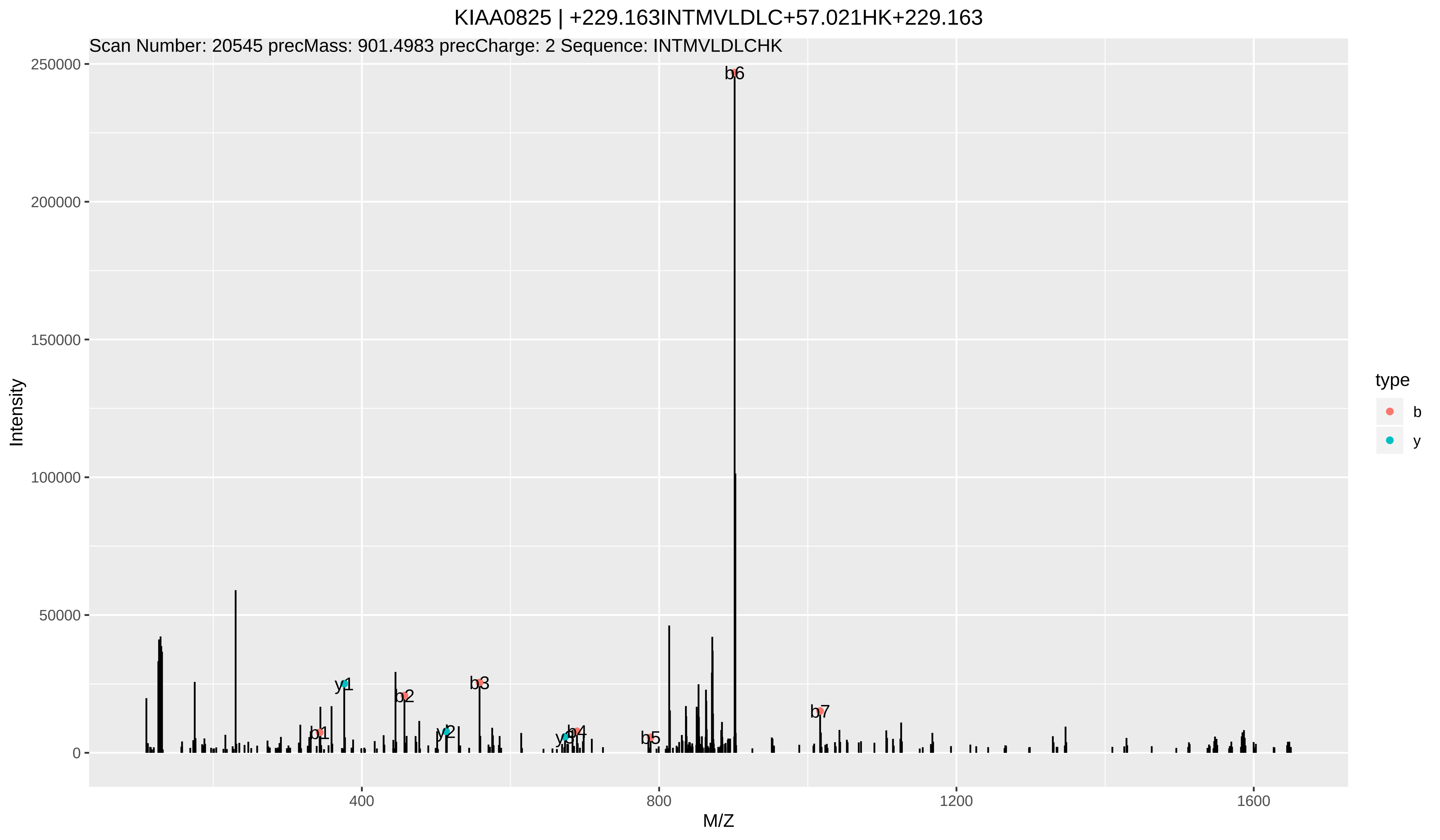

KIAA0825 | +229.163INTMVLDLC+57.021HK+229.163
Scan Number: 20545 precMass: 901.4983 precCharge: 2 Sequence: INTMVLDLCHK
Intensity
250000
200000
150000
100000
50000
0
M/Z
400
800
1200
1600
b6
y1
b2
b3
b1
y2
y3
b4
b5
b7
type
b
y

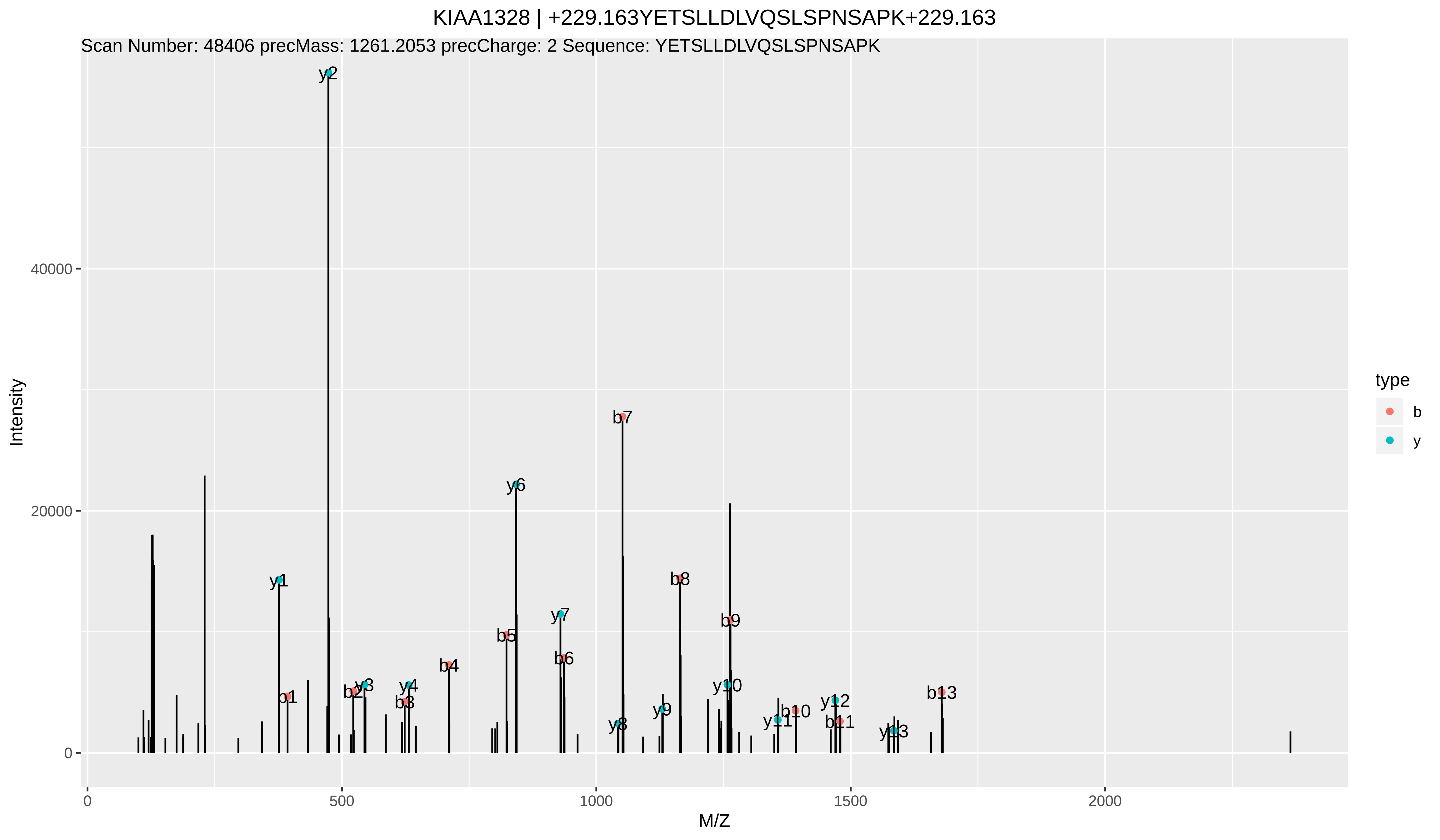
KIAA1328 | +229.163YETSLLDLVQSLSPNSAPK+229.163
Scan Number: 48406 precMass: 1261.2053 precCharge: 2 Sequence: YETSLLDLVQSLSPNSAPK
Intensity
M/Z
0
20000
40000
0
500
1000
1500
2000
y2
b7
y6
y1
b8
y7
b9
b5
b6
b4
y3
y4
b2
b1
b3
y10
y12
b13
y9
b10
y11
b11
y8
y13
type
b
y

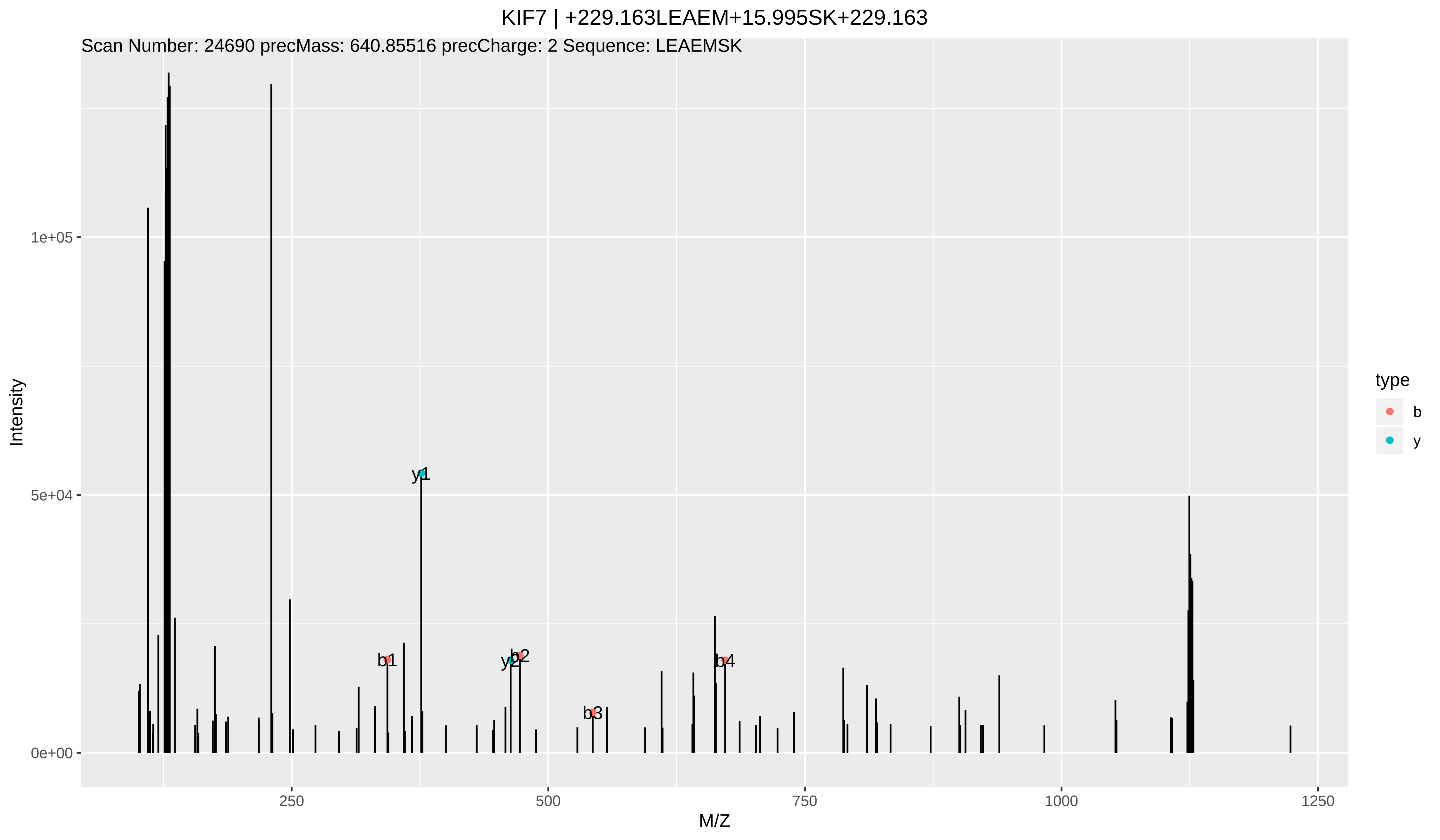

KIF7 | +229.163LEAEM+15.995SK+229.163
Scan Number: 24690 precMass: 640.85516 precCharge: 2 Sequence: LEAEMSK
Intensity
M/Z
0e+00
5e+04
1e+05
250
500
750
1000
1250
y1
b1
y2
b2
b3
b4
type
b
y

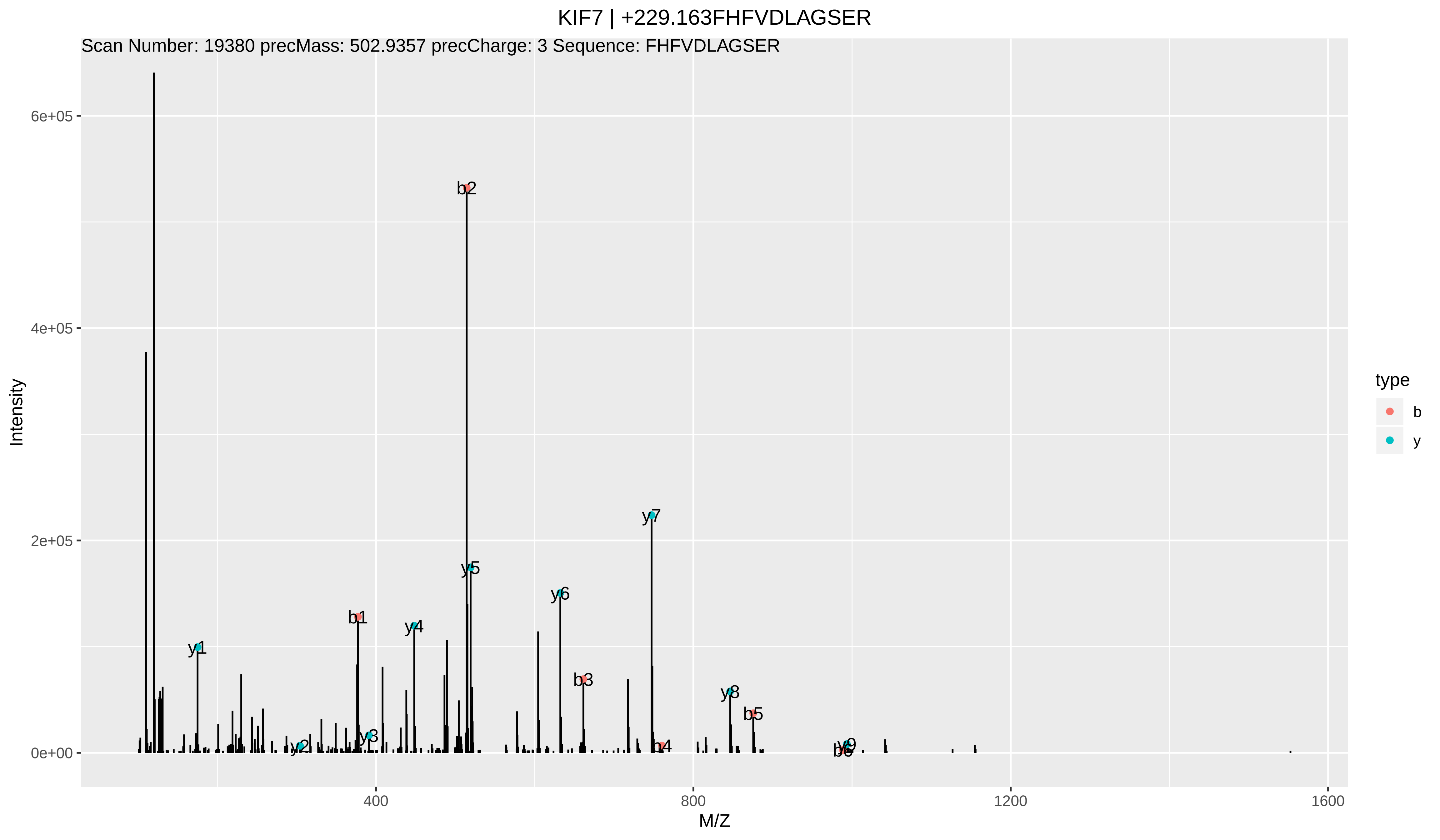
KIF7 | +229.163FHFVDLAGSER
Scan Number: 19380 precMass: 502.9357 precCharge: 3 Sequence: FHFVDLAGSER
Intensity
M/Z
6e+05
4e+05
2e+05
0e+00
400
800
1200
1600
b2
y7
y5
y6
b1
y4
y1
b3
y8
b5
y3
y2
b4
y9
b6
type
b
y

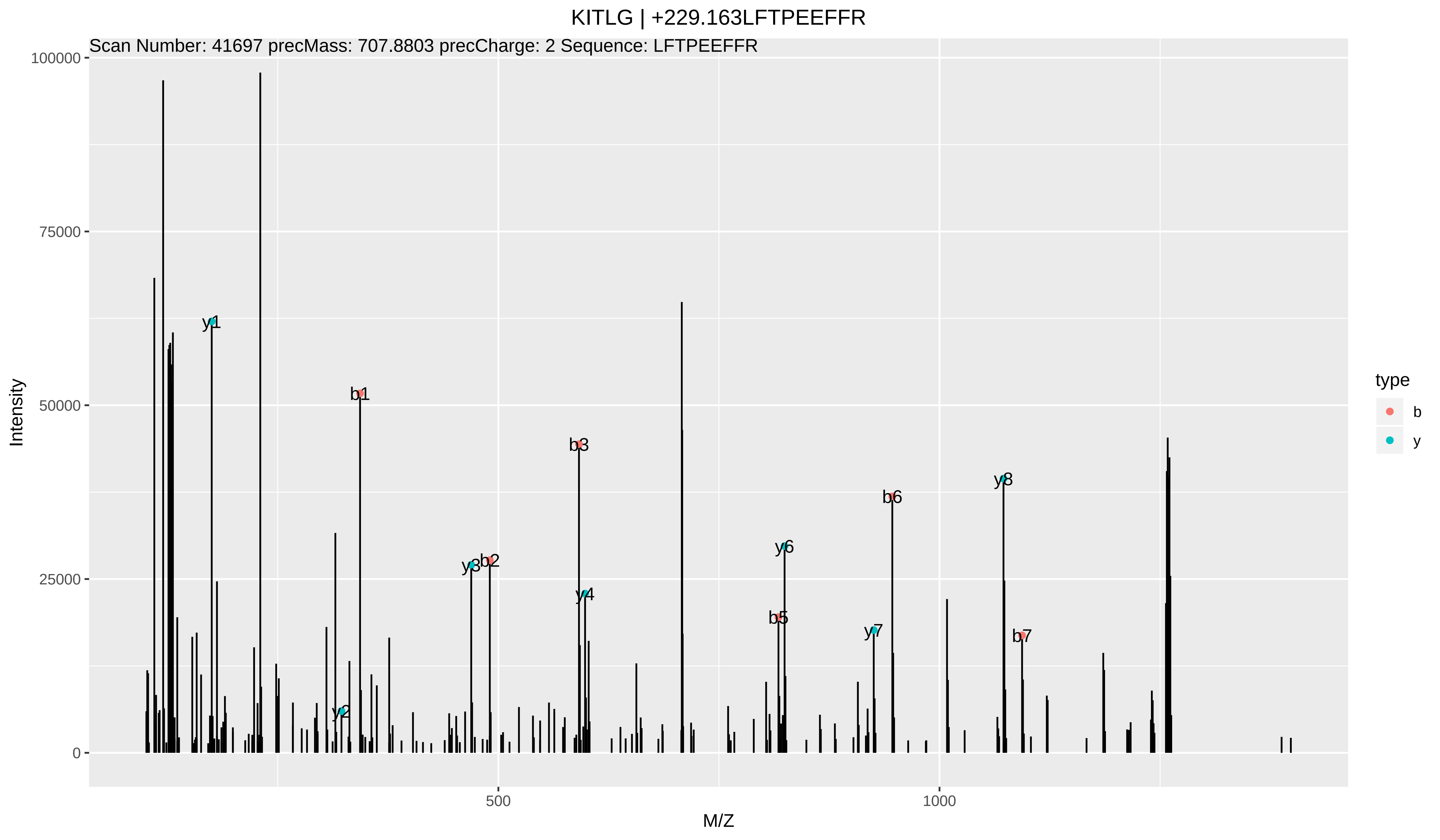

KITLG | +229.163LFTPEEFFR
Scan Number: 41697 precMass: 707.8803 precCharge: 2 Sequence: LFTPEEFFR
Intensity
100000
75000
50000
25000
0
M/Z
500
1000
y1
b1
b3
y8
b6
y6
y3
b2
y4
b5
y7
b7
y2
type
b
y

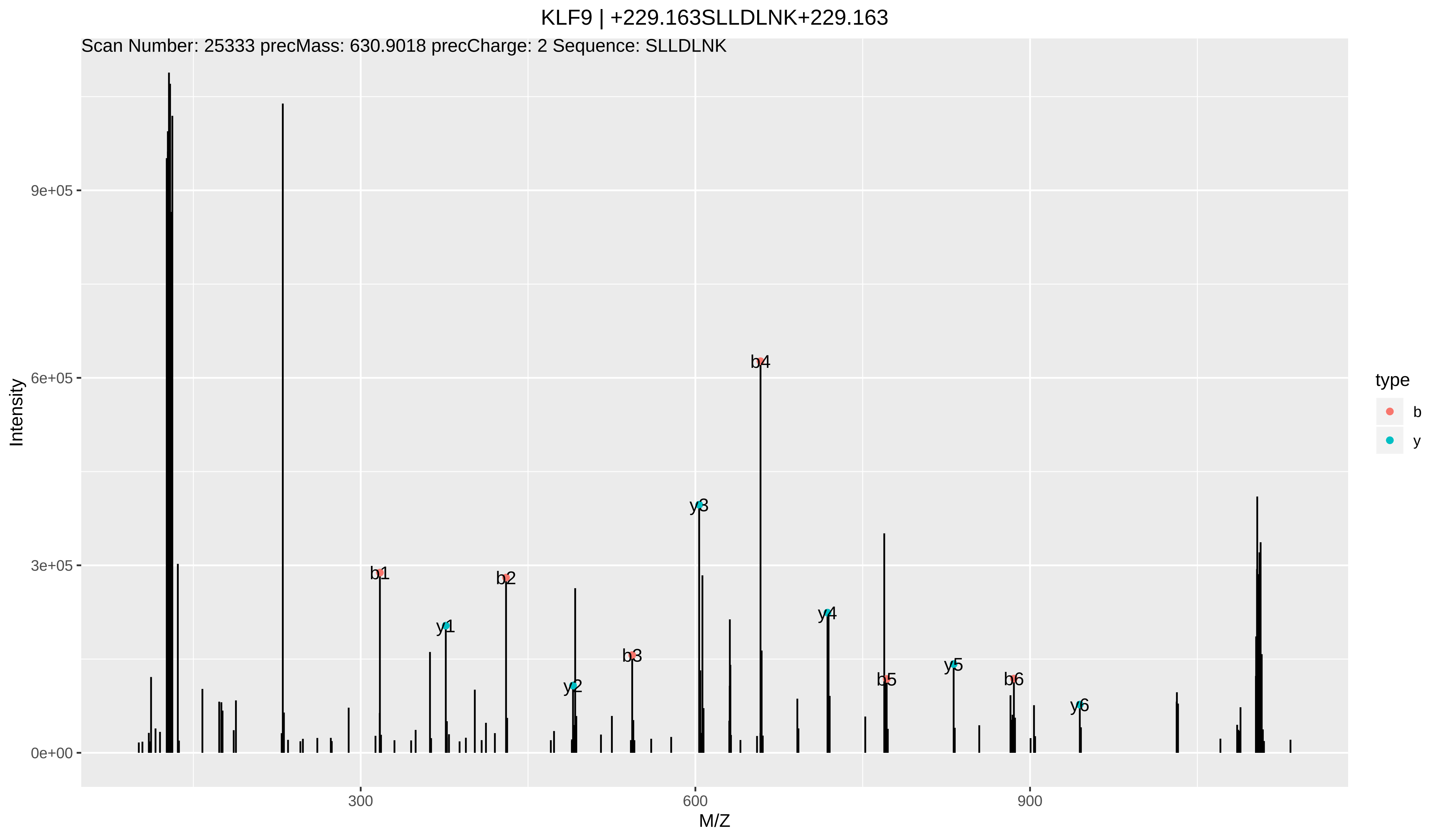
KLF9 | +229.163SLLDLNK+229.163
Scan Number: 25333 precMass: 630.9018 precCharge: 2 Sequence: SLLDLNK
Intensity
9e+05
6e+05
3e+05
0e+00
M/Z
300
600
900
b1
y1
b2
y2
b3
y3
b4
y4
b5
y5
b6
y6
type
b
y

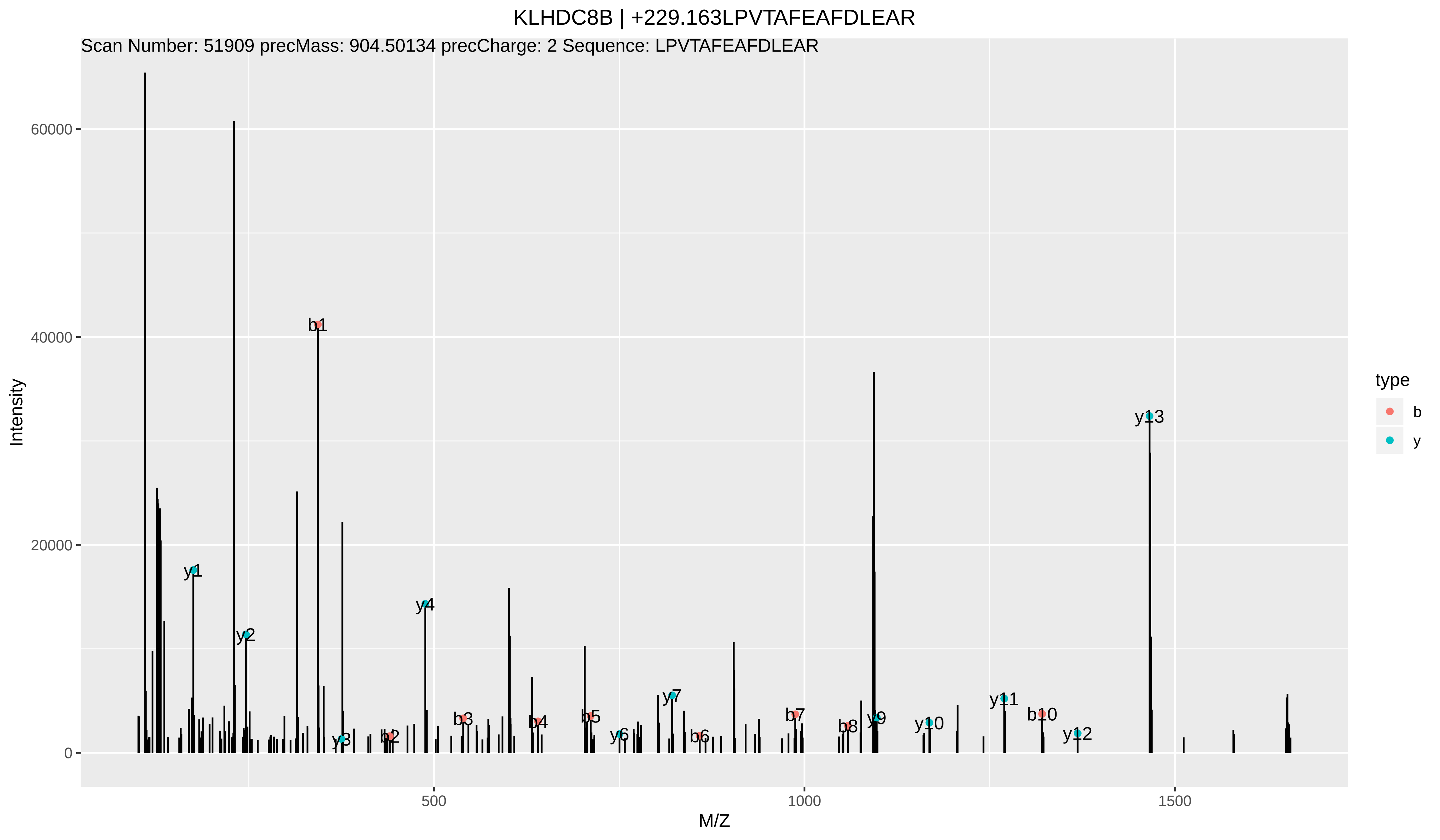
KLHDC8B | +229.163LPVTAFEAFDLEAR
Scan Number: 51909 precMass: 904.50134 precCharge: 2 Sequence: LPVTAFEAFDLEAR
Intensity
M/Z
type
b
y
60000
40000
20000
0
500
1000
1500
b1
y1
y2
y3
b2
y4
b3
b4
b5
y6
y7
b6
b7
b8
y9
y10
y11
b10
y12
y13

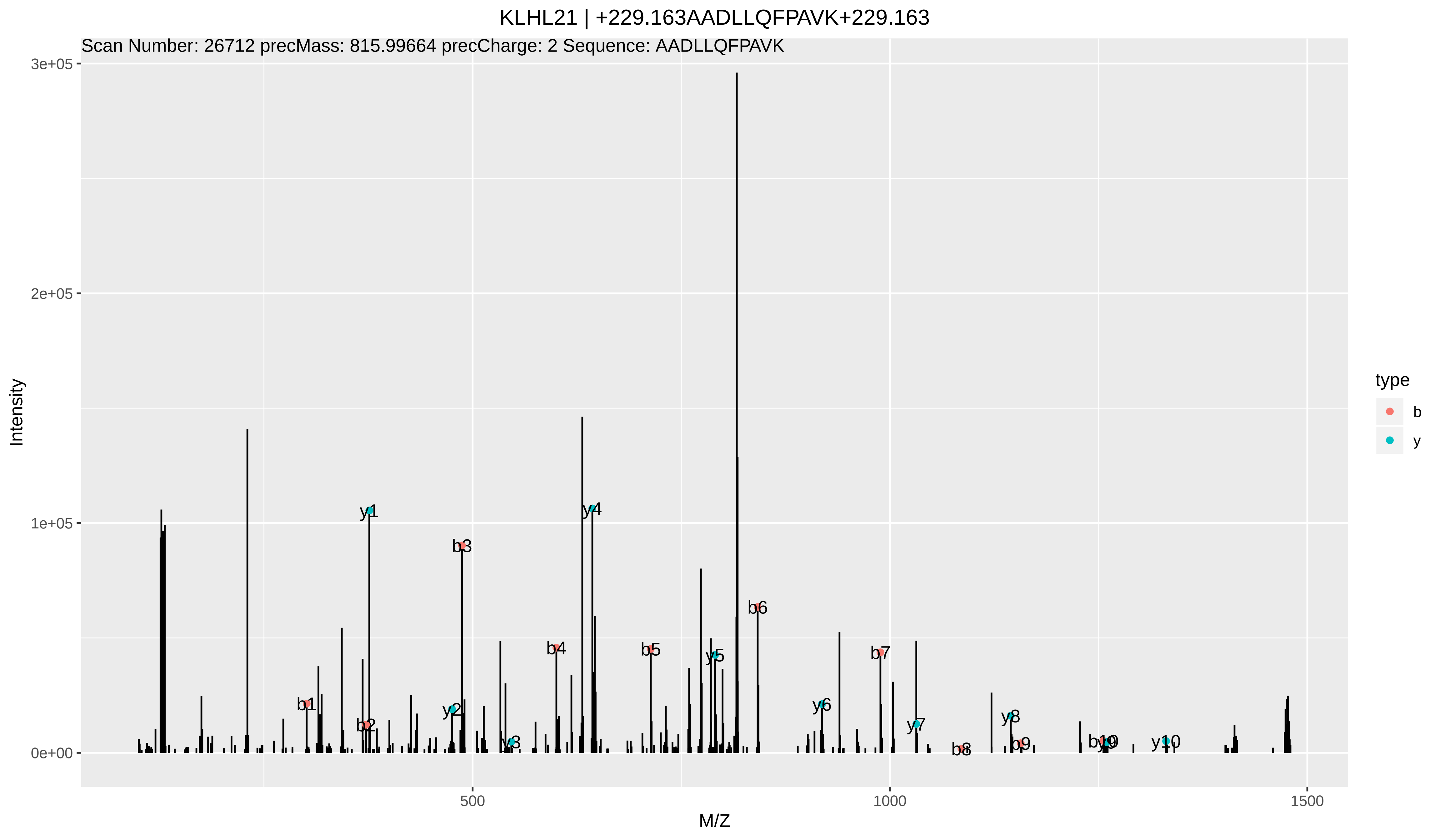

KLHL21 | +229.163AADLLQFPAVK+229.163
Scan Number: 26712 precMass: 815.99664 precCharge: 2 Sequence: AADLLQFPAVK
Intensity
M/Z
3e+05
2e+05
1e+05
0e+00
500
1000
1500
y1
y4
b3
b6
b4
b5
y5
b7
b1
y6
y2
b2
y7
y8
y3
b9
b10
y9
y10
b8
type
b
y

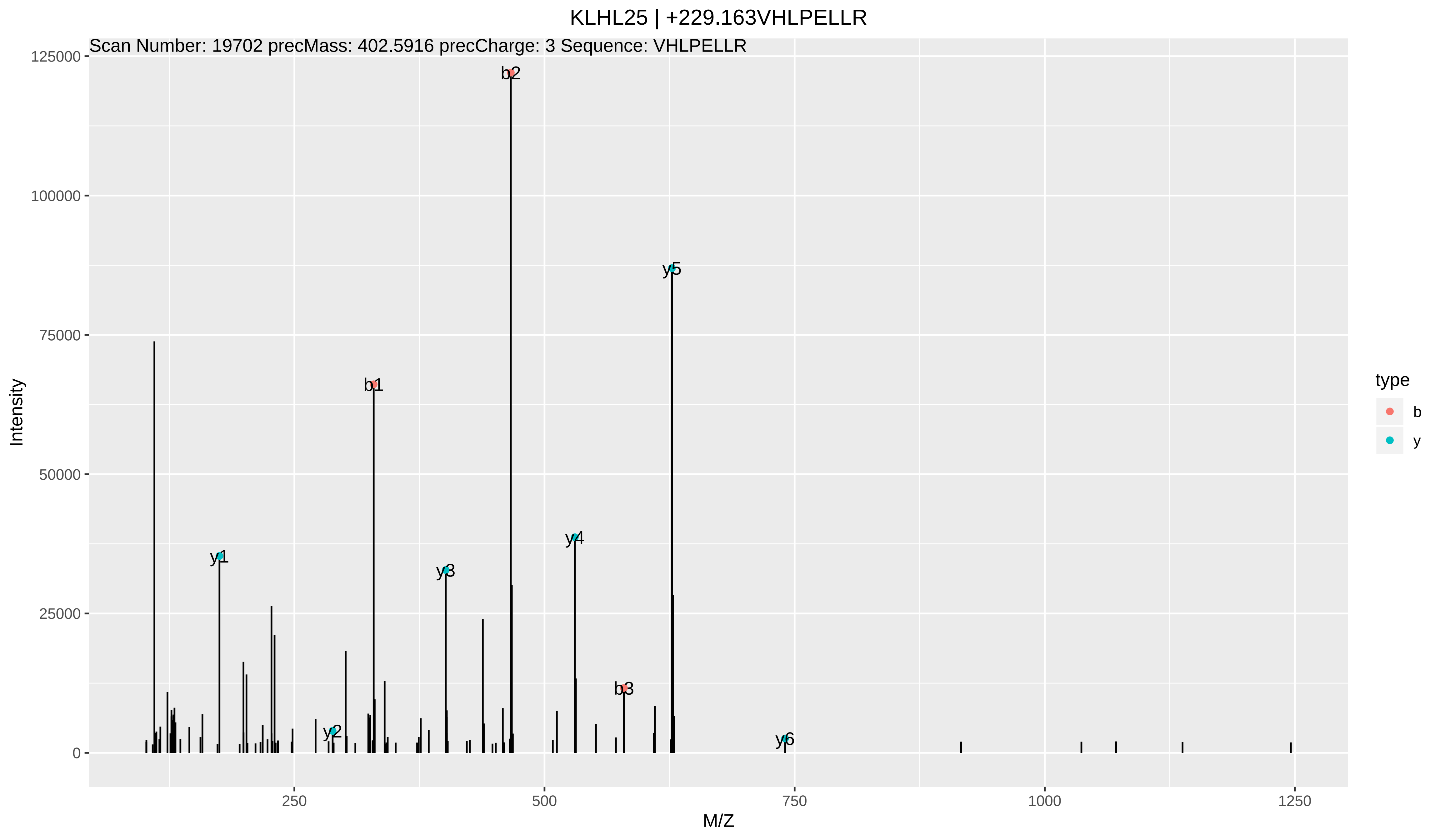

KLHL25 | +229.163VHLPELLR
Scan Number: 19702 precMass: 402.5916 precCharge: 3 Sequence: VHLPELLR
Intensity
M/Z
type
b
y
b1
b2
b3
y1
y2
y3
y4
y5
y6
0
25000
50000
75000
100000
125000
250
500
750
1000
1250

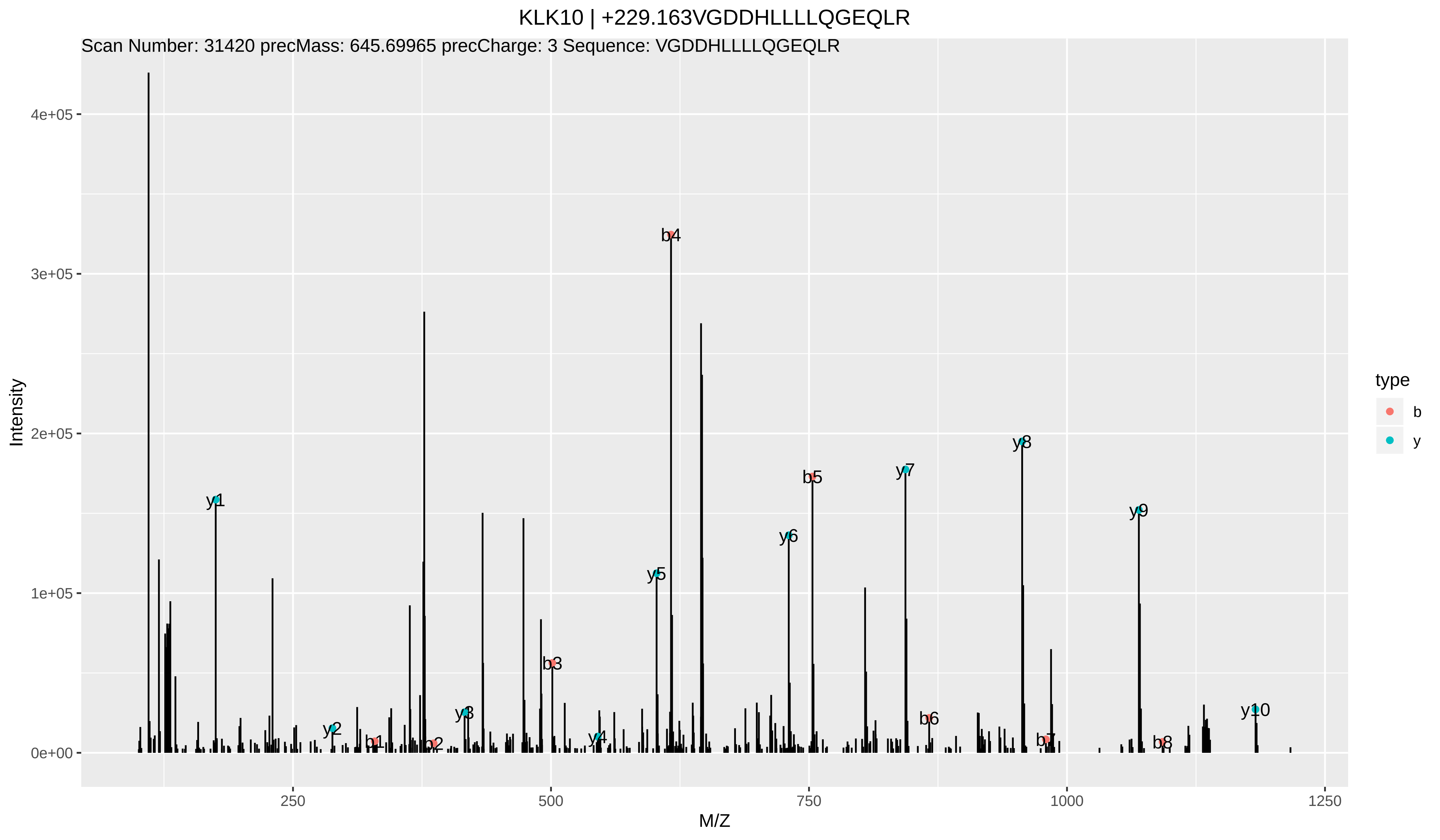

KLK10 | +229.163VGDDHLLLLQGEQLR
Scan Number: 31420 precMass: 645.69965 precCharge: 3 Sequence: VGDDHLLLLQGEQLR
Intensity
4e+05
3e+05
2e+05
1e+05
0e+00
M/Z
250
500
750
1000
1250
y1
y2
b1
b2
y3
b3
y4
y5
b4
y6
b5
y7
b6
y8
b7
y9
b8
y10
type
b
y

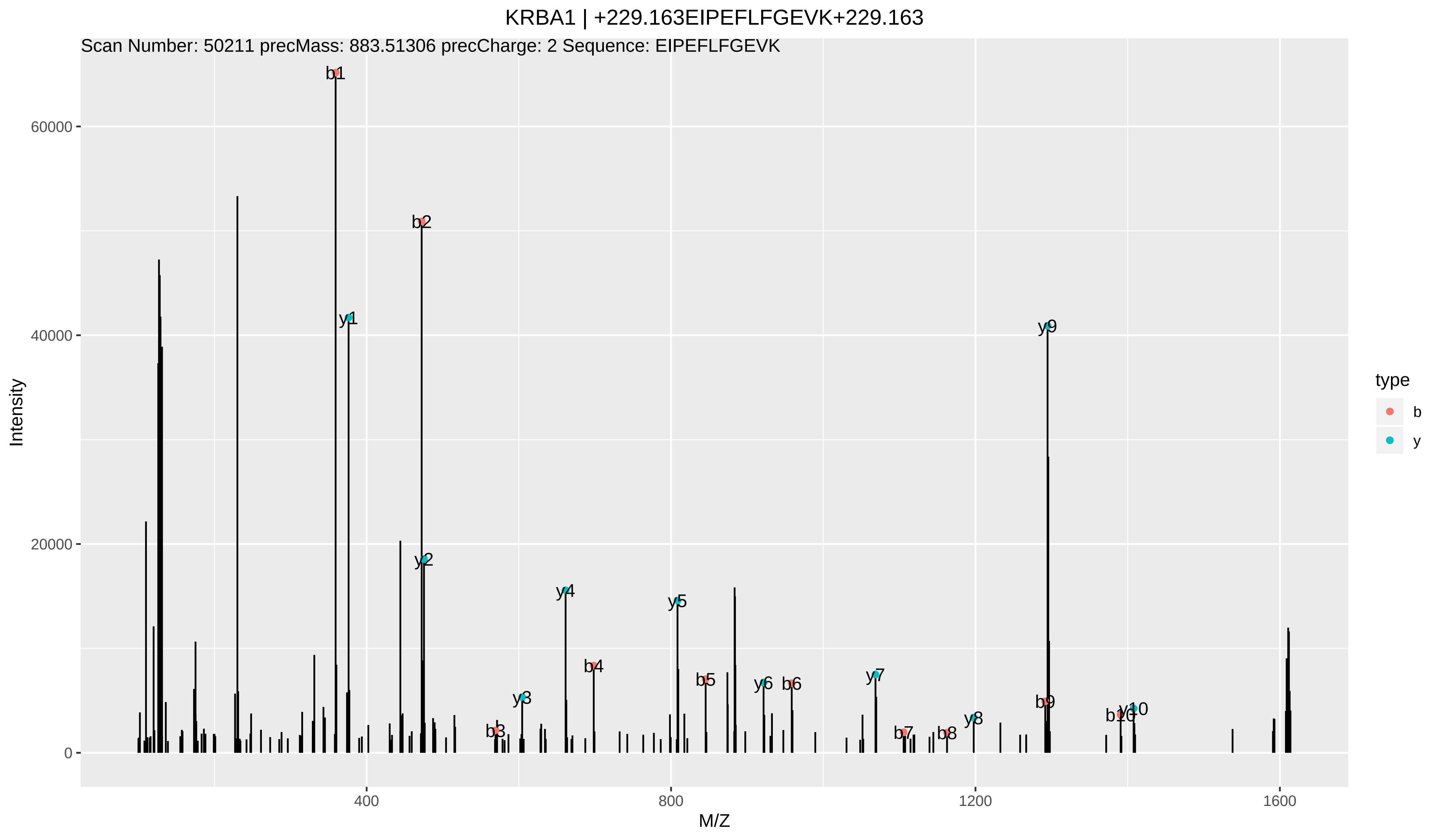
KRBA1 | +229.163EIPEFLFGEVK+229.163
Scan Number: 50211 precMass: 883.51306 precCharge: 2 Sequence: EIPEFLFGEVK
Intensity
M/Z
type
b
y
b1
b2
y1
y2
b3
y3
y4
b4
y5
b5
y6
b6
y7
b7
b8
y8
y9
b9
b10
y10
0
20000
40000
60000
400
800
1200
1600

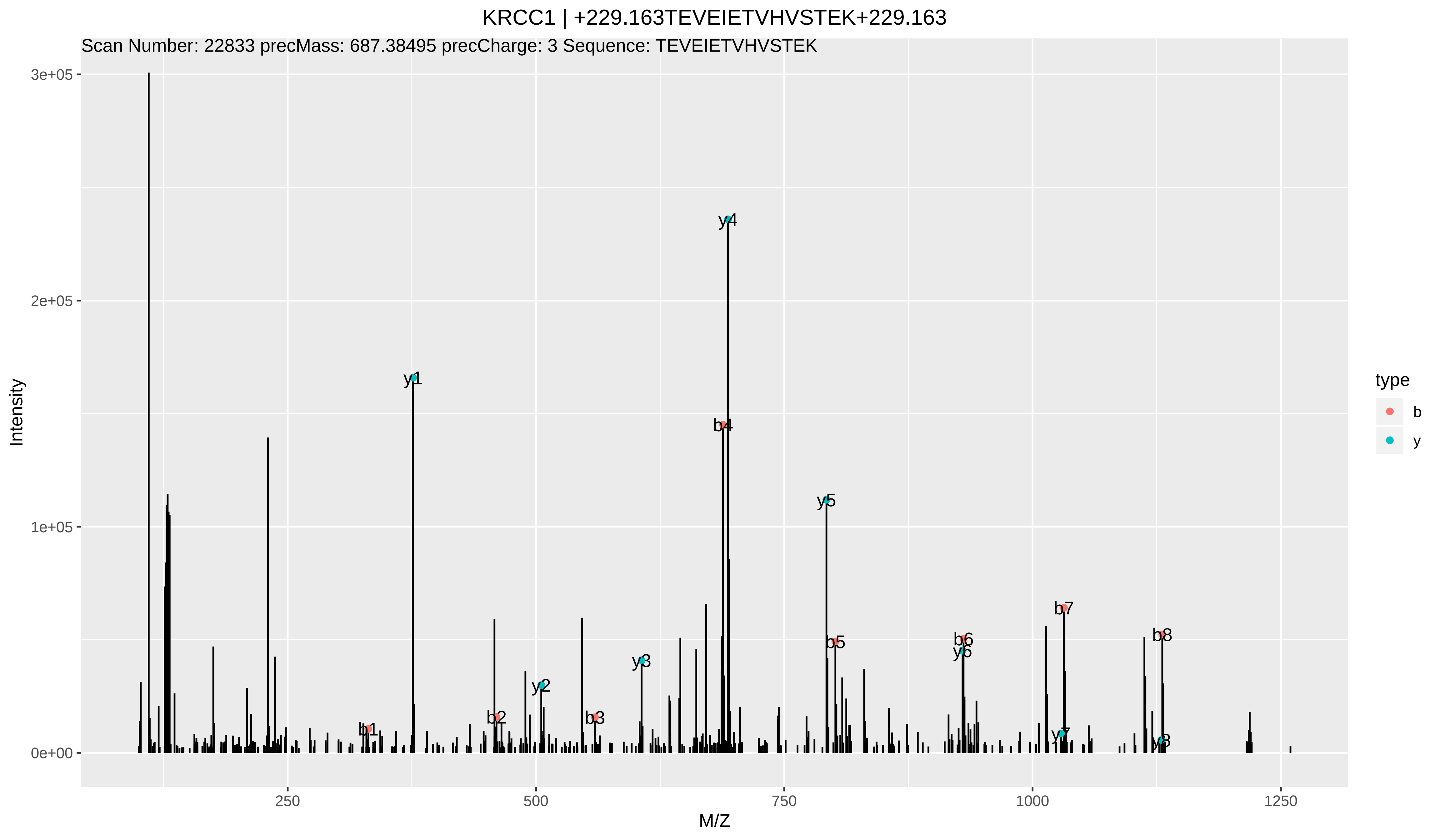

KRCC1 | +229.163TEVEIETVHVSTEK+229.163
Scan Number: 22833 precMass: 687.38495 precCharge: 3 Sequence: TEVEIETVHVSTEK
Intensity
3e+05
2e+05
1e+05
0e+00
M/Z
250
500
750
1000
1250
y1
y2
y3
y4
y5
y6
y7
y8
b1
b2
b3
b4
b5
b6
b7
b8
type
b
y

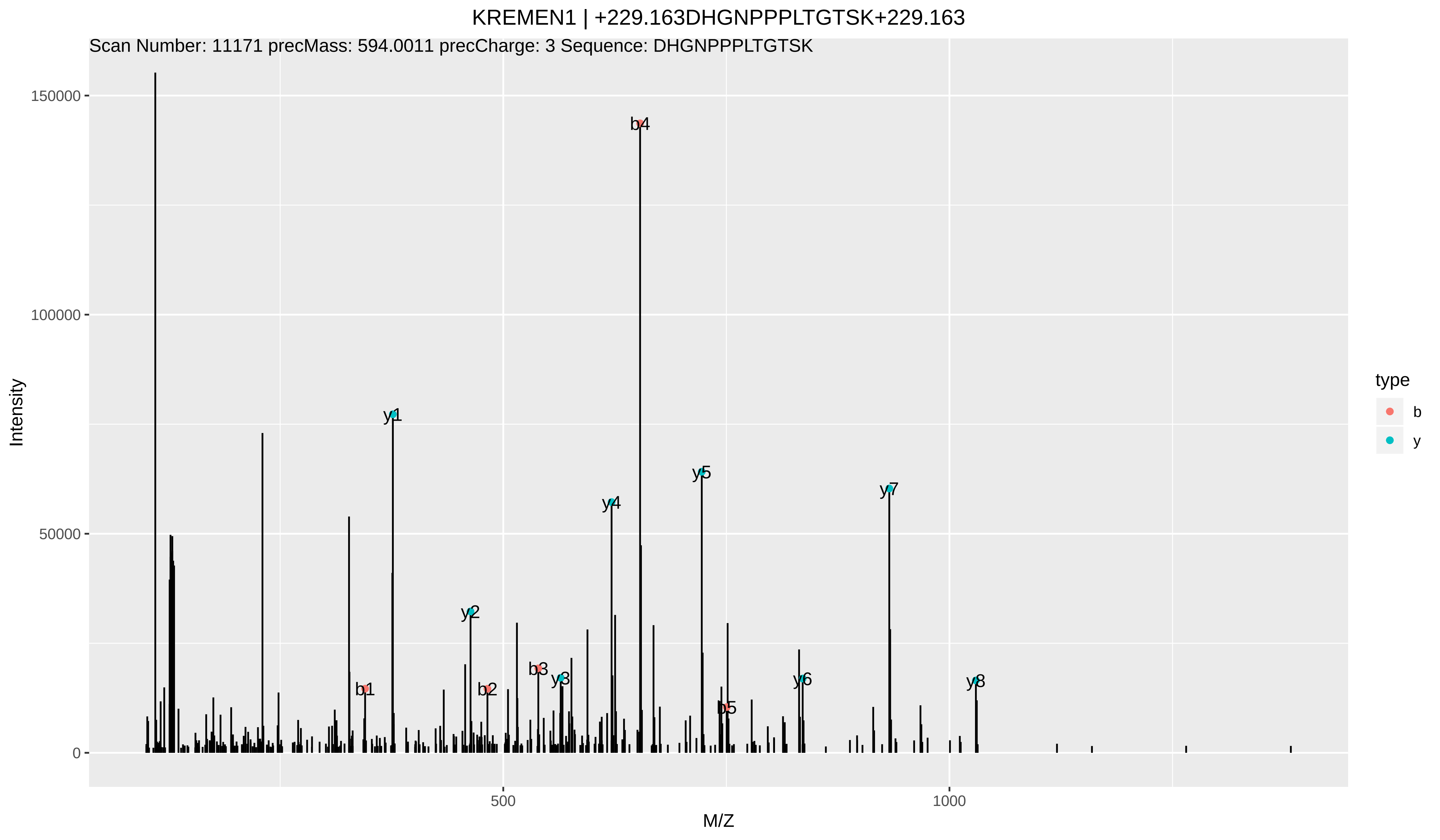

KREMEN1 | +229.163DHGNPPPLTGTSK+229.163
Scan Number: 11171 precMass: 594.0011 precCharge: 3 Sequence: DHGNPPPLTGTSK
Intensity
150000
100000
50000
0
500
1000
M/Z
b4
y1
y5
y7
y4
y2
b3
b1
b2
y3
b5
y6
y8
type
b
y

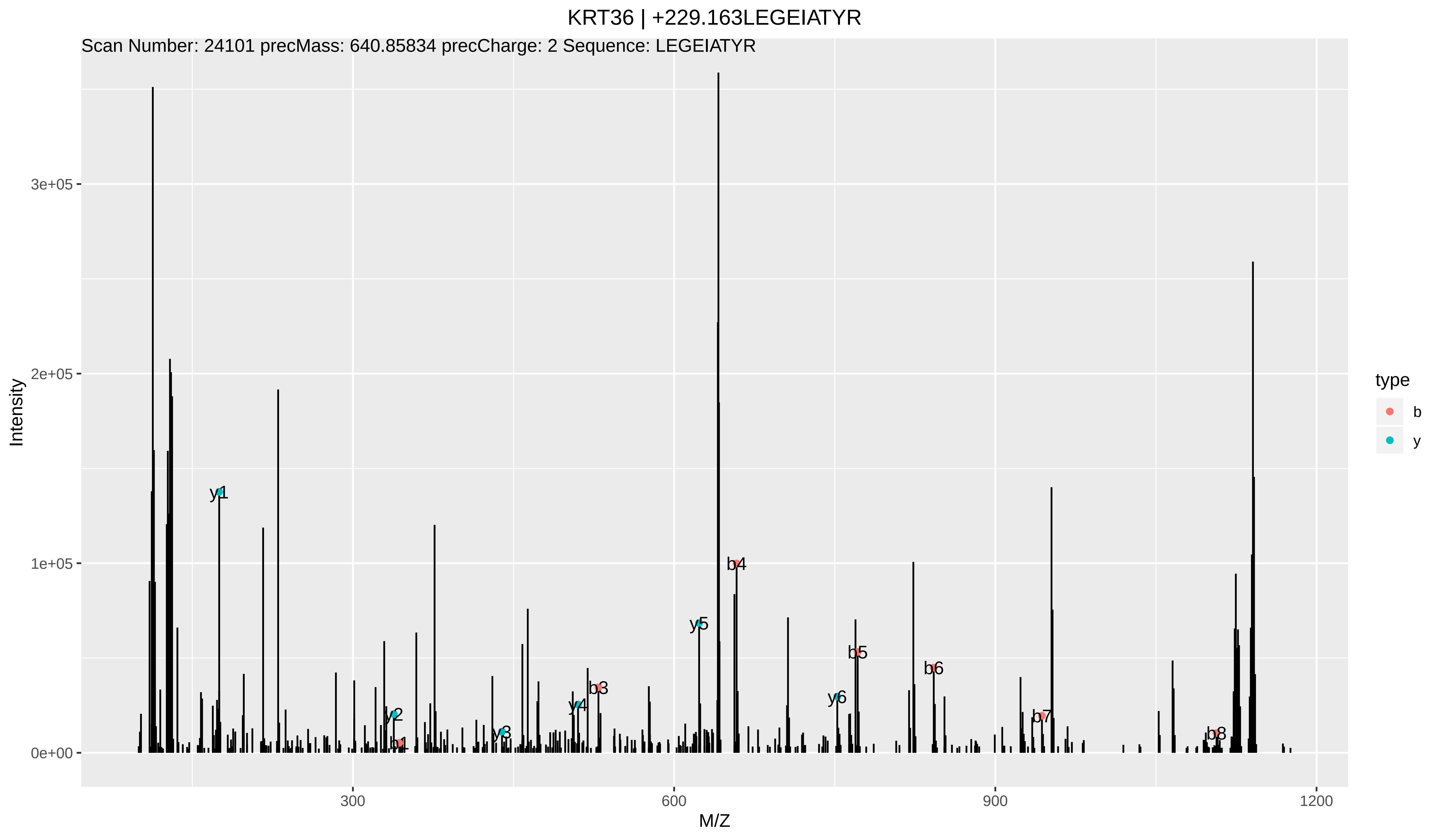

KRT36 | +229.163LEGEIATYR
Scan Number: 24101 precMass: 640.85834 precCharge: 2 Sequence: LEGEIATYR
Intensity
3e+05
2e+05
1e+05
0e+00
M/Z
300
600
900
1200
type
b
y
y1
y2
b1
y3
y4
b3
y5
b4
y6
b5
b6
b7
b8

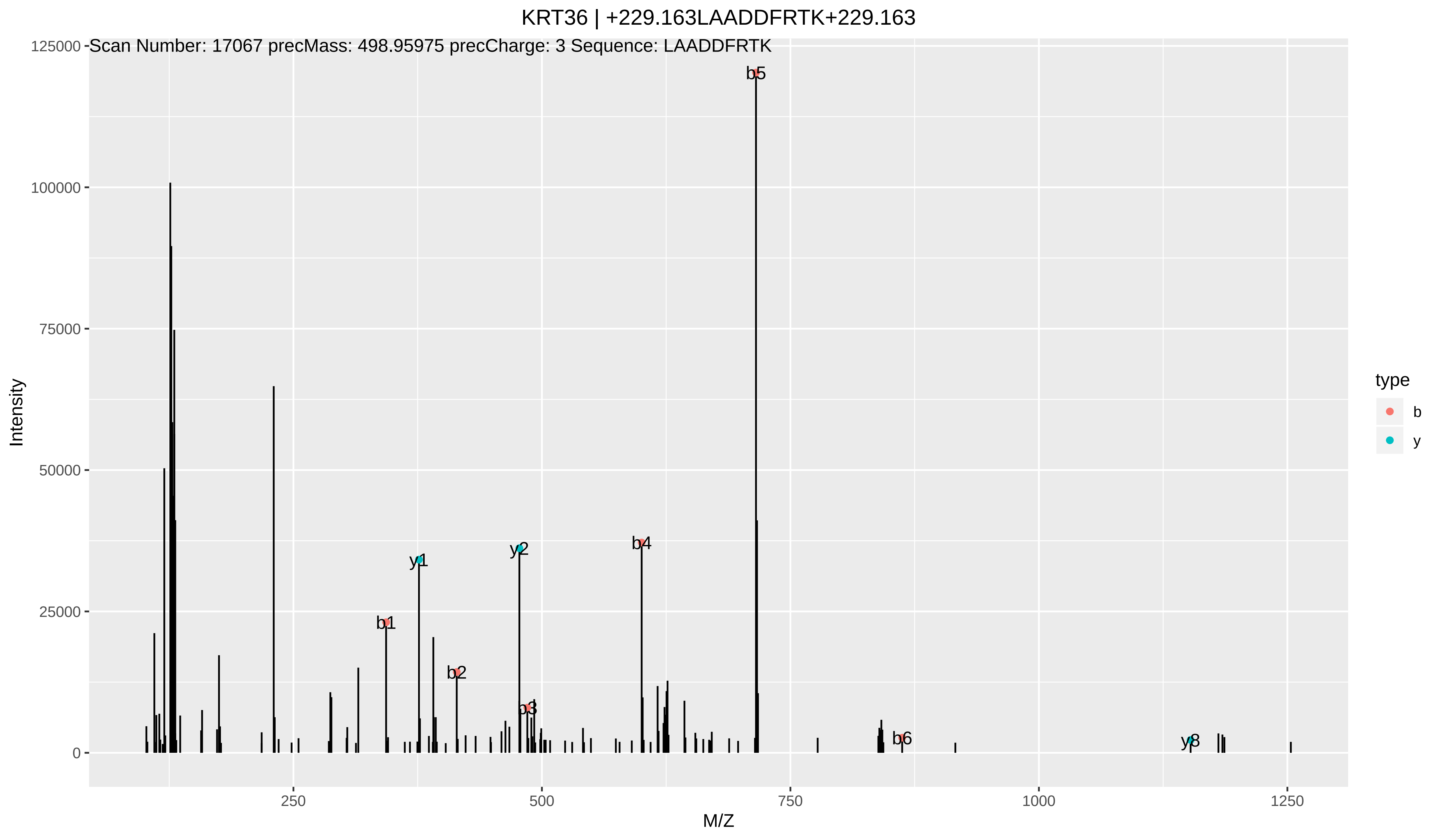

KRT36 | +229.163LAADDFRTK+229.163
Scan Number: 17067 precMass: 498.95975 precCharge: 3 Sequence: LAADDFRTK
Intensity
M/Z
125000
100000
75000
50000
25000
0
250
500
750
1000
1250
b5
b4
y2
y1
b1
b2
b3
b6
y8
type
b
y

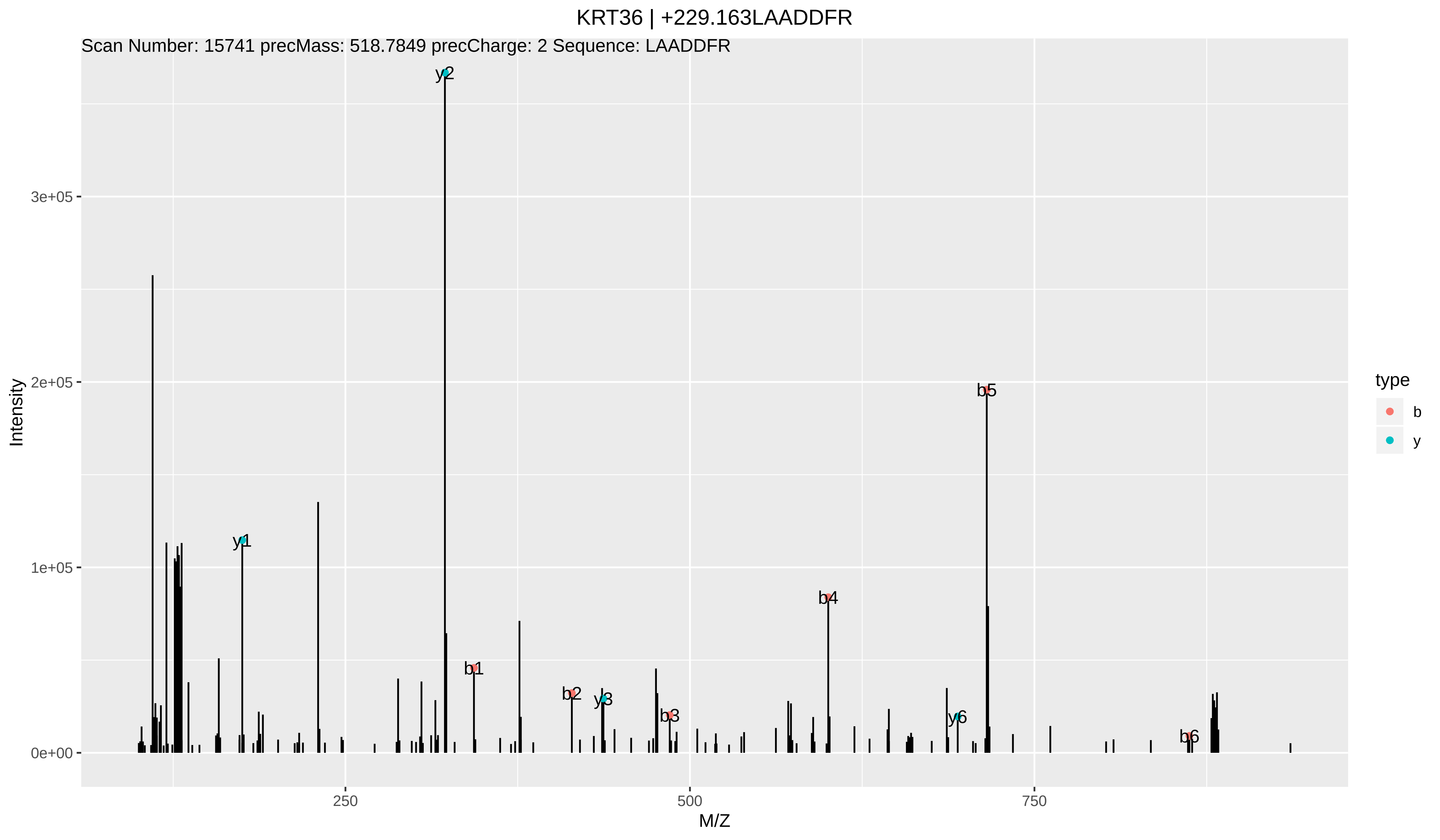
KRT36 | +229.163LAADDFR
Scan Number: 15741 precMass: 518.7849 precCharge: 2 Sequence: LAADDFR
Intensity
3e+05
2e+05
1e+05
0e+00
250
500
750
M/Z
y2
b5
y1
b4
b1
b2
y3
b3
y6
b6
type
b
y

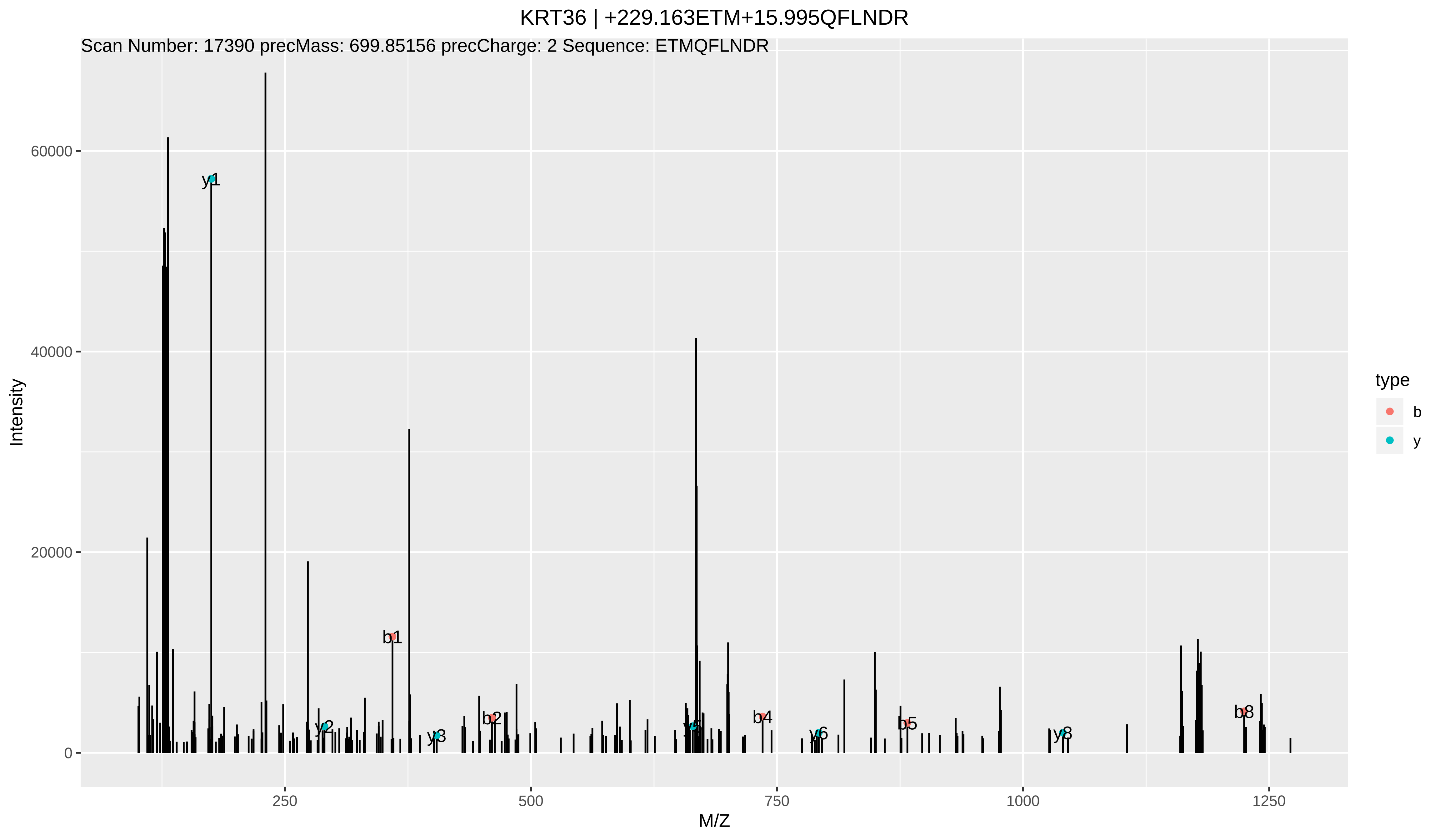
KRT36 | +229.163ETM+15.995QFLNDR
Scan Number: 17390 precMass: 699.85156 precCharge: 2 Sequence: ETMQFLNDR
Intensity
M/Z
y1
b1
y2
y3
b2
y5
b4
y6
b5
y8
b8
type
b
y
0
20000
40000
60000
250
500
750
1000
1250

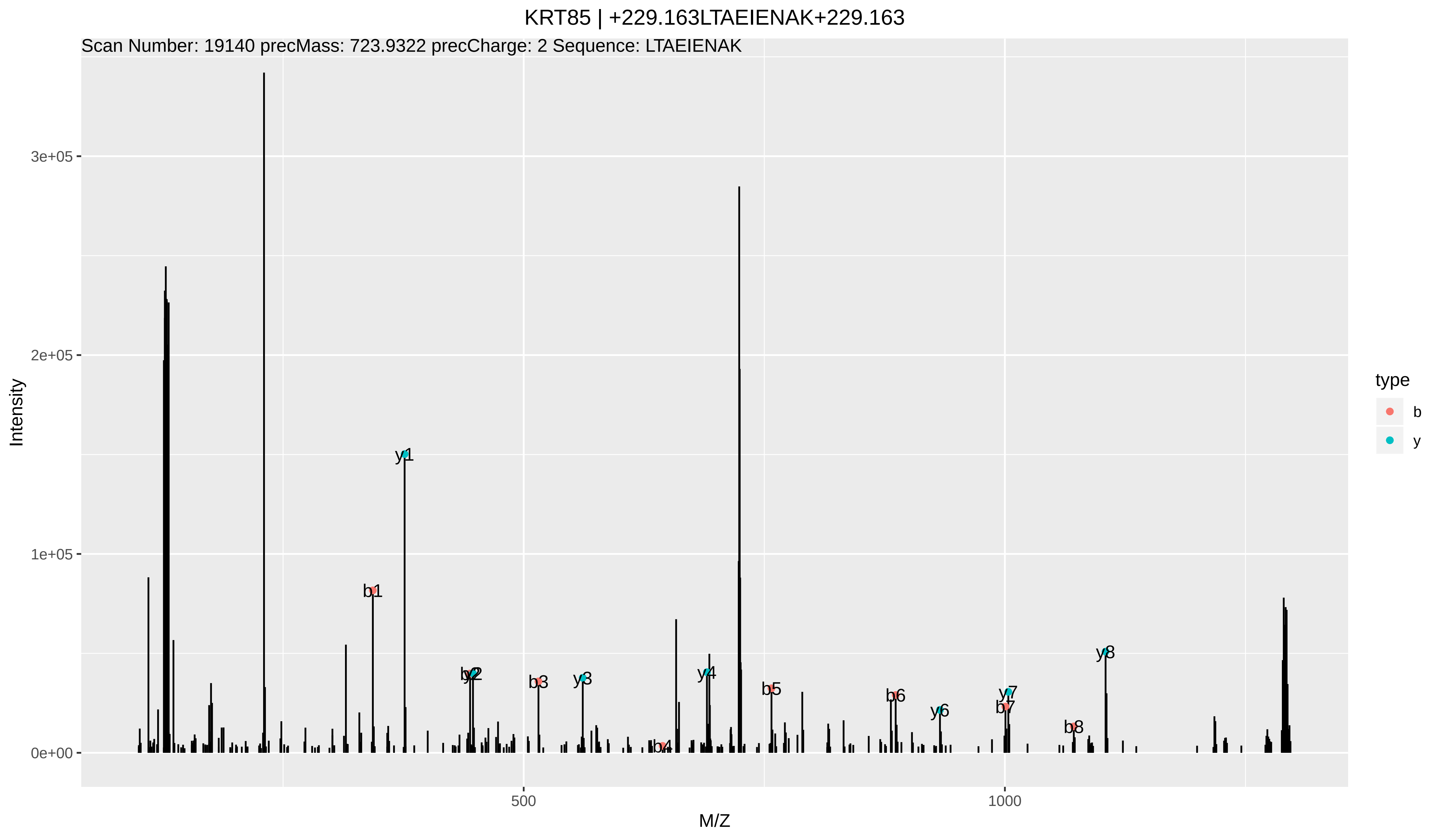

KRT85 | +229.163LTAEIENAK+229.163
Scan Number: 19140 precMass: 723.9322 precCharge: 2 Sequence: LTAEIENAK
Intensity
3e+05
2e+05
1e+05
0e+00
M/Z
500
1000
y1
b1
b2
y2
b3
y3
b4
y4
b5
b6
y6
y7
b7
b8
y8
type
b
y

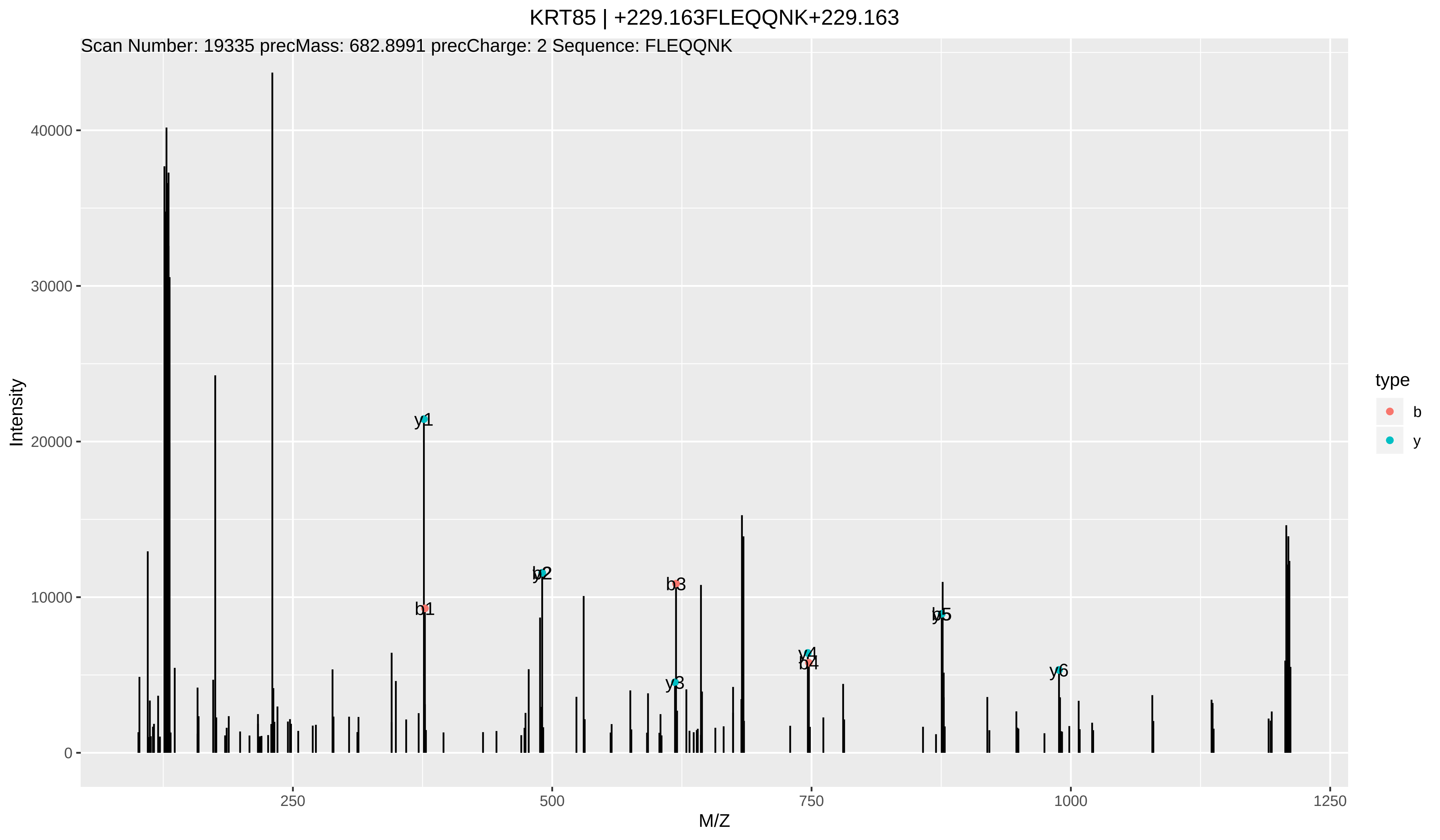

KRT85 | +229.163FLEQQNK+229.163
Scan Number: 19335 precMass: 682.8991 precCharge: 2 Sequence: FLEQQNK
Intensity
M/Z
type
b
y
y1
b1
b2
y2
b3
y3
y4
b4
b5
y5
y6
0
10000
20000
30000
40000
250
500
750
1000
1250

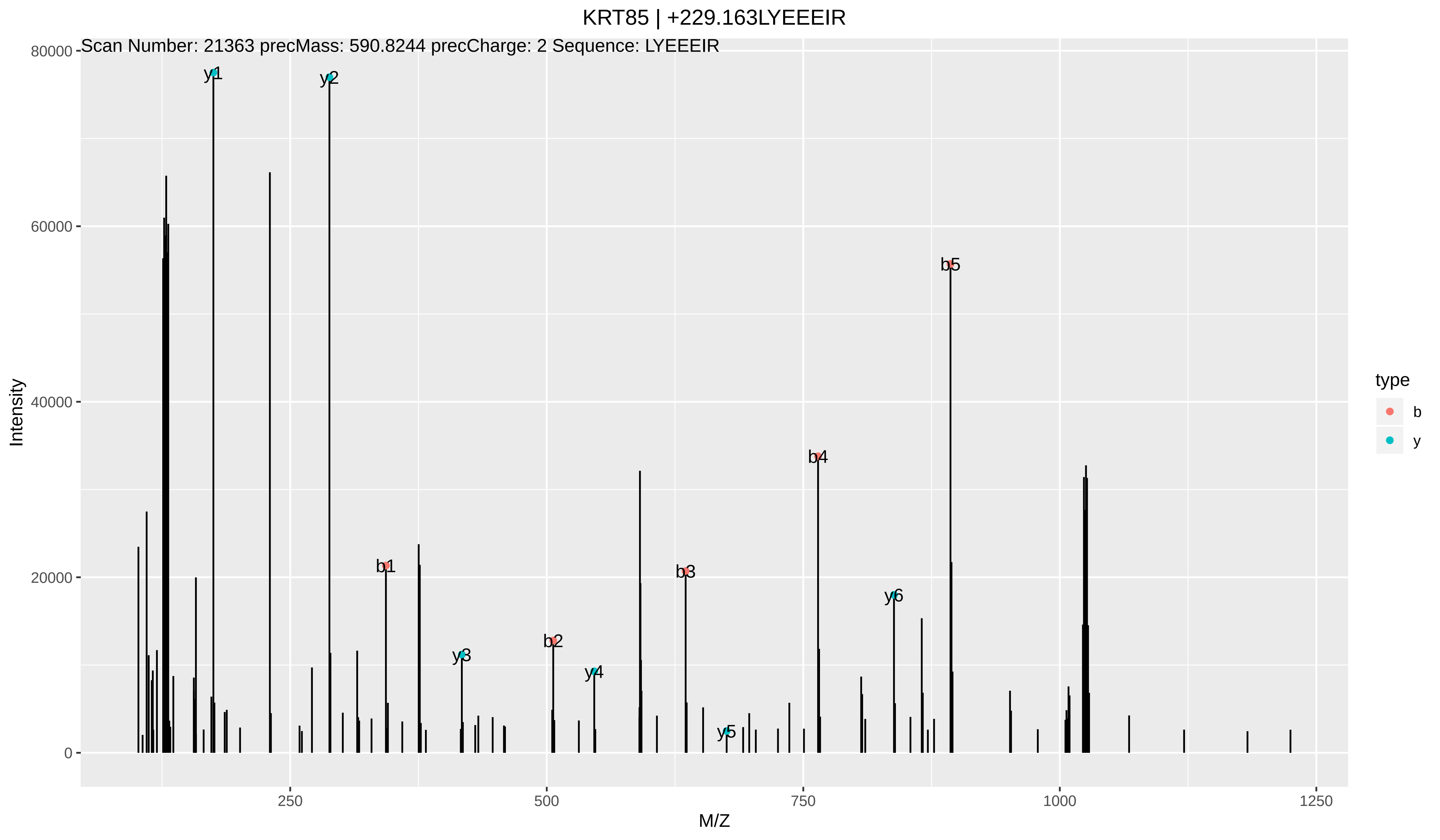
KRT85 | +229.163LYEEEIR
Scan Number: 21363 precMass: 590.8244 precCharge: 2 Sequence: LYEEEIR
Intensity
M/Z
y1
y2
b1
y3
b2
y4
b3
y5
b4
y6
b5
type
b
y
0
20000
40000
60000
80000
250
500
750
1000
1250

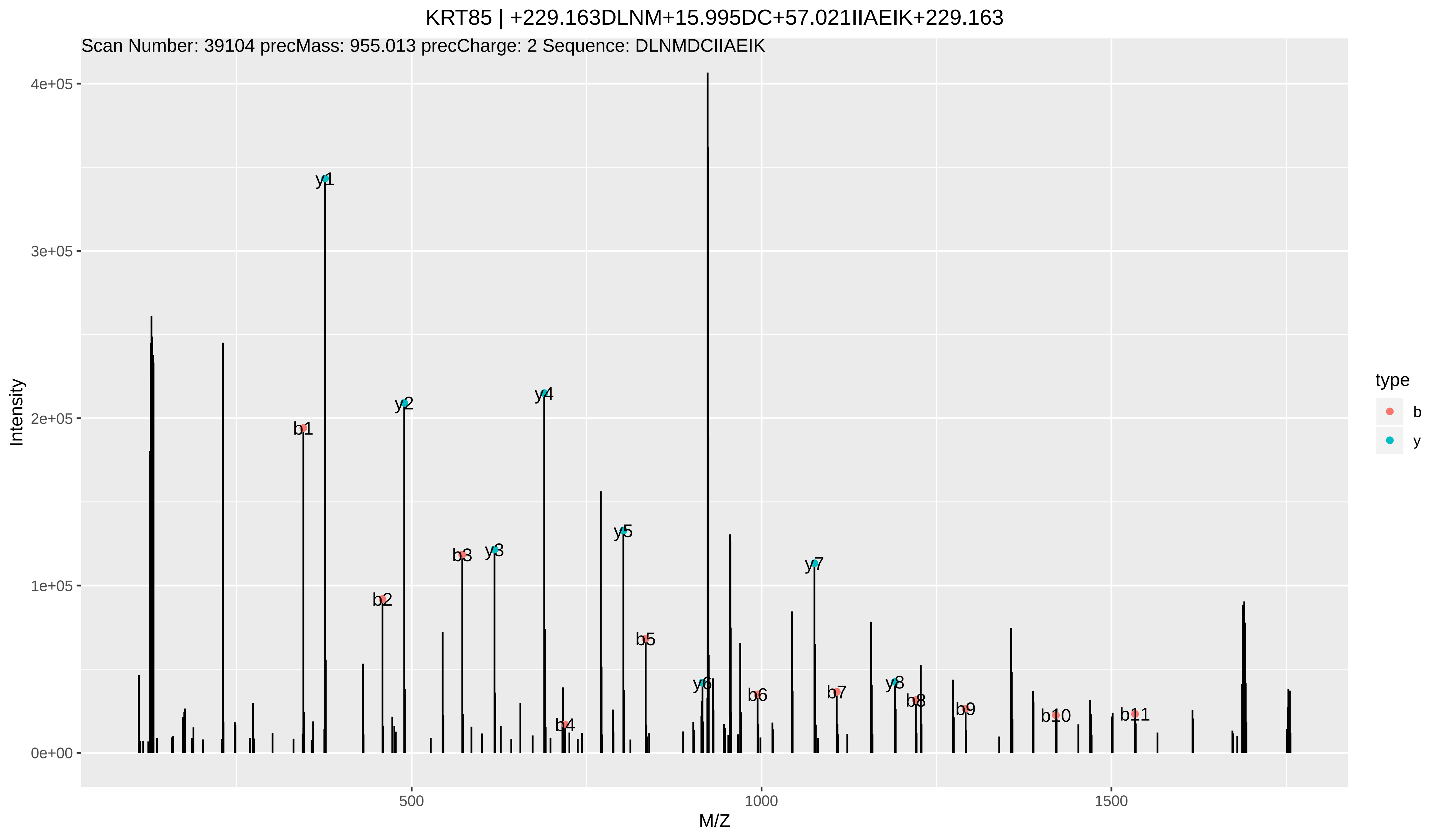
KRT85 | +229.163DLNM+15.995DC+57.021IIAEIK+229.163
Scan Number: 39104 precMass: 955.013 precCharge: 2 Sequence: DLNMDCIIAEIK
Intensity
4e+05
3e+05
2e+05
1e+05
0e+00
M/Z
500
1000
1500
type
b
y
y1
y2
y3
y4
y5
y6
y7
y8
b1
b2
b3
b4
b5
b6
b7
b8
b9
b10
b11

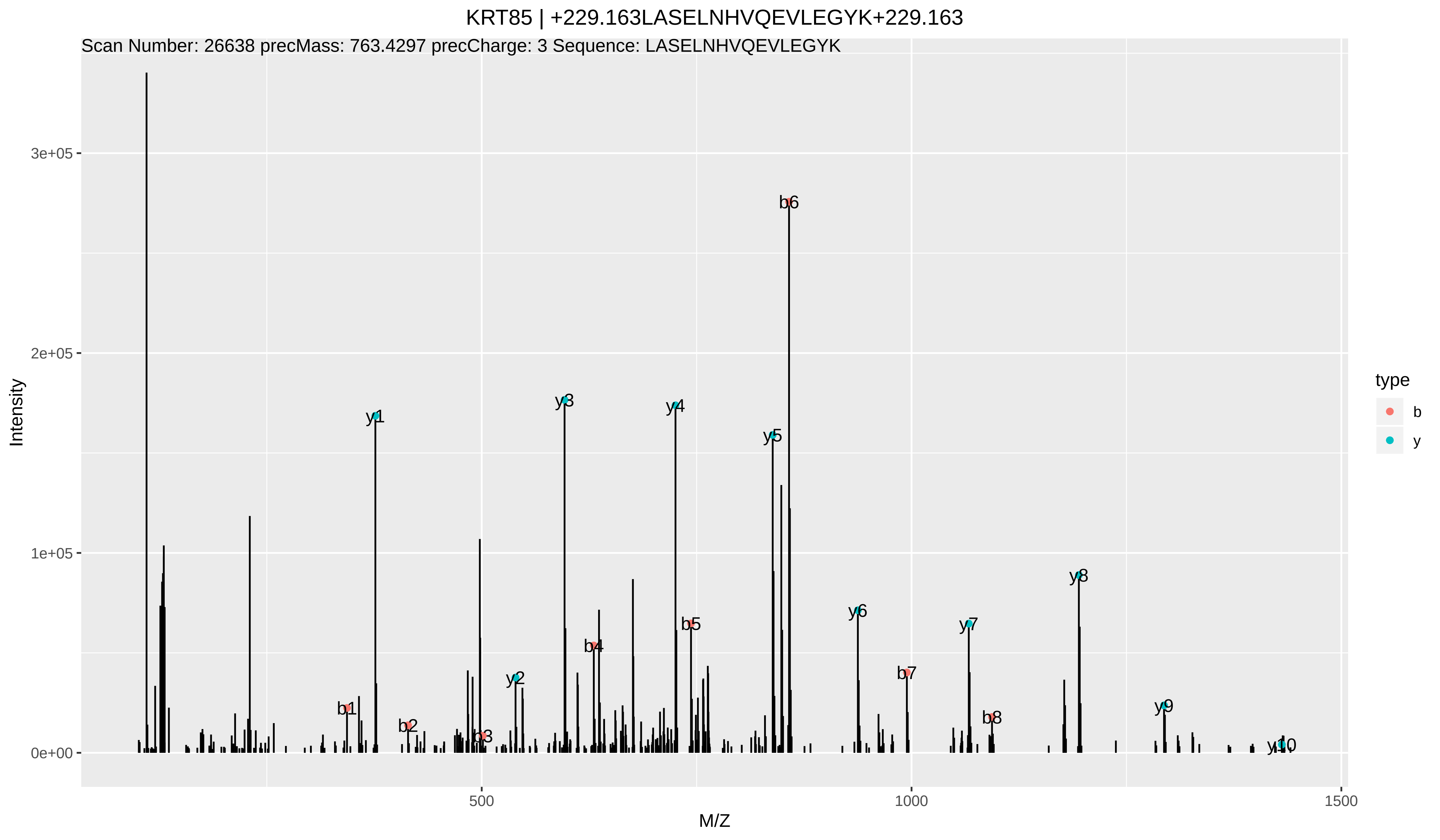
KRT85 | +229.163LASELNHVQEVLEGYK+229.163
Scan Number: 26638 precMass: 763.4297 precCharge: 3 Sequence: LASELNHVQEVLEGYK
Intensity
3e+05
2e+05
1e+05
0e+00
M/Z
500
1000
1500
type
b
y
y1
y2
y3
y4
y5
y6
y7
y8
y9
y10
b1
b2
b3
b4
b5
b6
b7
b8

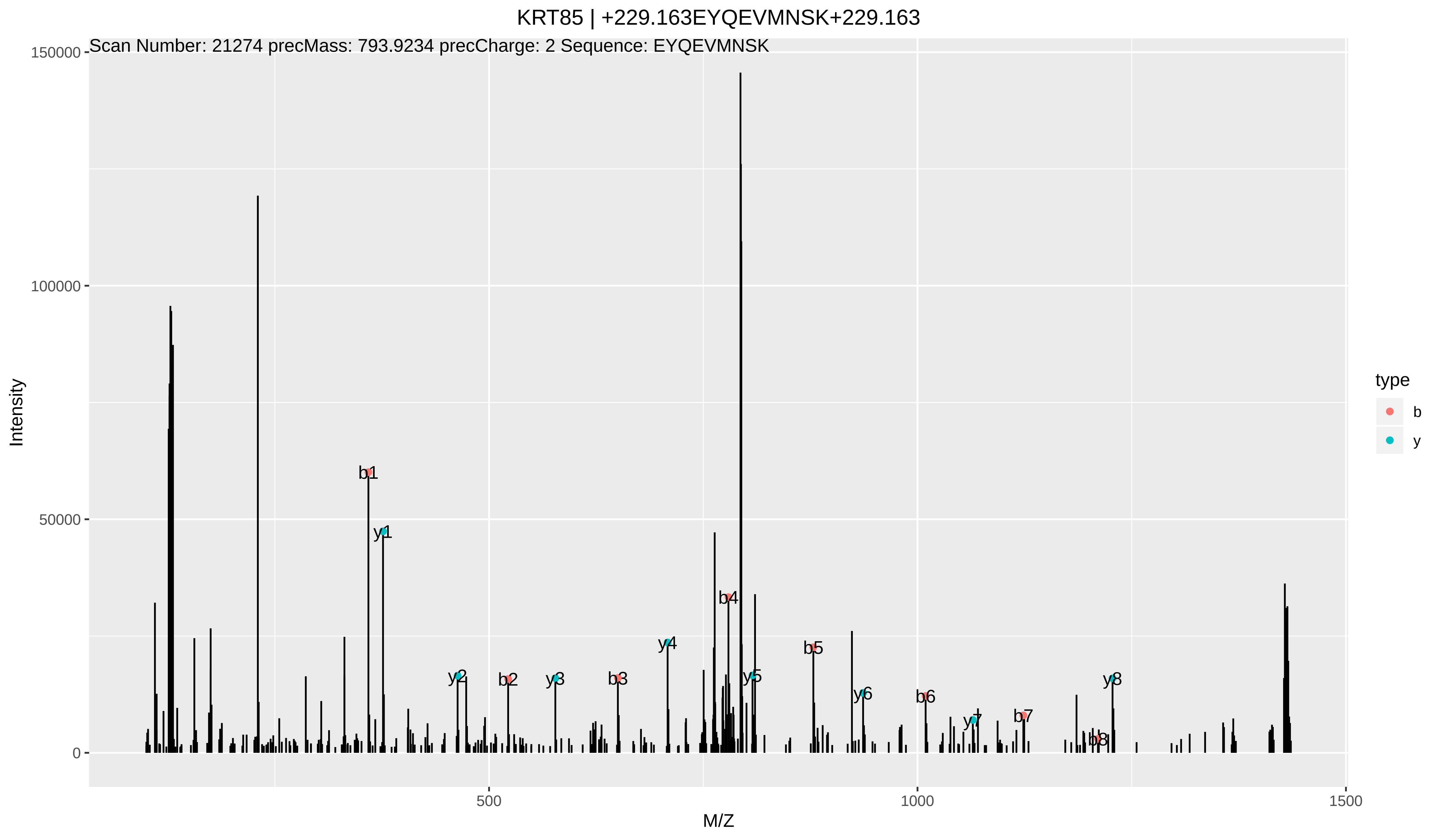

KRT85 | +229.163EYQEVMNSK+229.163
Scan Number: 21274 precMass: 793.9234 precCharge: 2 Sequence: EYQEVMNSK
Intensity
150000
100000
50000
0
M/Z
500
1000
1500
b1
y1
y2
b2
y3
b3
y4
b4
y5
b5
y6
b6
y7
b7
y8
b8
type
b
y

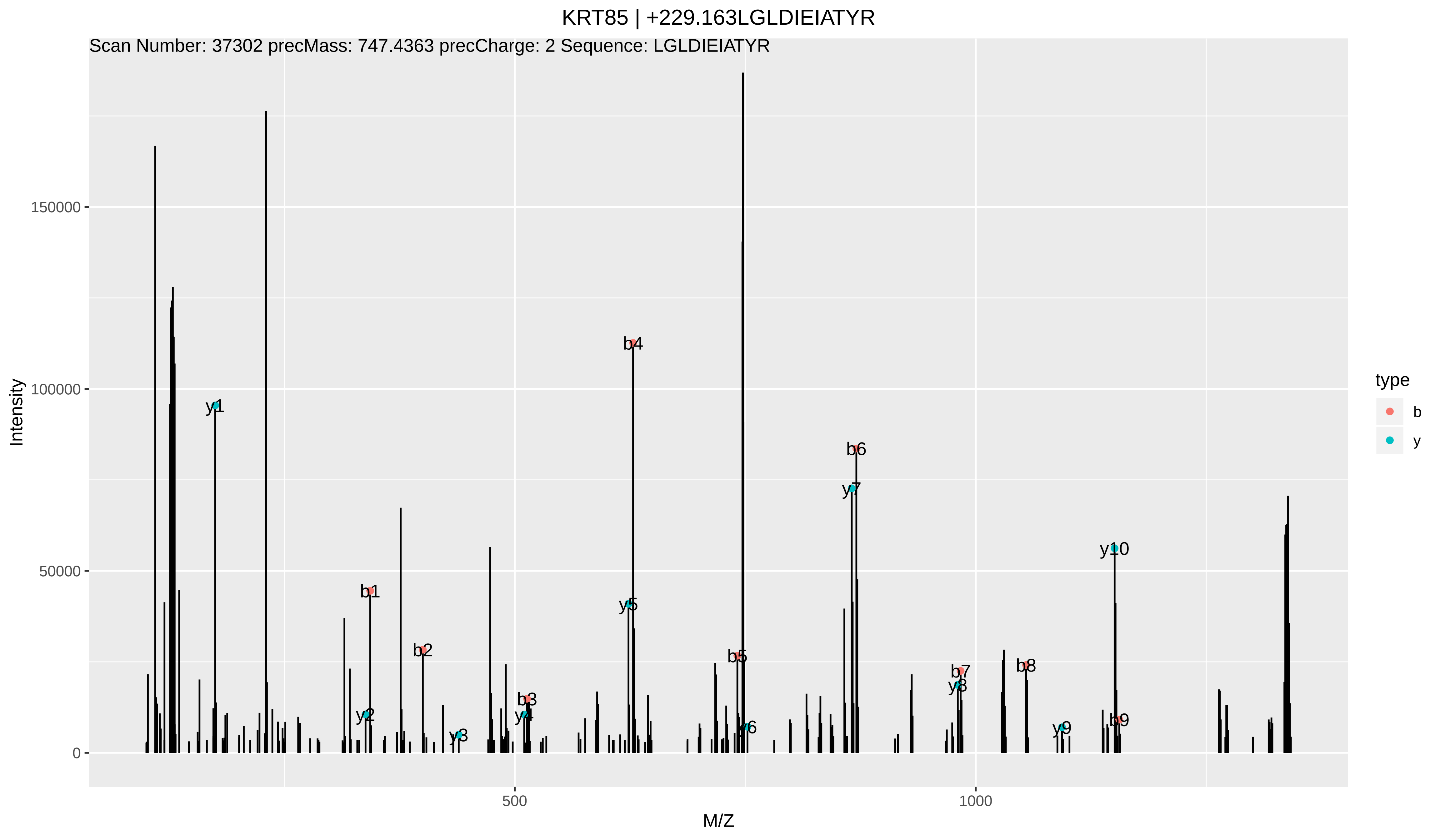
KRT85 | +229.163LGLDIEIATYR
Scan Number: 37302 precMass: 747.4363 precCharge: 2 Sequence: LGLDIEIATYR
Intensity
150000
100000
50000
0
M/Z
500
1000
type
b
y
y1
b1
y2
b2
y3
b3
y4
b4
y5
b5
y6
b6
y7
b7
y8
b8
y9
b9
y10

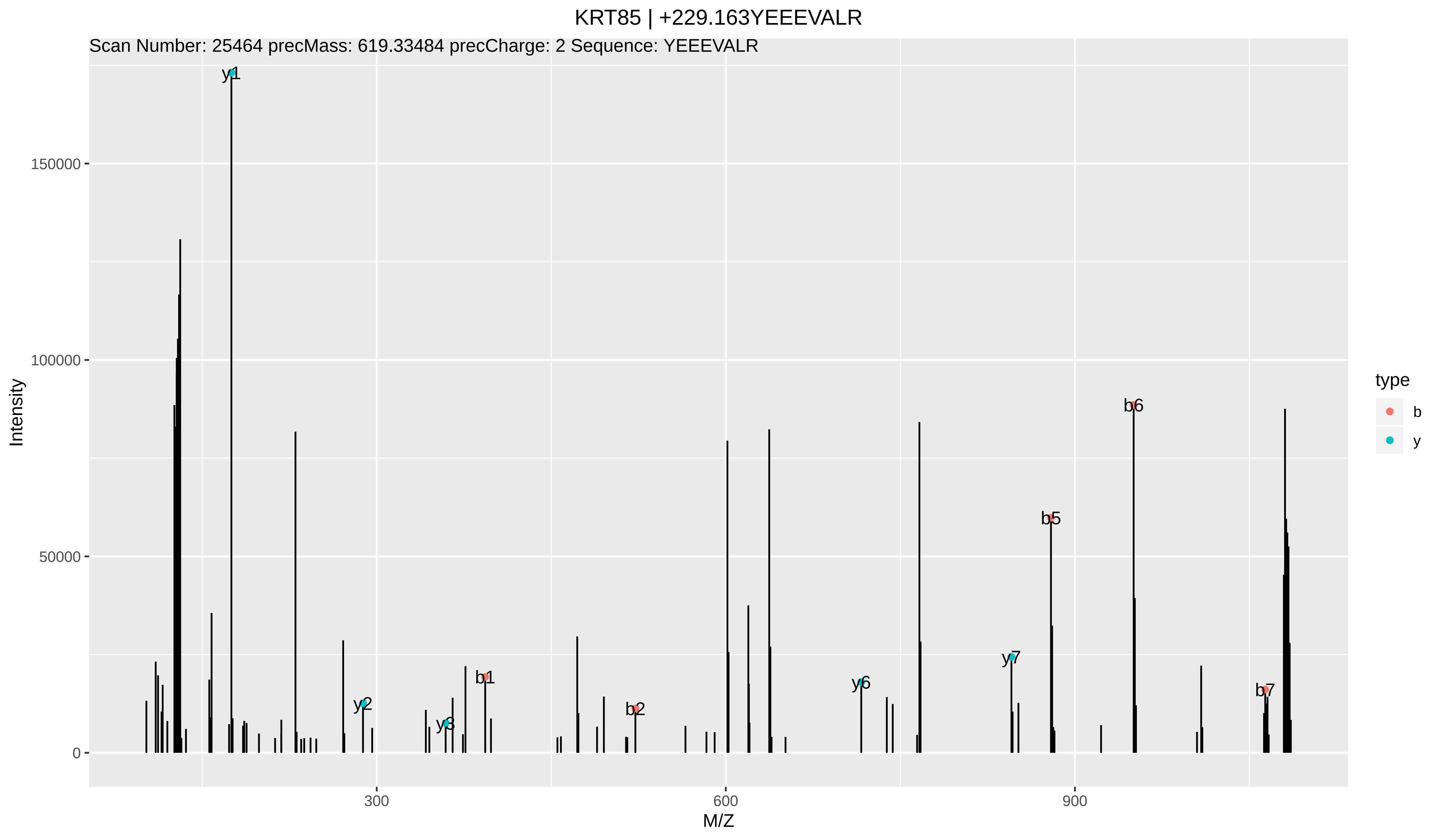

KRT85 | +229.163YEEEVALR
Scan Number: 25464 precMass: 619.33484 precCharge: 2 Sequence: YEEEVALR
Intensity
M/Z
150000
100000
50000
0
300
600
900
y1
y2
y3
b1
b2
y6
y7
b5
b6
b7
type
b
y

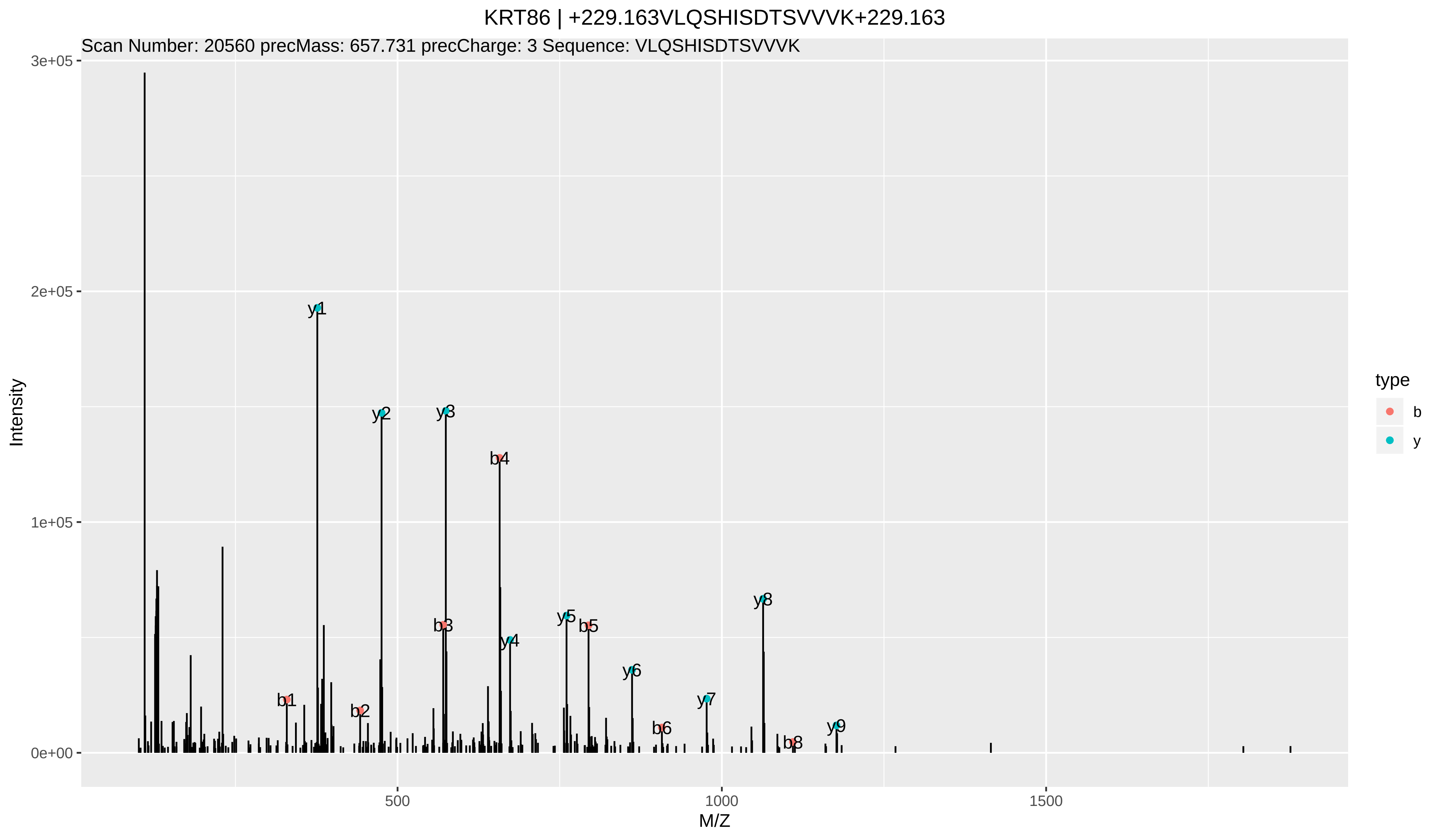
KRT86 | +229.163VLQSHISDTSVVVK+229.163
Scan Number: 20560 precMass: 657.731 precCharge: 3 Sequence: VLQSHISDTSVVVK
Intensity
3e+05
2e+05
1e+05
0e+00
M/Z
500
1000
1500
y1
y2
y3
b4
y8
y5
b3
b5
y4
y6
b1
b2
y7
b6
y9
b8
type
b
y

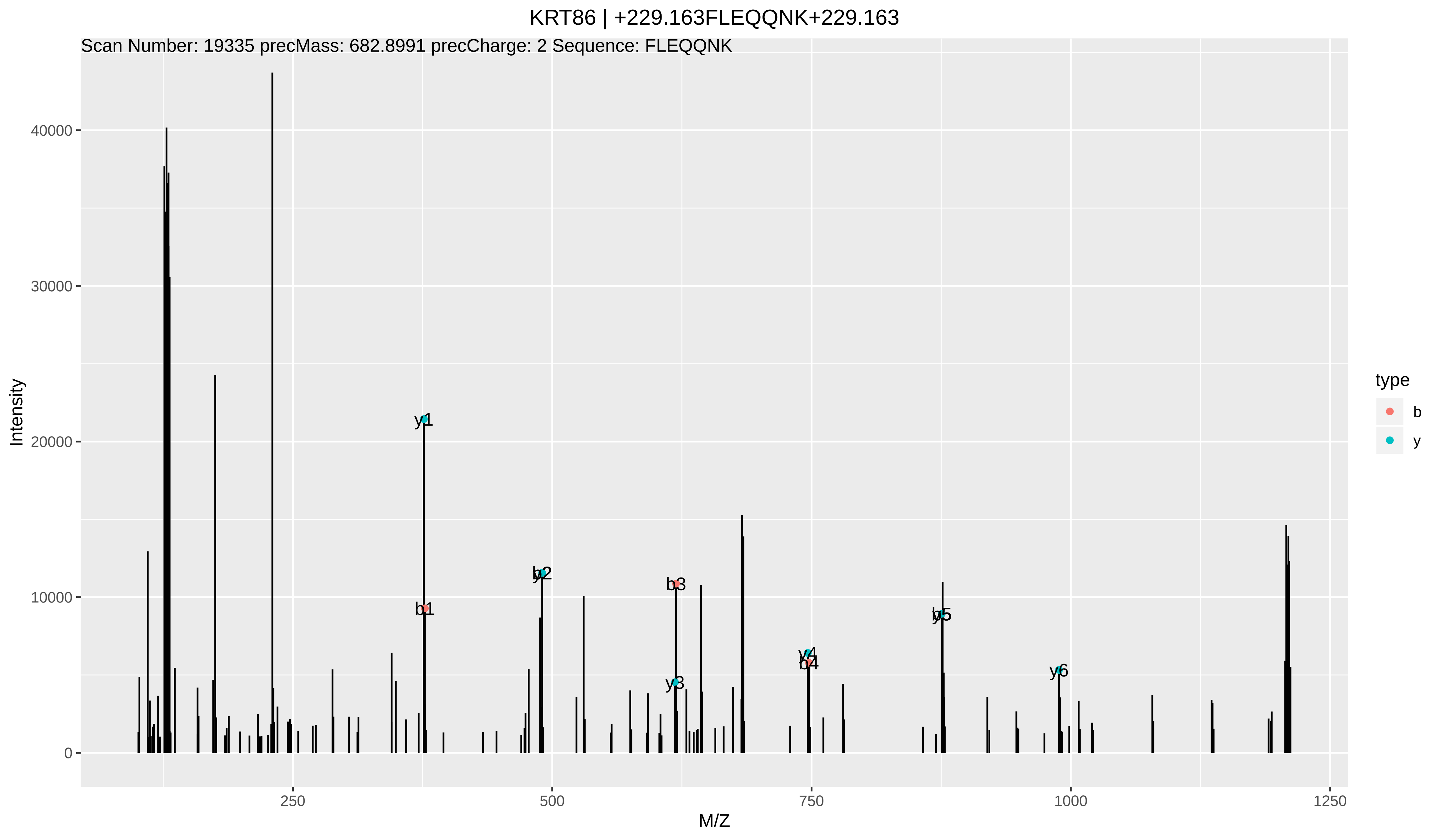

KRT86 | +229.163FLEQQNK+229.163
Scan Number: 19335 precMass: 682.8991 precCharge: 2 Sequence: FLEQQNK
Intensity
M/Z
type
b
y
y1
b1
b2
y2
b3
y3
y4
b4
b5
y5
y6
0
10000
20000
30000
40000
250
500
750
1000
1250

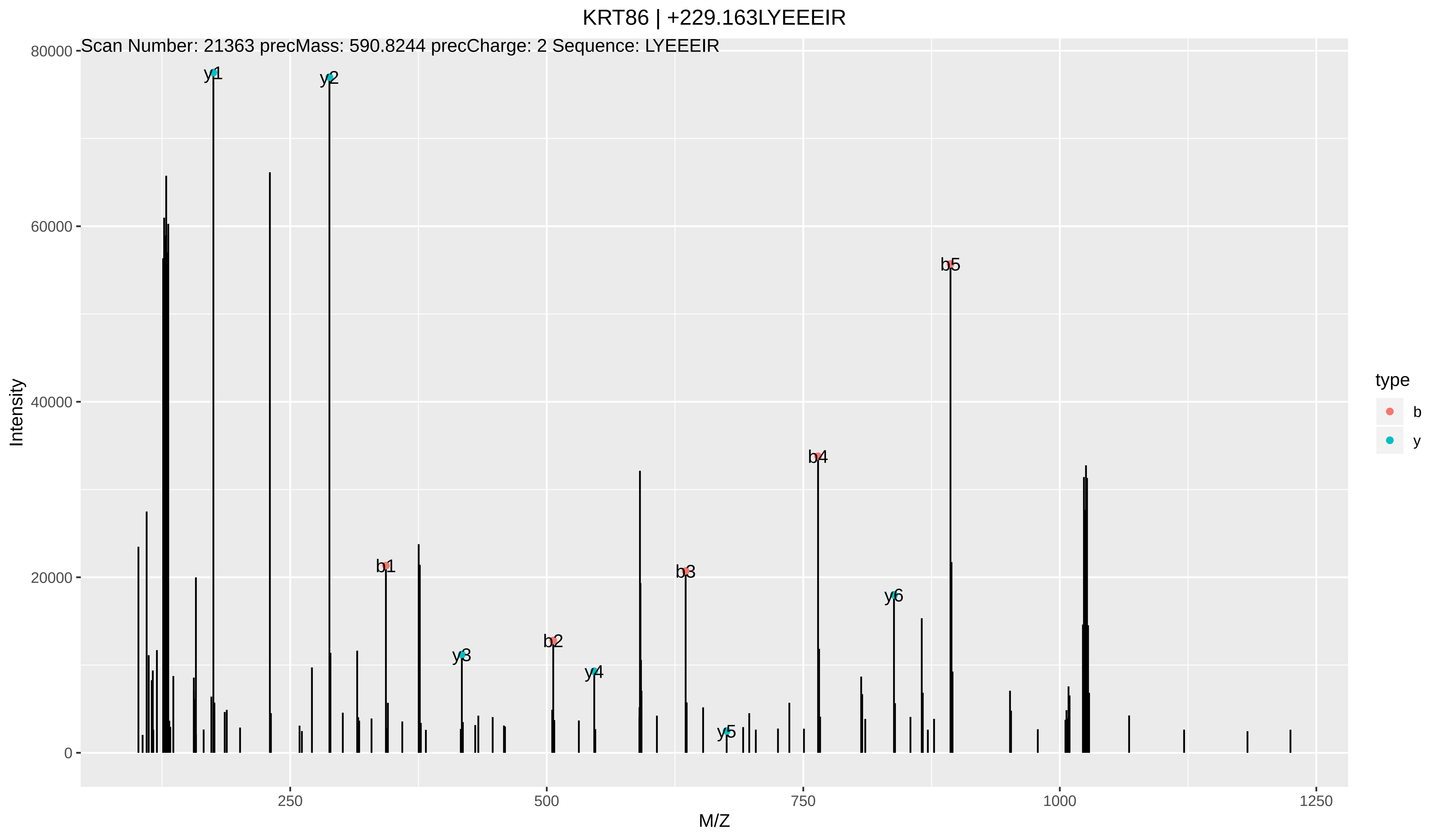

KRT86 | +229.163LYEEEIR
Scan Number: 21363 precMass: 590.8244 precCharge: 2 Sequence: LYEEEIR
Intensity
M/Z
type
b
y
y1
y2
b1
y3
b2
y4
b3
y5
b4
y6
b5

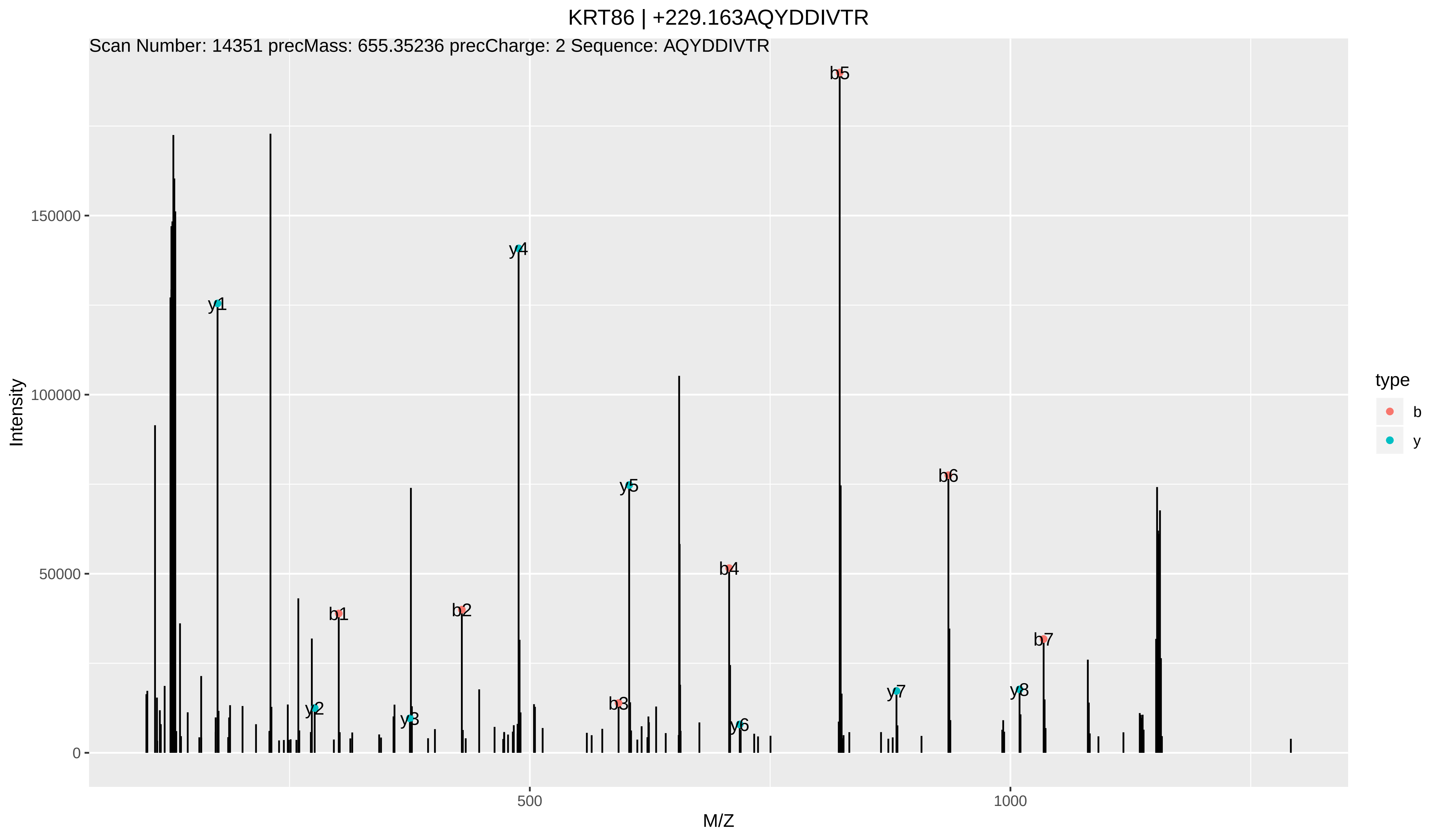

KRT86 | +229.163AQYDDIVTR
Scan Number: 14351 precMass: 655.35236 precCharge: 2 Sequence: AQYDDIVTR
Intensity
M/Z
150000
100000
50000
0
500
1000
b5
y4
y1
y5
b6
b4
b1
b2
b7
y7
y8
b3
y2
y3
y6
type
b
y

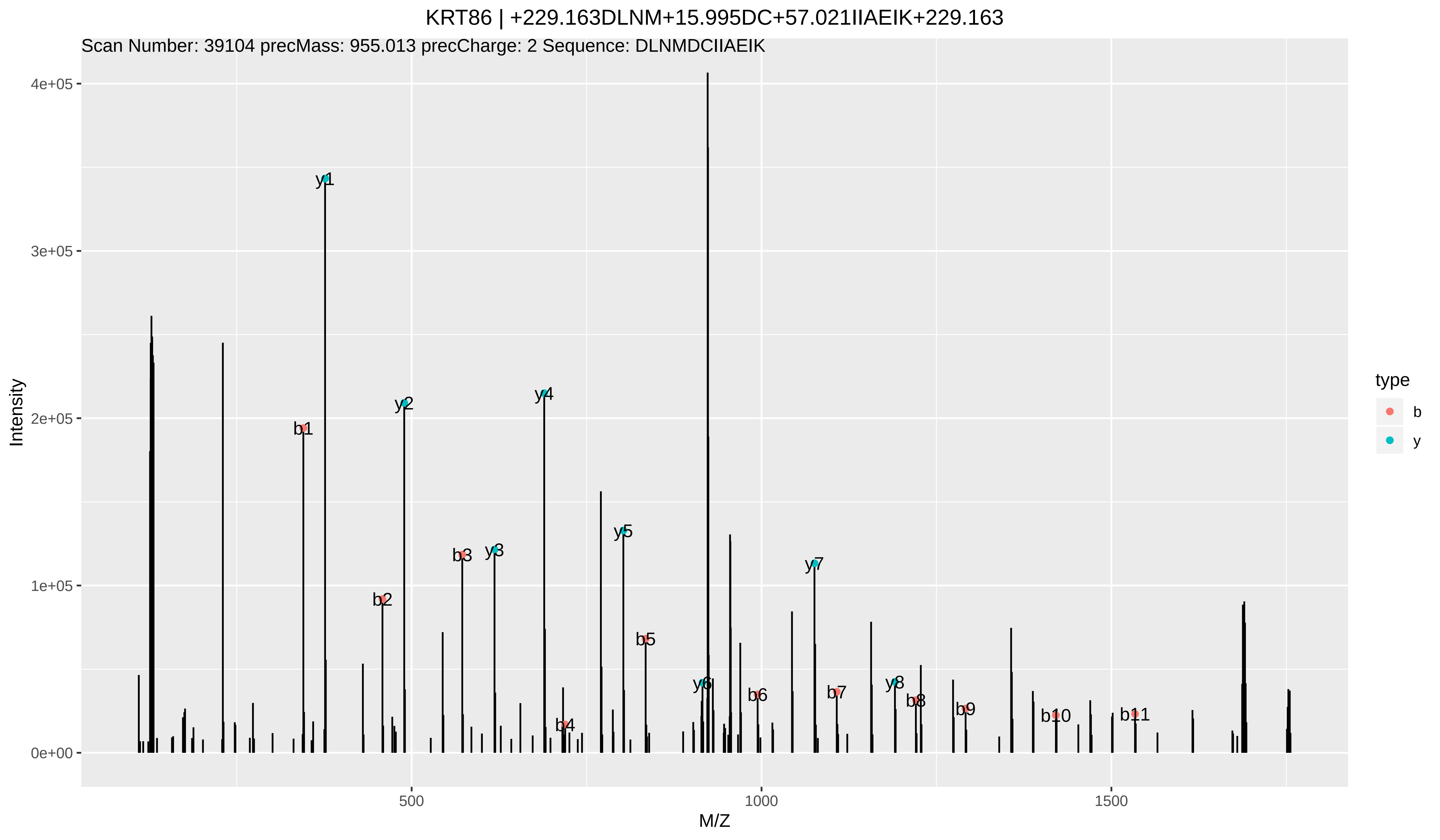

KRT86 | +229.163DLNM+15.995DC+57.021IIAEIK+229.163
Scan Number: 39104 precMass: 955.013 precCharge: 2 Sequence: DLNMDCIIAEIK
Intensity
4e+05
3e+05
2e+05
1e+05
0e+00
M/Z
500
1000
1500
y1
y2
y3
y4
y5
y6
y7
y8
b1
b2
b3
b4
b5
b6
b7
b8
b9
b10
b11
type
b
y

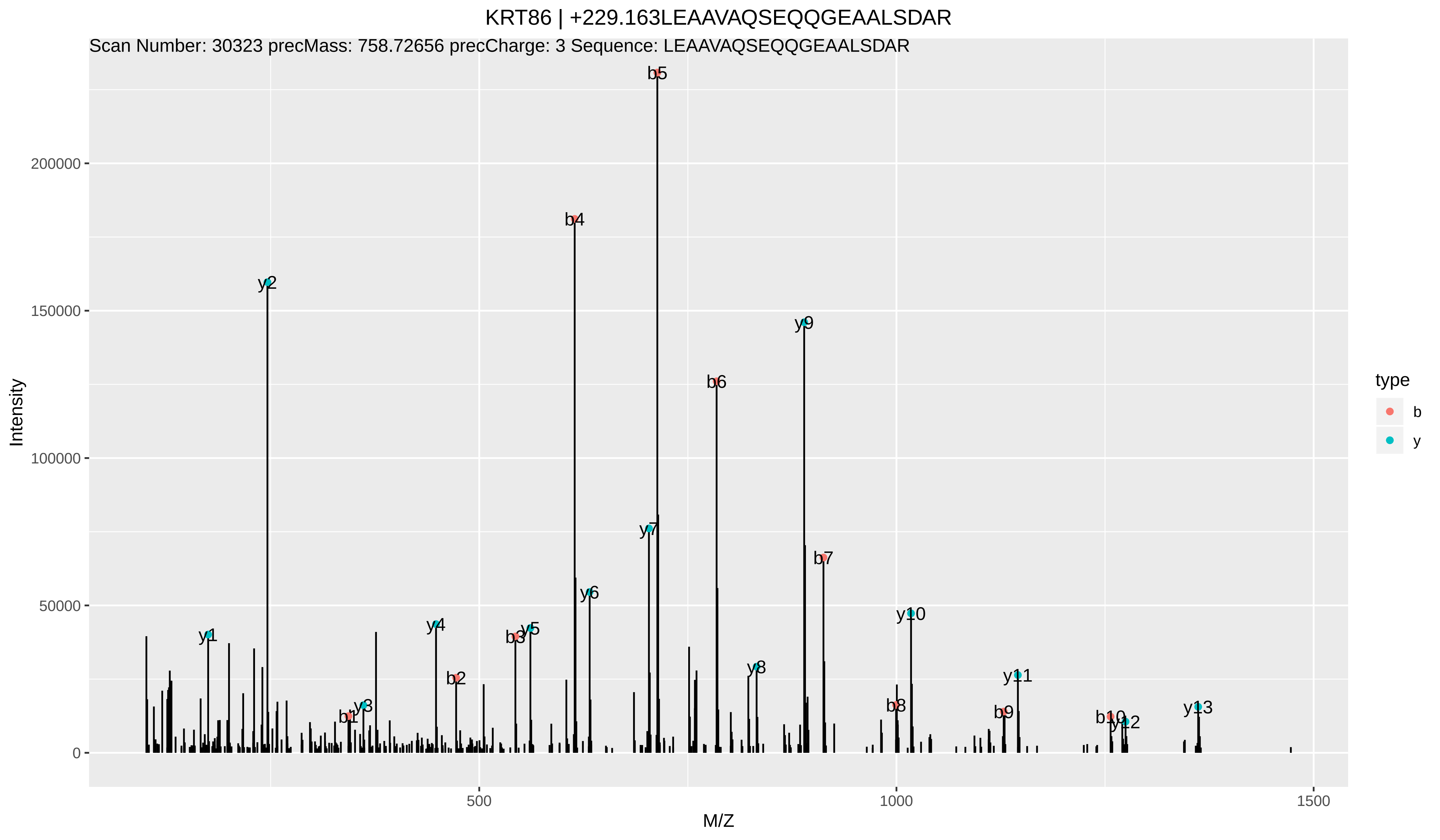
KRT86 | +229.163LEAAVAQSEQQGEAALSDAR
Scan Number: 30323 precMass: 758.72656 precCharge: 3 Sequence: LEAAVAQSEQQGEAALSDAR
Intensity
M/Z
type
b
y
b5
b4
y2
y9
b6
y7
b7
y6
y10
y1
y4
b3
y5
b2
y8
y11
y3
b1
b8
b9
b10
y12
y13
0
50000
100000
150000
200000
500
1000
1500

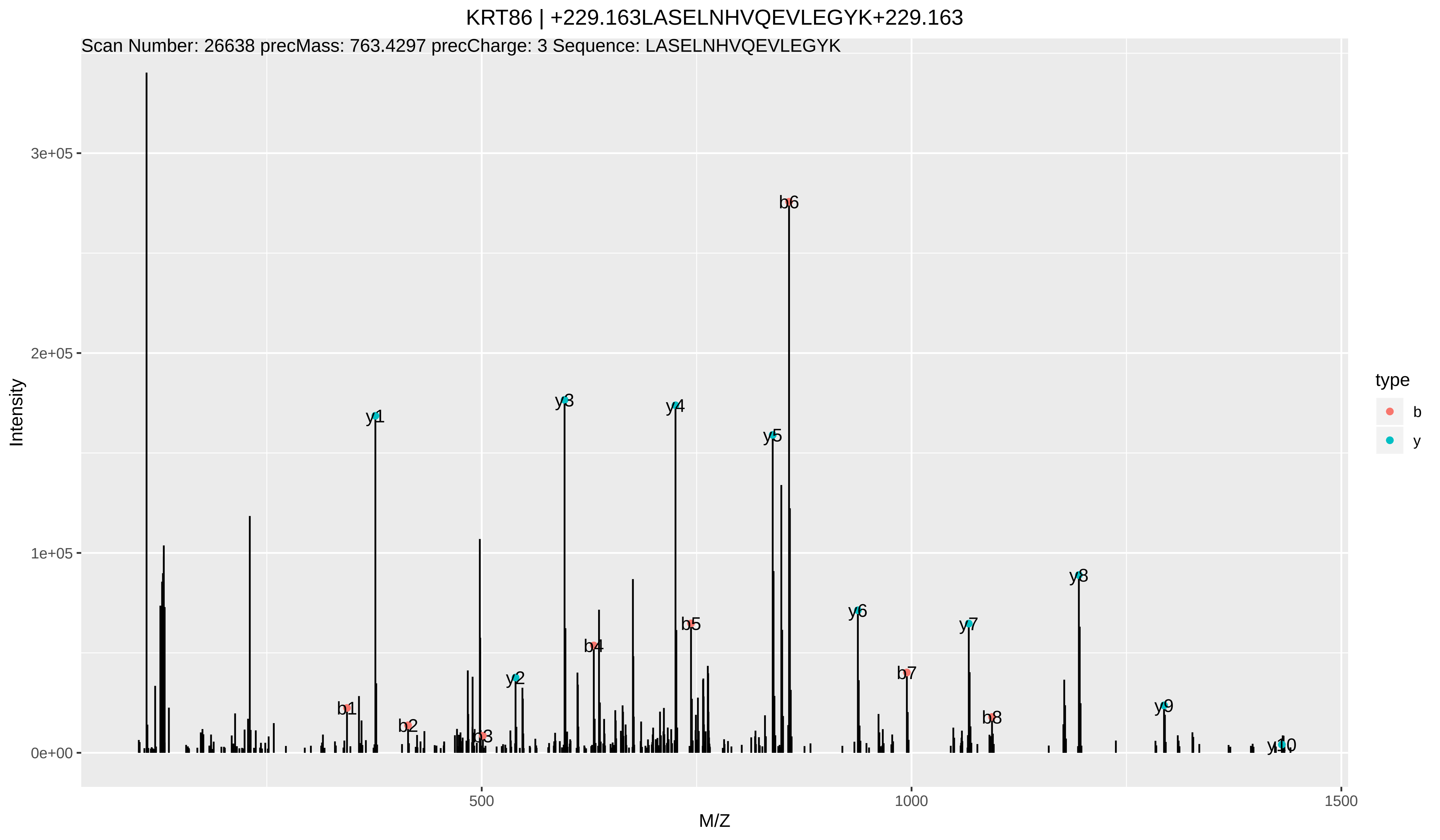

KRT86 | +229.163LASELNHVQEVLEGYK+229.163
Scan Number: 26638 precMass: 763.4297 precCharge: 3 Sequence: LASELNHVQEVLEGYK
Intensity
3e+05
2e+05
1e+05
0e+00
M/Z
500
1000
1500
b6
y1
y3
y4
y5
y2
b1
b2
b3
b4
b5
y6
b7
y7
y8
b8
y9
y10
type
b
y

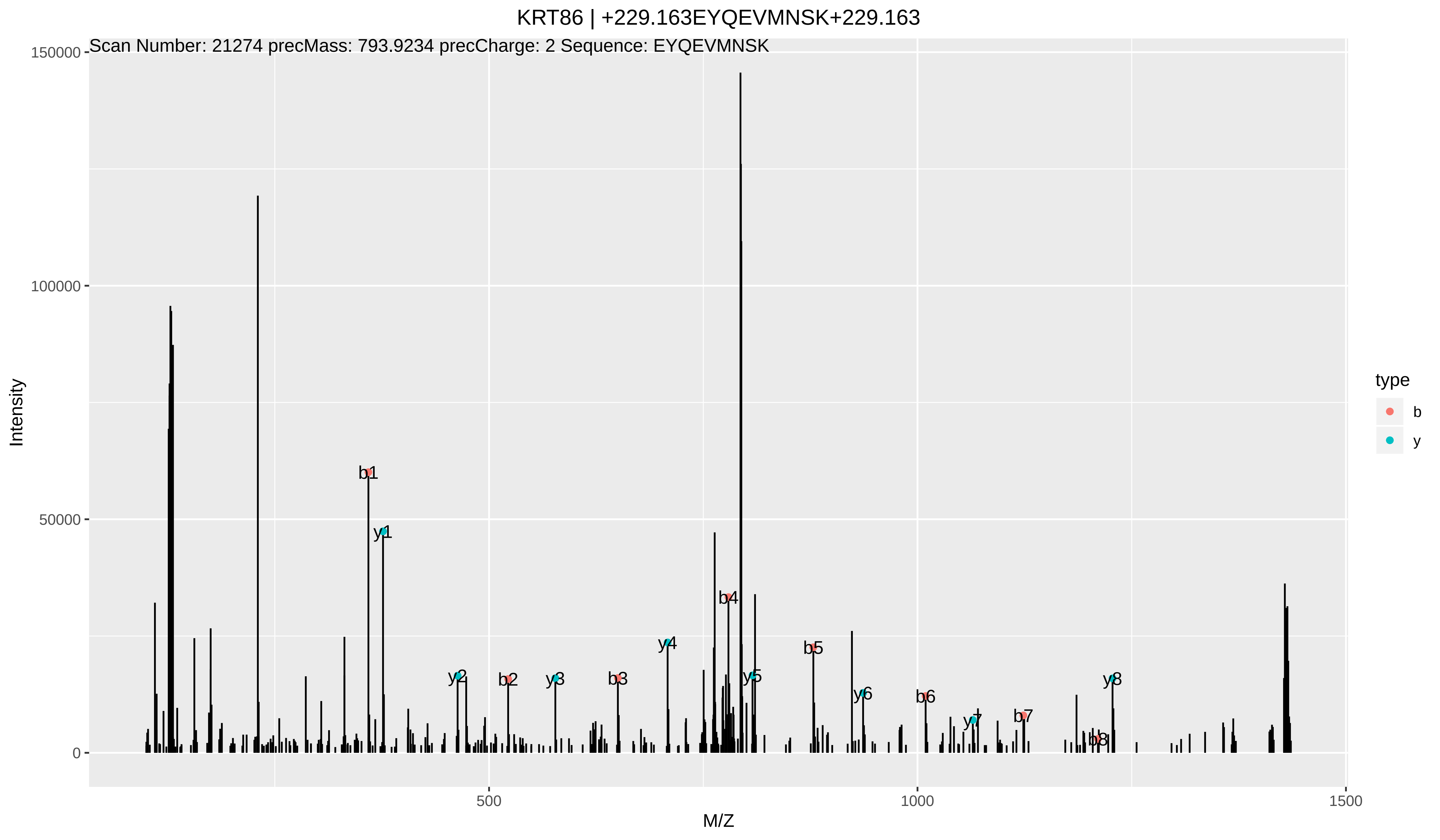
KRT86 | +229.163EYQEVMNSK+229.163
Scan Number: 21274 precMass: 793.9234 precCharge: 2 Sequence: EYQEVMNSK
Intensity
0
50000
100000
150000
M/Z
500
1000
1500
b1
y1
y2
b2
y3
b3
y4
b4
y5
b5
y6
b6
y7
b7
b8
y8
type
b
y

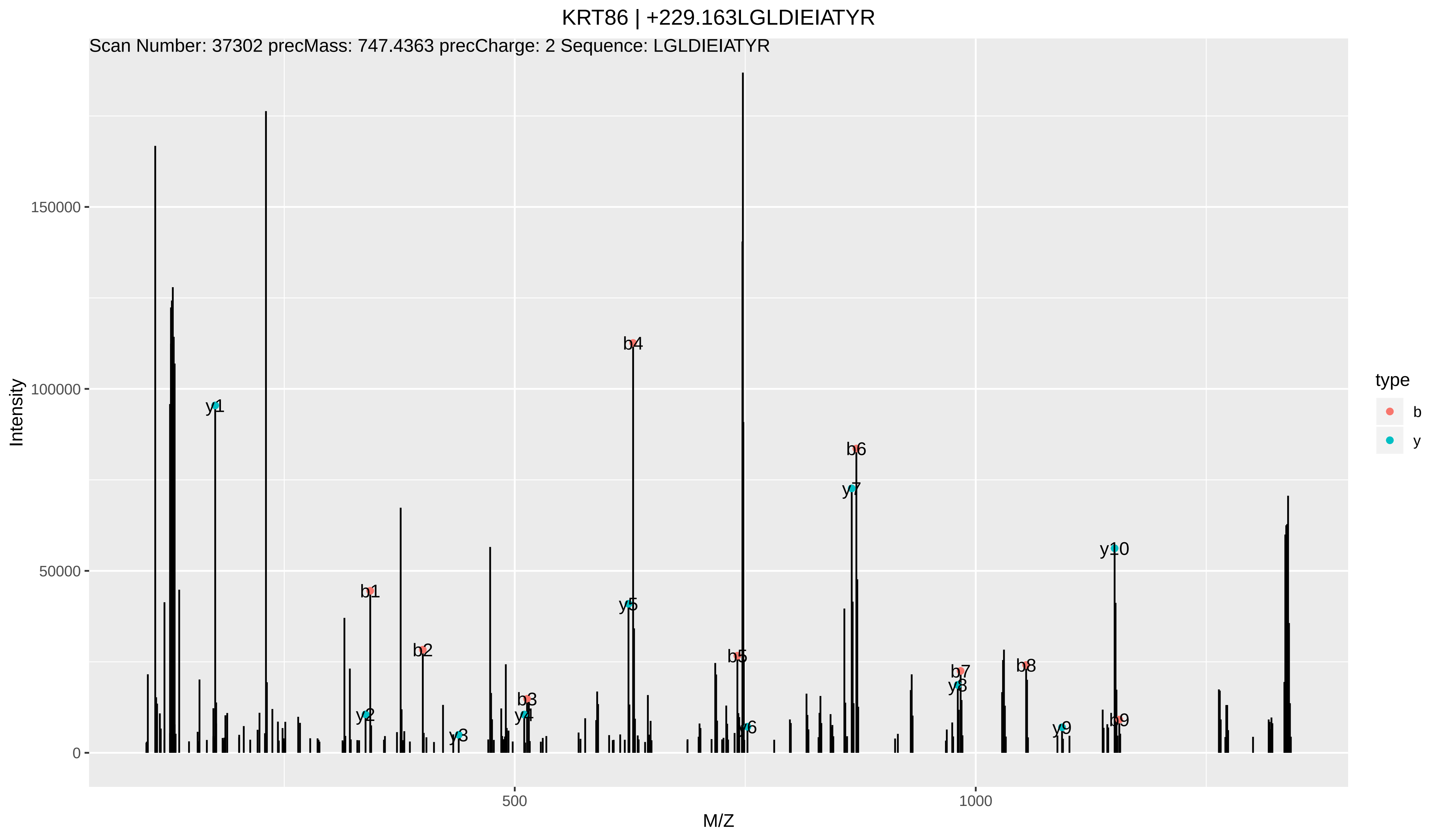

KRT86 | +229.163LGLDIEIATYR
Scan Number: 37302 precMass: 747.4363 precCharge: 2 Sequence: LGLDIEIATYR
Intensity
M/Z
type
b
y
y1
b1
y2
b2
y3
b3
y4
b4
y5
b5
y6
b6
y7
b7
y8
b8
y9
b9
y10
0
50000
100000
150000
500
1000

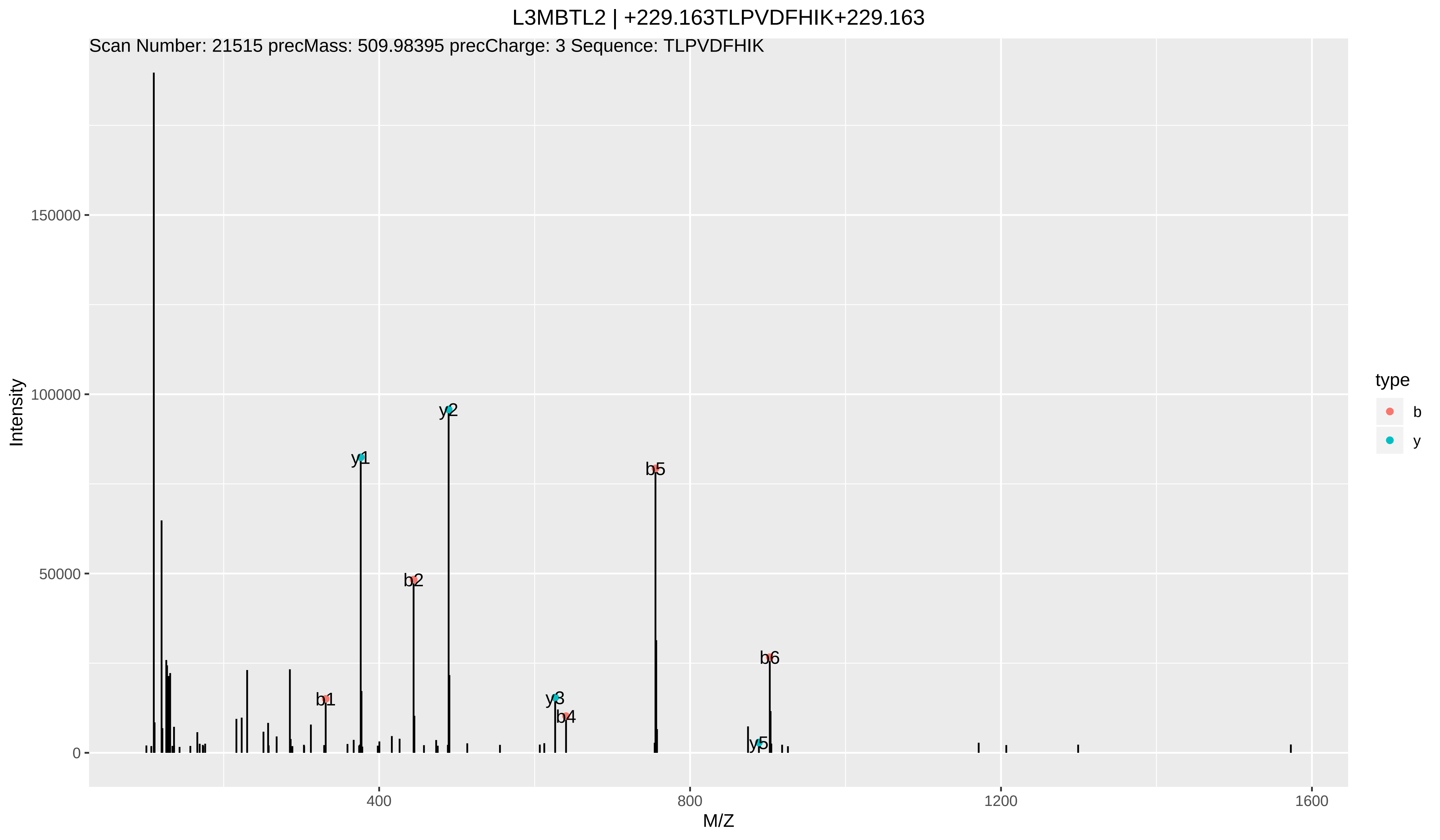

L3MBTL2 | +229.163TLPVDFHIK+229.163
Scan Number: 21515 precMass: 509.98395 precCharge: 3 Sequence: TLPVDFHIK
Intensity
150000
100000
50000
0
M/Z
400
800
1200
1600
y1
y2
b1
b2
b5
b6
y3
b4
y5
type
b
y

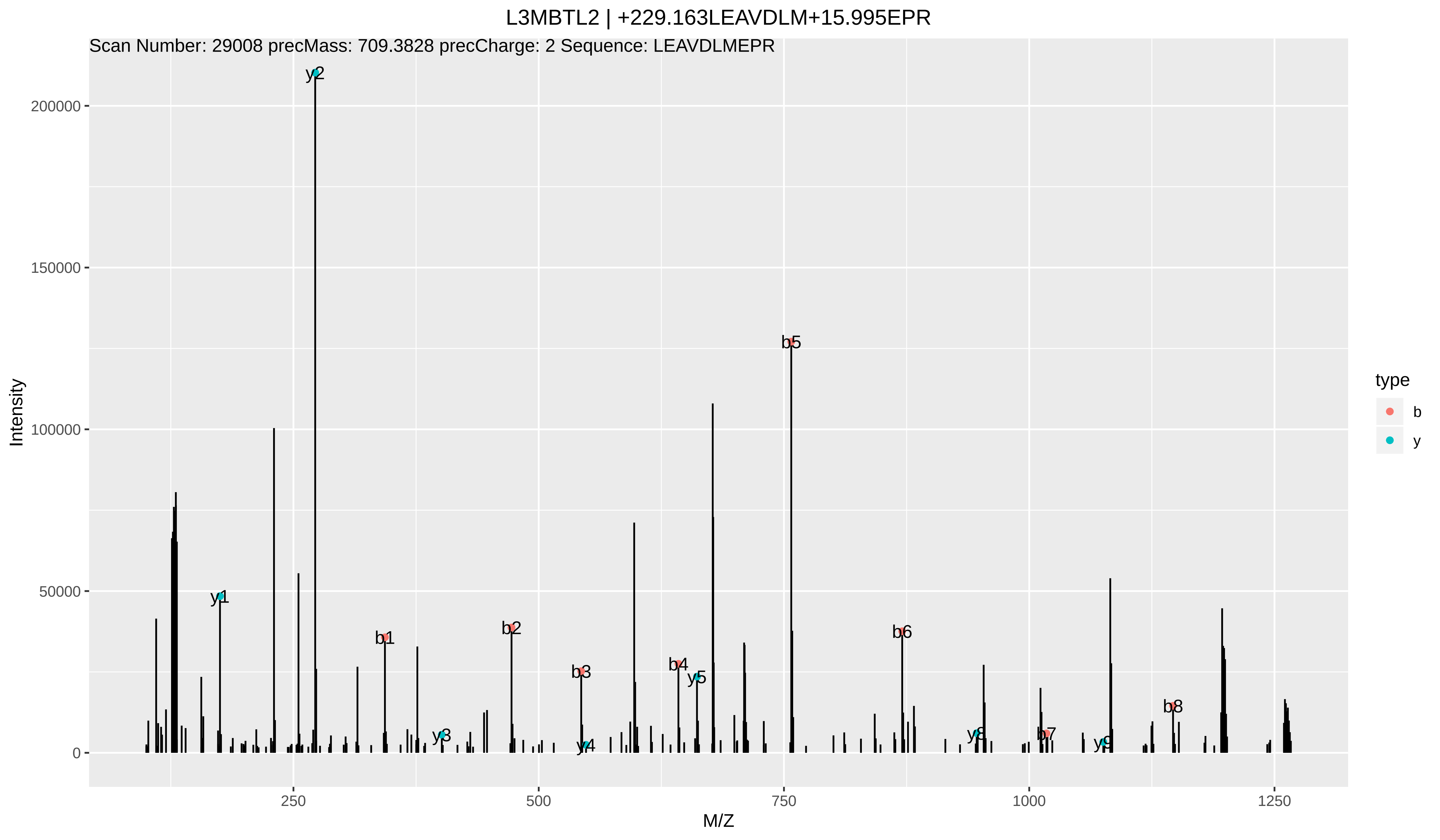

L3MBTL2 | +229.163LEAVDLM+15.995EPR
Scan Number: 29008 precMass: 709.3828 precCharge: 2 Sequence: LEAVDLMEPR
Intensity
M/Z
type
b
y
y1
y2
y3
y4
y5
y8
y9
b1
b2
b3
b4
b5
b6
b7
b8
0
50000
100000
150000
200000
250
500
750
1000
1250

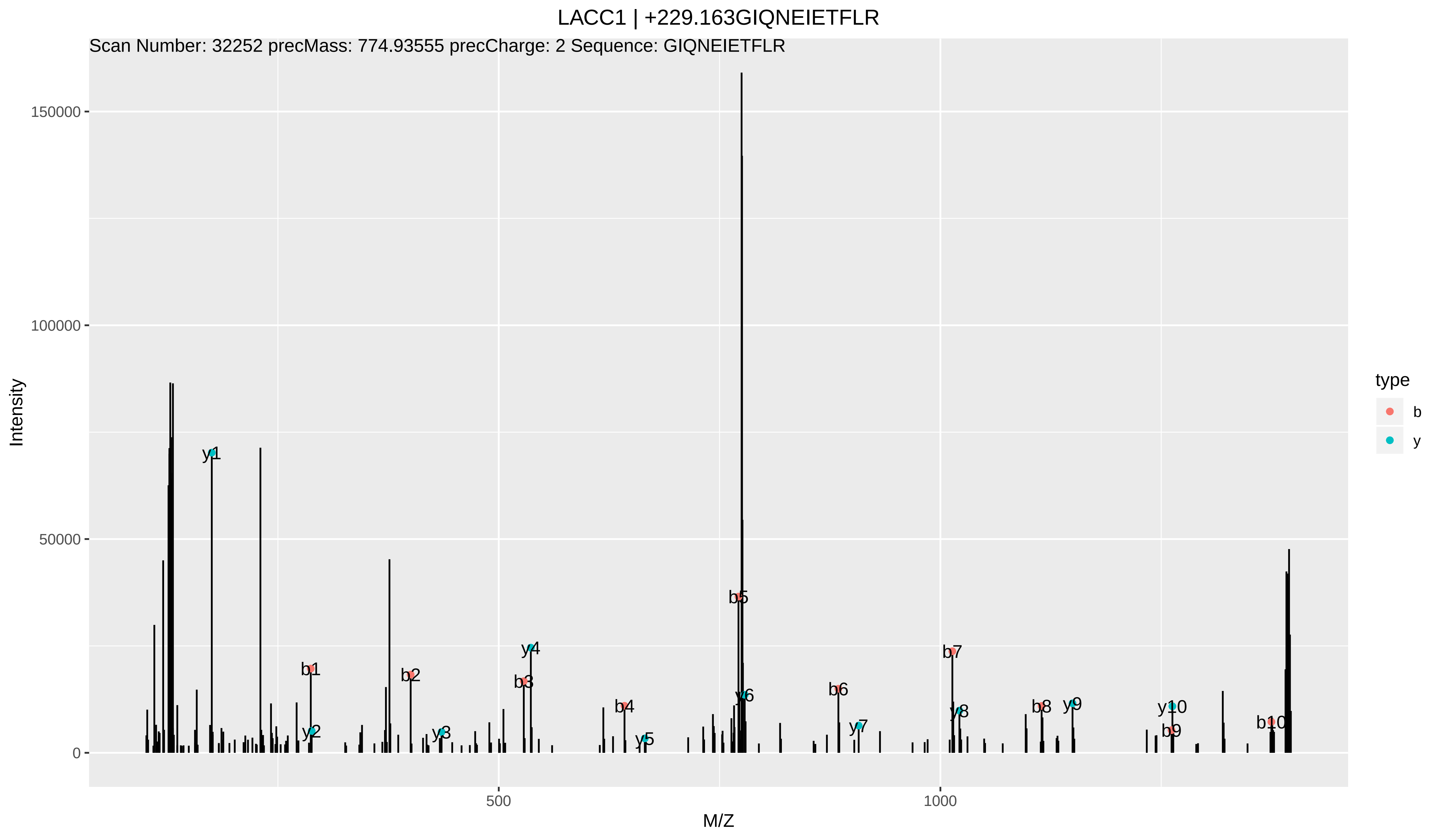

LACC1 | +229.163GIQNEIETFLR
Scan Number: 32252 precMass: 774.93555 precCharge: 2 Sequence: GIQNEIETFLR
Intensity
150000
100000
50000
0
500
1000
M/Z
type
b
y
y1
b1
y2
b2
y3
y4
b3
b4
y5
b5
y6
b6
y7
b7
y8
b8
y9
y10
b9
b10

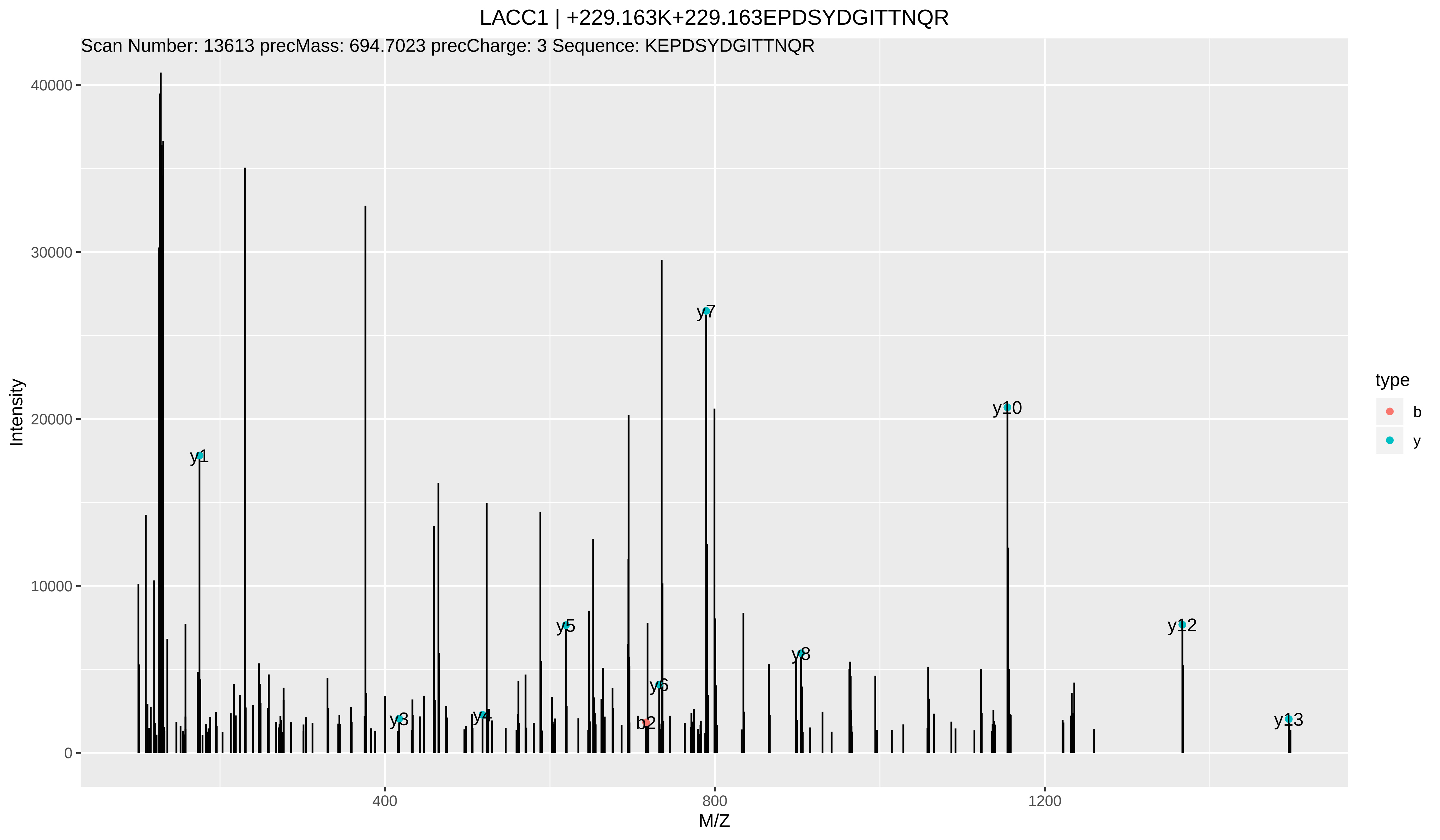
LACC1 | +229.163K+229.163EPDSYDGITTNQR
Scan Number: 13613 precMass: 694.7023 precCharge: 3 Sequence: KEPDSYDGITTNQR
Intensity
M/Z
0
10000
20000
30000
40000
400
800
1200
y1
y3
y4
y5
b2
y6
y7
y8
y10
y12
y13
type
b
y

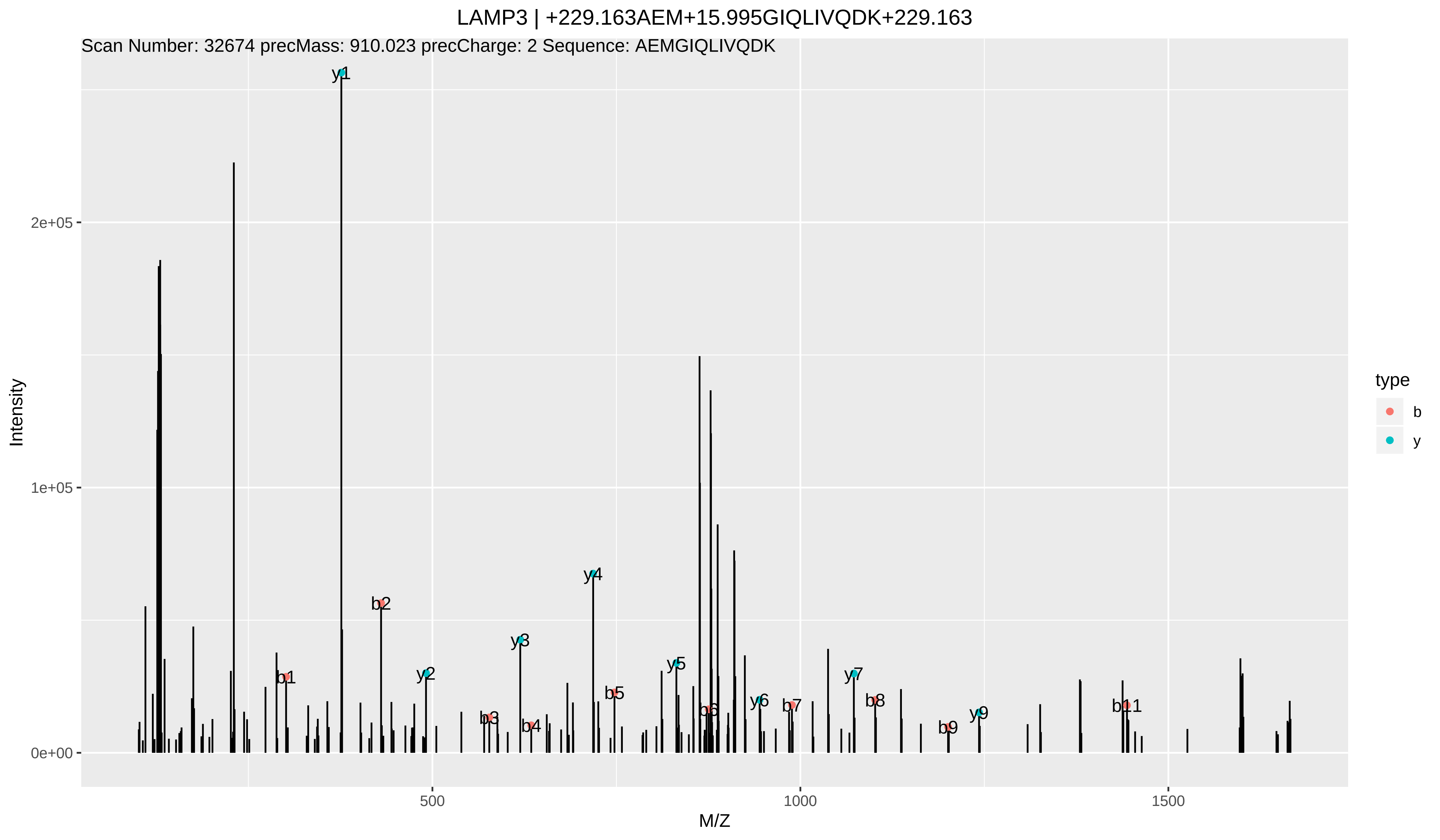

LAMP3 | +229.163AEM+15.995GIQLIVQDK+229.163
Scan Number: 32674 precMass: 910.023 precCharge: 2 Sequence: AEMGIQLIVQDK
Intensity
M/Z
2e+05
1e+05
0e+00
500
1000
1500
y1
b2
y4
y3
y2
b1
y5
b5
b3
b4
y6
b6
b7
y7
b8
b9
y9
b11
type
b
y

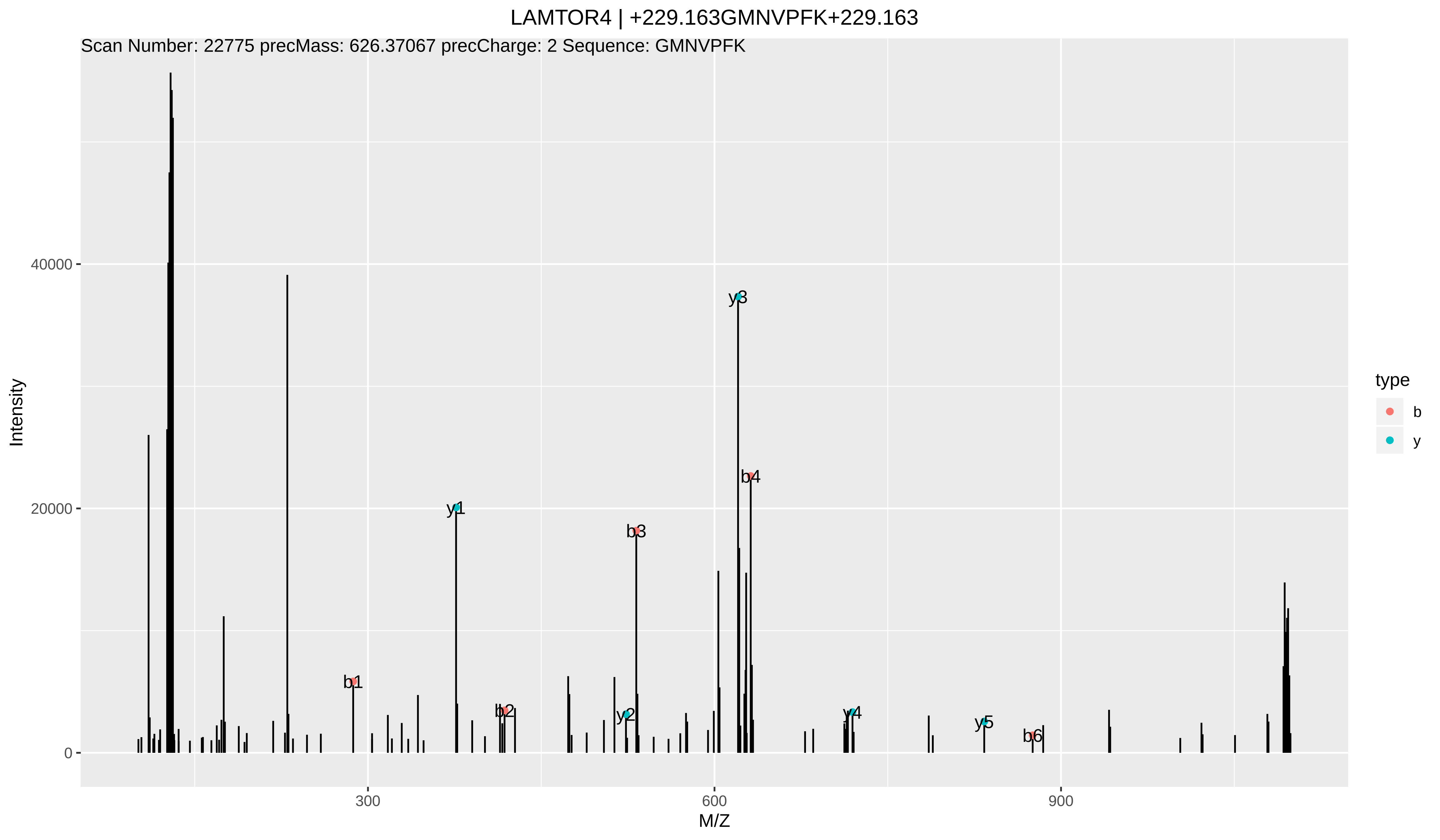

LAMTOR4 | +229.163GMNVPFK+229.163
Scan Number: 22775 precMass: 626.37067 precCharge: 2 Sequence: GMNVPFK
Intensity
M/Z
0
20000
40000
300
600
900
type
b
y
y1
y2
y3
y4
y5
b1
b2
b3
b4
b6

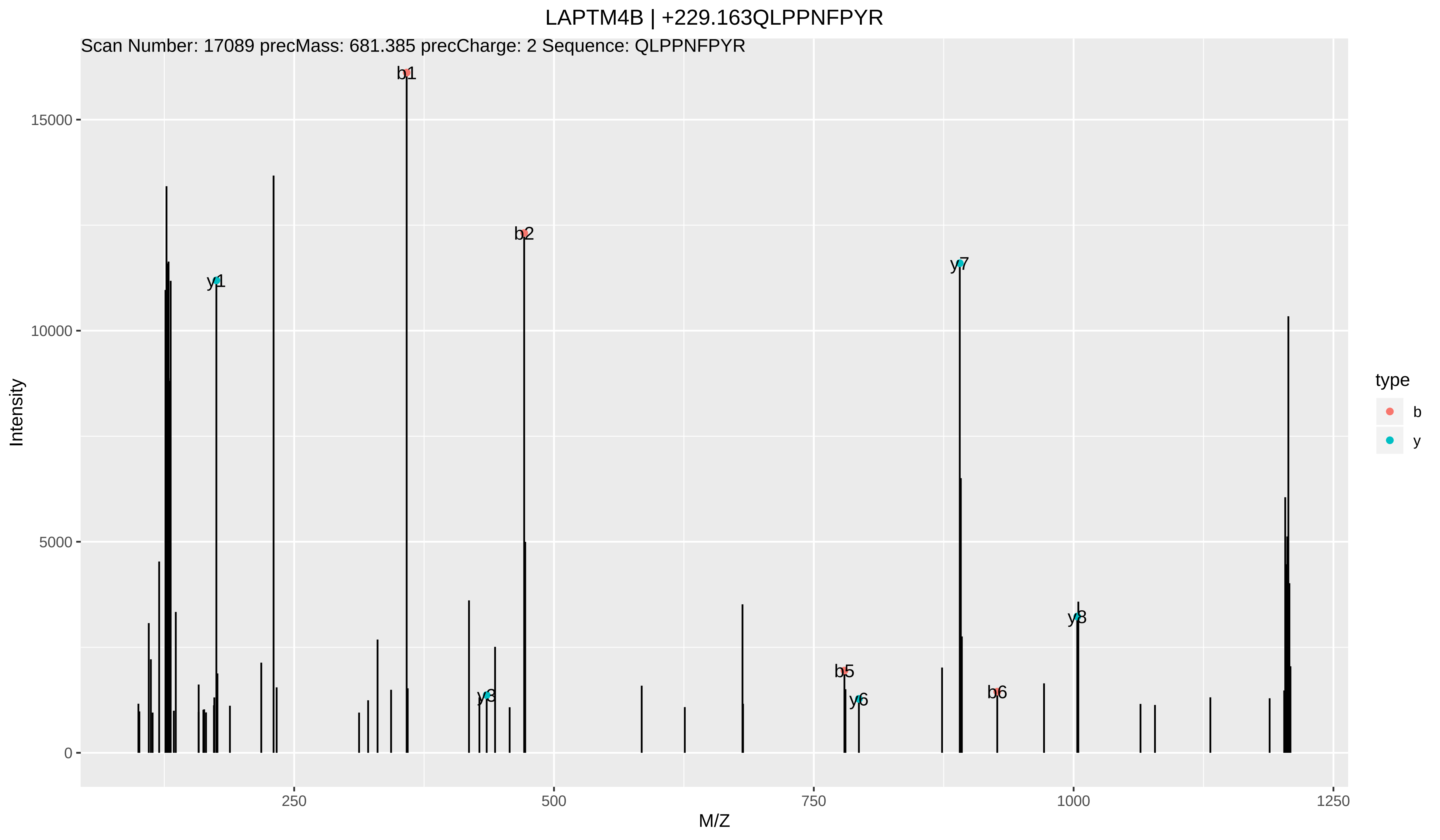

LAPTM4B | +229.163QLPPNFPYR
Scan Number: 17089 precMass: 681.385 precCharge: 2 Sequence: QLPPNFPYR
b1
b2
y1
y7
y3
y8
b5
y6
b6
Intensity
15000
10000
5000
0
250
500
750
1000
1250
M/Z
type
b
y

LCN1 | +229.163NNLEALEDFEK+229.163

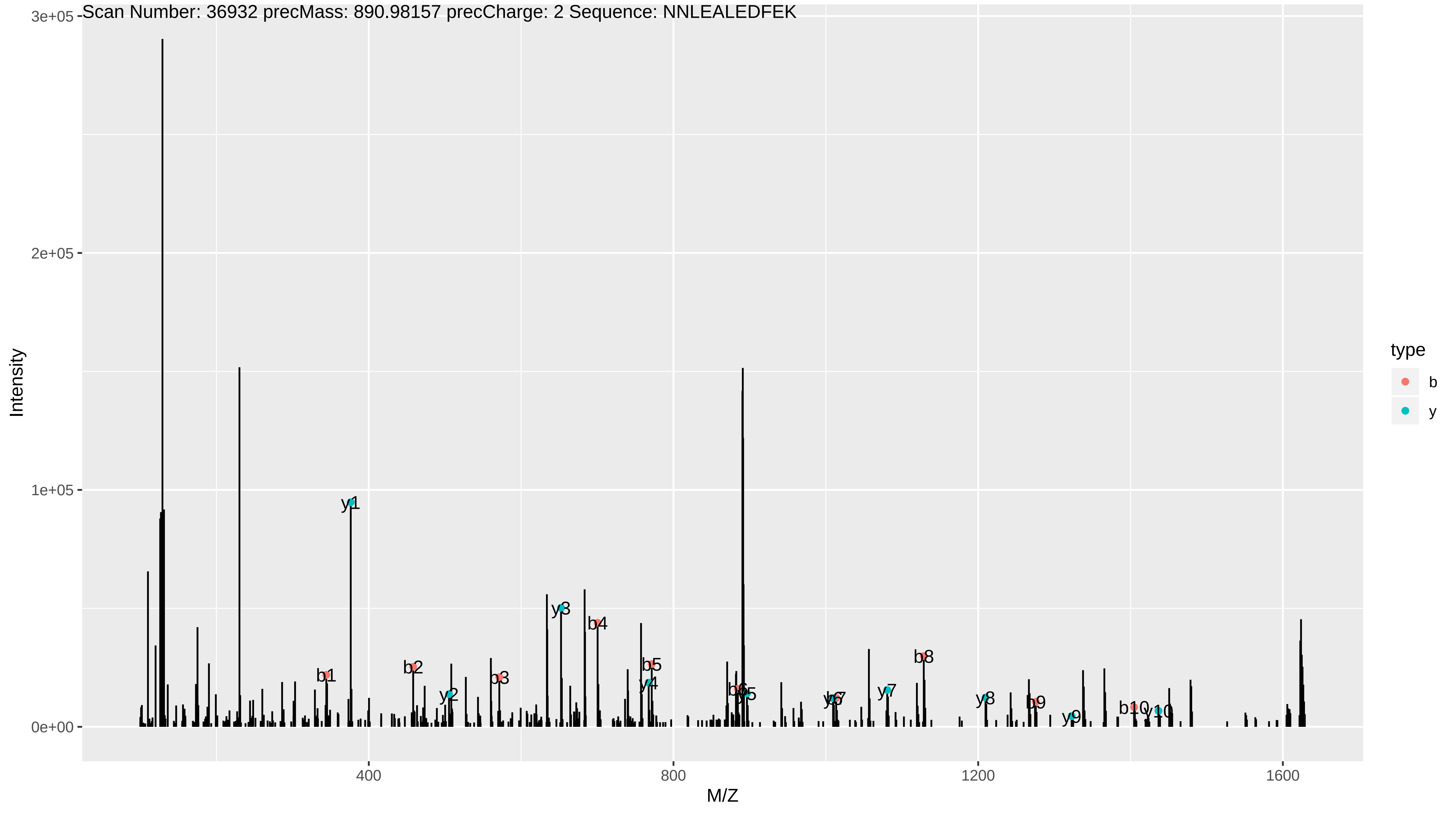

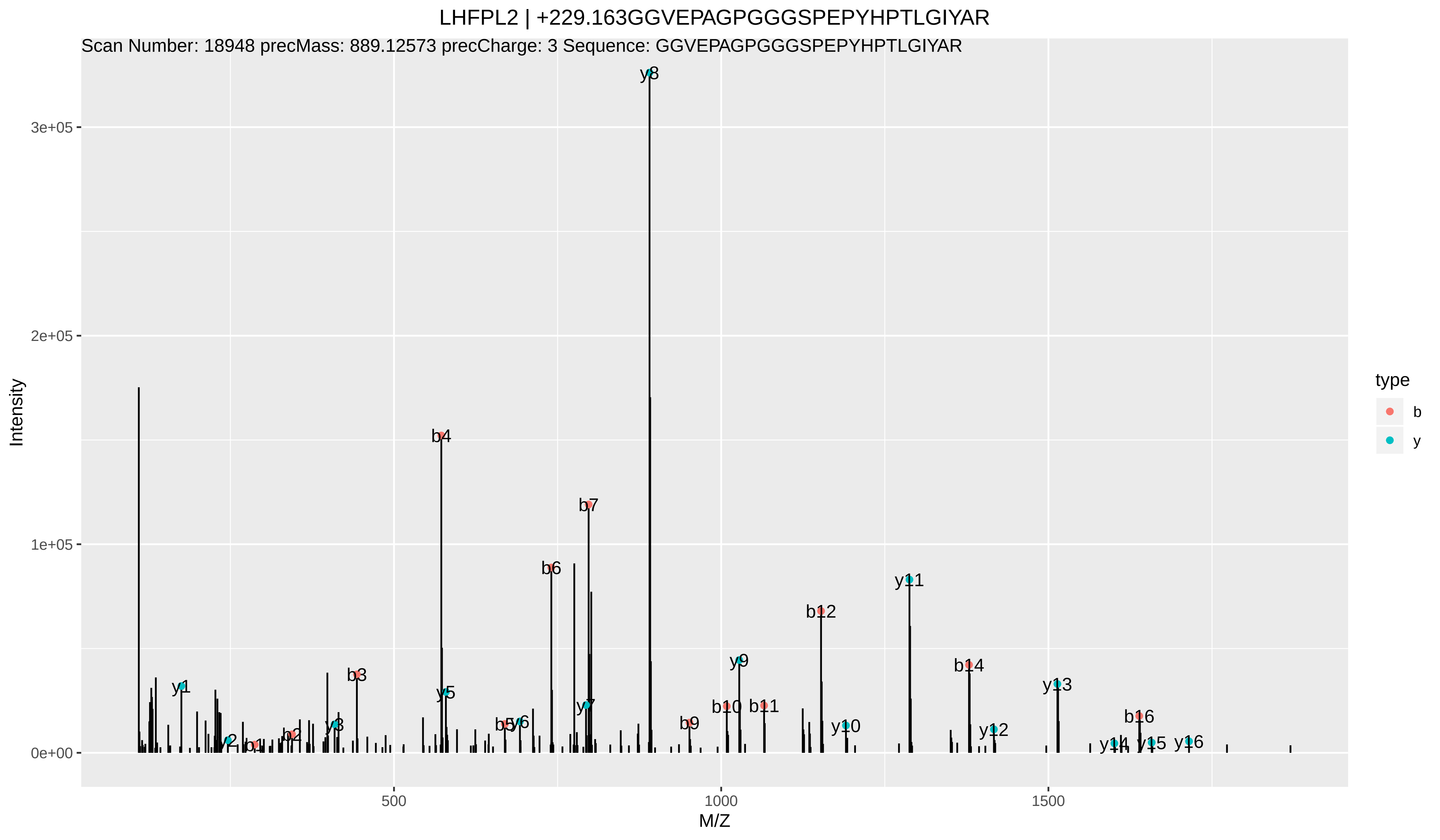

LHFPL2 | +229.163GGVEPAGPGGGSPEPYHPTLGIYAR
Scan Number: 18948 precMass: 889.12573 precCharge: 3 Sequence: GGVEPAGPGGGSPEPYHPTLGIYAR
Intensity
M/Z
3e+05
2e+05
1e+05
0e+00
500
1000
1500
type
b
y
y8
b4
b7
b6
y11
b12
y9
b14
b3
y1
y13
y5
y7
b10
b11
b16
b5
y6
b9
y3
y10
y12
b2
y2
b1
y14
y15
y16

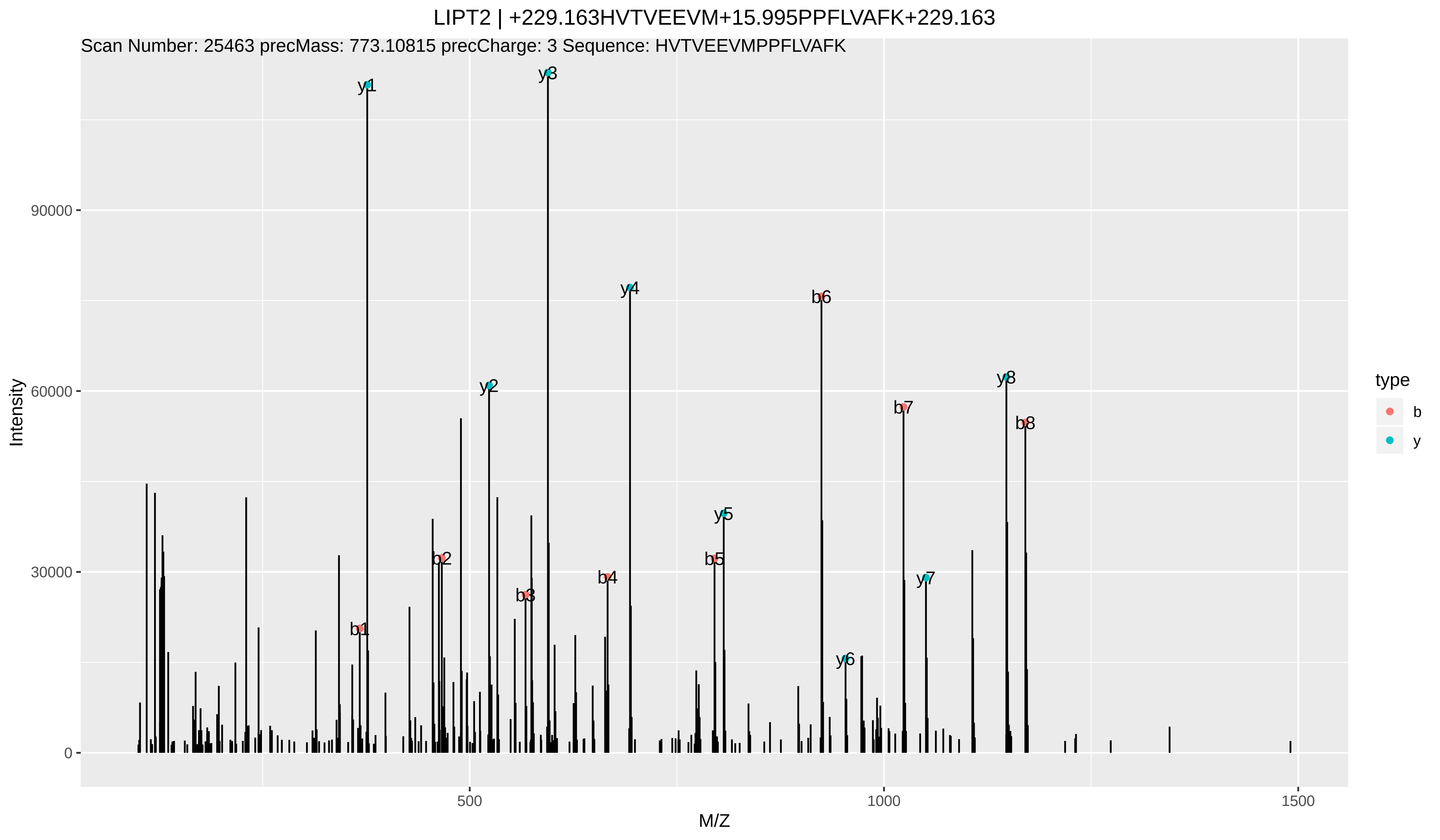
LIPT2 | +229.163HVTVEEVM+15.995PPFLVAFK+229.163
Scan Number: 25463 precMass: 773.10815 precCharge: 3 Sequence: HVTVEEVMPPFLVAFK
Intensity
90000
60000
30000
0
500
1000
1500
M/Z
y1
y2
y3
y4
y5
y6
y7
y8
b1
b2
b3
b4
b5
b6
b7
b8
type
b
y

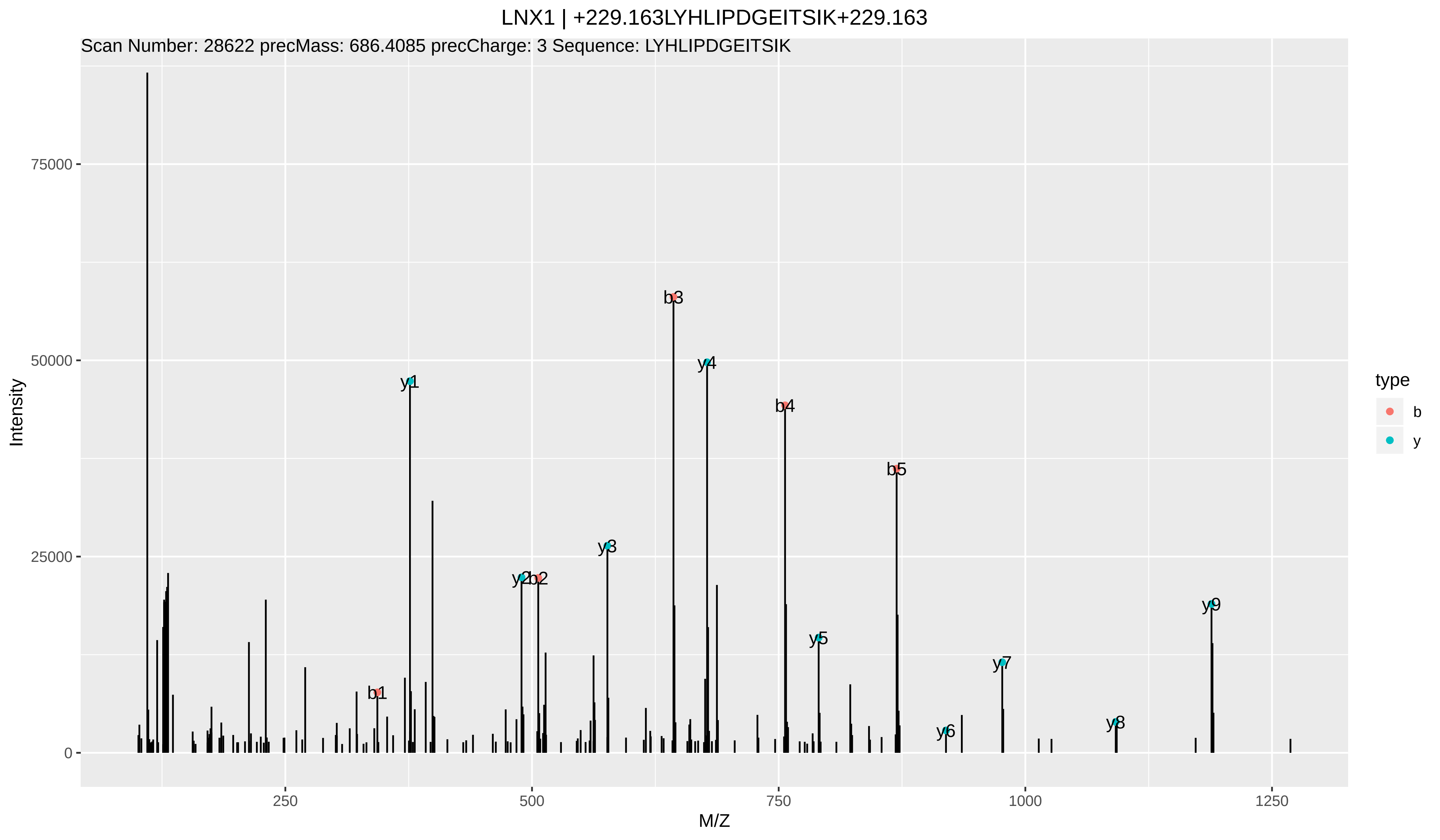
LNX1 | +229.163LYHLIPDGEITSIK+229.163
Scan Number: 28622 precMass: 686.4085 precCharge: 3 Sequence: LYHLIPDGEITSIK
Intensity
75000
50000
25000
0
250
500
750
1000
1250
M/Z
b1
y1
y2
b2
y3
b3
y4
b4
y5
b5
y6
y7
y8
y9
type
b
y

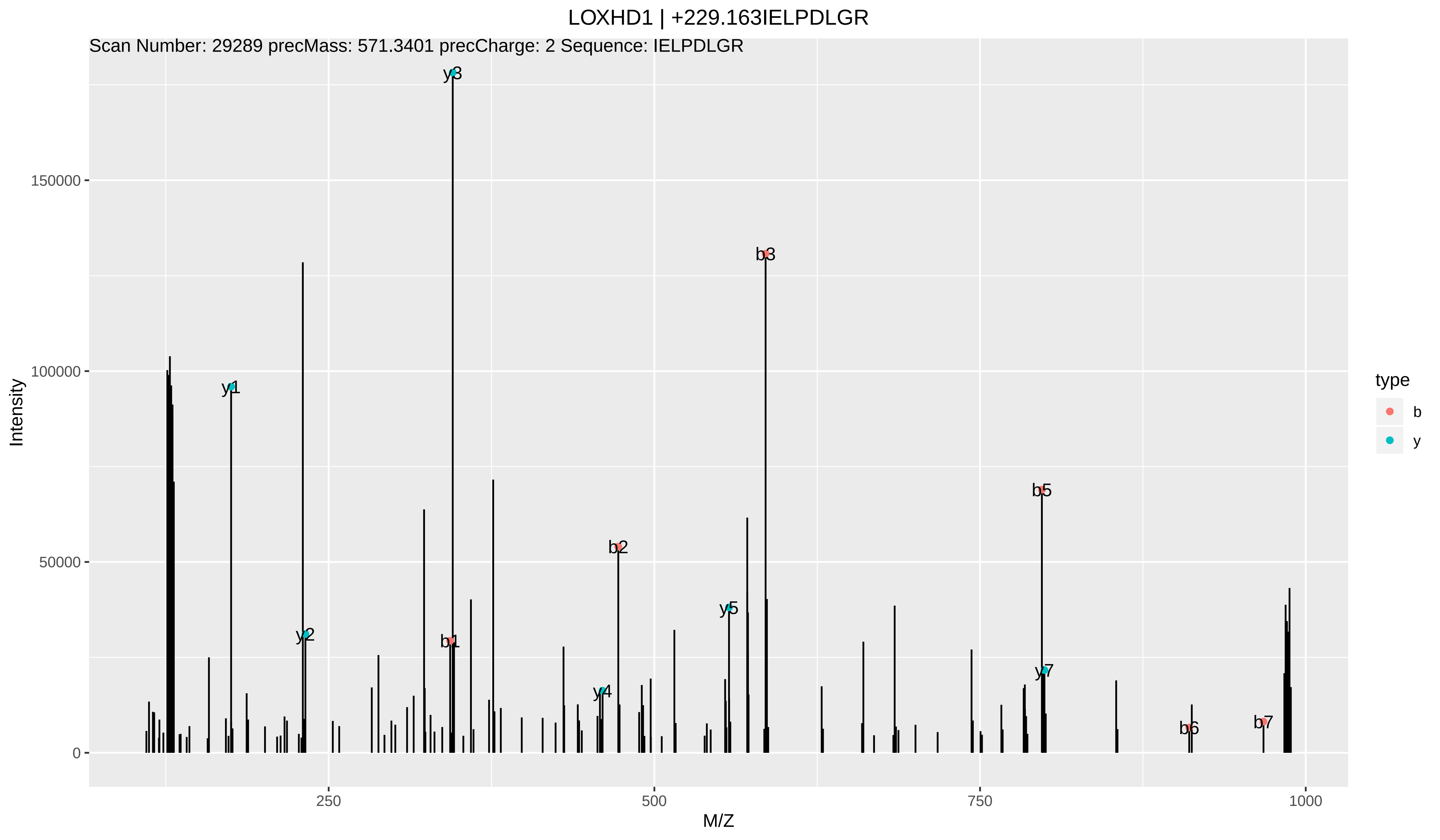

LOXHD1 | +229.163IELPDLGR
Scan Number: 29289 precMass: 571.3401 precCharge: 2 Sequence: IELPDLGR
Intensity
M/Z
0
50000
100000
150000
250
500
750
1000
y1
y2
y3
y4
y5
y7
b1
b2
b3
b5
b6
b7
type
b
y

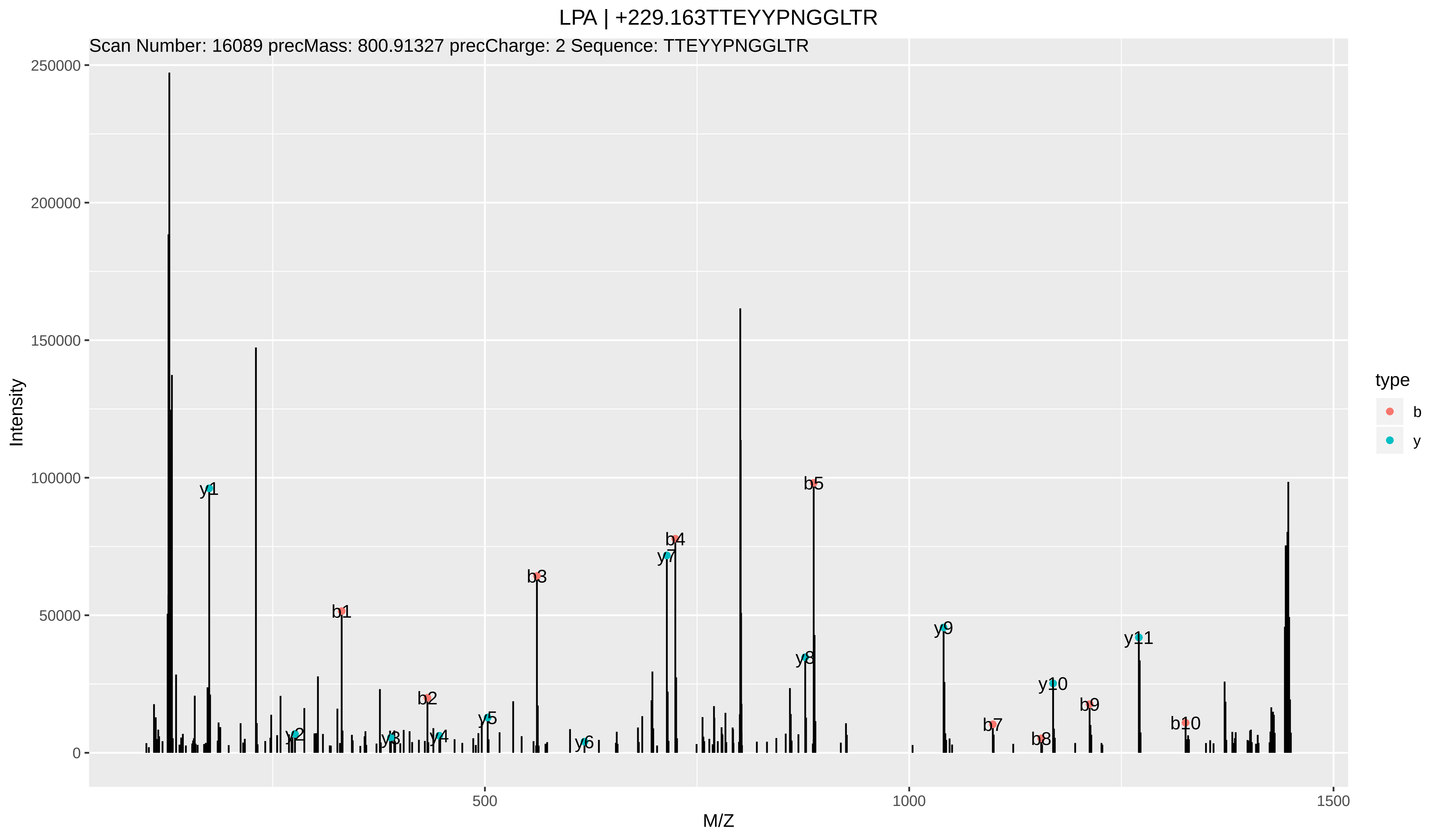

LPA | +229.163TTEYYPNGGLTR
Scan Number: 16089 precMass: 800.91327 precCharge: 2 Sequence: TTEYYPNGGLTR
Intensity
250000
200000
150000
100000
50000
0
M/Z
500
1000
1500
y1
b1
y2
y3
b2
y4
y5
b3
y6
y7
b4
y8
b5
y9
b7
b8
y10
b9
y11
b10
type
b
y

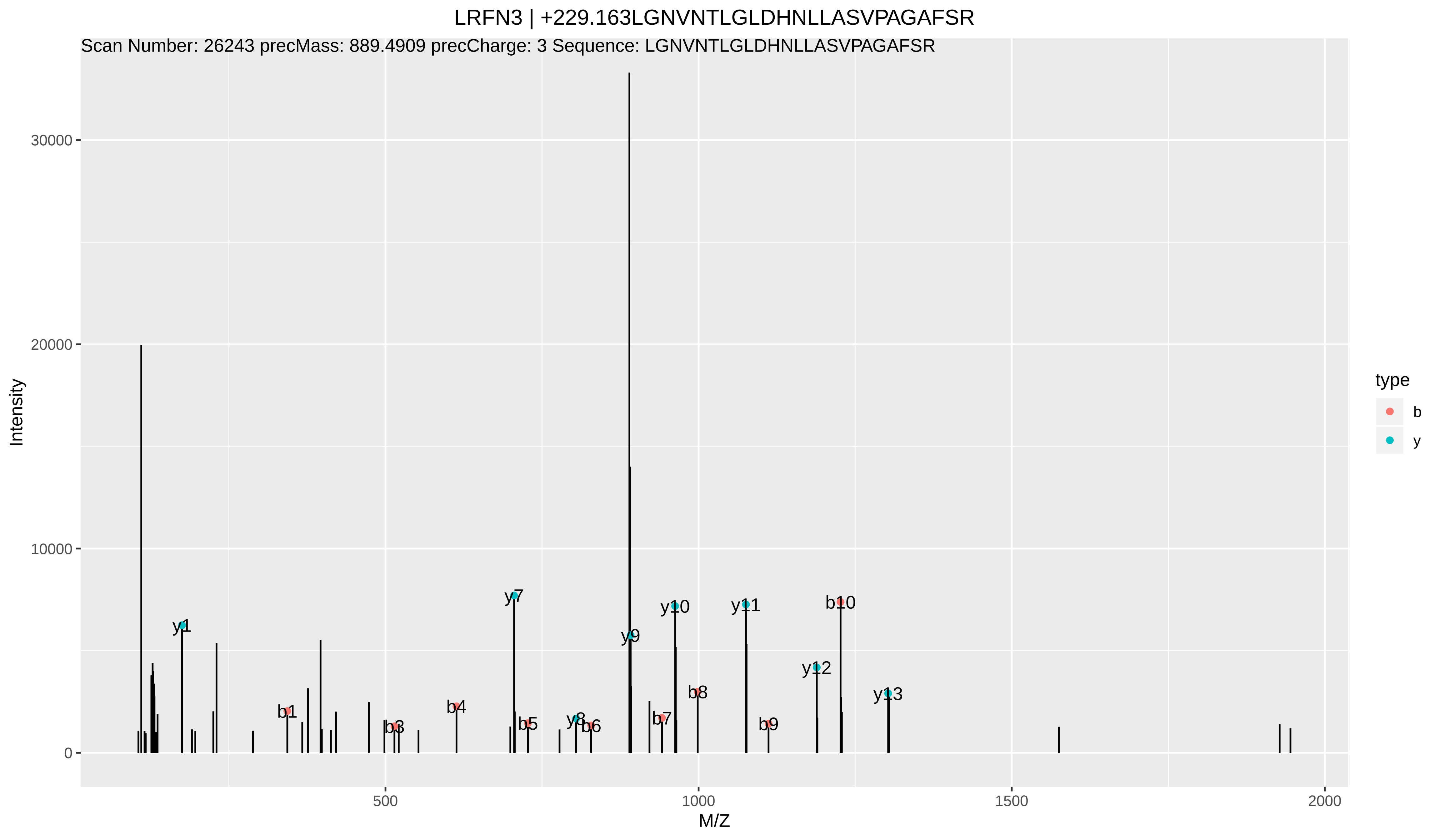
LRFN3 | +229.163LGNVNTLGLDHNLLASVPAGAFSR
Scan Number: 26243 precMass: 889.4909 precCharge: 3 Sequence: LGNVNTLGLDHNLLASVPAGAFSR
Intensity
M/Z
type
b
y
y1
b1
b3
b4
y7
b5
y8
b6
y9
b7
y10
b8
y11
b9
y12
b10
y13
0
10000
20000
30000
500
1000
1500
2000

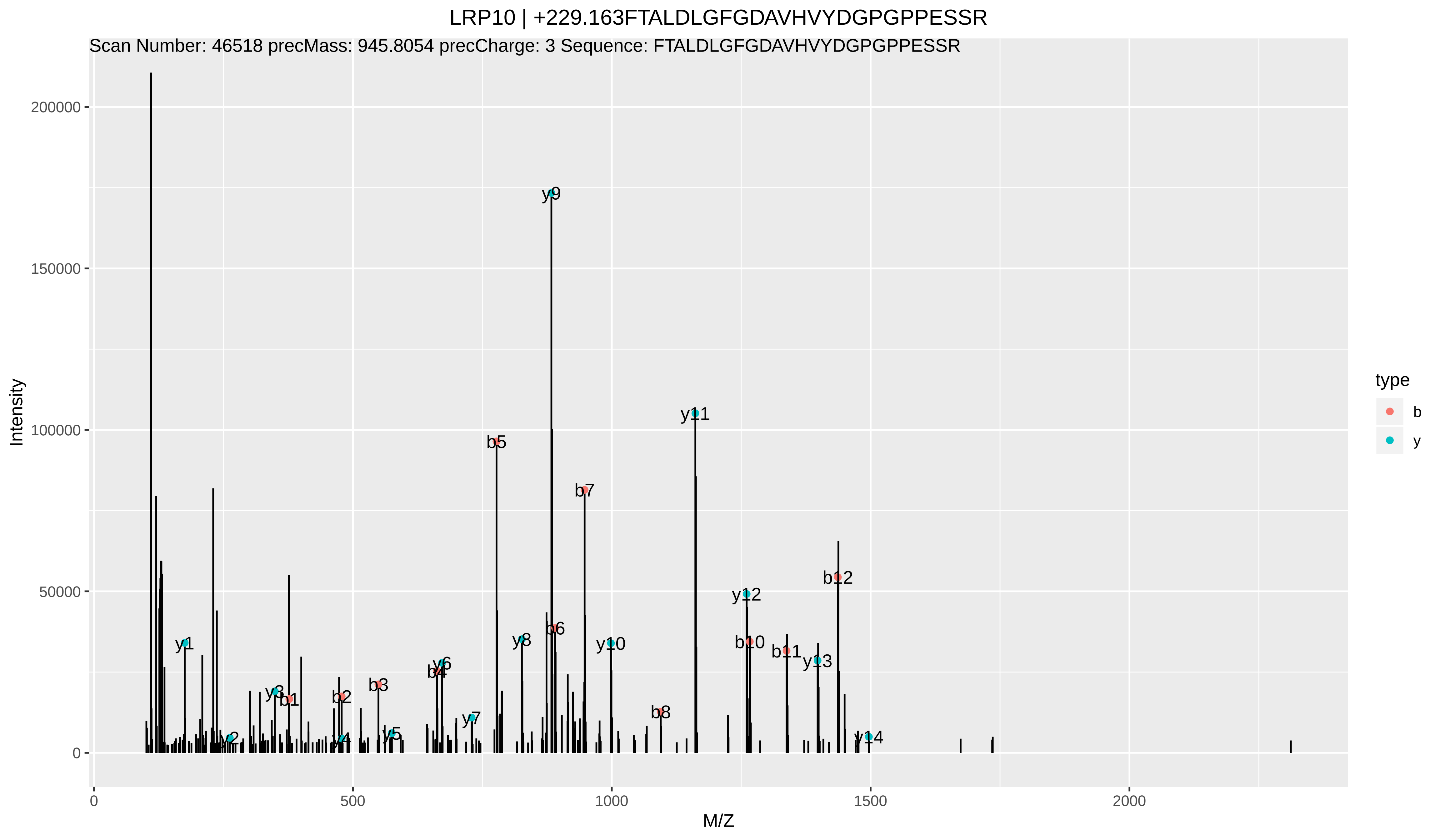

LRP10 | +229.163FTALDLGFGDAVHVYDGPGPPESSR
Scan Number: 46518 precMass: 945.8054 precCharge: 3 Sequence: FTALDLGFGDAVHVYDGPGPPESSR
Intensity
M/Z
type
b
y
y1
y2
y3
b1
b2
y4
b3
y5
b4
y6
y7
b5
y8
y9
b6
b7
y10
b8
y11
y12
b10
b11
y13
b12
y14

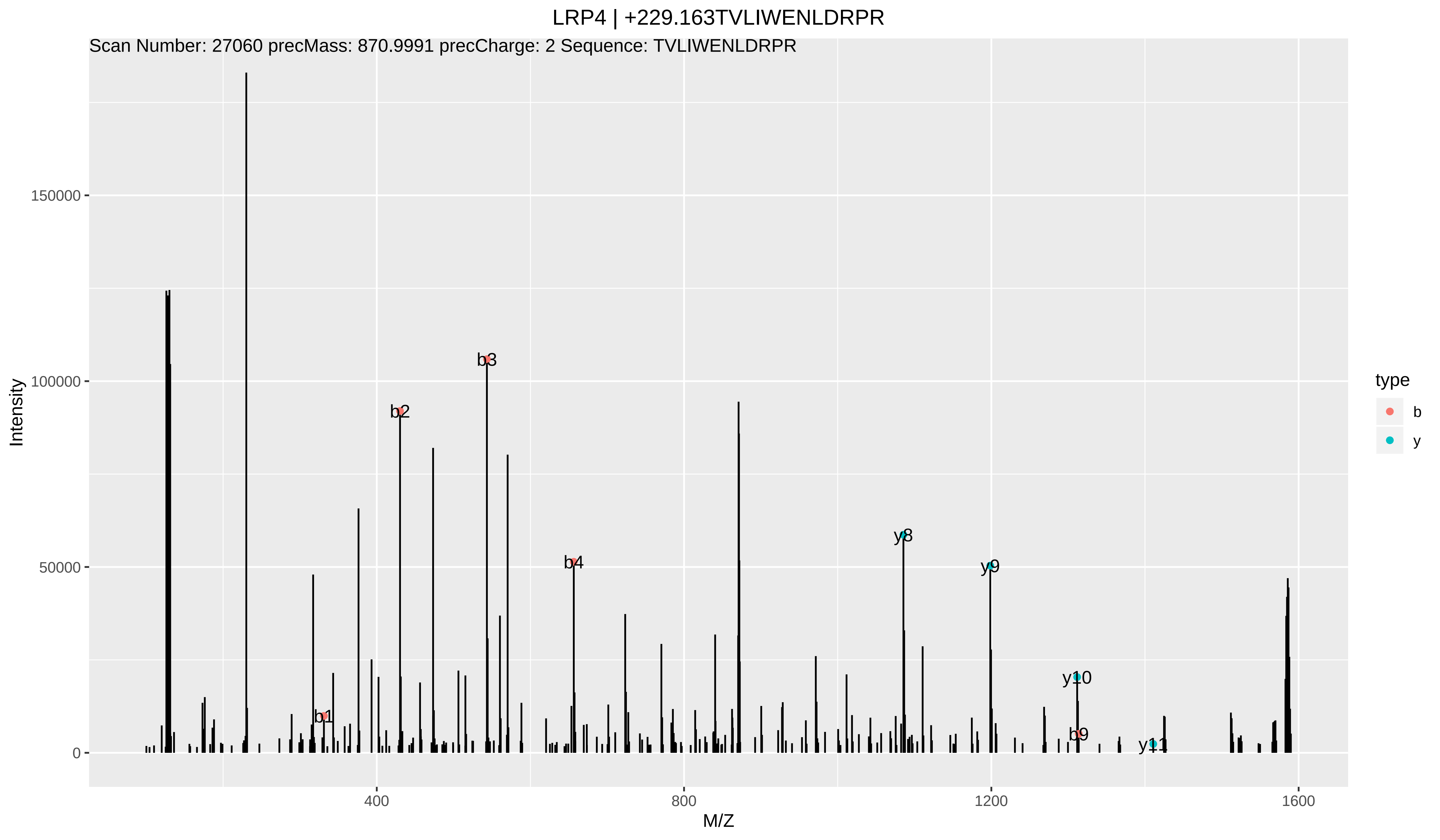

LRP4 | +229.163TVLIWENLDRPR
Scan Number: 27060 precMass: 870.9991 precCharge: 2 Sequence: TVLIWENLDRPR
Intensity
150000
100000
50000
0
400
800
1200
1600
M/Z
b1
b2
b3
b4
b9
y8
y9
y10
y11
type
b
y

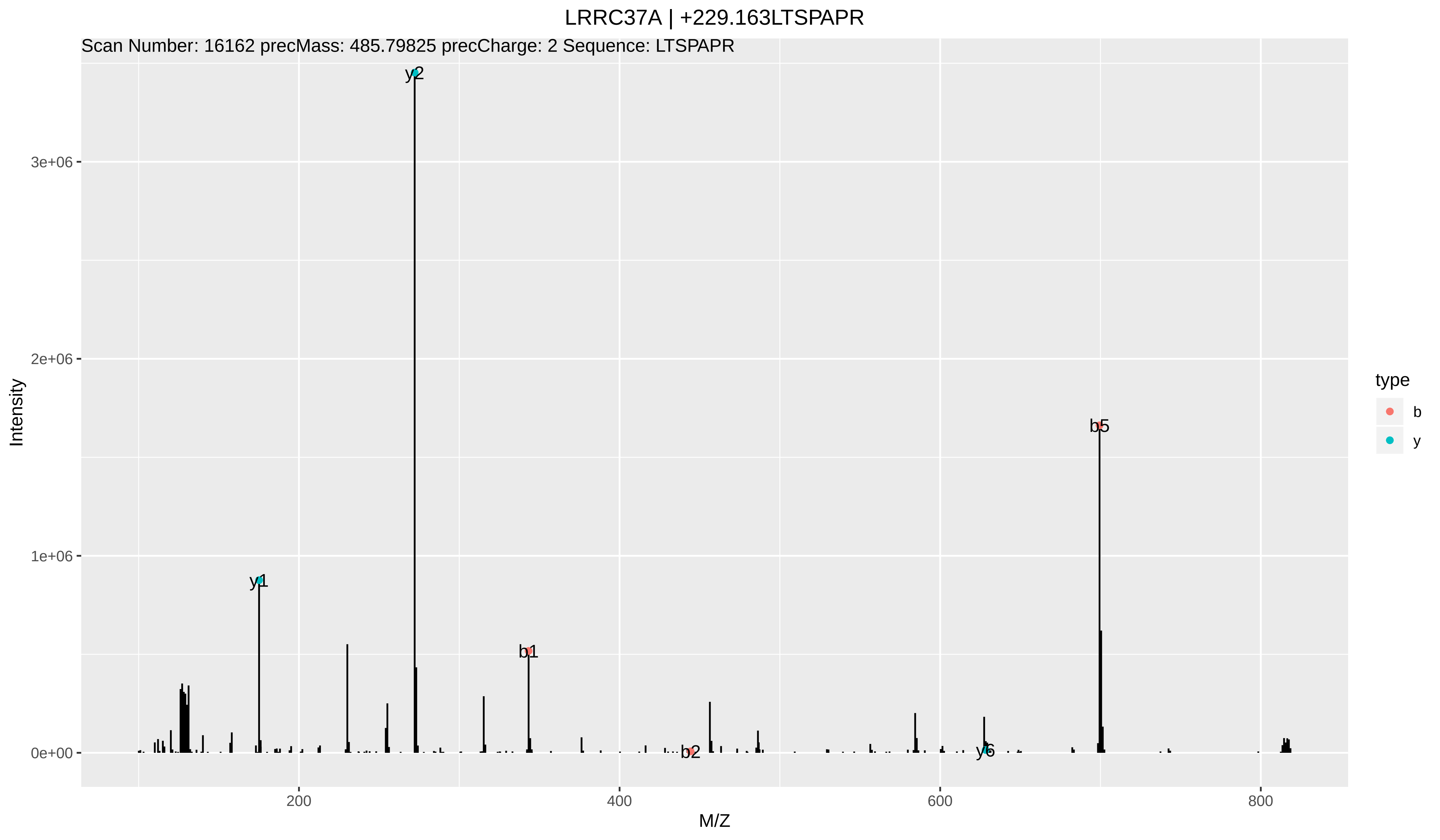

LRRC37A | +229.163LTSPAPR
Scan Number: 16162 precMass: 485.79825 precCharge: 2 Sequence: LTSPAPR
Intensity
3e+06
2e+06
1e+06
0e+00
M/Z
200
400
600
800
y2
y1
b1
b2
b5
y6
type
b
y

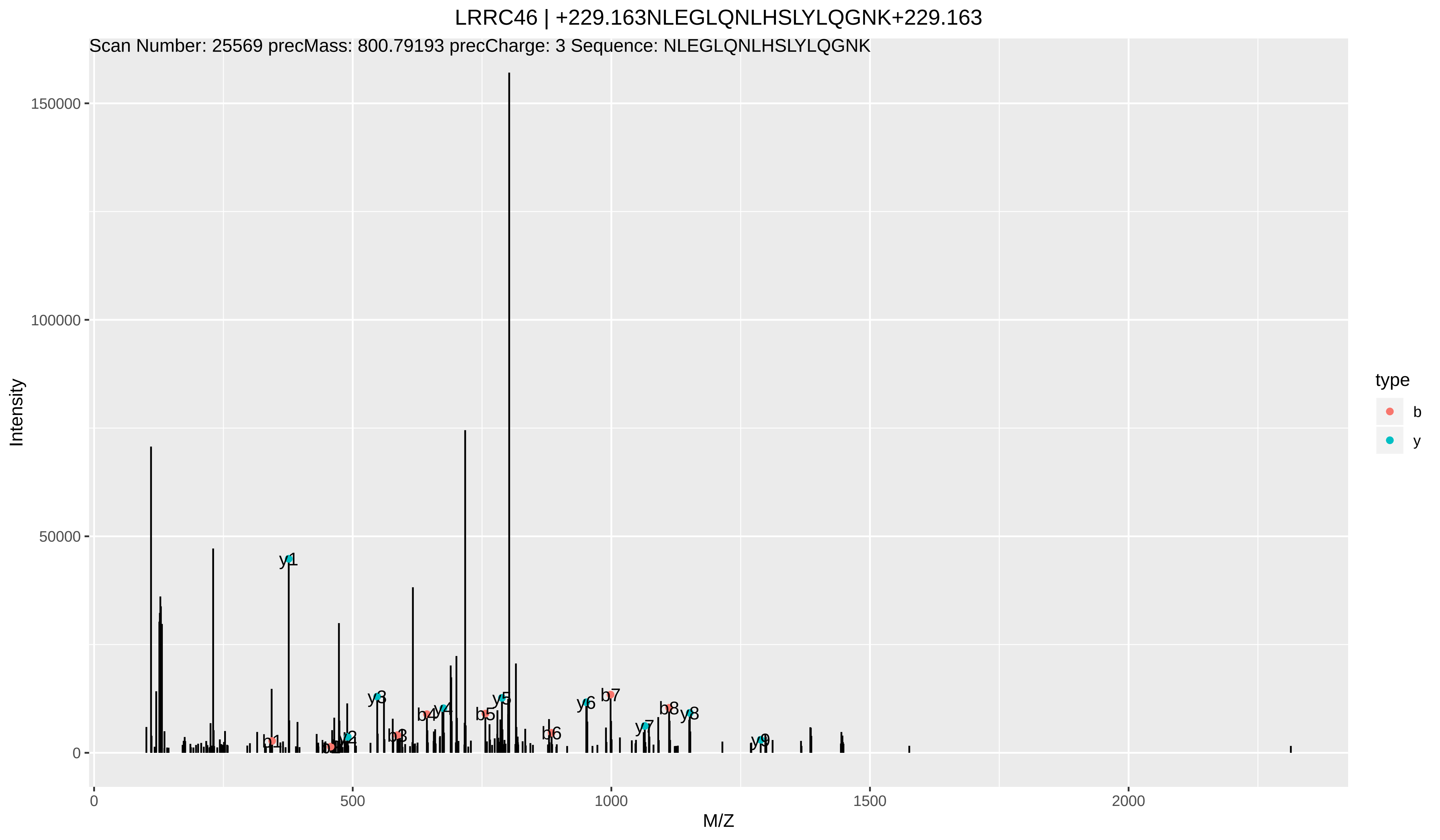
LRRC46 | +229.163NLEGLQNLHSLYLQGNK+229.163
Scan Number: 25569 precMass: 800.79193 precCharge: 3 Sequence: NLEGLQNLHSLYLQGNK
Intensity
M/Z
type
b
y
0
50000
100000
150000
0
500
1000
1500
2000
y1
y3
y4
y5
y6
y7
y8
y9
b1
b3
b4
b5
b6
b7
b8

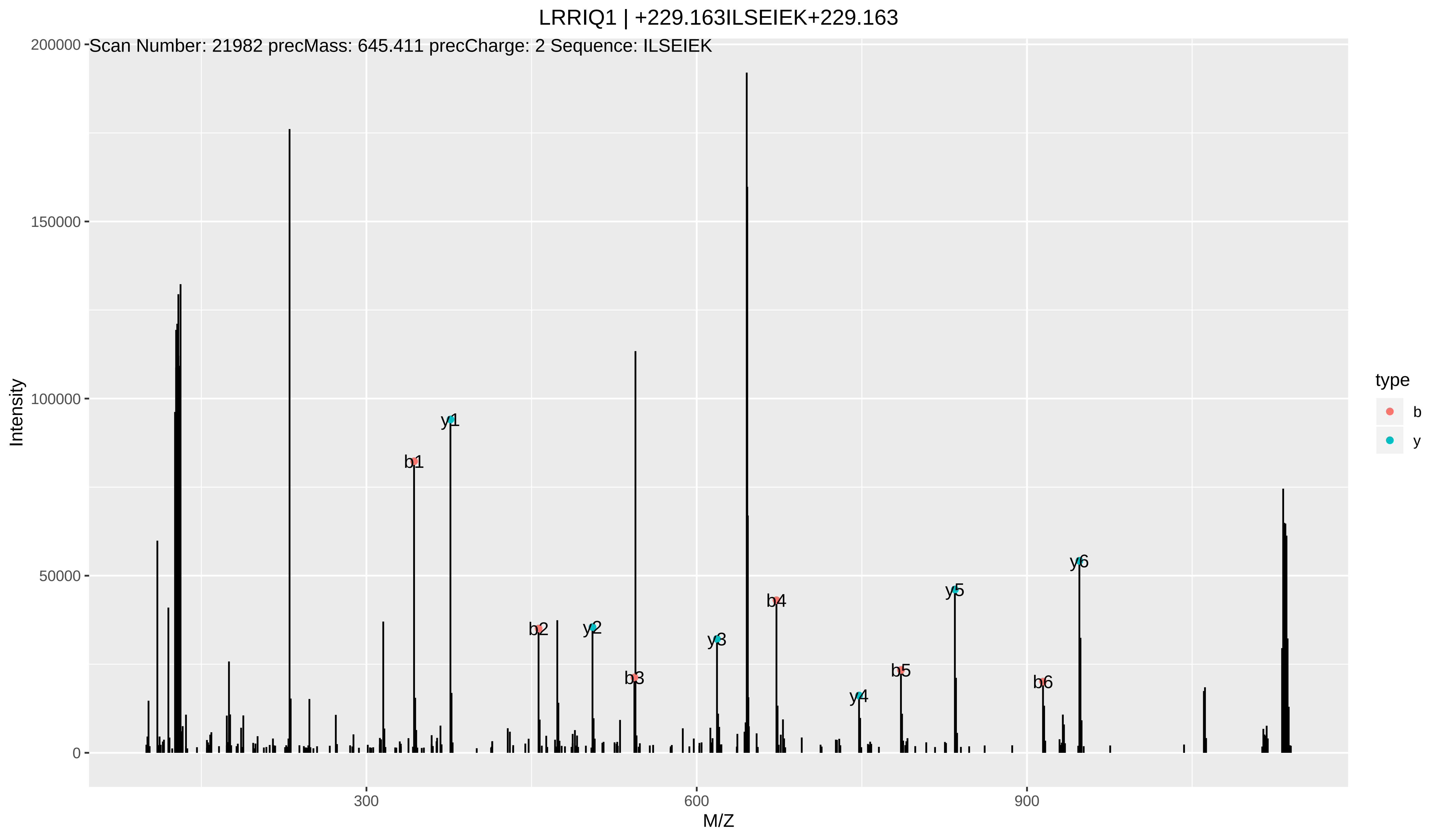
LRRIQ1 | +229.163ILSEIEK+229.163
Scan Number: 21982 precMass: 645.411 precCharge: 2 Sequence: ILSEIEK
Intensity
M/Z
200000
150000
100000
50000
0
300
600
900
b1
y1
b2
y2
b3
y3
b4
y4
b5
y5
b6
y6
type
b
y

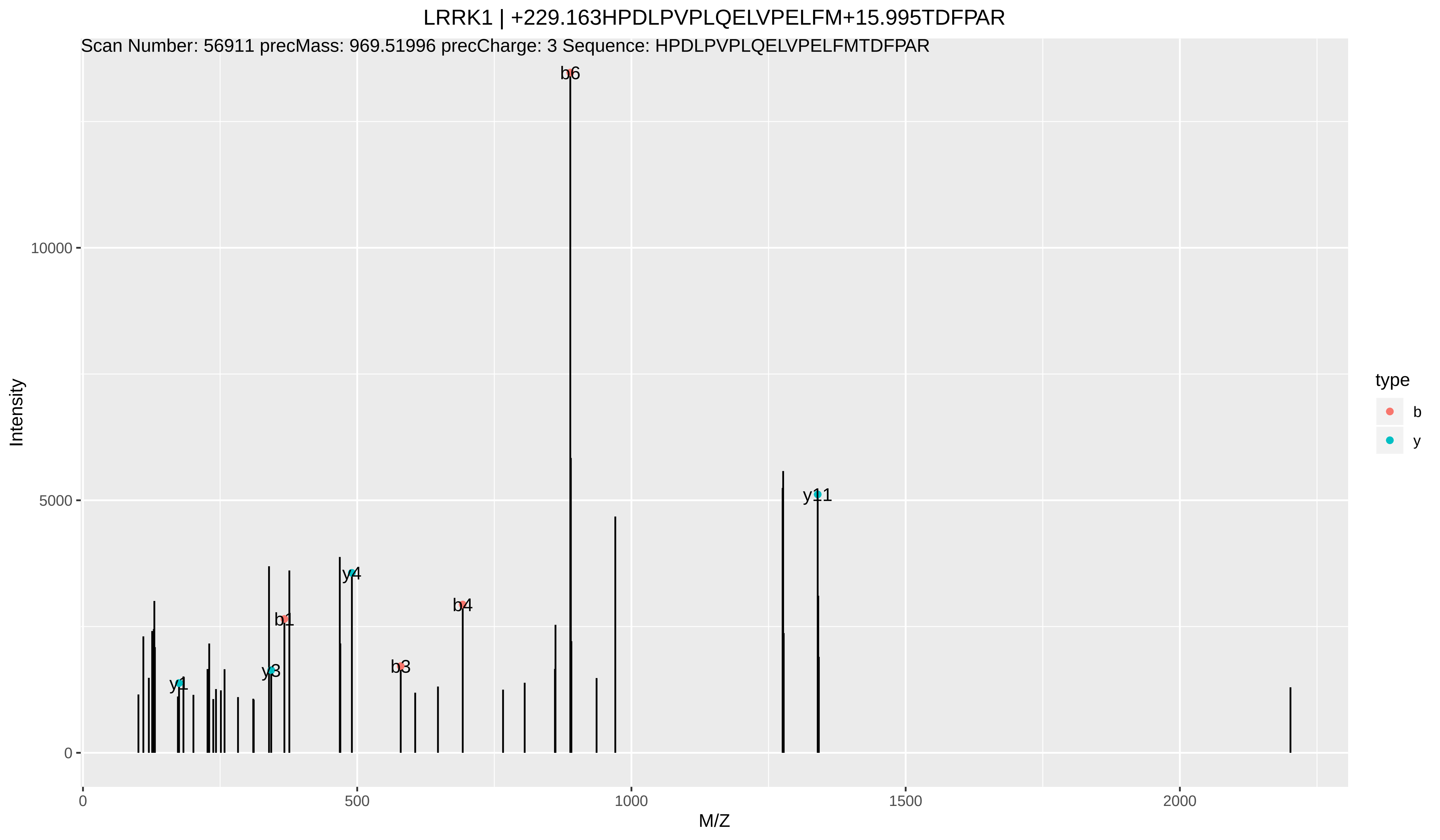

LRRK1 | +229.163HPDLPVPLQELVPELFM+15.995TDFPAR
Scan Number: 56911 precMass: 969.51996 precCharge: 3 Sequence: HPDLPVPLQELVPELFMTDFPAR
b6
y11
y4
b4
b1
b3
y3
y1
Intensity
10000
5000
0
0
500
1000
1500
2000
M/Z
type
b
y

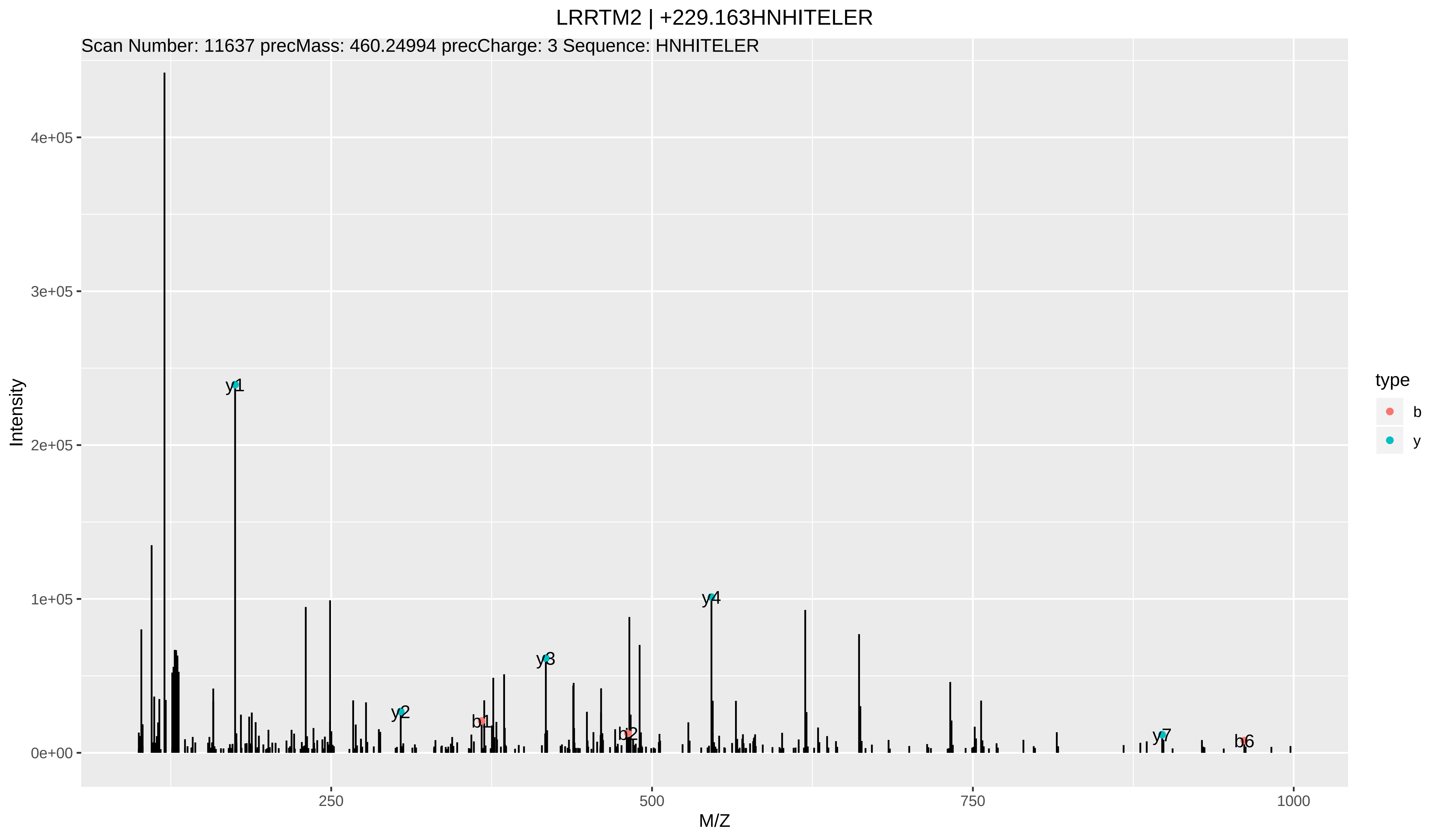
LRRTM2 | +229.163HNHITELER
Scan Number: 11637 precMass: 460.24994 precCharge: 3 Sequence: HNHITELER
Intensity
4e+05
3e+05
2e+05
1e+05
0e+00
M/Z
250
500
750
1000
y1
y2
b1
y3
b2
y4
y7
b6
type
b
y

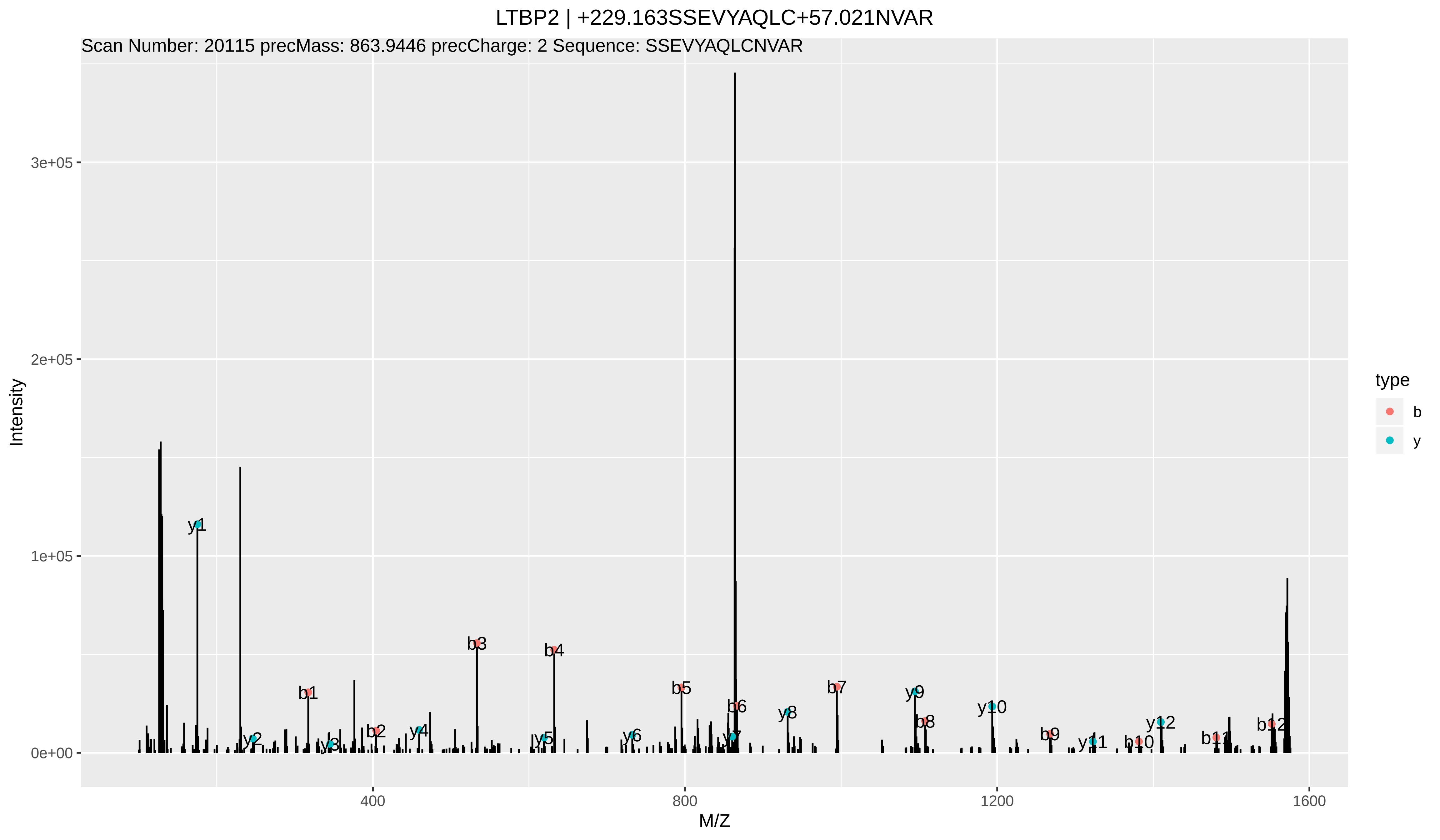

LTBP2 | +229.163SSEVYAQLC+57.021NVAR
Scan Number: 20115 precMass: 863.9446 precCharge: 2 Sequence: SSEVYAQLCNVAR
Intensity
M/Z
3e+05
2e+05
1e+05
0e+00
400
800
1200
1600
y1
b1
y2
y3
b2
y4
b3
b4
y5
y6
b5
y7
b6
y8
b7
y9
b8
y10
b9
y11
b10
y12
b11
b12
type
b
y

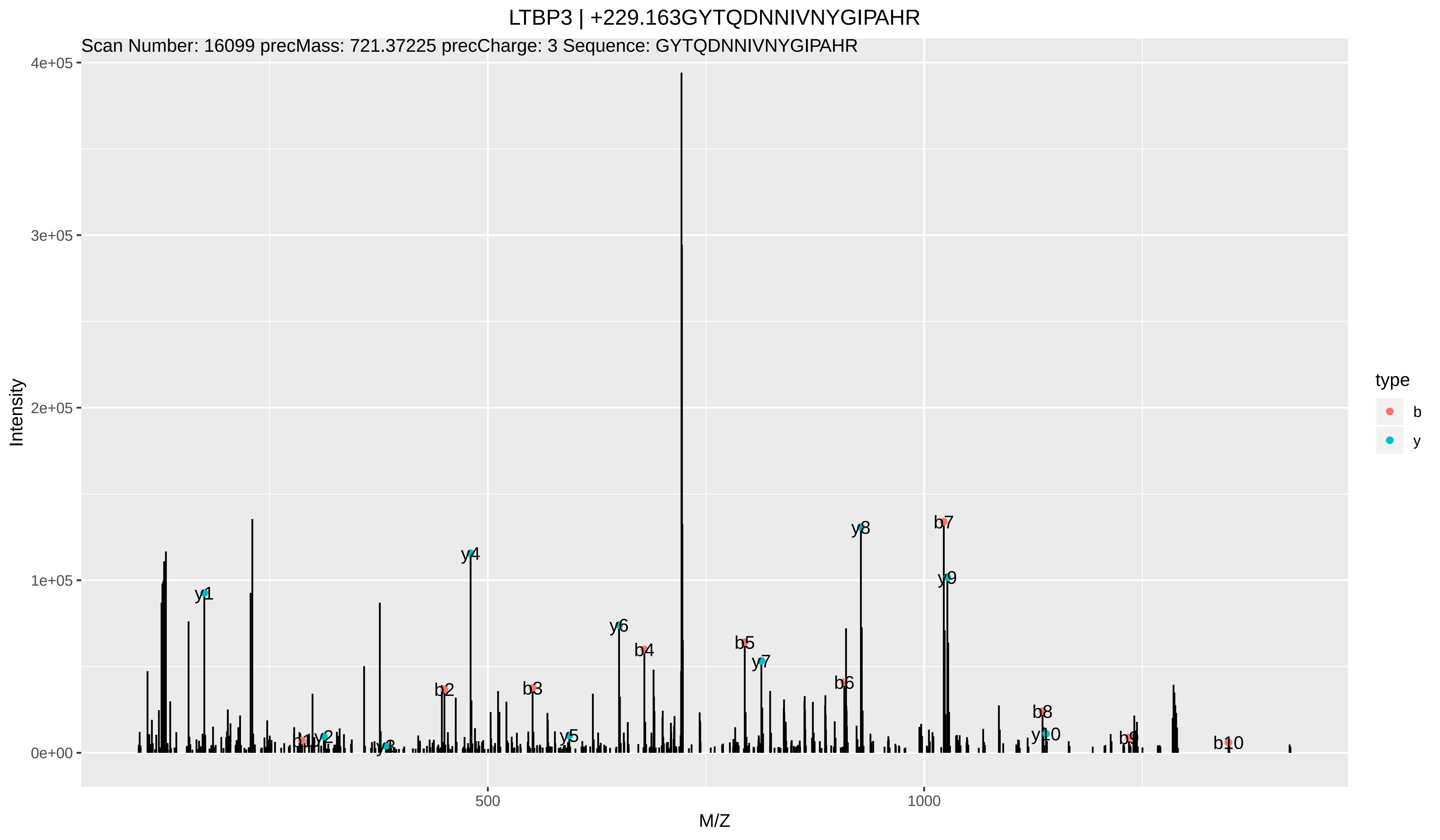
LTBP3 | +229.163GYTQDNNIVNYGIPAHR
Scan Number: 16099 precMass: 721.37225 precCharge: 3 Sequence: GYTQDNNIVNYGIPAHR
Intensity
M/Z
4e+05
3e+05
2e+05
1e+05
0e+00
500
1000
y1
y2
y3
y4
y5
y6
y7
y8
y9
y10
b1
b2
b3
b4
b5
b6
b7
b8
b9
b10
type
b
y

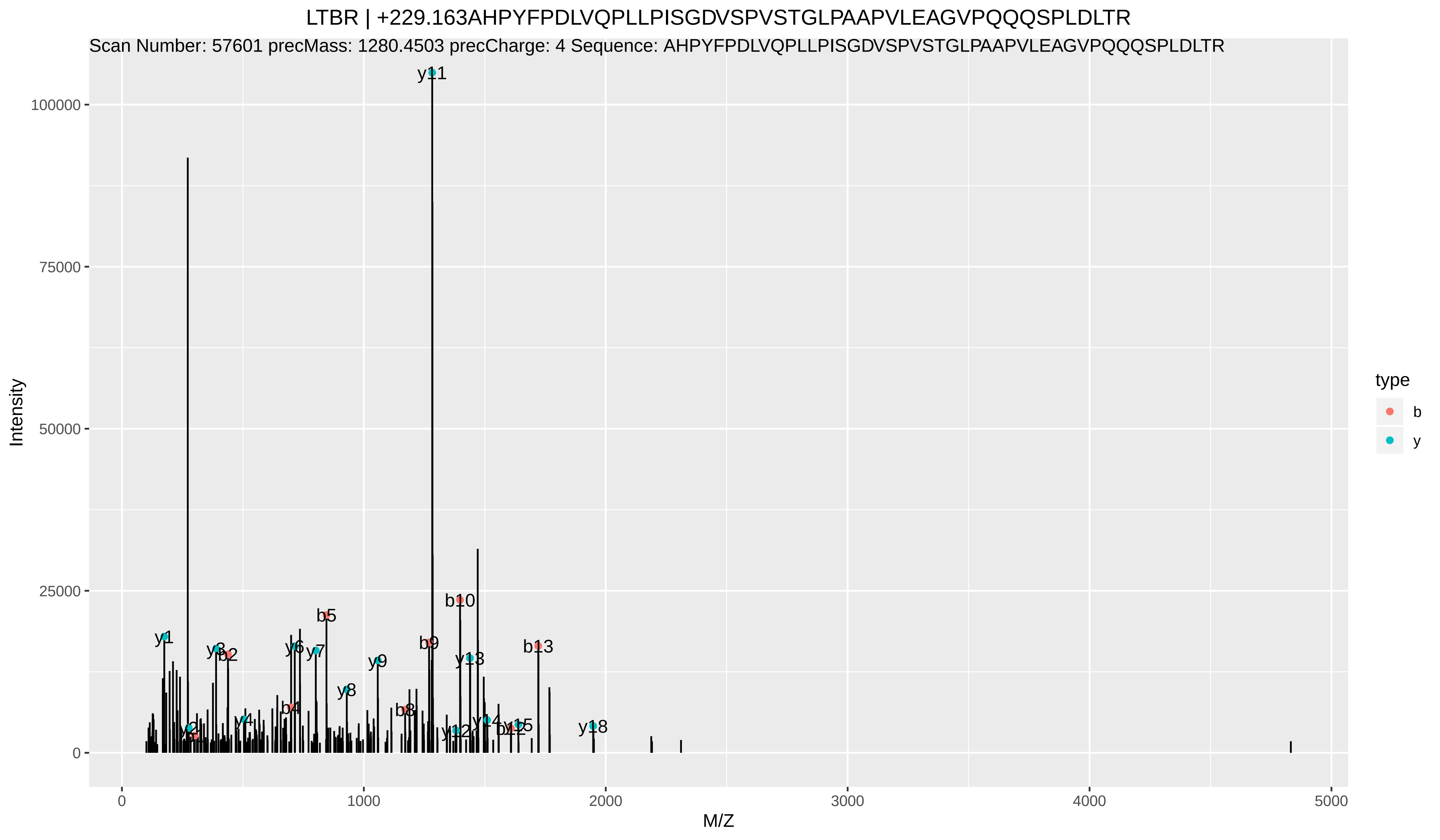
LTBR | +229.163AHPYFPDLVQPLLPISGDVSPVSTGLPAAPVLEAGVPQQQSPLDLTR
Scan Number: 57601 precMass: 1280.4503 precCharge: 4 Sequence: AHPYFPDLVQPLLPISGDVSPVSTGLPAAPVLEAGVPQQQSPLDLTR
Intensity
M/Z
type
b
y
0
25000
50000
75000
100000
0
1000
2000
3000
4000
5000
y1
y2
y3
b2
y4
b4
y6
y7
b5
y8
y9
b8
b9
y11
y12
b10
y13
y14
b12
y15
b13
y18

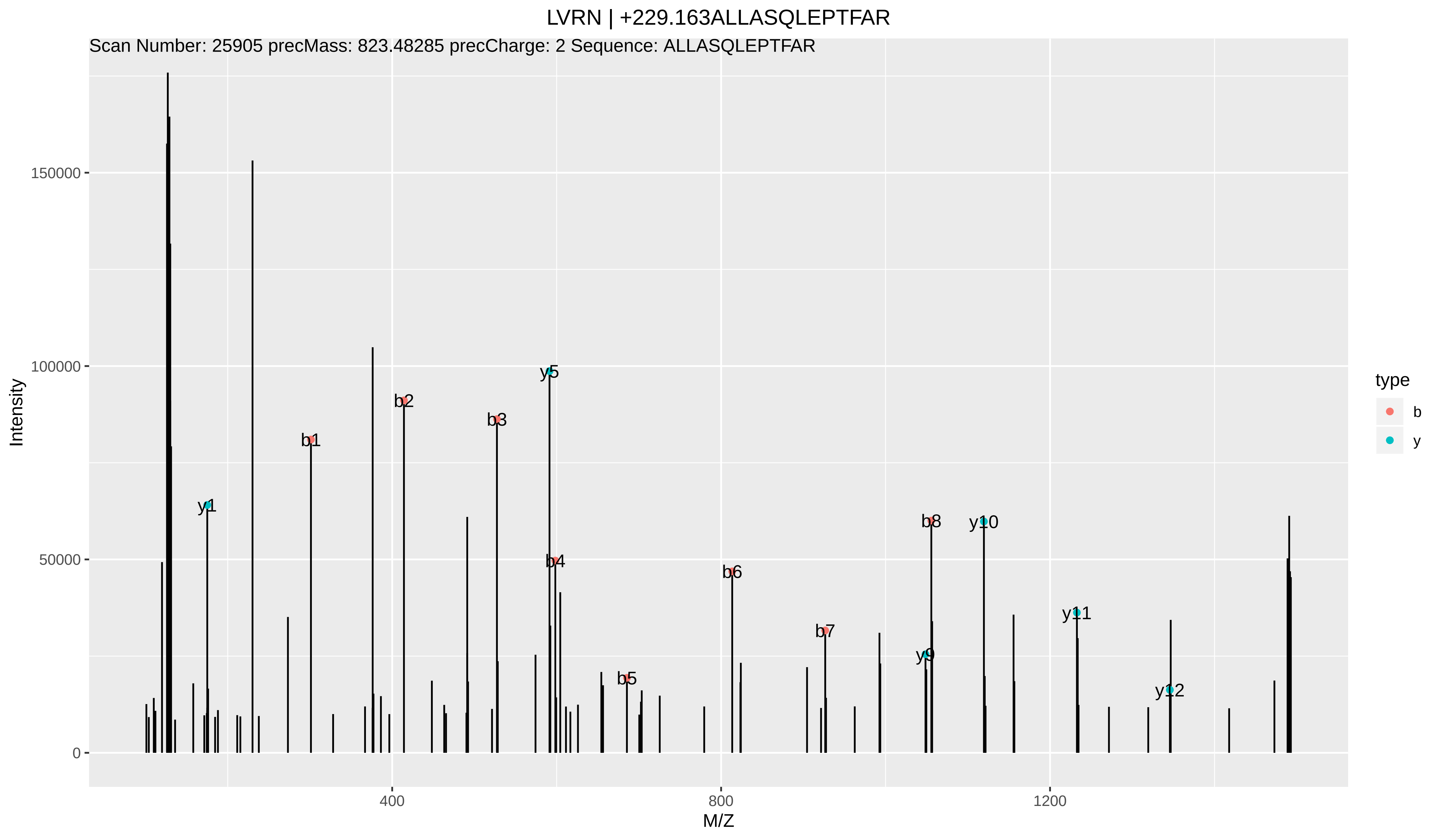

LVRN | +229.163ALLASQLEPTFAR
Scan Number: 25905 precMass: 823.48285 precCharge: 2 Sequence: ALLASQLEPTFAR
Intensity
150000
100000
50000
0
400
800
1200
M/Z
y1
b1
b2
b3
y5
b4
b5
b6
b7
y9
b8
y10
y11
y12
type
b
y

LYRM1 | +229.163NLTDTDLIK+229.163

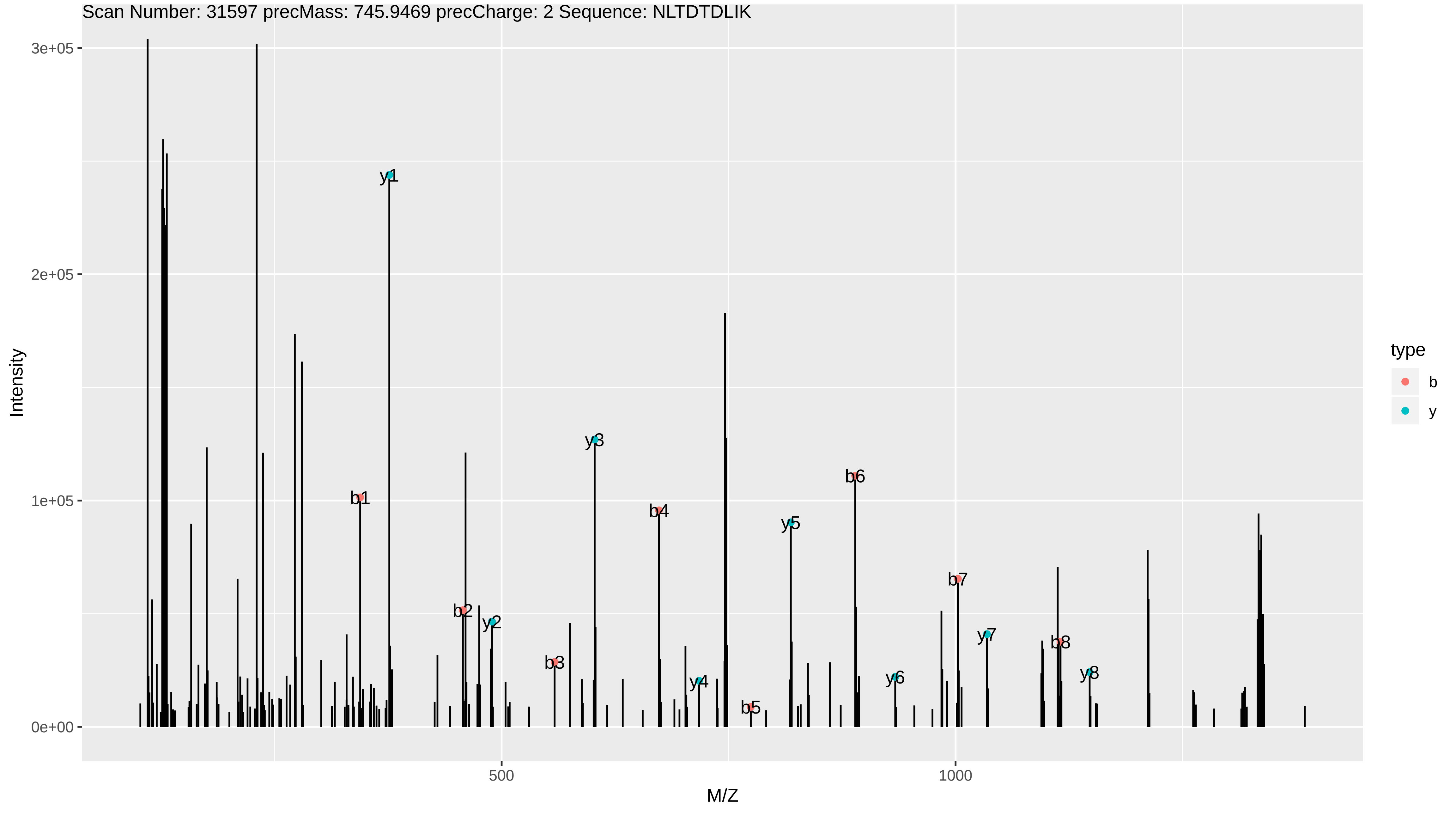

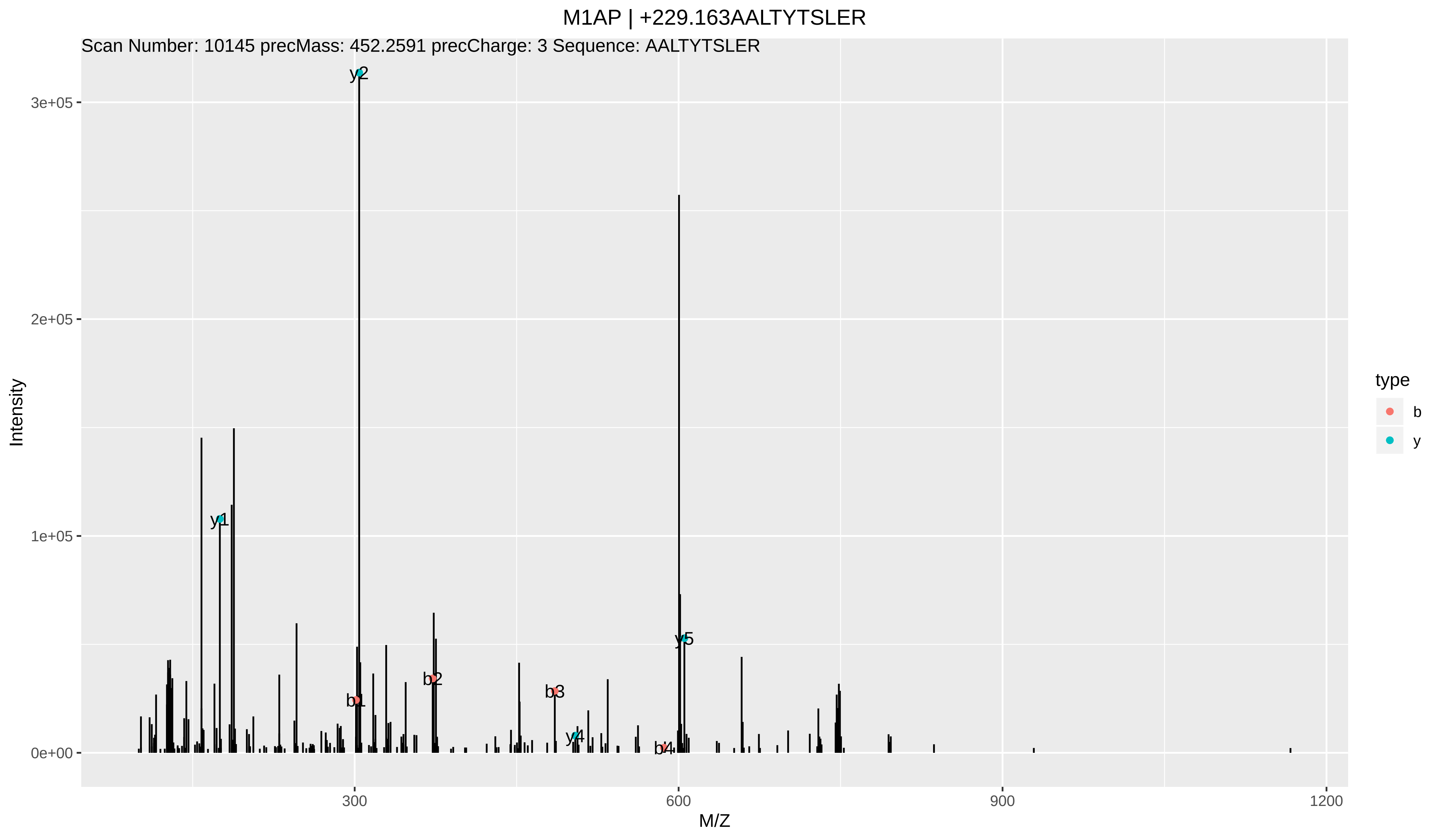
M1AP | +229.163AALTYTSLER
Scan Number: 10145 precMass: 452.2591 precCharge: 3 Sequence: AALTYTSLER
Intensity
M/Z
type
b
y
y1
y2
y4
y5
b1
b2
b3
b4
3e+05
2e+05
1e+05
0e+00
300
600
900
1200

MAGEF1 | +229.163ILFPDIIAR

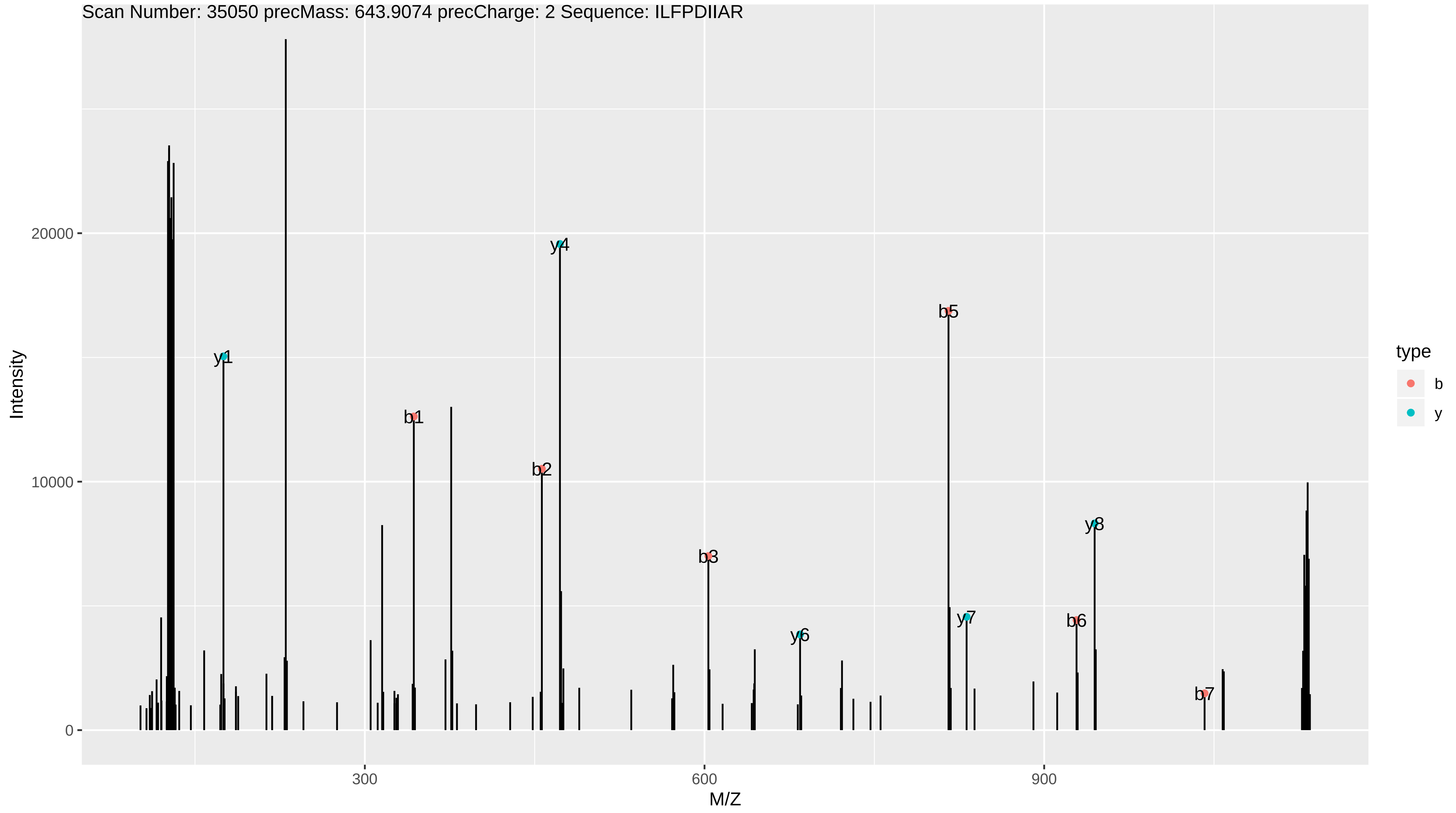

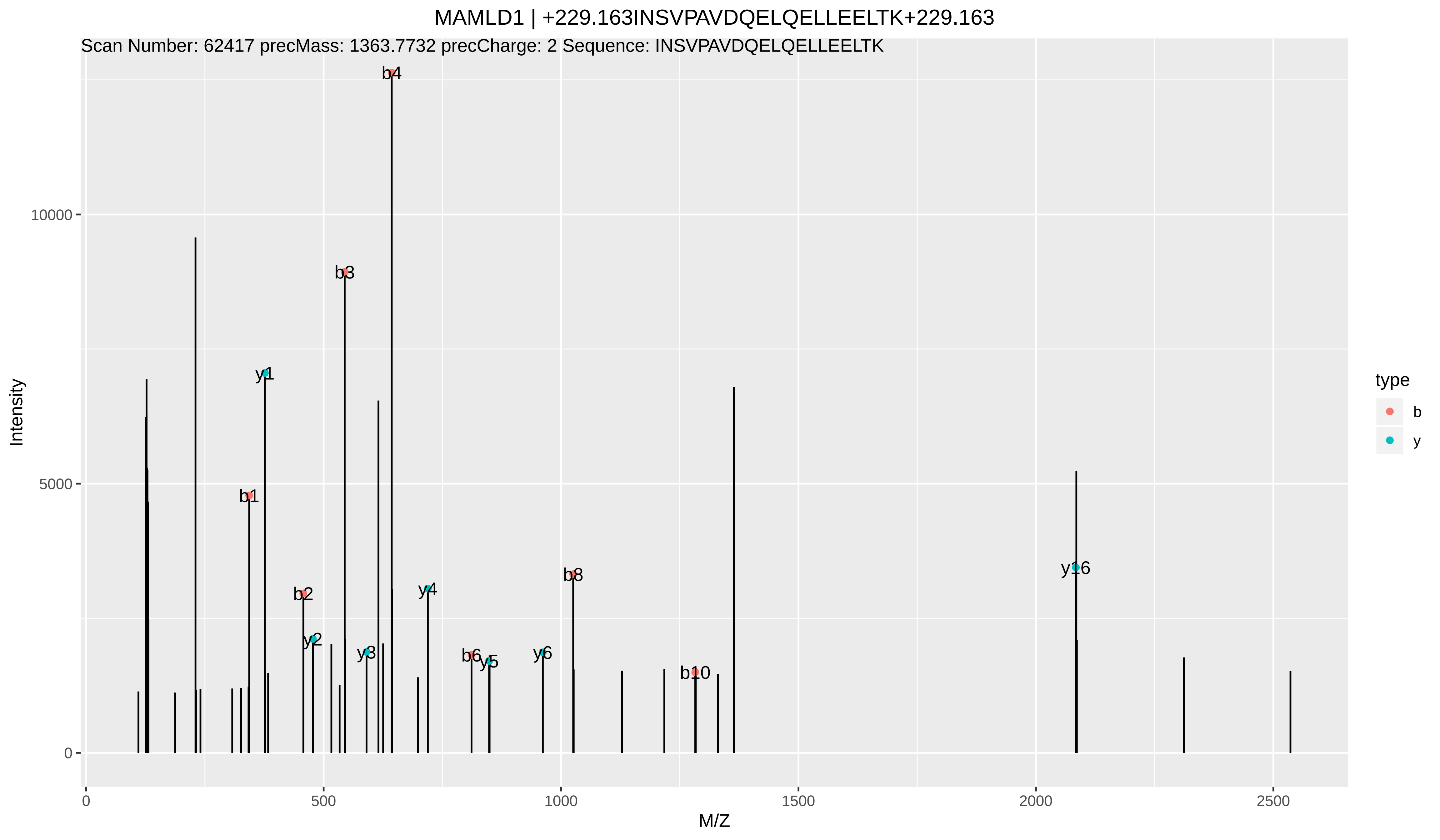

MAMLD1 | +229.163INSVPAVDQELQELLEELTK+229.163
Scan Number: 62417 precMass: 1363.7732 precCharge: 2 Sequence: INSVPAVDQELQELLEELTK
Intensity
M/Z
type
b
y
b4
b3
y1
b1
b2
y2
y3
y4
b6
y5
y6
b8
b10
y16
0
5000
10000
0
500
1000
1500
2000
2500

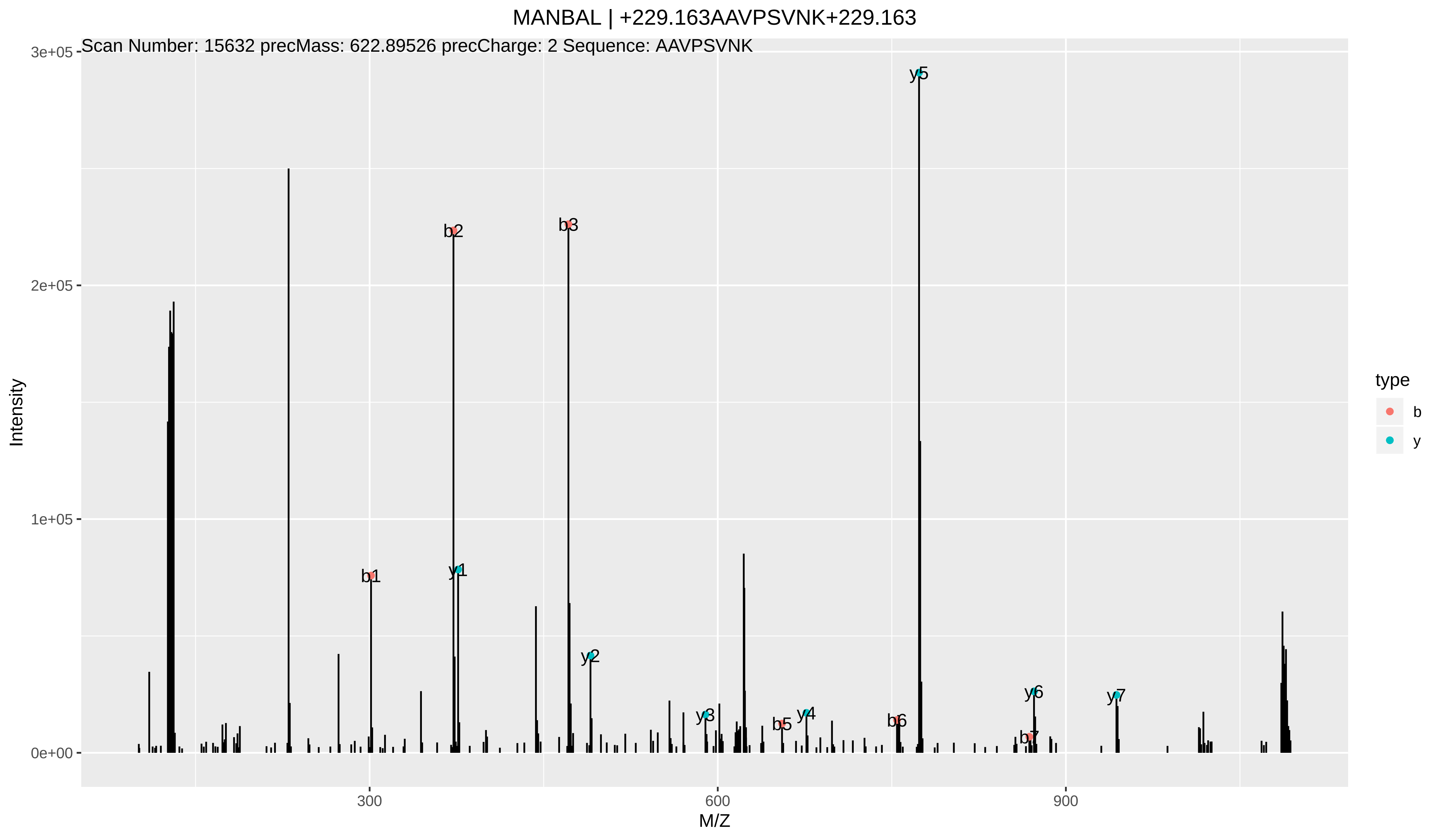

MANBAL | +229.163AAVPSVNK+229.163
Scan Number: 15632 precMass: 622.89526 precCharge: 2 Sequence: AAVPSVNK
Intensity
M/Z
3e+05
2e+05
1e+05
0e+00
300
600
900
y5
b2
b3
b1
y1
y2
y3
b5
y4
b6
y6
b7
y7
type
b
y

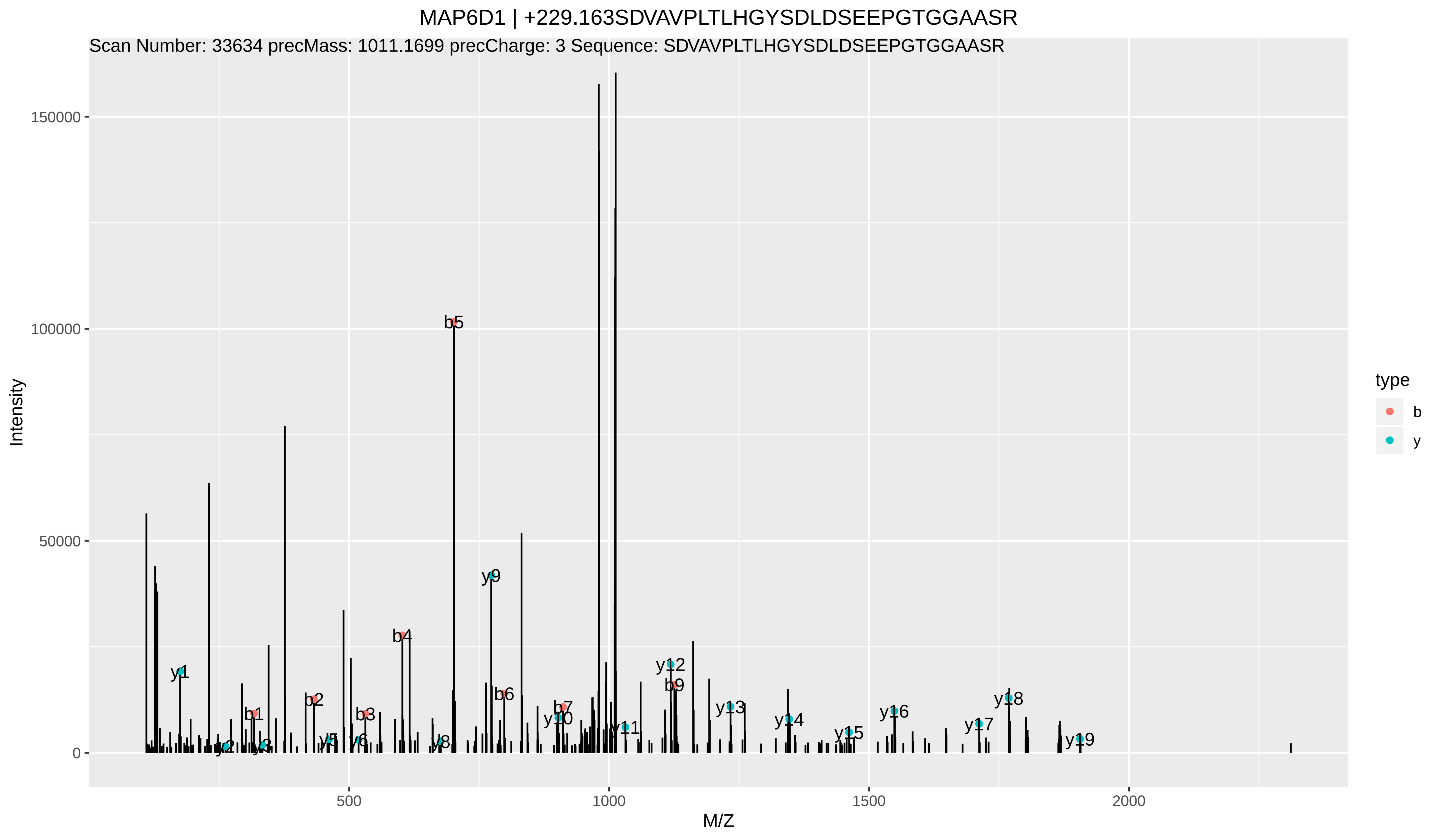

MAP6D1 | +229.163SDVAVPLTLHGYSDLDSEEPGTGGAASR
Scan Number: 33634 precMass: 1011.1699 precCharge: 3 Sequence: SDVAVPLTLHGYSDLDSEEPGTGGAASR
Intensity
150000
100000
50000
0
M/Z
500
1000
1500
2000
type
b
y
b1
b2
b3
b4
b5
b6
b7
b9
y1
y2
y3
y5
y6
y8
y9
y10
y11
y12
y13
y14
y15
y16
y17
y18
y19

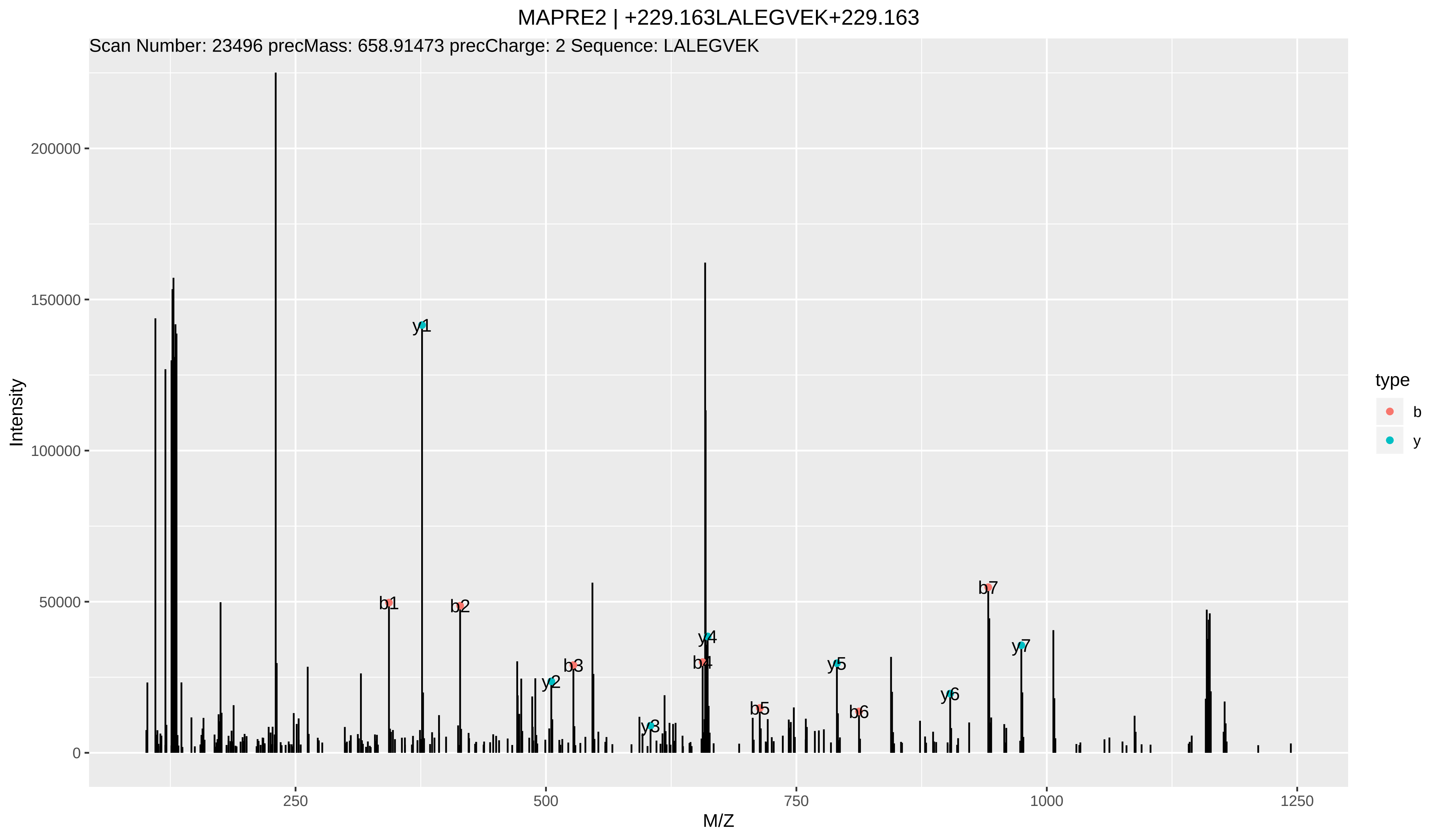
MAPRE2 | +229.163LALEGVEK+229.163
Scan Number: 23496 precMass: 658.91473 precCharge: 2 Sequence: LALEGVEK
Intensity
0
50000
100000
150000
200000
M/Z
250
500
750
1000
1250
y1
b1
b2
b3
y2
y3
y4
b4
b5
y5
b6
y6
b7
y7
type
b
y

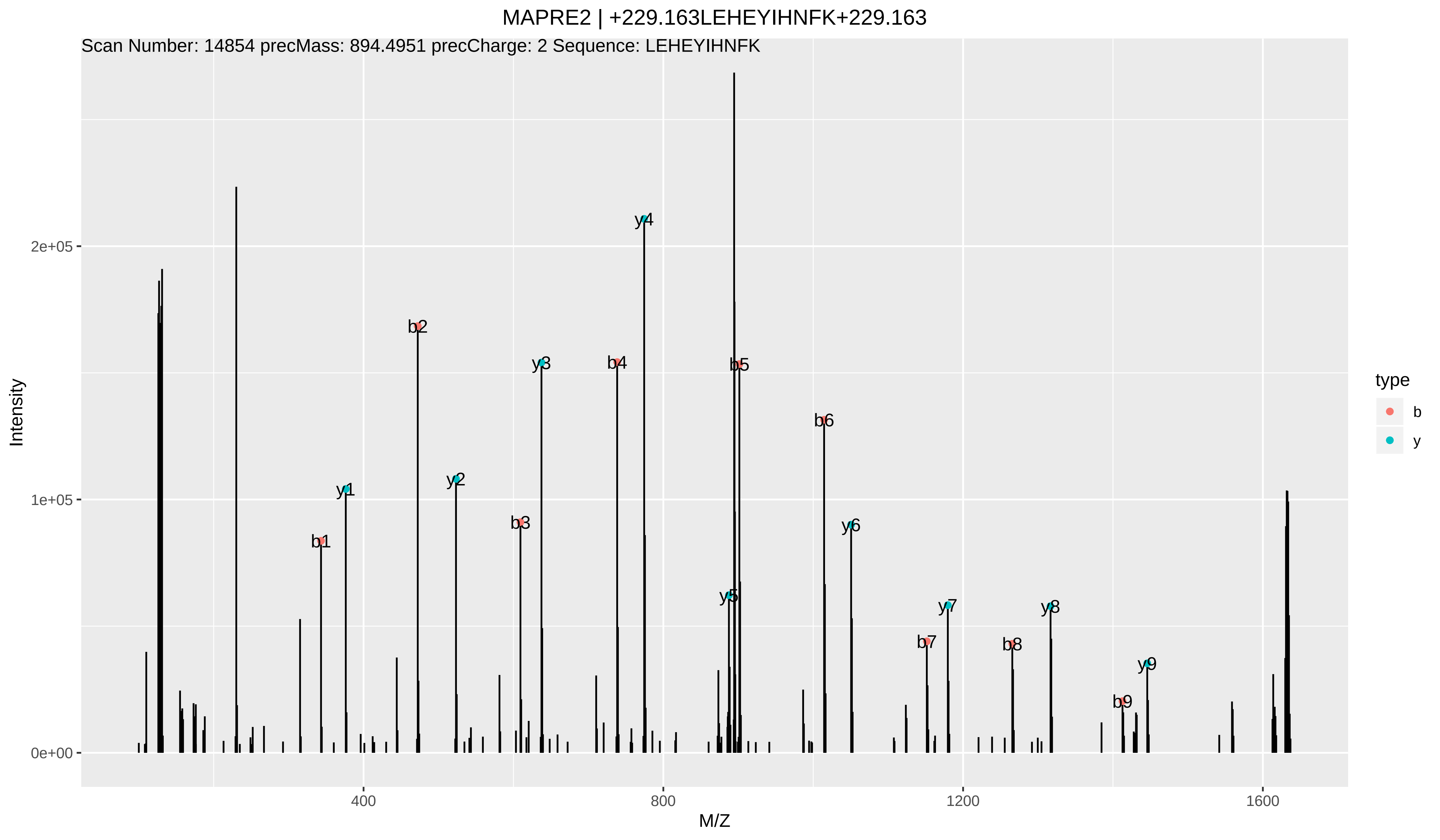
MAPRE2 | +229.163LEHEYIHNFK+229.163
Scan Number: 14854 precMass: 894.4951 precCharge: 2 Sequence: LEHEYIHNFK
Intensity
M/Z
2e+05
1e+05
0e+00
400
800
1200
1600
b1
y1
b2
y2
b3
y3
b4
y4
b5
y5
b6
y6
b7
y7
b8
y8
b9
y9
type
b
y

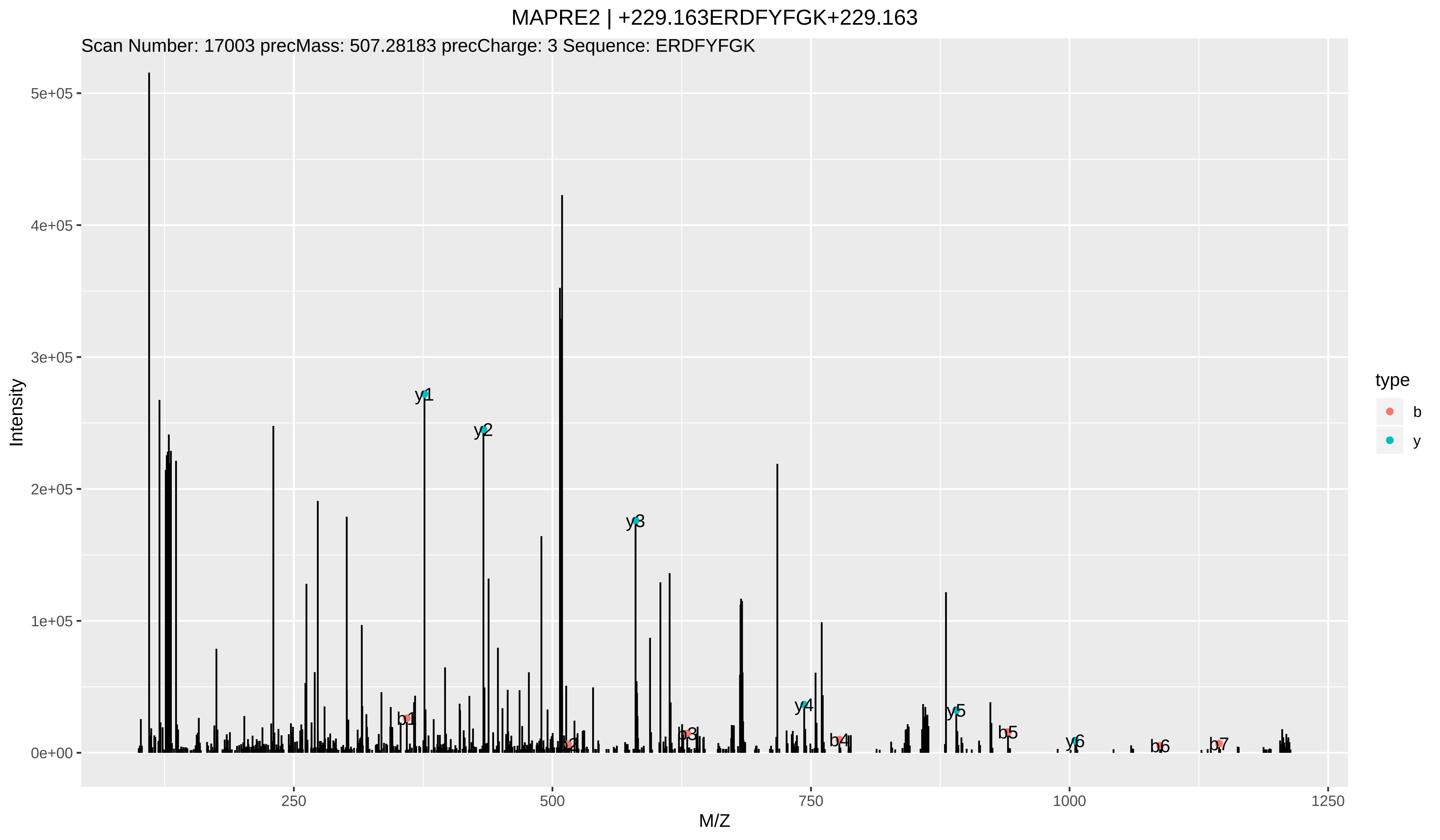
MAPRE2 | +229.163ERDFYFGK+229.163
Scan Number: 17003 precMass: 507.28183 precCharge: 3 Sequence: ERDFYFGK
Intensity
5e+05
4e+05
3e+05
2e+05
1e+05
0e+00
M/Z
250
500
750
1000
1250
y1
y2
y3
y4
y5
y6
b1
b3
b4
b5
b6
b7
type
b
y

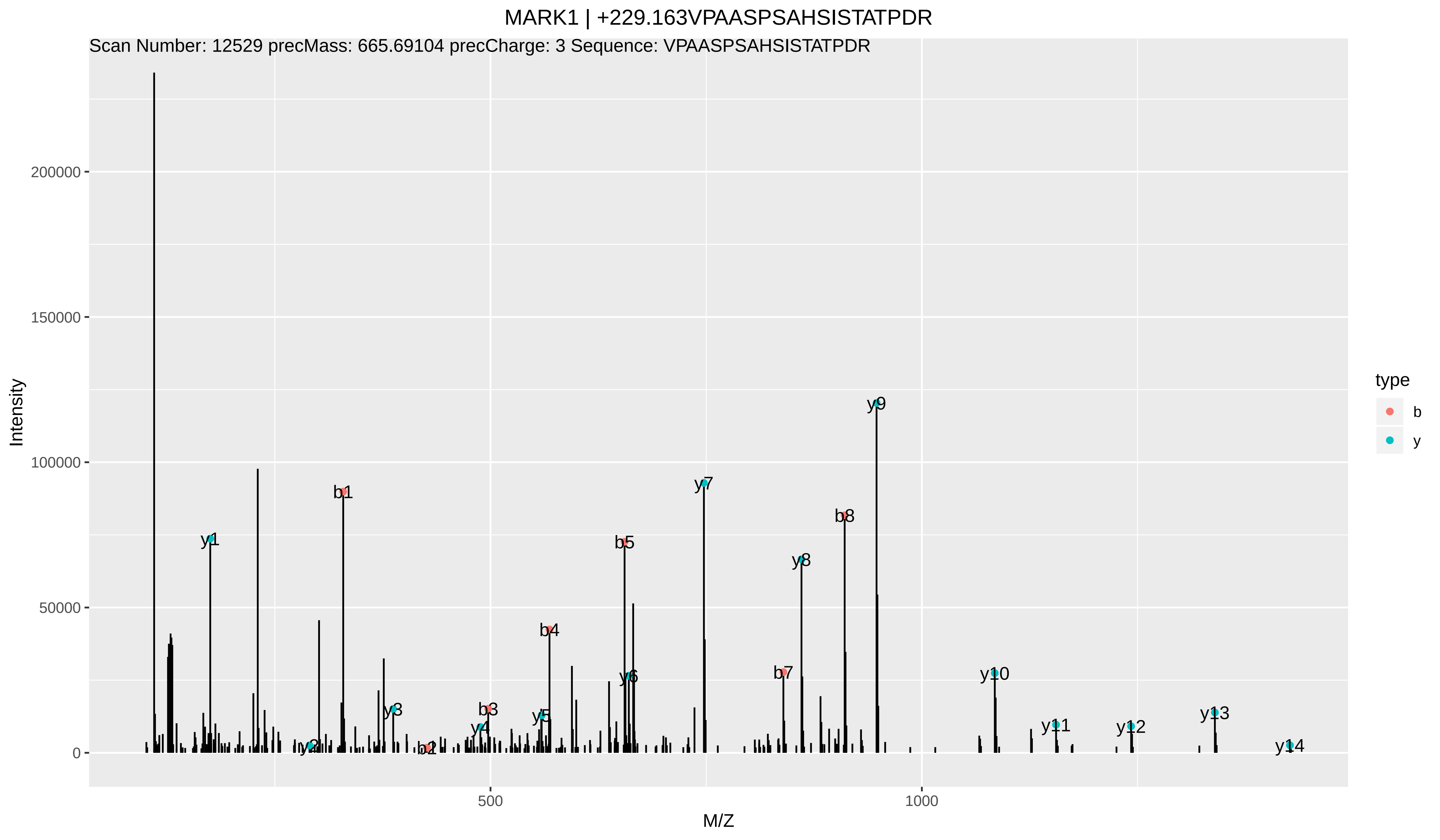
MARK1 | +229.163VPAASPSAHSISTATPDR
Scan Number: 12529 precMass: 665.69104 precCharge: 3 Sequence: VPAASPSAHSISTATPDR
Intensity
200000
150000
100000
50000
0
500
1000
M/Z
y1
y2
b1
y3
b2
y4
b3
y5
b4
b5
y6
y7
b7
y8
b8
y9
y10
y11
y12
y13
y14
type
b
y

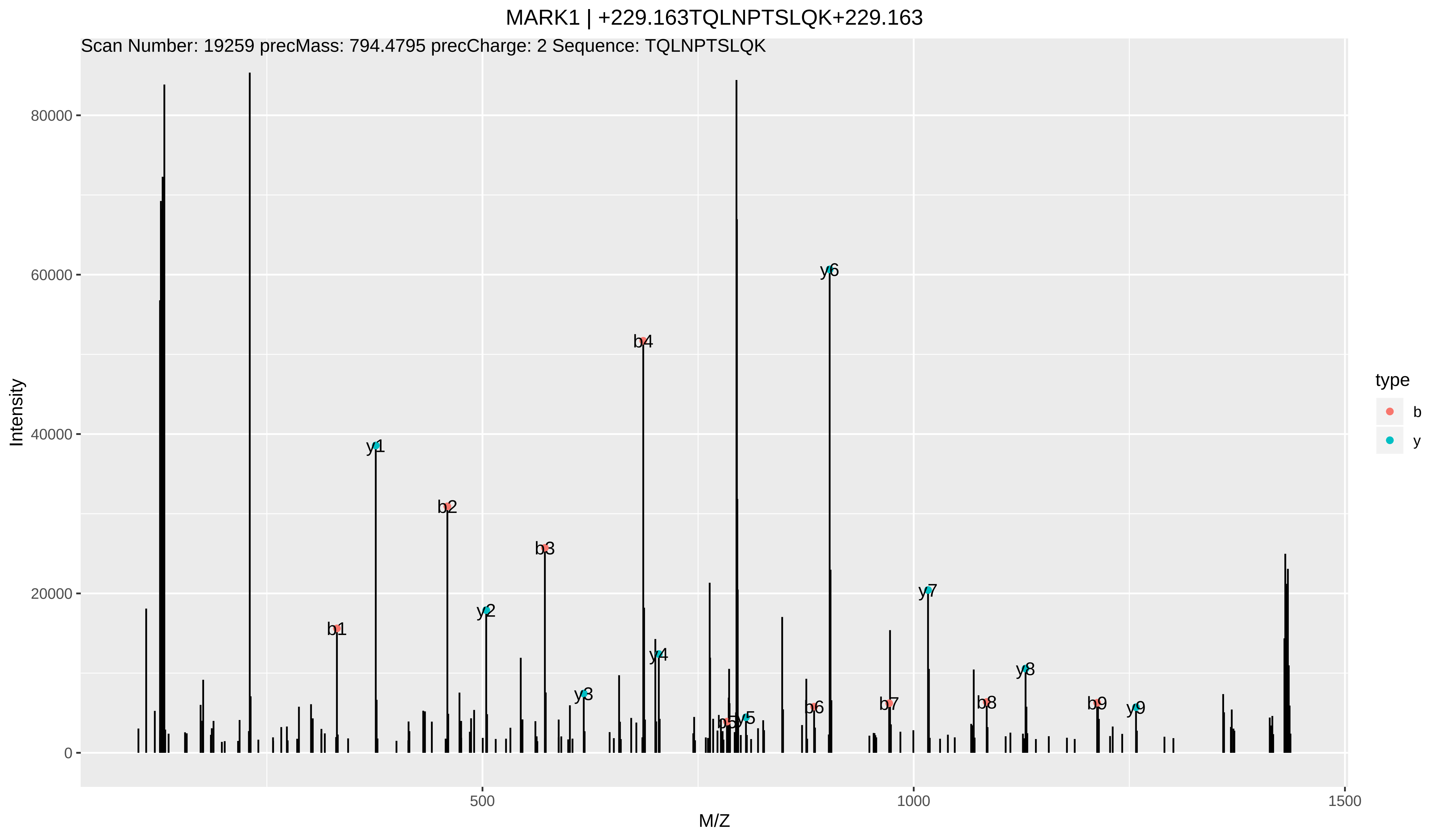

MARK1 | +229.163TQLNPTSLQK+229.163
Scan Number: 19259 precMass: 794.4795 precCharge: 2 Sequence: TQLNPTSLQK
Intensity
M/Z
type
b
y
b1
y1
b2
y2
b3
y3
b4
y4
b5
y5
b6
y6
b7
y7
b8
y8
b9
y9
0
20000
40000
60000
80000
500
1000
1500

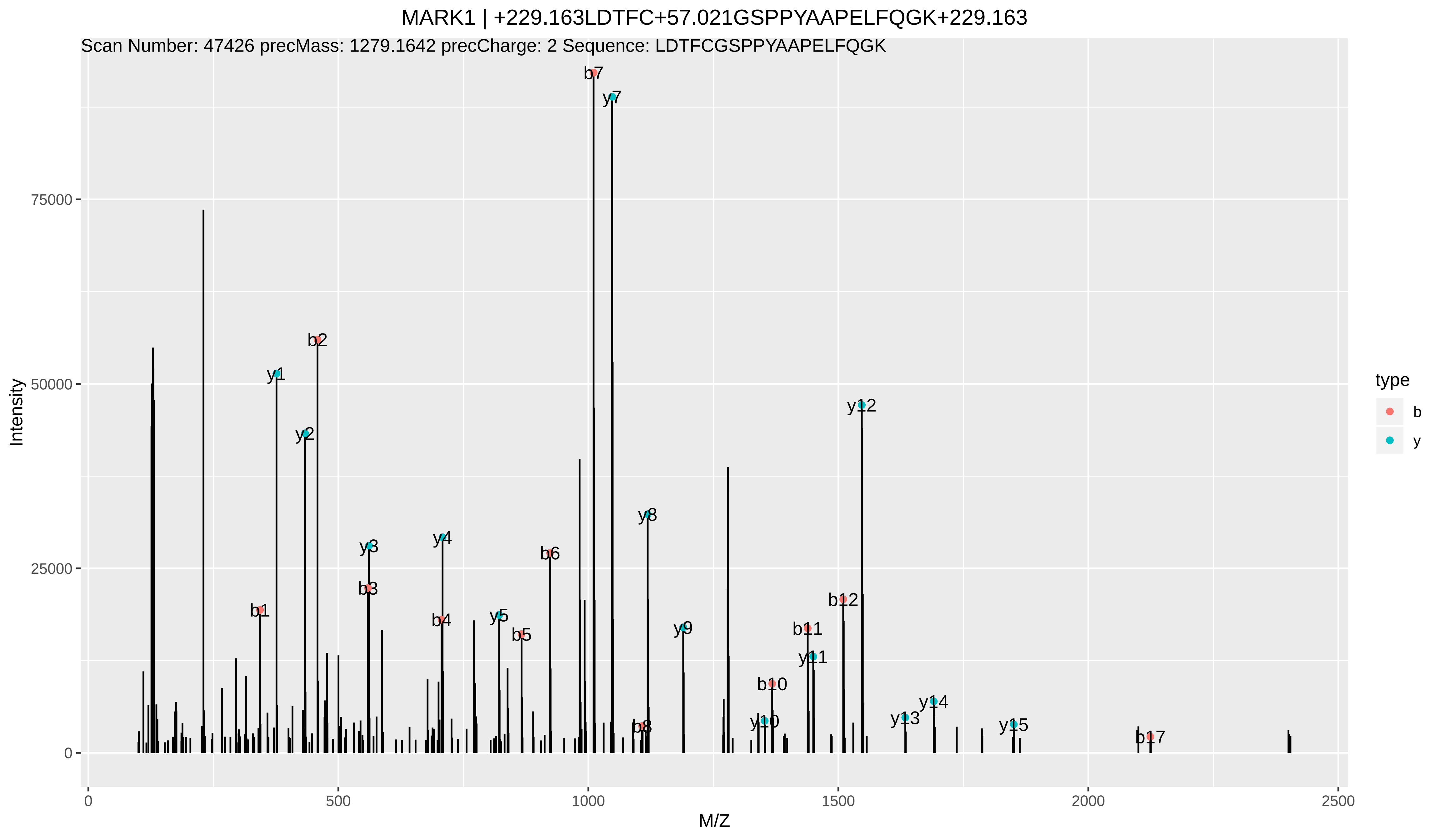
MARK1 | +229.163LDTFC+57.021GSPPYAAPELFQGK+229.163
Scan Number: 47426 precMass: 1279.1642 precCharge: 2 Sequence: LDTFCGSPPYAAPELFQGK
Intensity
75000
50000
25000
0
M/Z
0
500
1000
1500
2000
2500
type
b
y
b1
b2
b3
b4
b5
b6
b7
b8
b10
b11
b12
b17
y1
y2
y3
y4
y5
y7
y8
y9
y10
y11
y12
y13
y14
y15

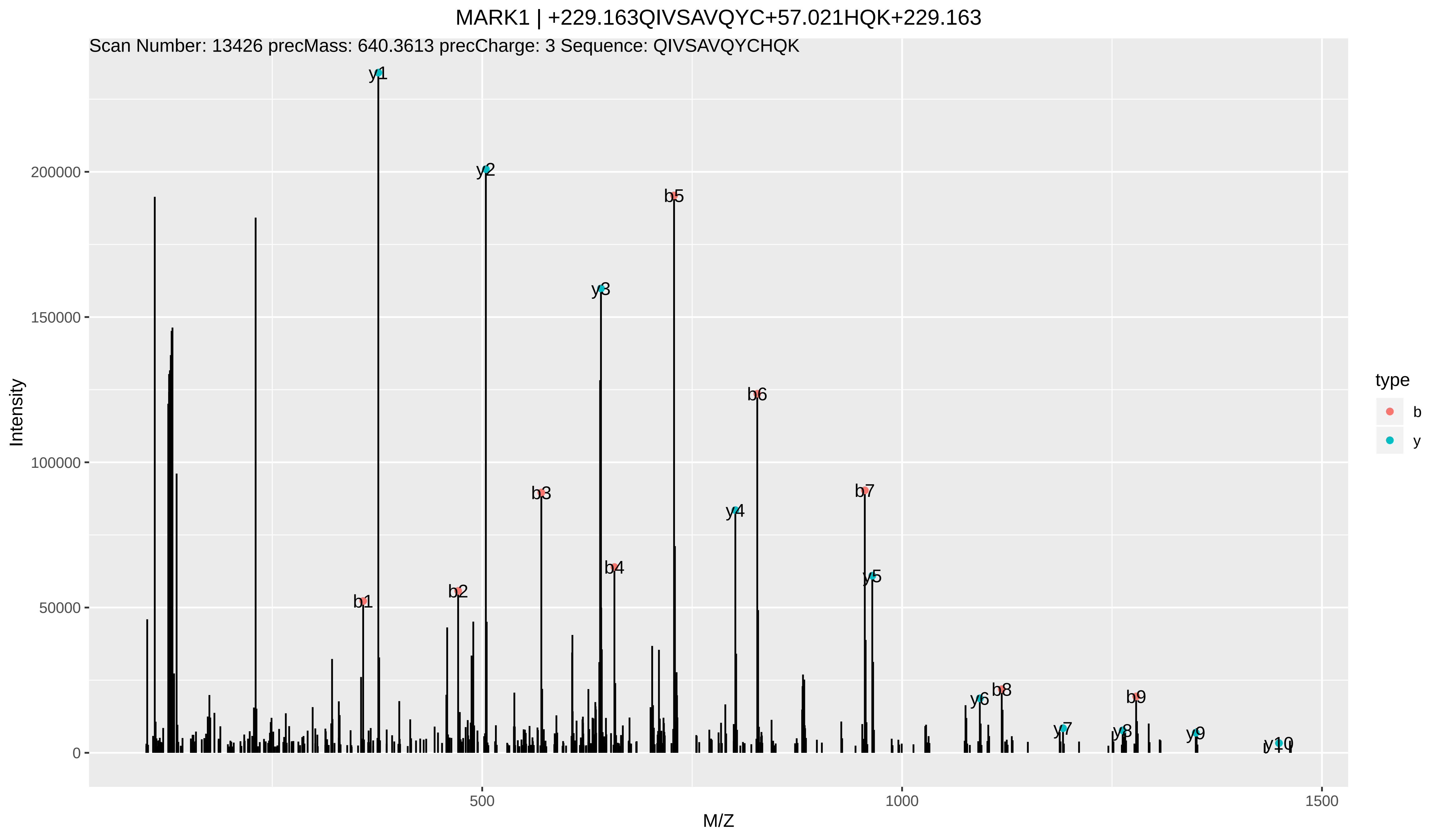

MARK1 | +229.163QIVSAVQYC+57.021HQK+229.163
Scan Number: 13426 precMass: 640.3613 precCharge: 3 Sequence: QIVSAVQYCHQK
Intensity
M/Z
0
50000
100000
150000
200000
500
1000
1500
y1
y2
b5
y3
b6
b7
y4
b3
b4
y5
b2
b1
b8
y6
b9
y7
y8
y9
y10
type
b
y

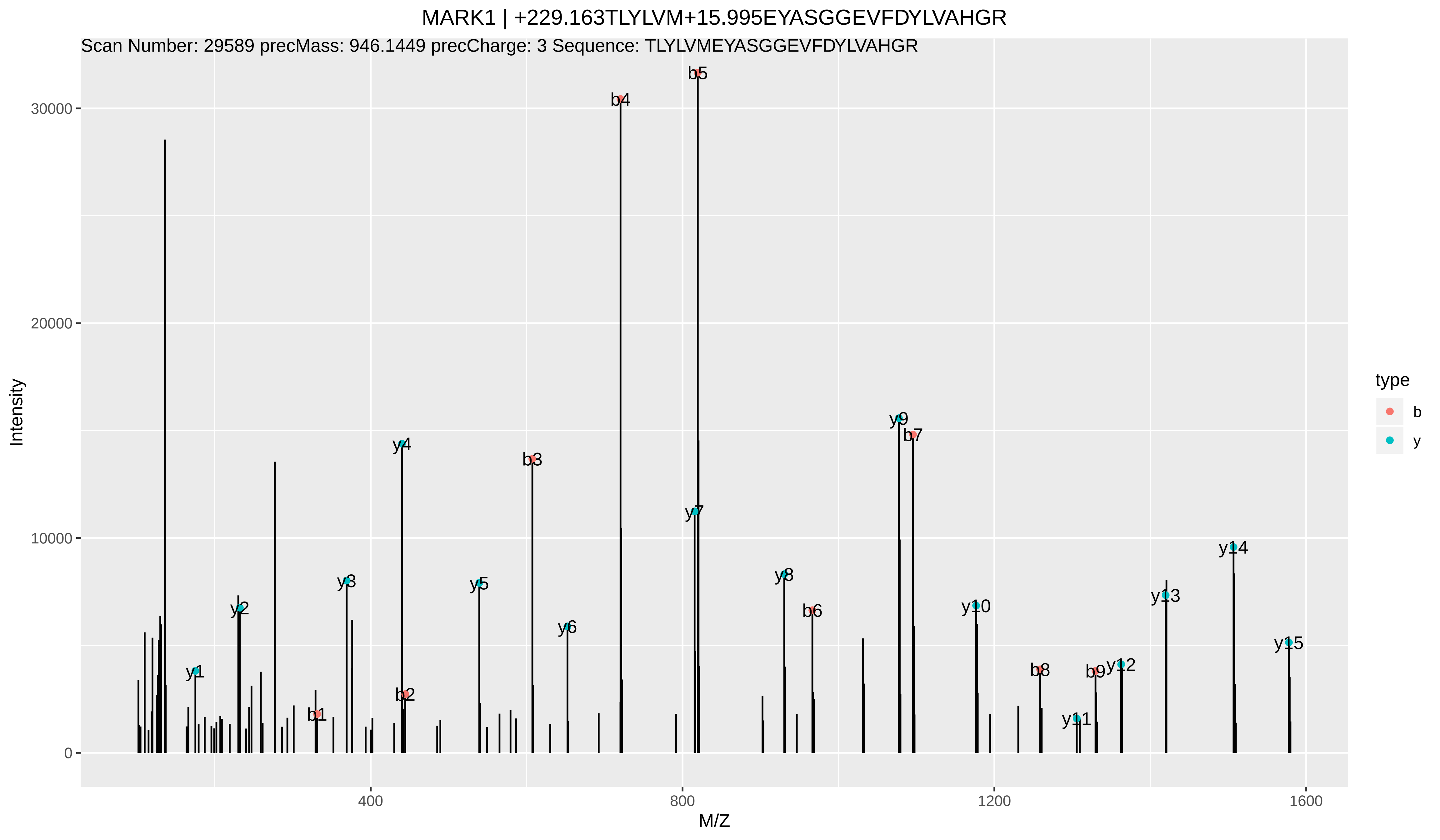
MARK1 | +229.163TLYLVM+15.995EYASGGEVFDYLVAHGR
Scan Number: 29589 precMass: 946.1449 precCharge: 3 Sequence: TLYLVMEYASGGEVFDYLVAHGR
Intensity
M/Z
type
b
y
0
10000
20000
30000
400
800
1200
1600
b1
b2
b3
b4
b5
b6
b7
b8
b9
y1
y2
y3
y4
y5
y6
y7
y8
y9
y10
y11
y12
y13
y14
y15

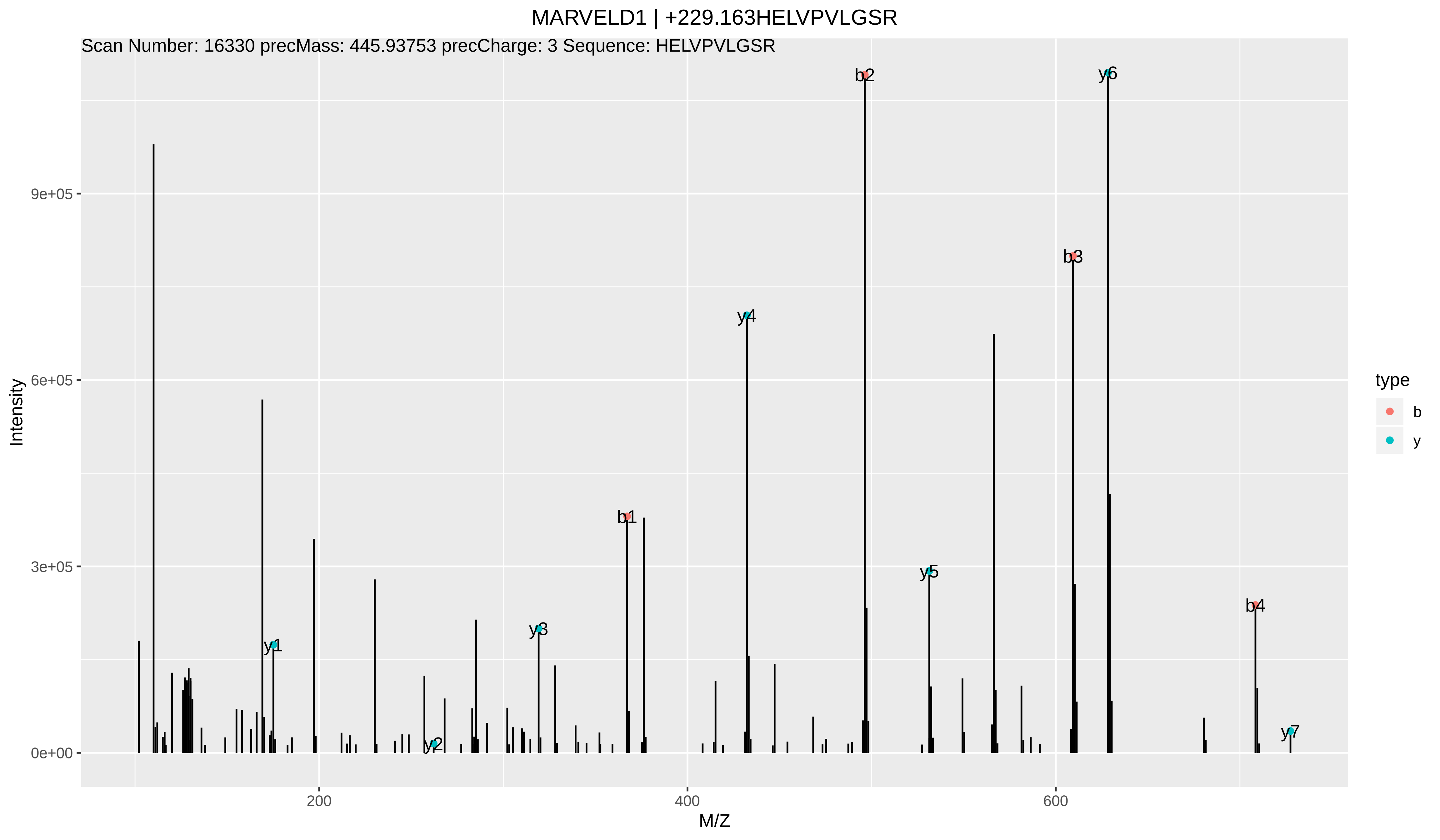

MARVELD1 | +229.163HELVPVLGSR
Scan Number: 16330 precMass: 445.93753 precCharge: 3 Sequence: HELVPVLGSR
Intensity
9e+05
6e+05
3e+05
0e+00
M/Z
200
400
600
b2
y6
b3
y4
b1
y5
b4
y1
y3
y2
y7
type
b
y

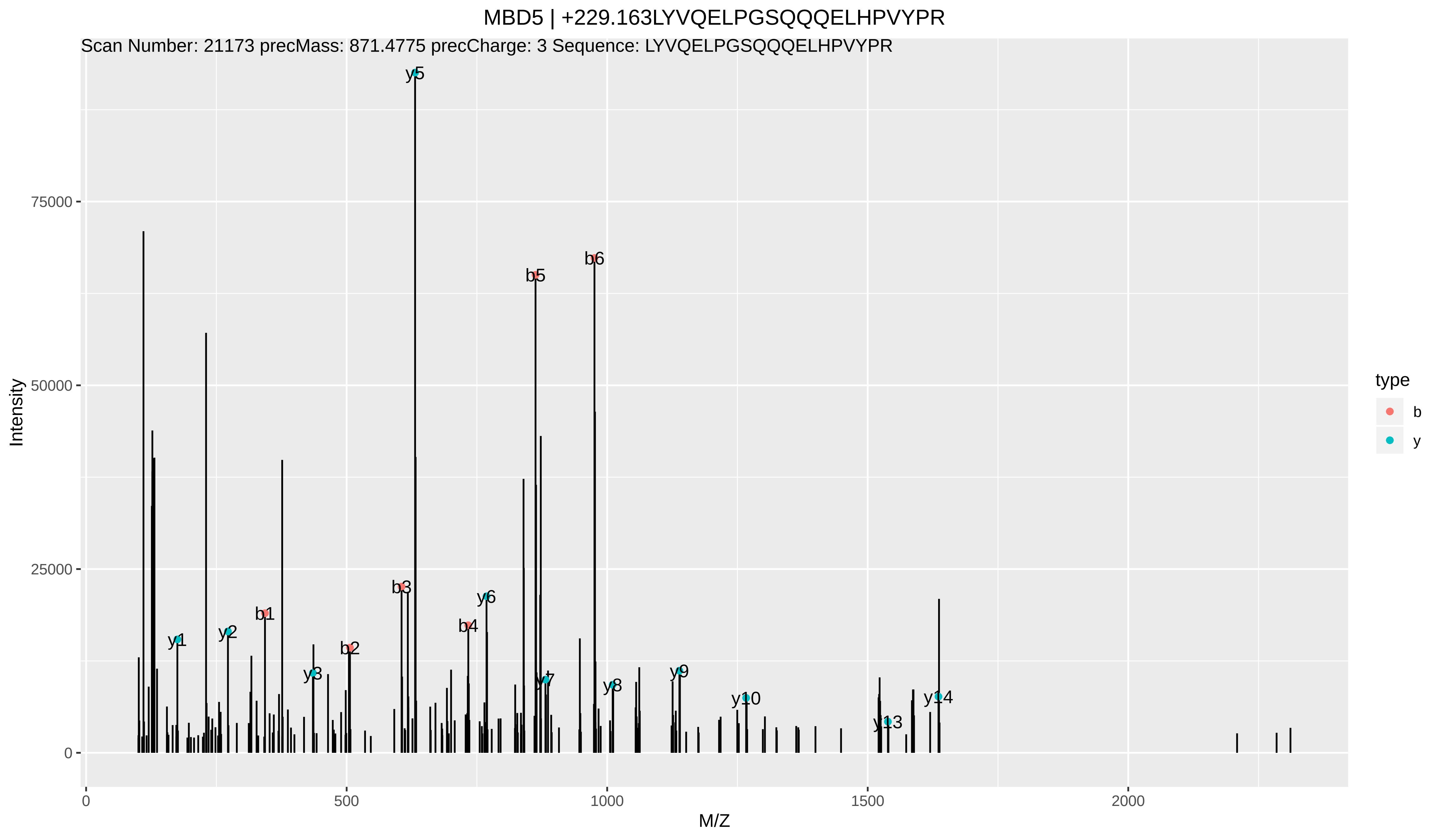

MBD5 | +229.163LYVQELPGSQQQELHPVYPR
Scan Number: 21173 precMass: 871.4775 precCharge: 3 Sequence: LYVQELPGSQQQELHPVYPR
y5
b6
b5
b3
y6
b1
b4
y2
y1
b2
y3
y9
y7
y8
y10
y14
y13
75000
50000
25000
0
0
500
1000
1500
2000
M/Z
Intensity
type
b
y

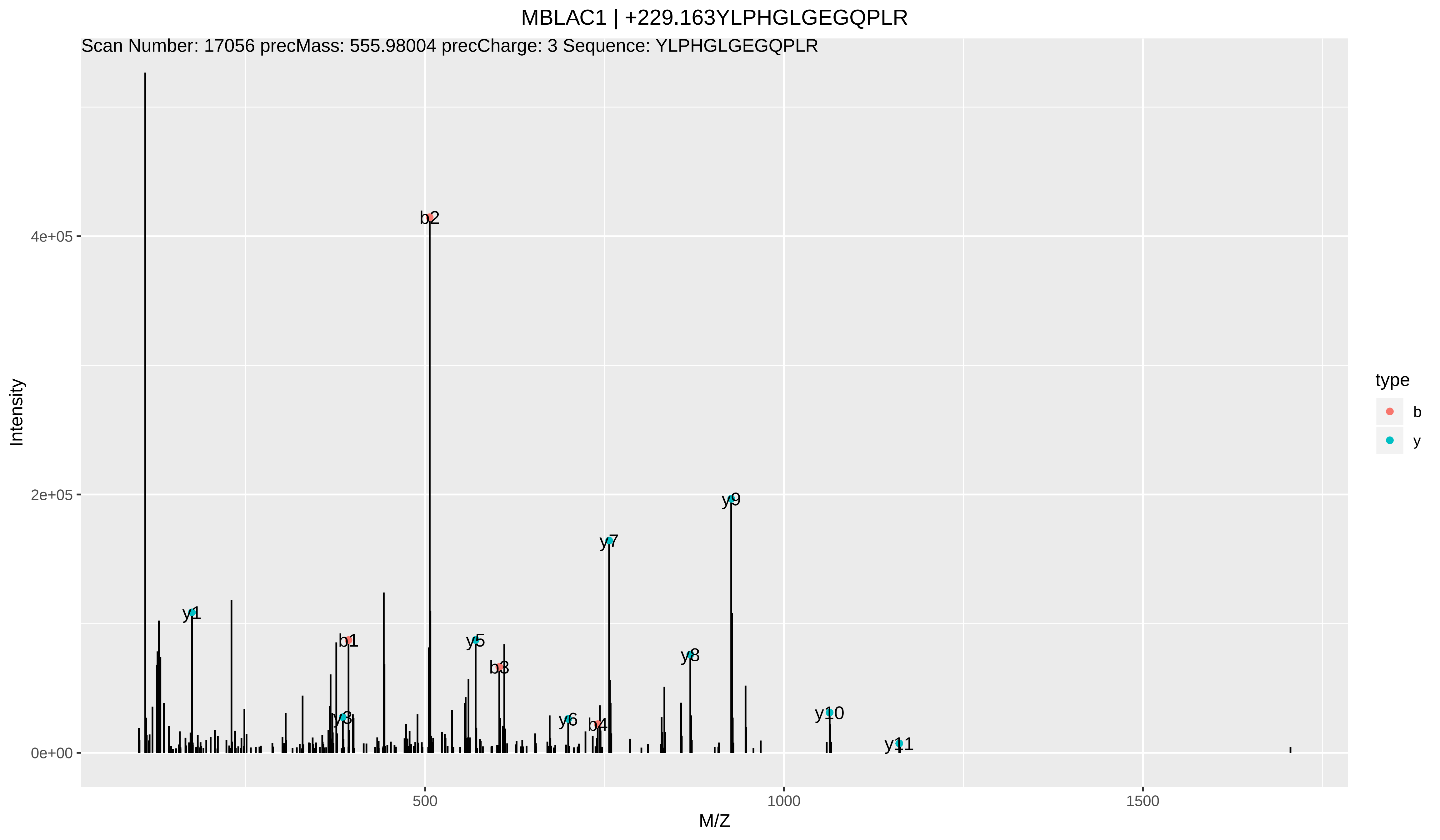

MBLAC1 | +229.163YLPHGLGEGQPLR
Scan Number: 17056 precMass: 555.98004 precCharge: 3 Sequence: YLPHGLGEGQPLR
Intensity
4e+05
2e+05
0e+00
M/Z
500
1000
1500
b2
y9
y7
y1
b1
y5
b3
y8
y3
y6
b4
y10
y11
type
b
y

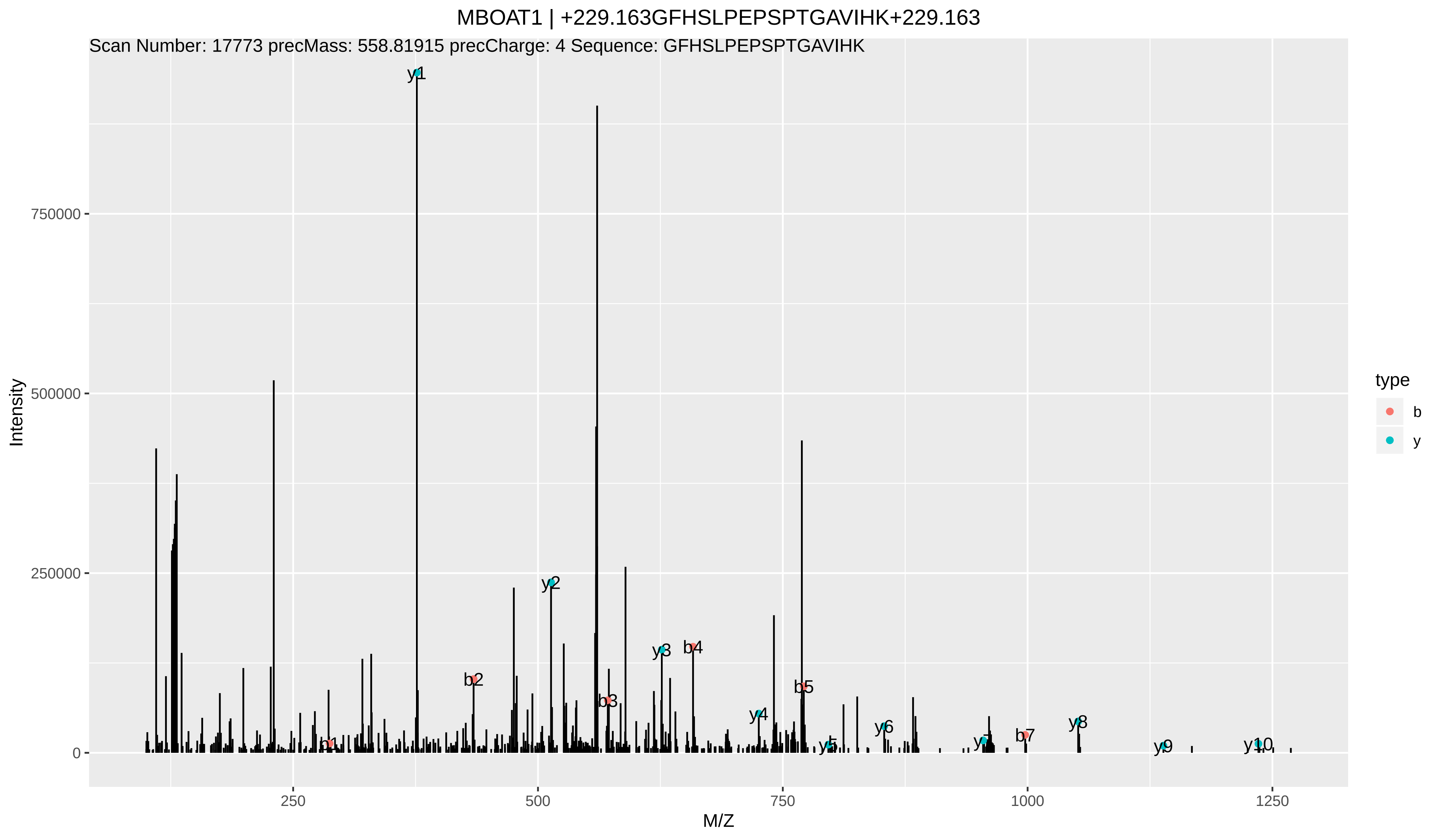

MBOAT1 | +229.163GFHSLPEPSPTGAVIHK+229.163
Scan Number: 17773 precMass: 558.81915 precCharge: 4 Sequence: GFHSLPEPSPTGAVIHK
Intensity
M/Z
type
b
y
y1
y2
y3
y4
y5
y6
y7
y8
y9
y10
b1
b2
b3
b4
b5
b7
750000
500000
250000
0
250
500
750
1000
1250

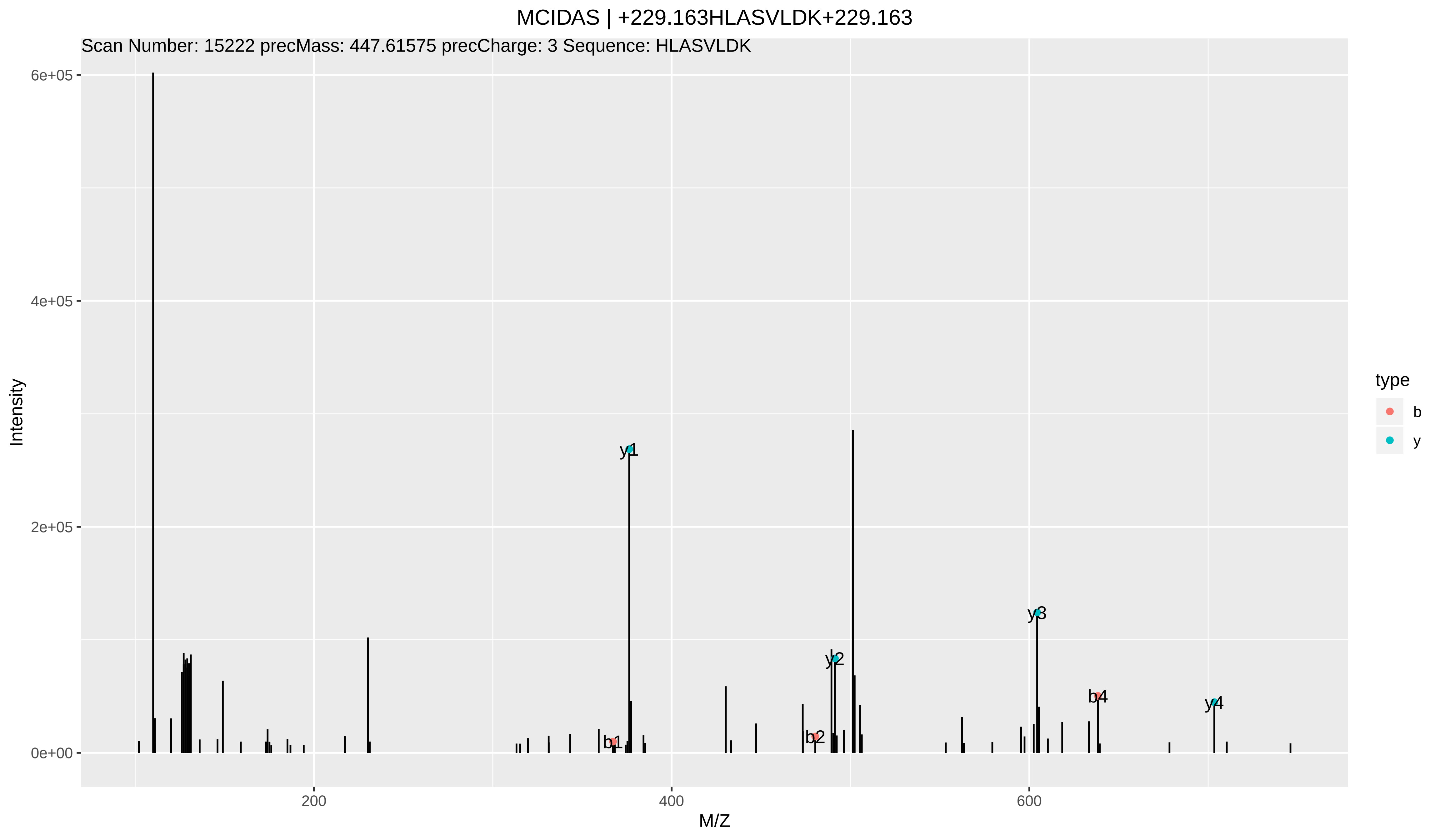

MCIDAS | +229.163HLASVLDK+229.163
Scan Number: 15222 precMass: 447.61575 precCharge: 3 Sequence: HLASVLDK
Intensity
6e+05
4e+05
2e+05
0e+00
M/Z
200
400
600
y1
y2
y3
y4
b1
b2
b4
type
b
y

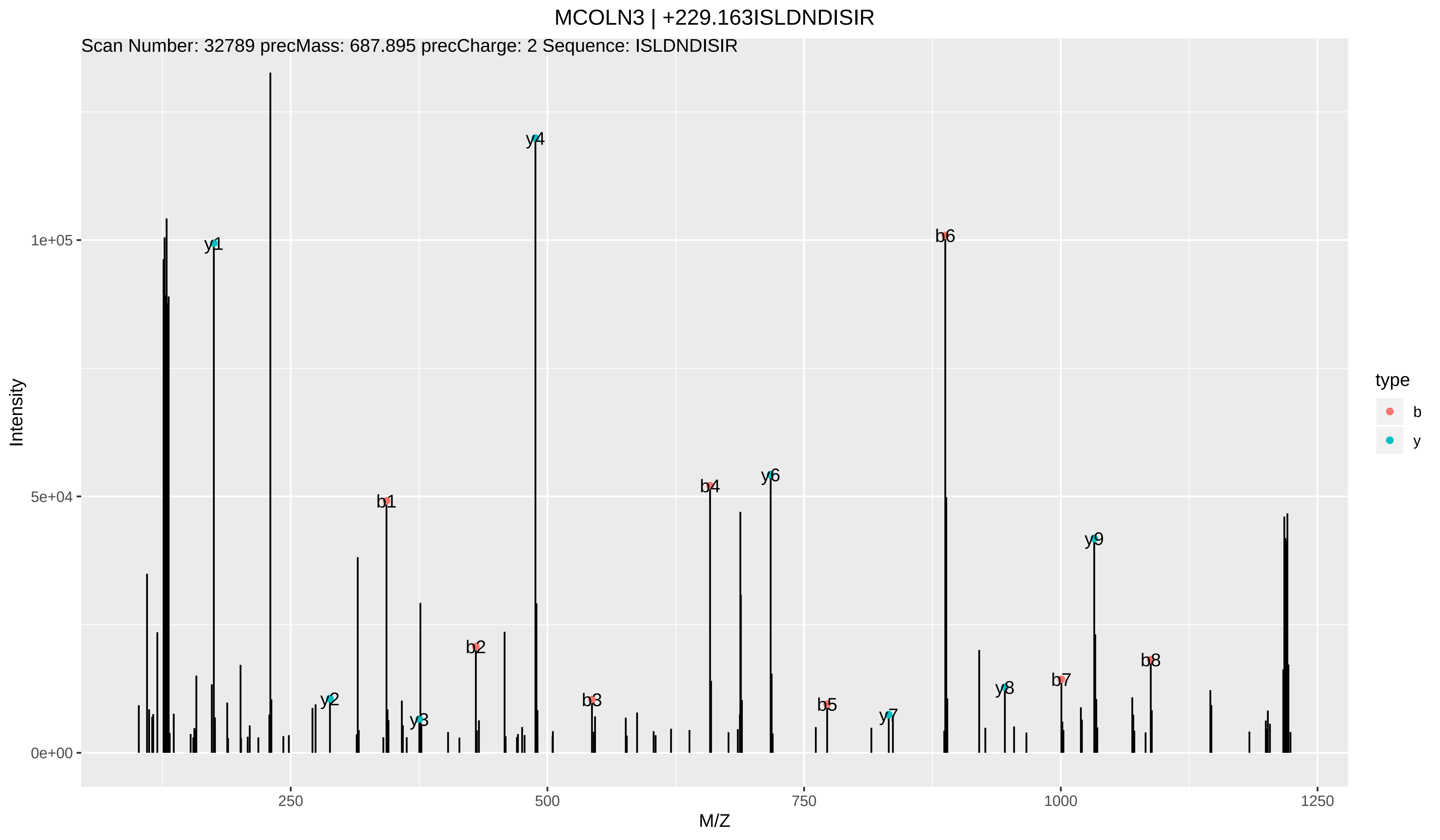

MCOLN3 | +229.163ISLDNDISIR
Scan Number: 32789 precMass: 687.895 precCharge: 2 Sequence: ISLDNDISIR
Intensity
1e+05
5e+04
0e+00
M/Z
250
500
750
1000
1250
y1
y2
y3
y4
y6
y7
y8
y9
b1
b2
b3
b4
b5
b6
b7
b8
type
b
y

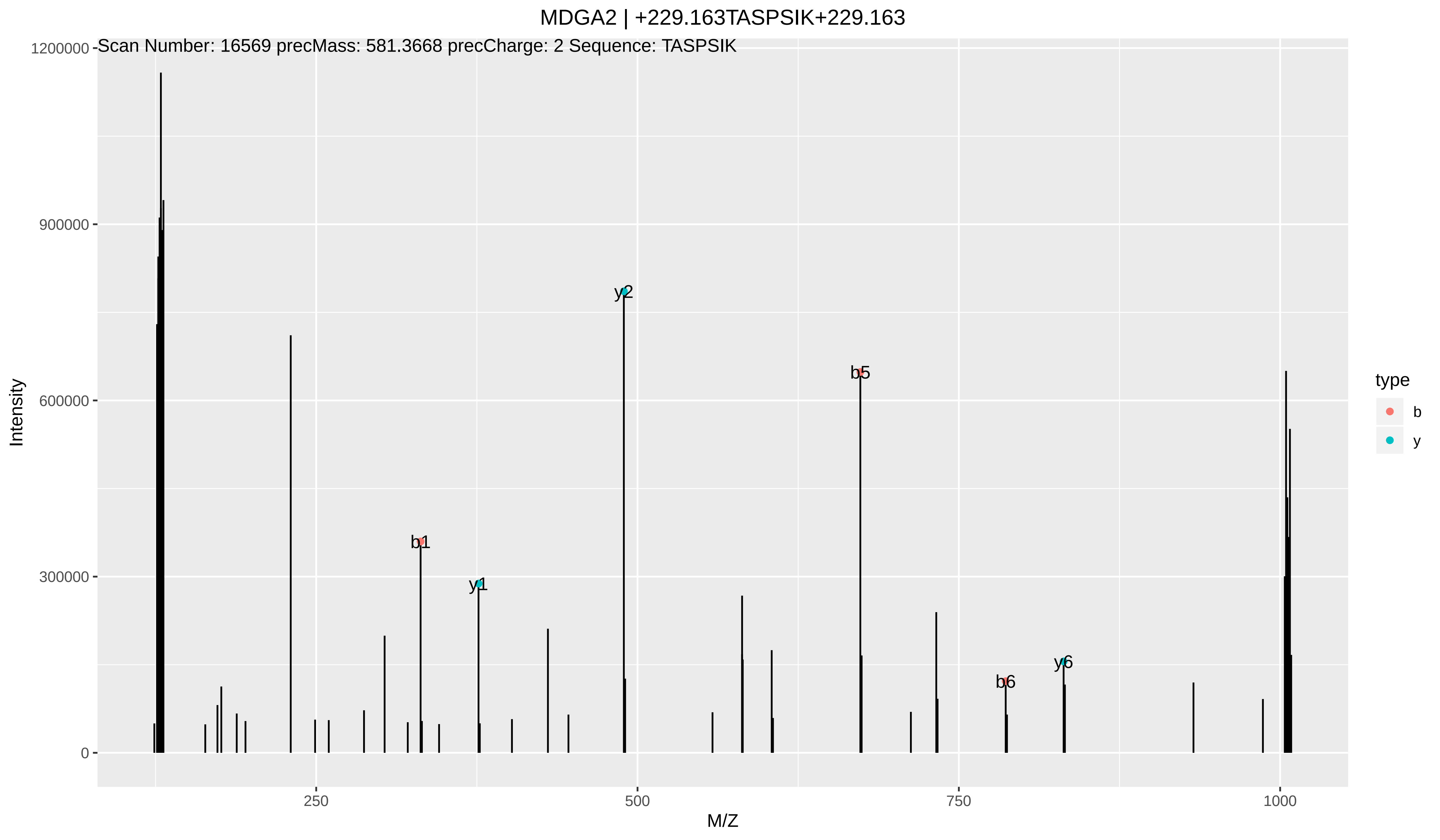

MDGA2 | +229.163TASPSIK+229.163
Scan Number: 16569 precMass: 581.3668 precCharge: 2 Sequence: TASPSIK
Intensity
M/Z
type
b
y
b1
y1
y2
b5
b6
y6
0
300000
600000
900000
1200000
250
500
750
1000

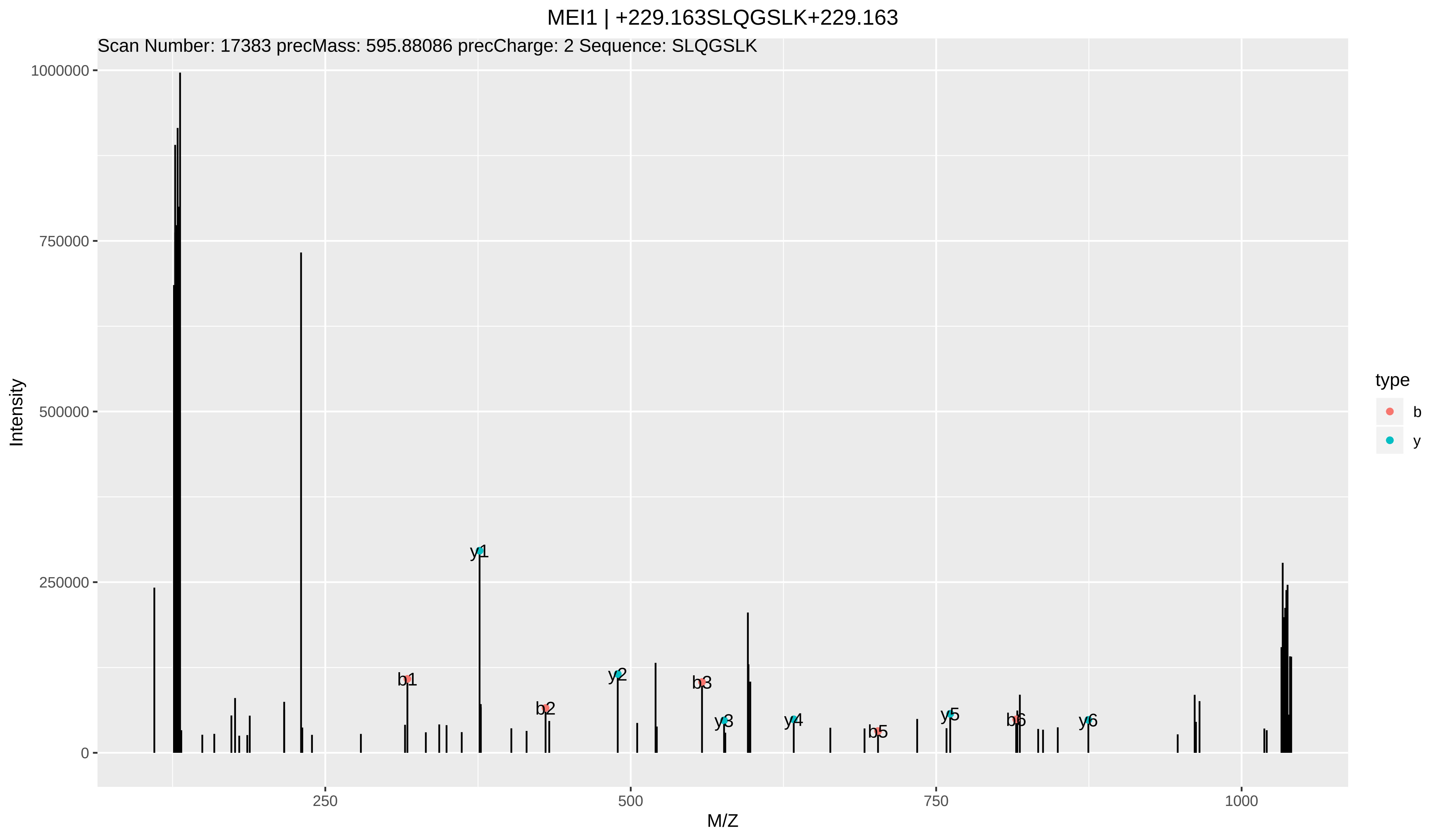

MEI1 | +229.163SLQGSLK+229.163
Scan Number: 17383 precMass: 595.88086 precCharge: 2 Sequence: SLQGSLK
Intensity
M/Z
1000000
750000
500000
250000
0
250
500
750
1000
y1
b1
b2
y2
b3
y3
y4
b5
y5
b6
y6
type
b
y

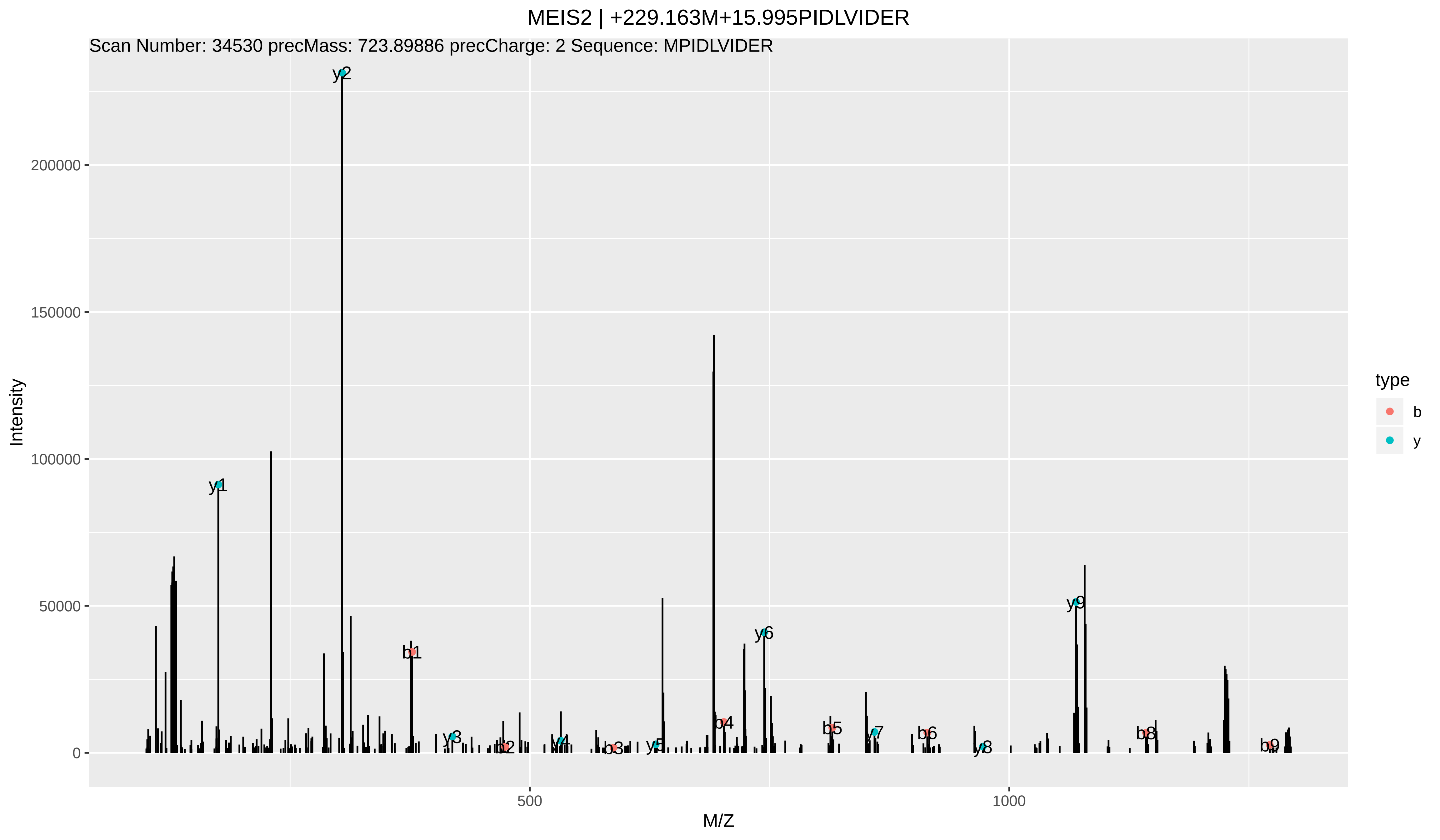
MEIS2 | +229.163M+15.995PIDLVIDER
Scan Number: 34530 precMass: 723.89886 precCharge: 2 Sequence: MPIDLVIDER
Intensity
M/Z
0
50000
100000
150000
200000
500
1000
y1
y2
b1
y3
b2
y4
b3
y5
b4
y6
b5
y7
b6
y8
y9
b8
b9
type
b
y

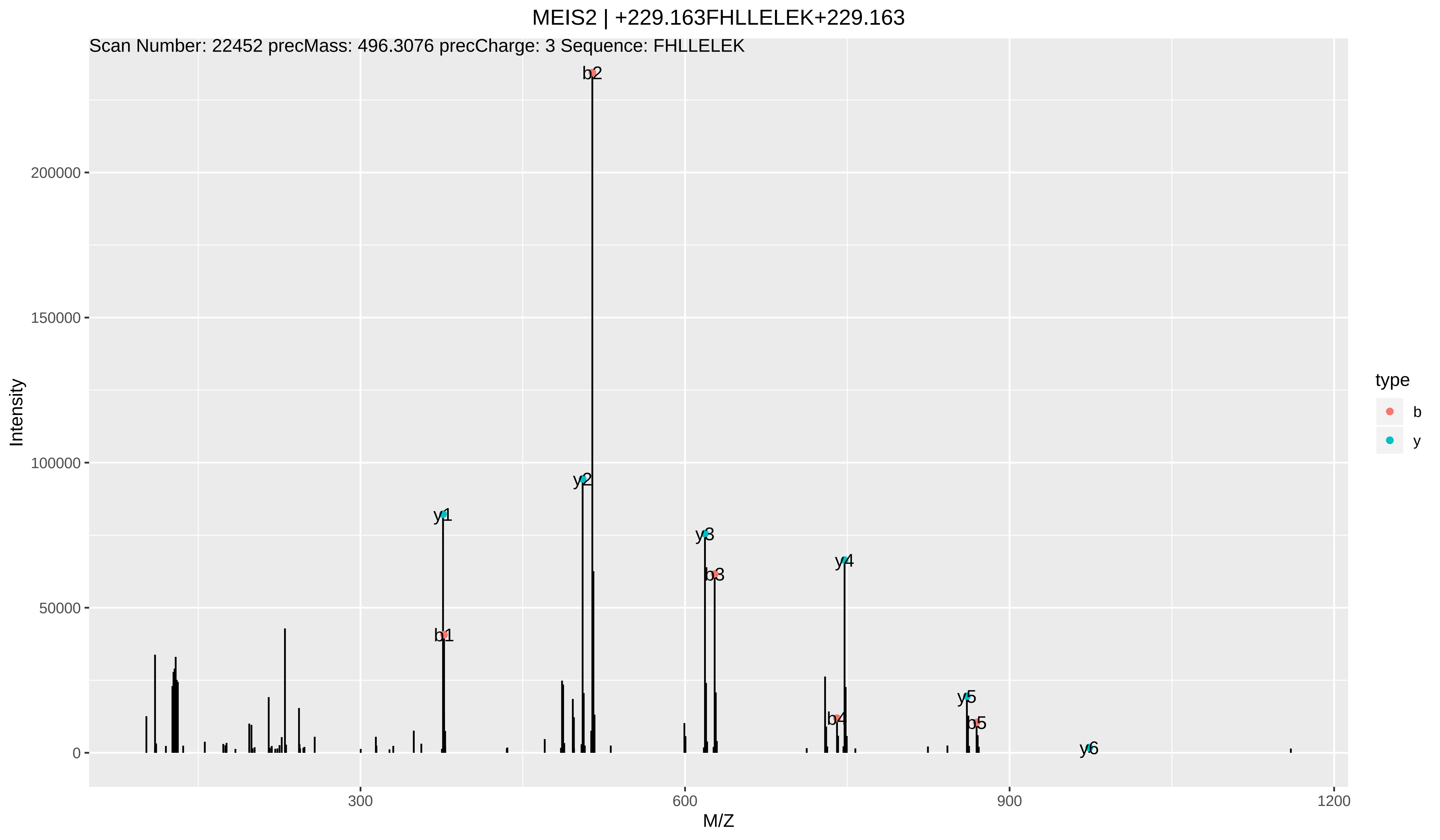

MEIS2 | +229.163FHLLELEK+229.163
Scan Number: 22452 precMass: 496.3076 precCharge: 3 Sequence: FHLLELEK
Intensity
M/Z
type
b
y
b1
b2
b3
b4
b5
y1
y2
y3
y4
y5
y6
0
50000
100000
150000
200000
300
600
900
1200

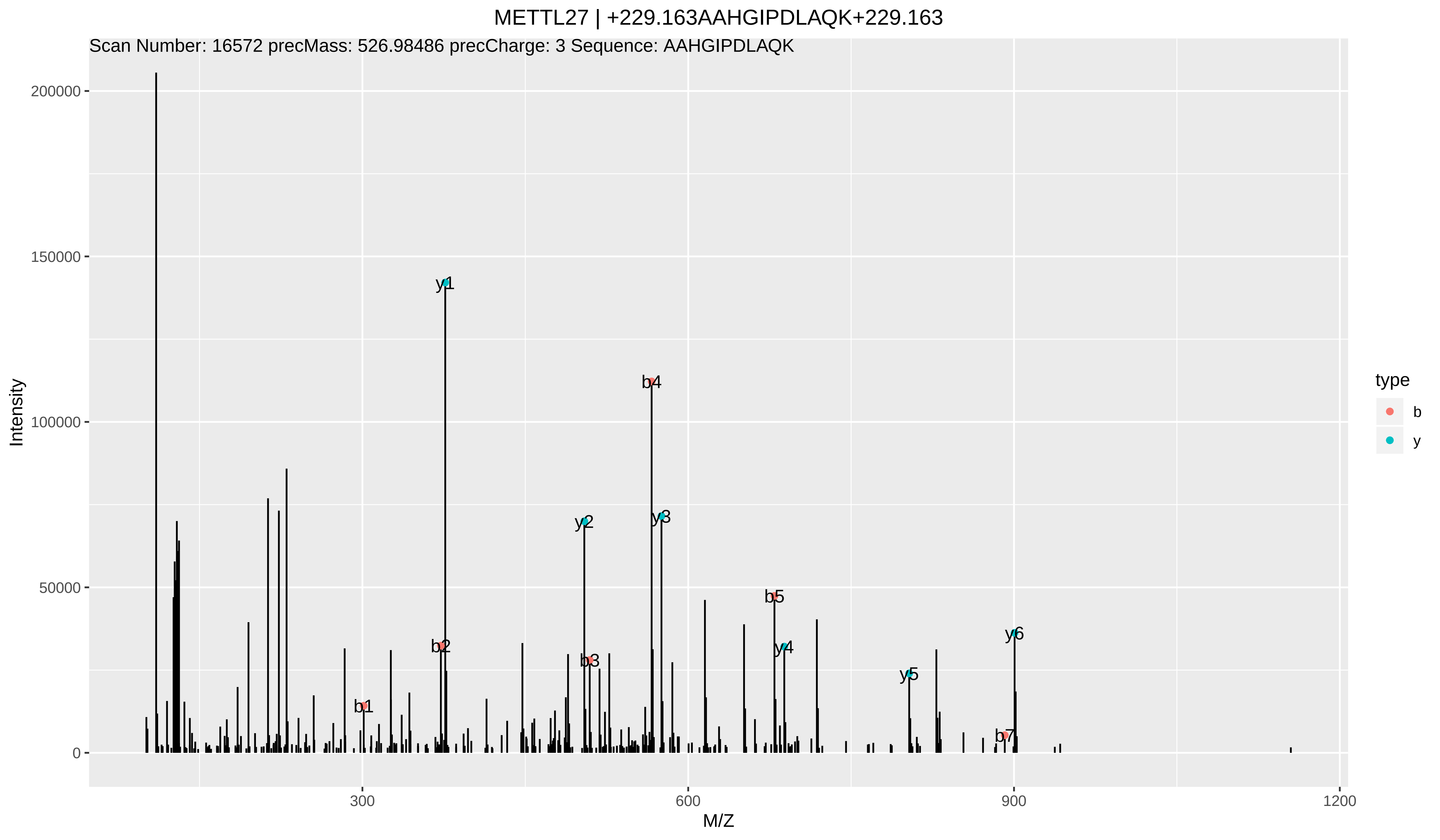
METTL27 | +229.163AAHGIPDLAQK+229.163
Scan Number: 16572 precMass: 526.98486 precCharge: 3 Sequence: AAHGIPDLAQK
Intensity
200000
150000
100000
50000
0
M/Z
300
600
900
1200
y1
b4
y2
y3
b5
y6
b2
y4
b3
y5
b1
b7
type
b
y

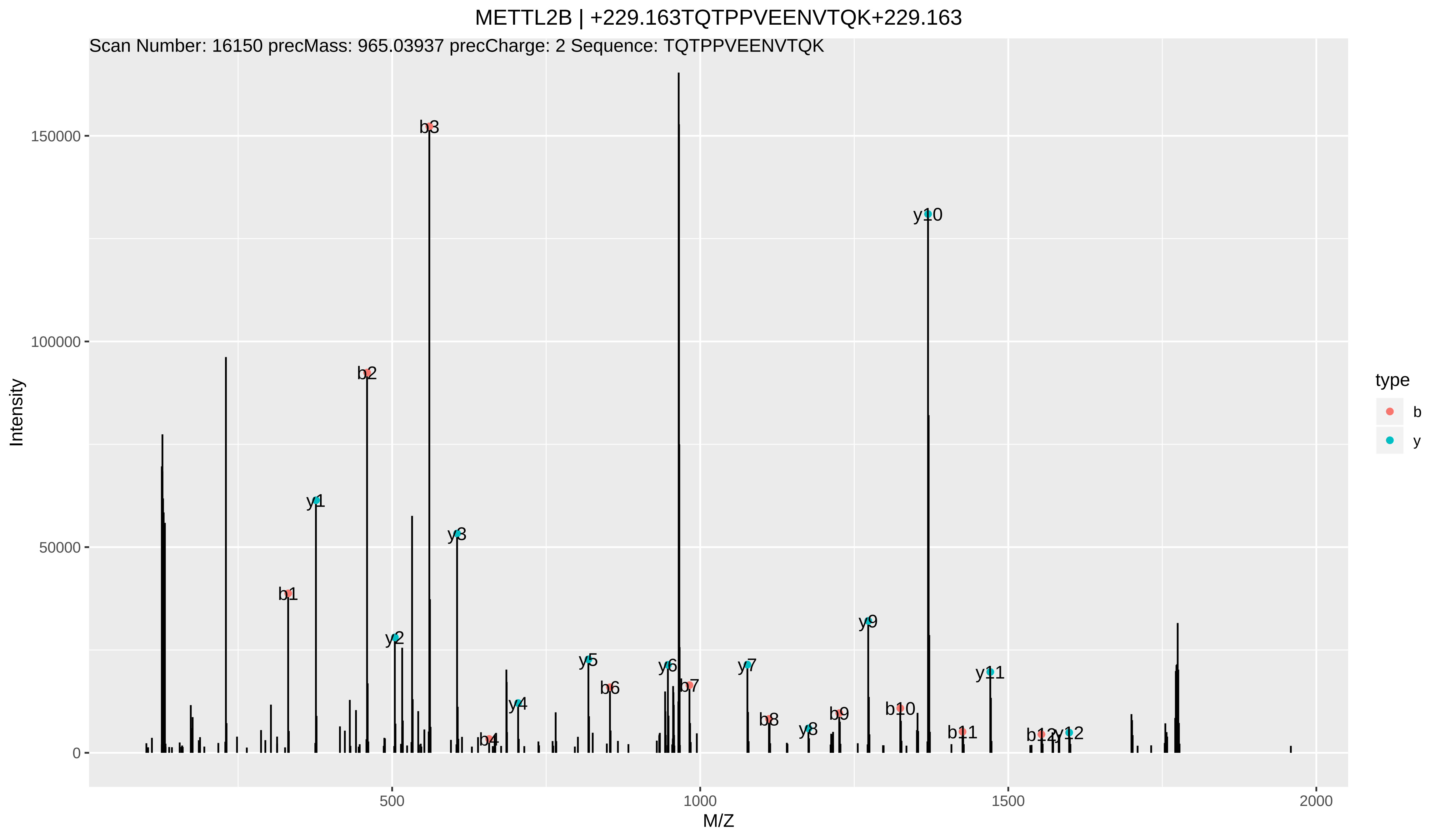

METTL2B | +229.163TQTPPVEENVTQK+229.163
Scan Number: 16150 precMass: 965.03937 precCharge: 2 Sequence: TQTPPVEENVTQK
Intensity
M/Z
0
50000
100000
150000
500
1000
1500
2000
b1
b2
b3
b4
b6
b7
b8
b9
b10
b11
b12
y1
y2
y3
y4
y5
y6
y7
y8
y9
y10
y11
y12
type
b
y

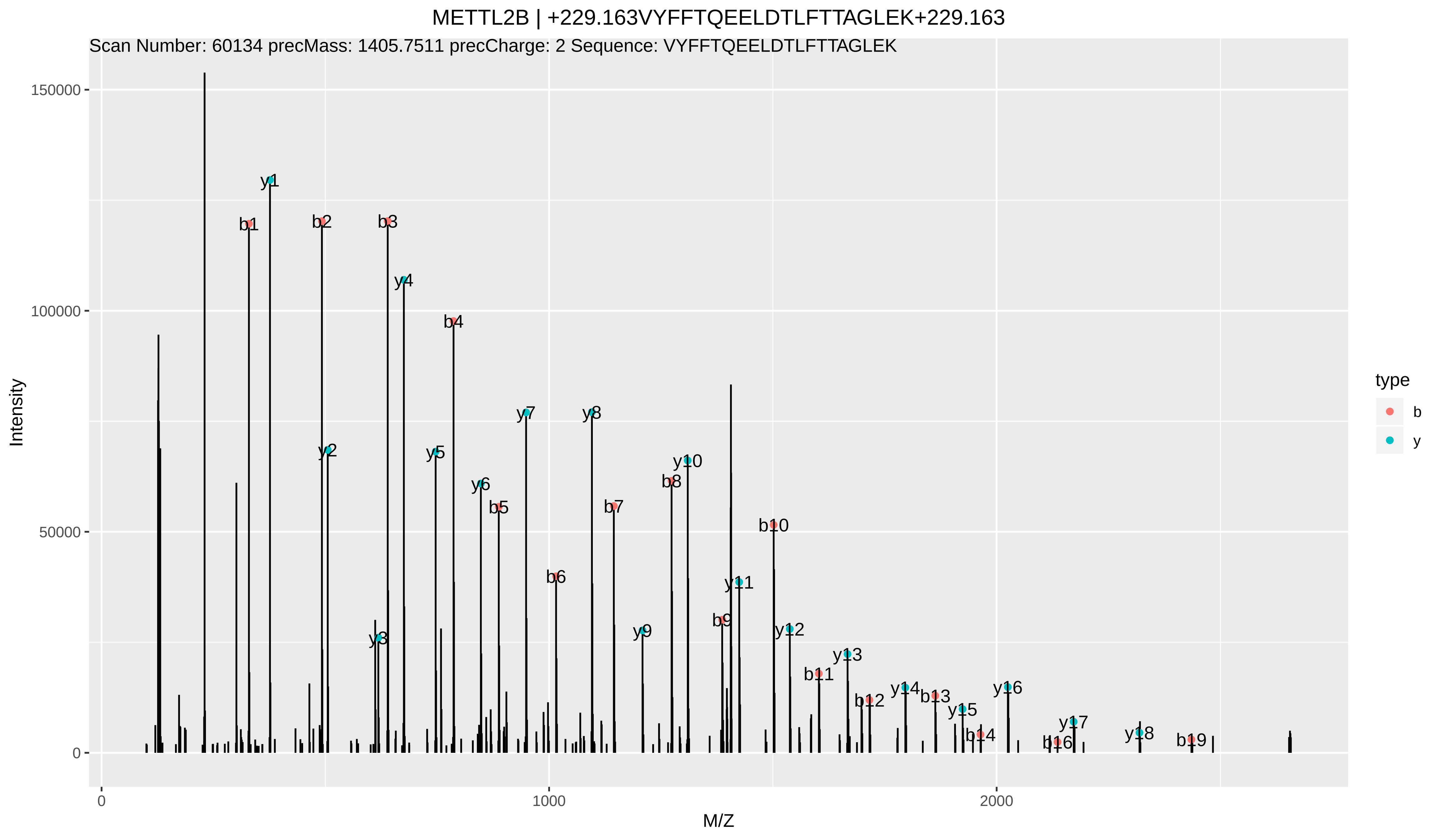

METTL2B | +229.163VYFFTQEELDTLFTTAGLEK+229.163
Scan Number: 60134 precMass: 1405.7511 precCharge: 2 Sequence: VYFFTQEELDTLFTTAGLEK
Intensity
150000
100000
50000
0
0
1000
2000
M/Z
y1
b1
b2
b3
y4
b4
y7
y8
y2
y5
y10
b8
y6
b5
b7
b10
b6
y11
b9
y3
y9
y12
y13
b11
y14
b13
y16
b12
y15
b14
y17
y18
b16
b19
type
b
y

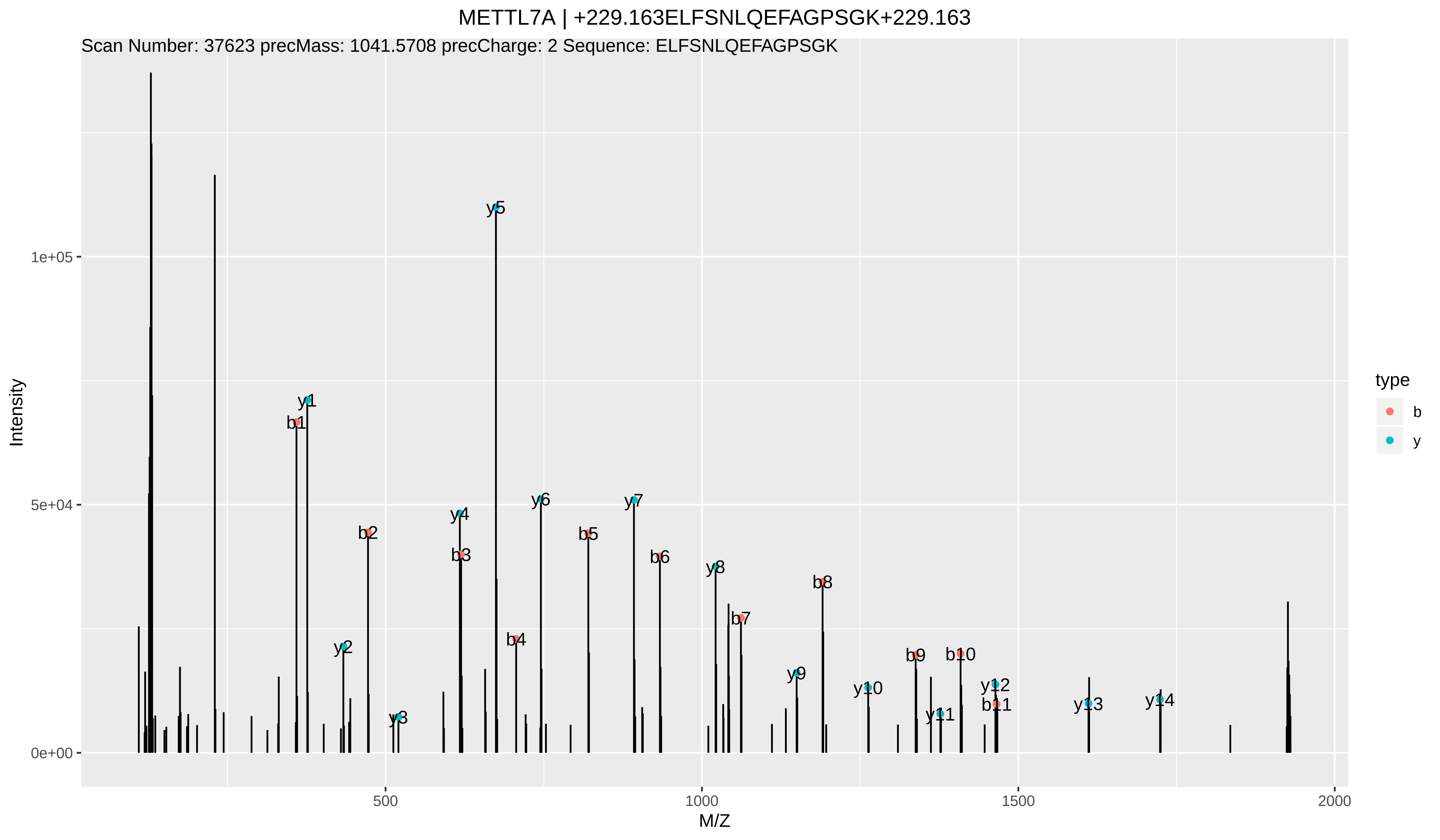
METTL7A | +229.163ELFSNLQEFAGPSGK+229.163
Scan Number: 37623 precMass: 1041.5708 precCharge: 2 Sequence: ELFSNLQEFAGPSGK
Intensity
M/Z
1e+05
5e+04
0e+00
500
1000
1500
2000
type
b
y
y5
y1
b1
y6
y7
y4
b2
b5
b3
b6
y8
b8
b7
b4
y2
b10
b9
y9
y10
y12
b11
y13
y14
y11
y3

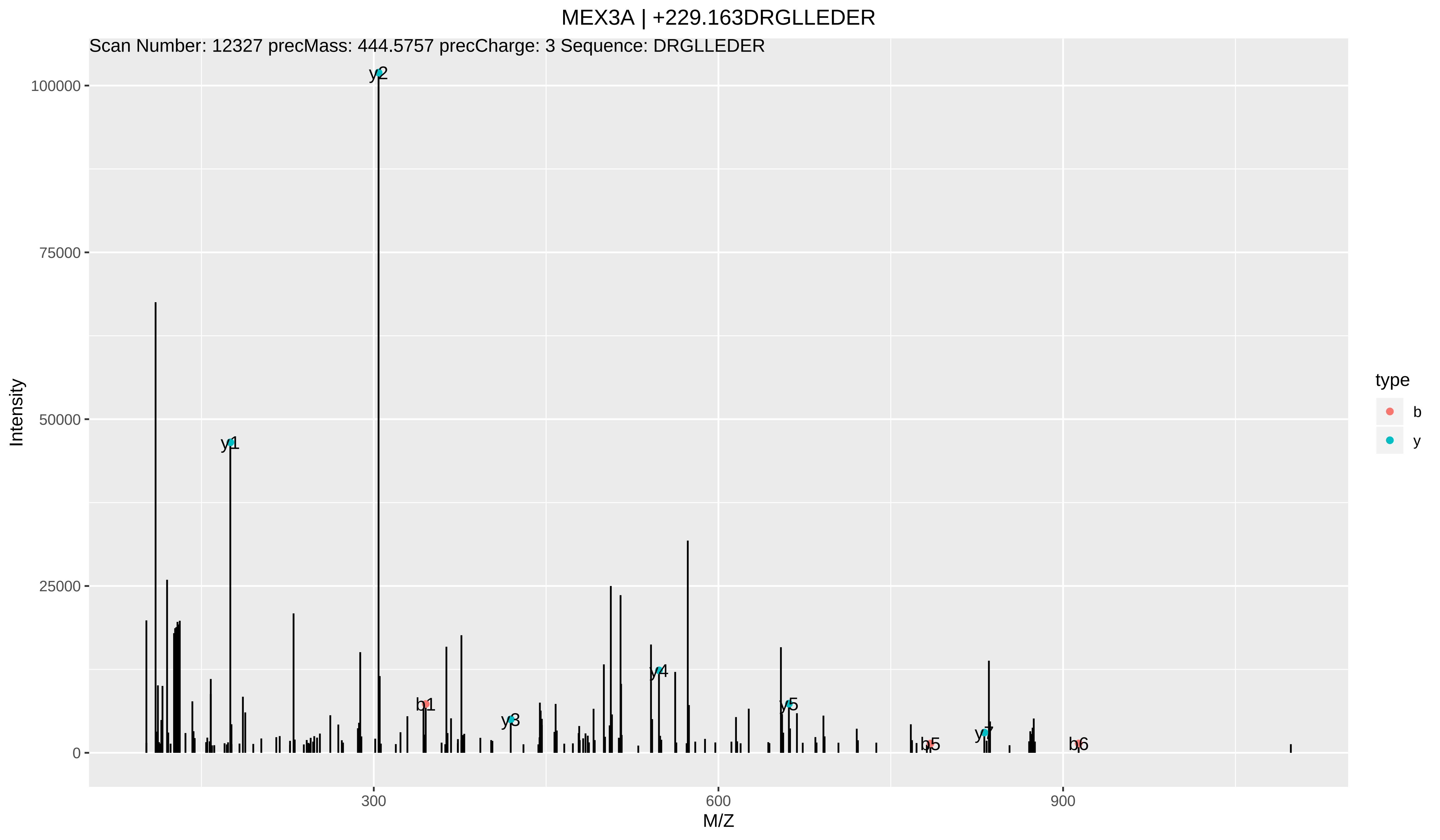

MEX3A | +229.163DRGLLEDER
Scan Number: 12327 precMass: 444.5757 precCharge: 3 Sequence: DRGLLEDER
Intensity
100000
75000
50000
25000
0
M/Z
300
600
900
y1
y2
b1
y3
y4
y5
b5
y7
b6
type
b
y

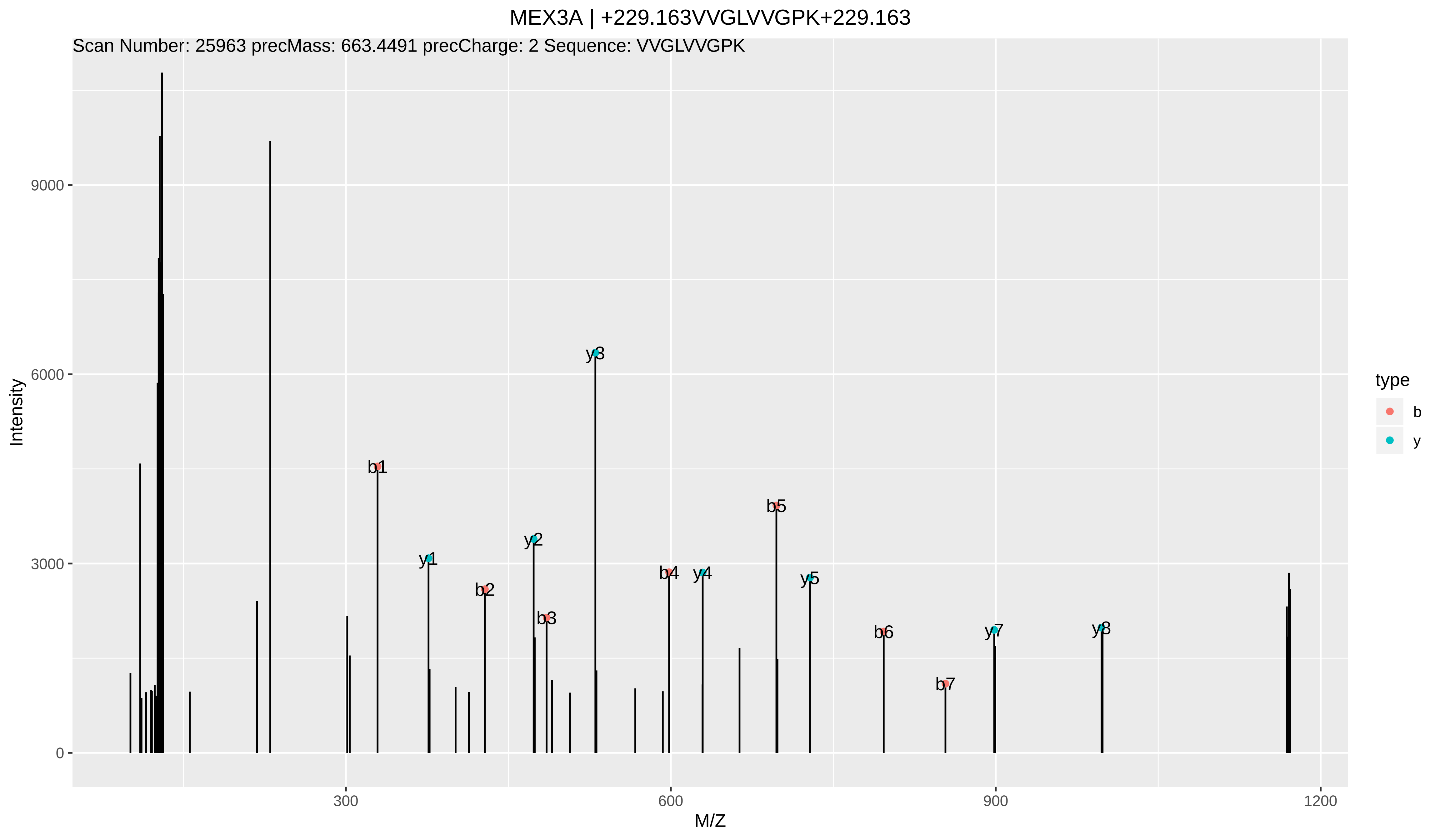
MEX3A | +229.163VVGLVVGPK+229.163
Scan Number: 25963 precMass: 663.4491 precCharge: 2 Sequence: VVGLVVGPK
Intensity
M/Z
0
3000
6000
9000
300
600
900
1200
b1
y1
b2
y2
b3
y3
b4
y4
b5
y5
b6
b7
y7
y8
type
b
y

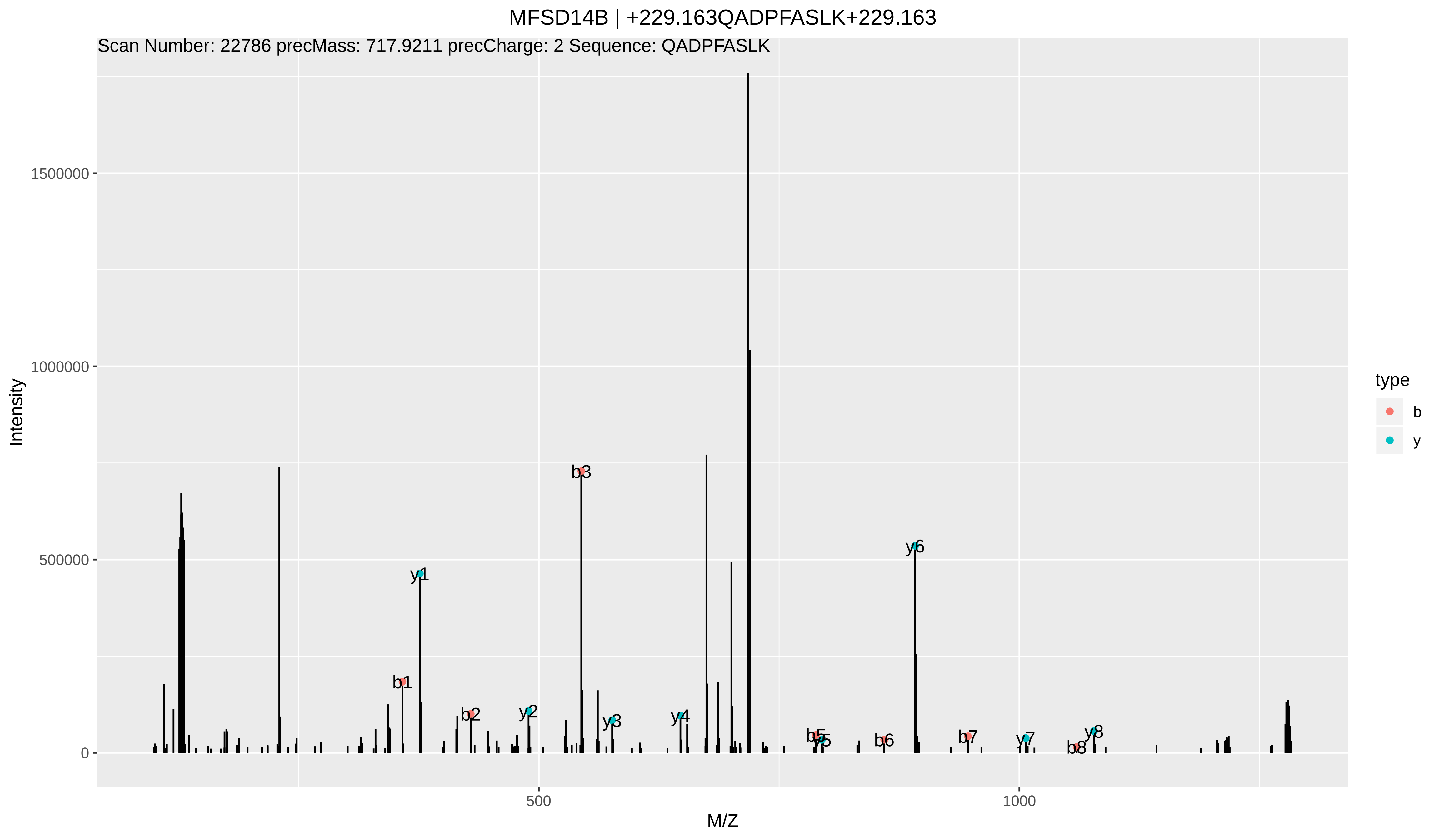
MFSD14B | +229.163QADPFASLK+229.163
Scan Number: 22786 precMass: 717.9211 precCharge: 2 Sequence: QADPFASLK
Intensity
1500000
1000000
500000
0
M/Z
500
1000
b1
y1
b2
y2
b3
y3
y4
b5
y5
b6
y6
b7
y7
b8
y8
type
b
y

MFSD4B | +229.163SSLTEPTAEVYNQYPSNALVFESSPFNTGSAHVK+229.163

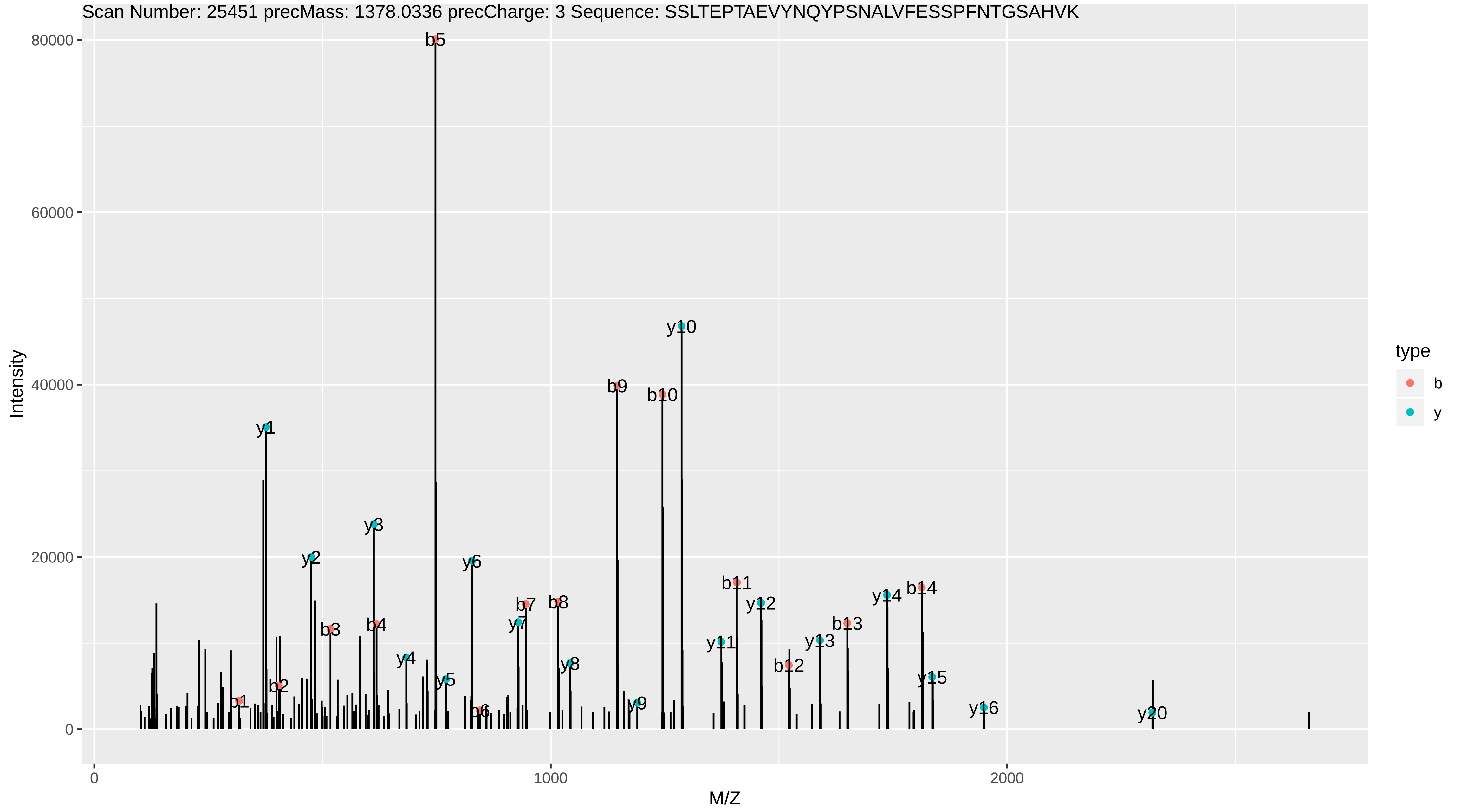

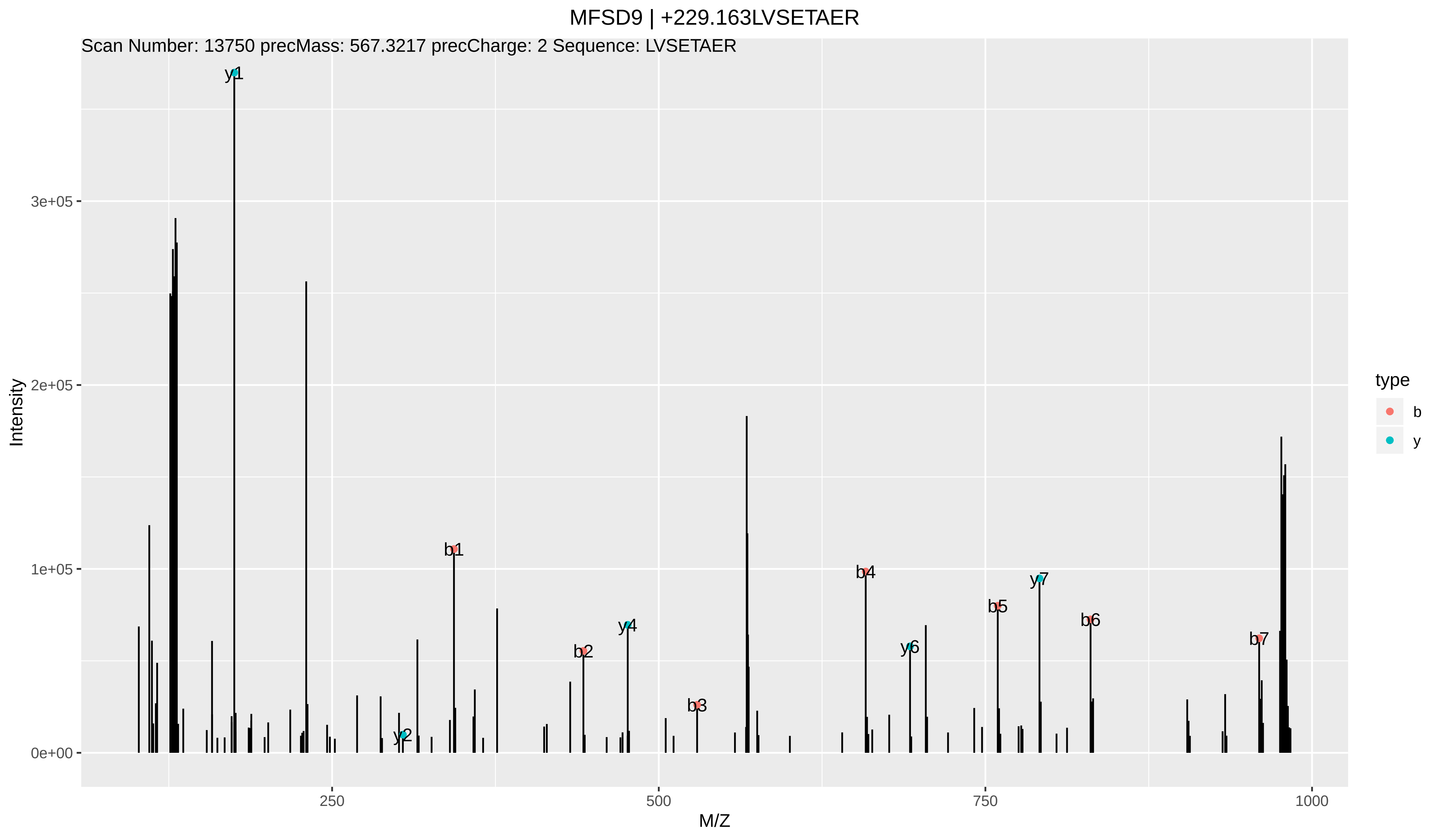
MFSD9 | +229.163LVSETAER
Scan Number: 13750 precMass: 567.3217 precCharge: 2 Sequence: LVSETAER
y1
b1
b2
y4
b3
b4
y6
b5
y7
b6
b7
y2
Intensity
3e+05
2e+05
1e+05
0e+00
M/Z
250
500
750
1000
type
b
y

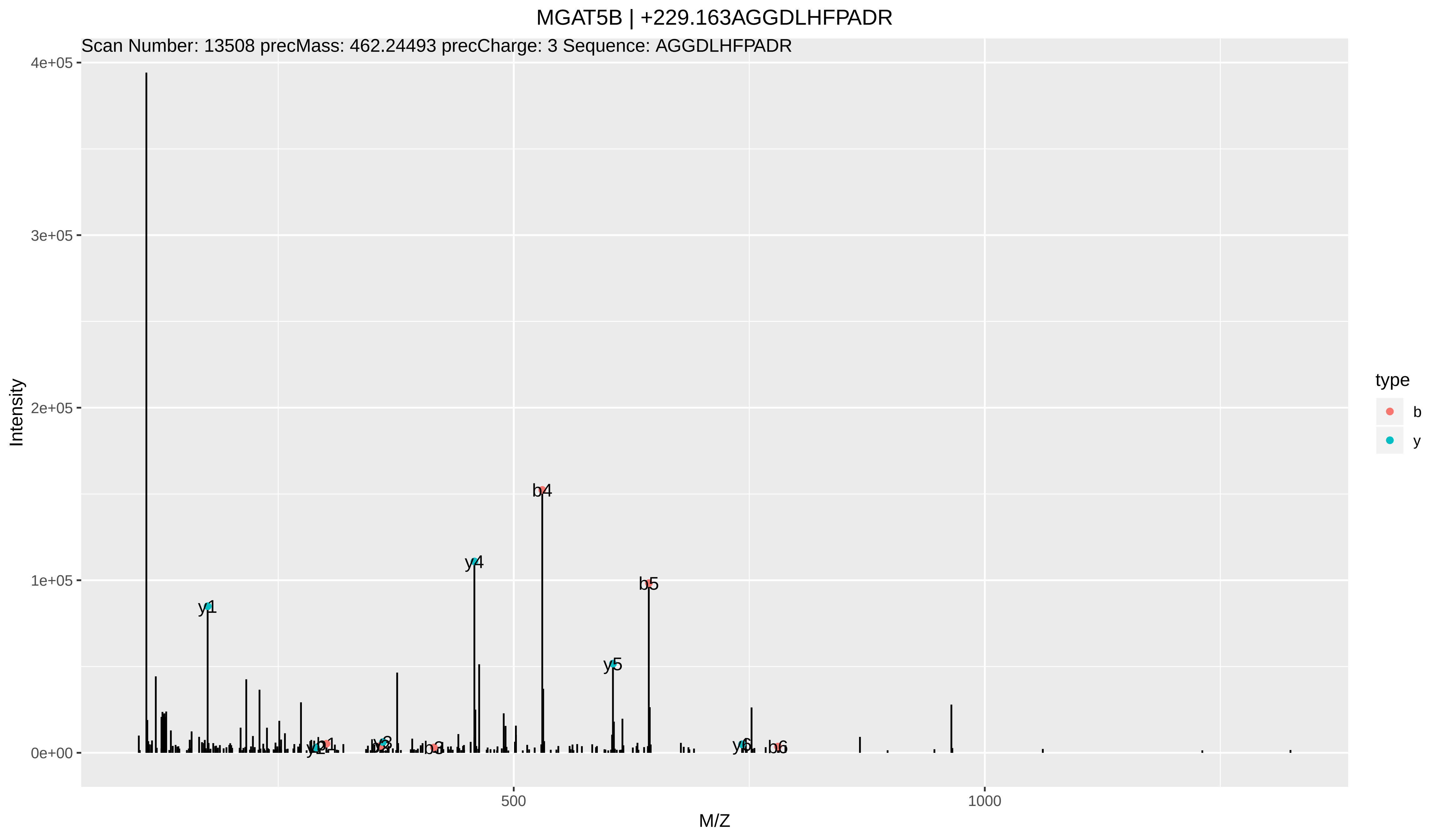

MGAT5B | +229.163AGGDLHFPADR
Scan Number: 13508 precMass: 462.24493 precCharge: 3 Sequence: AGGDLHFPADR
Intensity
4e+05
3e+05
2e+05
1e+05
0e+00
M/Z
500
1000
type
b
y
y1
y2
b1
y3
b2
b3
y4
b4
y5
b5
y6
b6

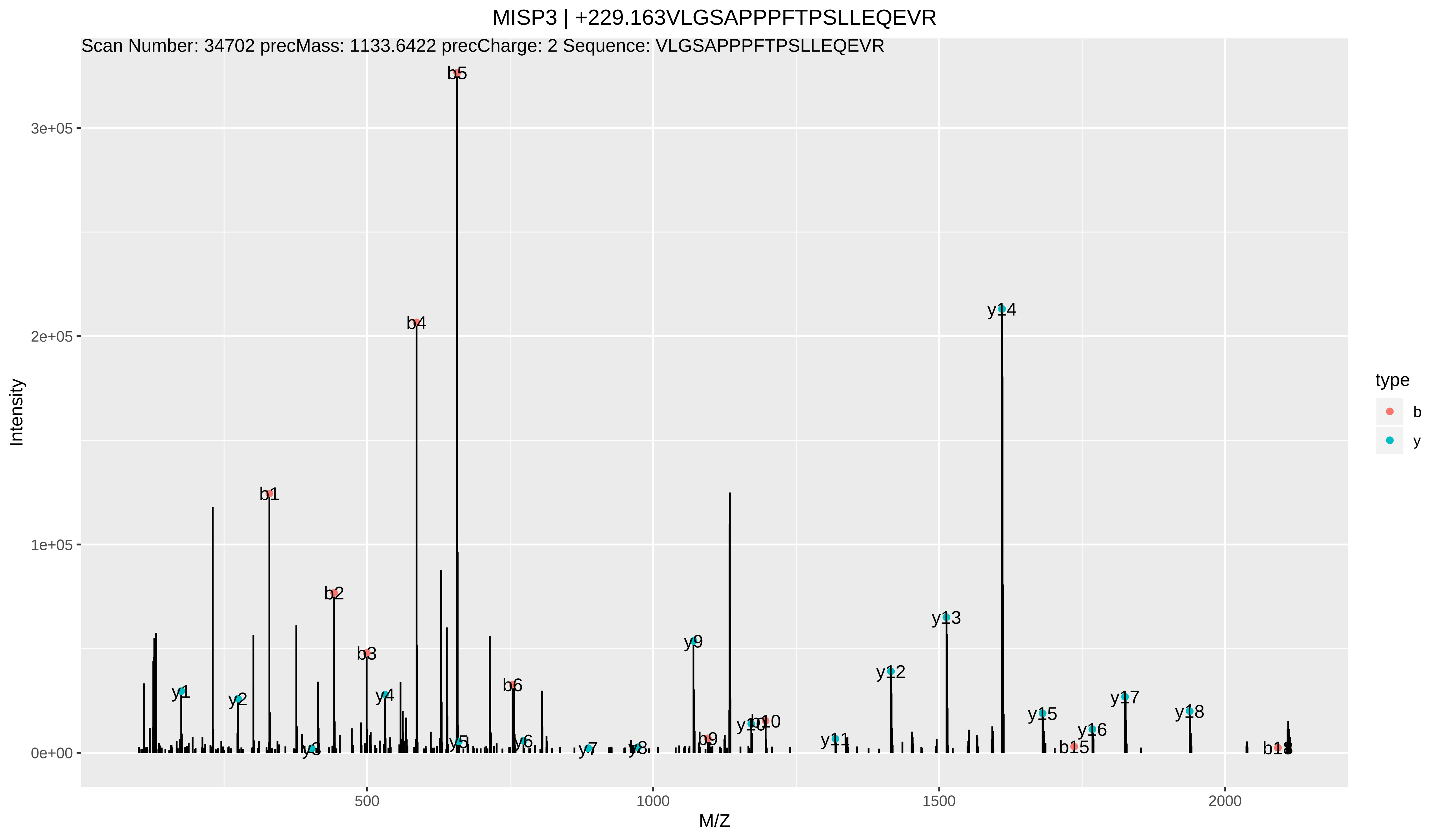

MISP3 | +229.163VLGSAPPPFTPSLLEQEVR
Scan Number: 34702 precMass: 1133.6422 precCharge: 2 Sequence: VLGSAPPPFTPSLLEQEVR
Intensity
M/Z
type
b
y
b1
b2
b3
b4
b5
b6
b9
b10
b15
b18
y1
y2
y3
y4
y5
y6
y7
y8
y9
y10
y11
y12
y13
y14
y15
y16
y17
y18
0e+00
1e+05
2e+05
3e+05
500
1000
1500
2000

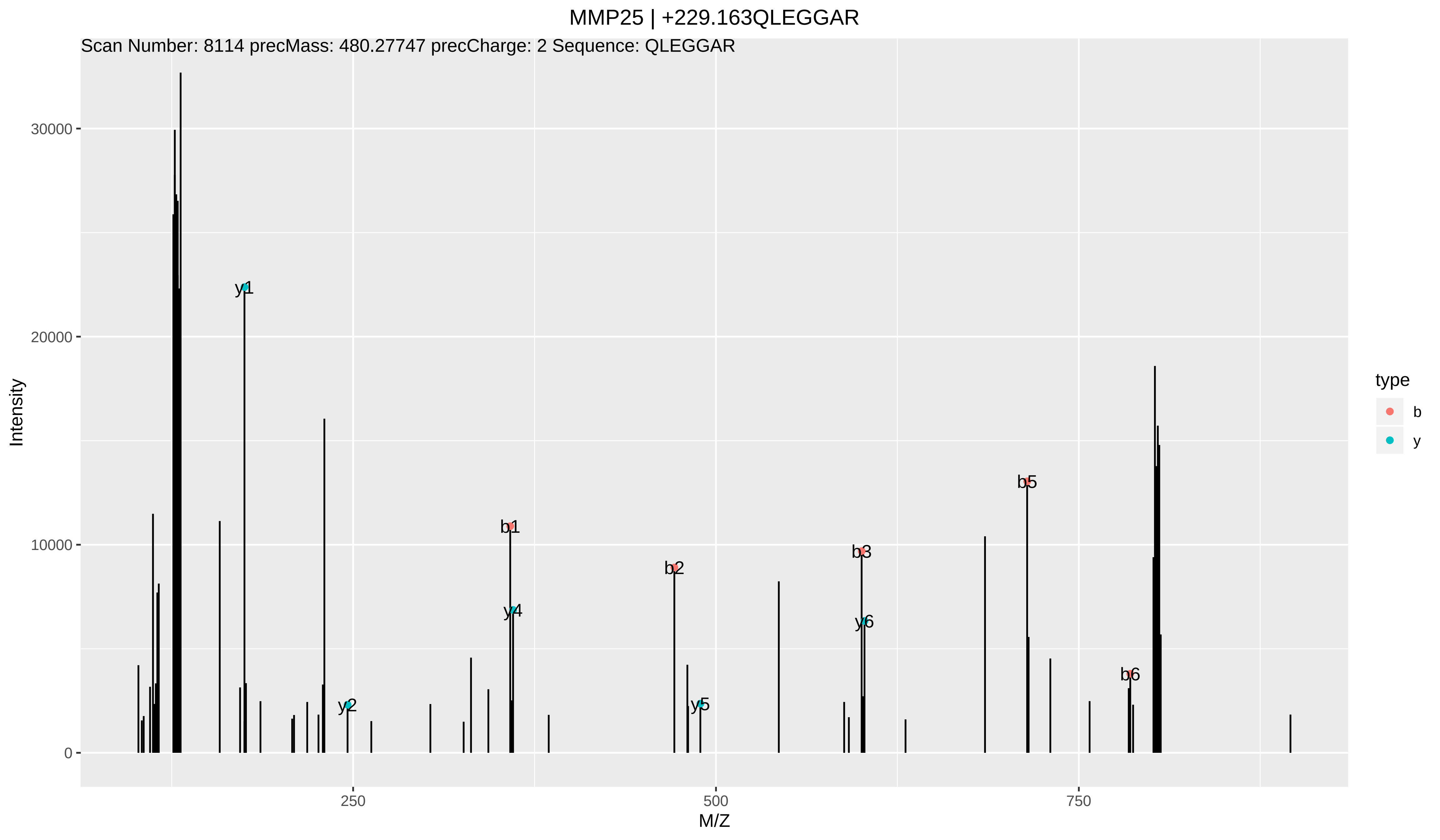
MMP25 | +229.163QLEGGAR
Scan Number: 8114 precMass: 480.27747 precCharge: 2 Sequence: QLEGGAR
Intensity
M/Z
0
10000
20000
30000
250
500
750
y1
y2
b1
y4
b2
y5
b3
y6
b5
b6
type
b
y

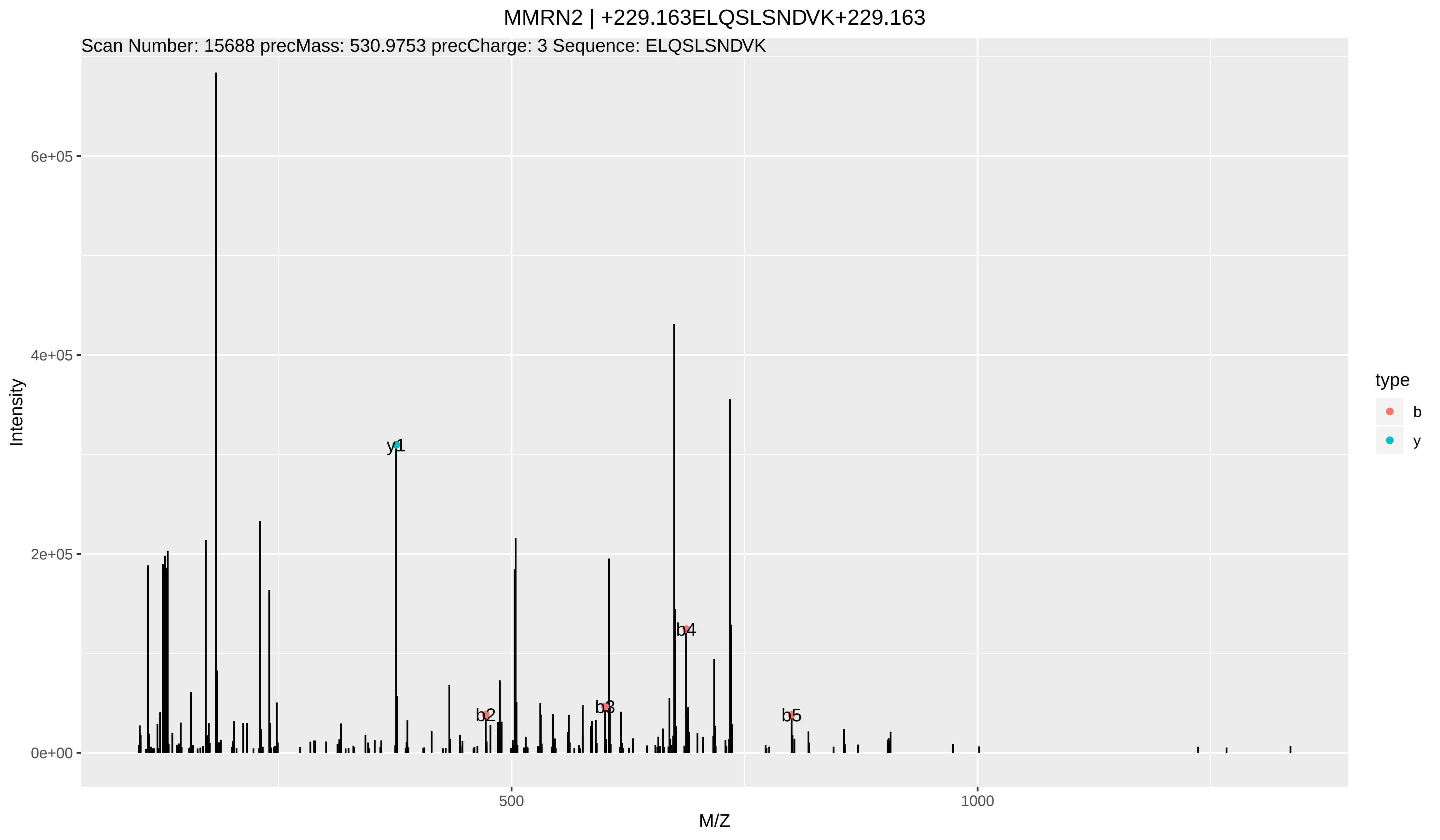

MMRN2 | +229.163ELQSLSNDVK+229.163
Scan Number: 15688 precMass: 530.9753 precCharge: 3 Sequence: ELQSLSNDVK
Intensity
6e+05
4e+05
2e+05
0e+00
M/Z
500
1000
y1
b2
b3
b4
b5
type
b
y

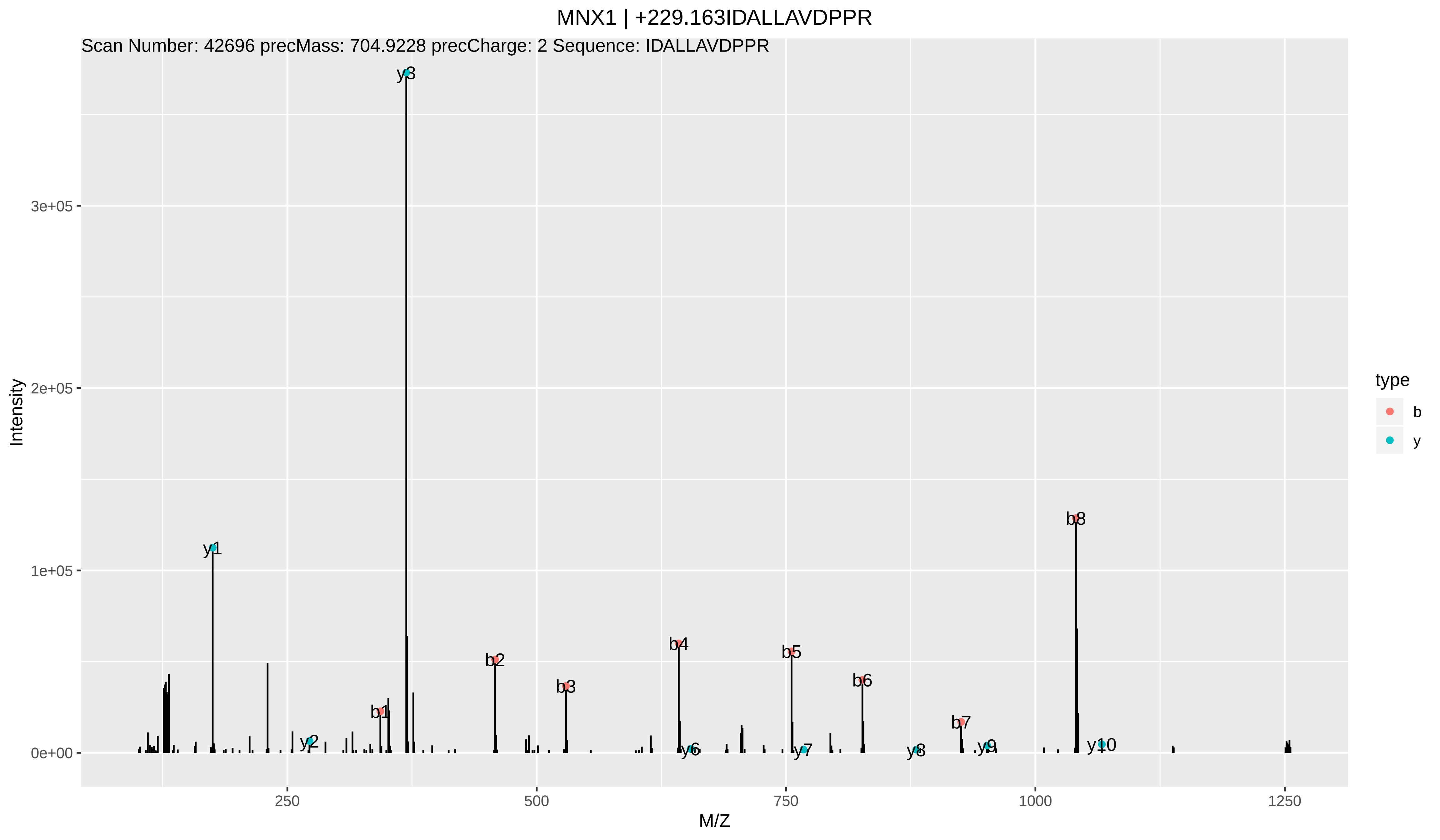

MNX1 | +229.163IDALLAVDPPR
Scan Number: 42696 precMass: 704.9228 precCharge: 2 Sequence: IDALLAVDPPR
y3
y1
b8
b4
b5
b2
b6
b3
b1
b7
y2
y6
y7
y8
y9
y10
Intensity
3e+05
2e+05
1e+05
0e+00
M/Z
250
500
750
1000
1250
type
b
y

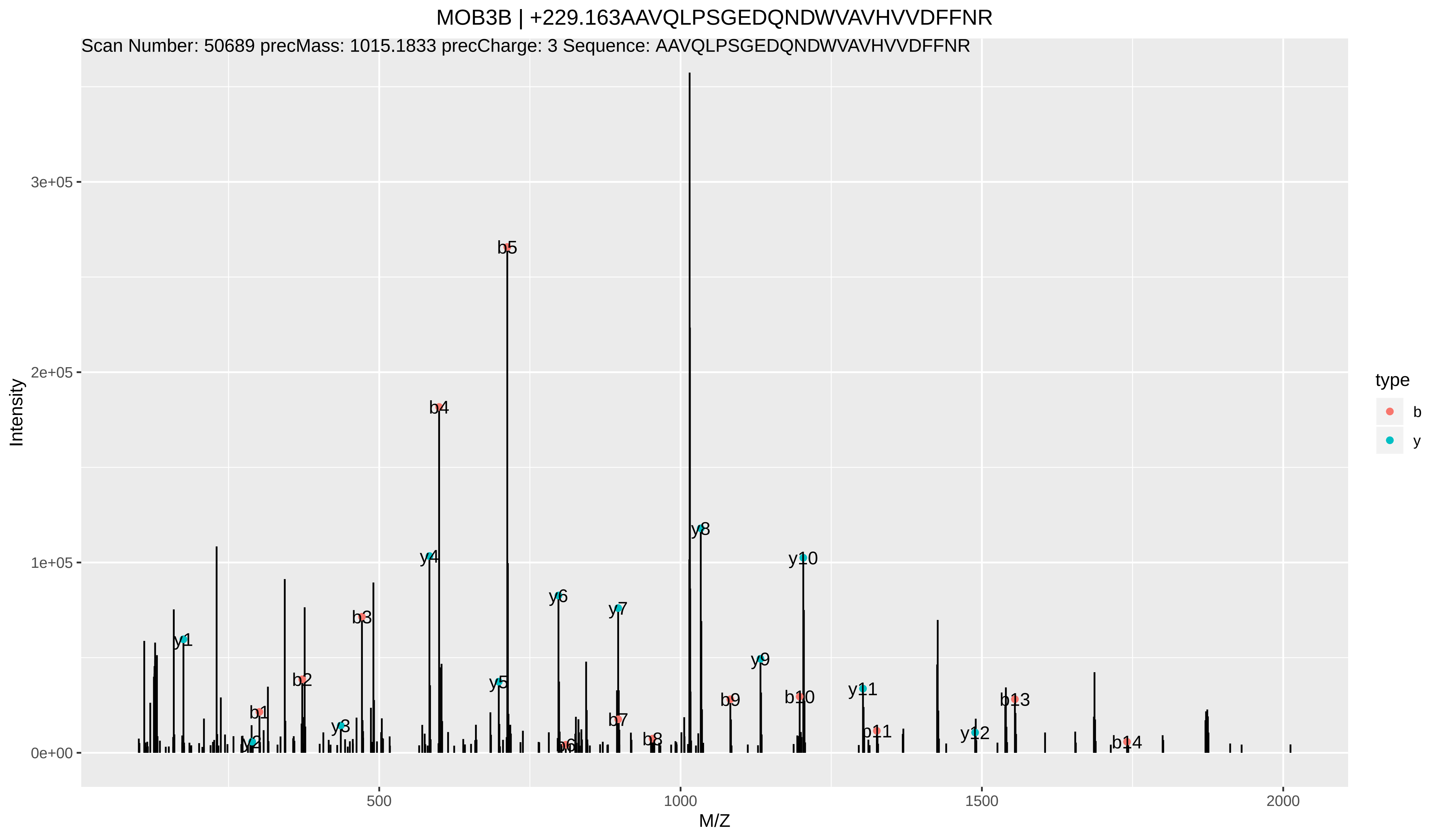

MOB3B | +229.163AAVQLPSGEDQNDWVAVHVVDFFNR
Scan Number: 50689 precMass: 1015.1833 precCharge: 3 Sequence: AAVQLPSGEDQNDWVAVHVVDFFNR
Intensity
M/Z
3e+05
2e+05
1e+05
0e+00
500
1000
1500
2000
type
b
y
b1
b2
b3
b4
b5
b6
b7
b8
b9
b10
b11
b13
b14
y1
y2
y3
y4
y5
y6
y7
y8
y9
y10
y11
y12

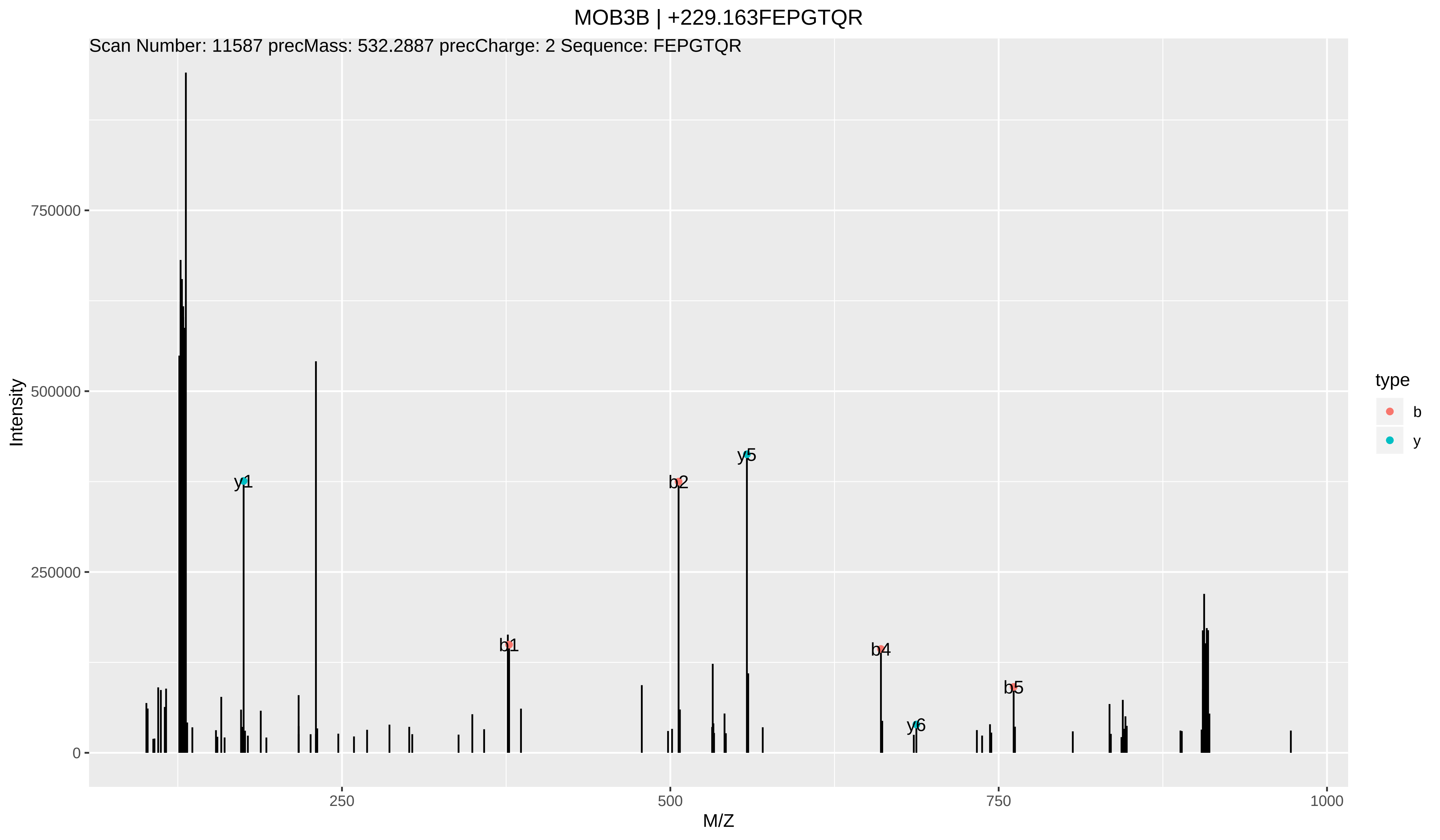
MOB3B | +229.163FEPGTQR
Scan Number: 11587 precMass: 532.2887 precCharge: 2 Sequence: FEPGTQR
Intensity
750000
500000
250000
0
y1
b1
b2
y5
b4
y6
b5
M/Z
250
500
750
1000
type
b
y

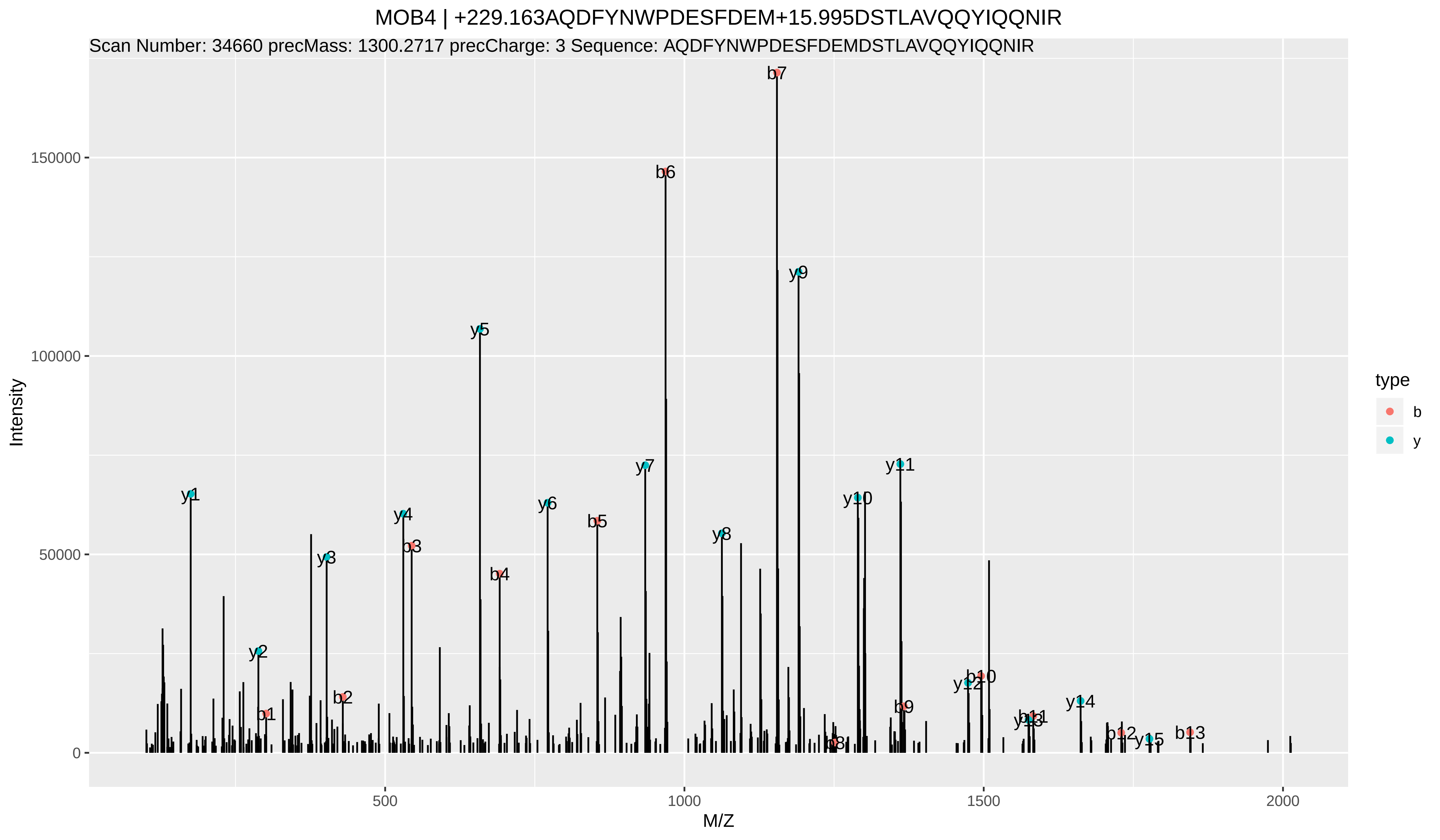

MOB4 | +229.163AQDFYNWPDESFDEM+15.995DSTLAVQQYIQQNIR
Scan Number: 34660 precMass: 1300.2717 precCharge: 3 Sequence: AQDFYNWPDESFDEMDSTLAVQQYIQQNIR
Intensity
0
50000
100000
150000
500
1000
1500
2000
M/Z
type
b
y
b7
b6
y9
y5
y11
y1
y10
y7
y6
y4
b5
y8
b3
y3
b4
y2
b10
y12
b2
b1
b9
y14
b11
y13
b12
b13
y15
b8

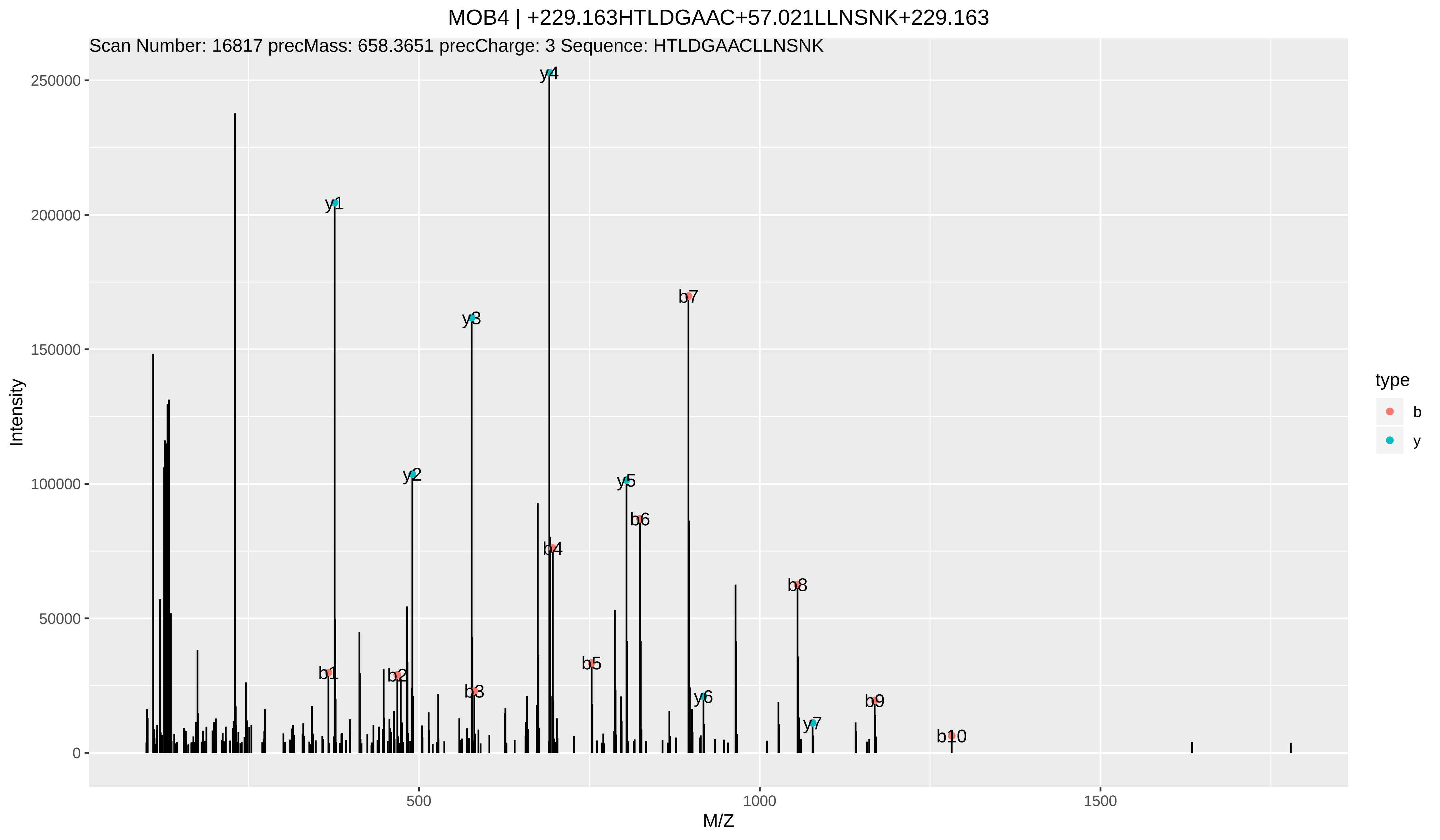
MOB4 | +229.163HTLDGAAC+57.021LLNSNK+229.163
Scan Number: 16817 precMass: 658.3651 precCharge: 3 Sequence: HTLDGAACLLNSNK
Intensity
250000
200000
150000
100000
50000
0
M/Z
500
1000
1500
y1
y2
y3
y4
y5
y6
y7
b1
b2
b3
b4
b5
b6
b7
b8
b9
b10
type
b
y

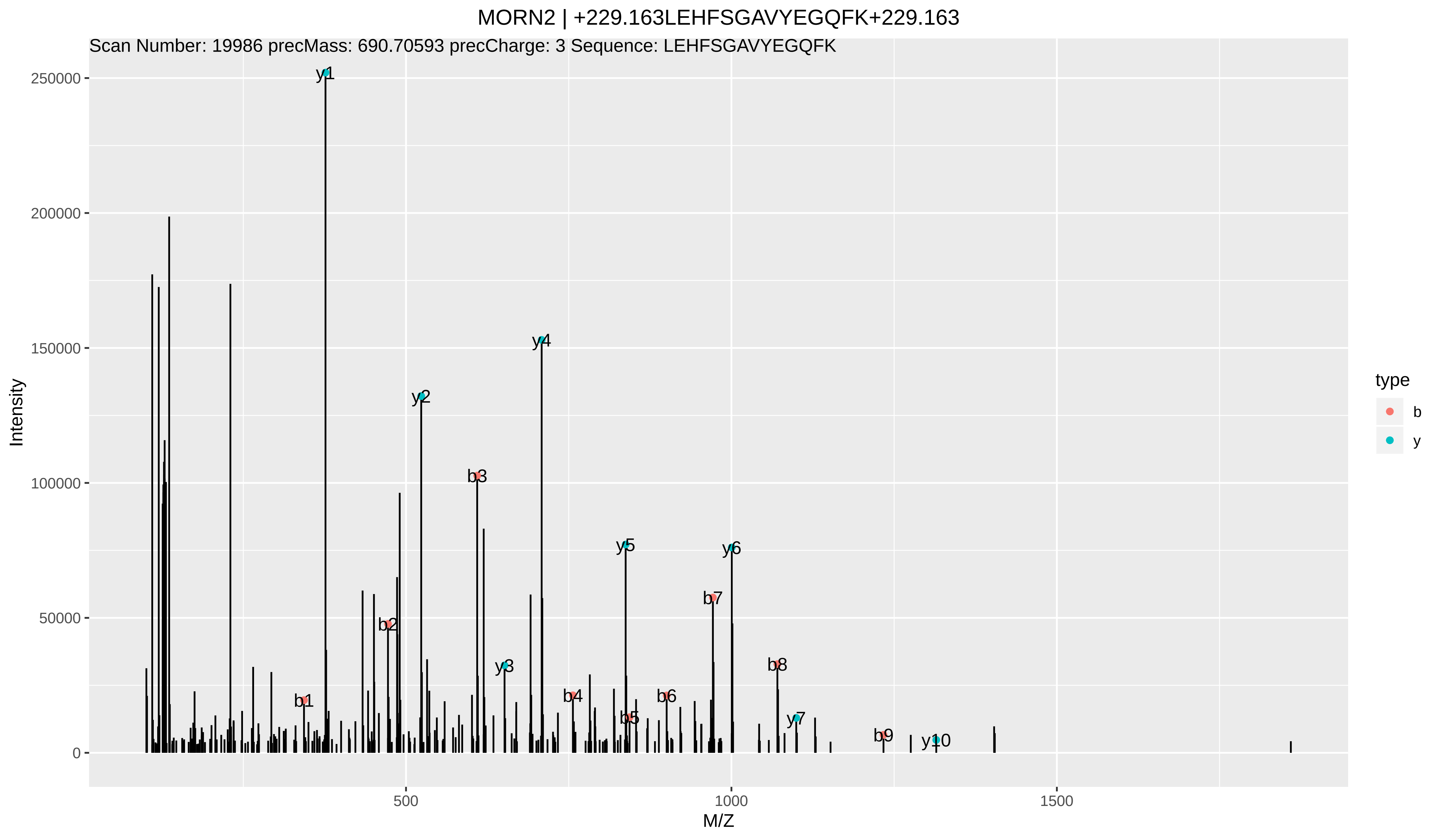

MORN2 | +229.163LEHFSGAVYEGQFK+229.163
Scan Number: 19986 precMass: 690.70593 precCharge: 3 Sequence: LEHFSGAVYEGQFK
Intensity
M/Z
0
50000
100000
150000
200000
250000
500
1000
1500
y1
y2
y3
y4
y5
y6
y7
y10
b1
b2
b3
b4
b5
b6
b7
b8
b9
type
b
y

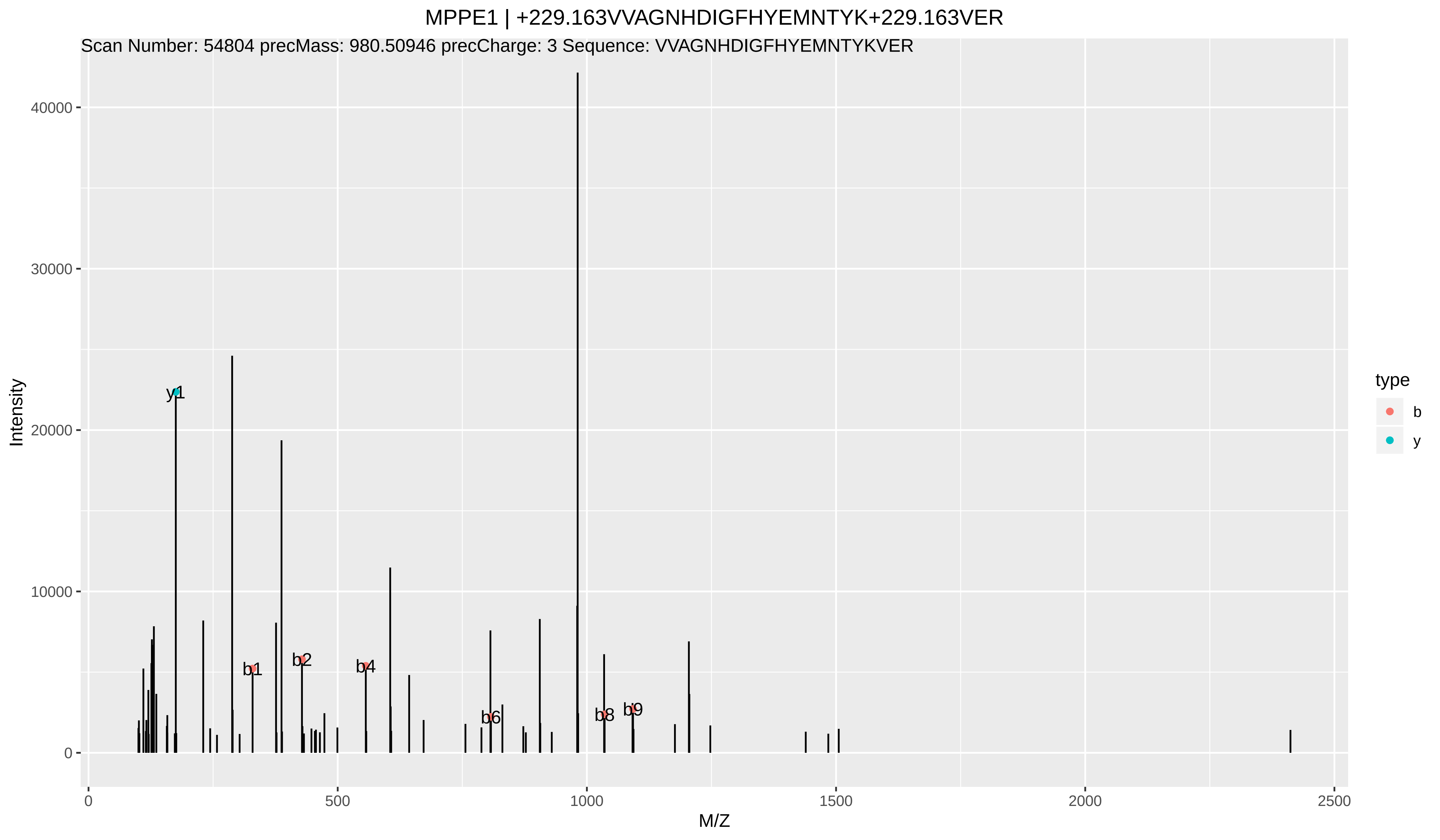
MPPE1 | +229.163VVAGNHDIGFHYEMNTYK+229.163VER
Scan Number: 54804 precMass: 980.50946 precCharge: 3 Sequence: VVAGNHDIGFHYEMNTYKVER
Intensity
M/Z
type
b
y
y1
b1
b2
b4
b6
b8
b9
0
10000
20000
30000
40000
500
1000
1500
2000
2500

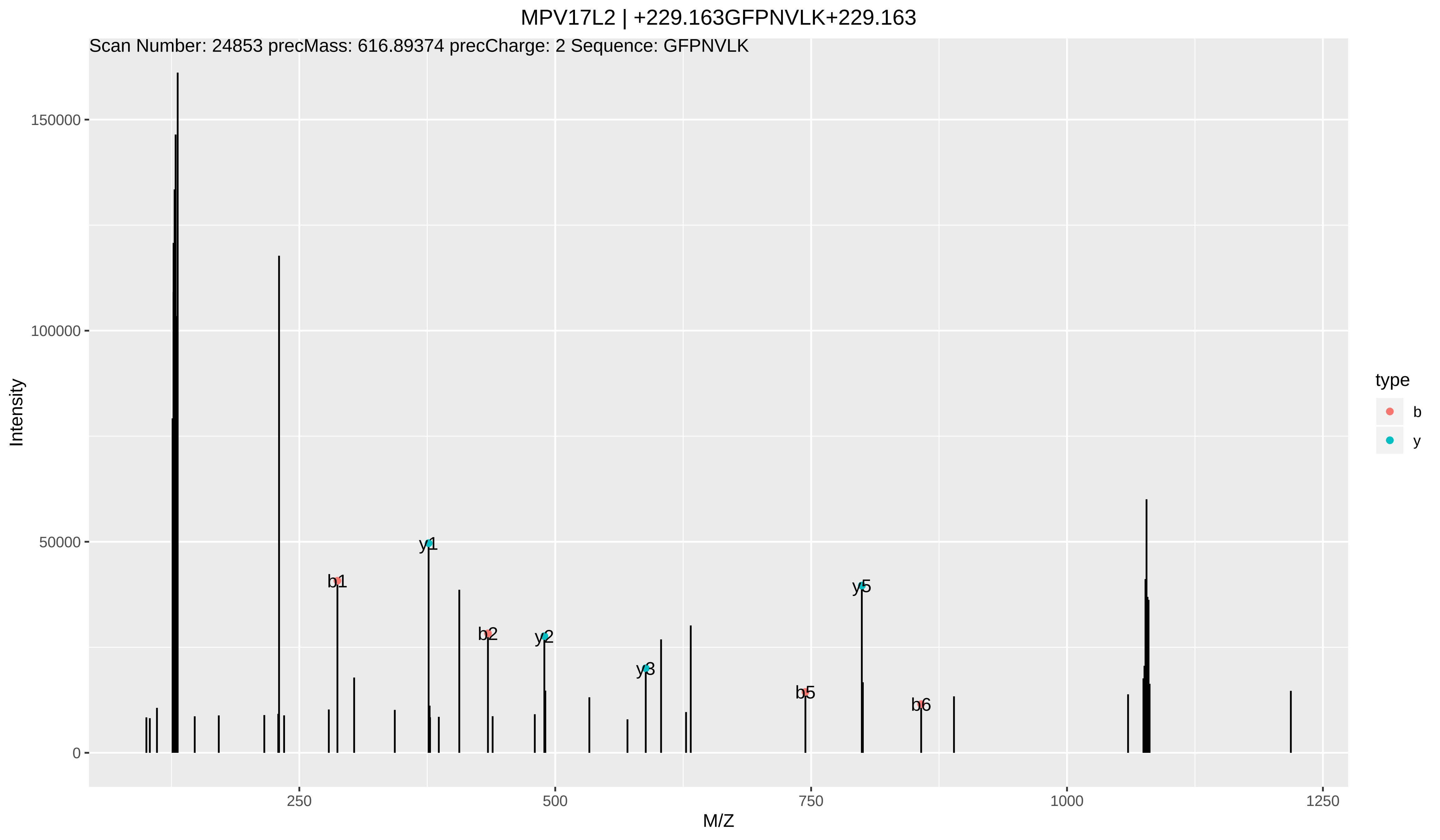
MPV17L2 | +229.163GFPNVLK+229.163
Scan Number: 24853 precMass: 616.89374 precCharge: 2 Sequence: GFPNVLK
Intensity
150000
100000
50000
0
M/Z
250
500
750
1000
1250
b1
y1
b2
y2
y3
b5
y5
b6
type
b
y

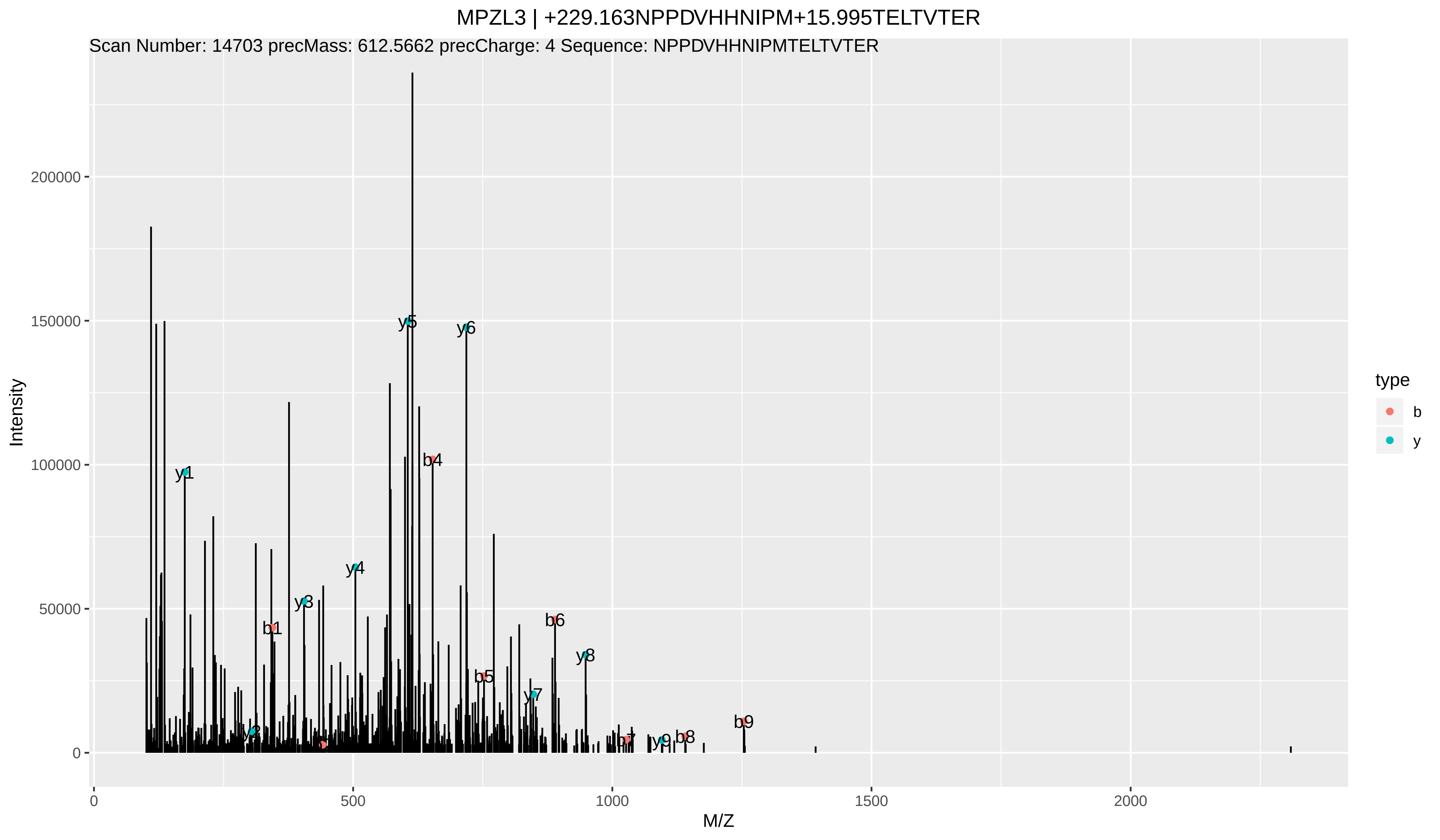
MPZL3 | +229.163NPPDVHHNIPM+15.995TELTVTER
Scan Number: 14703 precMass: 612.5662 precCharge: 4 Sequence: NPPDVHHNIPMTELTVTER
Intensity
M/Z
0
50000
100000
150000
200000
0
500
1000
1500
2000
y1
y2
y3
y4
y5
y6
y7
y8
y9
b1
b4
b5
b6
b7
b8
b9
type
b
y

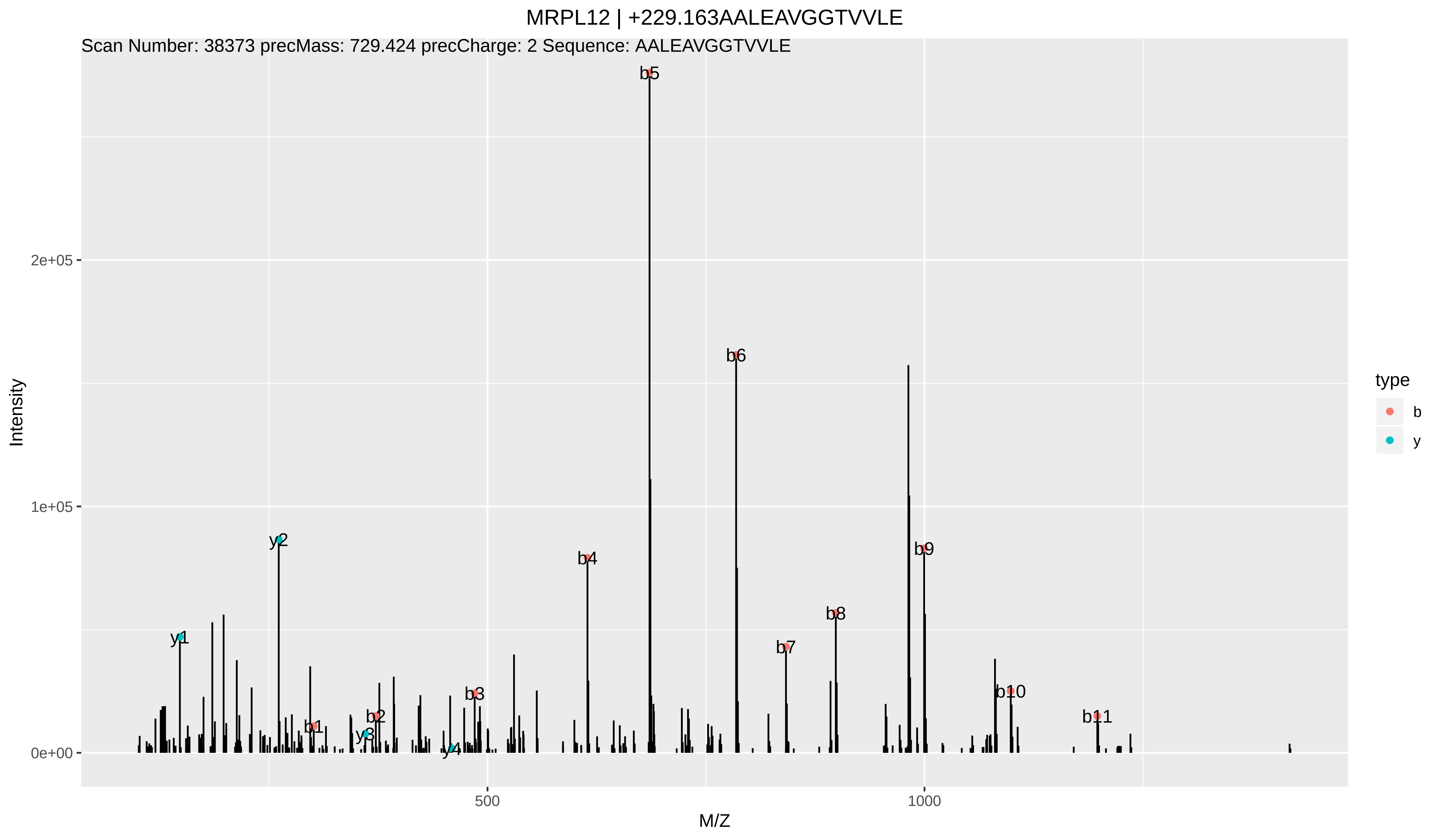

MRPL12 | +229.163AALEAVGGTVVLE
Scan Number: 38373 precMass: 729.424 precCharge: 2 Sequence: AALEAVGGTVVLE
b5
b6
b9
b4
y2
b8
b7
y1
b3
b10
b2
b11
b1
y3
y4
2e+05
1e+05
0e+00
Intensity
500
1000
M/Z
type
b
y

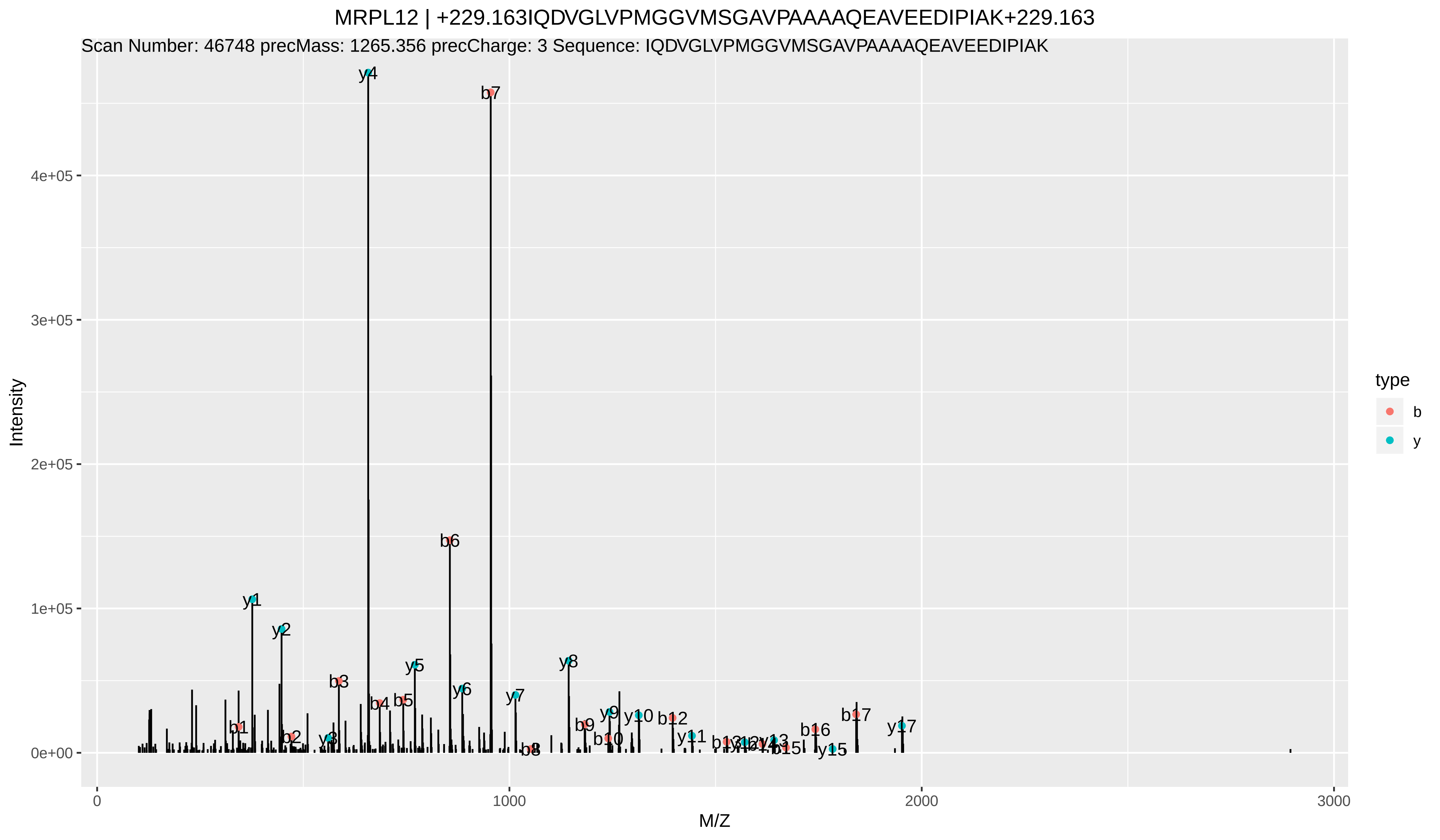
MRPL12 | +229.163IQDVGLVPMGGVMSGAVPAAAAQEAVEEDIPIAK+229.163
Scan Number: 46748 precMass: 1265.356 precCharge: 3 Sequence: IQDVGLVPMGGVMSGAVPAAAAQEAVEEDIPIAK
Intensity
M/Z
type
b
y
y4
b7
b6
y1
y2
y5
b3
y8
y6
b4
b5
y7
b17
y9
y10
b12
b9
b1
b2
y3
b10
y11
b16
y17
b15
y15
b8
0e+00
1e+05
2e+05
3e+05
4e+05
0
1000
2000
3000

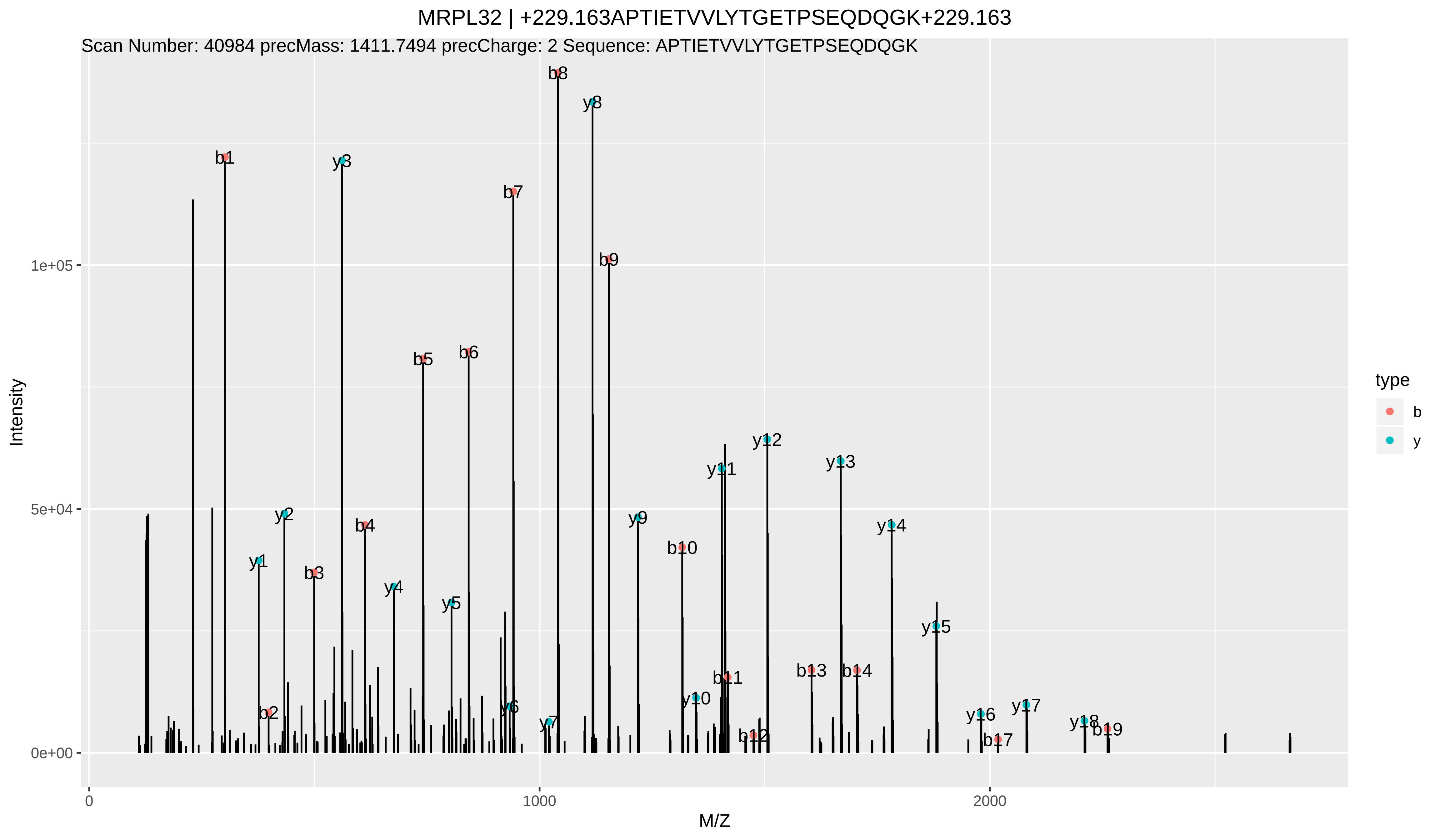

MRPL32 | +229.163APTIETVVLYTGETPSEQDQGK+229.163
Scan Number: 40984 precMass: 1411.7494 precCharge: 2 Sequence: APTIETVVLYTGETPSEQDQGK
Intensity
M/Z
0e+00
5e+04
1e+05
0
1000
2000
b8
y8
b1
y3
b7
b9
b5
b6
y12
y11
y13
y9
y2
b4
y14
b10
y1
b3
y4
y5
y15
b13
b14
b11
y10
y6
y17
y16
y7
y18
b19
b12
b17
type
b
y

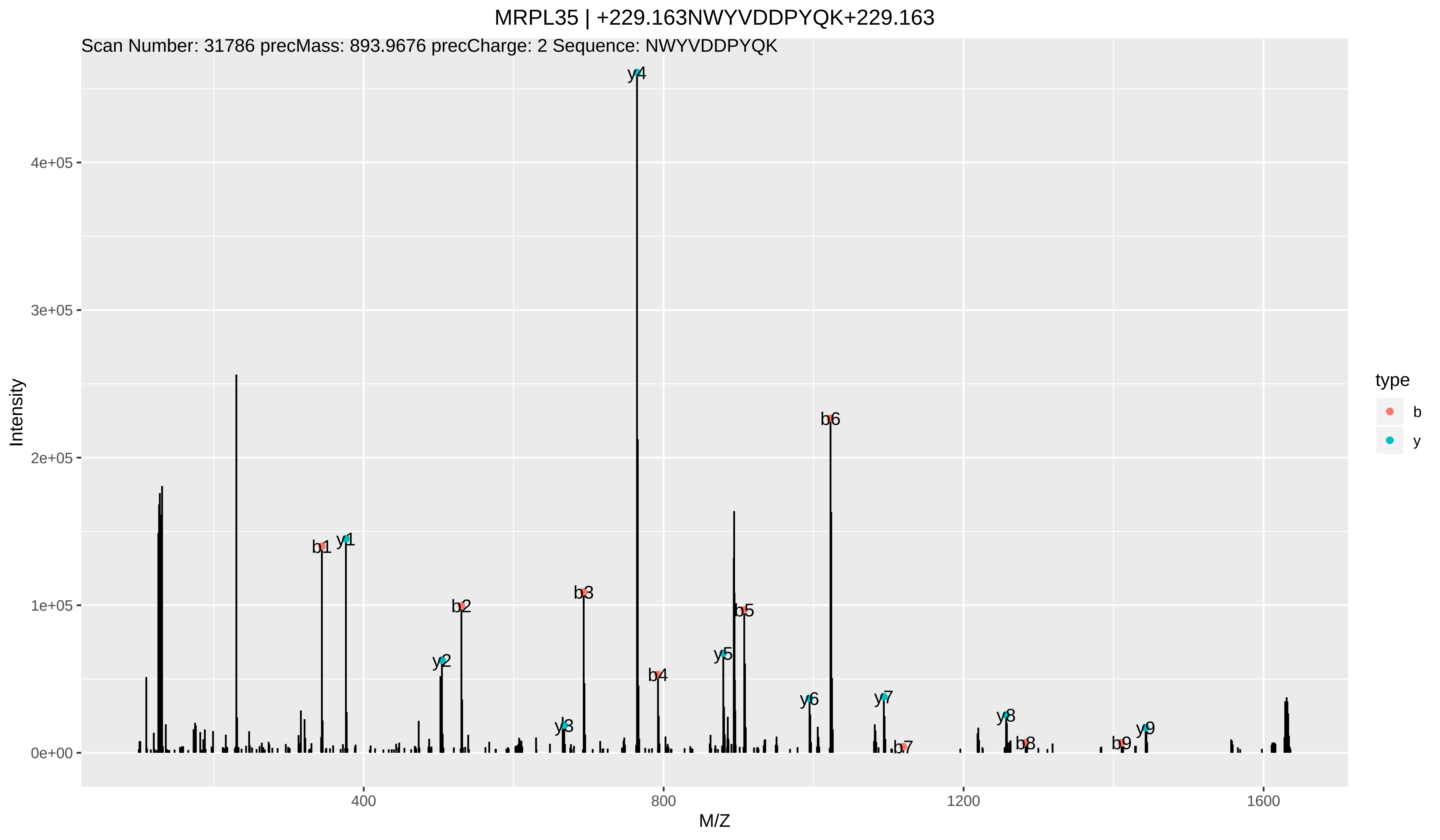
MRPL35 | +229.163NWYVDDPYQK+229.163
Scan Number: 31786 precMass: 893.9676 precCharge: 2 Sequence: NWYVDDPYQK
y4
b6
b1
y1
b2
b3
b5
y2
y5
b4
y6
y7
y3
y8
y9
b8
b9
b7
4e+05
3e+05
2e+05
1e+05
0e+00
Intensity
400
800
1200
1600
M/Z
type
b
y

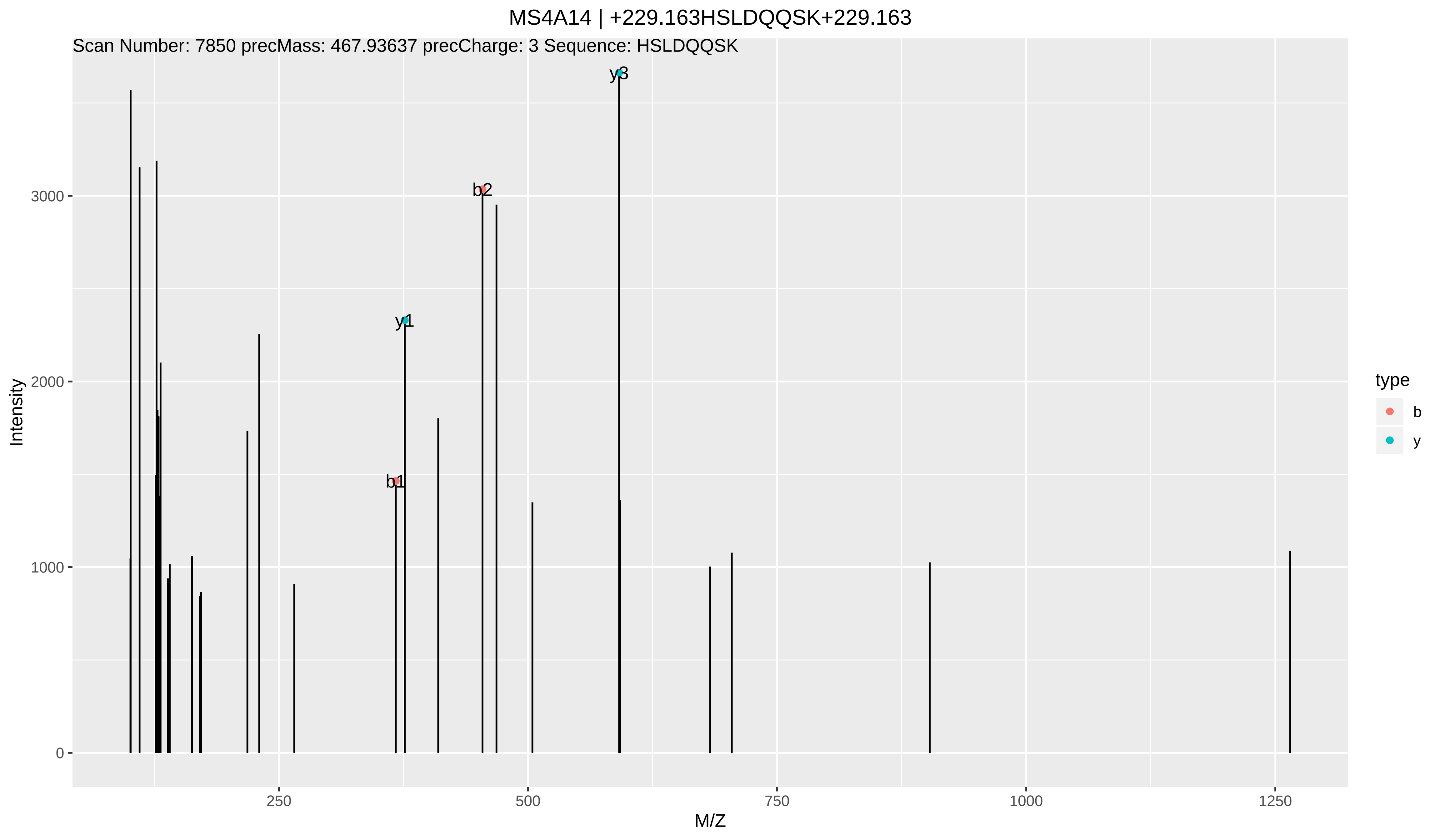

MS4A14 | +229.163HSLDQQSK+229.163
Scan Number: 7850 precMass: 467.93637 precCharge: 3 Sequence: HSLDQQSK
y3
b2
y1
b1
Intensity
3000
2000
1000
0
250
500
750
1000
1250
M/Z
type
b
y

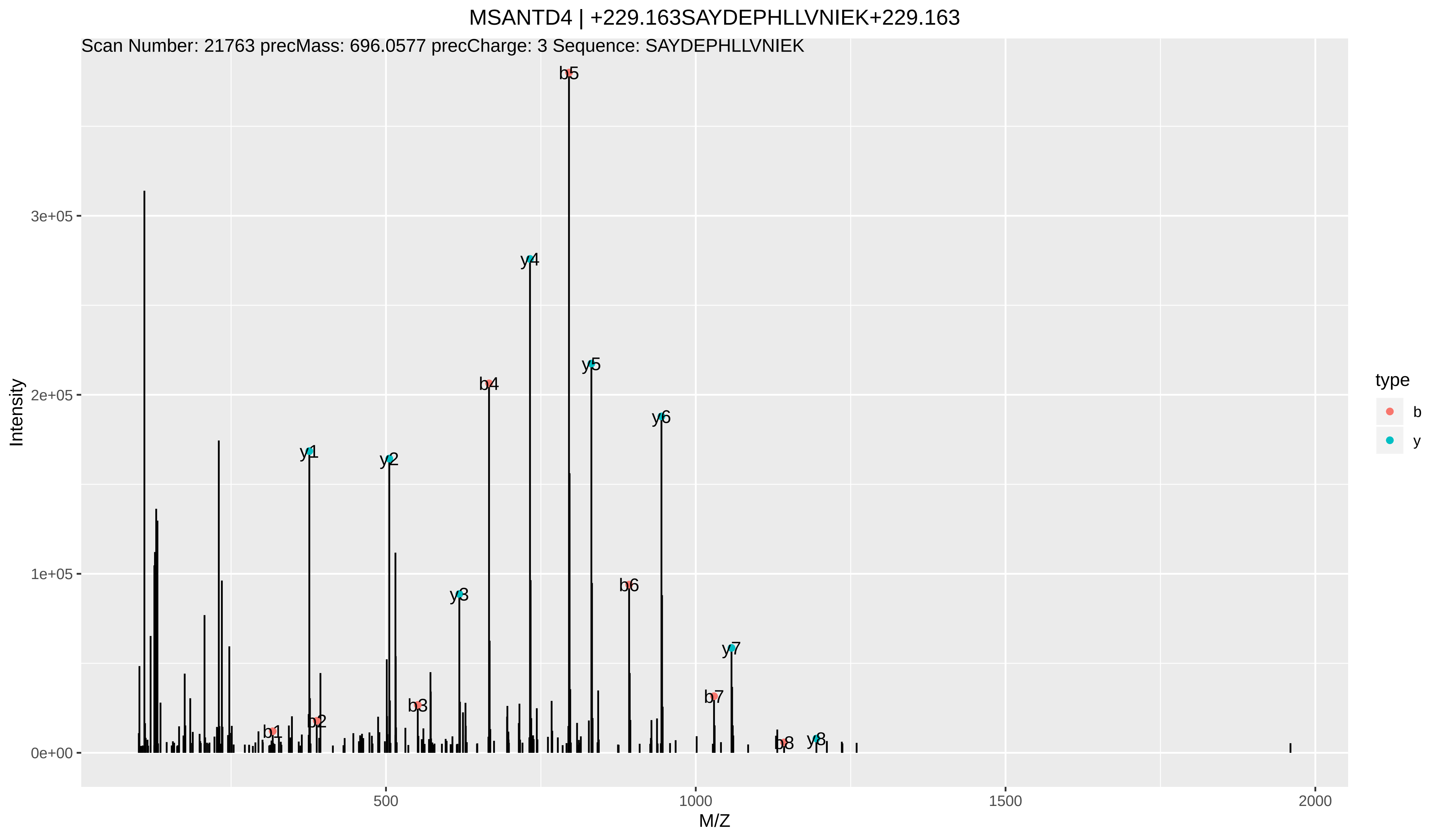
MSANTD4 | +229.163SAYDEPHLLVNIEK+229.163
Scan Number: 21763 precMass: 696.0577 precCharge: 3 Sequence: SAYDEPHLLVNIEK
Intensity
M/Z
type
b
y
b1
b2
b3
b4
b5
b6
b7
b8
y1
y2
y3
y4
y5
y6
y7
y8
0e+00
1e+05
2e+05
3e+05
500
1000
1500
2000

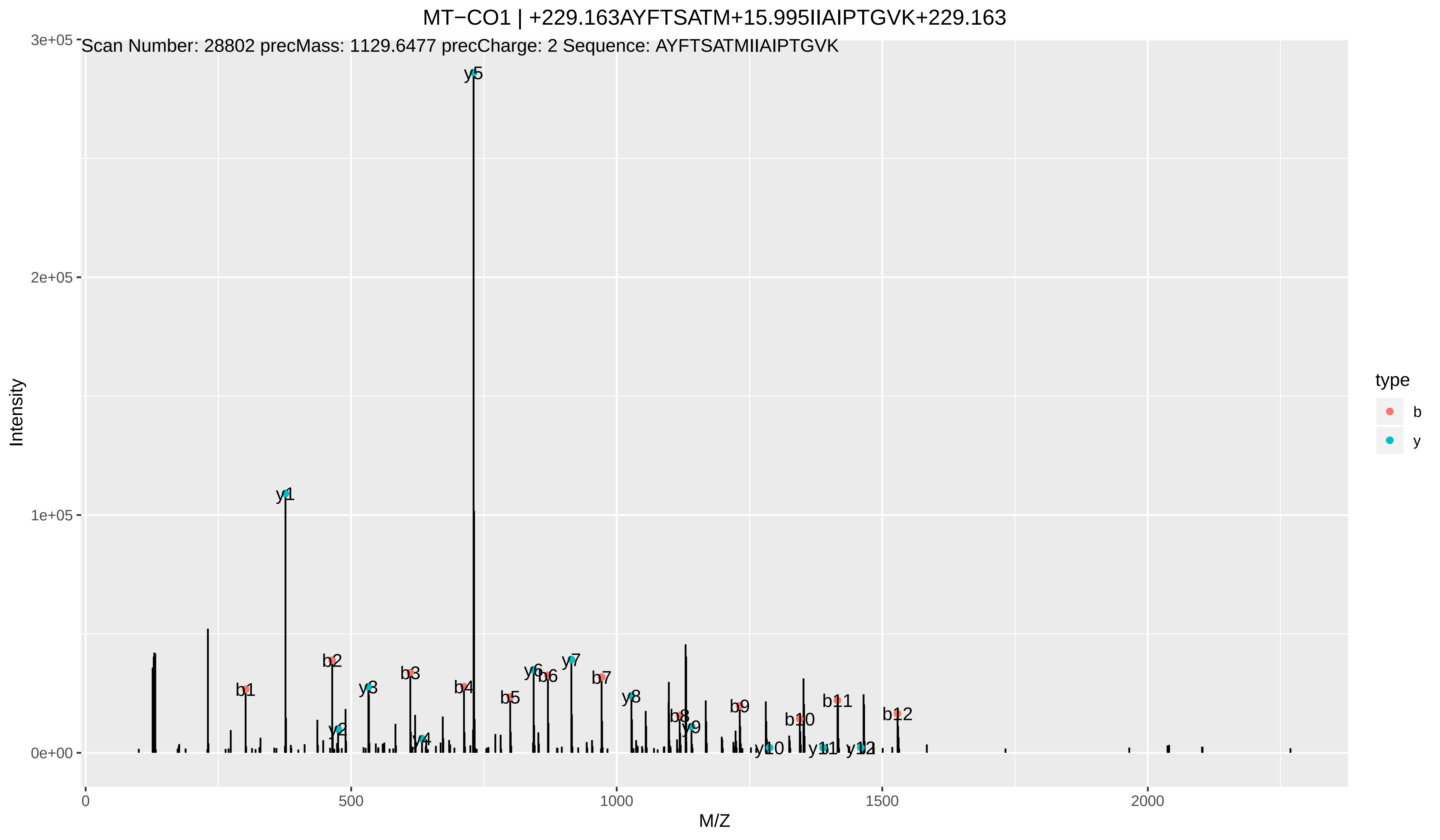

MT−CO1 | +229.163AYFTSATM+15.995IIAIPTGVK+229.163
Scan Number: 28802 precMass: 1129.6477 precCharge: 2 Sequence: AYFTSATMIIAIPTGVK
Intensity
M/Z
type
b
y
y5
y1
b1
b2
y2
y3
b3
y4
b4
b5
y6
b6
y7
b7
y8
b8
y9
b9
y10
b10
y11
b11
y12
b12

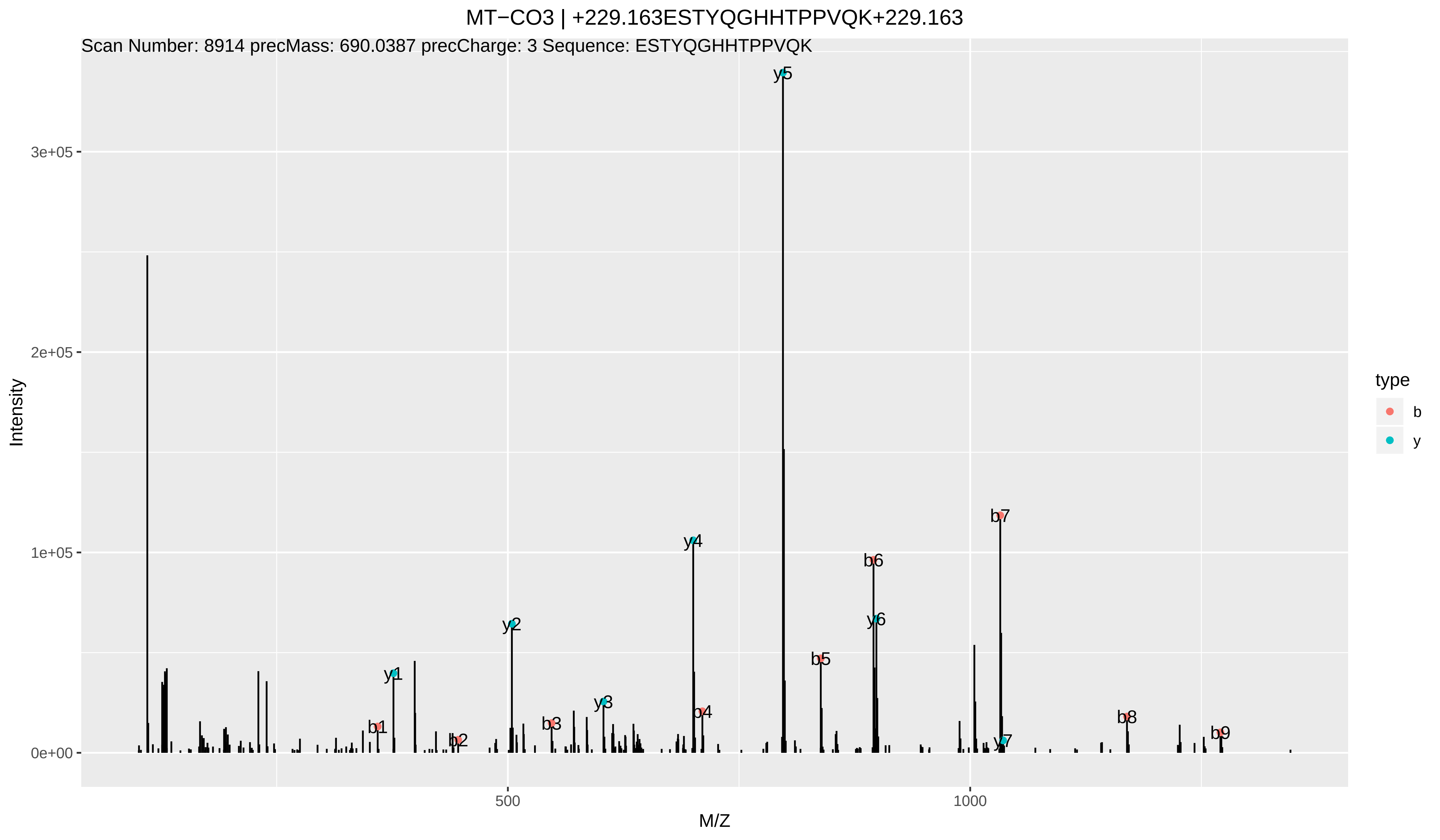
MT−CO3 | +229.163ESTYQGHHTPPVQK+229.163
Scan Number: 8914 precMass: 690.0387 precCharge: 3 Sequence: ESTYQGHHTPPVQK
Intensity
3e+05
2e+05
1e+05
0e+00
M/Z
500
1000
y5
b7
y4
b6
y6
y2
b5
y1
y3
b4
b1
b3
b8
b2
y7
b9
type
b
y

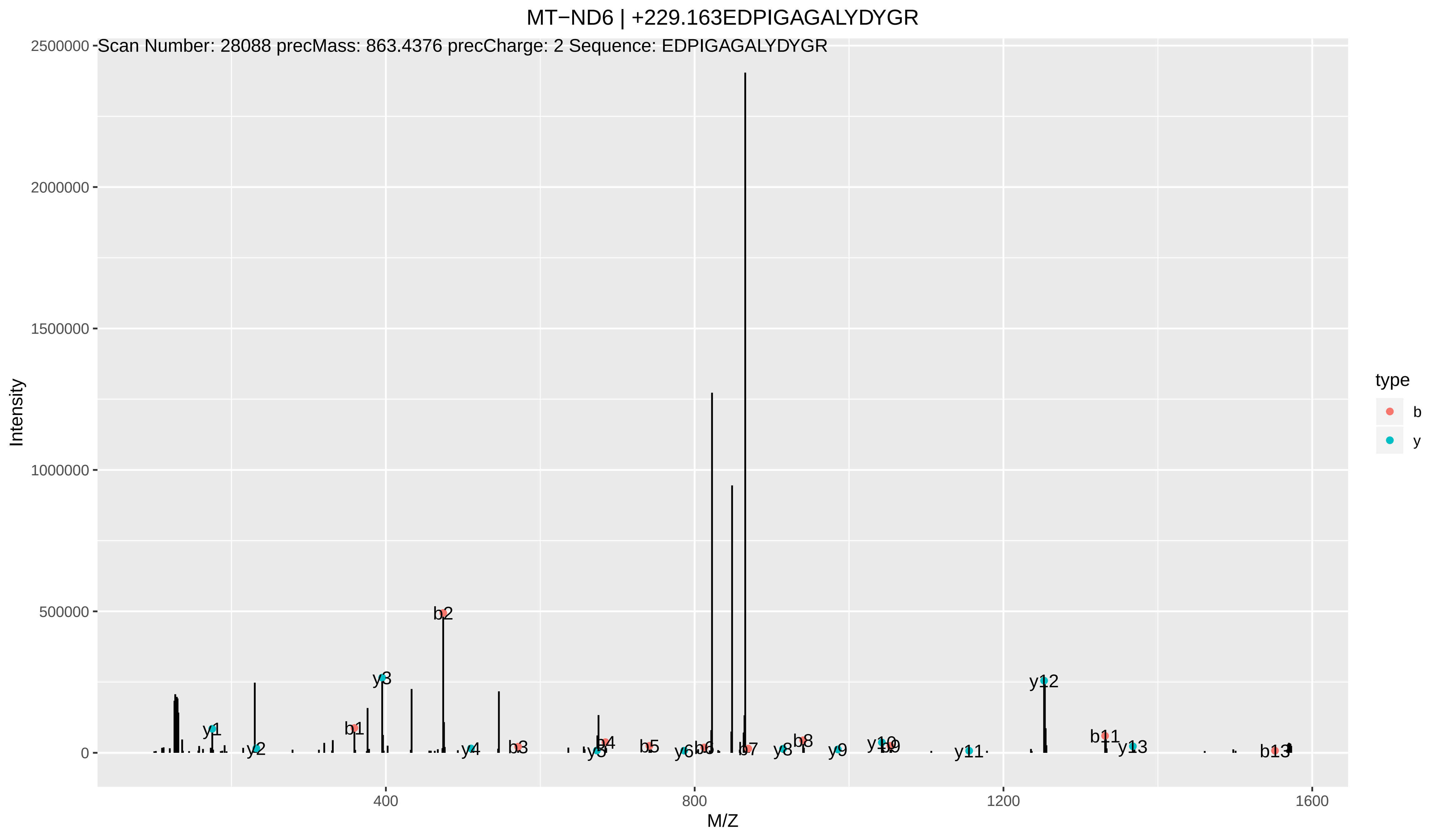

MT−ND6 | +229.163EDPIGAGALYDYGR
Scan Number: 28088 precMass: 863.4376 precCharge: 2 Sequence: EDPIGAGALYDYGR
Intensity
M/Z
type
b
y
0
500000
1000000
1500000
2000000
2500000
400
800
1200
1600
y1
y2
y3
y4
y5
y6
y8
y9
y10
y11
y12
y13
b1
b2
b3
b4
b5
b6
b7
b8
b9
b11
b13

MTF1 | +229.163YFTTLSDLRK+229.163

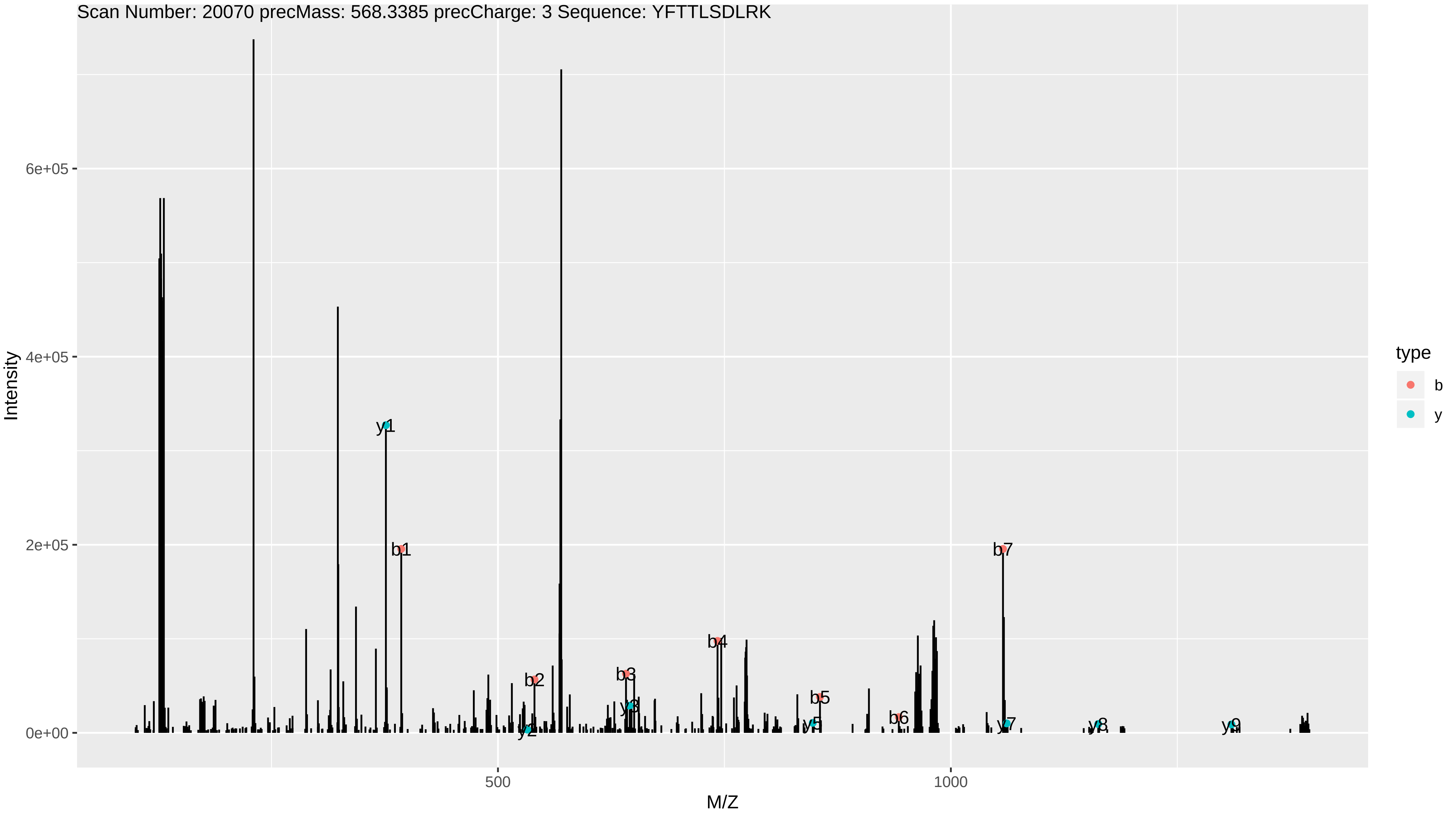

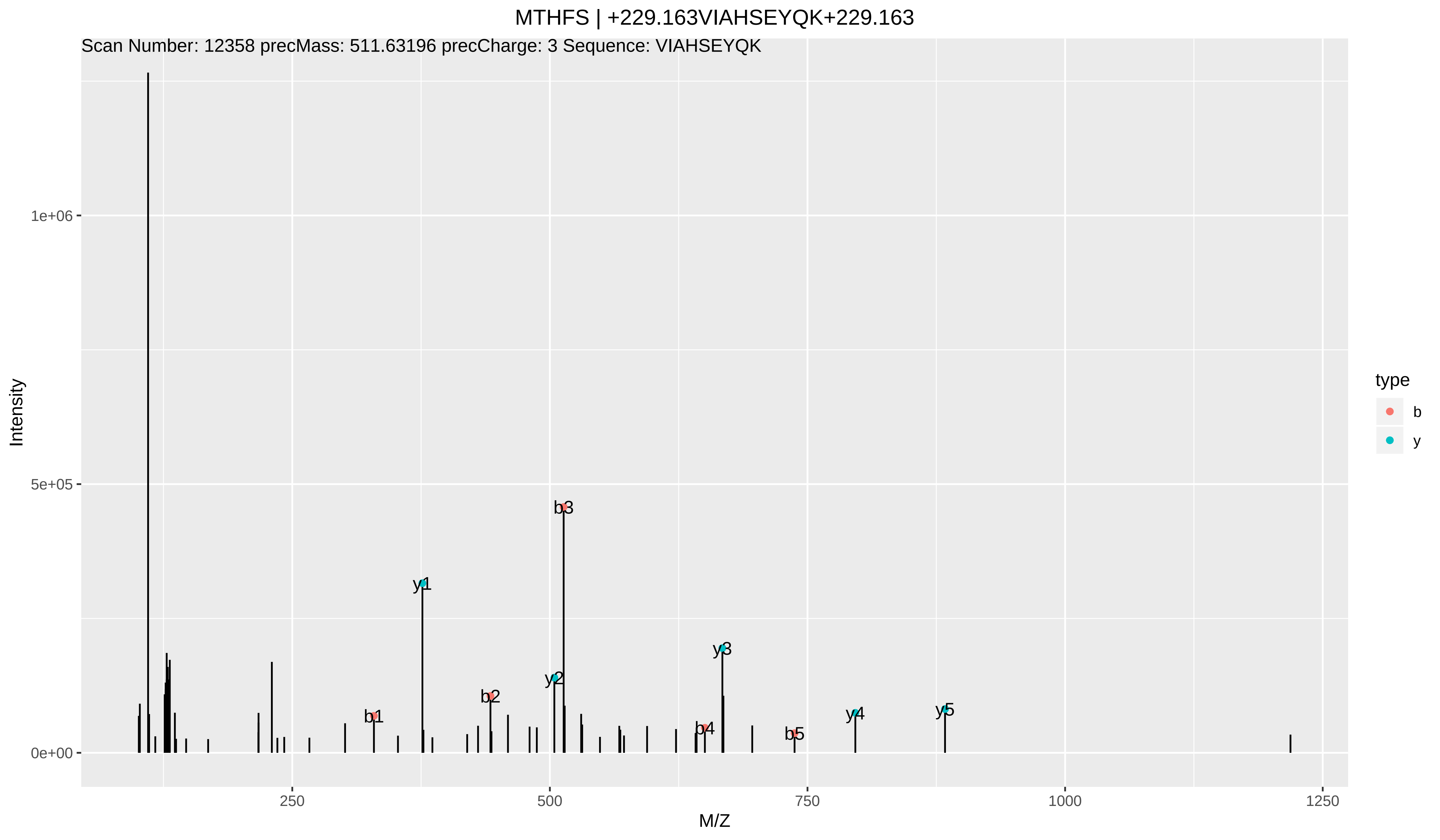
MTHFS | +229.163VIAHSEYQK+229.163
Scan Number: 12358 precMass: 511.63196 precCharge: 3 Sequence: VIAHSEYQK
Intensity
1e+06
5e+05
0e+00
M/Z
250
500
750
1000
1250
b1
b2
b3
b4
b5
y1
y2
y3
y4
y5
type
b
y

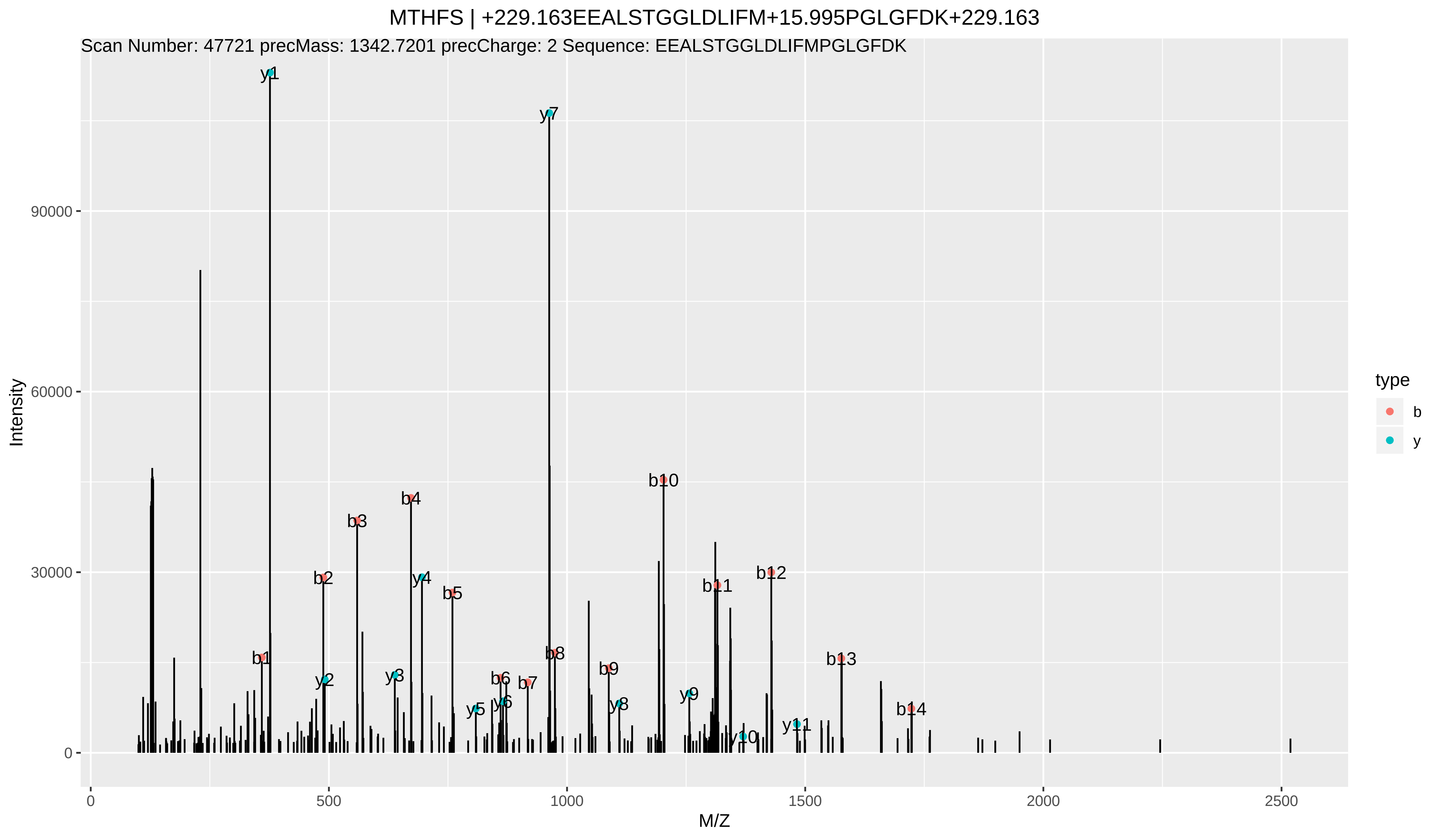

MTHFS | +229.163EEALSTGGLDLIFM+15.995PGLGFDK+229.163
Scan Number: 47721 precMass: 1342.7201 precCharge: 2 Sequence: EEALSTGGLDLIFMPGLGFDK
y1
y7
b10
b4
b3
b2
y4
b5
b12
b11
b13
b1
b8
b9
y2
y3
b6
b7
y6
y8
y9
y5
b14
y11
y10
Intensity
90000
60000
30000
0
M/Z
0
500
1000
1500
2000
2500
type
b
y

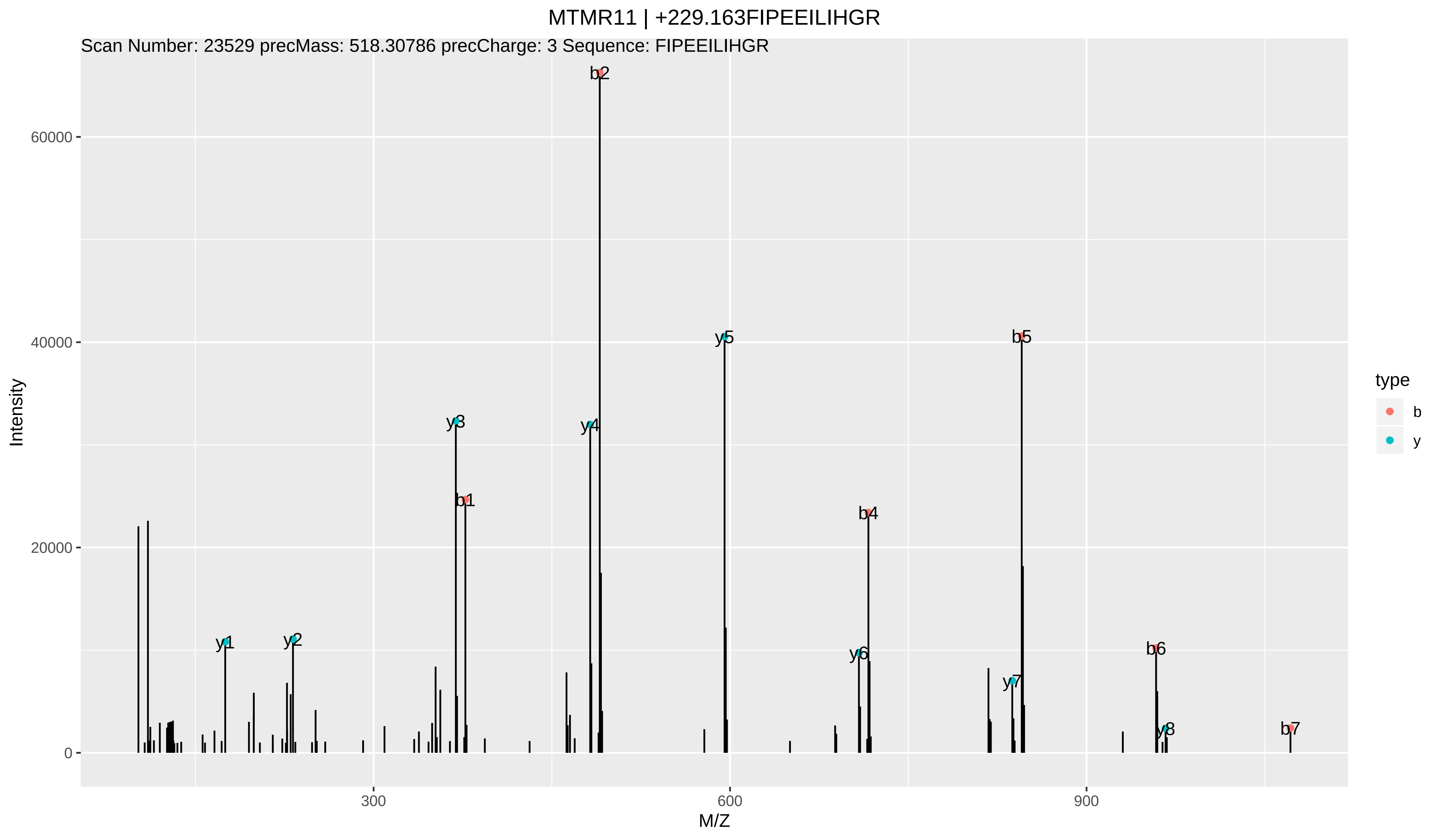
MTMR11 | +229.163FIPEEILIHGR
Scan Number: 23529 precMass: 518.30786 precCharge: 3 Sequence: FIPEEILIHGR
Intensity
M/Z
0
20000
40000
60000
300
600
900
b2
y5
b5
y3
y4
b1
b4
y1
y2
y6
b6
y7
y8
b7
type
b
y

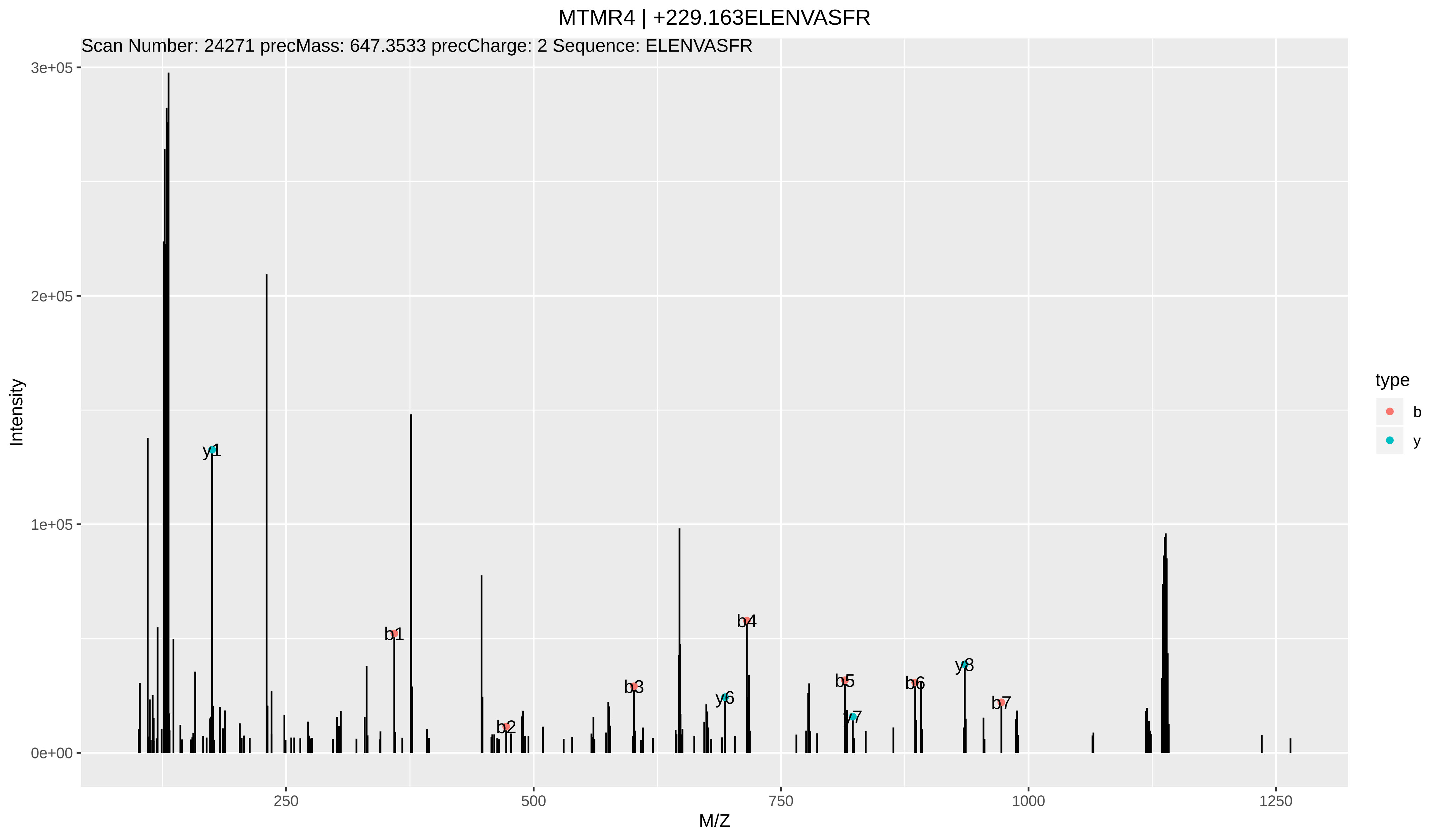
MTMR4 | +229.163ELENVASFR
Scan Number: 24271 precMass: 647.3533 precCharge: 2 Sequence: ELENVASFR
Intensity
M/Z
3e+05
2e+05
1e+05
0e+00
250
500
750
1000
1250
y1
b1
b2
b3
y6
b4
b5
y7
b6
y8
b7
type
b
y

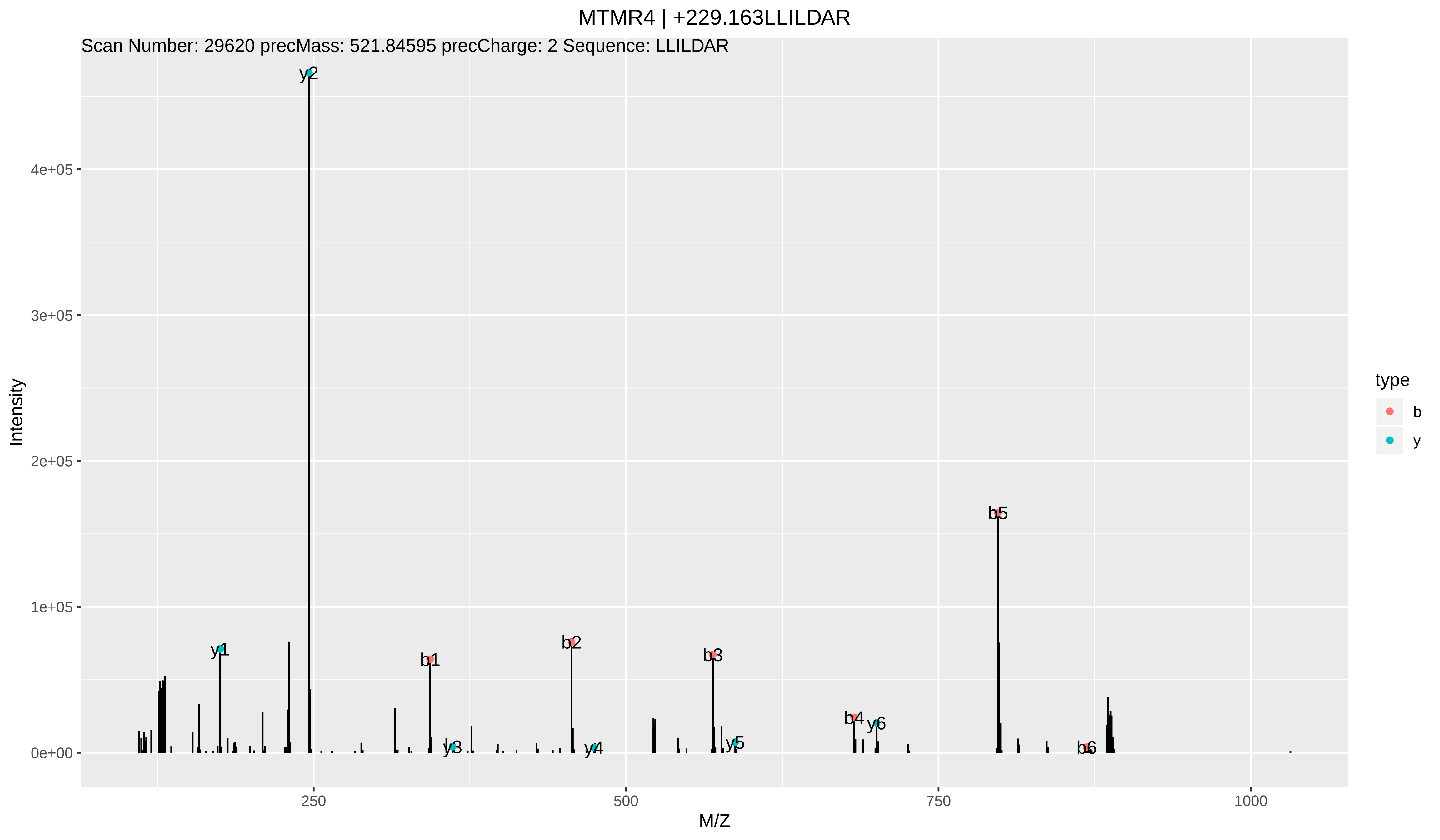

MTMR4 | +229.163LLILDAR
Scan Number: 29620 precMass: 521.84595 precCharge: 2 Sequence: LLILDAR
Intensity
M/Z
type
b
y
y2
b5
y1
b1
b2
b3
y3
y4
y5
b4
y6
b6
4e+05
3e+05
2e+05
1e+05
0e+00
250
500
750
1000

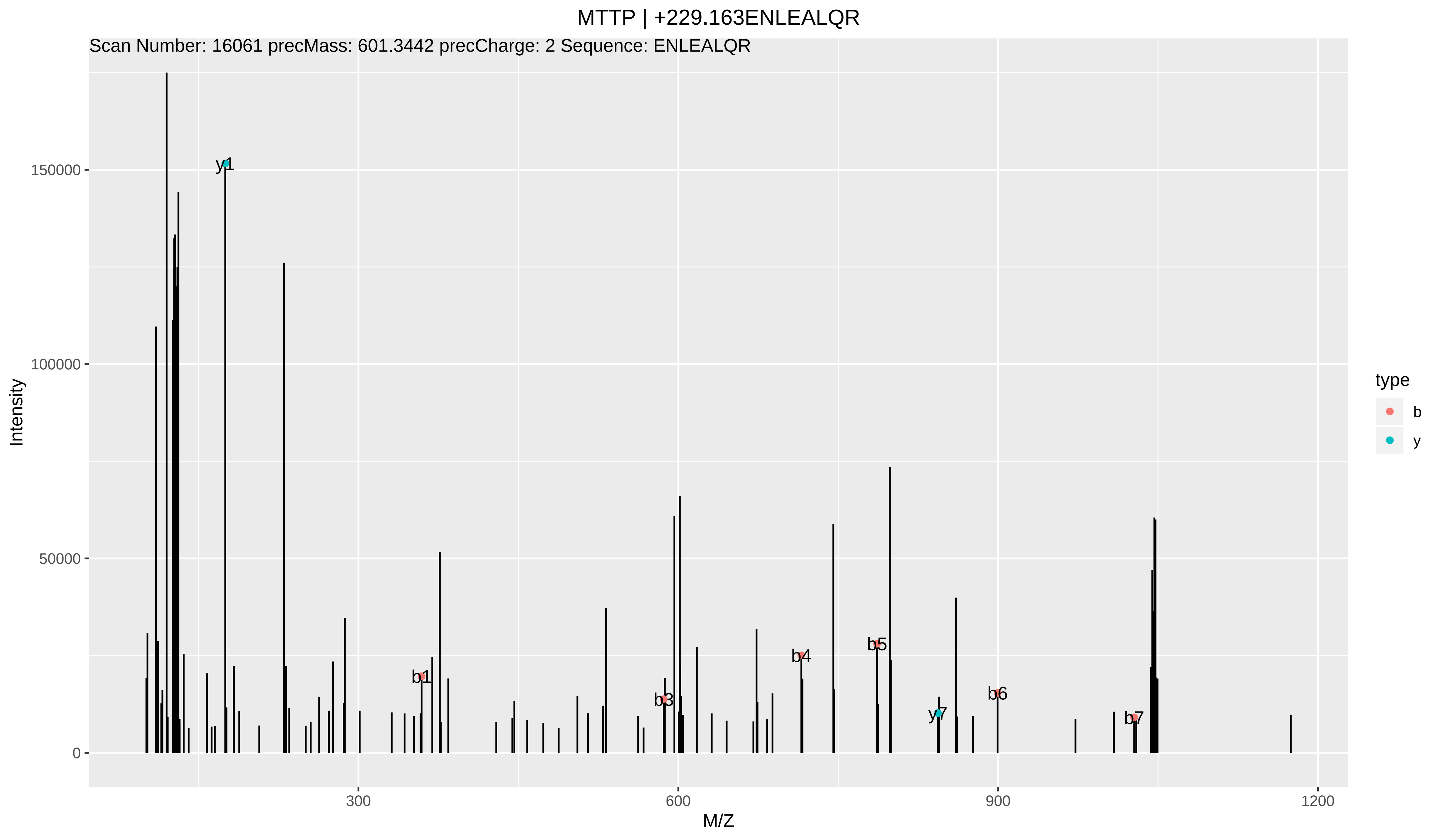
MTTP | +229.163ENLEALQR
Scan Number: 16061 precMass: 601.3442 precCharge: 2 Sequence: ENLEALQR
Intensity
M/Z
150000
100000
50000
0
300
600
900
1200
y1
b1
b3
b4
b5
y7
b6
b7
type
b
y

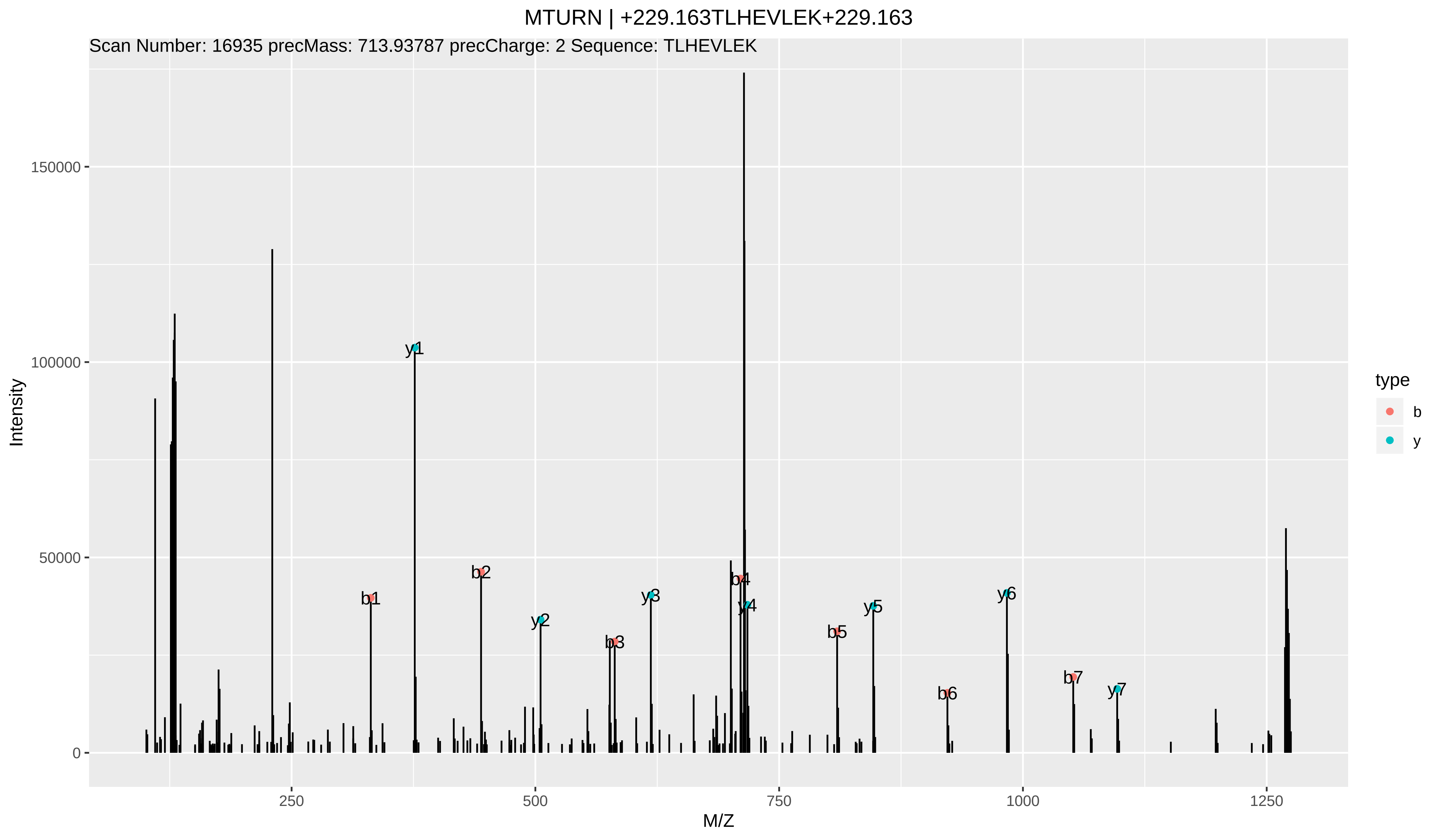

MTURN | +229.163TLHEVLEK+229.163
Scan Number: 16935 precMass: 713.93787 precCharge: 2 Sequence: TLHEVLEK
Intensity
150000
100000
50000
0
M/Z
250
500
750
1000
1250
y1
b1
b2
y2
b3
y3
b4
y4
b5
y5
b6
y6
b7
y7
type
b
y

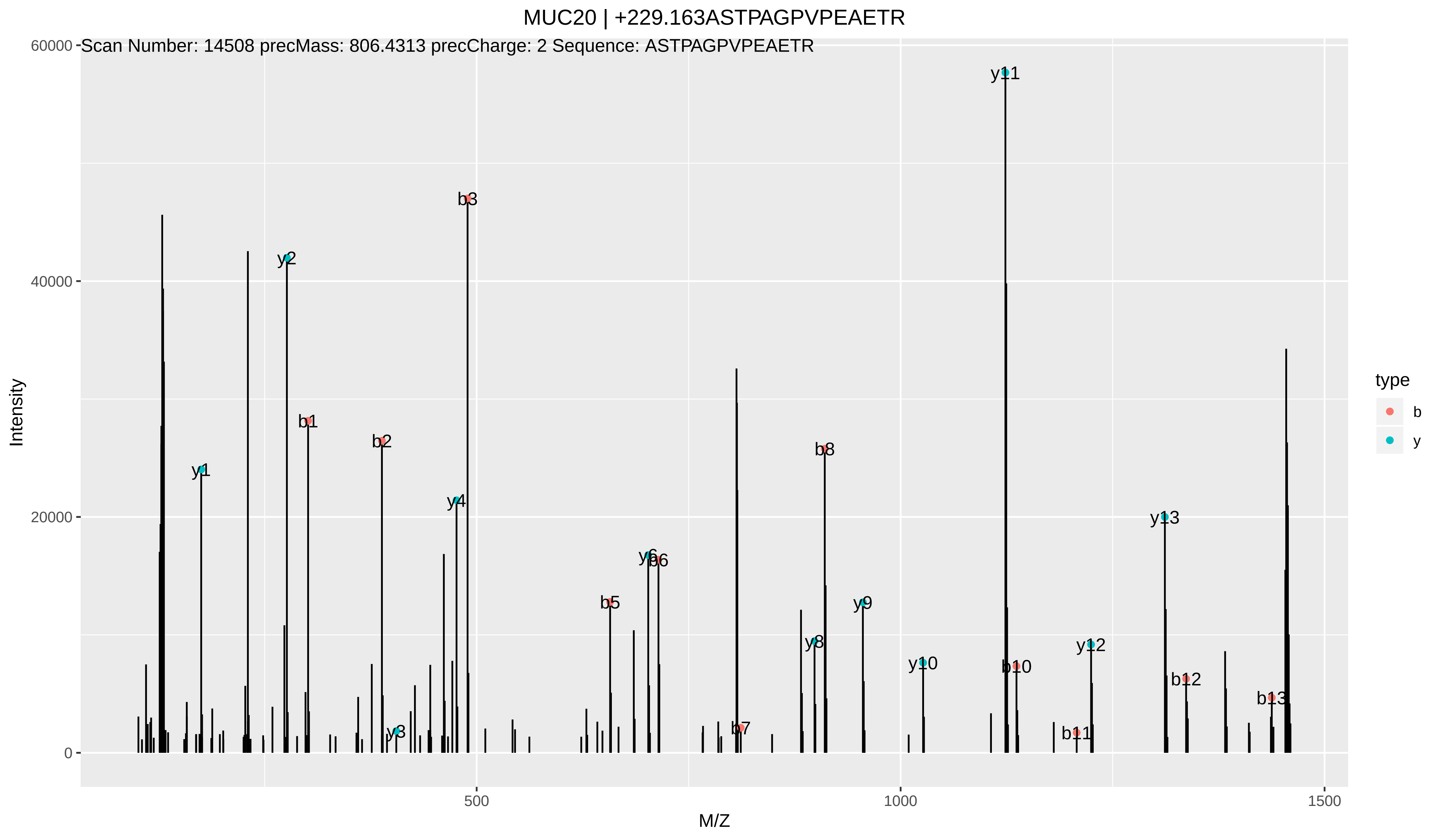
MUC20 | +229.163ASTPAGPVPEAETR
Scan Number: 14508 precMass: 806.4313 precCharge: 2 Sequence: ASTPAGPVPEAETR
Intensity
60000
40000
20000
0
M/Z
500
1000
1500
y11
b3
y2
b1
b2
y1
y4
y3
b5
y6
b6
b7
b8
y8
y9
y10
b10
b11
y12
y13
b12
b13
type
b
y

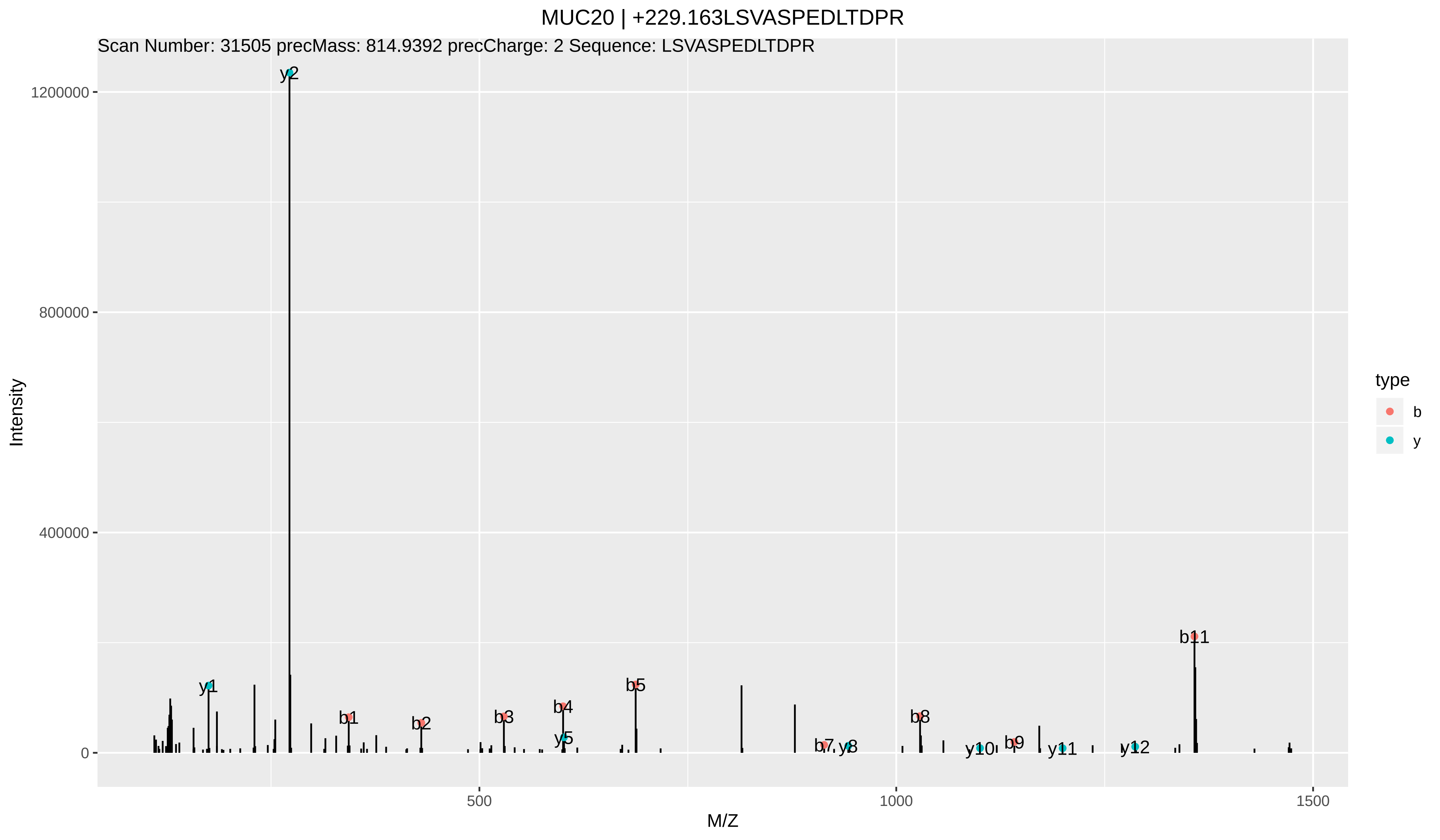

MUC20 | +229.163LSVASPEDLTDPR
Scan Number: 31505 precMass: 814.9392 precCharge: 2 Sequence: LSVASPEDLTDPR
Intensity
M/Z
1200000
800000
400000
0
500
1000
1500
y2
y1
b1
b2
b3
b4
y5
b5
b7
y8
b8
y10
b9
y11
y12
b11
type
b
y

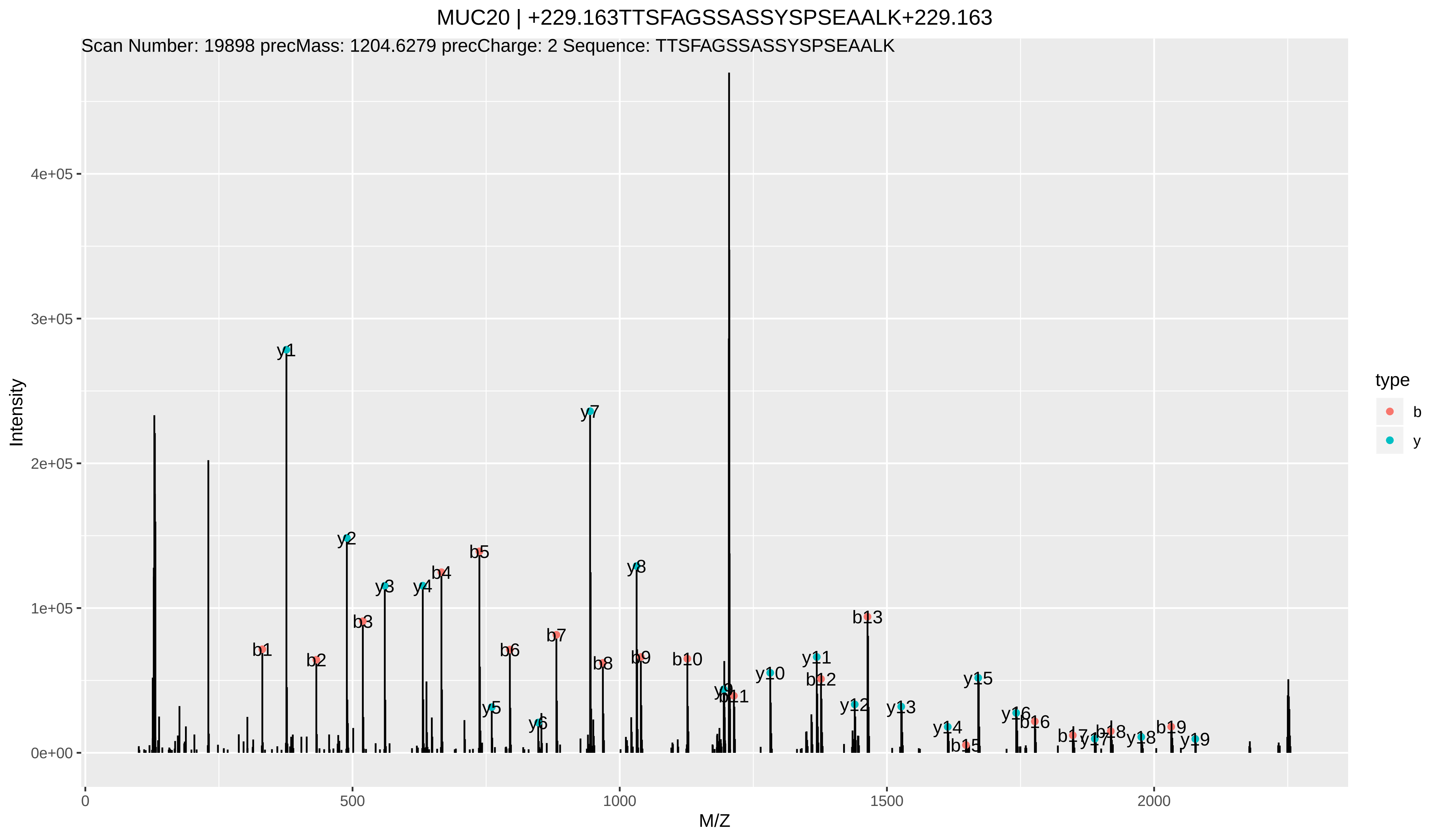
MUC20 | +229.163TTSFAGSSASSYSPSEAALK+229.163
Scan Number: 19898 precMass: 1204.6279 precCharge: 2 Sequence: TTSFAGSSASSYSPSEAALK
Intensity
4e+05
3e+05
2e+05
1e+05
0e+00
M/Z
0
500
1000
1500
2000
type
b
y
y1
y2
y3
y4
y5
y6
y7
y8
y10
y11
y12
y13
y14
y15
y16
y18
y19
b1
b2
b3
b4
b5
b6
b7
b8
b9
b10
b12
b13
b15
b16
b18
b19

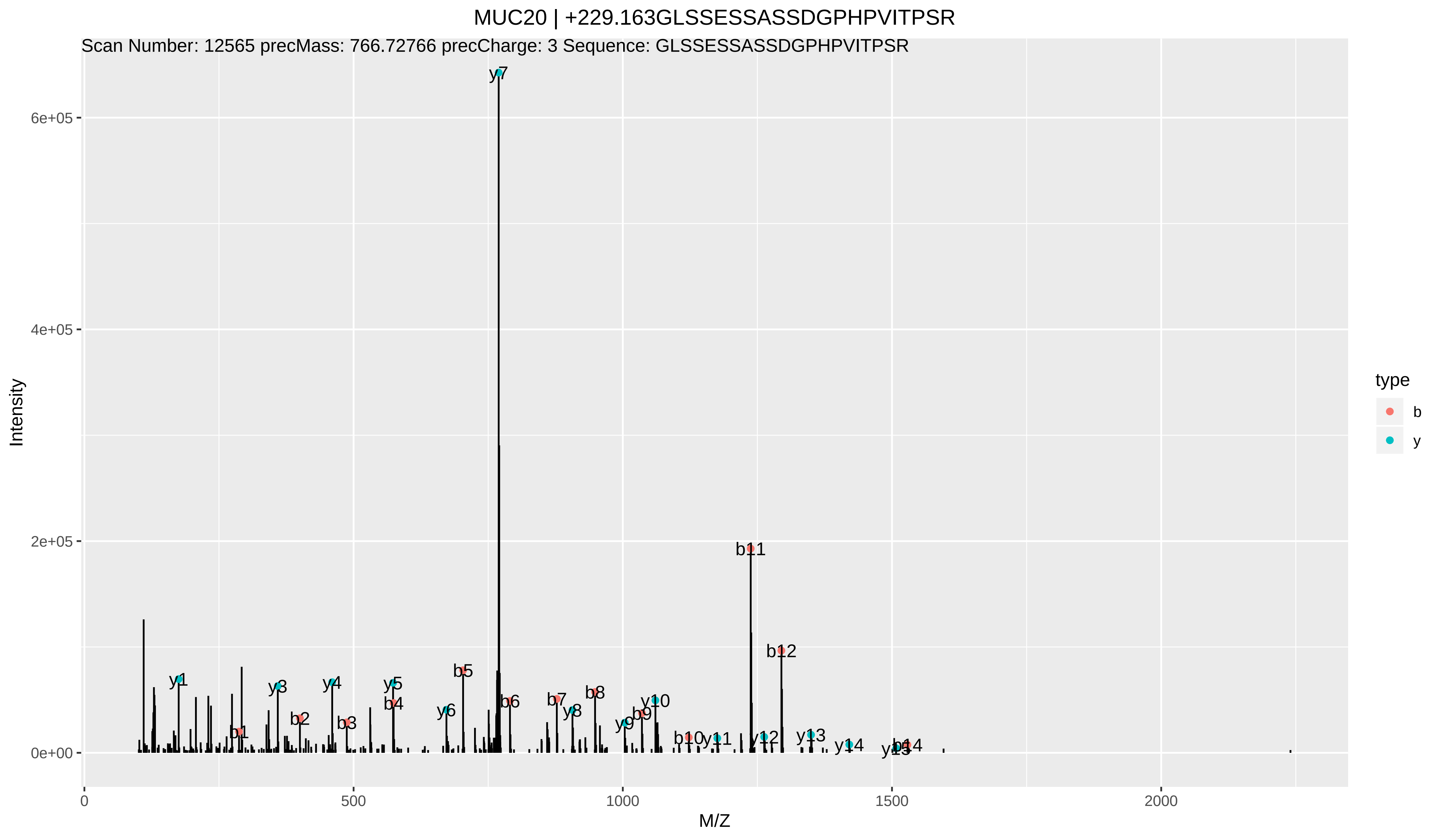

MUC20 | +229.163GLSSESSASSDGPHPVITPSR
Scan Number: 12565 precMass: 766.72766 precCharge: 3 Sequence: GLSSESSASSDGPHPVITPSR
Intensity
M/Z
type
b
y
y7
b11
b12
b5
y1
y3
y4
y5
b4
b2
b3
b1
y6
b6
b7
b8
y8
y9
b9
y10
b10
y11
y12
y13
y14
y15
b14
6e+05
4e+05
2e+05
0e+00
0
500
1000
1500
2000

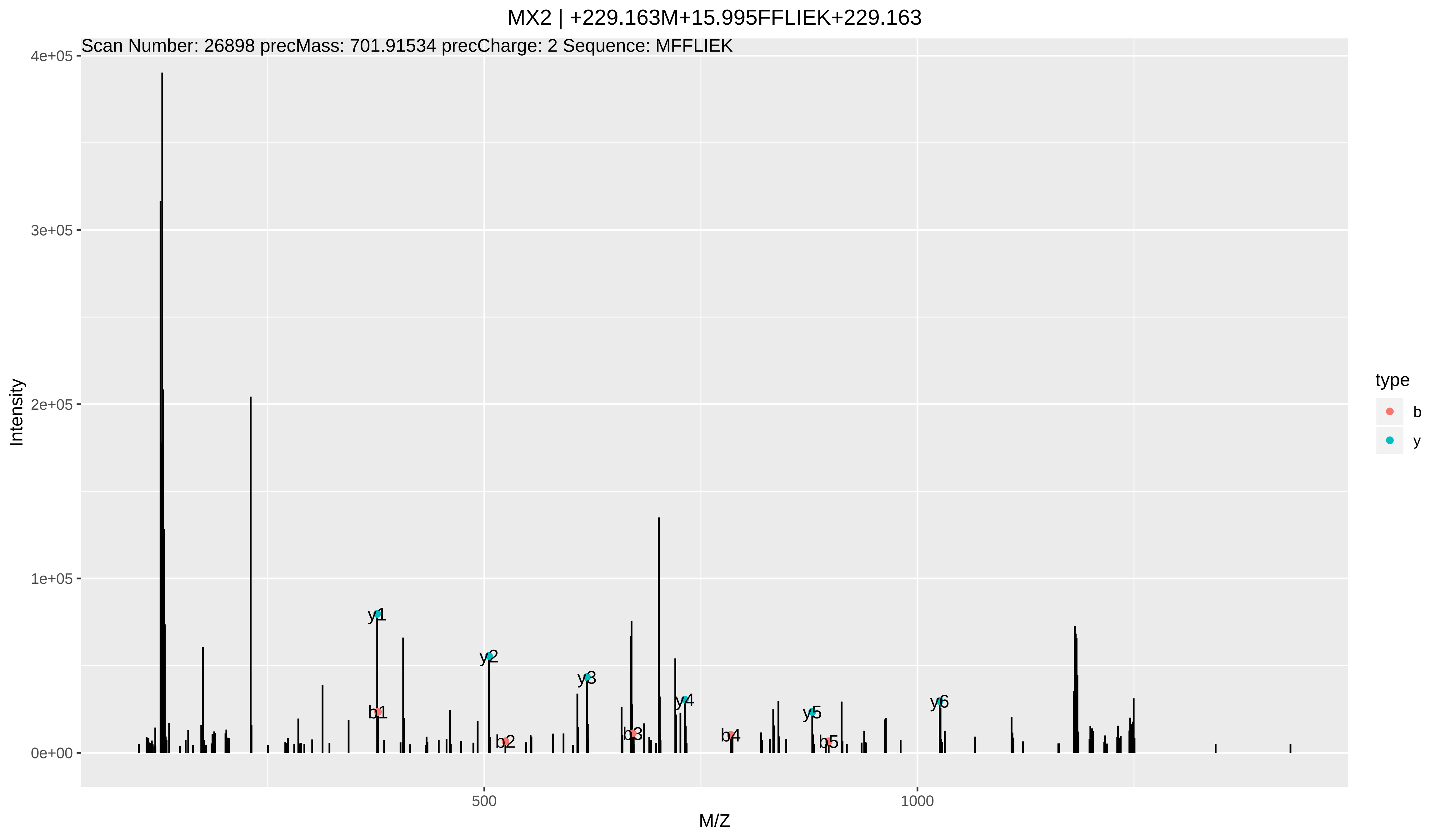

MX2 | +229.163M+15.995FFLIEK+229.163
Scan Number: 26898 precMass: 701.91534 precCharge: 2 Sequence: MFFLIEK
Intensity
4e+05
3e+05
2e+05
1e+05
0e+00
M/Z
500
1000
y1
y2
y3
y4
y5
y6
b1
b2
b3
b4
b5
type
b
y

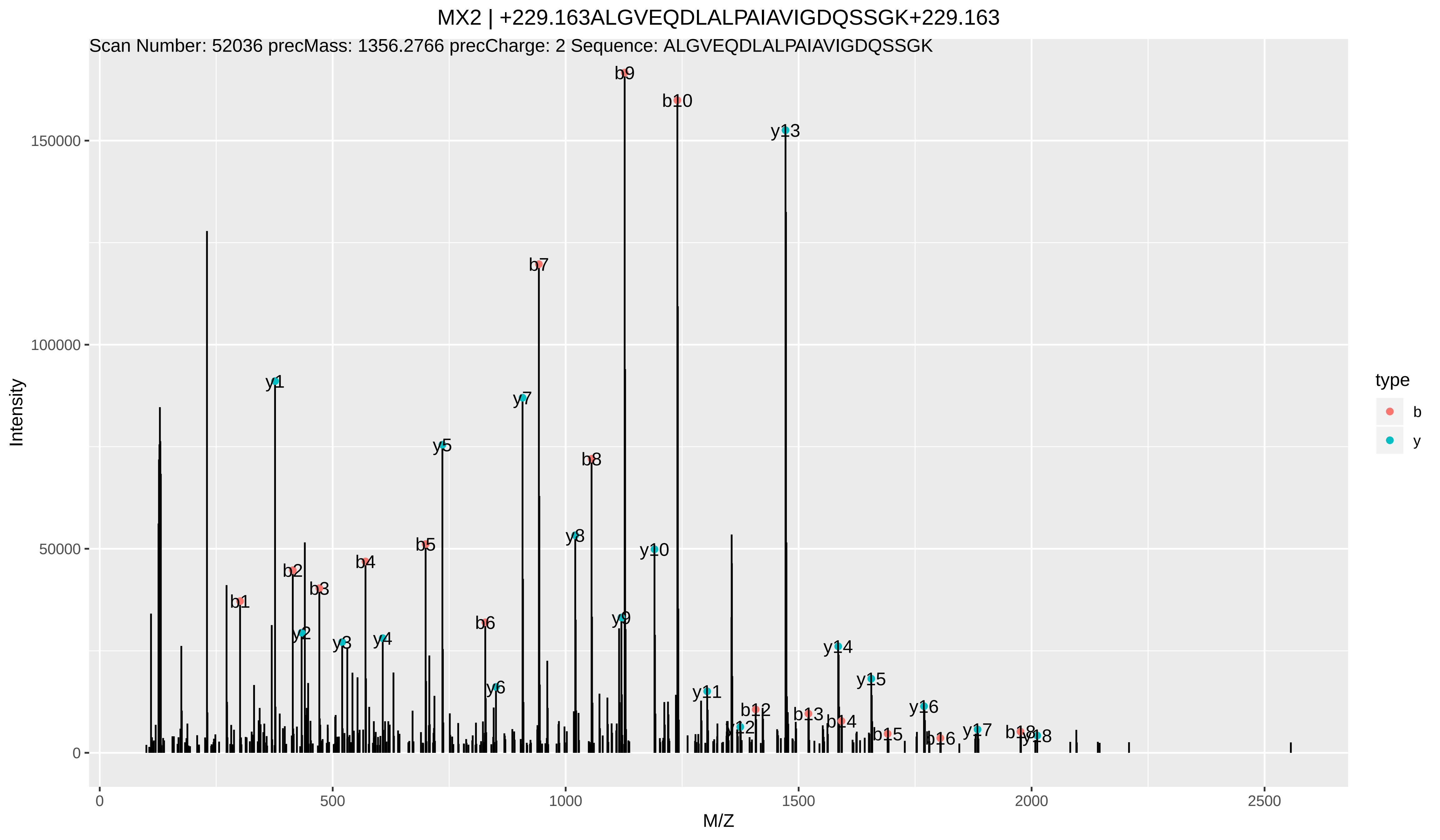
MX2 | +229.163ALGVEQDLALPAIAVIGDQSSGK+229.163
Scan Number: 52036 precMass: 1356.2766 precCharge: 2 Sequence: ALGVEQDLALPAIAVIGDQSSGK
Intensity
150000
100000
50000
0
M/Z
0
500
1000
1500
2000
2500
type
b
y
b1
b2
b3
b4
b5
b6
b7
b8
b9
b10
b12
b13
b14
b15
b16
b18
y1
y2
y3
y4
y5
y6
y7
y8
y9
y10
y11
y12
y13
y14
y15
y16
y17
y18

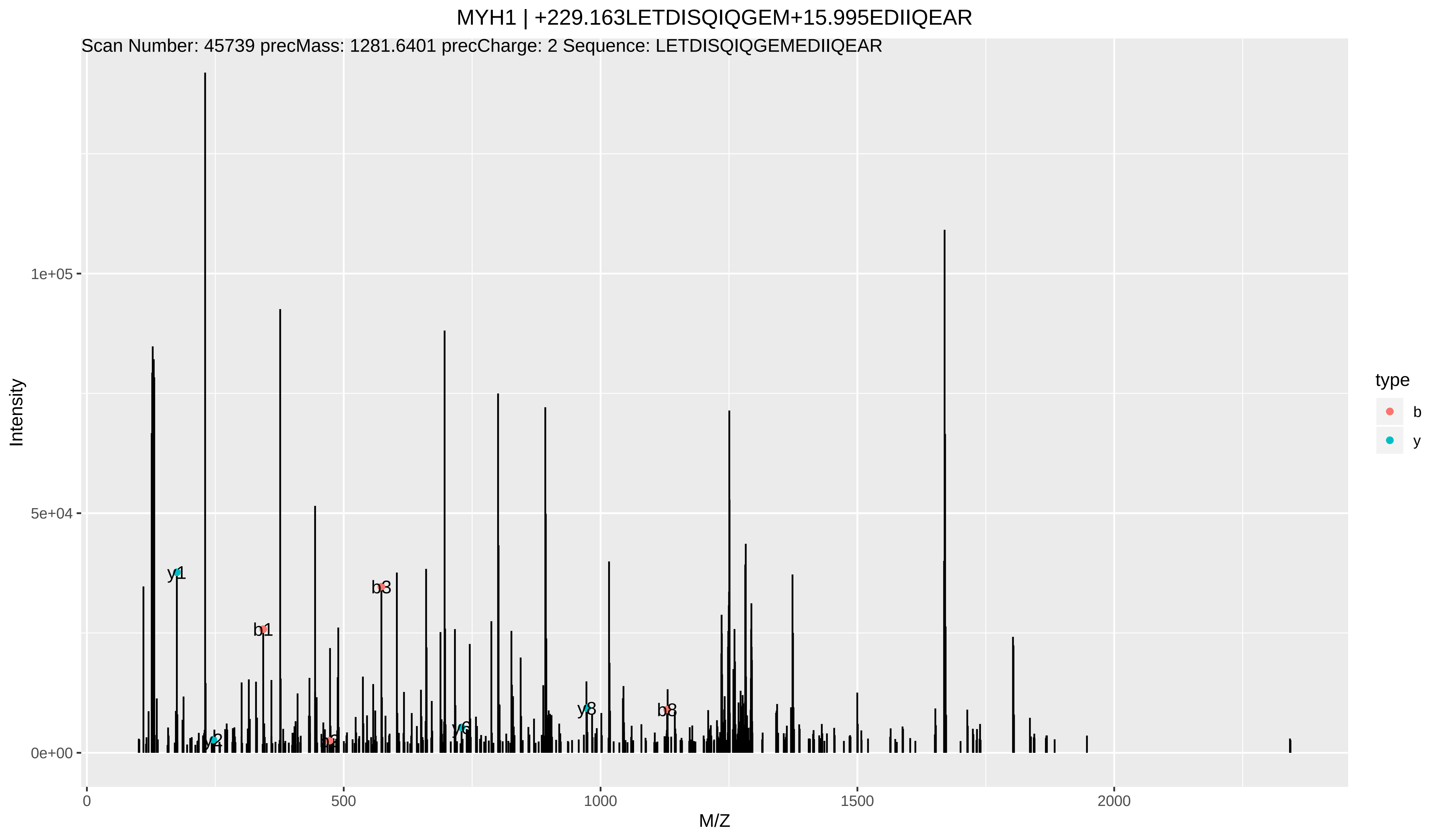

MYH1 | +229.163LETDISQIQGEM+15.995EDIIQEAR
Scan Number: 45739 precMass: 1281.6401 precCharge: 2 Sequence: LETDISQIQGEMEDIIQEAR
Intensity
1e+05
5e+04
0e+00
0
500
1000
1500
2000
M/Z
y1
b1
b3
y8
b8
type
b
y

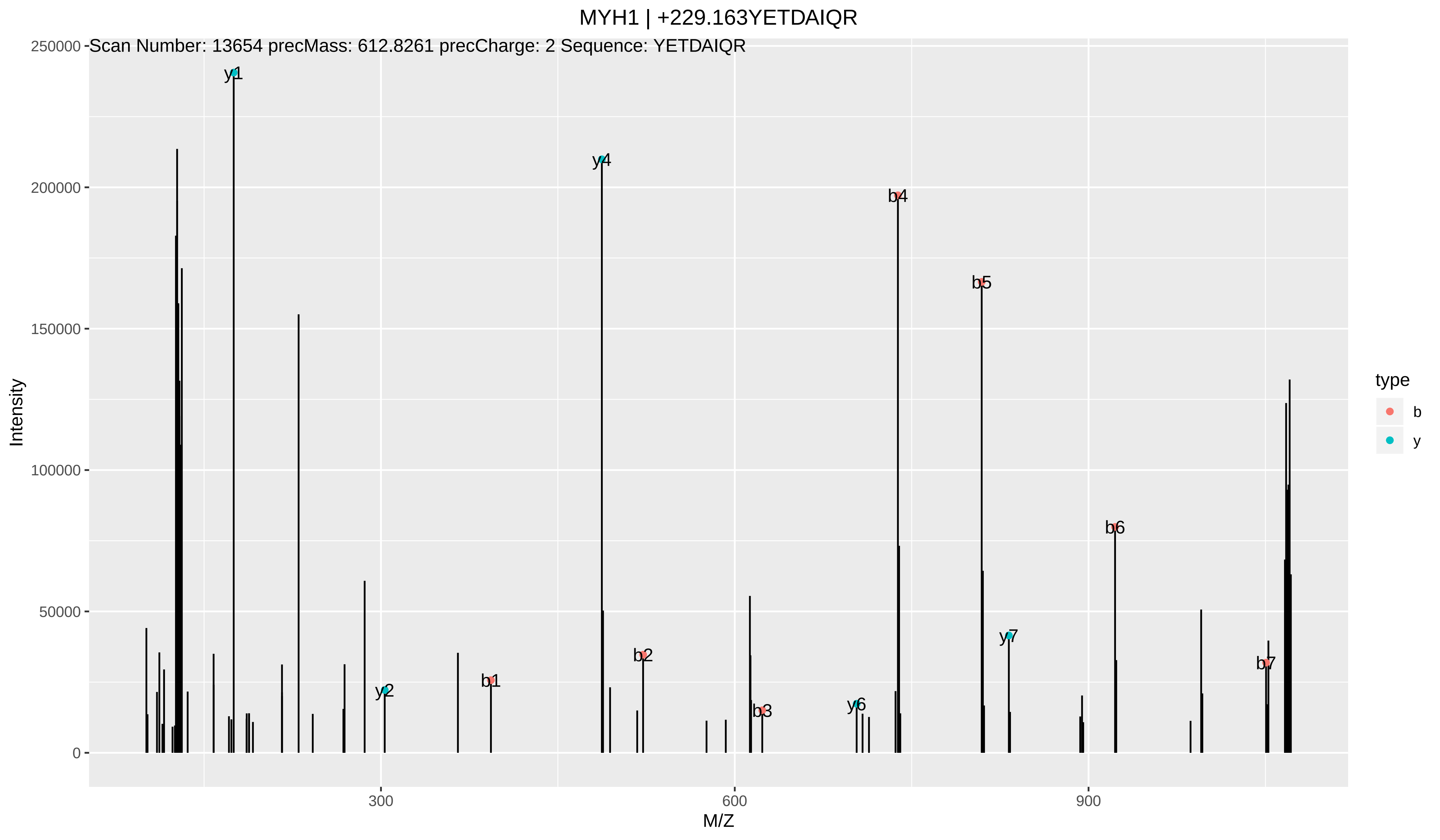

MYH1 | +229.163YETDAIQR
Scan Number: 13654 precMass: 612.8261 precCharge: 2 Sequence: YETDAIQR
Intensity
250000
200000
150000
100000
50000
0
M/Z
300
600
900
y1
y4
b4
b5
b6
y7
b2
b1
y2
b3
y6
b7
type
b
y

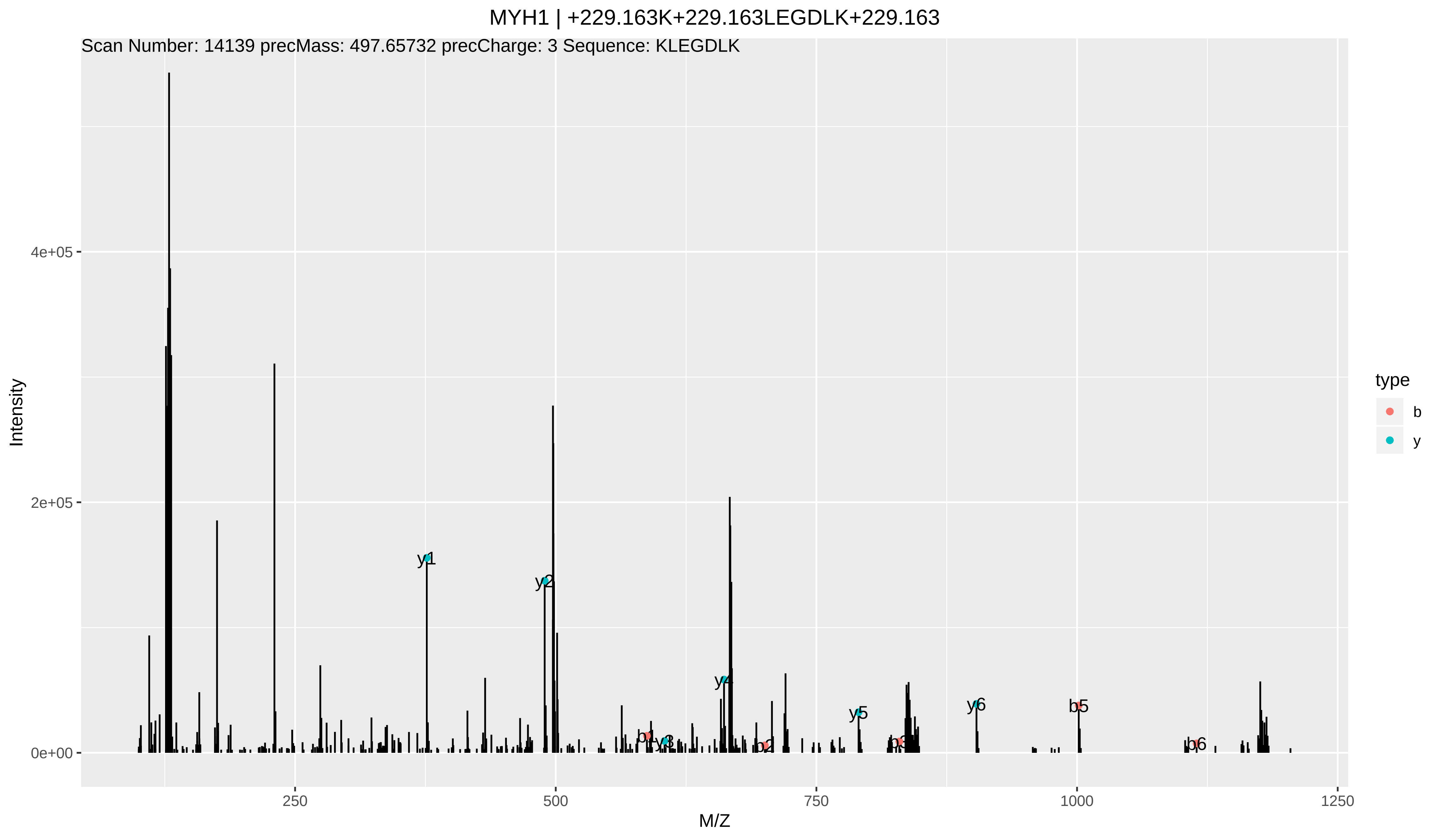

MYH1 | +229.163K+229.163LEGDLK+229.163
Scan Number: 14139 precMass: 497.65732 precCharge: 3 Sequence: KLEGDLK
Intensity
4e+05
2e+05
0e+00
250
500
750
1000
1250
M/Z
y1
y2
y3
y4
y5
y6
b1
b2
b3
b5
b6
type
b
y

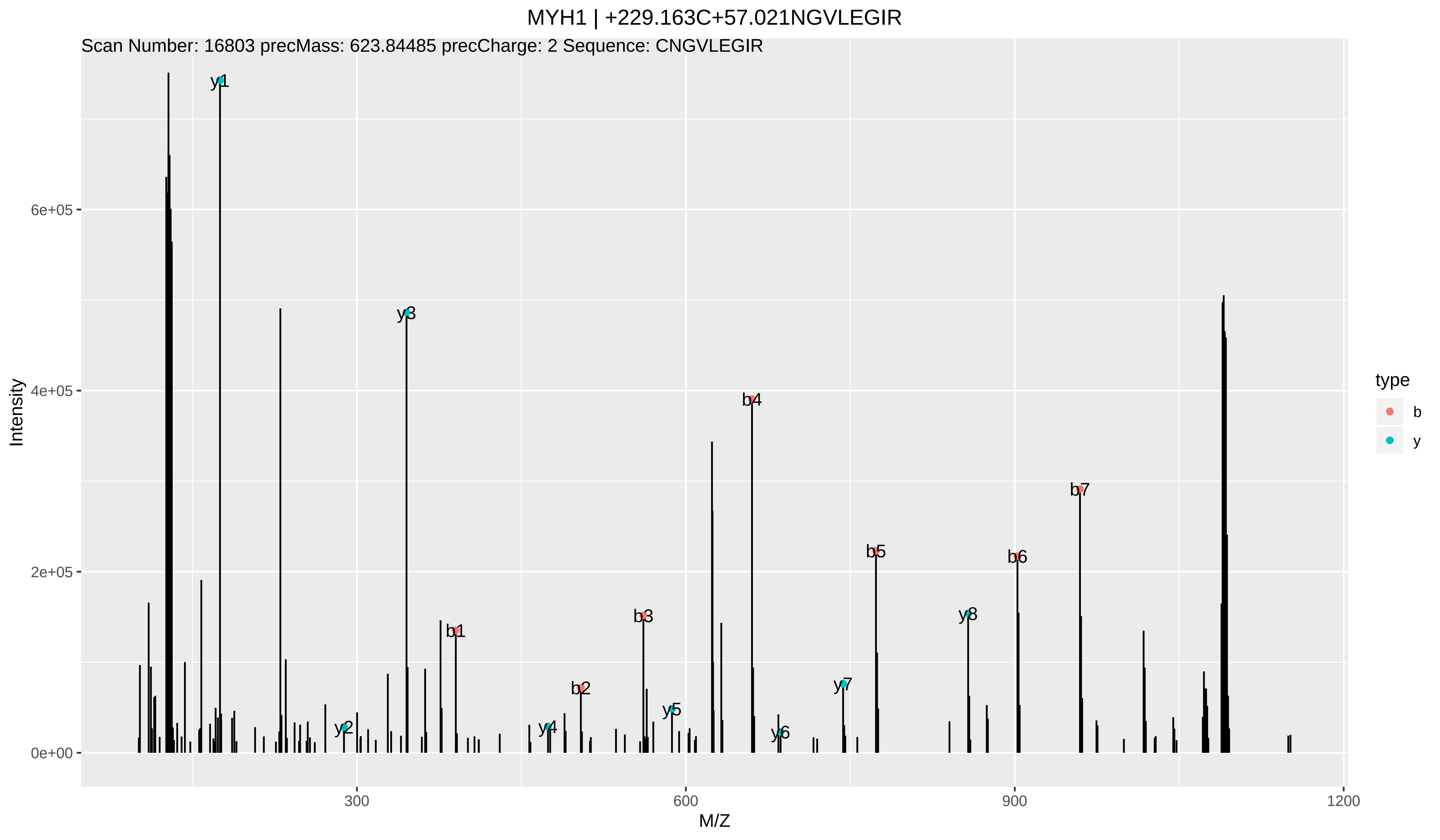

MYH1 | +229.163C+57.021NGVLEGIR
Scan Number: 16803 precMass: 623.84485 precCharge: 2 Sequence: CNGVLEGIR
Intensity
M/Z
type
b
y
y1
y2
y3
y4
y5
y6
y7
y8
b1
b2
b3
b4
b5
b6
b7
0e+00
2e+05
4e+05
6e+05
300
600
900
1200

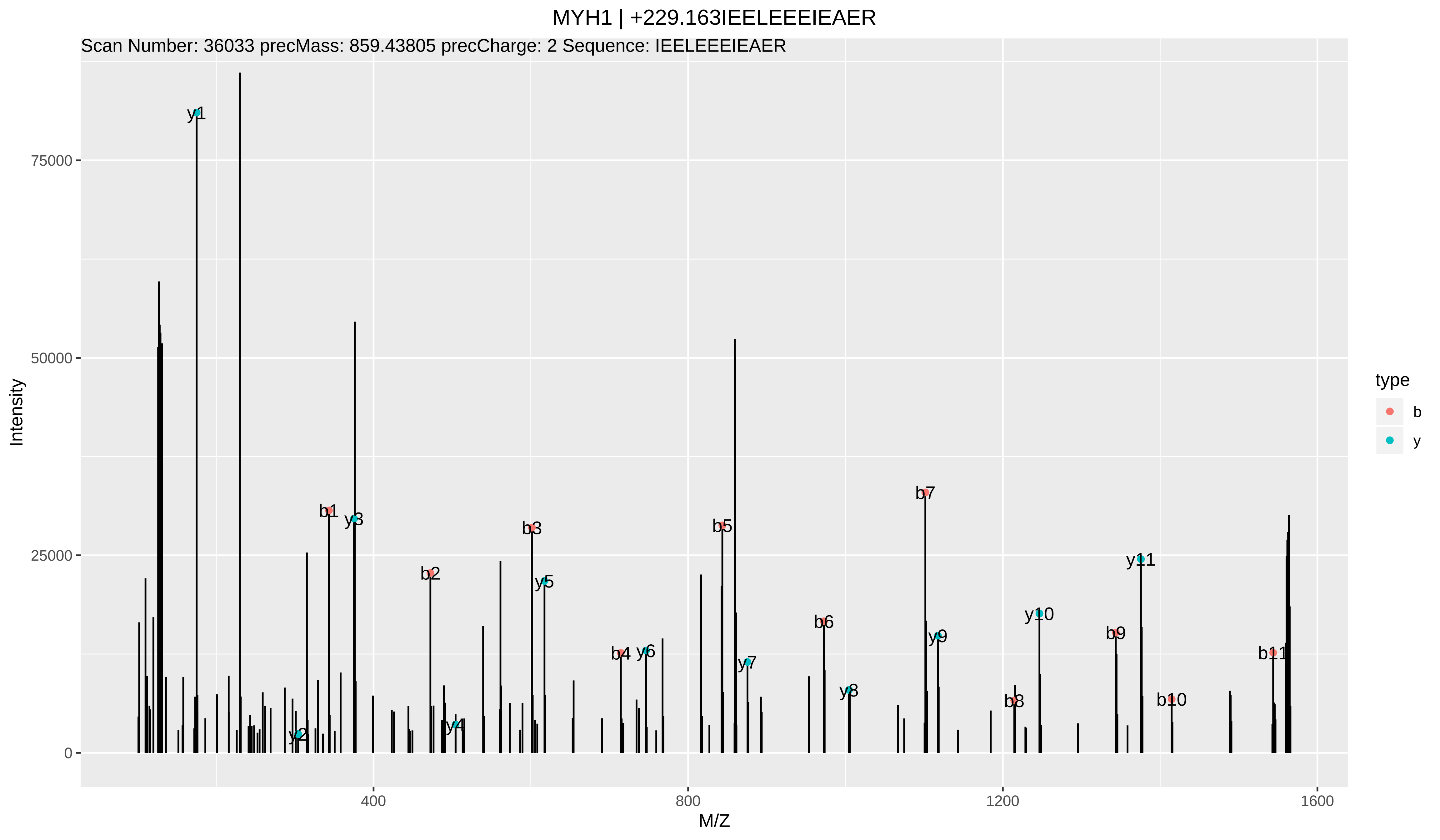
MYH1 | +229.163IEELEEEIEAER
Scan Number: 36033 precMass: 859.43805 precCharge: 2 Sequence: IEELEEEIEAER
Intensity
M/Z
type
b
y
y1
y2
y3
y4
y5
y6
y7
y8
y9
y10
y11
b1
b2
b3
b4
b5
b6
b7
b8
b9
b10
b11
0
25000
50000
75000
400
800
1200
1600

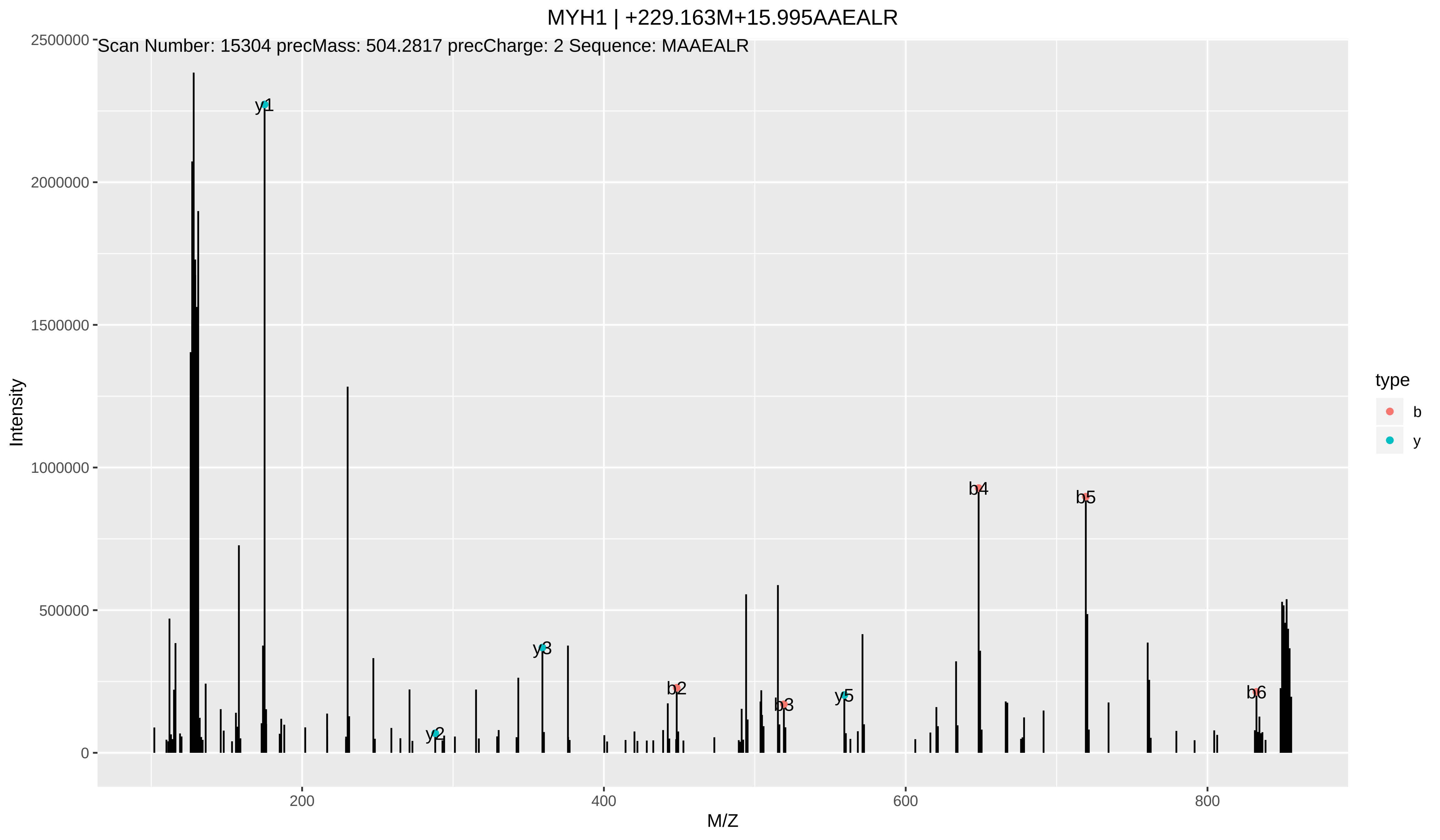

MYH1 | +229.163M+15.995AAEALR
Scan Number: 15304 precMass: 504.2817 precCharge: 2 Sequence: MAAEALR
Intensity
2500000
2000000
1500000
1000000
500000
0
M/Z
200
400
600
800
y1
y2
y3
y5
b2
b3
b4
b5
b6
type
b
y

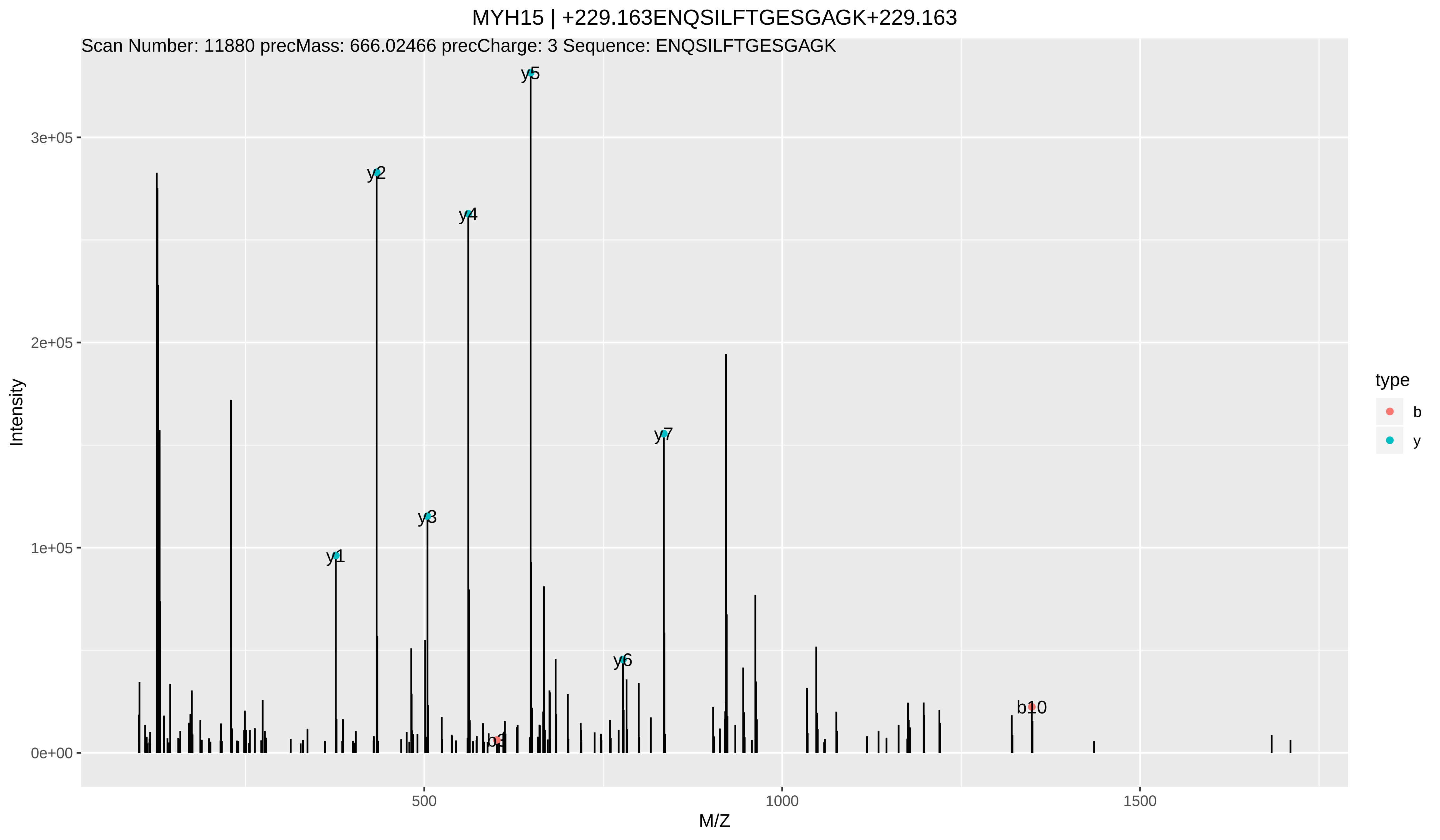

MYH15 | +229.163ENQSILFTGESGAGK+229.163
Scan Number: 11880 precMass: 666.02466 precCharge: 3 Sequence: ENQSILFTGESGAGK
Intensity
M/Z
y1
y2
y3
y4
y5
y6
y7
b10
type
b
y

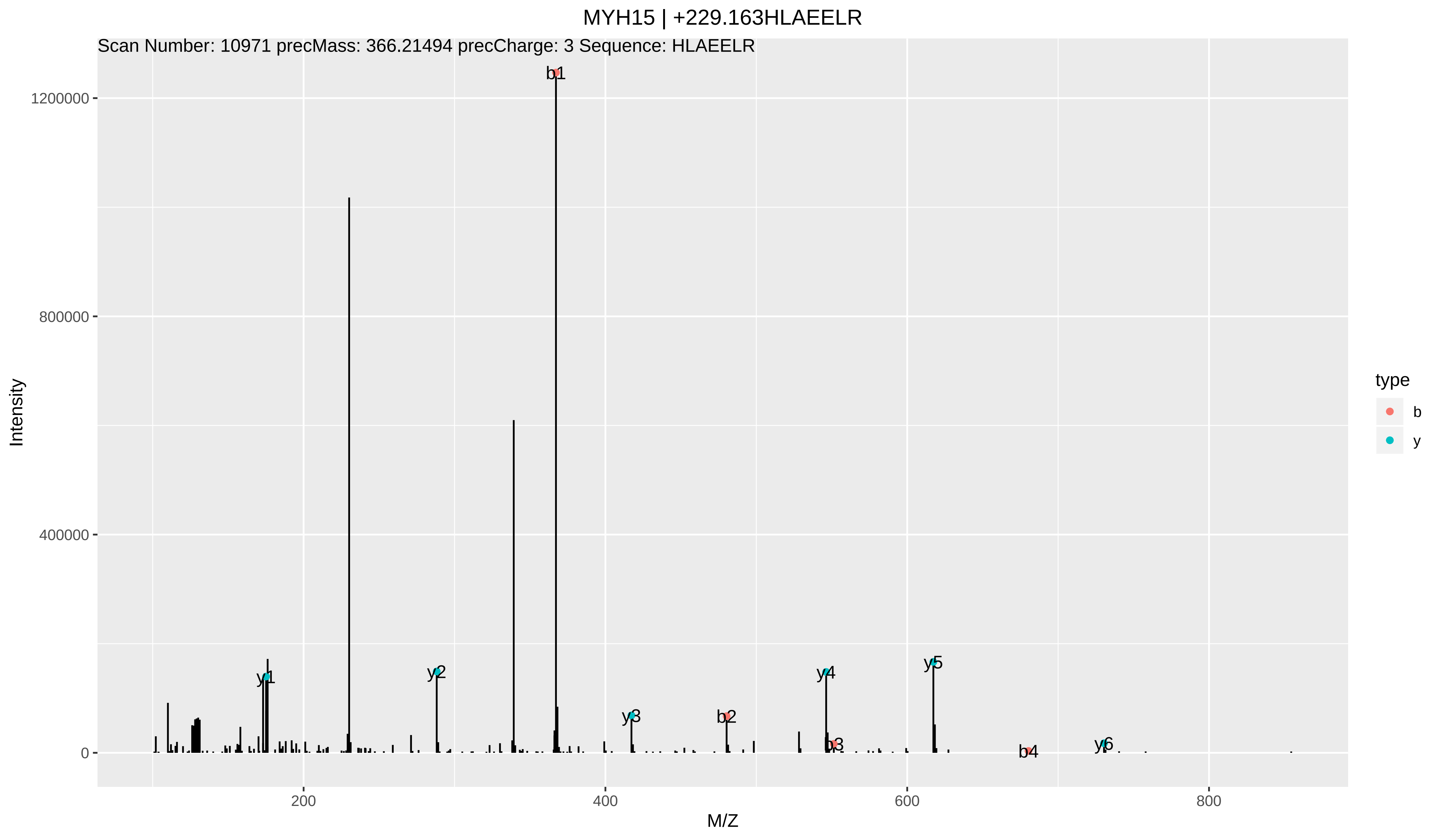
MYH15 | +229.163HLAEELR
Scan Number: 10971 precMass: 366.21494 precCharge: 3 Sequence: HLAEELR
Intensity
M/Z
0
400000
800000
1200000
200
400
600
800
b1
y1
y2
y3
b2
y4
b3
y5
b4
y6
type
b
y

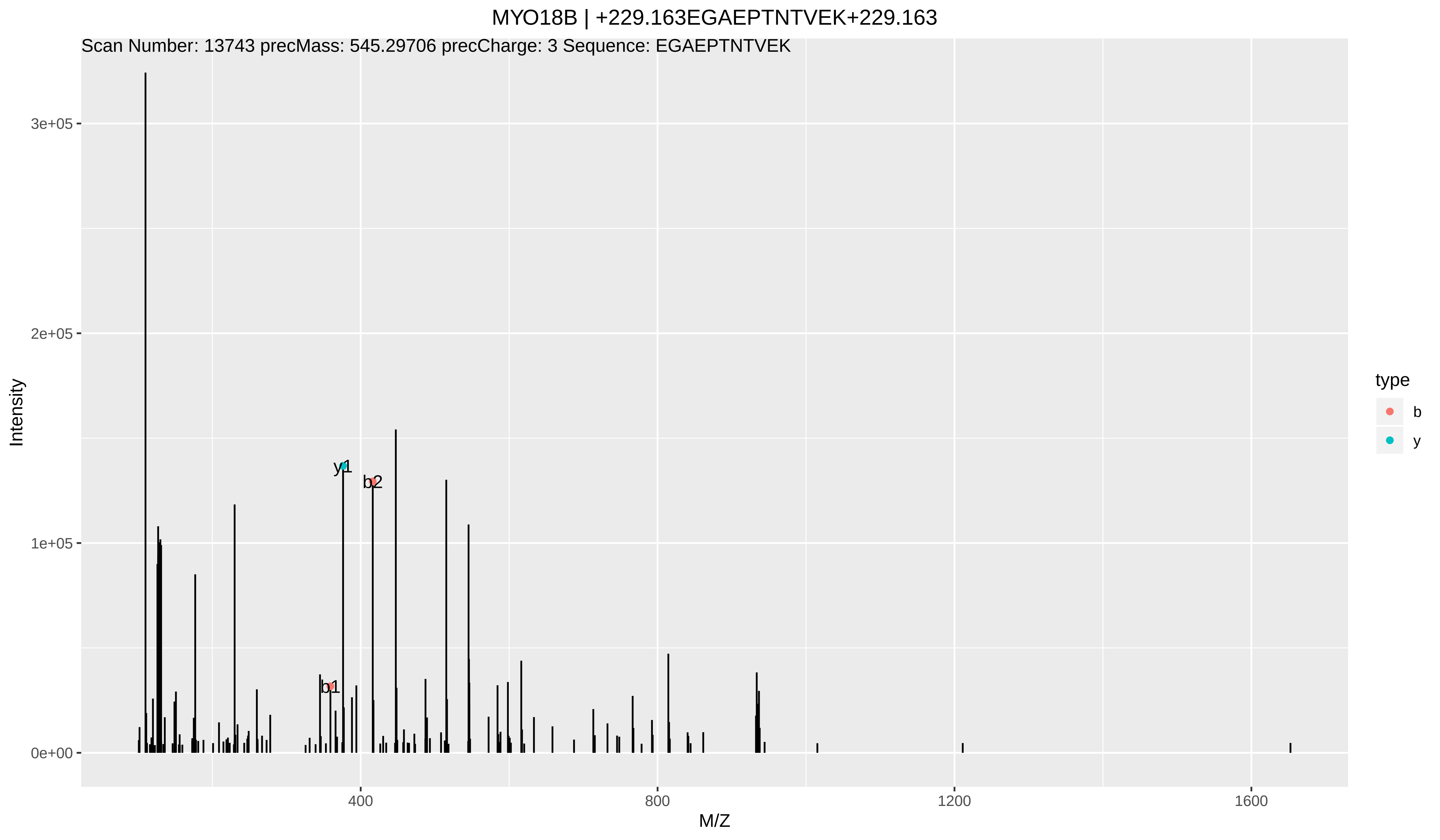
MYO18B | +229.163EGAEPTNTVEK+229.163
Scan Number: 13743 precMass: 545.29706 precCharge: 3 Sequence: EGAEPTNTVEK
Intensity
M/Z
3e+05
2e+05
1e+05
0e+00
400
800
1200
1600
y1
b2
b1
type
b
y

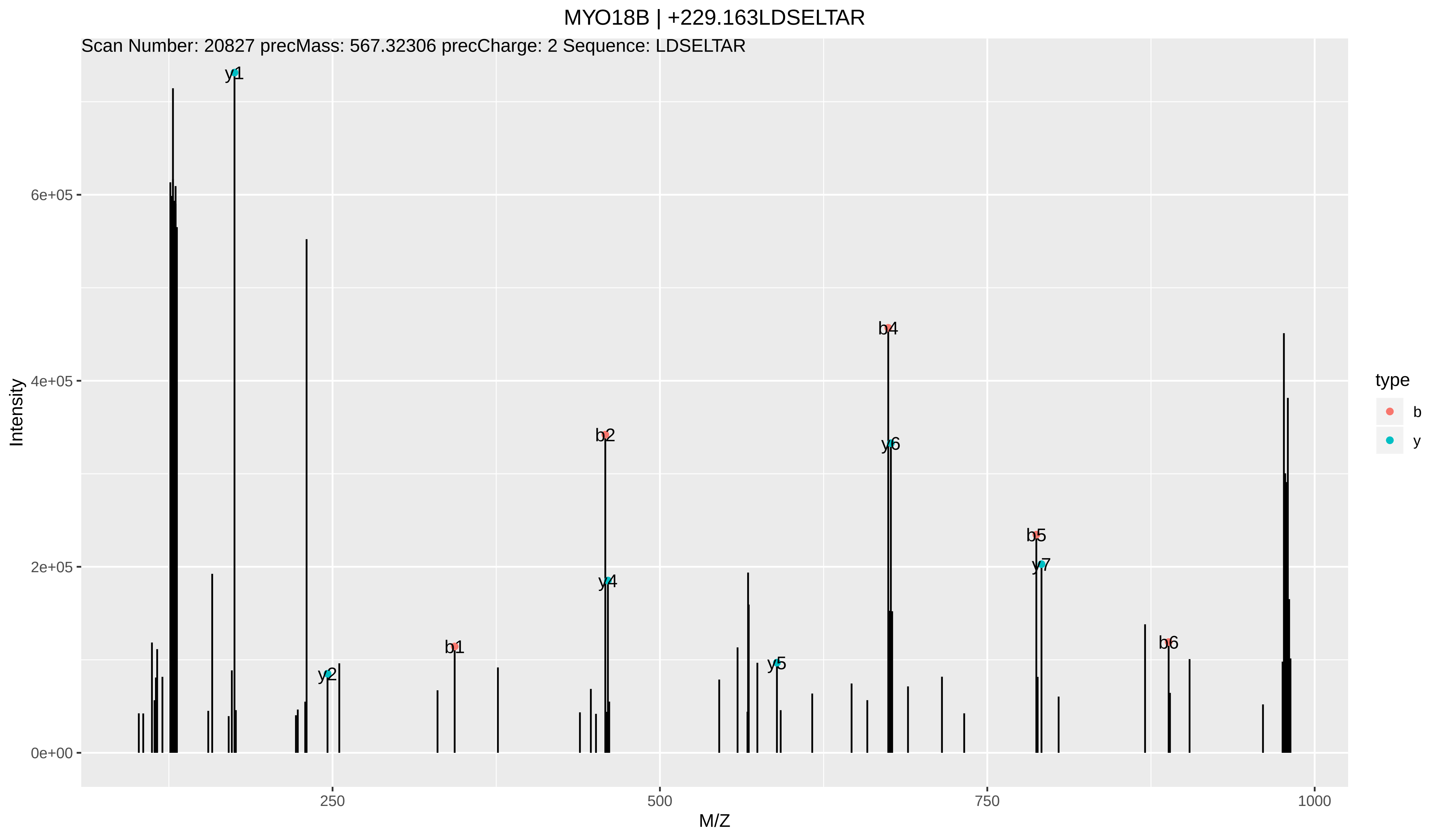
MYO18B | +229.163LDSELTAR
Scan Number: 20827 precMass: 567.32306 precCharge: 2 Sequence: LDSELTAR
y1
b4
b2
y6
b5
y7
y4
b1
b6
y5
y2
Intensity
6e+05
4e+05
2e+05
0e+00
250
500
750
1000
M/Z
type
b
y

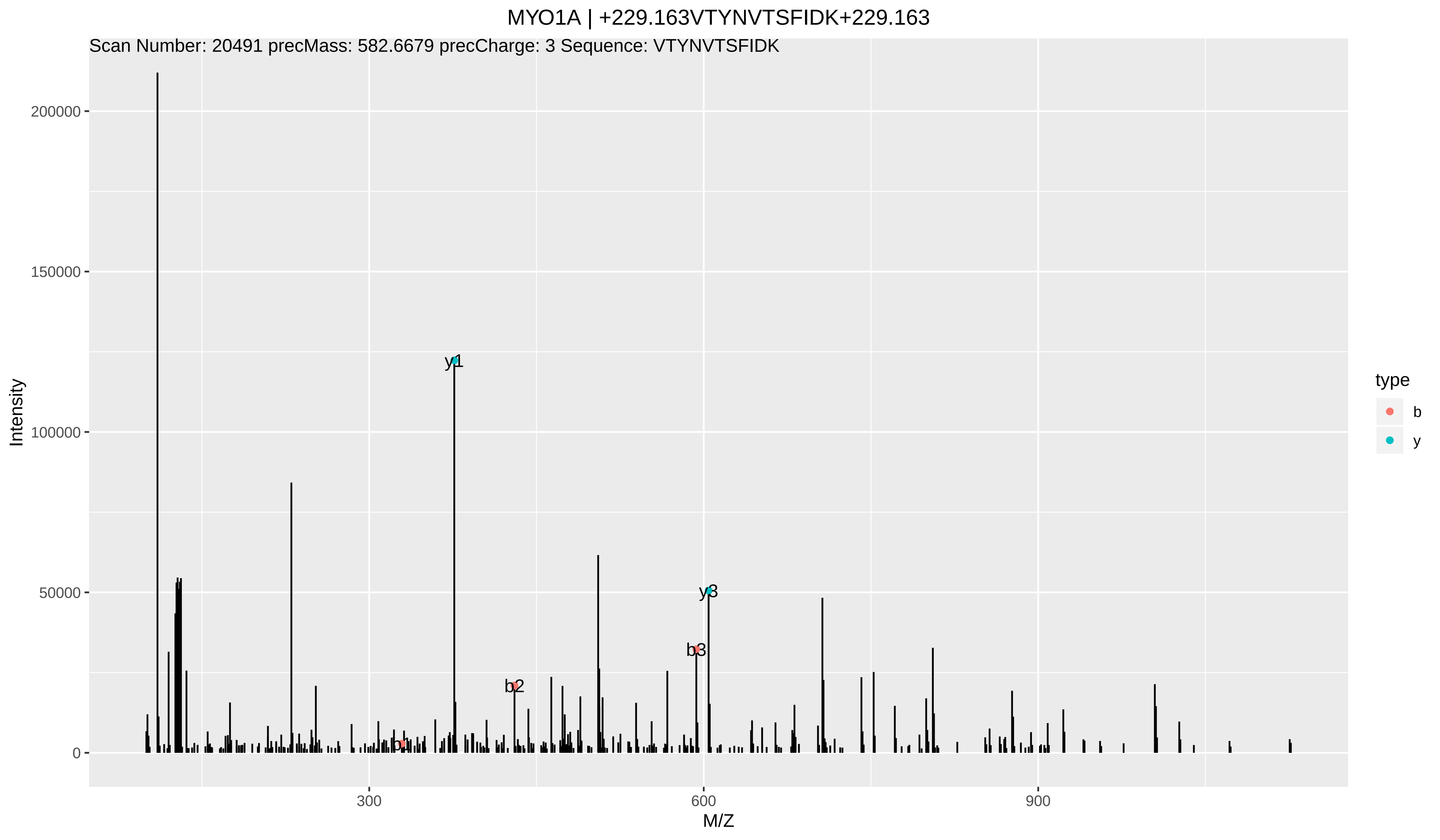

MYO1A | +229.163VTYNVTSFIDK+229.163
Scan Number: 20491 precMass: 582.6679 precCharge: 3 Sequence: VTYNVTSFIDK
Intensity
200000
150000
100000
50000
0
M/Z
300
600
900
y1
y3
b1
b2
b3
type
b
y

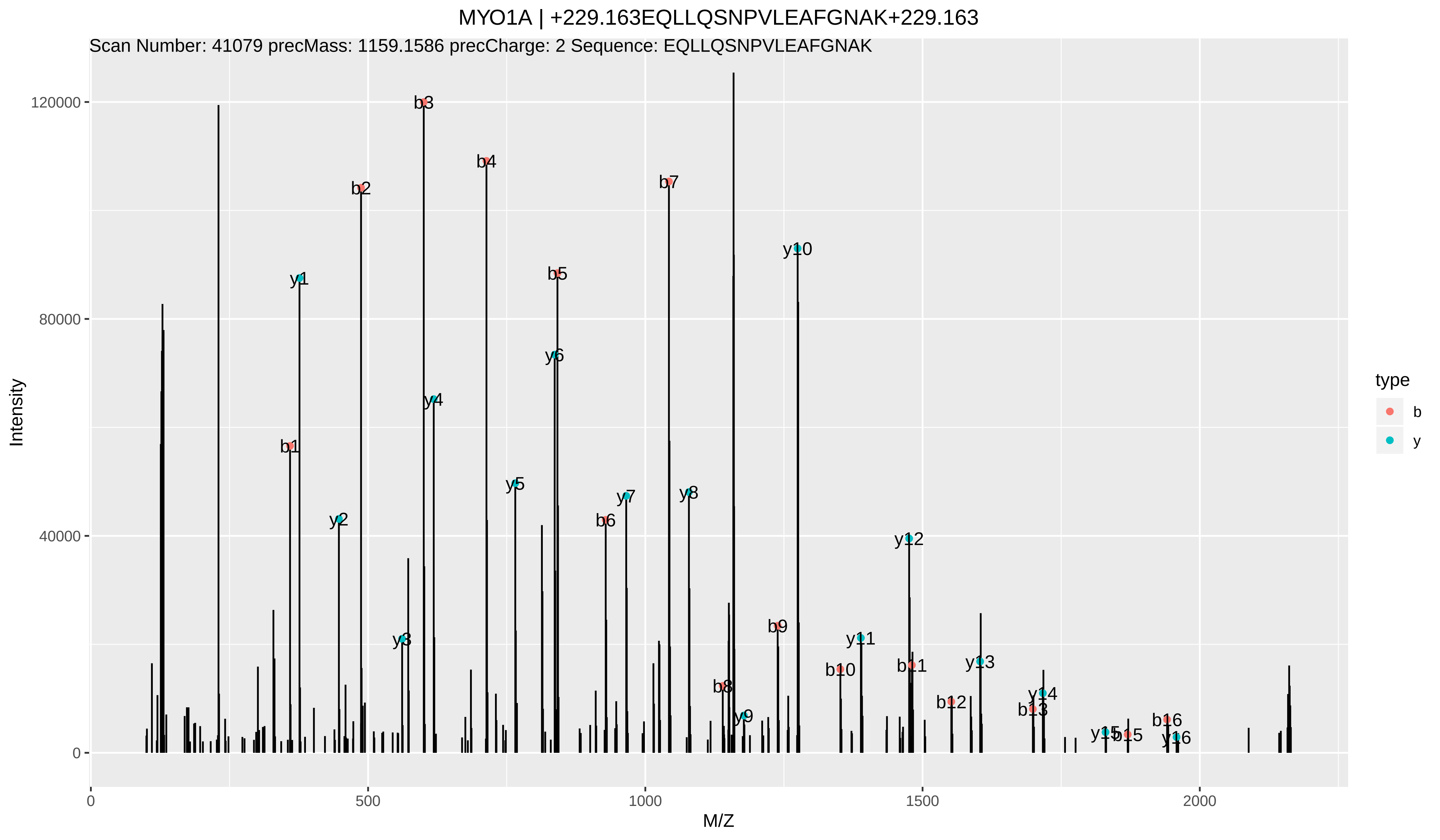

MYO1A | +229.163EQLLQSNPVLEAFGNAK+229.163
Scan Number: 41079 precMass: 1159.1586 precCharge: 2 Sequence: EQLLQSNPVLEAFGNAK
Intensity
M/Z
type
b
y
120000
80000
40000
0
0
500
1000
1500
2000
b1
b2
b3
b4
b5
b6
b7
b8
b9
b10
b11
b12
b13
b15
b16
y1
y2
y3
y4
y5
y6
y7
y8
y9
y10
y11
y12
y13
y14
y15
y16

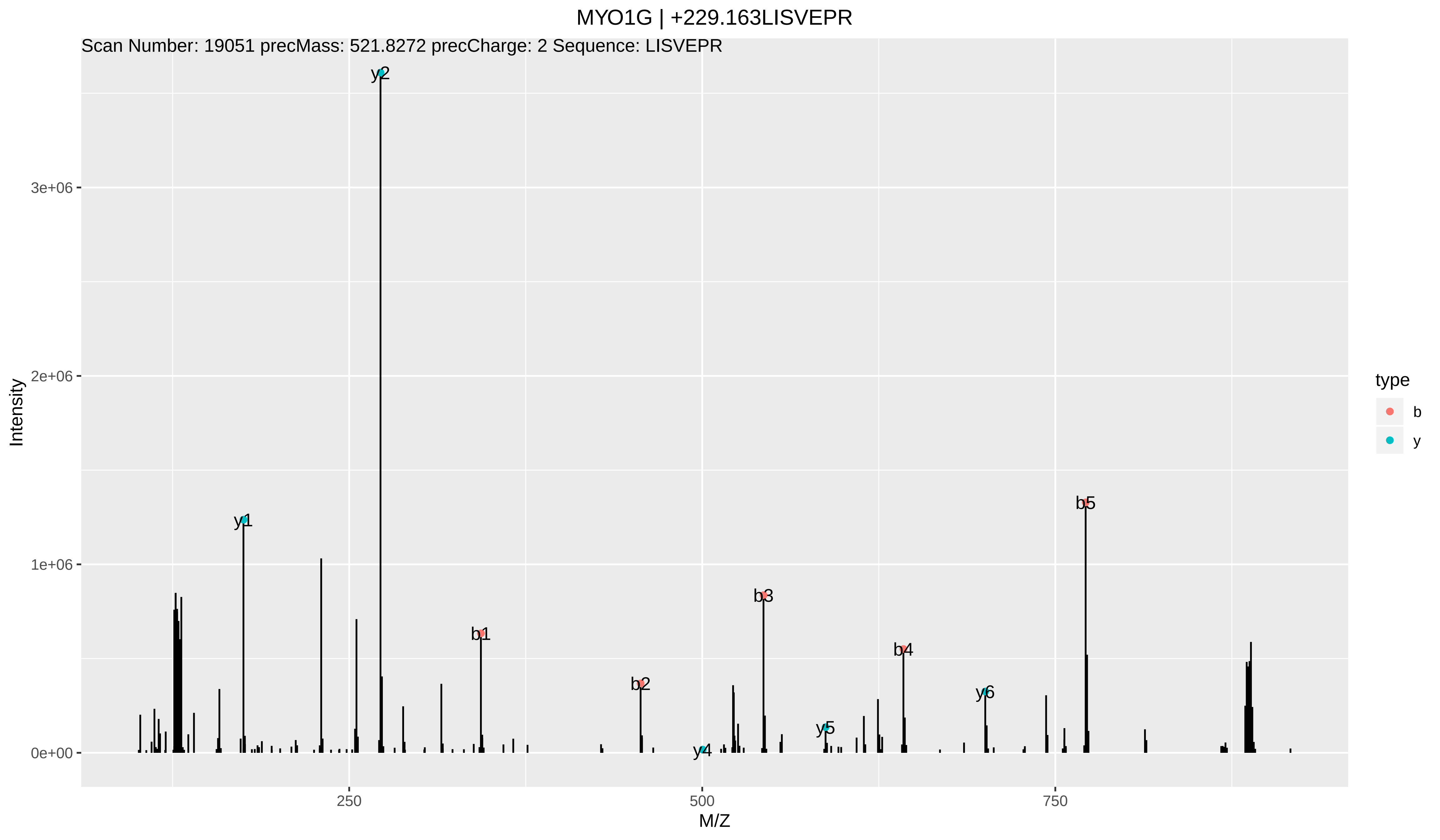

MYO1G | +229.163LISVEPR
Scan Number: 19051 precMass: 521.8272 precCharge: 2 Sequence: LISVEPR
y2
y1
b1
b2
y4
b3
y5
b4
y6
b5
Intensity
3e+06
2e+06
1e+06
0e+00
250
500
750
M/Z
type
b
y

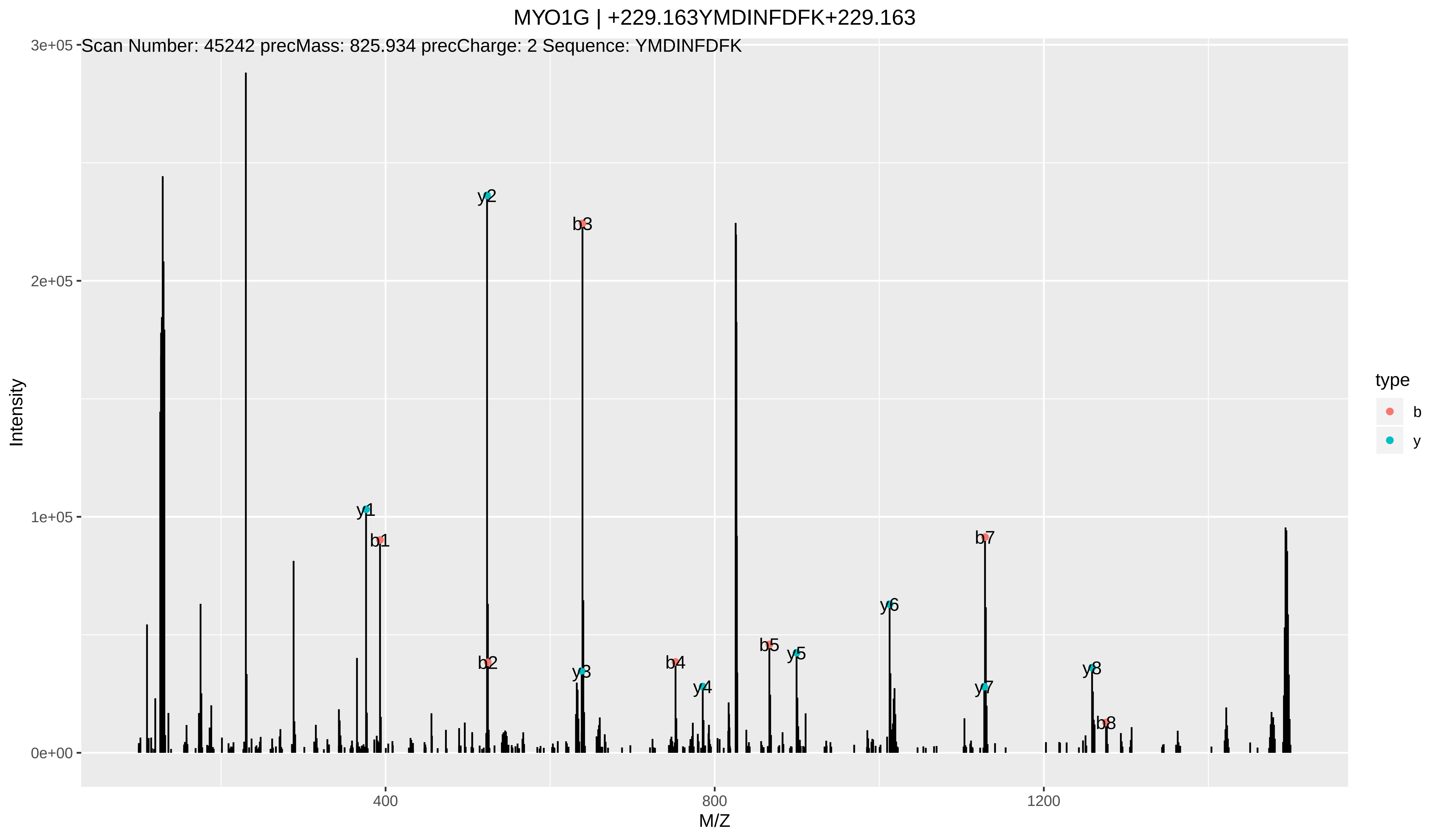
MYO1G | +229.163YMDINFDFK+229.163
Scan Number: 45242 precMass: 825.934 precCharge: 2 Sequence: YMDINFDFK
Intensity
M/Z
3e+05
2e+05
1e+05
0e+00
400
800
1200
y1
b1
y2
b2
b3
y3
b4
y4
b5
y5
y6
b7
y7
y8
b8
type
b
y

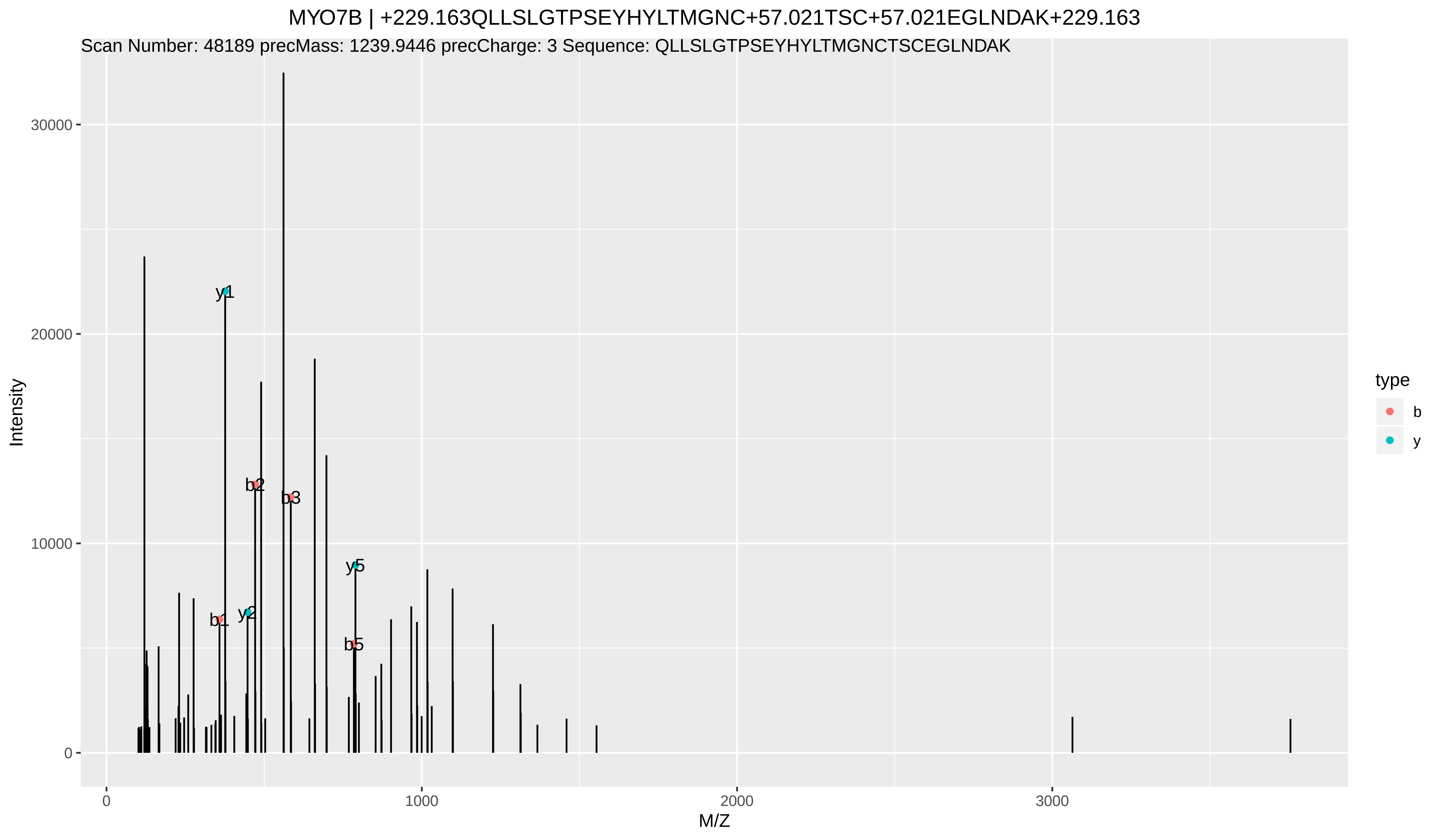

MYO7B | +229.163QLLSLGTPSEYHYLTMGNC+57.021TSC+57.021EGLNDAK+229.163
Scan Number: 48189 precMass: 1239.9446 precCharge: 3 Sequence: QLLSLGTPSEYHYLTMGNCTSCEGLNDAK
Intensity
M/Z
0
10000
20000
30000
1000
2000
3000
y1
b2
b3
y5
b1
y2
b5
type
b
y

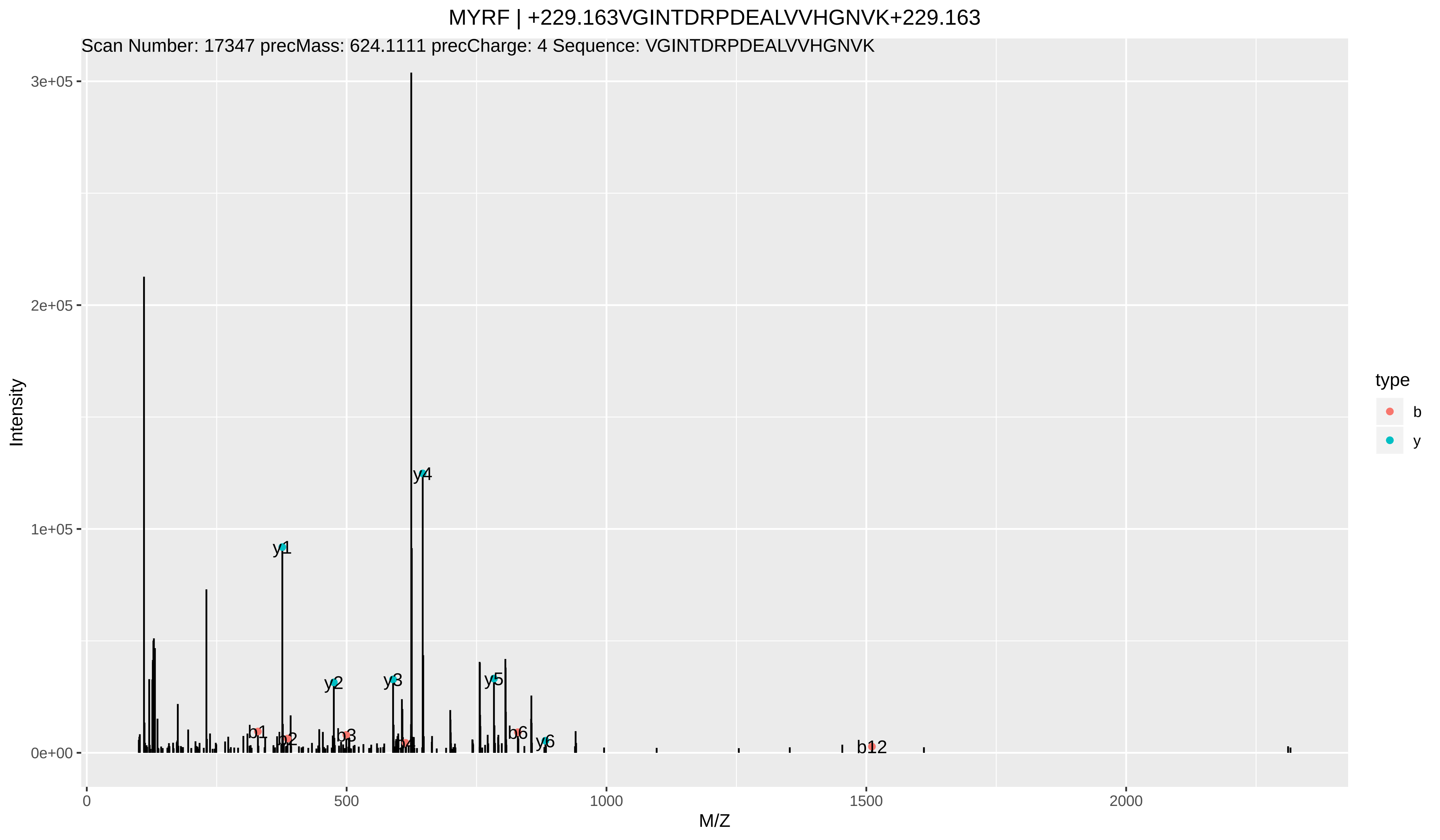

MYRF | +229.163VGINTDRPDEALVVHGNVK+229.163
Scan Number: 17347 precMass: 624.1111 precCharge: 4 Sequence: VGINTDRPDEALVVHGNVK
Intensity
M/Z
type
b
y
y1
y2
y3
y4
y5
y6
b1
b2
b3
b4
b6
b12

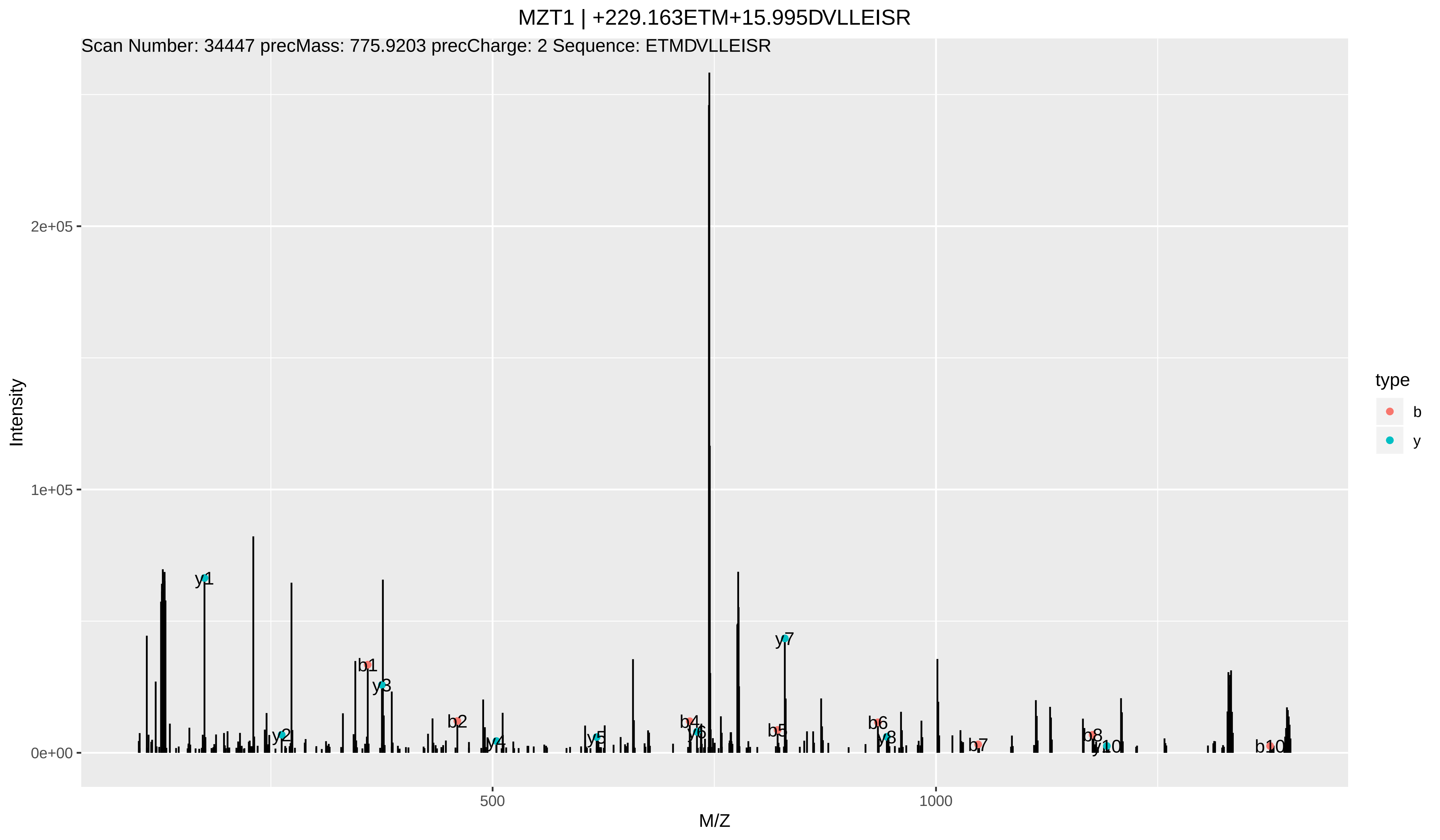
MZT1 | +229.163ETM+15.995DVLLEISR
Scan Number: 34447 precMass: 775.9203 precCharge: 2 Sequence: ETMDVLLEISR
Intensity
2e+05
1e+05
0e+00
M/Z
500
1000
y1
y2
b1
y3
b2
y4
y5
b4
y6
b5
y7
b6
y8
b7
b8
y10
b10
type
b
y

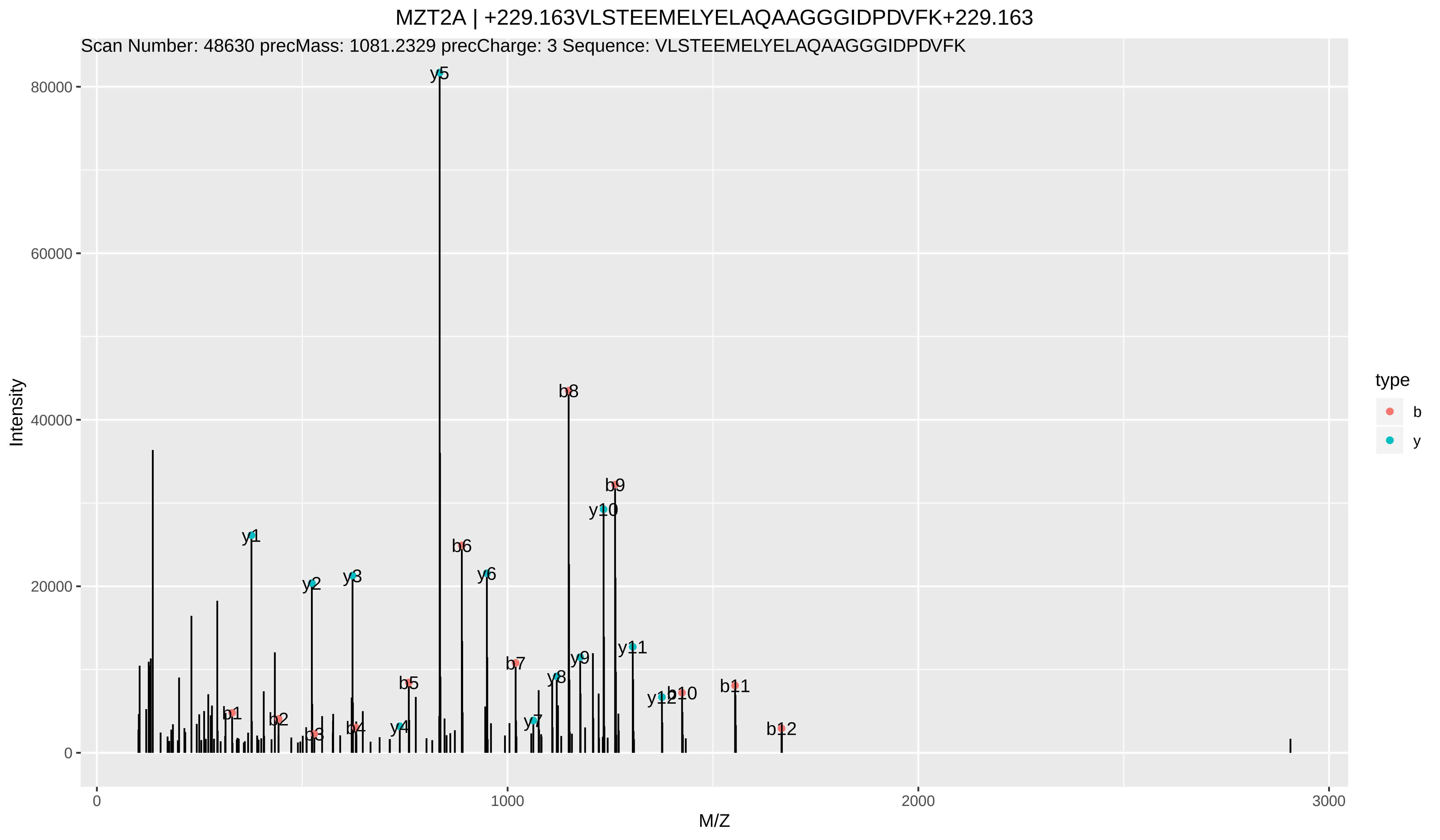
MZT2A | +229.163VLSTEEMELYELAQAAGGGIDPDVFK+229.163
Scan Number: 48630 precMass: 1081.2329 precCharge: 3 Sequence: VLSTEEMELYELAQAAGGGIDPDVFK
Intensity
M/Z
type
b
y
y5
b8
b9
y10
y1
b6
y2
y3
y6
y11
y9
b7
y8
b5
b11
y12
b10
b1
b2
y7
b3
b4
y4
b12
0
20000
40000
60000
80000
1000
2000
3000

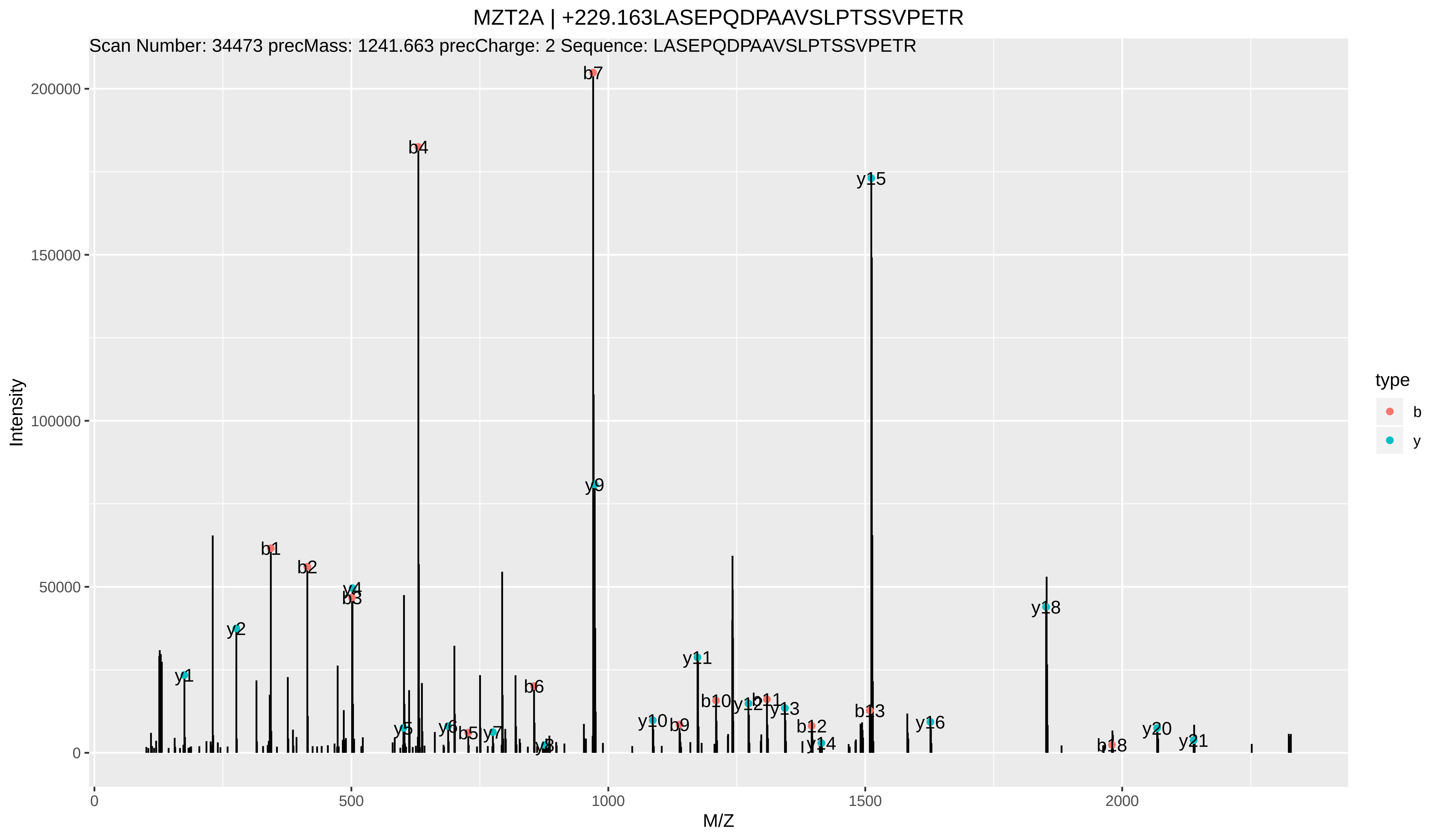
MZT2A | +229.163LASEPQDPAAVSLPTSSVPETR
Scan Number: 34473 precMass: 1241.663 precCharge: 2 Sequence: LASEPQDPAAVSLPTSSVPETR
Intensity
M/Z
type
b
y
b7
b4
y15
y9
b1
b2
y4
b3
y18
y2
y11
y1
b6
b10
y12
b11
y13
b13
y10
b9
y16
b12
y5
y6
b5
y7
y20
y14
y21
y8
b18
0
50000
100000
150000
200000
500
1000
1500
2000

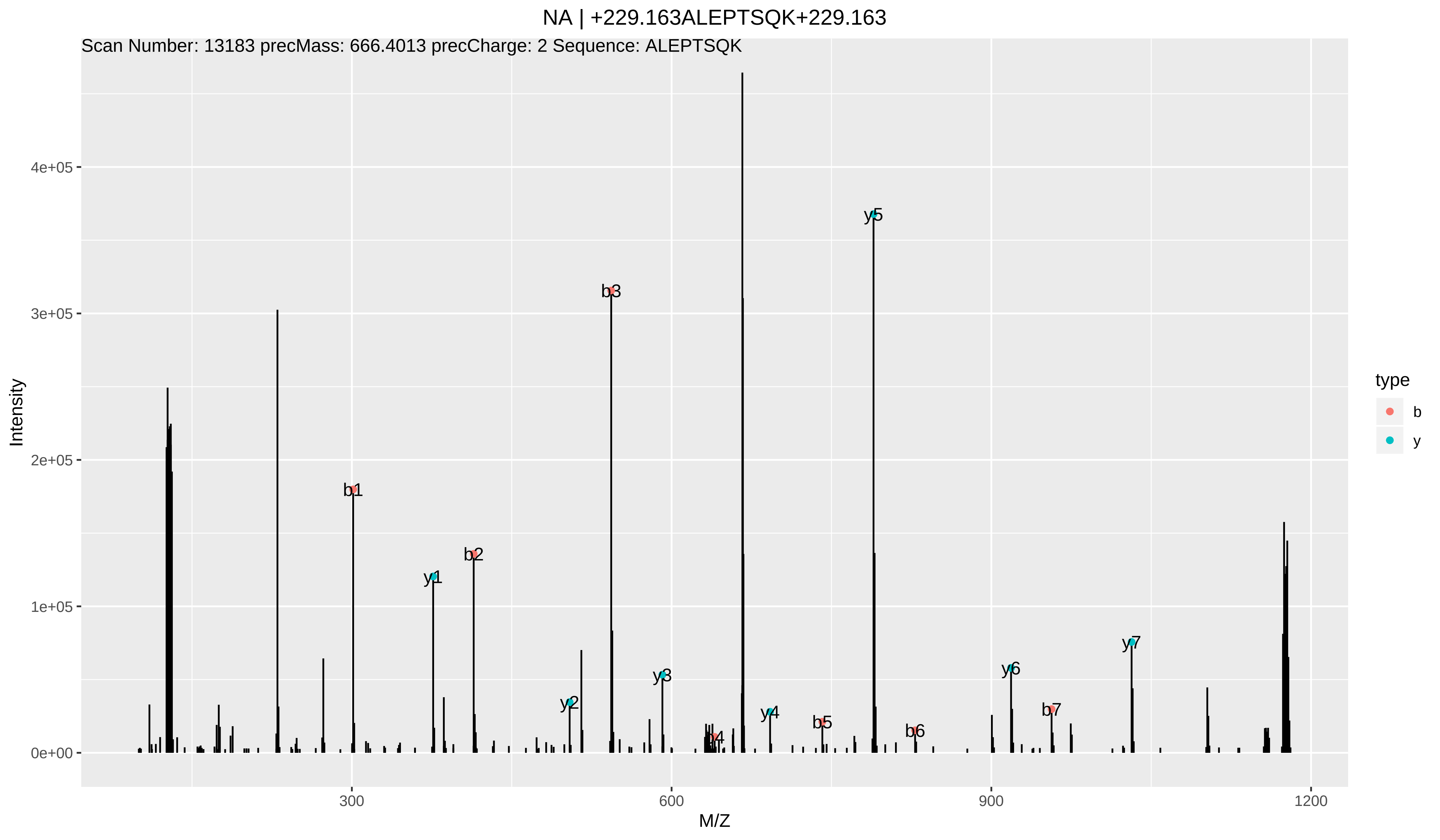
NA | +229.163ALEPTSQK+229.163
Scan Number: 13183 precMass: 666.4013 precCharge: 2 Sequence: ALEPTSQK
Intensity
0e+00
1e+05
2e+05
3e+05
4e+05
300
600
900
1200
M/Z
b1
b2
b3
b4
b5
b6
b7
y1
y2
y3
y4
y5
y6
y7
type
b
y

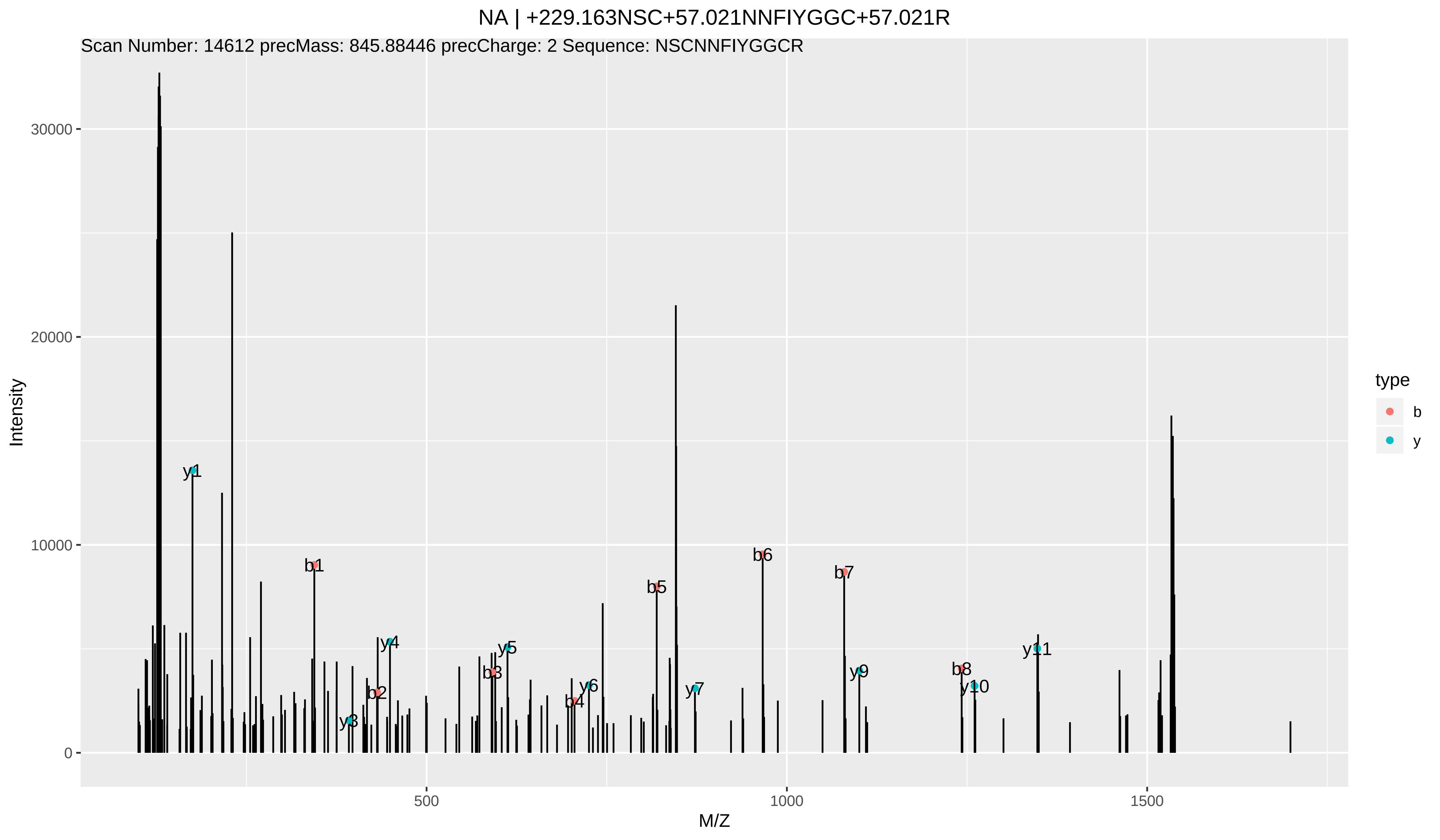
NA | +229.163NSC+57.021NNFIYGGC+57.021R
Scan Number: 14612 precMass: 845.88446 precCharge: 2 Sequence: NSCNNFIYGGCR
Intensity
M/Z
0
10000
20000
30000
500
1000
1500
y1
b1
y3
b2
y4
b3
y5
b4
y6
b5
y7
b6
b7
y9
b8
y10
y11
type
b
y

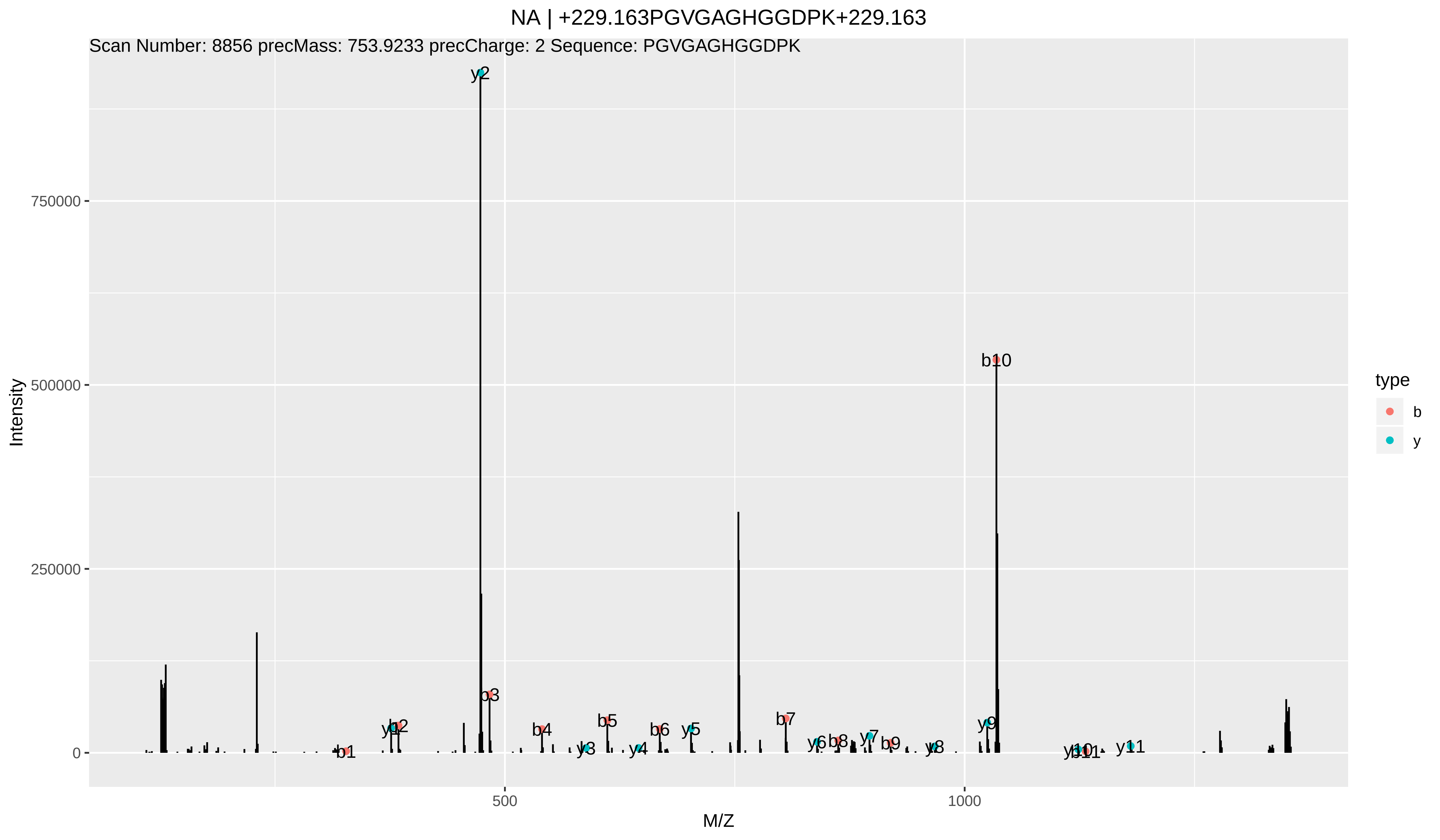
NA | +229.163PGVGAGHGGDPK+229.163
Scan Number: 8856 precMass: 753.9233 precCharge: 2 Sequence: PGVGAGHGGDPK
y2
b10
b3
b1
y1
b2
b4
y3
b5
y4
b6
y5
b7
y6
b8
y7
b9
y8
y9
y10
b11
y11
750000
500000
250000
0
500
1000
Intensity
M/Z
type
b
y

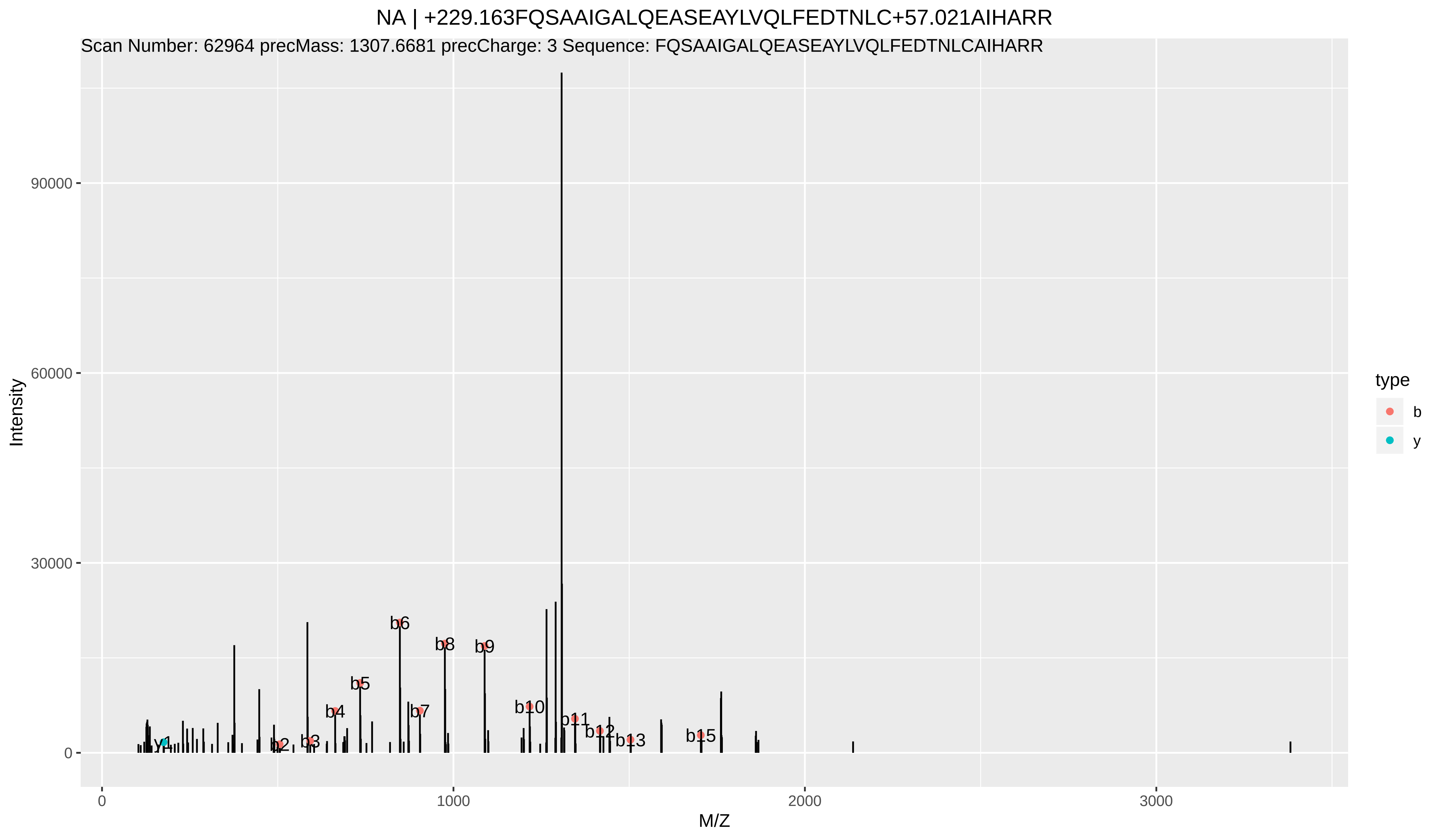

NA | +229.163FQSAAIGALQEASEAYLVQLFEDTNLC+57.021AIHARR
Scan Number: 62964 precMass: 1307.6681 precCharge: 3 Sequence: FQSAAIGALQEASEAYLVQLFEDTNLCAIHARR
Intensity
90000
60000
30000
0
M/Z
0
1000
2000
3000
type
b
y
y1
b2
b3
b4
b5
b6
b7
b8
b9
b10
b11
b12
b13
b15

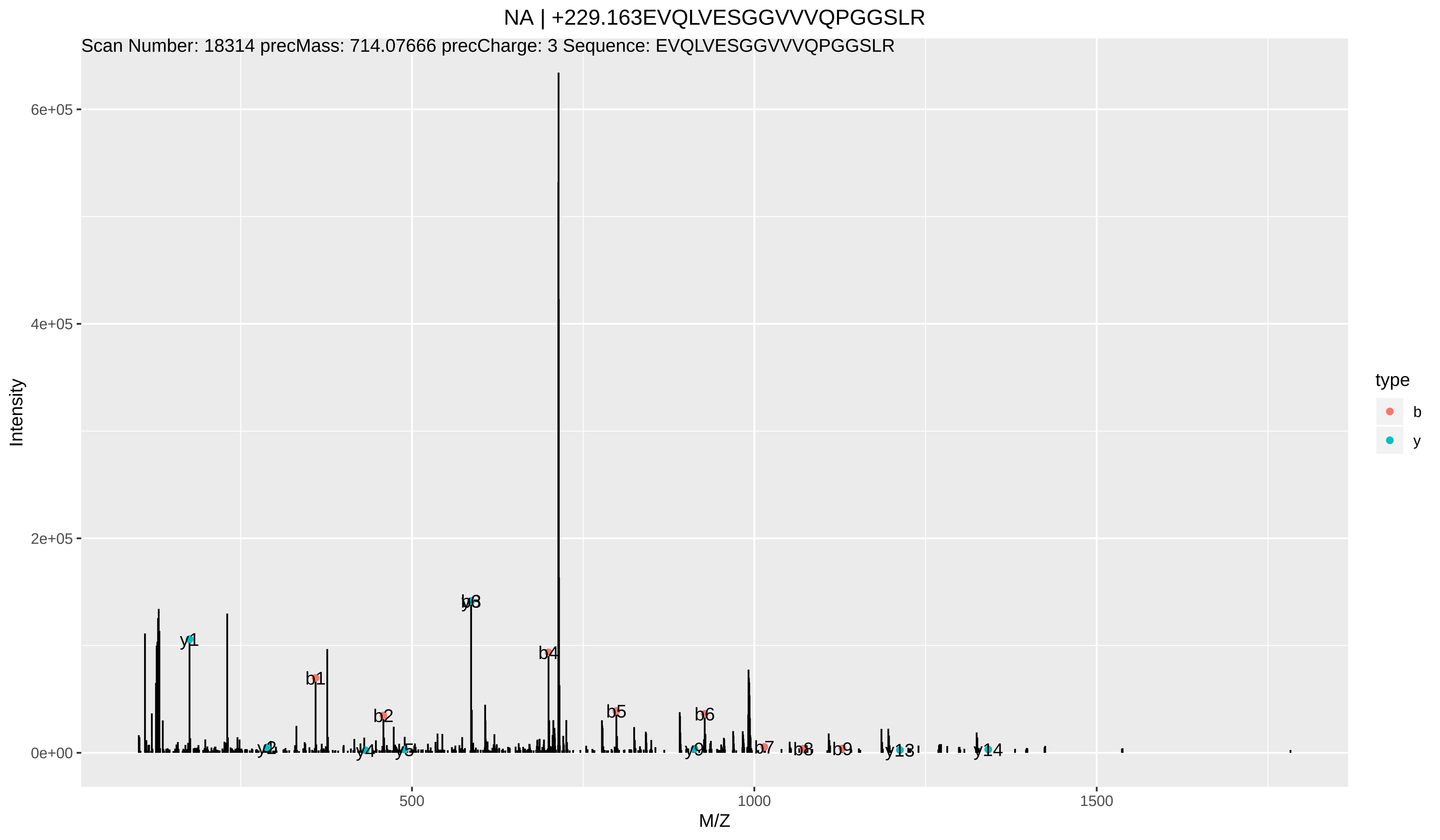

NA | +229.163EVQLVESGGVVVQPGGSLR
Scan Number: 18314 precMass: 714.07666 precCharge: 3 Sequence: EVQLVESGGVVVQPGGSLR
Intensity
6e+05
4e+05
2e+05
0e+00
M/Z
500
1000
1500
y1
b1
b2
y2
y4
y5
b3
y8
b4
b5
b6
y9
b7
b8
b9
y13
y14
type
b
y

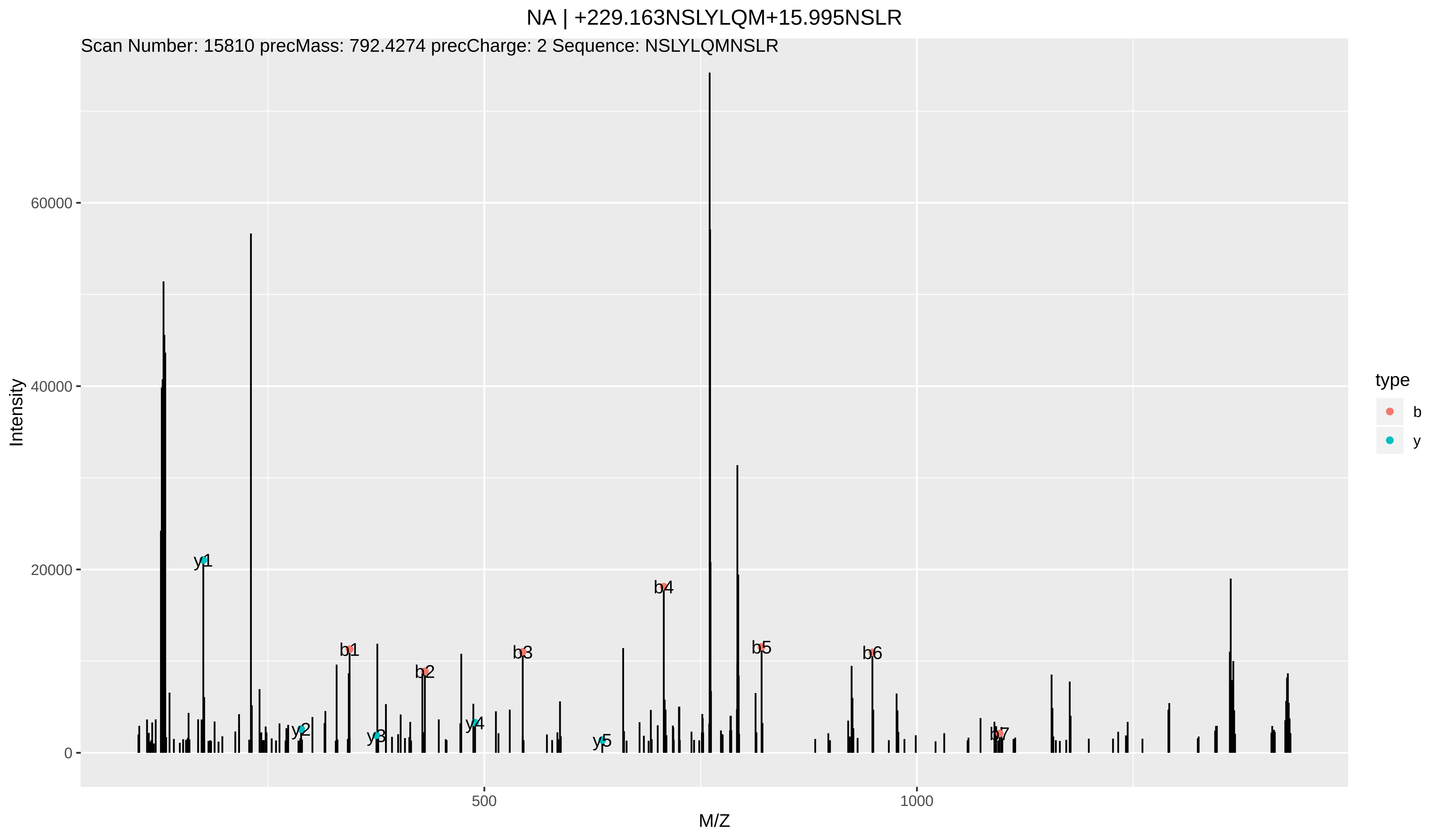

NA | +229.163NSLYLQM+15.995NSLR
Scan Number: 15810 precMass: 792.4274 precCharge: 2 Sequence: NSLYLQMNSLR
Intensity
60000
40000
20000
0
M/Z
500
1000
y1
y2
y3
y4
y5
b1
b2
b3
b4
b5
b6
b7
type
b
y

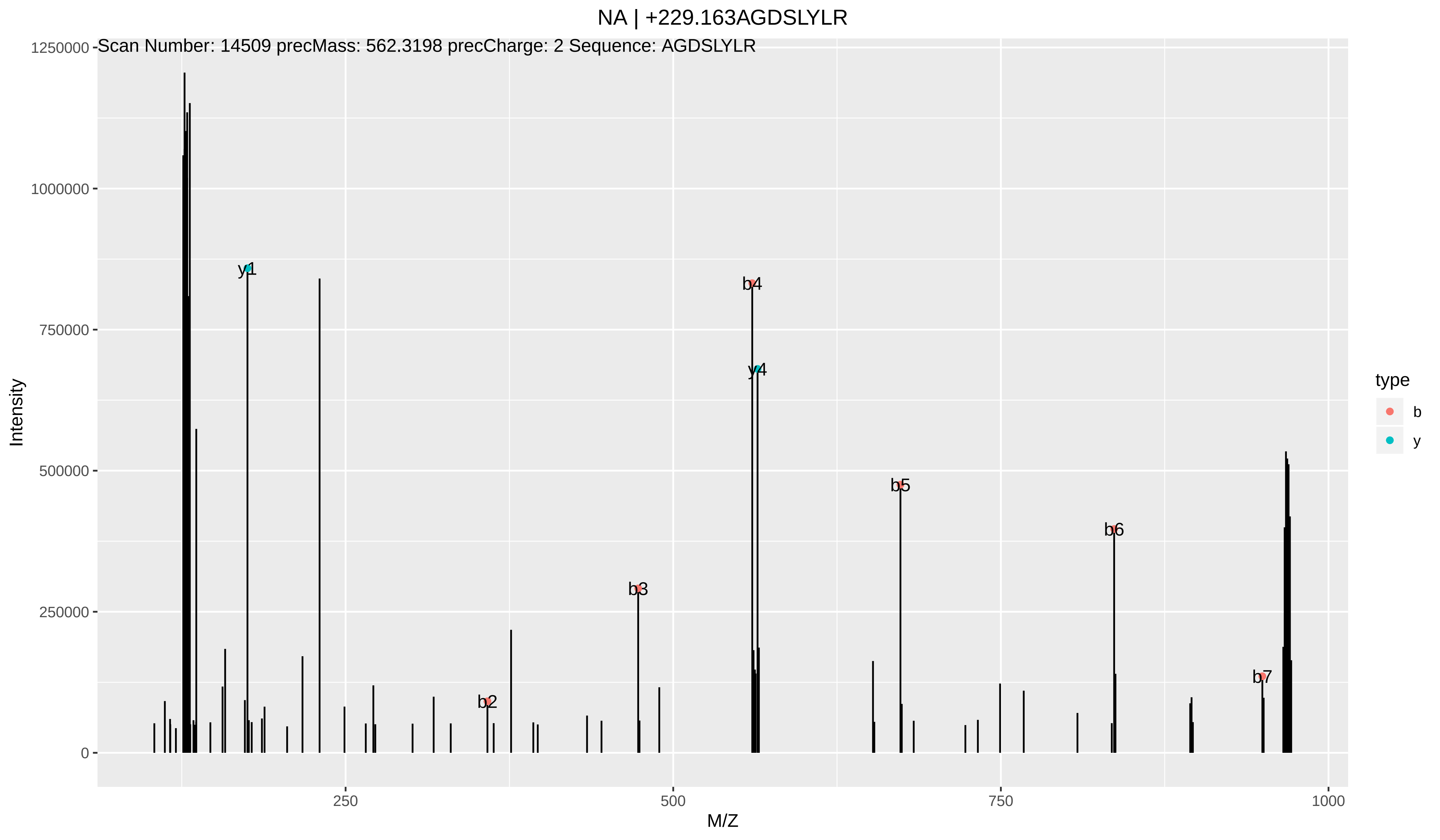

NA | +229.163AGDSLYLR
Scan Number: 14509 precMass: 562.3198 precCharge: 2 Sequence: AGDSLYLR
Intensity
1250000
1000000
750000
500000
250000
0
M/Z
250
500
750
1000
y1
b2
b3
b4
y4
b5
b6
b7
type
b
y

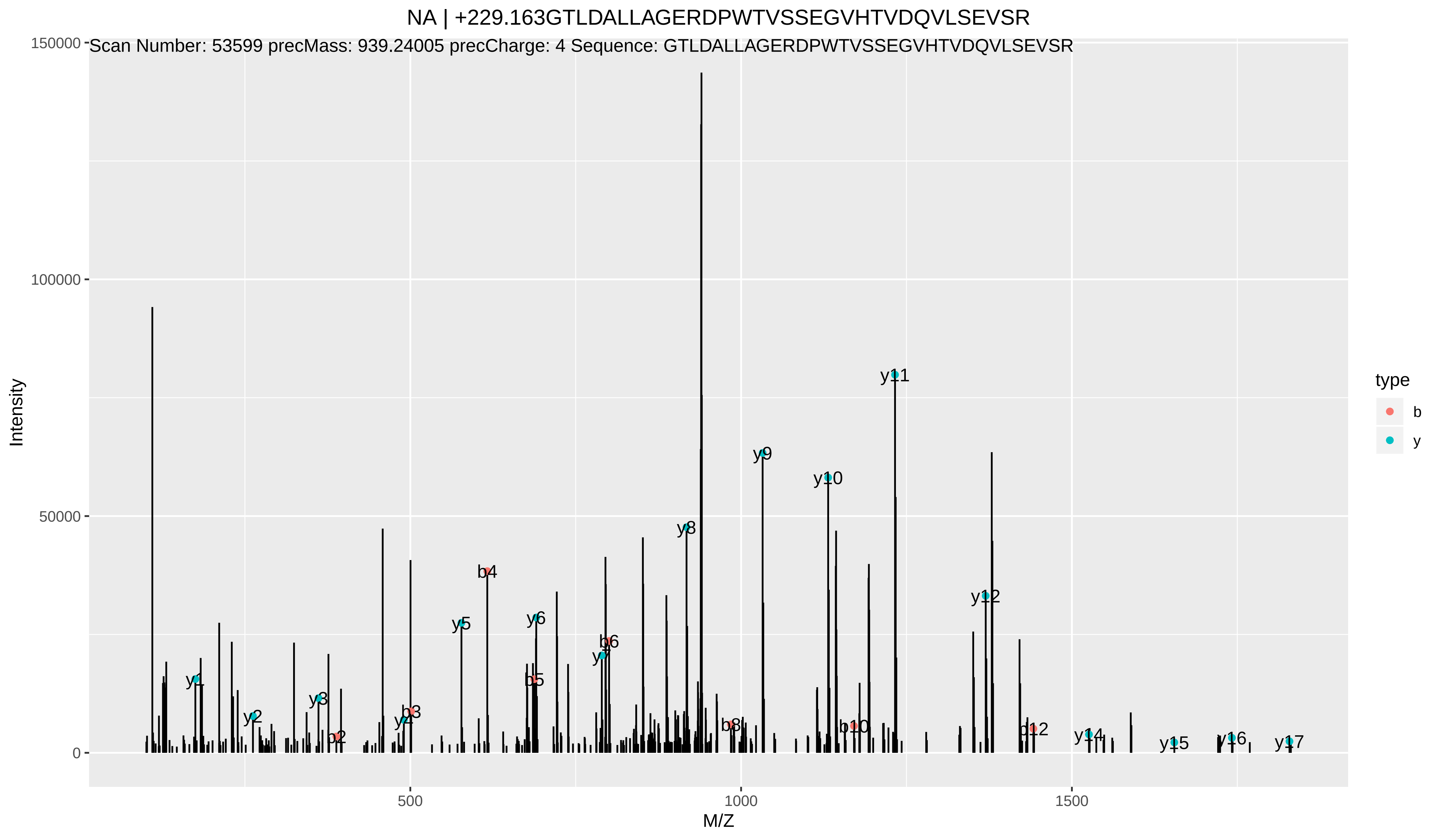

NA | +229.163GTLDALLAGERDPWTVSSEGVHTVDQVLSEVSR
Scan Number: 53599 precMass: 939.24005 precCharge: 4 Sequence: GTLDALLAGERDPWTVSSEGVHTVDQVLSEVSR
Intensity
M/Z
type
b
y
150000
100000
50000
0
500
1000
1500
y1
y2
y3
b2
y4
b3
y5
b4
y6
b5
y7
b6
y8
b8
y9
y10
b10
y11
y12
b12
y14
y15
y16
y17

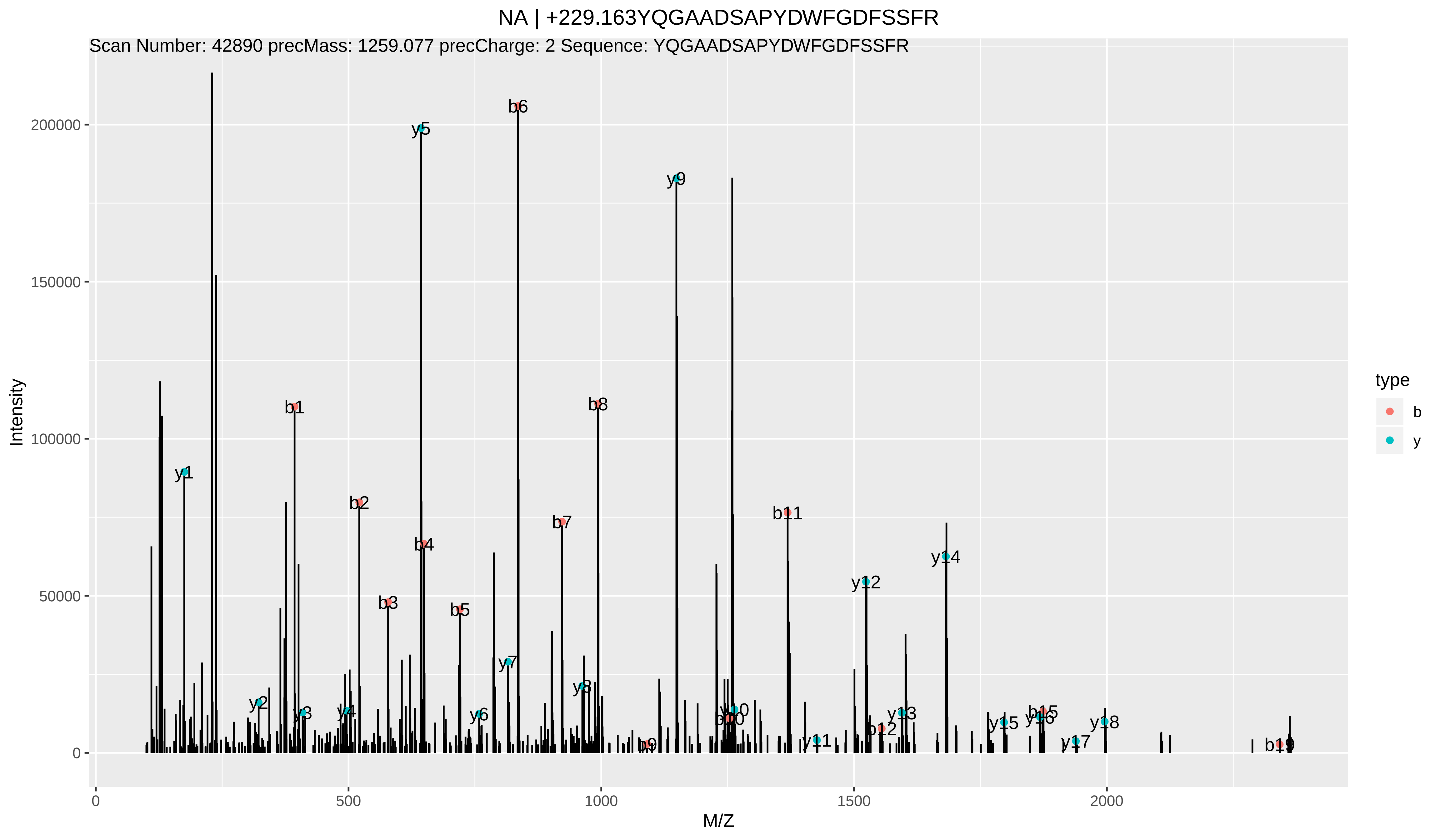
NA | +229.163YQGAADSAPYDWFGDFSSFR
Scan Number: 42890 precMass: 1259.077 precCharge: 2 Sequence: YQGAADSAPYDWFGDFSSFR
Intensity
M/Z
type
b
y
y1
y2
y3
y4
y5
y6
y7
y8
y9
y11
y12
y13
y14
y15
y16
y17
y18
b1
b2
b3
b4
b5
b6
b7
b8
b11
b12
b15
0
50000
100000
150000
200000
500
1000
1500
2000

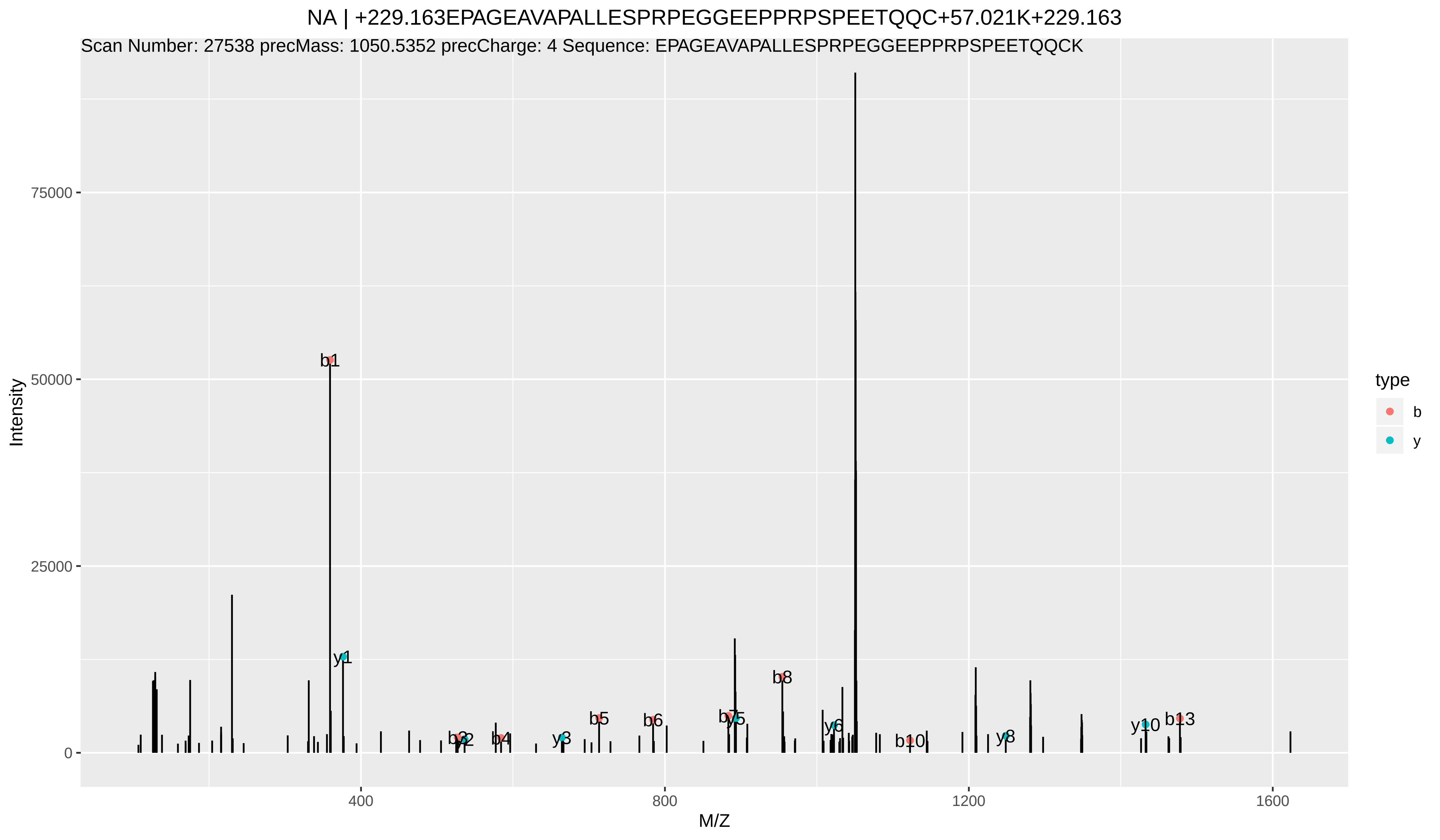

NA | +229.163EPAGEAVAPALLESPRPEGGEEPPRPSPEETQQC+57.021K+229.163
Scan Number: 27538 precMass: 1050.5352 precCharge: 4 Sequence: EPAGEAVAPALLESPRPEGGEEPPRPSPEETQQCK
Intensity
75000
50000
25000
0
400
800
1200
1600
M/Z
b1
y1
b2
y2
b4
y3
b5
b6
b7
y5
b8
y6
b10
y8
y10
b13
type
b
y

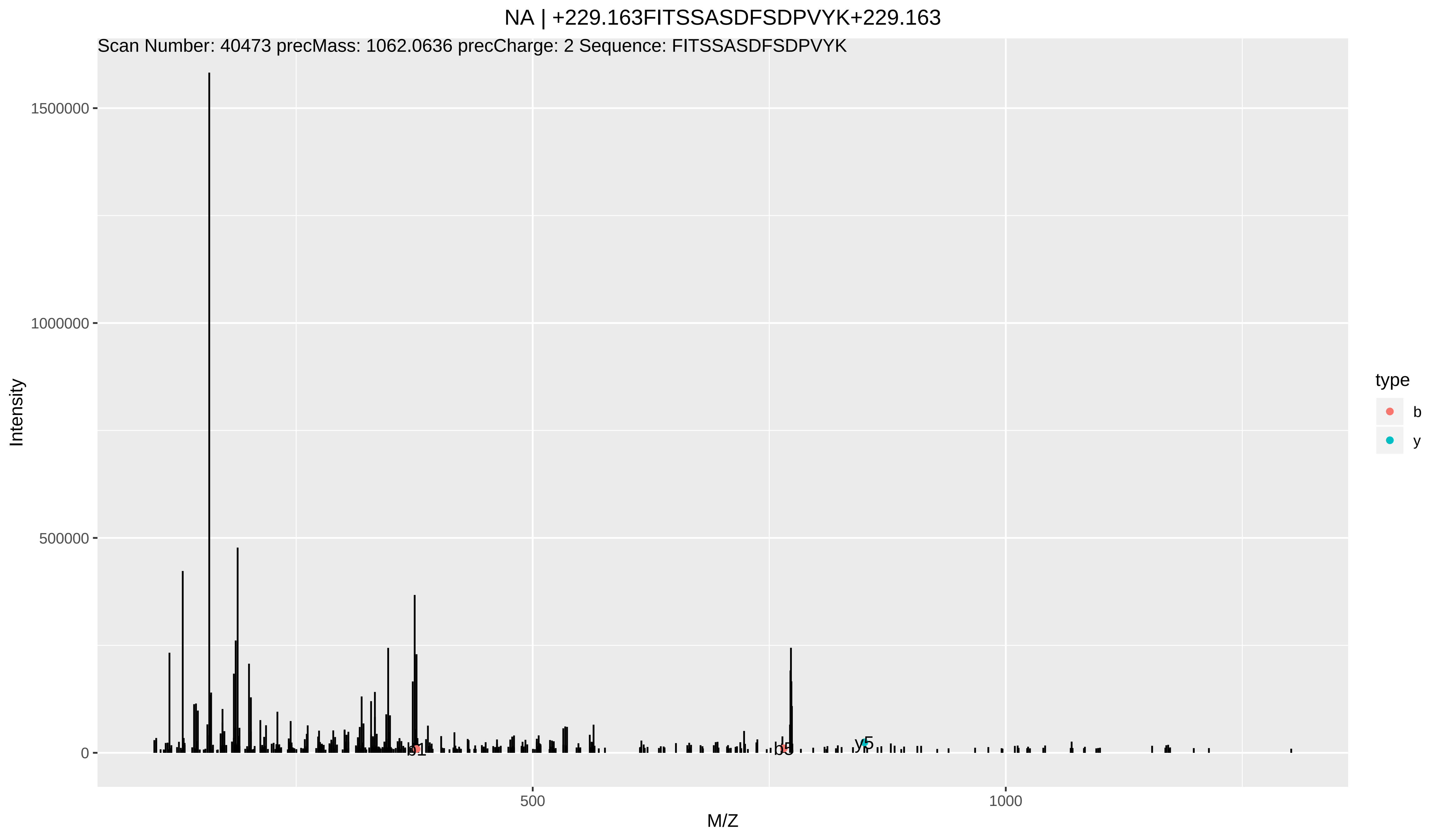
NA | +229.163FITSSASDFSDPVYK+229.163
Scan Number: 40473 precMass: 1062.0636 precCharge: 2 Sequence: FITSSASDFSDPVYK
1500000
1000000
500000
0
Intensity
500
1000
M/Z
b1
b5
y5
type
b
y

NAA60 | +229.163HGIGSLLLESLK+229.163

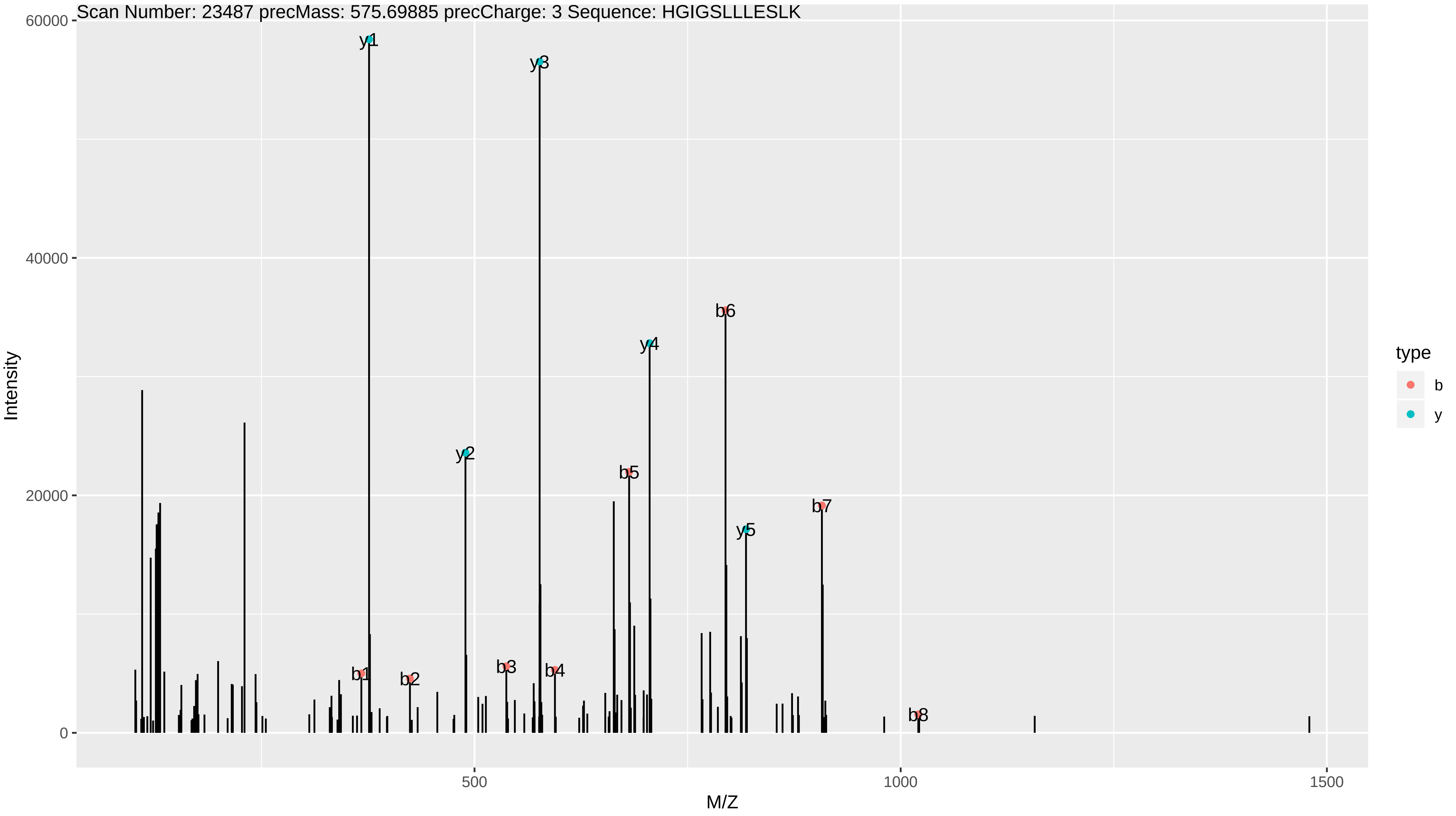

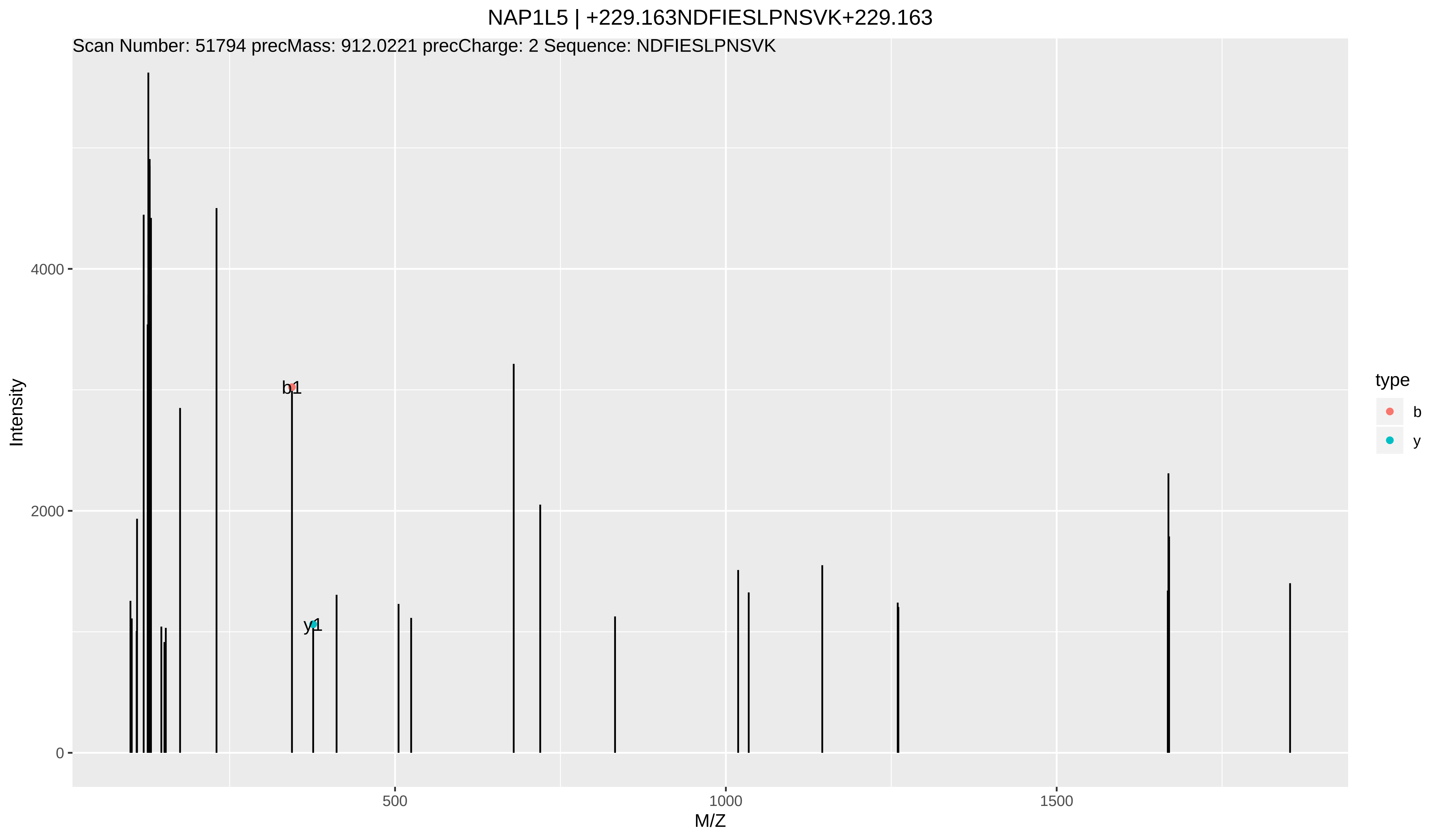

NAP1L5 | +229.163NDFIESLPNSVK+229.163
Scan Number: 51794 precMass: 912.0221 precCharge: 2 Sequence: NDFIESLPNSVK
Intensity
4000
2000
0
500
1000
1500
M/Z
b1
y1
type
b
y

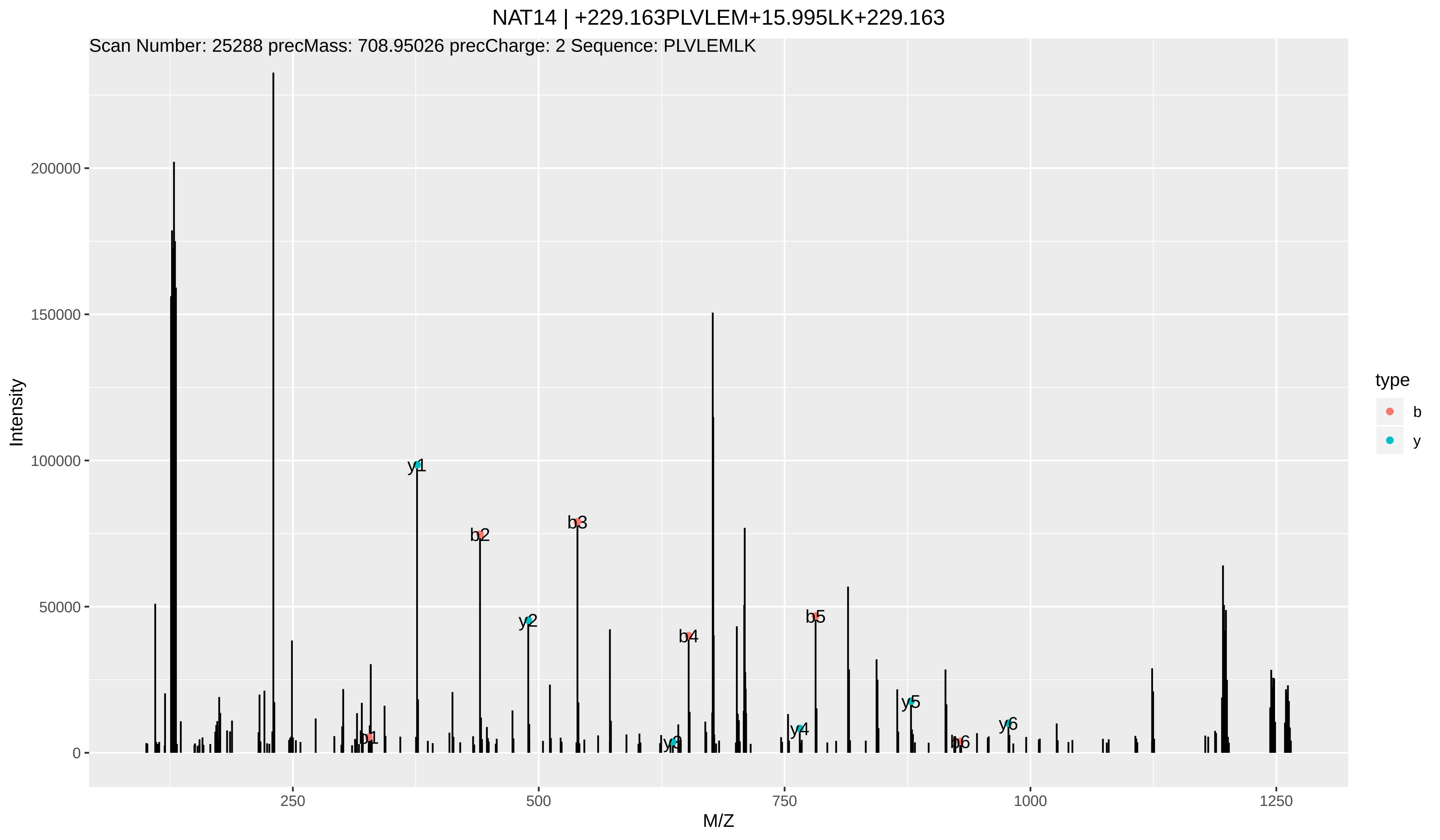
NAT14 | +229.163PLVLEM+15.995LK+229.163
Scan Number: 25288 precMass: 708.95026 precCharge: 2 Sequence: PLVLEMLK
Intensity
M/Z
type
b
y
y1
b2
y2
b3
b4
b5
y4
y5
b6
y6
b1
0
50000
100000
150000
200000
250
500
750
1000
1250

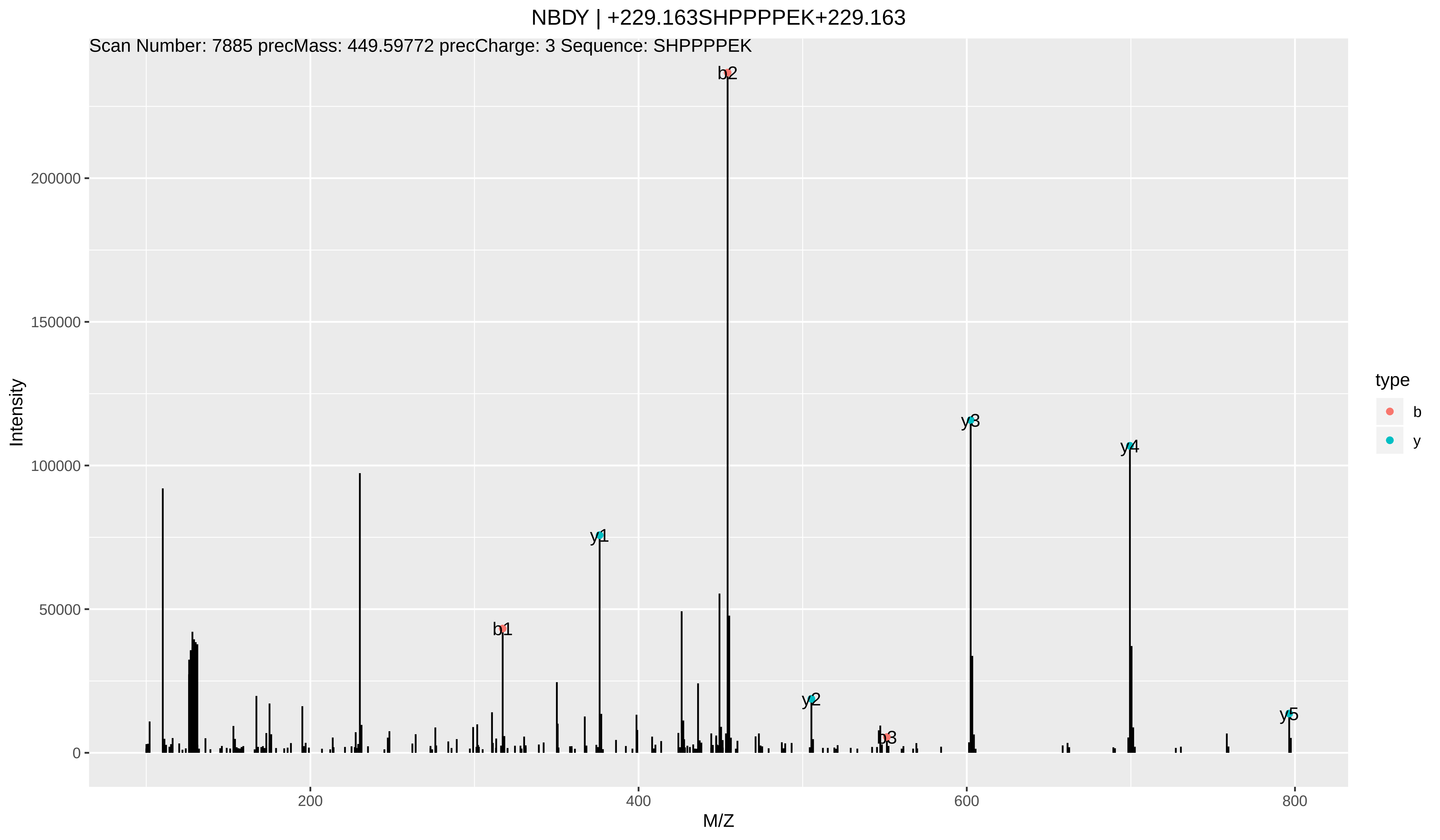

NBDY | +229.163SHPPPPEK+229.163
Scan Number: 7885 precMass: 449.59772 precCharge: 3 Sequence: SHPPPPEK
Intensity
M/Z
0
50000
100000
150000
200000
200
400
600
800
b1
b2
b3
y1
y2
y3
y4
y5
type
b
y

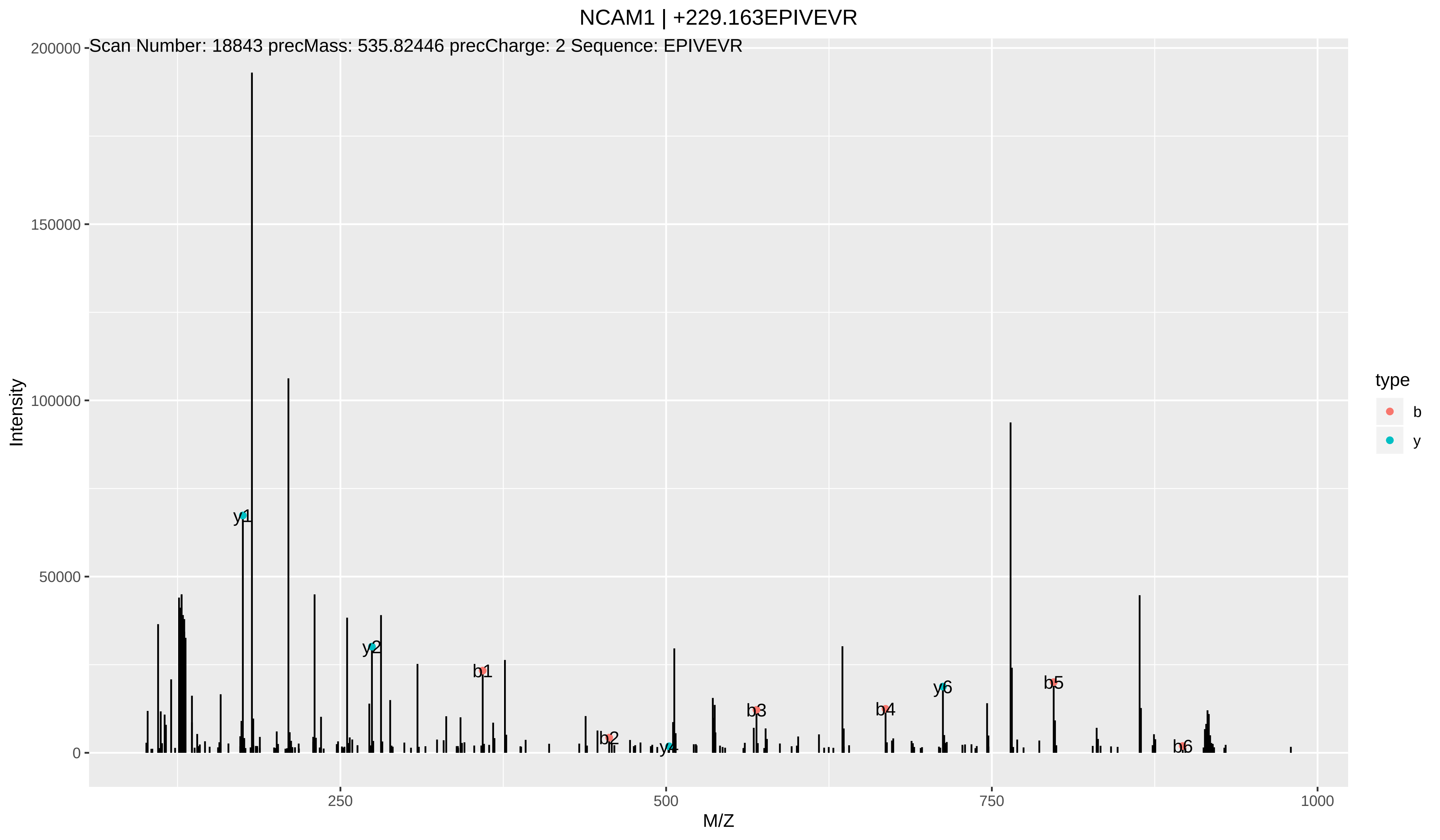

NCAM1 | +229.163EPIVEVR
Scan Number: 18843 precMass: 535.82446 precCharge: 2 Sequence: EPIVEVR
Intensity
200000
150000
100000
50000
0
M/Z
250
500
750
1000
y1
y2
b1
b2
y4
b3
b4
y6
b5
b6
type
b
y

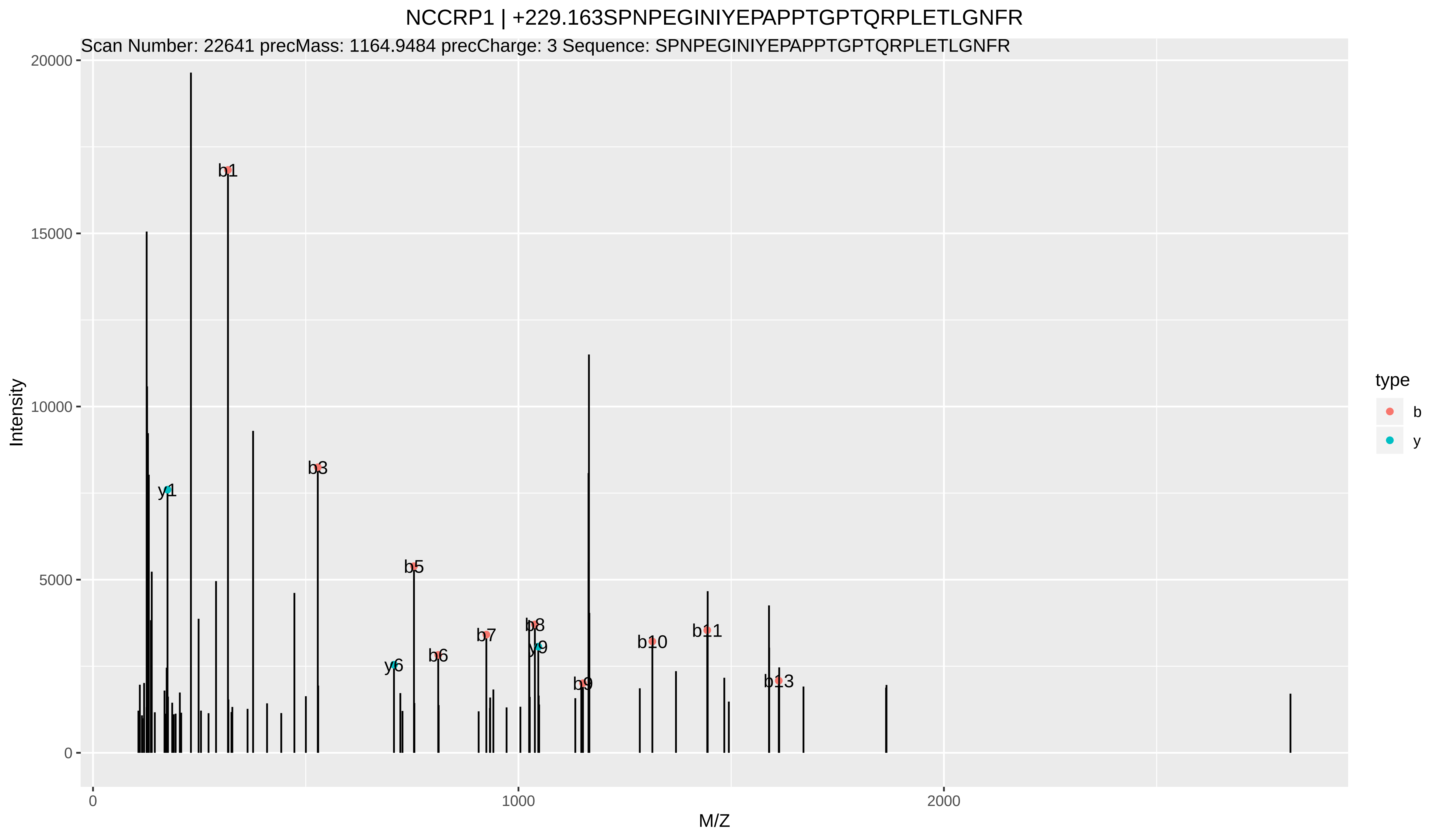
NCCRP1 | +229.163SPNPEGINIYEPAPPTGPTQRPLETLGNFR
Scan Number: 22641 precMass: 1164.9484 precCharge: 3 Sequence: SPNPEGINIYEPAPPTGPTQRPLETLGNFR
Intensity
M/Z
0
5000
10000
15000
20000
1000
2000
b1
b3
y1
b5
y6
b6
b7
b8
y9
b9
b10
b11
b13
type
b
y

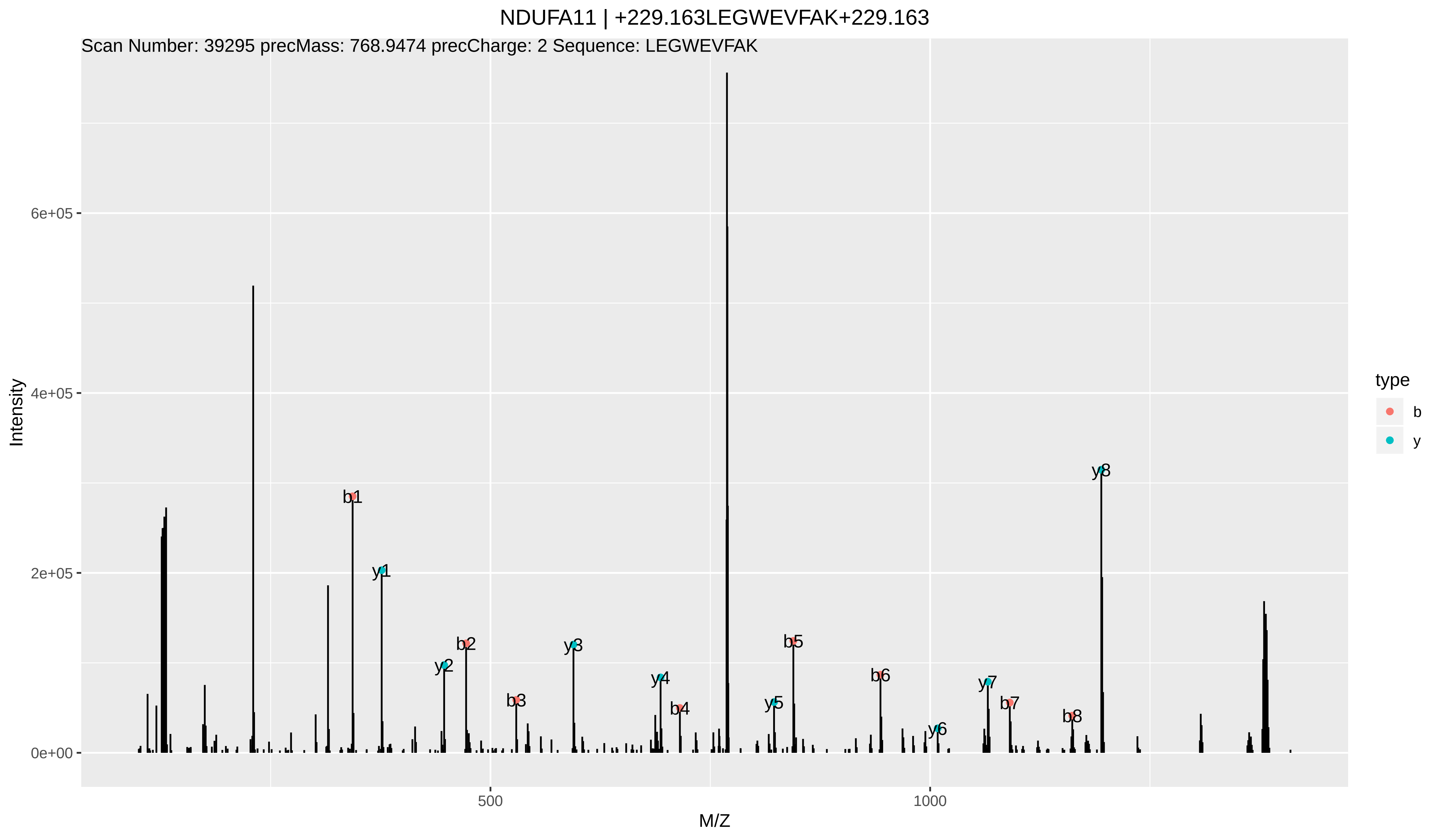

NDUFA11 | +229.163LEGWEVFAK+229.163
Scan Number: 39295 precMass: 768.9474 precCharge: 2 Sequence: LEGWEVFAK
Intensity
6e+05
4e+05
2e+05
0e+00
M/Z
500
1000
b1
y1
y2
b2
b3
y3
y4
b4
y5
b5
b6
y6
y7
b7
b8
y8
type
b
y

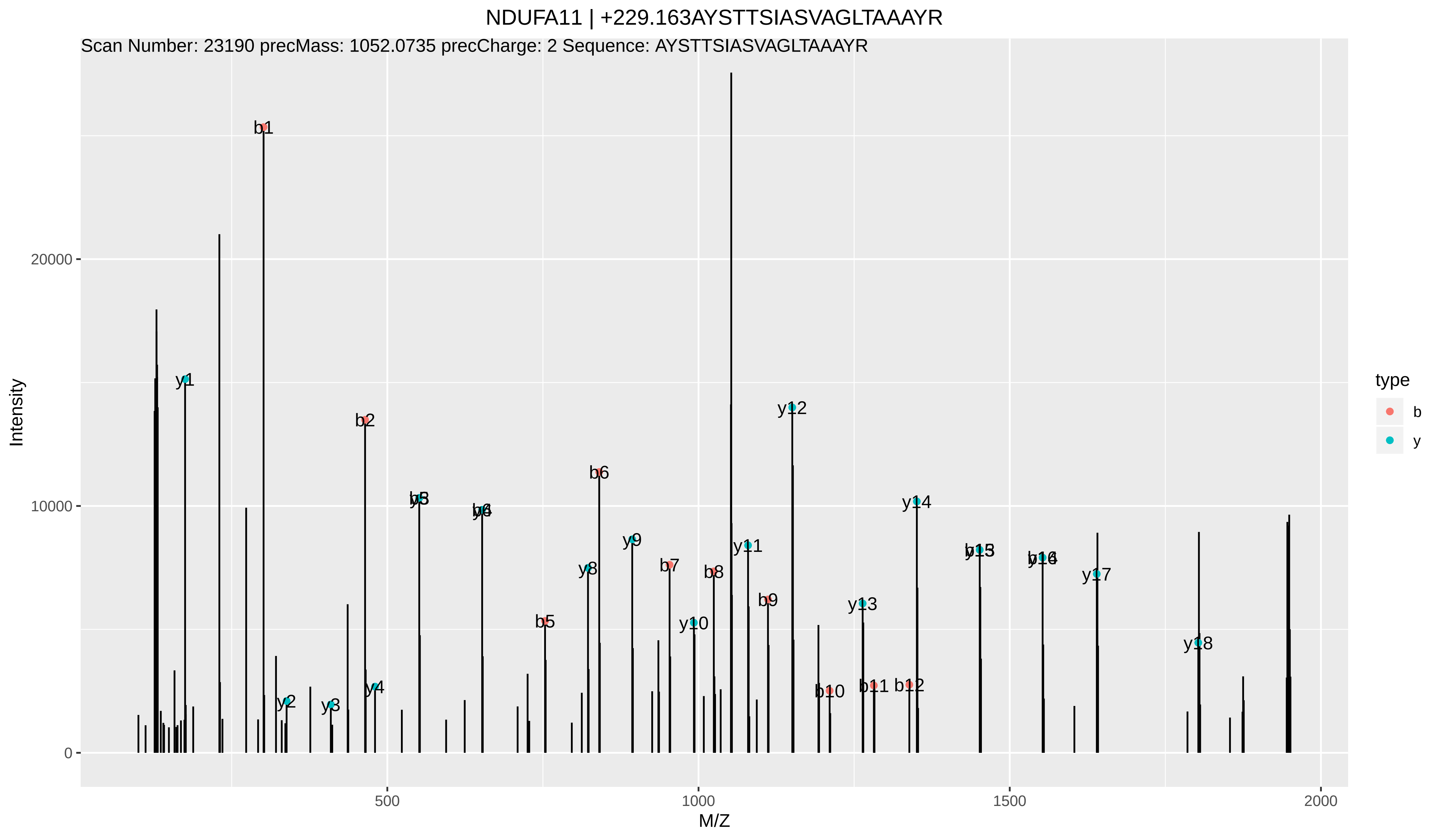
NDUFA11 | +229.163AYSTTSIASVAGLTAAAYR
Scan Number: 23190 precMass: 1052.0735 precCharge: 2 Sequence: AYSTTSIASVAGLTAAAYR
Intensity
M/Z
type
b
y
0
10000
20000
500
1000
1500
2000
b1
y1
b2
b6
y12
y14
y9
y11
y8
b7
b8
b9
b5
y10
y13
y17
y18
y2
y3
y4
b10
b11
b12

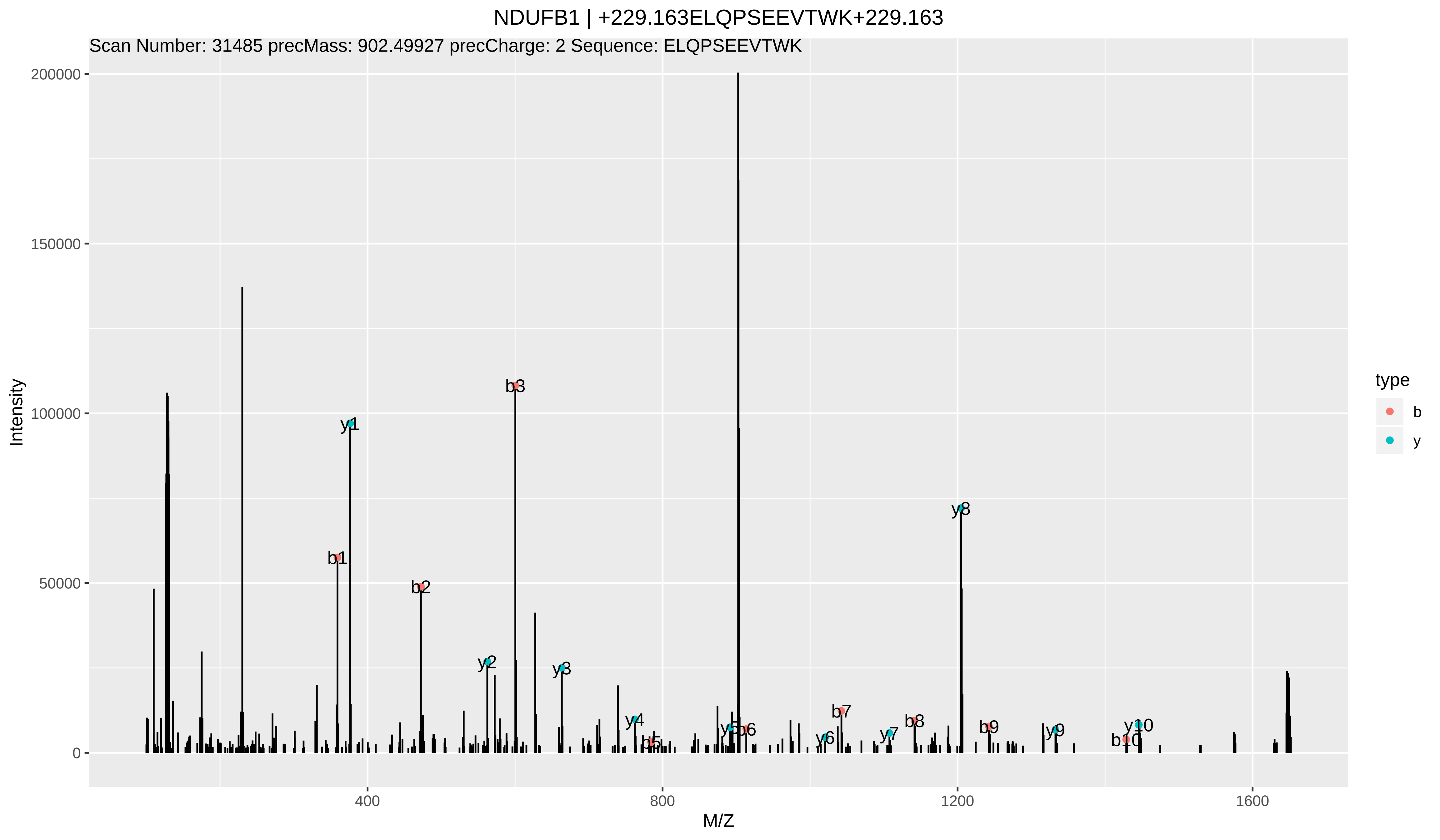

NDUFB1 | +229.163ELQPSEEVTWK+229.163
Scan Number: 31485 precMass: 902.49927 precCharge: 2 Sequence: ELQPSEEVTWK
Intensity
200000
150000
100000
50000
0
M/Z
400
800
1200
1600
b1
b2
b3
b5
b6
b7
b8
b9
b10
y1
y2
y3
y4
y5
y6
y7
y8
y9
y10
type
b
y

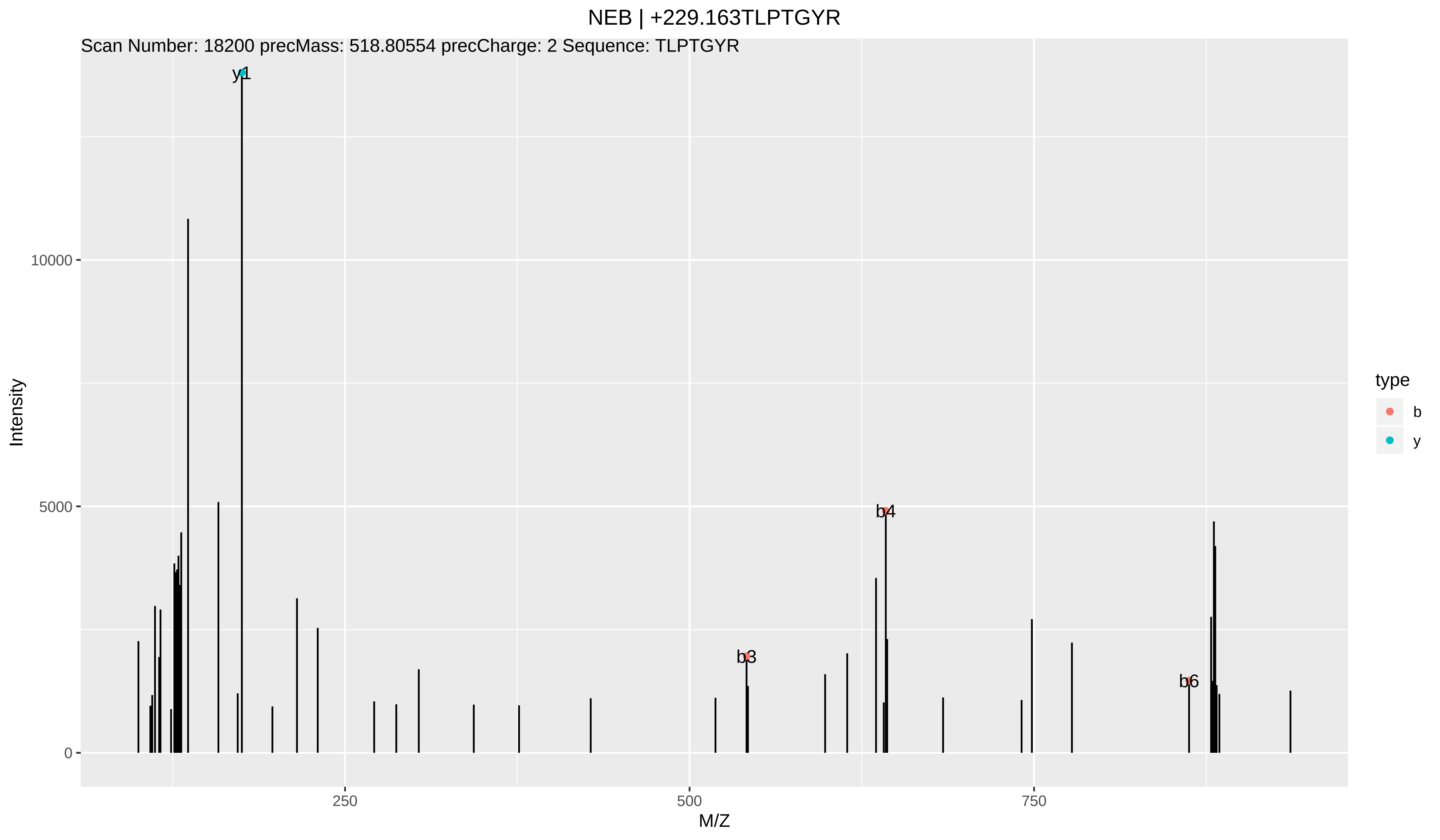

NEB | +229.163TLPTGYR
Scan Number: 18200 precMass: 518.80554 precCharge: 2 Sequence: TLPTGYR
y1
b4
b3
b6
Intensity
10000
5000
0
250
500
750
M/Z
type
b
y

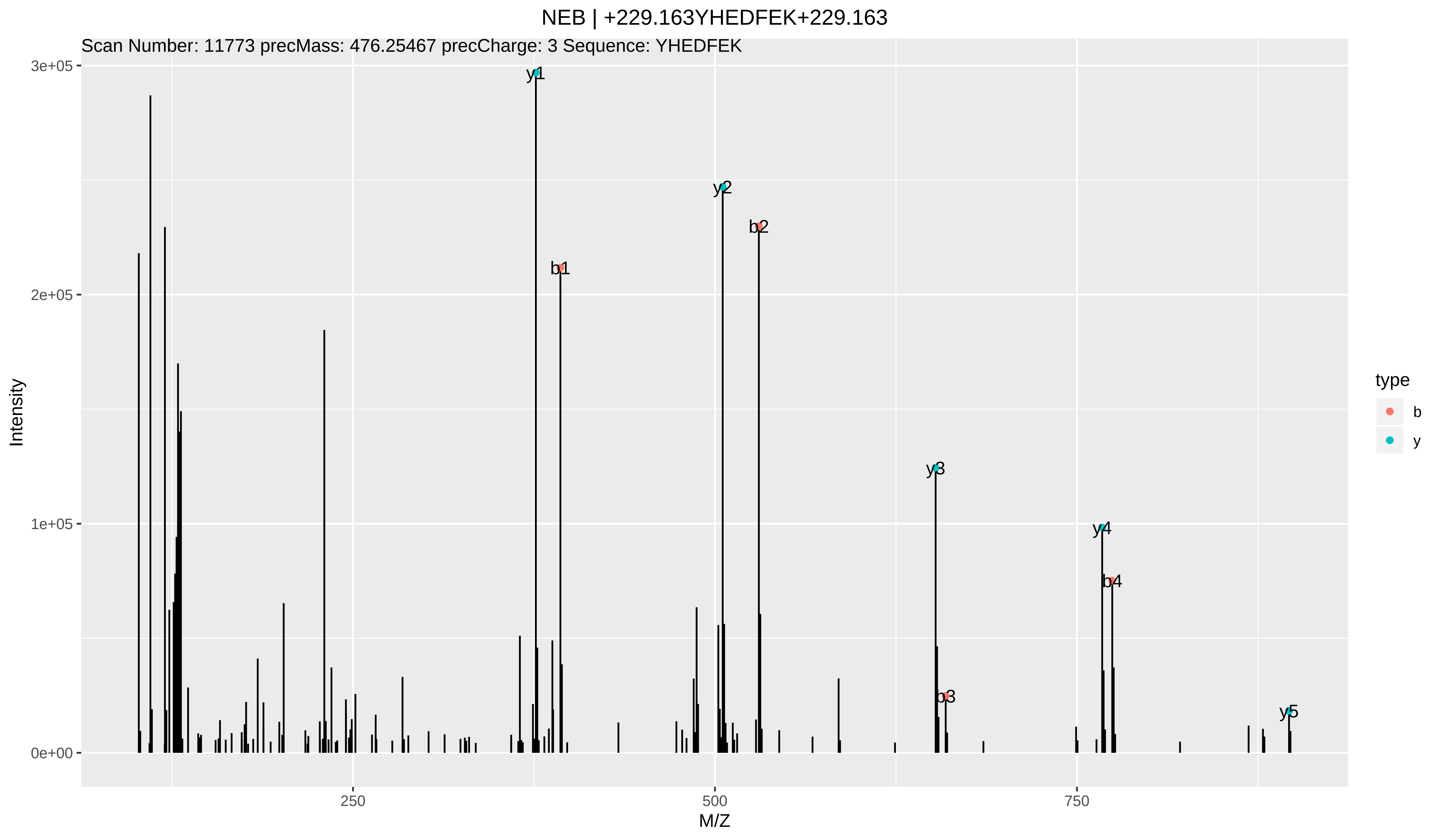

NEB | +229.163YHEDFEK+229.163
Scan Number: 11773 precMass: 476.25467 precCharge: 3 Sequence: YHEDFEK
Intensity
3e+05
2e+05
1e+05
0e+00
M/Z
250
500
750
y1
b1
y2
b2
y3
b3
y4
b4
y5
type
b
y

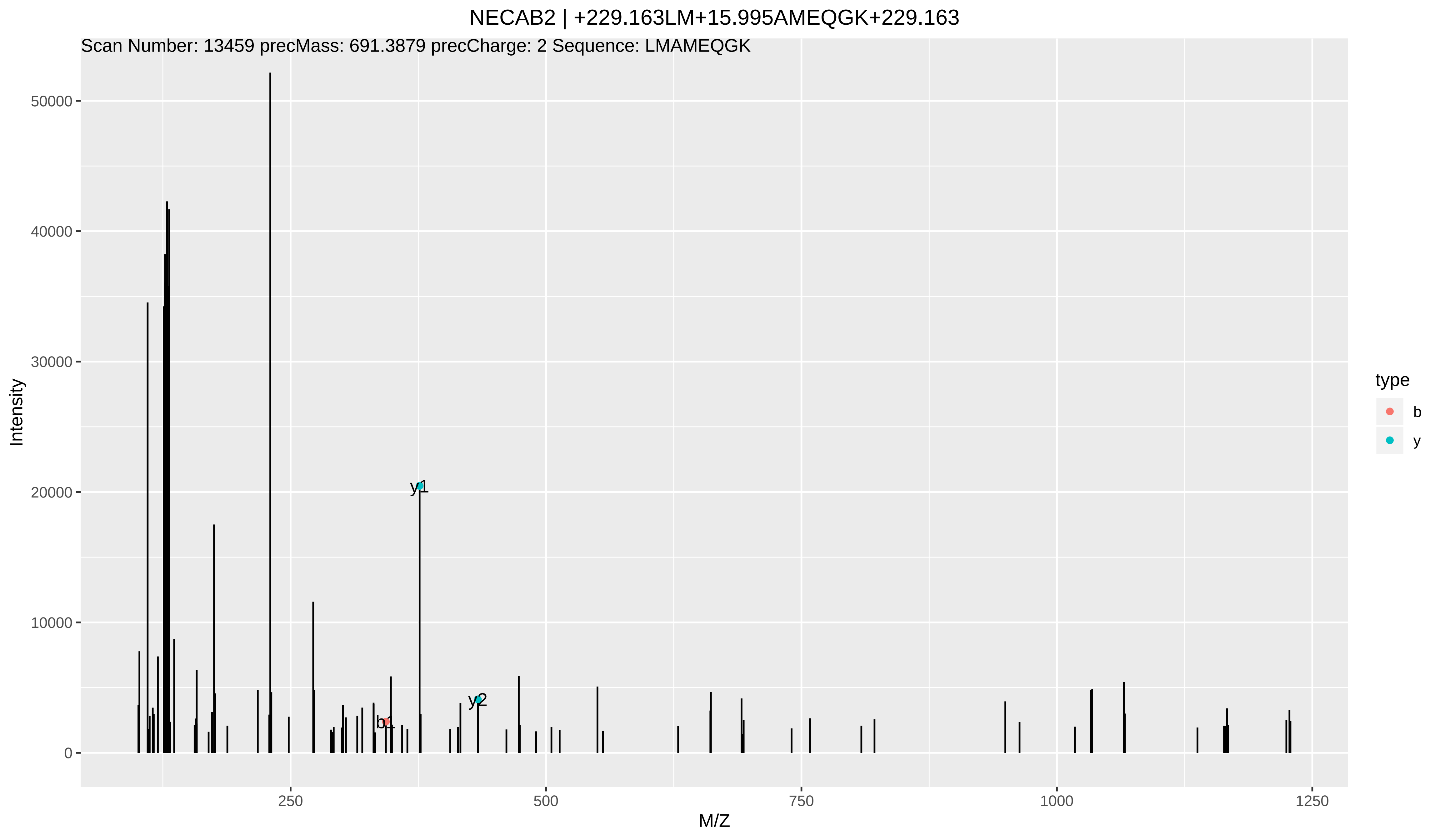
NECAB2 | +229.163LM+15.995AMEQGK+229.163
Scan Number: 13459 precMass: 691.3879 precCharge: 2 Sequence: LMAMEQGK
Intensity
M/Z
type
b
y
y1
y2
b1
0
10000
20000
30000
40000
50000
250
500
750
1000
1250

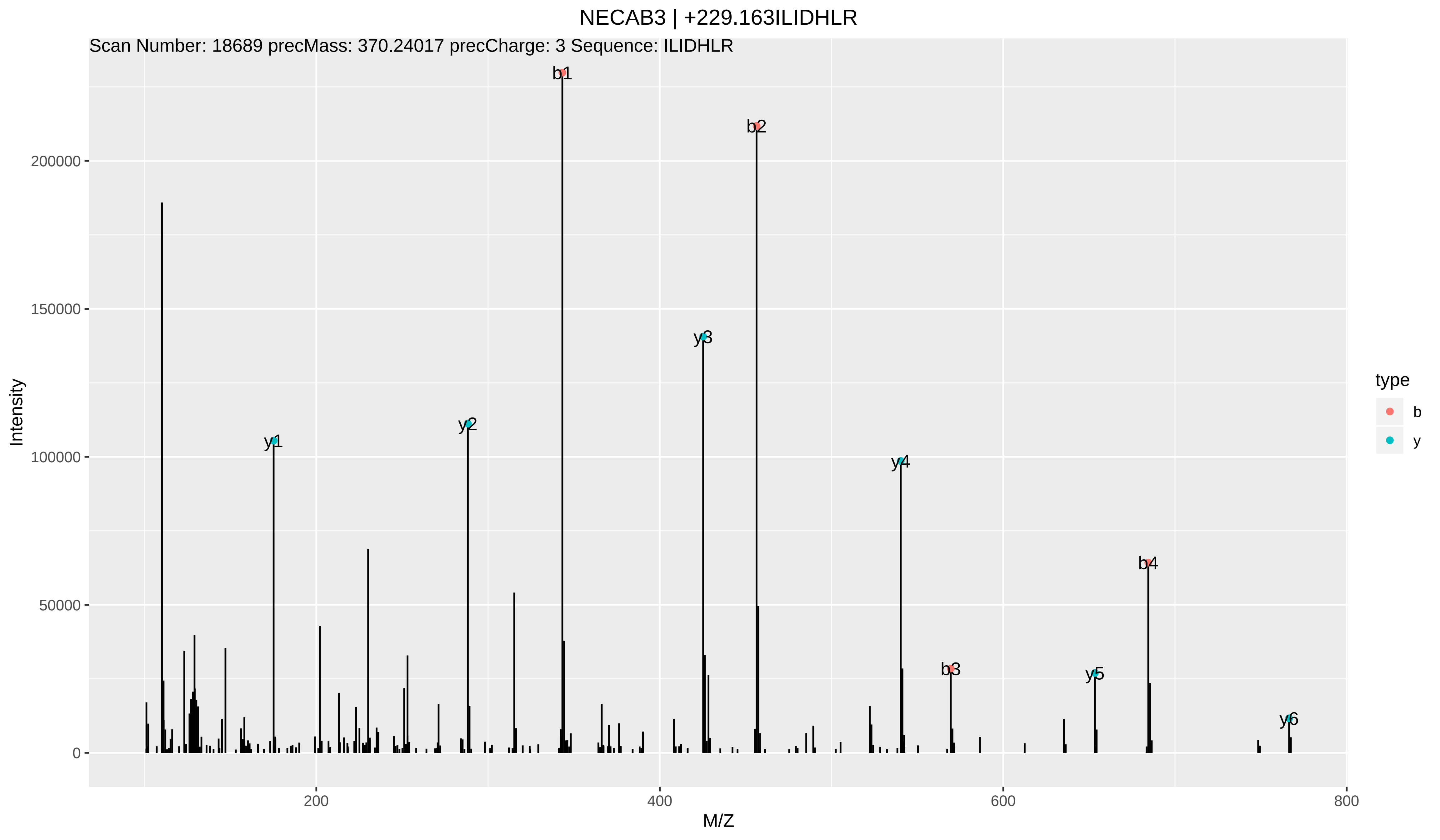

NECAB3 | +229.163ILIDHLR
Scan Number: 18689 precMass: 370.24017 precCharge: 3 Sequence: ILIDHLR
b1
b2
y3
y2
y1
y4
b4
b3
y5
y6
Intensity
200000
150000
100000
50000
0
M/Z
200
400
600
800
type
b
y

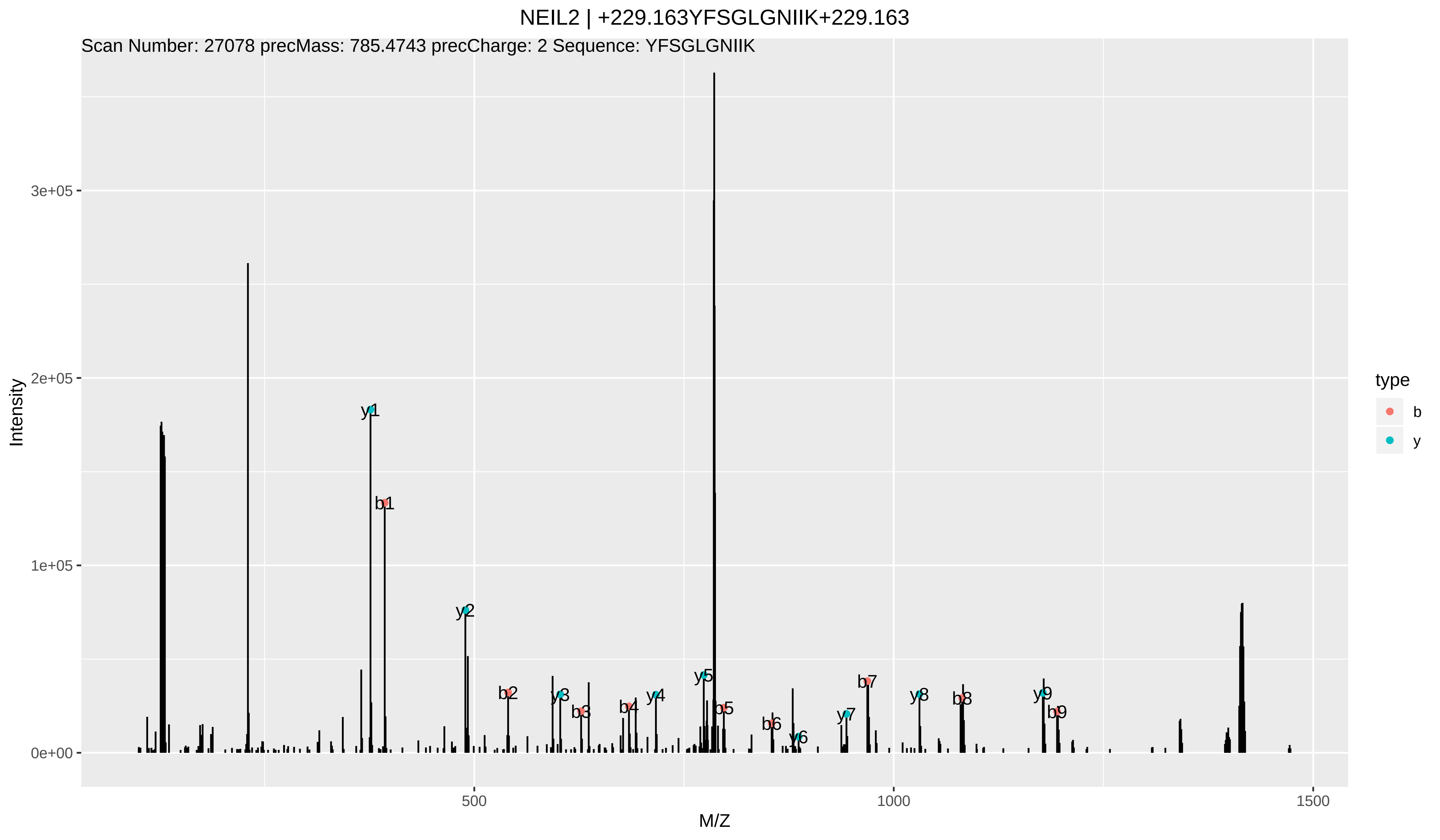

NEIL2 | +229.163YFSGLGNIIK+229.163
Scan Number: 27078 precMass: 785.4743 precCharge: 2 Sequence: YFSGLGNIIK
Intensity
M/Z
3e+05
2e+05
1e+05
0e+00
500
1000
1500
y1
b1
y2
b2
y3
b3
b4
y4
y5
b5
b6
y6
y7
b7
y8
b8
y9
b9
type
b
y

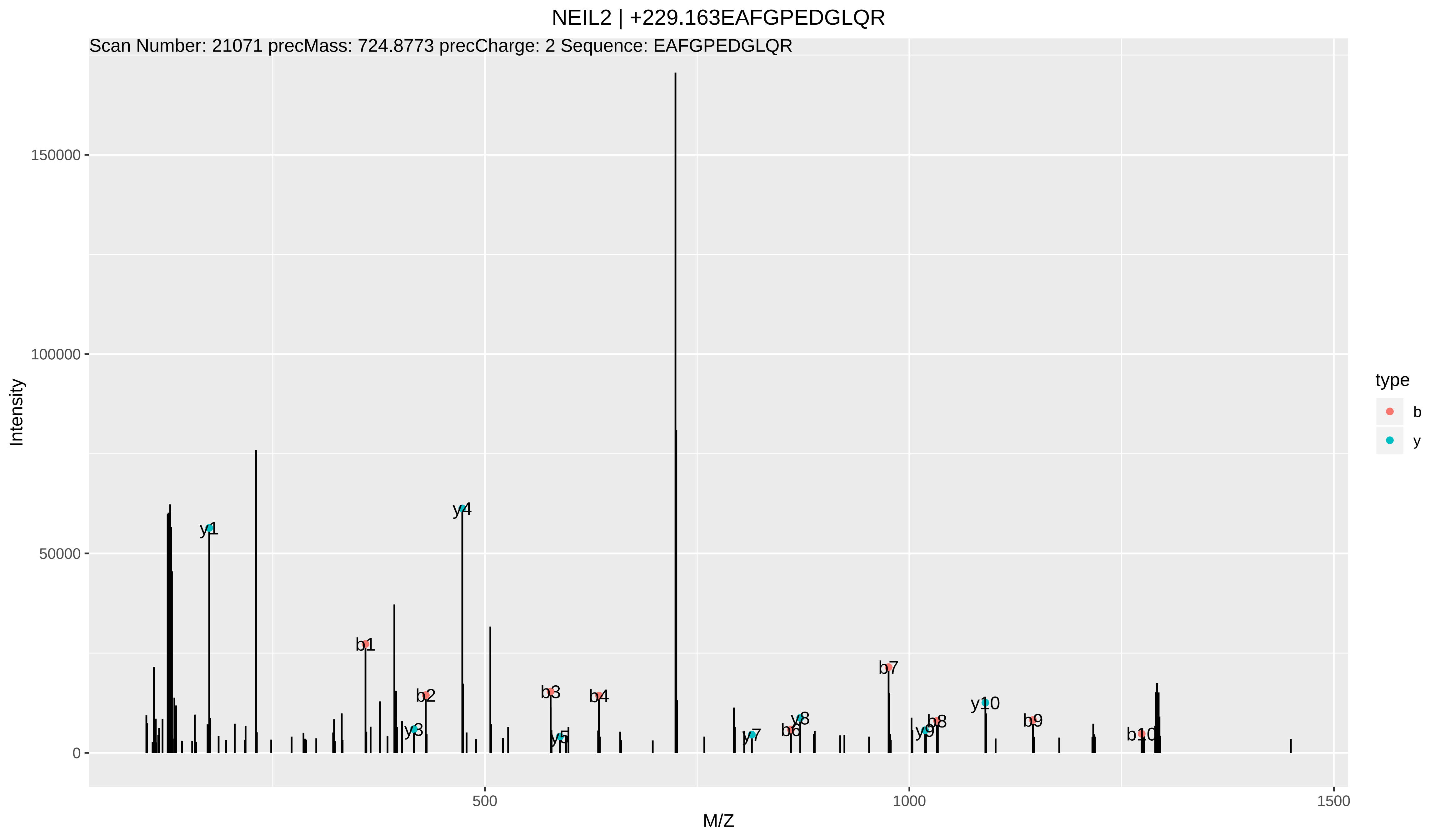

NEIL2 | +229.163EAFGPEDGLQR
Scan Number: 21071 precMass: 724.8773 precCharge: 2 Sequence: EAFGPEDGLQR
Intensity
M/Z
0
50000
100000
150000
500
1000
1500
y1
y4
b1
b2
y3
b3
y5
b4
y7
b6
y8
b7
y9
b8
y10
b9
b10
type
b
y

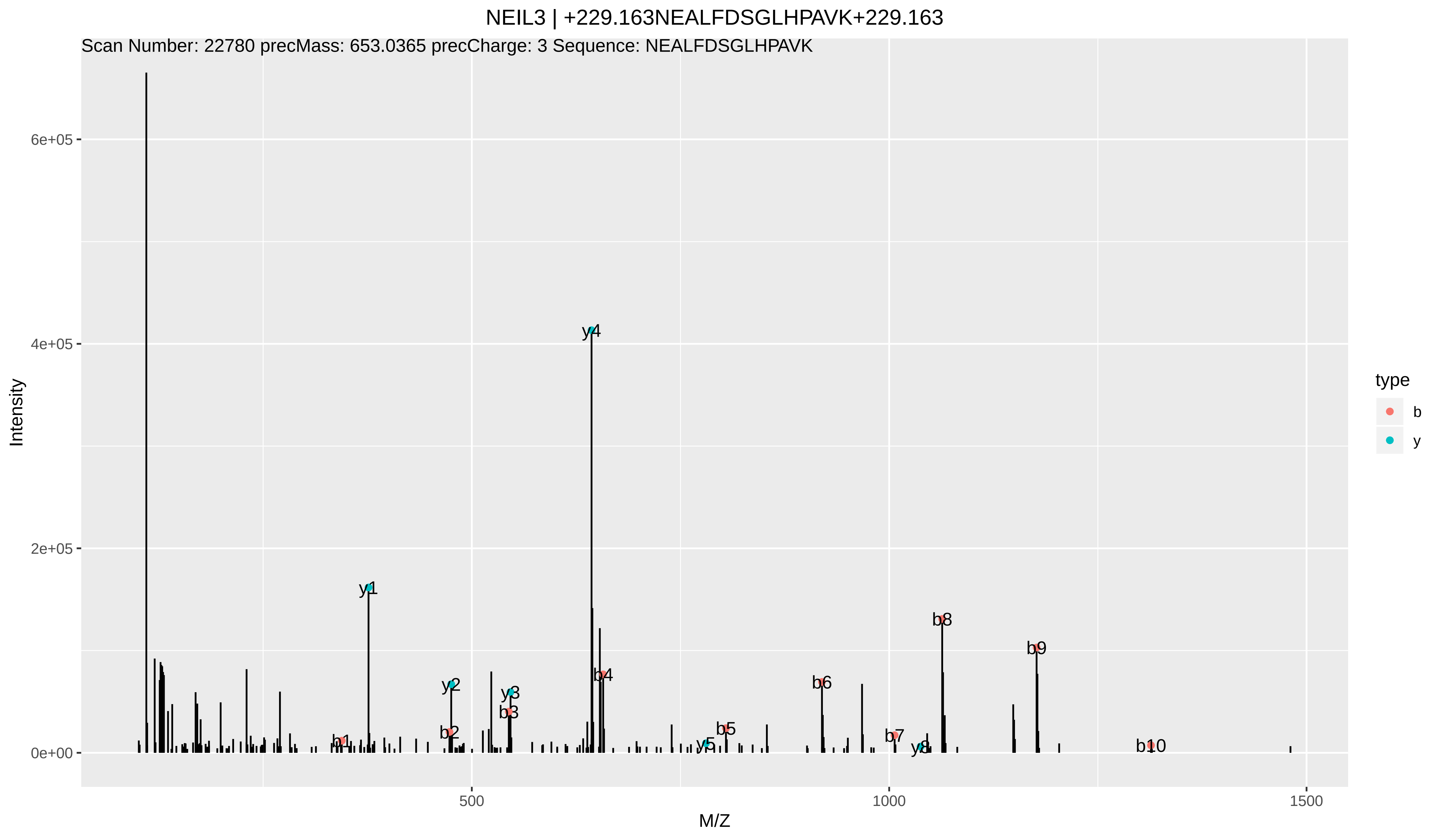

NEIL3 | +229.163NEALFDSGLHPAVK+229.163
Scan Number: 22780 precMass: 653.0365 precCharge: 3 Sequence: NEALFDSGLHPAVK
Intensity
6e+05
4e+05
2e+05
0e+00
M/Z
500
1000
1500
y1
y2
y3
y4
y5
y8
b1
b2
b3
b4
b5
b6
b7
b8
b9
b10
type
b
y

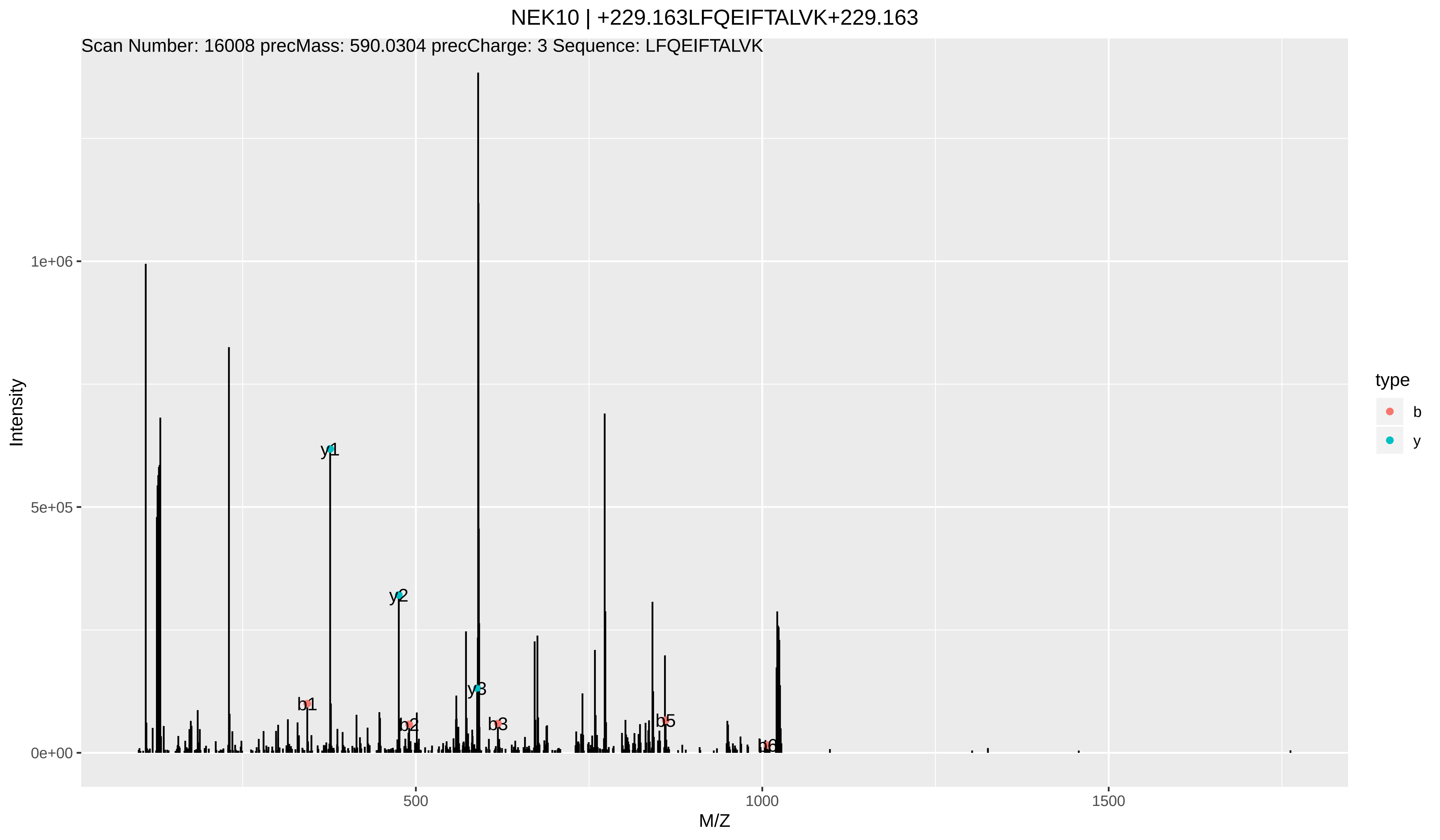

NEK10 | +229.163LFQEIFTALVK+229.163
Scan Number: 16008 precMass: 590.0304 precCharge: 3 Sequence: LFQEIFTALVK
Intensity
1e+06
5e+05
0e+00
M/Z
500
1000
1500
y1
y2
y3
b1
b2
b3
b5
b6
type
b
y

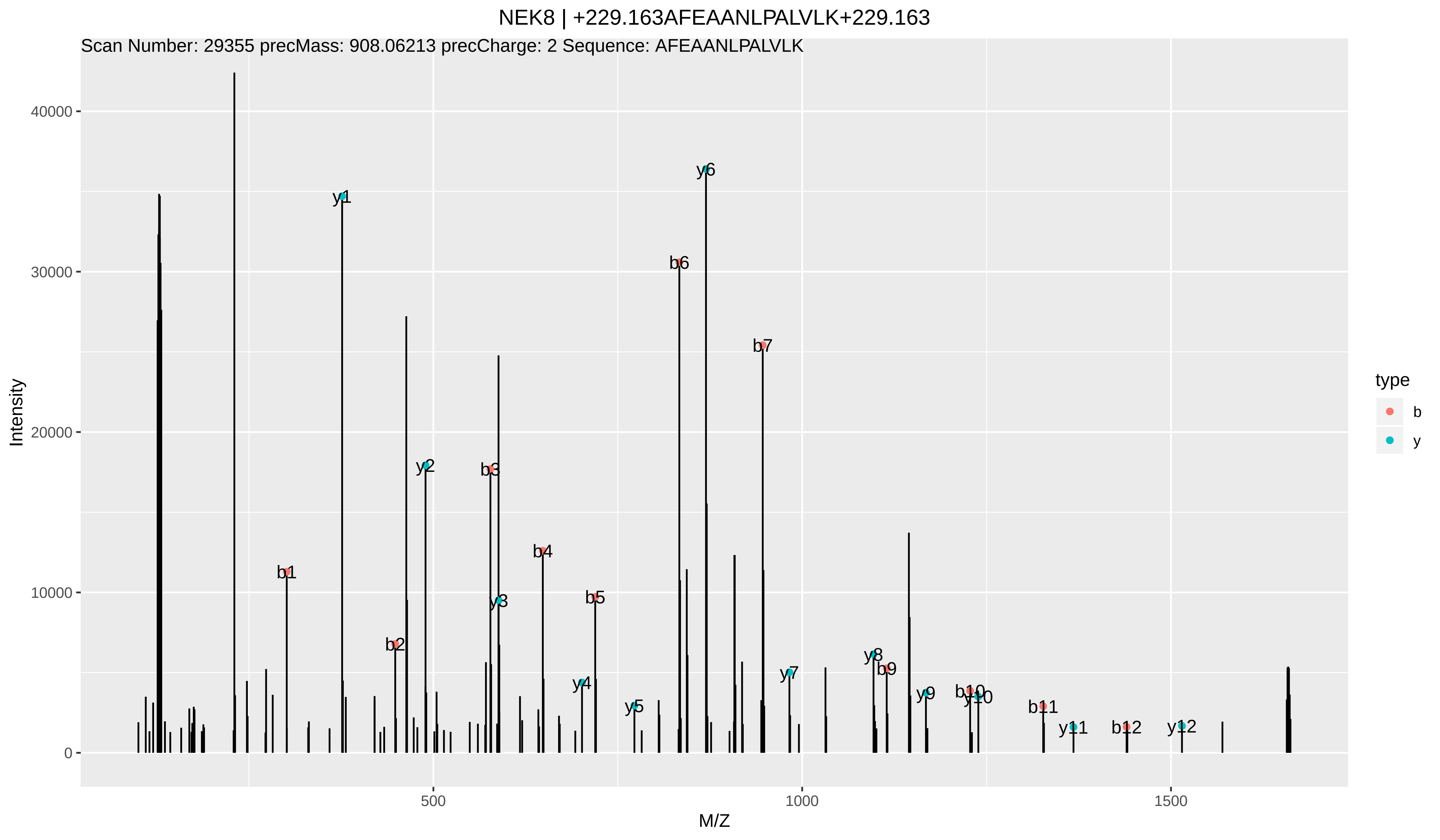

NEK8 | +229.163AFEAANLPALVLK+229.163
Scan Number: 29355 precMass: 908.06213 precCharge: 2 Sequence: AFEAANLPALVLK
Intensity
M/Z
0
10000
20000
30000
40000
500
1000
1500
type
b
y
y1
y2
y3
y4
y5
y6
y7
y8
y9
y10
y11
y12
b1
b2
b3
b4
b5
b6
b7
b9
b10
b11
b12

NETO1 | +229.163FDHIEVR

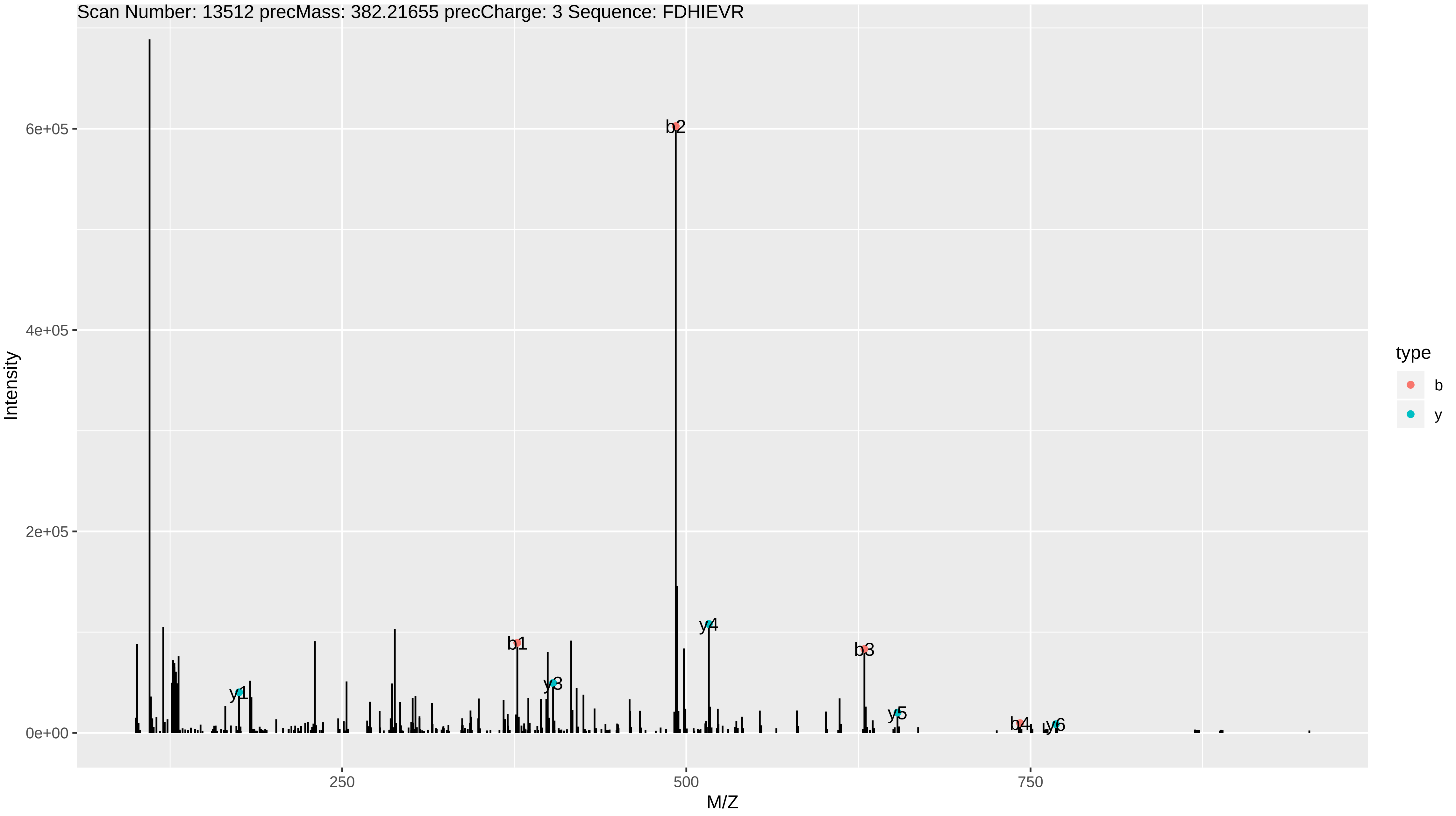

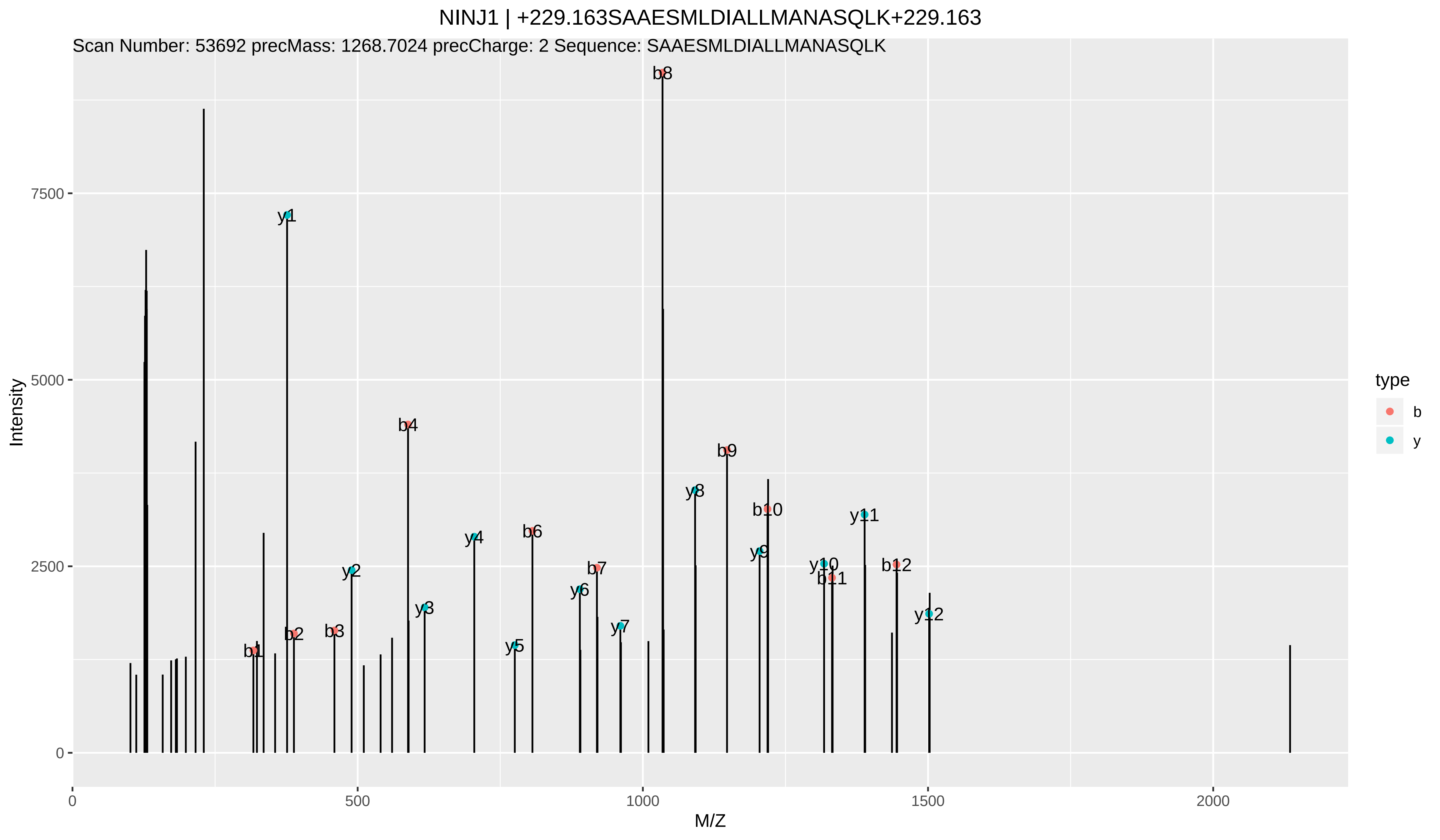
NINJ1 | +229.163SAAESMLDIALLMANASQLK+229.163
Scan Number: 53692 precMass: 1268.7024 precCharge: 2 Sequence: SAAESMLDIALLMANASQLK
Intensity
M/Z
type
b
y
b1
b2
b3
b4
b6
b7
b8
b9
b10
b11
b12
y1
y2
y3
y4
y5
y6
y7
y8
y9
y10
y11
y12
0
2500
5000
7500
0
500
1000
1500
2000

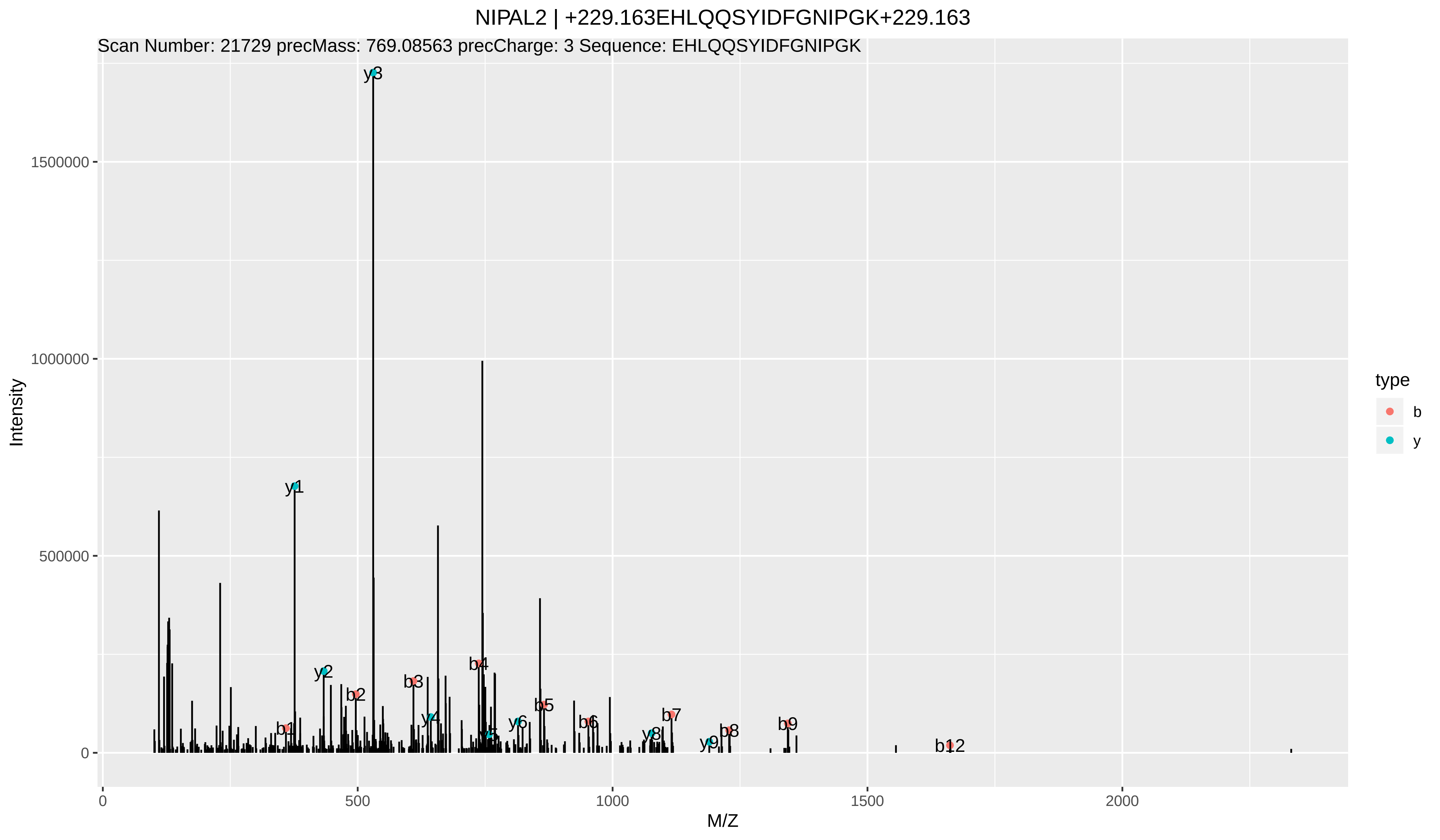
NIPAL2 | +229.163EHLQQSYIDFGNIPGK+229.163
Scan Number: 21729 precMass: 769.08563 precCharge: 3 Sequence: EHLQQSYIDFGNIPGK
Intensity
M/Z
type
b
y
y3
y1
y2
b1
b2
b3
b4
y4
b5
y6
b6
y8
b7
y9
b8
b9
b12
1500000
1000000
500000
0
0
500
1000
1500
2000

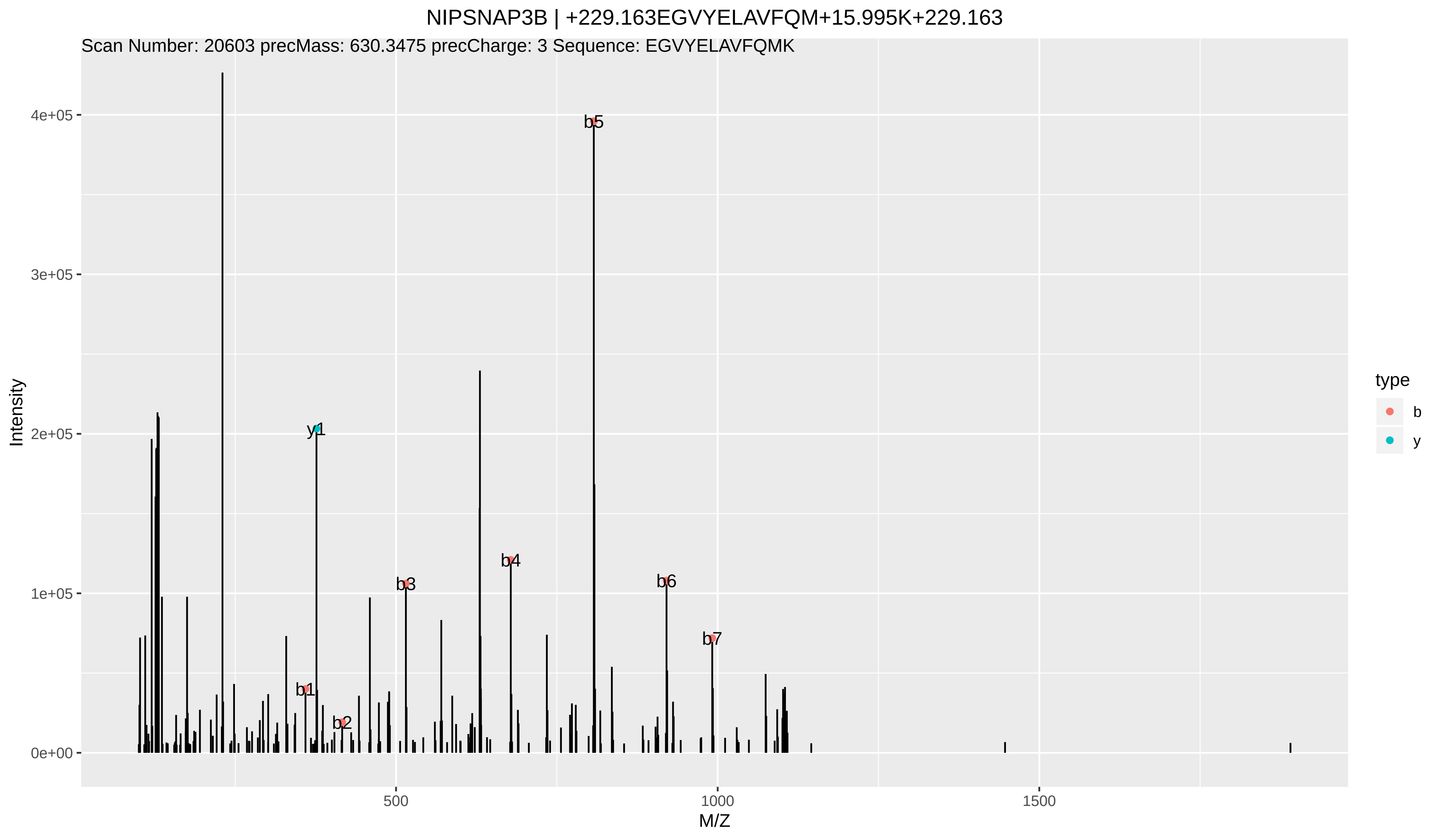
NIPSNAP3B | +229.163EGVYELAVFQM+15.995K+229.163
Scan Number: 20603 precMass: 630.3475 precCharge: 3 Sequence: EGVYELAVFQMK
Intensity
M/Z
4e+05
3e+05
2e+05
1e+05
0e+00
500
1000
1500
b5
y1
b4
b3
b6
b7
b1
b2
type
b
y

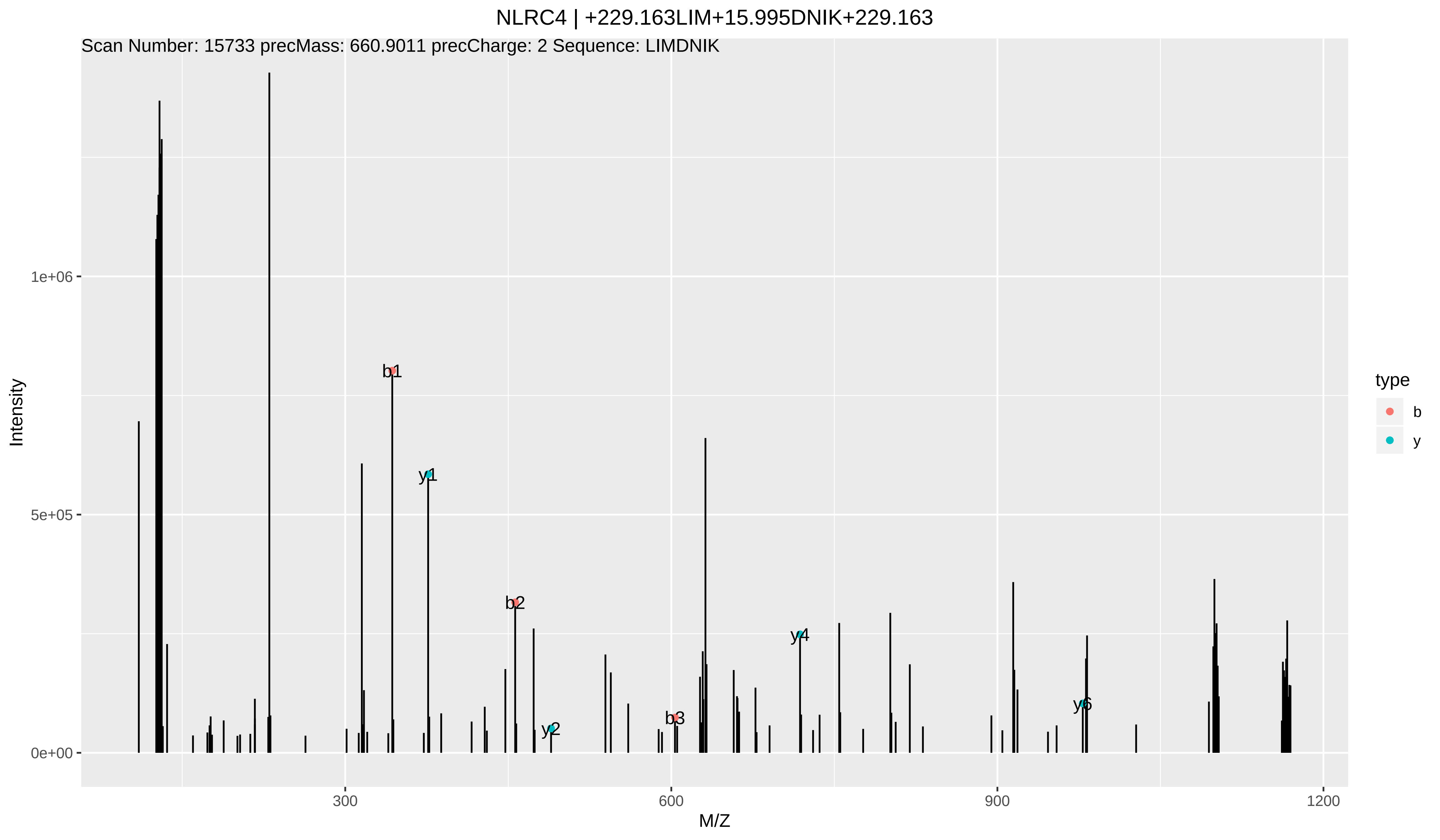
NLRC4 | +229.163LIM+15.995DNIK+229.163
Scan Number: 15733 precMass: 660.9011 precCharge: 2 Sequence: LIMDNIK
Intensity
1e+06
5e+05
0e+00
M/Z
300
600
900
1200
b1
y1
b2
y2
b3
y4
y6
type
b
y

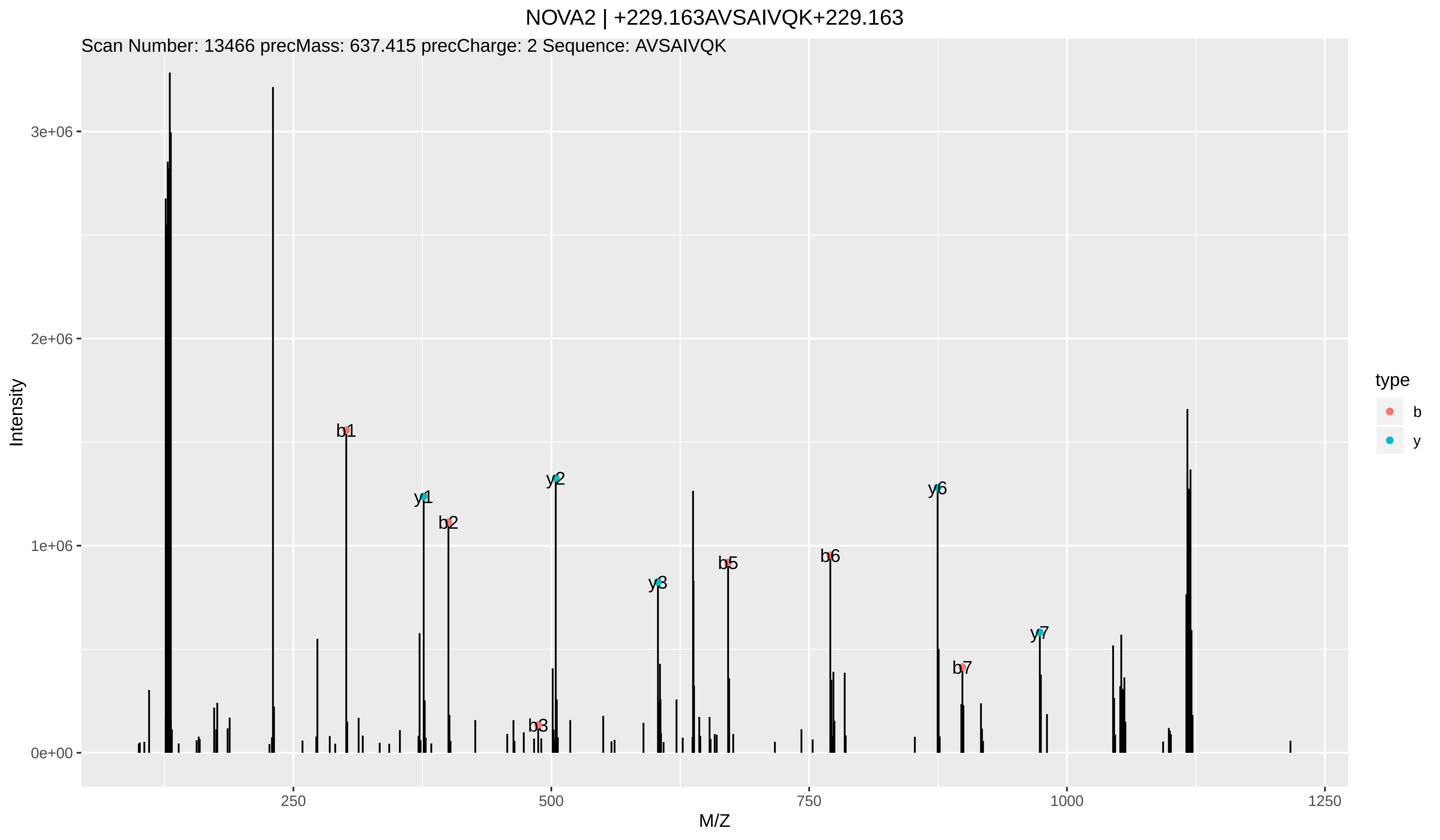

NOVA2 | +229.163AVSAIVQK+229.163
Scan Number: 13466 precMass: 637.415 precCharge: 2 Sequence: AVSAIVQK
Intensity
3e+06
2e+06
1e+06
0e+00
M/Z
250
500
750
1000
1250
b1
y1
b2
y2
b3
y3
b5
b6
y6
b7
y7
type
b
y

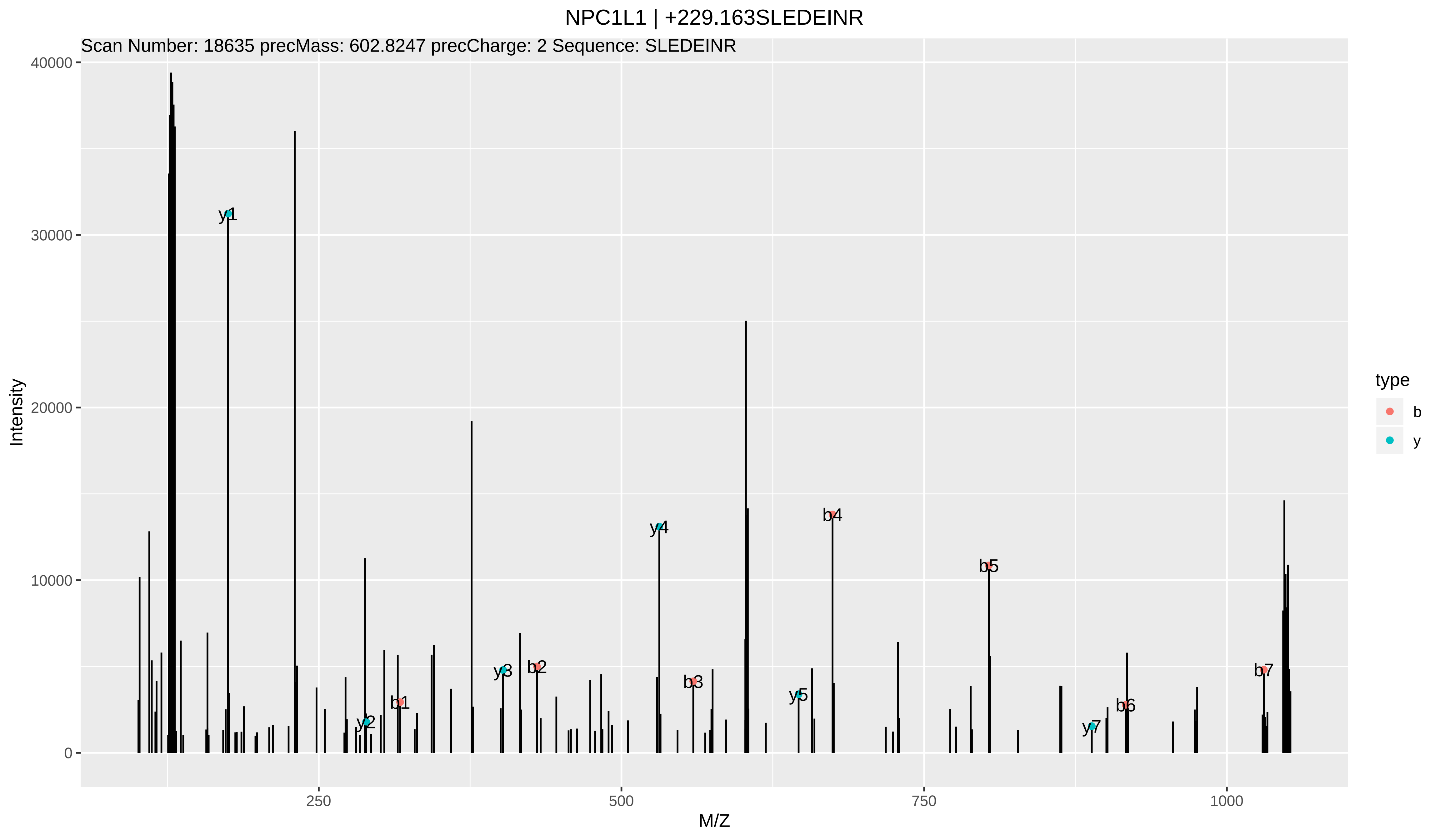

NPC1L1 | +229.163SLEDEINR
Scan Number: 18635 precMass: 602.8247 precCharge: 2 Sequence: SLEDEINR
Intensity
M/Z
40000
30000
20000
10000
0
250
500
750
1000
y1
y2
y3
y4
y5
y7
b1
b2
b3
b4
b5
b6
b7
type
b
y

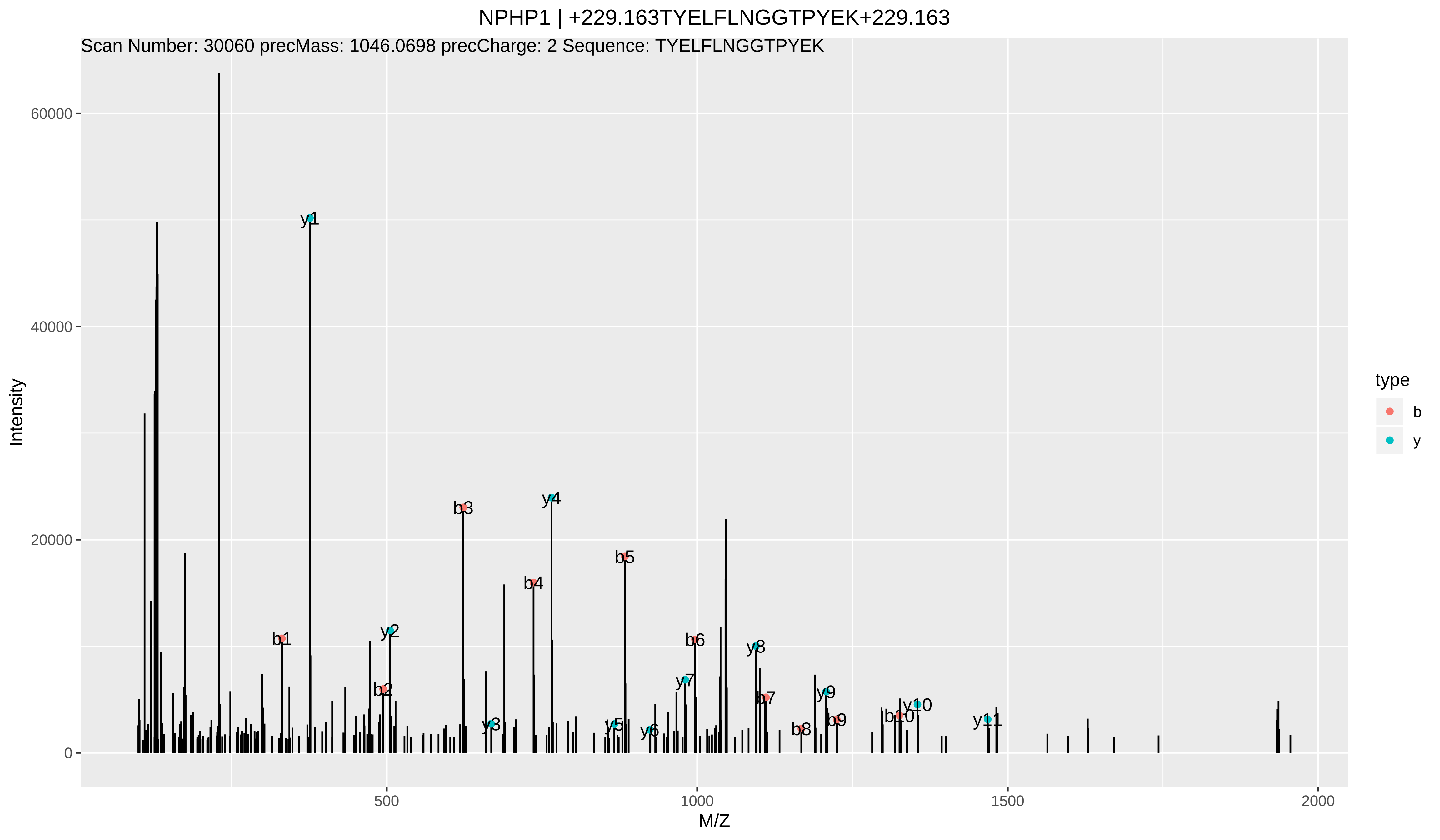

NPHP1 | +229.163TYELFLNGGTPYEK+229.163
Scan Number: 30060 precMass: 1046.0698 precCharge: 2 Sequence: TYELFLNGGTPYEK
Intensity
M/Z
0
20000
40000
60000
500
1000
1500
2000
y1
y2
y3
y4
y5
y6
y7
y8
y9
y10
y11
b1
b2
b3
b4
b5
b6
b7
b8
b9
b10
type
b
y

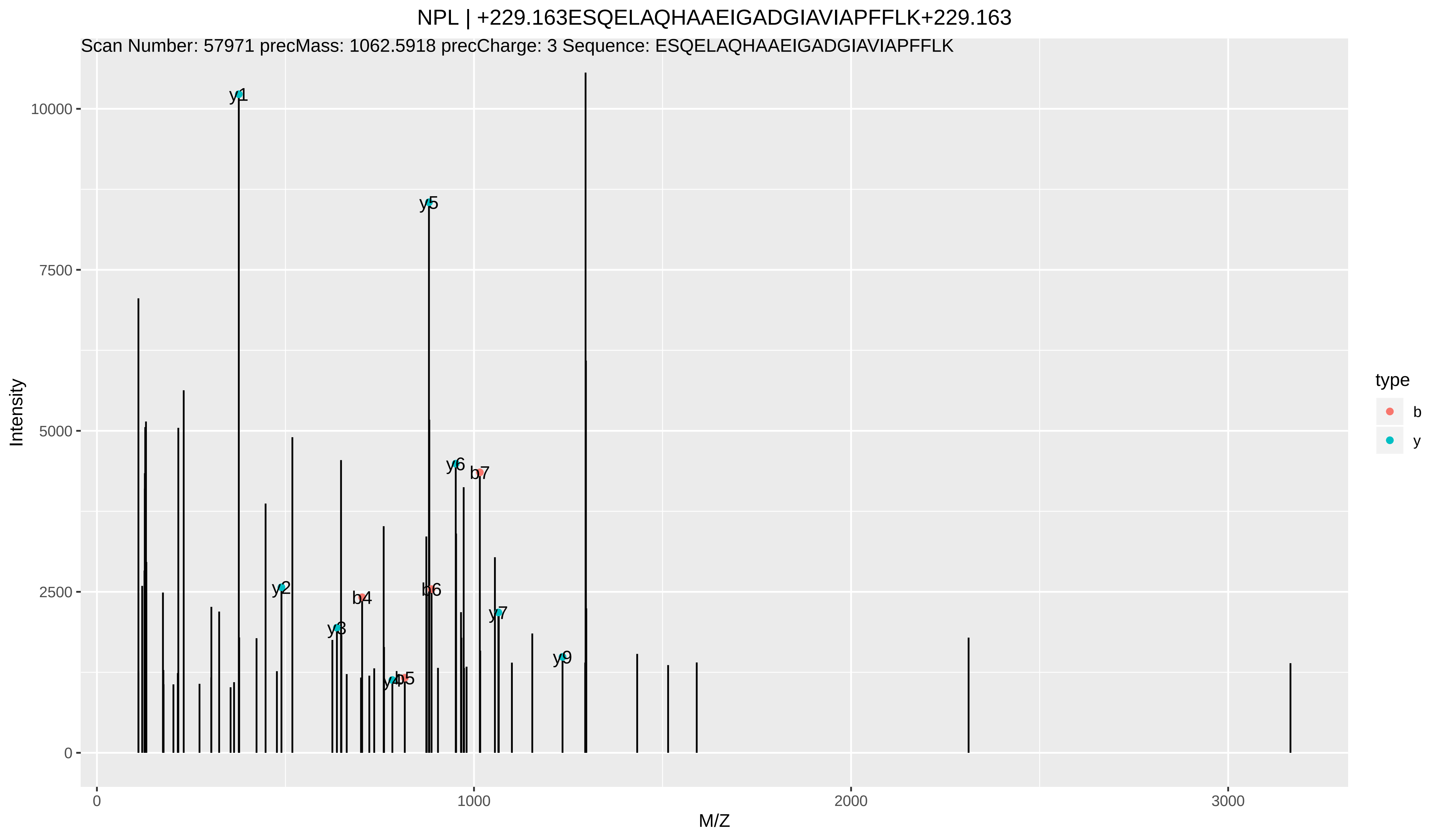

NPL | +229.163ESQELAQHAAEIGADGIAVIAPFFLK+229.163
Scan Number: 57971 precMass: 1062.5918 precCharge: 3 Sequence: ESQELAQHAAEIGADGIAVIAPFFLK
Intensity
M/Z
0
2500
5000
7500
10000
0
1000
2000
3000
y1
y2
y3
y4
y5
y6
y7
y9
b4
b5
b6
b7
type
b
y

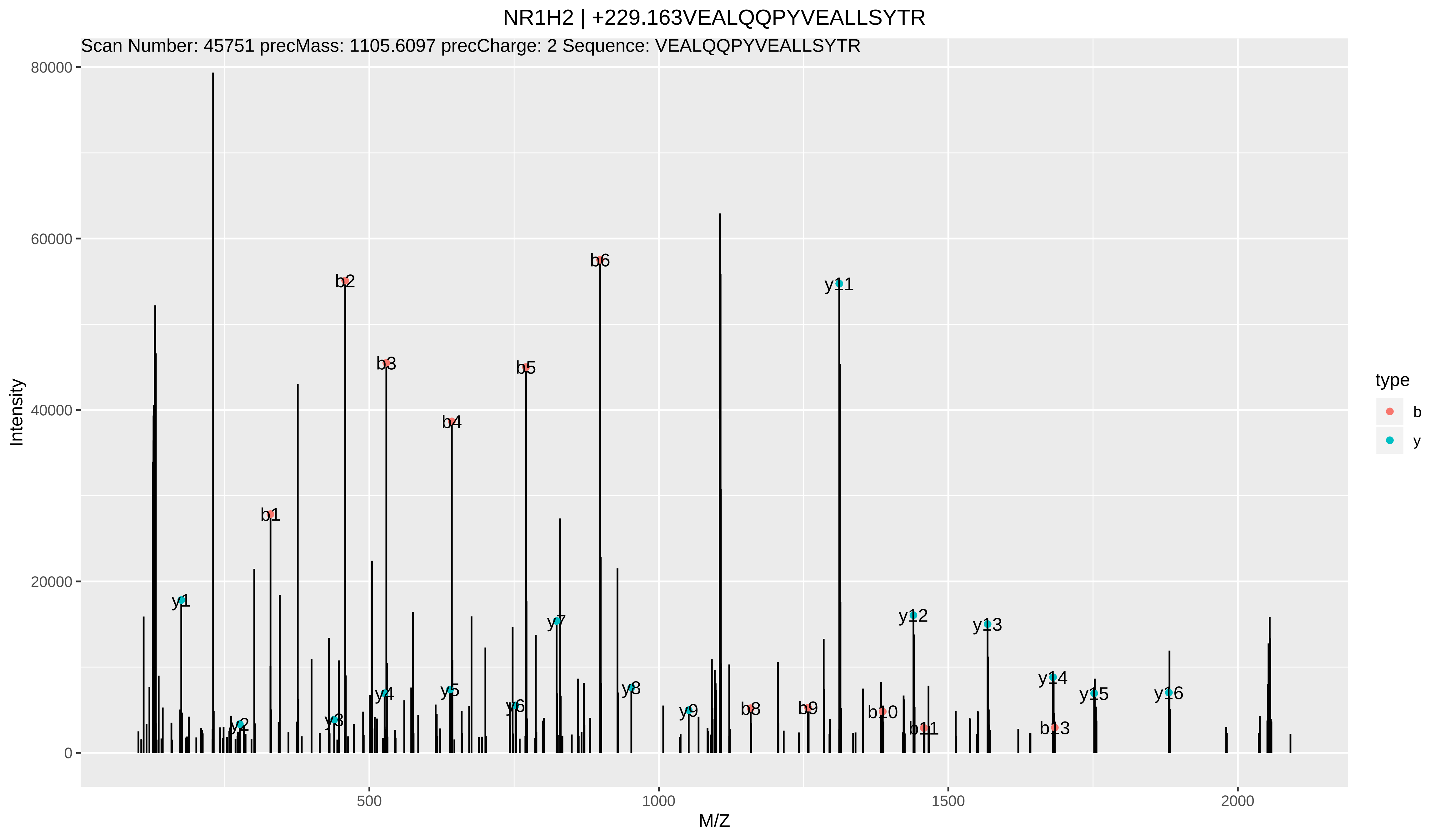

NR1H2 | +229.163VEALQQPYVEALLSYTR
Scan Number: 45751 precMass: 1105.6097 precCharge: 2 Sequence: VEALQQPYVEALLSYTR
Intensity
M/Z
0
20000
40000
60000
80000
500
1000
1500
2000
b1
b2
b3
b4
b5
b6
b8
b9
b10
b11
b13
y1
y2
y3
y4
y5
y6
y7
y8
y9
y11
y12
y13
y14
y15
y16
type
b
y

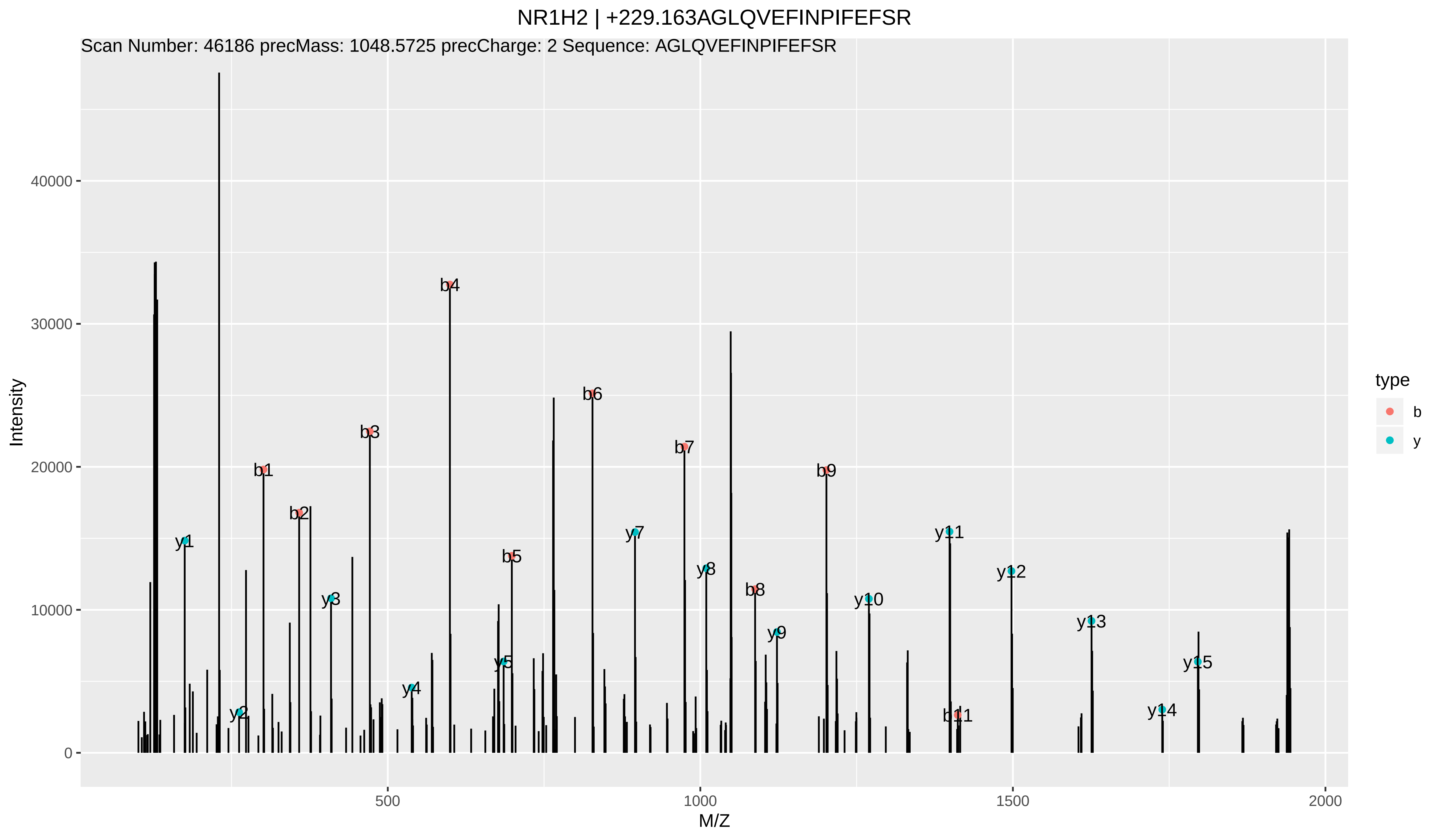

NR1H2 | +229.163AGLQVEFINPIFEFSR
Scan Number: 46186 precMass: 1048.5725 precCharge: 2 Sequence: AGLQVEFINPIFEFSR
Intensity
M/Z
0
10000
20000
30000
40000
500
1000
1500
2000
y1
y2
b1
b2
y3
b3
b4
y4
y5
b5
b6
y7
b7
y8
b8
y9
b9
y10
y11
b11
y12
y13
y14
y15
type
b
y

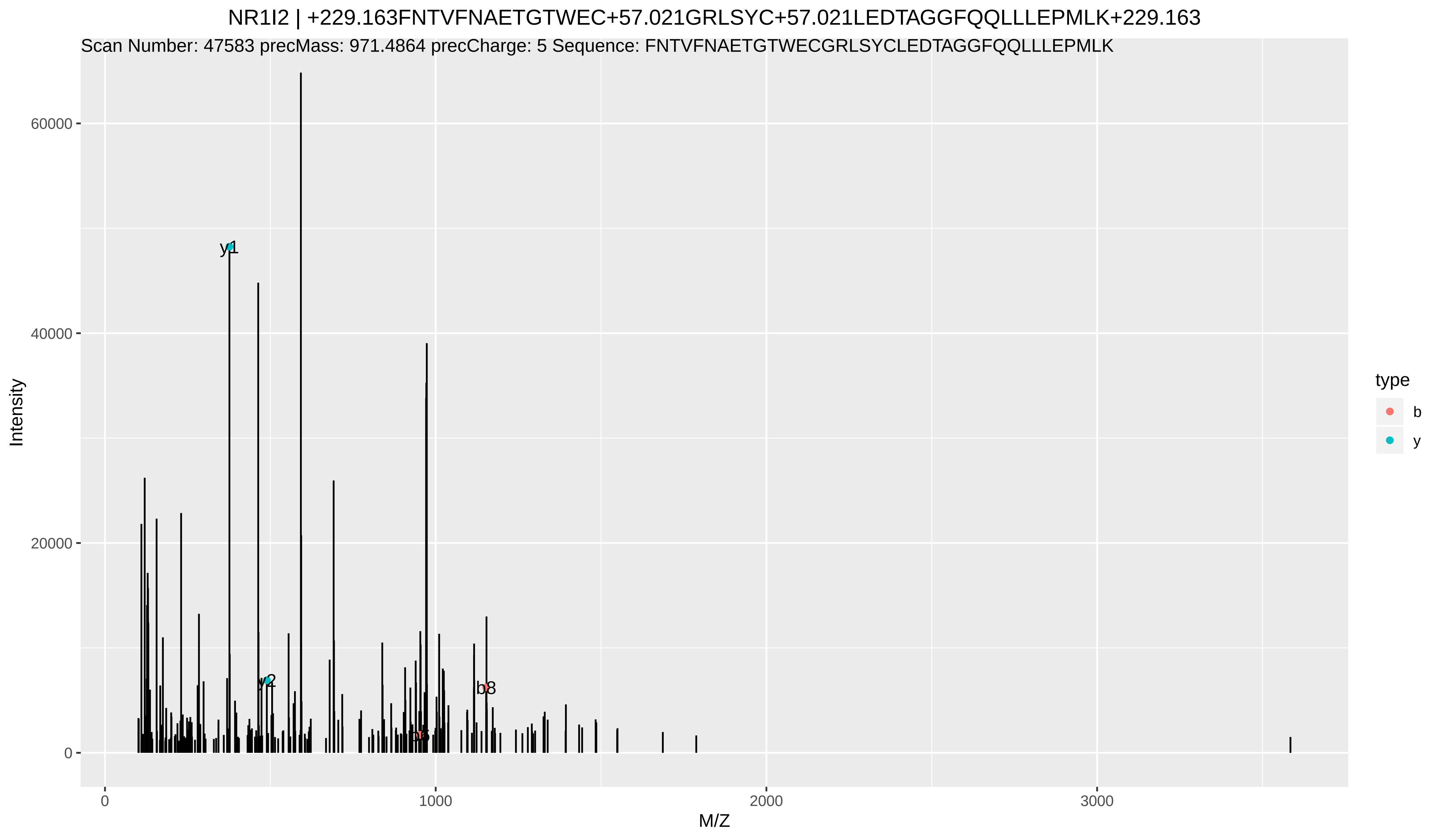
NR1I2 | +229.163FNTVFNAETGTWEC+57.021GRLSYC+57.021LEDTAGGFQQLLLEPMLK+229.163
Scan Number: 47583 precMass: 971.4864 precCharge: 5 Sequence: FNTVFNAETGTWECGRLSYCLEDTAGGFQQLLLEPMLK
Intensity
60000
40000
20000
0
0
1000
2000
3000
M/Z
y1
y2
b6
b8
type
b
y

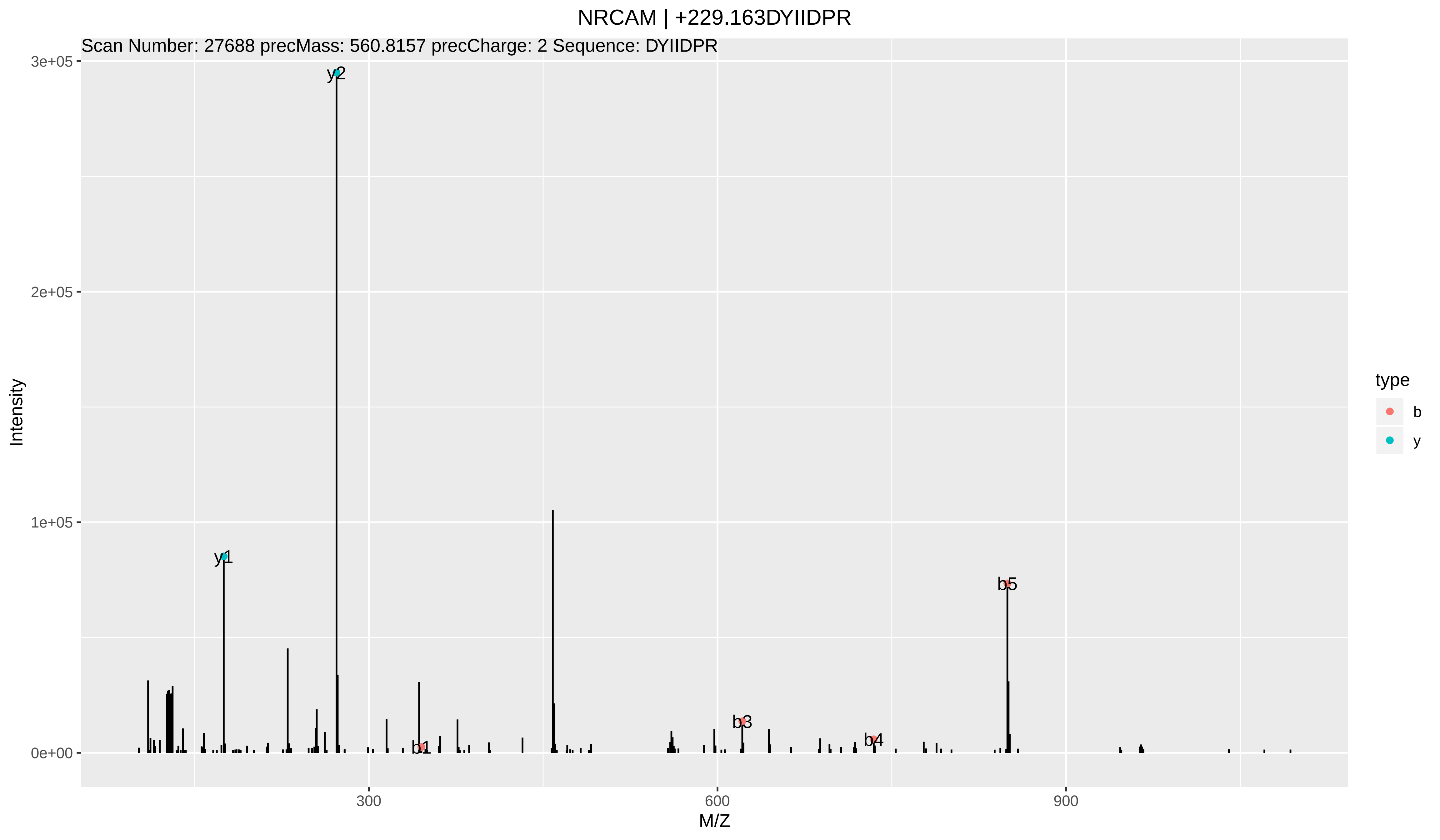
NRCAM | +229.163DYIIDPR
Scan Number: 27688 precMass: 560.8157 precCharge: 2 Sequence: DYIIDPR
Intensity
3e+05
2e+05
1e+05
0e+00
y2
y1
b1
b3
b4
b5
300
600
900
M/Z
type
b
y

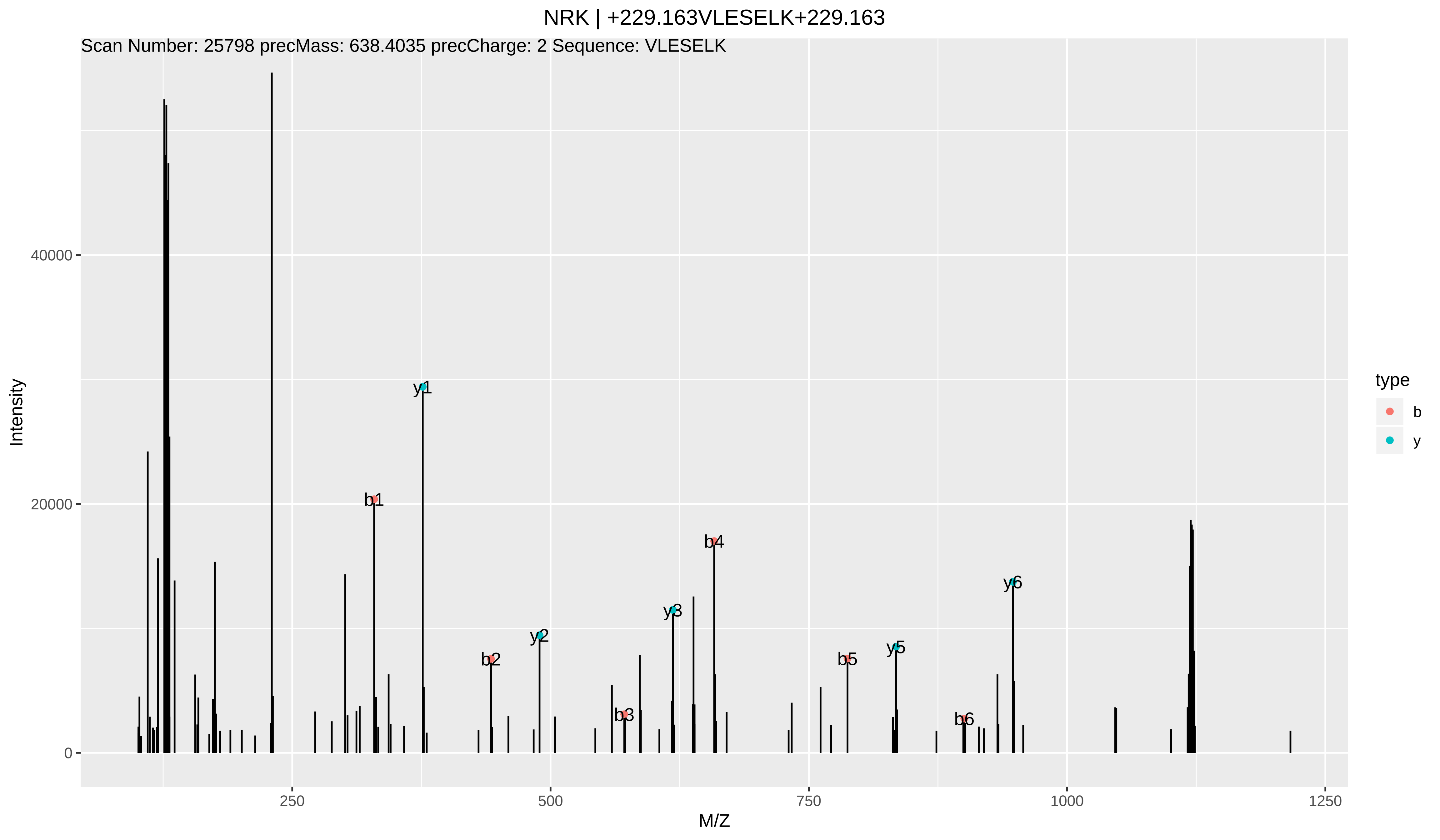

NRK | +229.163VLESELK+229.163
Scan Number: 25798 precMass: 638.4035 precCharge: 2 Sequence: VLESELK
Intensity
M/Z
0
20000
40000
250
500
750
1000
1250
b1
b2
b3
b4
b5
b6
y1
y2
y3
y5
y6
type
b
y

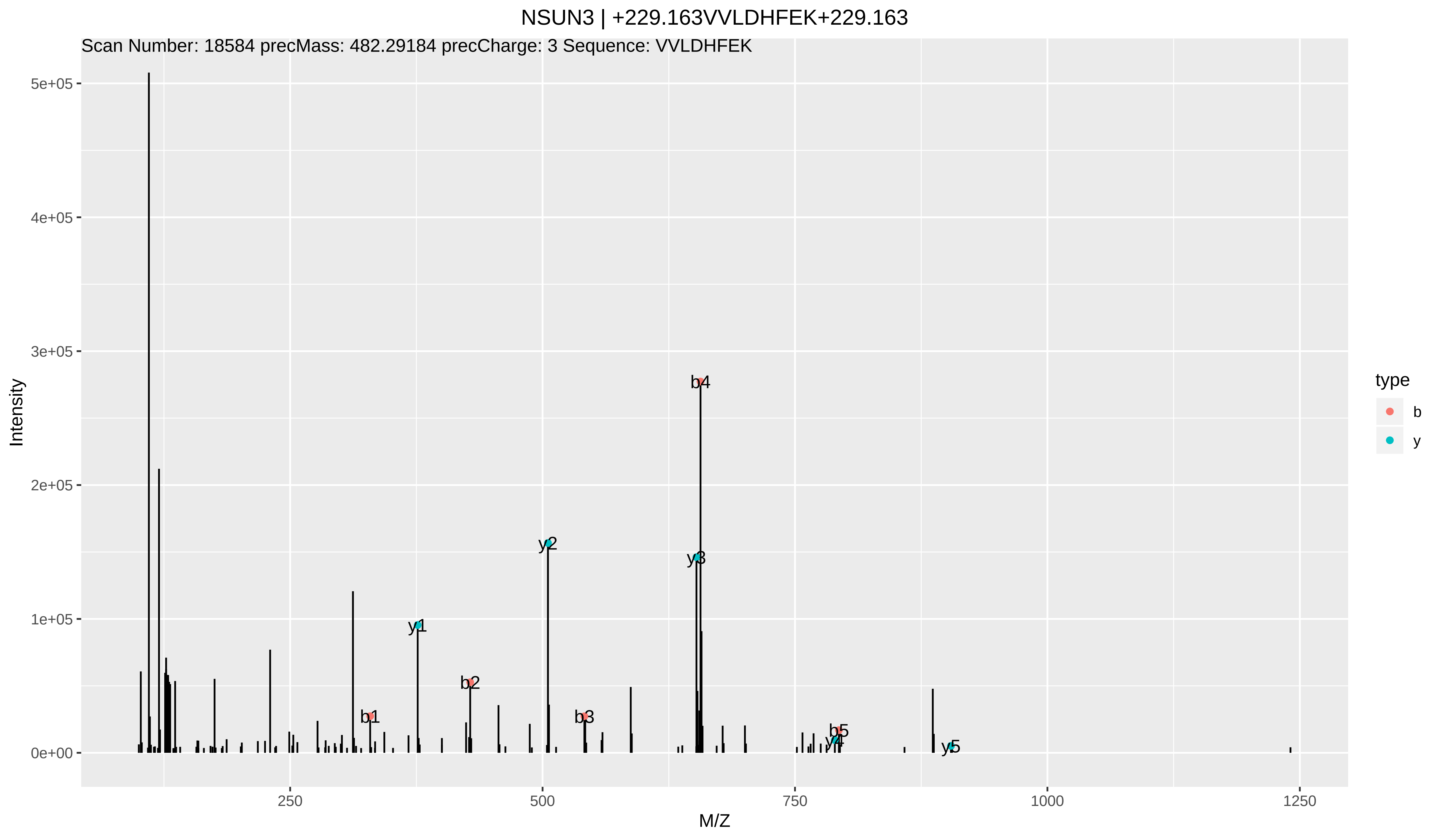
NSUN3 | +229.163VVLDHFEK+229.163
Scan Number: 18584 precMass: 482.29184 precCharge: 3 Sequence: VVLDHFEK
Intensity
5e+05
4e+05
3e+05
2e+05
1e+05
0e+00
M/Z
250
500
750
1000
1250
b1
b2
b3
b4
b5
y1
y2
y3
y4
y5
type
b
y

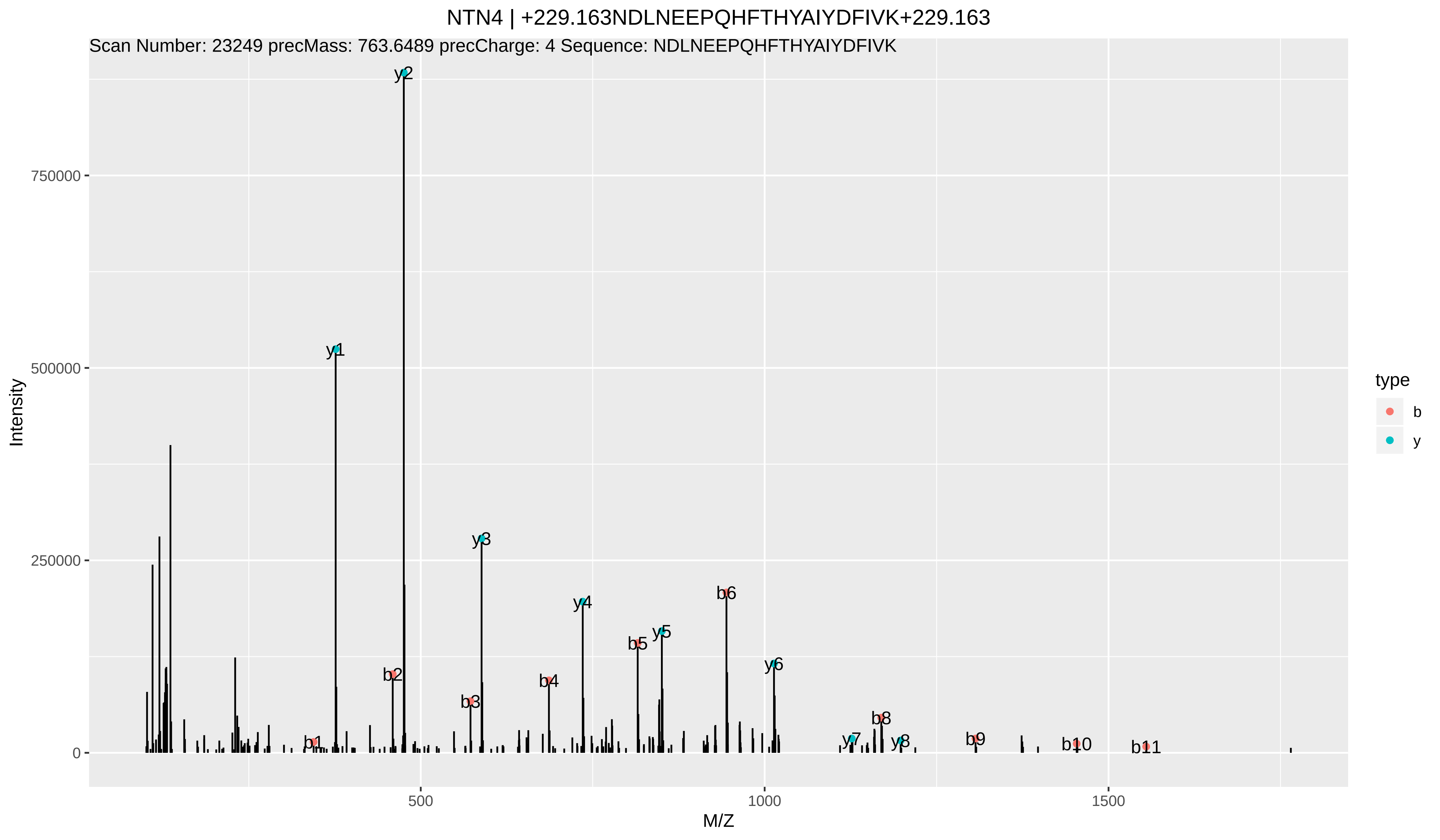
NTN4 | +229.163NDLNEEPQHFTHYAIYDFIVK+229.163
Scan Number: 23249 precMass: 763.6489 precCharge: 4 Sequence: NDLNEEPQHFTHYAIYDFIVK
Intensity
M/Z
750000
500000
250000
0
500
1000
1500
y1
y2
y3
y4
y5
y6
y7
y8
b1
b2
b3
b4
b5
b6
b8
b9
b10
b11
type
b
y

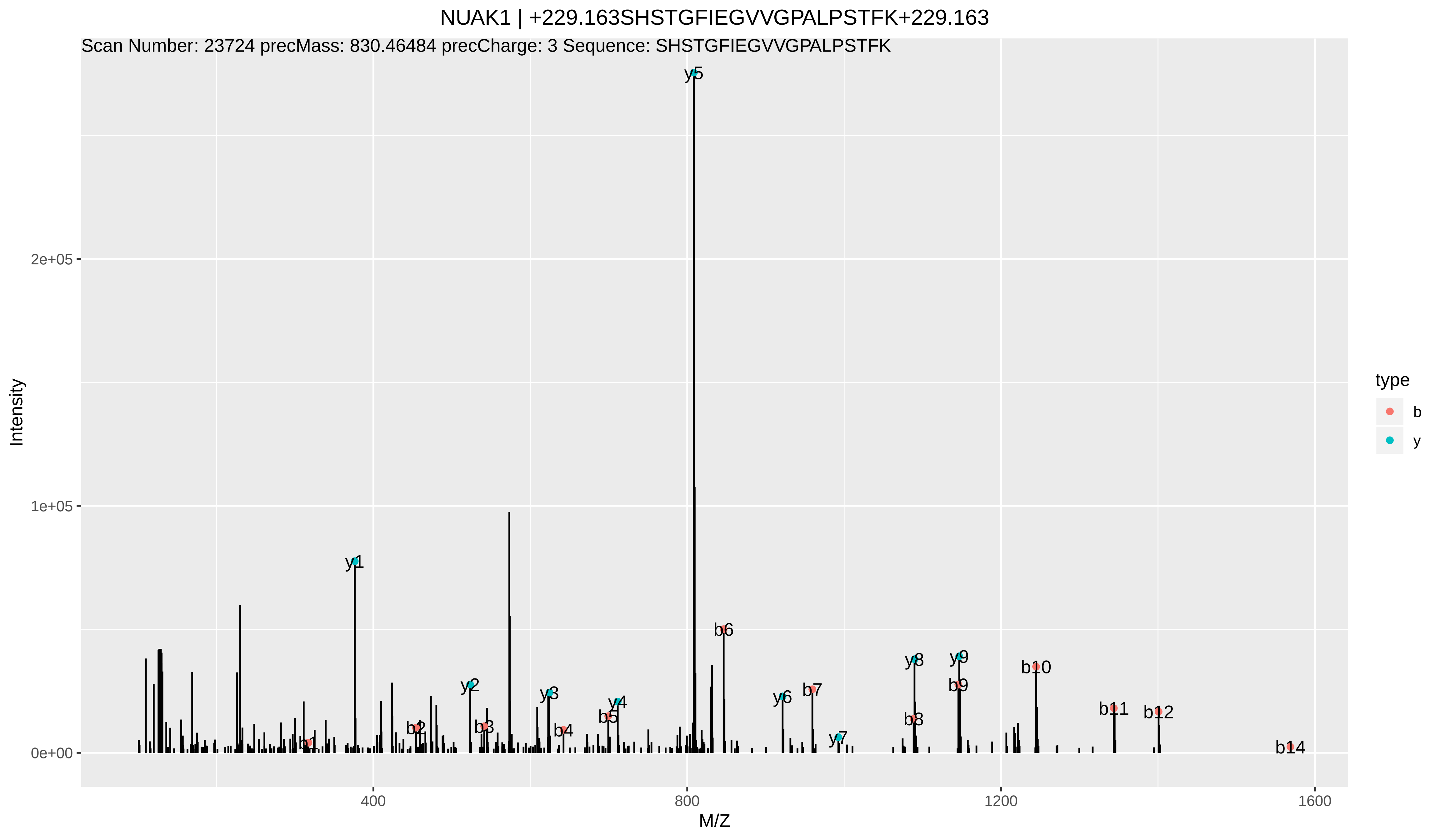

NUAK1 | +229.163SHSTGFIEGVVGPALPSTFK+229.163
Scan Number: 23724 precMass: 830.46484 precCharge: 3 Sequence: SHSTGFIEGVVGPALPSTFK
Intensity
M/Z
type
b
y
y5
y1
b6
y8
y9
b10
y2
b9
b7
y3
y6
y4
b5
b11
b12
b8
b2
b3
b4
y7
b1
b14
0e+00
1e+05
2e+05
400
800
1200
1600

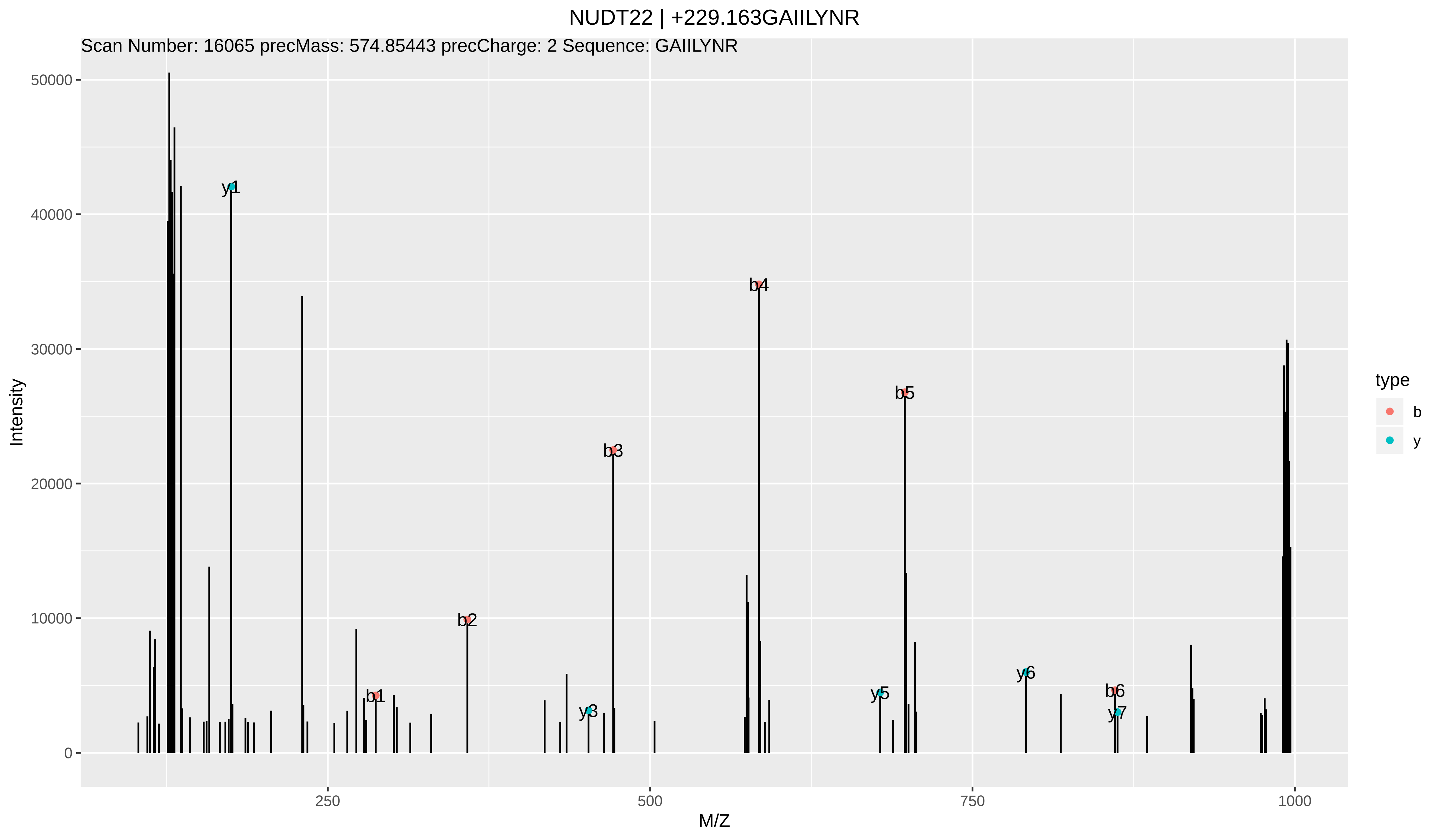
NUDT22 | +229.163GAIILYNR
Scan Number: 16065 precMass: 574.85443 precCharge: 2 Sequence: GAIILYNR
Intensity
M/Z
0
10000
20000
30000
40000
50000
250
500
750
1000
y1
b1
b2
b3
y3
b4
y5
b5
y6
b6
y7
type
b
y

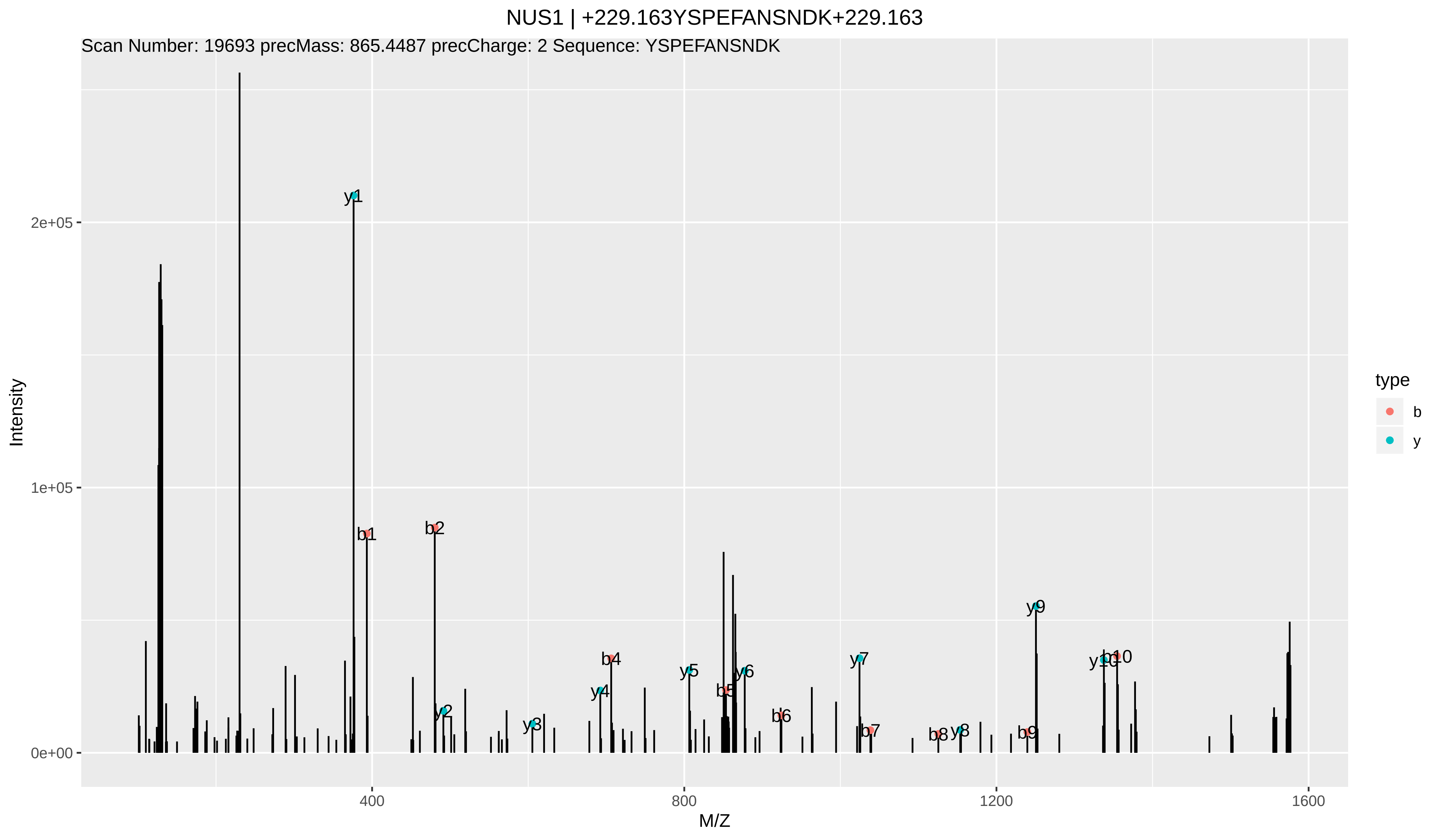

NUS1 | +229.163YSPEFANSNDK+229.163
Scan Number: 19693 precMass: 865.4487 precCharge: 2 Sequence: YSPEFANSNDK
Intensity
M/Z
2e+05
1e+05
0e+00
400
800
1200
1600
y1
b1
b2
y2
y3
y4
b4
y5
b5
y6
b6
y7
b7
b8
y8
b9
y9
y10
b10
type
b
y

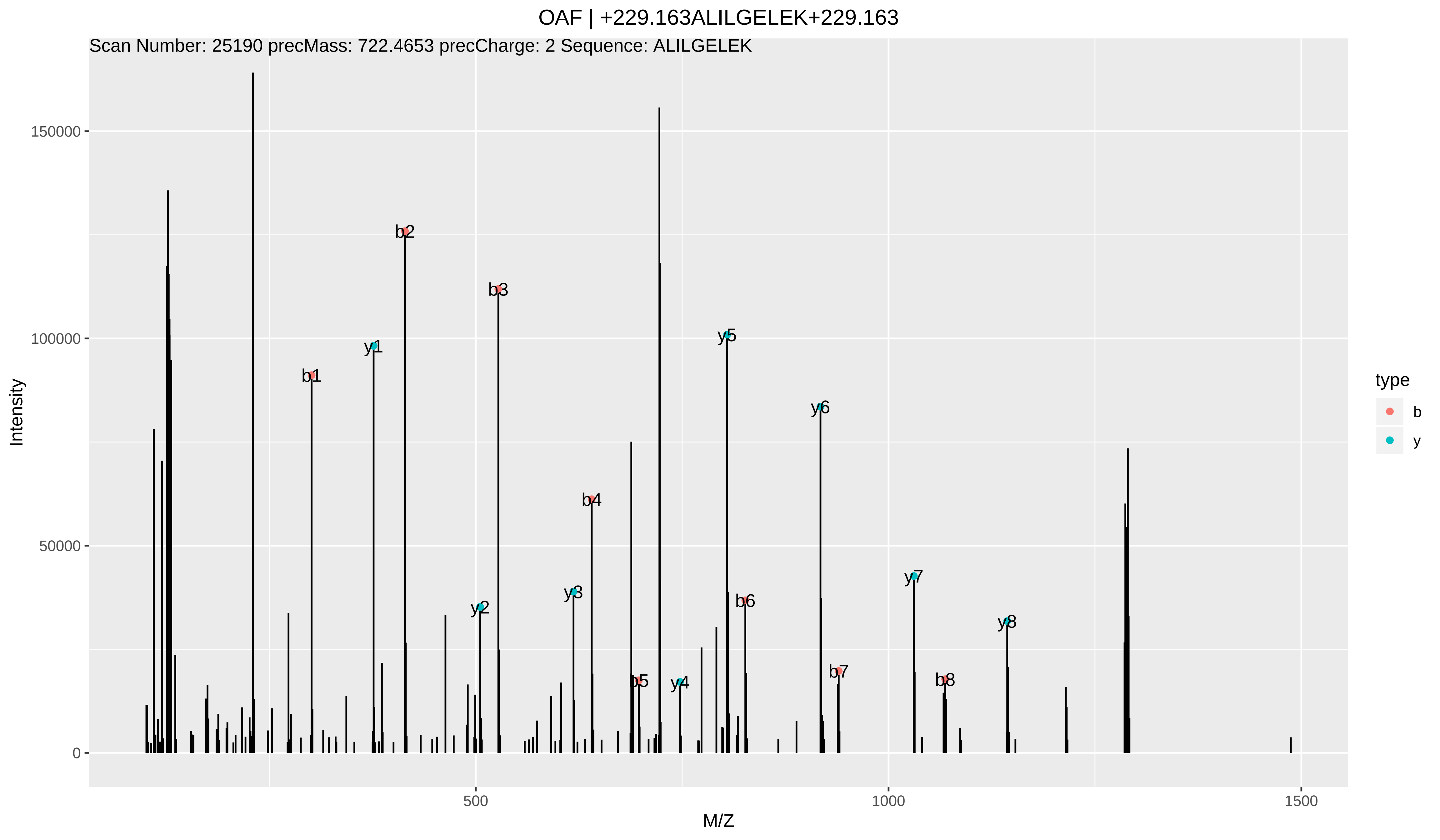
OAF | +229.163ALILGELEK+229.163
Scan Number: 25190 precMass: 722.4653 precCharge: 2 Sequence: ALILGELEK
Intensity
M/Z
0
50000
100000
150000
500
1000
1500
b1
b2
b3
b4
b5
b6
b7
b8
y1
y2
y3
y4
y5
y6
y7
y8
type
b
y

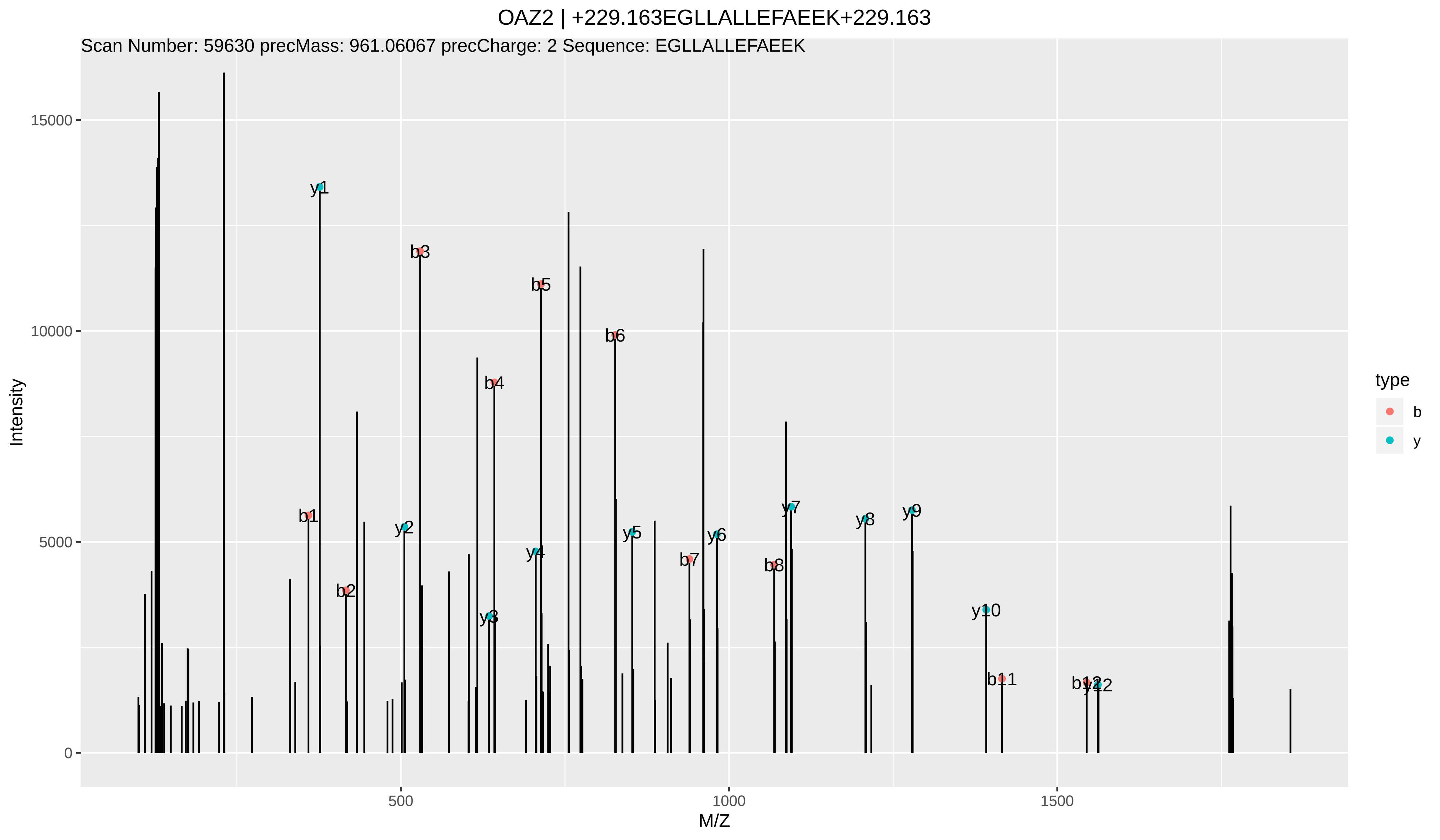

OAZ2 | +229.163EGLLALLEFAEEK+229.163
Scan Number: 59630 precMass: 961.06067 precCharge: 2 Sequence: EGLLALLEFAEEK
Intensity
M/Z
type
b
y
y1
b1
b2
b3
y2
y3
b4
y4
b5
b6
y5
b7
y6
b8
y7
y8
y9
y10
b11
b12
y12

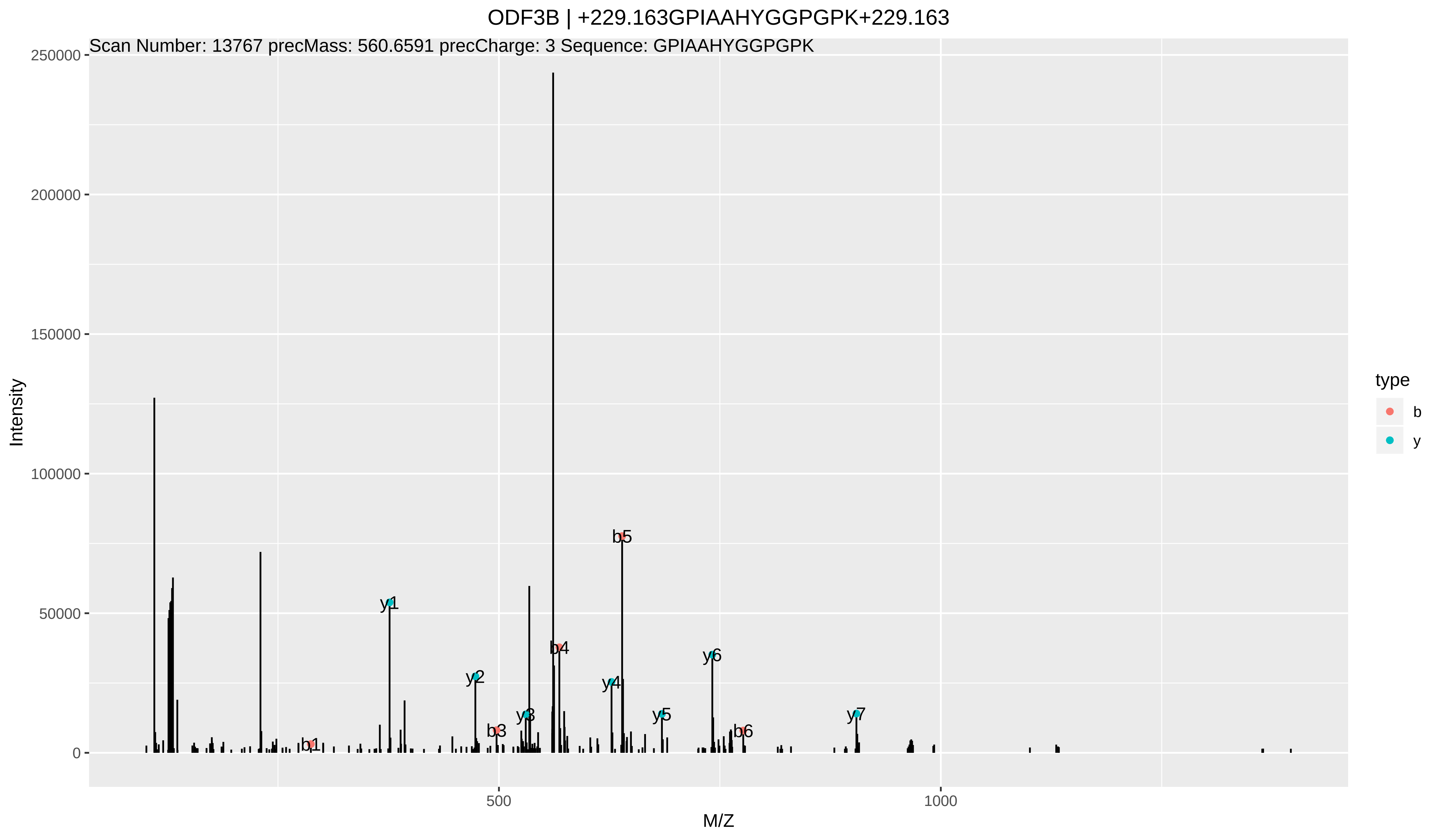
ODF3B | +229.163GPIAAHYGGPGPK+229.163
Scan Number: 13767 precMass: 560.6591 precCharge: 3 Sequence: GPIAAHYGGPGPK
Intensity
250000
200000
150000
100000
50000
0
M/Z
500
1000
type
b
y
b1
b3
b4
b5
b6
y1
y2
y3
y4
y5
y6
y7

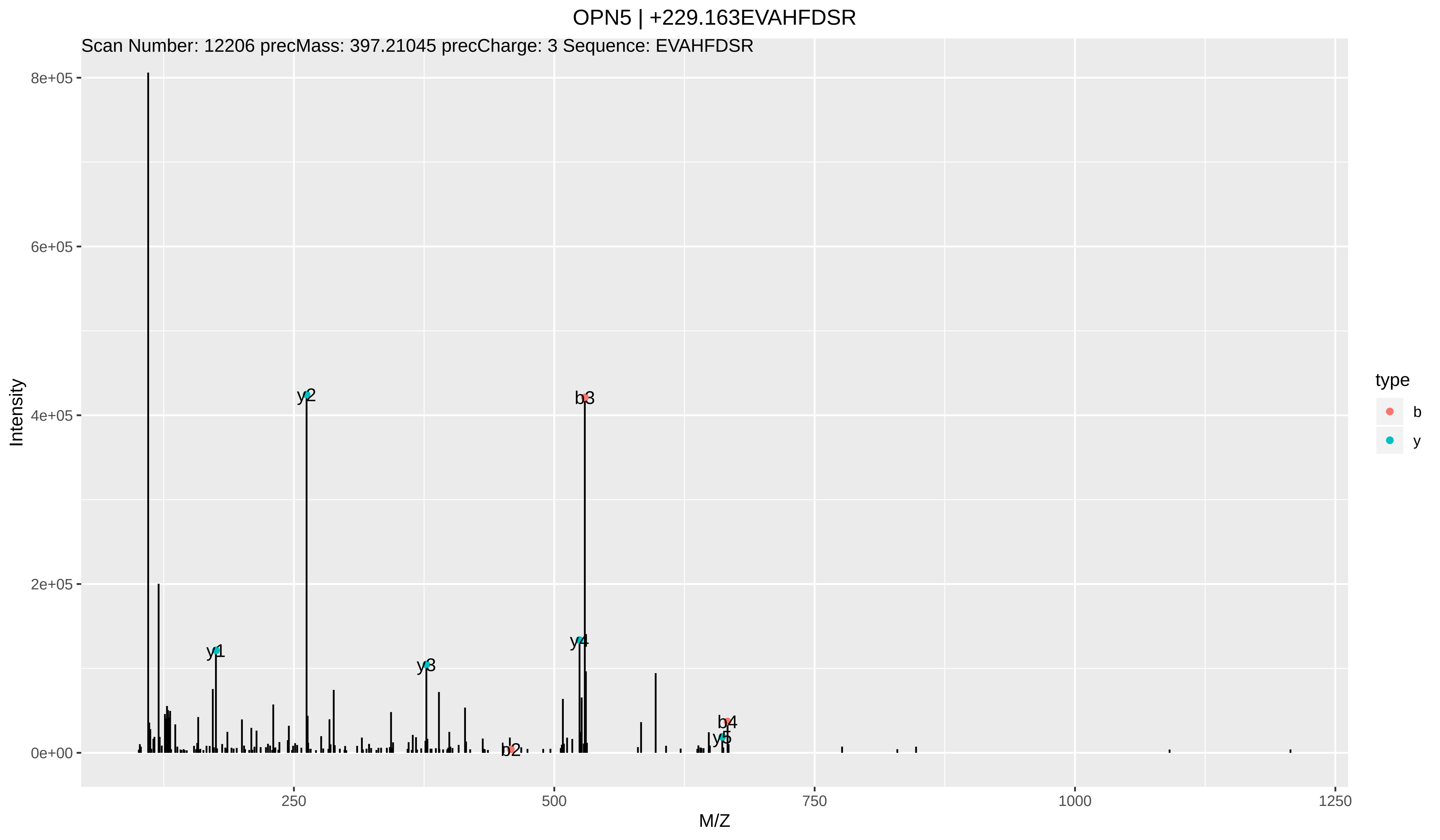
OPN5 | +229.163EVAHFDSR
Scan Number: 12206 precMass: 397.21045 precCharge: 3 Sequence: EVAHFDSR
Intensity
8e+05
6e+05
4e+05
2e+05
0e+00
M/Z
250
500
750
1000
1250
y1
y2
y3
y4
y5
b2
b3
b4
type
b
y

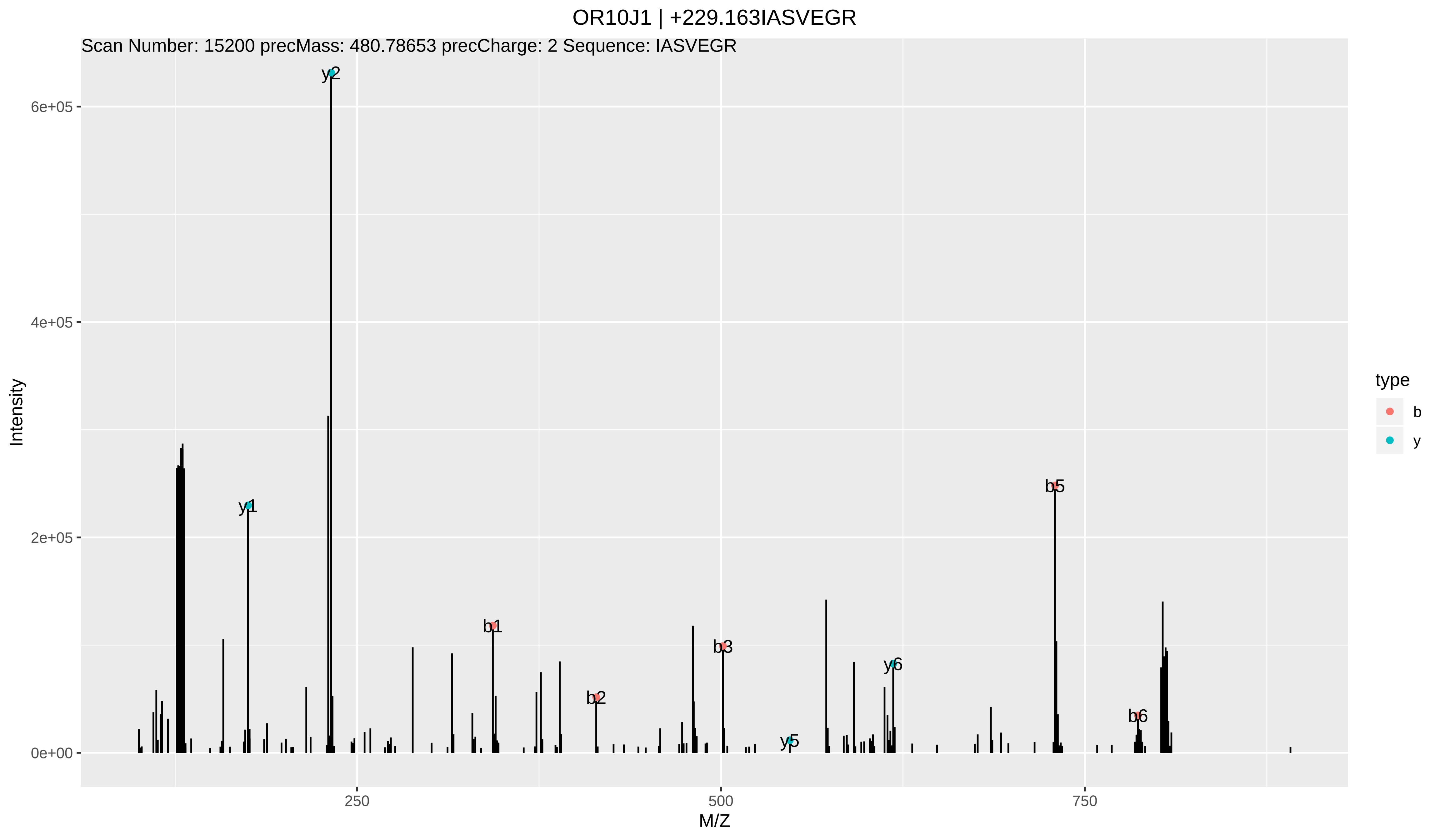

OR10J1 | +229.163IASVEGR
Scan Number: 15200 precMass: 480.78653 precCharge: 2 Sequence: IASVEGR
y2
y1
b1
b2
b3
y5
y6
b5
b6
Intensity
6e+05
4e+05
2e+05
0e+00
M/Z
250
500
750
type
b
y

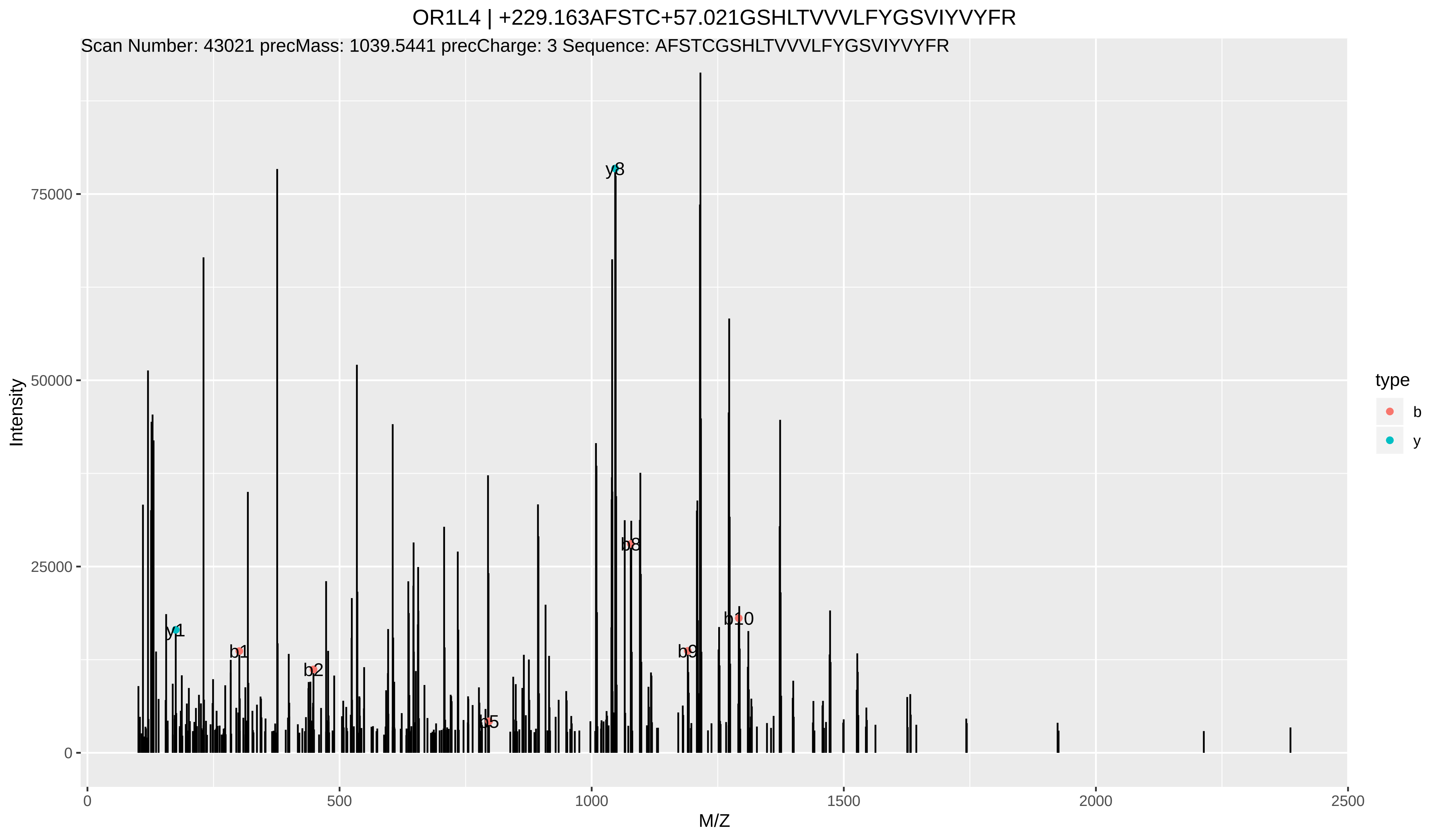

OR1L4 | +229.163AFSTC+57.021GSHLTVVVLFYGSVIYVYFR
Scan Number: 43021 precMass: 1039.5441 precCharge: 3 Sequence: AFSTCGSHLTVVVLFYGSVIYVYFR
Intensity
M/Z
y8
y1
b1
b2
b5
b8
b9
b10
type
b
y
0
25000
50000
75000
500
1000
1500
2000
2500

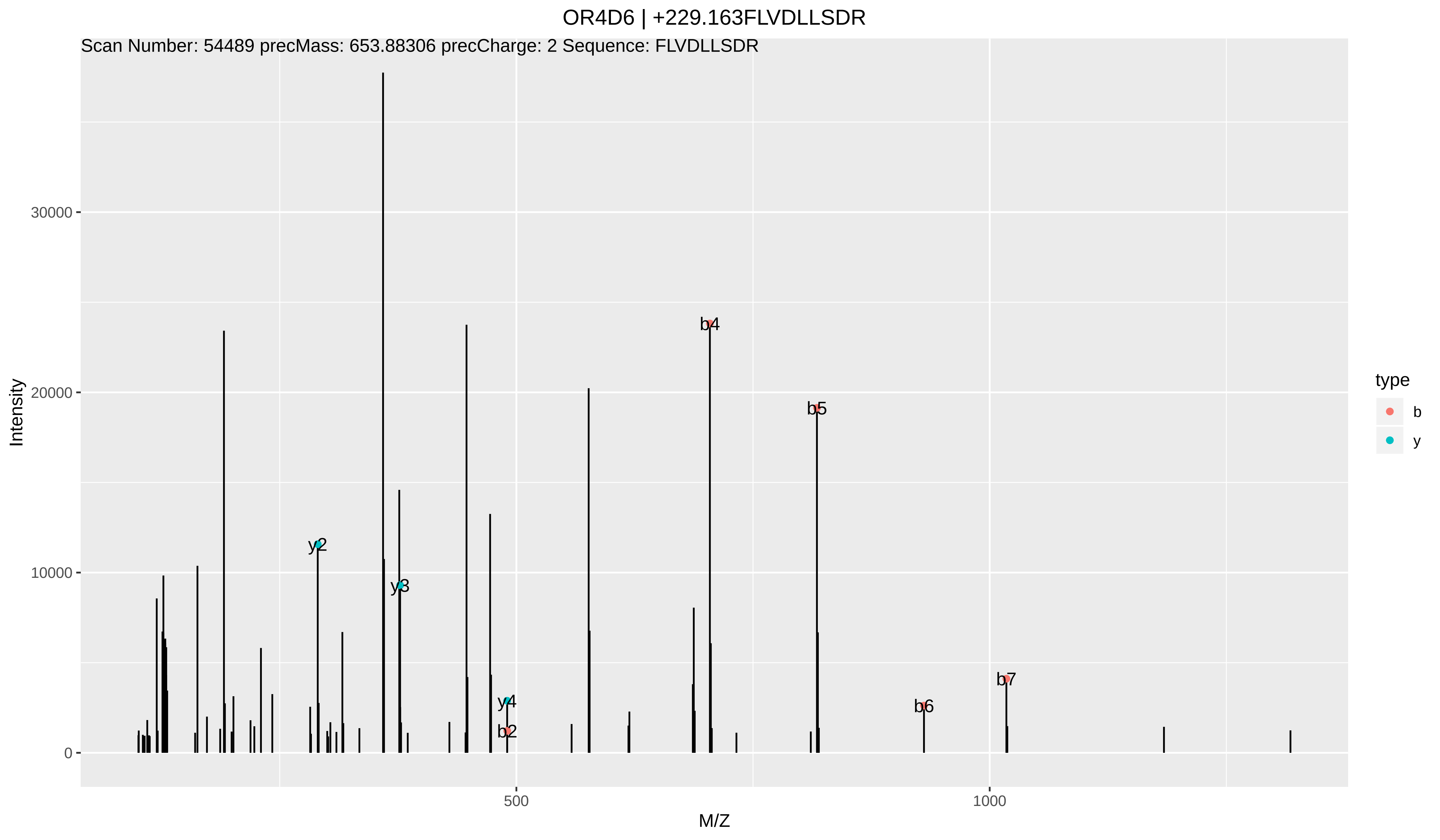

OR4D6 | +229.163FLVDLLSDR
Scan Number: 54489 precMass: 653.88306 precCharge: 2 Sequence: FLVDLLSDR
Intensity
M/Z
0
10000
20000
30000
500
1000
y2
y3
y4
b2
b4
b5
b6
b7
type
b
y

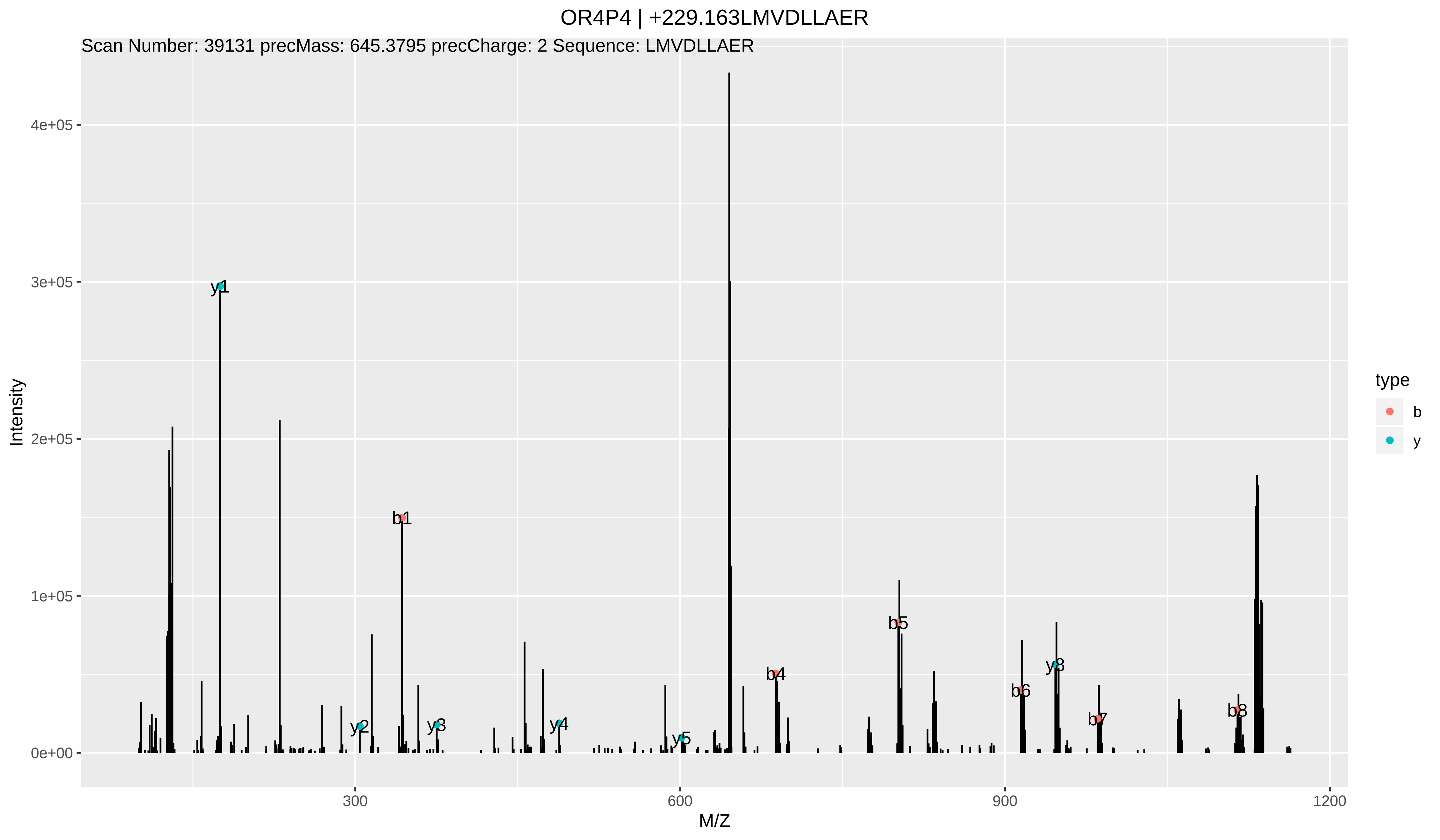
OR4P4 | +229.163LMVDLLAER
Scan Number: 39131 precMass: 645.3795 precCharge: 2 Sequence: LMVDLLAER
Intensity
4e+05
3e+05
2e+05
1e+05
0e+00
M/Z
300
600
900
1200
y1
b1
y2
y3
y4
y5
b4
b5
b6
y8
b7
b8
type
b
y

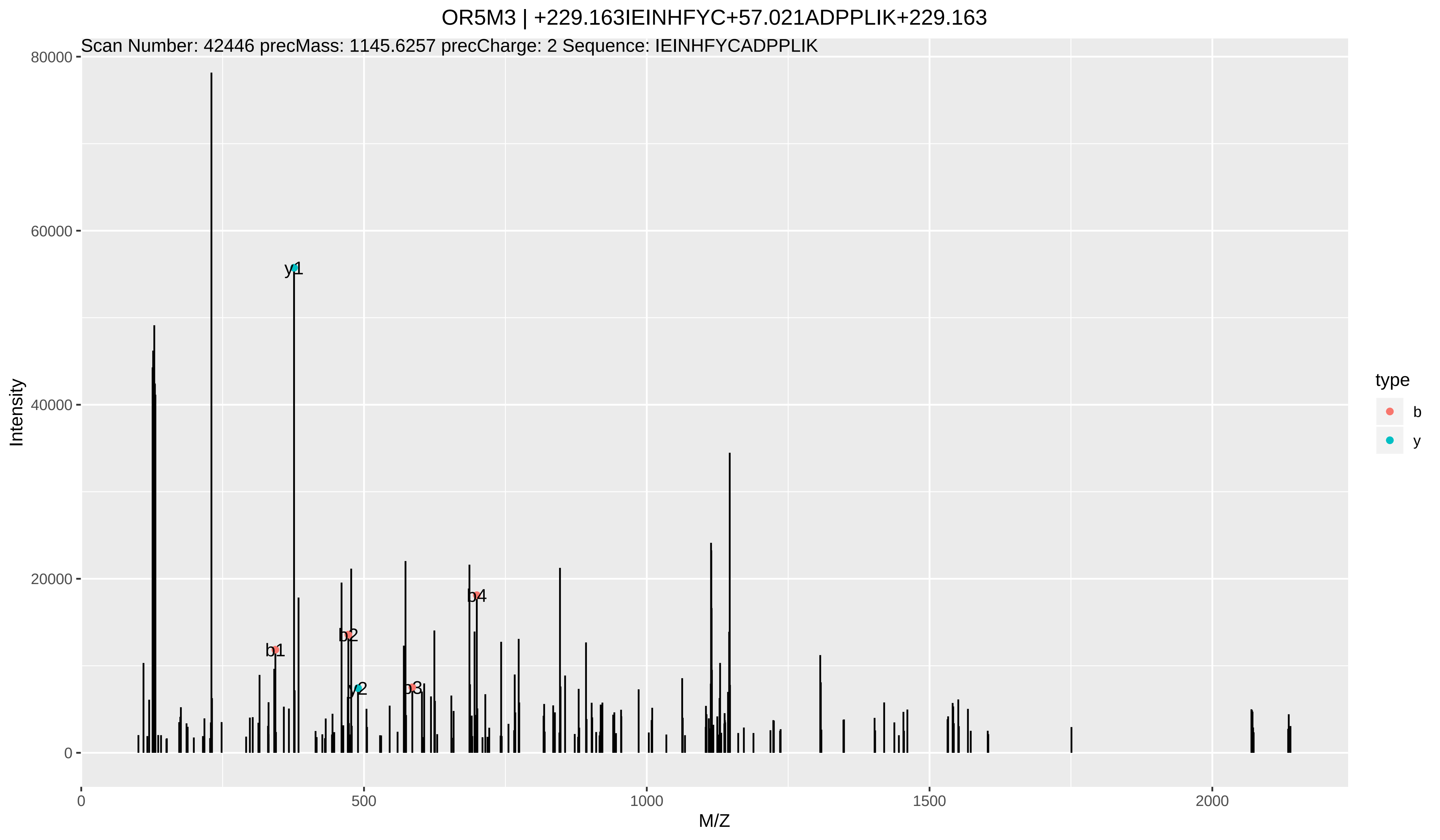

OR5M3 | +229.163IEINHFYC+57.021ADPPLIK+229.163
Scan Number: 42446 precMass: 1145.6257 precCharge: 2 Sequence: IEINHFYCADPPLIK
Intensity
M/Z
y1
y2
b1
b2
b3
b4
type
b
y
0
20000
40000
60000
80000
500
1000
1500
2000

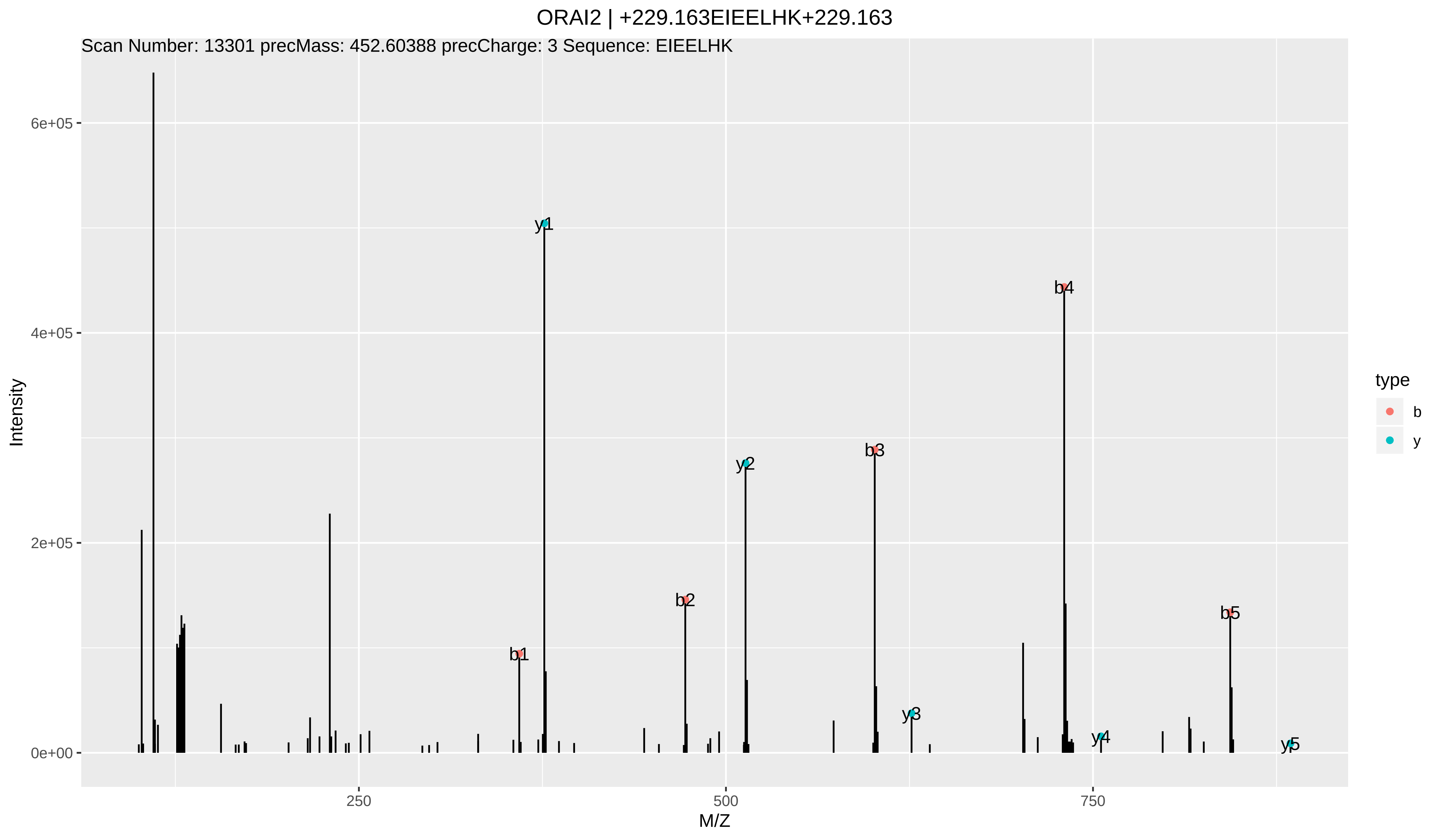
ORAI2 | +229.163EIEELHK+229.163
Scan Number: 13301 precMass: 452.60388 precCharge: 3 Sequence: EIEELHK
Intensity
M/Z
6e+05
4e+05
2e+05
0e+00
250
500
750
y1
b4
b3
y2
b2
b5
b1
y3
y4
y5
type
b
y

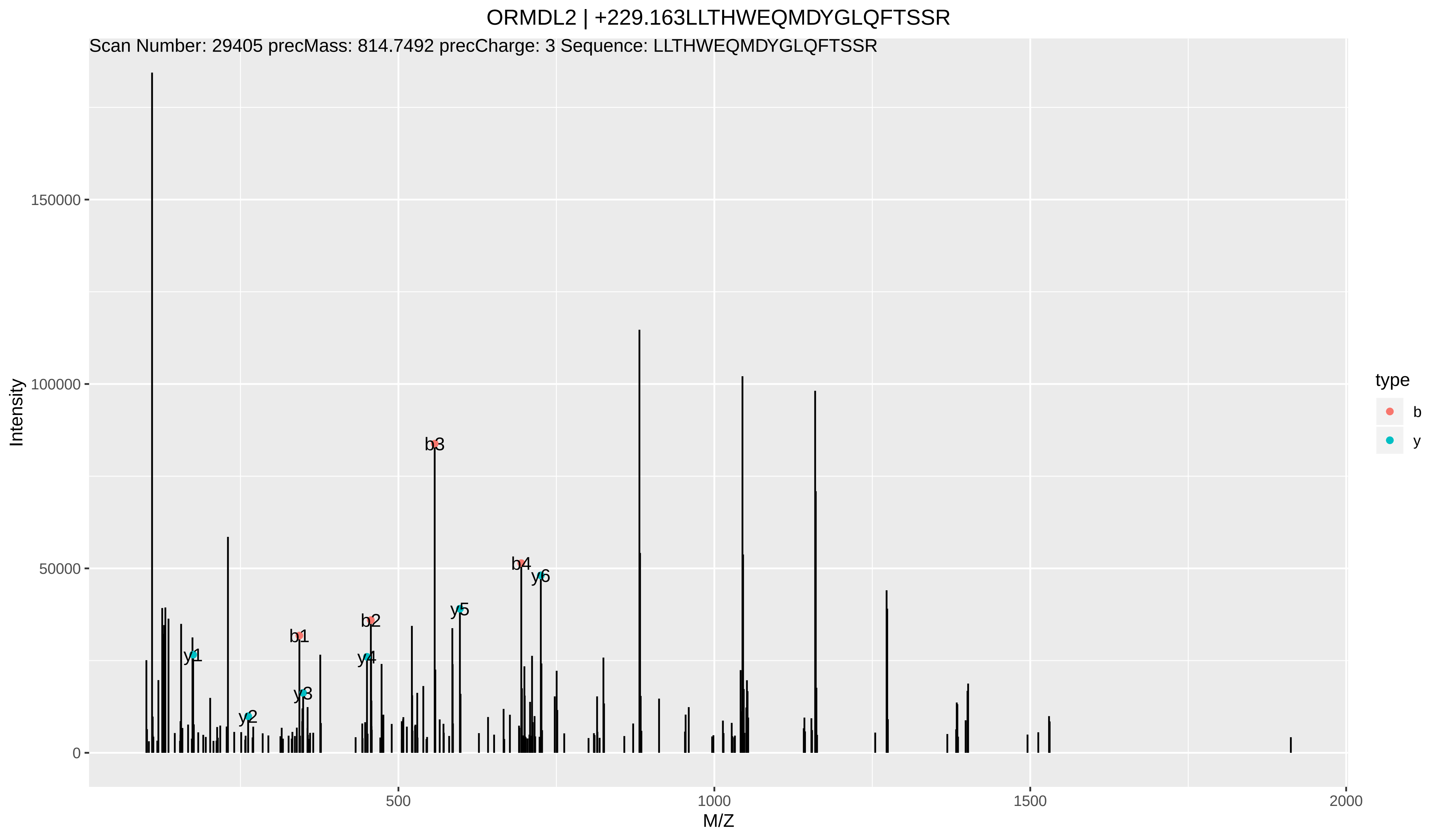
ORMDL2 | +229.163LLTHWEQMDYGLQFTSSR
Scan Number: 29405 precMass: 814.7492 precCharge: 3 Sequence: LLTHWEQMDYGLQFTSSR
Intensity
150000
100000
50000
0
500
1000
1500
2000
M/Z
type
b
y
b1
b2
b3
b4
y1
y2
y3
y4
y5
y6

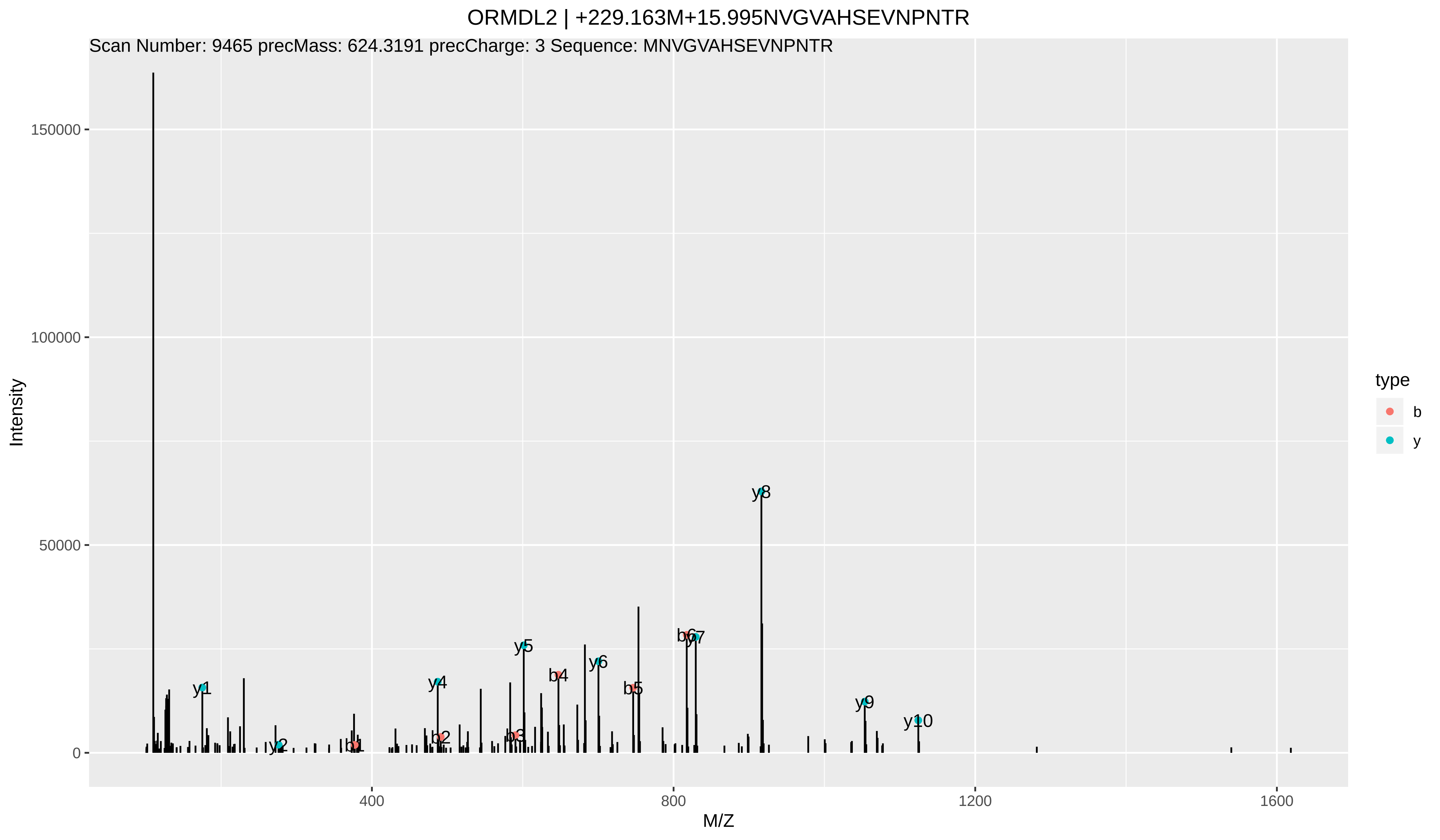

ORMDL2 | +229.163M+15.995NVGVAHSEVNPNTR
Scan Number: 9465 precMass: 624.3191 precCharge: 3 Sequence: MNVGVAHSEVNPNTR
Intensity
M/Z
type
b
y
y1
y2
b1
y4
b2
y5
b3
b4
y6
b5
b6
y7
y8
y9
y10
0
50000
100000
150000
400
800
1200
1600

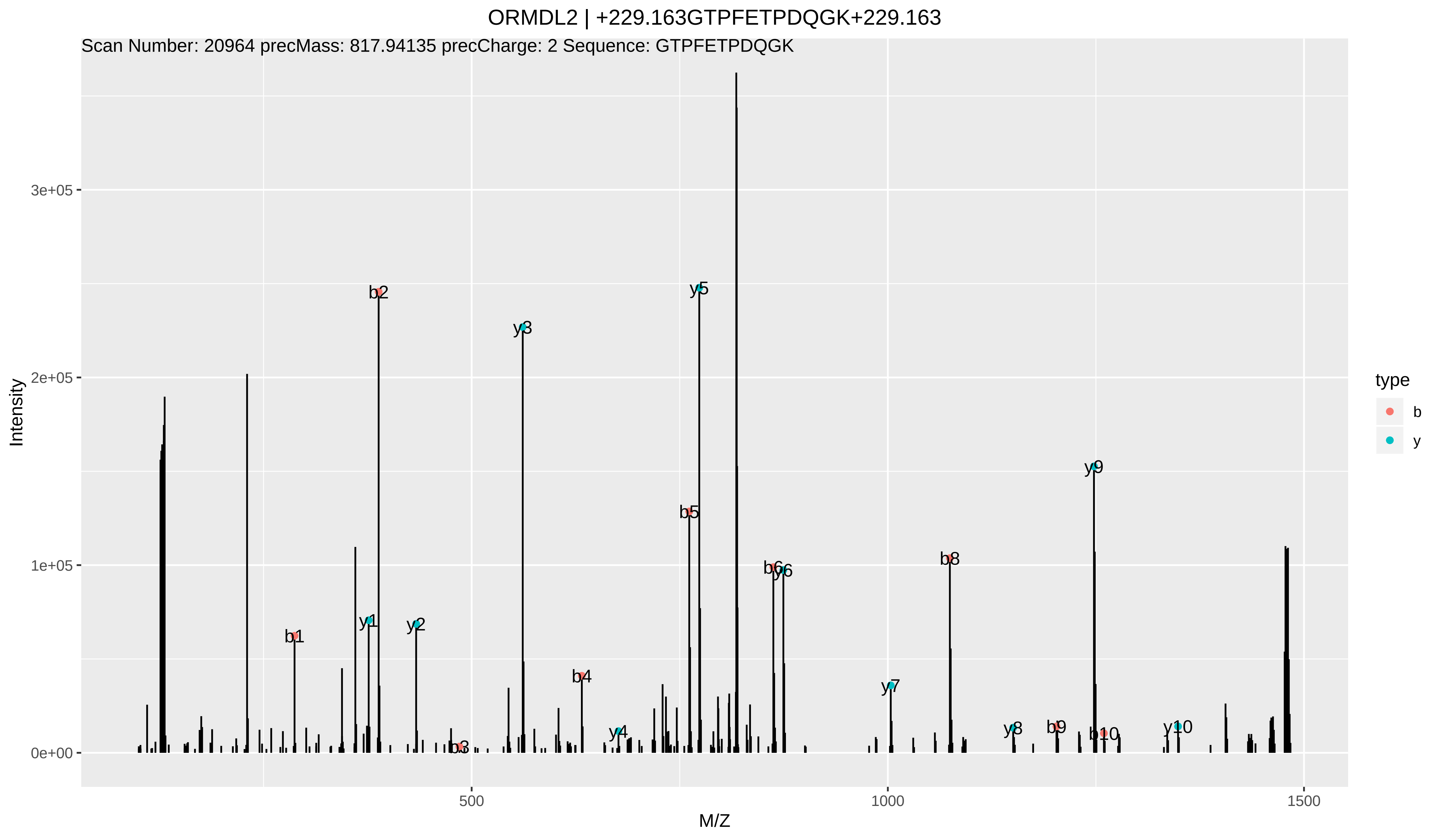

ORMDL2 | +229.163GTPFETPDQGK+229.163
Scan Number: 20964 precMass: 817.94135 precCharge: 2 Sequence: GTPFETPDQGK
Intensity
3e+05
2e+05
1e+05
0e+00
M/Z
500
1000
1500
b1
b2
b3
b4
b5
b6
b8
b9
b10
y1
y2
y3
y4
y5
y6
y7
y8
y9
y10
type
b
y

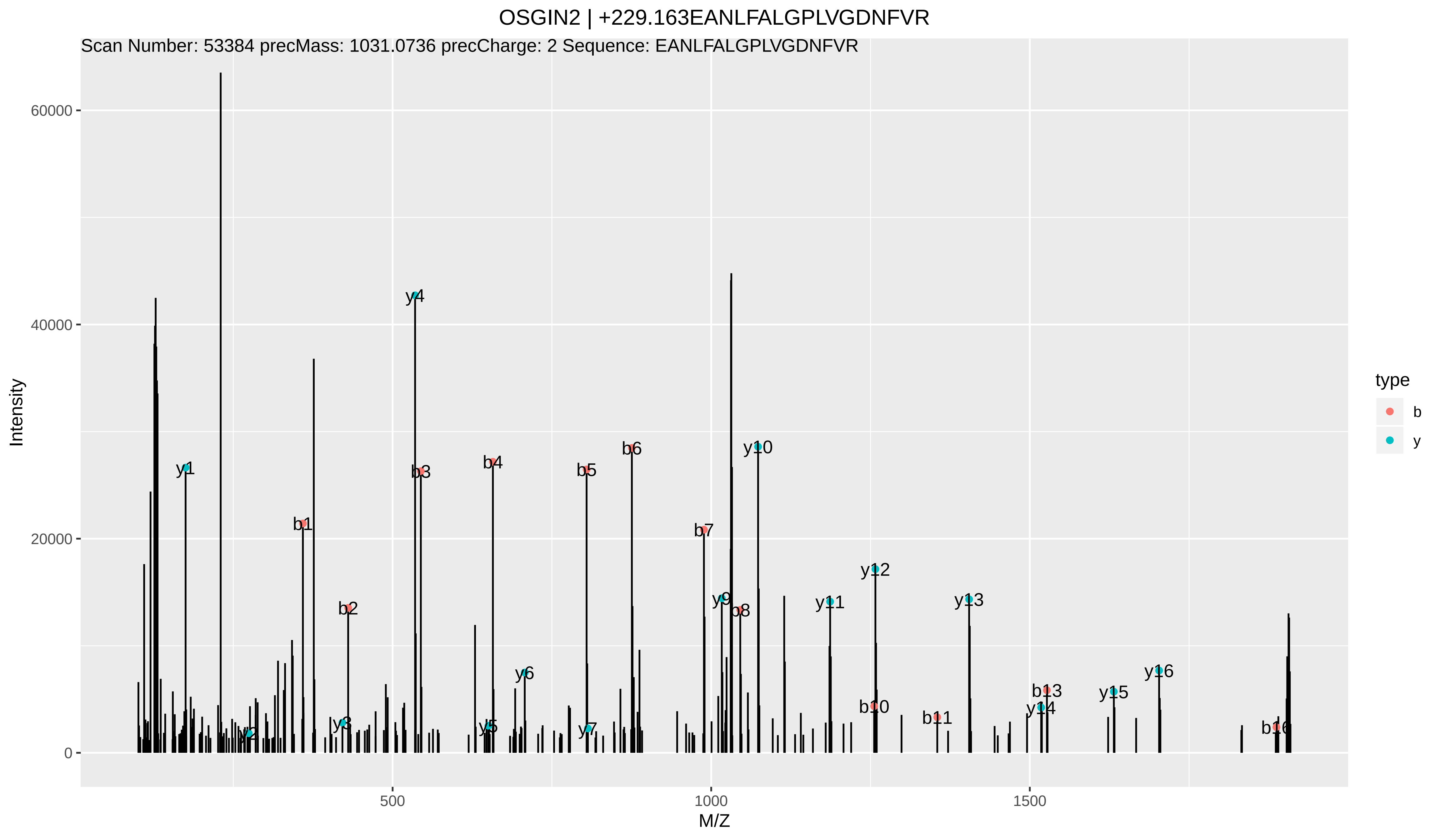

OSGIN2 | +229.163EANLFALGPLVGDNFVR
Scan Number: 53384 precMass: 1031.0736 precCharge: 2 Sequence: EANLFALGPLVGDNFVR
Intensity
M/Z
60000
40000
20000
0
500
1000
1500
type
b
y
y1
y2
y3
y4
y5
y6
y7
y9
y10
y11
y12
y13
y14
y15
y16
b1
b2
b3
b4
b5
b6
b7
b8
b10
b11
b13
b16

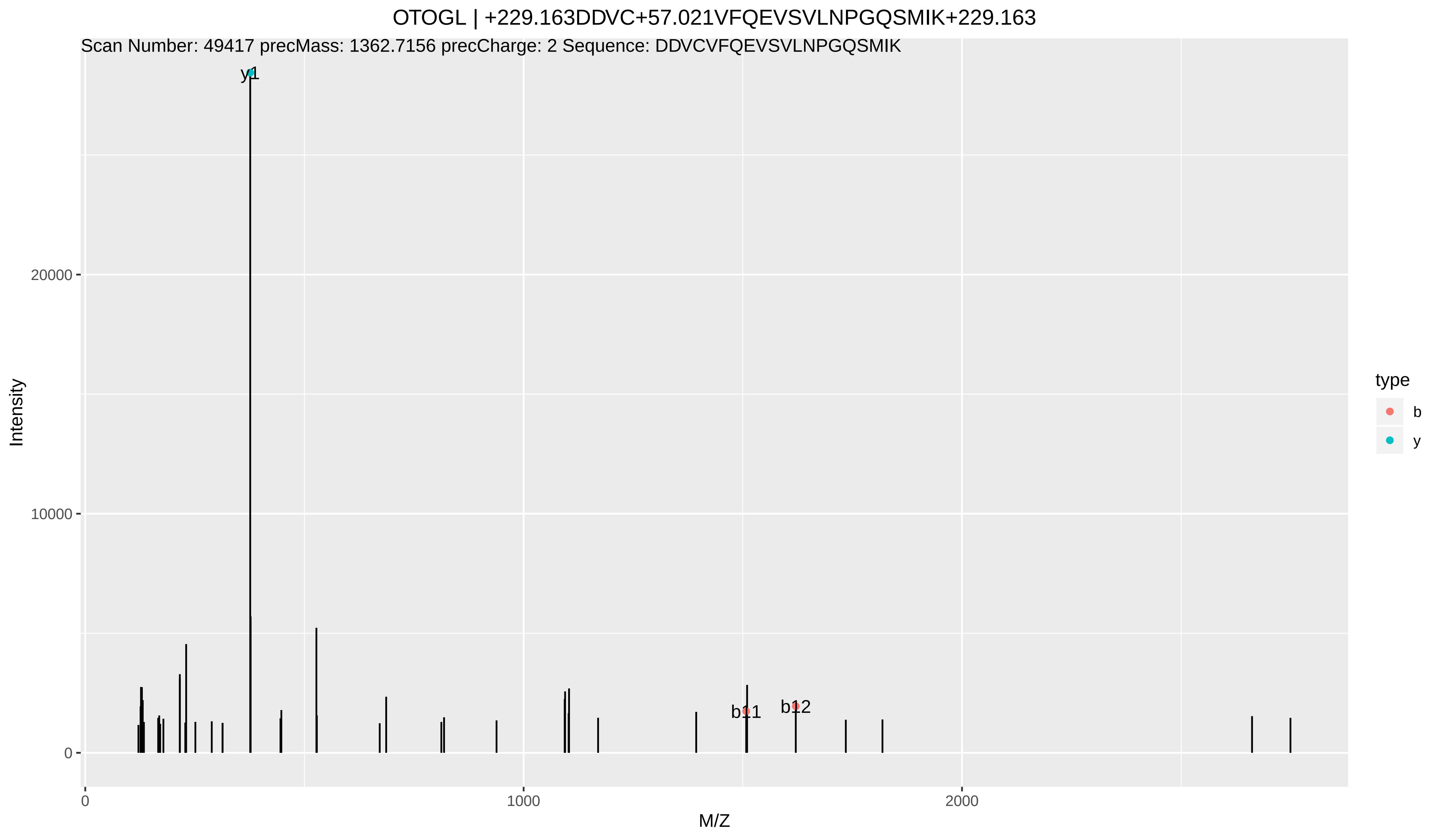

OTOGL | +229.163DDVC+57.021VFQEVSVLNPGQSMIK+229.163
Scan Number: 49417 precMass: 1362.7156 precCharge: 2 Sequence: DDVCVFQEVSVLNPGQSMIK
y1
b11
b12
20000
10000
0
0
1000
2000
M/Z
Intensity
type
b
y

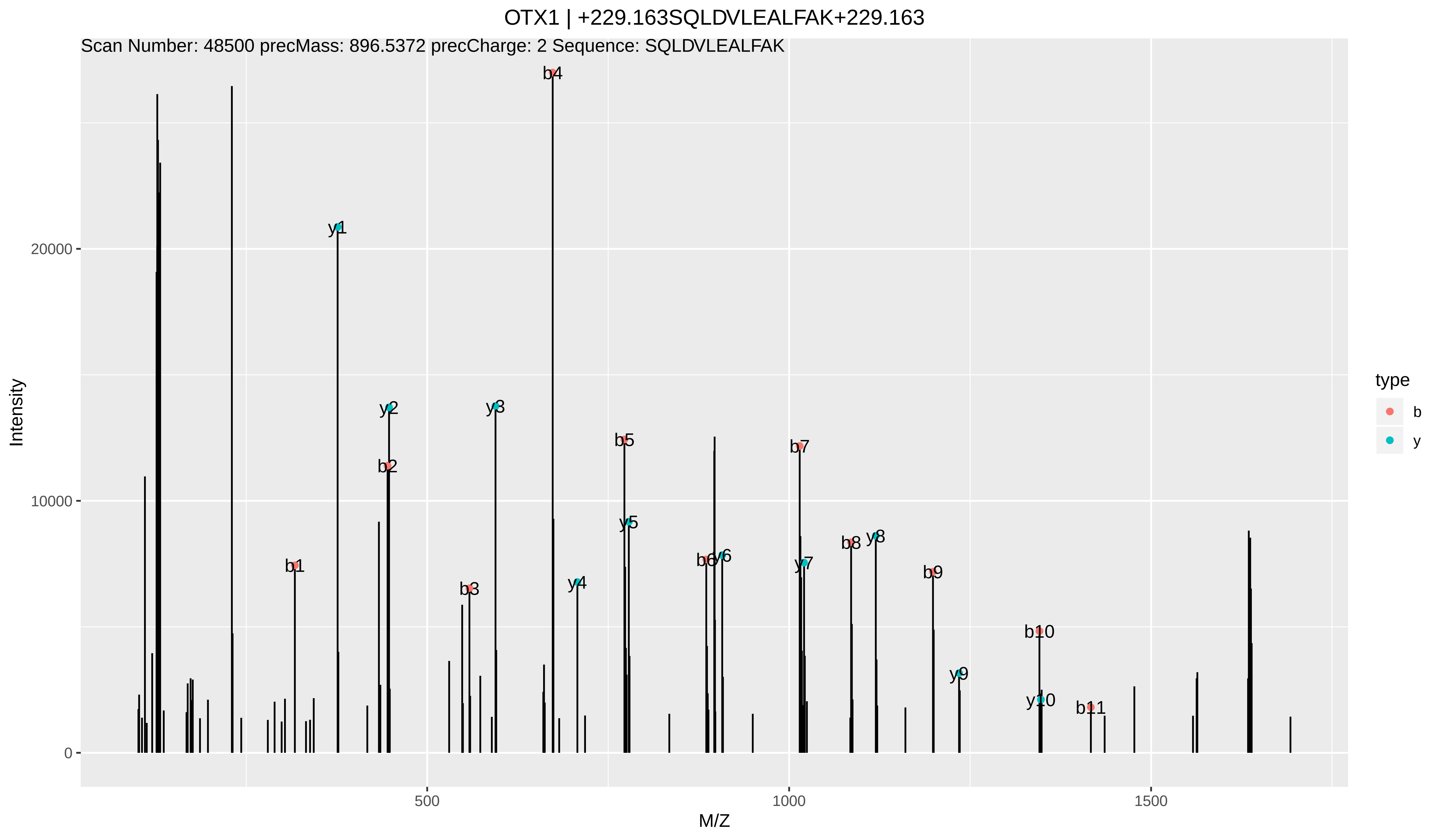
OTX1 | +229.163SQLDVLEALFAK+229.163
Scan Number: 48500 precMass: 896.5372 precCharge: 2 Sequence: SQLDVLEALFAK
Intensity
M/Z
0
10000
20000
500
1000
1500
b4
y1
y2
b2
y3
b5
b7
y5
b8
y8
b6
y6
y7
b9
b1
b3
y4
b10
y9
y10
b11
type
b
y

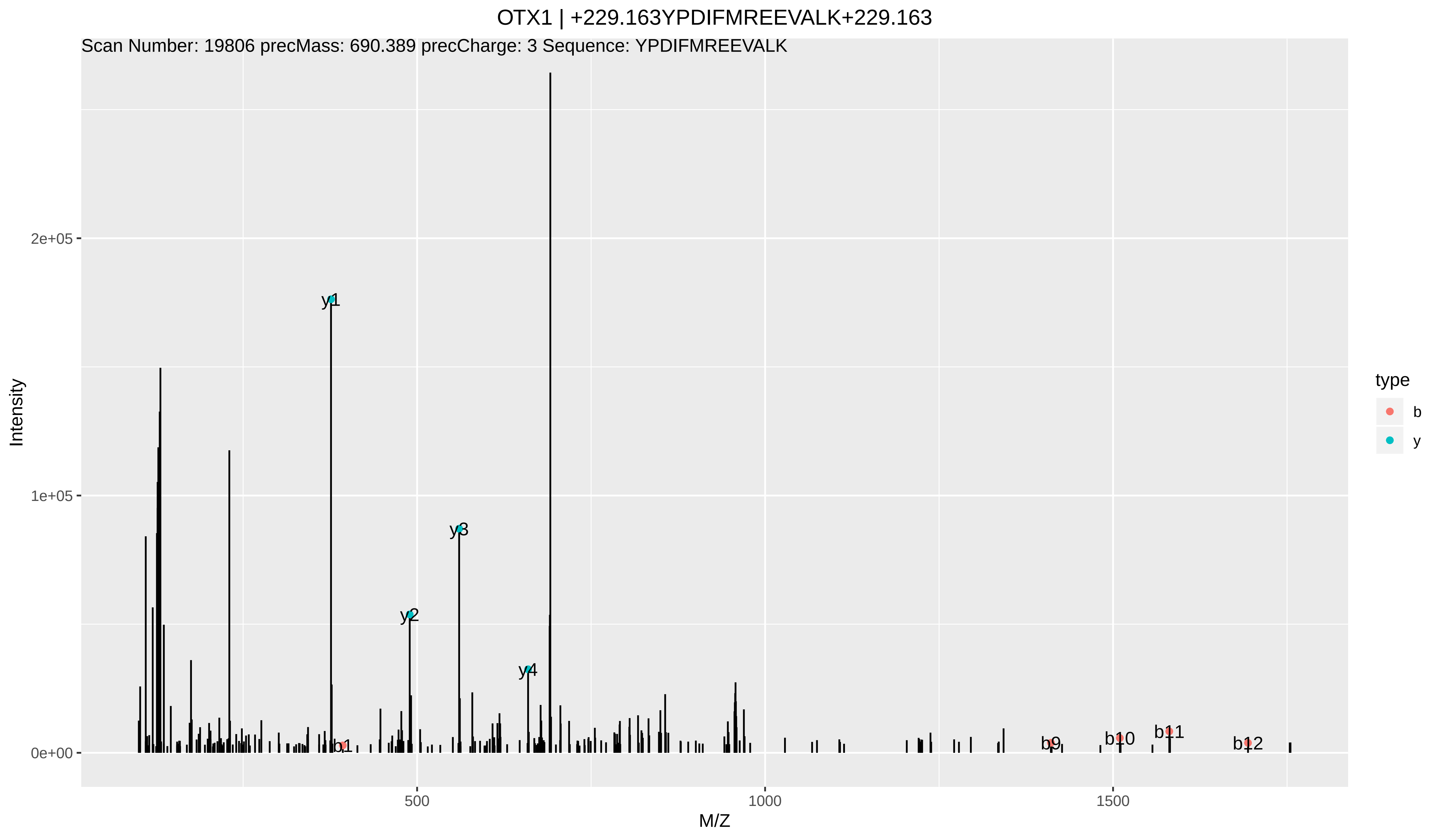

OTX1 | +229.163YPDIFMREEVALK+229.163
Scan Number: 19806 precMass: 690.389 precCharge: 3 Sequence: YPDIFMREEVALK
Intensity
M/Z
2e+05
1e+05
0e+00
500
1000
1500
y1
y2
y3
y4
b1
b9
b10
b11
b12
type
b
y

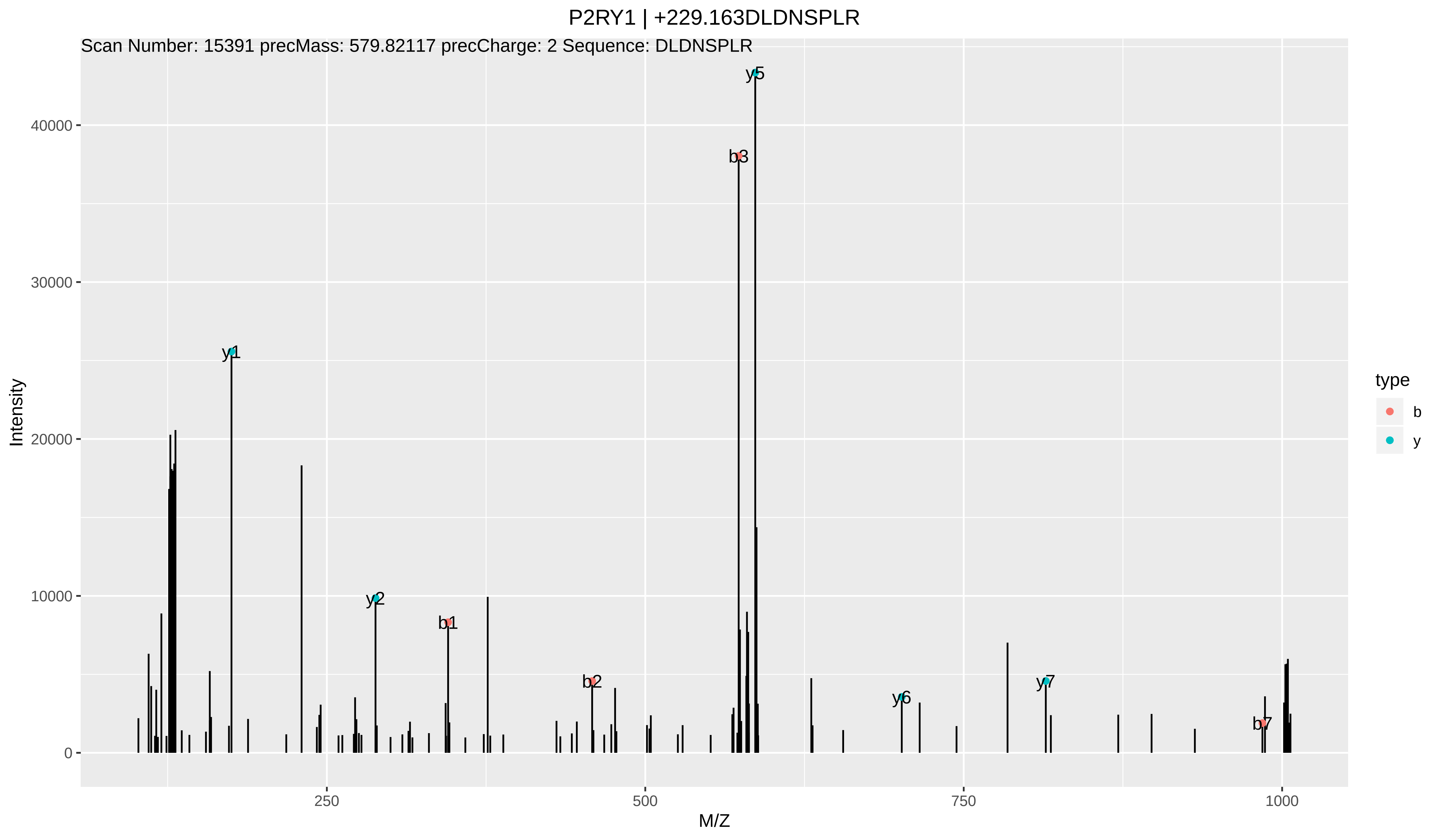
P2RY1 | +229.163DLDNSPLR
Scan Number: 15391 precMass: 579.82117 precCharge: 2 Sequence: DLDNSPLR
Intensity
M/Z
type
b
y
y5
b3
y1
y2
b1
b2
y6
y7
b7
0
10000
20000
30000
40000
250
500
750
1000

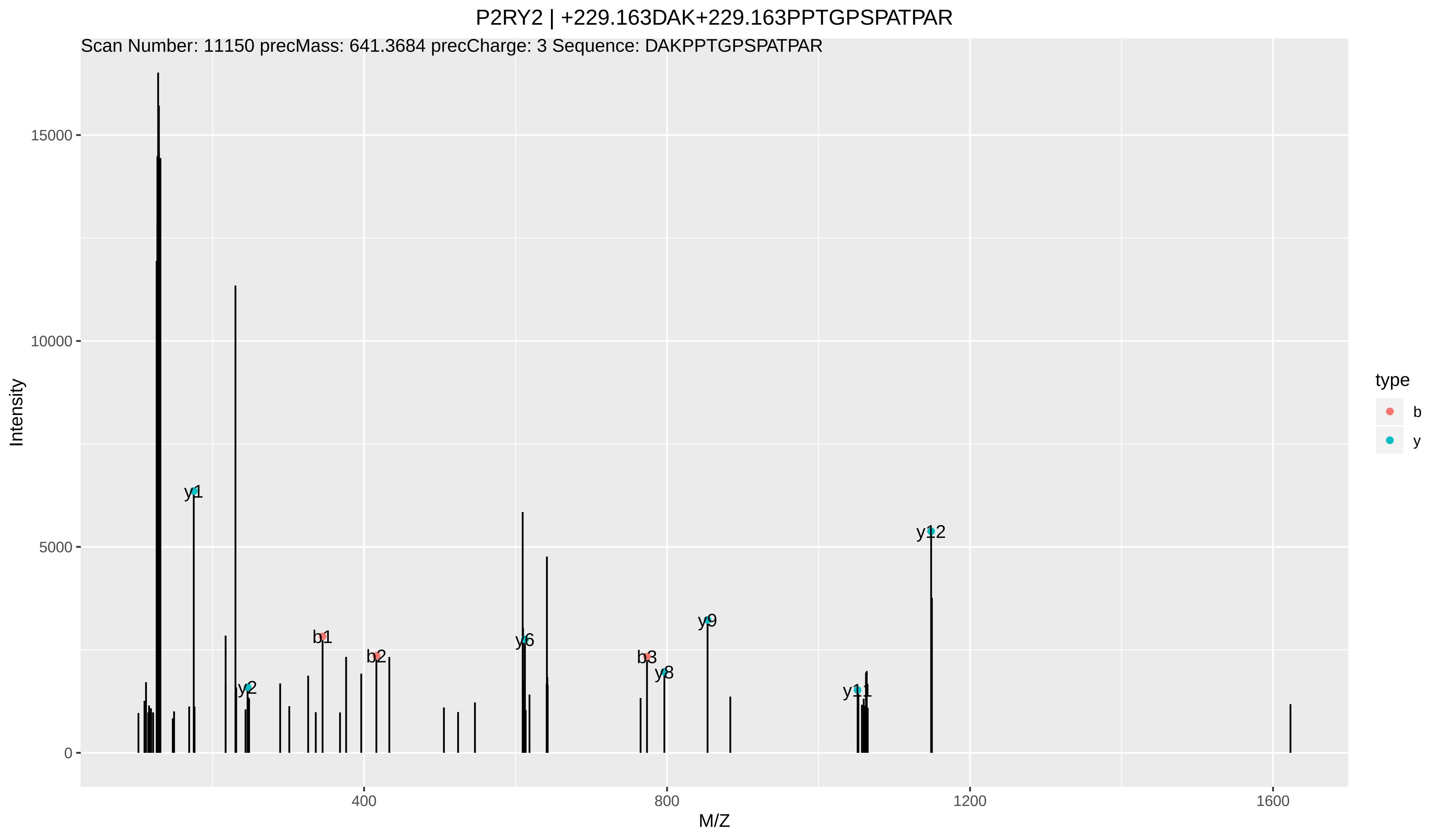
P2RY2 | +229.163DAK+229.163PPTGPSPATPAR
Scan Number: 11150 precMass: 641.3684 precCharge: 3 Sequence: DAKPPTGPSPATPAR
Intensity
15000
10000
5000
0
M/Z
400
800
1200
1600
y1
y2
b1
b2
y6
b3
y8
y9
y11
y12
type
b
y

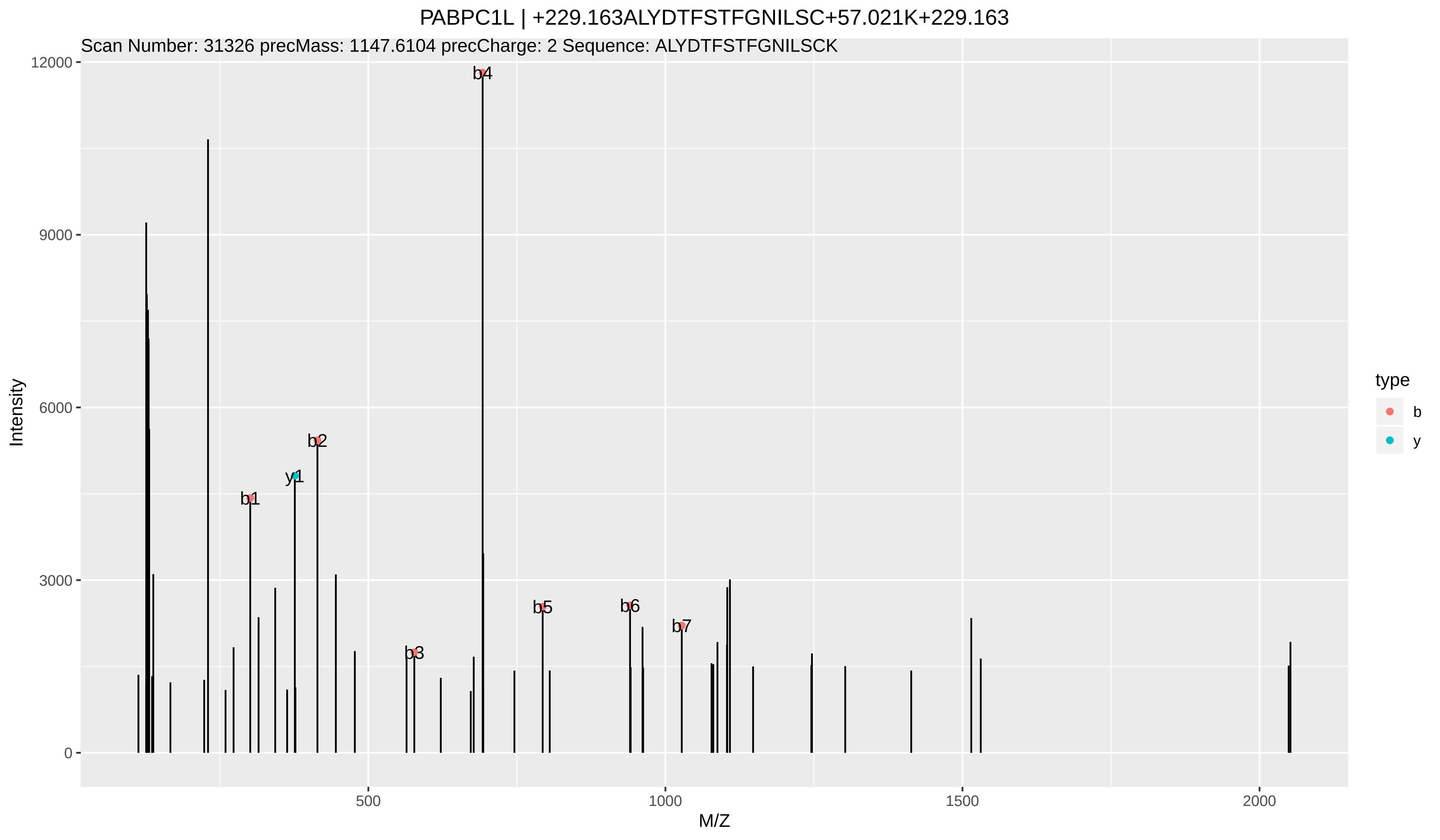
PABPC1L | +229.163ALYDTFSTFGNILSC+57.021K+229.163
Scan Number: 31326 precMass: 1147.6104 precCharge: 2 Sequence: ALYDTFSTFGNILSCK
Intensity
M/Z
0
3000
6000
9000
12000
500
1000
1500
2000
b1
y1
b2
b3
b4
b5
b6
b7
type
b
y

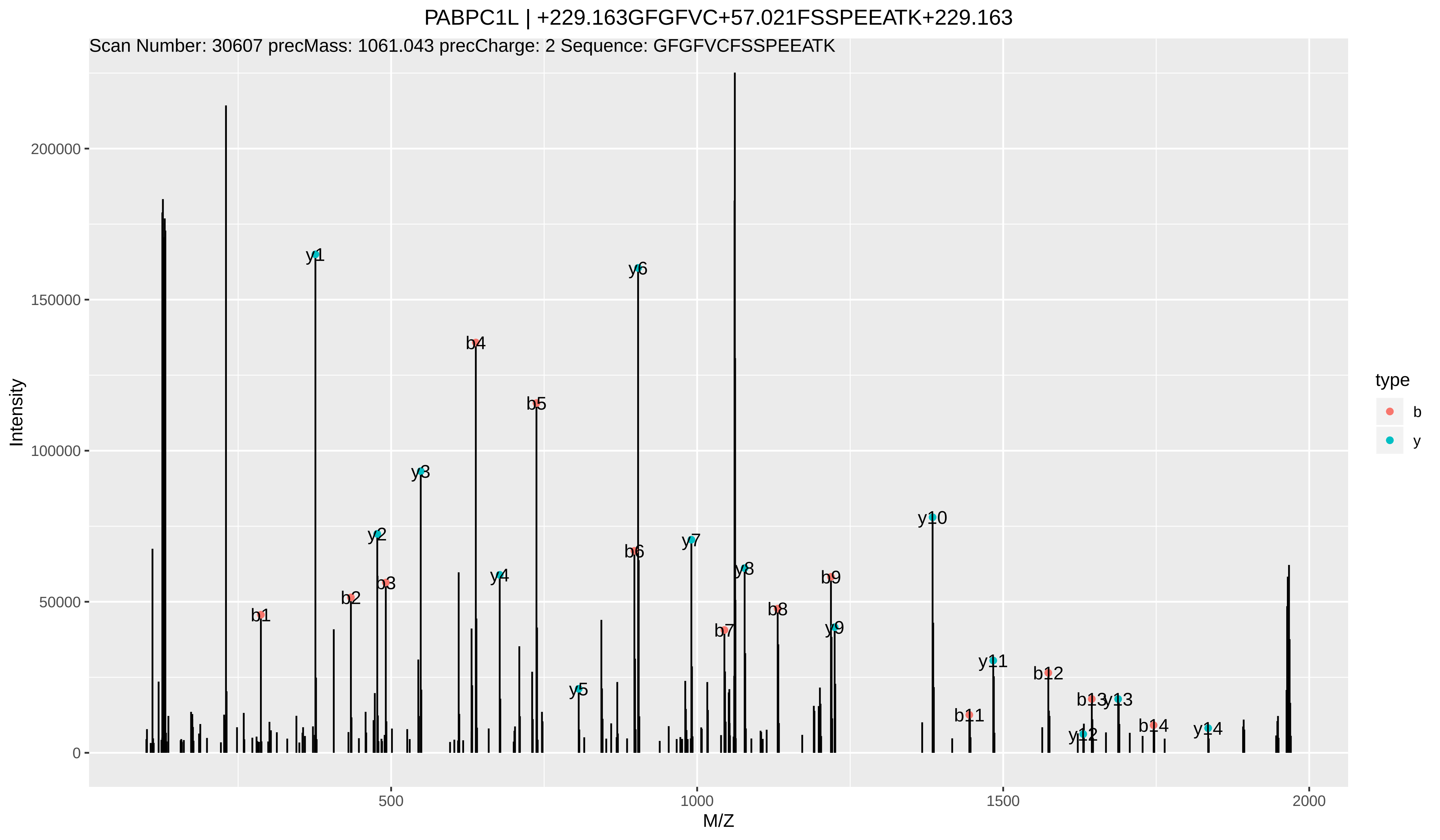
PABPC1L | +229.163GFGFVC+57.021FSSPEEATK+229.163
Scan Number: 30607 precMass: 1061.043 precCharge: 2 Sequence: GFGFVCFSSPEEATK
Intensity
M/Z
type
b
y
y1
y2
y3
y4
y5
y6
y7
y8
y9
y10
y11
y12
y13
y14
b1
b2
b3
b4
b5
b6
b7
b8
b9
b11
b12
b13
b14
0
50000
100000
150000
200000
500
1000
1500
2000

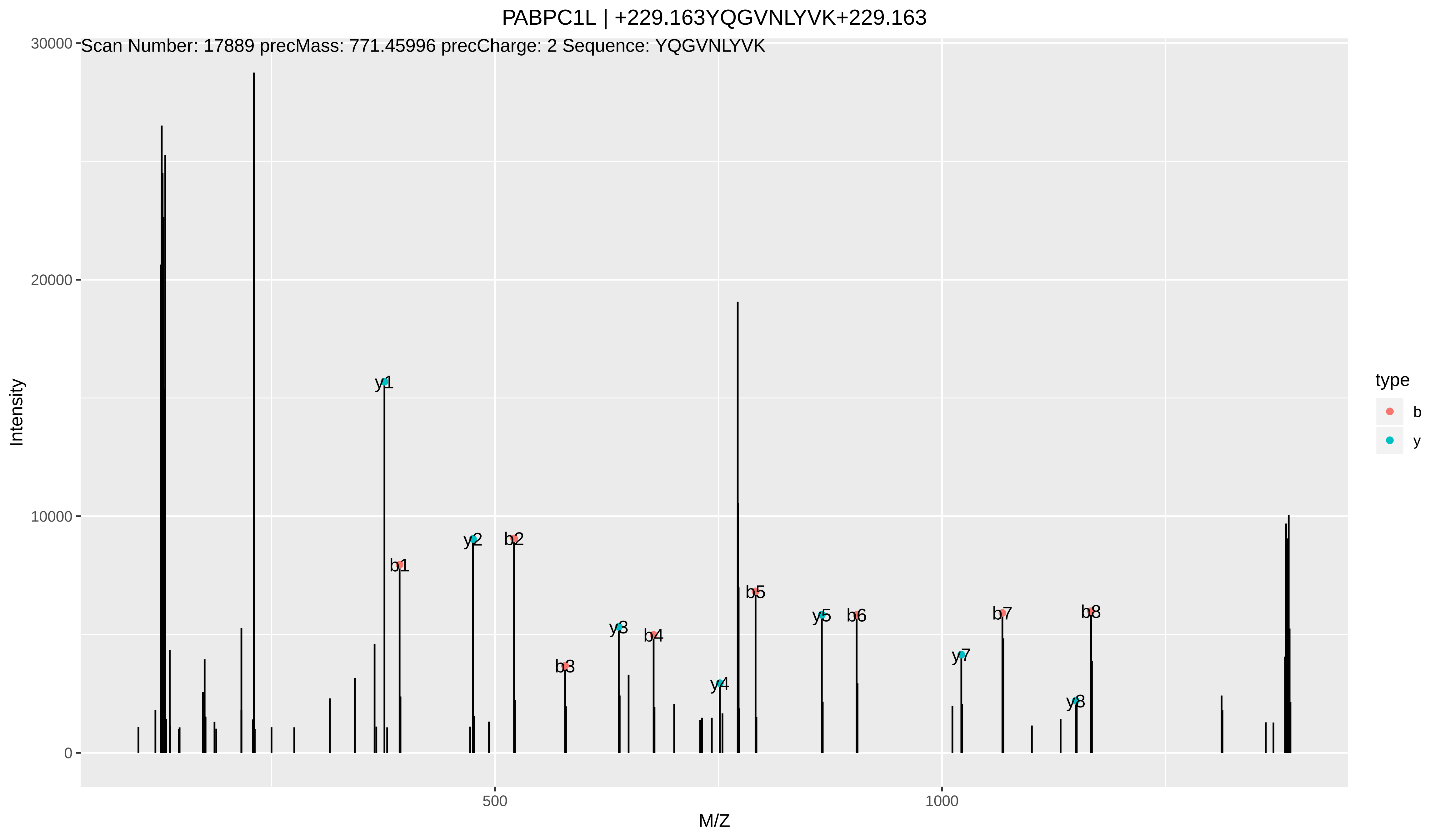

PABPC1L | +229.163YQGVNLYVK+229.163
Scan Number: 17889 precMass: 771.45996 precCharge: 2 Sequence: YQGVNLYVK
Intensity
M/Z
0
10000
20000
30000
500
1000
y1
b1
y2
b2
b3
y3
b4
y4
b5
y5
b6
y7
b7
y8
b8
type
b
y

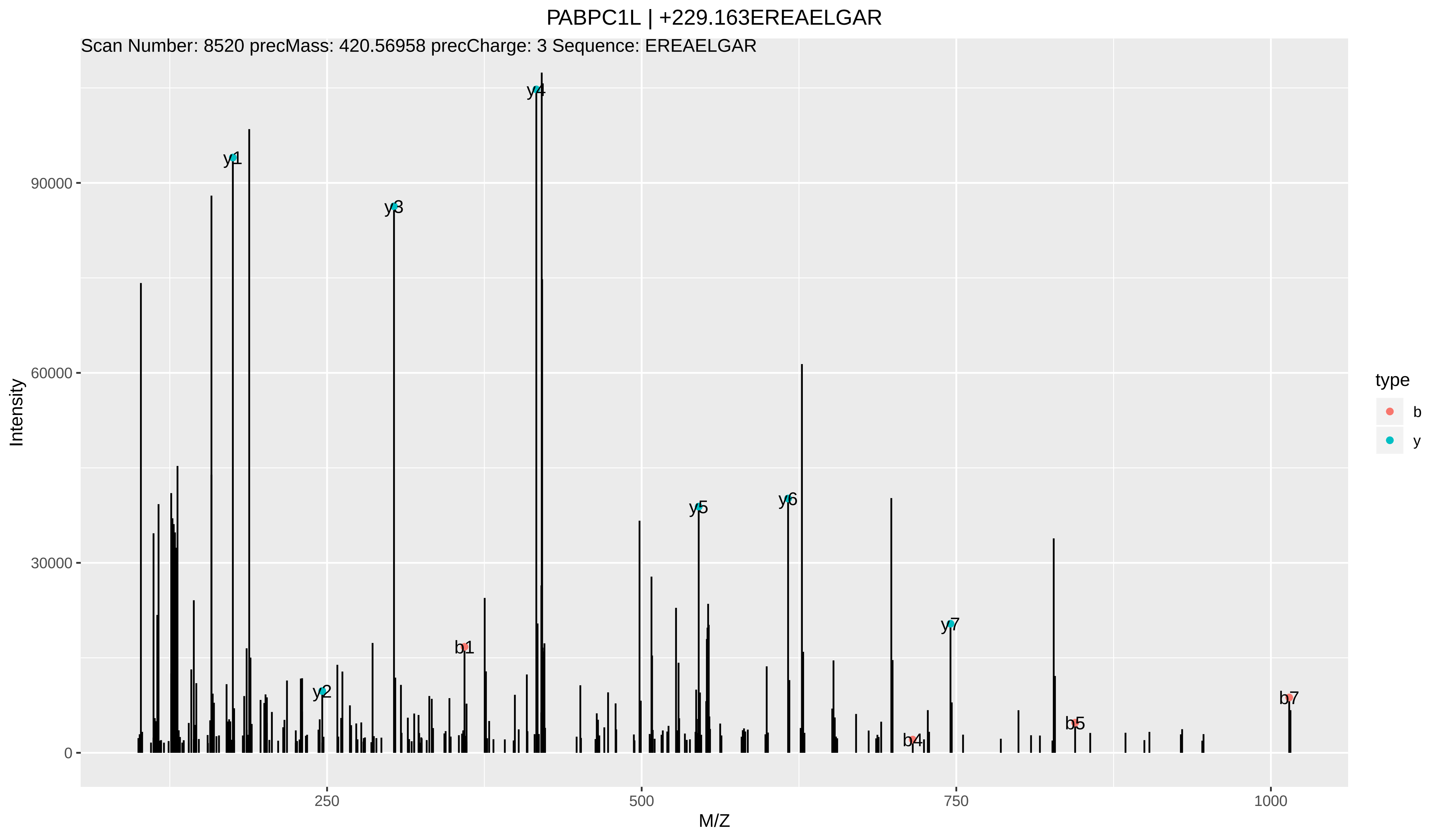

PABPC1L | +229.163EREAELGAR
Scan Number: 8520 precMass: 420.56958 precCharge: 3 Sequence: EREAELGAR
Intensity
90000
60000
30000
0
M/Z
250
500
750
1000
y1
y2
y3
y4
y5
y6
y7
b1
b4
b5
b7
type
b
y

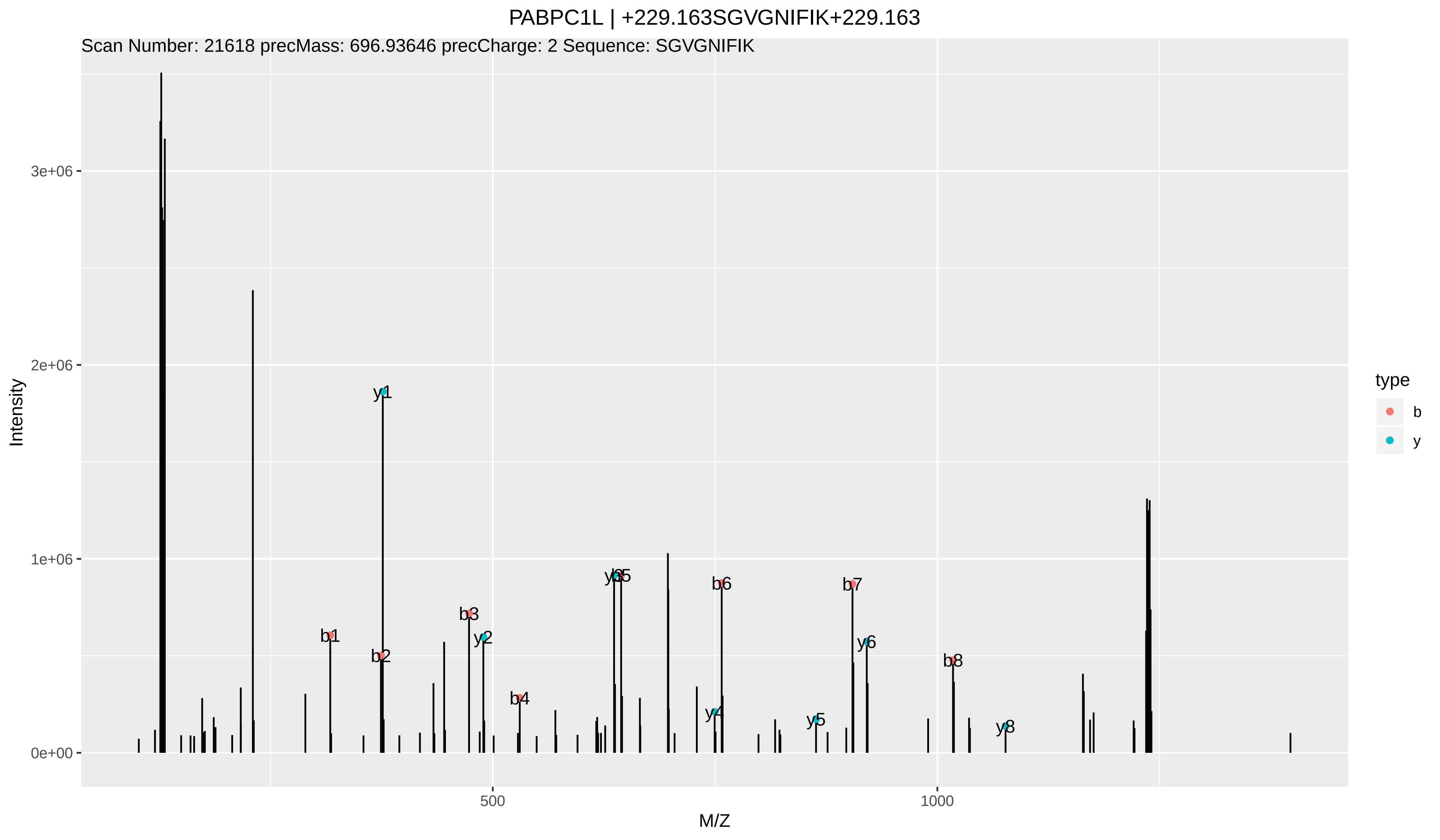

PABPC1L | +229.163SGVGNIFIK+229.163
Scan Number: 21618 precMass: 696.93646 precCharge: 2 Sequence: SGVGNIFIK
Intensity
3e+06
2e+06
1e+06
0e+00
M/Z
500
1000
y1
b1
b2
b3
y2
b4
y3
b5
b6
y4
y5
b7
y6
b8
y8
type
b
y

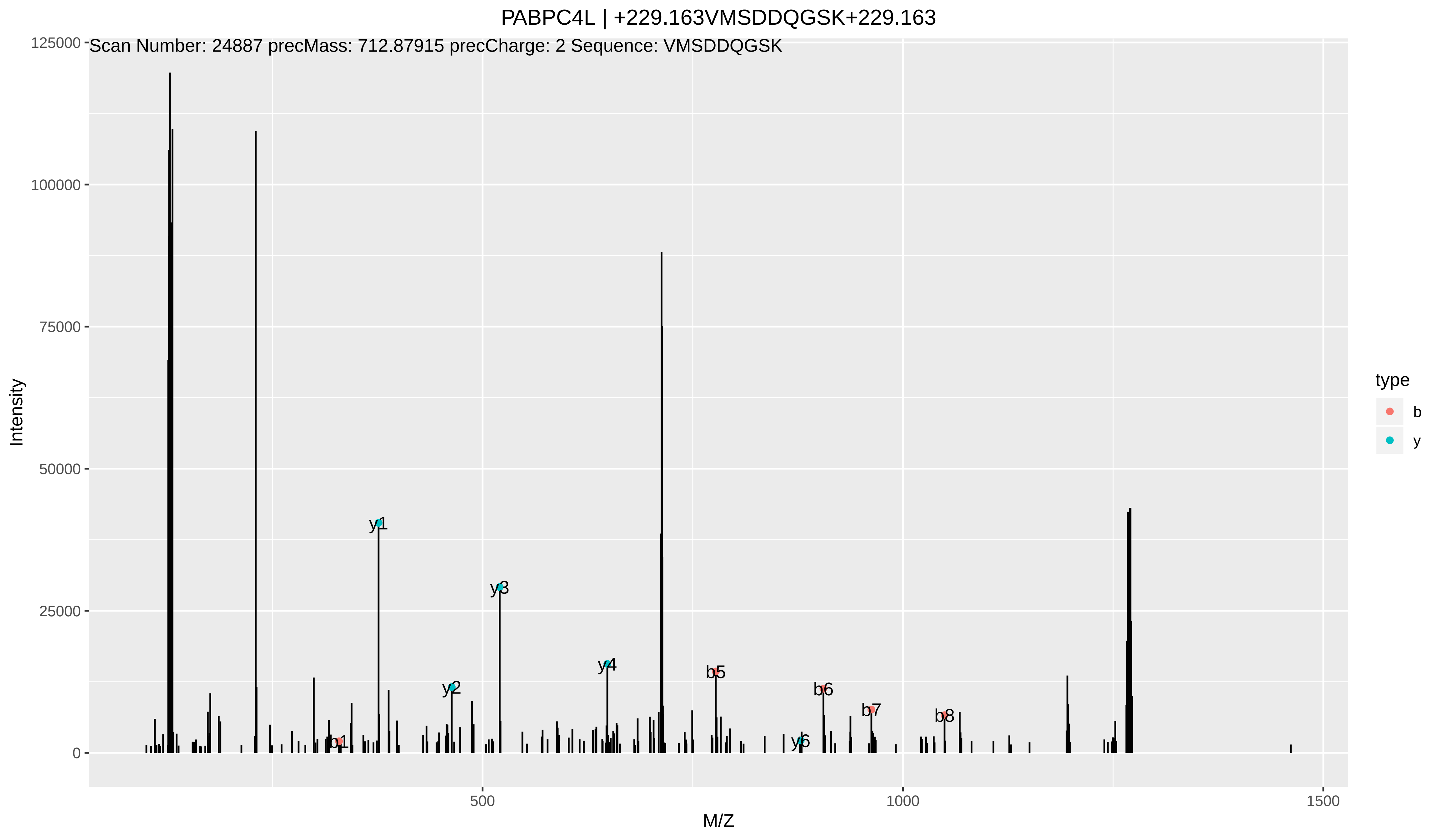
PABPC4L | +229.163VMSDDQGSK+229.163
Scan Number: 24887 precMass: 712.87915 precCharge: 2 Sequence: VMSDDQGSK
Intensity
125000
100000
75000
50000
25000
0
M/Z
500
1000
1500
y1
y2
y3
y4
y6
b1
b5
b6
b7
b8
type
b
y

PABPC4L | +229.163NLDDTIDDEK+229.163

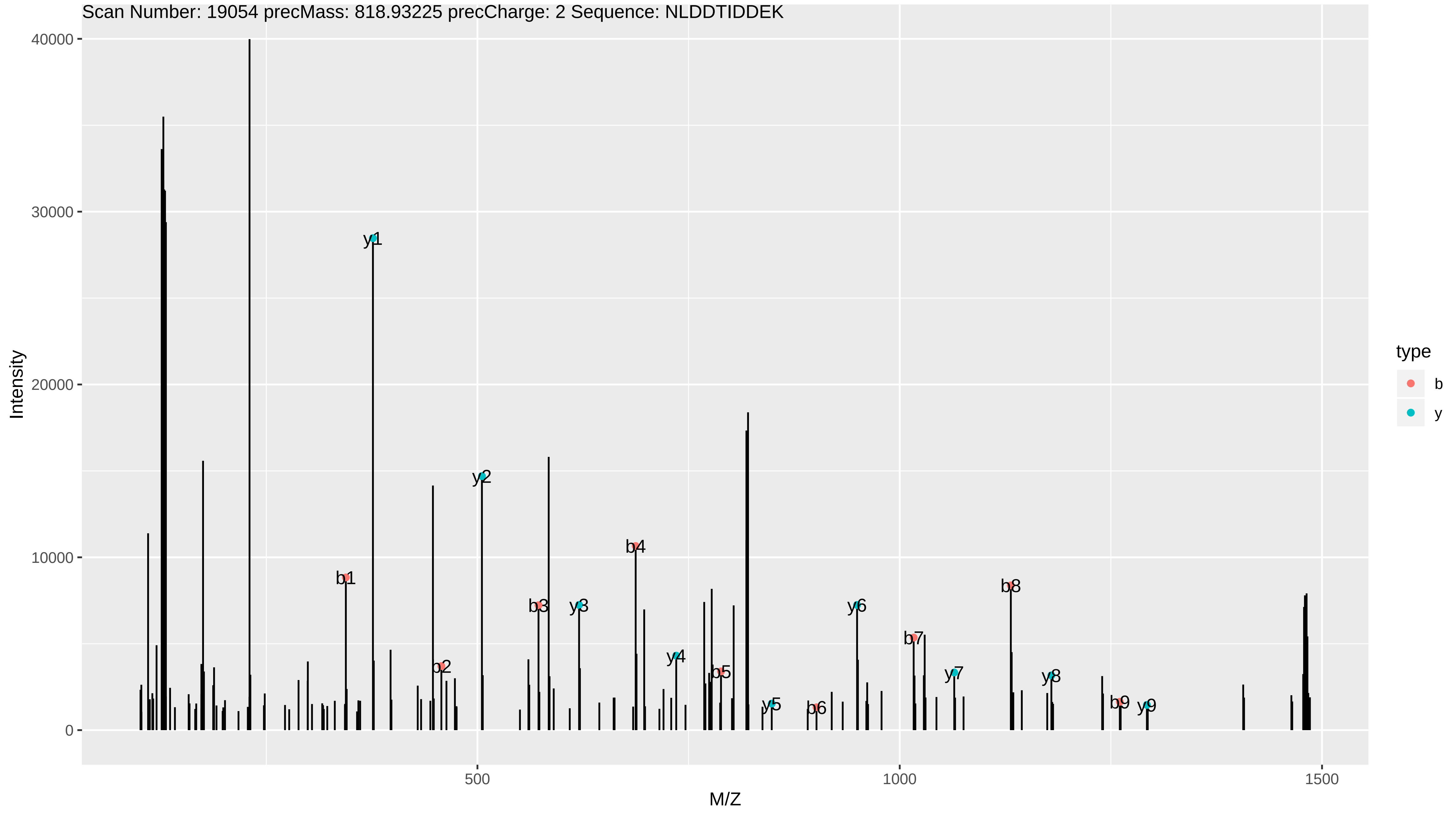

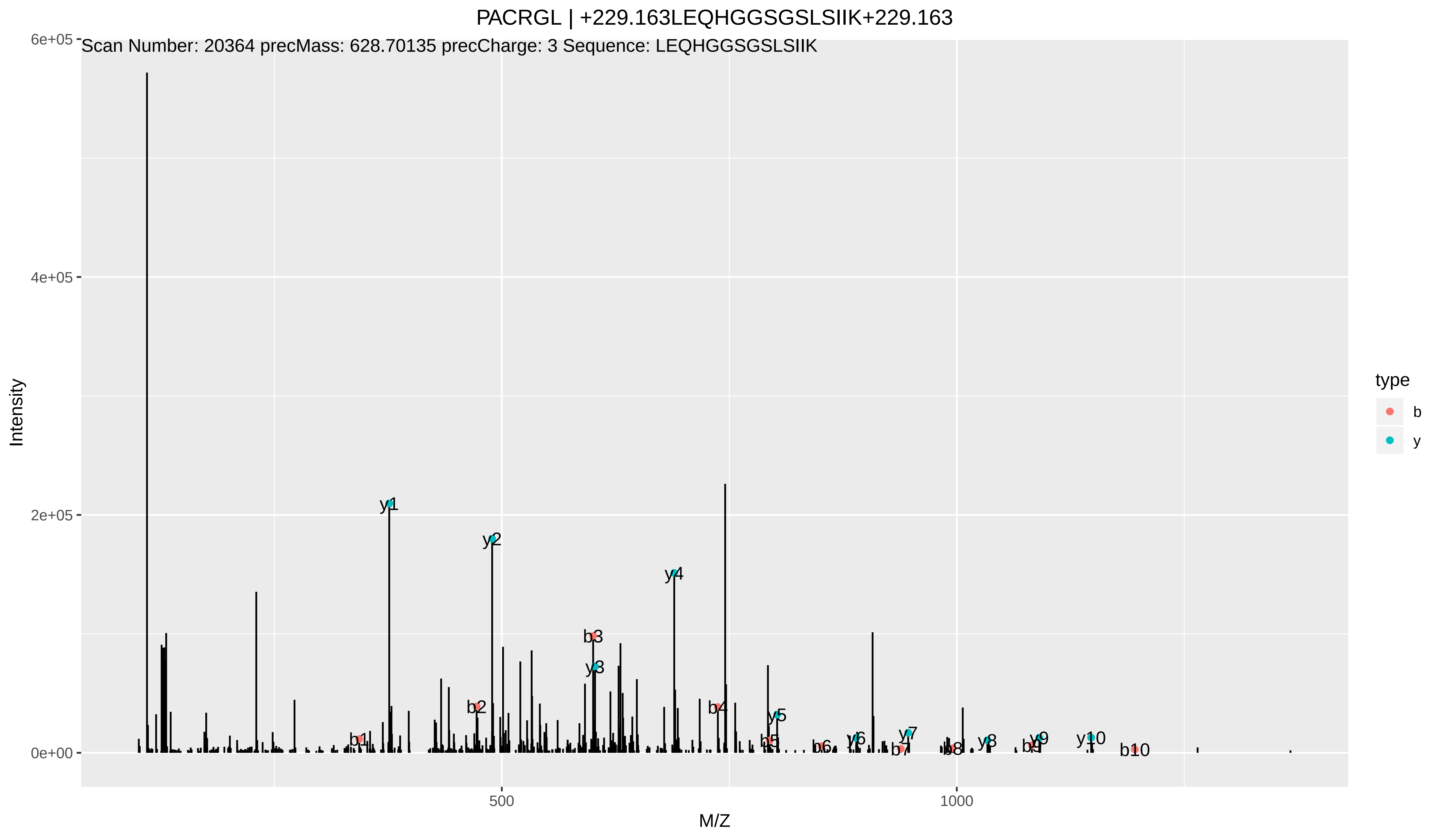

PACRGL | +229.163LEQHGGSGSLSIIK+229.163
Scan Number: 20364 precMass: 628.70135 precCharge: 3 Sequence: LEQHGGSGSLSIIK
Intensity
6e+05
4e+05
2e+05
0e+00
M/Z
500
1000
y1
y2
b3
y3
y4
b2
b4
y5
b1
b5
b6
y6
y7
b7
b8
y8
b9
y9
y10
b10
type
b
y

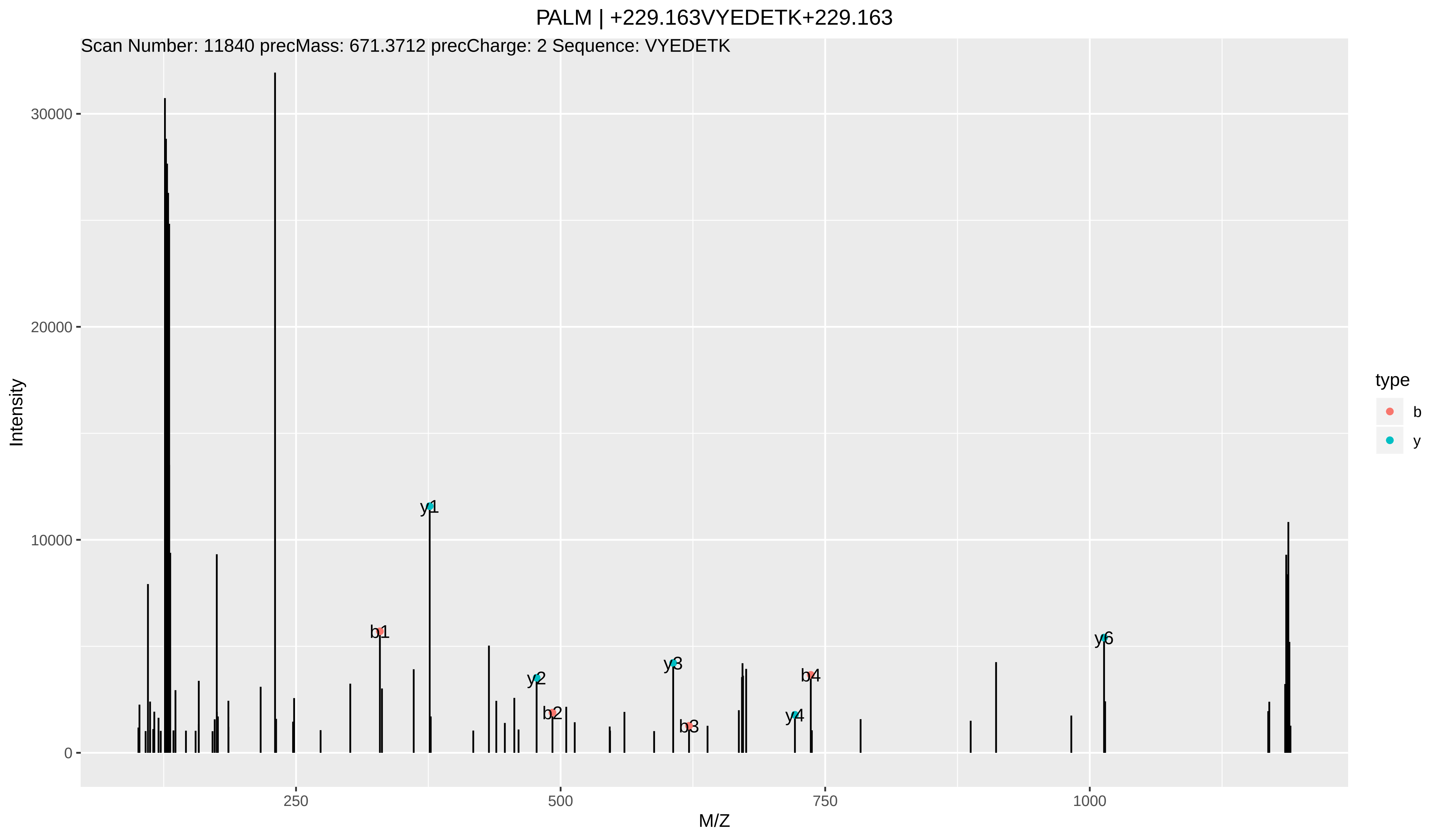

PALM | +229.163VYEDETK+229.163
Scan Number: 11840 precMass: 671.3712 precCharge: 2 Sequence: VYEDETK
Intensity
M/Z
30000
20000
10000
0
250
500
750
1000
y1
b1
y2
b2
y3
b3
y4
b4
y6
type
b
y

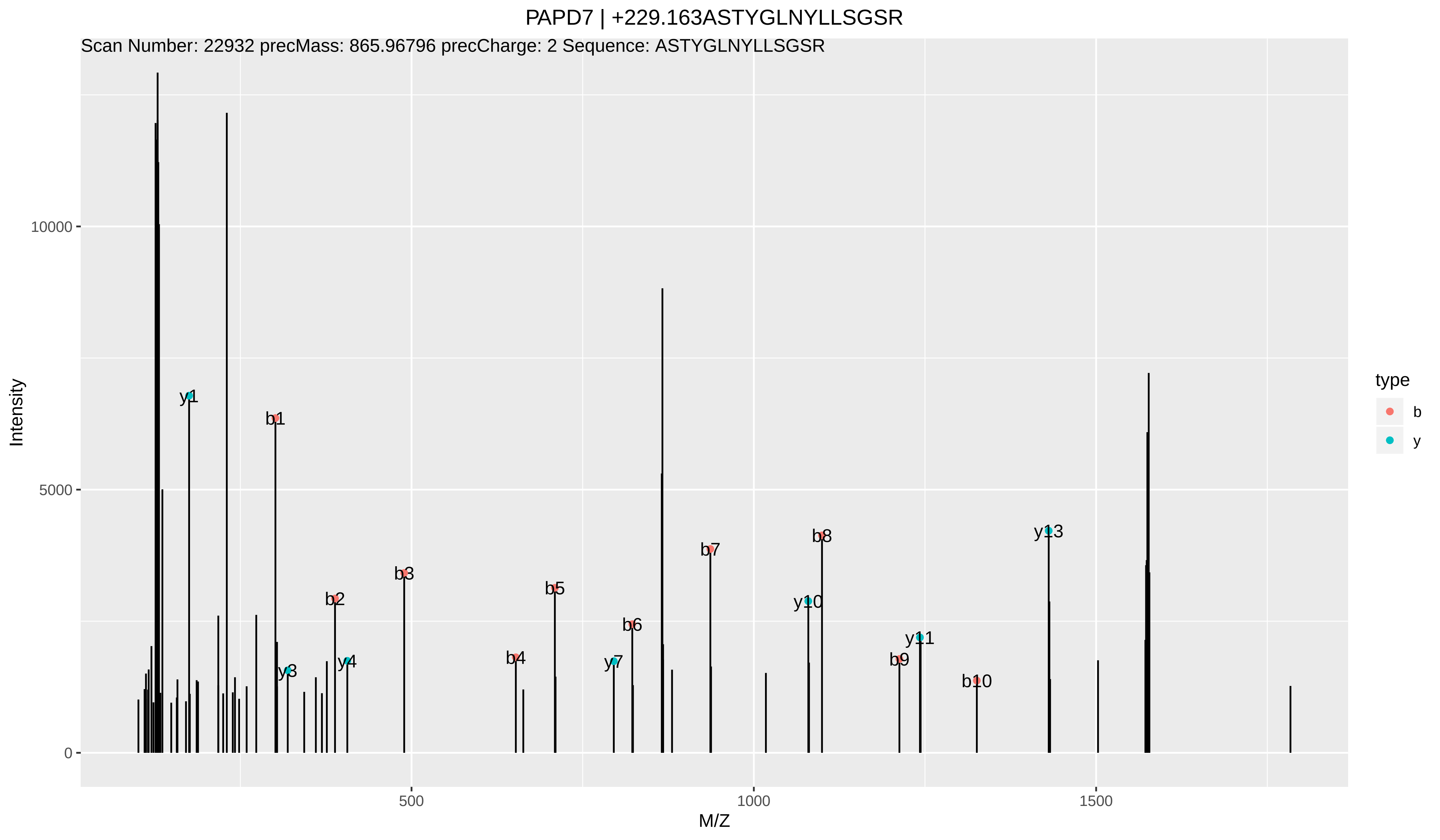
PAPD7 | +229.163ASTYGLNYLLSGSR
Scan Number: 22932 precMass: 865.96796 precCharge: 2 Sequence: ASTYGLNYLLSGSR
Intensity
10000
5000
0
500
1000
1500
M/Z
y1
b1
y3
b2
y4
b3
b4
b5
y7
b6
b7
y10
b8
b9
y11
b10
y13
type
b
y

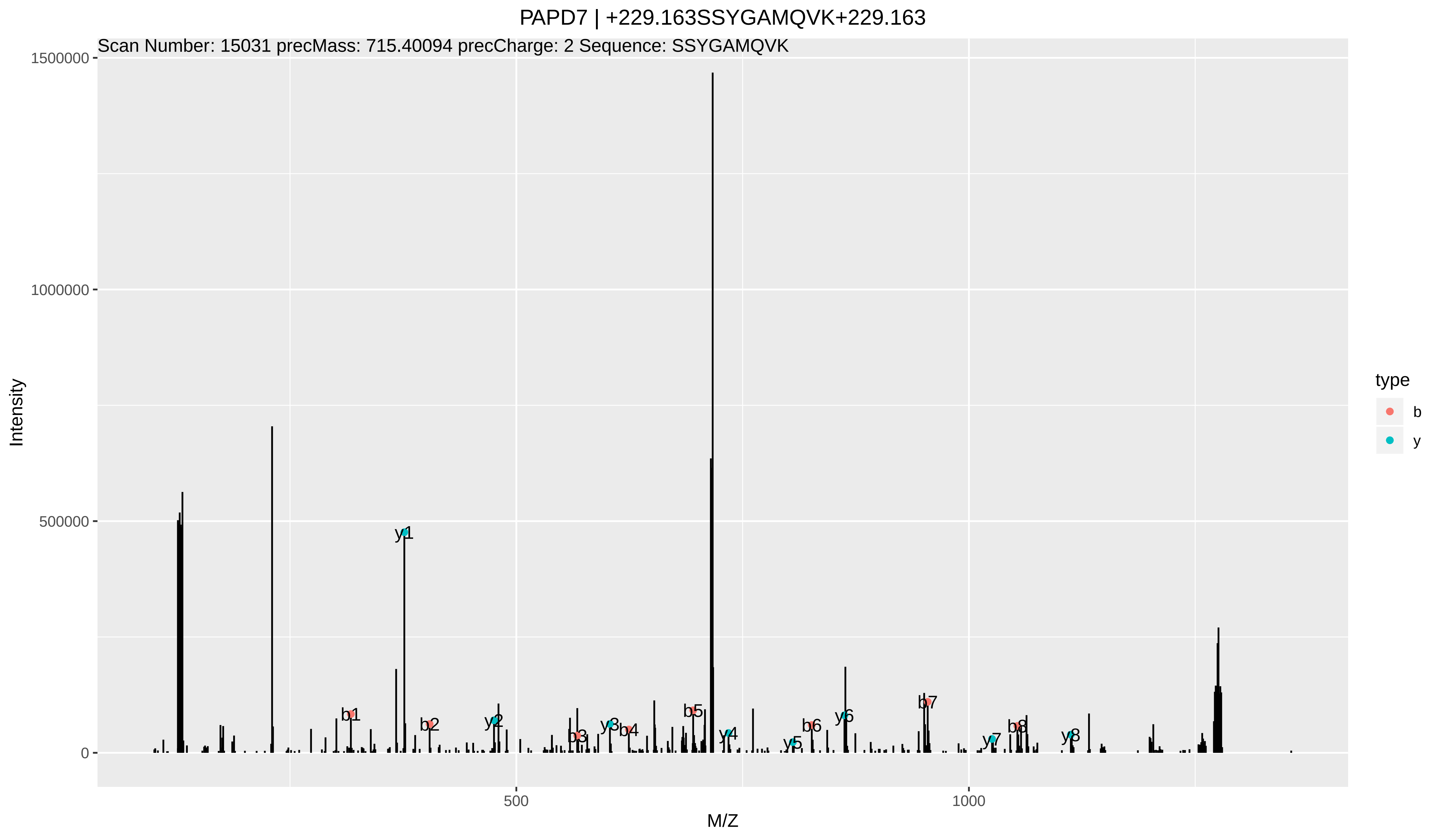

PAPD7 | +229.163SSYGAMQVK+229.163
Scan Number: 15031 precMass: 715.40094 precCharge: 2 Sequence: SSYGAMQVK
Intensity
1500000
1000000
500000
0
500
1000
M/Z
y1
b1
b2
y2
b3
y3
b4
b5
y4
y5
b6
y6
b7
y7
b8
y8
type
b
y

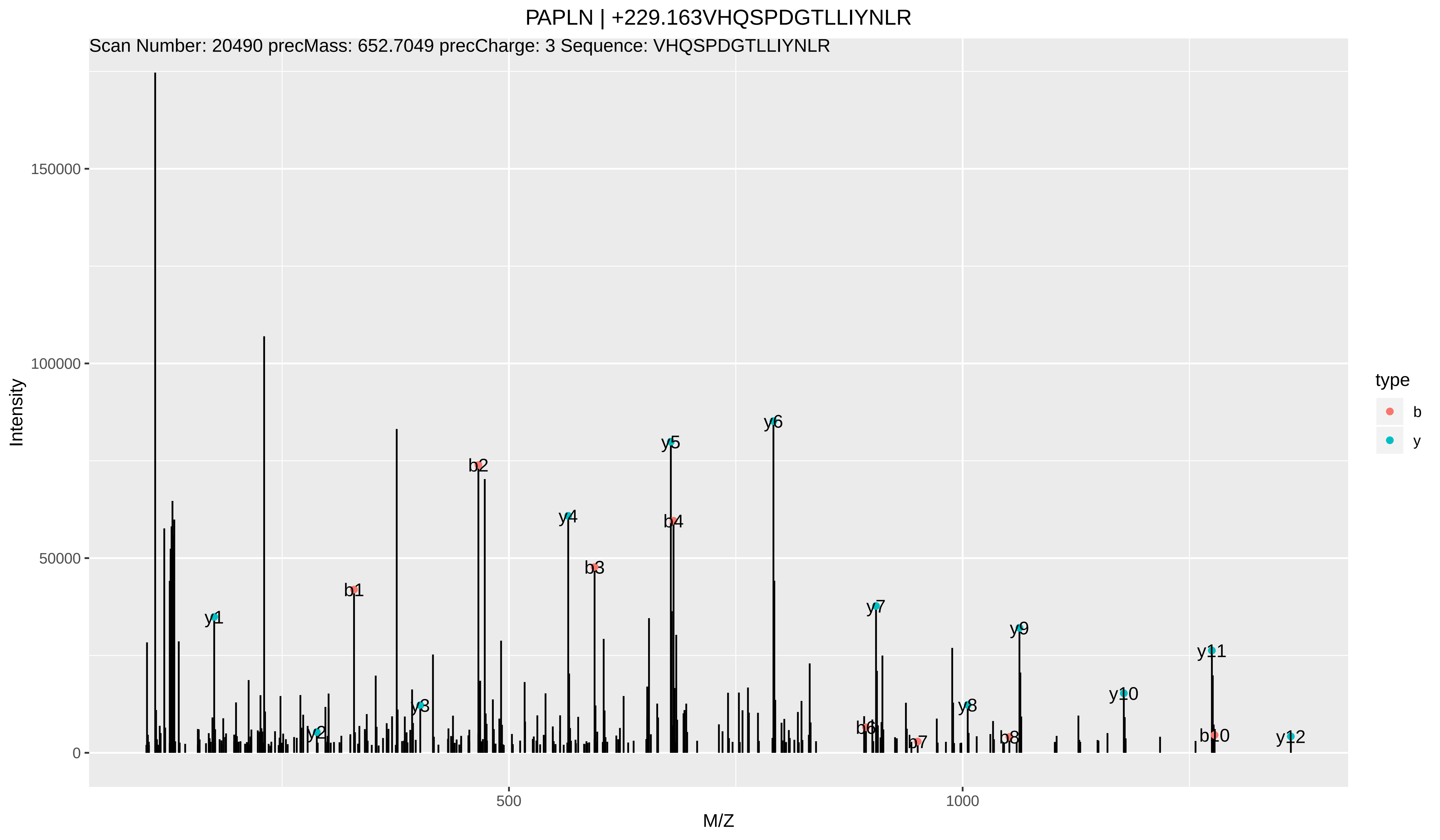
PAPLN | +229.163VHQSPDGTLLIYNLR
Scan Number: 20490 precMass: 652.7049 precCharge: 3 Sequence: VHQSPDGTLLIYNLR
Intensity
150000
100000
50000
0
500
1000
M/Z
y1
y2
b1
y3
b2
y4
b3
y5
b4
y6
b6
y7
b7
y8
b8
y9
y10
y11
b10
y12
type
b
y

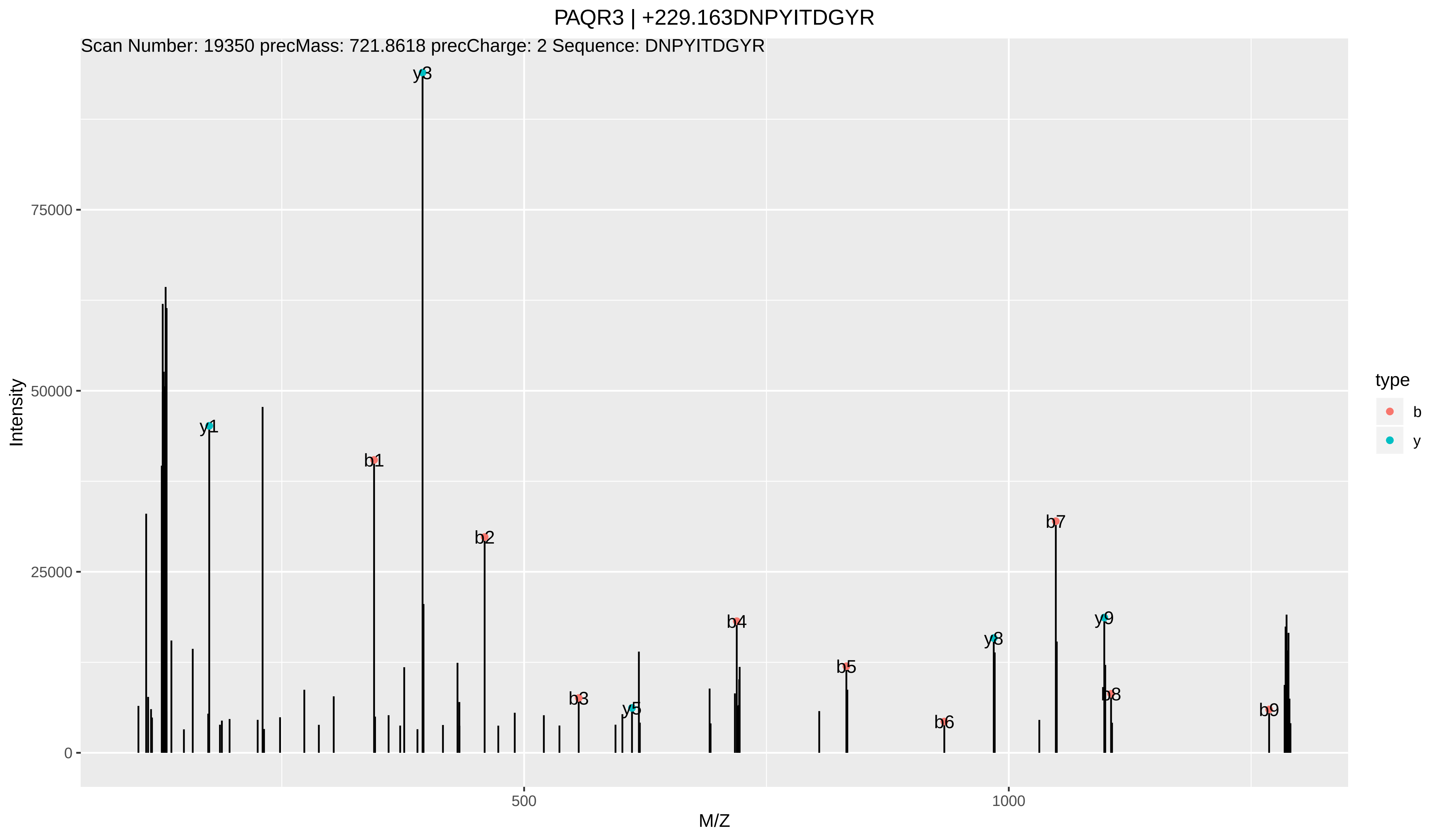
PAQR3 | +229.163DNPYITDGYR
Scan Number: 19350 precMass: 721.8618 precCharge: 2 Sequence: DNPYITDGYR
Intensity
M/Z
type
b
y
y3
y1
b1
b2
b3
y5
b4
b5
b6
y8
b7
y9
b8
b9
0
25000
50000
75000
500
1000

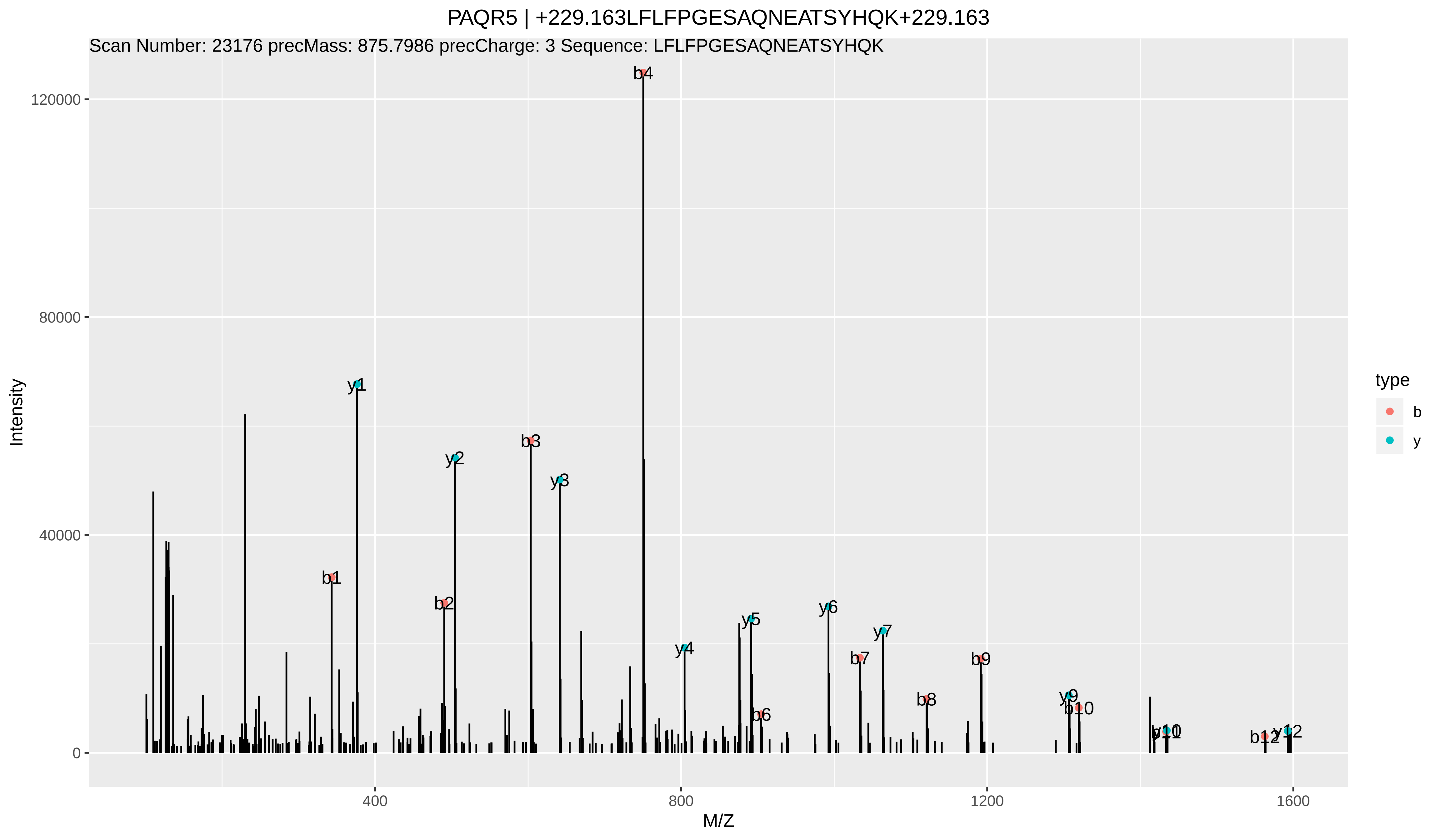
PAQR5 | +229.163LFLFPGESAQNEATSYHQK+229.163
Scan Number: 23176 precMass: 875.7986 precCharge: 3 Sequence: LFLFPGESAQNEATSYHQK
Intensity
M/Z
type
b
y
b1
b2
b3
b4
b6
b7
b8
b9
b10
b12
y1
y2
y3
y4
y5
y6
y7
y9
y10
y11
y12
0
40000
80000
120000
400
800
1200
1600

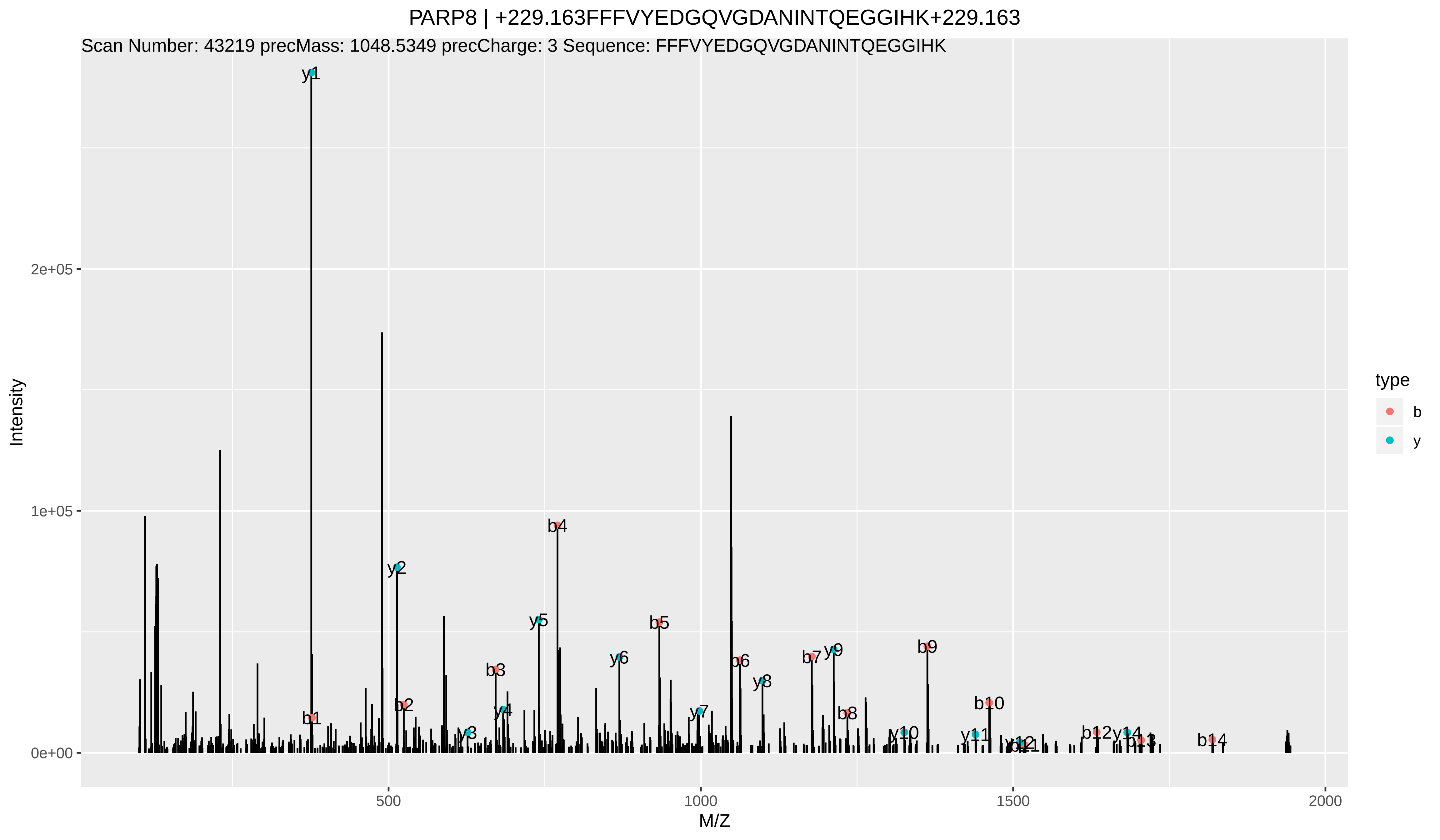
PARP8 | +229.163FFFVYEDGQVGDANINTQEGGIHK+229.163
Scan Number: 43219 precMass: 1048.5349 precCharge: 3 Sequence: FFFVYEDGQVGDANINTQEGGIHK
Intensity
2e+05
1e+05
0e+00
M/Z
500
1000
1500
2000
type
b
y
y1
y2
y3
y4
y5
y6
y7
y8
y9
y10
y11
y12
y14
b1
b2
b3
b4
b5
b6
b7
b8
b9
b10
b11
b12
b13
b14

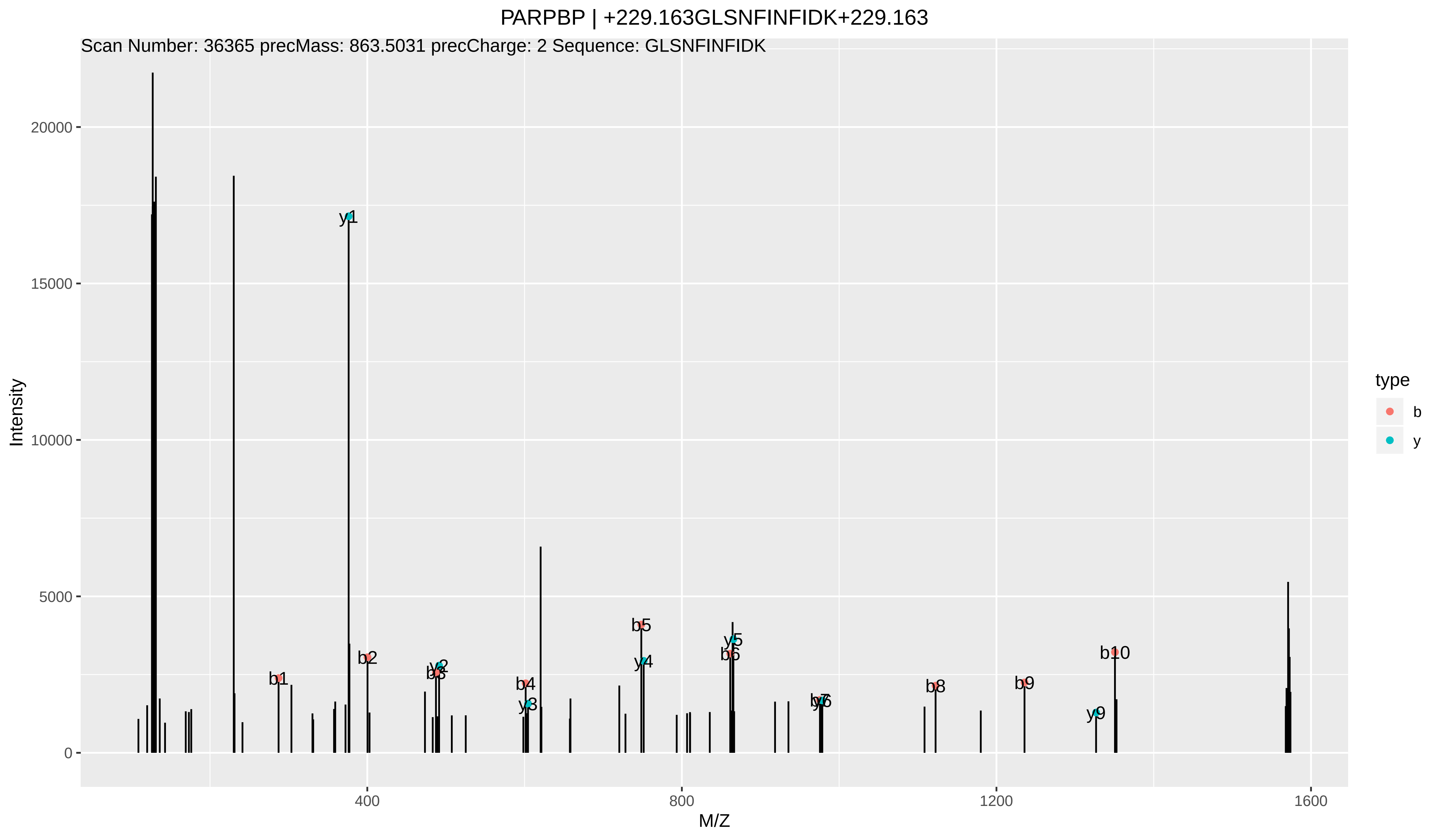

PARPBP | +229.163GLSNFINFIDK+229.163
Scan Number: 36365 precMass: 863.5031 precCharge: 2 Sequence: GLSNFINFIDK
Intensity
M/Z
type
b
y
y1
b1
b2
y2
b3
b4
y3
b5
y4
y5
b6
b7
y6
b8
b9
y9
b10
0
5000
10000
15000
20000
400
800
1200
1600

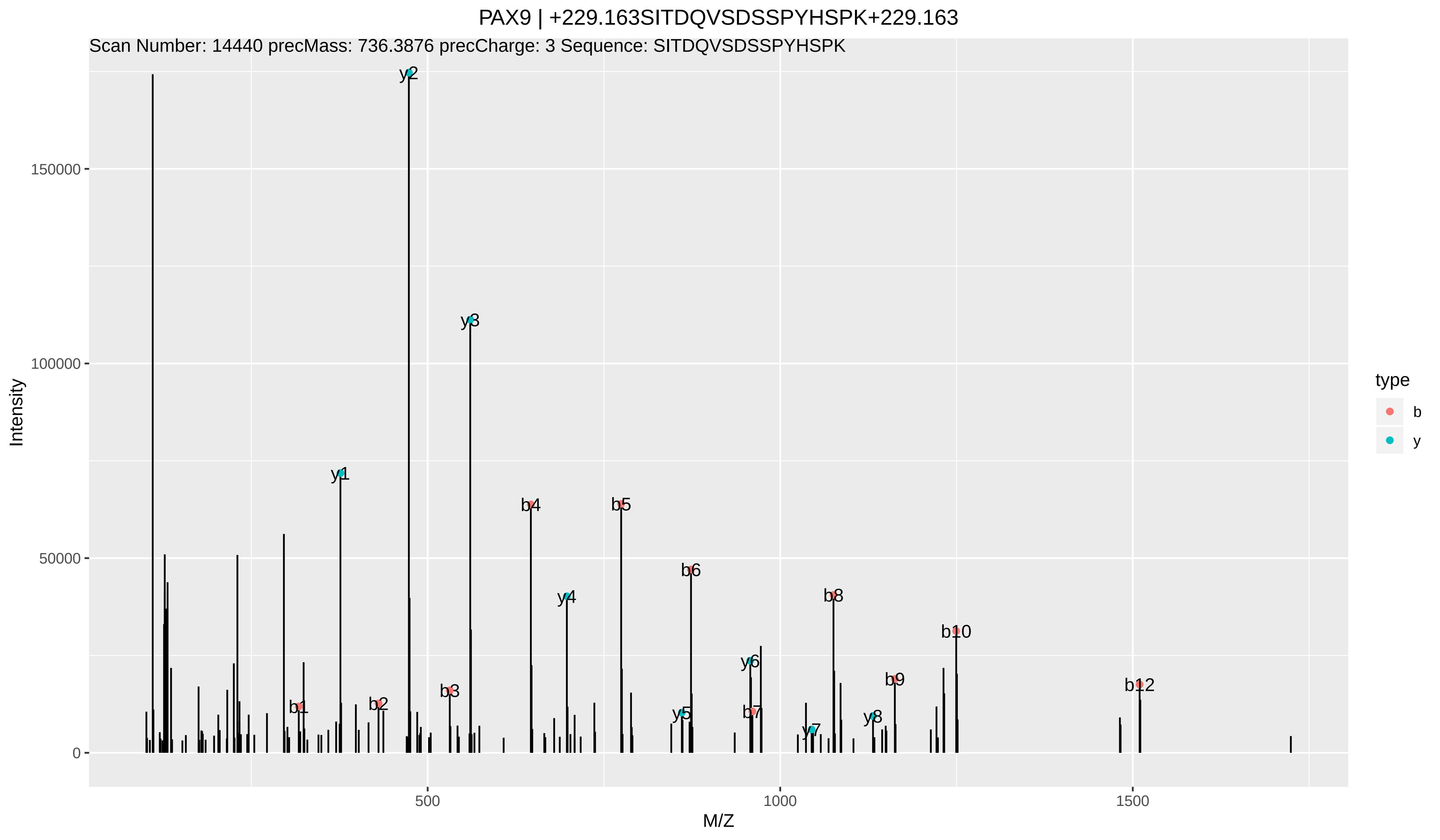
PAX9 | +229.163SITDQVSDSSPYHSPK+229.163
Scan Number: 14440 precMass: 736.3876 precCharge: 3 Sequence: SITDQVSDSSPYHSPK
Intensity
M/Z
0
50000
100000
150000
500
1000
1500
y2
y3
y1
b4
b5
b6
y4
b8
b10
y6
b9
b12
b3
b2
b1
y5
b7
y7
y8
type
b
y

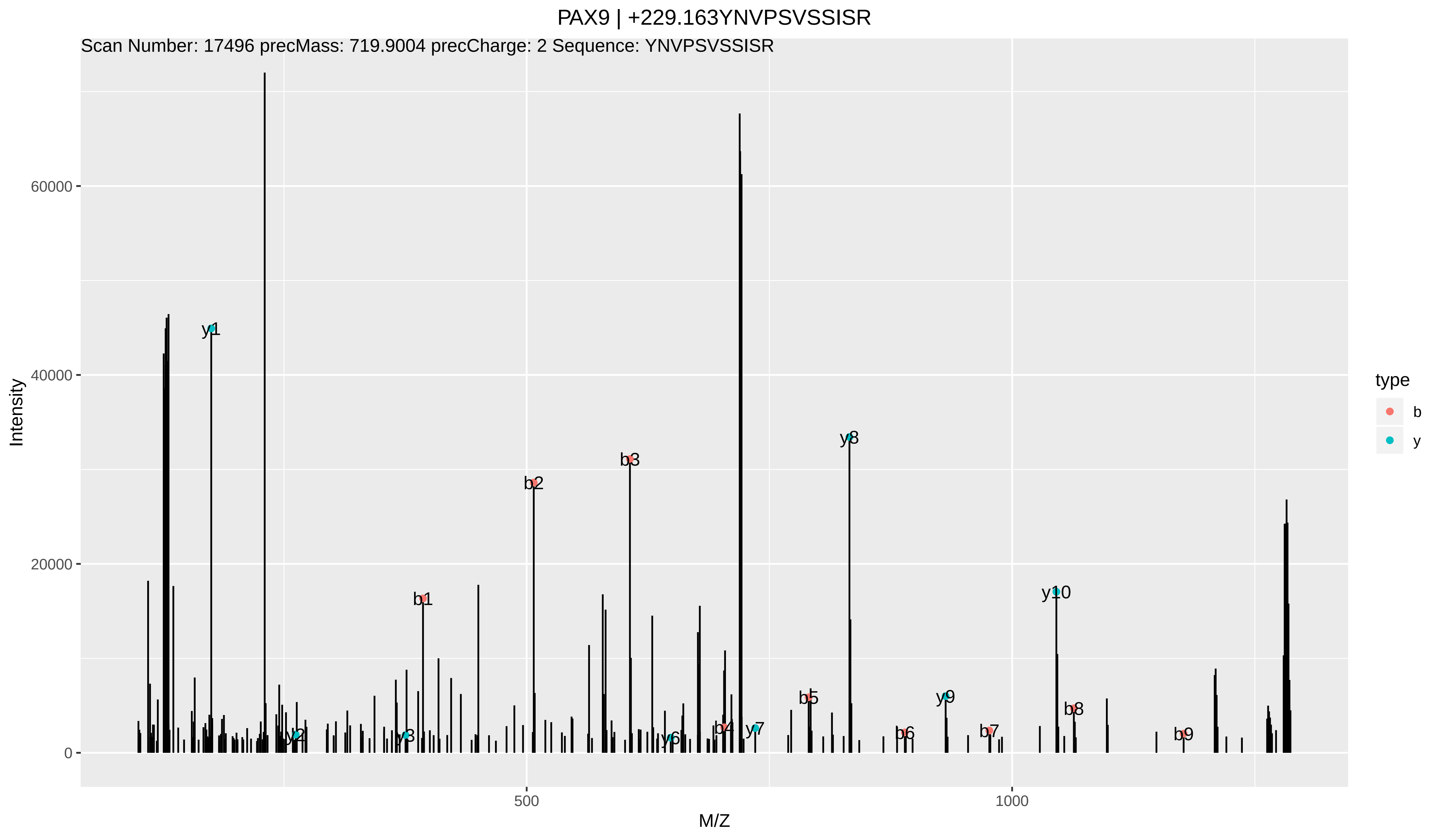

PAX9 | +229.163YNVPSVSSISR
Scan Number: 17496 precMass: 719.9004 precCharge: 2 Sequence: YNVPSVSSISR
Intensity
60000
40000
20000
0
M/Z
500
1000
y1
y2
y3
b1
b2
b3
y6
b4
y7
b5
y8
b6
y9
b7
y10
b8
b9
type
b
y

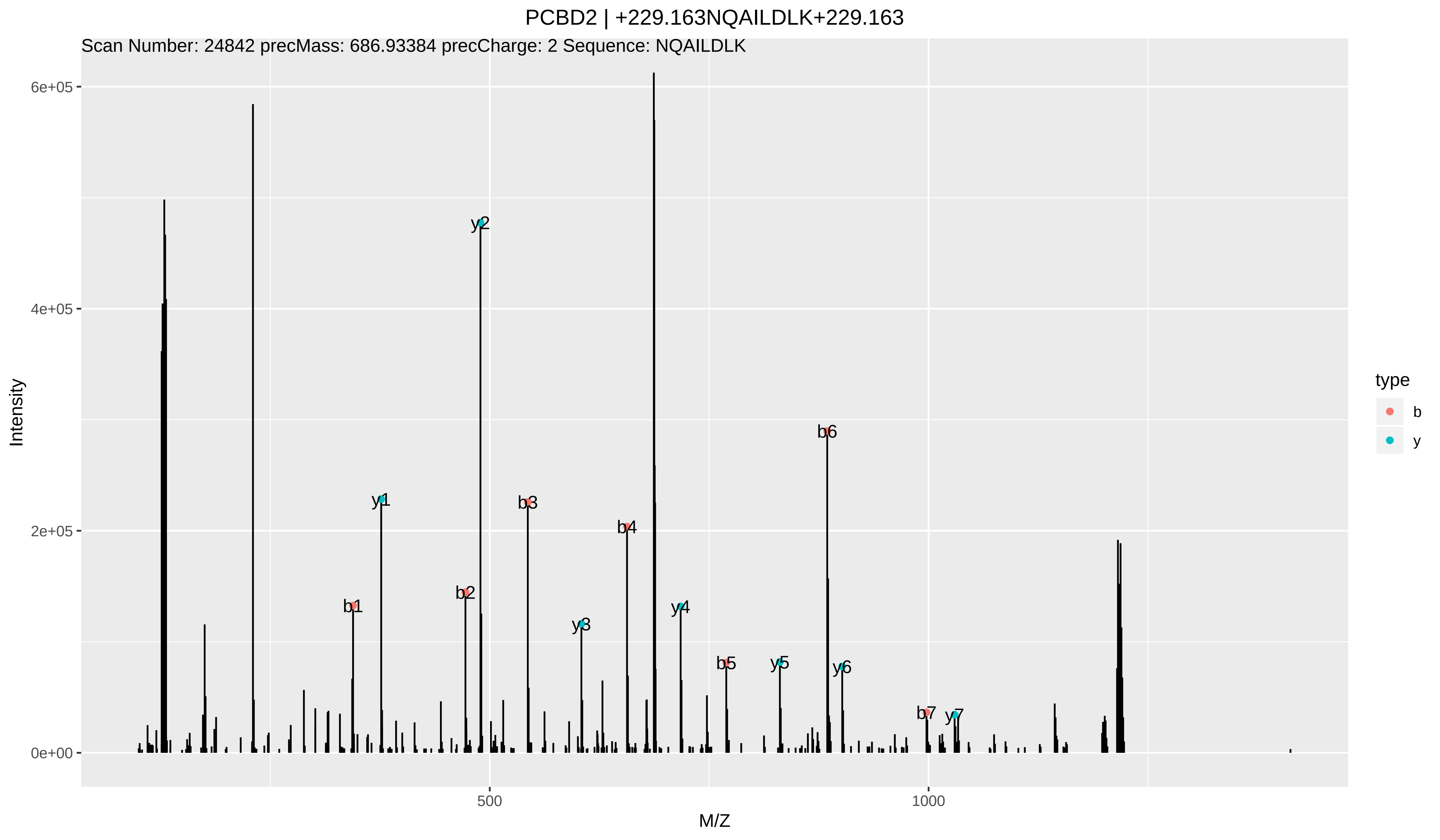
PCBD2 | +229.163NQAILDLK+229.163
Scan Number: 24842 precMass: 686.93384 precCharge: 2 Sequence: NQAILDLK
Intensity
M/Z
6e+05
4e+05
2e+05
0e+00
500
1000
y2
b6
y1
b3
b4
b2
b1
y4
y3
b5
y5
y6
b7
y7
type
b
y

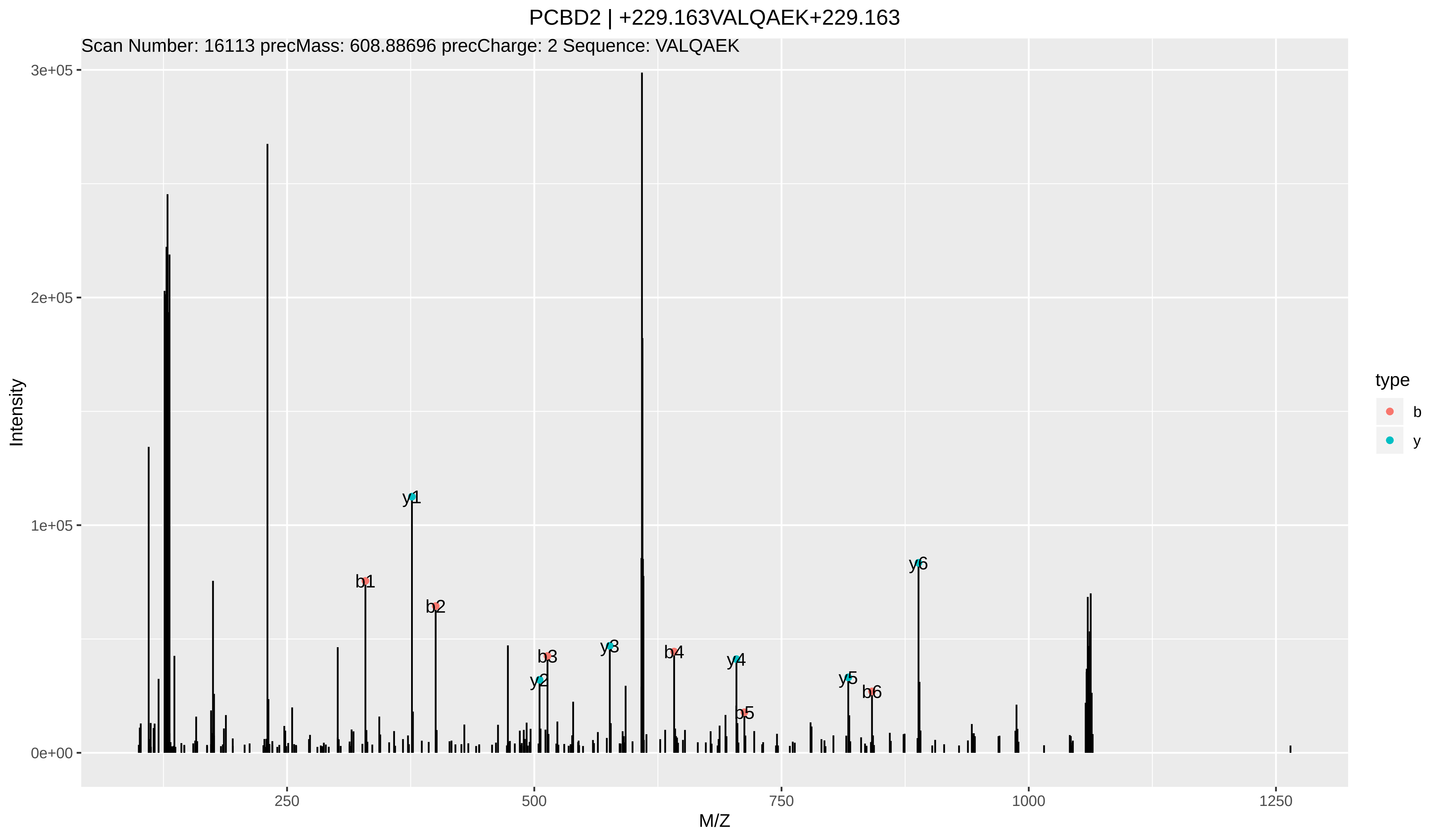

PCBD2 | +229.163VALQAEK+229.163
Scan Number: 16113 precMass: 608.88696 precCharge: 2 Sequence: VALQAEK
Intensity
3e+05
2e+05
1e+05
0e+00
M/Z
250
500
750
1000
1250
y1
b1
b2
y6
y3
b4
y4
b3
y2
y5
b6
b5
type
b
y

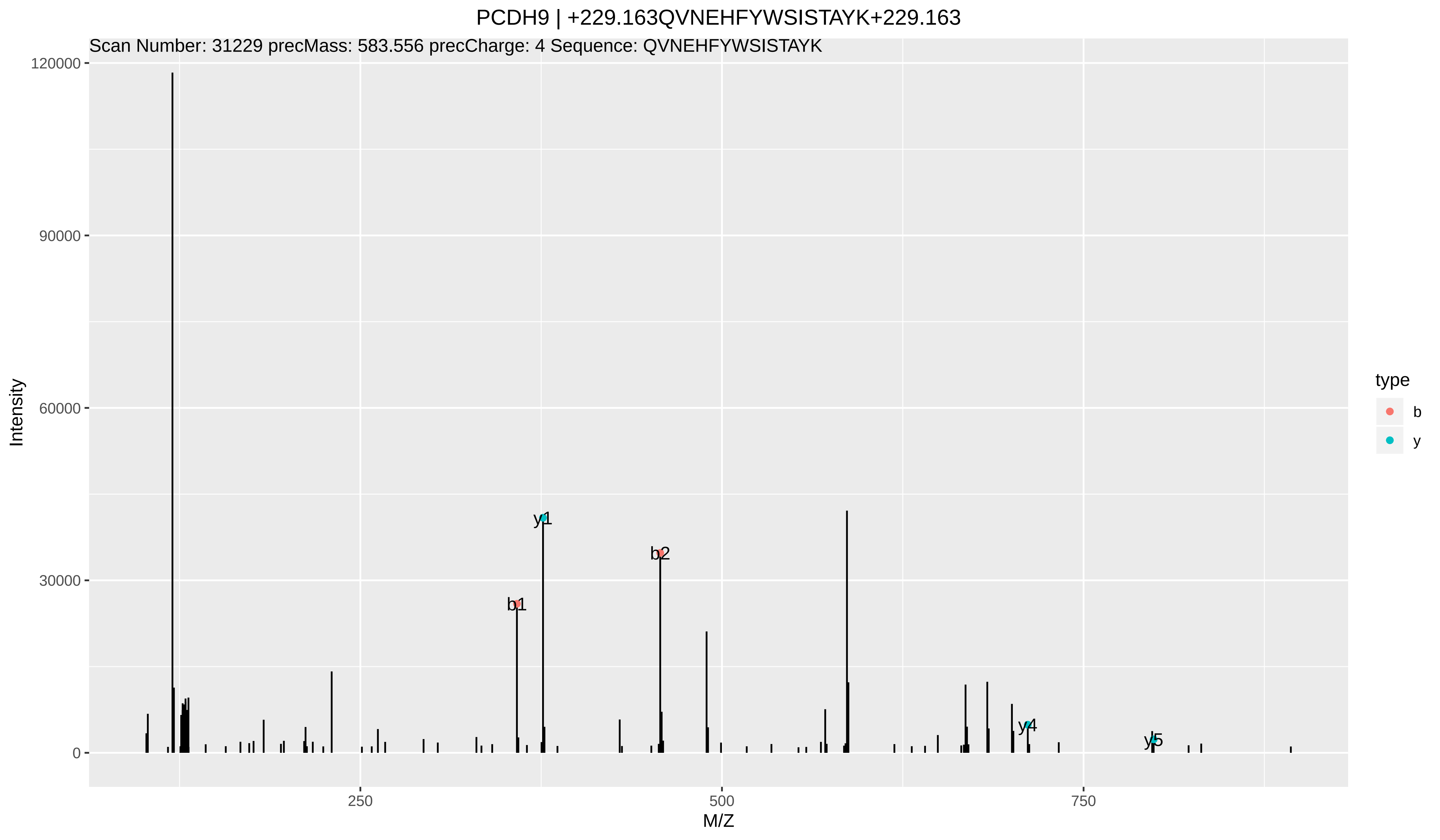
PCDH9 | +229.163QVNEHFYWSISTAYK+229.163
Scan Number: 31229 precMass: 583.556 precCharge: 4 Sequence: QVNEHFYWSISTAYK
Intensity
120000
90000
60000
30000
0
250
500
750
M/Z
y1
b2
b1
y4
y5
type
b
y

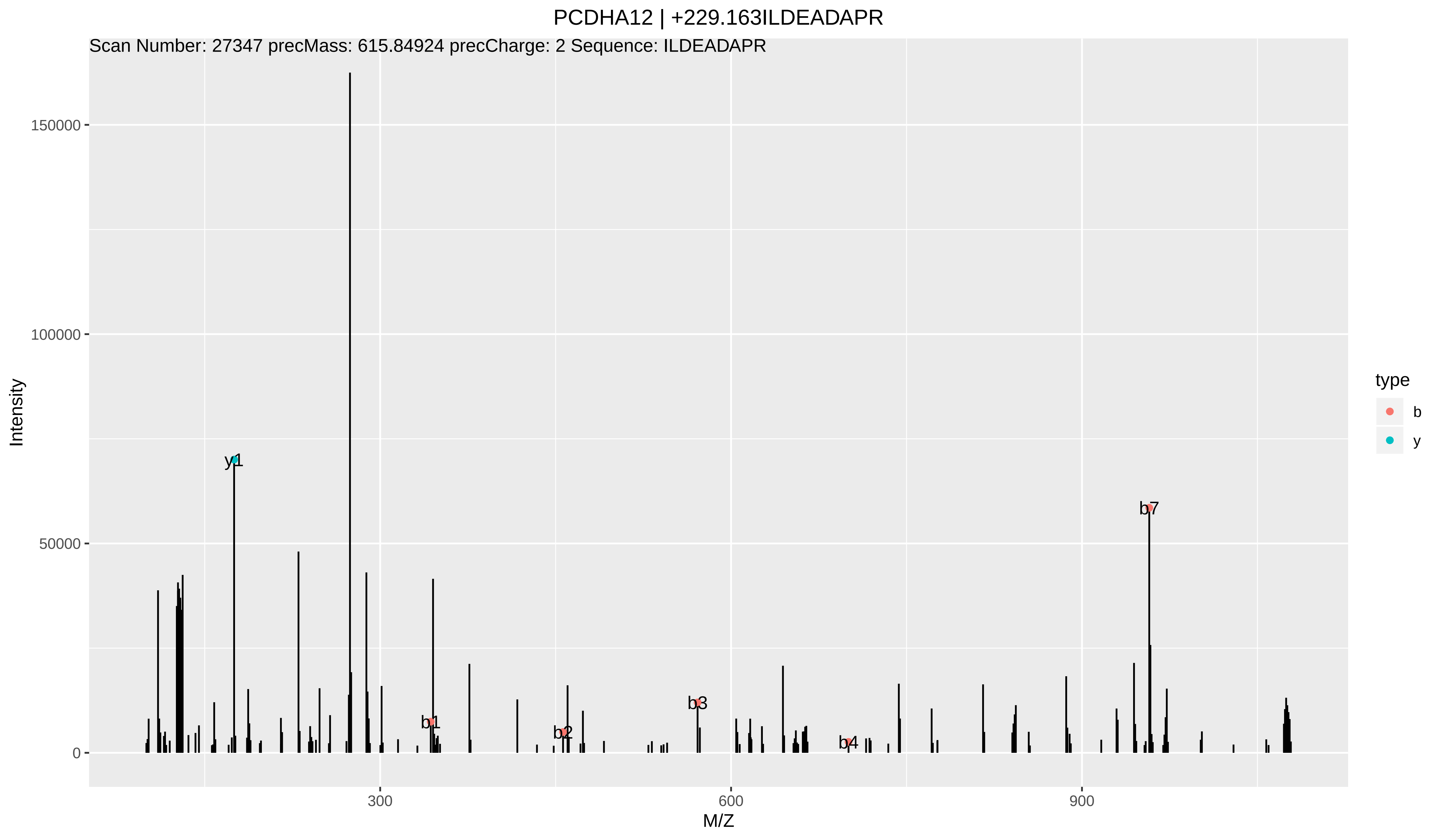
PCDHA12 | +229.163ILDEADAPR
Scan Number: 27347 precMass: 615.84924 precCharge: 2 Sequence: ILDEADAPR
Intensity
150000
100000
50000
0
300
600
900
M/Z
y1
b1
b2
b3
b4
b7
type
b
y

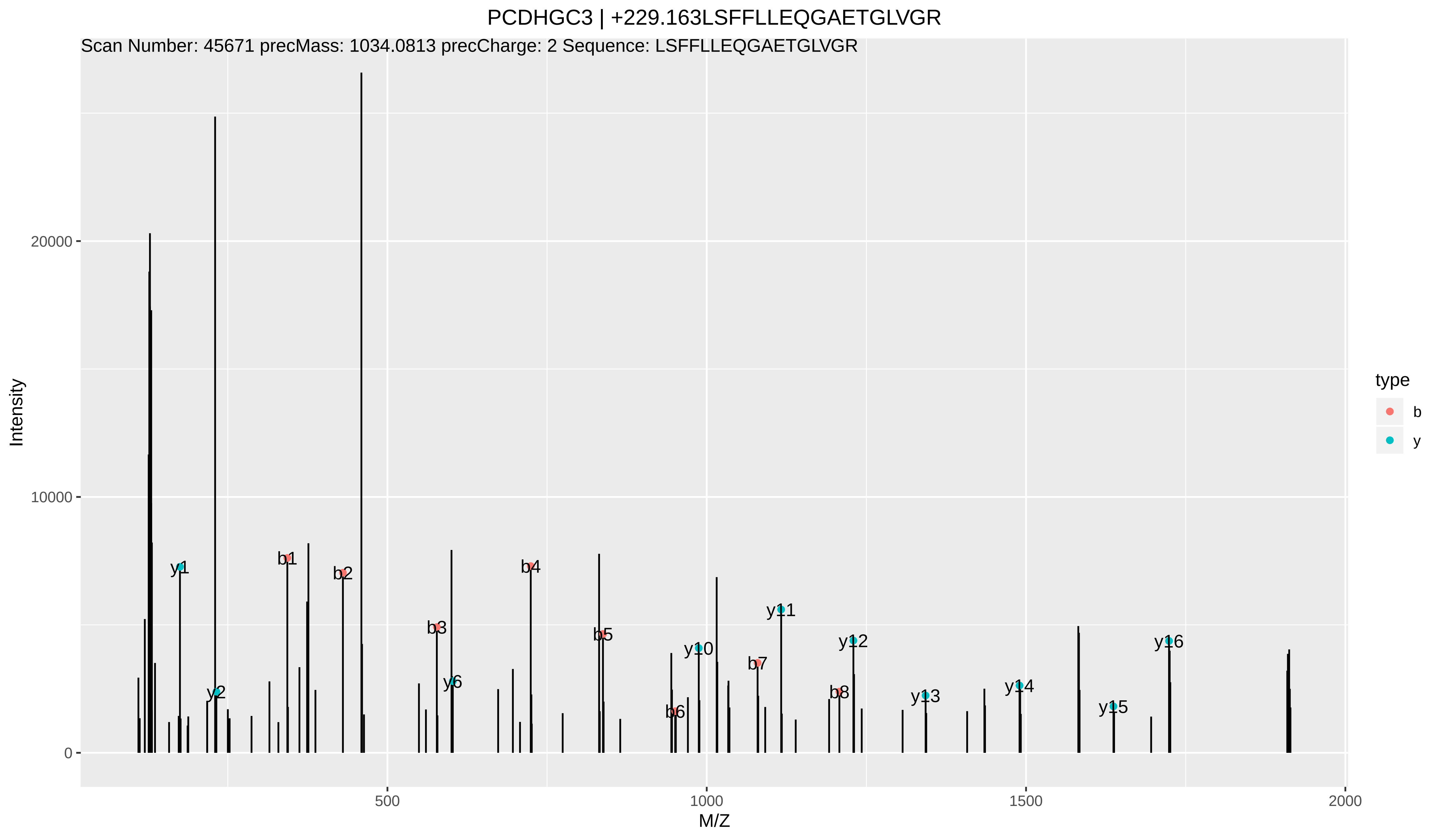

PCDHGC3 | +229.163LSFFLLEQGAETGLVGR
Scan Number: 45671 precMass: 1034.0813 precCharge: 2 Sequence: LSFFLLEQGAETGLVGR
Intensity
M/Z
type
b
y
y1
y2
b1
b2
b3
y6
b4
b5
b6
y10
b7
y11
b8
y12
y13
y14
y15
y16
0
10000
20000
500
1000
1500
2000

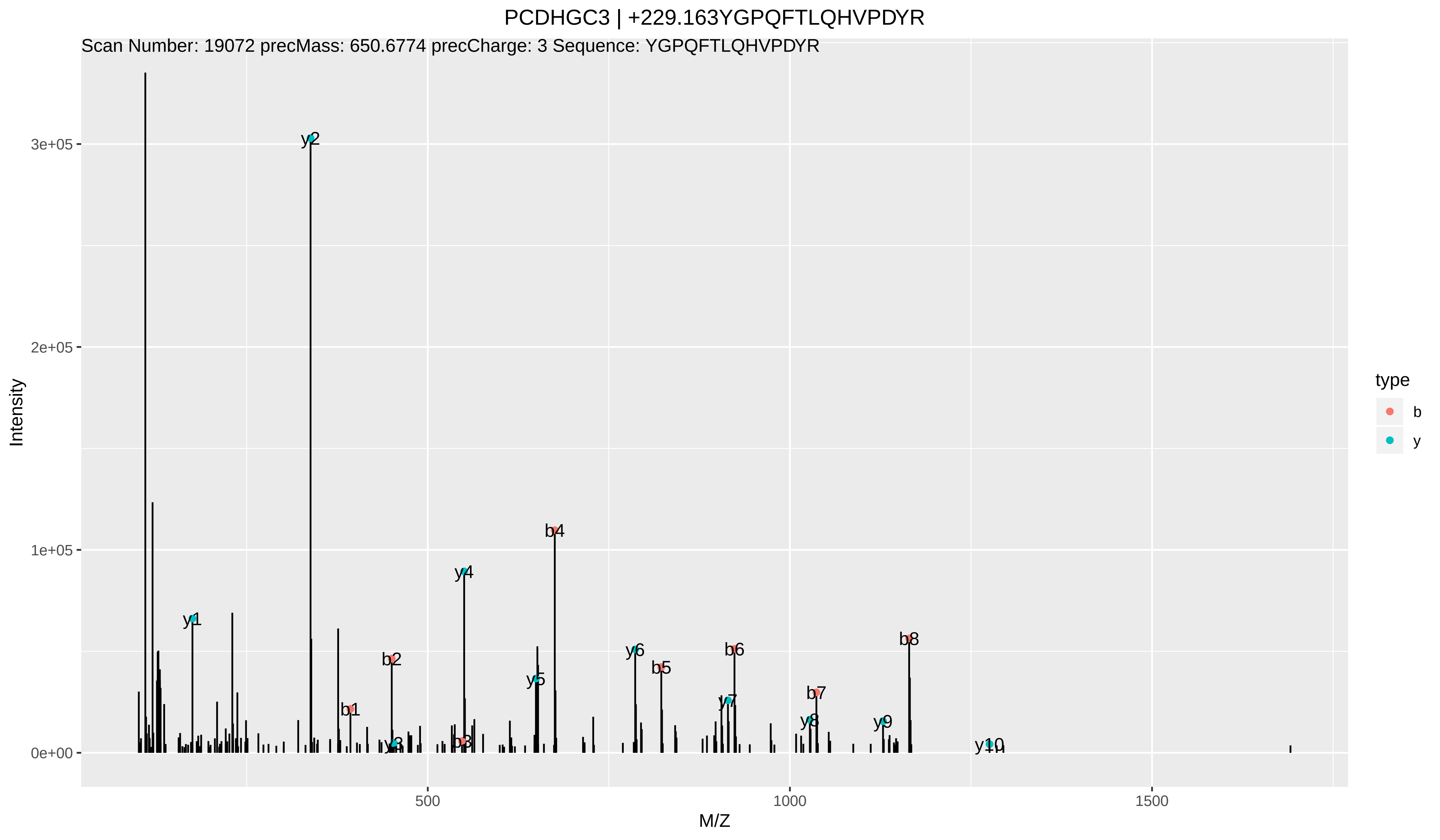
PCDHGC3 | +229.163YGPQFTLQHVPDYR
Scan Number: 19072 precMass: 650.6774 precCharge: 3 Sequence: YGPQFTLQHVPDYR
Intensity
M/Z
3e+05
2e+05
1e+05
0e+00
500
1000
1500
y2
b4
y4
y1
b2
b1
y3
b3
y5
y6
b5
b6
y7
b7
y8
y9
b8
y10
type
b
y

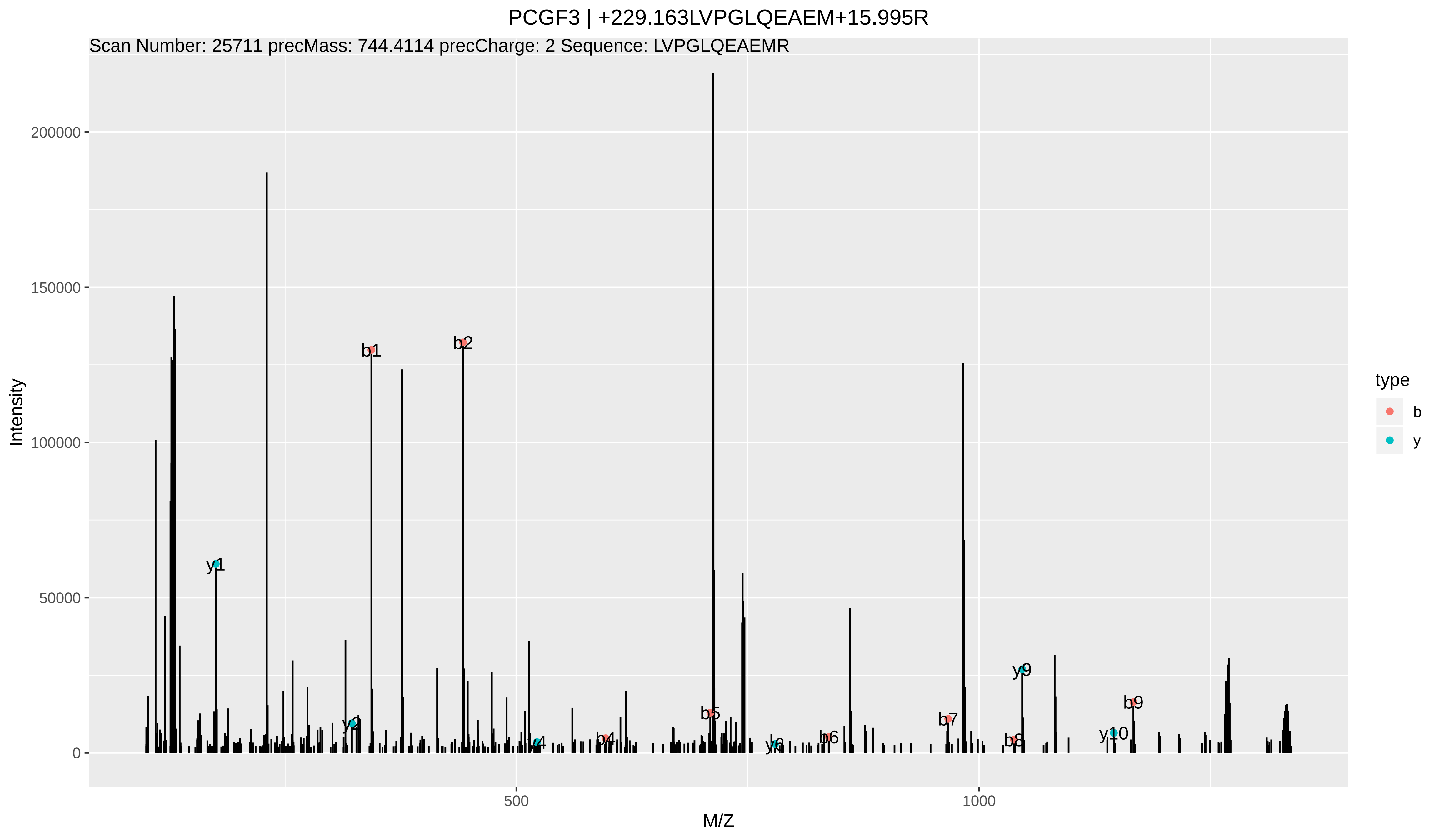

PCGF3 | +229.163LVPGLQEAEM+15.995R
Scan Number: 25711 precMass: 744.4114 precCharge: 2 Sequence: LVPGLQEAEMR
Intensity
M/Z
200000
150000
100000
50000
0
500
1000
y1
y2
b1
b2
y4
b4
b5
y6
b6
b7
b8
y9
y10
b9
type
b
y

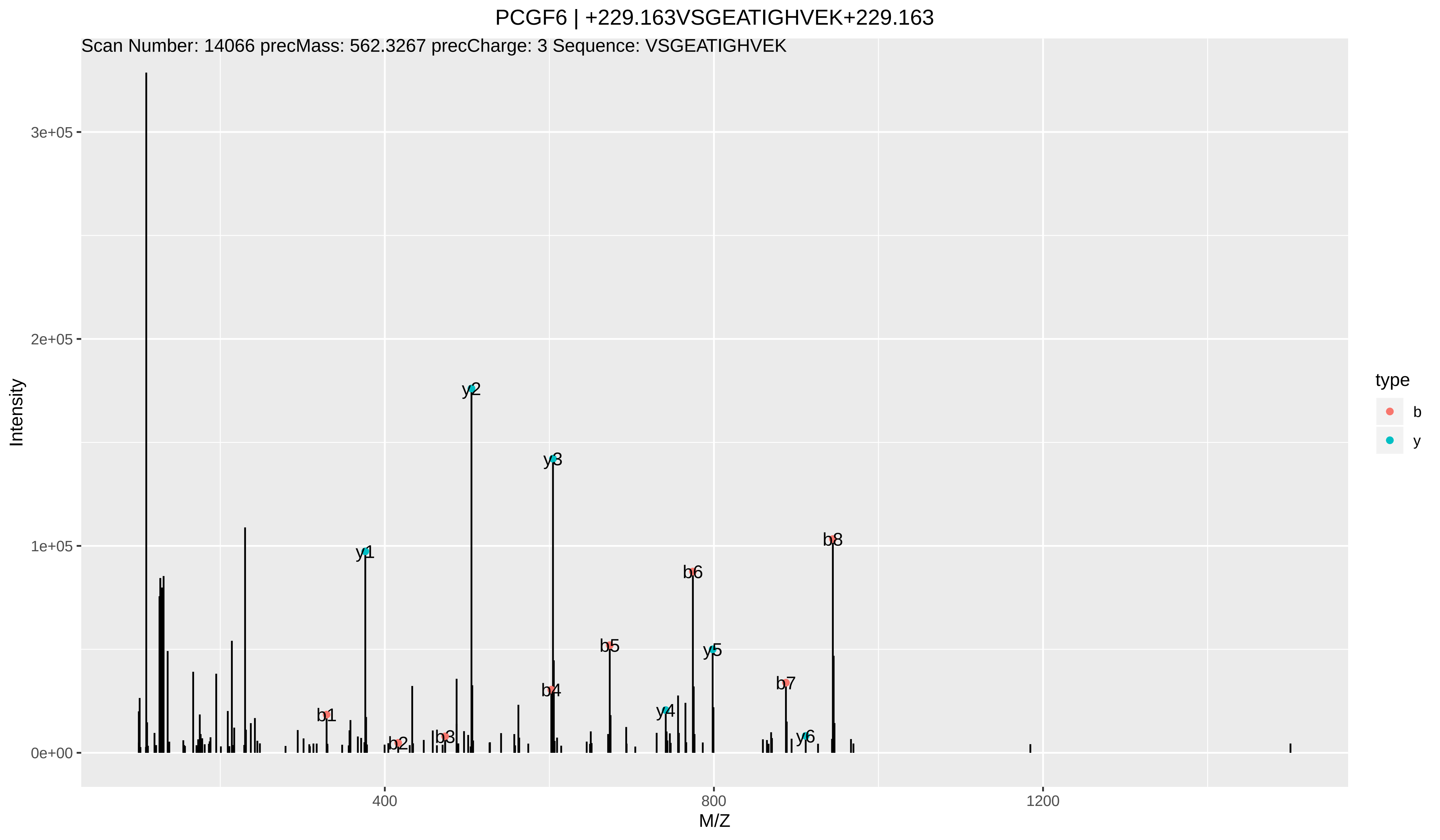

PCGF6 | +229.163VSGEATIGHVEK+229.163
Scan Number: 14066 precMass: 562.3267 precCharge: 3 Sequence: VSGEATIGHVEK
Intensity
3e+05
2e+05
1e+05
0e+00
M/Z
400
800
1200
y1
y2
y3
y4
y5
y6
b1
b2
b3
b4
b5
b6
b7
b8
type
b
y

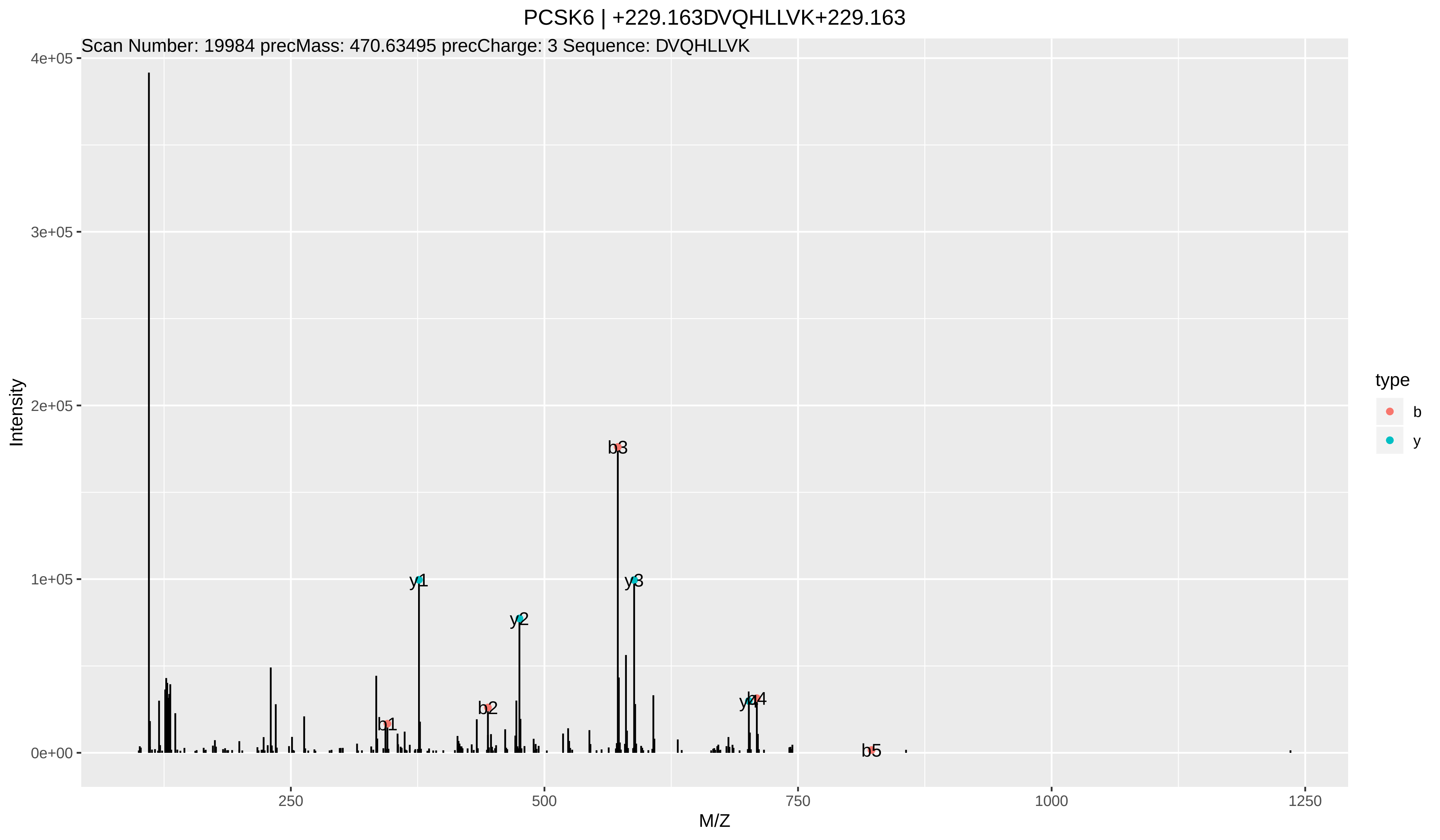

PCSK6 | +229.163DVQHLLVK+229.163
Scan Number: 19984 precMass: 470.63495 precCharge: 3 Sequence: DVQHLLVK
Intensity
4e+05
3e+05
2e+05
1e+05
0e+00
M/Z
250
500
750
1000
1250
b1
b2
b3
b4
b5
y1
y2
y3
y4
type
b
y

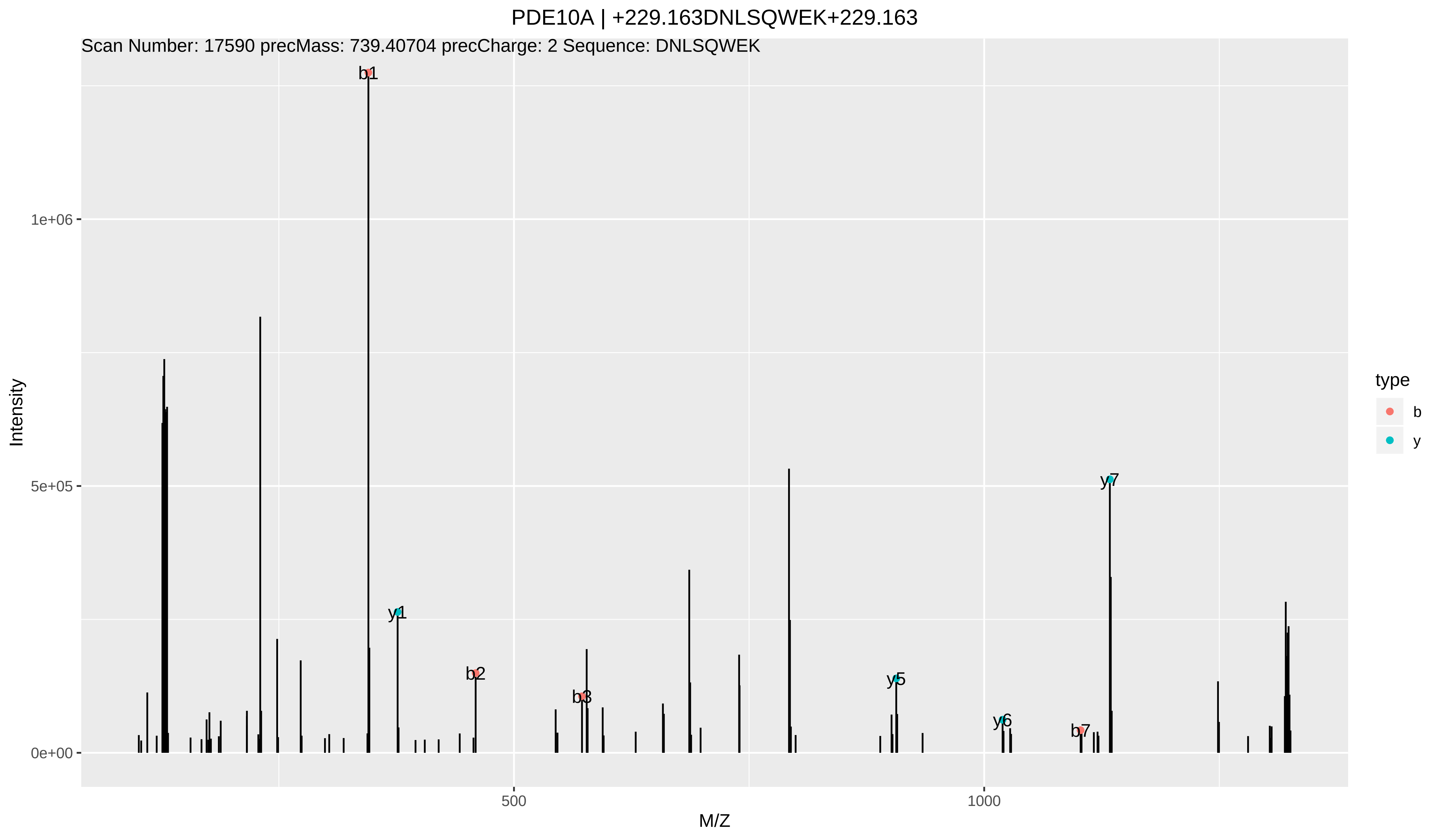

PDE10A | +229.163DNLSQWEK+229.163
Scan Number: 17590 precMass: 739.40704 precCharge: 2 Sequence: DNLSQWEK
b1
y1
b2
b3
y5
y6
b7
y7
1e+06
5e+05
0e+00
Intensity
500
1000
M/Z
type
b
y

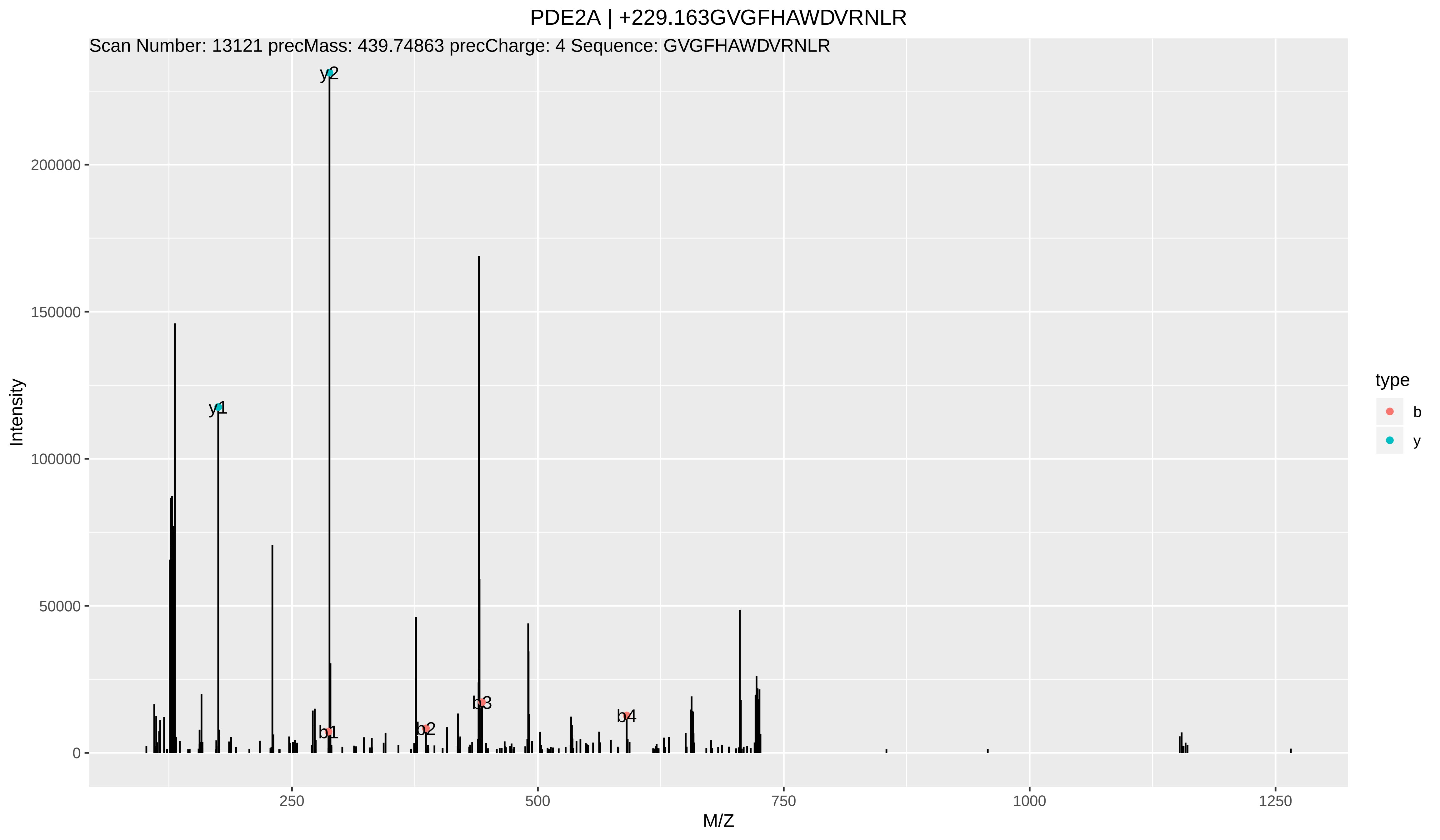
PDE2A | +229.163GVGFHAWDVRNLR
Scan Number: 13121 precMass: 439.74863 precCharge: 4 Sequence: GVGFHAWDVRNLR
y2
y1
b1
b2
b3
b4
Intensity
200000
150000
100000
50000
0
M/Z
250
500
750
1000
1250
type
b
y

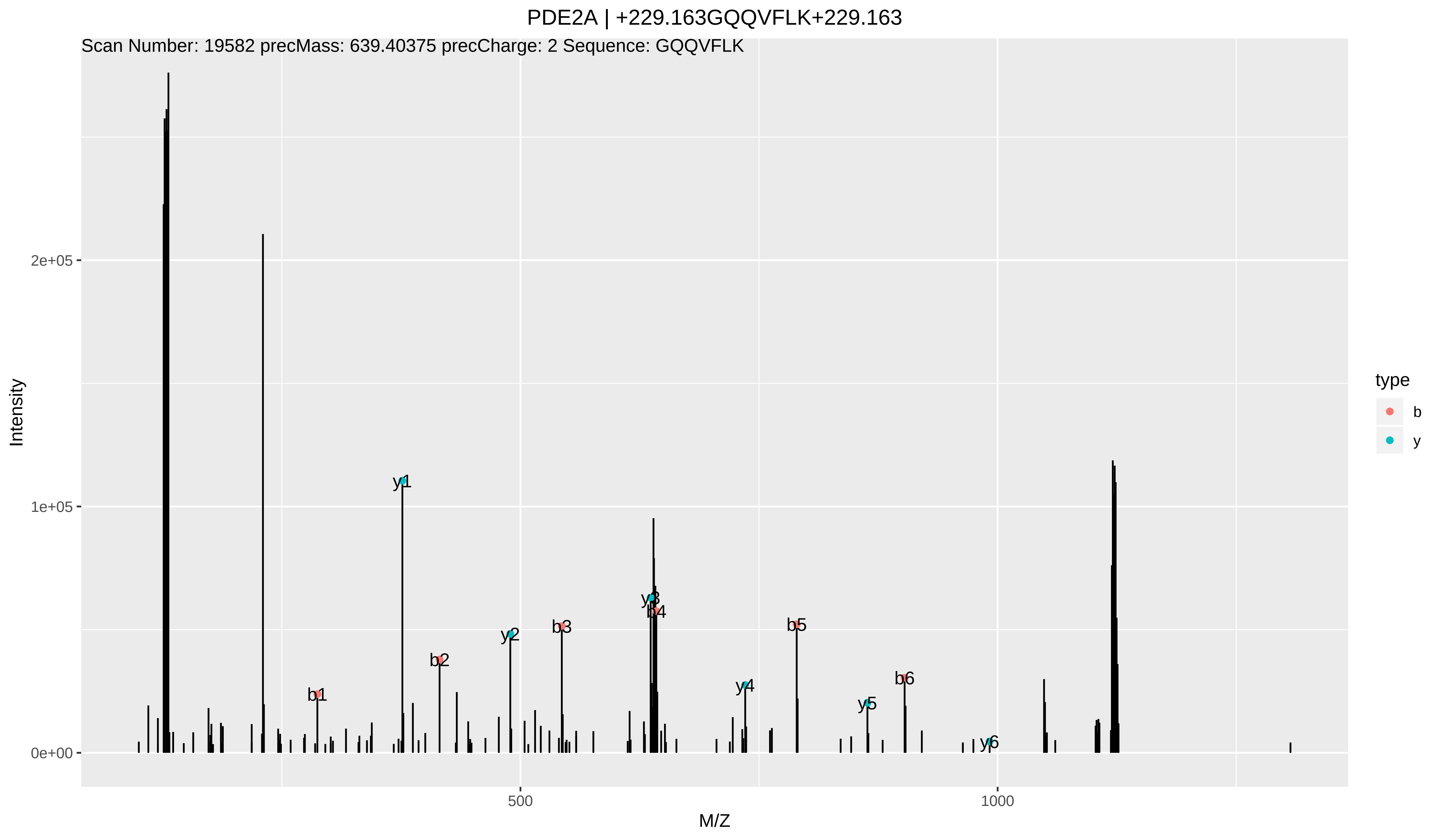
PDE2A | +229.163GQQVFLK+229.163
Scan Number: 19582 precMass: 639.40375 precCharge: 2 Sequence: GQQVFLK
Intensity
M/Z
2e+05
1e+05
0e+00
500
1000
y1
y2
y3
y4
y5
y6
b1
b2
b3
b4
b5
b6
type
b
y

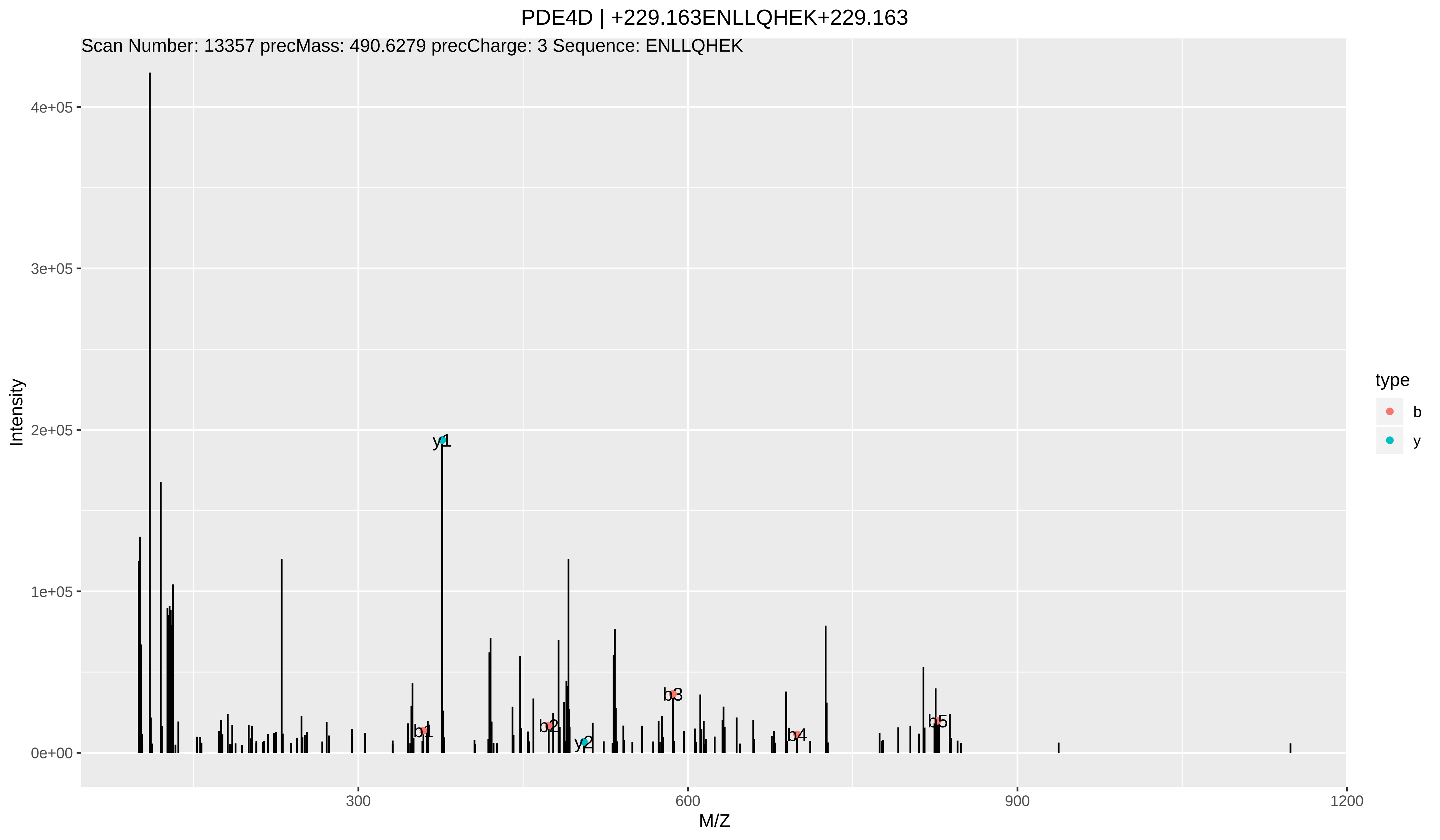
PDE4D | +229.163ENLLQHEK+229.163
Scan Number: 13357 precMass: 490.6279 precCharge: 3 Sequence: ENLLQHEK
Intensity
4e+05
3e+05
2e+05
1e+05
0e+00
y1
b1
b2
y2
b3
b4
b5
300
600
900
1200
M/Z
type
b
y

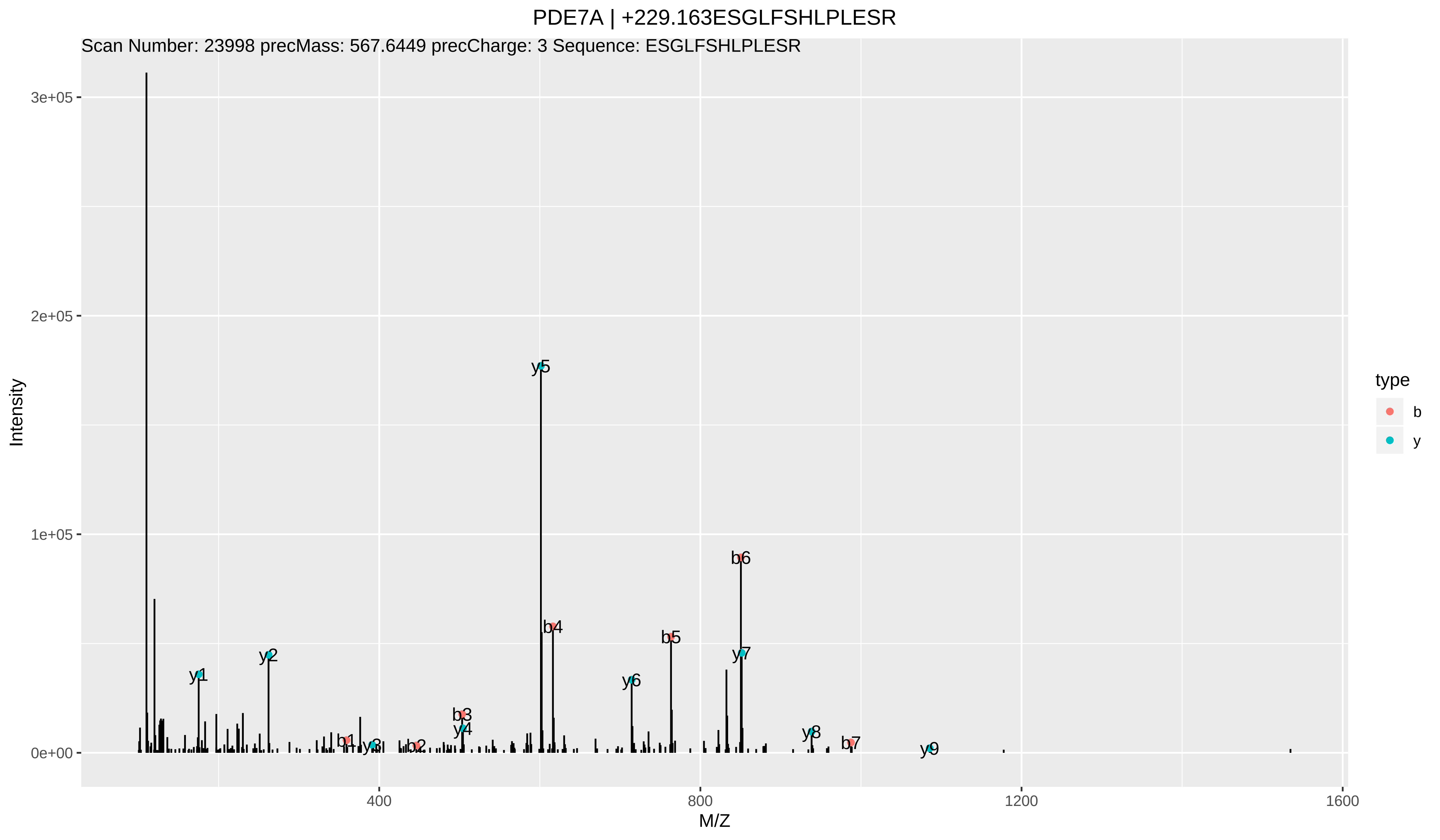
PDE7A | +229.163ESGLFSHLPLESR
Scan Number: 23998 precMass: 567.6449 precCharge: 3 Sequence: ESGLFSHLPLESR
Intensity
3e+05
2e+05
1e+05
0e+00
M/Z
400
800
1200
1600
y1
y2
b1
y3
b2
b3
y4
y5
b4
y6
b5
b6
y7
y8
b7
y9
type
b
y

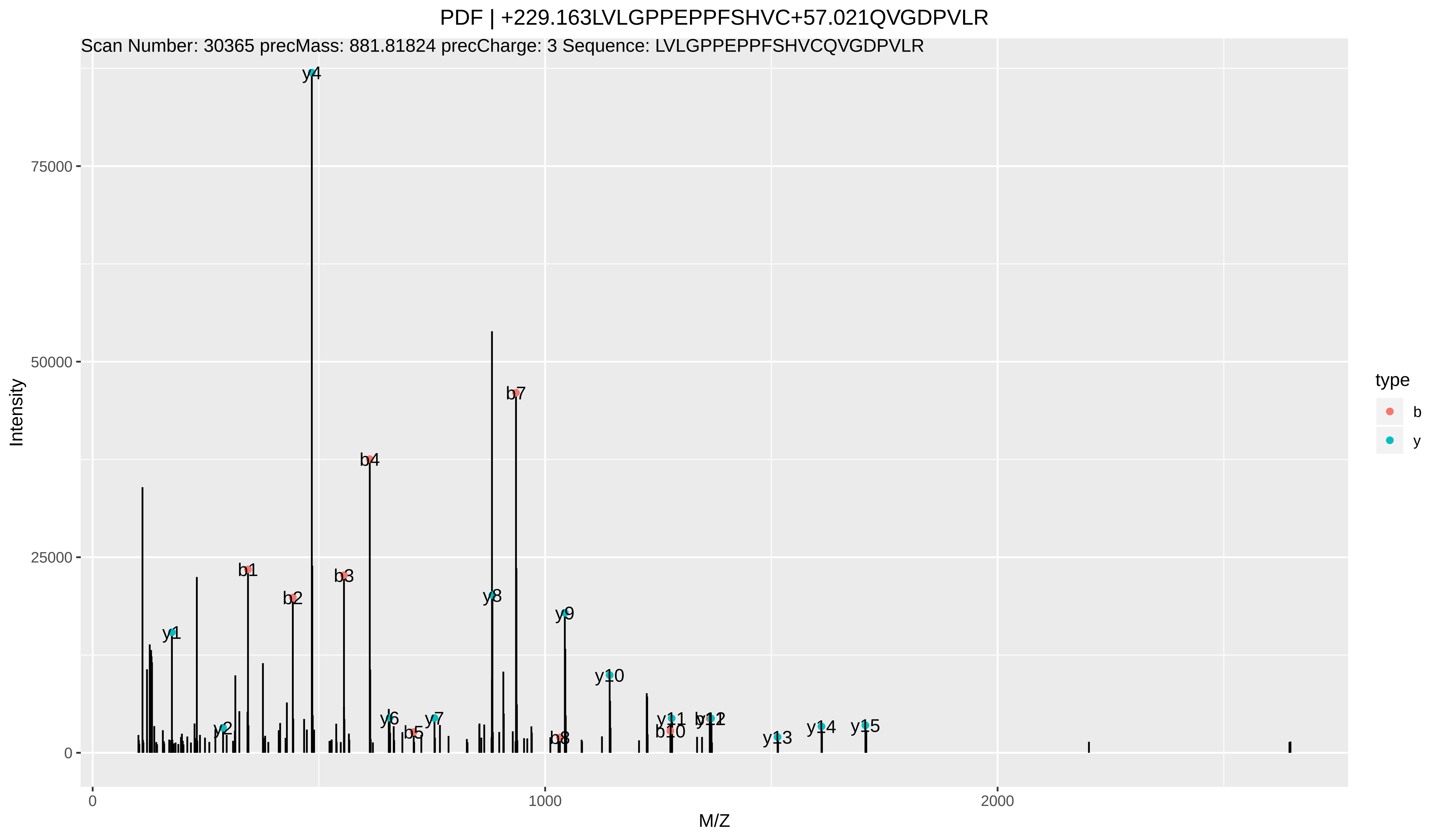
PDF | +229.163LVLGPPEPPFSHVC+57.021QVGDPVLR
Scan Number: 30365 precMass: 881.81824 precCharge: 3 Sequence: LVLGPPEPPFSHVCQVGDPVLR
Intensity
M/Z
0
25000
50000
75000
1000
2000
type
b
y
y1
y2
b1
b2
y4
b3
b4
y6
b5
y7
y8
b7
b8
y9
y10
y11
b10
b11
y12
y13
y14
y15

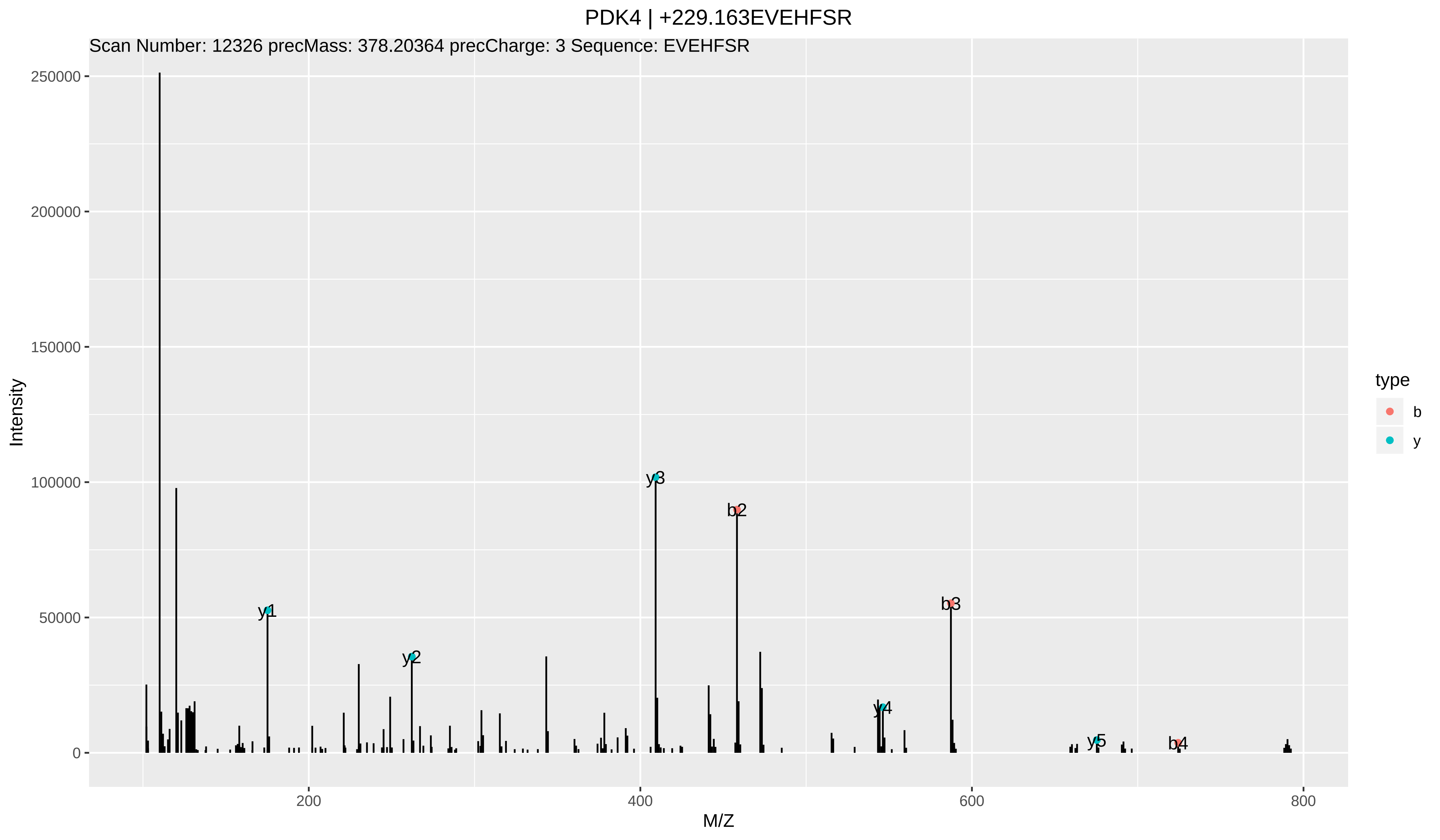

PDK4 | +229.163EVEHFSR
Scan Number: 12326 precMass: 378.20364 precCharge: 3 Sequence: EVEHFSR
Intensity
250000
200000
150000
100000
50000
0
200
400
600
800
M/Z
y1
y2
y3
b2
y4
b3
y5
b4
type
b
y

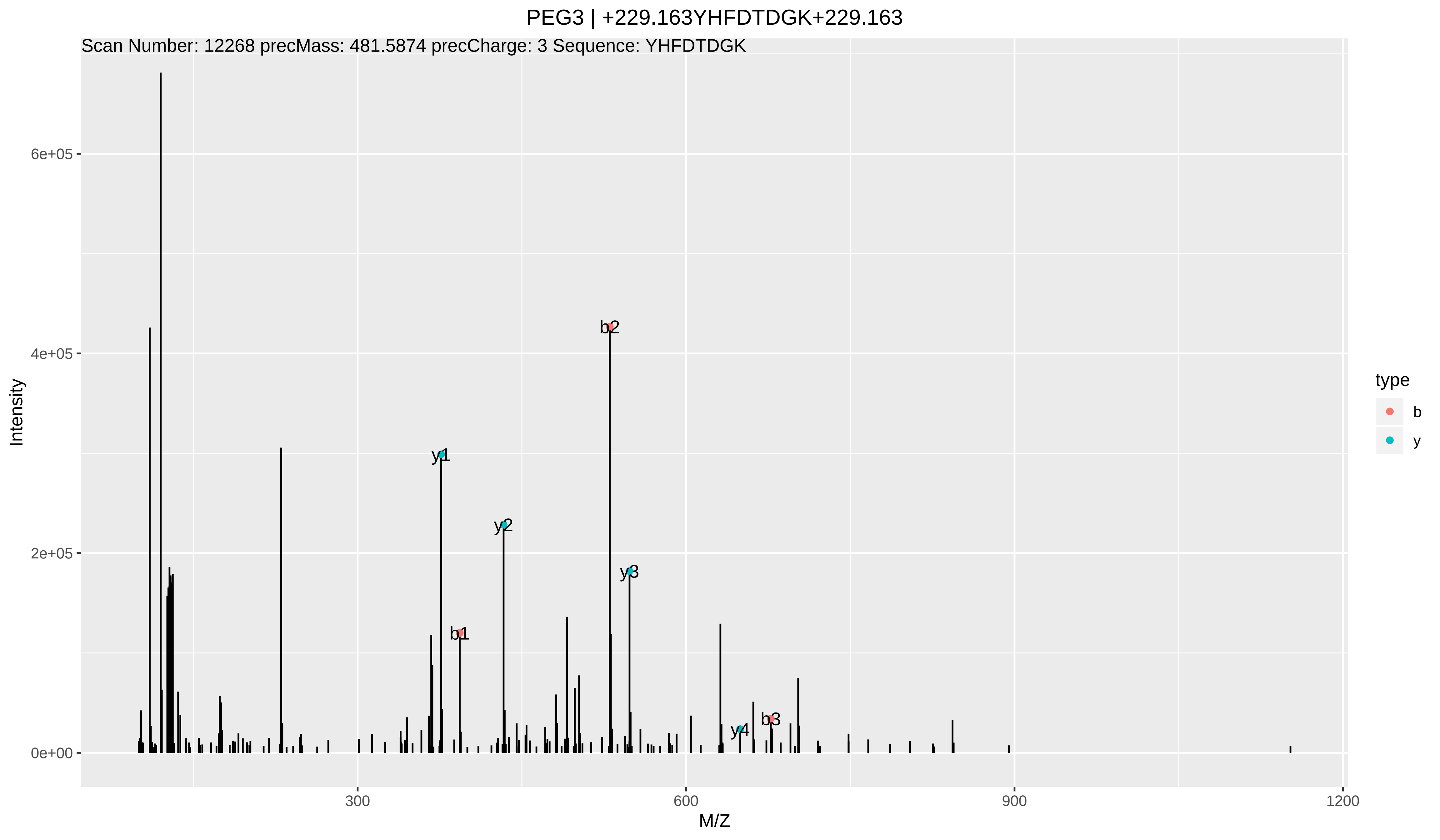

PEG3 | +229.163YHFDTDGK+229.163
Scan Number: 12268 precMass: 481.5874 precCharge: 3 Sequence: YHFDTDGK
Intensity
6e+05
4e+05
2e+05
0e+00
300
600
900
1200
M/Z
y1
y2
y3
y4
b1
b2
b3
type
b
y

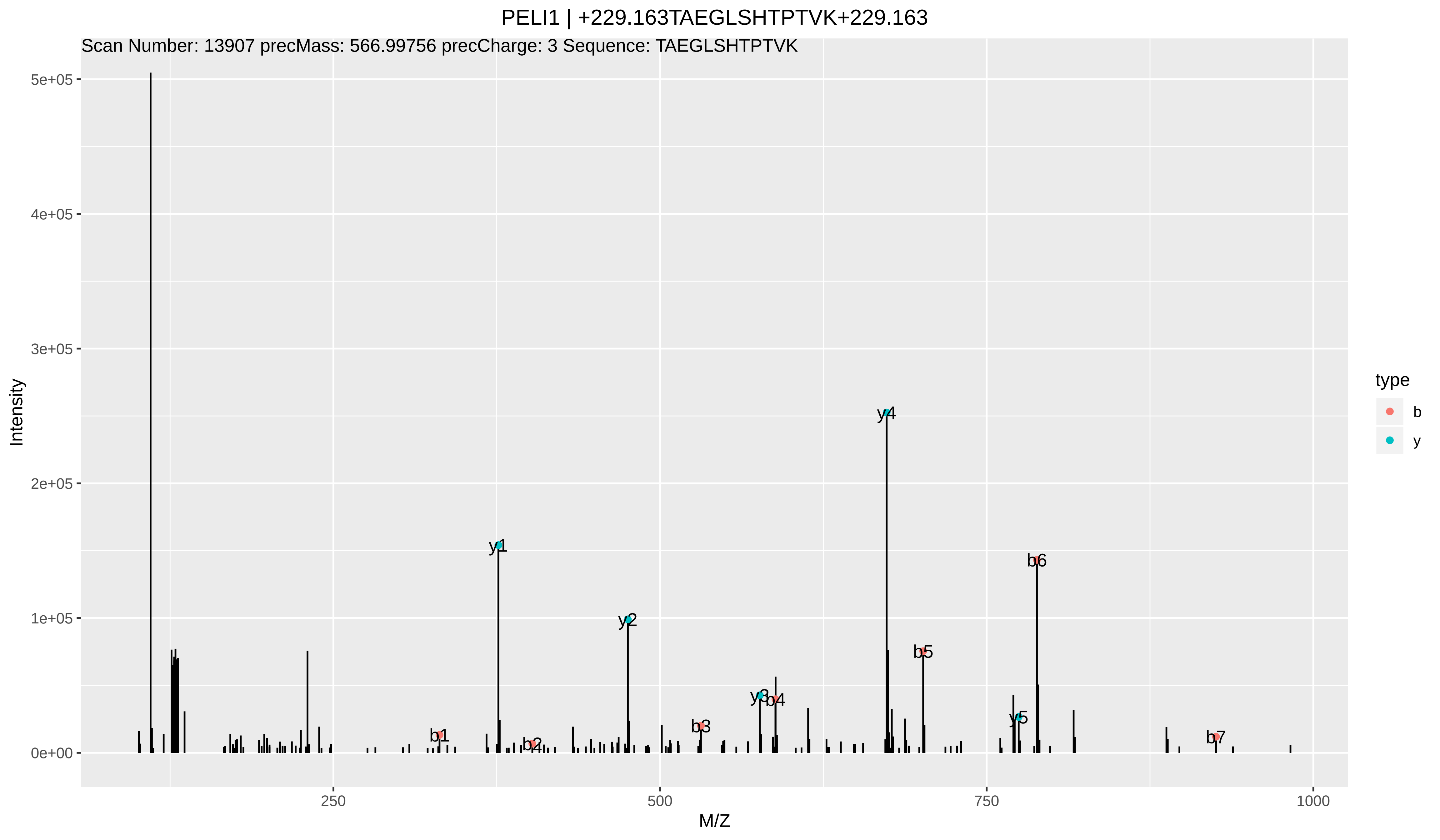

PELI1 | +229.163TAEGLSHTPTVK+229.163
Scan Number: 13907 precMass: 566.99756 precCharge: 3 Sequence: TAEGLSHTPTVK
Intensity
5e+05
4e+05
3e+05
2e+05
1e+05
0e+00
M/Z
250
500
750
1000
y1
y2
y3
y4
y5
b1
b2
b3
b4
b5
b6
b7
type
b
y

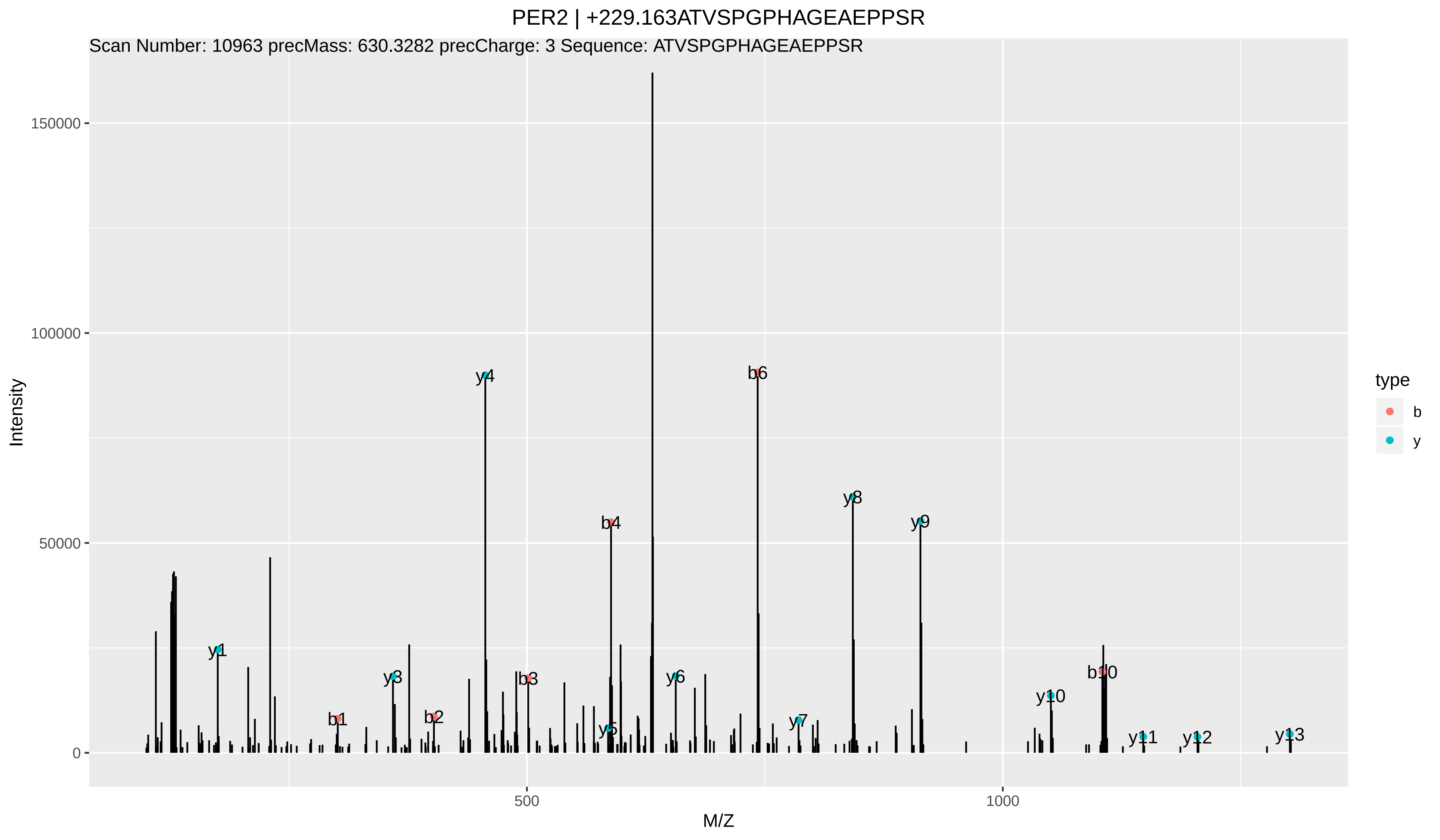
PER2 | +229.163ATVSPGPHAGEAEPPSR
Scan Number: 10963 precMass: 630.3282 precCharge: 3 Sequence: ATVSPGPHAGEAEPPSR
Intensity
150000
100000
50000
0
M/Z
500
1000
y1
b1
y3
b2
y4
b3
y5
b4
y6
b6
y7
y8
y9
y10
b10
y11
y12
y13
type
b
y

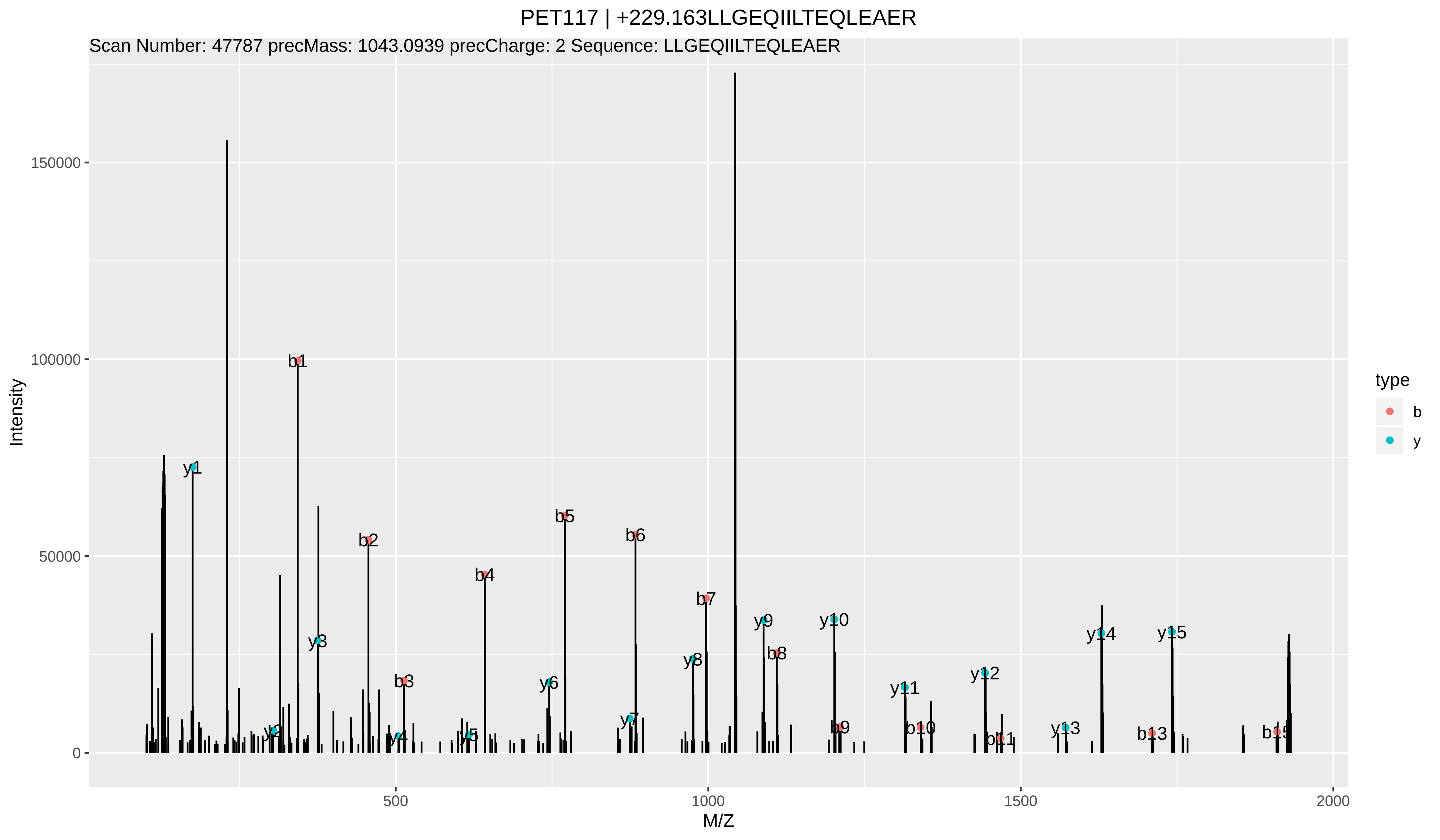

PET117 | +229.163LLGEQIILTEQLEAER
Scan Number: 47787 precMass: 1043.0939 precCharge: 2 Sequence: LLGEQIILTEQLEAER
Intensity
150000
100000
50000
0
M/Z
500
1000
1500
2000
type
b
y
y1
b1
y2
y3
b2
b3
y4
y5
b4
y6
b5
y7
b6
y8
b7
y9
b8
y10
b9
y11
b10
y12
b11
y13
y14
b13
y15
b15

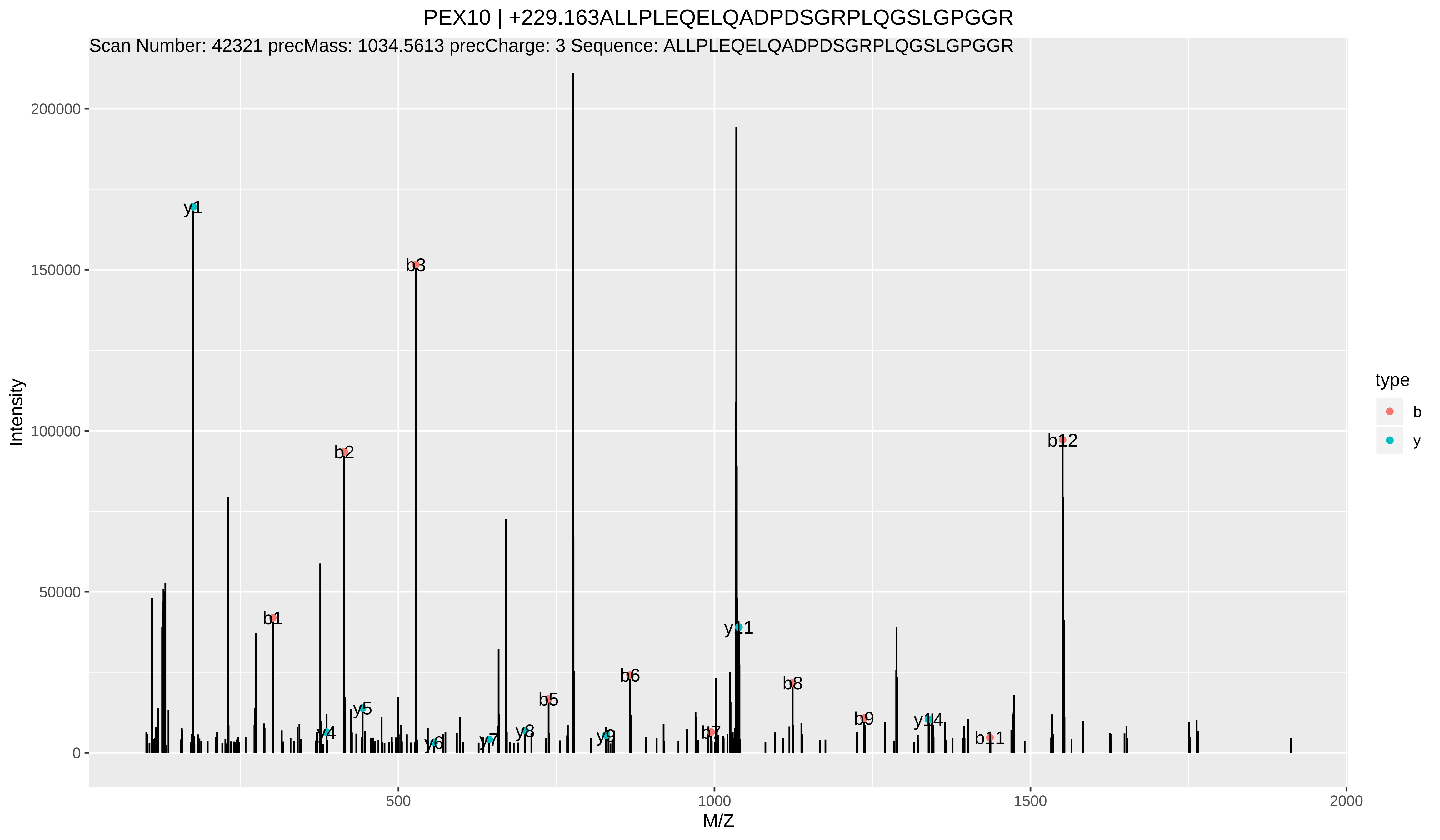
PEX10 | +229.163ALLPLEQELQADPDSGRPLQGSLGPGGR
Scan Number: 42321 precMass: 1034.5613 precCharge: 3 Sequence: ALLPLEQELQADPDSGRPLQGSLGPGGR
Intensity
M/Z
type
b
y
y1
b1
b2
b3
b5
b6
b7
b8
b9
b11
b12
y4
y5
y6
y7
y8
y9
y11
y14
0
50000
100000
150000
200000
500
1000
1500
2000

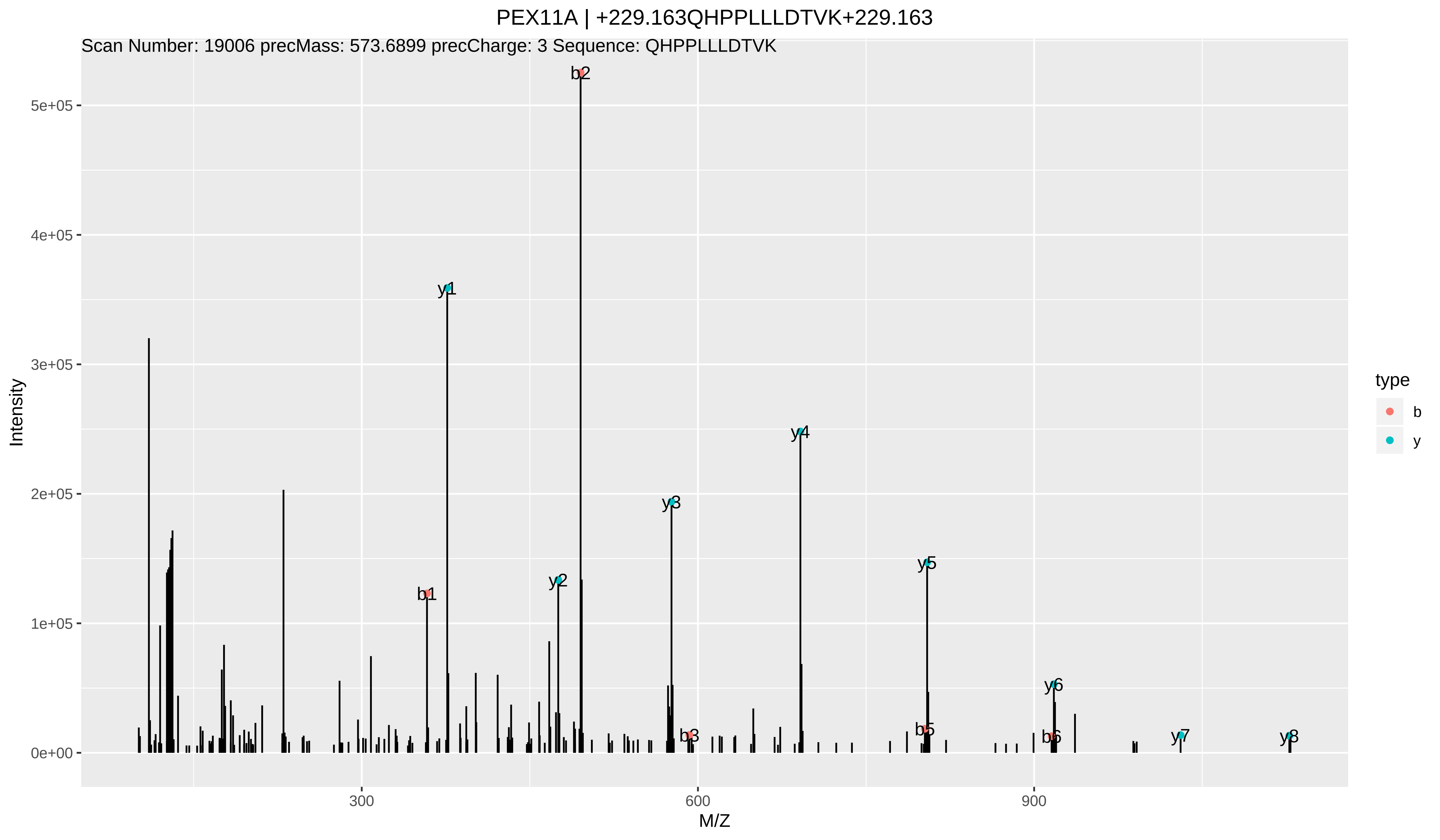

PEX11A | +229.163QHPPLLLDTVK+229.163
Scan Number: 19006 precMass: 573.6899 precCharge: 3 Sequence: QHPPLLLDTVK
b2
y1
y4
y3
y5
y2
b1
y6
b5
b3
b6
y7
y8
Intensity
0e+00
1e+05
2e+05
3e+05
4e+05
5e+05
M/Z
300
600
900
type
b
y

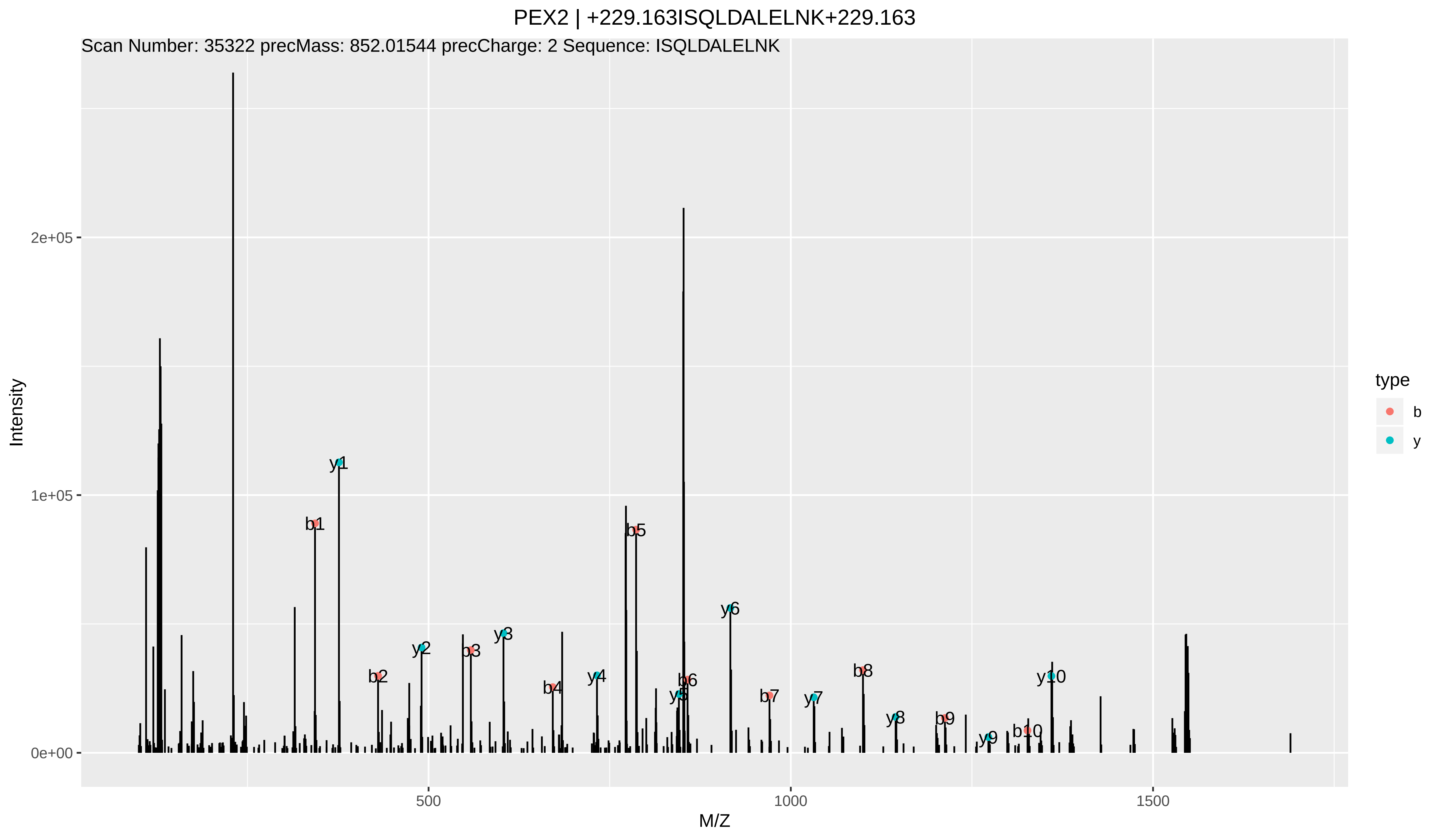
PEX2 | +229.163ISQLDALELNK+229.163
Scan Number: 35322 precMass: 852.01544 precCharge: 2 Sequence: ISQLDALELNK
Intensity
2e+05
1e+05
0e+00
M/Z
500
1000
1500
y1
b1
b5
y6
y2
y3
b3
b2
b4
y4
b6
y5
b7
y7
b8
y8
b9
y9
b10
y10
type
b
y

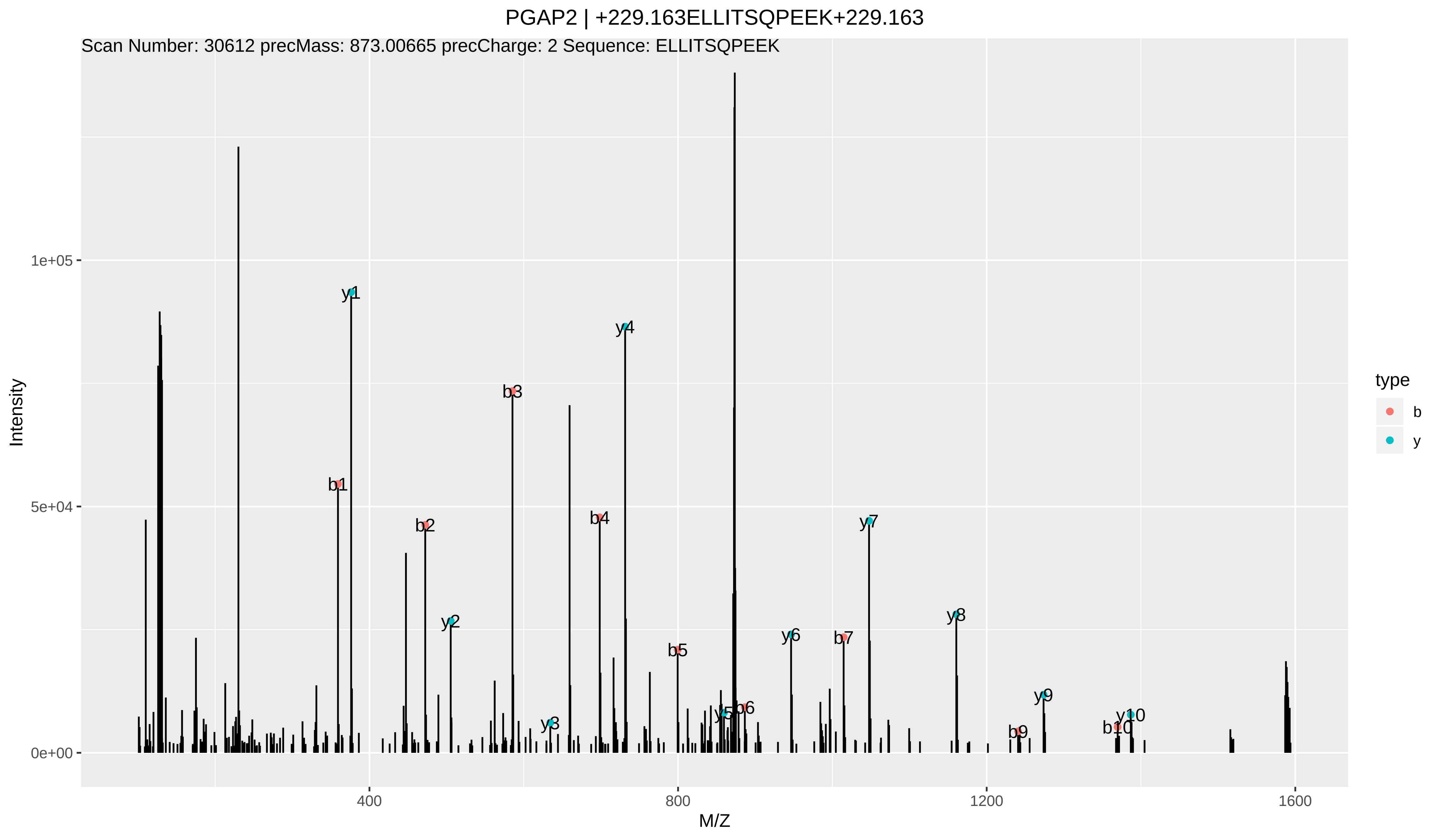
PGAP2 | +229.163ELLITSQPEEK+229.163
Scan Number: 30612 precMass: 873.00665 precCharge: 2 Sequence: ELLITSQPEEK
Intensity
1e+05
5e+04
0e+00
M/Z
400
800
1200
1600
y1
y4
b3
b1
b2
b4
y7
y2
y8
y6
b7
b5
y9
y3
y5
b6
y10
b9
b10
type
b
y

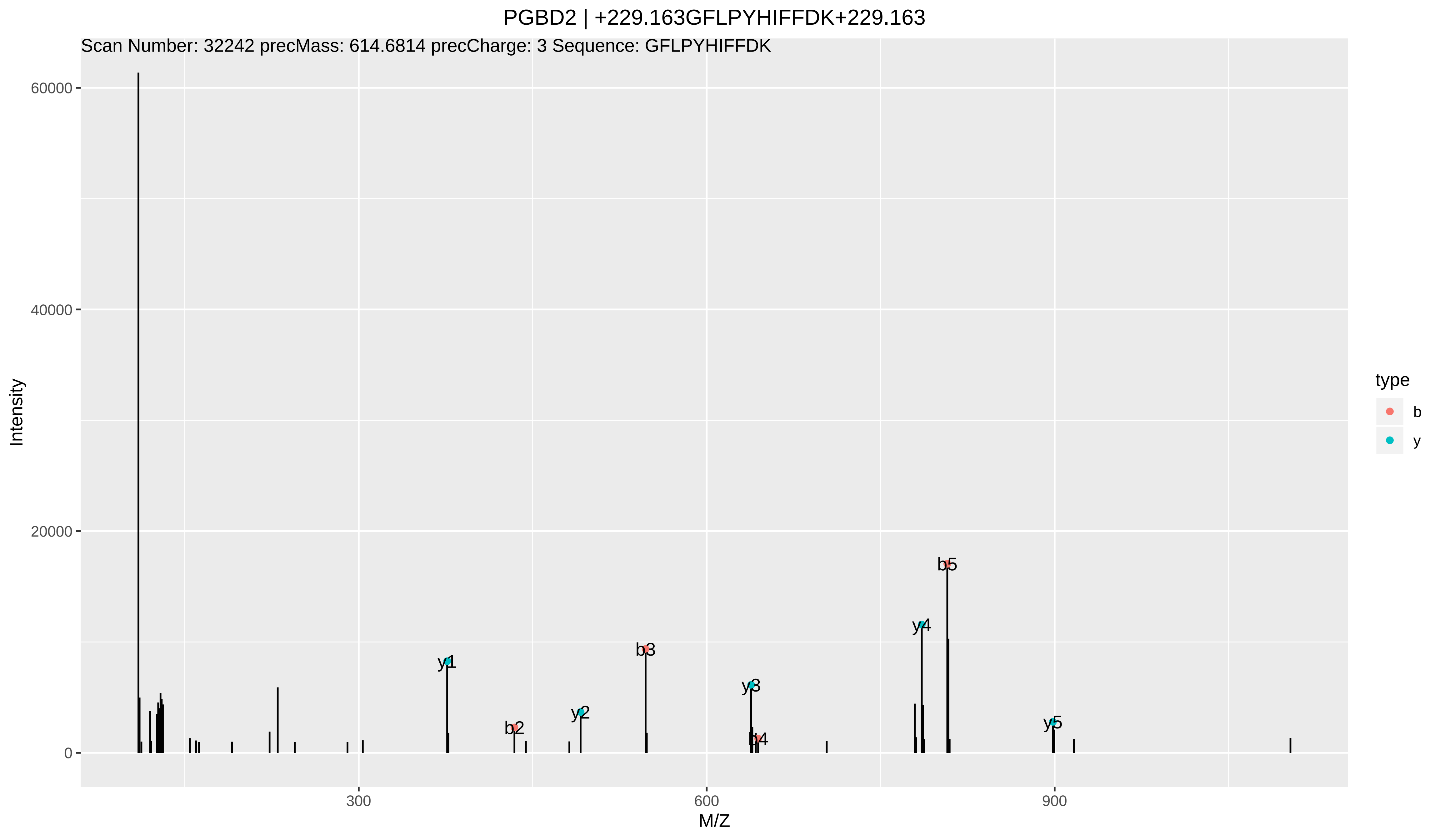
PGBD2 | +229.163GFLPYHIFFDK+229.163
Scan Number: 32242 precMass: 614.6814 precCharge: 3 Sequence: GFLPYHIFFDK
Intensity
60000
40000
20000
0
300
600
900
M/Z
y1
b2
y2
b3
y3
b4
y4
b5
y5
type
b
y

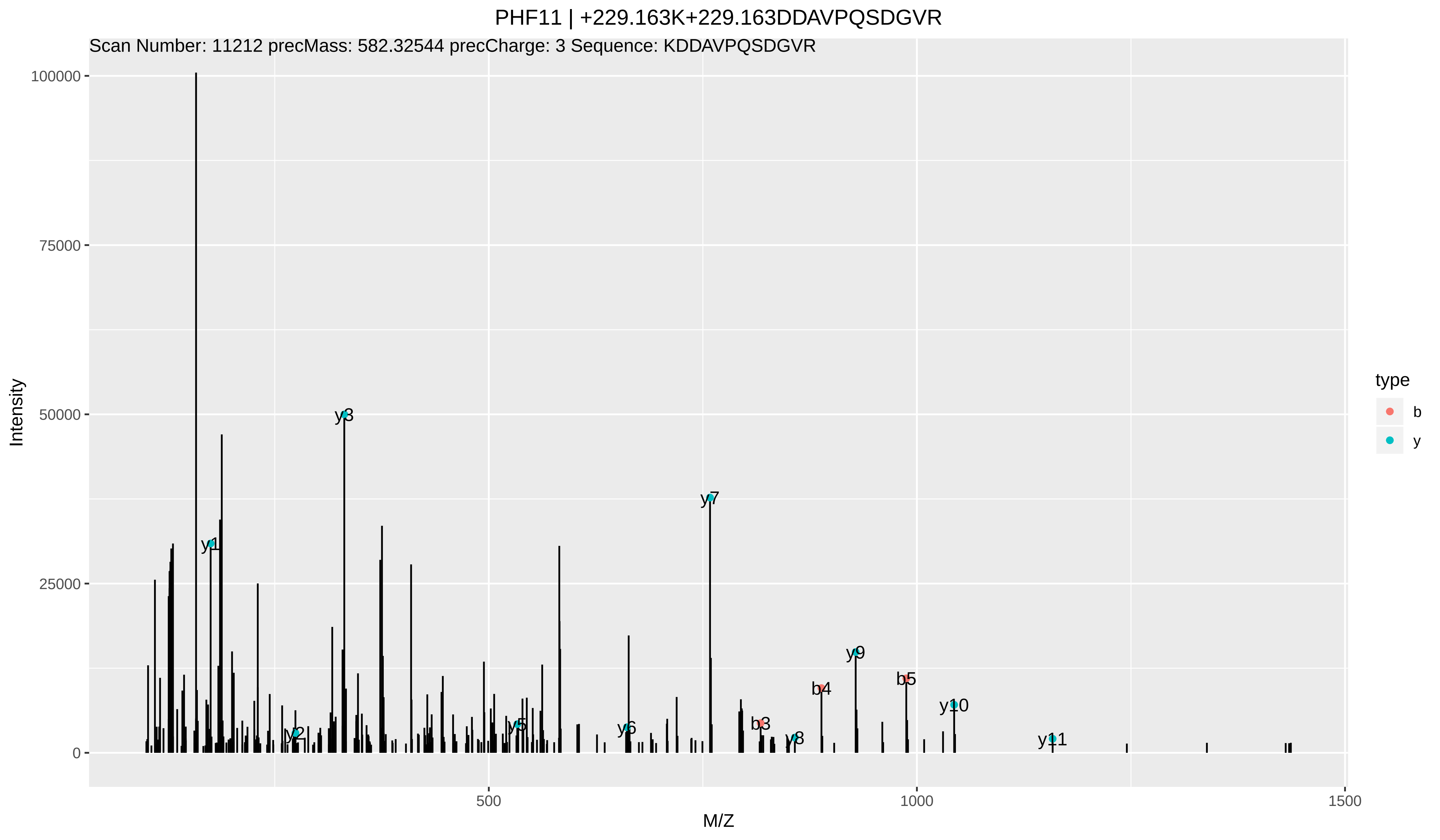

PHF11 | +229.163K+229.163DDAVPQSDGVR
Scan Number: 11212 precMass: 582.32544 precCharge: 3 Sequence: KDDAVPQSDGVR
Intensity
100000
75000
50000
25000
0
500
1000
1500
M/Z
y1
y2
y3
y5
y6
y7
y8
y9
y10
y11
b3
b4
b5
type
b
y

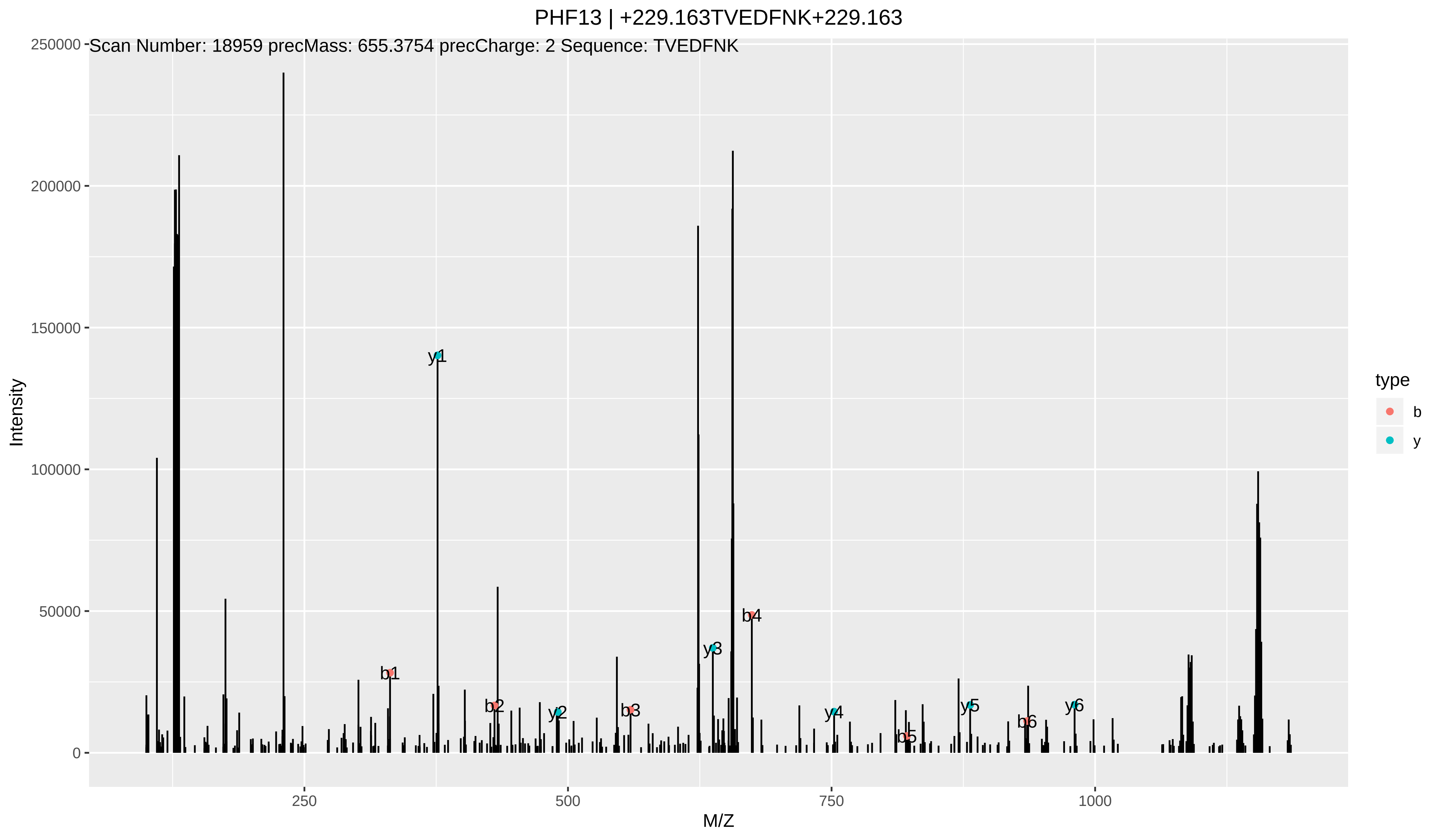
PHF13 | +229.163TVEDFNK+229.163
Scan Number: 18959 precMass: 655.3754 precCharge: 2 Sequence: TVEDFNK
Intensity
250000
200000
150000
100000
50000
0
M/Z
250
500
750
1000
y1
b1
b2
y2
b3
y3
b4
y4
b5
y5
b6
y6
type
b
y

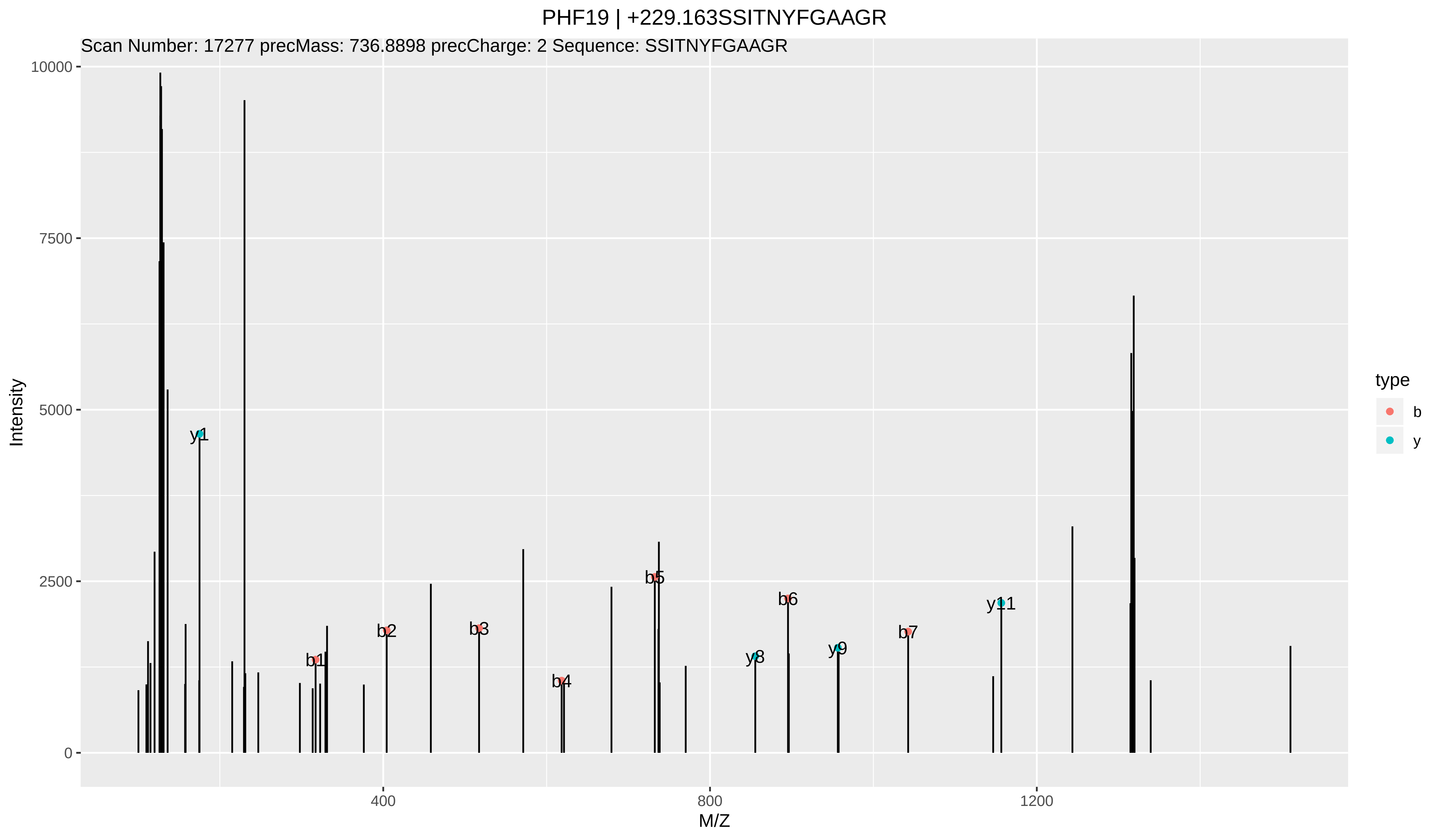

PHF19 | +229.163SSITNYFGAAGR
Scan Number: 17277 precMass: 736.8898 precCharge: 2 Sequence: SSITNYFGAAGR
Intensity
10000
7500
5000
2500
0
M/Z
400
800
1200
y1
b1
b2
b3
b4
b5
y8
b6
y9
b7
y11
type
b
y

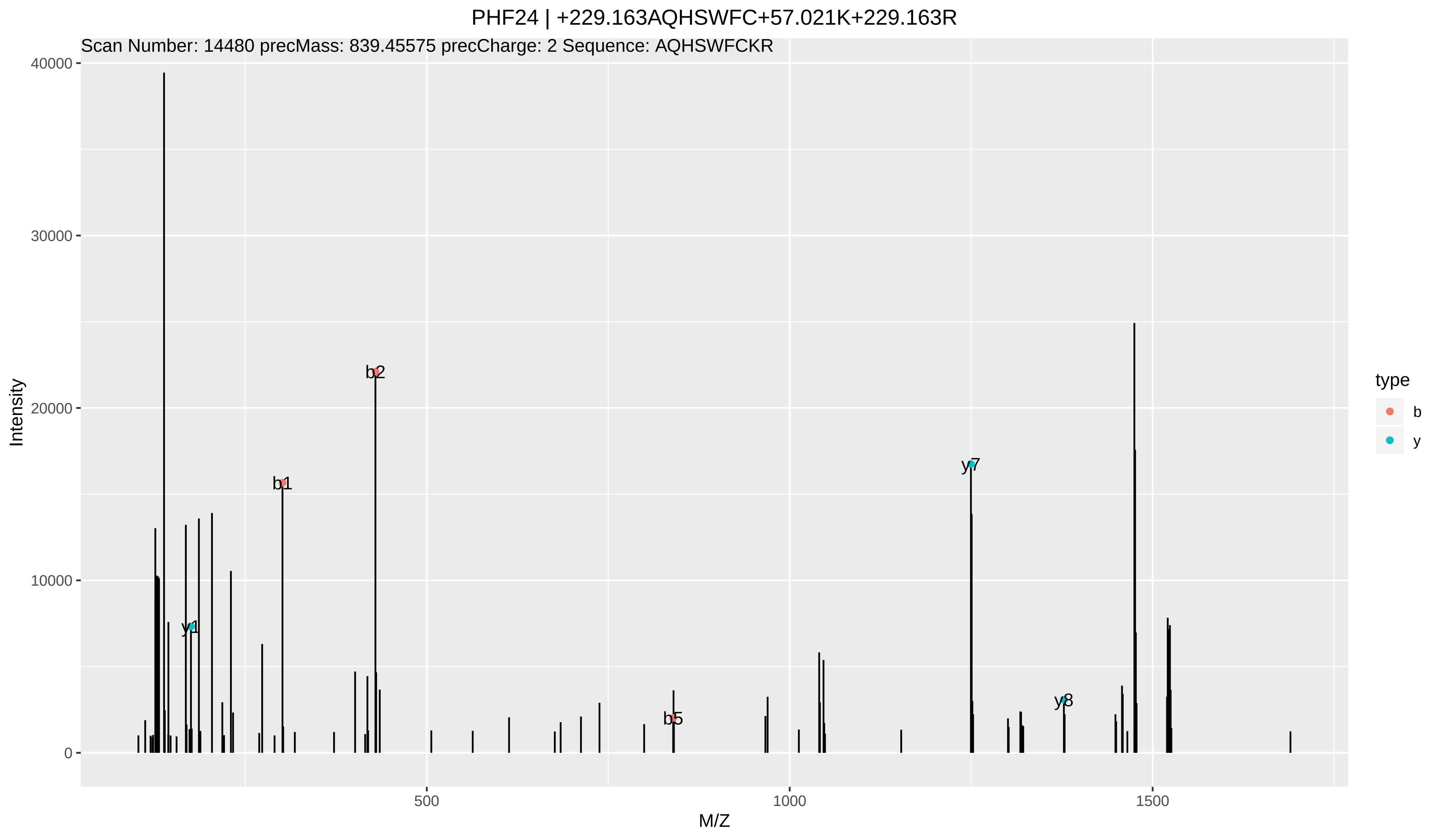
PHF24 | +229.163AQHSWFC+57.021K+229.163R
Scan Number: 14480 precMass: 839.45575 precCharge: 2 Sequence: AQHSWFCKR
Intensity
M/Z
40000
30000
20000
10000
0
500
1000
1500
b1
b2
b5
y1
y7
y8
type
b
y

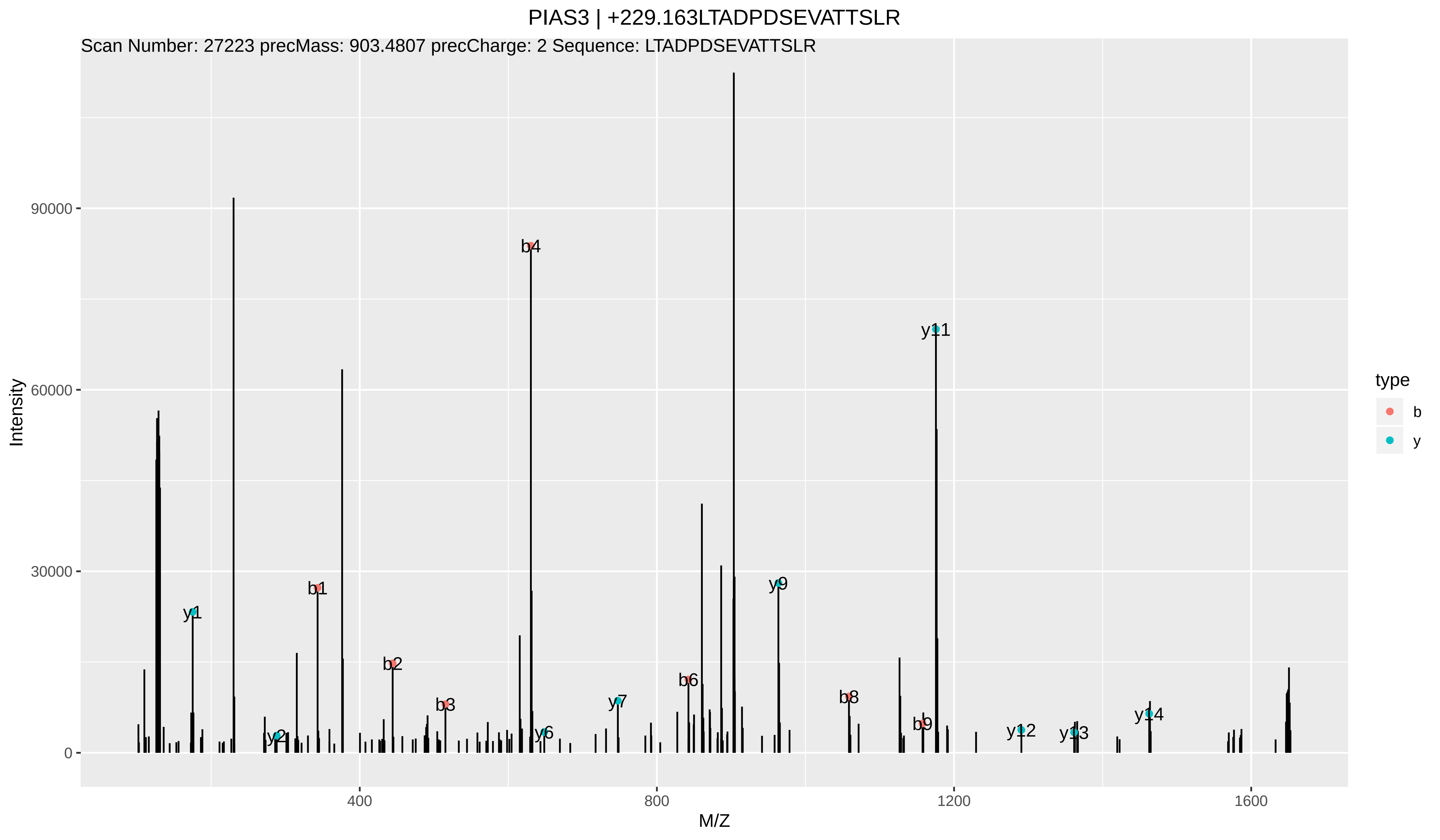
PIAS3 | +229.163LTADPDSEVATTSLR
Scan Number: 27223 precMass: 903.4807 precCharge: 2 Sequence: LTADPDSEVATTSLR
Intensity
M/Z
90000
60000
30000
0
400
800
1200
1600
y1
y2
b1
b2
b3
b4
y6
y7
b6
y9
b8
b9
y11
y12
y13
y14
type
b
y

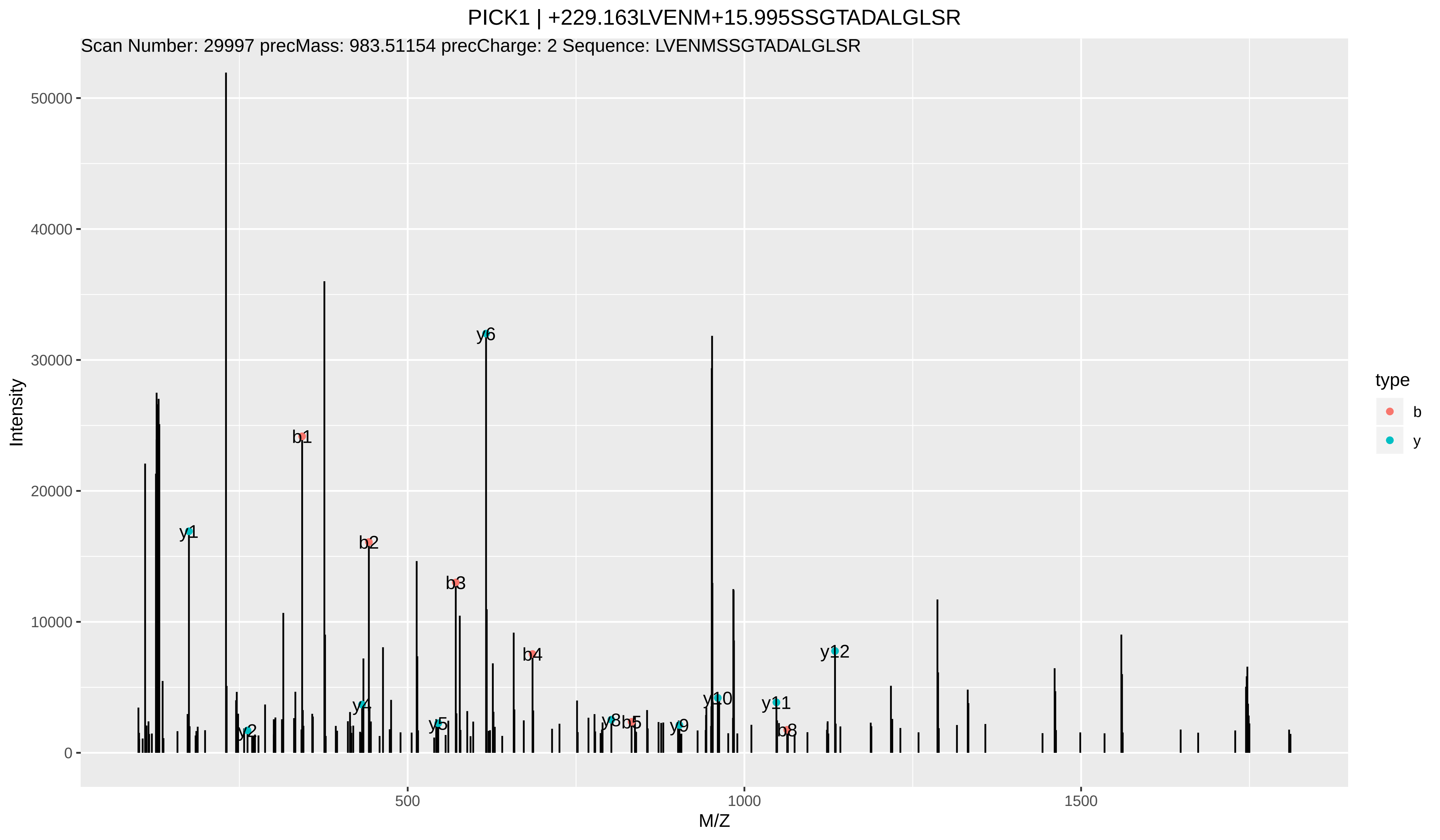
PICK1 | +229.163LVENM+15.995SSGTADALGLSR
Scan Number: 29997 precMass: 983.51154 precCharge: 2 Sequence: LVENMSSGTADALGLSR
Intensity
M/Z
type
b
y
0
10000
20000
30000
40000
50000
500
1000
1500
y1
y2
b1
y4
b2
y5
b3
y6
b4
y8
b5
y9
y10
y11
b8
y12

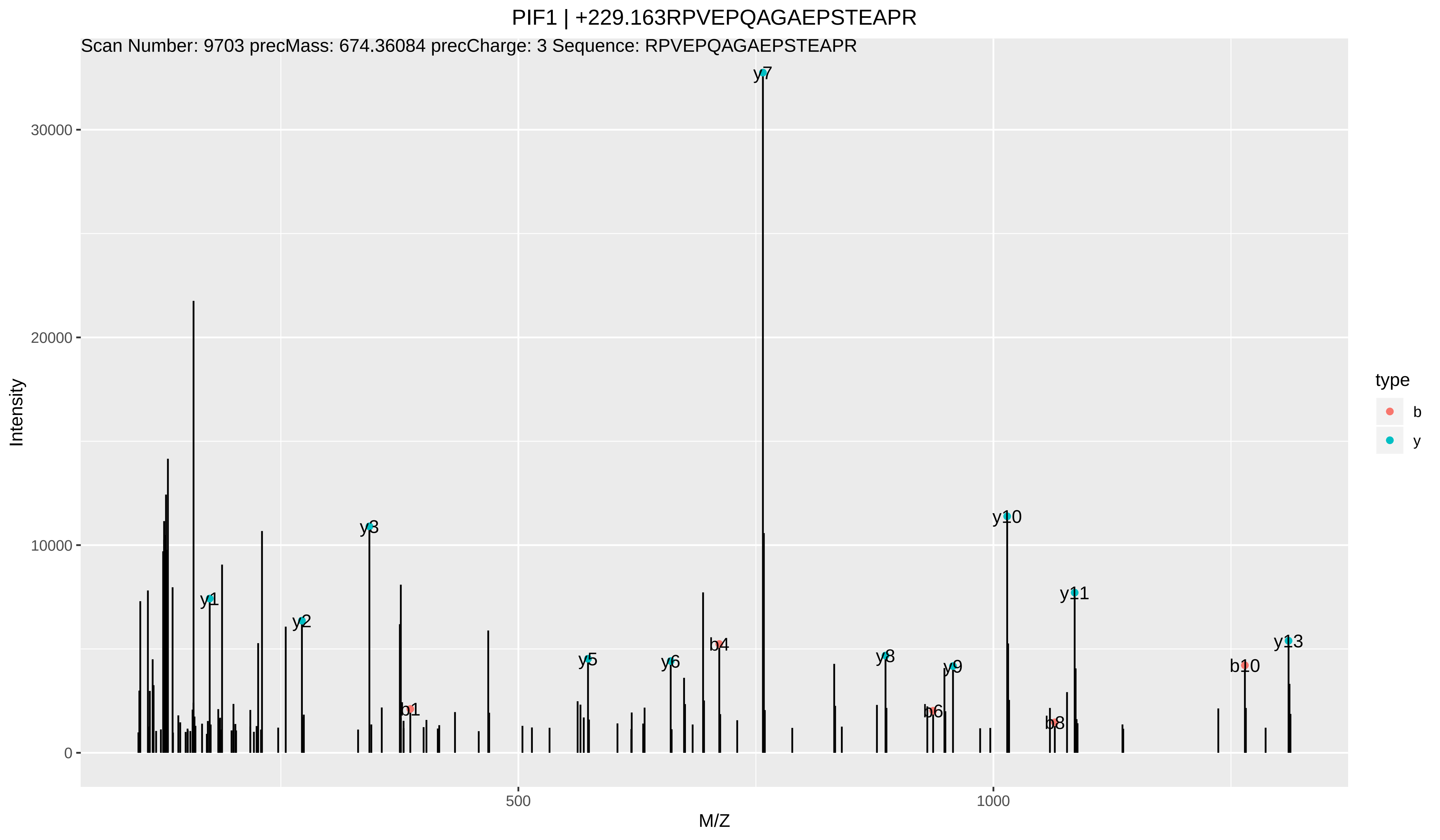
PIF1 | +229.163RPVEPQAGAEPSTEAPR
Scan Number: 9703 precMass: 674.36084 precCharge: 3 Sequence: RPVEPQAGAEPSTEAPR
Intensity
M/Z
30000
20000
10000
0
500
1000
y7
y3
y10
y1
y2
y11
y13
b4
y5
y6
y8
y9
b10
b1
b6
b8
type
b
y

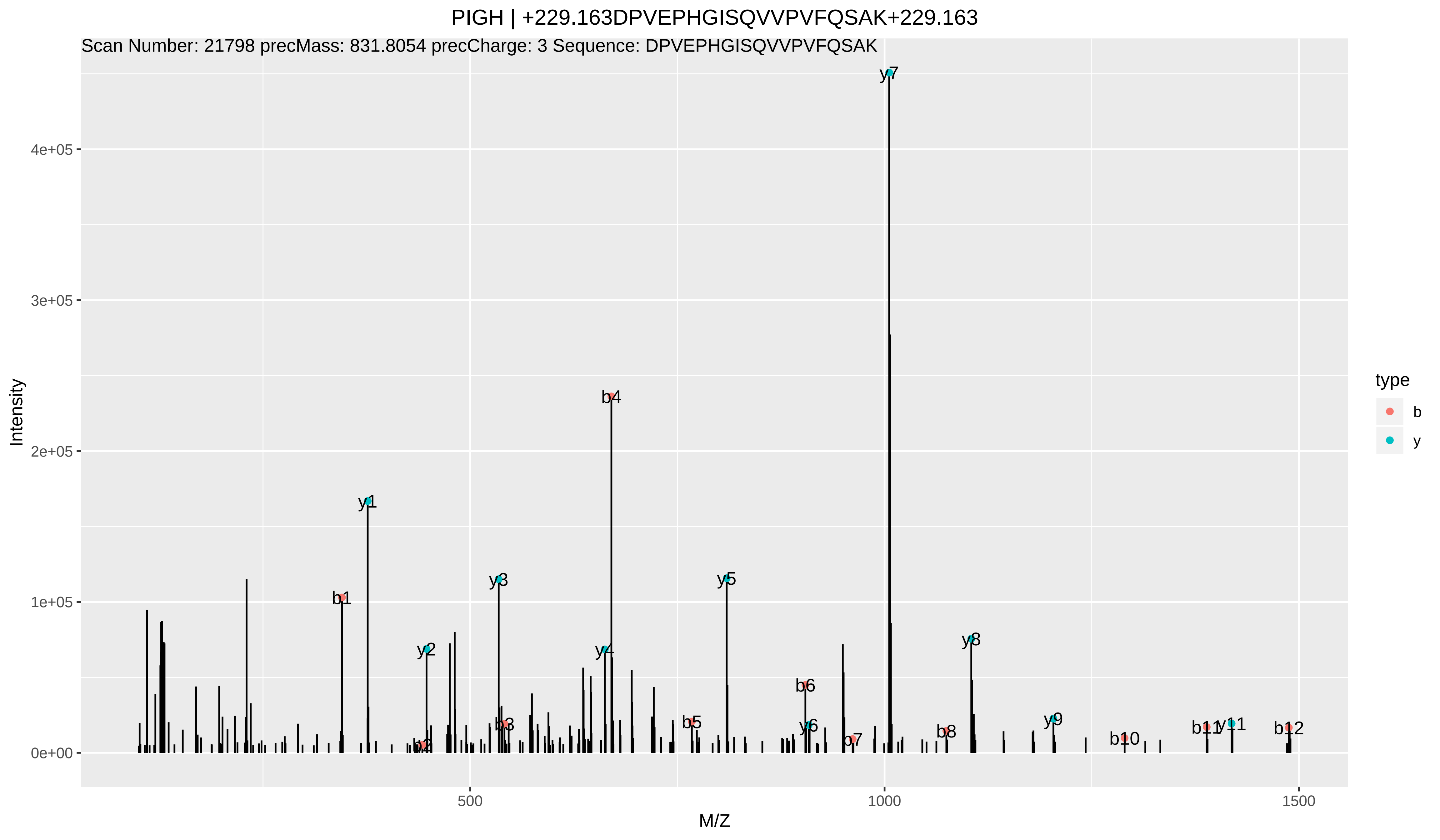
PIGH | +229.163DPVEPHGISQVVPVFQSAK+229.163
Scan Number: 21798 precMass: 831.8054 precCharge: 3 Sequence: DPVEPHGISQVVPVFQSAK
Intensity
4e+05
3e+05
2e+05
1e+05
0e+00
M/Z
500
1000
1500
type
b
y
y7
b4
y1
y3
y5
b1
y2
y4
y8
b6
b5
b3
y6
y9
b7
b2
b8
b10
b11
y11
b12

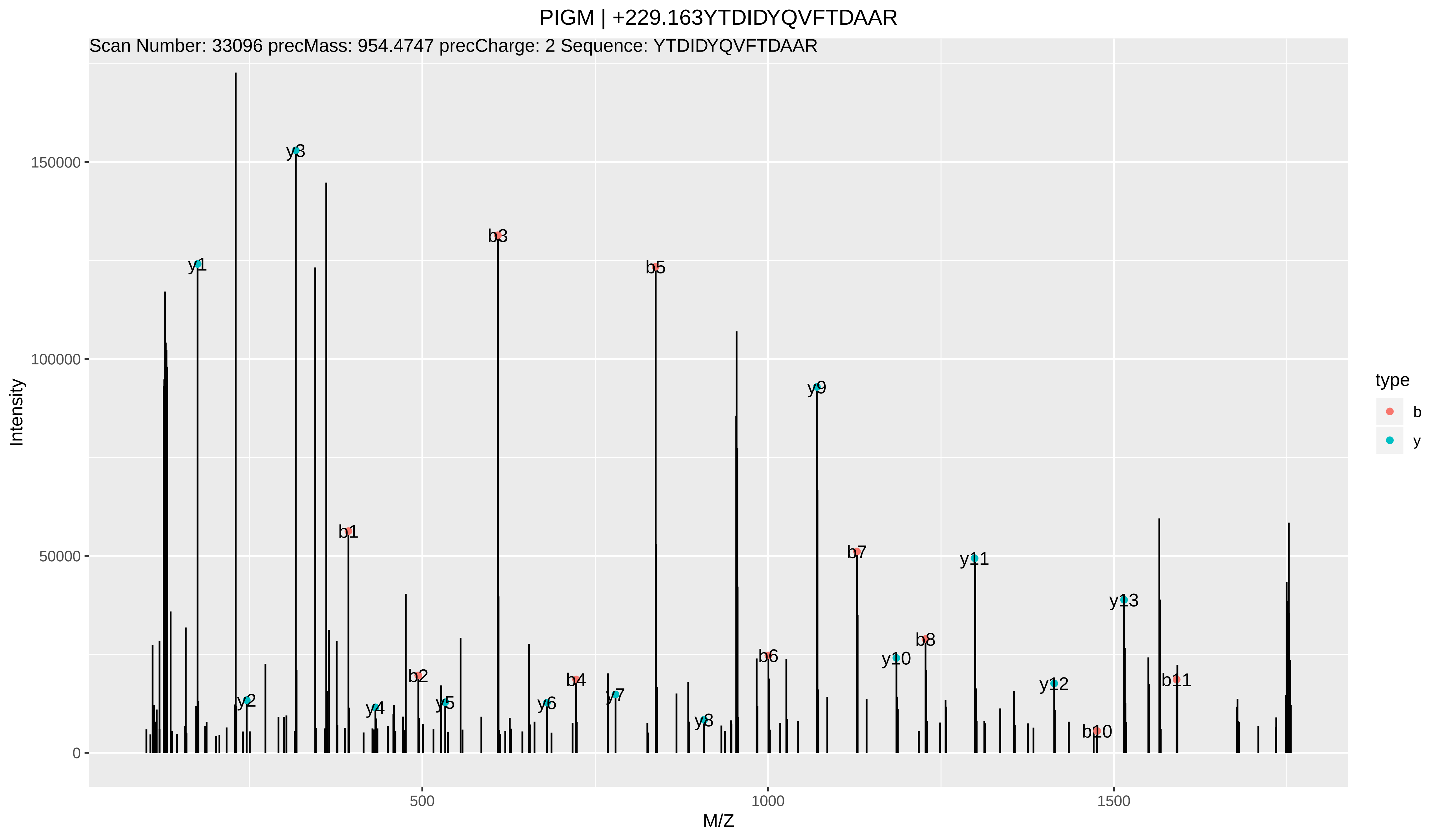
PIGM | +229.163YTDIDYQVFTDAAR
Scan Number: 33096 precMass: 954.4747 precCharge: 2 Sequence: YTDIDYQVFTDAAR
Intensity
150000
100000
50000
0
500
1000
1500
M/Z
y1
y2
y3
y4
y5
y6
y7
y8
y9
y10
y11
y12
y13
b1
b2
b3
b4
b5
b6
b7
b8
b10
b11
type
b
y

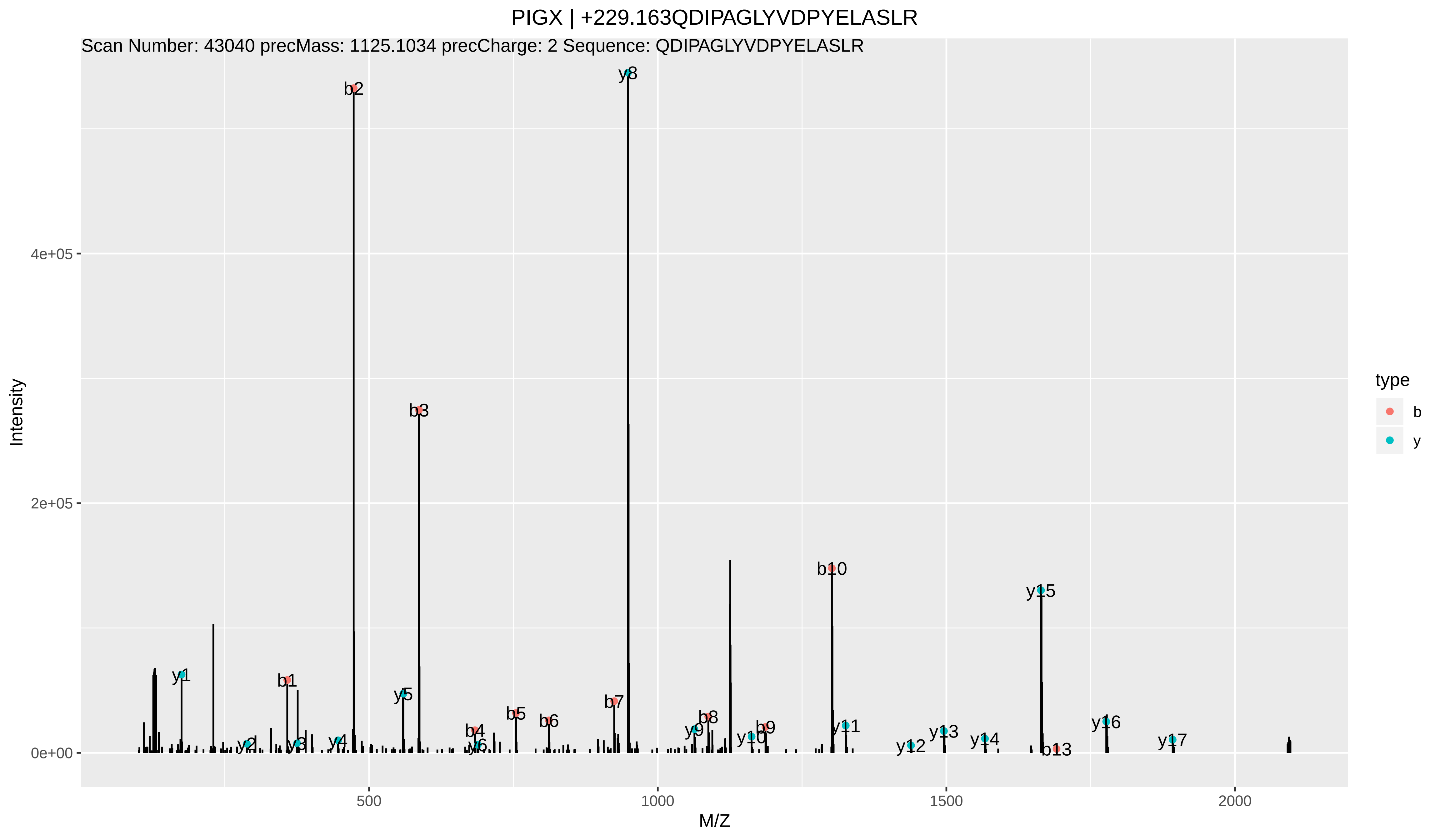
PIGX | +229.163QDIPAGLYVDPYELASLR
Scan Number: 43040 precMass: 1125.1034 precCharge: 2 Sequence: QDIPAGLYVDPYELASLR
Intensity
4e+05
2e+05
0e+00
M/Z
500
1000
1500
2000
type
b
y
y8
b2
b3
b10
y15
y1
b1
y5
b7
b5
b6
b8
y9
b4
y16
y11
b9
y13
y10
y14
y17
y2
y3
y4
y6
y12
b13

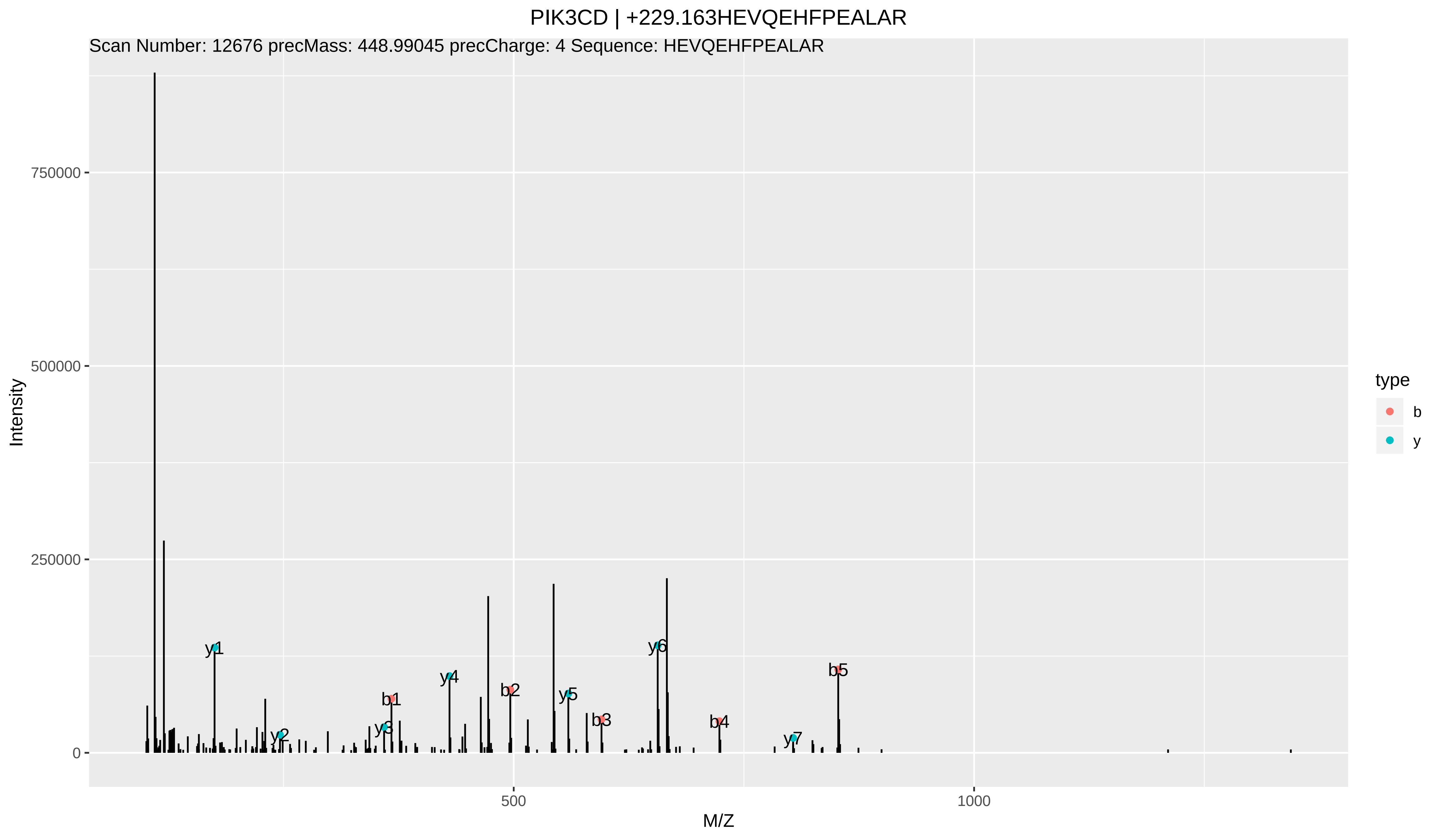
PIK3CD | +229.163HEVQEHFPEALAR
Scan Number: 12676 precMass: 448.99045 precCharge: 4 Sequence: HEVQEHFPEALAR
Intensity
750000
500000
250000
0
500
1000
M/Z
y1
y2
y3
b1
y4
b2
y5
b3
y6
b4
y7
b5
type
b
y

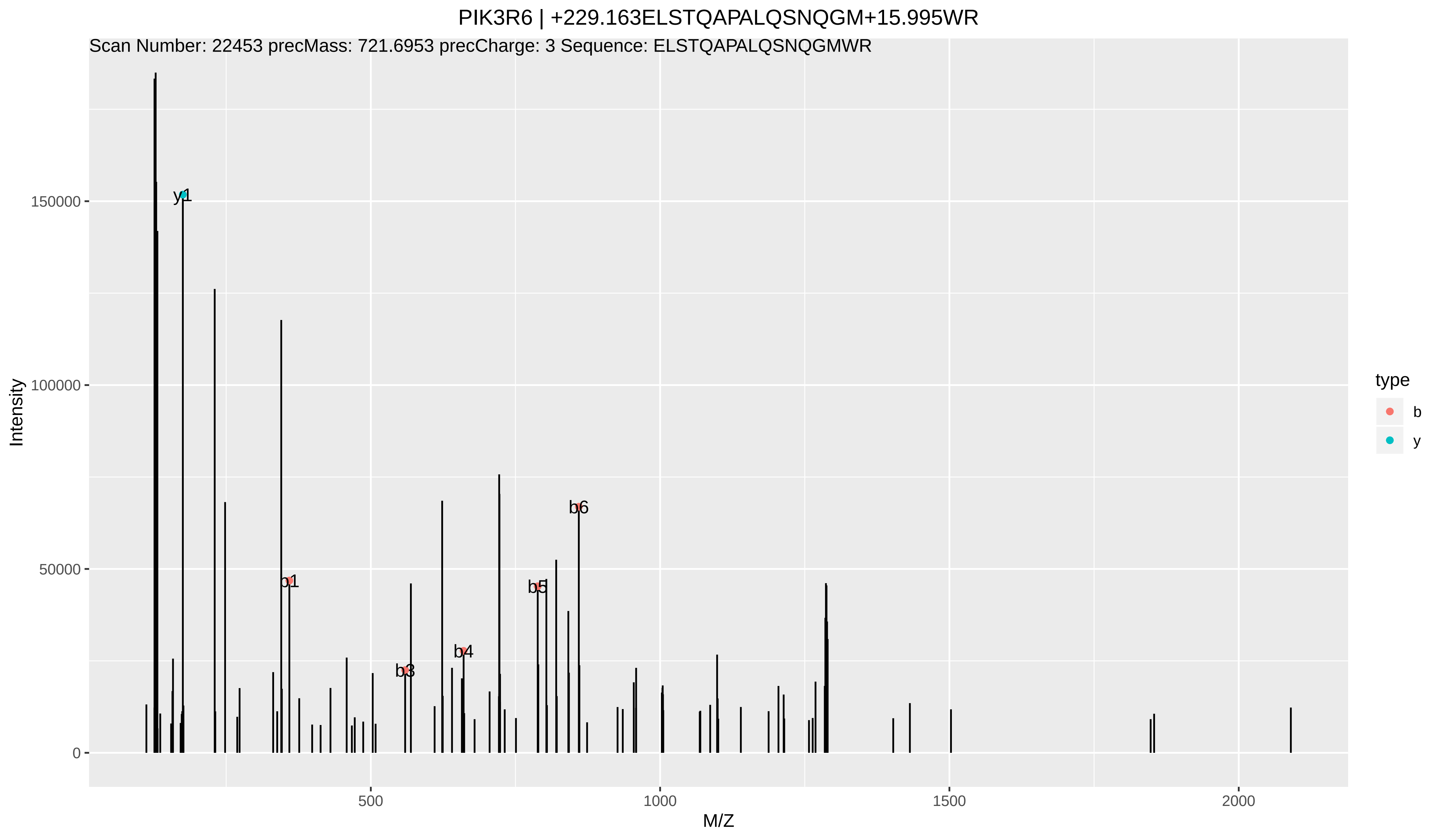
PIK3R6 | +229.163ELSTQAPALQSNQGM+15.995WR
Scan Number: 22453 precMass: 721.6953 precCharge: 3 Sequence: ELSTQAPALQSNQGMWR
y1
b1
b3
b4
b5
b6
150000
100000
50000
0
Intensity
500
1000
1500
2000
M/Z
type
b
y

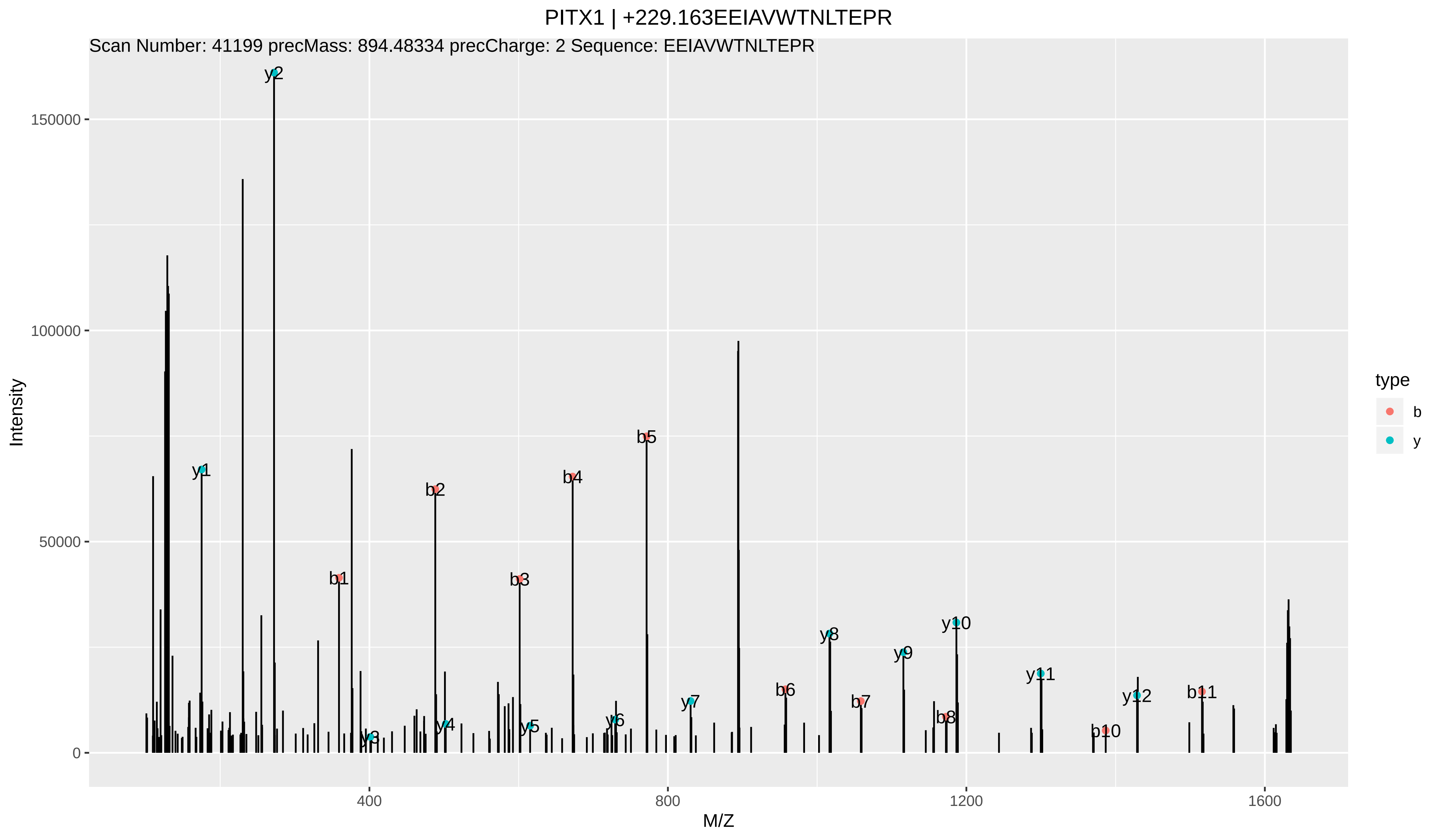

PITX1 | +229.163EEIAVWTNLTEPR
Scan Number: 41199 precMass: 894.48334 precCharge: 2 Sequence: EEIAVWTNLTEPR
Intensity
M/Z
type
b
y
y1
y2
y3
y4
y5
y6
y7
y8
y9
y10
y11
y12
b1
b2
b3
b4
b5
b6
b7
b8
b10
b11
150000
100000
50000
0
400
800
1200
1600

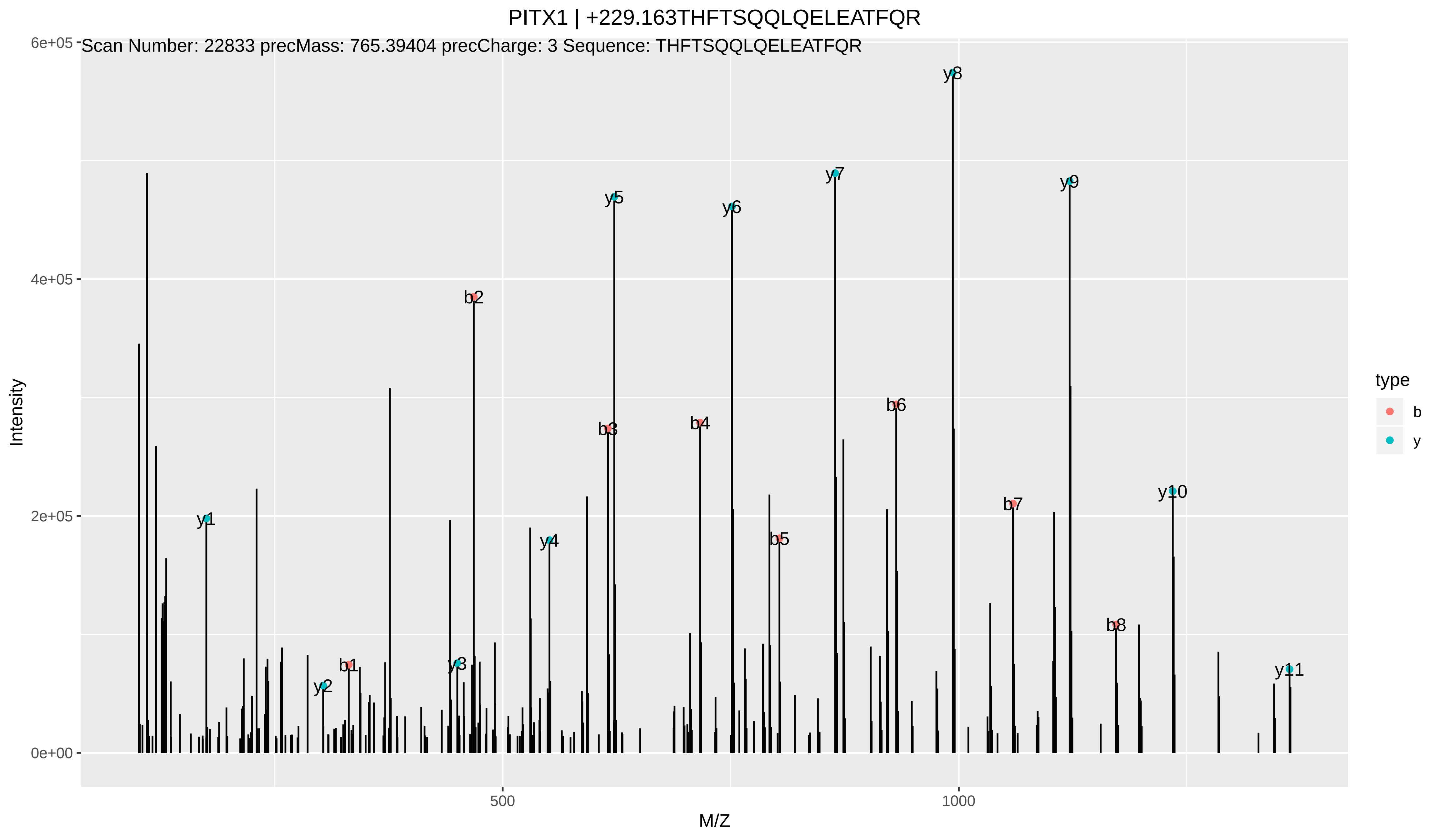

PITX1 | +229.163THFTSQQLQELEATFQR
Scan Number: 22833 precMass: 765.39404 precCharge: 3 Sequence: THFTSQQLQELEATFQR
Intensity
M/Z
6e+05
4e+05
2e+05
0e+00
500
1000
y1
y2
b1
y3
b2
y4
b3
y5
b4
y6
b5
y7
b6
y8
b7
y9
b8
y10
y11
type
b
y

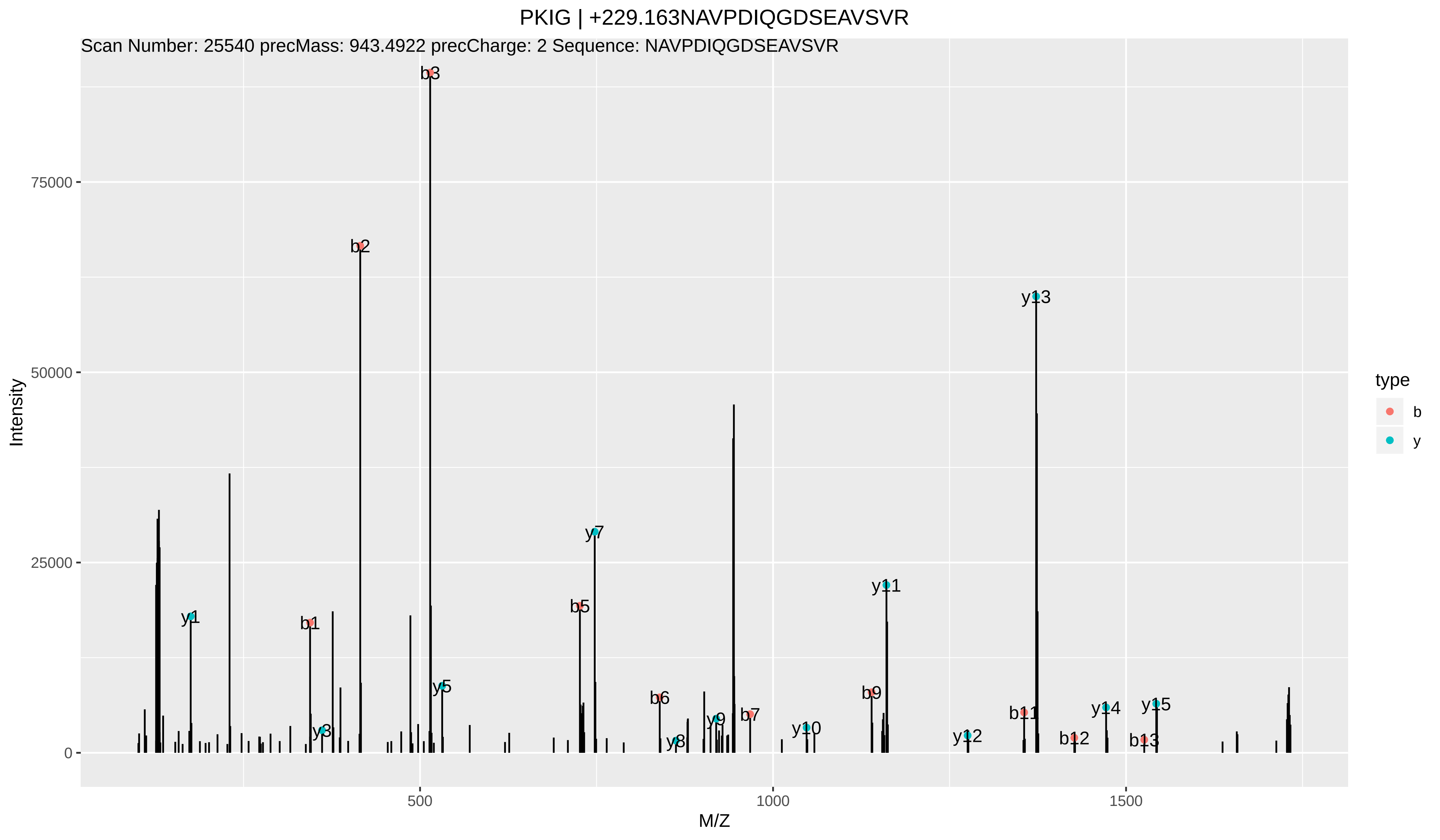

PKIG | +229.163NAVPDIQGDSEAVSVR
Scan Number: 25540 precMass: 943.4922 precCharge: 2 Sequence: NAVPDIQGDSEAVSVR
Intensity
M/Z
0
25000
50000
75000
500
1000
1500
b3
b2
y13
y7
y11
b5
y1
b1
y5
b9
b6
y15
y14
b11
b7
y9
y3
y10
y12
b12
b13
y8
type
b
y

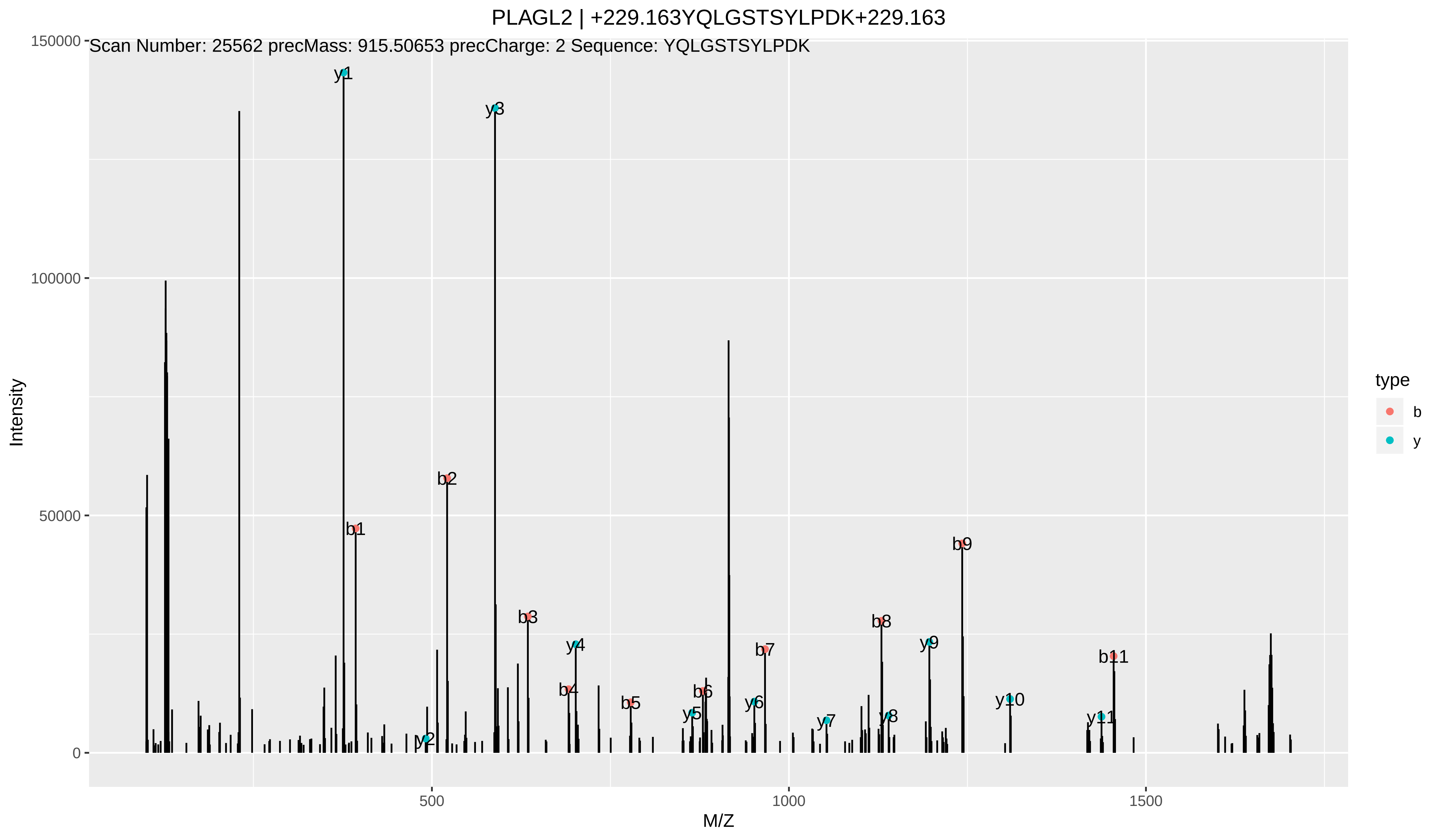
PLAGL2 | +229.163YQLGSTSYLPDK+229.163
Scan Number: 25562 precMass: 915.50653 precCharge: 2 Sequence: YQLGSTSYLPDK
Intensity
M/Z
type
b
y
150000
100000
50000
0
500
1000
1500
y1
y3
b2
b1
b9
b3
b8
y4
y9
b7
b11
b4
b6
b5
y6
y10
y5
y8
y11
y7
y2

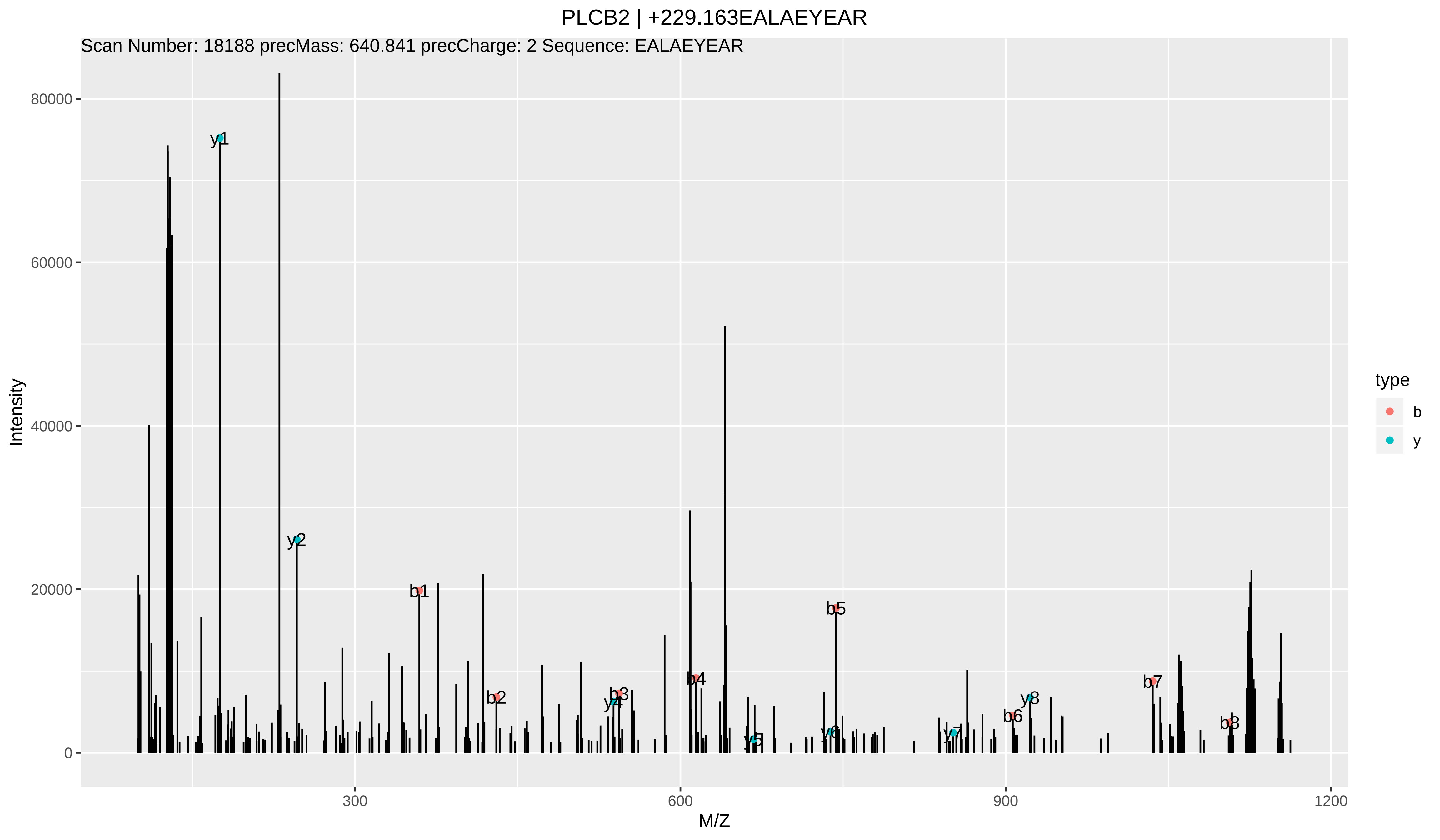

PLCB2 | +229.163EALAEYEAR
Scan Number: 18188 precMass: 640.841 precCharge: 2 Sequence: EALAEYEAR
Intensity
M/Z
type
b
y
y1
y2
b1
b2
b3
y4
b4
y5
y6
b5
y7
b6
y8
b7
b8
80000
60000
40000
20000
0
300
600
900
1200

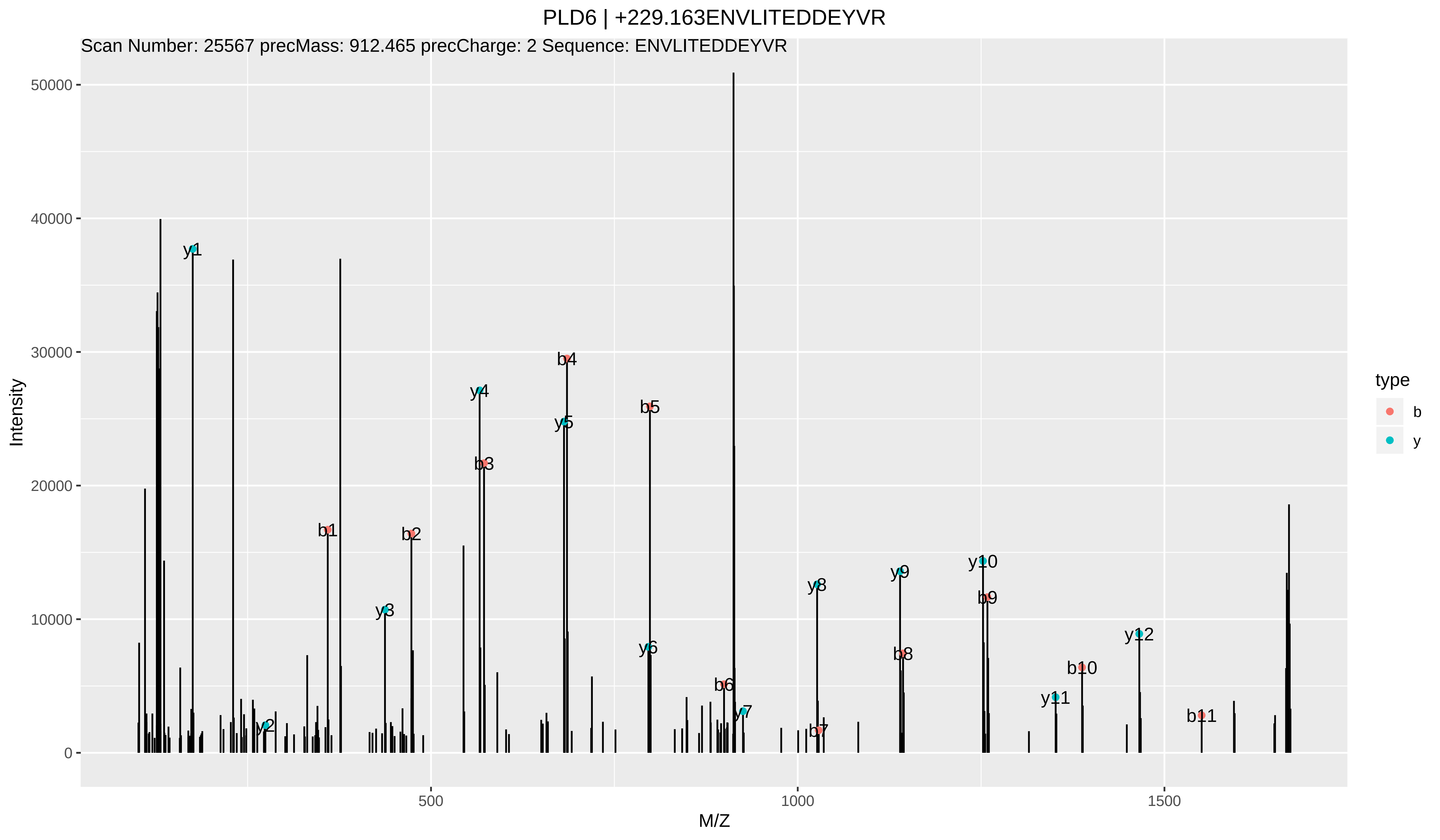
PLD6 | +229.163ENVLITEDDEYVR
Scan Number: 25567 precMass: 912.465 precCharge: 2 Sequence: ENVLITEDDEYVR
Intensity
M/Z
0
10000
20000
30000
40000
50000
500
1000
1500
y1
y2
b1
y3
b2
y4
b3
b4
y5
b5
y6
b6
y7
b7
y8
y9
b8
y10
b9
y11
b10
y12
b11
type
b
y

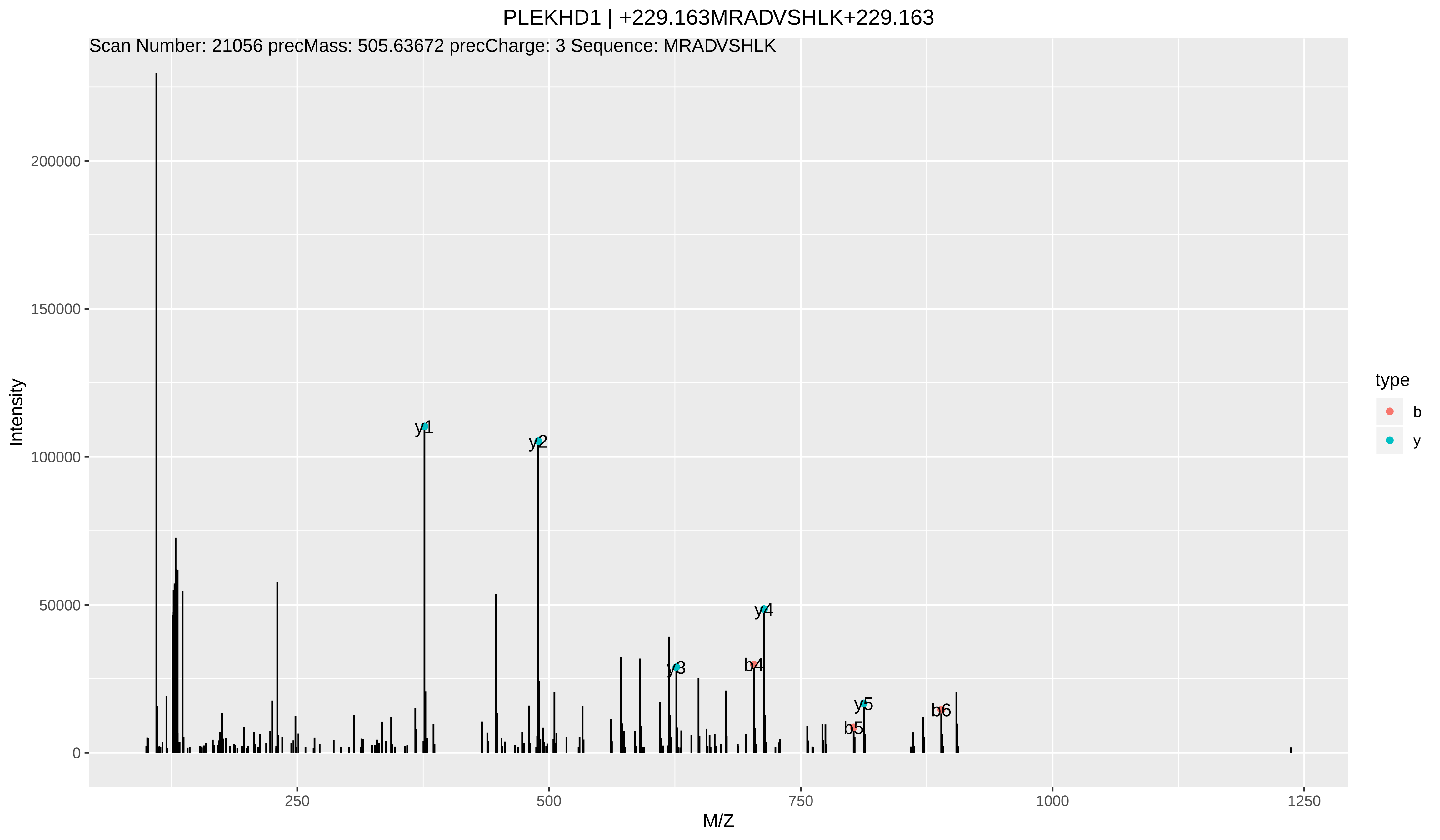

PLEKHD1 | +229.163MRADVSHLK+229.163
Scan Number: 21056 precMass: 505.63672 precCharge: 3 Sequence: MRADVSHLK
Intensity
0
50000
100000
150000
200000
M/Z
250
500
750
1000
1250
y1
y2
y3
y4
y5
b4
b5
b6
type
b
y

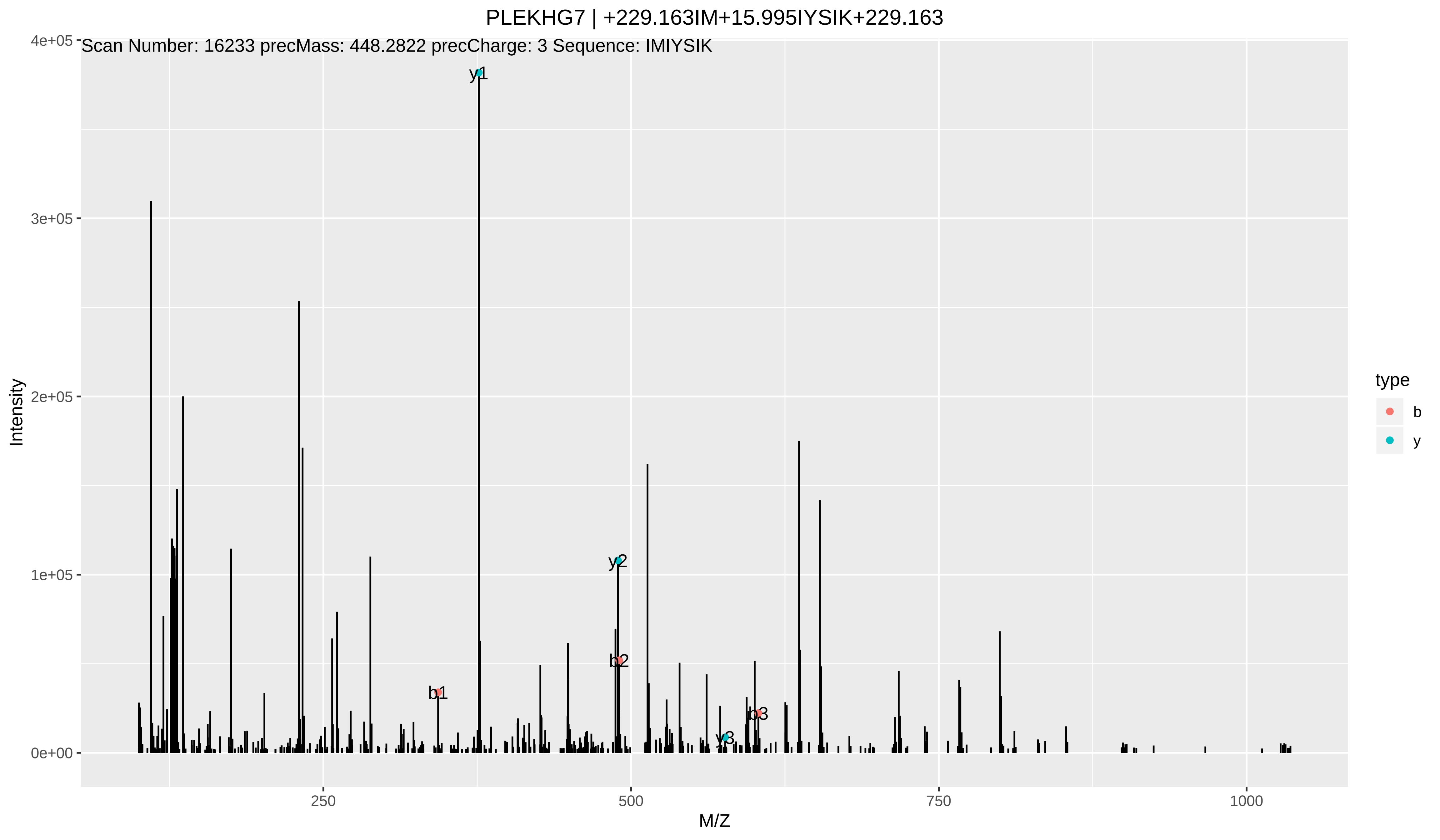
PLEKHG7 | +229.163IM+15.995IYSIK+229.163
Scan Number: 16233 precMass: 448.2822 precCharge: 3 Sequence: IMIYSIK
Intensity
4e+05
3e+05
2e+05
1e+05
0e+00
M/Z
250
500
750
1000
y1
y2
b2
b1
b3
y3
type
b
y

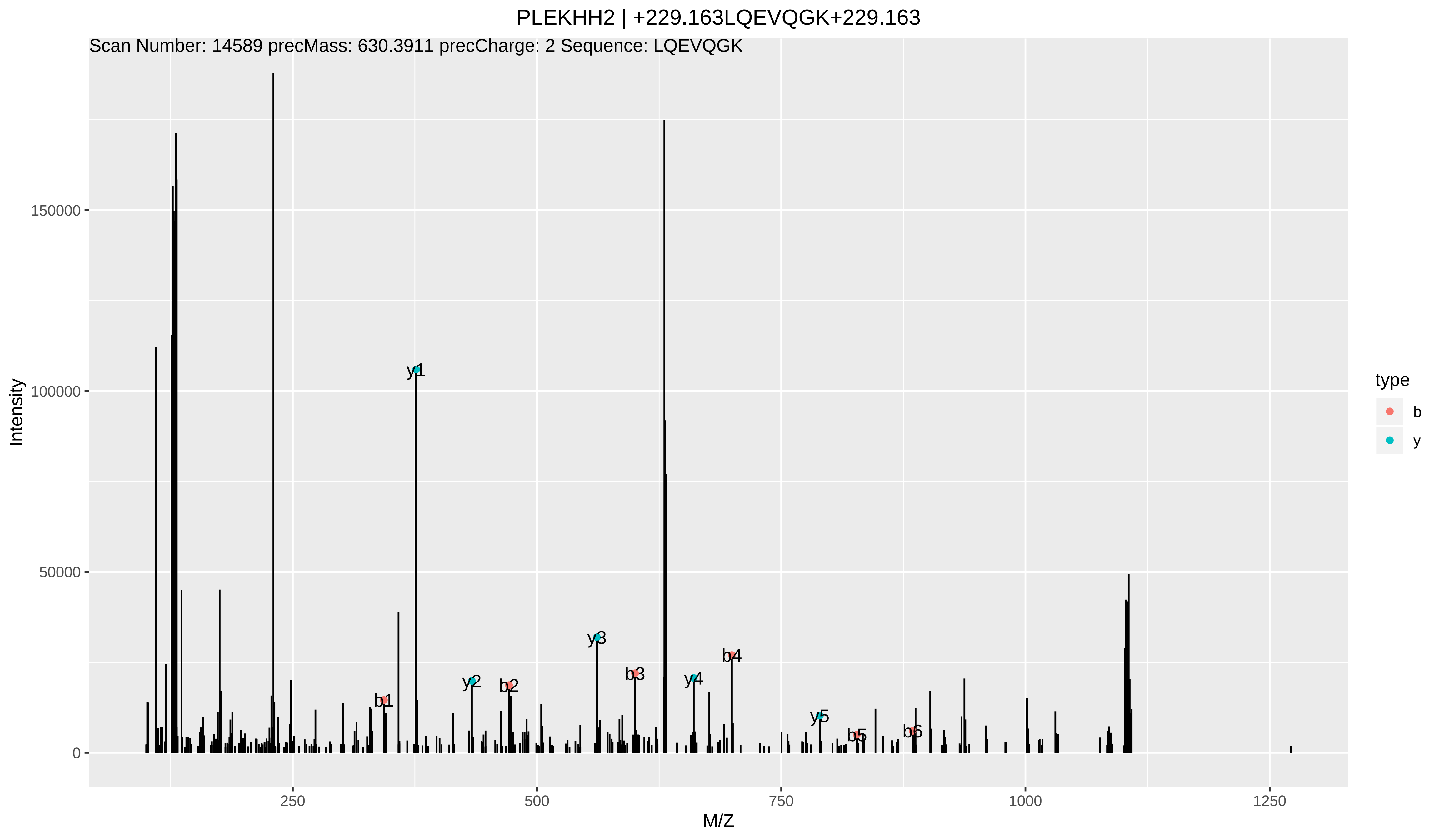
PLEKHH2 | +229.163LQEVQGK+229.163
Scan Number: 14589 precMass: 630.3911 precCharge: 2 Sequence: LQEVQGK
Intensity
150000
100000
50000
0
250
500
750
1000
1250
M/Z
y1
y2
y3
y4
y5
b1
b2
b3
b4
b5
b6
type
b
y

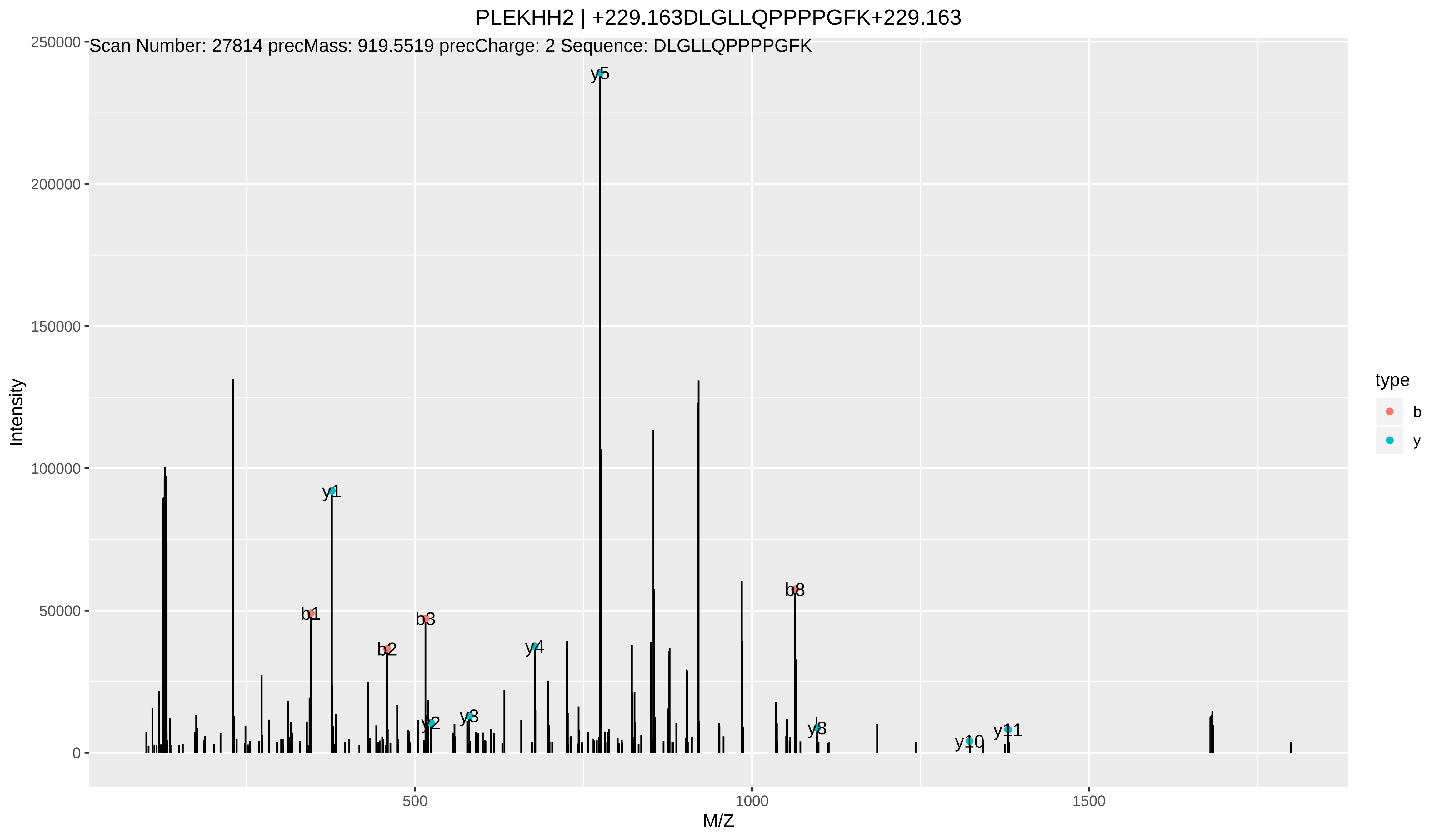
PLEKHH2 | +229.163DLGLLQPPPPGFK+229.163
Scan Number: 27814 precMass: 919.5519 precCharge: 2 Sequence: DLGLLQPPPPGFK
Intensity
M/Z
0
50000
100000
150000
200000
250000
500
1000
1500
y5
y1
b1
b2
b3
y2
y3
y4
b8
y8
y10
y11
type
b
y

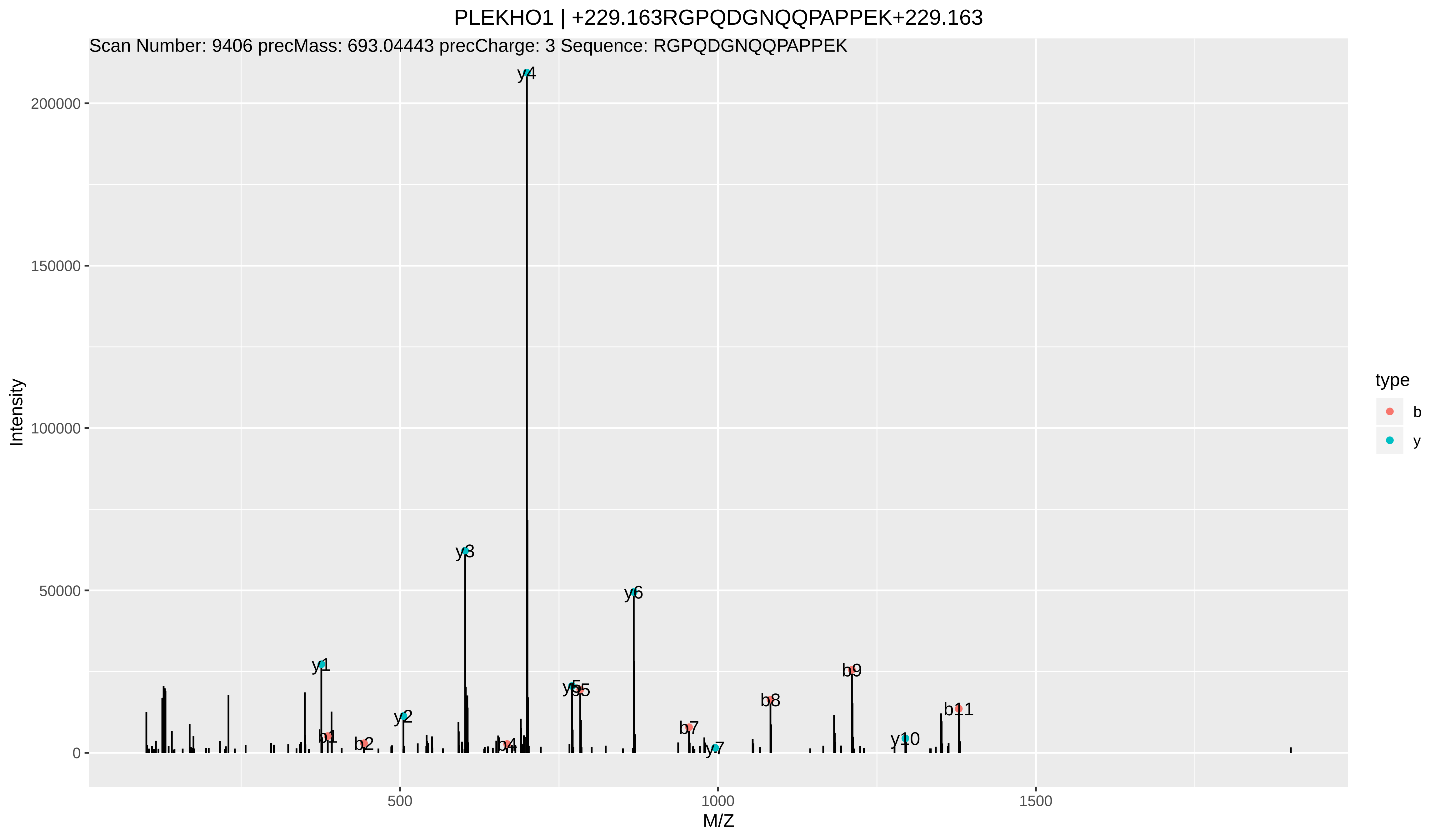
PLEKHO1 | +229.163RGPQDGNQQPAPPEK+229.163
Scan Number: 9406 precMass: 693.04443 precCharge: 3 Sequence: RGPQDGNQQPAPPEK
Intensity
M/Z
type
b
y
y4
y3
y6
y1
y5
b5
b9
b8
b11
y2
b7
b1
b2
b4
y7
y10
0
50000
100000
150000
200000
500
1000
1500

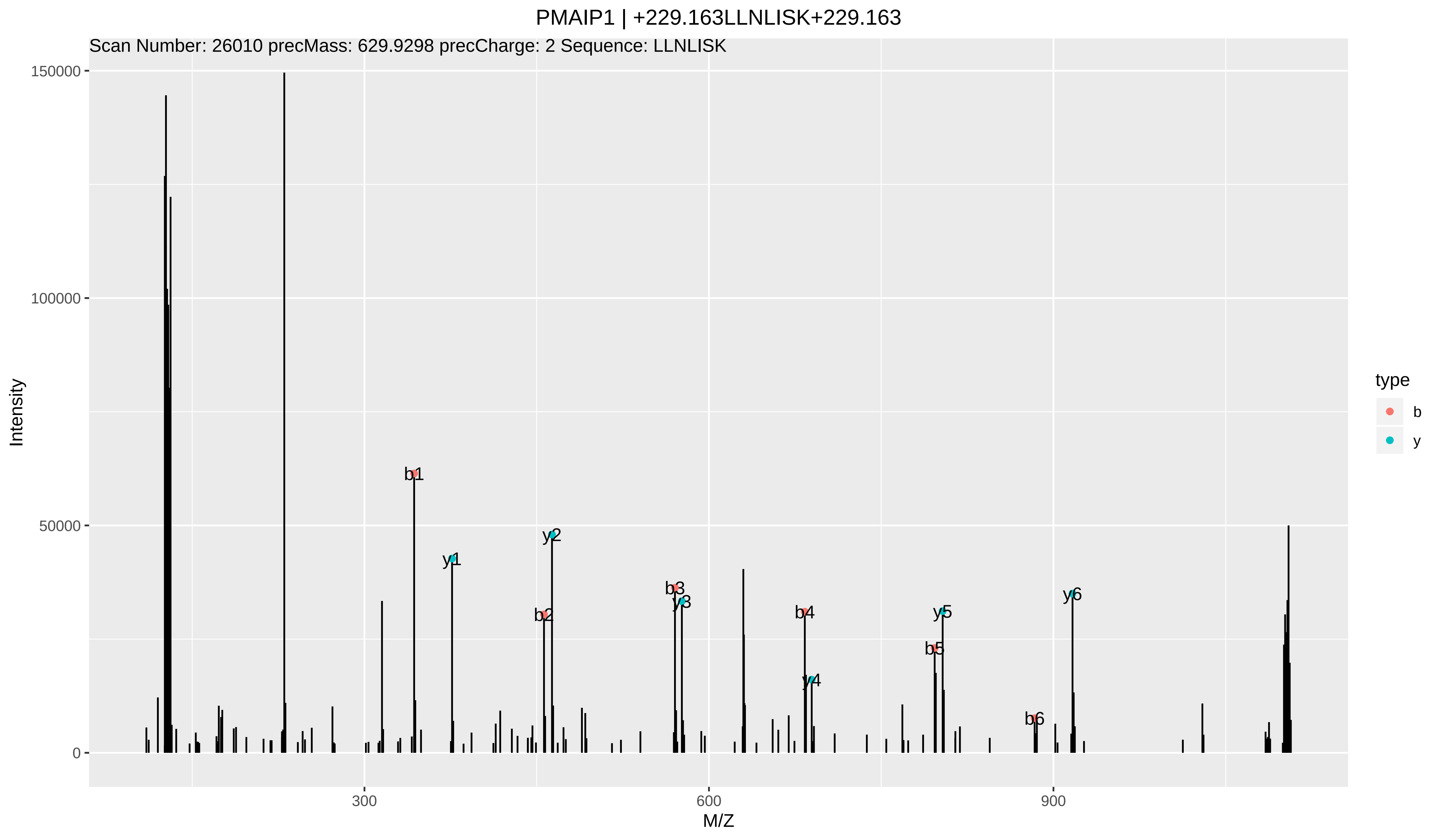

PMAIP1 | +229.163LLNLISK+229.163
Scan Number: 26010 precMass: 629.9298 precCharge: 2 Sequence: LLNLISK
Intensity
150000
100000
50000
0
300
600
900
M/Z
b1
y1
y2
b2
b3
y3
b4
y4
y5
b5
b6
y6
type
b
y

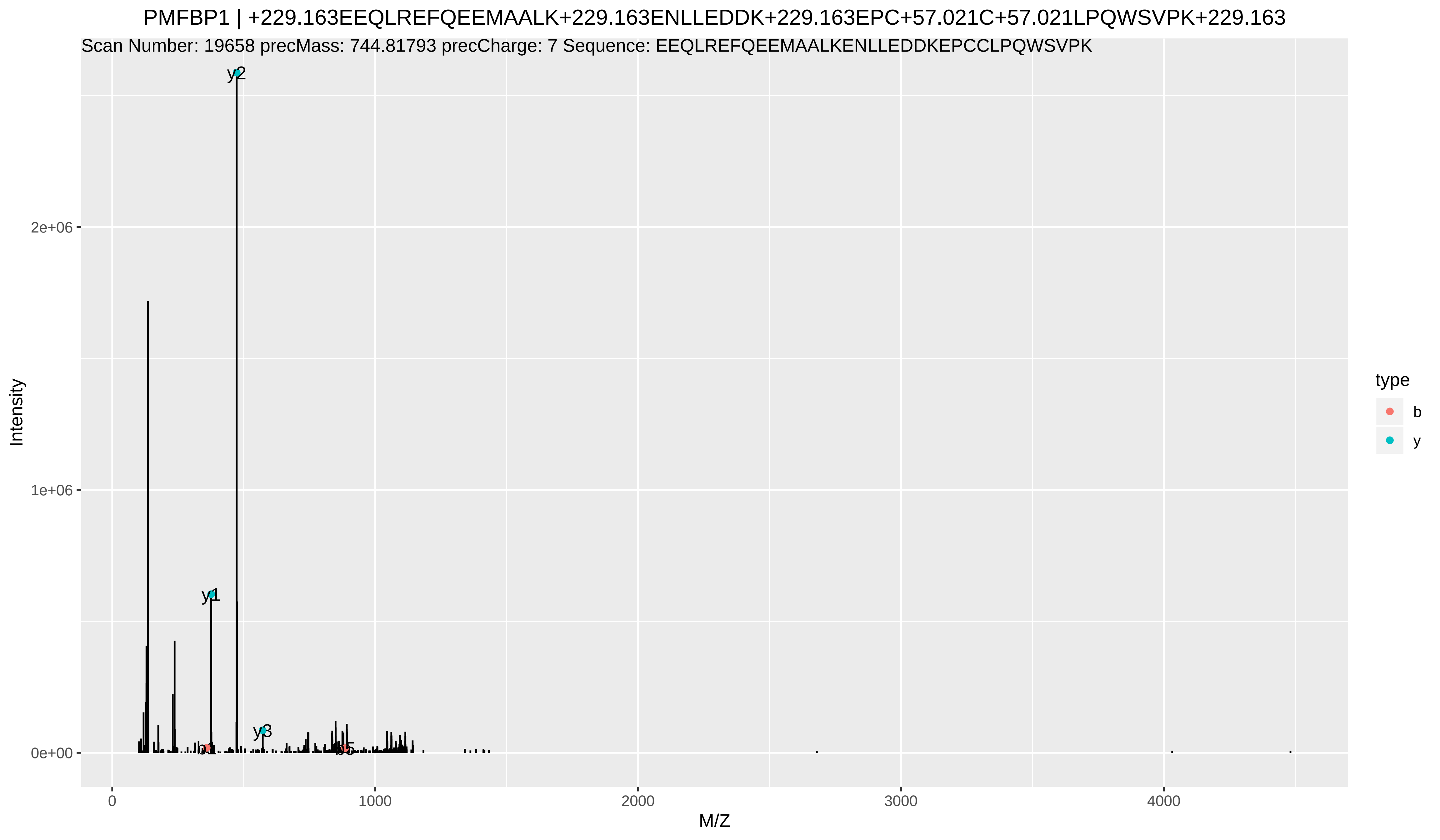

PMFBP1 | +229.163EEQLREFQEEMAALK+229.163ENLLEDDK+229.163EPC+57.021C+57.021LPQWSVPK+229.163
Scan Number: 19658 precMass: 744.81793 precCharge: 7 Sequence: EEQLREFQEEMAALKENLLEDDKEPCCLPQWSVPK
y2
y1
y3
b1
b5
Intensity
2e+06
1e+06
0e+00
0
1000
2000
3000
4000
M/Z
type
b
y

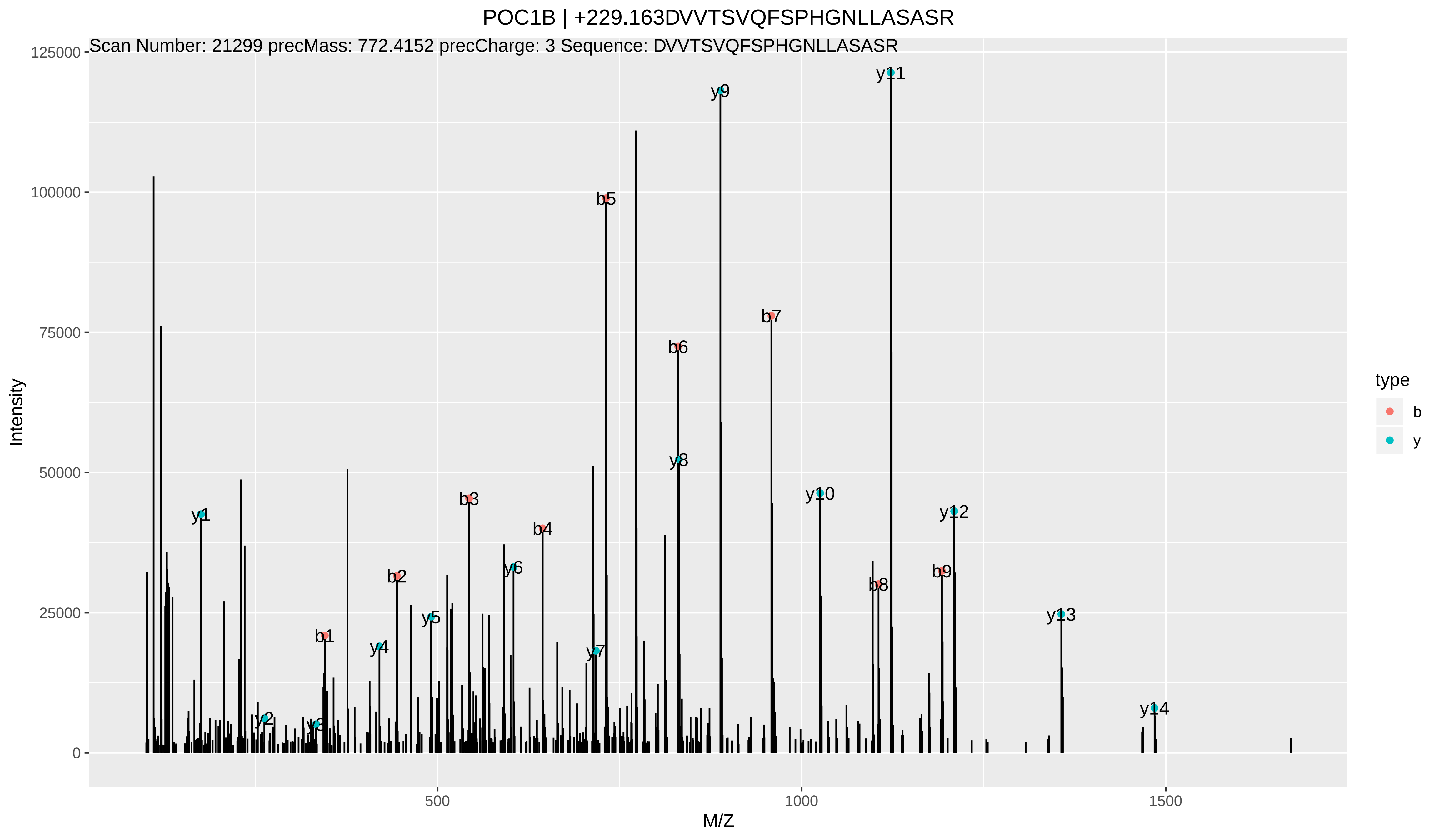

POC1B | +229.163DVVTSVQFSPHGNLLASASR
Scan Number: 21299 precMass: 772.4152 precCharge: 3 Sequence: DVVTSVQFSPHGNLLASASR
Intensity
M/Z
0
25000
50000
75000
100000
125000
500
1000
1500
y1
y2
y3
b1
y4
b2
y5
b3
y6
b4
y7
b5
b6
y8
y9
b7
y10
b8
y11
b9
y12
y13
y14
type
b
y

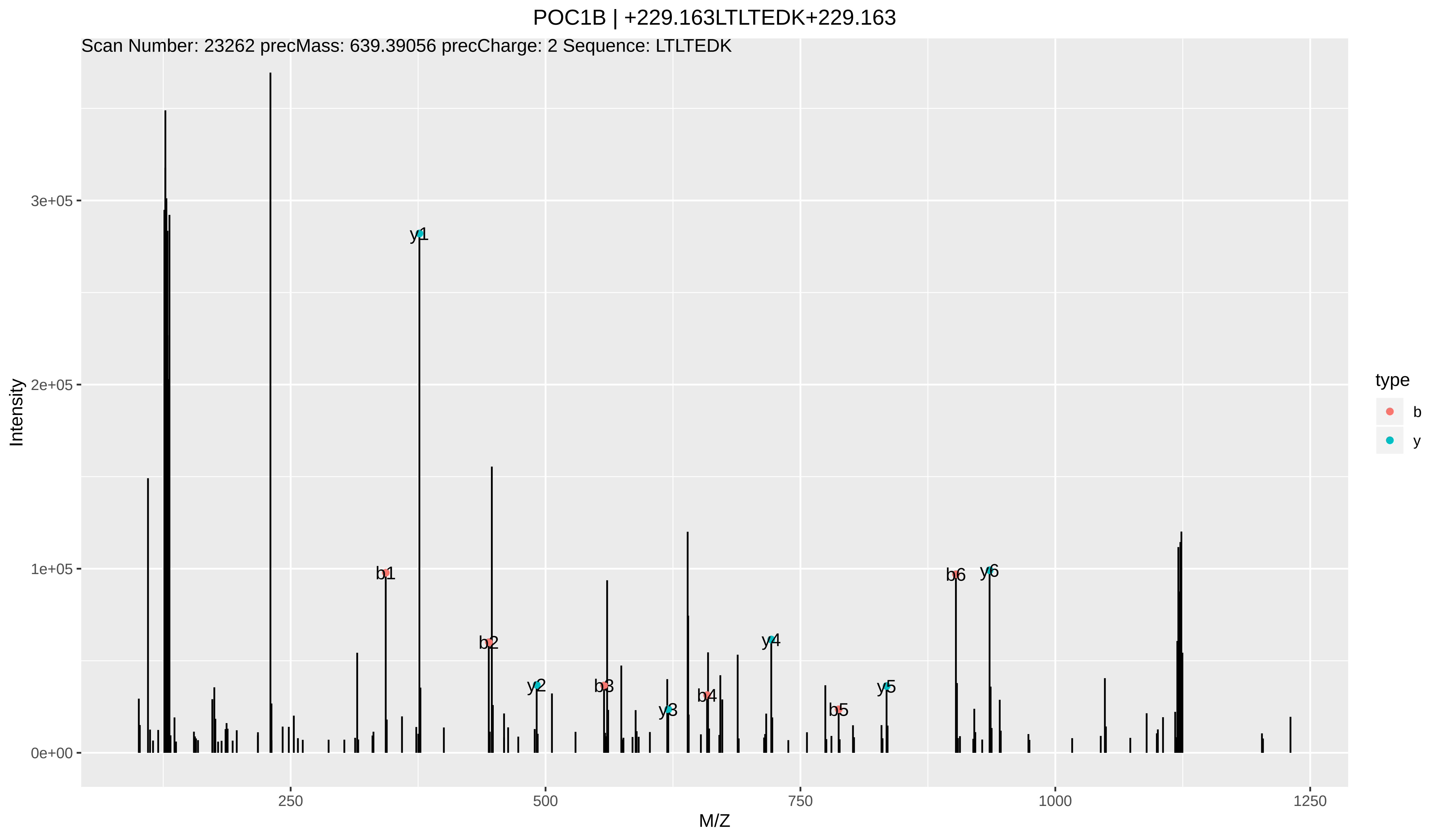

POC1B | +229.163LTLTEDK+229.163
Scan Number: 23262 precMass: 639.39056 precCharge: 2 Sequence: LTLTEDK
Intensity
3e+05
2e+05
1e+05
0e+00
M/Z
250
500
750
1000
1250
y1
b1
b2
y2
b3
y3
b4
y4
b5
y5
b6
y6
type
b
y

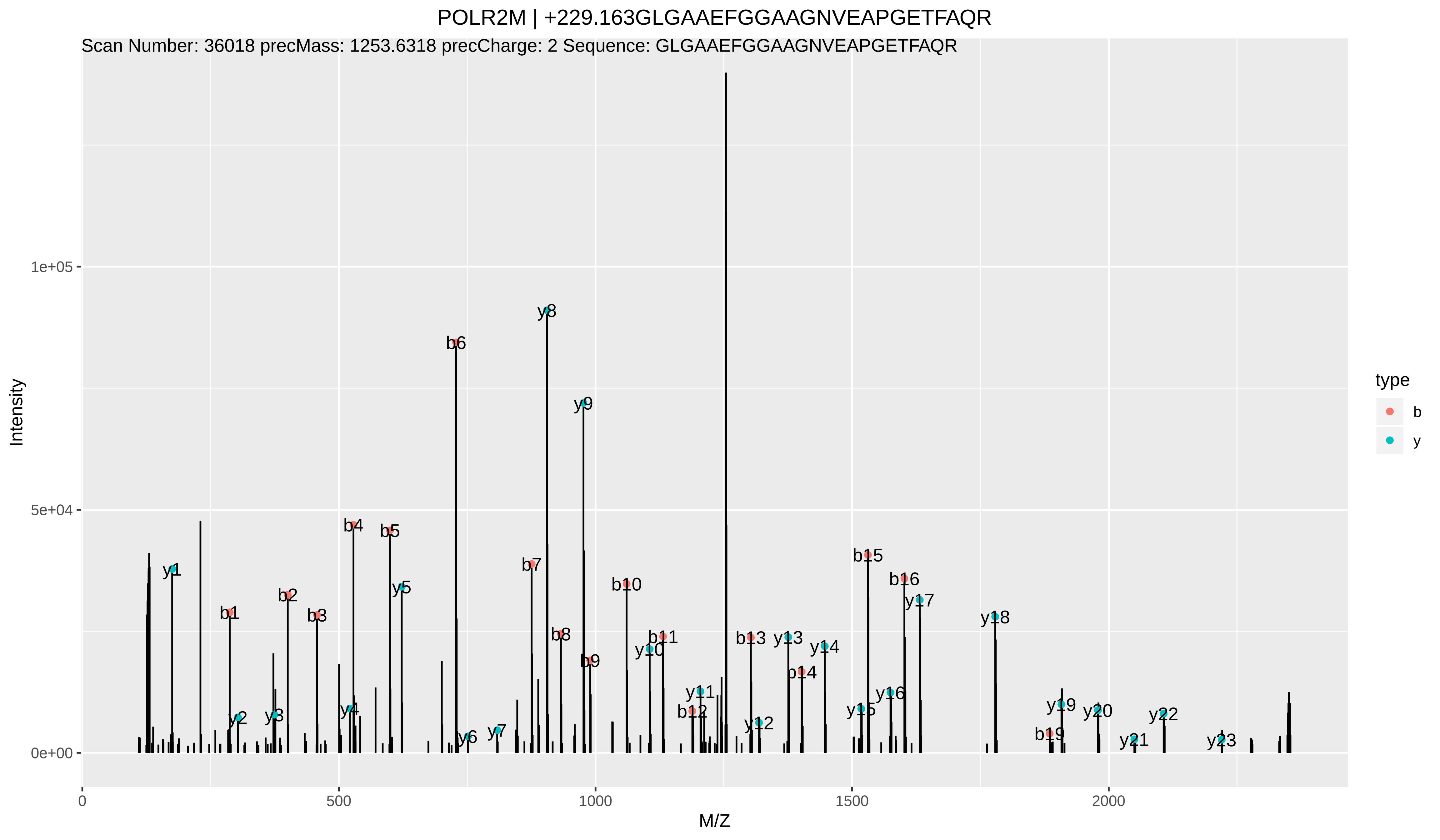
POLR2M | +229.163GLGAAEFGGAAGNVEAPGETFAQR
Scan Number: 36018 precMass: 1253.6318 precCharge: 2 Sequence: GLGAAEFGGAAGNVEAPGETFAQR
Intensity
M/Z
0e+00
5e+04
1e+05
0
500
1000
1500
2000
type
b
y
y1
b1
y2
b2
y3
b3
y4
b4
b5
y5
b6
y6
y7
b7
y8
b8
y9
b9
b10
y10
b11
y11
b12
b13
y12
y13
b14
y14
y15
b15
y16
b16
y17
y18
b19
y19
y20
y21
y22
y23

POLR2M | +229.163SSQTSQNQGLGRPTLEGDEETSEVEYTVNK+229.163

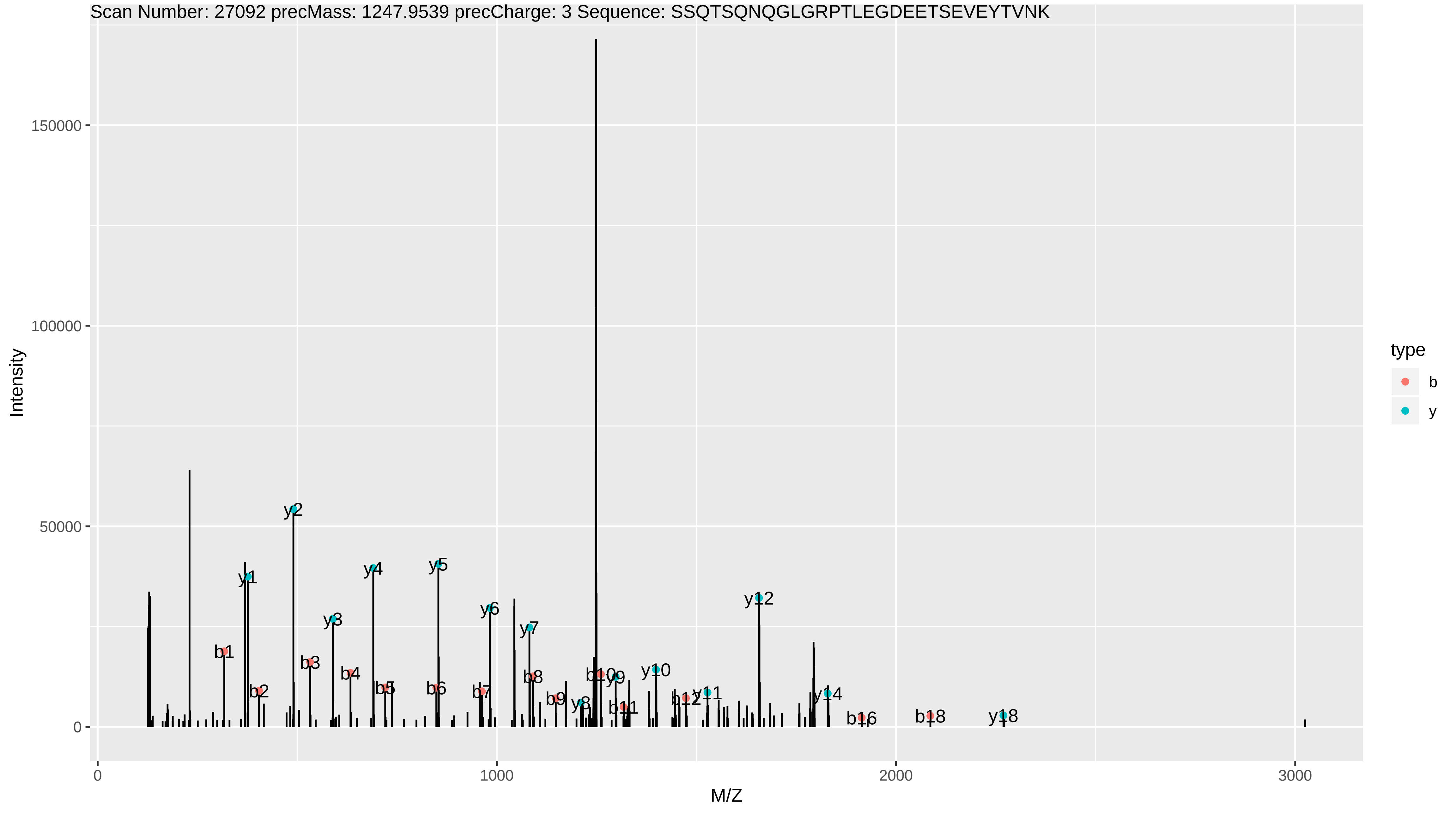

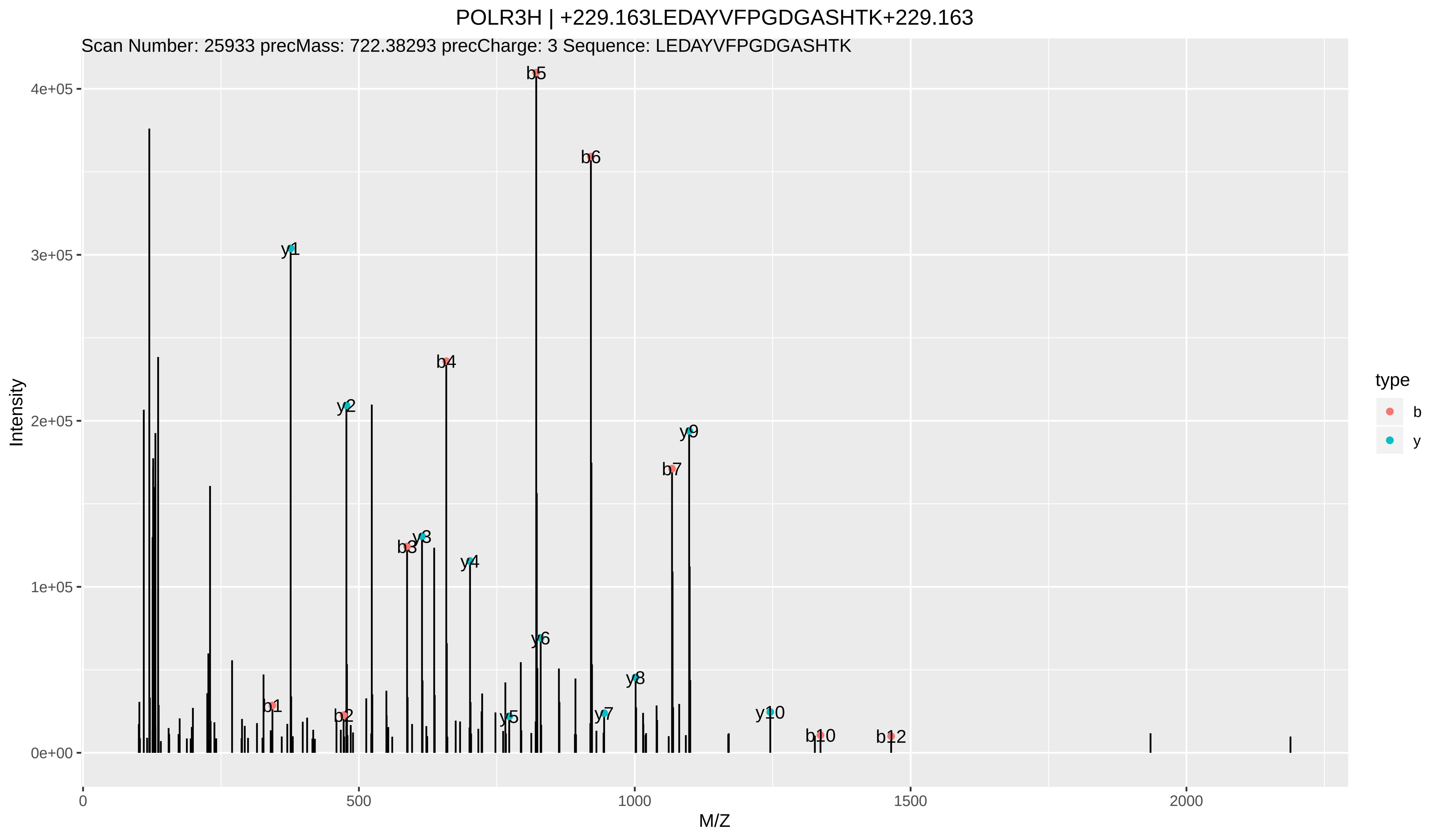

POLR3H | +229.163LEDAYVFPGDGASHTK+229.163
Scan Number: 25933 precMass: 722.38293 precCharge: 3 Sequence: LEDAYVFPGDGASHTK
Intensity
M/Z
4e+05
3e+05
2e+05
1e+05
0e+00
0
500
1000
1500
2000
b5
b6
y1
b4
y2
y9
b7
y3
b3
y4
y6
y8
b1
b2
y5
y7
y10
b10
b12
type
b
y

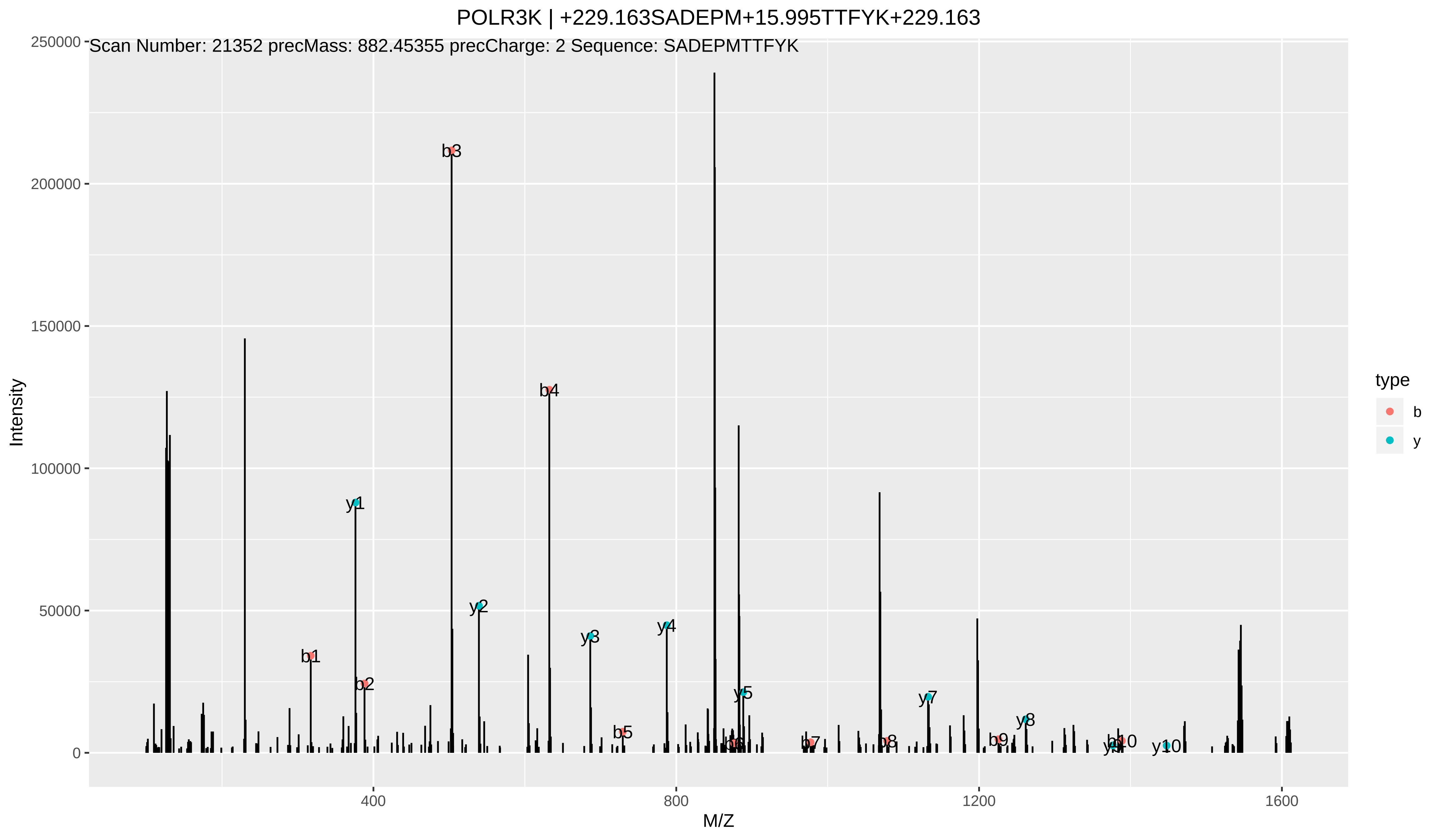
POLR3K | +229.163SADEPM+15.995TTFYK+229.163
Scan Number: 21352 precMass: 882.45355 precCharge: 2 Sequence: SADEPMTTFYK
Intensity
M/Z
type
b
y
b1
b2
b3
b4
b5
b6
b7
b8
b9
b10
y1
y2
y3
y4
y5
y7
y8
y9
y10
0
50000
100000
150000
200000
250000
400
800
1200
1600

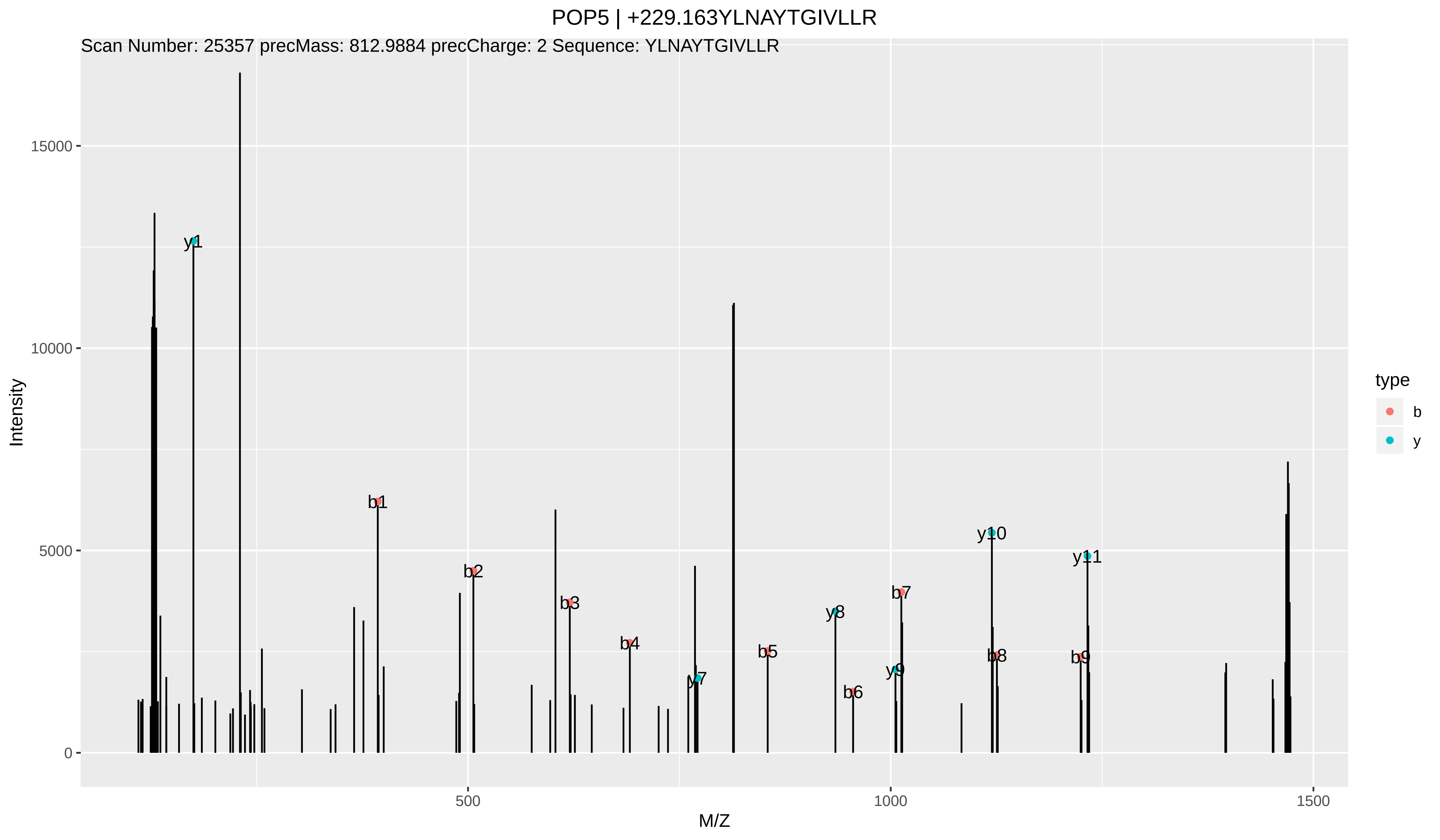

POP5 | +229.163YLNAYTGIVLLR
Scan Number: 25357 precMass: 812.9884 precCharge: 2 Sequence: YLNAYTGIVLLR
Intensity
M/Z
15000
10000
5000
0
500
1000
1500
y1
b1
b2
b3
b4
y7
b5
y8
b6
y9
b7
y10
b8
b9
y11
type
b
y

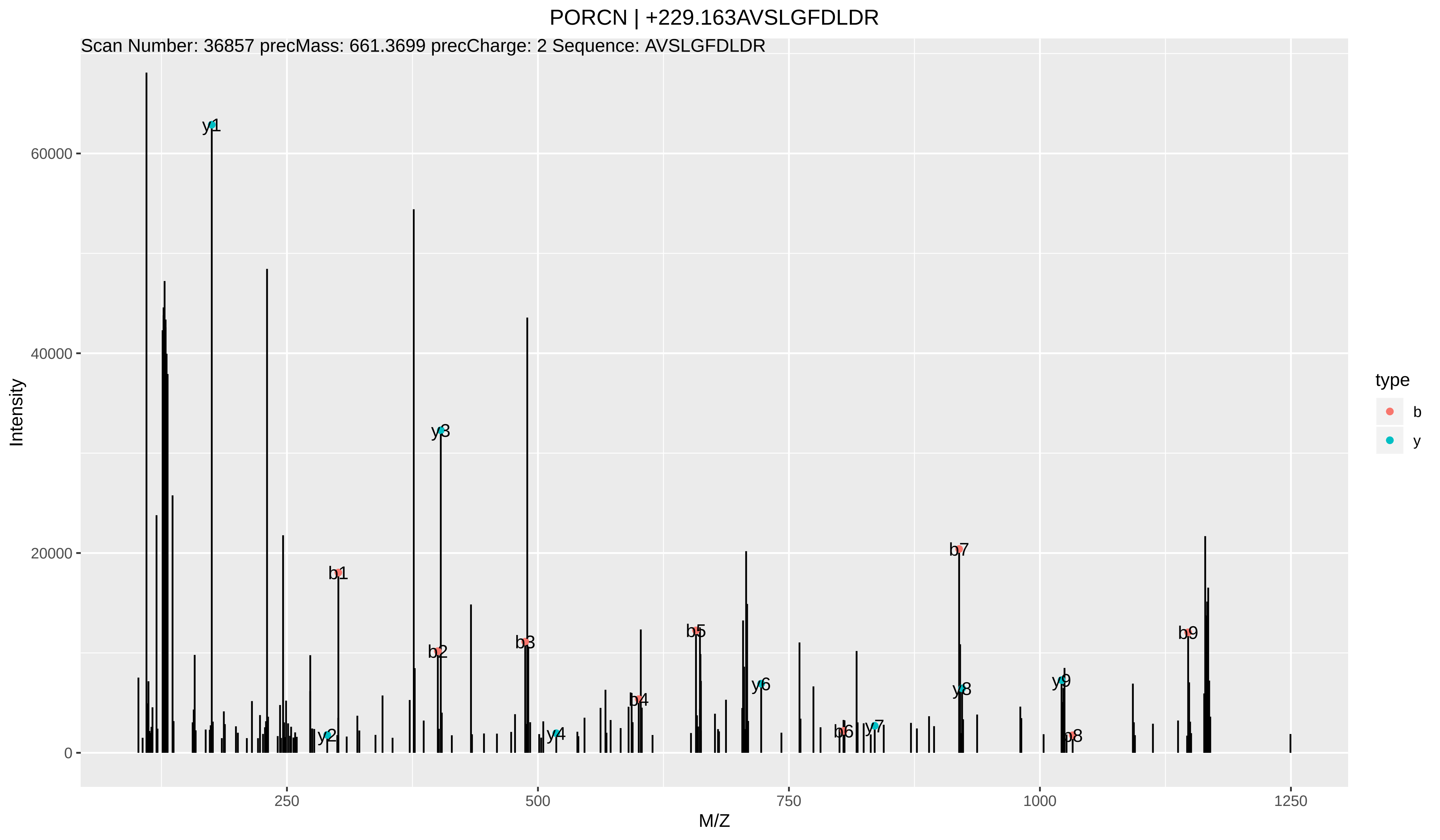
PORCN | +229.163AVSLGFDLDR
Scan Number: 36857 precMass: 661.3699 precCharge: 2 Sequence: AVSLGFDLDR
y1
y3
b1
b2
b3
y2
y4
b4
b5
y6
b6
y7
b7
y8
y9
b8
b9
Intensity
60000
40000
20000
0
250
500
750
1000
1250
M/Z
type
b
y

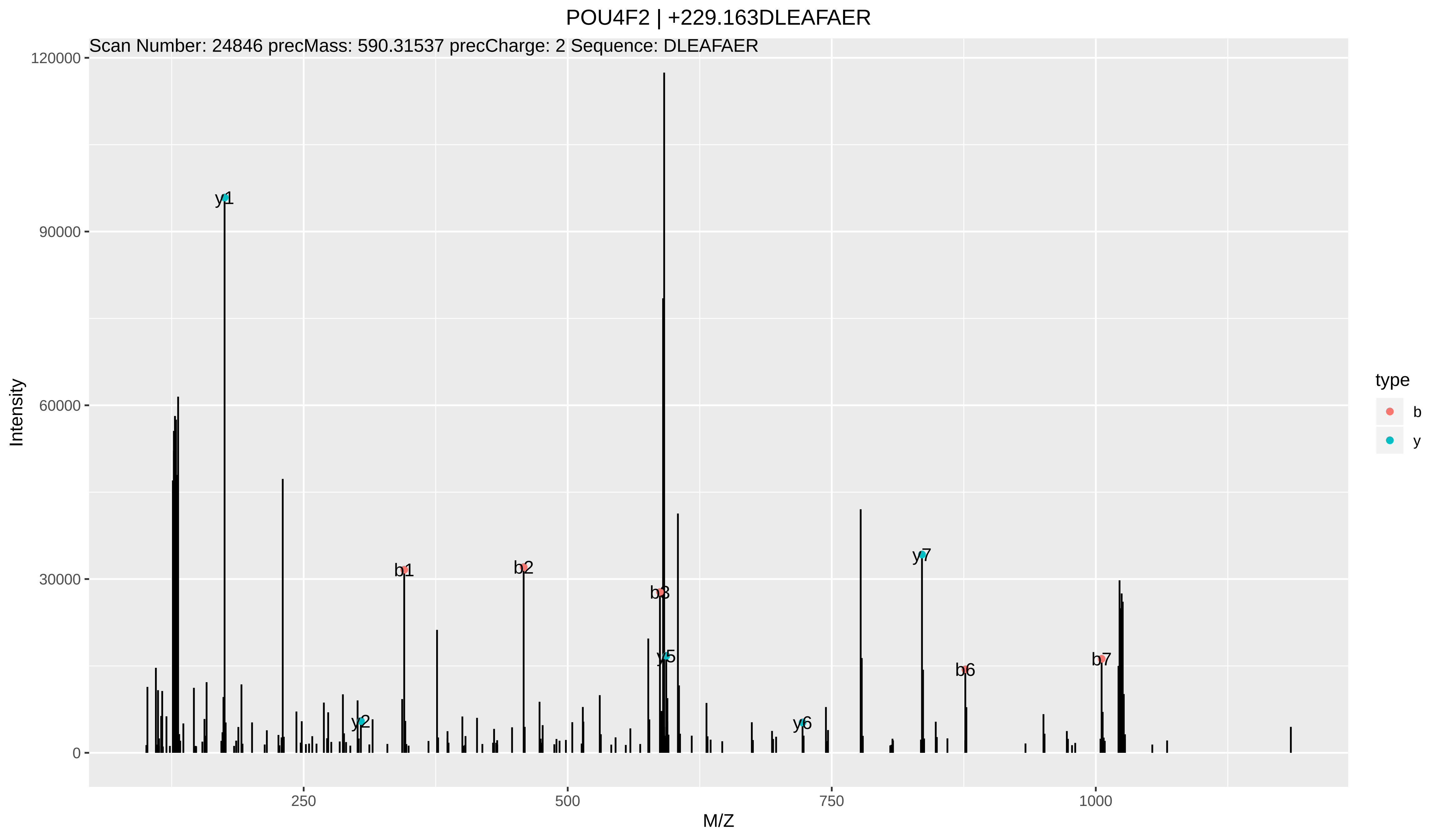
POU4F2 | +229.163DLEAFAER
Scan Number: 24846 precMass: 590.31537 precCharge: 2 Sequence: DLEAFAER
Intensity
M/Z
type
b
y
y1
y2
b1
b2
b3
y5
y6
y7
b6
b7
0
30000
60000
90000
120000
250
500
750
1000

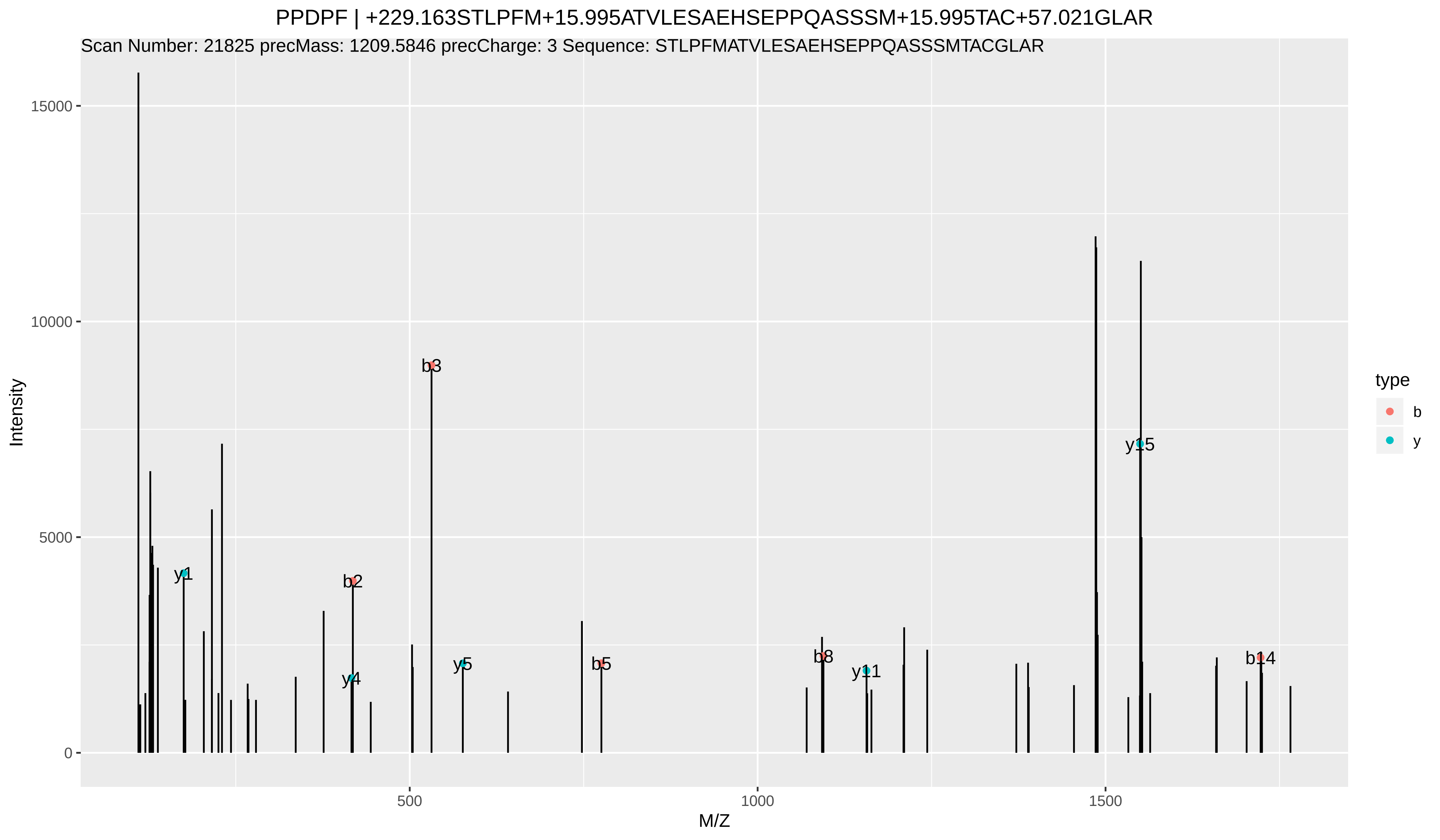

PPDPF | +229.163STLPFM+15.995ATVLESAEHSEPPQASSSM+15.995TAC+57.021GLAR
Scan Number: 21825 precMass: 1209.5846 precCharge: 3 Sequence: STLPFMATVLESAEHSEPPQASSSMTACGLAR
Intensity
M/Z
15000
10000
5000
0
500
1000
1500
y1
b2
y4
b3
y5
b5
b8
y11
y15
b14
type
b
y

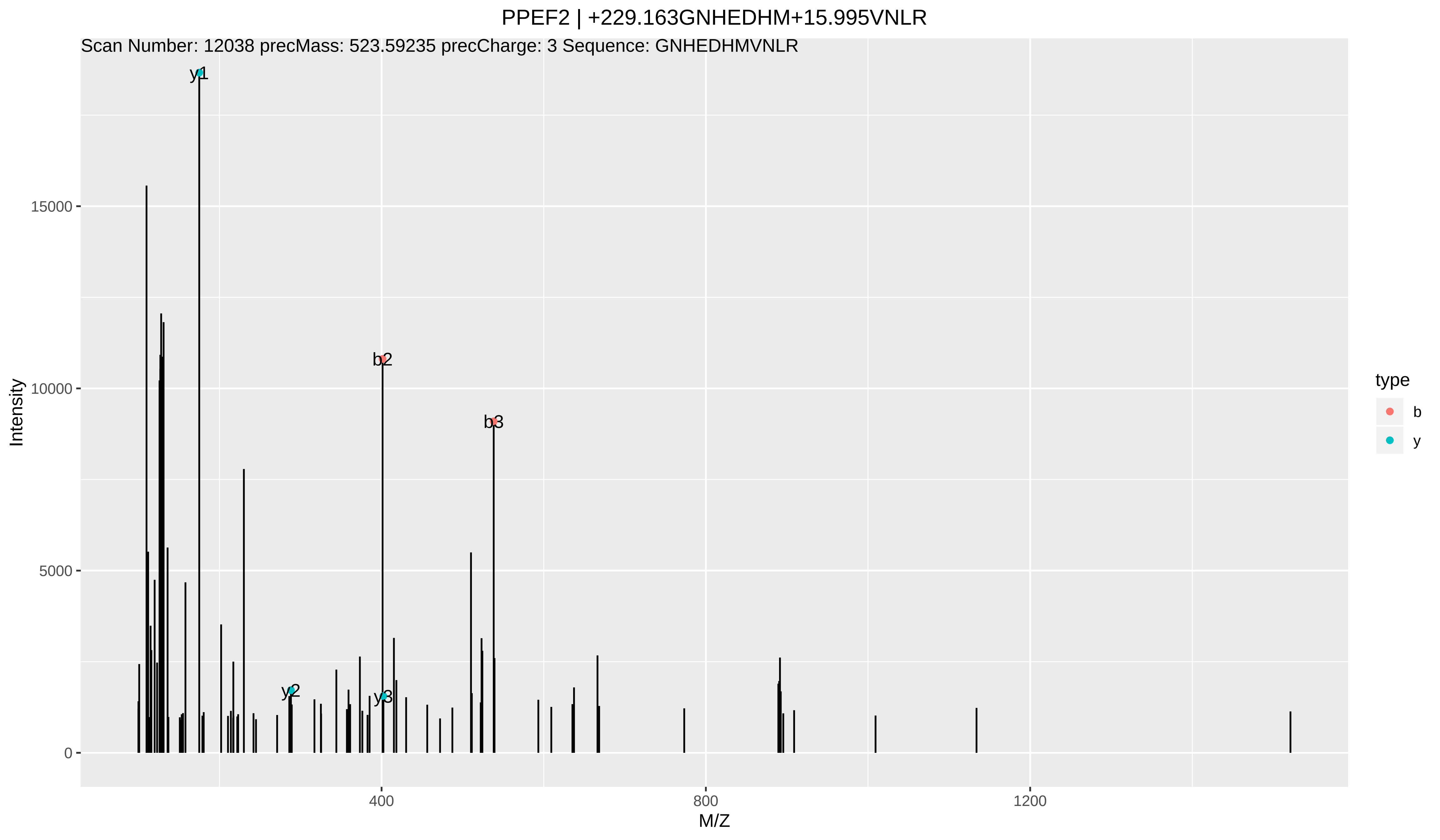
PPEF2 | +229.163GNHEDHM+15.995VNLR
Scan Number: 12038 precMass: 523.59235 precCharge: 3 Sequence: GNHEDHMVNLR
y1
b2
b3
y2
y3
Intensity
15000
10000
5000
0
400
800
1200
M/Z
type
b
y

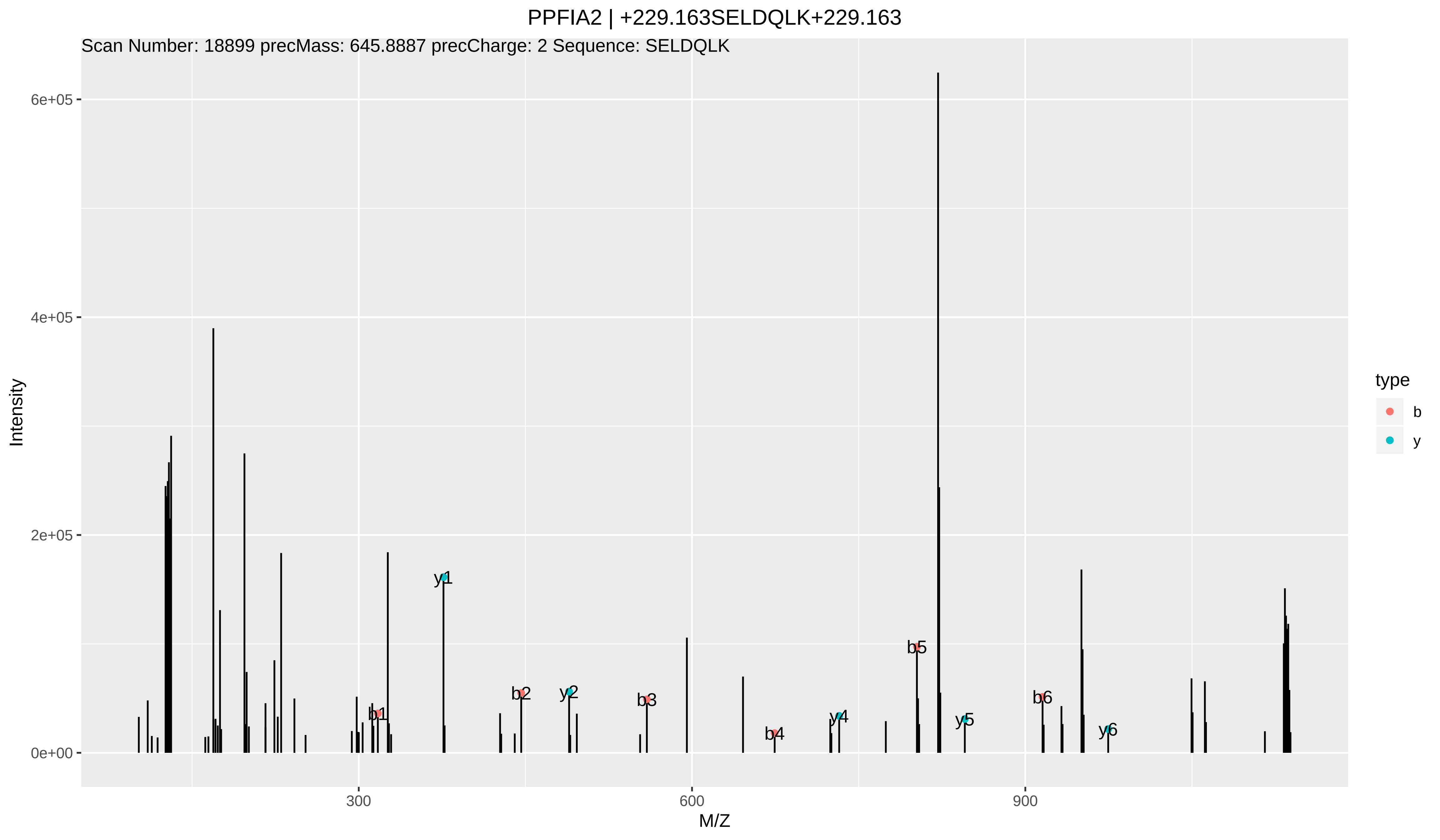

PPFIA2 | +229.163SELDQLK+229.163
Scan Number: 18899 precMass: 645.8887 precCharge: 2 Sequence: SELDQLK
Intensity
6e+05
4e+05
2e+05
0e+00
M/Z
300
600
900
y1
b1
b2
y2
b3
b4
y4
b5
y5
b6
y6
type
b
y

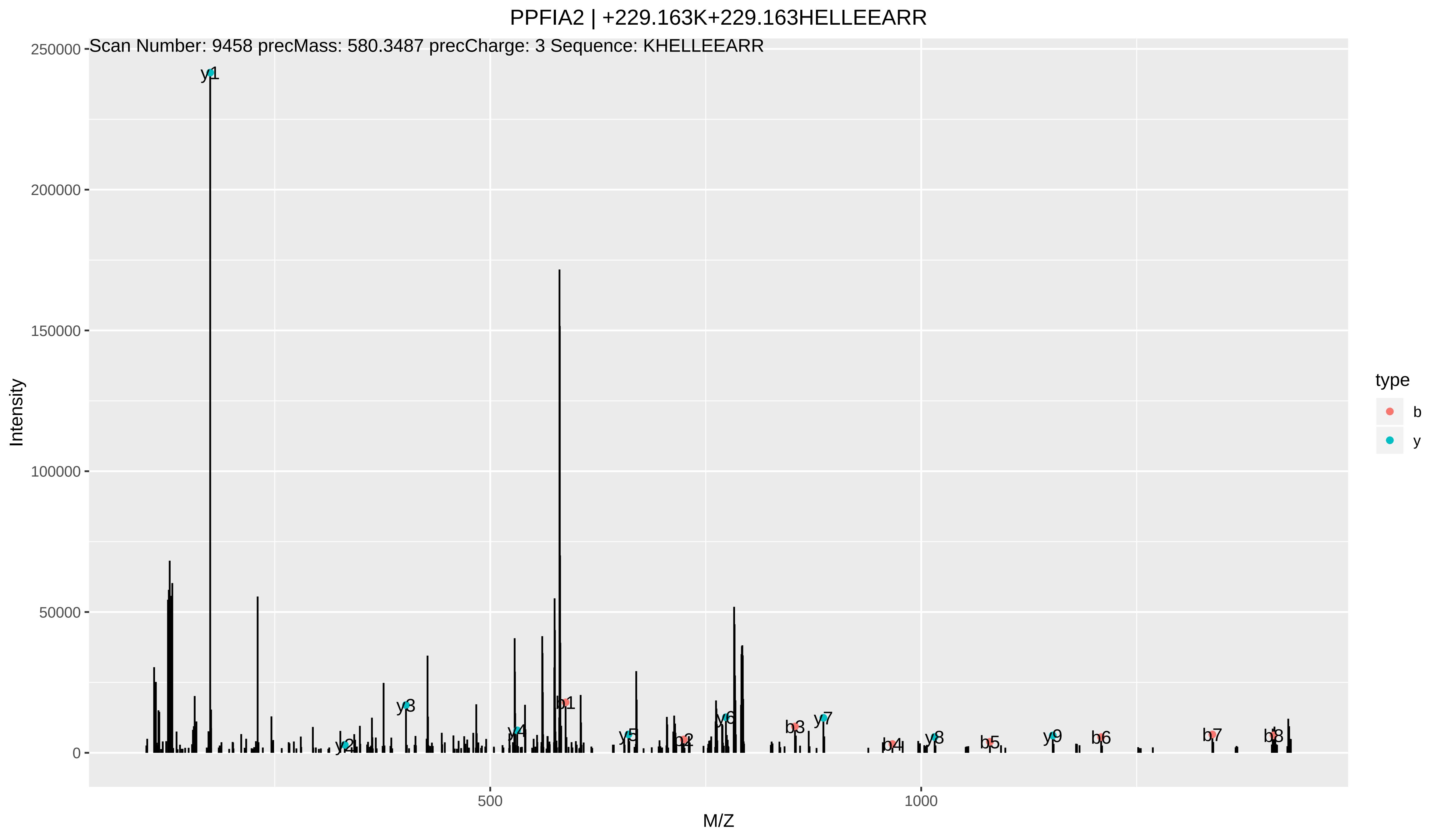
PPFIA2 | +229.163K+229.163HELLEEARR
Scan Number: 9458 precMass: 580.3487 precCharge: 3 Sequence: KHELLEEARR
Intensity
M/Z
0
50000
100000
150000
200000
250000
500
1000
y1
y2
y3
y4
y5
y6
y7
y8
y9
b1
b2
b3
b4
b5
b6
b7
b8
type
b
y

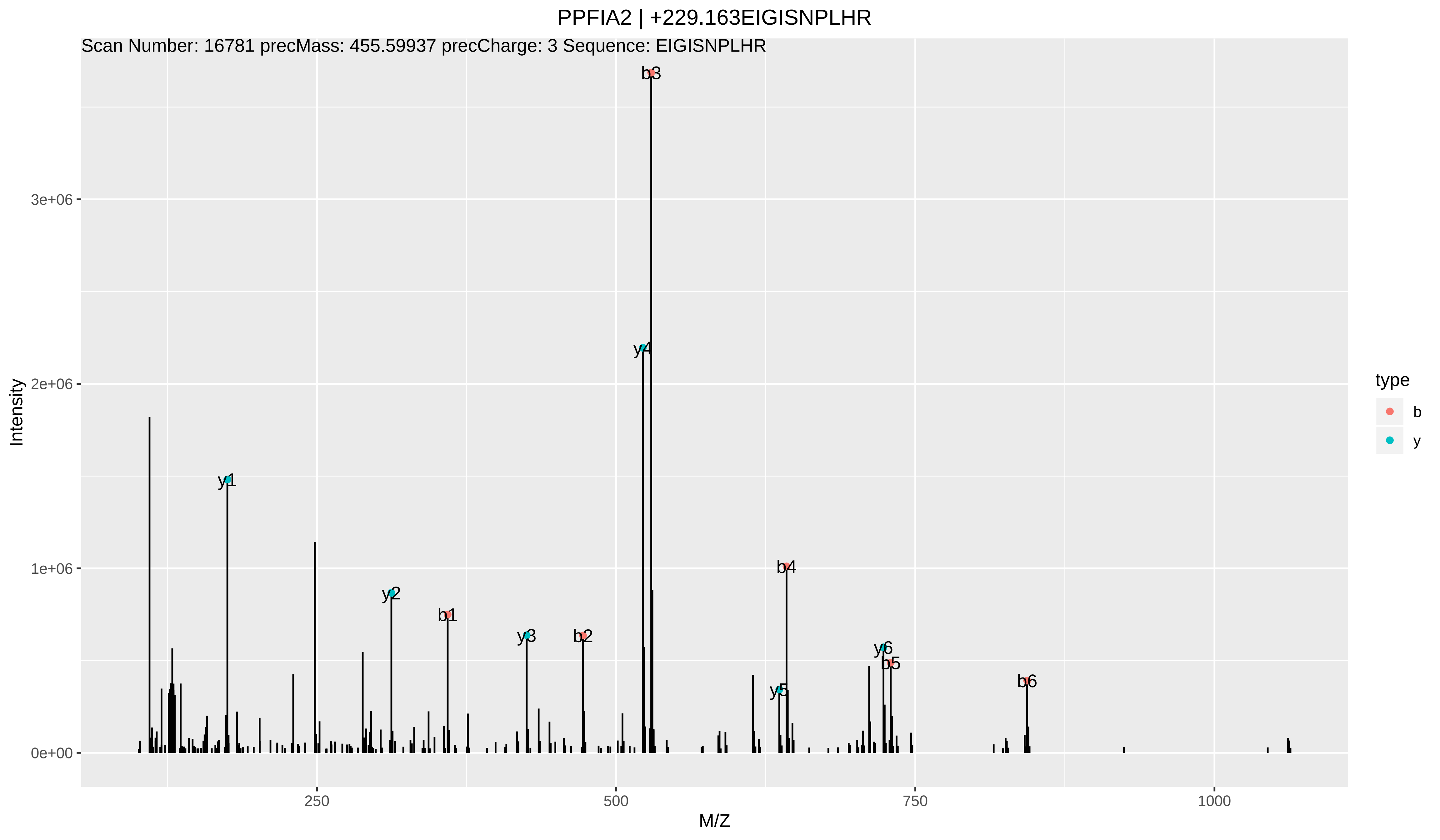

PPFIA2 | +229.163EIGISNPLHR
Scan Number: 16781 precMass: 455.59937 precCharge: 3 Sequence: EIGISNPLHR
Intensity
M/Z
0e+00
1e+06
2e+06
3e+06
250
500
750
1000
b3
y4
y1
y2
b1
y3
b2
b4
y6
b5
y5
b6
type
b
y

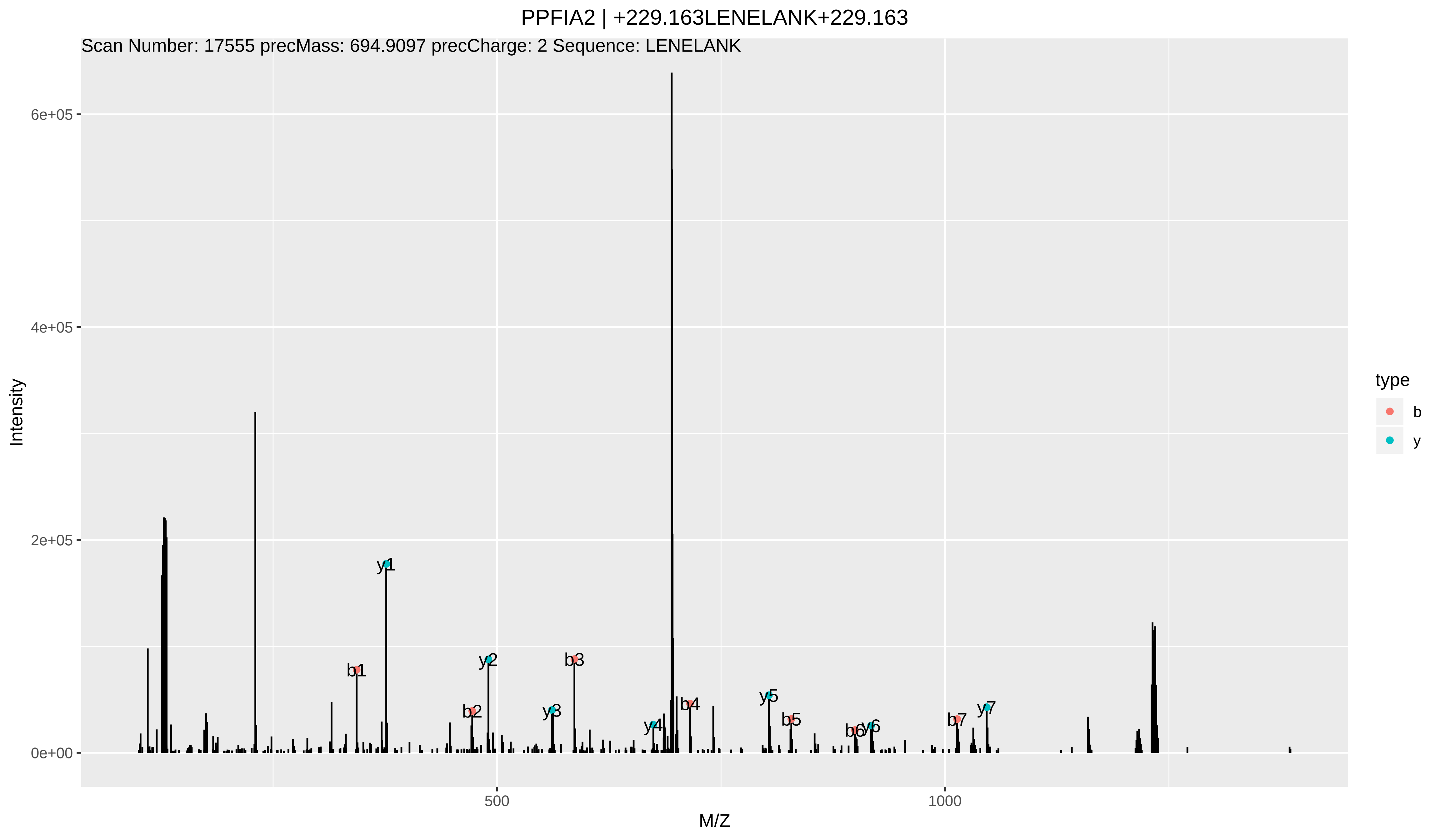
PPFIA2 | +229.163LENELANK+229.163
Scan Number: 17555 precMass: 694.9097 precCharge: 2 Sequence: LENELANK
Intensity
6e+05
4e+05
2e+05
0e+00
M/Z
500
1000
y1
b1
y2
b2
b3
y3
y4
b4
y5
b5
b6
y6
b7
y7
type
b
y

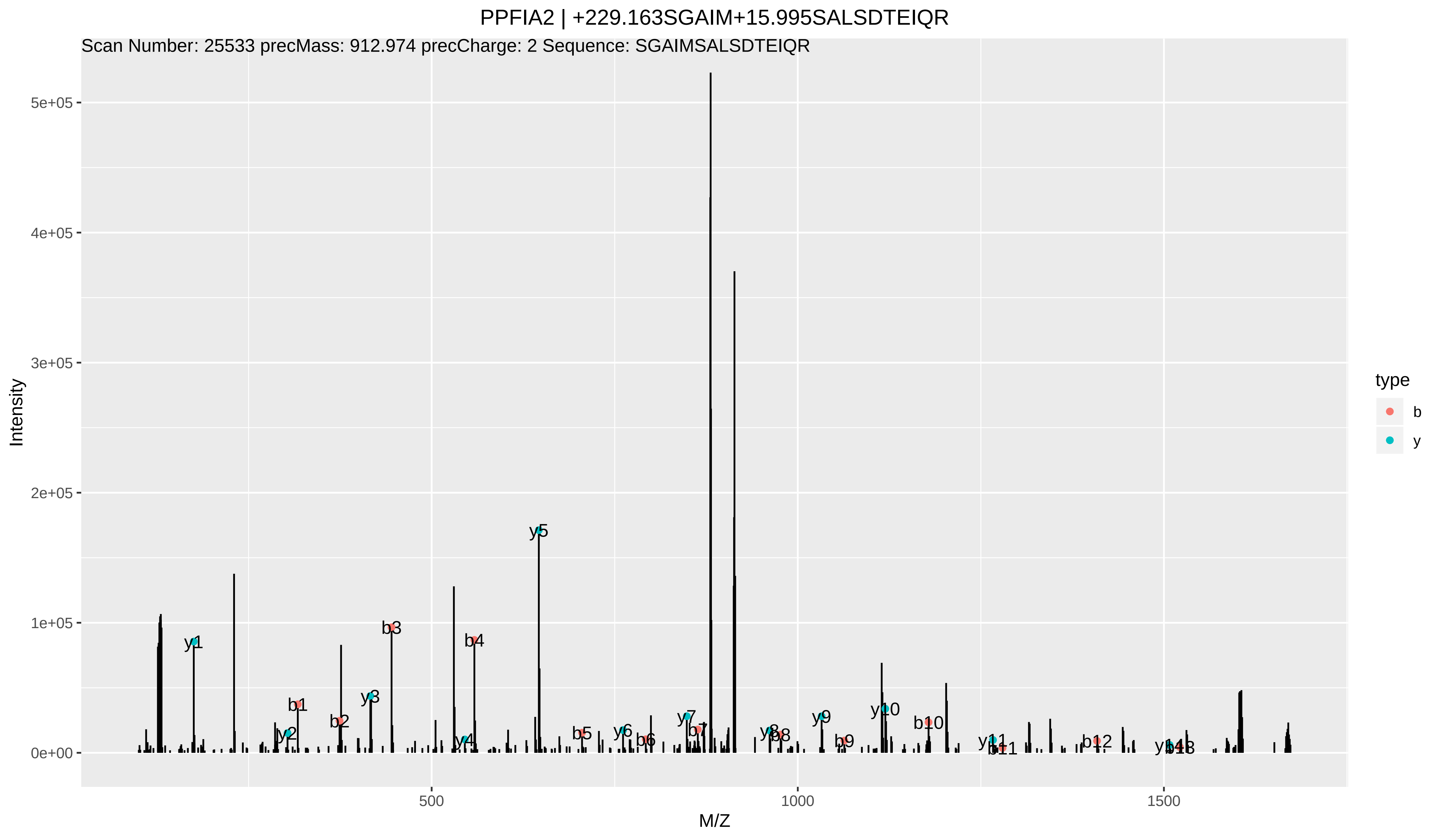

PPFIA2 | +229.163SGAIM+15.995SALSDTEIQR
Scan Number: 25533 precMass: 912.974 precCharge: 2 Sequence: SGAIMSALSDTEIQR
Intensity
M/Z
type
b
y

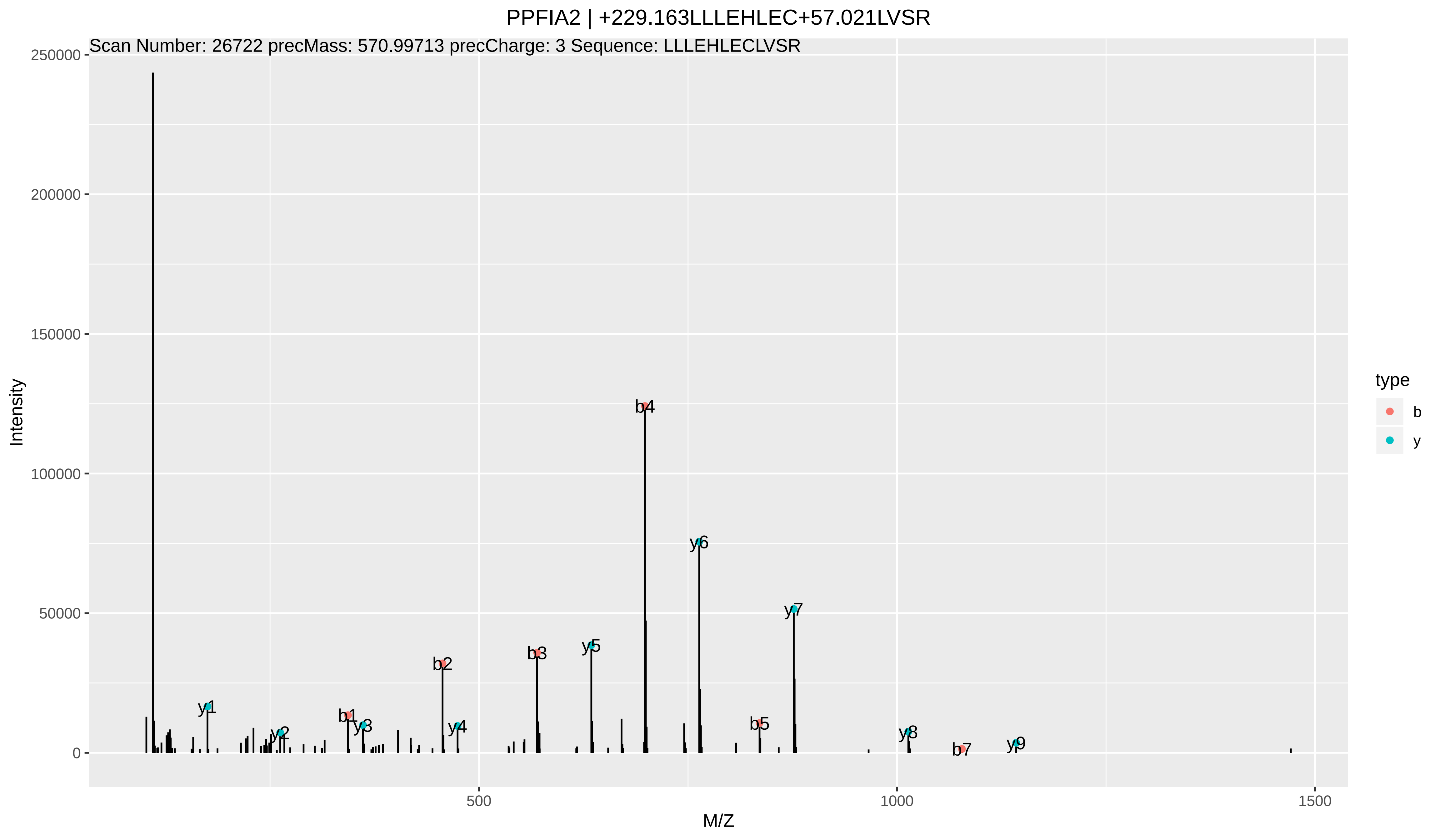

PPFIA2 | +229.163LLLEHLEC+57.021LVSR
Scan Number: 26722 precMass: 570.99713 precCharge: 3 Sequence: LLLEHLECLVSR
Intensity
M/Z
type
b
y
b1
b2
b3
b4
b5
b7
y1
y2
y3
y4
y5
y6
y7
y8
y9
0
50000
100000
150000
200000
250000
500
1000
1500

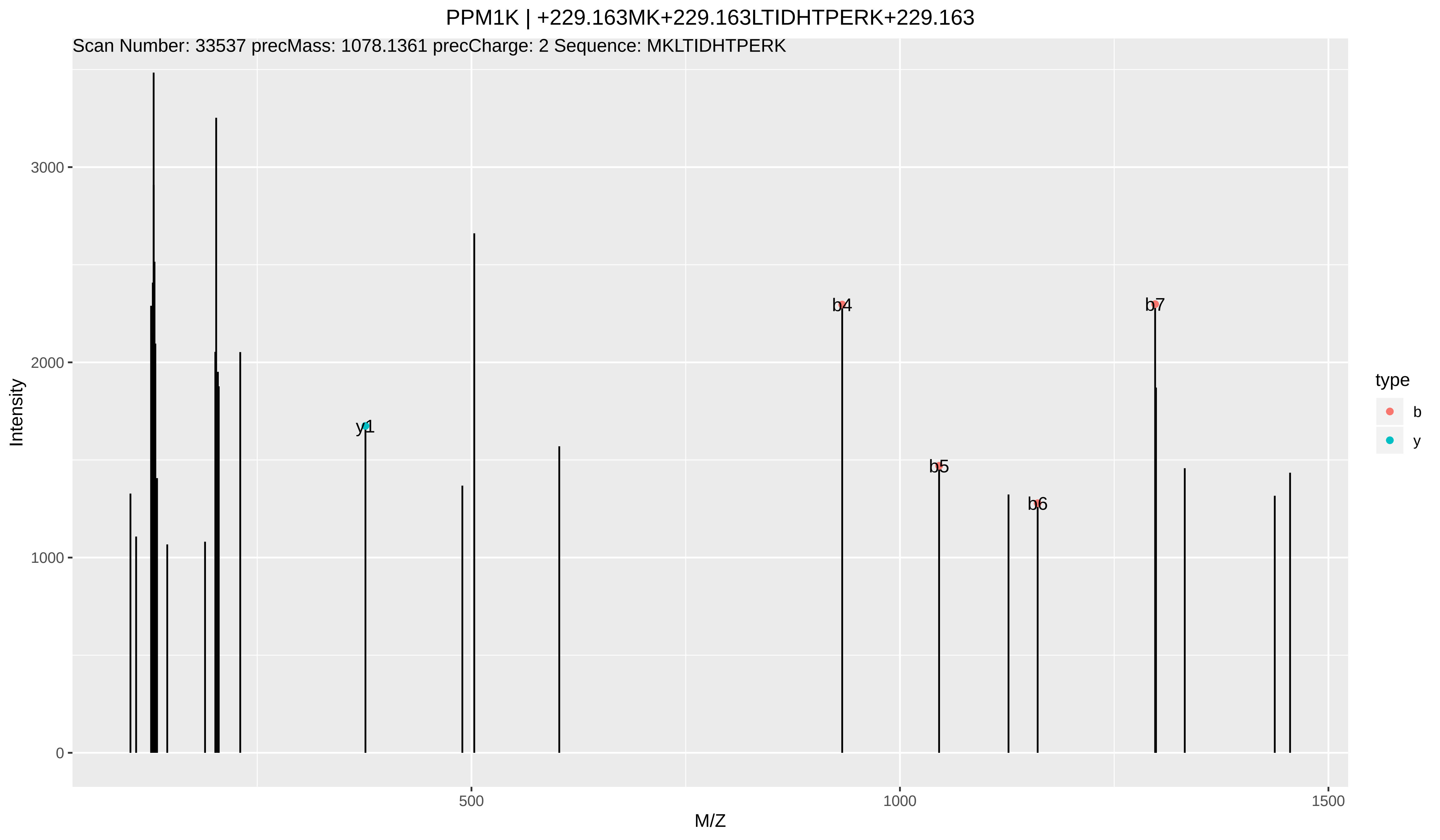
PPM1K | +229.163MK+229.163LTIDHTPERK+229.163
Scan Number: 33537 precMass: 1078.1361 precCharge: 2 Sequence: MKLTIDHTPERK
Intensity
M/Z
0
1000
2000
3000
500
1000
1500
y1
b4
b5
b6
b7
type
b
y

PPP1R15B | +229.163VTFLEEVTEYYISGDEDRK+229.163

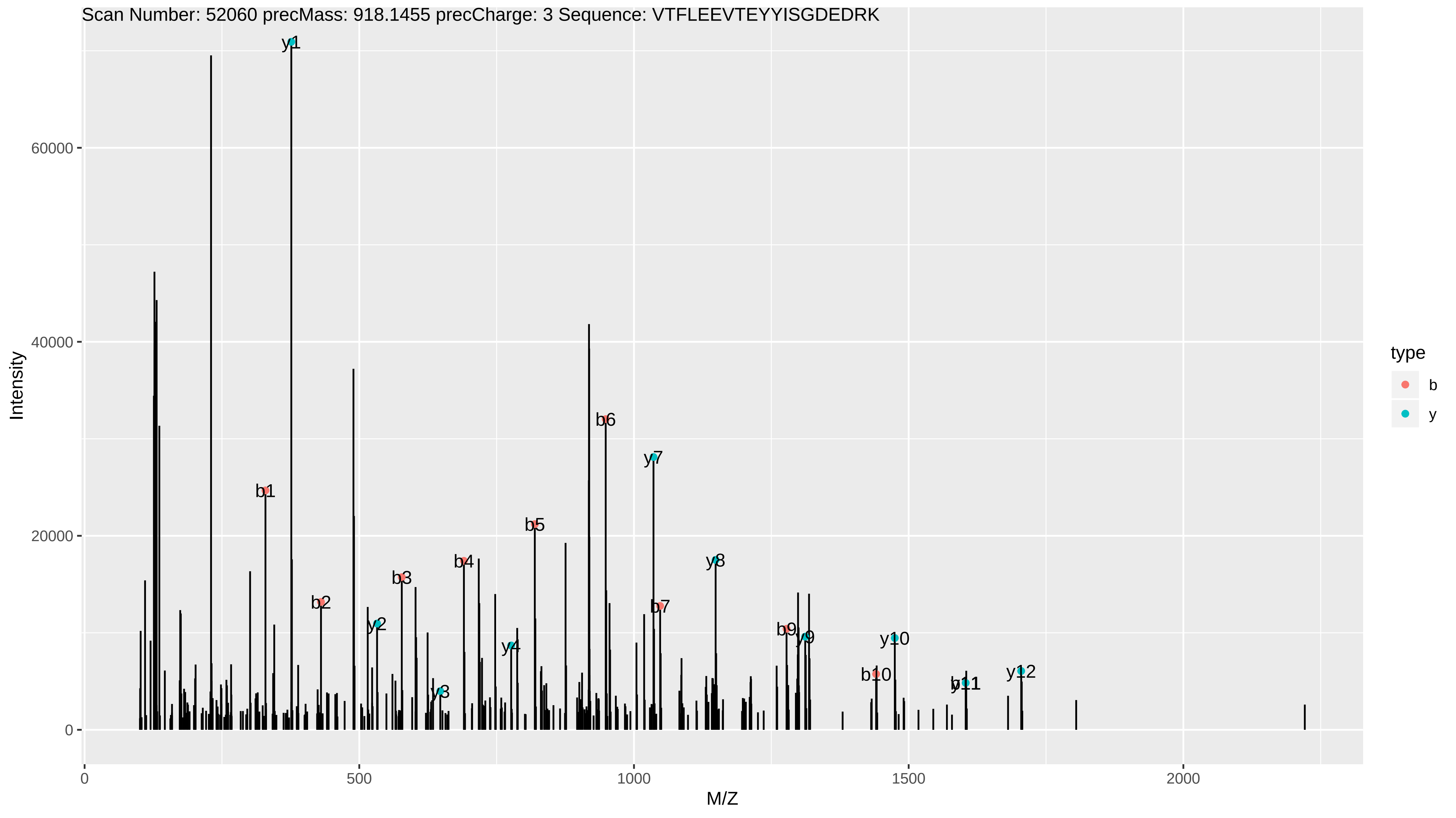

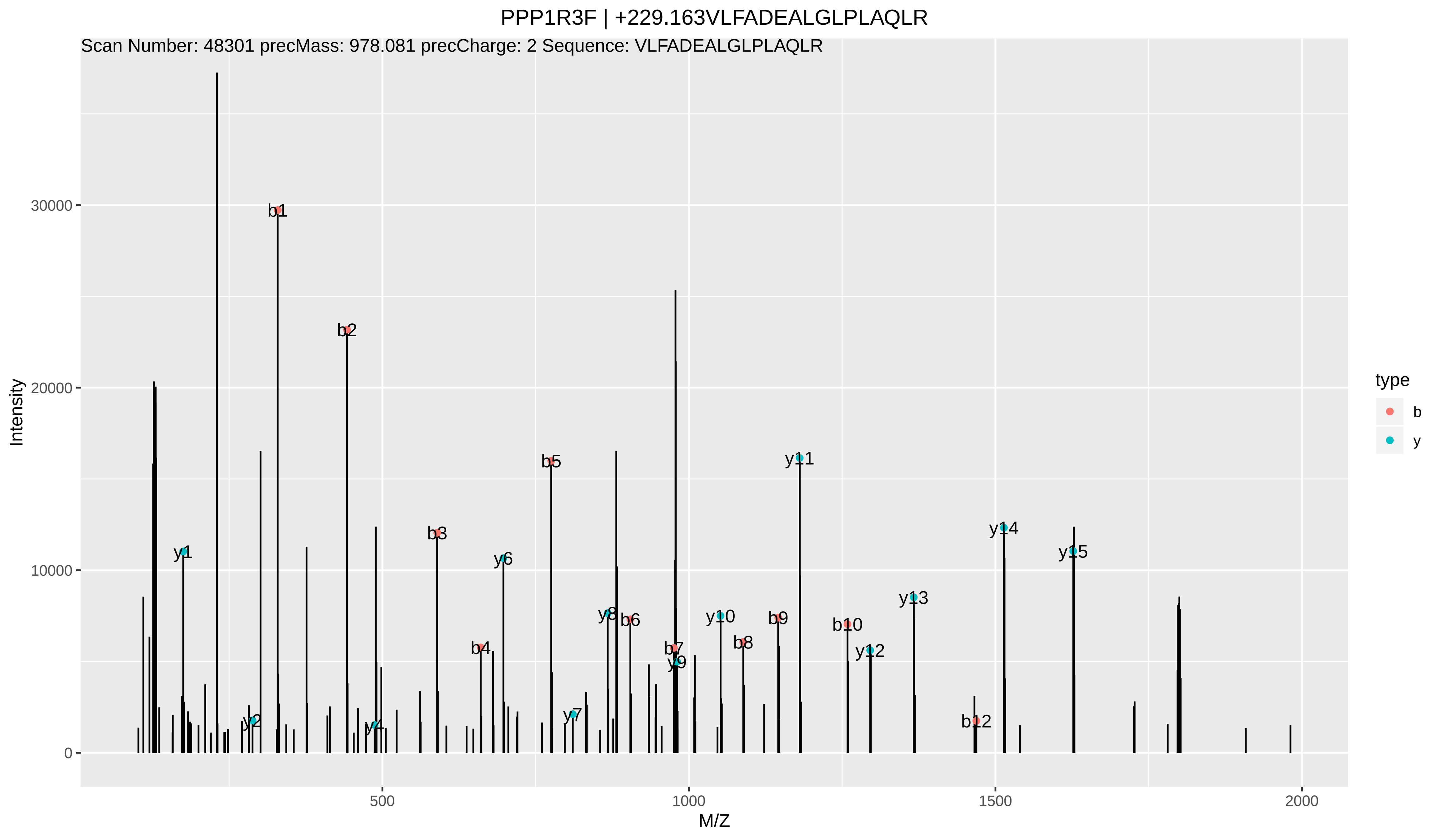
PPP1R3F | +229.163VLFADEALGLPLAQLR
Scan Number: 48301 precMass: 978.081 precCharge: 2 Sequence: VLFADEALGLPLAQLR
Intensity
M/Z
type
b
y
b1
b2
b3
b4
b5
b6
b7
b8
b9
b10
b12
y1
y2
y4
y6
y7
y8
y9
y10
y11
y12
y13
y14
y15
0
10000
20000
30000
500
1000
1500
2000

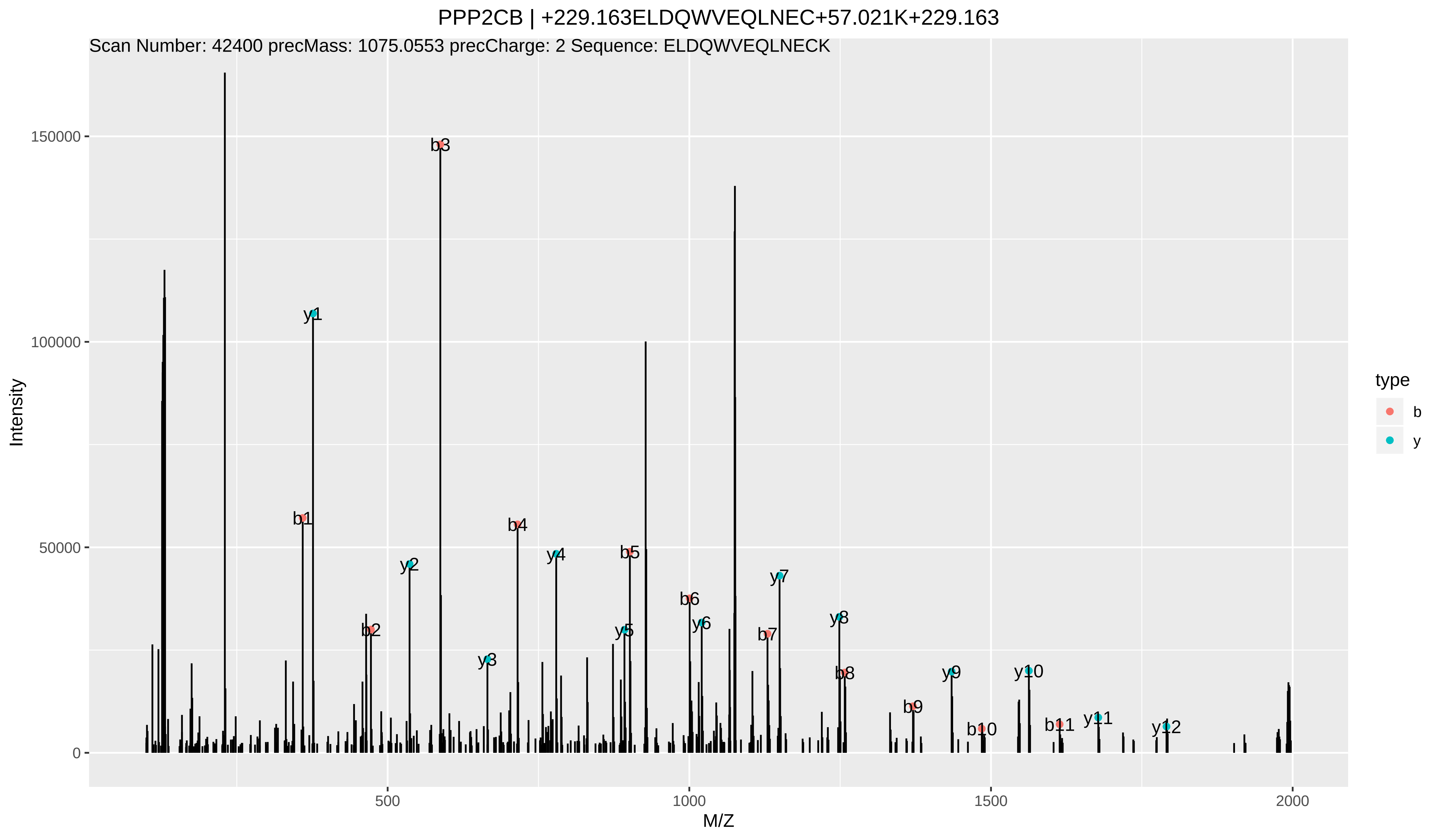

PPP2CB | +229.163ELDQWVEQLNEC+57.021K+229.163
Scan Number: 42400 precMass: 1075.0553 precCharge: 2 Sequence: ELDQWVEQLNECK
Intensity
150000
100000
50000
0
500
1000
1500
2000
M/Z
b1
b2
b3
b4
b5
b6
b7
b8
b9
b10
b11
y1
y2
y3
y4
y5
y6
y7
y8
y9
y10
y11
y12
type
b
y

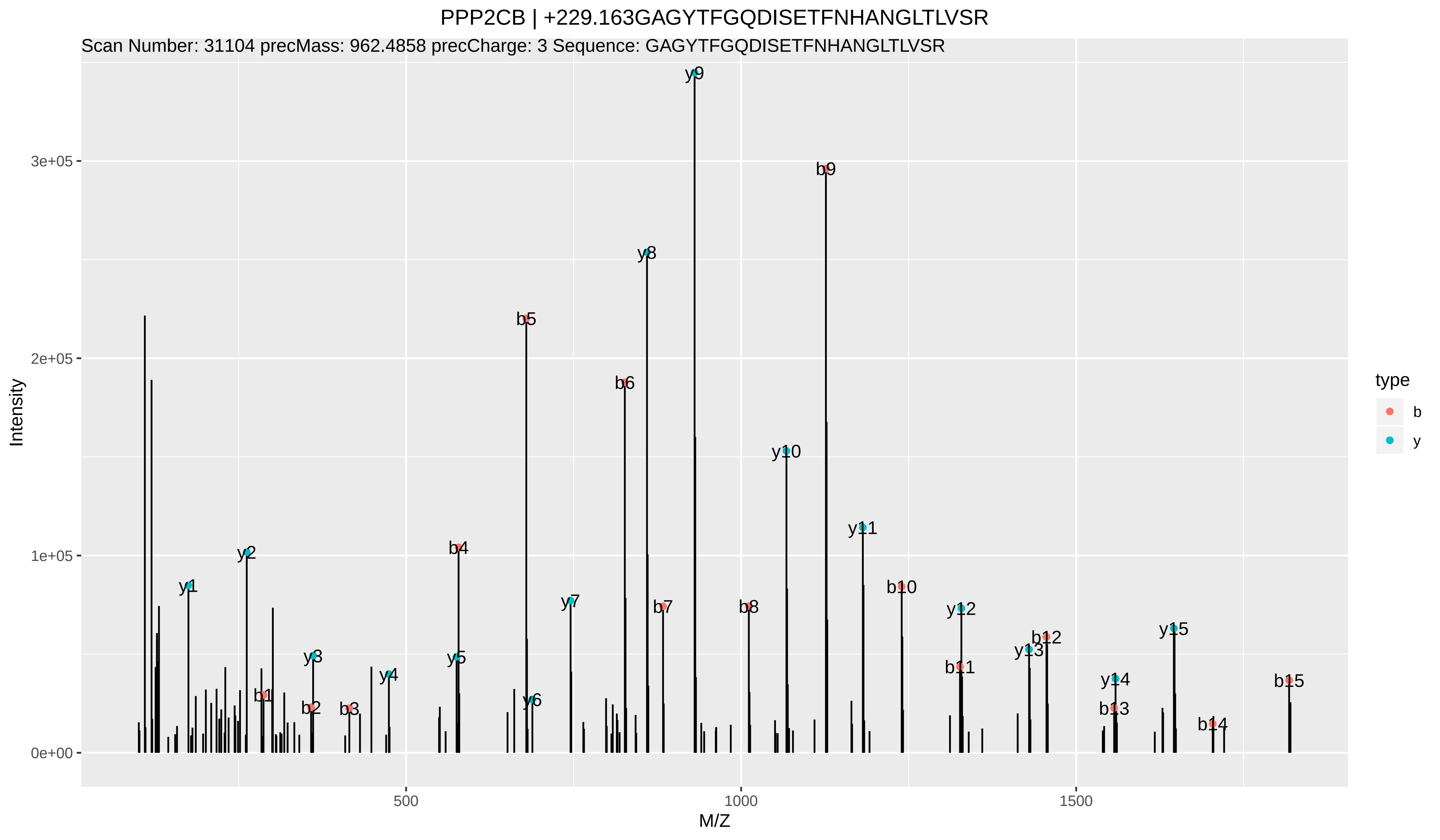
PPP2CB | +229.163GAGYTFGQDISETFNHANGLTLVSR
Scan Number: 31104 precMass: 962.4858 precCharge: 3 Sequence: GAGYTFGQDISETFNHANGLTLVSR
Intensity
M/Z
0e+00
1e+05
2e+05
3e+05
500
1000
1500
type
b
y
y1
y2
y3
y4
y5
y6
y7
y8
y9
y10
y11
y12
y13
y14
y15
b1
b2
b3
b4
b5
b6
b7
b8
b9
b10
b11
b12
b13
b14
b15

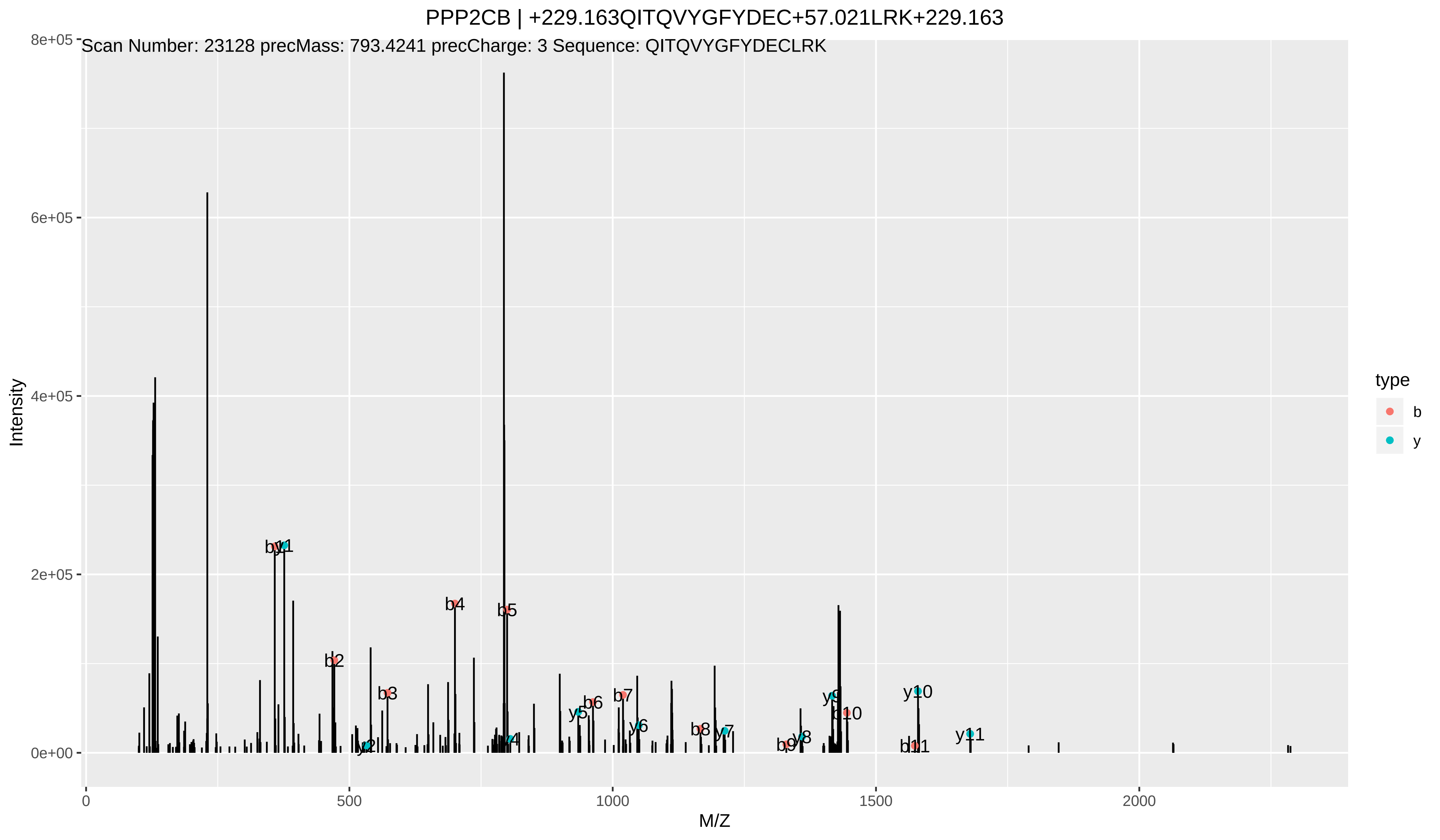

PPP2CB | +229.163QITQVYGFYDEC+57.021LRK+229.163
Scan Number: 23128 precMass: 793.4241 precCharge: 3 Sequence: QITQVYGFYDECLRK
Intensity
8e+05
6e+05
4e+05
2e+05
0e+00
M/Z
0
500
1000
1500
2000
b1
y1
b2
y2
b3
b4
b5
y4
y5
b6
b7
y6
b8
y7
b9
y8
y9
b10
y10
b11
y11
type
b
y

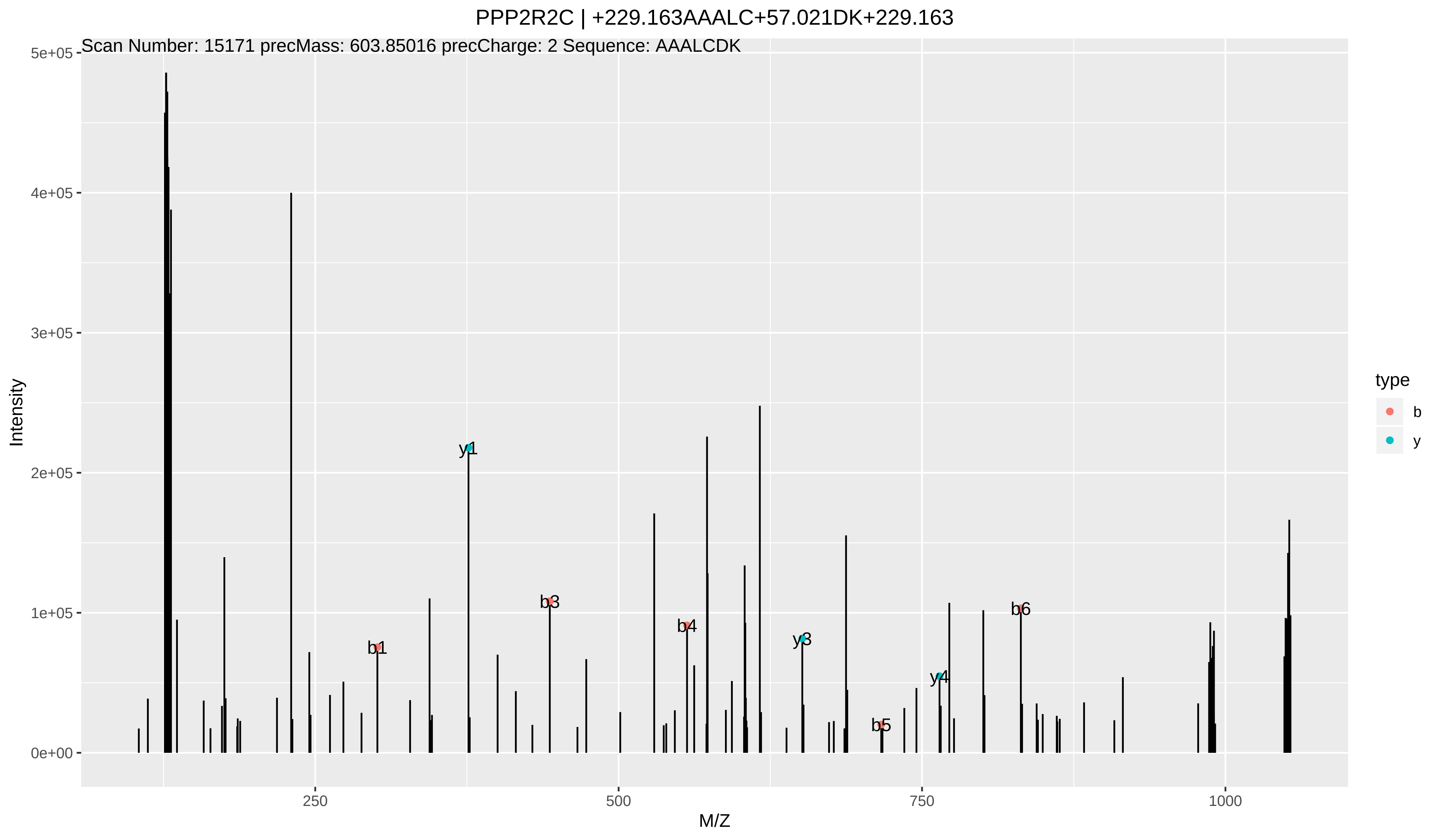

PPP2R2C | +229.163AAALC+57.021DK+229.163
Scan Number: 15171 precMass: 603.85016 precCharge: 2 Sequence: AAALCDK
Intensity
M/Z
5e+05
4e+05
3e+05
2e+05
1e+05
0e+00
250
500
750
1000
y1
b1
b3
b4
y3
b5
y4
b6
type
b
y

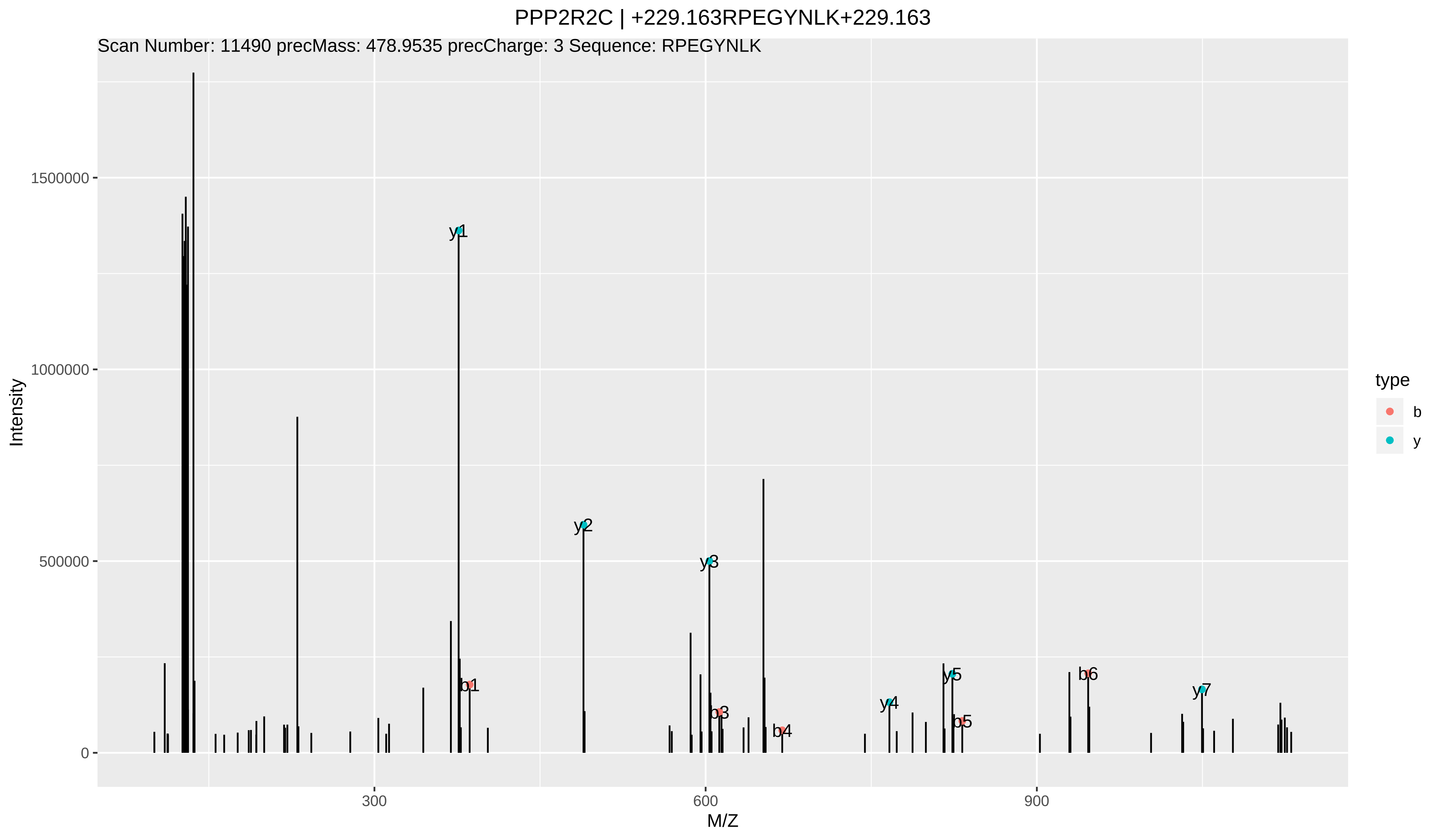
PPP2R2C | +229.163RPEGYNLK+229.163
Scan Number: 11490 precMass: 478.9535 precCharge: 3 Sequence: RPEGYNLK
Intensity
1500000
1000000
500000
0
300
600
900
M/Z
y1
y2
y3
y4
y5
y7
b1
b3
b4
b5
b6
type
b
y

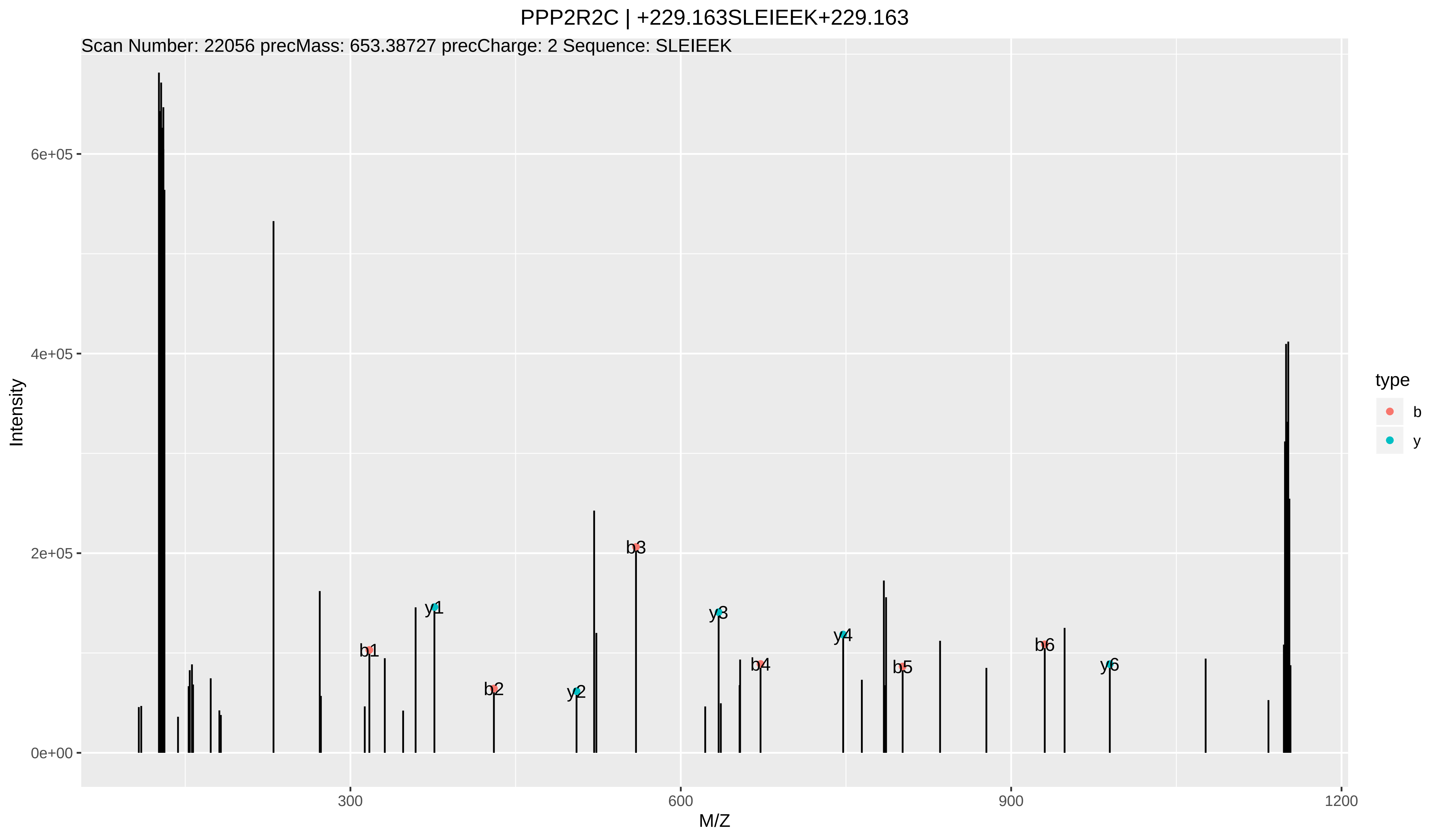

PPP2R2C | +229.163SLEIEEK+229.163
Scan Number: 22056 precMass: 653.38727 precCharge: 2 Sequence: SLEIEEK
Intensity
M/Z
6e+05
4e+05
2e+05
0e+00
300
600
900
1200
b1
y1
b2
y2
b3
y3
b4
y4
b5
b6
y6
type
b
y

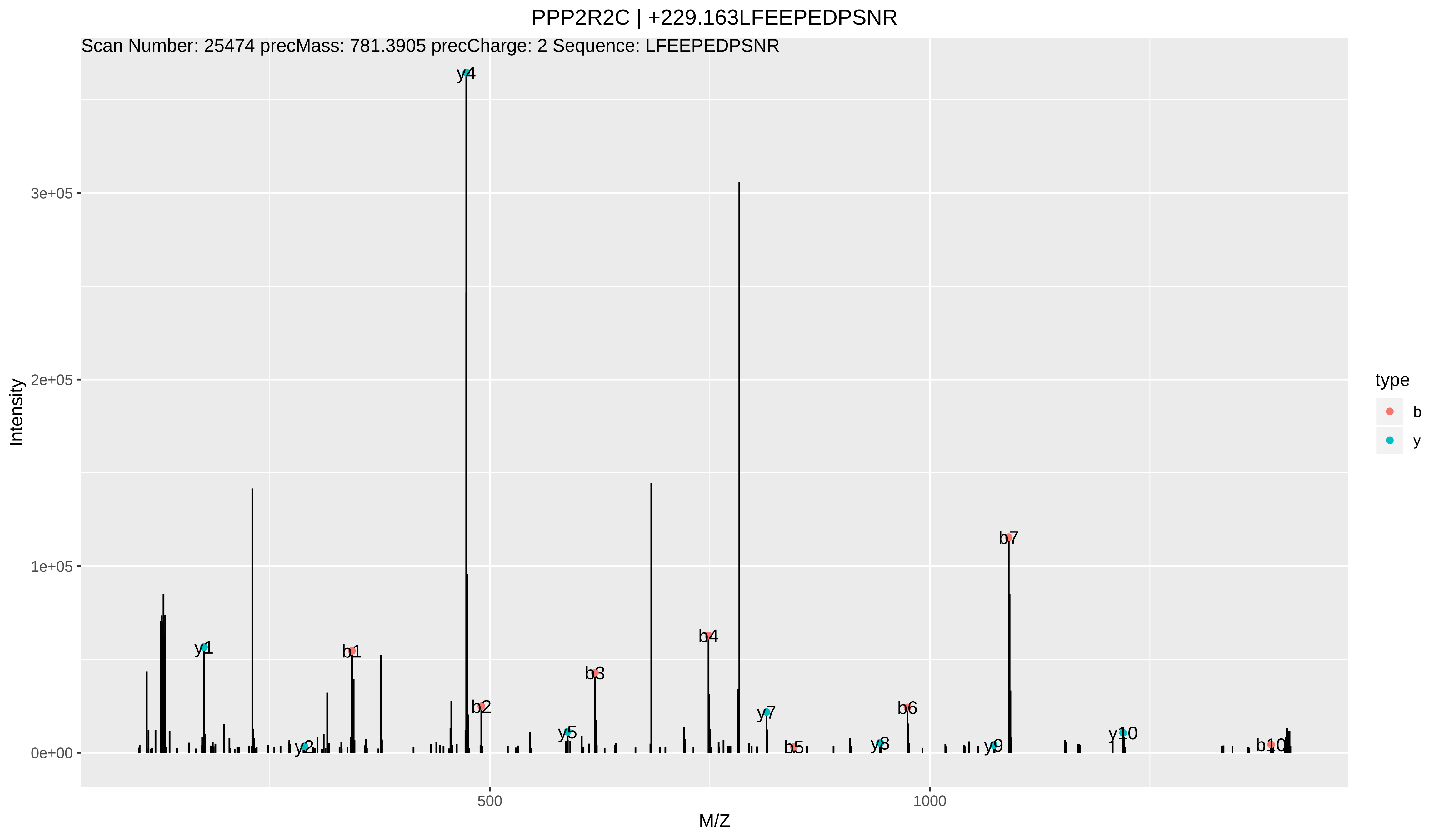
PPP2R2C | +229.163LFEEPEDPSNR
Scan Number: 25474 precMass: 781.3905 precCharge: 2 Sequence: LFEEPEDPSNR
Intensity
3e+05
2e+05
1e+05
0e+00
M/Z
500
1000
y4
b7
b4
y1
b1
b3
b2
y7
b6
y5
y10
y8
y9
b10
y2
b5
type
b
y

PPP2R2C | +229.163SFNIVDIK+229.163

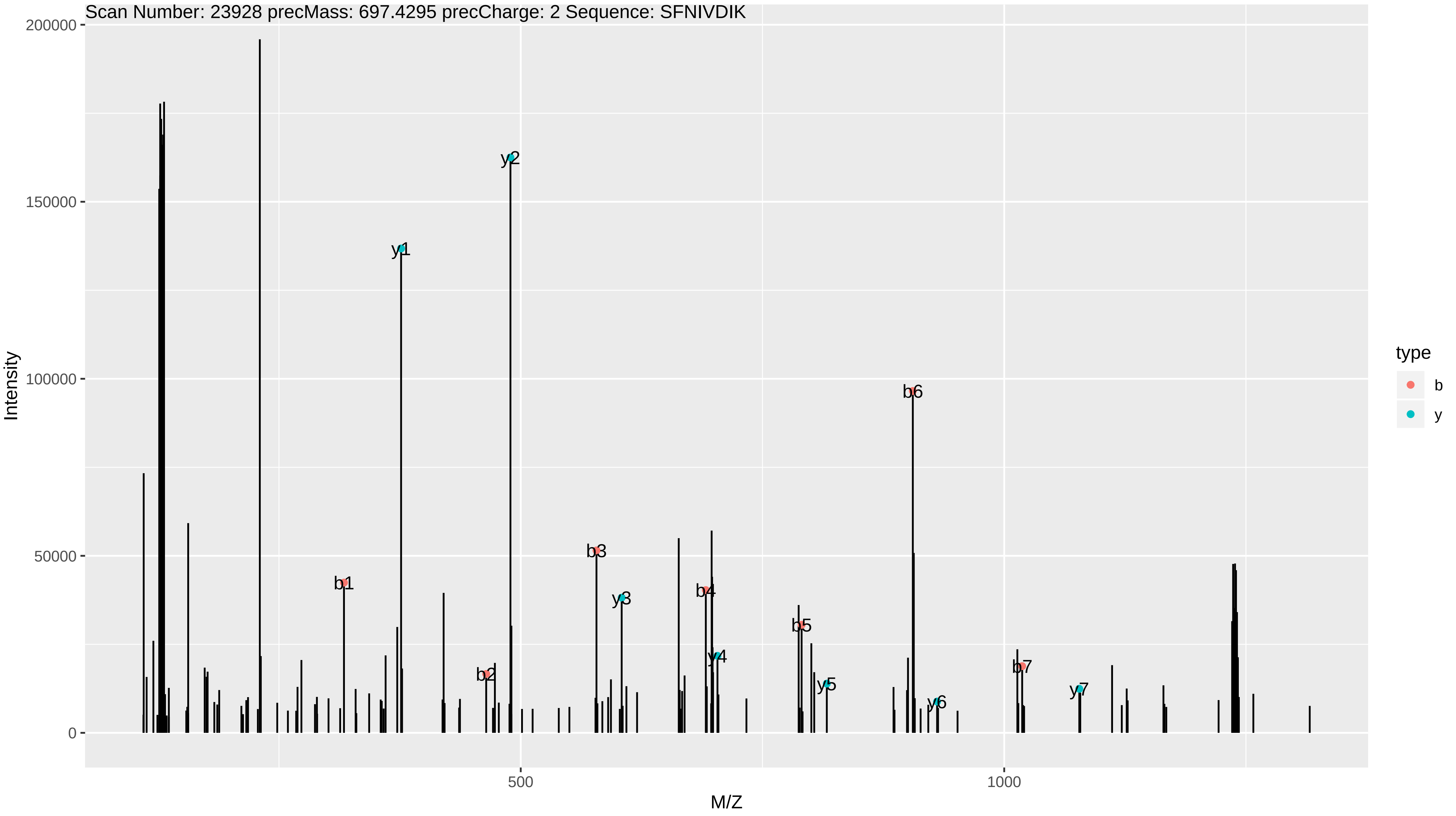

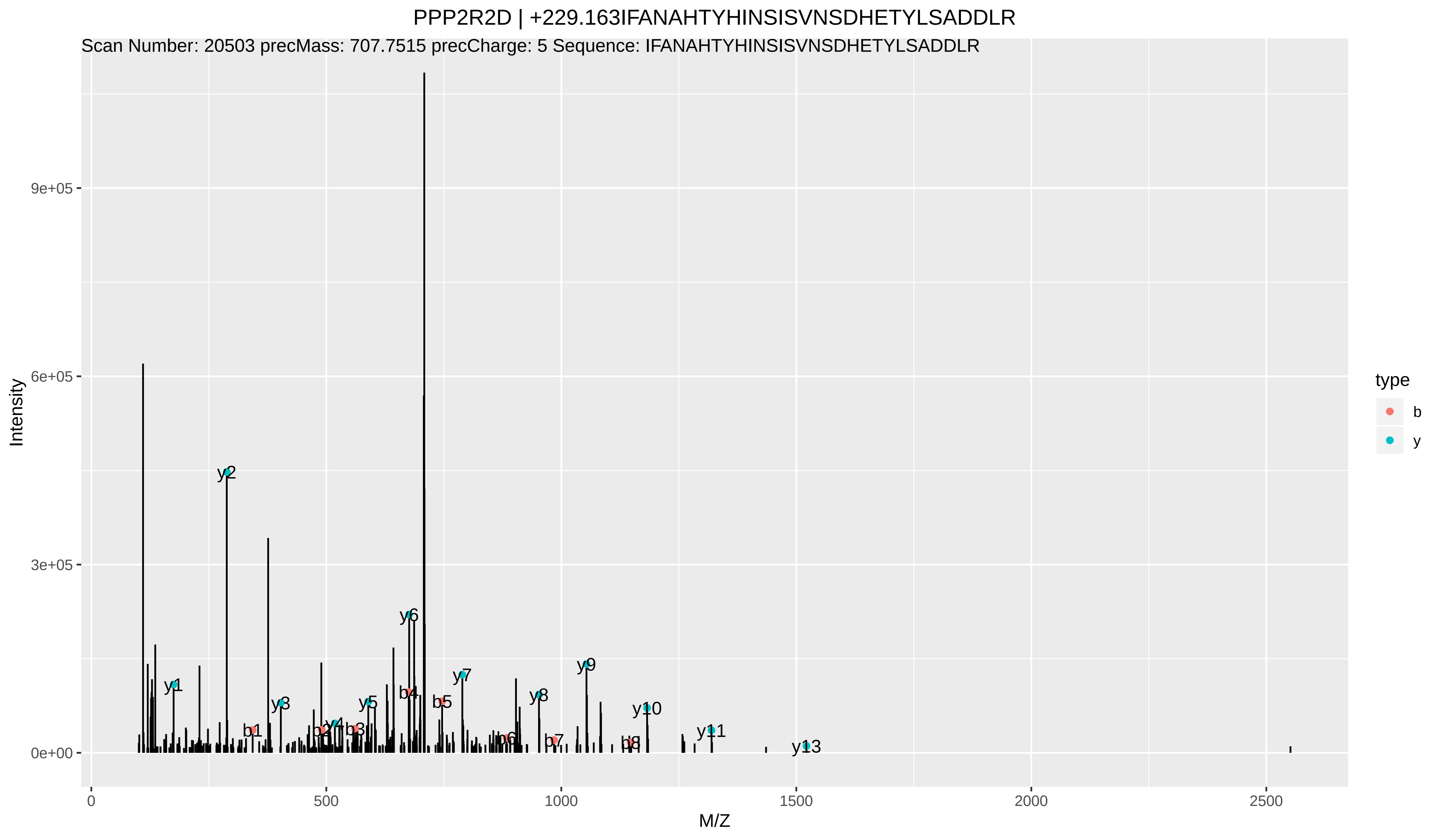

PPP2R2D | +229.163IFANAHTYHINSISVNSDHETYLSADDLR
Scan Number: 20503 precMass: 707.7515 precCharge: 5 Sequence: IFANAHTYHINSISVNSDHETYLSADDLR
Intensity
M/Z
0e+00
3e+05
6e+05
9e+05
0
500
1000
1500
2000
2500
type
b
y
y1
y2
y3
y4
y5
y6
y7
y8
y9
y10
y11
y13
b1
b2
b3
b4
b5
b6
b7
b8

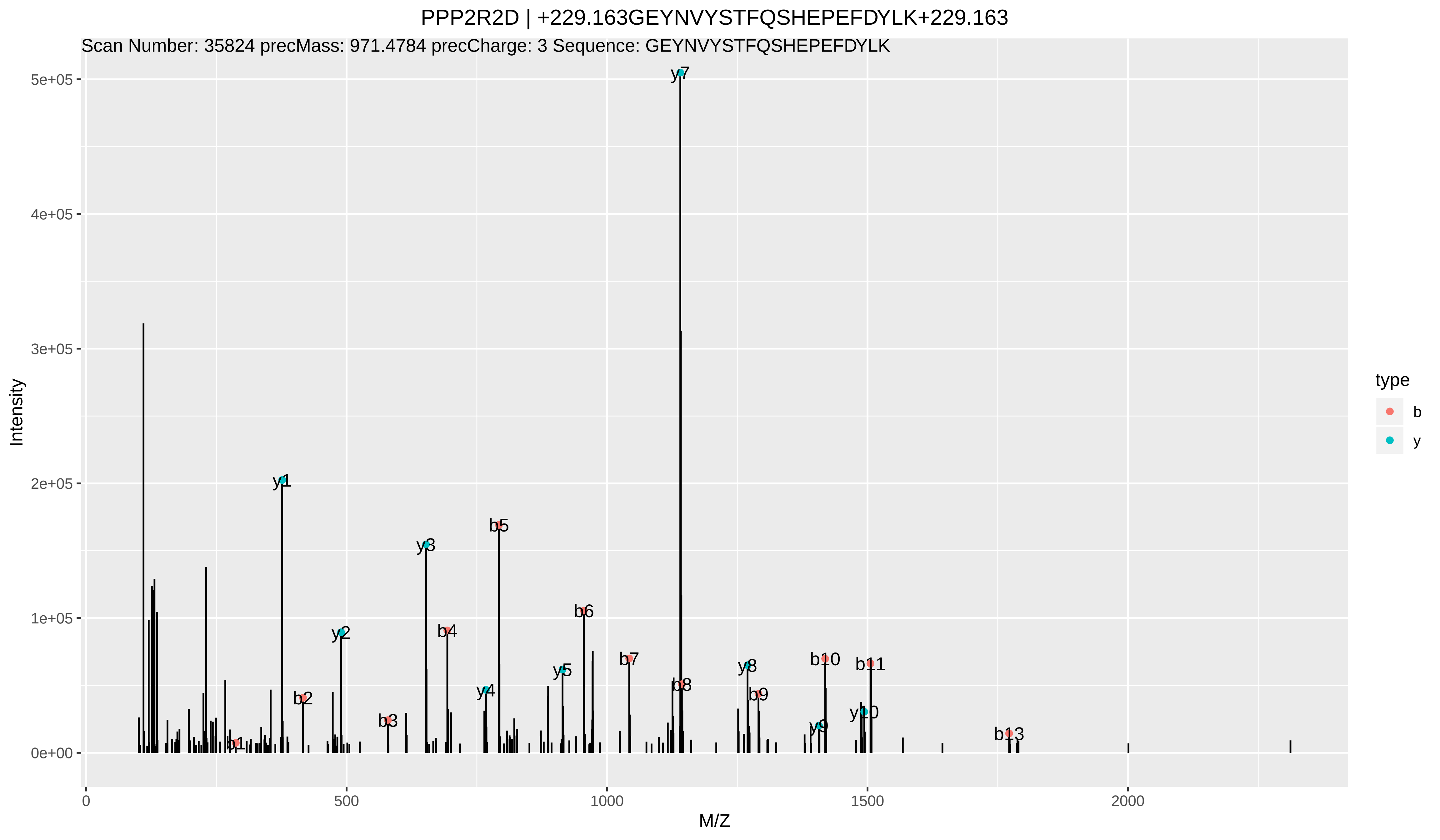
PPP2R2D | +229.163GEYNVYSTFQSHEPEFDYLK+229.163
Scan Number: 35824 precMass: 971.4784 precCharge: 3 Sequence: GEYNVYSTFQSHEPEFDYLK
Intensity
M/Z
type
b
y
y7
y1
b5
y3
b6
y2
b4
b7
b10
b11
y8
y5
b8
y4
b9
b2
y10
b3
y9
b13
b1
0e+00
1e+05
2e+05
3e+05
4e+05
5e+05
0
500
1000
1500
2000

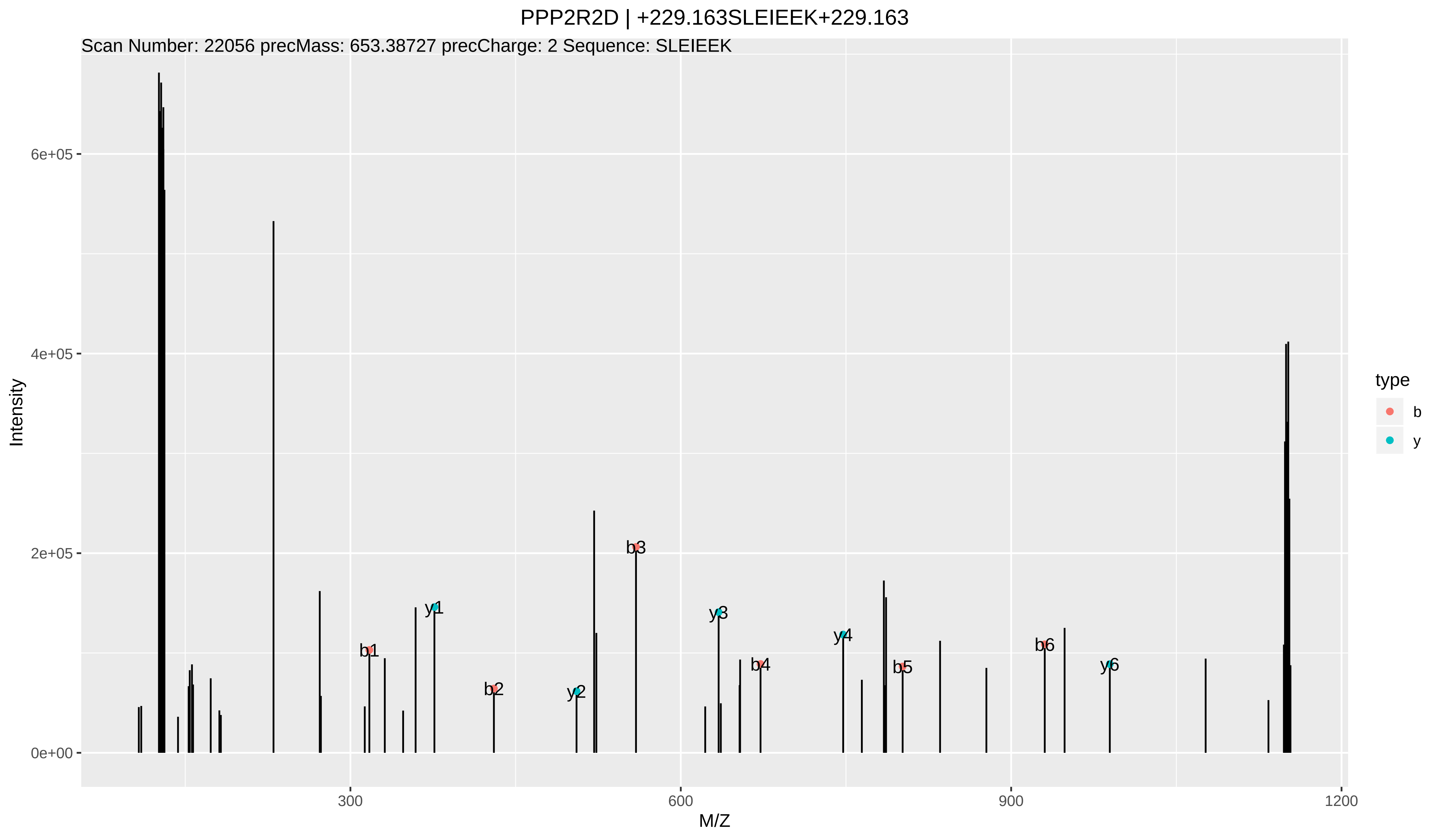

PPP2R2D | +229.163SLEIEEK+229.163
Scan Number: 22056 precMass: 653.38727 precCharge: 2 Sequence: SLEIEEK
Intensity
M/Z
6e+05
4e+05
2e+05
0e+00
300
600
900
1200
b1
b2
b3
b4
b5
b6
y1
y2
y3
y4
y6
type
b
y

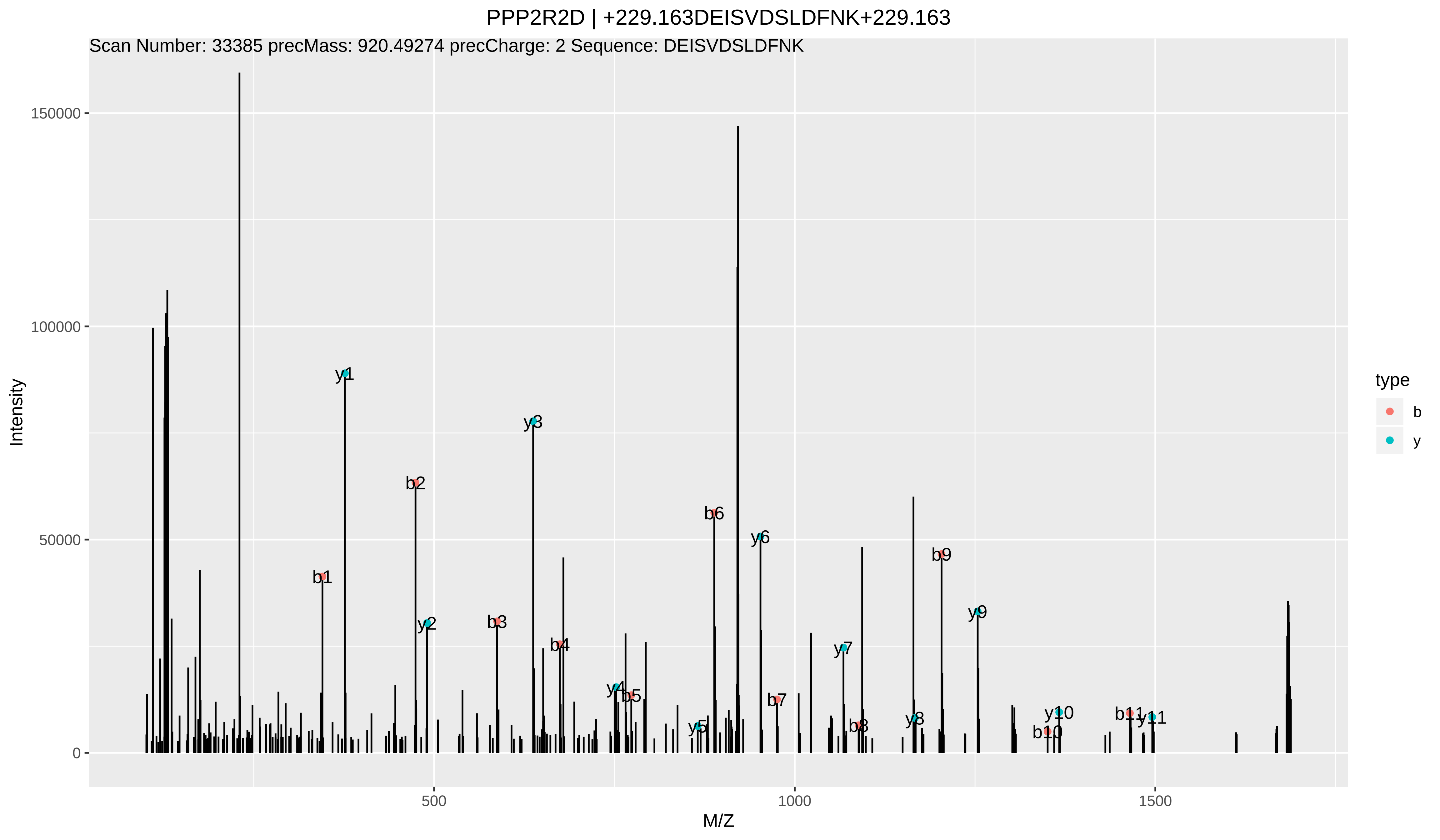
PPP2R2D | +229.163DEISVDSLDFNK+229.163
Scan Number: 33385 precMass: 920.49274 precCharge: 2 Sequence: DEISVDSLDFNK
Intensity
M/Z
150000
100000
50000
0
500
1000
1500
y1
y2
y3
y4
y5
y6
y7
y8
y9
y10
y11
b1
b2
b3
b4
b5
b6
b7
b8
b9
b10
b11
type
b
y

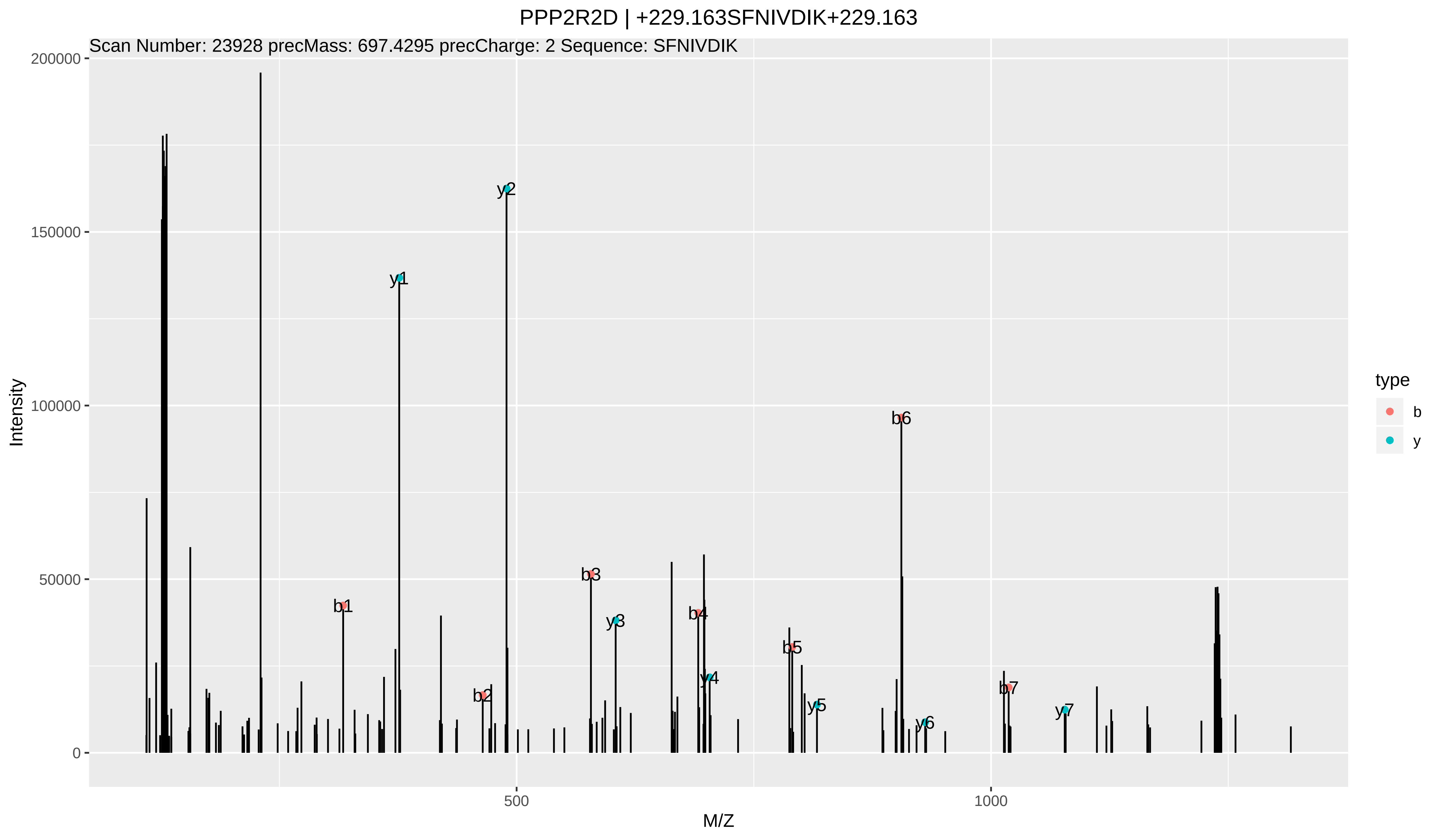
PPP2R2D | +229.163SFNIVDIK+229.163
Scan Number: 23928 precMass: 697.4295 precCharge: 2 Sequence: SFNIVDIK
Intensity
200000
150000
100000
50000
0
500
1000
M/Z
y2
y1
b6
b3
b1
y3
b4
b5
y4
b2
b7
y5
y7
y6
type
b
y

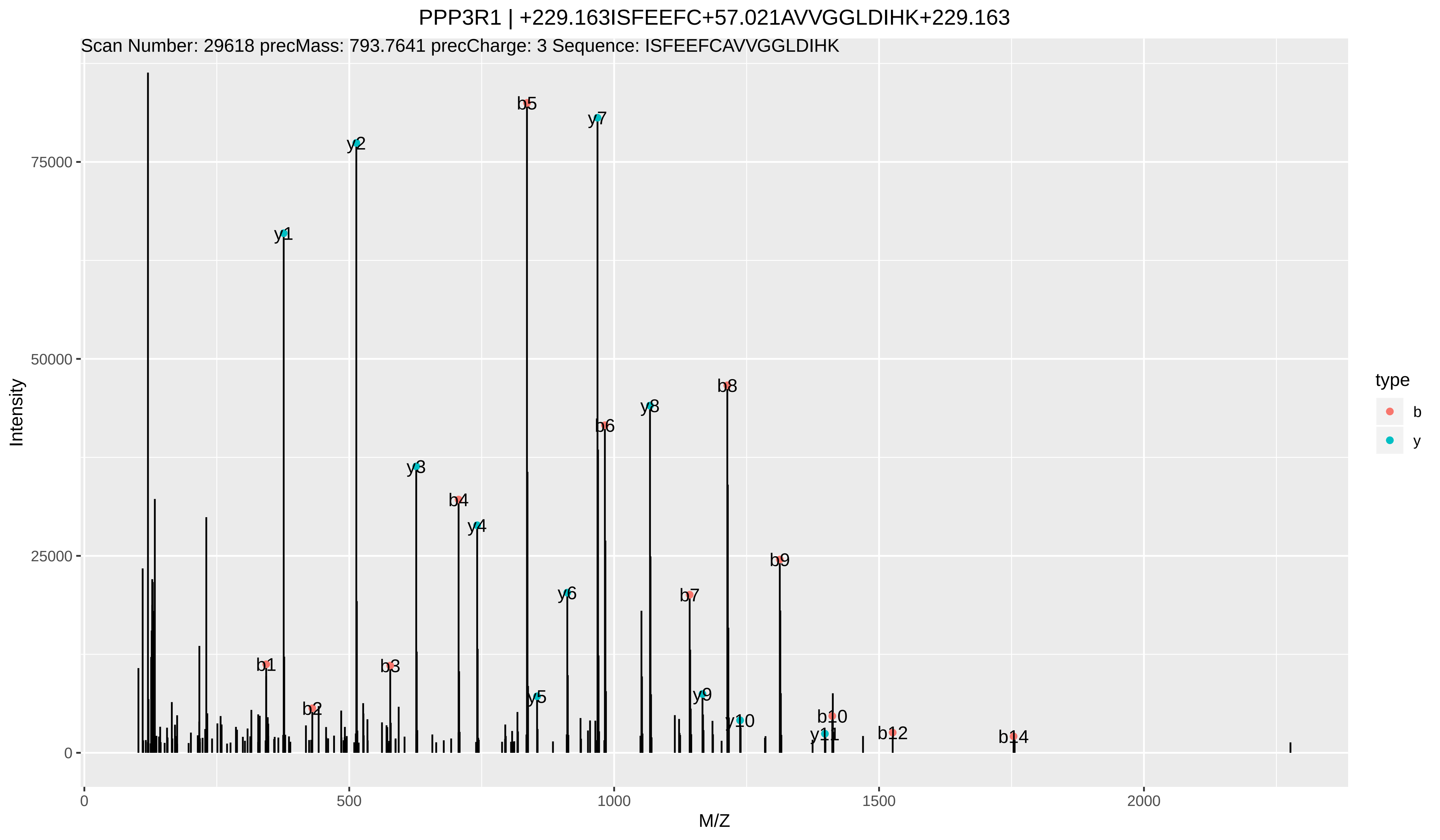
PPP3R1 | +229.163ISFEEFC+57.021AVVGGLDIHK+229.163
Scan Number: 29618 precMass: 793.7641 precCharge: 3 Sequence: ISFEEFCAVVGGLDIHK
Intensity
M/Z
type
b
y
b5
y7
y2
y1
b8
y8
b6
y3
b4
y4
b9
y6
b7
b1
b3
y5
y9
b2
y10
b10
y11
b12
b14
75000
50000
25000
0
0
500
1000
1500
2000

PPP3R1 | +229.163LDLDNSGSLSVEEFMSLPELQQNPLVQR

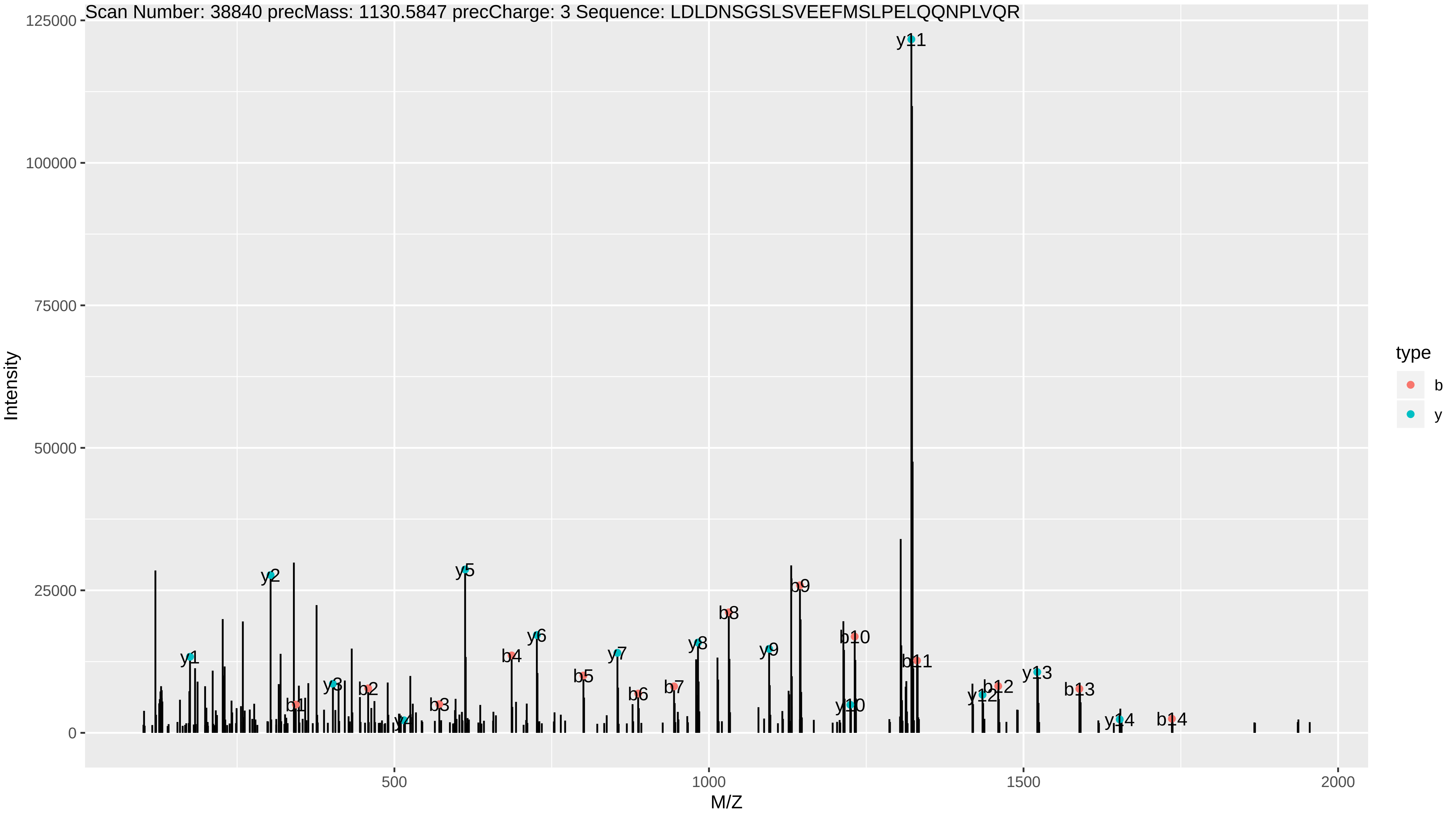

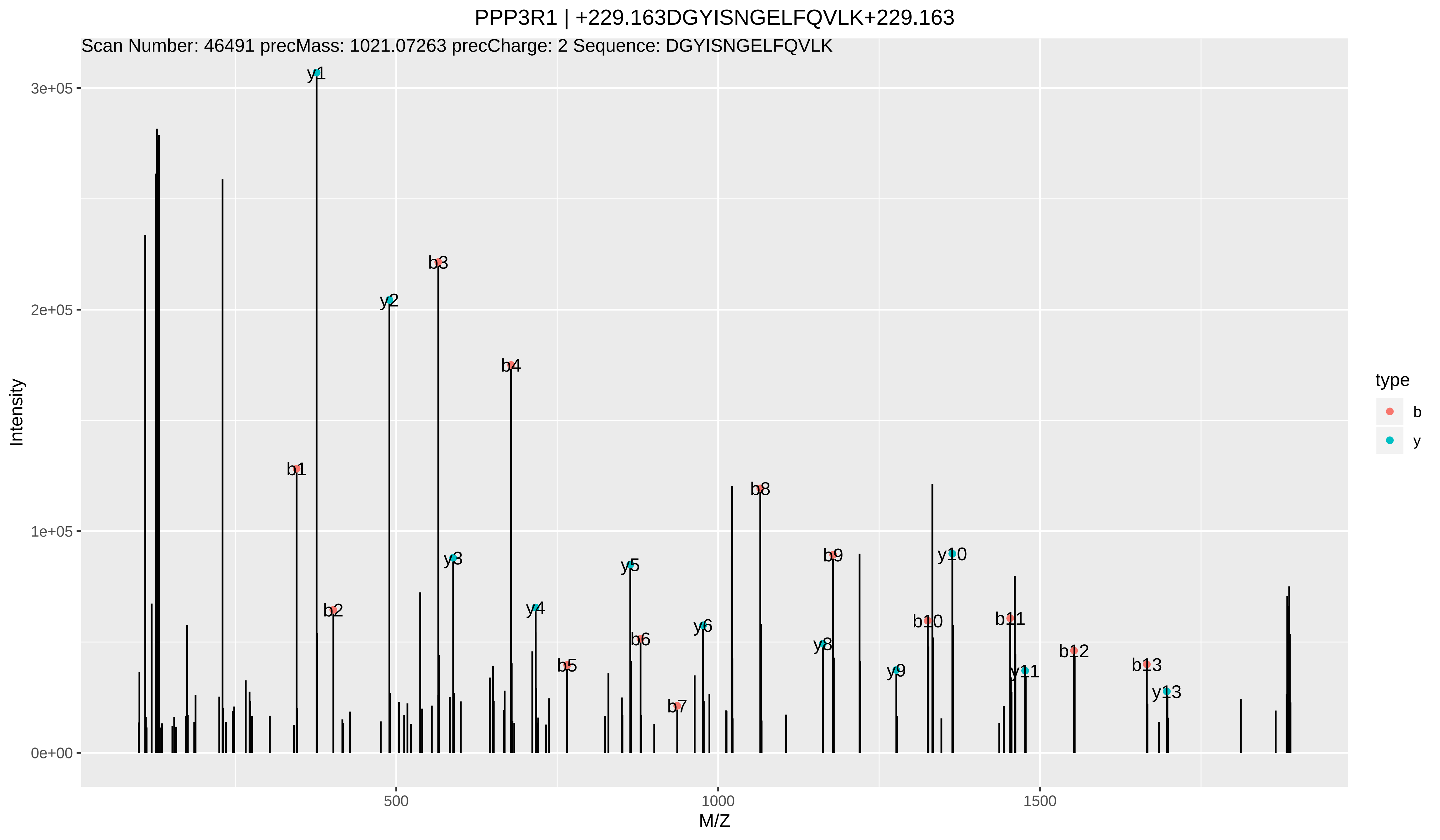

PPP3R1 | +229.163DGYISNGELFQVLK+229.163
Scan Number: 46491 precMass: 1021.07263 precCharge: 2 Sequence: DGYISNGELFQVLK
Intensity
M/Z
3e+05
2e+05
1e+05
0e+00
500
1000
1500
y1
y2
b3
b4
b1
y3
y5
b2
y4
b6
b5
b7
y6
b8
b9
y8
y9
b10
y10
b11
y11
b12
b13
y13
type
b
y

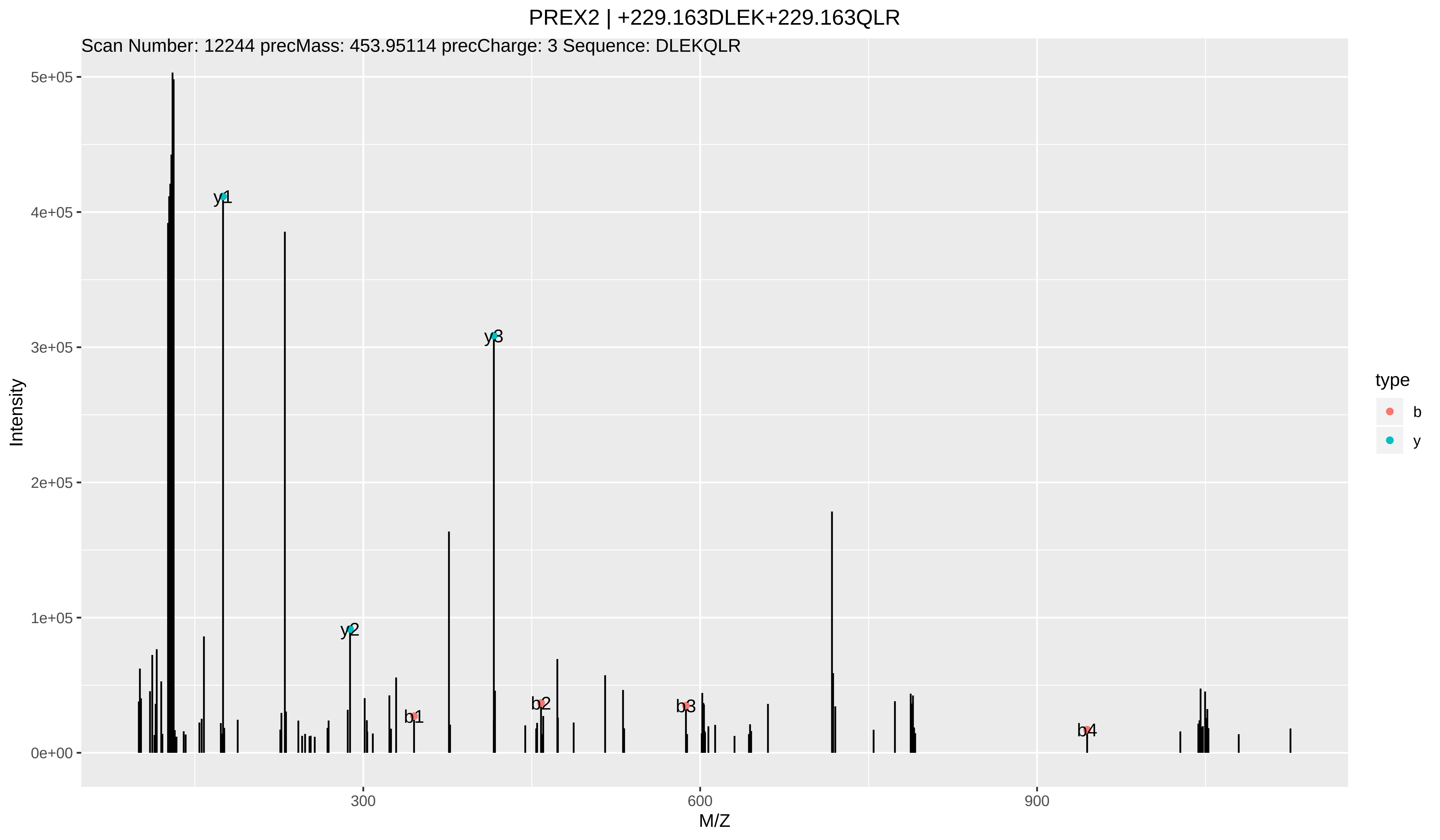

PREX2 | +229.163DLEK+229.163QLR
Scan Number: 12244 precMass: 453.95114 precCharge: 3 Sequence: DLEKQLR
Intensity
5e+05
4e+05
3e+05
2e+05
1e+05
0e+00
y1
y2
y3
b1
b2
b3
b4
300
600
900
M/Z
type
b
y

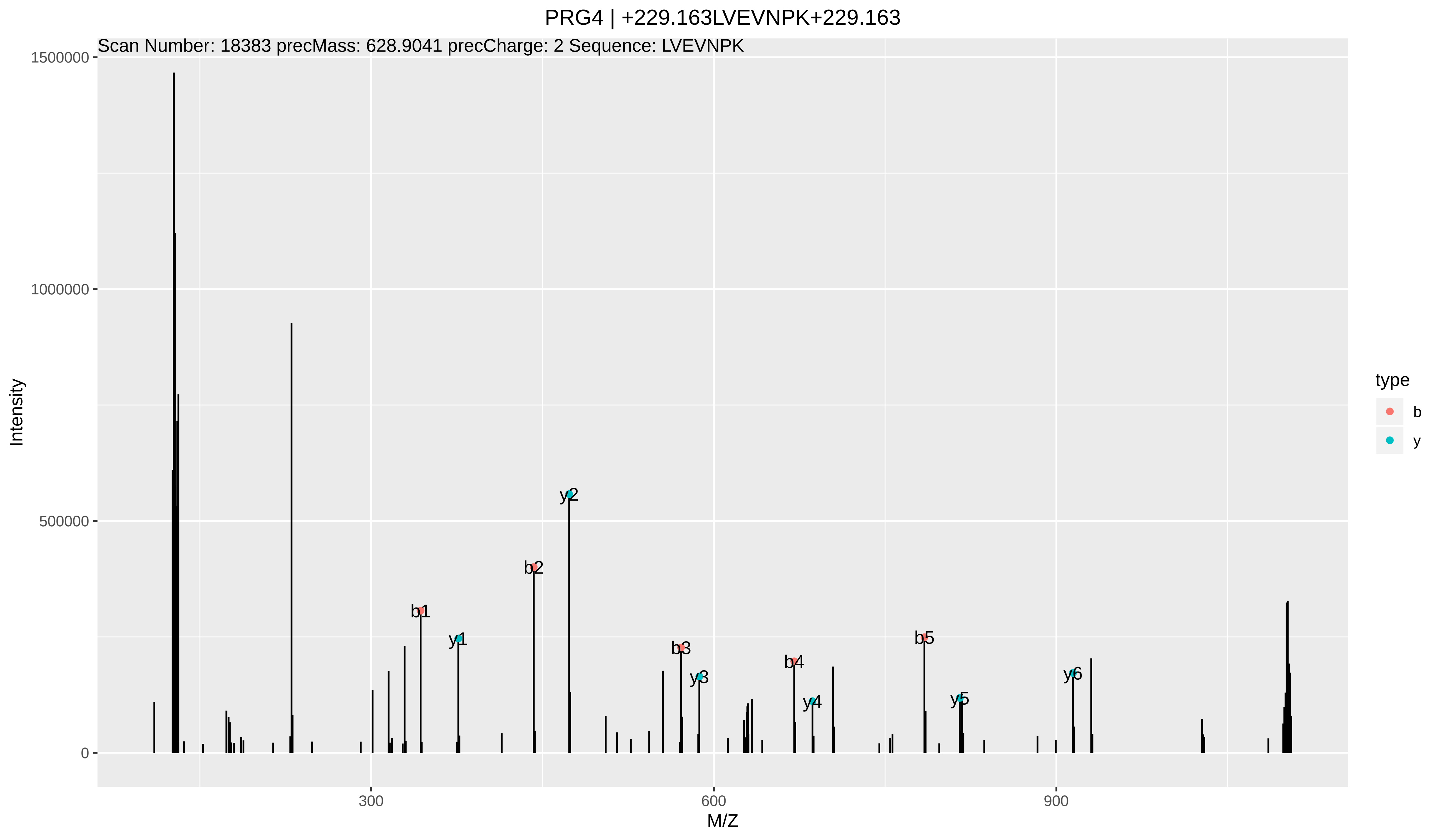
PRG4 | +229.163LVEVNPK+229.163
Scan Number: 18383 precMass: 628.9041 precCharge: 2 Sequence: LVEVNPK
Intensity
M/Z
type
b
y
b1
b2
b3
b4
b5
y1
y2
y3
y4
y5
y6
1500000
1000000
500000
0
300
600
900

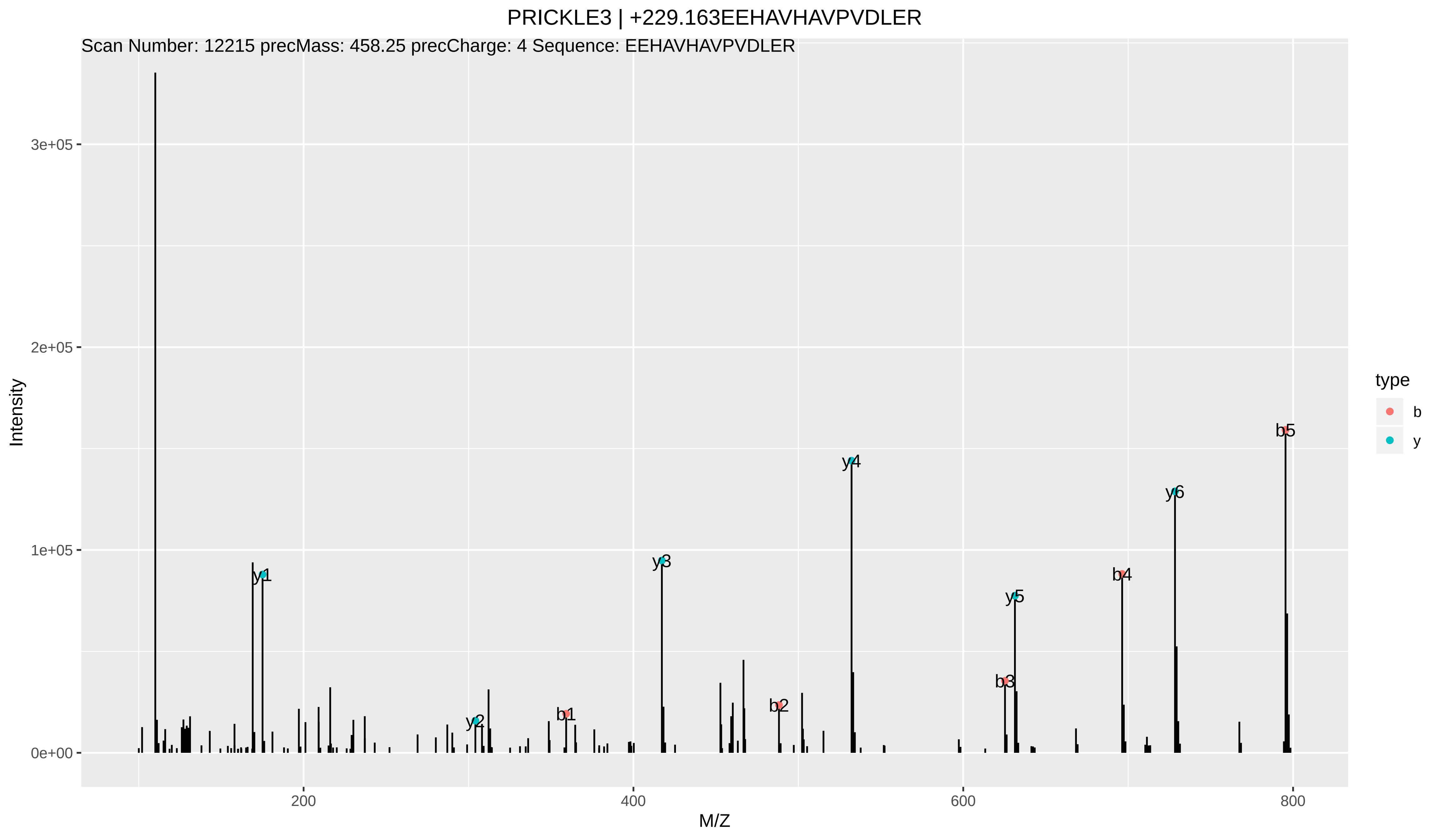
PRICKLE3 | +229.163EEHAVHAVPVDLER
Scan Number: 12215 precMass: 458.25 precCharge: 4 Sequence: EEHAVHAVPVDLER
Intensity
M/Z
type
b
y
y1
y2
b1
y3
b2
y4
b3
y5
b4
y6
b5
3e+05
2e+05
1e+05
0e+00
200
400
600
800

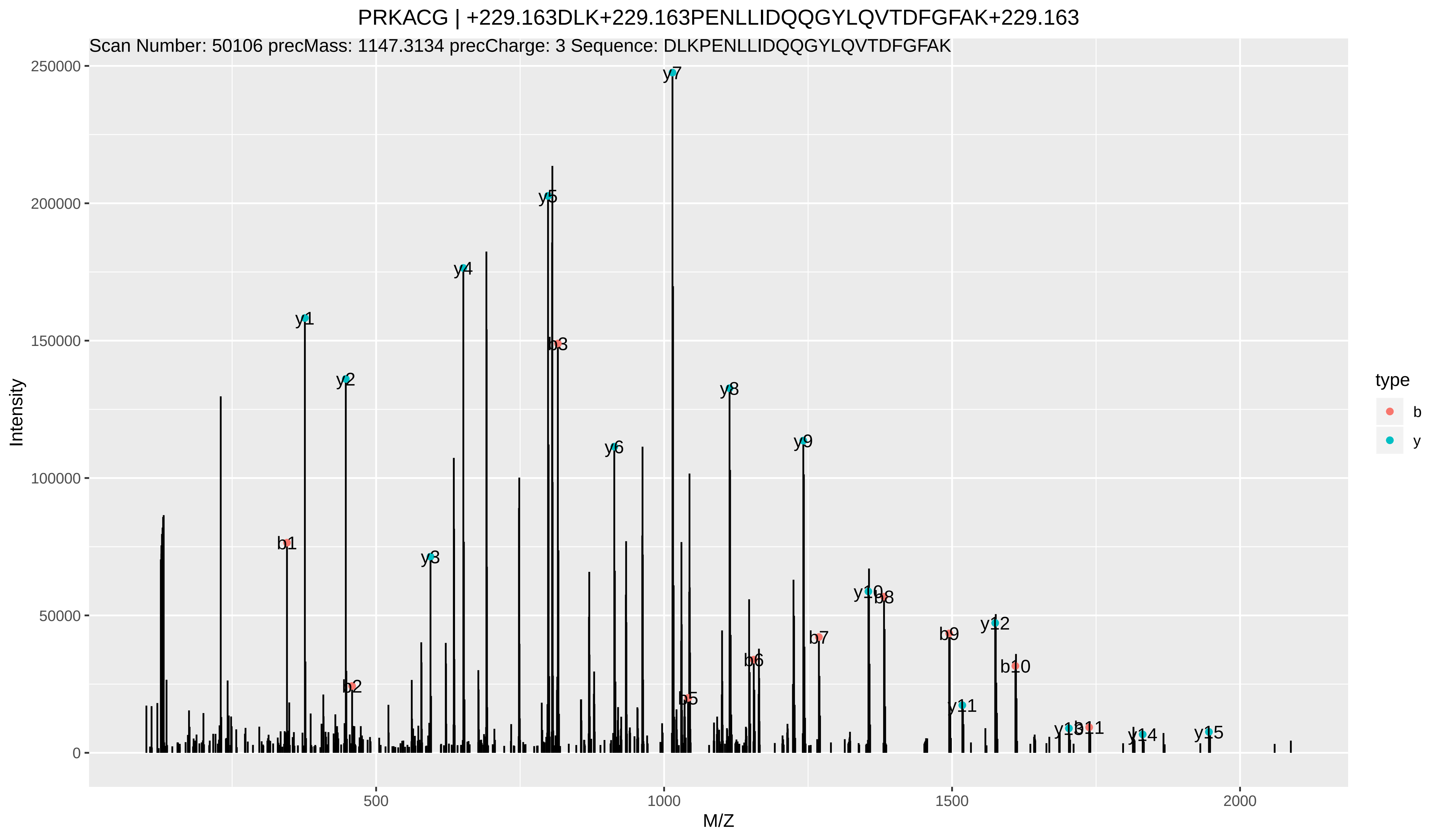
PRKACG | +229.163DLK+229.163PENLLIDQQGYLQVTDFGFAK+229.163
Scan Number: 50106 precMass: 1147.3134 precCharge: 3 Sequence: DLKPENLLIDQQGYLQVTDFGFAK
Intensity
M/Z
type
b
y
y1
y2
y3
y4
y5
y6
y7
y8
y9
y10
y11
y12
y13
y14
y15
b1
b2
b3
b5
b6
b7
b8
b9
b10
b11
0
50000
100000
150000
200000
250000
500
1000
1500
2000

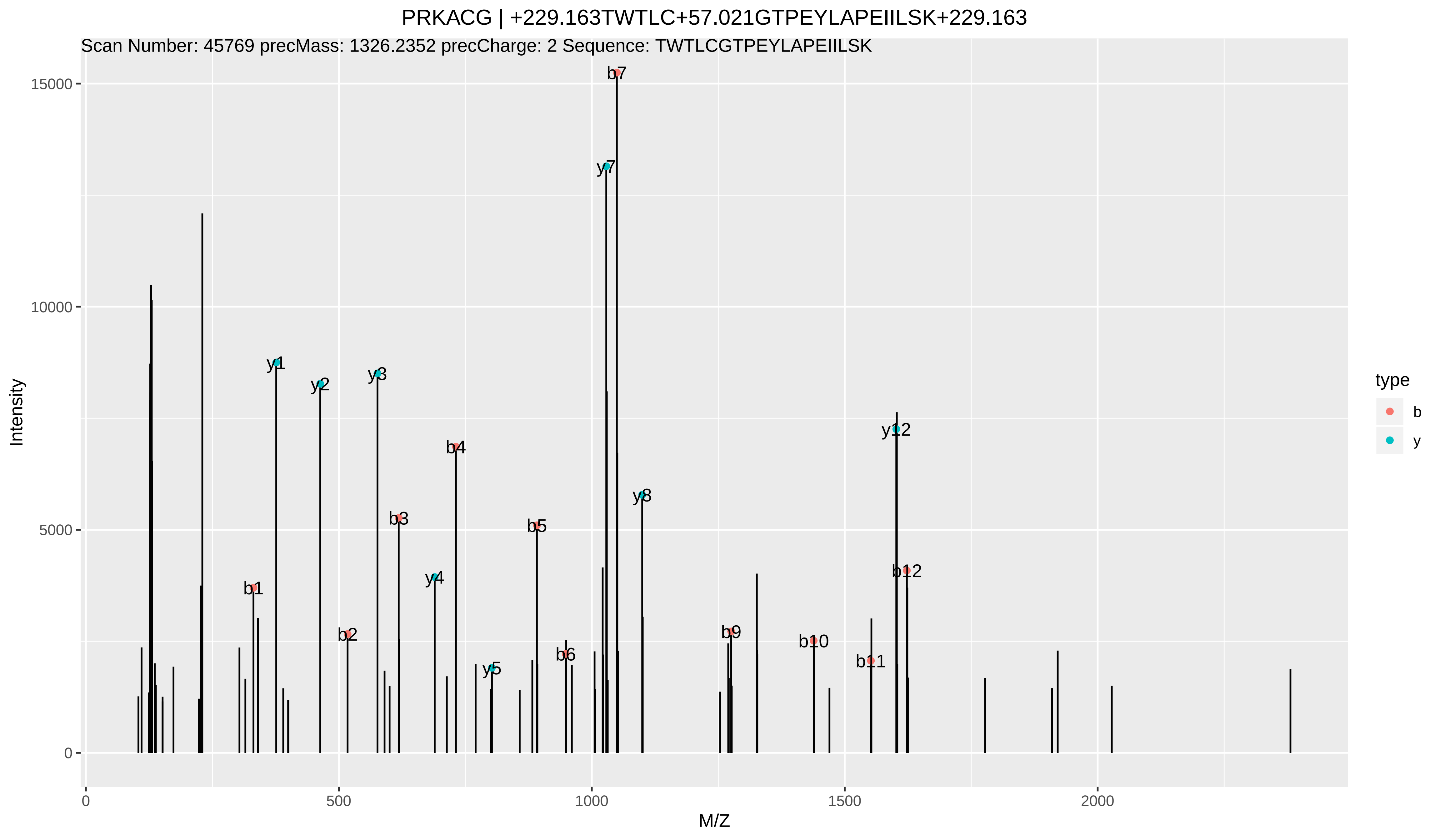

PRKACG | +229.163TWTLC+57.021GTPEYLAPEIILSK+229.163
Scan Number: 45769 precMass: 1326.2352 precCharge: 2 Sequence: TWTLCGTPEYLAPEIILSK
Intensity
M/Z
0
5000
10000
15000
0
500
1000
1500
2000
b7
y7
y1
y2
y3
b4
y12
y8
b3
b5
b12
y4
b1
b9
b2
b10
b6
b11
y5
type
b
y

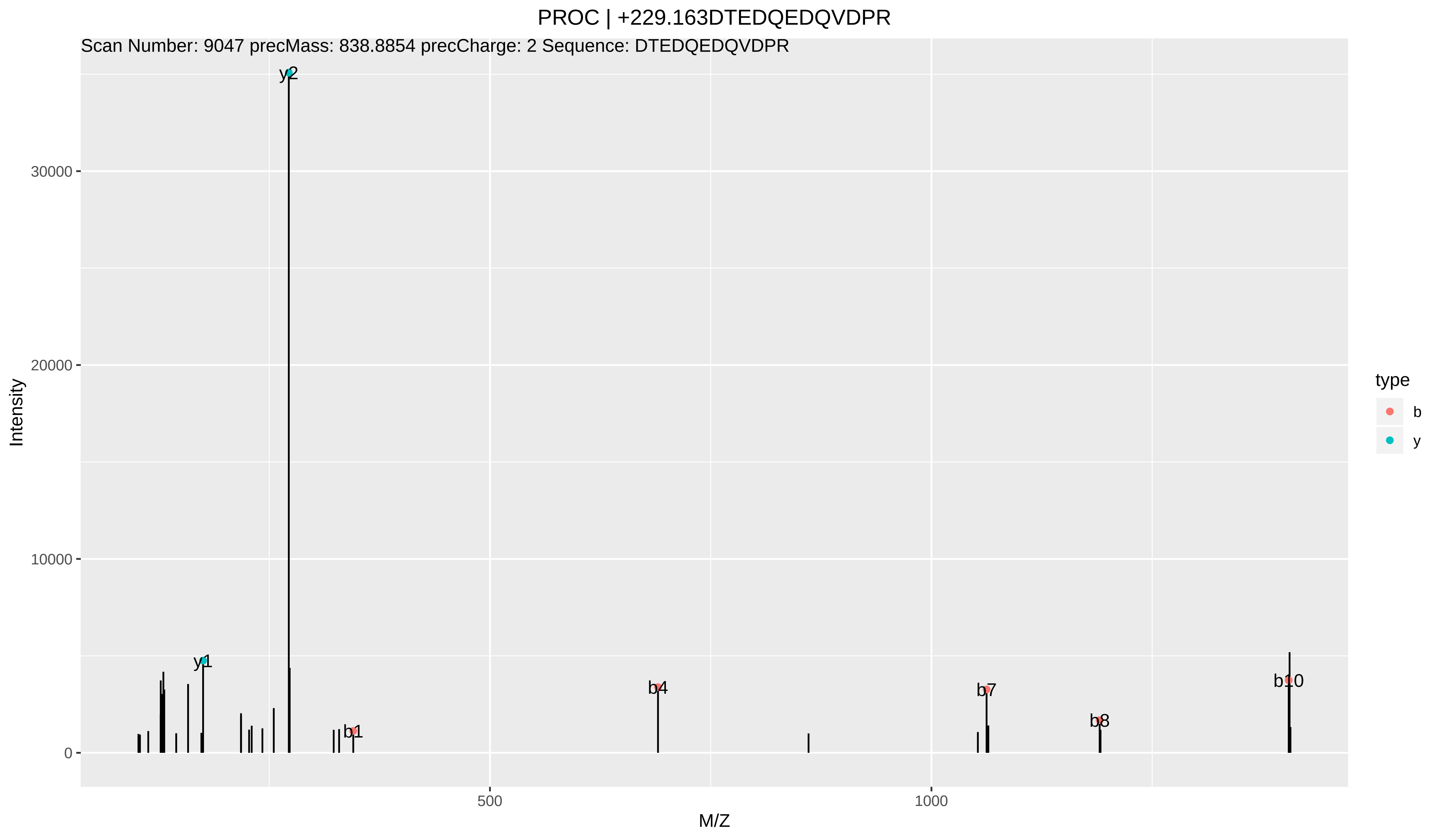

PROC | +229.163DTEDQEDQVDPR
Scan Number: 9047 precMass: 838.8854 precCharge: 2 Sequence: DTEDQEDQVDPR
y2
y1
b1
b4
b7
b8
b10
30000
20000
10000
0
500
1000
M/Z
Intensity
type
b
y

PROCR | +229.163HISAENTK+229.163

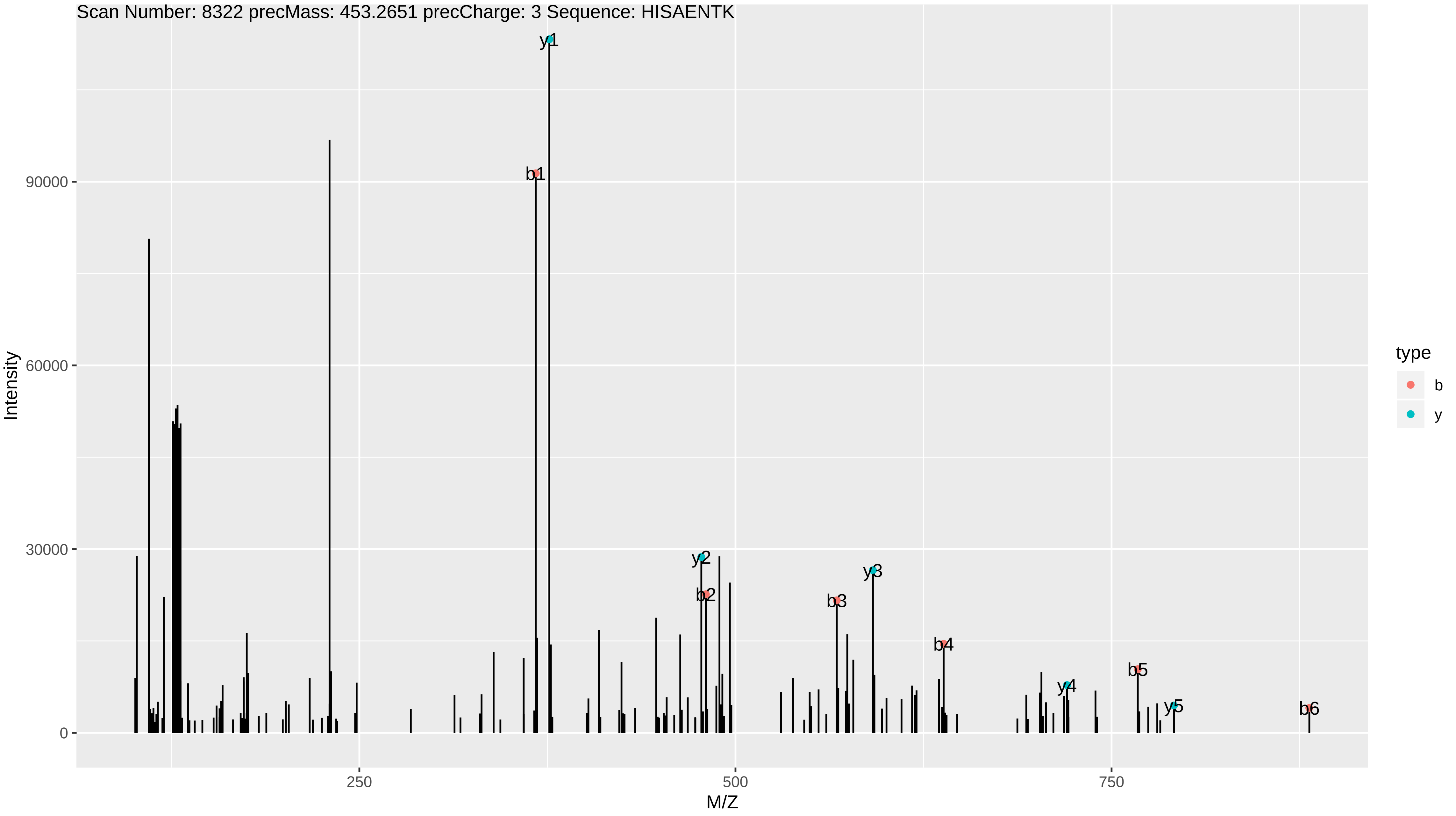

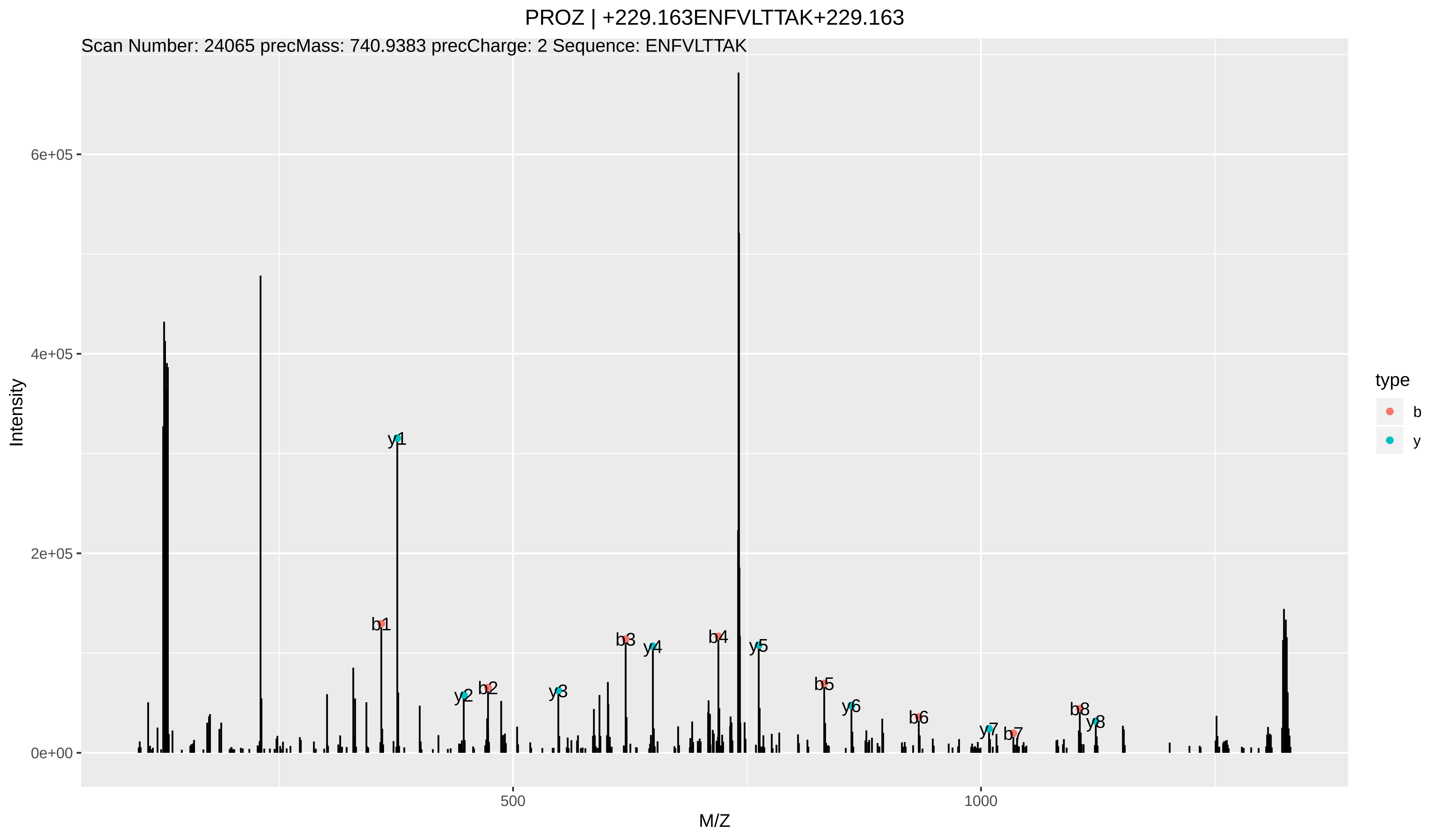

PROZ | +229.163ENFVLTTAK+229.163
Scan Number: 24065 precMass: 740.9383 precCharge: 2 Sequence: ENFVLTTAK
Intensity
6e+05
4e+05
2e+05
0e+00
M/Z
500
1000
y1
b1
y2
b2
y3
b3
y4
b4
y5
b5
y6
b6
y7
b7
b8
y8
type
b
y

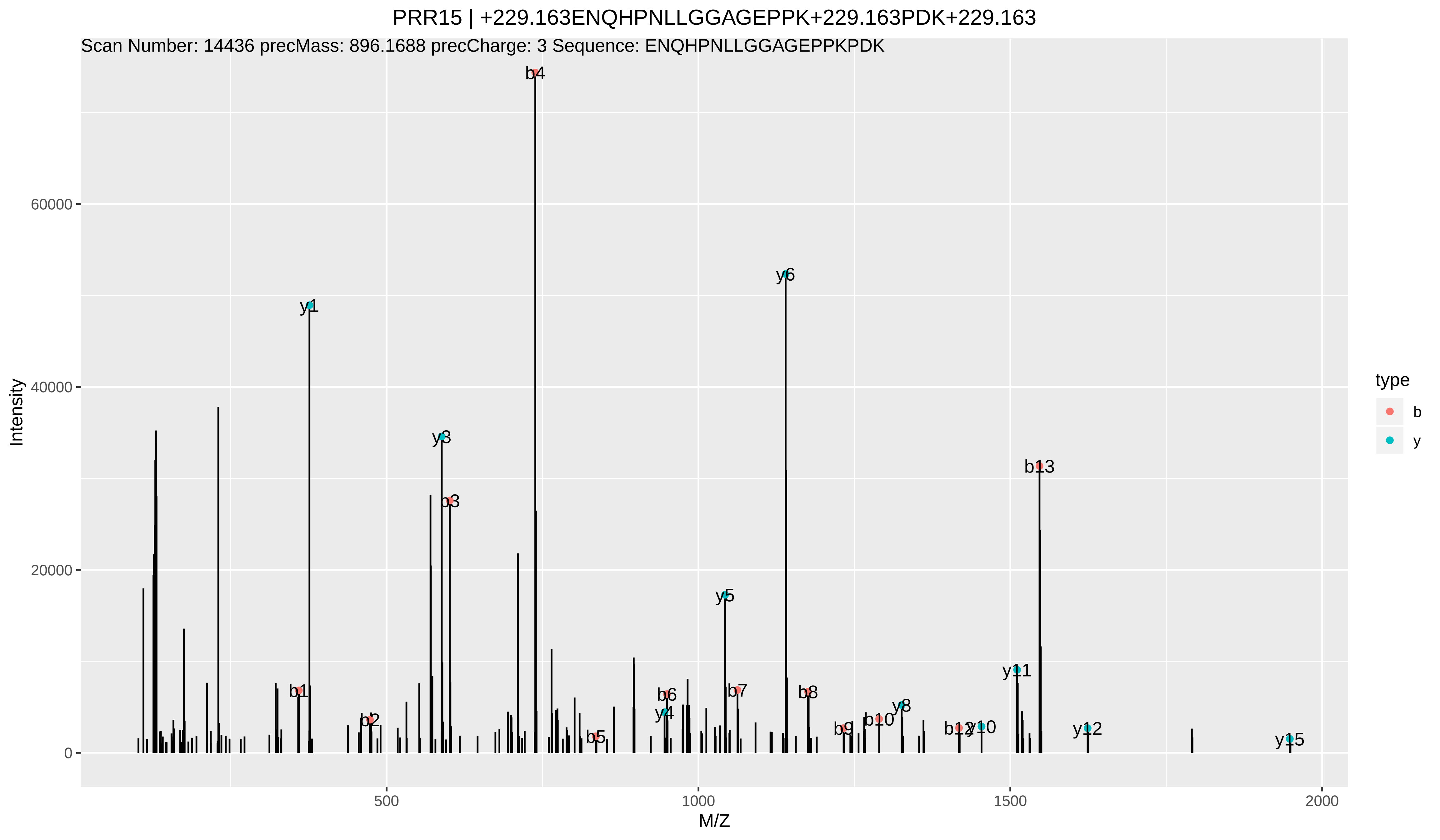
PRR15 | +229.163ENQHPNLLGGAGEPPK+229.163PDK+229.163
Scan Number: 14436 precMass: 896.1688 precCharge: 3 Sequence: ENQHPNLLGGAGEPPKPDK
Intensity
M/Z
0
20000
40000
60000
500
1000
1500
2000
b4
y6
y1
y3
b3
b13
y5
y11
b1
b6
b7
b8
y4
y8
b2
b10
b5
b9
b12
y10
y12
y15
type
b
y

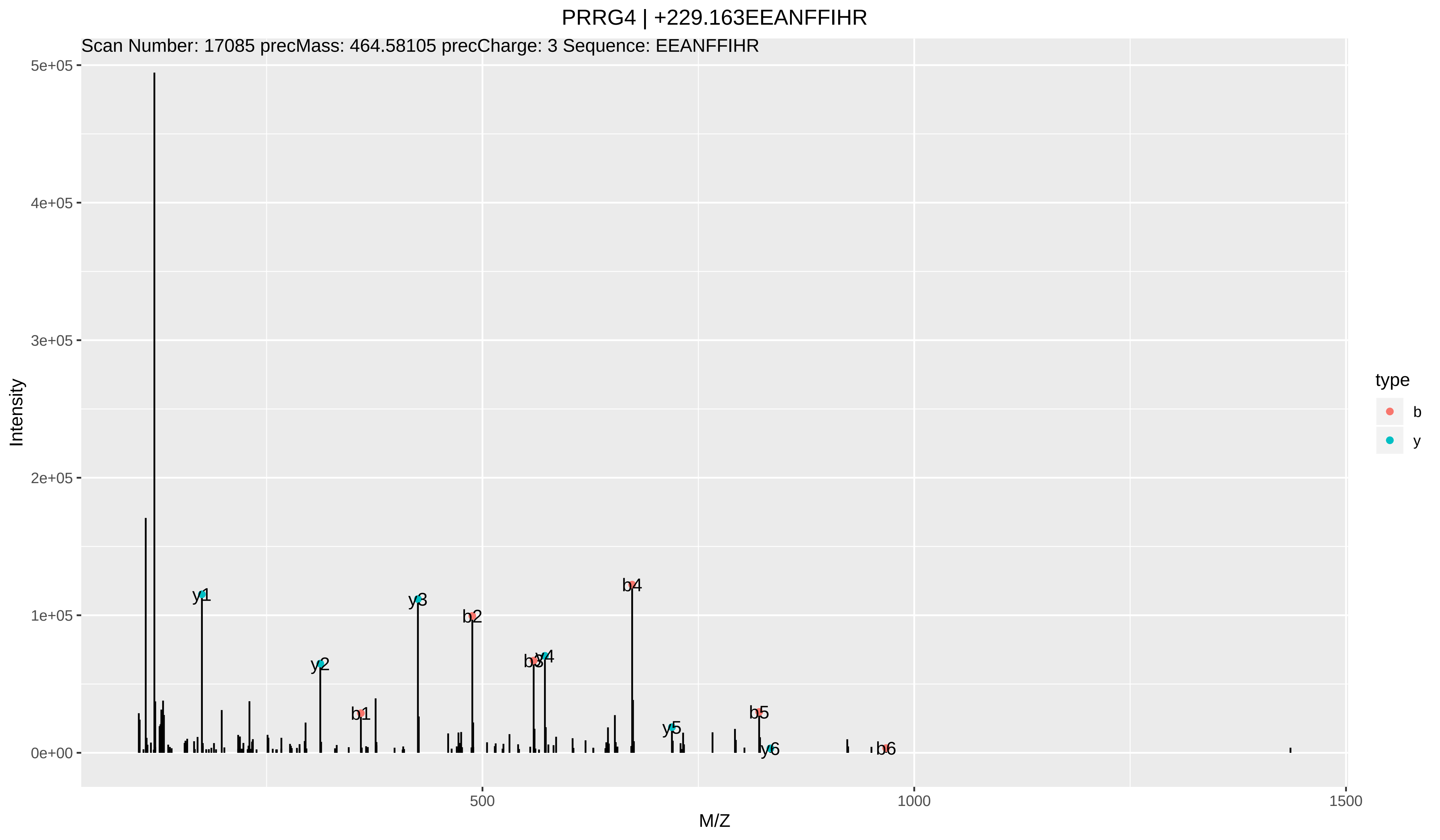
PRRG4 | +229.163EEANFFIHR
Scan Number: 17085 precMass: 464.58105 precCharge: 3 Sequence: EEANFFIHR
Intensity
M/Z
5e+05
4e+05
3e+05
2e+05
1e+05
0e+00
500
1000
1500
y1
y2
b1
y3
b2
b3
y4
b4
y5
b5
y6
b6
type
b
y

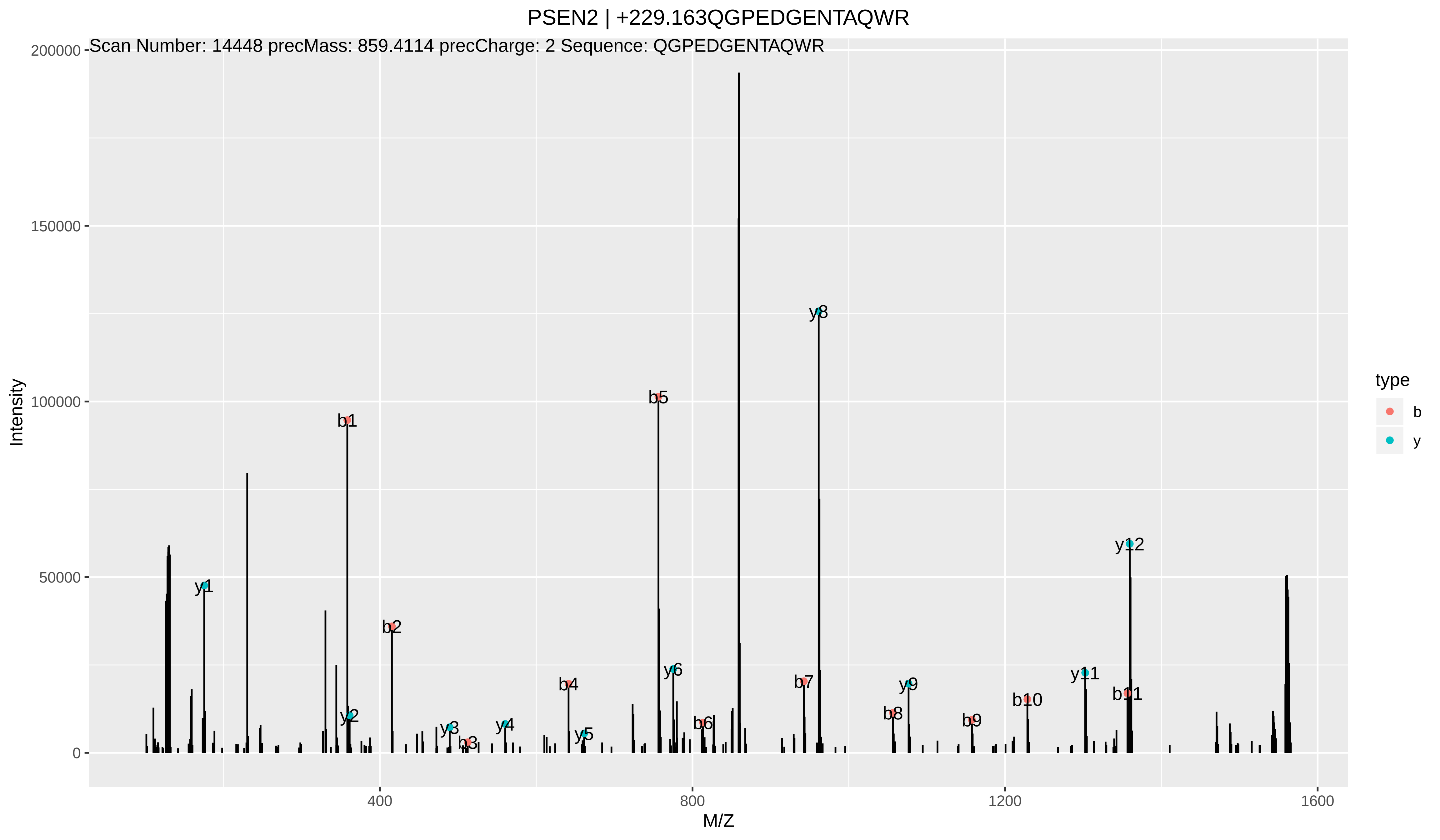

PSEN2 | +229.163QGPEDGENTAQWR
Scan Number: 14448 precMass: 859.4114 precCharge: 2 Sequence: QGPEDGENTAQWR
Intensity
M/Z
0
50000
100000
150000
200000
400
800
1200
1600
b1
b2
b3
b4
b5
b6
b7
b8
b9
b10
b11
y1
y2
y3
y4
y5
y6
y8
y9
y11
y12
type
b
y

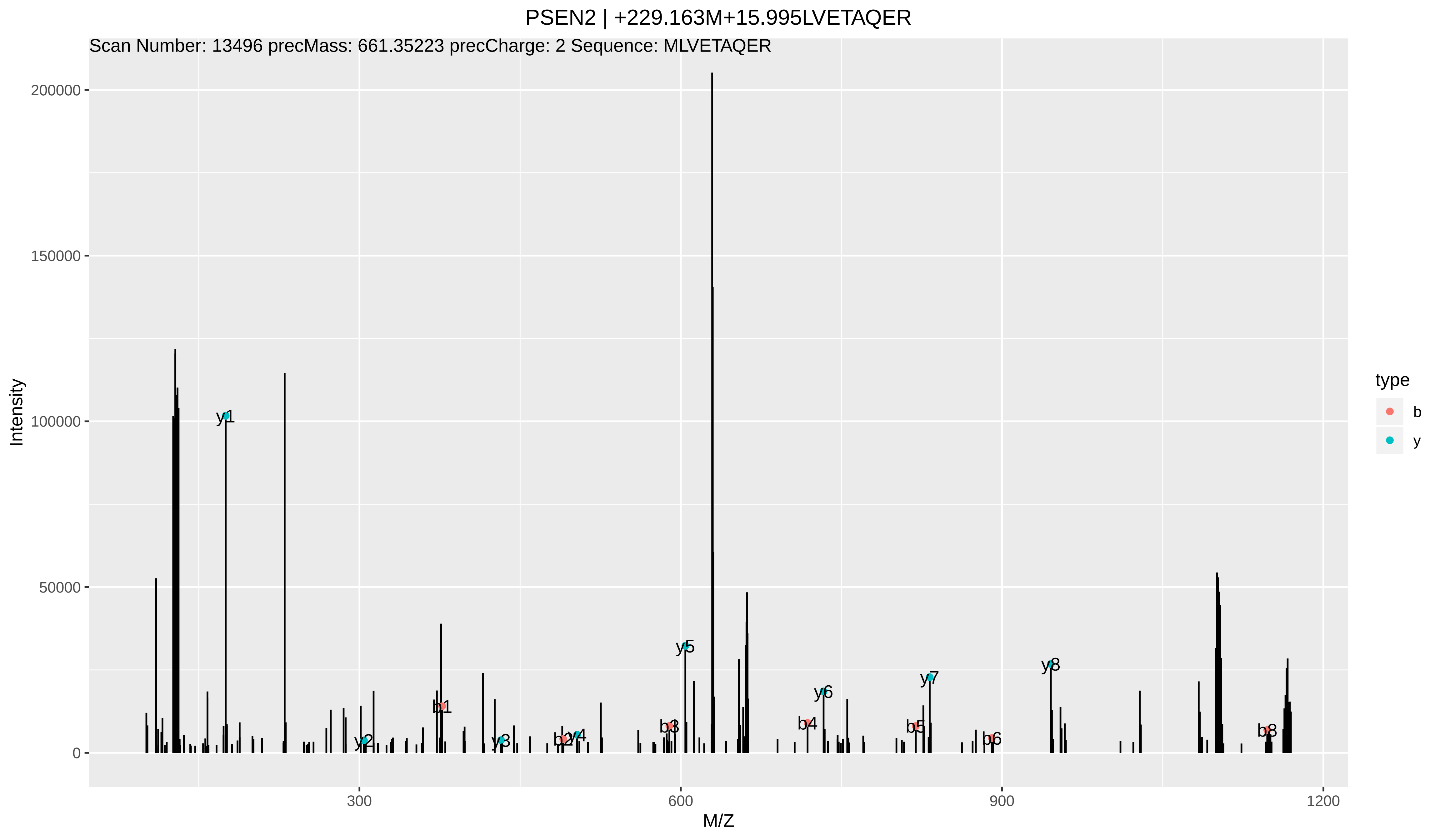
PSEN2 | +229.163M+15.995LVETAQER
Scan Number: 13496 precMass: 661.35223 precCharge: 2 Sequence: MLVETAQER
Intensity
M/Z
type
b
y
y1
y2
y3
y4
y5
y6
y7
y8
b1
b2
b3
b4
b5
b6
b8
0
50000
100000
150000
200000
300
600
900
1200

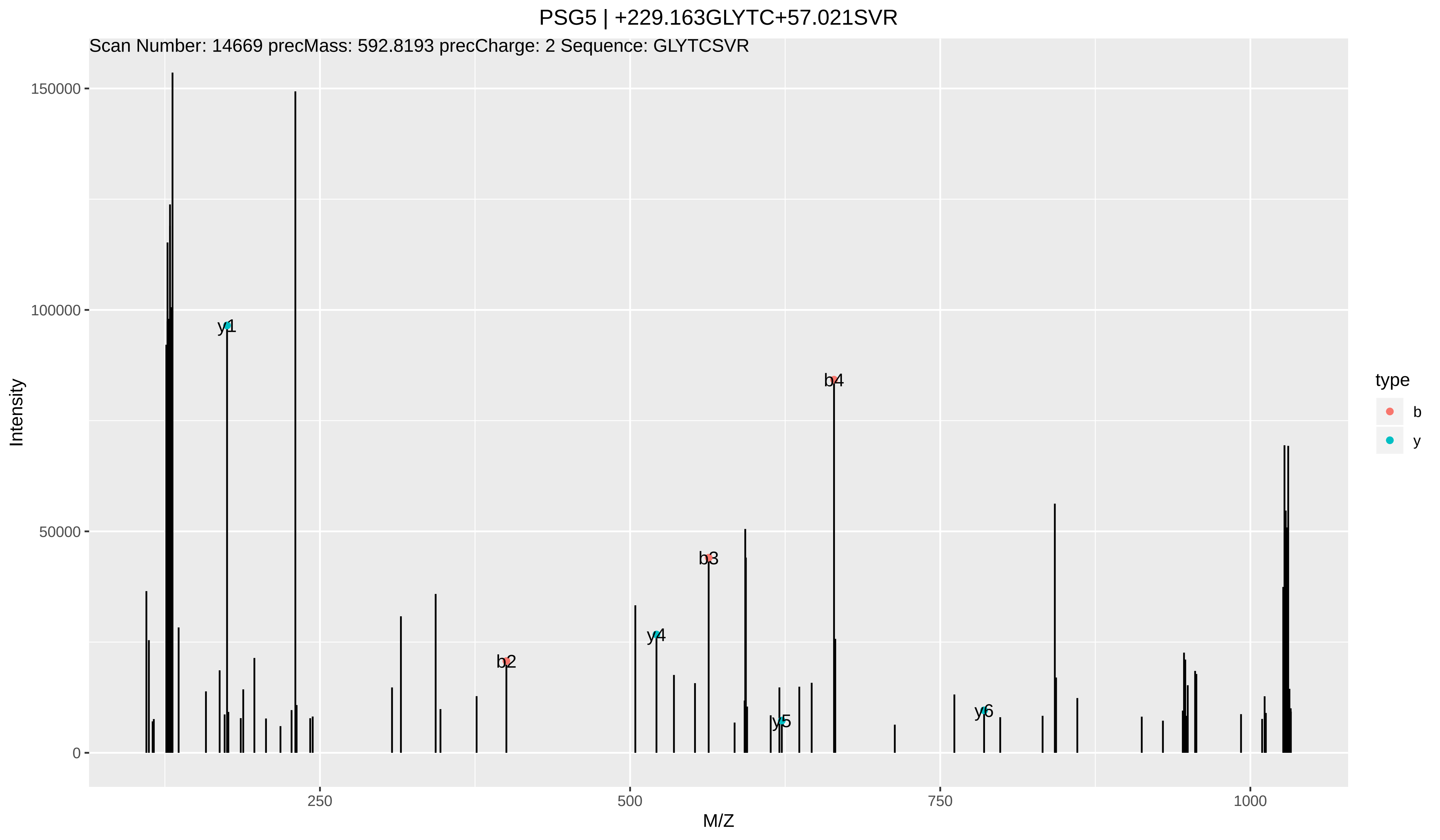

PSG5 | +229.163GLYTC+57.021SVR
Scan Number: 14669 precMass: 592.8193 precCharge: 2 Sequence: GLYTCSVR
Intensity
150000
100000
50000
0
M/Z
250
500
750
1000
y1
b4
b3
y4
b2
y5
y6
type
b
y

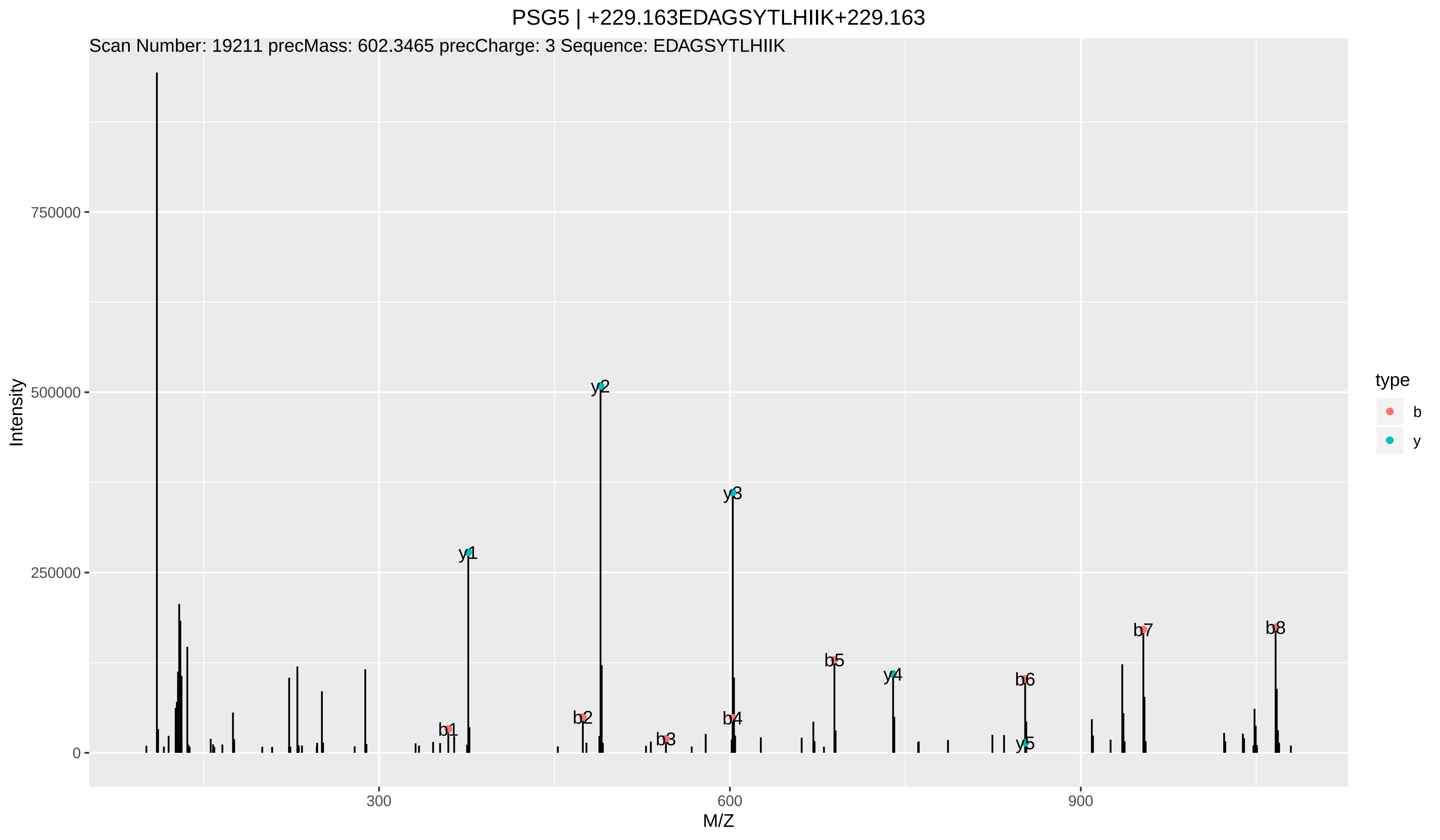
PSG5 | +229.163EDAGSYTLHIIK+229.163
Scan Number: 19211 precMass: 602.3465 precCharge: 3 Sequence: EDAGSYTLHIIK
Intensity
750000
500000
250000
0
300
600
900
M/Z
y1
y2
y3
y4
y5
b1
b2
b3
b4
b5
b6
b7
b8
type
b
y

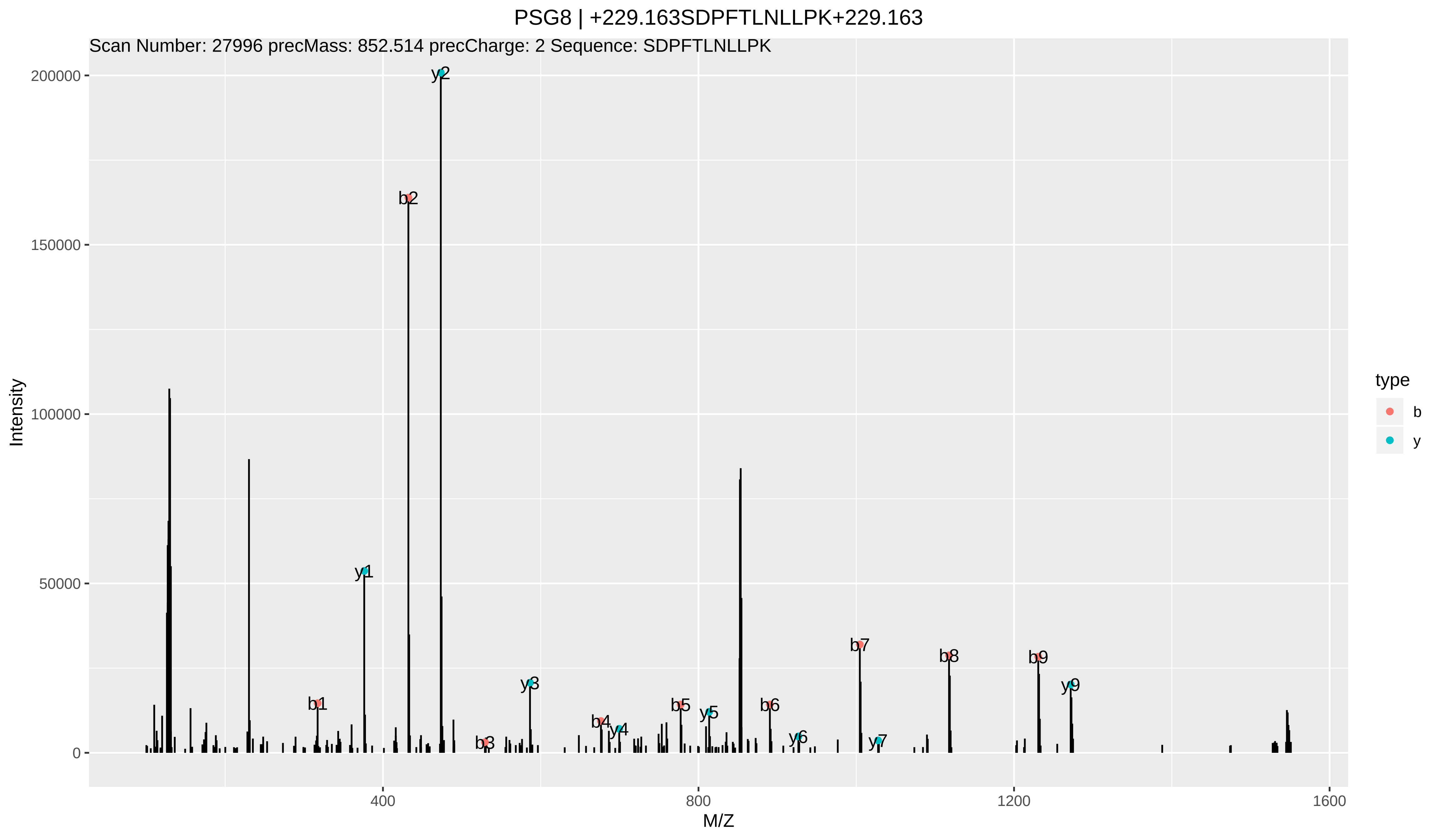

PSG8 | +229.163SDPFTLNLLPK+229.163
Scan Number: 27996 precMass: 852.514 precCharge: 2 Sequence: SDPFTLNLLPK
Intensity
M/Z
200000
150000
100000
50000
0
400
800
1200
1600
y2
b2
y1
b1
b3
y3
b4
y4
b5
y5
b6
y6
b7
y7
b8
b9
y9
type
b
y

PSG8 | +229.163SYPVTLNVLYGPDLPR

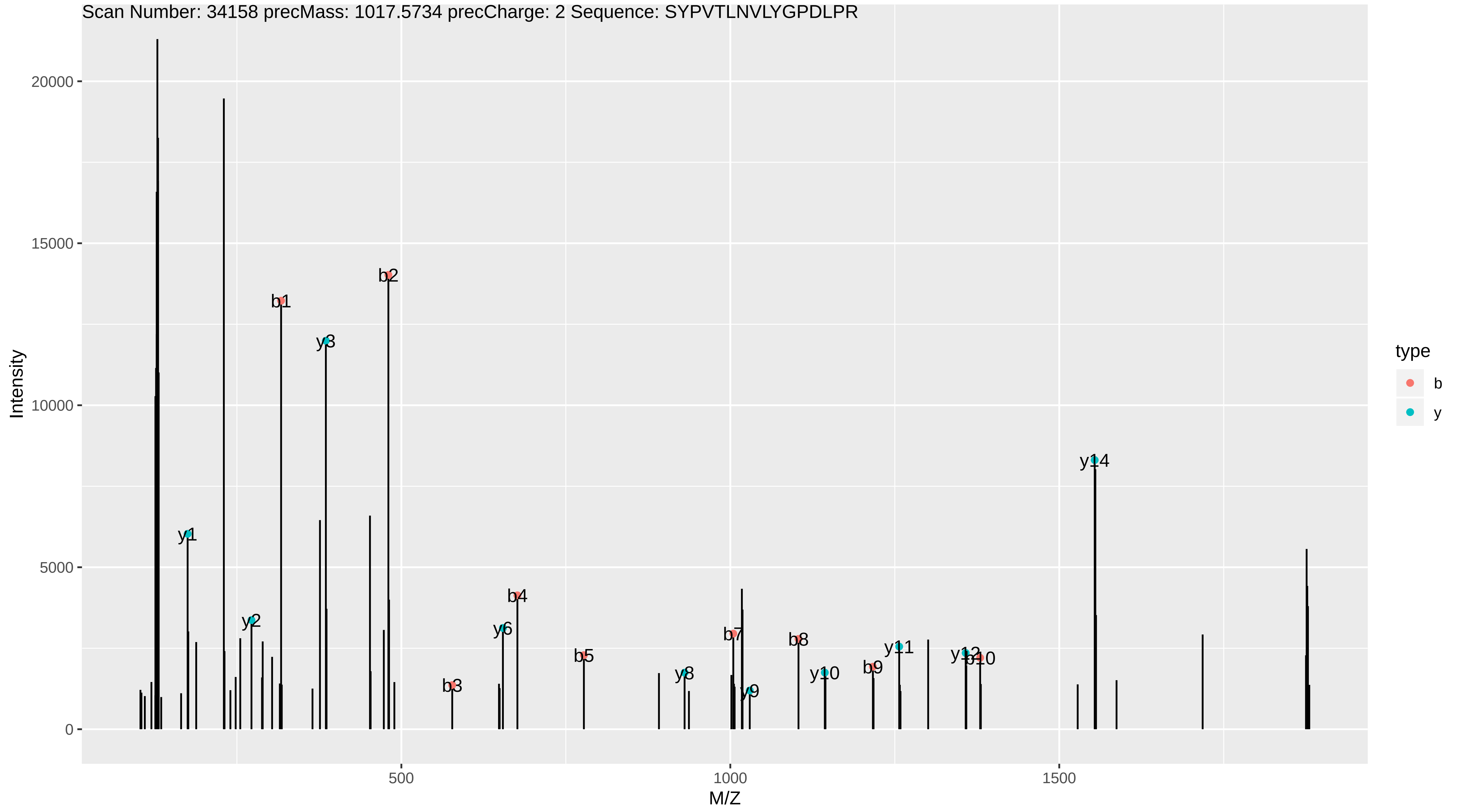

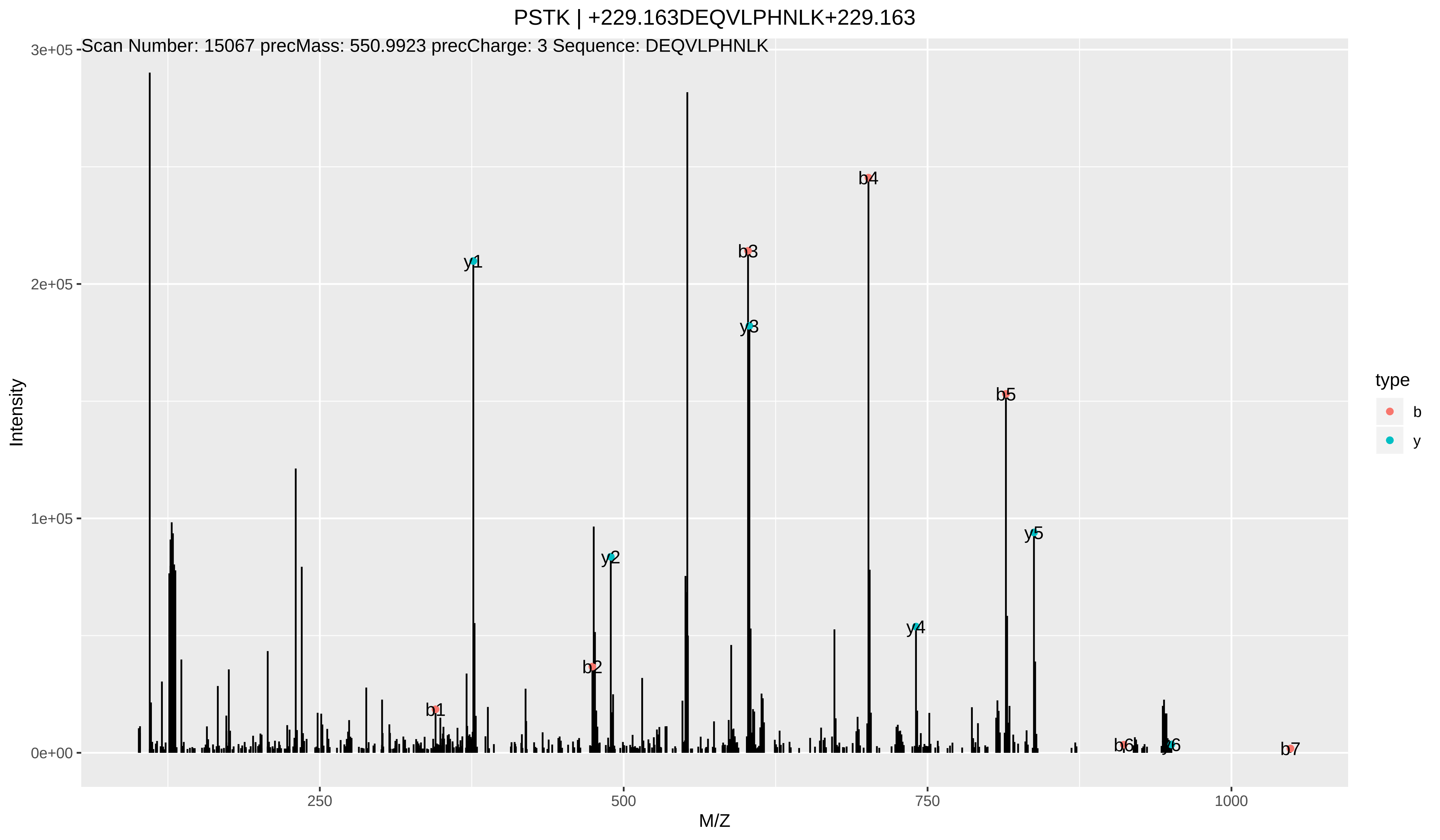

PSTK | +229.163DEQVLPHNLK+229.163
Scan Number: 15067 precMass: 550.9923 precCharge: 3 Sequence: DEQVLPHNLK
Intensity
3e+05
2e+05
1e+05
0e+00
M/Z
250
500
750
1000
y1
b3
b4
y3
b5
y5
y2
y4
b2
b1
b6
y6
b7
type
b
y

PSTPIP1 | +229.163DDELYEEVR

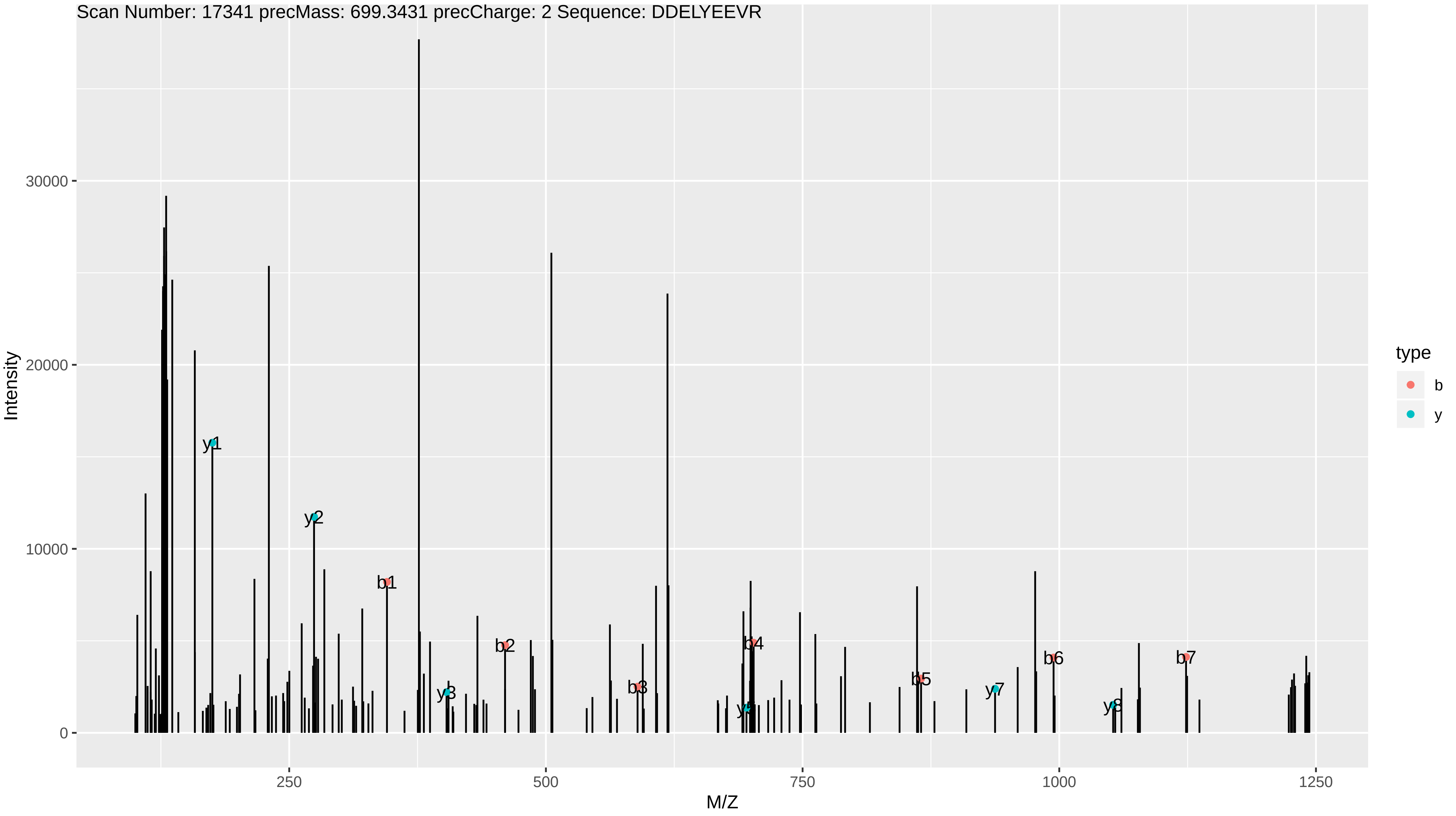

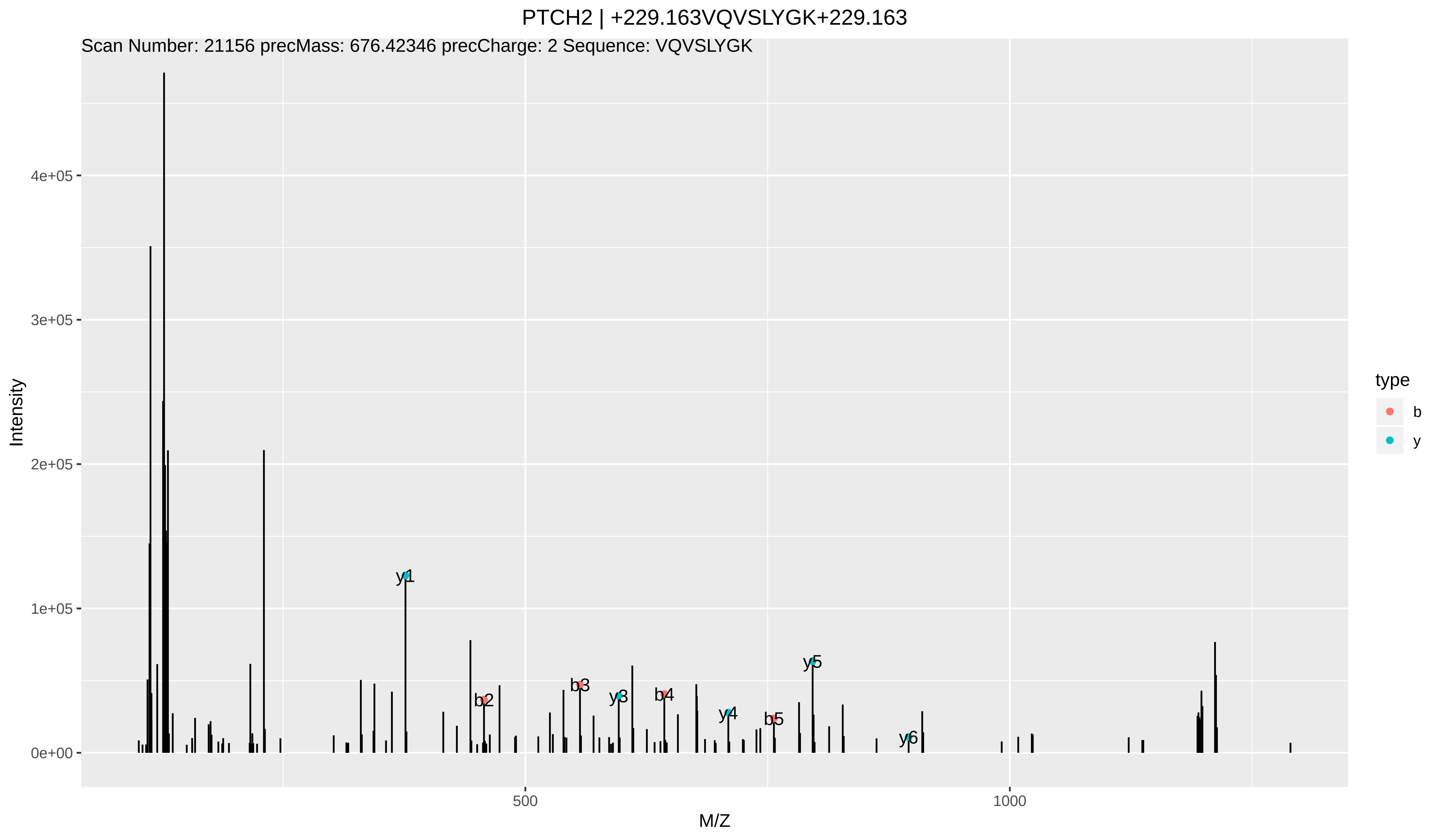
PTCH2 | +229.163VQVSLYGK+229.163
Scan Number: 21156 precMass: 676.42346 precCharge: 2 Sequence: VQVSLYGK
Intensity
M/Z
0e+00
1e+05
2e+05
3e+05
4e+05
500
1000
y1
b2
b3
y3
b4
y4
b5
y5
y6
type
b
y

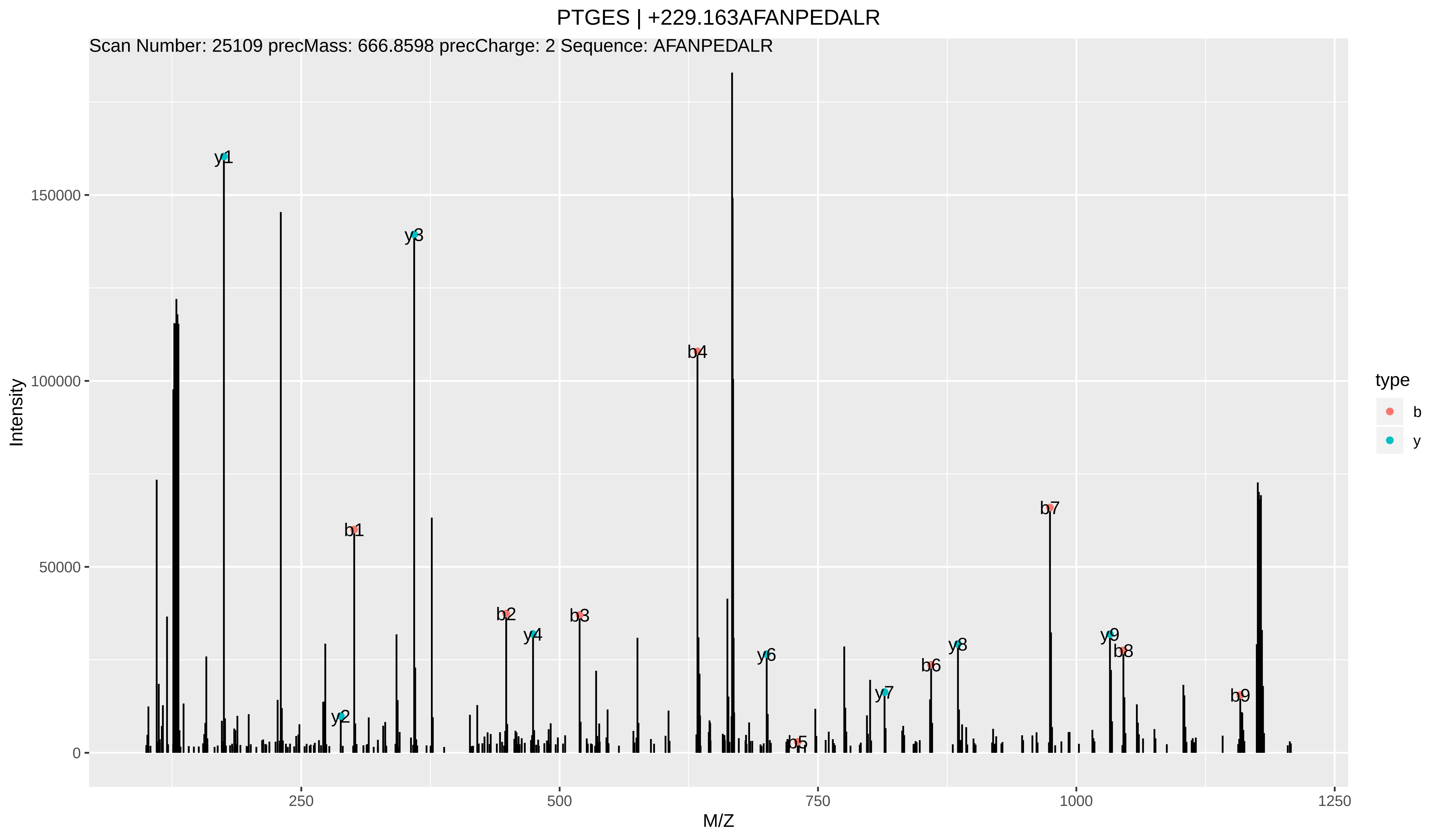

PTGES | +229.163AFANPEDALR
Scan Number: 25109 precMass: 666.8598 precCharge: 2 Sequence: AFANPEDALR
Intensity
M/Z
0
50000
100000
150000
250
500
750
1000
1250
y1
y2
y3
y4
y6
y7
y8
y9
b1
b2
b3
b4
b5
b6
b7
b8
b9
type
b
y

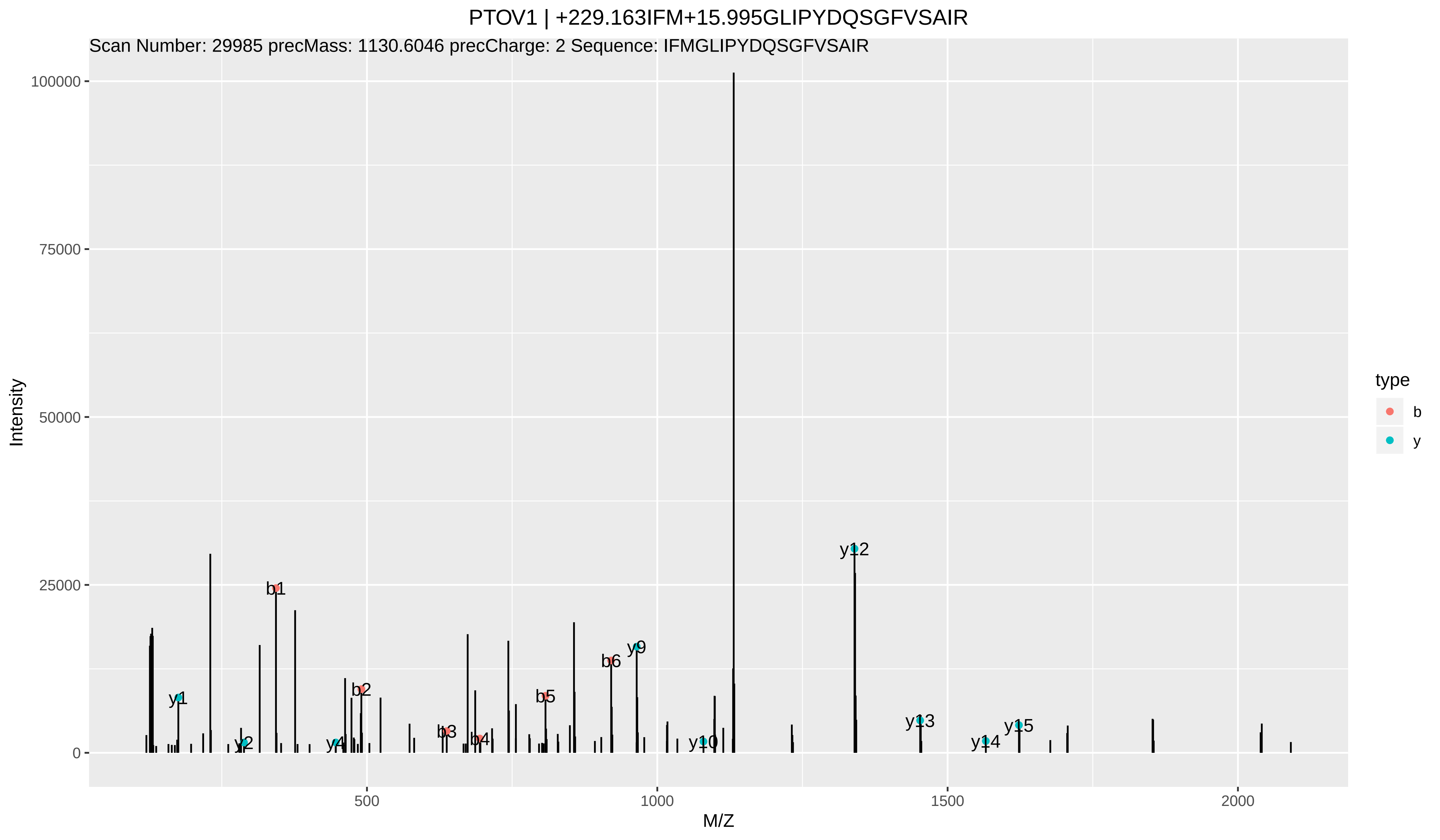

PTOV1 | +229.163IFM+15.995GLIPYDQSGFVSAIR
Scan Number: 29985 precMass: 1130.6046 precCharge: 2 Sequence: IFMGLIPYDQSGFVSAIR
Intensity
100000
75000
50000
25000
0
M/Z
500
1000
1500
2000
y1
y2
b1
y4
b2
b3
b4
b5
b6
y9
y10
y12
y13
y14
y15
type
b
y

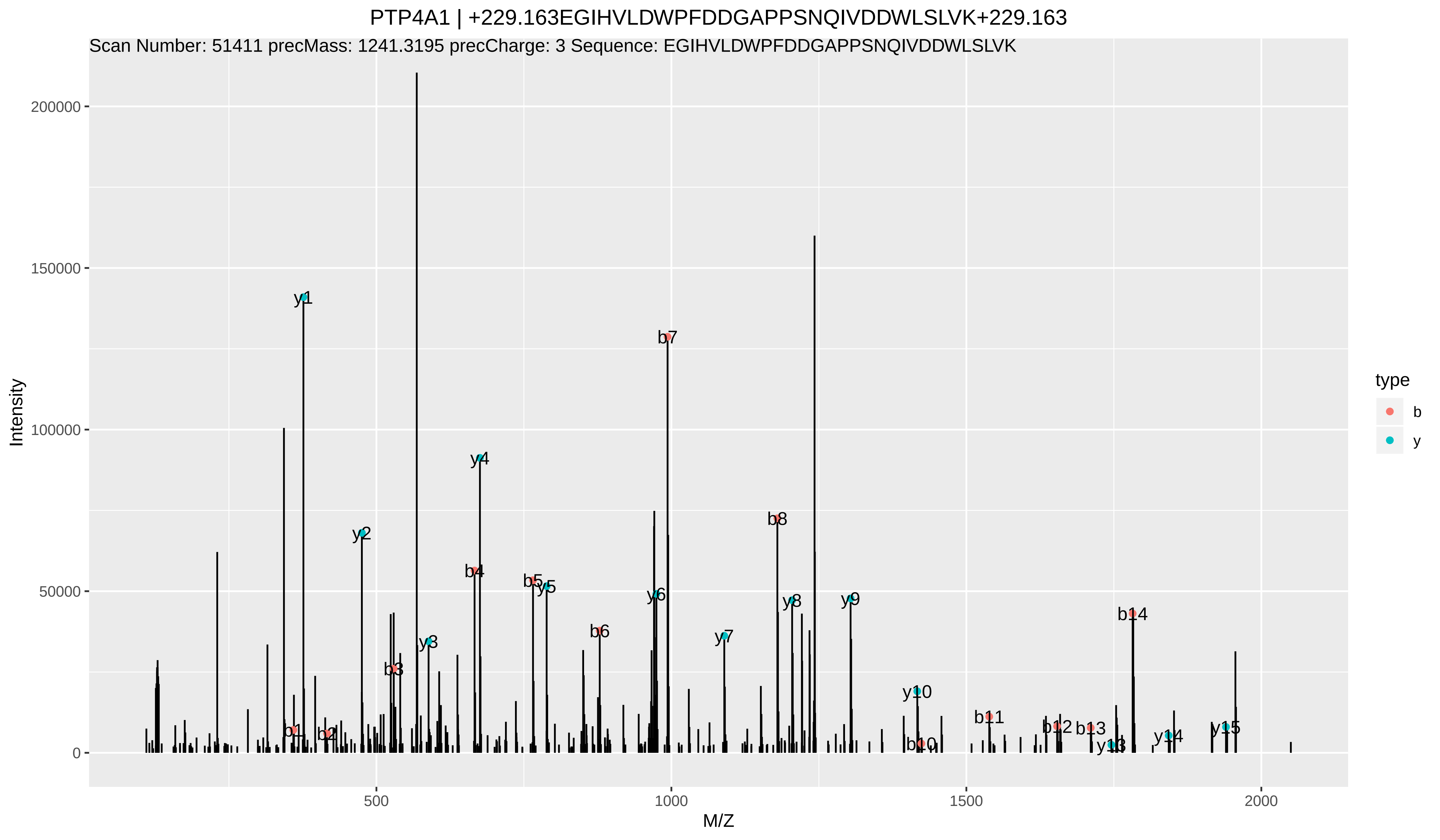

PTP4A1 | +229.163EGIHVLDWPFDDGAPPSNQIVDDWLSLVK+229.163
Scan Number: 51411 precMass: 1241.3195 precCharge: 3 Sequence: EGIHVLDWPFDDGAPPSNQIVDDWLSLVK
Intensity
0
50000
100000
150000
200000
M/Z
500
1000
1500
2000
y1
y2
y3
y4
y5
y6
y7
y8
y9
y10
y13
y14
y15
b1
b2
b3
b4
b5
b6
b7
b8
b10
b11
b12
b13
b14
type
b
y

PTP4A1 | +229.163FLITHNPTNATLNK+229.163

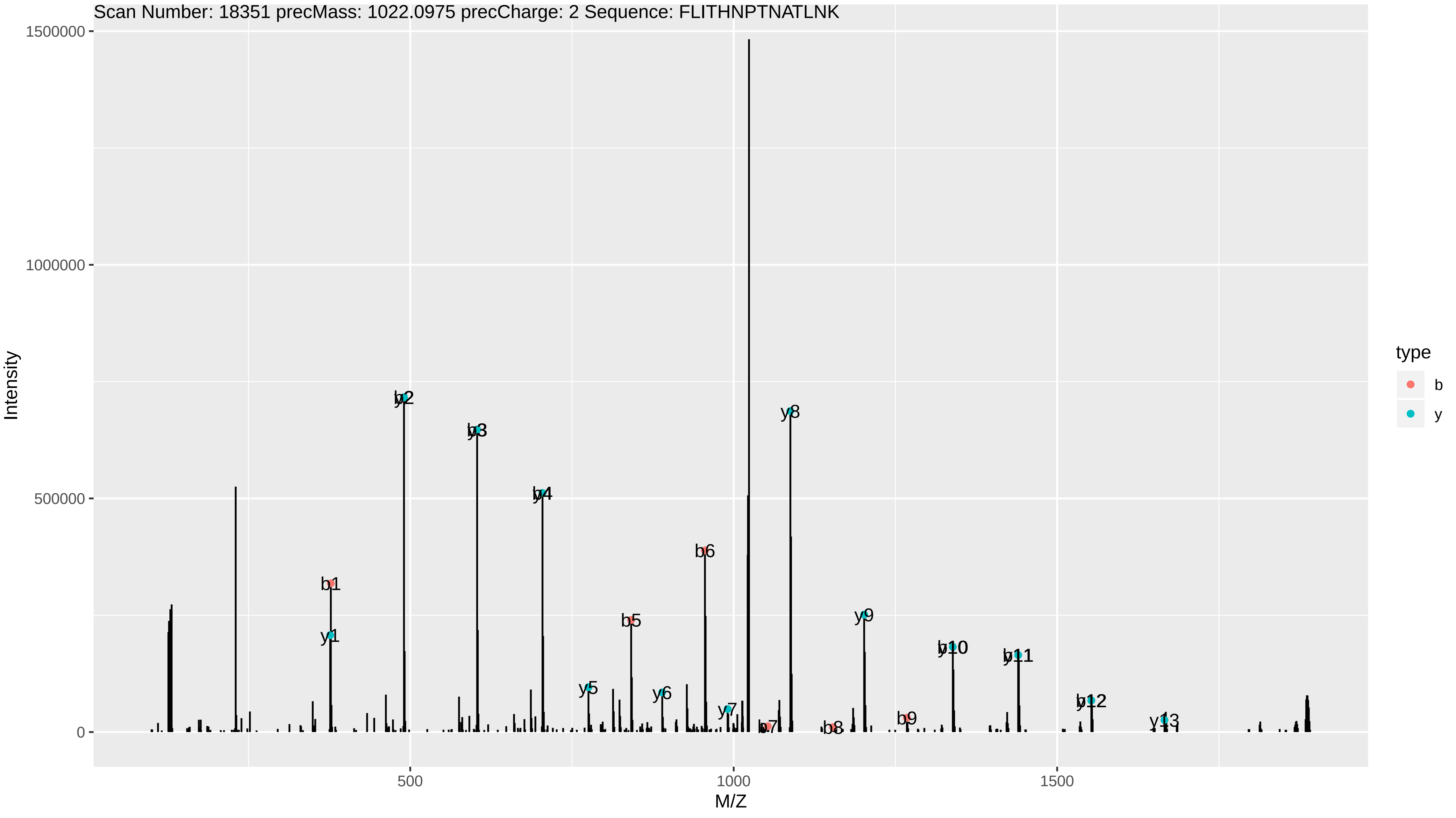

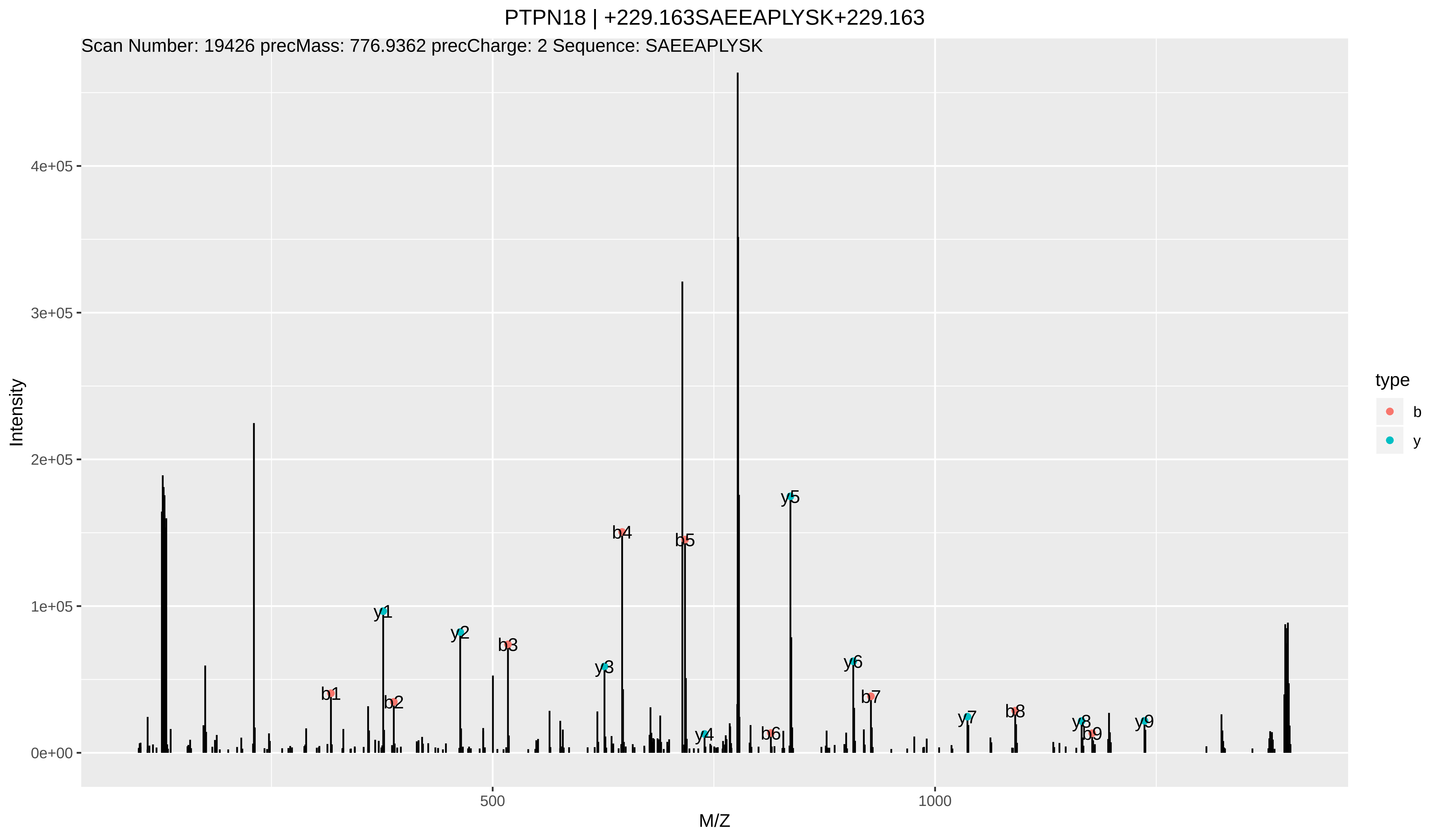
PTPN18 | +229.163SAEEAPLYSK+229.163
Scan Number: 19426 precMass: 776.9362 precCharge: 2 Sequence: SAEEAPLYSK
Intensity
4e+05
3e+05
2e+05
1e+05
0e+00
M/Z
500
1000
y1
y2
b3
y3
b4
b5
y4
b6
y5
y6
b7
y7
b8
y8
b9
y9
b1
b2
type
b
y

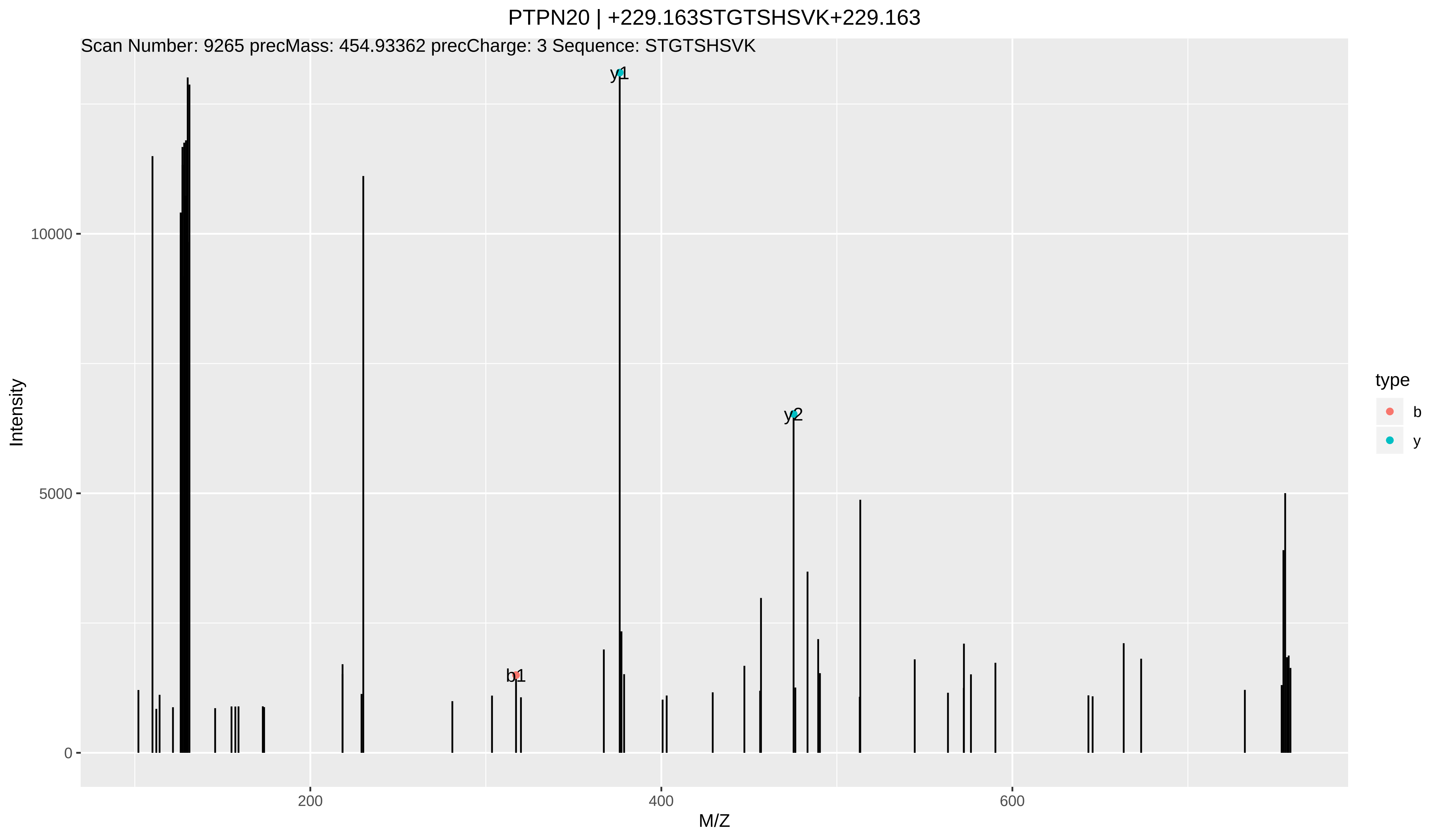
PTPN20 | +229.163STGTSHSVK+229.163
Scan Number: 9265 precMass: 454.93362 precCharge: 3 Sequence: STGTSHSVK
y1
y2
b1
10000
5000
0
200
400
600
Intensity
M/Z
type
b
y

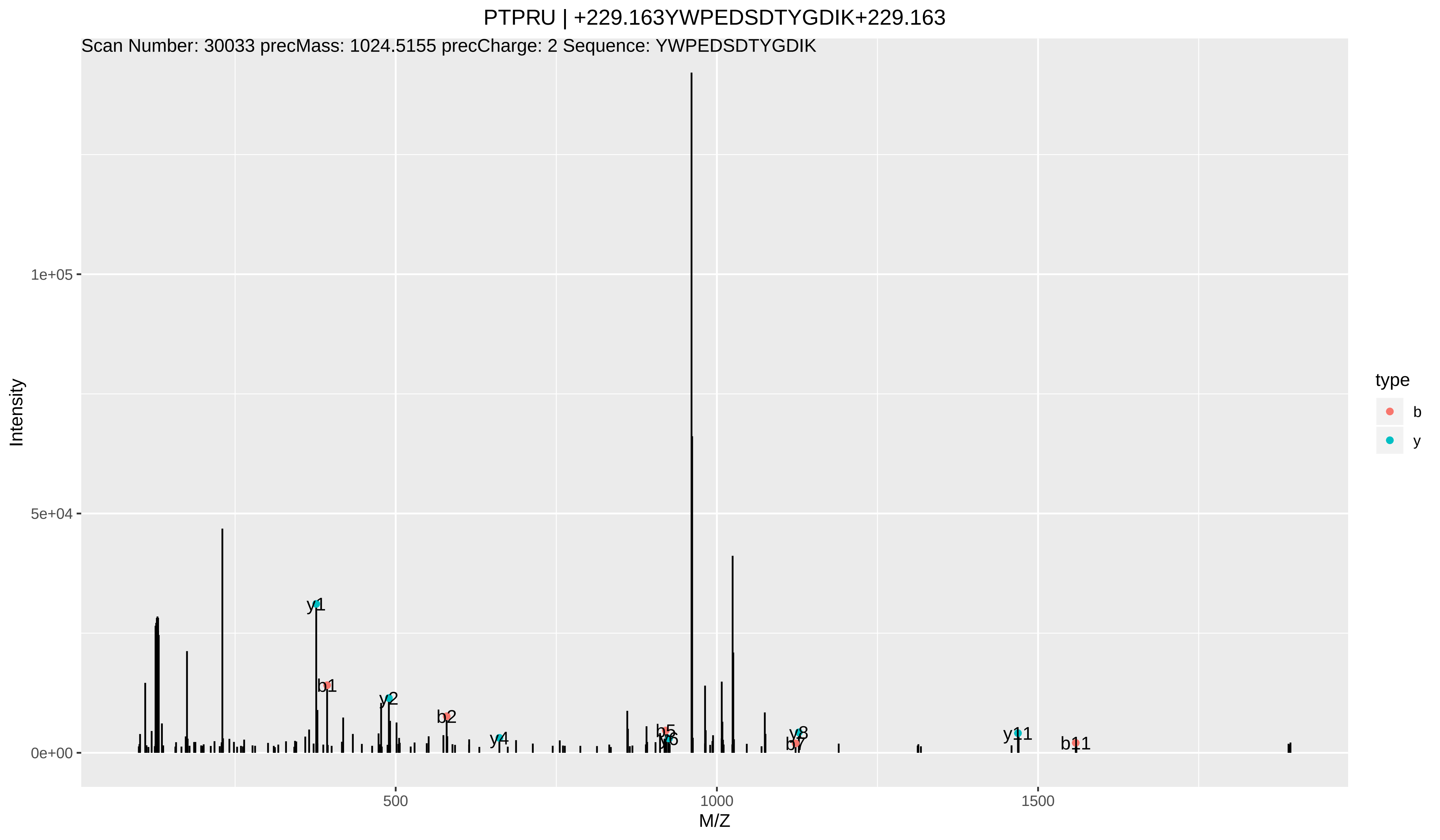

PTPRU | +229.163YWPEDSDTYGDIK+229.163
Scan Number: 30033 precMass: 1024.5155 precCharge: 2 Sequence: YWPEDSDTYGDIK
Intensity
1e+05
5e+04
0e+00
M/Z
500
1000
1500
y1
b1
y2
b2
y4
b5
y6
y8
b7
y11
b11
type
b
y

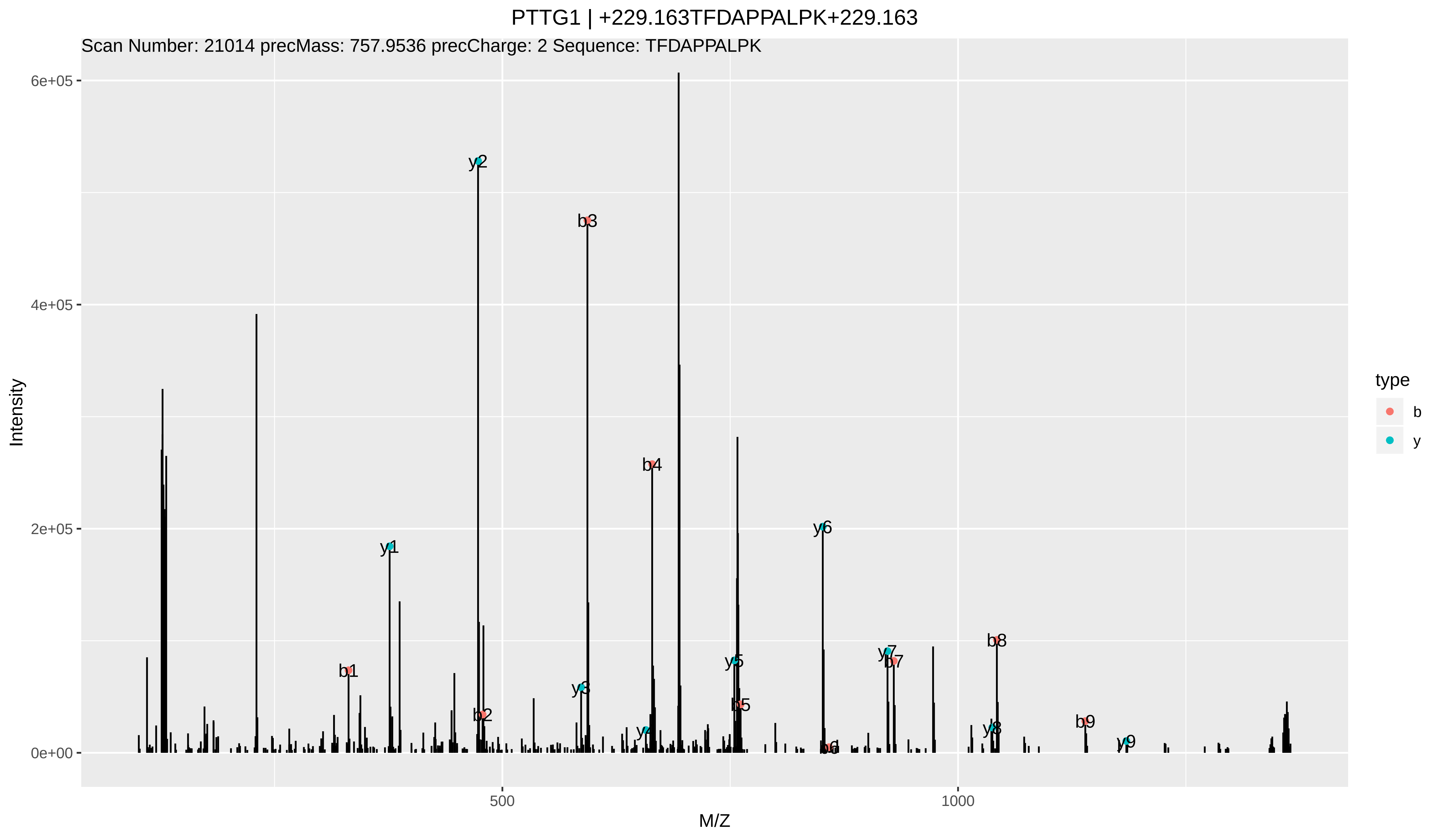
PTTG1 | +229.163TFDAPPALPK+229.163
Scan Number: 21014 precMass: 757.9536 precCharge: 2 Sequence: TFDAPPALPK
Intensity
6e+05
4e+05
2e+05
0e+00
M/Z
500
1000
y2
b3
b4
y6
y1
b1
b2
y3
y5
b5
y7
b7
b8
y8
b9
y9
type
b
y

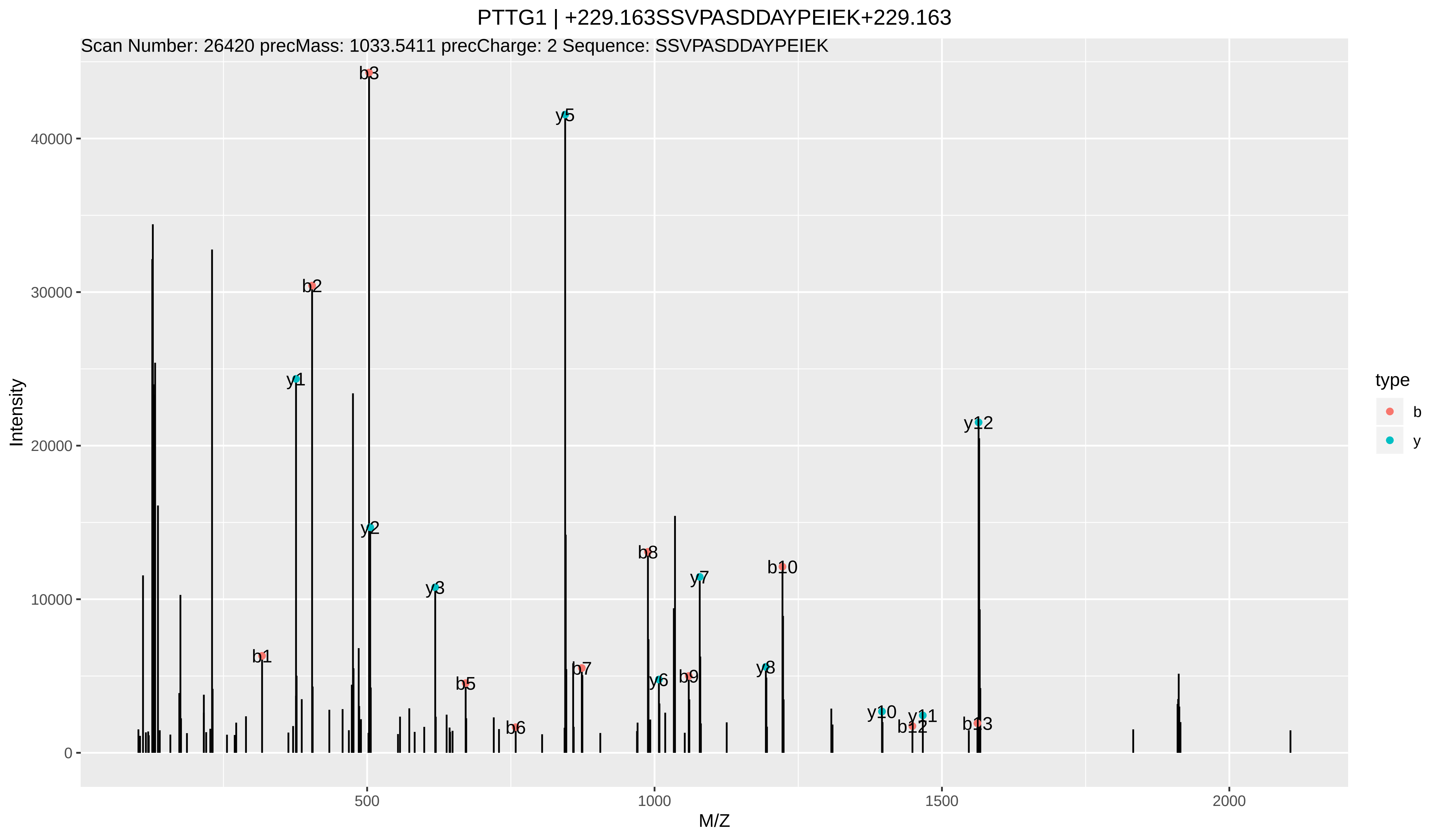

PTTG1 | +229.163SSVPASDDAYPEIEK+229.163
Scan Number: 26420 precMass: 1033.5411 precCharge: 2 Sequence: SSVPASDDAYPEIEK
Intensity
M/Z
0
10000
20000
30000
40000
500
1000
1500
2000
b3
y5
b2
y1
y12
y2
b8
b10
y7
y3
b1
b7
b5
y6
b9
y8
y10
y11
b12
b13
b6
type
b
y

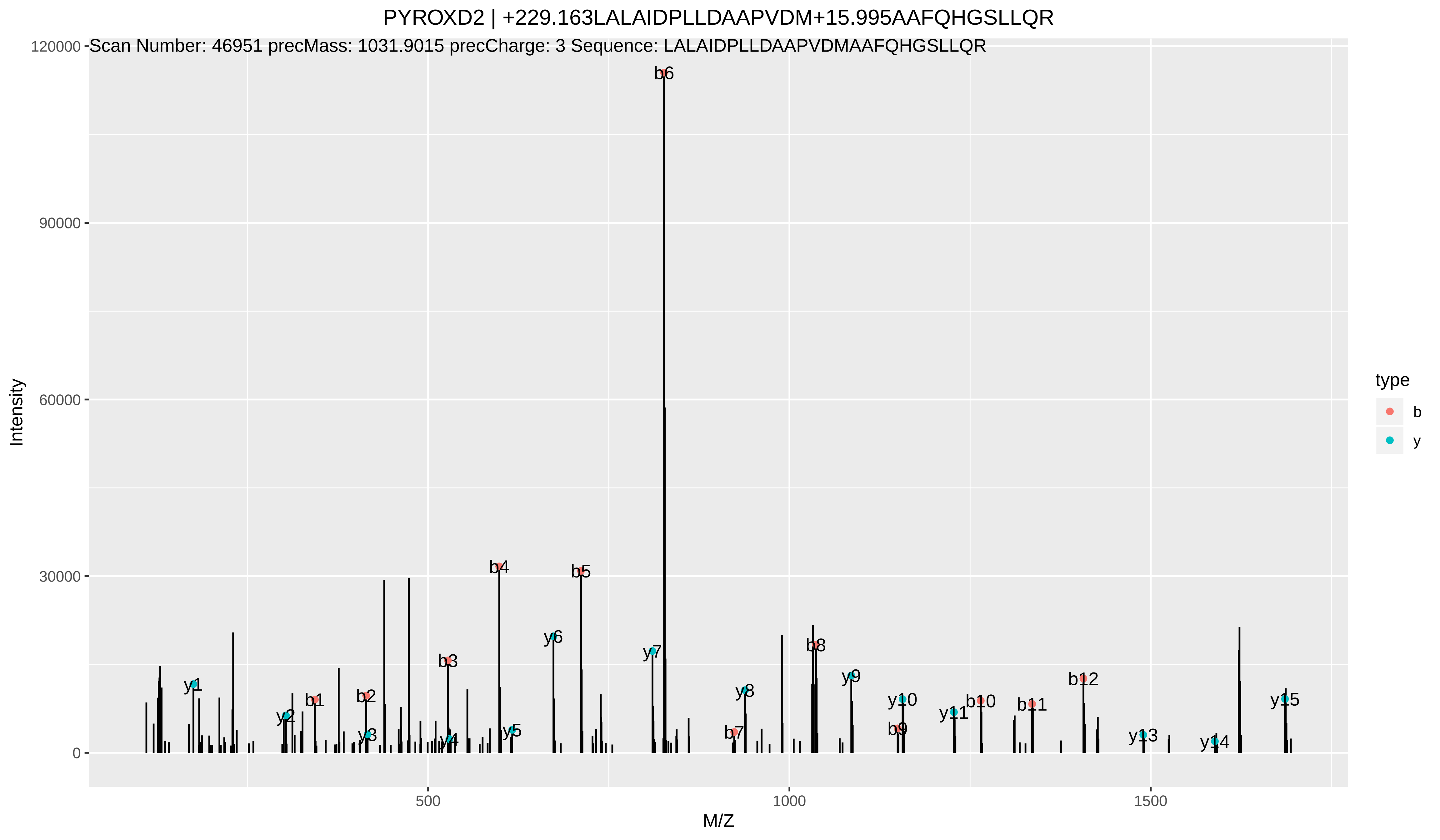

PYROXD2 | +229.163LALAIDPLLDAAPVDM+15.995AAFQHGSLLQR
Scan Number: 46951 precMass: 1031.9015 precCharge: 3 Sequence: LALAIDPLLDAAPVDMAAFQHGSLLQR
Intensity
M/Z
type
b
y
0
30000
60000
90000
120000
500
1000
1500
b1
b2
b3
b4
b5
b6
b7
b8
b9
b10
b11
b12
y1
y2
y3
y4
y5
y6
y7
y8
y9
y10
y11
y13
y14
y15

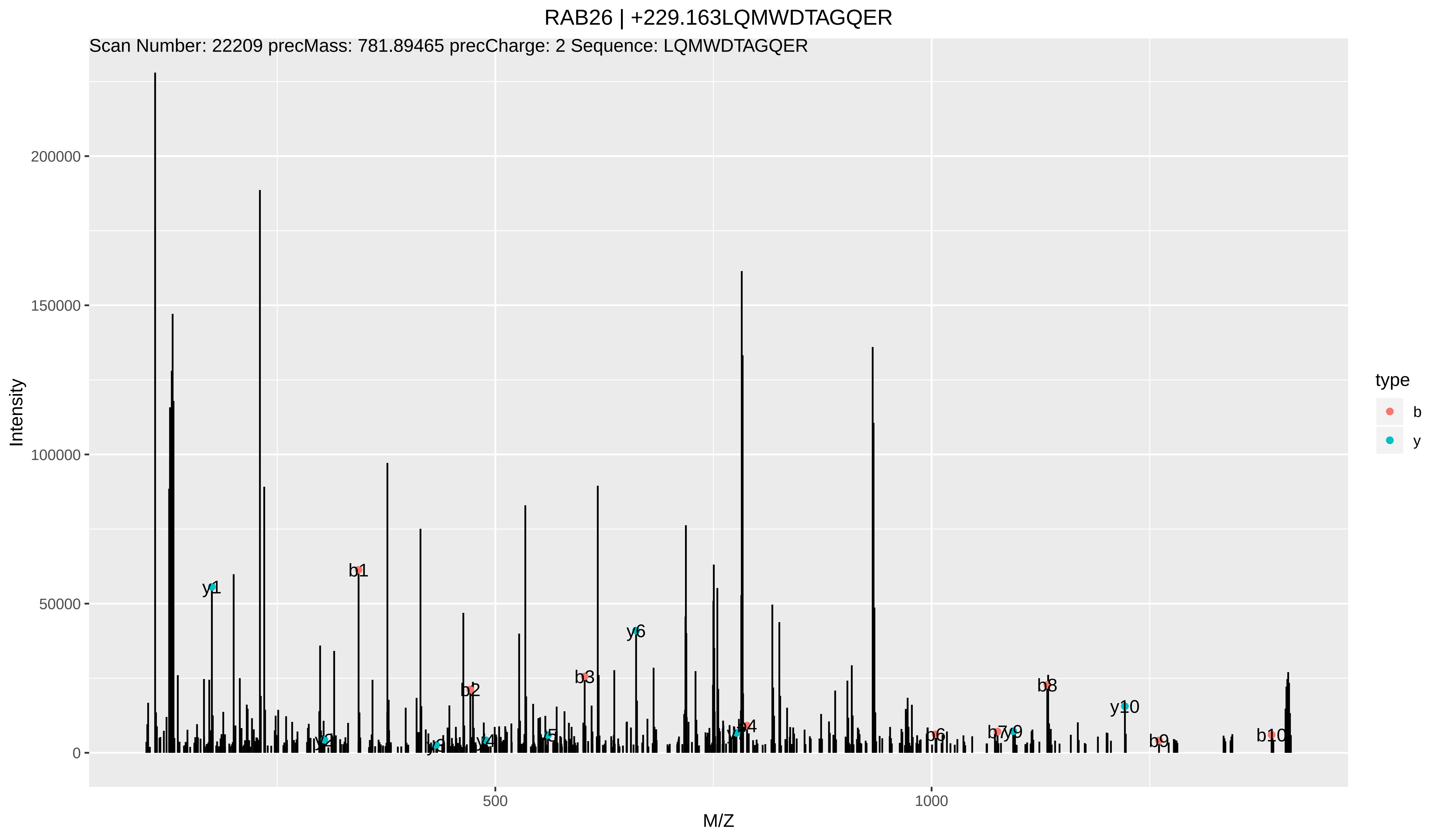
RAB26 | +229.163LQMWDTAGQER
Scan Number: 22209 precMass: 781.89465 precCharge: 2 Sequence: LQMWDTAGQER
Intensity
M/Z
200000
150000
100000
50000
0
500
1000
y1
b1
b2
b3
y6
b8
y10
b9
b10
b6
type
b
y

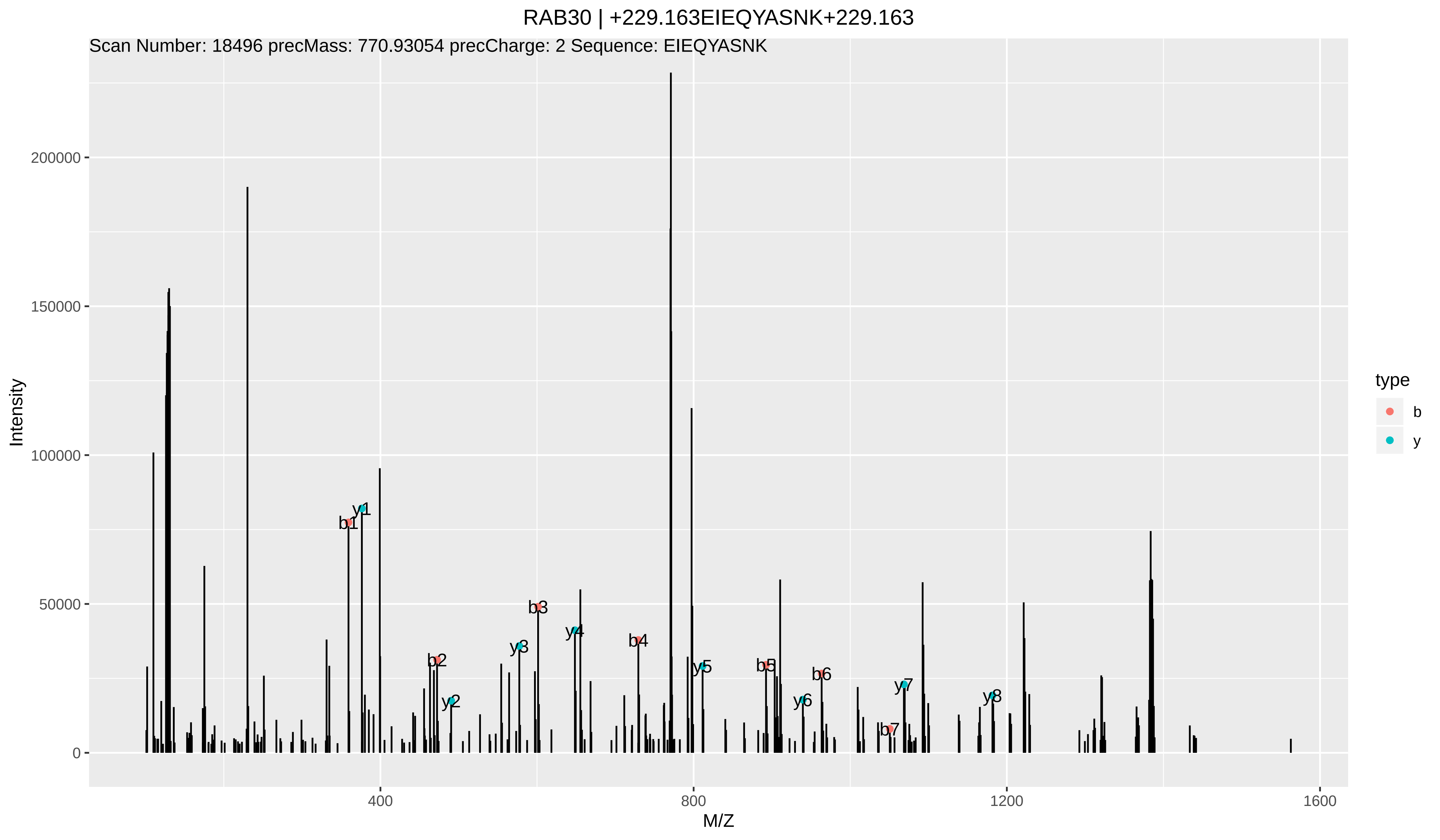
RAB30 | +229.163EIEQYASNK+229.163
Scan Number: 18496 precMass: 770.93054 precCharge: 2 Sequence: EIEQYASNK
Intensity
M/Z
type
b
y
b1
y1
b2
y2
b3
y3
b4
y4
b5
y5
b6
y6
b7
y7
y8
0
50000
100000
150000
200000
400
800
1200
1600

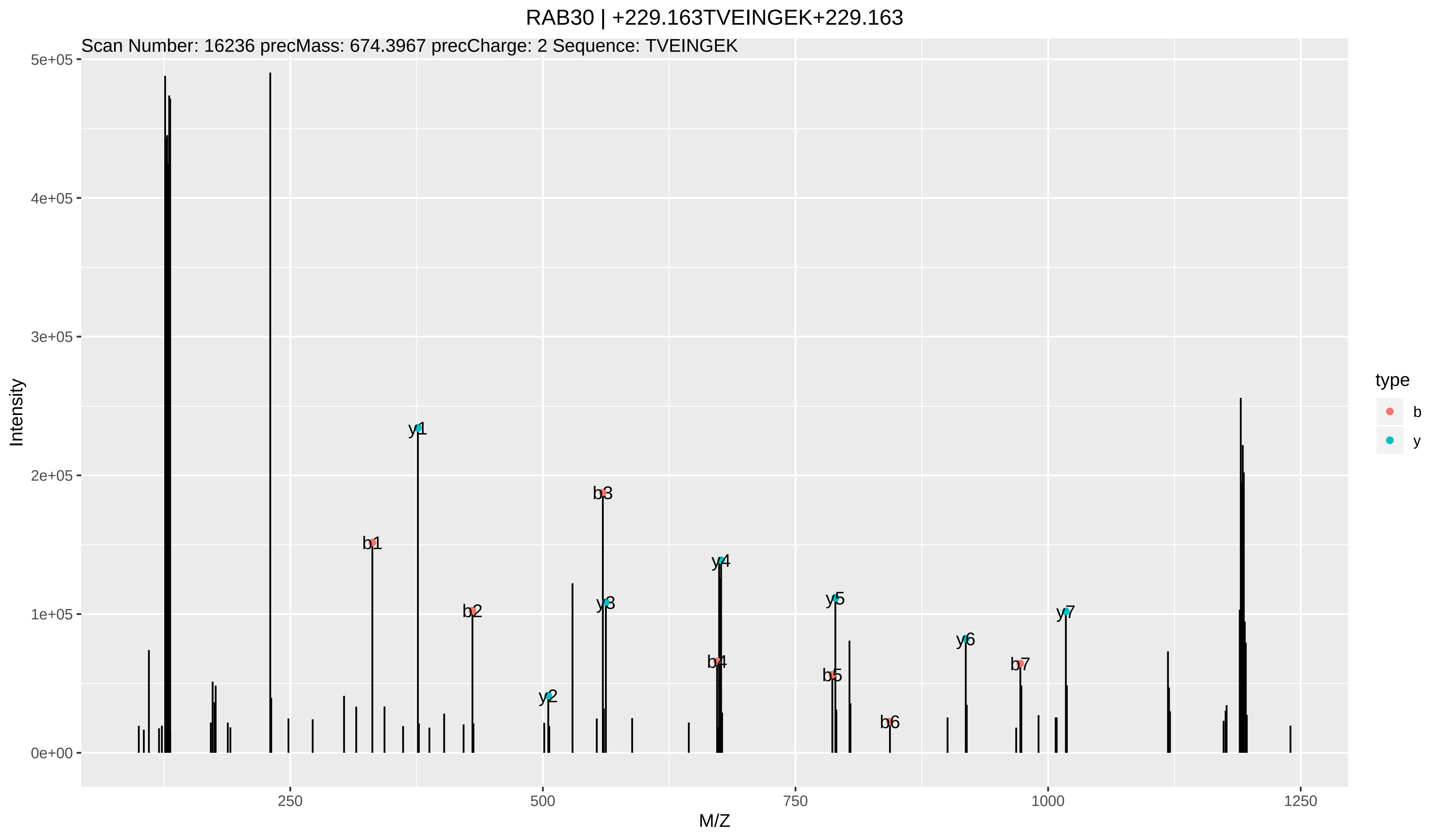

RAB30 | +229.163TVEINGEK+229.163
Scan Number: 16236 precMass: 674.3967 precCharge: 2 Sequence: TVEINGEK
Intensity
M/Z
0e+00
1e+05
2e+05
3e+05
4e+05
5e+05
250
500
750
1000
1250
y1
b1
b2
y2
b3
y3
y4
b4
y5
b5
b6
y6
b7
y7
type
b
y

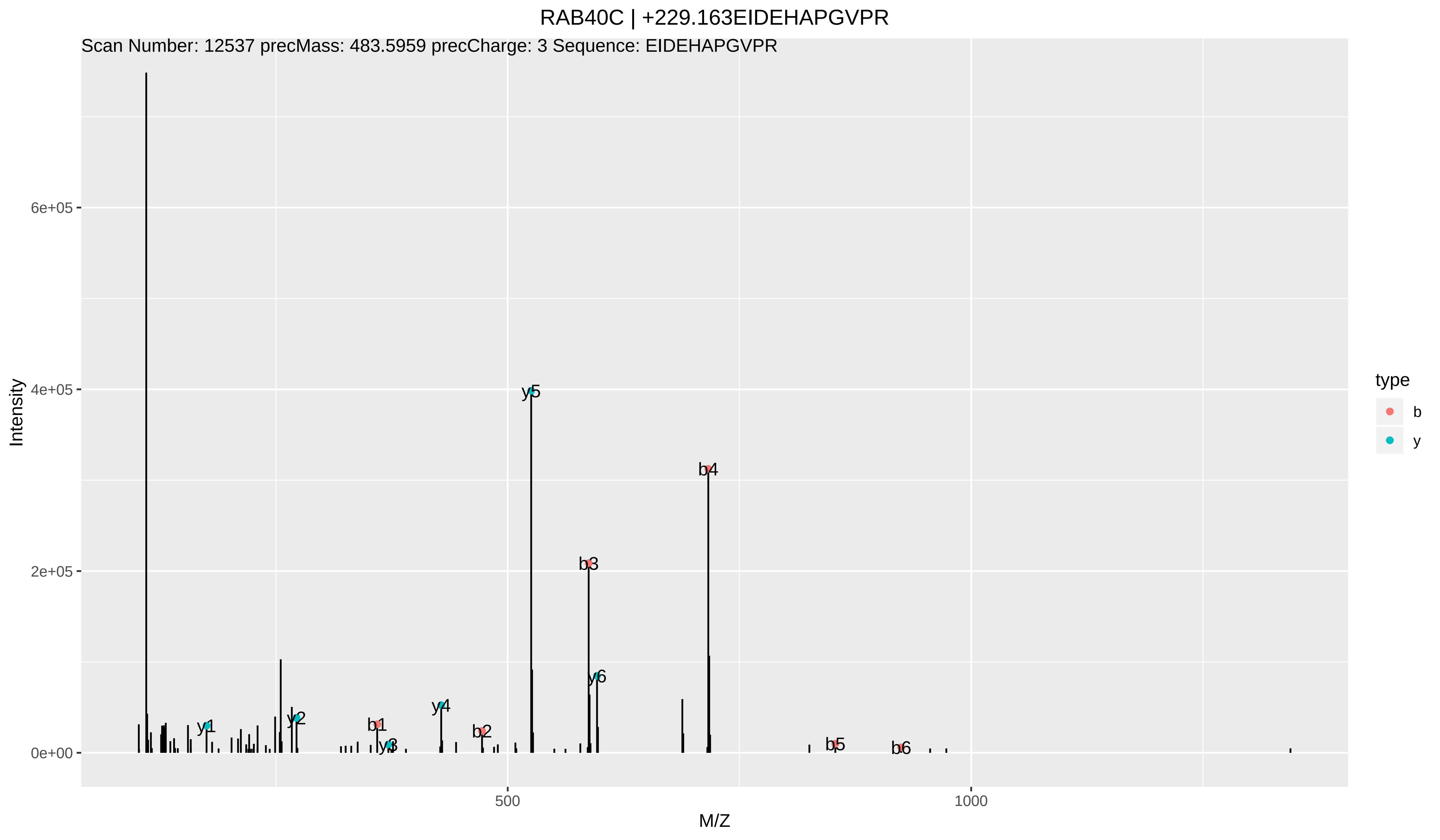

RAB40C | +229.163EIDEHAPGVPR
Scan Number: 12537 precMass: 483.5959 precCharge: 3 Sequence: EIDEHAPGVPR
Intensity
M/Z
0e+00
2e+05
4e+05
6e+05
500
1000
y1
y2
b1
y3
y4
b2
y5
b3
y6
b4
b5
b6
type
b
y

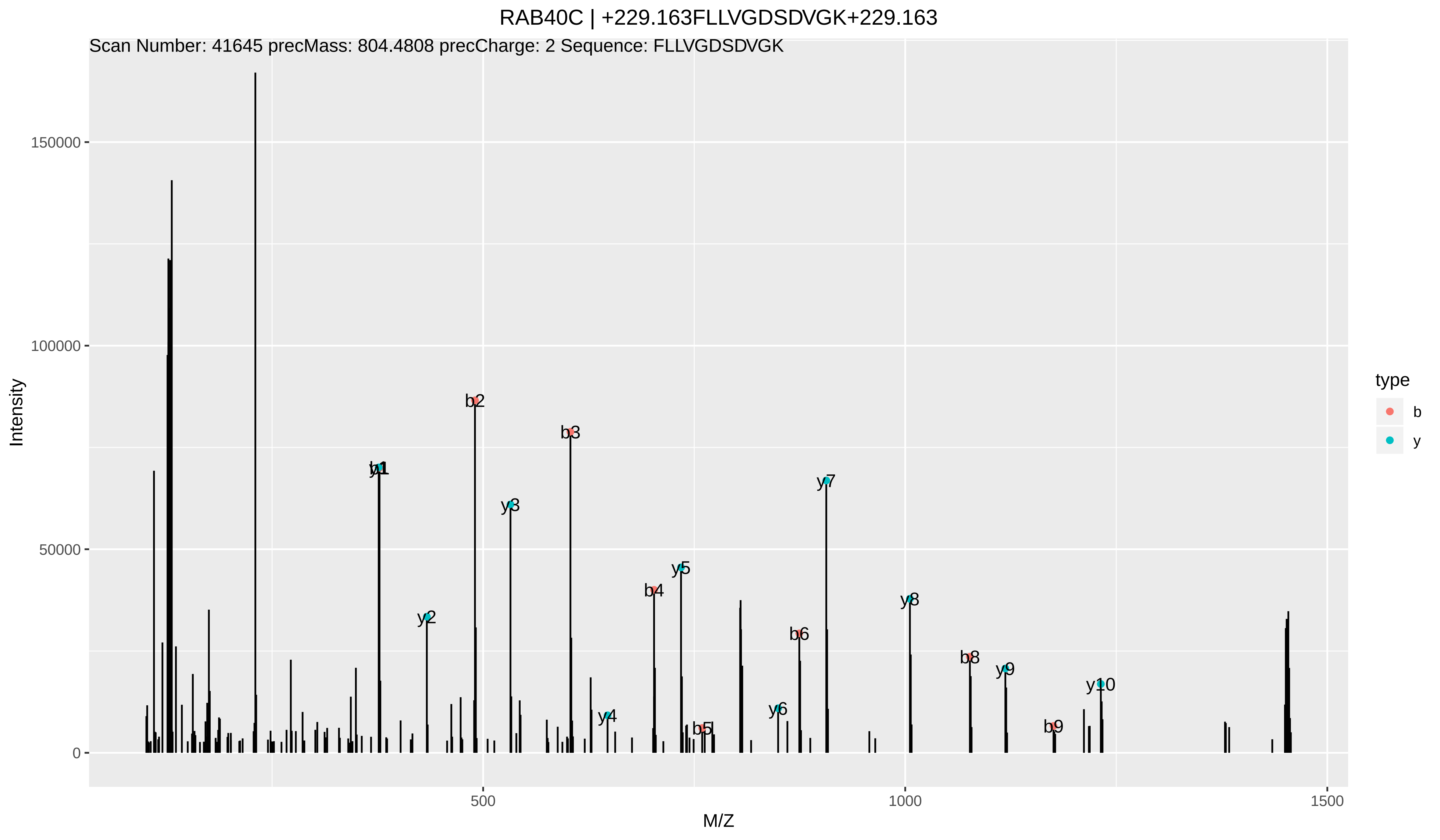

RAB40C | +229.163FLLVGDSDVGK+229.163
Scan Number: 41645 precMass: 804.4808 precCharge: 2 Sequence: FLLVGDSDVGK
Intensity
150000
100000
50000
0
500
1000
1500
M/Z
type
b
y
b1
y1
y2
b2
y3
b3
y4
b4
y5
b5
y6
b6
y7
y8
b8
y9
b9
y10

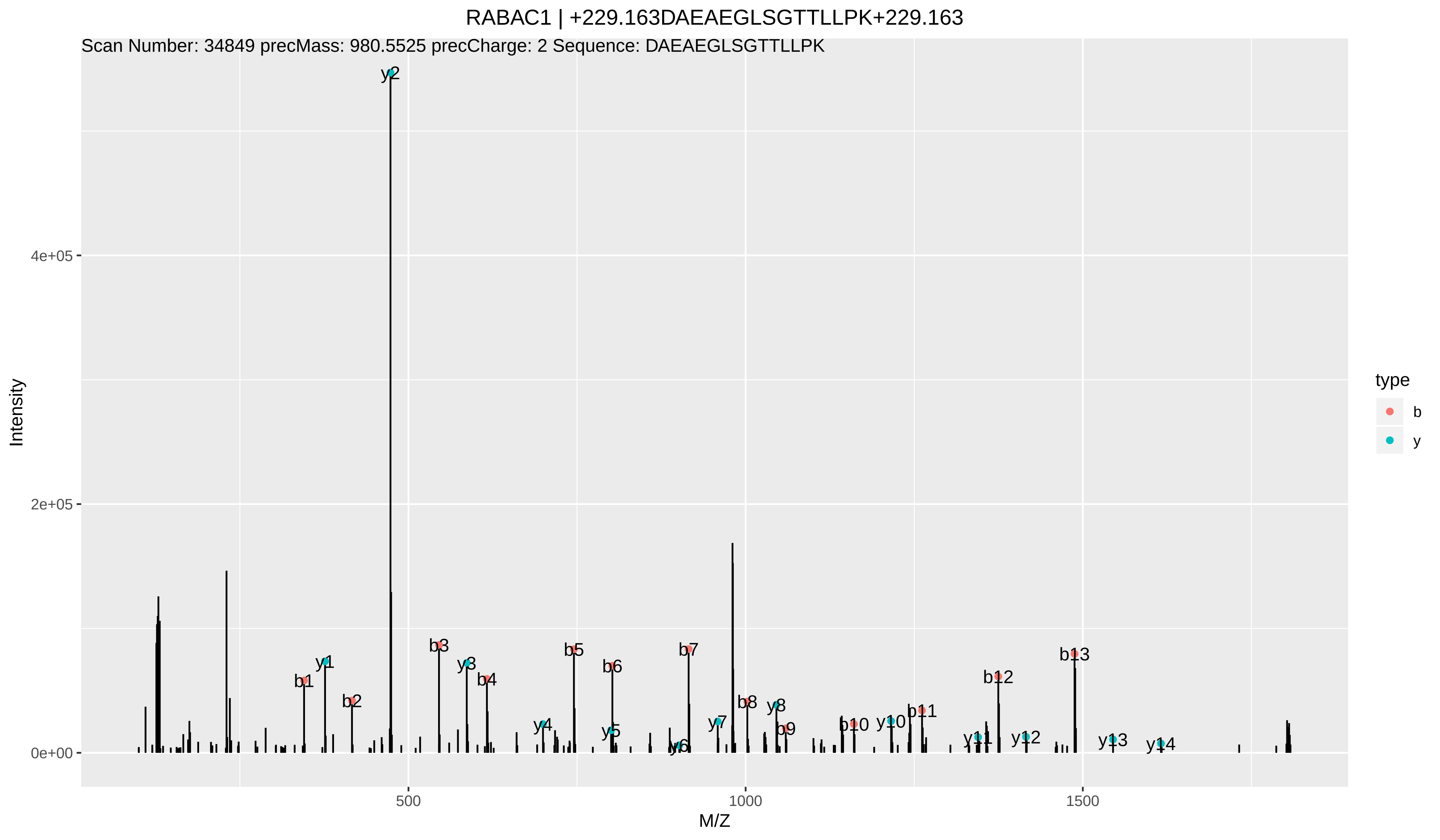
RABAC1 | +229.163DAEAEGLSGTTLLPK+229.163
Scan Number: 34849 precMass: 980.5525 precCharge: 2 Sequence: DAEAEGLSGTTLLPK
Intensity
4e+05
2e+05
0e+00
M/Z
500
1000
1500
y2
y1
b1
b2
b3
y3
b4
y4
b5
y5
b6
y6
b7
y7
b8
y8
b9
b10
y10
b11
y11
b12
y12
b13
y13
y14
type
b
y

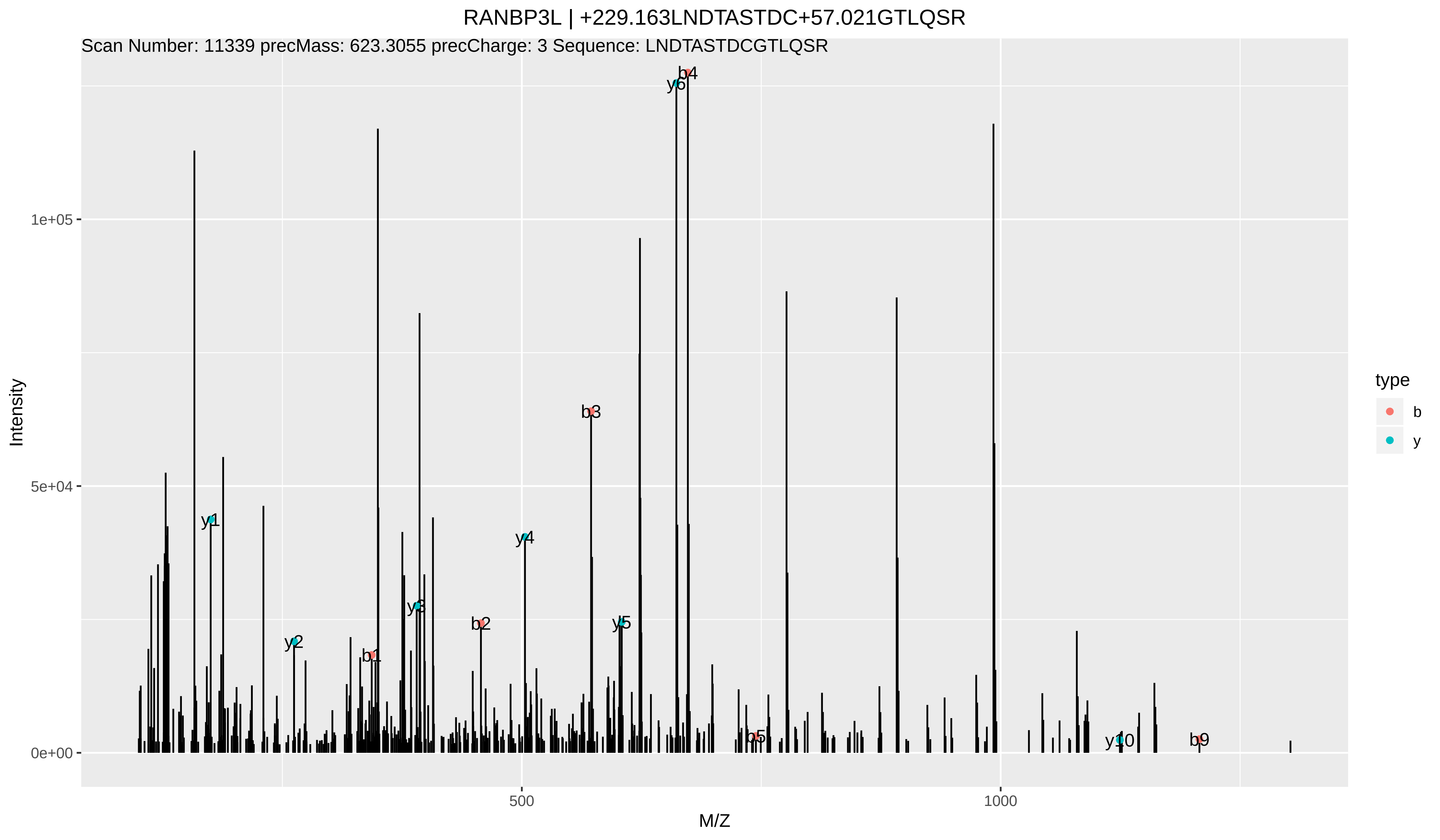

RANBP3L | +229.163LNDTASTDC+57.021GTLQSR
Scan Number: 11339 precMass: 623.3055 precCharge: 3 Sequence: LNDTASTDCGTLQSR
Intensity
M/Z
0e+00
5e+04
1e+05
500
1000
y1
y2
b1
y3
b2
y4
b3
y5
y6
b4
b5
y10
b9
type
b
y

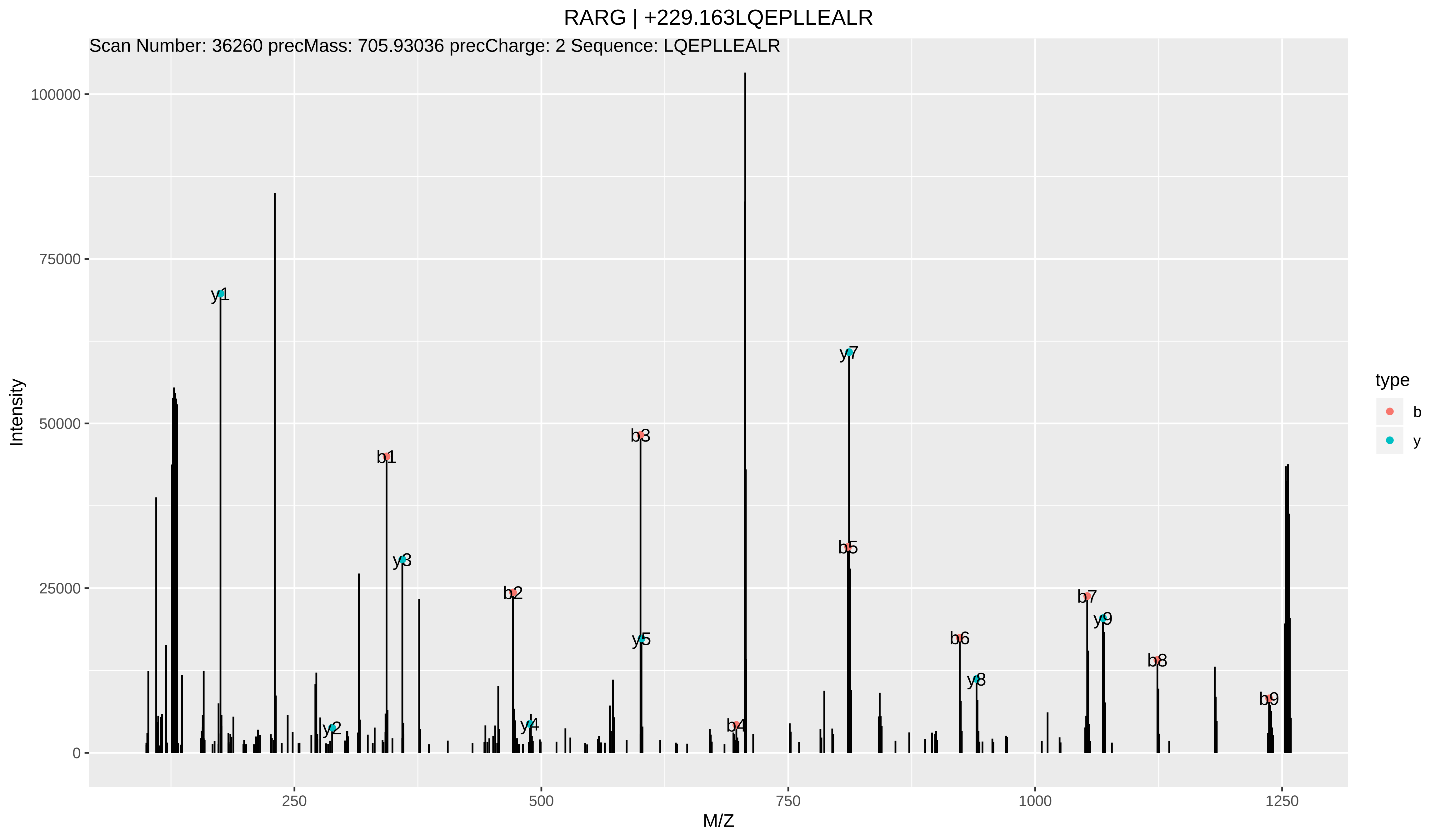

RARG | +229.163LQEPLLEALR
Scan Number: 36260 precMass: 705.93036 precCharge: 2 Sequence: LQEPLLEALR
Intensity
M/Z
100000
75000
50000
25000
0
250
500
750
1000
1250
y1
b1
y3
y2
b2
y4
b3
y5
b4
y7
b5
b6
y8
b7
y9
b8
b9
type
b
y

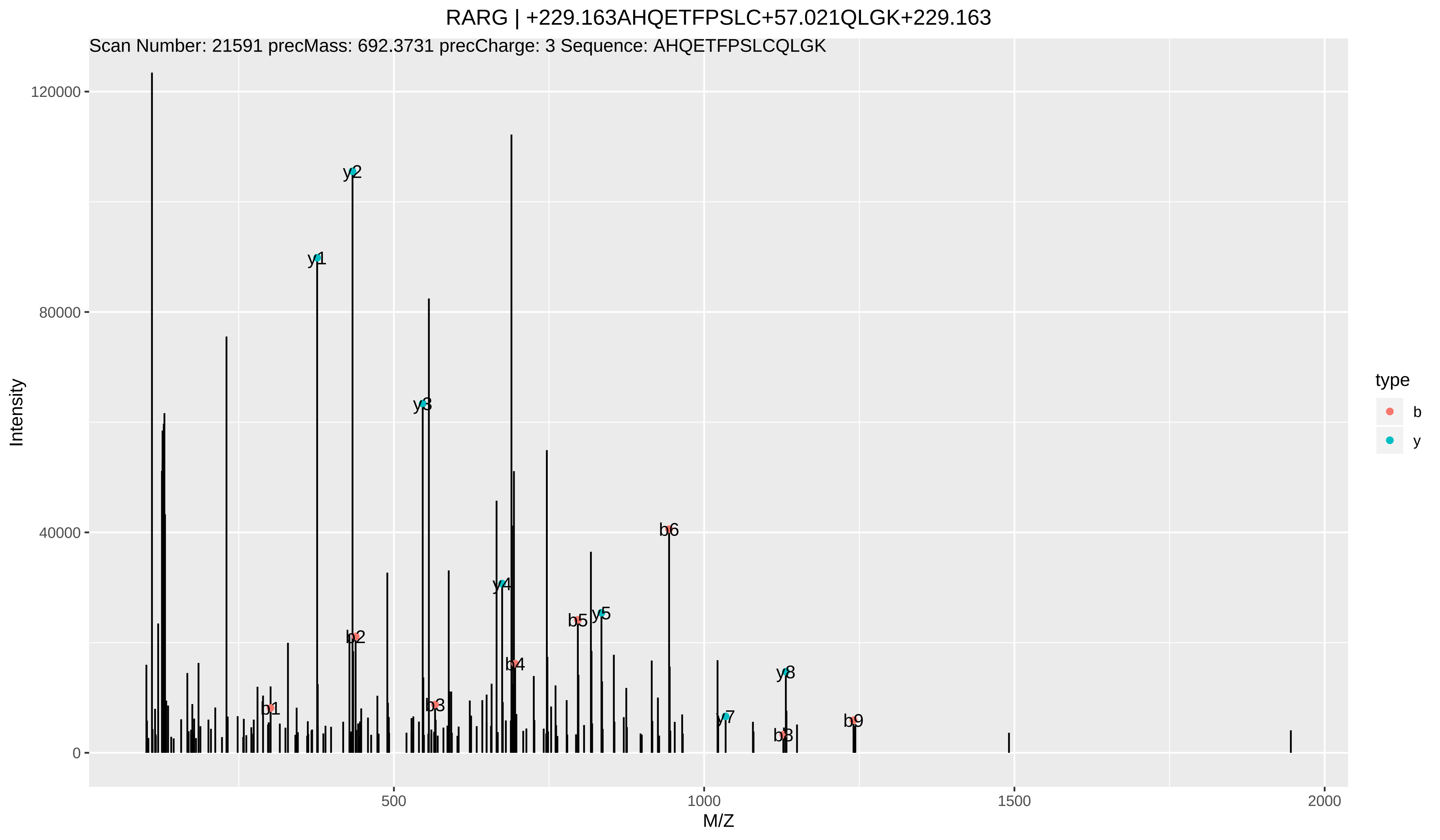

RARG | +229.163AHQETFPSLC+57.021QLGK+229.163
Scan Number: 21591 precMass: 692.3731 precCharge: 3 Sequence: AHQETFPSLCQLGK
Intensity
120000
80000
40000
0
M/Z
500
1000
1500
2000
y1
y2
y3
y4
y5
y7
y8
b1
b2
b3
b4
b5
b6
b8
b9
type
b
y

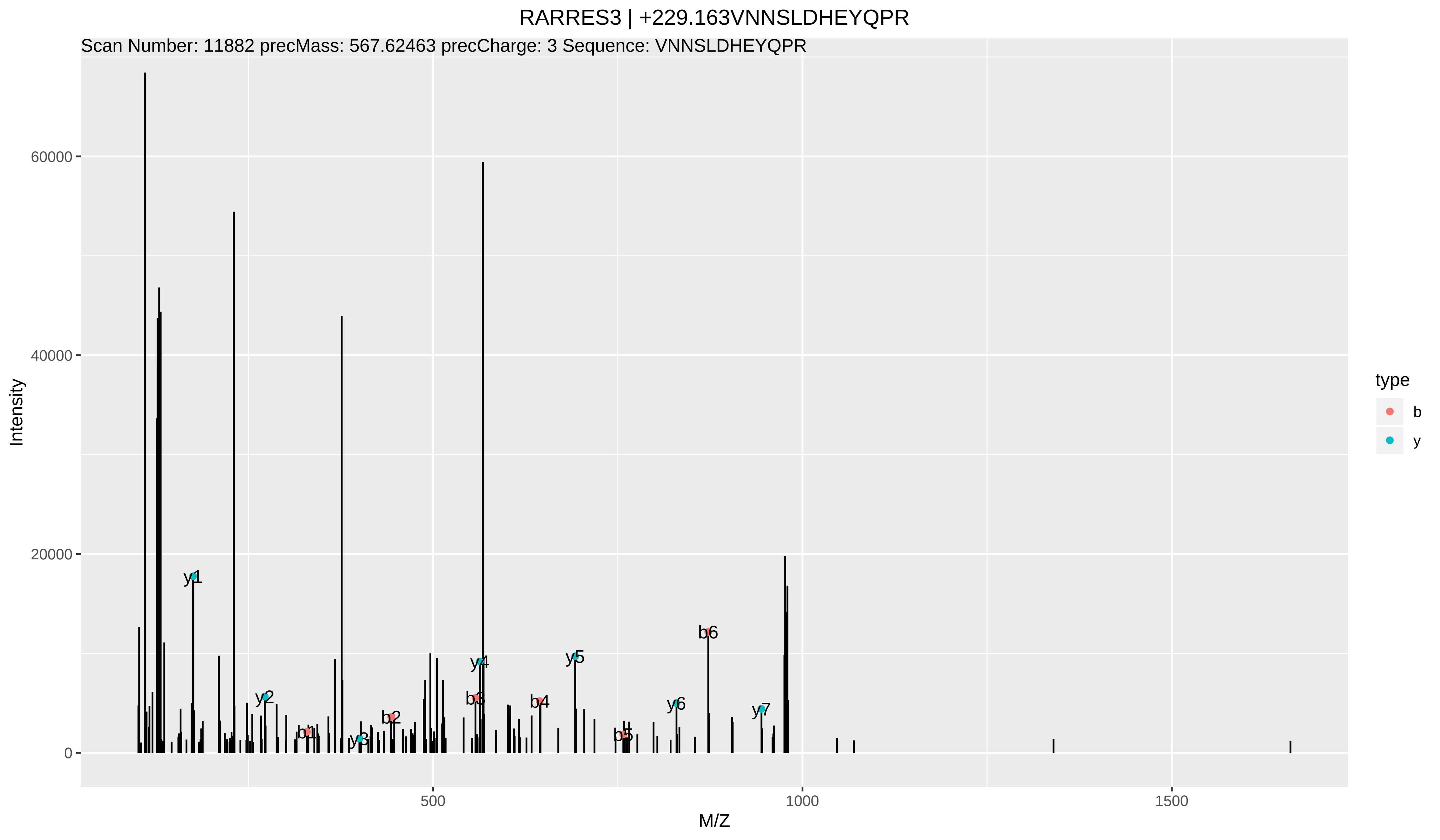

RARRES3 | +229.163VNNSLDHEYQPR
Scan Number: 11882 precMass: 567.62463 precCharge: 3 Sequence: VNNSLDHEYQPR
Intensity
60000
40000
20000
0
M/Z
500
1000
1500
y1
y2
b1
y3
b2
b3
y4
b4
y5
b5
y6
b6
y7
type
b
y

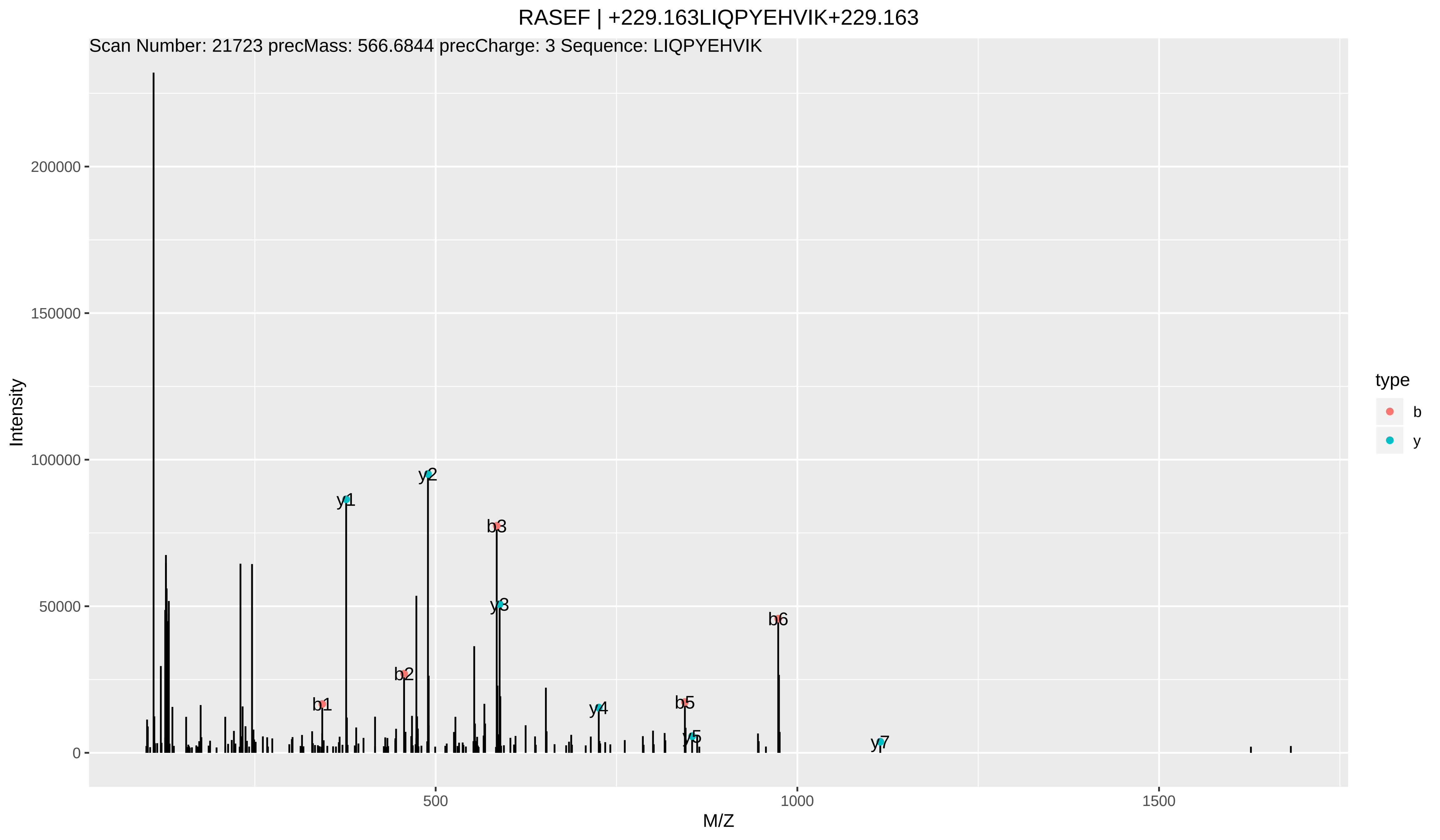

RASEF | +229.163LIQPYEHVIK+229.163
Scan Number: 21723 precMass: 566.6844 precCharge: 3 Sequence: LIQPYEHVIK
Intensity
200000
150000
100000
50000
0
M/Z
500
1000
1500
y1
y2
b3
y3
b6
b1
b2
y4
b5
y5
y7
type
b
y

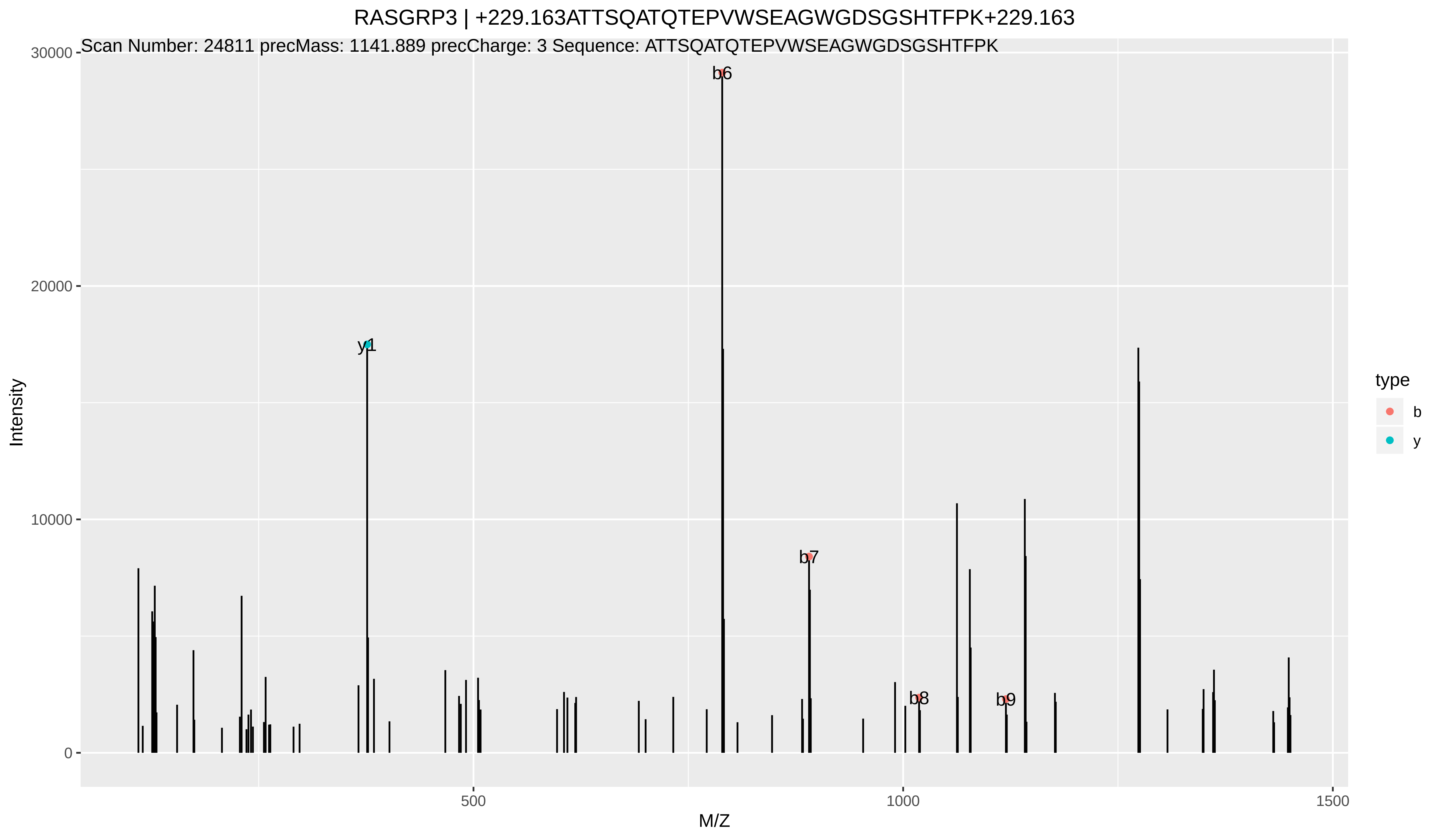
RASGRP3 | +229.163ATTSQATQTEPVWSEAGWGDSGSHTFPK+229.163
Scan Number: 24811 precMass: 1141.889 precCharge: 3 Sequence: ATTSQATQTEPVWSEAGWGDSGSHTFPK
Intensity
M/Z
0
10000
20000
30000
500
1000
1500
b6
y1
b7
b8
b9
type
b
y

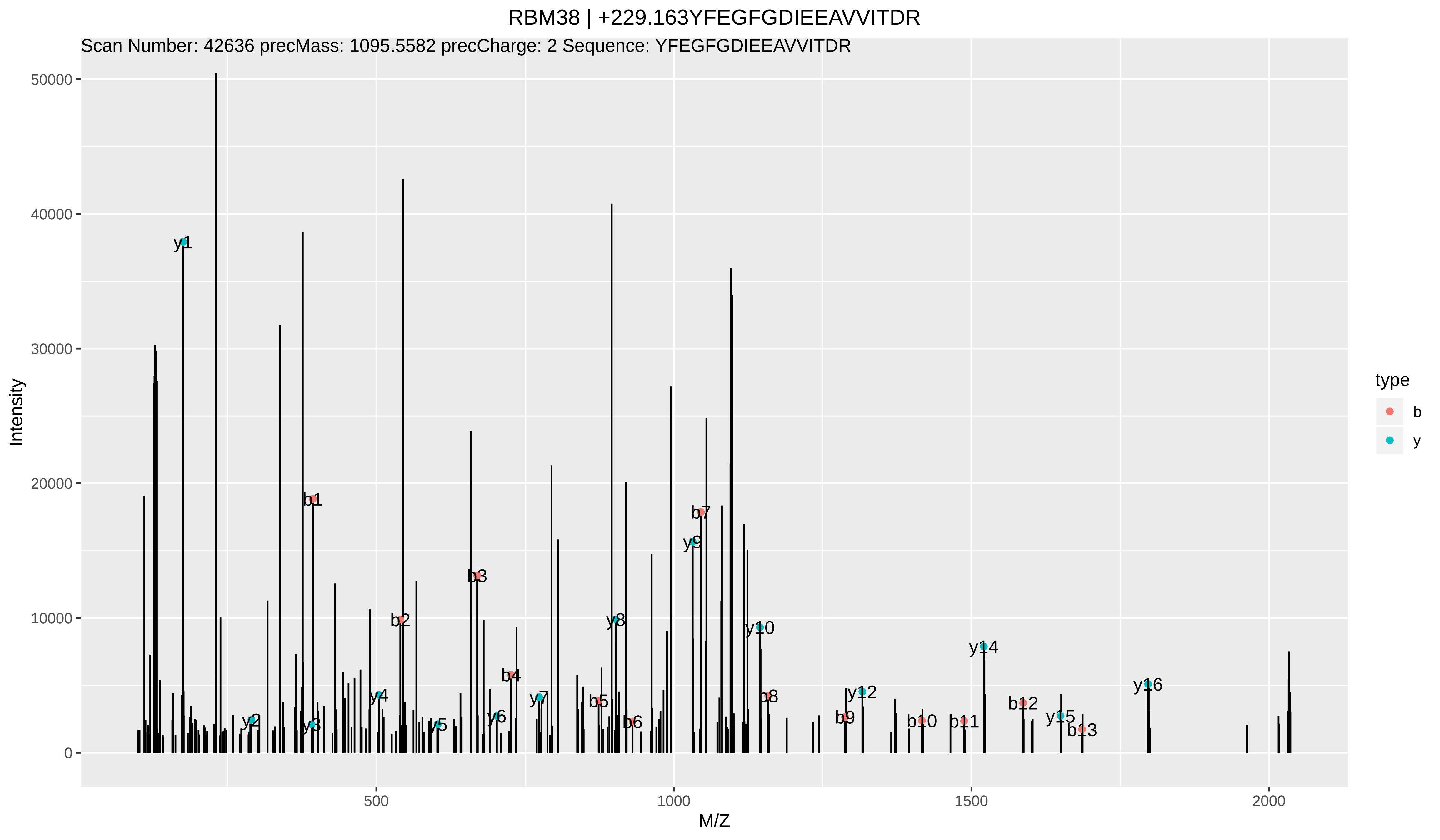
RBM38 | +229.163YFEGFGDIEEAVVITDR
Scan Number: 42636 precMass: 1095.5582 precCharge: 2 Sequence: YFEGFGDIEEAVVITDR
Intensity
M/Z
type
b
y
y1
b1
y2
y3
y4
b2
y5
b3
y6
b4
y7
b5
y8
b6
b7
y9
y10
b8
b9
y12
b10
b11
y14
b12
y15
b13
y16
0
10000
20000
30000
40000
50000
500
1000
1500
2000

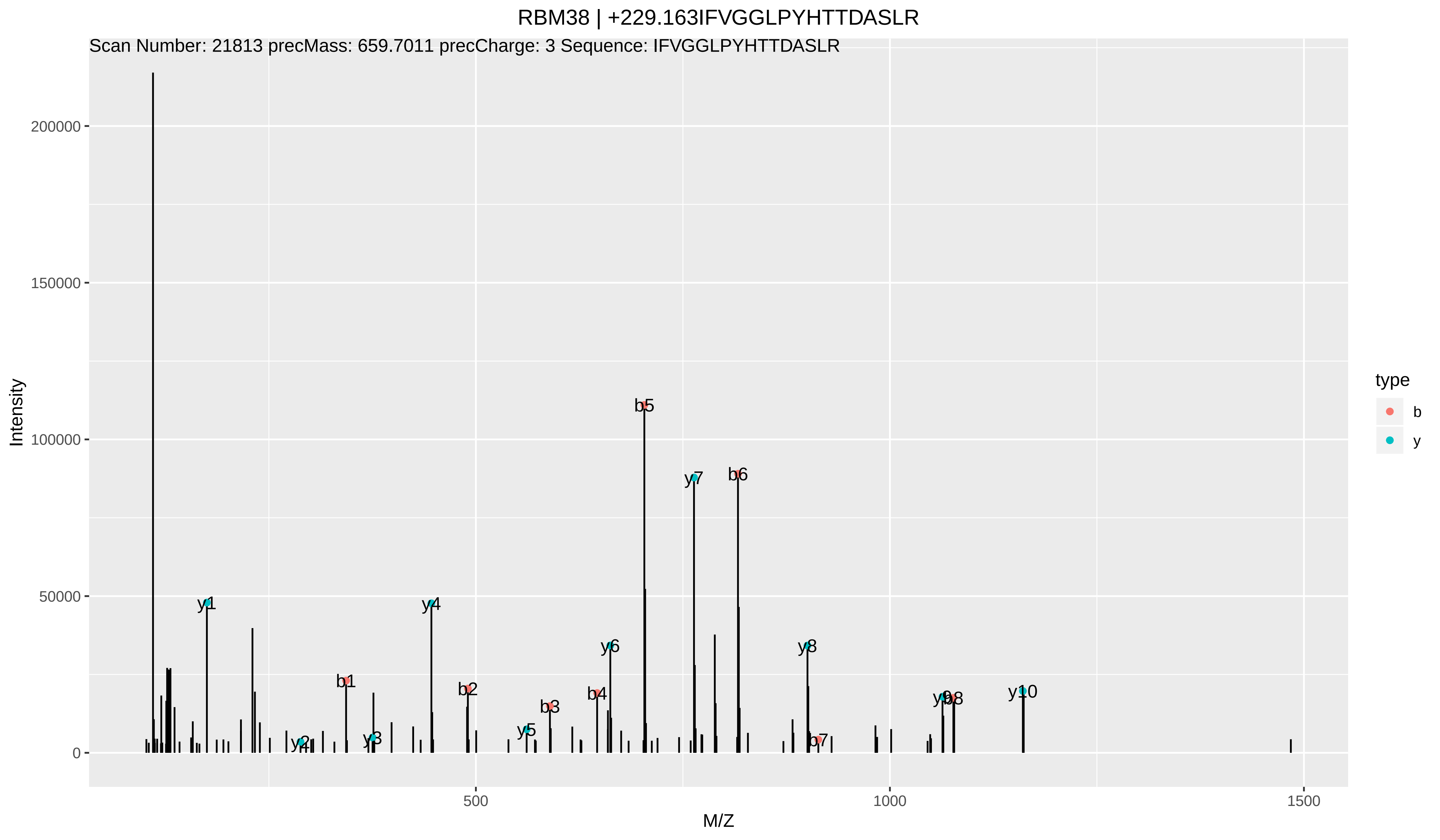

RBM38 | +229.163IFVGGLPYHTTDASLR
Scan Number: 21813 precMass: 659.7011 precCharge: 3 Sequence: IFVGGLPYHTTDASLR
Intensity
200000
150000
100000
50000
0
M/Z
500
1000
1500
y1
y2
y3
y4
y5
y6
y7
y8
y10
b1
b2
b3
b4
b5
b6
b7
b8
type
b
y

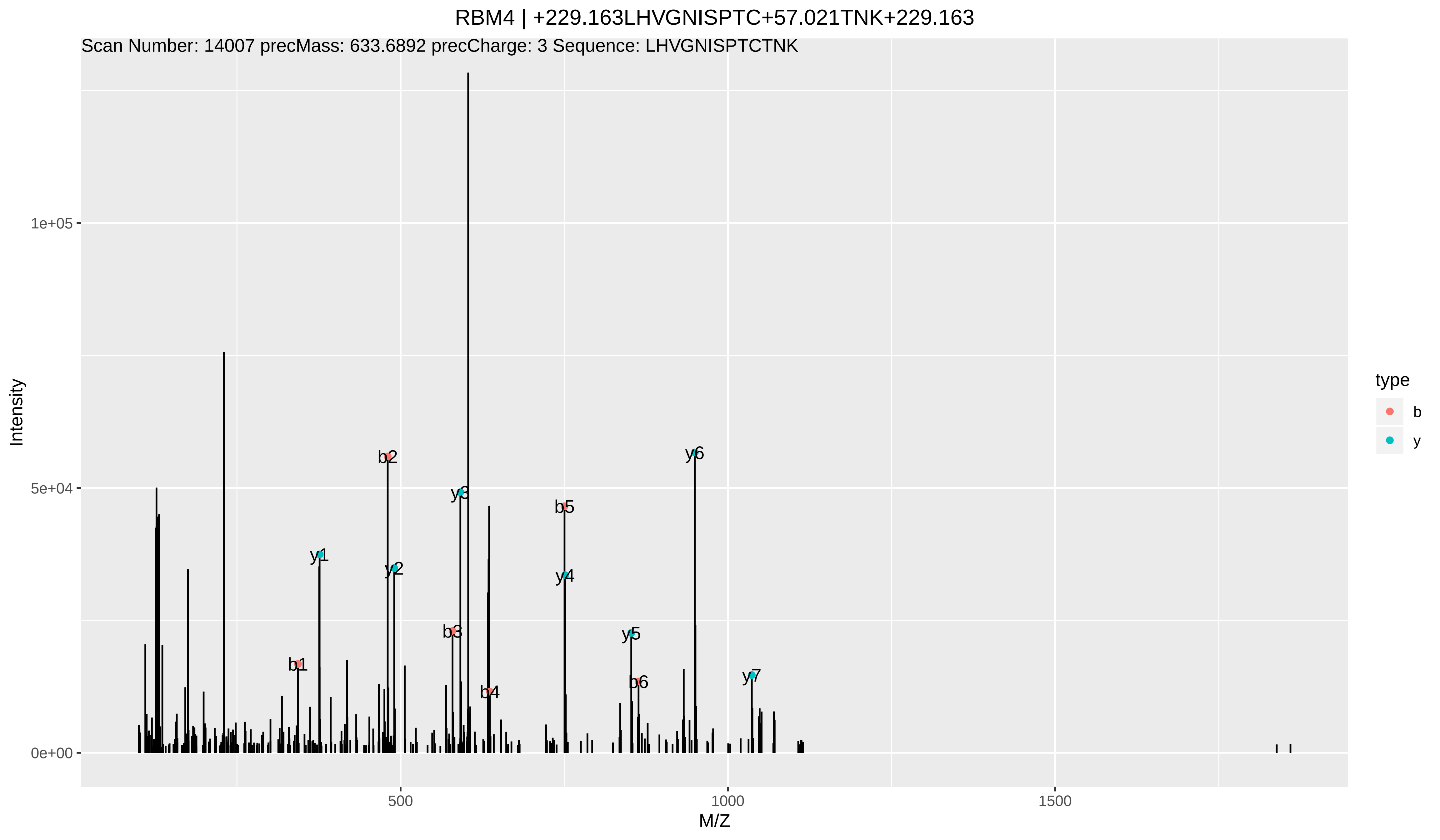
RBM4 | +229.163LHVGNISPTC+57.021TNK+229.163
Scan Number: 14007 precMass: 633.6892 precCharge: 3 Sequence: LHVGNISPTCTNK
Intensity
1e+05
5e+04
0e+00
500
1000
1500
M/Z
b1
b2
b3
b4
b5
b6
y1
y2
y3
y4
y5
y6
y7
type
b
y

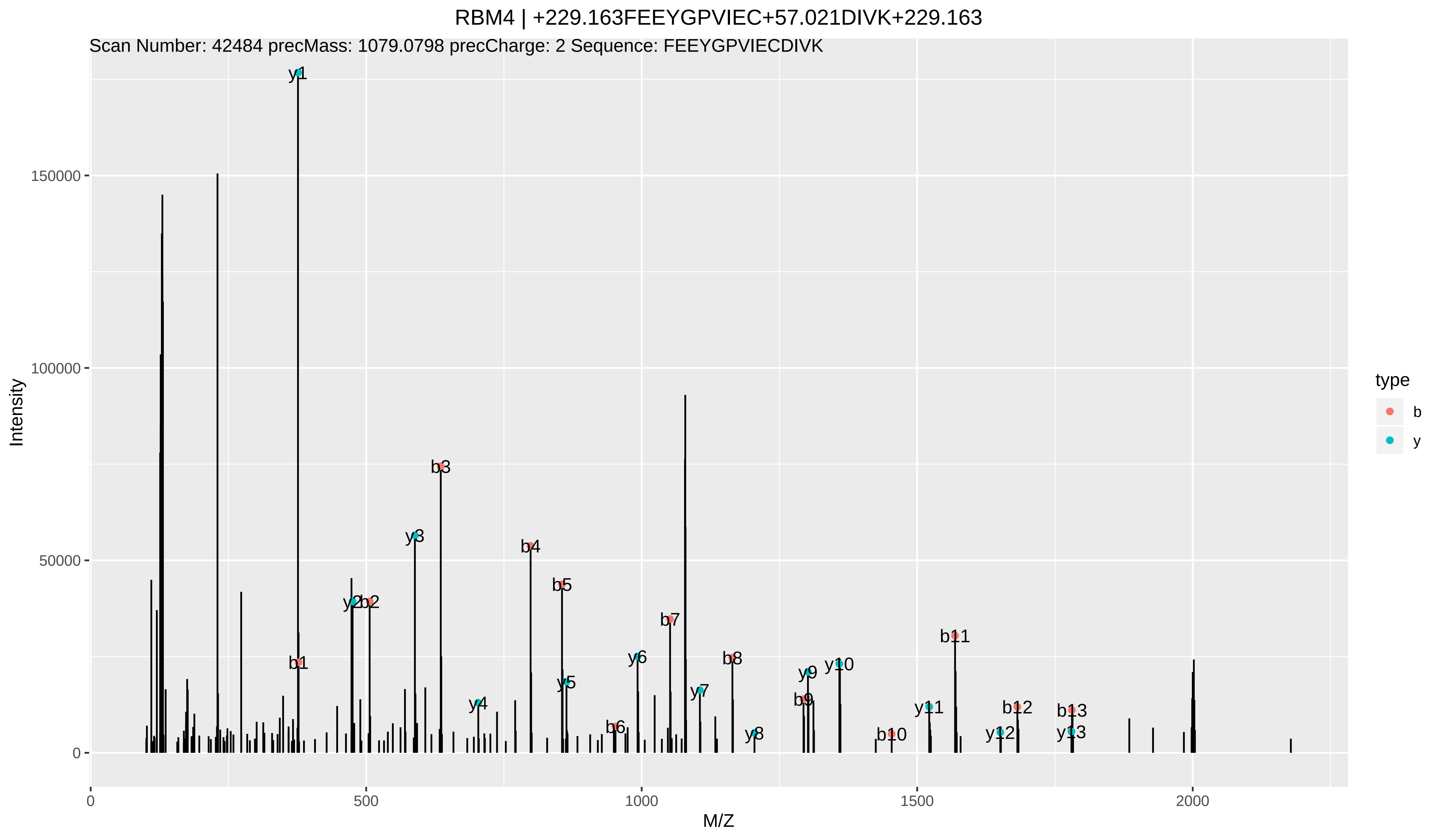
RBM4 | +229.163FEEYGPVIEC+57.021DIVK+229.163
Scan Number: 42484 precMass: 1079.0798 precCharge: 2 Sequence: FEEYGPVIECDIVK
Intensity
M/Z
type
b
y
y1
b1
y2
b2
y3
b3
y4
b4
y5
b5
y6
b6
y7
b7
y8
b8
y9
b9
y10
b10
y11
b11
y12
b12
y13
b13
0
50000
100000
150000
500
1000
1500
2000

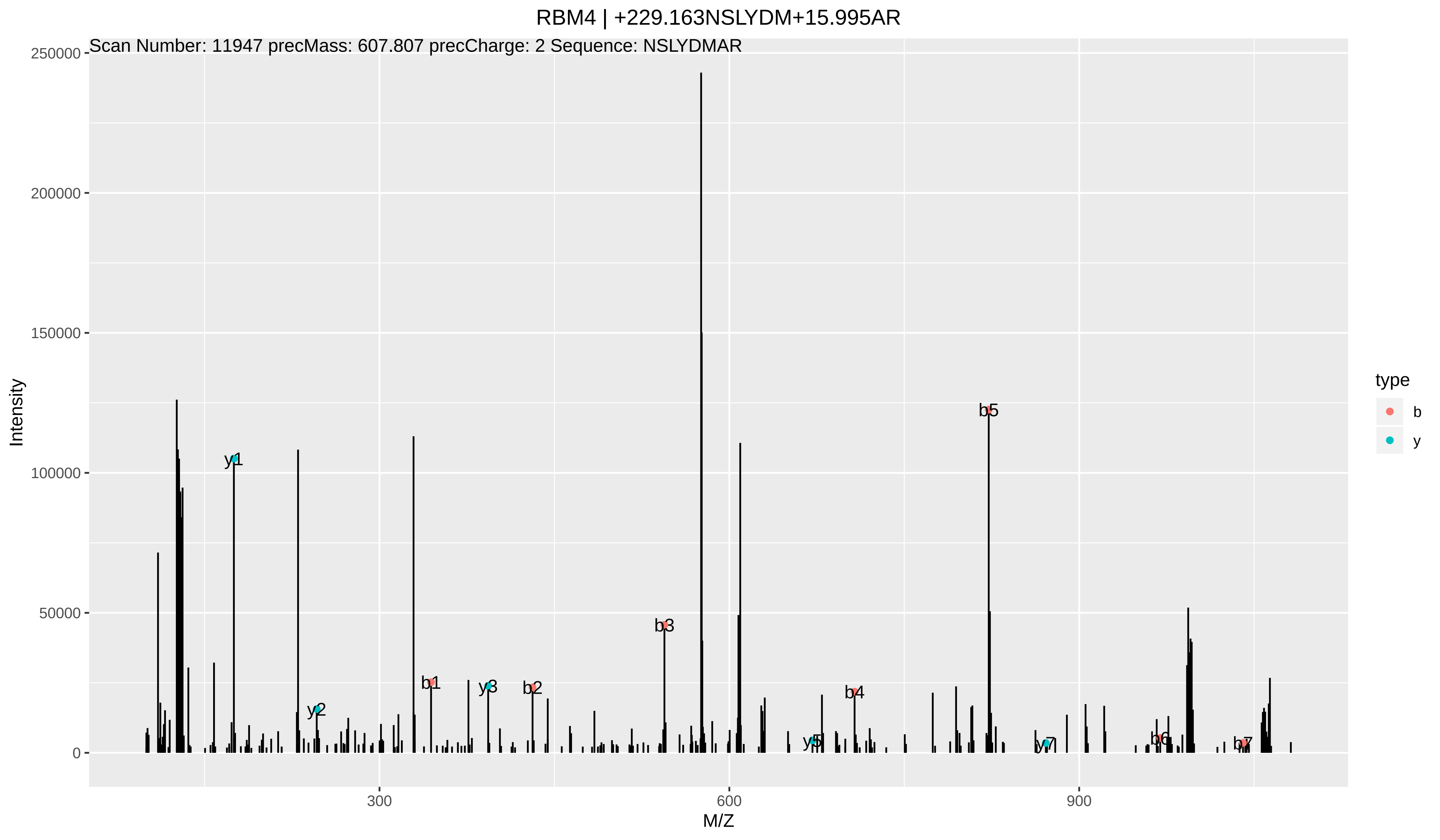
RBM4 | +229.163NSLYDM+15.995AR
Scan Number: 11947 precMass: 607.807 precCharge: 2 Sequence: NSLYDMAR
Intensity
250000
200000
150000
100000
50000
0
M/Z
300
600
900
y1
y2
b1
y3
b2
b3
y5
b4
b5
y7
b6
b7
type
b
y

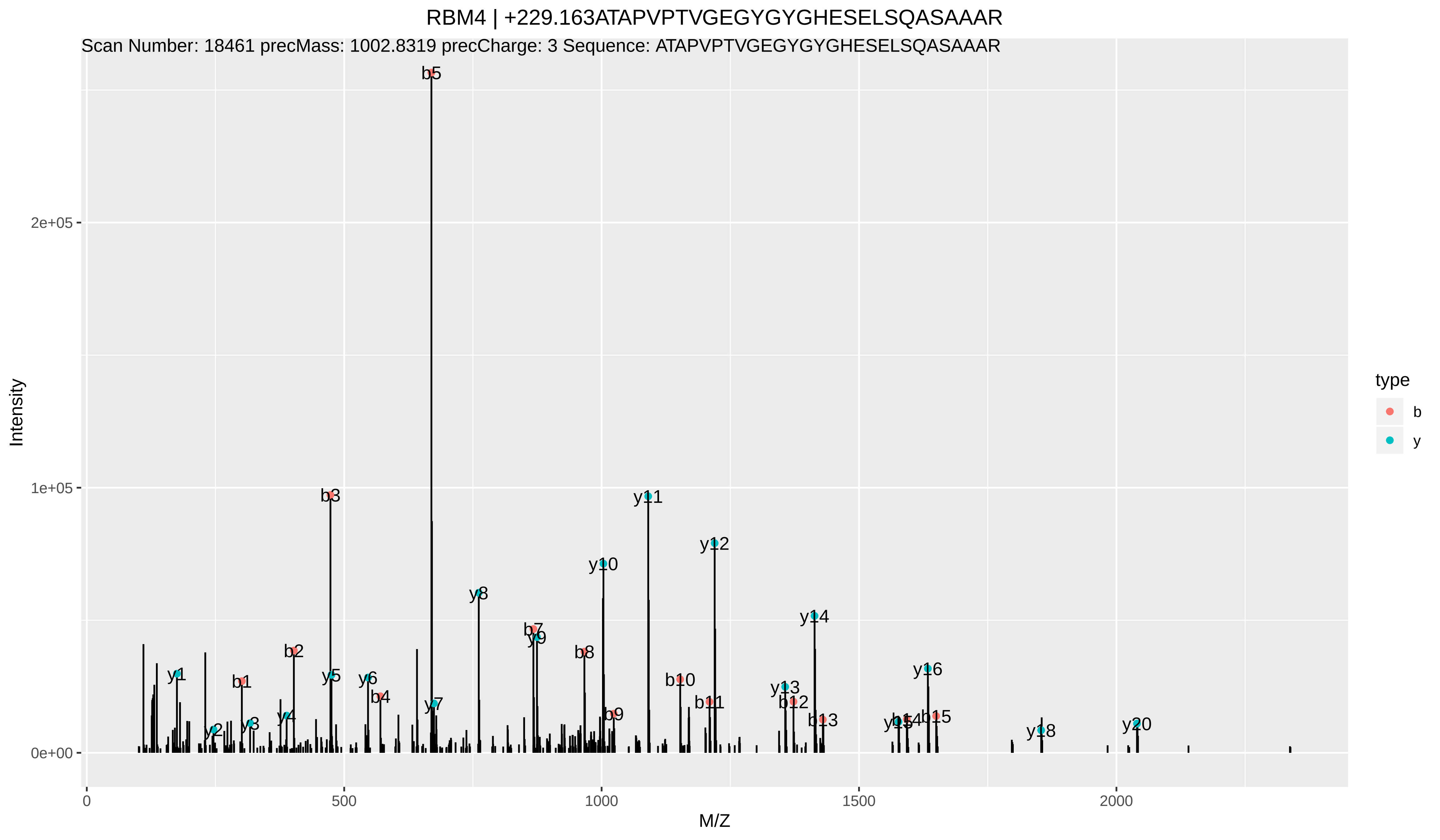
RBM4 | +229.163ATAPVPTVGEGYGYGHESELSQASAAAR
Scan Number: 18461 precMass: 1002.8319 precCharge: 3 Sequence: ATAPVPTVGEGYGYGHESELSQASAAAR
Intensity
M/Z
type
b
y
0e+00
1e+05
2e+05
0
500
1000
1500
2000
b5
b3
y11
y12
y10
y8
y14
b7
y9
b8
b2
y16
y1
b1
y5
y6
b10
y13
b4
b11
b12
y7
b9
y4
b15
b13
y15
b14
y20
y3
y2
y18

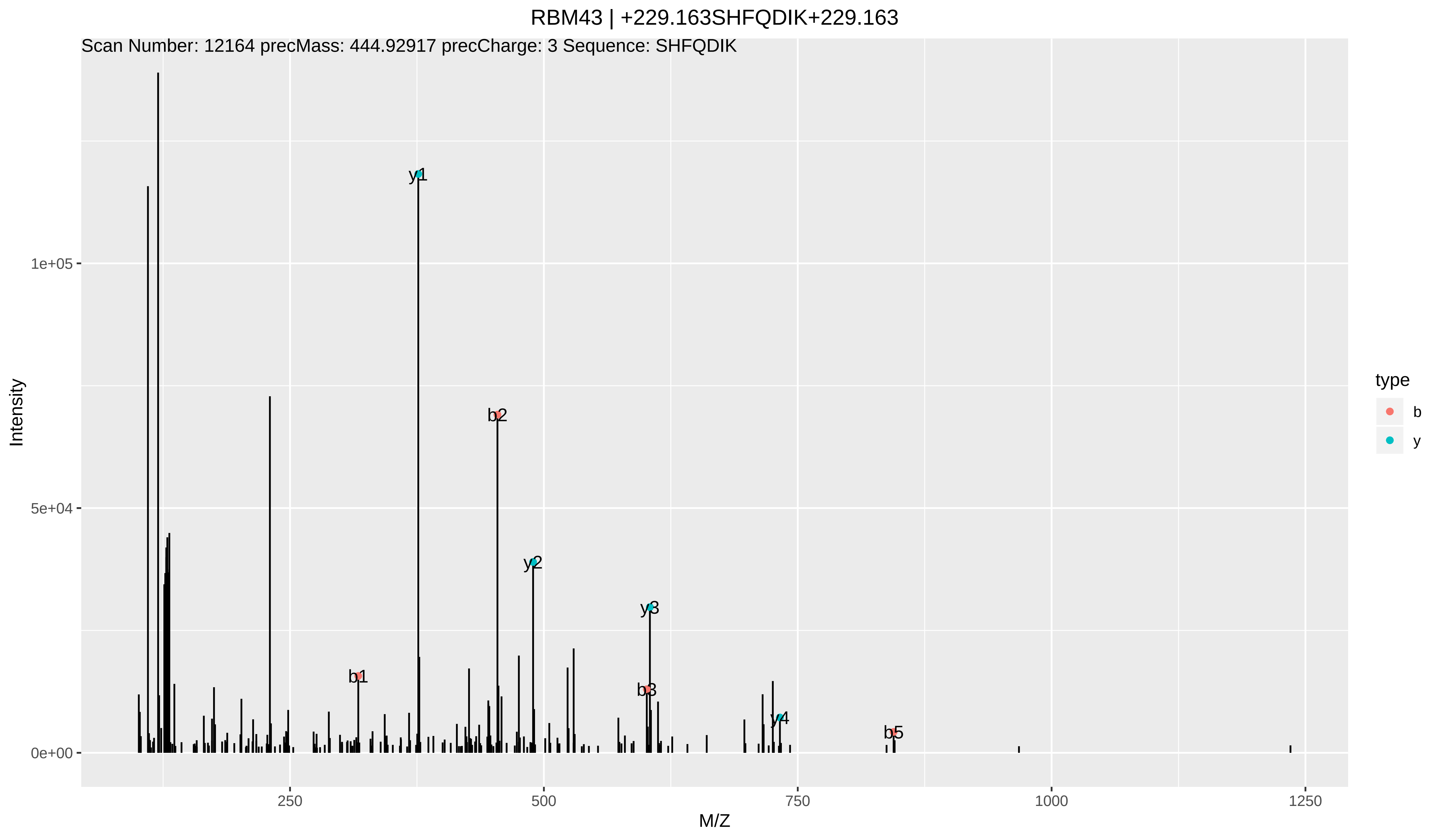

RBM43 | +229.163SHFQDIK+229.163
Scan Number: 12164 precMass: 444.92917 precCharge: 3 Sequence: SHFQDIK
Intensity
0e+00
5e+04
1e+05
M/Z
250
500
750
1000
1250
y1
b2
y2
y3
b1
b3
y4
b5
type
b
y

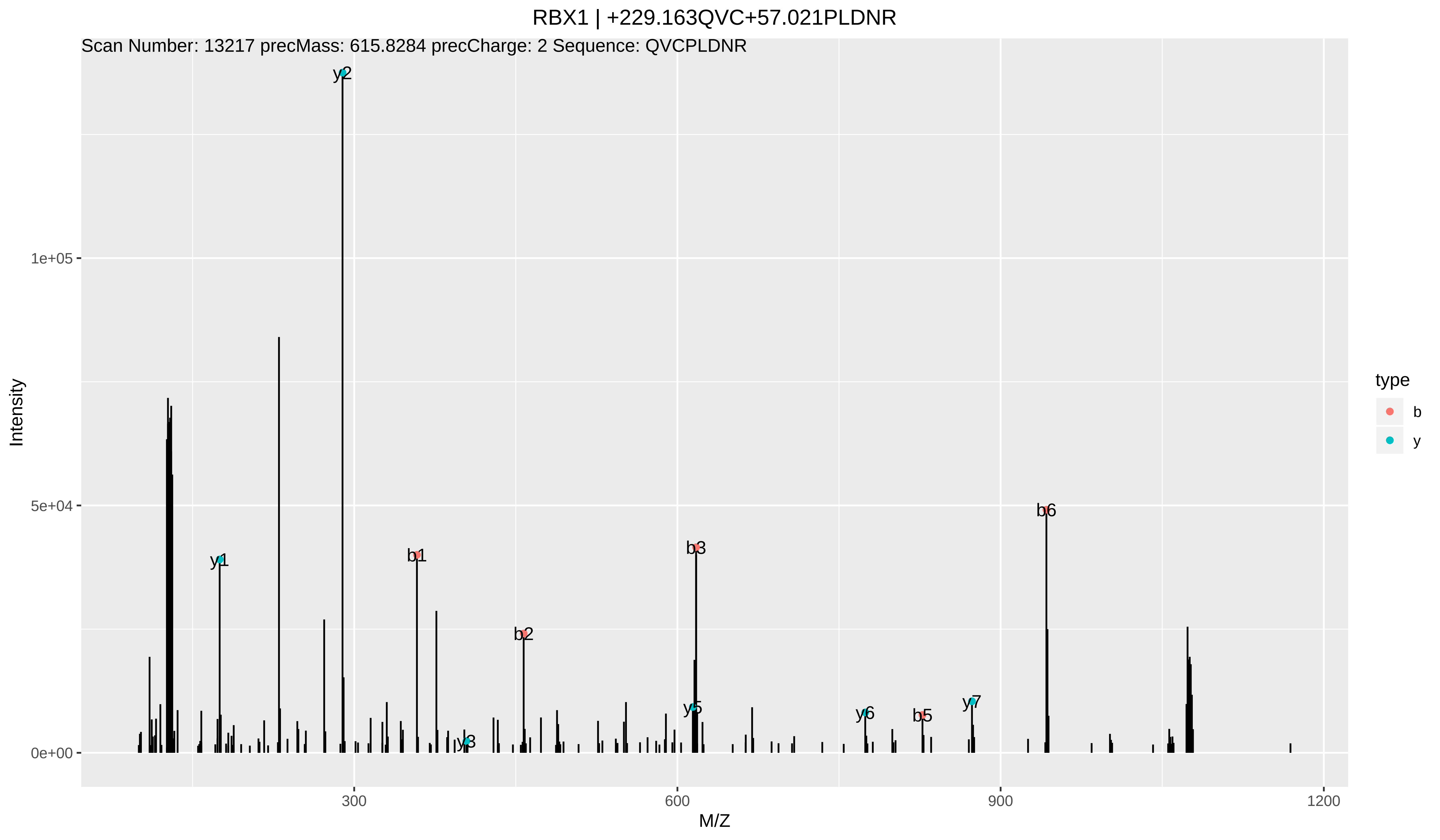
RBX1 | +229.163QVC+57.021PLDNR
Scan Number: 13217 precMass: 615.8284 precCharge: 2 Sequence: QVCPLDNR
Intensity
M/Z
0e+00
5e+04
1e+05
300
600
900
1200
y1
y2
y3
y5
y6
y7
b1
b2
b3
b5
b6
type
b
y

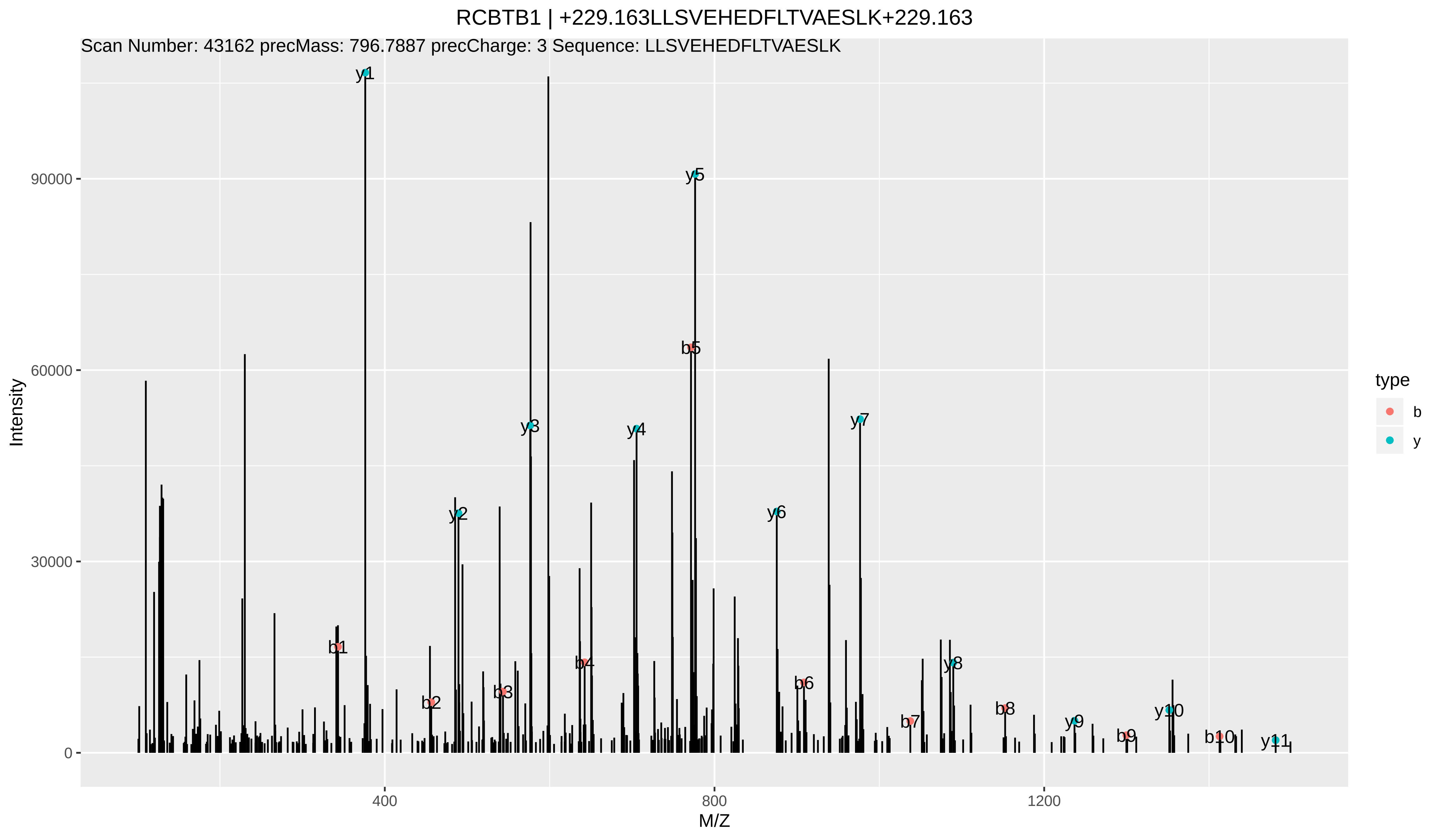
RCBTB1 | +229.163LLSVEHEDFLTVAESLK+229.163
Scan Number: 43162 precMass: 796.7887 precCharge: 3 Sequence: LLSVEHEDFLTVAESLK
Intensity
90000
60000
30000
0
M/Z
400
800
1200
type
b
y
y1
y2
y3
y4
y5
y6
y7
y8
y9
y10
y11
b1
b2
b3
b4
b5
b6
b7
b8
b9
b10

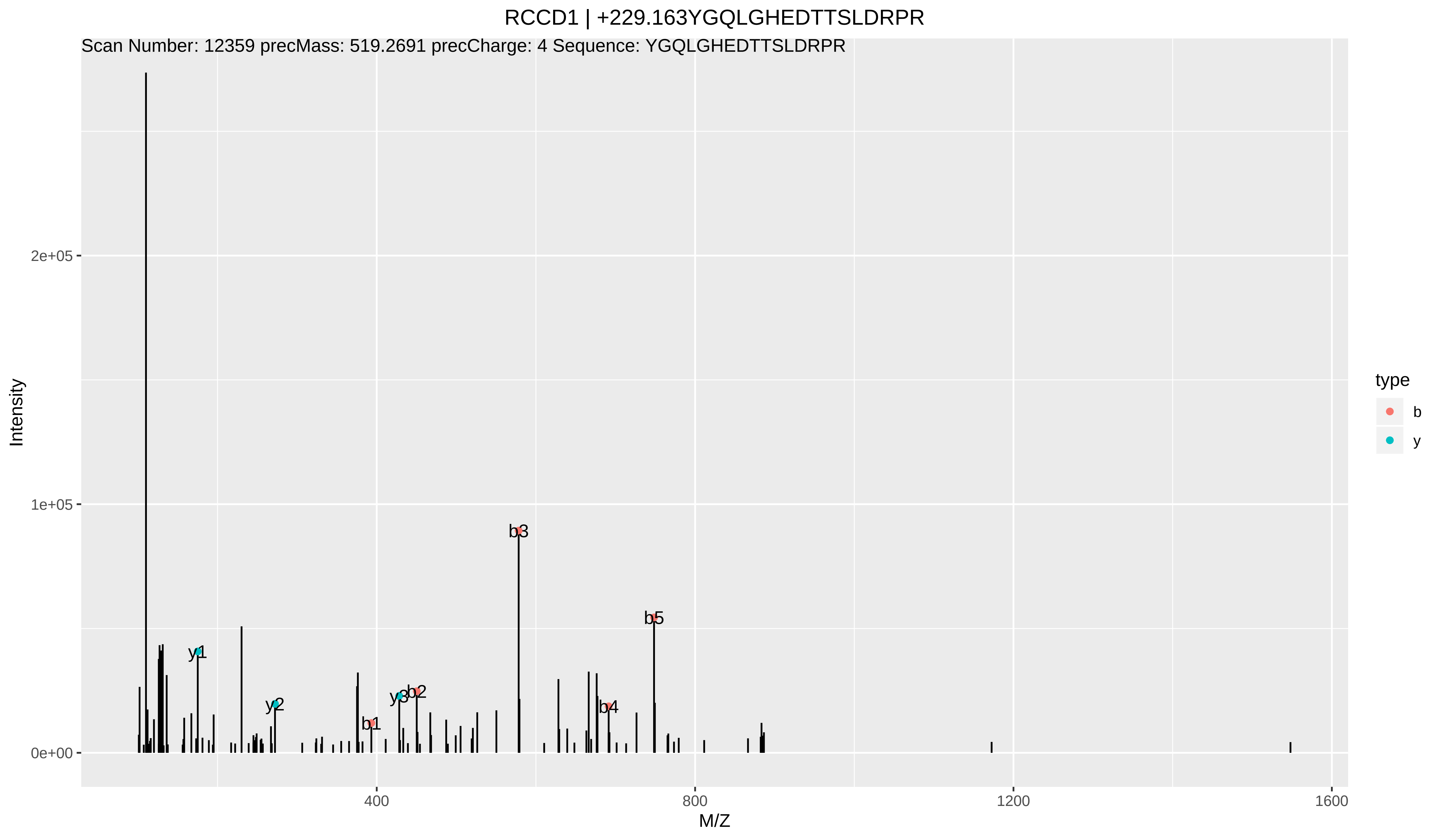
RCCD1 | +229.163YGQLGHEDTTSLDRPR
Scan Number: 12359 precMass: 519.2691 precCharge: 4 Sequence: YGQLGHEDTTSLDRPR
Intensity
2e+05
1e+05
0e+00
M/Z
400
800
1200
1600
b3
b5
y1
y2
y3
b2
b1
b4
type
b
y

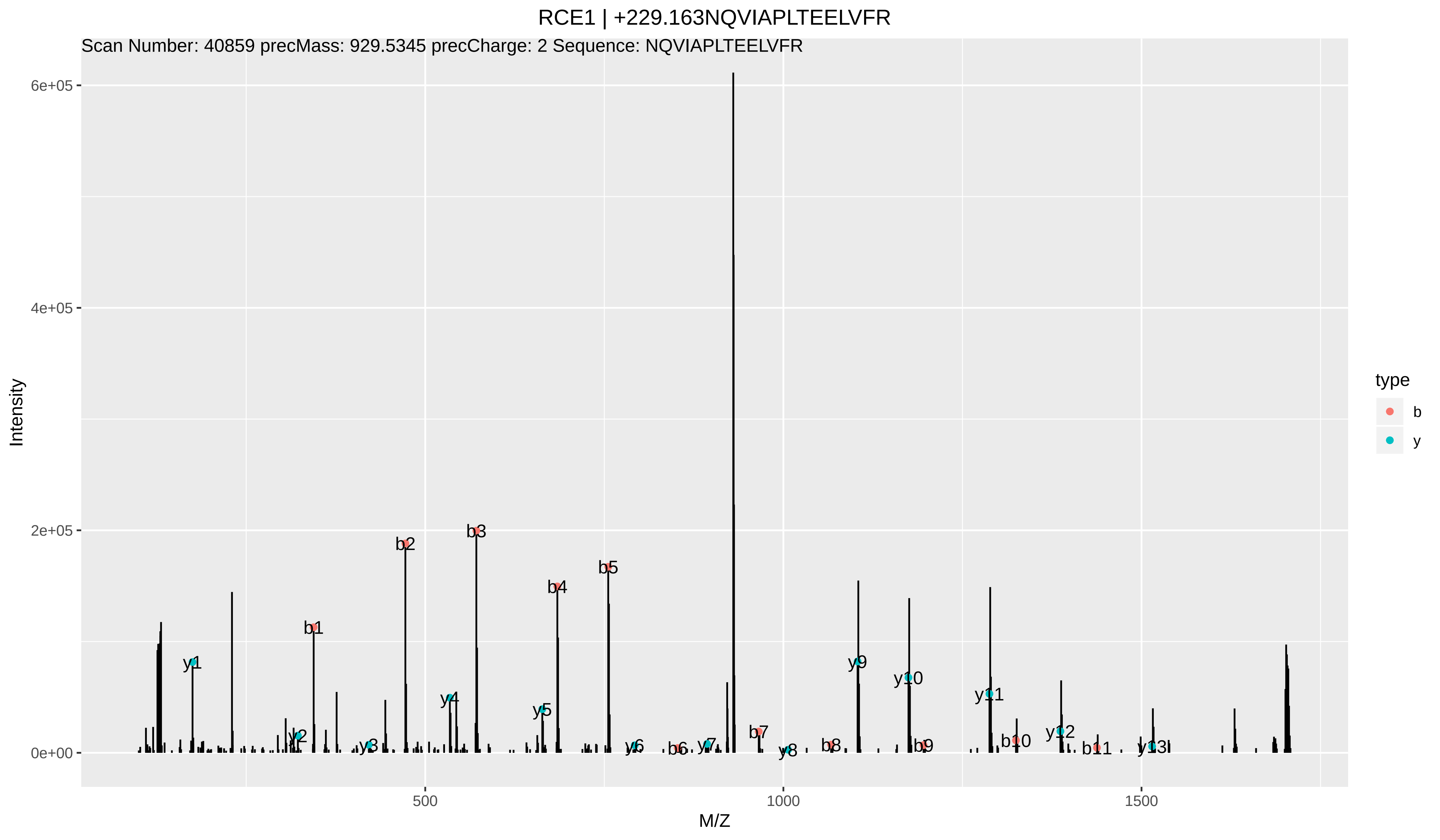

RCE1 | +229.163NQVIAPLTEELVFR
Scan Number: 40859 precMass: 929.5345 precCharge: 2 Sequence: NQVIAPLTEELVFR
Intensity
6e+05
4e+05
2e+05
0e+00
M/Z
500
1000
1500
type
b
y
y1
y2
y3
y4
y5
y6
y7
y8
y9
y10
y11
y12
y13
b1
b2
b3
b4
b5
b6
b7
b8
b9
b10
b11

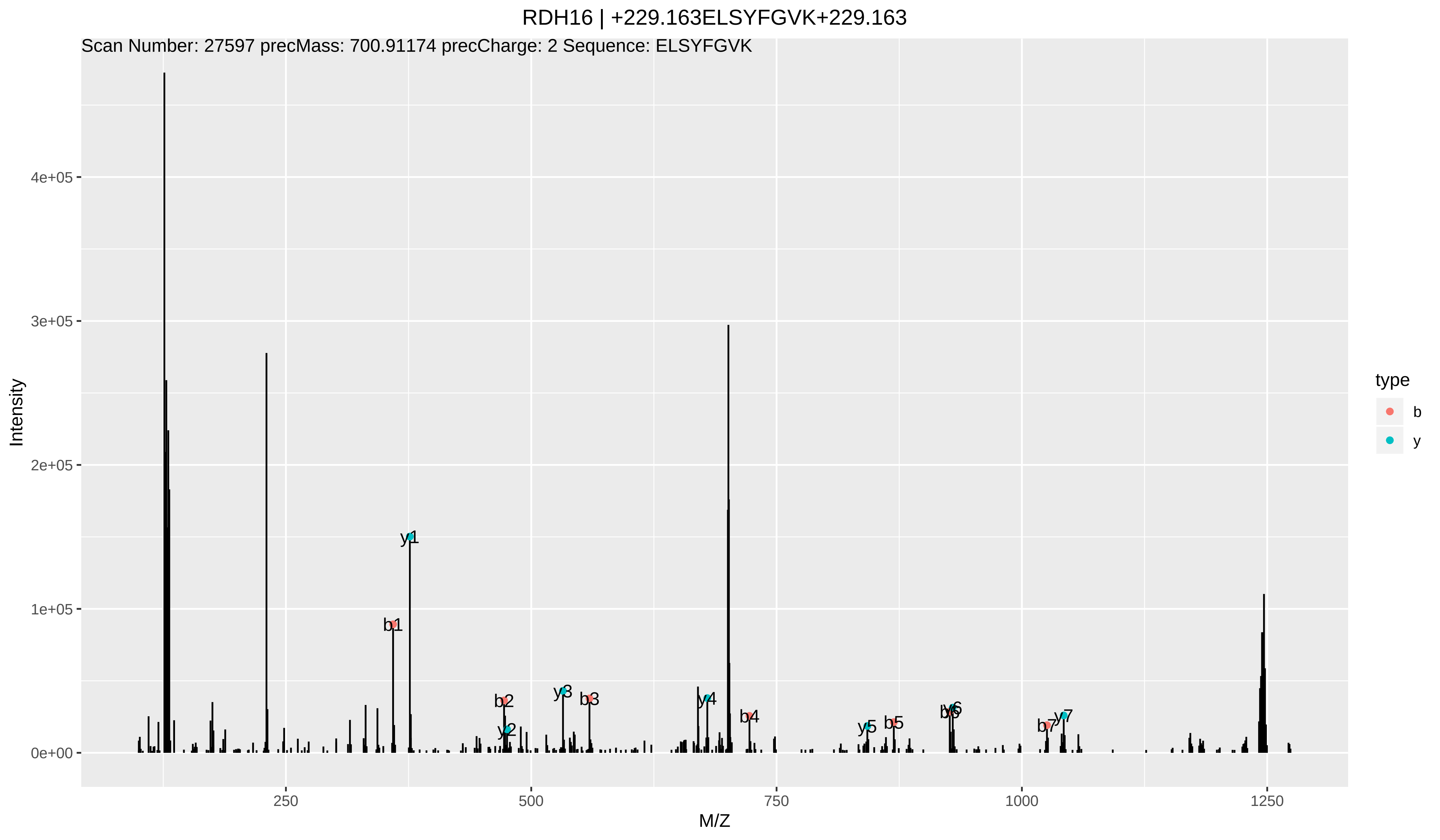
RDH16 | +229.163ELSYFGVK+229.163
Scan Number: 27597 precMass: 700.91174 precCharge: 2 Sequence: ELSYFGVK
Intensity
M/Z
0e+00
1e+05
2e+05
3e+05
4e+05
250
500
750
1000
1250
y1
b1
b2
y2
y3
b3
y4
b4
y5
b5
b6
y6
b7
y7
type
b
y

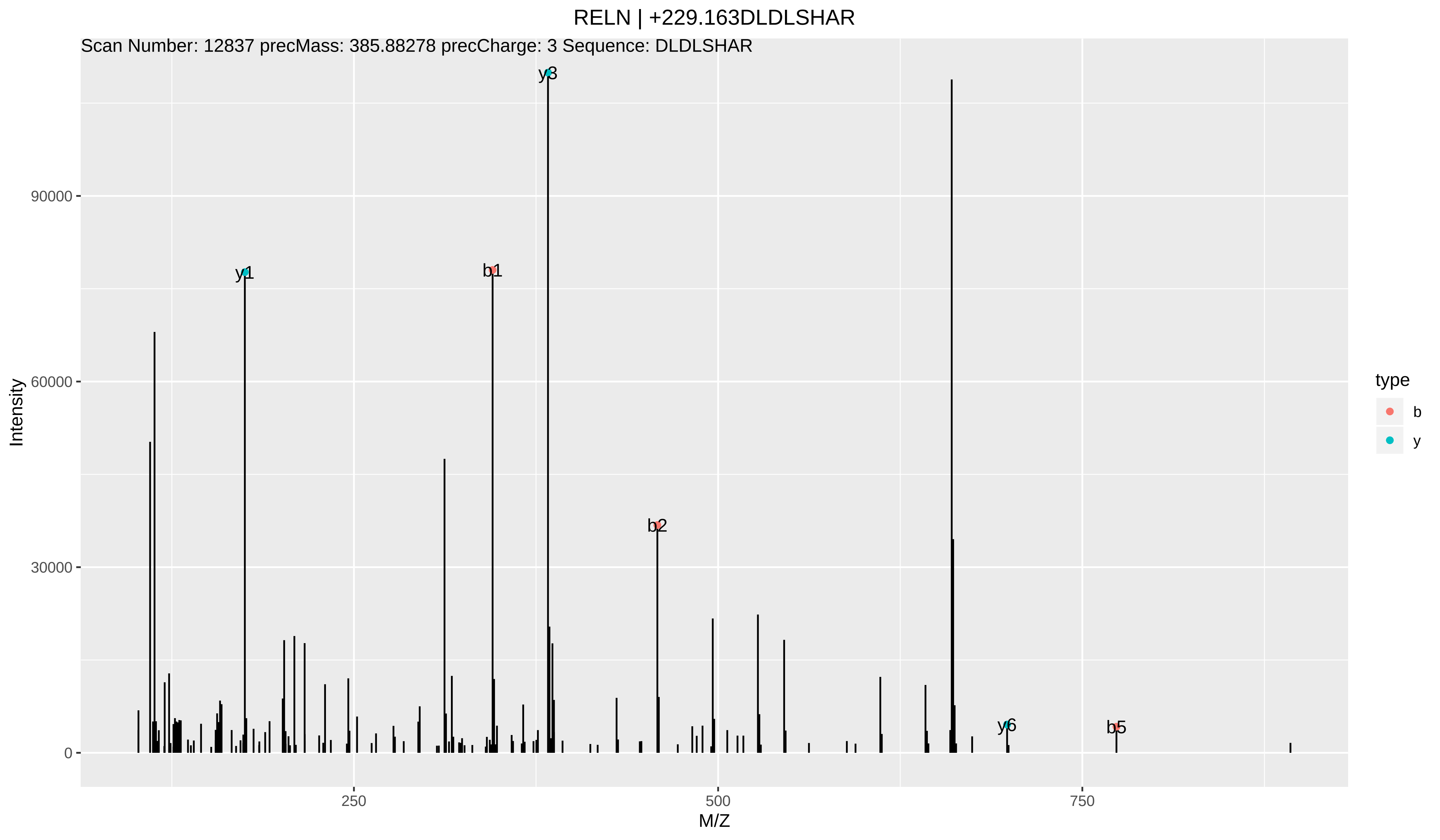
RELN | +229.163DLDLSHAR
Scan Number: 12837 precMass: 385.88278 precCharge: 3 Sequence: DLDLSHAR
Intensity
90000
60000
30000
0
M/Z
250
500
750
y1
b1
y3
b2
y6
b5
type
b
y

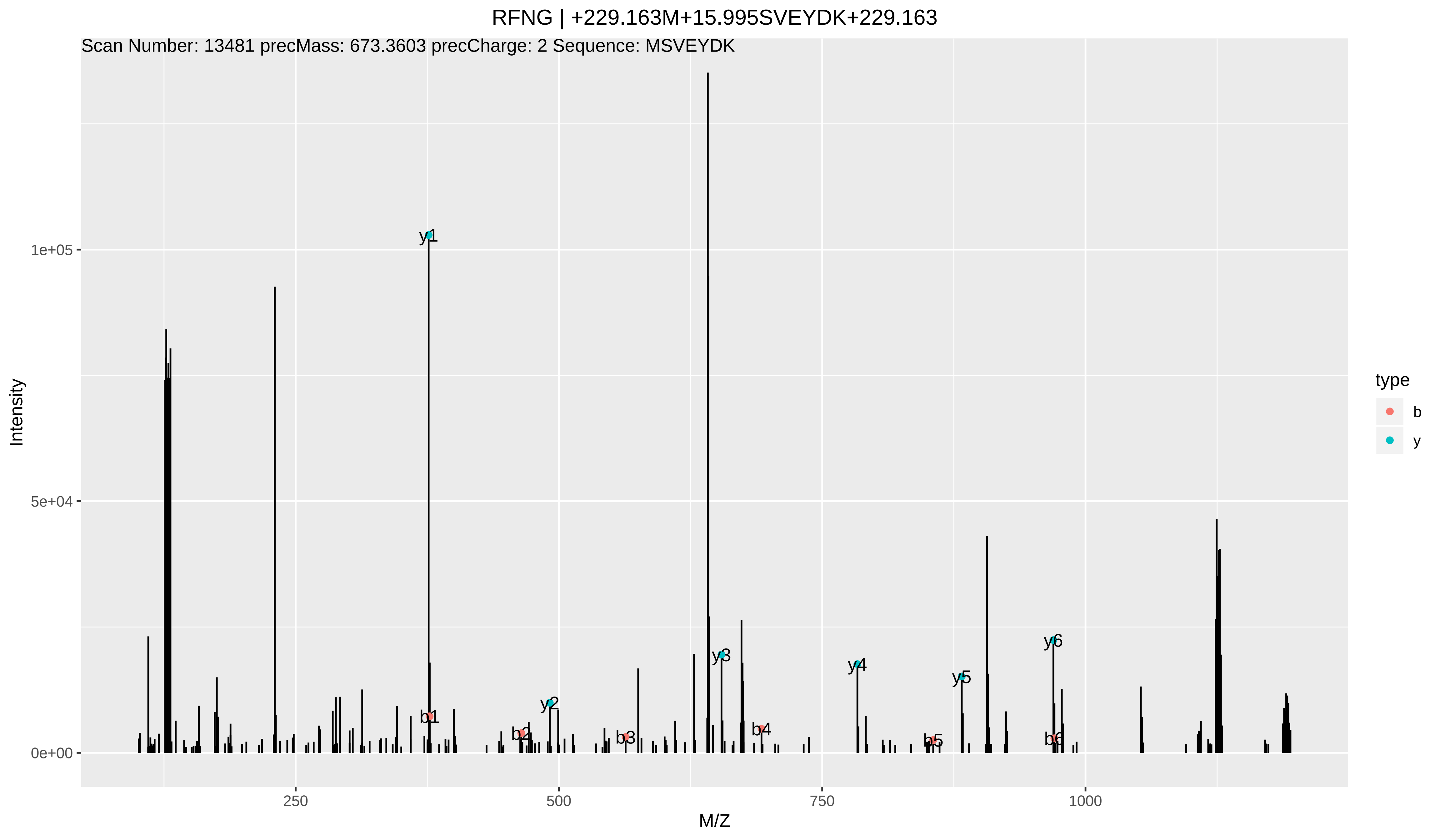
RFNG | +229.163M+15.995SVEYDK+229.163
Scan Number: 13481 precMass: 673.3603 precCharge: 2 Sequence: MSVEYDK
Intensity
M/Z
0e+00
5e+04
1e+05
250
500
750
1000
y1
y2
y3
y4
y5
y6
b1
b2
b3
b4
b5
b6
type
b
y

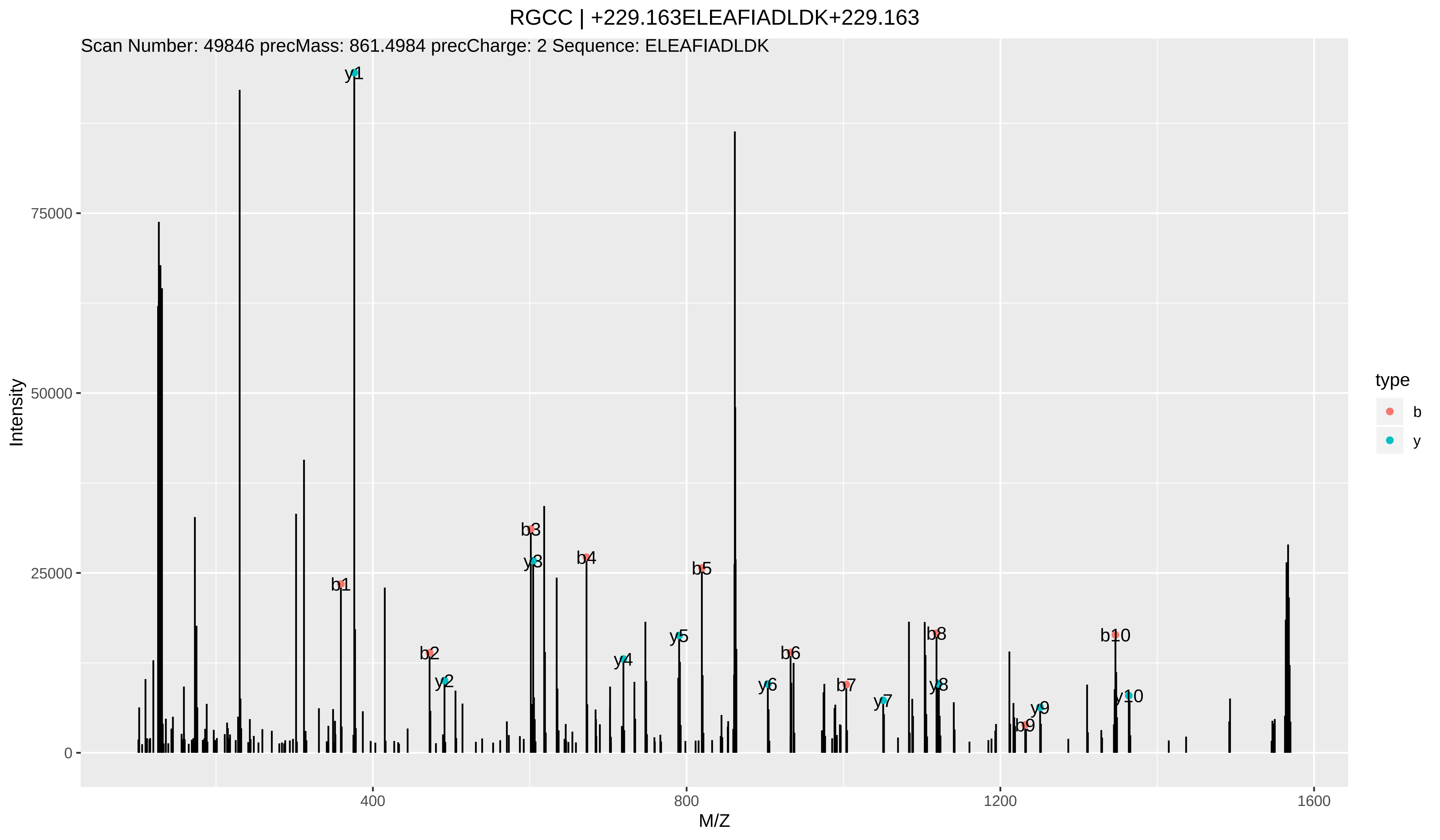

RGCC | +229.163ELEAFIADLDK+229.163
Scan Number: 49846 precMass: 861.4984 precCharge: 2 Sequence: ELEAFIADLDK
Intensity
M/Z
type
b
y
y1
b1
b2
y2
b3
y3
b4
y4
y5
b5
y6
b6
b7
y7
b8
y8
b9
y9
b10
y10
0
25000
50000
75000
400
800
1200
1600

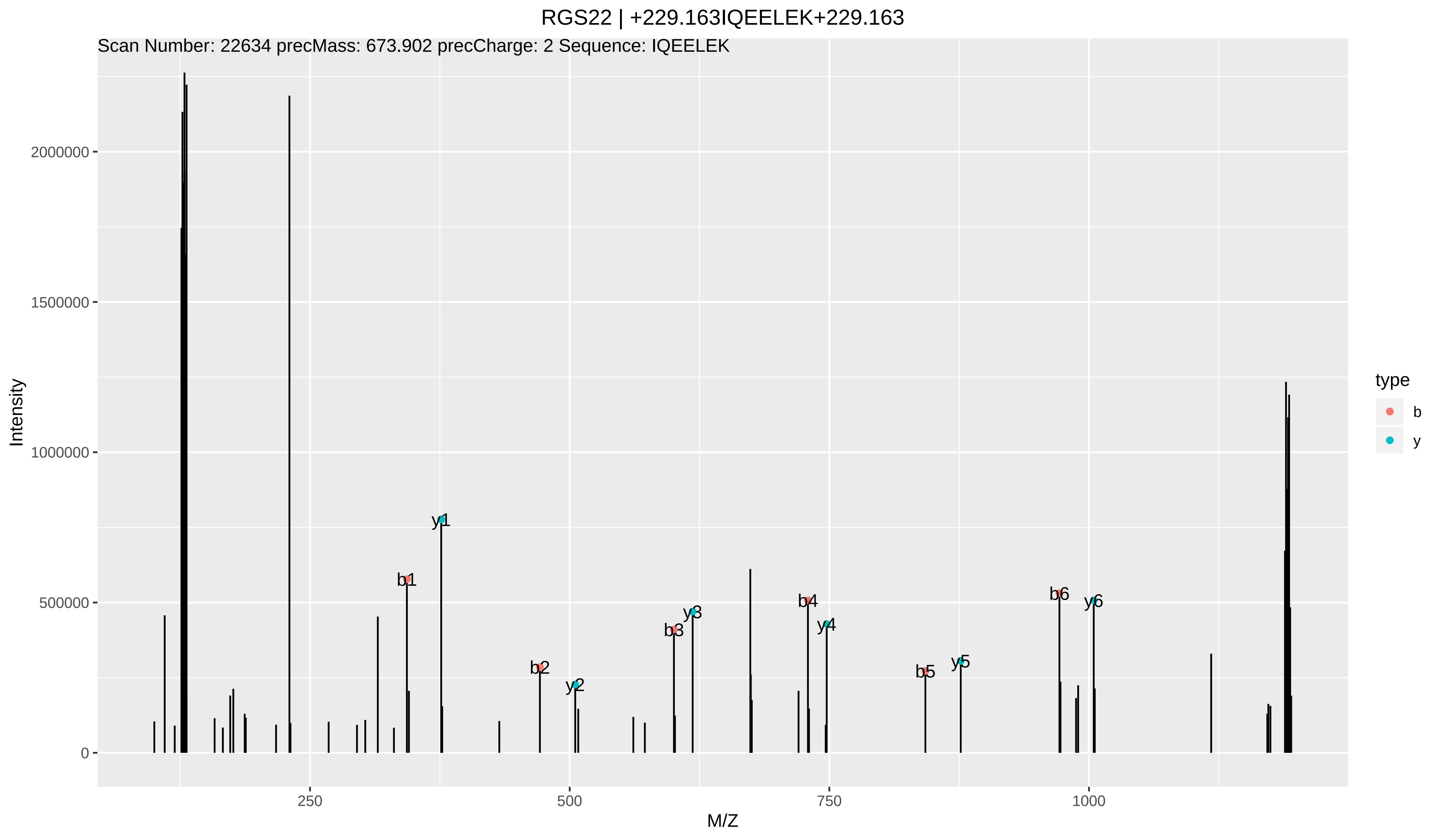

RGS22 | +229.163IQEELEK+229.163
Scan Number: 22634 precMass: 673.902 precCharge: 2 Sequence: IQEELEK
Intensity
2000000
1500000
1000000
500000
0
250
500
750
1000
M/Z
y1
b1
b2
y2
b3
y3
b4
y4
b5
y5
b6
y6
type
b
y

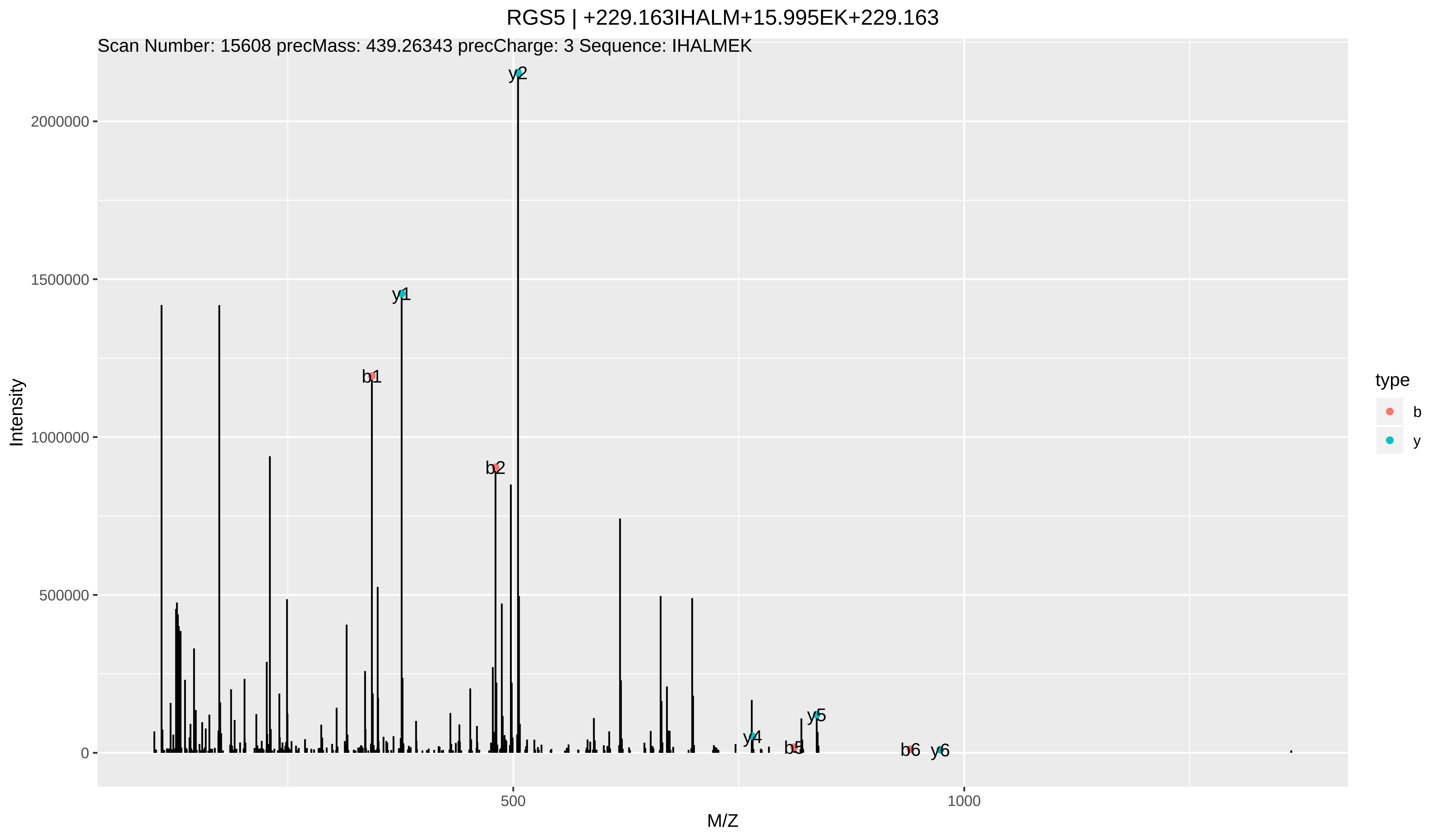
RGS5 | +229.163IHALM+15.995EK+229.163
Scan Number: 15608 precMass: 439.26343 precCharge: 3 Sequence: IHALMEK
y2
y1
b1
b2
y5
y4
b5
b6
y6
Intensity
2000000
1500000
1000000
500000
0
500
1000
M/Z
type
b
y

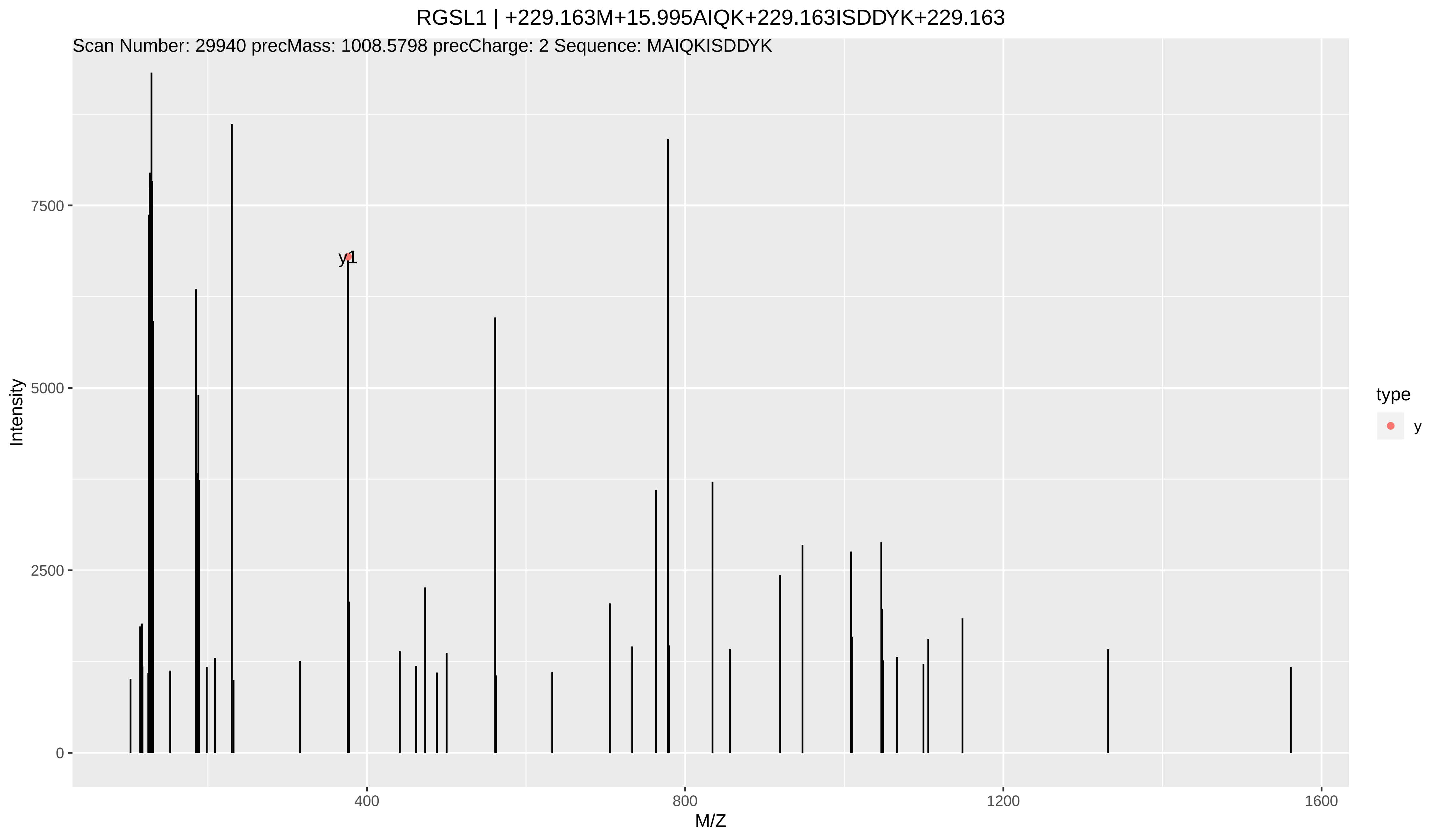

RGSL1 | +229.163M+15.995AIQK+229.163ISDDYK+229.163
Scan Number: 29940 precMass: 1008.5798 precCharge: 2 Sequence: MAIQKISDDYK
y1
Intensity
7500
5000
2500
0
400
800
1200
1600
M/Z
type
y

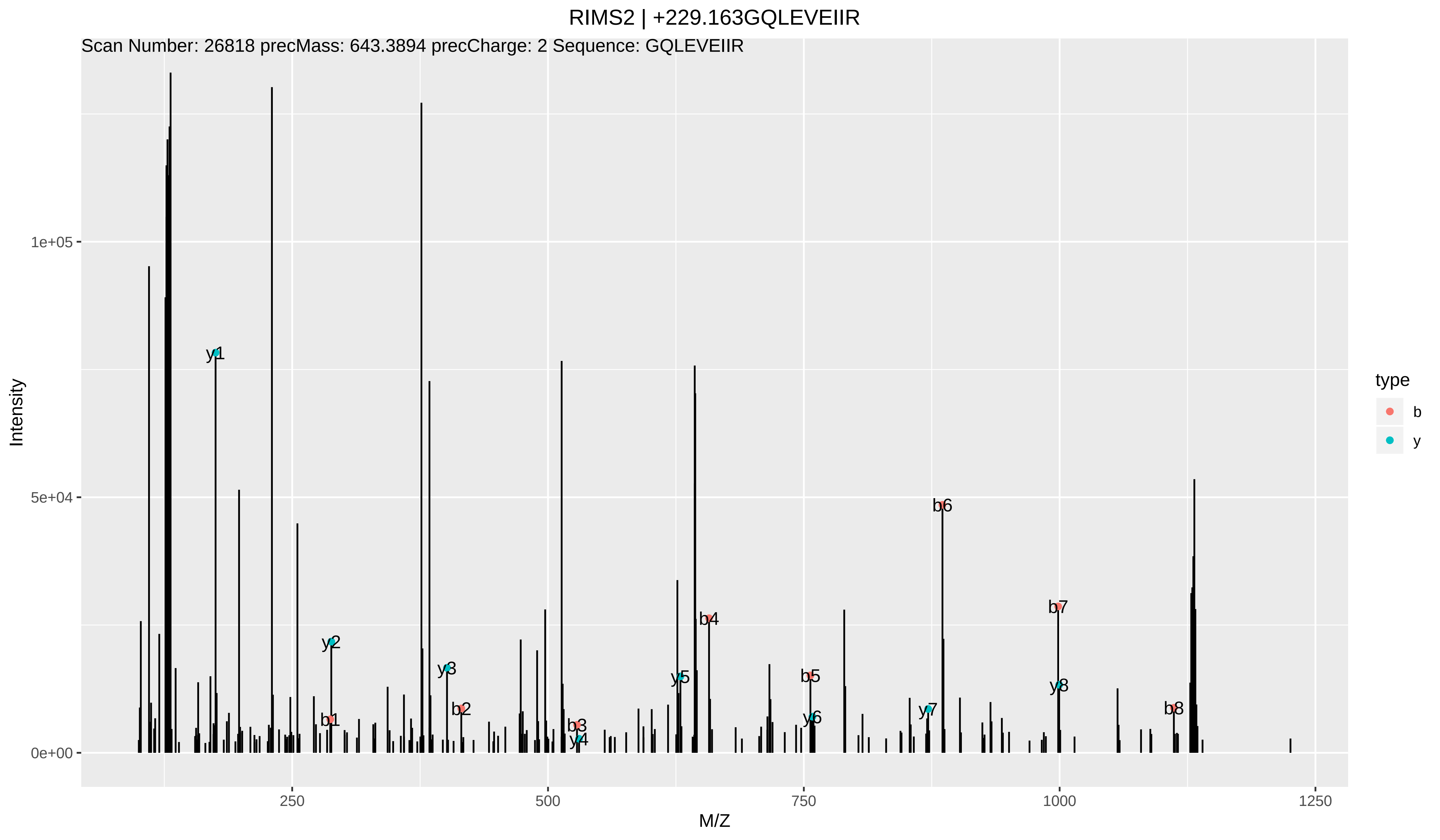

RIMS2 | +229.163GQLEVEIIR
Scan Number: 26818 precMass: 643.3894 precCharge: 2 Sequence: GQLEVEIIR
Intensity
1e+05
5e+04
0e+00
M/Z
250
500
750
1000
1250
y1
y2
b1
y3
b2
b3
y4
y5
b4
b5
y6
b6
y7
b7
y8
b8
type
b
y

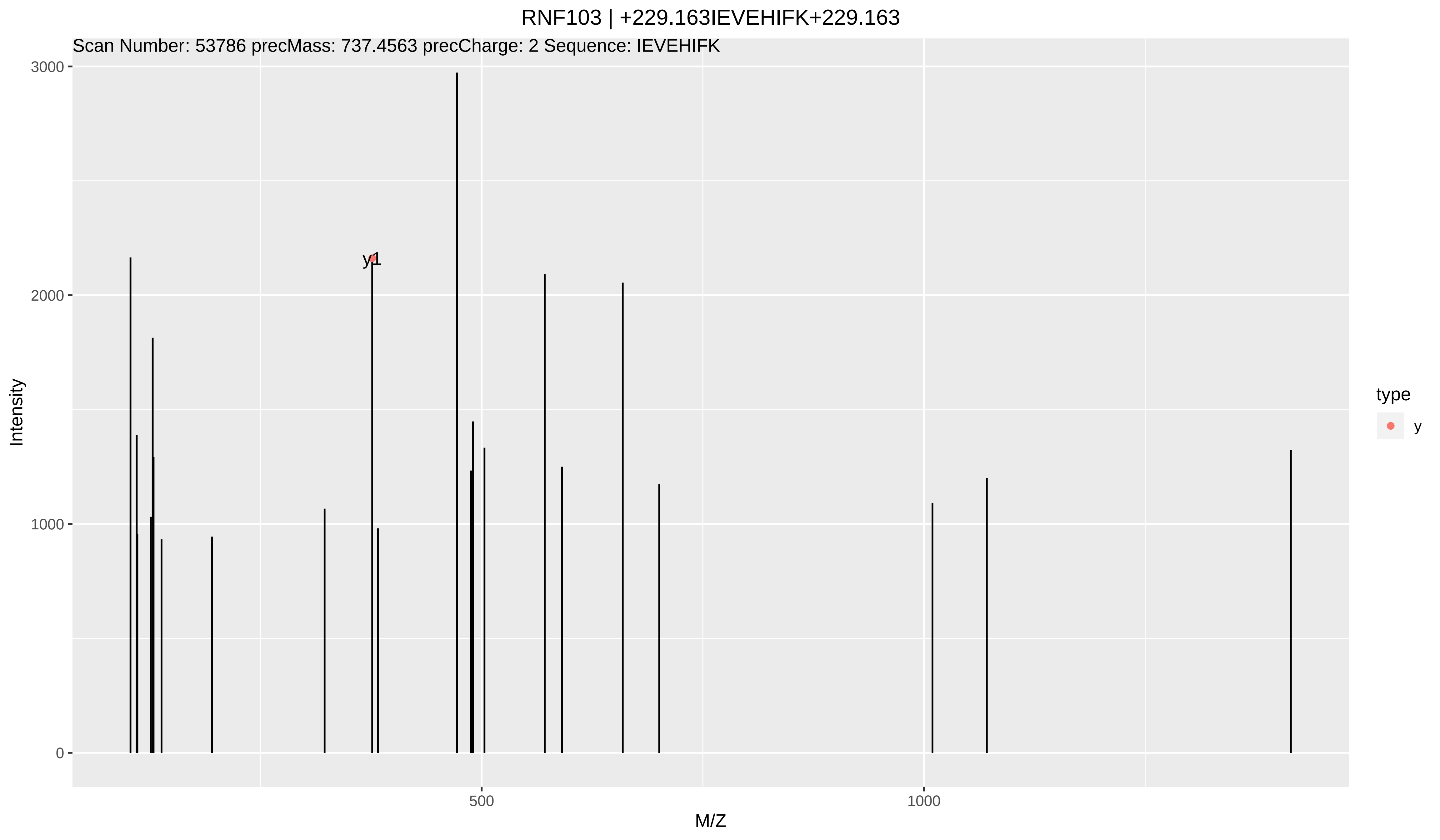
RNF103 | +229.163IEVEHIFK+229.163
Scan Number: 53786 precMass: 737.4563 precCharge: 2 Sequence: IEVEHIFK
Intensity
3000
2000
1000
0
y1
500
1000
M/Z
type
y

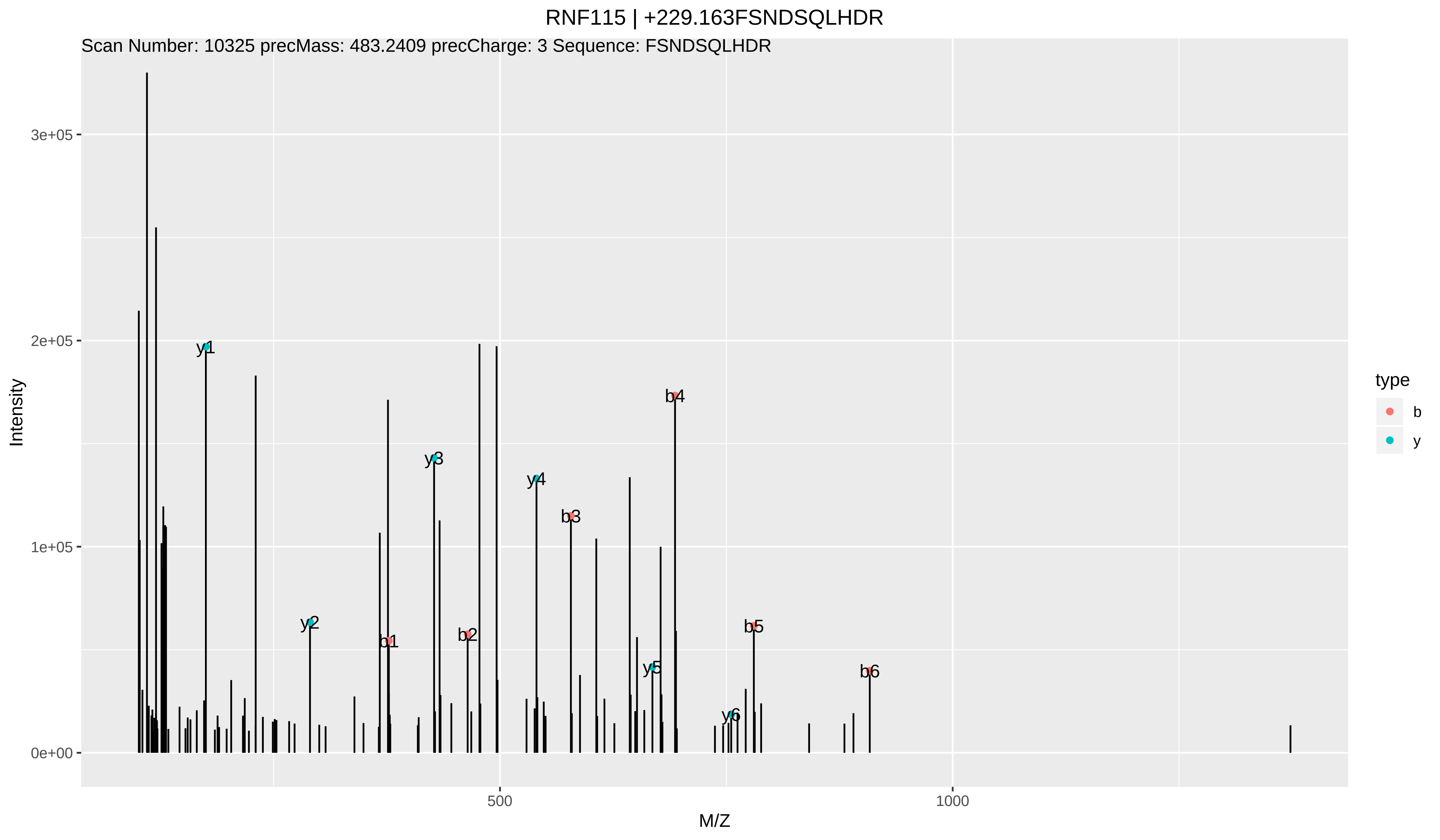

RNF115 | +229.163FSNDSQLHDR
Scan Number: 10325 precMass: 483.2409 precCharge: 3 Sequence: FSNDSQLHDR
Intensity
3e+05
2e+05
1e+05
0e+00
M/Z
500
1000
y1
y2
b1
y3
b2
y4
b3
y5
b4
y6
b5
b6
type
b
y

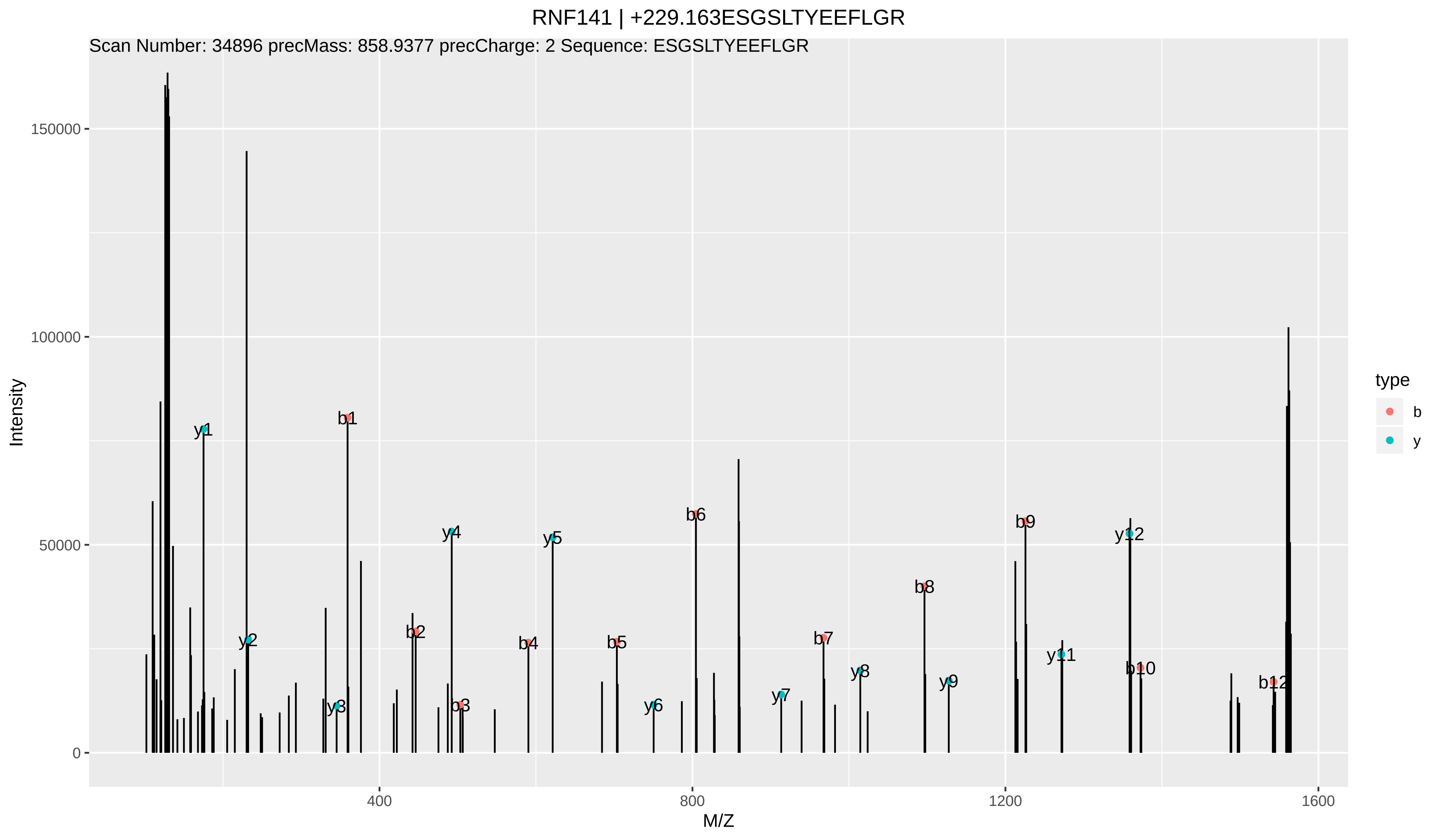

RNF141 | +229.163ESGSLTYEEFLGR
Scan Number: 34896 precMass: 858.9377 precCharge: 2 Sequence: ESGSLTYEEFLGR
Intensity
M/Z
150000
100000
50000
0
400
800
1200
1600
y1
b1
y2
y3
b2
y4
b3
b4
y5
b5
y6
b6
y7
b7
y8
b8
y9
b9
y11
y12
b10
b12
type
b
y

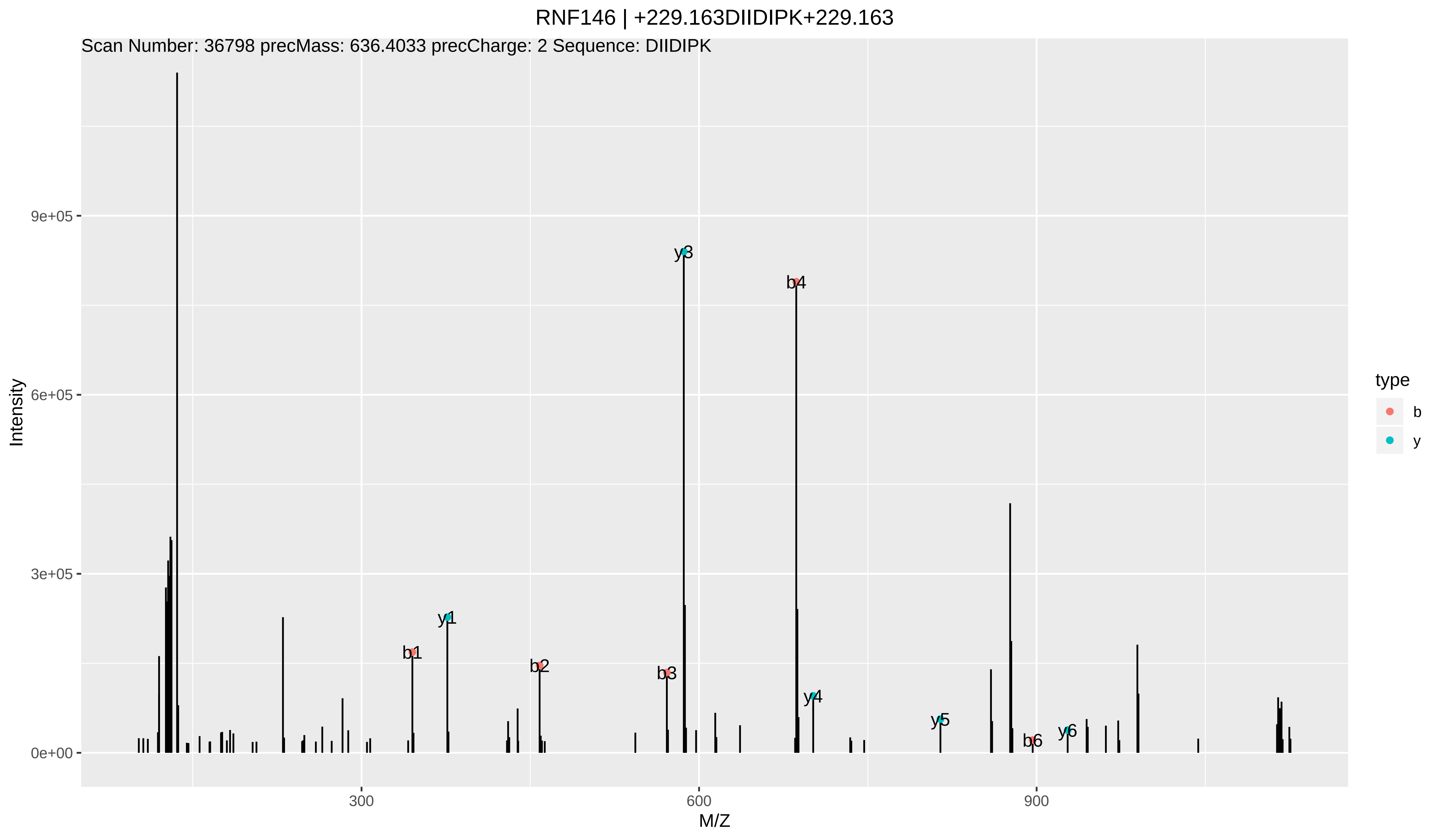
RNF146 | +229.163DIIDIPK+229.163
Scan Number: 36798 precMass: 636.4033 precCharge: 2 Sequence: DIIDIPK
Intensity
9e+05
6e+05
3e+05
0e+00
M/Z
300
600
900
y3
b4
y1
b1
b2
b3
y4
y5
b6
y6
type
b
y

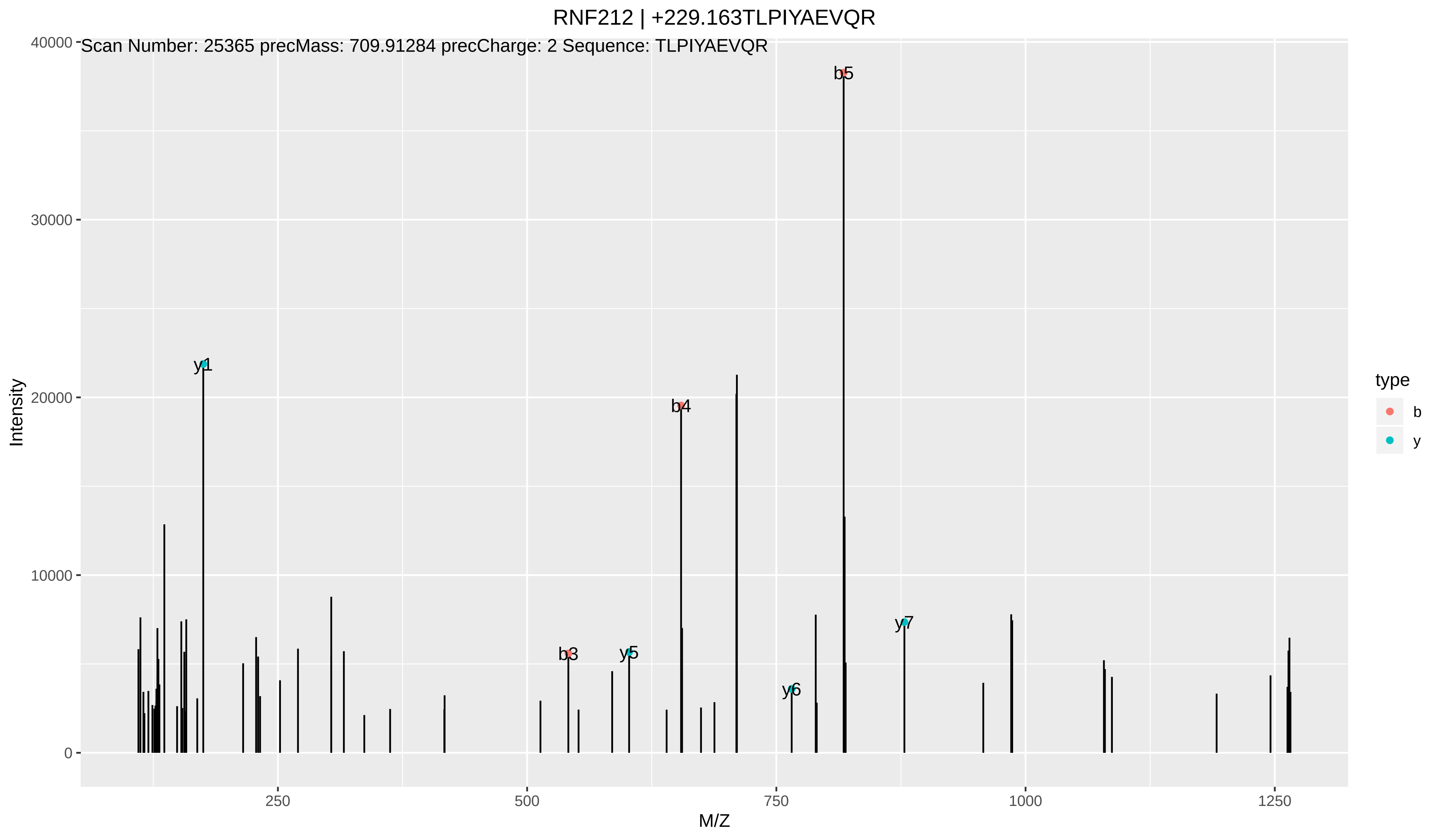
RNF212 | +229.163TLPIYAEVQR
Scan Number: 25365 precMass: 709.91284 precCharge: 2 Sequence: TLPIYAEVQR
Intensity
M/Z
0
10000
20000
30000
40000
250
500
750
1000
1250
y1
b3
y5
b4
y6
b5
y7
type
b
y

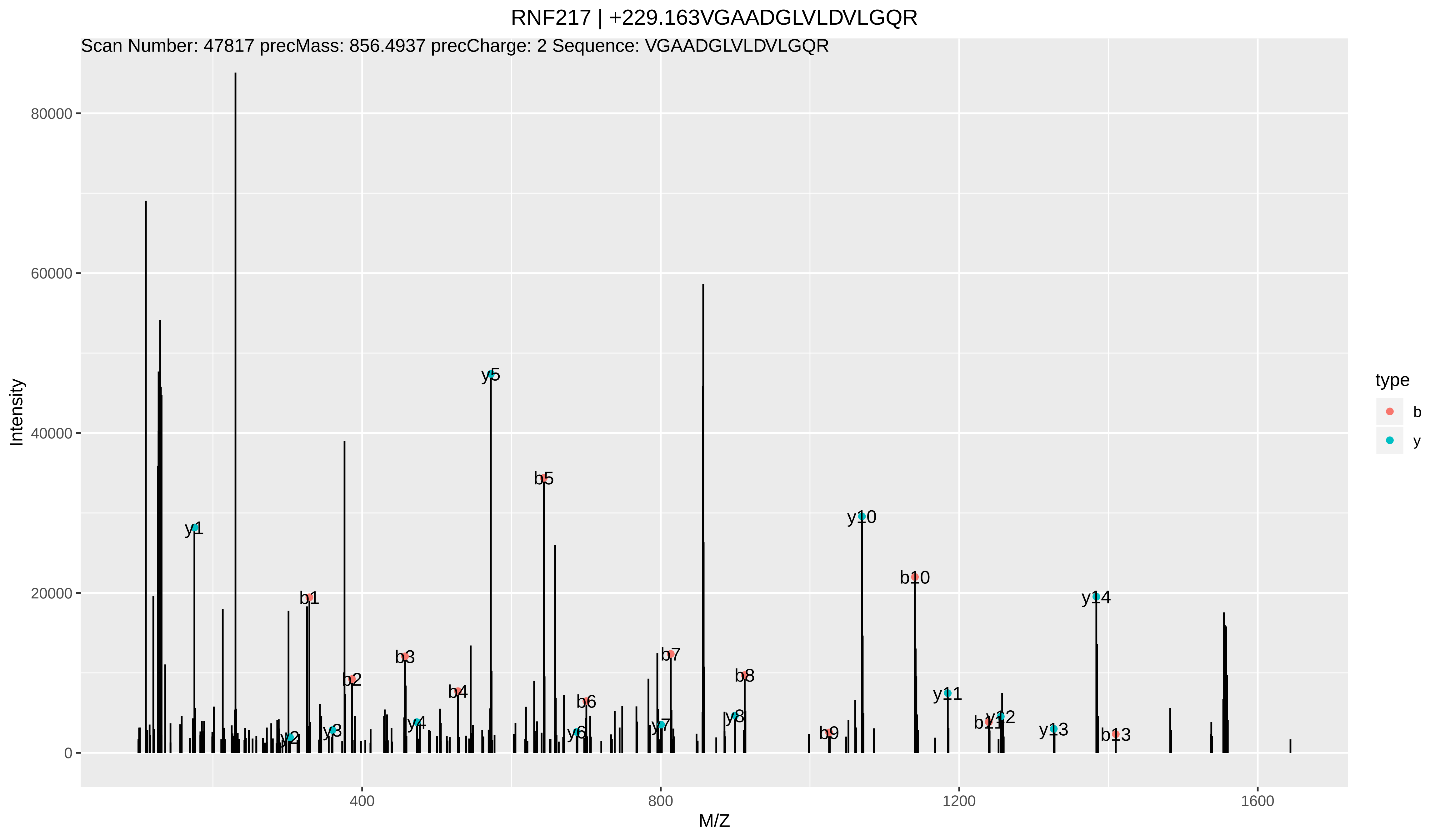
RNF217 | +229.163VGAADGLVLDVLGQR
Scan Number: 47817 precMass: 856.4937 precCharge: 2 Sequence: VGAADGLVLDVLGQR
Intensity
M/Z
type
b
y
y1
y2
b1
y3
b2
b3
y4
b4
y5
b5
y6
b6
y7
b7
y8
b8
b9
y10
b10
y11
b11
y12
y13
y14
b13
0
20000
40000
60000
80000
400
800
1200
1600

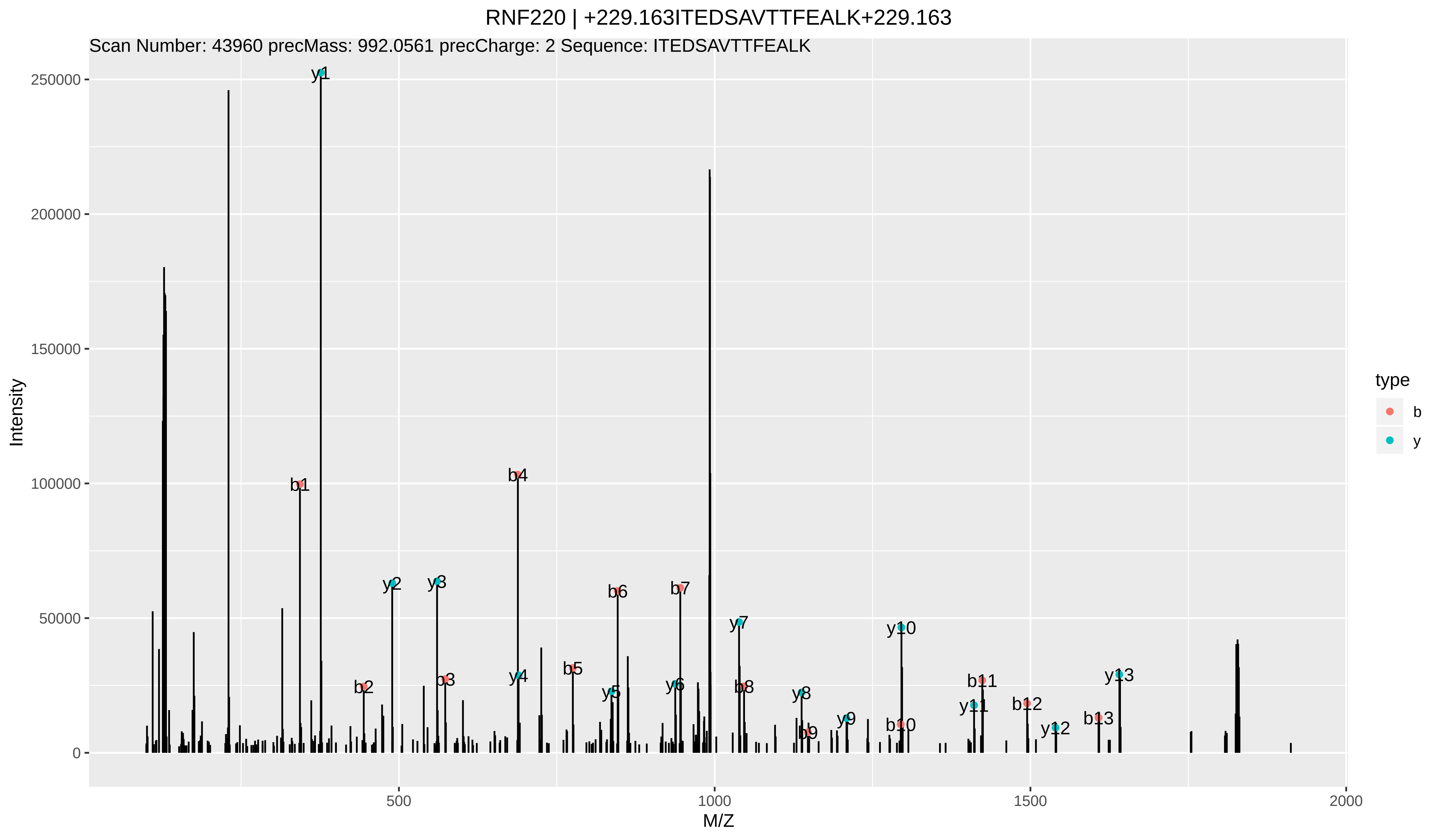

RNF220 | +229.163ITEDSAVTTFEALK+229.163
Scan Number: 43960 precMass: 992.0561 precCharge: 2 Sequence: ITEDSAVTTFEALK
Intensity
M/Z
type
b
y
y1
b1
b4
y2
y3
b6
b7
y7
y10
b2
b3
y4
b5
y5
y6
b8
y8
b9
y9
b10
y11
b11
b12
y12
b13
y13
0
50000
100000
150000
200000
250000
500
1000
1500
2000

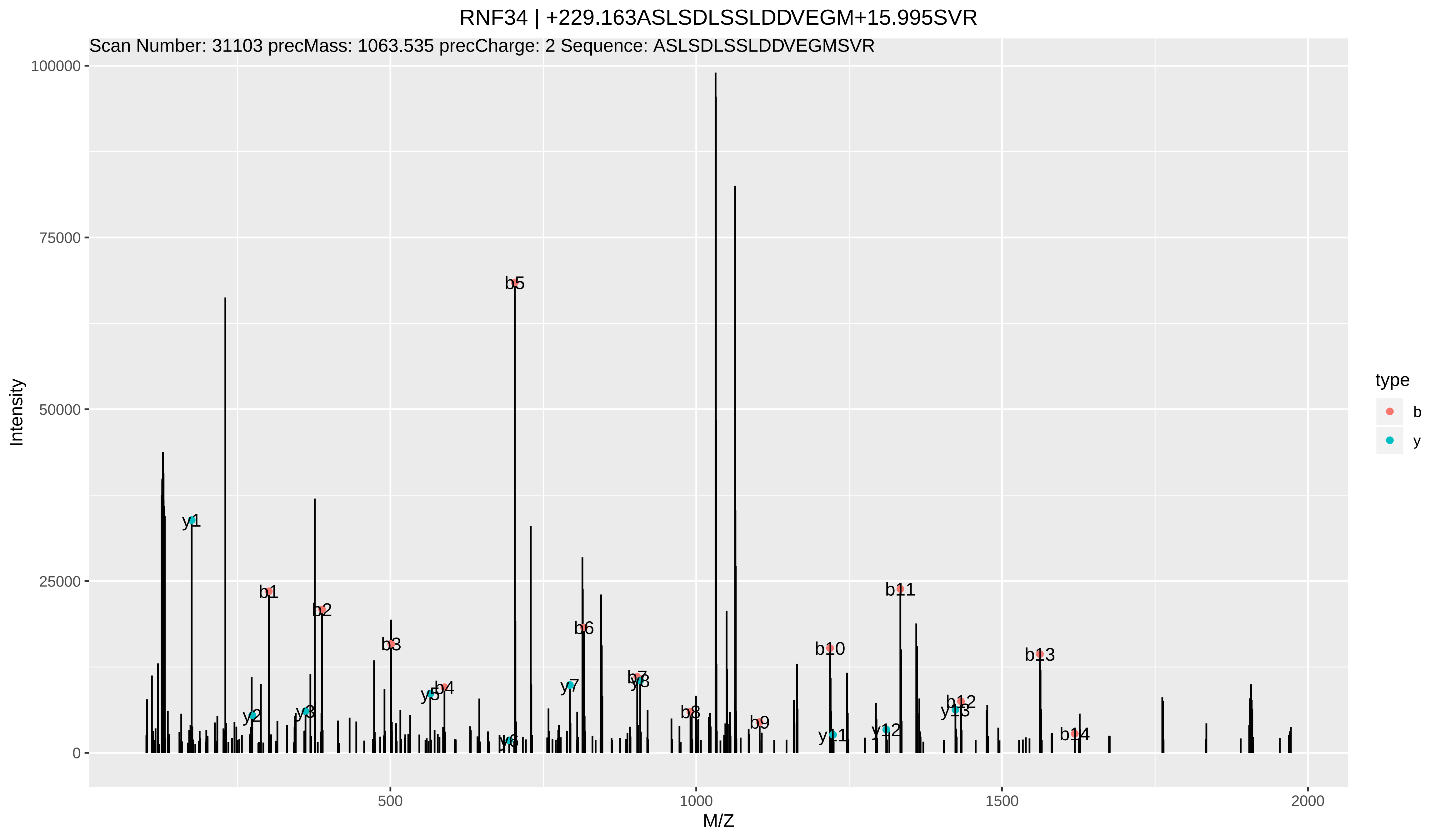
RNF34 | +229.163ASLSDLSSLDDVEGM+15.995SVR
Scan Number: 31103 precMass: 1063.535 precCharge: 2 Sequence: ASLSDLSSLDDVEGMSVR
Intensity
M/Z
100000
75000
50000
25000
0
500
1000
1500
2000
type
b
y
y1
b1
b2
y2
y3
b3
y5
b4
b5
y6
y7
b6
b7
y8
b8
b9
b10
y11
y12
b11
b12
y13
b13
b14

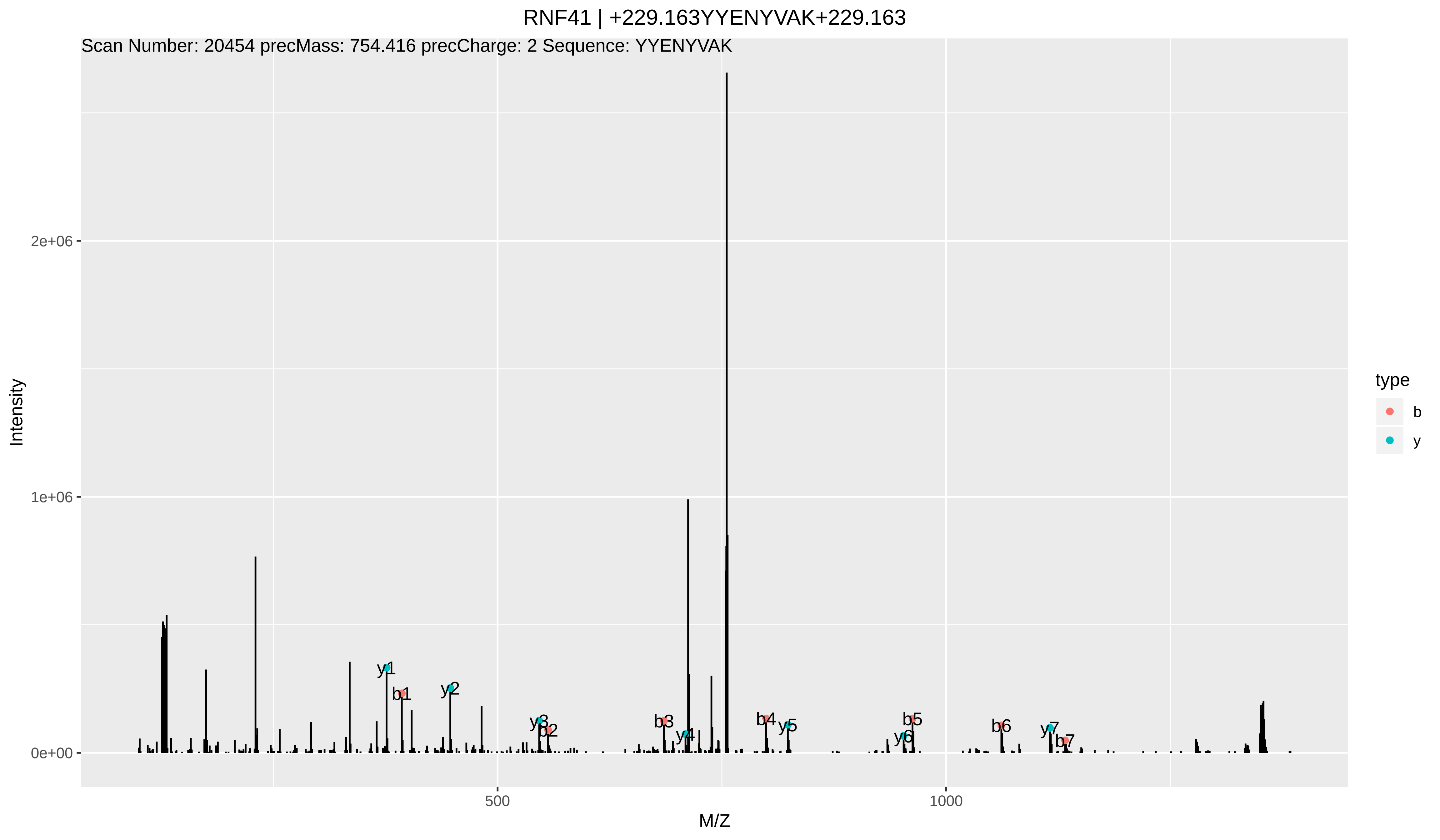
RNF41 | +229.163YYENYVAK+229.163
Scan Number: 20454 precMass: 754.416 precCharge: 2 Sequence: YYENYVAK
Intensity
2e+06
1e+06
0e+00
M/Z
500
1000
y1
b1
y2
y3
b2
b3
y4
b4
y5
b5
y6
b6
y7
b7
type
b
y

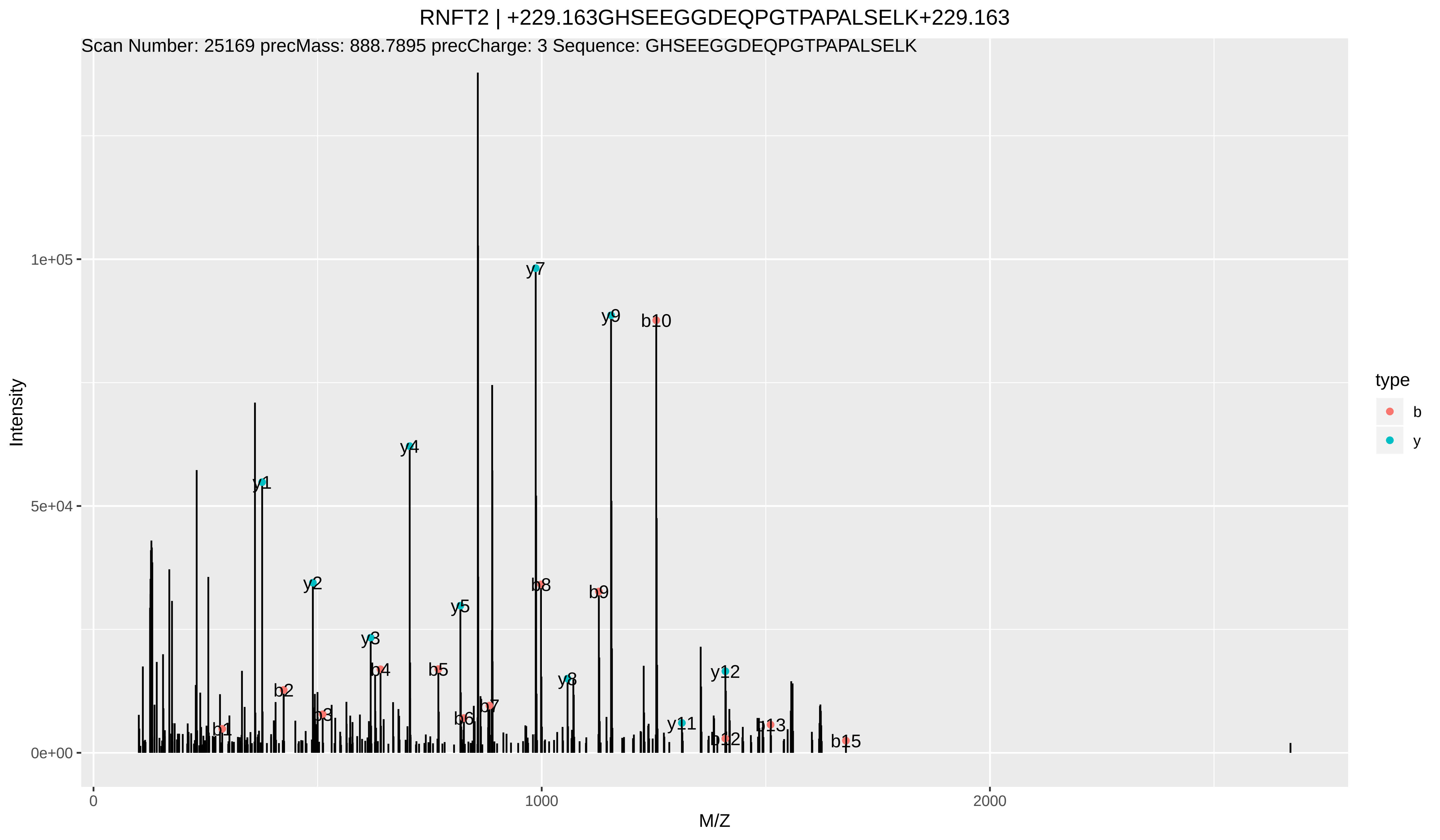

RNFT2 | +229.163GHSEEGGDEQPGTPAPALSELK+229.163
Scan Number: 25169 precMass: 888.7895 precCharge: 3 Sequence: GHSEEGGDEQPGTPAPALSELK
Intensity
1e+05
5e+04
0e+00
M/Z
0
1000
2000
type
b
y
y1
y2
y3
y4
y5
y7
y8
y9
y11
y12
b1
b2
b3
b4
b5
b6
b7
b8
b9
b10
b12
b13
b15

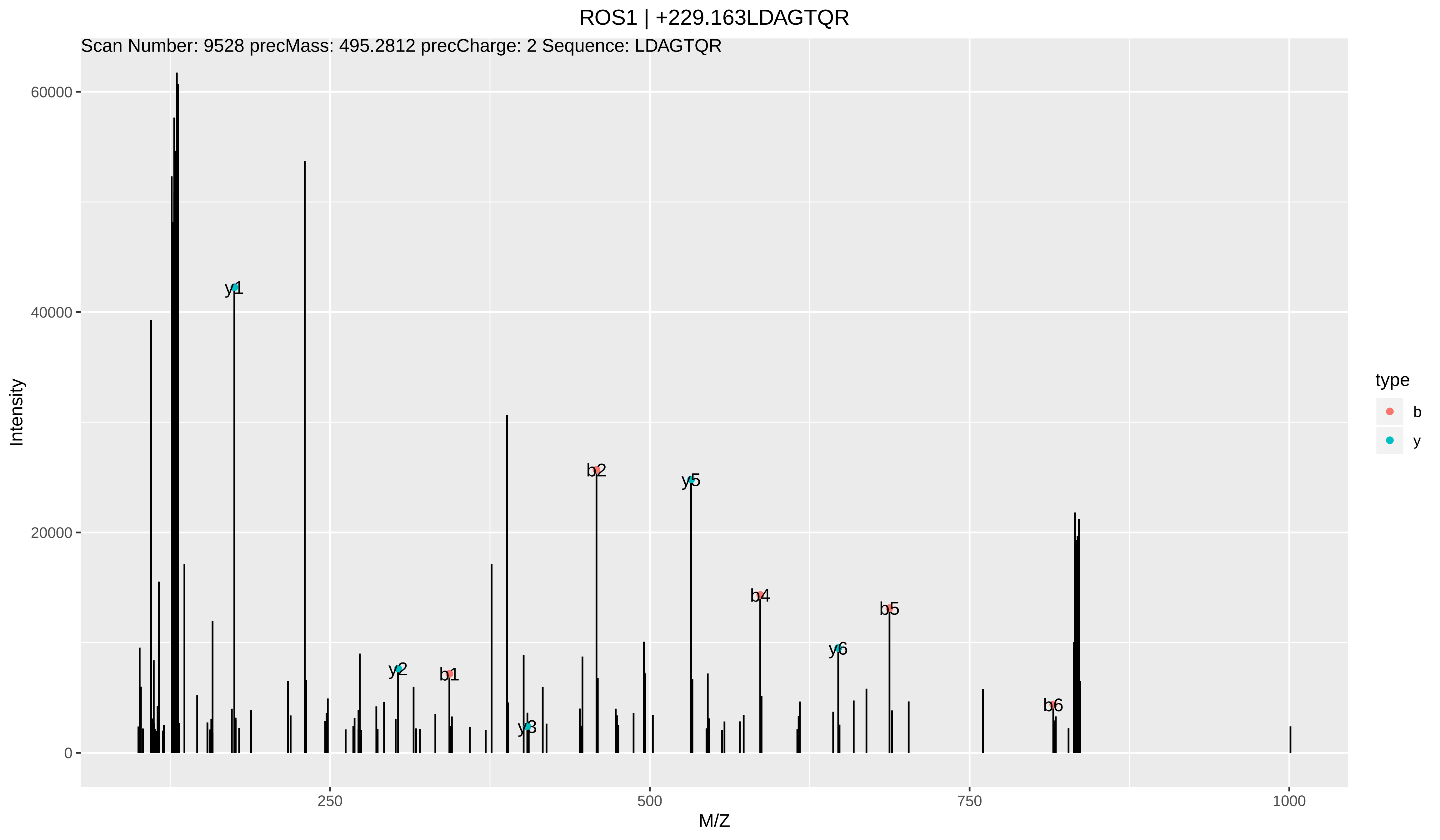

ROS1 | +229.163LDAGTQR
Scan Number: 9528 precMass: 495.2812 precCharge: 2 Sequence: LDAGTQR
Intensity
M/Z
0
20000
40000
60000
250
500
750
1000
y1
y2
b1
y3
b2
y5
b4
y6
b5
b6
type
b
y

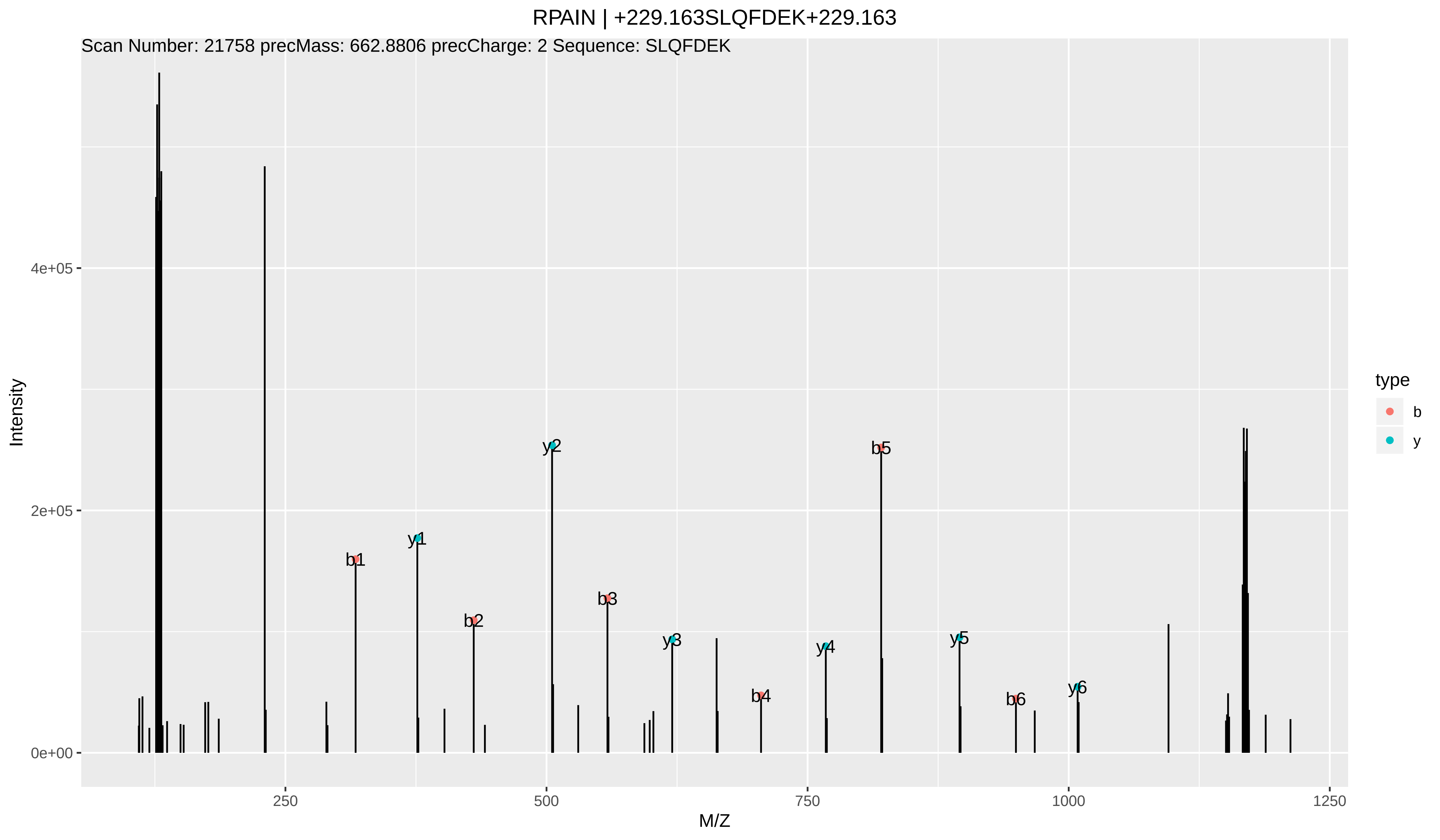
RPAIN | +229.163SLQFDEK+229.163
Scan Number: 21758 precMass: 662.8806 precCharge: 2 Sequence: SLQFDEK
Intensity
4e+05
2e+05
0e+00
M/Z
250
500
750
1000
1250
y2
b5
y1
b1
b3
b2
y3
y4
y5
b4
b6
y6
type
b
y

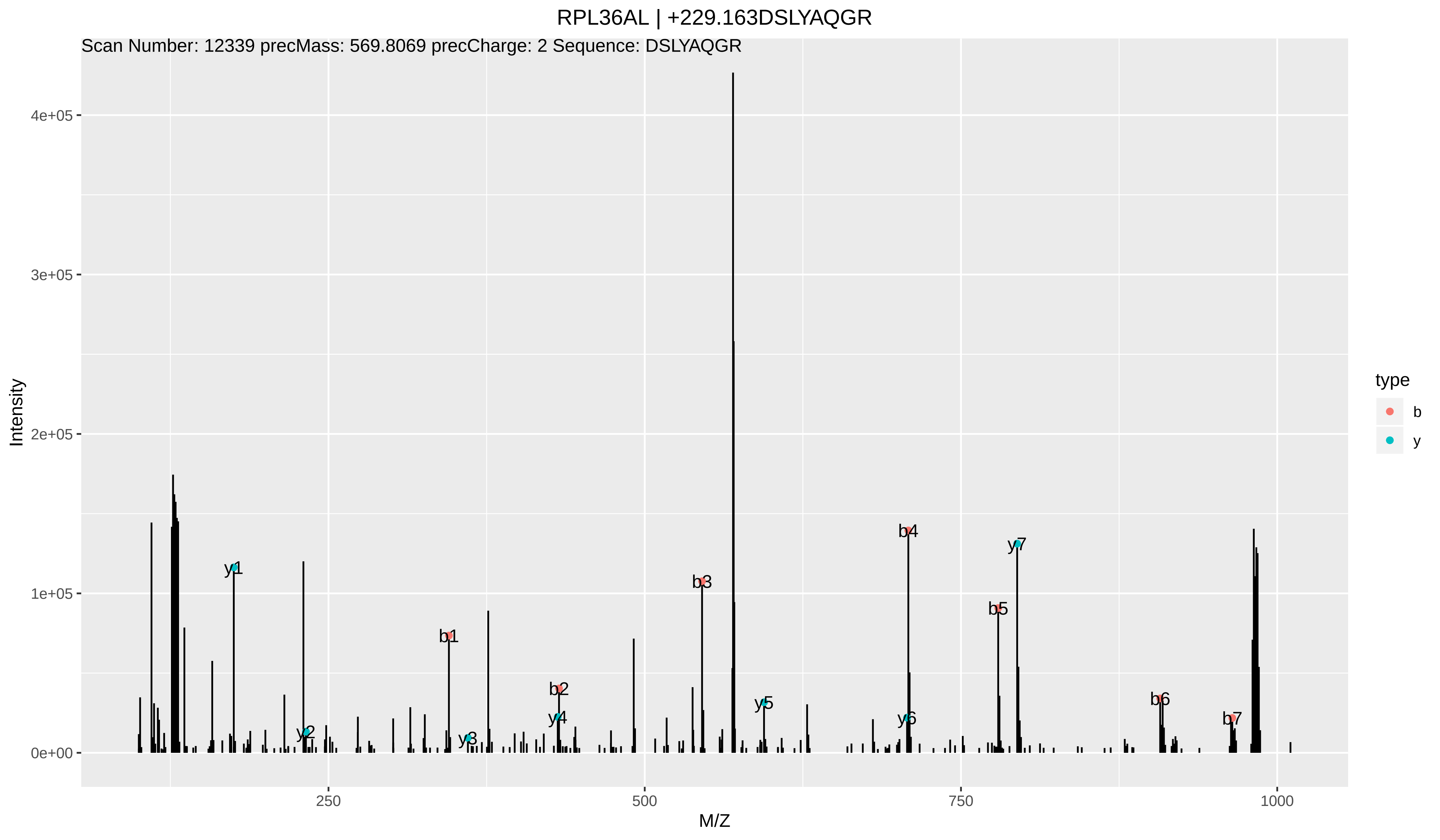
RPL36AL | +229.163DSLYAQGR
Scan Number: 12339 precMass: 569.8069 precCharge: 2 Sequence: DSLYAQGR
Intensity
4e+05
3e+05
2e+05
1e+05
0e+00
M/Z
250
500
750
1000
y1
y2
y3
y4
y5
y6
y7
b1
b2
b3
b4
b5
b6
b7
type
b
y

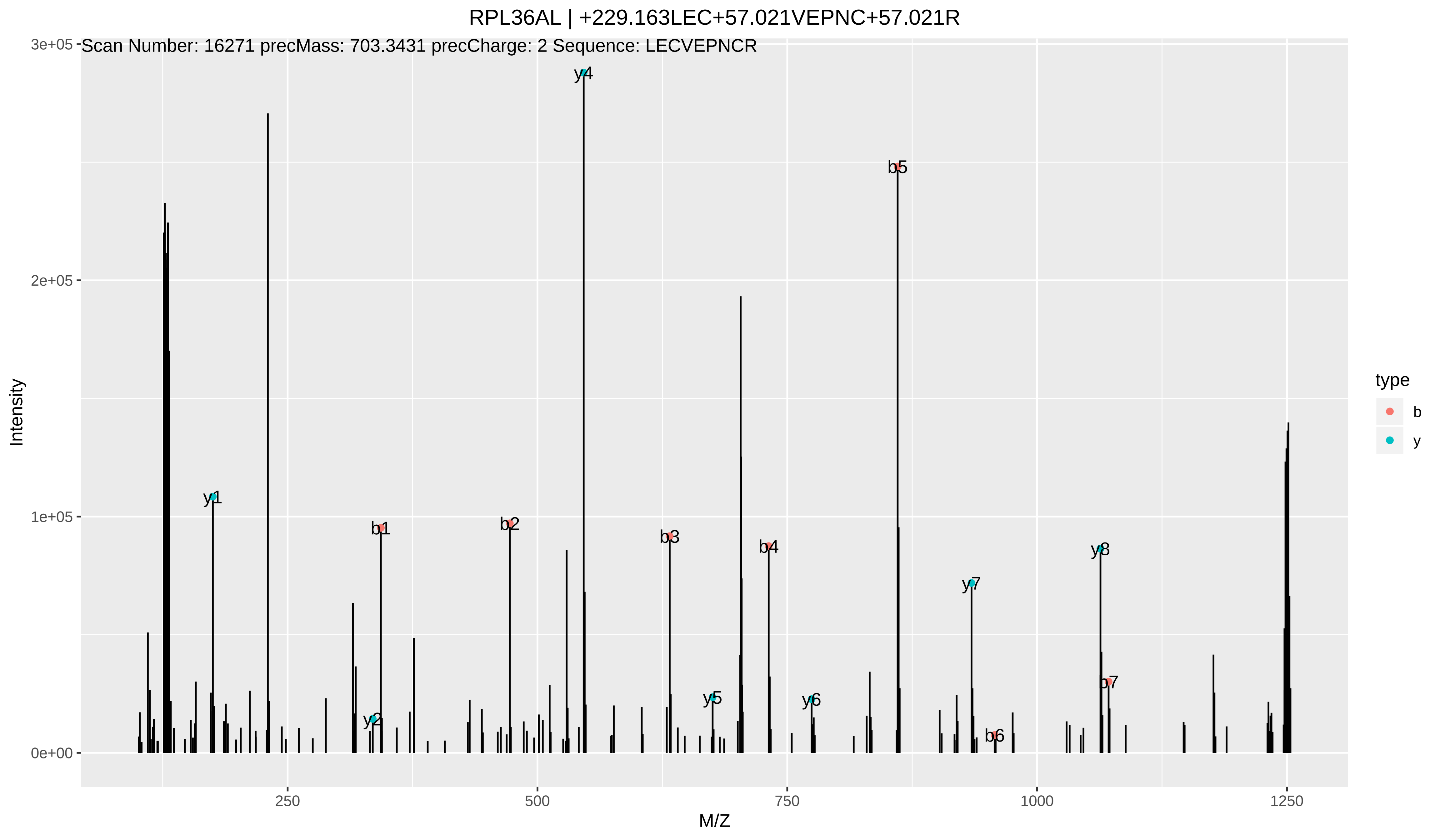

RPL36AL | +229.163LEC+57.021VEPNC+57.021R
Scan Number: 16271 precMass: 703.3431 precCharge: 2 Sequence: LECVEPNCR
y4
b5
y1
b1
b2
b3
b4
y8
y7
b7
y5
y6
y2
b6
3e+05
2e+05
1e+05
0e+00
250
500
750
1000
1250
Intensity
M/Z
type
b
y

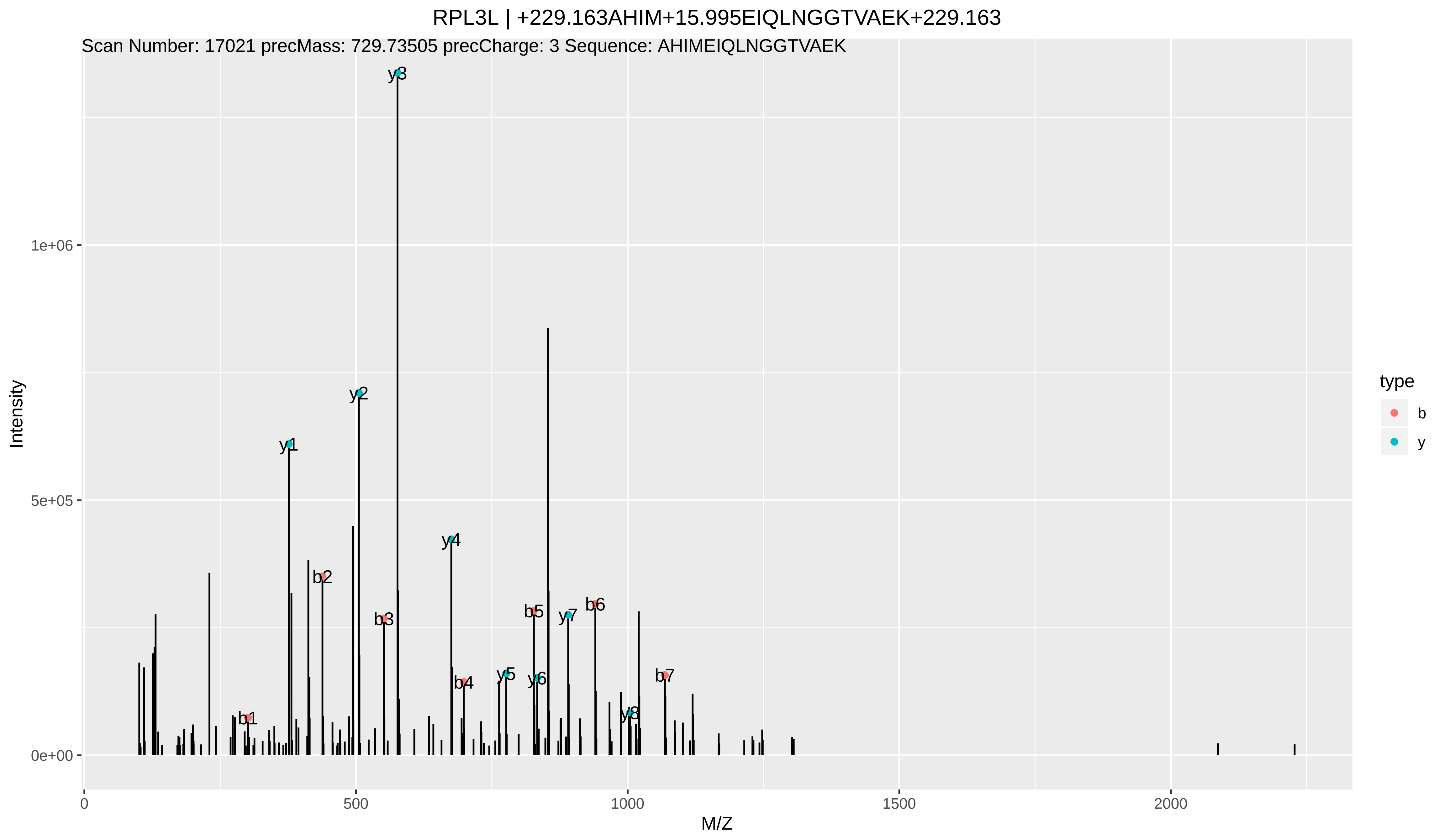

RPL3L | +229.163AHIM+15.995EIQLNGGTVAEK+229.163
Scan Number: 17021 precMass: 729.73505 precCharge: 3 Sequence: AHIMEIQLNGGTVAEK
y3
y2
y1
y4
b2
b3
b5
y7
b6
y5
y6
b4
b7
b1
y8
1e+06
5e+05
0e+00
Intensity
0
500
1000
1500
2000
M/Z
type
b
y

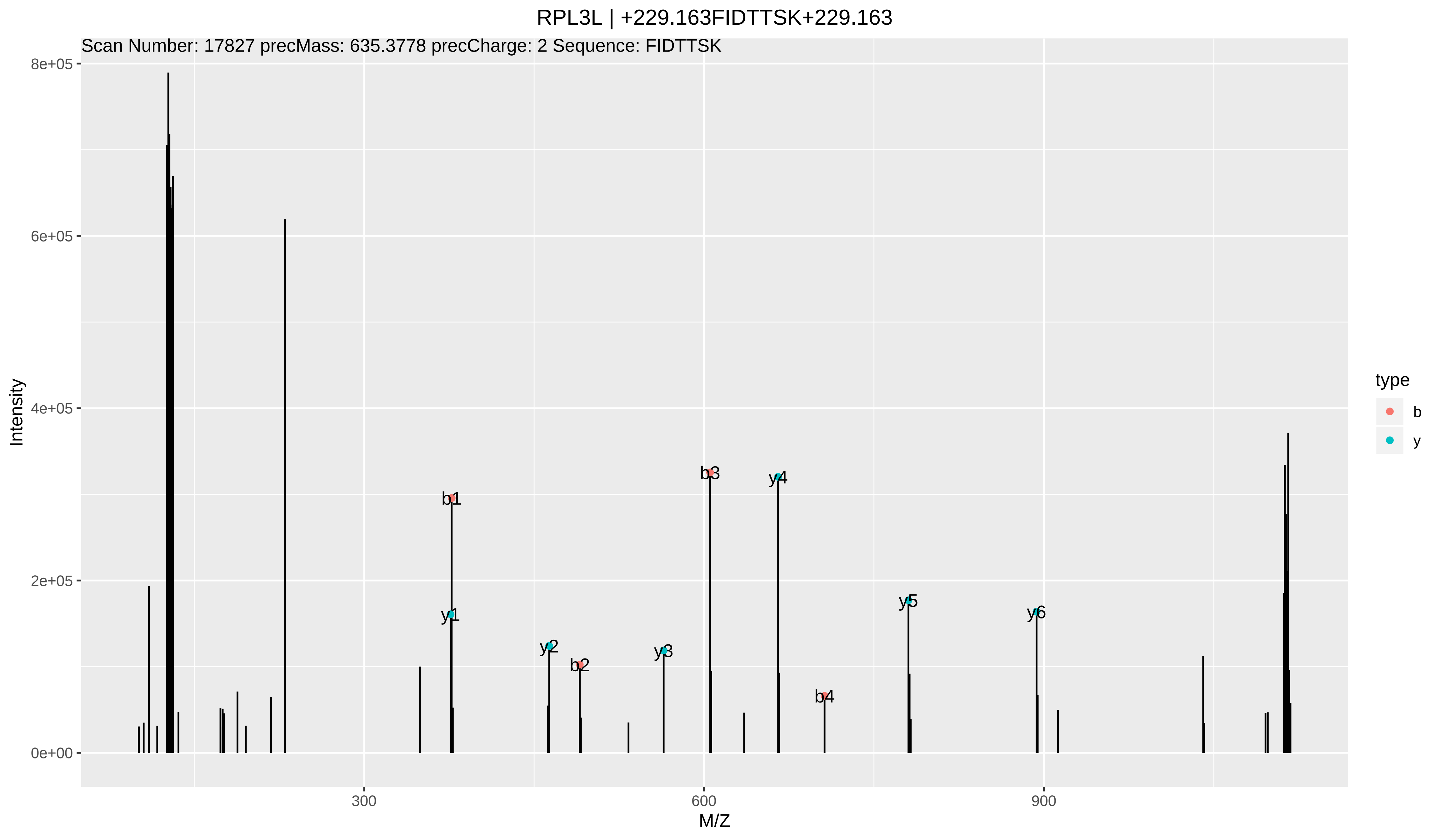

RPL3L | +229.163FIDTTSK+229.163
Scan Number: 17827 precMass: 635.3778 precCharge: 2 Sequence: FIDTTSK
8e+05
6e+05
4e+05
2e+05
0e+00
Intensity
300
600
900
M/Z
b1
y1
y2
b2
y3
b3
y4
b4
y5
y6
type
b
y

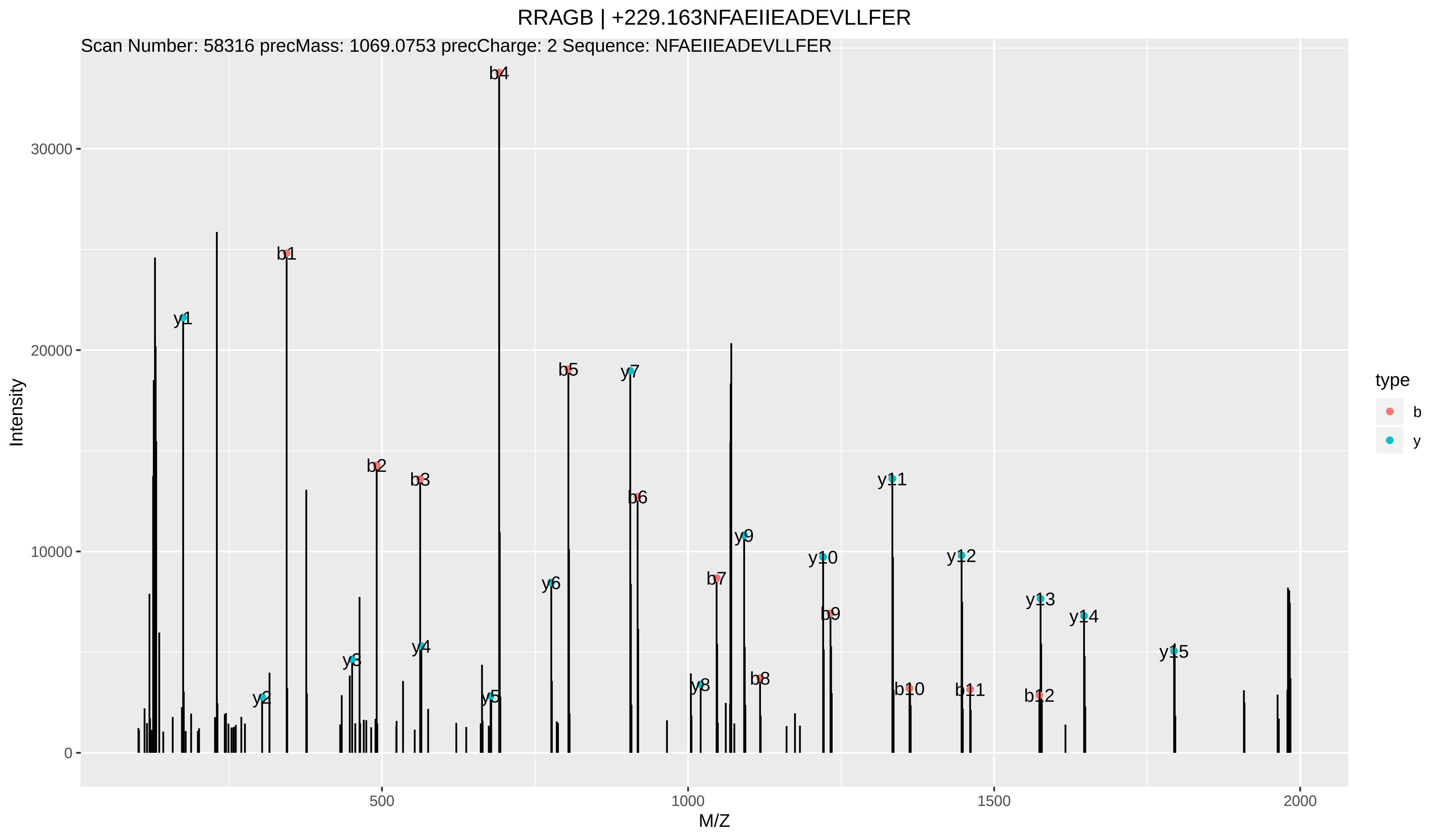

RRAGB | +229.163NFAEIIEADEVLLFER
Scan Number: 58316 precMass: 1069.0753 precCharge: 2 Sequence: NFAEIIEADEVLLFER
Intensity
M/Z
type
b
y
b1
b2
b3
b4
b5
b6
b7
b8
b9
b10
b11
b12
y1
y2
y3
y4
y5
y6
y7
y8
y9
y10
y11
y12
y13
y14
y15
0
10000
20000
30000
500
1000
1500
2000

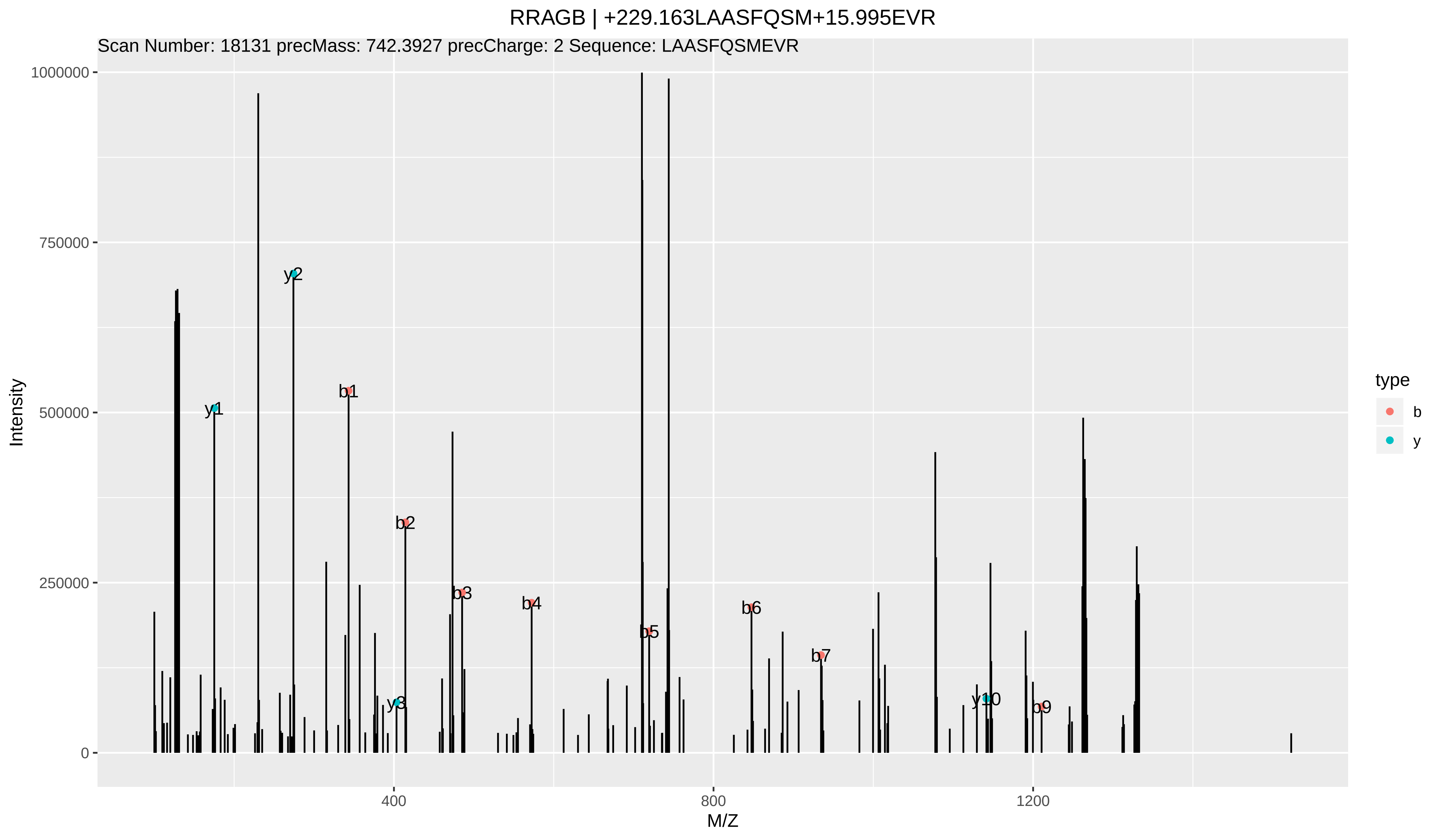

RRAGB | +229.163LAASFQSM+15.995EVR
Scan Number: 18131 precMass: 742.3927 precCharge: 2 Sequence: LAASFQSMEVR
Intensity
1000000
750000
500000
250000
0
400
800
1200
M/Z
y1
y2
b1
b2
b3
y3
b4
b5
b6
b7
y10
b9
type
b
y

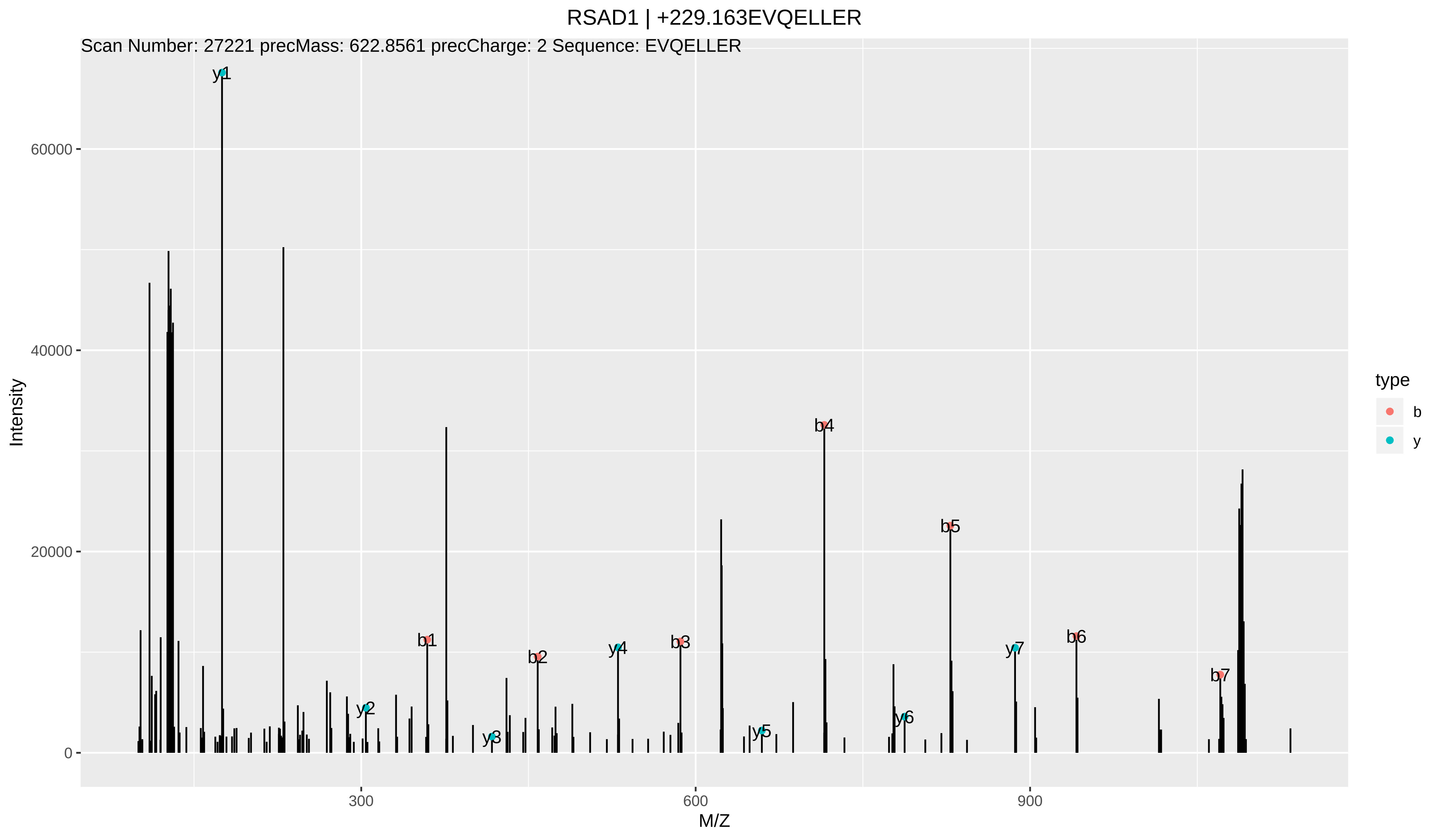
RSAD1 | +229.163EVQELLER
Scan Number: 27221 precMass: 622.8561 precCharge: 2 Sequence: EVQELLER
Intensity
M/Z
0
20000
40000
60000
300
600
900
y1
y2
y3
y4
y5
y6
y7
b1
b2
b3
b4
b5
b6
b7
type
b
y

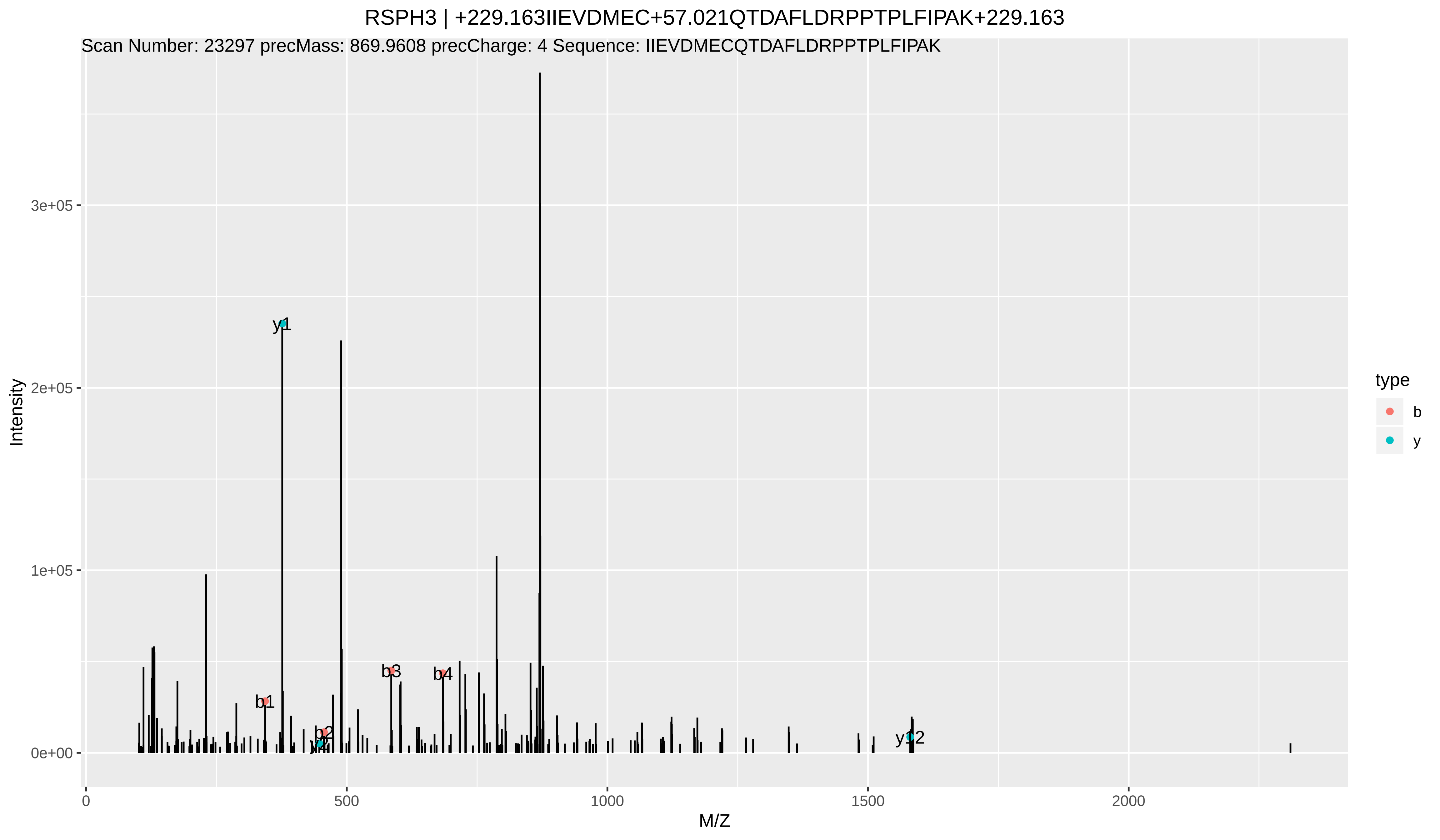
RSPH3 | +229.163IIEVDMEC+57.021QTDAFLDRPPTPLFIPAK+229.163
Scan Number: 23297 precMass: 869.9608 precCharge: 4 Sequence: IIEVDMECQTDAFLDRPPTPLFIPAK
Intensity
3e+05
2e+05
1e+05
0e+00
y1
b1
b2
y2
b3
b4
y12
0
500
1000
1500
2000
M/Z
type
b
y

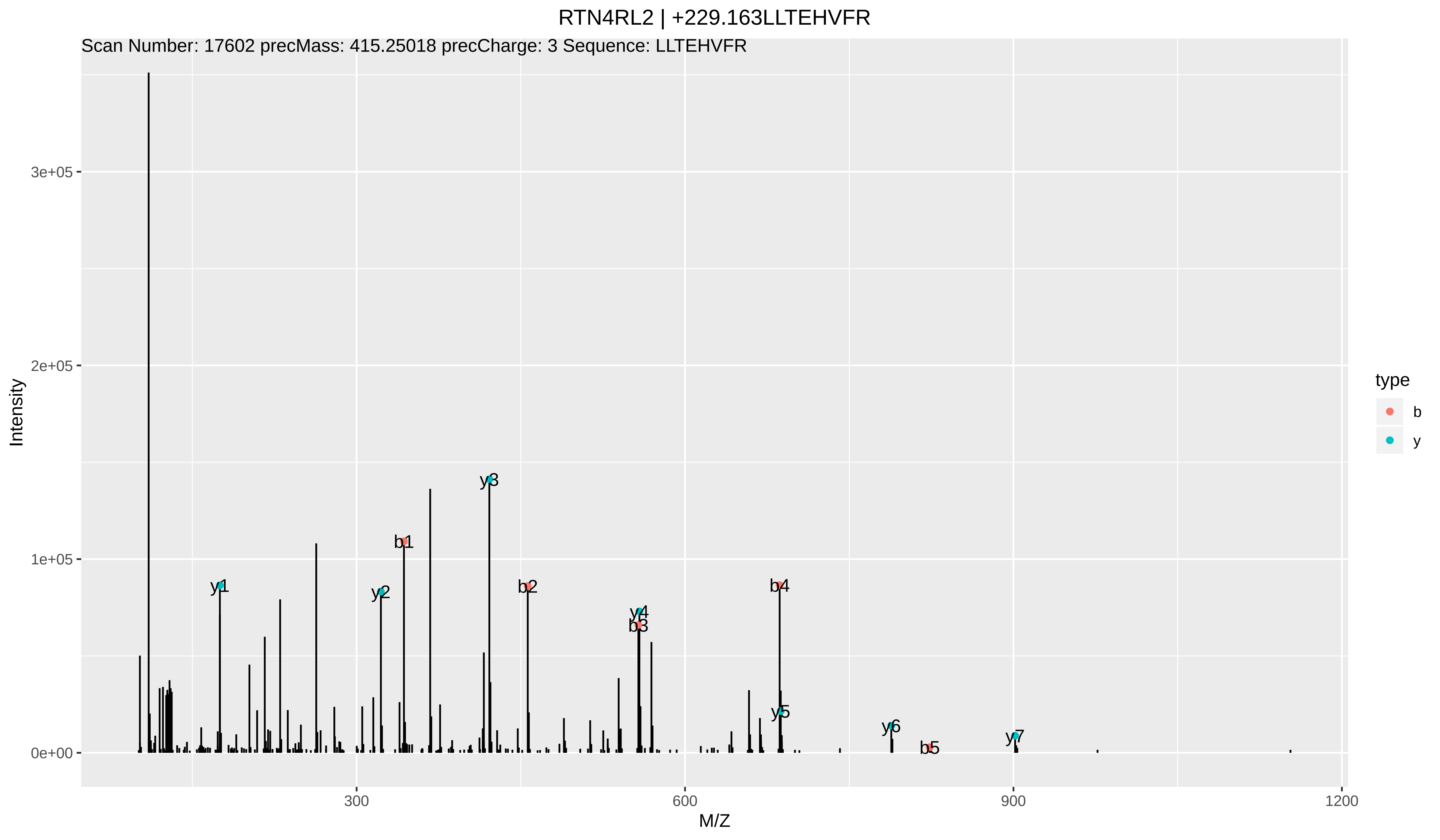
RTN4RL2 | +229.163LLTEHVFR
Scan Number: 17602 precMass: 415.25018 precCharge: 3 Sequence: LLTEHVFR
Intensity
3e+05
2e+05
1e+05
0e+00
M/Z
300
600
900
1200
y1
y2
b1
y3
b2
y4
b3
b4
y5
y6
b5
y7
type
b
y

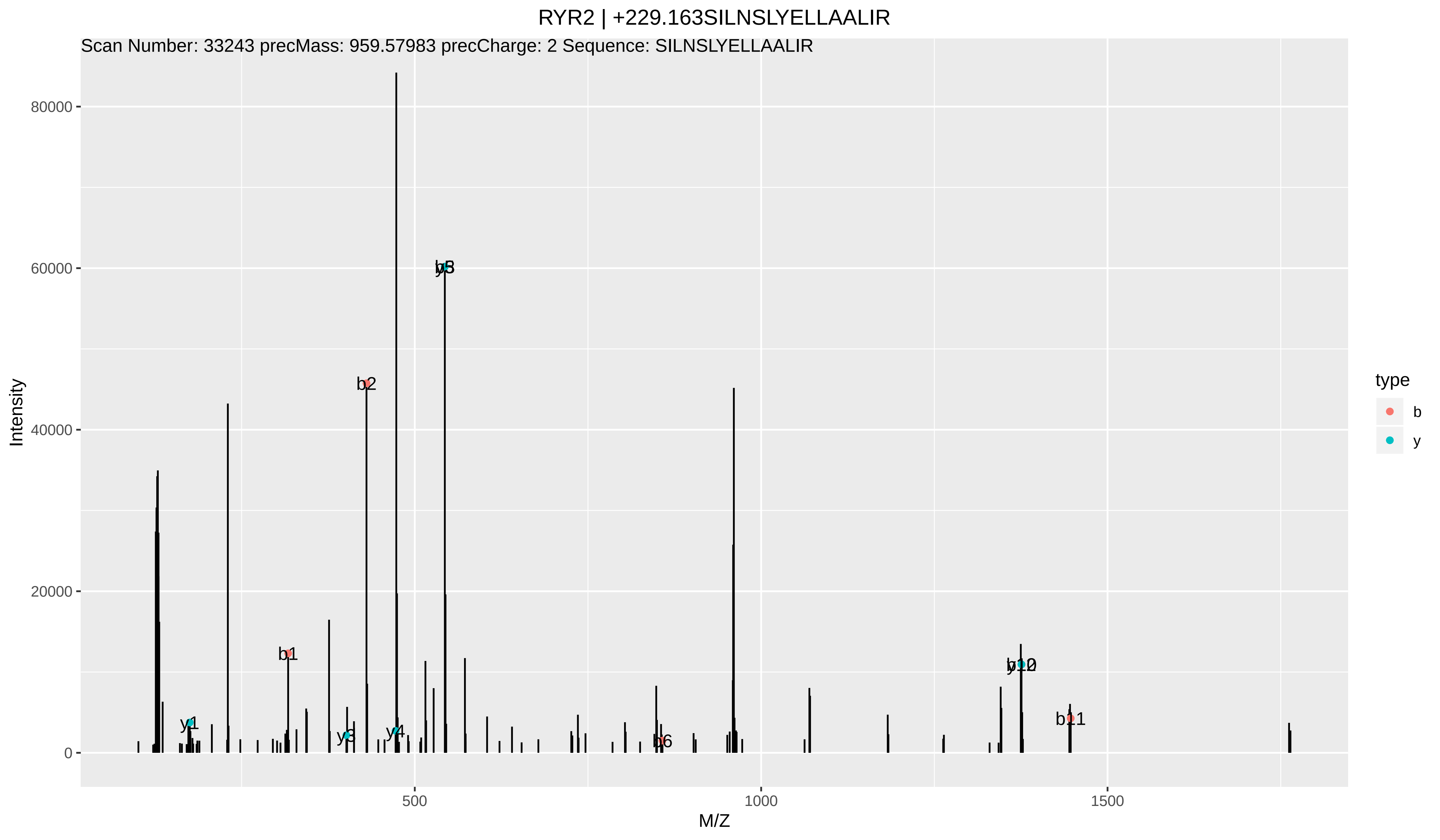
RYR2 | +229.163SILNSLYELLAALIR
Scan Number: 33243 precMass: 959.57983 precCharge: 2 Sequence: SILNSLYELLAALIR
Intensity
M/Z
type
b
y
b1
b2
b3
y5
b6
b11
b12
y12
y1
y3
y4

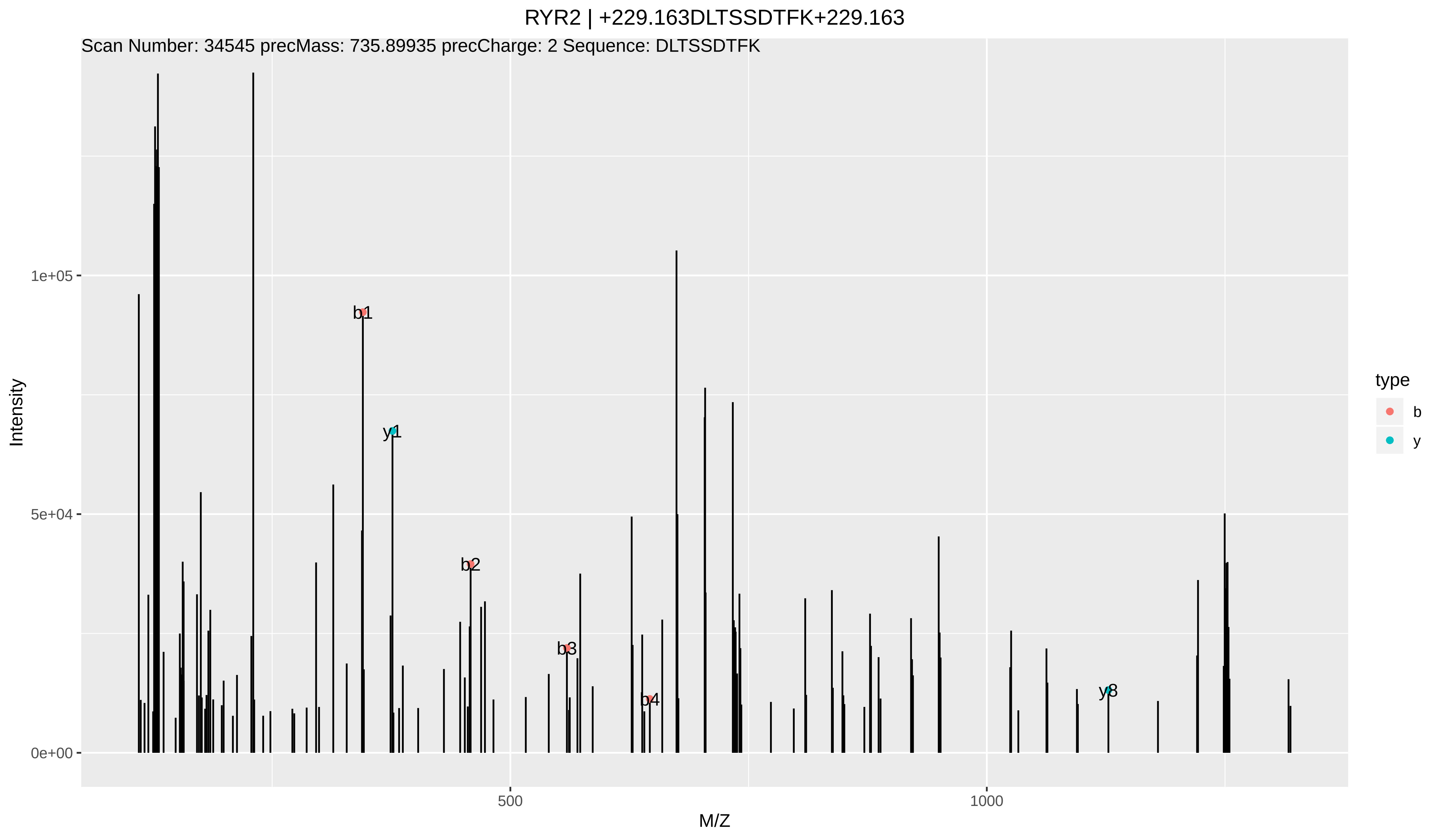

RYR2 | +229.163DLTSSDTFK+229.163
Scan Number: 34545 precMass: 735.89935 precCharge: 2 Sequence: DLTSSDTFK
Intensity
1e+05
5e+04
0e+00
M/Z
500
1000
b1
y1
b2
b3
b4
y8
type
b
y

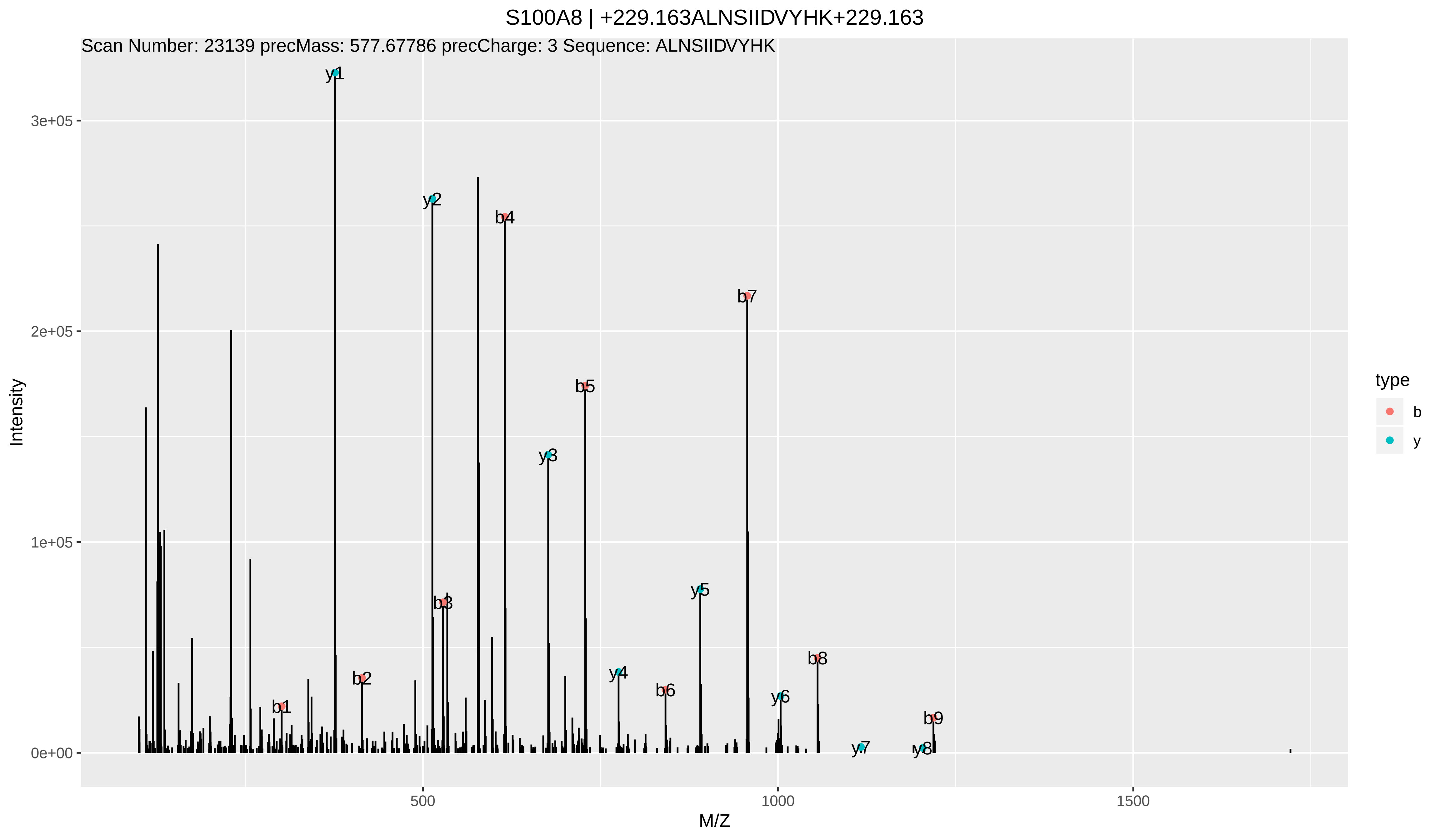
S100A8 | +229.163ALNSIIDVYHK+229.163
Scan Number: 23139 precMass: 577.67786 precCharge: 3 Sequence: ALNSIIDVYHK
y1
y2
b4
b7
b5
y3
y5
b3
b8
y4
b2
b6
y6
b1
b9
y7
y8
3e+05
2e+05
1e+05
0e+00
Intensity
500
1000
1500
M/Z
type
b
y

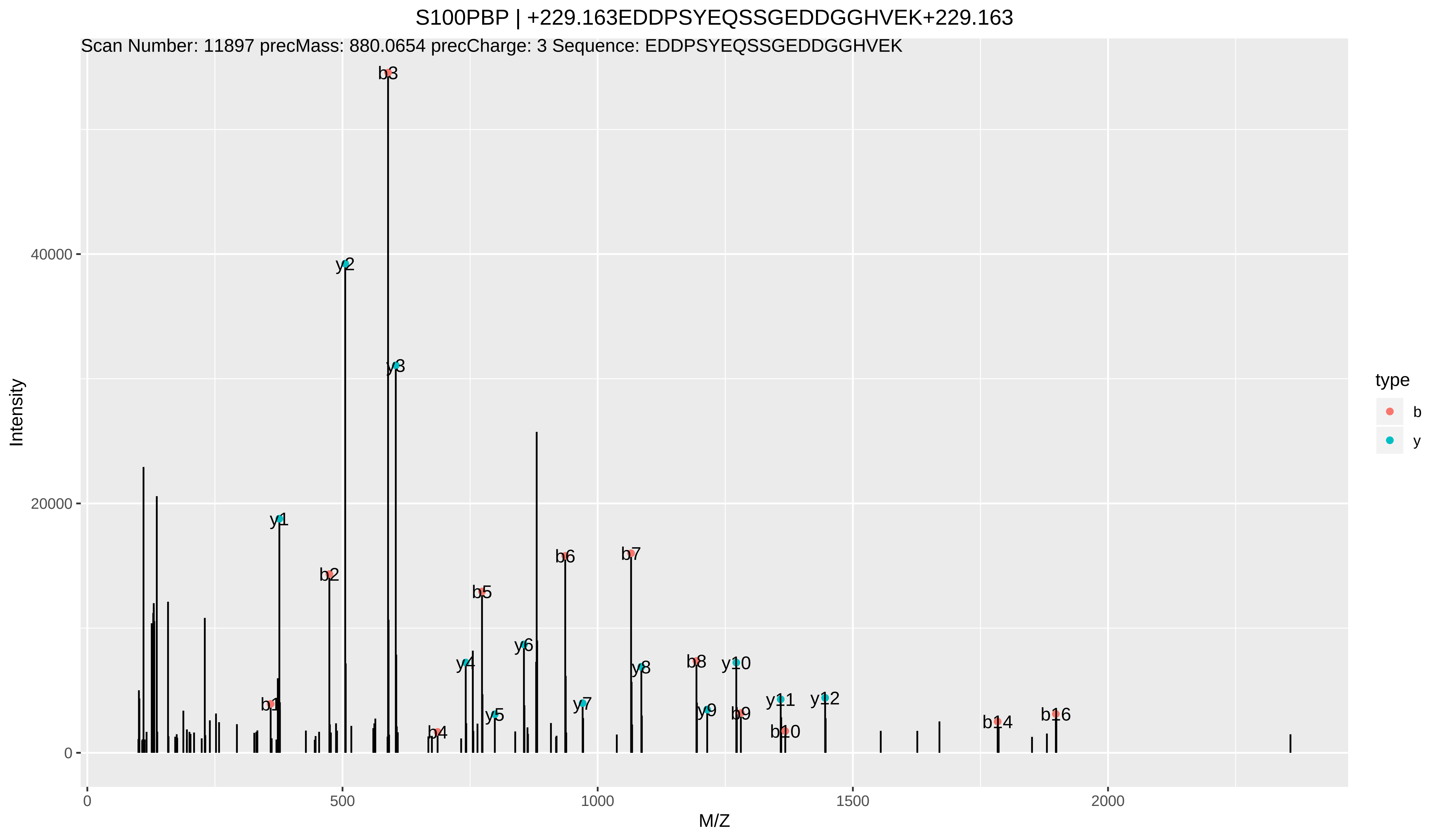

S100PBP | +229.163EDDPSYEQSSGEDDGGHVEK+229.163
Scan Number: 11897 precMass: 880.0654 precCharge: 3 Sequence: EDDPSYEQSSGEDDGGHVEK
Intensity
M/Z
type
b
y
b3
y2
y3
y1
b2
b6
b7
b5
y6
y4
y8
b8
y10
y11
y12
b1
y7
y9
b9
y5
b4
b10
b14
b16
0
20000
40000
0
500
1000
1500
2000

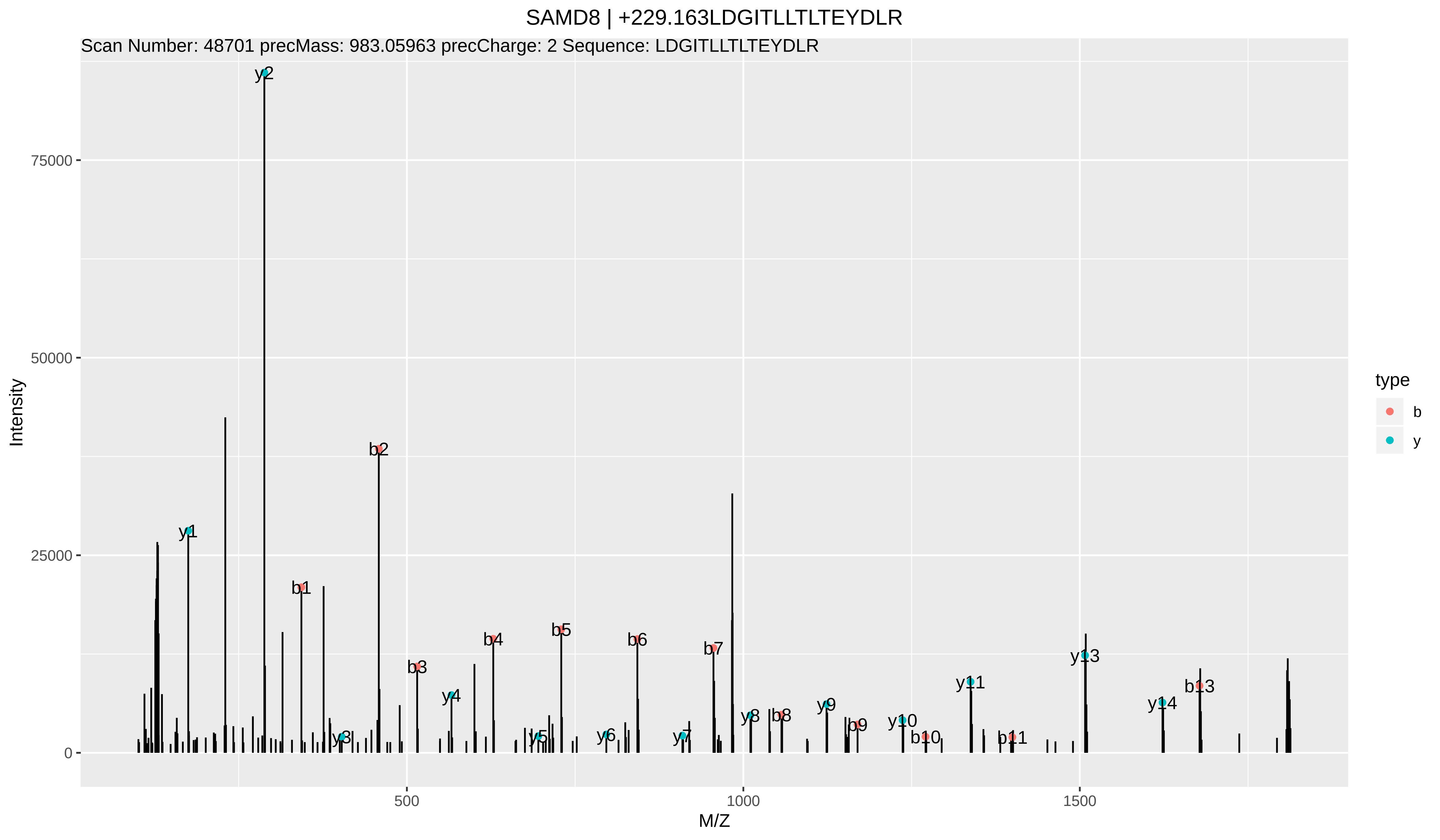
SAMD8 | +229.163LDGITLLTLTEYDLR
Scan Number: 48701 precMass: 983.05963 precCharge: 2 Sequence: LDGITLLTLTEYDLR
Intensity
M/Z
75000
50000
25000
0
500
1000
1500
y2
b2
y1
b1
b3
y4
b4
b5
b6
b7
y3
y5
y6
y7
y8
b8
y9
b9
y10
b10
y11
b11
y13
y14
b13
type
b
y

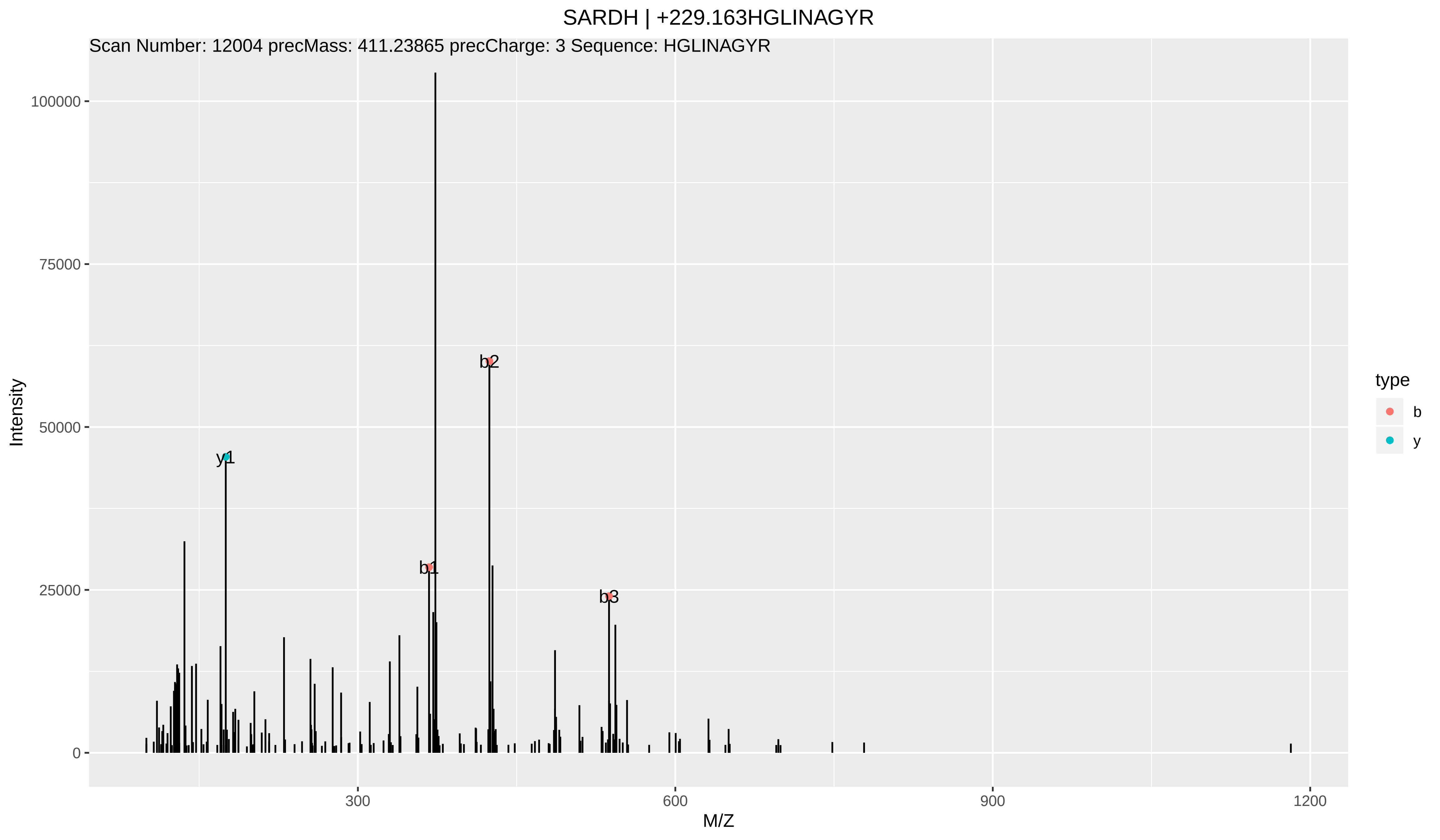

SARDH | +229.163HGLINAGYR
Scan Number: 12004 precMass: 411.23865 precCharge: 3 Sequence: HGLINAGYR
Intensity
100000
75000
50000
25000
0
M/Z
300
600
900
1200
y1
b1
b2
b3
type
b
y

SARM1 | +229.163EVAQGLC+57.021DAIR

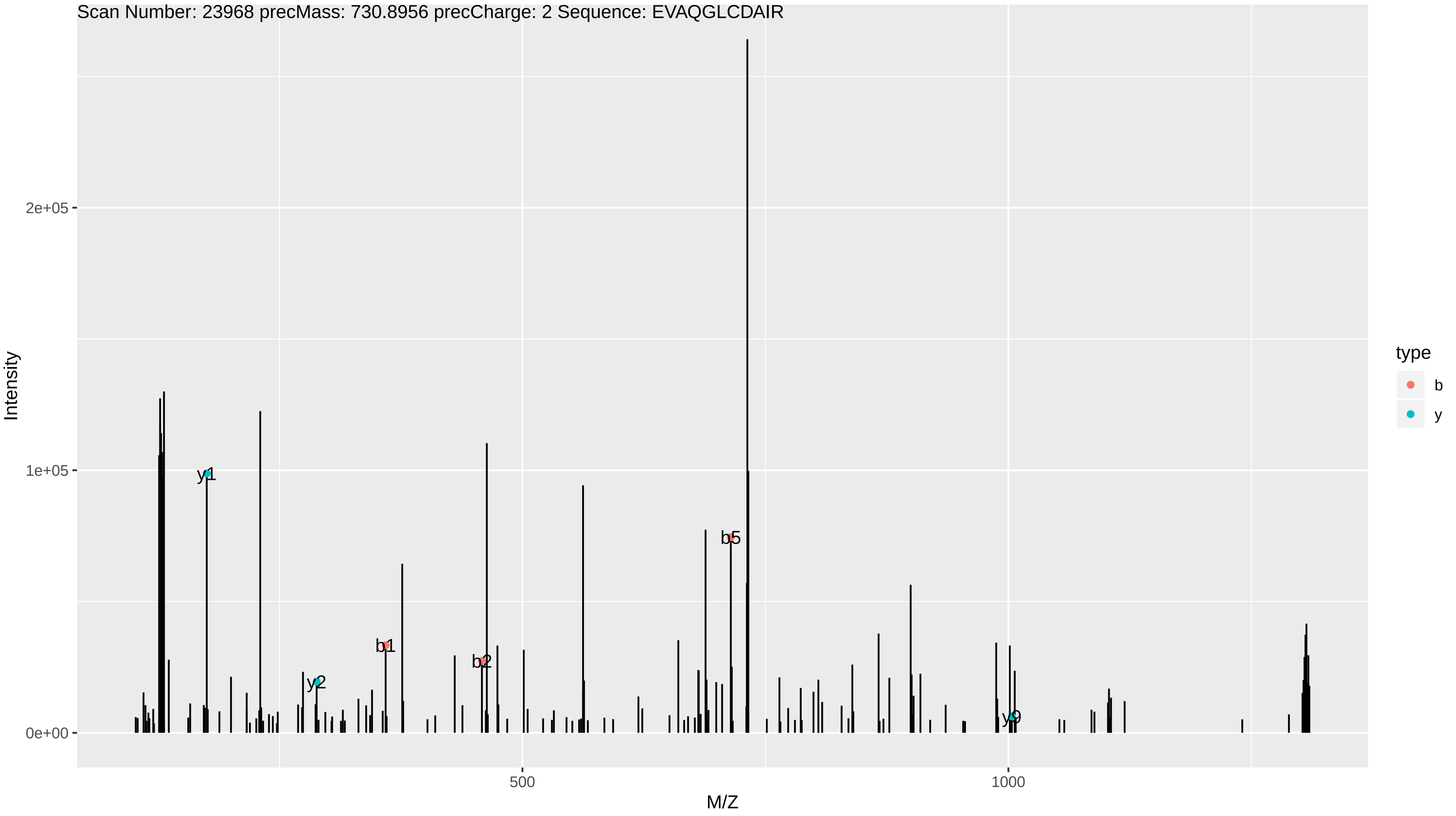

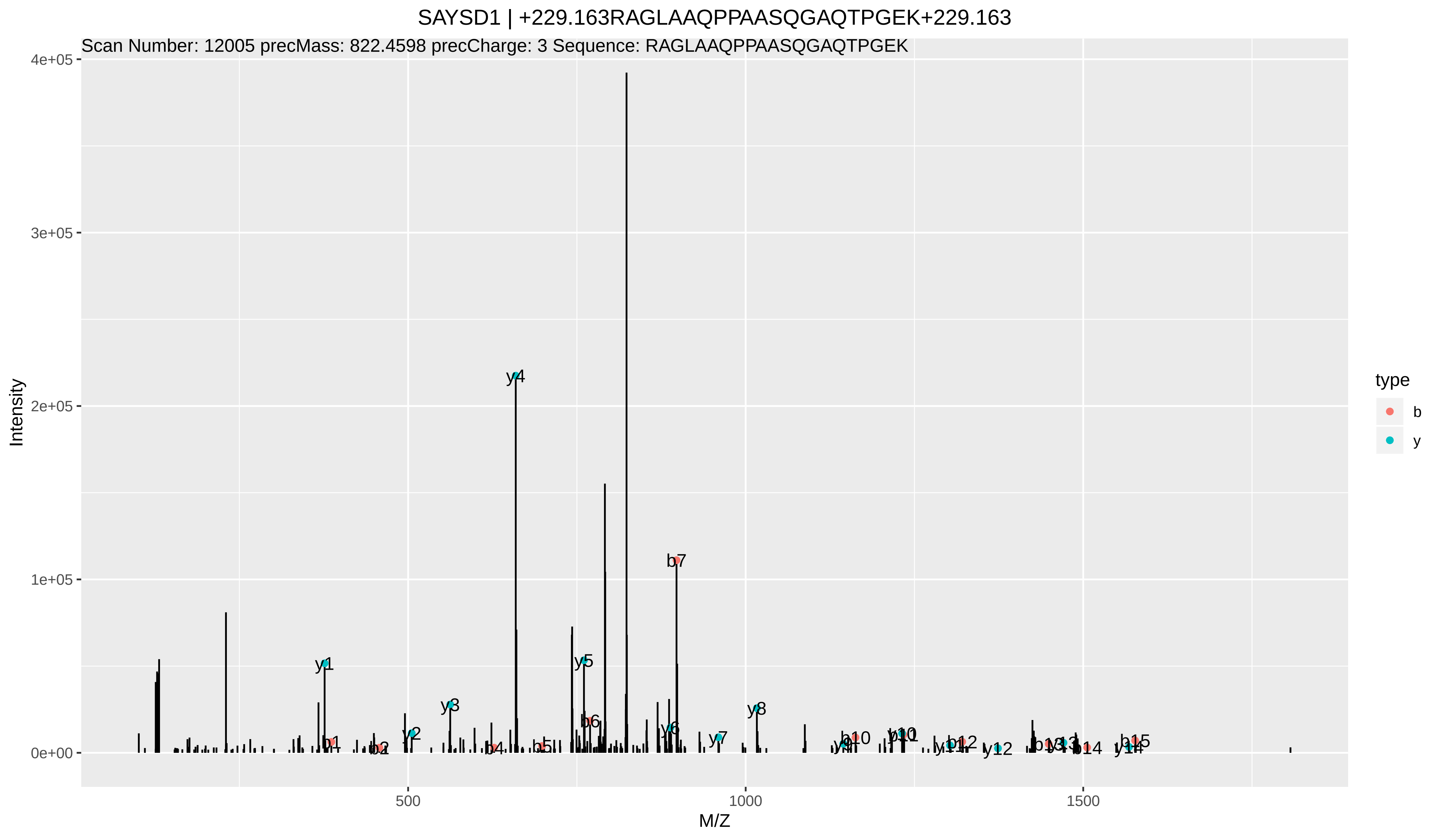

SAYSD1 | +229.163RAGLAAQPPAASQGAQTPGEK+229.163
Scan Number: 12005 precMass: 822.4598 precCharge: 3 Sequence: RAGLAAQPPAASQGAQTPGEK
Intensity
M/Z
type
b
y

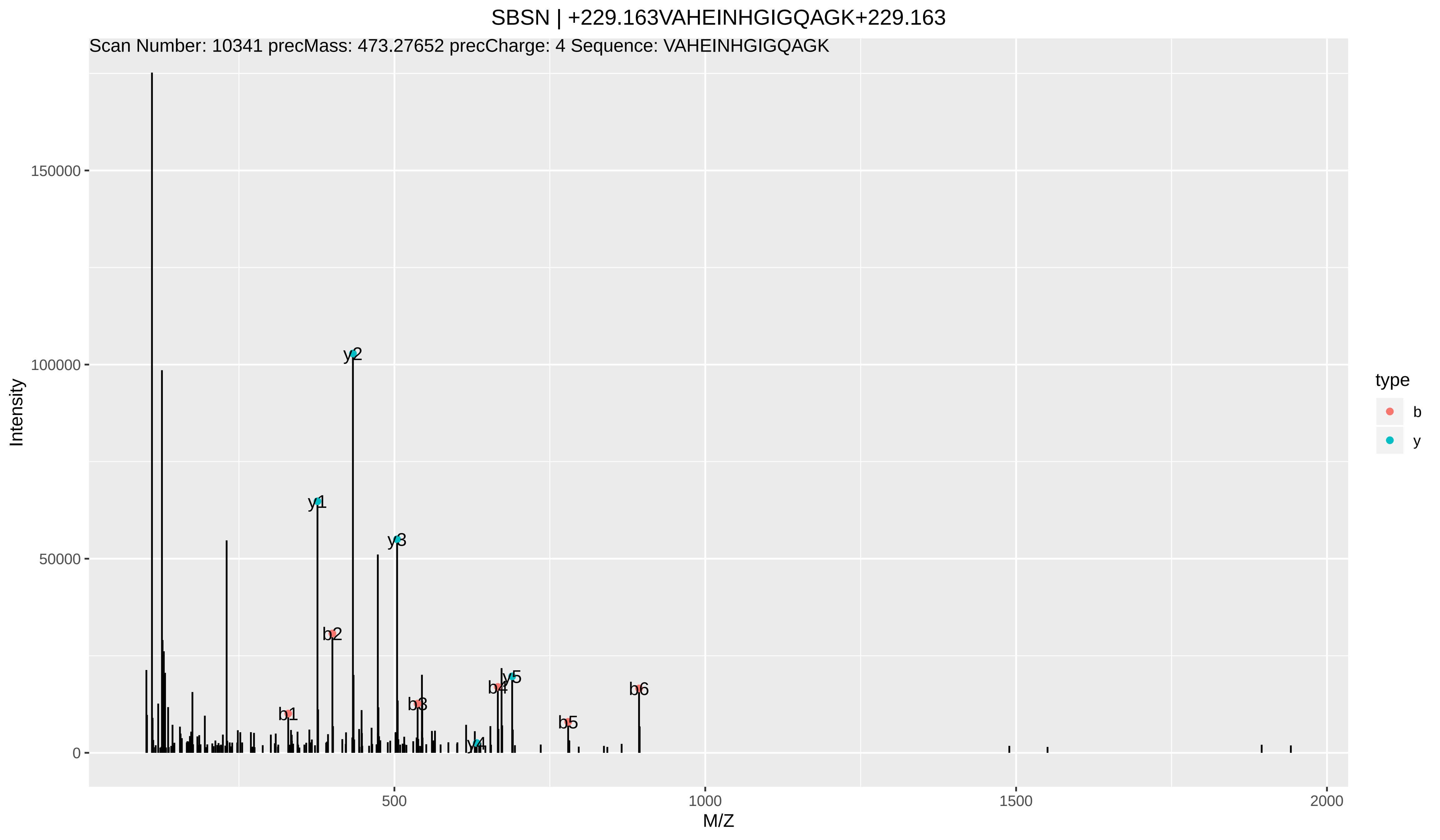

SBSN | +229.163VAHEINHGIGQAGK+229.163
Scan Number: 10341 precMass: 473.27652 precCharge: 4 Sequence: VAHEINHGIGQAGK
Intensity
150000
100000
50000
0
M/Z
500
1000
1500
2000
y1
y2
y3
y4
y5
b1
b2
b3
b4
b5
b6
type
b
y

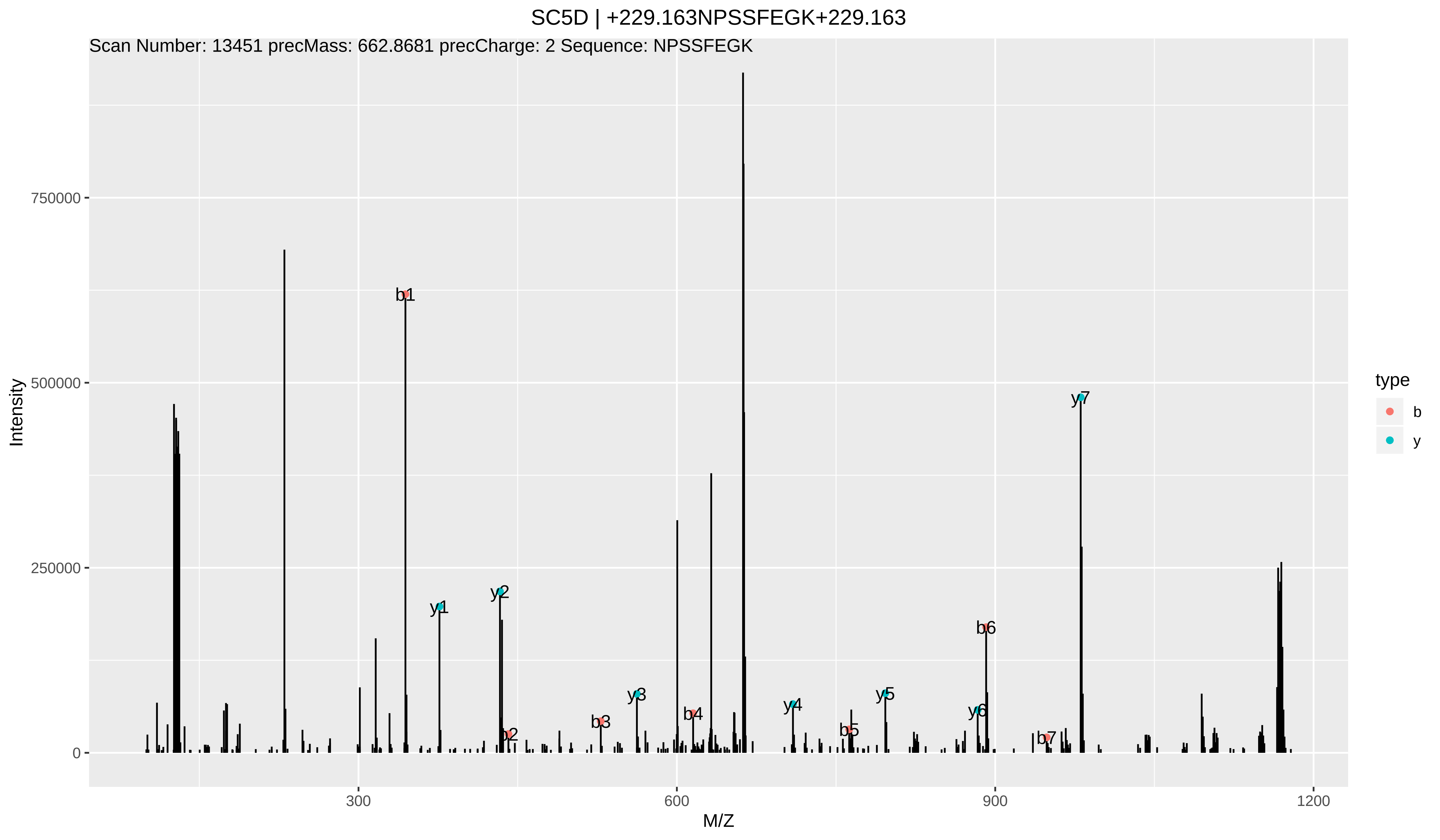

SC5D | +229.163NPSSFEGK+229.163
Scan Number: 13451 precMass: 662.8681 precCharge: 2 Sequence: NPSSFEGK
Intensity
750000
500000
250000
0
300
600
900
1200
M/Z
b1
y1
y2
b2
b3
y3
b4
y4
b5
y5
y6
b6
b7
y7
type
b
y

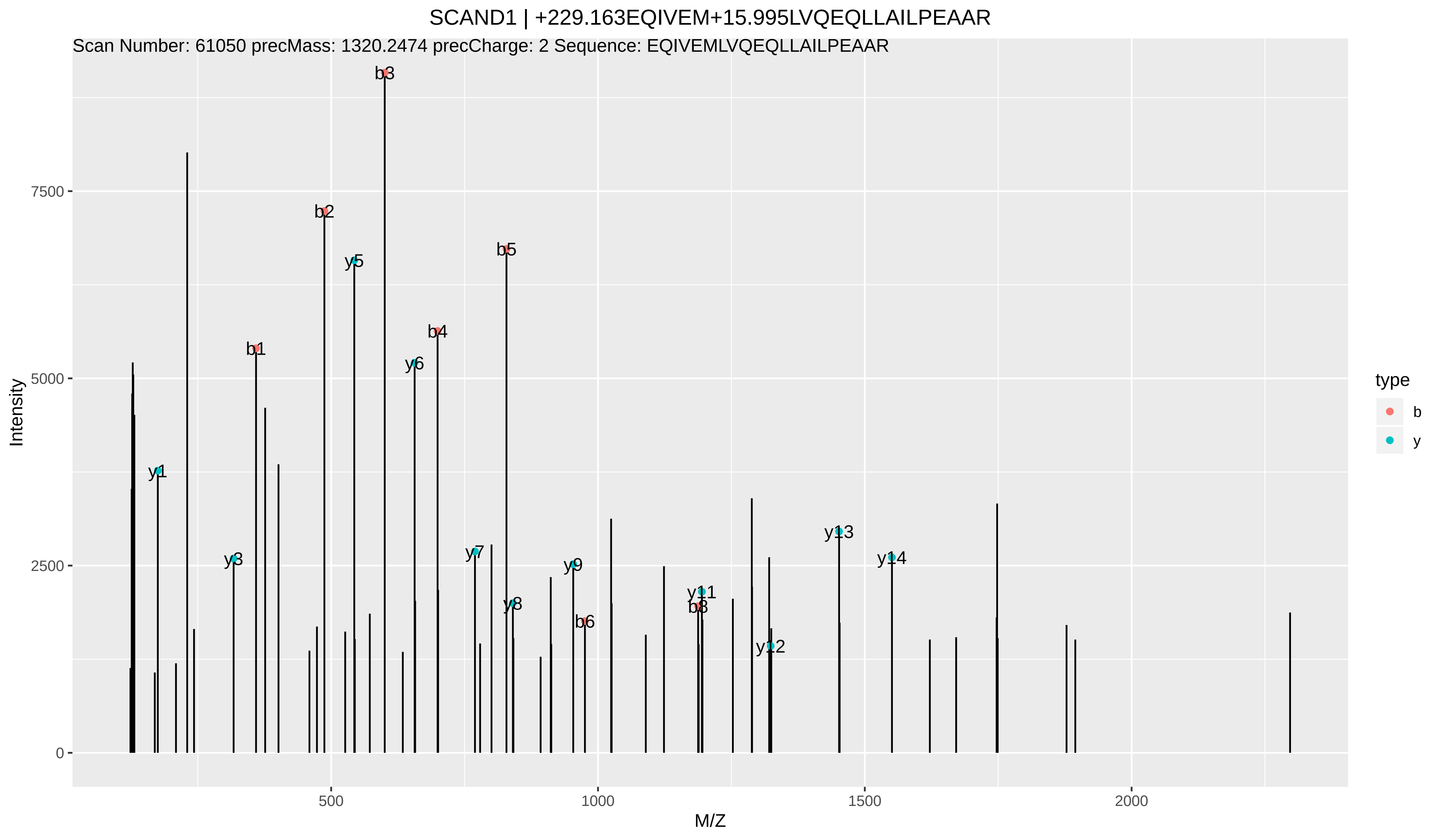
SCAND1 | +229.163EQIVEM+15.995LVQEQLLAILPEAAR
Scan Number: 61050 precMass: 1320.2474 precCharge: 2 Sequence: EQIVEMLVQEQLLAILPEAAR
Intensity
M/Z
0
2500
5000
7500
500
1000
1500
2000
b3
b2
y5
b5
b4
b1
y6
y1
y13
y14
y3
y7
y9
y11
b8
y8
b6
y12
type
b
y

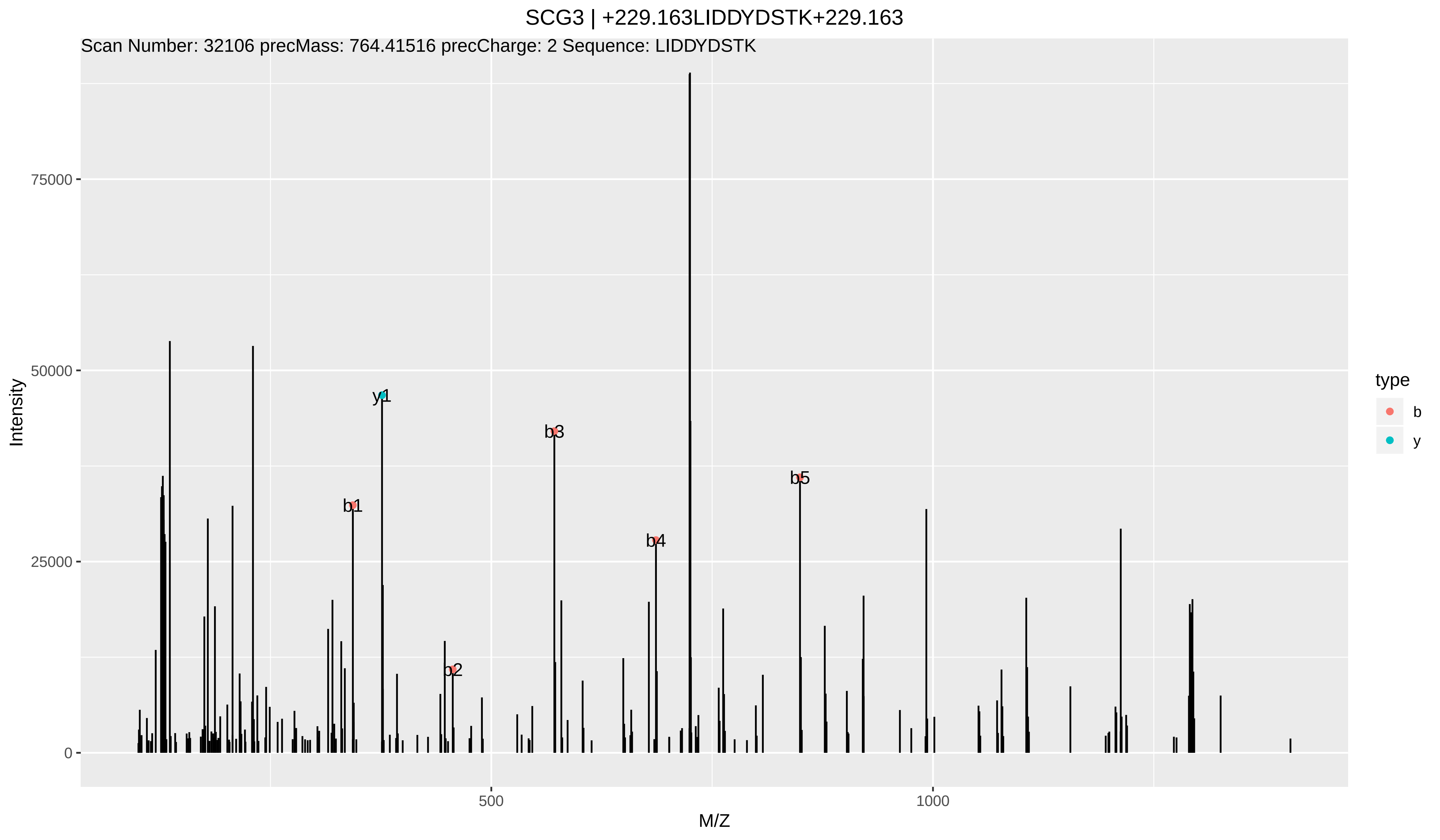

SCG3 | +229.163LIDDYDSTK+229.163
Scan Number: 32106 precMass: 764.41516 precCharge: 2 Sequence: LIDDYDSTK
Intensity
75000
50000
25000
0
M/Z
500
1000
y1
b1
b2
b3
b4
b5
type
b
y

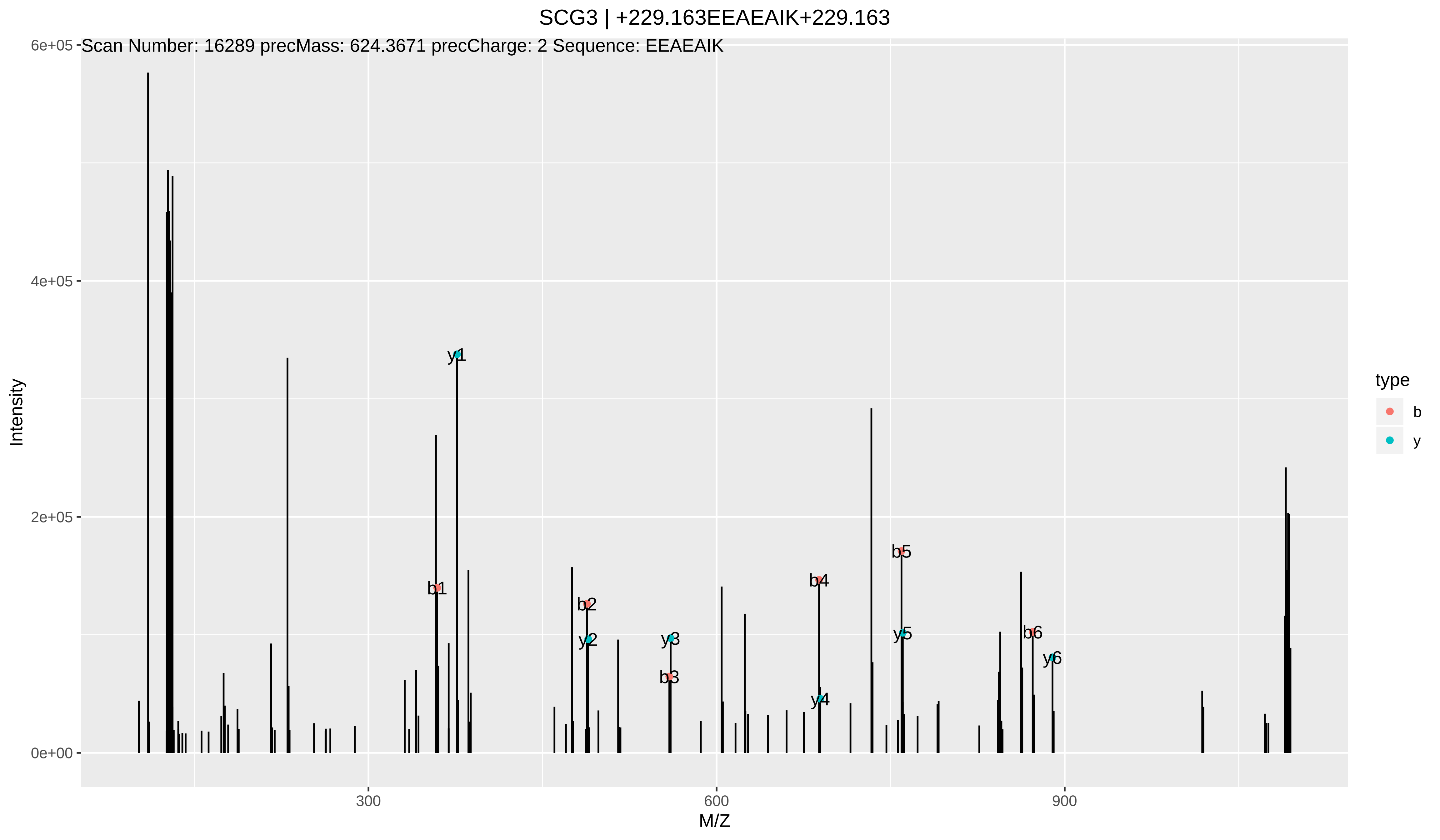
SCG3 | +229.163EEAEAIK+229.163
Scan Number: 16289 precMass: 624.3671 precCharge: 2 Sequence: EEAEAIK
Intensity
6e+05
4e+05
2e+05
0e+00
M/Z
300
600
900
y1
b1
b2
y2
y3
b3
b4
y4
b5
y5
b6
y6
type
b
y

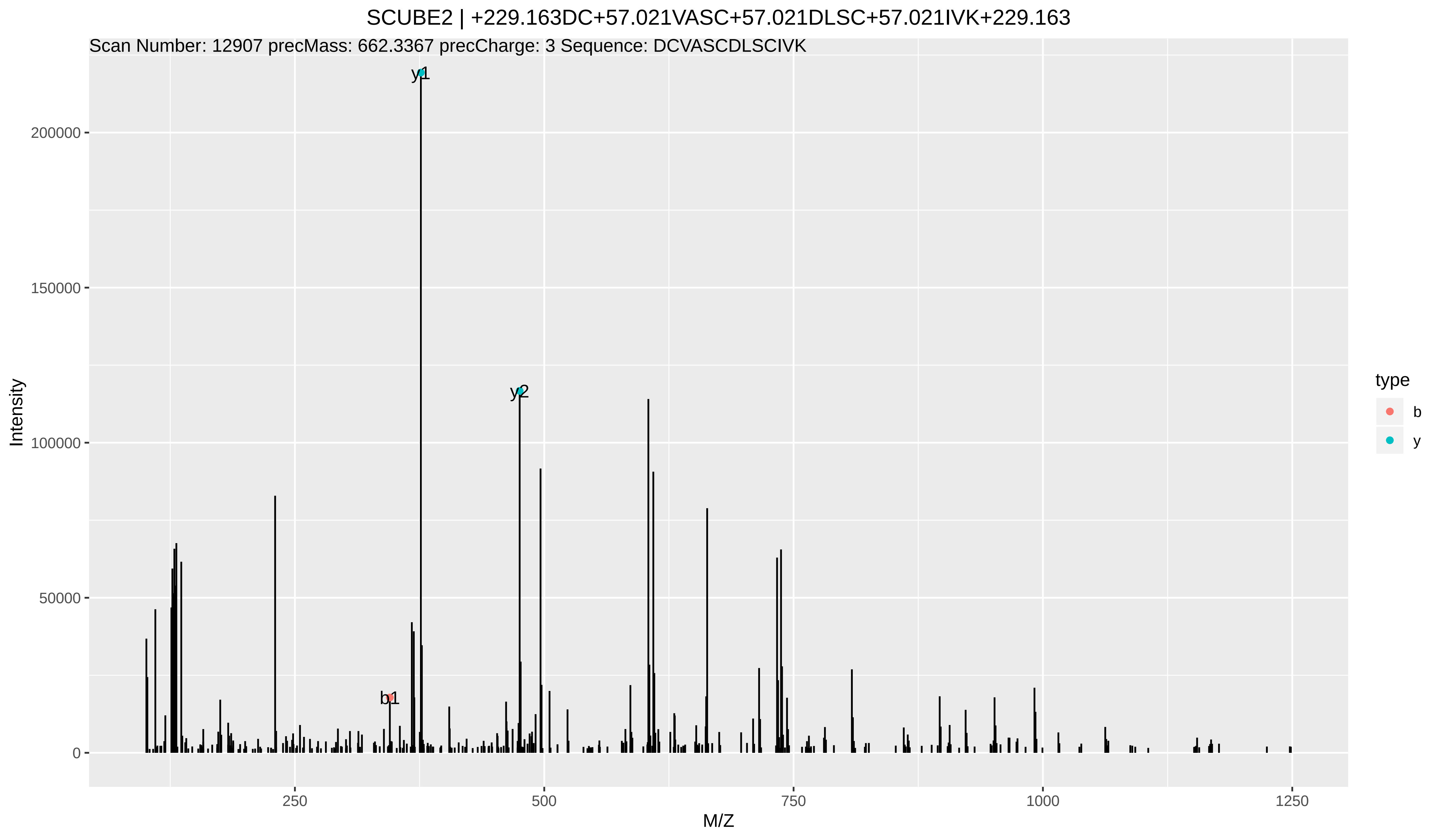
SCUBE2 | +229.163DC+57.021VASC+57.021DLSC+57.021IVK+229.163
Scan Number: 12907 precMass: 662.3367 precCharge: 3 Sequence: DCVASCDLSCIVK
y1
y2
b1
Intensity
200000
150000
100000
50000
0
250
500
750
1000
1250
M/Z
type
b
y

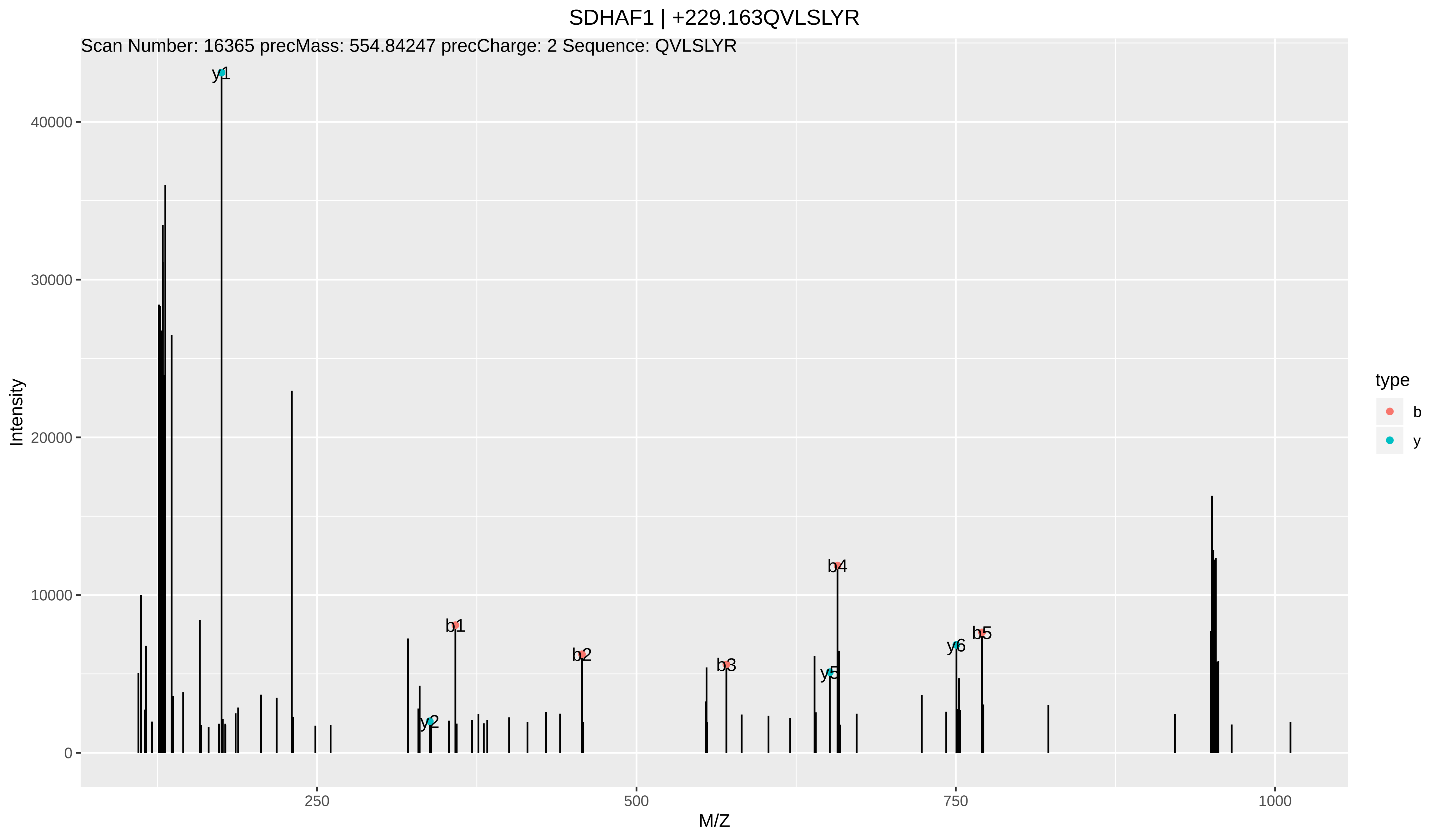
SDHAF1 | +229.163QVLSLYR
Scan Number: 16365 precMass: 554.84247 precCharge: 2 Sequence: QVLSLYR
y1
b1
b2
b3
b4
b5
y2
y5
y6
Intensity
40000
30000
20000
10000
0
250
500
750
1000
M/Z
type
b
y

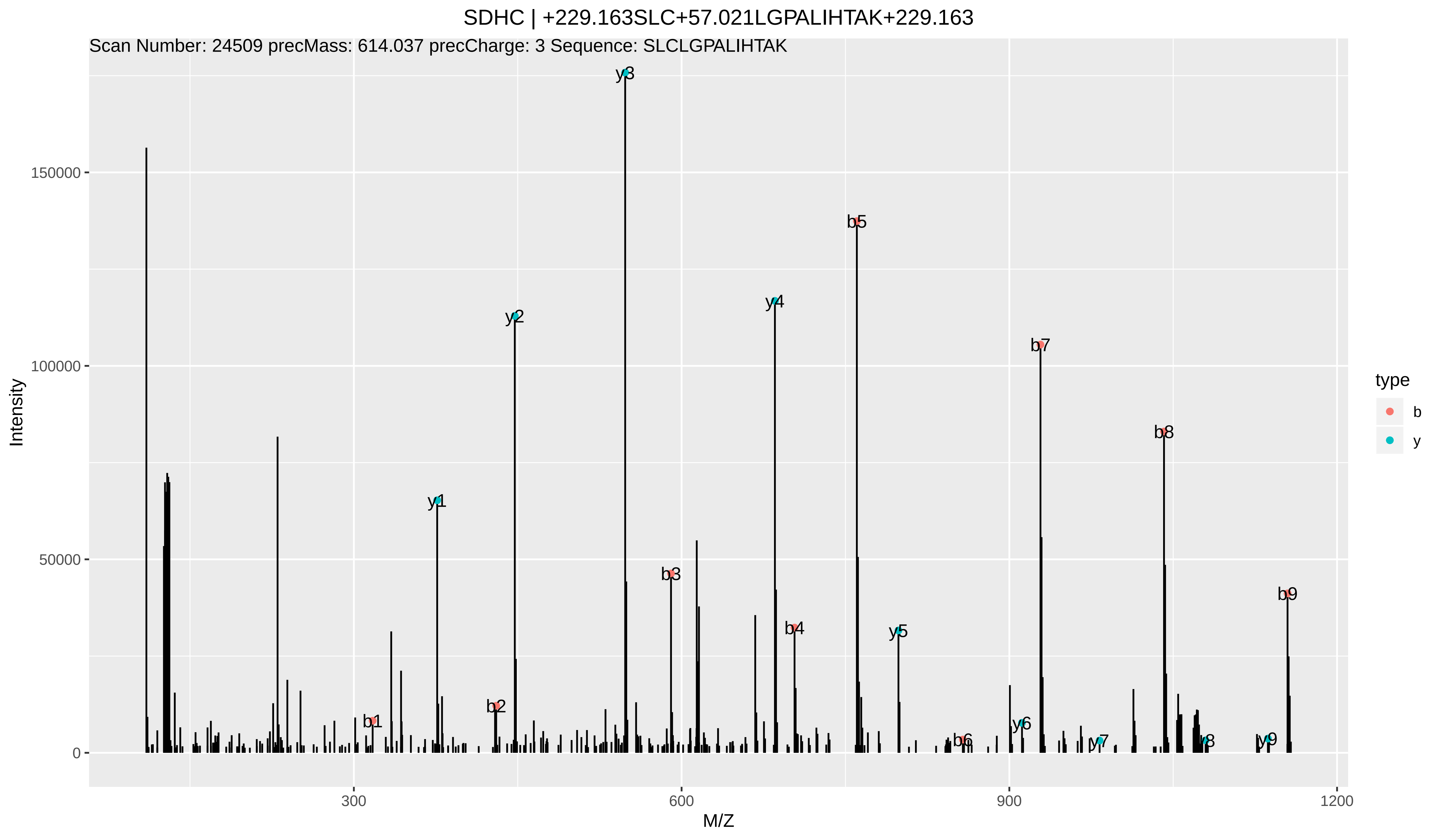
SDHC | +229.163SLC+57.021LGPALIHTAK+229.163
Scan Number: 24509 precMass: 614.037 precCharge: 3 Sequence: SLCLGPALIHTAK
Intensity
M/Z
type
b
y
y1
y2
y3
y4
y5
y6
y7
y8
y9
b1
b2
b3
b4
b5
b6
b7
b8
b9
0
50000
100000
150000
300
600
900
1200

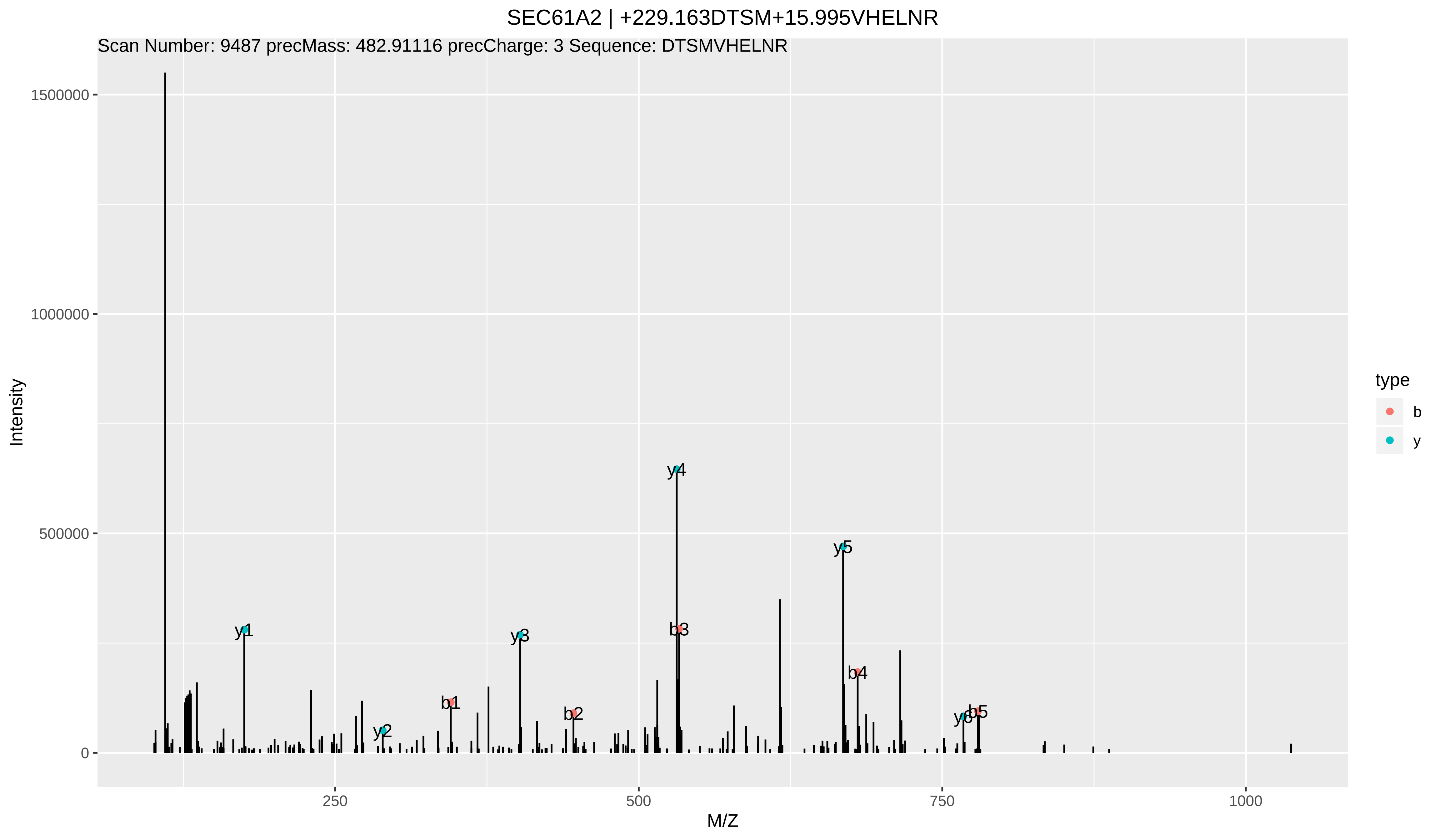
SEC61A2 | +229.163DTSM+15.995VHELNR
Scan Number: 9487 precMass: 482.91116 precCharge: 3 Sequence: DTSMVHELNR
Intensity
1500000
1000000
500000
0
M/Z
250
500
750
1000
y1
y2
b1
y3
b2
y4
b3
y5
b4
y6
b5
type
b
y

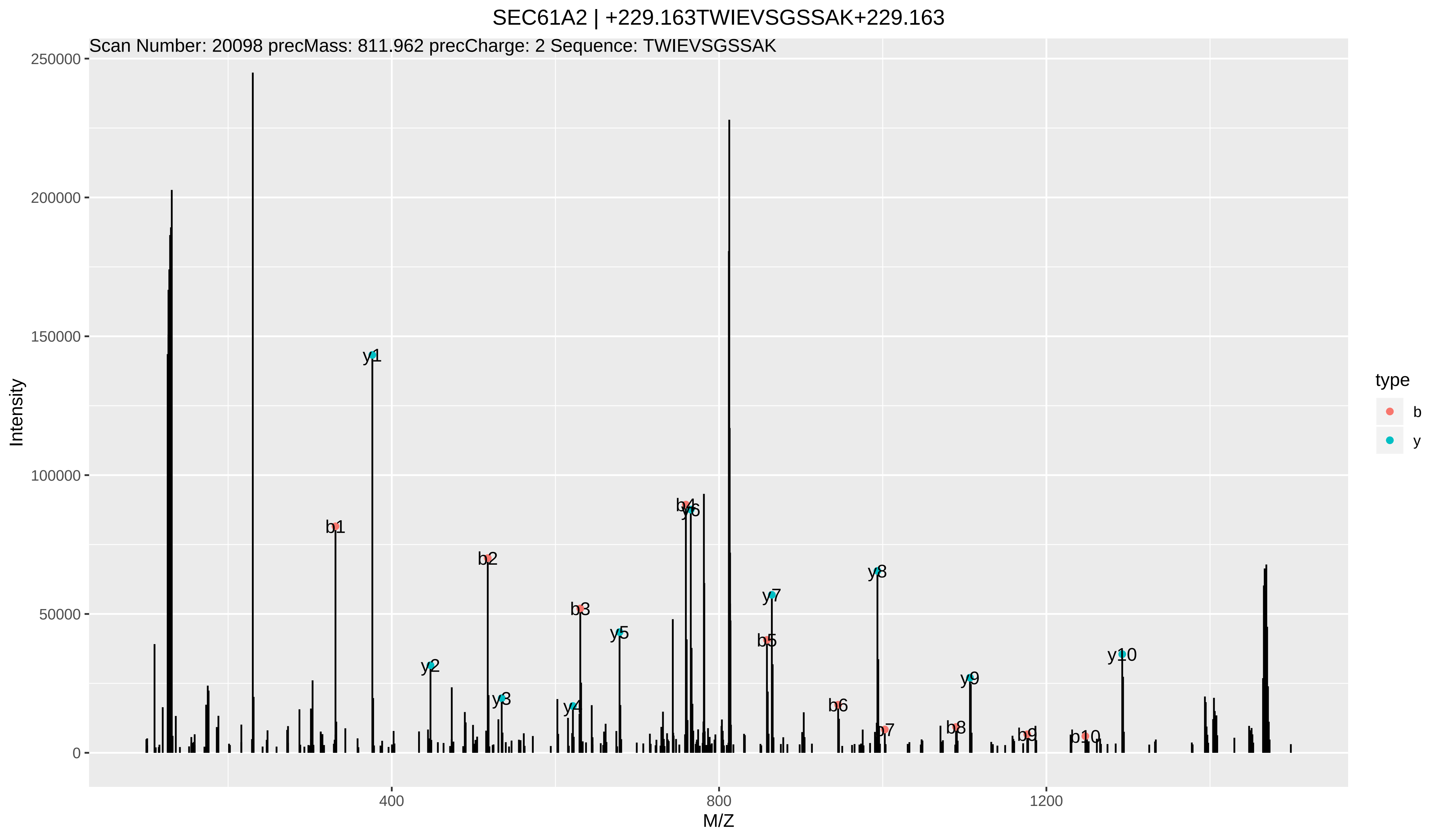

SEC61A2 | +229.163TWIEVSGSSAK+229.163
Scan Number: 20098 precMass: 811.962 precCharge: 2 Sequence: TWIEVSGSSAK
Intensity
M/Z
type
b
y

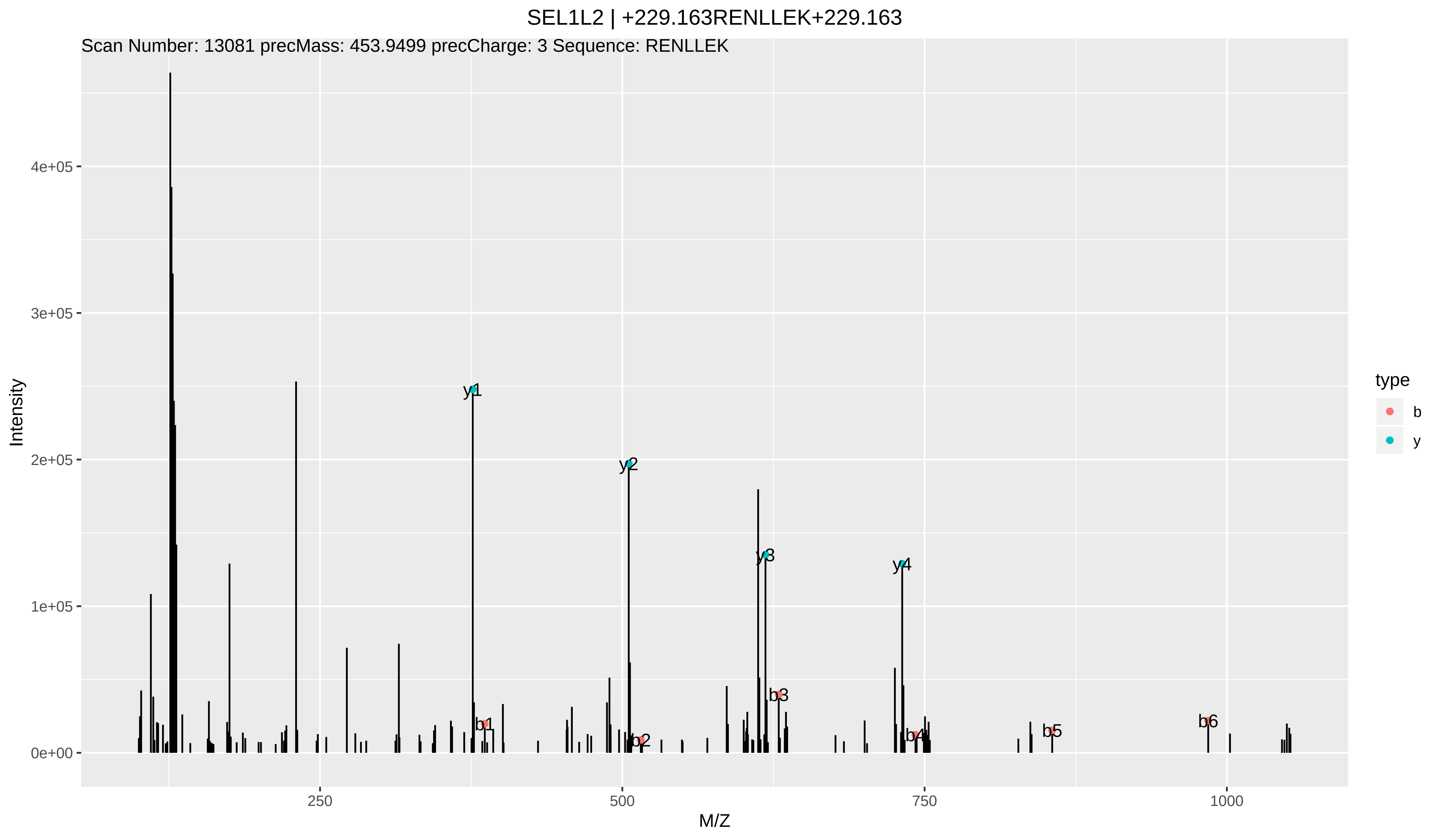
SEL1L2 | +229.163RENLLEK+229.163
Scan Number: 13081 precMass: 453.9499 precCharge: 3 Sequence: RENLLEK
Intensity
4e+05
3e+05
2e+05
1e+05
0e+00
M/Z
250
500
750
1000
y1
y2
y3
y4
b1
b2
b3
b4
b5
b6
type
b
y

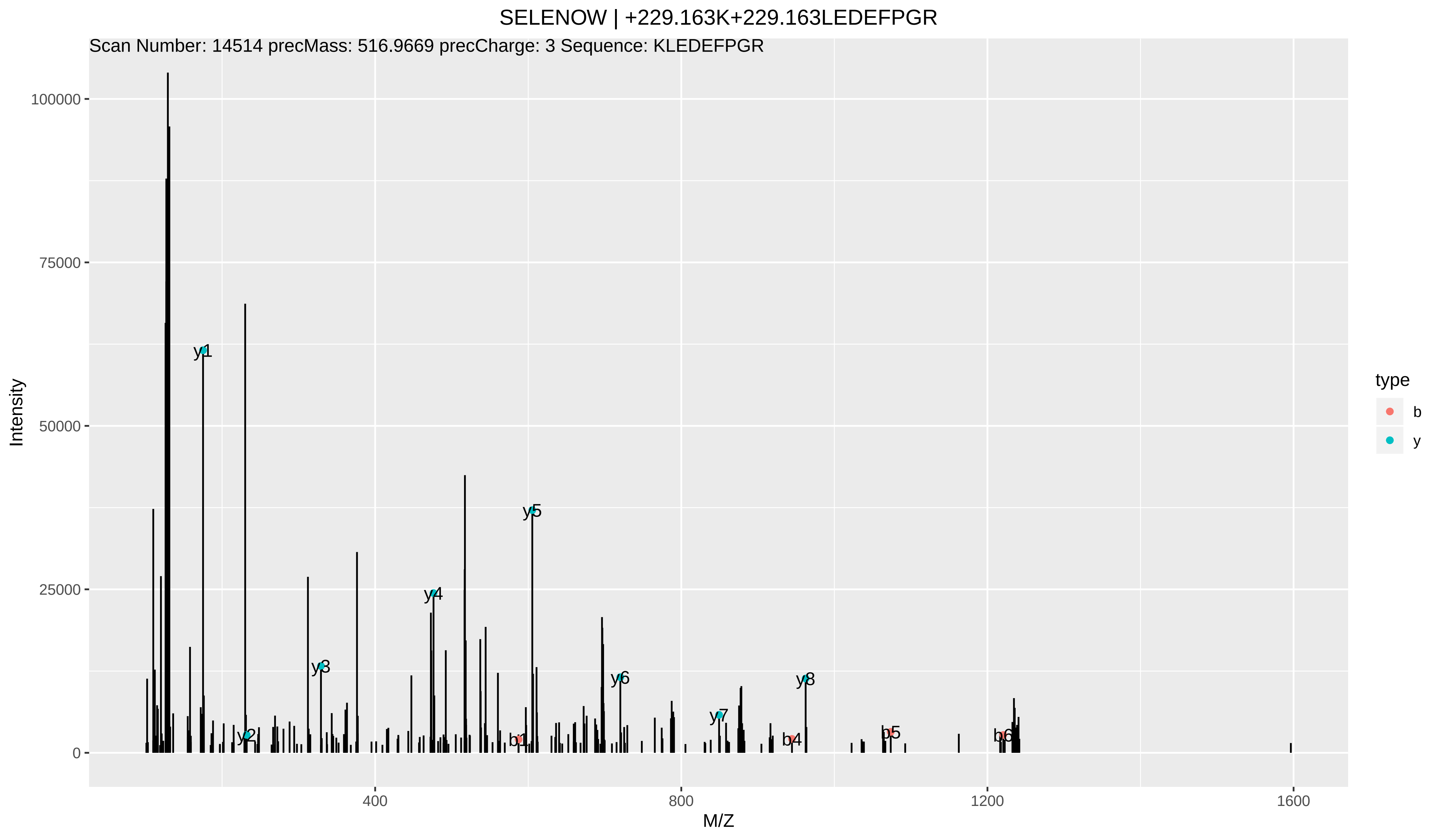

SELENOW | +229.163K+229.163LEDEFPGR
Scan Number: 14514 precMass: 516.9669 precCharge: 3 Sequence: KLEDEFPGR
Intensity
100000
75000
50000
25000
0
400
800
1200
1600
M/Z
y1
y2
y3
y4
y5
y6
y7
y8
b1
b4
b5
b6
type
b
y

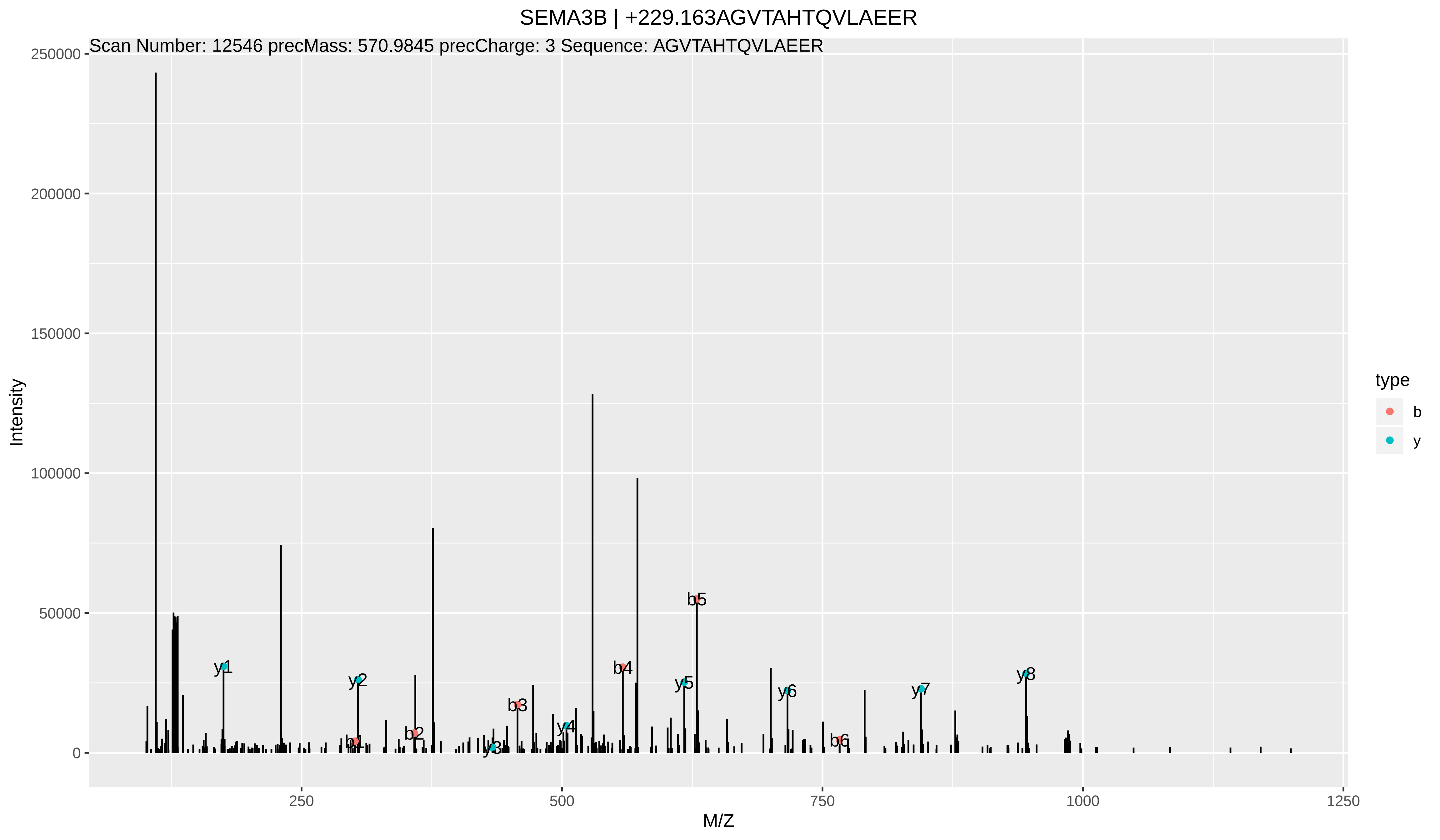
SEMA3B | +229.163AGVTAHTQVLAEER
Scan Number: 12546 precMass: 570.9845 precCharge: 3 Sequence: AGVTAHTQVLAEER
Intensity
M/Z
0
50000
100000
150000
200000
250000
250
500
750
1000
1250
y1
y2
b1
b2
y3
b3
y4
b4
y5
b5
y6
b6
y7
y8
type
b
y

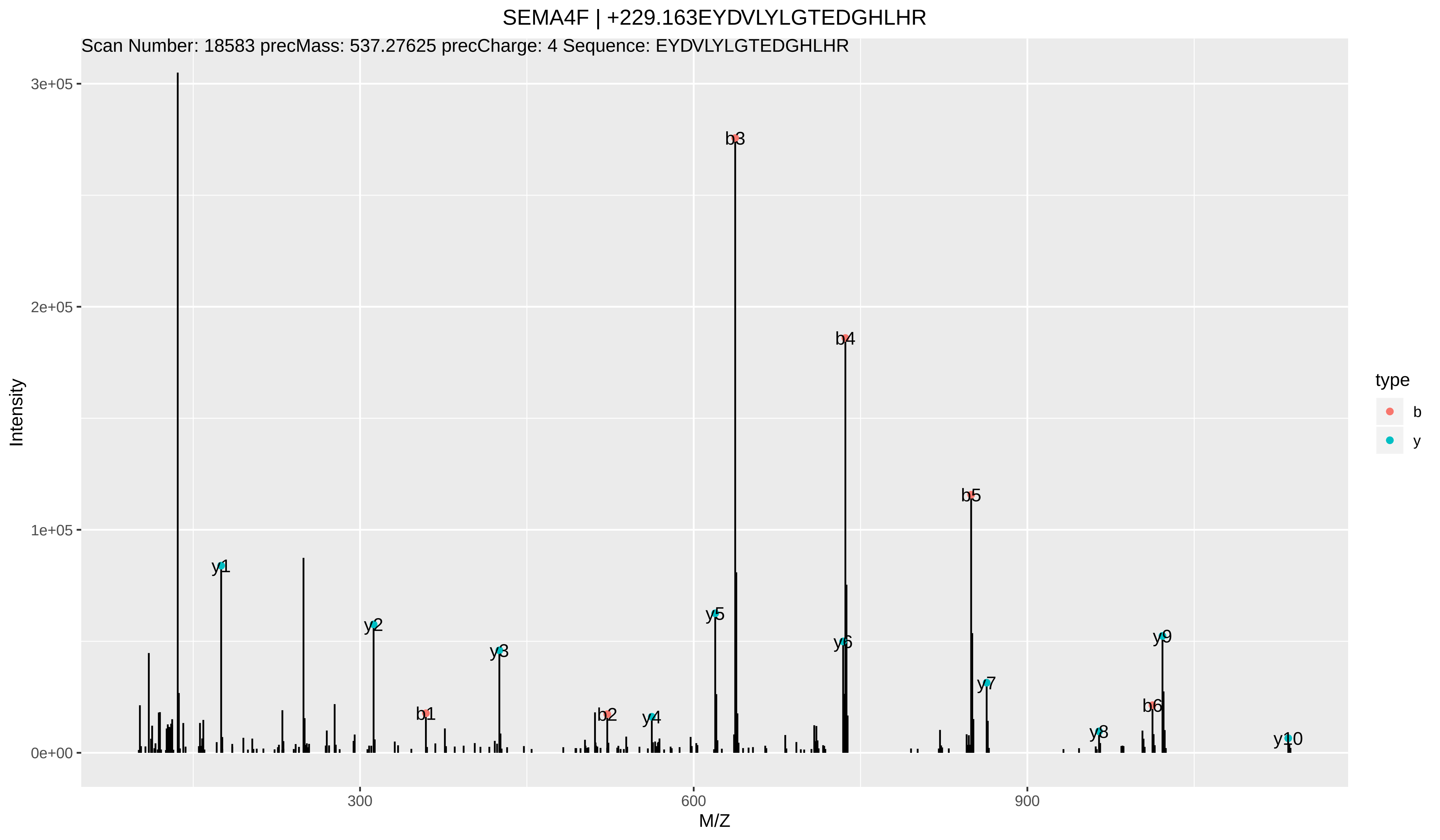

SEMA4F | +229.163EYDVLYLGTEDGHLHR
Scan Number: 18583 precMass: 537.27625 precCharge: 4 Sequence: EYDVLYLGTEDGHLHR
Intensity
M/Z
3e+05
2e+05
1e+05
0e+00
300
600
900
b3
b4
b5
b6
b1
b2
y1
y2
y3
y4
y5
y6
y7
y8
y9
y10
type
b
y

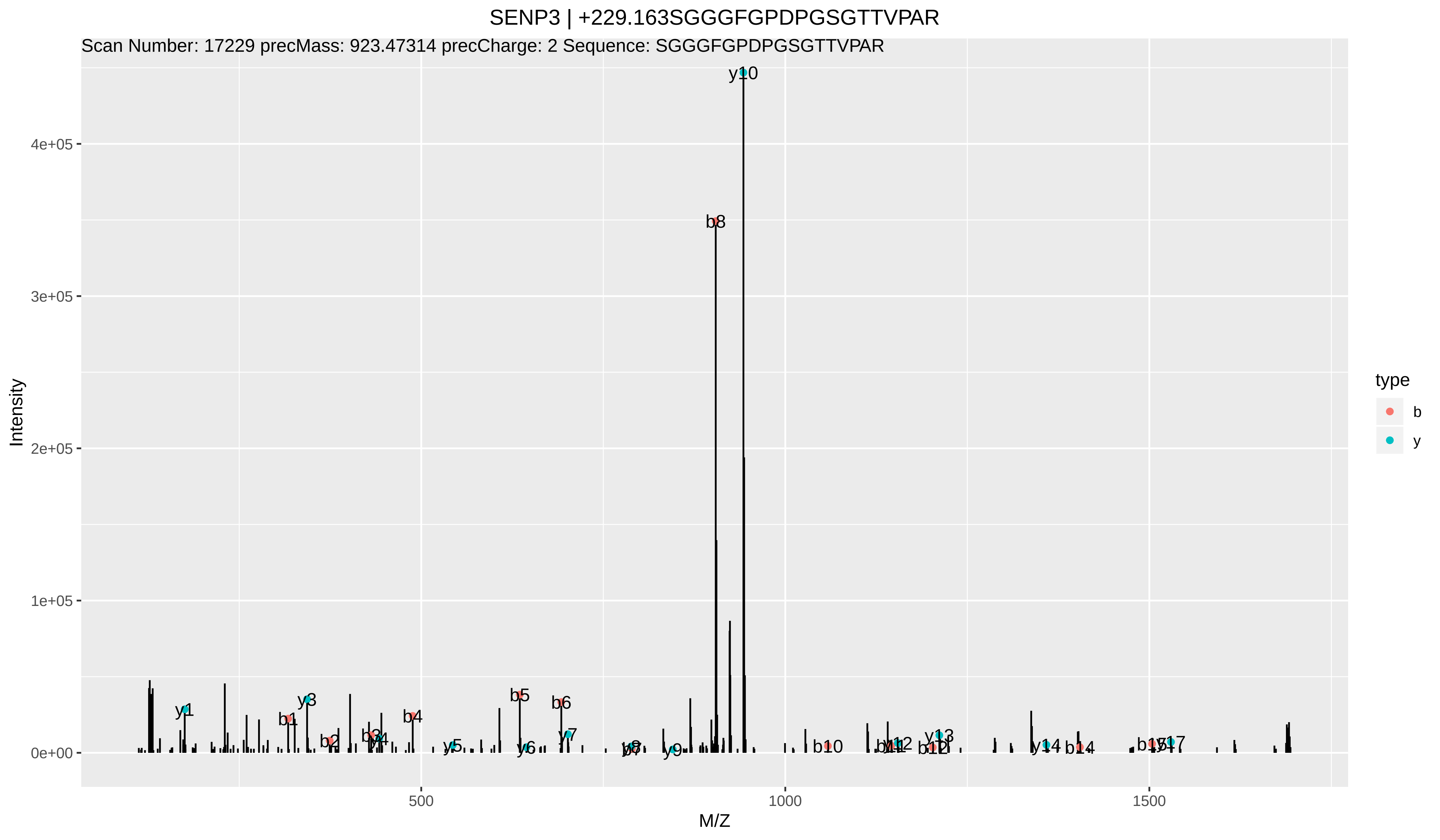
SENP3 | +229.163SGGGFGPDPGSGTTVPAR
Scan Number: 17229 precMass: 923.47314 precCharge: 2 Sequence: SGGGFGPDPGSGTTVPAR
Intensity
M/Z
type
b
y
4e+05
3e+05
2e+05
1e+05
0e+00
500
1000
1500
y10
b8
y1
b1
y3
b2
b3
y4
b4
y5
b5
y6
b6
y7
y8
b7
y9
b10
b11
y12
b12
y13
y14
b14
b15
y17

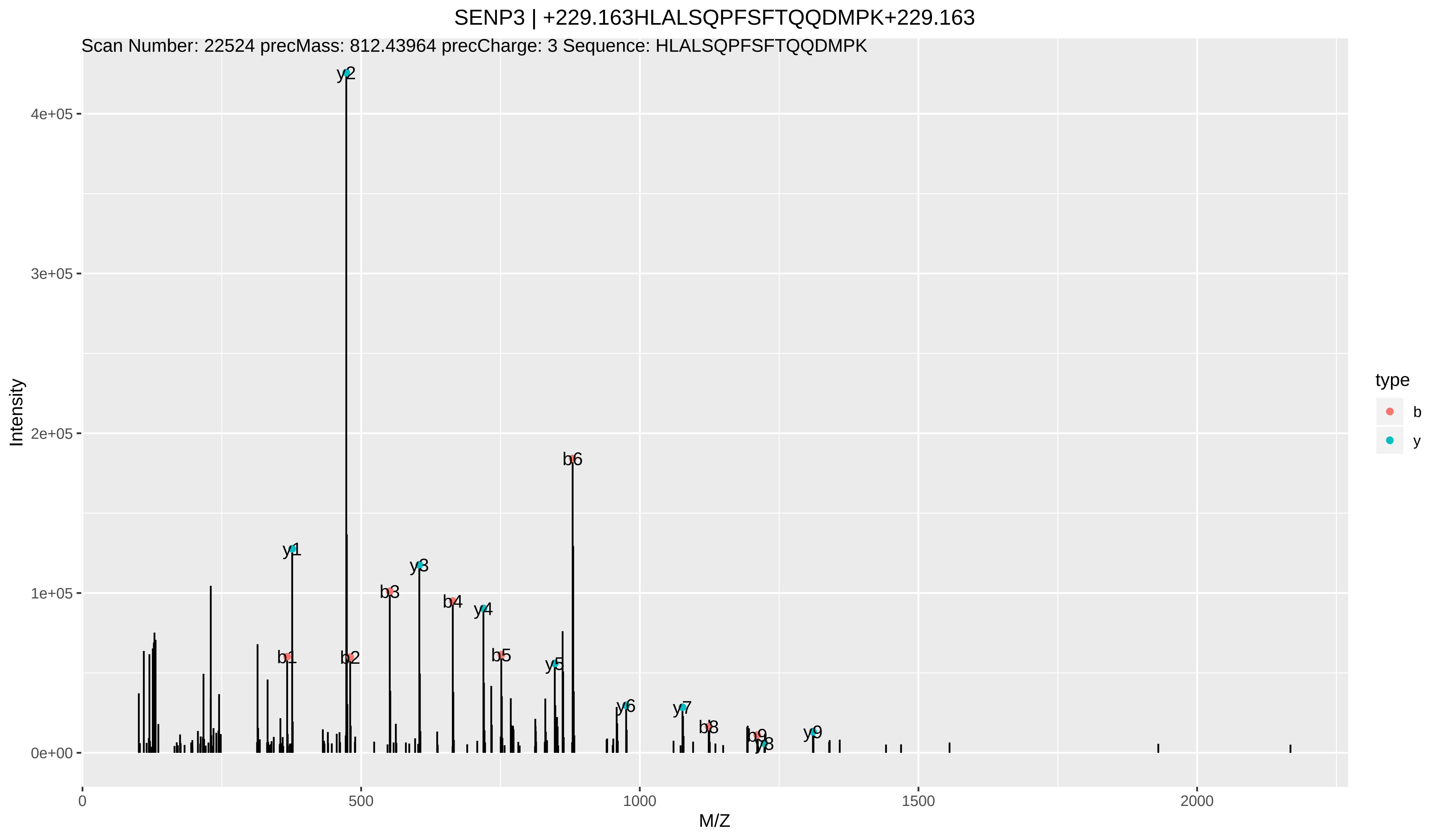

SENP3 | +229.163HLALSQPFSFTQQDMPK+229.163
Scan Number: 22524 precMass: 812.43964 precCharge: 3 Sequence: HLALSQPFSFTQQDMPK
y2
b6
y1
y3
b3
b4
y4
b5
b1
b2
y5
y6
y7
b8
b9
y8
y9
Intensity
4e+05
3e+05
2e+05
1e+05
0e+00
0
500
1000
1500
2000
M/Z
type
b
y

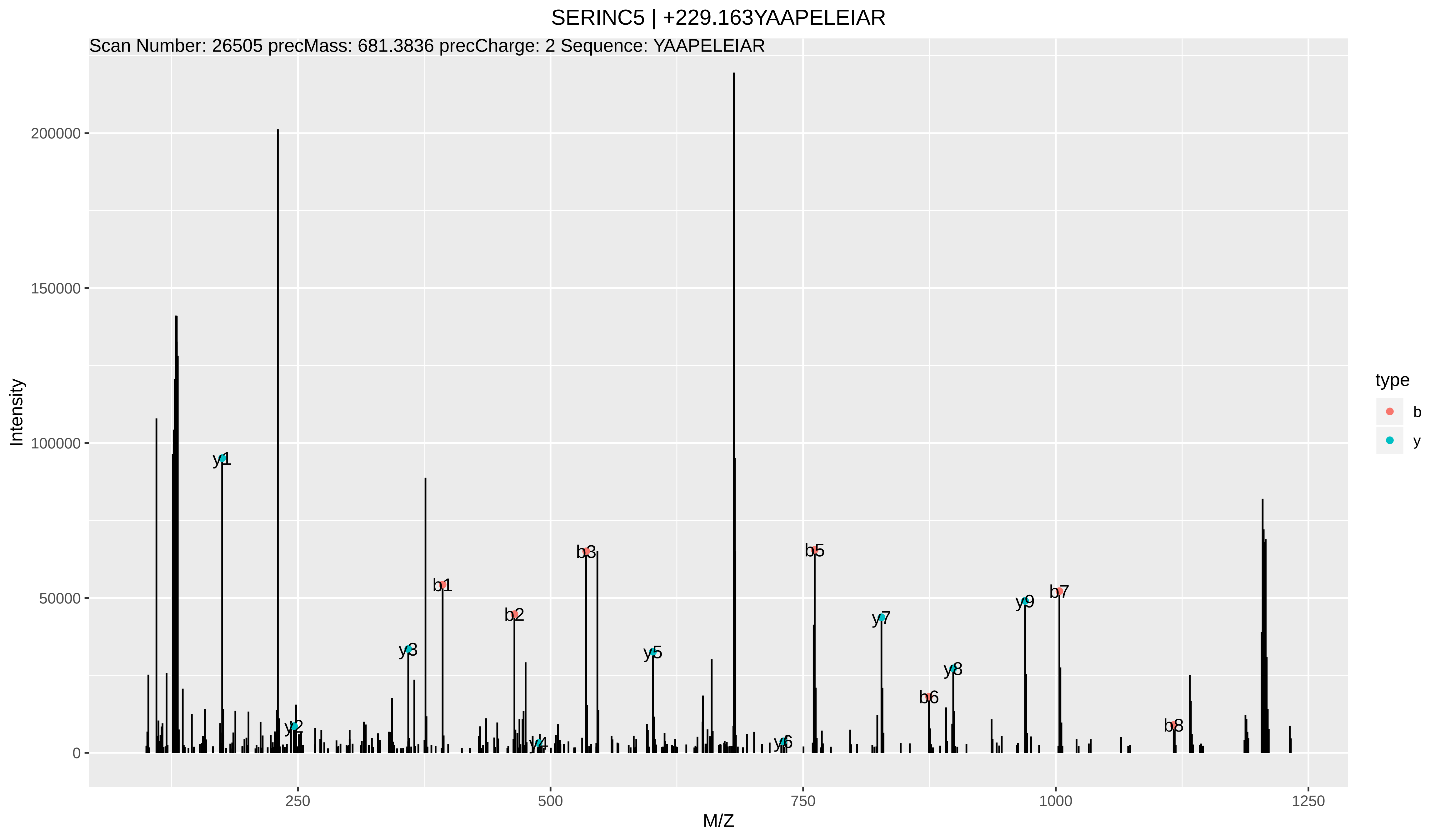
SERINC5 | +229.163YAAPELEIAR
Scan Number: 26505 precMass: 681.3836 precCharge: 2 Sequence: YAAPELEIAR
Intensity
200000
150000
100000
50000
0
M/Z
250
500
750
1000
1250
y1
y2
y3
y4
y5
y6
y7
y8
y9
b1
b2
b3
b5
b6
b7
b8
type
b
y

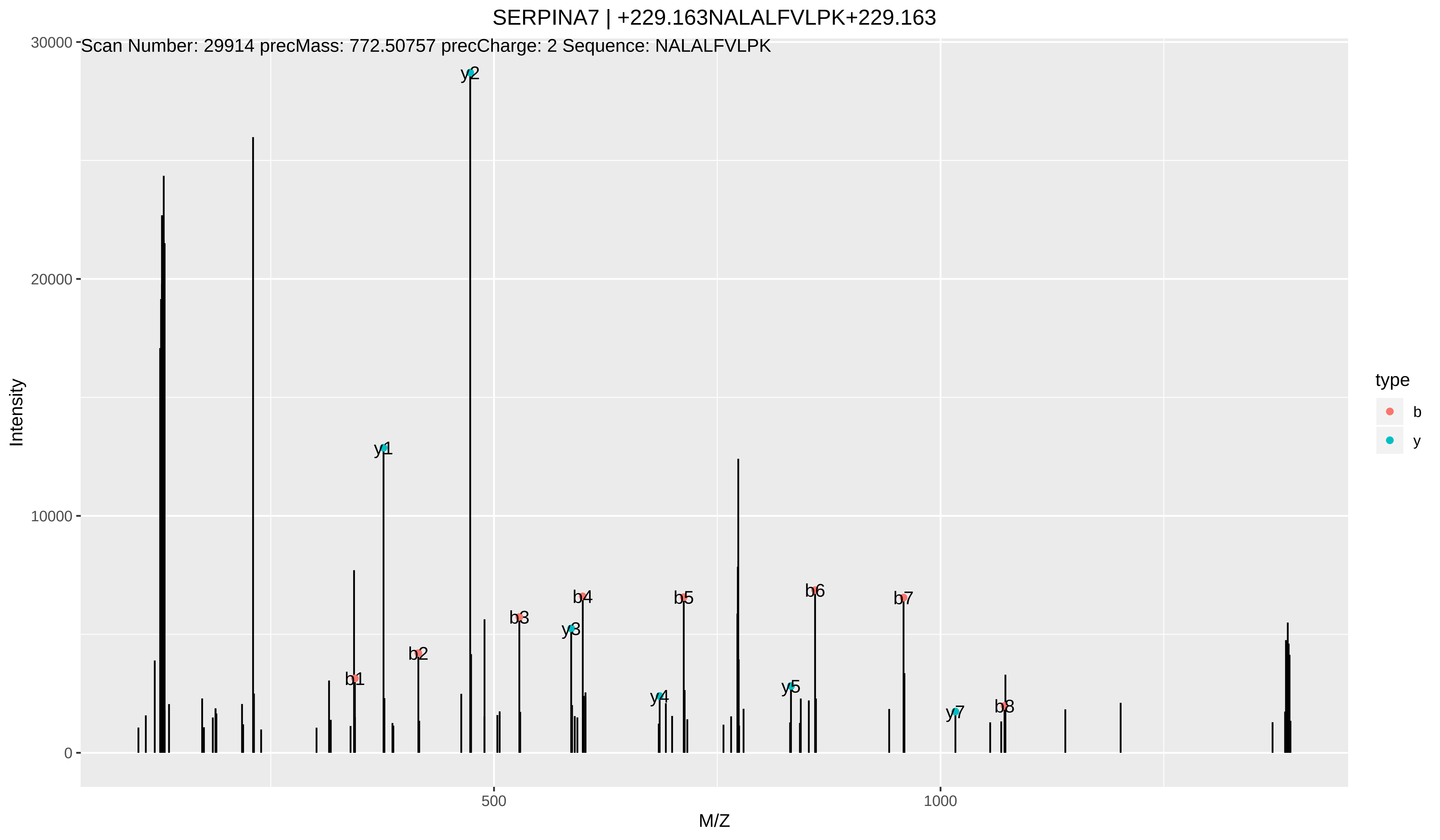

SERPINA7 | +229.163NALALFVLPK+229.163
Scan Number: 29914 precMass: 772.50757 precCharge: 2 Sequence: NALALFVLPK
Intensity
M/Z
type
b
y
0
10000
20000
30000
500
1000
y2
y1
b1
b2
b3
y3
b4
y4
b5
y5
b6
b7
y7
b8

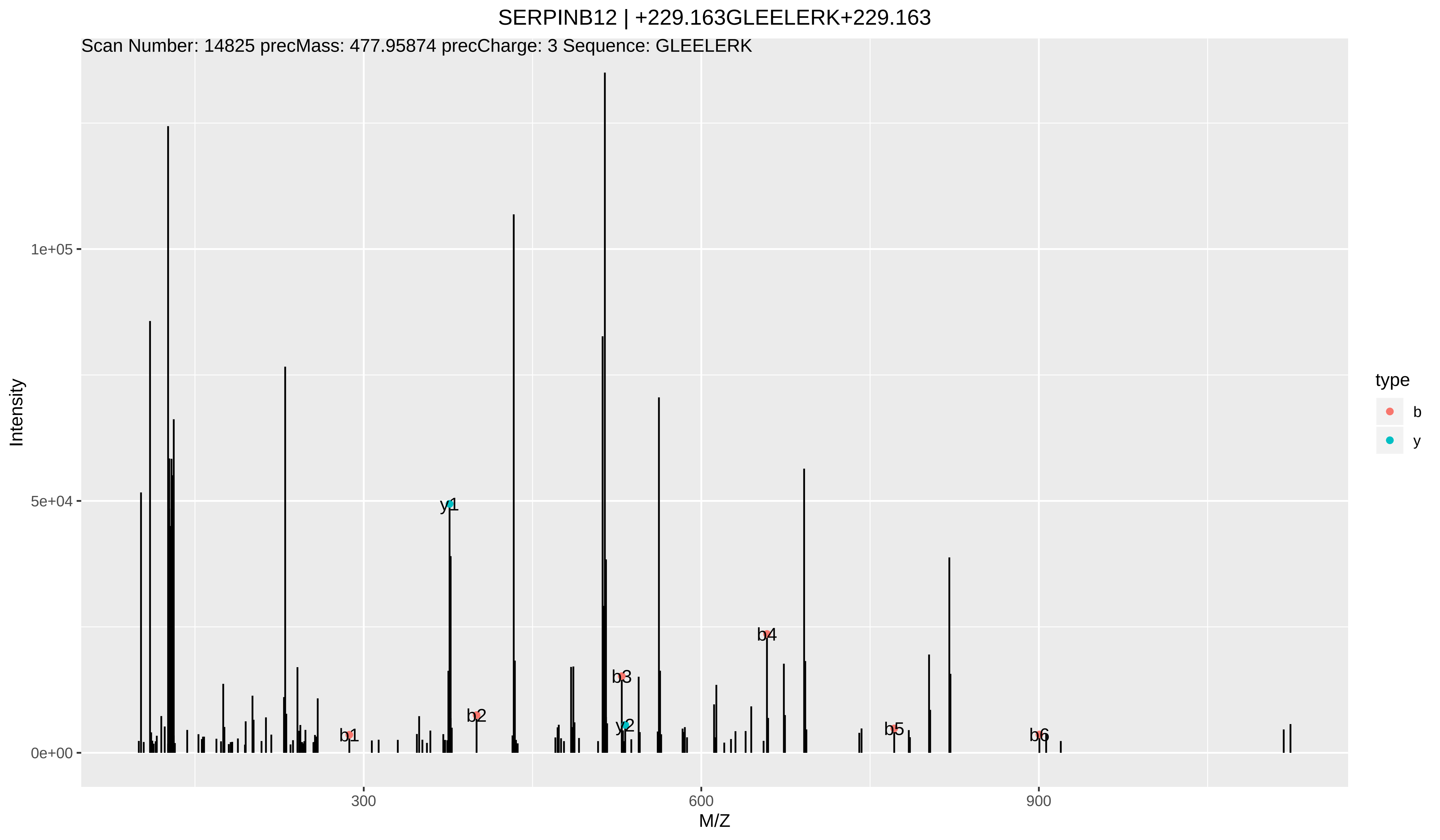
SERPINB12 | +229.163GLEELERK+229.163
Scan Number: 14825 precMass: 477.95874 precCharge: 3 Sequence: GLEELERK
Intensity
1e+05
5e+04
0e+00
M/Z
300
600
900
y1
b1
b2
b3
y2
b4
b5
b6
type
b
y

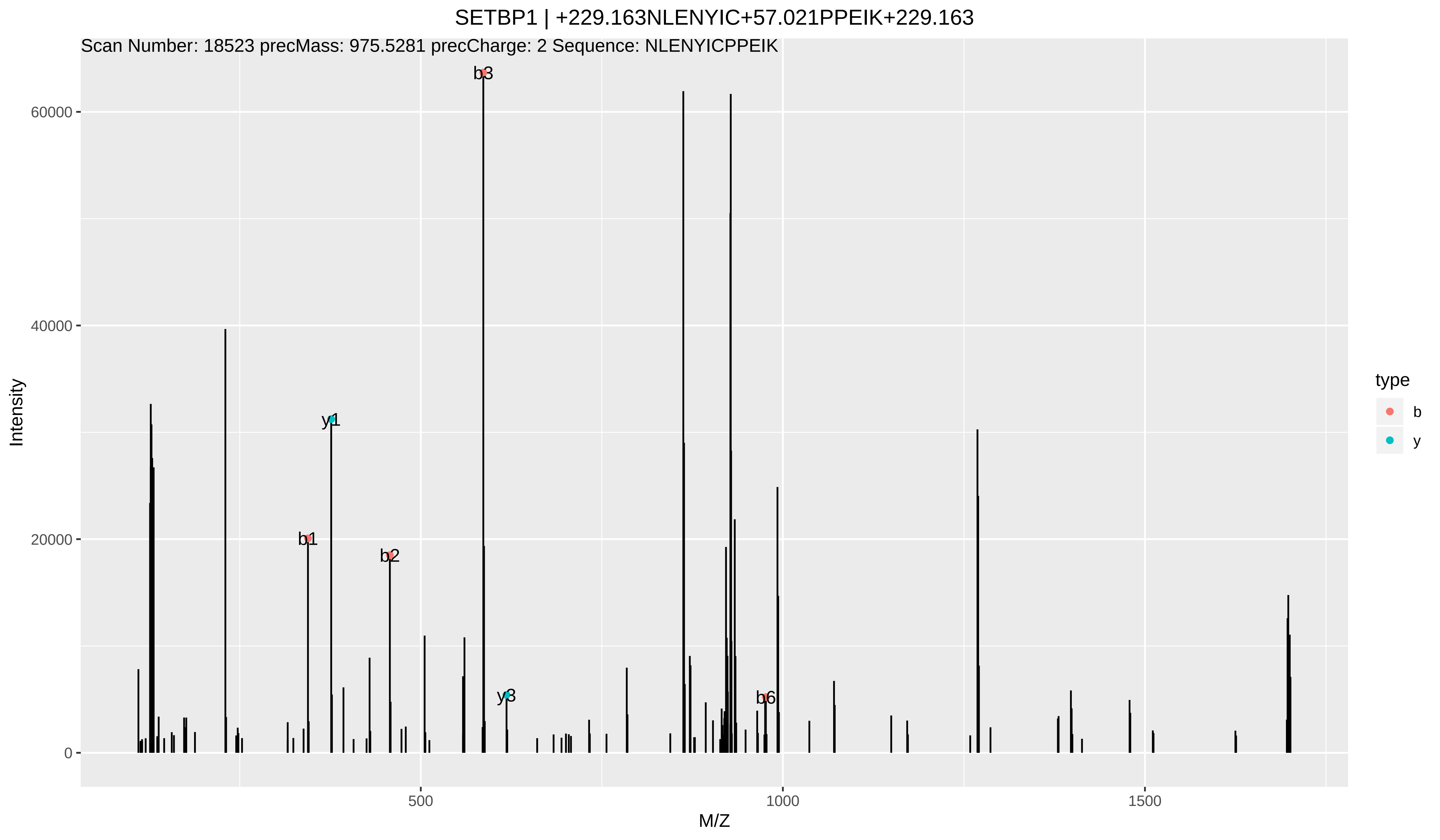
SETBP1 | +229.163NLENYIC+57.021PPEIK+229.163
Scan Number: 18523 precMass: 975.5281 precCharge: 2 Sequence: NLENYICPPEIK
b3
y1
b1
b2
y3
b6
60000
40000
20000
0
500
1000
1500
Intensity
M/Z
type
b
y

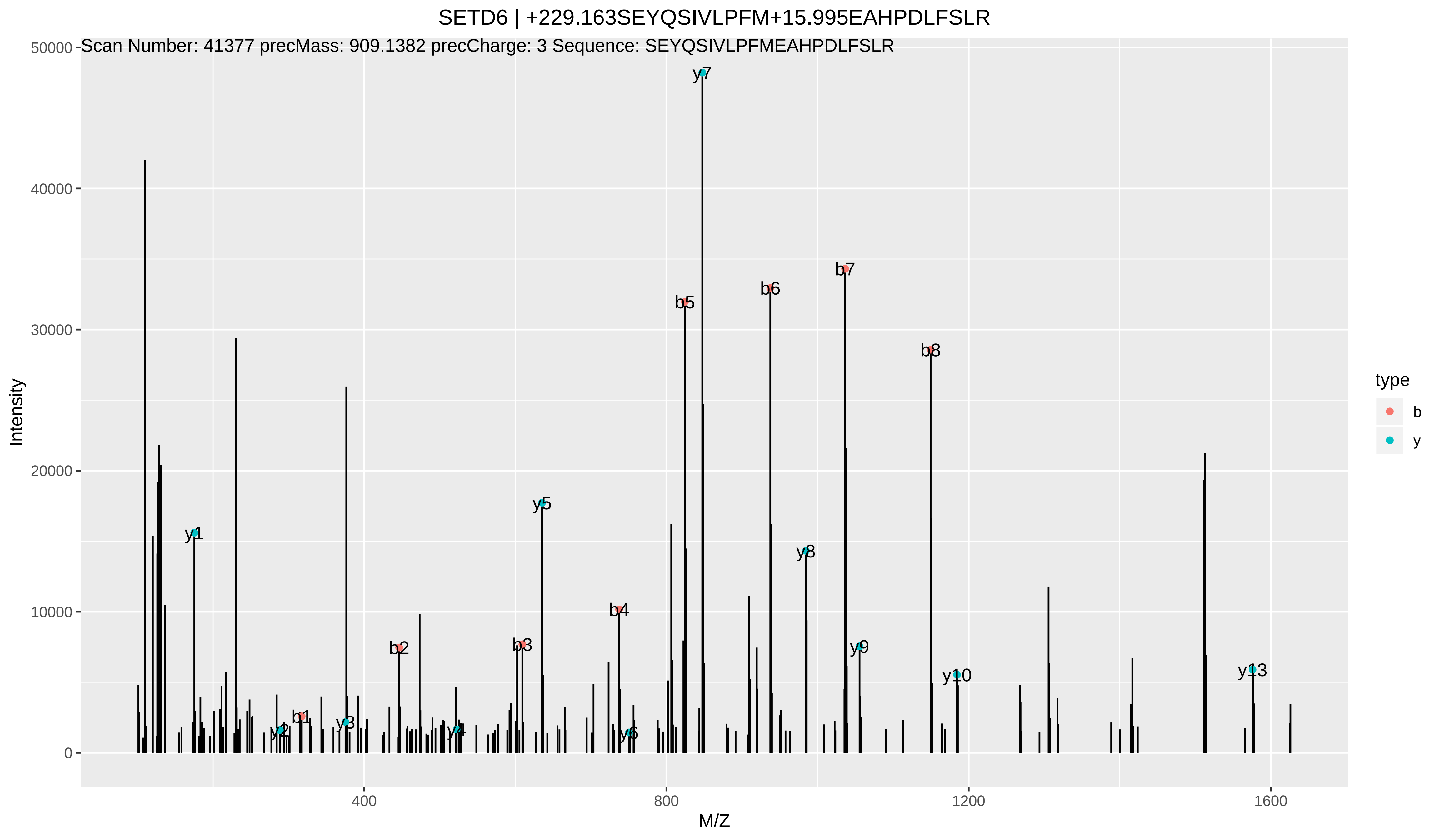
SETD6 | +229.163SEYQSIVLPFM+15.995EAHPDLFSLR
Scan Number: 41377 precMass: 909.1382 precCharge: 3 Sequence: SEYQSIVLPFMEAHPDLFSLR
Intensity
M/Z
type
b
y
y1
y2
b1
y3
b2
y4
b3
y5
b4
y6
b5
y7
b6
y8
b7
y9
b8
y10
y13
0
10000
20000
30000
40000
50000
400
800
1200
1600

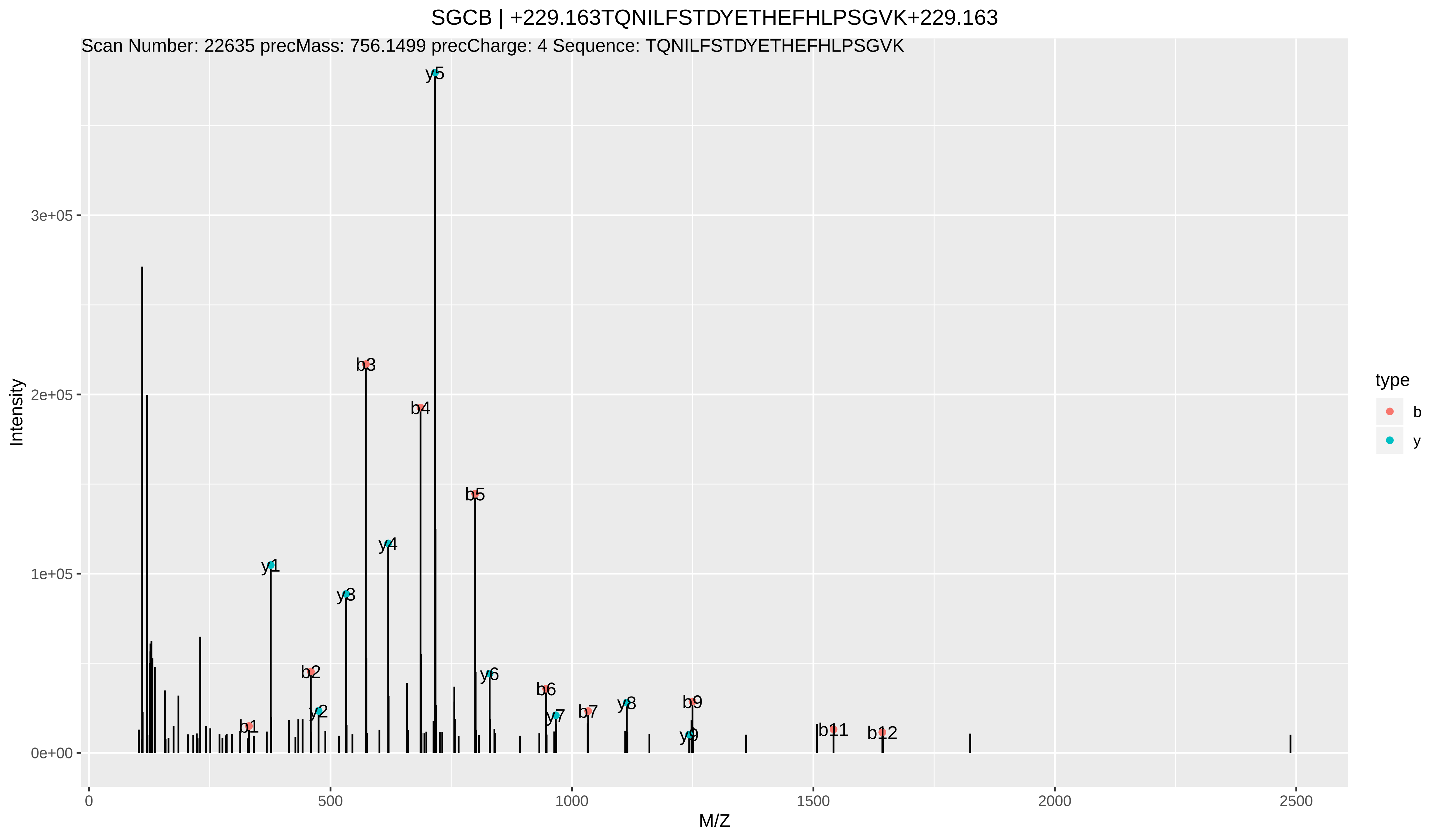

SGCB | +229.163TQNILFSTDYETHEFHLPSGVK+229.163
Scan Number: 22635 precMass: 756.1499 precCharge: 4 Sequence: TQNILFSTDYETHEFHLPSGVK
Intensity
M/Z
0e+00
1e+05
2e+05
3e+05
0
500
1000
1500
2000
2500
y5
b3
b4
b5
y4
y1
y3
b2
y2
b1
y6
b6
y7
b7
y8
b9
y9
b11
b12
type
b
y

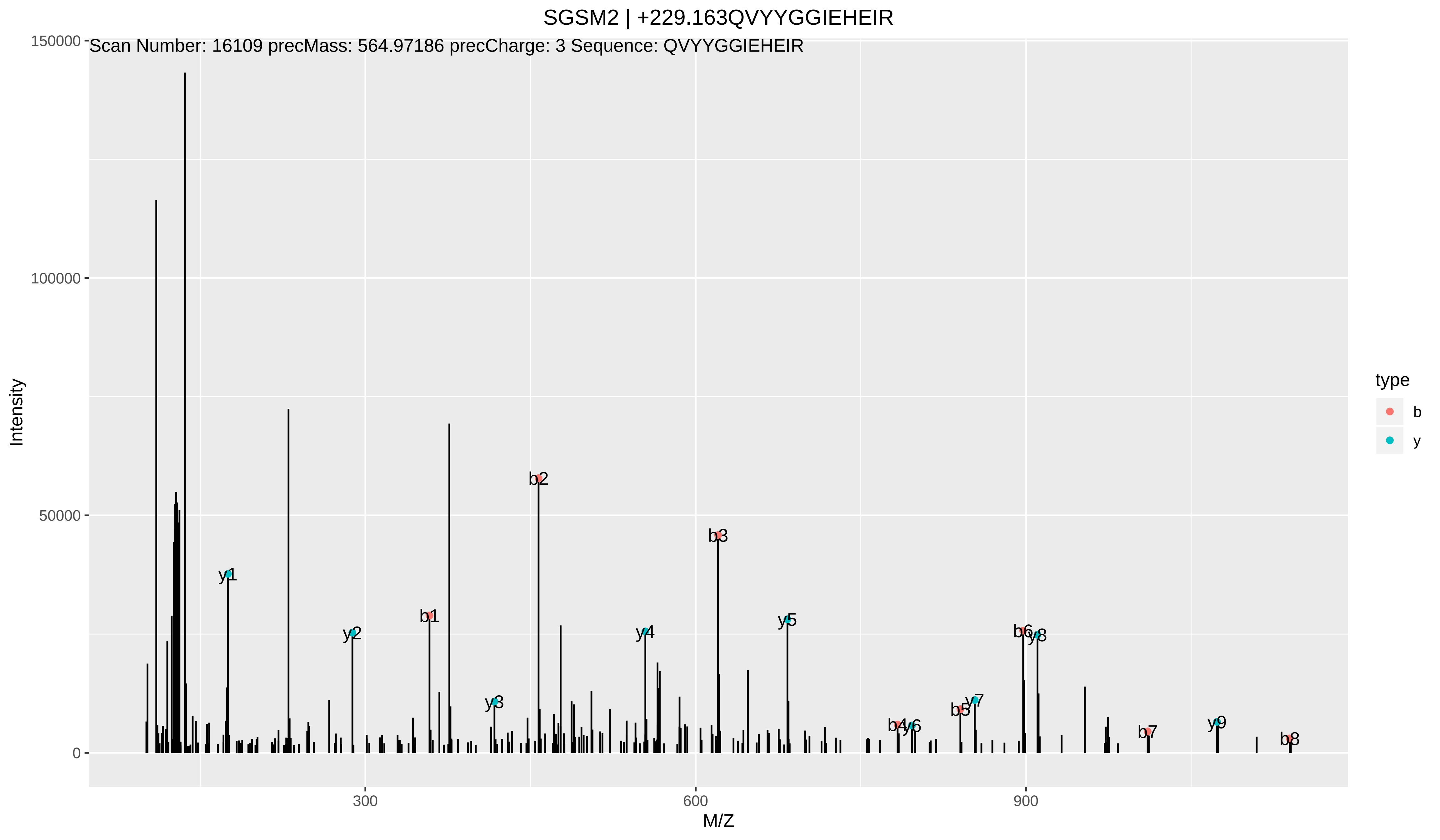
SGSM2 | +229.163QVYYGGIEHEIR
Scan Number: 16109 precMass: 564.97186 precCharge: 3 Sequence: QVYYGGIEHEIR
Intensity
M/Z
type
b
y
y1
y2
b1
y3
b2
y4
b3
y5
b4
y6
b5
y7
b6
y8
b7
y9
b8

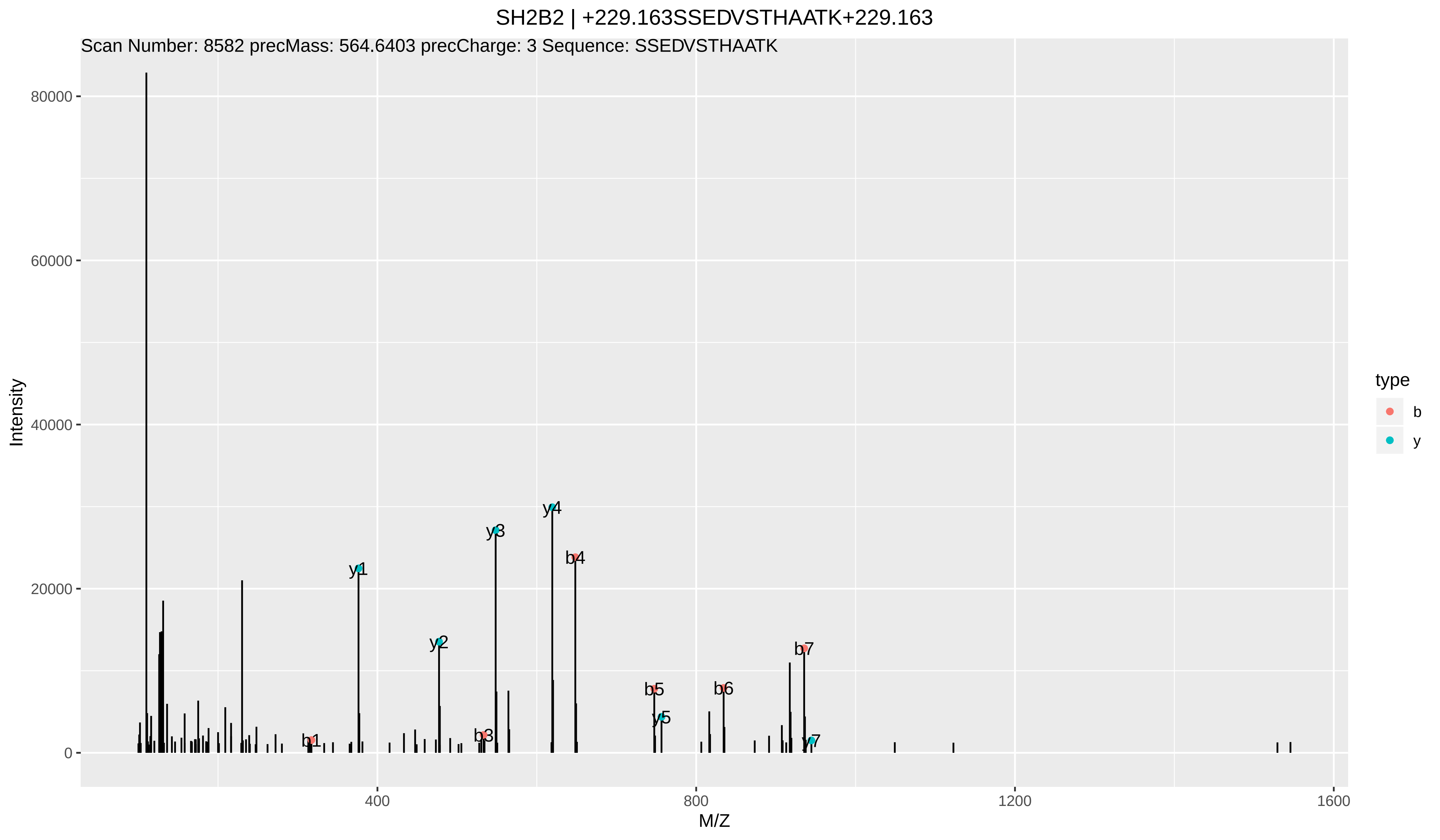
SH2B2 | +229.163SSEDVSTHAATK+229.163
Scan Number: 8582 precMass: 564.6403 precCharge: 3 Sequence: SSEDVSTHAATK
Intensity
80000
60000
40000
20000
0
M/Z
400
800
1200
1600
y1
y2
y3
y4
y5
y7
b1
b3
b4
b5
b6
b7
type
b
y

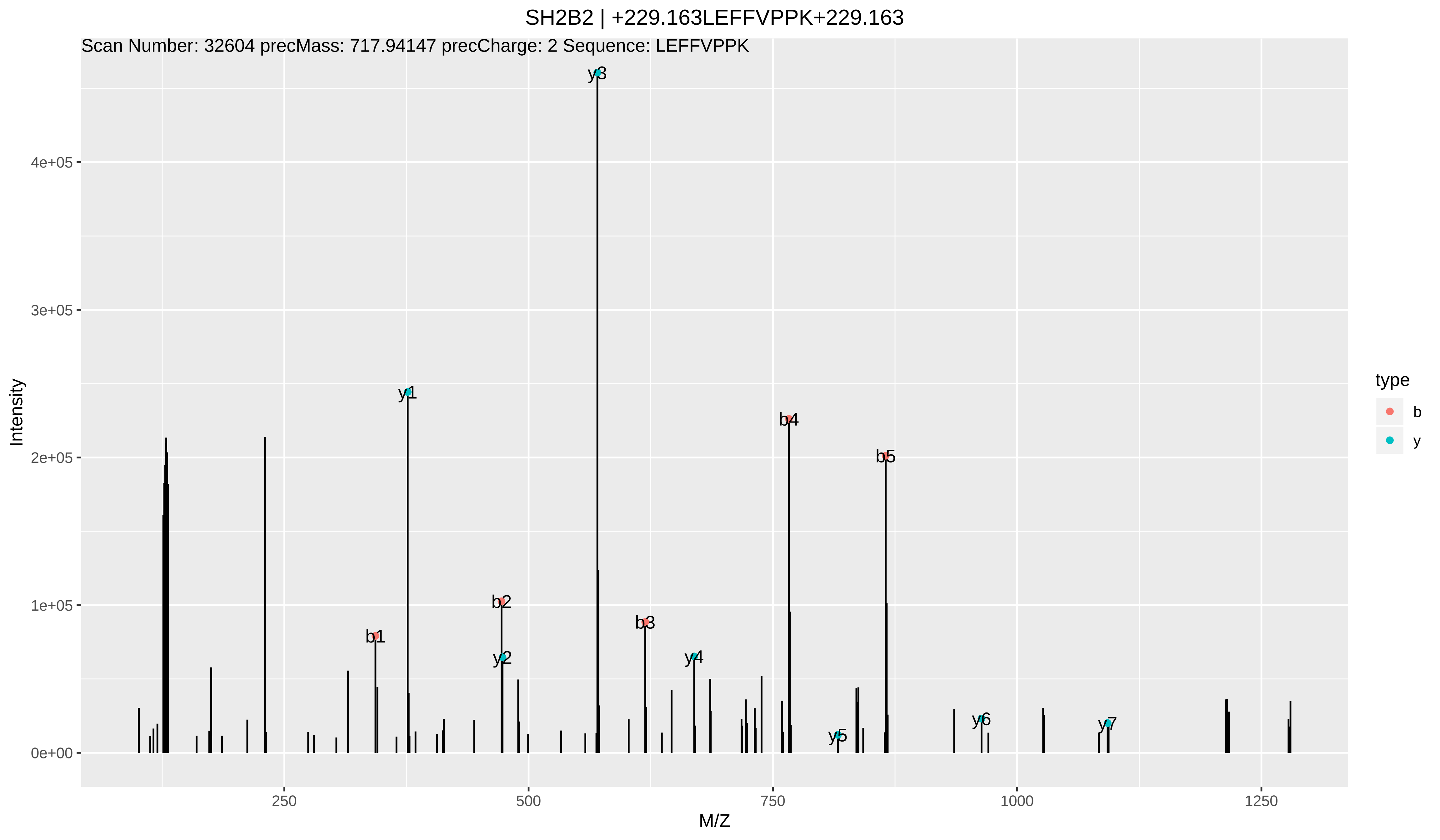

SH2B2 | +229.163LEFFVPPK+229.163
Scan Number: 32604 precMass: 717.94147 precCharge: 2 Sequence: LEFFVPPK
Intensity
4e+05
3e+05
2e+05
1e+05
0e+00
M/Z
250
500
750
1000
1250
y3
y1
b4
b5
b2
b3
b1
y2
y4
y5
y6
y7
type
b
y

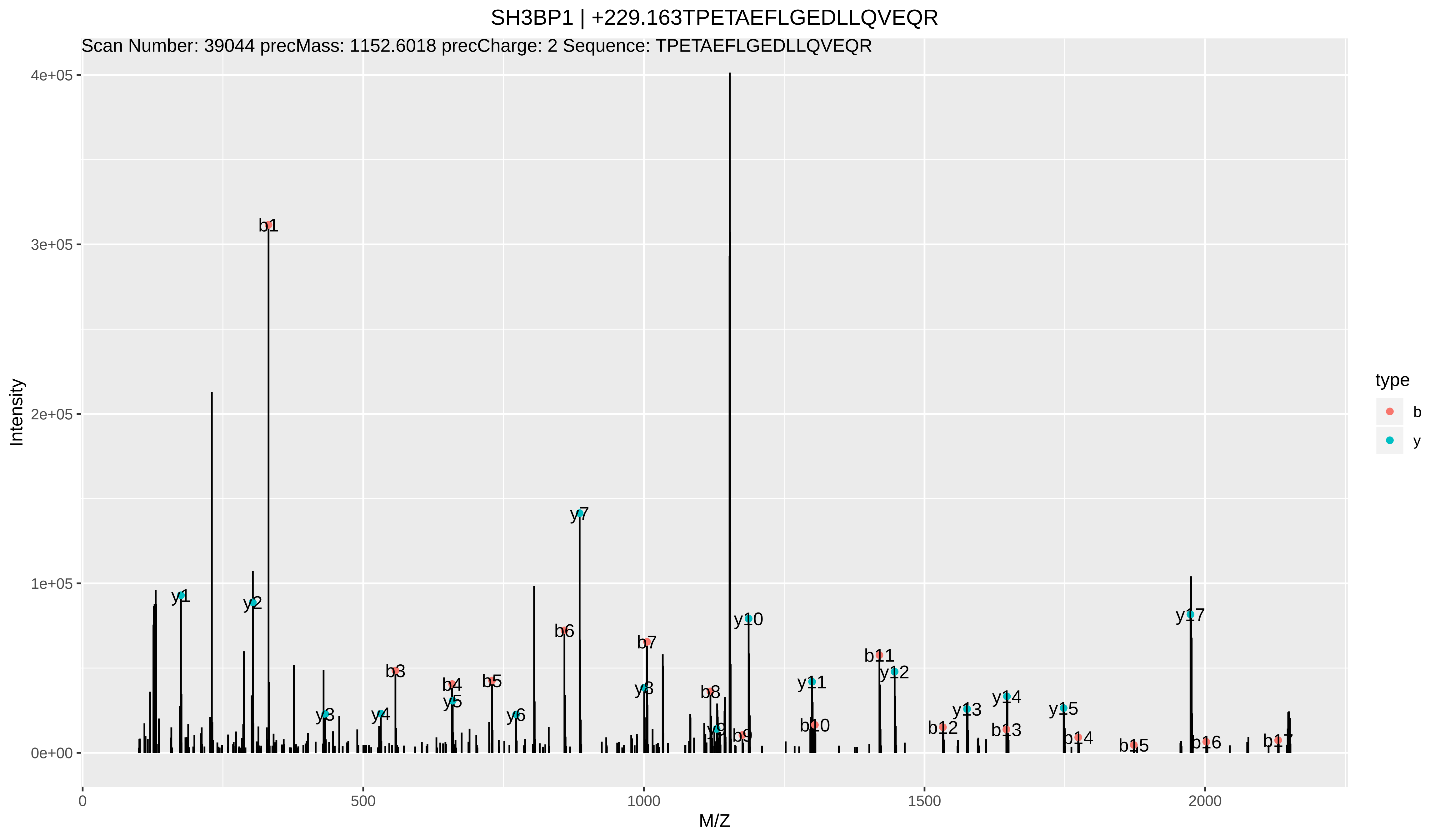

SH3BP1 | +229.163TPETAEFLGEDLLQVEQR
Scan Number: 39044 precMass: 1152.6018 precCharge: 2 Sequence: TPETAEFLGEDLLQVEQR
Intensity
M/Z
type
b
y
4e+05
3e+05
2e+05
1e+05
0e+00
0
500
1000
1500
2000
b1
y1
y2
y3
y4
b3
b4
y5
b5
y6
b6
y7
y8
b7
b8
y9
b9
y10
y11
b10
b11
y12
b12
y13
y14
b13
y15
b14
b15
y17
b16
b17

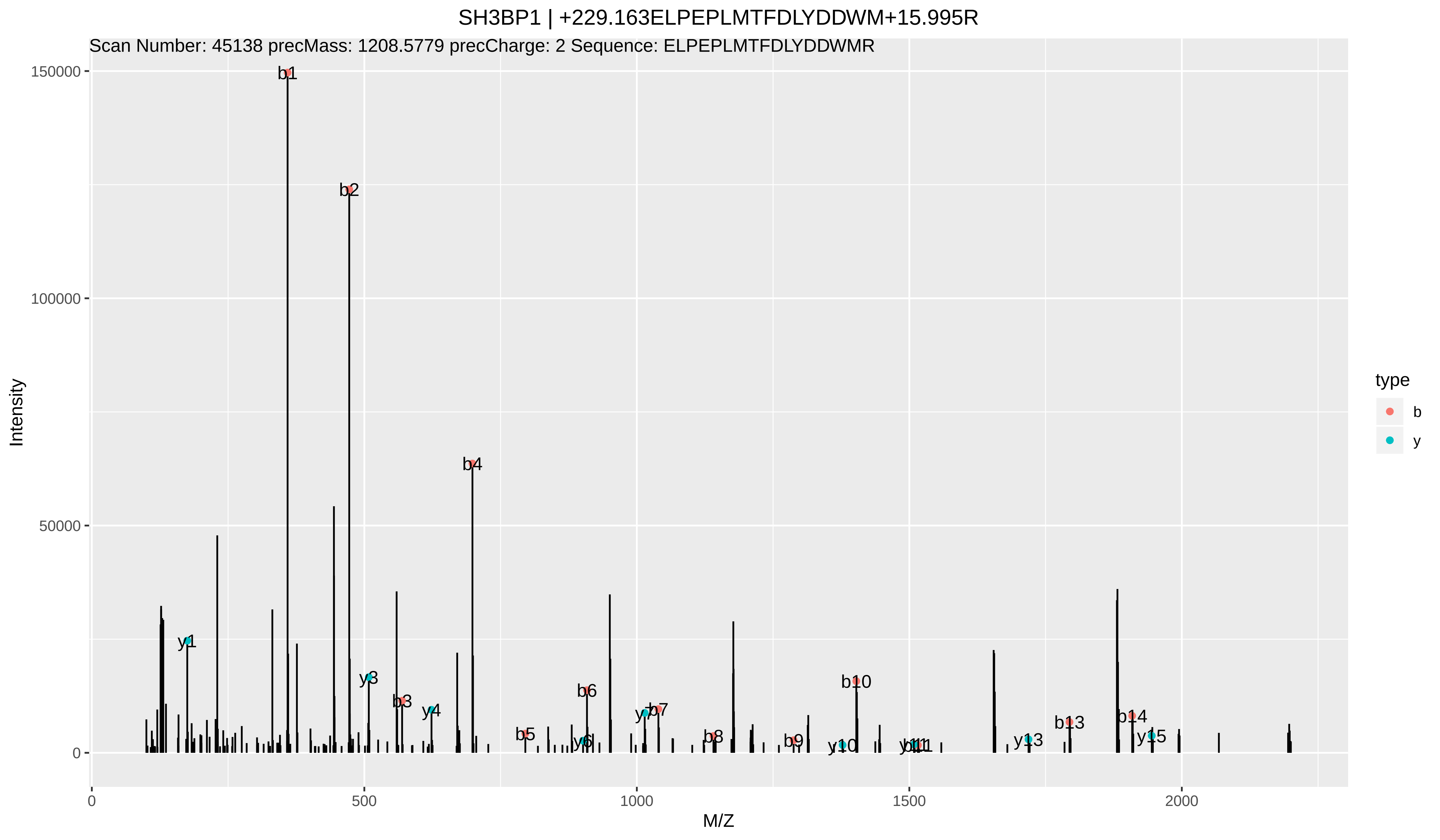
SH3BP1 | +229.163ELPEPLMTFDLYDDWM+15.995R
Scan Number: 45138 precMass: 1208.5779 precCharge: 2 Sequence: ELPEPLMTFDLYDDWMR
Intensity
M/Z
150000
100000
50000
0
0
500
1000
1500
2000
b1
b2
b3
b4
b5
b6
b7
b8
b9
b10
b13
b14
y1
y3
y4
y6
y7
y10
y13
y15
type
b
y

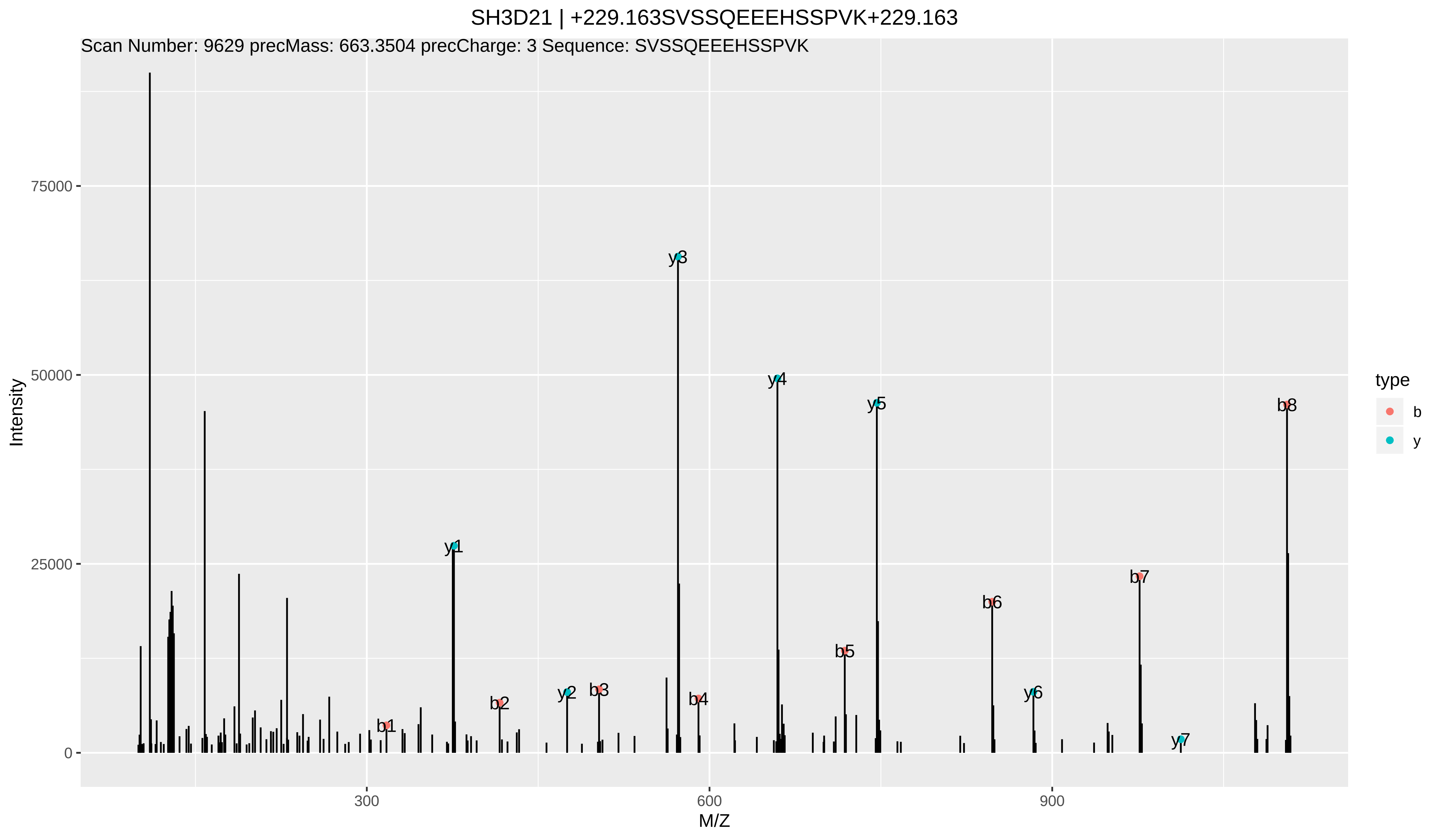
SH3D21 | +229.163SVSSQEEEHSSPVK+229.163
Scan Number: 9629 precMass: 663.3504 precCharge: 3 Sequence: SVSSQEEEHSSPVK
Intensity
M/Z
0
25000
50000
75000
300
600
900
y1
y2
y3
y4
y5
y6
y7
b1
b2
b3
b4
b5
b6
b7
b8
type
b
y

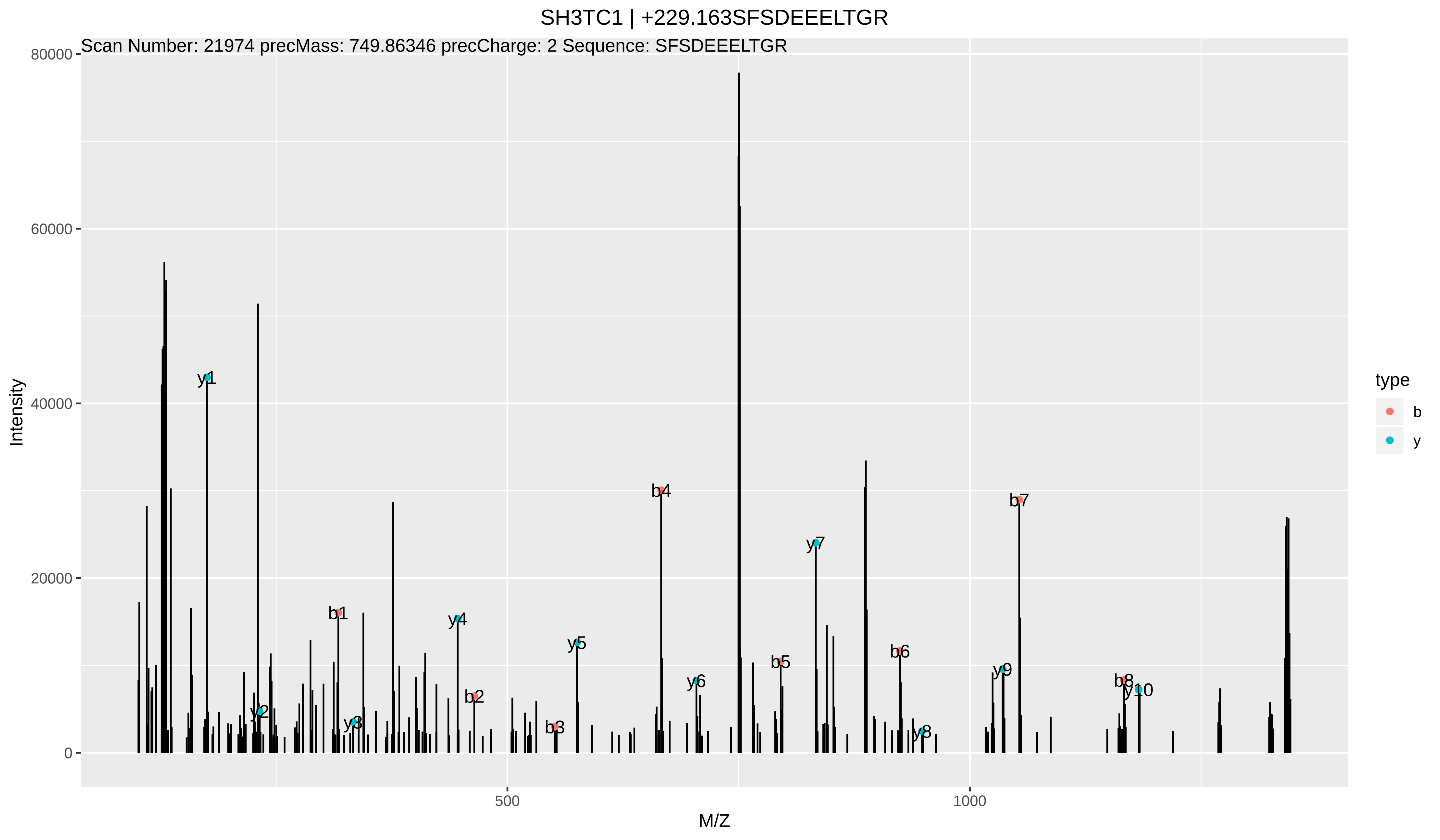
SH3TC1 | +229.163SFSDEEELTGR
Scan Number: 21974 precMass: 749.86346 precCharge: 2 Sequence: SFSDEEELTGR
Intensity
M/Z
80000
60000
40000
20000
0
500
1000
y1
y2
y3
y4
y5
y6
y7
y8
y9
y10
b1
b2
b3
b4
b5
b6
b7
b8
type
b
y

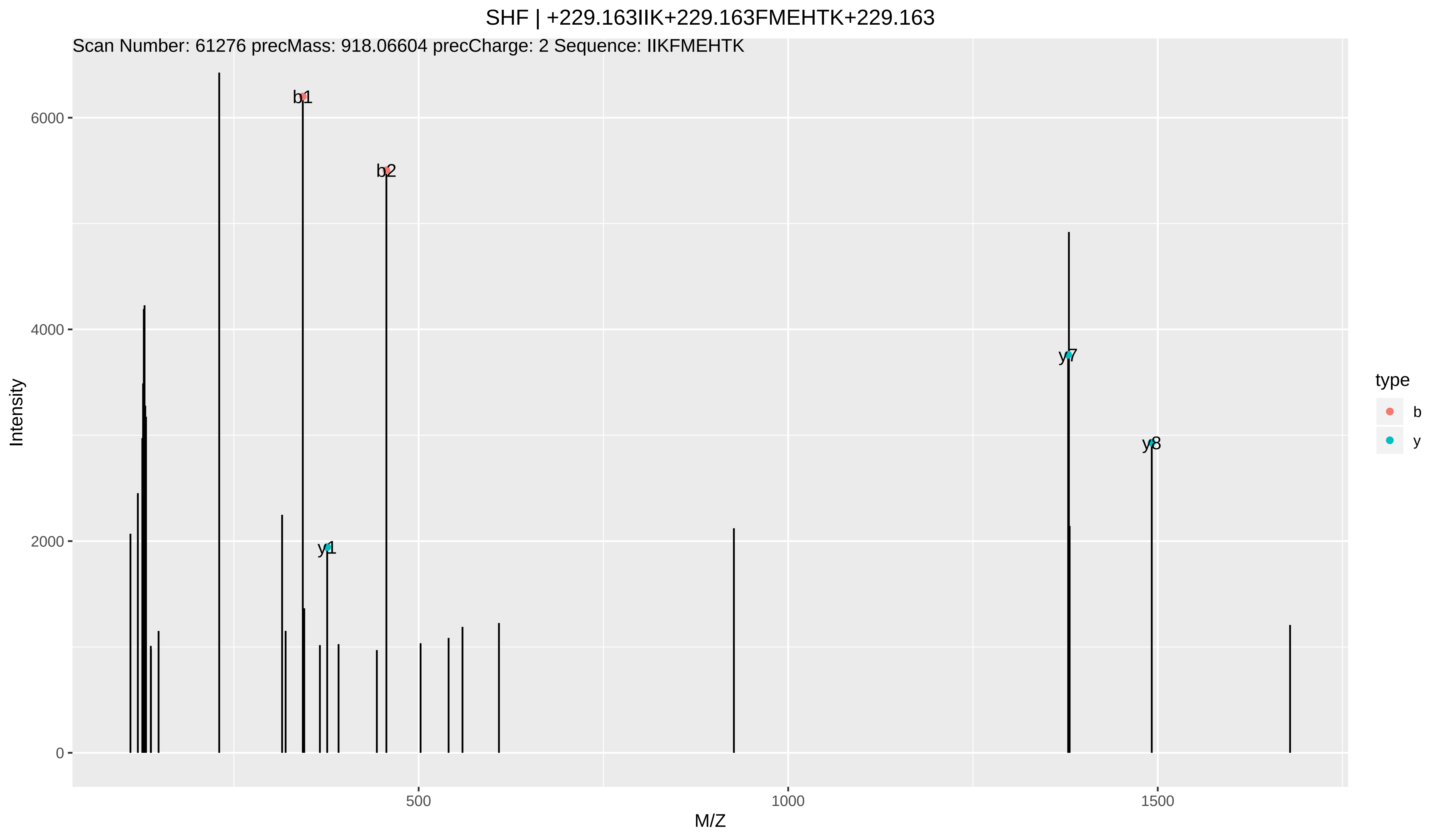

SHF | +229.163IIK+229.163FMEHTK+229.163
Scan Number: 61276 precMass: 918.06604 precCharge: 2 Sequence: IIKFMEHTK
Intensity
M/Z
b1
b2
y1
y7
y8
type
b
y
0
2000
4000
6000
500
1000
1500

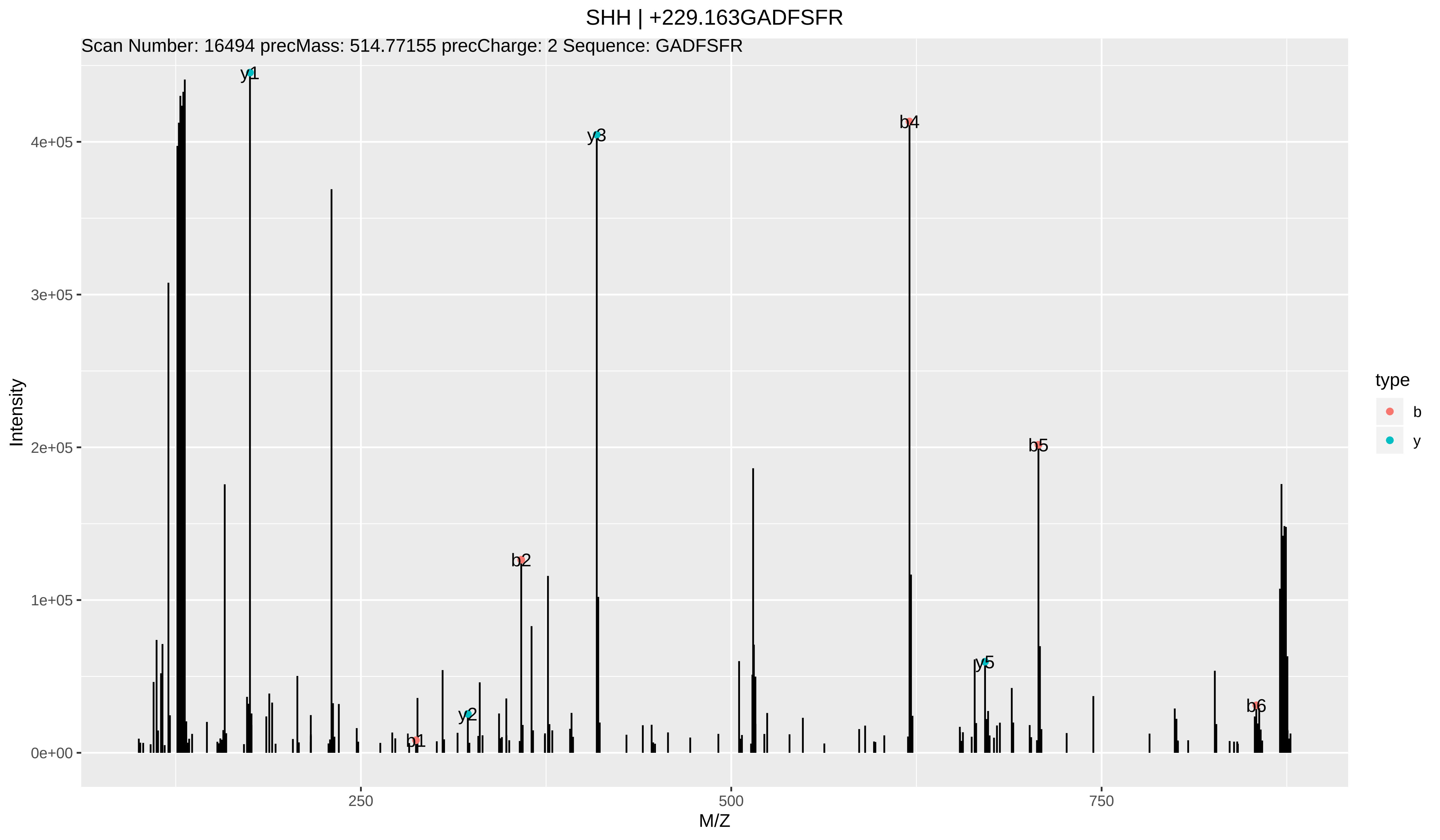

SHH | +229.163GADFSFR
Scan Number: 16494 precMass: 514.77155 precCharge: 2 Sequence: GADFSFR
y1
y3
b4
b5
b2
y5
y2
b1
b6
Intensity
4e+05
3e+05
2e+05
1e+05
0e+00
250
500
750
M/Z
type
b
y

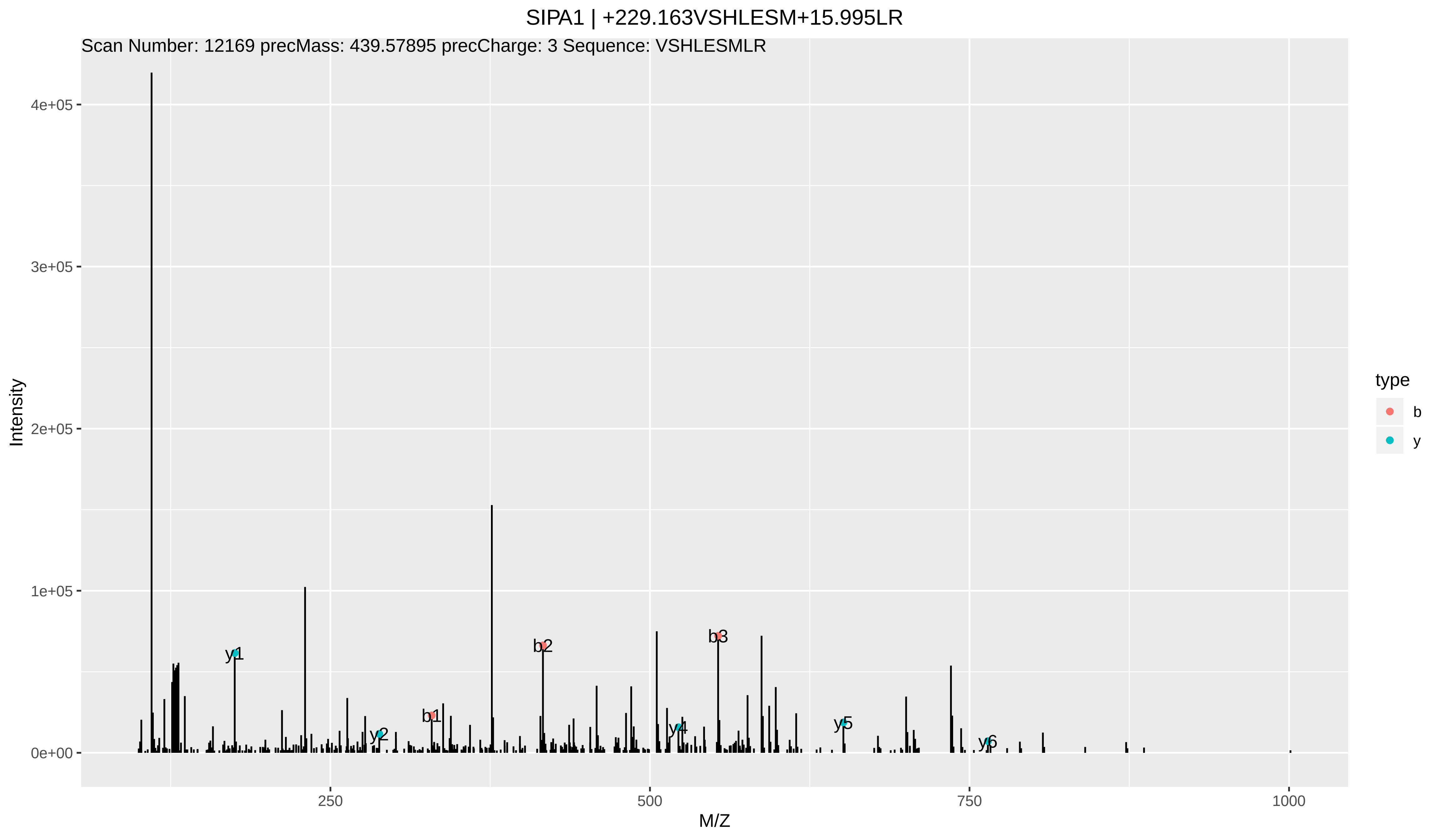

SIPA1 | +229.163VSHLESM+15.995LR
Scan Number: 12169 precMass: 439.57895 precCharge: 3 Sequence: VSHLESMLR
Intensity
4e+05
3e+05
2e+05
1e+05
0e+00
y1
y2
b1
b2
y4
b3
y5
y6
250
500
750
1000
M/Z
type
b
y

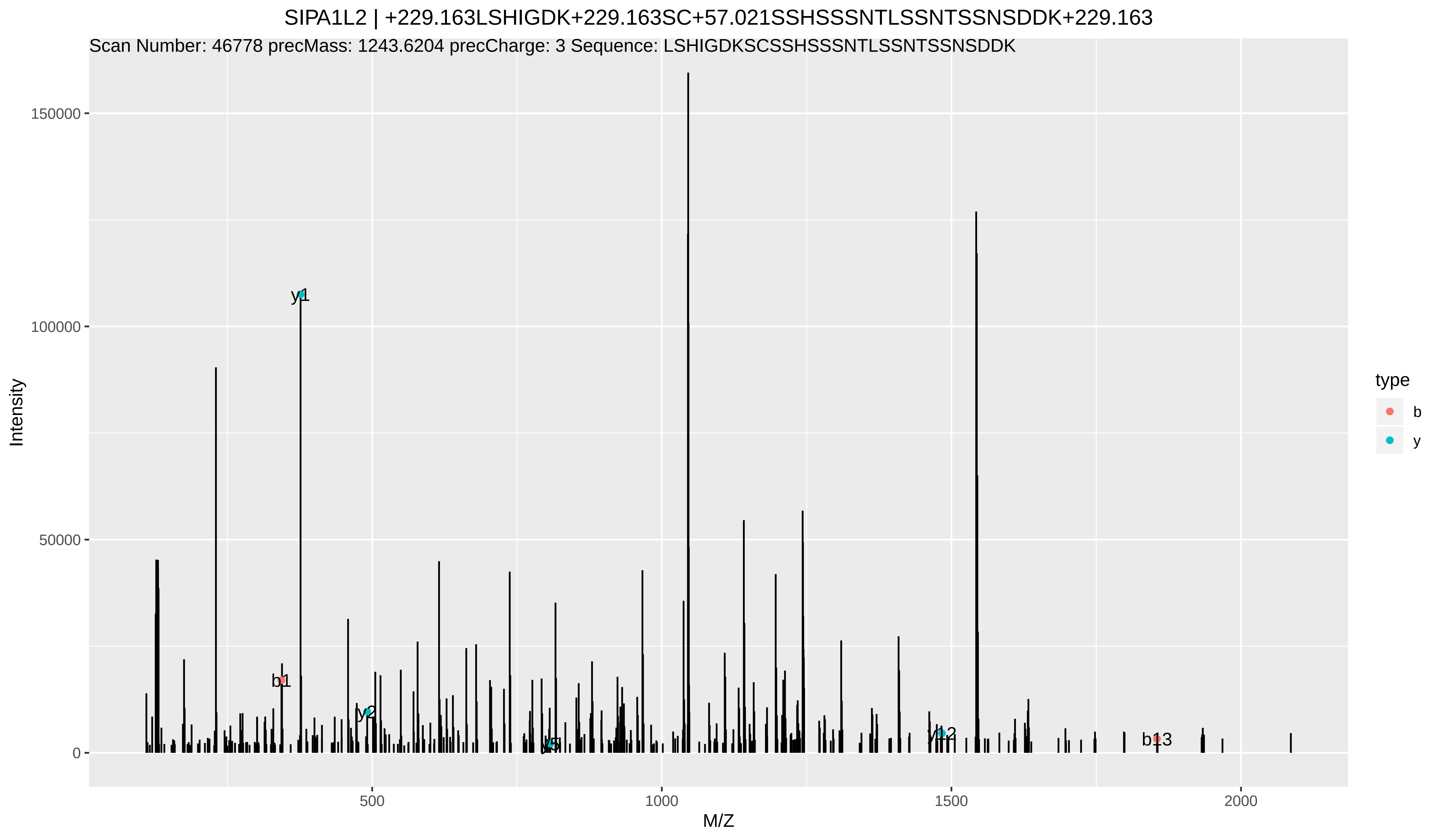
SIPA1L2 | +229.163LSHIGDK+229.163SC+57.021SSHSSSNTLSSNTSSNSDDK+229.163
Scan Number: 46778 precMass: 1243.6204 precCharge: 3 Sequence: LSHIGDKSCSSHSSSNTLSSNTSSNSDDK
Intensity
150000
100000
50000
0
500
1000
1500
2000
M/Z
y1
b1
y2
y5
y12
b13
type
b
y

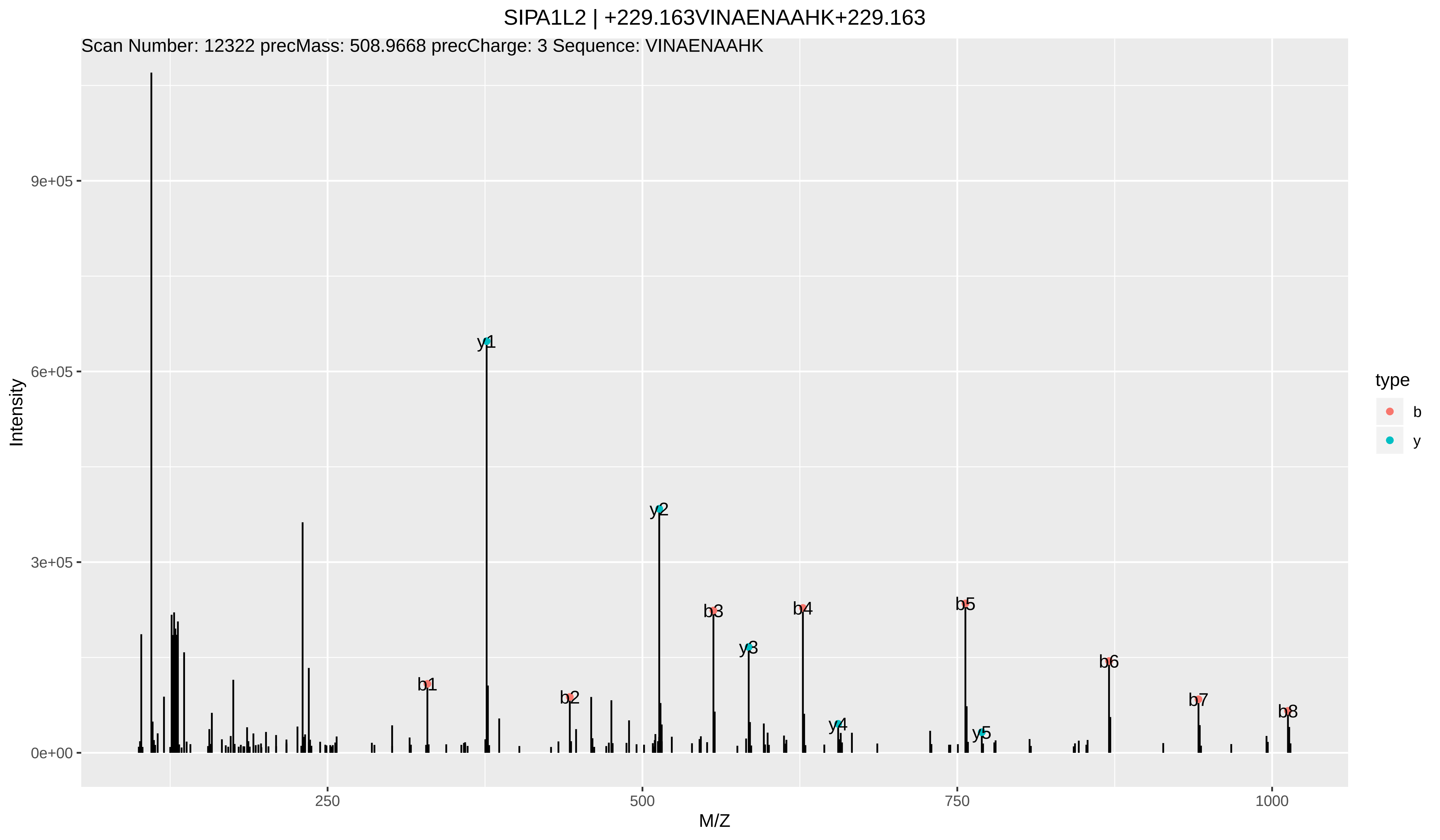
SIPA1L2 | +229.163VINAENAAHK+229.163
Scan Number: 12322 precMass: 508.9668 precCharge: 3 Sequence: VINAENAAHK
Intensity
9e+05
6e+05
3e+05
0e+00
M/Z
250
500
750
1000
y1
y2
y3
y4
y5
b1
b2
b3
b4
b5
b6
b7
b8
type
b
y

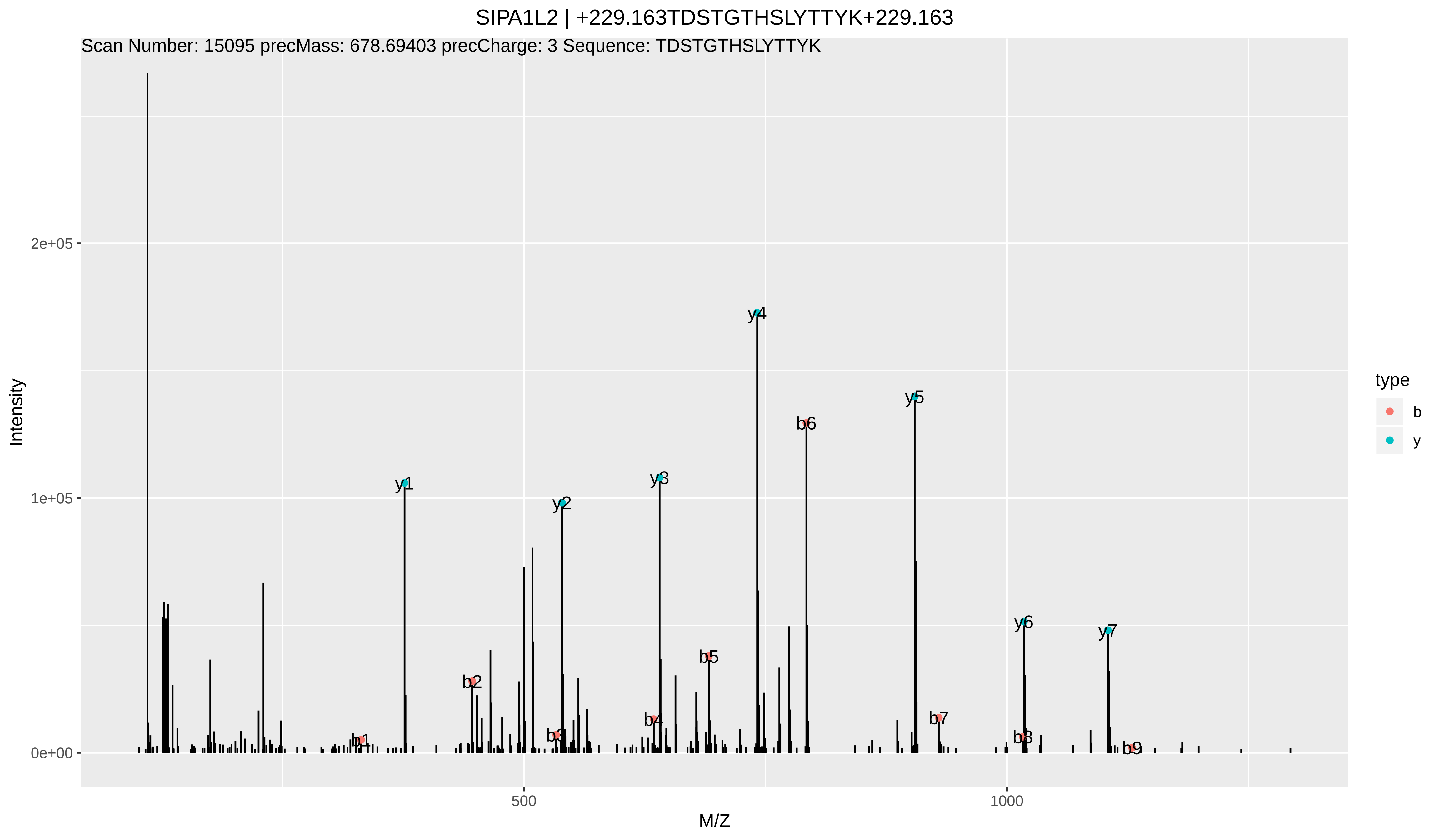

SIPA1L2 | +229.163TDSTGTHSLYTTYK+229.163
Scan Number: 15095 precMass: 678.69403 precCharge: 3 Sequence: TDSTGTHSLYTTYK
Intensity
2e+05
1e+05
0e+00
M/Z
500
1000
type
b
y
y1
y2
y3
y4
y5
y6
y7
b1
b2
b3
b4
b5
b6
b7
b8
b9

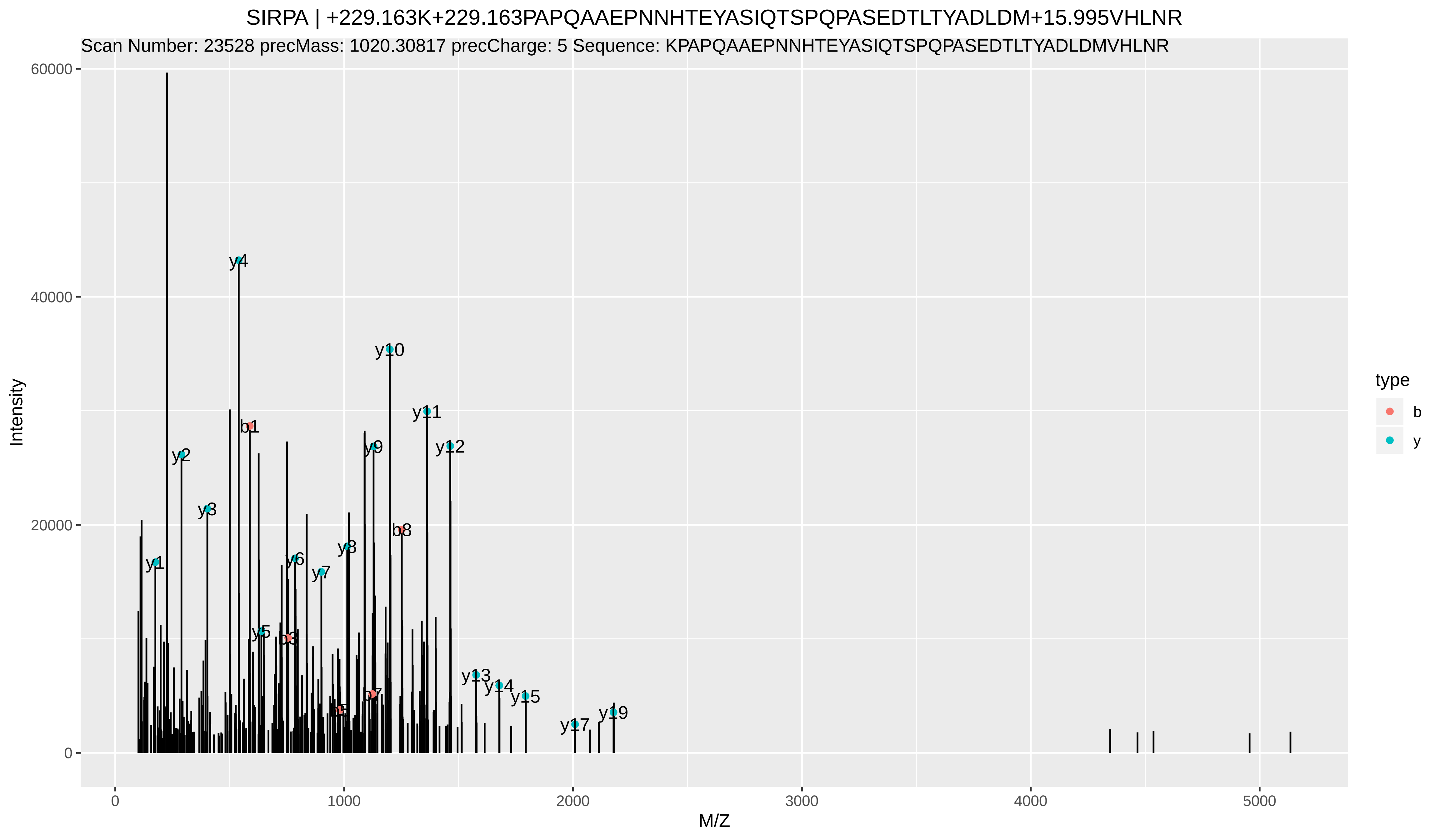

SIRPA | +229.163K+229.163PAPQAAEPNNHTEYASIQTSPQPASEDTLTYADLDM+15.995VHLNR
Scan Number: 23528 precMass: 1020.30817 precCharge: 5 Sequence: KPAPQAAEPNNHTEYASIQTSPQPASEDTLTYADLDMVHLNR
Intensity
60000
40000
20000
0
M/Z
0
1000
2000
3000
4000
5000
y1
y2
y3
y4
y5
y6
y7
y8
y9
y10
y11
y12
y13
y14
y15
y17
y19
b1
b8
b7
type
b
y

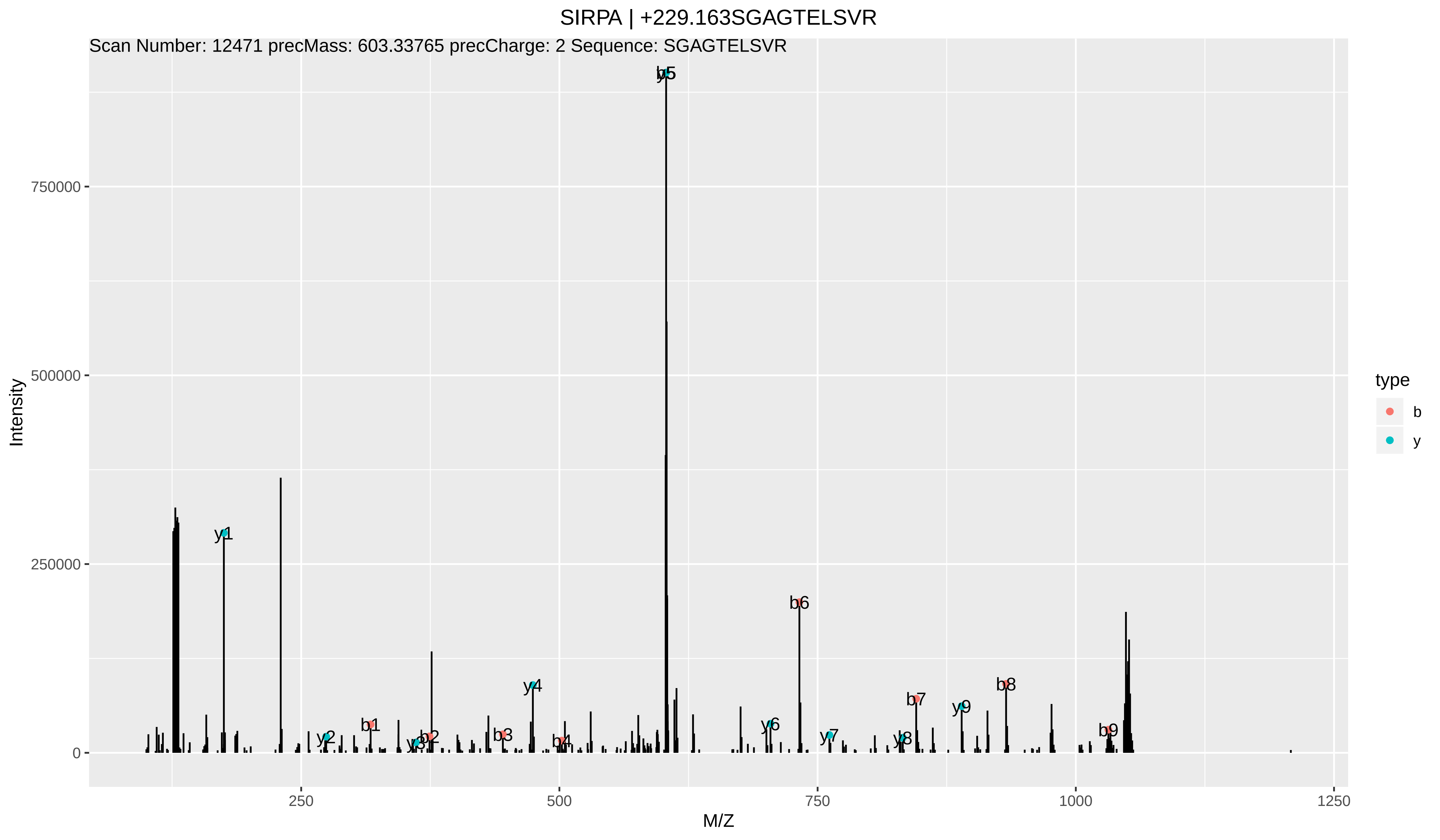

SIRPA | +229.163SGAGTELSVR
Scan Number: 12471 precMass: 603.33765 precCharge: 2 Sequence: SGAGTELSVR
Intensity
750000
500000
250000
0
M/Z
250
500
750
1000
1250
y1
y2
b1
y3
b2
b3
y4
b4
y5
b6
y6
y7
b7
y8
y9
b8
b9
type
b
y

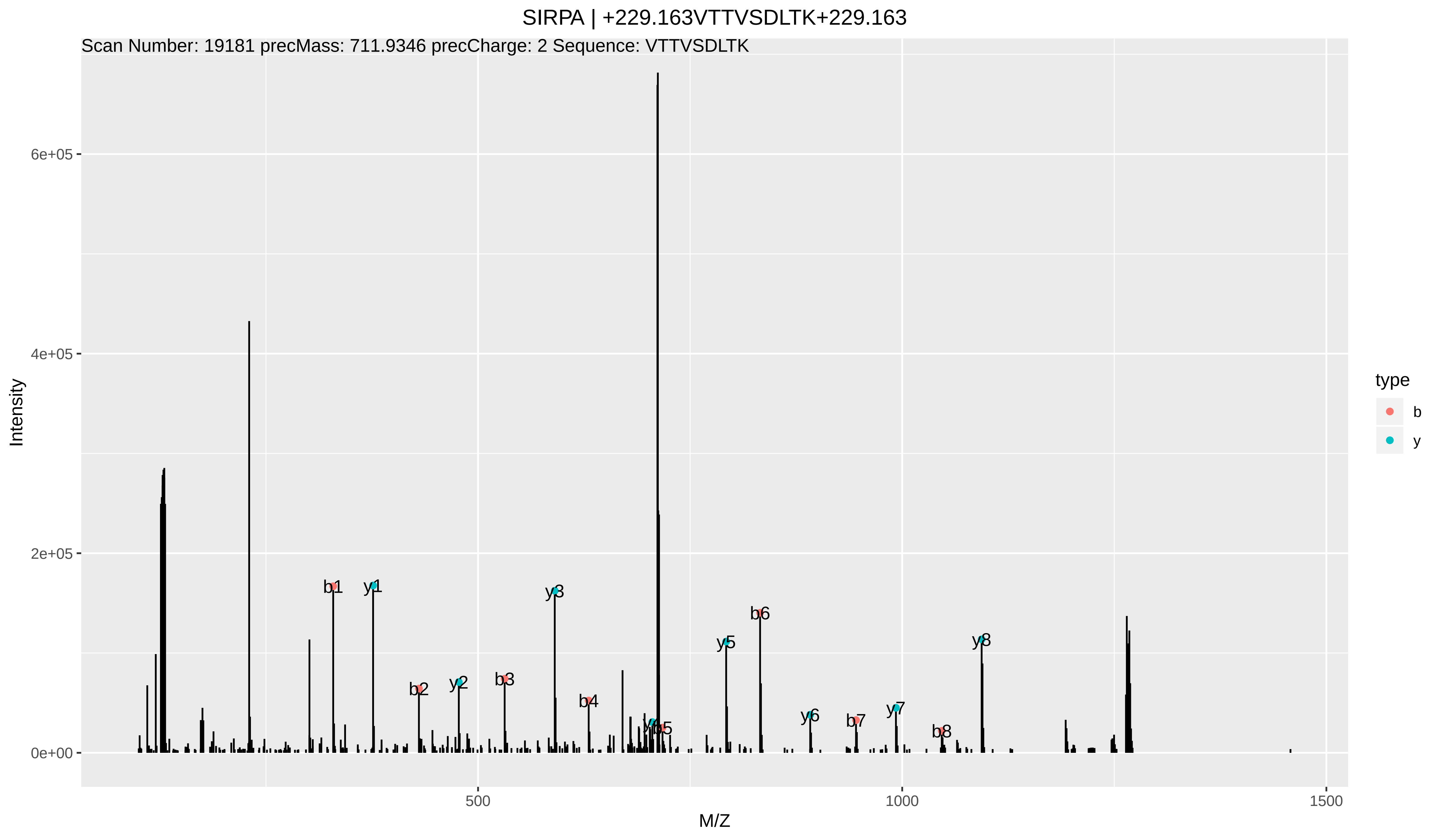

SIRPA | +229.163VTTVSDLTK+229.163
Scan Number: 19181 precMass: 711.9346 precCharge: 2 Sequence: VTTVSDLTK
Intensity
6e+05
4e+05
2e+05
0e+00
M/Z
500
1000
1500
b1
y1
b2
y2
b3
y3
b4
b5
y5
b6
y6
b7
y7
b8
y8
type
b
y

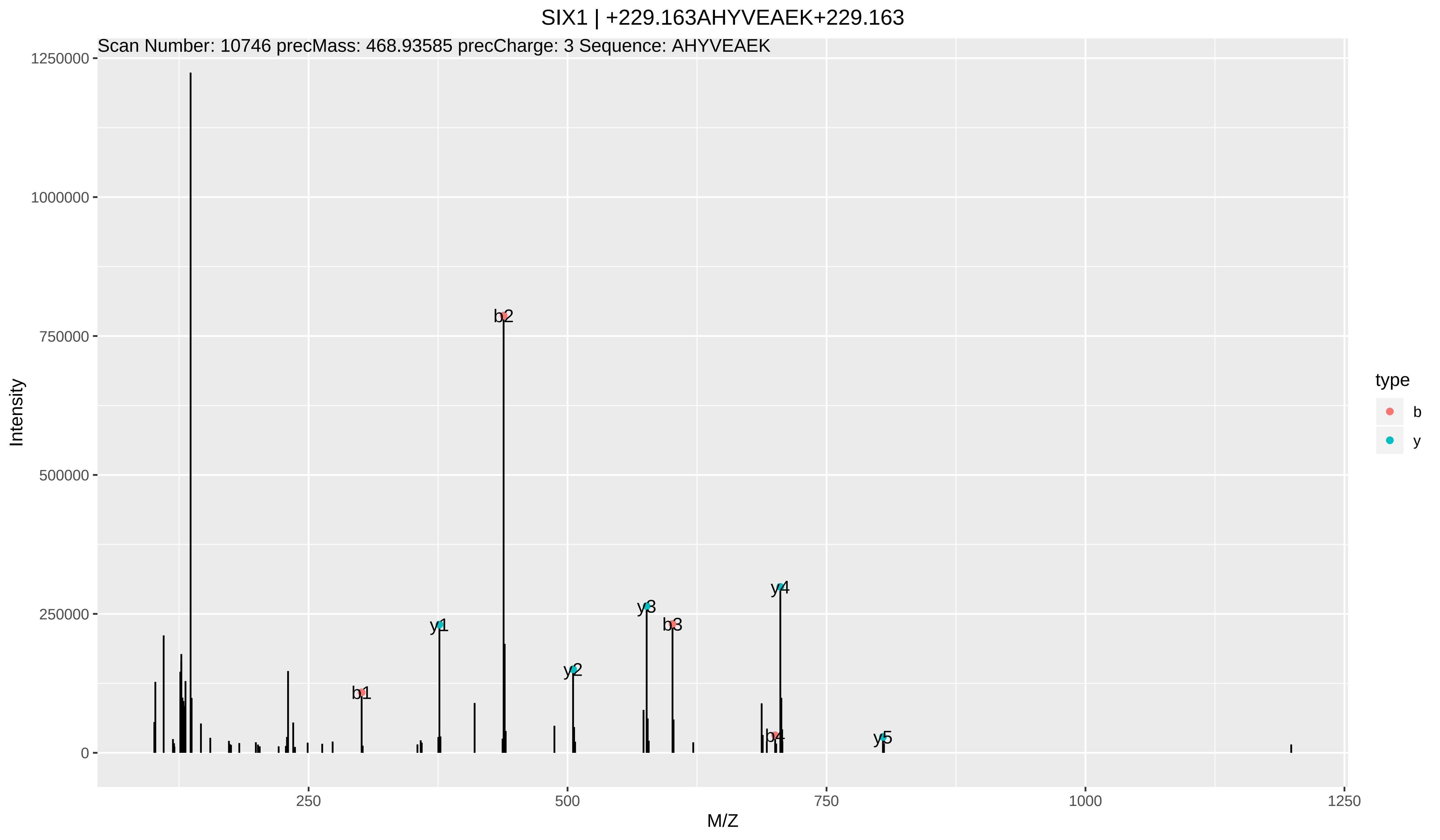

SIX1 | +229.163AHYVEAEK+229.163
Scan Number: 10746 precMass: 468.93585 precCharge: 3 Sequence: AHYVEAEK
Intensity
M/Z
0
250000
500000
750000
1000000
1250000
250
500
750
1000
1250
b1
y1
b2
y2
y3
b3
y4
b4
y5
type
b
y

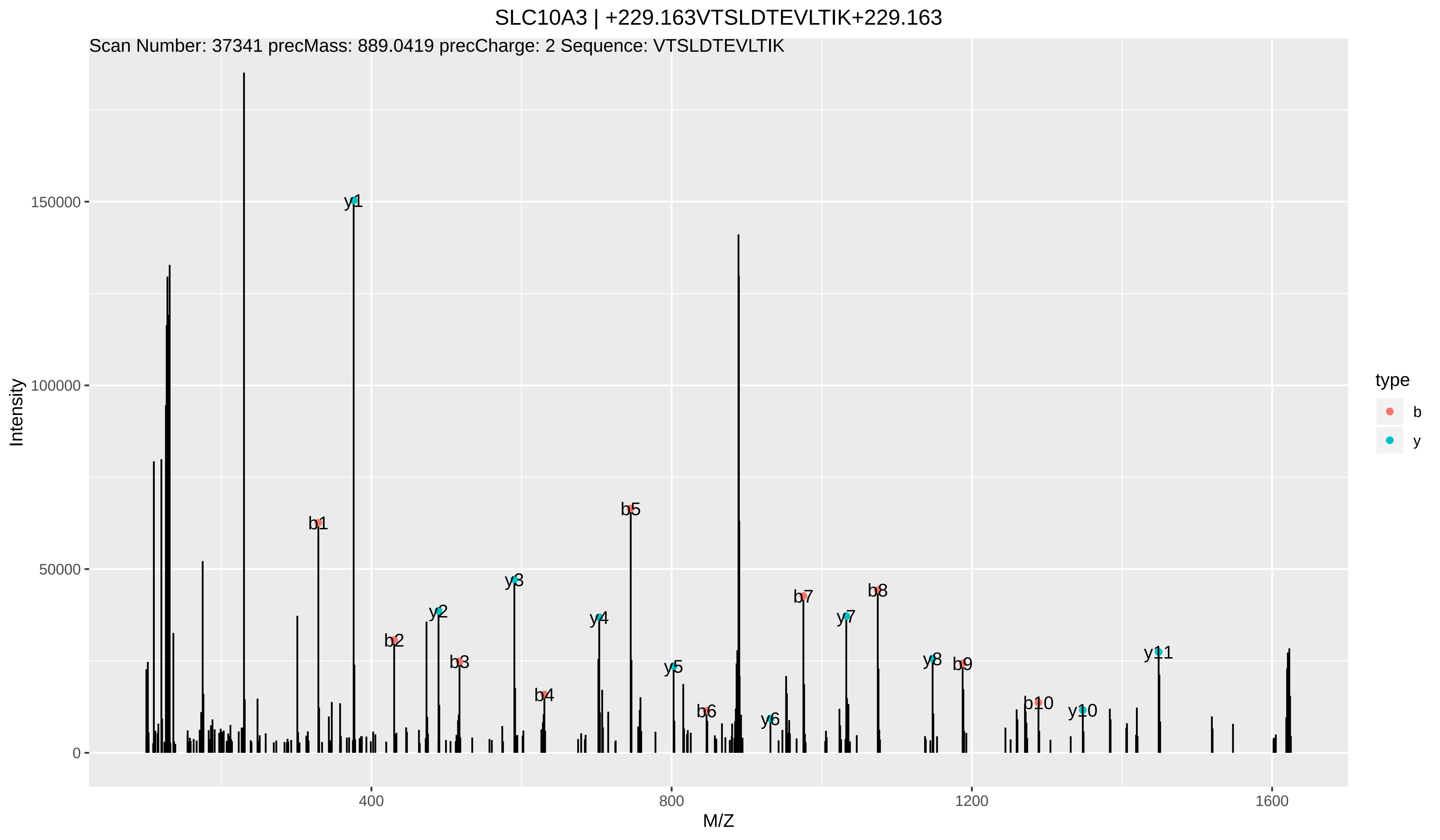
SLC10A3 | +229.163VTSLDTEVLTIK+229.163
Scan Number: 37341 precMass: 889.0419 precCharge: 2 Sequence: VTSLDTEVLTIK
Intensity
M/Z
type
b
y
y1
b1
b2
y2
b3
y3
b4
y4
b5
y5
b6
y6
b7
y7
b8
y8
b9
b10
y10
y11
0
50000
100000
150000
400
800
1200
1600

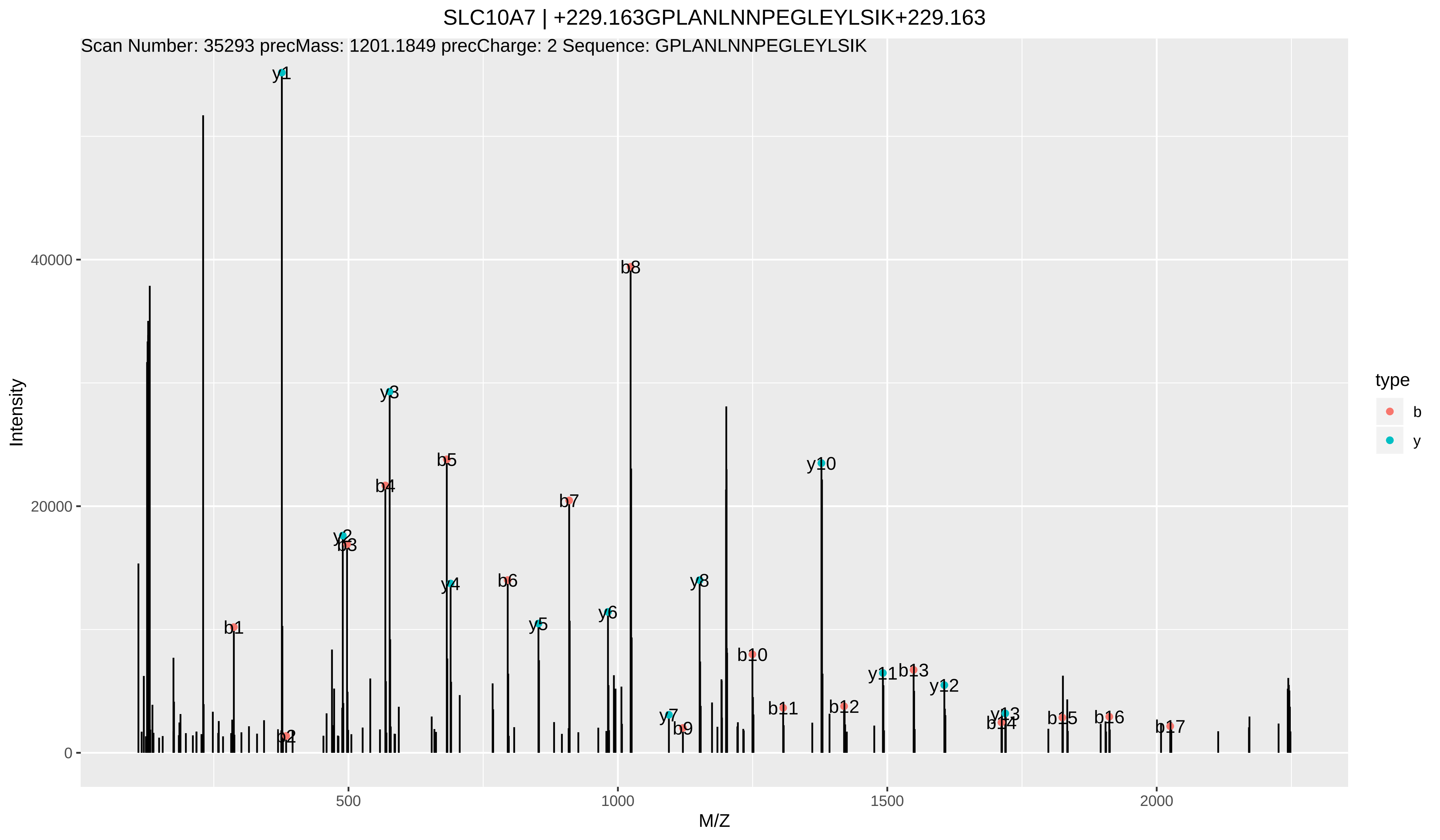

SLC10A7 | +229.163GPLANLNNPEGLEYLSIK+229.163
Scan Number: 35293 precMass: 1201.1849 precCharge: 2 Sequence: GPLANLNNPEGLEYLSIK
Intensity
M/Z
type
b
y

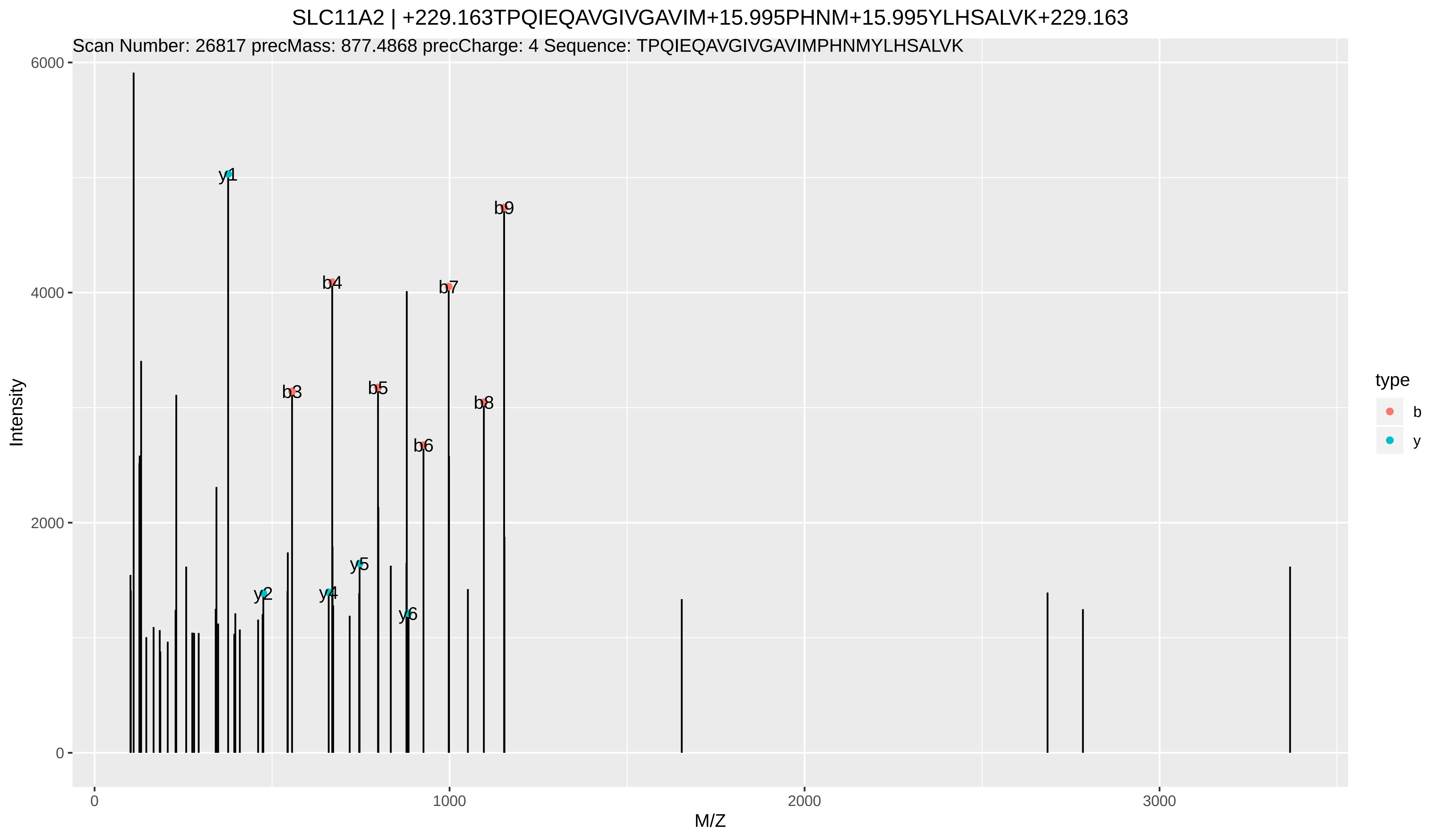

SLC11A2 | +229.163TPQIEQAVGIVGAVIM+15.995PHNM+15.995YLHSALVK+229.163
Scan Number: 26817 precMass: 877.4868 precCharge: 4 Sequence: TPQIEQAVGIVGAVIMPHNMYLHSALVK
Intensity
6000
4000
2000
0
M/Z
0
1000
2000
3000
y1
b9
b4
b7
b3
b5
b8
b6
y2
y4
y5
y6
type
b
y

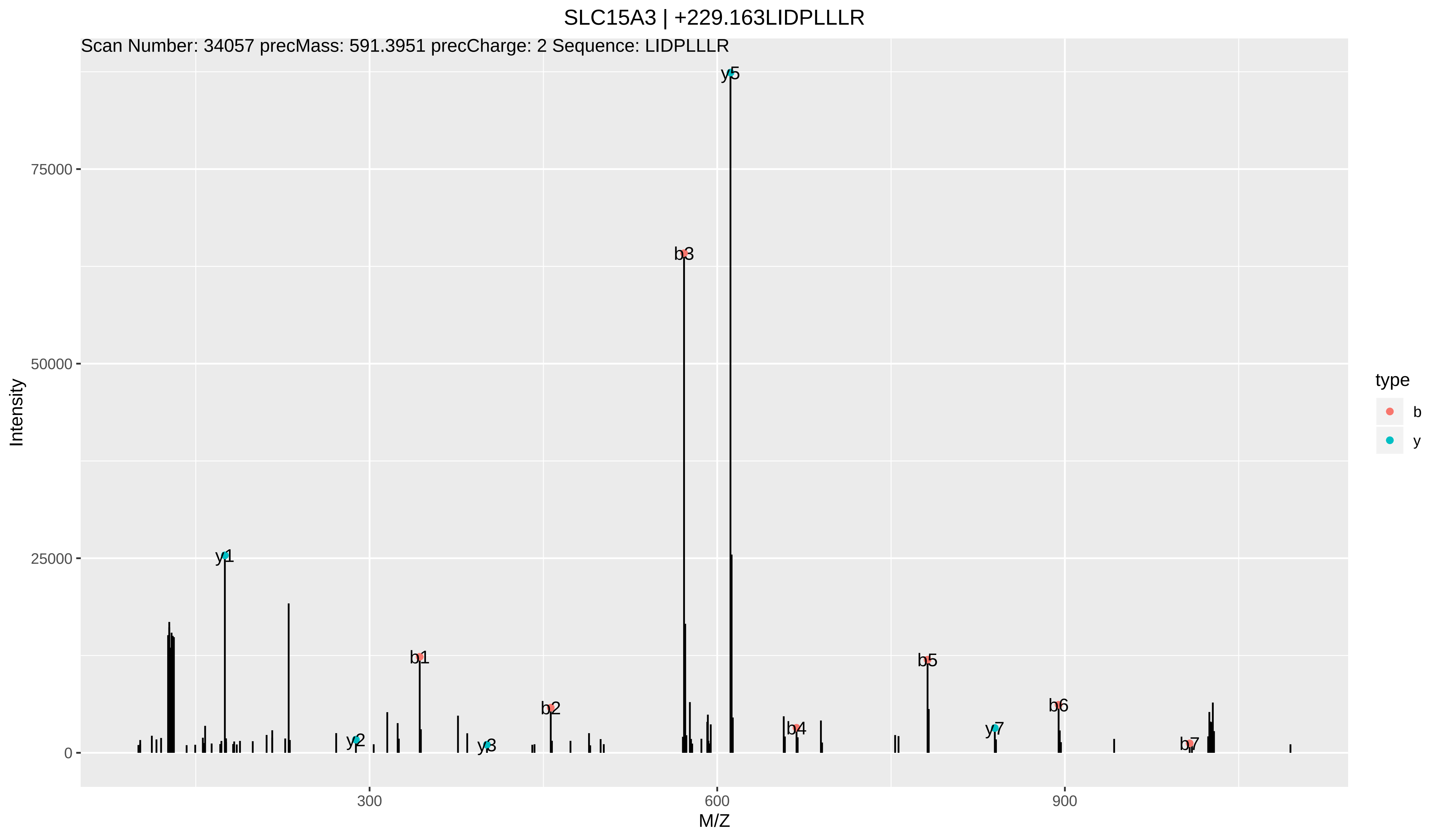
SLC15A3 | +229.163LIDPLLLR
Scan Number: 34057 precMass: 591.3951 precCharge: 2 Sequence: LIDPLLLR
Intensity
M/Z
75000
50000
25000
0
300
600
900
y5
b3
y1
b1
b5
b2
b6
y7
b4
y2
y3
b7
type
b
y

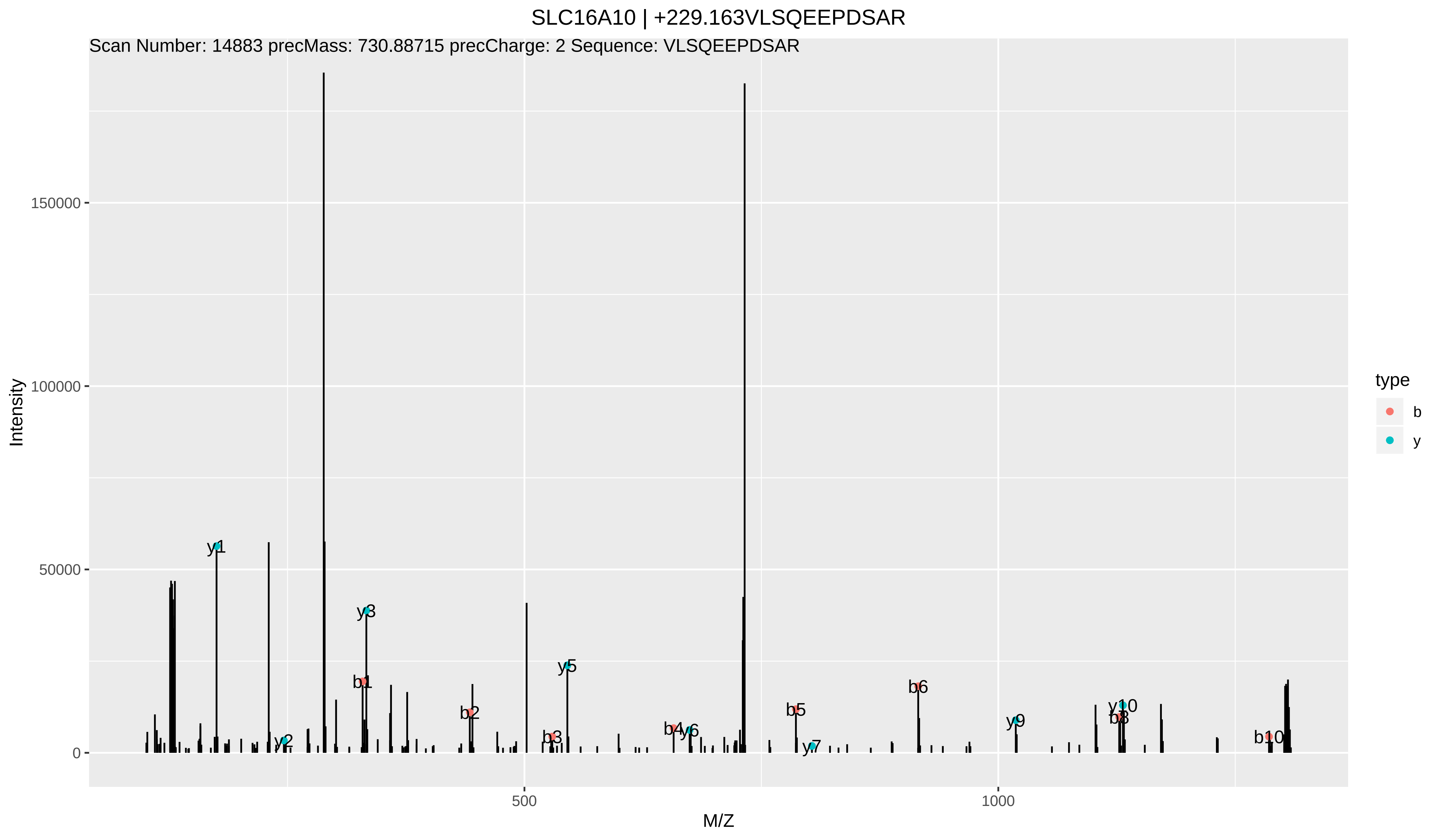

SLC16A10 | +229.163VLSQEEPDSAR
Scan Number: 14883 precMass: 730.88715 precCharge: 2 Sequence: VLSQEEPDSAR
Intensity
150000
100000
50000
0
500
1000
M/Z
y1
y2
y3
b1
b2
b3
y5
b4
y6
b5
y7
b6
y9
y10
b8
b10
type
b
y

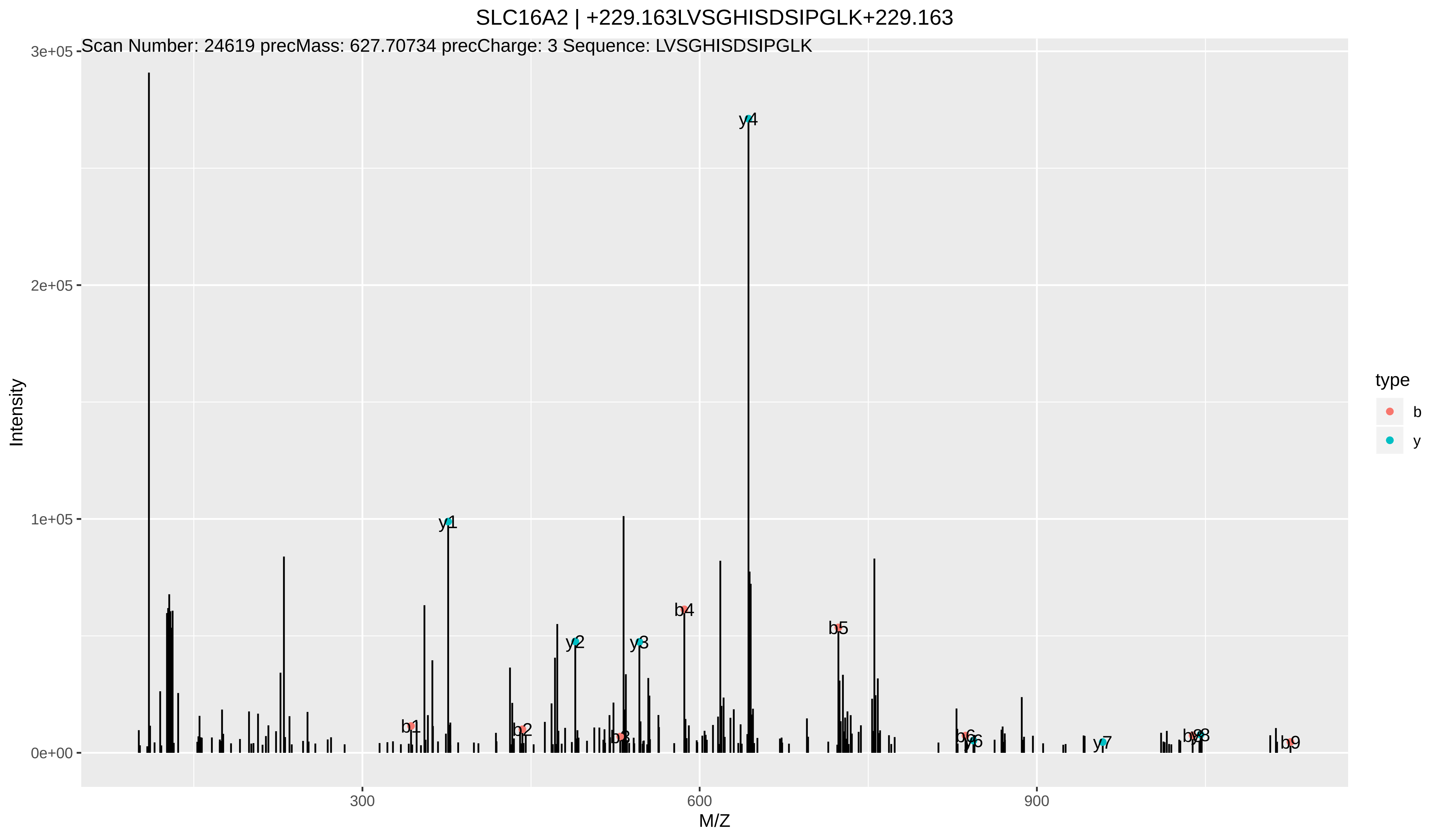
SLC16A2 | +229.163LVSGHISDSIPGLK+229.163
Scan Number: 24619 precMass: 627.70734 precCharge: 3 Sequence: LVSGHISDSIPGLK
Intensity
3e+05
2e+05
1e+05
0e+00
M/Z
300
600
900
type
b
y
y4
y1
b4
b5
y2
y3
b1
b2
b3
b6
y6
y7
b9

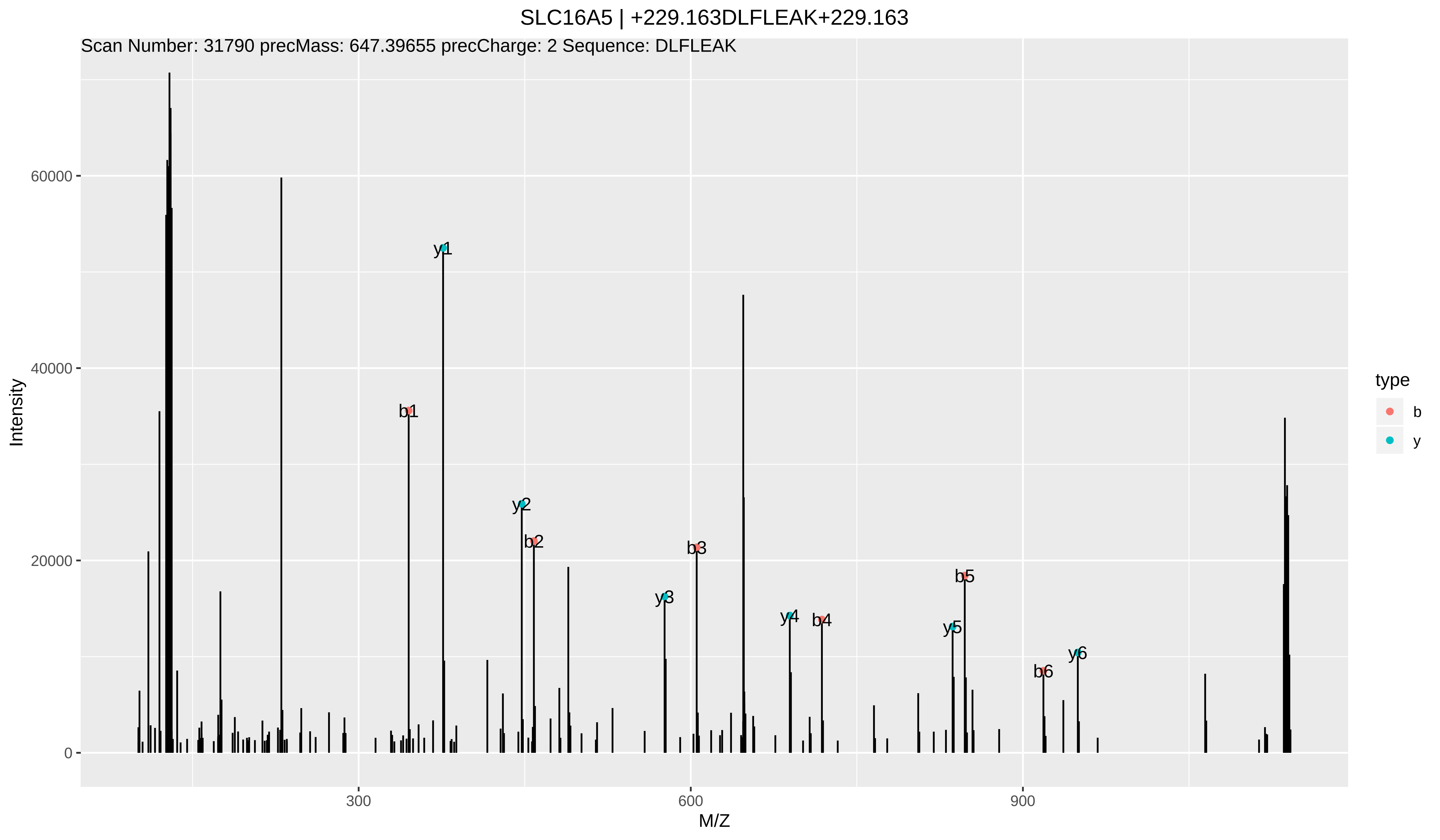
SLC16A5 | +229.163DLFLEAK+229.163
Scan Number: 31790 precMass: 647.39655 precCharge: 2 Sequence: DLFLEAK
Intensity
M/Z
type
b
y
b1
y1
y2
b2
y3
b3
y4
b4
y5
b5
b6
y6
0
20000
40000
60000
300
600
900

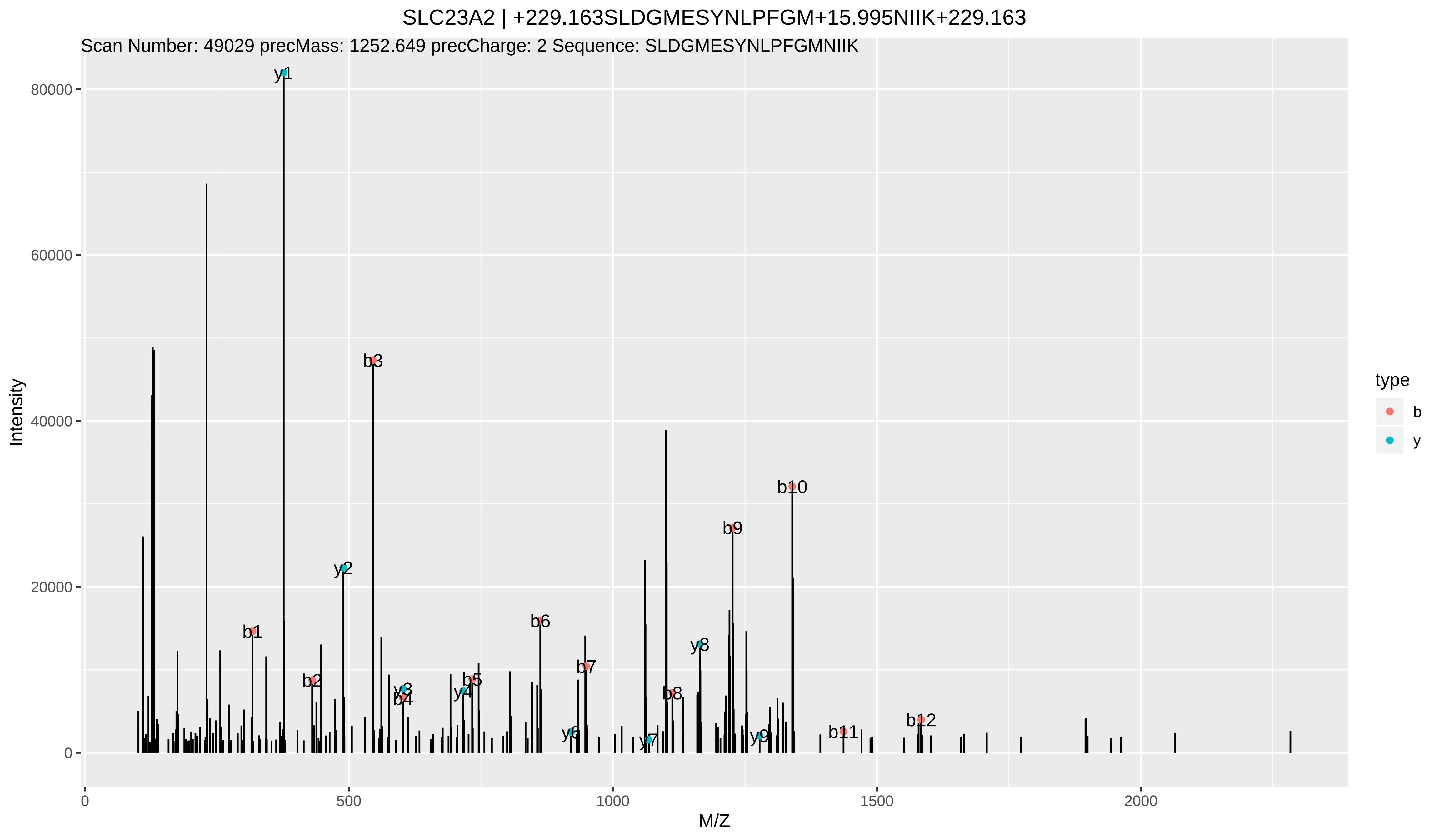

SLC23A2 | +229.163SLDGMESYNLPFGM+15.995NIIK+229.163
Scan Number: 49029 precMass: 1252.649 precCharge: 2 Sequence: SLDGMESYNLPFGMNIIK
Intensity
M/Z
type
b
y
y1
b3
b10
b9
y2
b6
b1
y8
b7
b2
y3
b5
b4
y4
b8
b12
b11
y6
y9
y7
80000
60000
40000
20000
0
0
500
1000
1500
2000

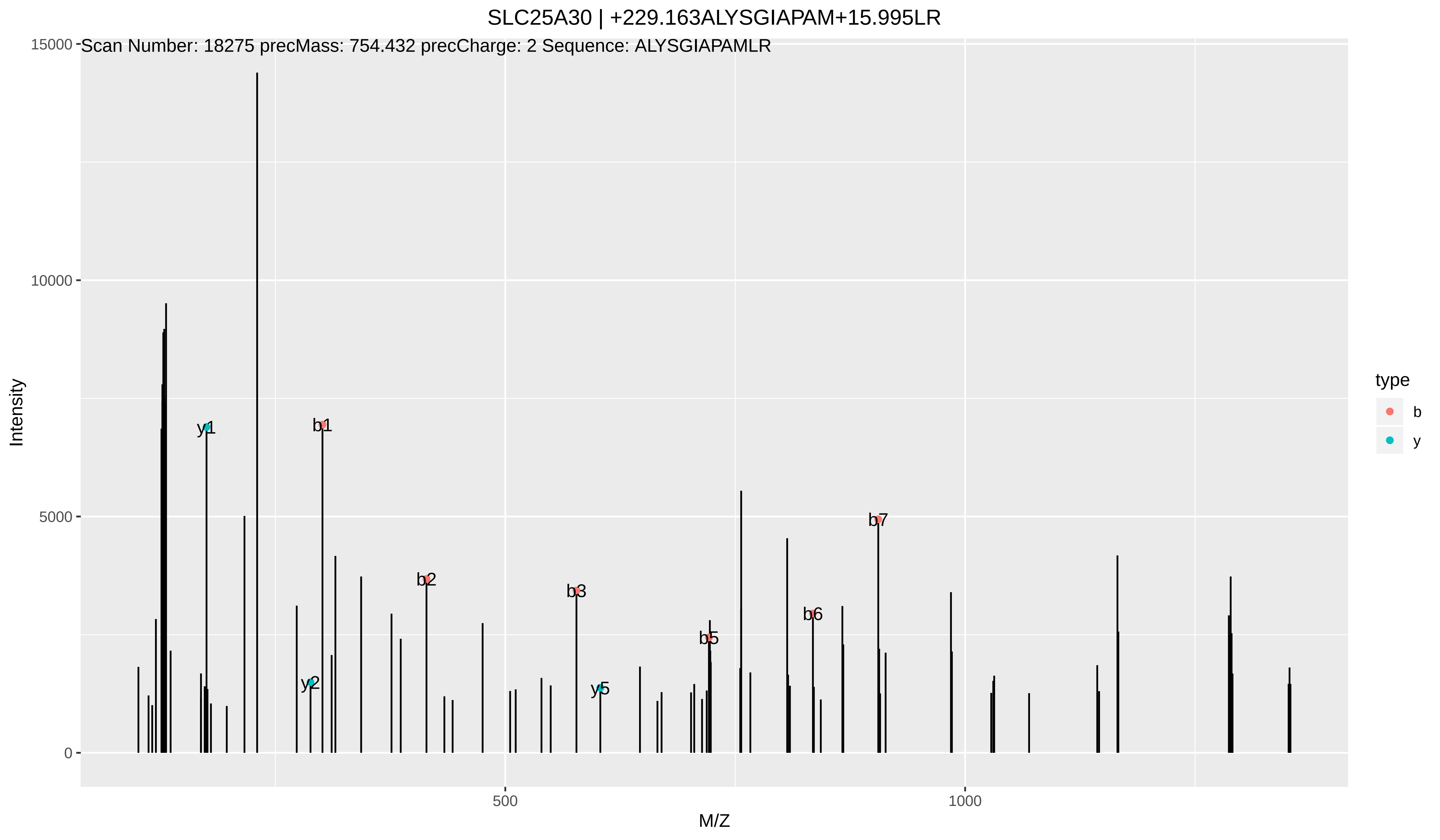
SLC25A30 | +229.163ALYSGIAPAM+15.995LR
Scan Number: 18275 precMass: 754.432 precCharge: 2 Sequence: ALYSGIAPAMLR
Intensity
15000
10000
5000
0
M/Z
500
1000
y1
b1
y2
b2
b3
y5
b5
b6
b7
type
b
y

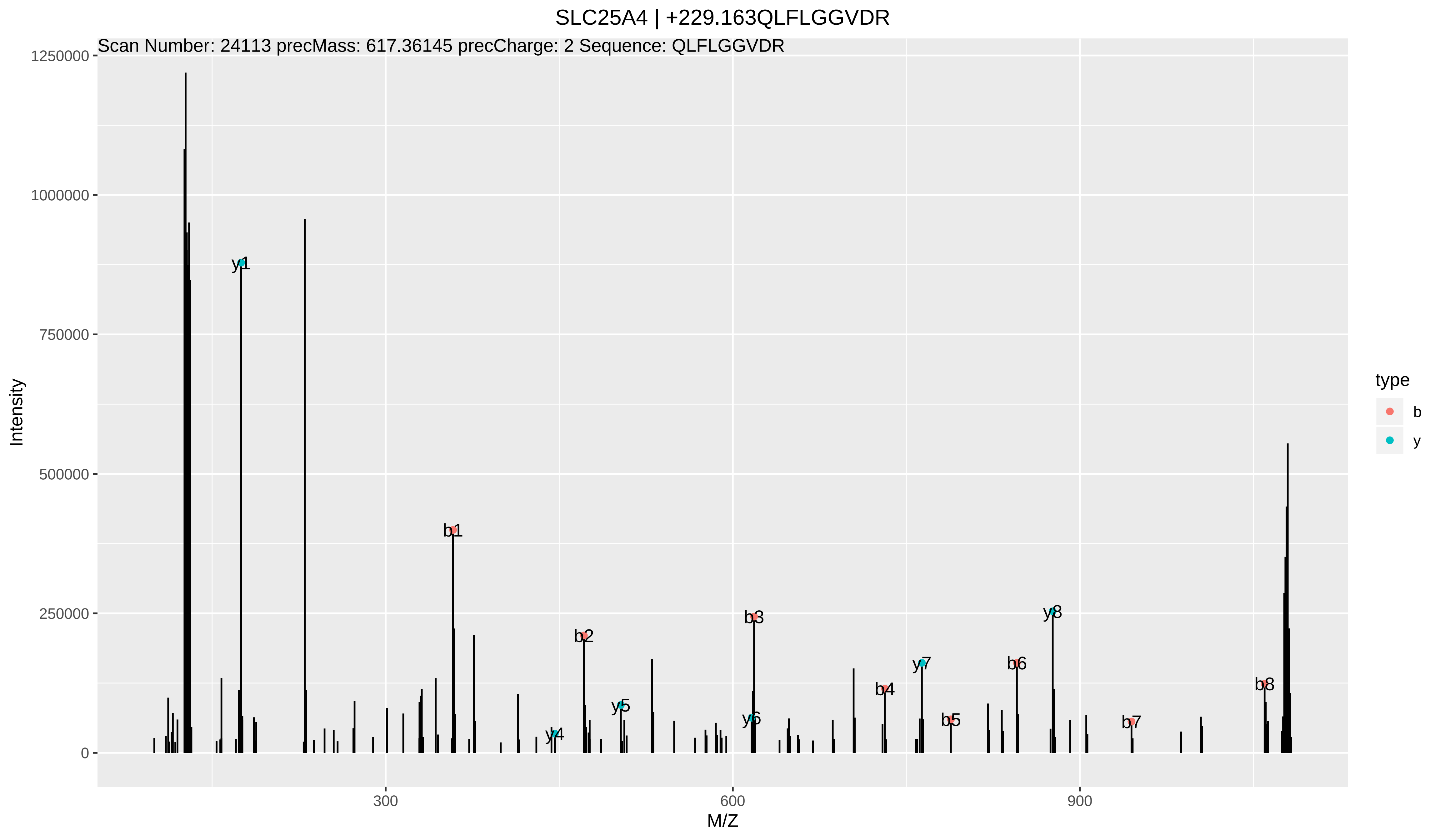

SLC25A4 | +229.163QLFLGGVDR
Scan Number: 24113 precMass: 617.36145 precCharge: 2 Sequence: QLFLGGVDR
Intensity
1250000
1000000
750000
500000
250000
0
M/Z
300
600
900
y1
b1
b2
y4
y5
y6
b3
b4
y7
b5
b6
y8
b7
b8
type
b
y

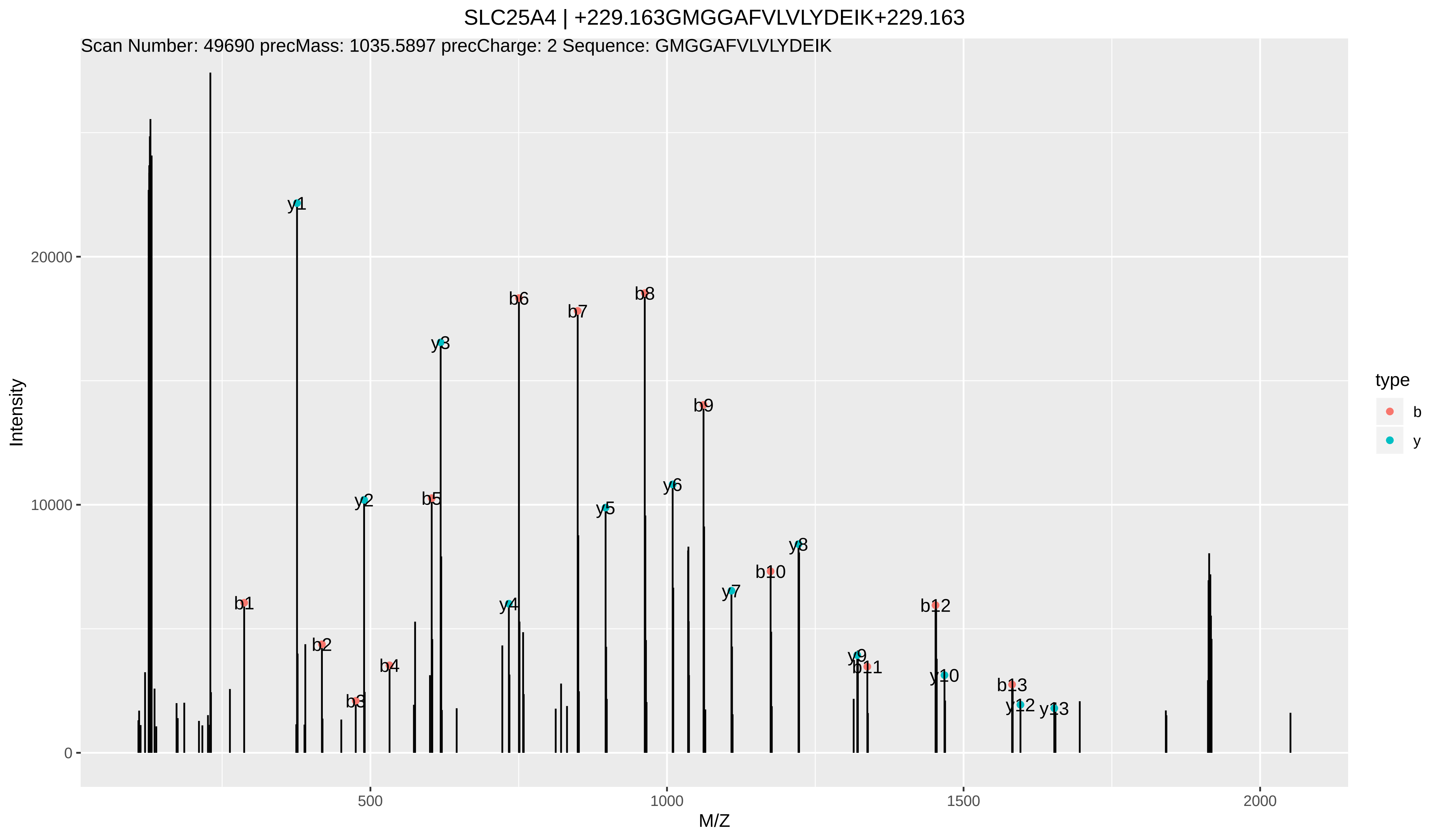
SLC25A4 | +229.163GMGGAFVLVLYDEIK+229.163
Scan Number: 49690 precMass: 1035.5897 precCharge: 2 Sequence: GMGGAFVLVLYDEIK
Intensity
M/Z
0
10000
20000
500
1000
1500
2000
type
b
y
y1
y2
y3
y4
y5
y6
y7
y8
y9
y10
y12
y13
b1
b2
b3
b4
b5
b6
b7
b8
b9
b10
b11
b12
b13

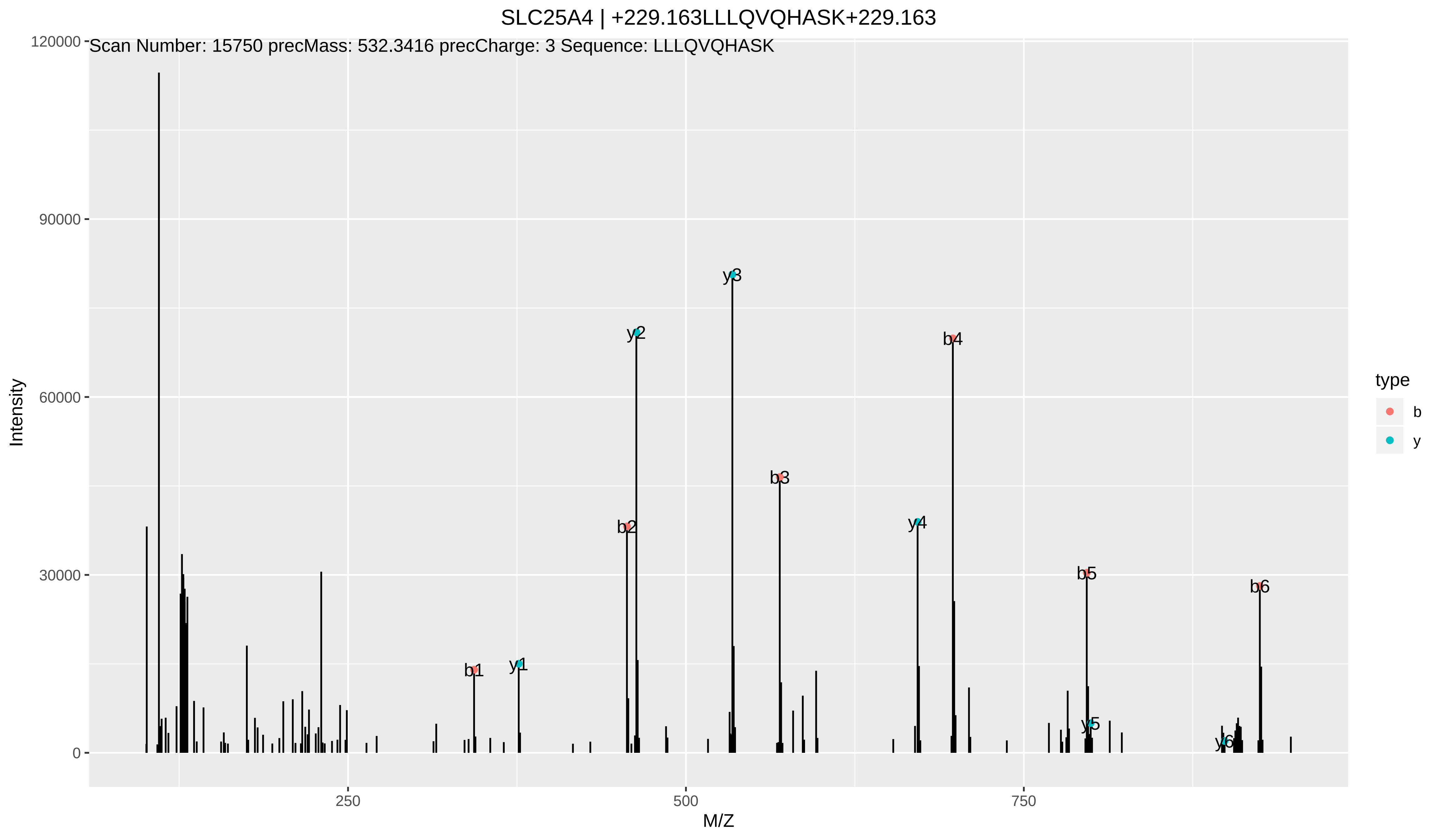

SLC25A4 | +229.163LLLQVQHASK+229.163
Scan Number: 15750 precMass: 532.3416 precCharge: 3 Sequence: LLLQVQHASK
Intensity
M/Z
120000
90000
60000
30000
0
250
500
750
y3
y2
b4
b3
b2
y4
b5
b6
b1
y1
y5
y6
type
b
y

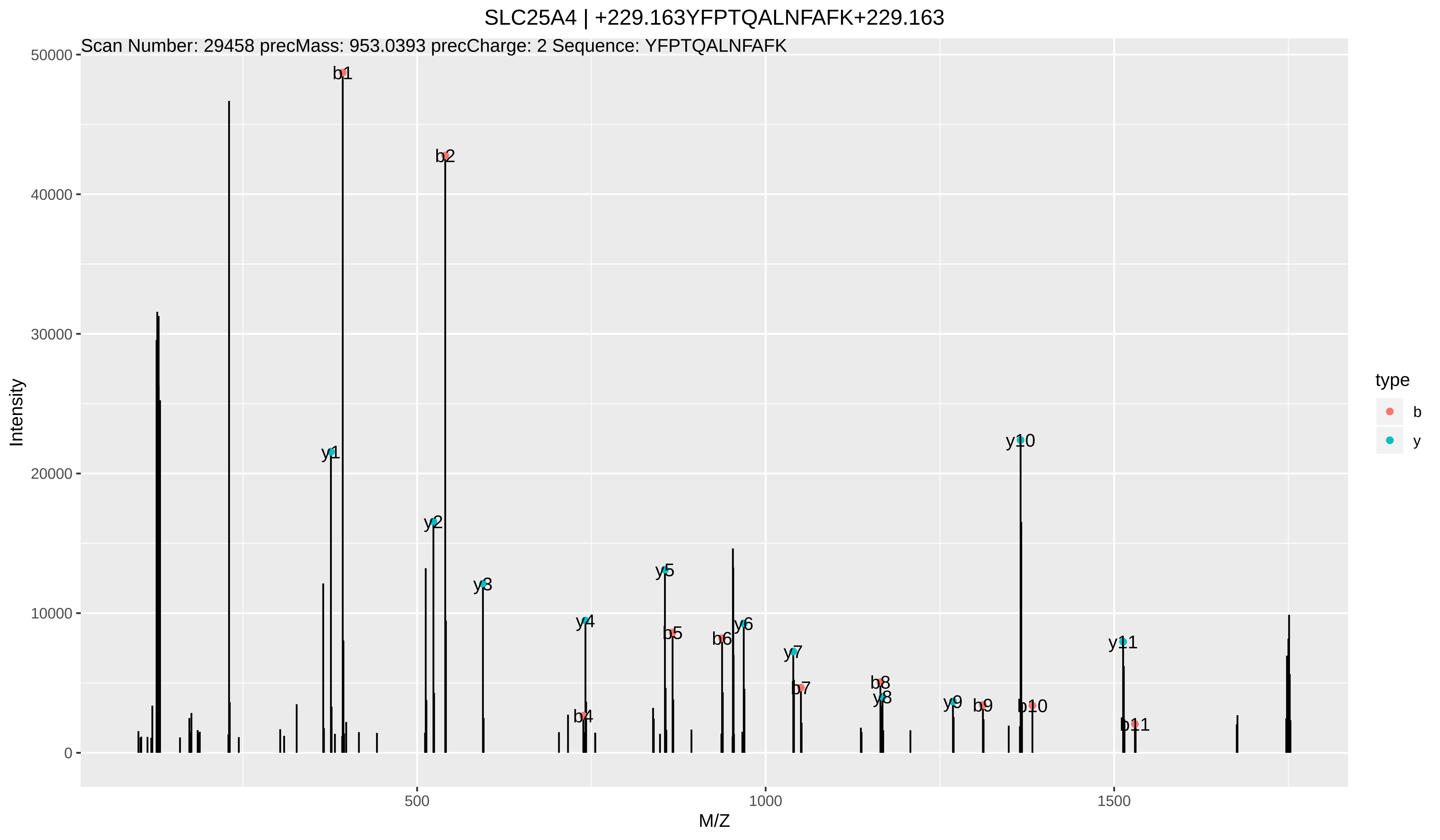
SLC25A4 | +229.163YFPTQALNFAFK+229.163
Scan Number: 29458 precMass: 953.0393 precCharge: 2 Sequence: YFPTQALNFAFK
Intensity
M/Z
50000
40000
30000
20000
10000
0
500
1000
1500
b1
b2
y1
y2
y3
y4
b4
y5
b5
b6
y6
y7
b7
b8
y8
y9
b9
y10
b10
y11
b11
type
b
y

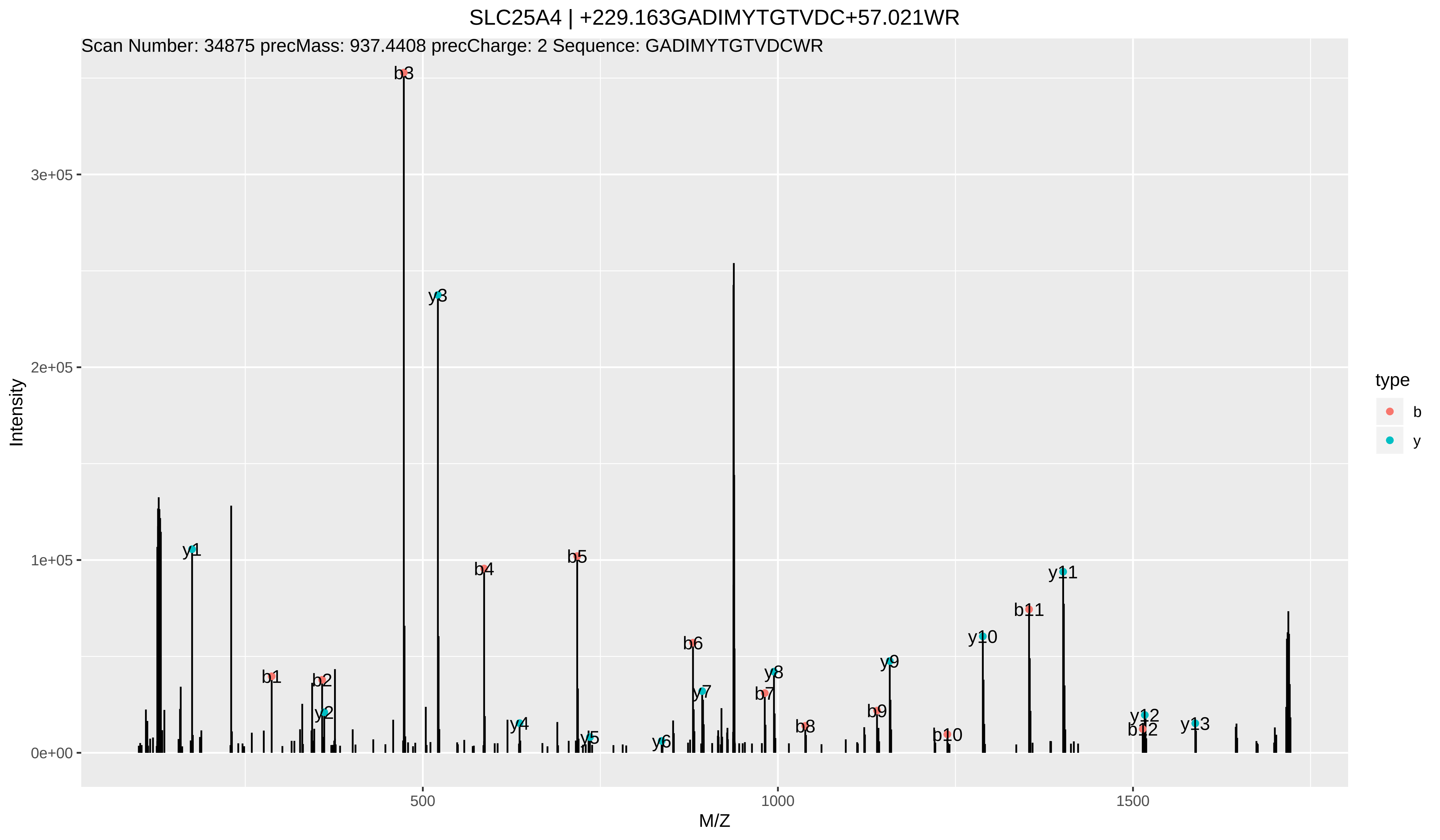

SLC25A4 | +229.163GADIMYTGTVDC+57.021WR
Scan Number: 34875 precMass: 937.4408 precCharge: 2 Sequence: GADIMYTGTVDCWR
Intensity
M/Z
0e+00
1e+05
2e+05
3e+05
500
1000
1500
b1
b2
b3
b4
b5
b6
b7
b8
b9
b10
b11
b12
y1
y2
y3
y4
y5
y6
y7
y8
y9
y10
y11
y12
y13
type
b
y

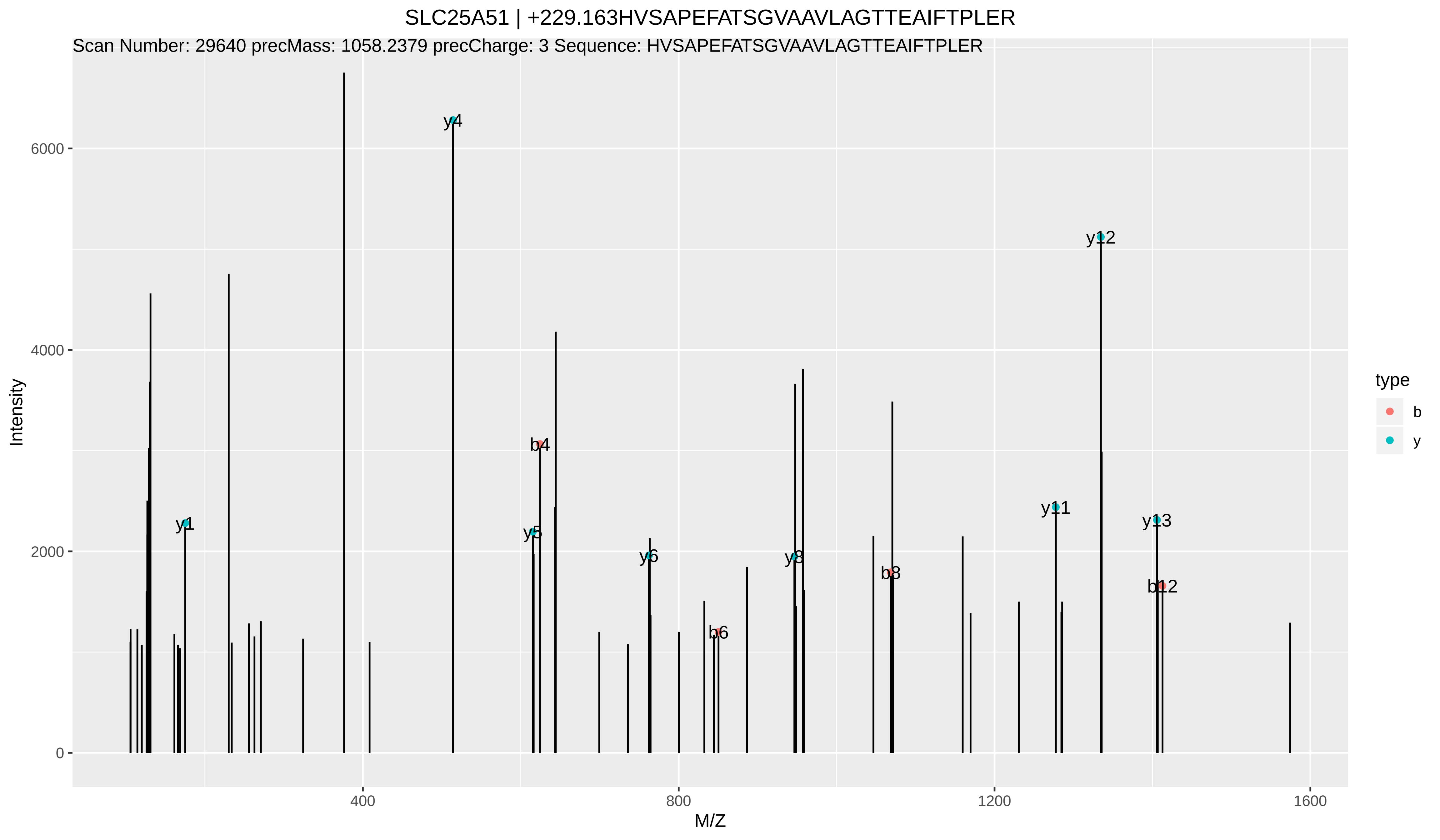

SLC25A51 | +229.163HVSAPEFATSGVAAVLAGTTEAIFTPLER
Scan Number: 29640 precMass: 1058.2379 precCharge: 3 Sequence: HVSAPEFATSGVAAVLAGTTEAIFTPLER
Intensity
M/Z
type
b
y
y1
y4
y5
b4
y6
b6
y8
b8
y11
y12
y13
b12
0
2000
4000
6000
400
800
1200
1600

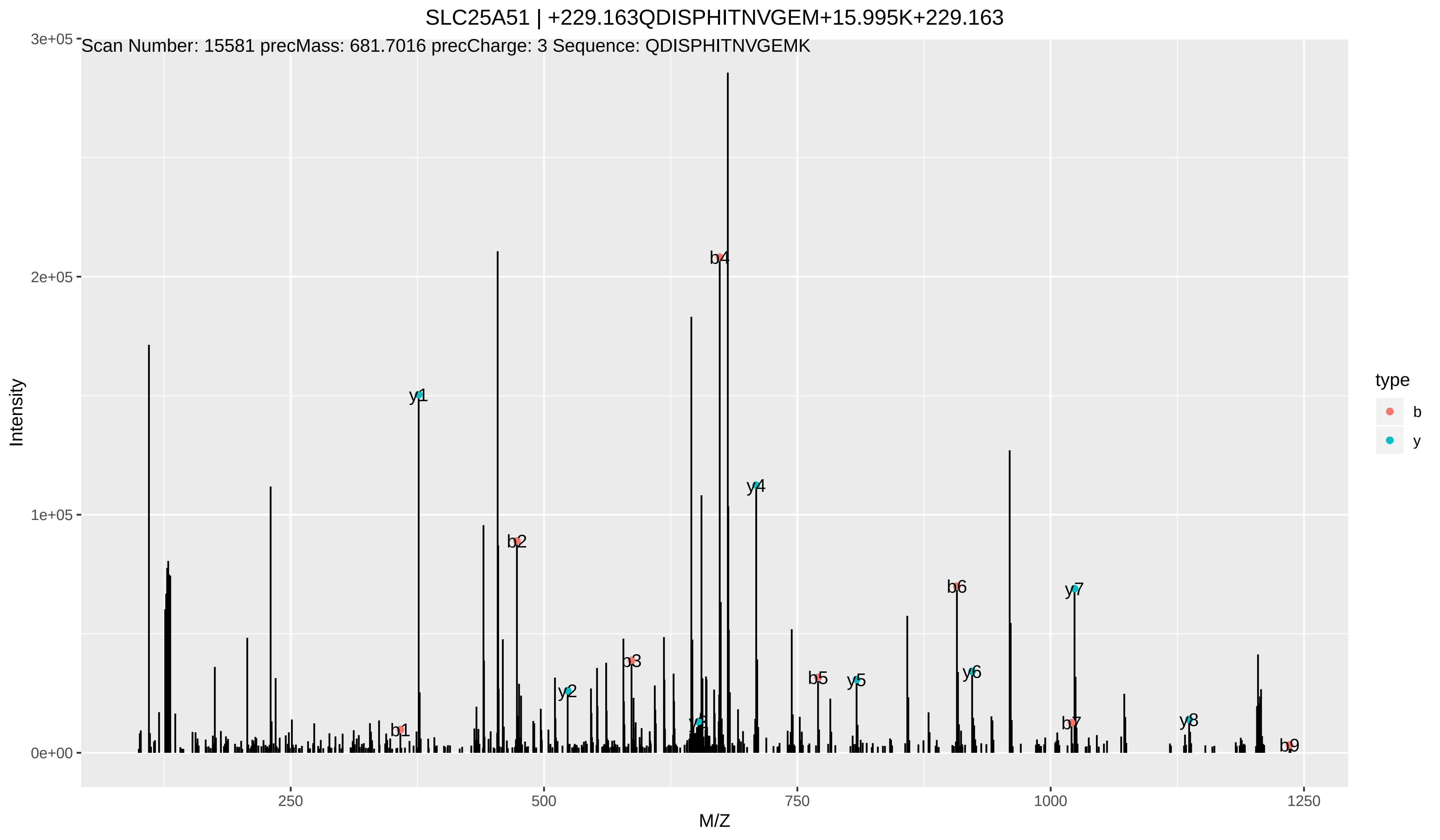

SLC25A51 | +229.163QDISPHITNVGEM+15.995K+229.163
Scan Number: 15581 precMass: 681.7016 precCharge: 3 Sequence: QDISPHITNVGEMK
Intensity
M/Z
3e+05
2e+05
1e+05
0e+00
250
500
750
1000
1250
b1
b2
b3
b4
b5
b6
b7
b9
y1
y2
y3
y4
y5
y6
y7
y8
type
b
y

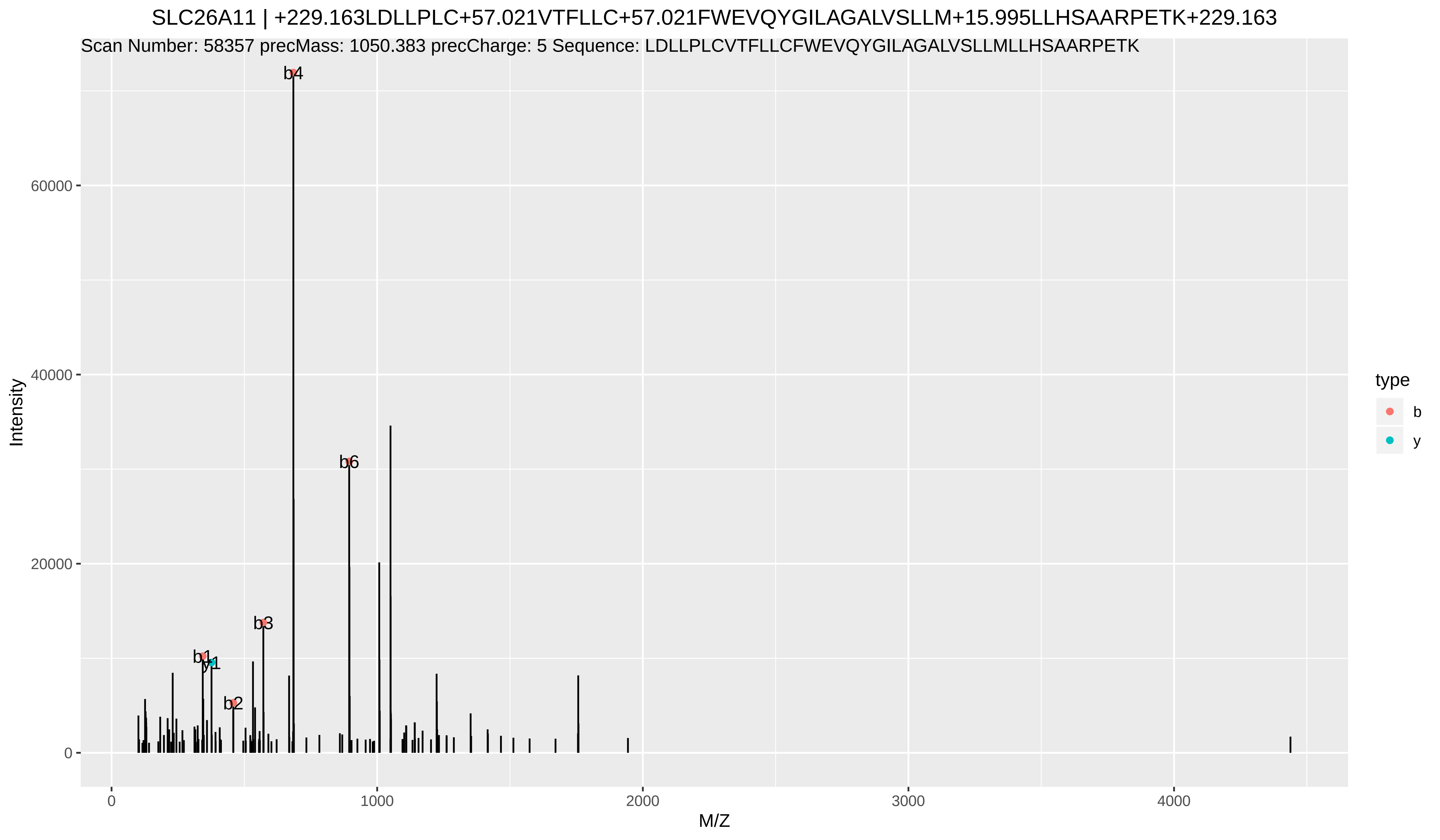

SLC26A11 | +229.163LDLLPLC+57.021VTFLLC+57.021FWEVQYGILAGALVSLLM+15.995LLHSAARPETK+229.163
Scan Number: 58357 precMass: 1050.383 precCharge: 5 Sequence: LDLLPLCVTFLLCFWEVQYGILAGALVSLLMLLHSAARPETK
b4
b6
b3
b1
y1
b2
Intensity
60000
40000
20000
0
0
1000
2000
3000
4000
M/Z
type
b
y

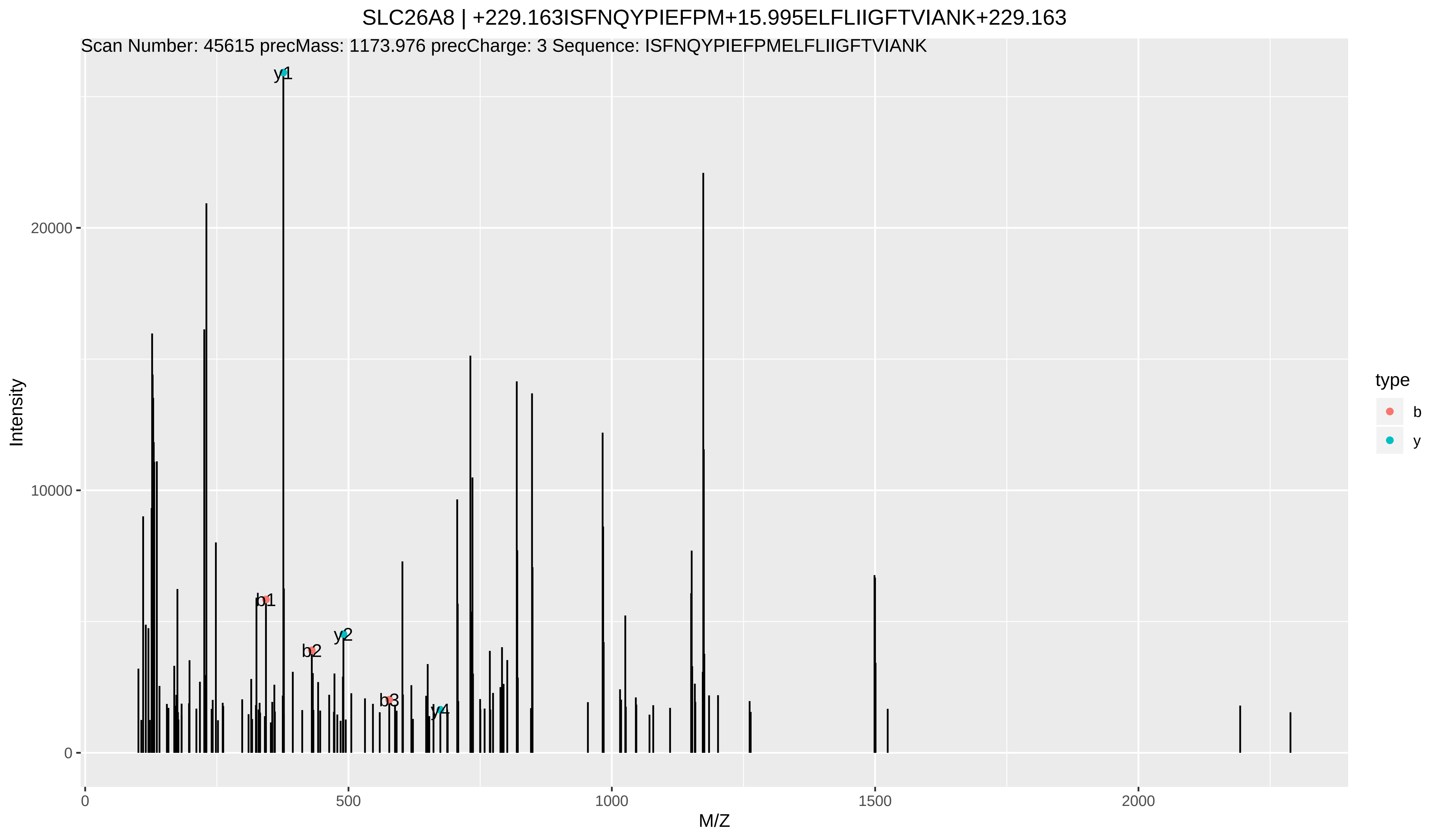
SLC26A8 | +229.163ISFNQYPIEFPM+15.995ELFLIIGFTVIANK+229.163
Scan Number: 45615 precMass: 1173.976 precCharge: 3 Sequence: ISFNQYPIEFPMELFLIIGFTVIANK
y1
b1
y2
b2
b3
y4
Intensity
20000
10000
0
M/Z
0
500
1000
1500
2000
type
b
y

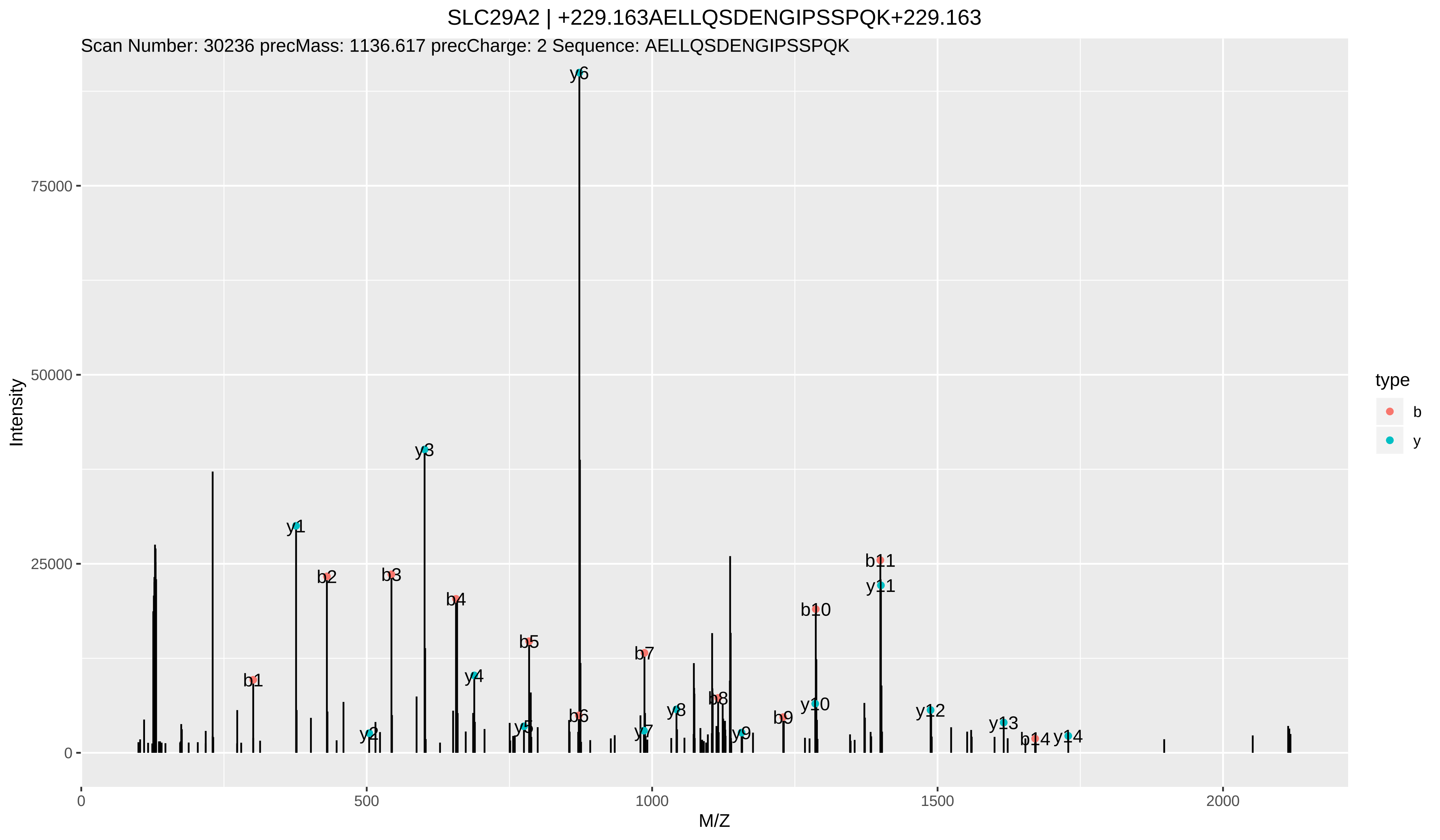
SLC29A2 | +229.163AELLQSDENGIPSSPQK+229.163
Scan Number: 30236 precMass: 1136.617 precCharge: 2 Sequence: AELLQSDENGIPSSPQK
Intensity
M/Z
type
b
y
y6
y3
y1
b2
b3
b4
b5
b7
b1
y4
b11
y11
b10
y10
y12
y13
b14
y14
y2
y5
b6
y7
y8
b8
y9
b9
0
25000
50000
75000
500
1000
1500
2000

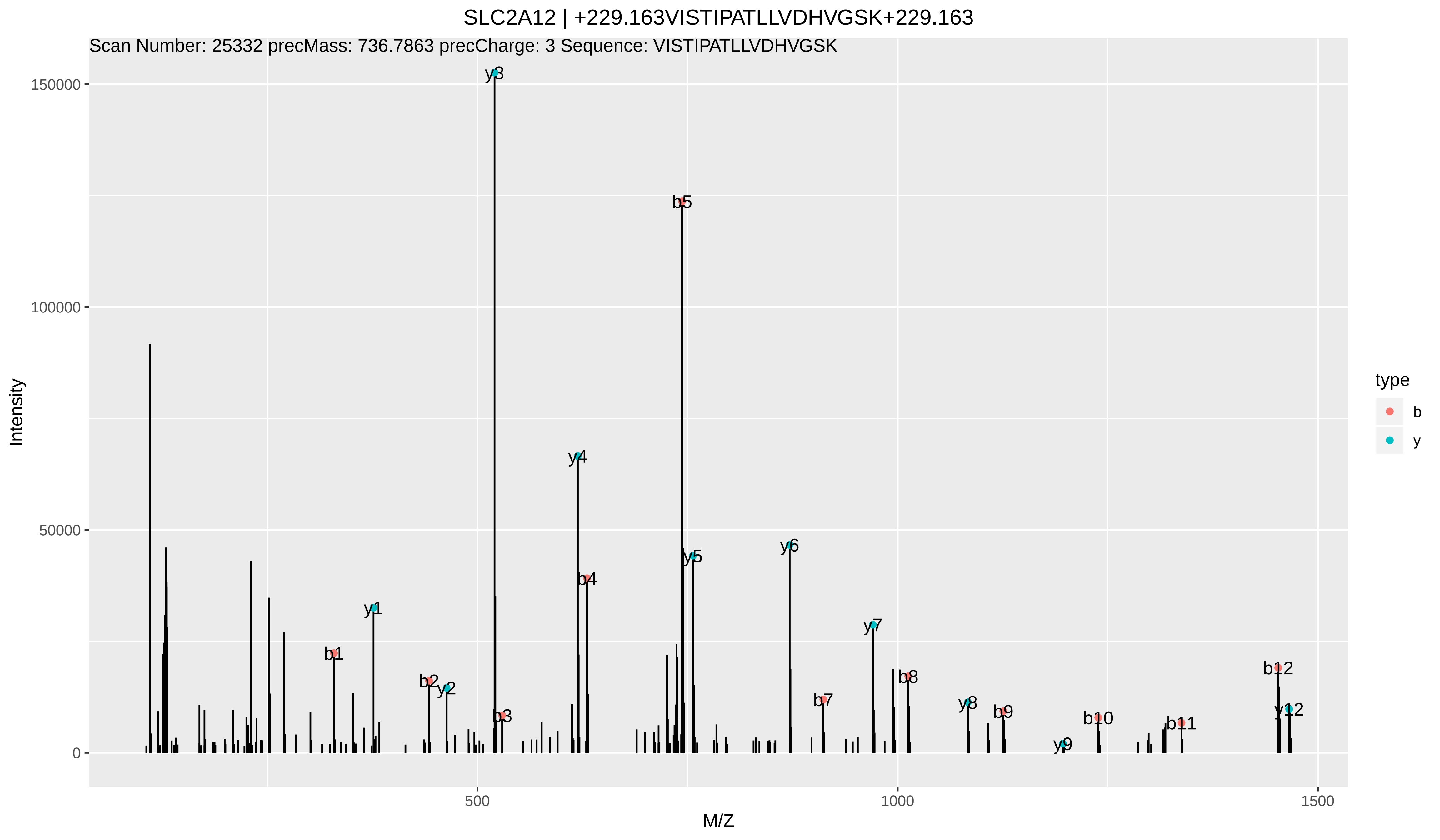
SLC2A12 | +229.163VISTIPATLLVDHVGSK+229.163
Scan Number: 25332 precMass: 736.7863 precCharge: 3 Sequence: VISTIPATLLVDHVGSK
Intensity
M/Z
0
50000
100000
150000
500
1000
1500
y3
b5
y4
y5
y6
b4
y1
y7
b1
b2
y2
b3
b7
b8
y8
b9
b10
b11
b12
y12
y9
type
b
y

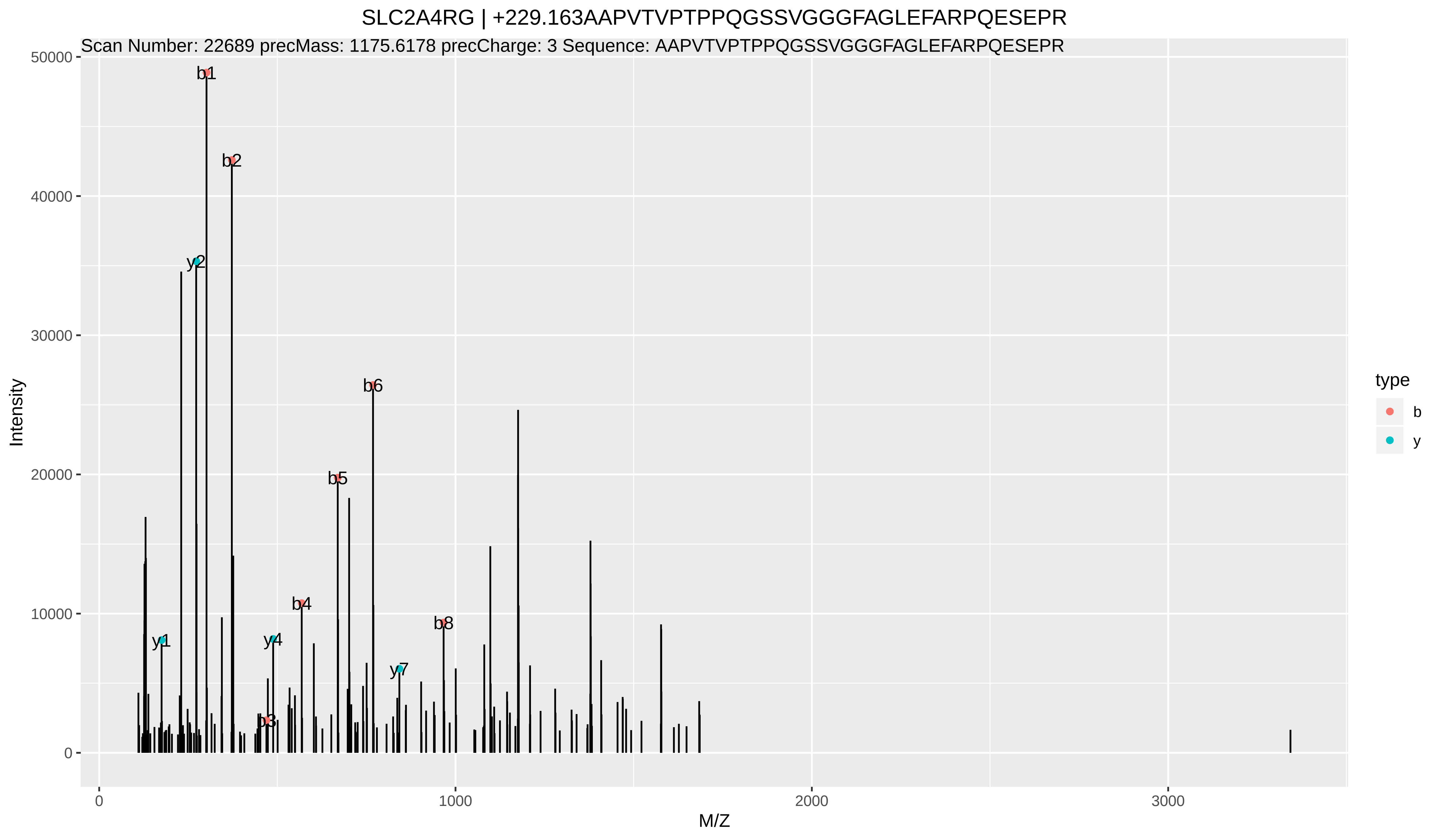
SLC2A4RG | +229.163AAPVTVPTPPQGSSVGGGFAGLEFARPQESEPR
Scan Number: 22689 precMass: 1175.6178 precCharge: 3 Sequence: AAPVTVPTPPQGSSVGGGFAGLEFARPQESEPR
Intensity
M/Z
0
10000
20000
30000
40000
50000
0
1000
2000
3000
b1
b2
y2
b6
b5
b4
y1
y4
b3
y7
b8
type
b
y

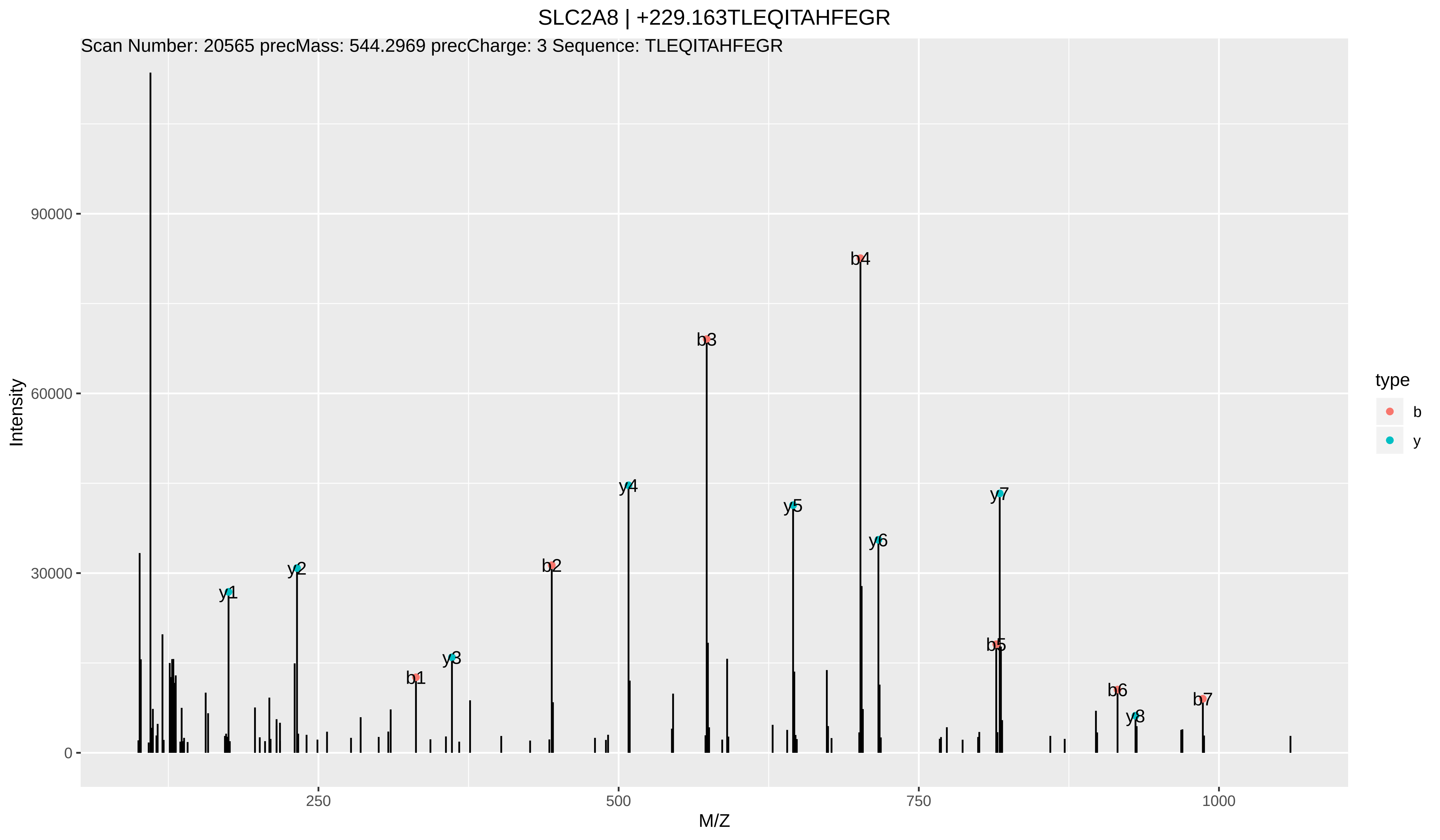

SLC2A8 | +229.163TLEQITAHFEGR
Scan Number: 20565 precMass: 544.2969 precCharge: 3 Sequence: TLEQITAHFEGR
Intensity
90000
60000
30000
0
M/Z
250
500
750
1000
y1
y2
b1
y3
b2
y4
b3
y5
b4
y6
y7
b5
b6
y8
b7
type
b
y

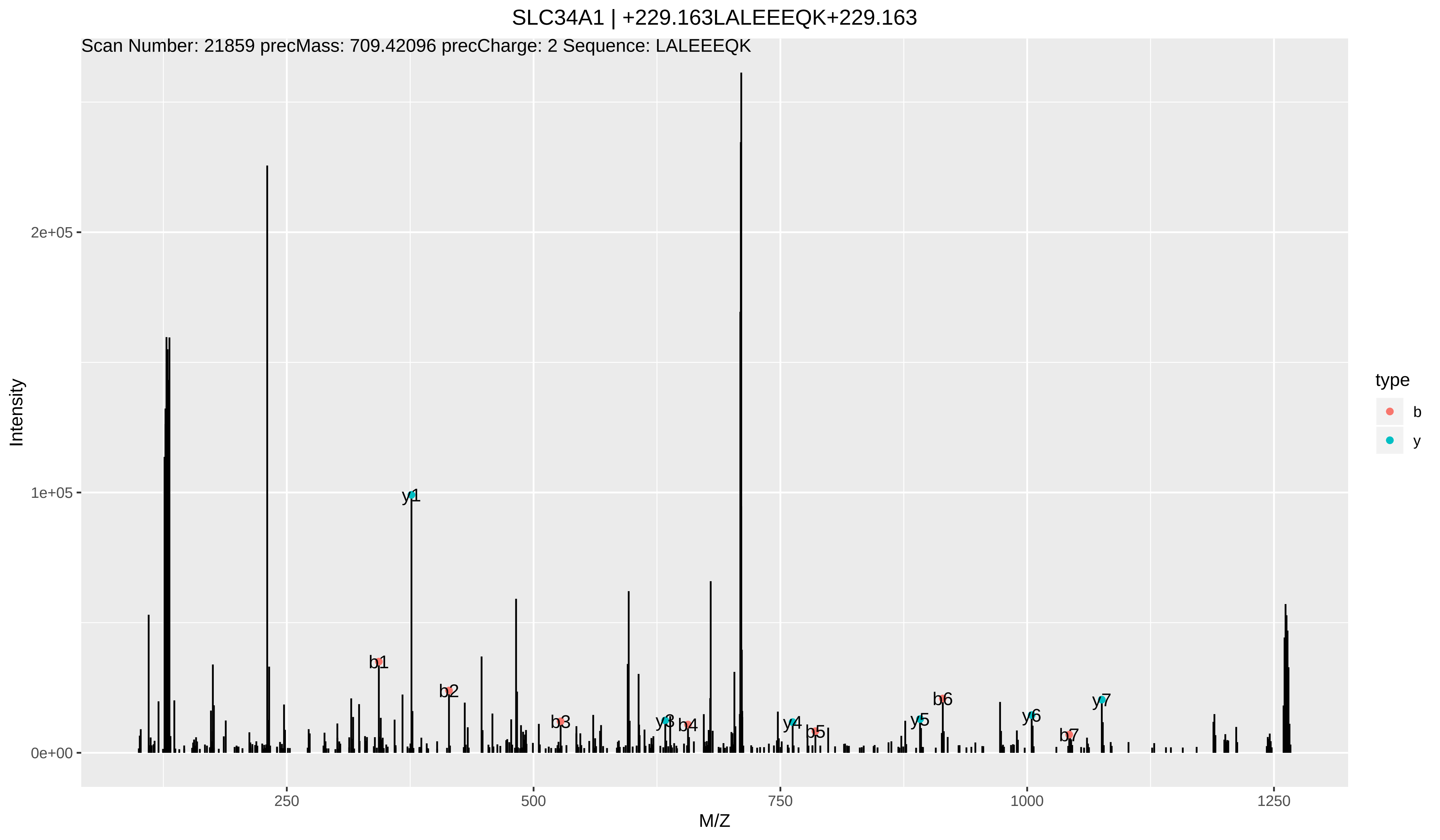
SLC34A1 | +229.163LALEEEQK+229.163
Scan Number: 21859 precMass: 709.42096 precCharge: 2 Sequence: LALEEEQK
Intensity
2e+05
1e+05
0e+00
M/Z
250
500
750
1000
1250
y1
b1
b2
b3
y3
b4
y4
b5
y5
b6
y6
b7
y7
type
b
y

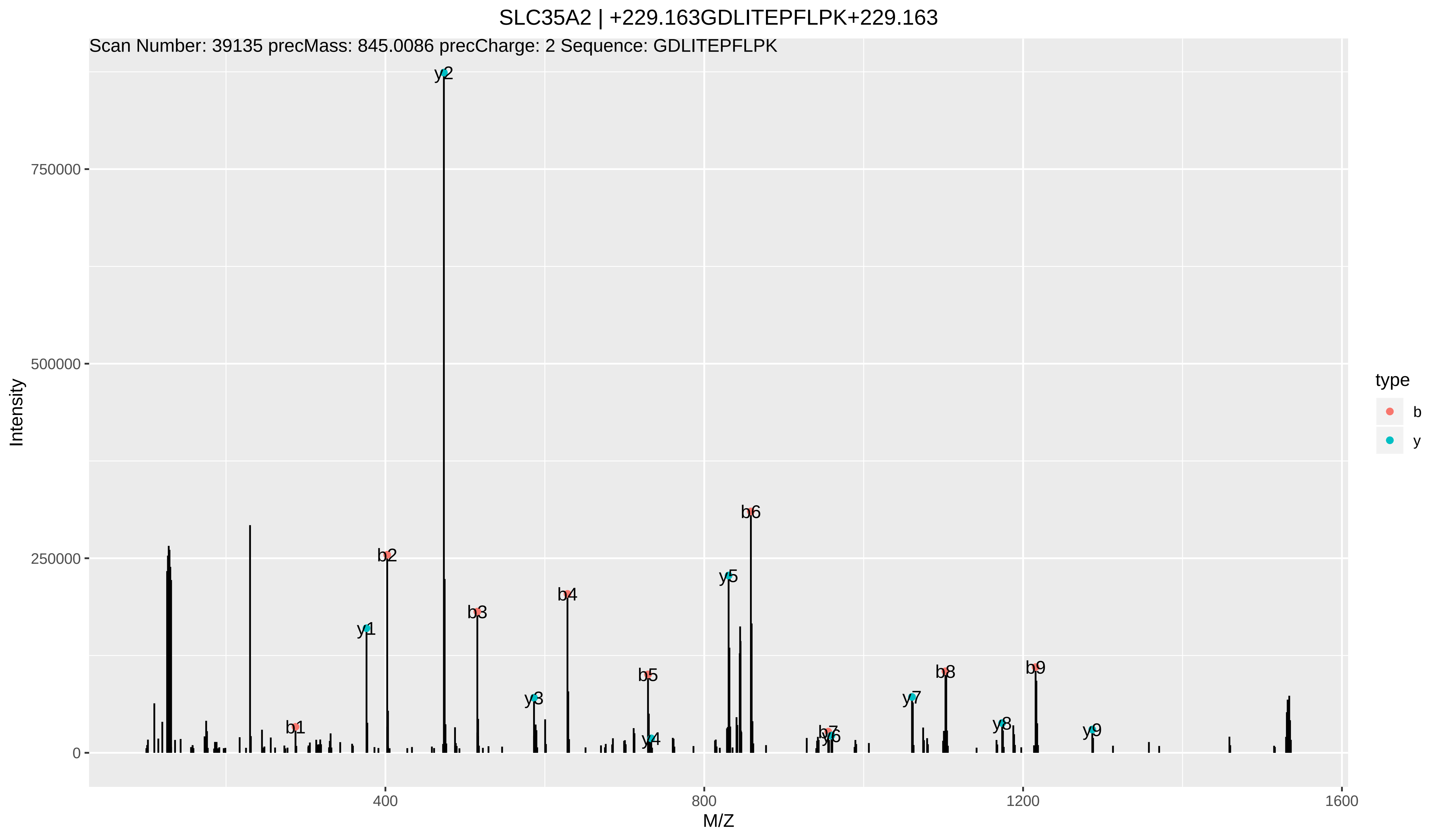

SLC35A2 | +229.163GDLITEPFLPK+229.163
Scan Number: 39135 precMass: 845.0086 precCharge: 2 Sequence: GDLITEPFLPK
Intensity
M/Z
750000
500000
250000
0
400
800
1200
1600
y2
b6
b2
y5
b4
b3
y1
b5
y3
y7
b8
b9
b1
y4
b7
y6
y8
y9
type
b
y

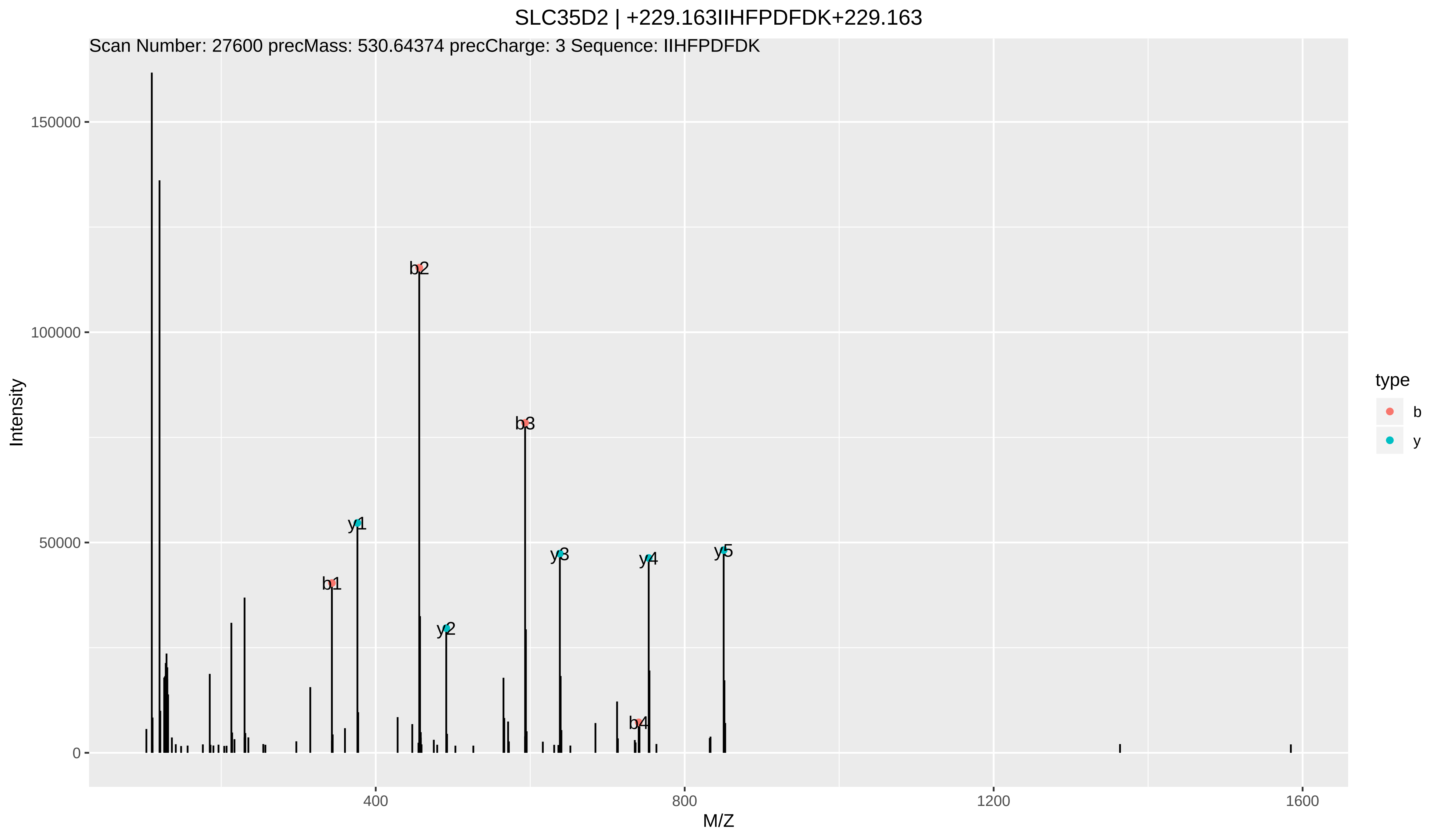

SLC35D2 | +229.163IIHFPDFDK+229.163
Scan Number: 27600 precMass: 530.64374 precCharge: 3 Sequence: IIHFPDFDK
Intensity
150000
100000
50000
0
400
800
1200
1600
M/Z
b2
b3
y1
b1
y3
y4
y5
y2
b4
type
b
y

SLC35E3 | +229.163LDIFAPK+229.163

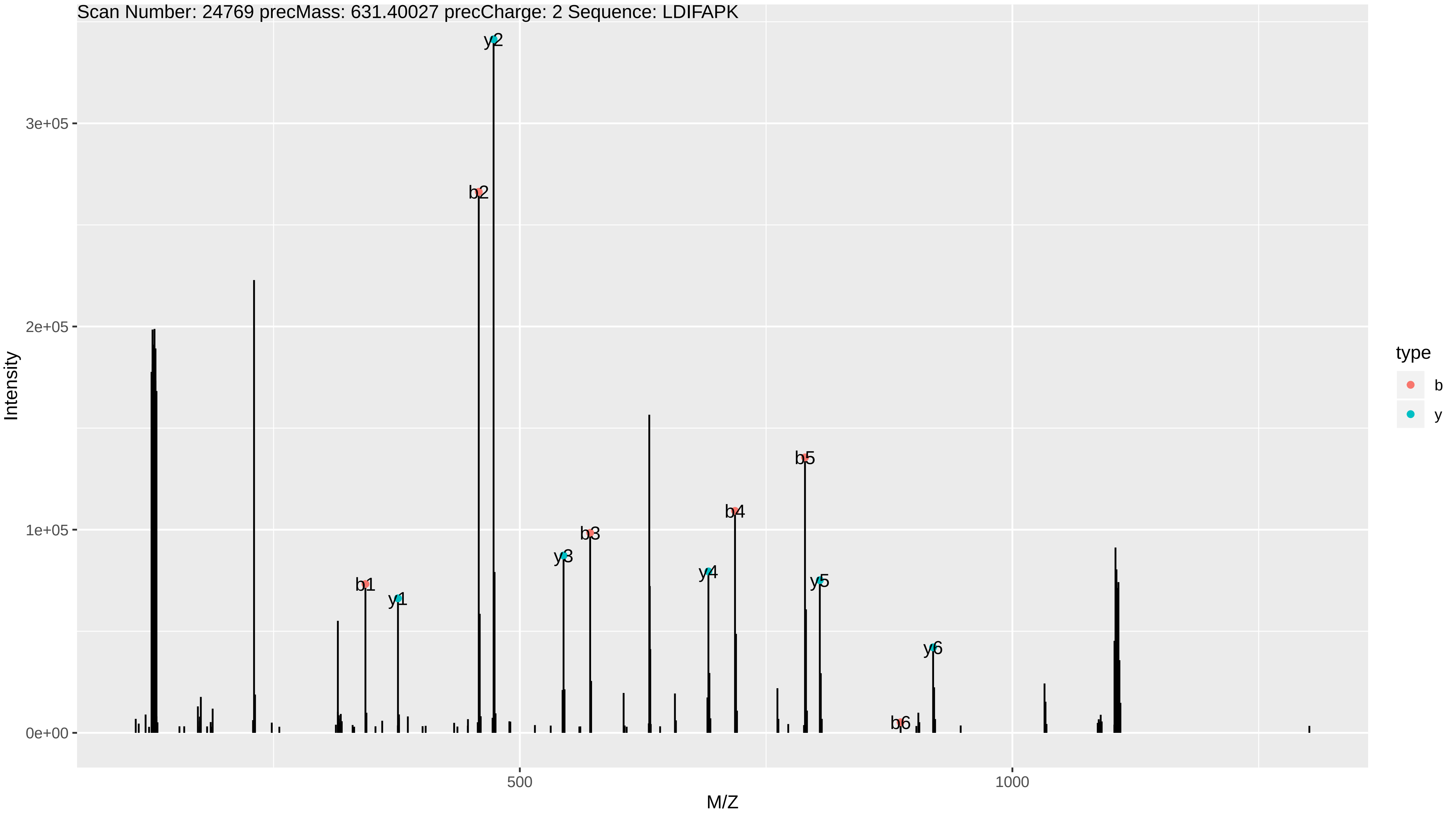

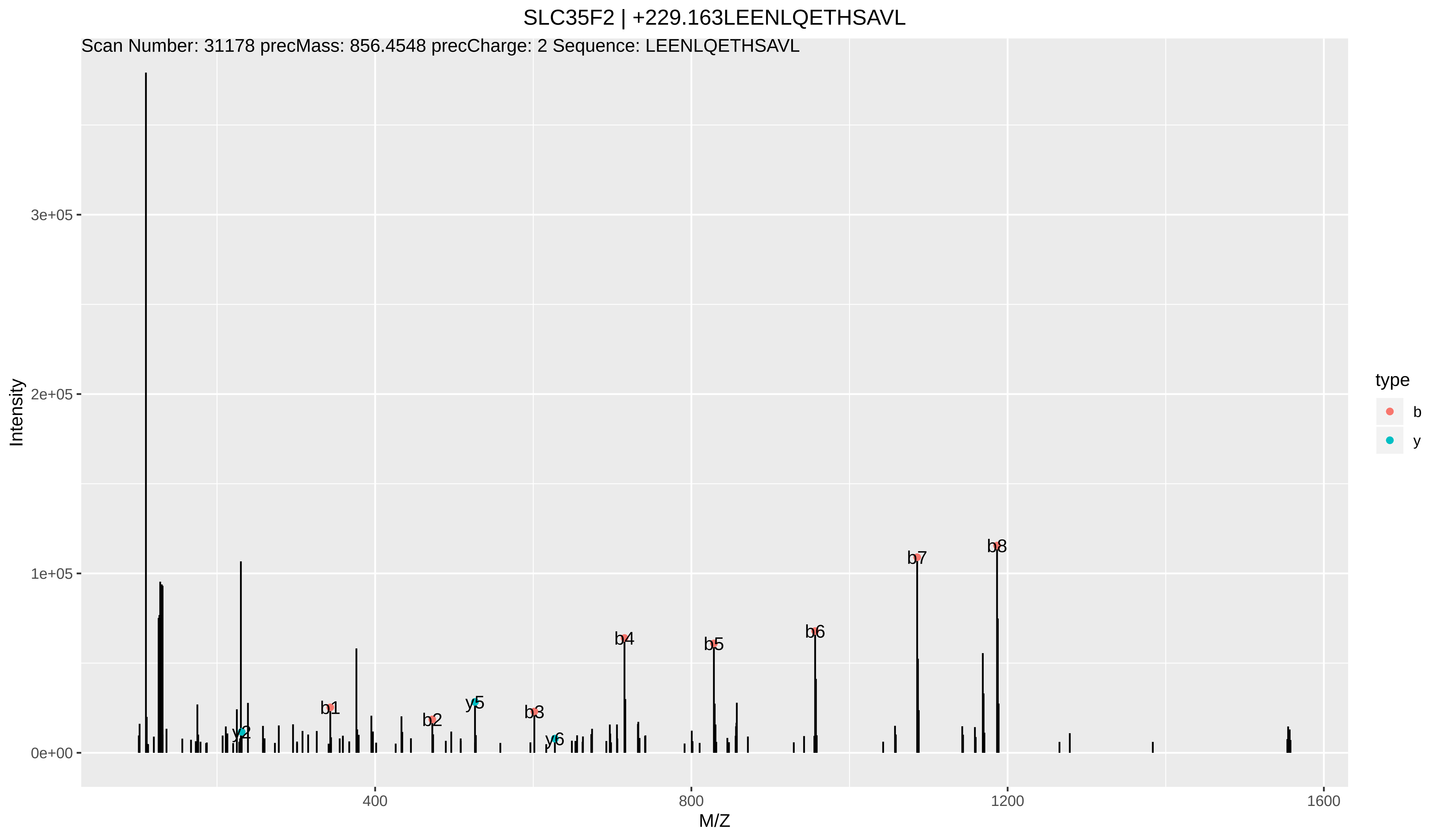
SLC35F2 | +229.163LEENLQETHSAVL
Scan Number: 31178 precMass: 856.4548 precCharge: 2 Sequence: LEENLQETHSAVL
Intensity
M/Z
3e+05
2e+05
1e+05
0e+00
400
800
1200
1600
b1
b2
b3
b4
b5
b6
b7
b8
y2
y5
y6
type
b
y

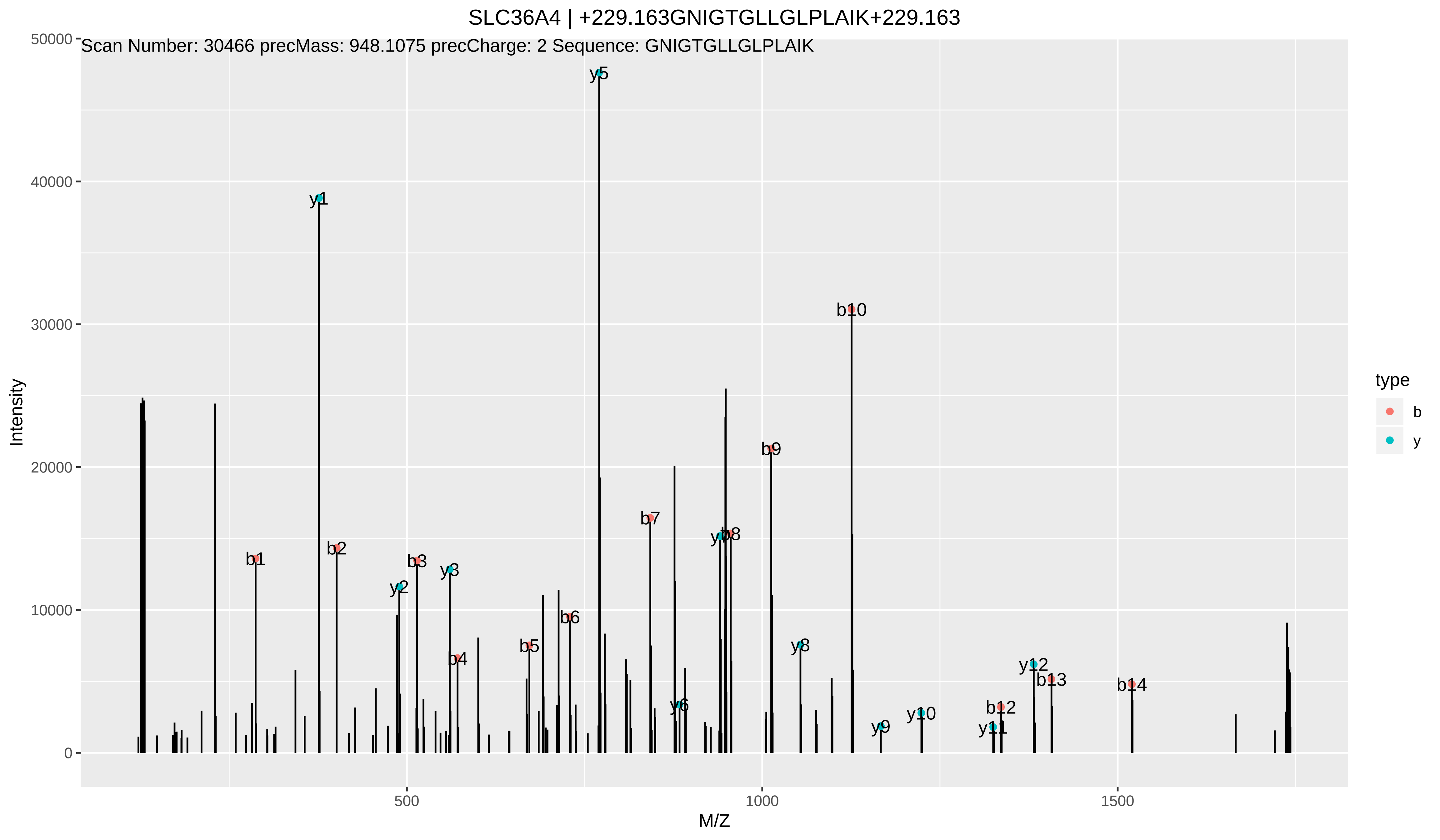
SLC36A4 | +229.163GNIGTGLLGLPLAIK+229.163
Scan Number: 30466 precMass: 948.1075 precCharge: 2 Sequence: GNIGTGLLGLPLAIK
Intensity
M/Z
0
10000
20000
30000
40000
50000
500
1000
1500
y5
y1
b10
b9
b7
b8
b1
b2
b3
y3
y2
b4
b5
b6
y6
y8
y12
b13
b14
b12
y10
y9
y11
type
b
y

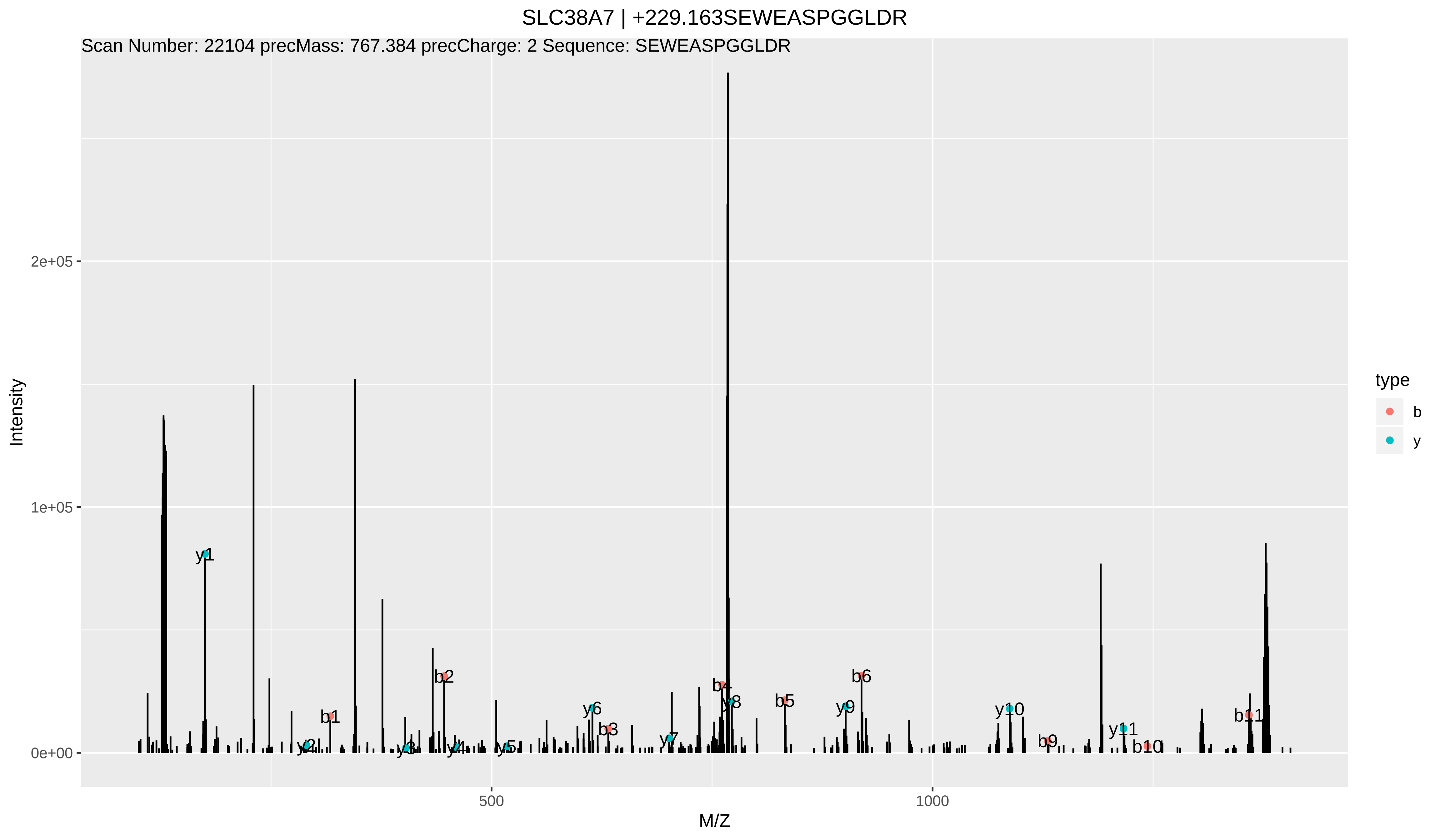
SLC38A7 | +229.163SEWEASPGGLDR
Scan Number: 22104 precMass: 767.384 precCharge: 2 Sequence: SEWEASPGGLDR
Intensity
2e+05
1e+05
0e+00
M/Z
500
1000
y1
y2
b1
y3
b2
y4
y5
y6
b3
y7
b4
y8
b5
y9
b6
y10
b9
y11
b10
b11
type
b
y

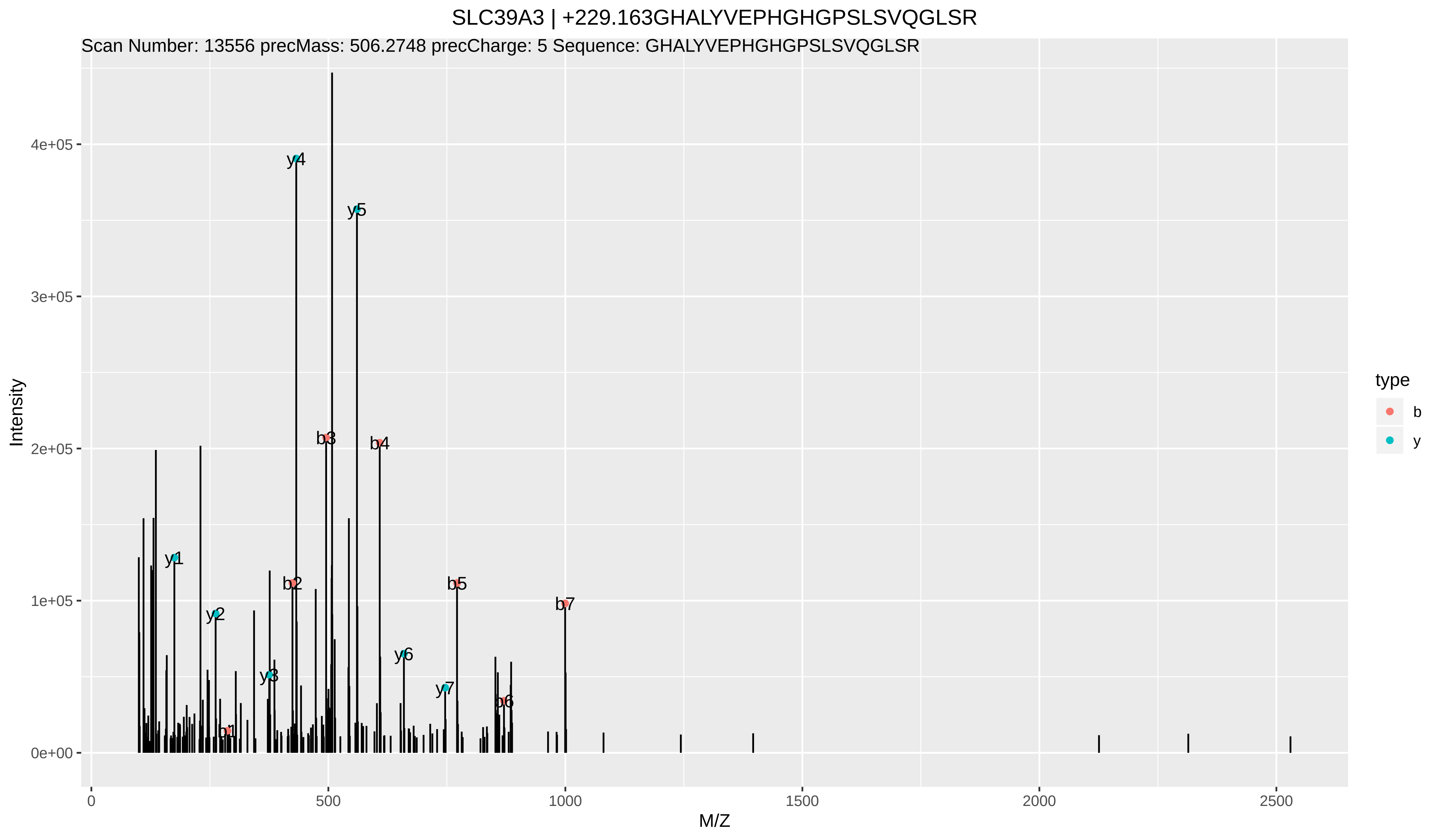
SLC39A3 | +229.163GHALYVEPHGHGPSLSVQGLSR
Scan Number: 13556 precMass: 506.2748 precCharge: 5 Sequence: GHALYVEPHGHGPSLSVQGLSR
Intensity
4e+05
3e+05
2e+05
1e+05
0e+00
M/Z
0
500
1000
1500
2000
2500
y1
y2
y3
y4
y5
y6
y7
b1
b2
b3
b4
b5
b6
b7
type
b
y

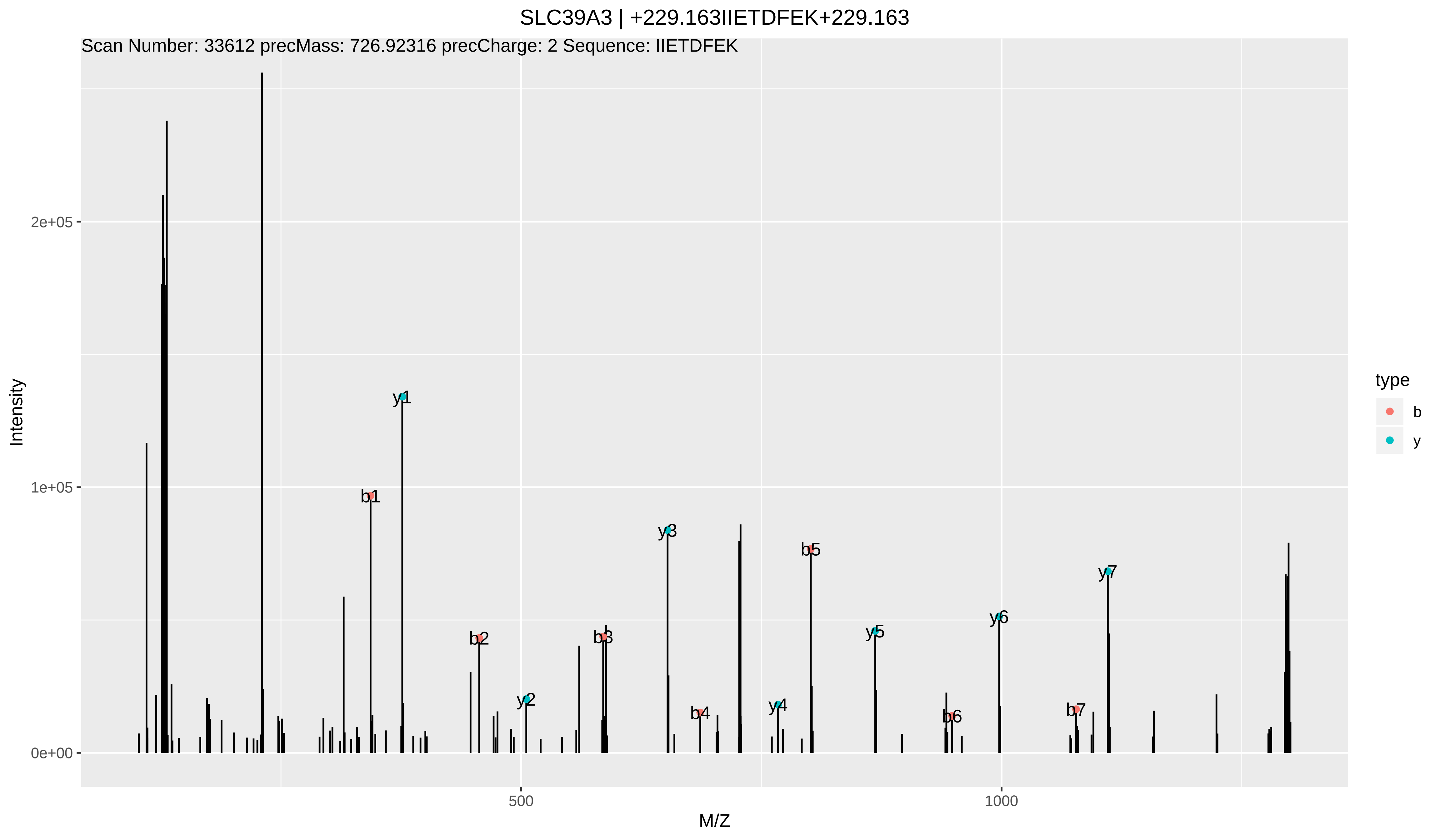
SLC39A3 | +229.163IIETDFEK+229.163
Scan Number: 33612 precMass: 726.92316 precCharge: 2 Sequence: IIETDFEK
Intensity
2e+05
1e+05
0e+00
M/Z
500
1000
y1
b1
y3
b5
y7
b2
b3
y6
y5
y2
b4
y4
b6
b7
type
b
y

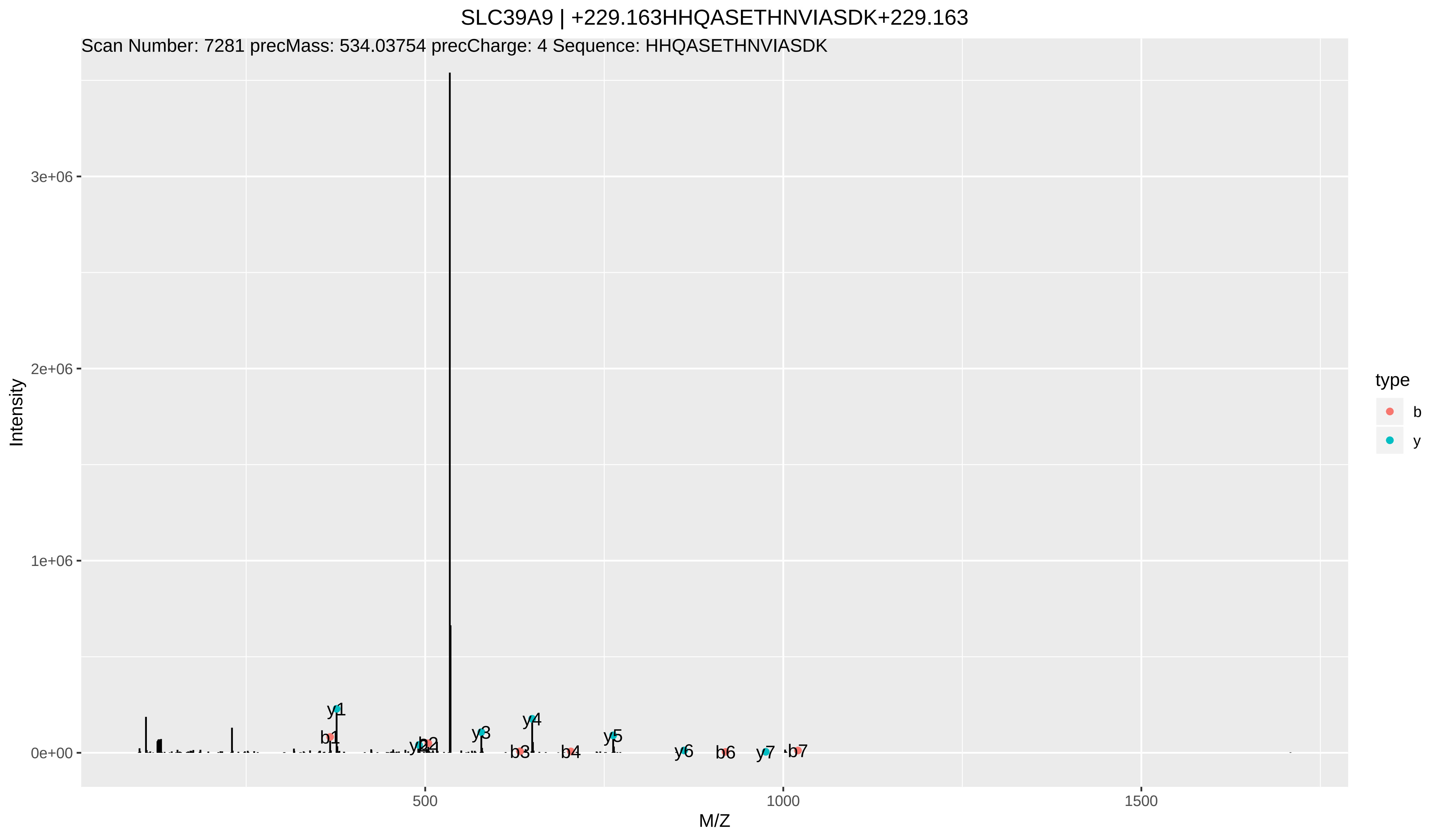
SLC39A9 | +229.163HHQASETHNVIASDK+229.163
Scan Number: 7281 precMass: 534.03754 precCharge: 4 Sequence: HHQASETHNVIASDK
Intensity
3e+06
2e+06
1e+06
0e+00
M/Z
500
1000
1500
y1
b1
y2
b2
y3
y4
b3
b4
y5
y6
b6
y7
b7
type
b
y

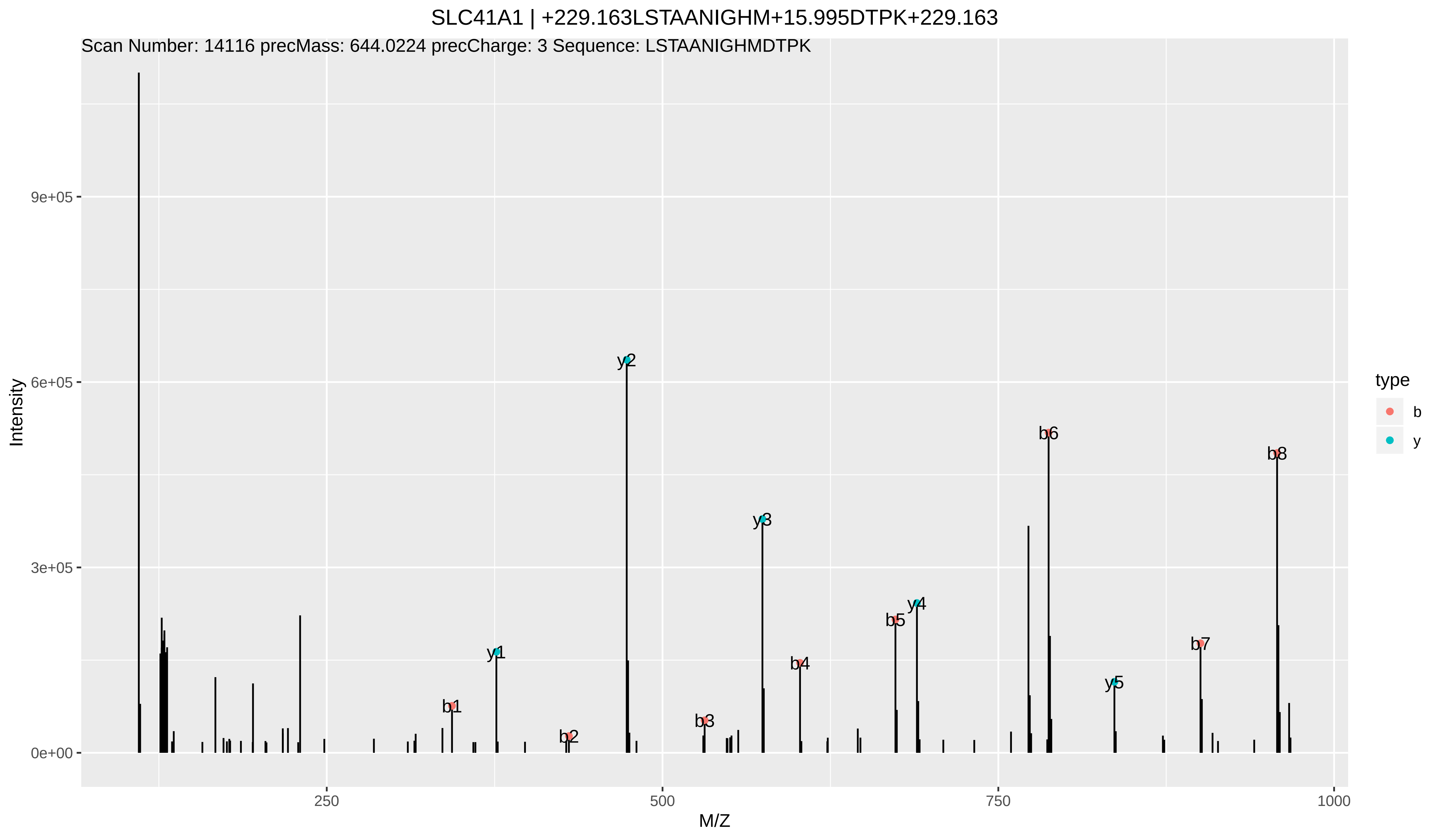

SLC41A1 | +229.163LSTAANIGHM+15.995DTPK+229.163
Scan Number: 14116 precMass: 644.0224 precCharge: 3 Sequence: LSTAANIGHMDTPK
Intensity
9e+05
6e+05
3e+05
0e+00
M/Z
250
500
750
1000
type
b
y
y2
b6
b8
y3
y4
b5
b7
y1
b4
y5
b1
b3
b2

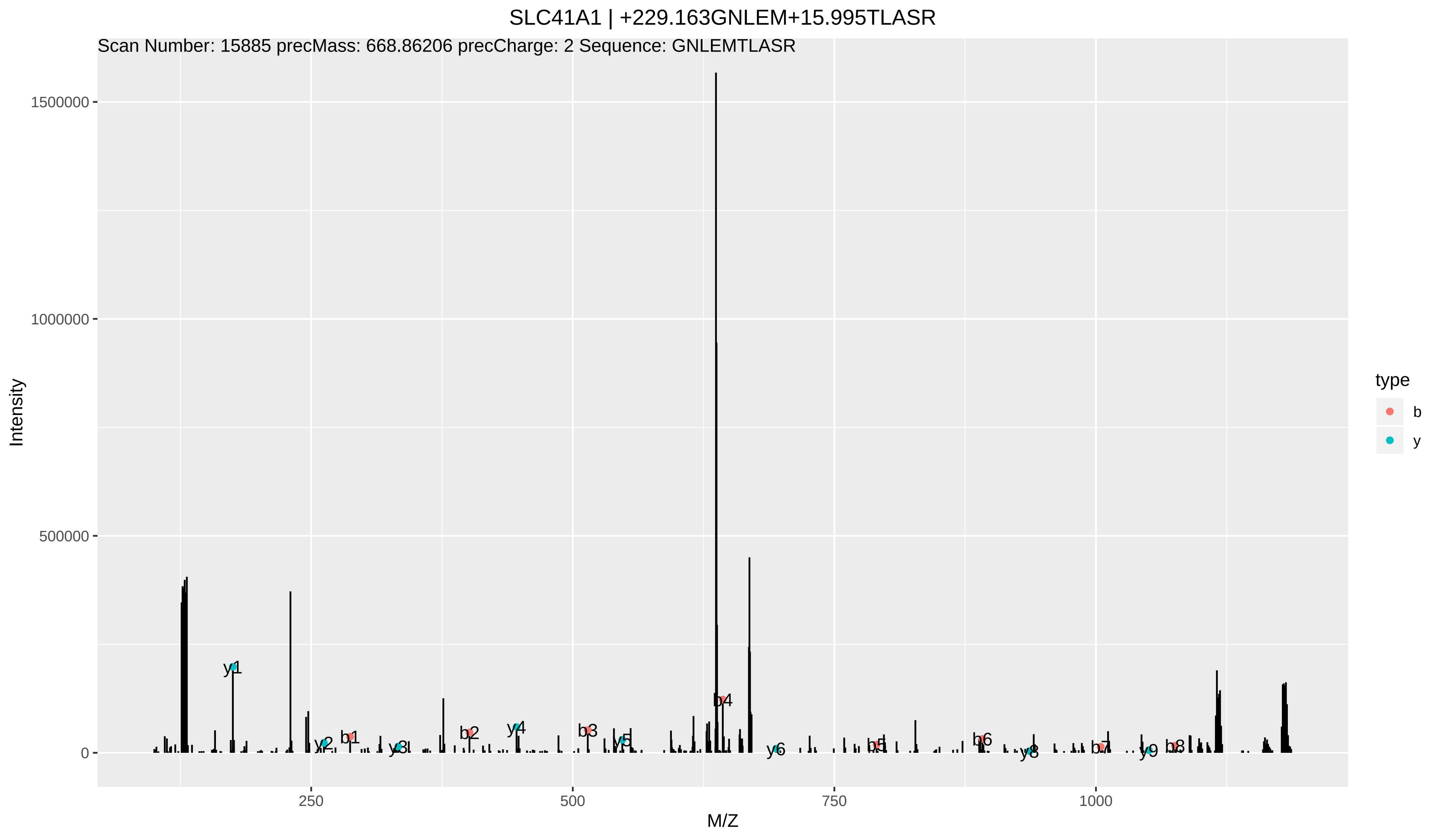

SLC41A1 | +229.163GNLEM+15.995TLASR
Scan Number: 15885 precMass: 668.86206 precCharge: 2 Sequence: GNLEMTLASR
Intensity
1500000
1000000
500000
0
250
500
750
1000
M/Z
y1
y2
b1
y3
b2
y4
b3
y5
b4
y6
b5
b6
y8
b7
y9
b8
type
b
y

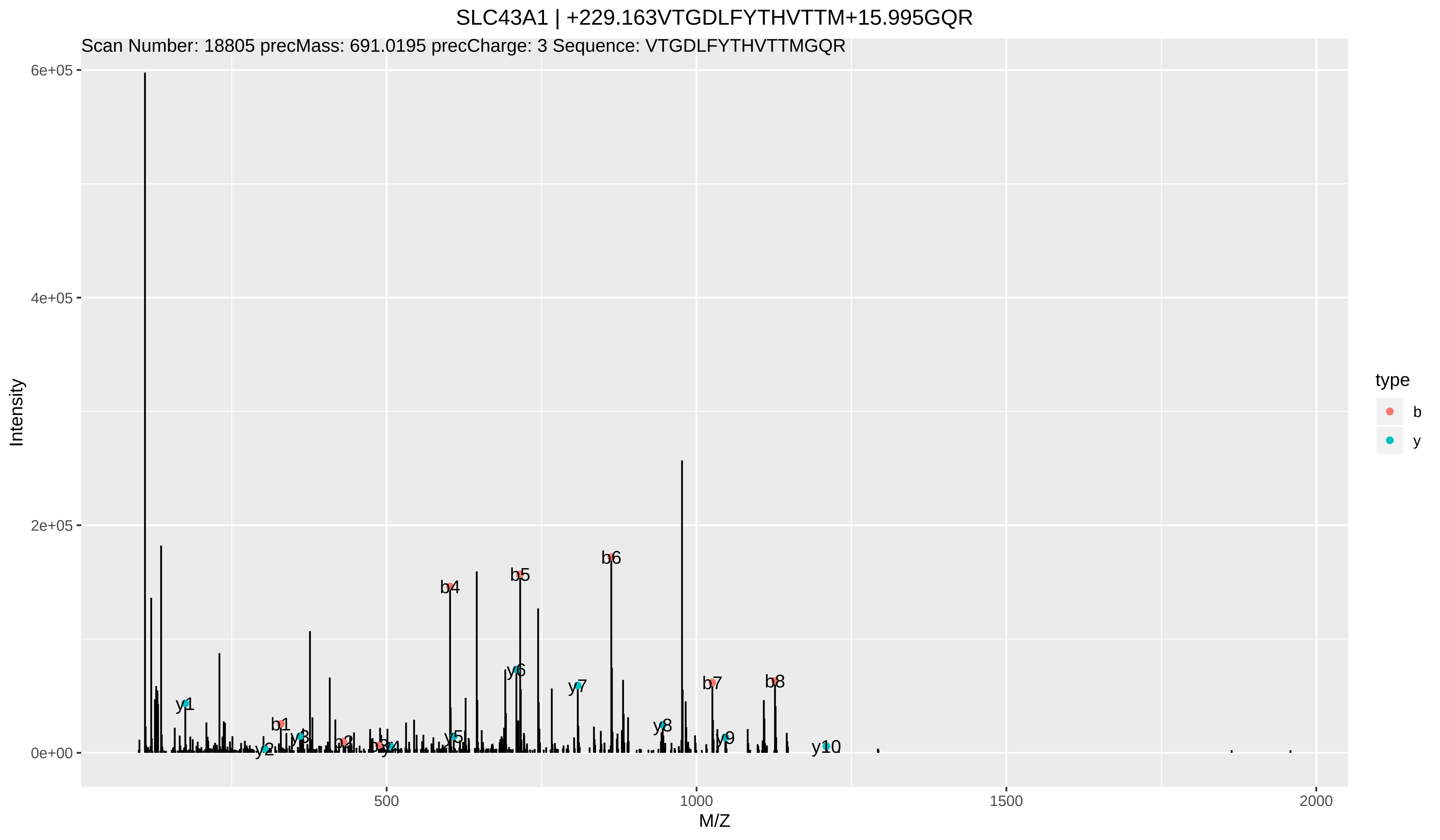

SLC43A1 | +229.163VTGDLFYTHVTTM+15.995GQR
Scan Number: 18805 precMass: 691.0195 precCharge: 3 Sequence: VTGDLFYTHVTTMGQR
Intensity
M/Z
6e+05
4e+05
2e+05
0e+00
500
1000
1500
2000
type
b
y
b1
b2
b3
b4
b5
b6
b7
b8
y1
y2
y3
y4
y5
y6
y7
y8
y9
y10

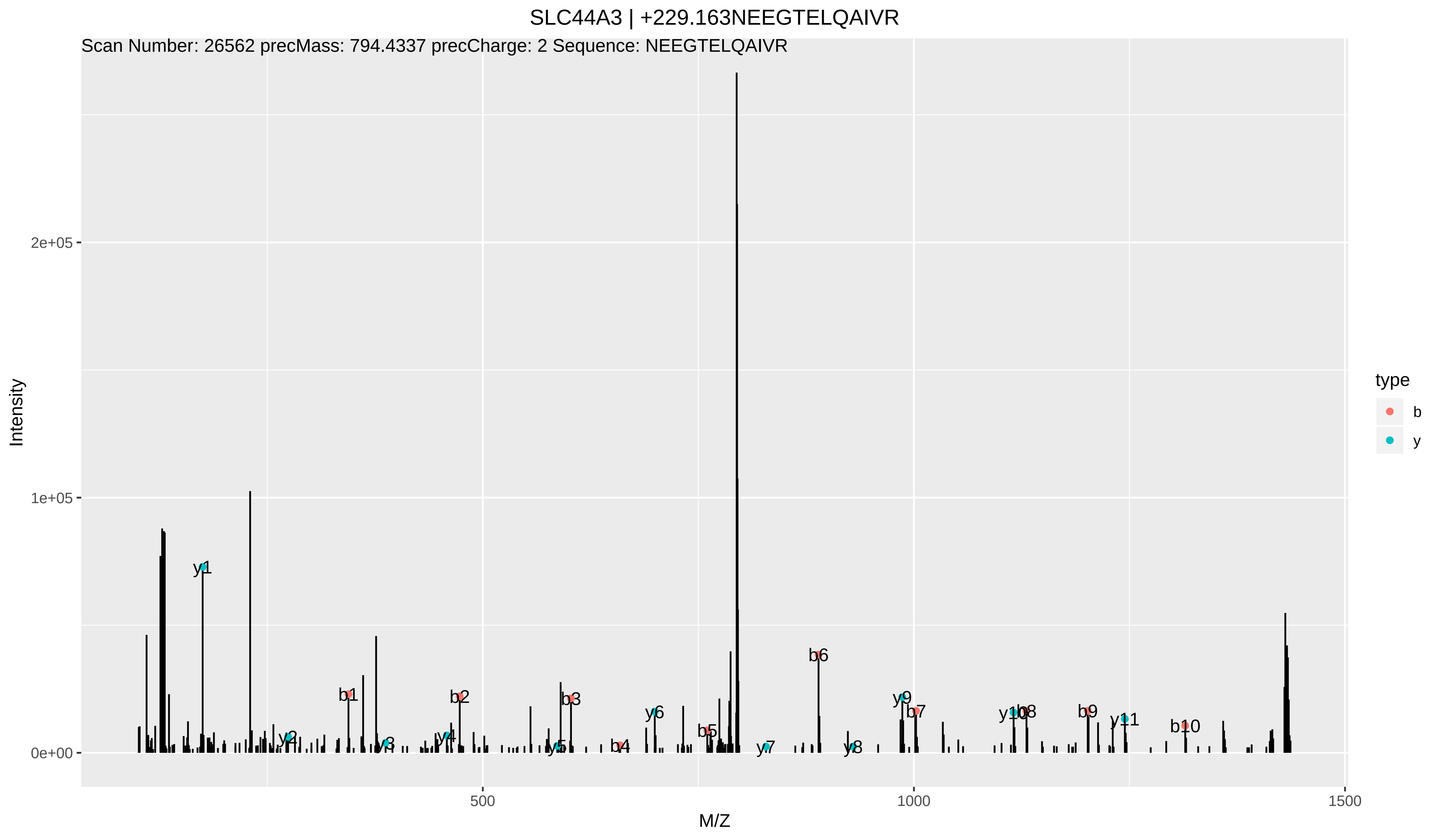

SLC44A3 | +229.163NEEGTELQAIVR
Scan Number: 26562 precMass: 794.4337 precCharge: 2 Sequence: NEEGTELQAIVR
Intensity
2e+05
1e+05
0e+00
M/Z
500
1000
1500
y1
y2
y3
y4
y5
y6
y7
y8
y9
y10
y11
b1
b2
b3
b4
b5
b6
b7
b8
b9
b10
type
b
y

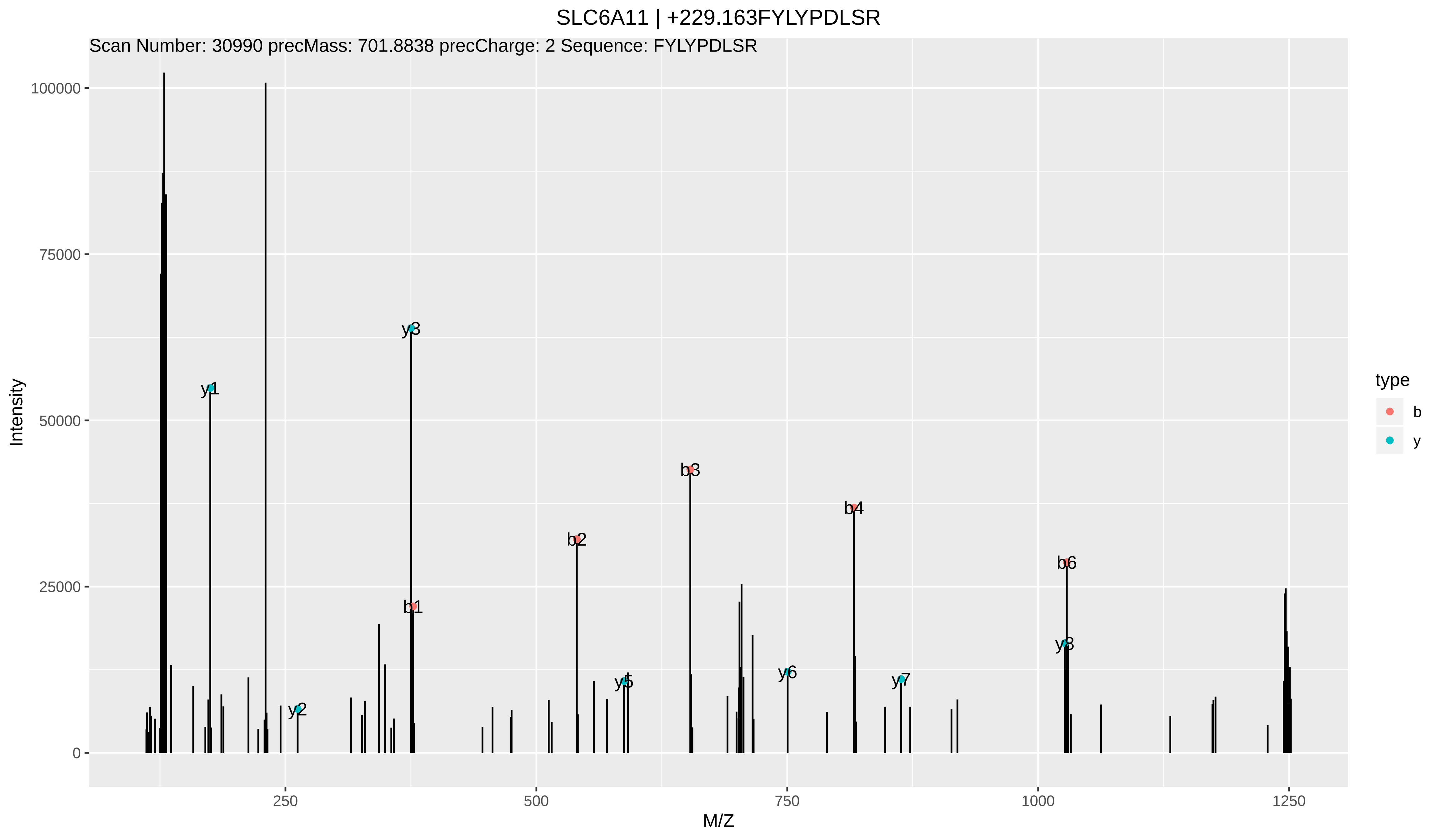

SLC6A11 | +229.163FYLYPDLSR
Scan Number: 30990 precMass: 701.8838 precCharge: 2 Sequence: FYLYPDLSR
Intensity
M/Z
100000
75000
50000
25000
0
250
500
750
1000
1250
y1
y2
y3
y5
y6
y7
y8
b1
b2
b3
b4
b6
type
b
y

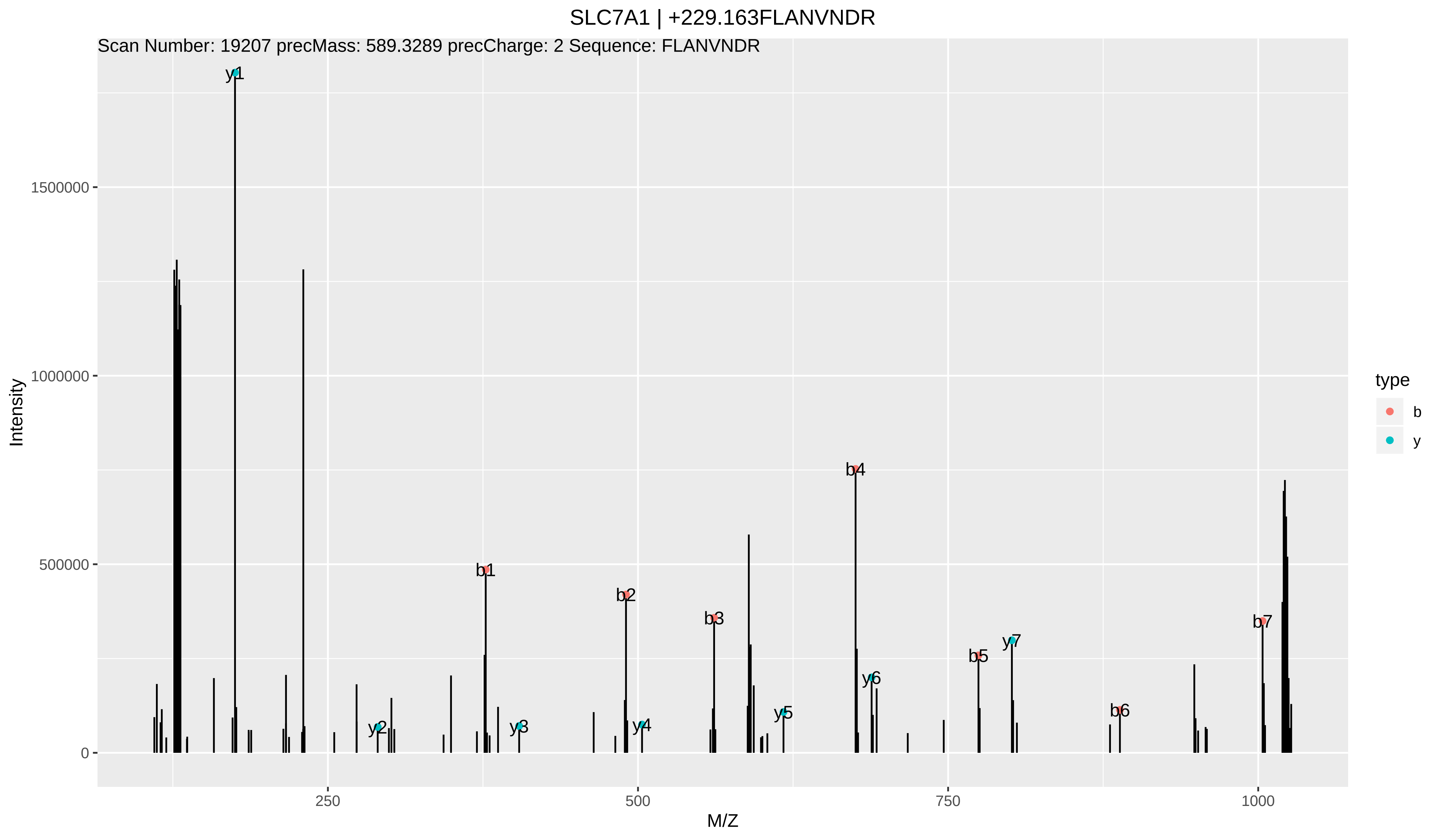
SLC7A1 | +229.163FLANVNDR
Scan Number: 19207 precMass: 589.3289 precCharge: 2 Sequence: FLANVNDR
y1
b1
b2
b3
b4
b5
b6
b7
y2
y3
y4
y5
y6
y7
Intensity
1500000
1000000
500000
0
250
500
750
1000
M/Z
type
b
y

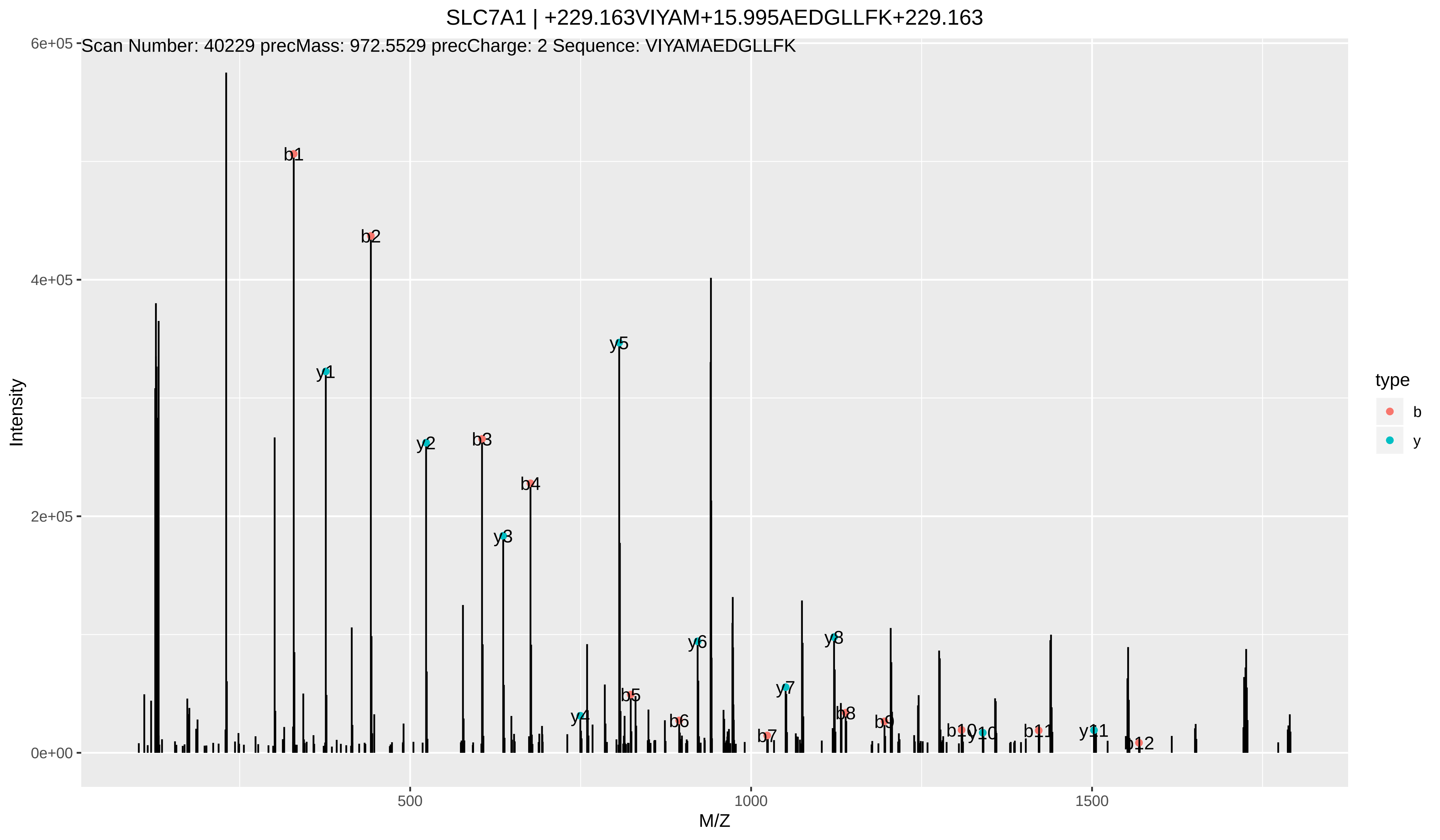

SLC7A1 | +229.163VIYAM+15.995AEDGLLFK+229.163
Scan Number: 40229 precMass: 972.5529 precCharge: 2 Sequence: VIYAMAEDGLLFK
Intensity
M/Z
0e+00
2e+05
4e+05
6e+05
500
1000
1500
type
b
y
b1
b2
b3
b4
b5
b6
b7
b8
b9
b10
b11
b12
y1
y2
y3
y4
y5
y6
y7
y8
y10
y11

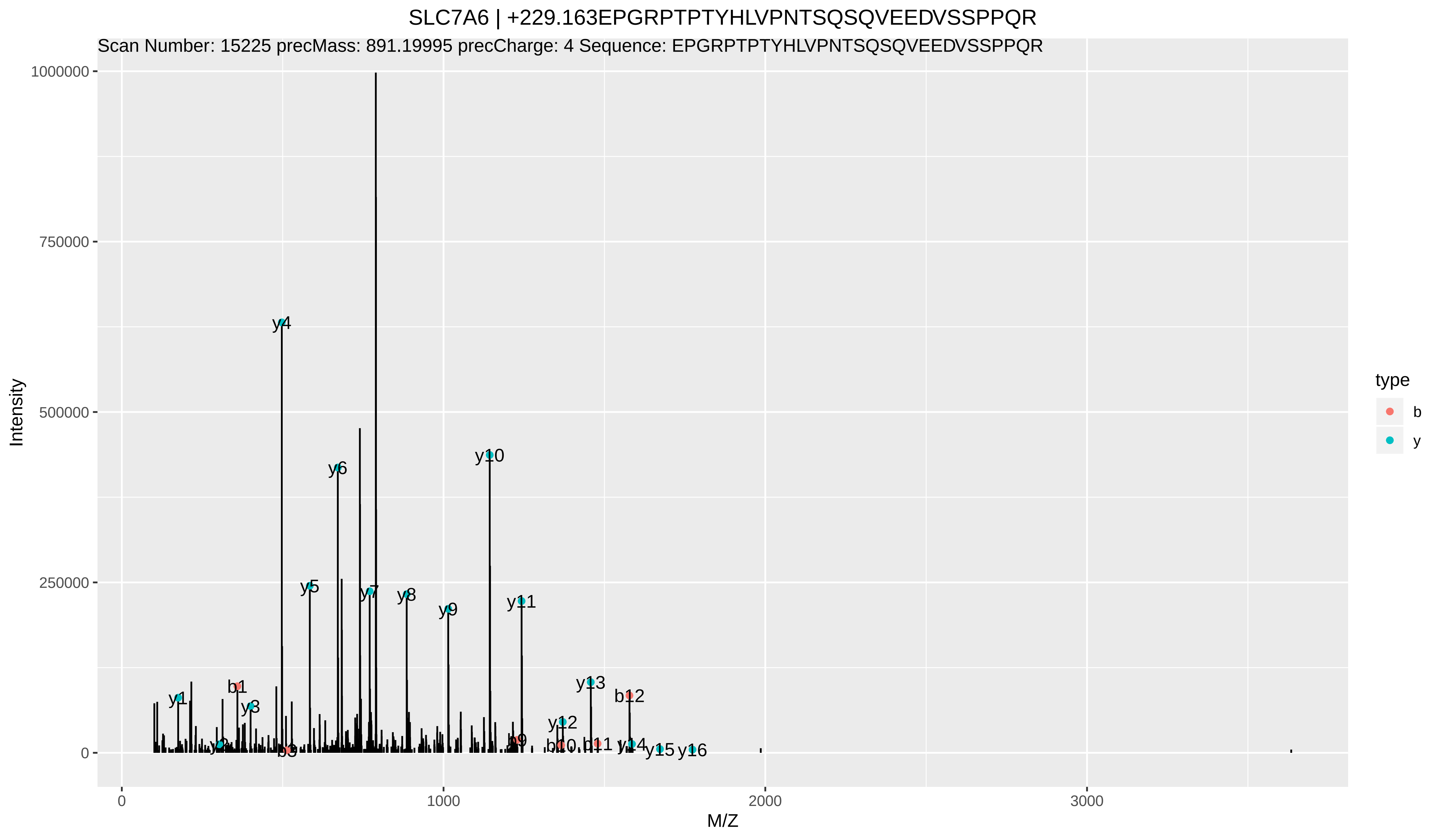

SLC7A6 | +229.163EPGRPTPTYHLVPNTSQSQVEEDVSSPPQR
Scan Number: 15225 precMass: 891.19995 precCharge: 4 Sequence: EPGRPTPTYHLVPNTSQSQVEEDVSSPPQR
Intensity
M/Z
1000000
750000
500000
250000
0
0
1000
2000
3000
type
b
y
y1
y2
y3
y4
y5
y6
y7
y8
y9
y10
y11
y12
y13
y14
y15
y16
b1
b3
b9
b10
b11
b12

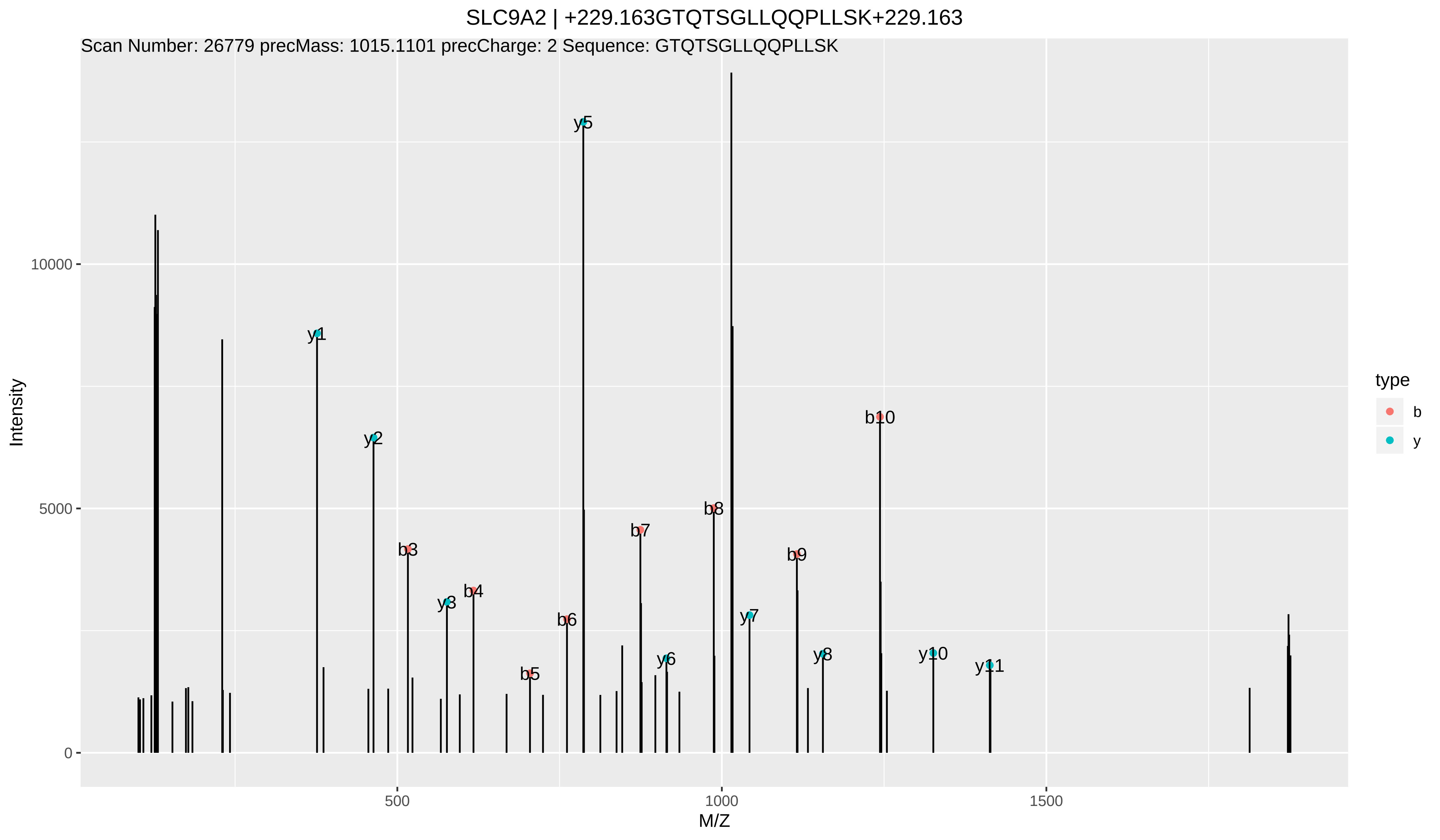
SLC9A2 | +229.163GTQTSGLLQQPLLSK+229.163
Scan Number: 26779 precMass: 1015.1101 precCharge: 2 Sequence: GTQTSGLLQQPLLSK
Intensity
M/Z
0
5000
10000
500
1000
1500
y1
y2
b3
y3
b4
b5
b6
y5
b7
y6
b8
y7
b9
y8
b10
y10
y11
type
b
y

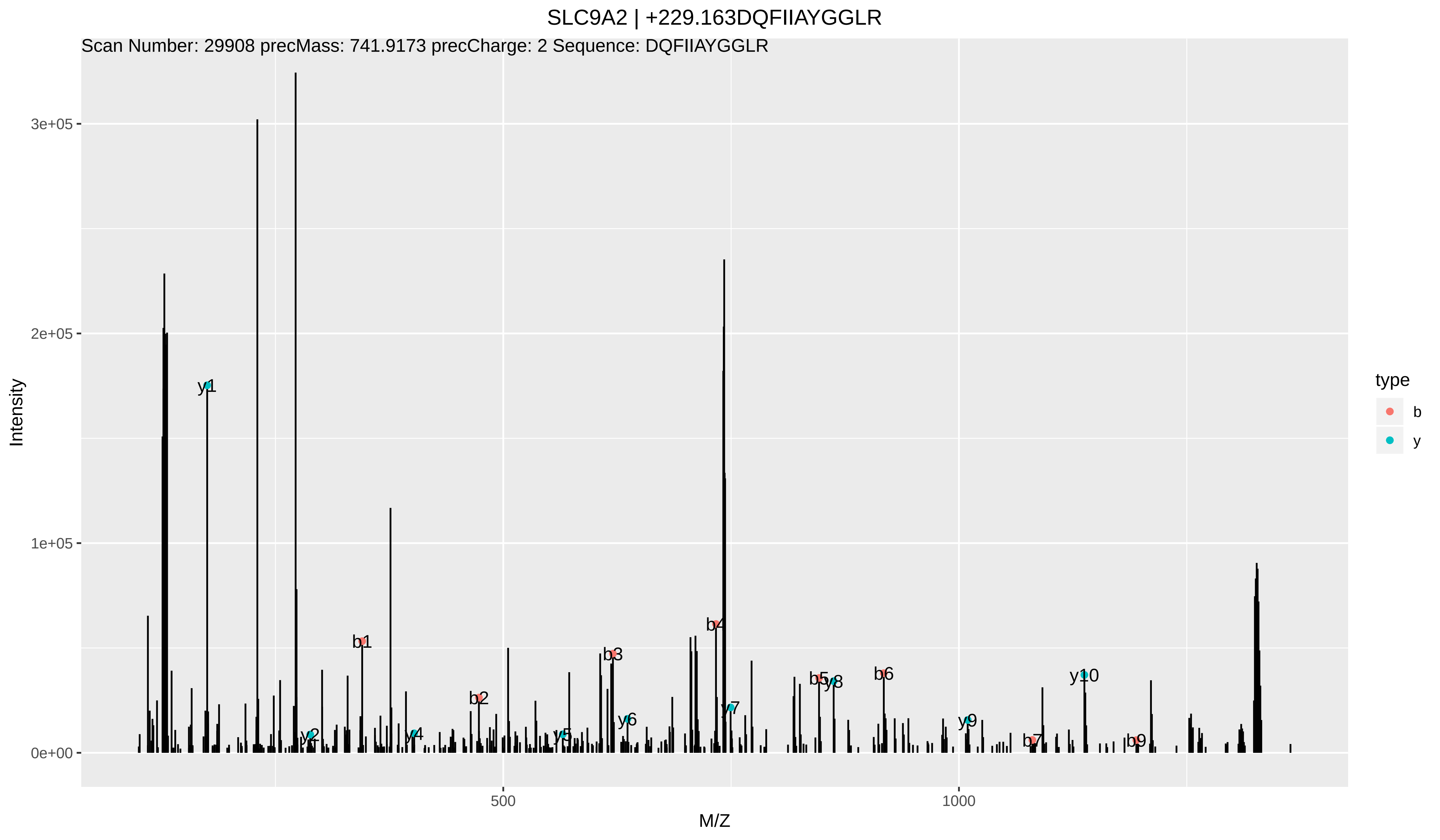
SLC9A2 | +229.163DQFIIAYGGLR
Scan Number: 29908 precMass: 741.9173 precCharge: 2 Sequence: DQFIIAYGGLR
Intensity
M/Z
type
b
y
y1
y2
y4
y5
y6
y7
y8
y9
y10
b1
b2
b3
b4
b5
b6
b7
b9
0e+00
1e+05
2e+05
3e+05
500
1000

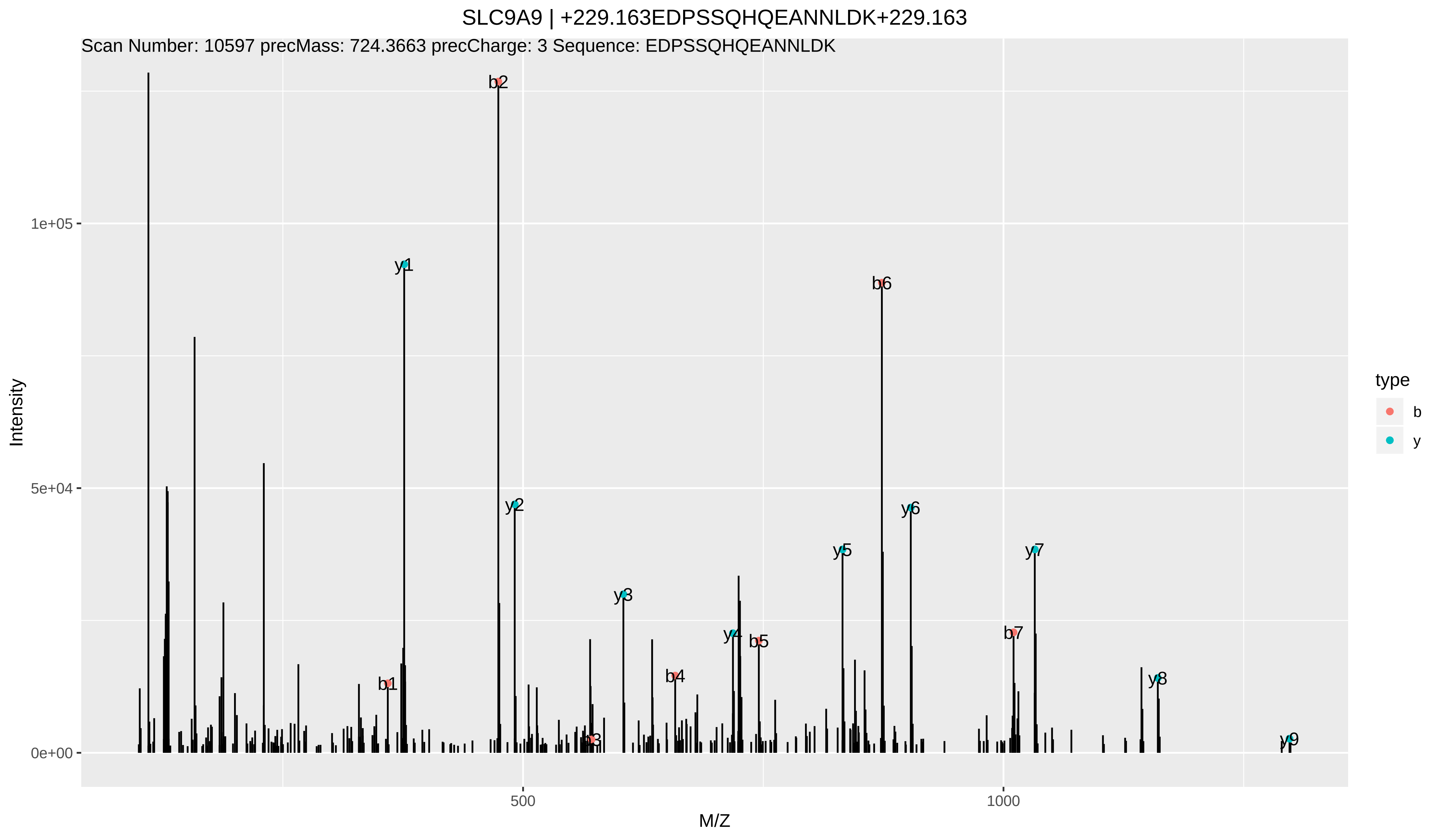
SLC9A9 | +229.163EDPSSQHQEANNLDK+229.163
Scan Number: 10597 precMass: 724.3663 precCharge: 3 Sequence: EDPSSQHQEANNLDK
Intensity
M/Z
type
b
y
b1
b2
b3
b4
b5
b6
b7
y1
y2
y3
y4
y5
y6
y7
y8
y9
0e+00
5e+04
1e+05
500
1000

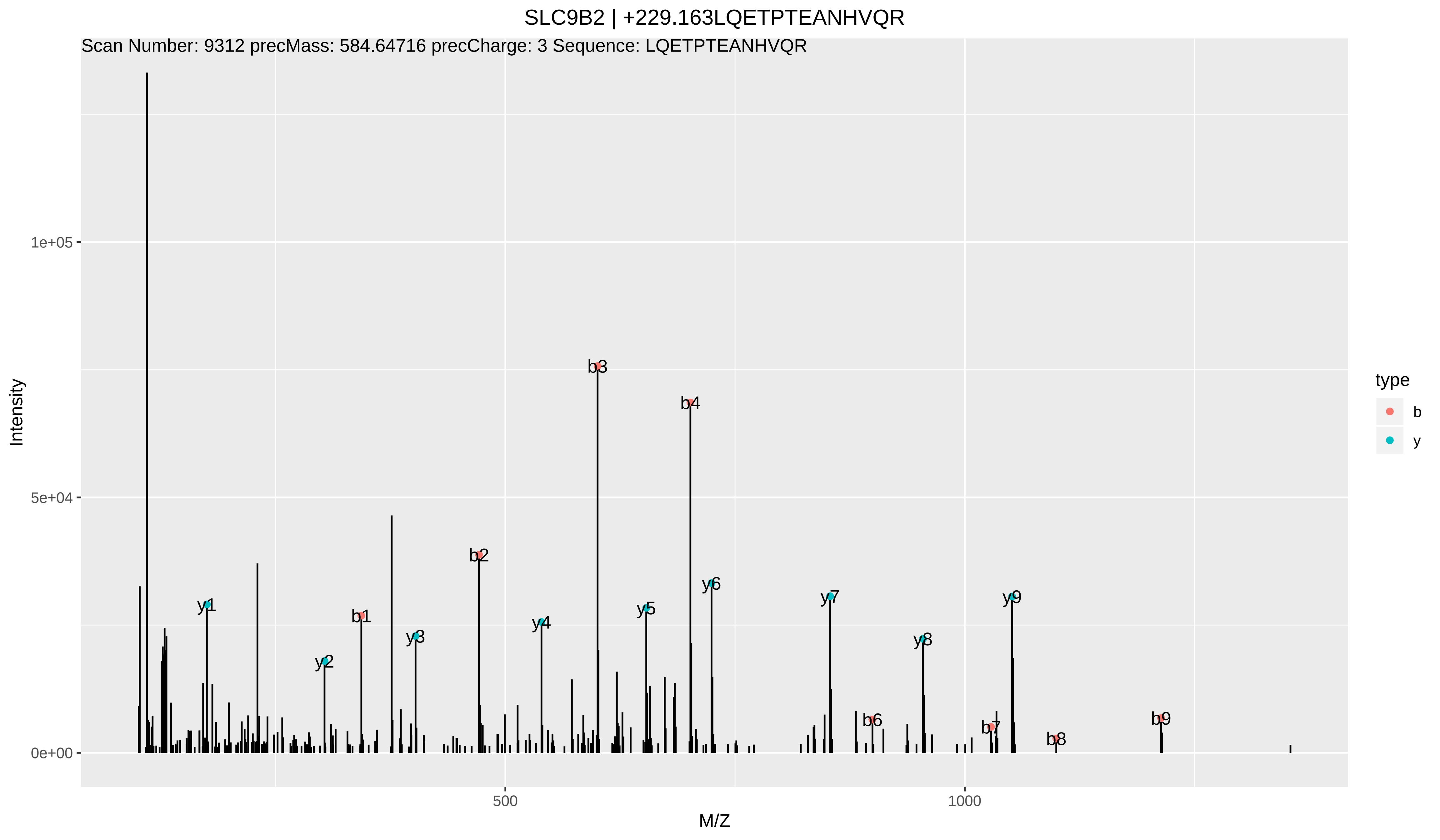

SLC9B2 | +229.163LQETPTEANHVQR
Scan Number: 9312 precMass: 584.64716 precCharge: 3 Sequence: LQETPTEANHVQR
Intensity
1e+05
5e+04
0e+00
M/Z
500
1000
b3
b4
b2
y6
y1
y5
y7
y9
b1
y4
y3
y8
y2
b6
b9
b7
b8
type
b
y

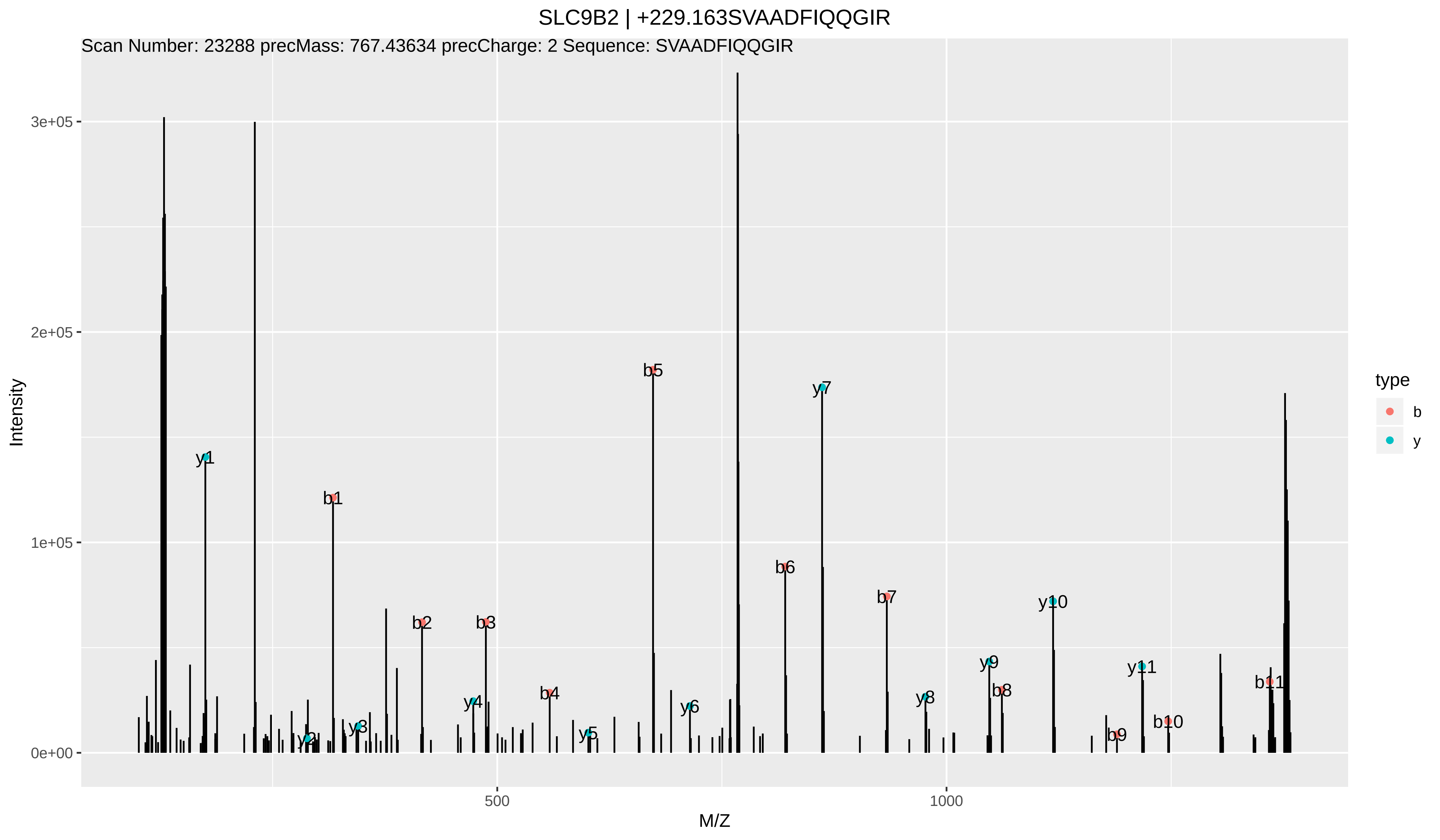

SLC9B2 | +229.163SVAADFIQQGIR
Scan Number: 23288 precMass: 767.43634 precCharge: 2 Sequence: SVAADFIQQGIR
Intensity
M/Z
3e+05
2e+05
1e+05
0e+00
500
1000
type
b
y
y1
b1
y2
y3
b2
b3
y4
b4
y5
b5
y6
b6
y7
b7
y8
y9
b8
y10
b9
y11
b10
b11

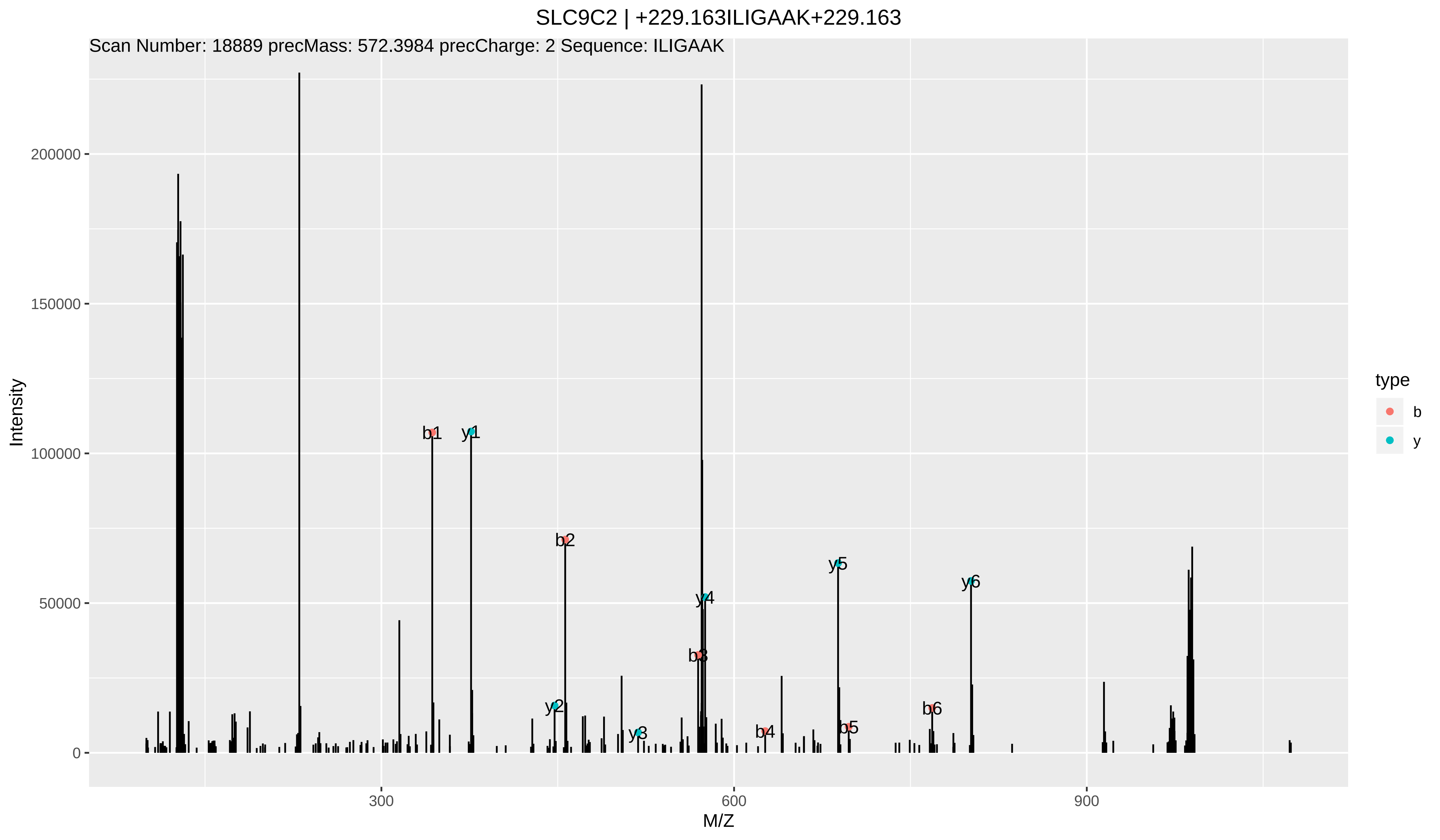
SLC9C2 | +229.163ILIGAAK+229.163
Scan Number: 18889 precMass: 572.3984 precCharge: 2 Sequence: ILIGAAK
Intensity
200000
150000
100000
50000
0
M/Z
300
600
900
b1
y1
b2
y2
y3
b3
y4
b4
y5
b5
b6
y6
type
b
y

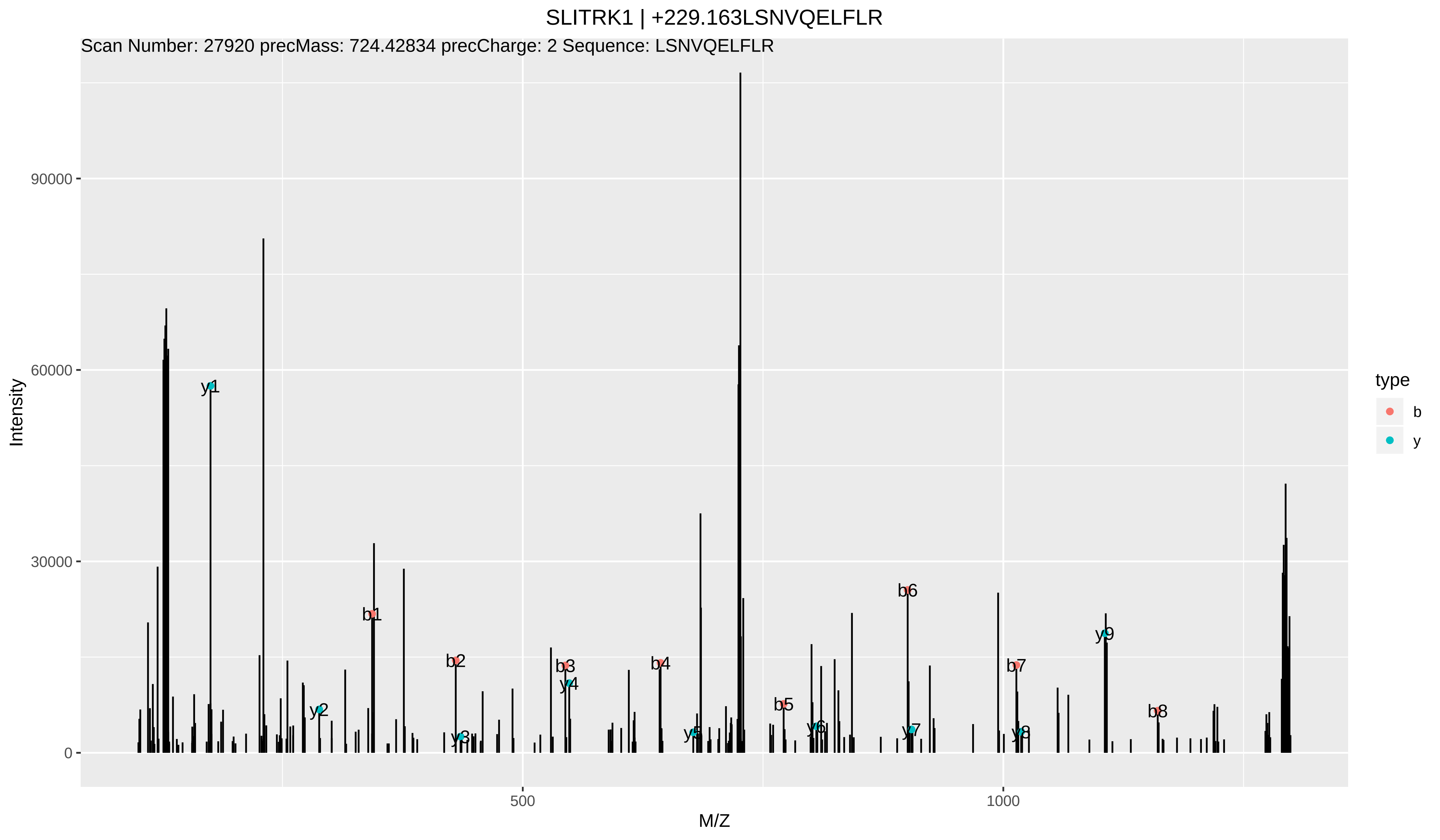
SLITRK1 | +229.163LSNVQELFLR
Scan Number: 27920 precMass: 724.42834 precCharge: 2 Sequence: LSNVQELFLR
Intensity
90000
60000
30000
0
500
1000
M/Z
y1
b1
b2
y2
y3
b3
y4
b4
y5
b5
y6
b6
y7
b7
y8
y9
b8
type
b
y

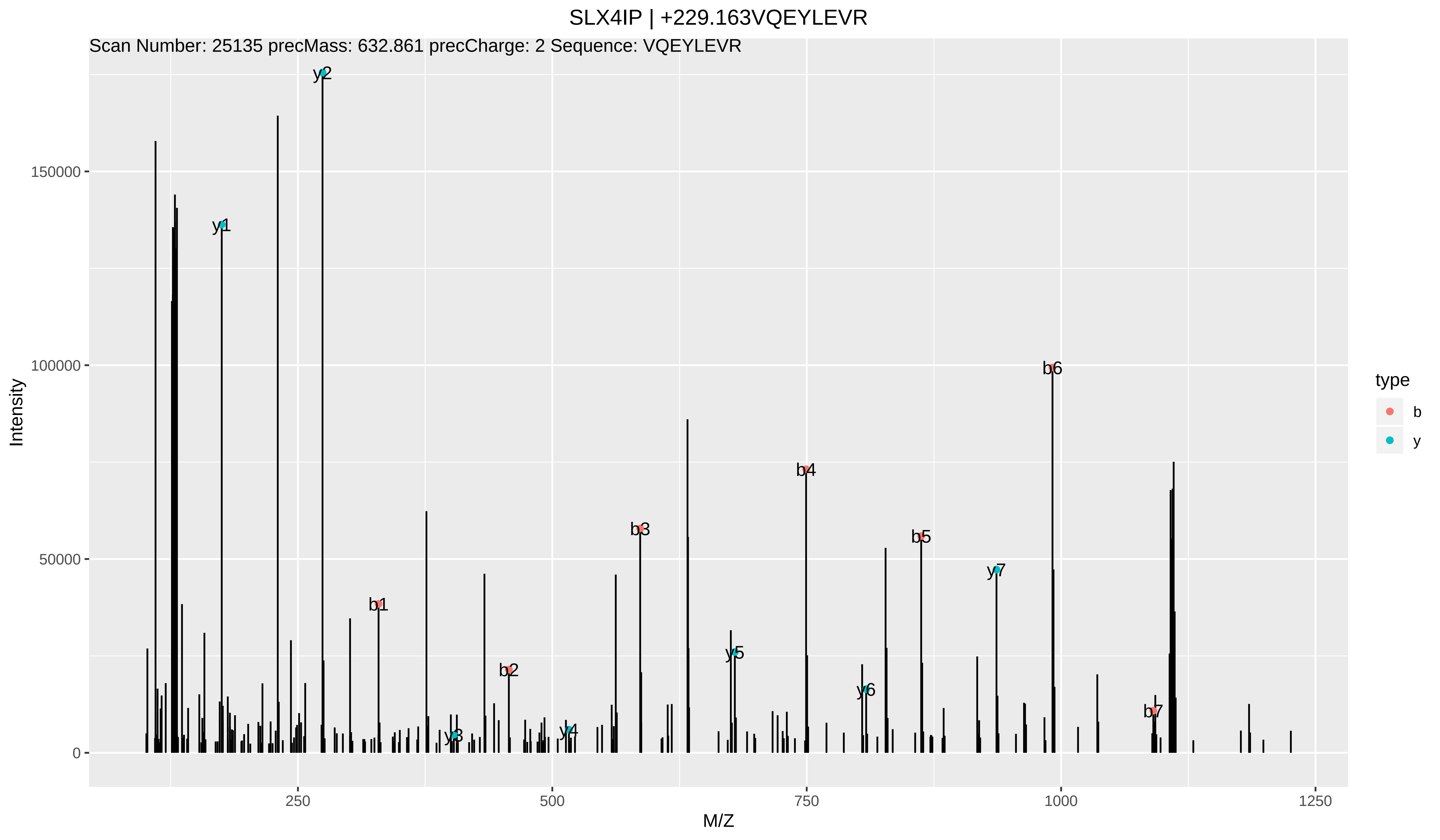

SLX4IP | +229.163VQEYLEVR
Scan Number: 25135 precMass: 632.861 precCharge: 2 Sequence: VQEYLEVR
Intensity
M/Z
type
b
y
y1
y2
y3
y4
y5
y6
y7
b1
b2
b3
b4
b5
b6
b7
0
50000
100000
150000
250
500
750
1000
1250

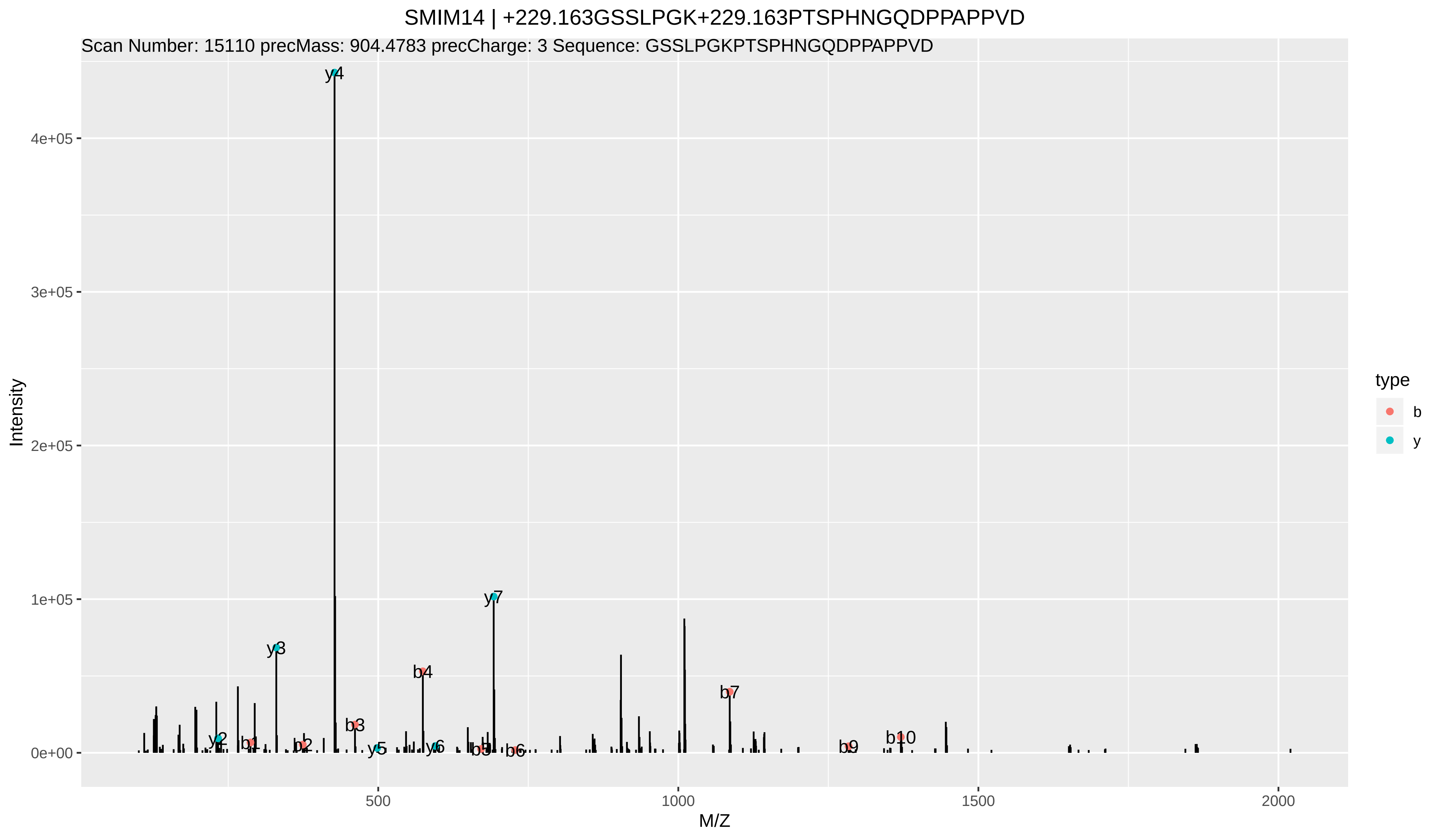

SMIM14 | +229.163GSSLPGK+229.163PTSPHNGQDPPAPPVD
Scan Number: 15110 precMass: 904.4783 precCharge: 3 Sequence: GSSLPGKPTSPHNGQDPPAPPVD
Intensity
M/Z
4e+05
3e+05
2e+05
1e+05
0e+00
500
1000
1500
2000
y4
y7
y3
b4
b7
b3
y2
b1
b2
y5
y6
b5
b6
b9
b10
type
b
y

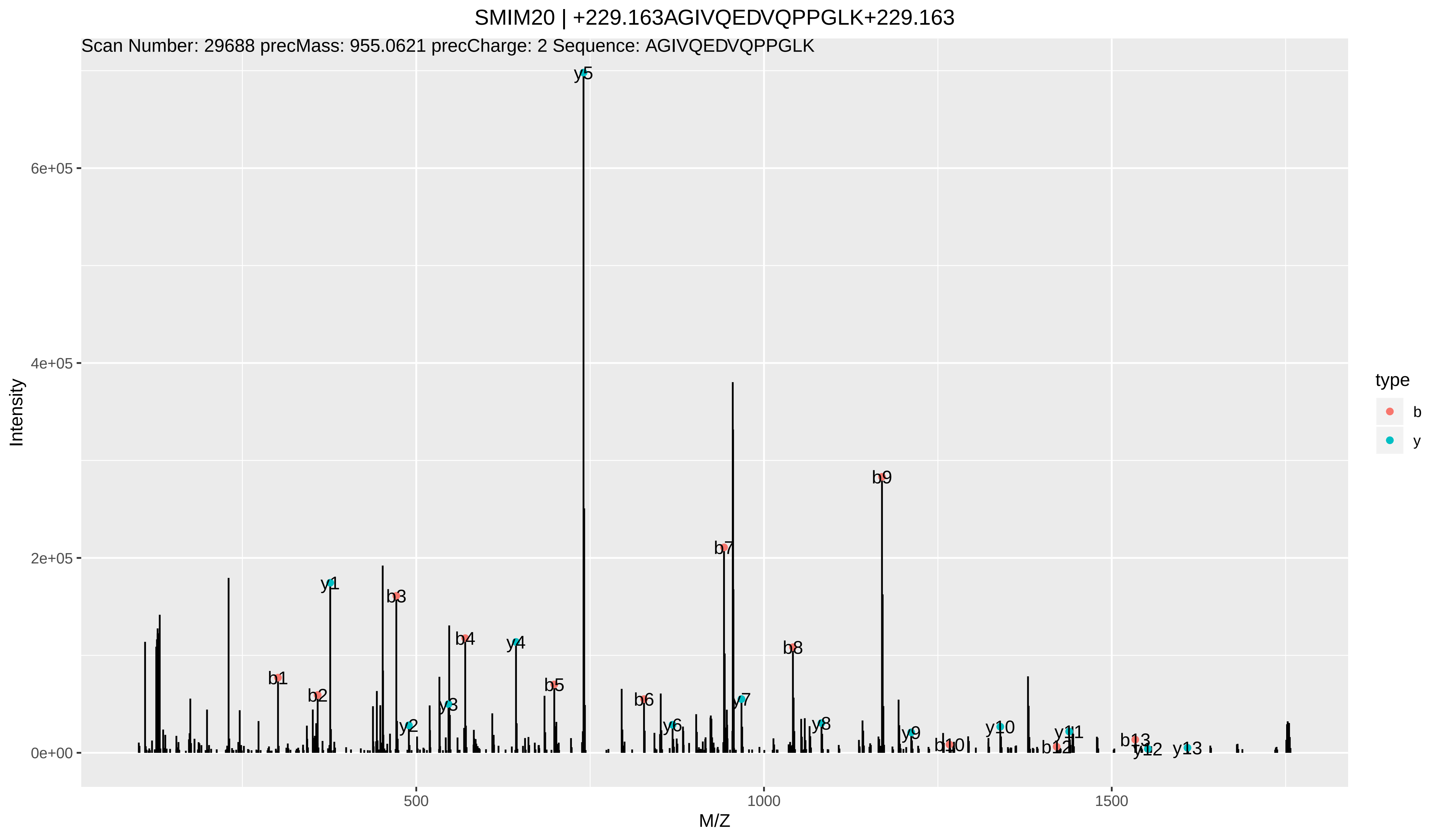

SMIM20 | +229.163AGIVQEDVQPPGLK+229.163
Scan Number: 29688 precMass: 955.0621 precCharge: 2 Sequence: AGIVQEDVQPPGLK
Intensity
M/Z
6e+05
4e+05
2e+05
0e+00
500
1000
1500
type
b
y
y5
b9
b7
y1
b3
b4
y4
b8
b1
b2
b5
b6
y7
y3
y2
y6
y8
y9
b10
y10
y11
b12
b13
y12
y13

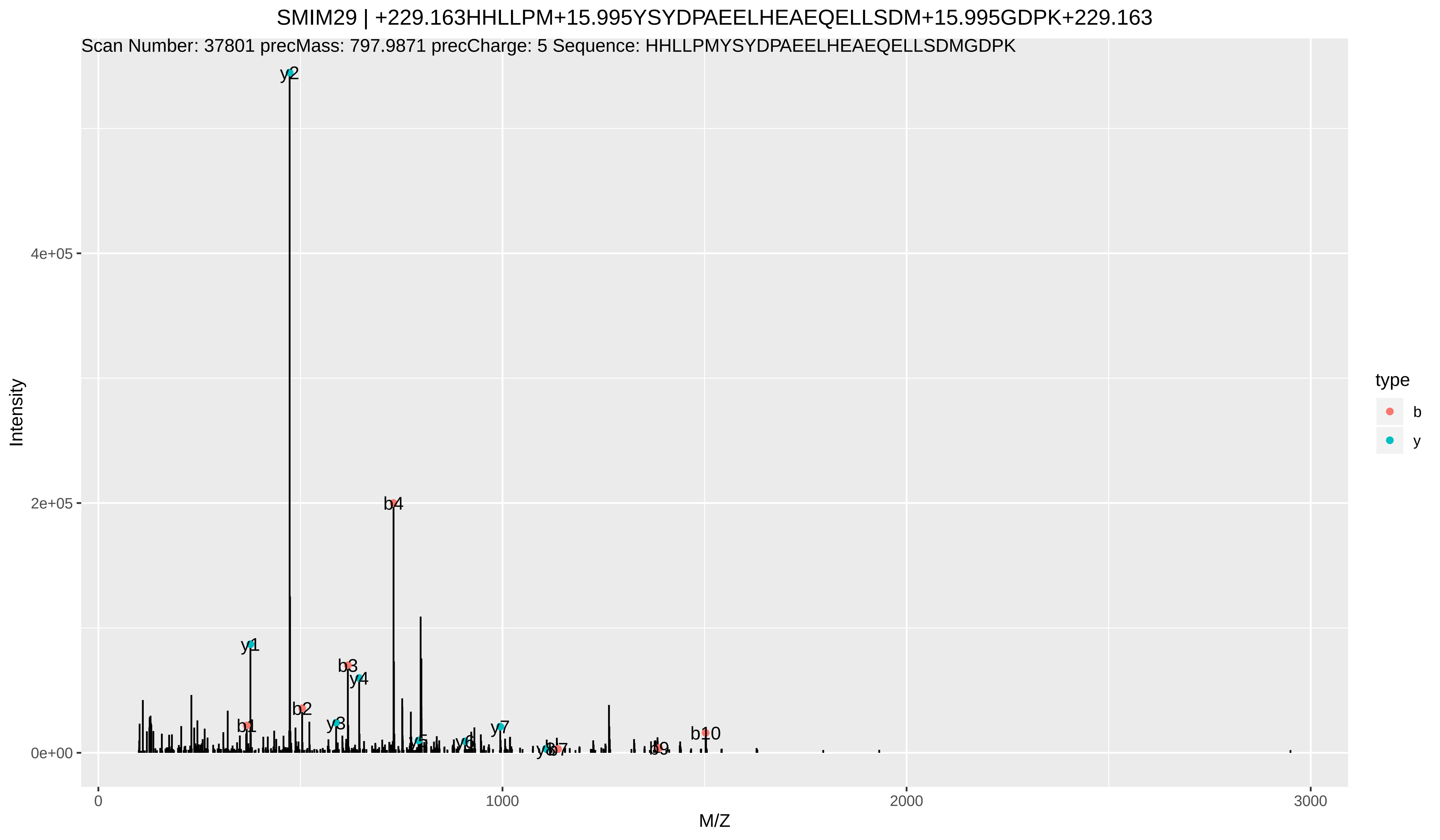

SMIM29 | +229.163HHLLPM+15.995YSYDPAEELHEAEQELLSDM+15.995GDPK+229.163
Scan Number: 37801 precMass: 797.9871 precCharge: 5 Sequence: HHLLPMYSYDPAEELHEAEQELLSDMGDPK
Intensity
M/Z
type
b
y

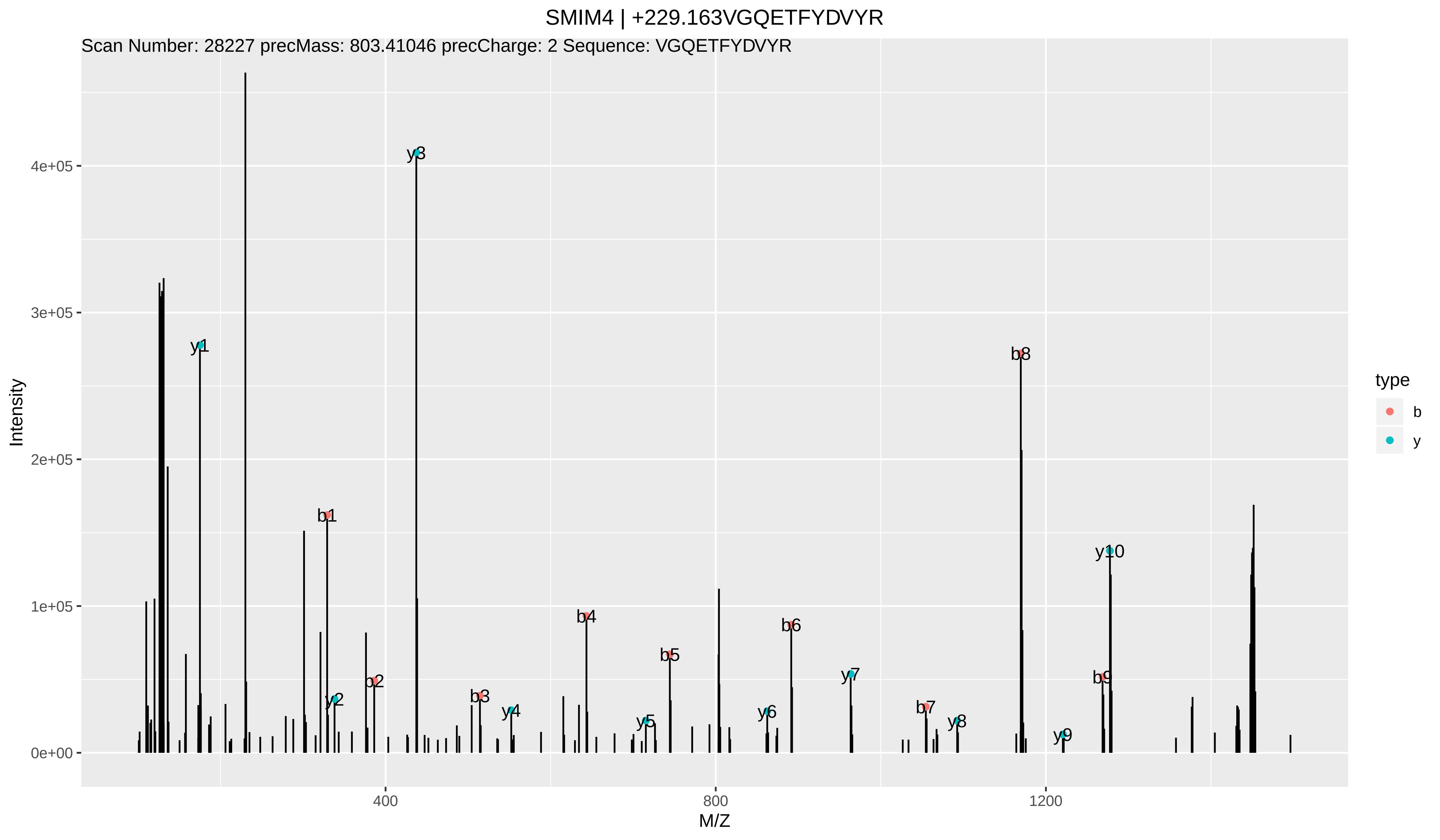

SMIM4 | +229.163VGQETFYDVYR
Scan Number: 28227 precMass: 803.41046 precCharge: 2 Sequence: VGQETFYDVYR
Intensity
M/Z
0e+00
1e+05
2e+05
3e+05
4e+05
400
800
1200
y1
y2
y3
y4
y5
y6
y7
y8
y9
y10
b1
b2
b3
b4
b5
b6
b7
b8
b9
type
b
y

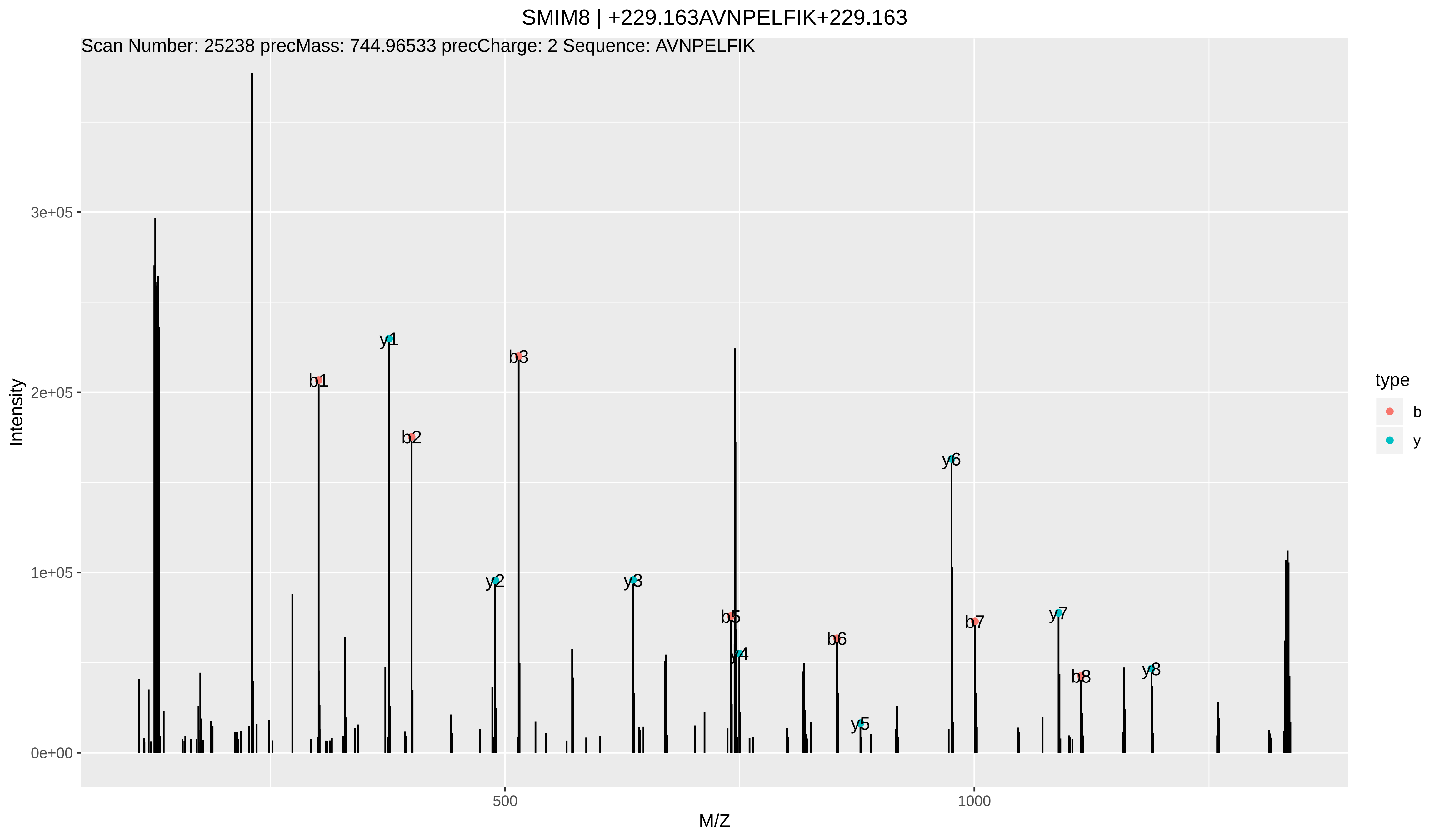
SMIM8 | +229.163AVNPELFIK+229.163
Scan Number: 25238 precMass: 744.96533 precCharge: 2 Sequence: AVNPELFIK
Intensity
3e+05
2e+05
1e+05
0e+00
M/Z
500
1000
b1
y1
b3
b2
y2
y3
b5
y4
b6
y5
y6
b7
y7
b8
y8
type
b
y

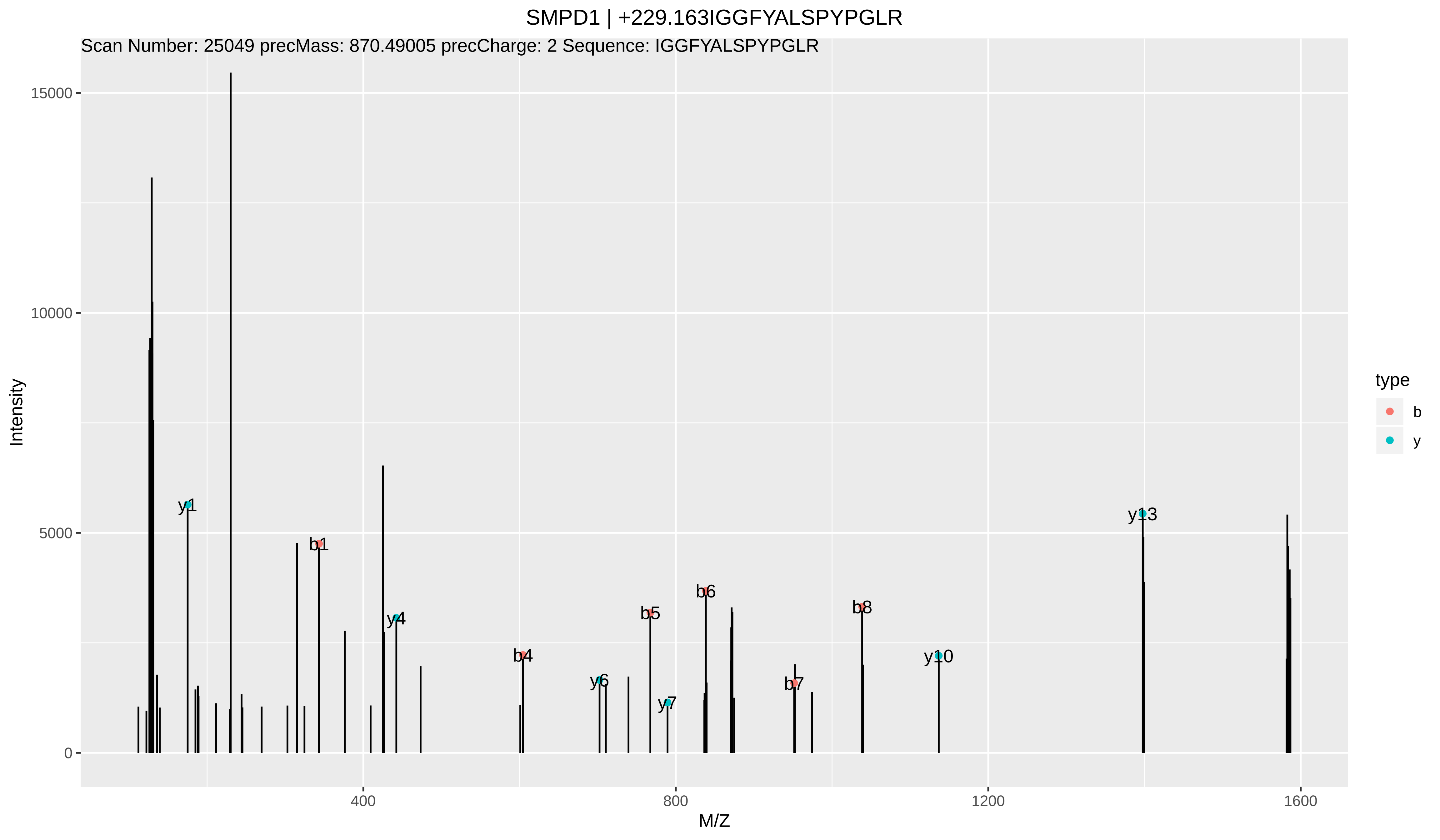
SMPD1 | +229.163IGGFYALSPYPGLR
Scan Number: 25049 precMass: 870.49005 precCharge: 2 Sequence: IGGFYALSPYPGLR
Intensity
M/Z
y1
b1
y4
b4
y6
b5
y7
b6
b7
b8
y10
y13
type
b
y
0
5000
10000
15000
400
800
1200
1600

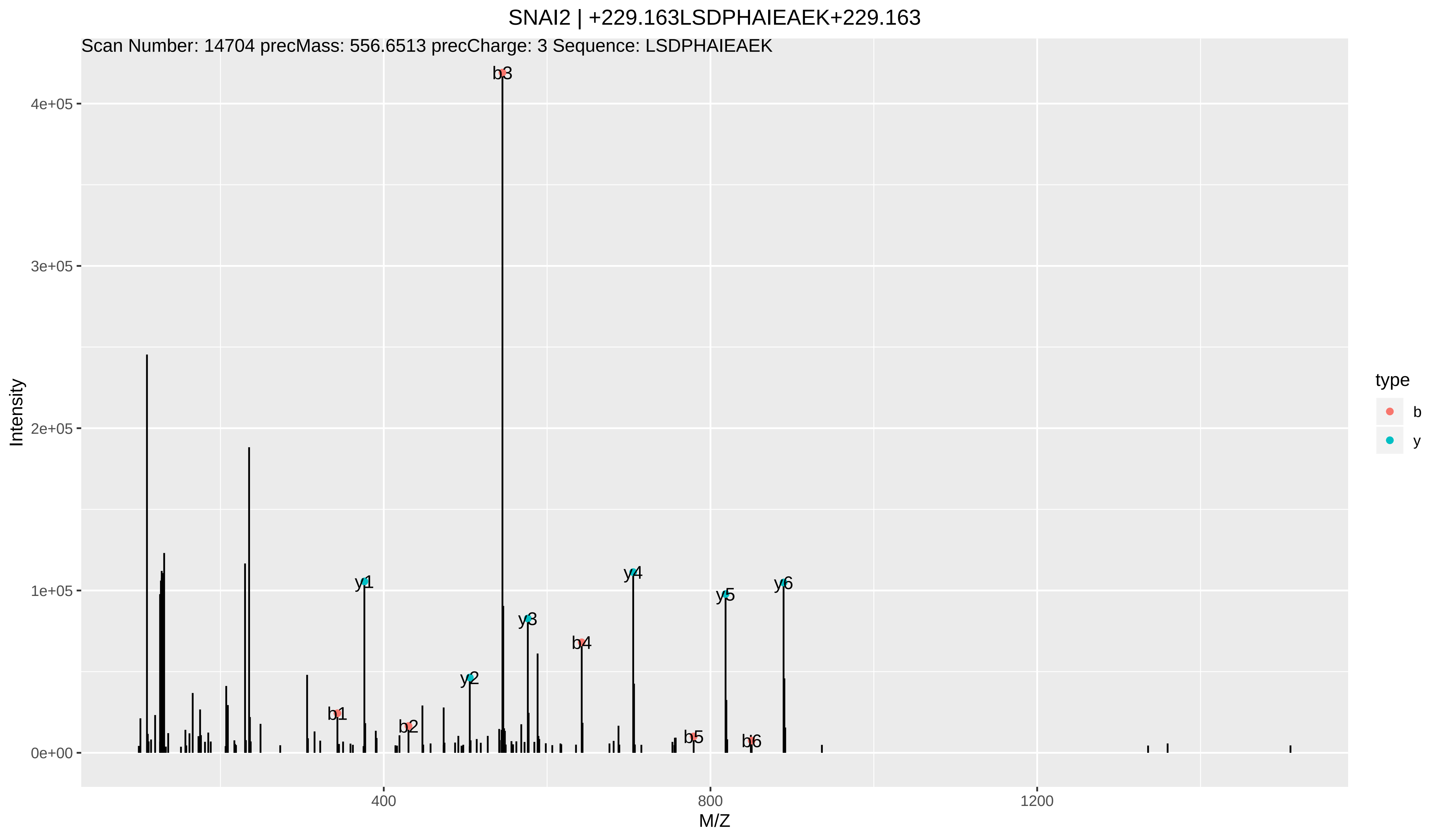
SNAI2 | +229.163LSDPHAIEAEK+229.163
Scan Number: 14704 precMass: 556.6513 precCharge: 3 Sequence: LSDPHAIEAEK
Intensity
M/Z
type
b
y
b1
b2
b3
b4
b5
b6
y1
y2
y3
y4
y5
y6
0e+00
1e+05
2e+05
3e+05
4e+05
400
800
1200

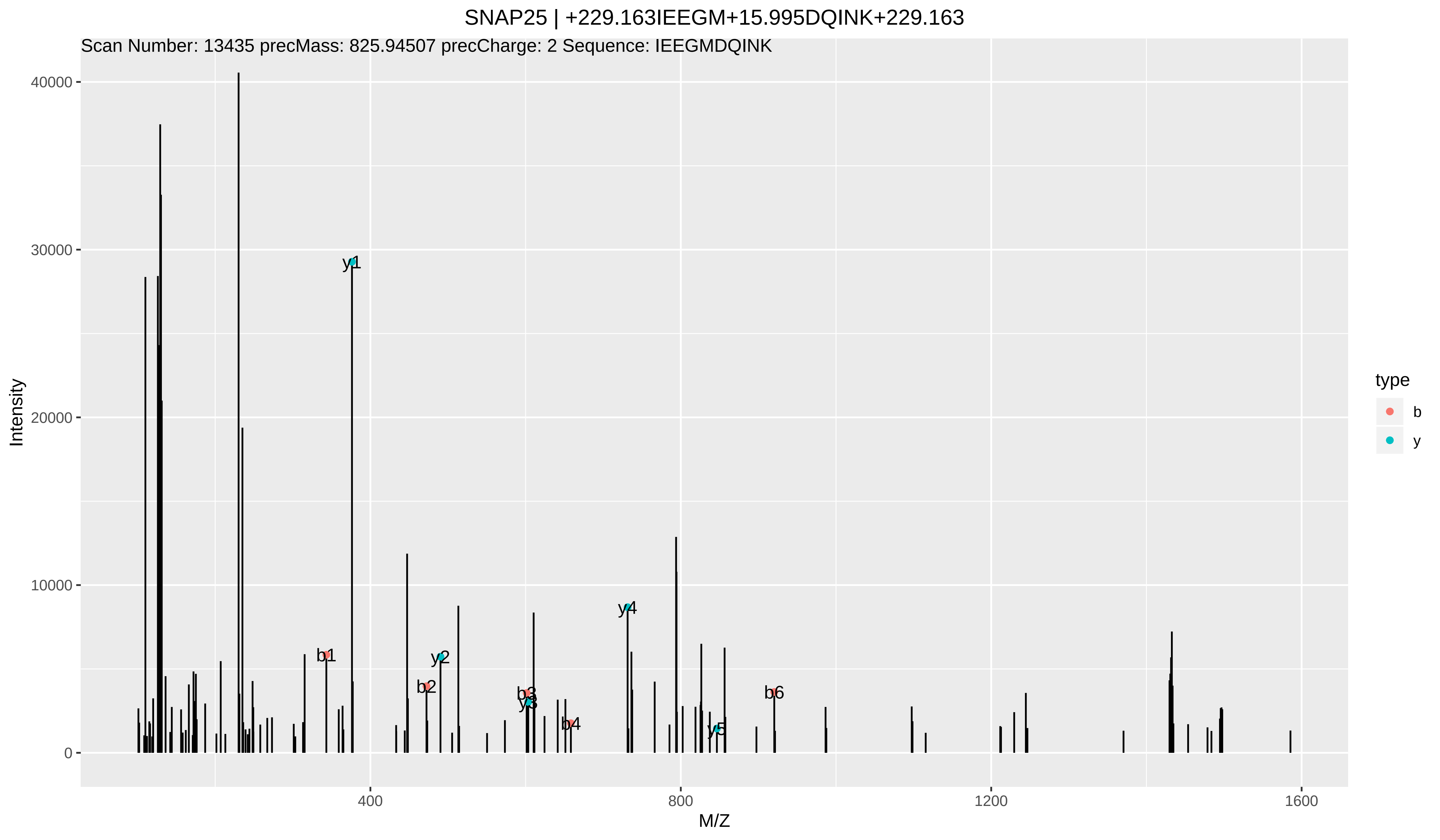
SNAP25 | +229.163IEEGM+15.995DQINK+229.163
Scan Number: 13435 precMass: 825.94507 precCharge: 2 Sequence: IEEGMDQINK
Intensity
40000
30000
20000
10000
0
400
800
1200
1600
M/Z
y1
y2
y3
y4
y5
b1
b2
b3
b4
b6
type
b
y

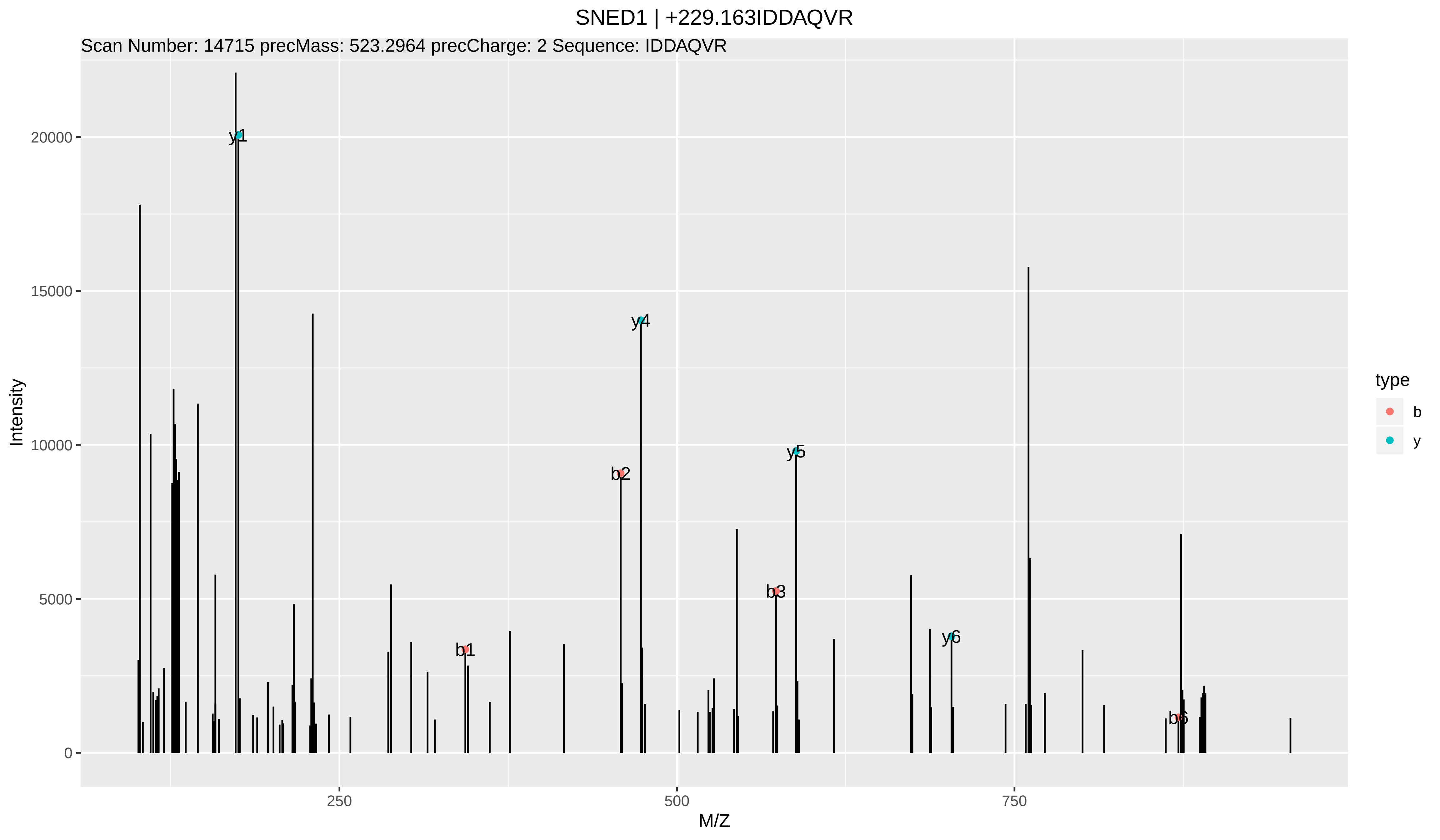

SNED1 | +229.163IDDAQVR
Scan Number: 14715 precMass: 523.2964 precCharge: 2 Sequence: IDDAQVR
Intensity
M/Z
type
b
y
y1
y4
y5
y6
b1
b2
b3
b6
0
5000
10000
15000
20000
250
500
750

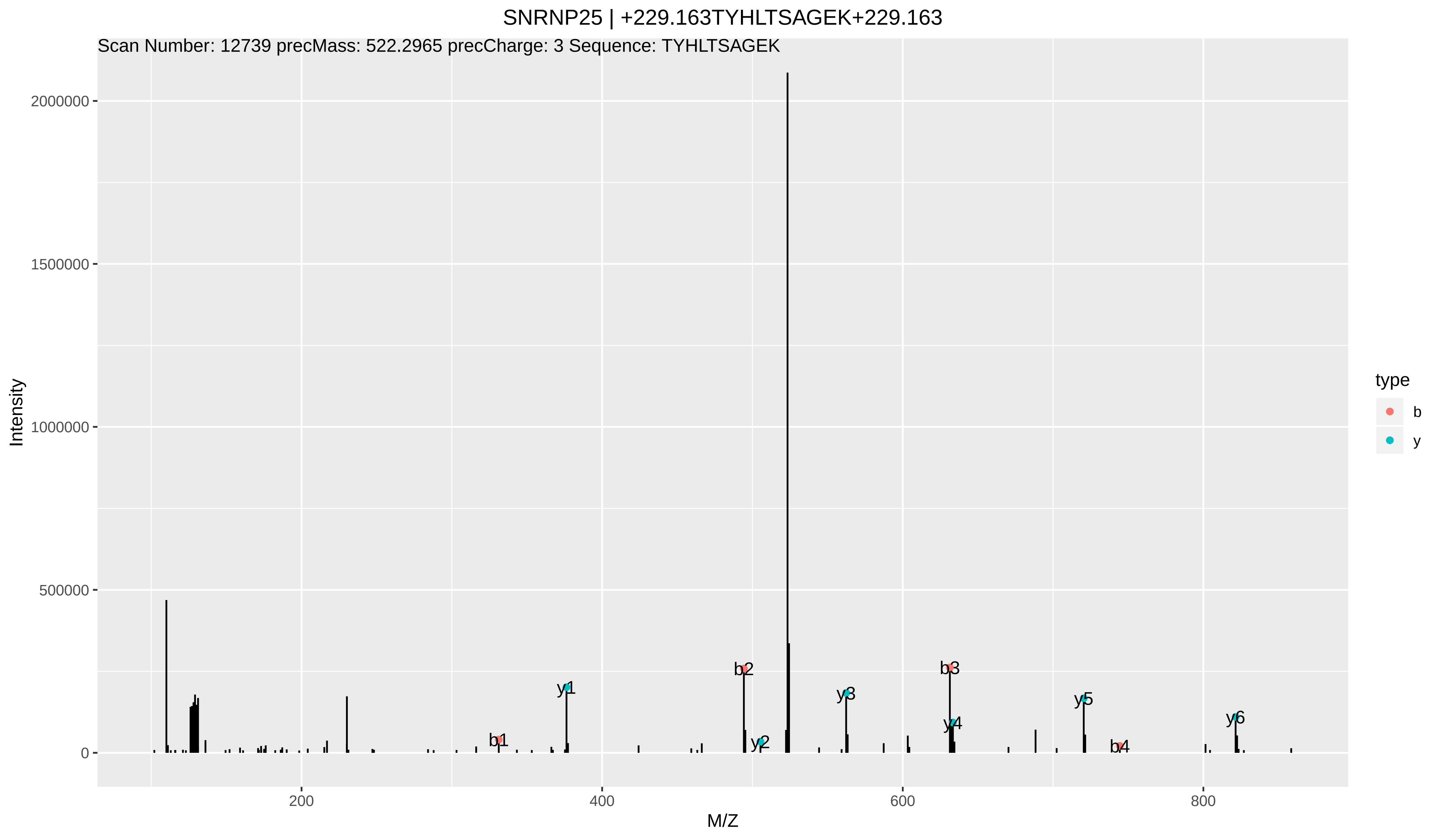
SNRNP25 | +229.163TYHLTSAGEK+229.163
Scan Number: 12739 precMass: 522.2965 precCharge: 3 Sequence: TYHLTSAGEK
Intensity
M/Z
2000000
1500000
1000000
500000
0
200
400
600
800
b1
y1
b2
y2
y3
b3
y4
y5
b4
y6
type
b
y

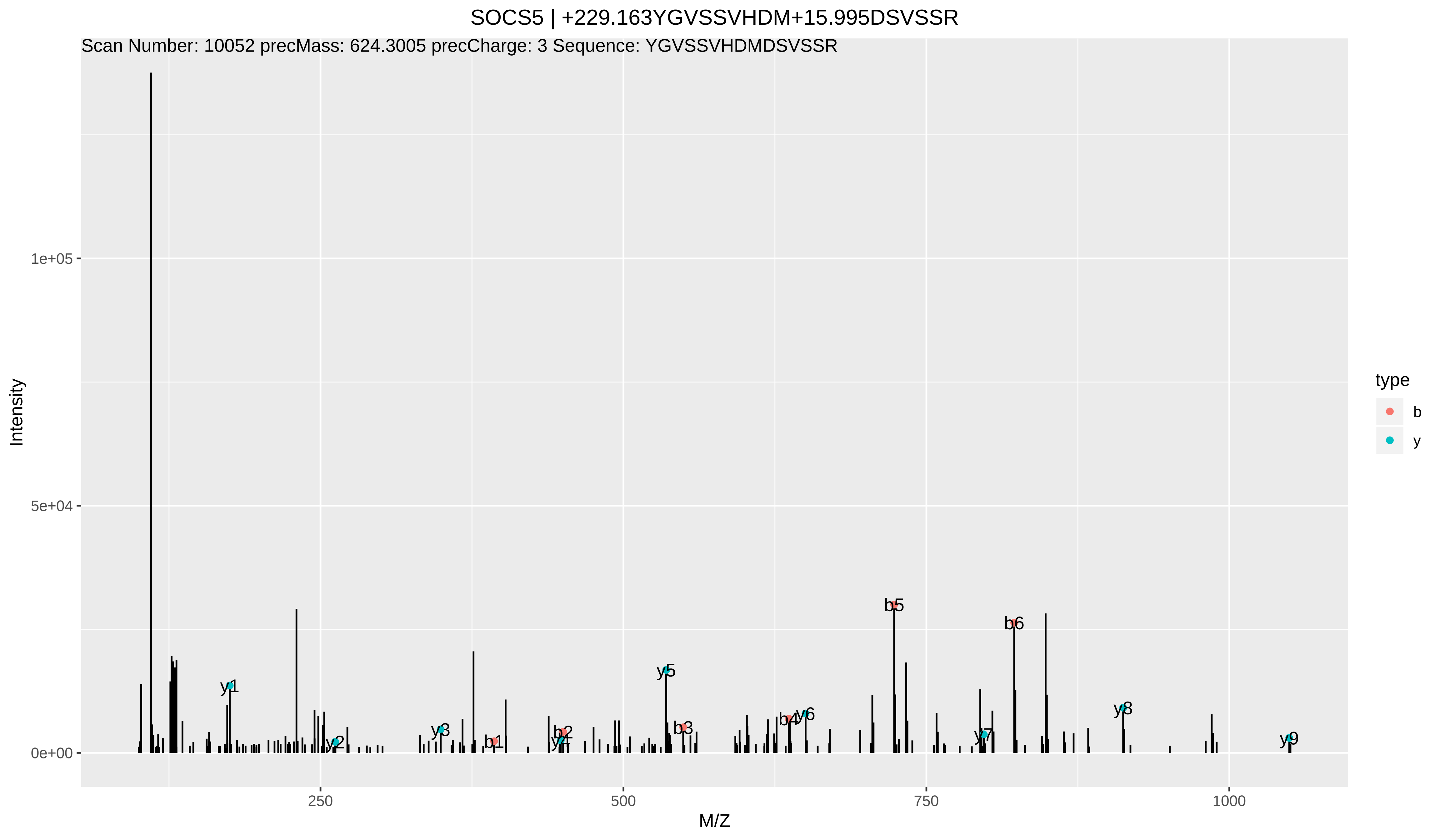

SOCS5 | +229.163YGVSSVHDM+15.995DSVSSR
Scan Number: 10052 precMass: 624.3005 precCharge: 3 Sequence: YGVSSVHDMDSVSSR
Intensity
1e+05
5e+04
0e+00
M/Z
250
500
750
1000
y1
y2
y3
b1
b2
y4
y5
b3
b4
y6
b5
y7
b6
y8
y9
type
b
y

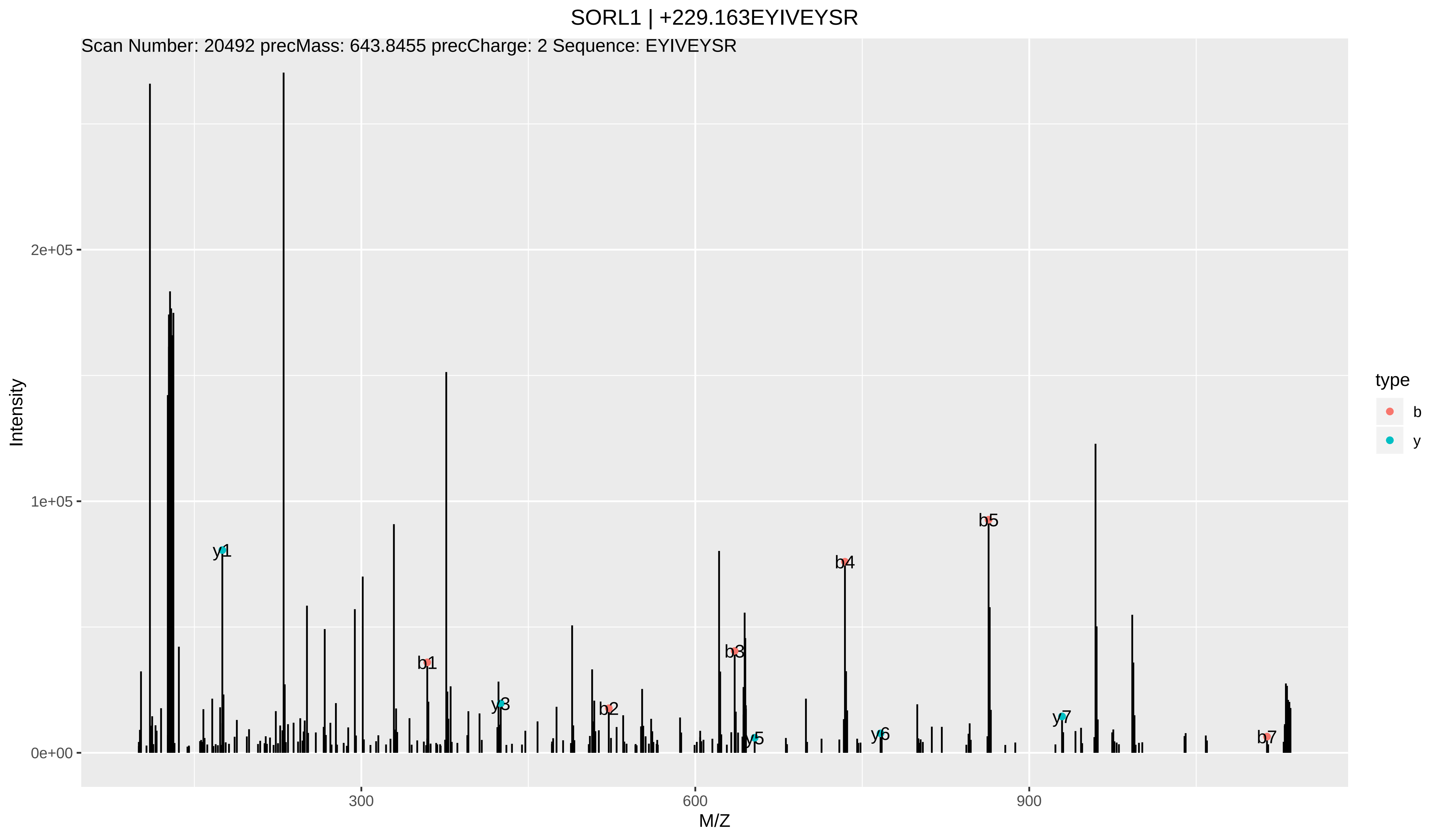
SORL1 | +229.163EYIVEYSR
Scan Number: 20492 precMass: 643.8455 precCharge: 2 Sequence: EYIVEYSR
Intensity
M/Z
0e+00
1e+05
2e+05
300
600
900
y1
b1
y3
b2
b3
y5
b4
y6
b5
y7
b7
type
b
y

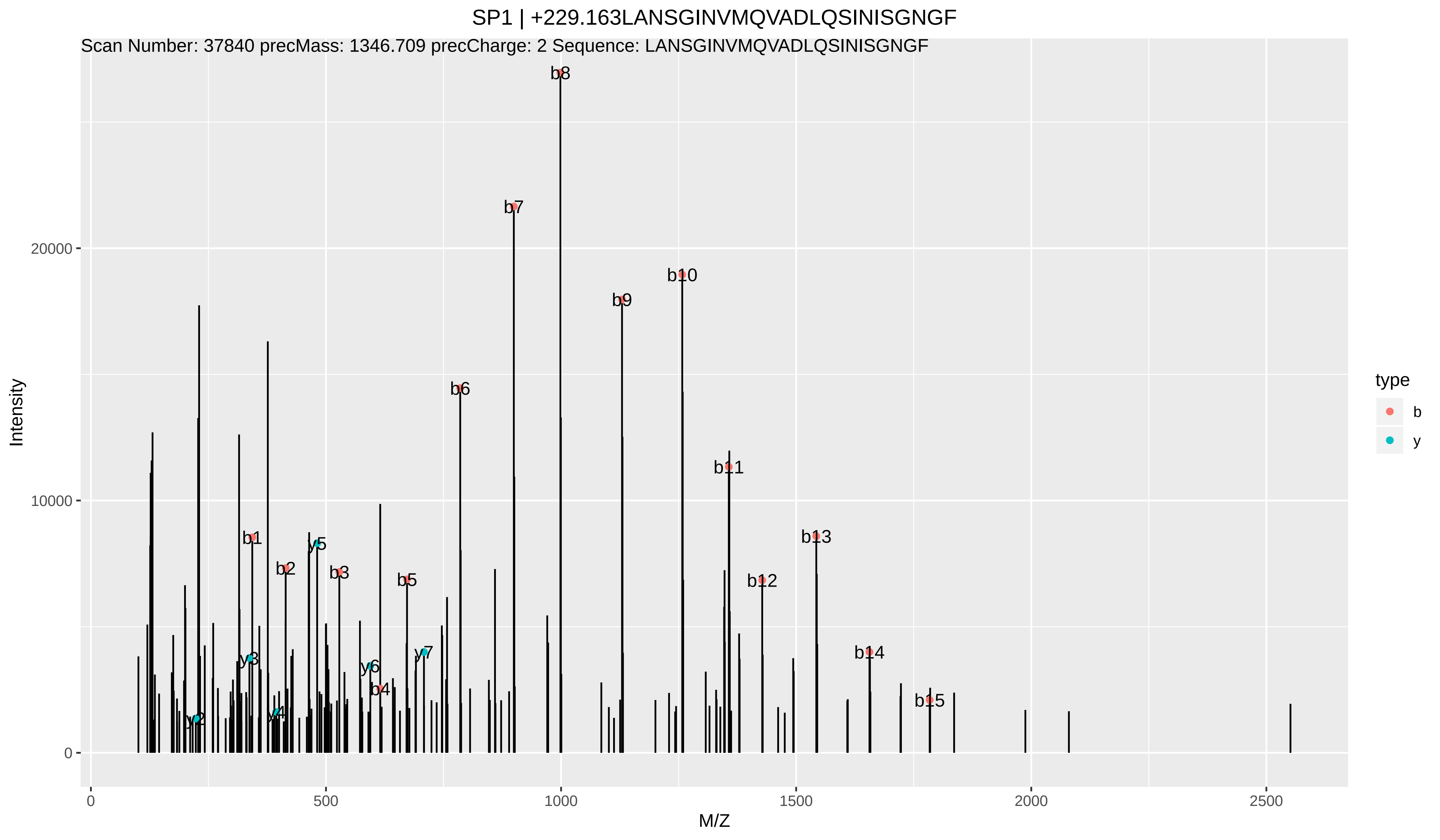

SP1 | +229.163LANSGINVMQVADLQSINISGNGF
Scan Number: 37840 precMass: 1346.709 precCharge: 2 Sequence: LANSGINVMQVADLQSINISGNGF
Intensity
M/Z
type
b
y
0
10000
20000
0
500
1000
1500
2000
2500
b1
b2
b3
b4
b5
b6
b7
b8
b9
b10
b11
b12
b13
b14
b15
y2
y3
y4
y5
y6
y7

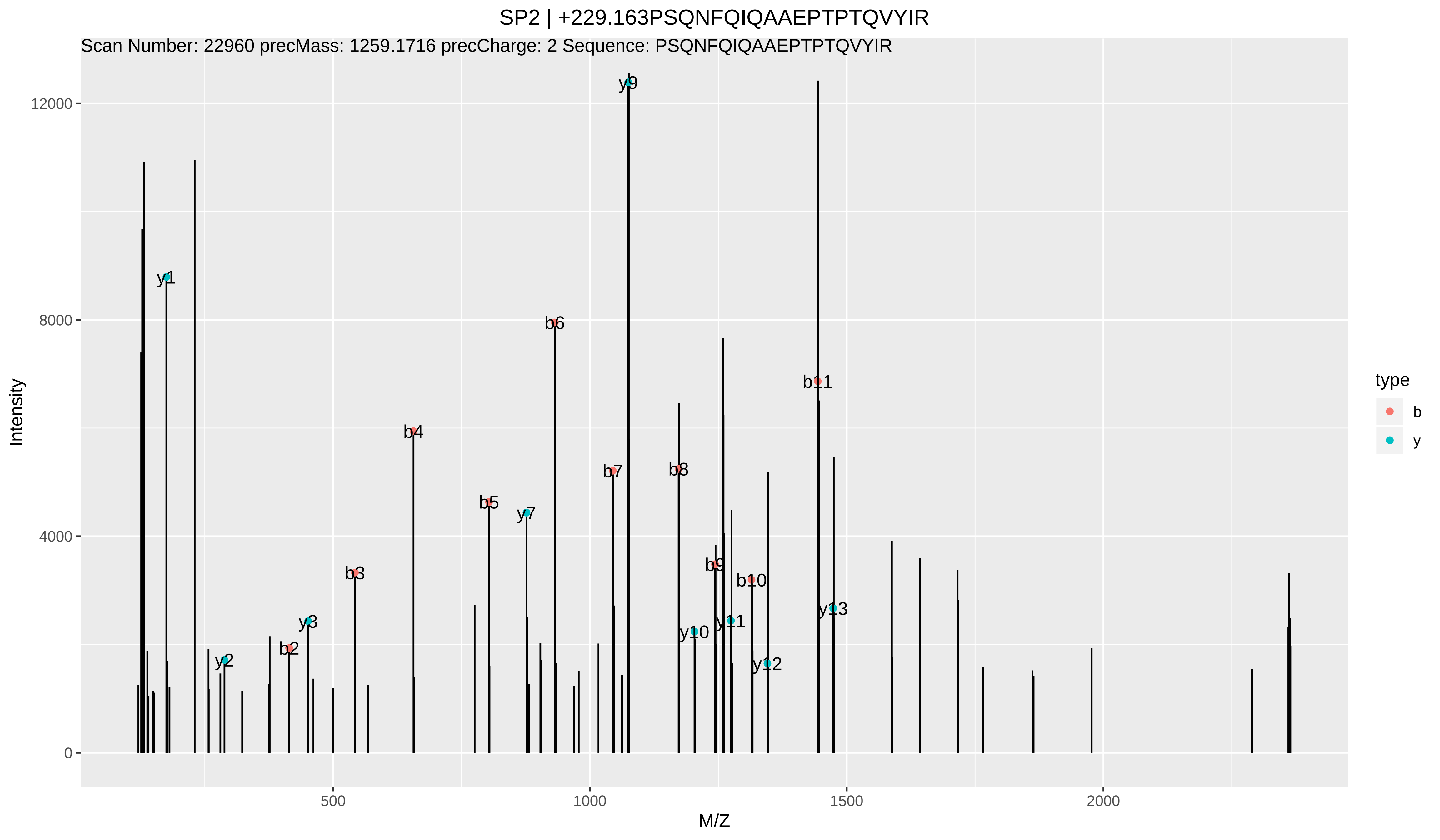
SP2 | +229.163PSQNFQIQAAEPTPTQVYIR
Scan Number: 22960 precMass: 1259.1716 precCharge: 2 Sequence: PSQNFQIQAAEPTPTQVYIR
Intensity
M/Z
type
b
y
y1
y2
y3
y7
y9
y10
y11
y12
y13
b2
b3
b4
b5
b6
b7
b8
b9
b10
b11
0
4000
8000
12000
500
1000
1500
2000

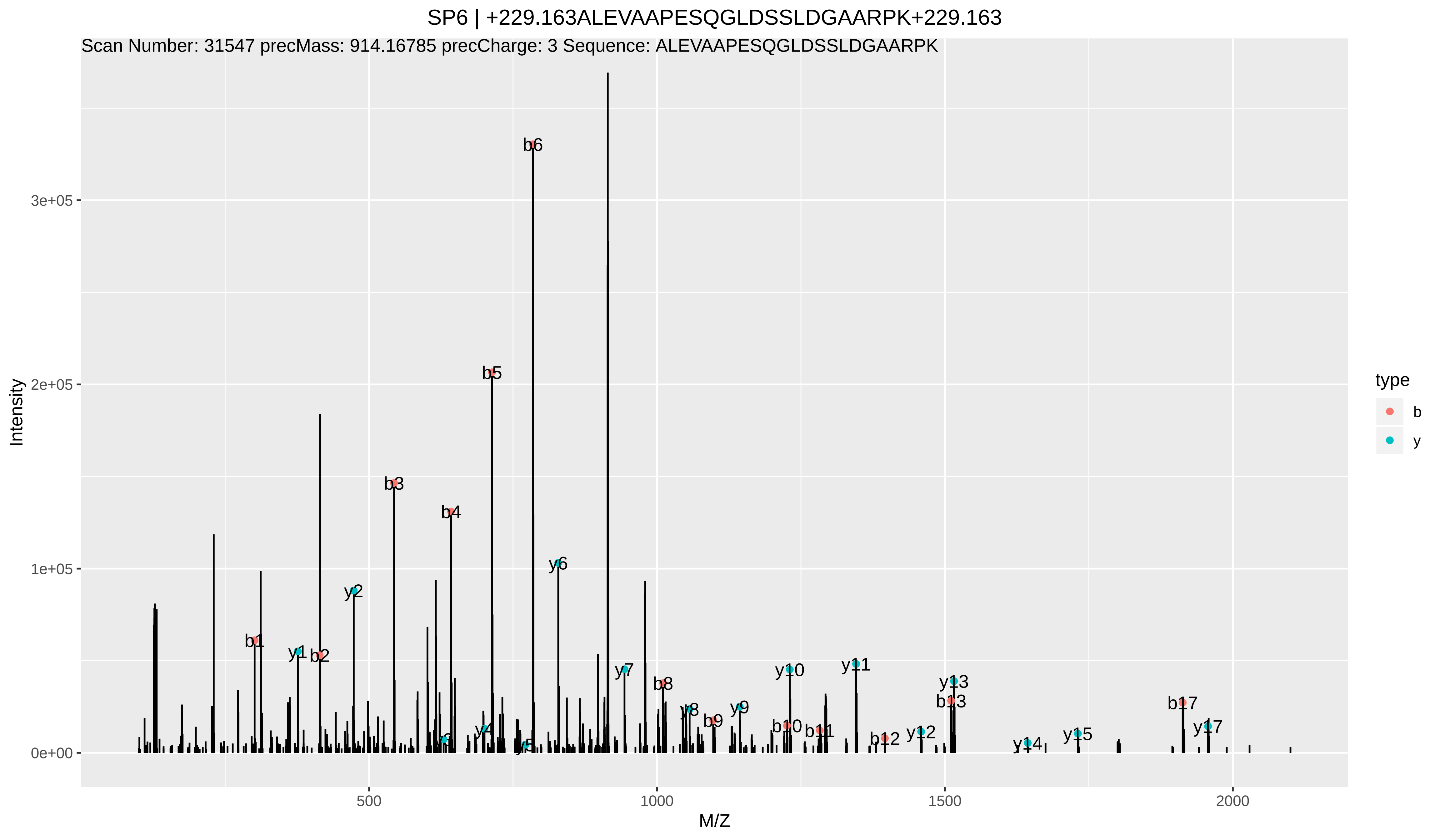
SP6 | +229.163ALEVAAPESQGLDSSLDGAARPK+229.163
Scan Number: 31547 precMass: 914.16785 precCharge: 3 Sequence: ALEVAAPESQGLDSSLDGAARPK
Intensity
M/Z
type
b
y
b1
b2
b3
b4
b5
b6
b8
b9
b10
b11
b12
b13
b17
y1
y2
y4
y6
y7
y8
y9
y10
y11
y12
y13
y14
y15
y17
3e+05
2e+05
1e+05
0e+00
500
1000
1500
2000

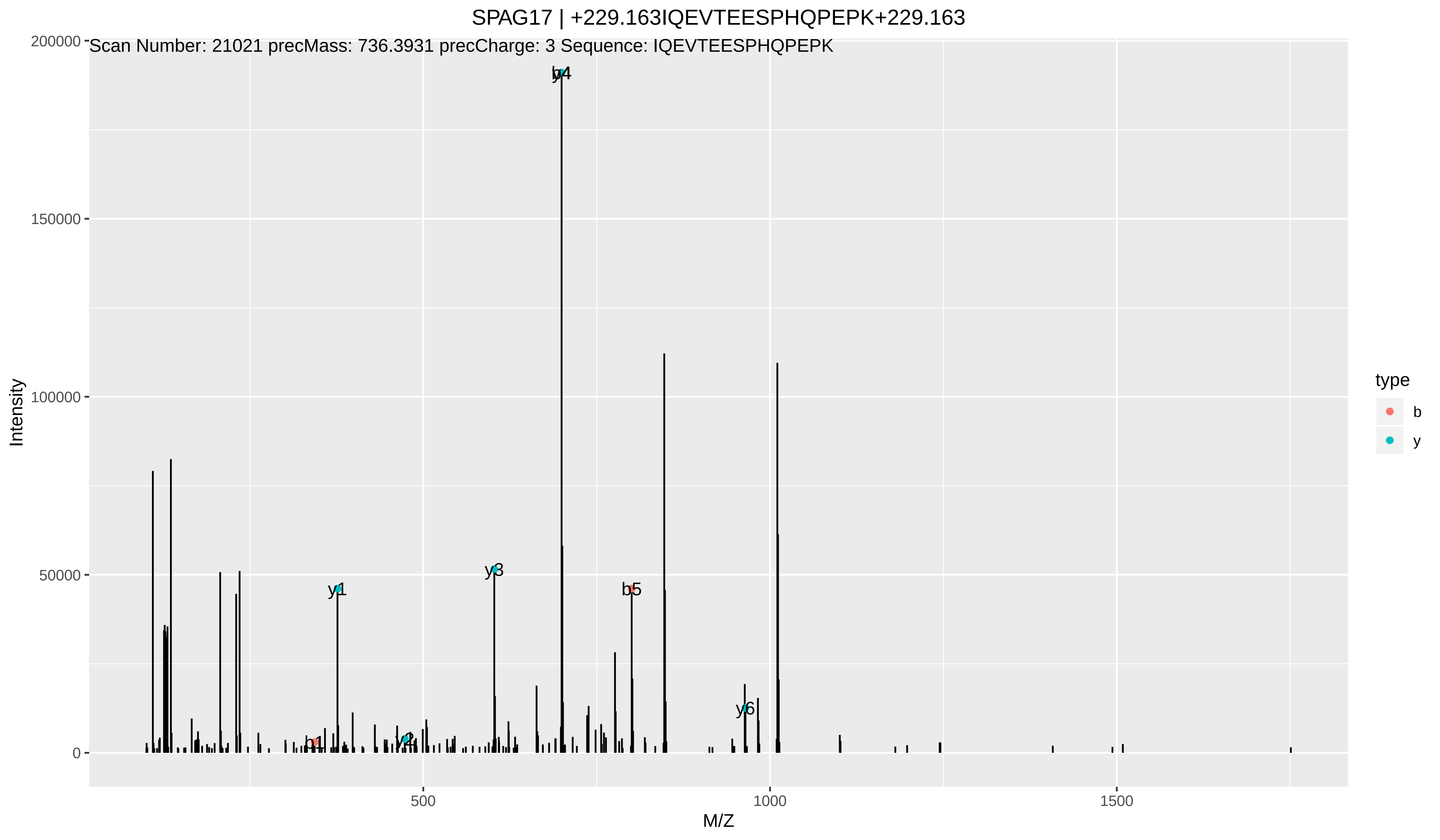

SPAG17 | +229.163IQEVTEESPHQPEPK+229.163
Scan Number: 21021 precMass: 736.3931 precCharge: 3 Sequence: IQEVTEESPHQPEPK
200000
150000
100000
50000
0
Intensity
500
1000
1500
M/Z
b4
y4
y3
y1
b5
y6
b1
y2
type
b
y

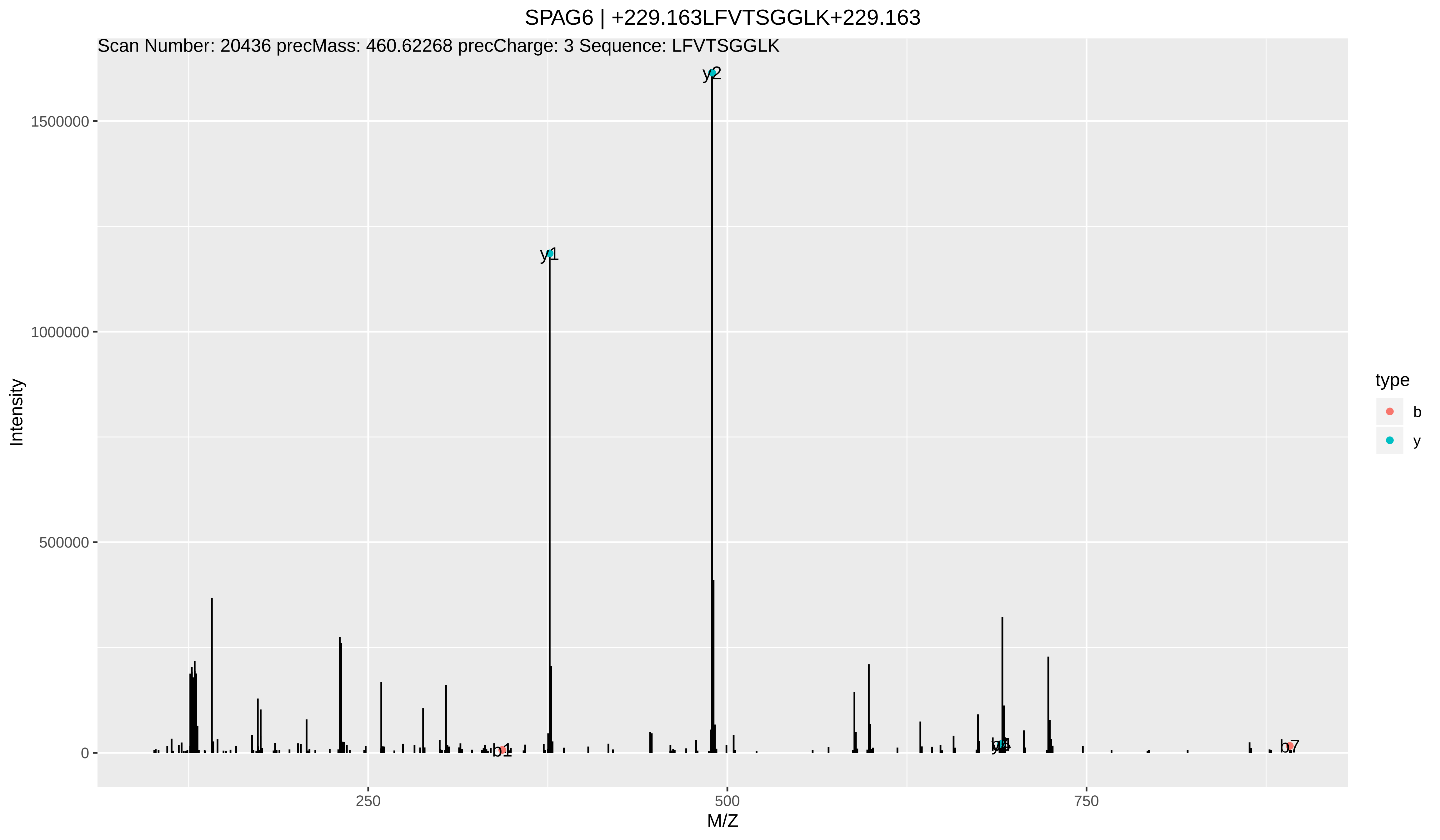

SPAG6 | +229.163LFVTSGGLK+229.163
Scan Number: 20436 precMass: 460.62268 precCharge: 3 Sequence: LFVTSGGLK
y2
y1
b1
b7
Intensity
1500000
1000000
500000
0
250
500
750
M/Z
type
b
y

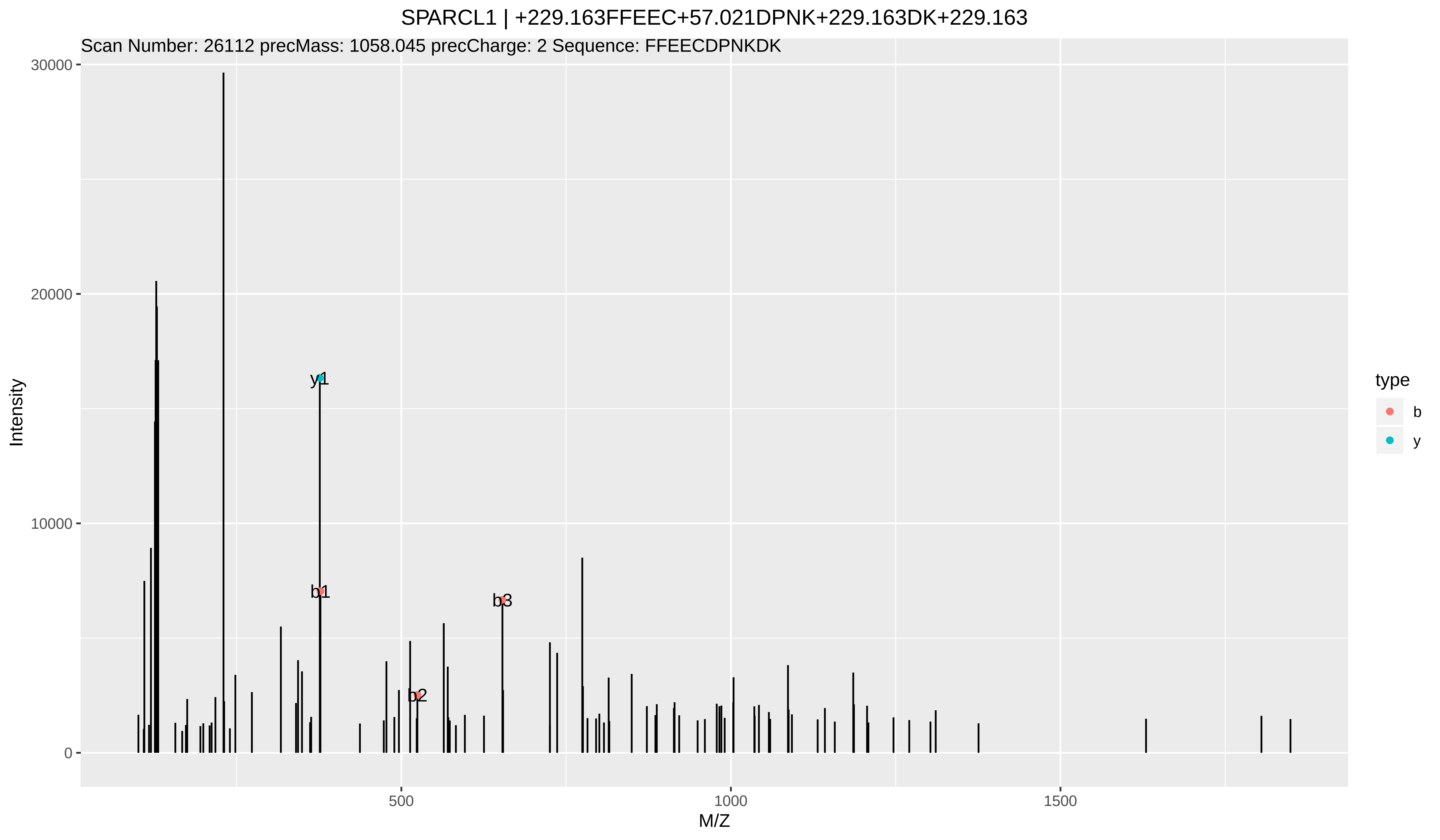
SPARCL1 | +229.163FFEEC+57.021DPNK+229.163DK+229.163
Scan Number: 26112 precMass: 1058.045 precCharge: 2 Sequence: FFEECDPNKDK
Intensity
M/Z
30000
20000
10000
0
500
1000
1500
y1
b1
b2
b3
type
b
y

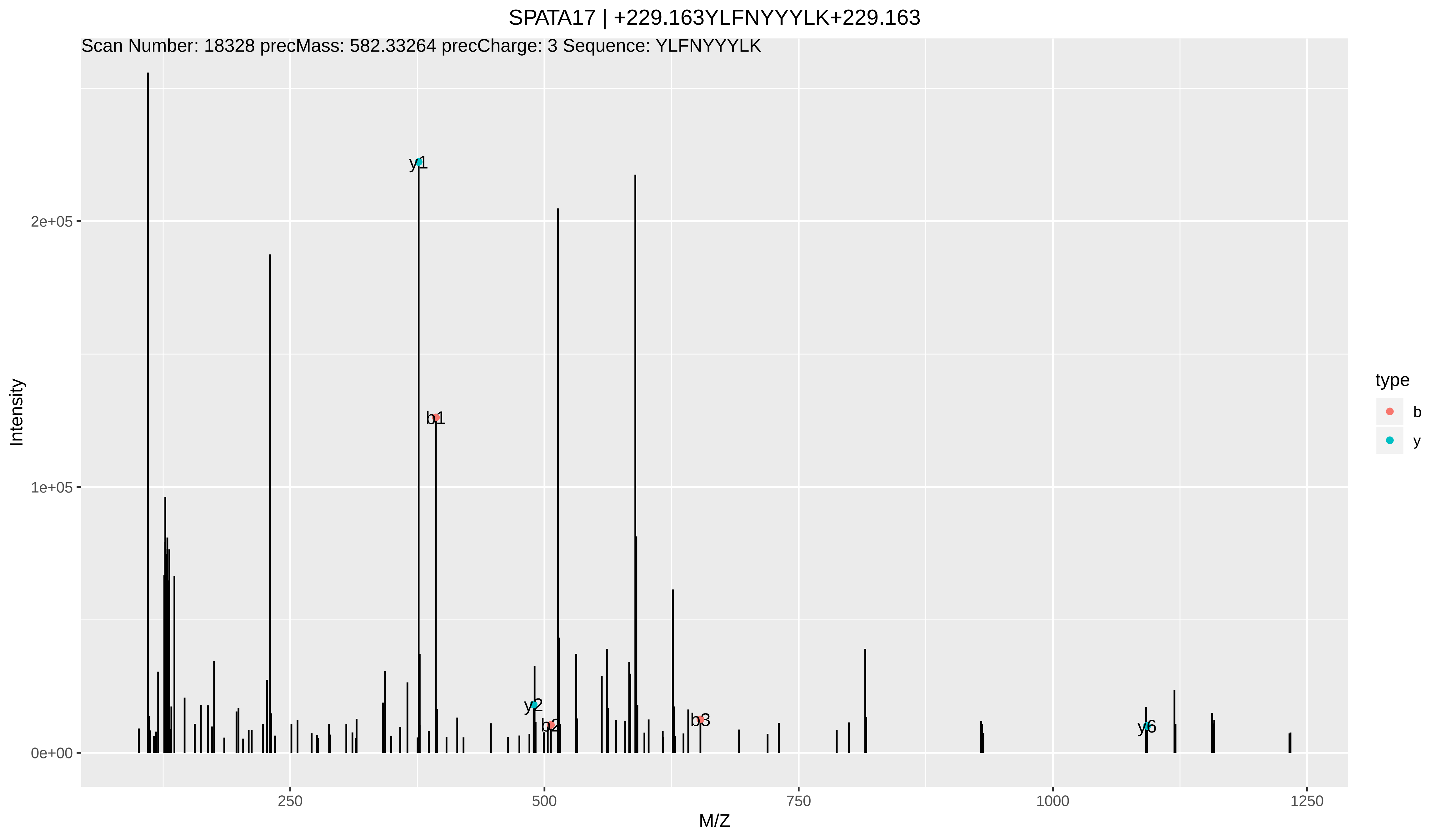
SPATA17 | +229.163YLFNYYYLK+229.163
Scan Number: 18328 precMass: 582.33264 precCharge: 3 Sequence: YLFNYYYLK
y1
b1
y2
b2
b3
y6
2e+05
1e+05
0e+00
Intensity
250
500
750
1000
1250
M/Z
type
b
y

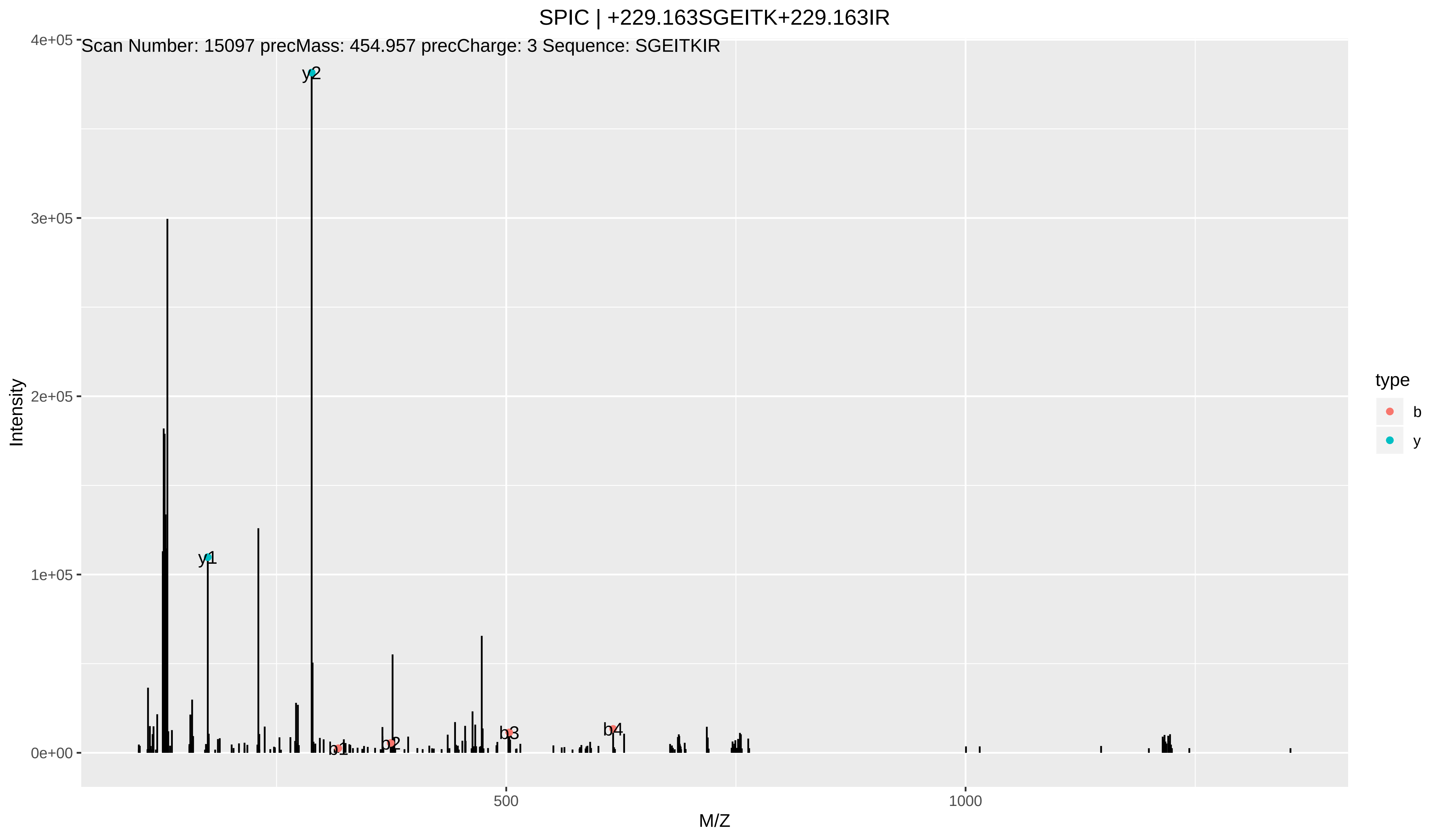
SPIC | +229.163SGEITK+229.163IR
Scan Number: 15097 precMass: 454.957 precCharge: 3 Sequence: SGEITKIR
Intensity
M/Z
4e+05
3e+05
2e+05
1e+05
0e+00
500
1000
y2
y1
b1
b2
b3
b4
type
b
y

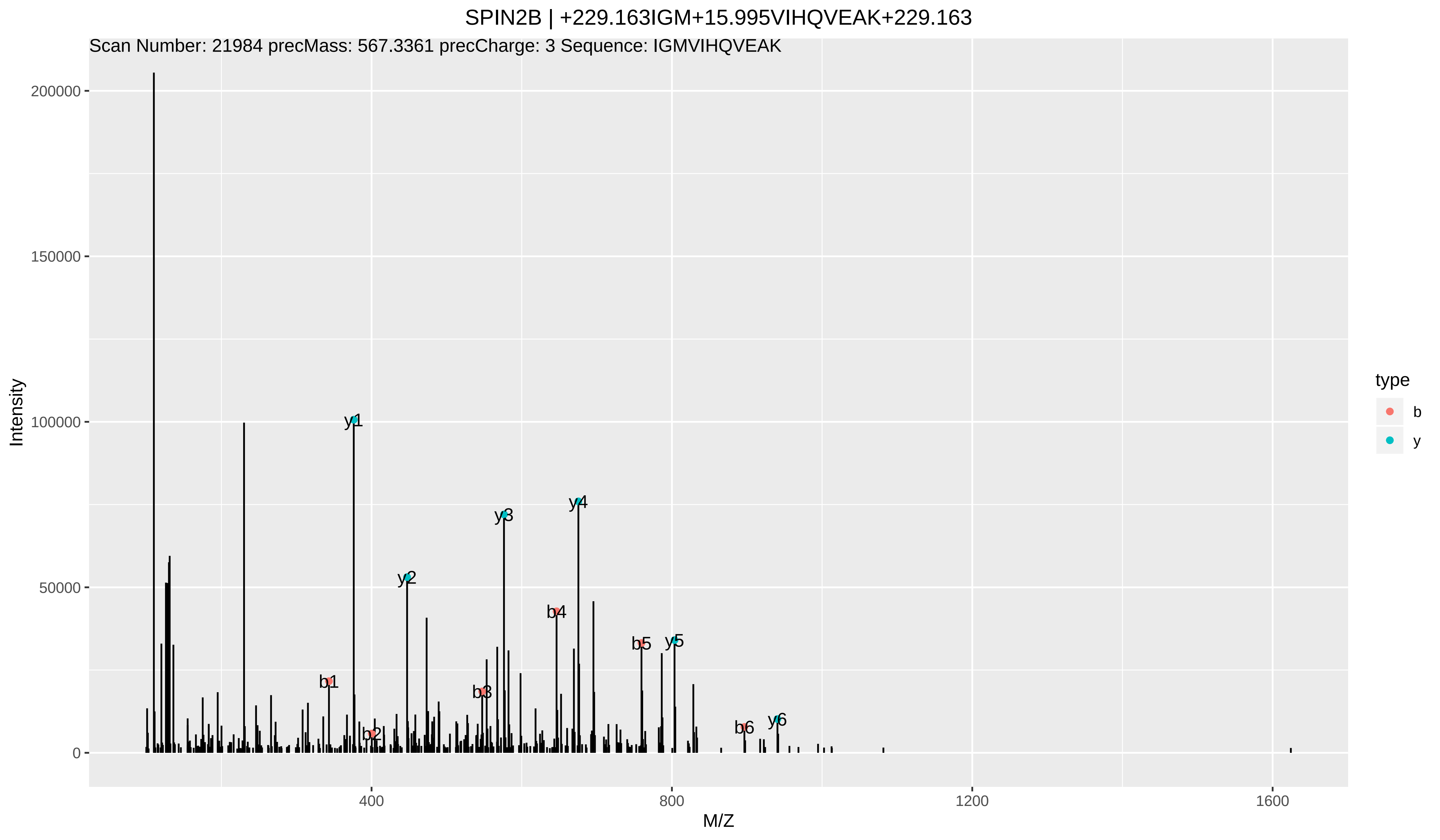
SPIN2B | +229.163IGM+15.995VIHQVEAK+229.163
Scan Number: 21984 precMass: 567.3361 precCharge: 3 Sequence: IGMVIHQVEAK
Intensity
200000
150000
100000
50000
0
M/Z
400
800
1200
1600
y1
y2
y3
y4
y5
y6
b1
b2
b3
b4
b5
b6
type
b
y

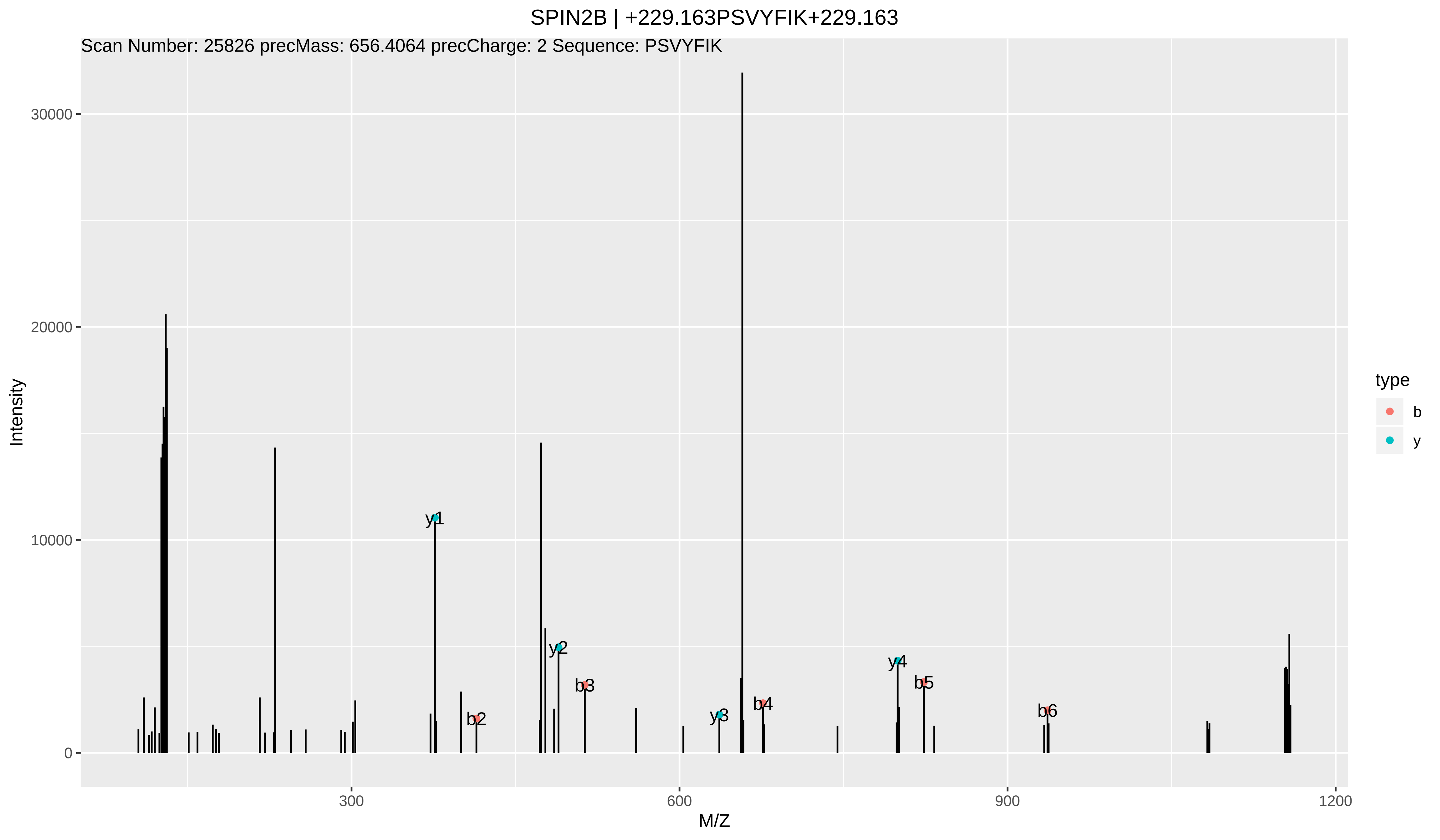

SPIN2B | +229.163PSVYFIK+229.163
Scan Number: 25826 precMass: 656.4064 precCharge: 2 Sequence: PSVYFIK
Intensity
M/Z
30000
20000
10000
0
300
600
900
1200
y1
b2
y2
b3
y3
b4
y4
b5
b6
type
b
y

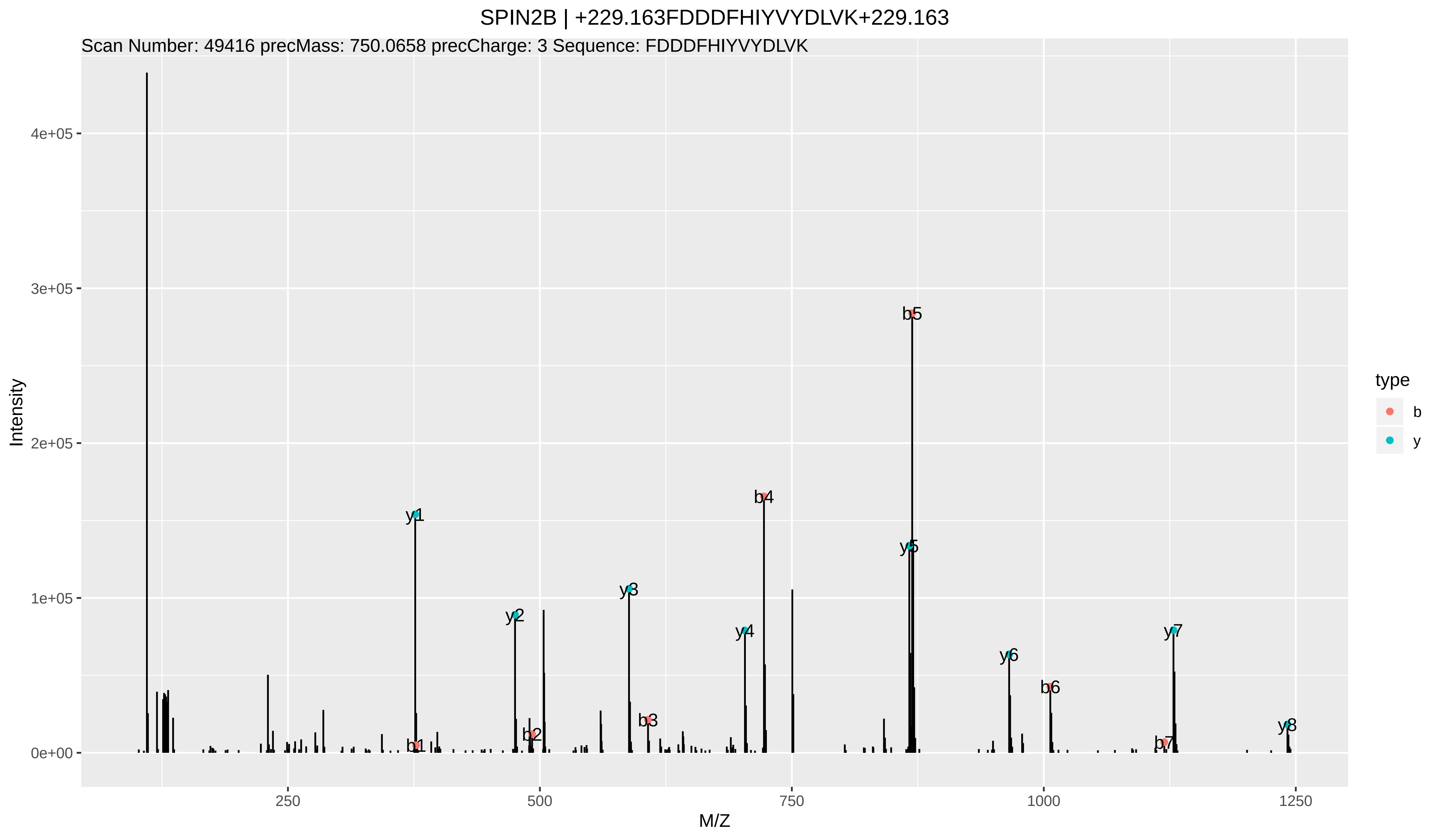
SPIN2B | +229.163FDDDFHIYVYDLVK+229.163
Scan Number: 49416 precMass: 750.0658 precCharge: 3 Sequence: FDDDFHIYVYDLVK
Intensity
M/Z
4e+05
3e+05
2e+05
1e+05
0e+00
250
500
750
1000
1250
type
b
y
y1
b1
y2
b2
y3
b3
y4
b4
y5
b5
y6
b6
y7
b7
y8

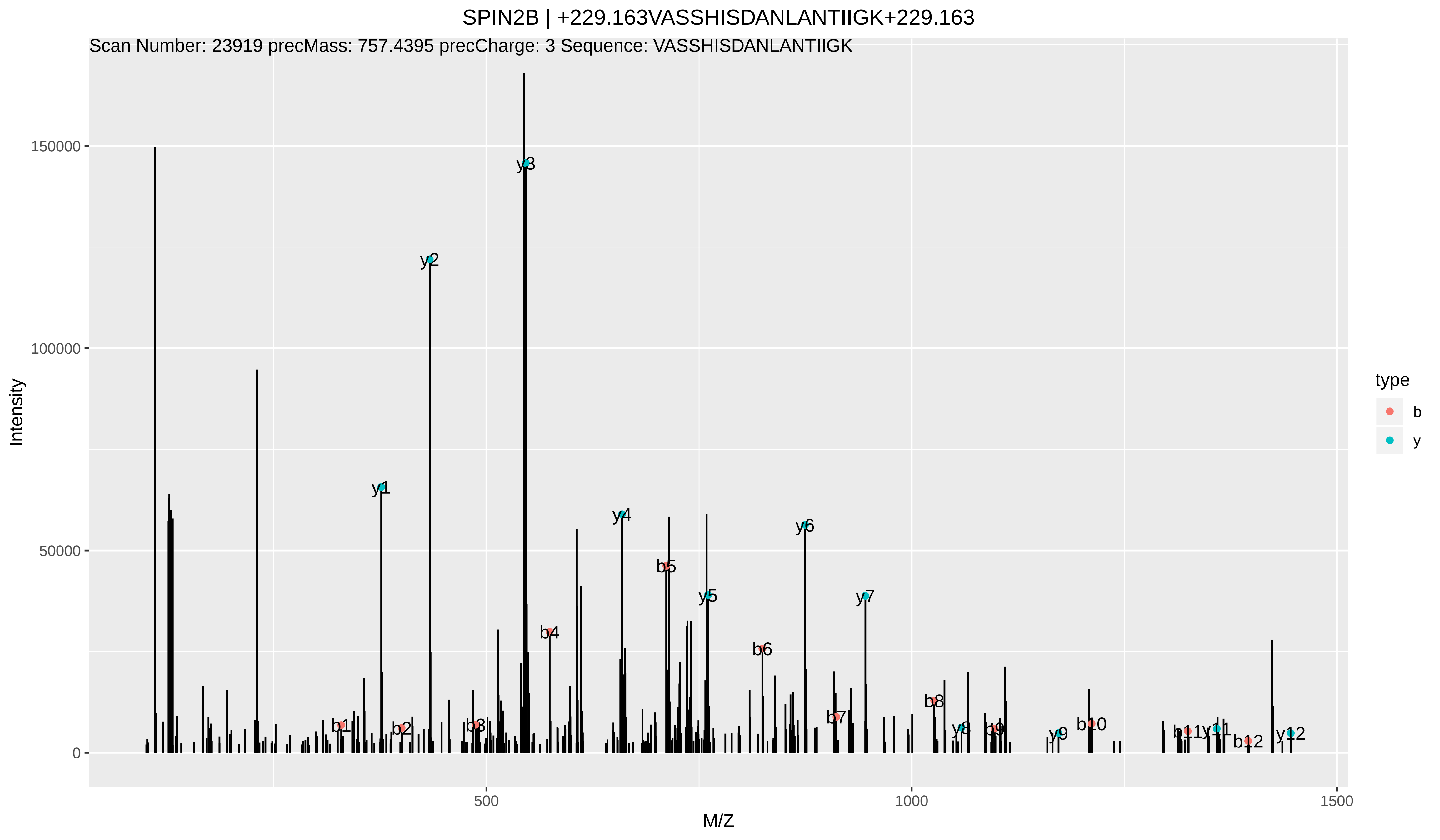
SPIN2B | +229.163VASSHISDANLANTIIGK+229.163
Scan Number: 23919 precMass: 757.4395 precCharge: 3 Sequence: VASSHISDANLANTIIGK
Intensity
M/Z
type
b
y
y1
y2
y3
y4
y5
y6
y7
y8
y9
y11
y12
b1
b2
b3
b4
b5
b6
b7
b8
b9
b10
b11
b12
0
50000
100000
150000
500
1000
1500

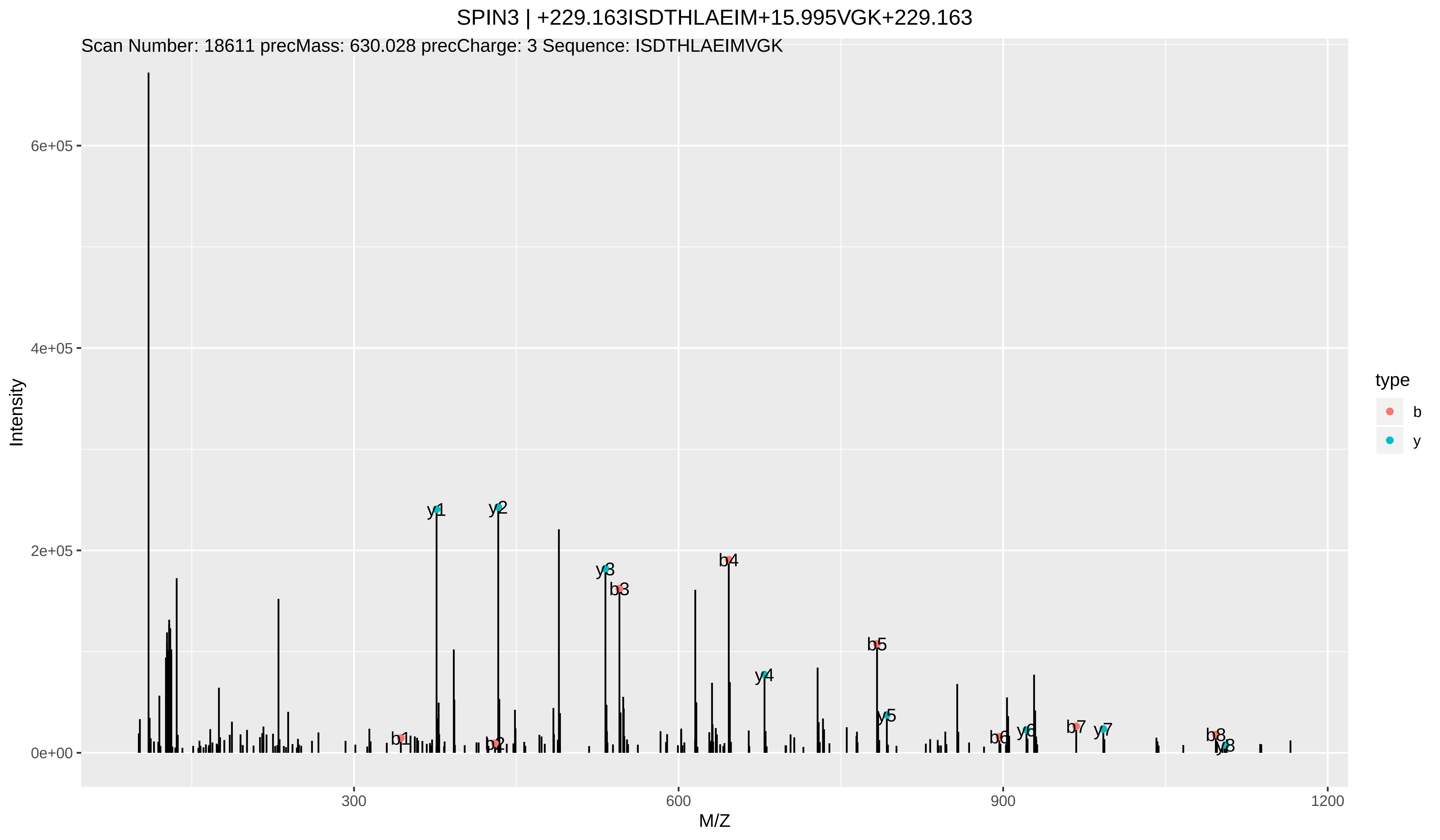
SPIN3 | +229.163ISDTHLAEIM+15.995VGK+229.163
Scan Number: 18611 precMass: 630.028 precCharge: 3 Sequence: ISDTHLAEIMVGK
Intensity
M/Z
0e+00
2e+05
4e+05
6e+05
300
600
900
1200
type
b
y
y1
y2
y3
b3
b4
y4
b5
y5
b1
b2
b6
y6
b7
y7
b8
y8

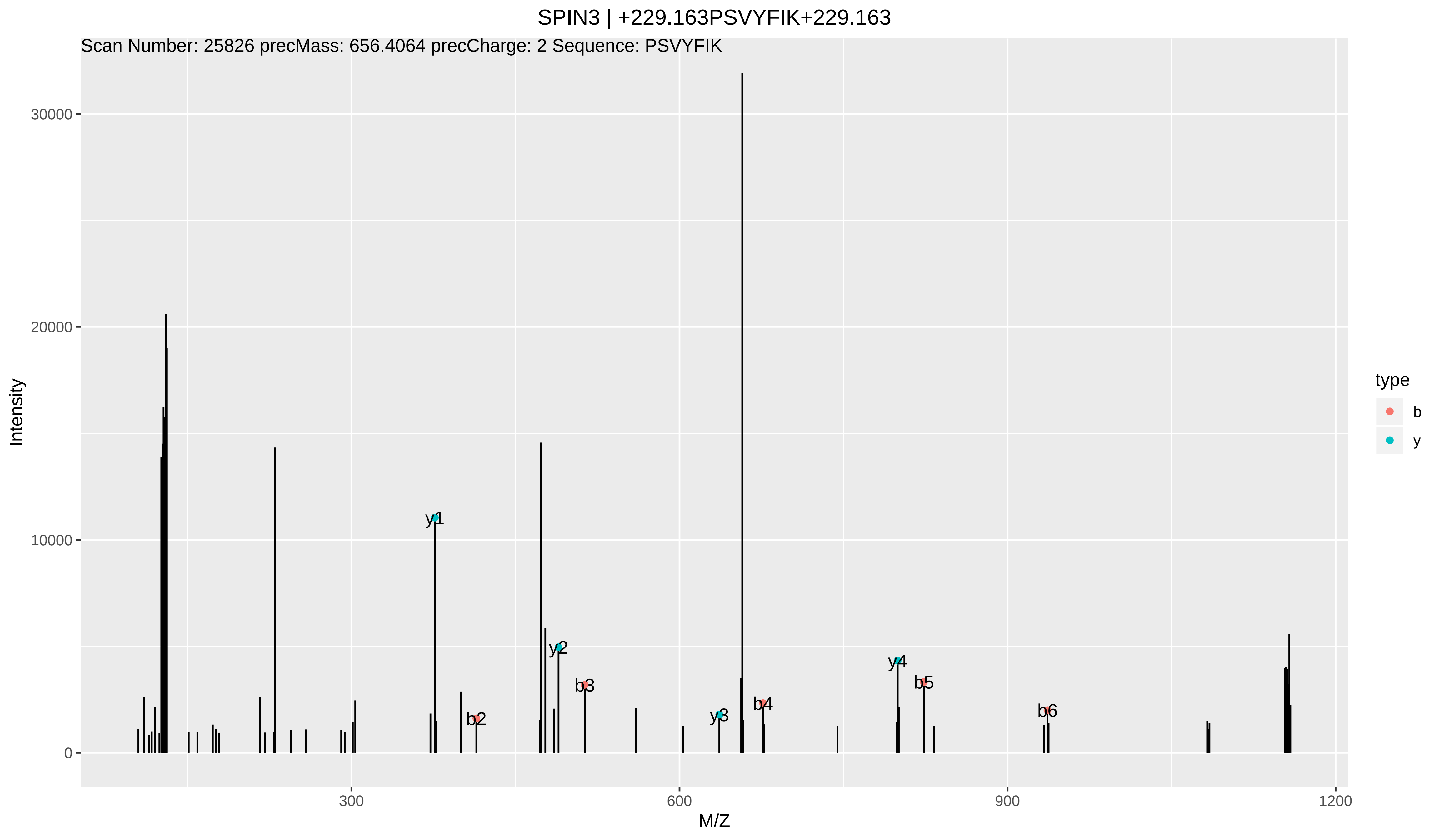
SPIN3 | +229.163PSVYFIK+229.163
Scan Number: 25826 precMass: 656.4064 precCharge: 2 Sequence: PSVYFIK
Intensity
30000
20000
10000
0
300
600
900
1200
M/Z
y1
b2
y2
b3
y3
b4
y4
b5
b6
type
b
y

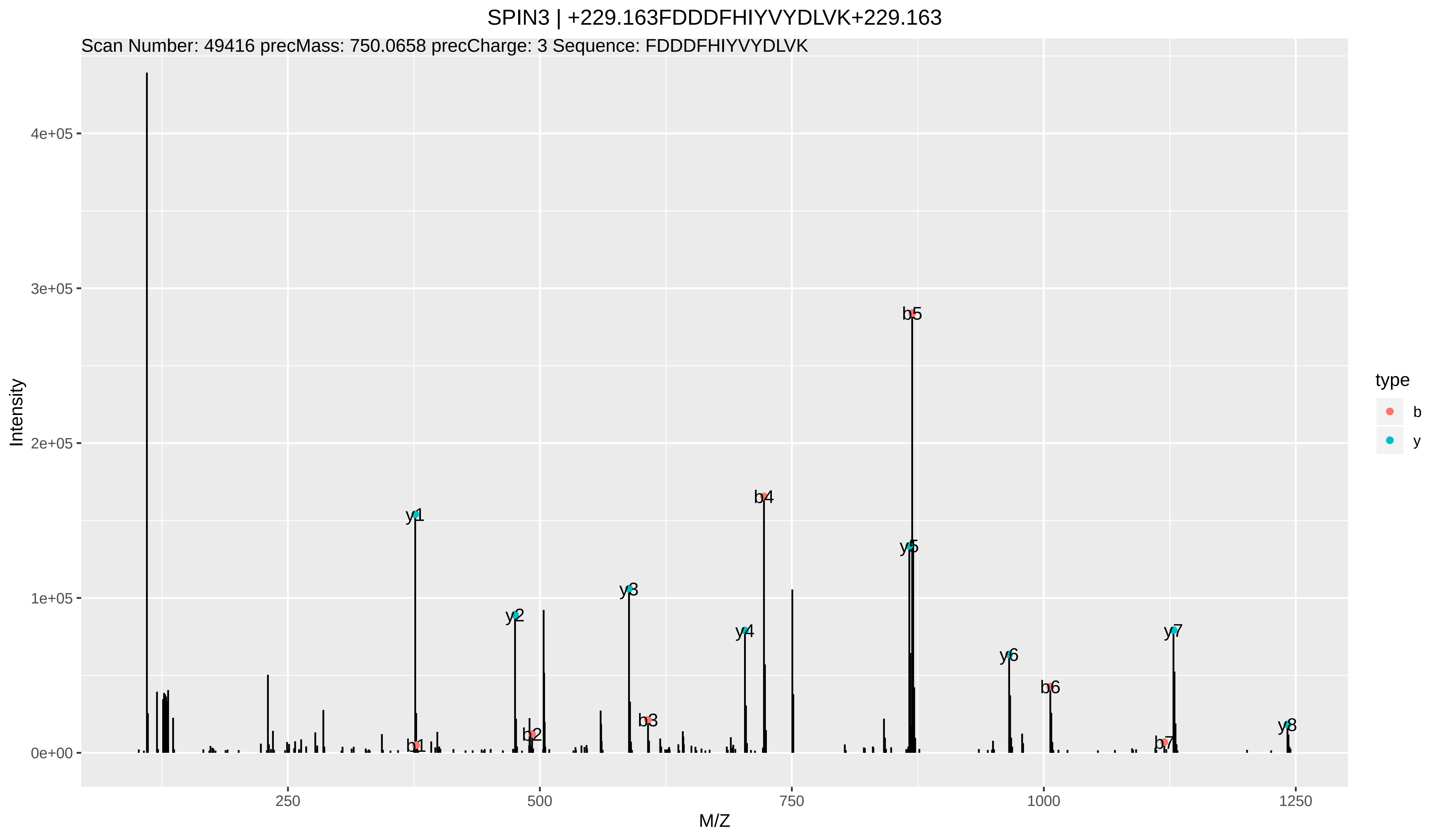
SPIN3 | +229.163FDDDFHIYVYDLVK+229.163
Scan Number: 49416 precMass: 750.0658 precCharge: 3 Sequence: FDDDFHIYVYDLVK
Intensity
M/Z
type
b
y
b1
b2
b3
b4
b5
b6
b7
y1
y2
y3
y4
y5
y6
y7
y8
0e+00
1e+05
2e+05
3e+05
4e+05
250
500
750
1000
1250

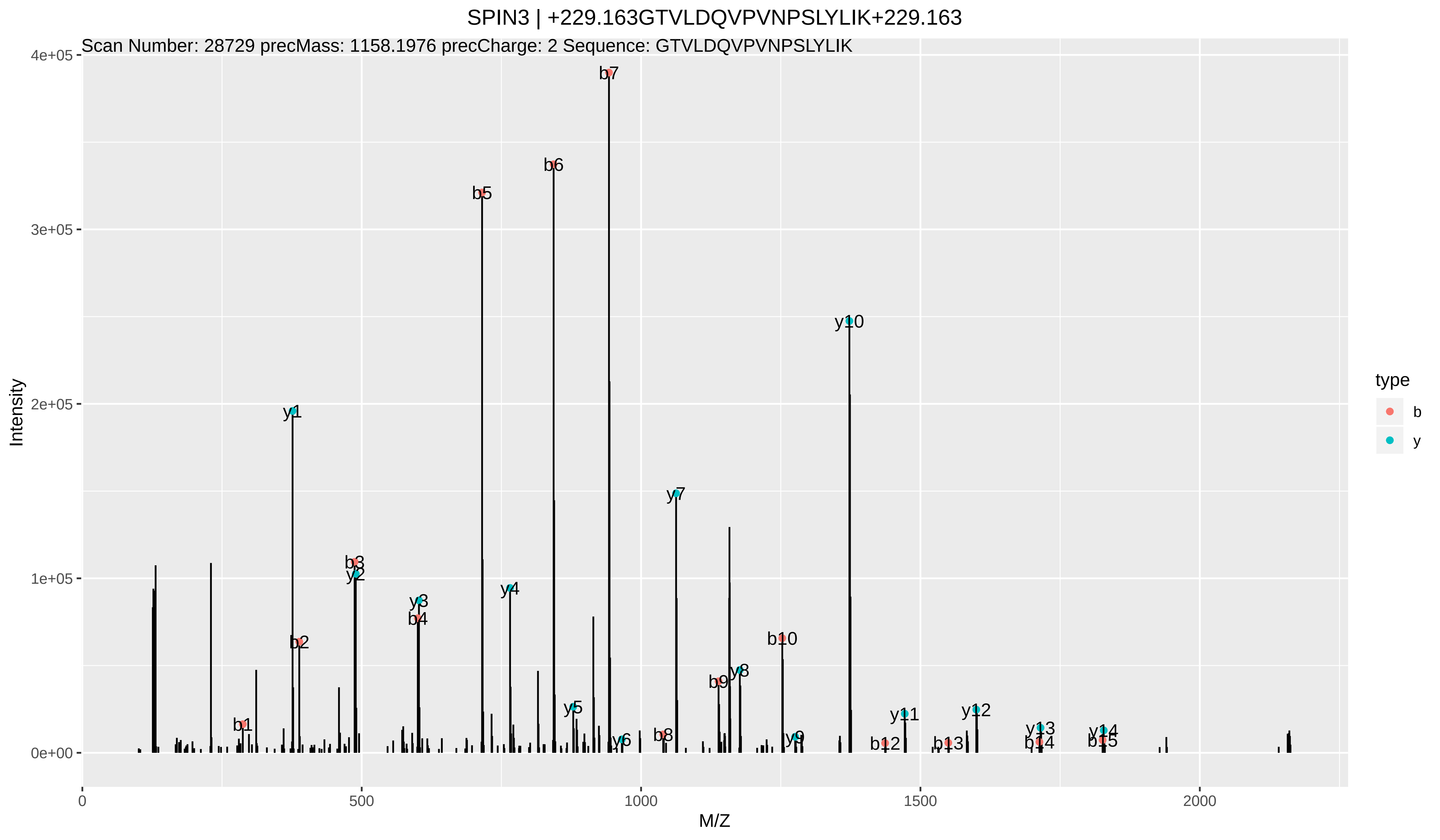

SPIN3 | +229.163GTVLDQVPVNPSLYLIK+229.163
Scan Number: 28729 precMass: 1158.1976 precCharge: 2 Sequence: GTVLDQVPVNPSLYLIK
Intensity
0e+00
1e+05
2e+05
3e+05
4e+05
M/Z
0
500
1000
1500
2000
type
b
y
b1
b2
b3
b4
b5
b6
b7
b8
b9
b10
b12
b13
b14
b15
y1
y2
y3
y4
y5
y6
y7
y8
y9
y10
y11
y12
y13
y14

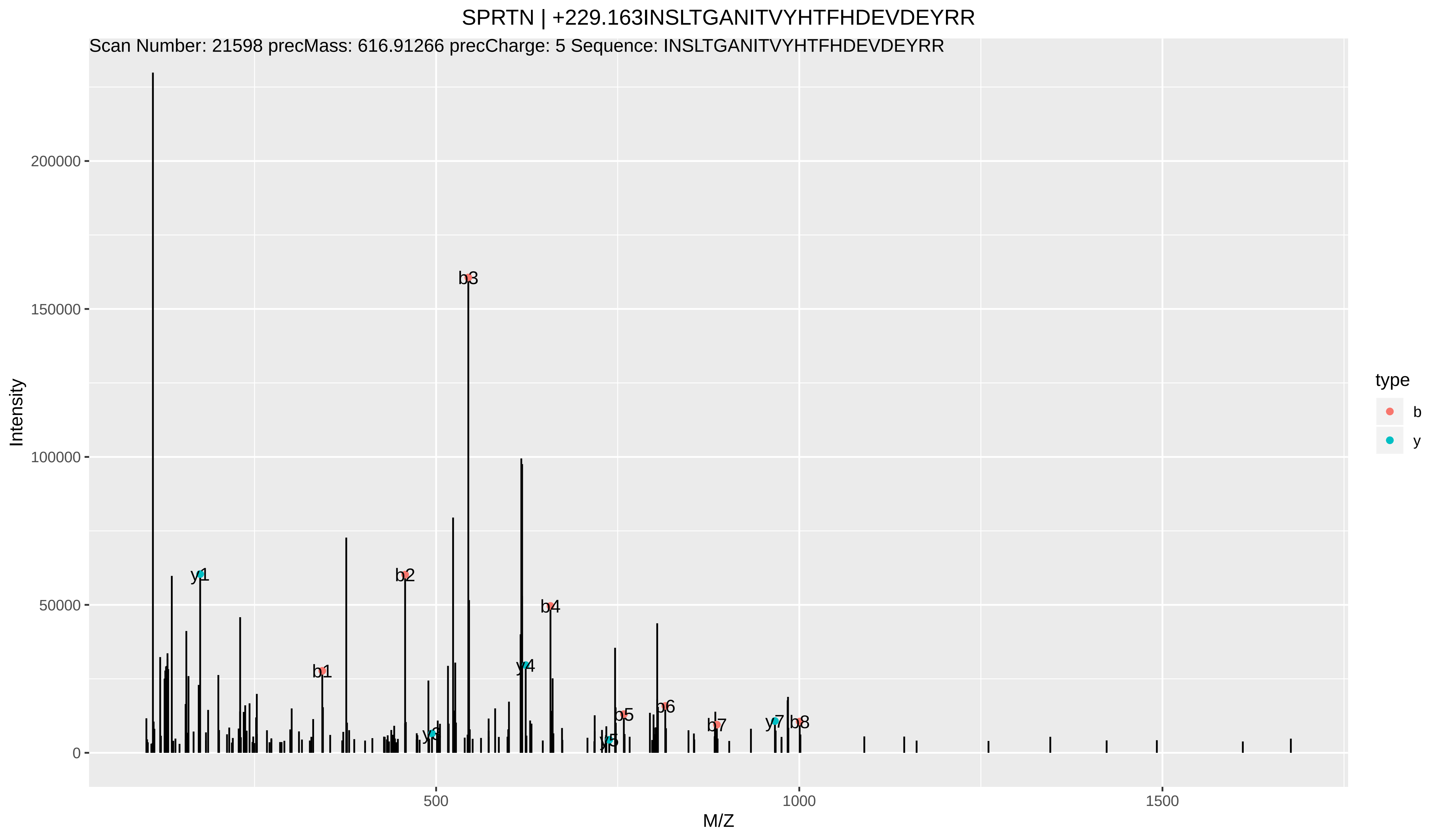
SPRTN | +229.163INSLTGANITVYHTFHDEVDEYRR
Scan Number: 21598 precMass: 616.91266 precCharge: 5 Sequence: INSLTGANITVYHTFHDEVDEYRR
Intensity
M/Z
type
b
y
b1
b2
b3
b4
b5
b6
b7
b8
y1
y4
y5
y7
0
50000
100000
150000
200000
500
1000
1500

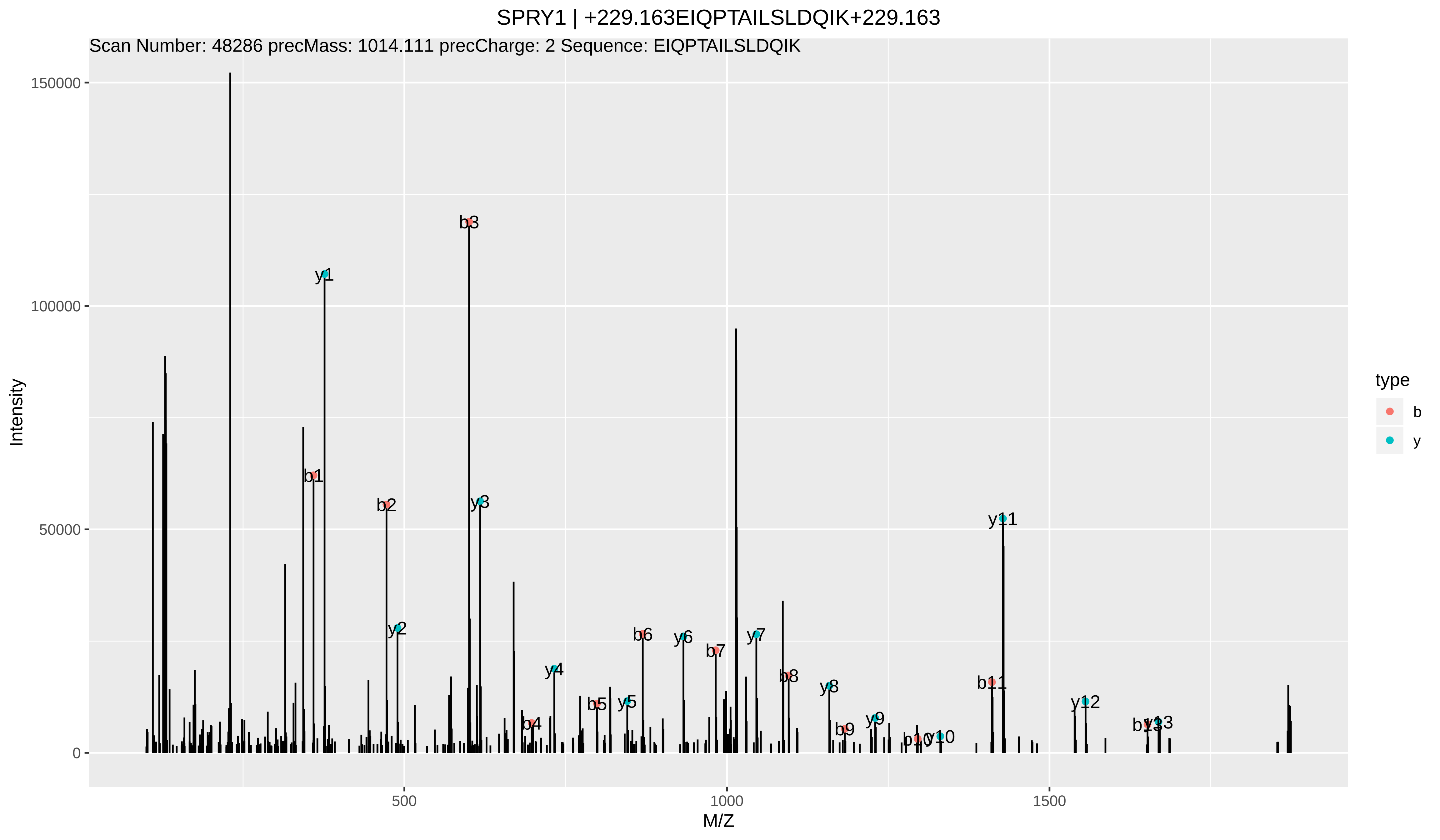
SPRY1 | +229.163EIQPTAILSLDQIK+229.163
Scan Number: 48286 precMass: 1014.111 precCharge: 2 Sequence: EIQPTAILSLDQIK
Intensity
150000
100000
50000
0
500
1000
1500
M/Z
type
b
y
b1
b2
b3
b4
b5
b6
b7
b8
b9
b10
b11
b13
y1
y2
y3
y4
y5
y6
y7
y8
y9
y10
y11
y12
y13

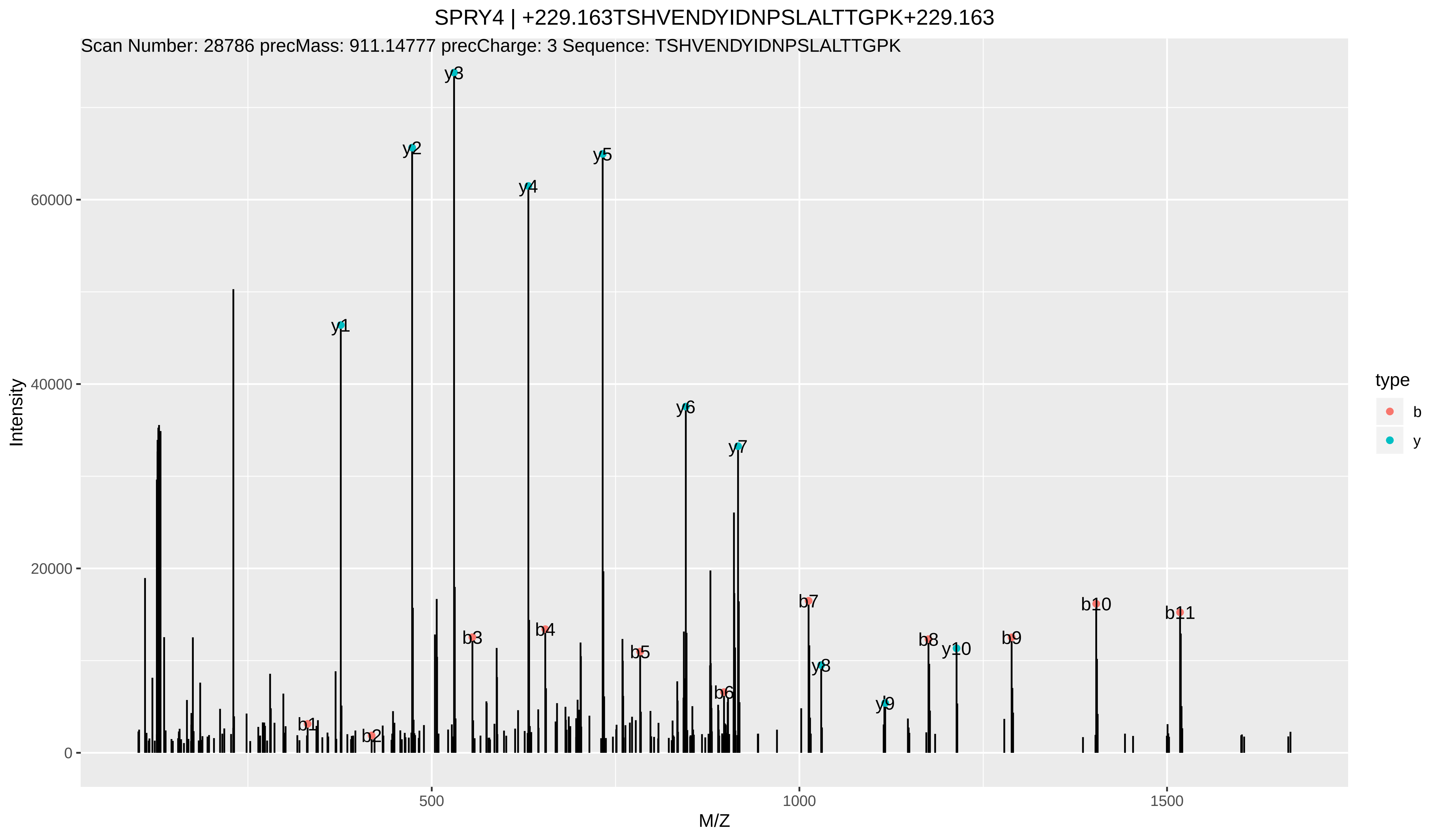
SPRY4 | +229.163TSHVENDYIDNPSLALTTGPK+229.163
Scan Number: 28786 precMass: 911.14777 precCharge: 3 Sequence: TSHVENDYIDNPSLALTTGPK
Intensity
M/Z
type
b
y
y1
y2
y3
y4
y5
y6
y7
y8
y9
y10
b1
b2
b3
b4
b5
b6
b7
b8
b9
b10
b11
0
20000
40000
60000
500
1000
1500

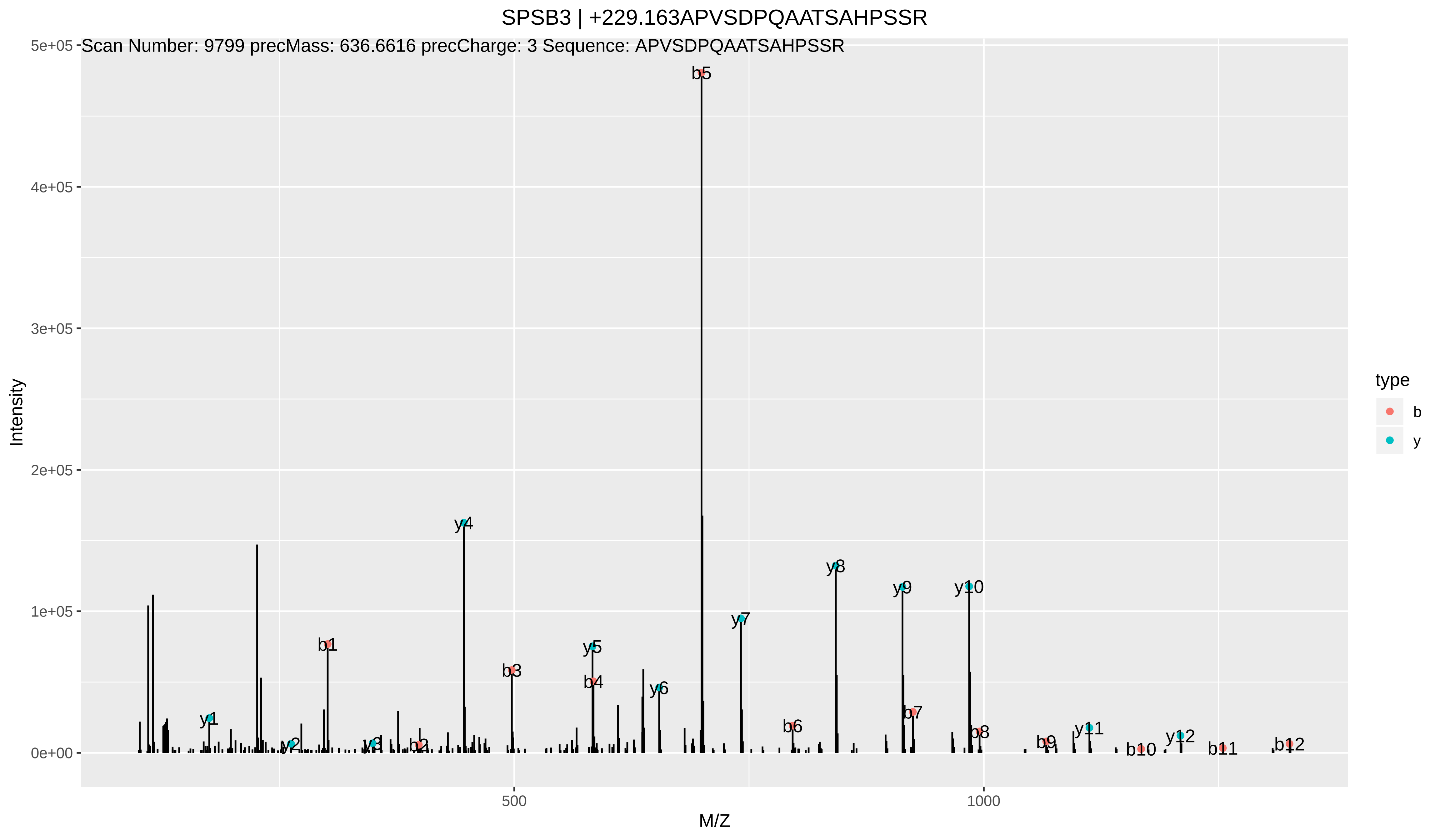
SPSB3 | +229.163APVSDPQAATSAHPSSR
Scan Number: 9799 precMass: 636.6616 precCharge: 3 Sequence: APVSDPQAATSAHPSSR
Intensity
M/Z
0e+00
1e+05
2e+05
3e+05
4e+05
5e+05
500
1000
b5
y4
y8
y9
y10
y7
b1
y5
b3
b4
y6
y1
y2
y3
b2
b6
b7
b8
b9
y11
b10
y12
b11
b12
type
b
y

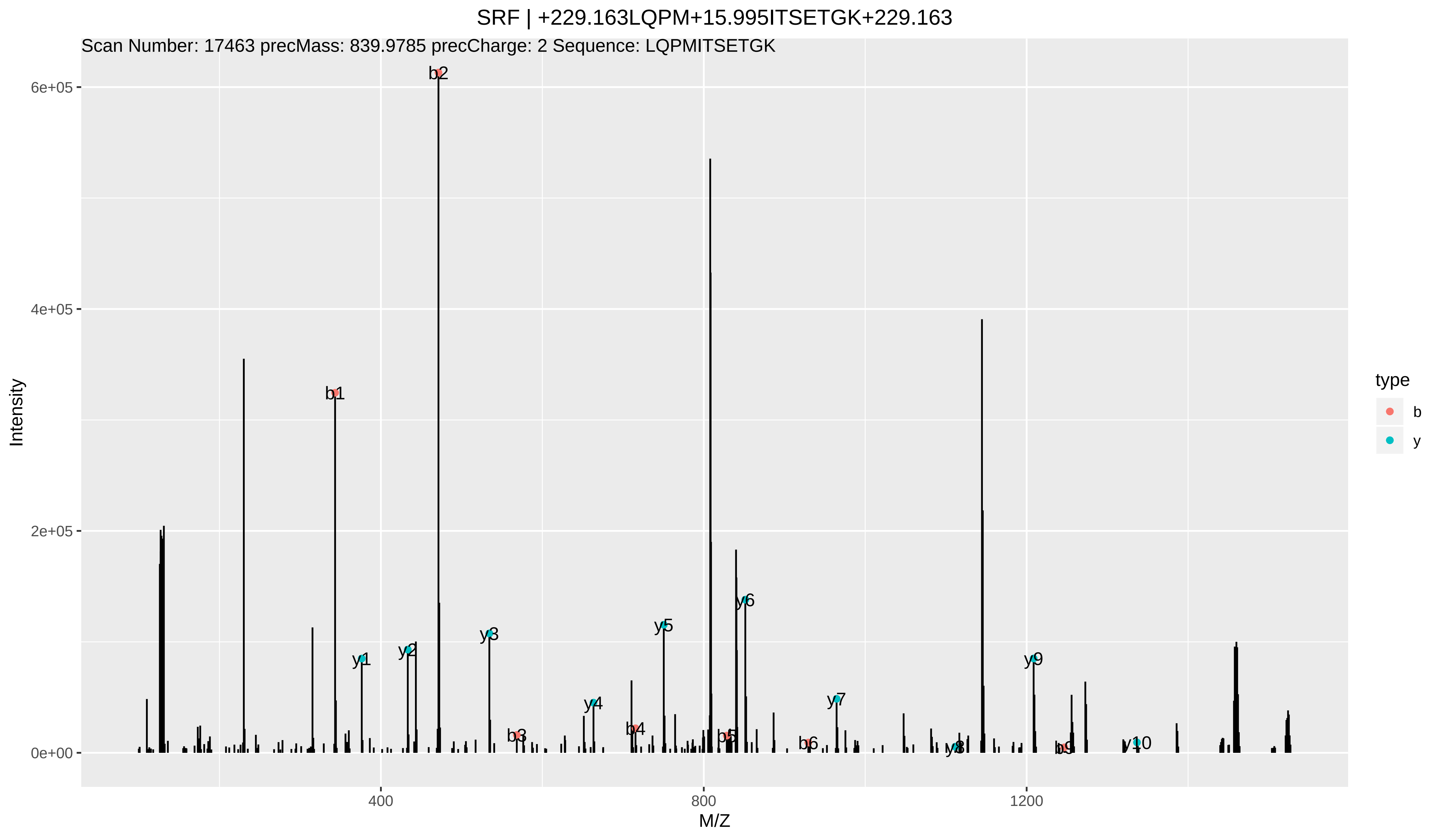

SRF | +229.163LQPM+15.995ITSETGK+229.163
Scan Number: 17463 precMass: 839.9785 precCharge: 2 Sequence: LQPMITSETGK
Intensity
M/Z
6e+05
4e+05
2e+05
0e+00
400
800
1200
b2
b1
y1
y2
y3
b3
y4
b4
y5
b5
y6
b6
y7
y8
y9
b9
y10
type
b
y

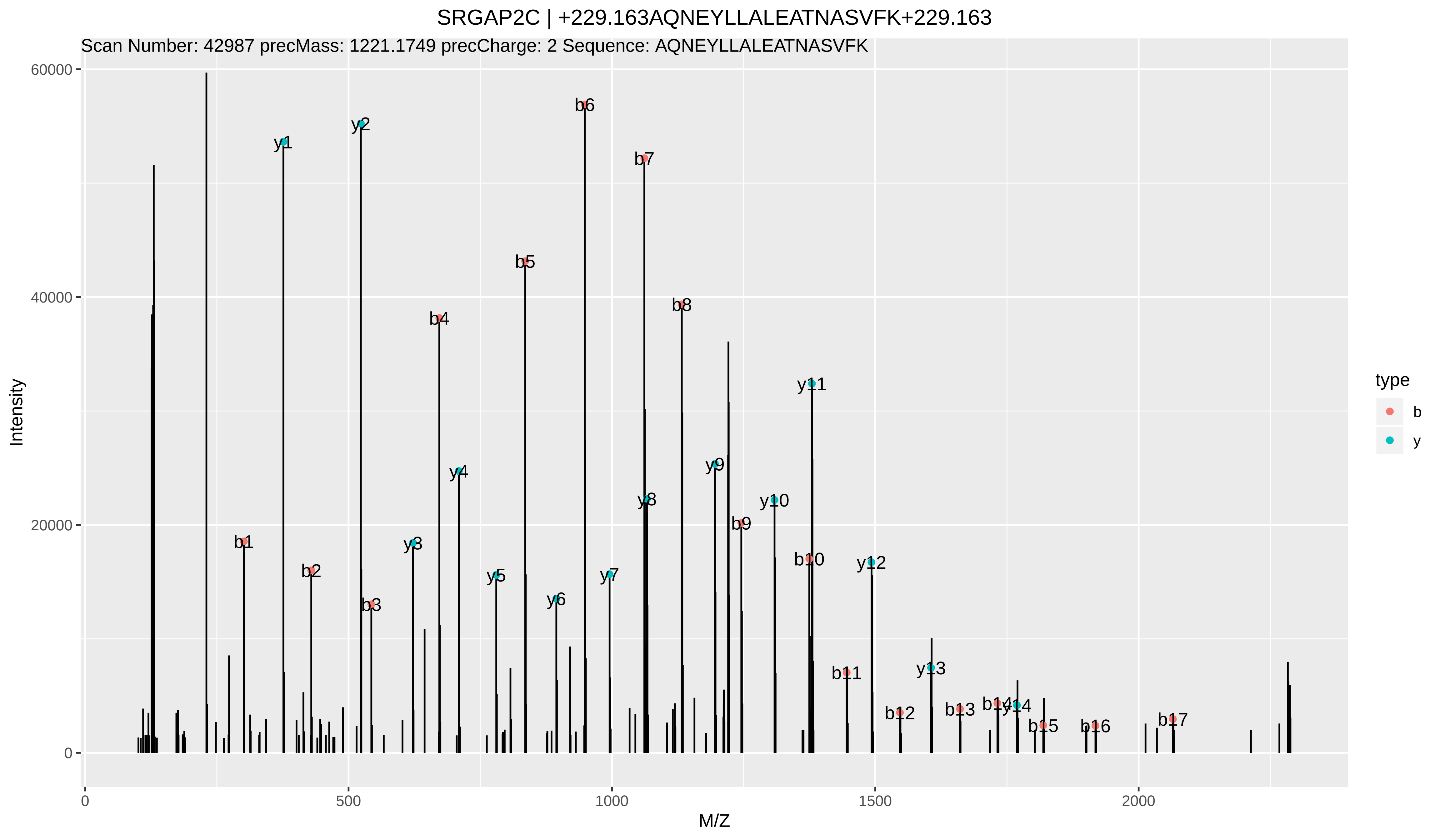

SRGAP2C | +229.163AQNEYLLALEATNASVFK+229.163
Scan Number: 42987 precMass: 1221.1749 precCharge: 2 Sequence: AQNEYLLALEATNASVFK
Intensity
M/Z
type
b
y
b1
b2
b3
b4
b5
b6
b7
b8
b9
b10
b11
b12
b13
b14
b15
b16
b17
y1
y2
y3
y4
y5
y6
y7
y8
y9
y10
y11
y12
y13
y14

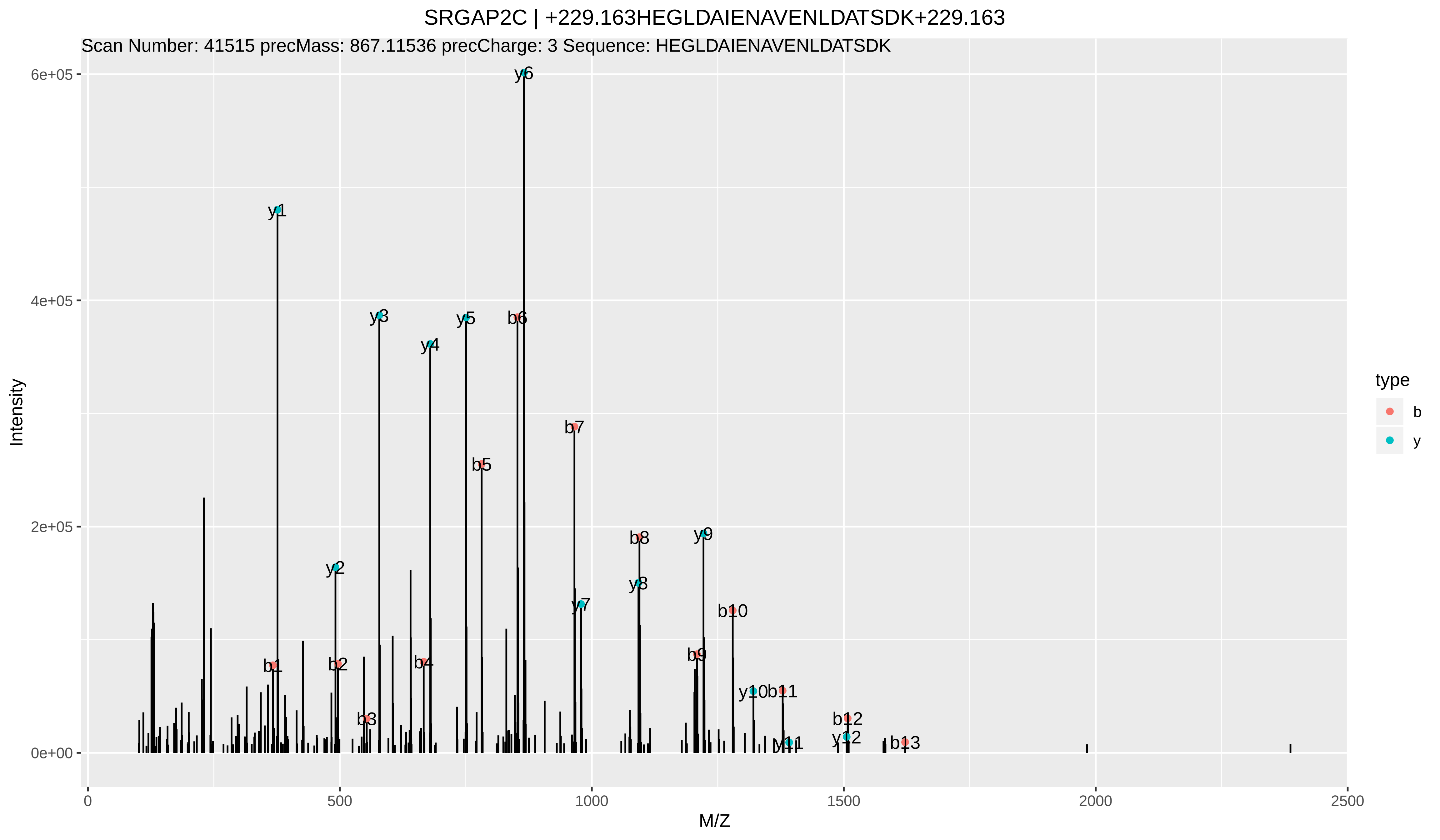

SRGAP2C | +229.163HEGLDAIENAVENLDATSDK+229.163
Scan Number: 41515 precMass: 867.11536 precCharge: 3 Sequence: HEGLDAIENAVENLDATSDK
Intensity
M/Z
6e+05
4e+05
2e+05
0e+00
0
500
1000
1500
2000
2500
type
b
y
y1
y2
y3
y4
y5
y6
y7
y8
y9
y10
y11
y12
b1
b2
b3
b4
b5
b6
b7
b8
b9
b10
b11
b12
b13

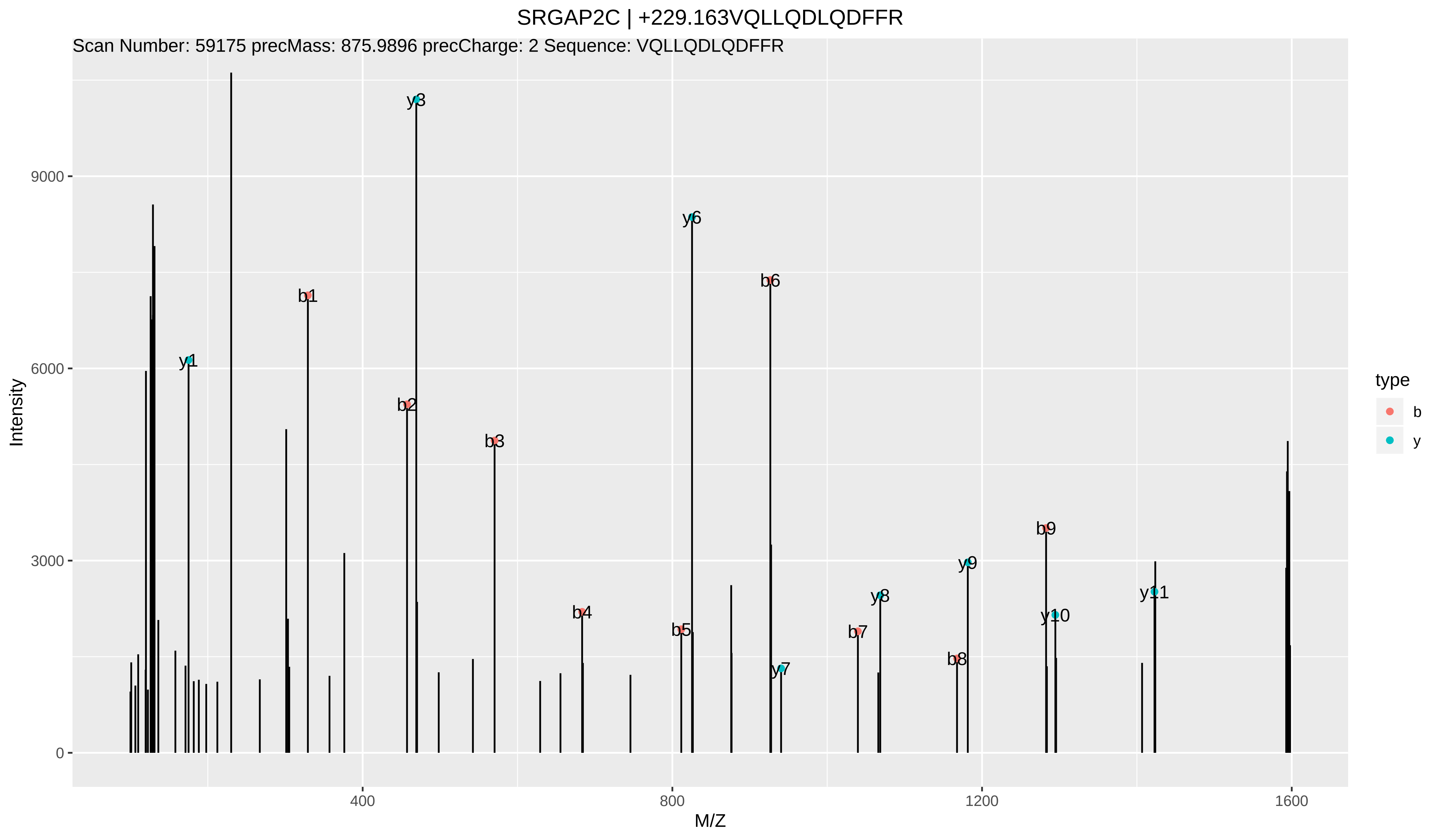

SRGAP2C | +229.163VQLLQDLQDFFR
Scan Number: 59175 precMass: 875.9896 precCharge: 2 Sequence: VQLLQDLQDFFR
Intensity
M/Z
type
b
y
y1
b1
b2
y3
b3
b4
b5
y6
b6
y7
b7
y8
b8
y9
b9
y10
y11

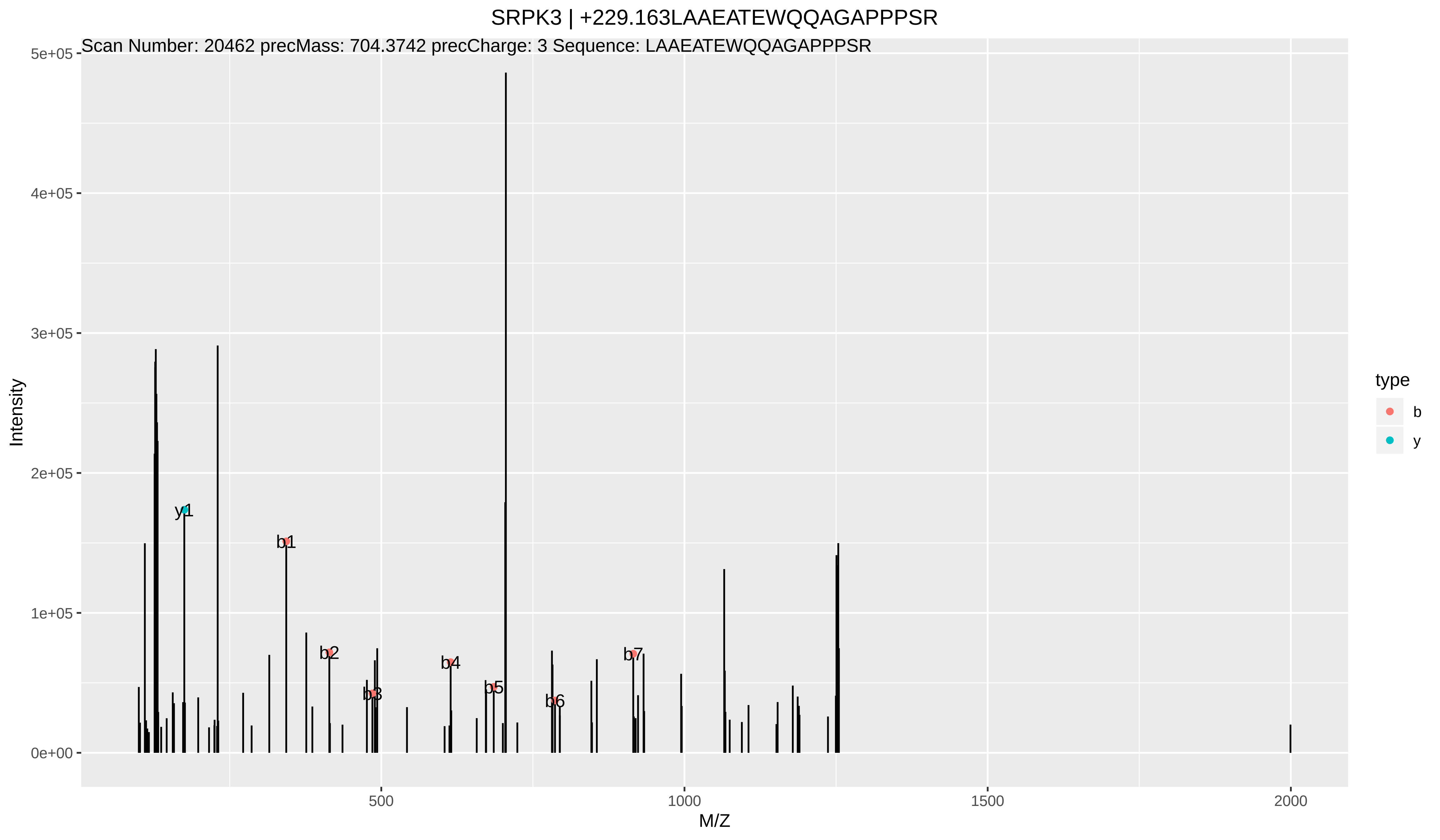
SRPK3 | +229.163LAAEATEWQQAGAPPPSR
Scan Number: 20462 precMass: 704.3742 precCharge: 3 Sequence: LAAEATEWQQAGAPPPSR
Intensity
M/Z
0e+00
1e+05
2e+05
3e+05
4e+05
5e+05
500
1000
1500
2000
y1
b1
b2
b3
b4
b5
b6
b7
type
b
y

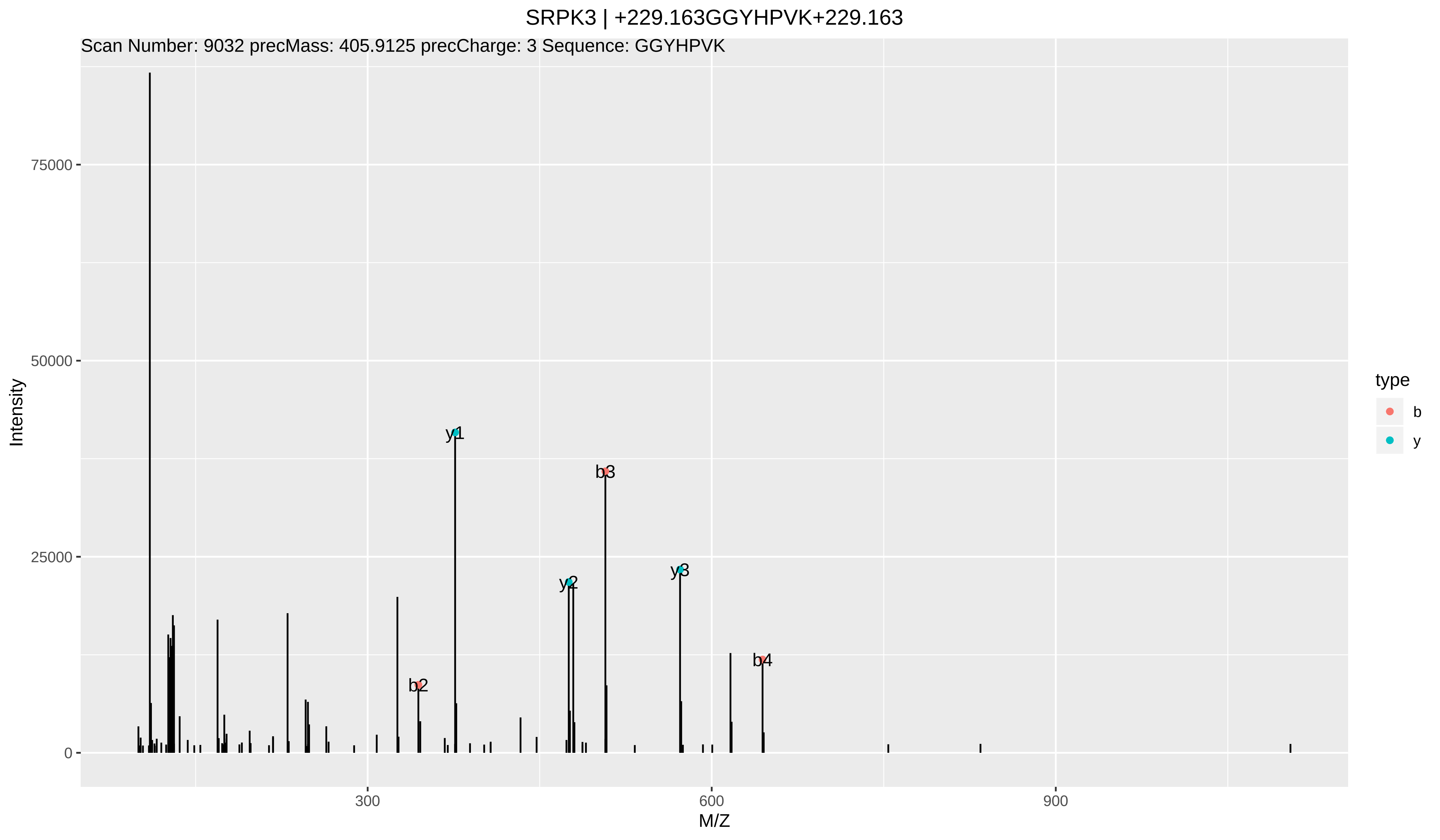

SRPK3 | +229.163GGYHPVK+229.163
Scan Number: 9032 precMass: 405.9125 precCharge: 3 Sequence: GGYHPVK
Intensity
75000
50000
25000
0
M/Z
300
600
900
y1
b3
y2
y3
b4
b2
type
b
y

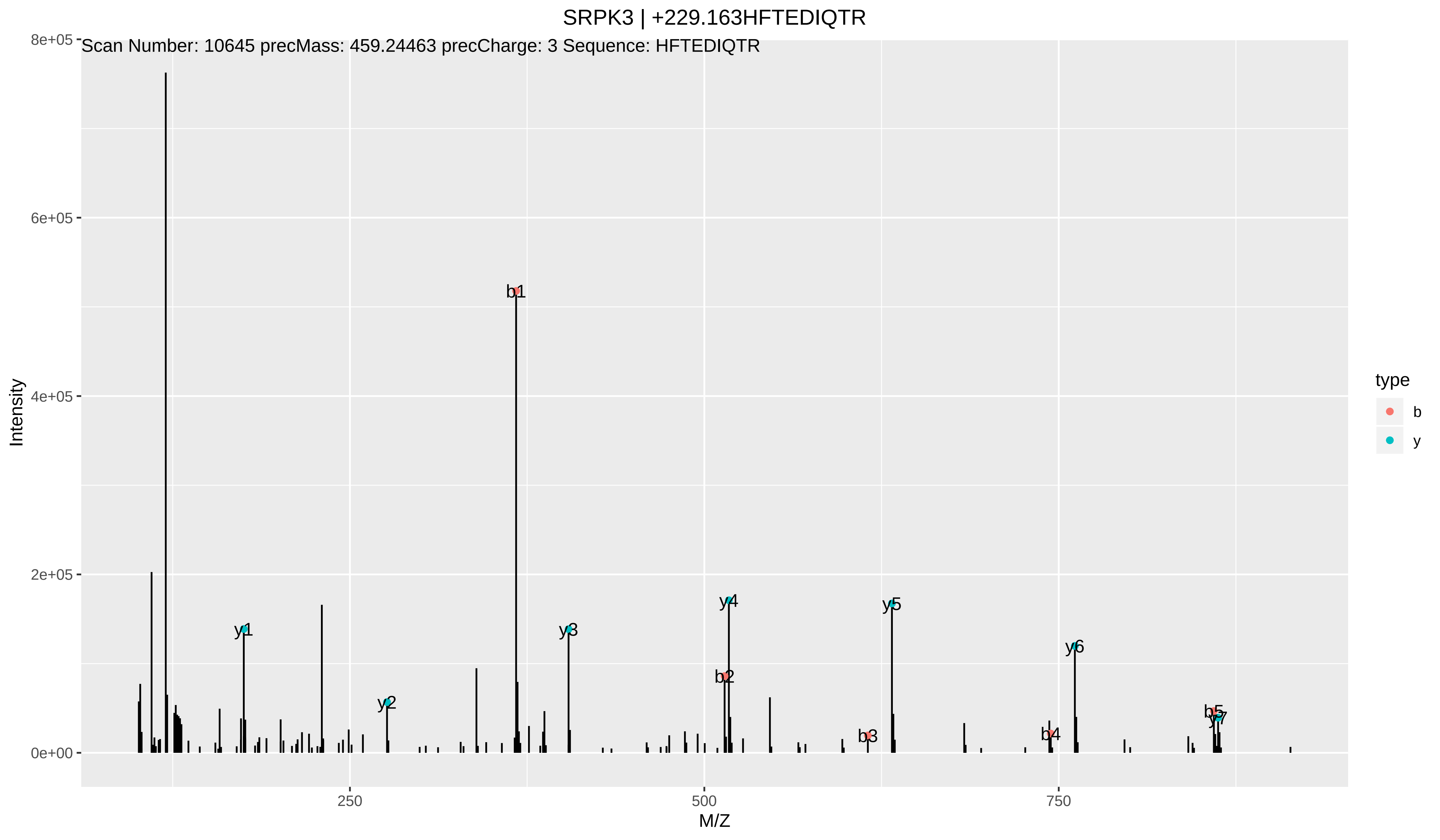

SRPK3 | +229.163HFTEDIQTR
Scan Number: 10645 precMass: 459.24463 precCharge: 3 Sequence: HFTEDIQTR
Intensity
M/Z
0e+00
2e+05
4e+05
6e+05
8e+05
250
500
750
b1
b2
b3
b4
b5
y1
y2
y3
y4
y5
y6
y7
type
b
y

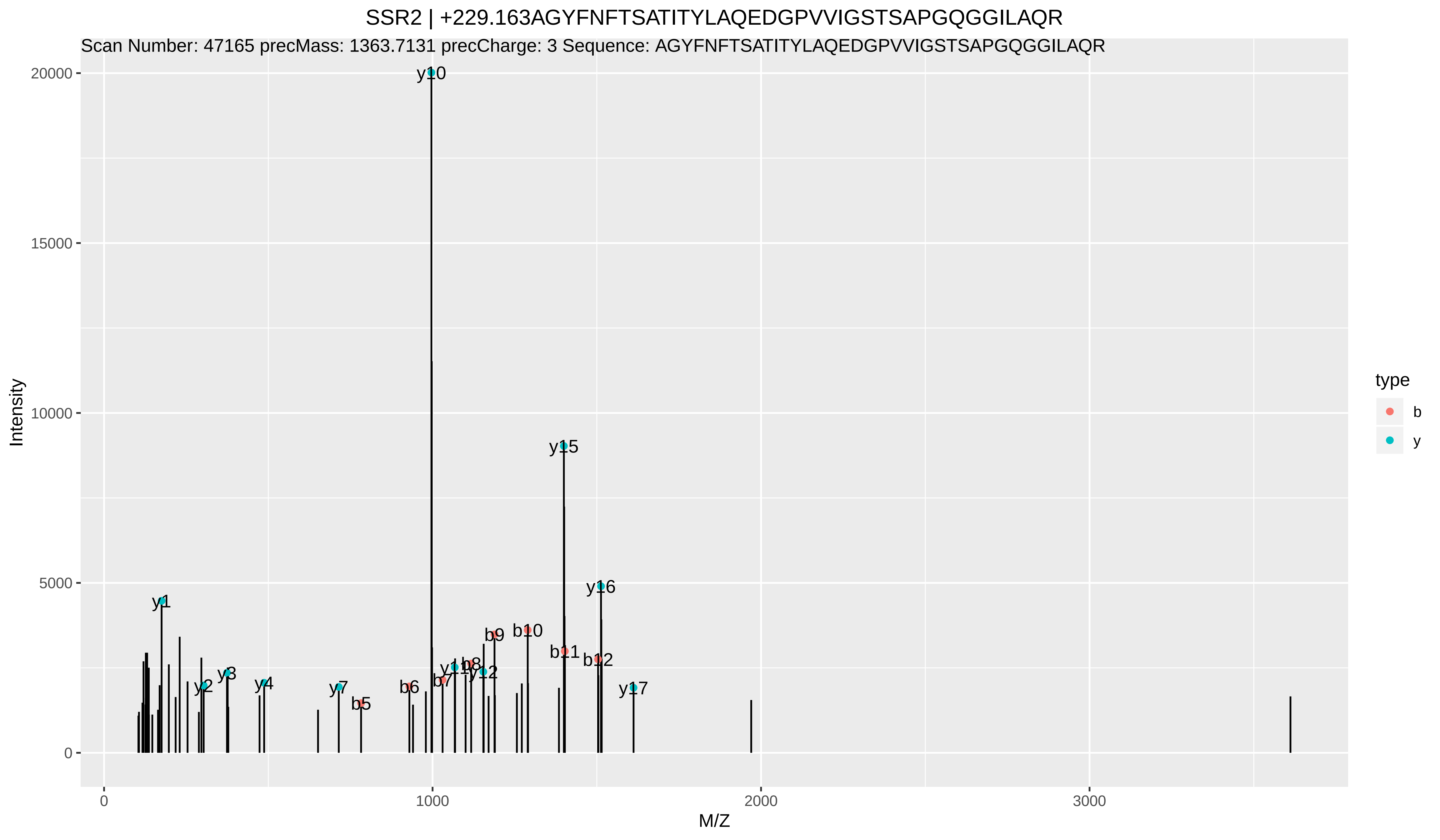
SSR2 | +229.163AGYFNFTSATITYLAQEDGPVVIGSTSAPGQGGILAQR
Scan Number: 47165 precMass: 1363.7131 precCharge: 3 Sequence: AGYFNFTSATITYLAQEDGPVVIGSTSAPGQGGILAQR
Intensity
M/Z
type
b
y
y10
y15
y16
y1
b9
b10
b11
b12
y2
y3
y4
y7
b5
b6
b7
y11
b8
y12
y17
0
5000
10000
15000
20000
1000
2000
3000

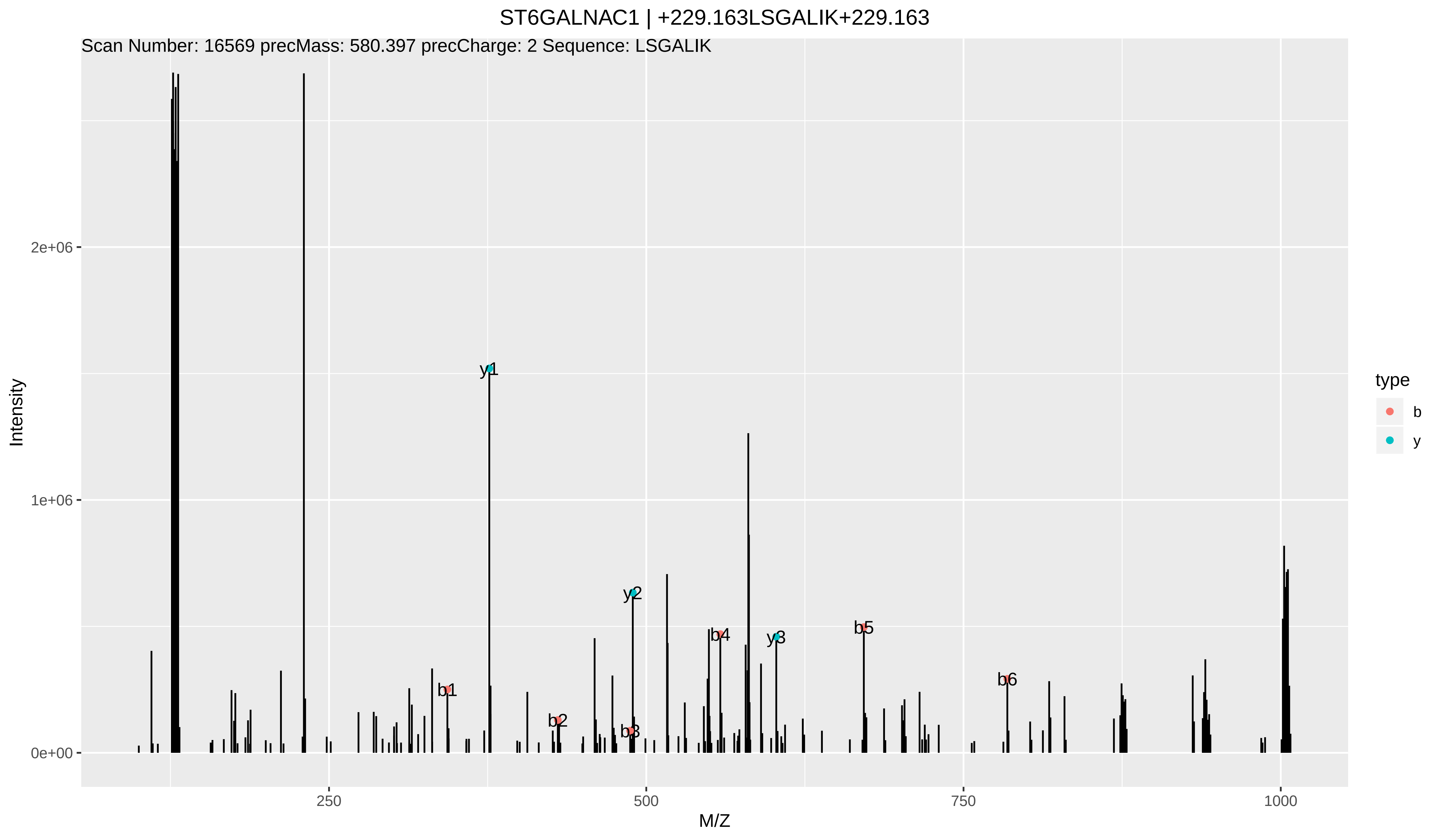

ST6GALNAC1 | +229.163LSGALIK+229.163
Scan Number: 16569 precMass: 580.397 precCharge: 2 Sequence: LSGALIK
Intensity
2e+06
1e+06
0e+00
y1
y2
y3
b1
b2
b3
b4
b5
b6
250
500
750
1000
M/Z
type
b
y

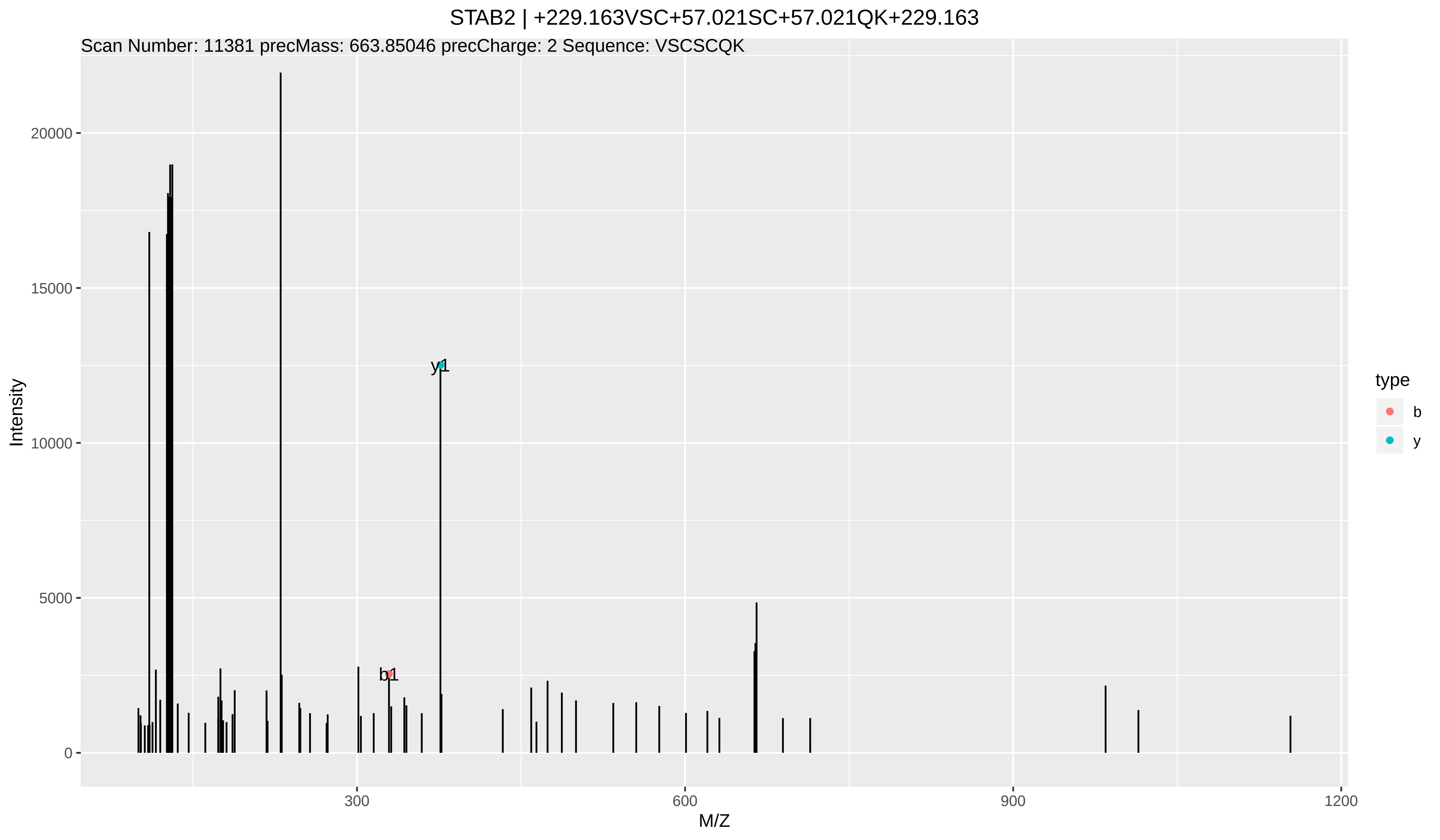

STAB2 | +229.163VSC+57.021SC+57.021QK+229.163
Scan Number: 11381 precMass: 663.85046 precCharge: 2 Sequence: VSCSCQK
Intensity
20000
15000
10000
5000
0
300
600
900
1200
M/Z
y1
b1
type
b
y

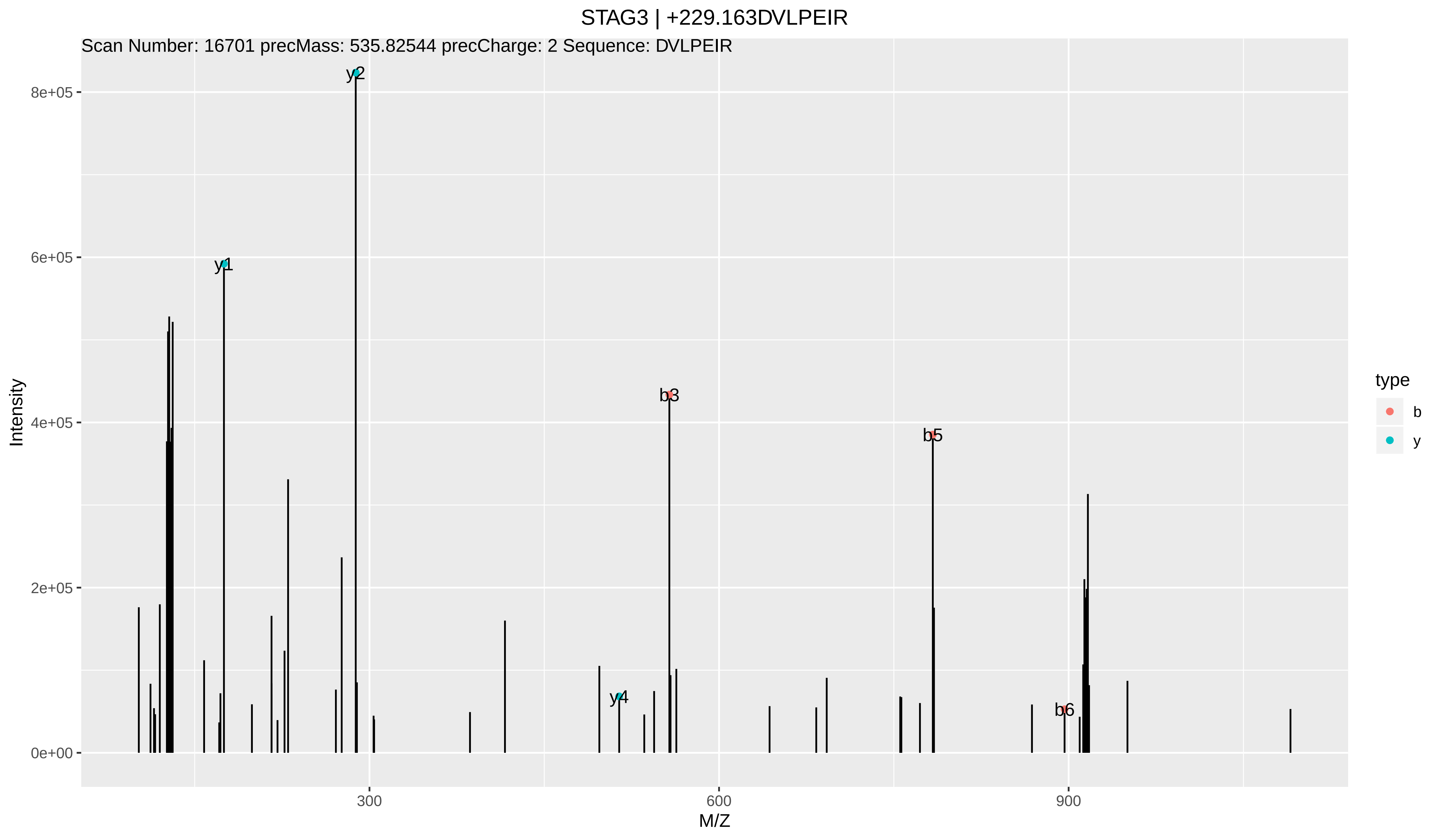

STAG3 | +229.163DVLPEIR
Scan Number: 16701 precMass: 535.82544 precCharge: 2 Sequence: DVLPEIR
y2
y1
b3
b5
y4
b6
8e+05
6e+05
4e+05
2e+05
0e+00
Intensity
300
600
900
M/Z
type
b
y

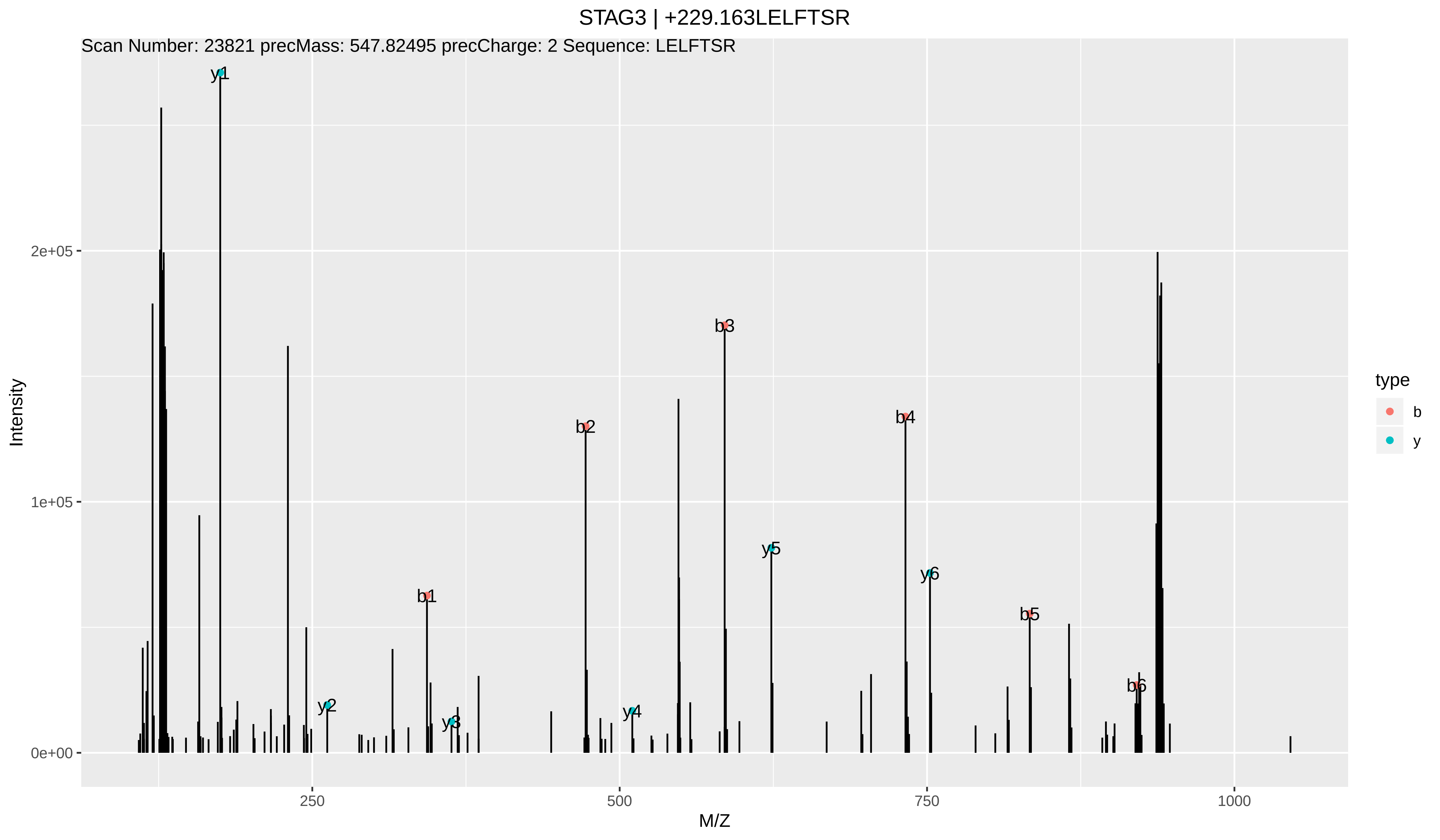
STAG3 | +229.163LELFTSR
Scan Number: 23821 precMass: 547.82495 precCharge: 2 Sequence: LELFTSR
y1
b1
b2
b3
b4
b5
b6
y2
y3
y4
y5
y6
Intensity
2e+05
1e+05
0e+00
250
500
750
1000
M/Z
type
b
y

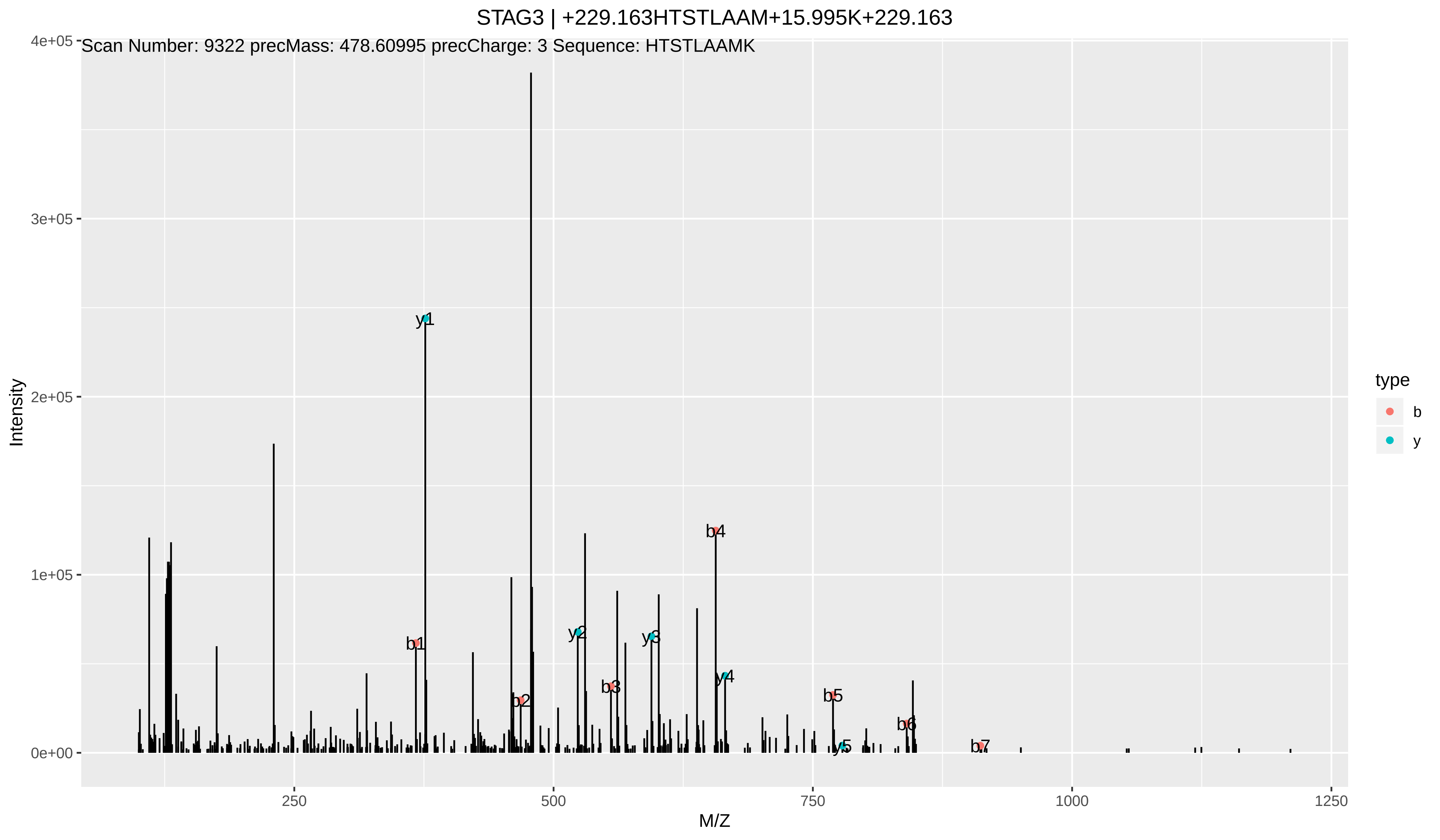
STAG3 | +229.163HTSTLAAM+15.995K+229.163
Scan Number: 9322 precMass: 478.60995 precCharge: 3 Sequence: HTSTLAAMK
Intensity
M/Z
y1
b1
b2
y2
b3
y3
b4
y4
b5
y5
b6
b7
type
b
y
4e+05
3e+05
2e+05
1e+05
0e+00
250
500
750
1000
1250

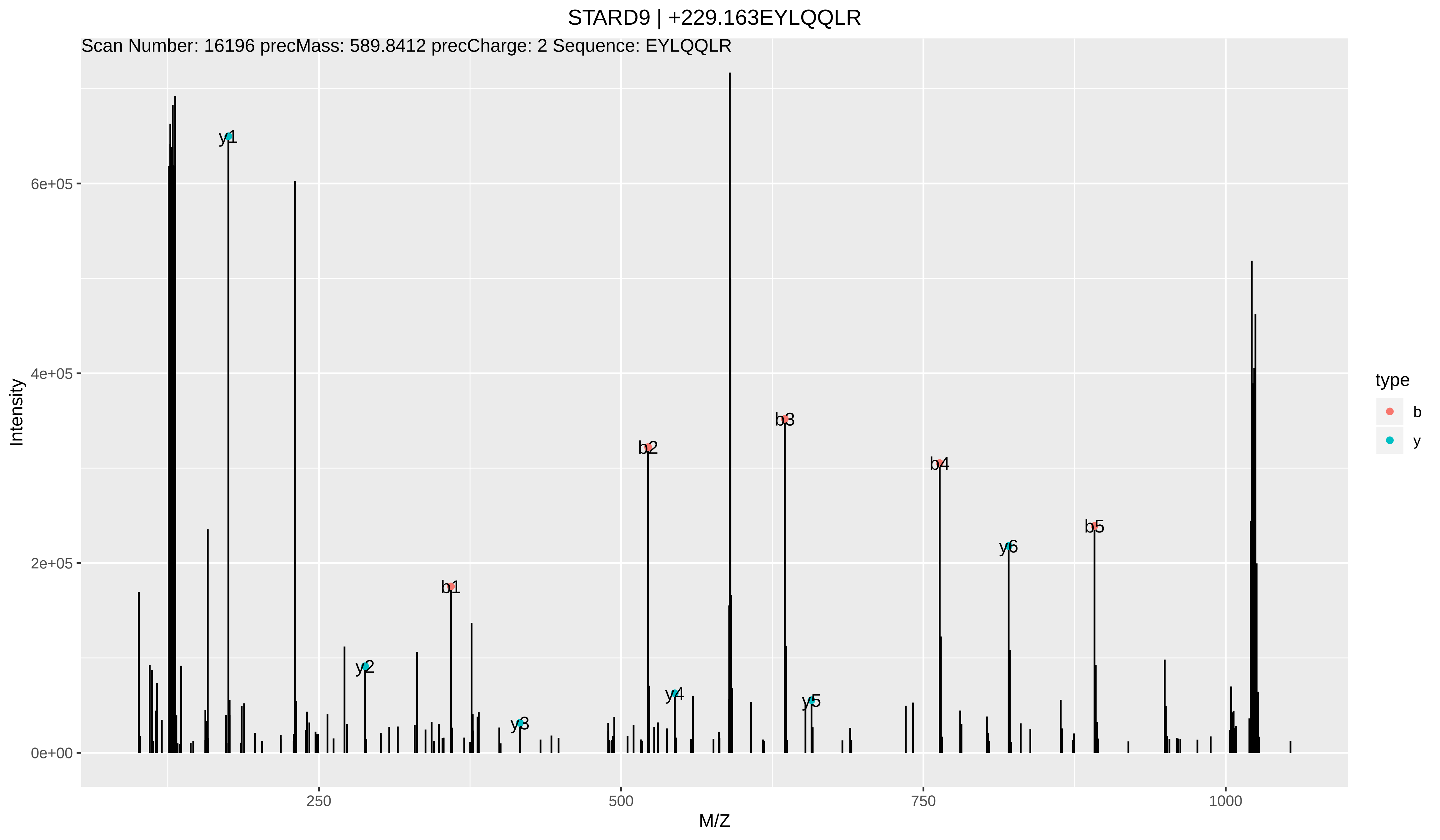
STARD9 | +229.163EYLQQLR
Scan Number: 16196 precMass: 589.8412 precCharge: 2 Sequence: EYLQQLR
Intensity
M/Z
0e+00
2e+05
4e+05
6e+05
250
500
750
1000
y1
y2
y3
y4
y5
y6
b1
b2
b3
b4
b5
type
b
y

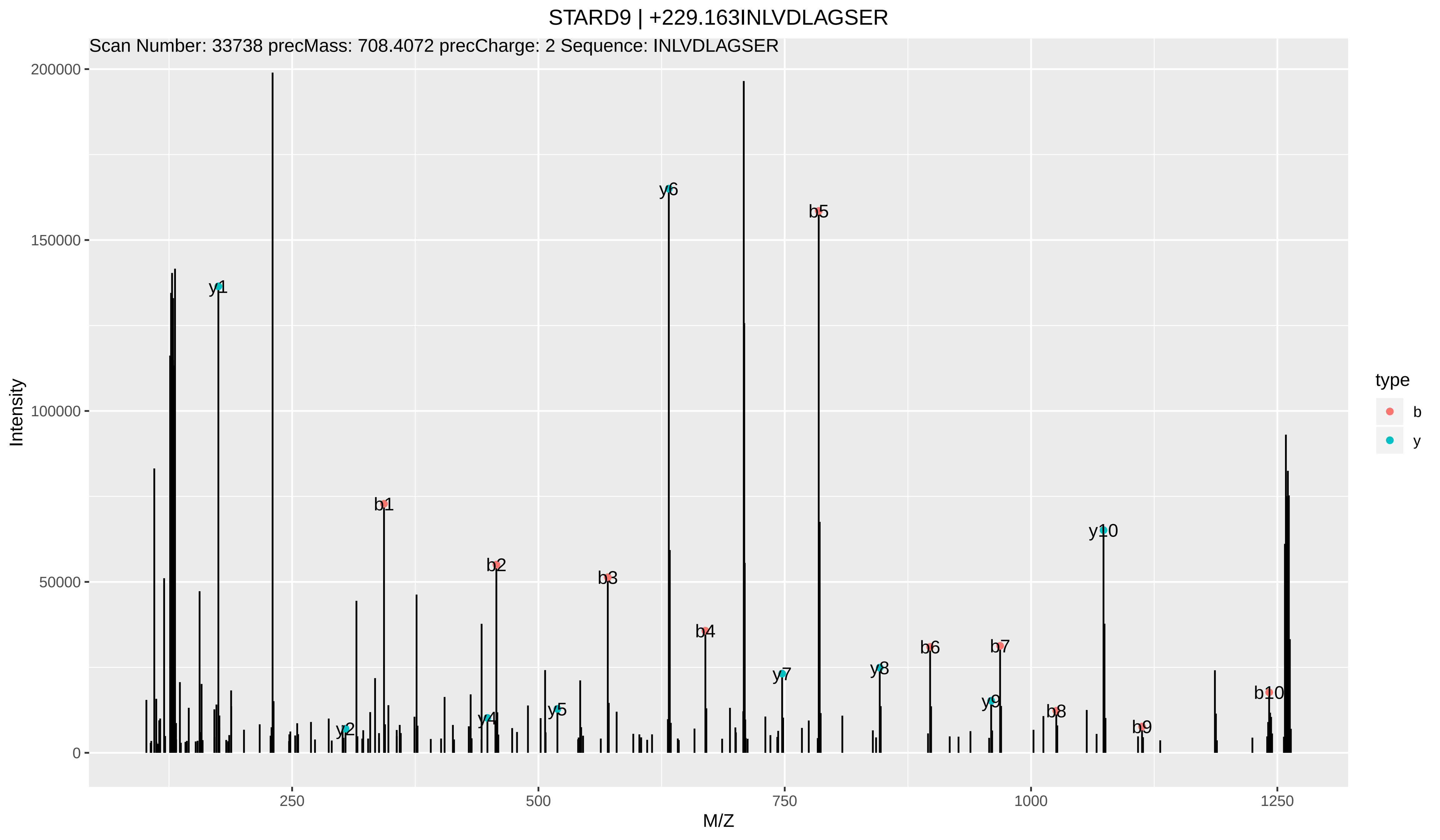

STARD9 | +229.163INLVDLAGSER
Scan Number: 33738 precMass: 708.4072 precCharge: 2 Sequence: INLVDLAGSER
Intensity
M/Z
200000
150000
100000
50000
0
250
500
750
1000
1250
y1
y2
y4
y5
y6
y7
y8
y9
y10
b1
b2
b3
b4
b5
b6
b7
b8
b9
b10
type
b
y

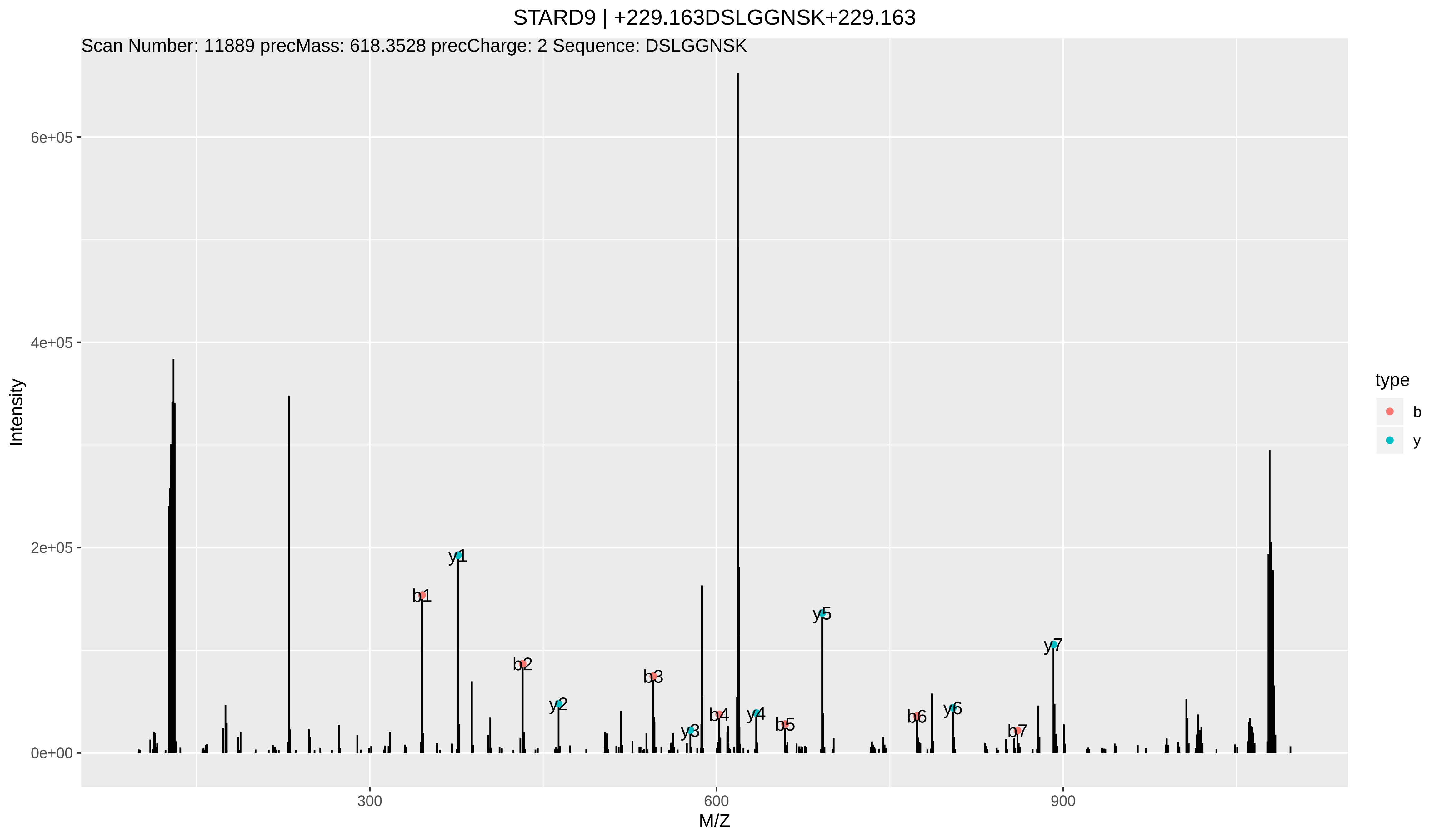
STARD9 | +229.163DSLGGNSK+229.163
Scan Number: 11889 precMass: 618.3528 precCharge: 2 Sequence: DSLGGNSK
Intensity
6e+05
4e+05
2e+05
0e+00
M/Z
300
600
900
y1
b1
b2
y2
b3
y3
b4
y4
b5
y5
b6
y6
b7
y7
type
b
y

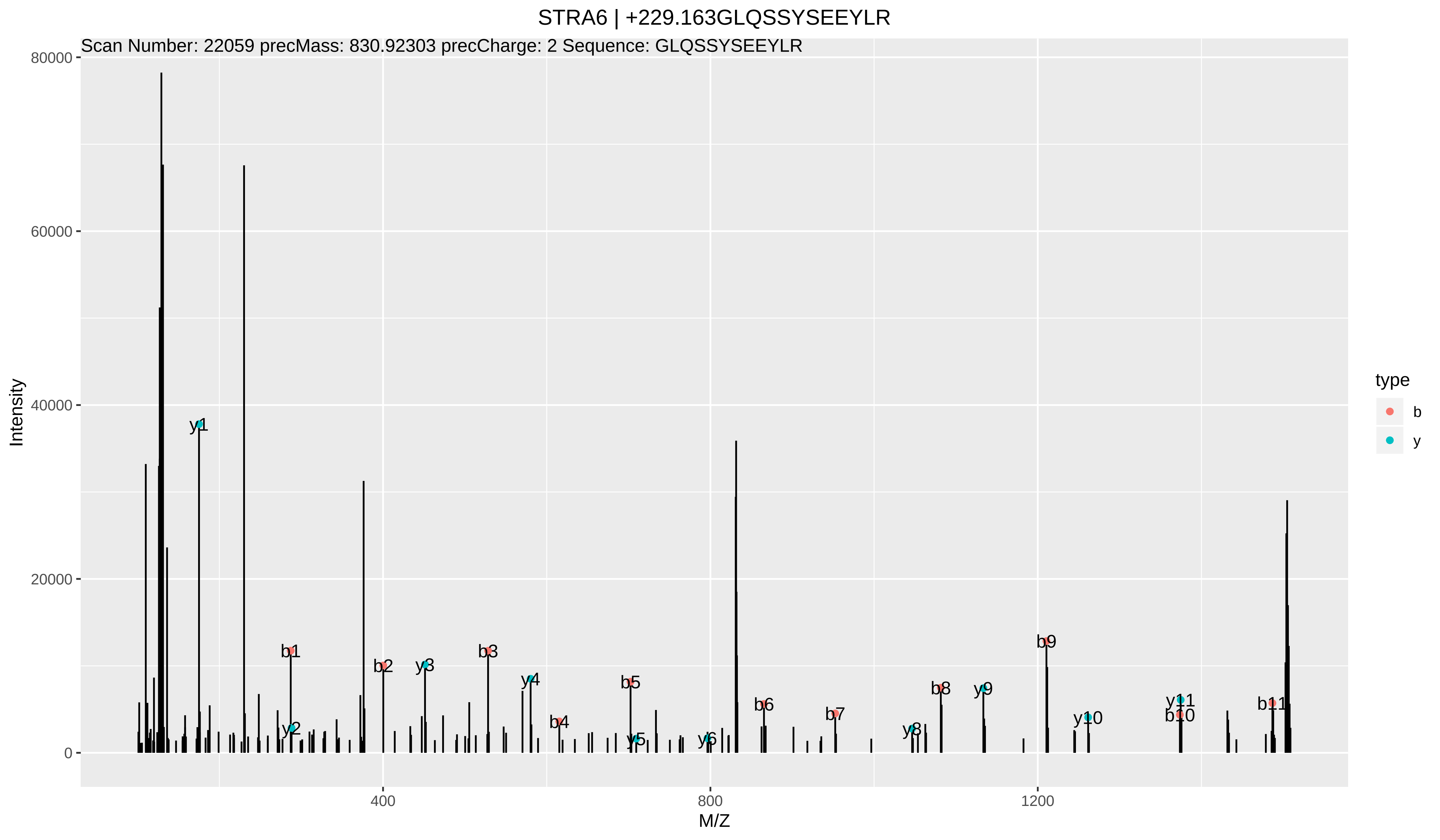

STRA6 | +229.163GLQSSYSEEYLR
Scan Number: 22059 precMass: 830.92303 precCharge: 2 Sequence: GLQSSYSEEYLR
Intensity
M/Z
80000
60000
40000
20000
0
400
800
1200
y1
y2
y3
y4
y5
y6
y8
y9
y10
y11
b1
b2
b3
b4
b5
b6
b7
b8
b9
b10
b11
type
b
y

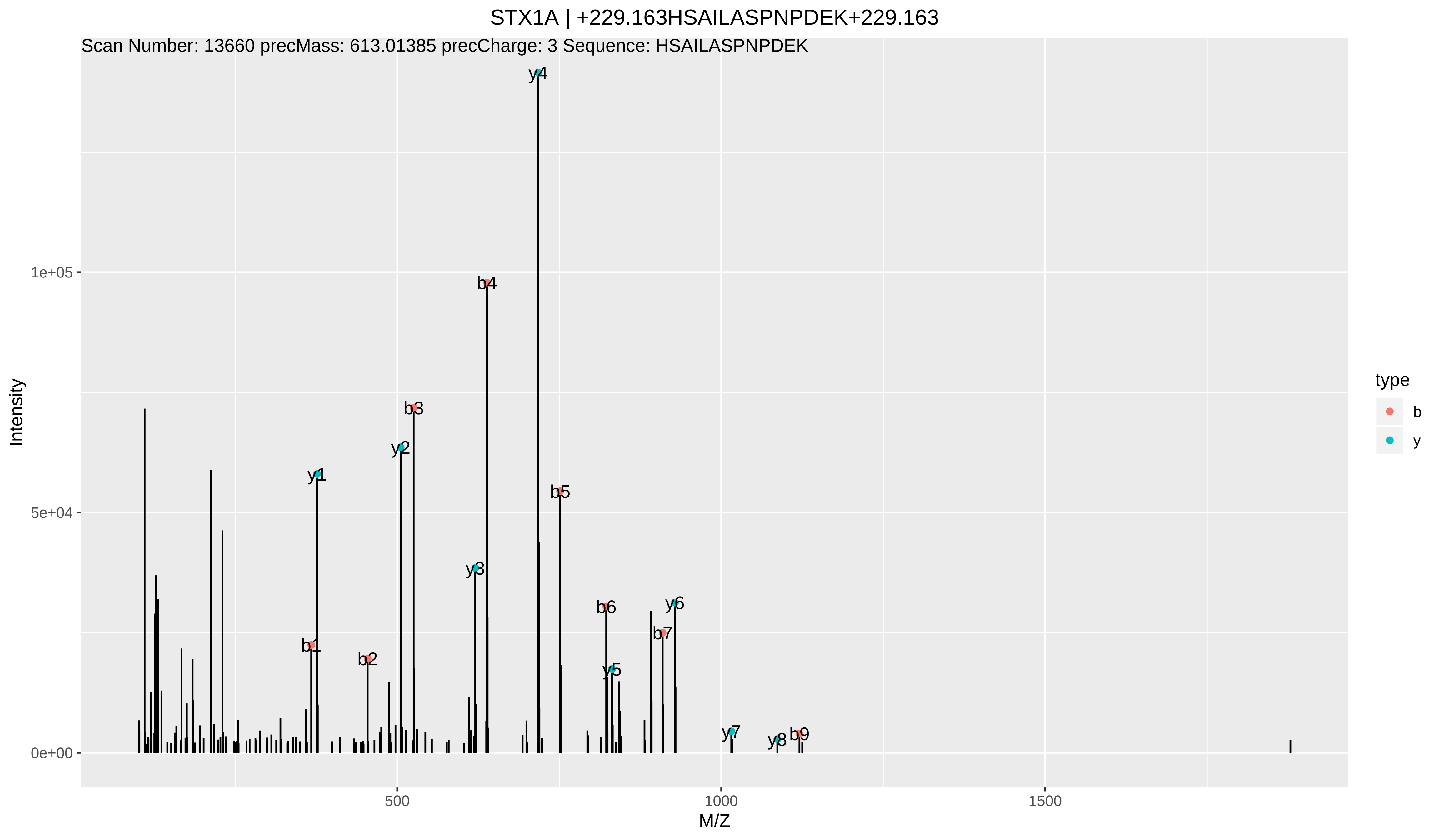

STX1A | +229.163HSAILASPNPDEK+229.163
Scan Number: 13660 precMass: 613.01385 precCharge: 3 Sequence: HSAILASPNPDEK
Intensity
1e+05
5e+04
0e+00
M/Z
500
1000
1500
y4
b4
b3
y2
y1
b5
y3
b6
y6
b7
b1
b2
y5
y7
y8
b9
type
b
y

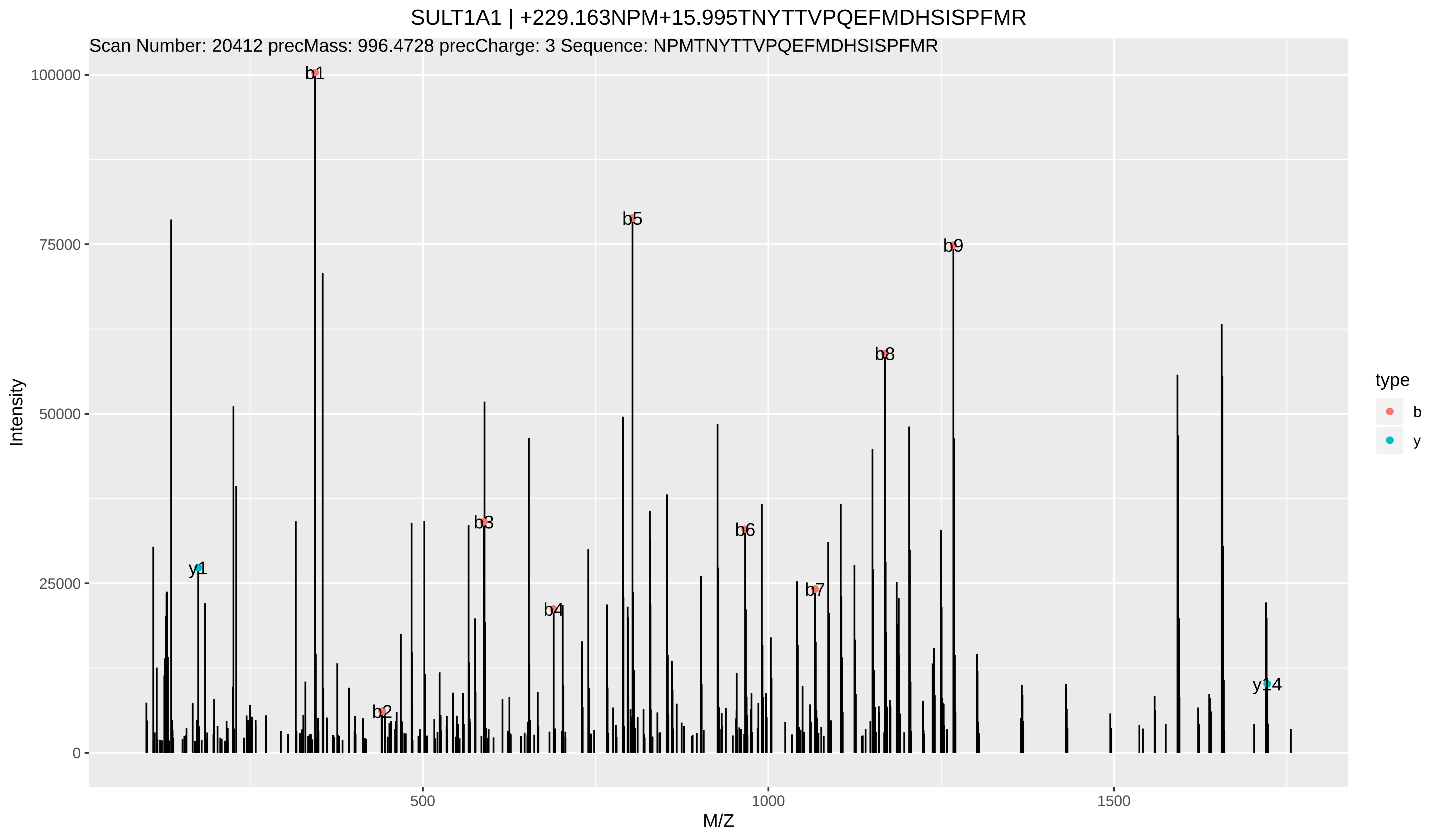

SULT1A1 | +229.163NPM+15.995TNYTTVPQEFMDHSISPFMR
Scan Number: 20412 precMass: 996.4728 precCharge: 3 Sequence: NPMTNYTTVPQEFMDHSISPFMR
Intensity
100000
75000
50000
25000
0
M/Z
500
1000
1500
b1
b2
b3
b4
b5
b6
b7
b8
b9
y1
y14
type
b
y

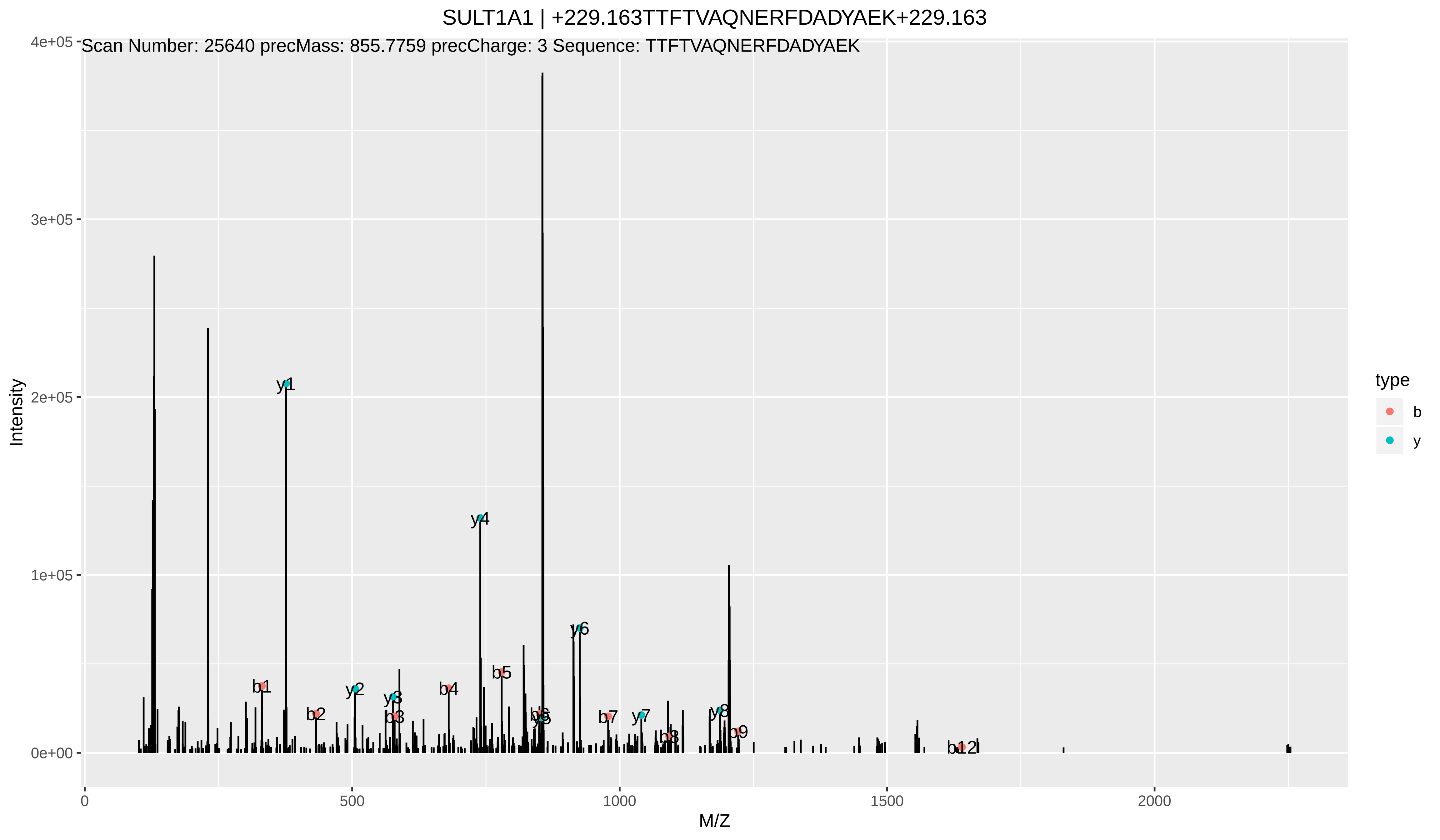
SULT1A1 | +229.163TTFTVAQNERFDADYAEK+229.163
Scan Number: 25640 precMass: 855.7759 precCharge: 3 Sequence: TTFTVAQNERFDADYAEK
Intensity
M/Z
0e+00
1e+05
2e+05
3e+05
4e+05
0
500
1000
1500
2000
y1
y2
y3
y4
y5
y6
y7
y8
b1
b2
b3
b4
b5
b6
b7
b8
b9
b12
type
b
y

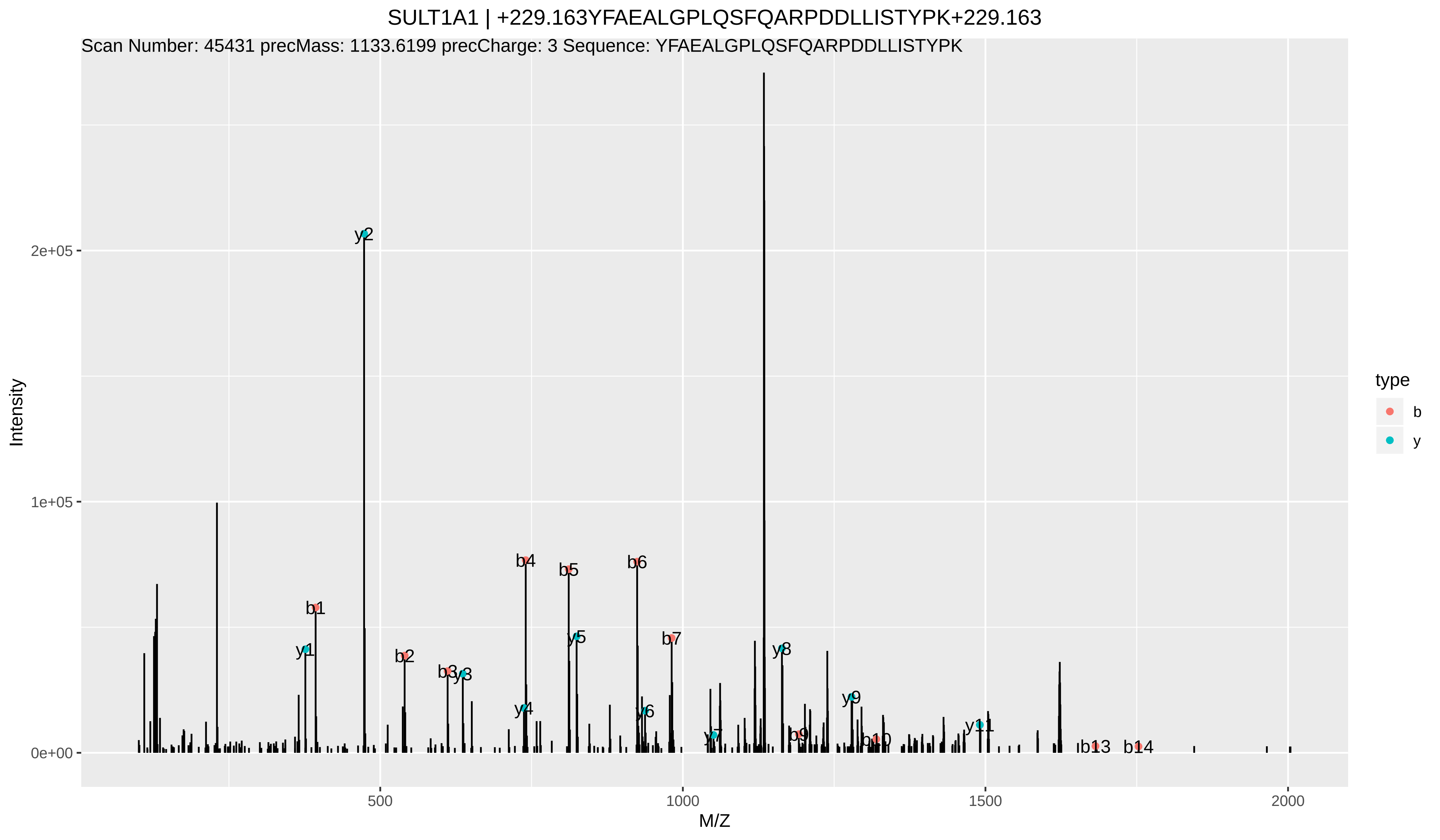

SULT1A1 | +229.163YFAEALGPLQSFQARPDDLLISTYPK+229.163
Scan Number: 45431 precMass: 1133.6199 precCharge: 3 Sequence: YFAEALGPLQSFQARPDDLLISTYPK
Intensity
M/Z
type
b
y
2e+05
1e+05
0e+00
500
1000
1500
2000
y2
b1
y1
b2
b3
y3
b4
b5
b6
y5
b7
y4
y6
y7
y8
b9
y9
b10
y11
b13
b14

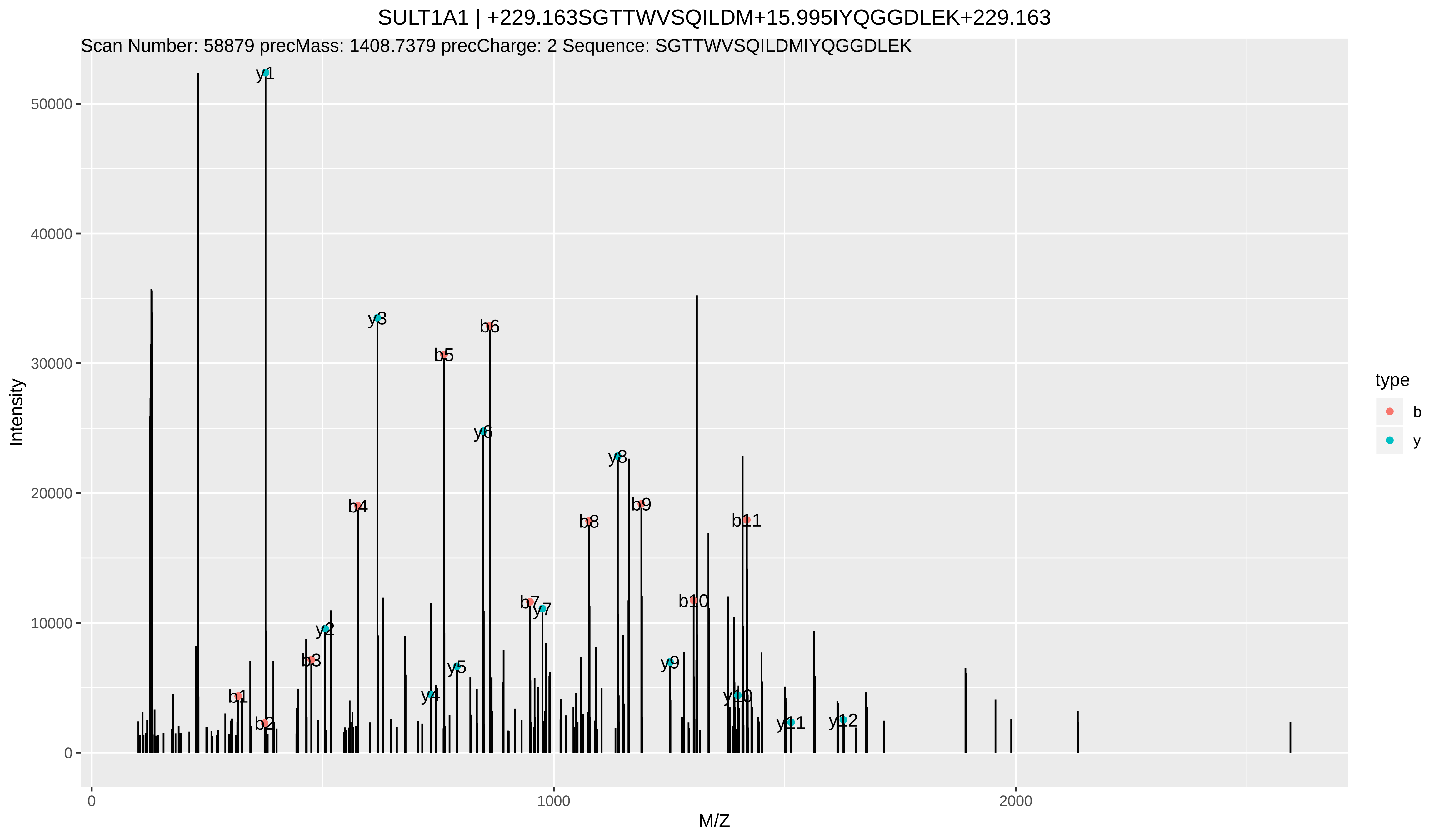
SULT1A1 | +229.163SGTTWVSQILDM+15.995IYQGGDLEK+229.163
Scan Number: 58879 precMass: 1408.7379 precCharge: 2 Sequence: SGTTWVSQILDMIYQGGDLEK
Intensity
M/Z
type
b
y
y1
y2
y3
y4
y5
y6
y7
y8
y9
y10
y11
y12
b1
b2
b3
b4
b5
b6
b7
b8
b9
b10
b11
0
10000
20000
30000
40000
50000
1000
2000

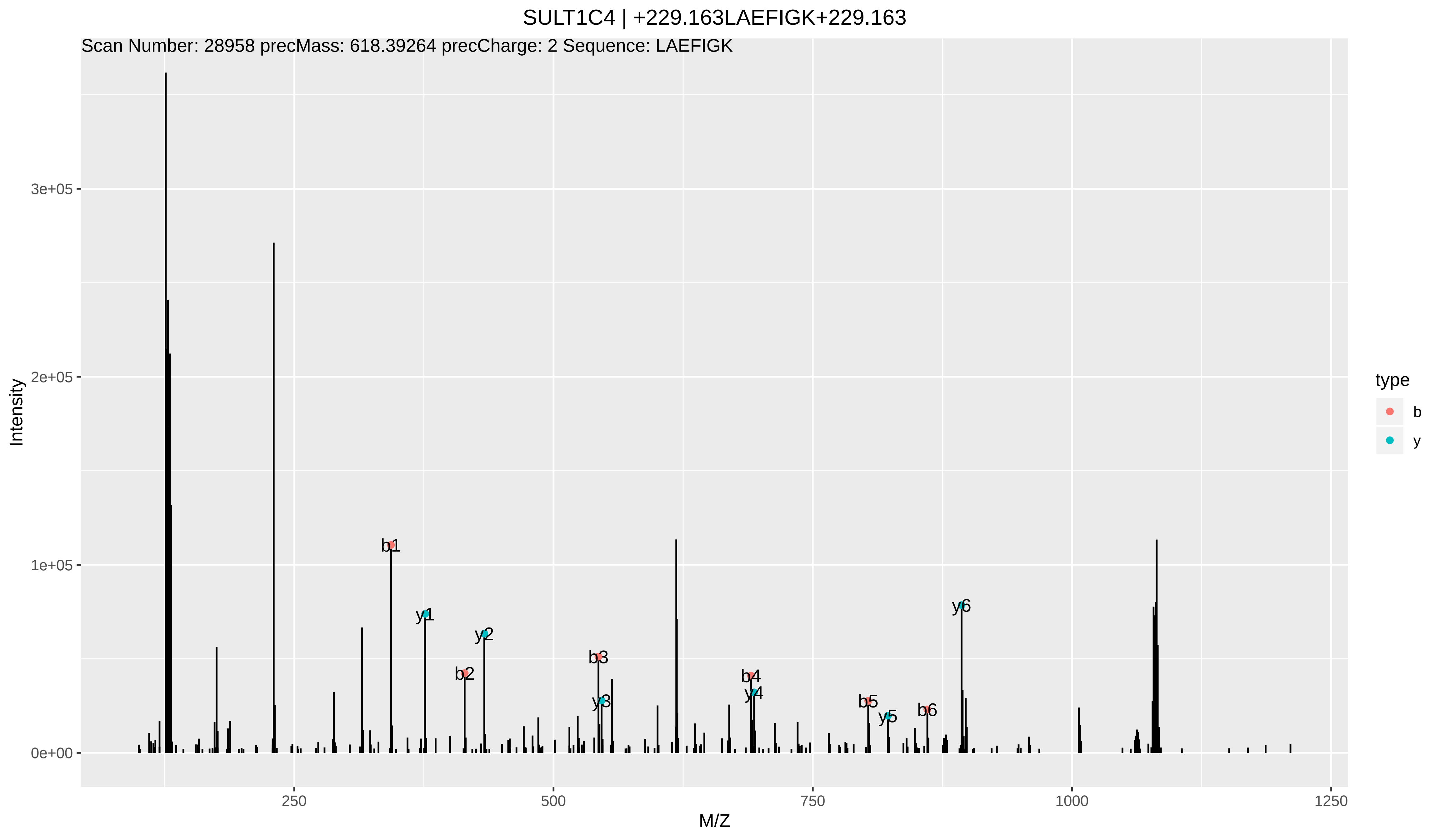

SULT1C4 | +229.163LAEFIGK+229.163
Scan Number: 28958 precMass: 618.39264 precCharge: 2 Sequence: LAEFIGK
Intensity
3e+05
2e+05
1e+05
0e+00
M/Z
250
500
750
1000
1250
b1
y1
y2
b2
b3
y3
b4
y4
b5
y5
b6
y6
type
b
y

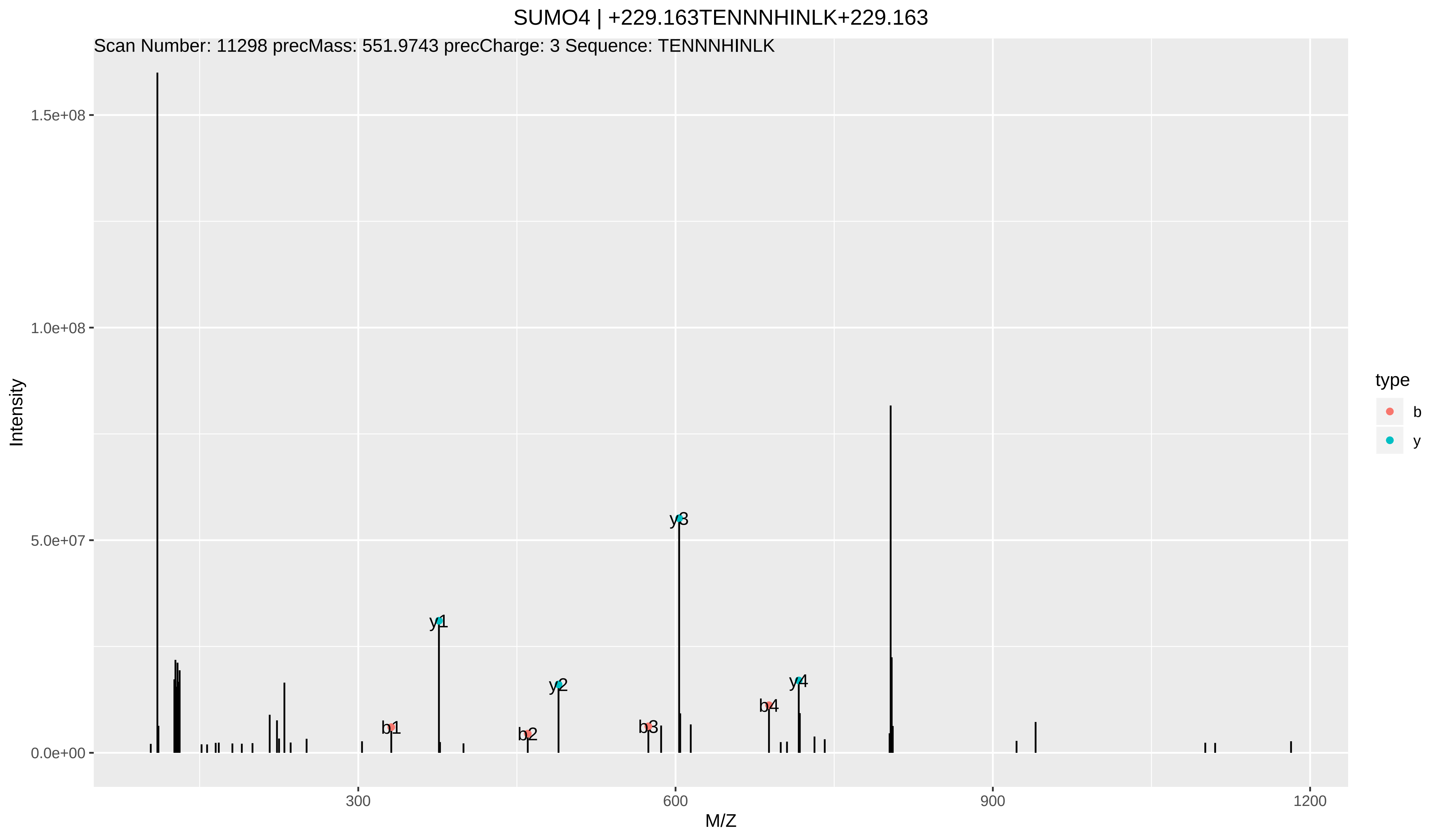

SUMO4 | +229.163TENNNHINLK+229.163
Scan Number: 11298 precMass: 551.9743 precCharge: 3 Sequence: TENNNHINLK
Intensity
1.5e+08
1.0e+08
5.0e+07
0.0e+00
M/Z
300
600
900
1200
y1
y2
y3
y4
b1
b2
b3
b4
type
b
y

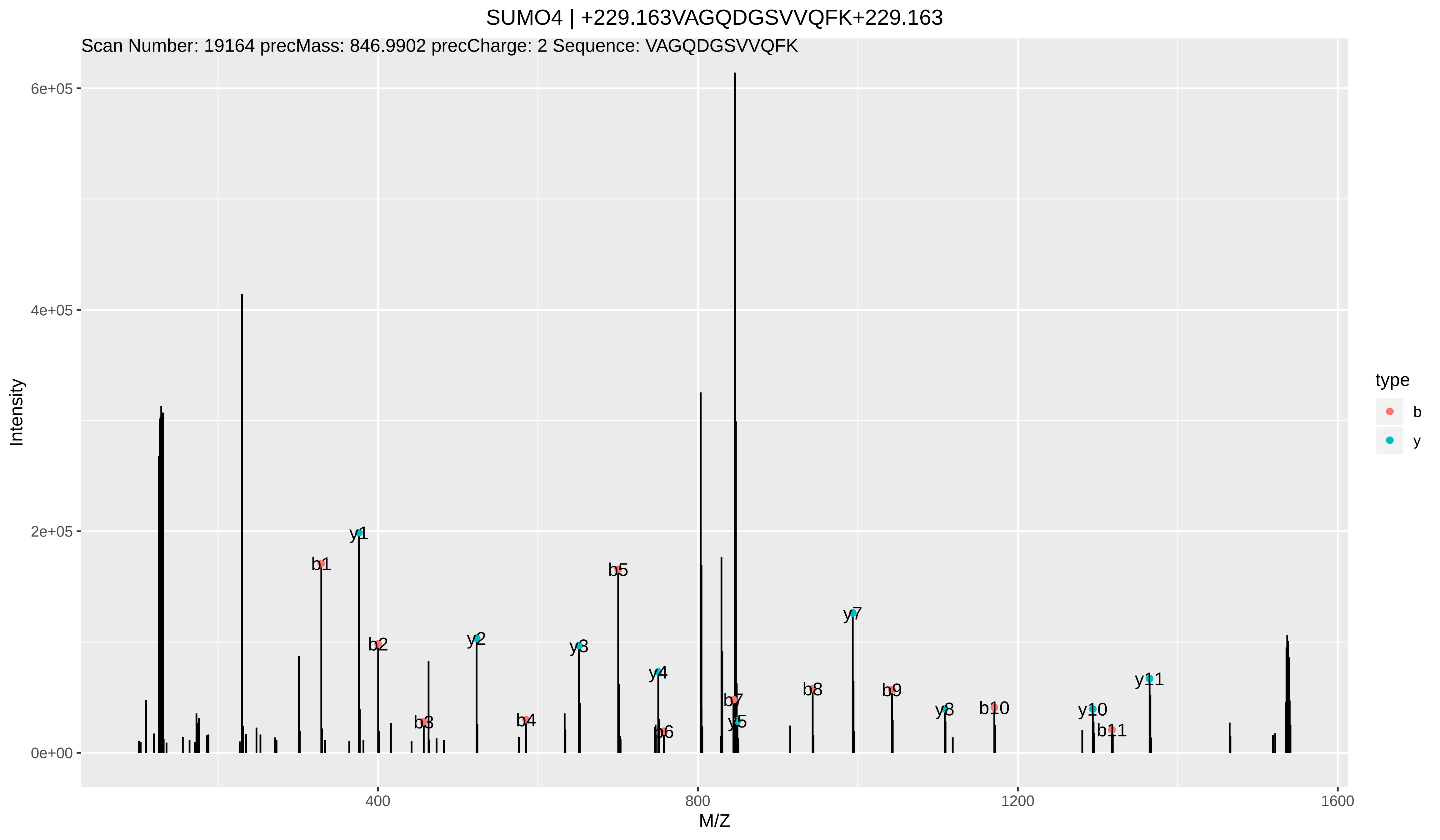

SUMO4 | +229.163VAGQDGSVVQFK+229.163
Scan Number: 19164 precMass: 846.9902 precCharge: 2 Sequence: VAGQDGSVVQFK
Intensity
M/Z
6e+05
4e+05
2e+05
0e+00
400
800
1200
1600
y1
b1
b5
b2
y2
y3
y4
b3
b4
b6
b7
y5
y7
b8
b9
y8
b10
y10
b11
y11
type
b
y

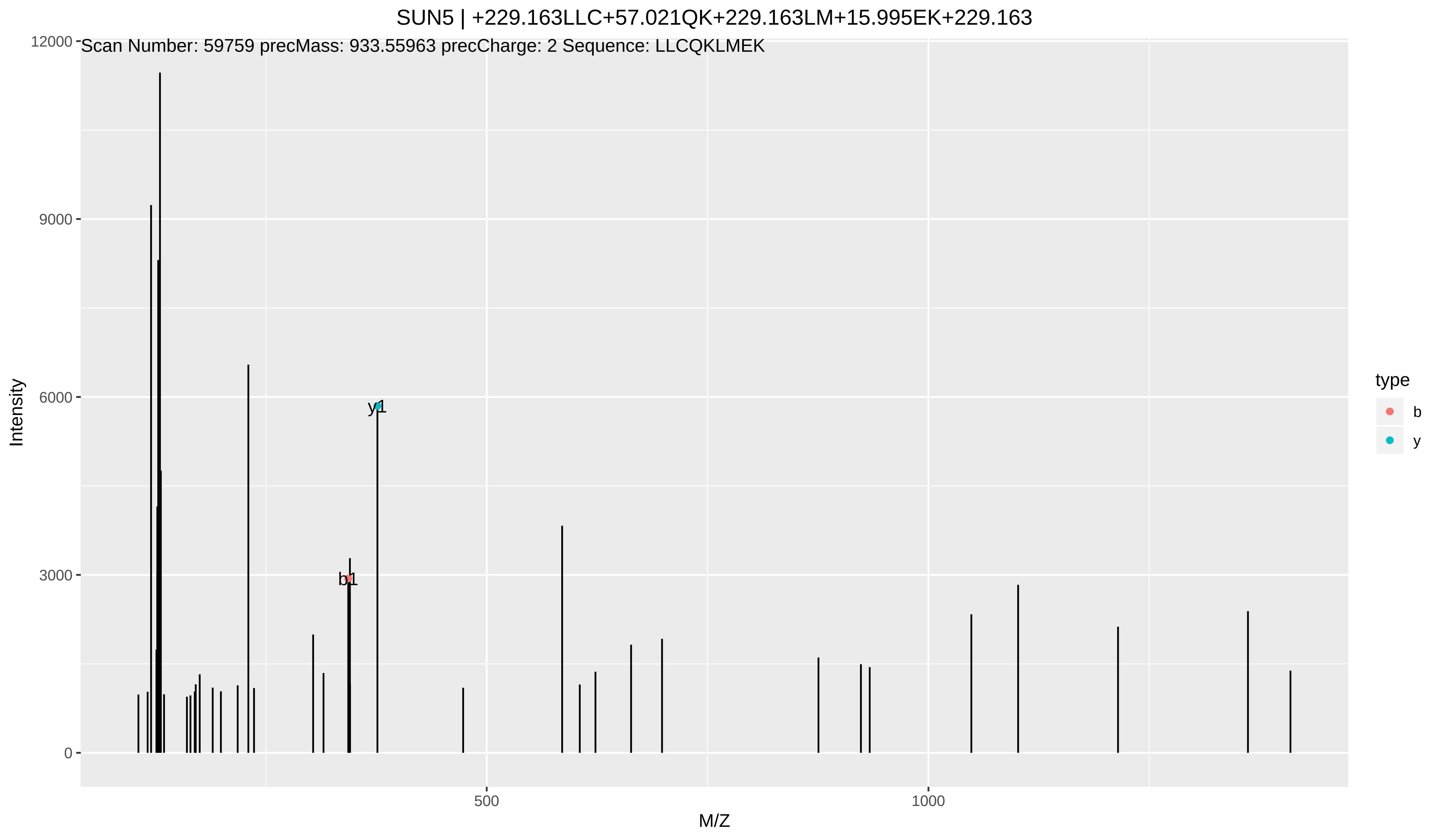
SUN5 | +229.163LLC+57.021QK+229.163LM+15.995EK+229.163
Scan Number: 59759 precMass: 933.55963 precCharge: 2 Sequence: LLCQKLMEK
Intensity
12000
9000
6000
3000
0
500
1000
M/Z
y1
b1
type
b
y

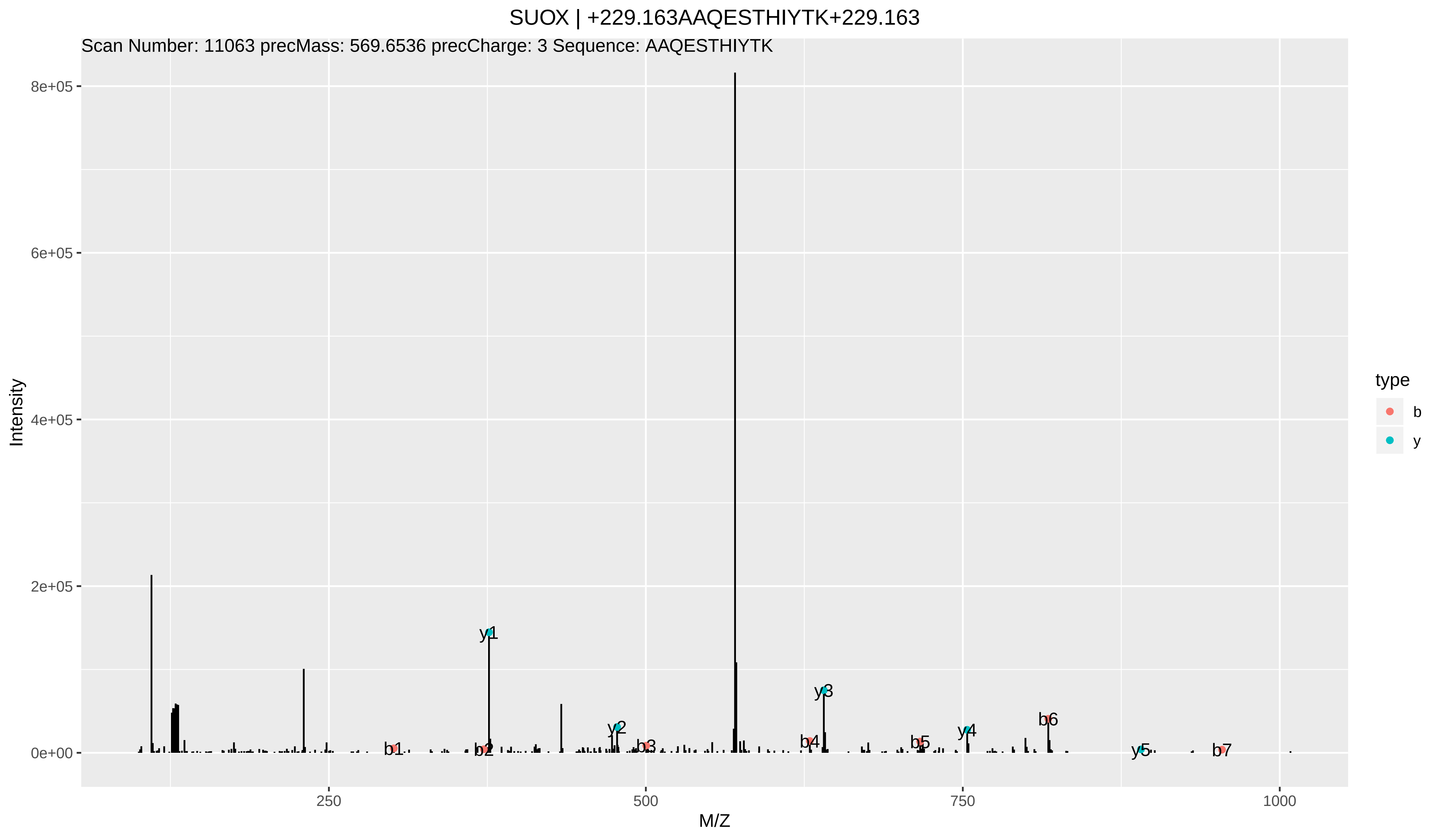

SUOX | +229.163AAQESTHIYTK+229.163
Scan Number: 11063 precMass: 569.6536 precCharge: 3 Sequence: AAQESTHIYTK
Intensity
8e+05
6e+05
4e+05
2e+05
0e+00
M/Z
250
500
750
1000
y1
y2
y3
y4
y5
b1
b2
b3
b4
b5
b6
b7
type
b
y

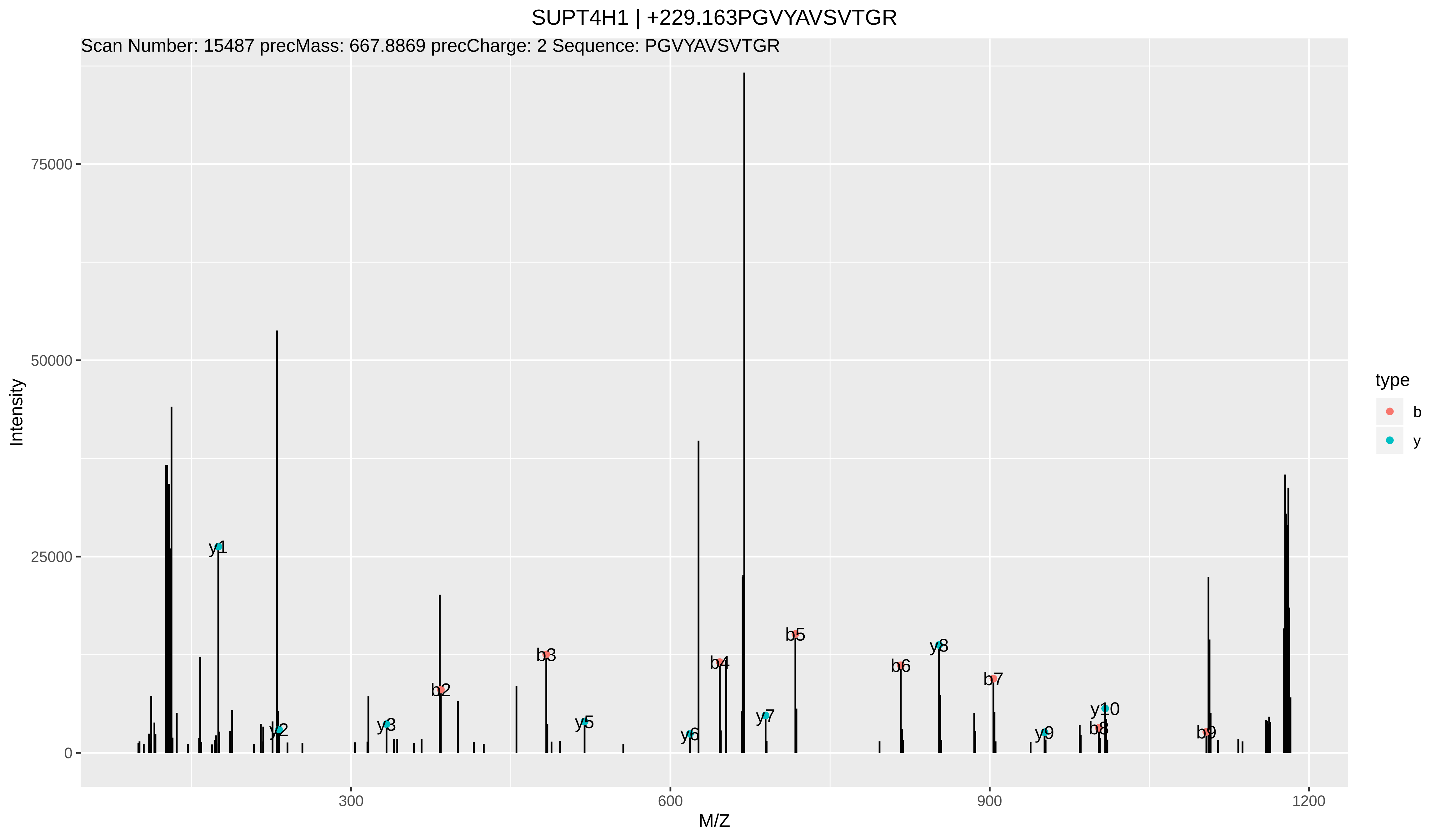

SUPT4H1 | +229.163PGVYAVSVTGR
Scan Number: 15487 precMass: 667.8869 precCharge: 2 Sequence: PGVYAVSVTGR
Intensity
M/Z
type
b
y
y1
y2
y3
b2
b3
y5
y6
b4
y7
b5
b6
y8
b7
y9
b8
y10
b9
75000
50000
25000
0
300
600
900
1200

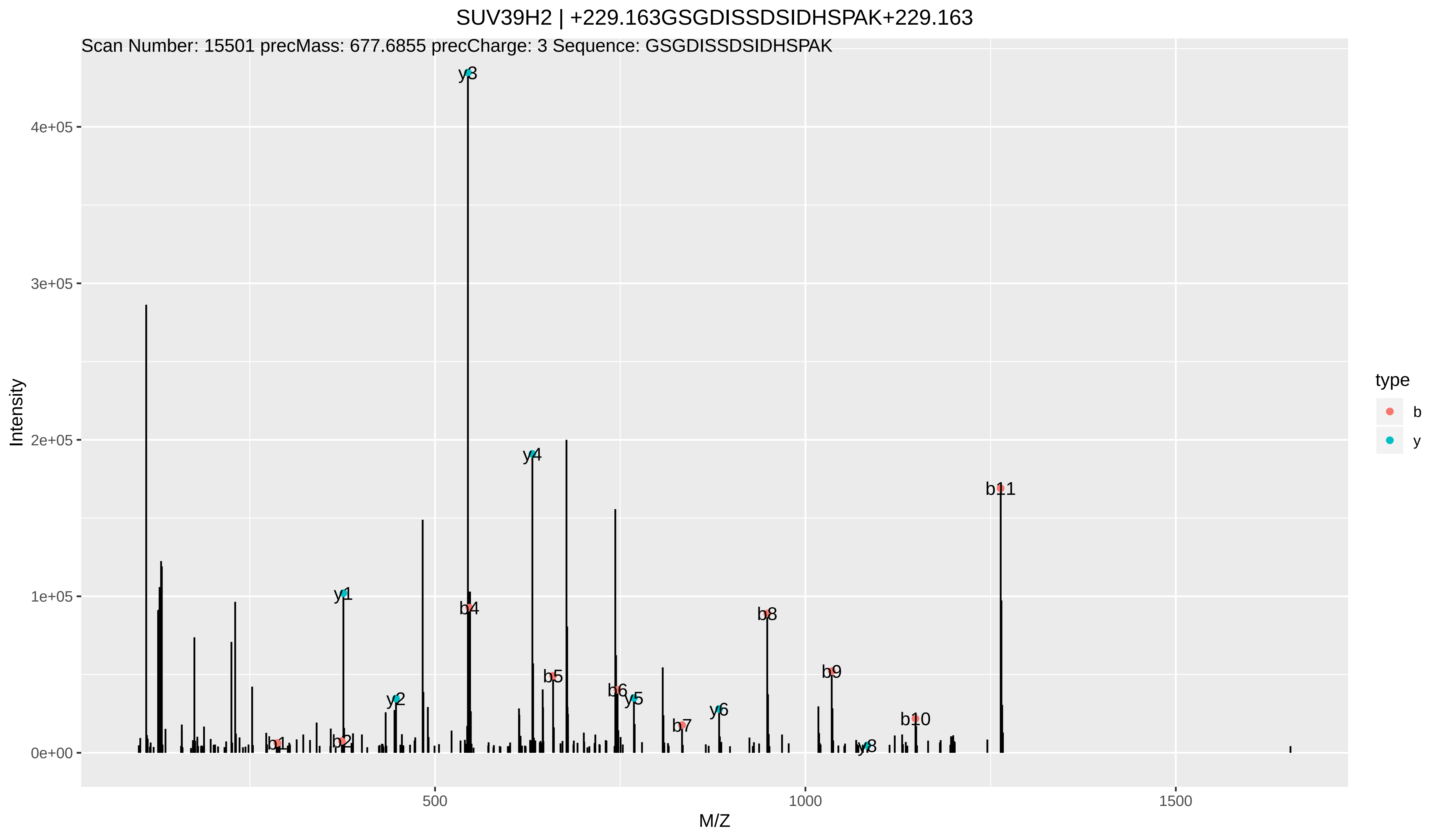
SUV39H2 | +229.163GSGDISSDSIDHSPAK+229.163
Scan Number: 15501 precMass: 677.6855 precCharge: 3 Sequence: GSGDISSDSIDHSPAK
Intensity
M/Z
0e+00
1e+05
2e+05
3e+05
4e+05
500
1000
1500
y3
y4
b11
y1
b4
b8
b9
b5
b6
y5
y2
y6
b10
b7
b1
b2
y8
type
b
y

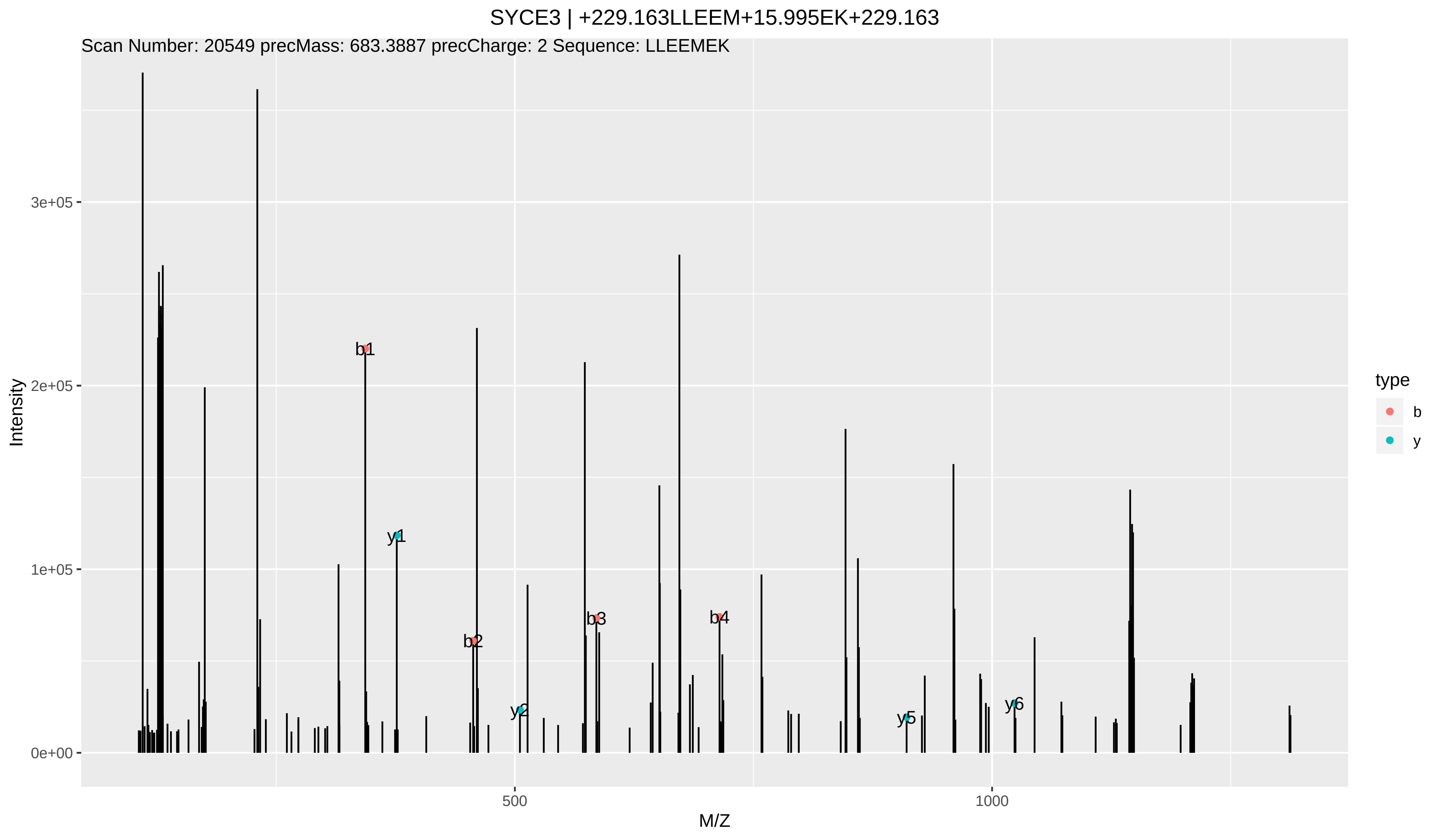

SYCE3 | +229.163LLEEM+15.995EK+229.163
Scan Number: 20549 precMass: 683.3887 precCharge: 2 Sequence: LLEEMEK
Intensity
M/Z
3e+05
2e+05
1e+05
0e+00
500
1000
b1
y1
b2
y2
b3
b4
y5
y6
type
b
y

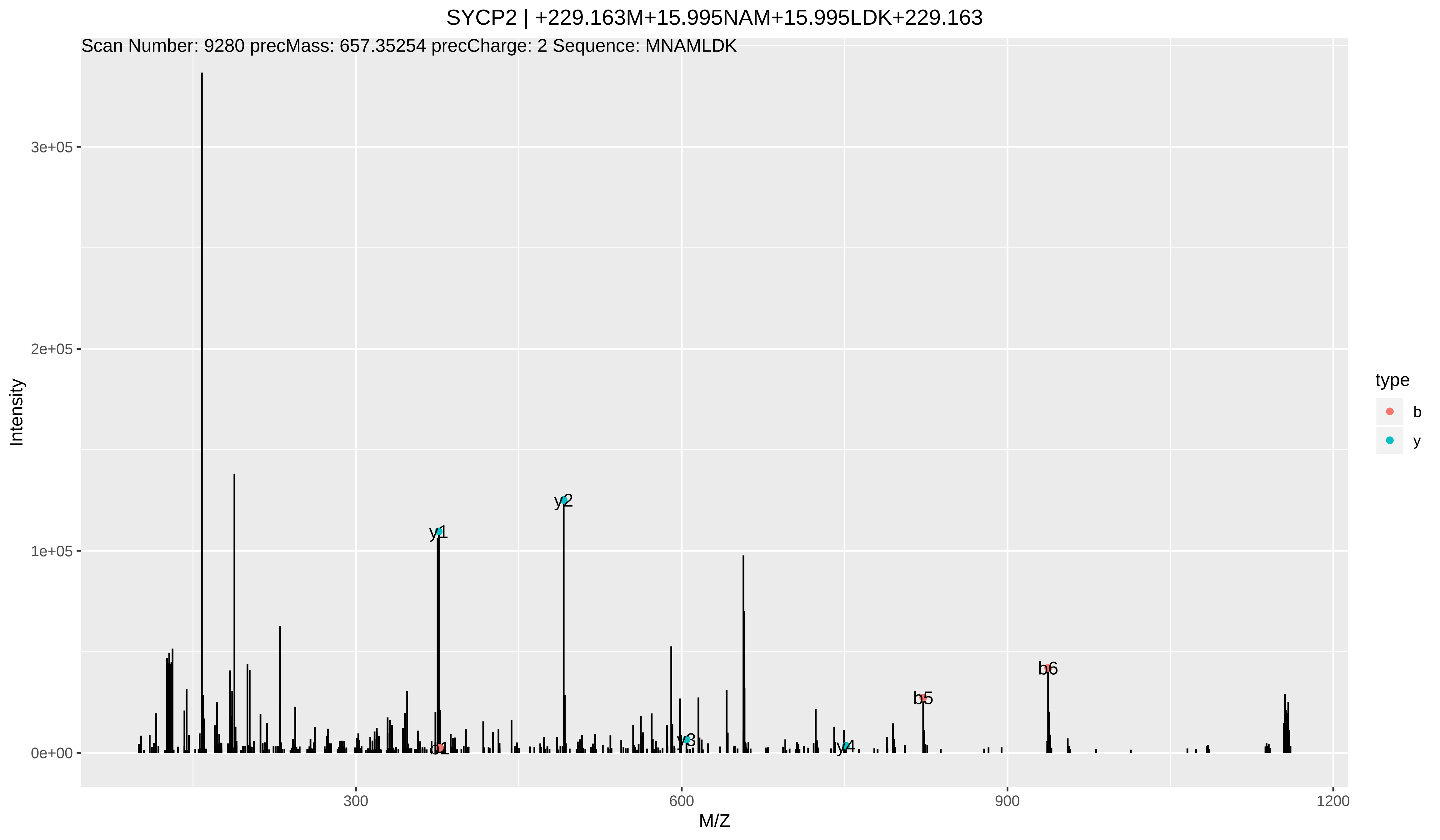

SYCP2 | +229.163M+15.995NAM+15.995LDK+229.163
Scan Number: 9280 precMass: 657.35254 precCharge: 2 Sequence: MNAMLDK
Intensity
M/Z
3e+05
2e+05
1e+05
0e+00
300
600
900
1200
y1
y2
y3
y4
b1
b5
b6
type
b
y

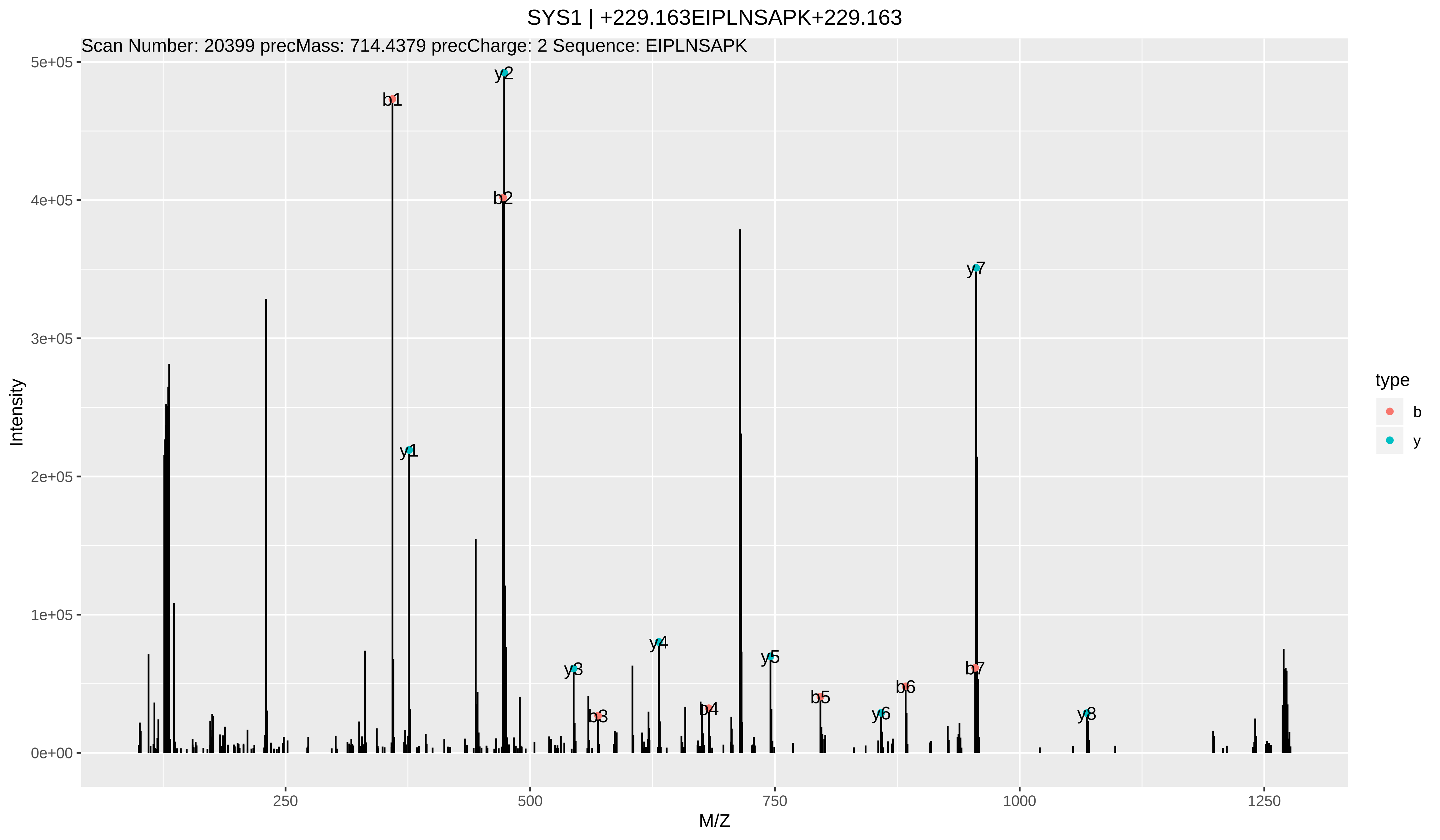

SYS1 | +229.163EIPLNSAPK+229.163
Scan Number: 20399 precMass: 714.4379 precCharge: 2 Sequence: EIPLNSAPK
Intensity
M/Z
0e+00
1e+05
2e+05
3e+05
4e+05
5e+05
250
500
750
1000
1250
b1
b2
b3
b4
b5
b6
b7
y1
y2
y3
y4
y5
y6
y7
y8
type
b
y

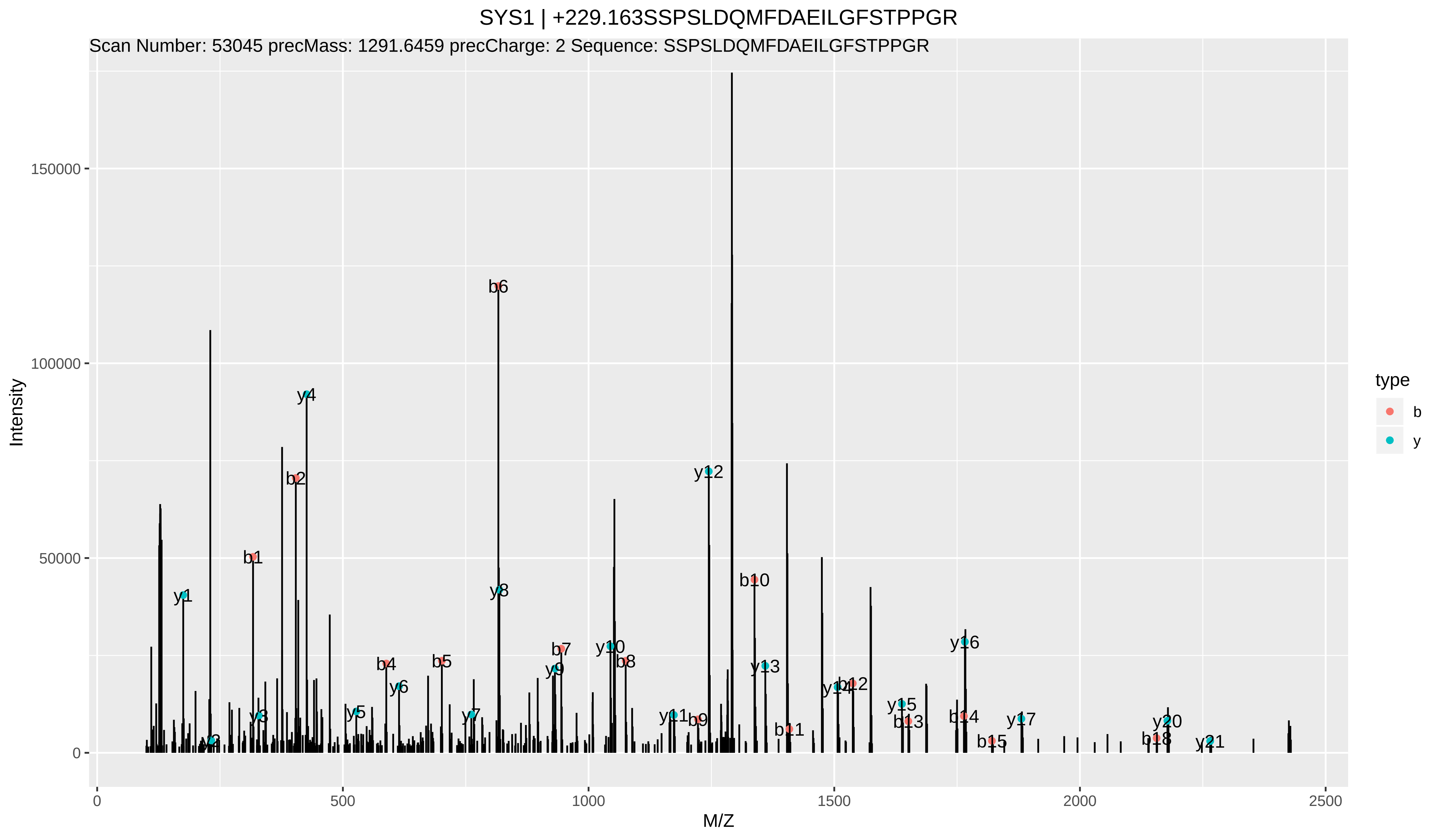

SYS1 | +229.163SSPSLDQMFDAEILGFSTPPGR
Scan Number: 53045 precMass: 1291.6459 precCharge: 2 Sequence: SSPSLDQMFDAEILGFSTPPGR
Intensity
M/Z
type
b
y
0
50000
100000
150000
500
1000
1500
2000
2500
b6
y4
b2
b1
y1
y8
y12
b10
y16
y10
b7
b8
b4
b5
y9
y13
y6
y5
y3
y7
y11
b9
b11
y14
b12
y15
b13
b14
y17
y20
b15
b18
y21

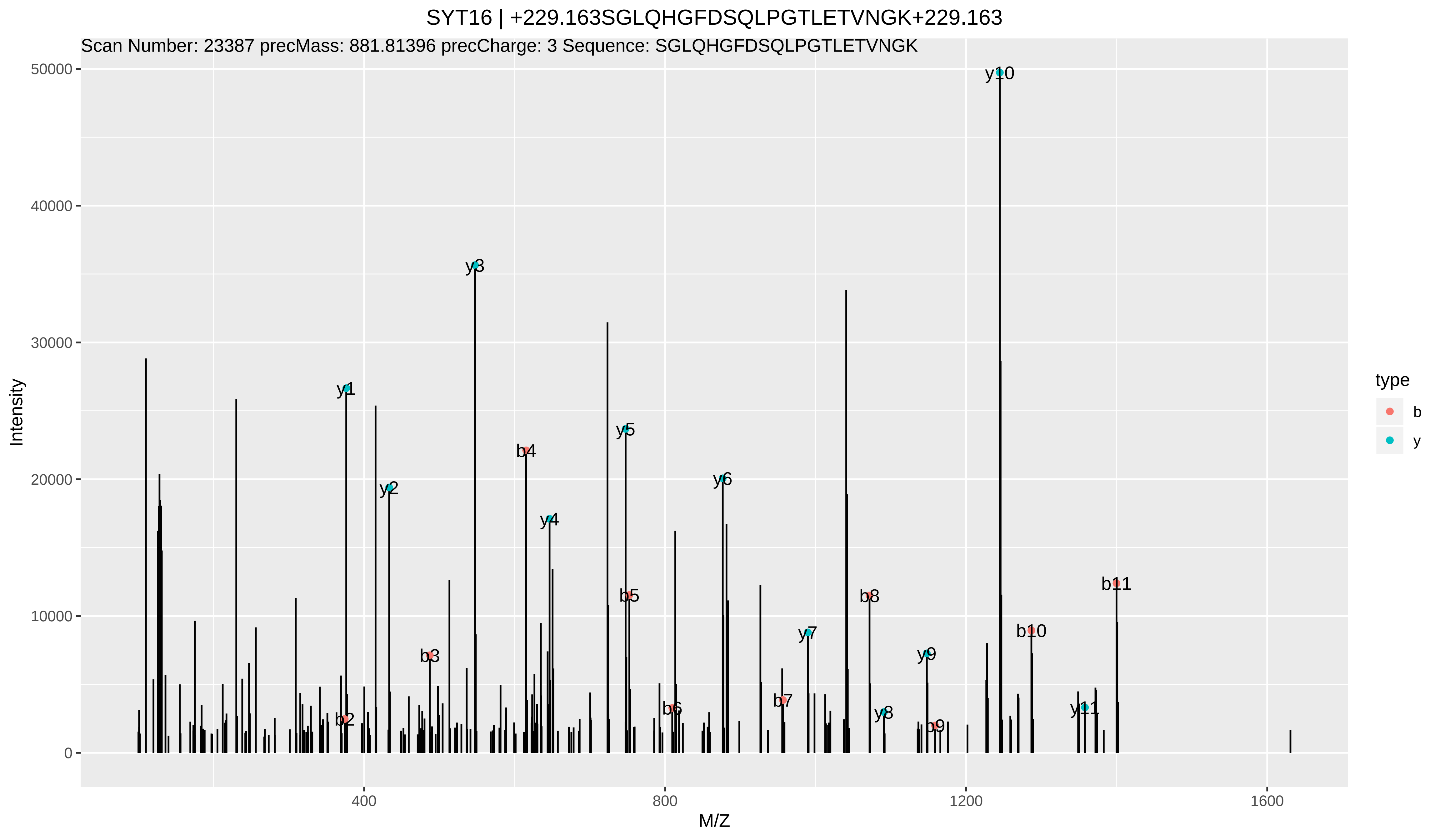
SYT16 | +229.163SGLQHGFDSQLPGTLETVNGK+229.163
Scan Number: 23387 precMass: 881.81396 precCharge: 3 Sequence: SGLQHGFDSQLPGTLETVNGK
Intensity
M/Z
type
b
y
y10
y3
y1
y2
b4
y4
y5
b5
y6
b3
b2
b6
b7
y7
b8
y8
y9
b9
b10
y11
b11
0
10000
20000
30000
40000
50000
400
800
1200
1600

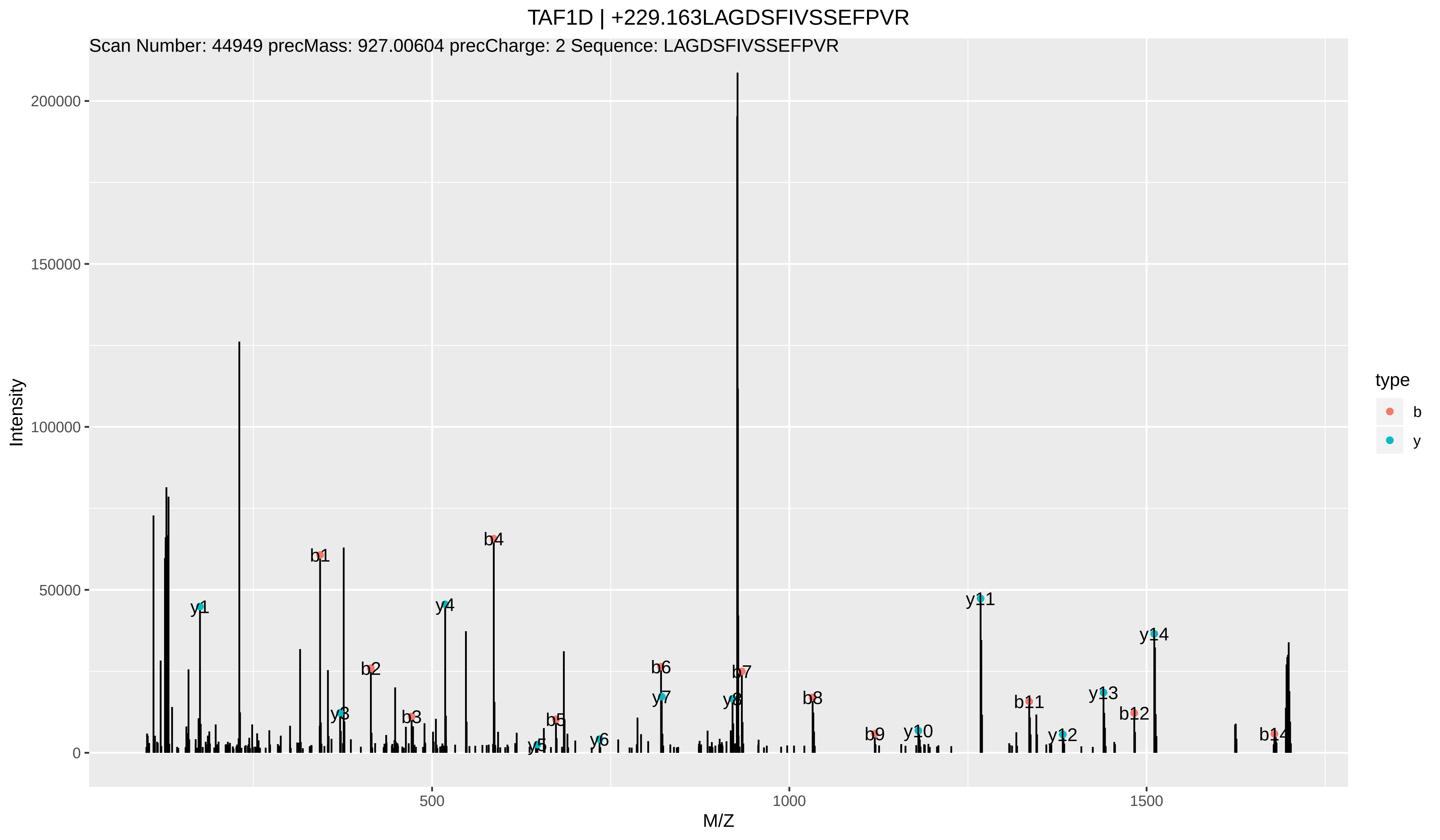
TAF1D | +229.163LAGDSFIVSSEFPVR
Scan Number: 44949 precMass: 927.00604 precCharge: 2 Sequence: LAGDSFIVSSEFPVR
Intensity
M/Z
type
b
y
y1
b1
b2
y3
b3
y4
b4
y5
b5
y6
b6
y7
b7
y8
b8
b9
y10
y11
b11
y12
y13
b12
y14
b14
0
50000
100000
150000
200000
500
1000
1500

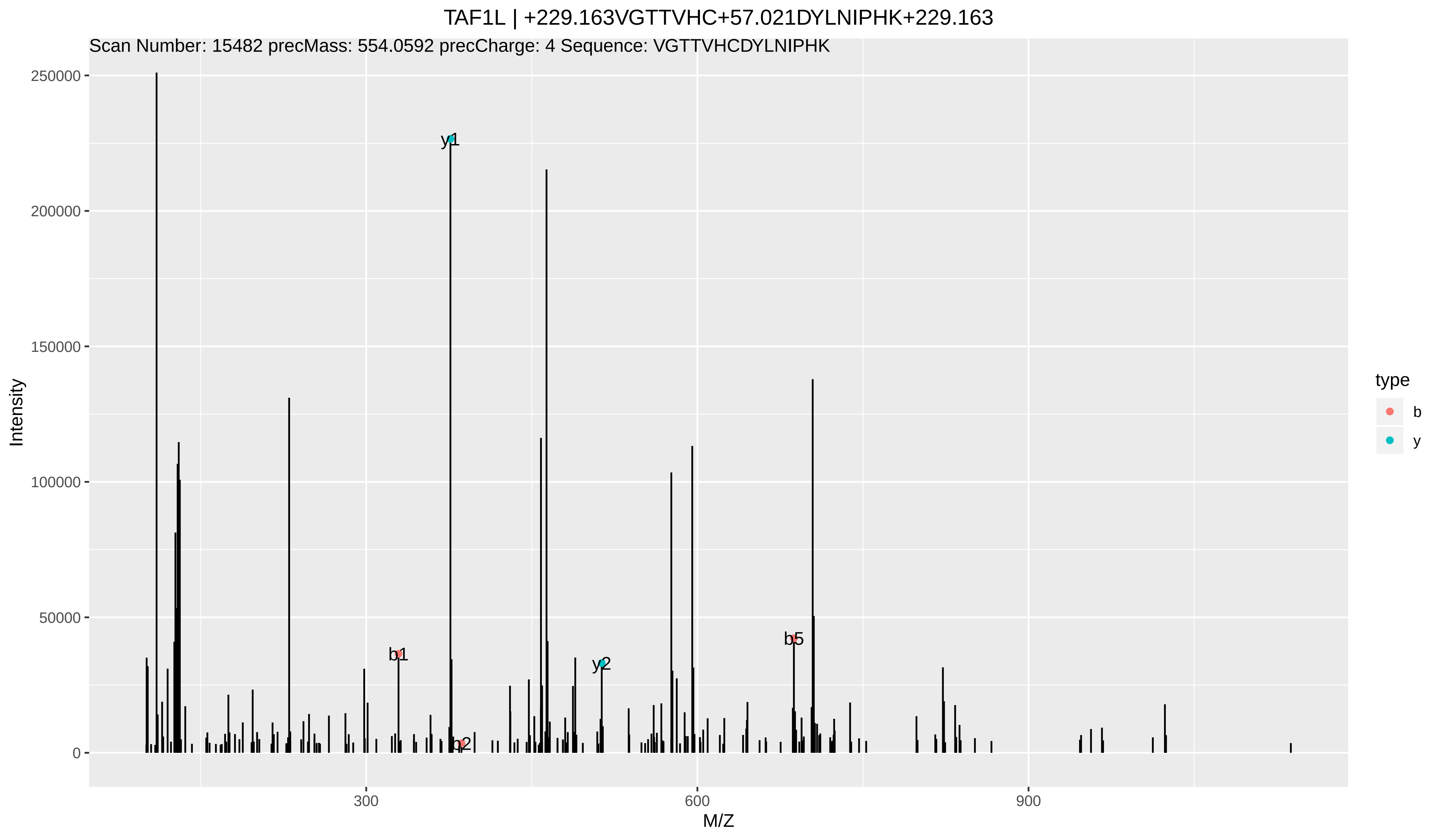

TAF1L | +229.163VGTTVHC+57.021DYLNIPHK+229.163
Scan Number: 15482 precMass: 554.0592 precCharge: 4 Sequence: VGTTVHCDYLNIPHK
Intensity
250000
200000
150000
100000
50000
0
M/Z
300
600
900
y1
b1
b2
y2
b5
type
b
y

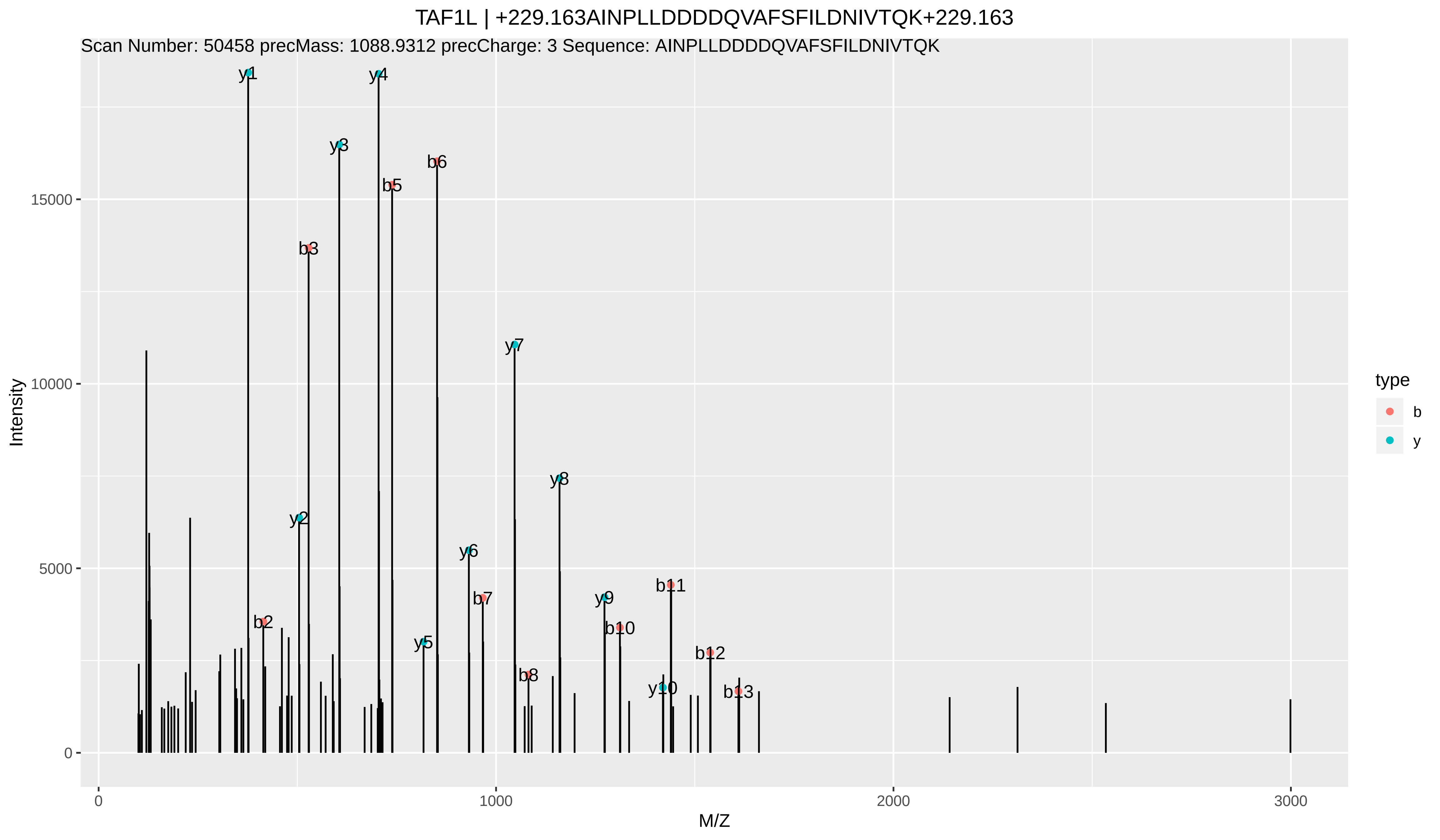

TAF1L | +229.163AINPLLDDDDQVAFSFILDNIVTQK+229.163
Scan Number: 50458 precMass: 1088.9312 precCharge: 3 Sequence: AINPLLDDDDQVAFSFILDNIVTQK
y1
y4
y3
b6
b5
b3
y7
y8
y2
y6
b11
b7
y9
b2
b10
y5
b12
b8
y10
b13
Intensity
15000
10000
5000
0
M/Z
1000
2000
3000
type
b
y

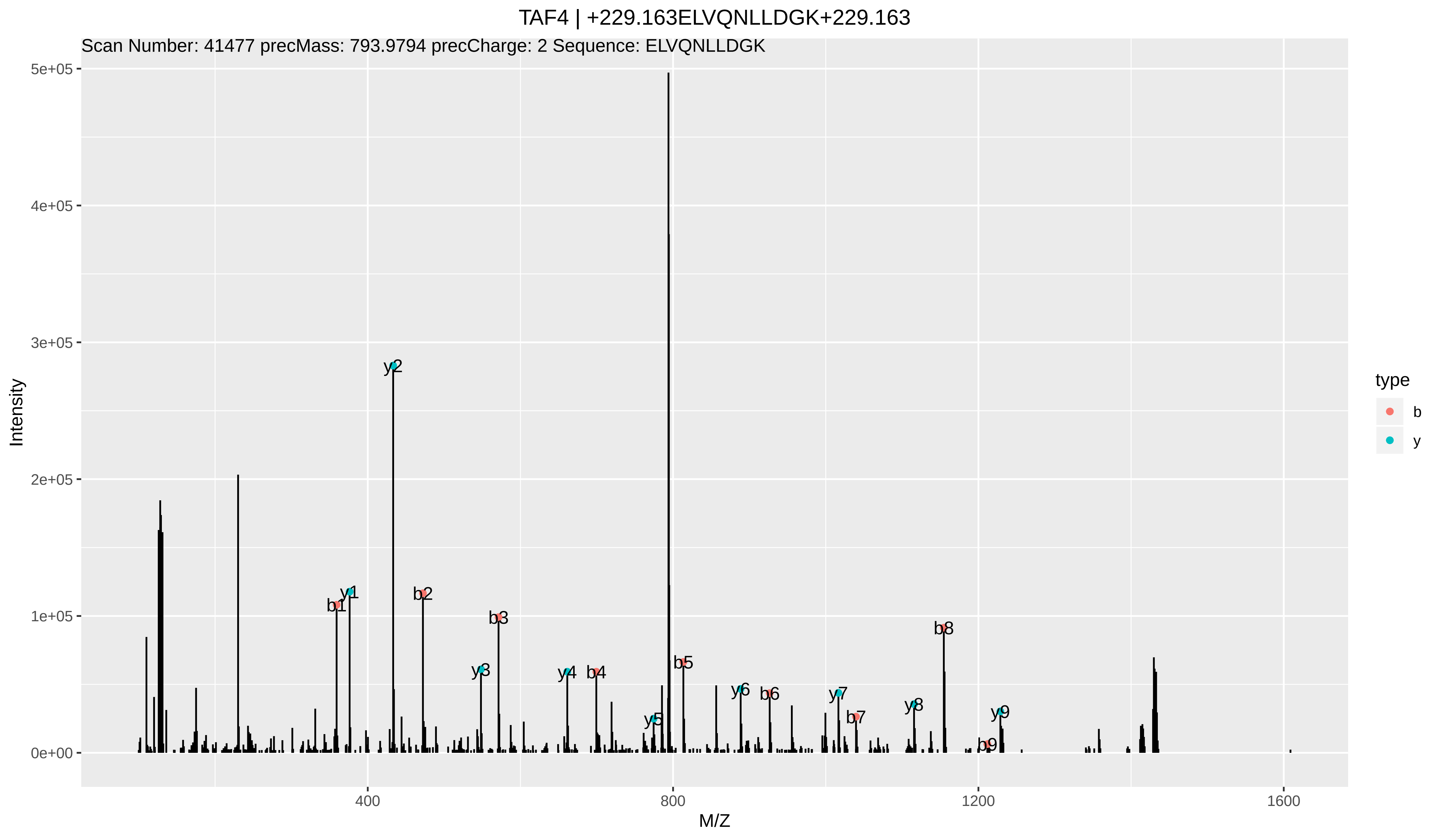
TAF4 | +229.163ELVQNLLDGK+229.163
Scan Number: 41477 precMass: 793.9794 precCharge: 2 Sequence: ELVQNLLDGK
Intensity
M/Z
0e+00
1e+05
2e+05
3e+05
4e+05
5e+05
400
800
1200
1600
y2
y1
b1
b2
b3
y3
y4
b4
y5
b5
y6
b6
y7
b7
y8
b8
b9
y9
type
b
y

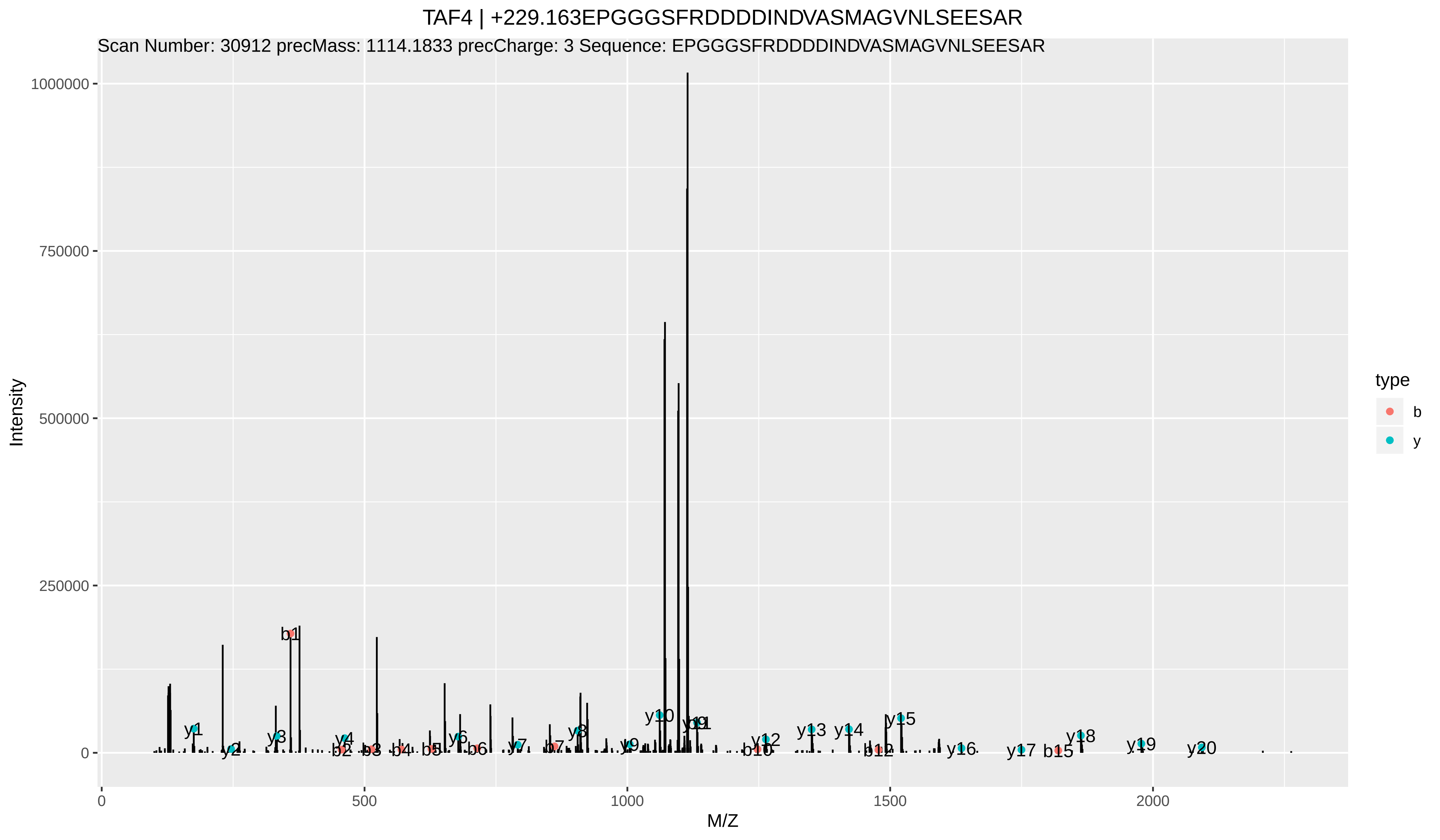
TAF4 | +229.163EPGGGSFRDDDDINDVASMAGVNLSEESAR
Scan Number: 30912 precMass: 1114.1833 precCharge: 3 Sequence: EPGGGSFRDDDDINDVASMAGVNLSEESAR
Intensity
M/Z
type
b
y
1000000
750000
500000
250000
0
0
500
1000
1500
2000

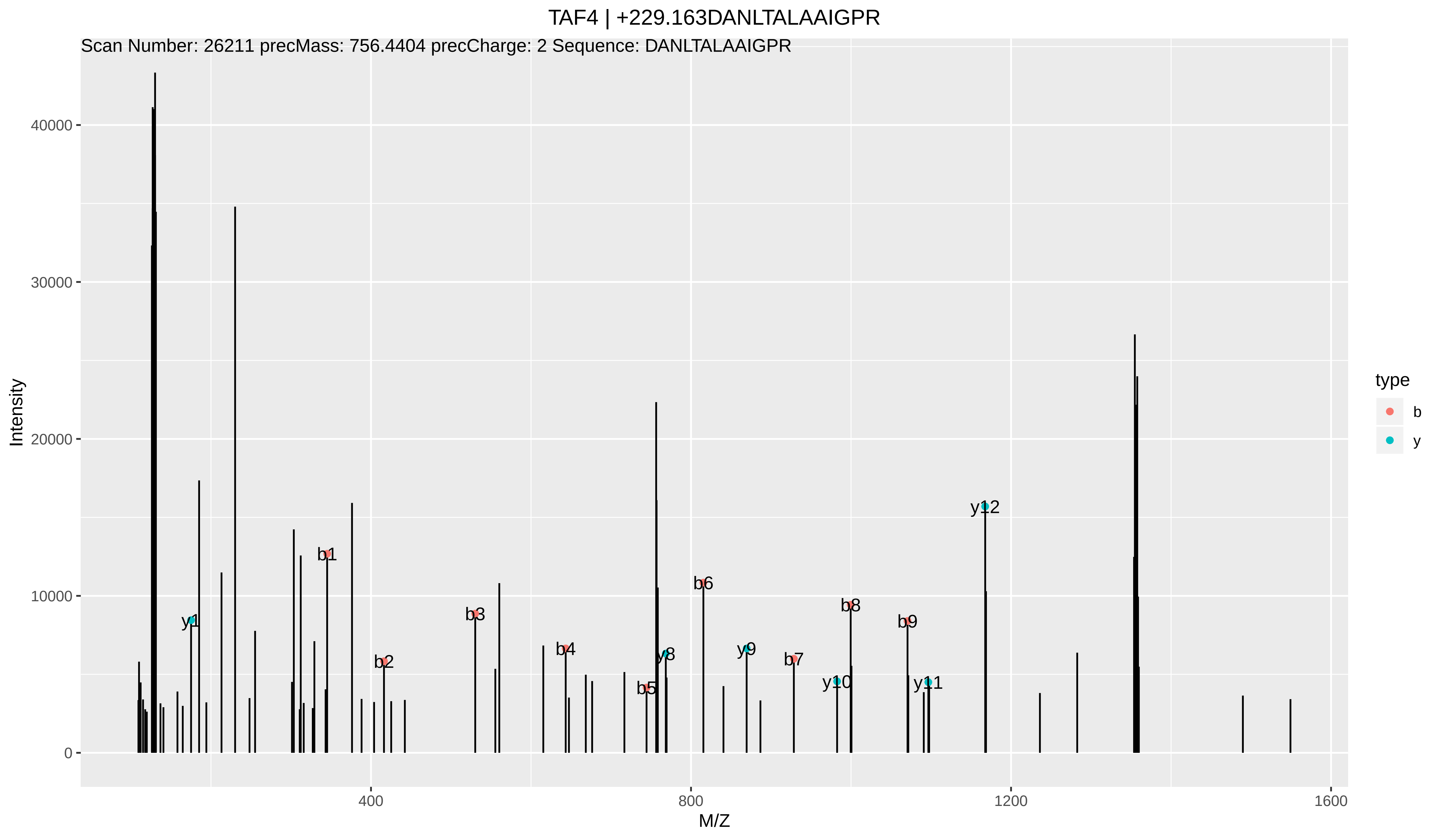
TAF4 | +229.163DANLTALAAIGPR
Scan Number: 26211 precMass: 756.4404 precCharge: 2 Sequence: DANLTALAAIGPR
Intensity
0
10000
20000
30000
40000
M/Z
400
800
1200
1600
type
b
y
y1
b1
b2
b3
b4
b5
y8
b6
y9
b7
y10
b8
b9
y11
y12

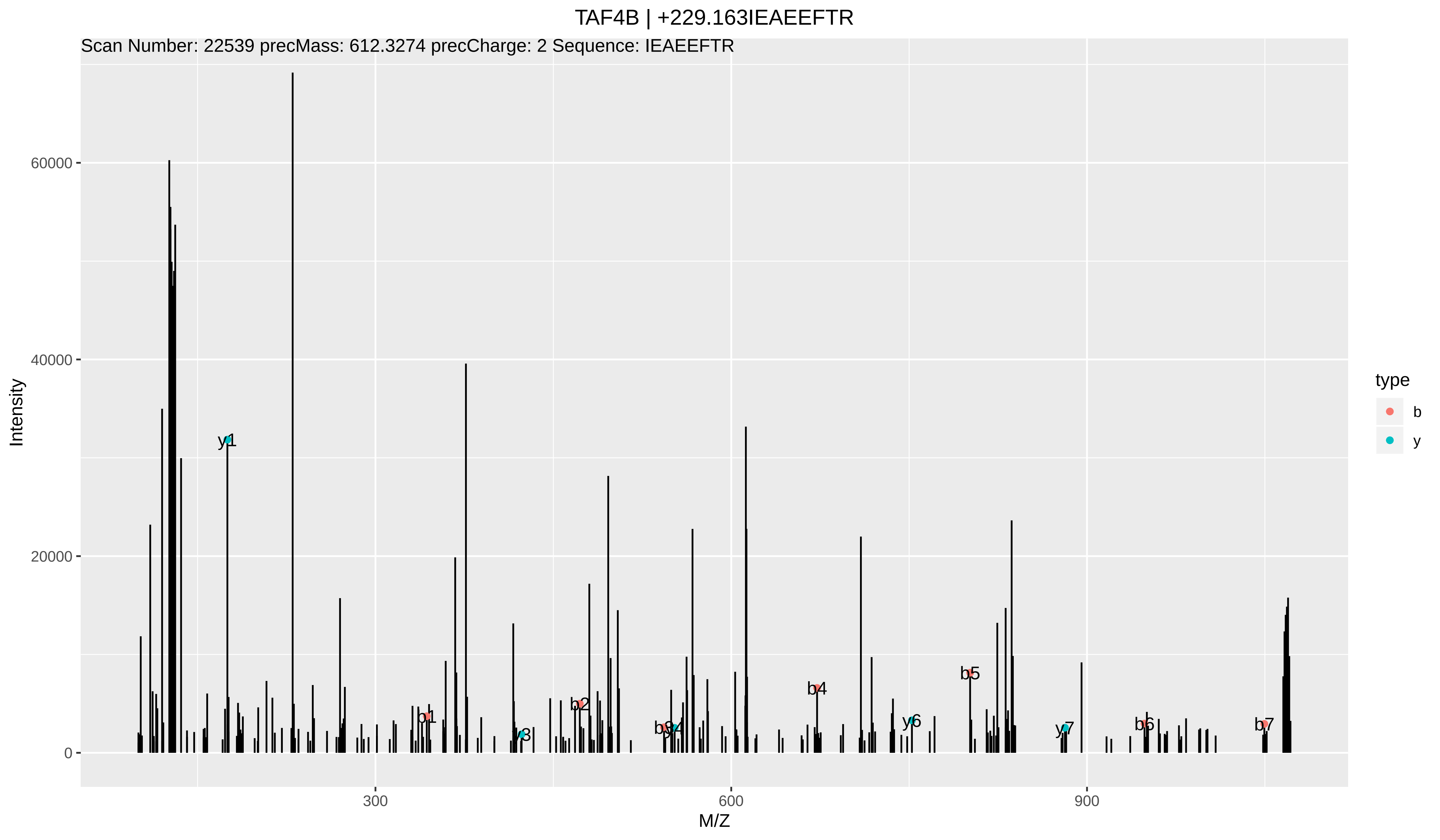

TAF4B | +229.163IEAEEFTR
Scan Number: 22539 precMass: 612.3274 precCharge: 2 Sequence: IEAEEFTR
Intensity
M/Z
type
b
y
y1
b1
y3
b2
b3
y4
b4
y6
b5
y7
b6
b7
0
20000
40000
60000
300
600
900

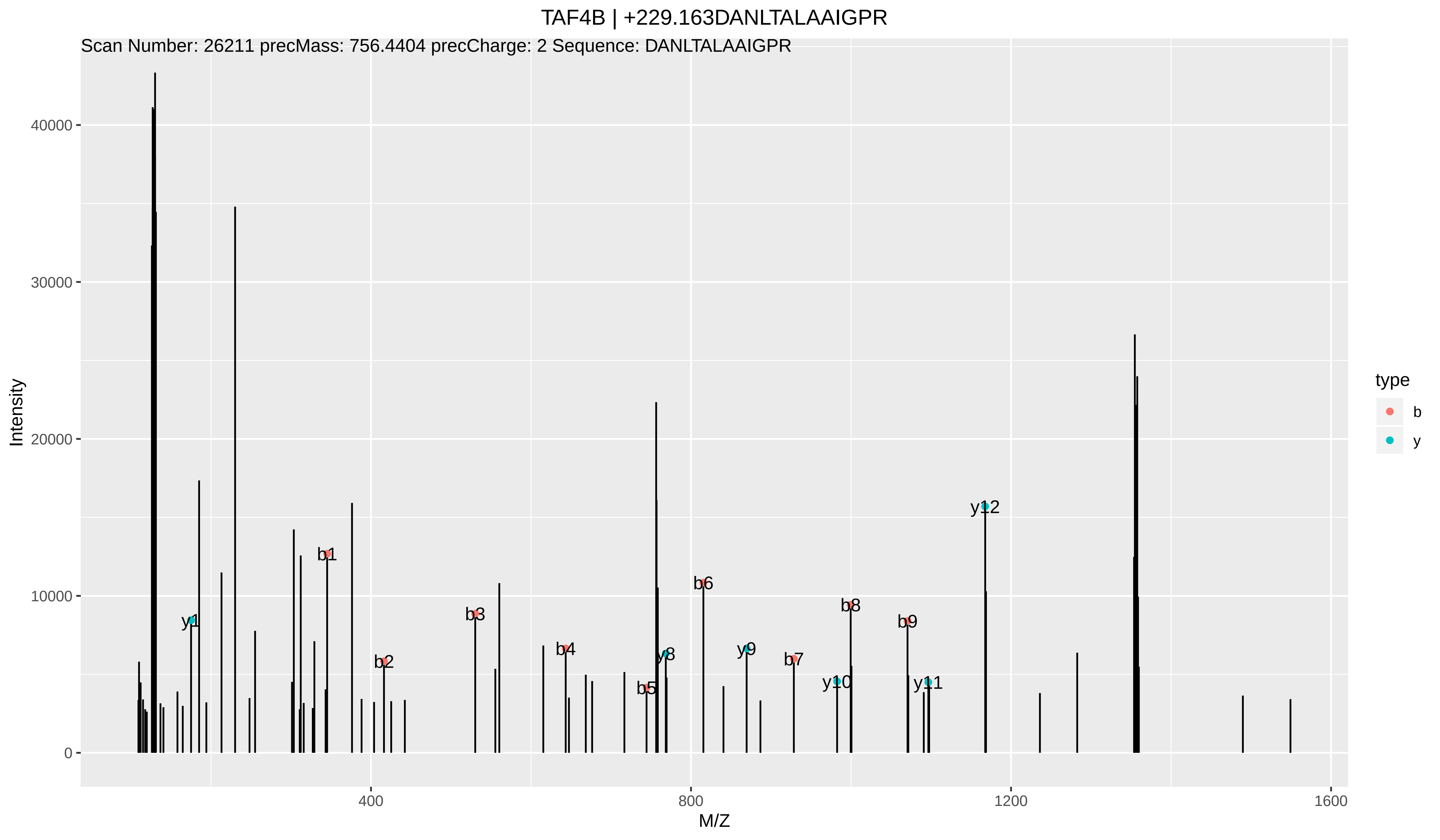
TAF4B | +229.163DANLTALAAIGPR
Scan Number: 26211 precMass: 756.4404 precCharge: 2 Sequence: DANLTALAAIGPR
Intensity
0
10000
20000
30000
40000
M/Z
400
800
1200
1600
type
b
y
y1
b1
b2
b3
b4
b5
y8
b6
y9
b7
y10
b8
b9
y11
y12

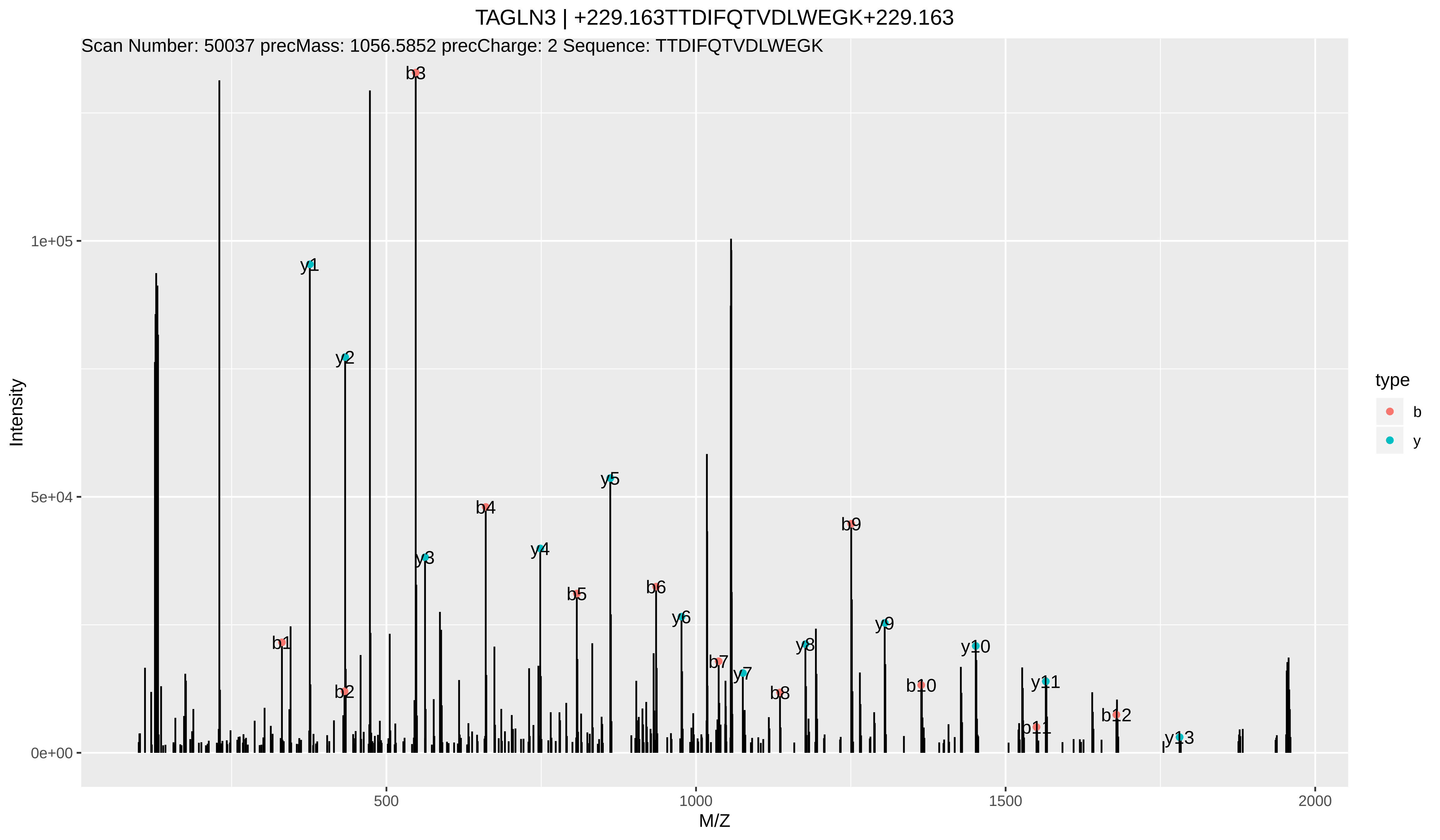
TAGLN3 | +229.163TTDIFQTVDLWEGK+229.163
Scan Number: 50037 precMass: 1056.5852 precCharge: 2 Sequence: TTDIFQTVDLWEGK
Intensity
M/Z
0e+00
5e+04
1e+05
500
1000
1500
2000
b3
y1
y2
y3
b4
y4
b5
y5
b6
y6
b1
b2
b7
y7
b8
y8
b9
y9
b10
y10
b11
y11
b12
y13
type
b
y

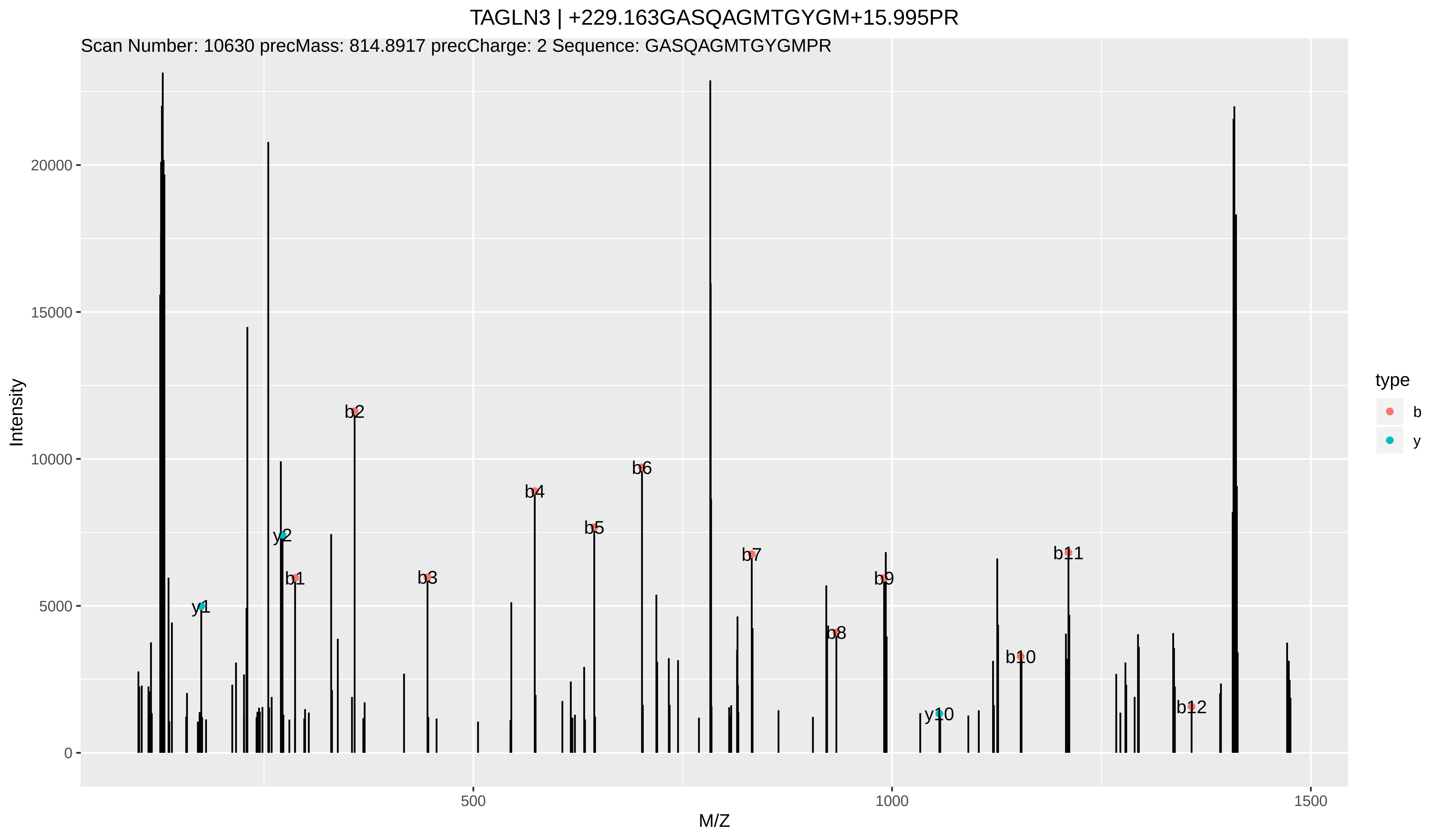
TAGLN3 | +229.163GASQAGMTGYGM+15.995PR
Scan Number: 10630 precMass: 814.8917 precCharge: 2 Sequence: GASQAGMTGYGMPR
Intensity
M/Z
0
5000
10000
15000
20000
500
1000
1500
y1
y2
b1
b2
b3
b4
b5
b6
b7
b8
b9
y10
b10
b11
b12
type
b
y

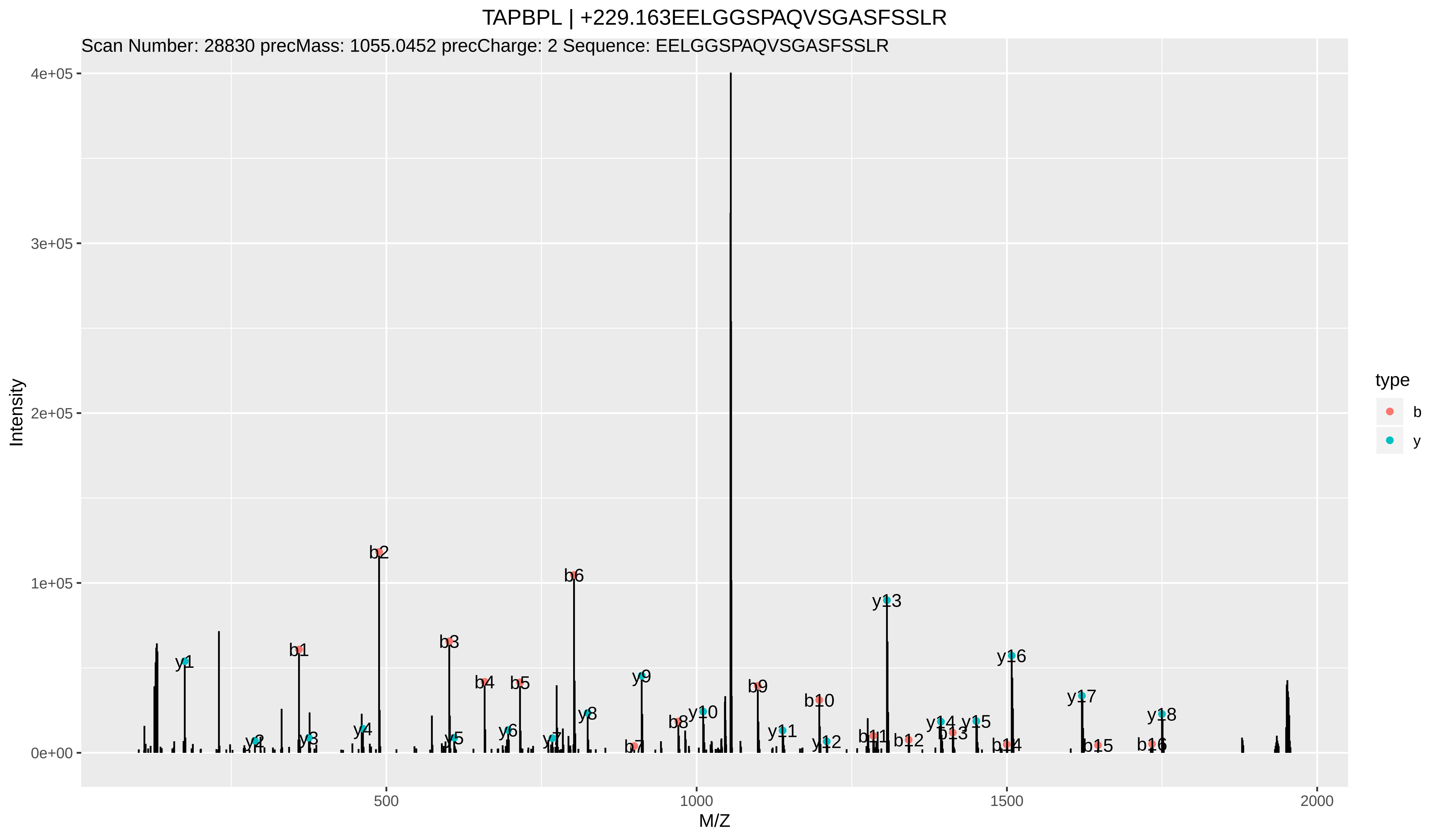

TAPBPL | +229.163EELGGSPAQVSGASFSSLR
Scan Number: 28830 precMass: 1055.0452 precCharge: 2 Sequence: EELGGSPAQVSGASFSSLR
Intensity
M/Z
type
b
y

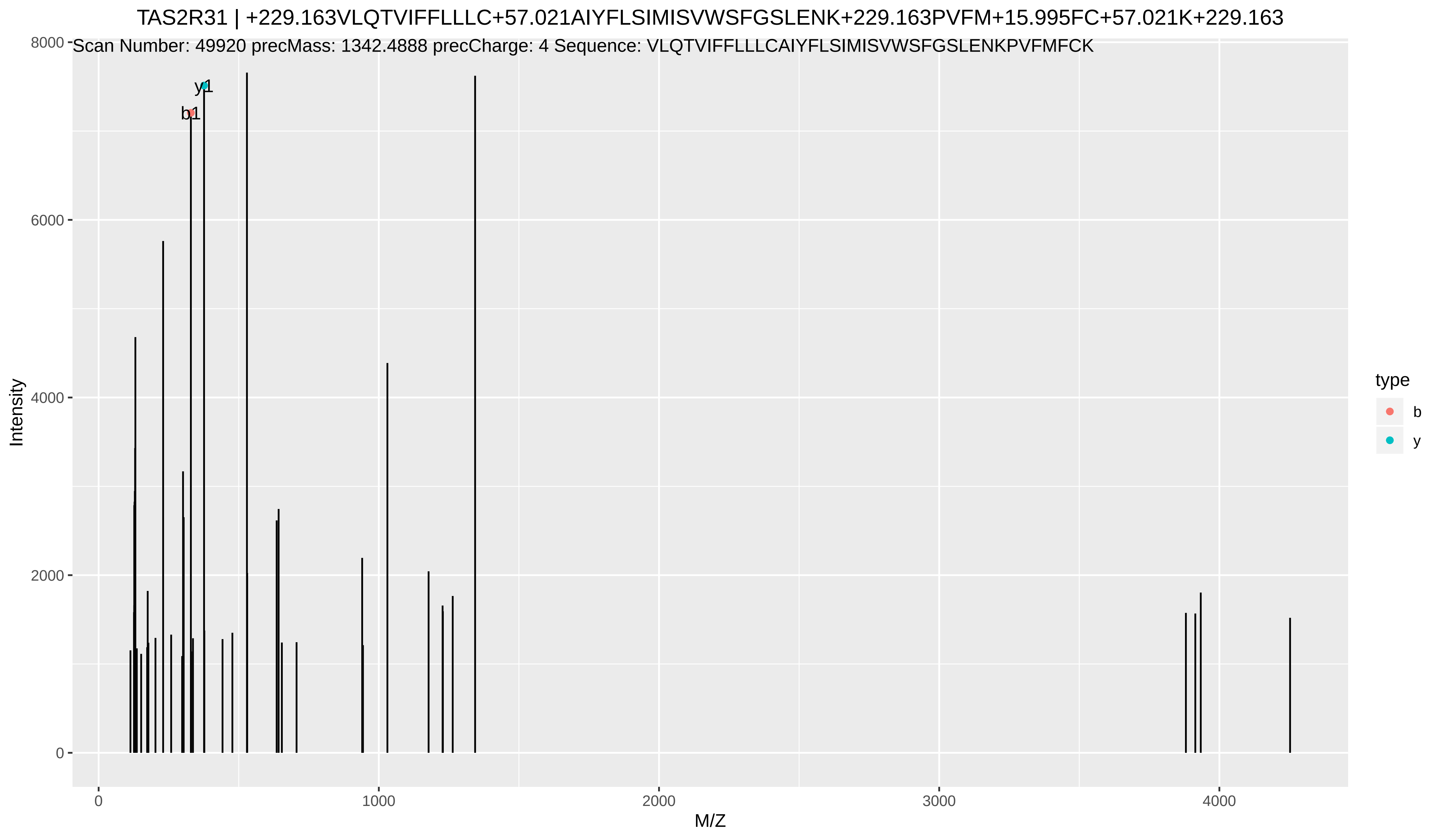

TAS2R31 | +229.163VLQTVIFFLLLC+57.021AIYFLSIMISVWSFGSLENK+229.163PVFM+15.995FC+57.021K+229.163
Scan Number: 49920 precMass: 1342.4888 precCharge: 4 Sequence: VLQTVIFFLLLCAIYFLSIMISVWSFGSLENKPVFMFCK
y1
b1
Intensity
M/Z
0
2000
4000
6000
8000
0
1000
2000
3000
4000
type
b
y

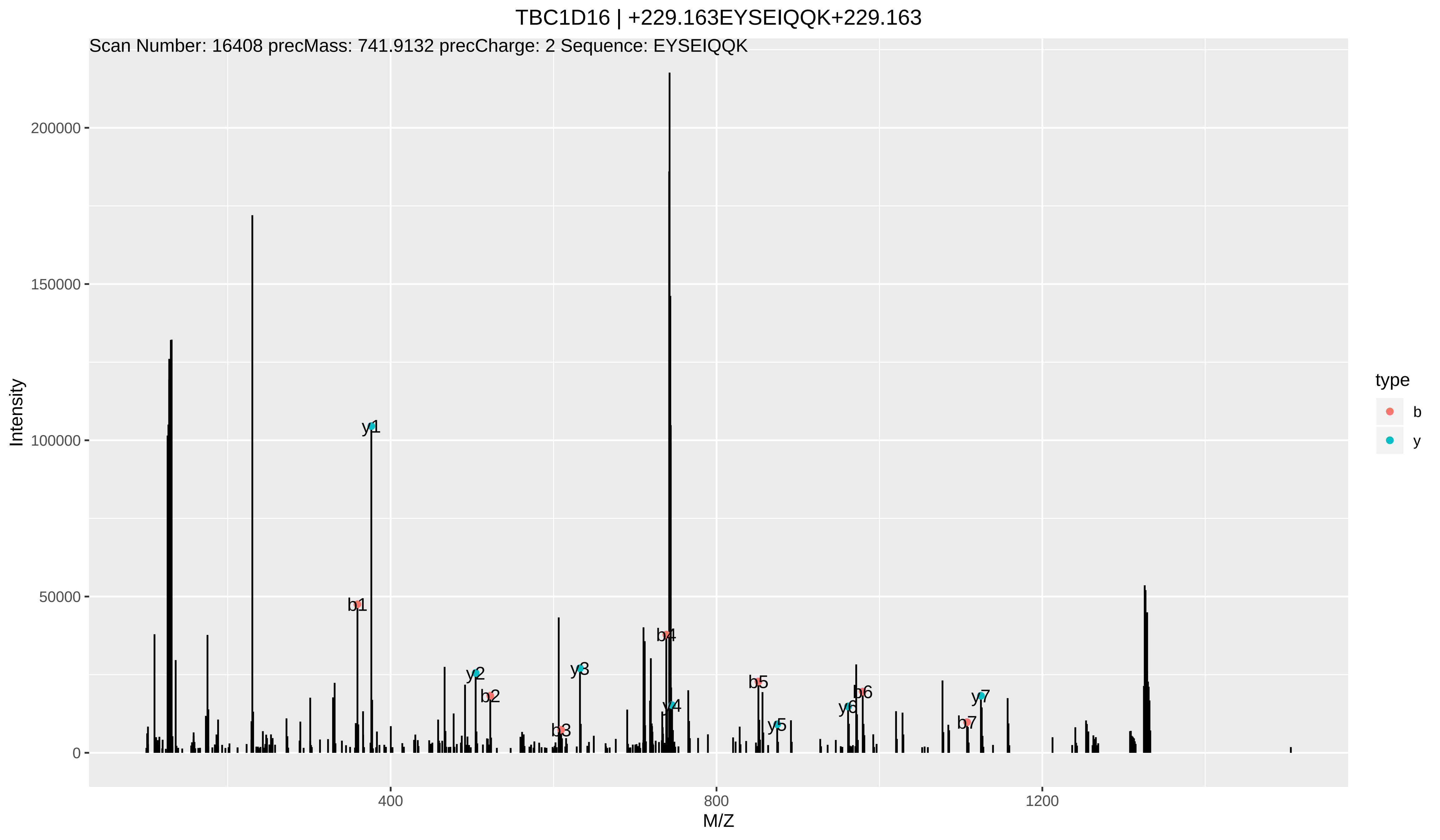

TBC1D16 | +229.163EYSEIQQK+229.163
Scan Number: 16408 precMass: 741.9132 precCharge: 2 Sequence: EYSEIQQK
Intensity
200000
150000
100000
50000
0
M/Z
400
800
1200
y1
b1
y2
b2
b3
y3
b4
y4
b5
y5
y6
b6
b7
y7
type
b
y

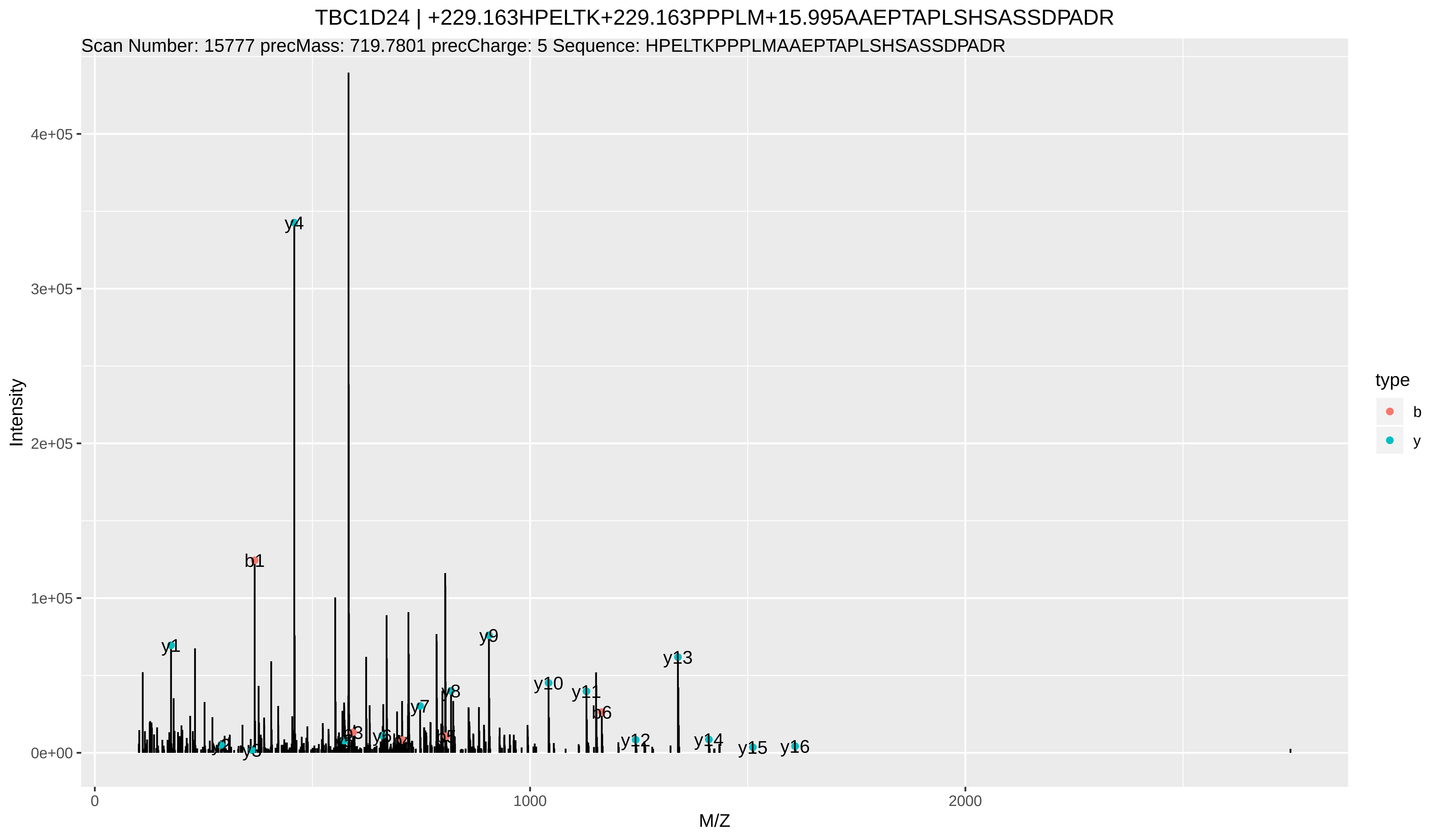

TBC1D24 | +229.163HPELTK+229.163PPPLM+15.995AAEPTAPLSHSASSDPADR
Scan Number: 15777 precMass: 719.7801 precCharge: 5 Sequence: HPELTKPPPLMAAEPTAPLSHSASSDPADR
Intensity
M/Z
type
b
y
y1
y2
y3
y4
b1
b3
y6
y7
y8
y9
y10
y11
b6
y12
y13
y14
y15
y16
0e+00
1e+05
2e+05
3e+05
4e+05
0
1000
2000

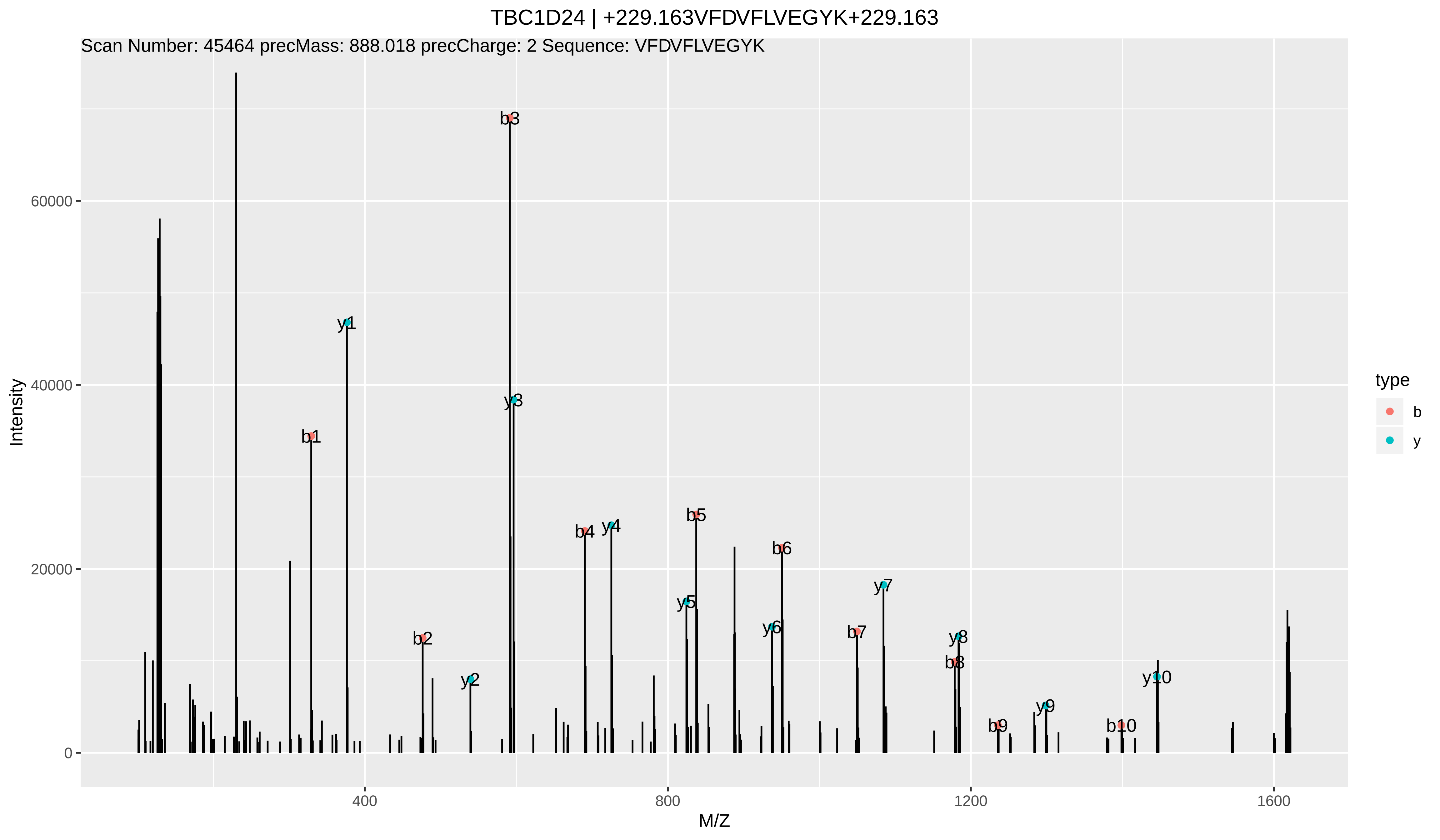

TBC1D24 | +229.163VFDVFLVEGYK+229.163
Scan Number: 45464 precMass: 888.018 precCharge: 2 Sequence: VFDVFLVEGYK
Intensity
M/Z
type
b
y
b1
b2
b3
b4
b5
b6
b7
b8
b9
b10
y1
y2
y3
y4
y5
y6
y7
y8
y9
y10
0
20000
40000
60000
400
800
1200
1600

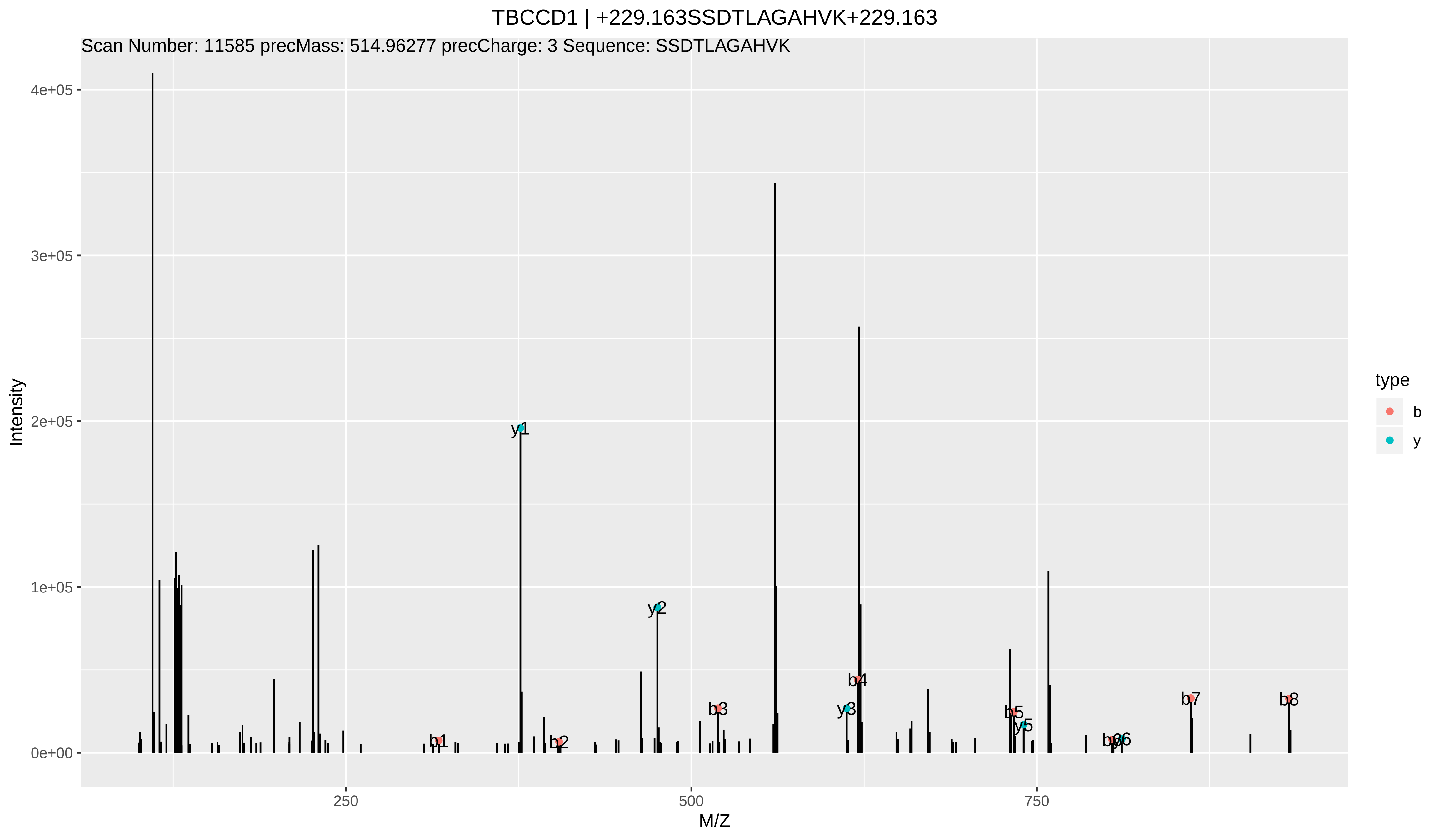

TBCCD1 | +229.163SSDTLAGAHVK+229.163
Scan Number: 11585 precMass: 514.96277 precCharge: 3 Sequence: SSDTLAGAHVK
Intensity
M/Z
type
b
y

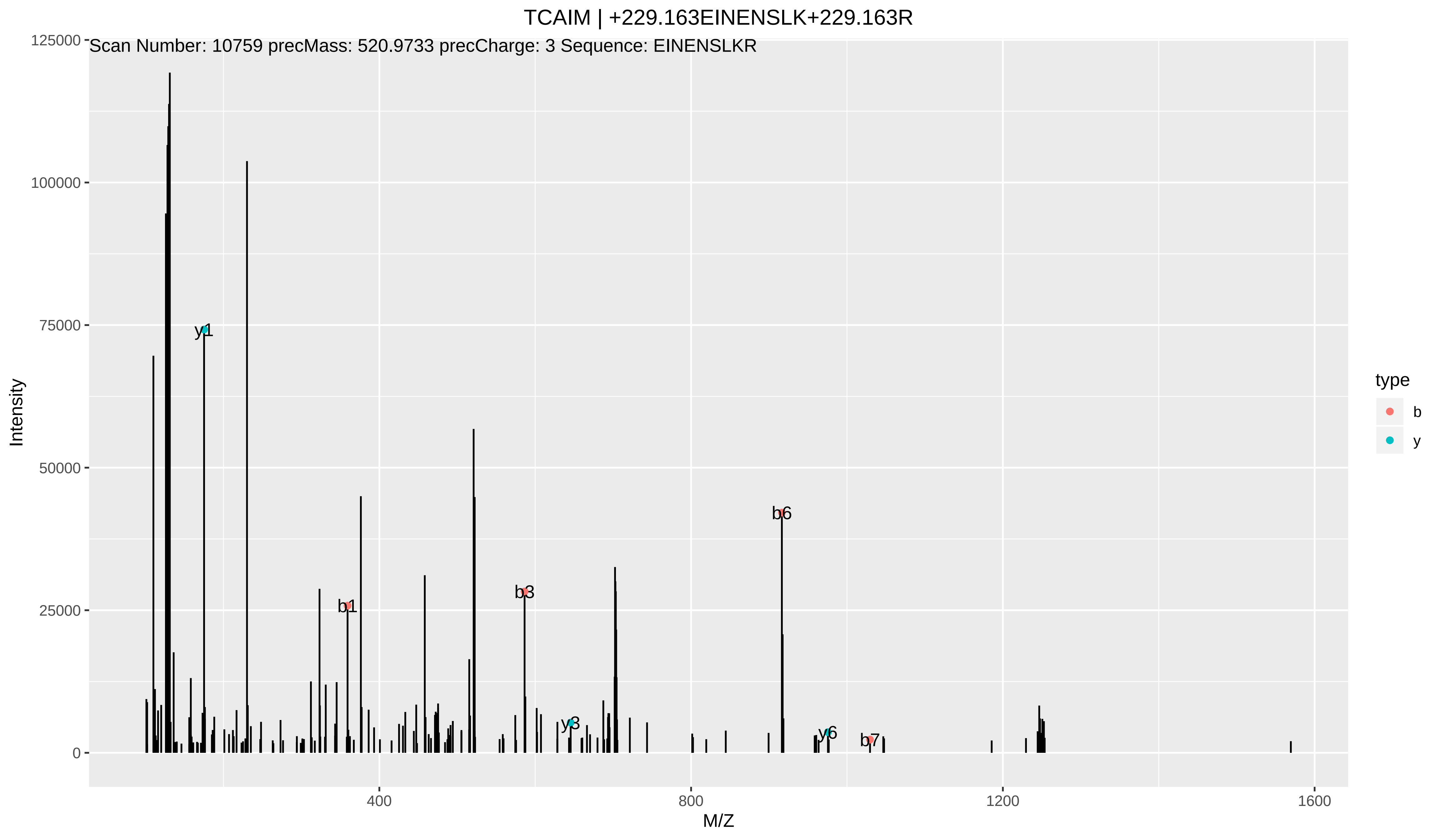
TCAIM | +229.163EINENSLK+229.163R
Scan Number: 10759 precMass: 520.9733 precCharge: 3 Sequence: EINENSLKR
Intensity
M/Z
125000
100000
75000
50000
25000
0
400
800
1200
1600
y1
b1
b3
y3
b6
y6
b7
type
b
y

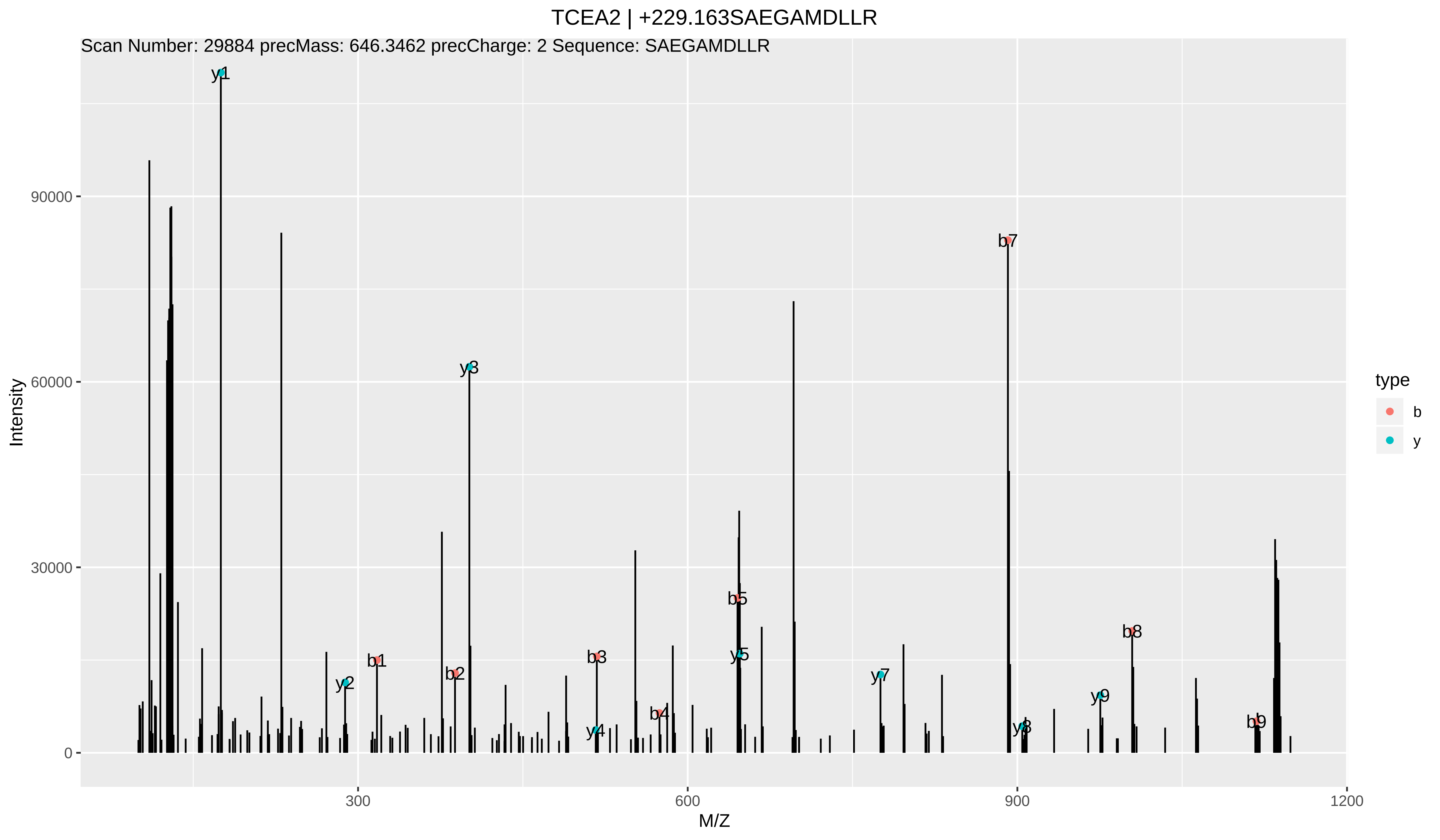
TCEA2 | +229.163SAEGAMDLLR
Scan Number: 29884 precMass: 646.3462 precCharge: 2 Sequence: SAEGAMDLLR
Intensity
M/Z
type
b
y
y1
y2
y3
y4
y5
y7
y8
y9
b1
b2
b3
b4
b5
b7
b8
b9
0
30000
60000
90000
300
600
900
1200

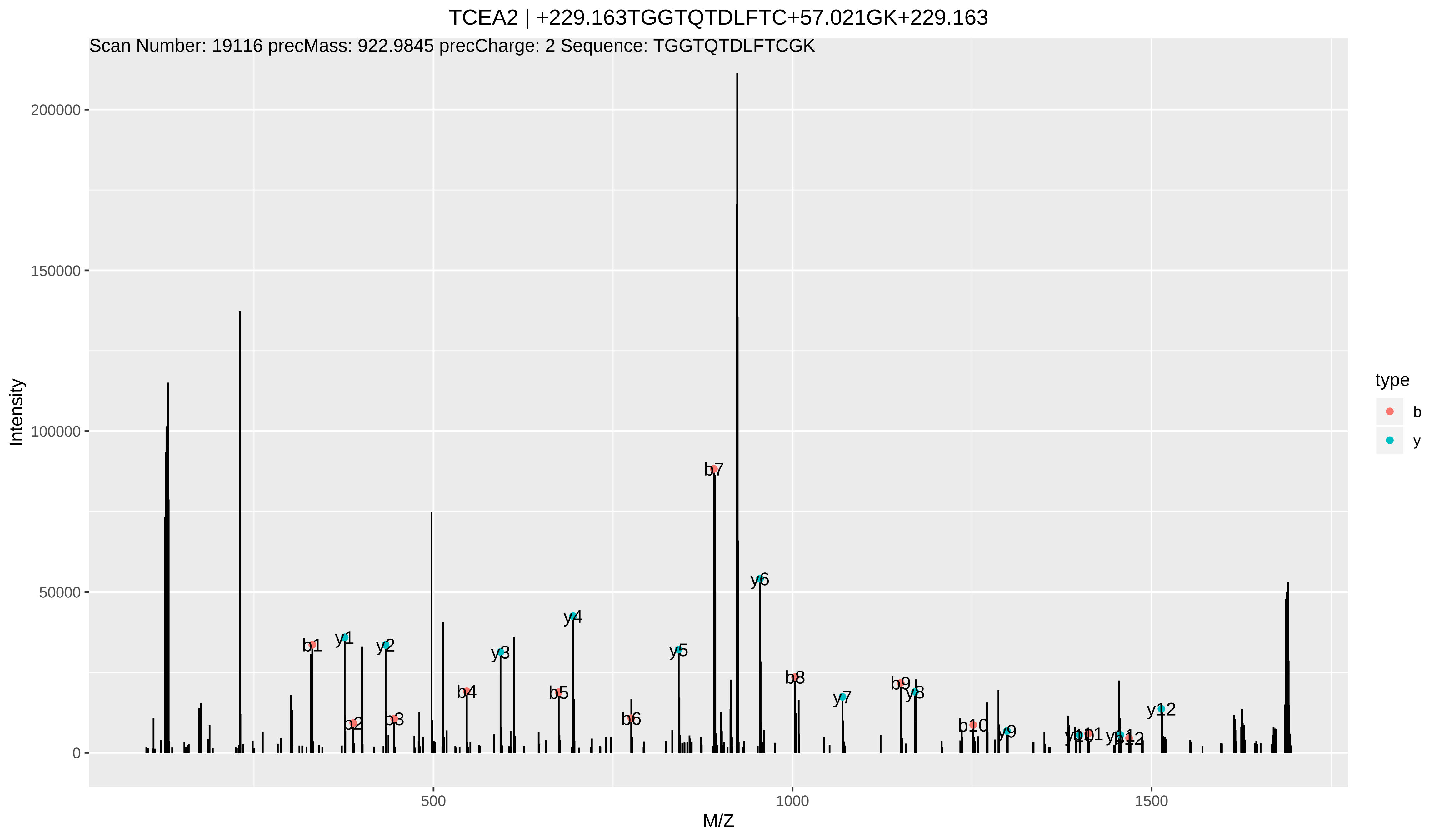
TCEA2 | +229.163TGGTQTDLFTC+57.021GK+229.163
Scan Number: 19116 precMass: 922.9845 precCharge: 2 Sequence: TGGTQTDLFTCGK
Intensity
M/Z
type
b
y
0
50000
100000
150000
200000
500
1000
1500
b1
y1
y2
b2
b3
y3
b4
b5
y4
b6
y5
b7
y6
b8
y7
b9
y8
b10
y9

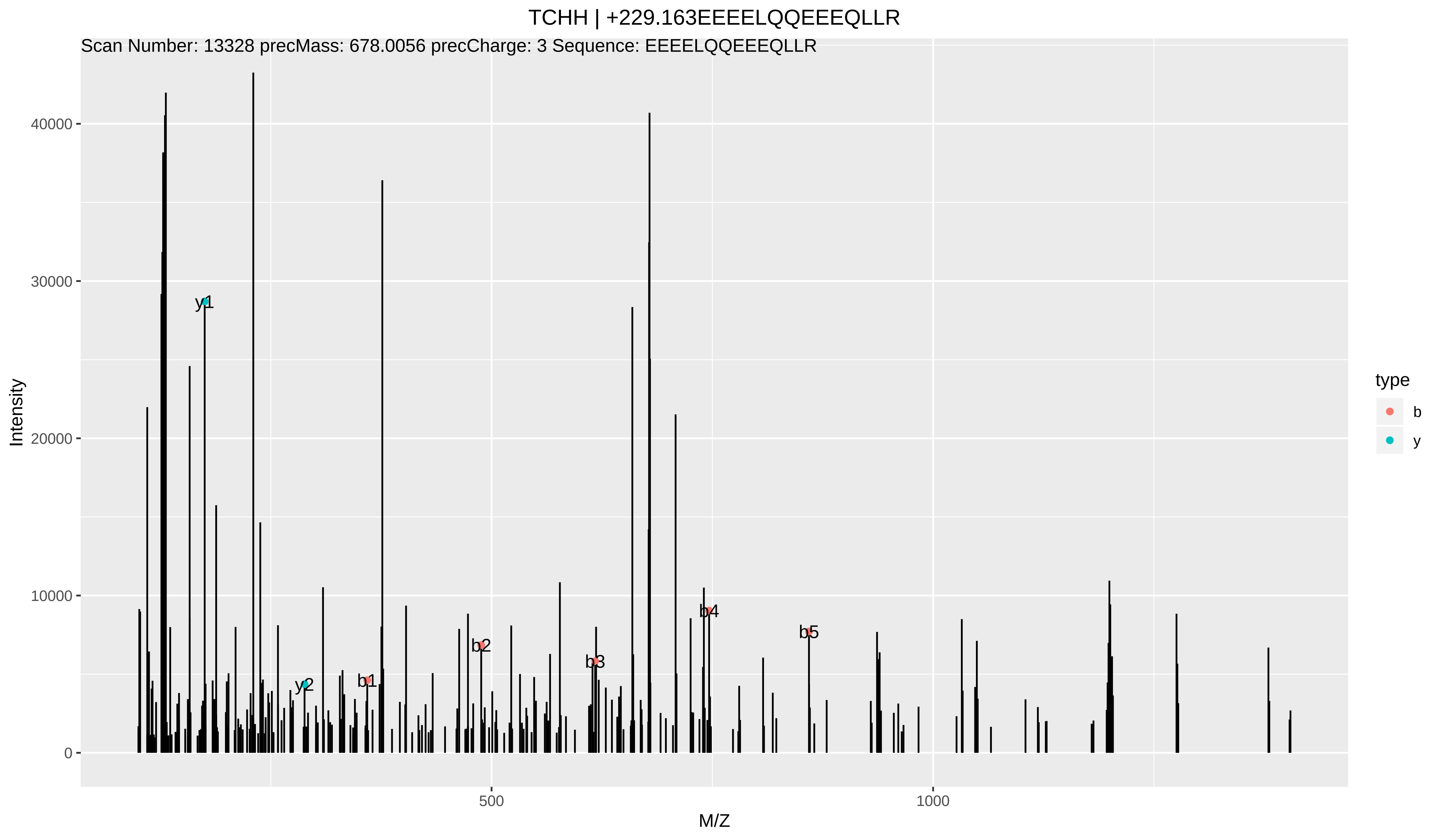
TCHH | +229.163EEEELQQEEEQLLR
Scan Number: 13328 precMass: 678.0056 precCharge: 3 Sequence: EEEELQQEEEQLLR
Intensity
M/Z
y1
y2
b1
b2
b3
b4
b5
type
b
y
0
10000
20000
30000
40000
500
1000

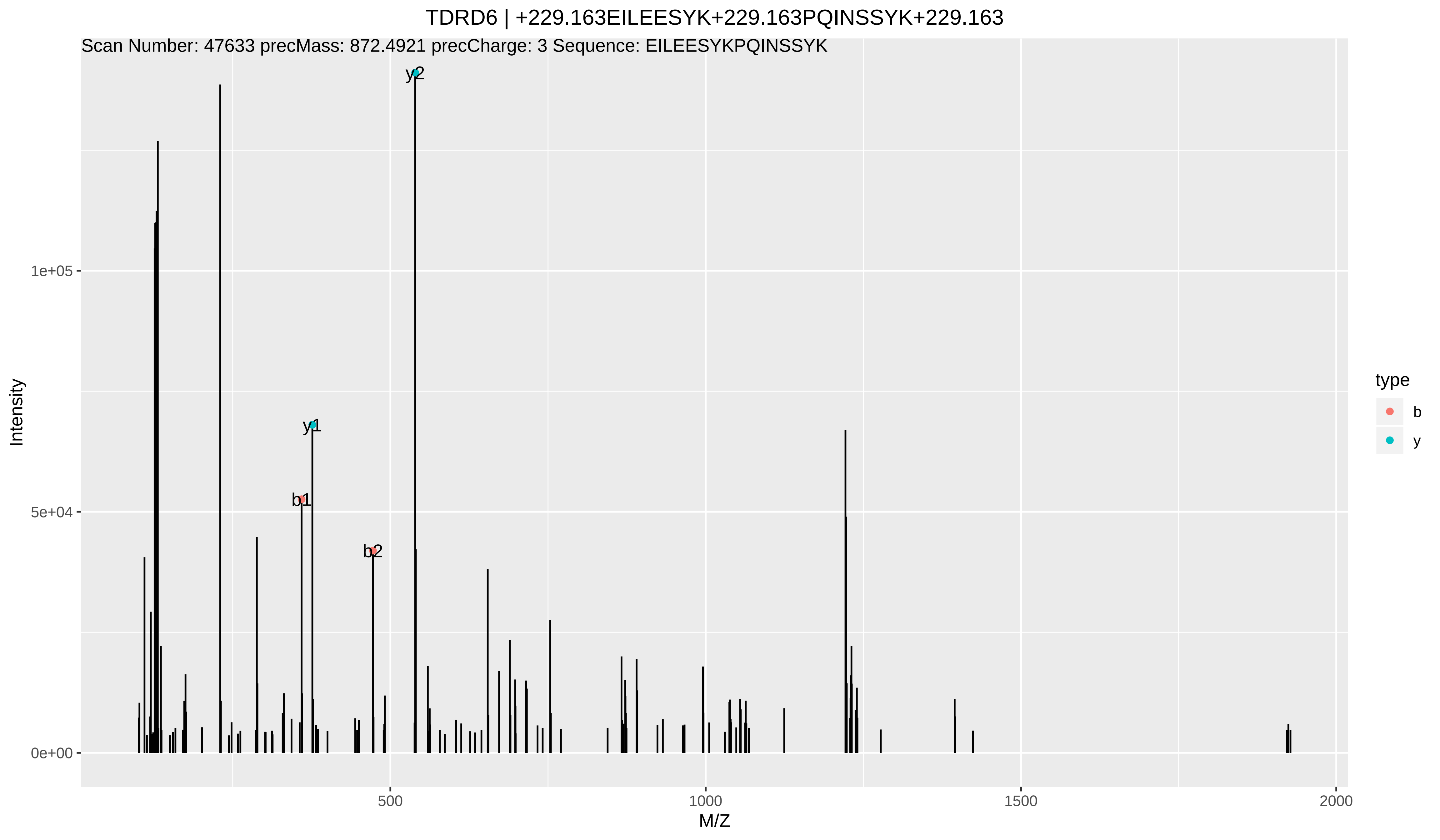

TDRD6 | +229.163EILEESYK+229.163PQINSSYK+229.163
Scan Number: 47633 precMass: 872.4921 precCharge: 3 Sequence: EILEESYKPQINSSYK
y2
y1
b1
b2
1e+05
5e+04
0e+00
Intensity
500
1000
1500
2000
M/Z
type
b
y

TECPR1 | +229.163TGVQPHEPK+229.163

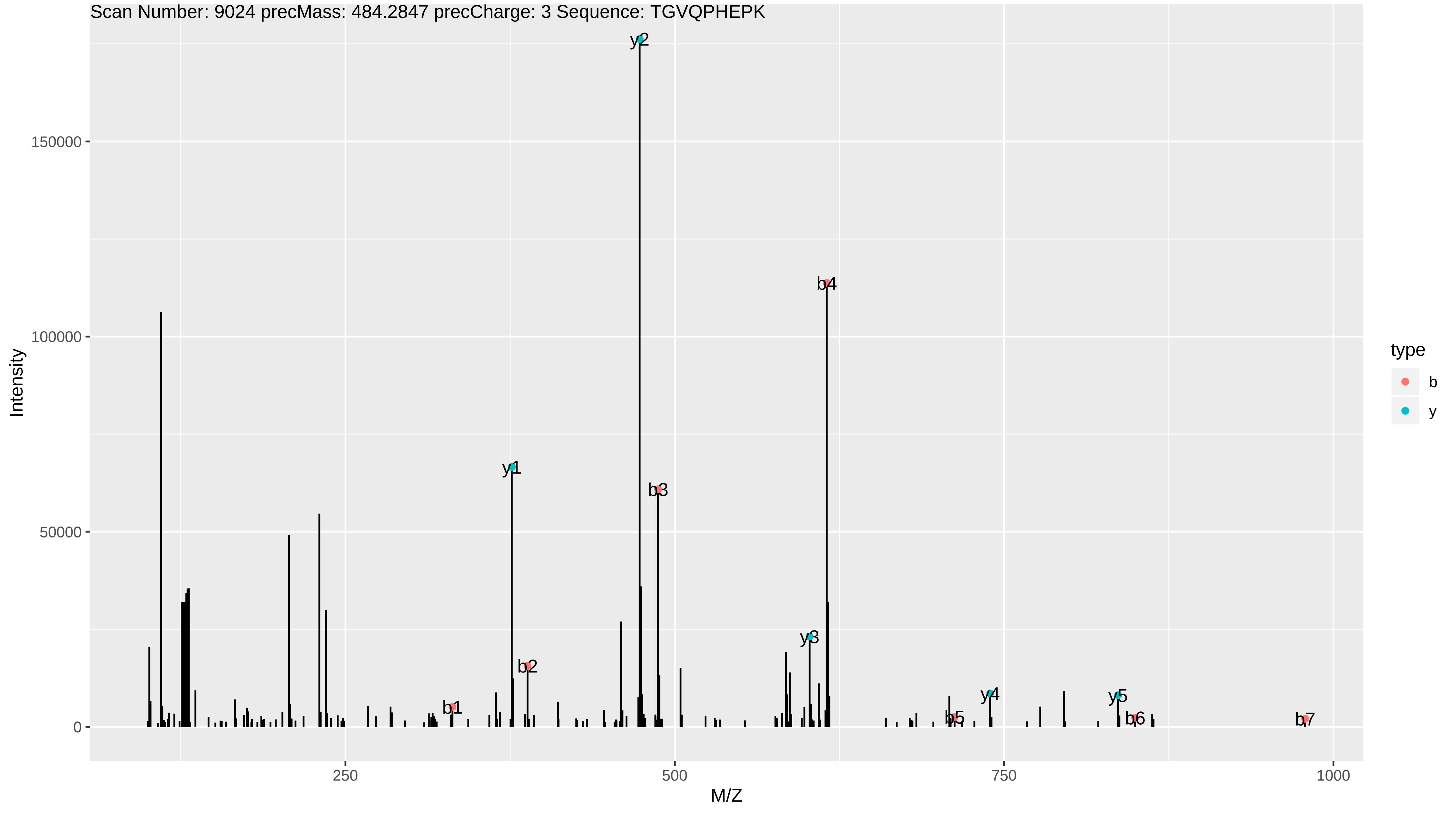

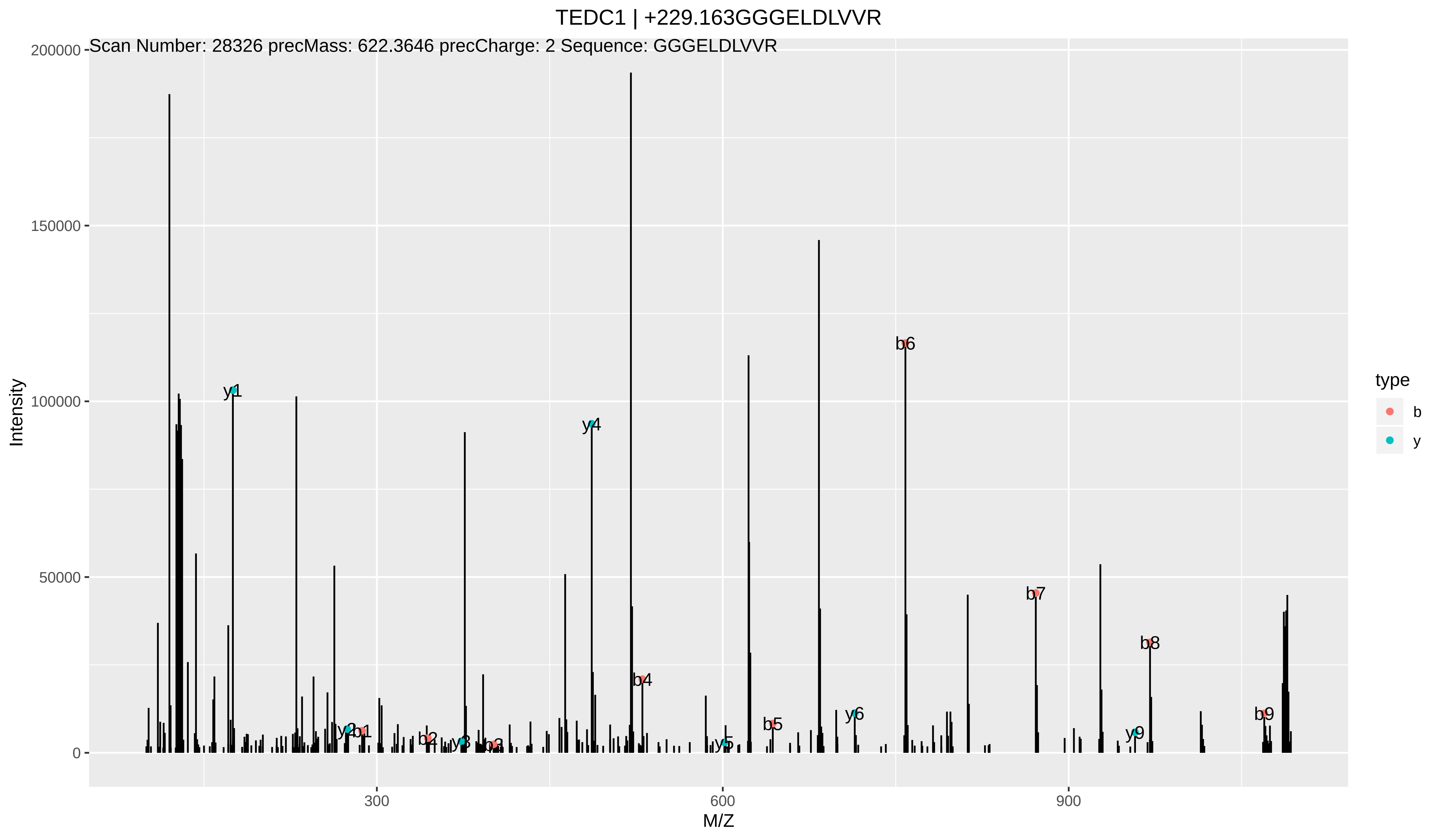

TEDC1 | +229.163GGGELDLVVR
Scan Number: 28326 precMass: 622.3646 precCharge: 2 Sequence: GGGELDLVVR
Intensity
200000
150000
100000
50000
0
M/Z
300
600
900
y1
y2
b1
b2
y3
b3
y4
b4
y5
b5
y6
b6
b7
y9
b8
b9
type
b
y

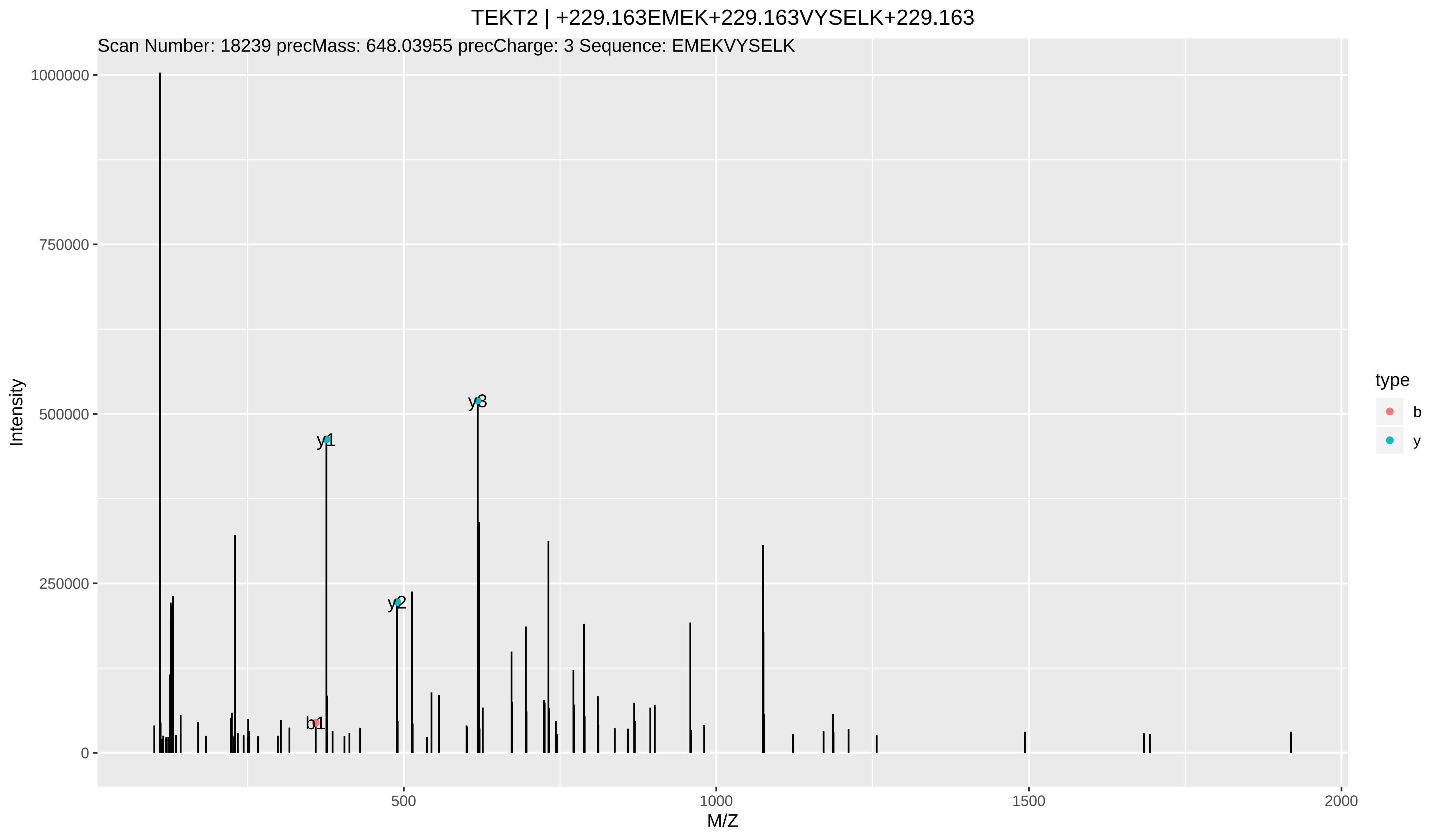

TEKT2 | +229.163EMEK+229.163VYSELK+229.163
Scan Number: 18239 precMass: 648.03955 precCharge: 3 Sequence: EMEKVYSELK
Intensity
1000000
750000
500000
250000
0
M/Z
500
1000
1500
2000
y1
y2
y3
b1
type
b
y

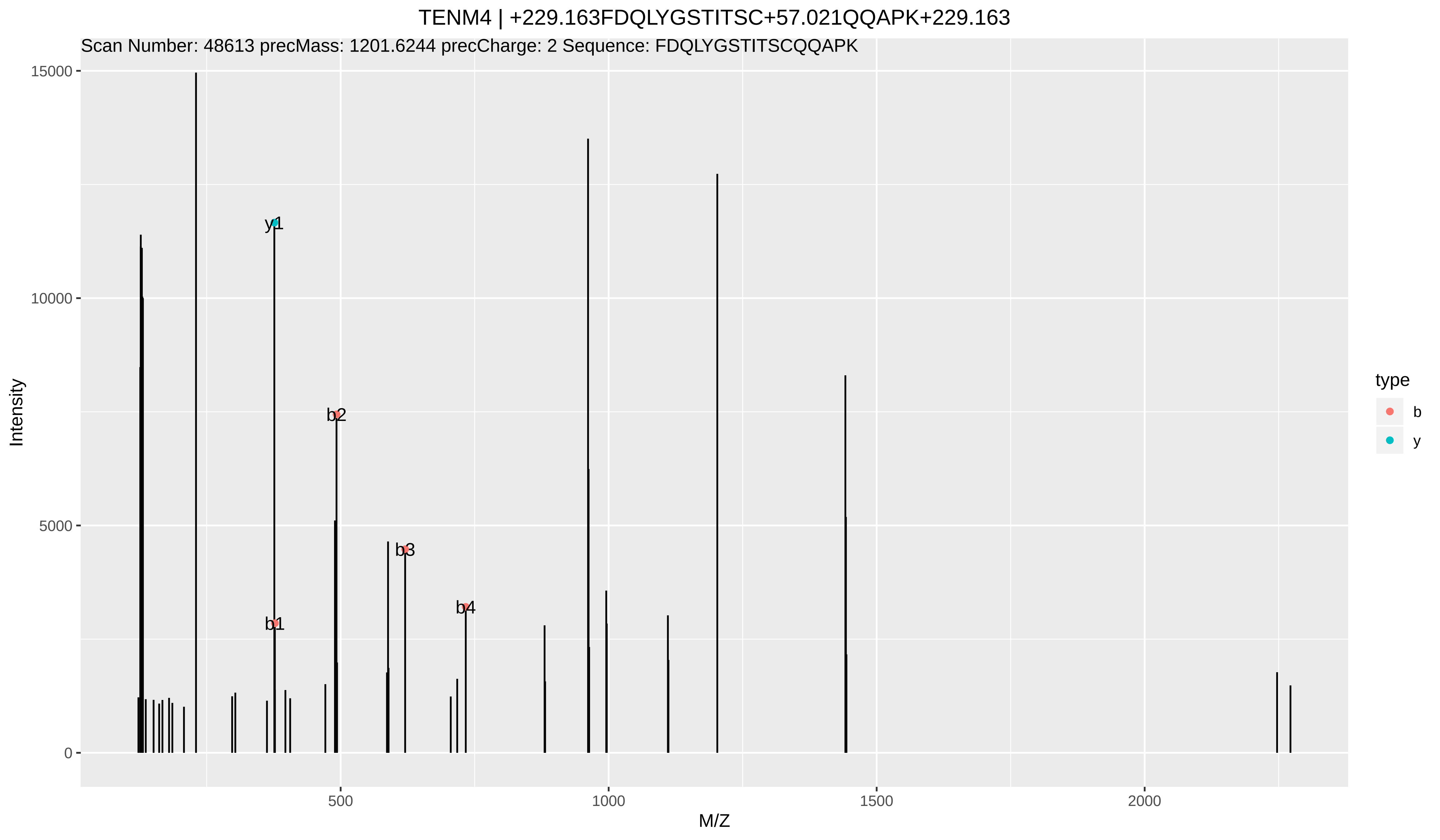

TENM4 | +229.163FDQLYGSTITSC+57.021QQAPK+229.163
Scan Number: 48613 precMass: 1201.6244 precCharge: 2 Sequence: FDQLYGSTITSCQQAPK
Intensity
15000
10000
5000
0
M/Z
500
1000
1500
2000
y1
b1
b2
b3
b4
type
b
y

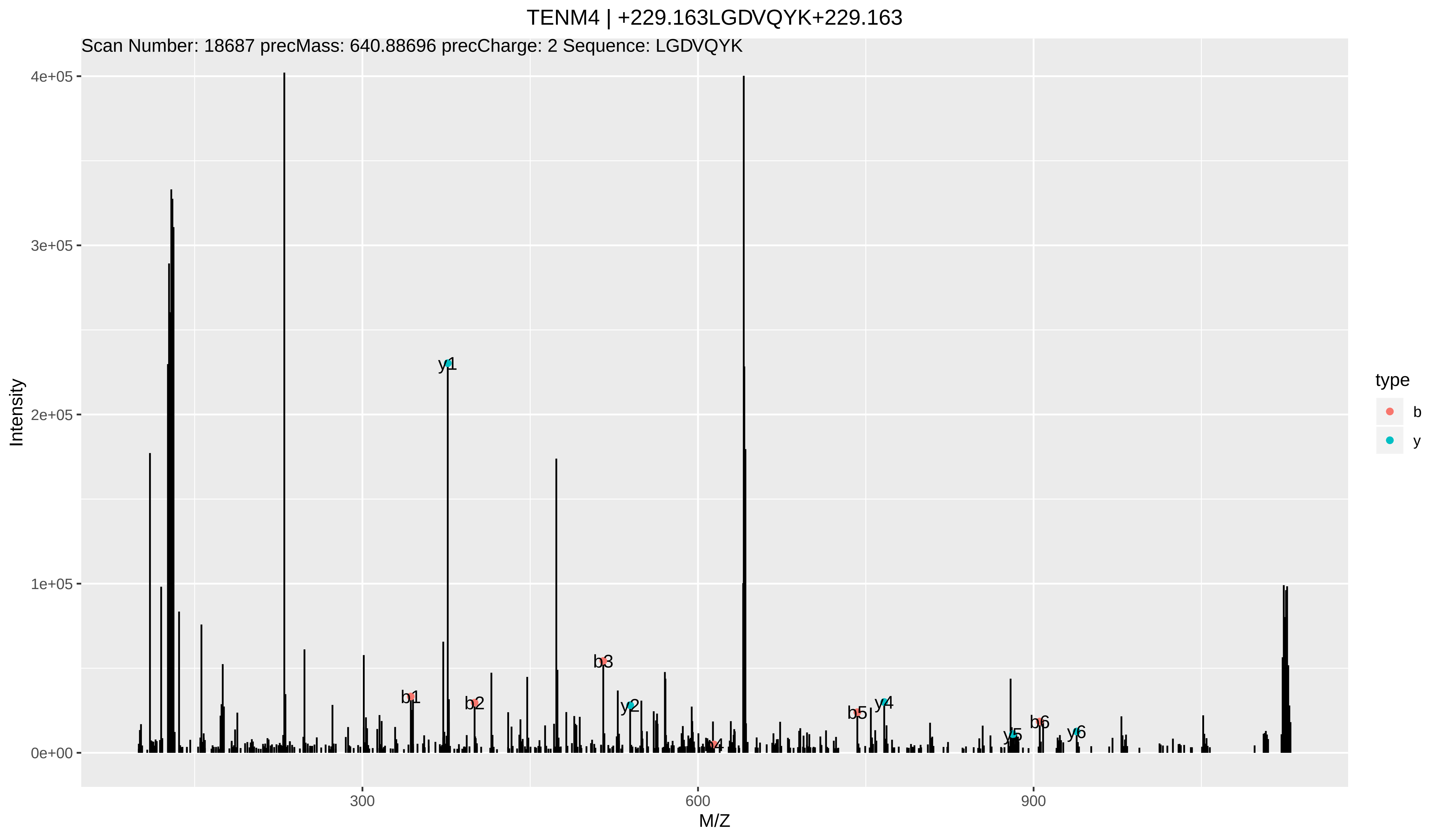
TENM4 | +229.163LGDVQYK+229.163
Scan Number: 18687 precMass: 640.88696 precCharge: 2 Sequence: LGDVQYK
Intensity
4e+05
3e+05
2e+05
1e+05
0e+00
M/Z
300
600
900
y1
b1
b2
b3
y2
b4
b5
y4
y5
b6
y6
type
b
y

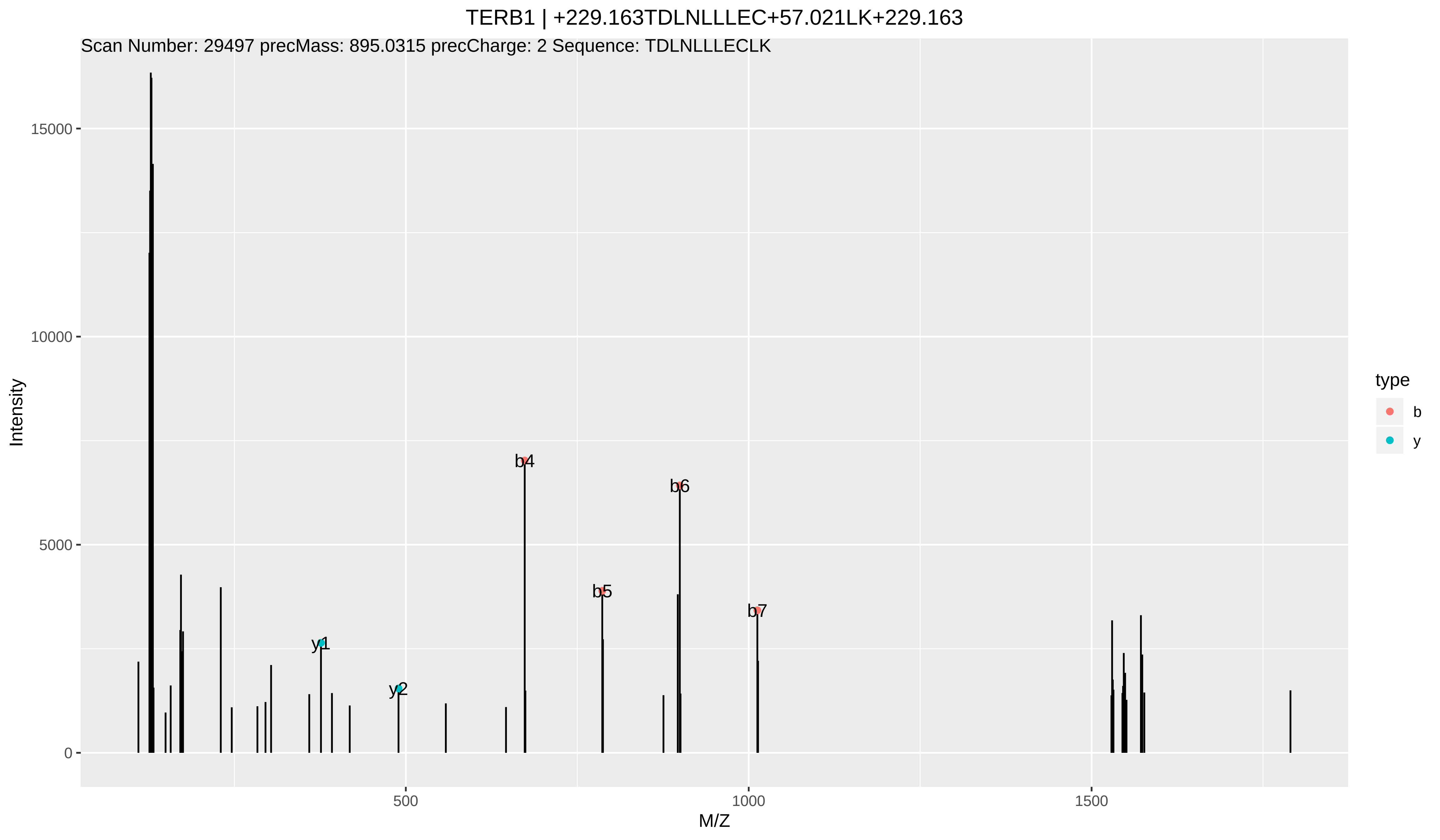
TERB1 | +229.163TDLNLLLEC+57.021LK+229.163
Scan Number: 29497 precMass: 895.0315 precCharge: 2 Sequence: TDLNLLLECLK
Intensity
M/Z
type
b
y
b4
b5
b6
b7
y1
y2
0
5000
10000
15000
500
1000
1500

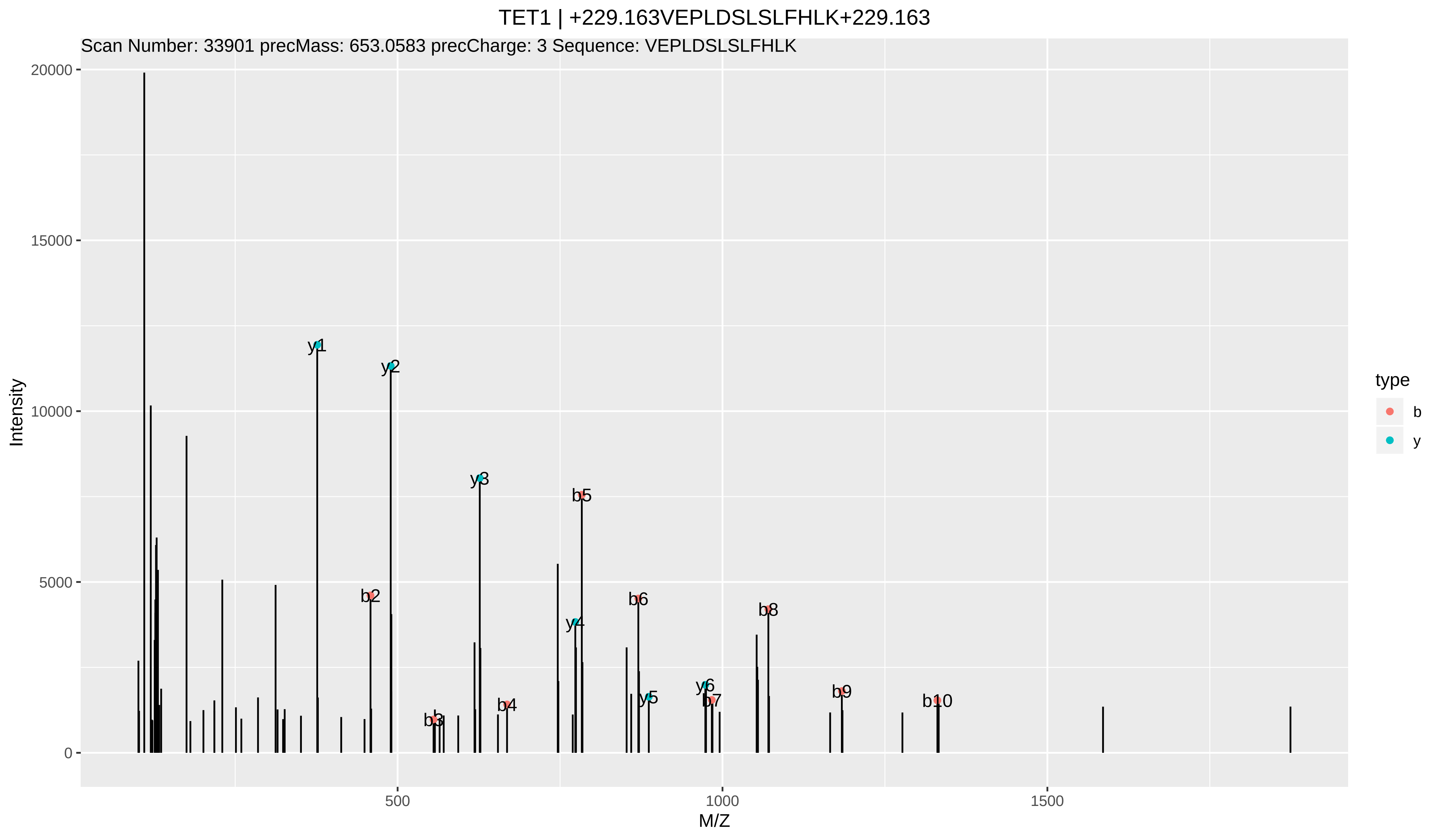
TET1 | +229.163VEPLDSLSLFHLK+229.163
Scan Number: 33901 precMass: 653.0583 precCharge: 3 Sequence: VEPLDSLSLFHLK
Intensity
20000
15000
10000
5000
0
M/Z
500
1000
1500
y1
y2
y3
y4
y5
y6
b2
b3
b4
b5
b6
b7
b8
b9
b10
type
b
y

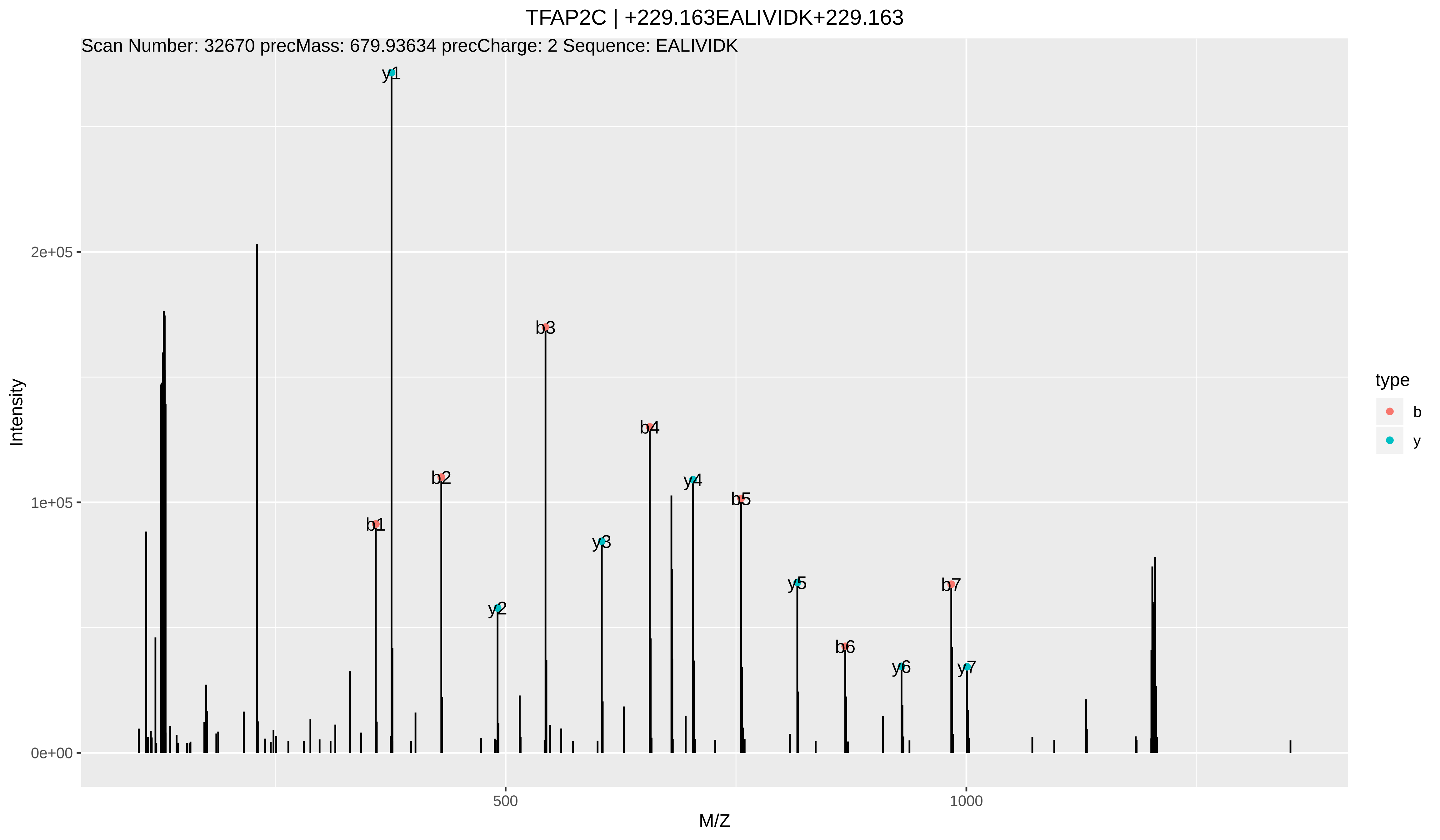

TFAP2C | +229.163EALIVIDK+229.163
Scan Number: 32670 precMass: 679.93634 precCharge: 2 Sequence: EALIVIDK
y1
b3
b4
b2
y4
b5
b1
y3
y5
b7
y2
b6
y6
y7
2e+05
1e+05
0e+00
Intensity
500
1000
M/Z
type
b
y

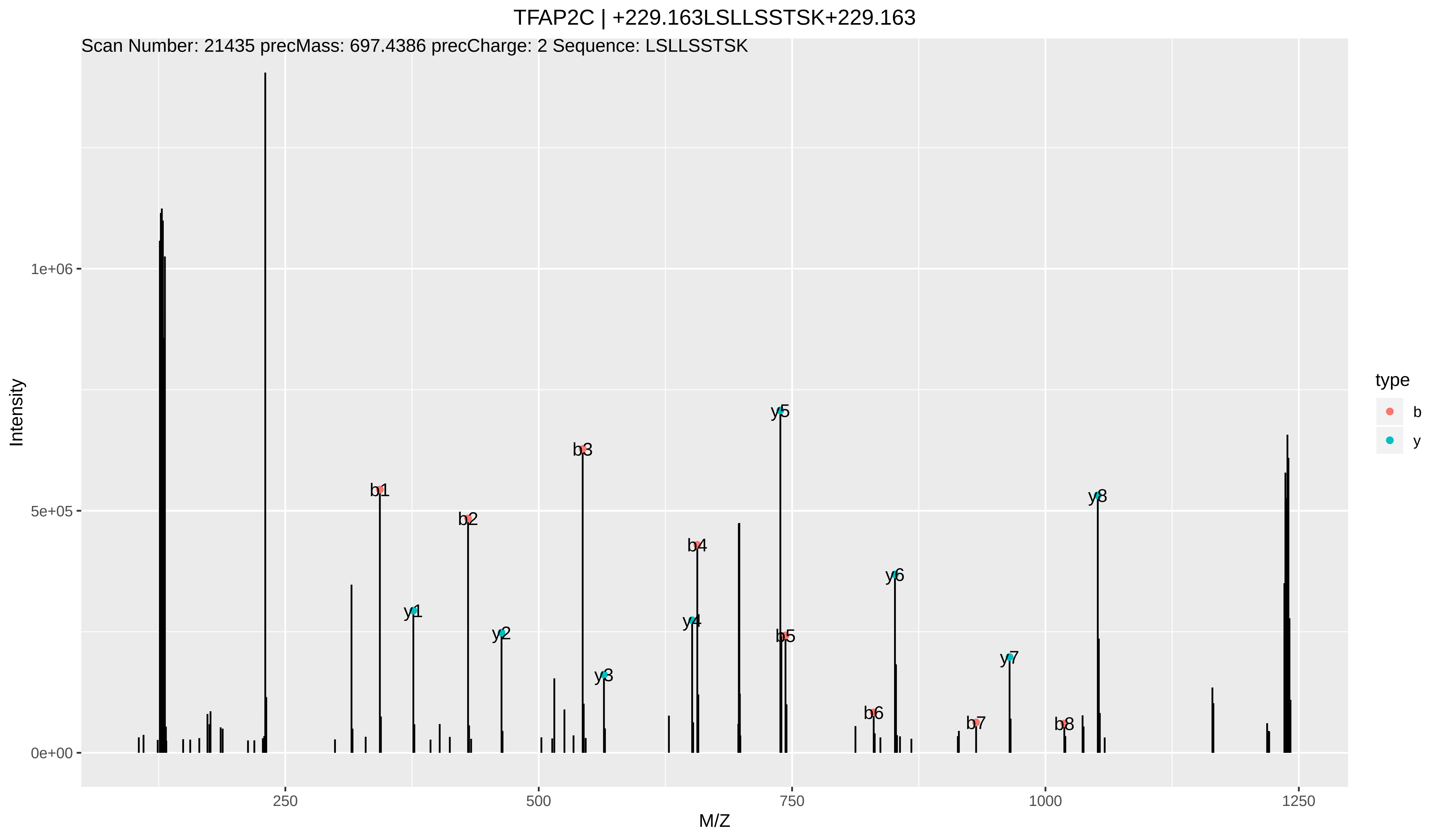
TFAP2C | +229.163LSLLSSTSK+229.163
Scan Number: 21435 precMass: 697.4386 precCharge: 2 Sequence: LSLLSSTSK
Intensity
1e+06
5e+05
0e+00
250
500
750
1000
1250
M/Z
b1
b2
b3
b4
b5
b6
b7
b8
y1
y2
y3
y4
y5
y6
y7
y8
type
b
y

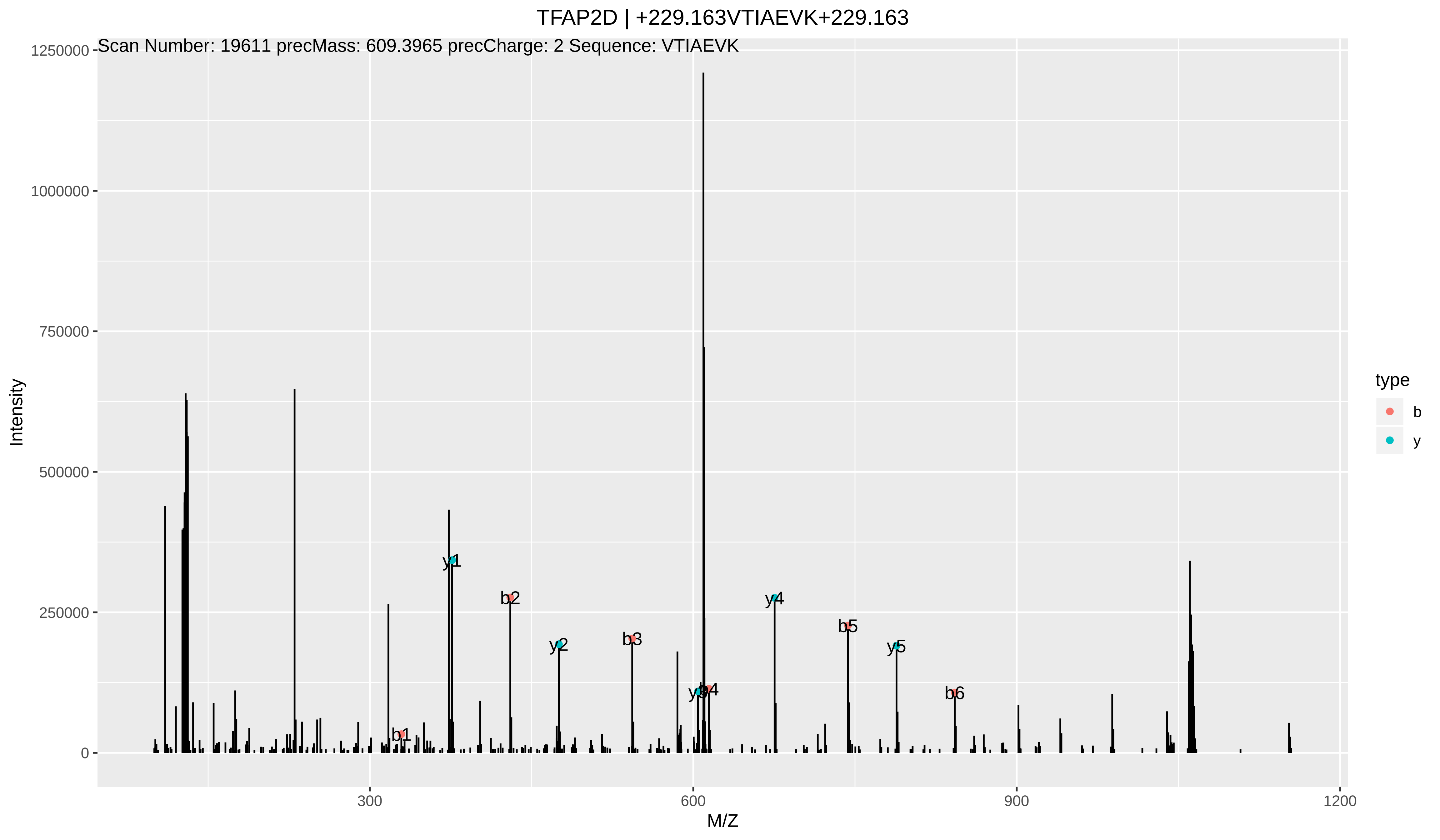

TFAP2D | +229.163VTIAEVK+229.163
Scan Number: 19611 precMass: 609.3965 precCharge: 2 Sequence: VTIAEVK
Intensity
1250000
1000000
750000
500000
250000
0
300
600
900
1200
M/Z
y1
b2
y2
b3
y3
b4
y4
b5
y5
b6
b1
type
b
y

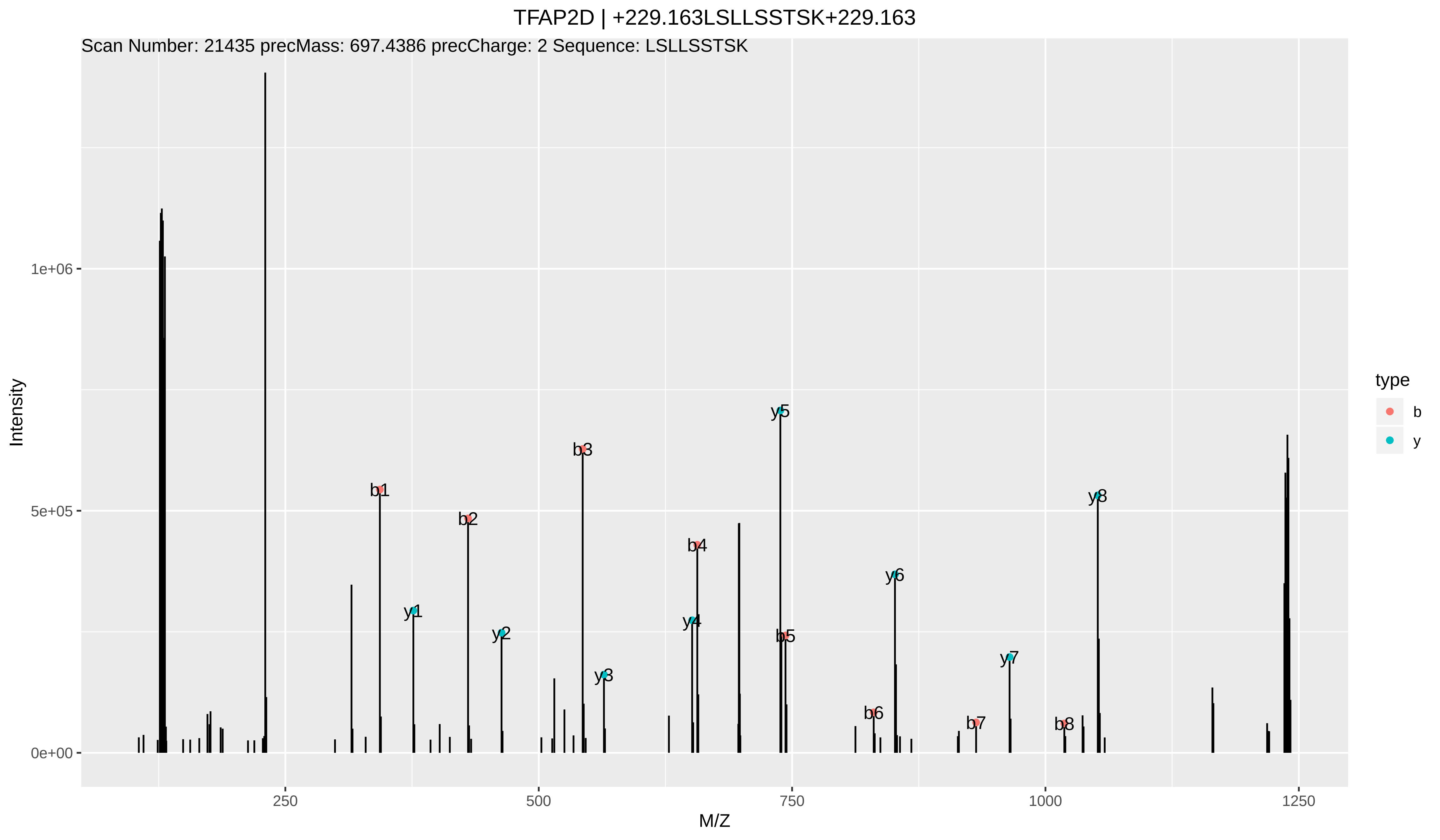

TFAP2D | +229.163LSLLSSTSK+229.163
Scan Number: 21435 precMass: 697.4386 precCharge: 2 Sequence: LSLLSSTSK
Intensity
M/Z
type
b
y
b1
b2
b3
b4
b5
b6
b7
b8
y1
y2
y3
y4
y5
y6
y7
y8
1e+06
5e+05
0e+00
250
500
750
1000
1250

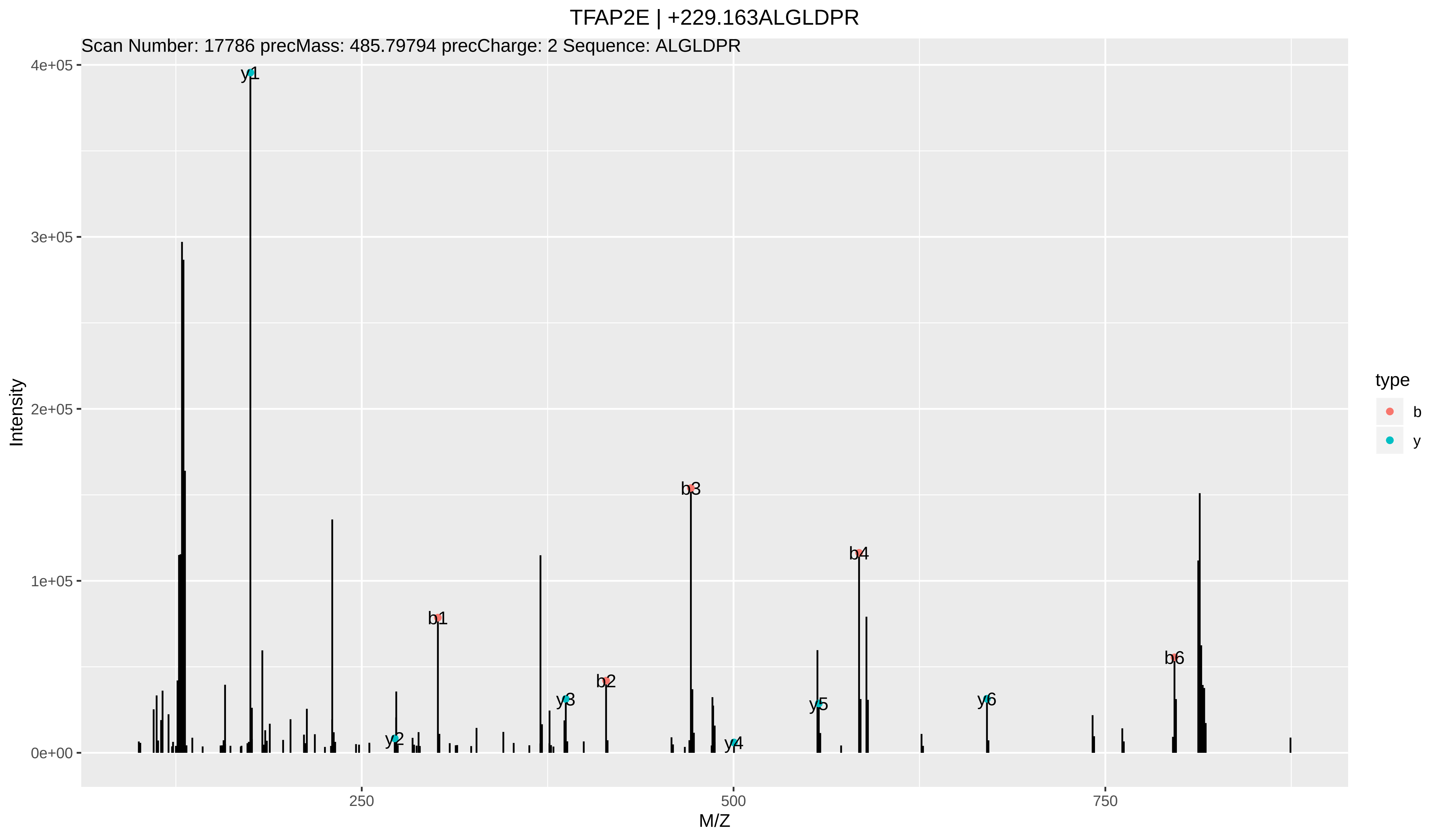

TFAP2E | +229.163ALGLDPR
Scan Number: 17786 precMass: 485.79794 precCharge: 2 Sequence: ALGLDPR
Intensity
4e+05
3e+05
2e+05
1e+05
0e+00
M/Z
250
500
750
y1
y2
y3
y4
y5
y6
b1
b2
b3
b4
b6
type
b
y

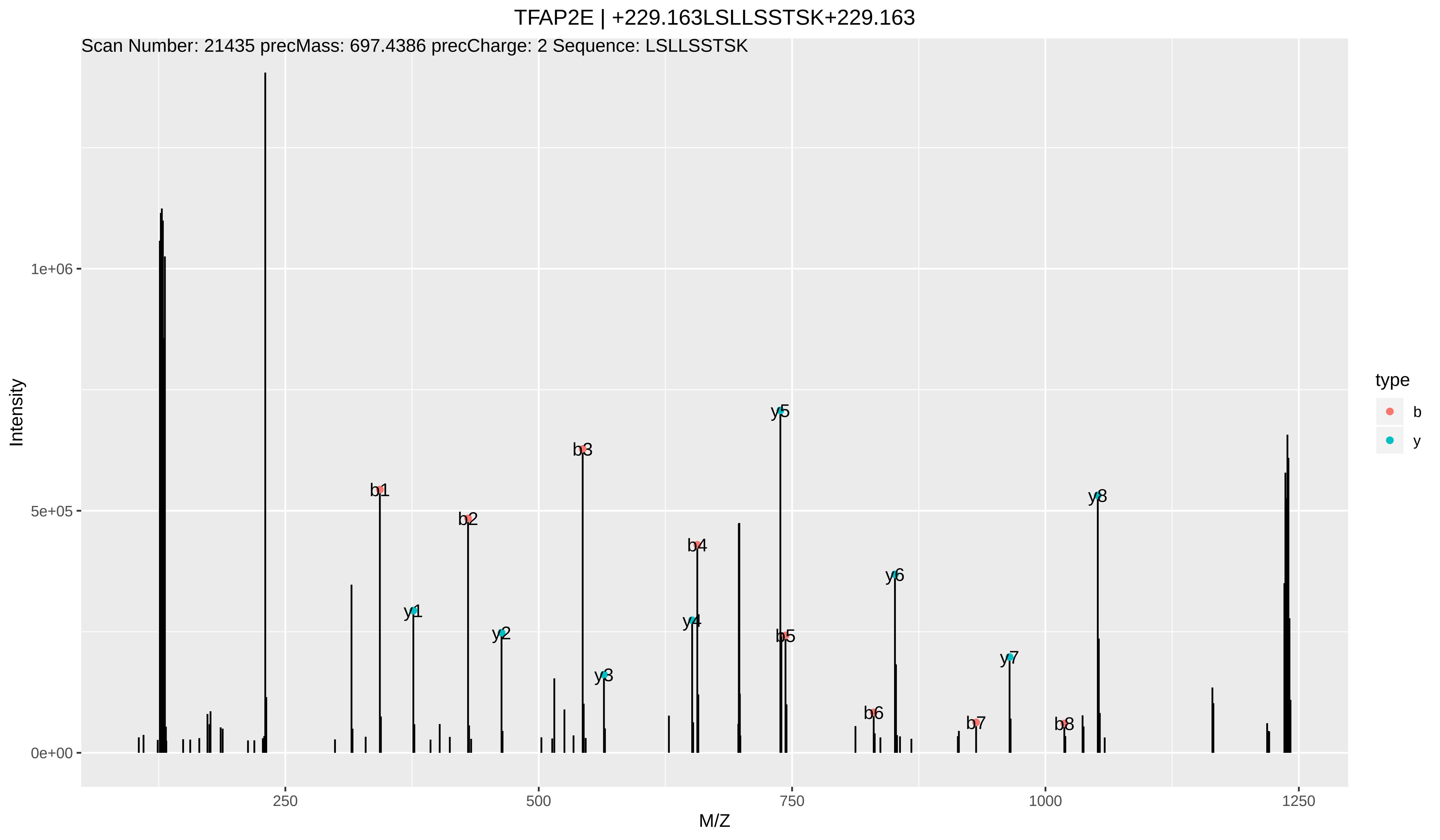

TFAP2E | +229.163LSLLSSTSK+229.163
Scan Number: 21435 precMass: 697.4386 precCharge: 2 Sequence: LSLLSSTSK
Intensity
M/Z
type
b
y
b1
b2
b3
b4
b5
b6
b7
b8
y1
y2
y3
y4
y5
y6
y7
y8
0e+00
5e+05
1e+06
250
500
750
1000
1250

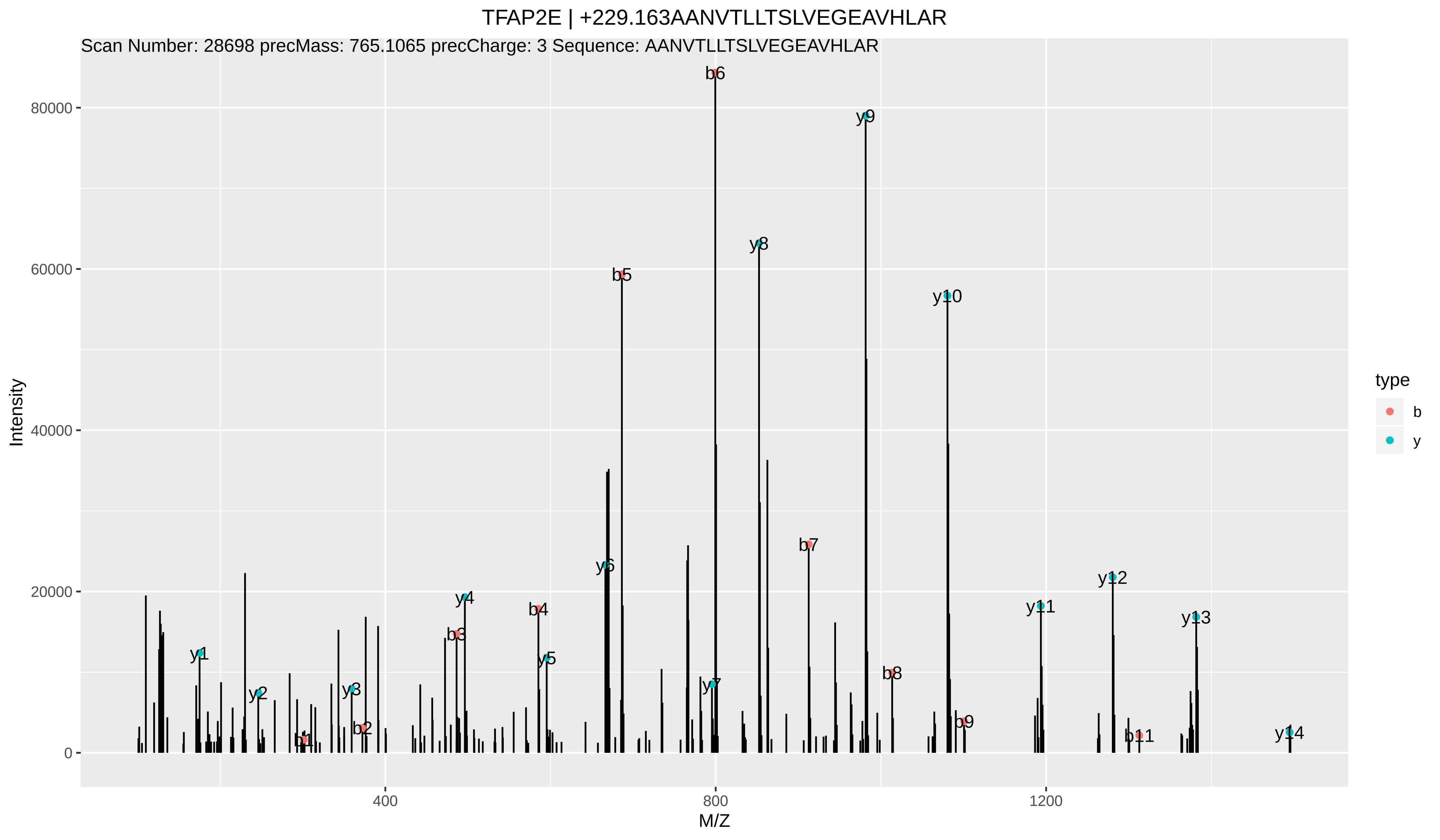
TFAP2E | +229.163AANVTLLTSLVEGEAVHLAR
Scan Number: 28698 precMass: 765.1065 precCharge: 3 Sequence: AANVTLLTSLVEGEAVHLAR
Intensity
M/Z
type
b
y
b1
b2
b3
b4
b5
b6
b7
b8
b9
b11
y1
y2
y3
y4
y5
y6
y7
y8
y9
y10
y11
y12
y13
y14
0
20000
40000
60000
80000
400
800
1200

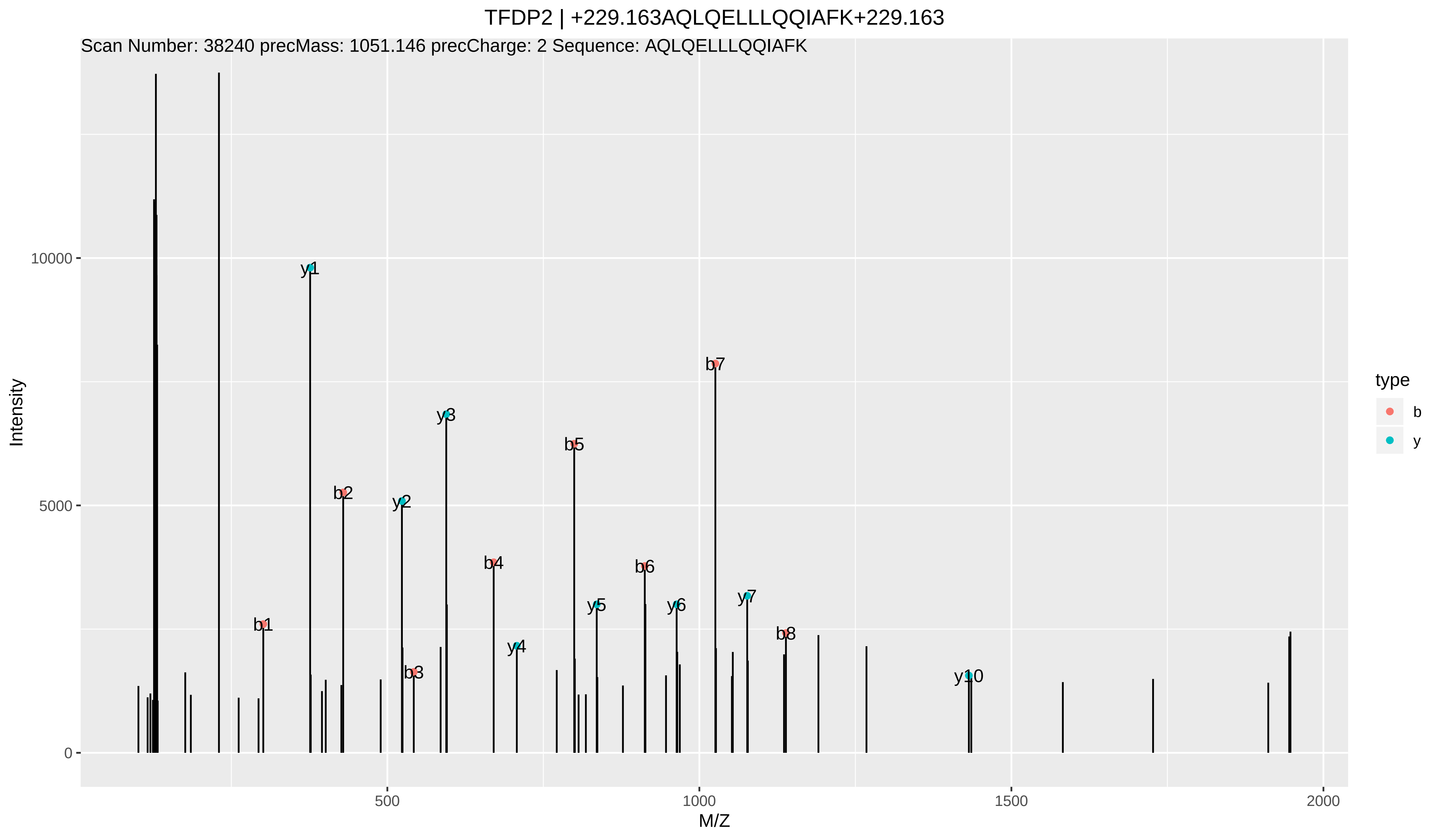

TFDP2 | +229.163AQLQELLLQQIAFK+229.163
Scan Number: 38240 precMass: 1051.146 precCharge: 2 Sequence: AQLQELLLQQIAFK
Intensity
M/Z
0
5000
10000
500
1000
1500
2000
b1
y1
b2
y2
b3
y3
b4
y4
b5
y5
b6
y6
b7
y7
b8
y10
type
b
y

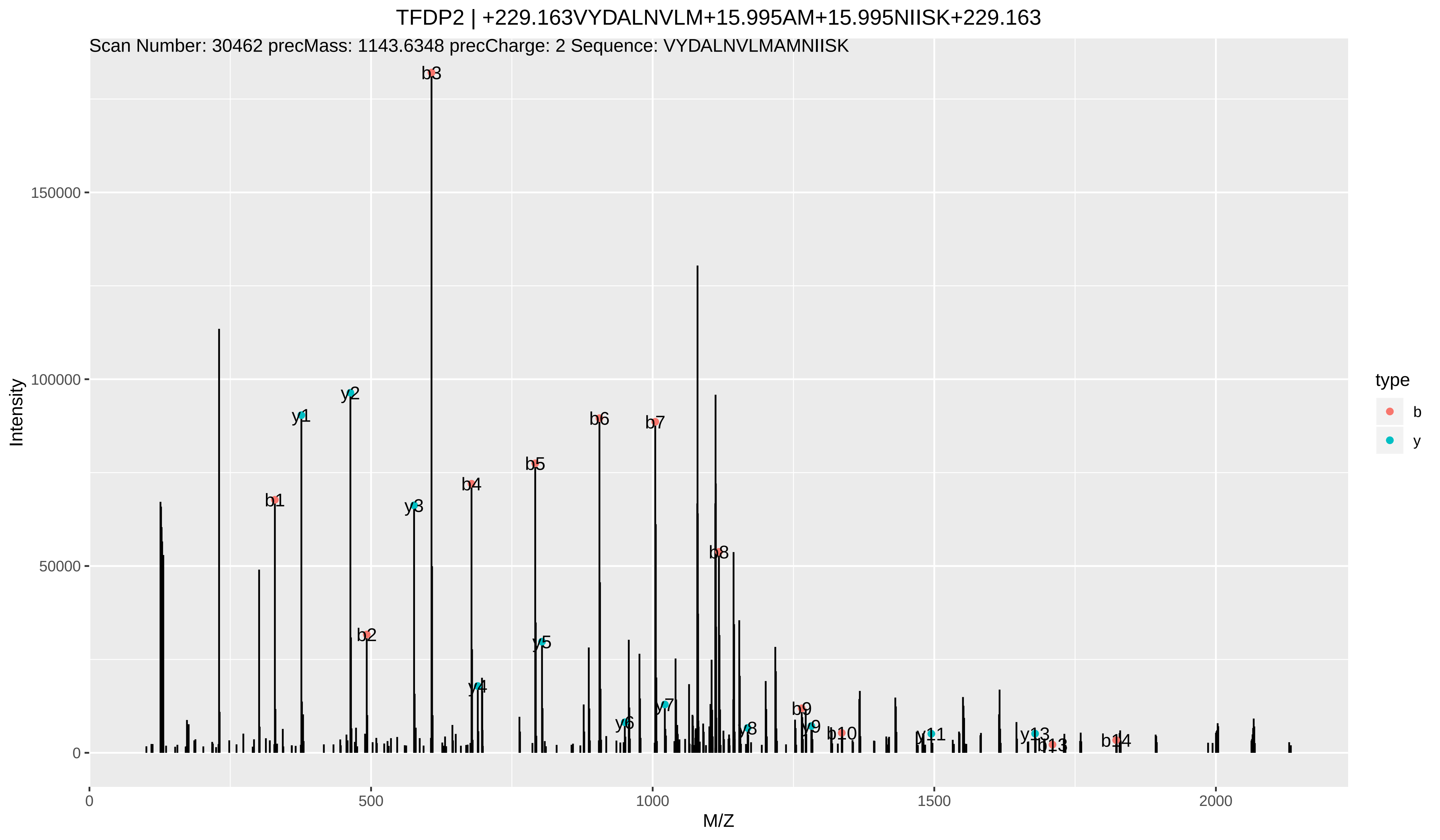

TFDP2 | +229.163VYDALNVLM+15.995AM+15.995NIISK+229.163
Scan Number: 30462 precMass: 1143.6348 precCharge: 2 Sequence: VYDALNVLMAMNIISK
Intensity
M/Z
type
b
y
b1
b2
b3
b4
b5
b6
b7
b8
b9
b10
b13
b14
y1
y2
y3
y4
y5
y6
y7
y8
y9
y11
y13
0
50000
100000
150000
500
1000
1500
2000

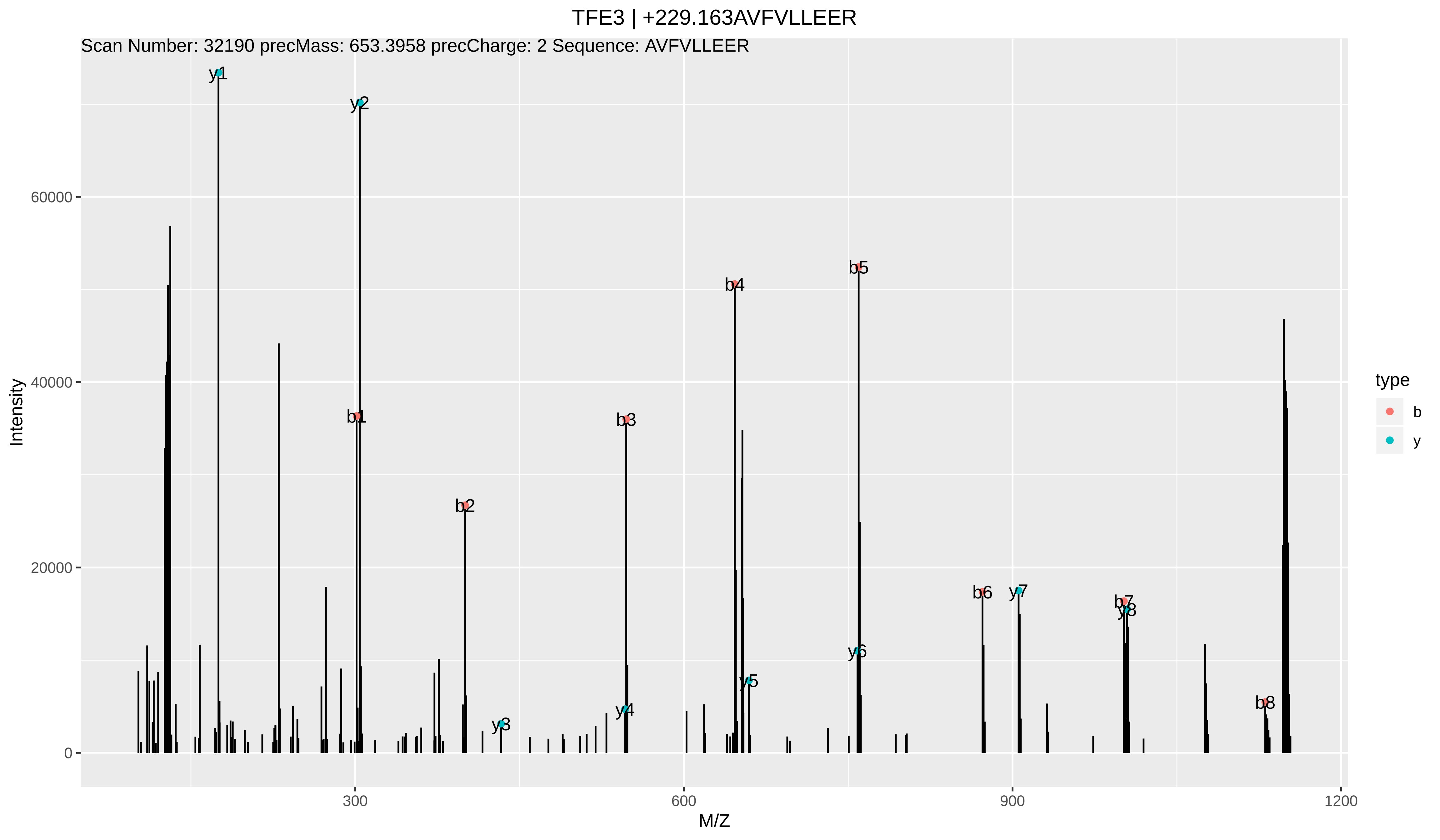
TFE3 | +229.163AVFVLLEER
Scan Number: 32190 precMass: 653.3958 precCharge: 2 Sequence: AVFVLLEER
y1
y2
b1
b2
y3
b3
y4
b4
y5
b5
y6
b6
y7
b7
y8
b8
Intensity
M/Z
60000
40000
20000
0
300
600
900
1200
type
b
y

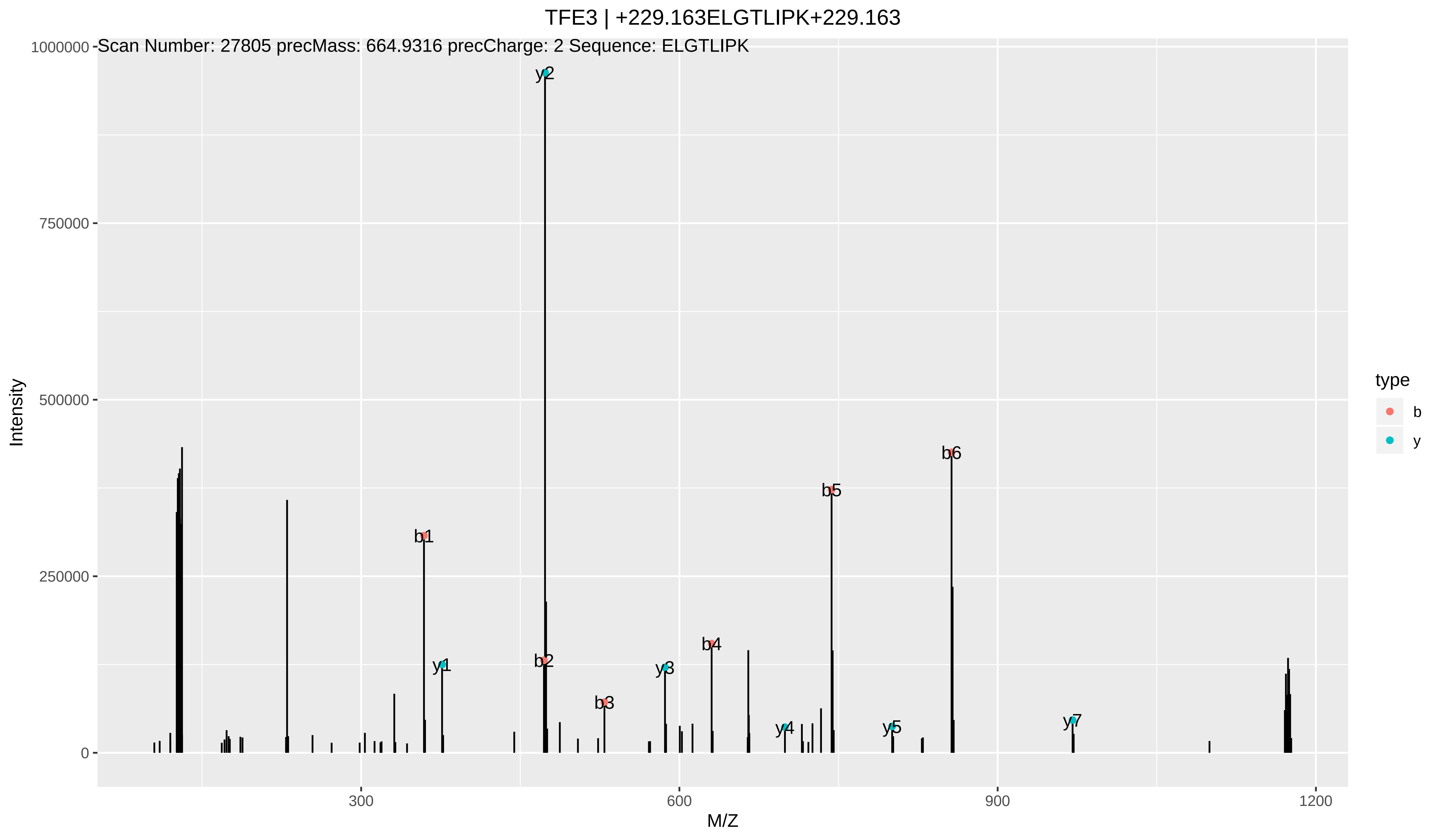

TFE3 | +229.163ELGTLIPK+229.163
Scan Number: 27805 precMass: 664.9316 precCharge: 2 Sequence: ELGTLIPK
Intensity
1000000
750000
500000
250000
0
M/Z
300
600
900
1200
y2
b6
b5
b1
b4
y1
b2
y3
b3
y7
y4
y5
type
b
y

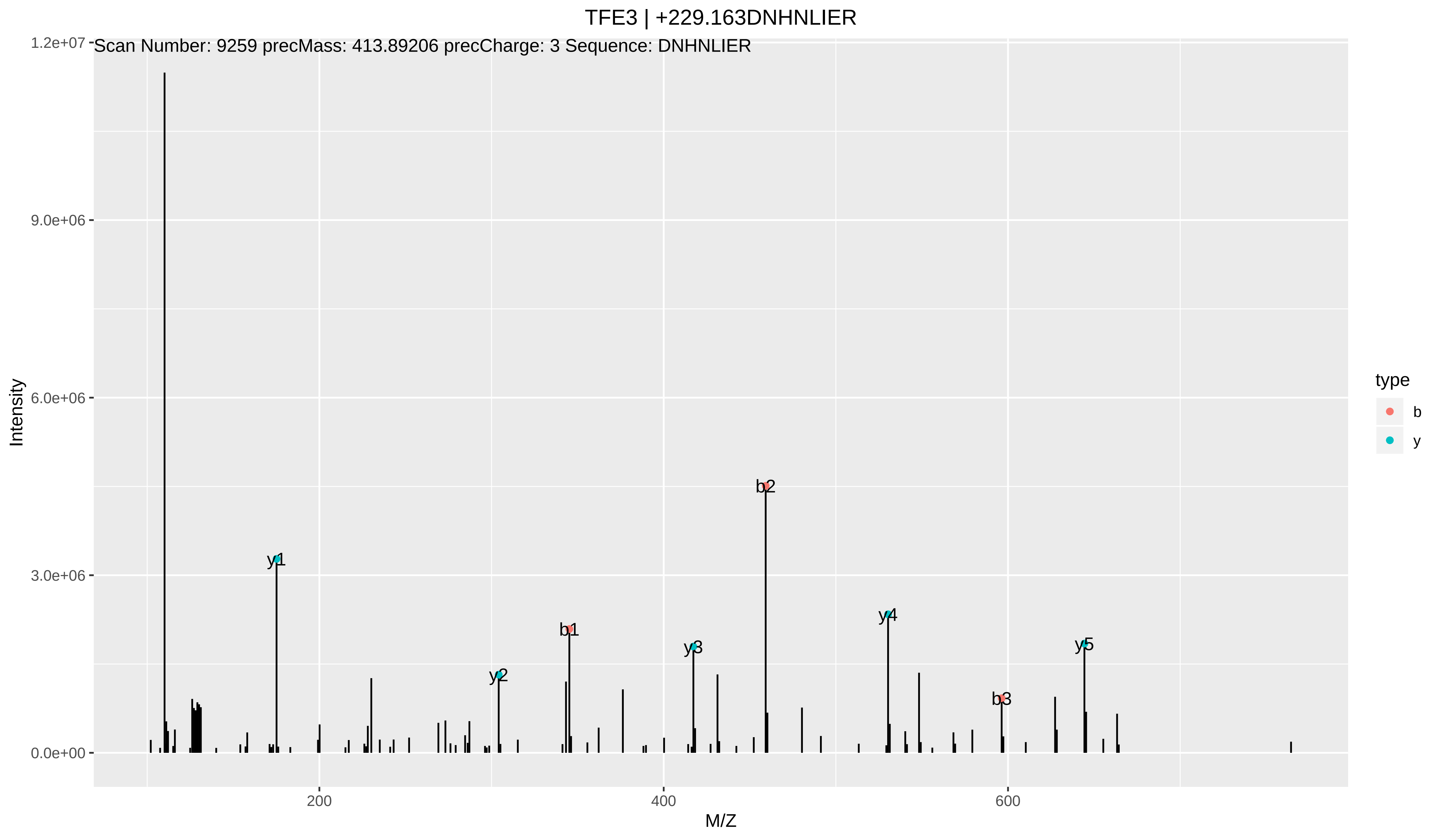
TFE3 | +229.163DNHNLIER
Scan Number: 9259 precMass: 413.89206 precCharge: 3 Sequence: DNHNLIER
Intensity
M/Z
1.2e+07
9.0e+06
6.0e+06
3.0e+06
0.0e+00
200
400
600
y1
y2
b1
y3
b2
y4
b3
y5
type
b
y

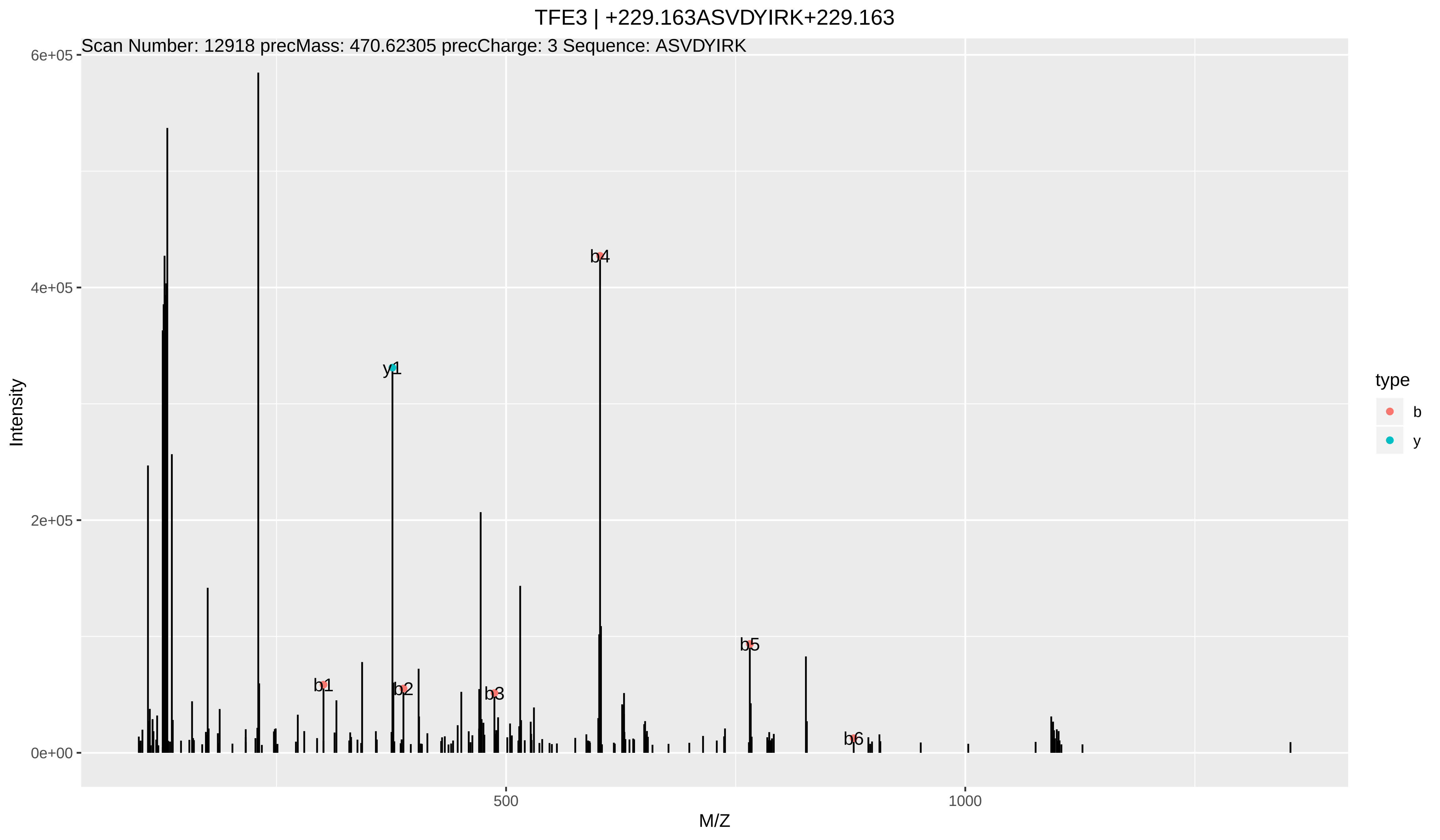
TFE3 | +229.163ASVDYIRK+229.163
Scan Number: 12918 precMass: 470.62305 precCharge: 3 Sequence: ASVDYIRK
Intensity
6e+05
4e+05
2e+05
0e+00
M/Z
500
1000
y1
b1
b2
b3
b4
b5
b6
type
b
y

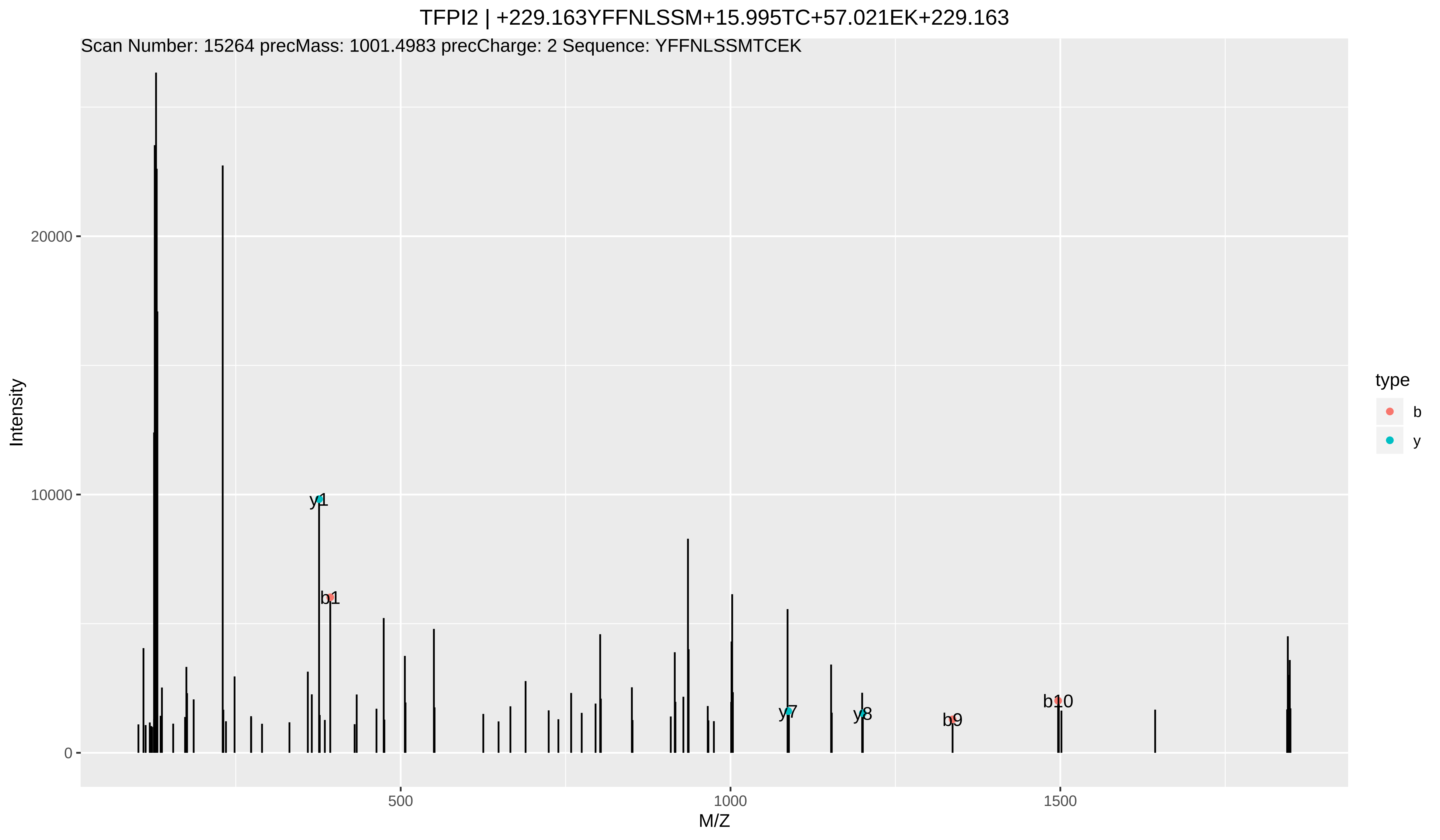

TFPI2 | +229.163YFFNLSSM+15.995TC+57.021EK+229.163
Scan Number: 15264 precMass: 1001.4983 precCharge: 2 Sequence: YFFNLSSMTCEK
Intensity
M/Z
20000
10000
0
500
1000
1500
y1
b1
y7
y8
b9
b10
type
b
y

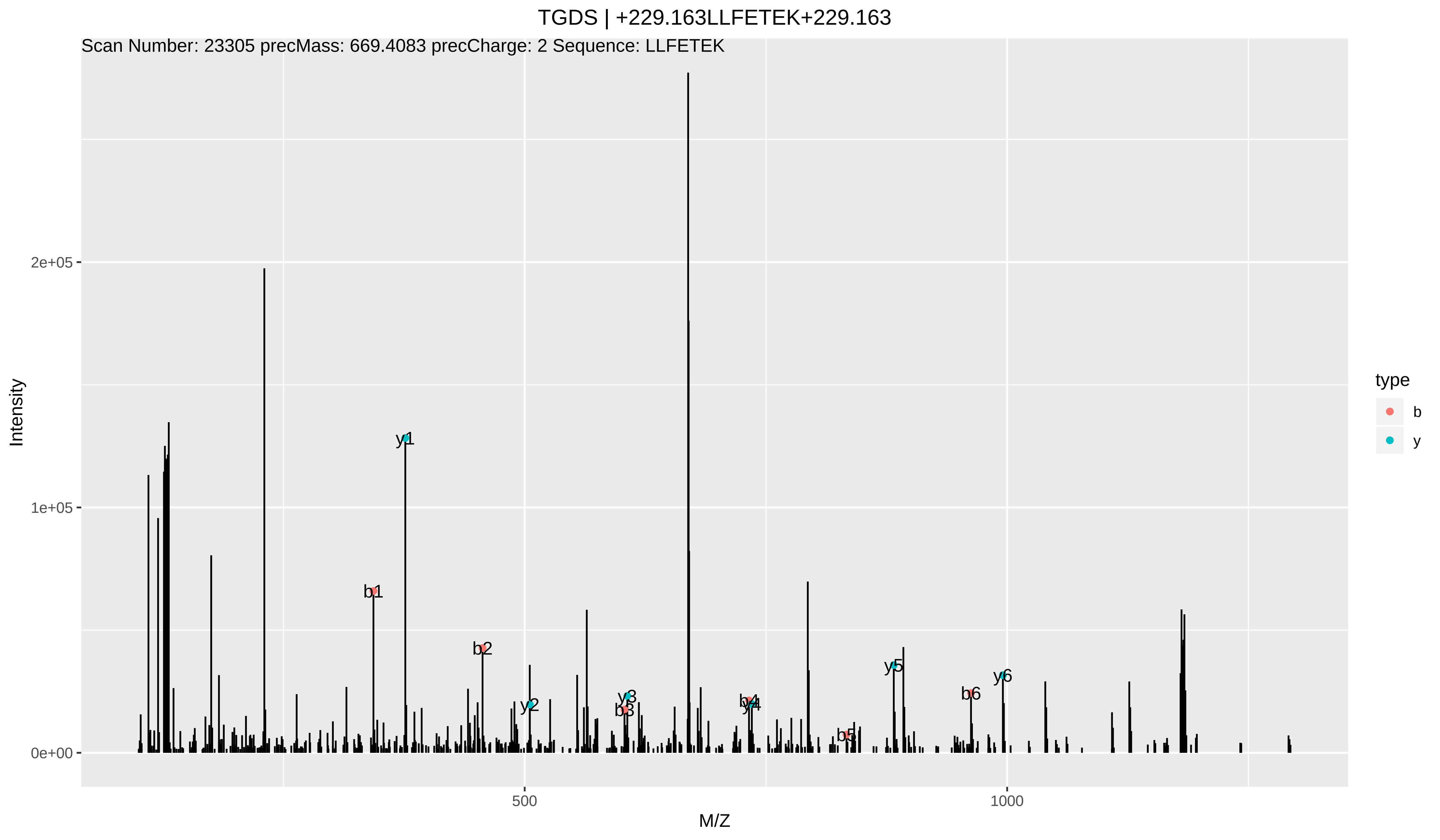

TGDS | +229.163LLFETEK+229.163
Scan Number: 23305 precMass: 669.4083 precCharge: 2 Sequence: LLFETEK
Intensity
2e+05
1e+05
0e+00
M/Z
500
1000
y1
b1
b2
y2
y3
b3
b4
y4
b5
y5
b6
y6
type
b
y

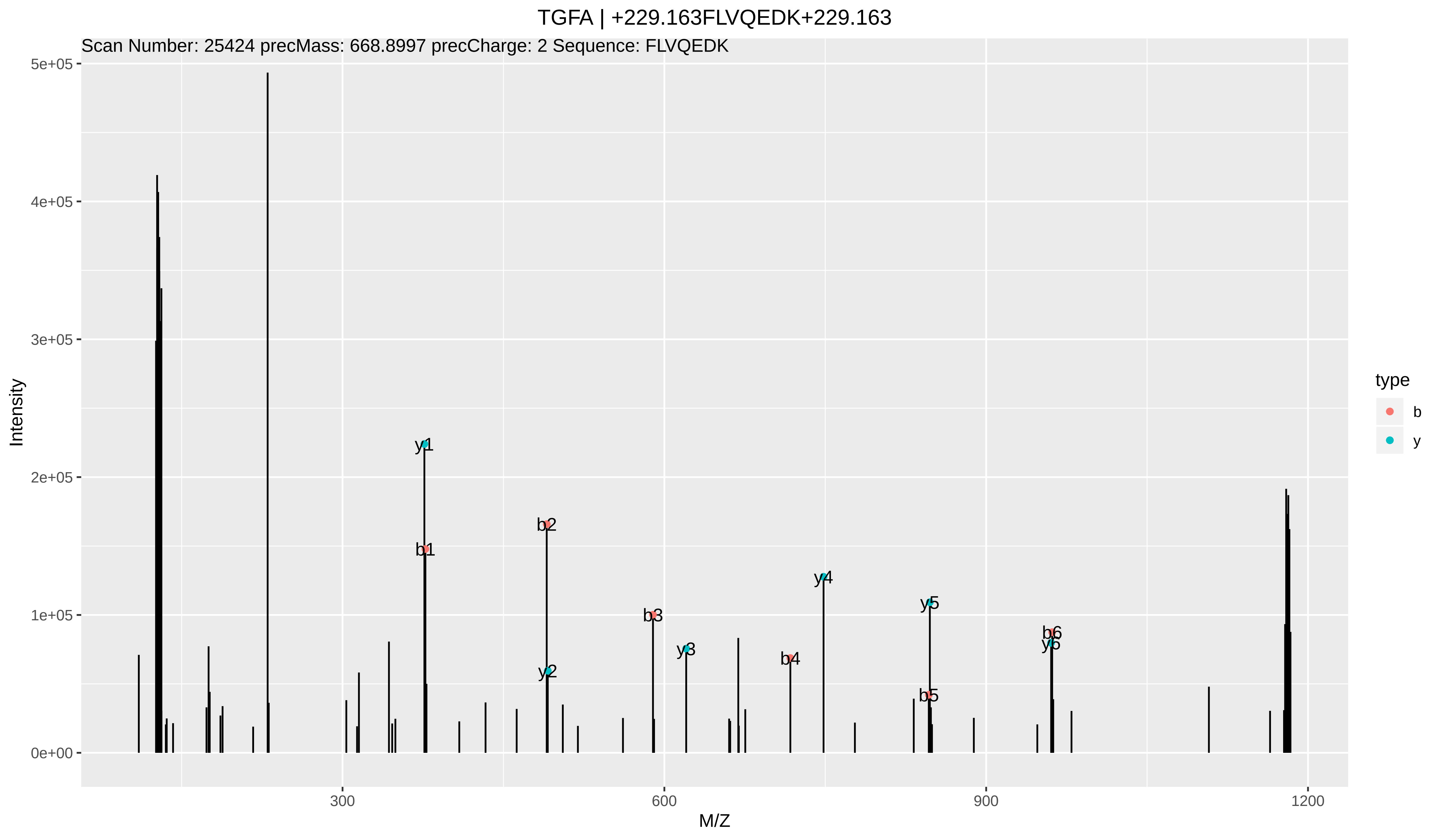
TGFA | +229.163FLVQEDK+229.163
Scan Number: 25424 precMass: 668.8997 precCharge: 2 Sequence: FLVQEDK
Intensity
0e+00
1e+05
2e+05
3e+05
4e+05
5e+05
M/Z
300
600
900
1200
y1
b1
b2
y2
b3
y3
b4
y4
y5
b5
b6
y6
type
b
y

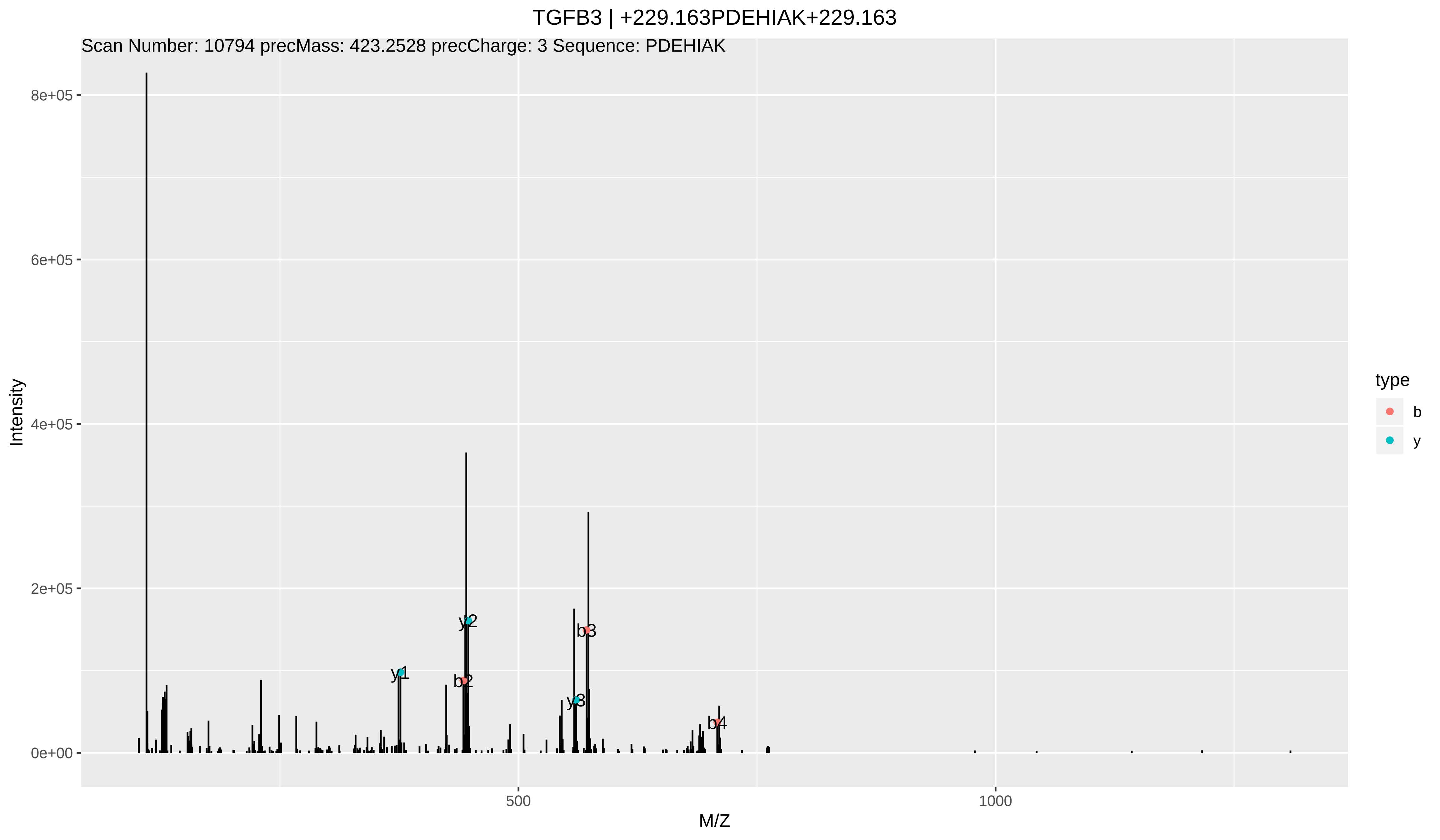

TGFB3 | +229.163PDEHIAK+229.163
Scan Number: 10794 precMass: 423.2528 precCharge: 3 Sequence: PDEHIAK
Intensity
8e+05
6e+05
4e+05
2e+05
0e+00
M/Z
500
1000
y1
y2
b2
b3
y3
b4
type
b
y

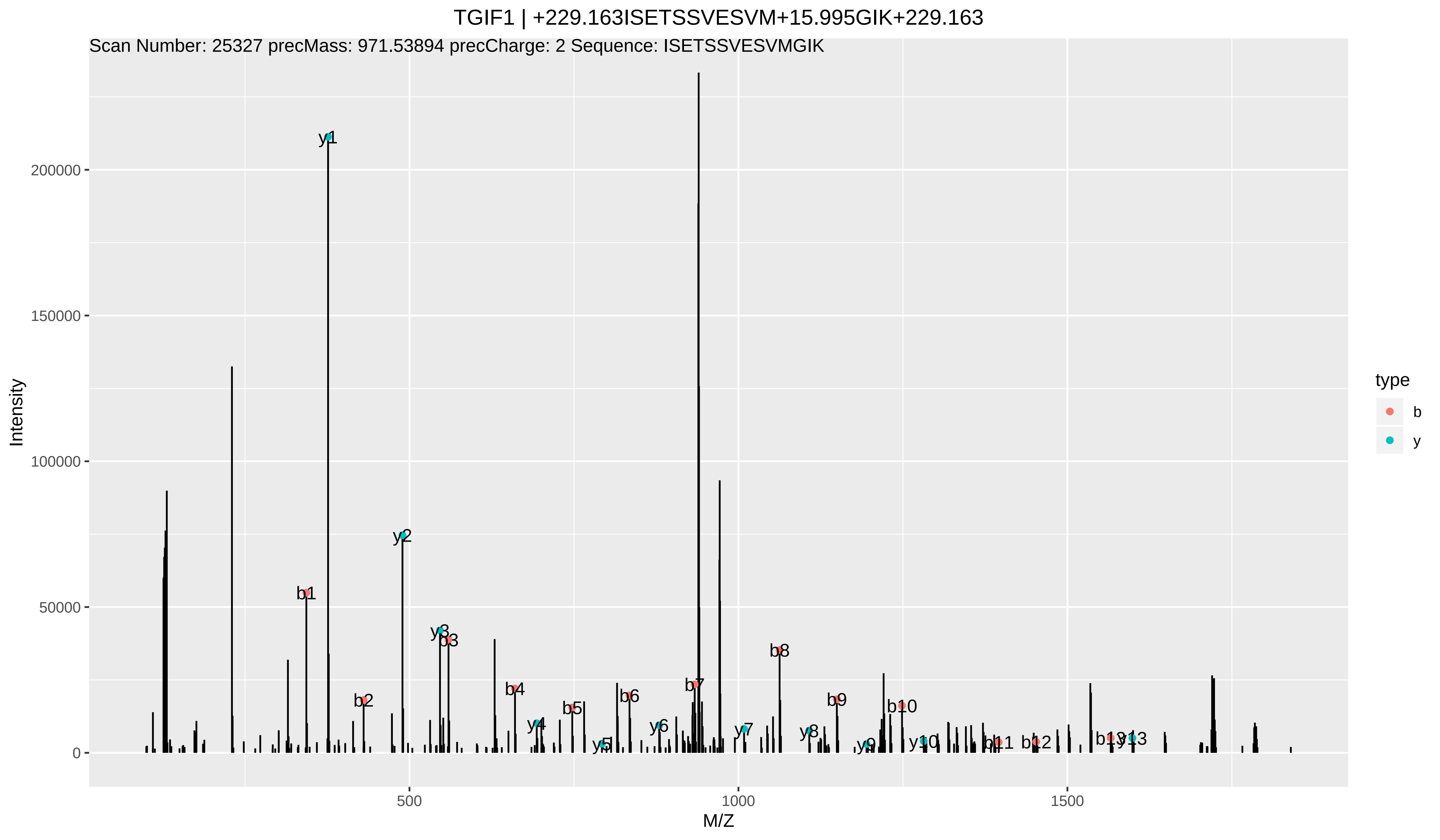

TGIF1 | +229.163ISETSSVESVM+15.995GIK+229.163
Scan Number: 25327 precMass: 971.53894 precCharge: 2 Sequence: ISETSSVESVMGIK
Intensity
M/Z
type
b
y
y1
y2
b1
b2
y3
b3
b4
y4
b5
y5
b6
y6
b7
y7
b8
y8
b9
y9
b10
y10
b11
b12
b13
y13
0
50000
100000
150000
200000
500
1000
1500

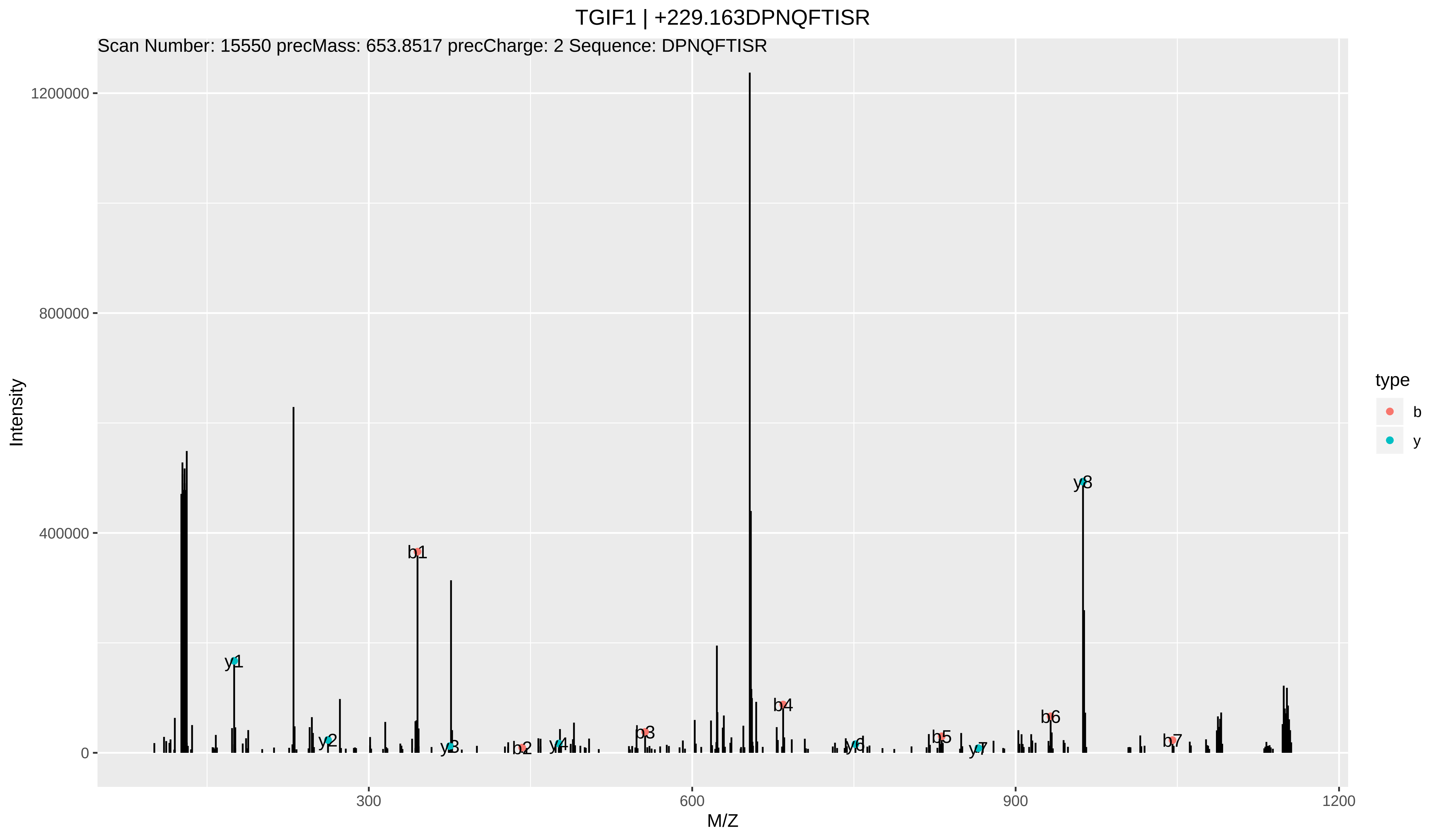

TGIF1 | +229.163DPNQFTISR
Scan Number: 15550 precMass: 653.8517 precCharge: 2 Sequence: DPNQFTISR
Intensity
1200000
800000
400000
0
300
600
900
1200
M/Z
y1
y2
b1
y3
b2
y4
b3
b4
y6
b5
y7
b6
y8
b7
type
b
y

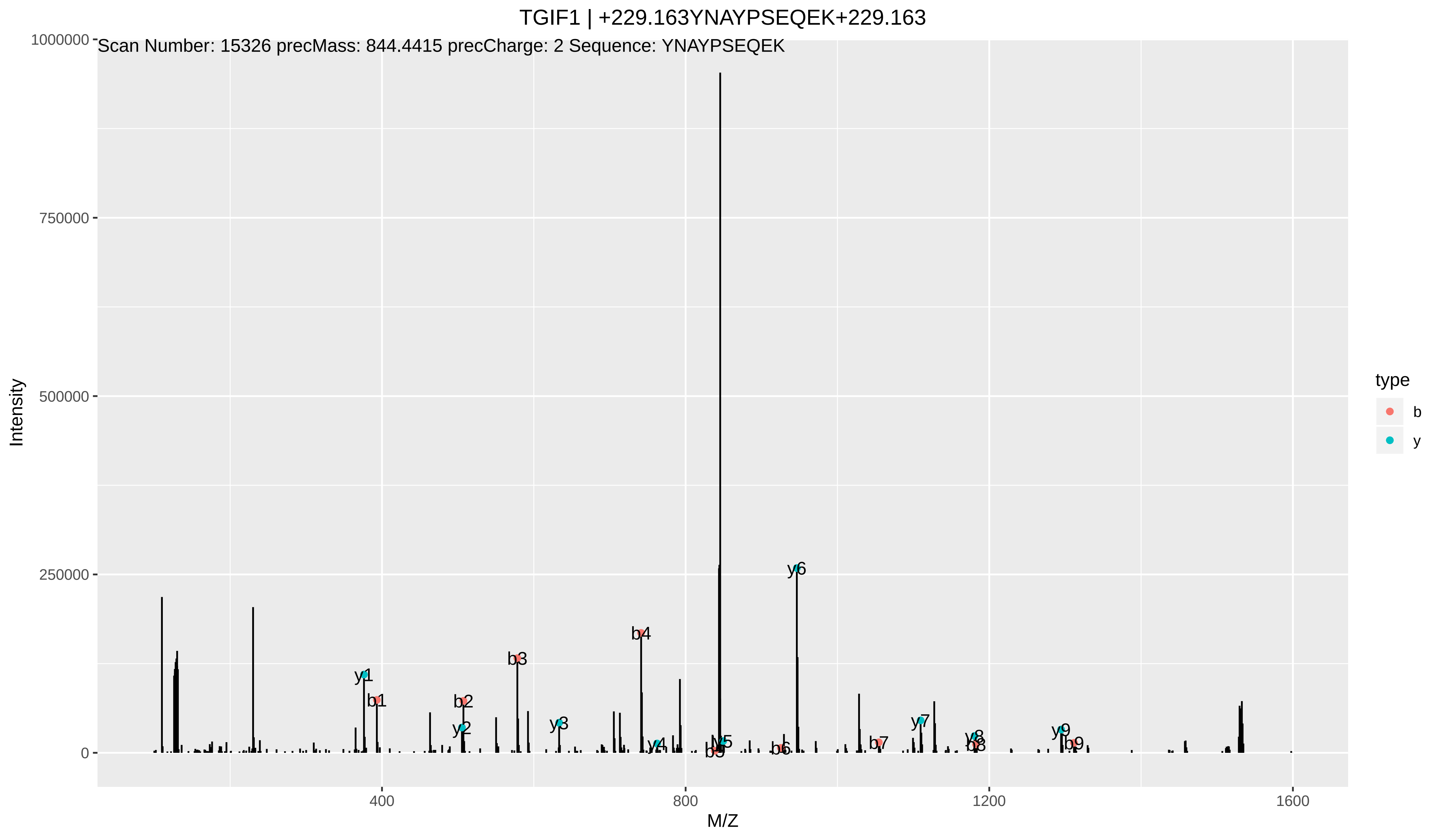

TGIF1 | +229.163YNAYPSEQEK+229.163
Scan Number: 15326 precMass: 844.4415 precCharge: 2 Sequence: YNAYPSEQEK
Intensity
M/Z
1000000
750000
500000
250000
0
400
800
1200
1600
y1
b1
b2
y2
b3
y3
b4
y4
y5
b5
y6
b6
b7
y7
y8
b8
y9
b9
type
b
y

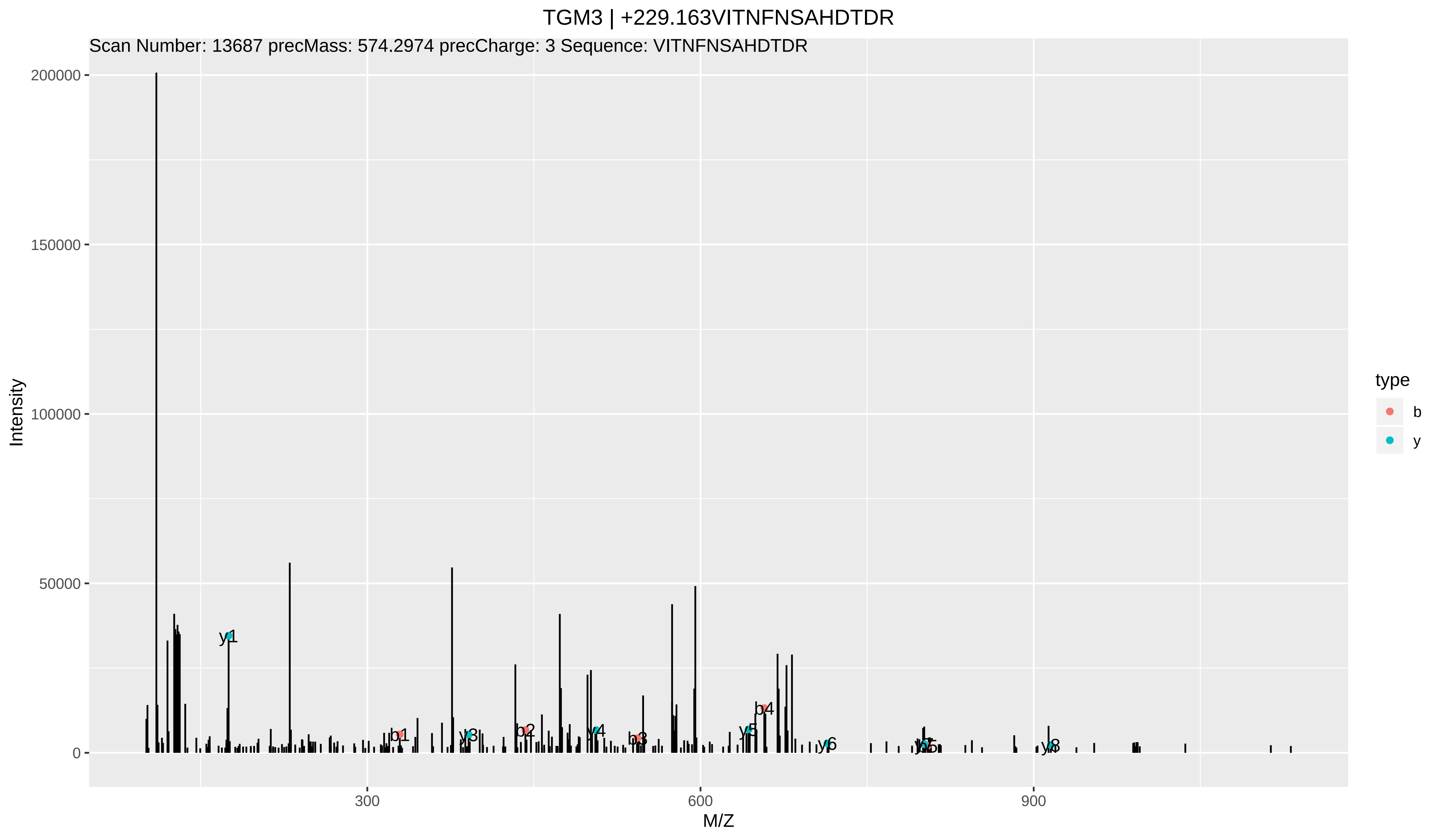
TGM3 | +229.163VITNFNSAHDTDR
Scan Number: 13687 precMass: 574.2974 precCharge: 3 Sequence: VITNFNSAHDTDR
Intensity
200000
150000
100000
50000
0
300
600
900
M/Z
y1
b1
y3
b2
y4
b3
y5
b4
y6
y8
type
b
y

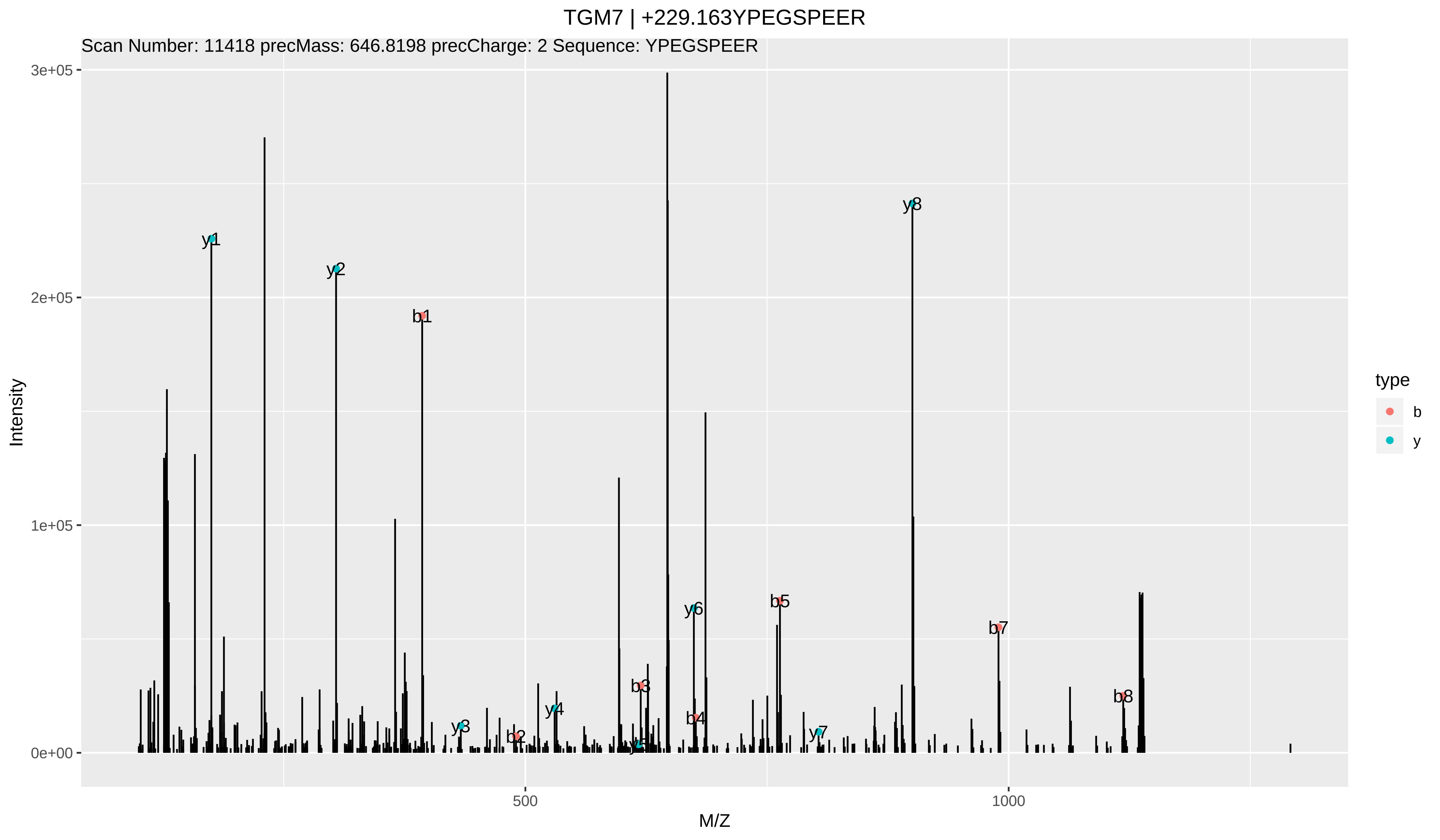

TGM7 | +229.163YPEGSPEER
Scan Number: 11418 precMass: 646.8198 precCharge: 2 Sequence: YPEGSPEER
Intensity
3e+05
2e+05
1e+05
0e+00
M/Z
500
1000
y1
y2
b1
y3
b2
y4
b3
y5
y6
b4
b5
y7
y8
b7
b8
type
b
y

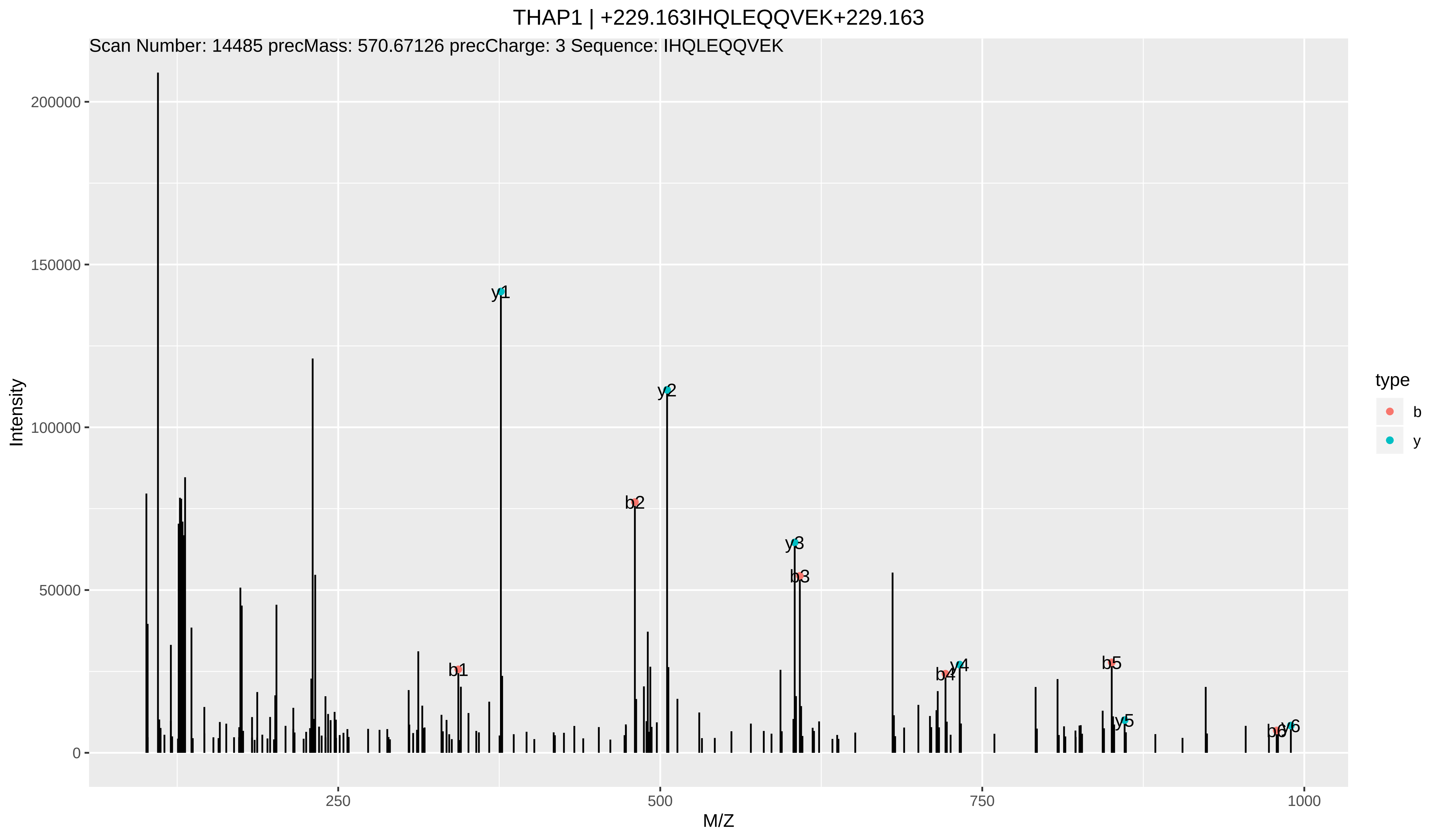

THAP1 | +229.163IHQLEQQVEK+229.163
Scan Number: 14485 precMass: 570.67126 precCharge: 3 Sequence: IHQLEQQVEK
Intensity
200000
150000
100000
50000
0
250
500
750
1000
M/Z
y1
y2
b2
y3
b3
b1
b4
y4
b5
y5
b6
y6
type
b
y

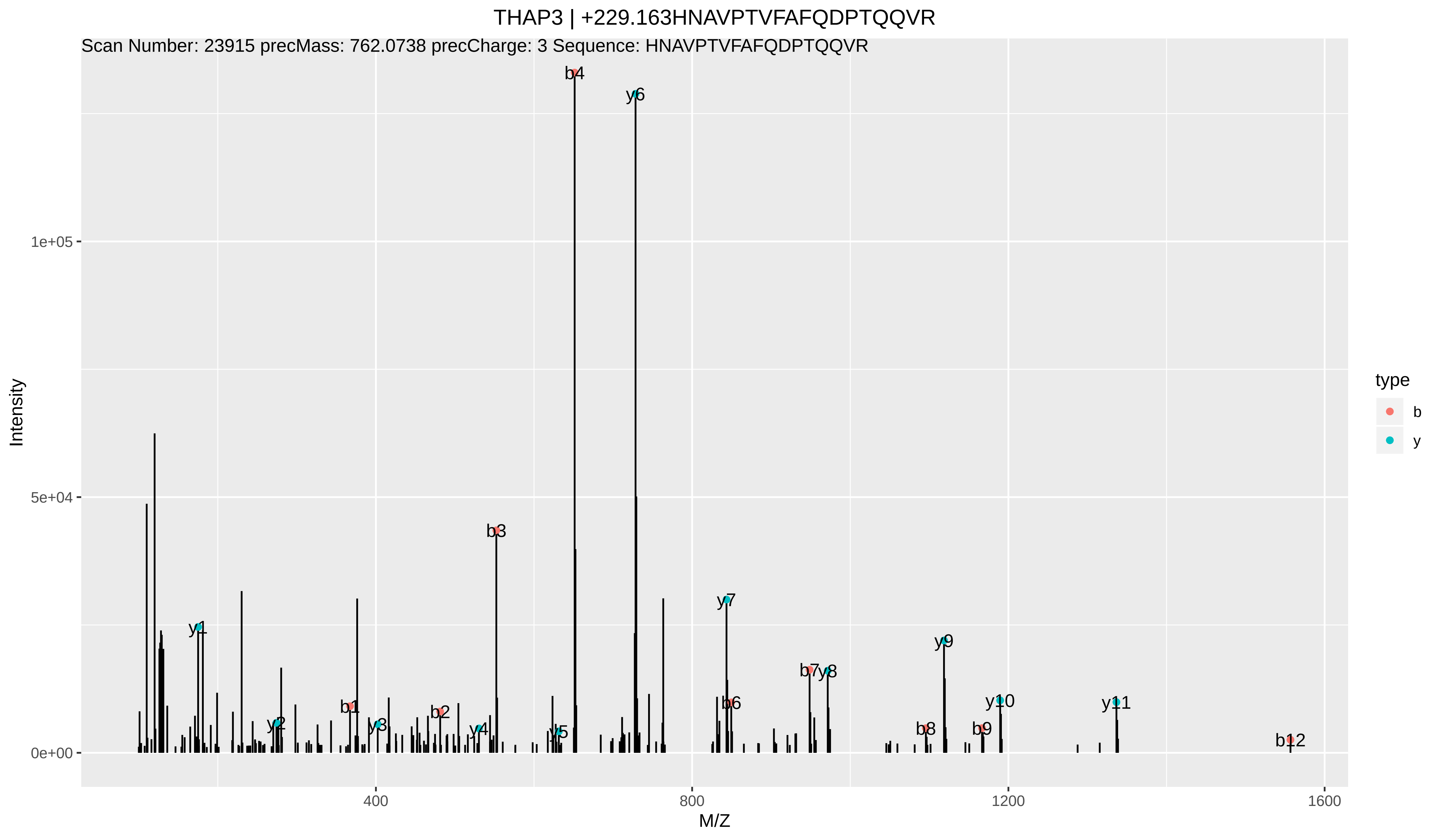

THAP3 | +229.163HNAVPTVFAFQDPTQQVR
Scan Number: 23915 precMass: 762.0738 precCharge: 3 Sequence: HNAVPTVFAFQDPTQQVR
b4
y6
b3
y7
y1
y9
b7
y8
b6
y10
y11
b1
b2
y2
y3
y4
y5
b8
b9
b12
Intensity
1e+05
5e+04
0e+00
400
800
1200
1600
M/Z
type
b
y

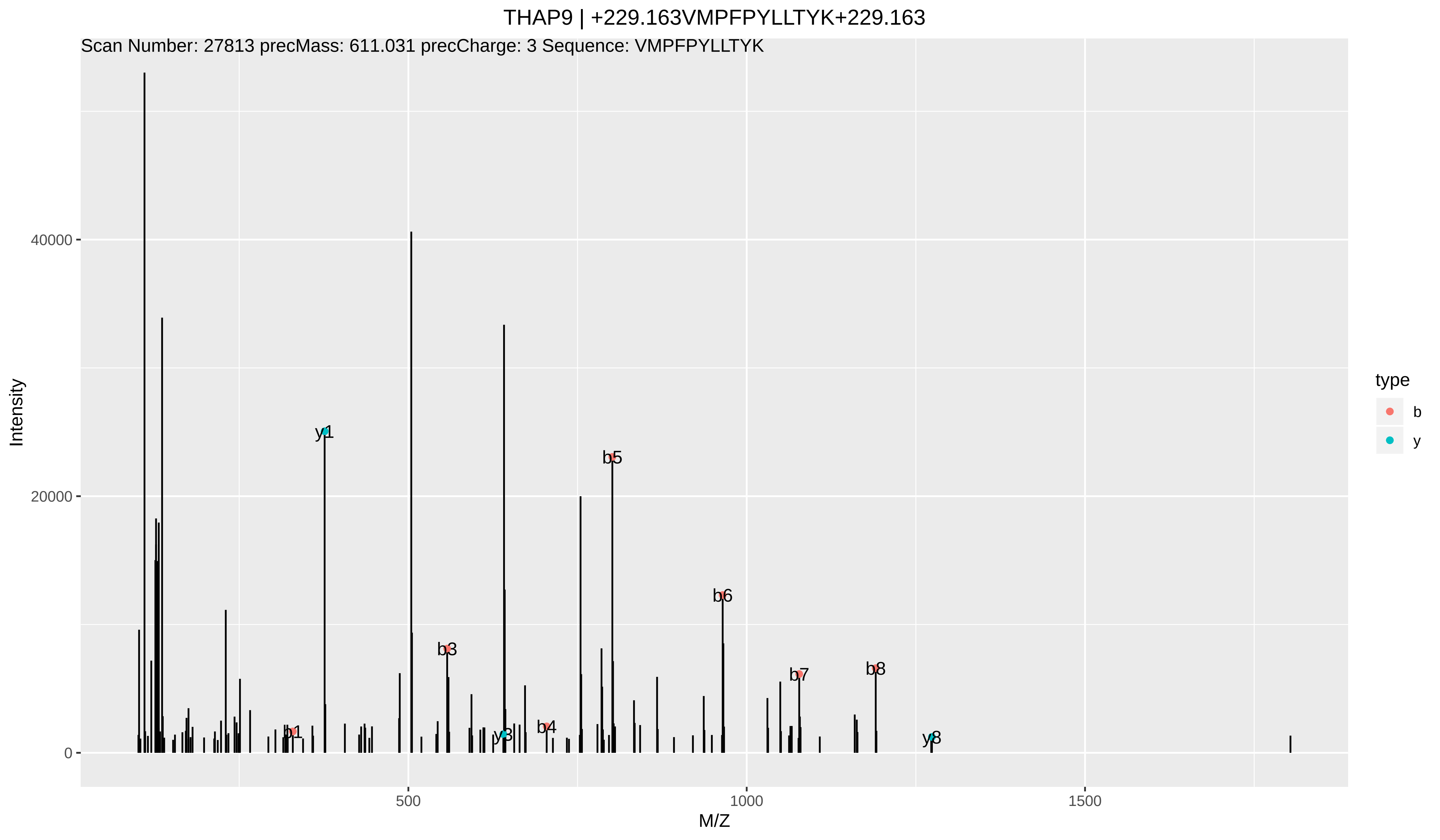

THAP9 | +229.163VMPFPYLLTYK+229.163
Scan Number: 27813 precMass: 611.031 precCharge: 3 Sequence: VMPFPYLLTYK
Intensity
40000
20000
0
500
1000
1500
M/Z
y1
b5
b6
b3
b7
b8
b1
y3
b4
y8
type
b
y

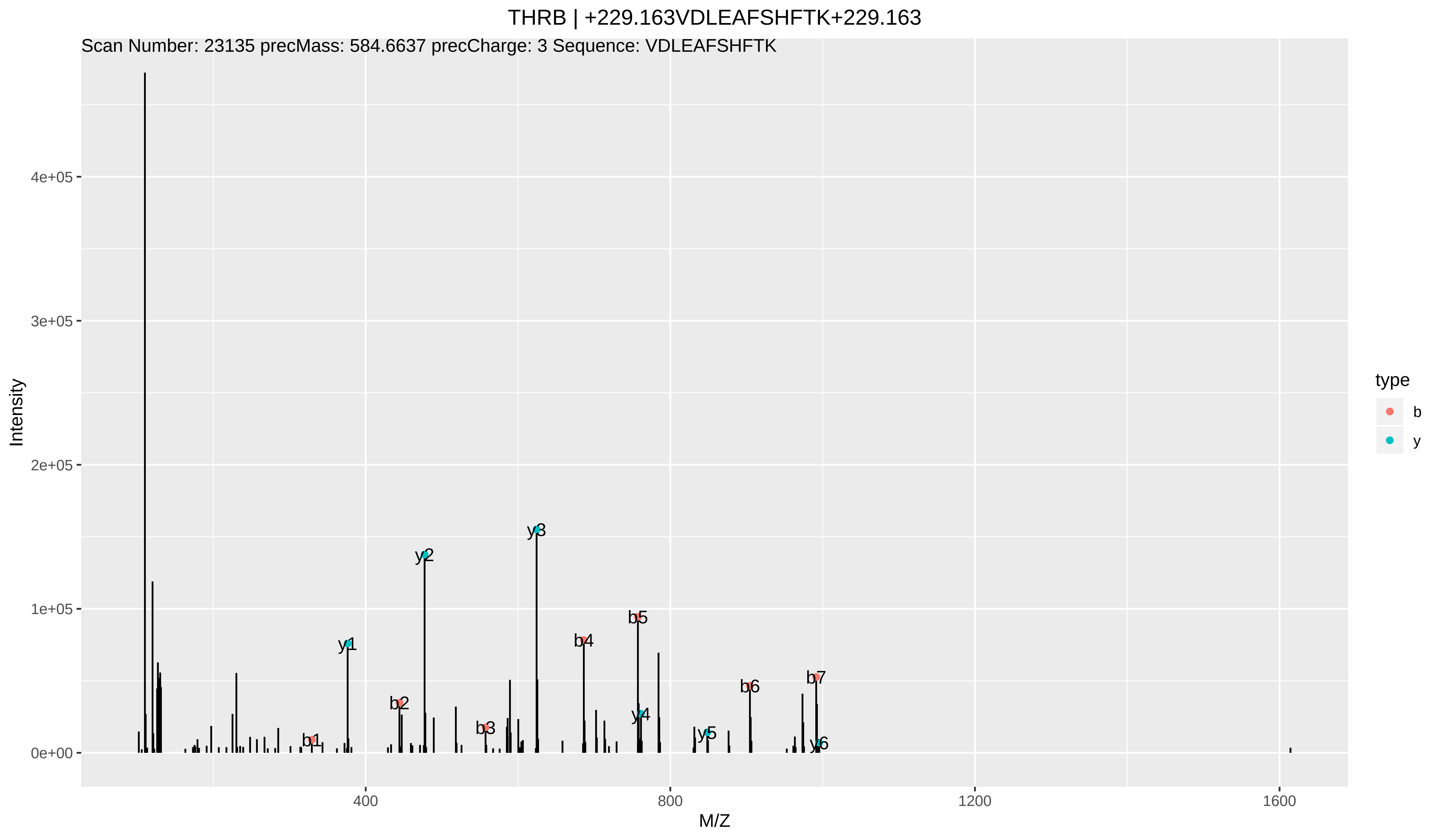
THRB | +229.163VDLEAFSHFTK+229.163
Scan Number: 23135 precMass: 584.6637 precCharge: 3 Sequence: VDLEAFSHFTK
Intensity
4e+05
3e+05
2e+05
1e+05
0e+00
400
800
1200
1600
M/Z
y1
y2
y3
y4
y5
y6
b1
b2
b3
b4
b5
b6
b7
type
b
y

TICAM2 | +229.163HSVDTSPGYHESDSK+229.163

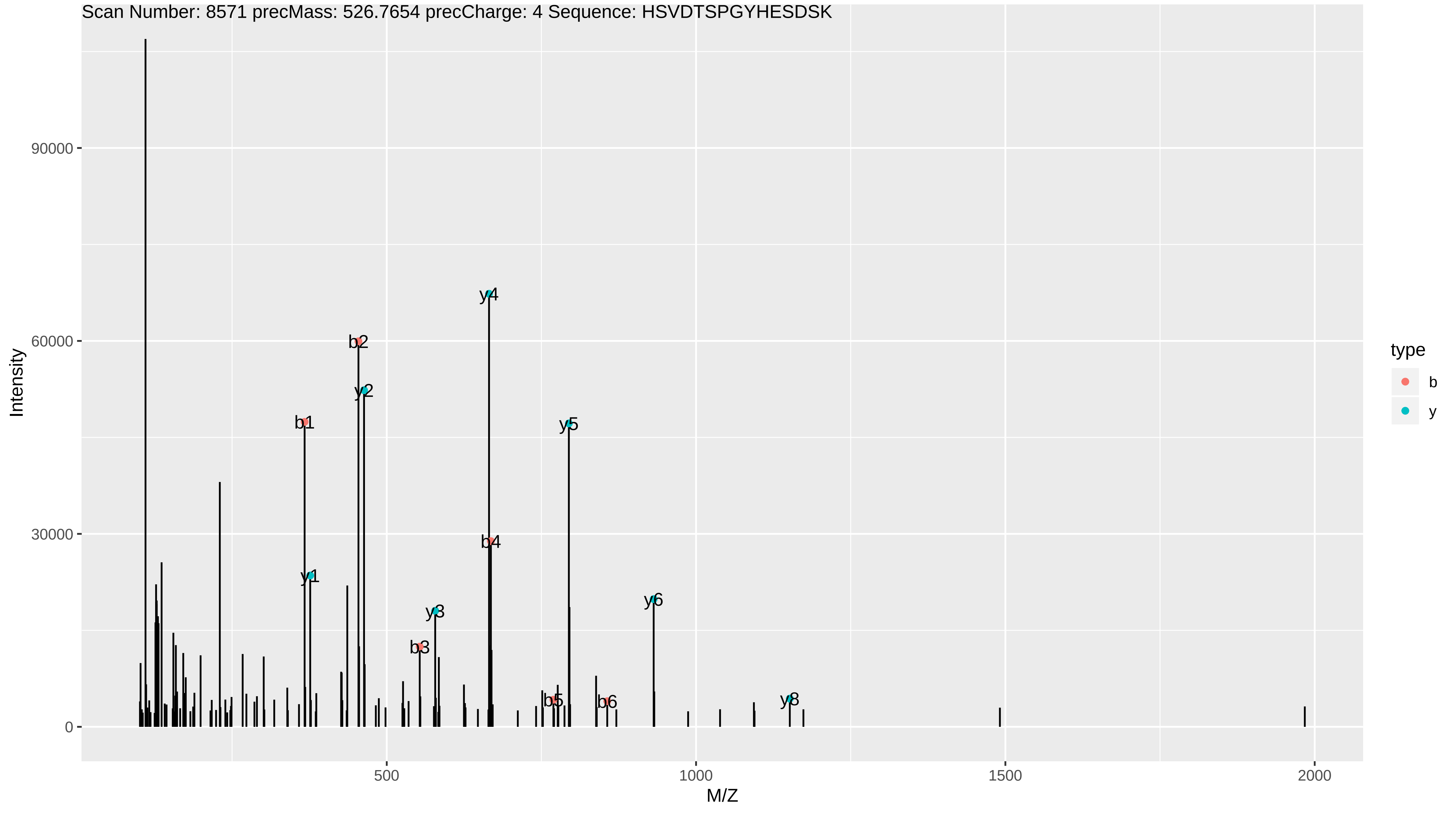

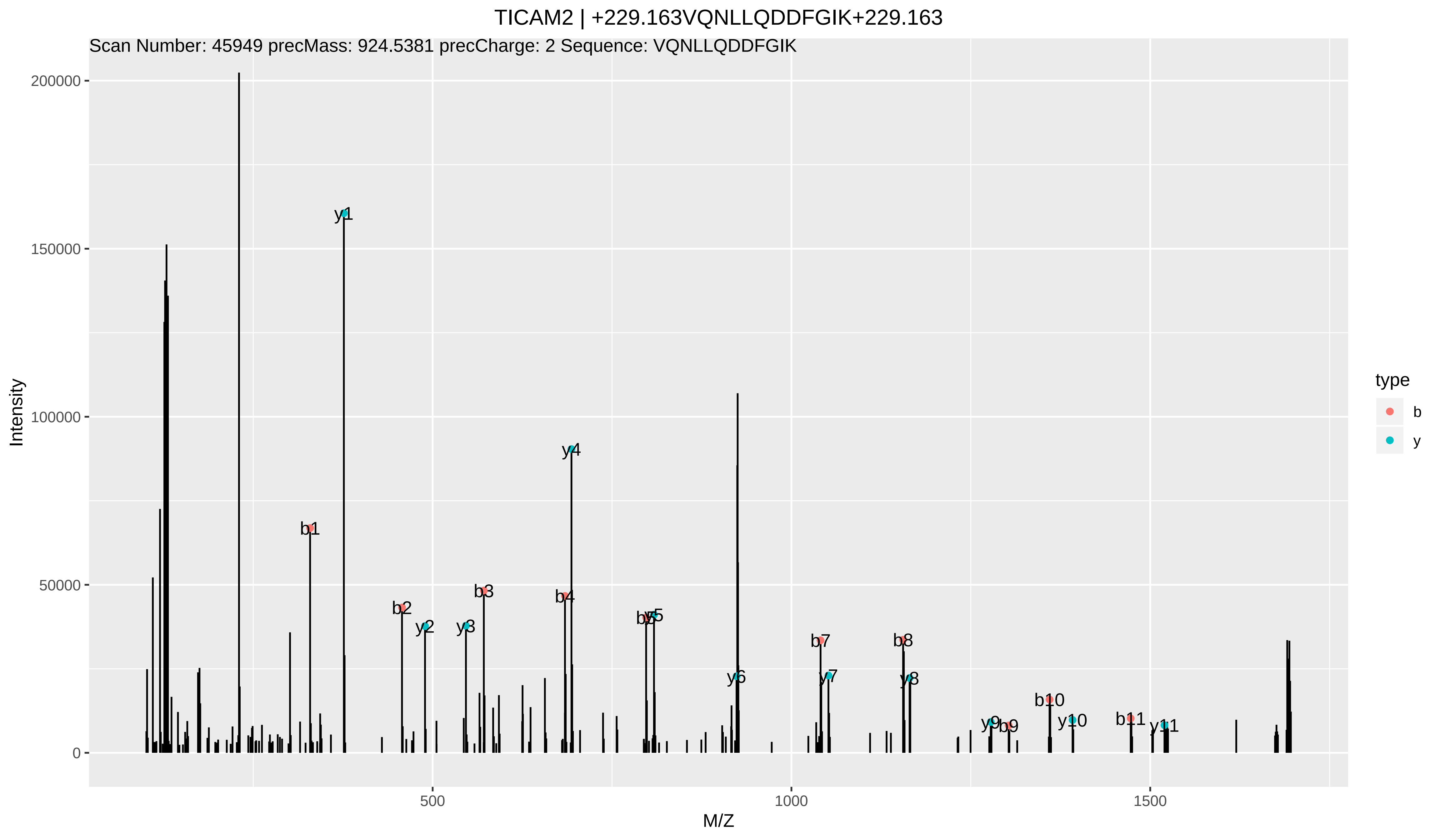

TICAM2 | +229.163VQNLLQDDFGIK+229.163
Scan Number: 45949 precMass: 924.5381 precCharge: 2 Sequence: VQNLLQDDFGIK
Intensity
M/Z
type
b
y
0
50000
100000
150000
200000
500
1000
1500
y1
b1
b2
y2
y3
b3
b4
y4
b5
y5
y6
b7
y7
b8
y8
y9
b9
b10
y10
b11
y11

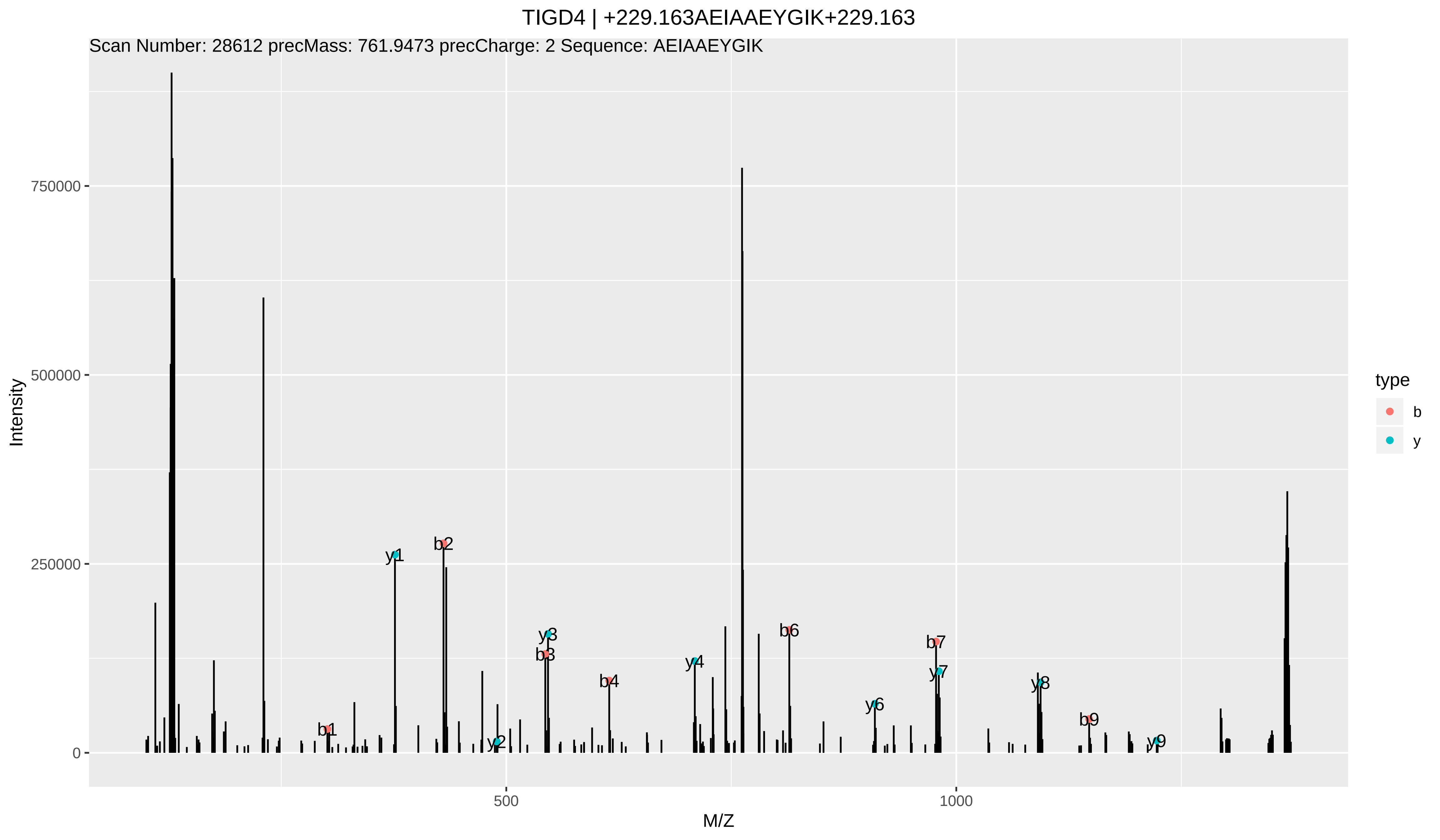
TIGD4 | +229.163AEIAAEYGIK+229.163
Scan Number: 28612 precMass: 761.9473 precCharge: 2 Sequence: AEIAAEYGIK
Intensity
750000
500000
250000
0
M/Z
500
1000
type
b
y
b1
y1
b2
y2
y3
b3
b4
y4
b6
y6
b7
y7
y8
b9
y9

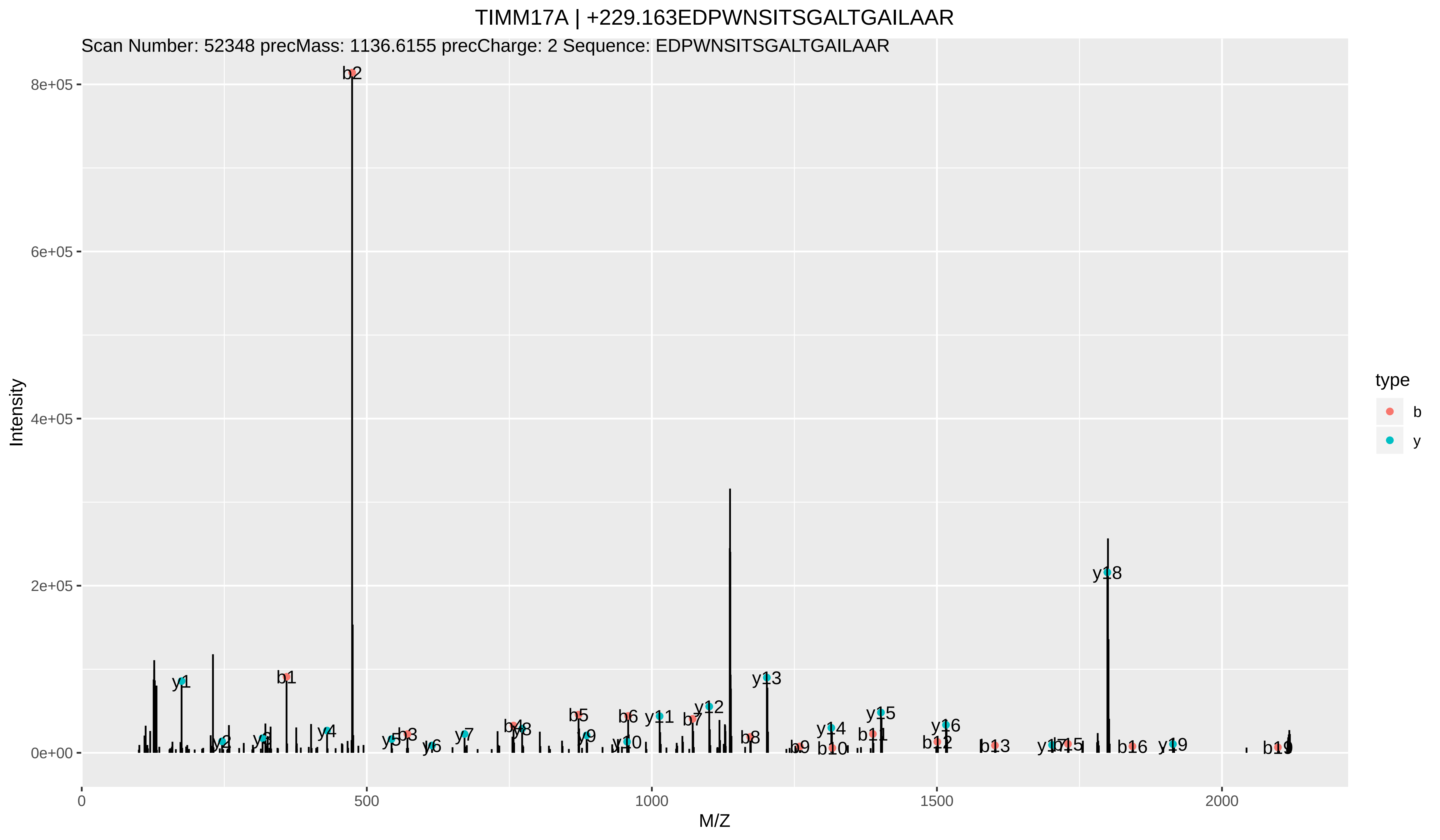
TIMM17A | +229.163EDPWNSITSGALTGAILAAR
Scan Number: 52348 precMass: 1136.6155 precCharge: 2 Sequence: EDPWNSITSGALTGAILAAR
Intensity
M/Z
type
b
y

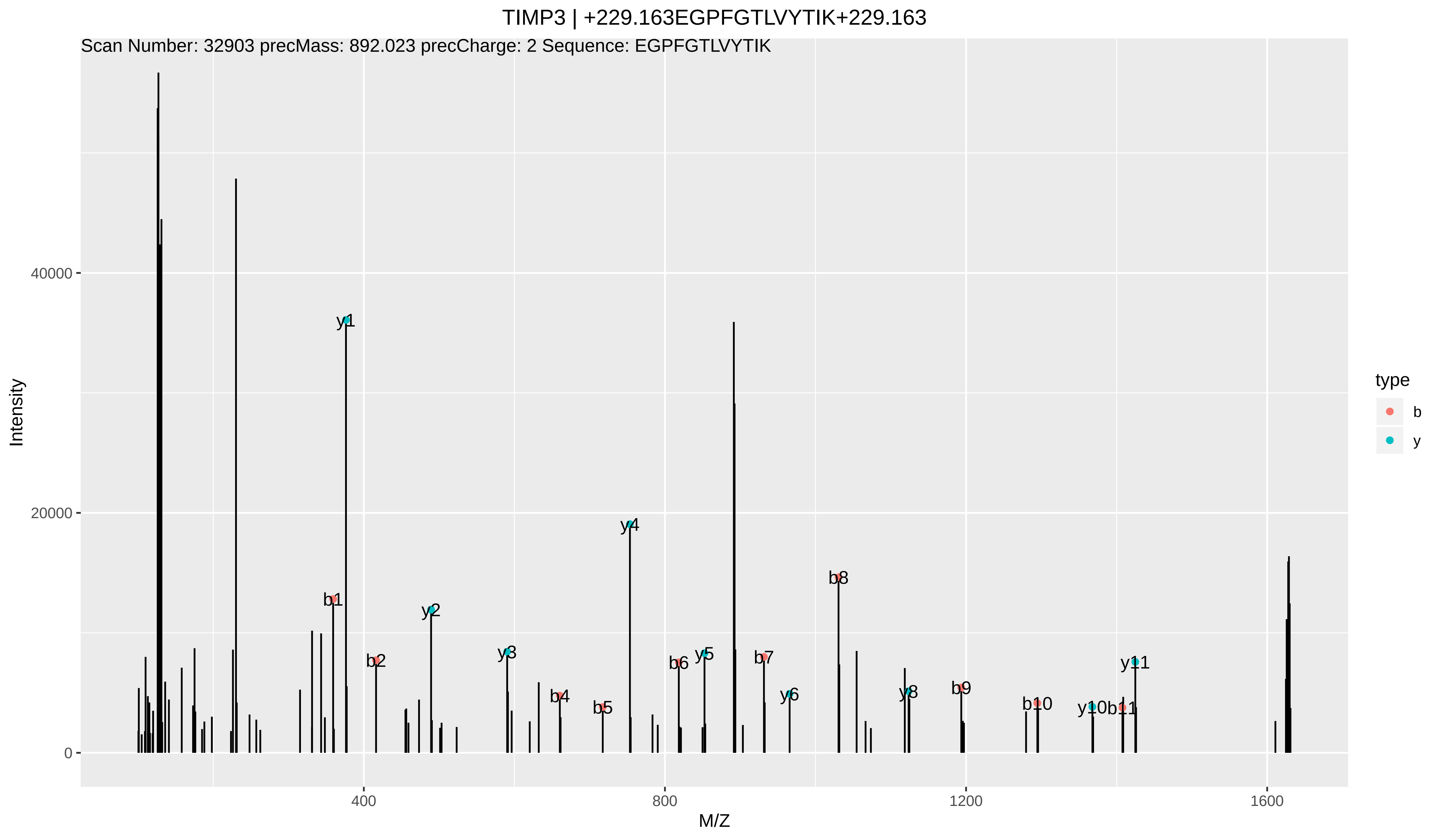
TIMP3 | +229.163EGPFGTLVYTIK+229.163
Scan Number: 32903 precMass: 892.023 precCharge: 2 Sequence: EGPFGTLVYTIK
Intensity
M/Z
0
20000
40000
400
800
1200
1600
type
b
y
y1
b1
y2
b2
y3
b4
b5
y4
b6
y5
b7
y6
b8
y8
b9
b10
y10
b11
y11

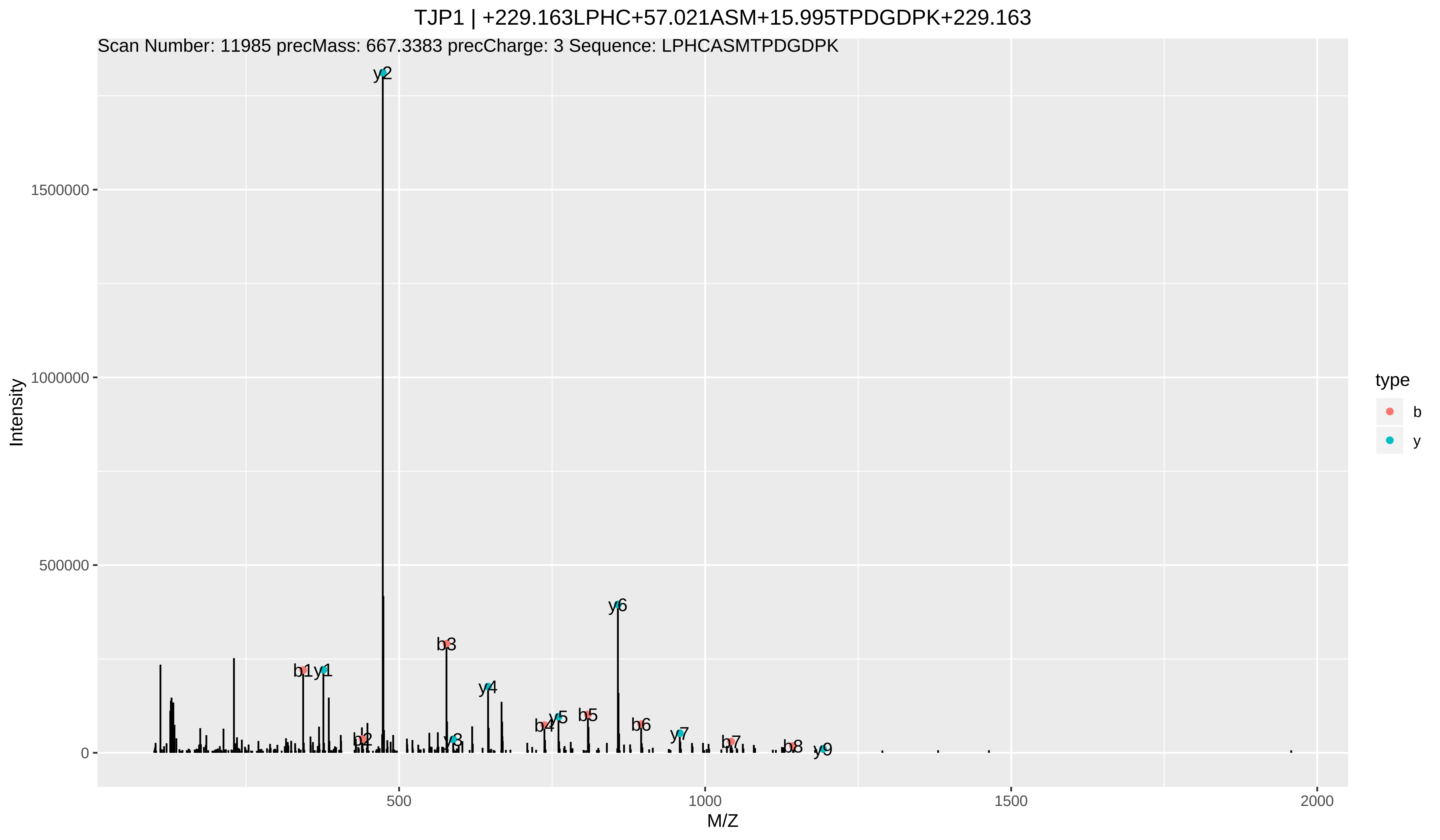

TJP1 | +229.163LPHC+57.021ASM+15.995TPDGDPK+229.163
Scan Number: 11985 precMass: 667.3383 precCharge: 3 Sequence: LPHCASMTPDGDPK
Intensity
M/Z
type
b
y
y2
y6
b3
b1
y1
y4
b5
y5
b4
b6
y7
b2
y3
b7
b8
y9
1500000
1000000
500000
0
500
1000
1500
2000

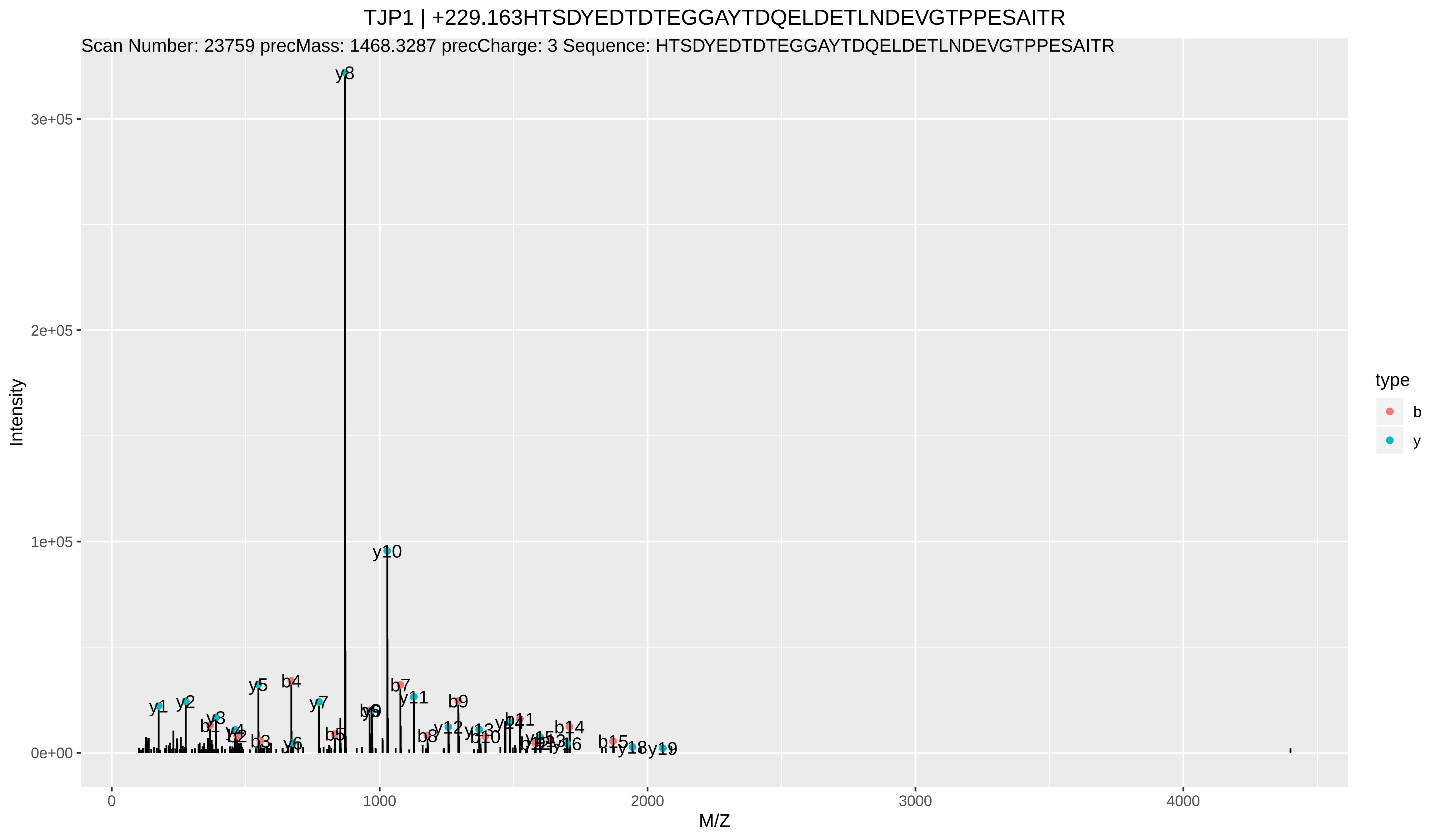
TJP1 | +229.163HTSDYEDTDTEGGAYTDQELDETLNDEVGTPPESAITR
Scan Number: 23759 precMass: 1468.3287 precCharge: 3 Sequence: HTSDYEDTDTEGGAYTDQELDETLNDEVGTPPESAITR
Intensity
M/Z
type
b
y
3e+05
2e+05
1e+05
0e+00
0
1000
2000
3000
4000
y8
y10
b4
y5
b7
y11
y7
b9
y2
y1
y3
b1
y4
b2
b3
y6
b5
b8
y12
y13
b10
b11
b14
y16
b15
y18
y19

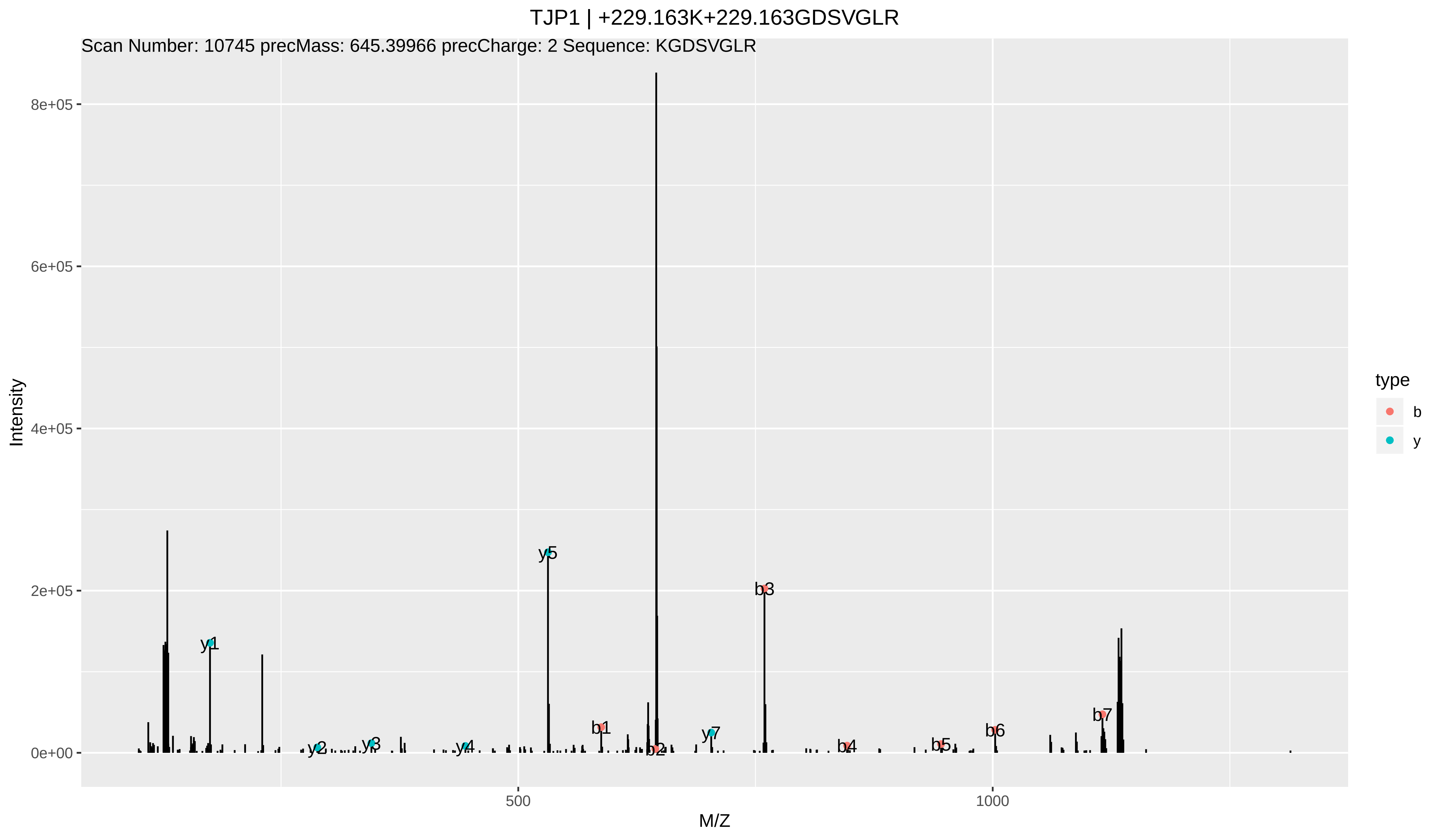

TJP1 | +229.163K+229.163GDSVGLR
Scan Number: 10745 precMass: 645.39966 precCharge: 2 Sequence: KGDSVGLR
Intensity
8e+05
6e+05
4e+05
2e+05
0e+00
M/Z
500
1000
y1
y2
y3
y4
y5
y7
b1
b2
b3
b4
b5
b6
b7
type
b
y

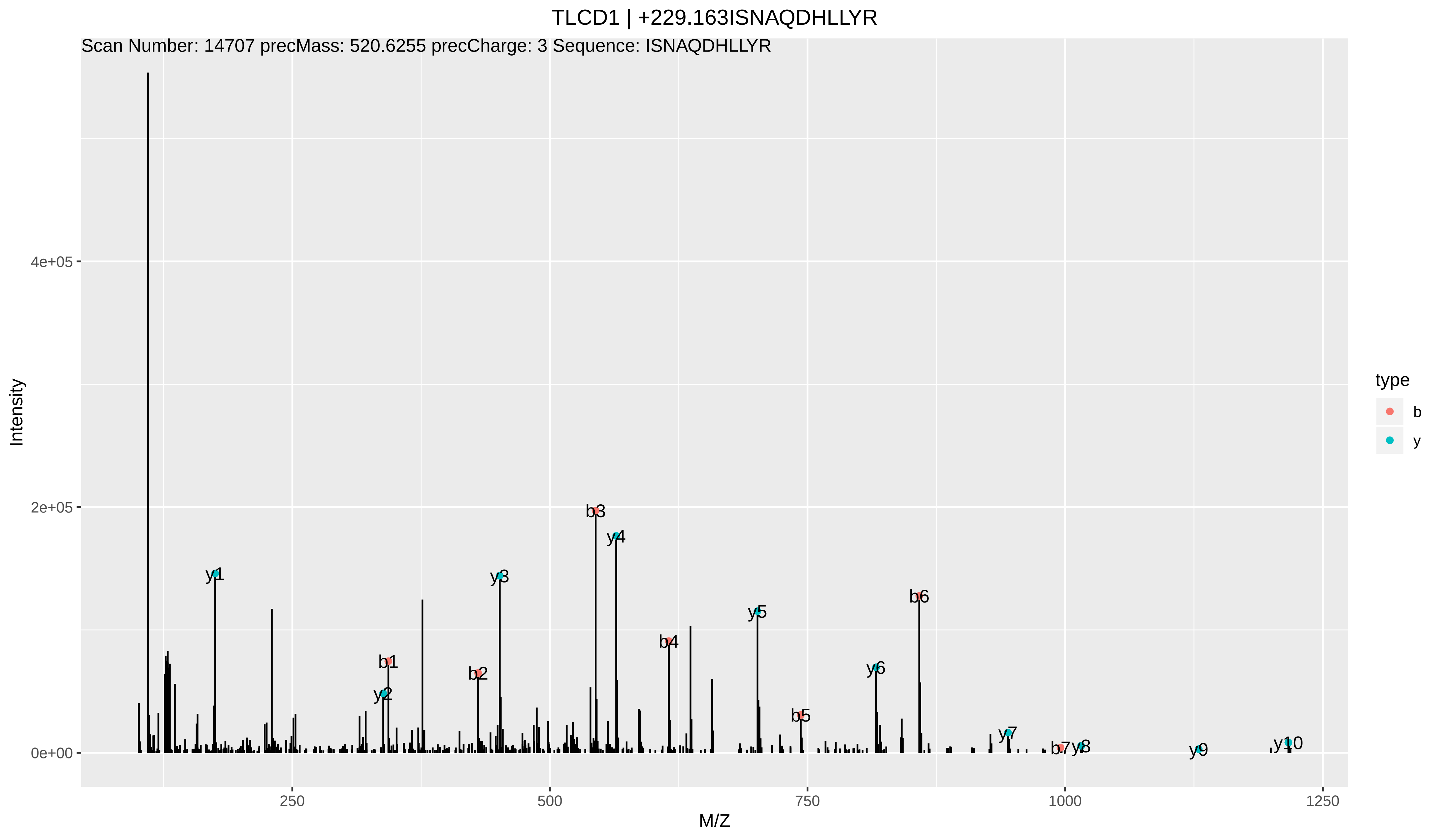

TLCD1 | +229.163ISNAQDHLLYR
Scan Number: 14707 precMass: 520.6255 precCharge: 3 Sequence: ISNAQDHLLYR
Intensity
4e+05
2e+05
0e+00
M/Z
250
500
750
1000
1250
y1
b1
y2
b2
y3
b3
y4
b4
y5
b5
y6
b6
y7
b7
y8
y9
y10
type
b
y

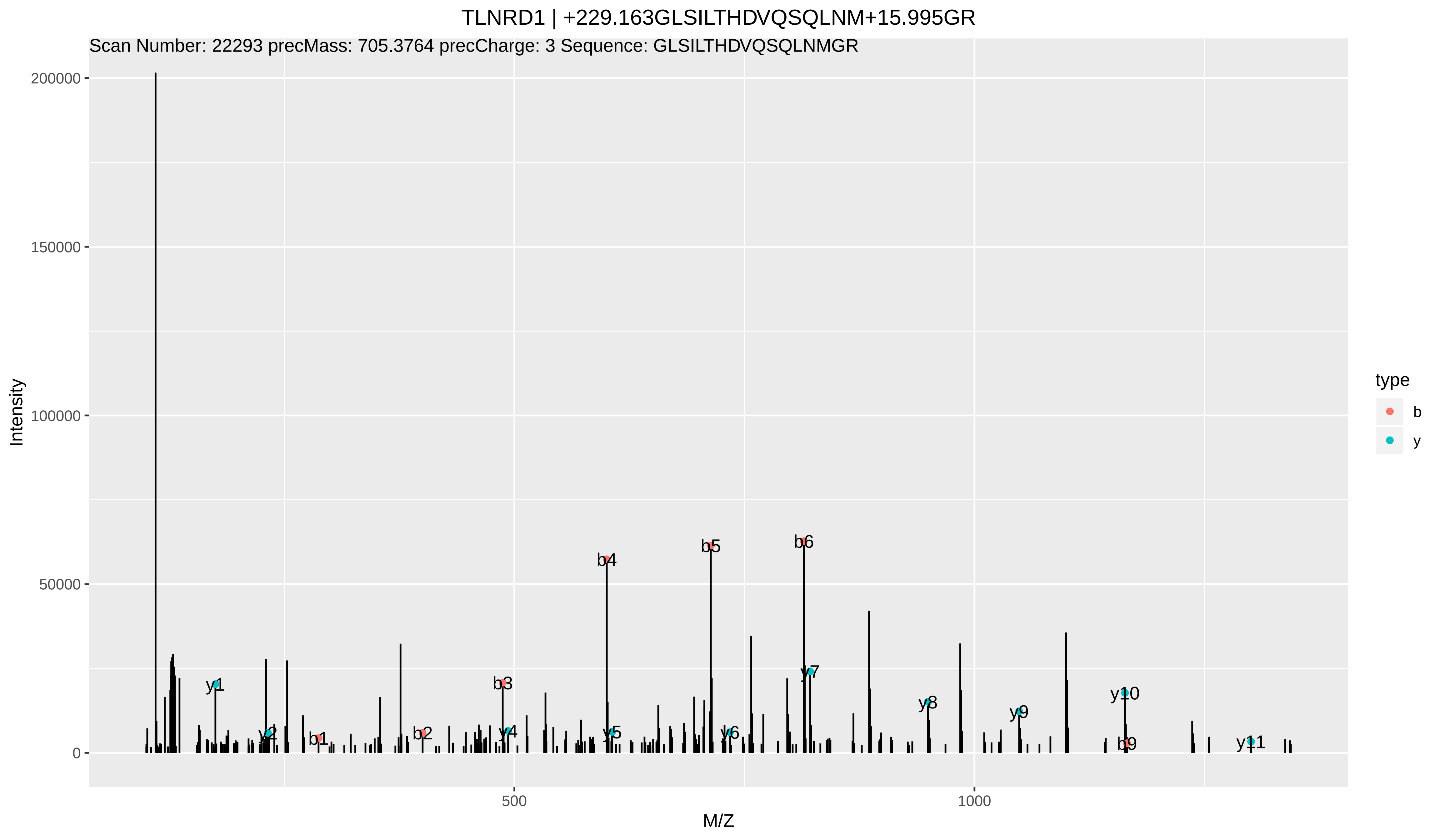
TLNRD1 | +229.163GLSILTHDVQSQLNM+15.995GR
Scan Number: 22293 precMass: 705.3764 precCharge: 3 Sequence: GLSILTHDVQSQLNMGR
Intensity
M/Z
type
b
y
y1
y2
b1
b2
b3
y4
b4
y5
b5
y6
b6
y7
y8
y9
y10
b9
y11

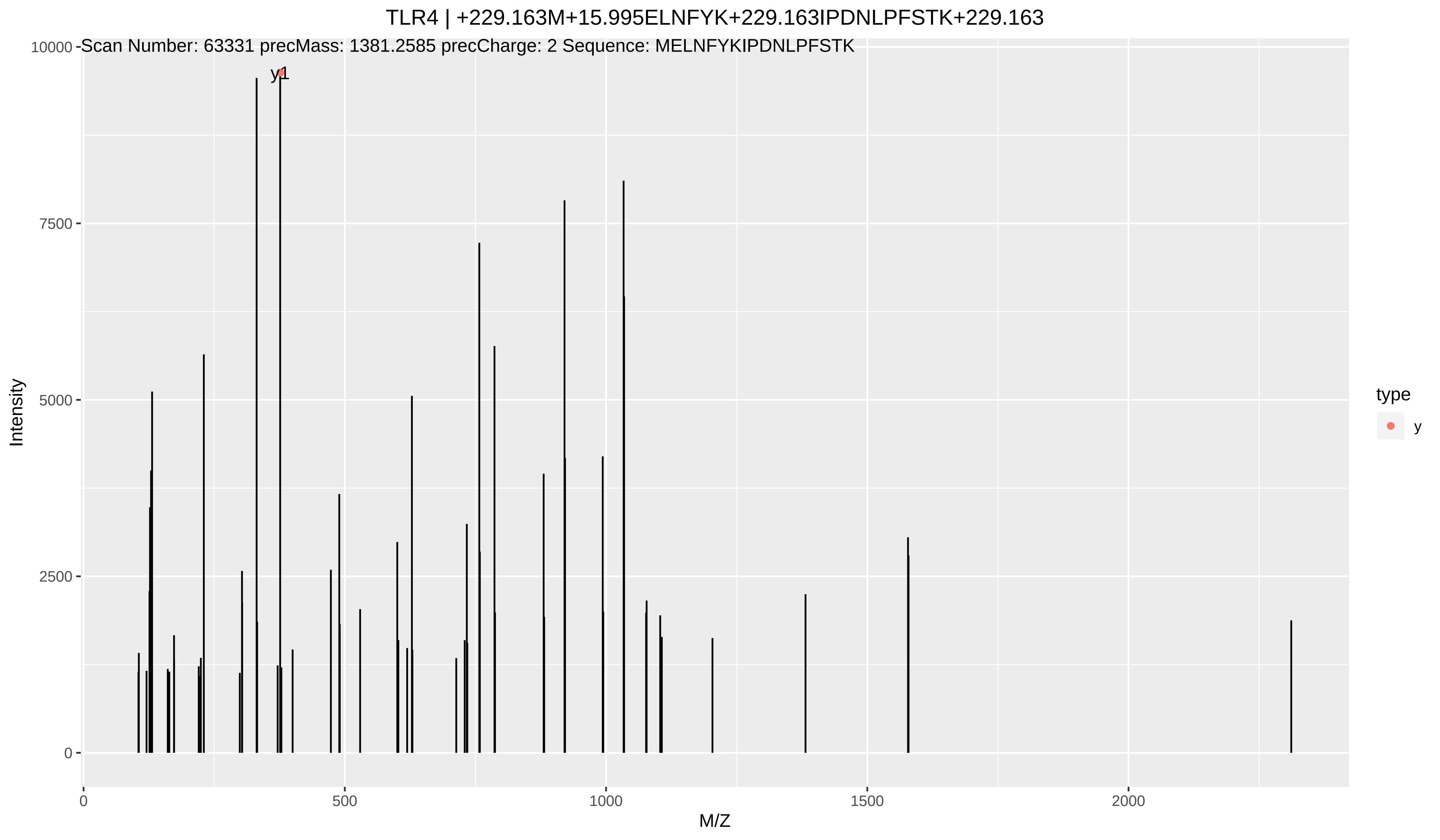

TLR4 | +229.163M+15.995ELNFYK+229.163IPDNLPFSTK+229.163
Scan Number: 63331 precMass: 1381.2585 precCharge: 2 Sequence: MELNFYKIPDNLPFSTK
y1
Intensity
10000
7500
5000
2500
0
0
500
1000
1500
2000
M/Z
type
y

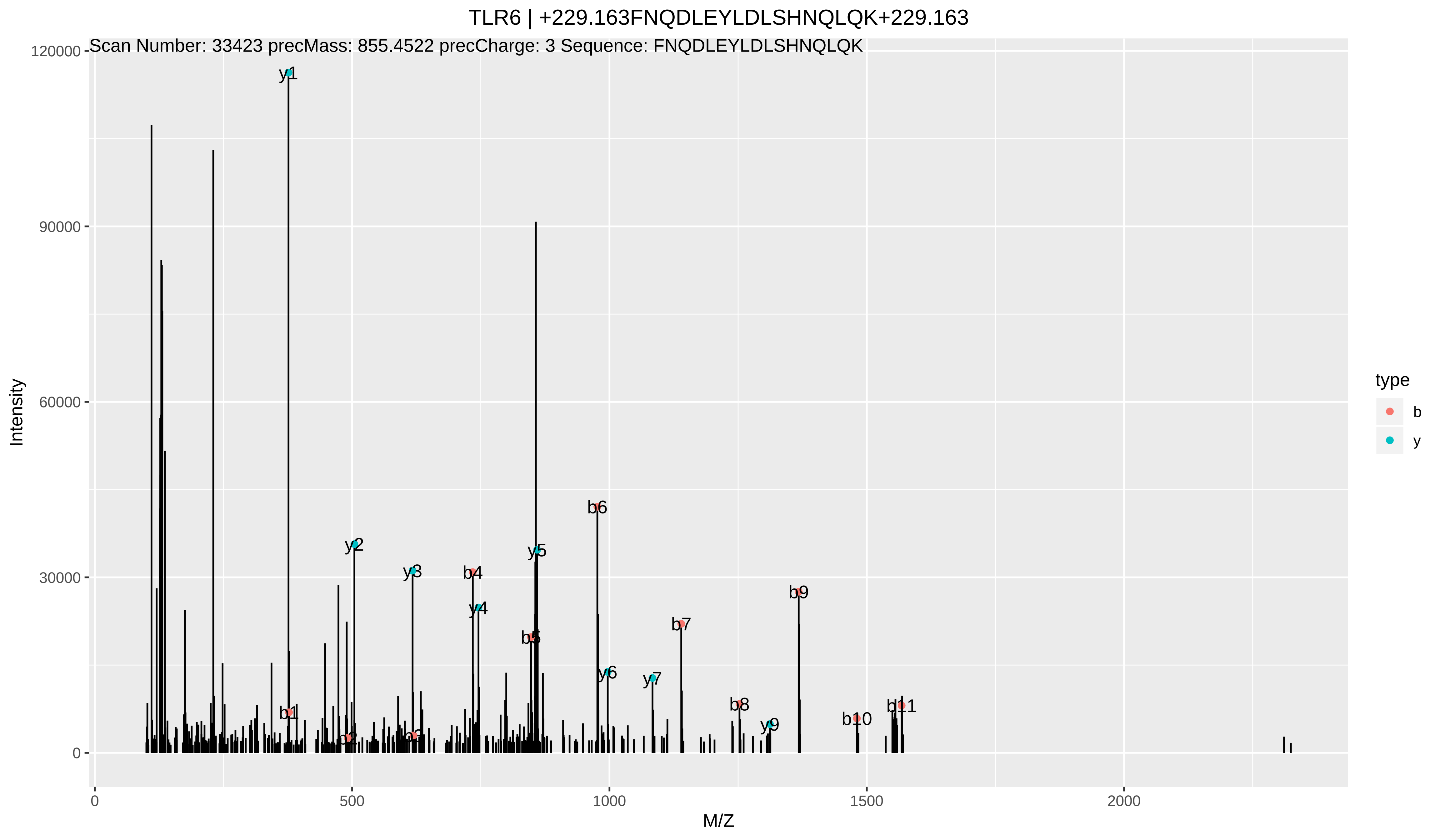
TLR6 | +229.163FNQDLEYLDLSHNQLQK+229.163
Scan Number: 33423 precMass: 855.4522 precCharge: 3 Sequence: FNQDLEYLDLSHNQLQK
Intensity
M/Z
type
b
y
120000
90000
60000
30000
0
0
500
1000
1500
2000
y1
y2
y3
y4
y5
y6
y7
y9
b1
b4
b5
b6
b7
b8
b9
b10
b11

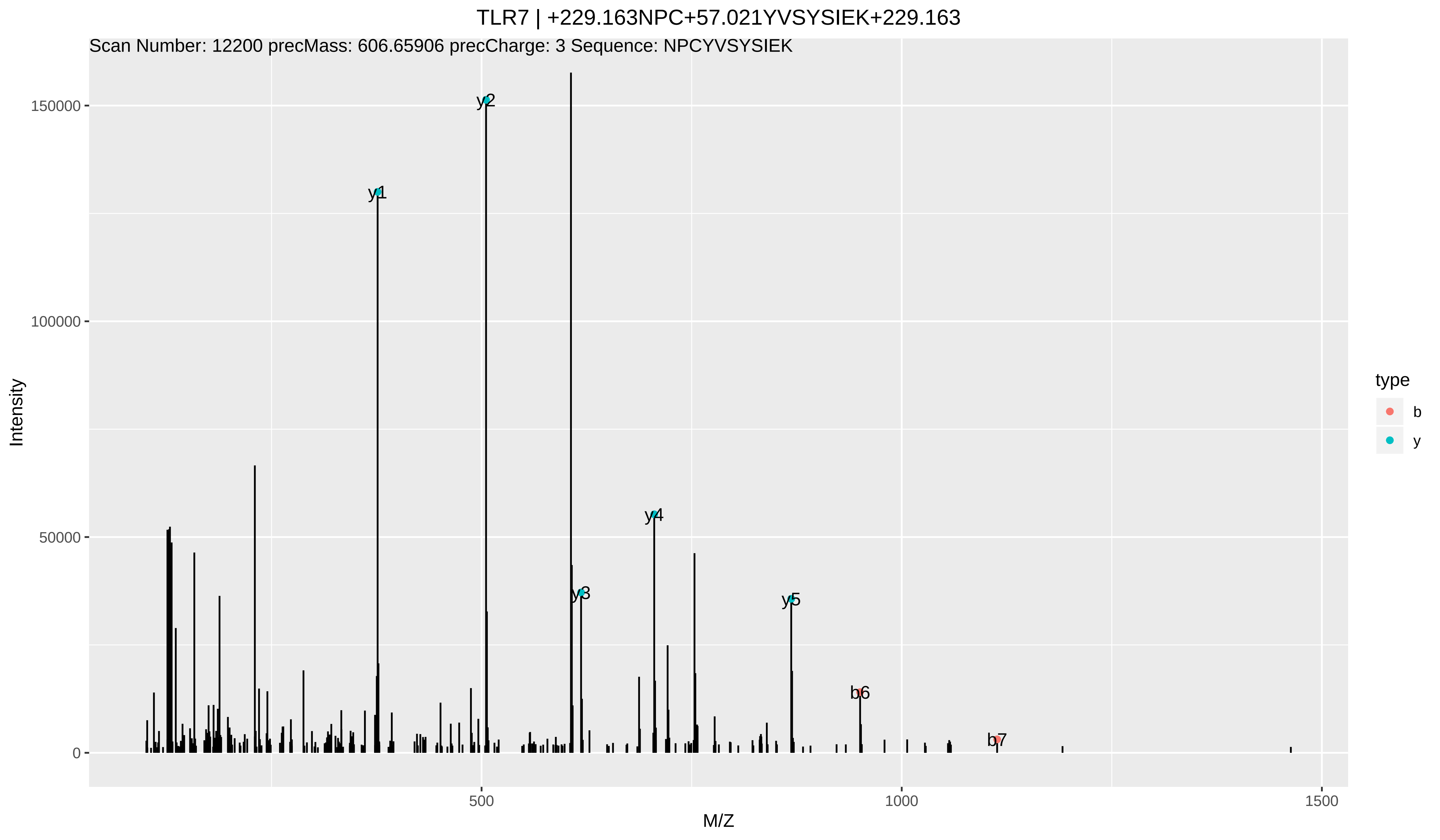
TLR7 | +229.163NPC+57.021YVSYSIEK+229.163
Scan Number: 12200 precMass: 606.65906 precCharge: 3 Sequence: NPCYVSYSIEK
Intensity
M/Z
150000
100000
50000
0
500
1000
1500
y1
y2
y3
y4
y5
b6
b7
type
b
y

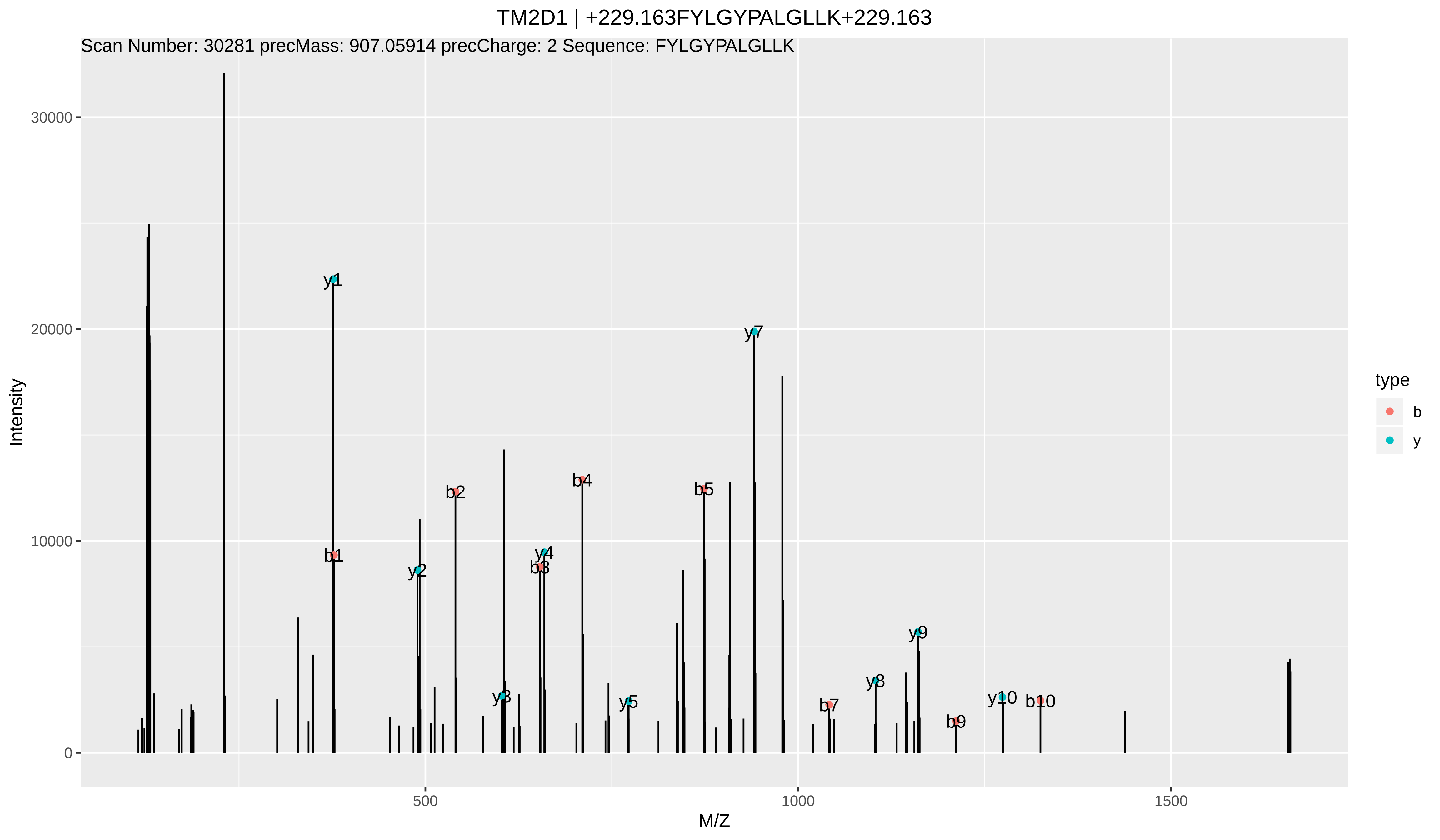

TM2D1 | +229.163FYLGYPALGLLK+229.163
Scan Number: 30281 precMass: 907.05914 precCharge: 2 Sequence: FYLGYPALGLLK
Intensity
M/Z
30000
20000
10000
0
500
1000
1500
y1
b1
y2
b2
y3
b3
y4
b4
y5
b5
y7
b7
y8
y9
b9
y10
b10
type
b
y

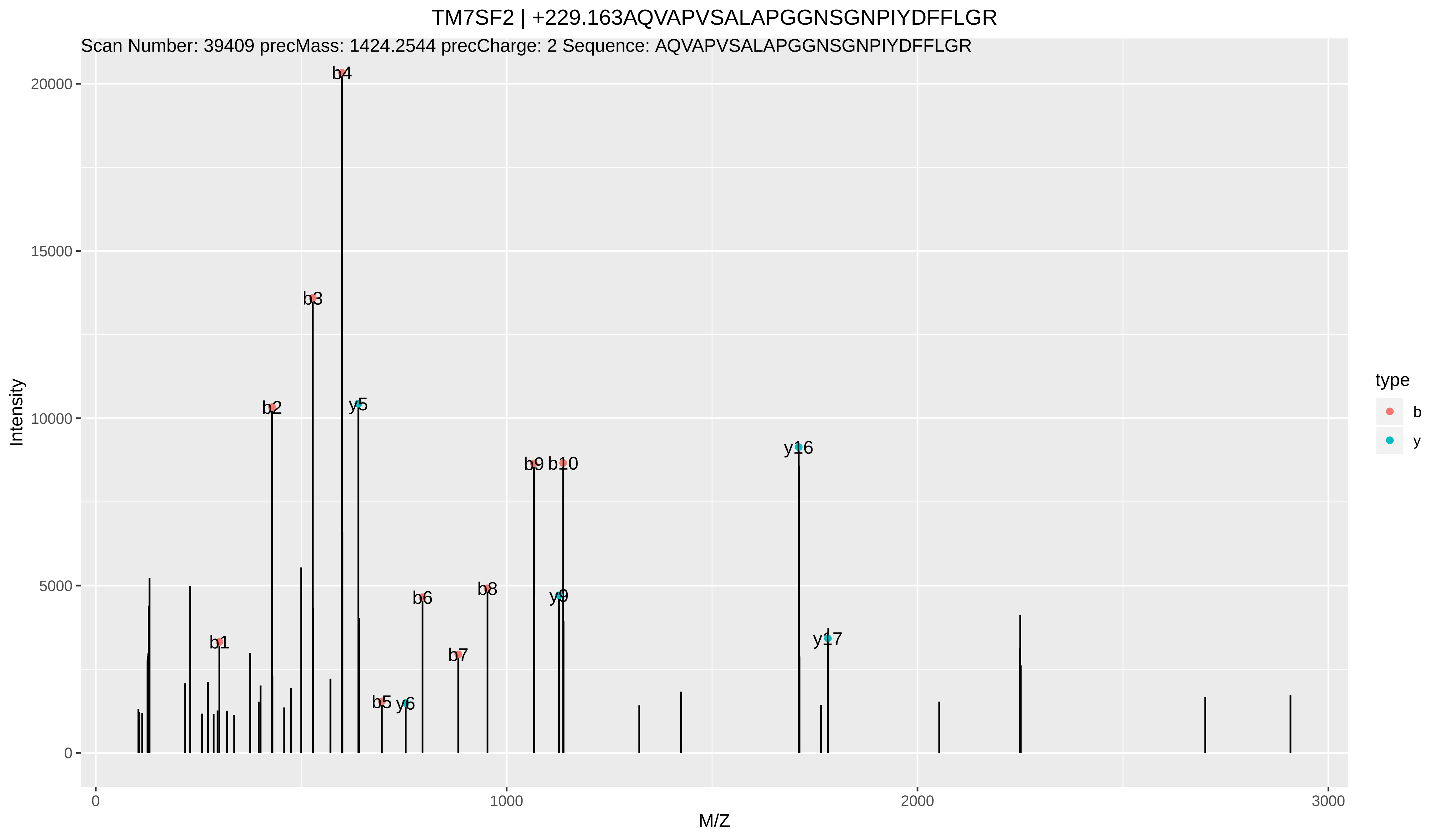

TM7SF2 | +229.163AQVAPVSALAPGGNSGNPIYDFFLGR
Scan Number: 39409 precMass: 1424.2544 precCharge: 2 Sequence: AQVAPVSALAPGGNSGNPIYDFFLGR
Intensity
M/Z
type
b
y
b1
b2
b3
b4
b5
b6
b7
b8
b9
b10
y5
y6
y9
y16
y17
0
5000
10000
15000
20000
1000
2000
3000

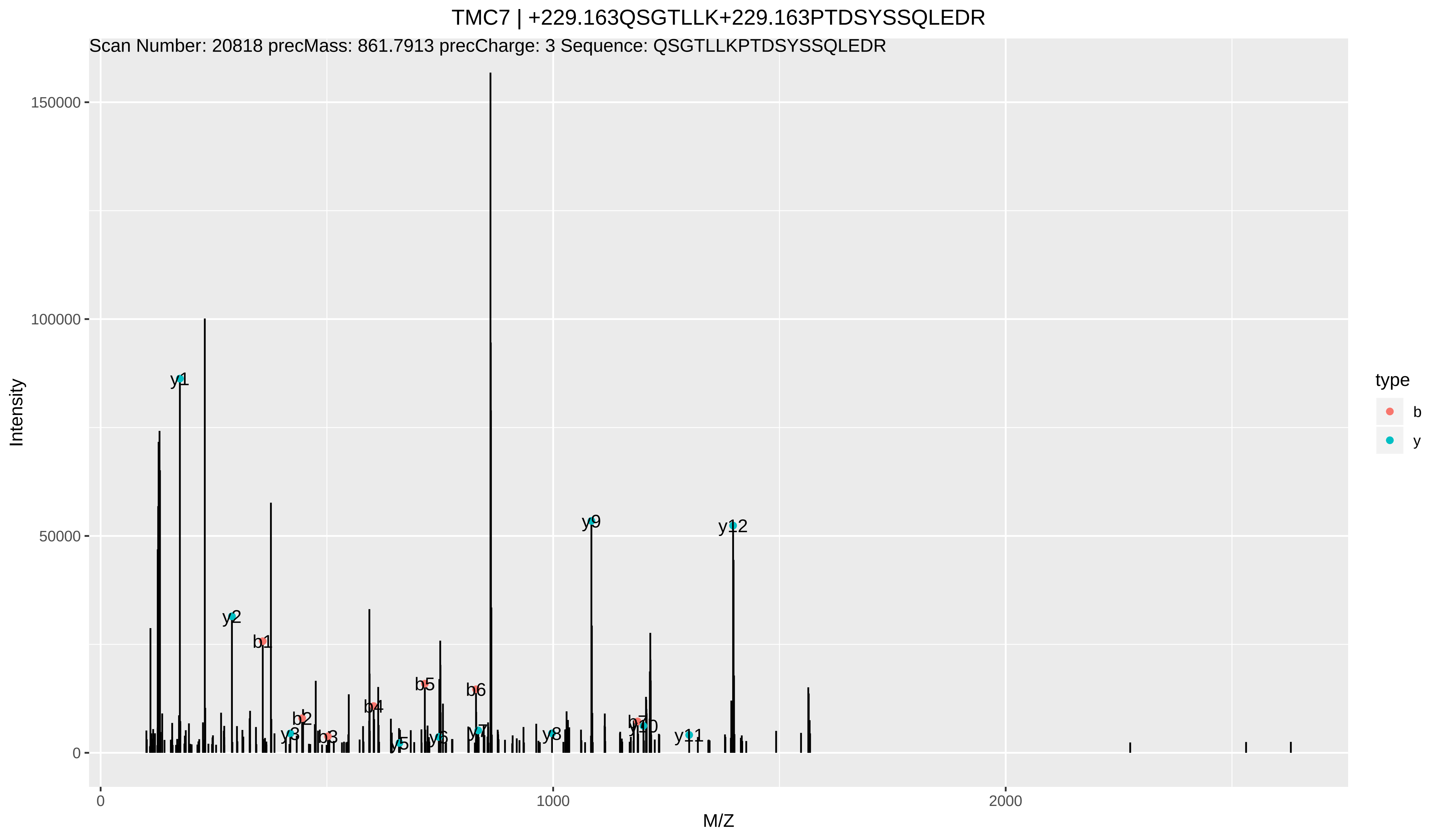

TMC7 | +229.163QSGTLLK+229.163PTDSYSSQLEDR
Scan Number: 20818 precMass: 861.7913 precCharge: 3 Sequence: QSGTLLKPTDSYSSQLEDR
Intensity
M/Z
type
b
y
y1
y2
b1
y3
b2
b3
b4
y5
b5
y6
b6
y7
y8
y9
b7
y10
y11
y12
0
50000
100000
150000
0
1000
2000

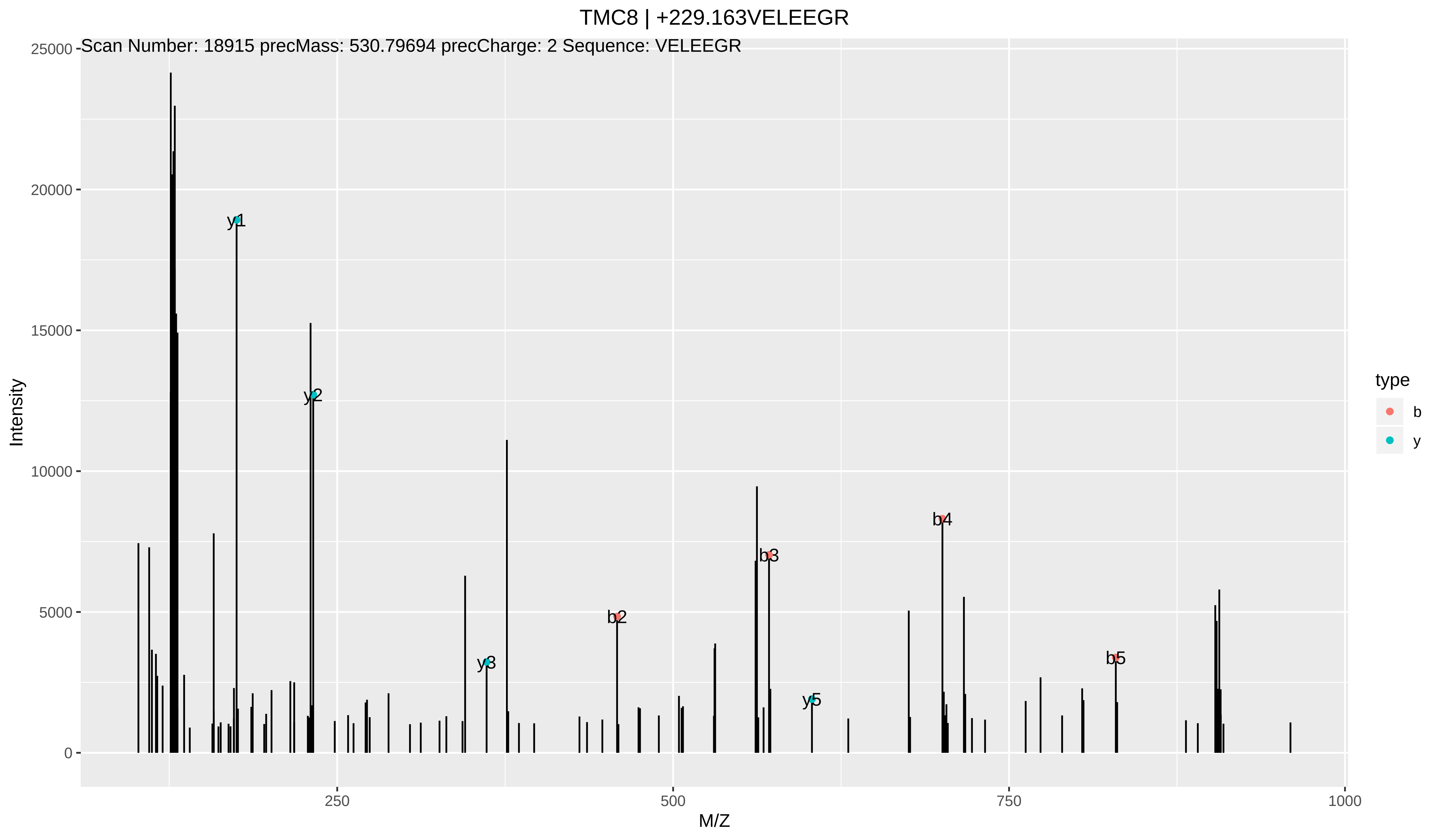

TMC8 | +229.163VELEEGR
Scan Number: 18915 precMass: 530.79694 precCharge: 2 Sequence: VELEEGR
Intensity
M/Z
type
b
y
y1
y2
y3
y5
b2
b3
b4
b5
25000
20000
15000
10000
5000
0
250
500
750
1000

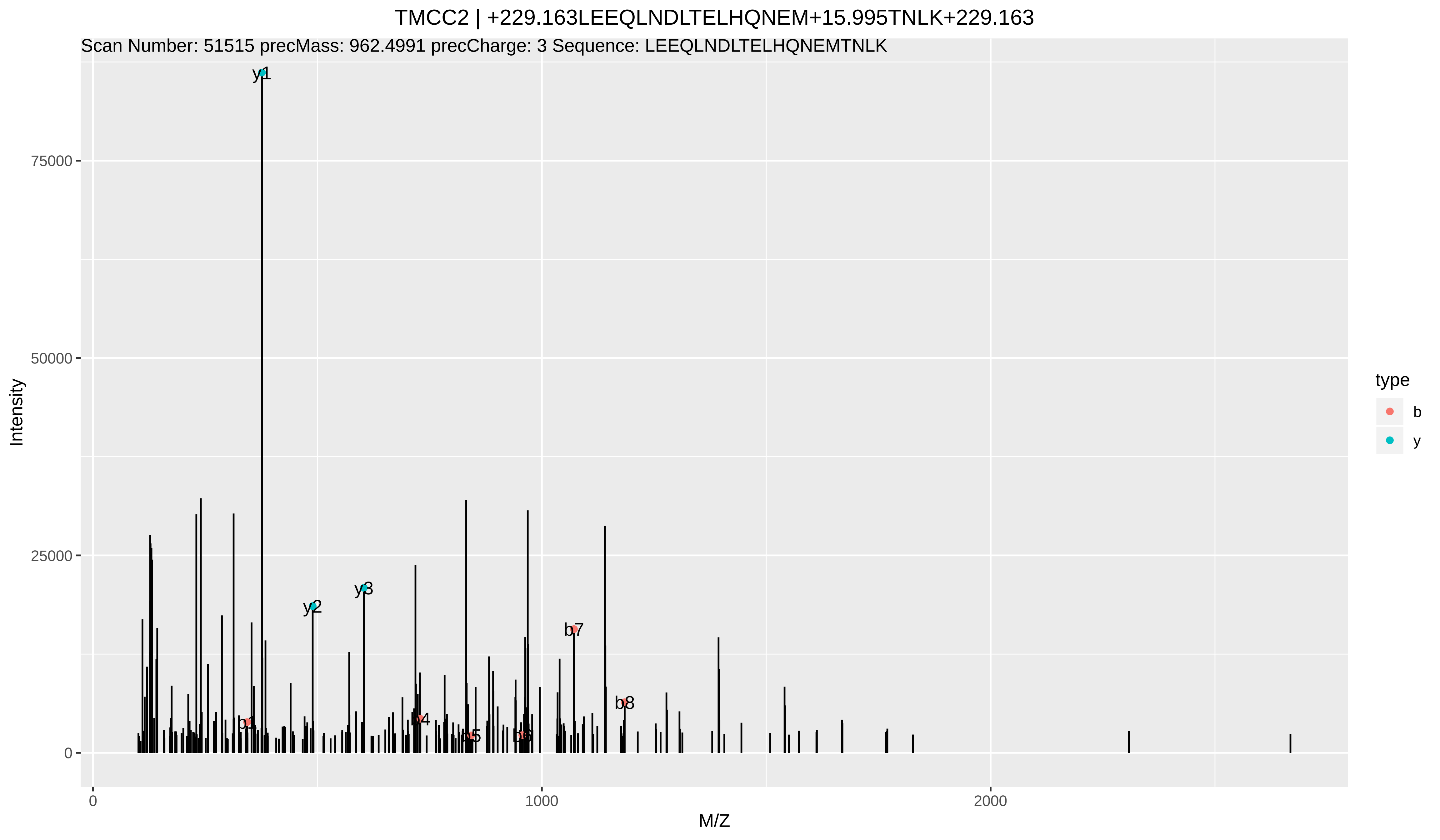
TMCC2 | +229.163LEEQLNDLTELHQNEM+15.995TNLK+229.163
Scan Number: 51515 precMass: 962.4991 precCharge: 3 Sequence: LEEQLNDLTELHQNEMTNLK
y1
y2
y3
b1
b4
b5
b6
b7
b8
Intensity
75000
50000
25000
0
M/Z
0
1000
2000
type
b
y

TMCC2 | +229.163FGSADNIAHLK+229.163

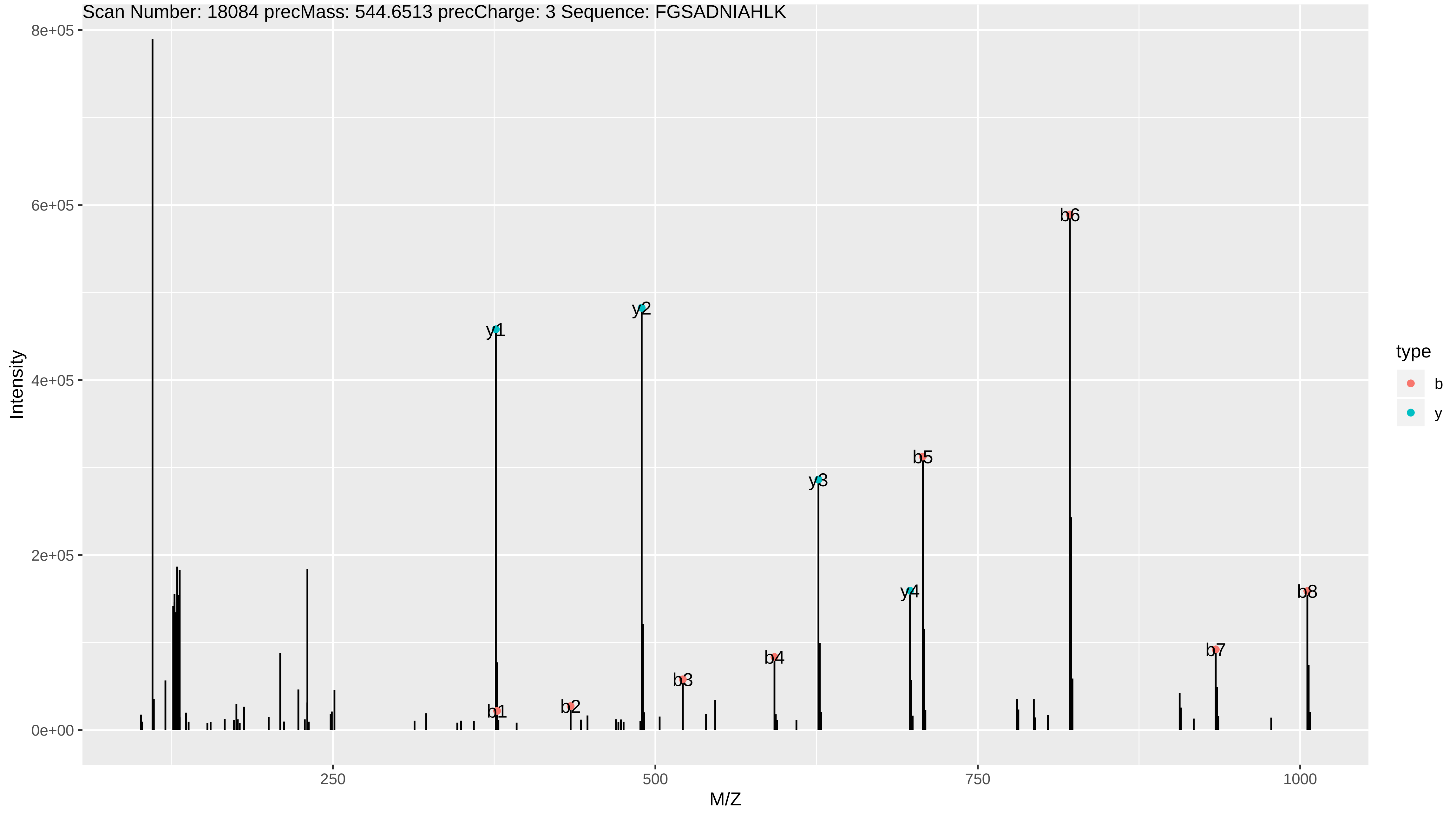

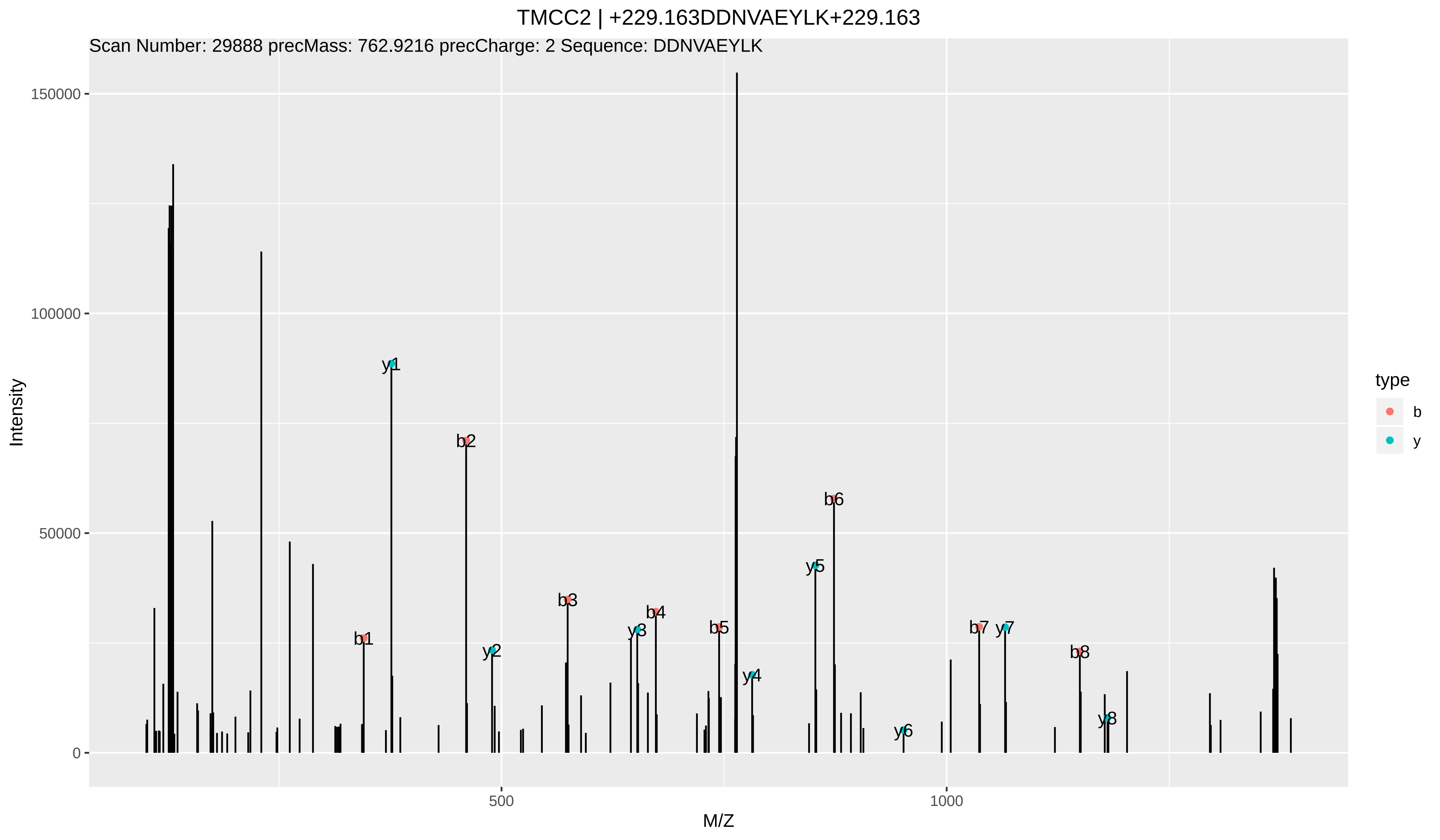
TMCC2 | +229.163DDNVAEYLK+229.163
Scan Number: 29888 precMass: 762.9216 precCharge: 2 Sequence: DDNVAEYLK
Intensity
150000
100000
50000
0
500
1000
M/Z
y1
b2
b6
y5
b3
b4
y3
b5
b1
y2
y4
b7
y7
b8
y8
y6
type
b
y

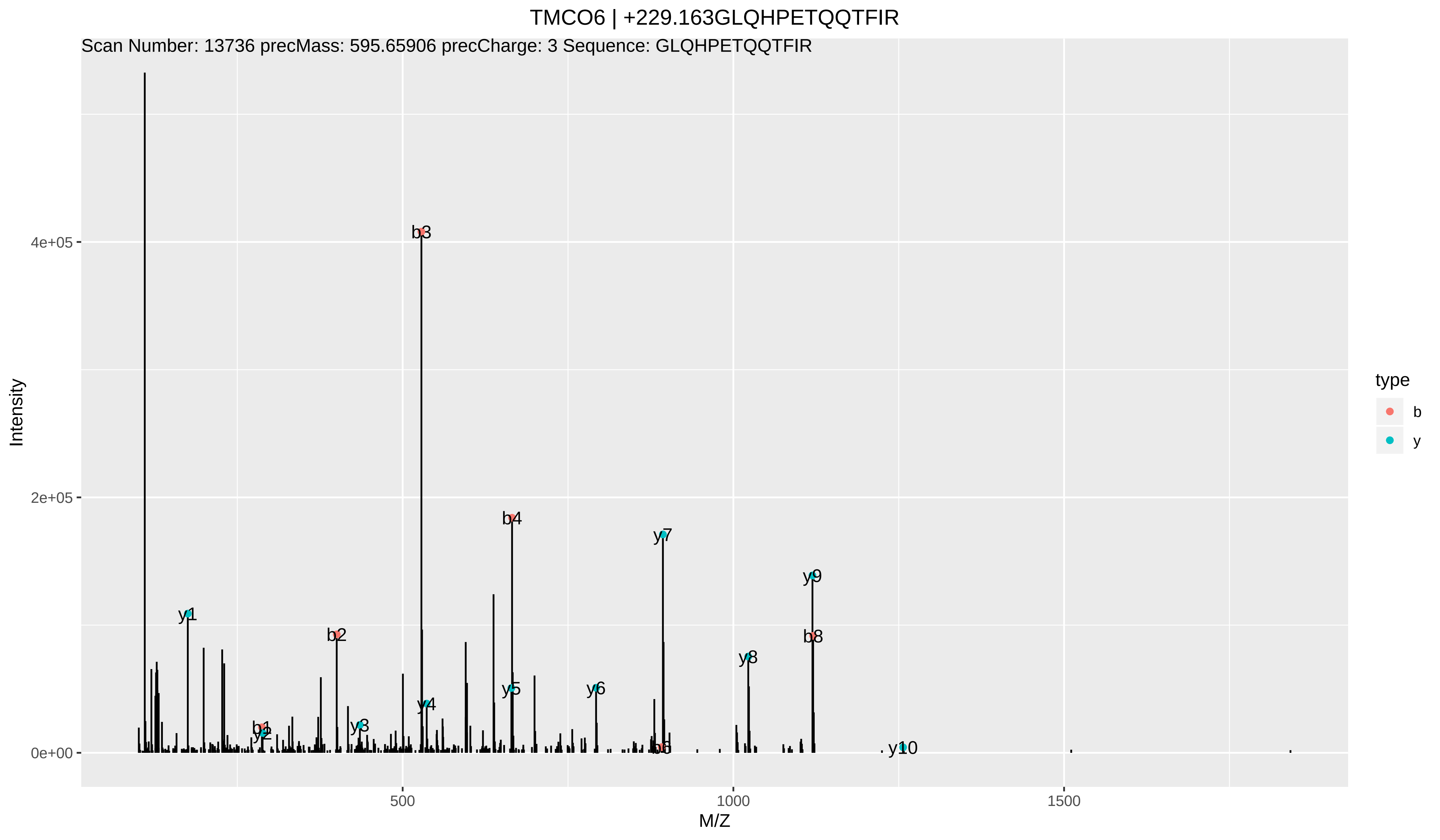

TMCO6 | +229.163GLQHPETQQTFIR
Scan Number: 13736 precMass: 595.65906 precCharge: 3 Sequence: GLQHPETQQTFIR
Intensity
4e+05
2e+05
0e+00
M/Z
500
1000
1500
b3
b4
y7
y9
y1
b2
b8
y8
y5
y6
y4
y3
b1
y2
b6
y10
type
b
y

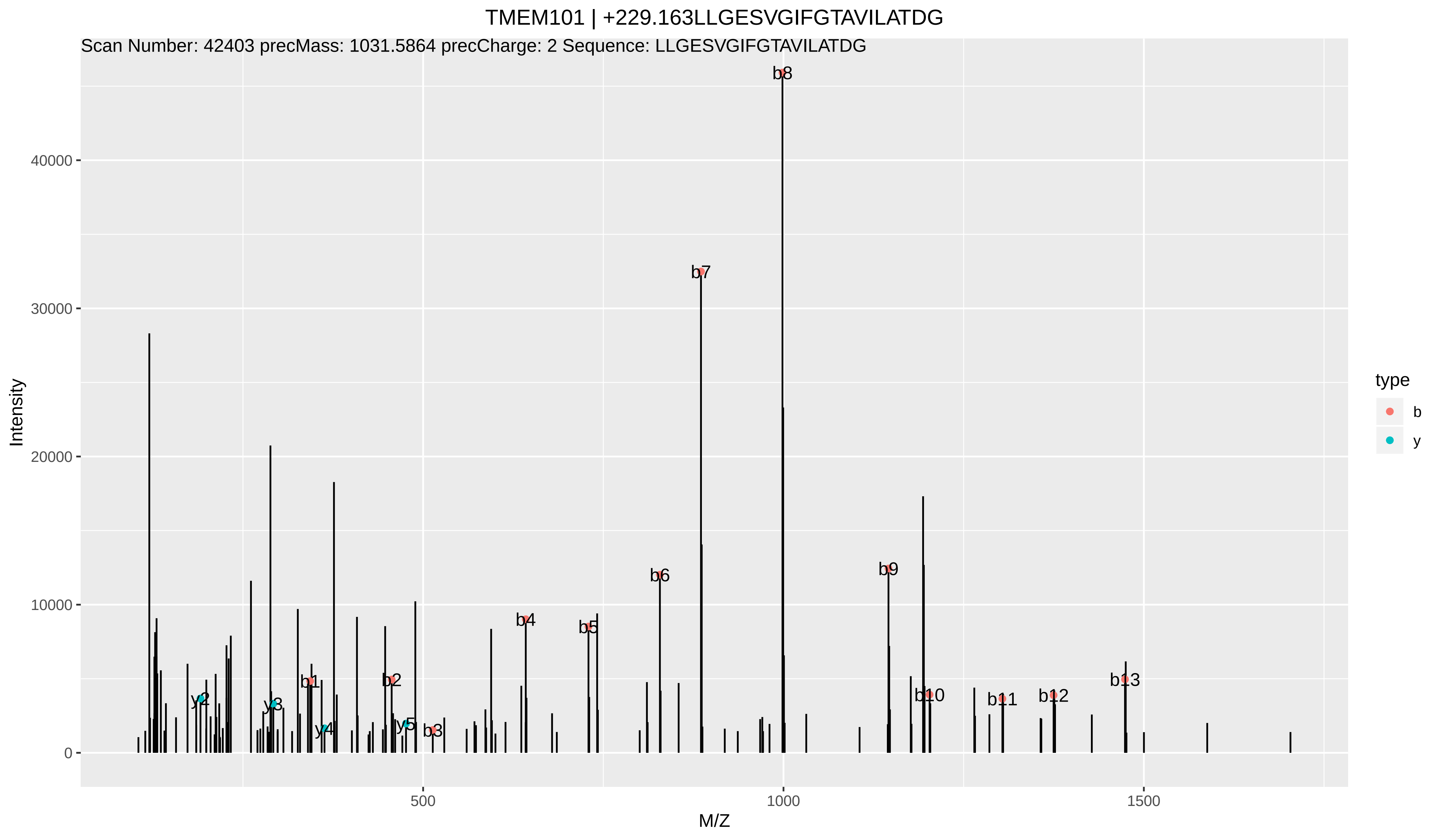

TMEM101 | +229.163LLGESVGIFGTAVILATDG
Scan Number: 42403 precMass: 1031.5864 precCharge: 2 Sequence: LLGESVGIFGTAVILATDG
Intensity
M/Z
type
b
y

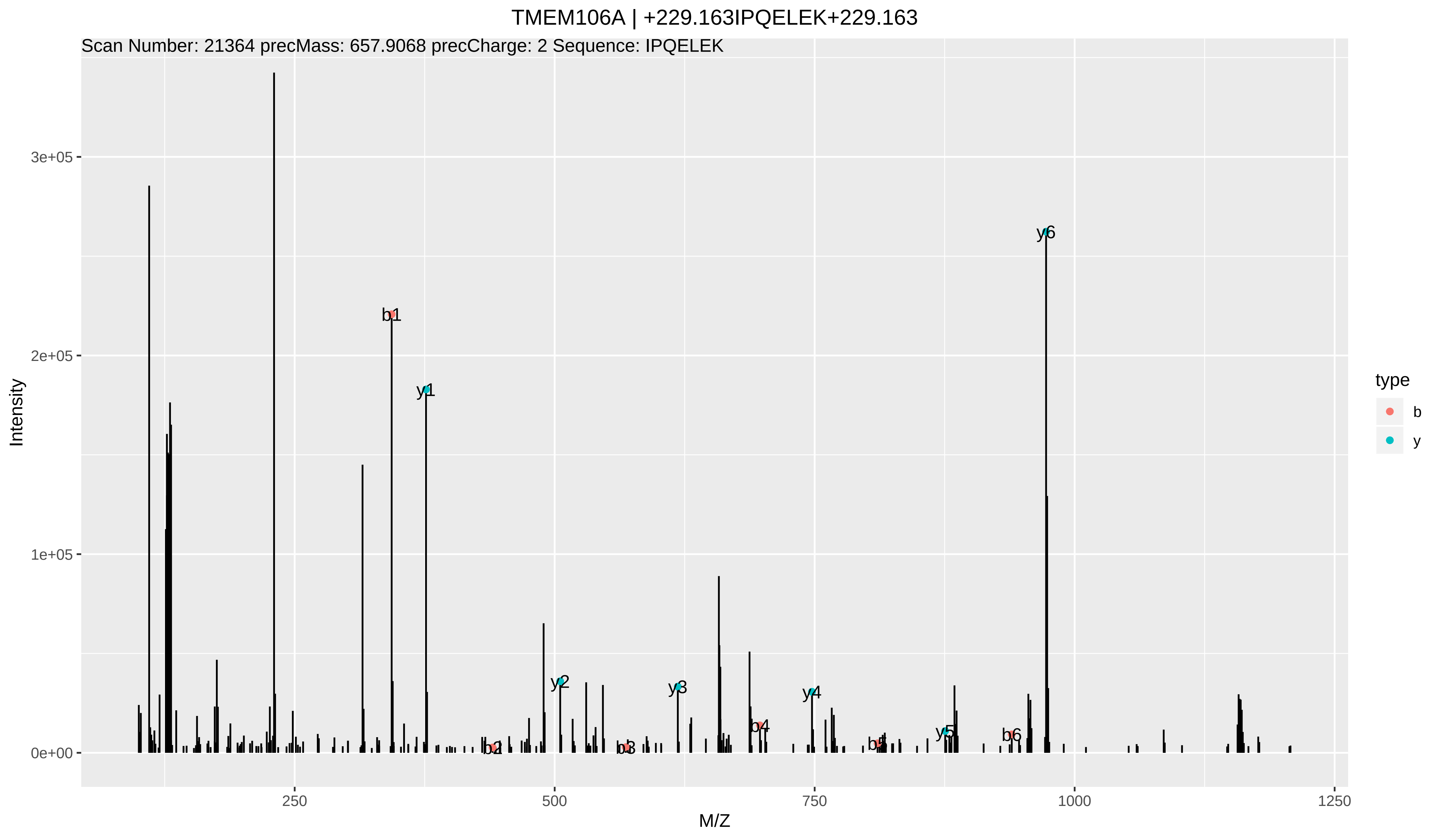
TMEM106A | +229.163IPQELEK+229.163
Scan Number: 21364 precMass: 657.9068 precCharge: 2 Sequence: IPQELEK
Intensity
M/Z
type
b
y
b1
y1
b2
y2
b3
y3
b4
y4
b5
y5
b6
y6
3e+05
2e+05
1e+05
0e+00
250
500
750
1000
1250

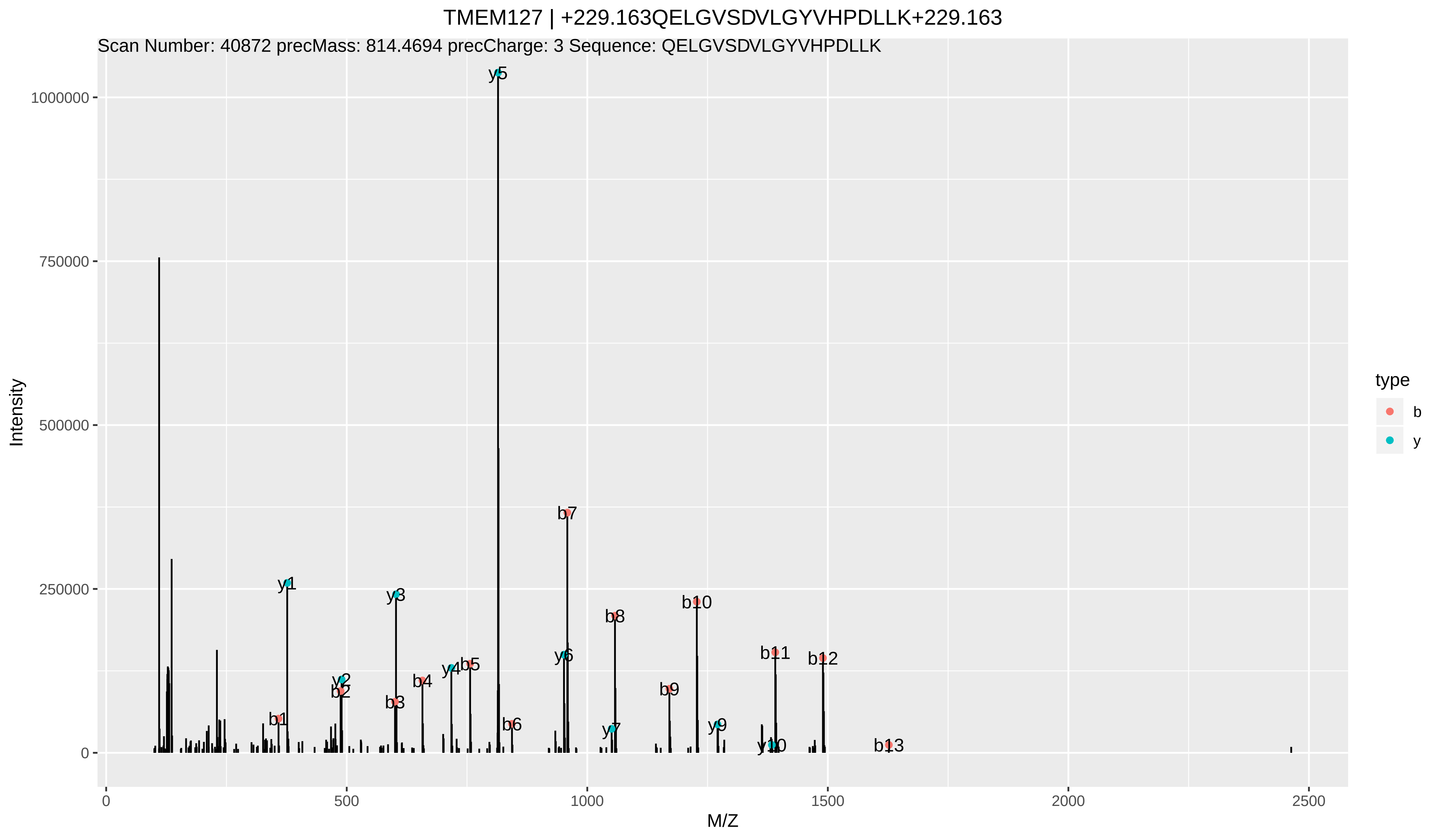

TMEM127 | +229.163QELGVSDVLGYVHPDLLK+229.163
Scan Number: 40872 precMass: 814.4694 precCharge: 3 Sequence: QELGVSDVLGYVHPDLLK
Intensity
1000000
750000
500000
250000
0
M/Z
0
500
1000
1500
2000
2500
y5
b7
y1
y3
b10
b8
y6
y4
b5
b11
b12
y2
b2
b4
b9
b3
b1
b6
y7
y9
y10
b13
type
b
y

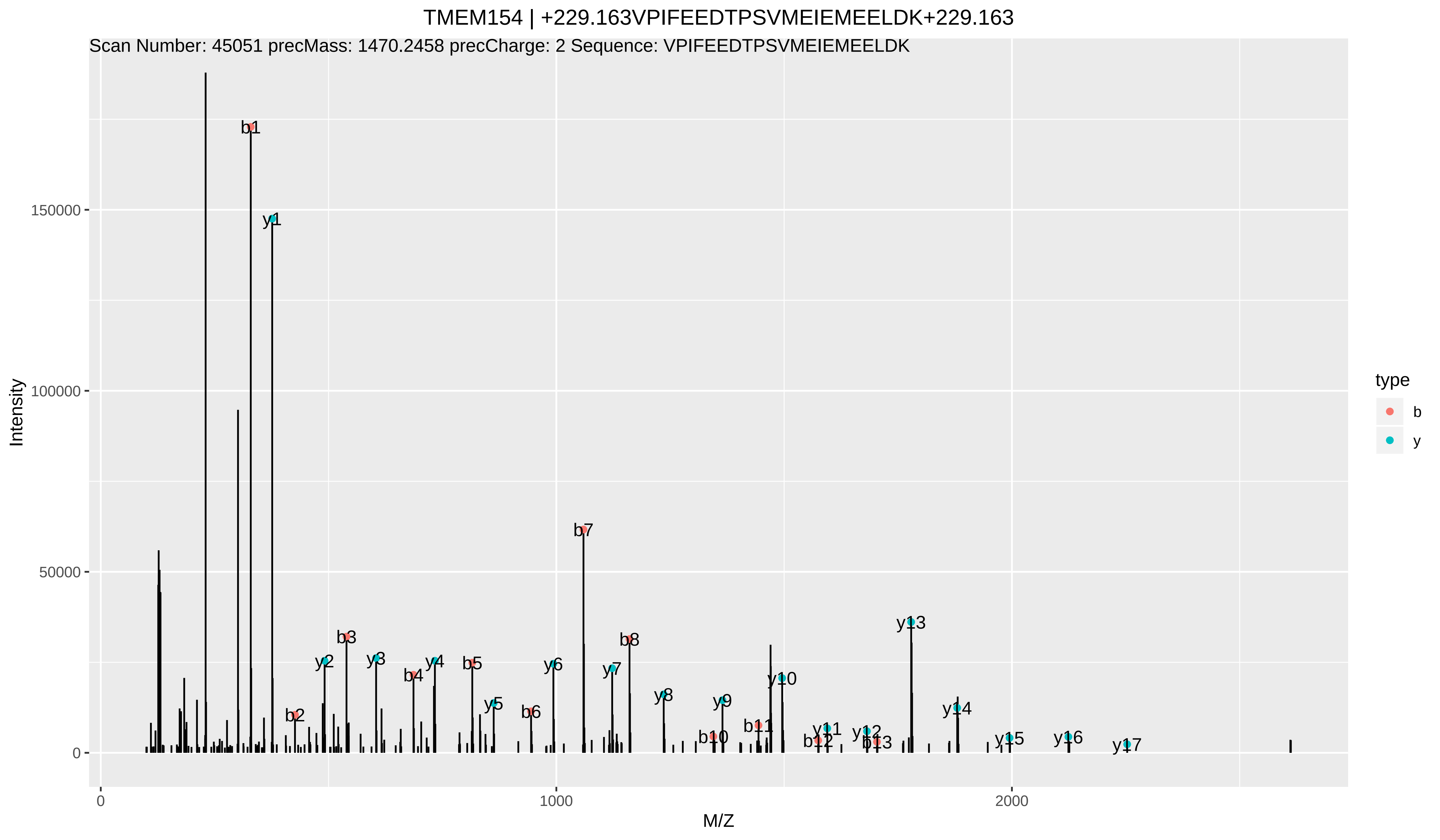
TMEM154 | +229.163VPIFEEDTPSVMEIEMEELDK+229.163
Scan Number: 45051 precMass: 1470.2458 precCharge: 2 Sequence: VPIFEEDTPSVMEIEMEELDK
Intensity
M/Z
type
b
y
0
50000
100000
150000
1000
2000
b1
y1
b2
y2
b3
y3
b4
y4
b5
y5
b6
y6
b7
y7
b8
y8
y9
b10
y10
b11
y11
b12
y12
b13
y13
y14
y15
y16
y17

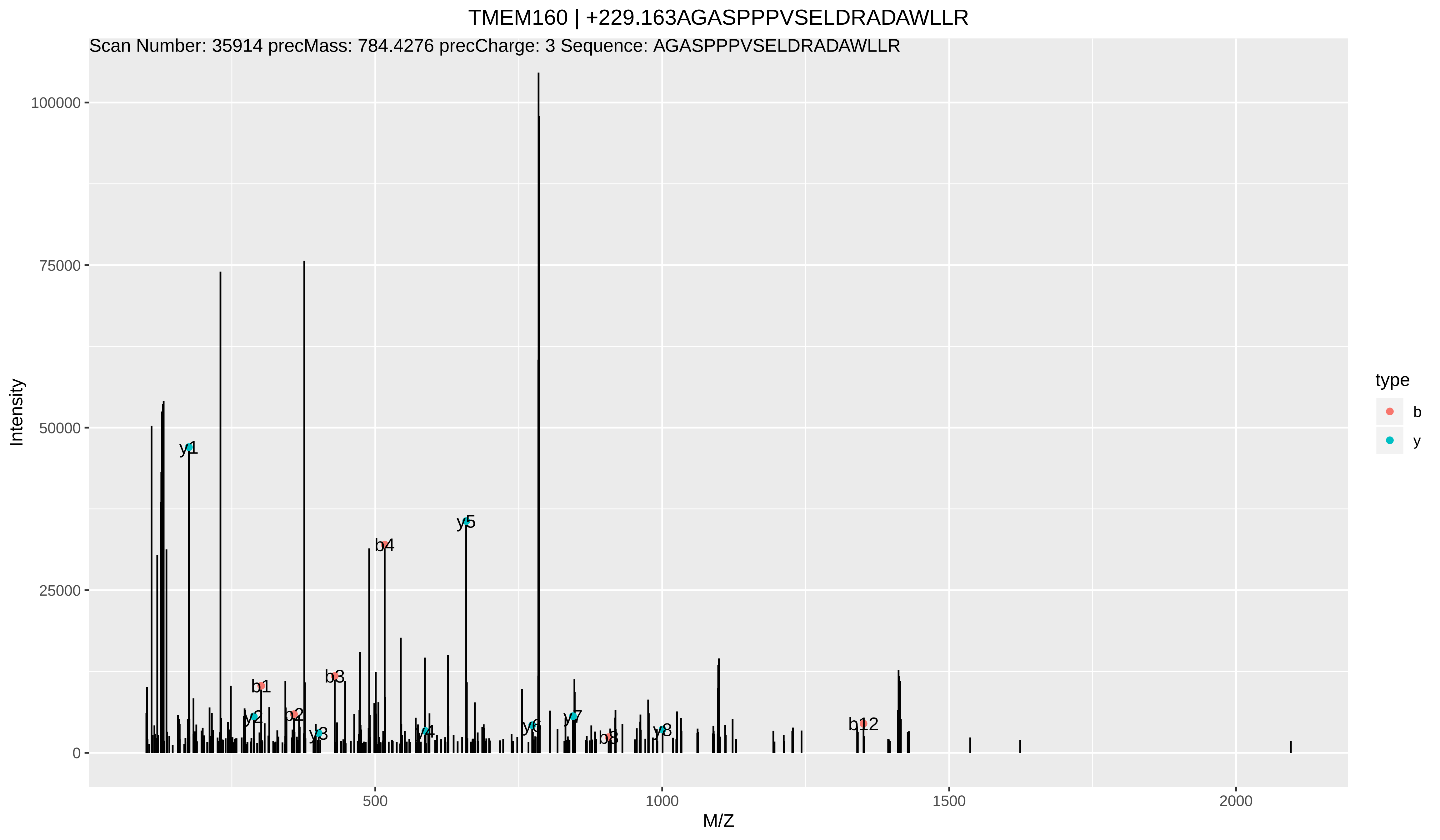

TMEM160 | +229.163AGASPPPVSELDRADAWLLR
Scan Number: 35914 precMass: 784.4276 precCharge: 3 Sequence: AGASPPPVSELDRADAWLLR
Intensity
100000
75000
50000
25000
0
M/Z
500
1000
1500
2000
y1
y2
y3
y4
y5
y6
y7
y8
b1
b2
b3
b4
b8
b12
type
b
y

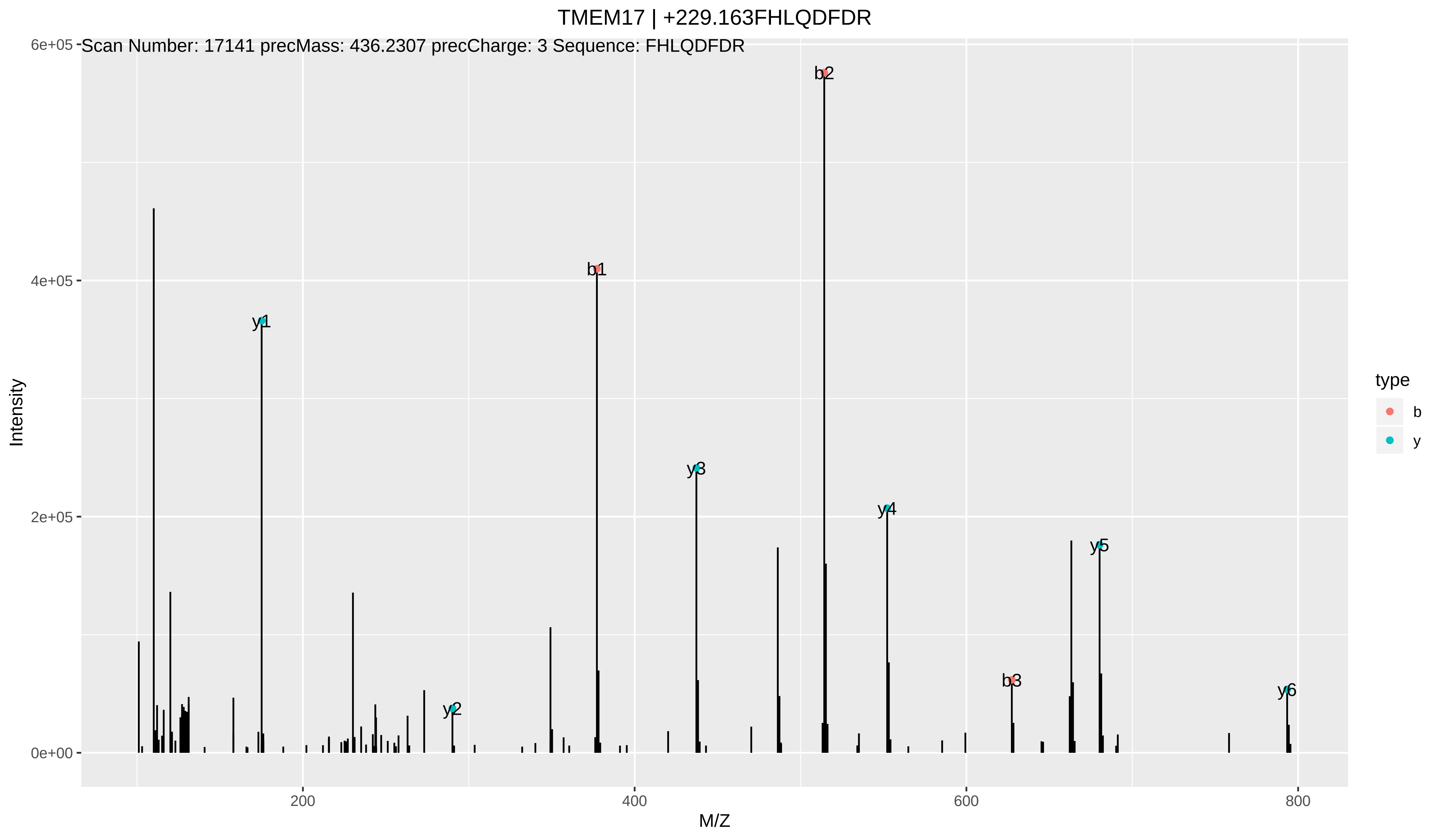

TMEM17 | +229.163FHLQDFDR
Scan Number: 17141 precMass: 436.2307 precCharge: 3 Sequence: FHLQDFDR
Intensity
M/Z
0e+00
2e+05
4e+05
6e+05
200
400
600
800
y1
y2
y3
y4
y5
y6
b1
b2
b3
type
b
y

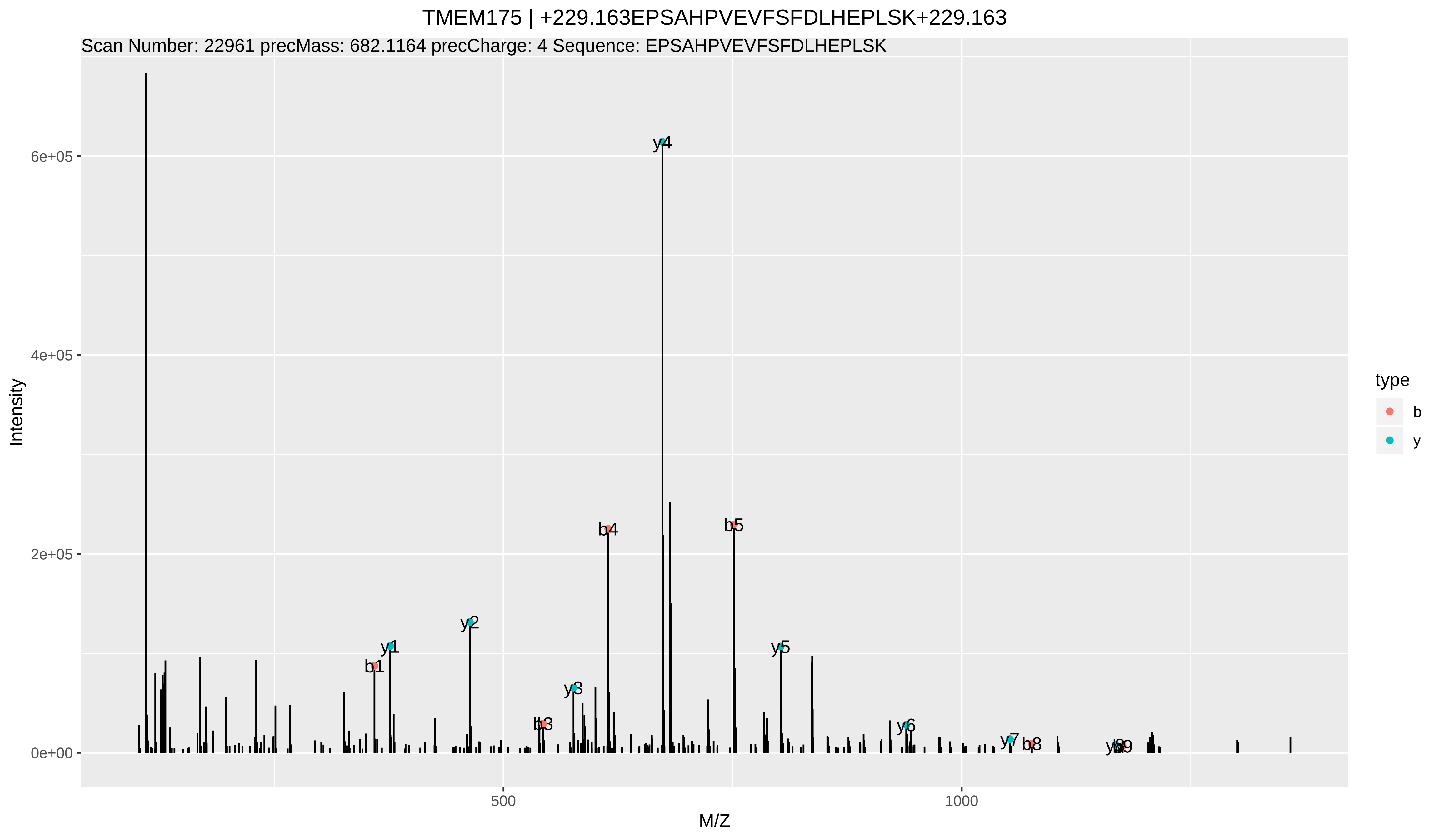
TMEM175 | +229.163EPSAHPVEVFSFDLHEPLSK+229.163
Scan Number: 22961 precMass: 682.1164 precCharge: 4 Sequence: EPSAHPVEVFSFDLHEPLSK
Intensity
M/Z
type
b
y
6e+05
4e+05
2e+05
0e+00
500
1000
y4
b4
b5
y2
y1
b1
y5
y3
b3
y6
y7
b8
y8
b9

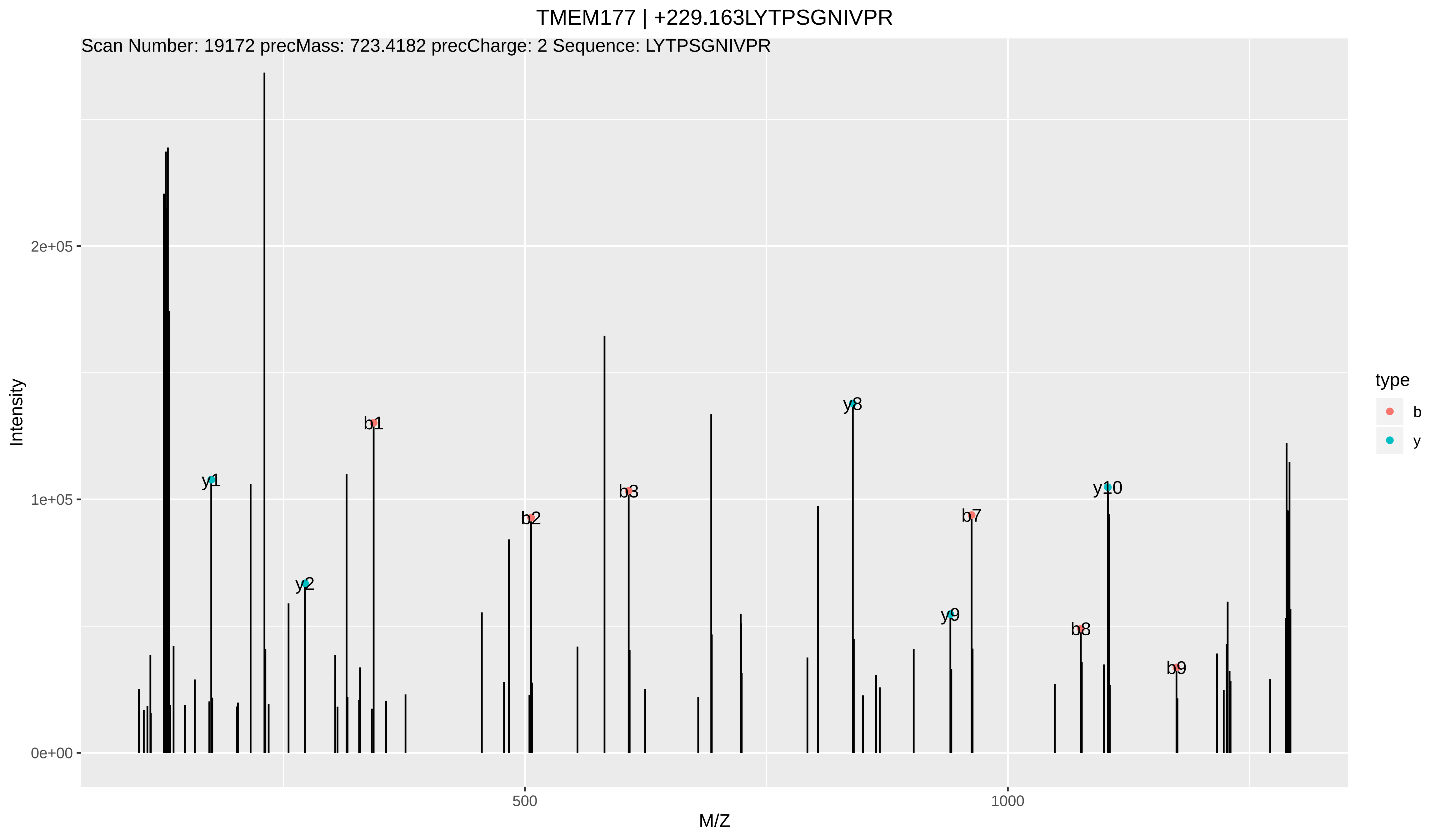
TMEM177 | +229.163LYTPSGNIVPR
Scan Number: 19172 precMass: 723.4182 precCharge: 2 Sequence: LYTPSGNIVPR
Intensity
2e+05
1e+05
0e+00
M/Z
500
1000
y1
y2
b1
b2
b3
y8
y9
b7
y10
b8
b9
type
b
y

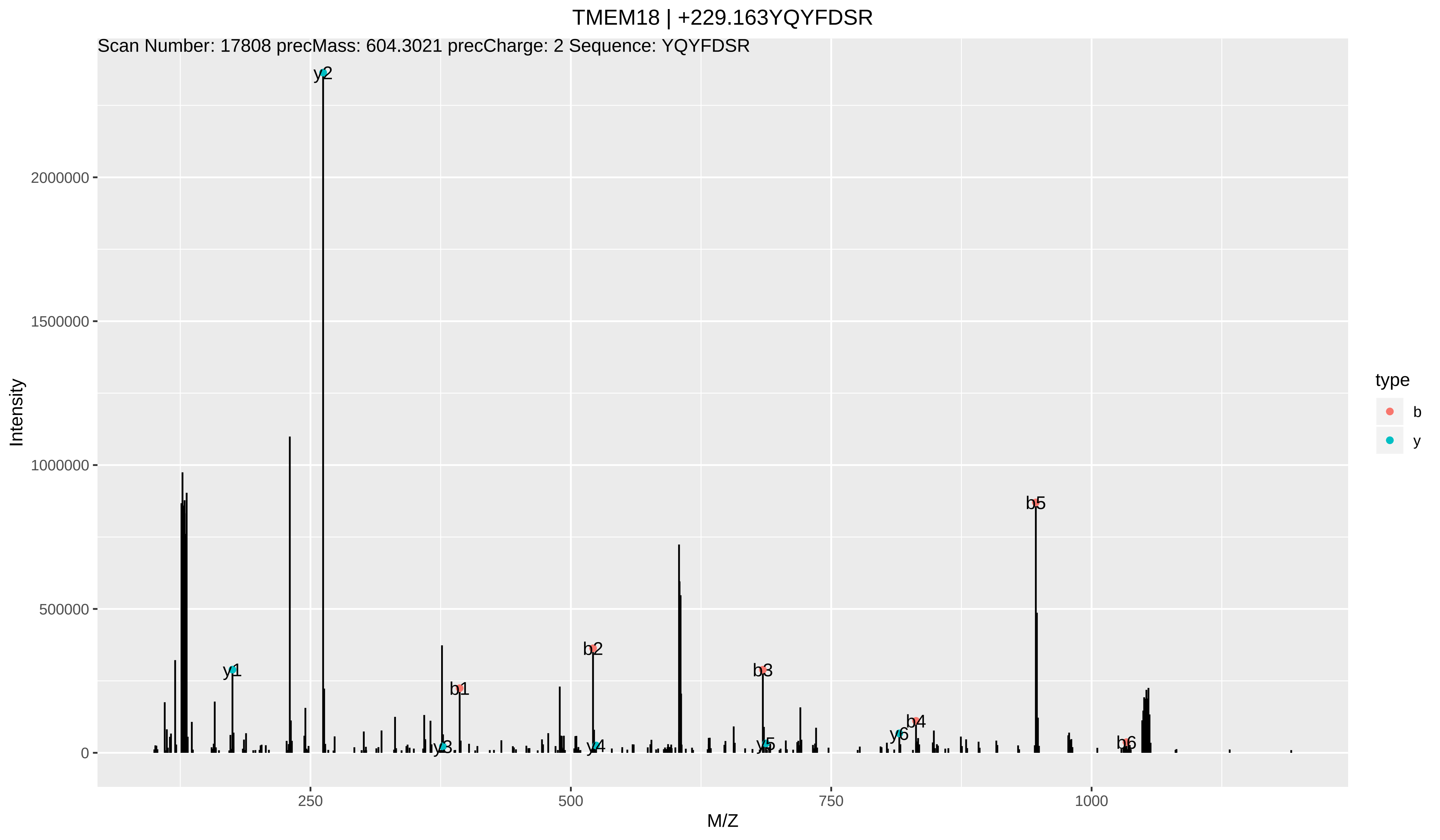

TMEM18 | +229.163YQYFDSR
Scan Number: 17808 precMass: 604.3021 precCharge: 2 Sequence: YQYFDSR
Intensity
M/Z
2000000
1500000
1000000
500000
0
250
500
750
1000
y2
y1
b1
y3
b2
y4
b3
y5
y6
b4
b5
b6
type
b
y

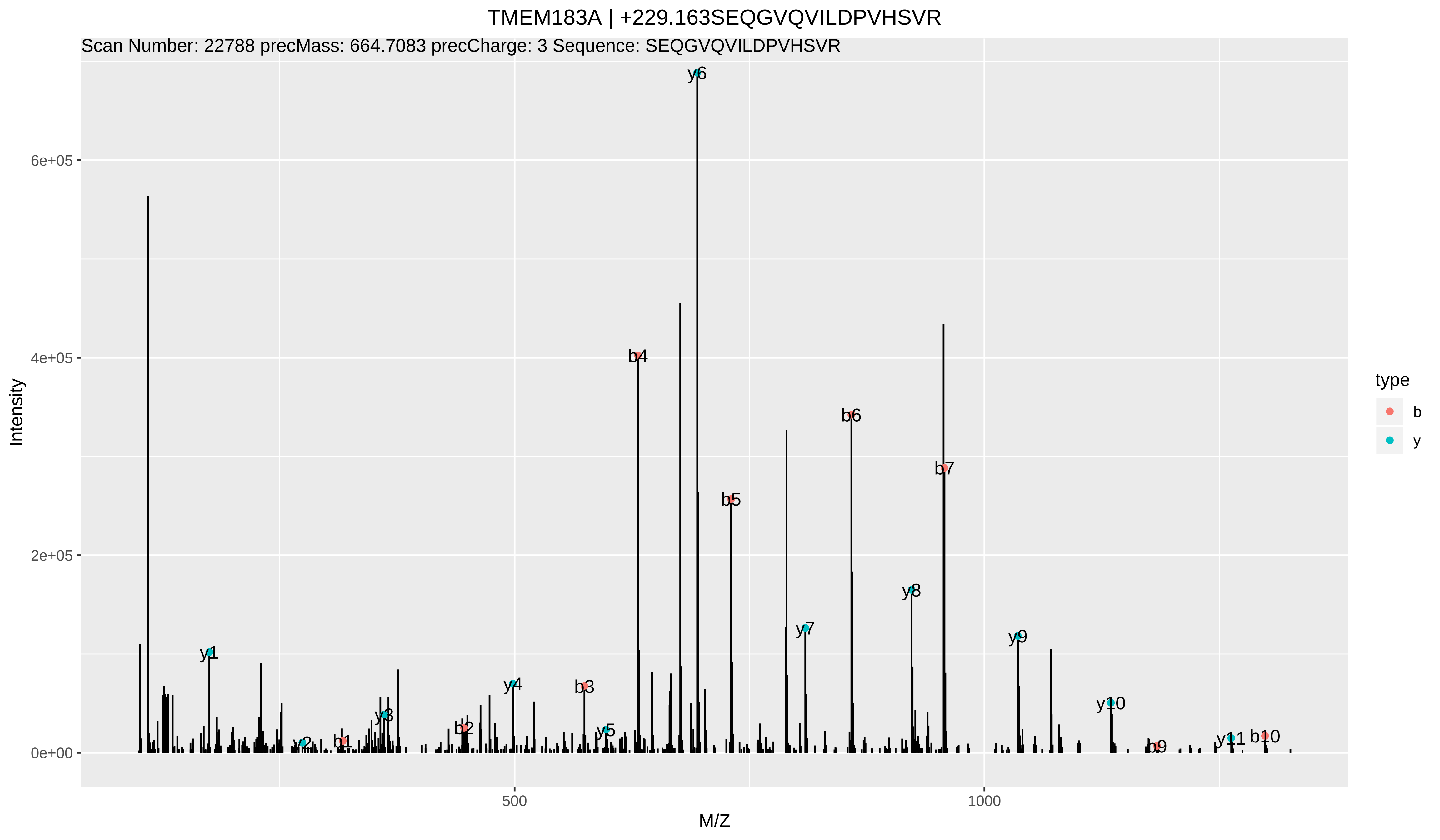

TMEM183A | +229.163SEQGVQVILDPVHSVR
Scan Number: 22788 precMass: 664.7083 precCharge: 3 Sequence: SEQGVQVILDPVHSVR
Intensity
M/Z
type
b
y

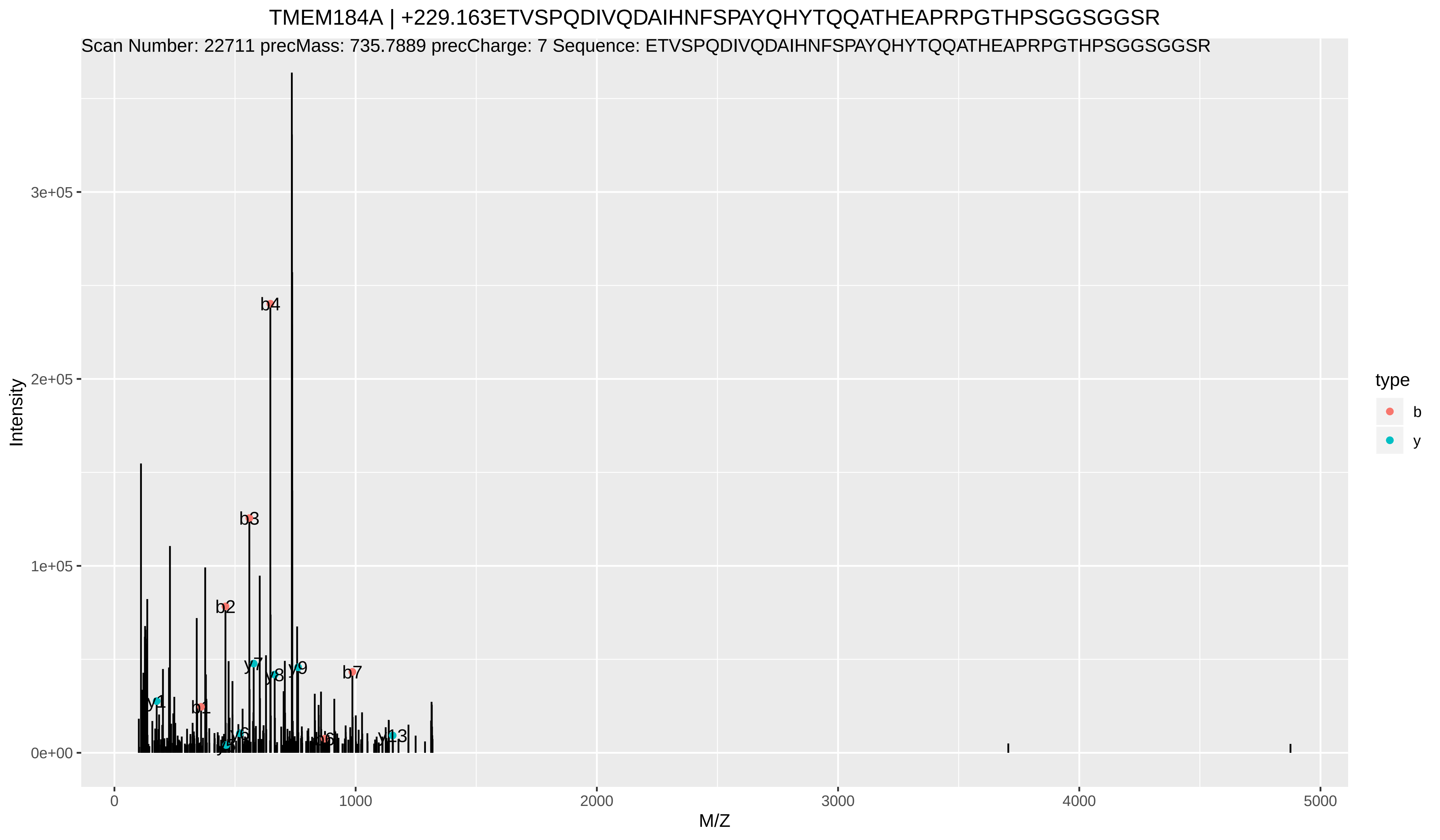
TMEM184A | +229.163ETVSPQDIVQDAIHNFSPAYQHYTQQATHEAPRPGTHPSGGSGGSR
Scan Number: 22711 precMass: 735.7889 precCharge: 7 Sequence: ETVSPQDIVQDAIHNFSPAYQHYTQQATHEAPRPGTHPSGGSGGSR
Intensity
M/Z
type
b
y
b4
b3
b2
b1
b7
y1
y7
y8
y9
y13
0e+00
1e+05
2e+05
3e+05
0
1000
2000
3000
4000
5000

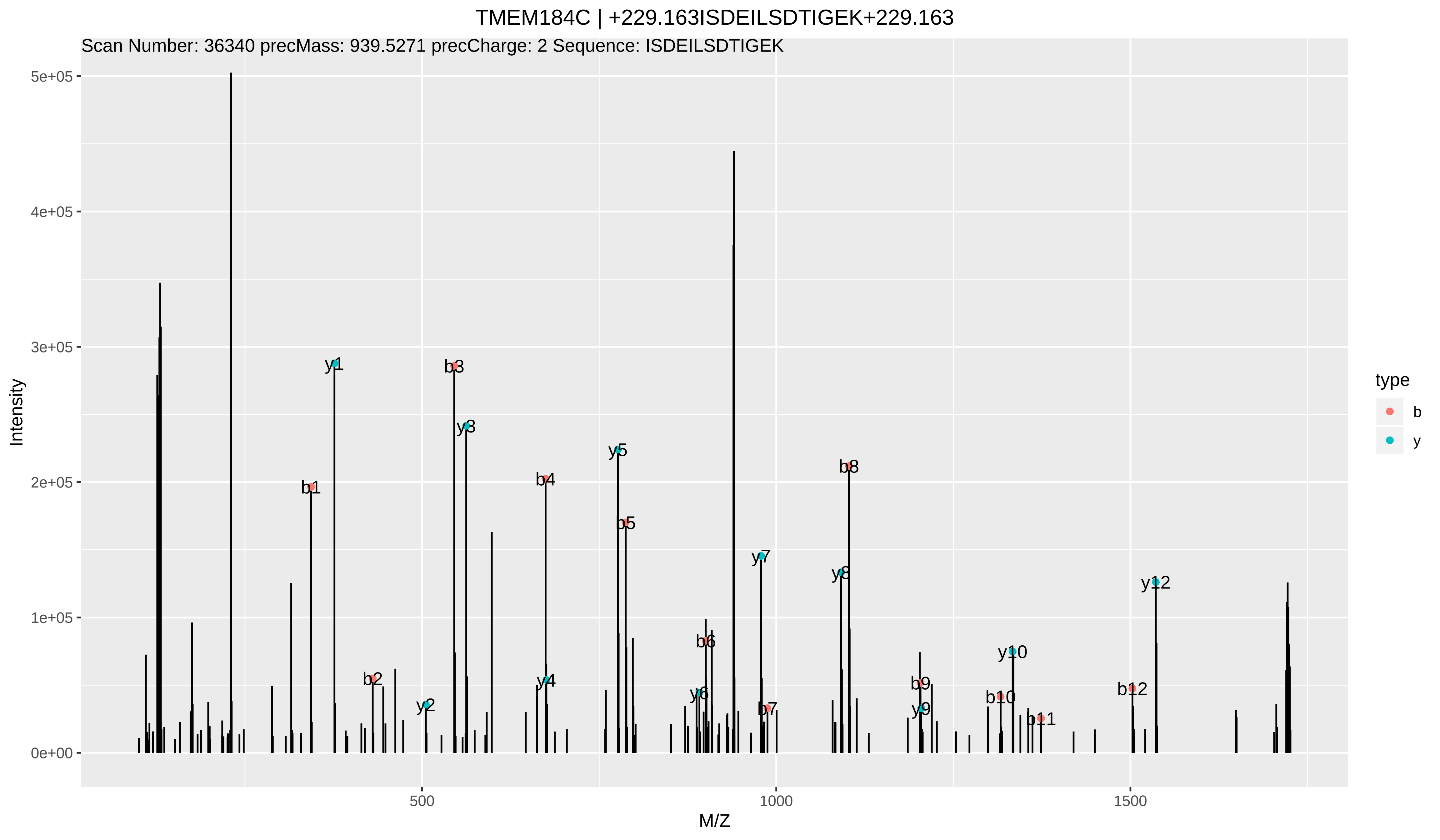

TMEM184C | +229.163ISDEILSDTIGEK+229.163
Scan Number: 36340 precMass: 939.5271 precCharge: 2 Sequence: ISDEILSDTIGEK
Intensity
M/Z
type
b
y

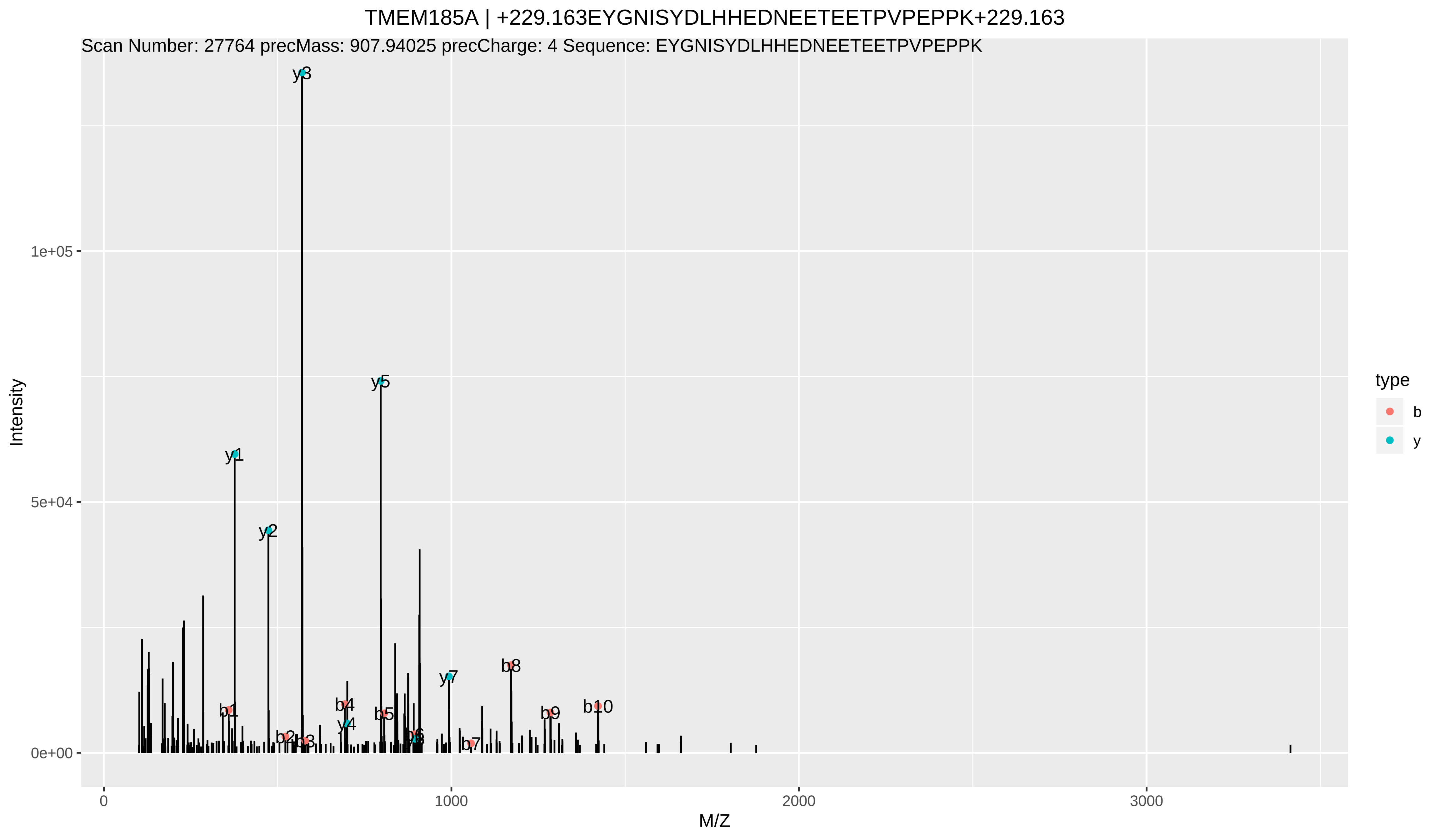
TMEM185A | +229.163EYGNISYDLHHEDNEETEETPVPEPPK+229.163
Scan Number: 27764 precMass: 907.94025 precCharge: 4 Sequence: EYGNISYDLHHEDNEETEETPVPEPPK
Intensity
M/Z
0e+00
5e+04
1e+05
0
1000
2000
3000
y3
y5
y1
y2
y7
y4
b8
b10
b9
b1
b4
b5
b2
b3
b7
type
b
y

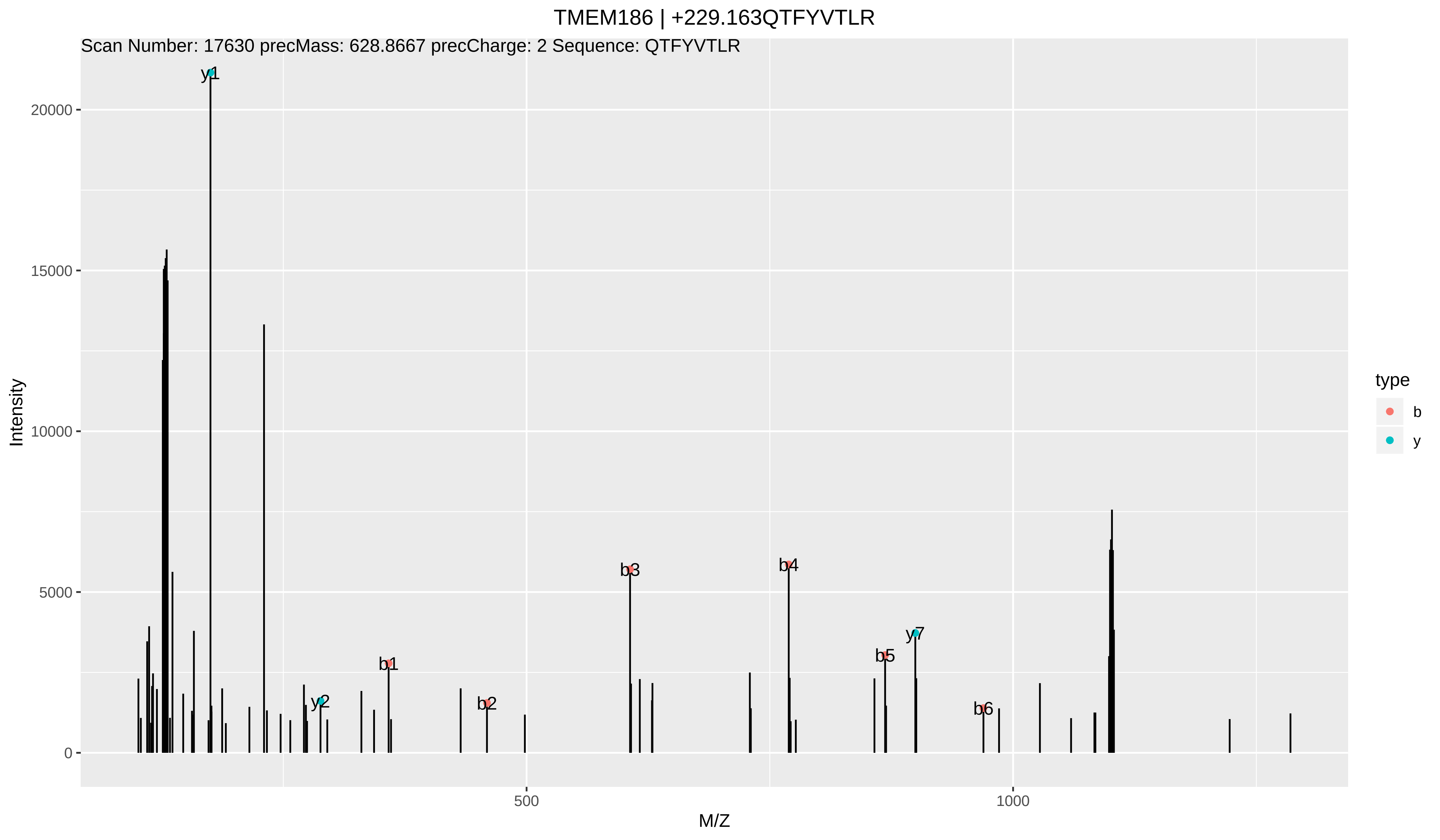
TMEM186 | +229.163QTFYVTLR
Scan Number: 17630 precMass: 628.8667 precCharge: 2 Sequence: QTFYVTLR
y1
y2
b1
b2
b3
b4
b5
y7
b6
Intensity
20000
15000
10000
5000
0
500
1000
M/Z
type
b
y

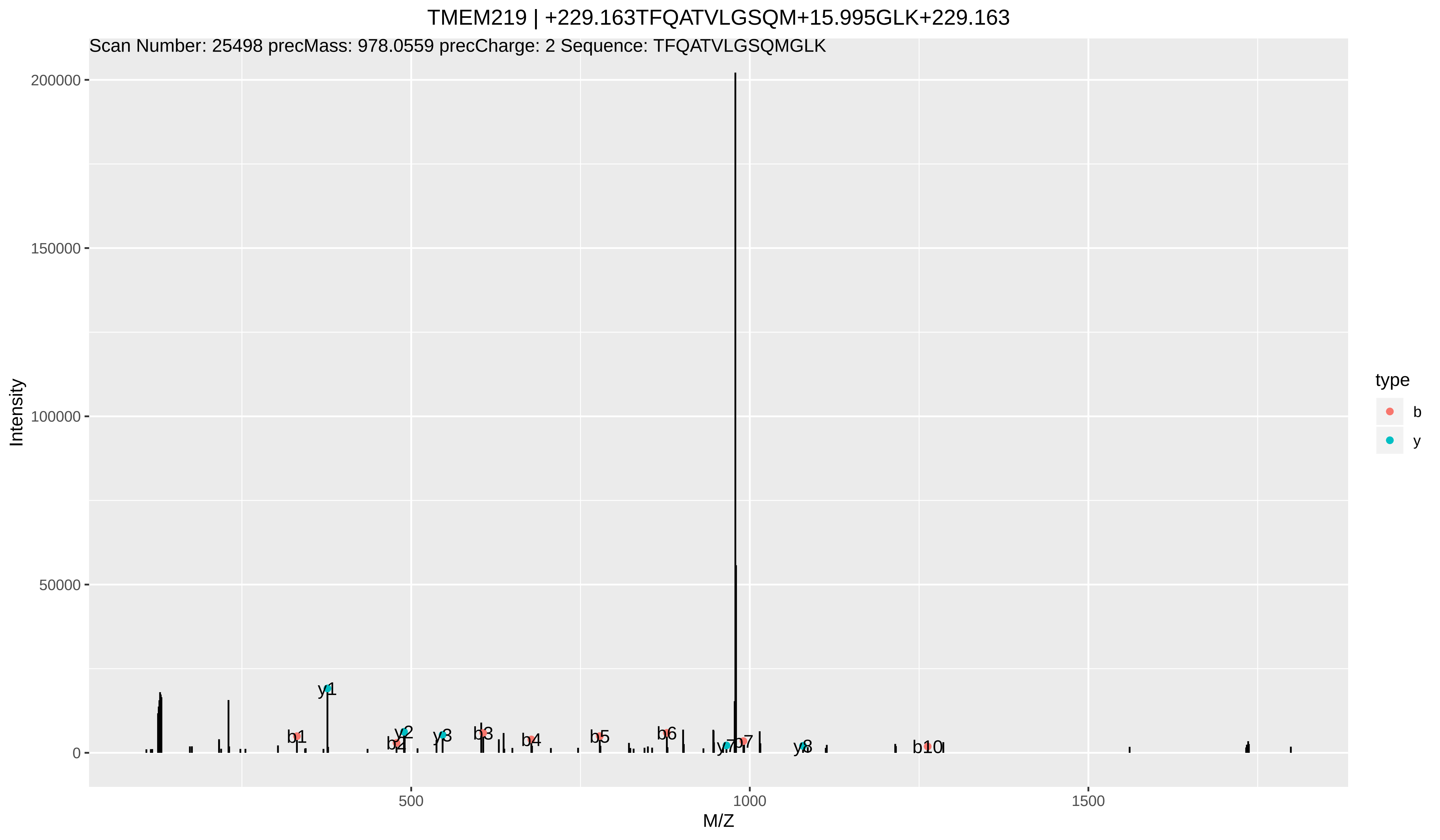

TMEM219 | +229.163TFQATVLGSQM+15.995GLK+229.163
Scan Number: 25498 precMass: 978.0559 precCharge: 2 Sequence: TFQATVLGSQMGLK
Intensity
M/Z
type
b
y

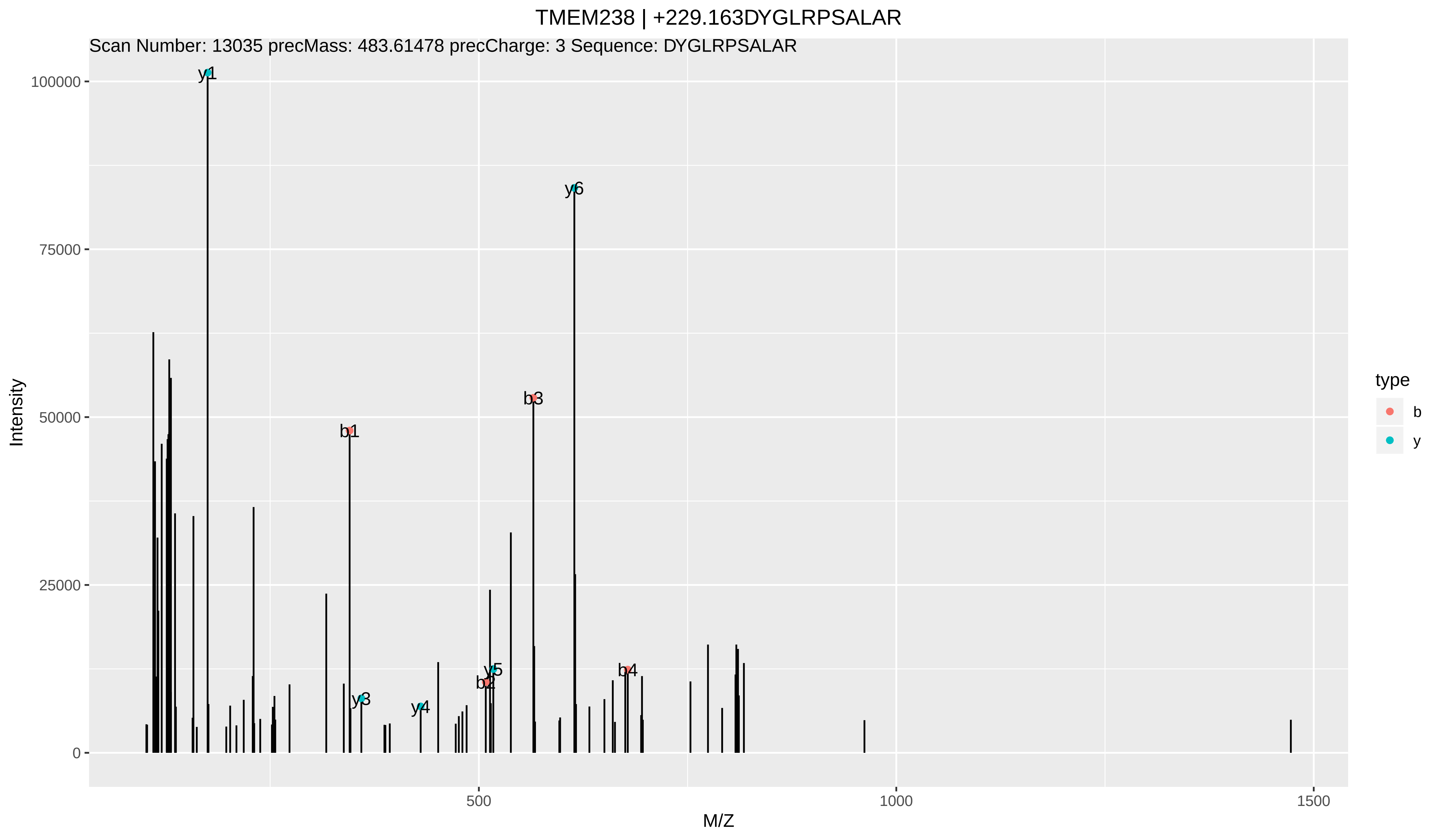

TMEM238 | +229.163DYGLRPSALAR
Scan Number: 13035 precMass: 483.61478 precCharge: 3 Sequence: DYGLRPSALAR
Intensity
100000
75000
50000
25000
0
500
1000
1500
M/Z
y1
y6
b3
b1
y5
b2
b4
y3
y4
type
b
y

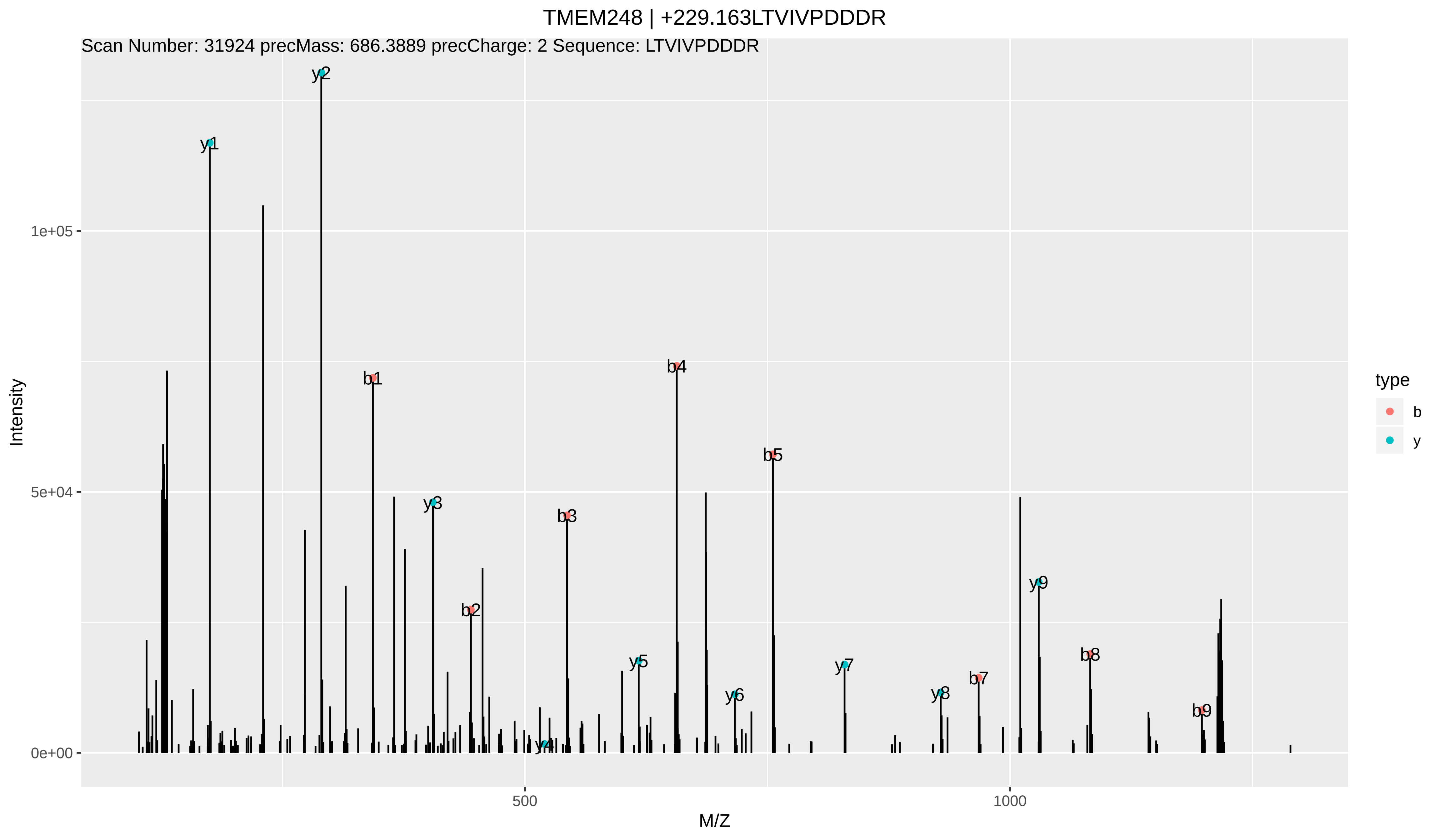

TMEM248 | +229.163LTVIVPDDDR
Scan Number: 31924 precMass: 686.3889 precCharge: 2 Sequence: LTVIVPDDDR
y1
y2
y3
y4
y5
y6
y7
y8
y9
b1
b2
b3
b4
b5
b7
b8
b9
Intensity
1e+05
5e+04
0e+00
M/Z
500
1000
type
b
y

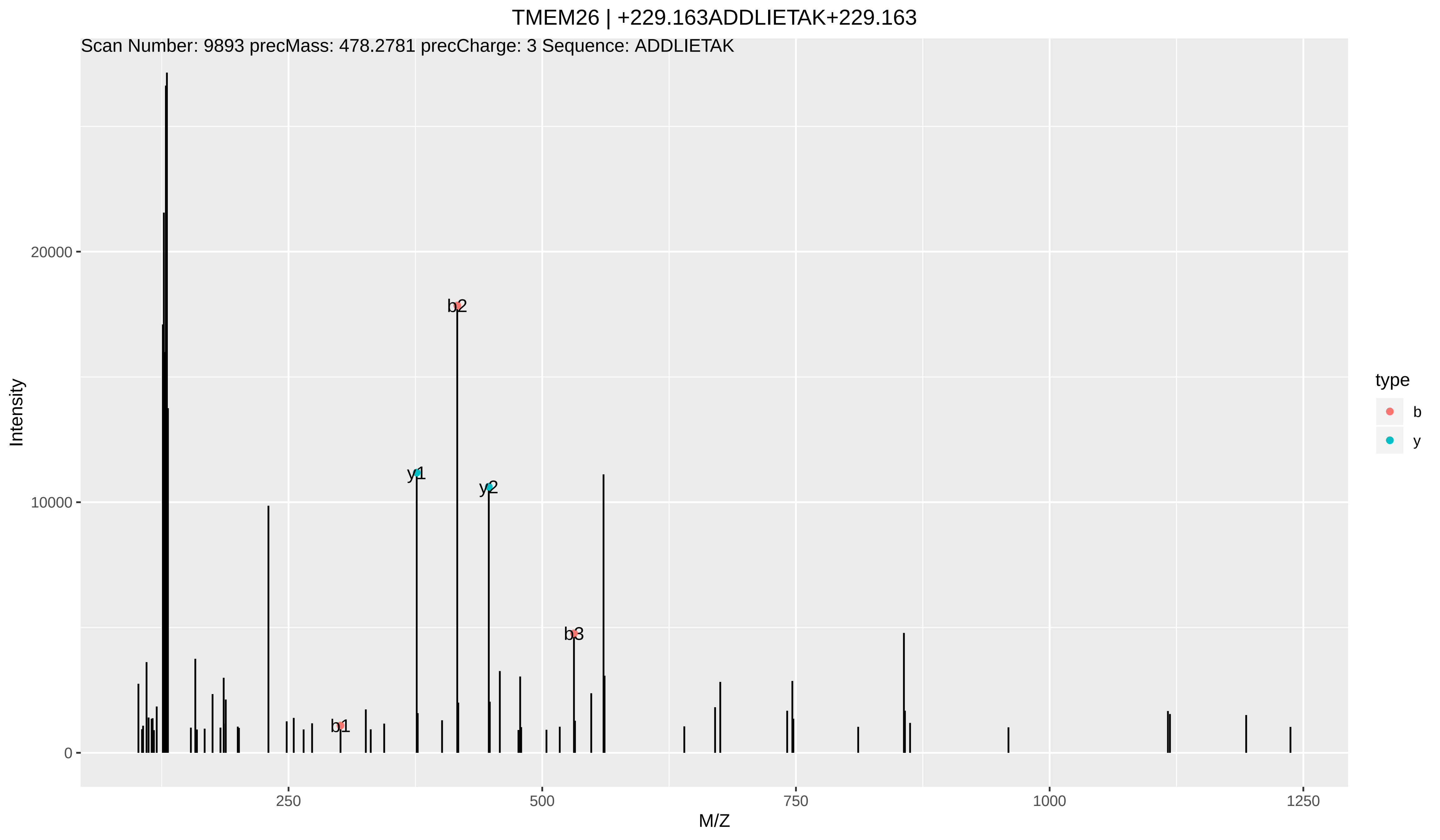

TMEM26 | +229.163ADDLIETAK+229.163
Scan Number: 9893 precMass: 478.2781 precCharge: 3 Sequence: ADDLIETAK
Intensity
M/Z
0
10000
20000
250
500
750
1000
1250
b1
y1
b2
y2
b3
type
b
y

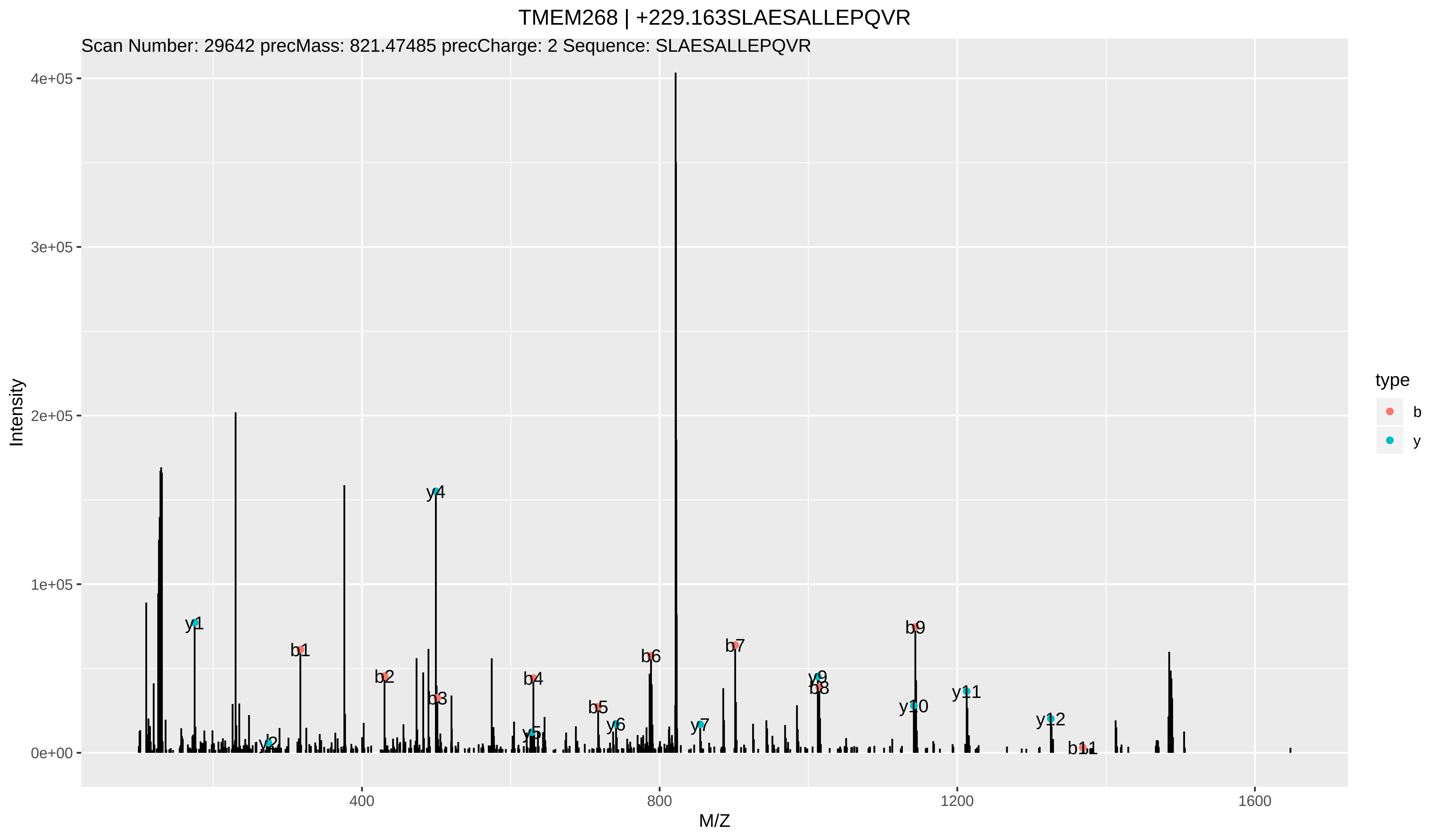
TMEM268 | +229.163SLAESALLEPQVR
Scan Number: 29642 precMass: 821.47485 precCharge: 2 Sequence: SLAESALLEPQVR
Intensity
M/Z
type
b
y
y1
y2
y4
y5
y6
y7
y9
y10
y11
y12
b1
b2
b3
b4
b5
b6
b7
b8
b9
b11
0e+00
1e+05
2e+05
3e+05
4e+05
400
800
1200
1600

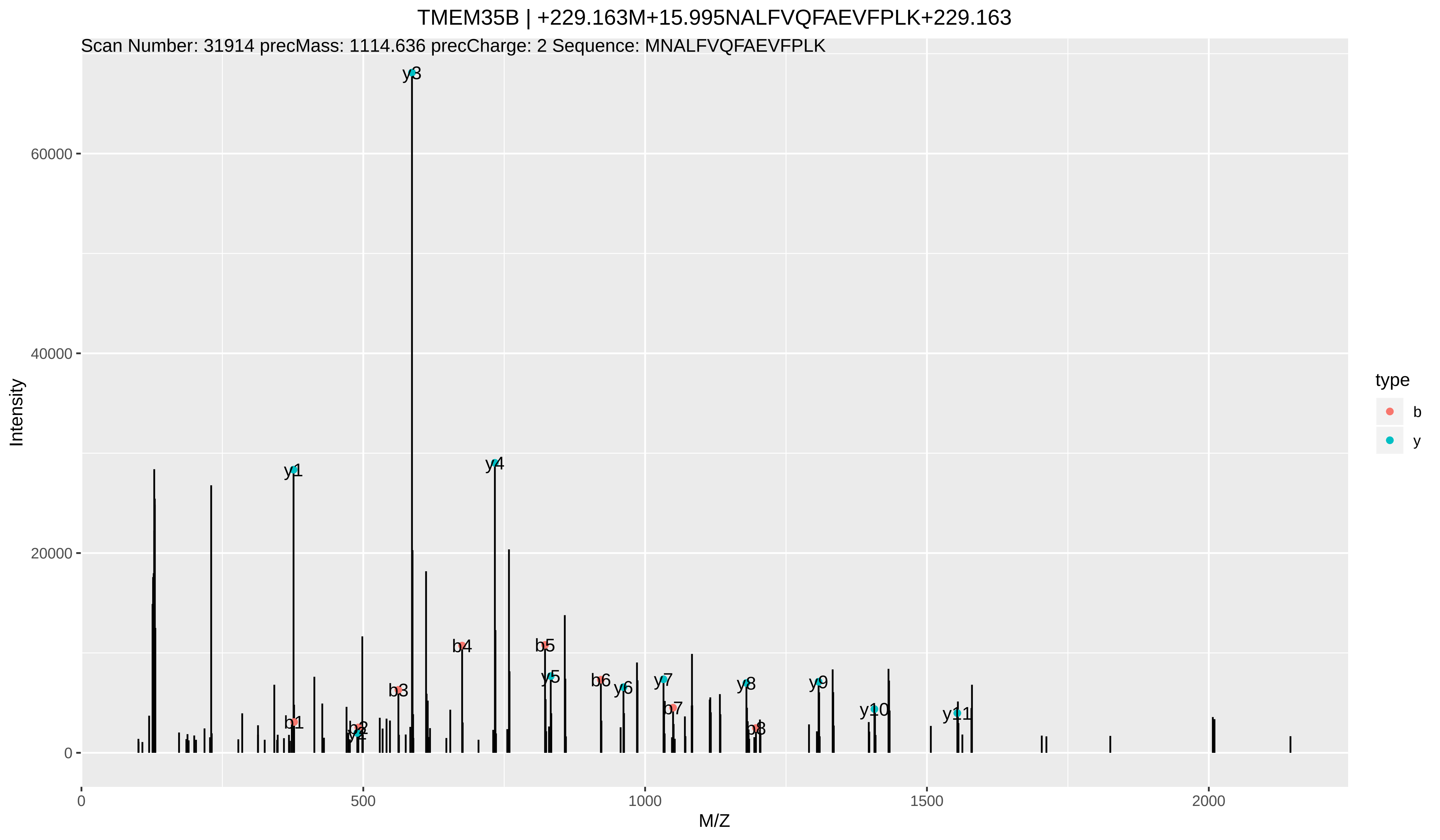
TMEM35B | +229.163M+15.995NALFVQFAEVFPLK+229.163
Scan Number: 31914 precMass: 1114.636 precCharge: 2 Sequence: MNALFVQFAEVFPLK
y3
y1
y4
b4
b5
y5
b6
y6
y7
b7
y8
y9
y10
y11
b3
b1
b2
y2
b8
Intensity
60000
40000
20000
0
M/Z
0
500
1000
1500
2000
type
b
y

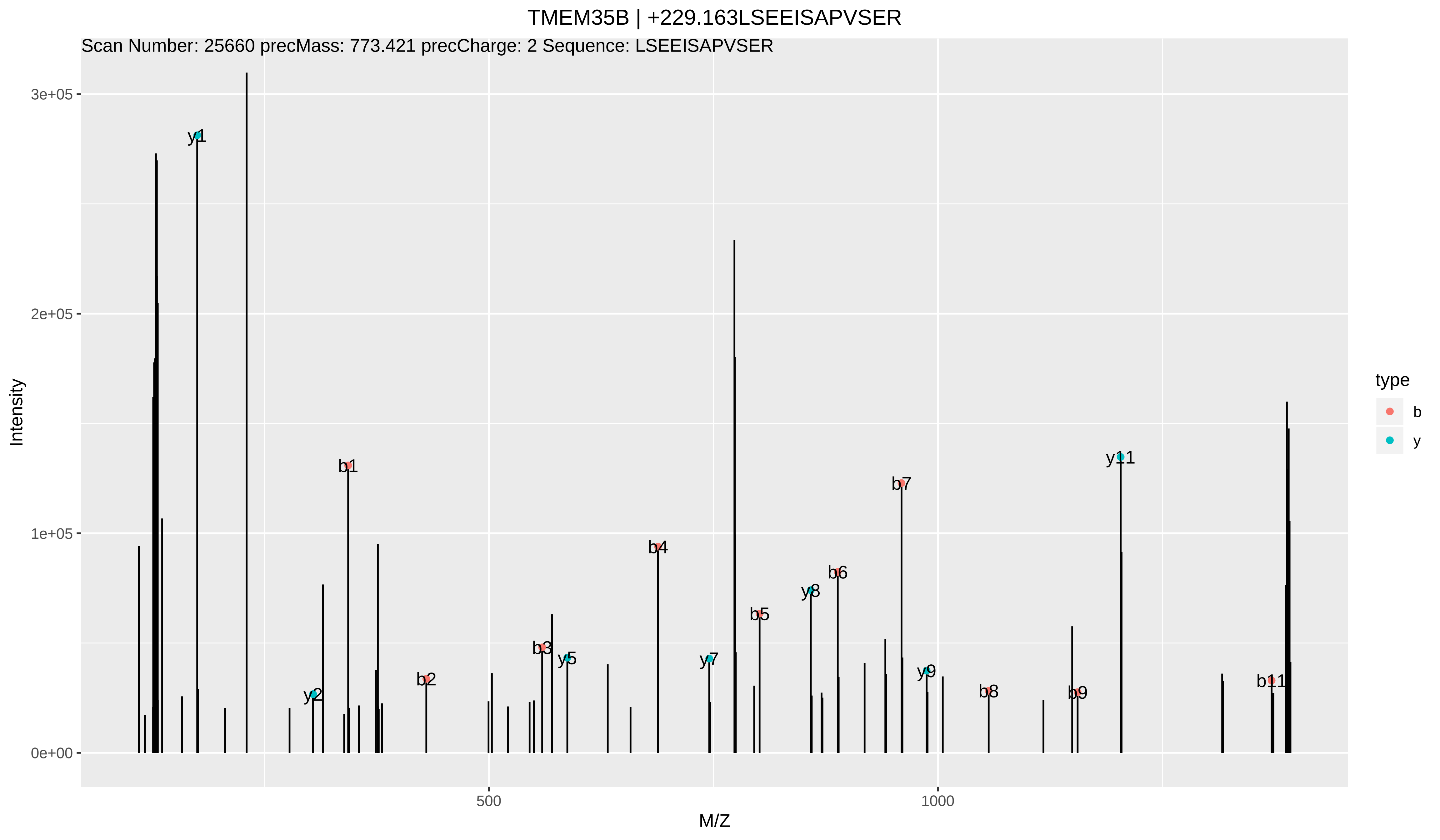
TMEM35B | +229.163LSEEISAPVSER
Scan Number: 25660 precMass: 773.421 precCharge: 2 Sequence: LSEEISAPVSER
Intensity
M/Z
3e+05
2e+05
1e+05
0e+00
500
1000
y1
b1
y2
b2
b3
y5
b4
y7
b5
y8
b6
b7
y9
b8
b9
y11
b11
type
b
y

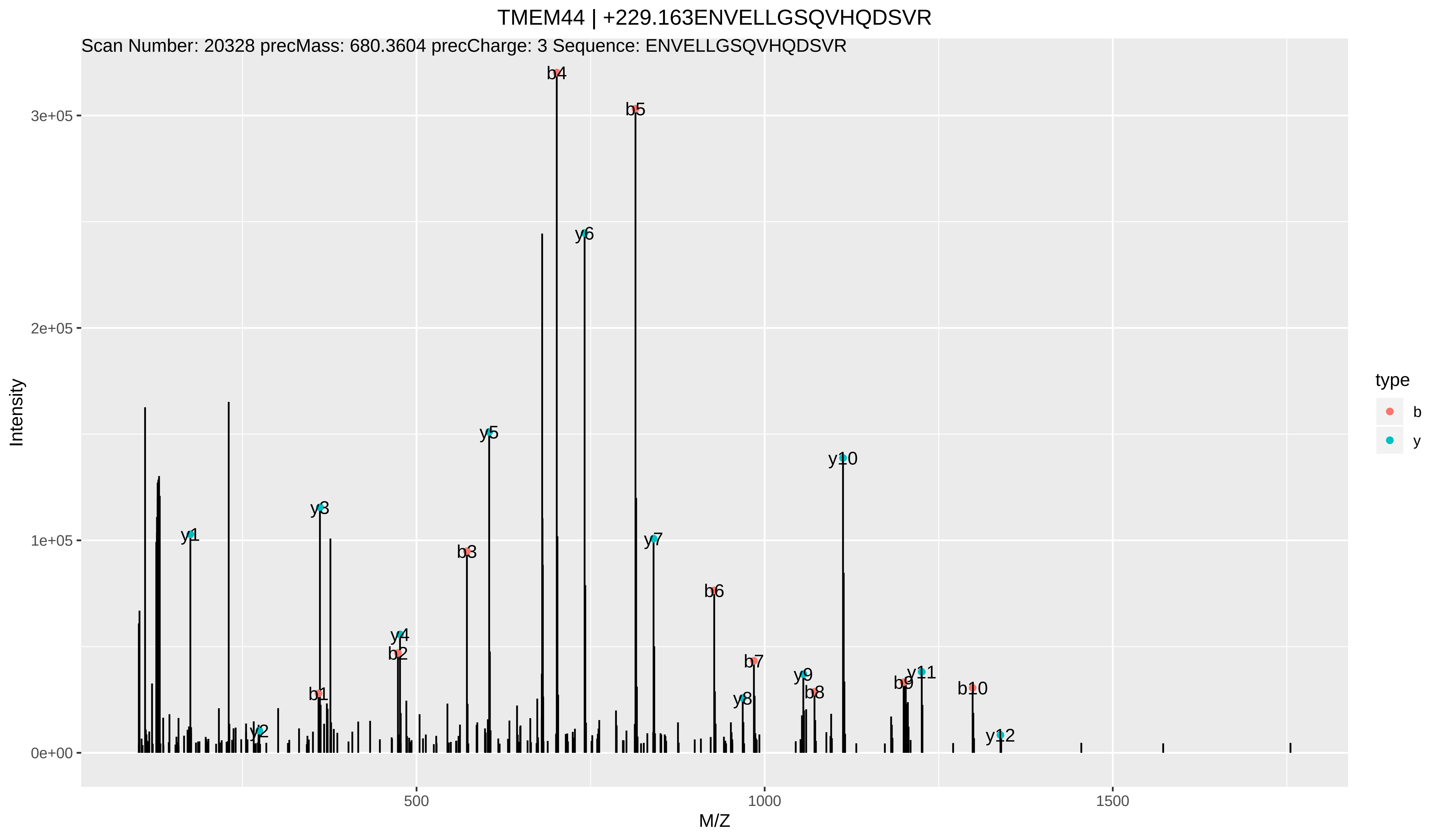

TMEM44 | +229.163ENVELLGSQVHQDSVR
Scan Number: 20328 precMass: 680.3604 precCharge: 3 Sequence: ENVELLGSQVHQDSVR
Intensity
M/Z
type
b
y
b4
b5
y6
y5
y10
y3
y1
y7
b3
b6
y4
b2
b7
y9
y11
b9
b8
b10
b1
y8
y2
y12
3e+05
2e+05
1e+05
0e+00
500
1000
1500

TMEM79 | +229.163LDYPDHAR

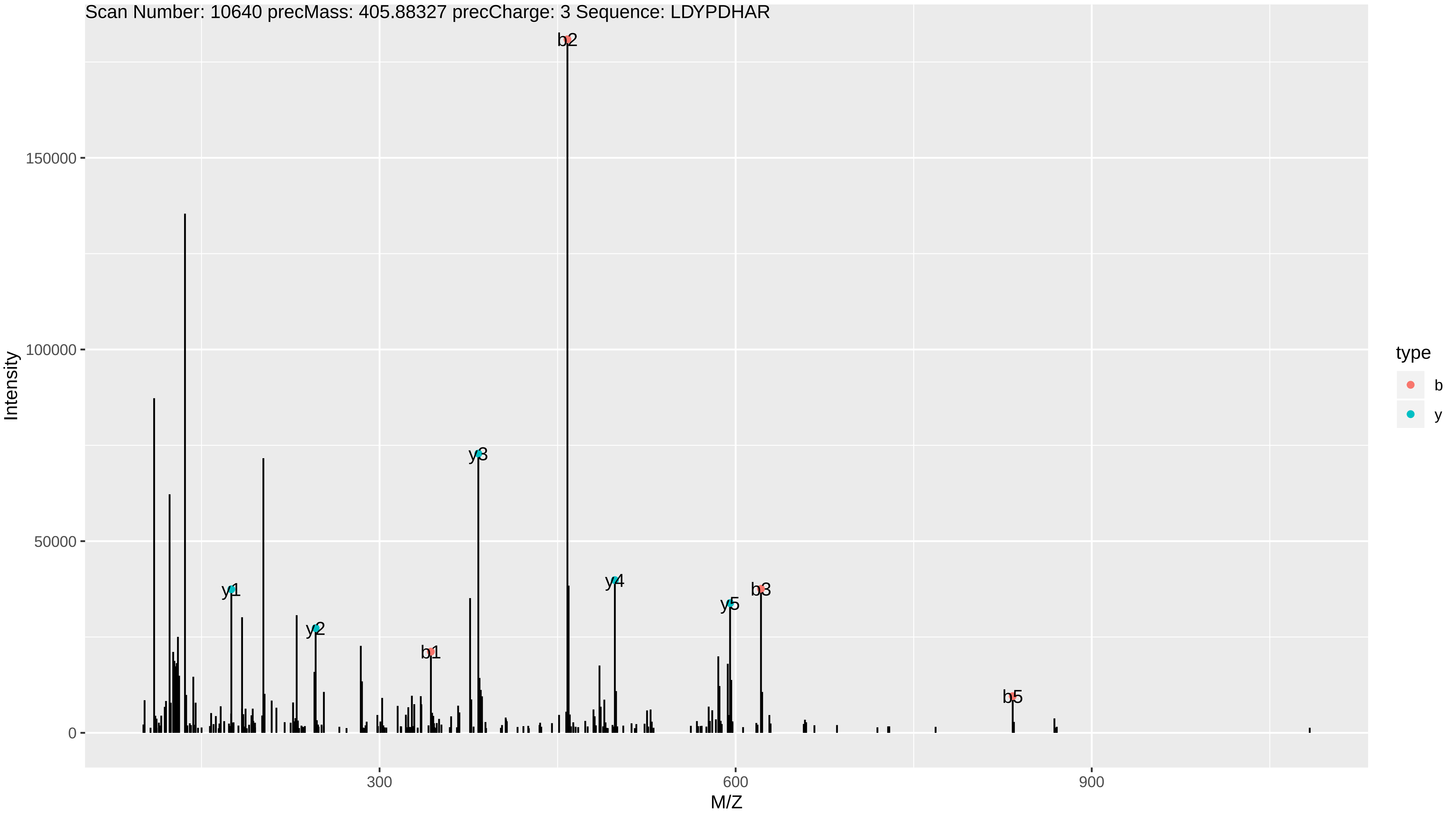

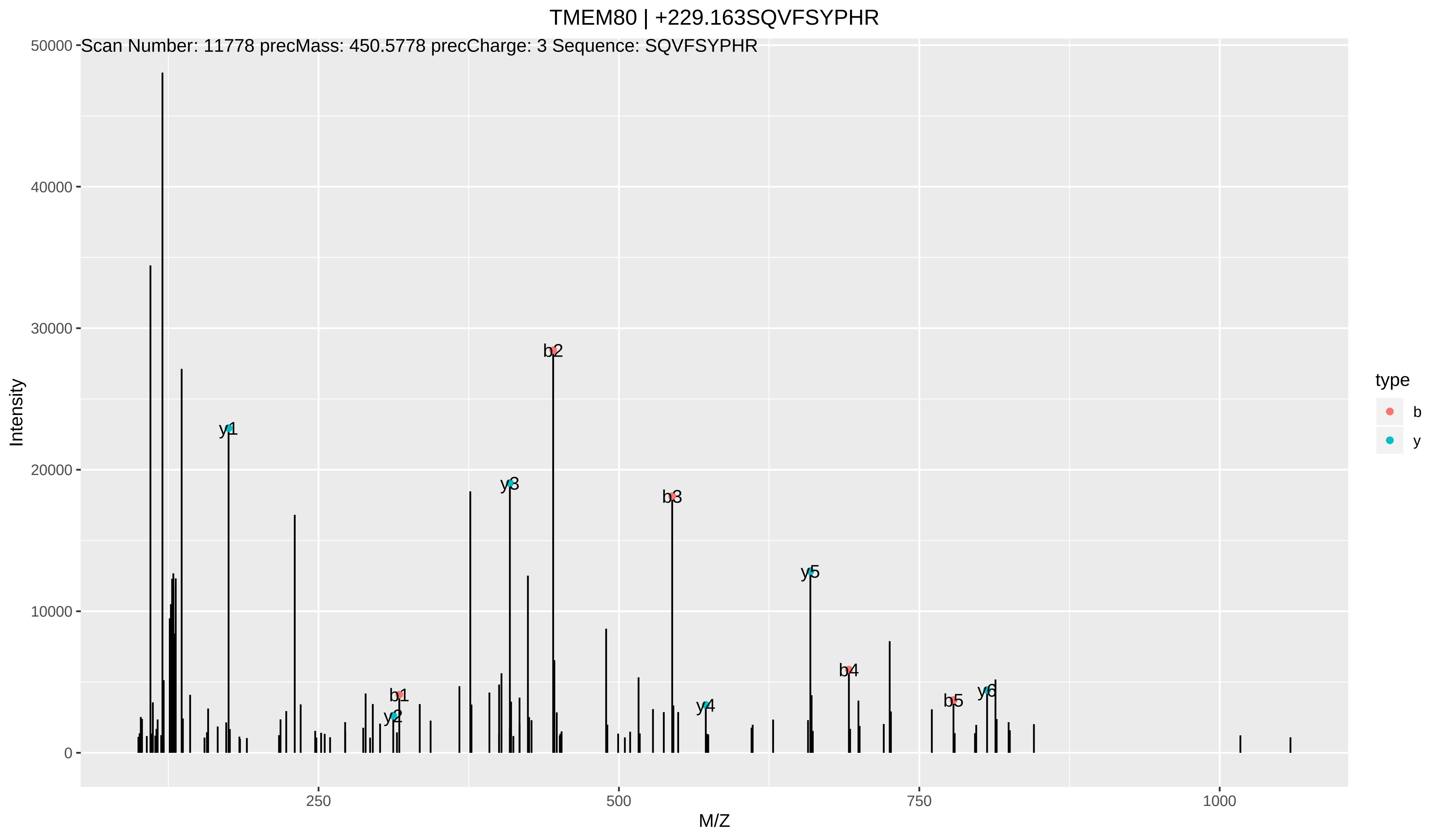
TMEM80 | +229.163SQVFSYPHR
Scan Number: 11778 precMass: 450.5778 precCharge: 3 Sequence: SQVFSYPHR
Intensity
M/Z
type
b
y
y1
y2
y3
y4
y5
y6
b1
b2
b3
b4
b5
0
10000
20000
30000
40000
50000
250
500
750
1000

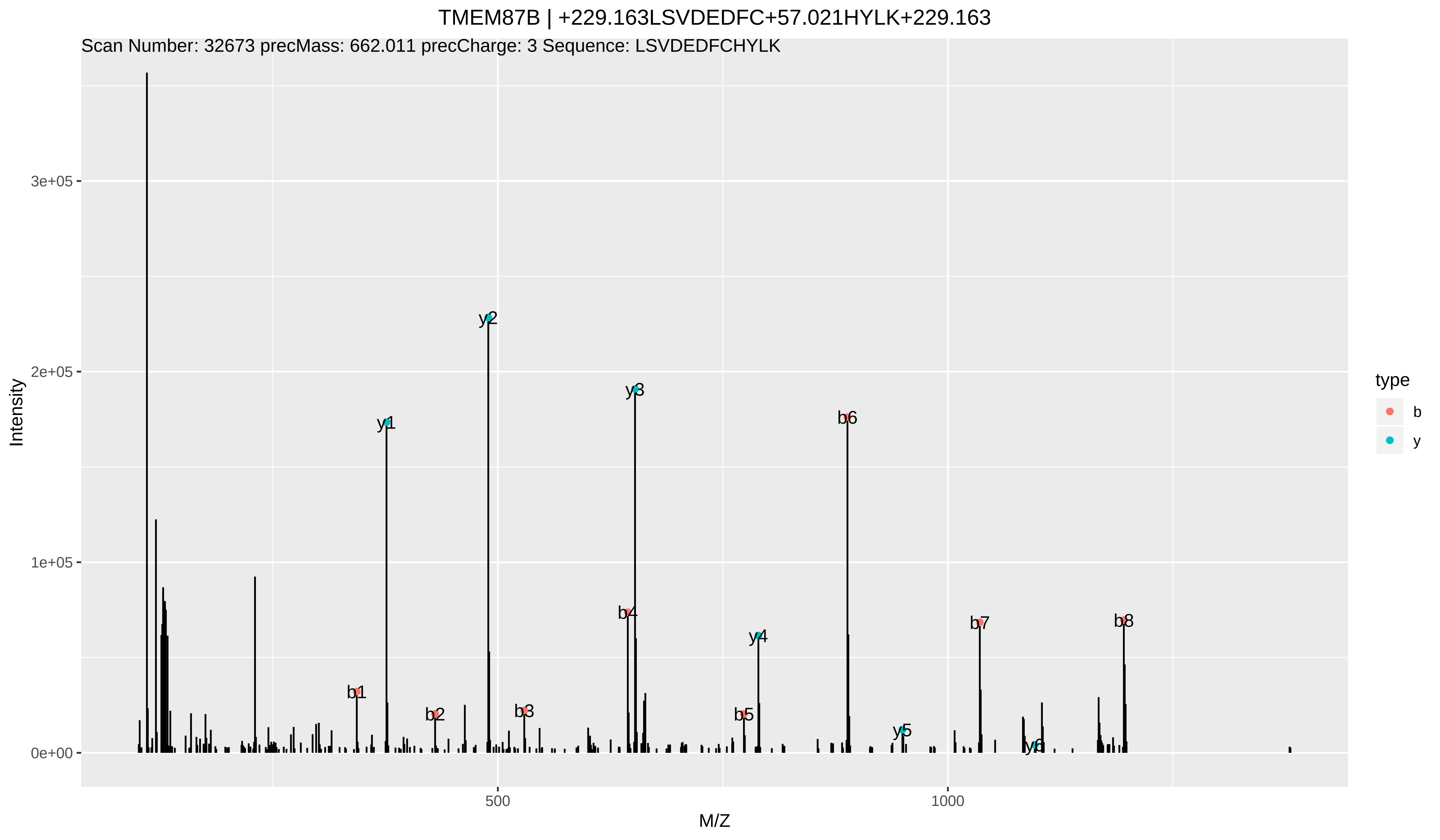
TMEM87B | +229.163LSVDEDFC+57.021HYLK+229.163
Scan Number: 32673 precMass: 662.011 precCharge: 3 Sequence: LSVDEDFCHYLK
Intensity
3e+05
2e+05
1e+05
0e+00
M/Z
500
1000
y1
y2
y3
y4
y5
y6
b1
b2
b3
b4
b5
b6
b7
b8
type
b
y

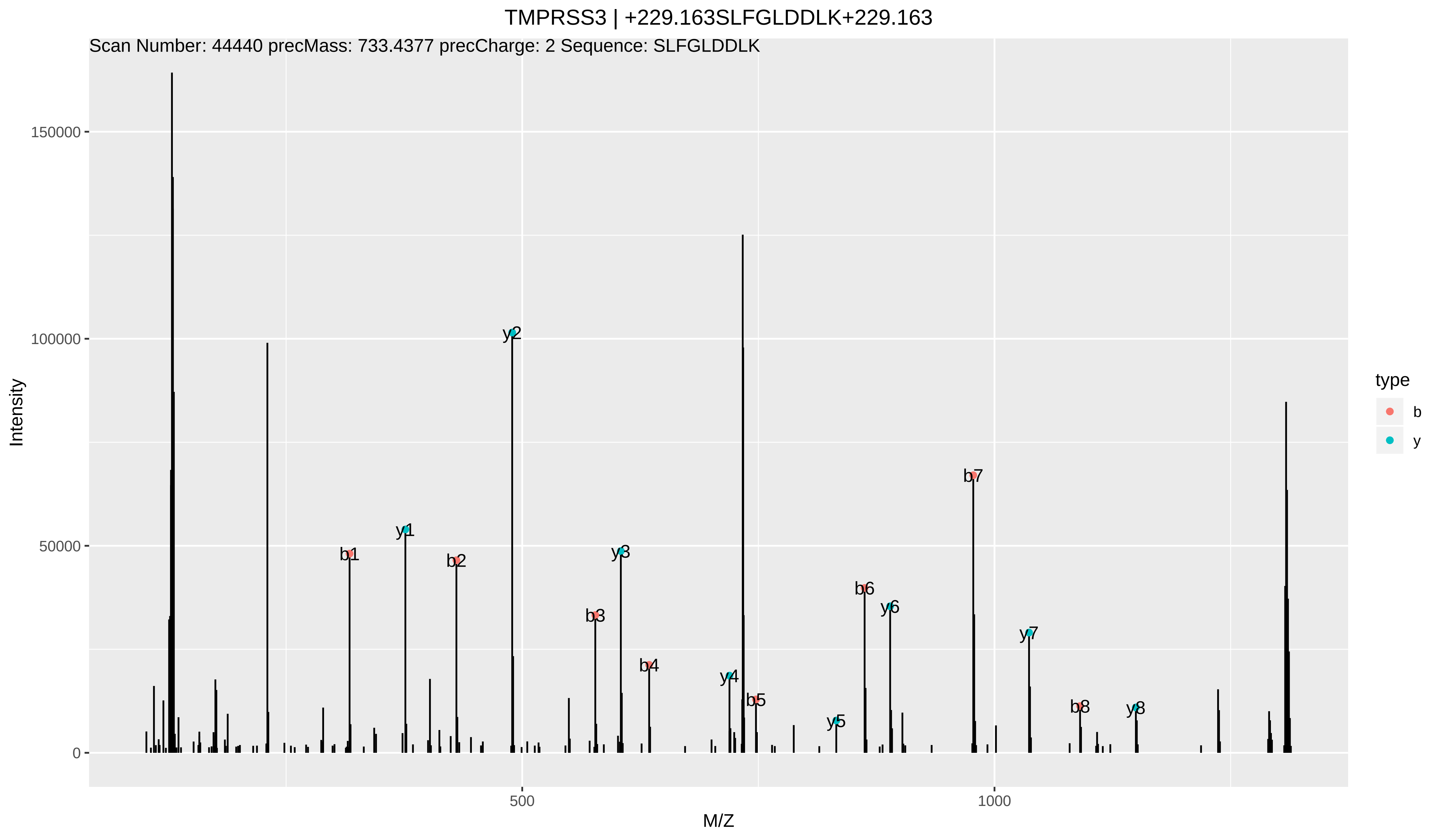
TMPRSS3 | +229.163SLFGLDDLK+229.163
Scan Number: 44440 precMass: 733.4377 precCharge: 2 Sequence: SLFGLDDLK
Intensity
150000
100000
50000
0
500
1000
M/Z
b1
y1
b2
y2
b3
y3
b4
y4
b5
y5
b6
y6
b7
y7
b8
y8
type
b
y

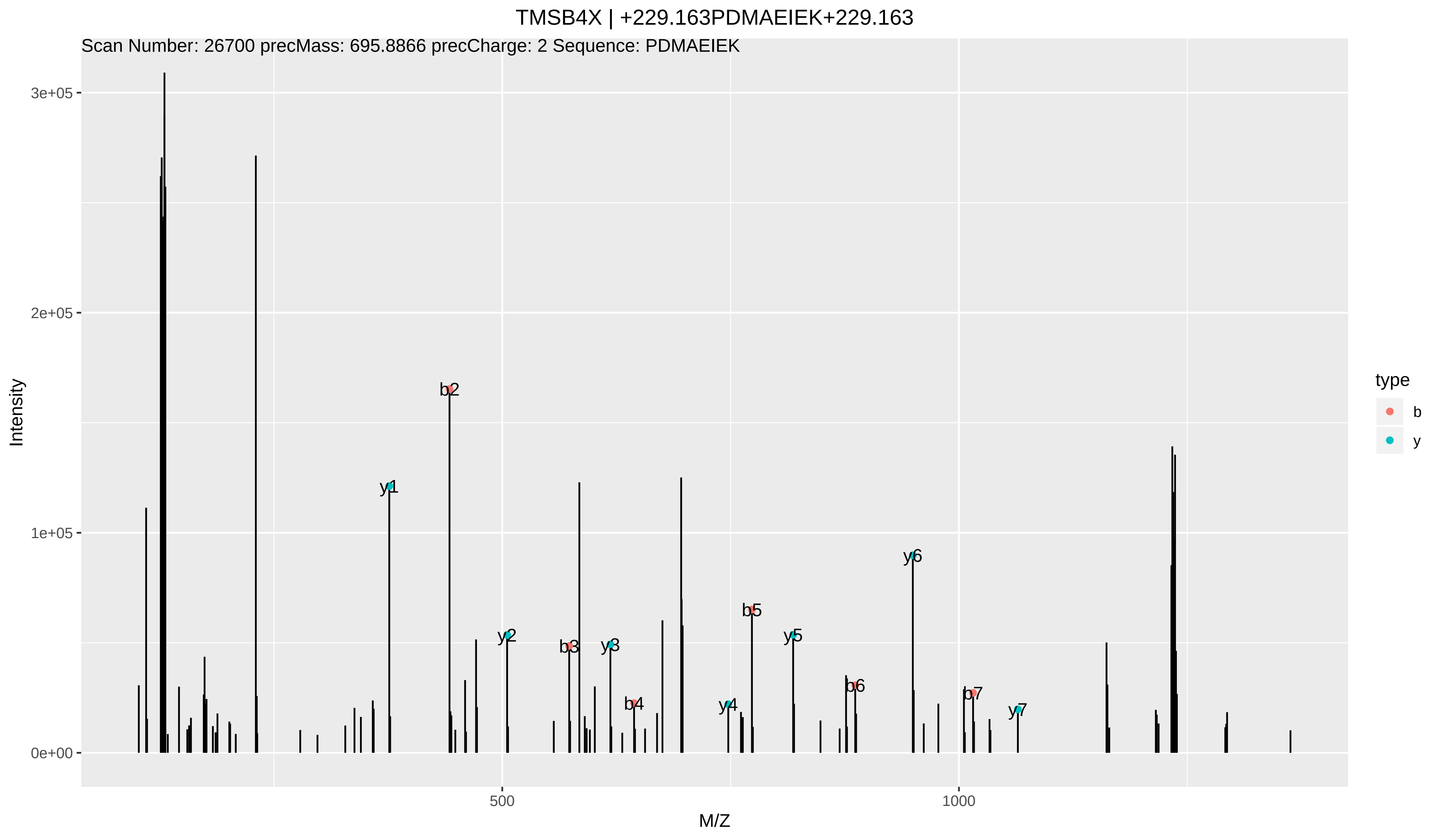
TMSB4X | +229.163PDMAEIEK+229.163
Scan Number: 26700 precMass: 695.8866 precCharge: 2 Sequence: PDMAEIEK
Intensity
3e+05
2e+05
1e+05
0e+00
M/Z
500
1000
y1
b2
y2
b3
y3
b4
y4
b5
y5
b6
y6
b7
y7
type
b
y

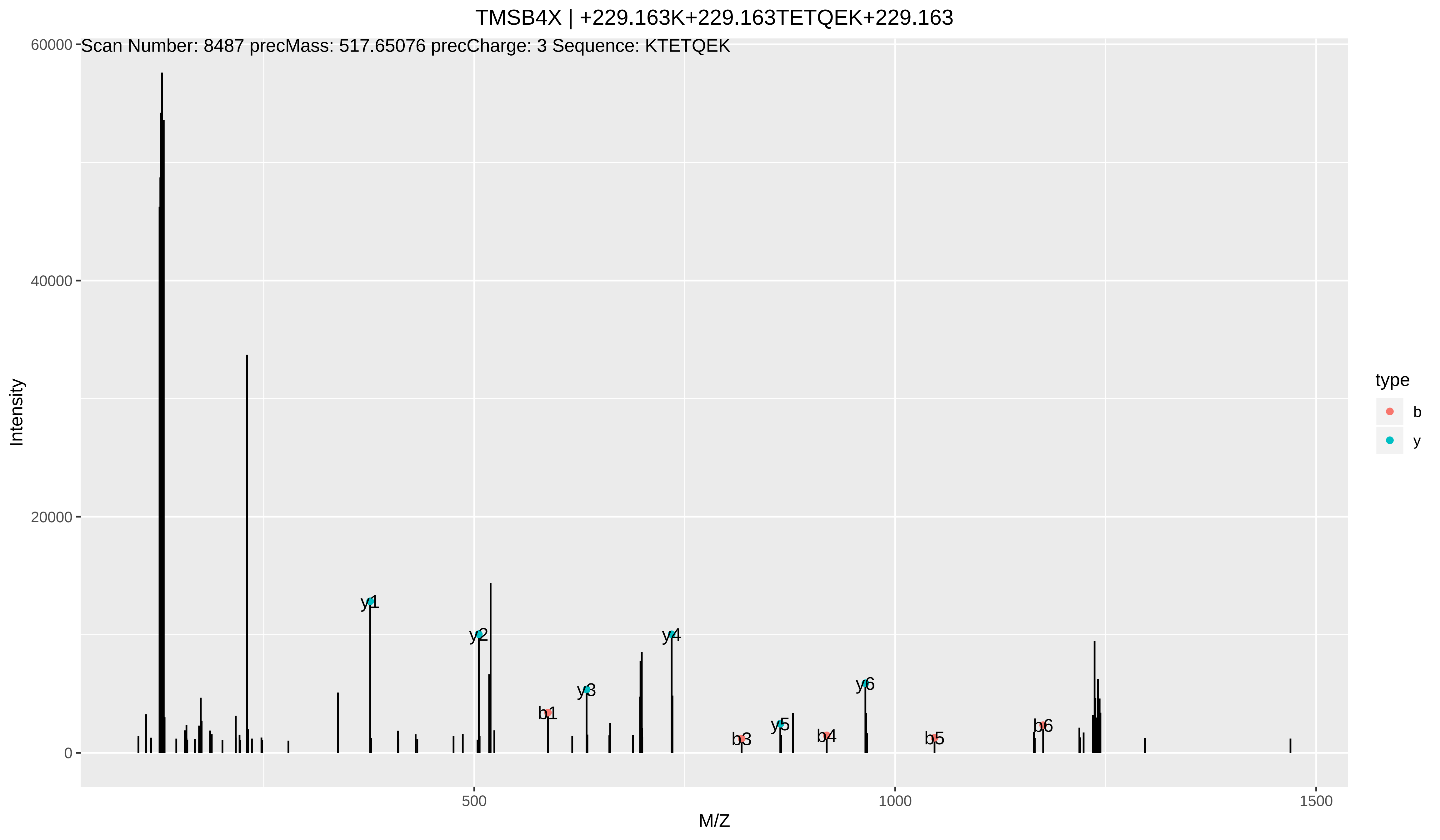

TMSB4X | +229.163K+229.163TETQEK+229.163
Scan Number: 8487 precMass: 517.65076 precCharge: 3 Sequence: KTETQEK
Intensity
M/Z
60000
40000
20000
0
500
1000
1500
y1
y2
b1
y3
y4
b3
y5
b4
y6
b5
b6
type
b
y

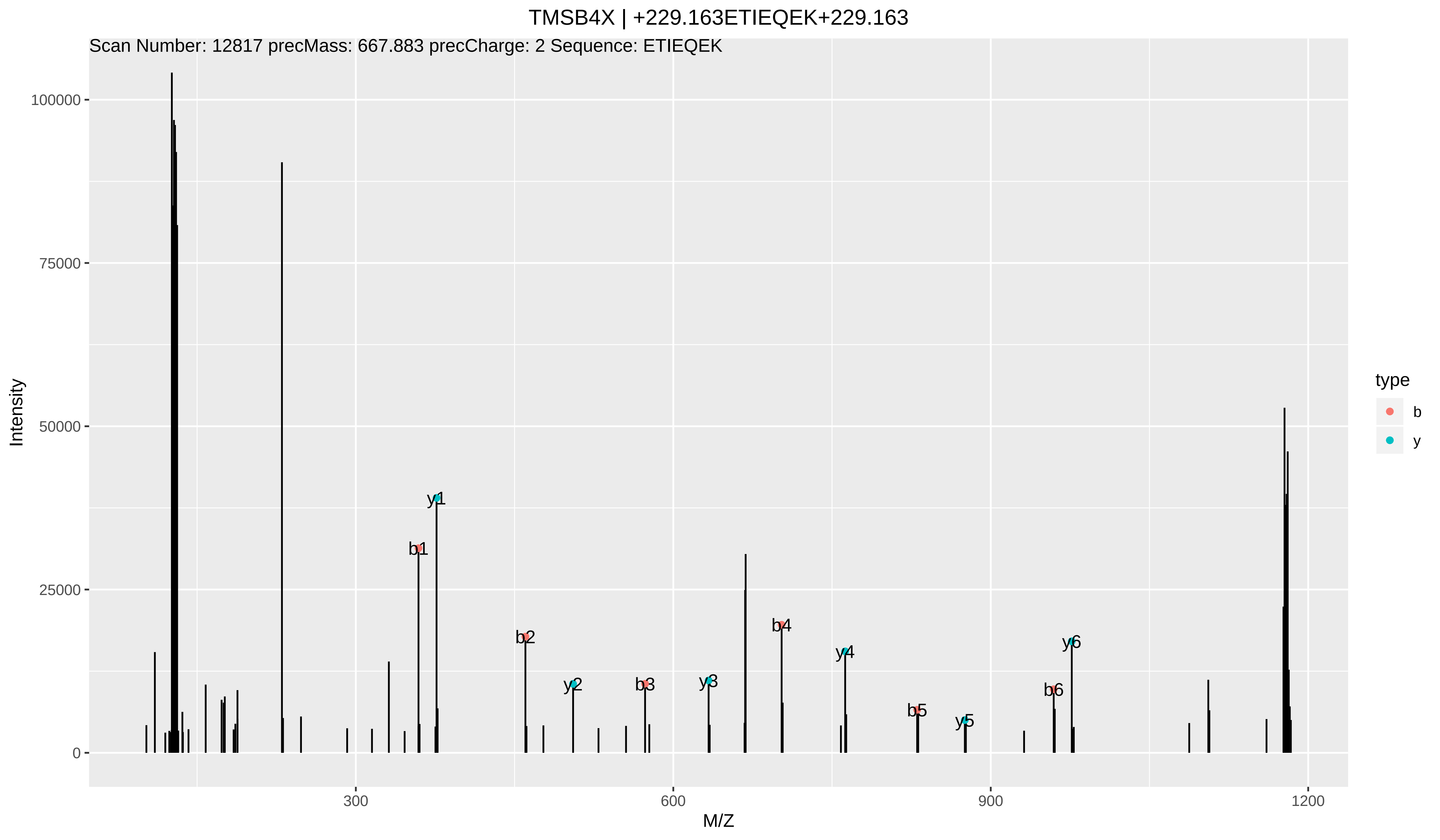

TMSB4X | +229.163ETIEQEK+229.163
Scan Number: 12817 precMass: 667.883 precCharge: 2 Sequence: ETIEQEK
Intensity
100000
75000
50000
25000
0
300
600
900
1200
M/Z
y1
b1
b2
y2
b3
y3
b4
y4
b5
y5
b6
y6
type
b
y

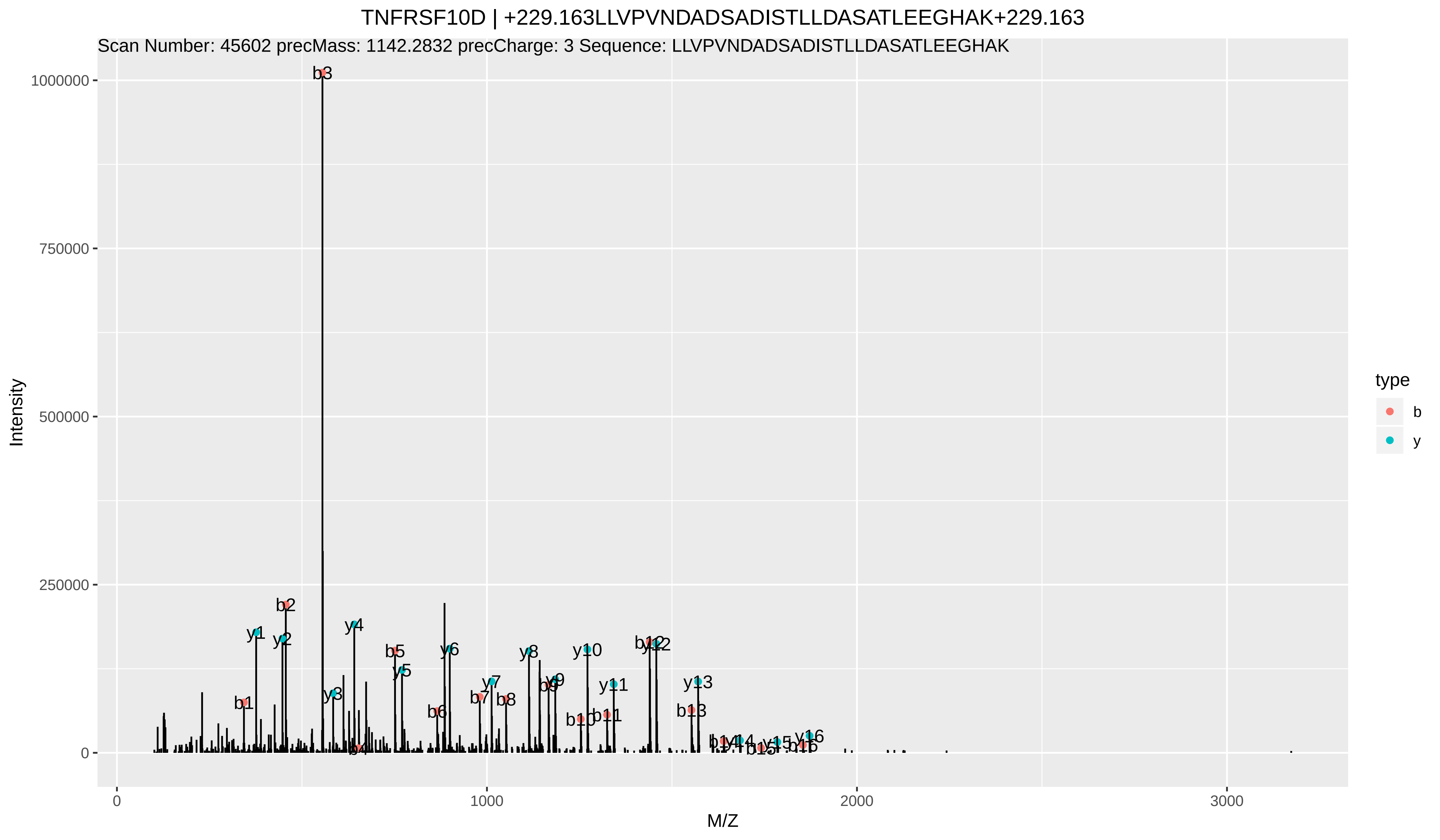

TNFRSF10D | +229.163LLVPVNDADSADISTLLDASATLEEGHAK+229.163
Scan Number: 45602 precMass: 1142.2832 precCharge: 3 Sequence: LLVPVNDADSADISTLLDASATLEEGHAK
Intensity
M/Z
type
b
y

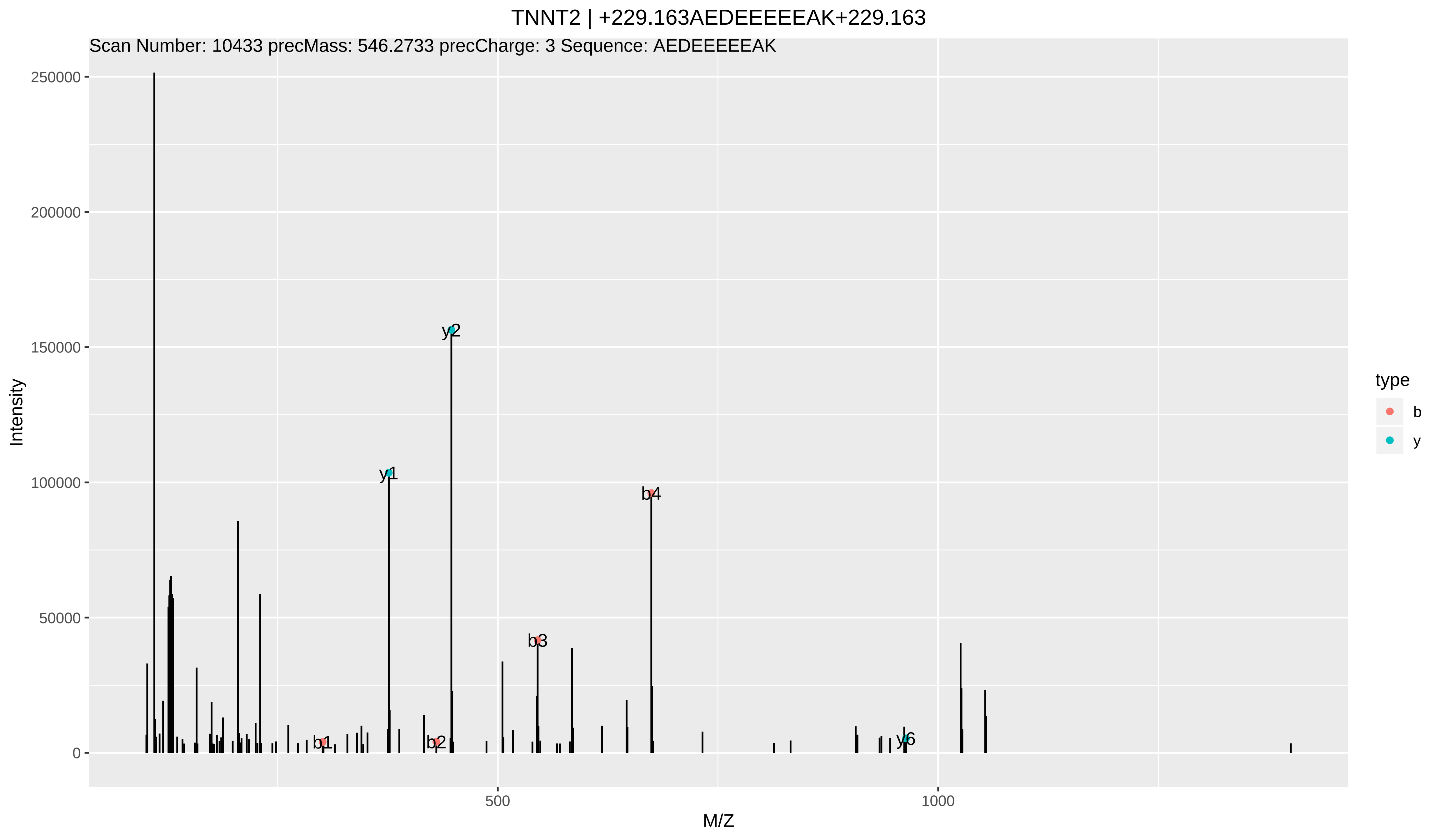
TNNT2 | +229.163AEDEEEEEAK+229.163
Scan Number: 10433 precMass: 546.2733 precCharge: 3 Sequence: AEDEEEEEAK
Intensity
M/Z
0
50000
100000
150000
200000
250000
500
1000
y1
y2
y6
b1
b2
b3
b4
type
b
y

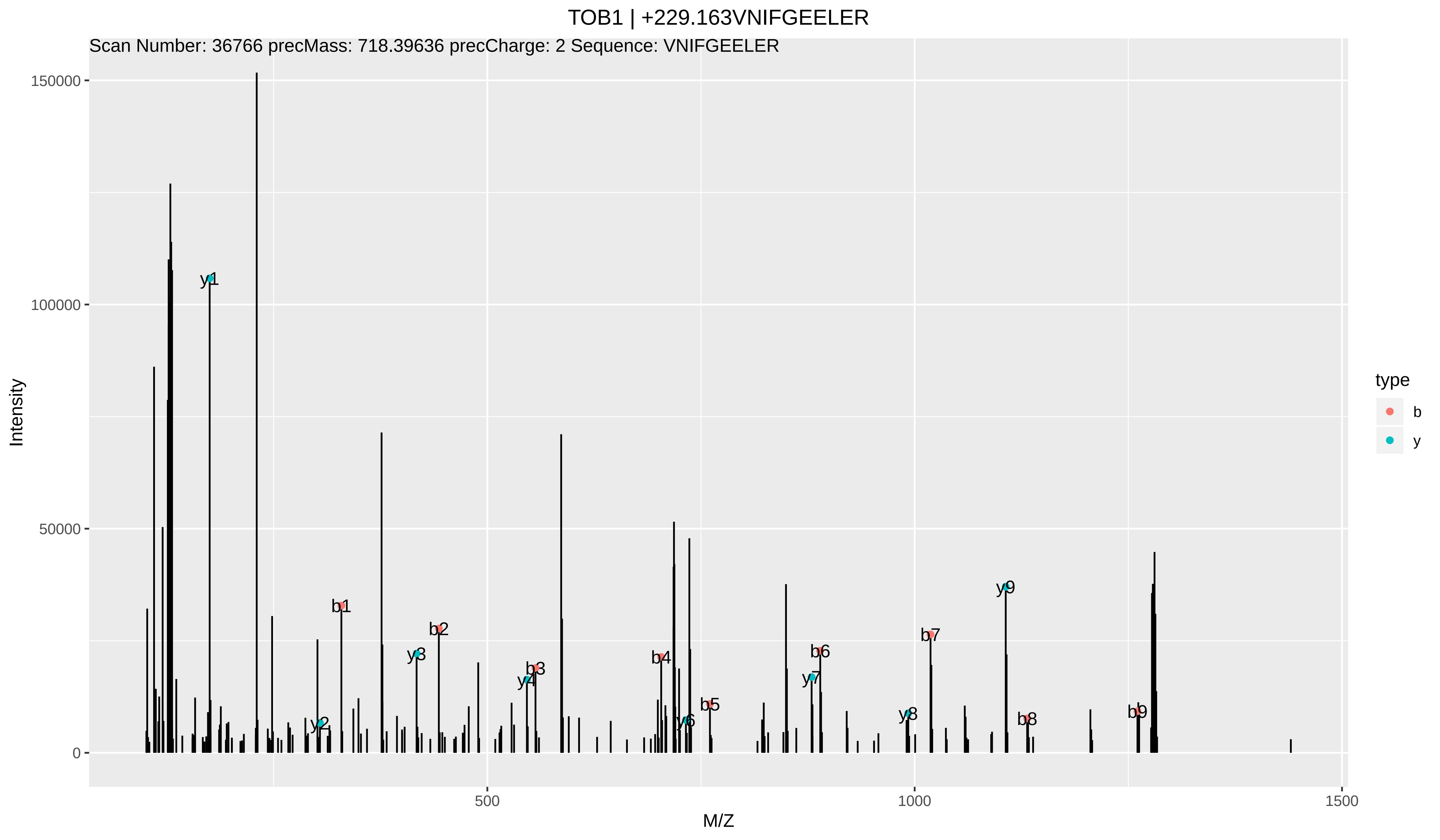
TOB1 | +229.163VNIFGEELER
Scan Number: 36766 precMass: 718.39636 precCharge: 2 Sequence: VNIFGEELER
Intensity
150000
100000
50000
0
M/Z
500
1000
1500
y1
y2
y3
y4
y6
y7
y8
y9
b1
b2
b3
b4
b5
b6
b7
b8
b9
type
b
y

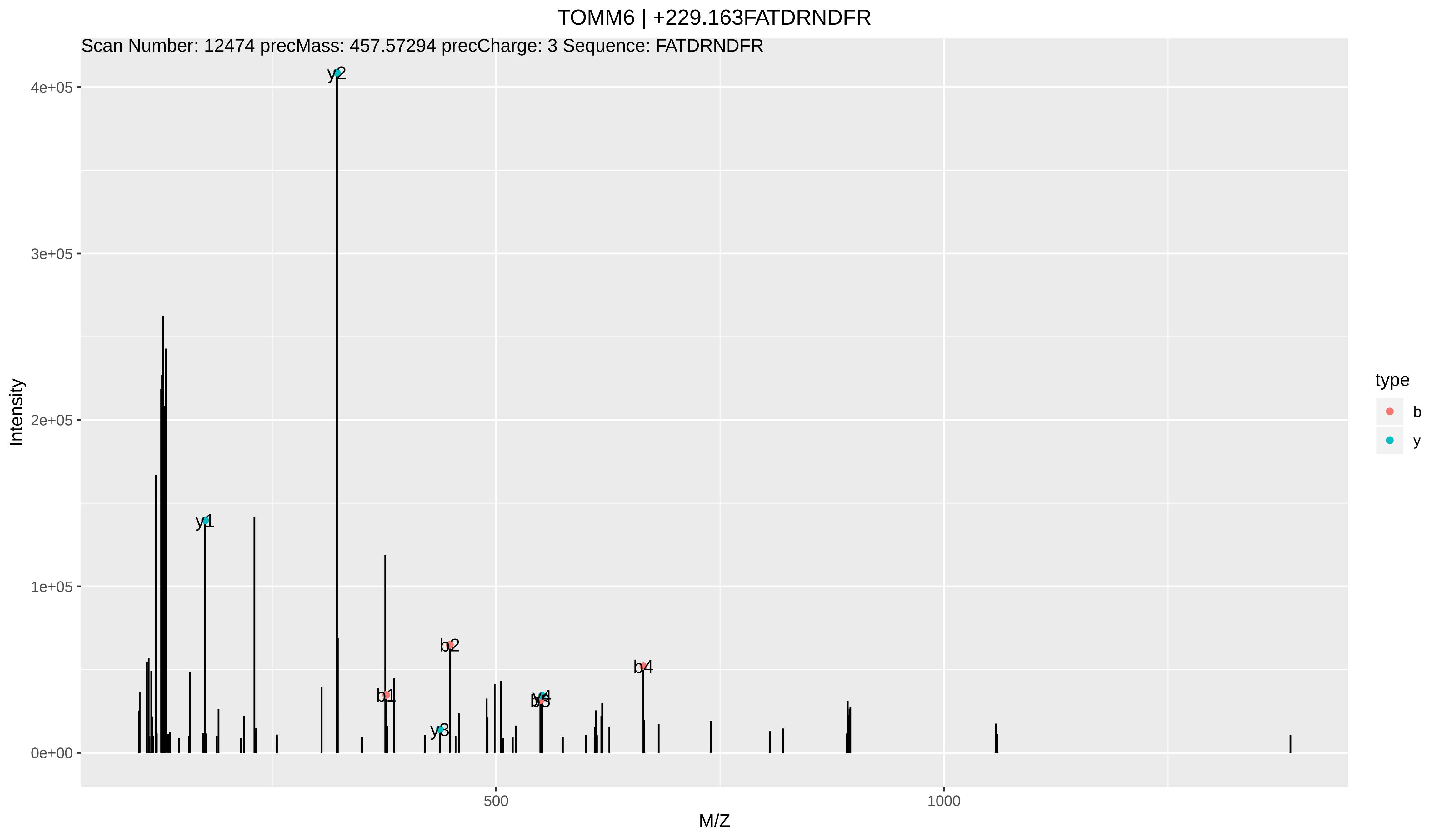

TOMM6 | +229.163FATDRNDFR
Scan Number: 12474 precMass: 457.57294 precCharge: 3 Sequence: FATDRNDFR
Intensity
M/Z
type
b
y
y1
y2
y3
y4
b1
b2
b3
b4
0e+00
1e+05
2e+05
3e+05
4e+05
500
1000

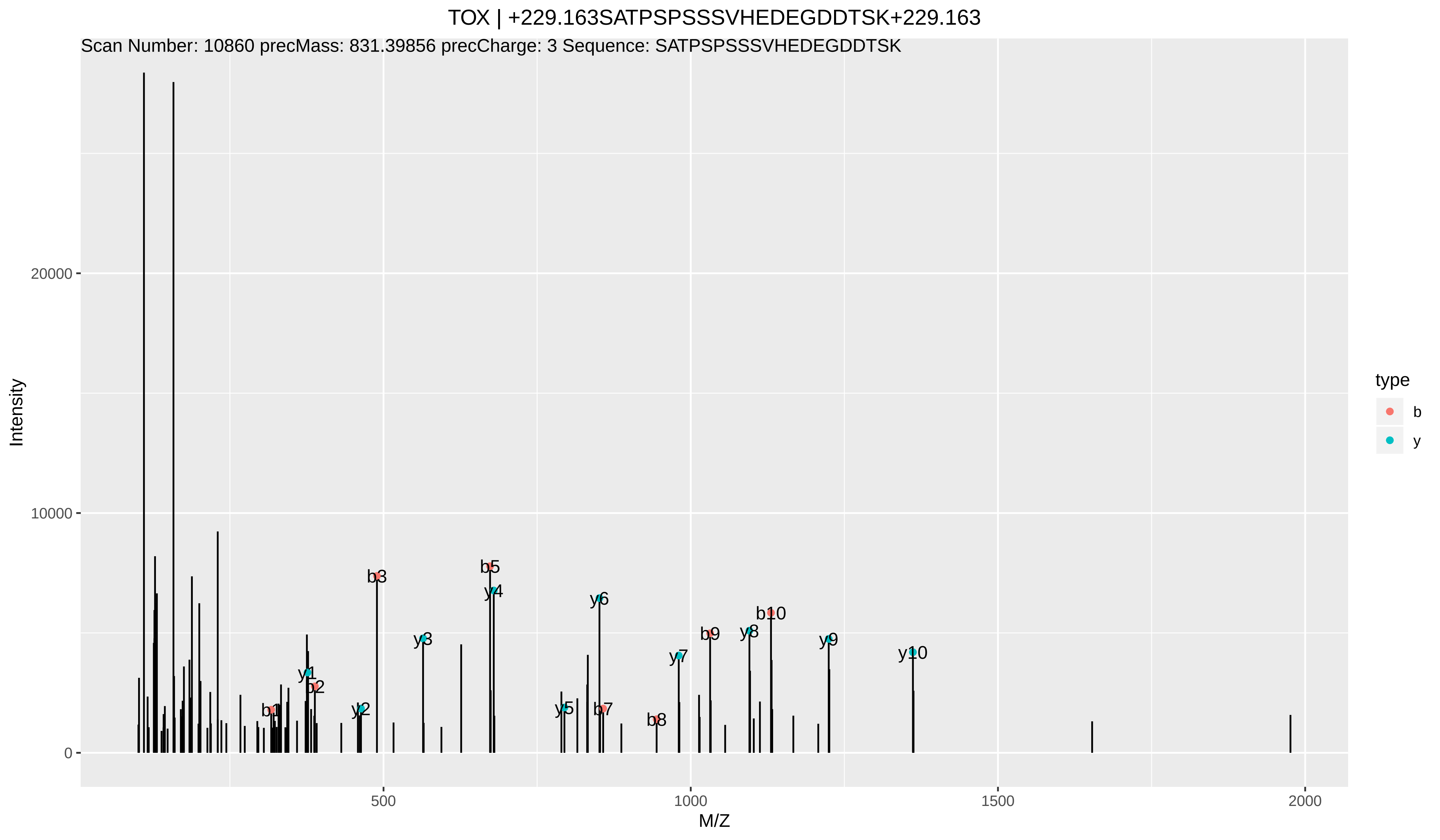

TOX | +229.163SATPSPSSSVHEDEGDDTSK+229.163
Scan Number: 10860 precMass: 831.39856 precCharge: 3 Sequence: SATPSPSSSVHEDEGDDTSK
Intensity
20000
10000
0
M/Z
500
1000
1500
2000
b1
b2
b3
b5
b7
b8
b9
b10
y1
y2
y3
y4
y5
y6
y7
y8
y9
y10
type
b
y

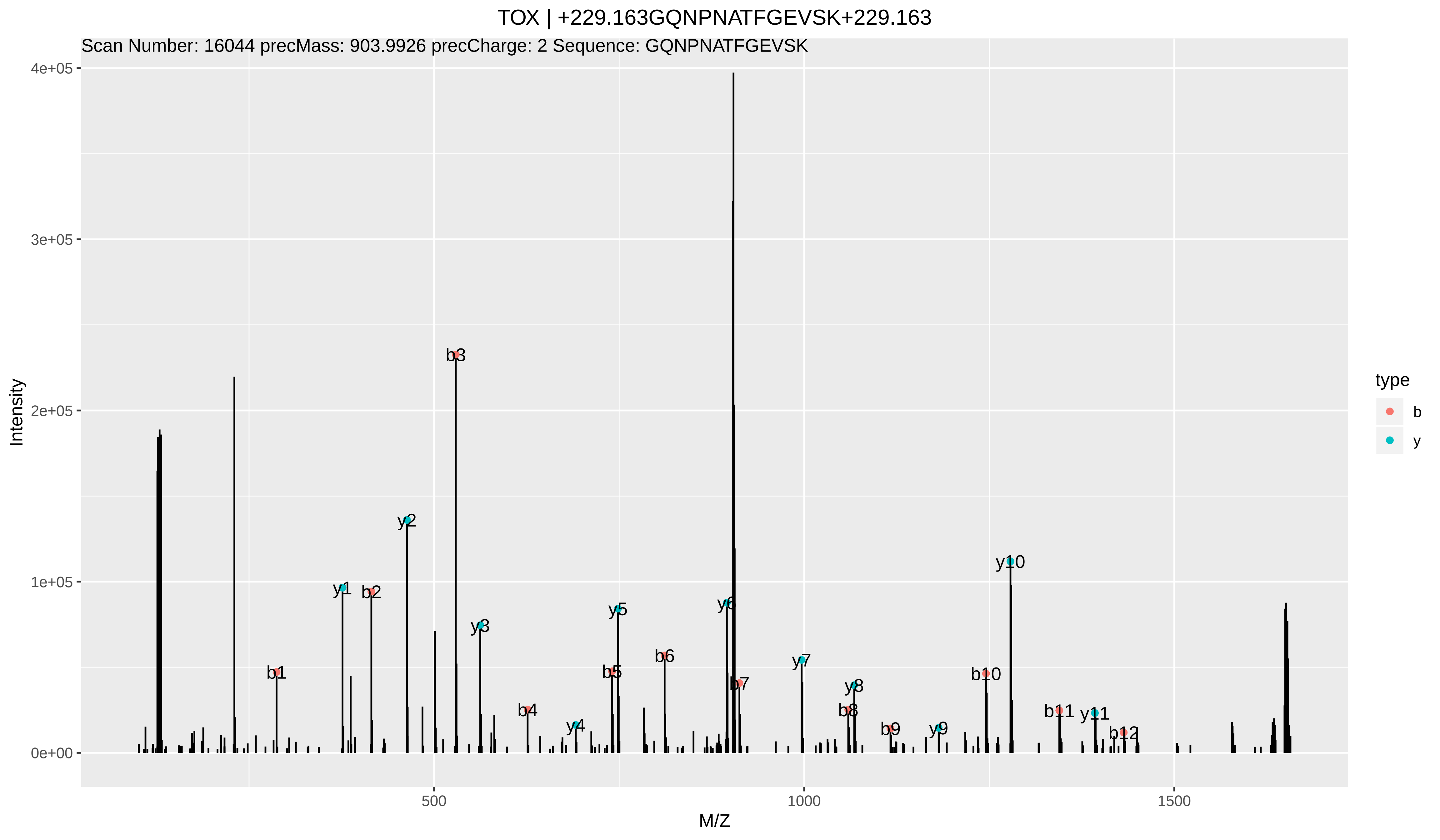
TOX | +229.163GQNPNATFGEVSK+229.163
Scan Number: 16044 precMass: 903.9926 precCharge: 2 Sequence: GQNPNATFGEVSK
Intensity
M/Z
type
b
y
b1
y1
b2
y2
b3
y3
b4
y4
b5
y5
b6
y6
b7
y7
b8
y8
b9
y9
b10
y10
b11
y11
b12
0e+00
1e+05
2e+05
3e+05
4e+05
500
1000
1500

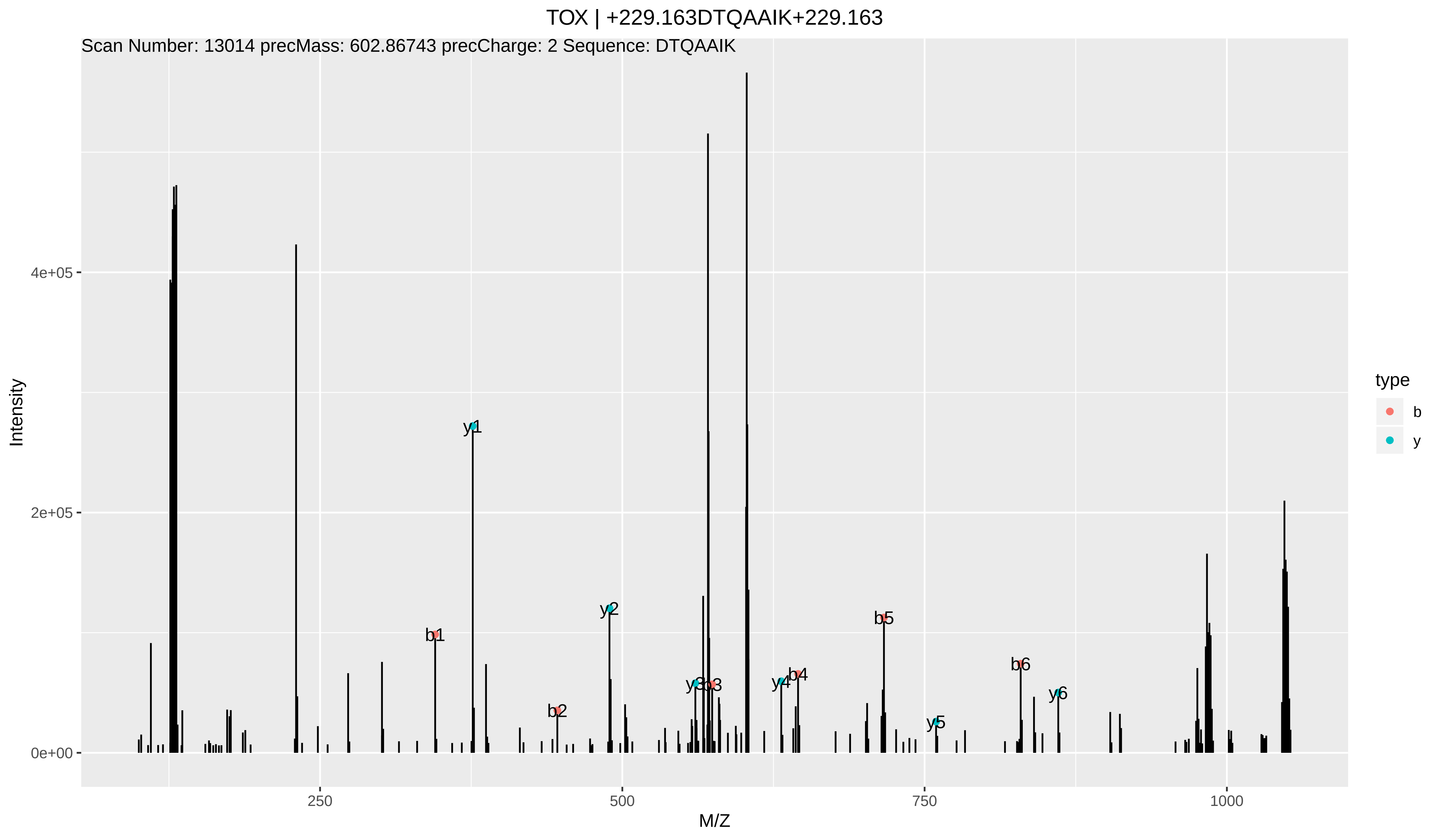

TOX | +229.163DTQAAIK+229.163
Scan Number: 13014 precMass: 602.86743 precCharge: 2 Sequence: DTQAAIK
Intensity
4e+05
2e+05
0e+00
M/Z
250
500
750
1000
y1
b1
b2
y2
y3
b3
y4
b4
b5
y5
b6
y6
type
b
y

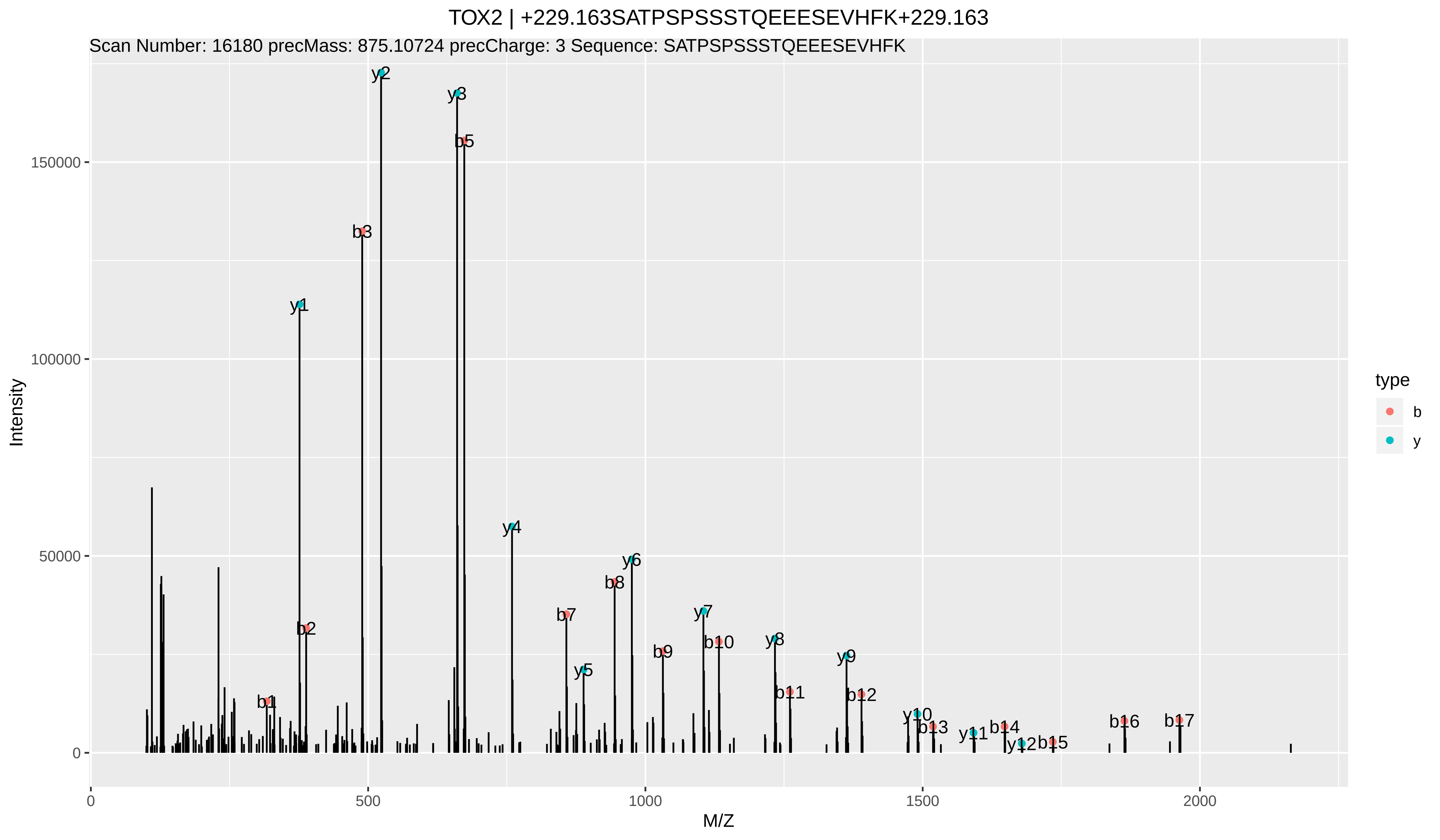

TOX2 | +229.163SATPSPSSSTQEEESEVHFK+229.163
Scan Number: 16180 precMass: 875.10724 precCharge: 3 Sequence: SATPSPSSSTQEEESEVHFK
Intensity
M/Z
150000
100000
50000
0
0
500
1000
1500
2000
y1
y2
y3
y4
y5
y6
y7
y8
y9
y10
y11
y12
b1
b2
b3
b5
b7
b8
b9
b10
b11
b12
b13
b14
b15
b16
b17
type
b
y

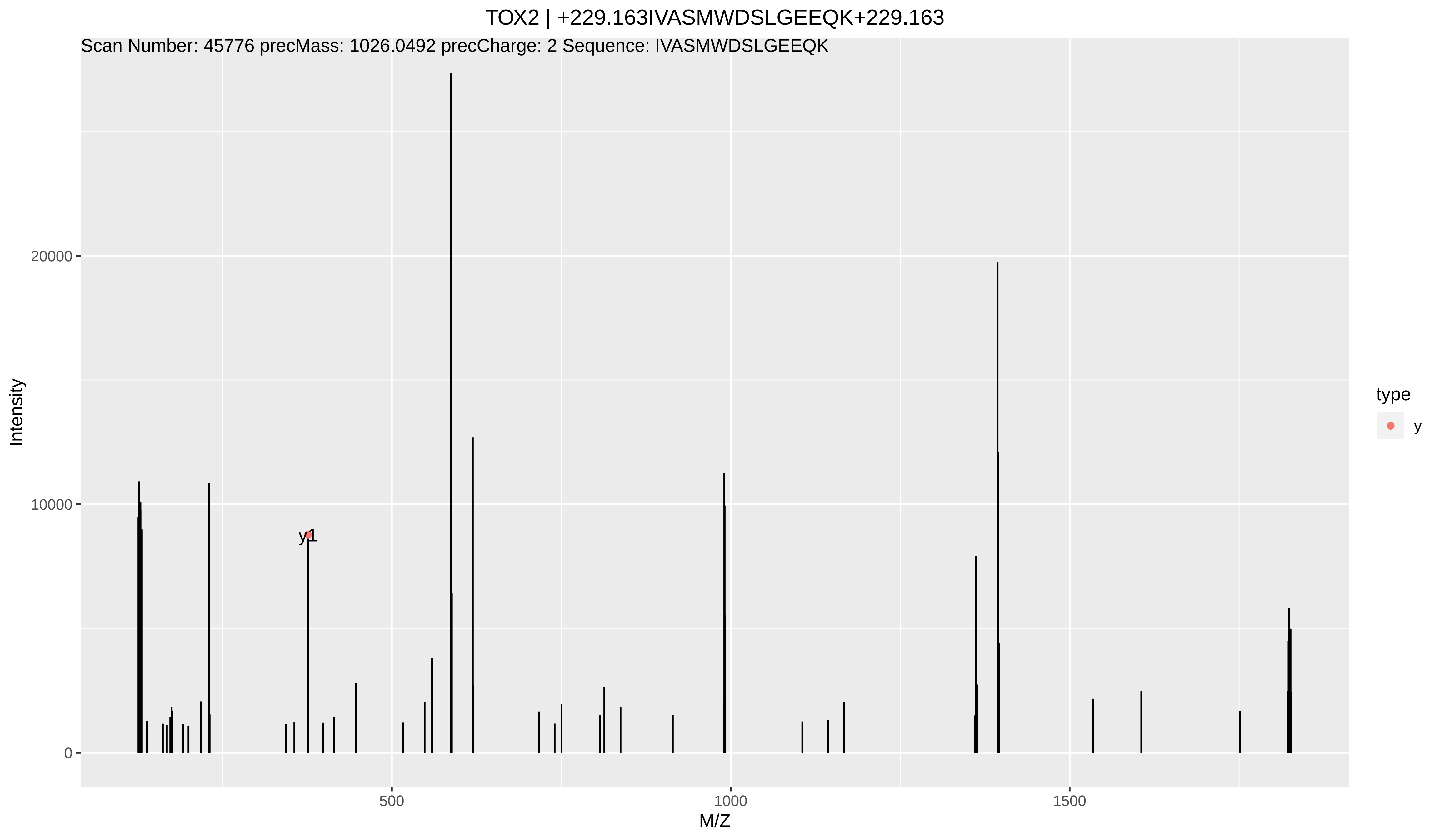

TOX2 | +229.163IVASMWDSLGEEQK+229.163
Scan Number: 45776 precMass: 1026.0492 precCharge: 2 Sequence: IVASMWDSLGEEQK
Intensity
20000
10000
0
y1
500
1000
1500
M/Z
type
y

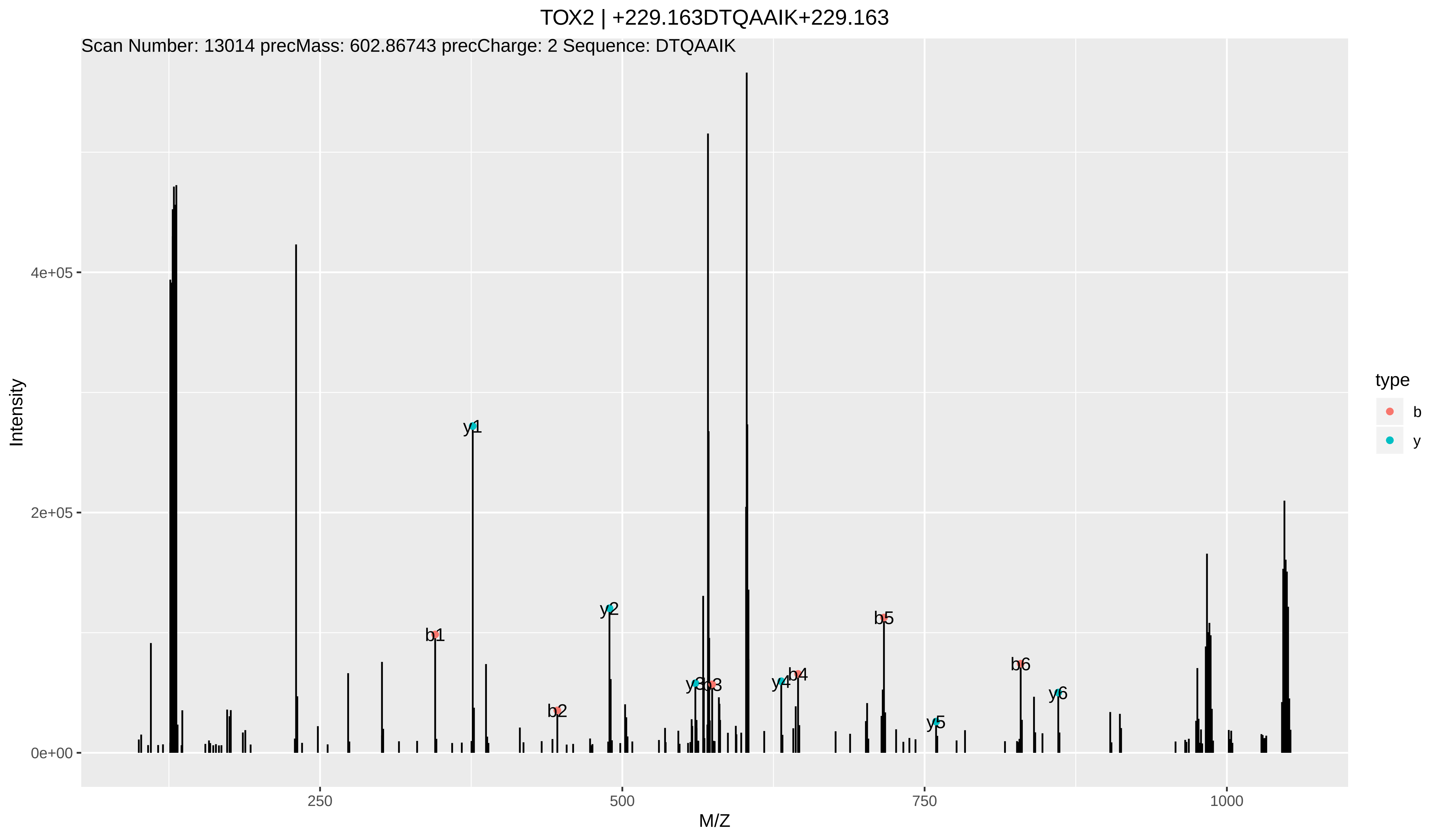
TOX2 | +229.163DTQAAIK+229.163
Scan Number: 13014 precMass: 602.86743 precCharge: 2 Sequence: DTQAAIK
Intensity
4e+05
2e+05
0e+00
250
500
750
1000
M/Z
y1
y2
b1
b2
y3
b3
y4
b4
b5
y5
b6
y6
type
b
y

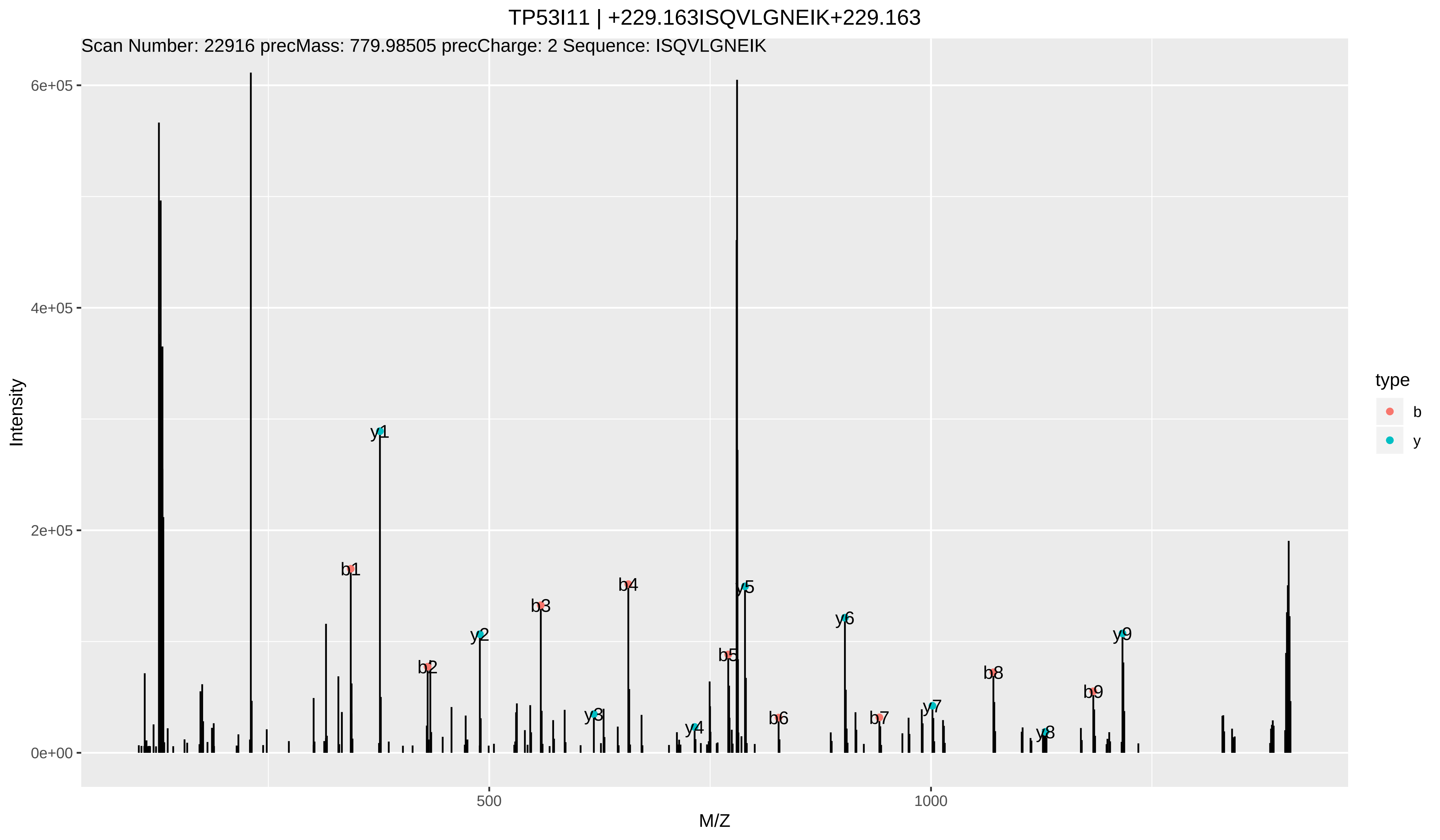
TP53I11 | +229.163ISQVLGNEIK+229.163
Scan Number: 22916 precMass: 779.98505 precCharge: 2 Sequence: ISQVLGNEIK
Intensity
6e+05
4e+05
2e+05
0e+00
M/Z
500
1000
y1
b1
b2
y2
b3
y3
b4
y4
b5
y5
b6
y6
b7
y7
b8
y8
b9
y9
type
b
y

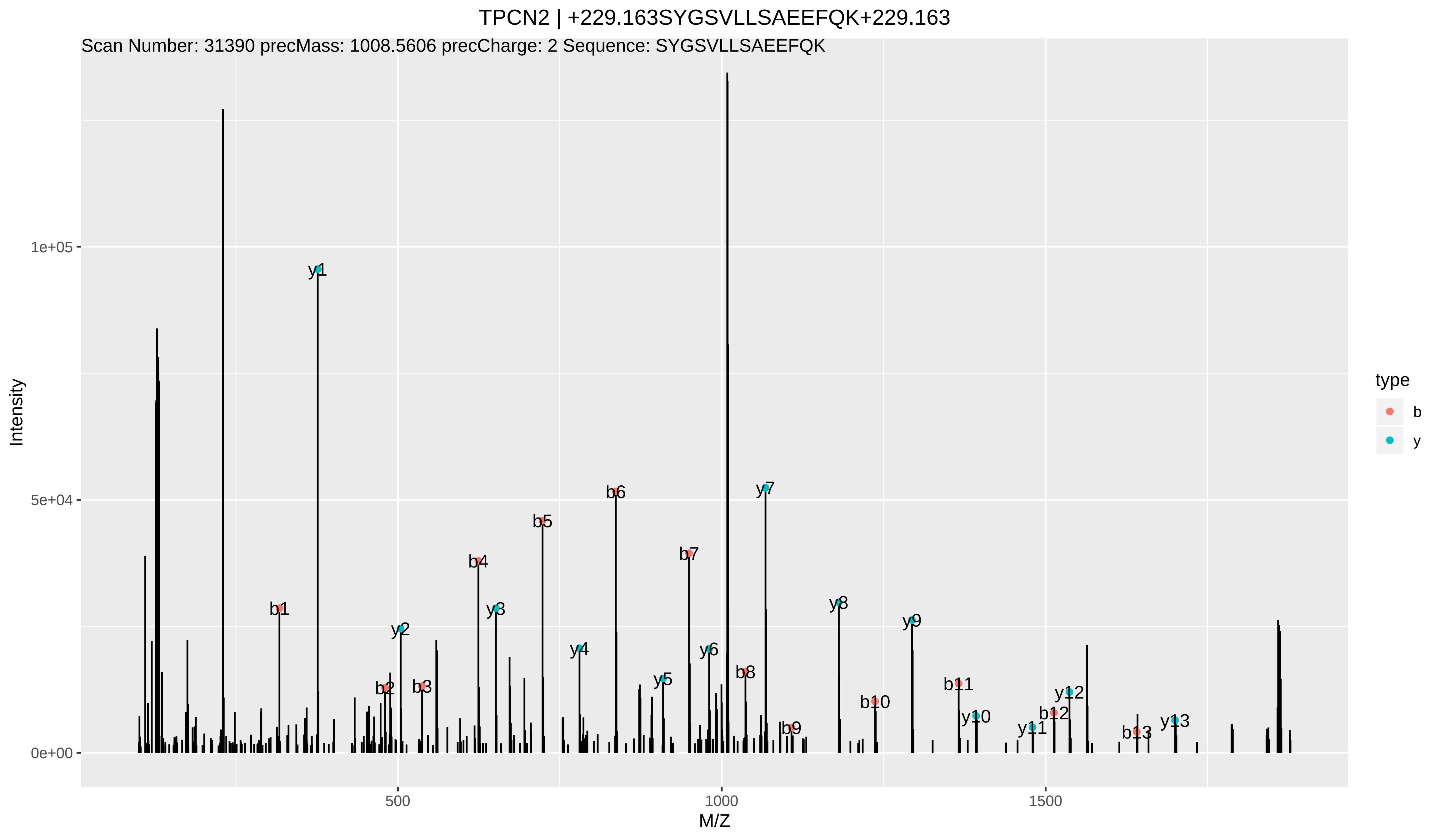

TPCN2 | +229.163SYGSVLLSAEEFQK+229.163
Scan Number: 31390 precMass: 1008.5606 precCharge: 2 Sequence: SYGSVLLSAEEFQK
Intensity
1e+05
5e+04
0e+00
M/Z
500
1000
1500
type
b
y
y1
y2
y3
y4
y5
y6
y7
y8
y9
y10
y11
y12
y13
b1
b2
b3
b4
b5
b6
b7
b8
b9
b10
b11
b12
b13

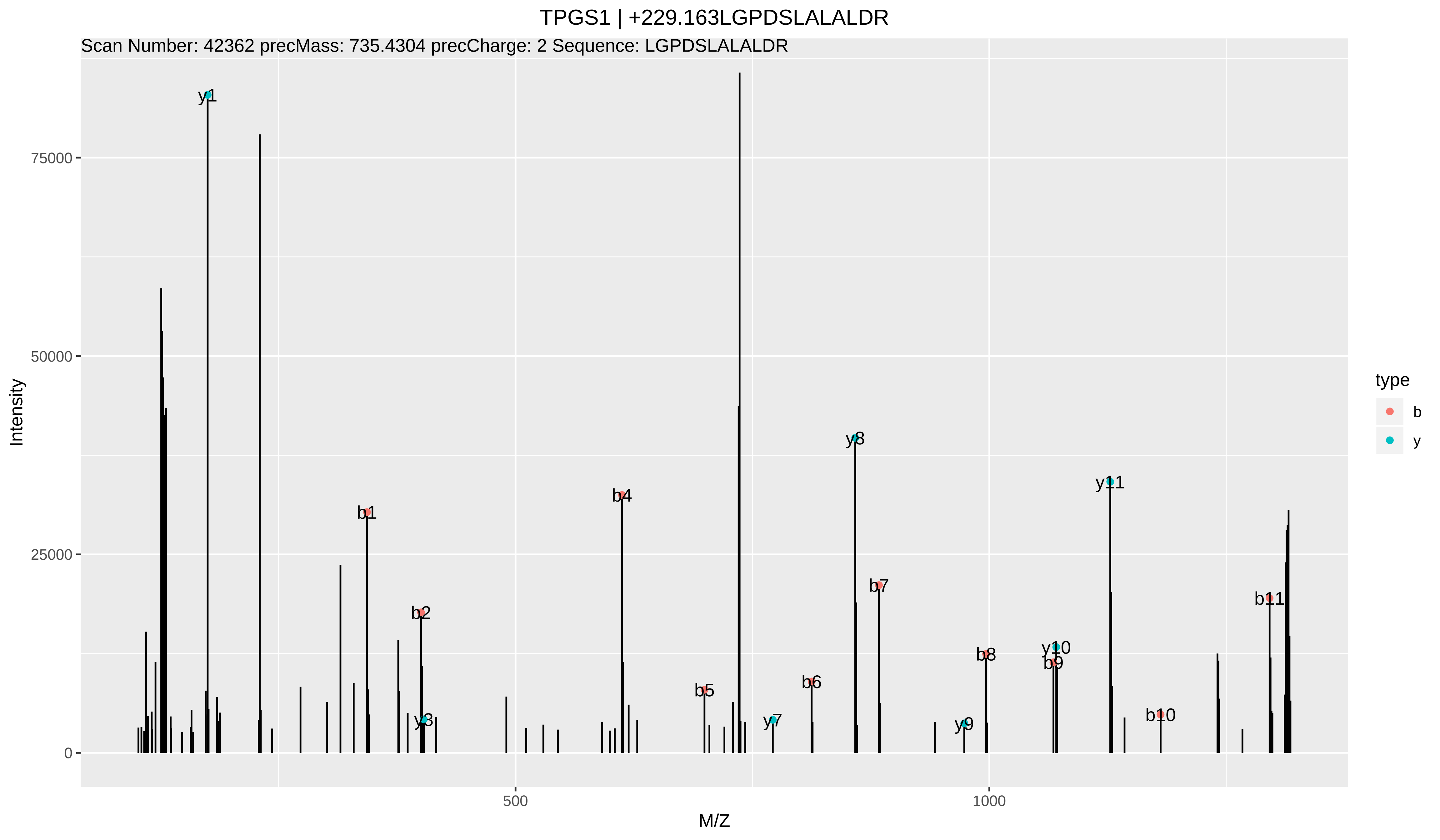
TPGS1 | +229.163LGPDSLALALDR
Scan Number: 42362 precMass: 735.4304 precCharge: 2 Sequence: LGPDSLALALDR
Intensity
M/Z
75000
50000
25000
0
500
1000
y1
b1
b2
y3
b4
b5
y7
b6
y8
b7
y9
b8
y10
b9
y11
b10
b11
type
b
y

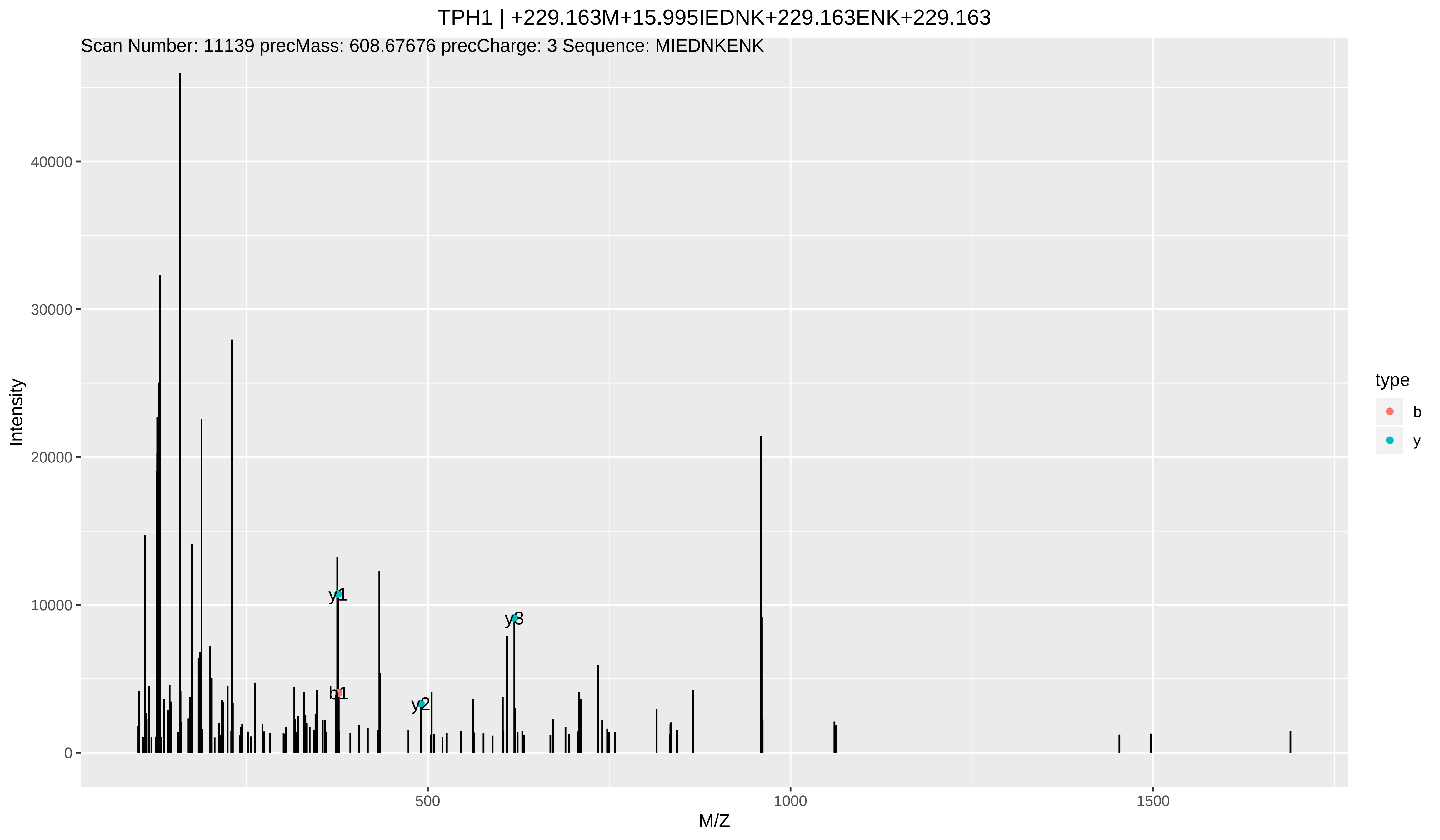

TPH1 | +229.163M+15.995IEDNK+229.163ENK+229.163
Scan Number: 11139 precMass: 608.67676 precCharge: 3 Sequence: MIEDNKENK
Intensity
40000
30000
20000
10000
0
y1
b1
y2
y3
500
1000
1500
M/Z
type
b
y

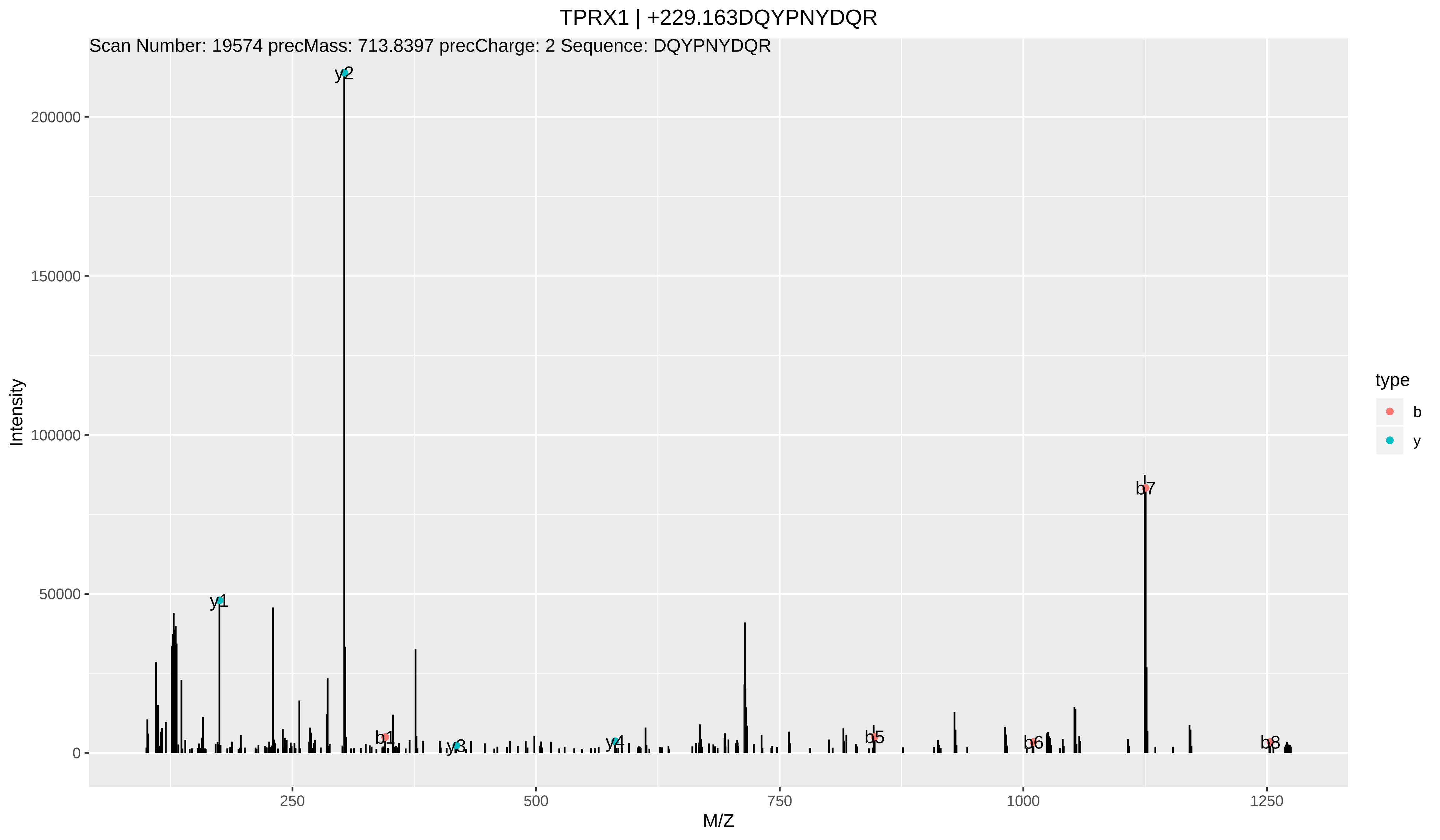

TPRX1 | +229.163DQYPNYDQR
Scan Number: 19574 precMass: 713.8397 precCharge: 2 Sequence: DQYPNYDQR
Intensity
M/Z
0
50000
100000
150000
200000
250
500
750
1000
1250
y1
y2
y3
y4
b1
b5
b6
b7
b8
type
b
y

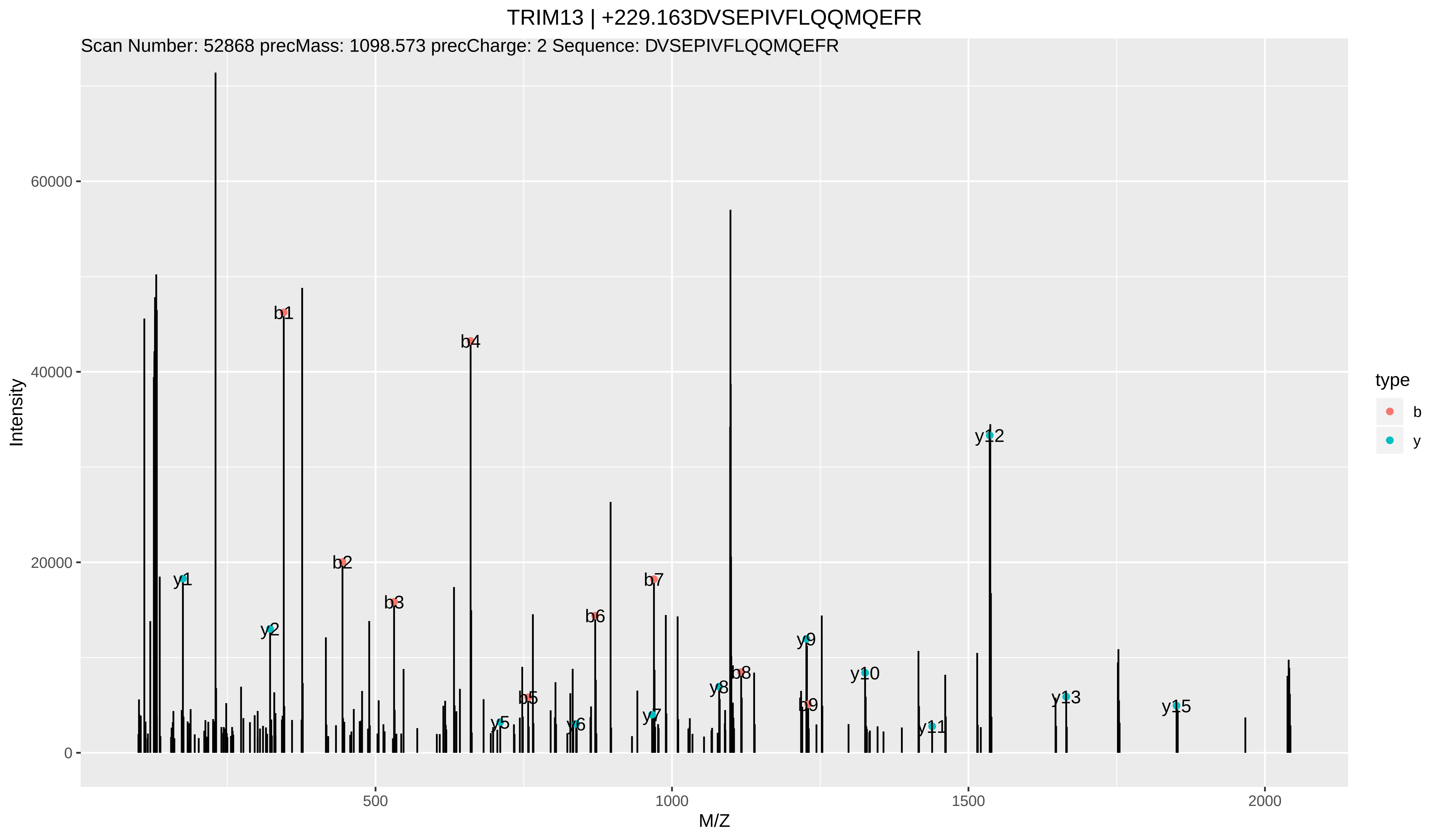
TRIM13 | +229.163DVSEPIVFLQQMQEFR
Scan Number: 52868 precMass: 1098.573 precCharge: 2 Sequence: DVSEPIVFLQQMQEFR
Intensity
60000
40000
20000
0
M/Z
500
1000
1500
2000
type
b
y
b1
b2
b3
b4
b5
b6
b7
b8
b9
y1
y2
y5
y6
y7
y8
y9
y10
y11
y12
y13
y15

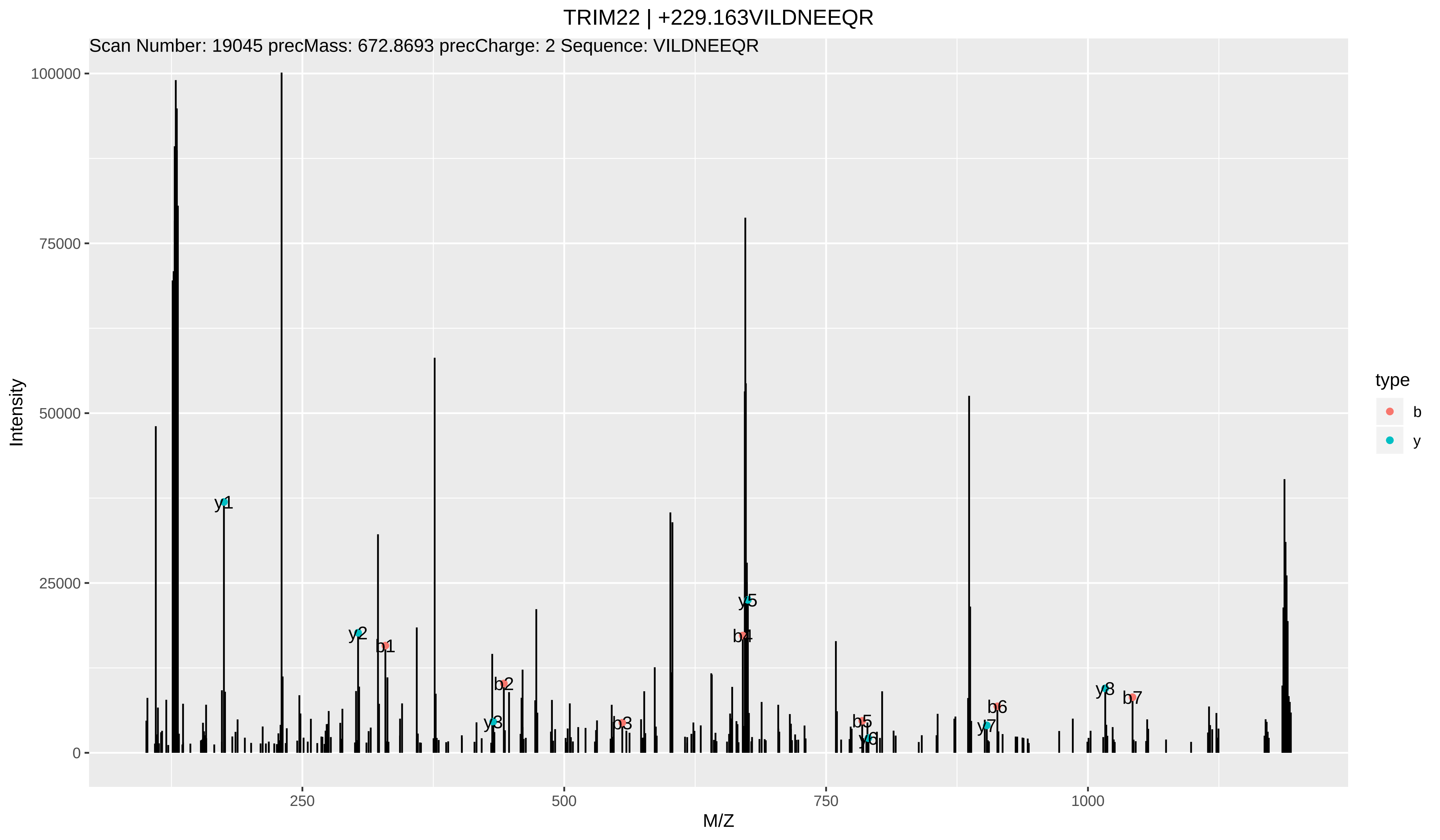

TRIM22 | +229.163VILDNEEQR
Scan Number: 19045 precMass: 672.8693 precCharge: 2 Sequence: VILDNEEQR
Intensity
M/Z
100000
75000
50000
25000
0
250
500
750
1000
y1
y2
b1
y3
b2
b3
y5
b4
b5
y6
y7
b6
y8
b7
type
b
y

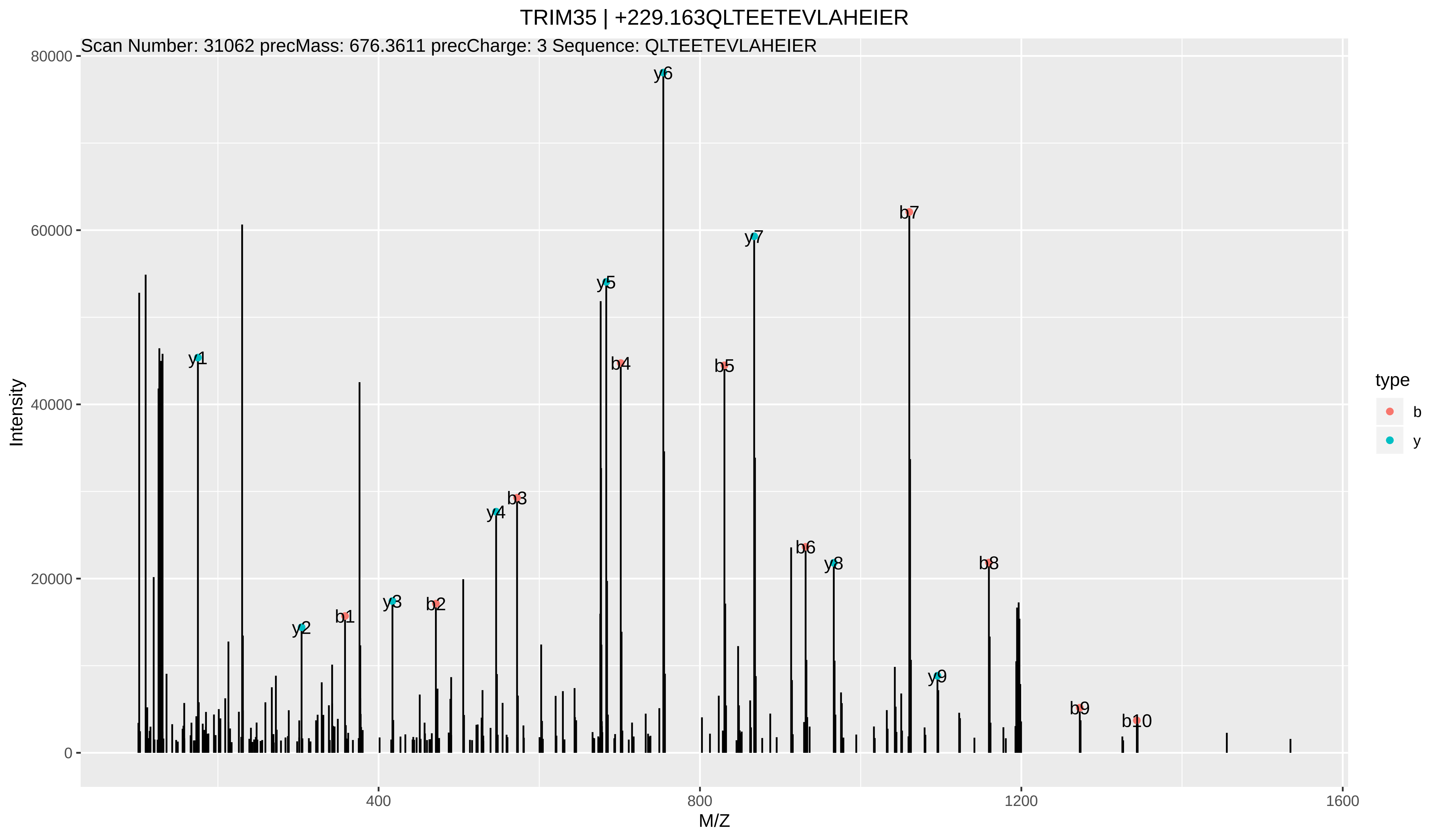

TRIM35 | +229.163QLTEETEVLAHEIER
Scan Number: 31062 precMass: 676.3611 precCharge: 3 Sequence: QLTEETEVLAHEIER
Intensity
M/Z
type
b
y
y1
y2
b1
y3
b2
y4
b3
y5
b4
y6
b5
y7
b6
y8
b7
y9
b8
b9
b10

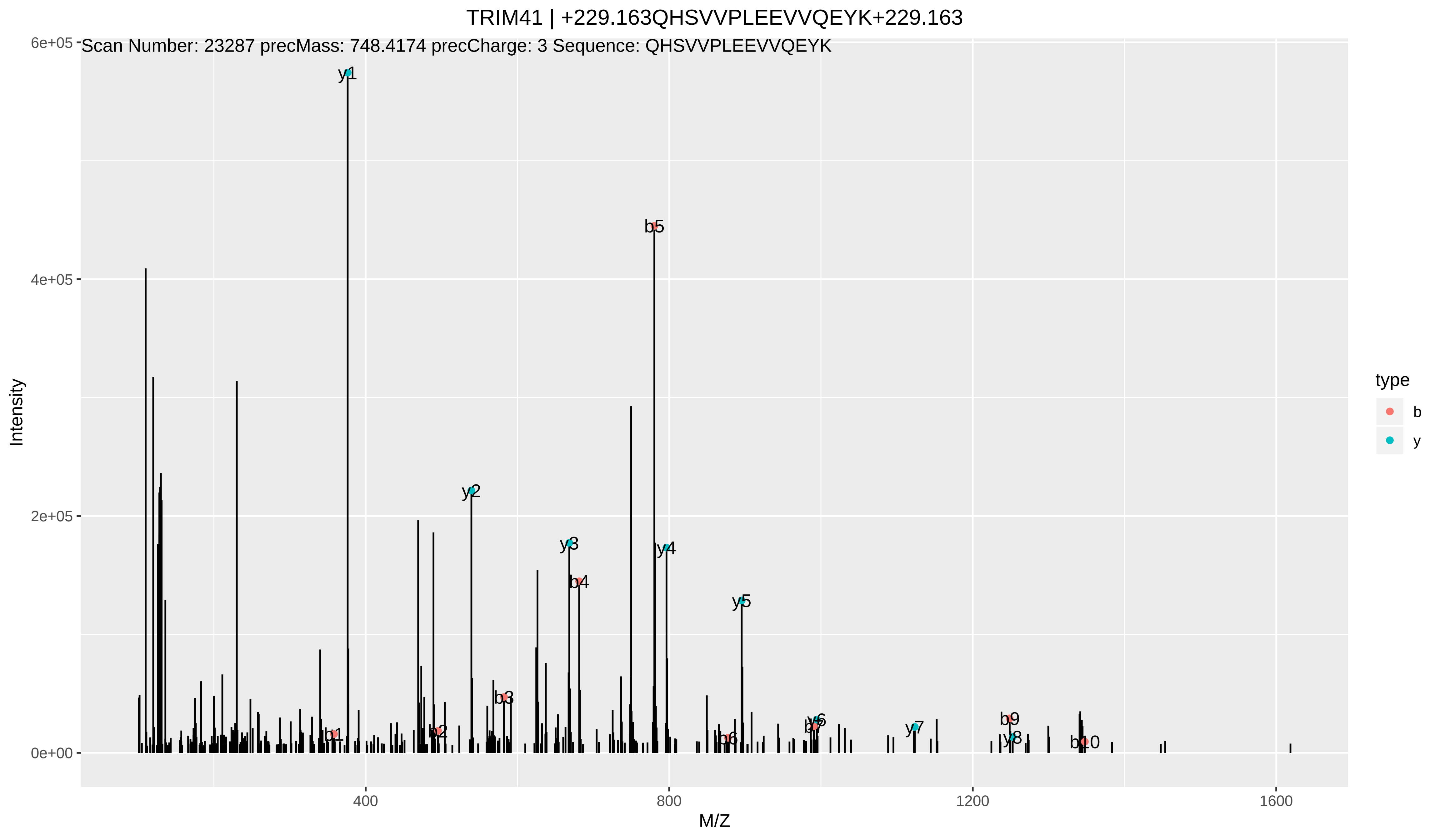

TRIM41 | +229.163QHSVVPLEEVVQEYK+229.163
Scan Number: 23287 precMass: 748.4174 precCharge: 3 Sequence: QHSVVPLEEVVQEYK
Intensity
M/Z
type
b
y

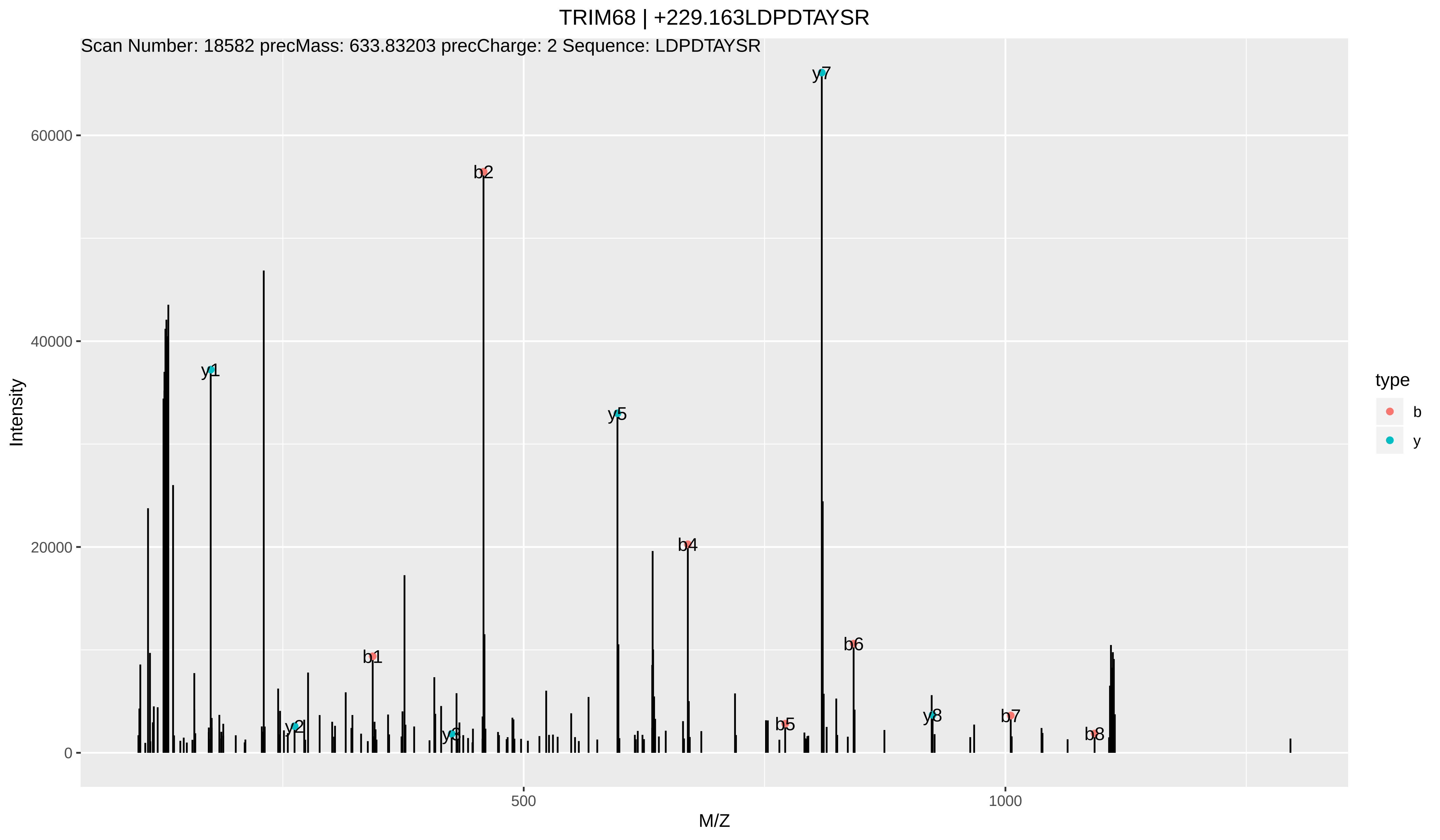
TRIM68 | +229.163LDPDTAYSR
Scan Number: 18582 precMass: 633.83203 precCharge: 2 Sequence: LDPDTAYSR
Intensity
M/Z
60000
40000
20000
0
500
1000
y7
b2
y1
y5
b4
b6
b1
y2
y3
b5
y8
b7
b8
type
b
y

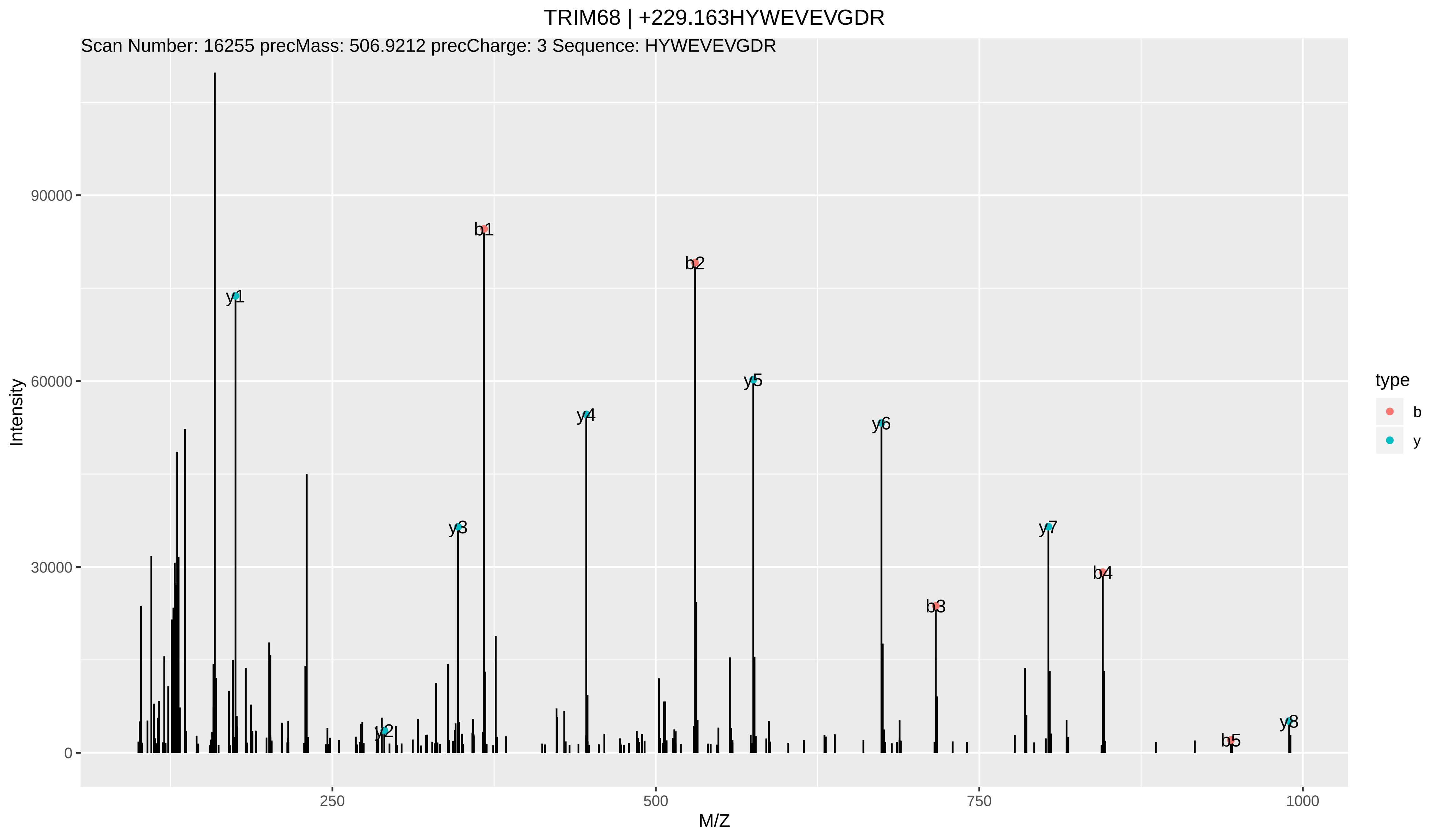

TRIM68 | +229.163HYWEVEVGDR
Scan Number: 16255 precMass: 506.9212 precCharge: 3 Sequence: HYWEVEVGDR
Intensity
M/Z
0
30000
60000
90000
250
500
750
1000
b1
b2
b3
b4
b5
y1
y2
y3
y4
y5
y6
y7
y8
type
b
y

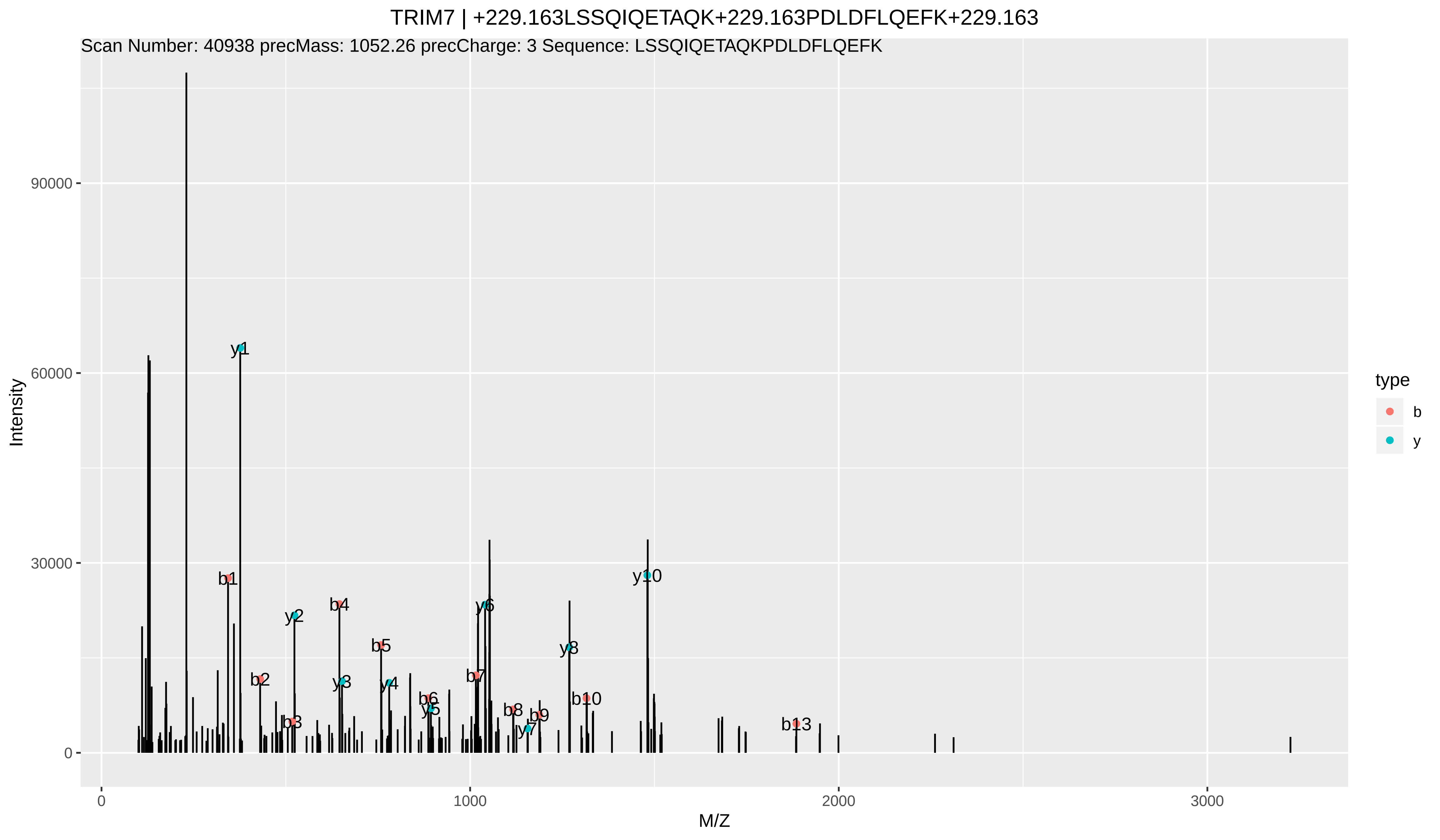

TRIM7 | +229.163LSSQIQETAQK+229.163PDLDFLQEFK+229.163
Scan Number: 40938 precMass: 1052.26 precCharge: 3 Sequence: LSSQIQETAQKPDLDFLQEFK
Intensity
90000
60000
30000
0
0
1000
2000
3000
M/Z
y1
b1
y2
b2
b3
b4
y3
b5
y4
b6
y5
b7
y6
b8
y7
b9
y8
b10
y10
b13
type
b
y

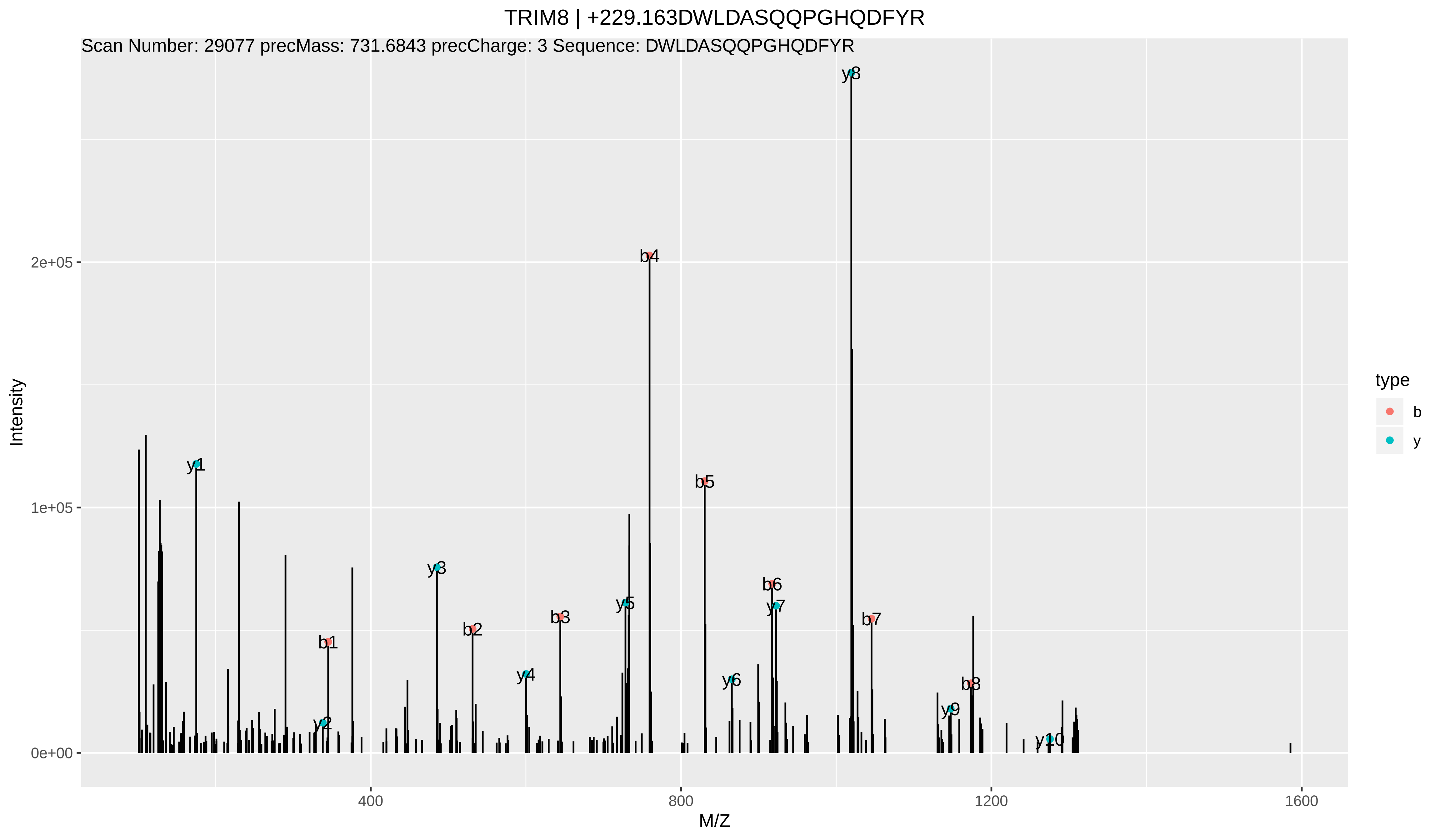
TRIM8 | +229.163DWLDASQQPGHQDFYR
Scan Number: 29077 precMass: 731.6843 precCharge: 3 Sequence: DWLDASQQPGHQDFYR
Intensity
M/Z
0e+00
1e+05
2e+05
400
800
1200
1600
type
b
y
y8
b4
b5
y1
y3
b6
y7
y5
b7
b3
b2
b1
y4
y6
b8
y9
y2
y10

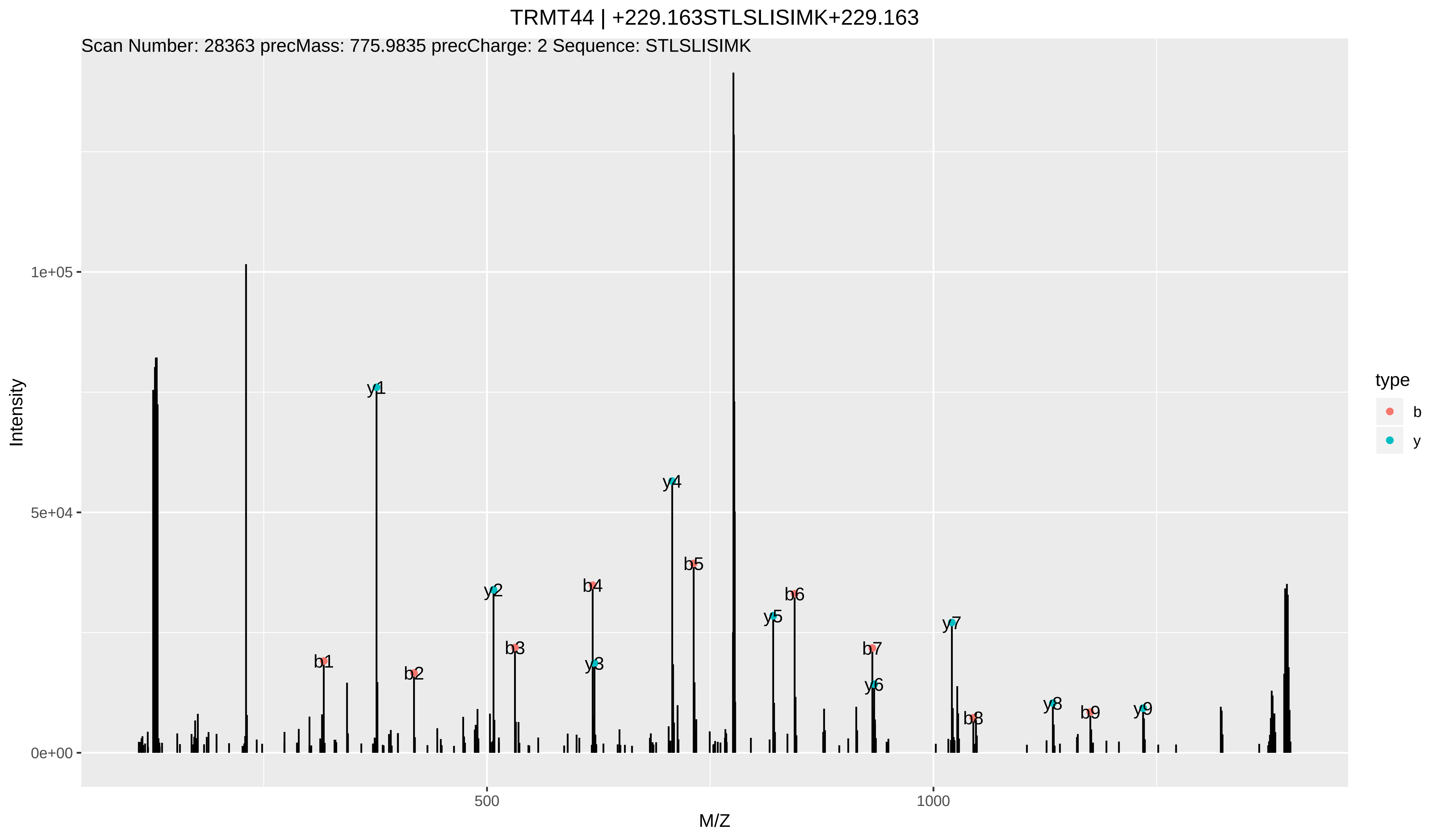
TRMT44 | +229.163STLSLISIMK+229.163
Scan Number: 28363 precMass: 775.9835 precCharge: 2 Sequence: STLSLISIMK
Intensity
1e+05
5e+04
0e+00
M/Z
500
1000
y1
y4
b5
y2
b4
b6
y5
y7
b3
b7
b1
y3
b2
y6
y8
b9
y9
b8
type
b
y

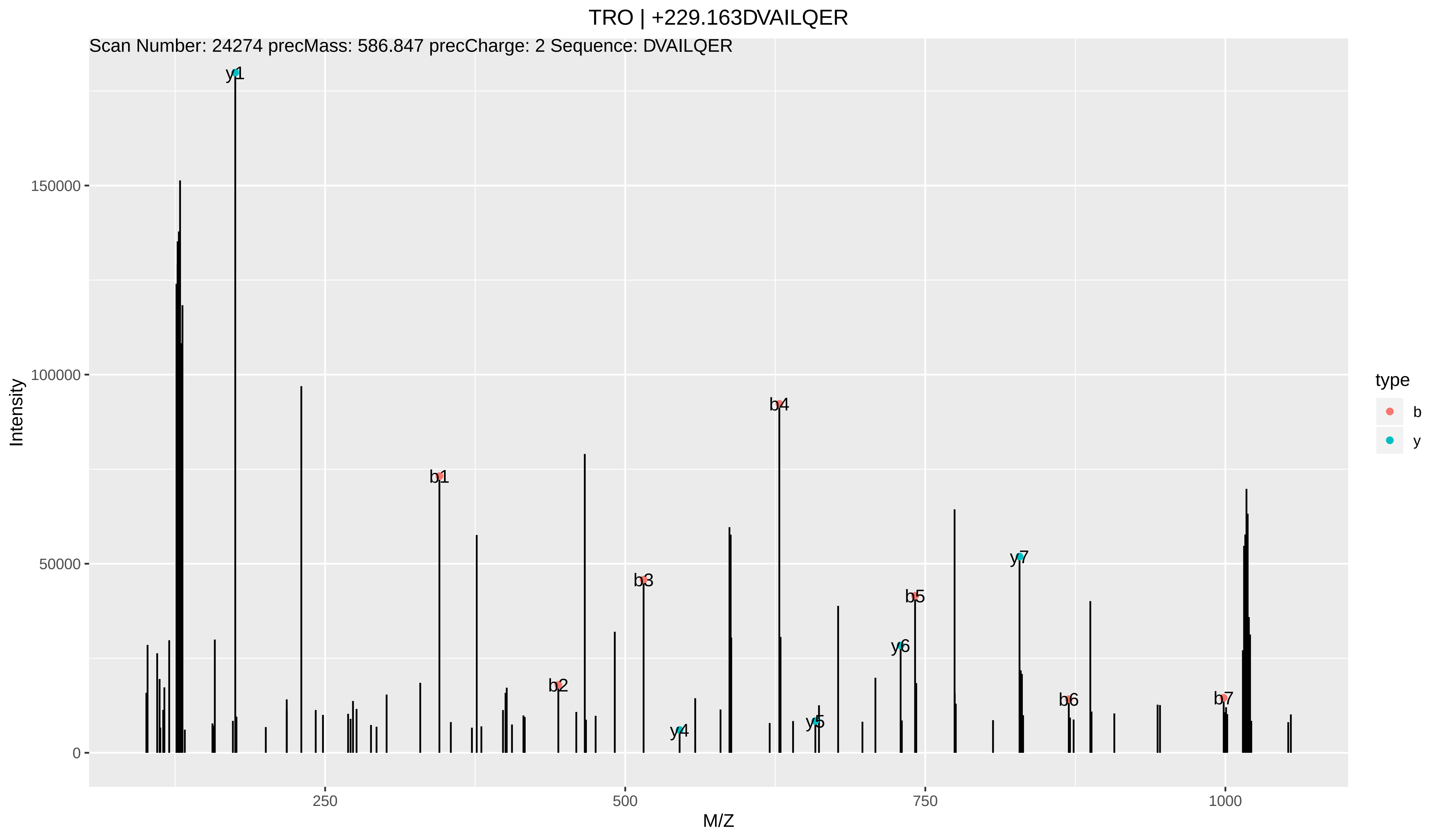

TRO | +229.163DVAILQER
Scan Number: 24274 precMass: 586.847 precCharge: 2 Sequence: DVAILQER
y1
b4
b1
y7
b3
b5
y6
b2
b6
b7
y5
y4
150000
100000
50000
0
250
500
750
1000
Intensity
M/Z
type
b
y

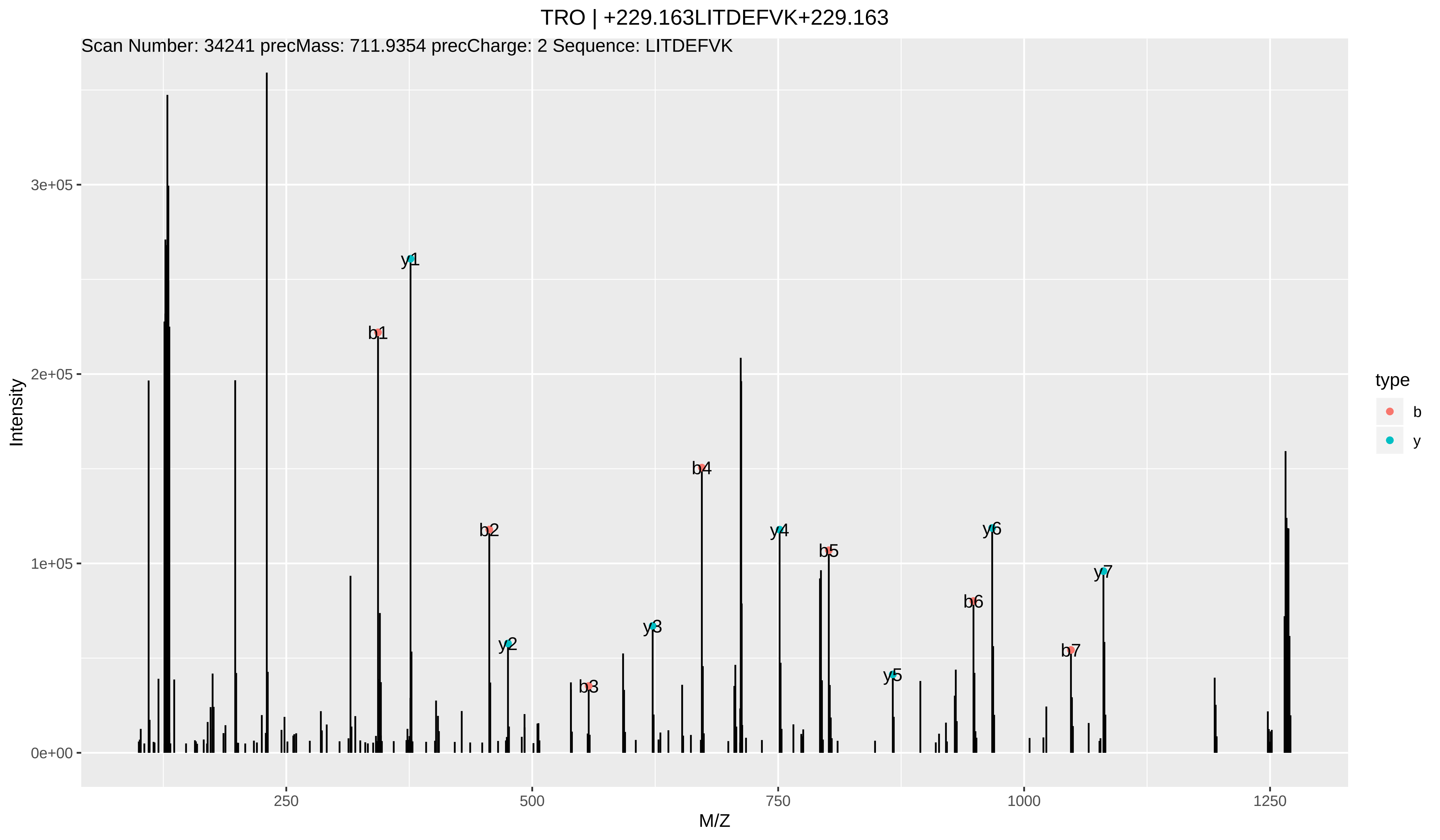

TRO | +229.163LITDEFVK+229.163
Scan Number: 34241 precMass: 711.9354 precCharge: 2 Sequence: LITDEFVK
Intensity
M/Z
0e+00
1e+05
2e+05
3e+05
250
500
750
1000
1250
y1
b1
b2
y2
b3
y3
b4
y4
b5
y5
b6
y6
b7
y7
type
b
y

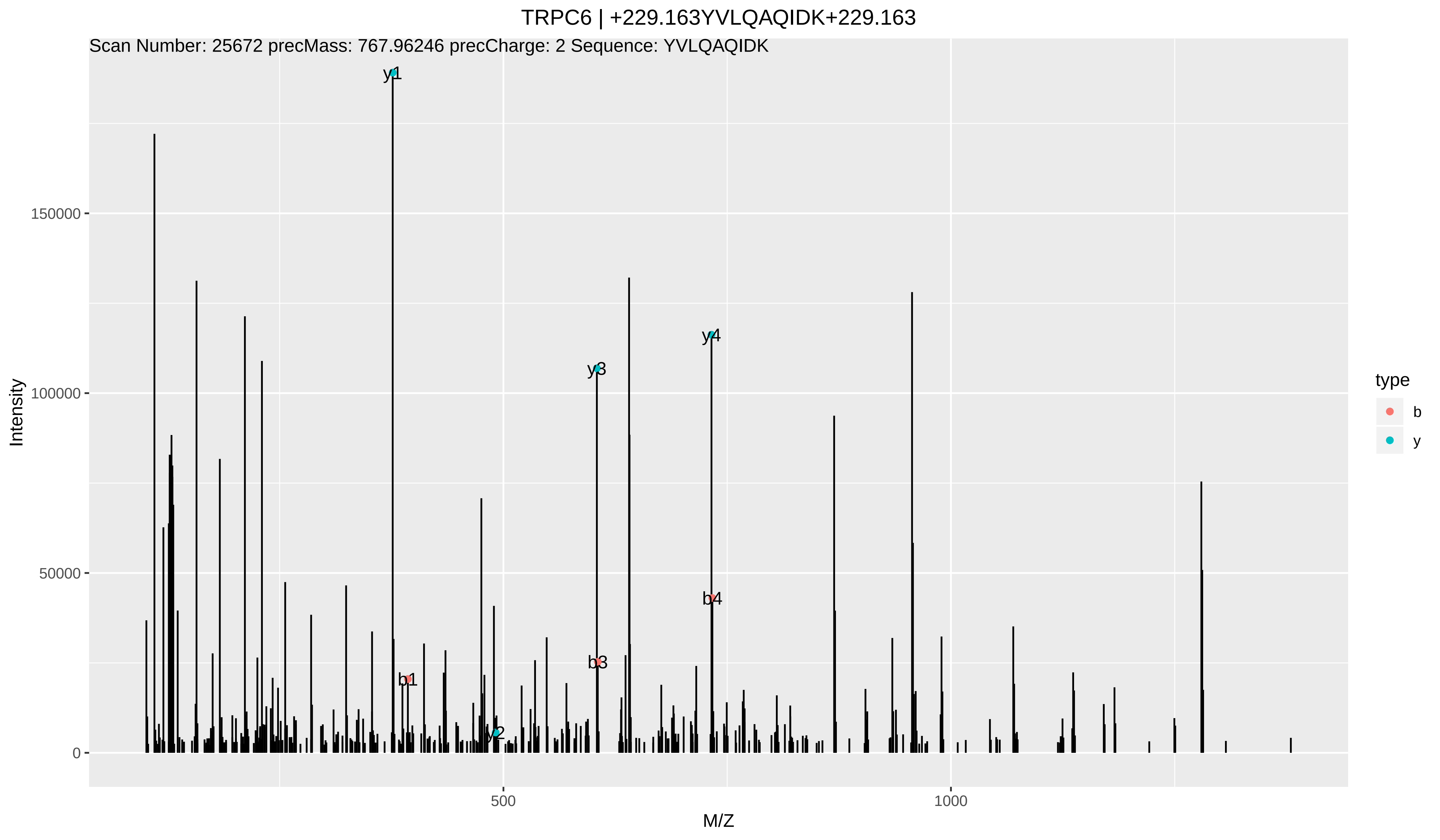

TRPC6 | +229.163YVLQAQIDK+229.163
Scan Number: 25672 precMass: 767.96246 precCharge: 2 Sequence: YVLQAQIDK
y1
y2
y3
y4
b1
b3
b4
Intensity
150000
100000
50000
0
500
1000
M/Z
type
b
y

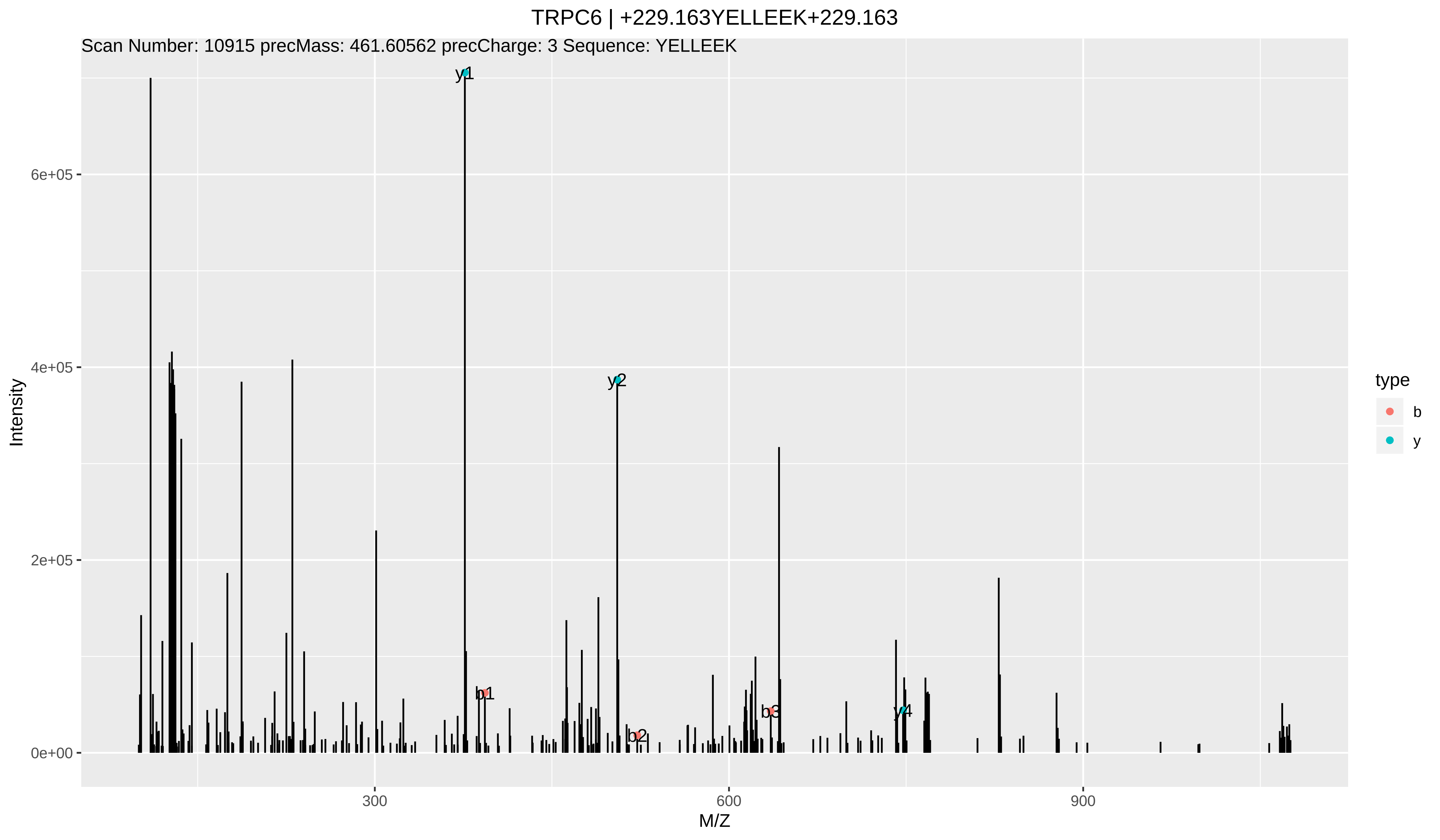
TRPC6 | +229.163YELLEEK+229.163
Scan Number: 10915 precMass: 461.60562 precCharge: 3 Sequence: YELLEEK
Intensity
6e+05
4e+05
2e+05
0e+00
M/Z
300
600
900
y1
y2
b1
b2
b3
y4
type
b
y

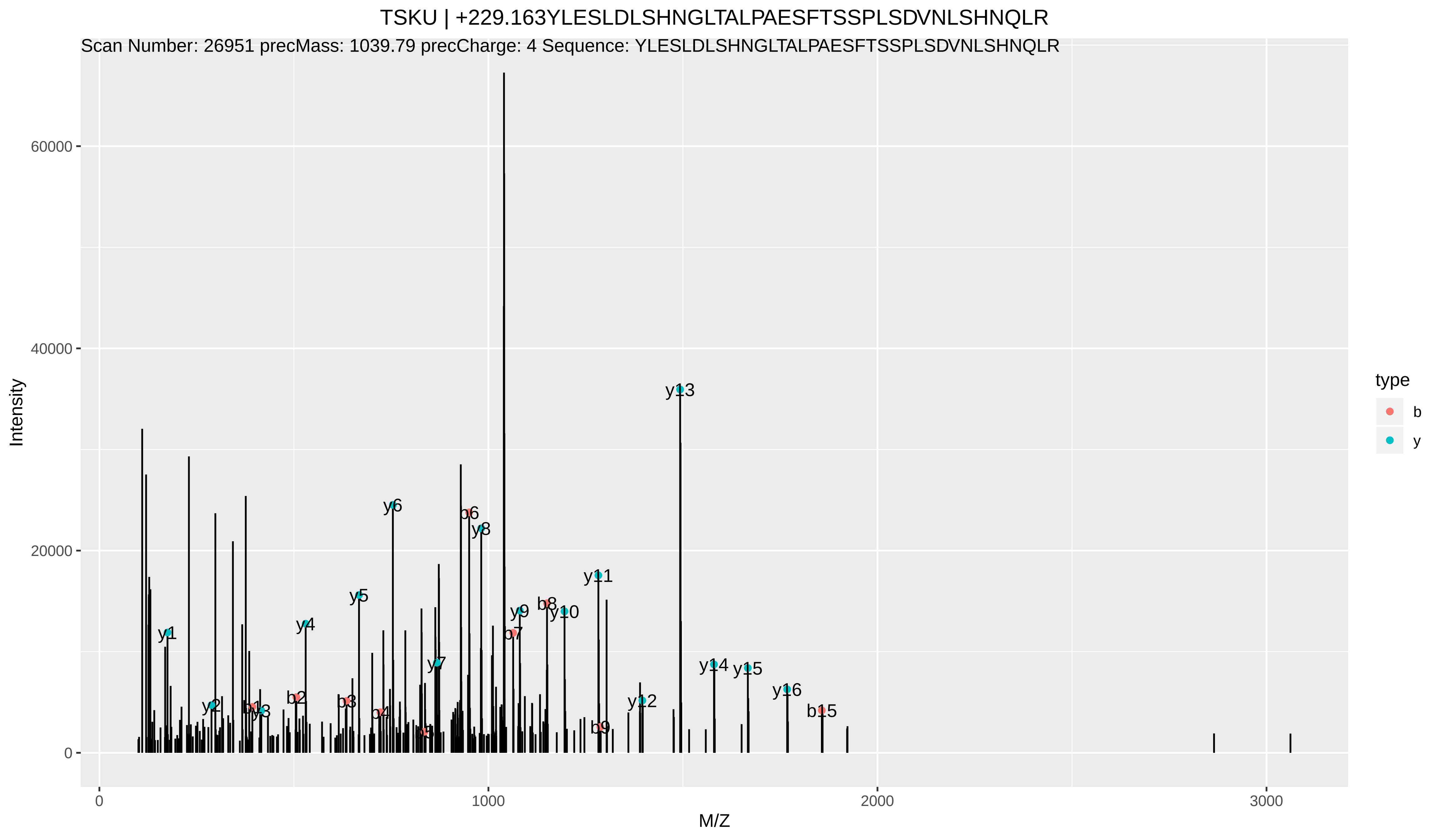

TSKU | +229.163YLESLDLSHNGLTALPAESFTSSPLSDVNLSHNQLR
Scan Number: 26951 precMass: 1039.79 precCharge: 4 Sequence: YLESLDLSHNGLTALPAESFTSSPLSDVNLSHNQLR
Intensity
M/Z
type
b
y
0
20000
40000
60000
1000
2000
3000
y1
y2
y3
y4
y5
y6
y7
y8
y9
y10
y11
y12
y13
y14
y15
y16
b2
b3
b4
b6
b7
b8
b9
b15

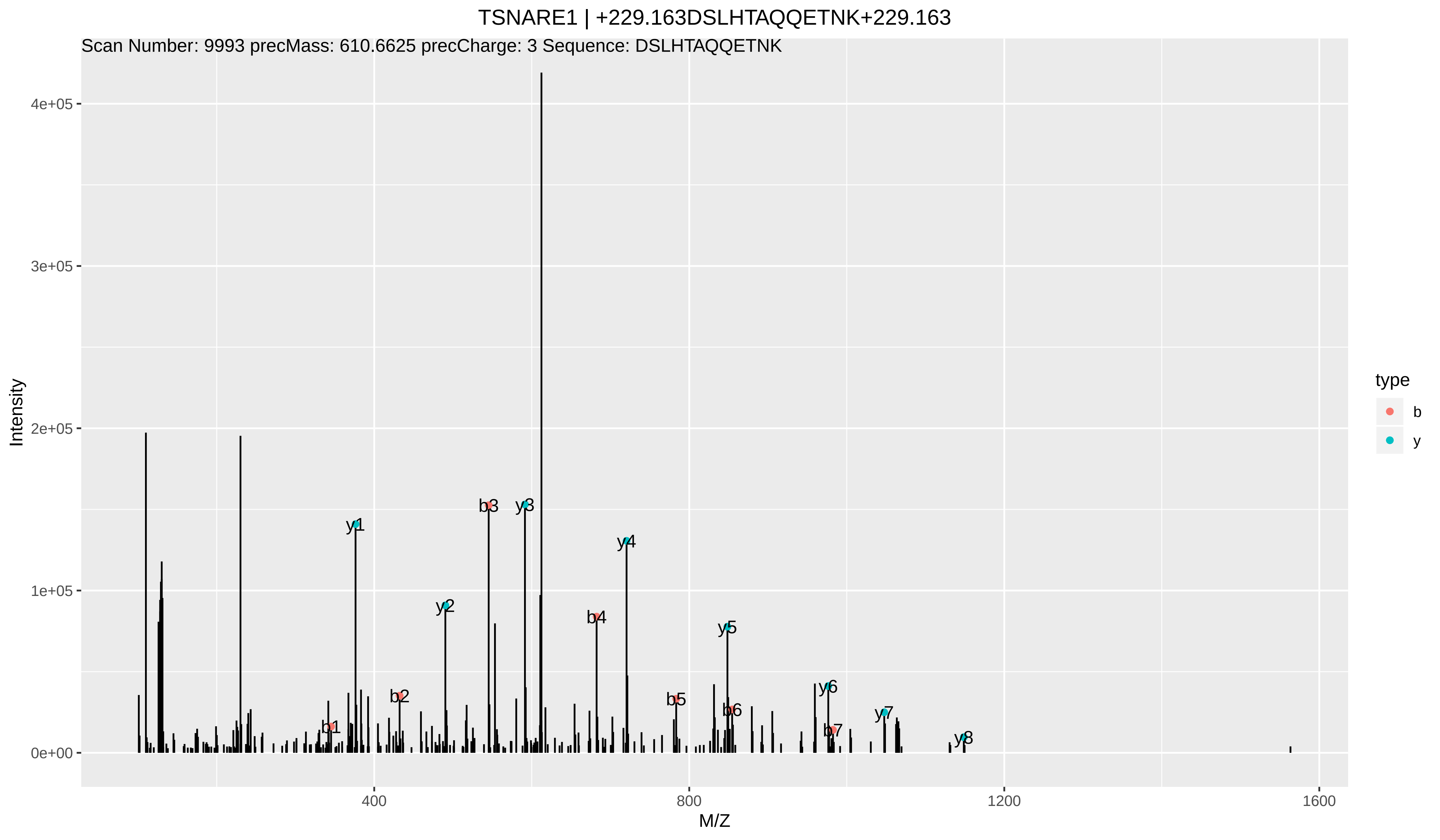

TSNARE1 | +229.163DSLHTAQQETNK+229.163
Scan Number: 9993 precMass: 610.6625 precCharge: 3 Sequence: DSLHTAQQETNK
Intensity
4e+05
3e+05
2e+05
1e+05
0e+00
M/Z
400
800
1200
1600
y1
b1
b2
y2
b3
y3
b4
y4
b5
y5
b6
y6
b7
y7
y8
type
b
y

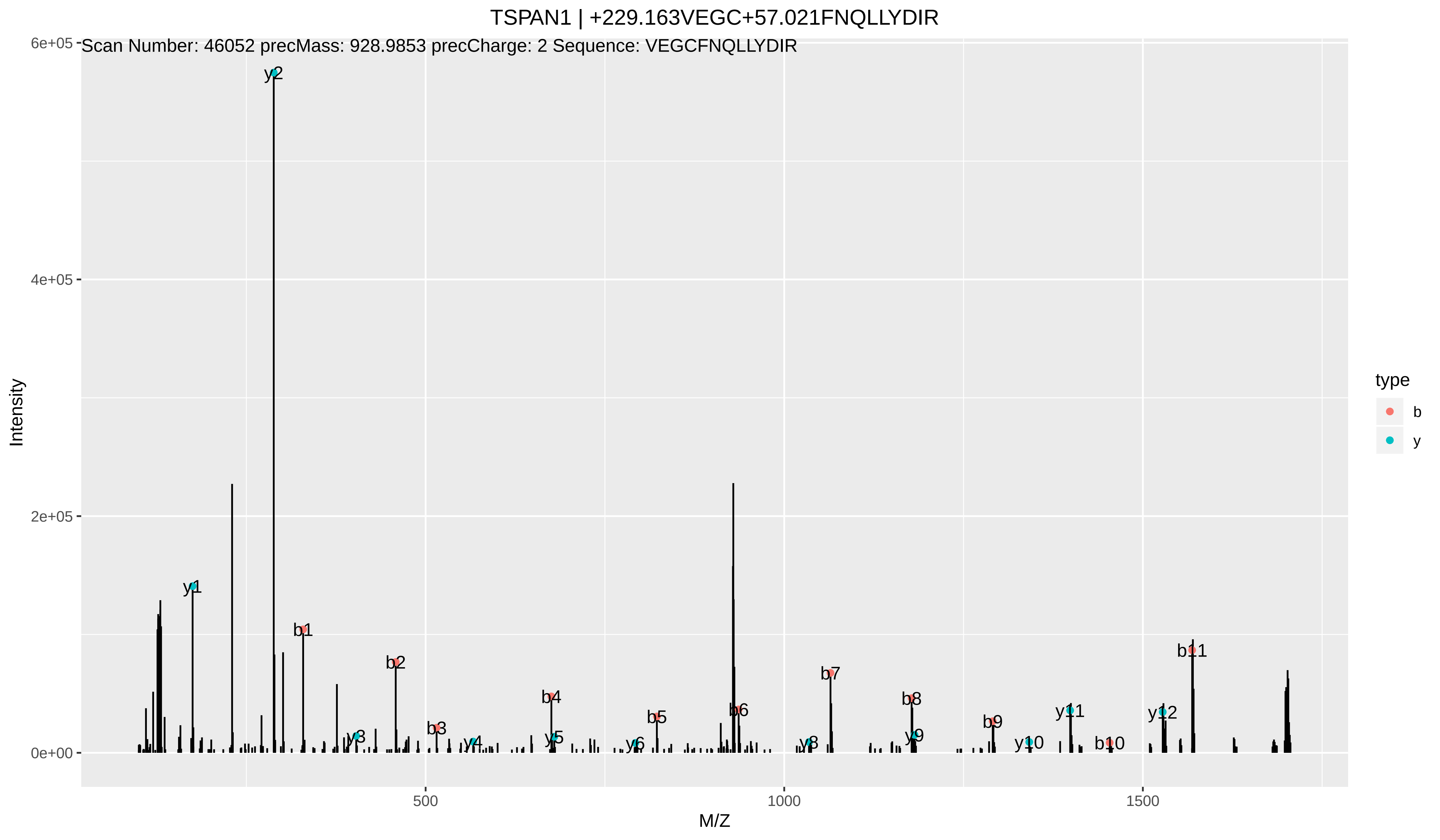

TSPAN1 | +229.163VEGC+57.021FNQLLYDIR
Scan Number: 46052 precMass: 928.9853 precCharge: 2 Sequence: VEGCFNQLLYDIR
Intensity
M/Z
type
b
y

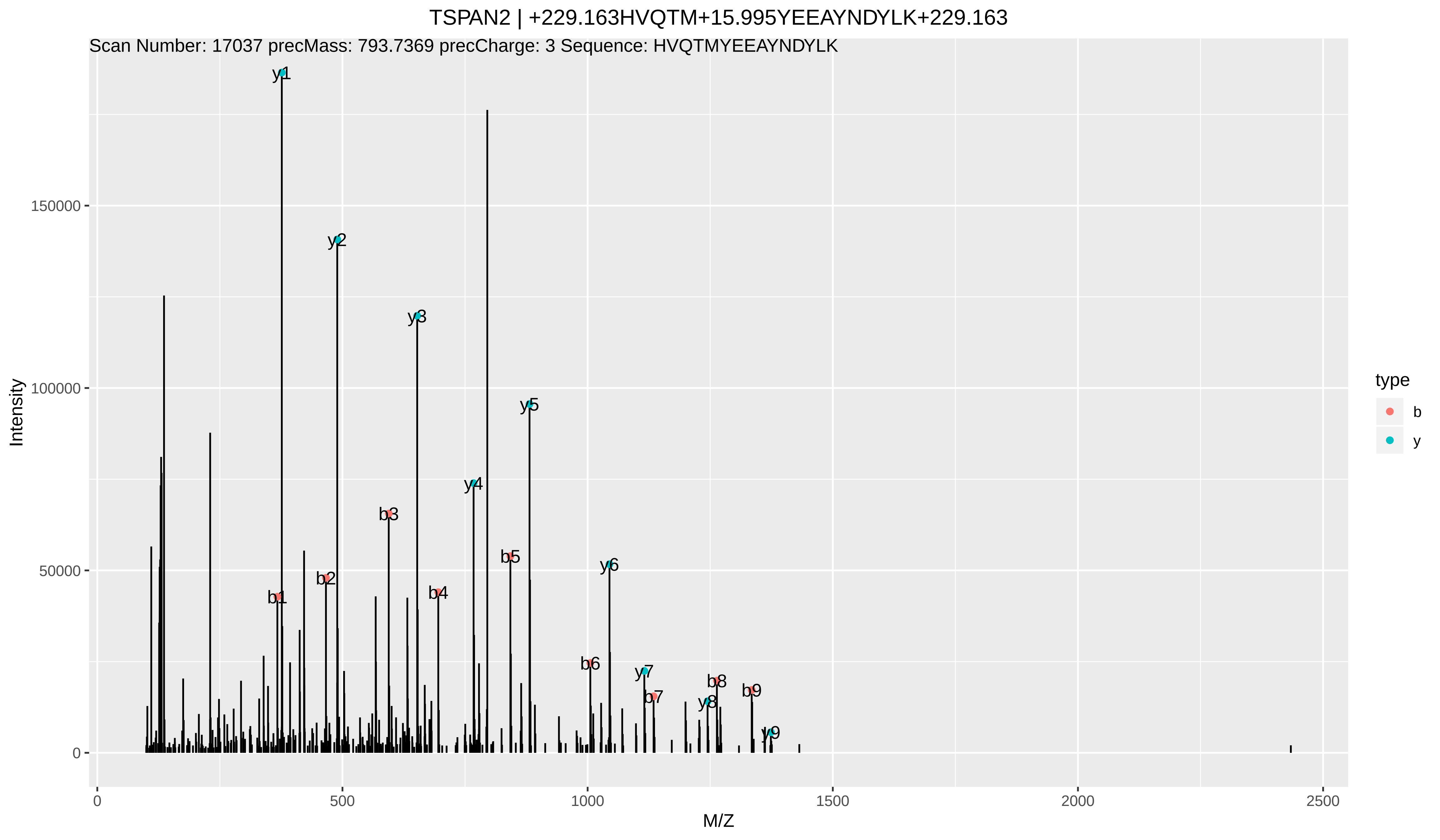
TSPAN2 | +229.163HVQTM+15.995YEEAYNDYLK+229.163
Scan Number: 17037 precMass: 793.7369 precCharge: 3 Sequence: HVQTMYEEAYNDYLK
Intensity
M/Z
type
b
y
y1
y2
y3
y4
y5
y6
y7
y8
y9
b1
b2
b3
b4
b5
b6
b7
b8
b9
0
50000
100000
150000
0
500
1000
1500
2000
2500

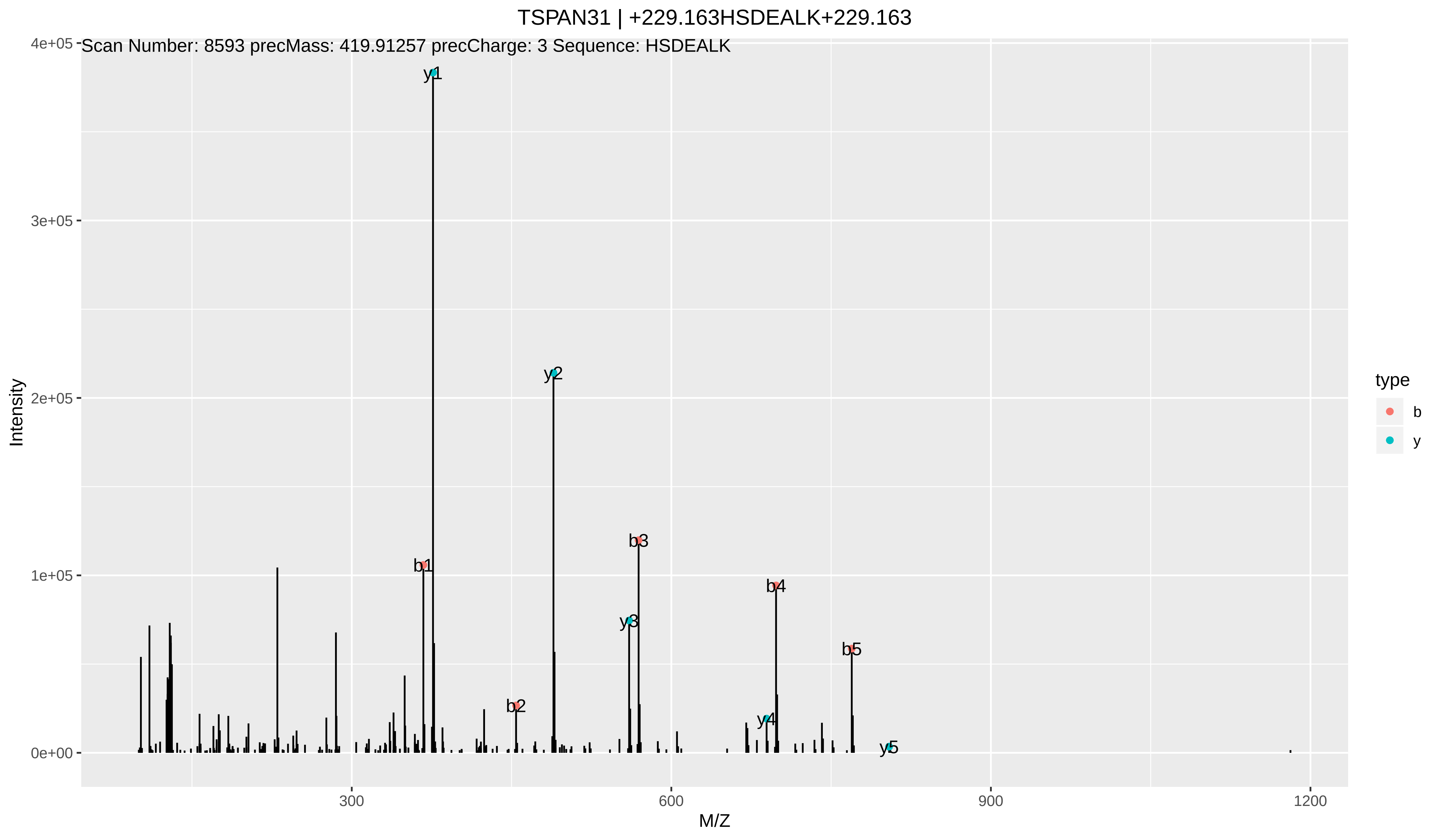

TSPAN31 | +229.163HSDEALK+229.163
Scan Number: 8593 precMass: 419.91257 precCharge: 3 Sequence: HSDEALK
Intensity
M/Z
4e+05
3e+05
2e+05
1e+05
0e+00
300
600
900
1200
y1
y2
b3
b1
b4
y3
b5
b2
y4
y5
type
b
y

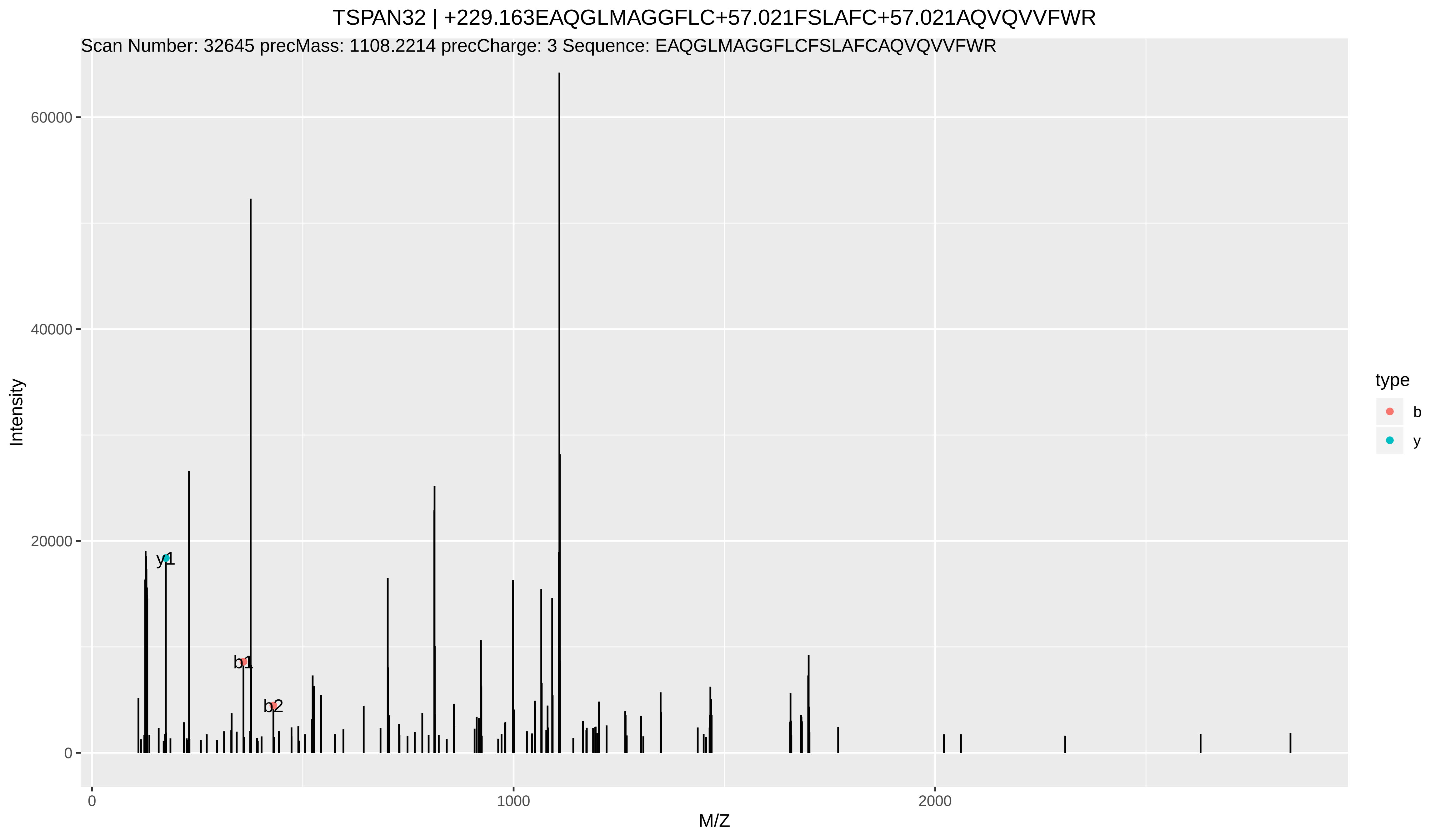
TSPAN32 | +229.163EAQGLMAGGFLC+57.021FSLAFC+57.021AQVQVVFWR
Scan Number: 32645 precMass: 1108.2214 precCharge: 3 Sequence: EAQGLMAGGFLCFSLAFCAQVQVVFWR
Intensity
M/Z
y1
b1
b2
type
b
y
0
20000
40000
60000
0
1000
2000

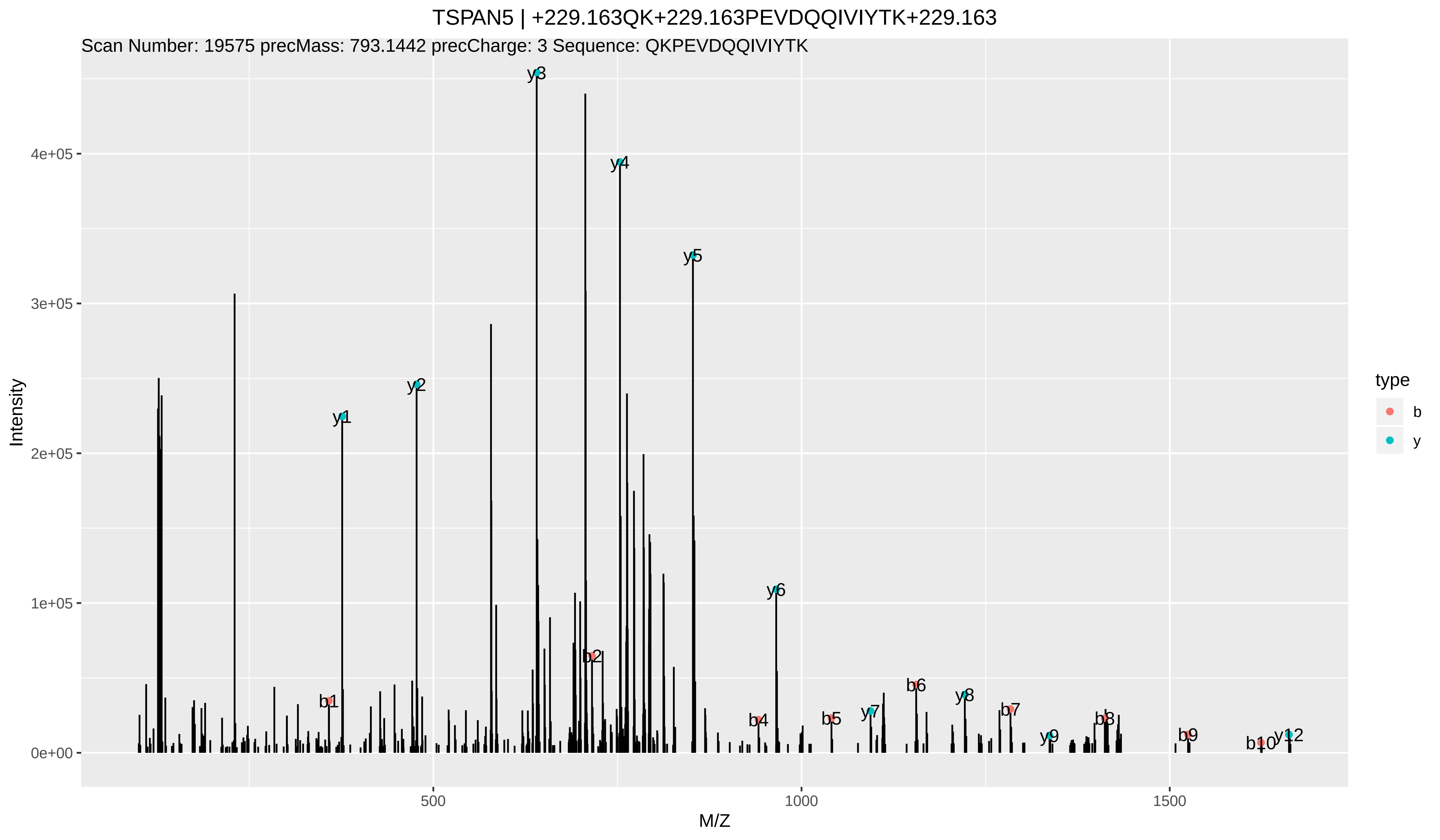
TSPAN5 | +229.163QK+229.163PEVDQQIVIYTK+229.163
Scan Number: 19575 precMass: 793.1442 precCharge: 3 Sequence: QKPEVDQQIVIYTK
Intensity
M/Z
type
b
y
y1
y2
y3
y4
y5
y6
y7
y8
y9
y12
b1
b2
b4
b5
b6
b7
b8
b9
b10
0e+00
1e+05
2e+05
3e+05
4e+05
500
1000
1500

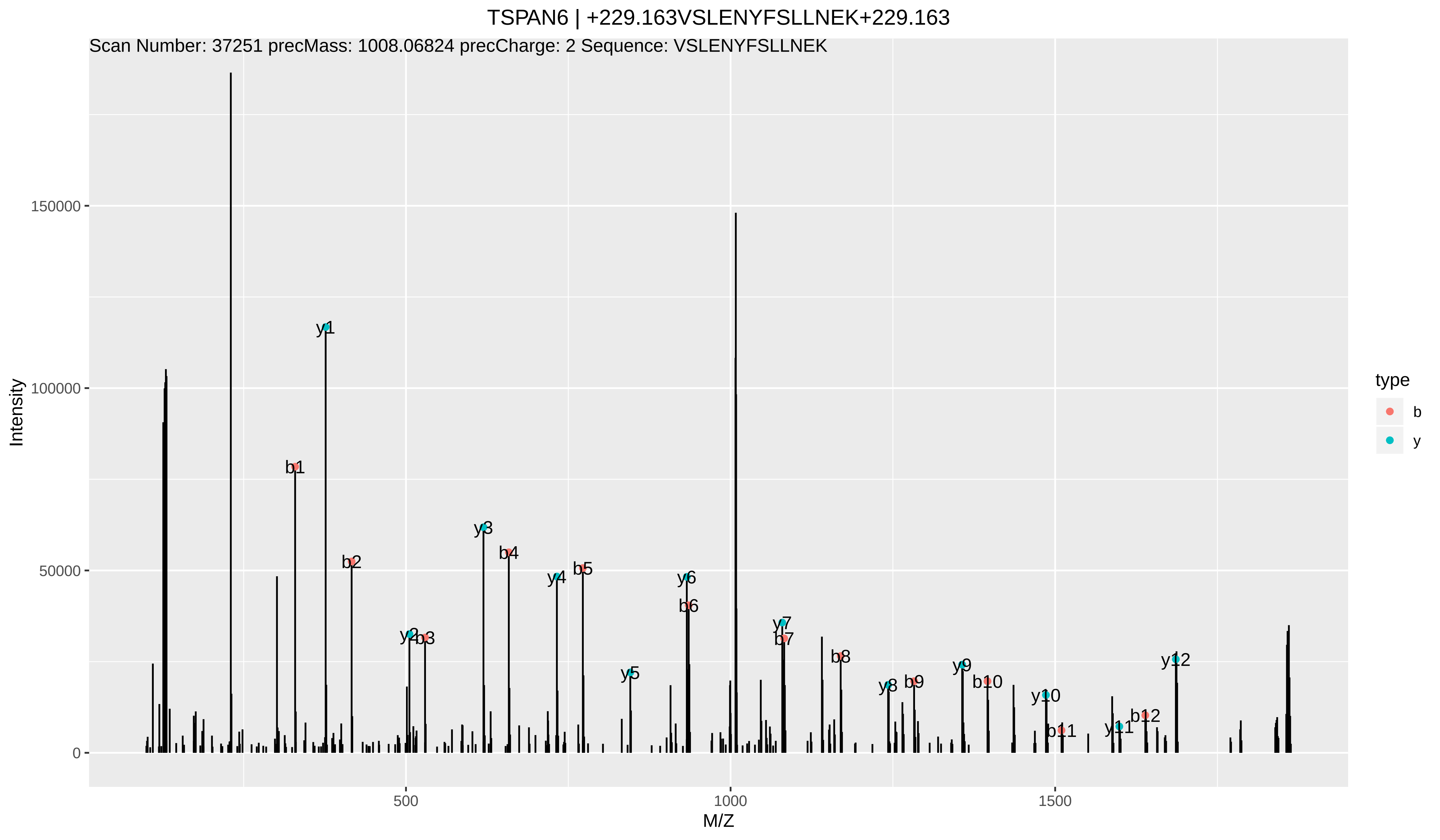

TSPAN6 | +229.163VSLENYFSLLNEK+229.163
Scan Number: 37251 precMass: 1008.06824 precCharge: 2 Sequence: VSLENYFSLLNEK
Intensity
150000
100000
50000
0
500
1000
1500
M/Z
type
b
y
y1
b1
y3
b4
b2
y4
b5
y6
b6
y2
b3
y7
b7
b8
y5
y9
y8
b9
b10
y12
y10
b12
y11
b11

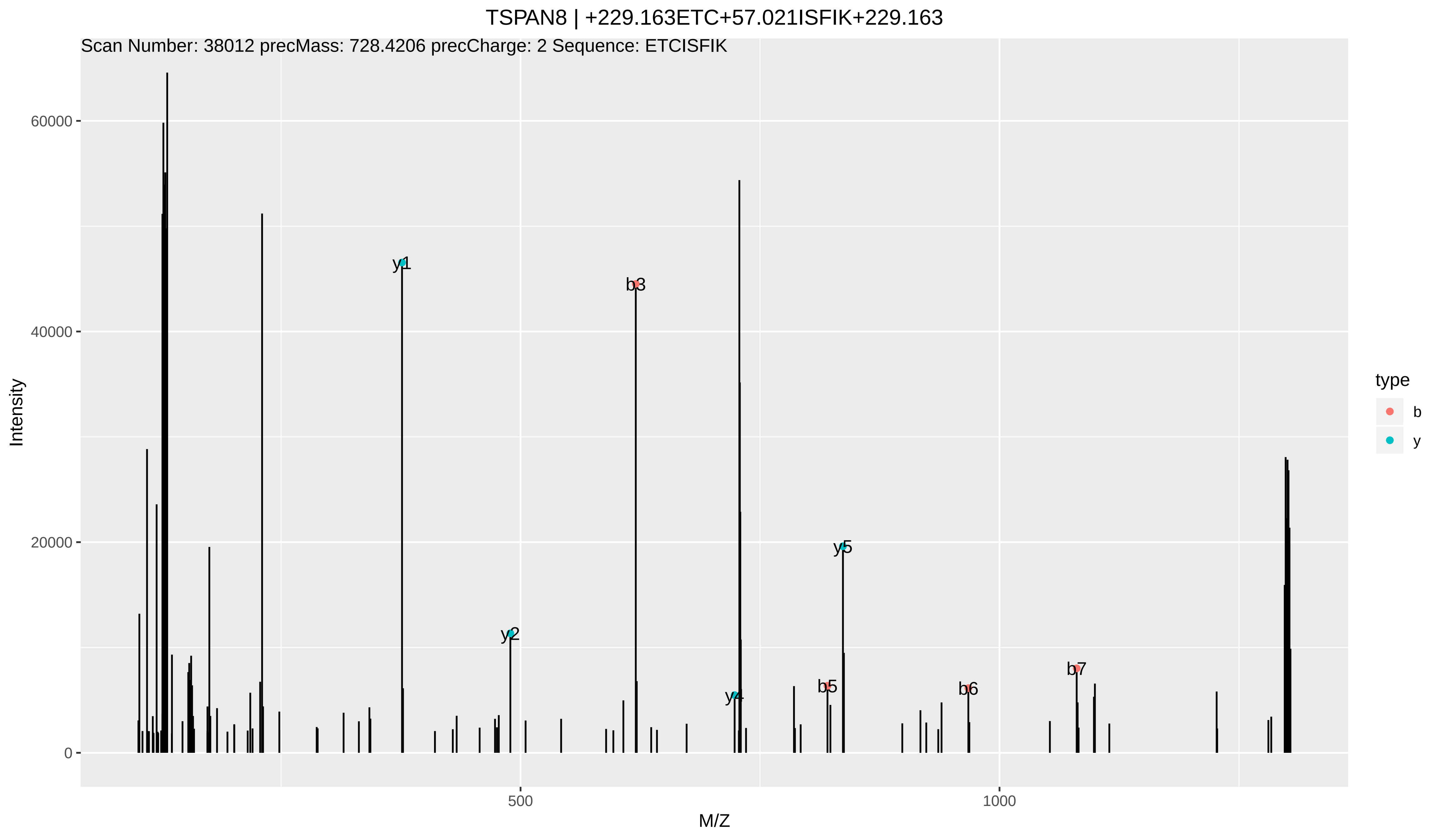

TSPAN8 | +229.163ETC+57.021ISFIK+229.163
Scan Number: 38012 precMass: 728.4206 precCharge: 2 Sequence: ETCISFIK
Intensity
60000
40000
20000
0
500
1000
M/Z
y1
b3
y5
y2
b7
b5
y4
b6
type
b
y

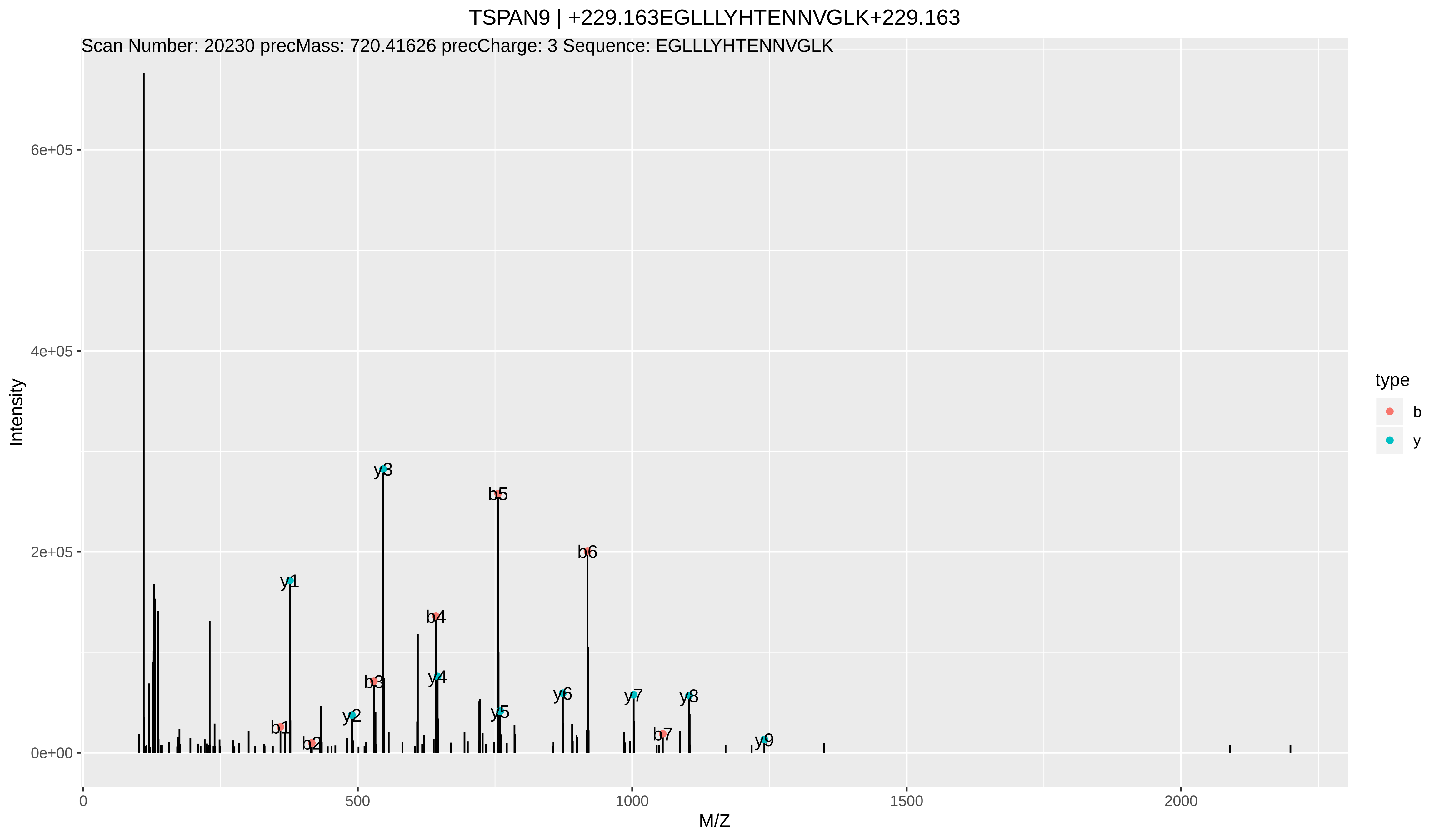
TSPAN9 | +229.163EGLLLYHTENNVGLK+229.163
Scan Number: 20230 precMass: 720.41626 precCharge: 3 Sequence: EGLLLYHTENNVGLK
Intensity
M/Z
type
b
y
y1
y2
y3
y4
y5
y6
y7
y8
y9
b1
b2
b3
b4
b5
b6
b7
0e+00
2e+05
4e+05
6e+05
0
500
1000
1500
2000

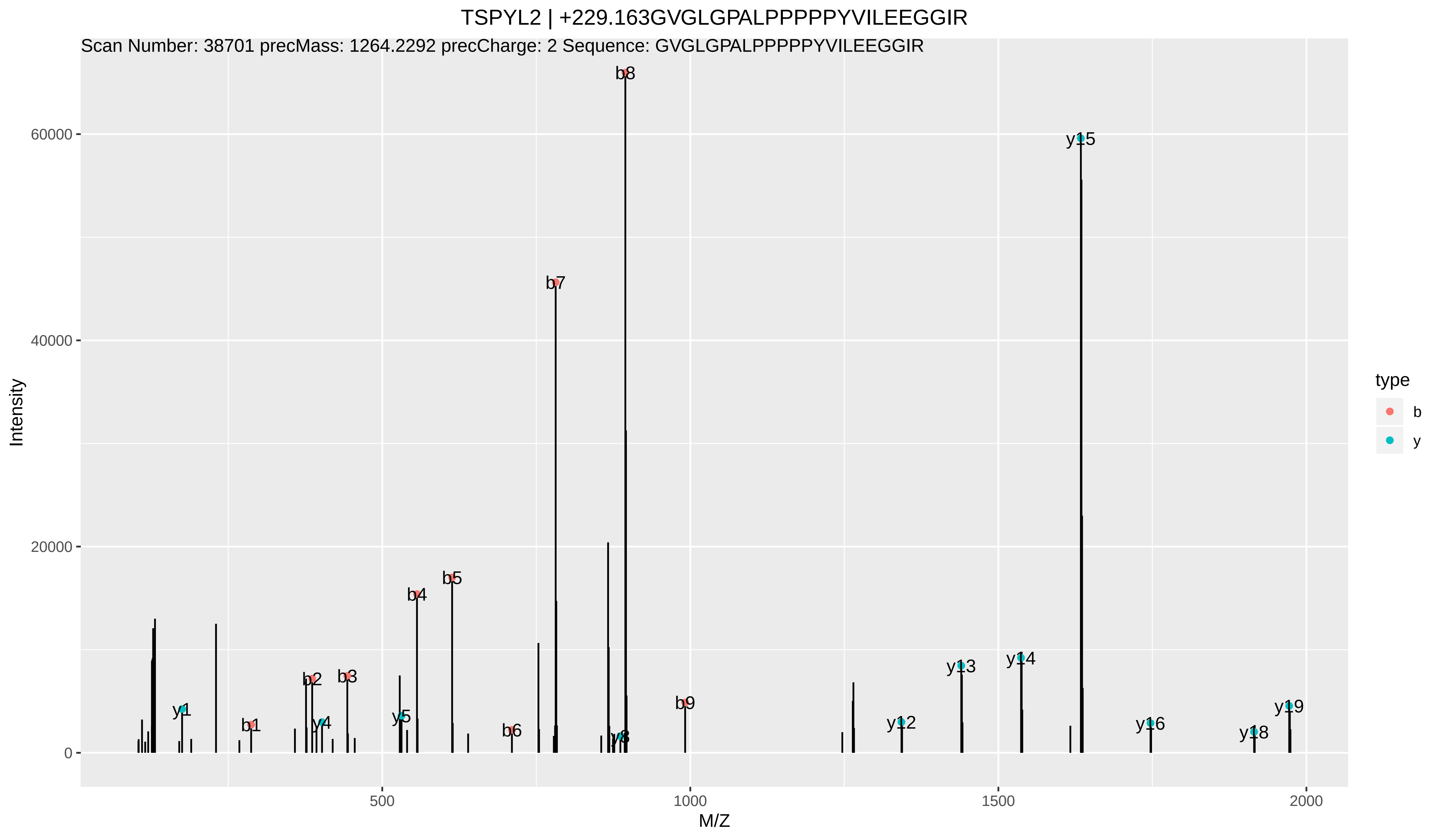
TSPYL2 | +229.163GVGLGPALPPPPPYVILEEGGIR
Scan Number: 38701 precMass: 1264.2292 precCharge: 2 Sequence: GVGLGPALPPPPPYVILEEGGIR
Intensity
M/Z
0
20000
40000
60000
500
1000
1500
2000
b8
y15
b7
b5
b4
b2
b3
b9
y1
b1
y4
y5
b6
y8
y12
y13
y14
y16
y18
y19
type
b
y

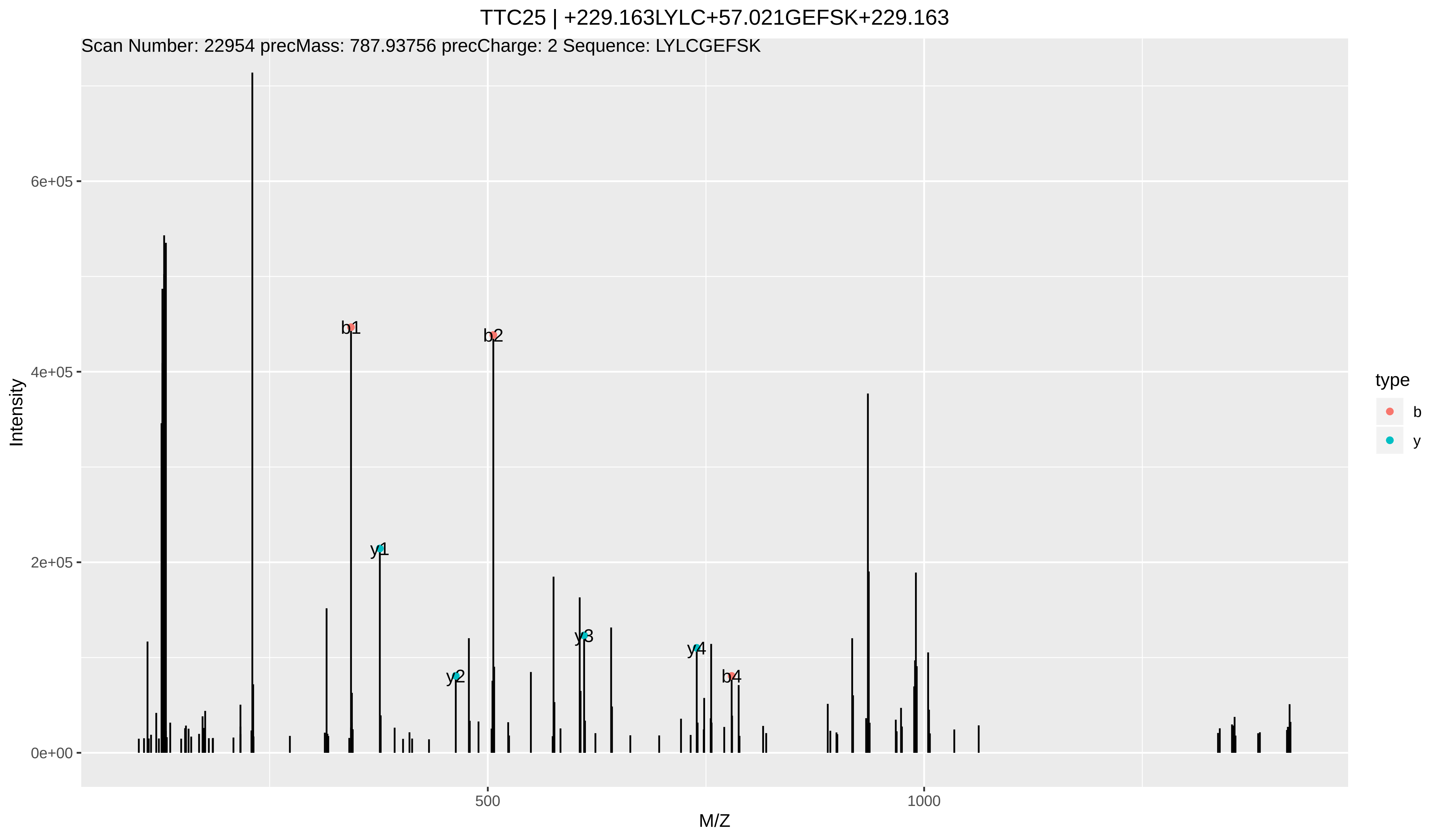
TTC25 | +229.163LYLC+57.021GEFSK+229.163
Scan Number: 22954 precMass: 787.93756 precCharge: 2 Sequence: LYLCGEFSK
Intensity
6e+05
4e+05
2e+05
0e+00
500
1000
M/Z
b1
b2
y1
y2
y3
y4
b4
type
b
y

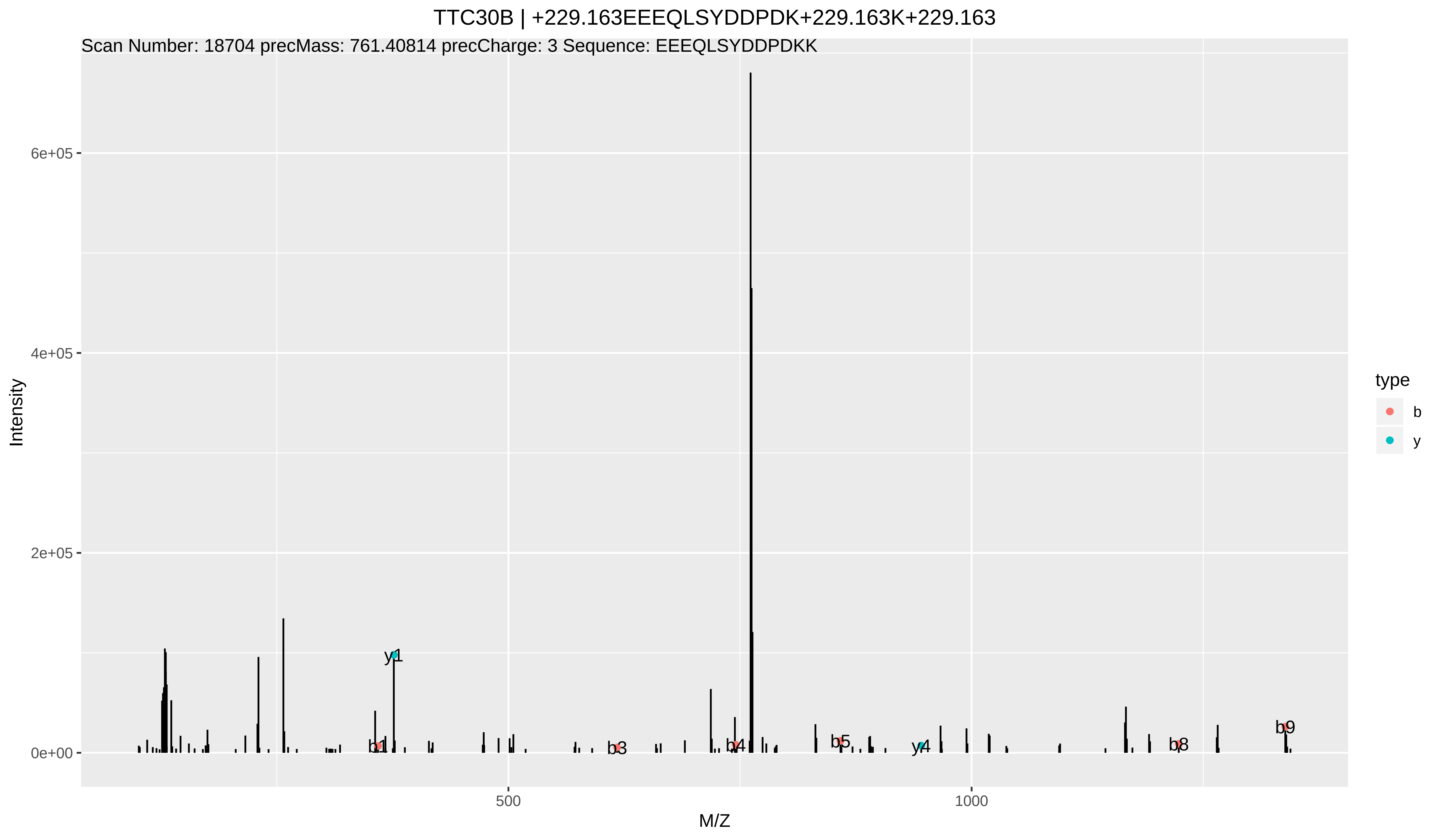
TTC30B | +229.163EEEQLSYDDPDK+229.163K+229.163
Scan Number: 18704 precMass: 761.40814 precCharge: 3 Sequence: EEEQLSYDDPDKK
Intensity
6e+05
4e+05
2e+05
0e+00
M/Z
500
1000
y1
b1
b3
b4
b5
y4
b8
b9
type
b
y

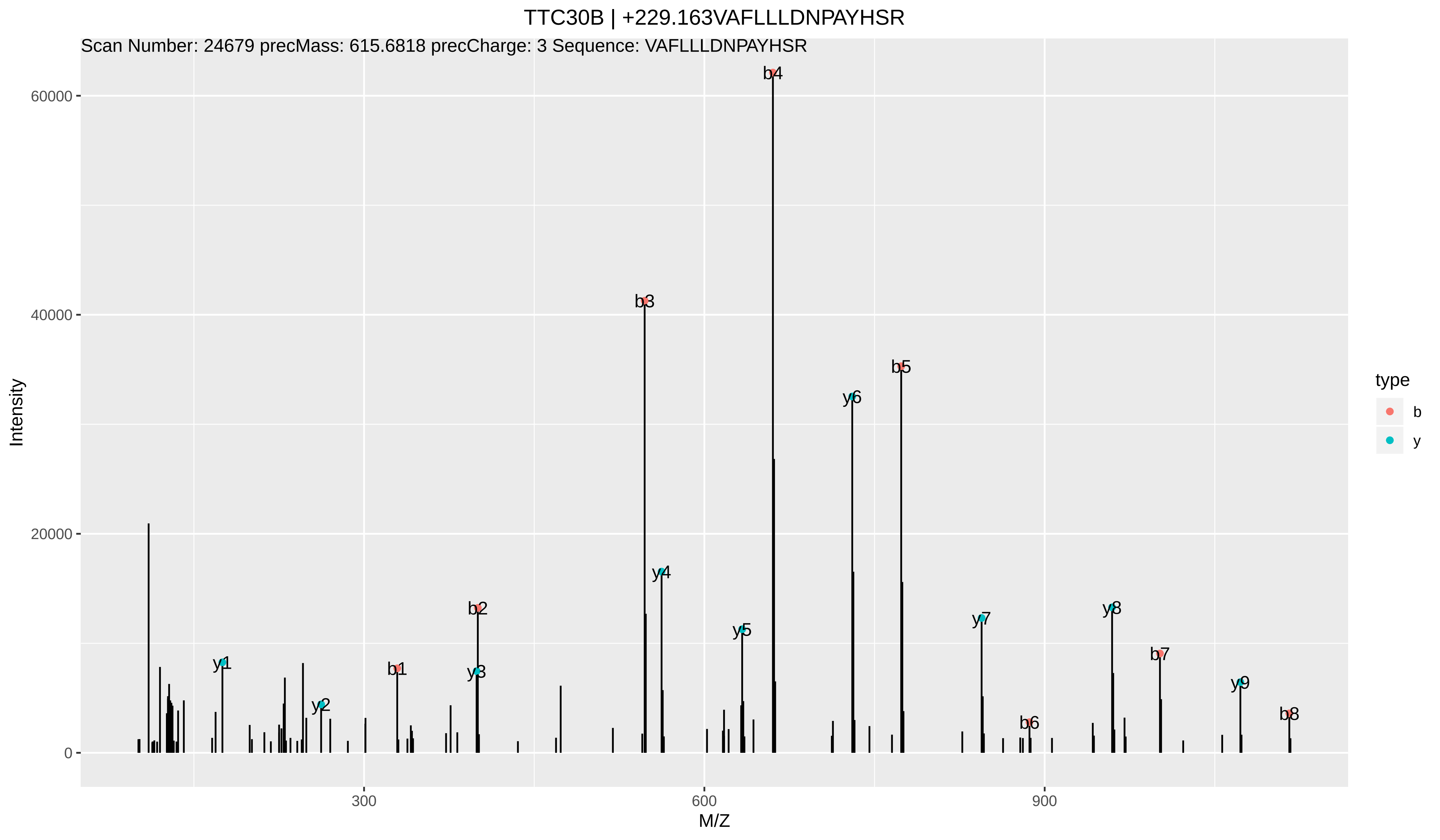

TTC30B | +229.163VAFLLLLDNPAYHSR
Scan Number: 24679 precMass: 615.6818 precCharge: 3 Sequence: VAFLLLLDNPAYHSR
Intensity
M/Z
0
20000
40000
60000
300
600
900
b4
b3
b5
y6
y4
b2
y8
y7
y5
y1
b1
y3
b7
y9
y2
b8
b6
type
b
y

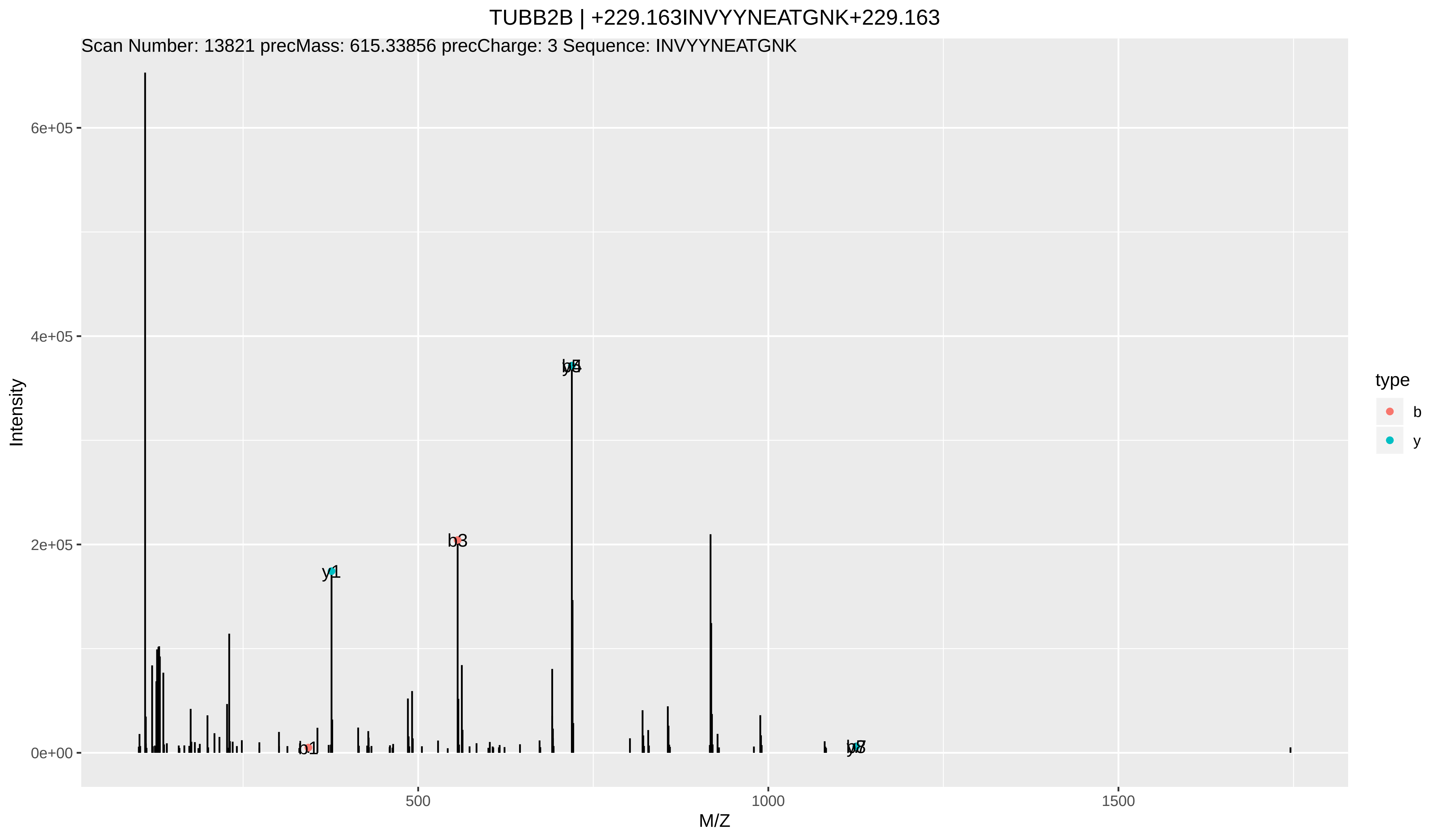

TUBB2B | +229.163INVYYNEATGNK+229.163
Scan Number: 13821 precMass: 615.33856 precCharge: 3 Sequence: INVYYNEATGNK
Intensity
6e+05
4e+05
2e+05
0e+00
M/Z
500
1000
1500
y1
b1
b3
type
b
y

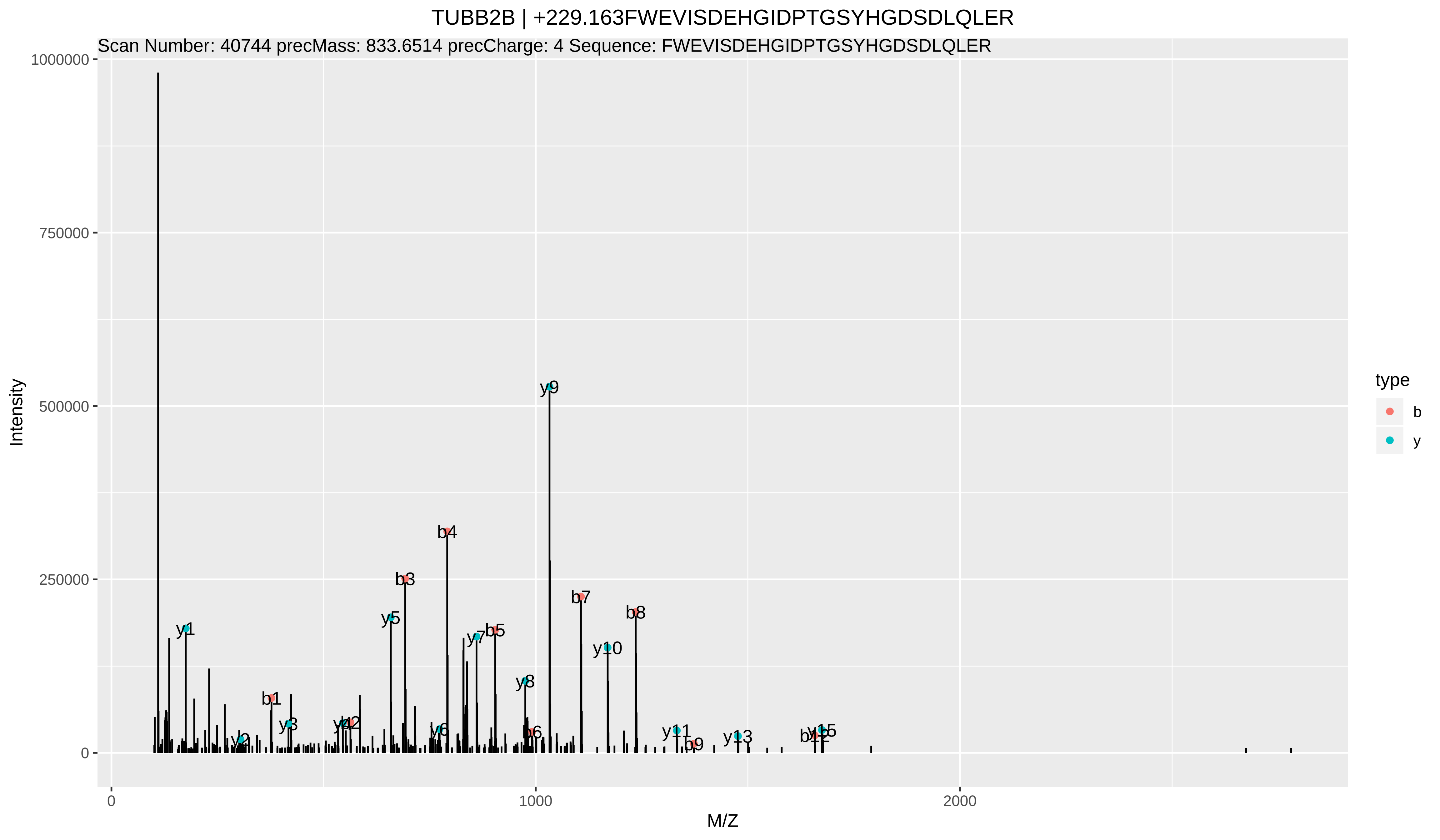
TUBB2B | +229.163FWEVISDEHGIDPTGSYHGDSDLQLER
Scan Number: 40744 precMass: 833.6514 precCharge: 4 Sequence: FWEVISDEHGIDPTGSYHGDSDLQLER
Intensity
M/Z
1000000
750000
500000
250000
0
0
1000
2000
y1
y2
y3
y5
y6
y7
y8
y9
y10
y11
y13
y15
b1
b3
b4
b5
b6
b7
b8
b9
b12
type
b
y

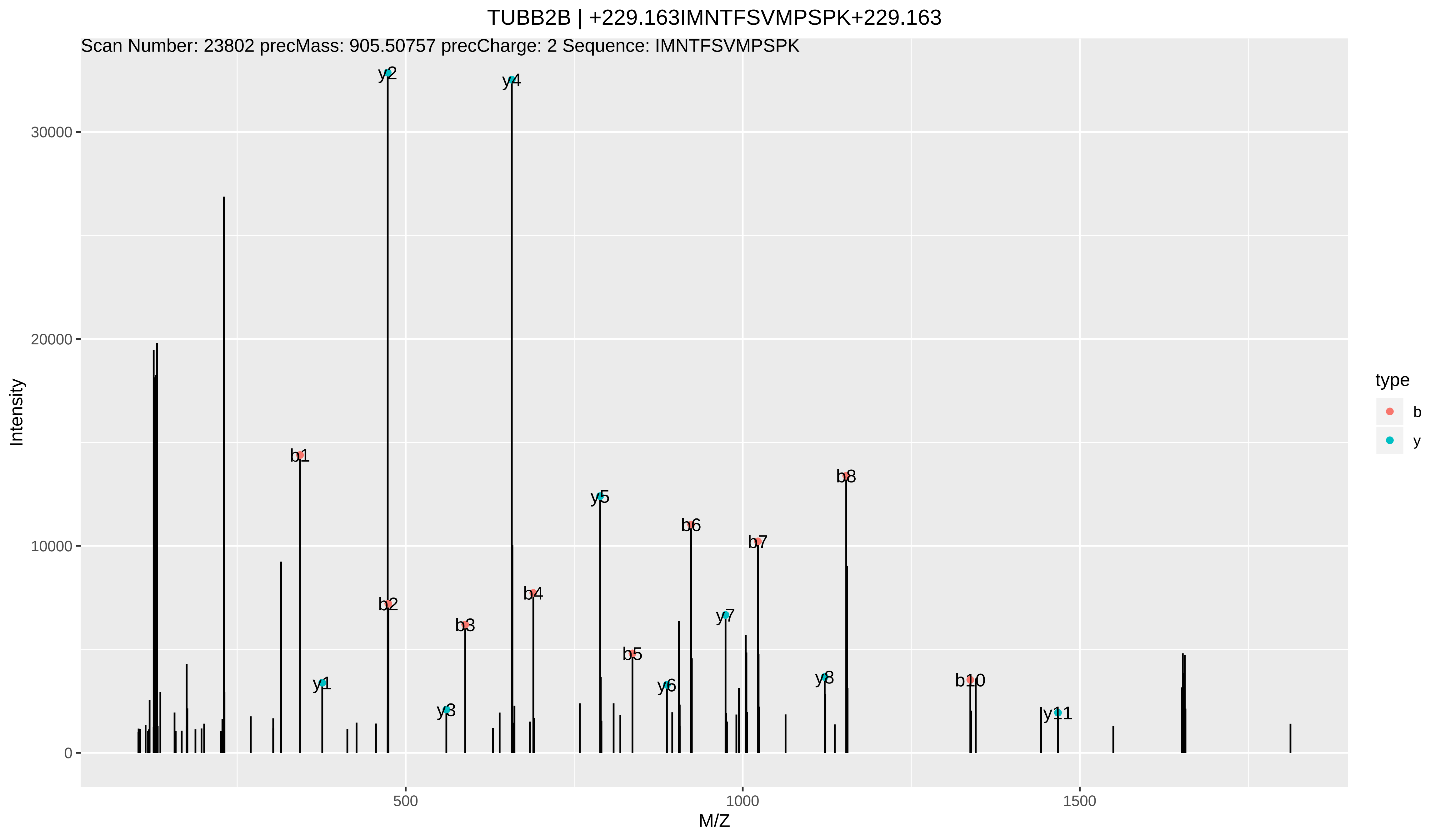

TUBB2B | +229.163IMNTFSVMPSPK+229.163
Scan Number: 23802 precMass: 905.50757 precCharge: 2 Sequence: IMNTFSVMPSPK
Intensity
30000
20000
10000
0
M/Z
500
1000
1500
y2
y4
b1
y5
b6
b7
b8
b2
b3
b4
y7
b5
y1
y6
y8
b10
y3
y11
type
b
y

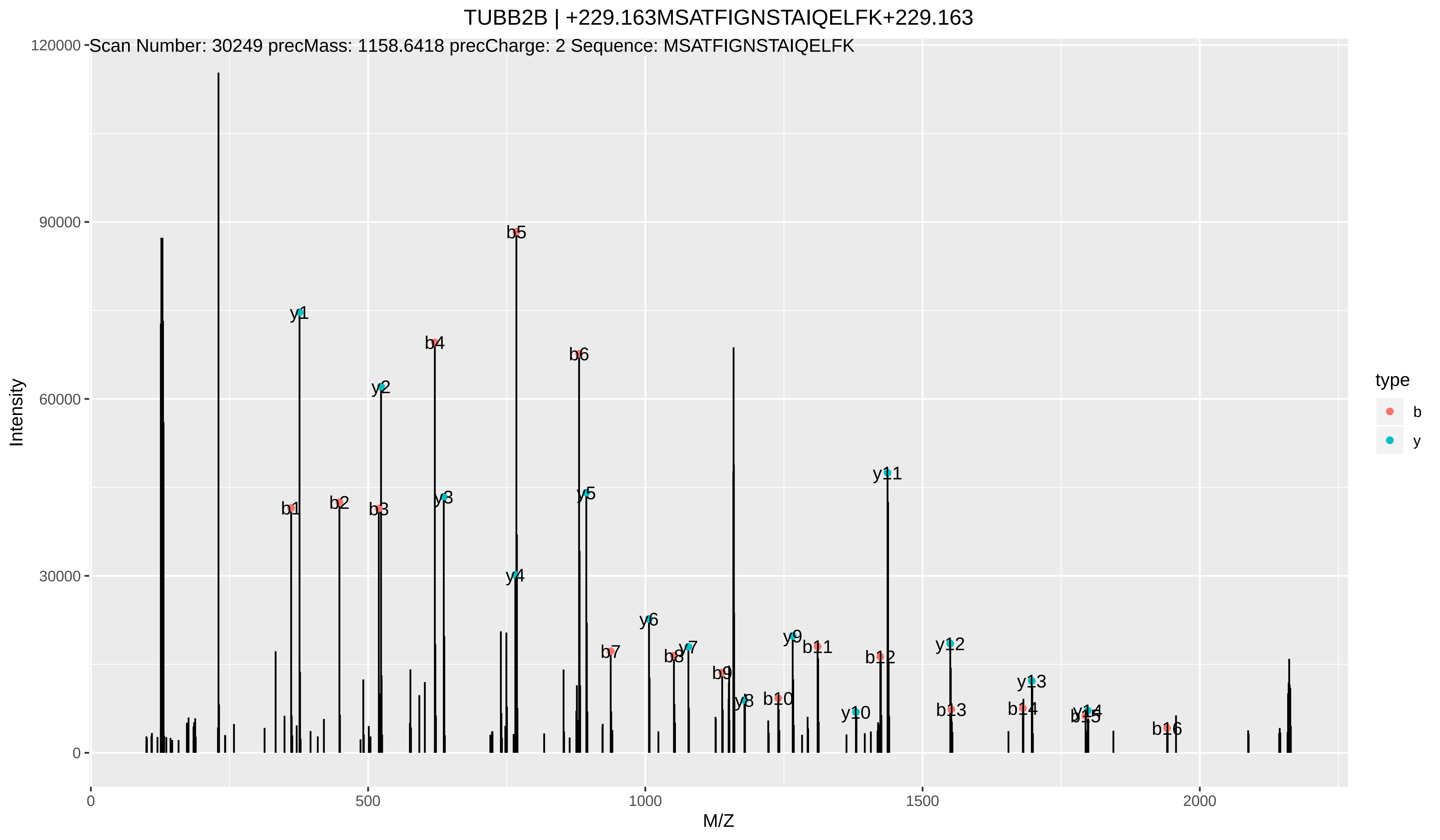

TUBB2B | +229.163MSATFIGNSTAIQELFK+229.163
Scan Number: 30249 precMass: 1158.6418 precCharge: 2 Sequence: MSATFIGNSTAIQELFK
Intensity
M/Z
type
b
y
b1
b2
b3
b4
b5
b6
b7
b8
b9
b10
b11
b12
b13
b14
b15
b16
y1
y2
y3
y4
y5
y6
y7
y8
y9
y10
y11
y12
y13
y14

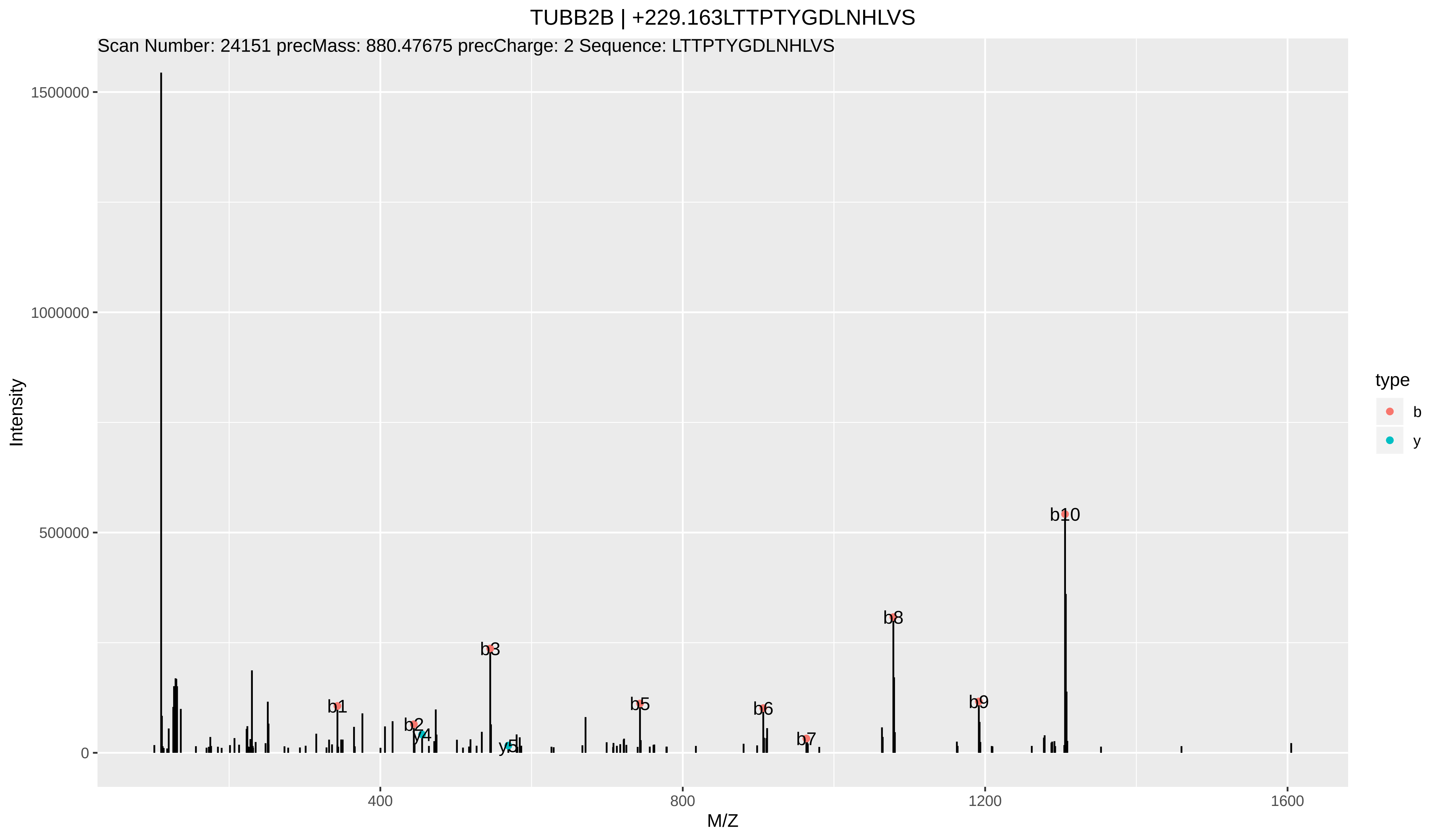
TUBB2B | +229.163LTTPTYGDLNHLVS
Scan Number: 24151 precMass: 880.47675 precCharge: 2 Sequence: LTTPTYGDLNHLVS
Intensity
1500000
1000000
500000
0
M/Z
400
800
1200
1600
b1
b2
y4
b3
y5
b5
b6
b7
b8
b9
b10
type
b
y

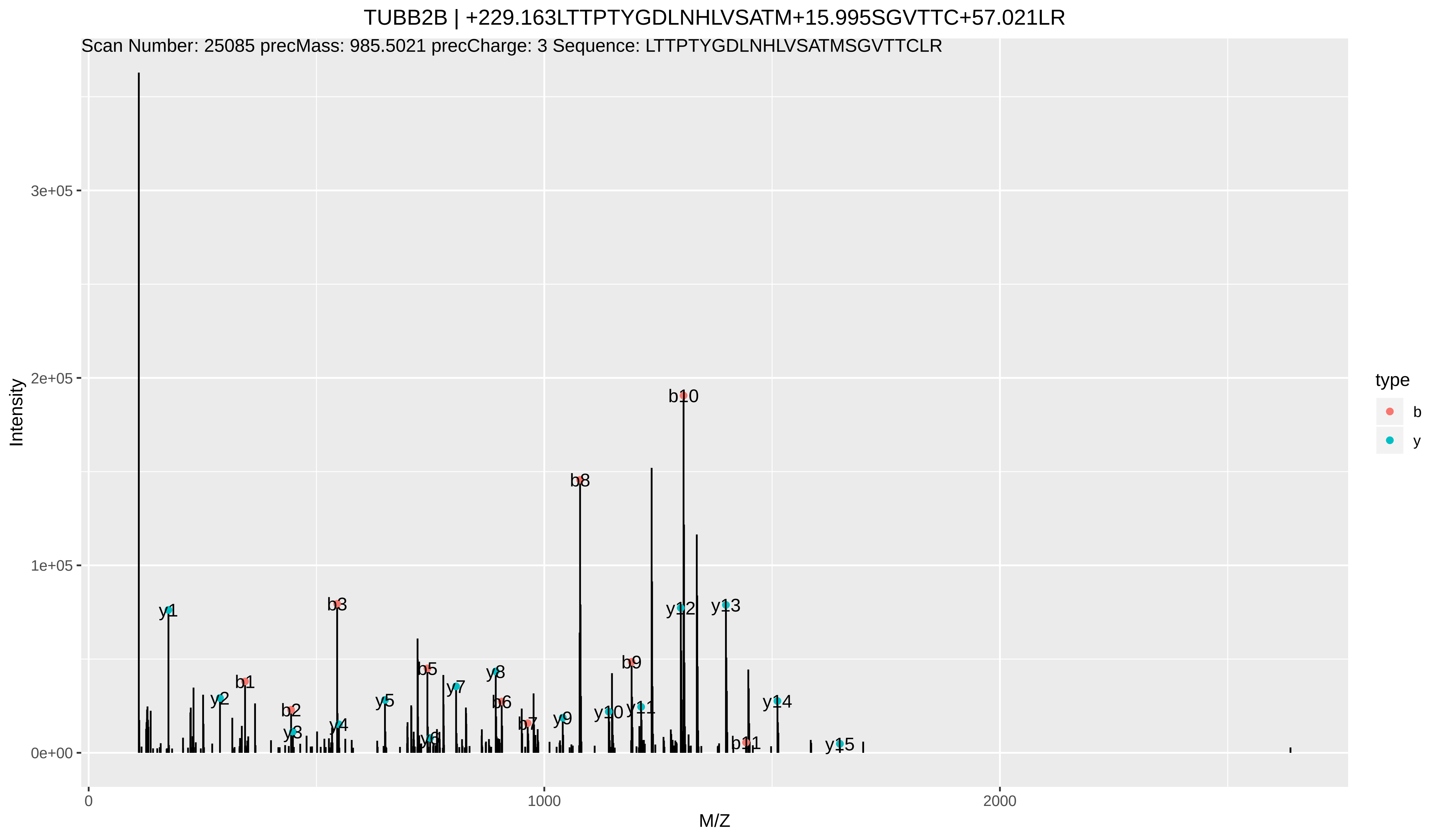

TUBB2B | +229.163LTTPTYGDLNHLVSATM+15.995SGVTTC+57.021LR
Scan Number: 25085 precMass: 985.5021 precCharge: 3 Sequence: LTTPTYGDLNHLVSATMSGVTTCLR
Intensity
M/Z
type
b
y
0e+00
1e+05
2e+05
3e+05
0
1000
2000
y1
y2
b1
b2
y3
b3
y4
y5
b5
y6
y7
y8
b6
b7
y9
b8
y10
b9
y11
y12
b10
y13
b11
y14
y15

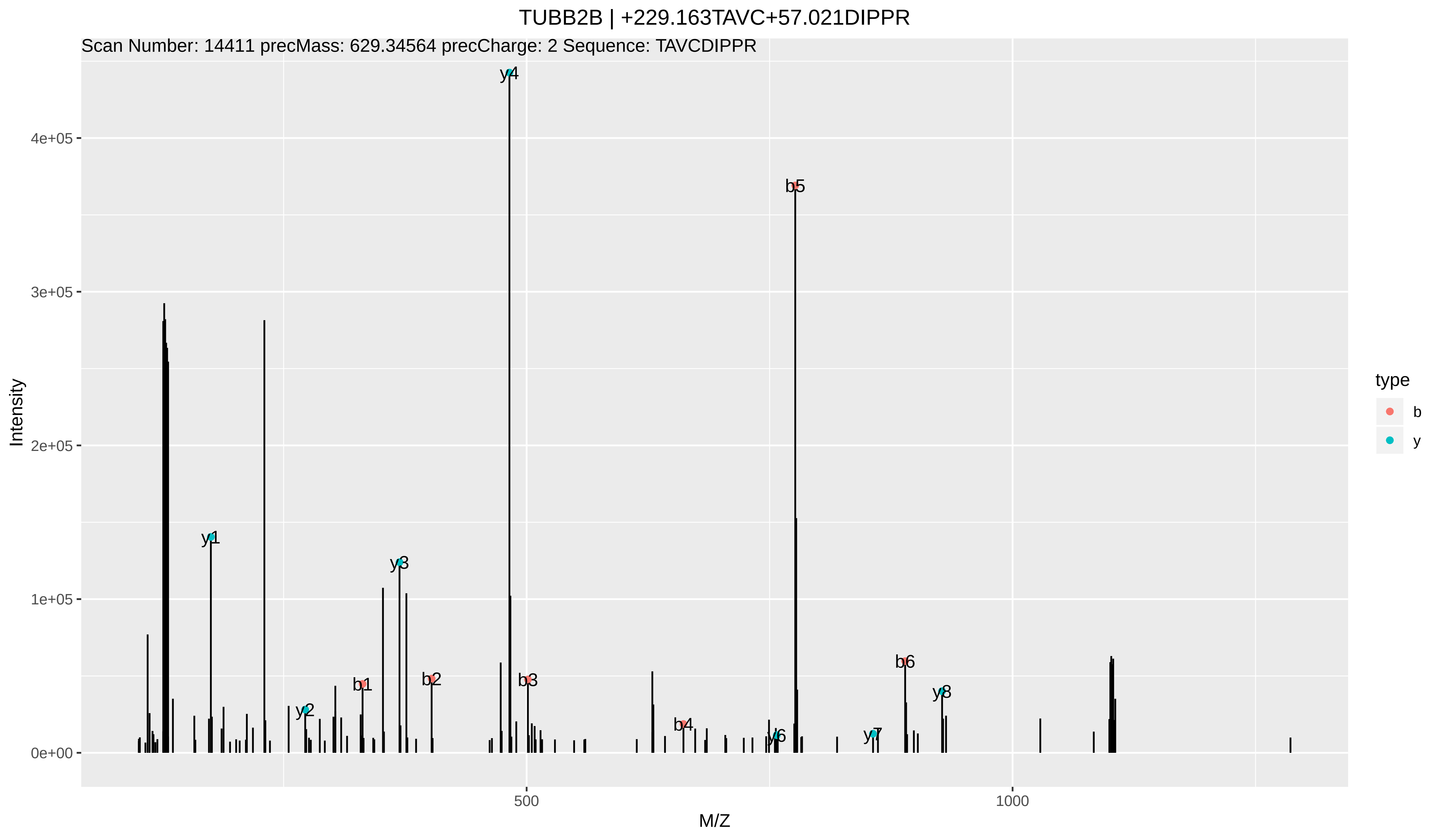

TUBB2B | +229.163TAVC+57.021DIPPR
Scan Number: 14411 precMass: 629.34564 precCharge: 2 Sequence: TAVCDIPPR
y4
b5
y1
y3
b1
b2
b3
y2
b4
y6
y7
b6
y8
4e+05
3e+05
2e+05
1e+05
0e+00
Intensity
500
1000
M/Z
type
b
y

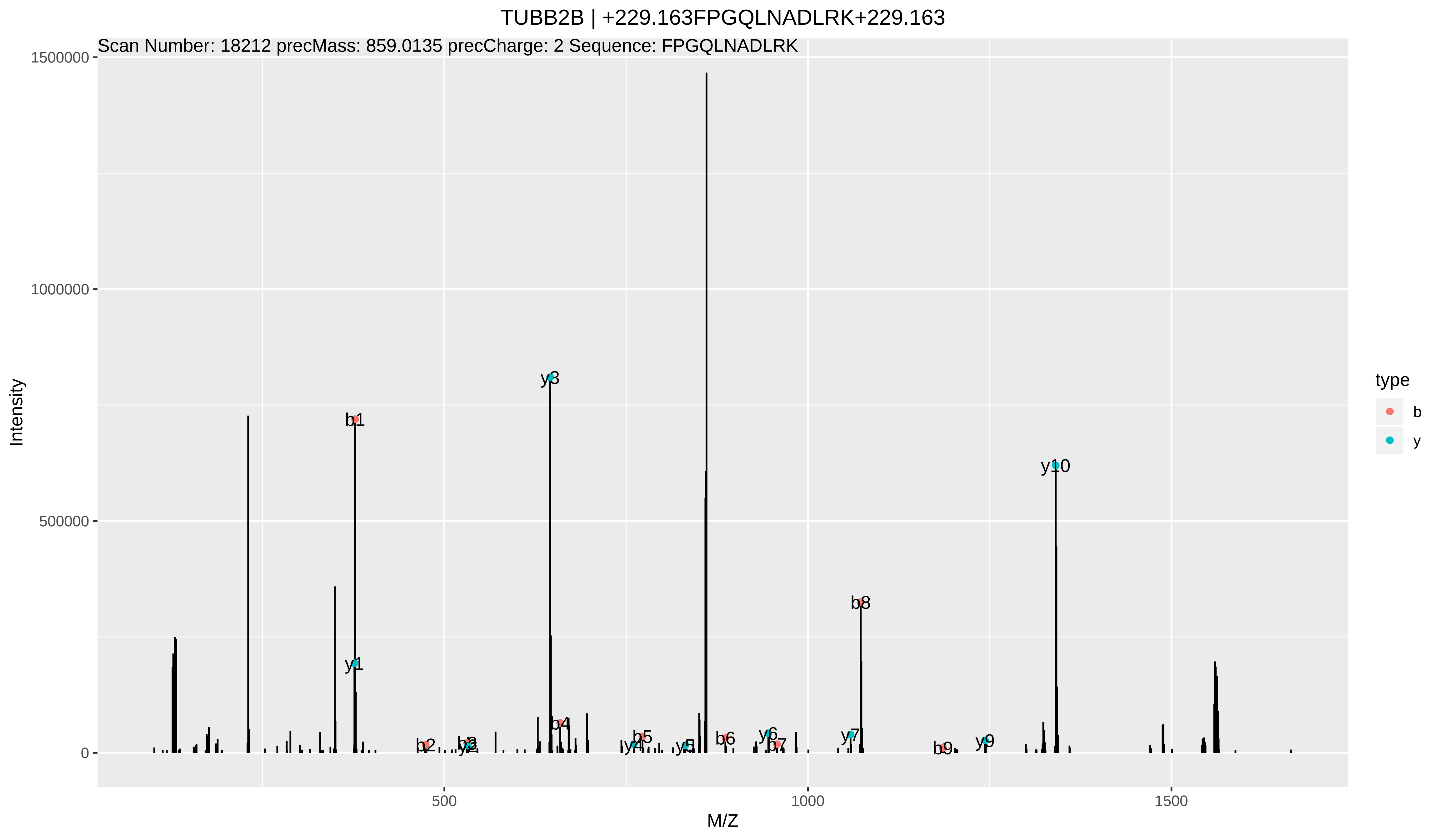

TUBB2B | +229.163FPGQLNADLRK+229.163
Scan Number: 18212 precMass: 859.0135 precCharge: 2 Sequence: FPGQLNADLRK
Intensity
1500000
1000000
500000
0
500
1000
1500
M/Z
y3
b1
y1
y10
b8
b4
b5
b6
y6
y7
b7
y9
b9
b2
b3
y2
y4
y5
type
b
y

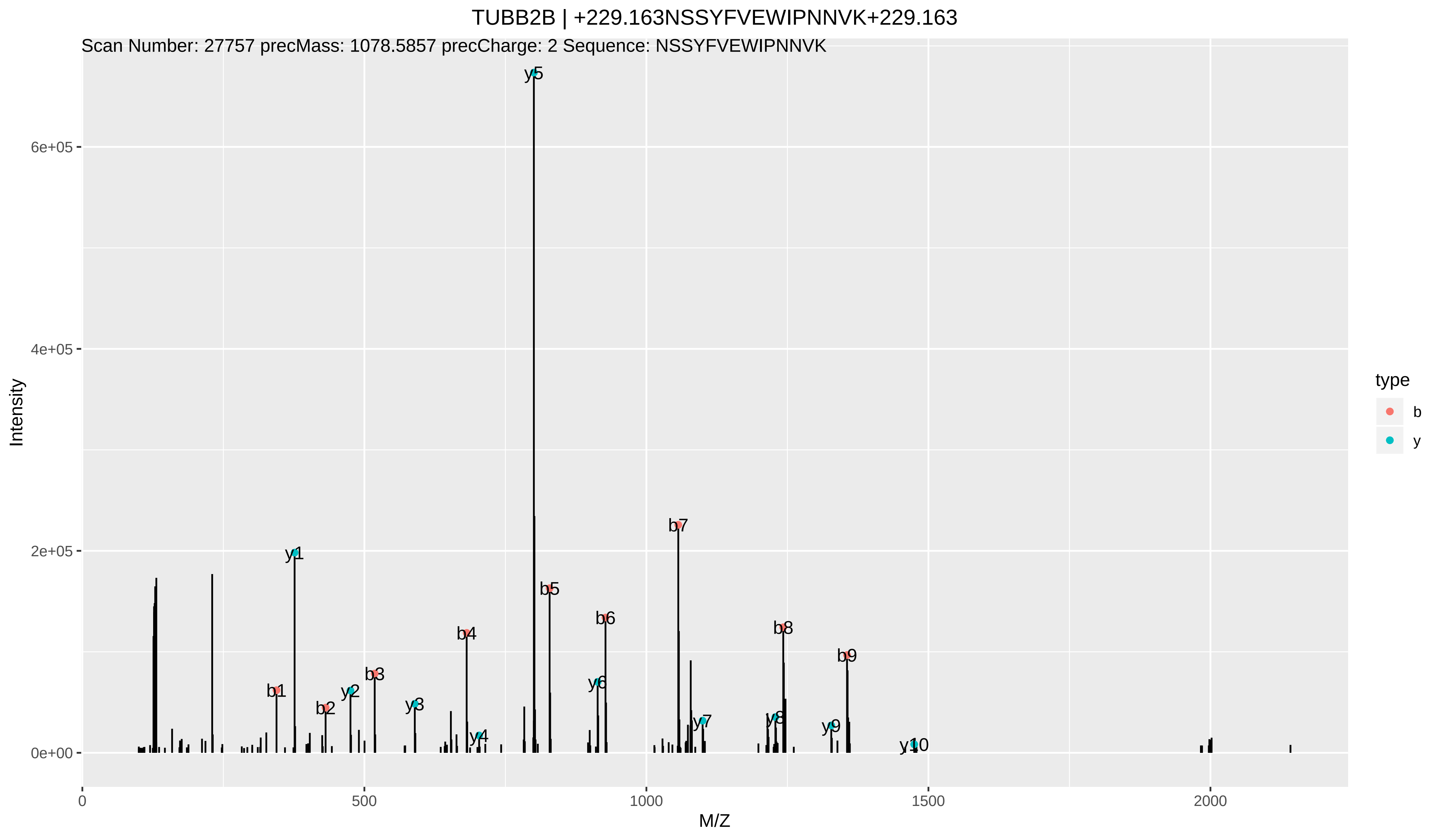

TUBB2B | +229.163NSSYFVEWIPNNVK+229.163
Scan Number: 27757 precMass: 1078.5857 precCharge: 2 Sequence: NSSYFVEWIPNNVK
Intensity
M/Z
6e+05
4e+05
2e+05
0e+00
0
500
1000
1500
2000
type
b
y
y5
b7
y1
b5
b6
b8
b4
b9
b3
y6
b1
y2
b2
y3
y7
y8
y9
y4
y10

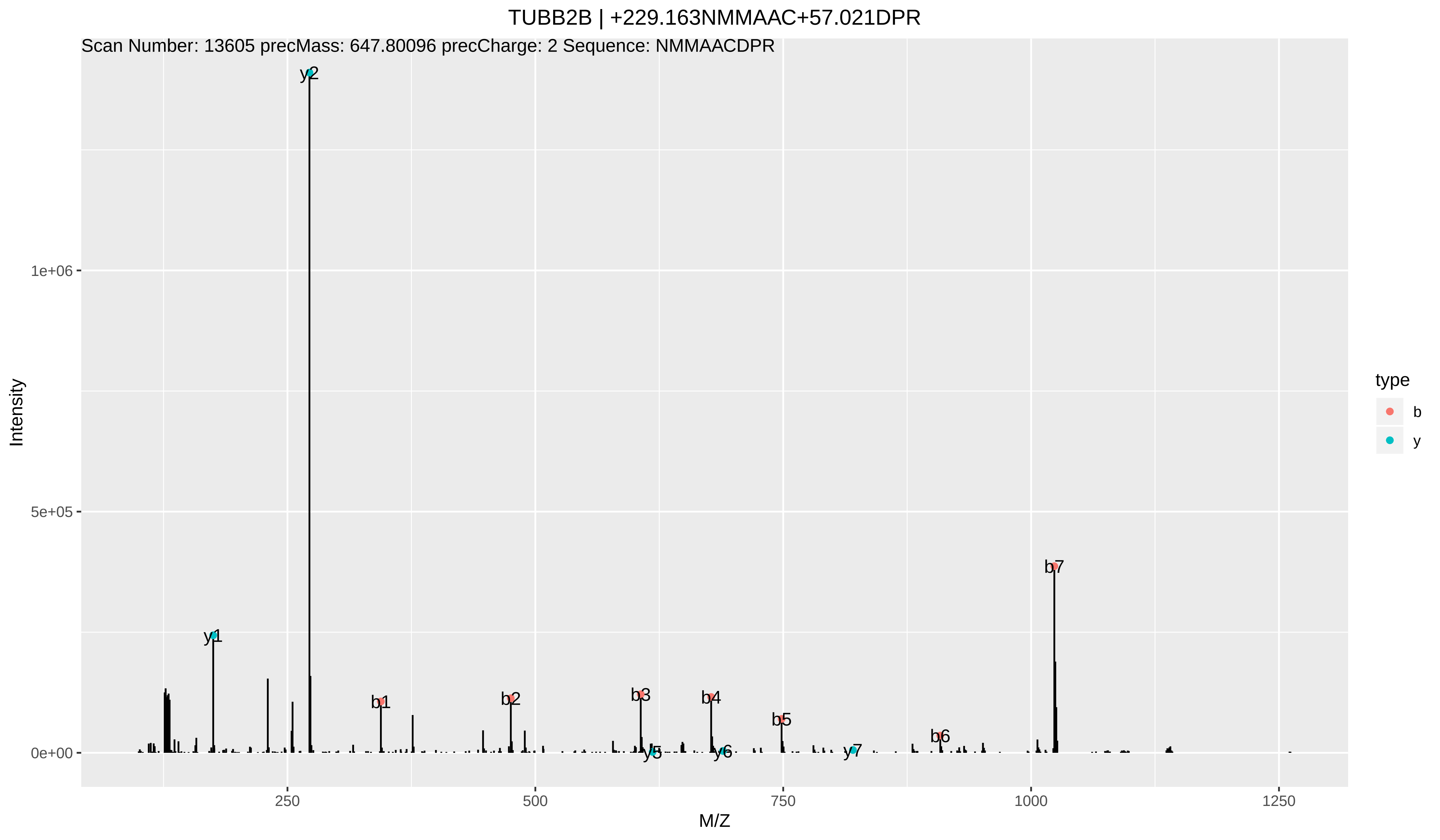

TUBB2B | +229.163NMMAAC+57.021DPR
Scan Number: 13605 precMass: 647.80096 precCharge: 2 Sequence: NMMAACDPR
Intensity
1e+06
5e+05
0e+00
M/Z
250
500
750
1000
1250
y2
y1
b1
b2
b3
y5
b4
y6
b5
y7
b6
b7
type
b
y

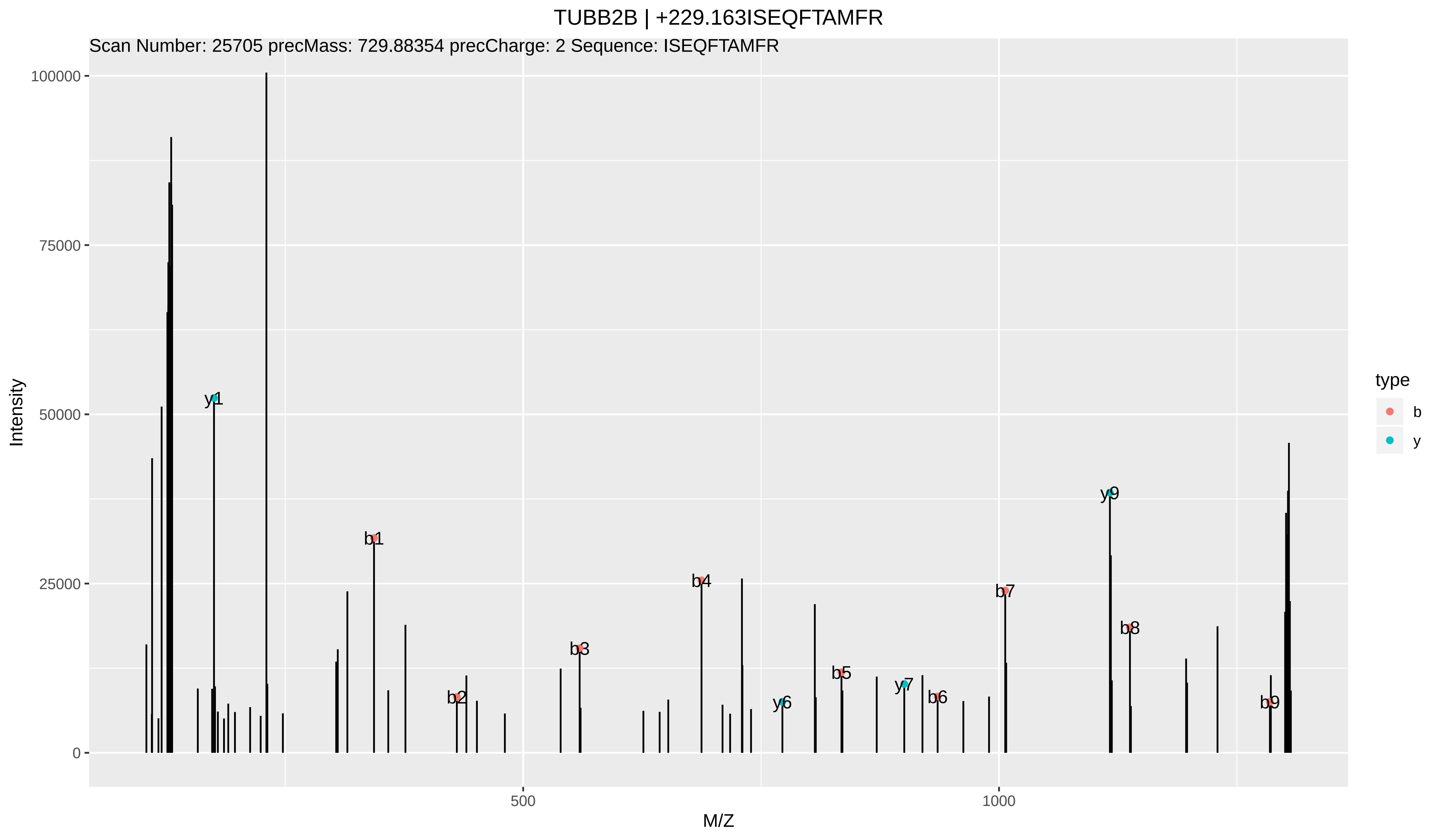
TUBB2B | +229.163ISEQFTAMFR
Scan Number: 25705 precMass: 729.88354 precCharge: 2 Sequence: ISEQFTAMFR
Intensity
M/Z
100000
75000
50000
25000
0
500
1000
y1
b1
b2
b3
b4
y6
b5
y7
b6
b7
y9
b8
b9
type
b
y

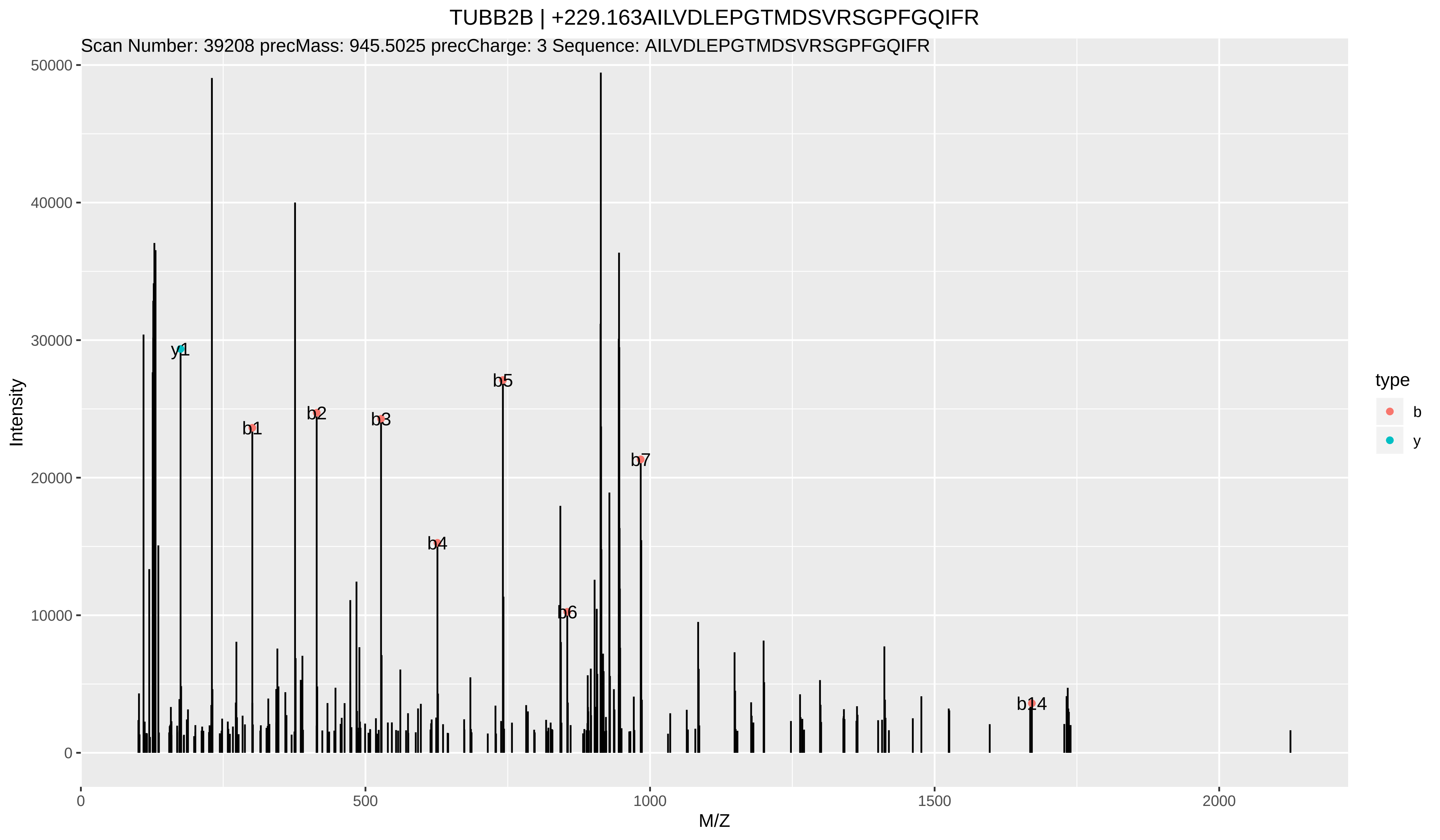
TUBB2B | +229.163AILVDLEPGTMDSVRSGPFGQIFR
Scan Number: 39208 precMass: 945.5025 precCharge: 3 Sequence: AILVDLEPGTMDSVRSGPFGQIFR
Intensity
M/Z
type
b
y
y1
b1
b2
b3
b4
b5
b6
b7
b14
0
10000
20000
30000
40000
50000
500
1000
1500
2000

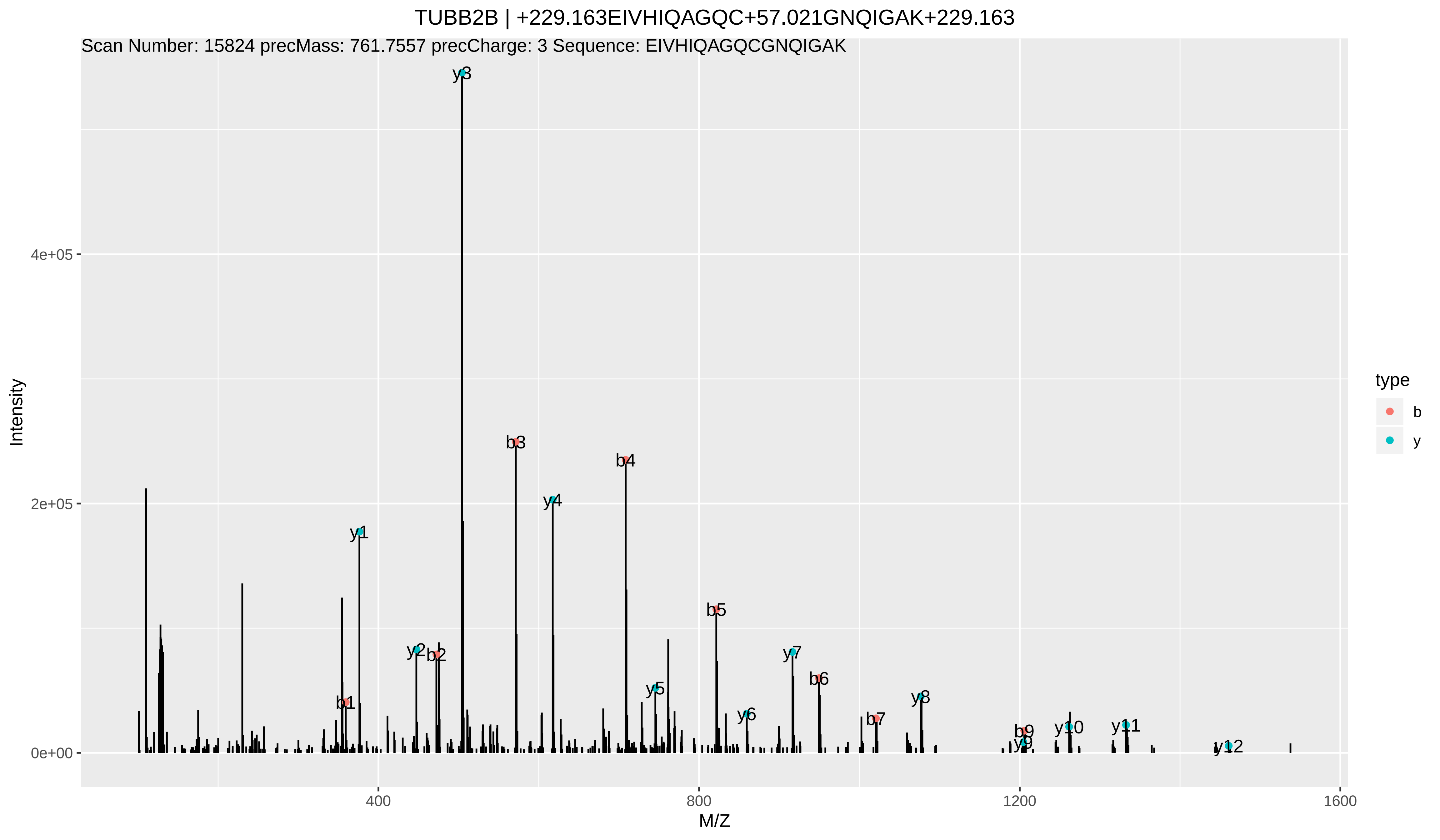

TUBB2B | +229.163EIVHIQAGQC+57.021GNQIGAK+229.163
Scan Number: 15824 precMass: 761.7557 precCharge: 3 Sequence: EIVHIQAGQCGNQIGAK
Intensity
M/Z
type
b
y
y3
b3
b4
y4
y1
b5
y2
b2
y7
b6
y5
y8
b1
y6
b7
b9
y10
y11
y9
y12
4e+05
2e+05
0e+00
400
800
1200
1600

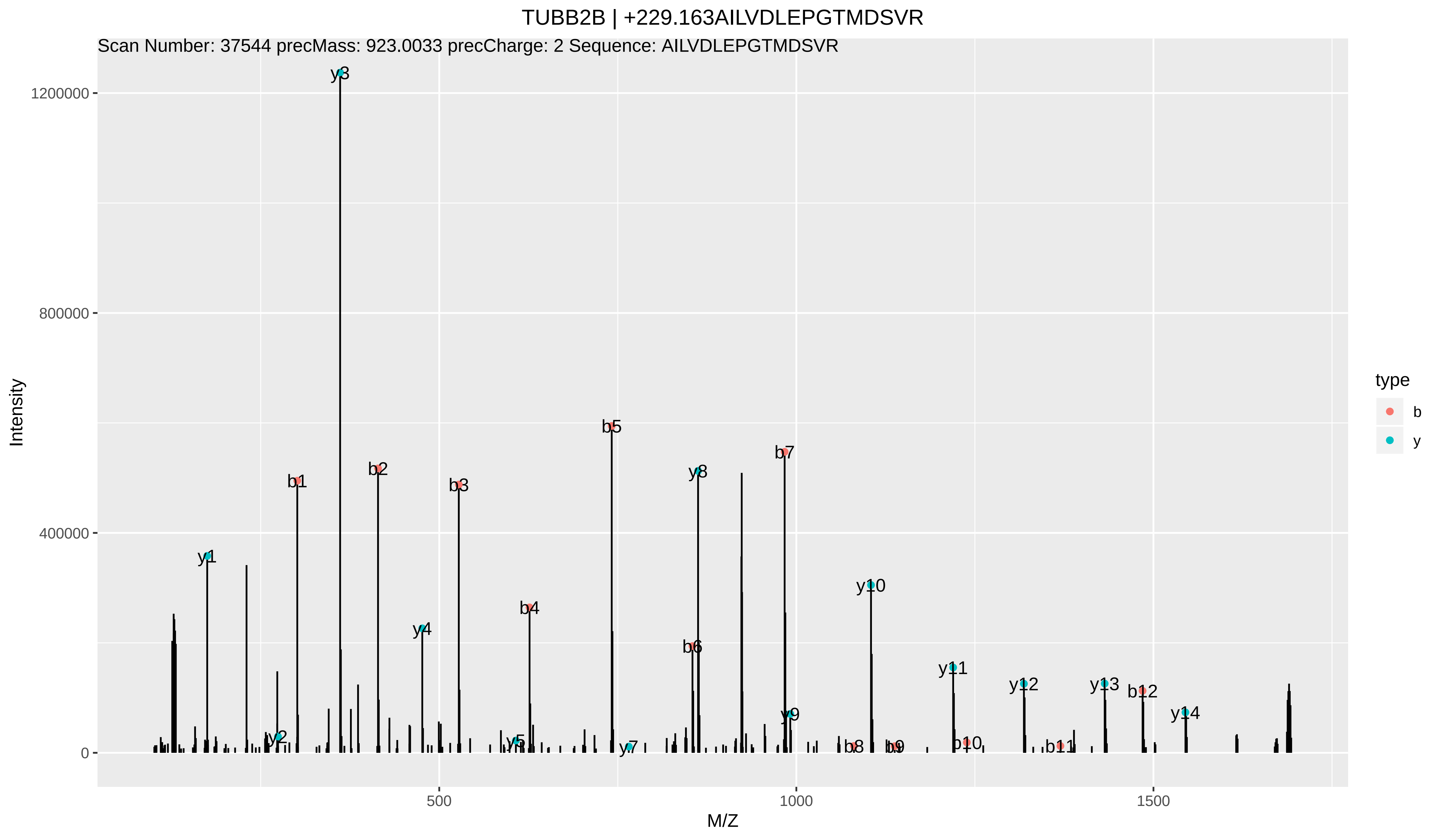

TUBB2B | +229.163AILVDLEPGTMDSVR
Scan Number: 37544 precMass: 923.0033 precCharge: 2 Sequence: AILVDLEPGTMDSVR
y3
b5
b7
y8
b2
b1
b3
y1
y10
b4
y4
b6
y11
y12
y13
b12
y14
y9
y2
y5
y7
b8
b9
b10
b11
1200000
800000
400000
0
Intensity
500
1000
1500
M/Z
type
b
y

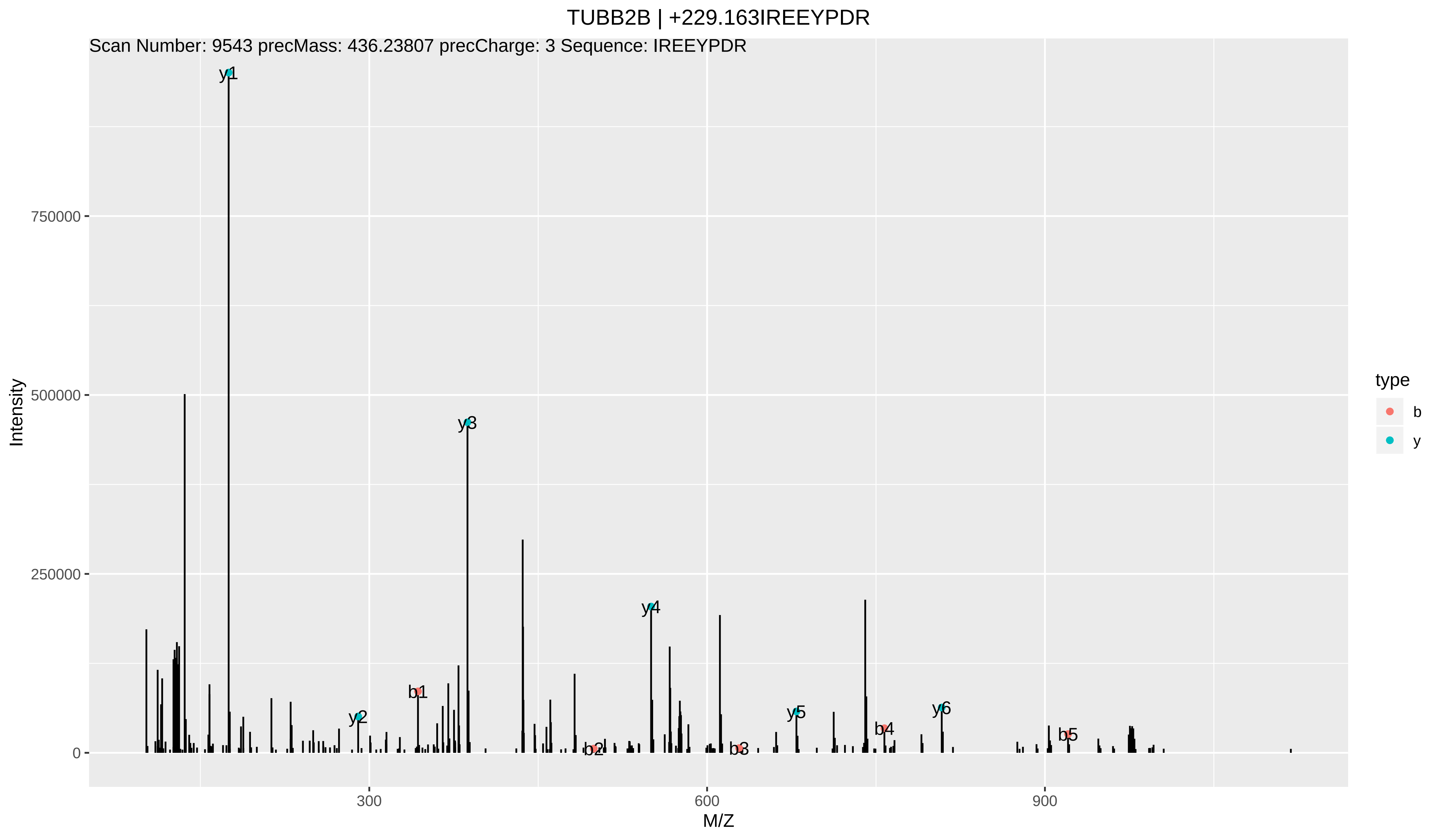
TUBB2B | +229.163IREEYPDR
Scan Number: 9543 precMass: 436.23807 precCharge: 3 Sequence: IREEYPDR
y1
y3
y4
y2
b1
y5
y6
b4
b5
b2
b3
Intensity
750000
500000
250000
0
300
600
900
M/Z
type
b
y

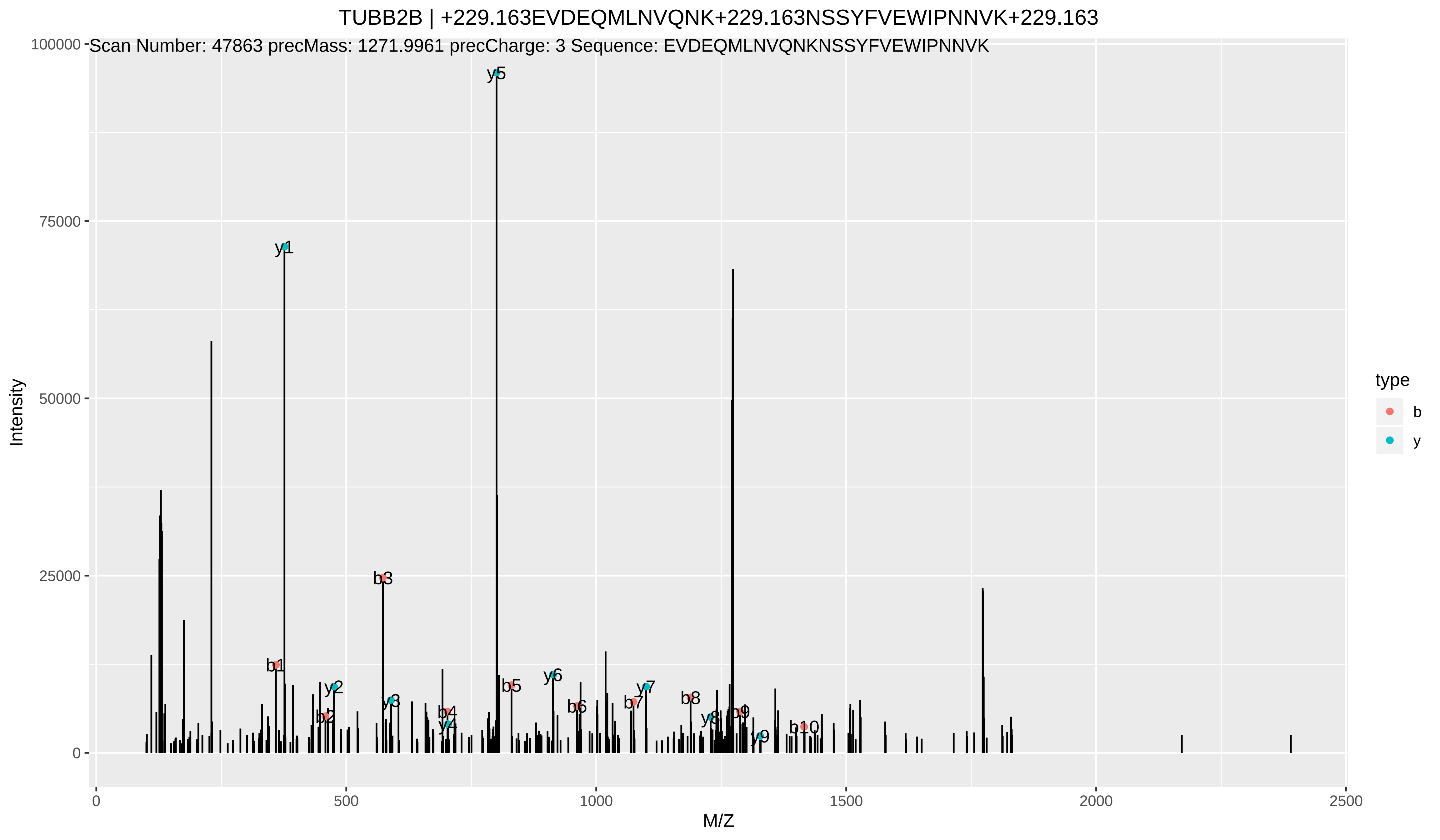

TUBB2B | +229.163EVDEQMLNVQNK+229.163NSSYFVEWIPNNVK+229.163
Scan Number: 47863 precMass: 1271.9961 precCharge: 3 Sequence: EVDEQMLNVQNKNSSYFVEWIPNNVK
Intensity
M/Z
type
b
y
y5
y1
b3
b1
y2
b2
y3
b4
y4
b5
y6
b6
b7
y7
b8
y8
b9
y9
b10

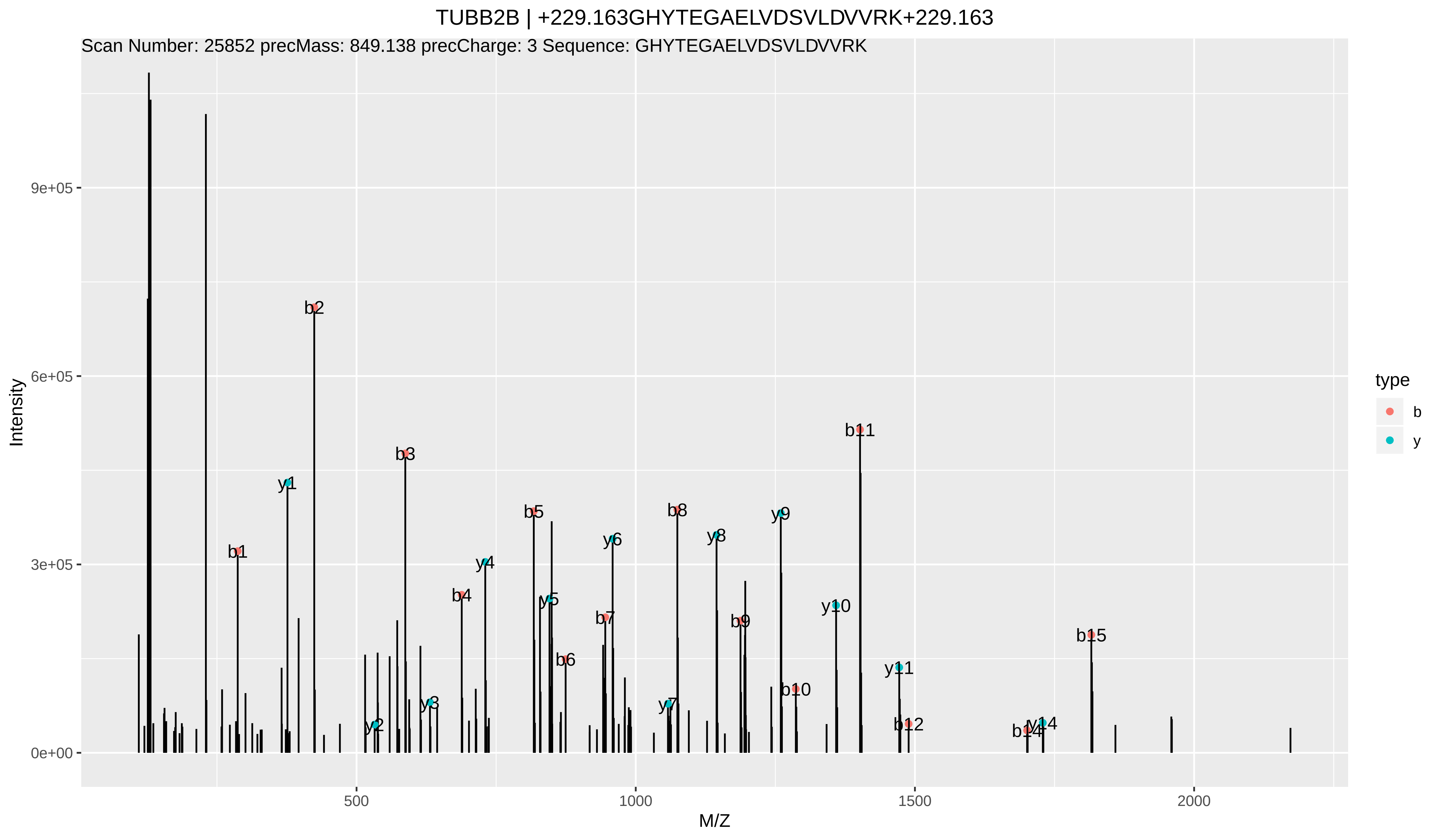
TUBB2B | +229.163GHYTEGAELVDSVLDVVRK+229.163
Scan Number: 25852 precMass: 849.138 precCharge: 3 Sequence: GHYTEGAELVDSVLDVVRK
Intensity
9e+05
6e+05
3e+05
0e+00
M/Z
500
1000
1500
2000
type
b
y
b1
b2
b3
b4
b5
b6
b7
b8
b9
b10
b11
b12
b14
b15
y1
y2
y3
y4
y5
y6
y7
y8
y9
y10
y11
y14

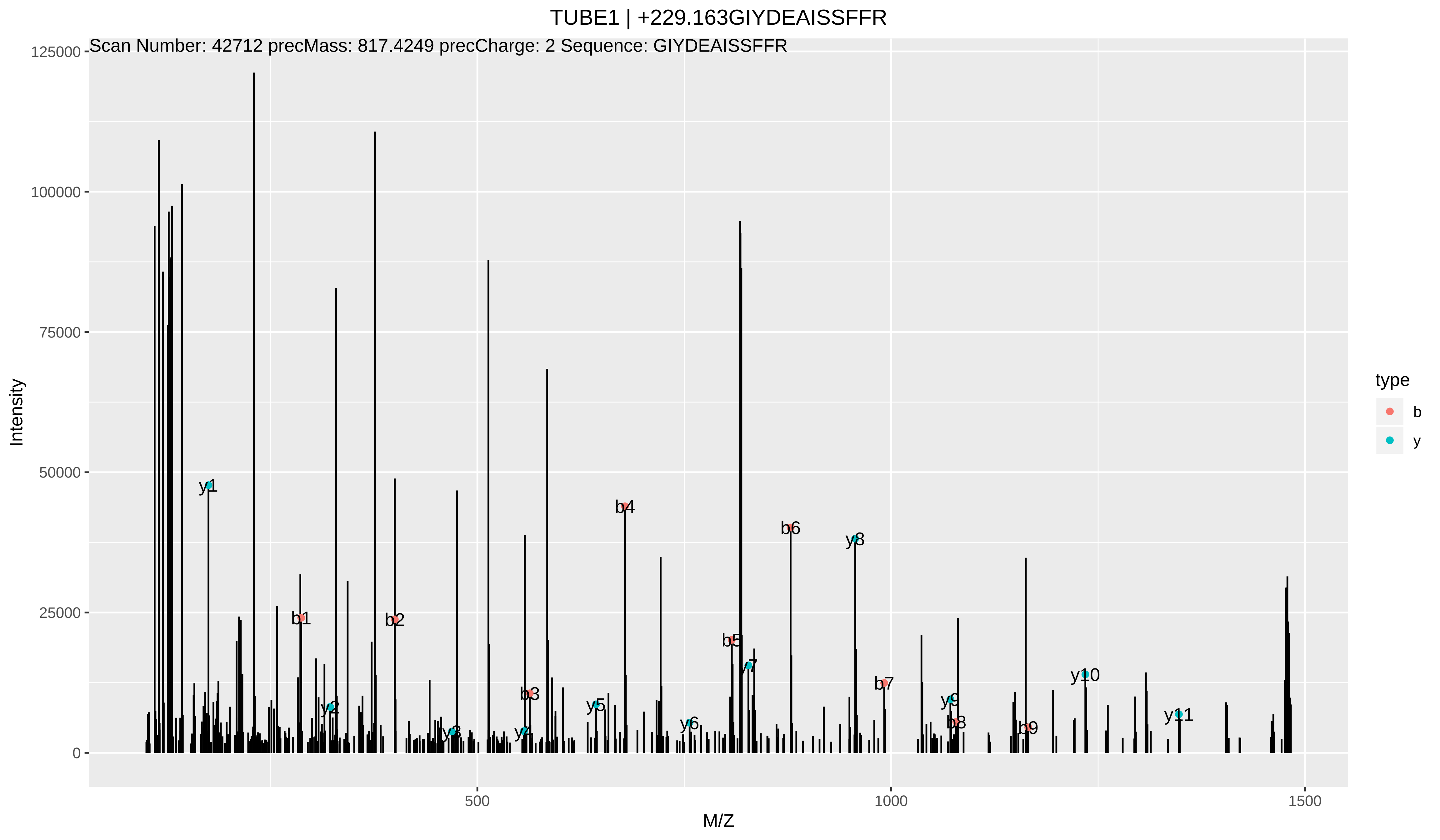

TUBE1 | +229.163GIYDEAISSFFR
Scan Number: 42712 precMass: 817.4249 precCharge: 2 Sequence: GIYDEAISSFFR
Intensity
M/Z
0
25000
50000
75000
100000
125000
500
1000
1500
y1
b1
y2
b2
y3
y4
b3
y5
b4
y6
b5
y7
b6
y8
b7
y9
b8
b9
y10
y11
type
b
y

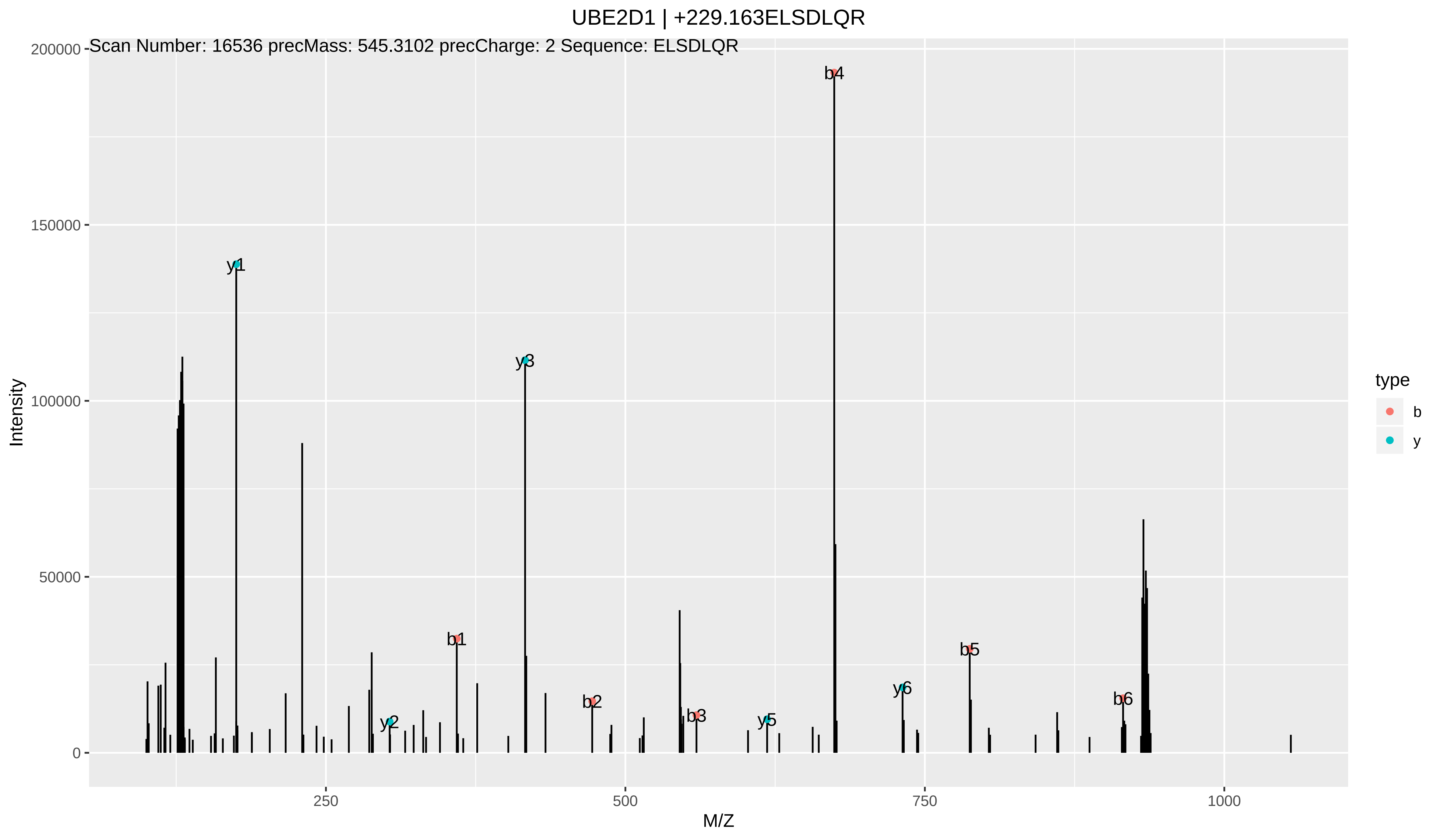

UBE2D1 | +229.163ELSDLQR
Scan Number: 16536 precMass: 545.3102 precCharge: 2 Sequence: ELSDLQR
Intensity
200000
150000
100000
50000
0
M/Z
250
500
750
1000
y1
y2
y3
y5
y6
b1
b2
b3
b4
b5
b6
type
b
y

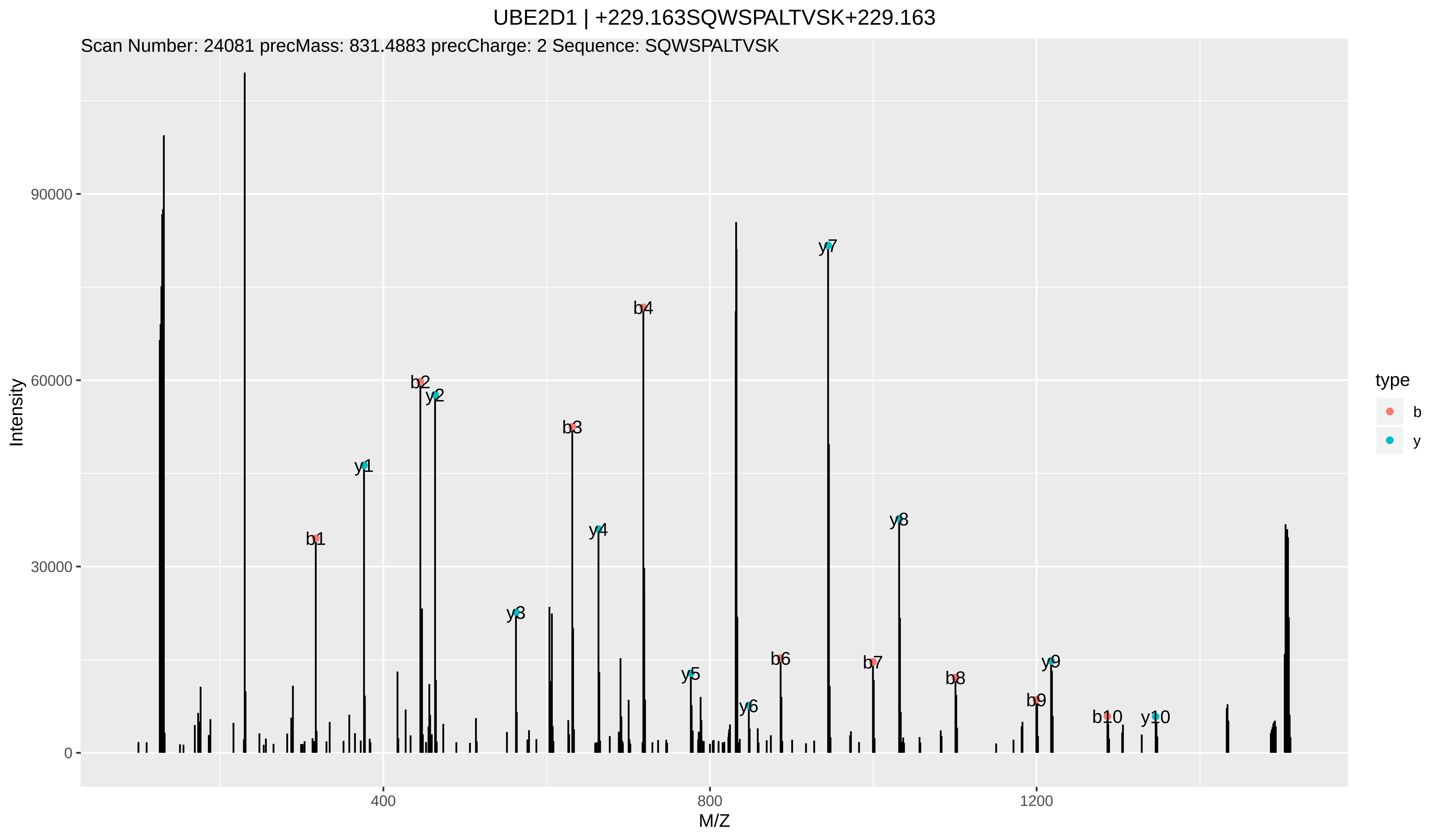
UBE2D1 | +229.163SQWSPALTVSK+229.163
Scan Number: 24081 precMass: 831.4883 precCharge: 2 Sequence: SQWSPALTVSK
Intensity
90000
60000
30000
0
400
800
1200
M/Z
b1
y1
b2
y2
y3
b3
y4
b4
y5
y6
b6
y7
b7
y8
b8
b9
y9
b10
y10
type
b
y

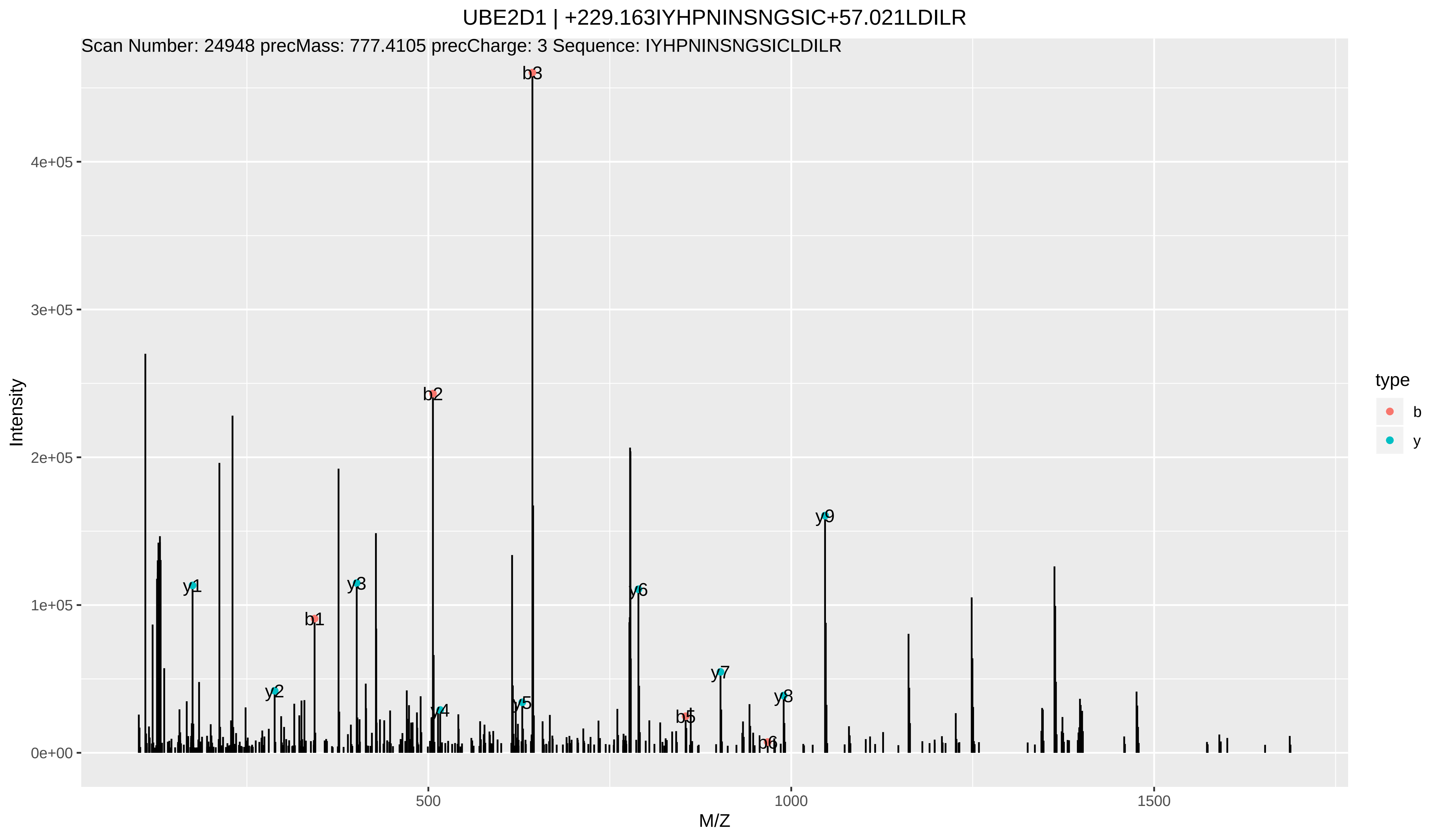

UBE2D1 | +229.163IYHPNINSNGSIC+57.021LDILR
Scan Number: 24948 precMass: 777.4105 precCharge: 3 Sequence: IYHPNINSNGSICLDILR
Intensity
4e+05
3e+05
2e+05
1e+05
0e+00
M/Z
500
1000
1500
b3
b2
y9
y1
y3
y6
b1
y7
y2
y8
y4
y5
b5
b6
type
b
y

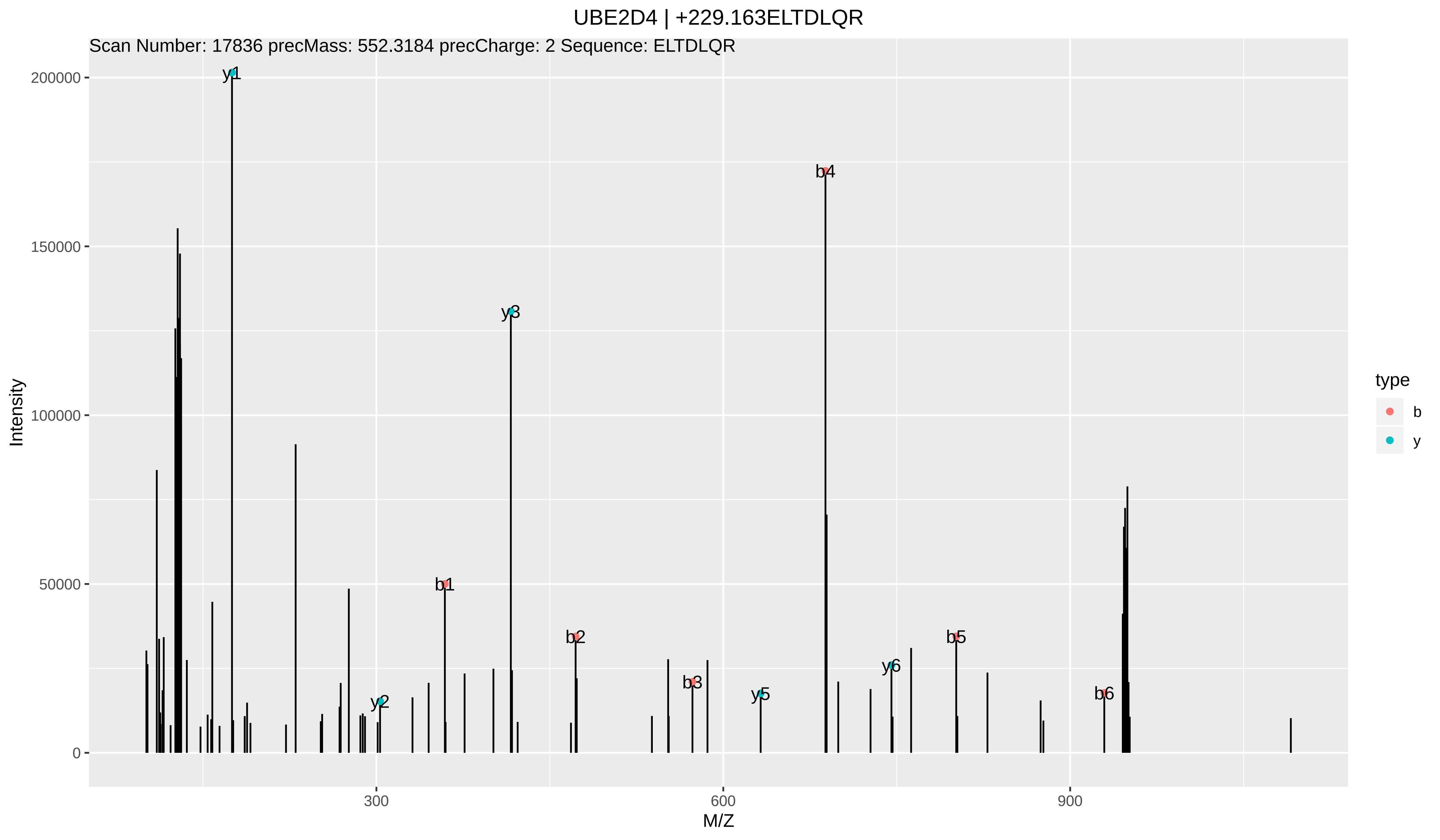
UBE2D4 | +229.163ELTDLQR
Scan Number: 17836 precMass: 552.3184 precCharge: 2 Sequence: ELTDLQR
Intensity
M/Z
200000
150000
100000
50000
0
300
600
900
y1
b4
y3
b1
b2
b3
y2
y5
y6
b5
b6
type
b
y

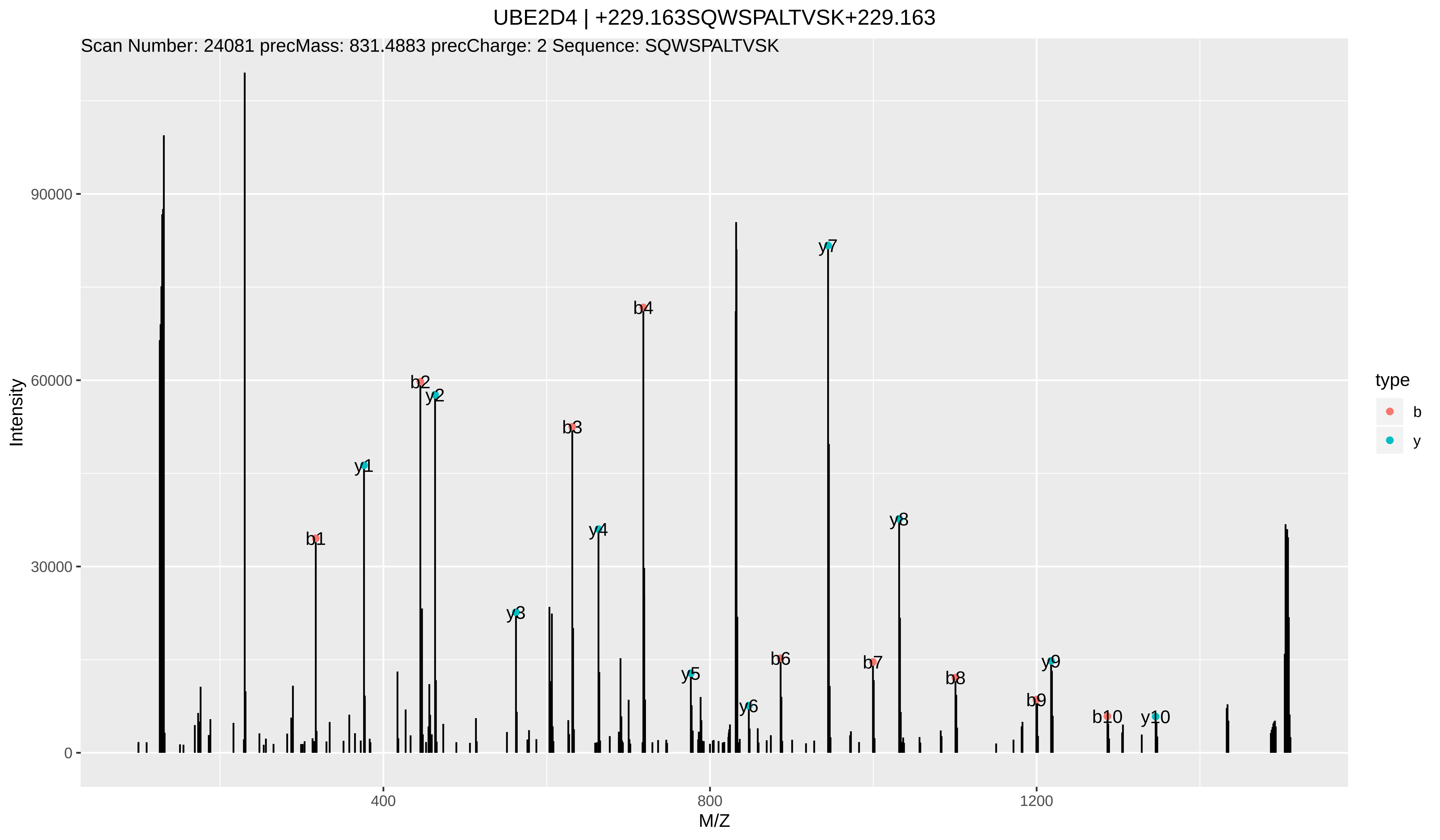
UBE2D4 | +229.163SQWSPALTVSK+229.163
Scan Number: 24081 precMass: 831.4883 precCharge: 2 Sequence: SQWSPALTVSK
Intensity
M/Z
0
30000
60000
90000
400
800
1200
b1
y1
b2
y2
y3
b3
y4
b4
y5
y6
b6
y7
b7
y8
b8
b9
y9
b10
y10
type
b
y

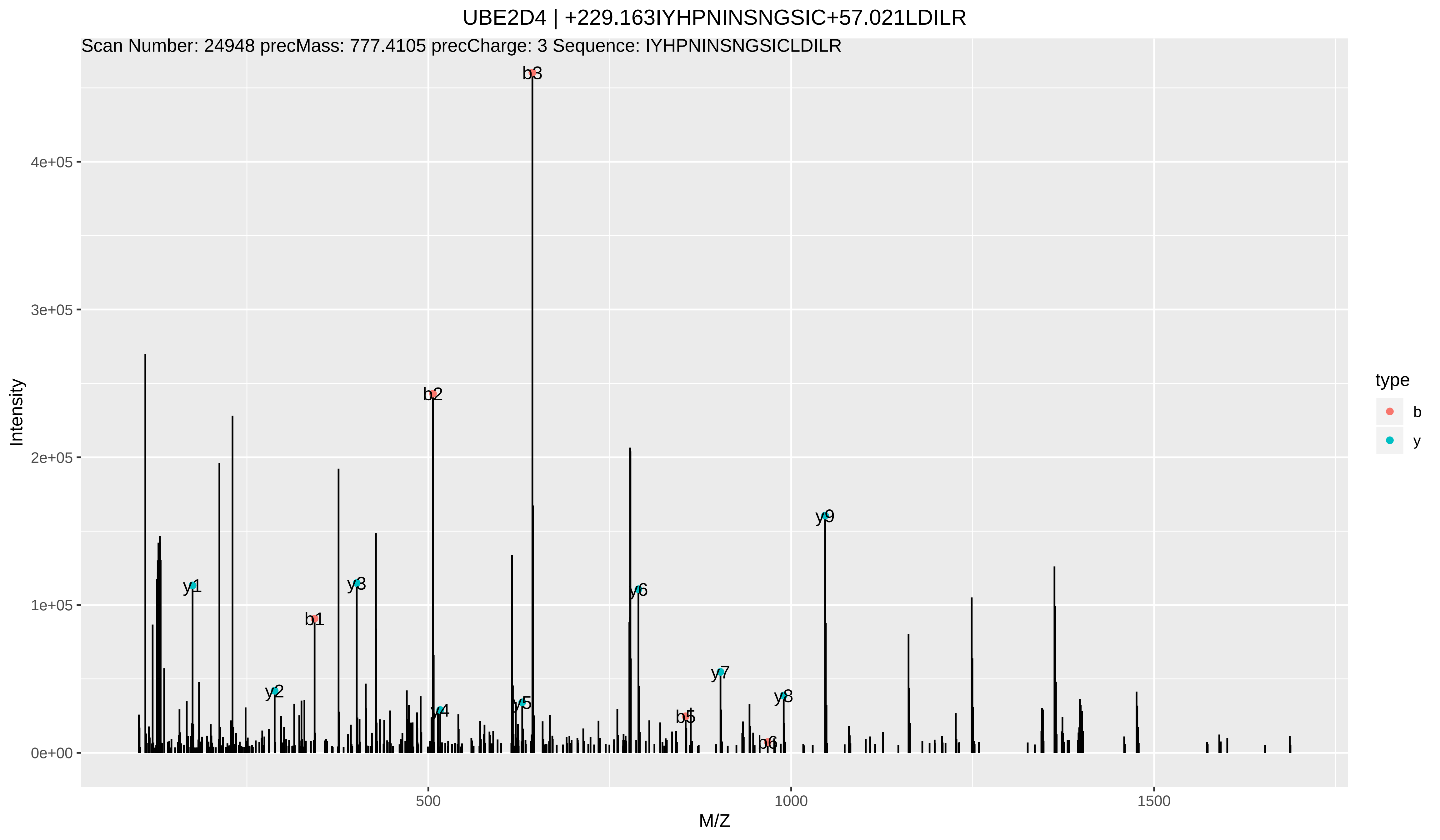
UBE2D4 | +229.163IYHPNINSNGSIC+57.021LDILR
Scan Number: 24948 precMass: 777.4105 precCharge: 3 Sequence: IYHPNINSNGSICLDILR
b3
b2
y9
y1
y3
y6
b1
y7
y2
y8
y4
y5
b5
b6
4e+05
3e+05
2e+05
1e+05
0e+00
Intensity
500
1000
1500
M/Z
type
b
y

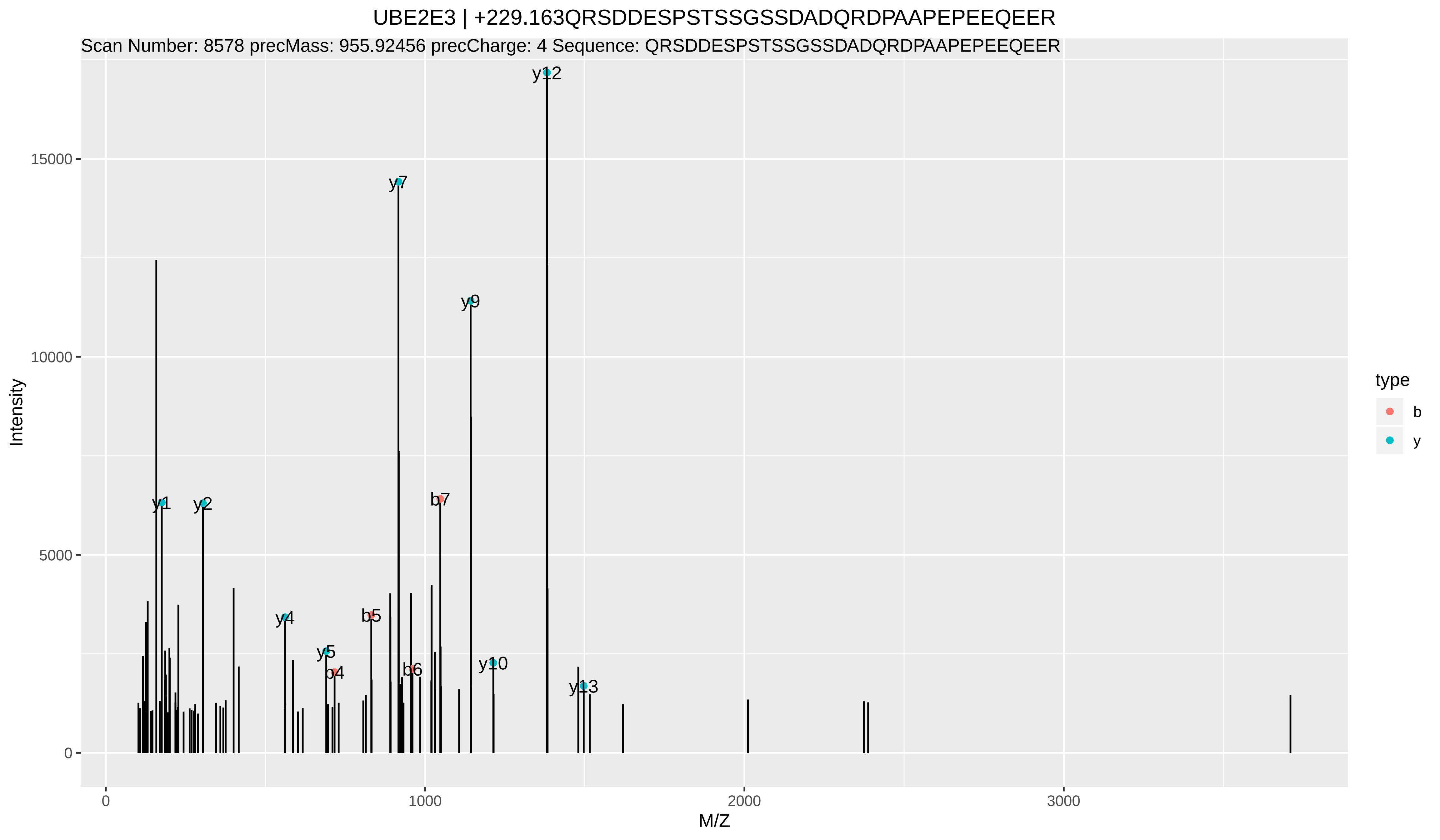
UBE2E3 | +229.163QRSDDESPSTSSGSSDADQRDPAAPEPEEQEER
Scan Number: 8578 precMass: 955.92456 precCharge: 4 Sequence: QRSDDESPSTSSGSSDADQRDPAAPEPEEQEER
Intensity
M/Z
type
b
y
y1
y2
y4
y5
b4
b5
b6
y7
b7
y9
y10
y12
y13
0
5000
10000
15000
1000
2000
3000

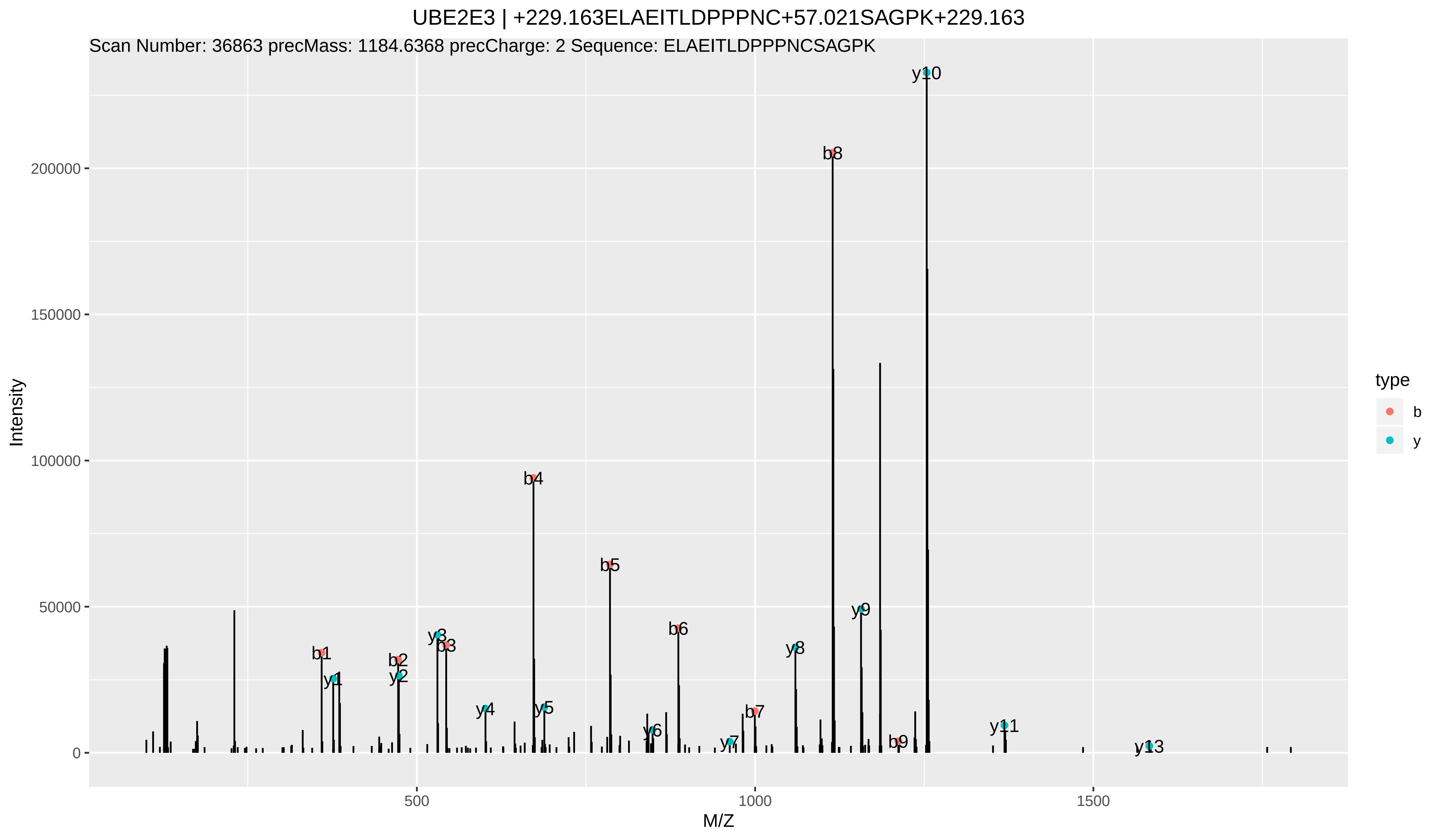
UBE2E3 | +229.163ELAEITLDPPPNC+57.021SAGPK+229.163
Scan Number: 36863 precMass: 1184.6368 precCharge: 2 Sequence: ELAEITLDPPPNCSAGPK
Intensity
M/Z
type
b
y
y10
b8
b4
b5
y9
b6
y3
b3
y8
b1
b2
y2
y1
y4
y5
b7
y11
y6
y7
b9
y13
0
50000
100000
150000
200000
500
1000
1500

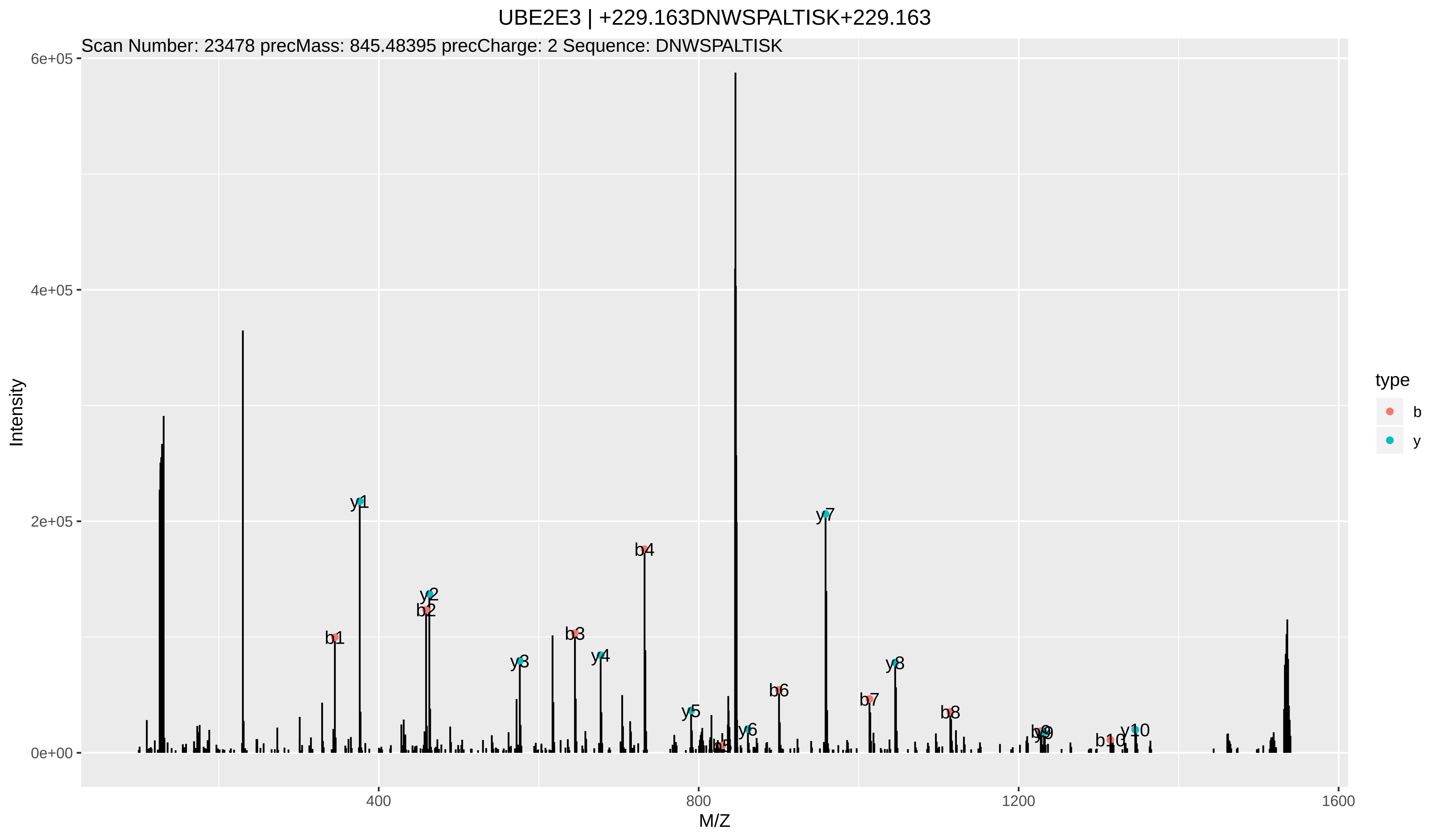

UBE2E3 | +229.163DNWSPALTISK+229.163
Scan Number: 23478 precMass: 845.48395 precCharge: 2 Sequence: DNWSPALTISK
Intensity
M/Z
type
b
y

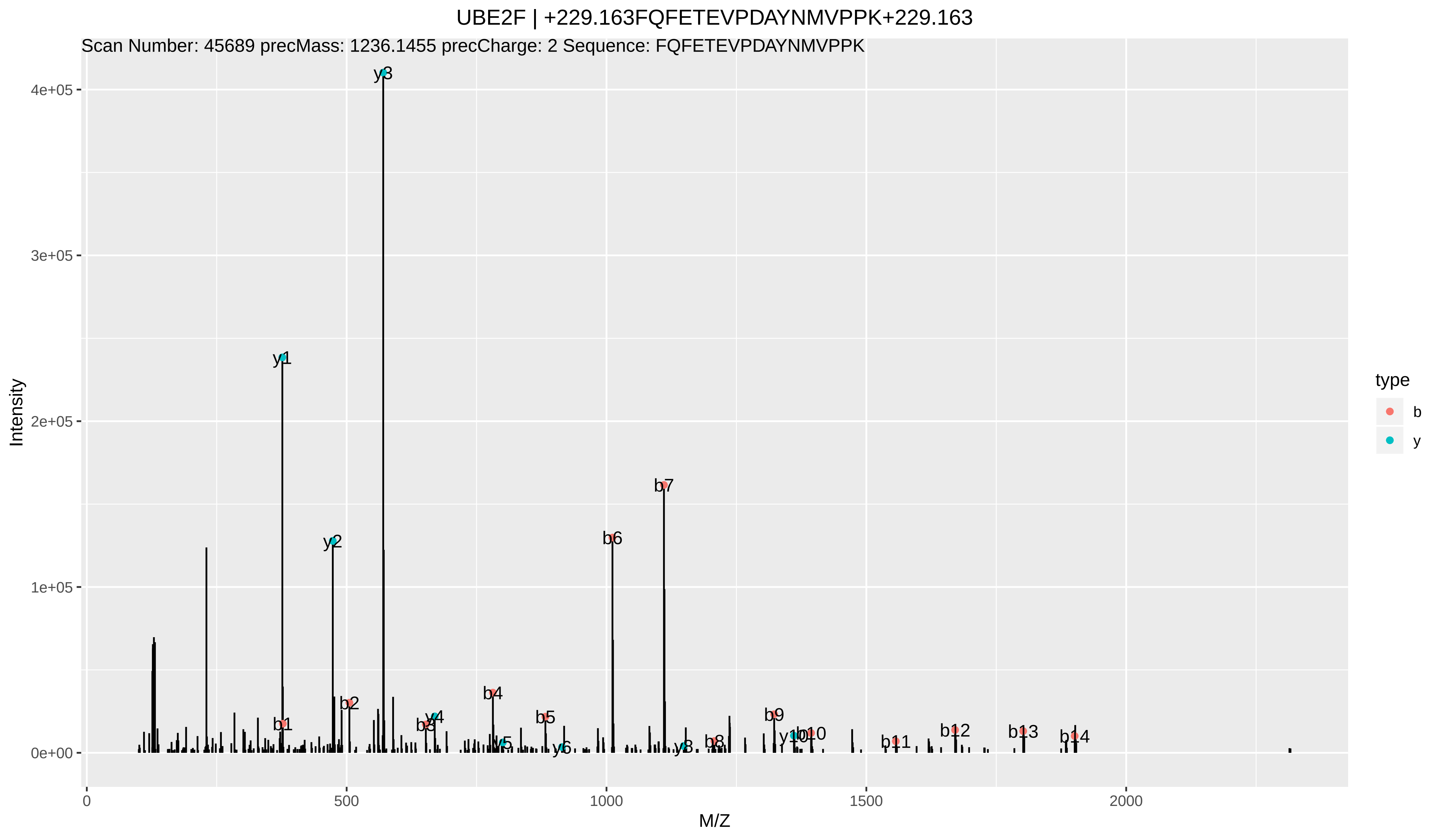

UBE2F | +229.163FQFETEVPDAYNMVPPK+229.163
Scan Number: 45689 precMass: 1236.1455 precCharge: 2 Sequence: FQFETEVPDAYNMVPPK
Intensity
M/Z
type
b
y
y3
y1
y2
b7
b6
b4
b2
b1
b3
y4
b5
b9
b10
y10
b12
b13
b14
b11
b8
y8
y6
y5
0e+00
1e+05
2e+05
3e+05
4e+05
0
500
1000
1500
2000

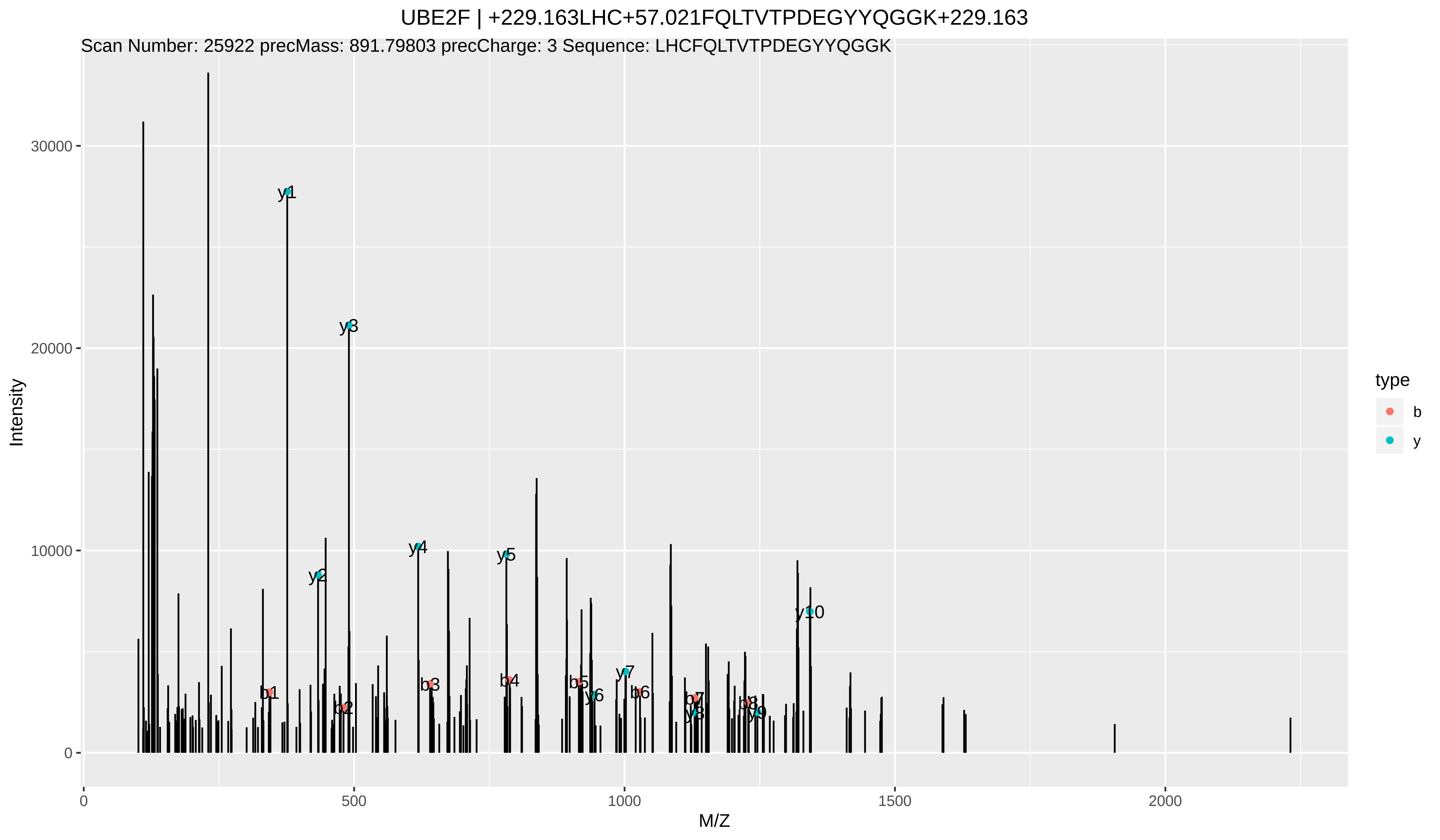

UBE2F | +229.163LHC+57.021FQLTVTPDEGYYQGGK+229.163
Scan Number: 25922 precMass: 891.79803 precCharge: 3 Sequence: LHCFQLTVTPDEGYYQGGK
Intensity
M/Z
type
b
y

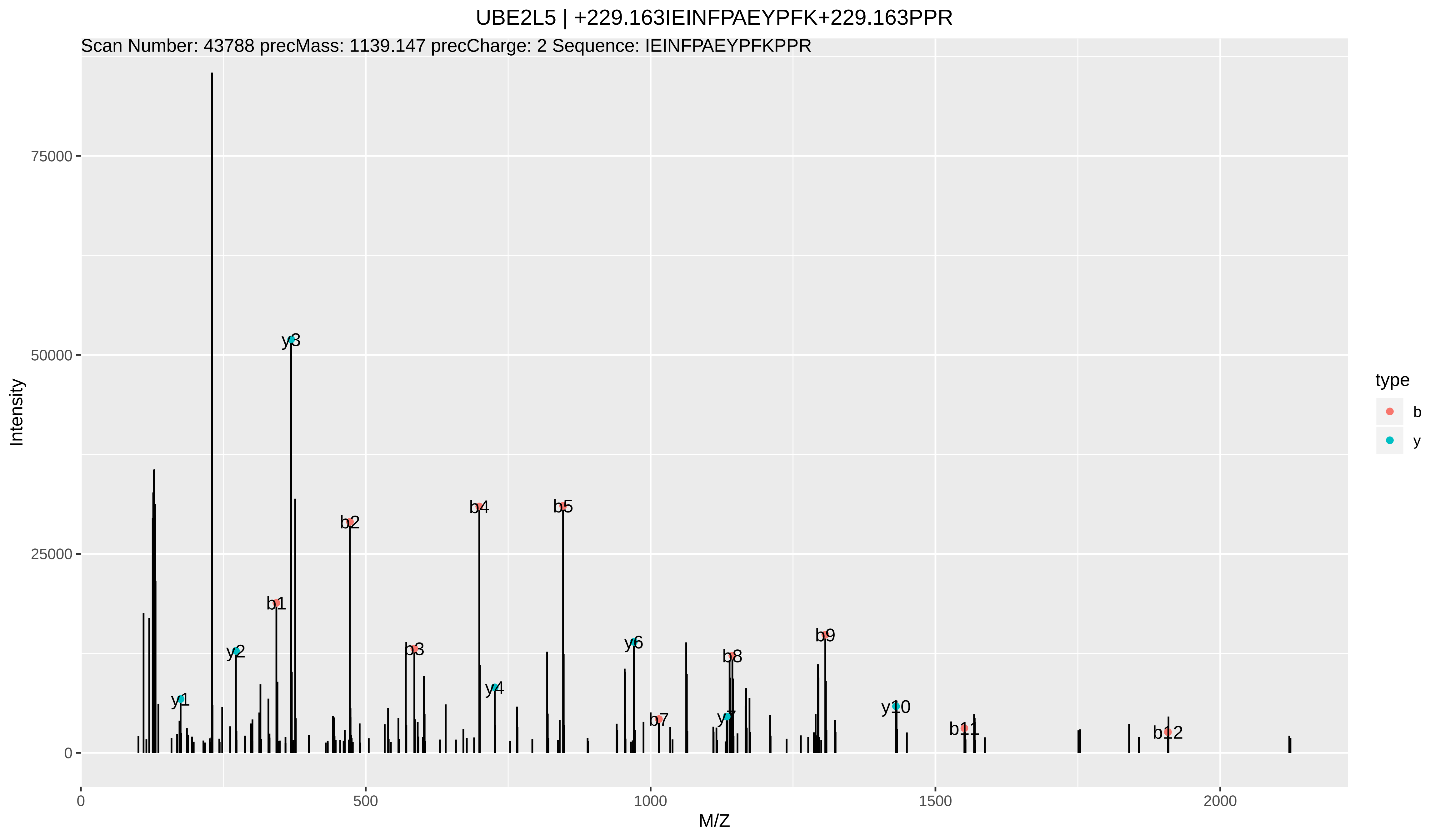

UBE2L5 | +229.163IEINFPAEYPFK+229.163PPR
Scan Number: 43788 precMass: 1139.147 precCharge: 2 Sequence: IEINFPAEYPFKPPR
Intensity
75000
50000
25000
0
0
500
1000
1500
2000
M/Z
y3
b1
b2
b3
b4
b5
b7
b8
b9
b11
b12
y1
y2
y4
y6
y7
y10
type
b
y

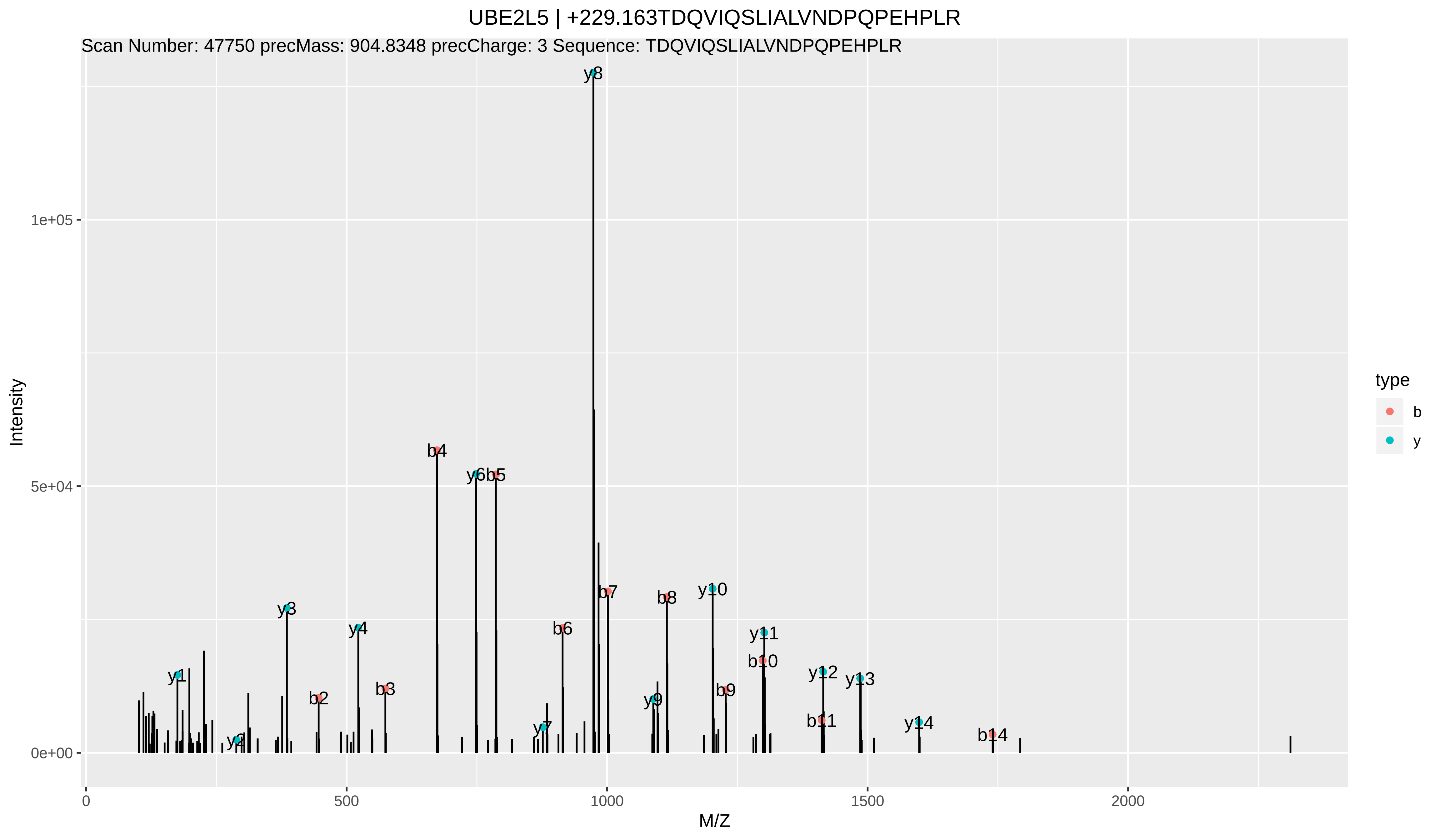

UBE2L5 | +229.163TDQVIQSLIALVNDPQPEHPLR
Scan Number: 47750 precMass: 904.8348 precCharge: 3 Sequence: TDQVIQSLIALVNDPQPEHPLR
Intensity
M/Z
type
b
y
0e+00
5e+04
1e+05
0
500
1000
1500
2000
y1
y2
y3
y4
y6
y7
y8
y9
y10
y11
y12
y13
y14
b2
b3
b4
b5
b6
b7
b8
b9
b10
b11
b14

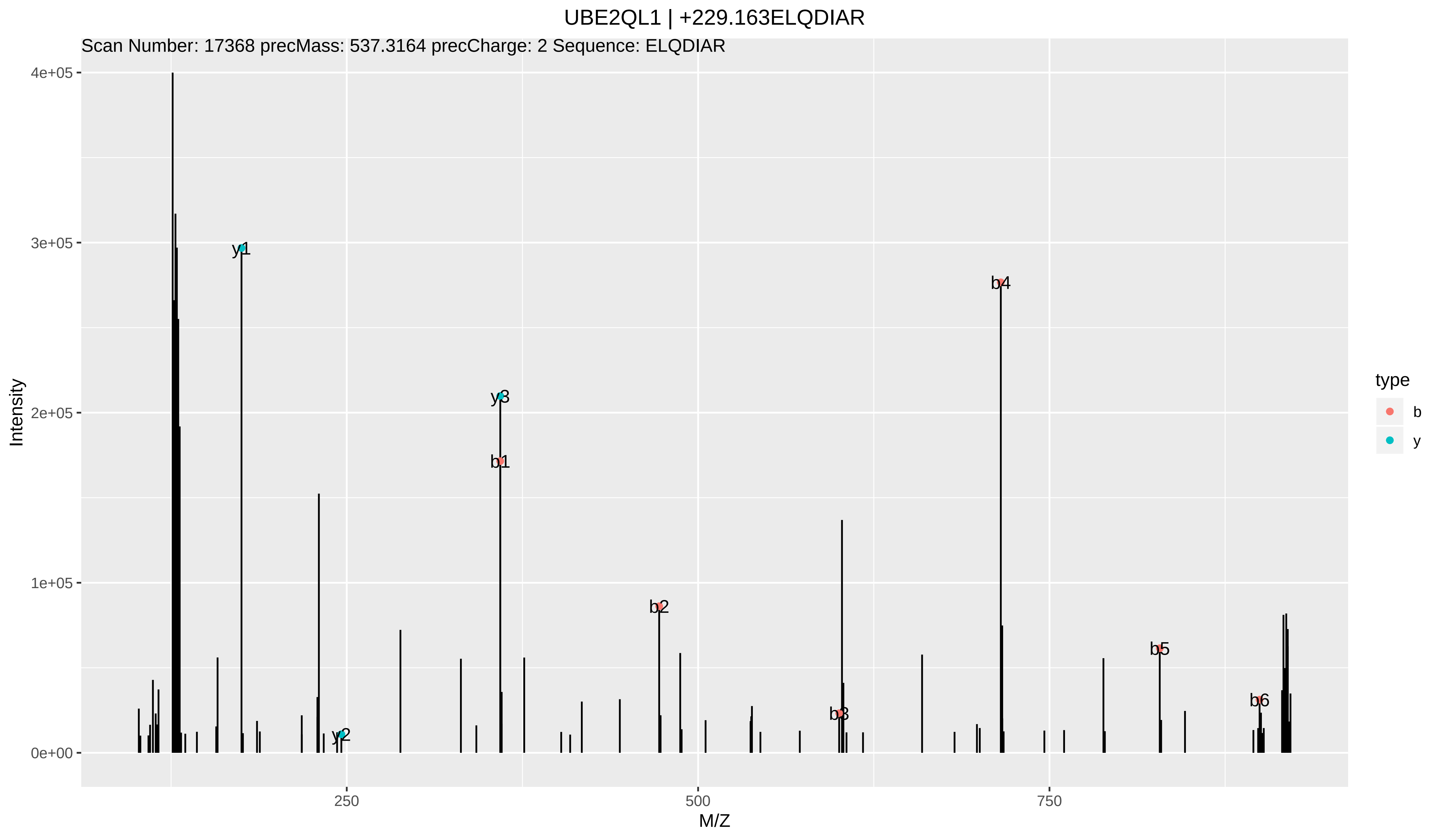

UBE2QL1 | +229.163ELQDIAR
Scan Number: 17368 precMass: 537.3164 precCharge: 2 Sequence: ELQDIAR
Intensity
4e+05
3e+05
2e+05
1e+05
0e+00
M/Z
250
500
750
y1
y2
y3
b1
b2
b3
b4
b5
b6
type
b
y

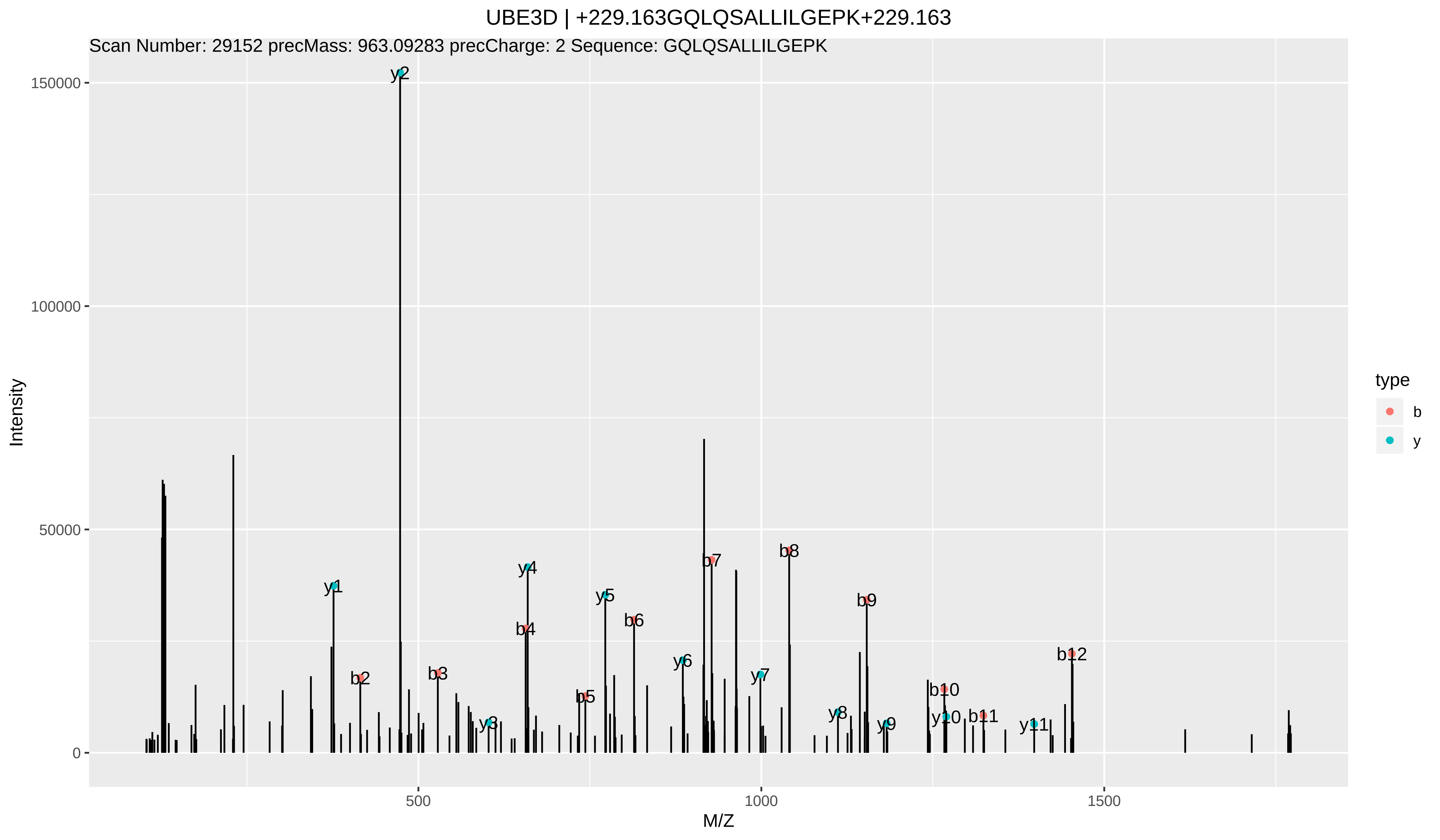
UBE3D | +229.163GQLQSALLILGEPK+229.163
Scan Number: 29152 precMass: 963.09283 precCharge: 2 Sequence: GQLQSALLILGEPK
Intensity
M/Z
0
50000
100000
150000
500
1000
1500
y1
y2
y3
y4
y5
y6
y7
y8
y9
y10
y11
b2
b3
b4
b5
b6
b7
b8
b9
b10
b11
b12
type
b
y

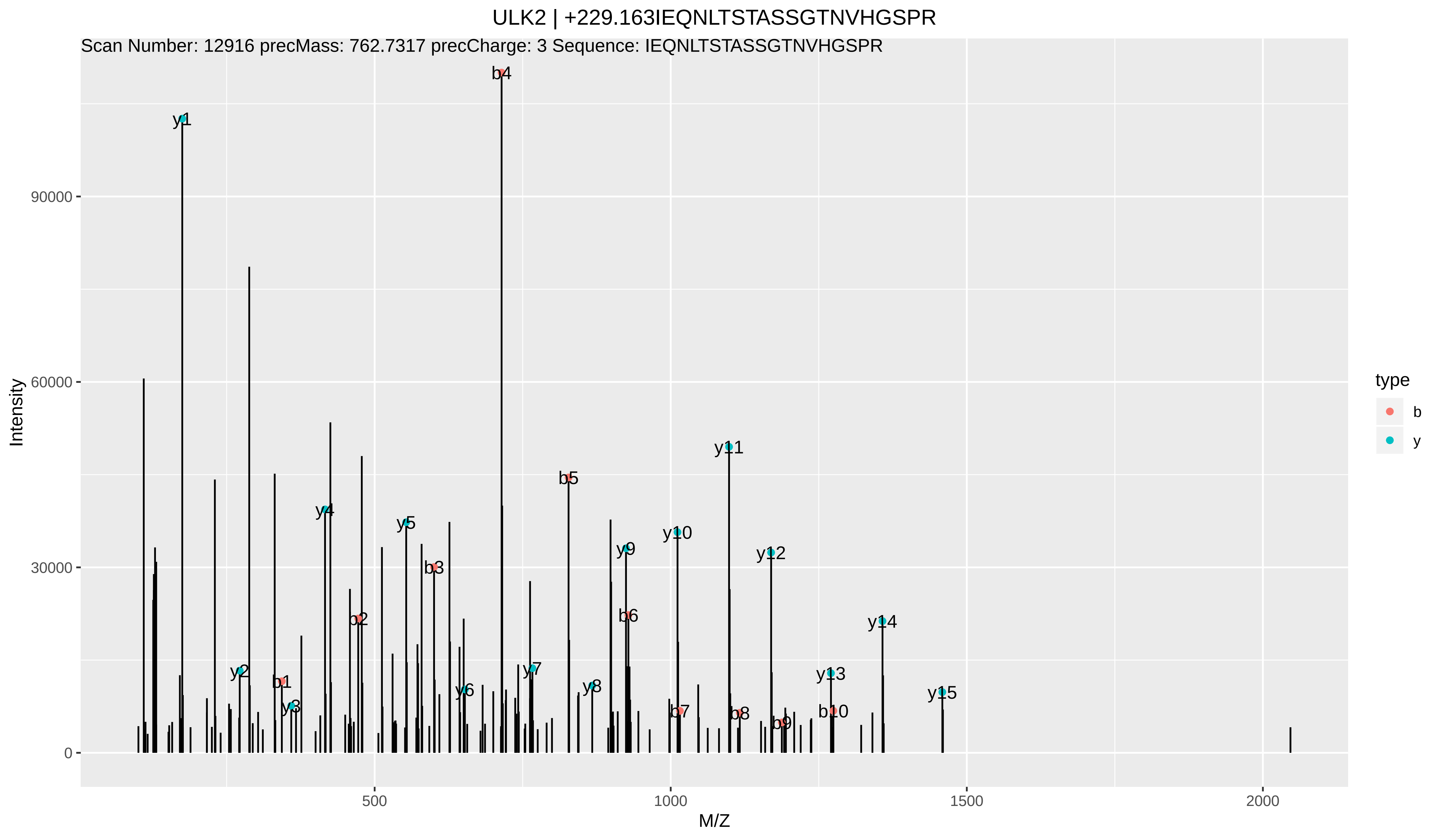

ULK2 | +229.163IEQNLTSTASSGTNVHGSPR
Scan Number: 12916 precMass: 762.7317 precCharge: 3 Sequence: IEQNLTSTASSGTNVHGSPR
Intensity
M/Z
0
30000
60000
90000
500
1000
1500
2000
type
b
y
b1
b2
b3
b4
b5
b6
b7
b8
b9
b10
y1
y2
y3
y4
y5
y6
y7
y8
y9
y10
y11
y12
y13
y14
y15

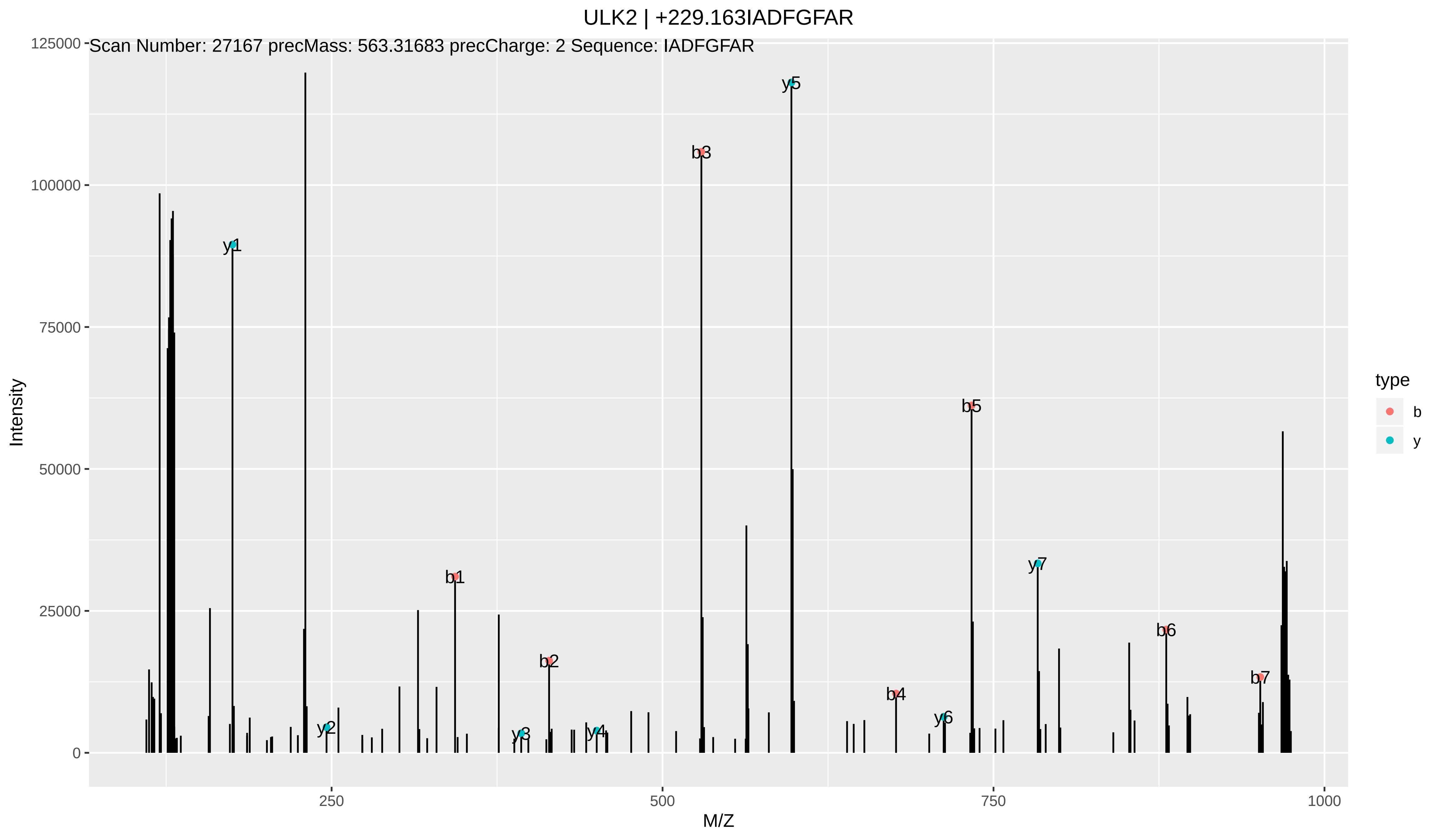
ULK2 | +229.163IADFGFAR
Scan Number: 27167 precMass: 563.31683 precCharge: 2 Sequence: IADFGFAR
Intensity
M/Z
0
25000
50000
75000
100000
125000
250
500
750
1000
y1
y2
y3
y4
y5
y6
y7
b1
b2
b3
b4
b5
b6
b7
type
b
y

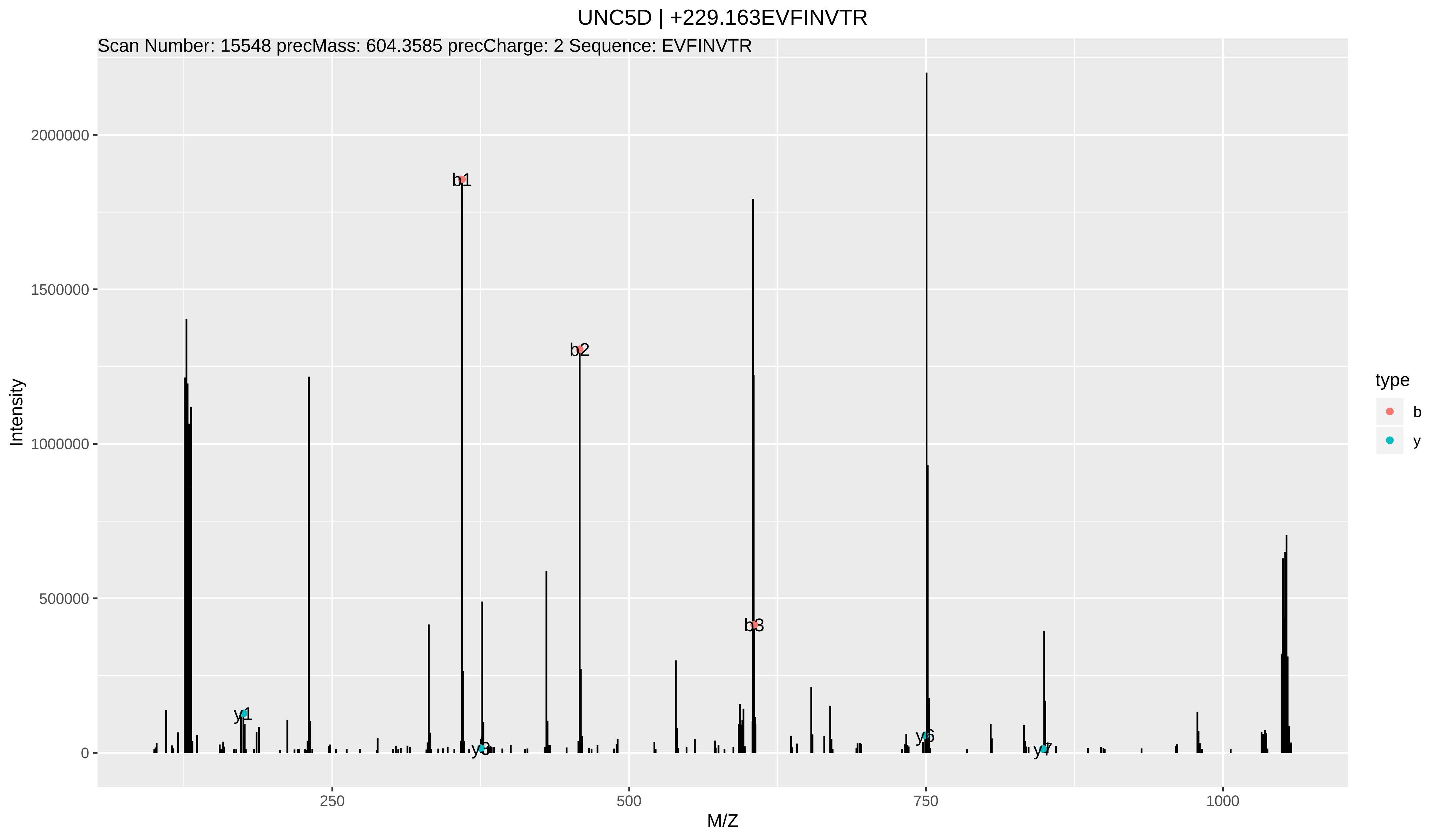

UNC5D | +229.163EVFINVTR
Scan Number: 15548 precMass: 604.3585 precCharge: 2 Sequence: EVFINVTR
Intensity
M/Z
2000000
1500000
1000000
500000
0
250
500
750
1000
b1
b2
b3
y1
y3
y6
y7
type
b
y

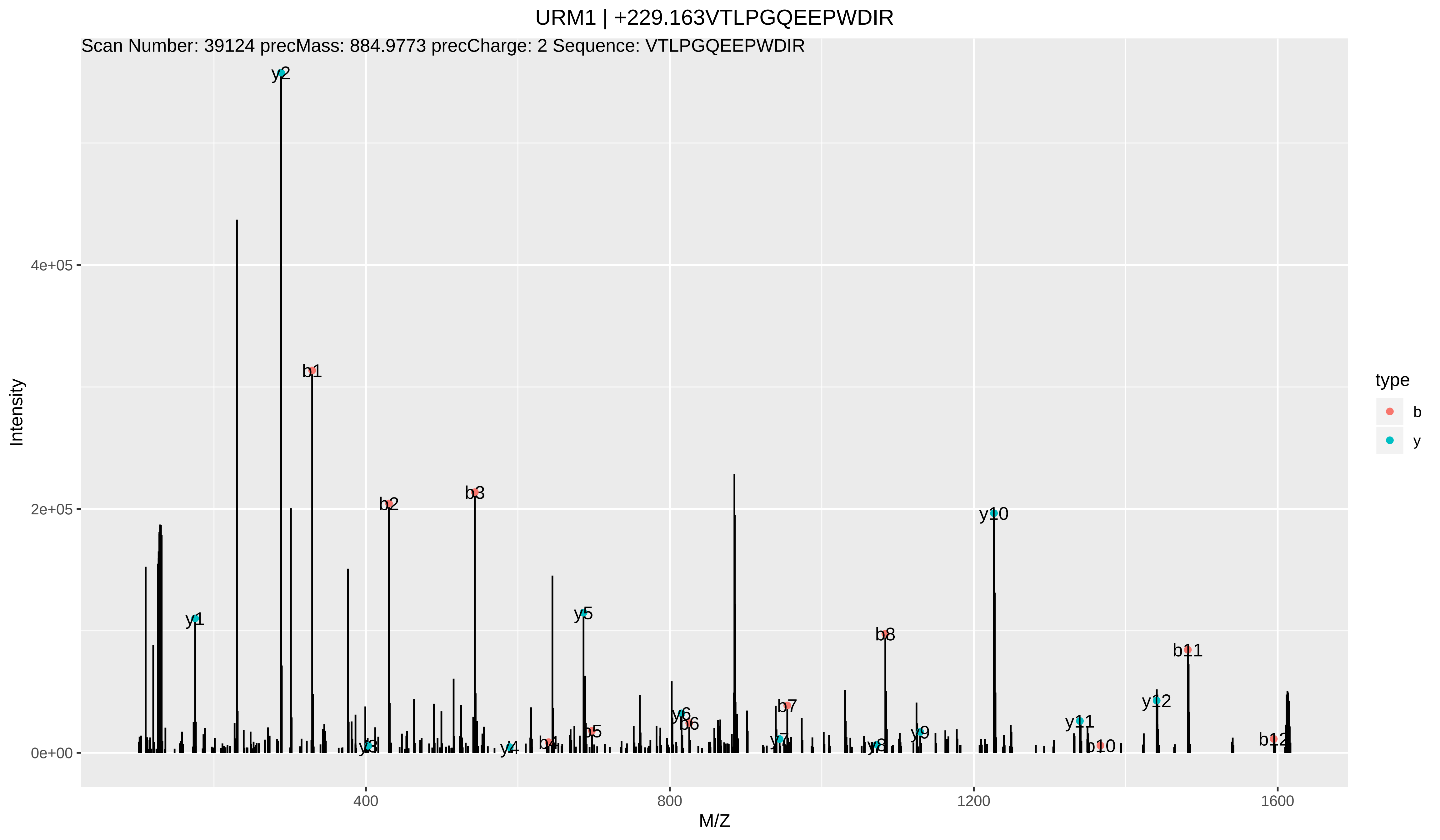

URM1 | +229.163VTLPGQEEPWDIR
Scan Number: 39124 precMass: 884.9773 precCharge: 2 Sequence: VTLPGQEEPWDIR
Intensity
M/Z
type
b
y

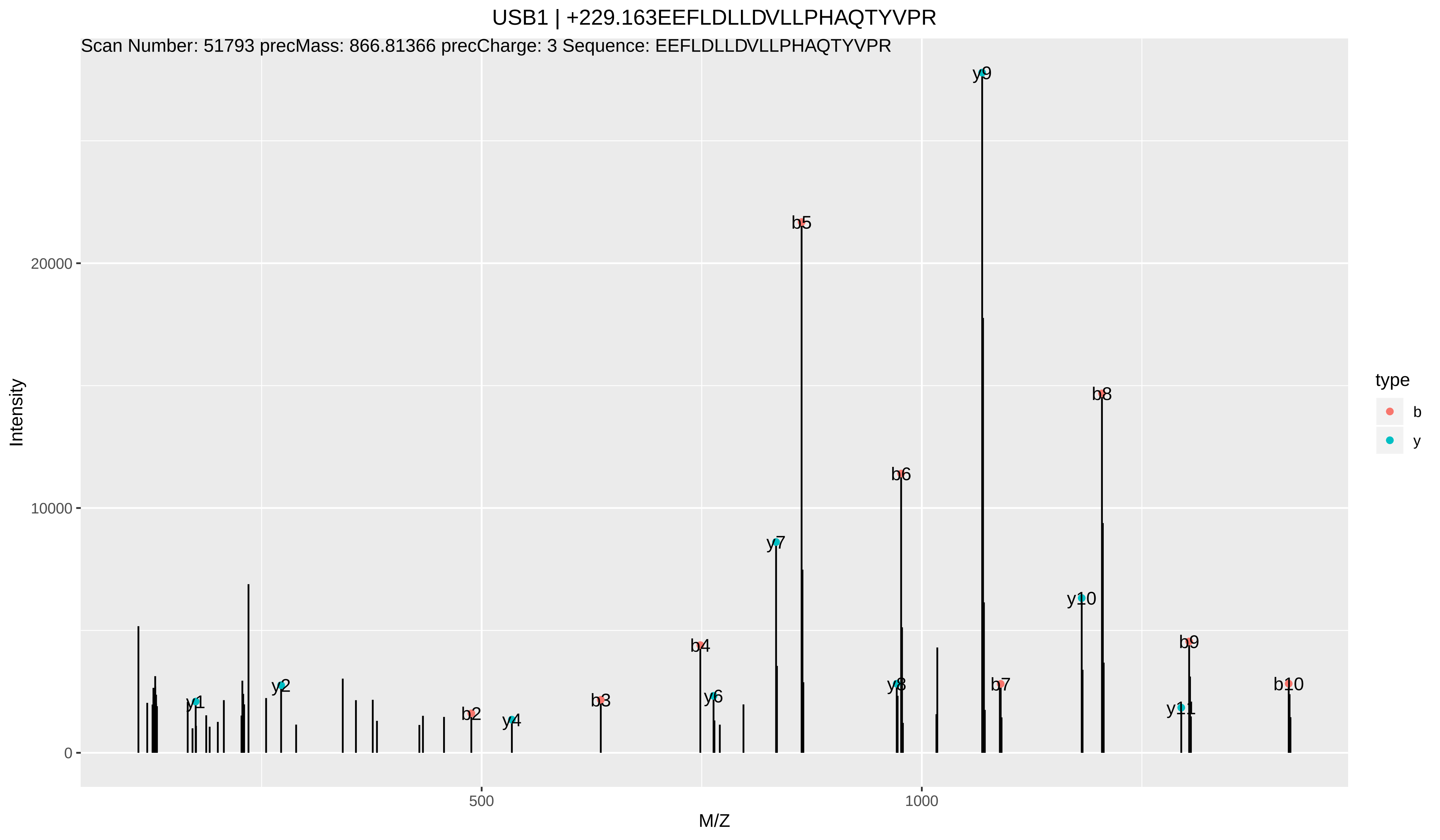

USB1 | +229.163EEFLDLLDVLLPHAQTYVPR
Scan Number: 51793 precMass: 866.81366 precCharge: 3 Sequence: EEFLDLLDVLLPHAQTYVPR
Intensity
M/Z
type
b
y
y9
b5
b8
b6
y7
y10
b4
b9
y2
y8
b7
b10
y1
y6
b3
b2
y4
y11
0
10000
20000
500
1000

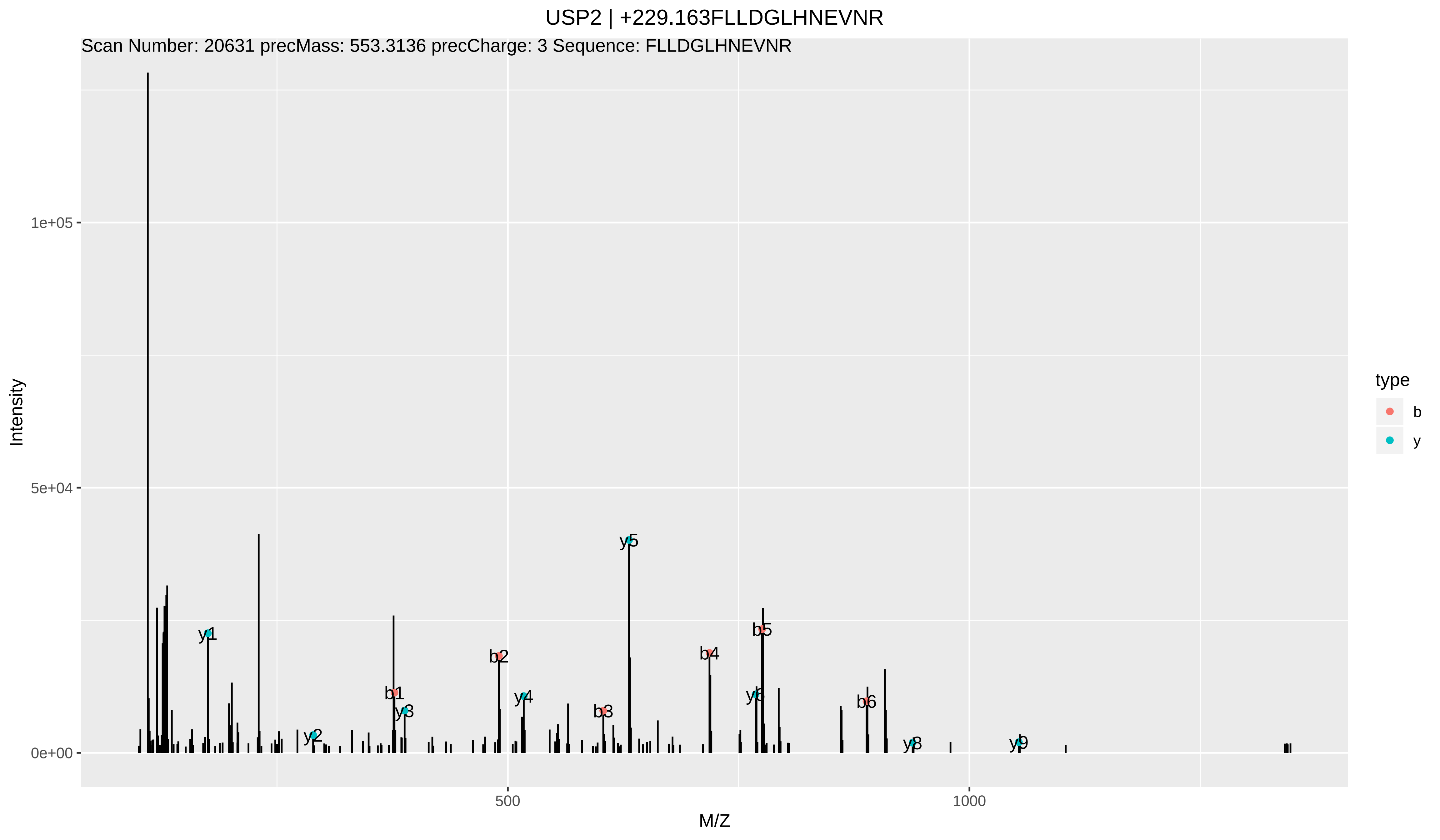

USP2 | +229.163FLLDGLHNEVNR
Scan Number: 20631 precMass: 553.3136 precCharge: 3 Sequence: FLLDGLHNEVNR
Intensity
1e+05
5e+04
0e+00
M/Z
500
1000
type
b
y
y1
y2
y3
y4
y5
y6
y8
y9
b1
b2
b3
b4
b5
b6

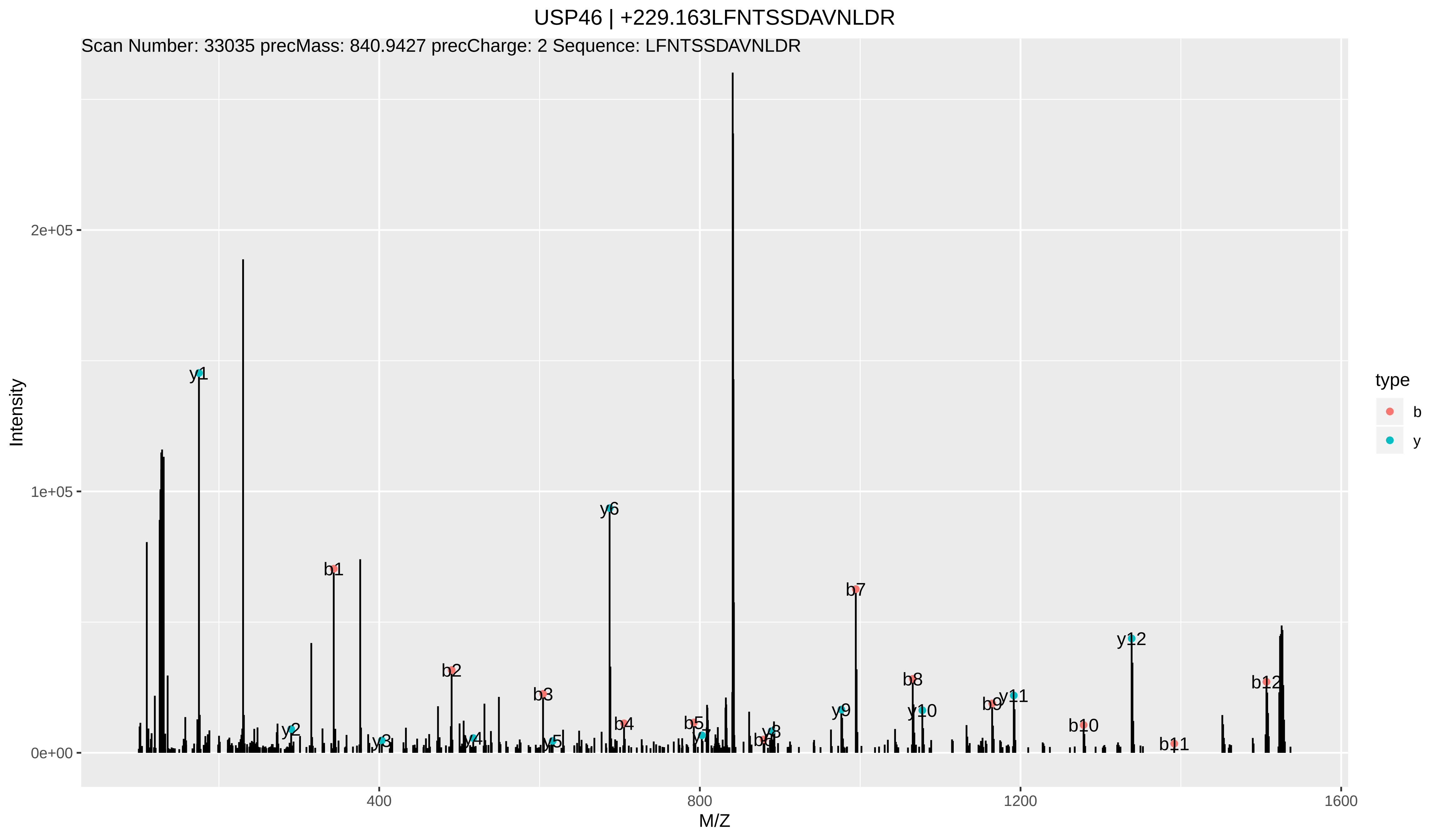
USP46 | +229.163LFNTSSDAVNLDR
Scan Number: 33035 precMass: 840.9427 precCharge: 2 Sequence: LFNTSSDAVNLDR
Intensity
2e+05
1e+05
0e+00
400
800
1200
1600
M/Z
y1
b1
y2
b2
y3
y4
b3
y5
y6
b4
b5
y7
b6
y8
y9
b7
b8
y10
b9
y11
b10
y12
b11
b12
type
b
y

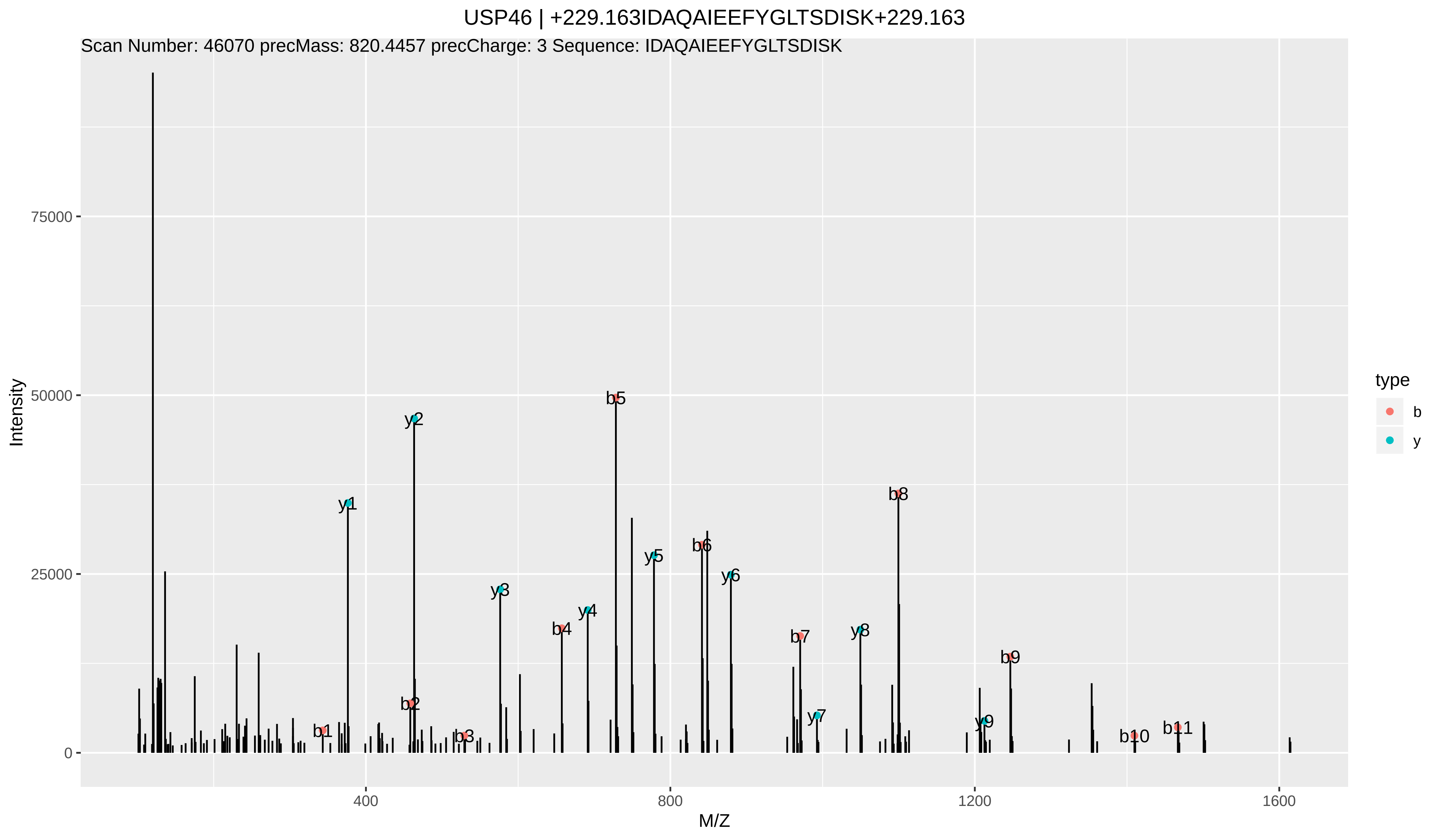
USP46 | +229.163IDAQAIEEFYGLTSDISK+229.163
Scan Number: 46070 precMass: 820.4457 precCharge: 3 Sequence: IDAQAIEEFYGLTSDISK
Intensity
M/Z
75000
50000
25000
0
400
800
1200
1600
y1
y2
b2
b1
b3
y3
b4
y4
b5
y5
b6
y6
b7
y7
y8
b8
y9
b9
b10
b11
type
b
y

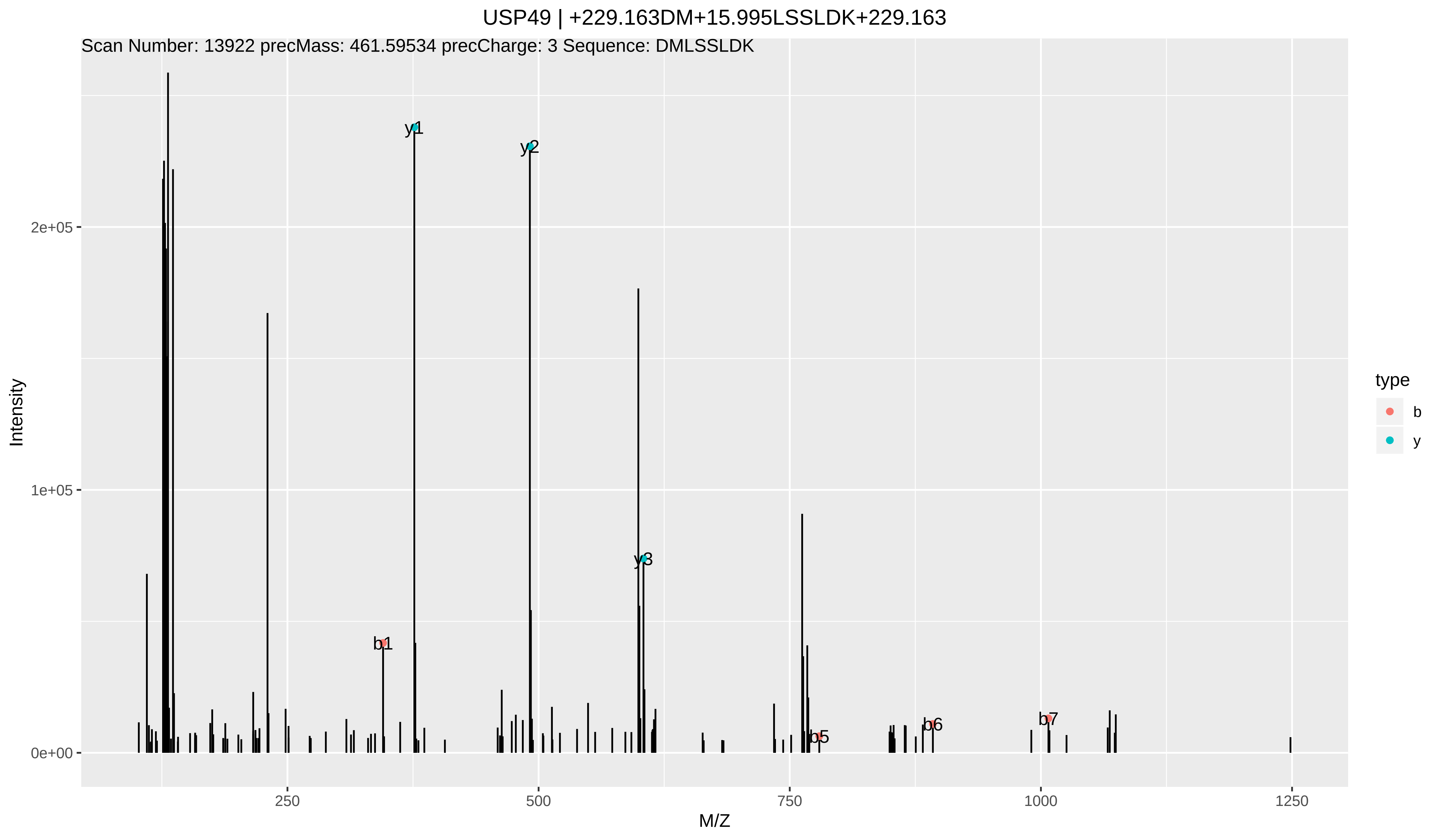
USP49 | +229.163DM+15.995LSSLDK+229.163
Scan Number: 13922 precMass: 461.59534 precCharge: 3 Sequence: DMLSSLDK
Intensity
M/Z
0e+00
1e+05
2e+05
250
500
750
1000
1250
y1
y2
y3
b1
b5
b6
b7
type
b
y

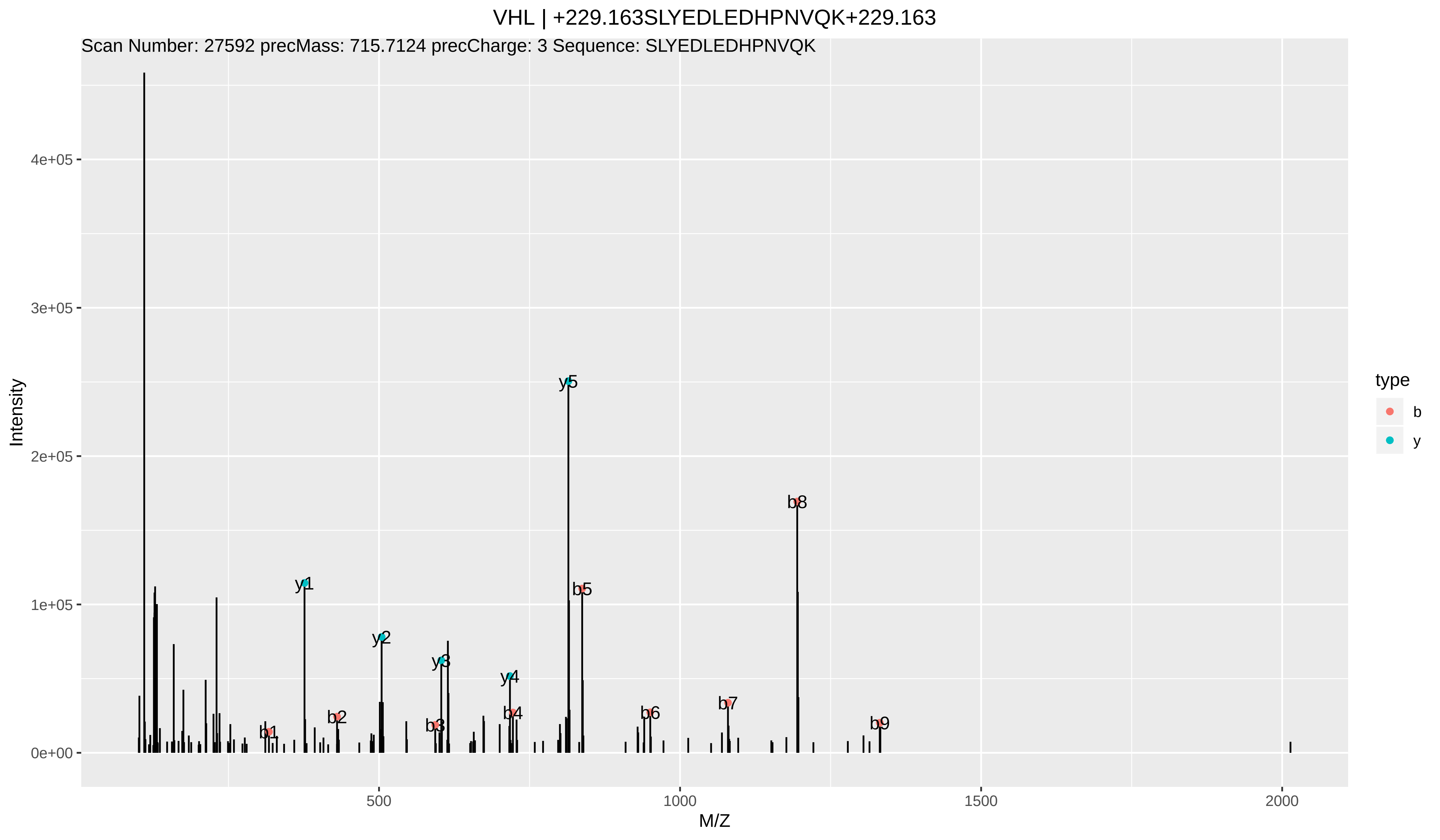

VHL | +229.163SLYEDLEDHPNVQK+229.163
Scan Number: 27592 precMass: 715.7124 precCharge: 3 Sequence: SLYEDLEDHPNVQK
Intensity
4e+05
3e+05
2e+05
1e+05
0e+00
M/Z
500
1000
1500
2000
y1
y2
y3
y4
y5
b1
b2
b3
b4
b5
b6
b7
b8
b9
type
b
y

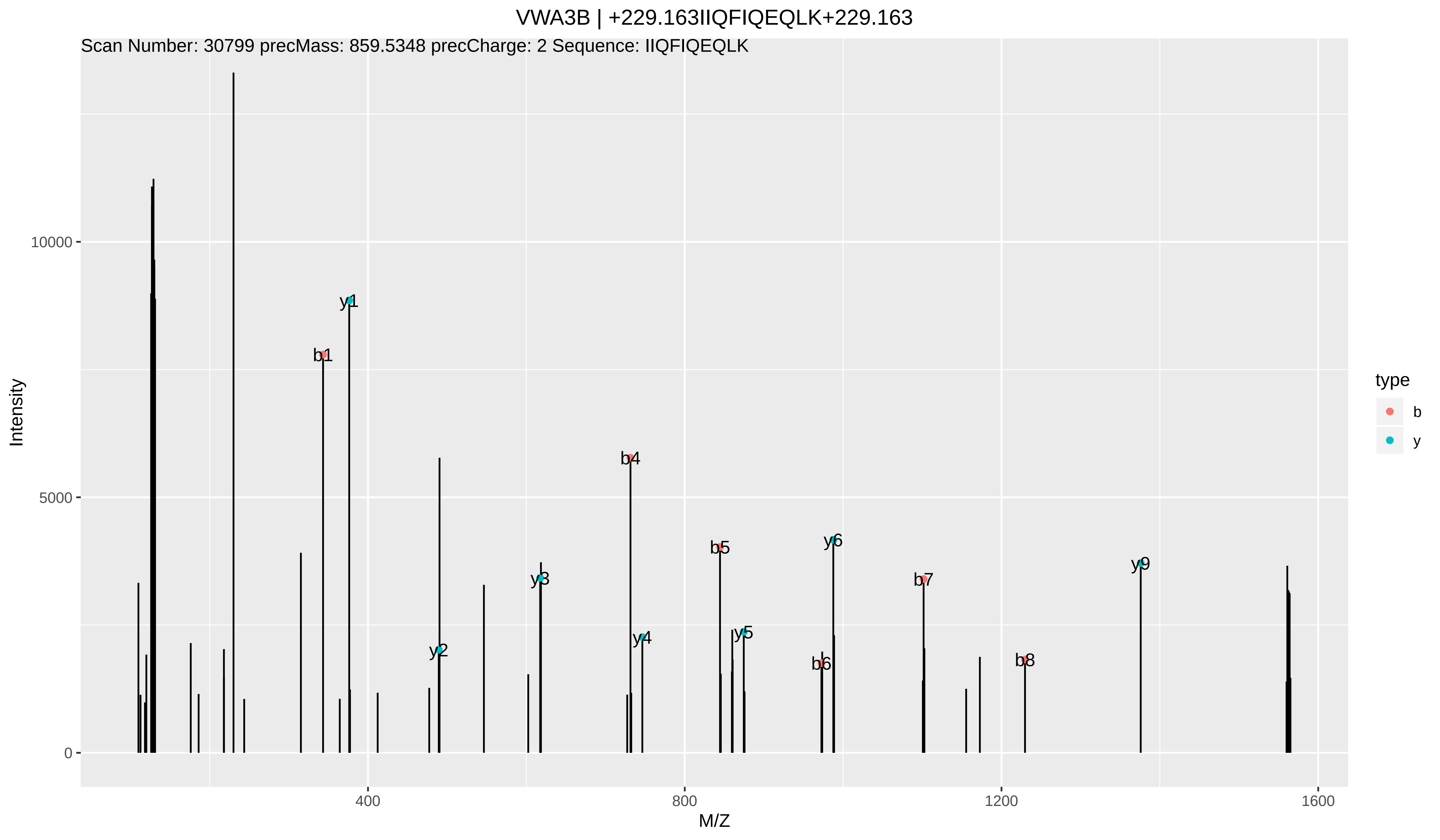

VWA3B | +229.163IIQFIQEQLK+229.163
Scan Number: 30799 precMass: 859.5348 precCharge: 2 Sequence: IIQFIQEQLK
Intensity
10000
5000
0
400
800
1200
1600
M/Z
y1
b1
b4
y2
y3
y4
b5
y5
b6
y6
b7
b8
y9
type
b
y

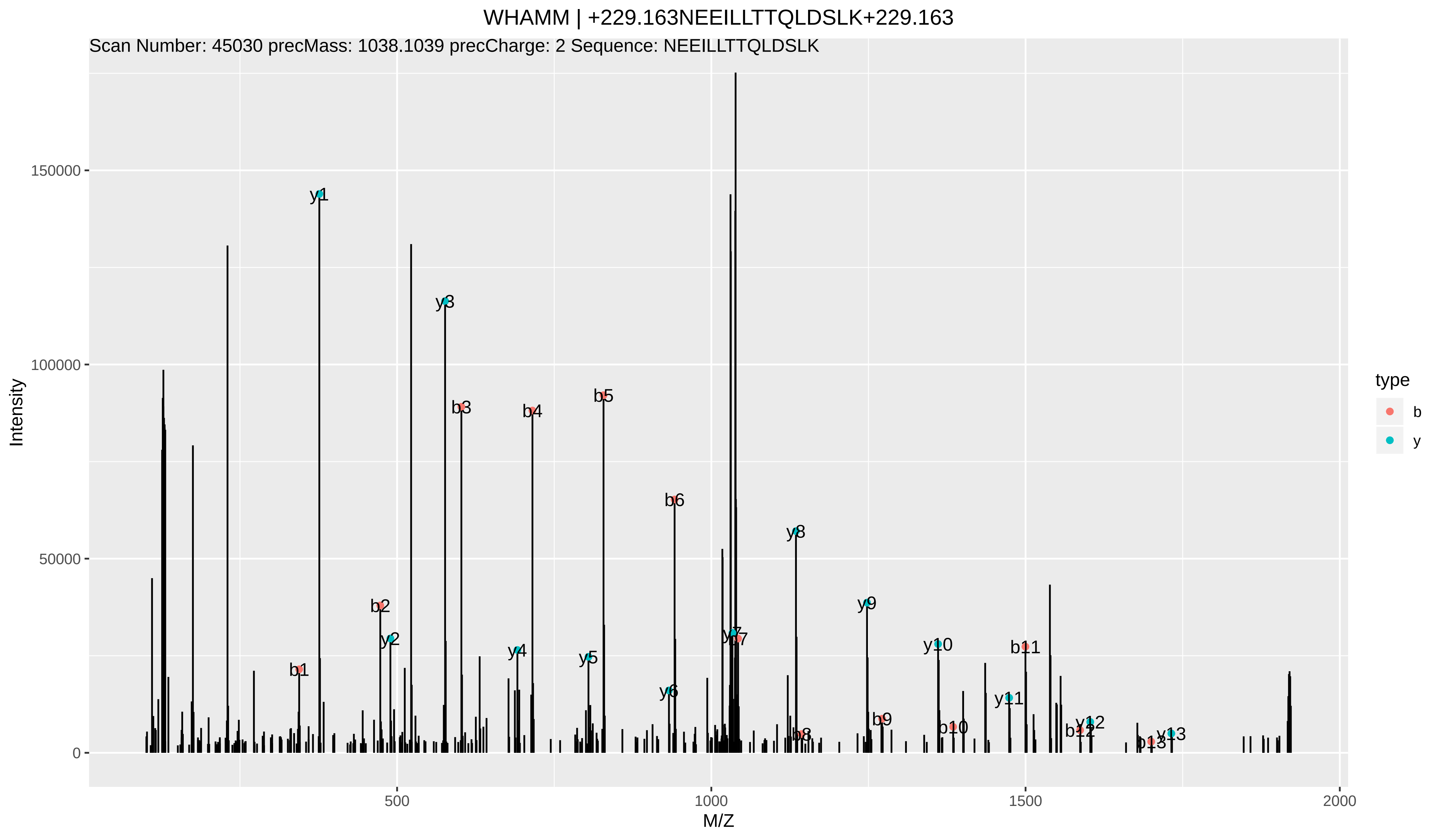

WHAMM | +229.163NEEILLTTQLDSLK+229.163
Scan Number: 45030 precMass: 1038.1039 precCharge: 2 Sequence: NEEILLTTQLDSLK
Intensity
M/Z
0
50000
100000
150000
500
1000
1500
2000
y1
y2
y3
y4
y5
y6
y7
y8
y9
y10
y11
y12
y13
b1
b2
b3
b4
b5
b6
b7
b8
b9
b10
b11
b12
b13
type
b
y

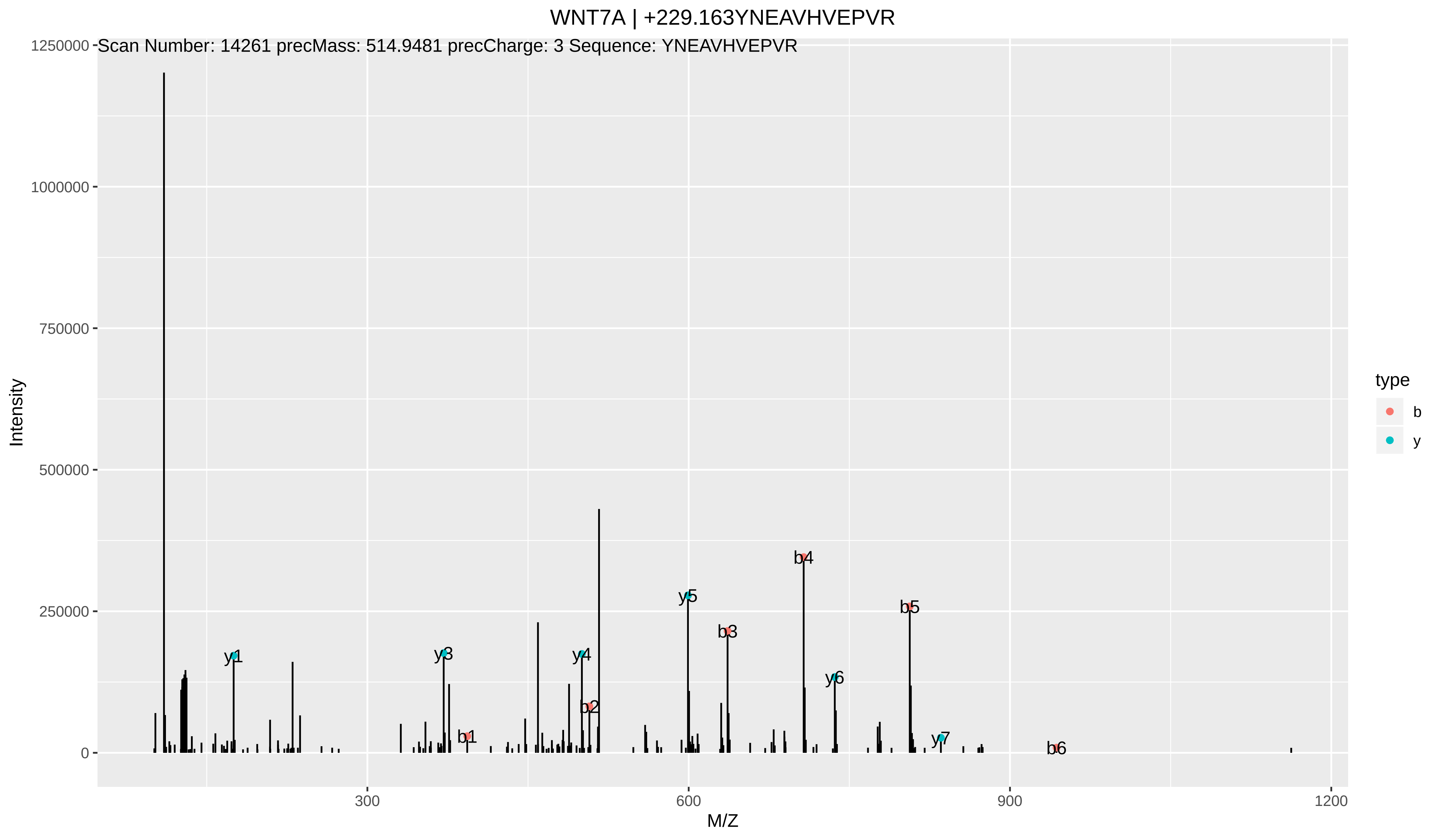
WNT7A | +229.163YNEAVHVEPVR
Scan Number: 14261 precMass: 514.9481 precCharge: 3 Sequence: YNEAVHVEPVR
Intensity
M/Z
1250000
1000000
750000
500000
250000
0
300
600
900
1200
y1
y3
b1
y4
b2
y5
b3
b4
y6
b5
y7
b6
type
b
y

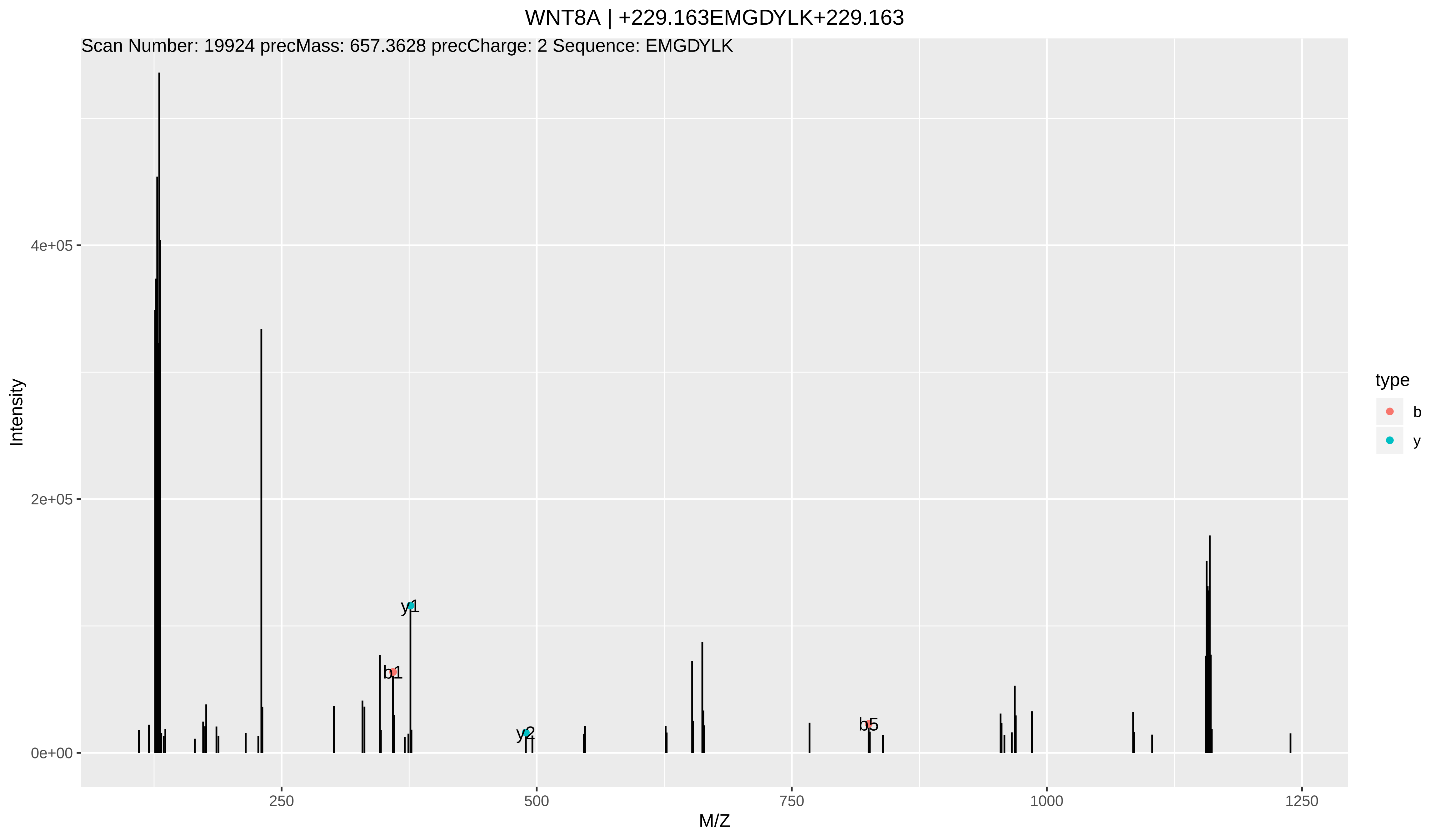
WNT8A | +229.163EMGDYLK+229.163
Scan Number: 19924 precMass: 657.3628 precCharge: 2 Sequence: EMGDYLK
Intensity
4e+05
2e+05
0e+00
M/Z
250
500
750
1000
1250
y1
b1
y2
b5
type
b
y

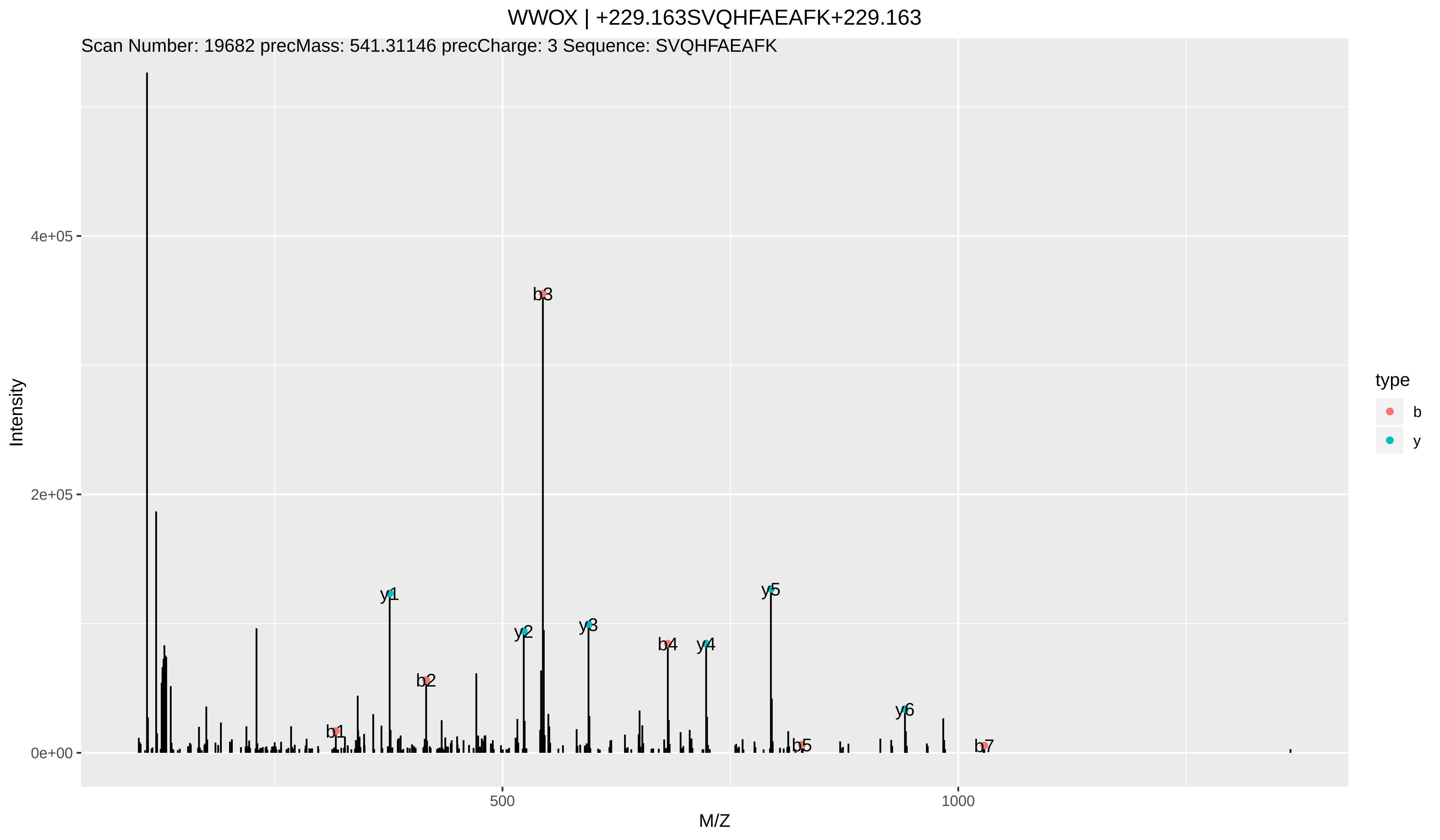
WWOX | +229.163SVQHFAEAFK+229.163
Scan Number: 19682 precMass: 541.31146 precCharge: 3 Sequence: SVQHFAEAFK
Intensity
4e+05
2e+05
0e+00
M/Z
500
1000
b3
y1
y2
y3
b4
y4
y5
b2
b1
b5
y6
b7
type
b
y

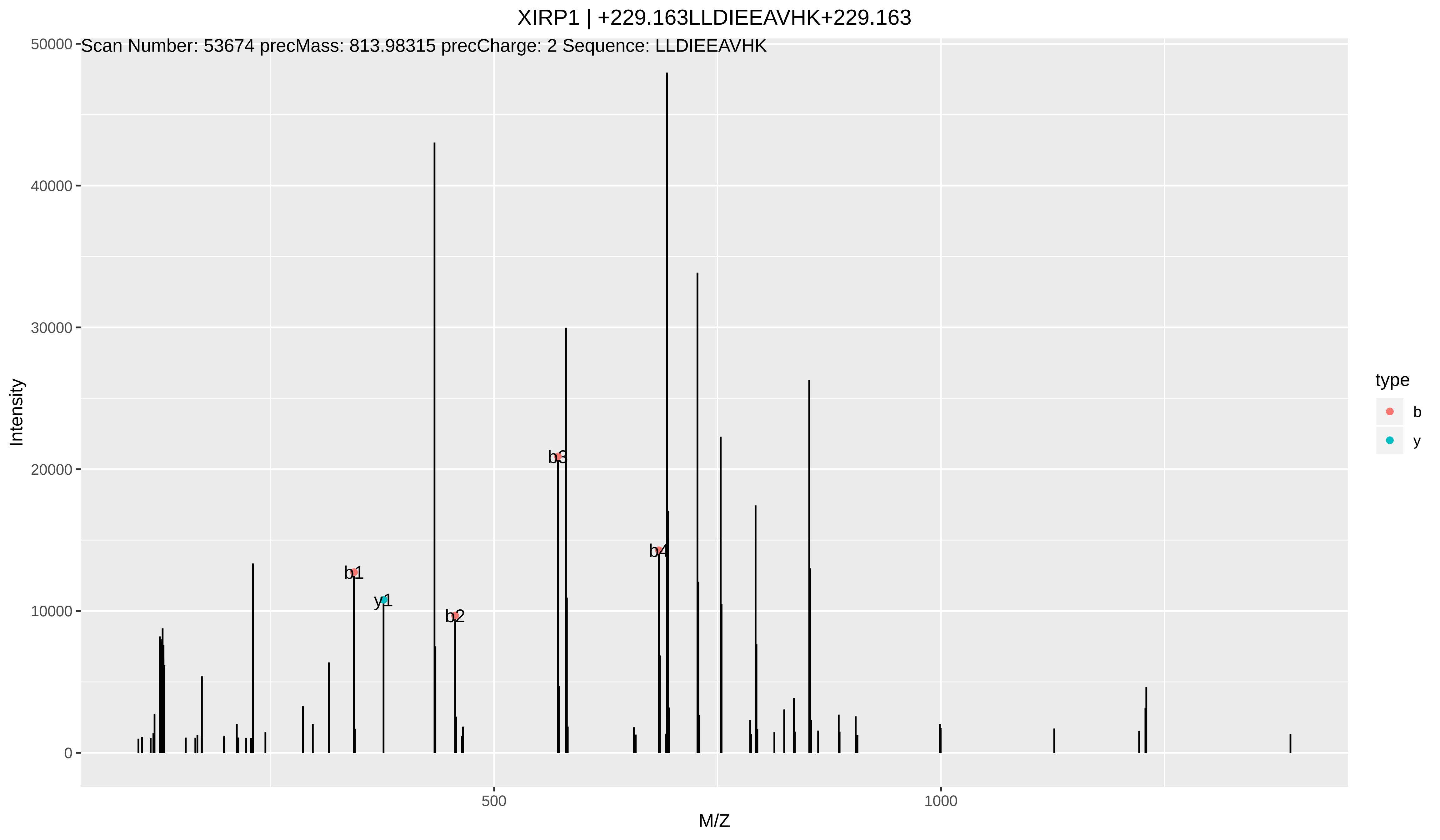

XIRP1 | +229.163LLDIEEAVHK+229.163
Scan Number: 53674 precMass: 813.98315 precCharge: 2 Sequence: LLDIEEAVHK
Intensity
M/Z
50000
40000
30000
20000
10000
0
500
1000
b1
y1
b2
b3
b4
type
b
y

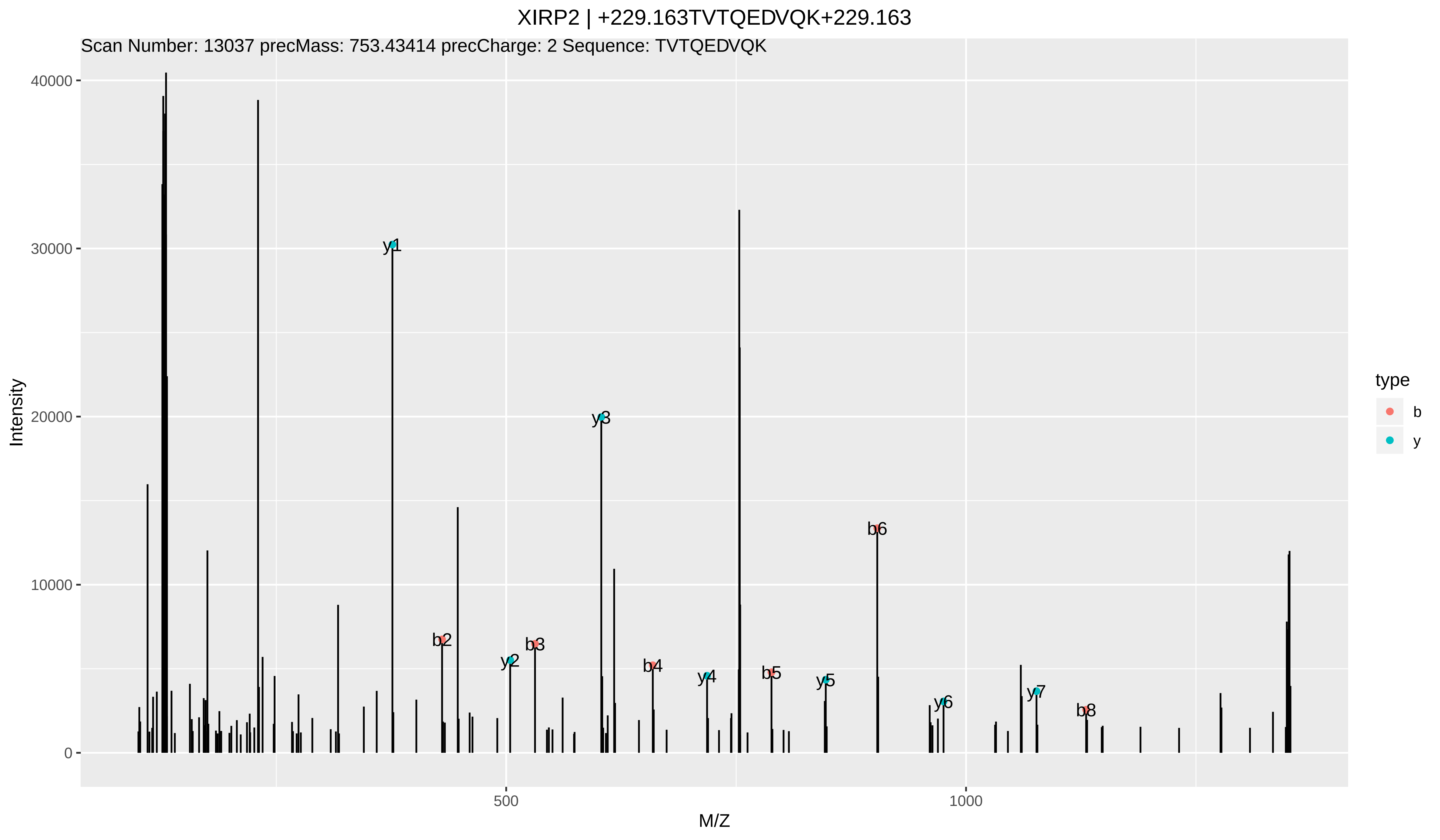
XIRP2 | +229.163TVTQEDVQK+229.163
Scan Number: 13037 precMass: 753.43414 precCharge: 2 Sequence: TVTQEDVQK
Intensity
0
10000
20000
30000
40000
M/Z
500
1000
y1
b2
y2
b3
y3
b4
y4
b5
y5
b6
y6
y7
b8
type
b
y

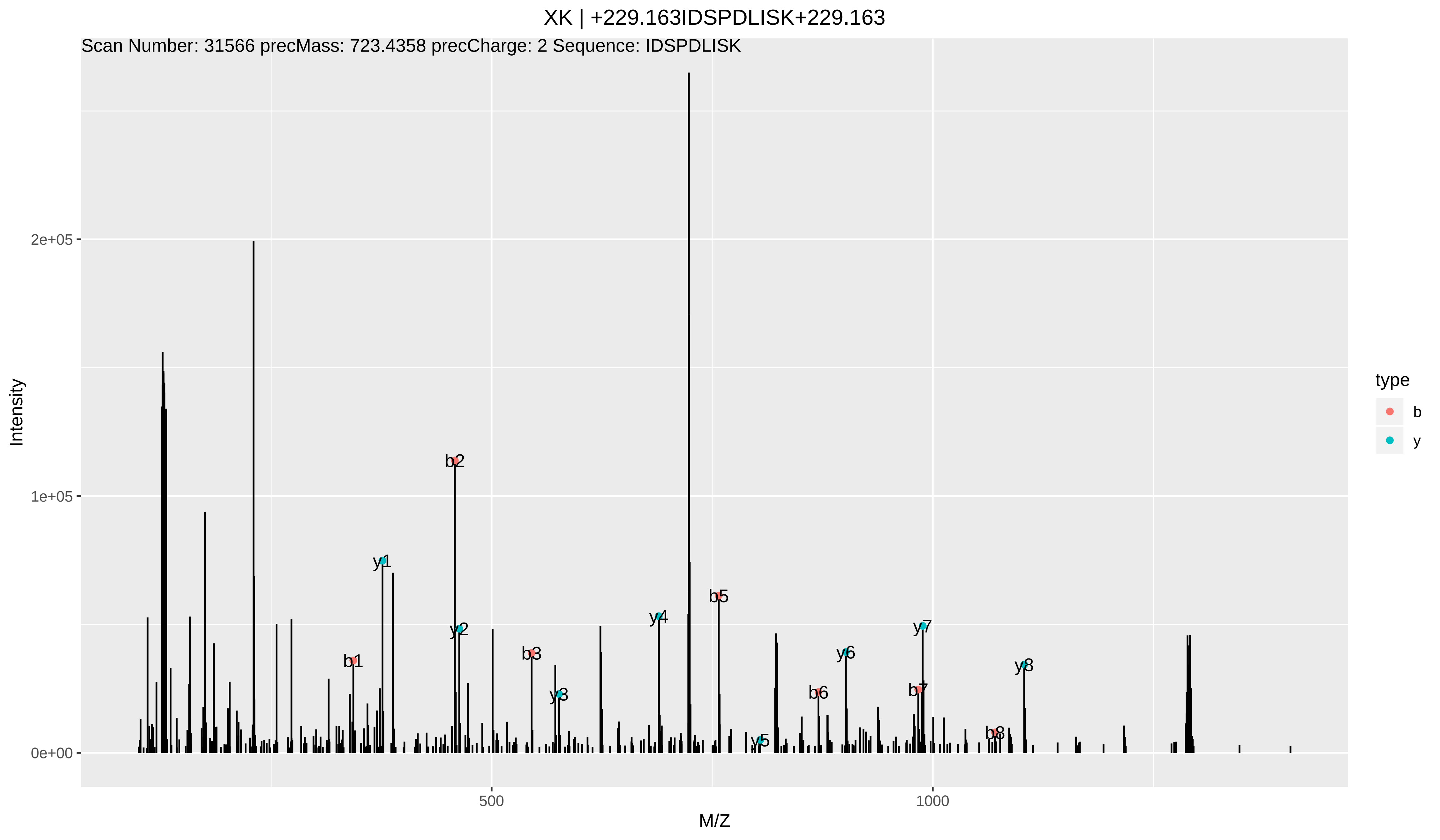
XK | +229.163IDSPDLISK+229.163
Scan Number: 31566 precMass: 723.4358 precCharge: 2 Sequence: IDSPDLISK
Intensity
M/Z
type
b
y
0e+00
1e+05
2e+05
500
1000
b1
b2
b3
b5
b6
b7
b8
y1
y2
y3
y4
y5
y6
y7
y8

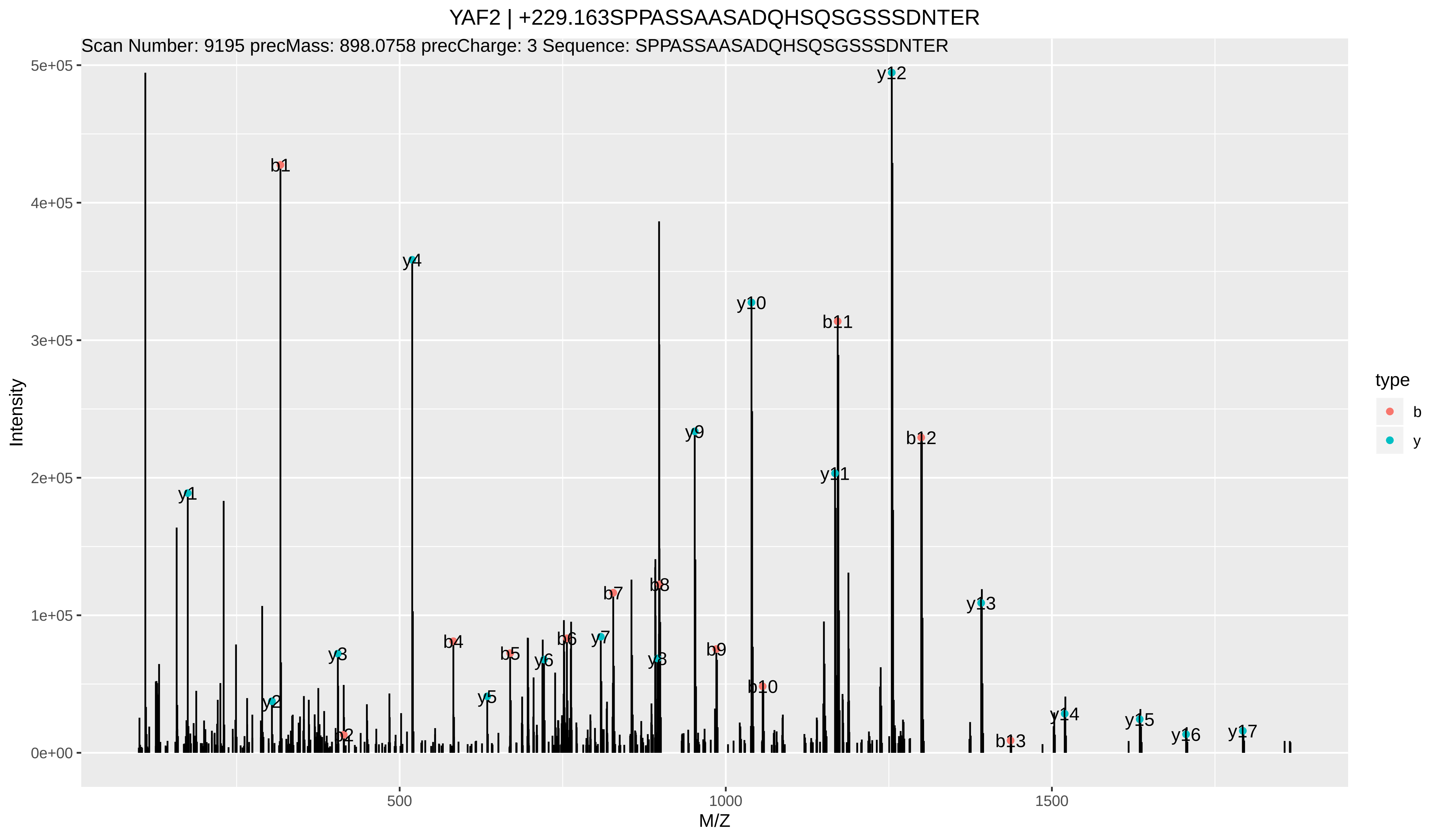

YAF2 | +229.163SPPASSAASADQHSQSGSSSDNTER
Scan Number: 9195 precMass: 898.0758 precCharge: 3 Sequence: SPPASSAASADQHSQSGSSSDNTER
Intensity
M/Z
type
b
y

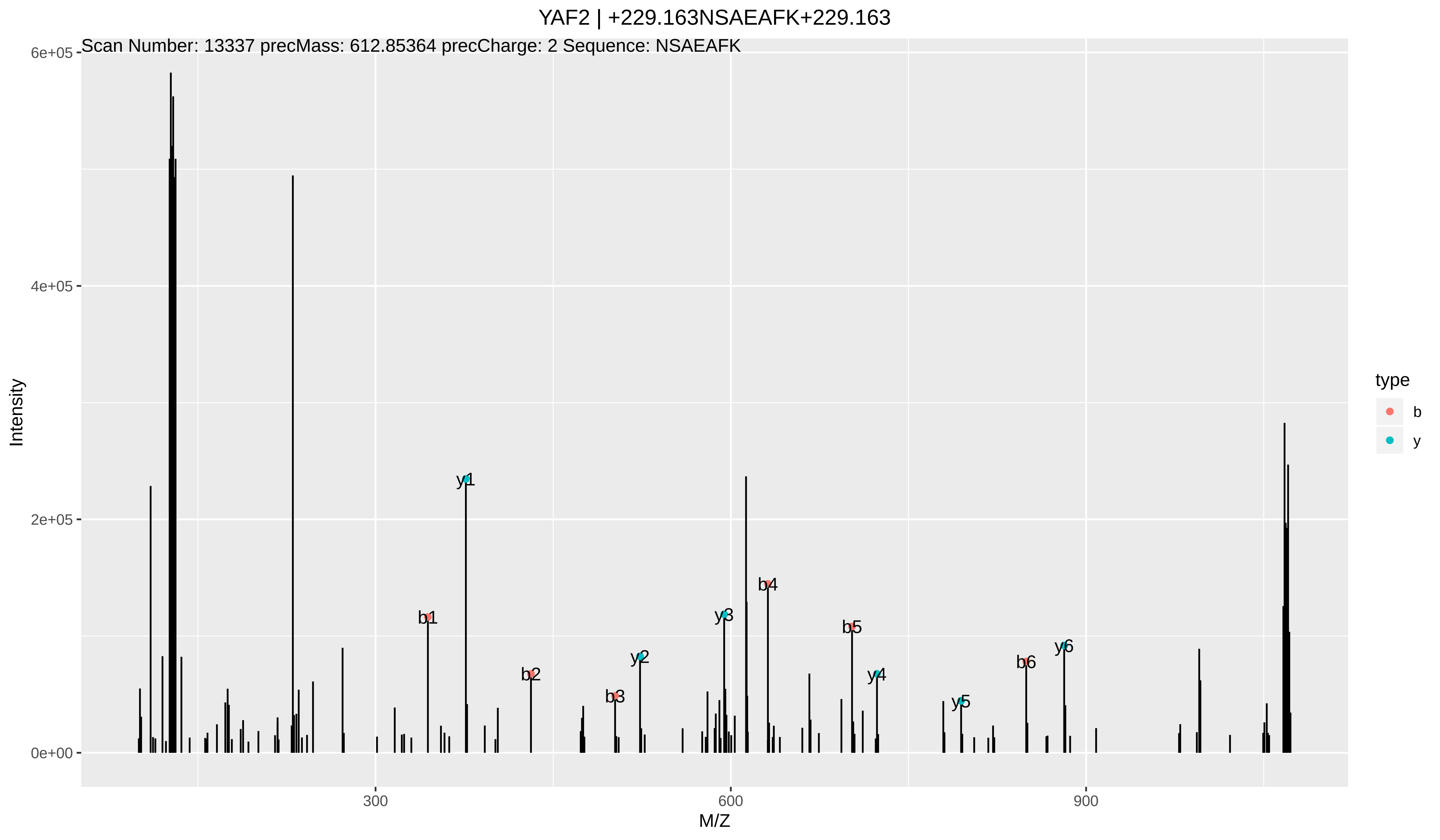

YAF2 | +229.163NSAEAFK+229.163
Scan Number: 13337 precMass: 612.85364 precCharge: 2 Sequence: NSAEAFK
Intensity
6e+05
4e+05
2e+05
0e+00
M/Z
300
600
900
y1
b1
b2
y2
b3
y3
b4
b5
y4
y5
b6
y6
type
b
y

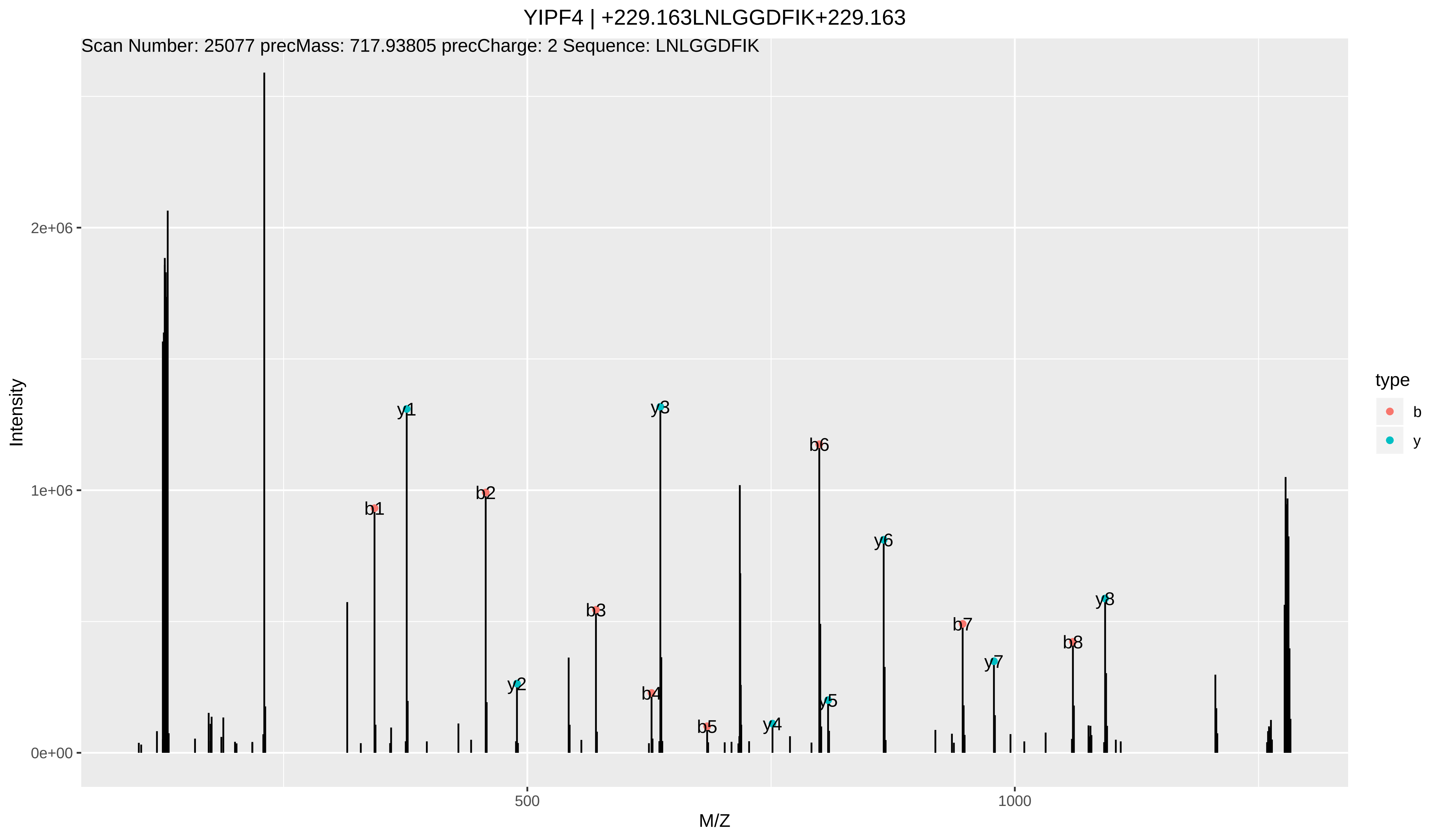
YIPF4 | +229.163LNLGGDFIK+229.163
Scan Number: 25077 precMass: 717.93805 precCharge: 2 Sequence: LNLGGDFIK
Intensity
M/Z
2e+06
1e+06
0e+00
500
1000
b1
y1
b2
y2
b3
y3
b4
b5
y4
b6
y5
y6
b7
y7
b8
y8
type
b
y

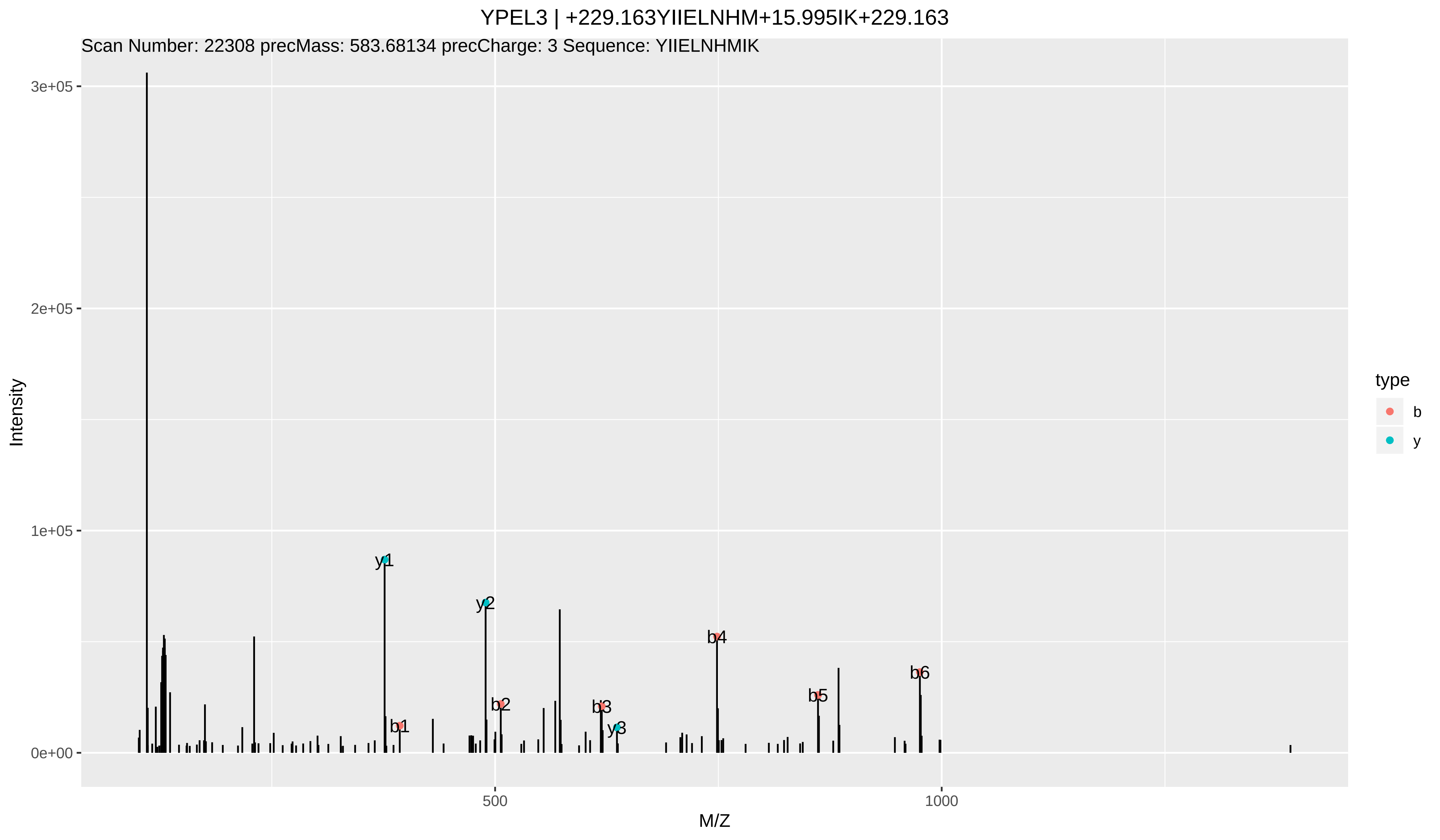

YPEL3 | +229.163YIIELNHM+15.995IK+229.163
Scan Number: 22308 precMass: 583.68134 precCharge: 3 Sequence: YIIELNHMIK
Intensity
3e+05
2e+05
1e+05
0e+00
M/Z
500
1000
y1
y2
y3
b1
b2
b3
b4
b5
b6
type
b
y

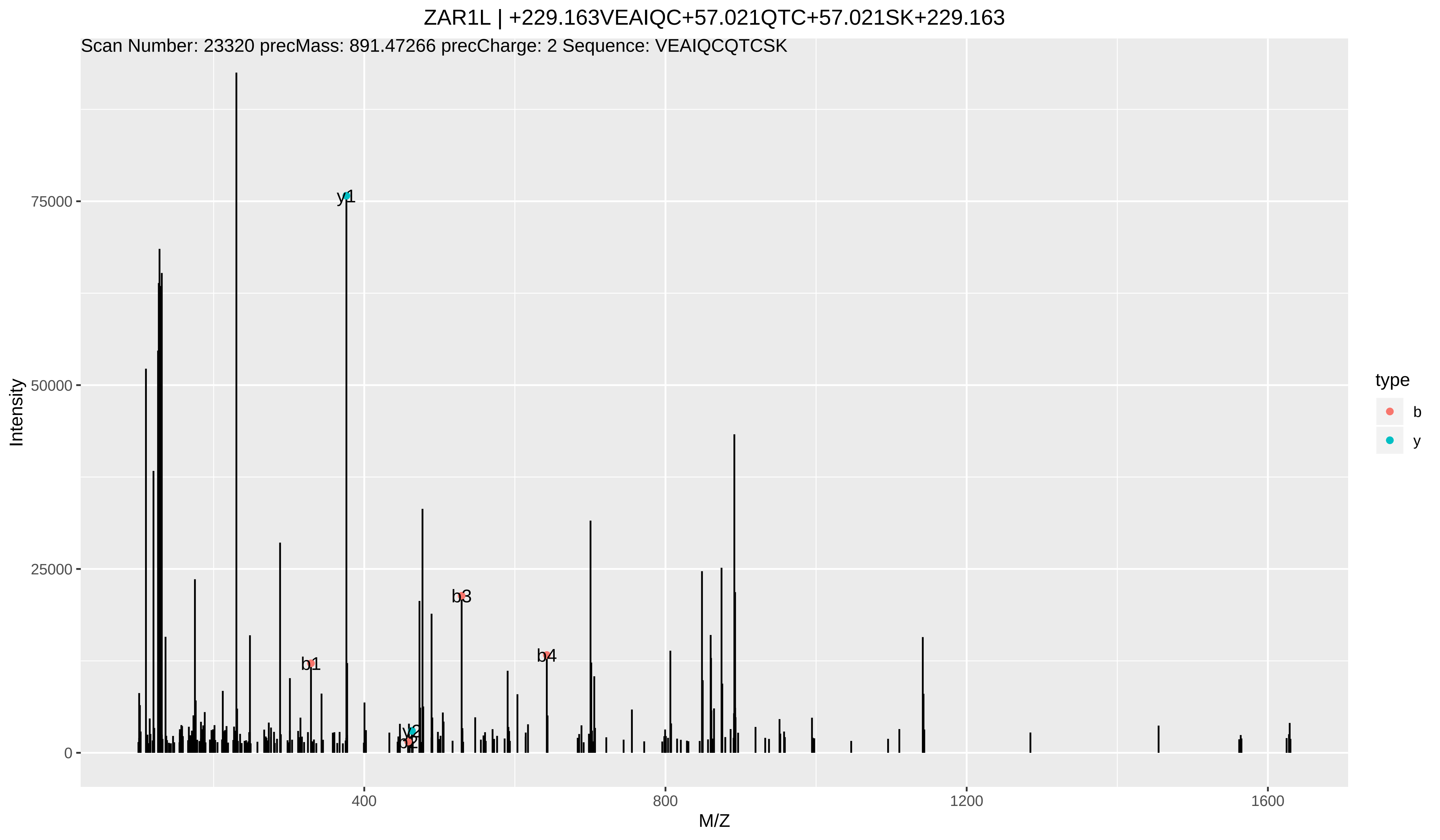
ZAR1L | +229.163VEAIQC+57.021QTC+57.021SK+229.163
Scan Number: 23320 precMass: 891.47266 precCharge: 2 Sequence: VEAIQCQTCSK
y1
b1
b2
y2
b3
b4
Intensity
75000
50000
25000
0
400
800
1200
1600
M/Z
type
b
y

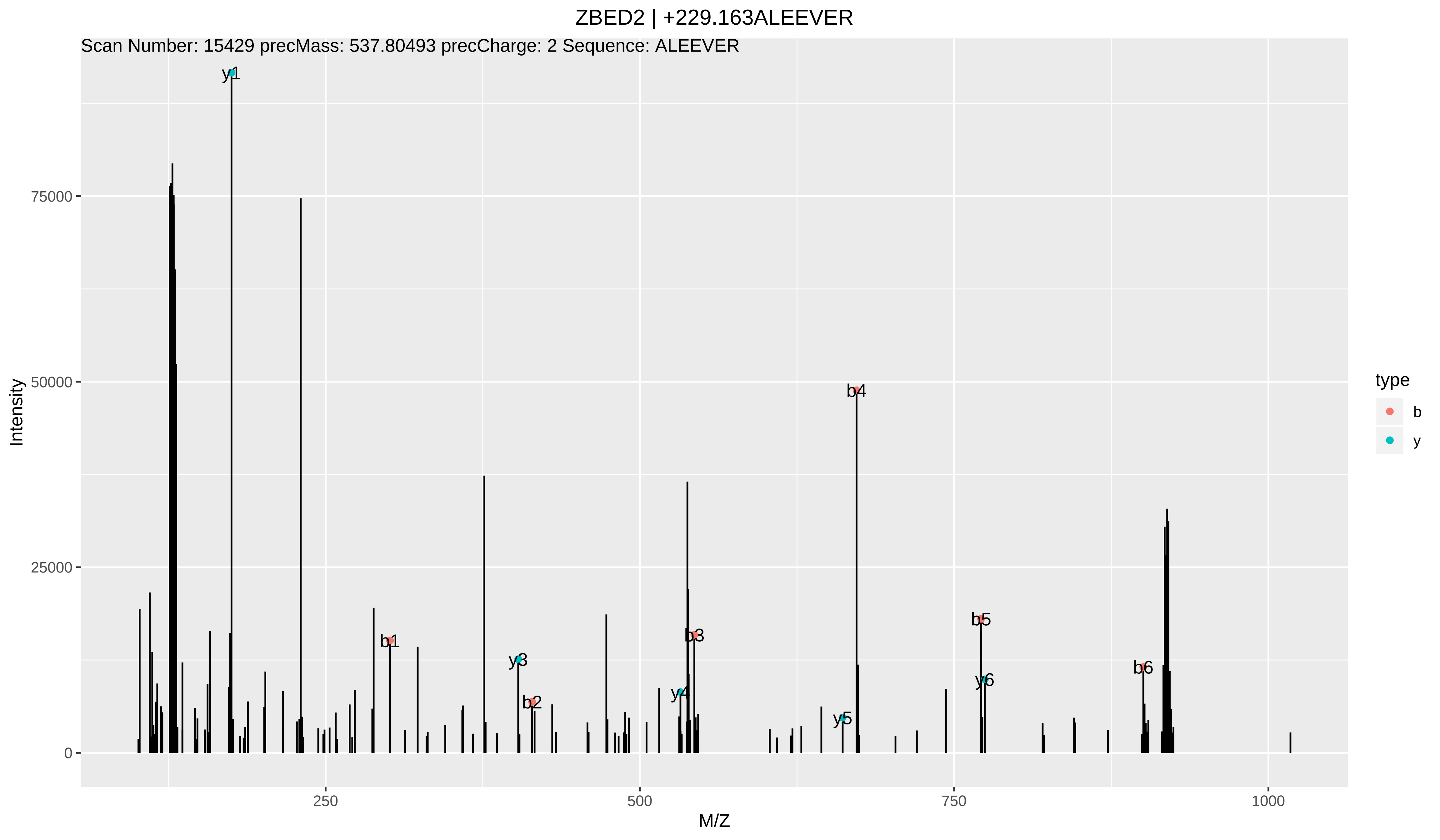

ZBED2 | +229.163ALEEVER
Scan Number: 15429 precMass: 537.80493 precCharge: 2 Sequence: ALEEVER
y1
b1
y3
b2
y4
b3
y5
b4
b5
y6
b6
Intensity
0
25000
50000
75000
M/Z
250
500
750
1000
type
b
y

ZBED3 | +229.163ELAVEQGER

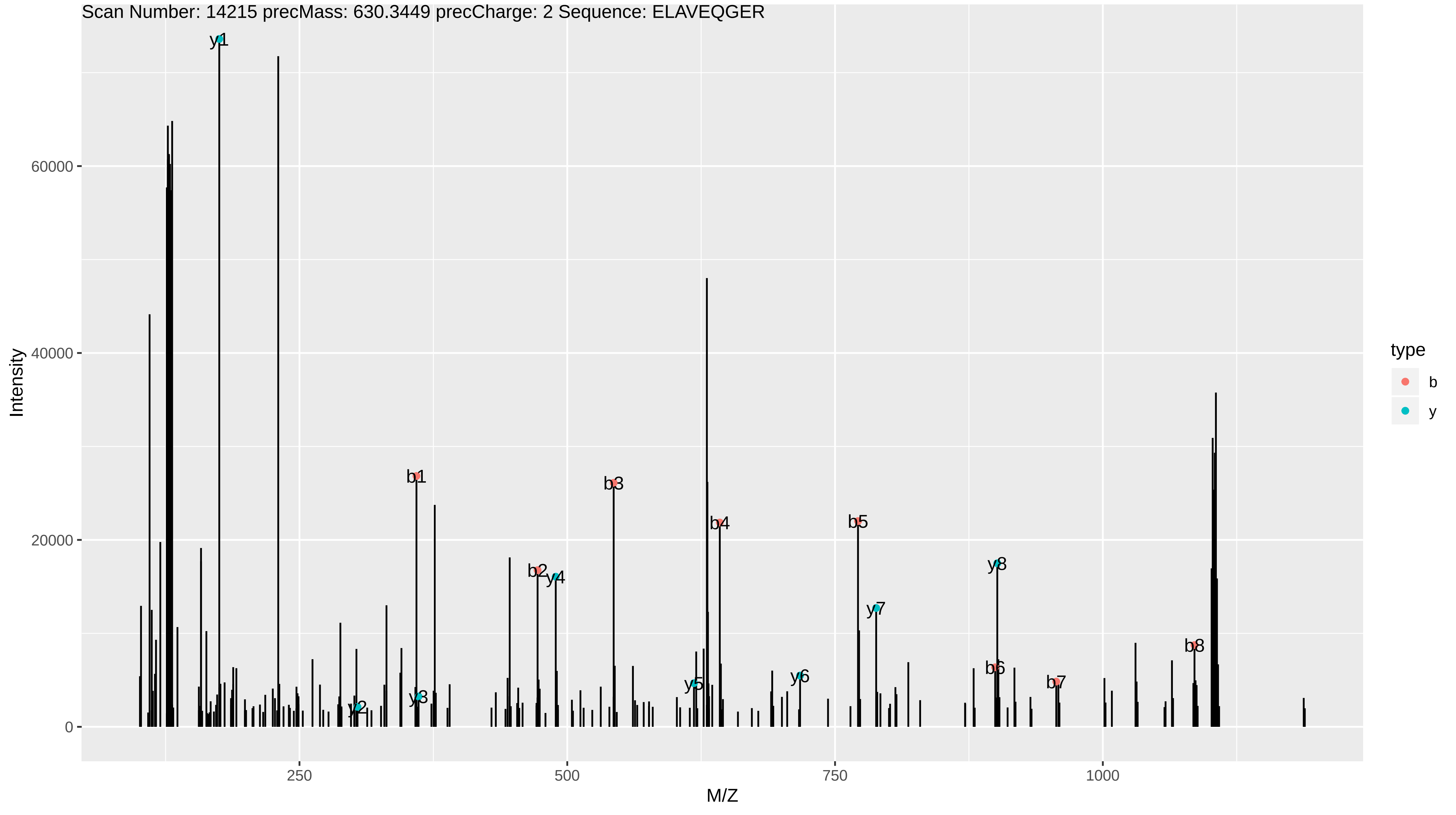

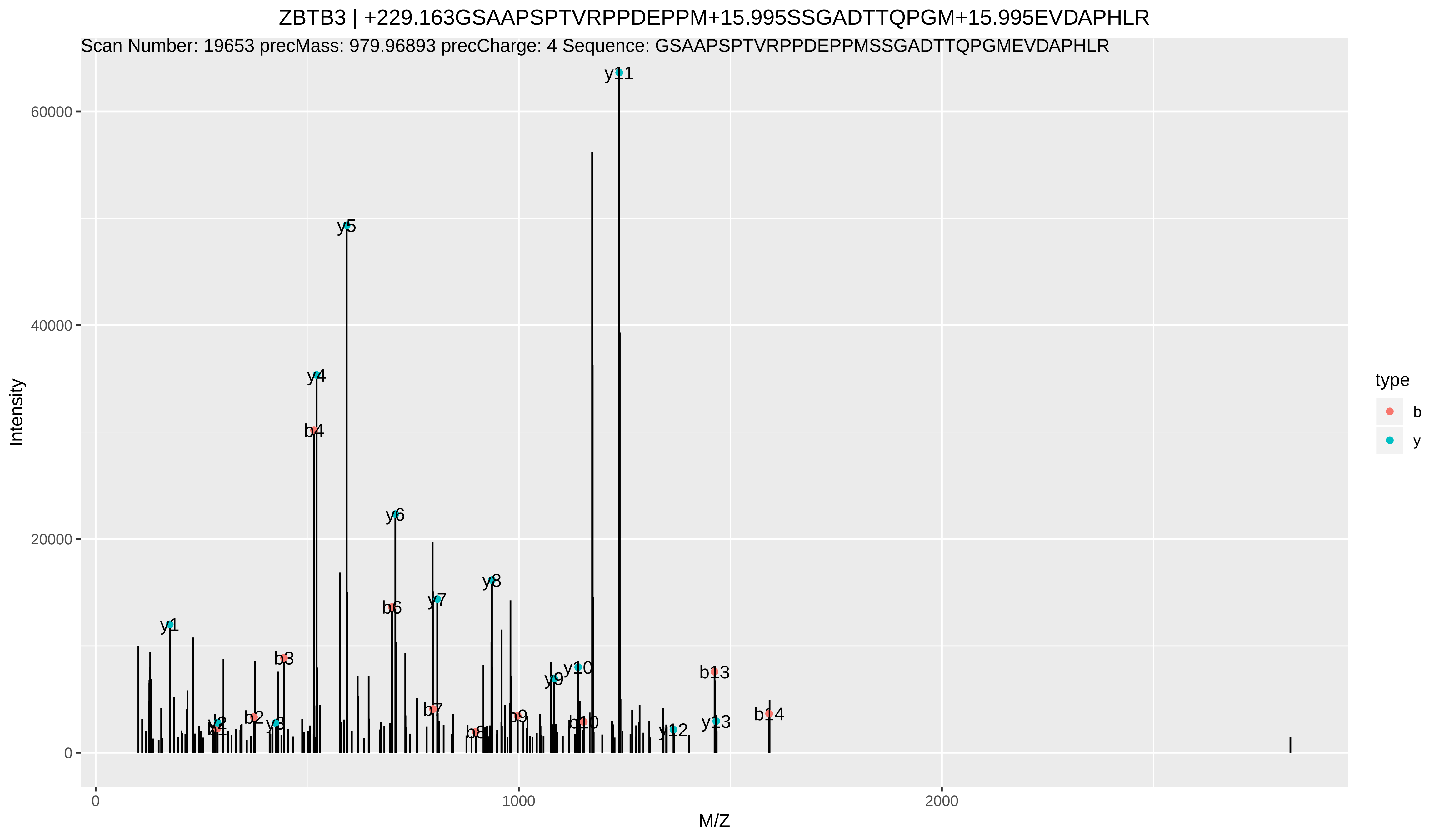

ZBTB3 | +229.163GSAAPSPTVRPPDEPPM+15.995SSGADTTQPGM+15.995EVDAPHLR
Scan Number: 19653 precMass: 979.96893 precCharge: 4 Sequence: GSAAPSPTVRPPDEPPMSSGADTTQPGMEVDAPHLR
Intensity
M/Z
type
b
y
y1
y2
b1
b2
y3
b3
b4
y4
y5
y6
b6
b7
y7
b8
y8
b9
y9
y10
b10
y11
y12
b13
y13
b14
0
20000
40000
60000
1000
2000

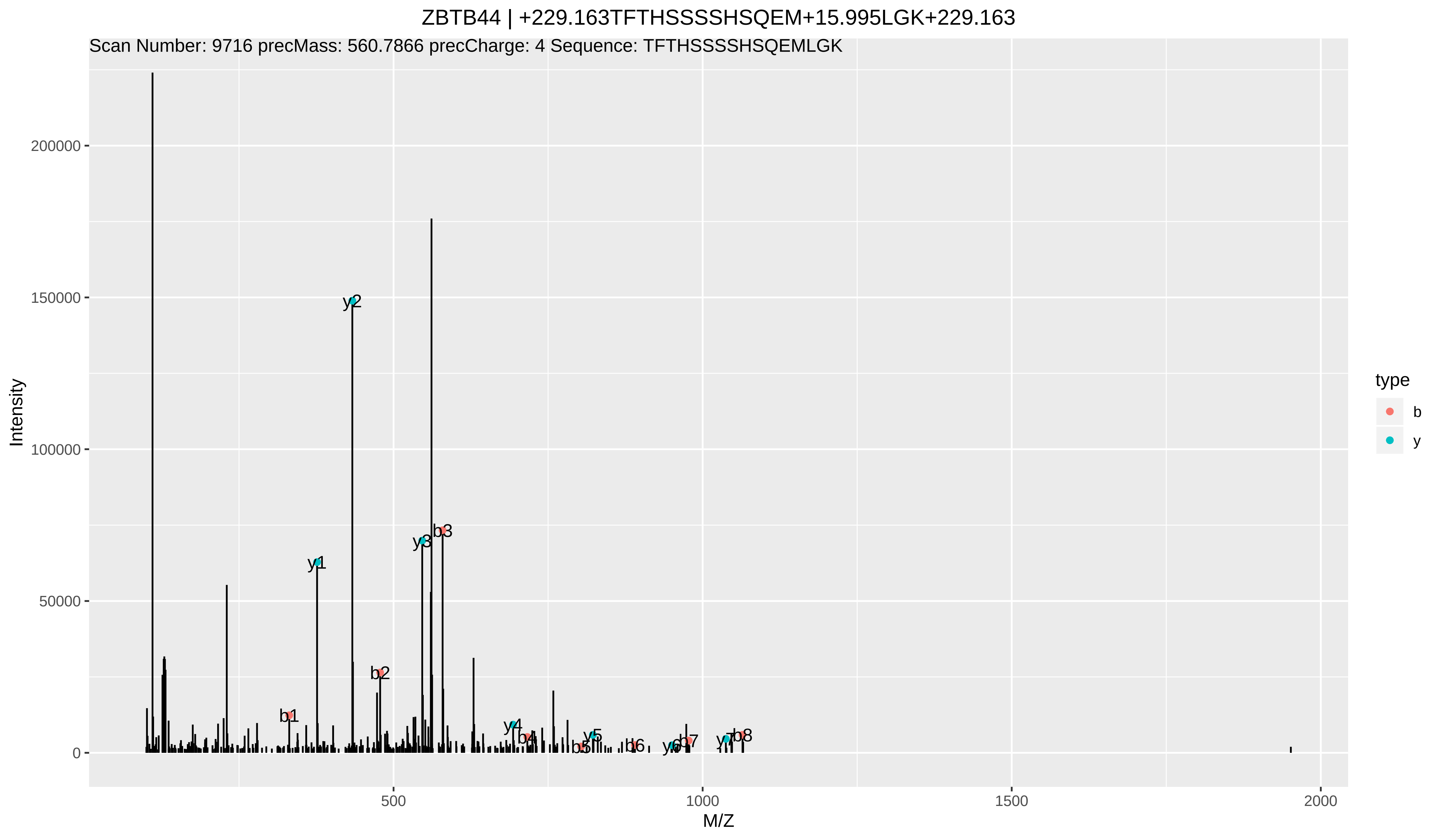
ZBTB44 | +229.163TFTHSSSSHSQEM+15.995LGK+229.163
Scan Number: 9716 precMass: 560.7866 precCharge: 4 Sequence: TFTHSSSSHSQEMLGK
Intensity
M/Z
200000
150000
100000
50000
0
500
1000
1500
2000
type
b
y
y1
y2
y3
y4
y5
y6
y7
b1
b2
b3
b4
b5
b6
b7
b8

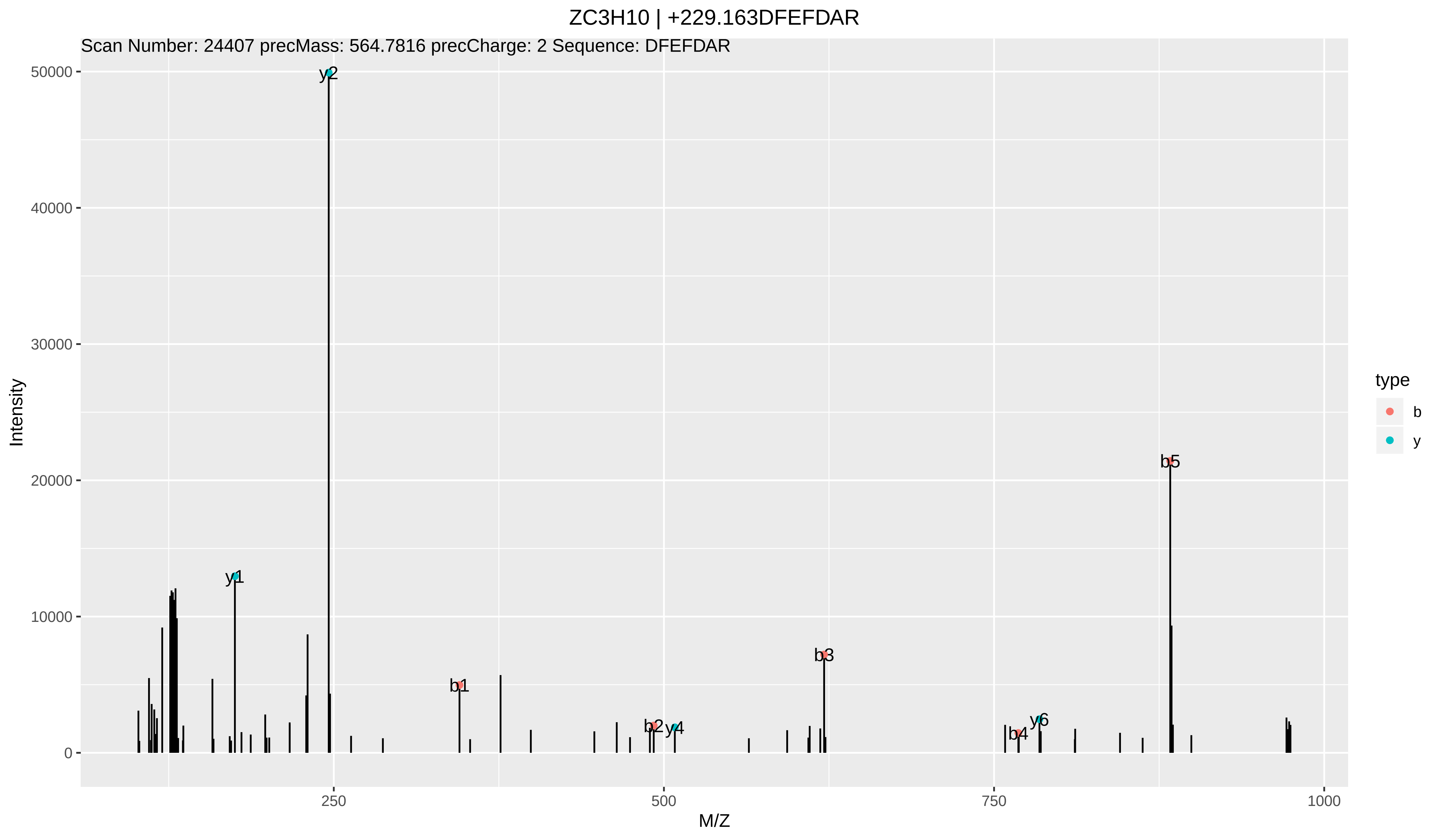

ZC3H10 | +229.163DFEFDAR
Scan Number: 24407 precMass: 564.7816 precCharge: 2 Sequence: DFEFDAR
Intensity
M/Z
y2
y1
b1
b2
y4
b3
b4
y6
b5
type
b
y
0
10000
20000
30000
40000
50000
250
500
750
1000

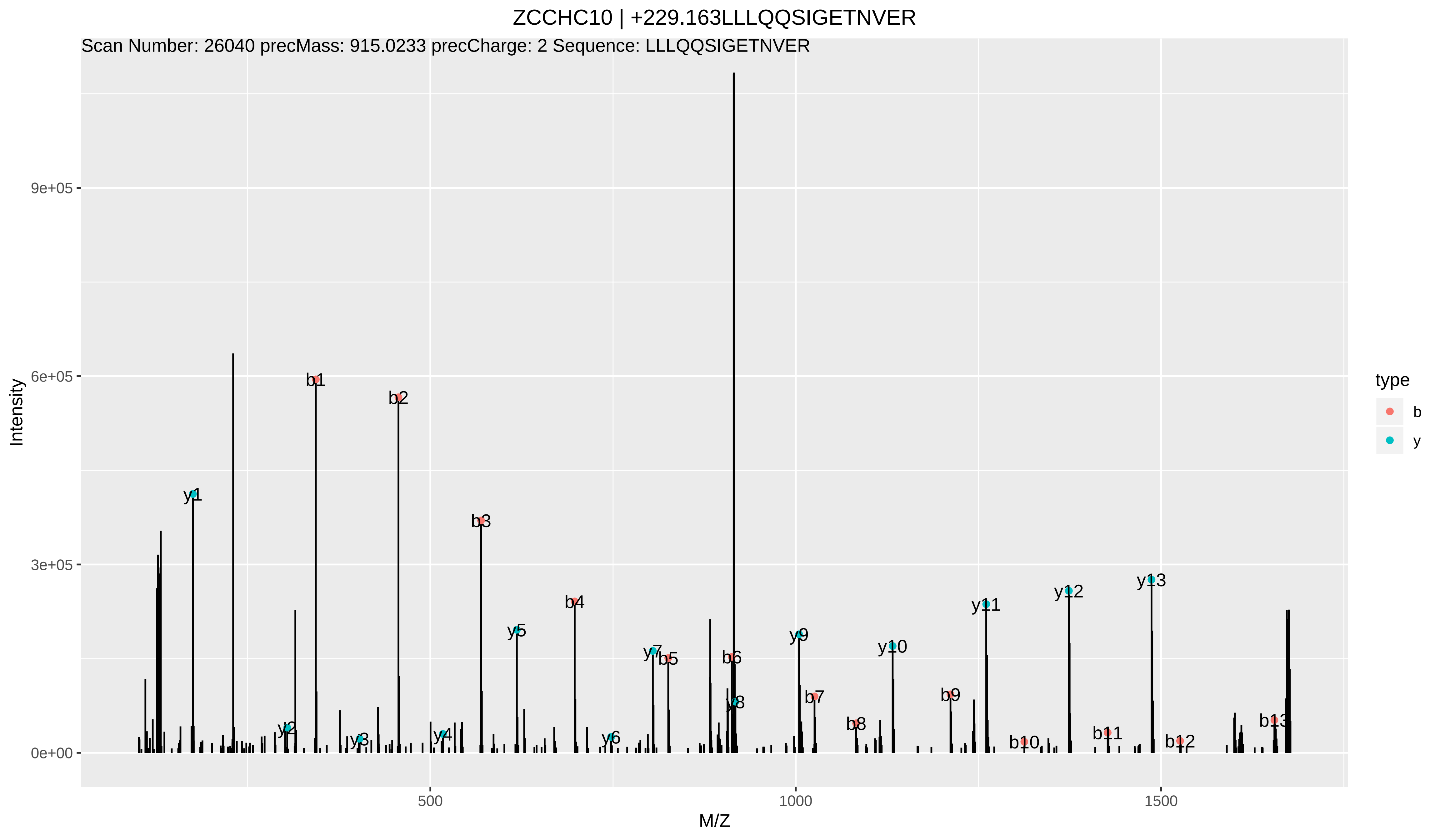

ZCCHC10 | +229.163LLLQQSIGETNVER
Scan Number: 26040 precMass: 915.0233 precCharge: 2 Sequence: LLLQQSIGETNVER
Intensity
9e+05
6e+05
3e+05
0e+00
M/Z
500
1000
1500
type
b
y
y1
b1
b2
b3
y2
y3
y4
y5
b4
y6
y7
b5
b6
y8
y9
b7
b8
y10
b9
y11
b10
y12
b11
y13
b12
b13

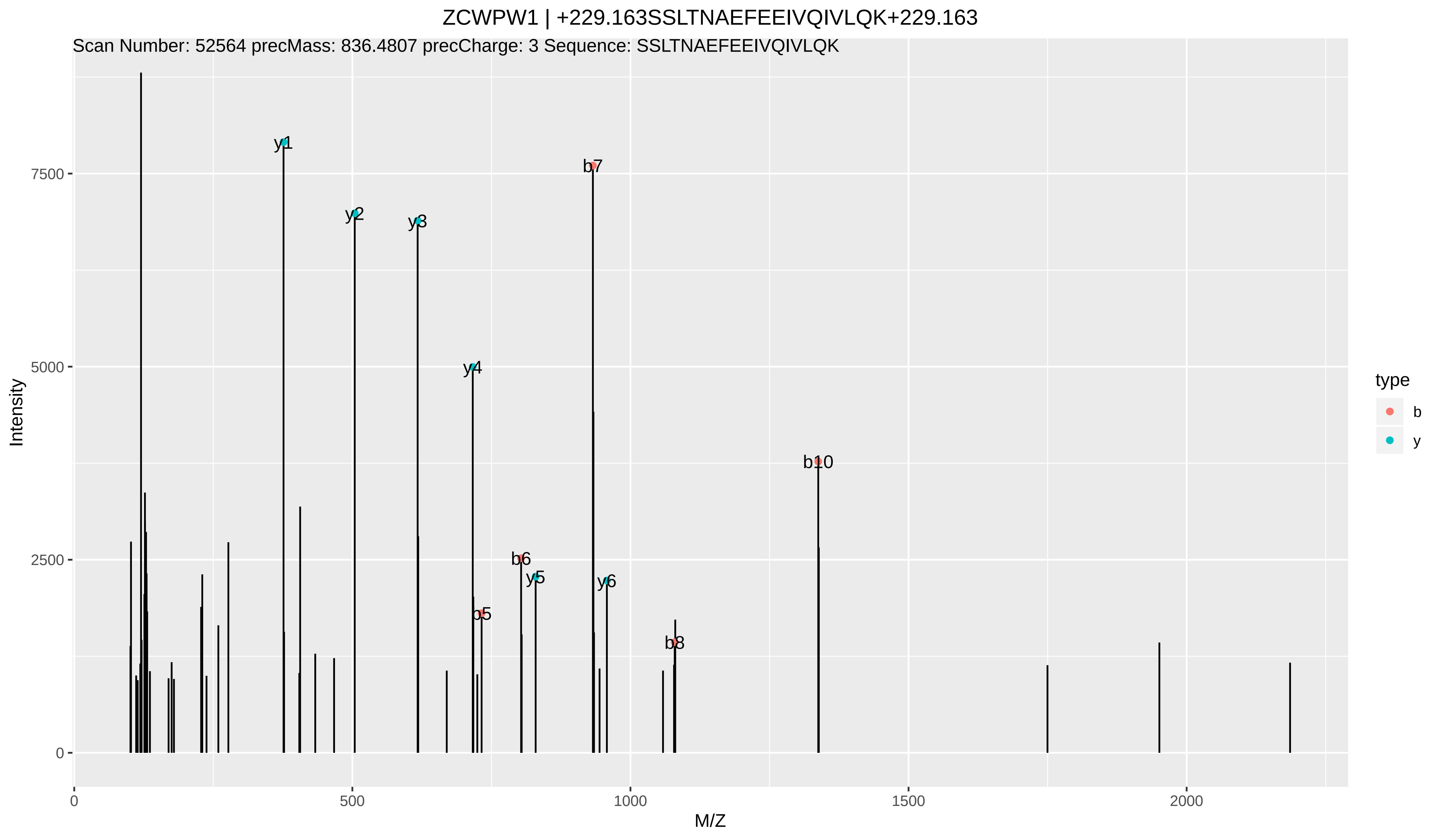

ZCWPW1 | +229.163SSLTNAEFEEIVQIVLQK+229.163
Scan Number: 52564 precMass: 836.4807 precCharge: 3 Sequence: SSLTNAEFEEIVQIVLQK
Intensity
M/Z
type
b
y
y1
y2
y3
y4
y5
y6
b5
b6
b7
b8
b10

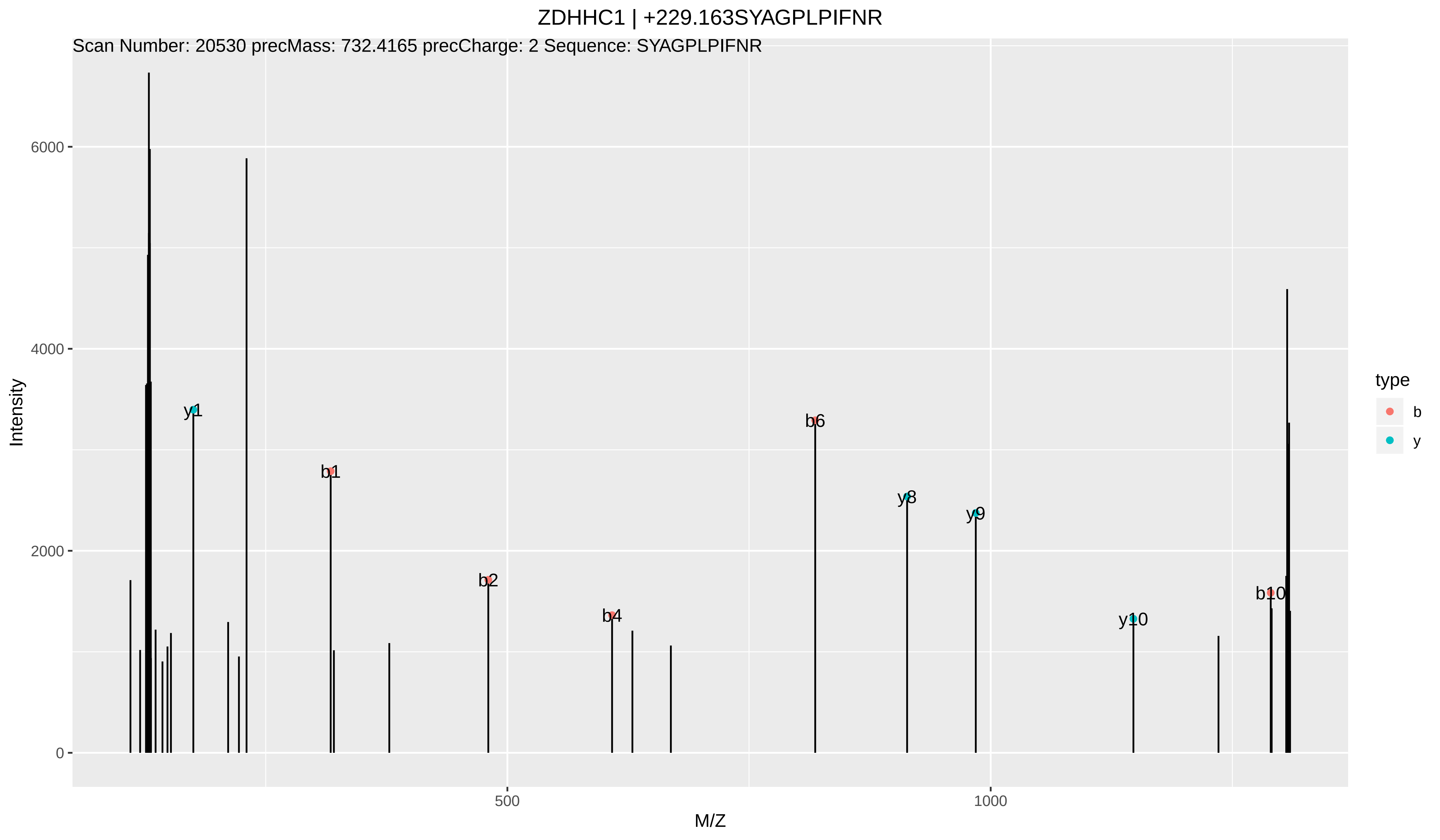

ZDHHC1 | +229.163SYAGPLPIFNR
Scan Number: 20530 precMass: 732.4165 precCharge: 2 Sequence: SYAGPLPIFNR
Intensity
6000
4000
2000
0
500
1000
M/Z
y1
b1
b2
b4
b6
y8
y9
y10
b10
type
b
y

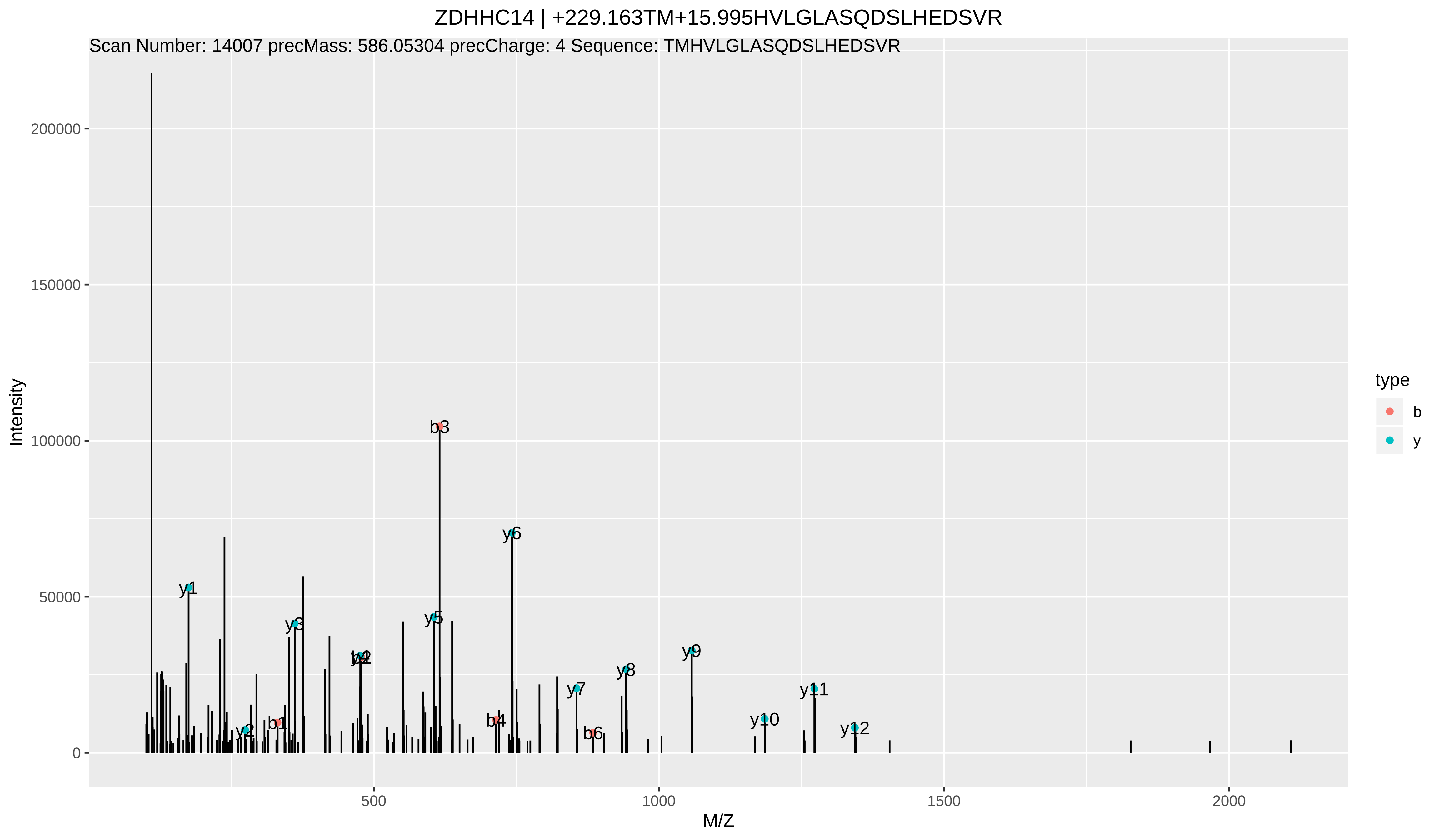

ZDHHC14 | +229.163TM+15.995HVLGLASQDSLHEDSVR
Scan Number: 14007 precMass: 586.05304 precCharge: 4 Sequence: TMHVLGLASQDSLHEDSVR
Intensity
M/Z
type
b
y
b3
y6
y1
y5
y3
y4
y9
y8
y7
y11
b4
b1
y2
y10
b6
y12

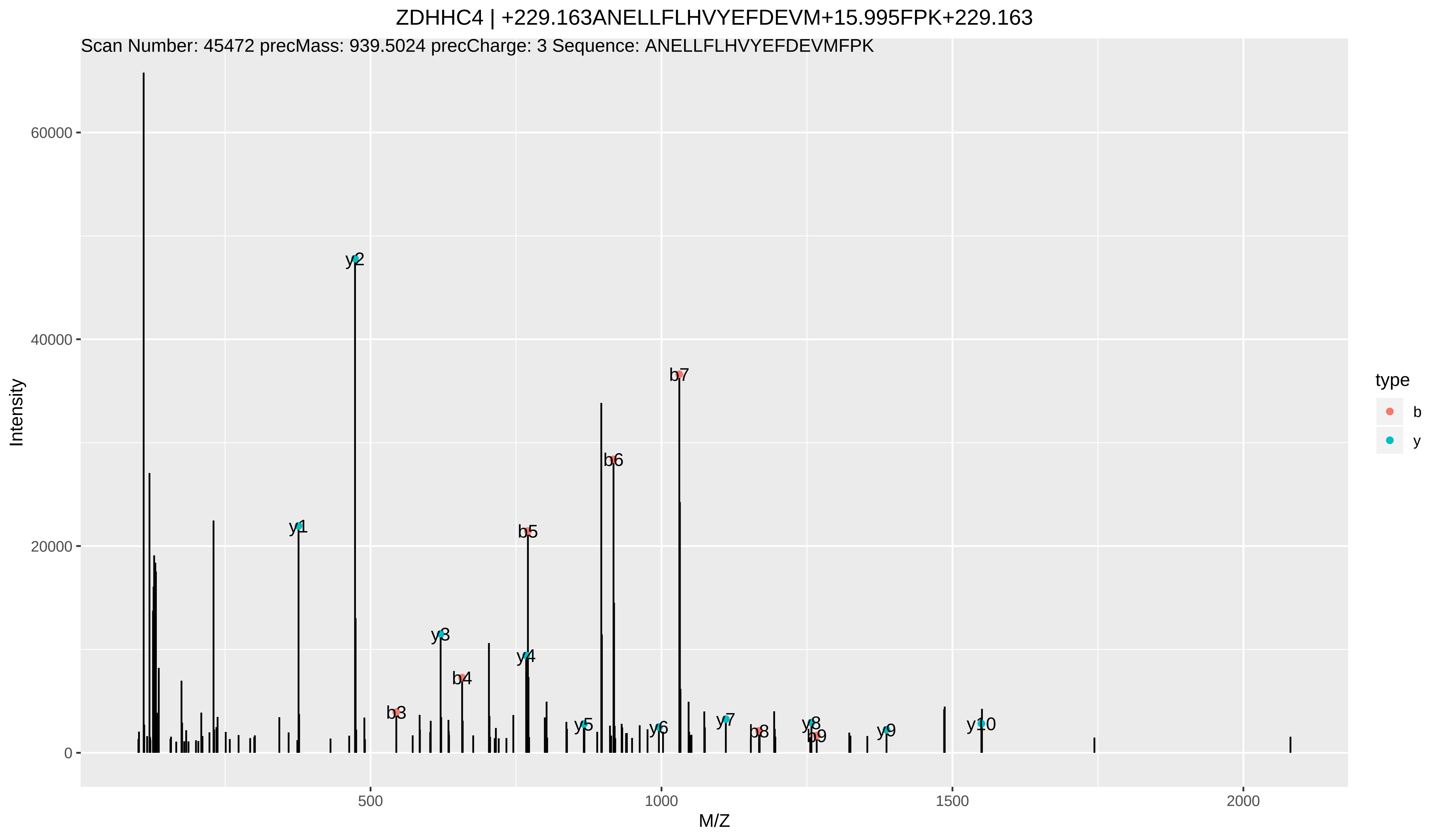

ZDHHC4 | +229.163ANELLFLHVYEFDEVM+15.995FPK+229.163
Scan Number: 45472 precMass: 939.5024 precCharge: 3 Sequence: ANELLFLHVYEFDEVMFPK
Intensity
M/Z
0
20000
40000
60000
500
1000
1500
2000
y1
y2
y3
y4
y5
y6
y7
y8
y9
y10
b3
b4
b5
b6
b7
b8
b9
type
b
y

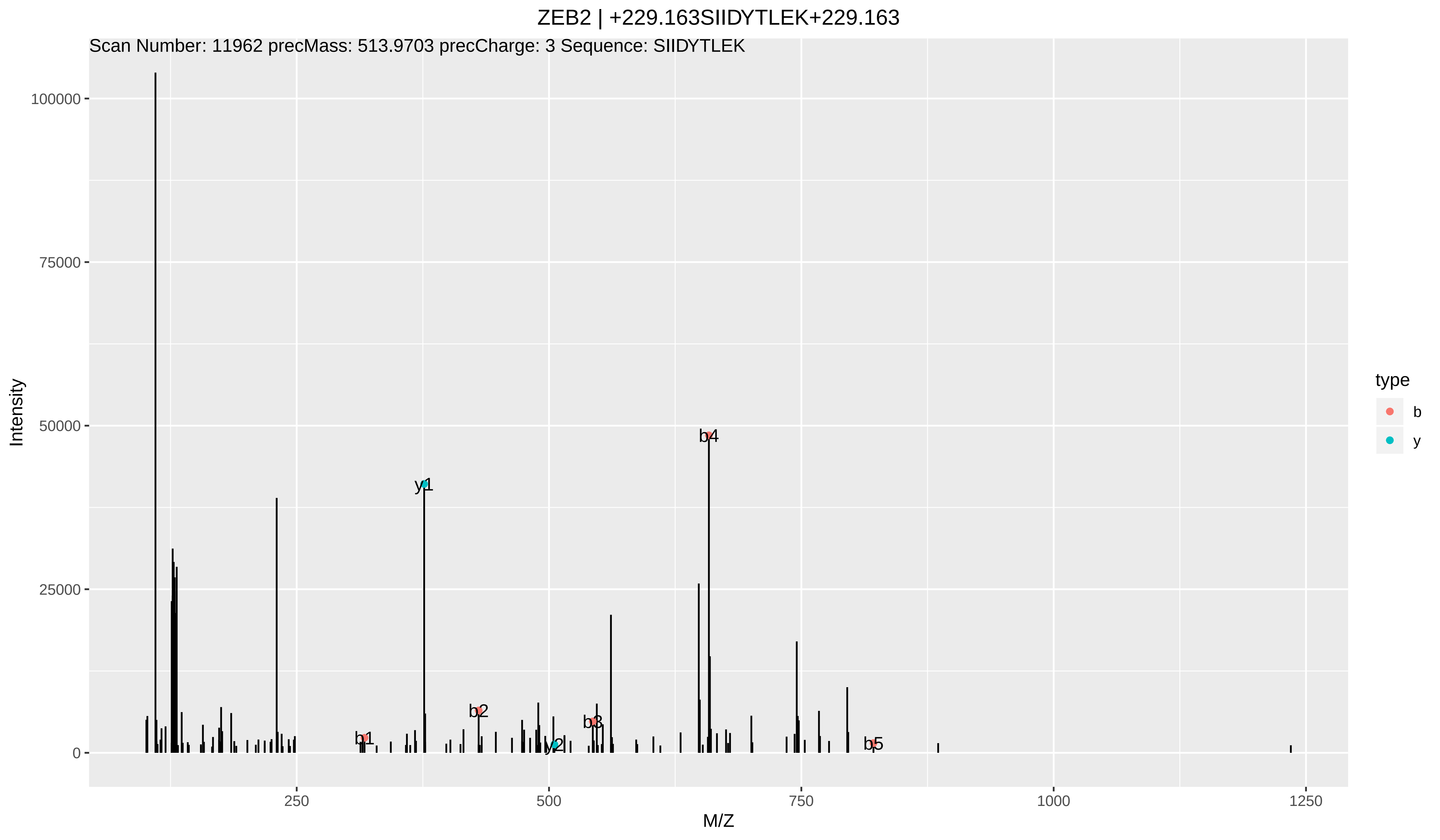
ZEB2 | +229.163SIIDYTLEK+229.163
Scan Number: 11962 precMass: 513.9703 precCharge: 3 Sequence: SIIDYTLEK
Intensity
100000
75000
50000
25000
0
250
500
750
1000
1250
M/Z
y1
b1
b2
y2
b3
b4
b5
type
b
y

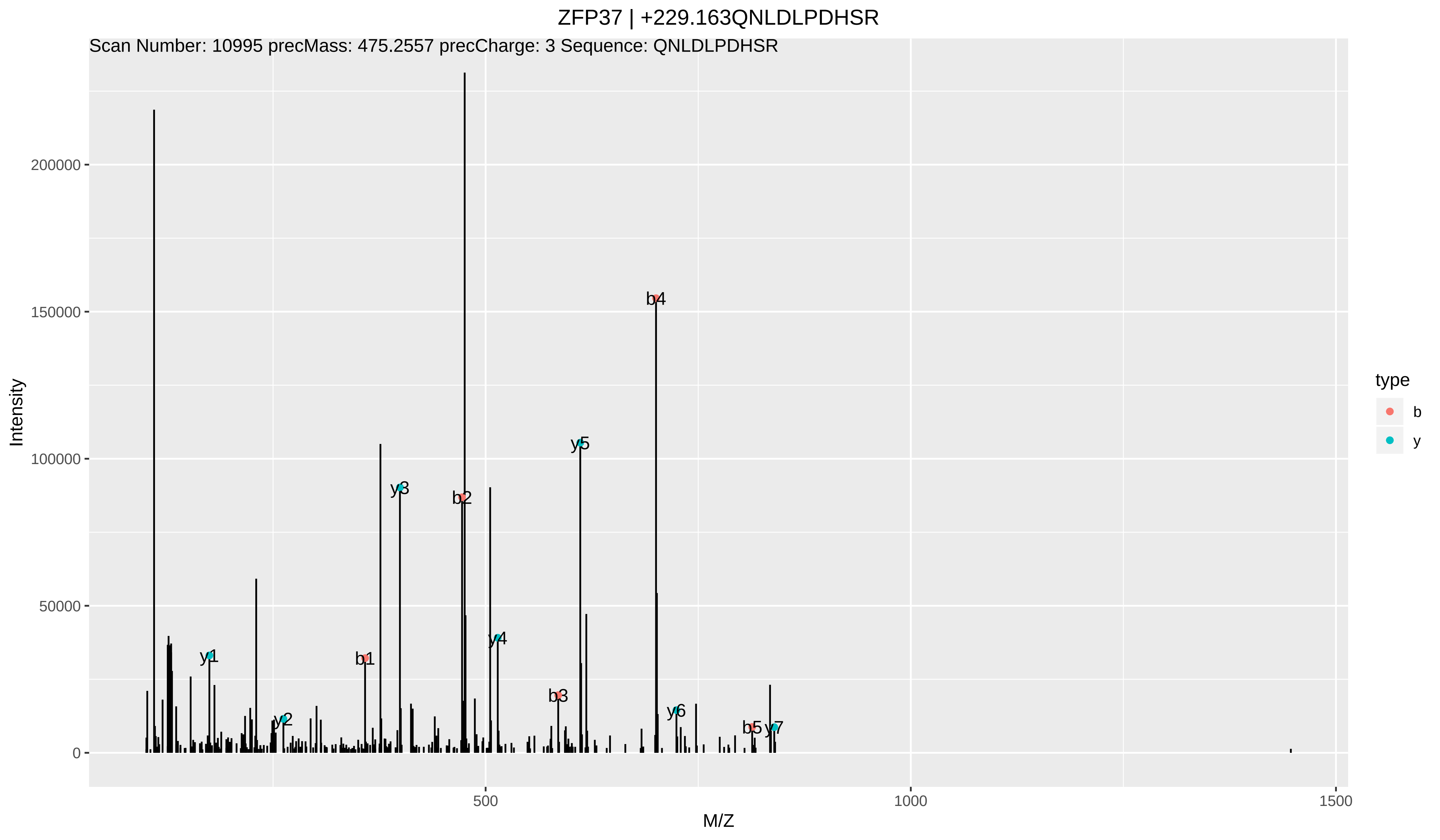
ZFP37 | +229.163QNLDLPDHSR
Scan Number: 10995 precMass: 475.2557 precCharge: 3 Sequence: QNLDLPDHSR
Intensity
200000
150000
100000
50000
0
M/Z
500
1000
1500
type
b
y
y1
y2
b1
y3
b2
y4
b3
y5
b4
y6
b5
y7

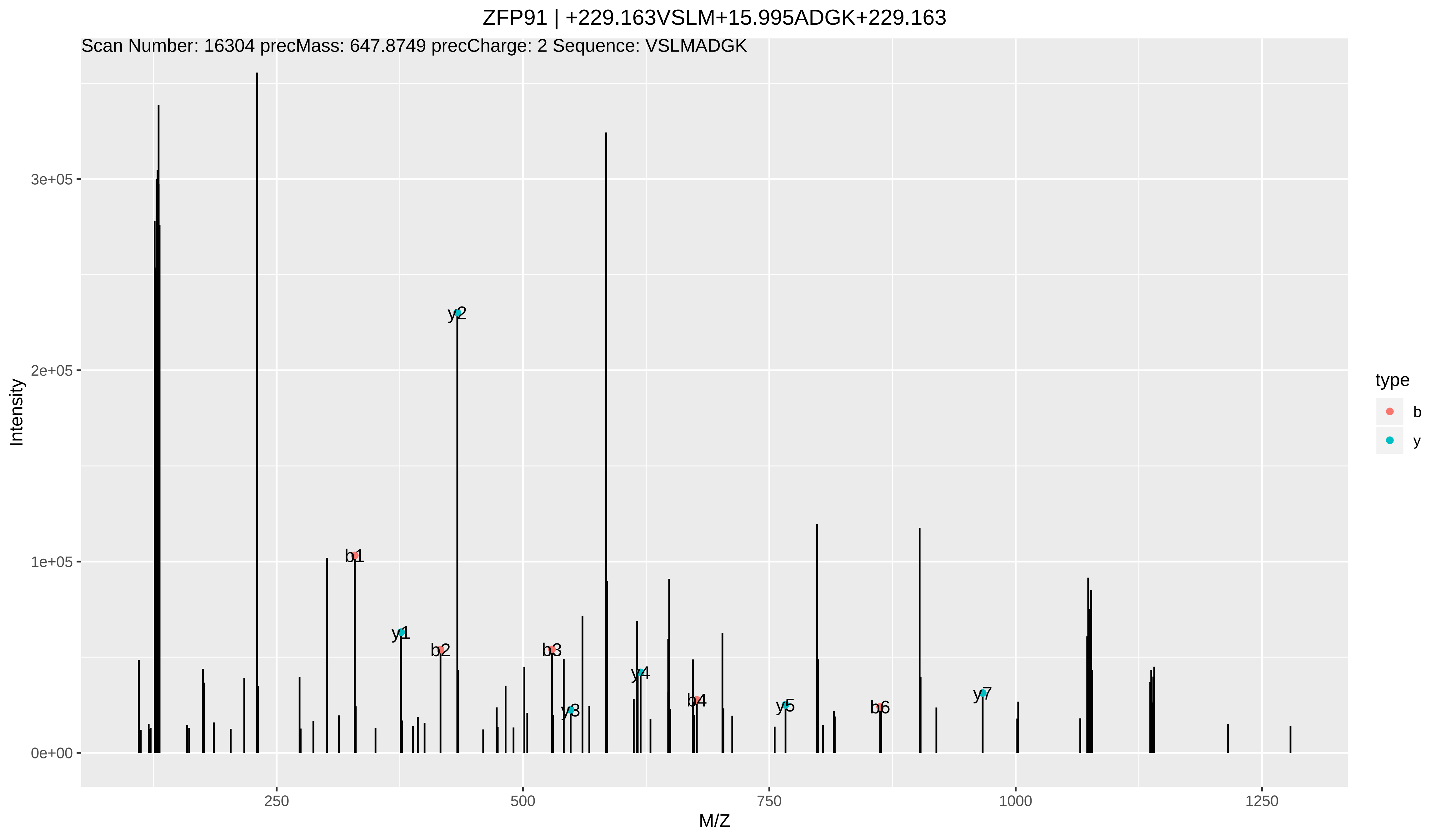
ZFP91 | +229.163VSLM+15.995ADGK+229.163
Scan Number: 16304 precMass: 647.8749 precCharge: 2 Sequence: VSLMADGK
Intensity
M/Z
type
b
y
b1
b2
b3
b4
b6
y1
y2
y3
y4
y5
y7
3e+05
2e+05
1e+05
0e+00
250
500
750
1000
1250

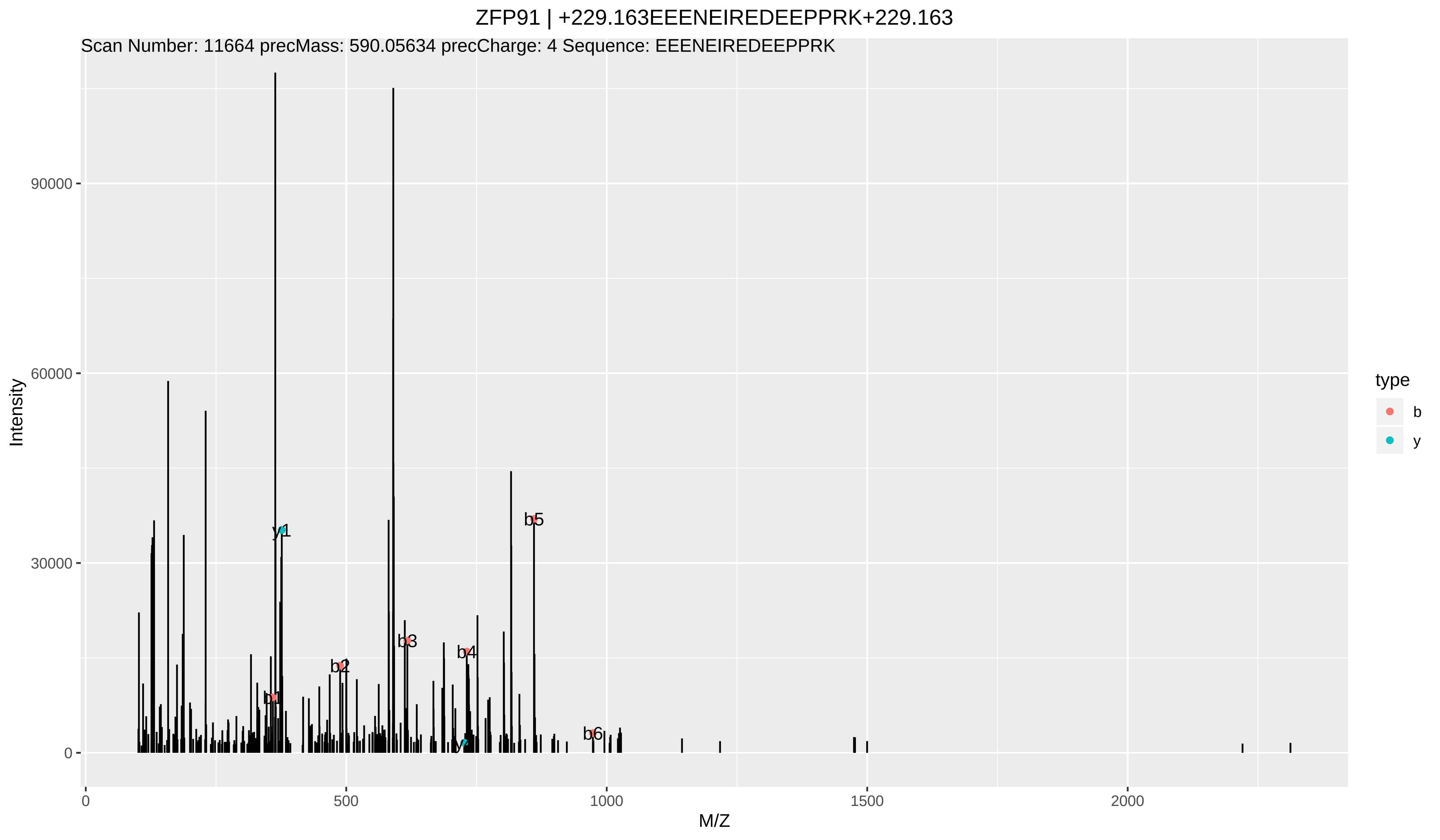
ZFP91 | +229.163EEENEIREDEEPPRK+229.163
Scan Number: 11664 precMass: 590.05634 precCharge: 4 Sequence: EEENEIREDEEPPRK
Intensity
90000
60000
30000
0
0
500
1000
1500
2000
M/Z
y1
b1
b2
b3
b4
y
b5
b6
type
b
y

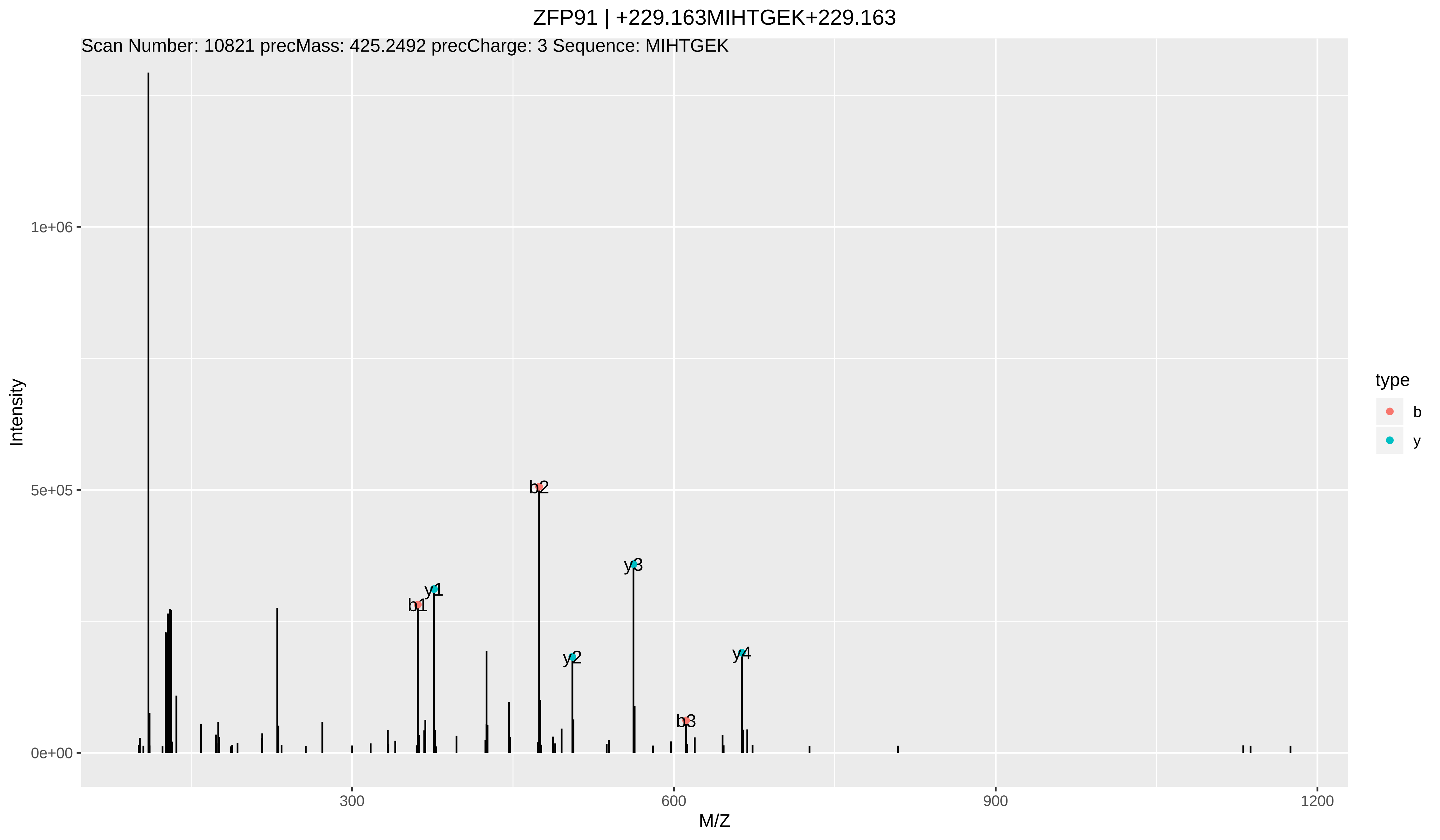
ZFP91 | +229.163MIHTGEK+229.163
Scan Number: 10821 precMass: 425.2492 precCharge: 3 Sequence: MIHTGEK
Intensity
M/Z
type
b
y
b1
y1
b2
y2
y3
b3
y4
1e+06
5e+05
0e+00
300
600
900
1200

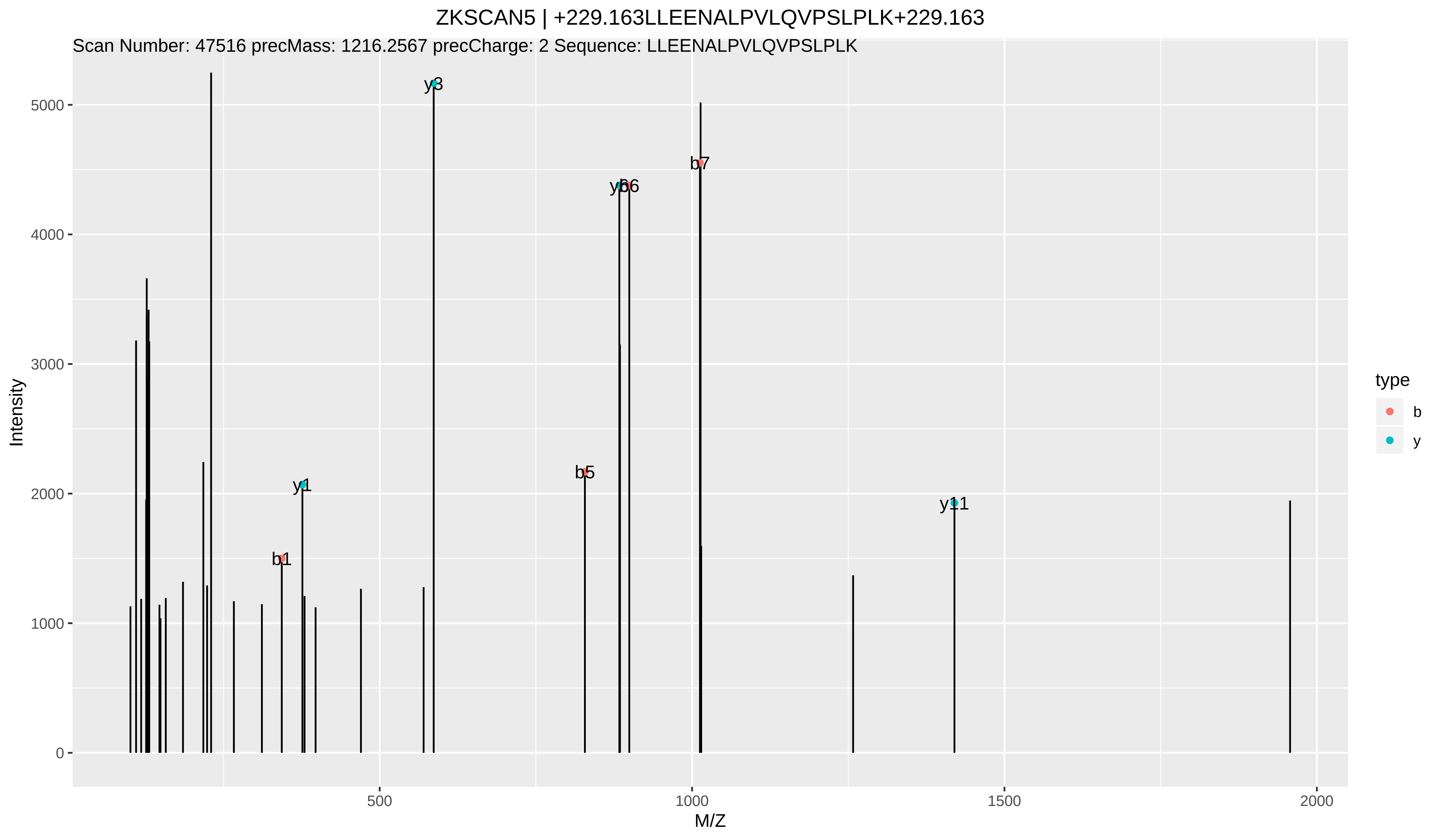
ZKSCAN5 | +229.163LLEENALPVLQVPSLPLK+229.163
Scan Number: 47516 precMass: 1216.2567 precCharge: 2 Sequence: LLEENALPVLQVPSLPLK
Intensity
M/Z
type
b
y
y3
b7
b6
b5
y1
b1
y11
0
1000
2000
3000
4000
5000
500
1000
1500
2000

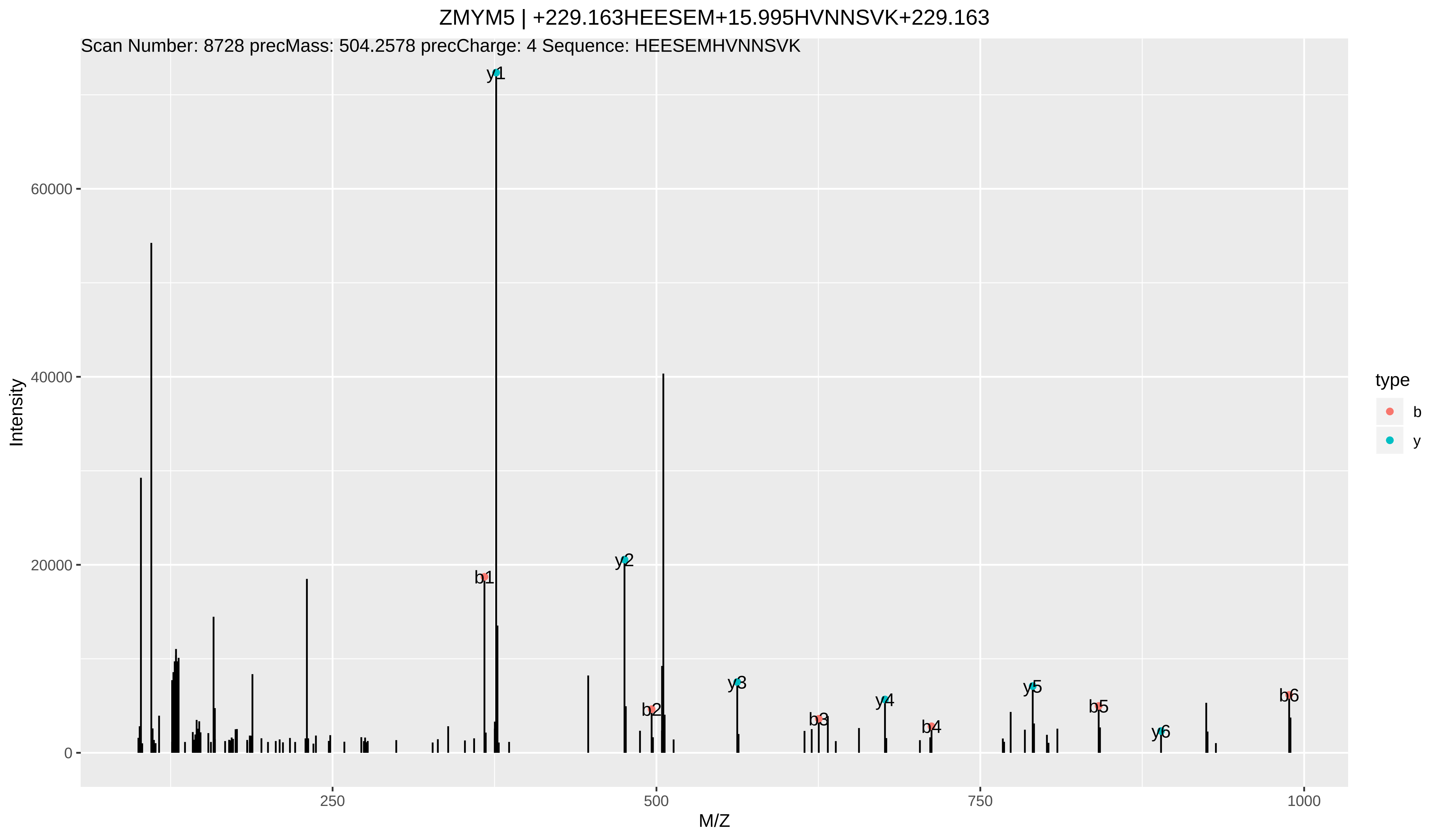
ZMYM5 | +229.163HEESEM+15.995HVNNSVK+229.163
Scan Number: 8728 precMass: 504.2578 precCharge: 4 Sequence: HEESEMHVNNSVK
Intensity
M/Z
type
b
y
y1
b1
y2
b2
y3
b3
y4
b4
y5
b5
y6
b6
0
20000
40000
60000
250
500
750
1000

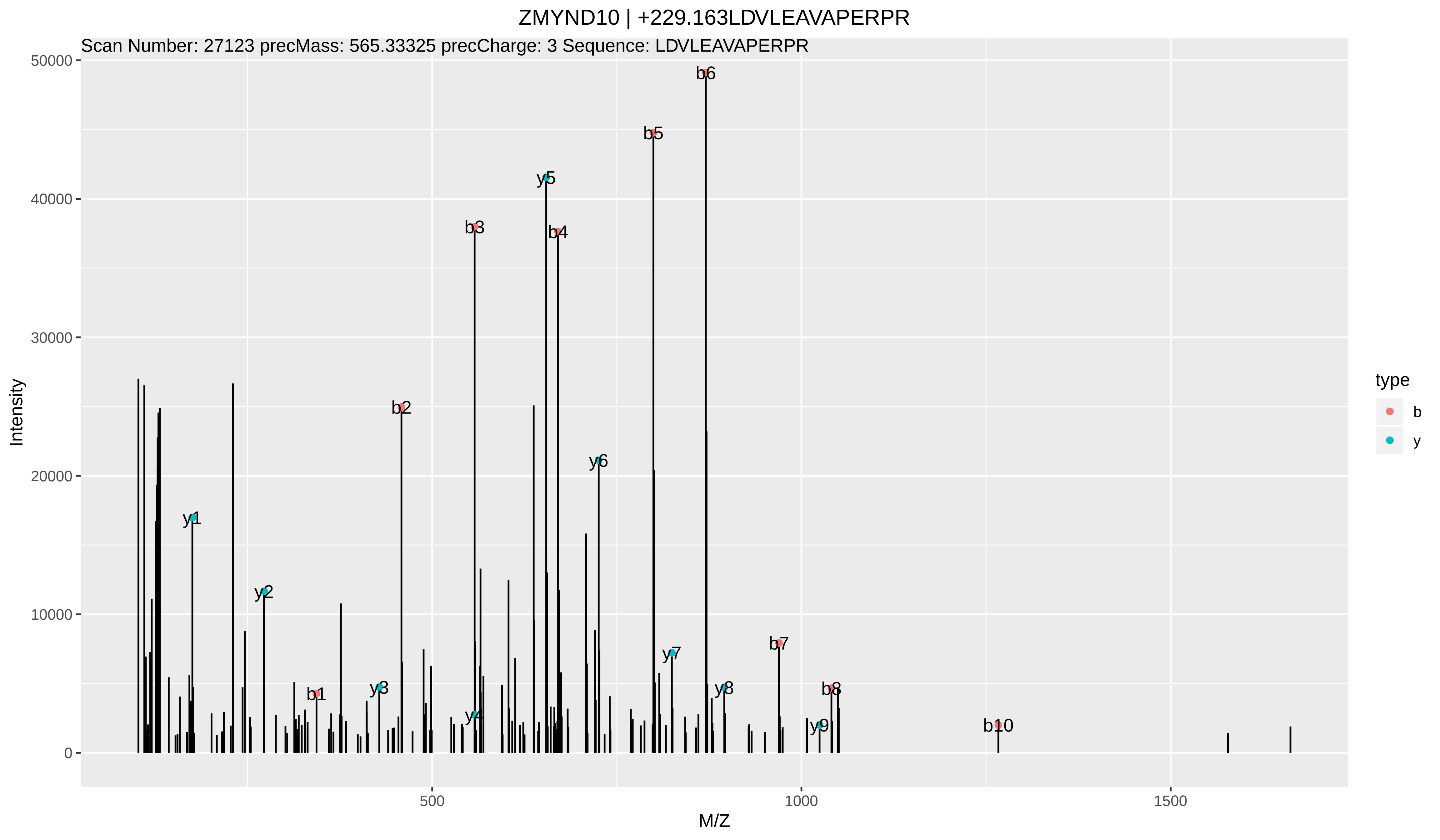
ZMYND10 | +229.163LDVLEAVAPERPR
Scan Number: 27123 precMass: 565.33325 precCharge: 3 Sequence: LDVLEAVAPERPR
Intensity
M/Z
type
b
y
b6
b5
y5
b3
b4
b2
y6
y1
y2
b7
y7
y8
b8
y3
b1
y4
y9
b10
0
10000
20000
30000
40000
50000
500
1000
1500

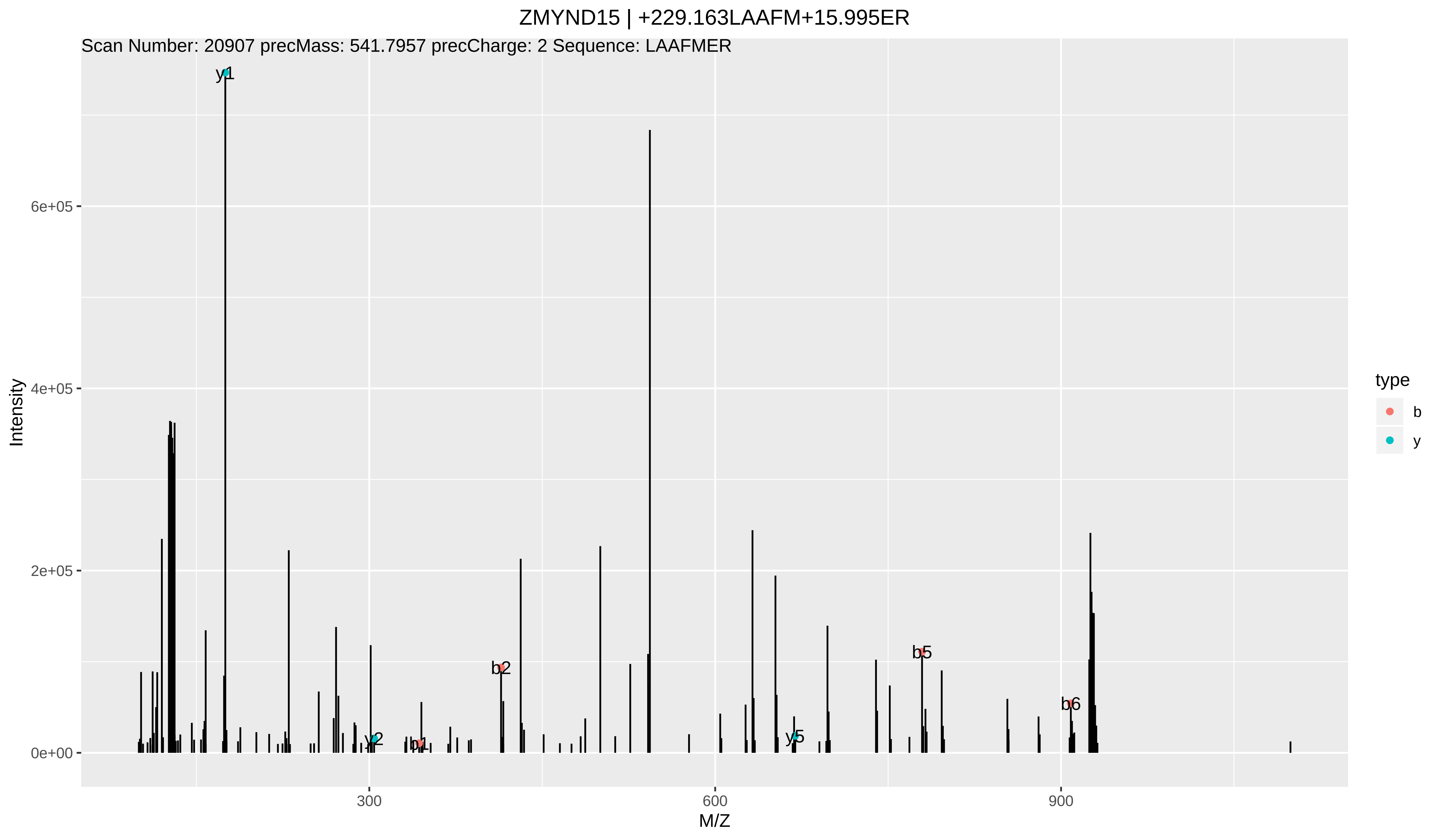
ZMYND15 | +229.163LAAFM+15.995ER
Scan Number: 20907 precMass: 541.7957 precCharge: 2 Sequence: LAAFMER
Intensity
6e+05
4e+05
2e+05
0e+00
M/Z
300
600
900
y1
y2
b1
b2
y5
b5
b6
type
b
y

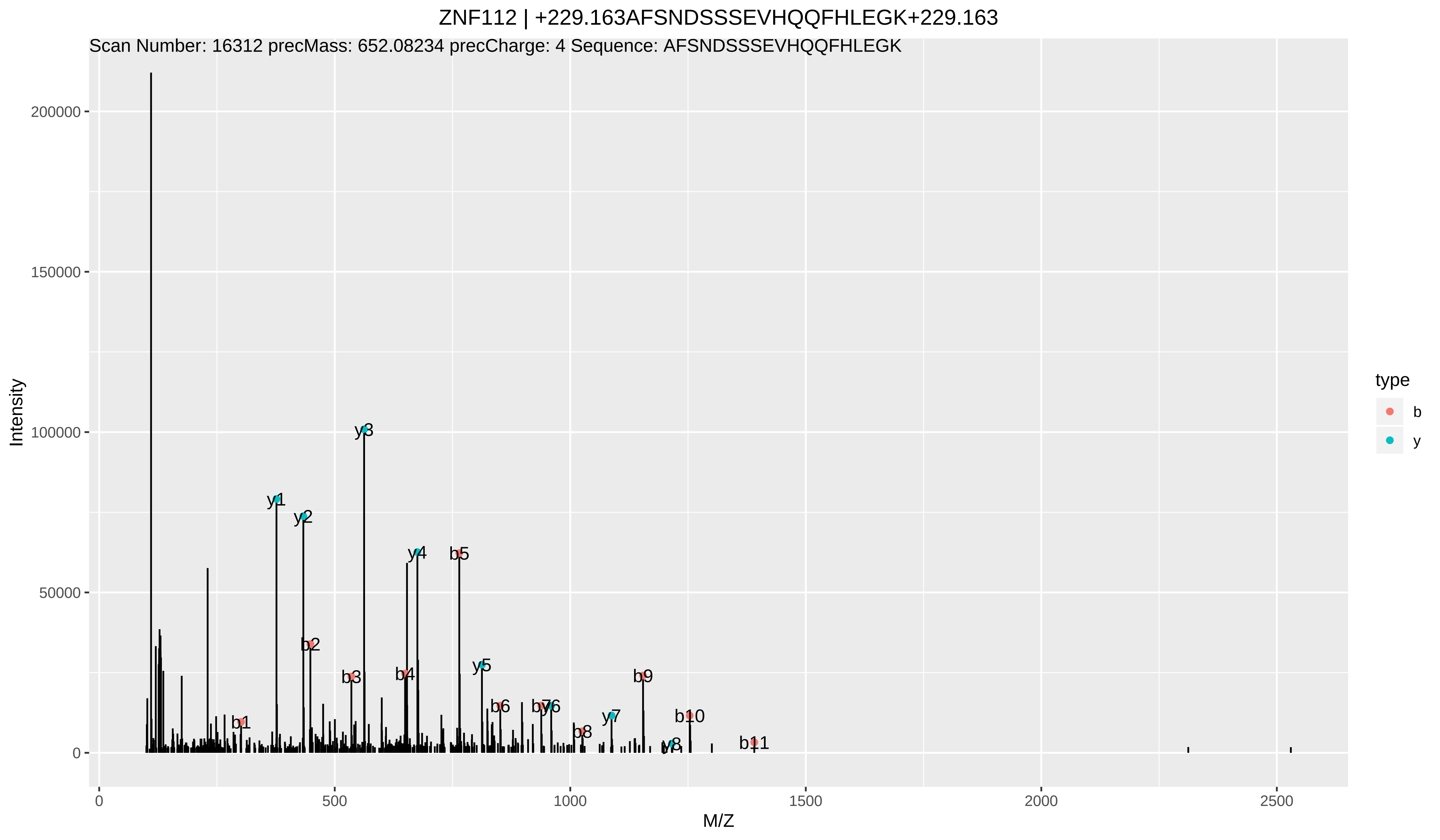

ZNF112 | +229.163AFSNDSSSEVHQQFHLEGK+229.163
Scan Number: 16312 precMass: 652.08234 precCharge: 4 Sequence: AFSNDSSSEVHQQFHLEGK
Intensity
M/Z
type
b
y
y1
y2
y3
y4
y5
y6
y7
y8
b1
b2
b3
b4
b5
b6
b7
b8
b9
b10
b11
0
50000
100000
150000
200000
500
1000
1500
2000
2500

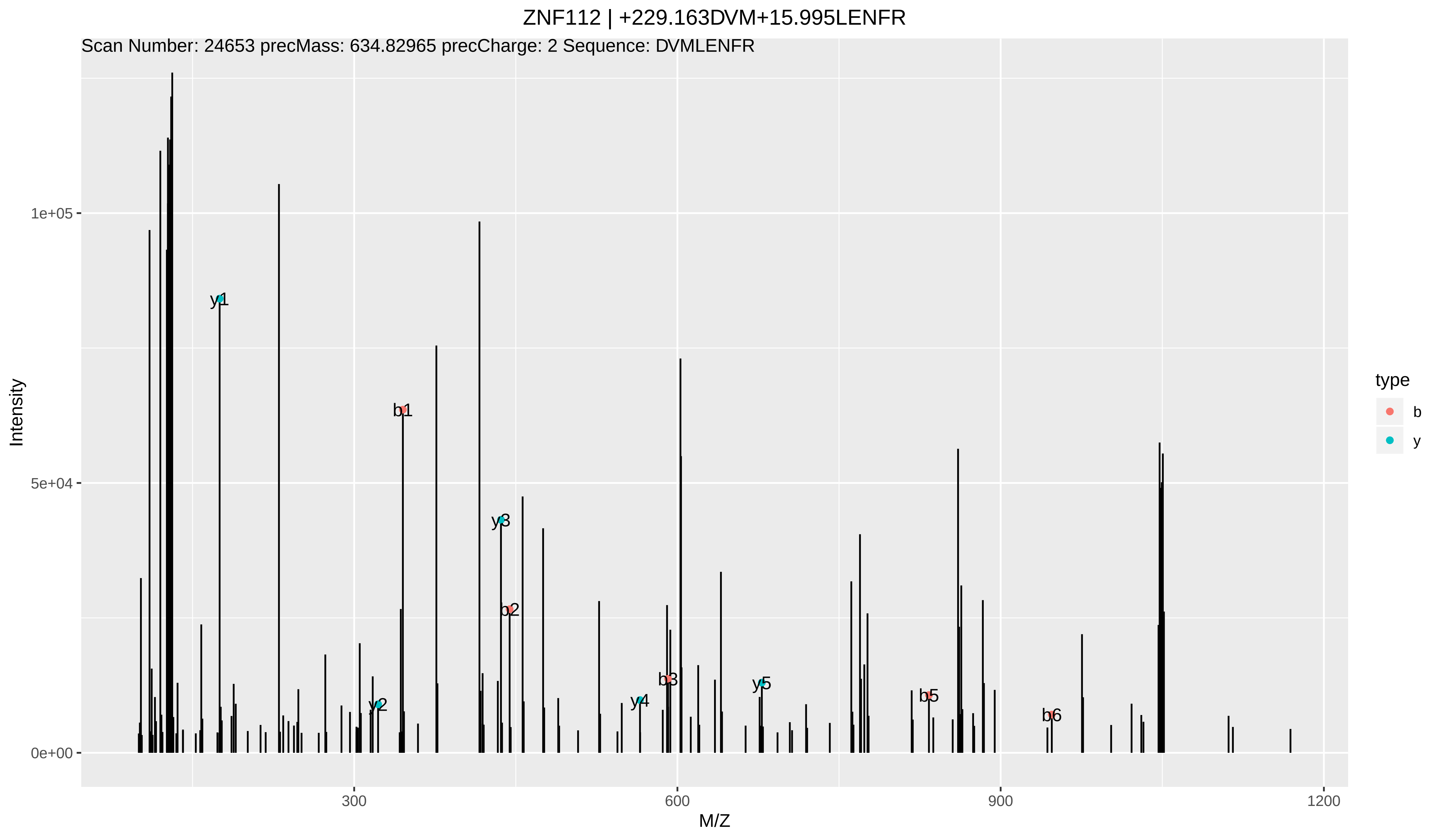
ZNF112 | +229.163DVM+15.995LENFR
Scan Number: 24653 precMass: 634.82965 precCharge: 2 Sequence: DVMLENFR
Intensity
M/Z
1e+05
5e+04
0e+00
300
600
900
1200
y1
b1
y3
b2
y2
y4
b3
y5
b5
b6
type
b
y

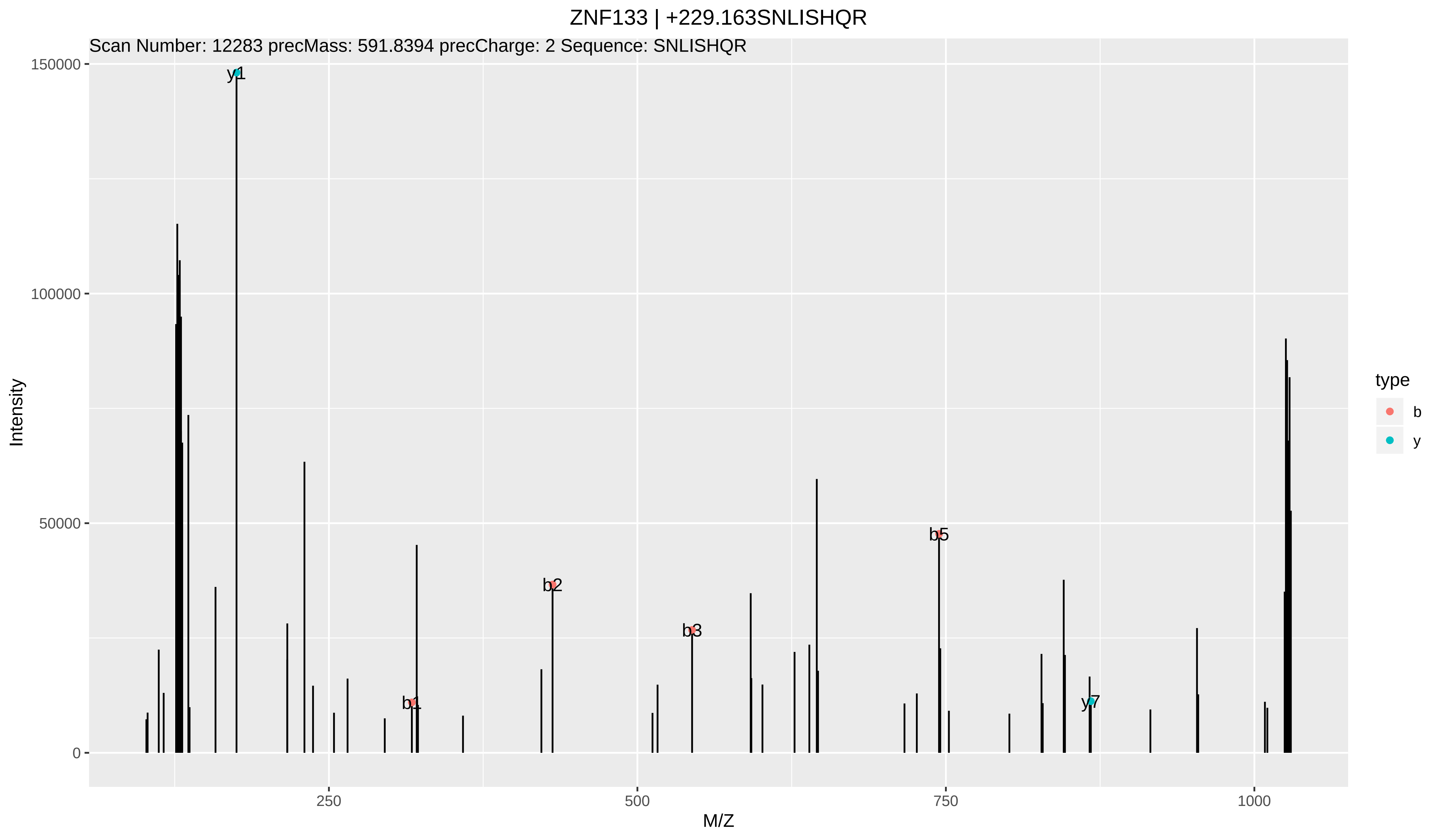

ZNF133 | +229.163SNLISHQR
Scan Number: 12283 precMass: 591.8394 precCharge: 2 Sequence: SNLISHQR
Intensity
150000
100000
50000
0
M/Z
250
500
750
1000
y1
b1
b2
b3
b5
y7
type
b
y

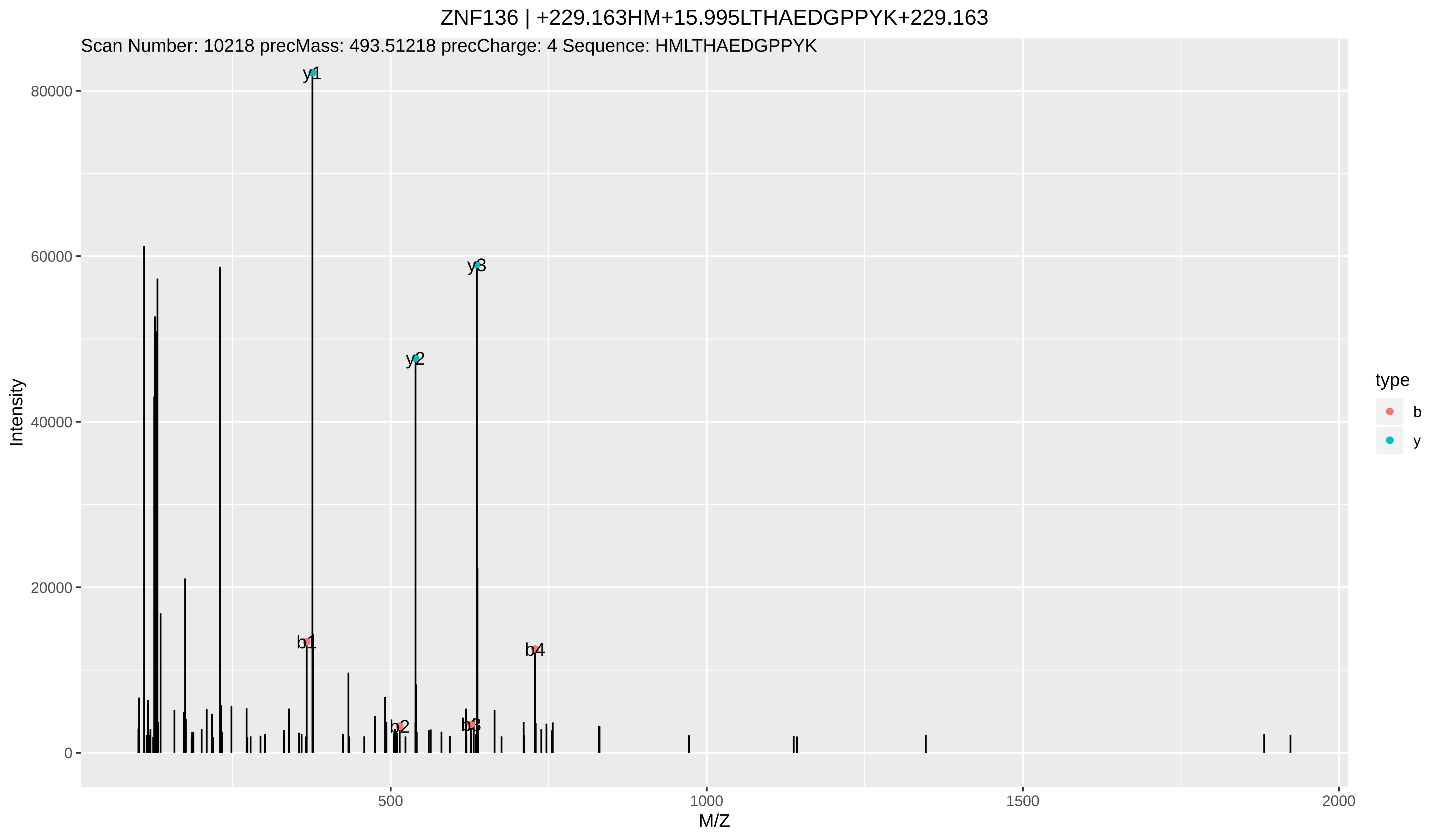
ZNF136 | +229.163HM+15.995LTHAEDGPPYK+229.163
Scan Number: 10218 precMass: 493.51218 precCharge: 4 Sequence: HMLTHAEDGPPYK
Intensity
M/Z
type
b
y
y1
y2
y3
b1
b2
b3
b4
0
20000
40000
60000
80000
500
1000
1500
2000

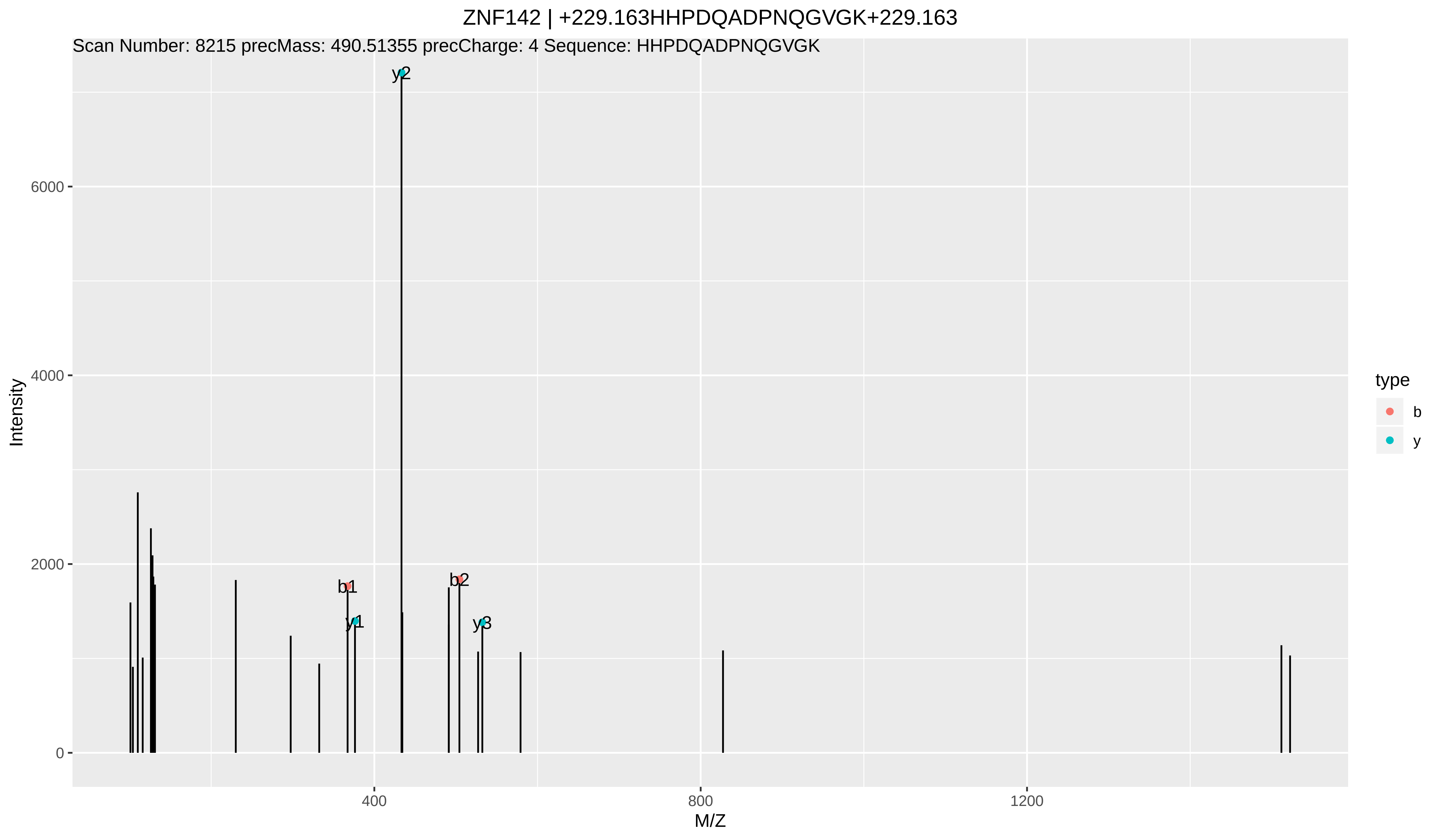
ZNF142 | +229.163HHPDQADPNQGVGK+229.163
Scan Number: 8215 precMass: 490.51355 precCharge: 4 Sequence: HHPDQADPNQGVGK
Intensity
6000
4000
2000
0
400
800
1200
M/Z
y2
b1
y1
b2
y3
type
b
y

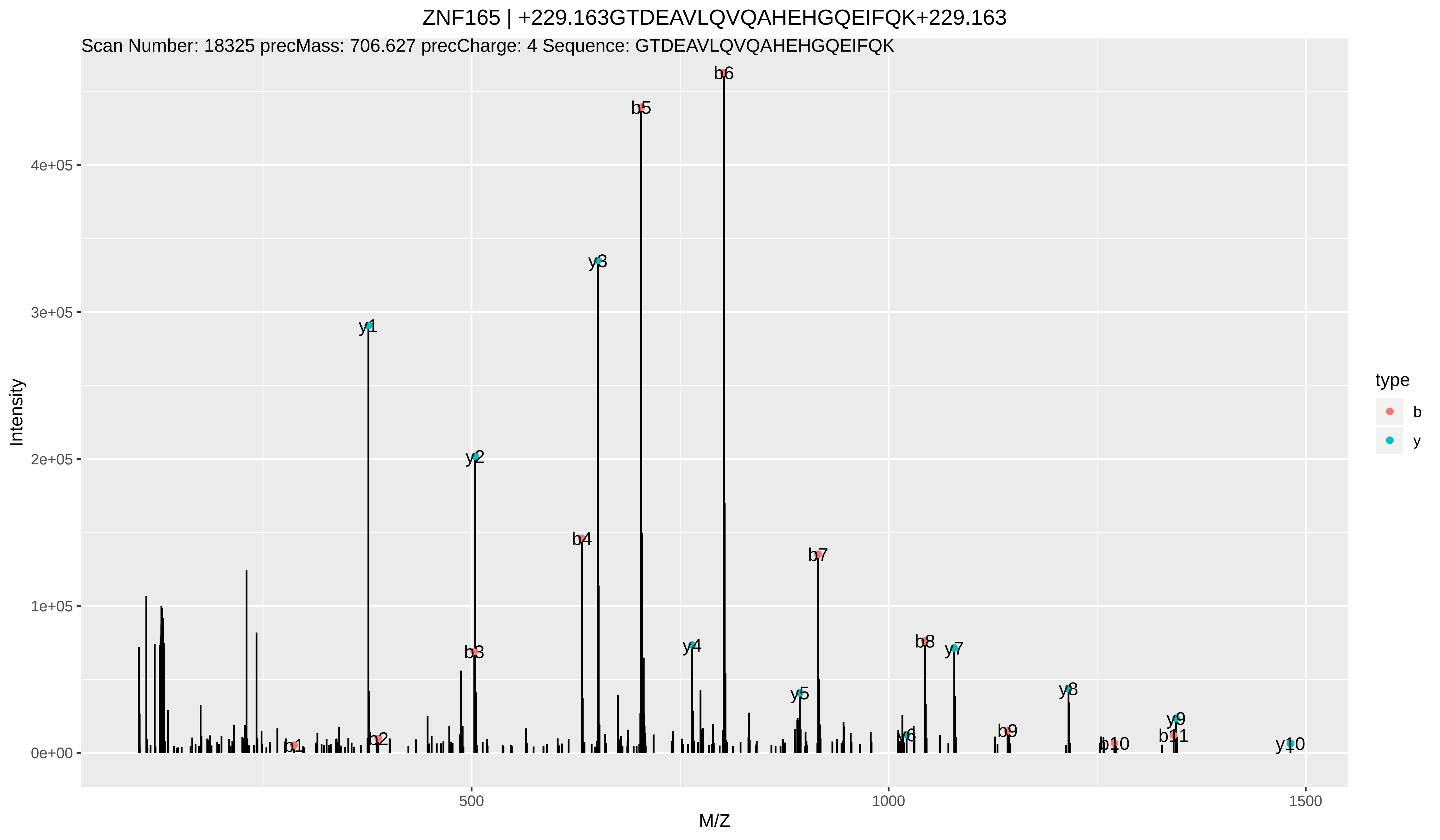
ZNF165 | +229.163GTDEAVLQVQAHEHGQEIFQK+229.163
Scan Number: 18325 precMass: 706.627 precCharge: 4 Sequence: GTDEAVLQVQAHEHGQEIFQK
Intensity
M/Z
type
b
y
b1
b2
b3
b4
b5
b6
b7
b8
b9
b10
b11
y1
y2
y3
y4
y5
y6
y7
y8
y9
y10
0e+00
1e+05
2e+05
3e+05
4e+05
500
1000
1500

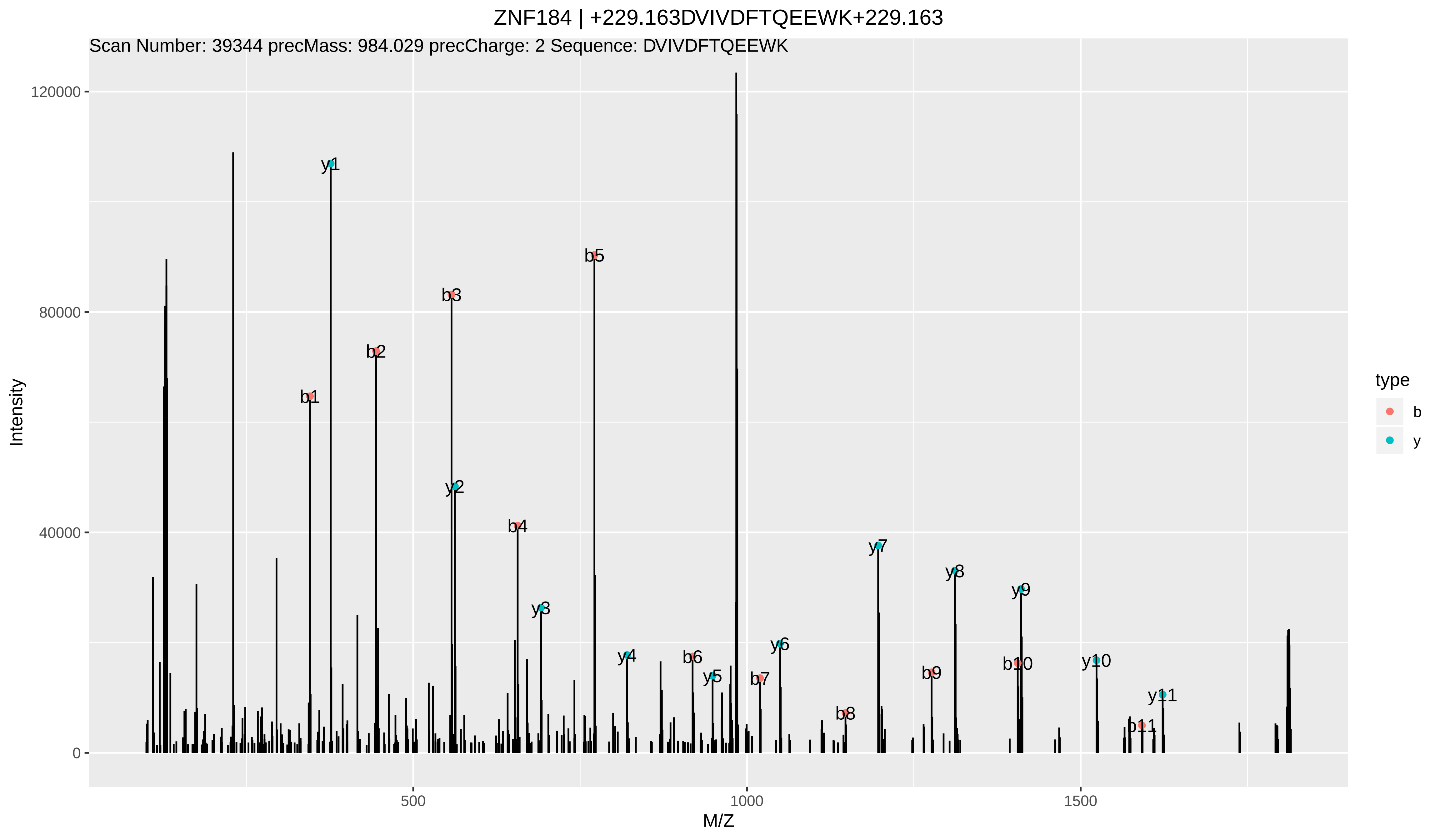
ZNF184 | +229.163DVIVDFTQEEWK+229.163
Scan Number: 39344 precMass: 984.029 precCharge: 2 Sequence: DVIVDFTQEEWK
Intensity
M/Z
120000
80000
40000
0
500
1000
1500
type
b
y
y1
b1
b2
b3
y2
b4
y3
b5
y4
b6
y5
b7
y6
b8
y7
b9
y8
b10
y9
y10
b11
y11

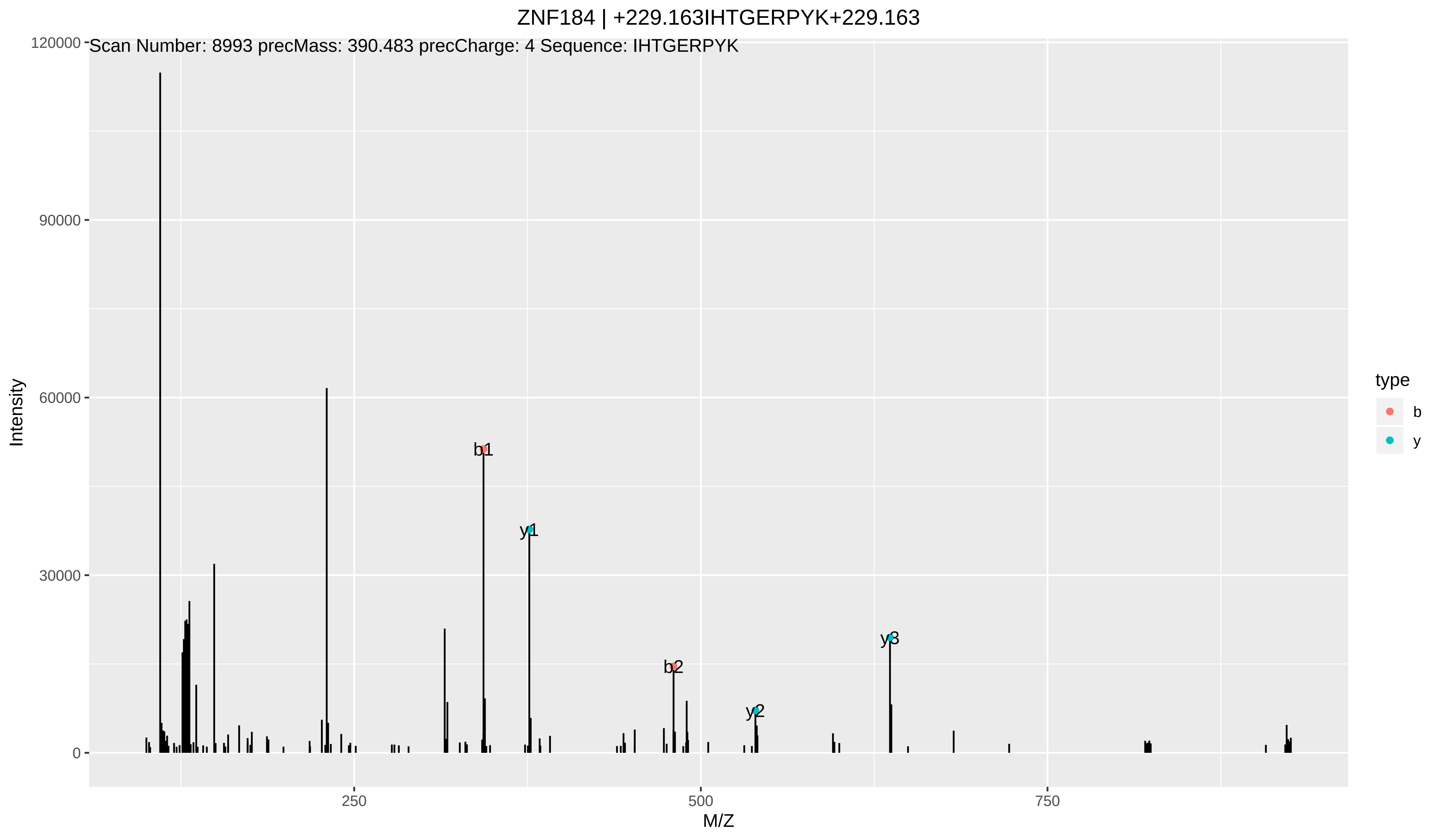

ZNF184 | +229.163IHTGERPYK+229.163
Scan Number: 8993 precMass: 390.483 precCharge: 4 Sequence: IHTGERPYK
Intensity
M/Z
120000
90000
60000
30000
0
250
500
750
b1
y1
b2
y2
y3
type
b
y

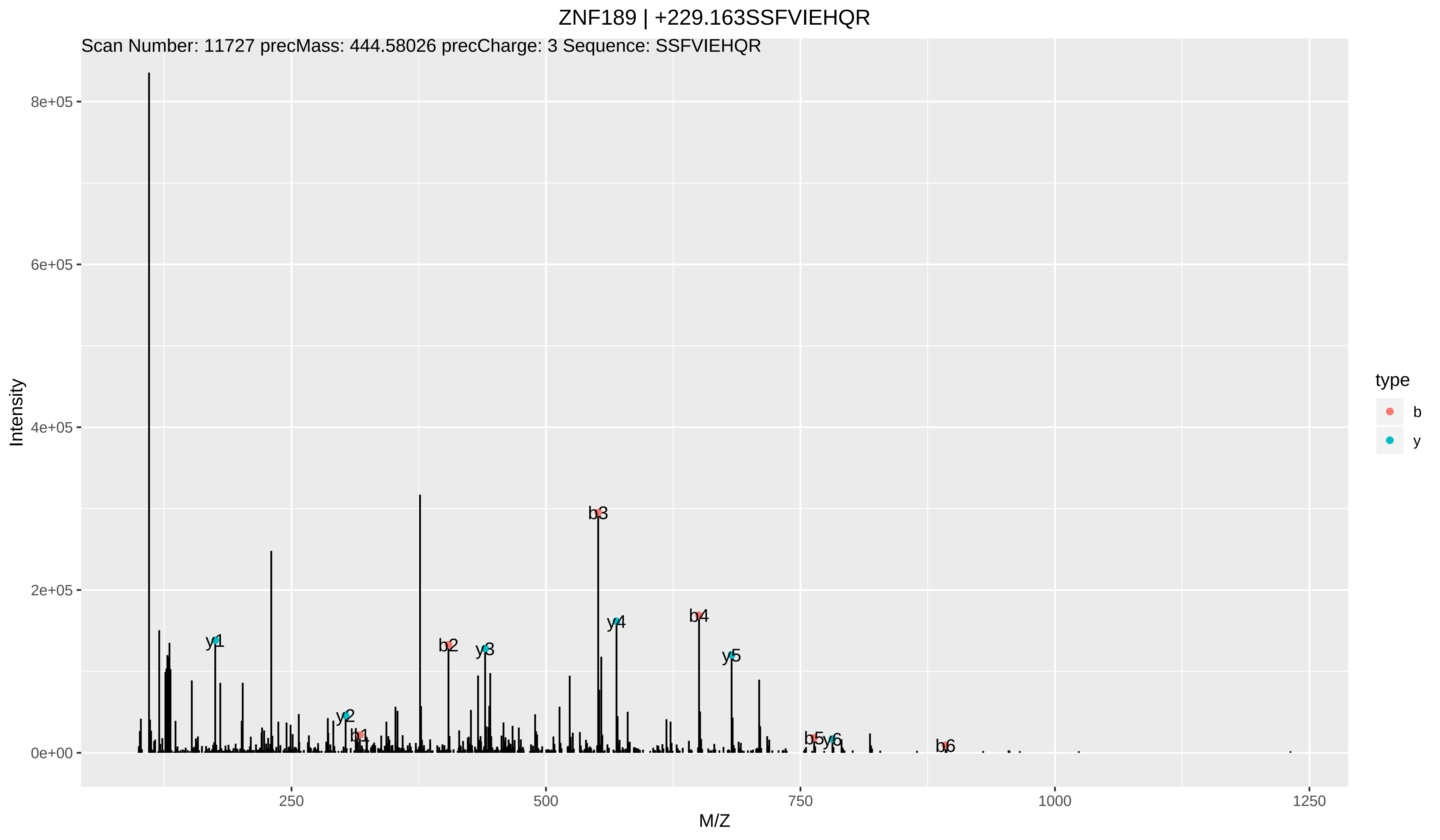

ZNF189 | +229.163SSFVIEHQR
Scan Number: 11727 precMass: 444.58026 precCharge: 3 Sequence: SSFVIEHQR
Intensity
8e+05
6e+05
4e+05
2e+05
0e+00
y1
y2
b1
b2
y3
b3
y4
b4
y5
b5
y6
b6
250
500
750
1000
1250
M/Z
type
b
y

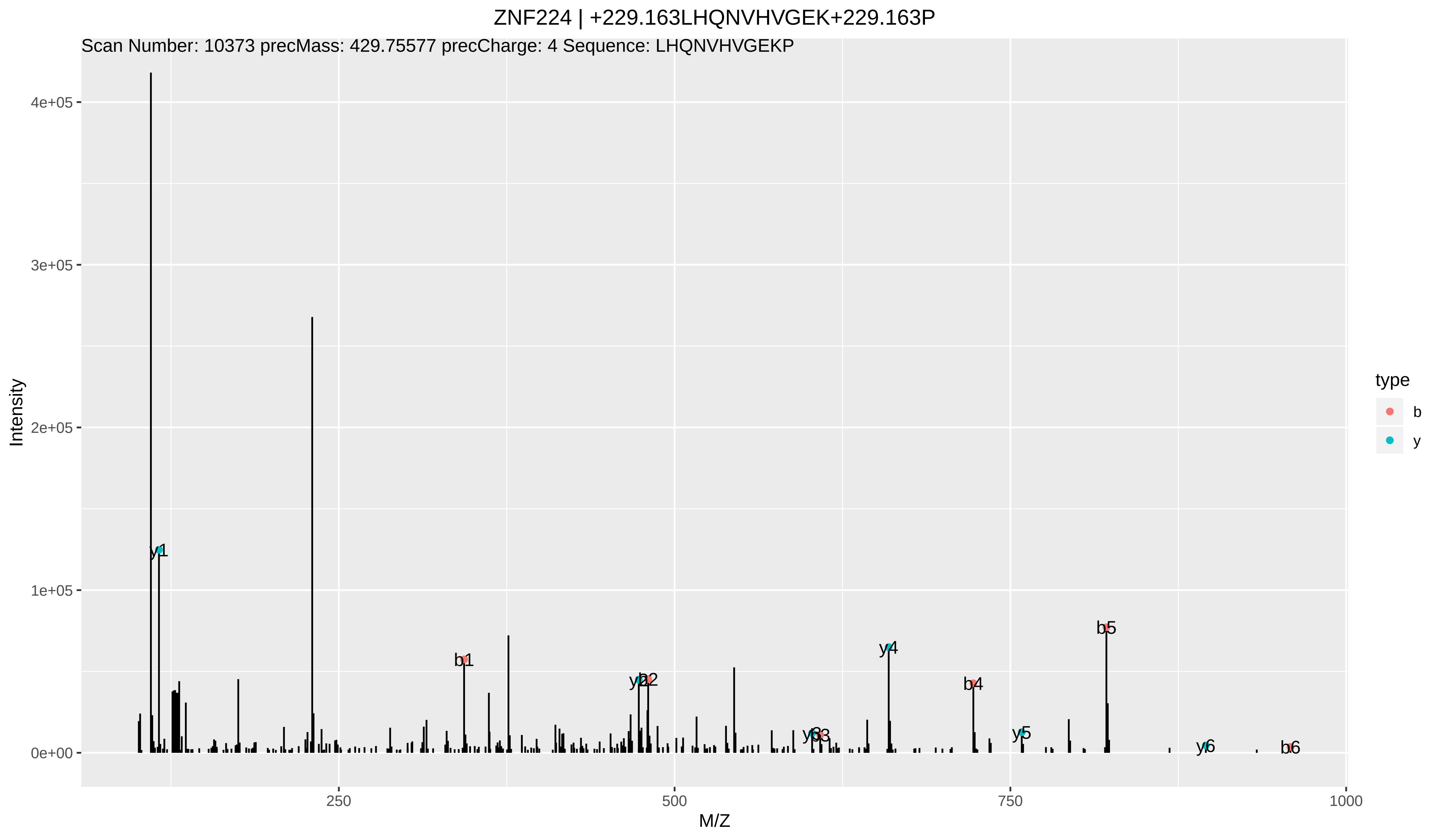

ZNF224 | +229.163LHQNVHVGEK+229.163P
Scan Number: 10373 precMass: 429.75577 precCharge: 4 Sequence: LHQNVHVGEKP
Intensity
M/Z
type
b
y
y1
b1
y2
b2
y3
b3
y4
b4
y5
b5
y6
b6
4e+05
3e+05
2e+05
1e+05
0e+00
250
500
750
1000

ZNF224 | +229.163DVM+15.995LENFR

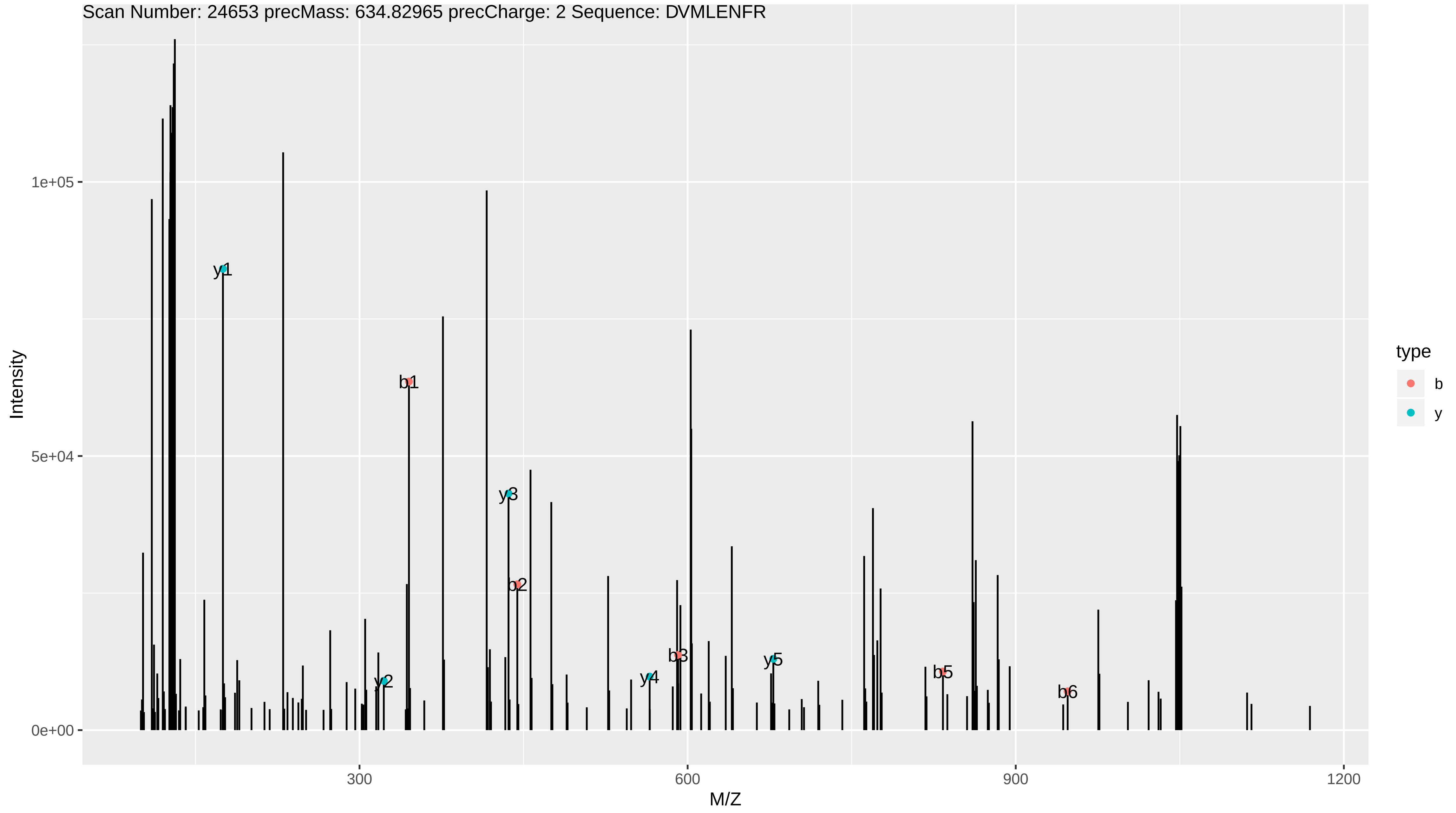

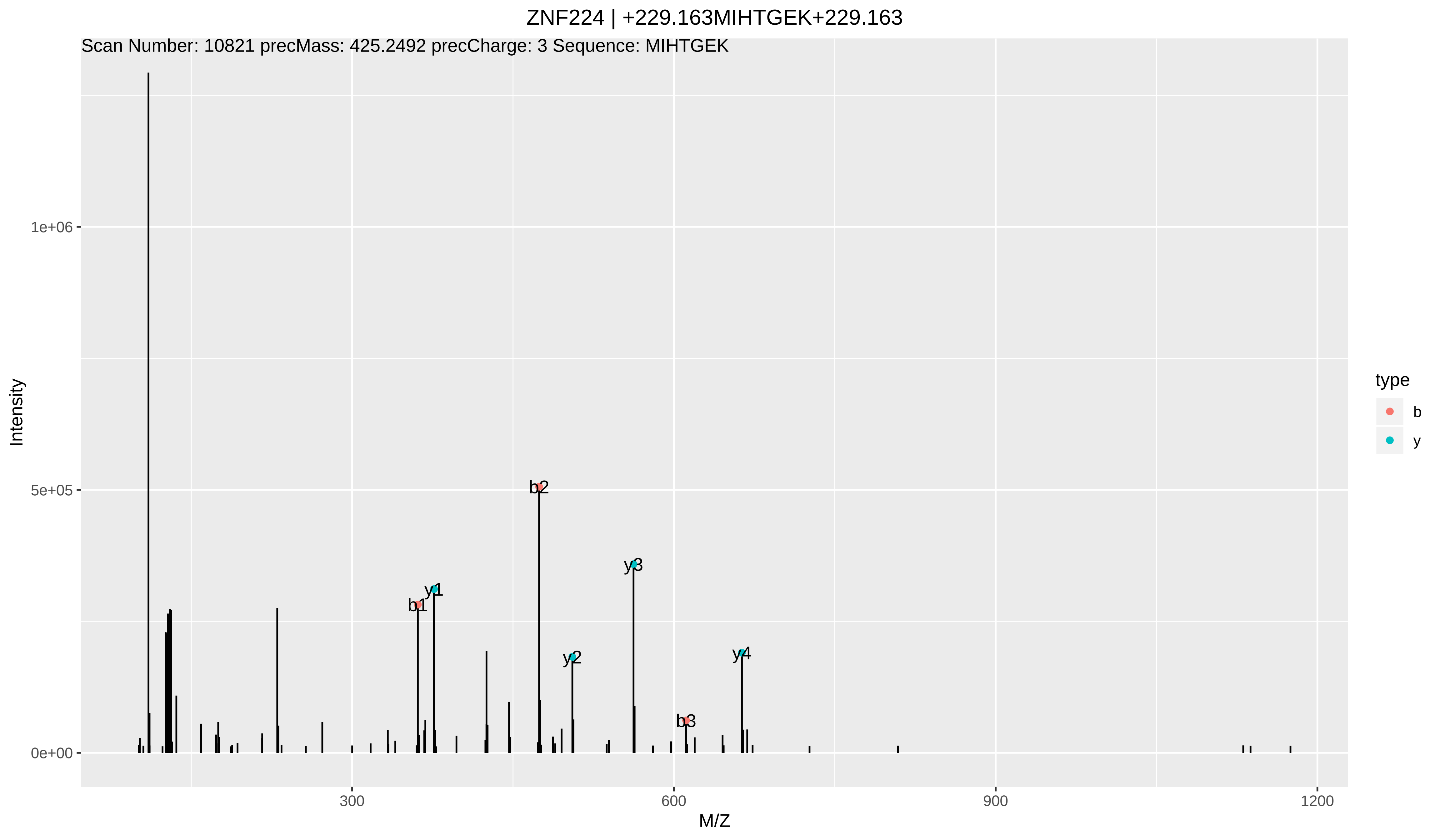

ZNF224 | +229.163MIHTGEK+229.163
Scan Number: 10821 precMass: 425.2492 precCharge: 3 Sequence: MIHTGEK
Intensity
1e+06
5e+05
0e+00
M/Z
300
600
900
1200
b1
y1
b2
y2
y3
b3
y4
type
b
y

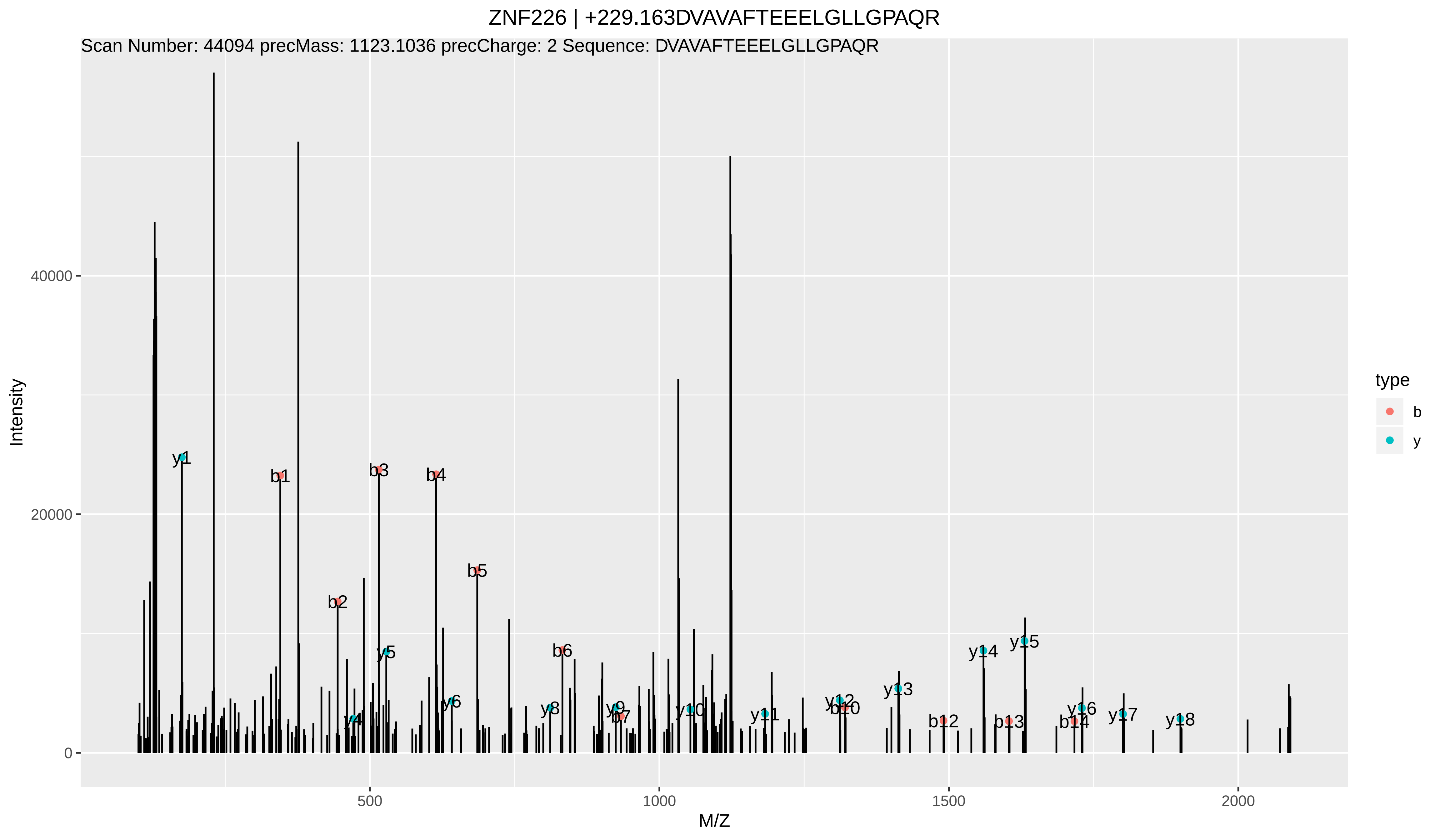
ZNF226 | +229.163DVAVAFTEEELGLLGPAQR
Scan Number: 44094 precMass: 1123.1036 precCharge: 2 Sequence: DVAVAFTEEELGLLGPAQR
Intensity
M/Z
type
b
y
y1
b1
b3
b4
b2
b5
y5
b6
y4
y6
y8
y9
b7
y10
y11
y12
b10
y13
b12
y14
y15
b13
b14
y16
y17
y18
40000
20000
0
500
1000
1500
2000

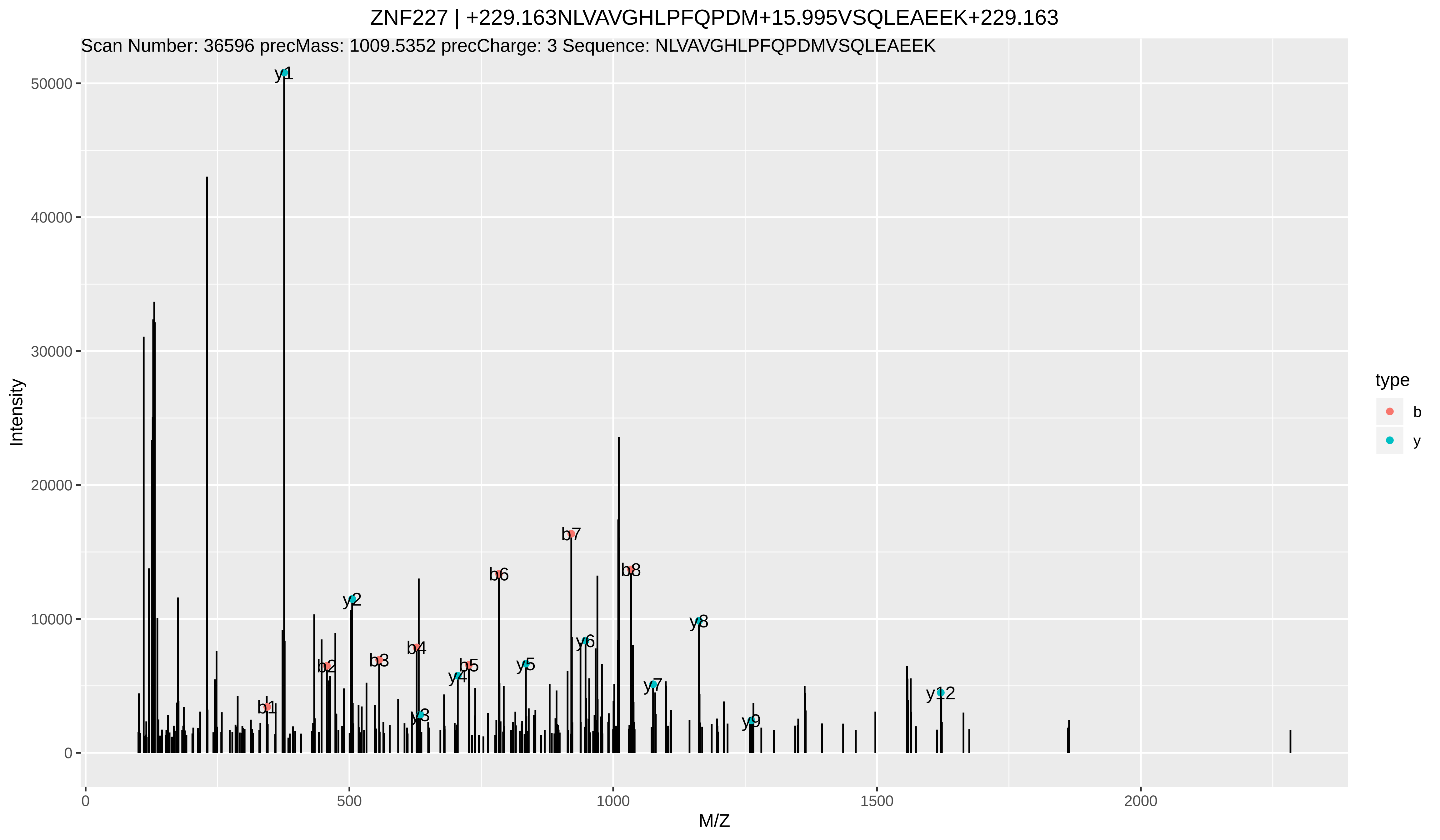
ZNF227 | +229.163NLVAVGHLPFQPDM+15.995VSQLEAEEK+229.163
Scan Number: 36596 precMass: 1009.5352 precCharge: 3 Sequence: NLVAVGHLPFQPDMVSQLEAEEK
Intensity
M/Z
type
b
y
y1
y2
y3
y4
y5
y6
y7
y8
y9
y12
b1
b2
b3
b4
b5
b6
b7
b8
0
10000
20000
30000
40000
50000
0
500
1000
1500
2000

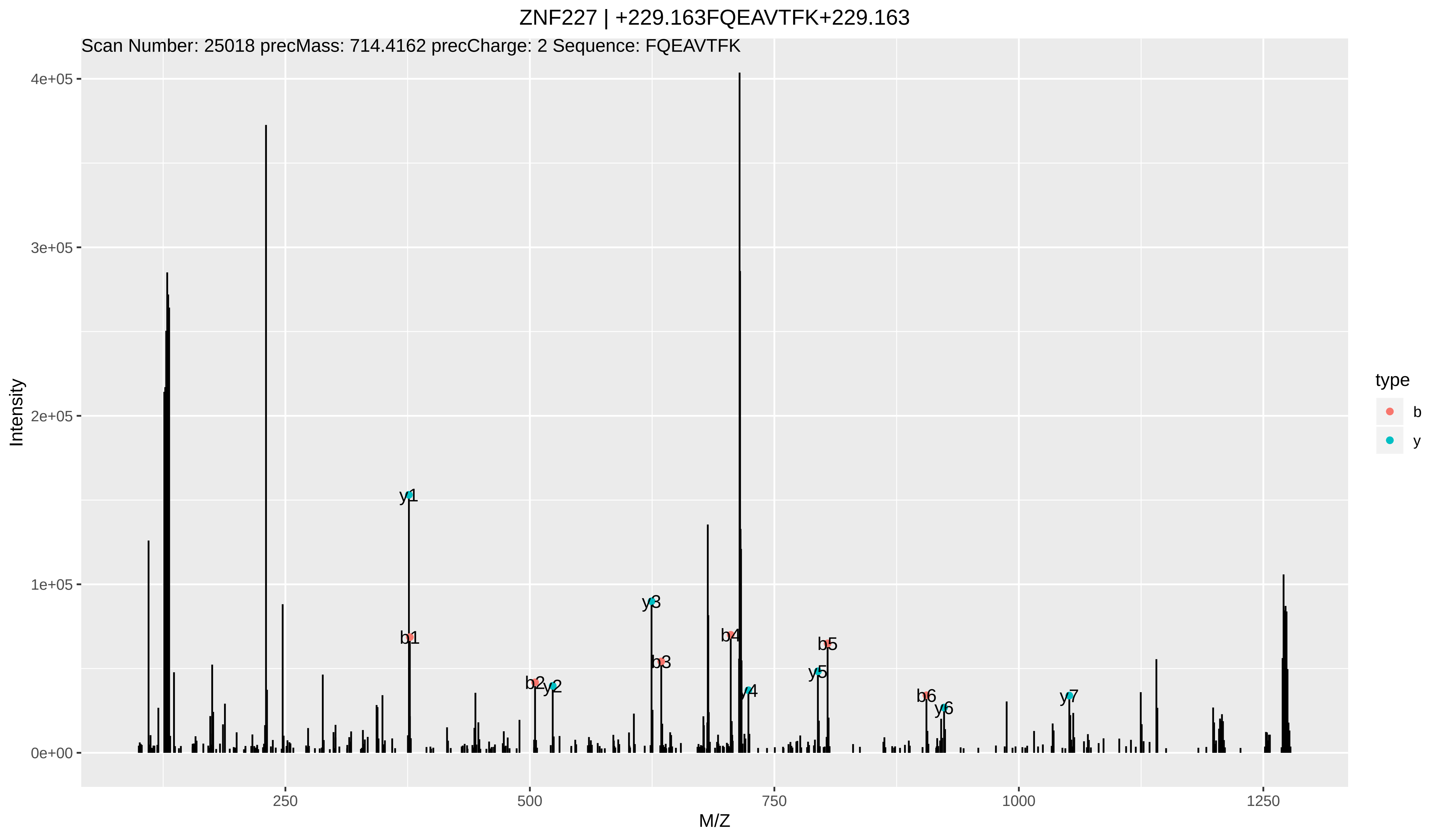
ZNF227 | +229.163FQEAVTFK+229.163
Scan Number: 25018 precMass: 714.4162 precCharge: 2 Sequence: FQEAVTFK
Intensity
M/Z
type
b
y
y1
b1
b2
y2
y3
b3
b4
y4
y5
b5
b6
y6
y7
0e+00
1e+05
2e+05
3e+05
4e+05
250
500
750
1000
1250

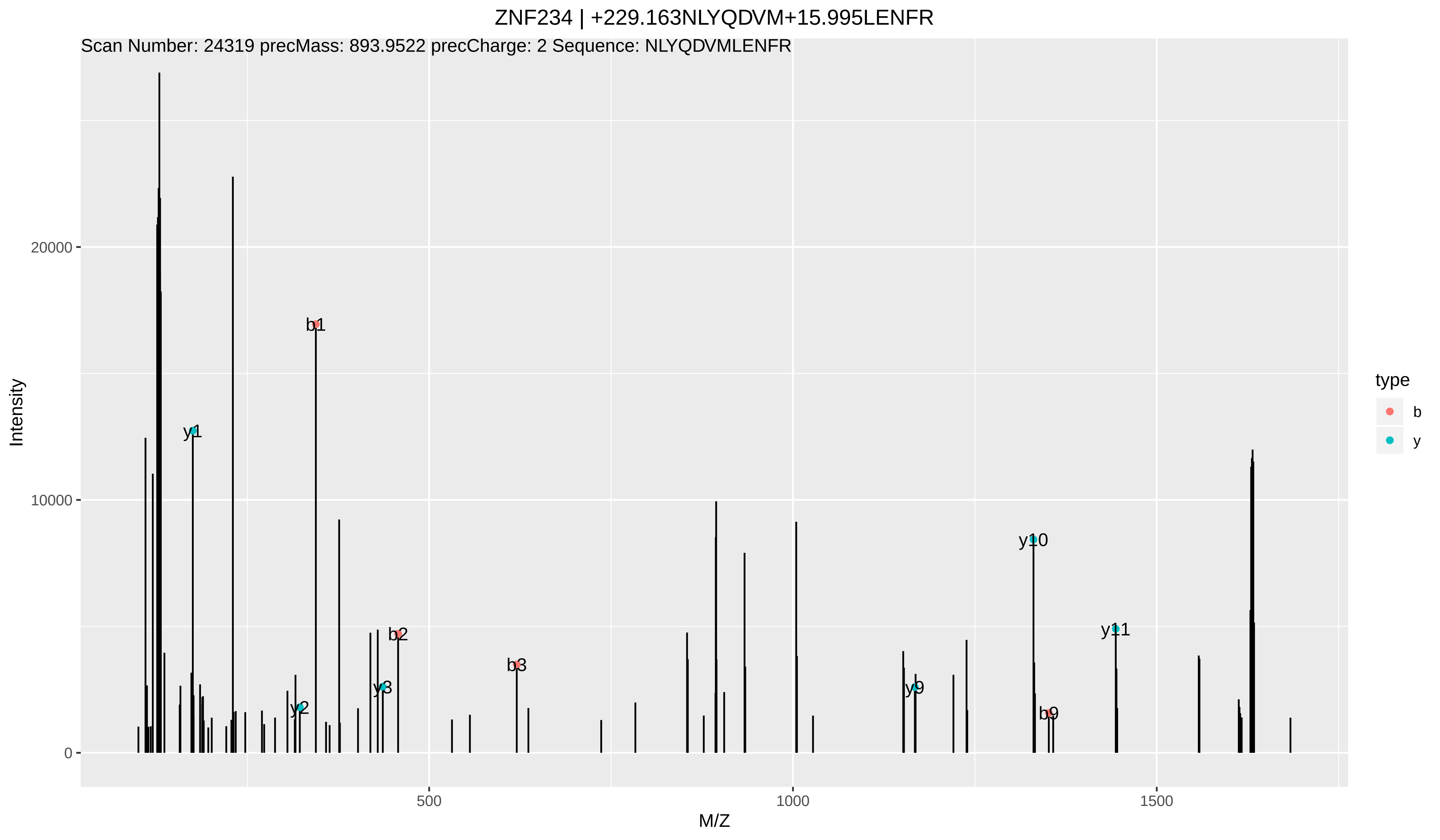

ZNF234 | +229.163NLYQDVM+15.995LENFR
Scan Number: 24319 precMass: 893.9522 precCharge: 2 Sequence: NLYQDVMLENFR
Intensity
20000
10000
0
500
1000
1500
M/Z
b1
y1
y2
y3
b2
b3
y9
y10
b9
y11
type
b
y

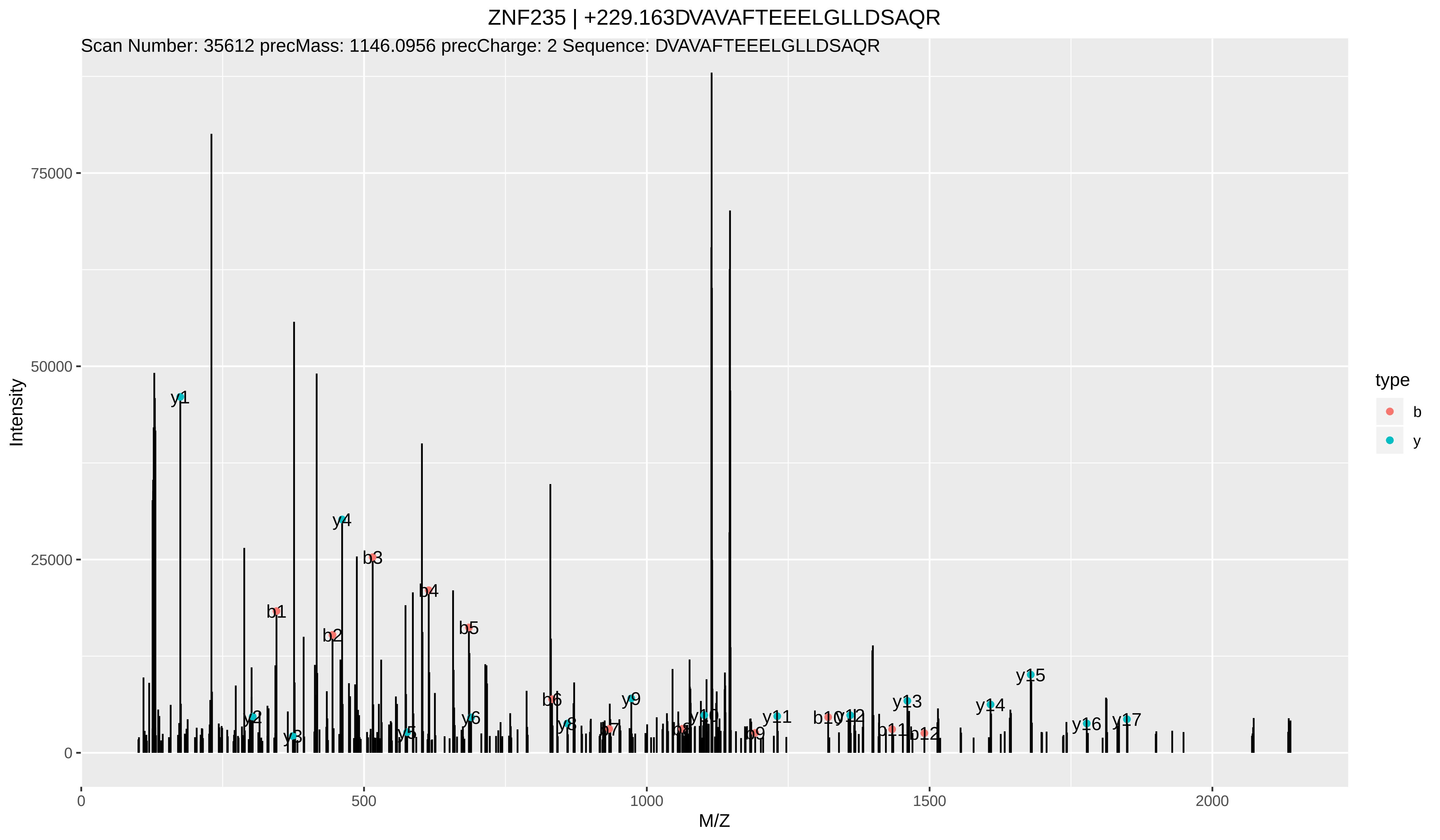
ZNF235 | +229.163DVAVAFTEEELGLLDSAQR
Scan Number: 35612 precMass: 1146.0956 precCharge: 2 Sequence: DVAVAFTEEELGLLDSAQR
Intensity
M/Z
type
b
y
y1
y2
y3
y4
y5
y6
y8
y9
y10
y11
y12
y13
y14
y15
y16
y17
b1
b2
b3
b4
b5
b6
b7
b8
b9
b10
b11
b12
0
25000
50000
75000
500
1000
1500
2000

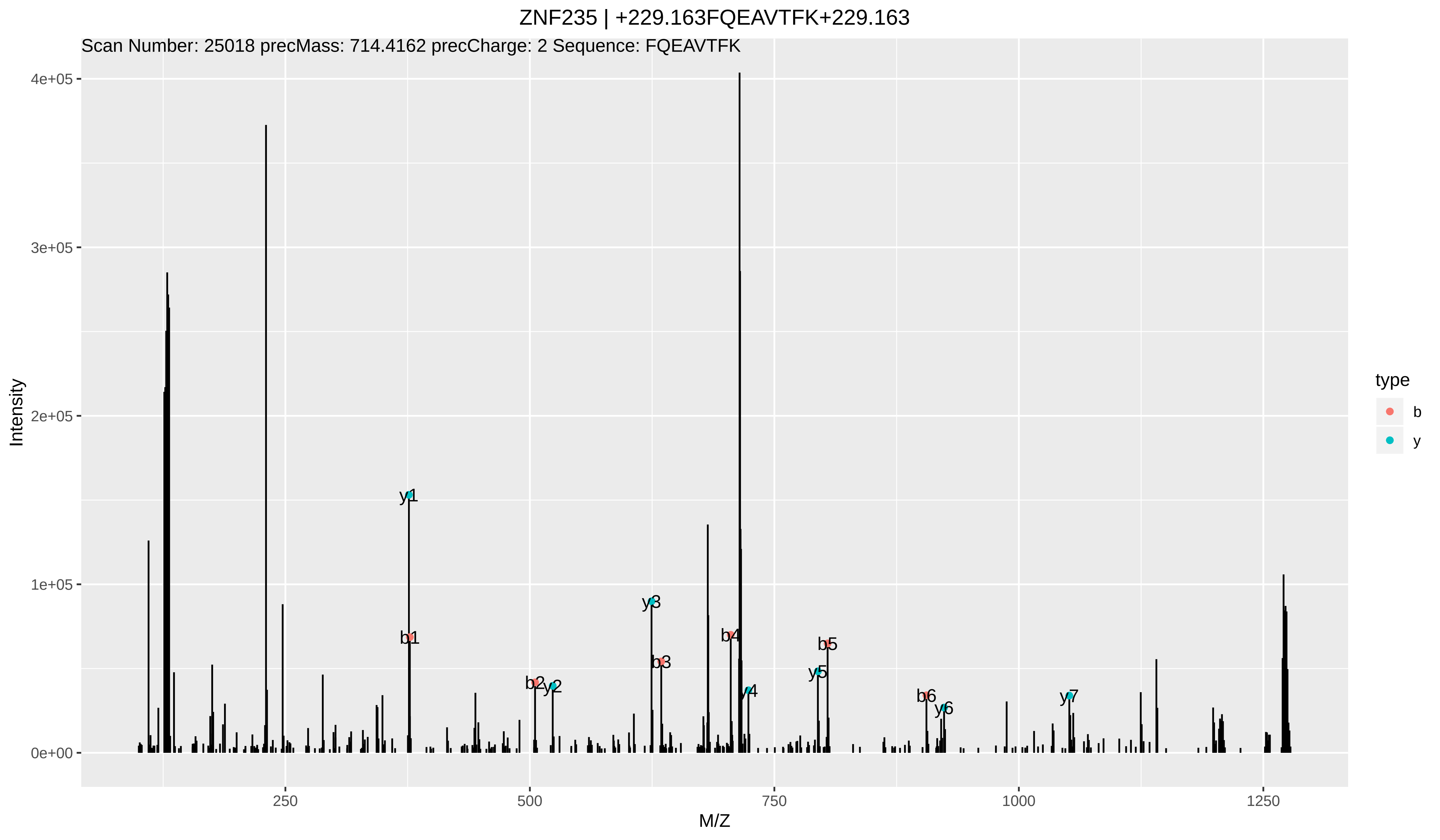
ZNF235 | +229.163FQEAVTFK+229.163
Scan Number: 25018 precMass: 714.4162 precCharge: 2 Sequence: FQEAVTFK
Intensity
M/Z
4e+05
3e+05
2e+05
1e+05
0e+00
250
500
750
1000
1250
y1
b1
b2
y2
y3
b3
b4
y4
y5
b5
b6
y6
y7
type
b
y

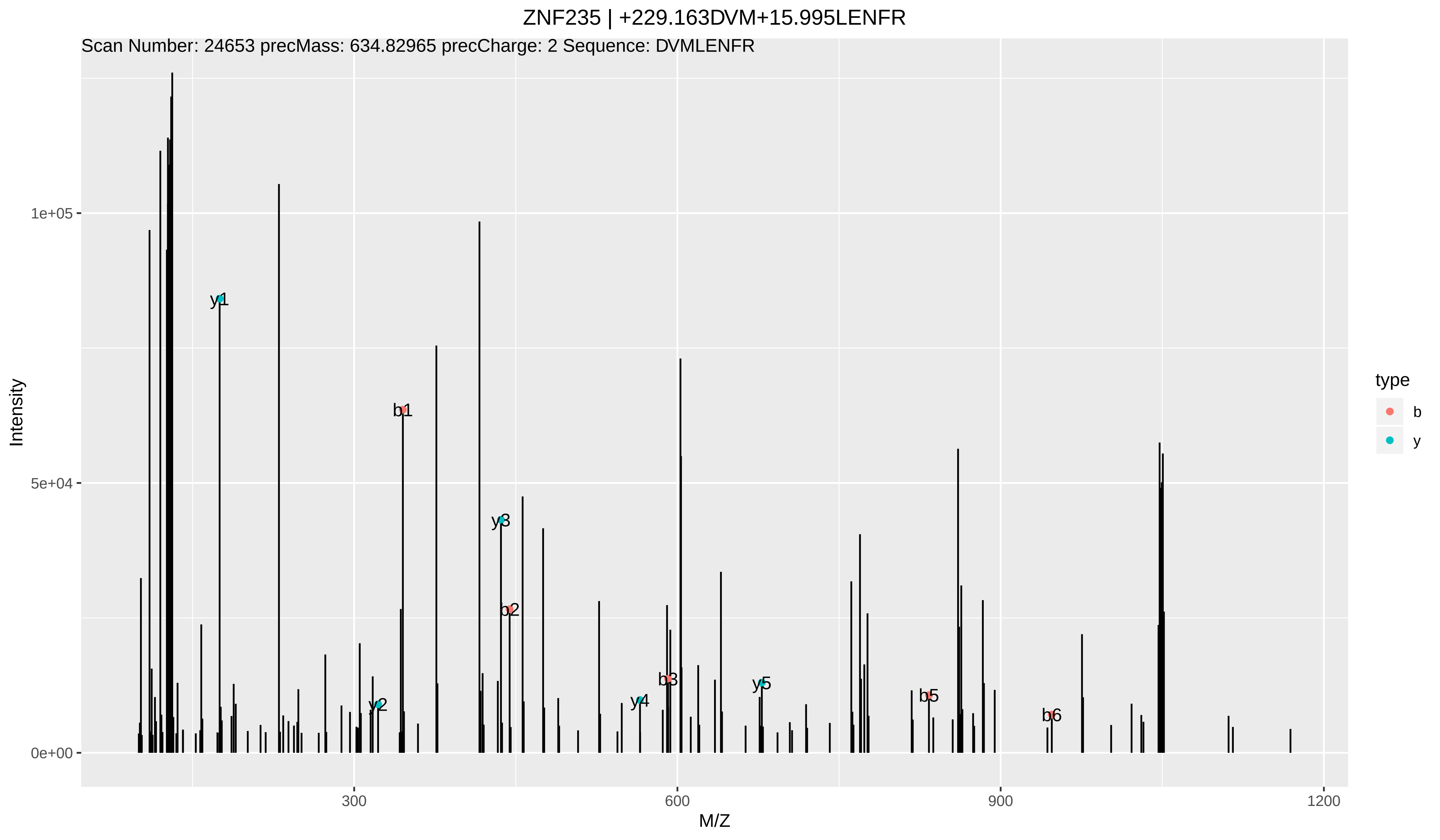
ZNF235 | +229.163DVM+15.995LENFR
Scan Number: 24653 precMass: 634.82965 precCharge: 2 Sequence: DVMLENFR
Intensity
M/Z
1e+05
5e+04
0e+00
300
600
900
1200
y1
b1
y3
b2
y2
y4
b3
y5
b5
b6
type
b
y

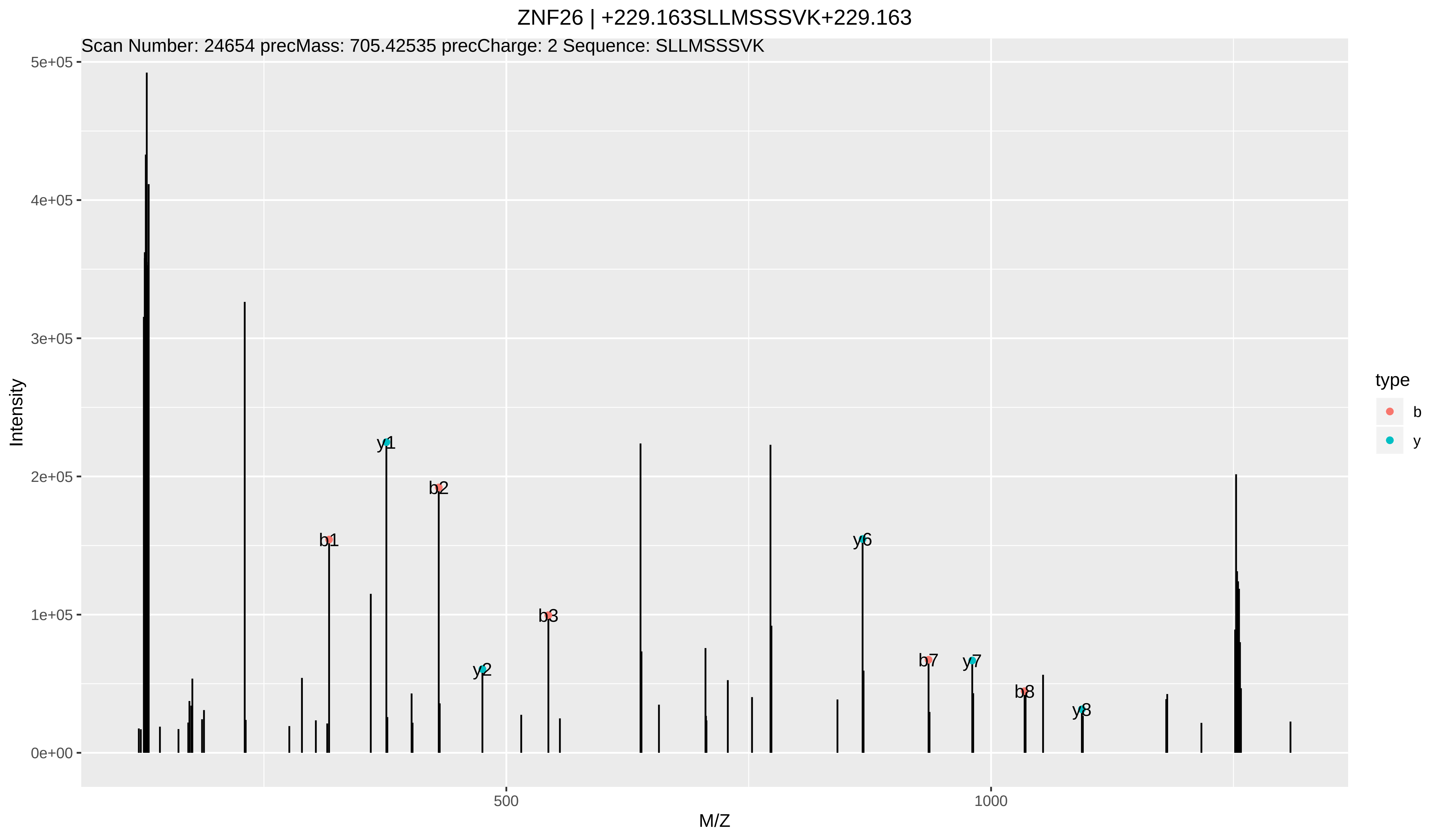

ZNF26 | +229.163SLLMSSSVK+229.163
Scan Number: 24654 precMass: 705.42535 precCharge: 2 Sequence: SLLMSSSVK
Intensity
5e+05
4e+05
3e+05
2e+05
1e+05
0e+00
500
1000
M/Z
y1
b2
b1
y6
b3
y2
b7
y7
b8
y8
type
b
y

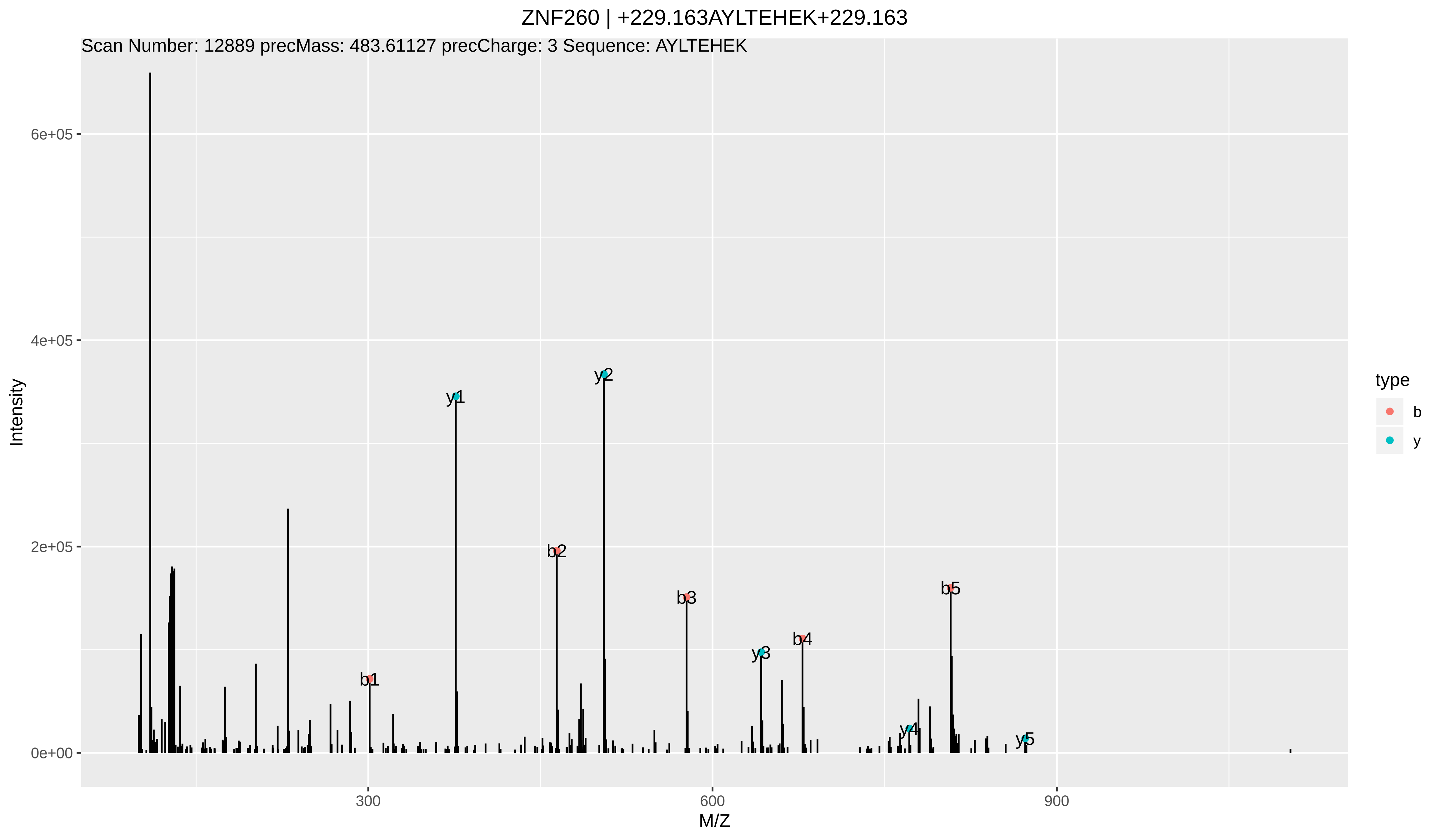

ZNF260 | +229.163AYLTEHEK+229.163
Scan Number: 12889 precMass: 483.61127 precCharge: 3 Sequence: AYLTEHEK
Intensity
6e+05
4e+05
2e+05
0e+00
M/Z
300
600
900
y1
y2
b1
b2
b3
y3
b4
y4
b5
y5
type
b
y

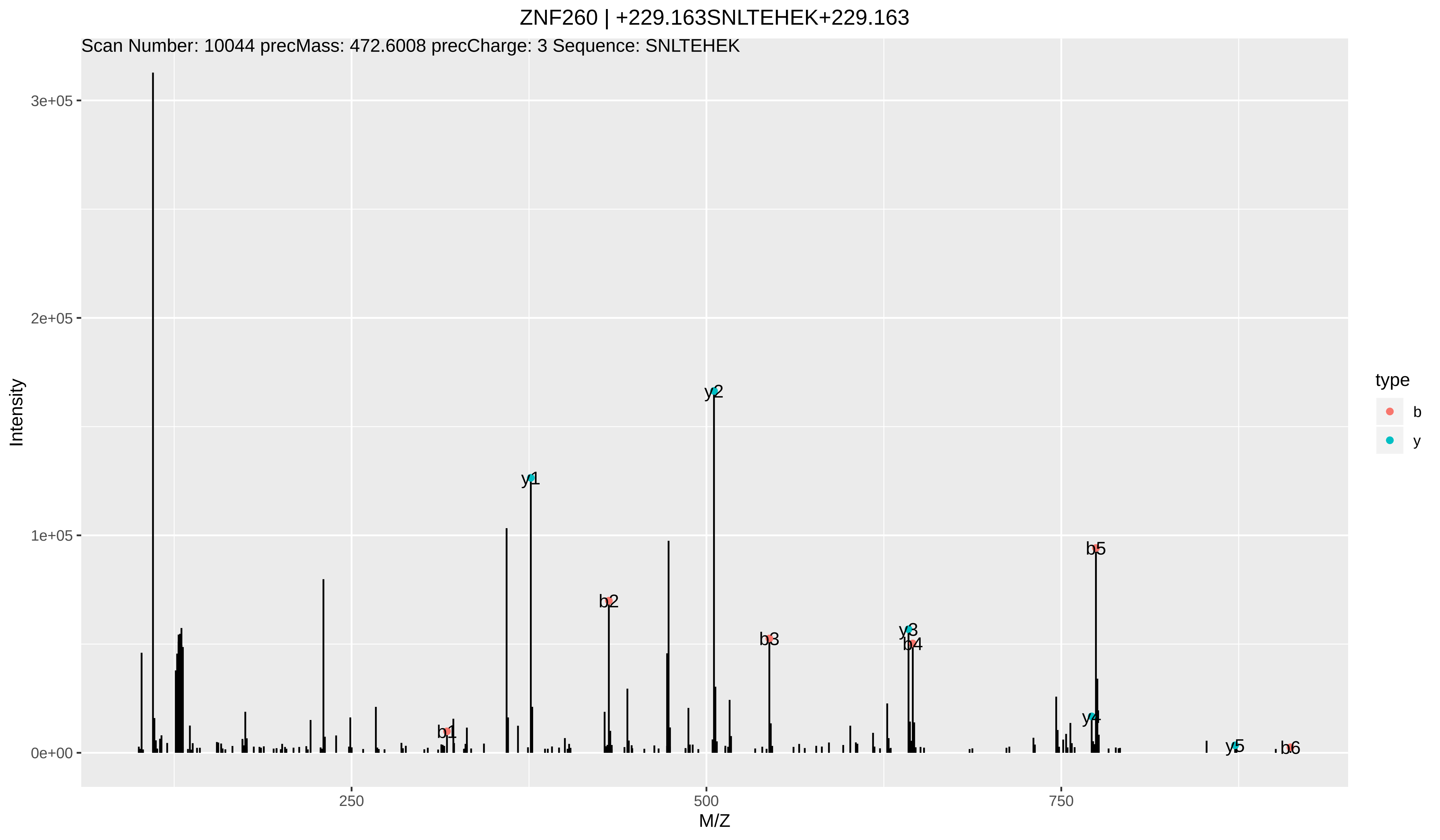
ZNF260 | +229.163SNLTEHEK+229.163
Scan Number: 10044 precMass: 472.6008 precCharge: 3 Sequence: SNLTEHEK
Intensity
3e+05
2e+05
1e+05
0e+00
250
500
750
M/Z
y1
y2
y3
y4
y5
b1
b2
b3
b4
b5
b6
type
b
y

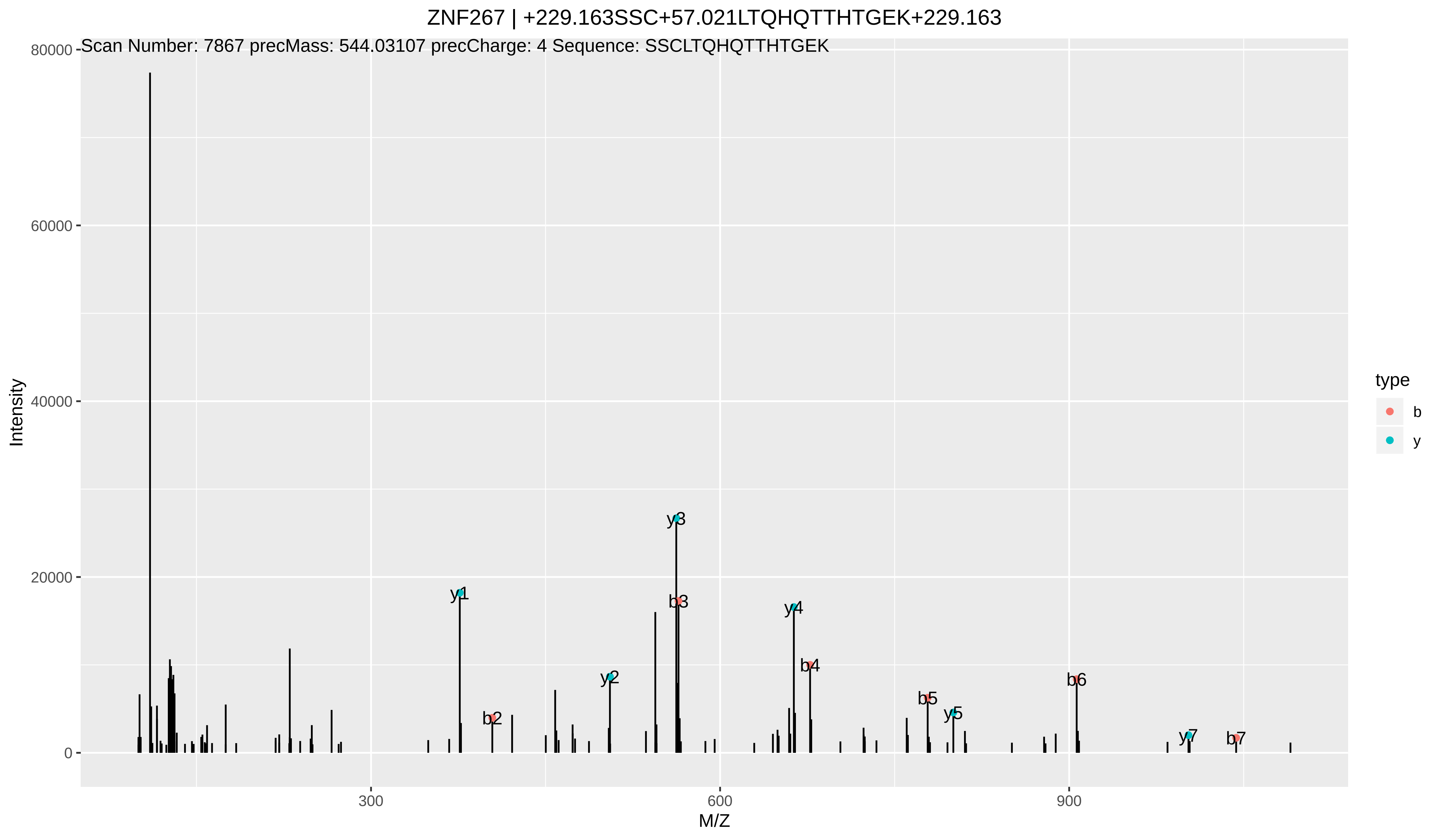

ZNF267 | +229.163SSC+57.021LTQHQTTHTGEK+229.163
Scan Number: 7867 precMass: 544.03107 precCharge: 4 Sequence: SSCLTQHQTTHTGEK
Intensity
M/Z
0
20000
40000
60000
80000
300
600
900
y1
y2
y3
y4
y5
y7
b2
b3
b4
b5
b6
b7
type
b
y

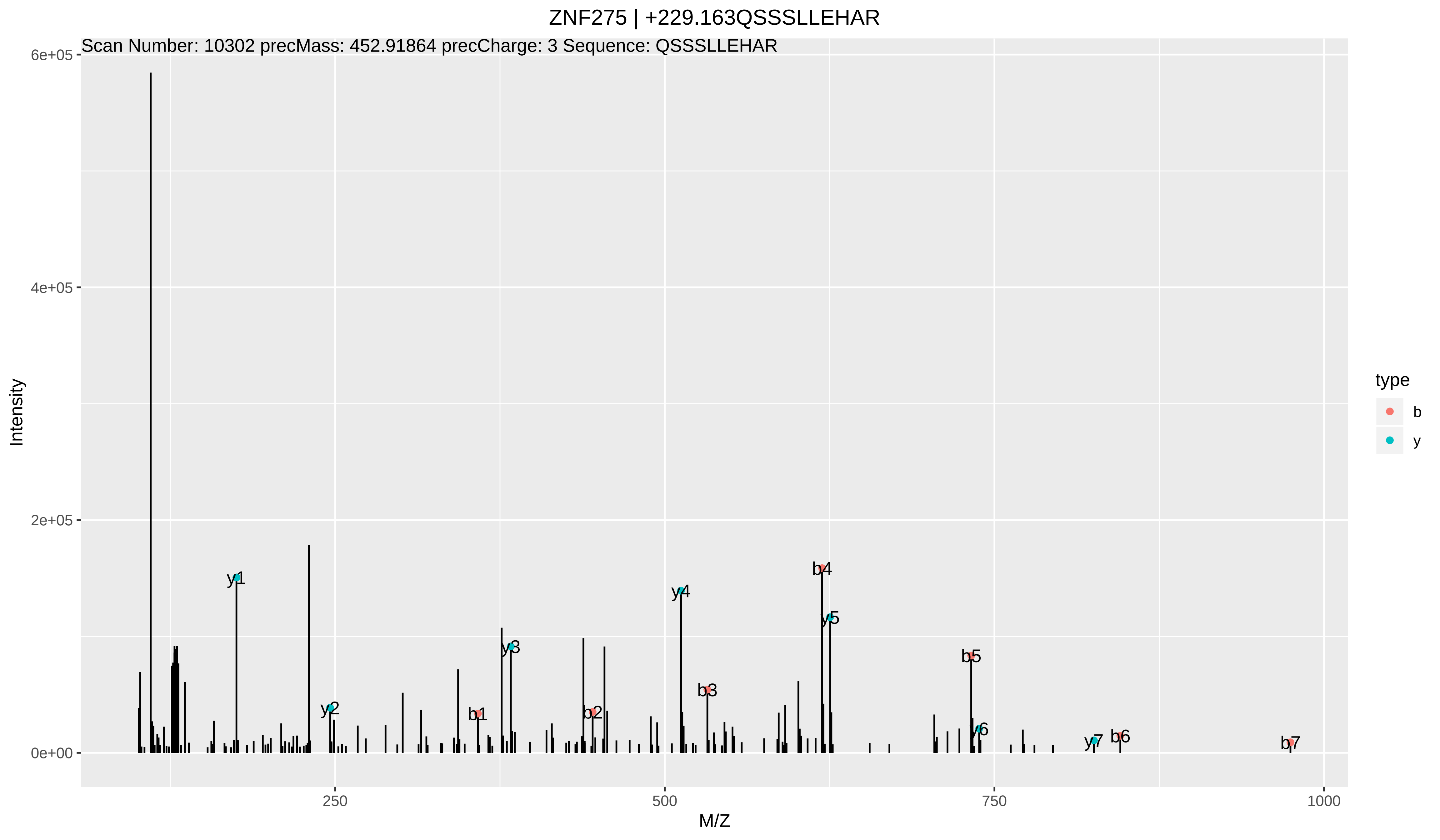

ZNF275 | +229.163QSSSLLEHAR
Scan Number: 10302 precMass: 452.91864 precCharge: 3 Sequence: QSSSLLEHAR
Intensity
M/Z
6e+05
4e+05
2e+05
0e+00
250
500
750
1000
y1
y2
b1
y3
b2
y4
b3
b4
y5
b5
y6
y7
b6
b7
type
b
y

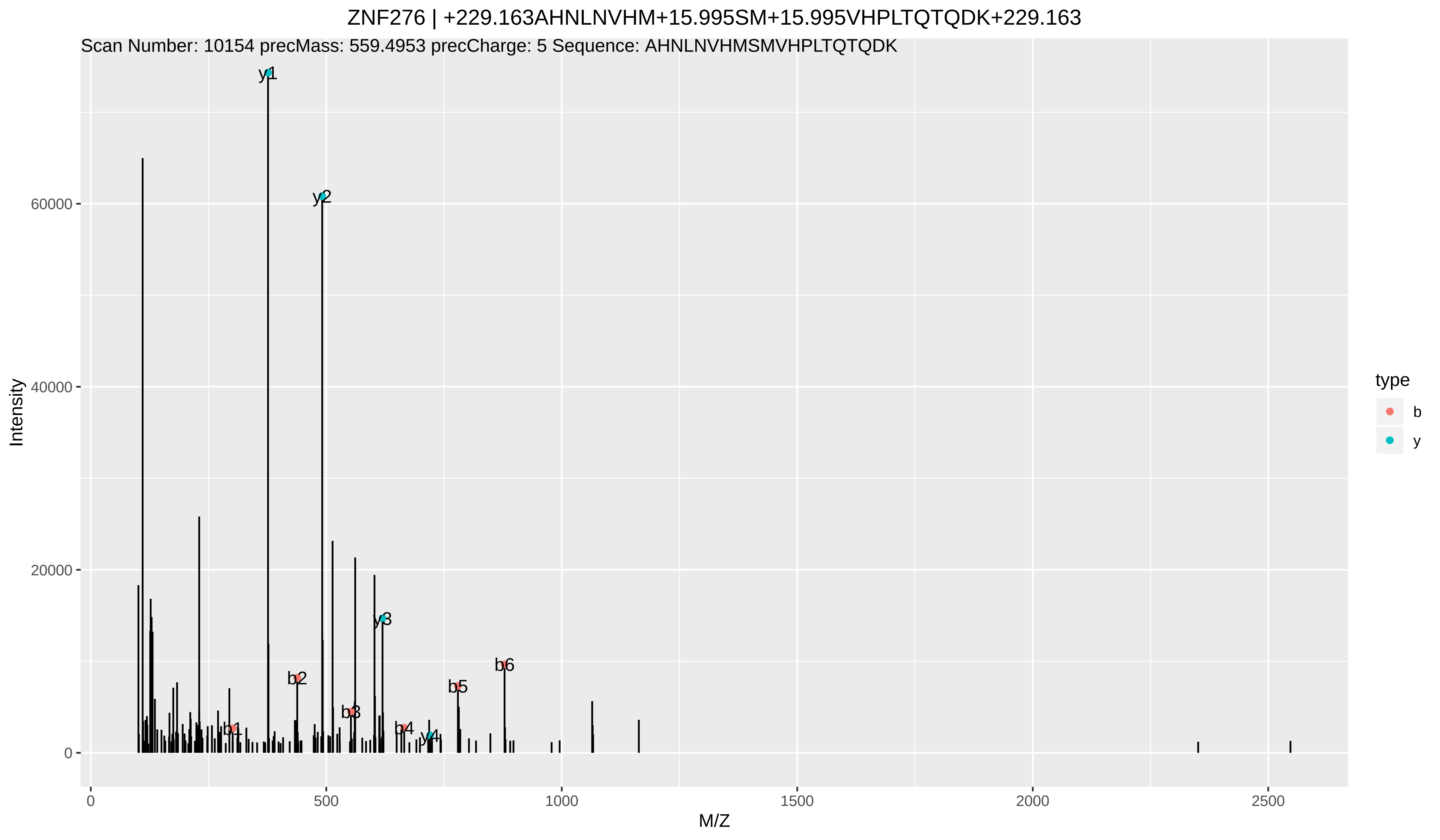
ZNF276 | +229.163AHNLNVHM+15.995SM+15.995VHPLTQTQDK+229.163
Scan Number: 10154 precMass: 559.4953 precCharge: 5 Sequence: AHNLNVHMSMVHPLTQTQDK
y1
y2
y3
y4
b1
b2
b3
b4
b5
b6
Intensity
M/Z
type
b
y
0
20000
40000
60000
0
500
1000
1500
2000
2500

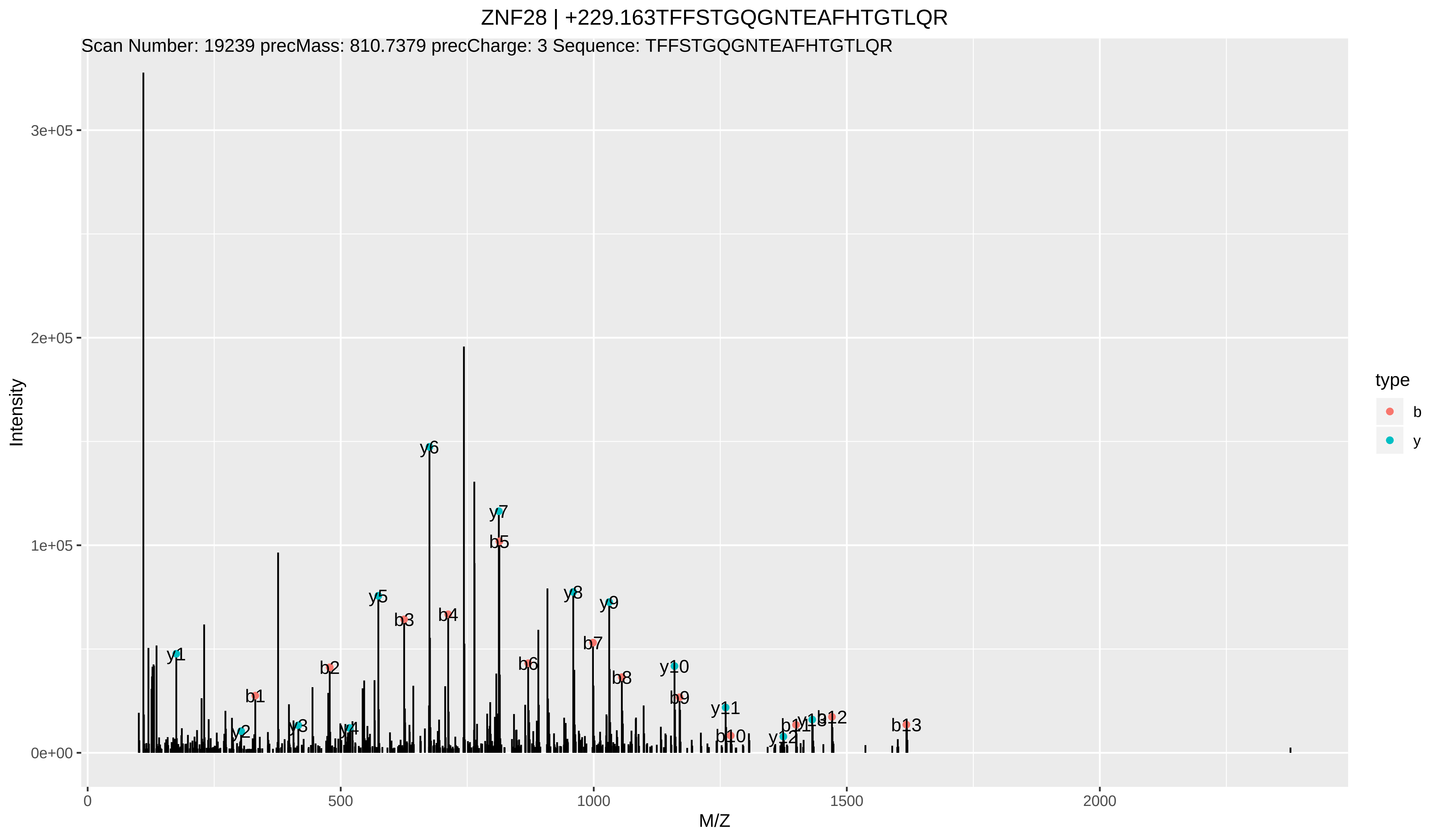
ZNF28 | +229.163TFFSTGQGNTEAFHTGTLQR
Scan Number: 19239 precMass: 810.7379 precCharge: 3 Sequence: TFFSTGQGNTEAFHTGTLQR
Intensity
M/Z
type
b
y
0e+00
1e+05
2e+05
3e+05
0
500
1000
1500
2000
y1
y2
y3
y4
y5
y6
y7
y8
y9
y10
y11
y12
b1
b2
b3
b4
b5
b6
b7
b8
b9
b10
b11
b12
b13

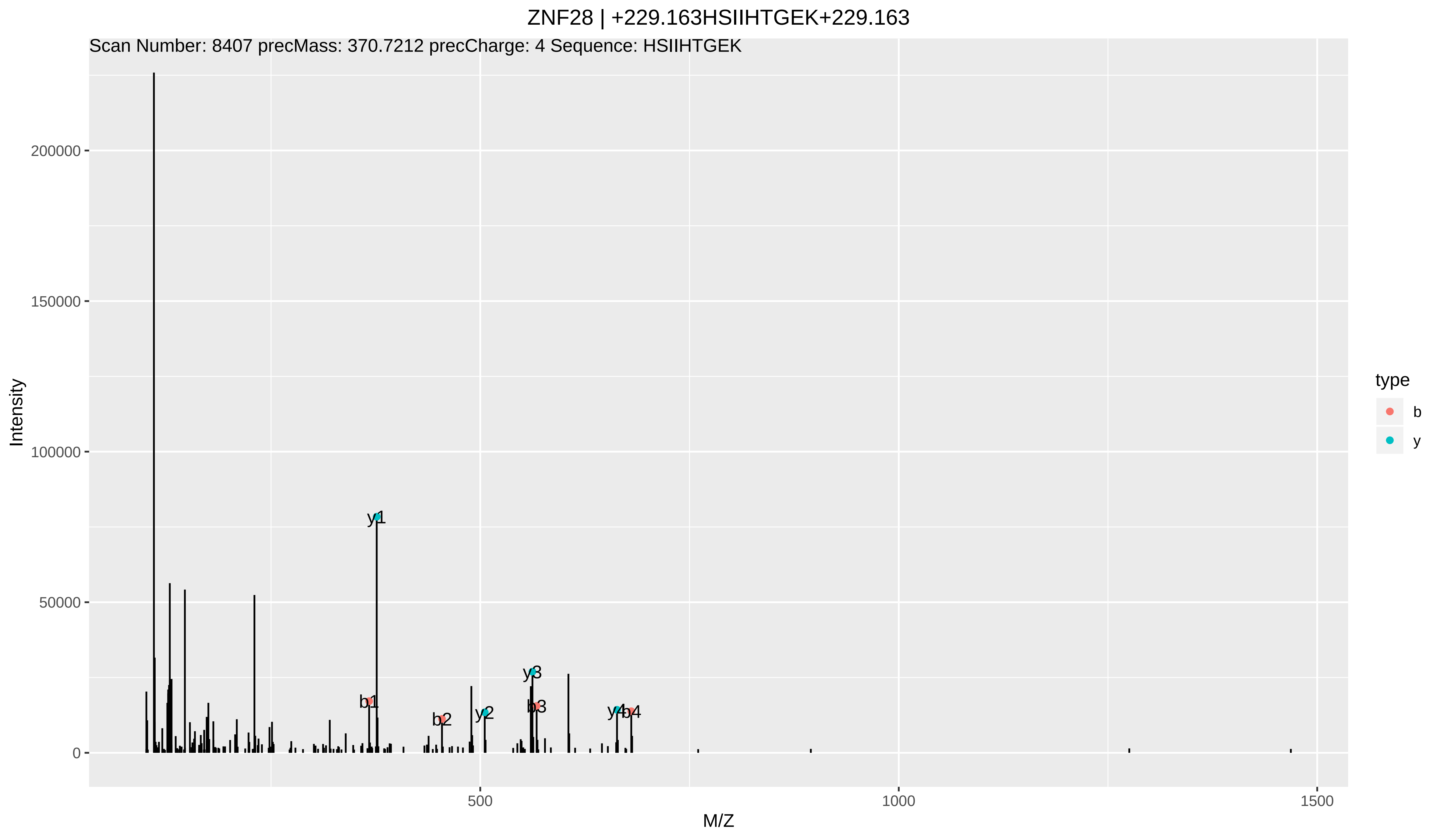

ZNF28 | +229.163HSIIHTGEK+229.163
Scan Number: 8407 precMass: 370.7212 precCharge: 4 Sequence: HSIIHTGEK
Intensity
200000
150000
100000
50000
0
M/Z
500
1000
1500
y1
b1
b2
y2
y3
b3
y4
b4
type
b
y

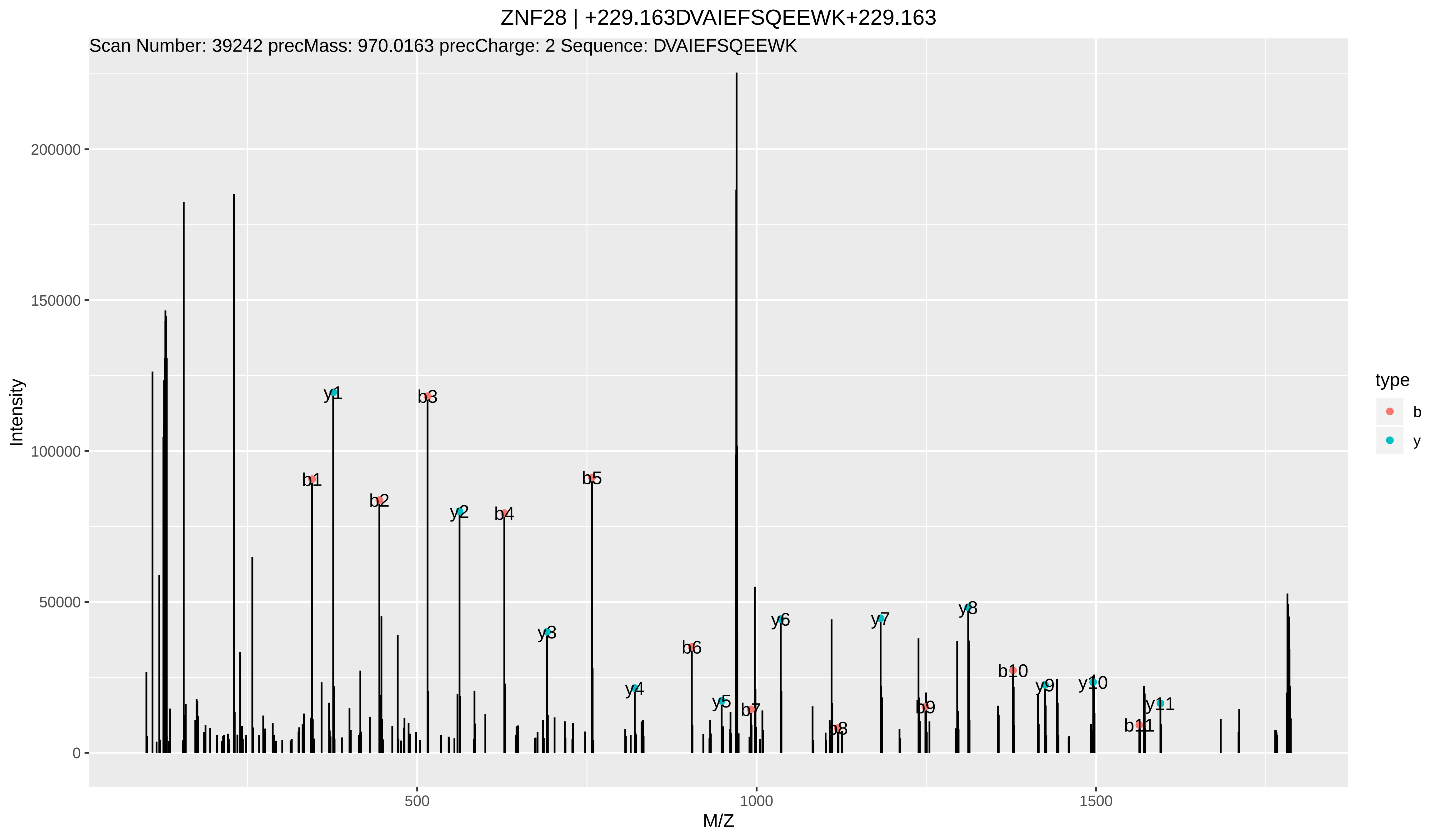

ZNF28 | +229.163DVAIEFSQEEWK+229.163
Scan Number: 39242 precMass: 970.0163 precCharge: 2 Sequence: DVAIEFSQEEWK
Intensity
M/Z
type
b
y
y1
b1
b2
b3
y2
b4
b5
y3
y4
b6
y5
b7
y6
b8
y7
b9
y8
b10
y9
y10
b11
y11

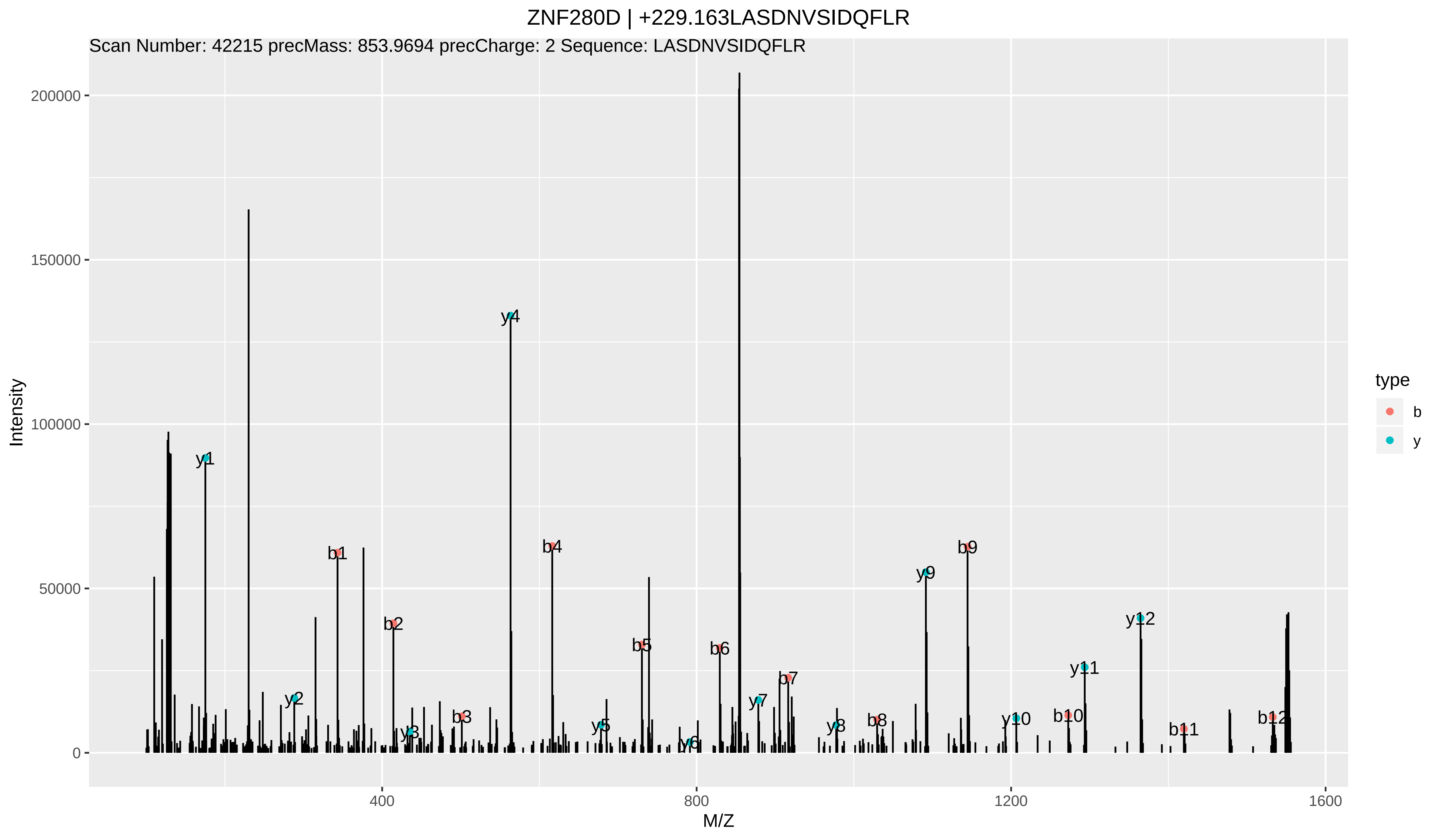
ZNF280D | +229.163LASDNVSIDQFLR
Scan Number: 42215 precMass: 853.9694 precCharge: 2 Sequence: LASDNVSIDQFLR
Intensity
M/Z
0
50000
100000
150000
200000
400
800
1200
1600
y1
y2
y3
y4
y5
y6
y7
y8
y9
y10
y11
y12
b1
b2
b3
b4
b5
b6
b7
b8
b9
b10
b11
b12
type
b
y

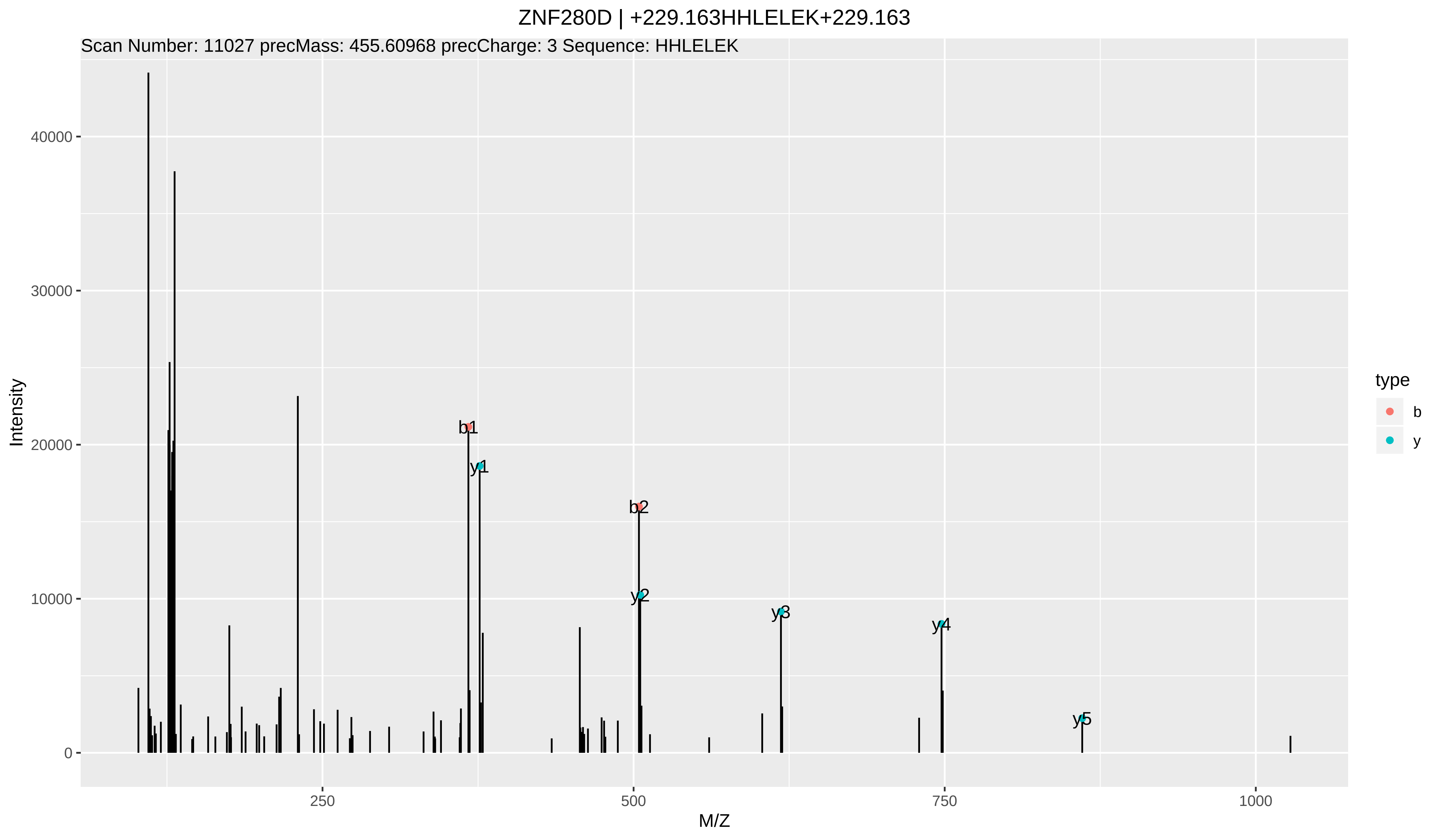

ZNF280D | +229.163HHLELEK+229.163
Scan Number: 11027 precMass: 455.60968 precCharge: 3 Sequence: HHLELEK
Intensity
M/Z
0
10000
20000
30000
40000
250
500
750
1000
b1
y1
b2
y2
y3
y4
y5
type
b
y

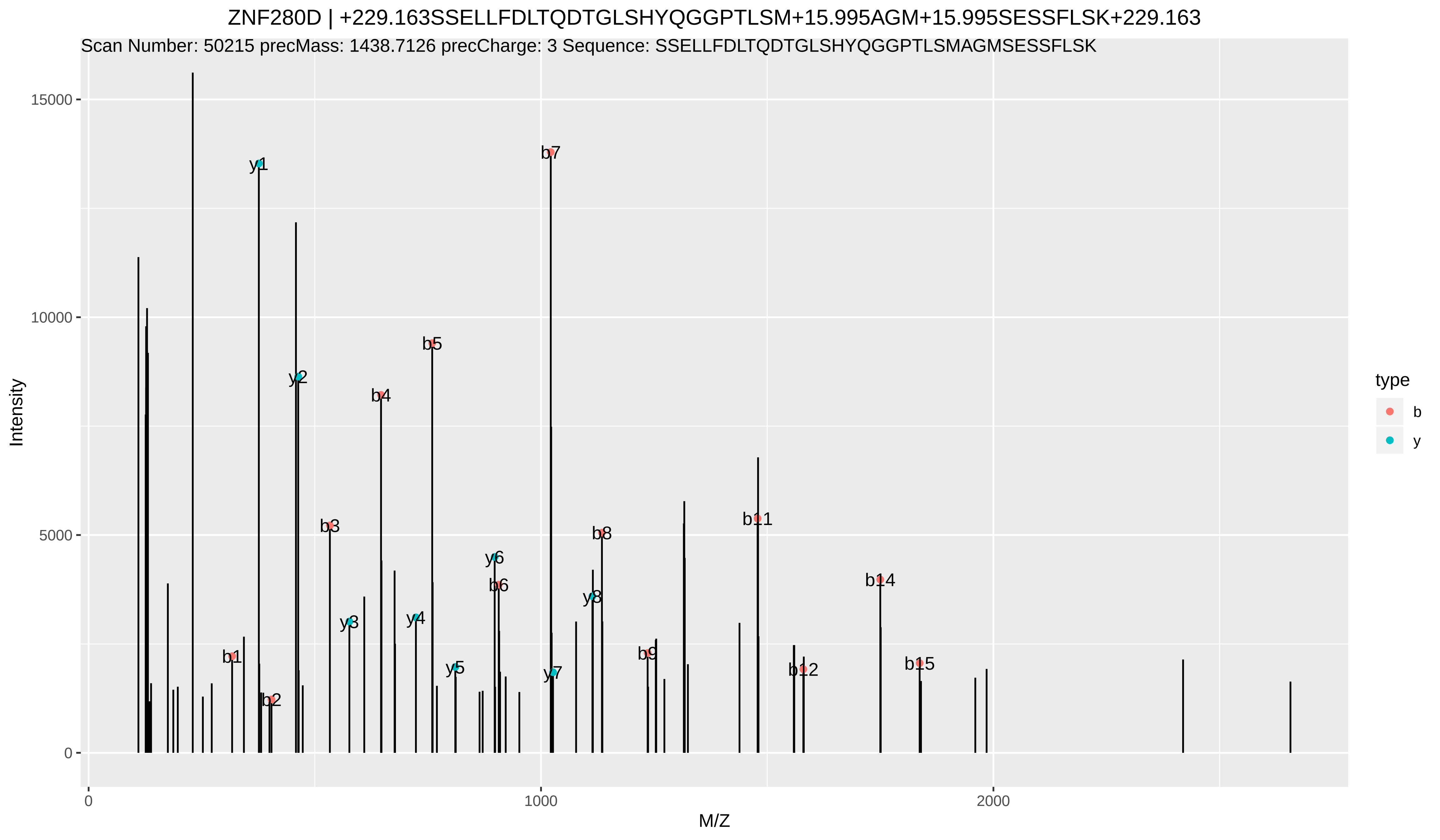

ZNF280D | +229.163SSELLFDLTQDTGLSHYQGGPTLSM+15.995AGM+15.995SESSFLSK+229.163
Scan Number: 50215 precMass: 1438.7126 precCharge: 3 Sequence: SSELLFDLTQDTGLSHYQGGPTLSMAGMSESSFLSK
Intensity
M/Z
type
b
y
y1
y2
y3
y4
y5
y6
y7
y8
b1
b2
b3
b4
b5
b6
b7
b8
b9
b11
b12
b14
b15
15000
10000
5000
0
1000
2000

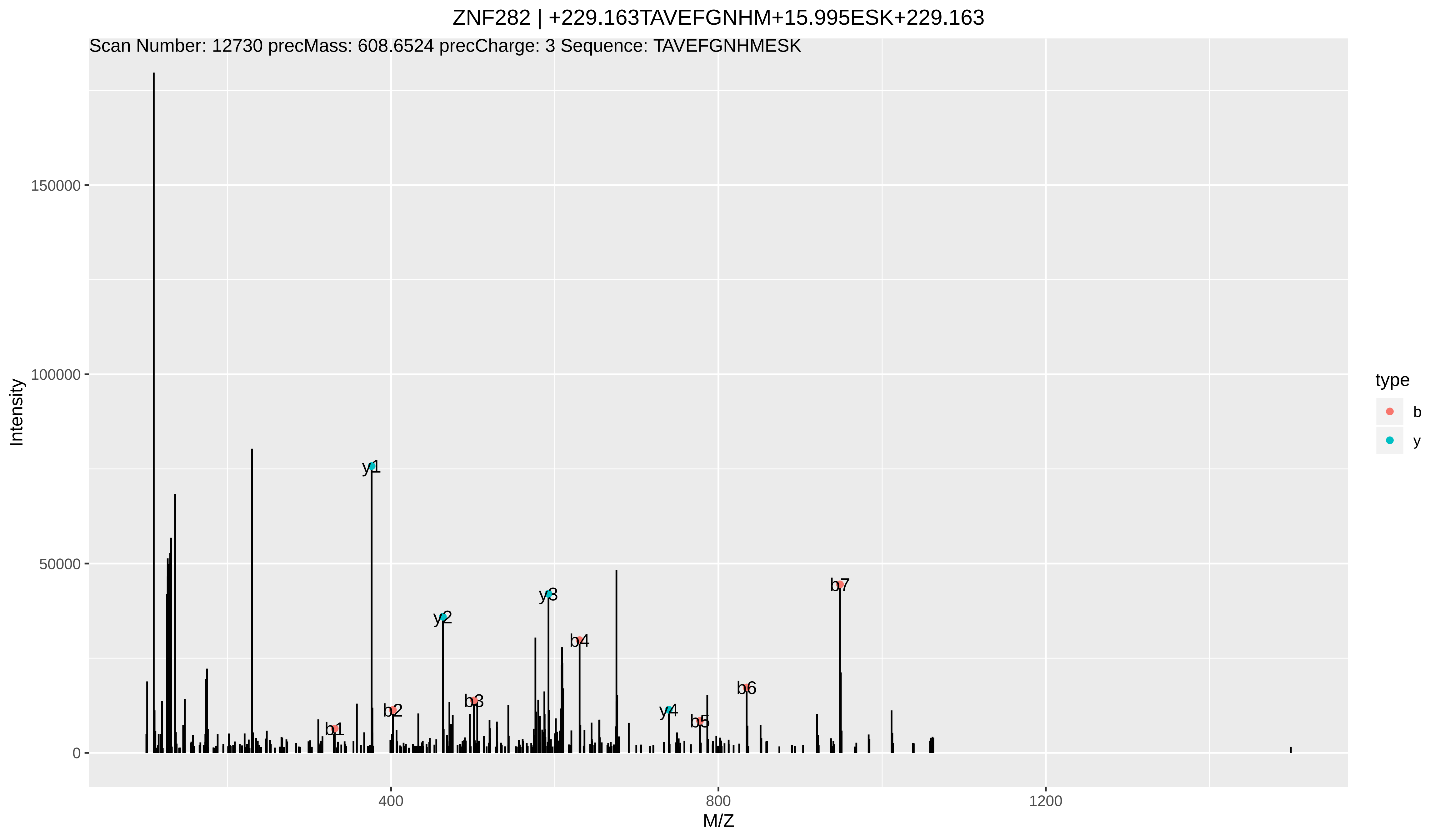
ZNF282 | +229.163TAVEFGNHM+15.995ESK+229.163
Scan Number: 12730 precMass: 608.6524 precCharge: 3 Sequence: TAVEFGNHMESK
Intensity
150000
100000
50000
0
400
800
1200
M/Z
type
b
y
y1
y2
y3
y4
b1
b2
b3
b4
b5
b6
b7

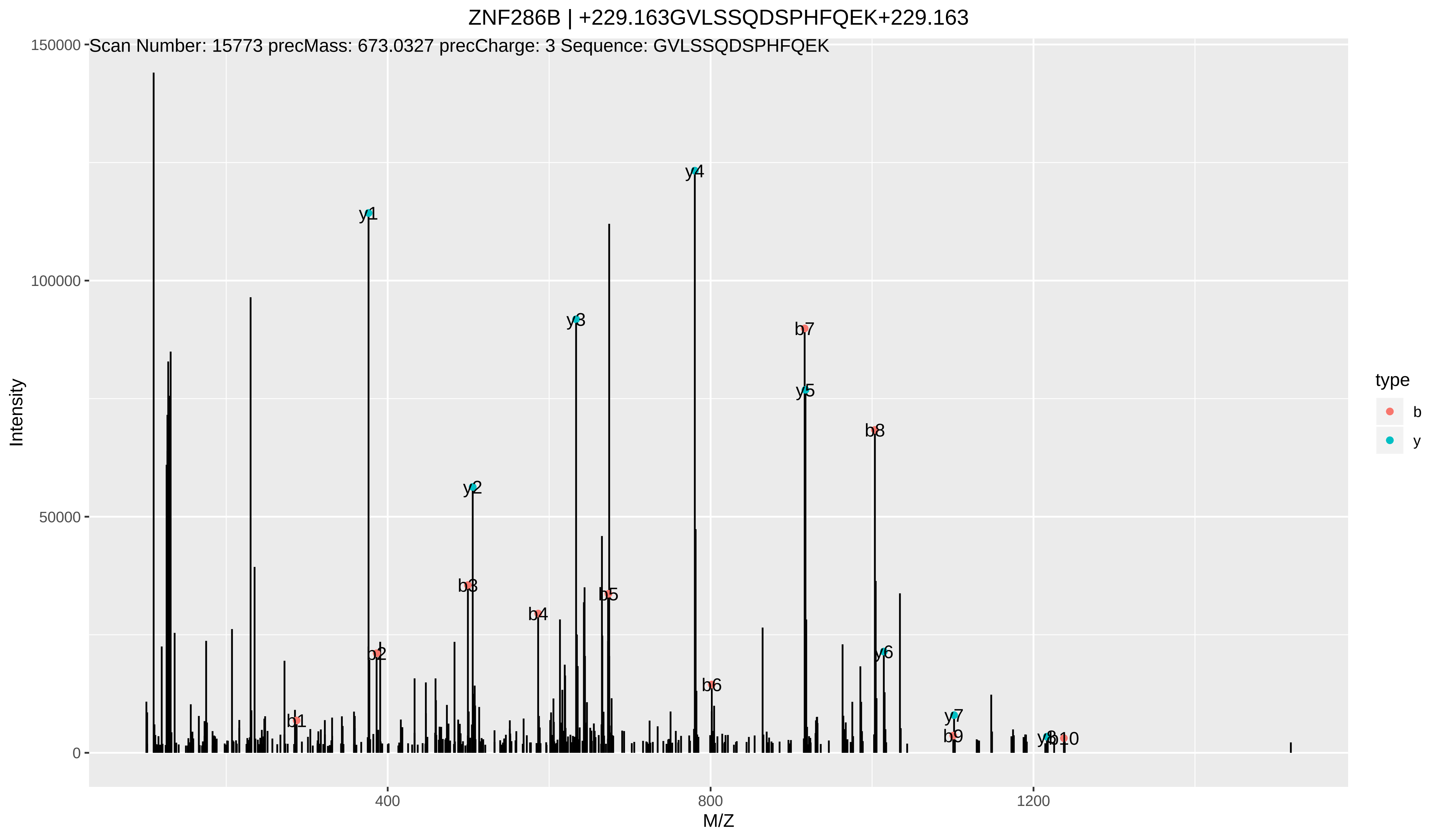

ZNF286B | +229.163GVLSSQDSPHFQEK+229.163
Scan Number: 15773 precMass: 673.0327 precCharge: 3 Sequence: GVLSSQDSPHFQEK
Intensity
M/Z
type
b
y
y1
y2
y3
y4
y5
y6
y7
y8
b1
b2
b3
b4
b5
b6
b7
b8
b9
b10
0
50000
100000
150000
400
800
1200

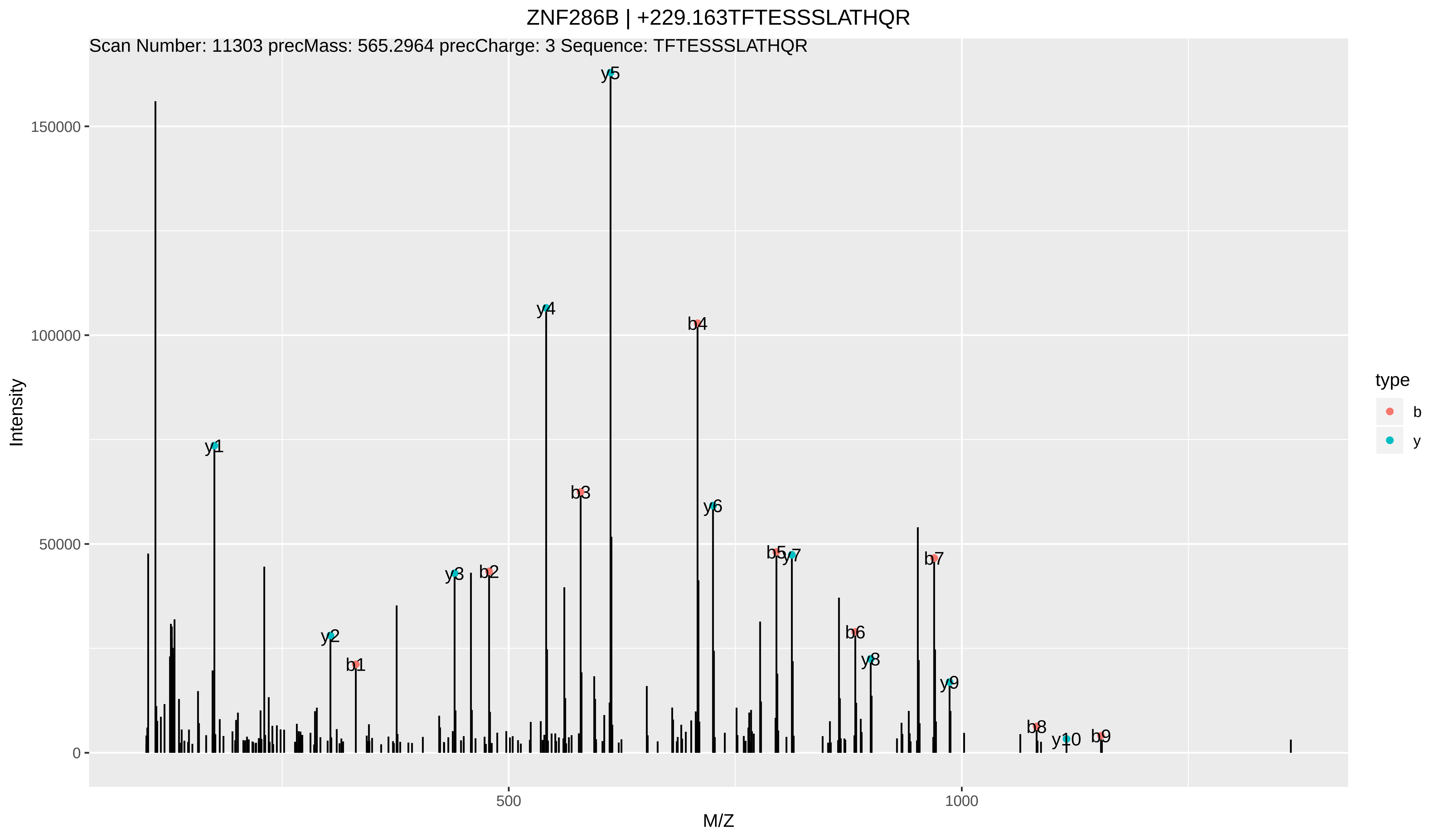
ZNF286B | +229.163TFTESSSLATHQR
Scan Number: 11303 precMass: 565.2964 precCharge: 3 Sequence: TFTESSSLATHQR
Intensity
M/Z
type
b
y
y5
y4
b4
y1
b3
y6
b5
y7
b7
y3
b2
y2
b1
b6
y8
y9
b8
y10
b9
0
50000
100000
150000
500
1000

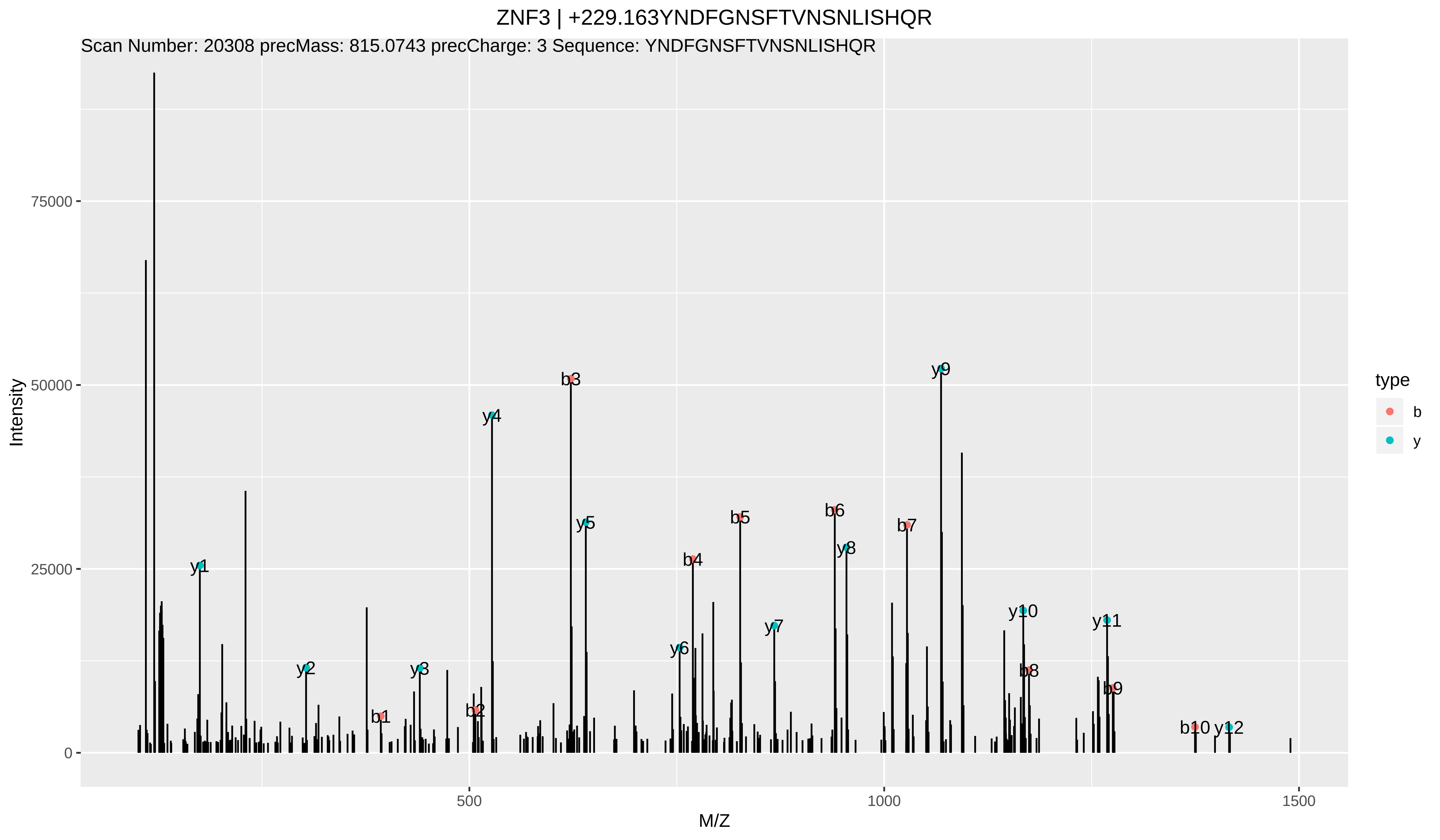
ZNF3 | +229.163YNDFGNSFTVNSNLISHQR
Scan Number: 20308 precMass: 815.0743 precCharge: 3 Sequence: YNDFGNSFTVNSNLISHQR
Intensity
75000
50000
25000
0
M/Z
500
1000
1500
type
b
y
y1
y2
y3
y4
y5
y6
y7
y8
y9
y10
y11
y12
b1
b2
b3
b4
b5
b6
b7
b8
b9
b10

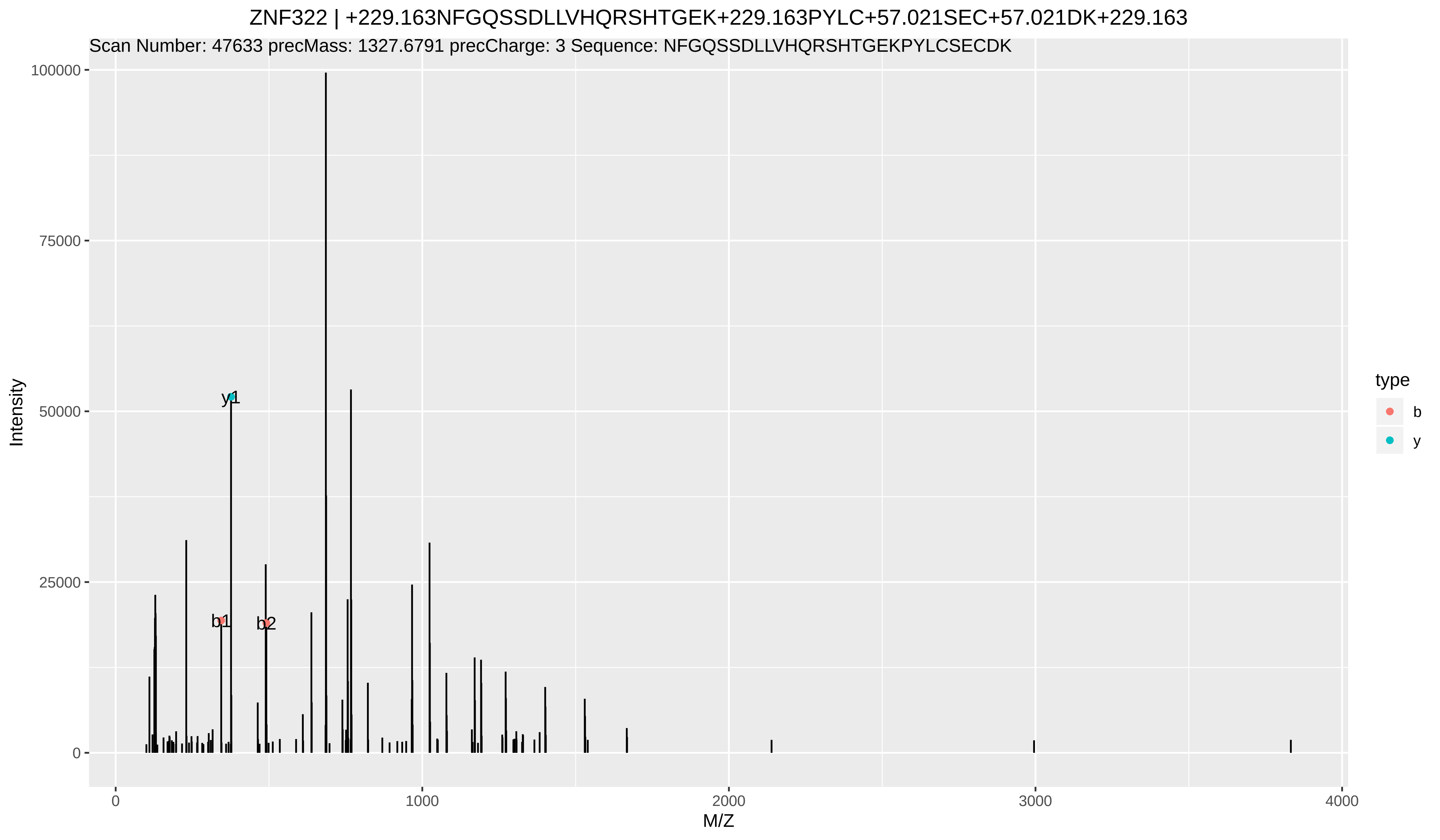

ZNF322 | +229.163NFGQSSDLLVHQRSHTGEK+229.163PYLC+57.021SEC+57.021DK+229.163
Scan Number: 47633 precMass: 1327.6791 precCharge: 3 Sequence: NFGQSSDLLVHQRSHTGEKPYLCSECDK
Intensity
100000
75000
50000
25000
0
0
1000
2000
3000
4000
M/Z
y1
b1
b2
type
b
y

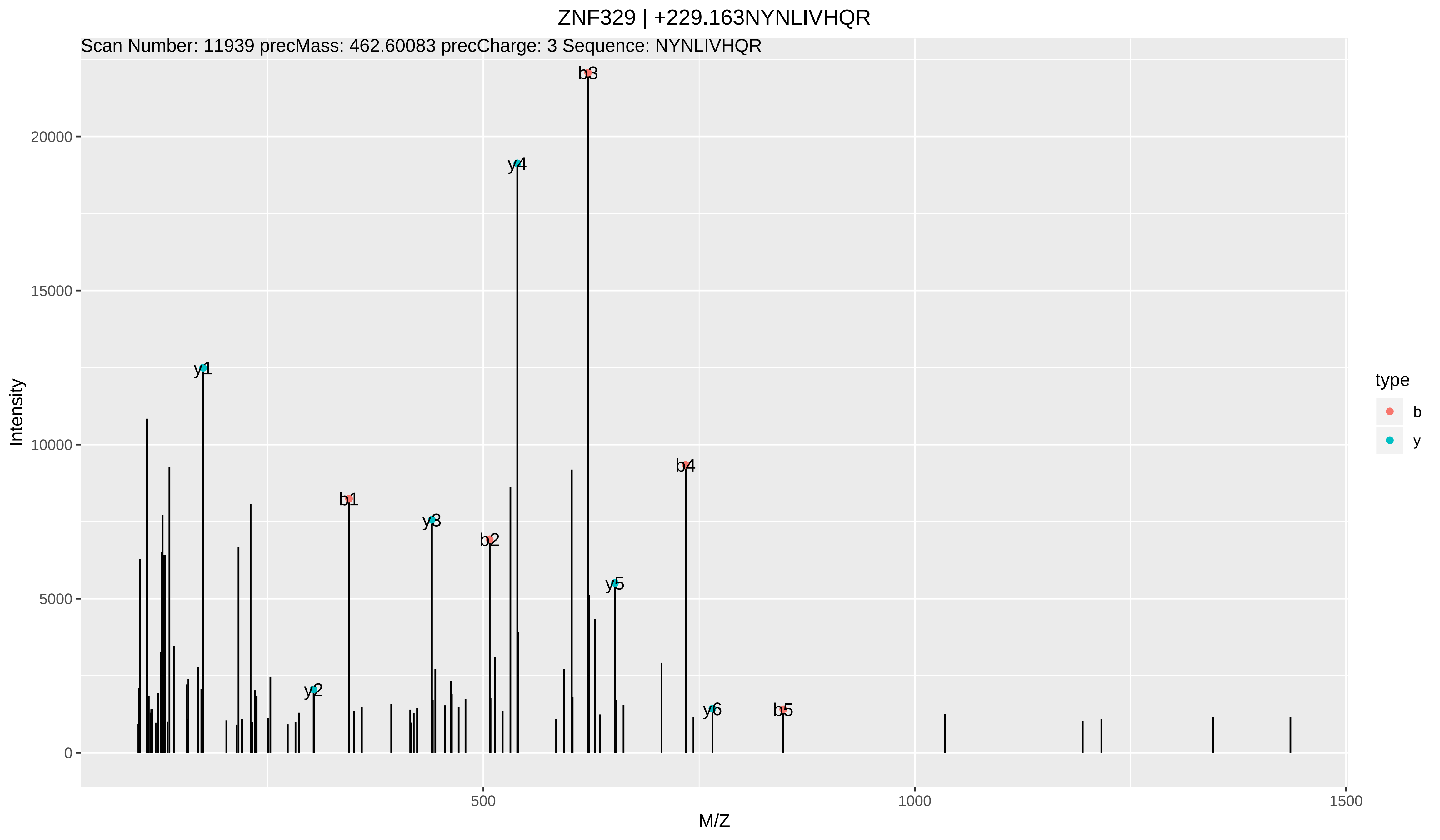

ZNF329 | +229.163NYNLIVHQR
Scan Number: 11939 precMass: 462.60083 precCharge: 3 Sequence: NYNLIVHQR
Intensity
M/Z
0
5000
10000
15000
20000
500
1000
1500
b3
y4
y1
b4
b1
y3
b2
y5
y2
y6
b5
type
b
y

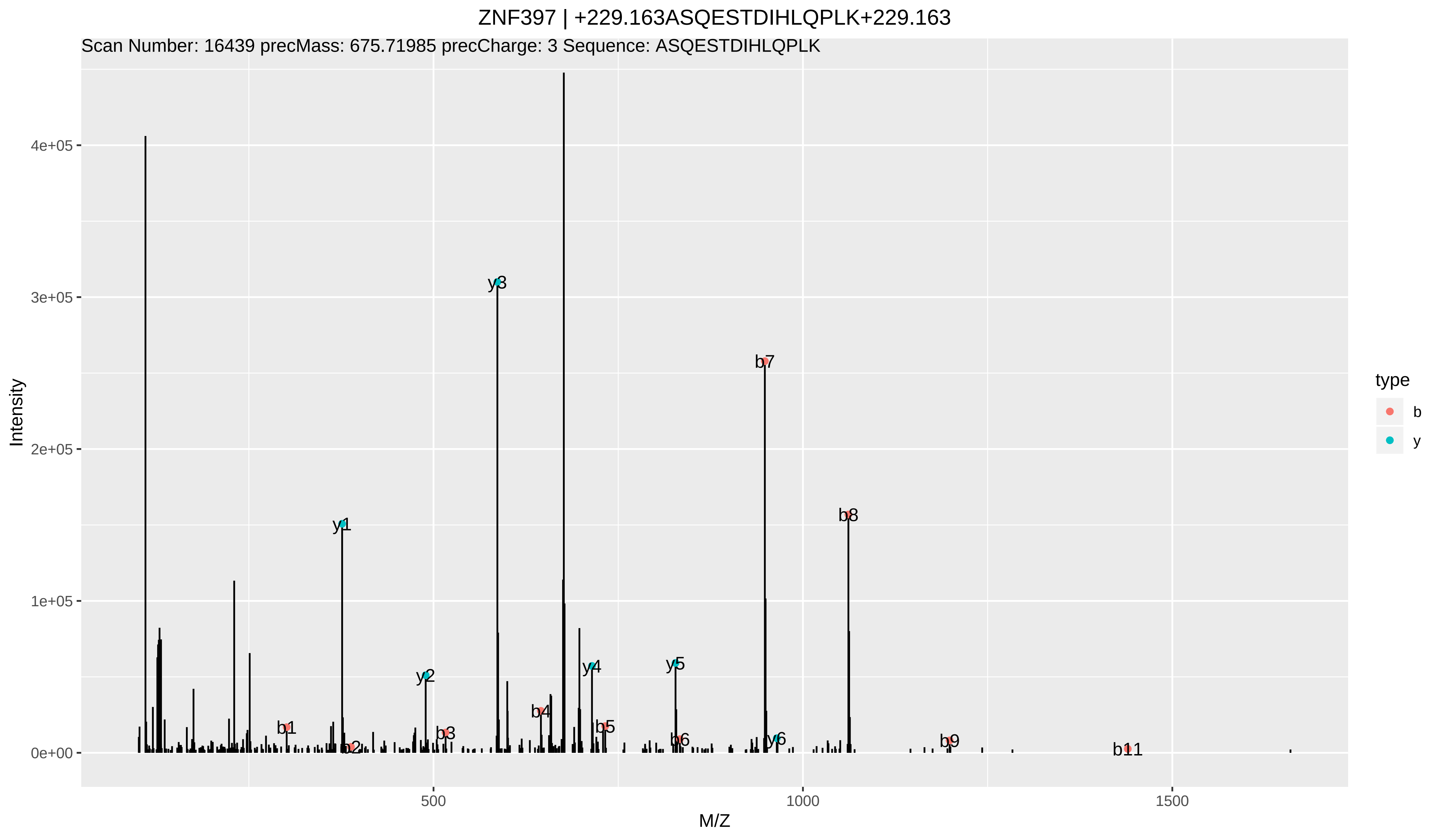

ZNF397 | +229.163ASQESTDIHLQPLK+229.163
Scan Number: 16439 precMass: 675.71985 precCharge: 3 Sequence: ASQESTDIHLQPLK
Intensity
M/Z
0e+00
1e+05
2e+05
3e+05
4e+05
500
1000
1500
y1
y2
y3
y4
y5
y6
b1
b2
b3
b4
b5
b6
b7
b8
b9
b11
type
b
y

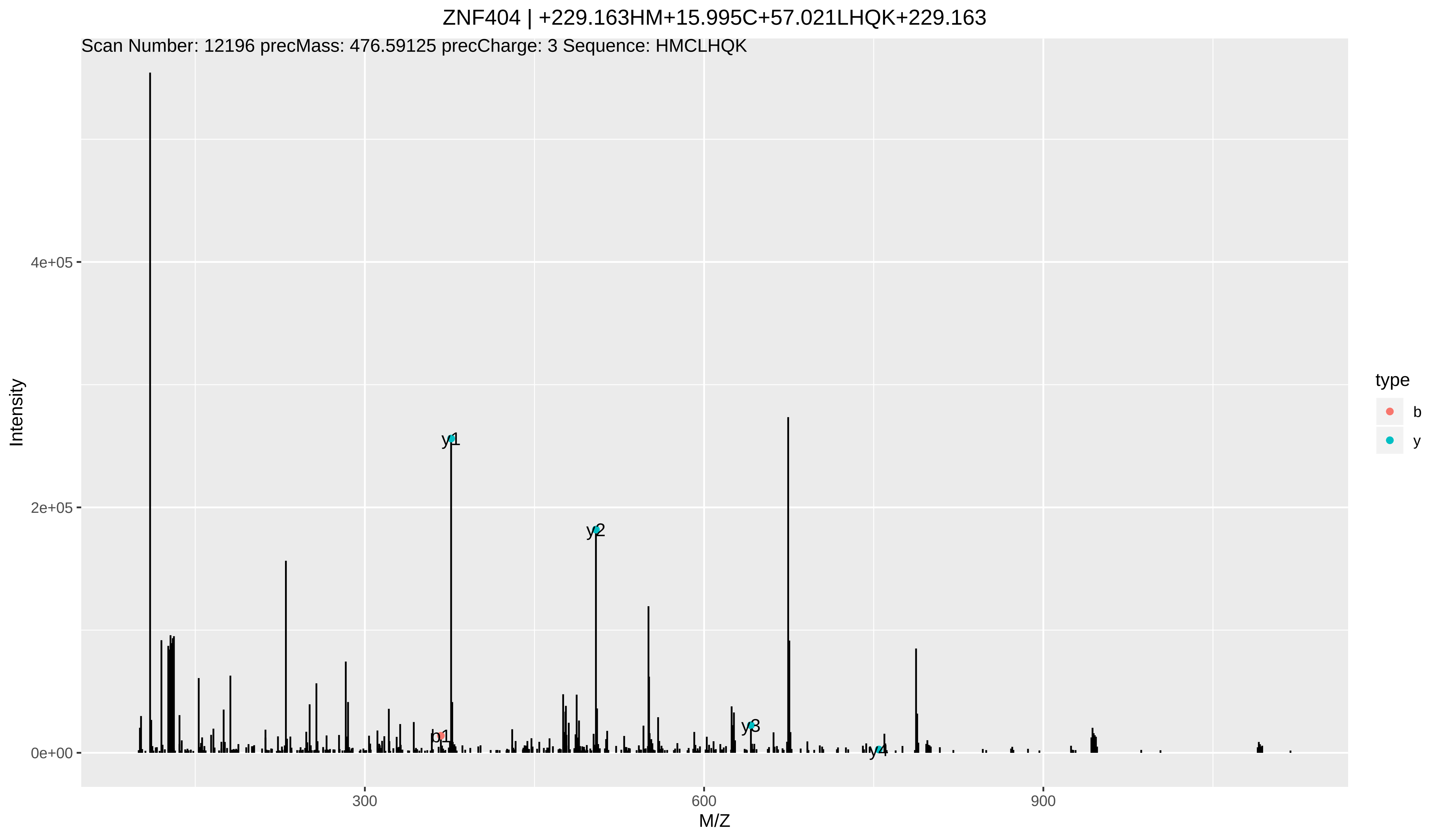

ZNF404 | +229.163HM+15.995C+57.021LHQK+229.163
Scan Number: 12196 precMass: 476.59125 precCharge: 3 Sequence: HMCLHQK
Intensity
4e+05
2e+05
0e+00
300
600
900
M/Z
y1
y2
y3
y4
b1
type
b
y

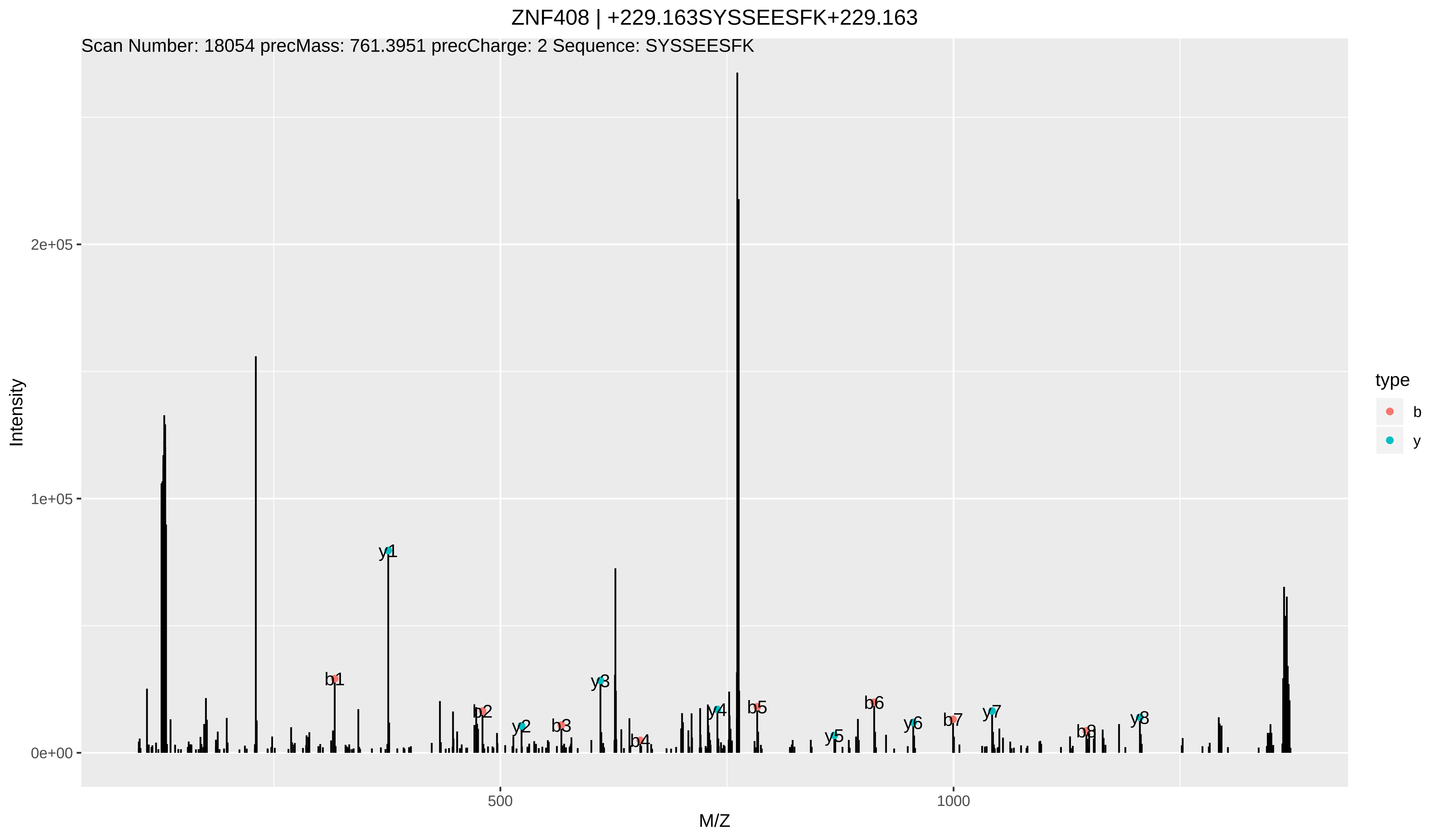

ZNF408 | +229.163SYSSEEESFK+229.163
Scan Number: 18054 precMass: 761.3951 precCharge: 2 Sequence: SYSSEESFK
Intensity
2e+05
1e+05
0e+00
M/Z
500
1000
type
b
y
y1
b1
b2
y2
b3
y3
b4
y4
b5
y5
b6
y6
b7
y7
b8
y8

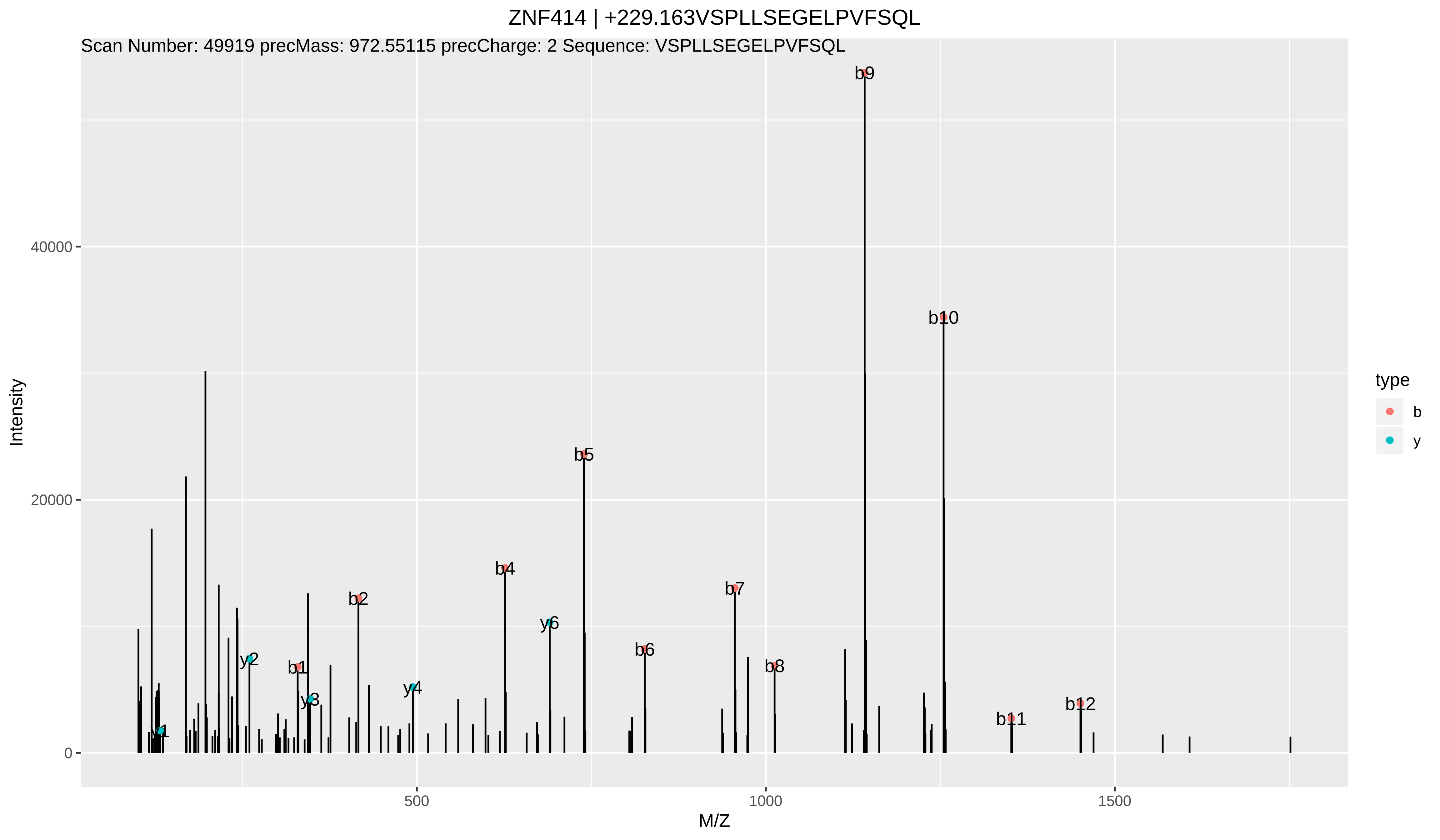
ZNF414 | +229.163VSPLLSEGELPVFSQL
Scan Number: 49919 precMass: 972.55115 precCharge: 2 Sequence: VSPLLSEGELPVFSQL
Intensity
M/Z
0
20000
40000
500
1000
1500
b9
b10
b5
b4
b2
b7
y6
b6
b8
y2
b1
y4
y3
b12
b11
y1
type
b
y

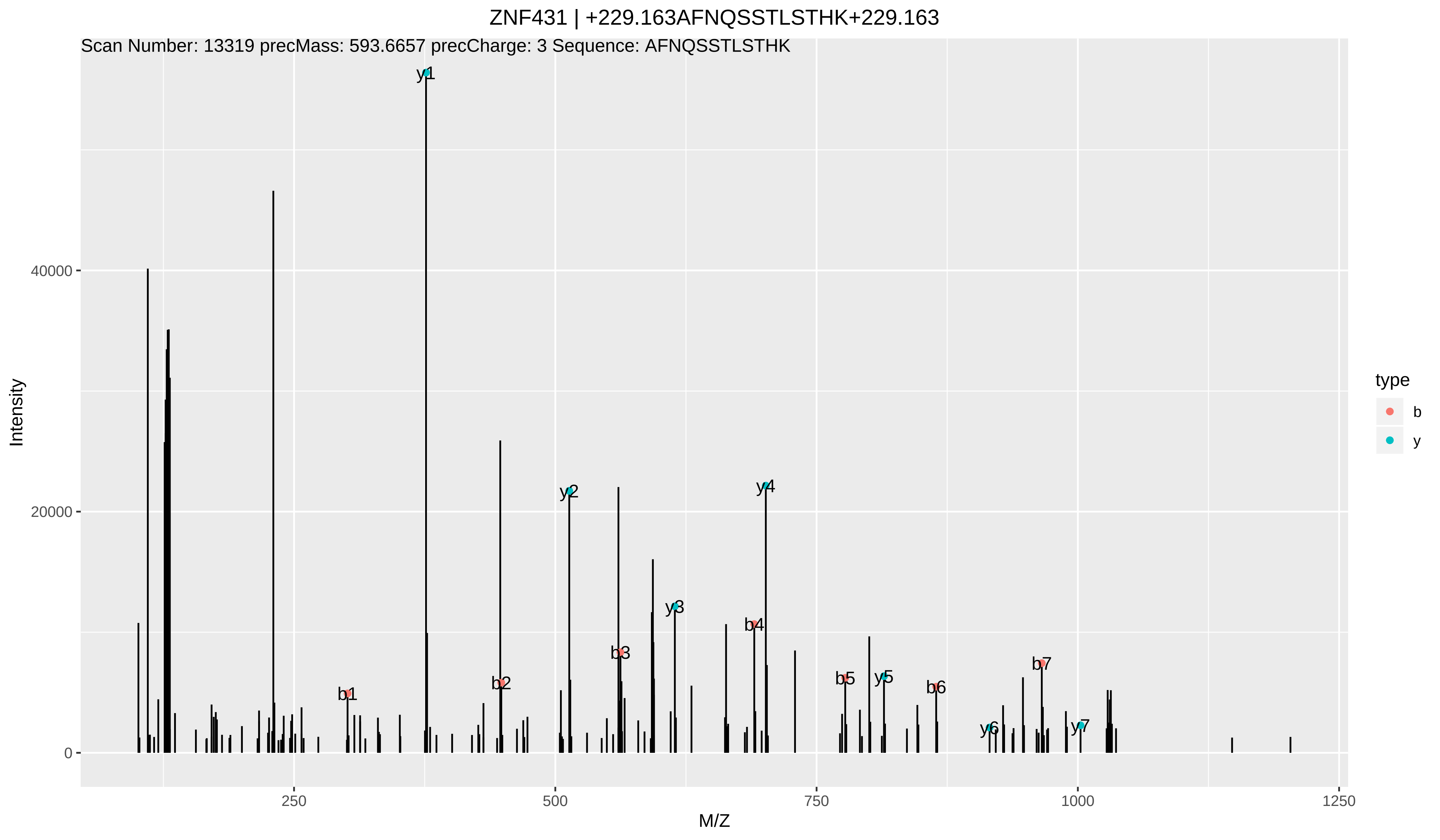

ZNF431 | +229.163AFNQSSTLSTHK+229.163
Scan Number: 13319 precMass: 593.6657 precCharge: 3 Sequence: AFNQSSTLSTHK
Intensity
M/Z
type
b
y
y1
y2
y3
y4
y5
y6
y7
b1
b2
b3
b4
b5
b6
b7
0
20000
40000
250
500
750
1000
1250

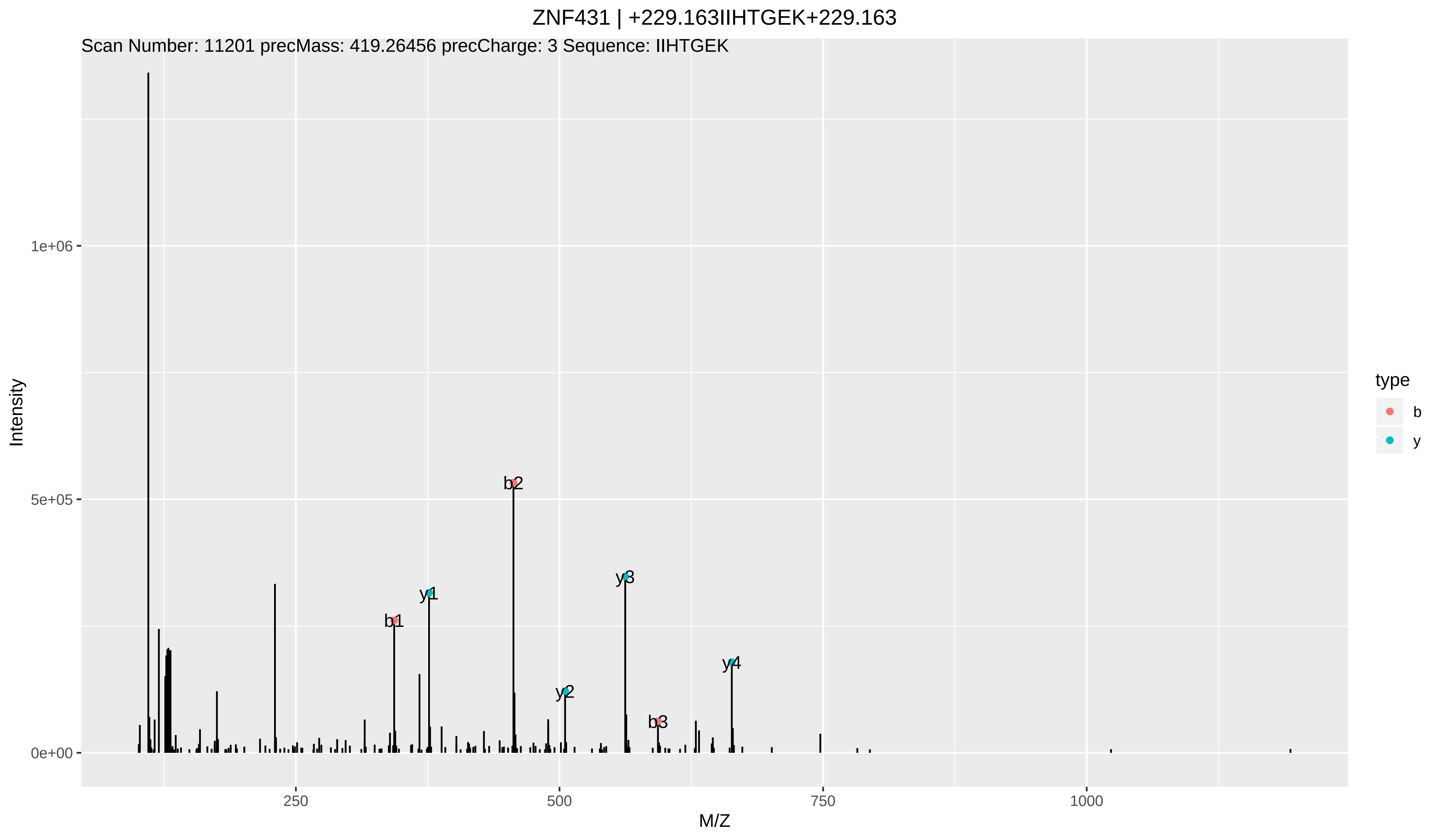
ZNF431 | +229.163IIHTGEK+229.163
Scan Number: 11201 precMass: 419.26456 precCharge: 3 Sequence: IIHTGEK
Intensity
1e+06
5e+05
0e+00
M/Z
250
500
750
1000
b1
y1
b2
y2
y3
b3
y4
type
b
y

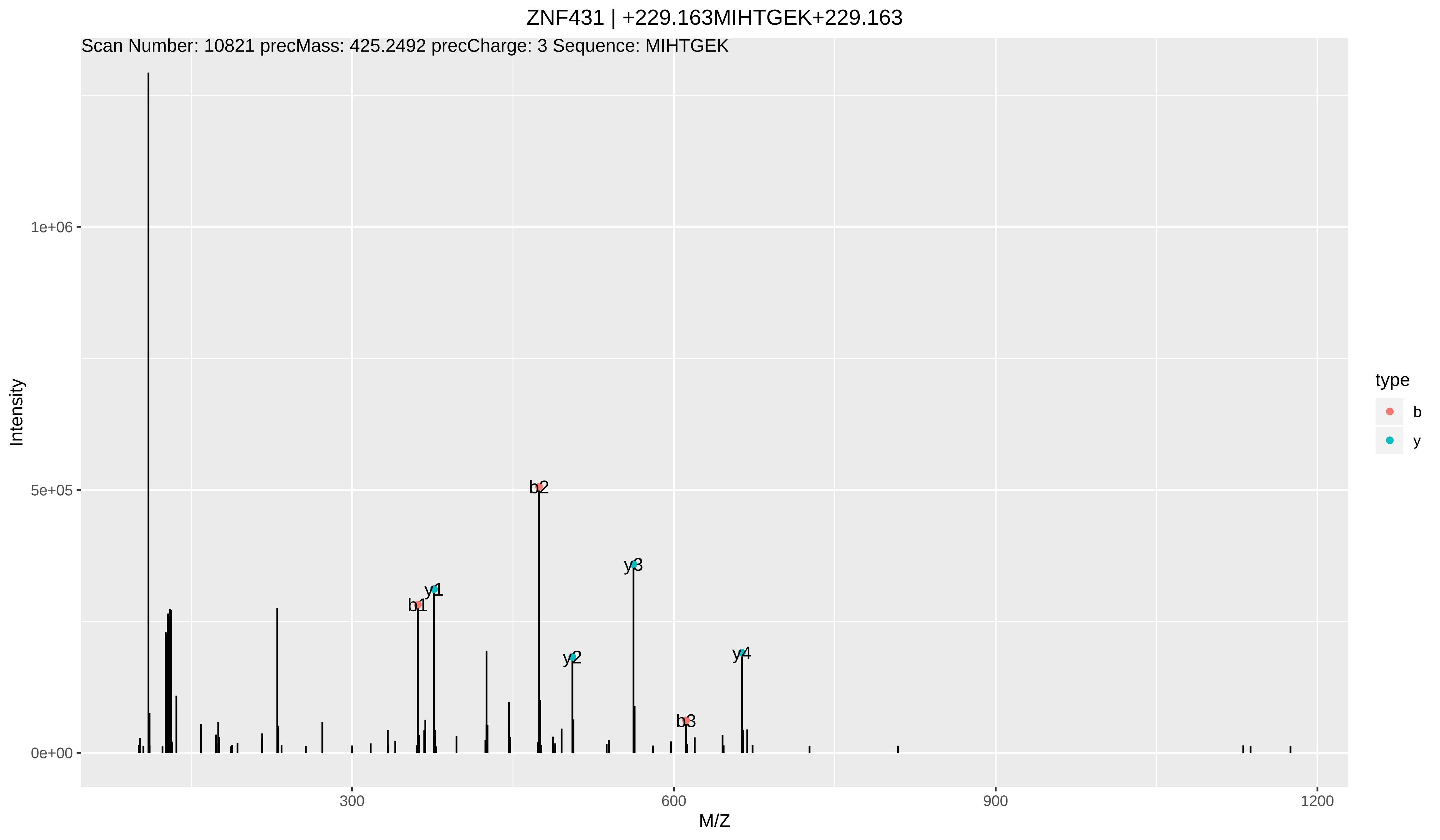
ZNF431 | +229.163MIHTGEK+229.163
Scan Number: 10821 precMass: 425.2492 precCharge: 3 Sequence: MIHTGEK
Intensity
1e+06
5e+05
0e+00
M/Z
300
600
900
1200
b1
y1
b2
y2
y3
b3
y4
type
b
y

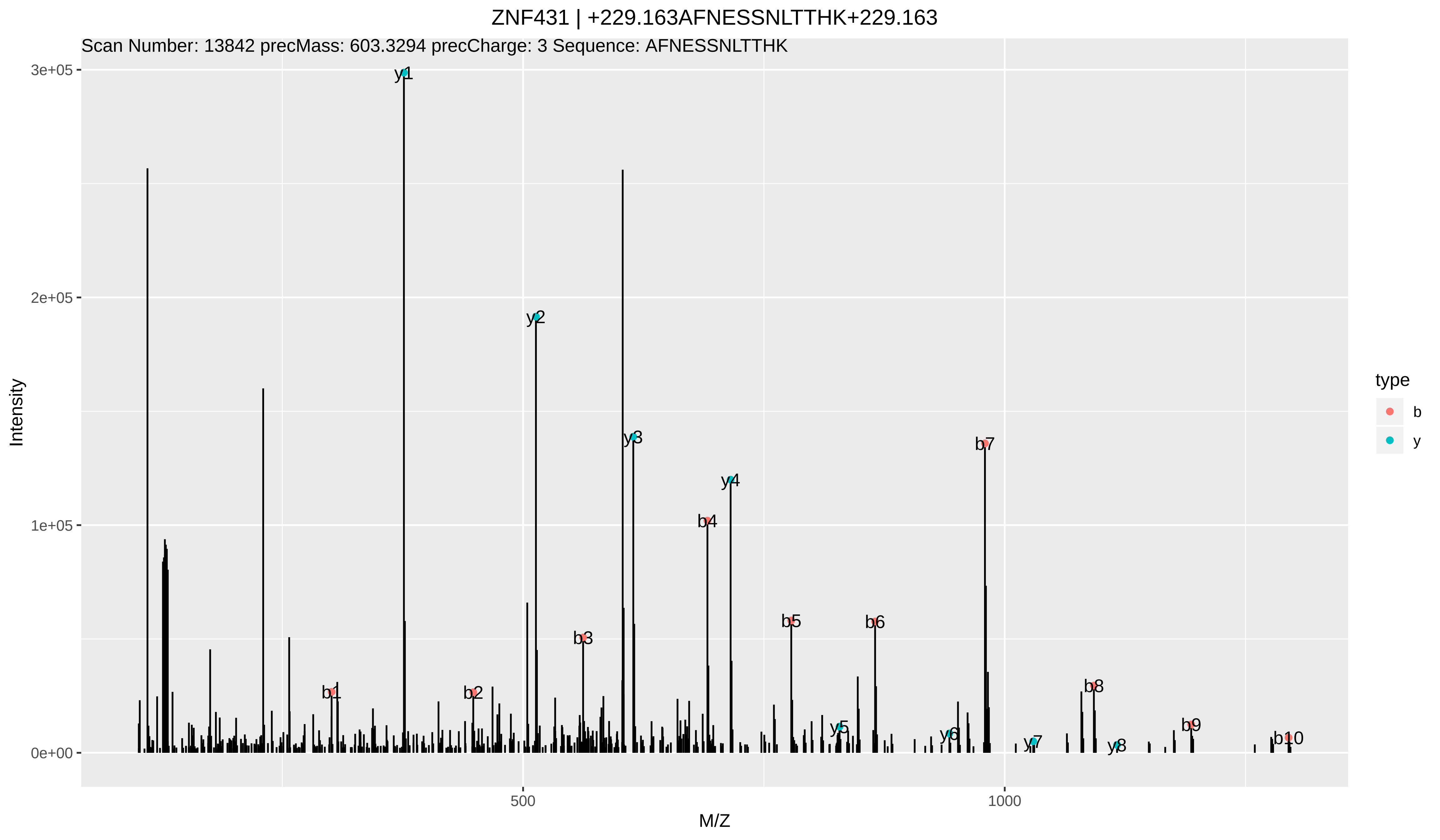
ZNF431 | +229.163AFNESSNLTTHK+229.163
Scan Number: 13842 precMass: 603.3294 precCharge: 3 Sequence: AFNESSNLTTHK
Intensity
M/Z
3e+05
2e+05
1e+05
0e+00
500
1000
y1
y2
y3
y4
y5
y6
y7
y8
b1
b2
b3
b4
b5
b6
b7
b8
b9
b10
type
b
y

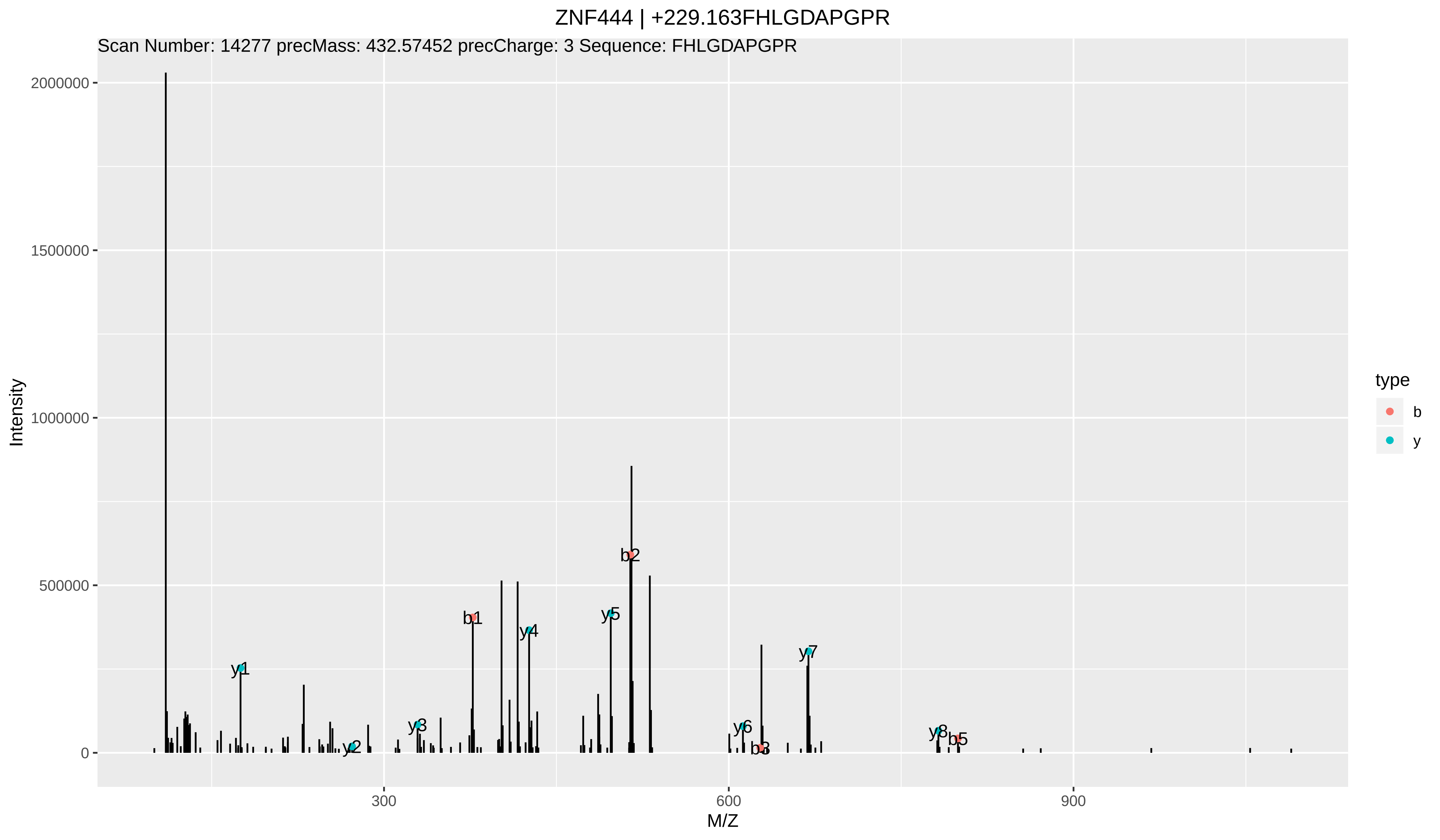

ZNF444 | +229.163FHLGDAPGPR
Scan Number: 14277 precMass: 432.57452 precCharge: 3 Sequence: FHLGDAPGPR
Intensity
2000000
1500000
1000000
500000
0
M/Z
300
600
900
y1
y2
y3
b1
y4
y5
b2
y6
b3
y7
y8
b5
type
b
y

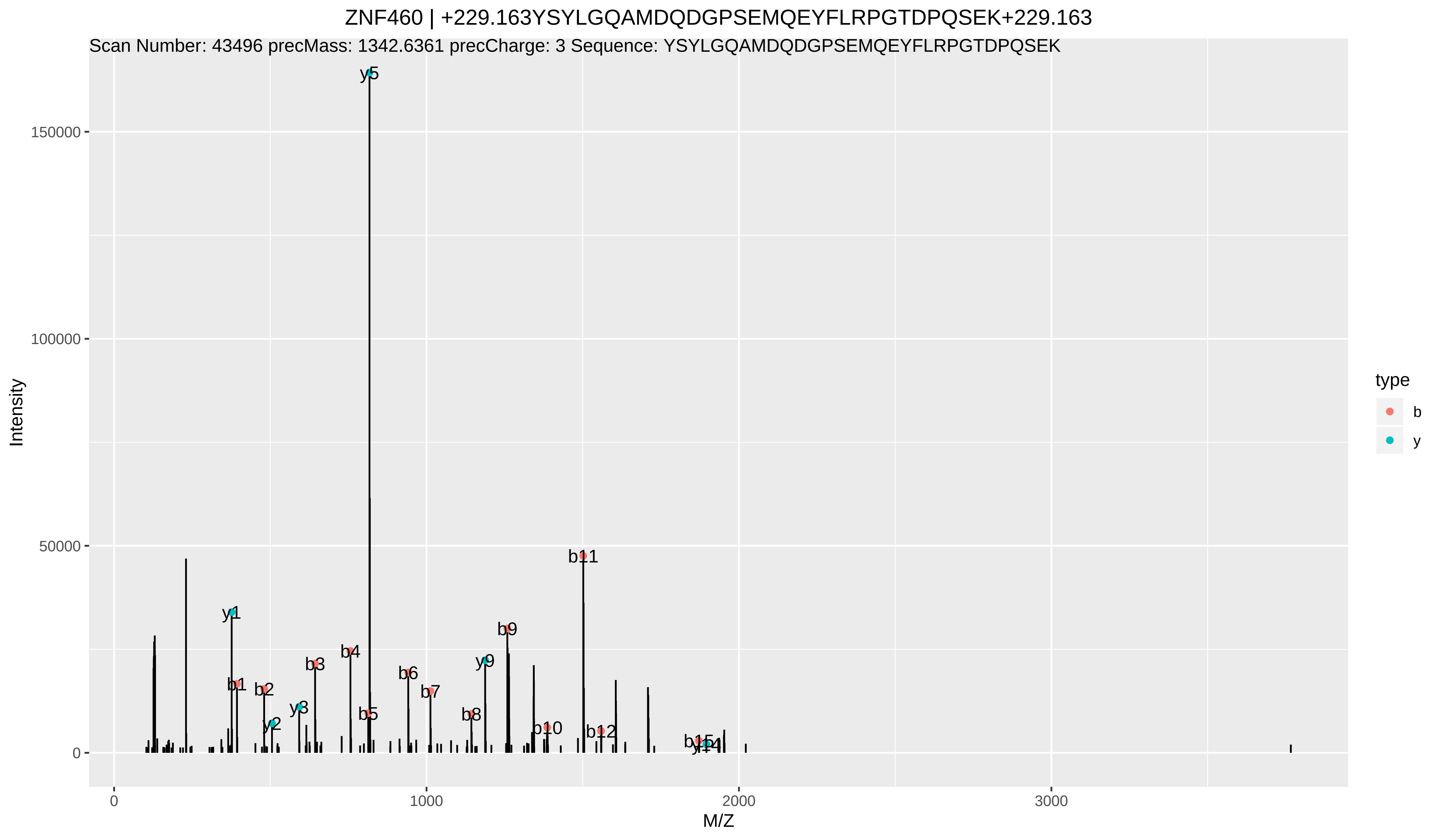

ZNF460 | +229.163YSYLGQAMDQDGPSEMQEYFLRPGTDPQSEK+229.163
Scan Number: 43496 precMass: 1342.6361 precCharge: 3 Sequence: YSYLGQAMDQDGPSEMQEYFLRPGTDPQSEK
Intensity
M/Z
type
b
y
y5
b11
y1
b9
b4
b3
y9
b6
b1
b2
b7
y3
b5
b8
y2
b10
b12
0
50000
100000
150000
1000
2000
3000

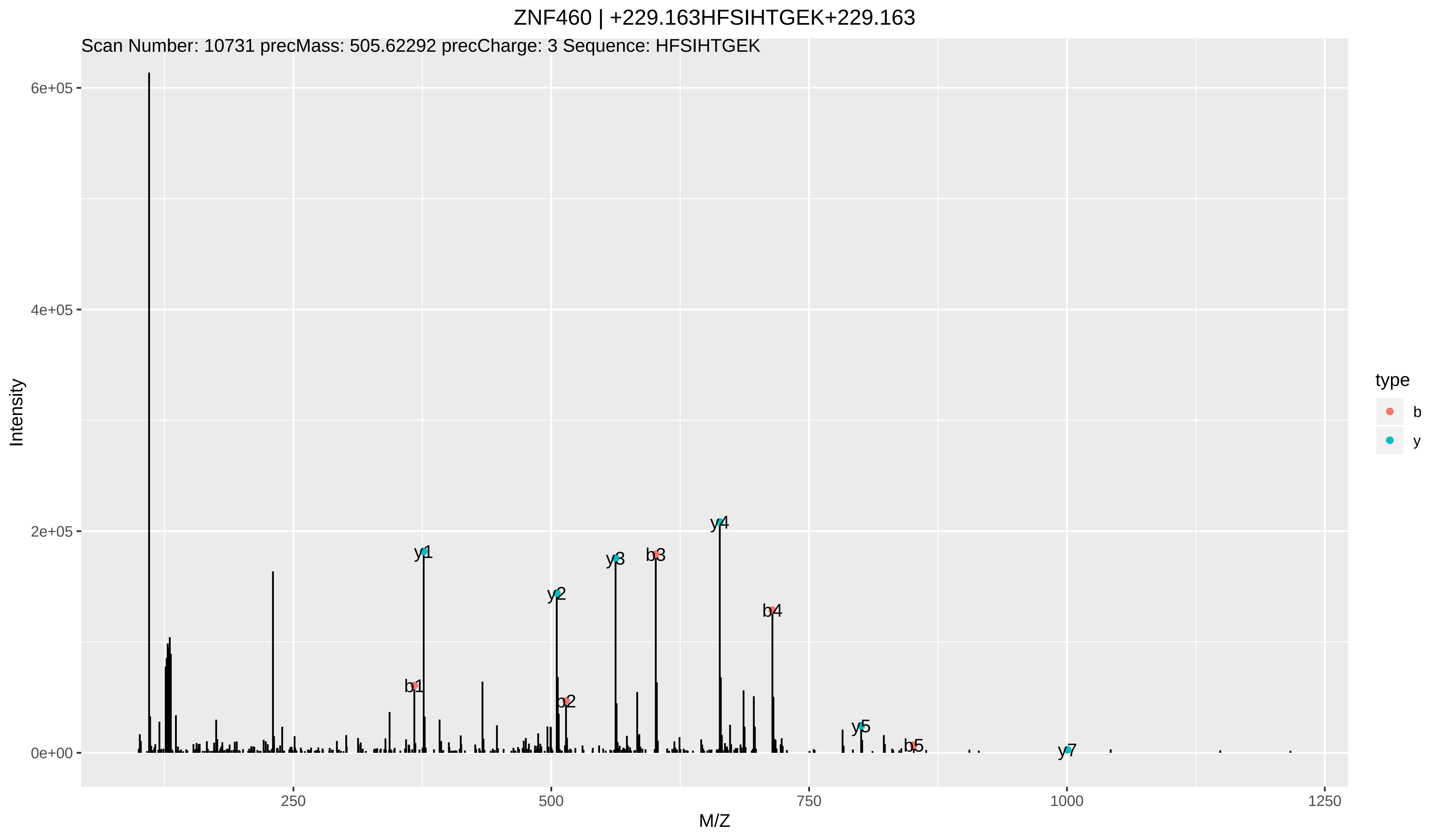
ZNF460 | +229.163HFSIHTGEK+229.163
Scan Number: 10731 precMass: 505.62292 precCharge: 3 Sequence: HFSIHTGEK
Intensity
6e+05
4e+05
2e+05
0e+00
M/Z
250
500
750
1000
1250
y1
y2
y3
b3
y4
b4
b1
b2
y5
b5
y7
type
b
y

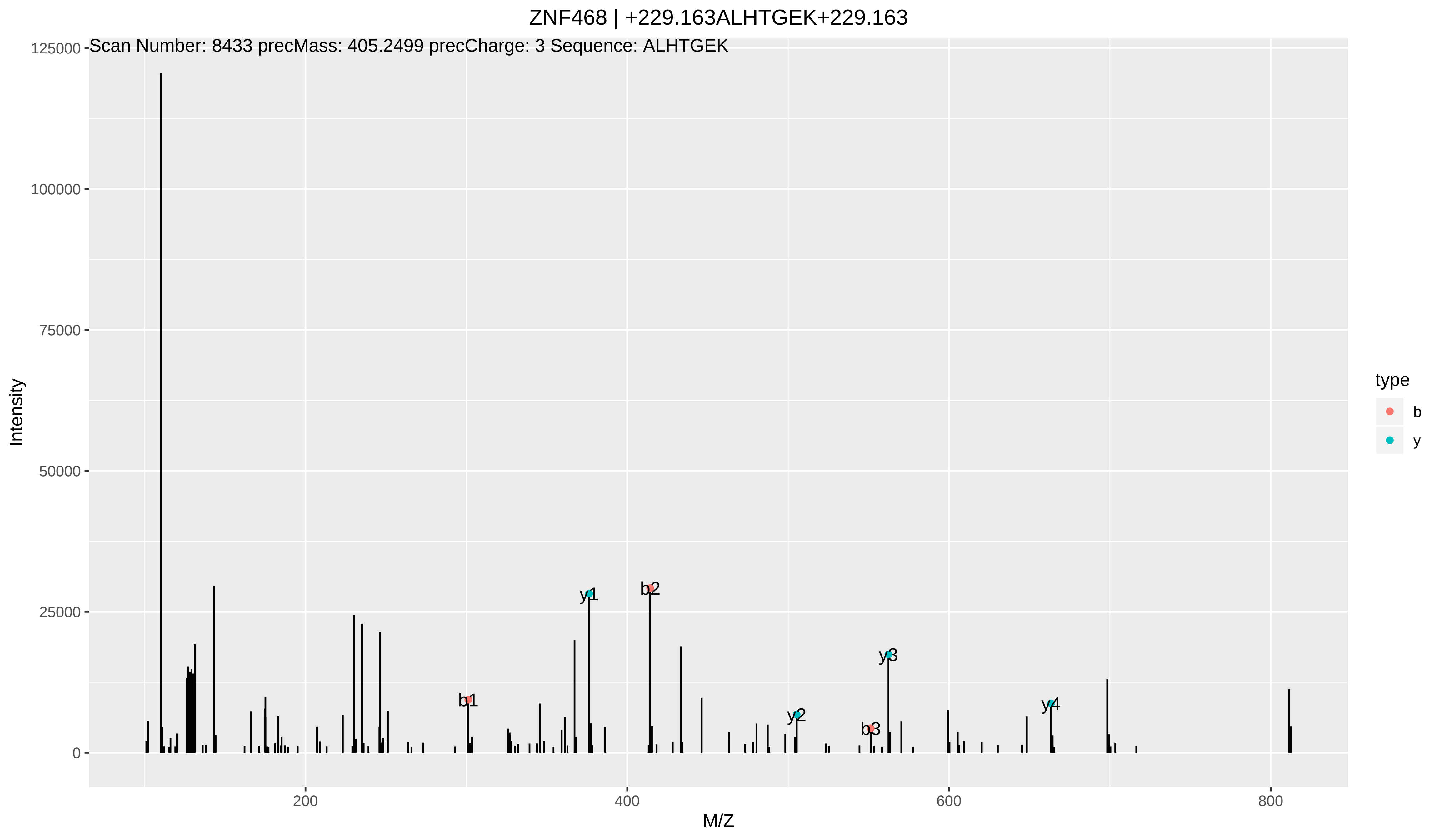
ZNF468 | +229.163ALHTGEK+229.163
Scan Number: 8433 precMass: 405.2499 precCharge: 3 Sequence: ALHTGEK
Intensity
125000
100000
75000
50000
25000
0
M/Z
200
400
600
800
b1
b2
b3
y1
y2
y3
y4
type
b
y

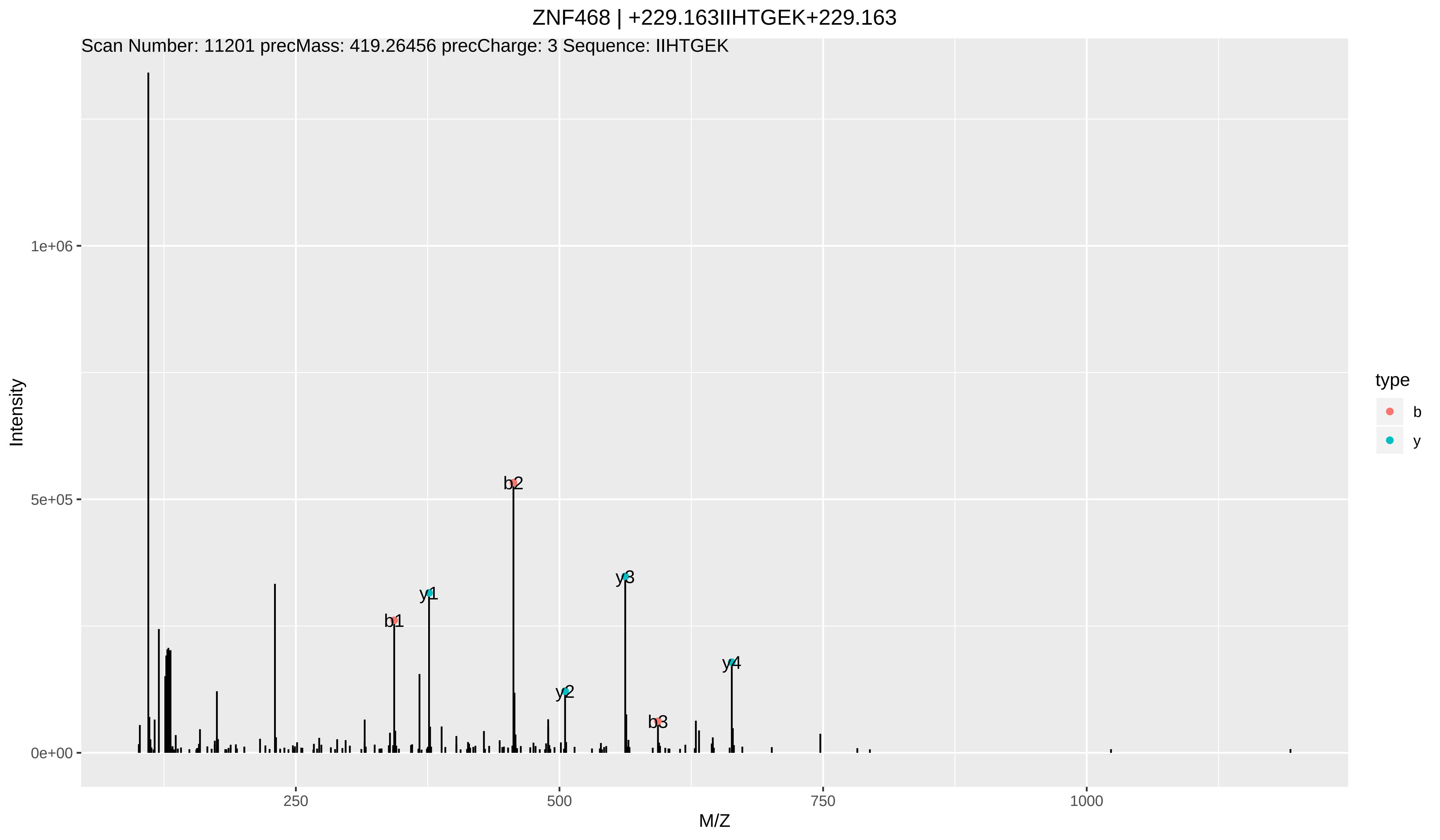
ZNF468 | +229.163IIHTGEK+229.163
Scan Number: 11201 precMass: 419.26456 precCharge: 3 Sequence: IIHTGEK
Intensity
1e+06
5e+05
0e+00
b1
y1
b2
y2
y3
b3
y4
250
500
750
1000
M/Z
type
b
y

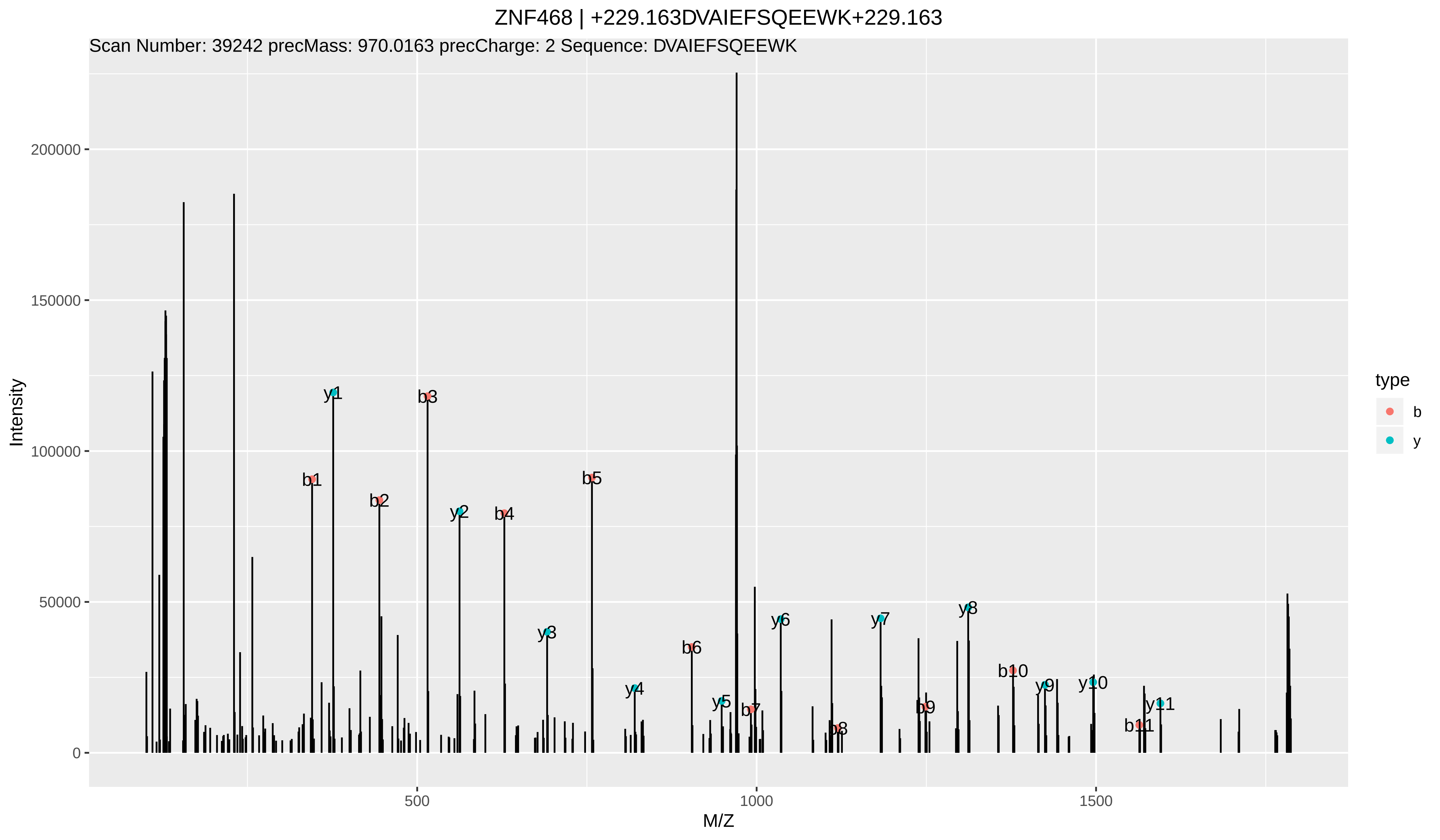
ZNF468 | +229.163DVAIEFSQEEWK+229.163
Scan Number: 39242 precMass: 970.0163 precCharge: 2 Sequence: DVAIEFSQEEWK
Intensity
M/Z
0
50000
100000
150000
200000
500
1000
1500
y1
b3
b1
b2
y2
b4
b5
y3
y4
b6
y5
b7
y6
b8
y7
b9
y8
b10
y9
y10
b11
y11
type
b
y

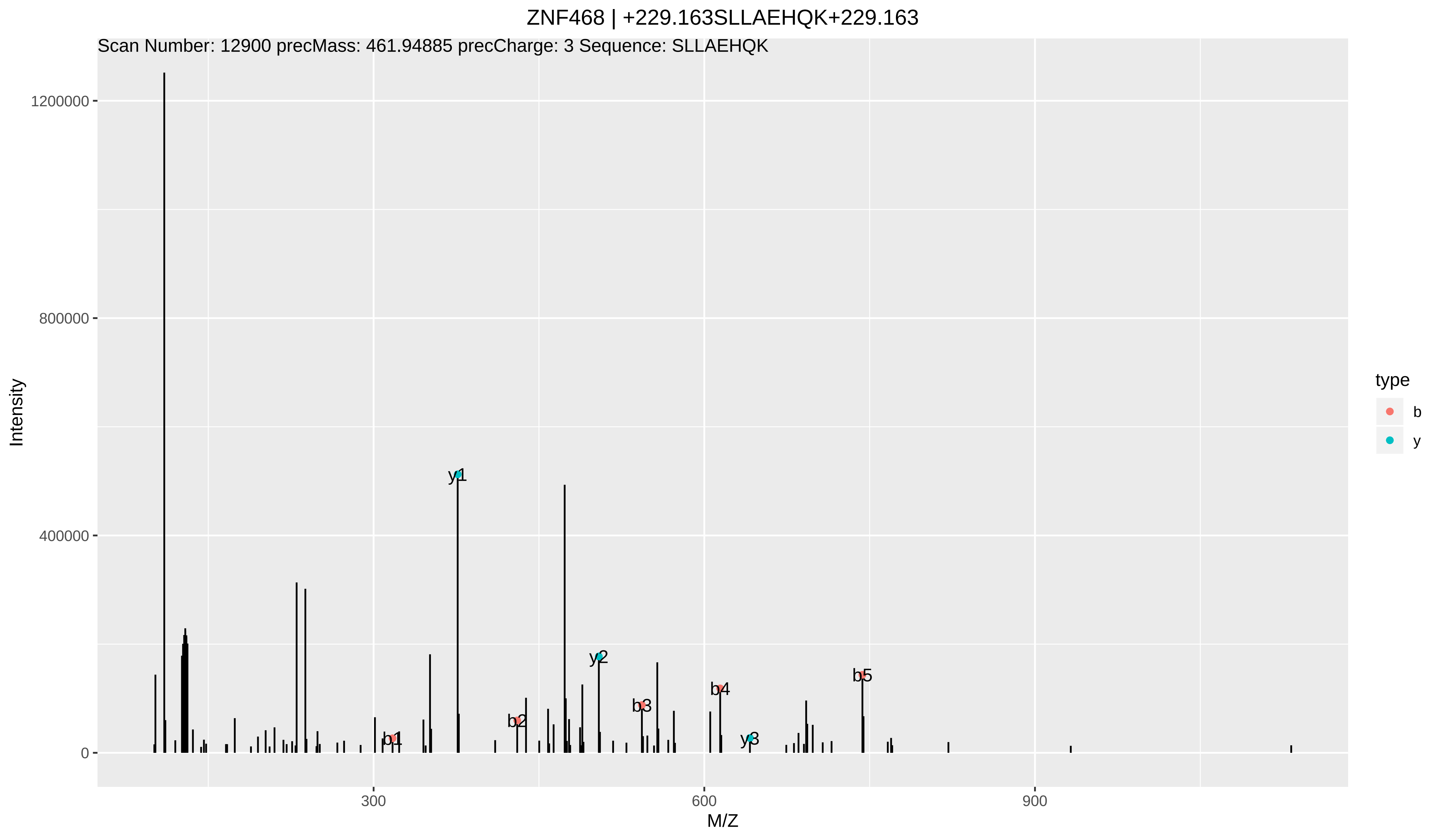
ZNF468 | +229.163SLLAEHQK+229.163
Scan Number: 12900 precMass: 461.94885 precCharge: 3 Sequence: SLLAEHQK
Intensity
1200000
800000
400000
0
300
600
900
M/Z
y1
y2
y3
b1
b2
b3
b4
b5
type
b
y

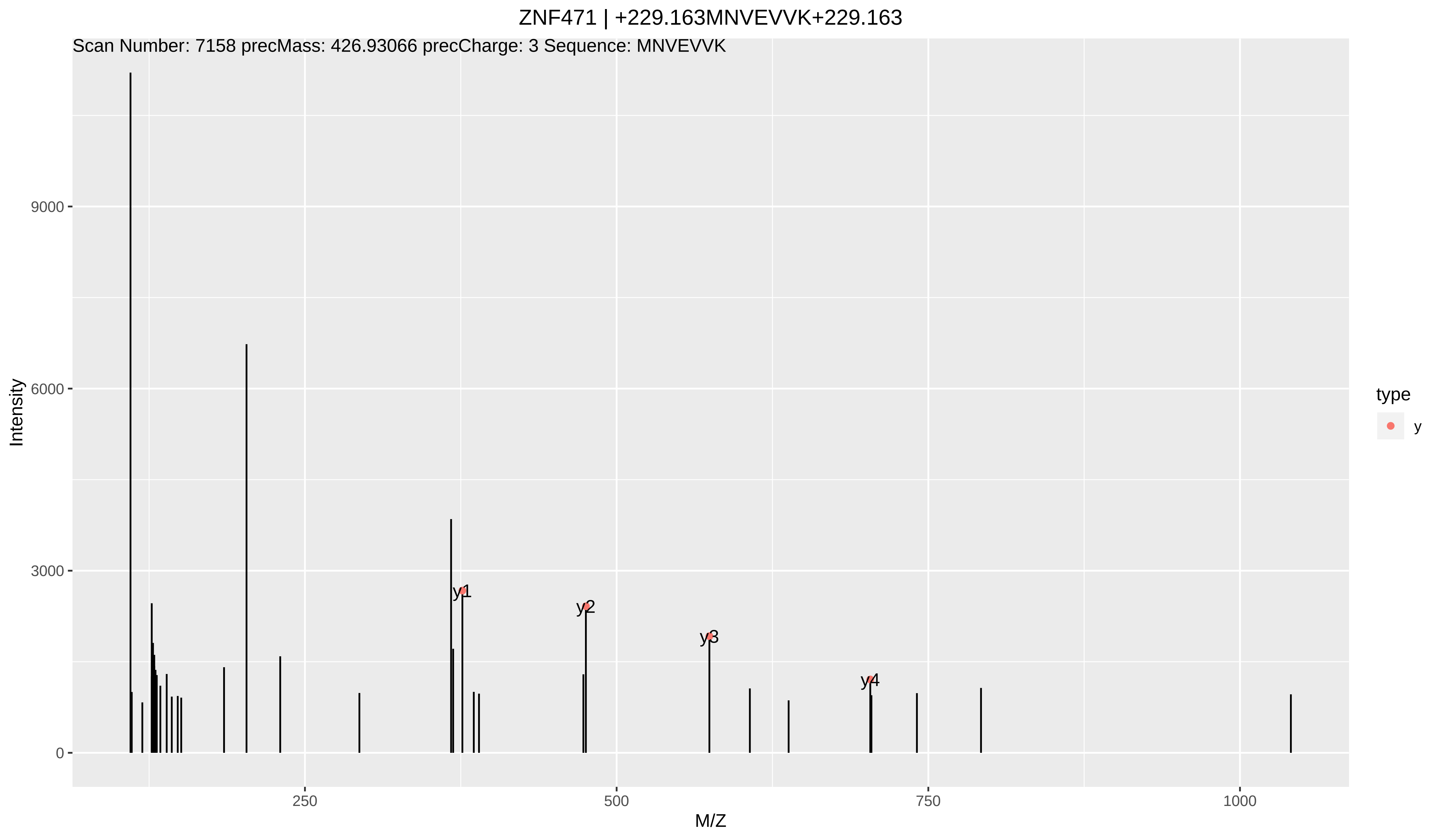

ZNF471 | +229.163MNVEVVK+229.163
Scan Number: 7158 precMass: 426.93066 precCharge: 3 Sequence: MNVEVVK
Intensity
9000
6000
3000
0
y1
y2
y3
y4
250
500
750
1000
M/Z
type
y

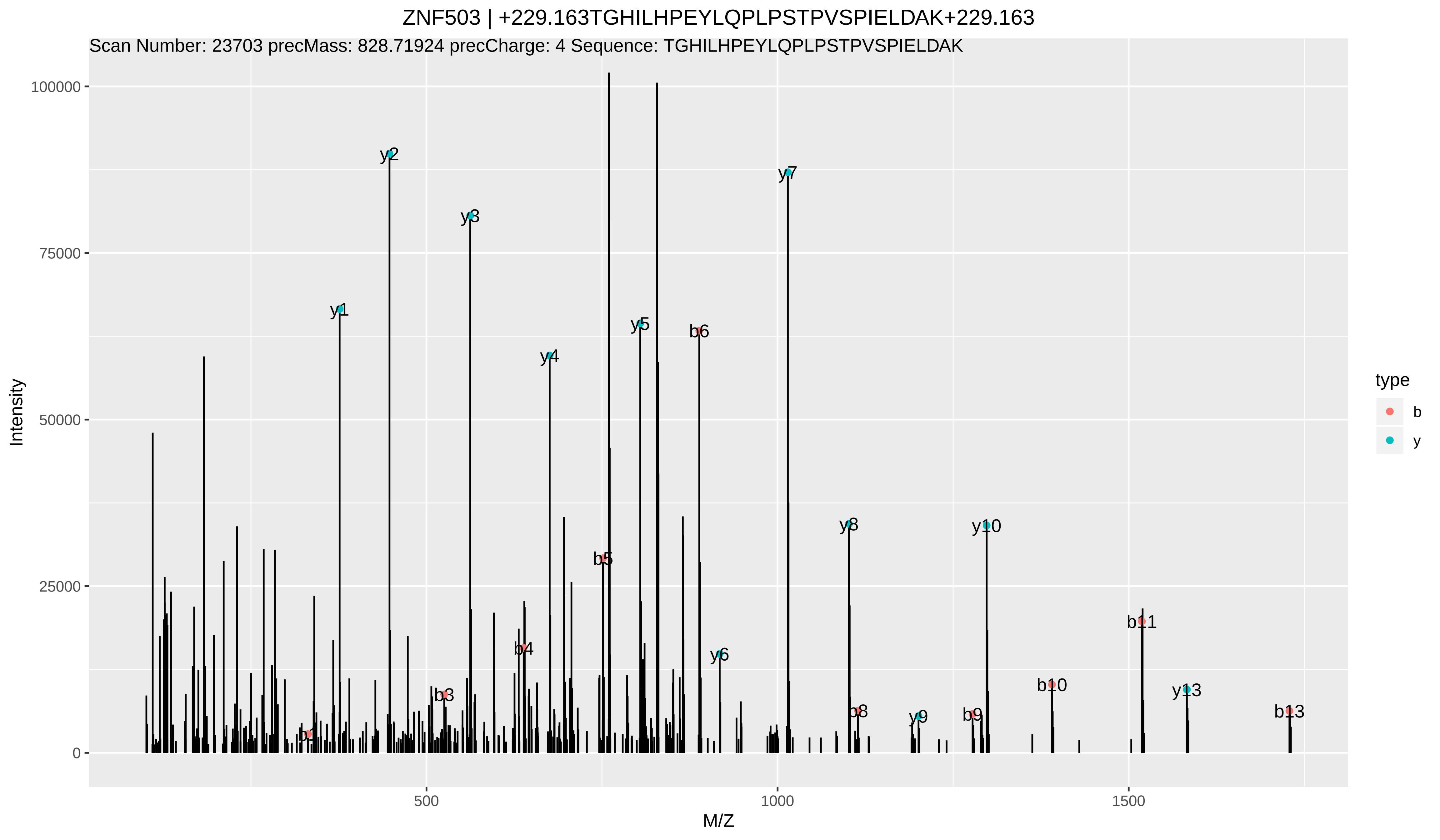

ZNF503 | +229.163TGHILHPEYLQPLPSTPVSPIELDAK+229.163
Scan Number: 23703 precMass: 828.71924 precCharge: 4 Sequence: TGHILHPEYLQPLPSTPVSPIELDAK
Intensity
100000
75000
50000
25000
0
500
1000
1500
M/Z
y1
y2
y3
y4
y5
y6
y7
y8
y9
y10
y13
b3
b4
b5
b6
b8
b9
b10
b11
b13
type
b
y

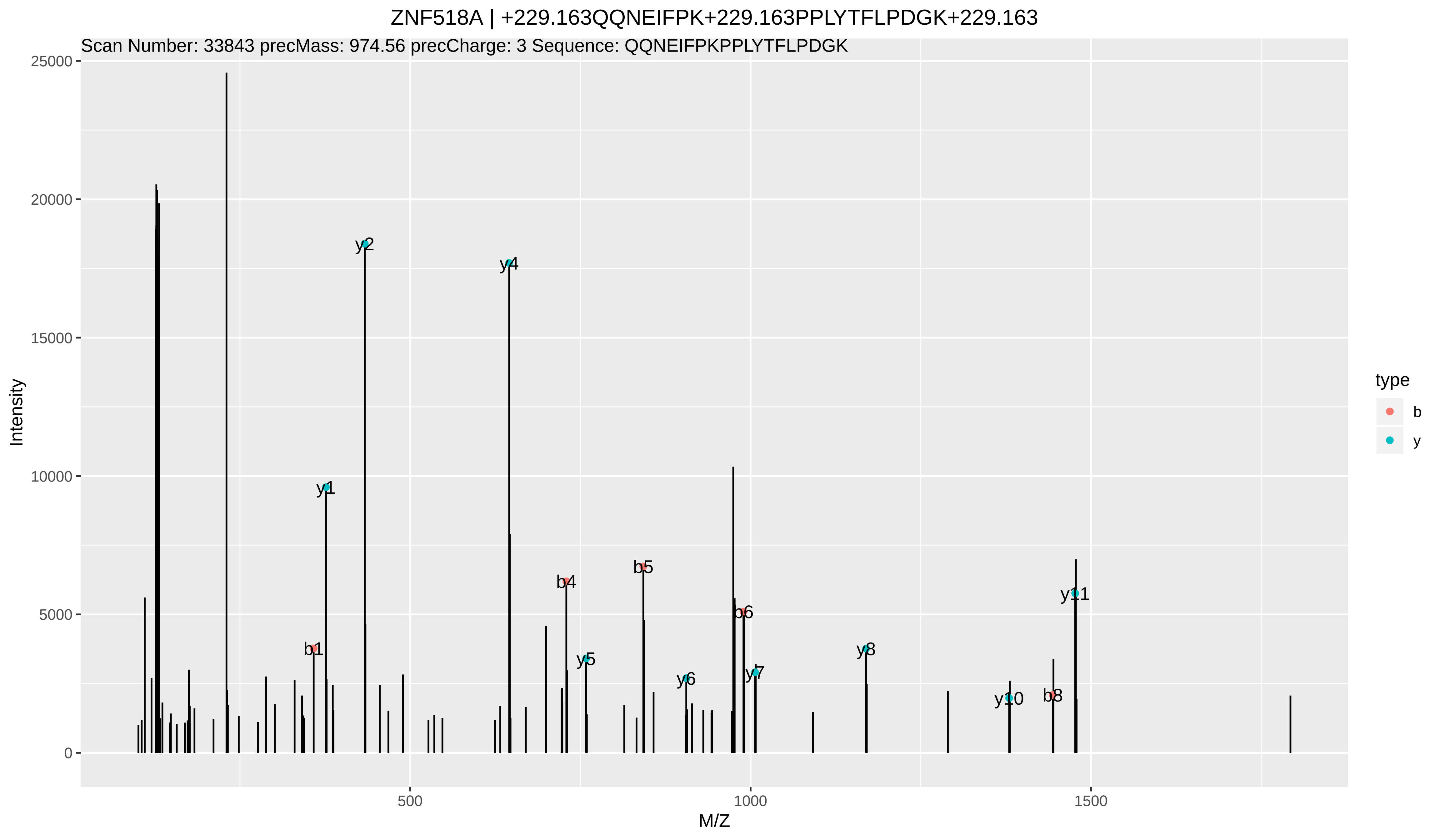
ZNF518A | +229.163QQNEIFPK+229.163PPLYTFLPDGK+229.163
Scan Number: 33843 precMass: 974.56 precCharge: 3 Sequence: QQNEIFPKPPLYTFLPDGK
Intensity
M/Z
type
b
y
y1
y2
y4
y5
y6
y7
y8
y10
y11
b1
b4
b5
b6
b8
0
5000
10000
15000
20000
25000
500
1000
1500

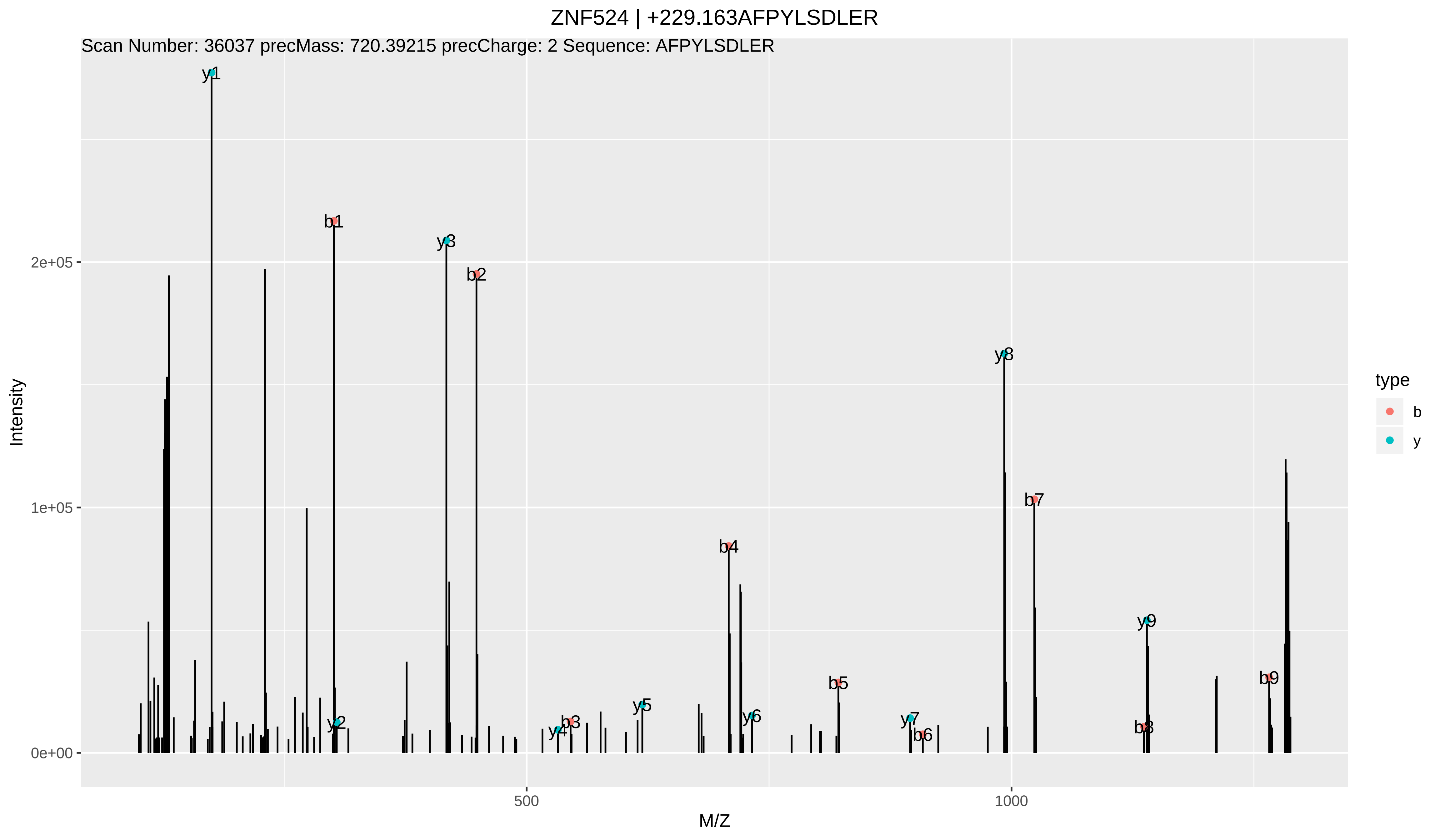
ZNF524 | +229.163AFPYLSDLER
Scan Number: 36037 precMass: 720.39215 precCharge: 2 Sequence: AFPYLSDLER
Intensity
M/Z
type
b
y
y1
b1
y3
b2
y8
b7
b4
y9
b5
b9
y5
y6
y7
b6
y2
b3
y4
b8
2e+05
1e+05
0e+00
500
1000

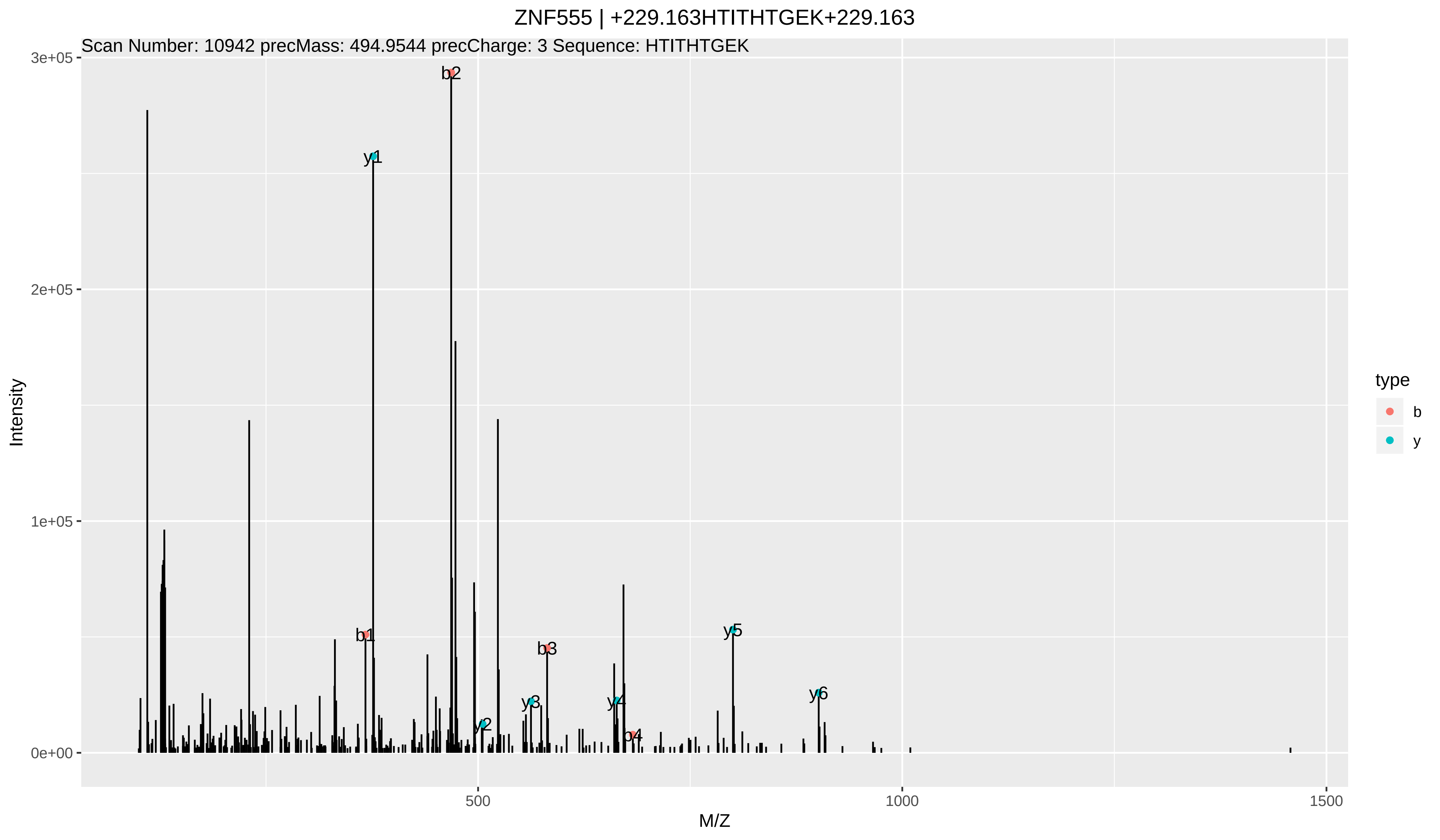

ZNF555 | +229.163HTITHTGEK+229.163
Scan Number: 10942 precMass: 494.9544 precCharge: 3 Sequence: HTITHTGEK
Intensity
M/Z
3e+05
2e+05
1e+05
0e+00
500
1000
1500
b2
y1
b1
b3
y5
y6
y3
y4
y2
b4
type
b
y

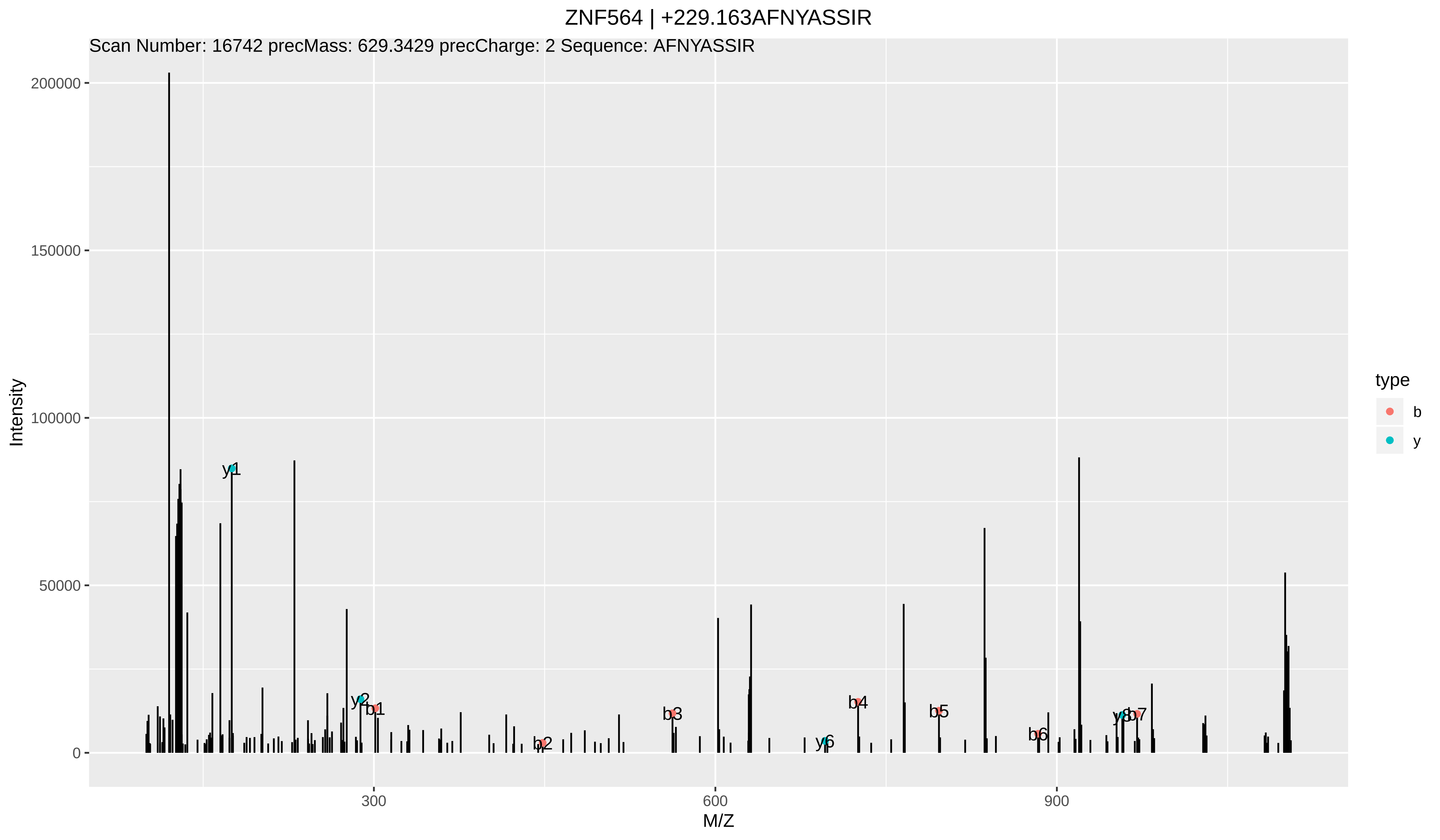

ZNF564 | +229.163AFNYASSIR
Scan Number: 16742 precMass: 629.3429 precCharge: 2 Sequence: AFNYASSIR
Intensity
M/Z
200000
150000
100000
50000
0
300
600
900
y1
y2
b1
b2
b3
y6
b4
b5
b6
y8
b7
type
b
y

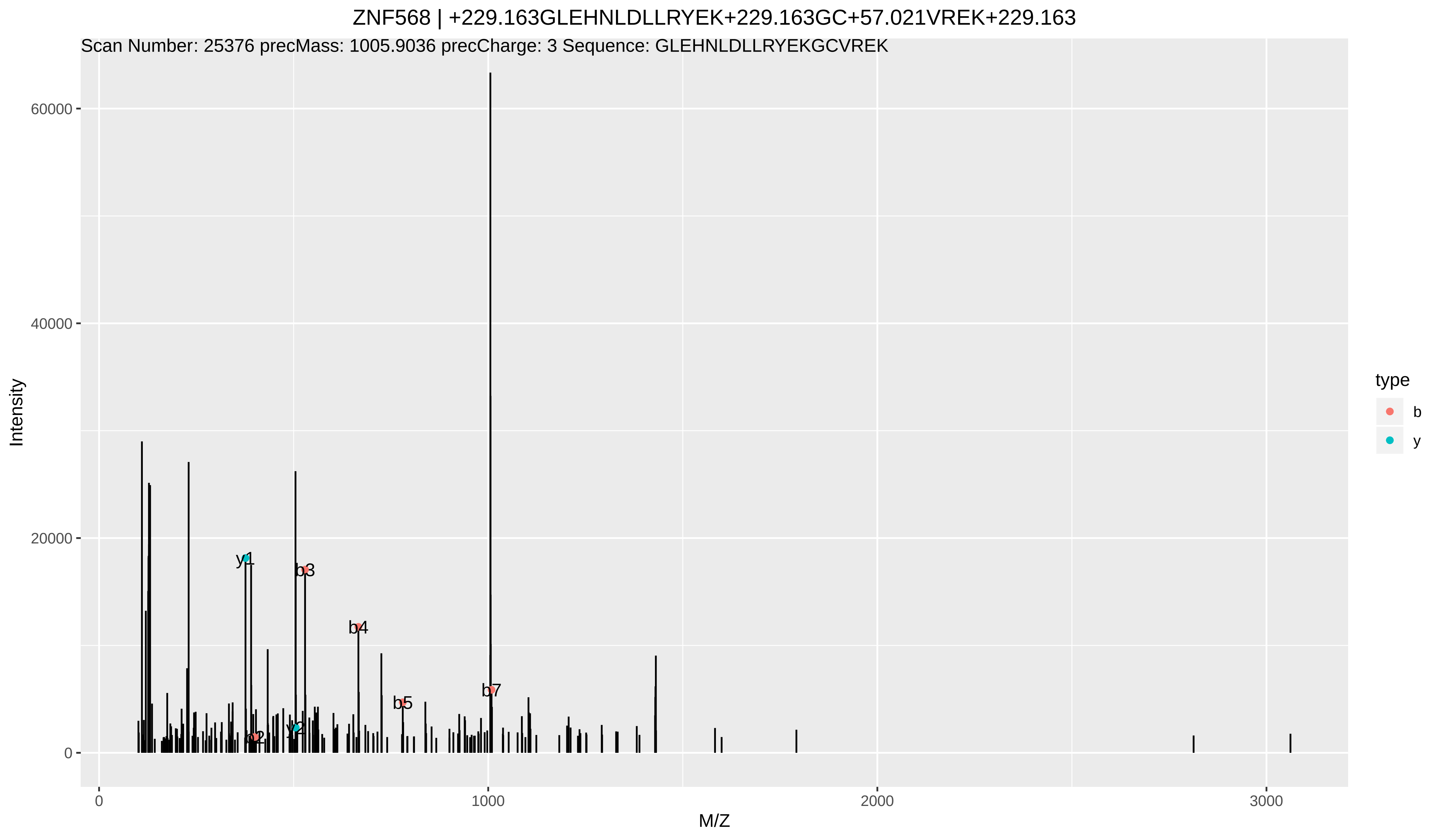

ZNF568 | +229.163GLEHNLDLLRYEK+229.163GC+57.021VREK+229.163
Scan Number: 25376 precMass: 1005.9036 precCharge: 3 Sequence: GLEHNLDLLRYEKGCVREK
Intensity
M/Z
60000
40000
20000
0
0
1000
2000
3000
y1
b3
b4
b5
b7
y2
b2
type
b
y

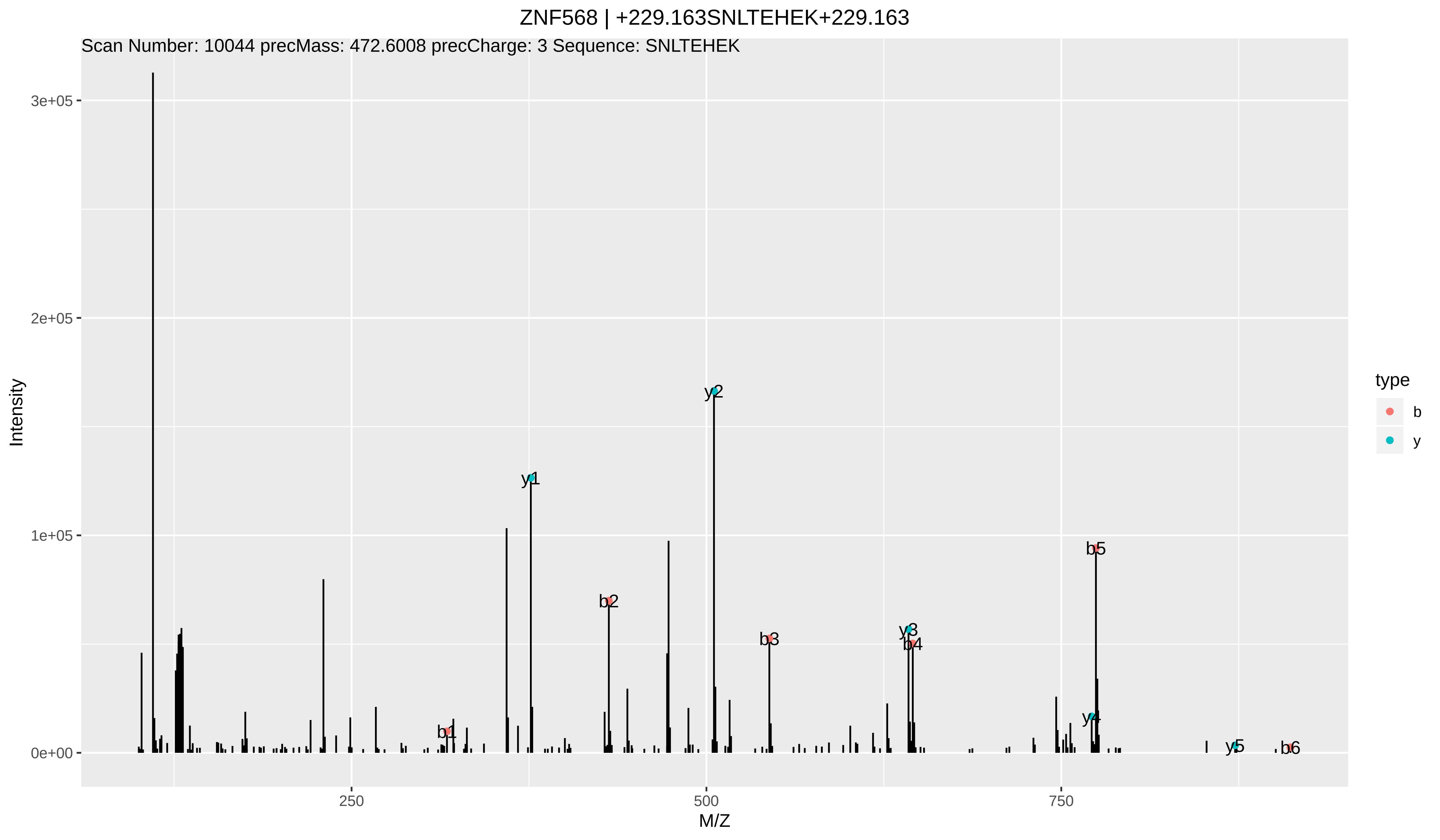
ZNF568 | +229.163SNLTEHEK+229.163
Scan Number: 10044 precMass: 472.6008 precCharge: 3 Sequence: SNLTEHEK
Intensity
3e+05
2e+05
1e+05
0e+00
250
500
750
M/Z
y1
y2
y3
y4
y5
b1
b2
b3
b4
b5
b6
type
b
y

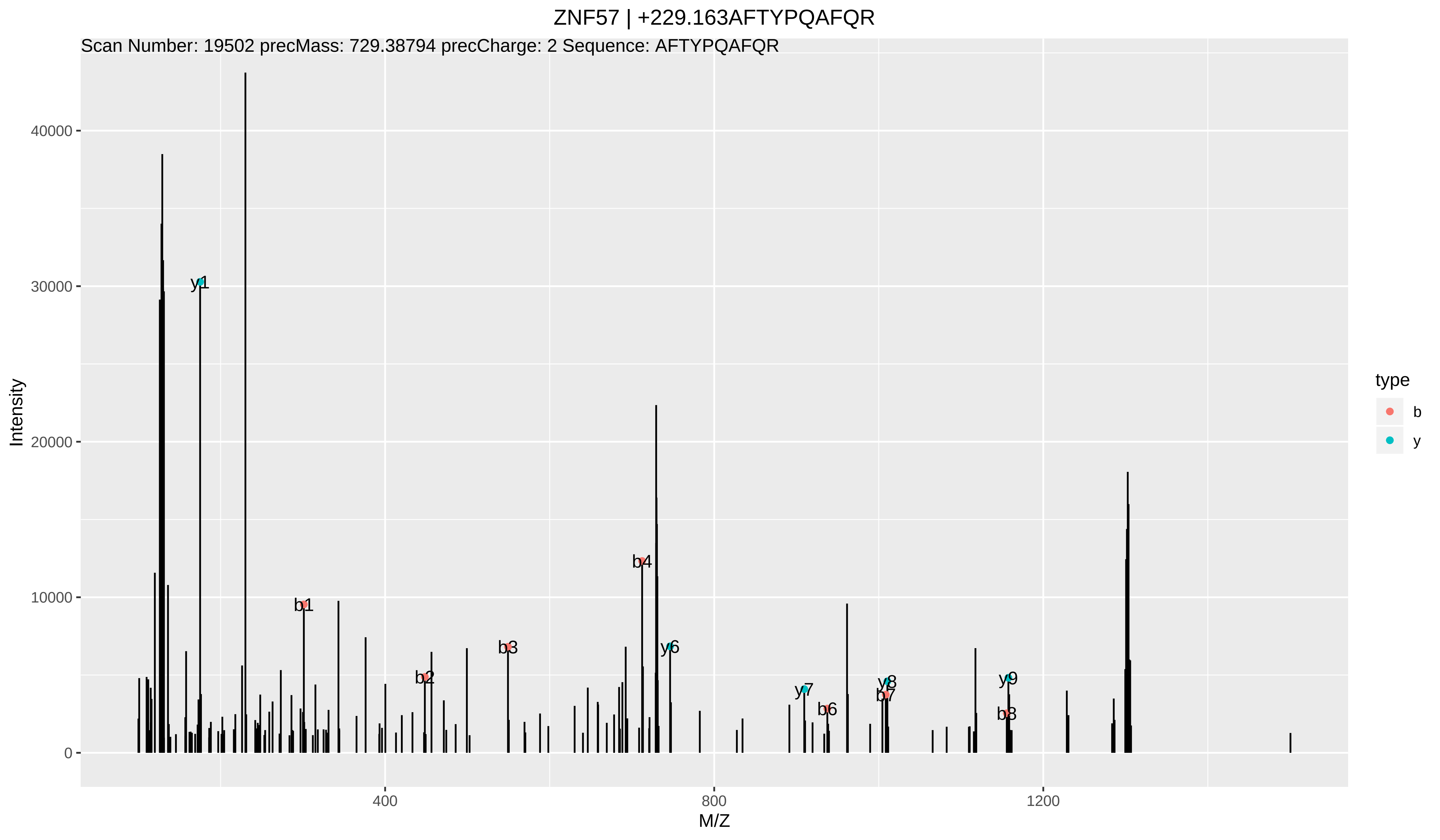
ZNF57 | +229.163AFTYPQAFQR
Scan Number: 19502 precMass: 729.38794 precCharge: 2 Sequence: AFTYPQAFQR
Intensity
M/Z
40000
30000
20000
10000
0
400
800
1200
y1
b1
b2
b3
b4
y6
y7
b6
y8
b7
y9
b8
type
b
y

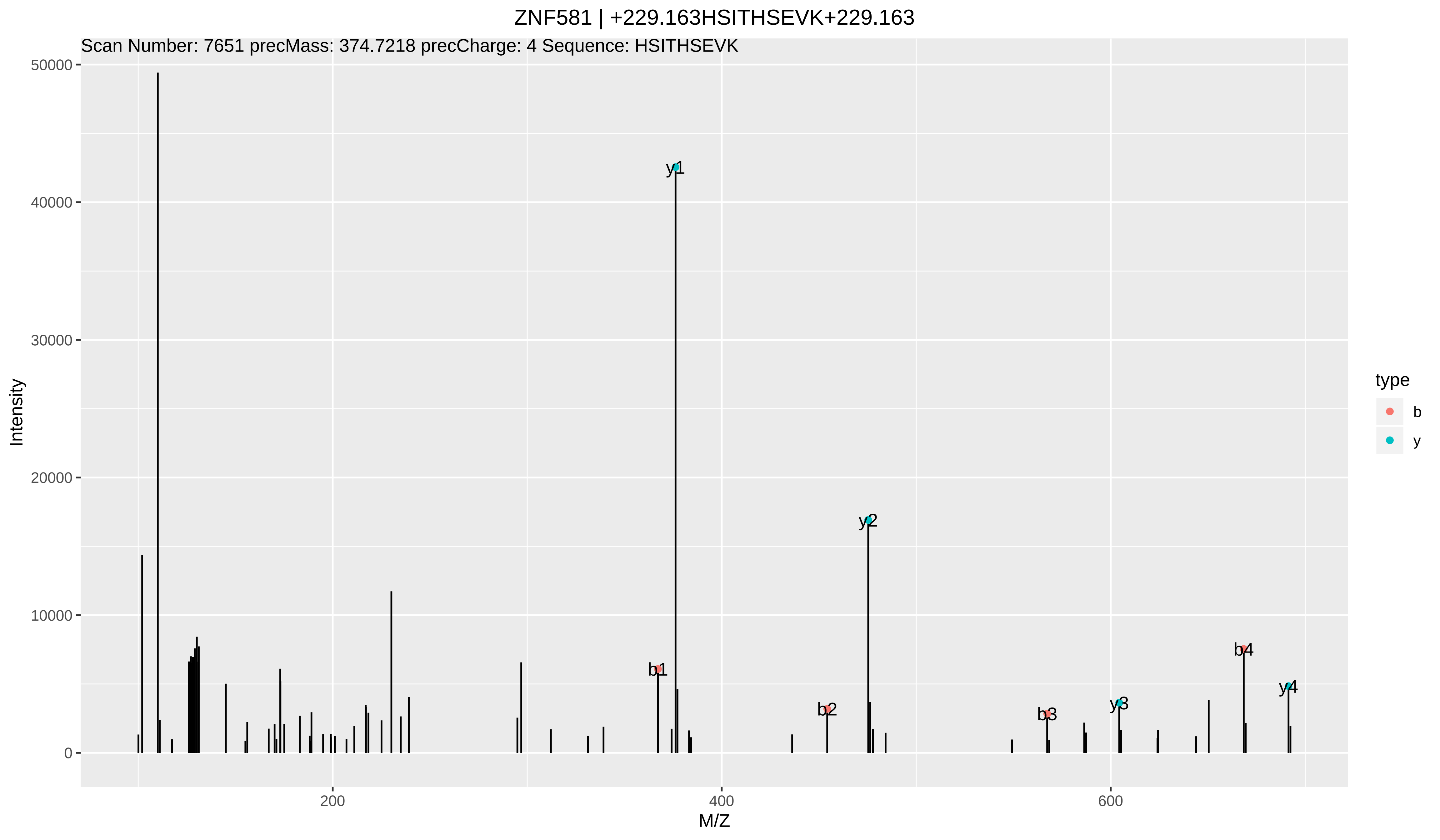

ZNF581 | +229.163HSITHSEVK+229.163
Scan Number: 7651 precMass: 374.7218 precCharge: 4 Sequence: HSITHSEVK
Intensity
M/Z
0
10000
20000
30000
40000
50000
200
400
600
y1
y2
y3
y4
b1
b2
b3
b4
type
b
y

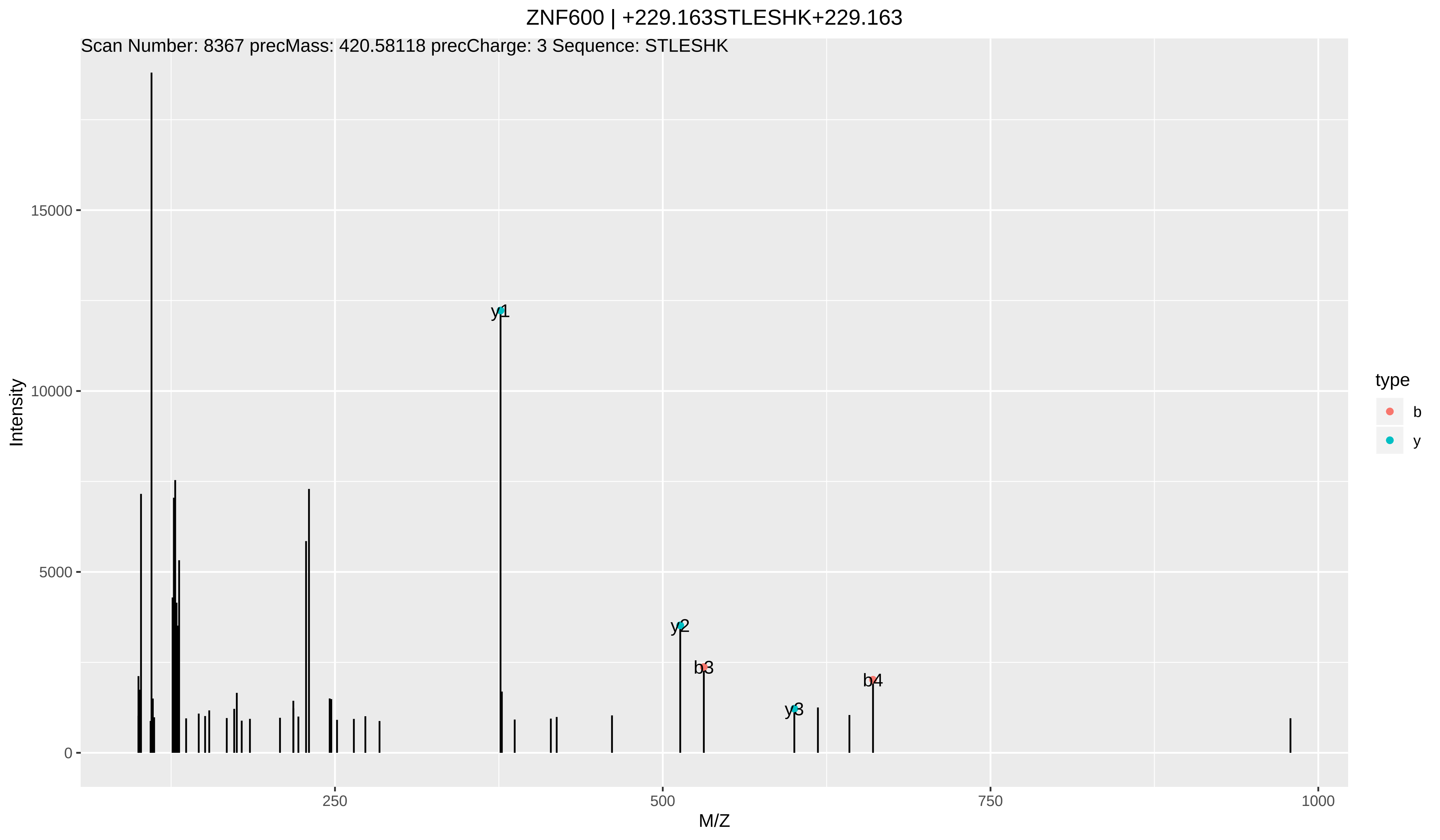

ZNF600 | +229.163STLESHK+229.163
Scan Number: 8367 precMass: 420.58118 precCharge: 3 Sequence: STLESHK
Intensity
M/Z
15000
10000
5000
0
250
500
750
1000
y1
y2
b3
y3
b4
type
b
y

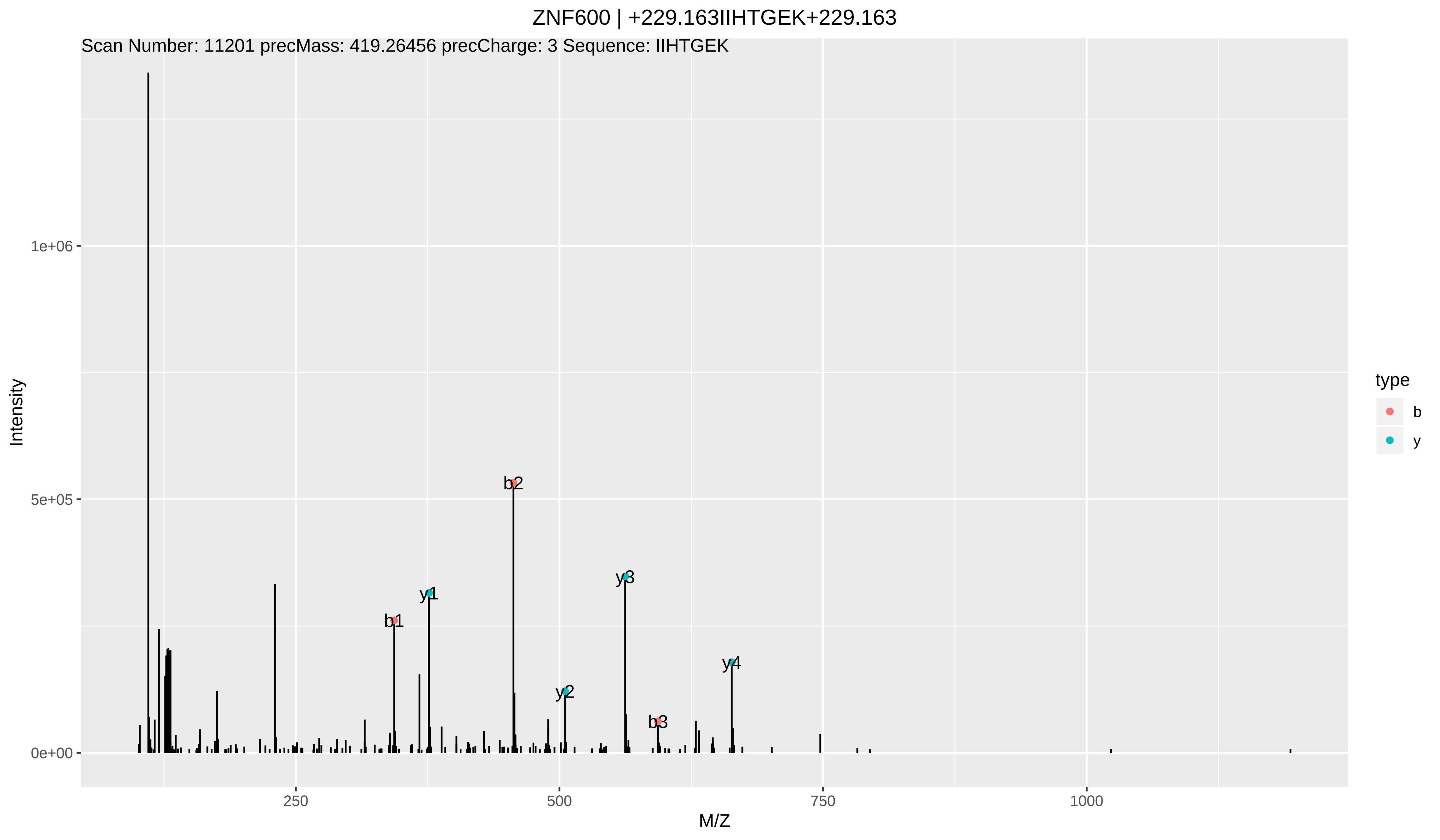

ZNF600 | +229.163IIHTGEK+229.163
Scan Number: 11201 precMass: 419.26456 precCharge: 3 Sequence: IIHTGEK
Intensity
1e+06
5e+05
0e+00
M/Z
250
500
750
1000
b1
y1
b2
y2
y3
b3
y4
type
b
y

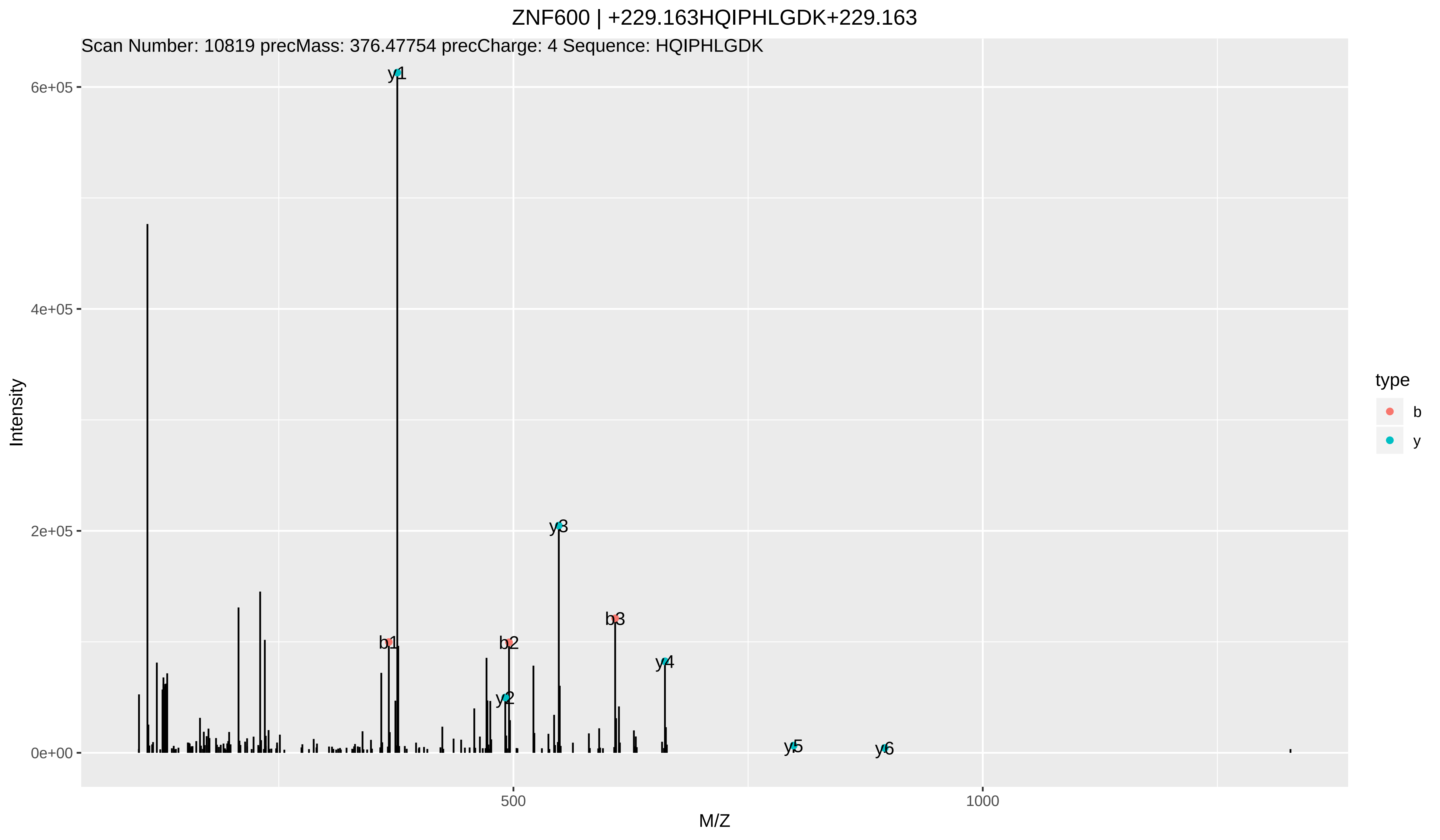
ZNF600 | +229.163HQIPHLGDK+229.163
Scan Number: 10819 precMass: 376.47754 precCharge: 4 Sequence: HQIPHLGDK
Intensity
6e+05
4e+05
2e+05
0e+00
M/Z
500
1000
y1
y2
y3
y4
y5
y6
b1
b2
b3
type
b
y

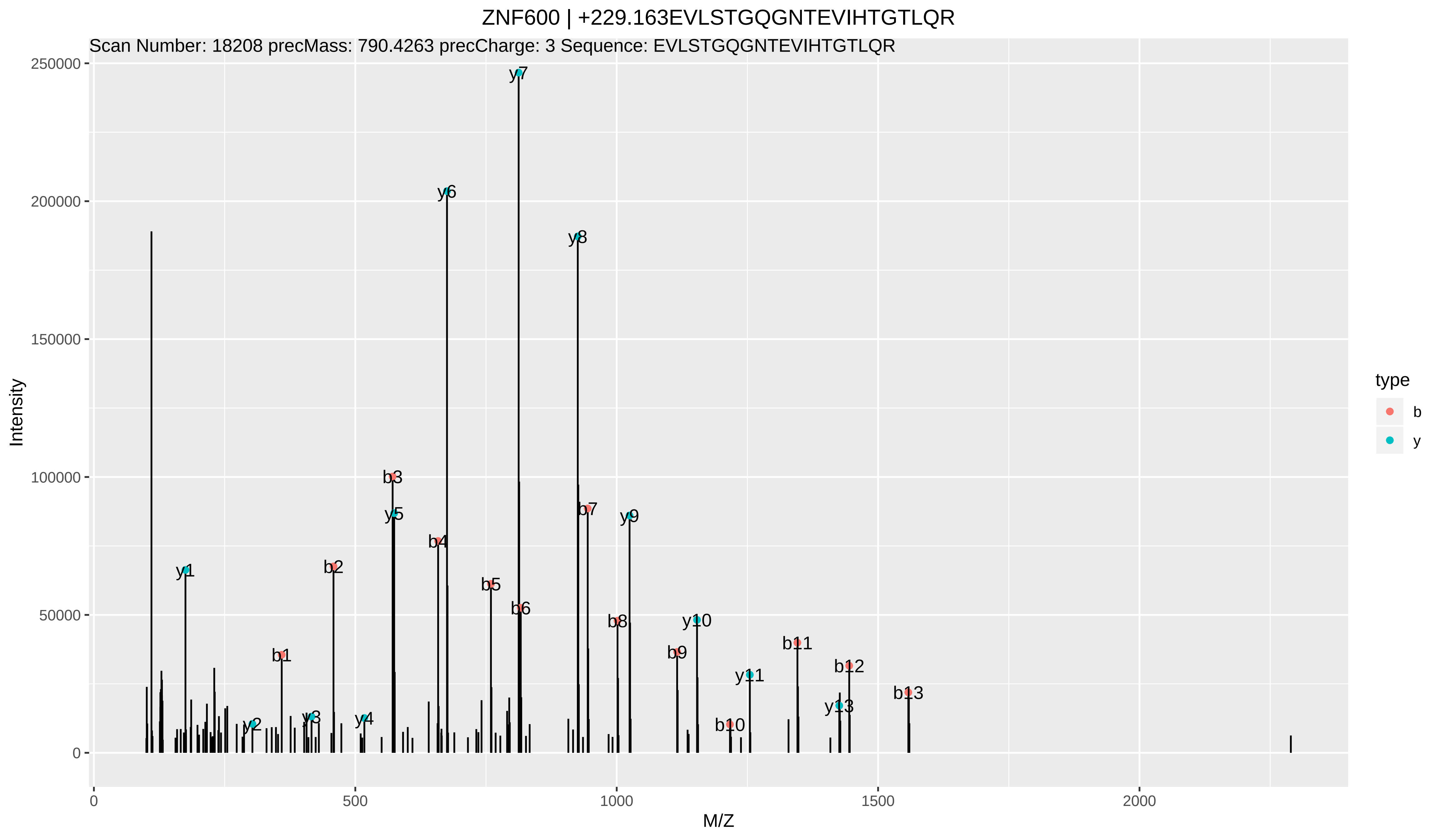
ZNF600 | +229.163EVLSTGQGNTEVIHTGTLQR
Scan Number: 18208 precMass: 790.4263 precCharge: 3 Sequence: EVLSTGQGNTEVIHTGTLQR
Intensity
M/Z
type
b
y
y7
y6
y8
b3
y5
b7
y9
b4
y1
b2
b5
b6
b8
y10
b1
b9
b11
b12
y11
b13
y13
y2
y3
y4
b10
0
50000
100000
150000
200000
250000
500
1000
1500
2000

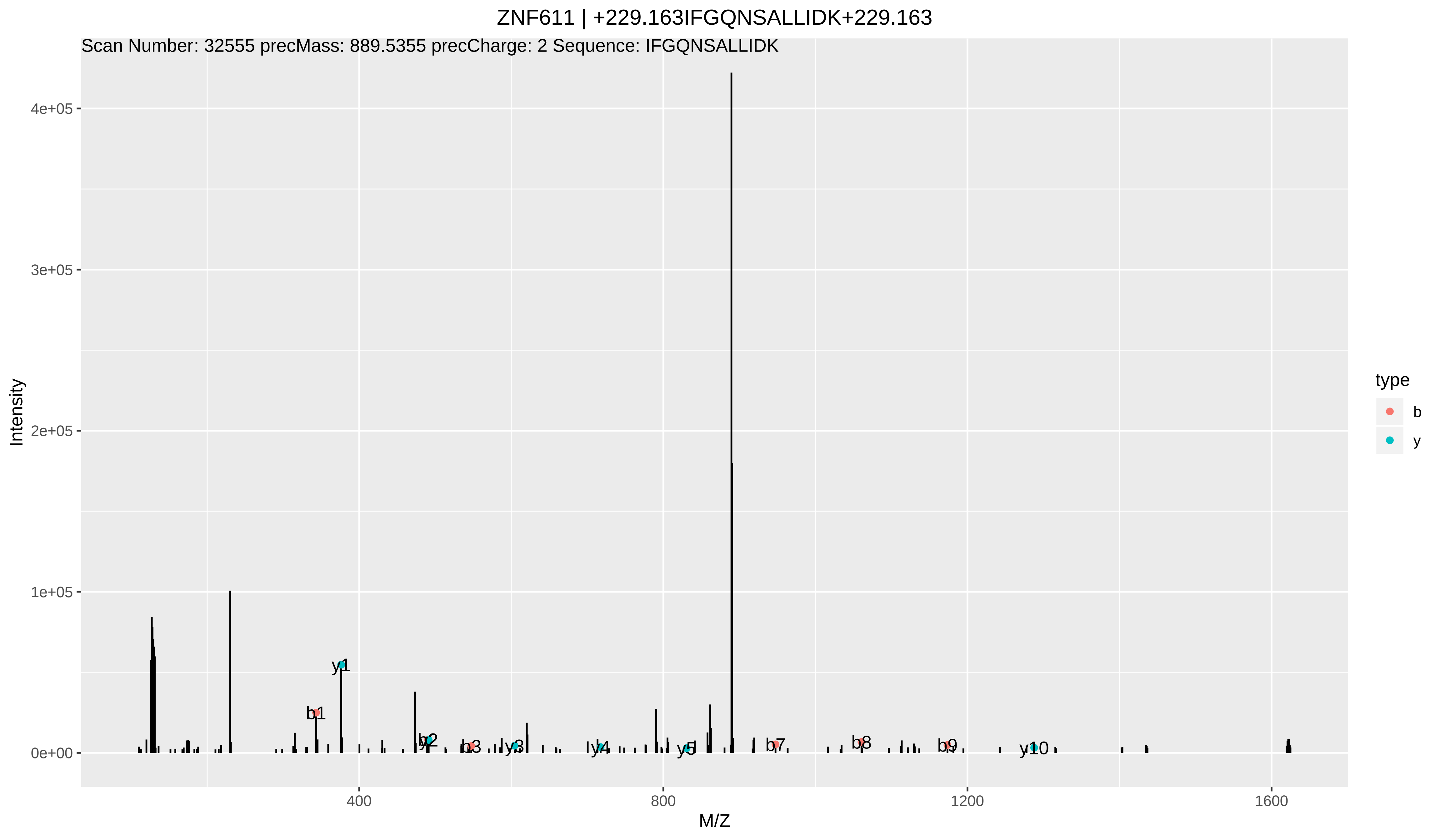
ZNF611 | +229.163IFGQNSALLIDK+229.163
Scan Number: 32555 precMass: 889.5355 precCharge: 2 Sequence: IFGQNSALLIDK
Intensity
M/Z
type
b
y
y1
b1
y2
b2
b3
y3
y4
y5
b7
b8
b9
y10
4e+05
3e+05
2e+05
1e+05
0e+00
400
800
1200
1600

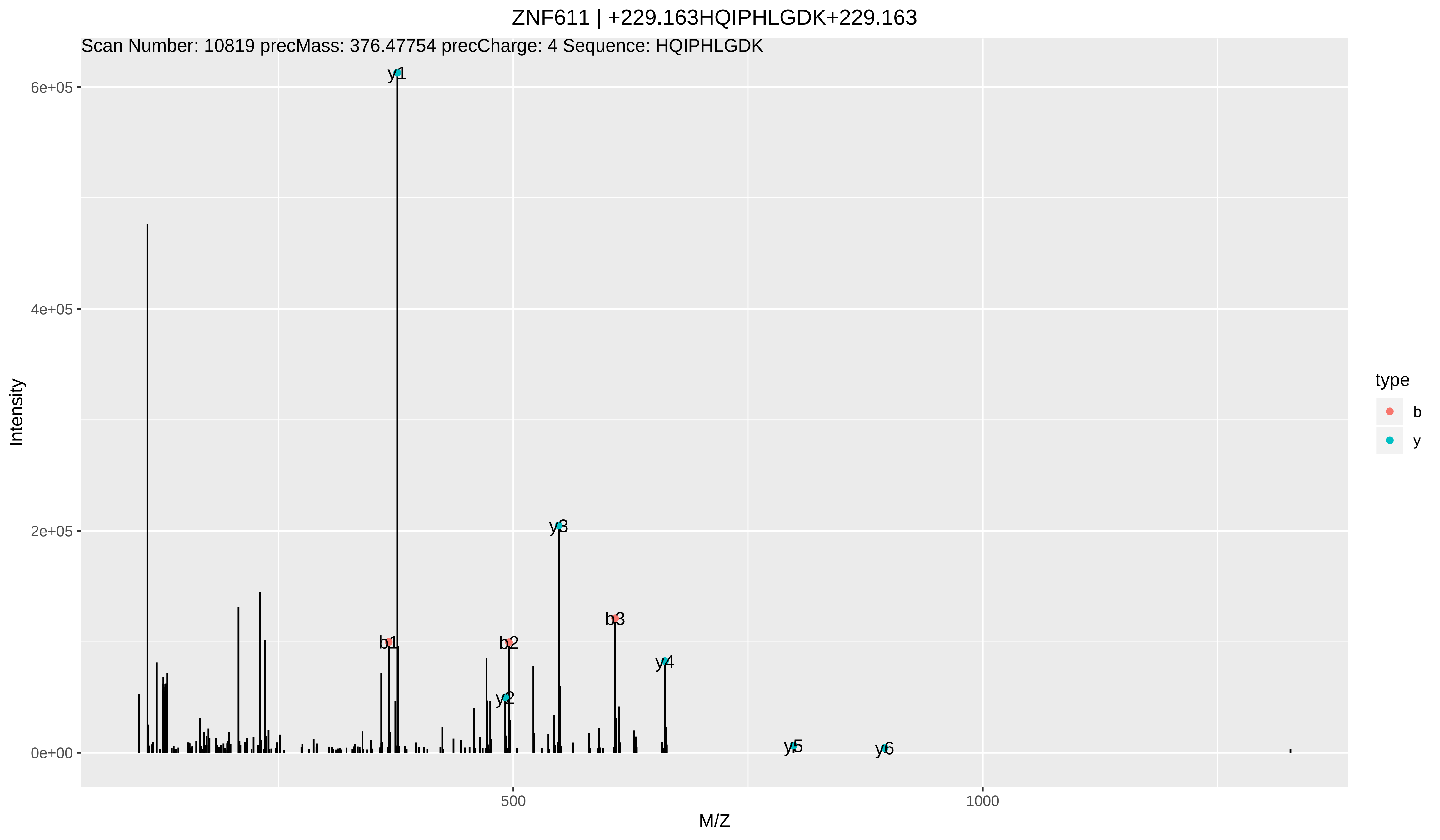
ZNF611 | +229.163HQIPHLGDK+229.163
Scan Number: 10819 precMass: 376.47754 precCharge: 4 Sequence: HQIPHLGDK
Intensity
6e+05
4e+05
2e+05
0e+00
M/Z
500
1000
y1
b1
b2
y2
y3
b3
y4
y5
y6
type
b
y

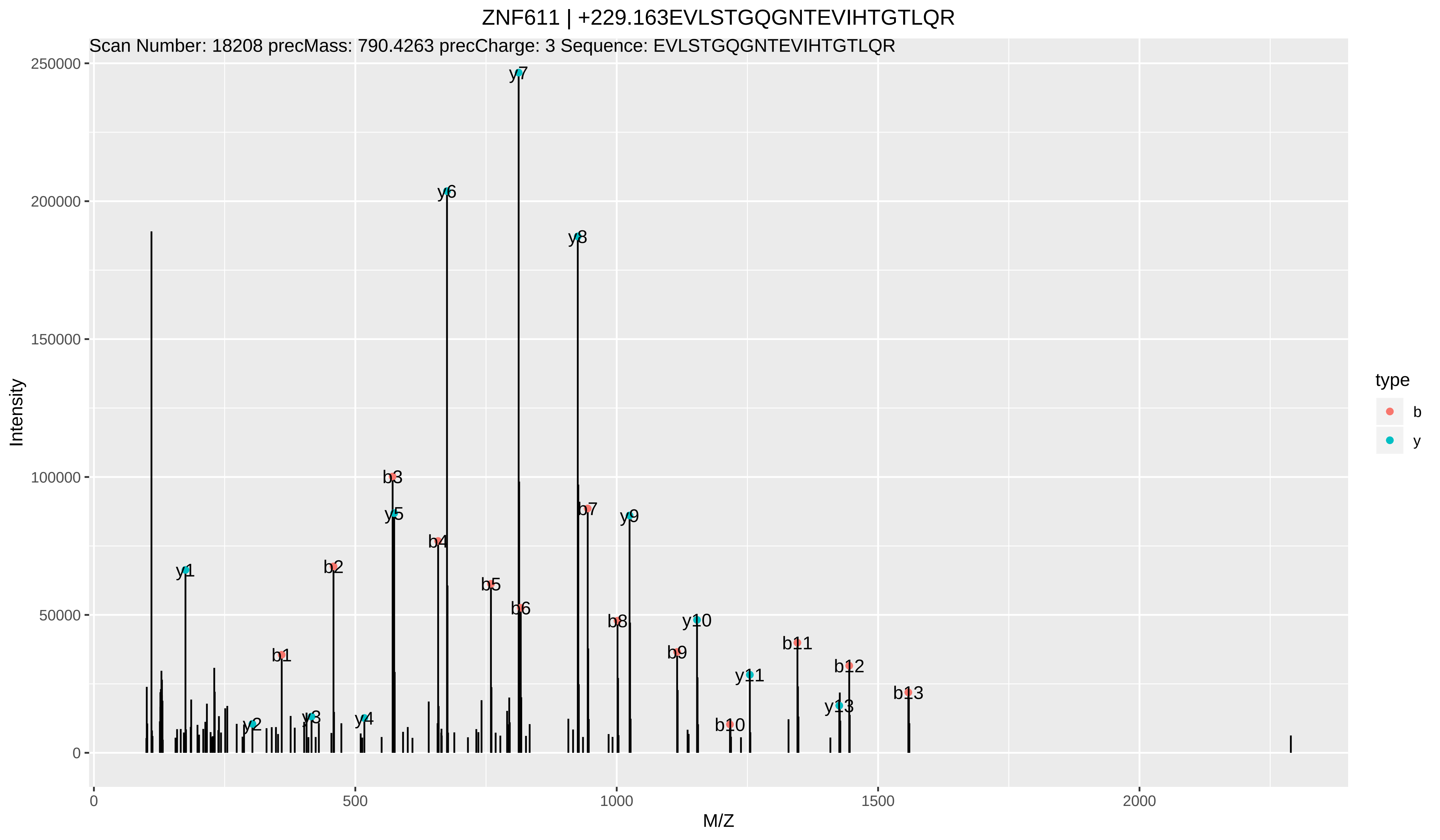
ZNF611 | +229.163EVLSTGQGNTEVIHTGTLQR
Scan Number: 18208 precMass: 790.4263 precCharge: 3 Sequence: EVLSTGQGNTEVIHTGTLQR
Intensity
M/Z
0
50000
100000
150000
200000
250000
0
500
1000
1500
2000
y1
y2
y3
y4
y5
y6
y7
y8
y9
y10
y11
y13
b1
b2
b3
b4
b5
b6
b7
b8
b9
b10
b11
b12
b13
type
b
y

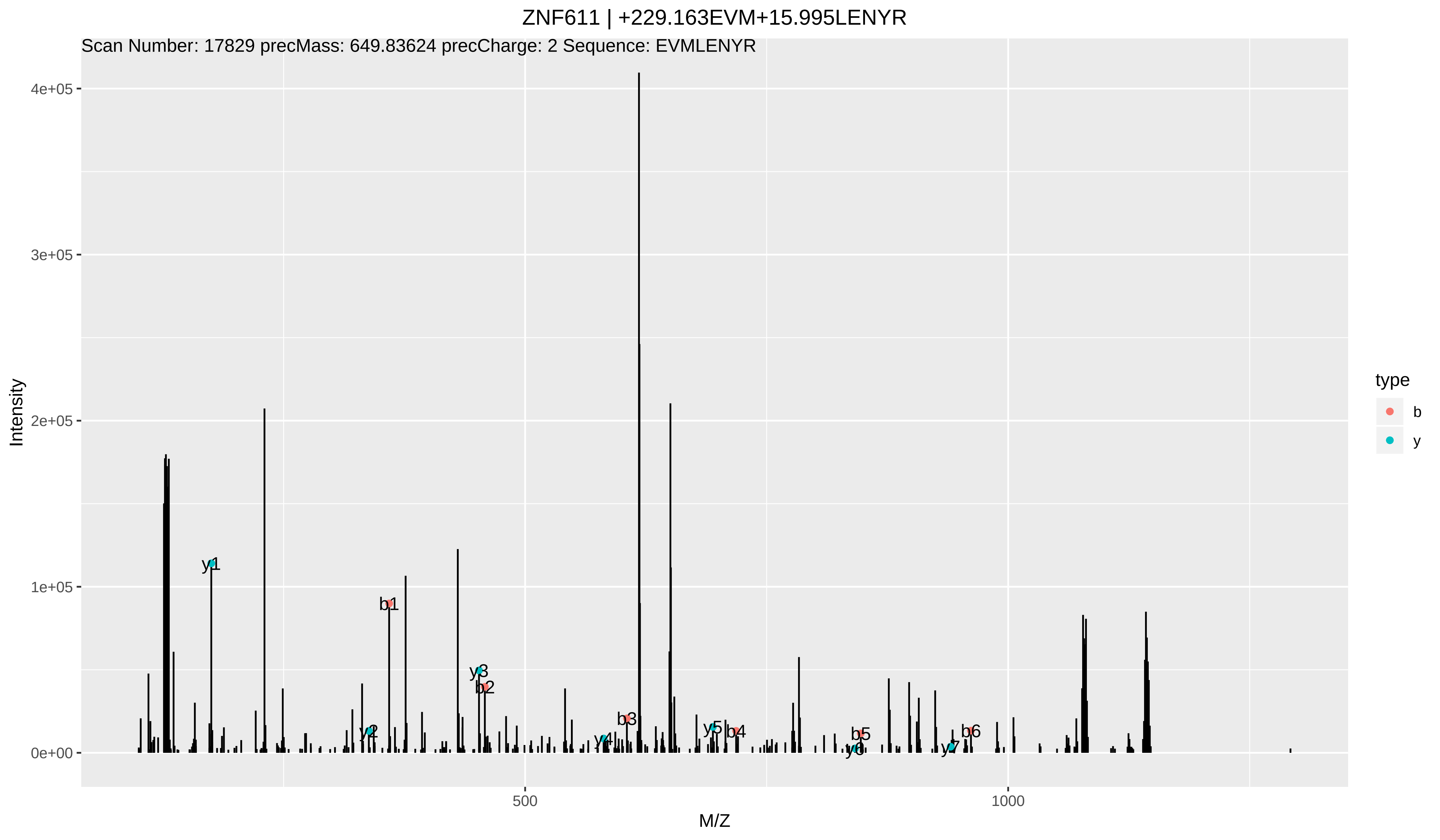
ZNF611 | +229.163EVM+15.995LENYR
Scan Number: 17829 precMass: 649.83624 precCharge: 2 Sequence: EVMLENYR
Intensity
4e+05
3e+05
2e+05
1e+05
0e+00
M/Z
500
1000
y1
b1
y2
y3
b2
y4
b3
y5
b4
y6
b5
y7
b6
type
b
y

ZNF614 | +229.163VDSHLQEHSPNQR

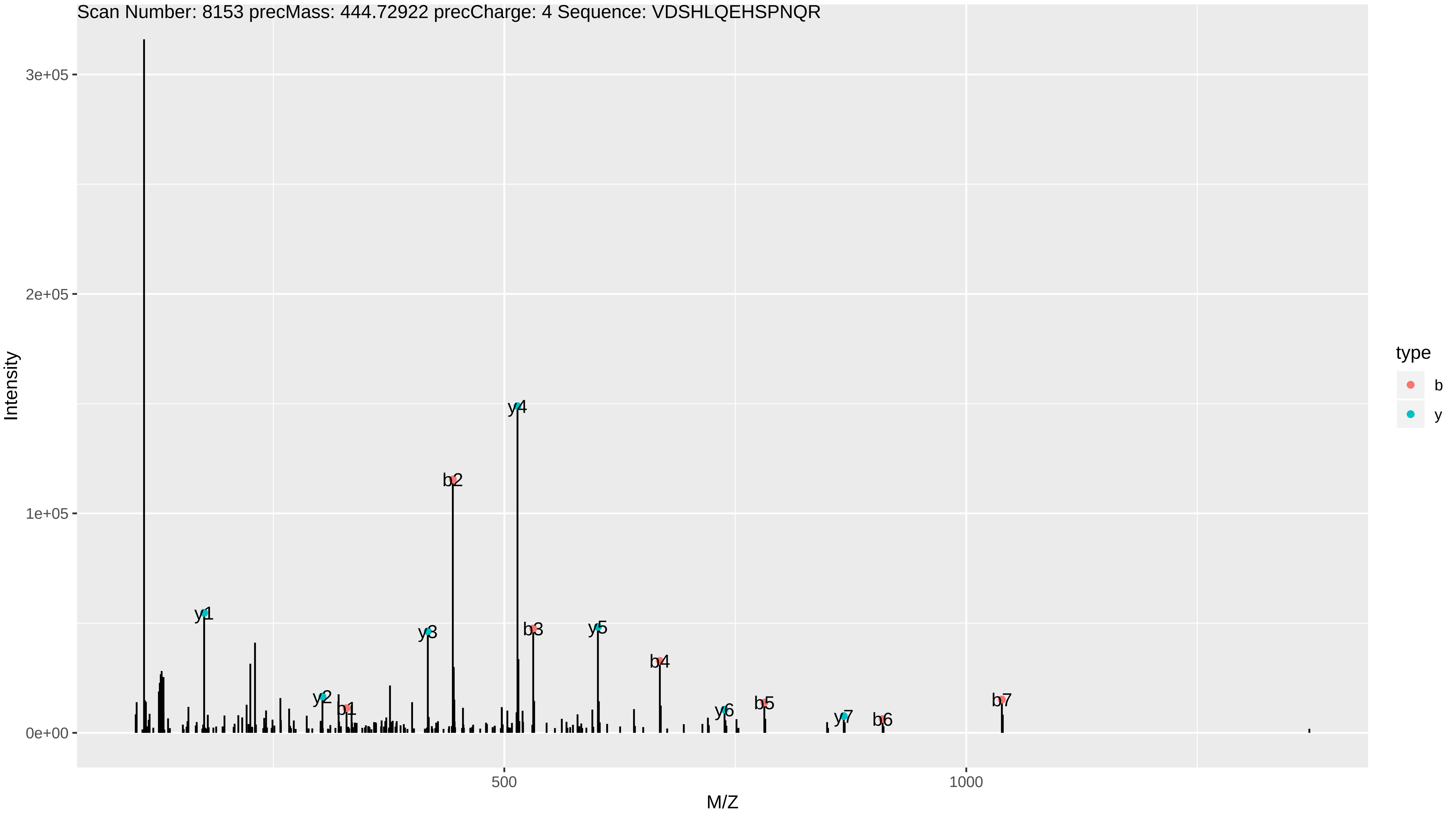

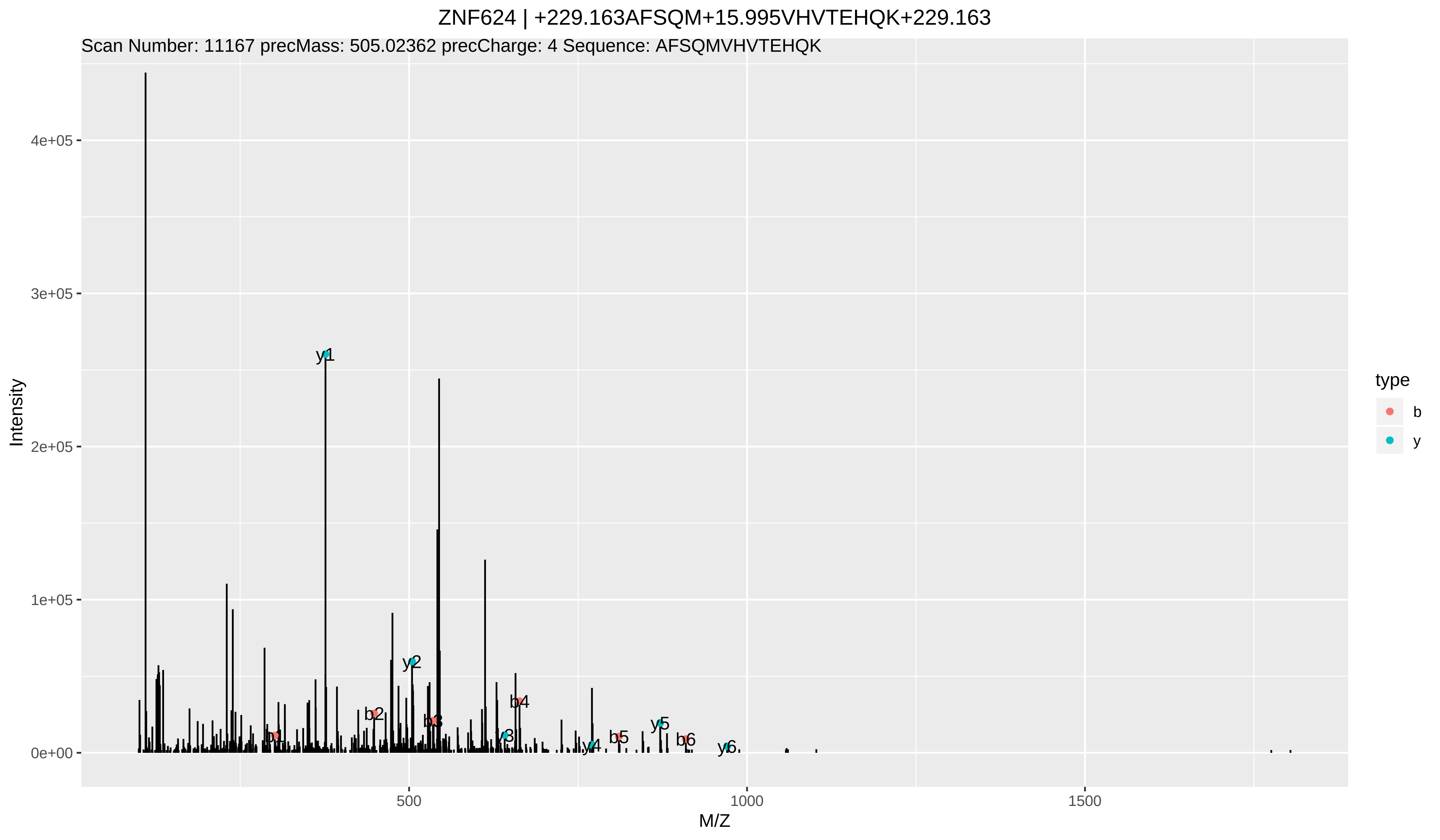
ZNF624 | +229.163AFSQM+15.995VHVTEHQK+229.163
Scan Number: 11167 precMass: 505.02362 precCharge: 4 Sequence: AFSQMVHVTEHQK
Intensity
4e+05
3e+05
2e+05
1e+05
0e+00
M/Z
500
1000
1500
y1
y2
b1
b2
b3
b4
y3
y4
b5
y5
b6
y6
type
b
y

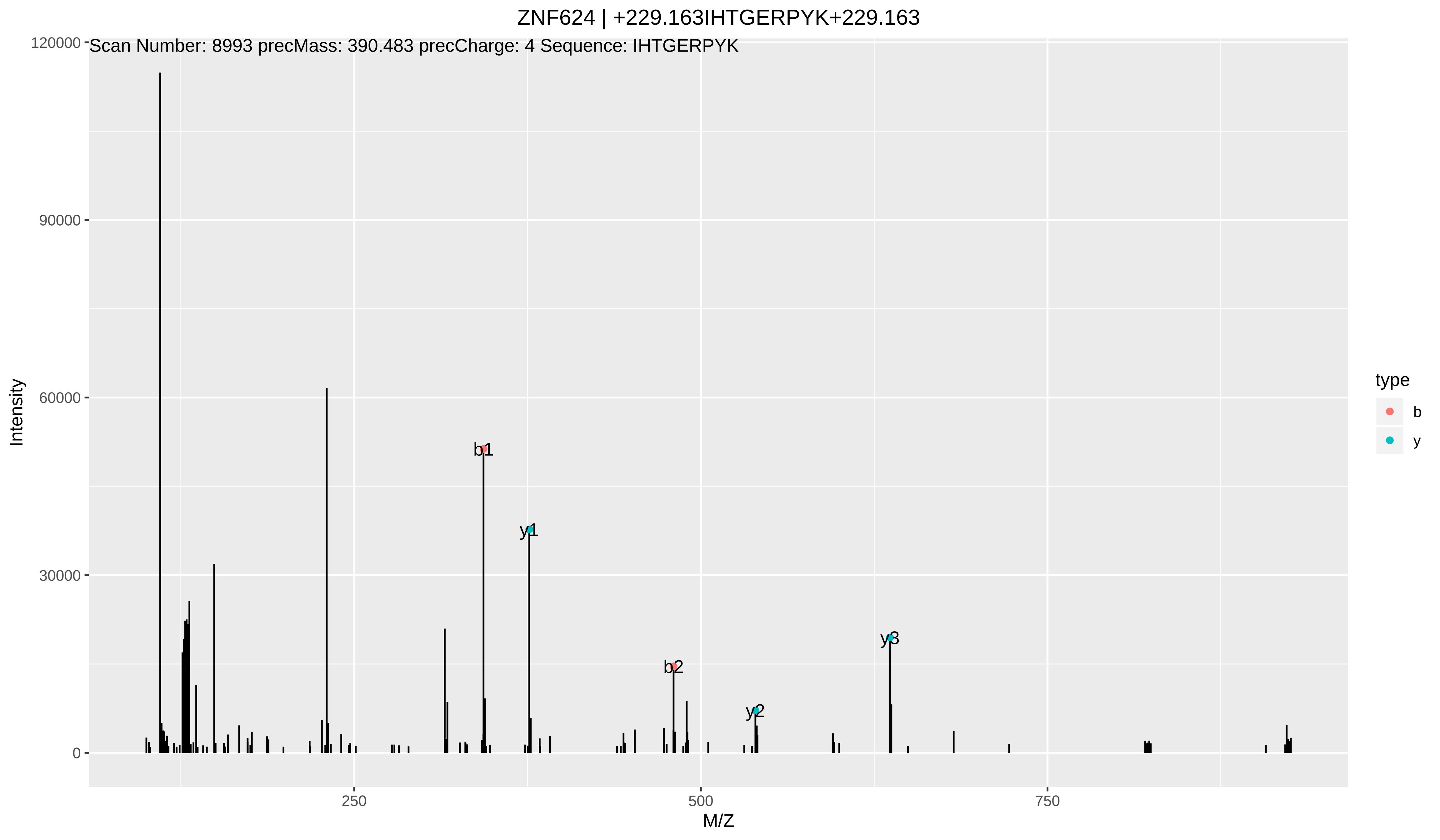

ZNF624 | +229.163IHTGERPYK+229.163
Scan Number: 8993 precMass: 390.483 precCharge: 4 Sequence: IHTGERPYK
Intensity
120000
90000
60000
30000
0
M/Z
250
500
750
b1
y1
b2
y2
y3
type
b
y

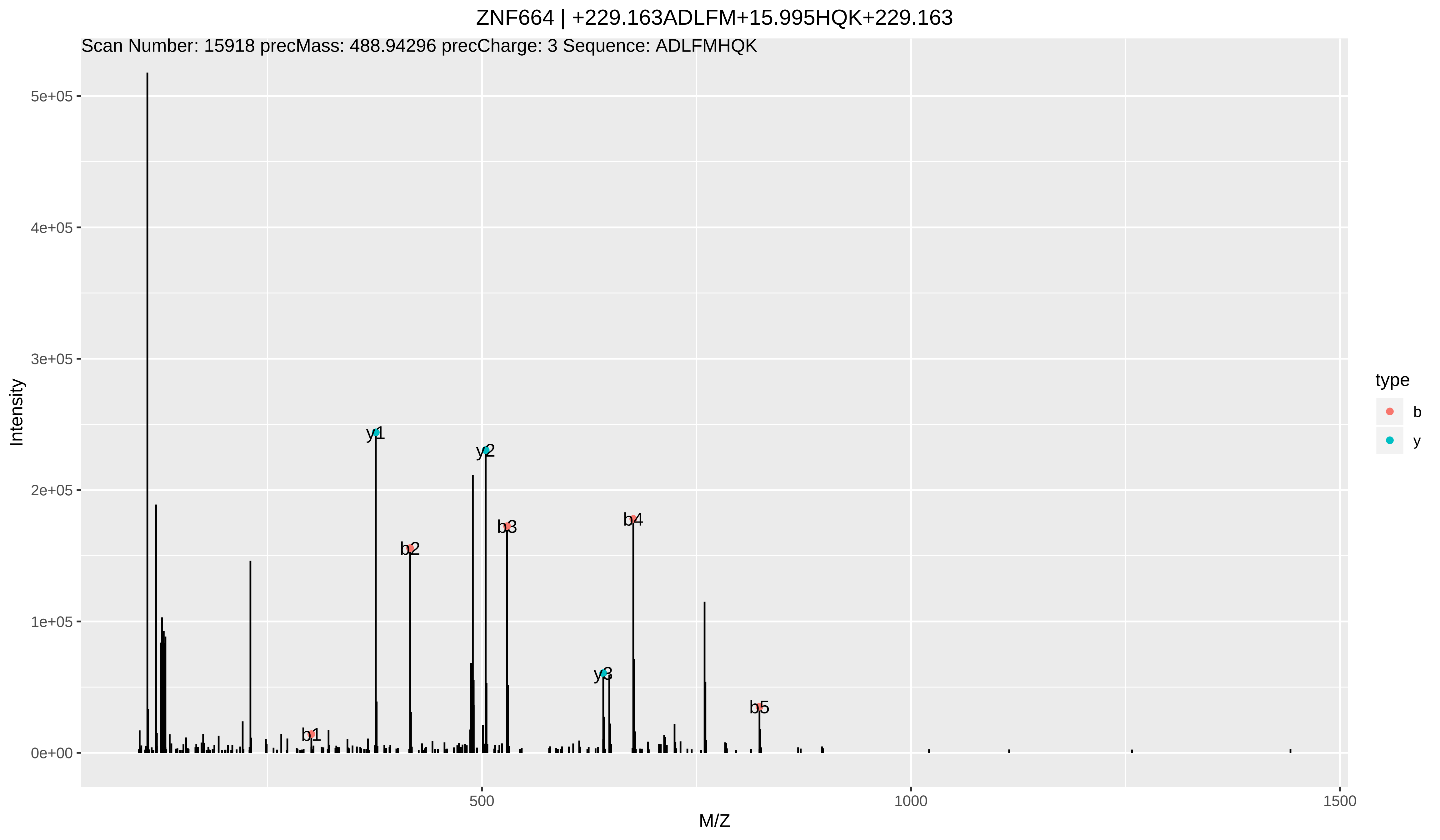

ZNF664 | +229.163ADLFM+15.995HQK+229.163
Scan Number: 15918 precMass: 488.94296 precCharge: 3 Sequence: ADLFMHQK
Intensity
M/Z
5e+05
4e+05
3e+05
2e+05
1e+05
0e+00
500
1000
1500
y1
y2
y3
b1
b2
b3
b4
b5
type
b
y

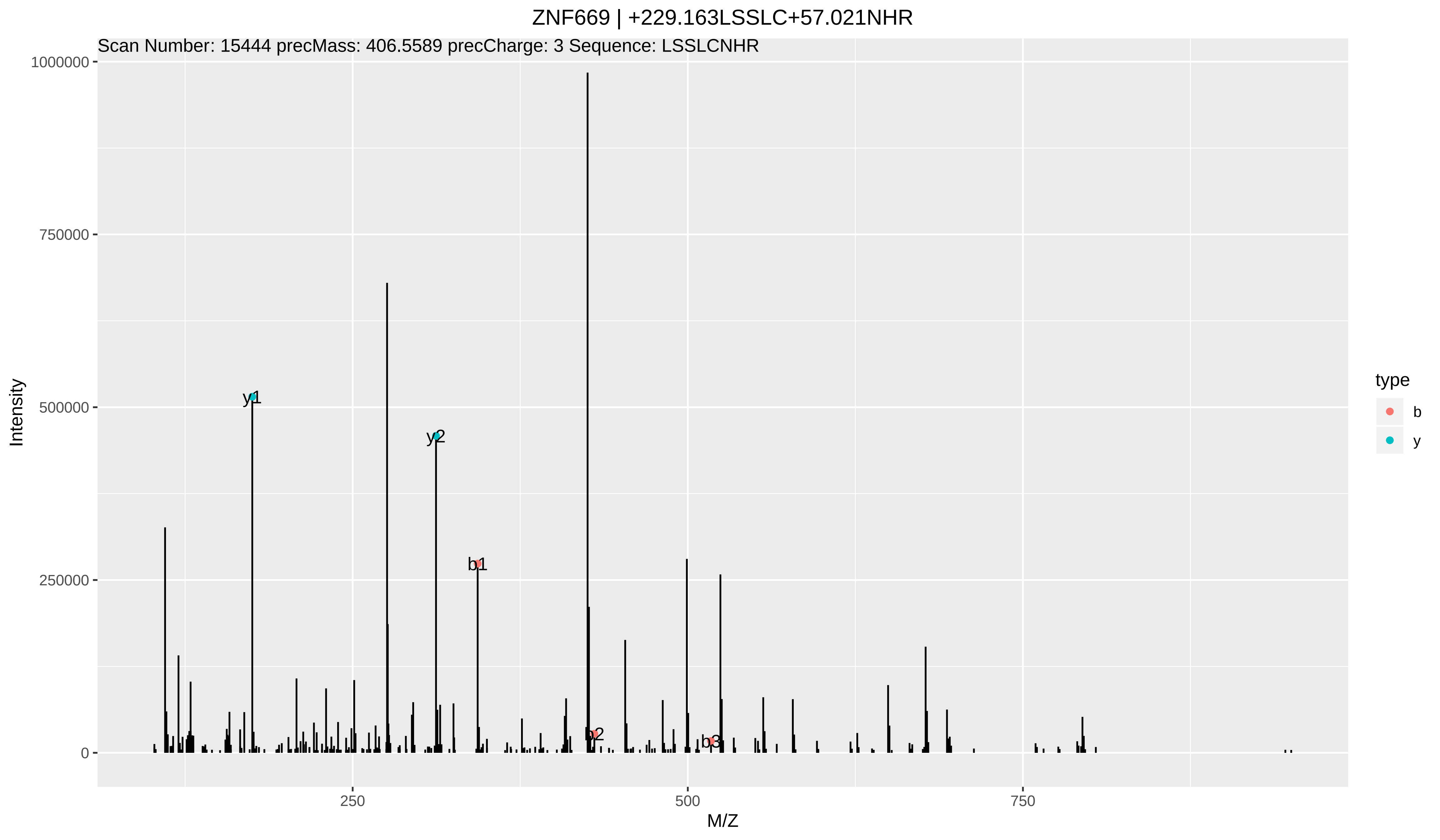

ZNF669 | +229.163LSSLC+57.021NHR
Scan Number: 15444 precMass: 406.5589 precCharge: 3 Sequence: LSSLCNHR
Intensity
1000000
750000
500000
250000
0
M/Z
250
500
750
y1
y2
b1
b2
b3
type
b
y

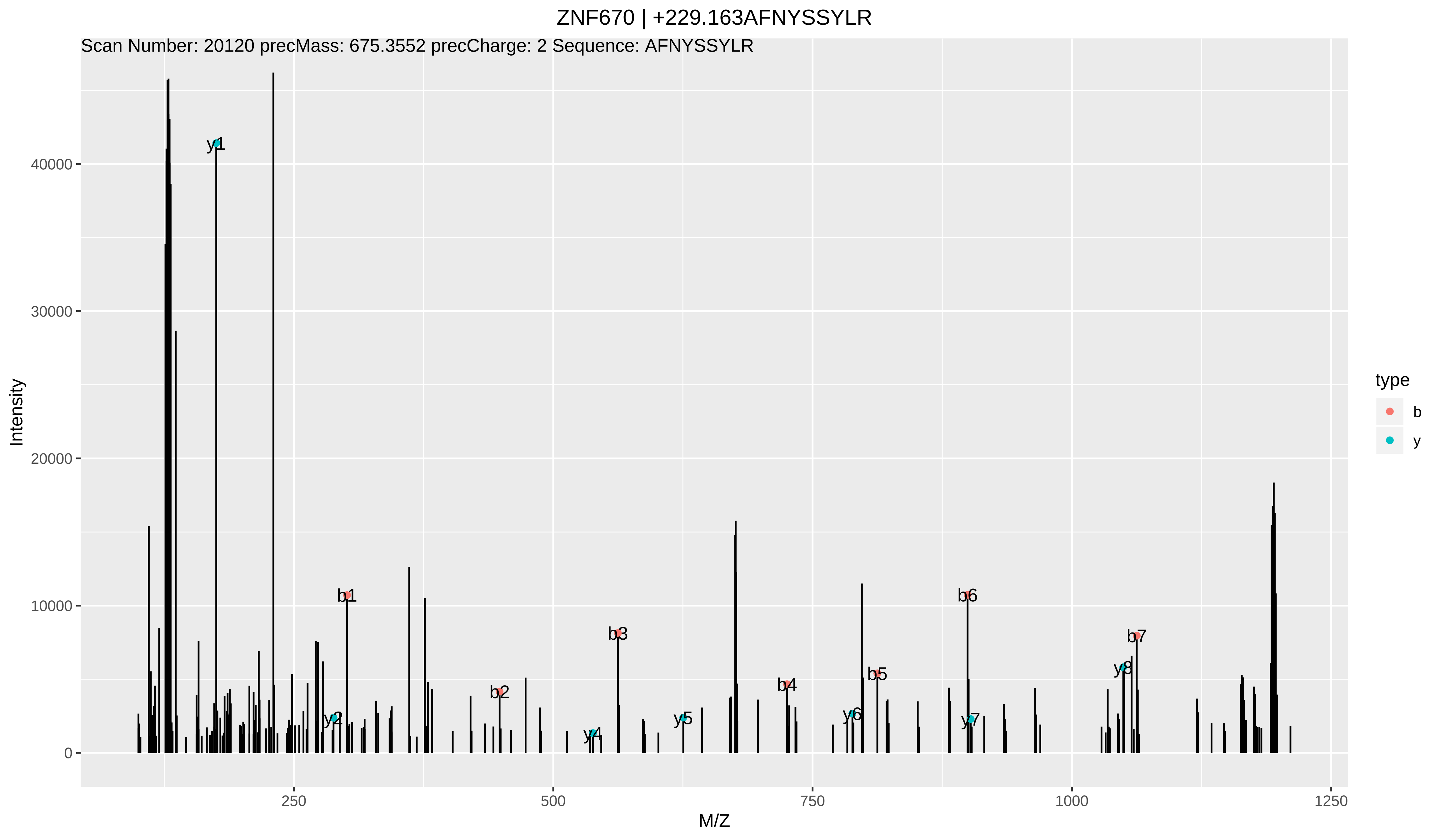
ZNF670 | +229.163AFNYSSYLR
Scan Number: 20120 precMass: 675.3552 precCharge: 2 Sequence: AFNYSSYLR
Intensity
M/Z
0
10000
20000
30000
40000
250
500
750
1000
1250
y1
y2
y4
y5
y6
y7
y8
b1
b2
b3
b4
b5
b6
b7
type
b
y

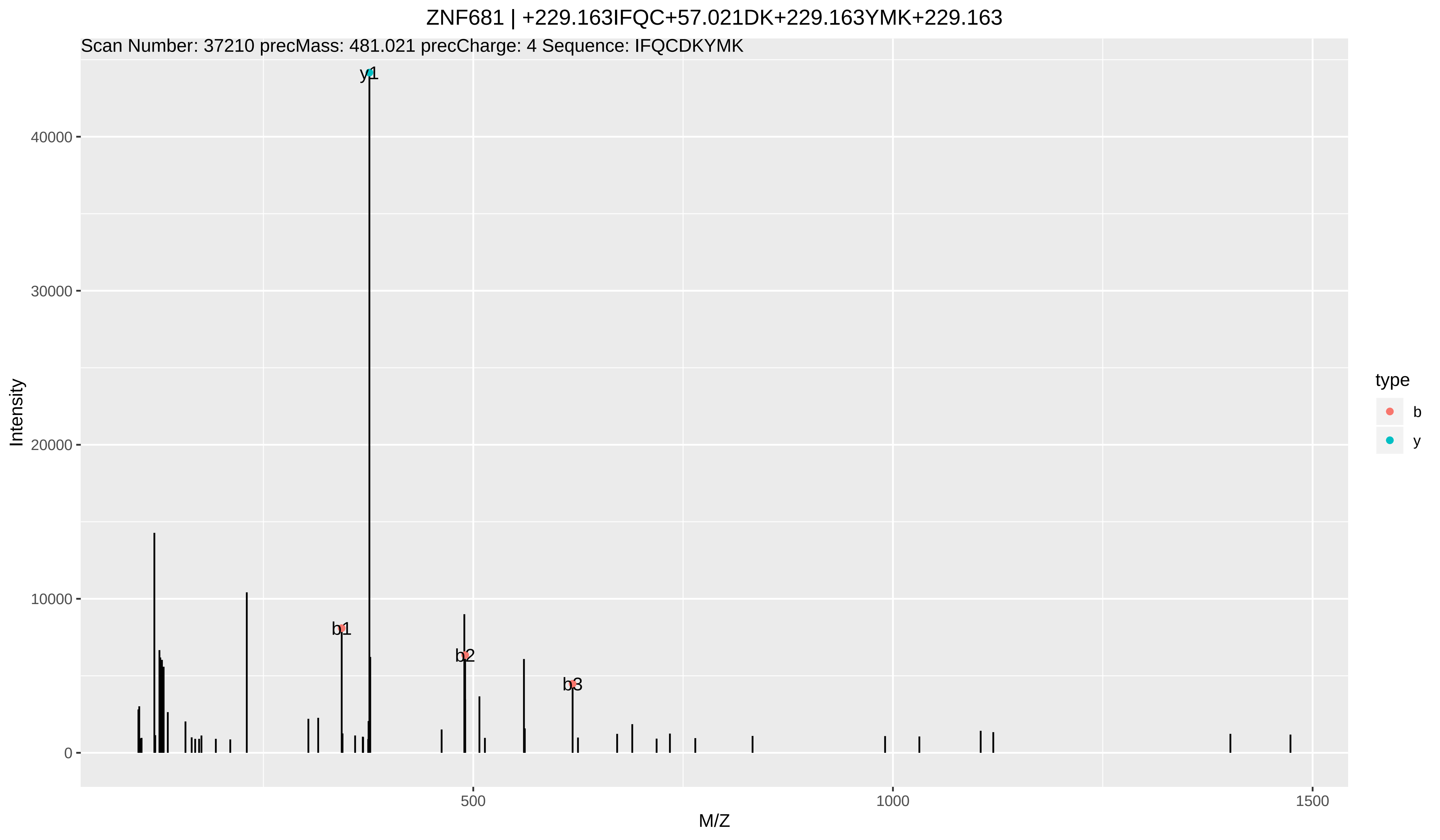

ZNF681 | +229.163IFQC+57.021DK+229.163YMK+229.163
Scan Number: 37210 precMass: 481.021 precCharge: 4 Sequence: IFQCDKYMK
y1
b1
b2
b3
Intensity
40000
30000
20000
10000
0
500
1000
1500
M/Z
type
b
y

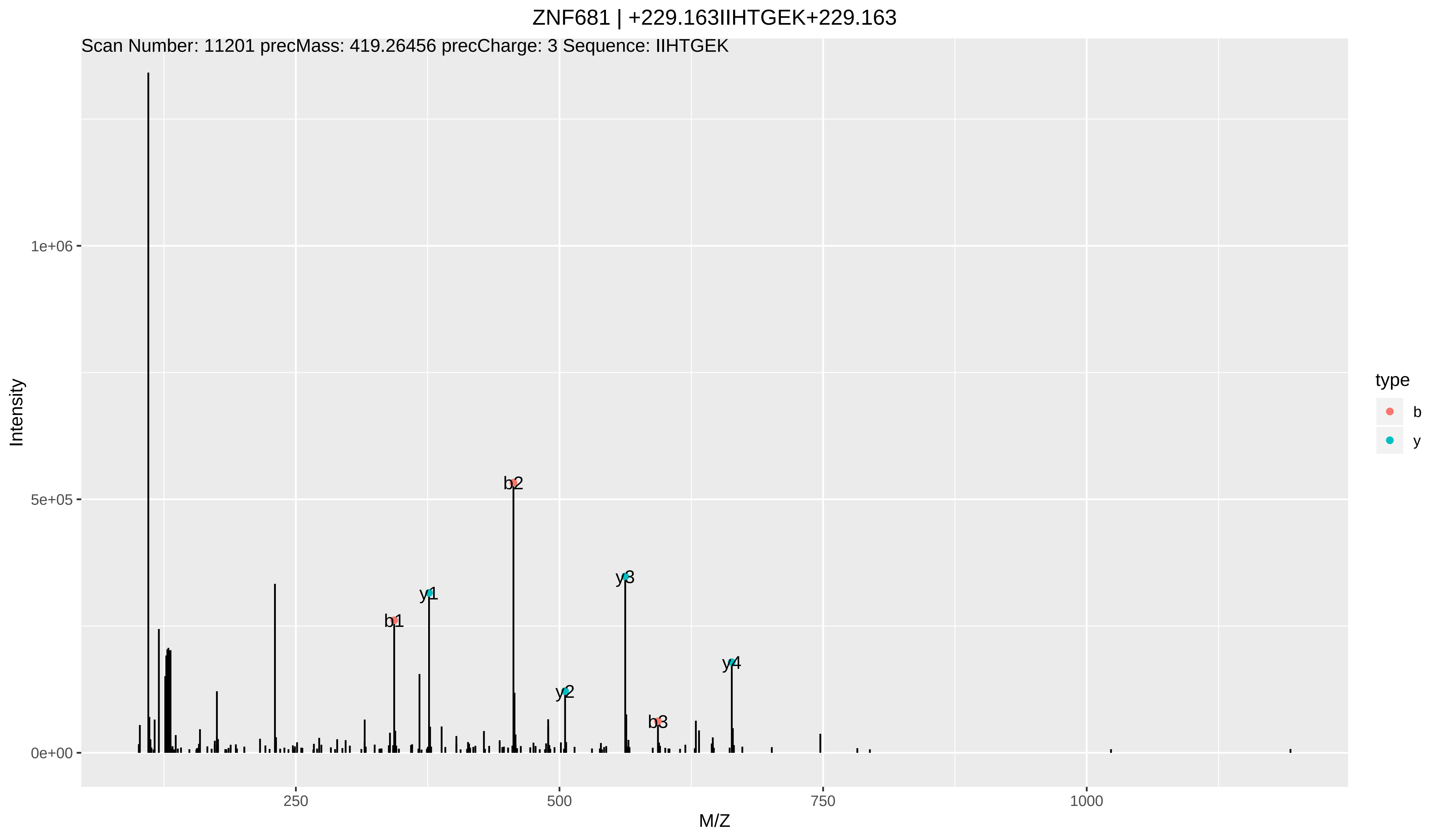
ZNF681 | +229.163IIHTGEK+229.163
Scan Number: 11201 precMass: 419.26456 precCharge: 3 Sequence: IIHTGEK
Intensity
1e+06
5e+05
0e+00
M/Z
250
500
750
1000
b1
y1
b2
y2
y3
b3
y4
type
b
y

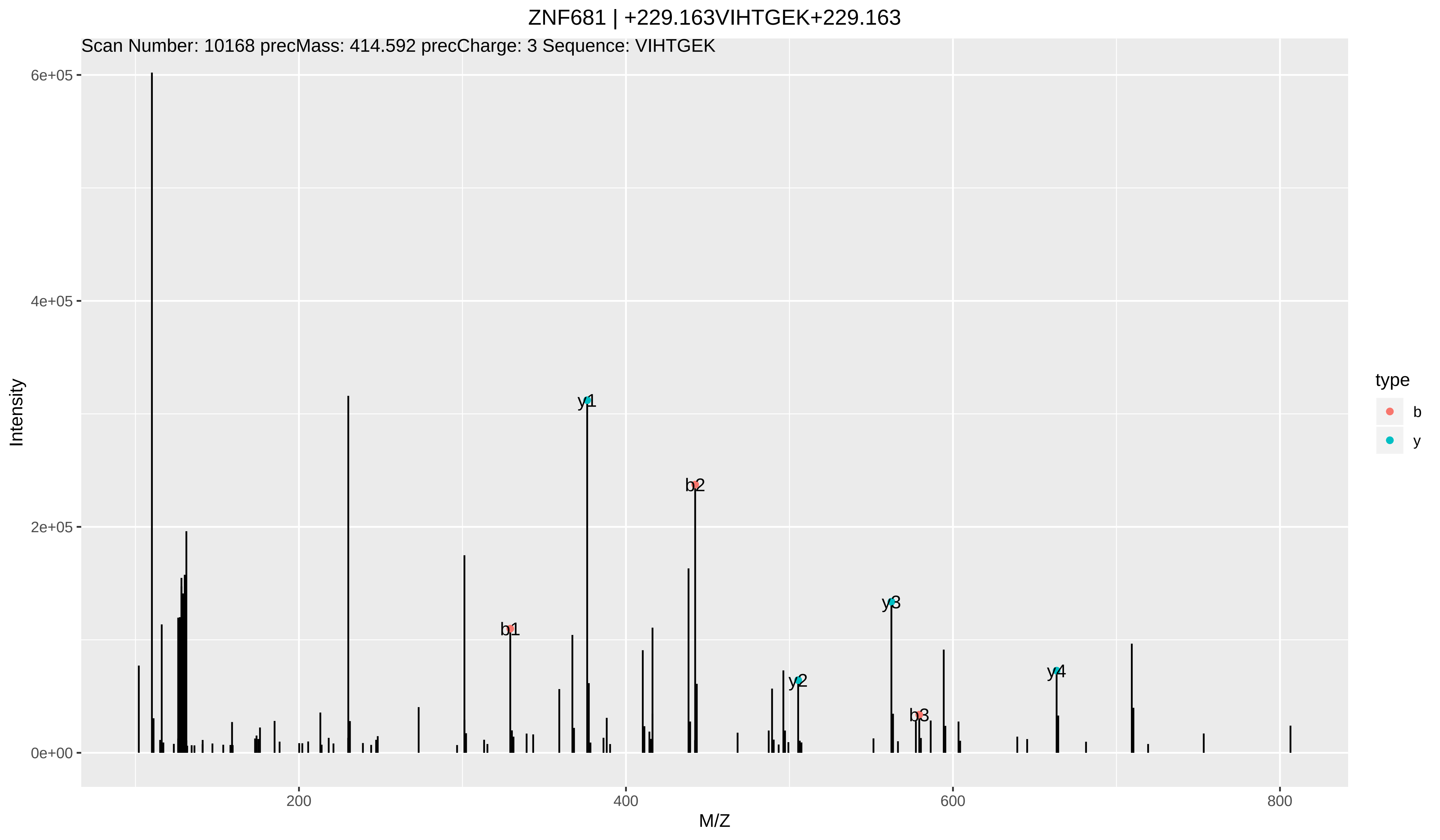

ZNF681 | +229.163VIHTGEK+229.163
Scan Number: 10168 precMass: 414.592 precCharge: 3 Sequence: VIHTGEK
Intensity
6e+05
4e+05
2e+05
0e+00
M/Z
200
400
600
800
y1
b2
y3
b1
y2
y4
b3
type
b
y

ZNF688 | +229.163EEEPEEVPR

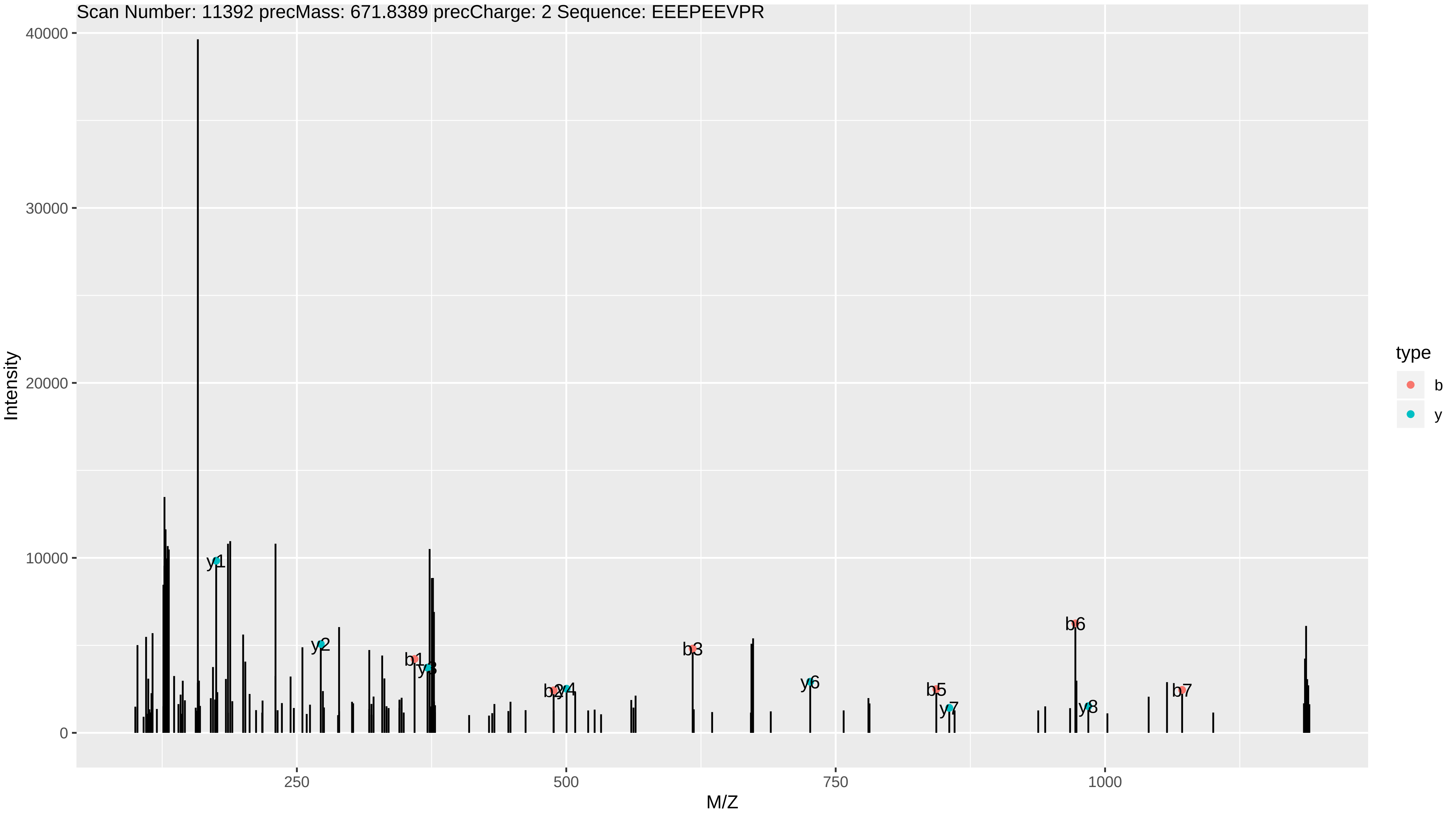

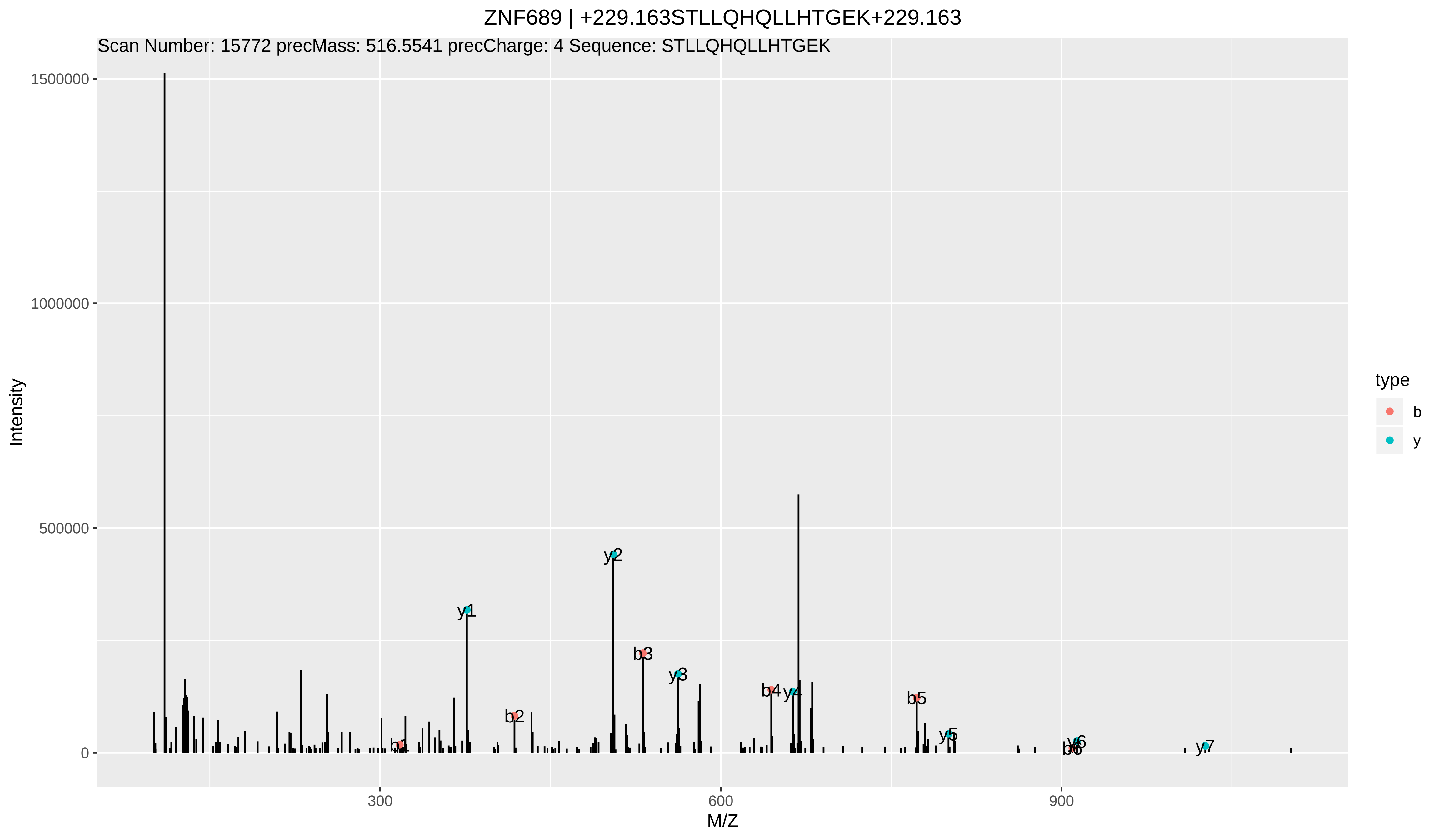
ZNF689 | +229.163STLLQHQLLHTGEK+229.163
Scan Number: 15772 precMass: 516.5541 precCharge: 4 Sequence: STLLQHQLLHTGEK
Intensity
1500000
1000000
500000
0
M/Z
300
600
900
y1
y2
b3
y3
b4
y4
b5
y5
b2
b1
y6
b6
y7
type
b
y

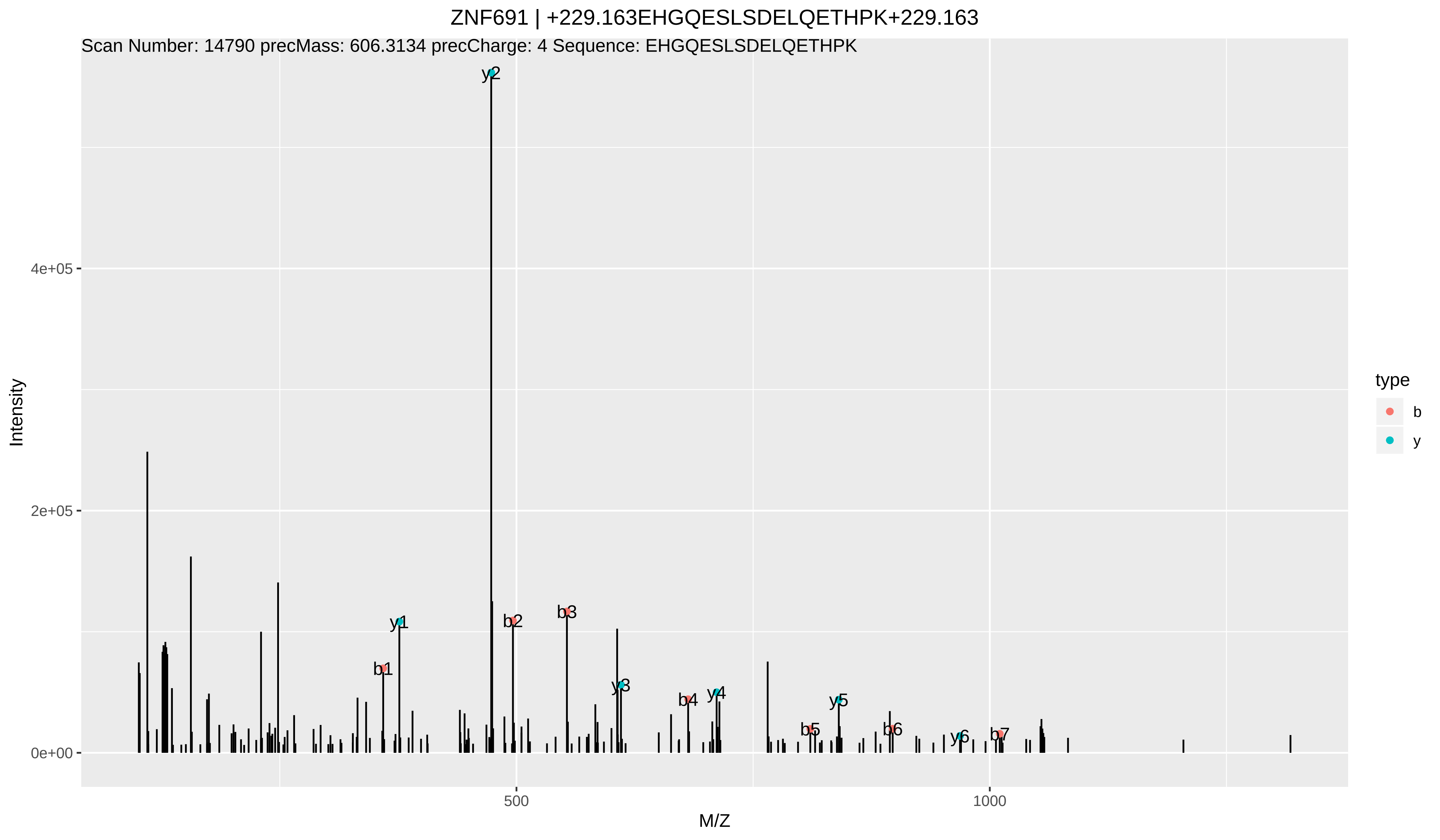
ZNF691 | +229.163EHGQESLSDELQETHPK+229.163
Scan Number: 14790 precMass: 606.3134 precCharge: 4 Sequence: EHGQESLSDELQETHPK
Intensity
M/Z
4e+05
2e+05
0e+00
500
1000
y2
y1
b1
b2
b3
y3
b4
y4
y5
b5
b6
y6
b7
type
b
y

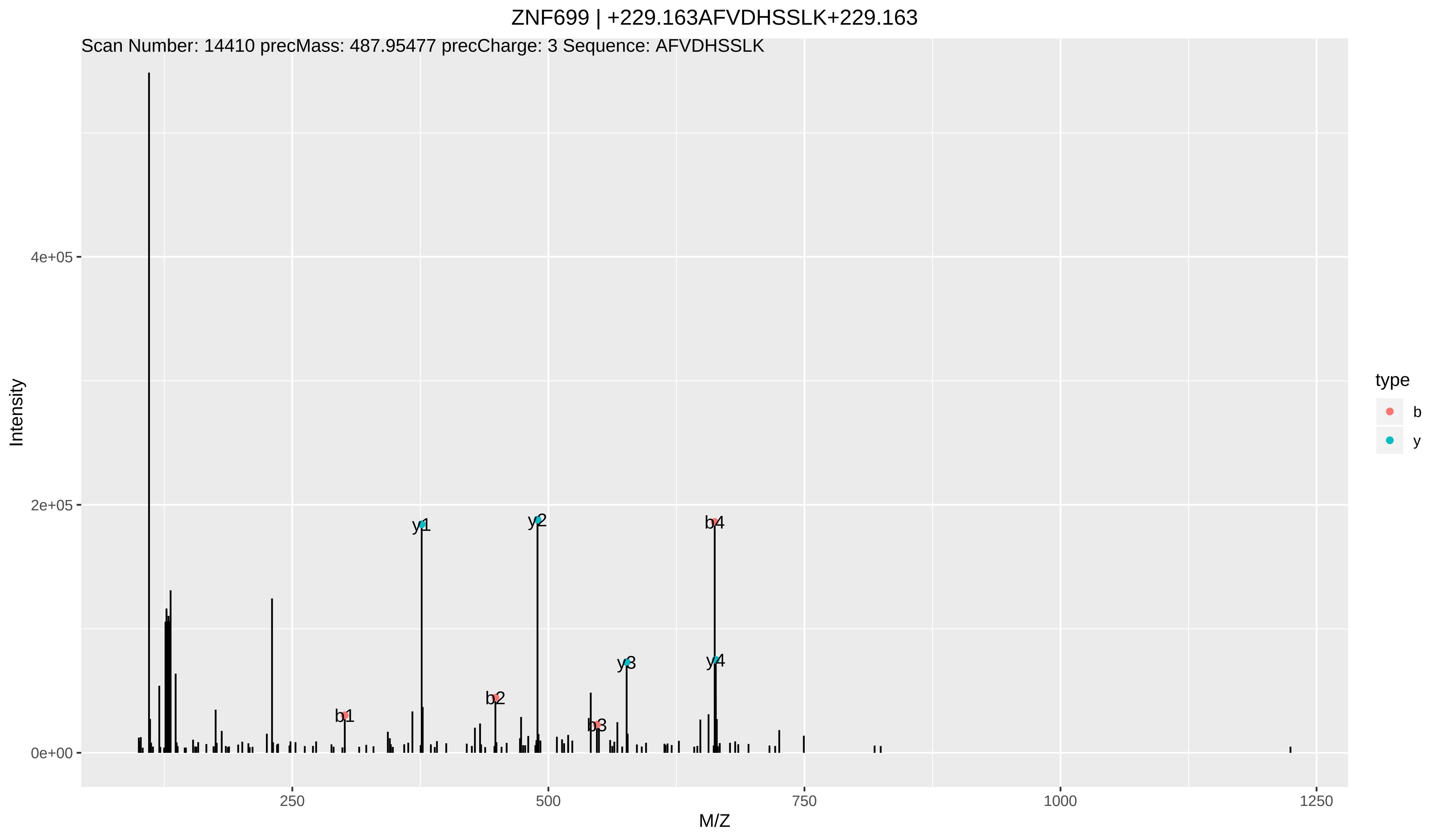
ZNF699 | +229.163AFVDHSSLK+229.163
Scan Number: 14410 precMass: 487.95477 precCharge: 3 Sequence: AFVDHSSLK
Intensity
4e+05
2e+05
0e+00
M/Z
250
500
750
1000
1250
y1
y2
b4
y3
y4
b2
b1
b3
type
b
y

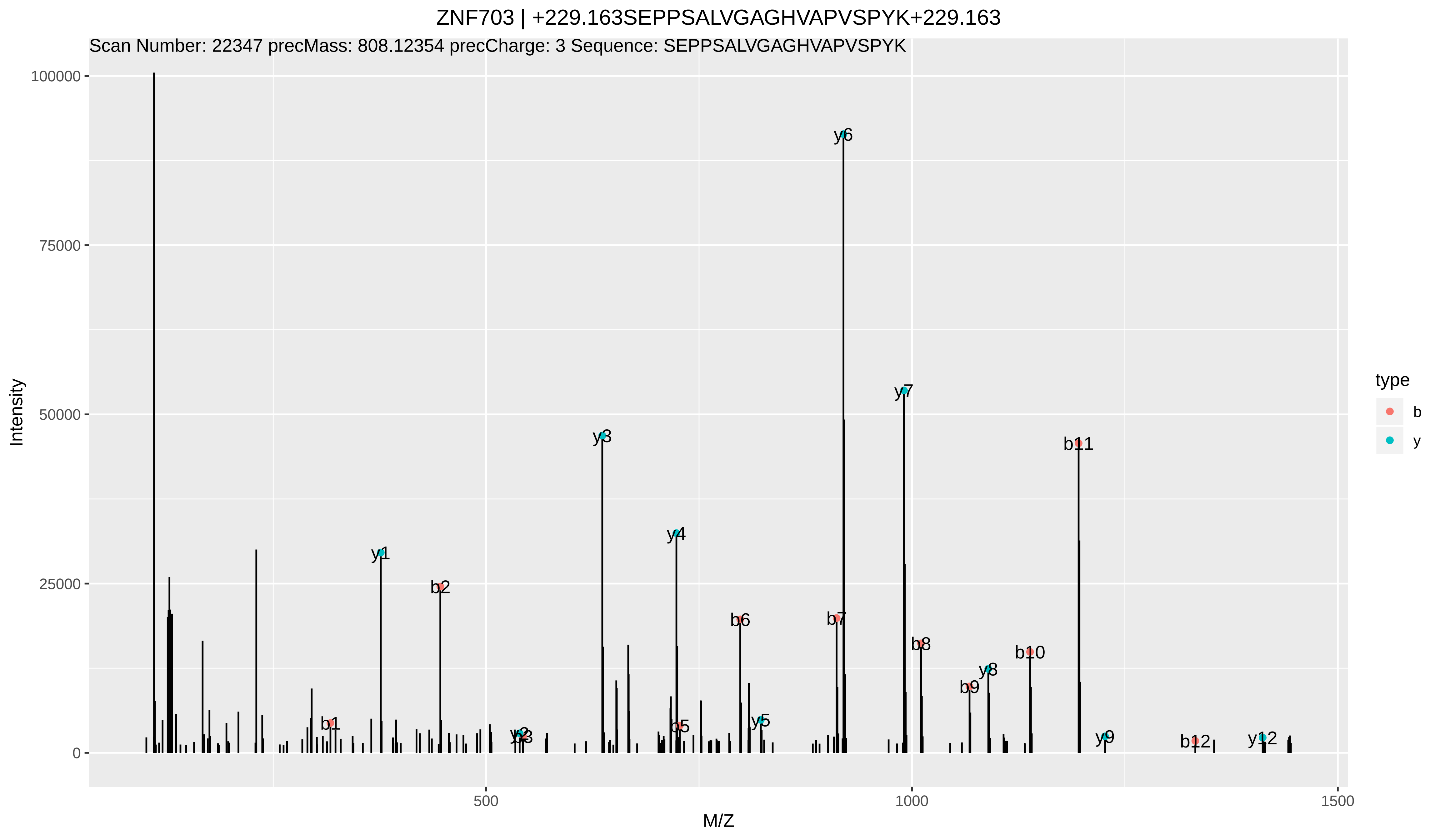
ZNF703 | +229.163SEPPSALVGAGHVAPVSPYK+229.163
Scan Number: 22347 precMass: 808.12354 precCharge: 3 Sequence: SEPPSALVGAGHVAPVSPYK
Intensity
100000
75000
50000
25000
0
500
1000
1500
M/Z
y6
y7
y3
b11
y4
y1
b2
b6
b7
b8
b10
y8
b9
b1
y5
b5
y2
b3
y9
b12
y12
type
b
y

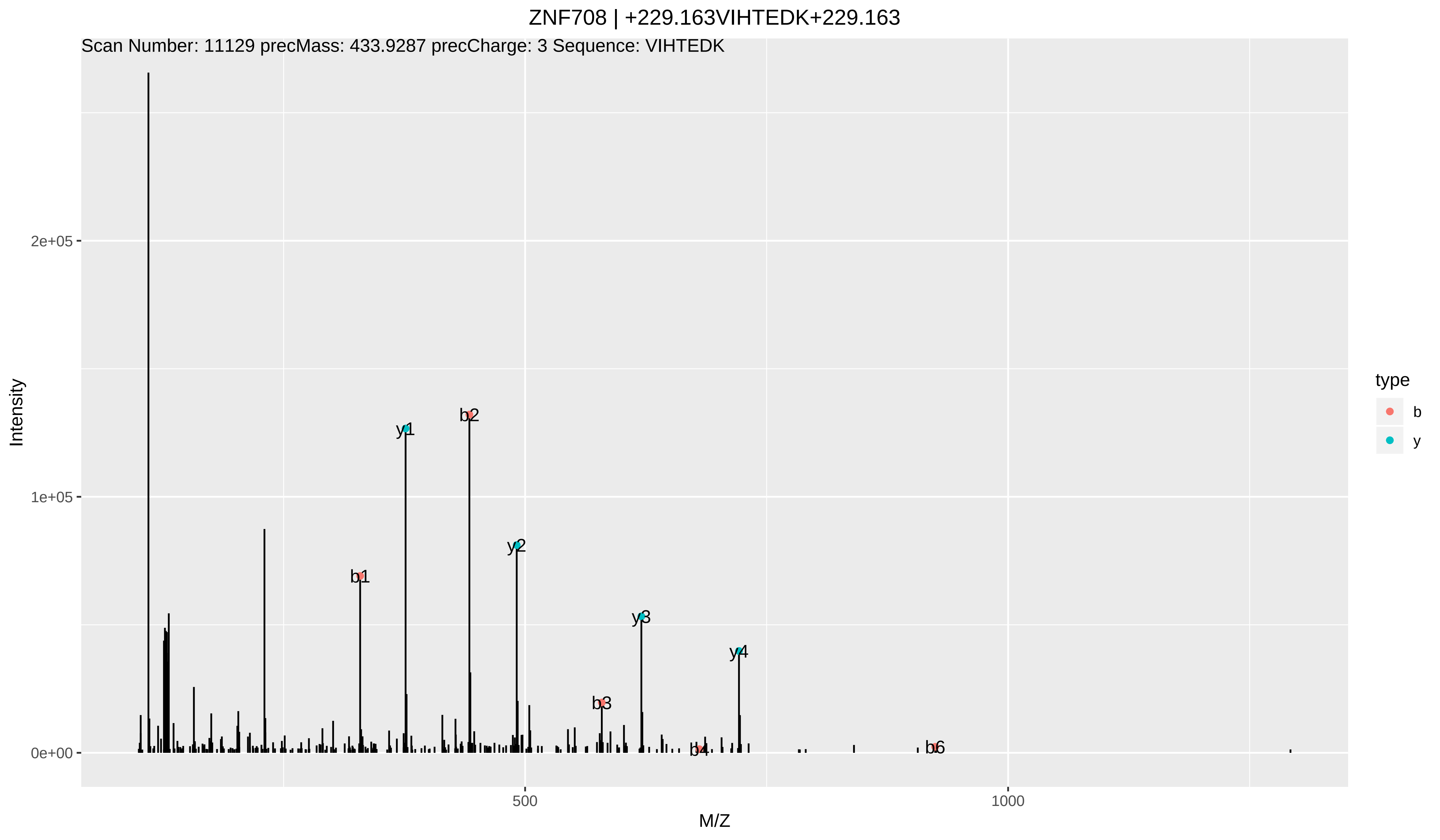
ZNF708 | +229.163VIHTEDK+229.163
Scan Number: 11129 precMass: 433.9287 precCharge: 3 Sequence: VIHTEDK
Intensity
2e+05
1e+05
0e+00
M/Z
500
1000
type
b
y
y1
b2
y2
b1
y3
y4
b3
b4
b6

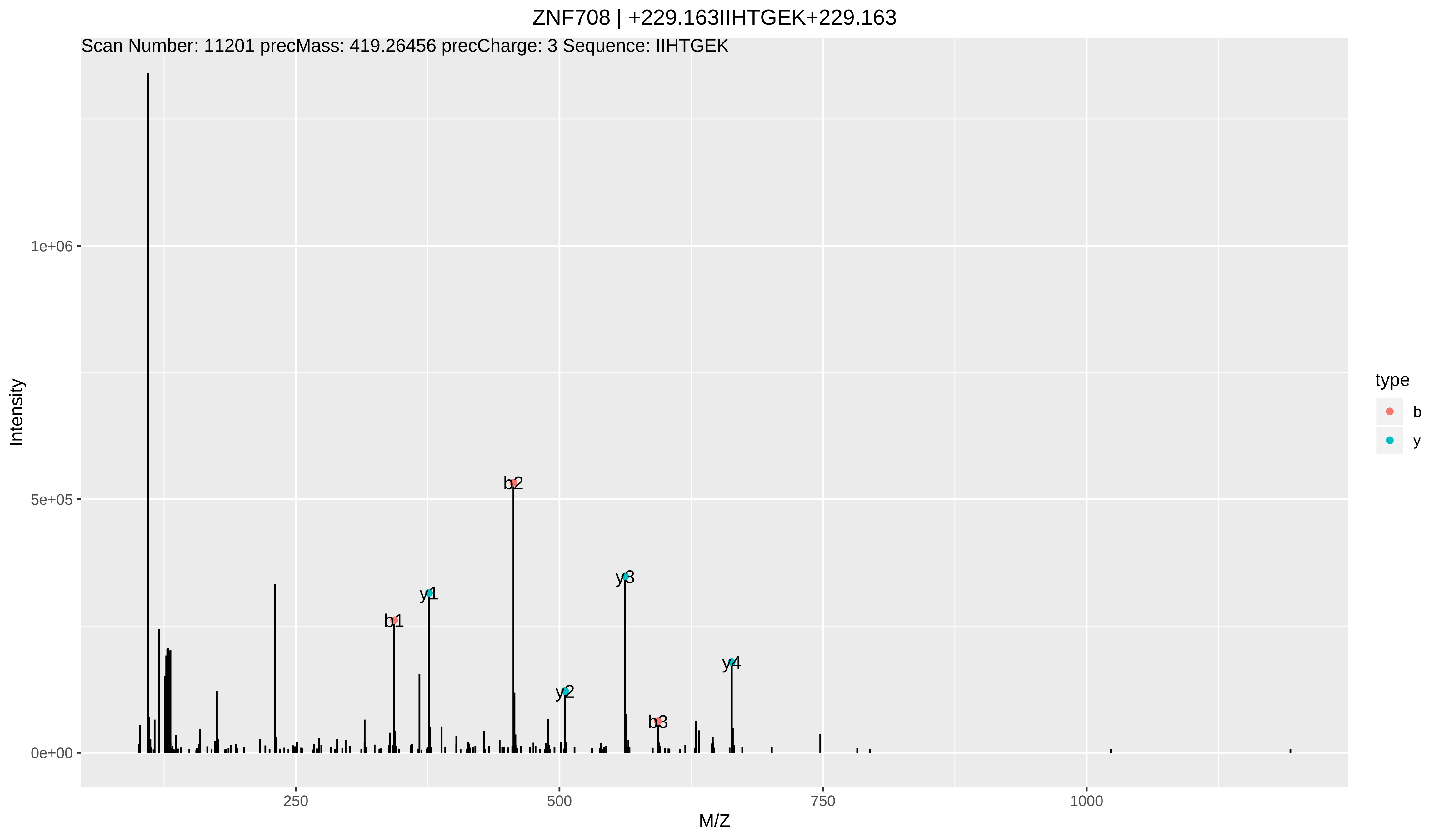

ZNF708 | +229.163IIHTGEK+229.163
Scan Number: 11201 precMass: 419.26456 precCharge: 3 Sequence: IIHTGEK
Intensity
1e+06
5e+05
0e+00
M/Z
250
500
750
1000
b1
b2
b3
y1
y2
y3
y4
type
b
y

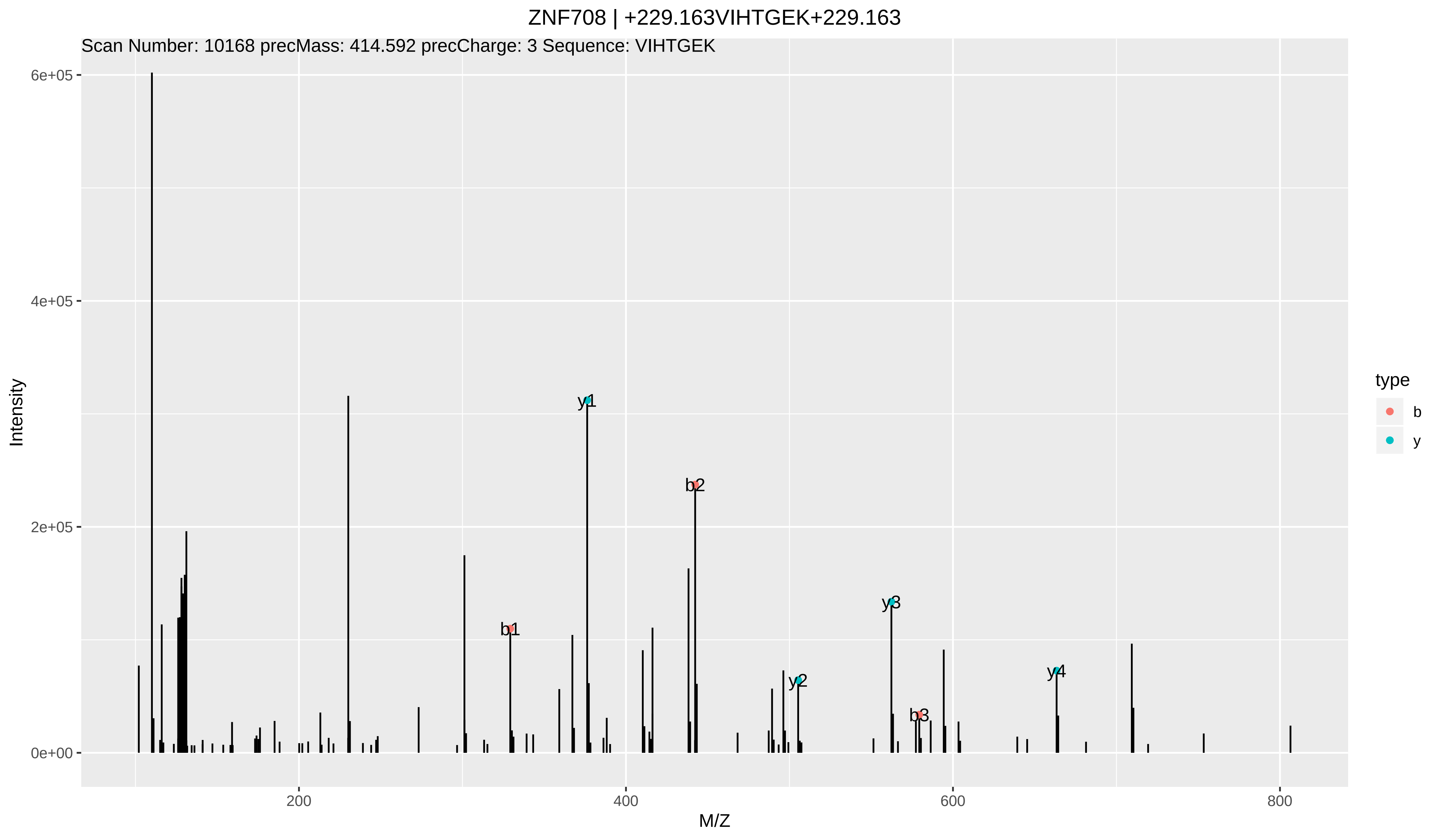
ZNF708 | +229.163VIHTGEK+229.163
Scan Number: 10168 precMass: 414.592 precCharge: 3 Sequence: VIHTGEK
Intensity
6e+05
4e+05
2e+05
0e+00
M/Z
200
400
600
800
y1
b2
y3
b1
y4
y2
b3
type
b
y

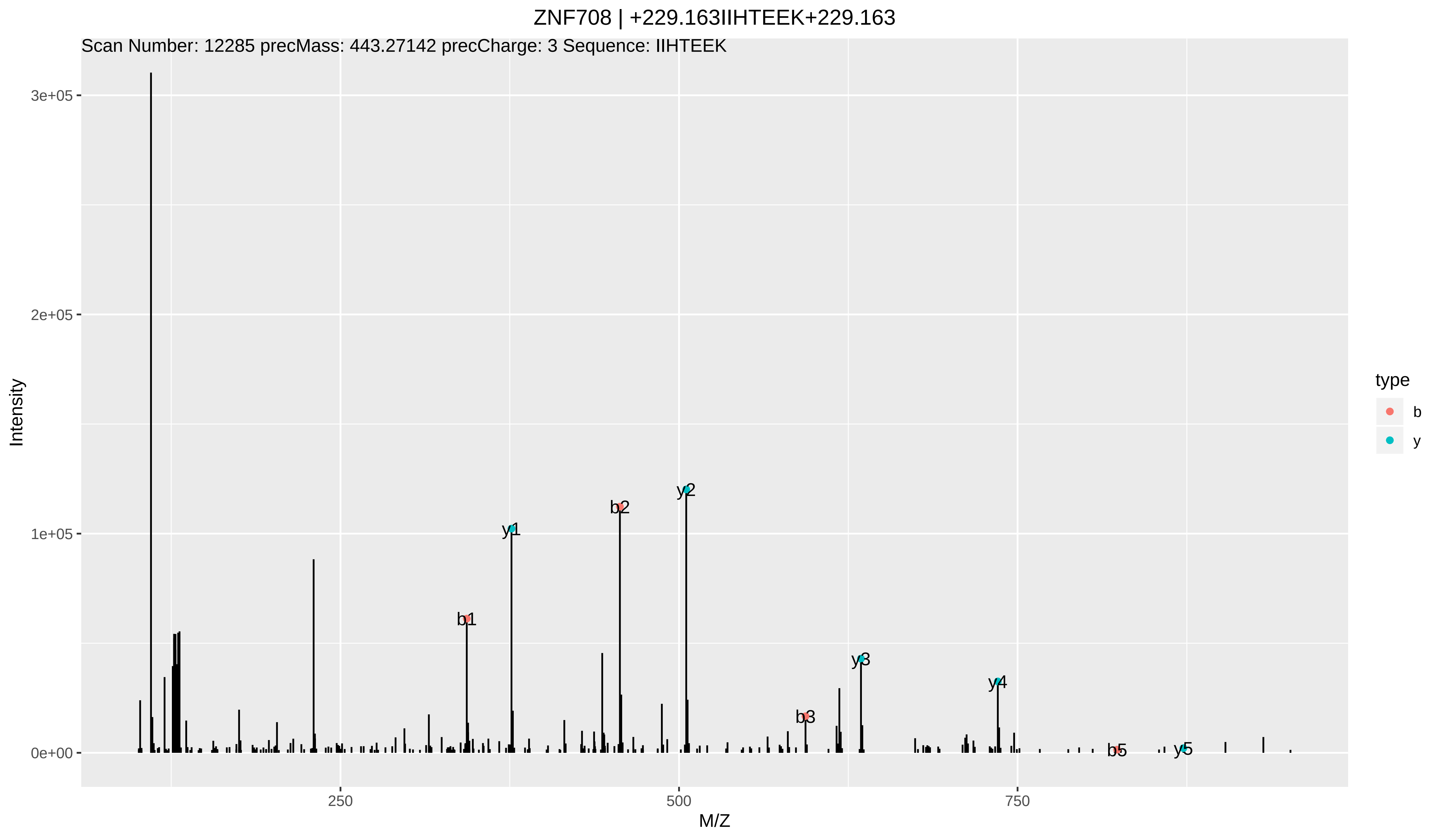
ZNF708 | +229.163IIHTEEK+229.163
Scan Number: 12285 precMass: 443.27142 precCharge: 3 Sequence: IIHTEEK
Intensity
3e+05
2e+05
1e+05
0e+00
M/Z
250
500
750
b1
y1
b2
y2
b3
y3
y4
b5
y5
type
b
y

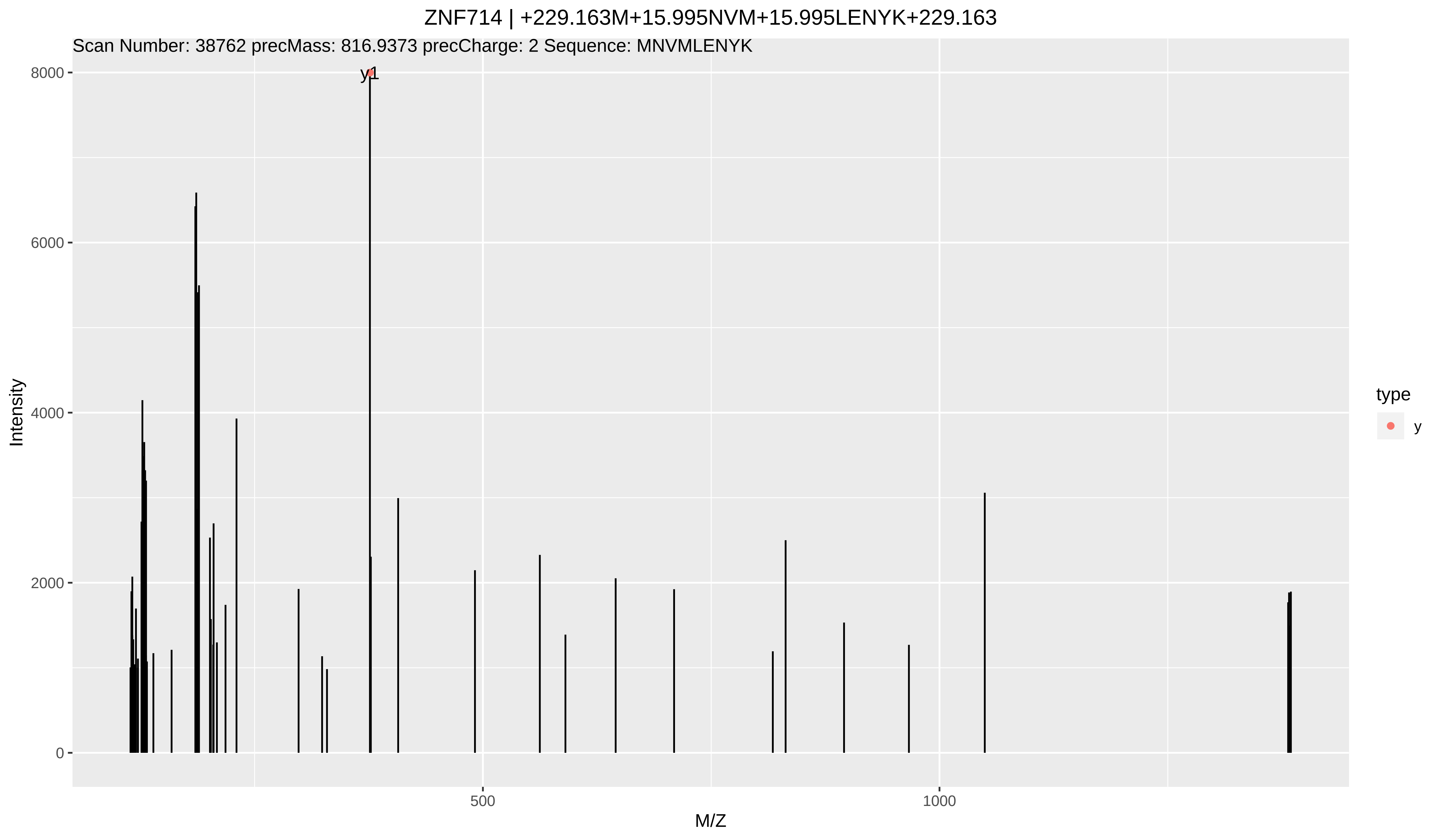
ZNF714 | +229.163M+15.995NVM+15.995LENYK+229.163
Scan Number: 38762 precMass: 816.9373 precCharge: 2 Sequence: MNVMLENYK
y1
Intensity
8000
6000
4000
2000
0
500
1000
M/Z
type
y

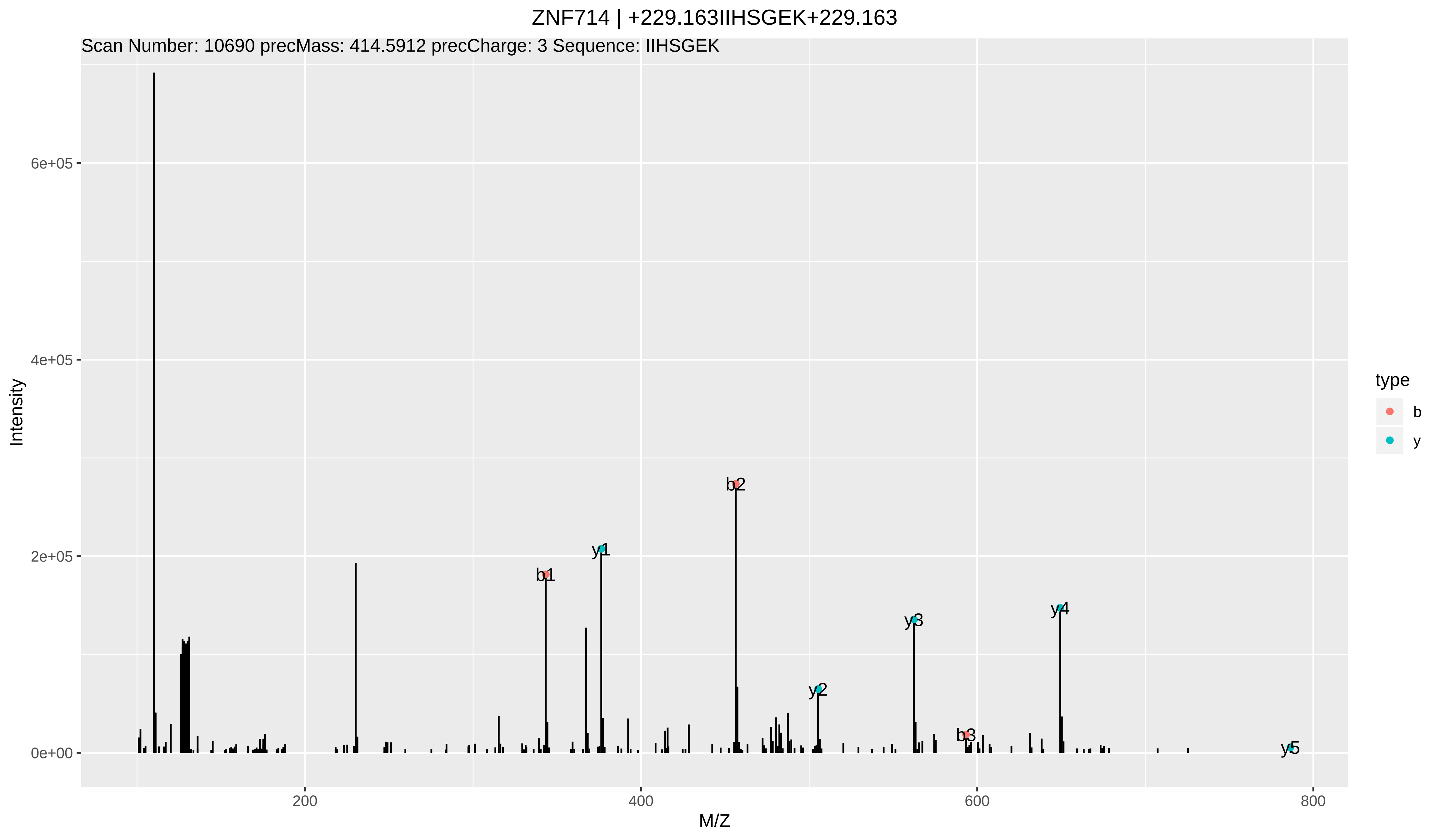
ZNF714 | +229.163IIHSGEK+229.163
Scan Number: 10690 precMass: 414.5912 precCharge: 3 Sequence: IIHSGEK
Intensity
6e+05
4e+05
2e+05
0e+00
M/Z
200
400
600
800
b1
y1
b2
y2
y3
b3
y4
y5
type
b
y

ZNF714 | +229.163IIHTGEK+229.163

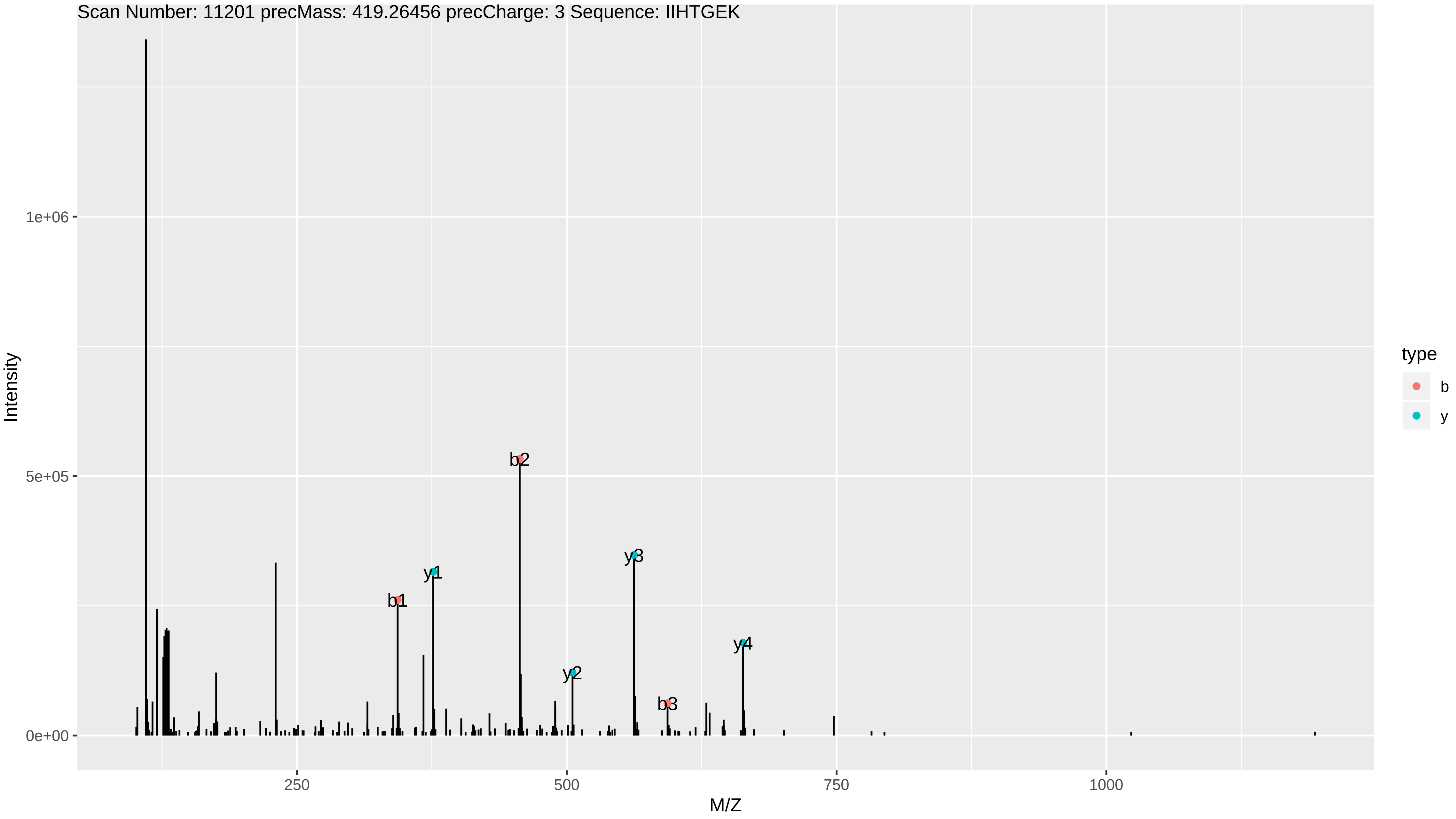

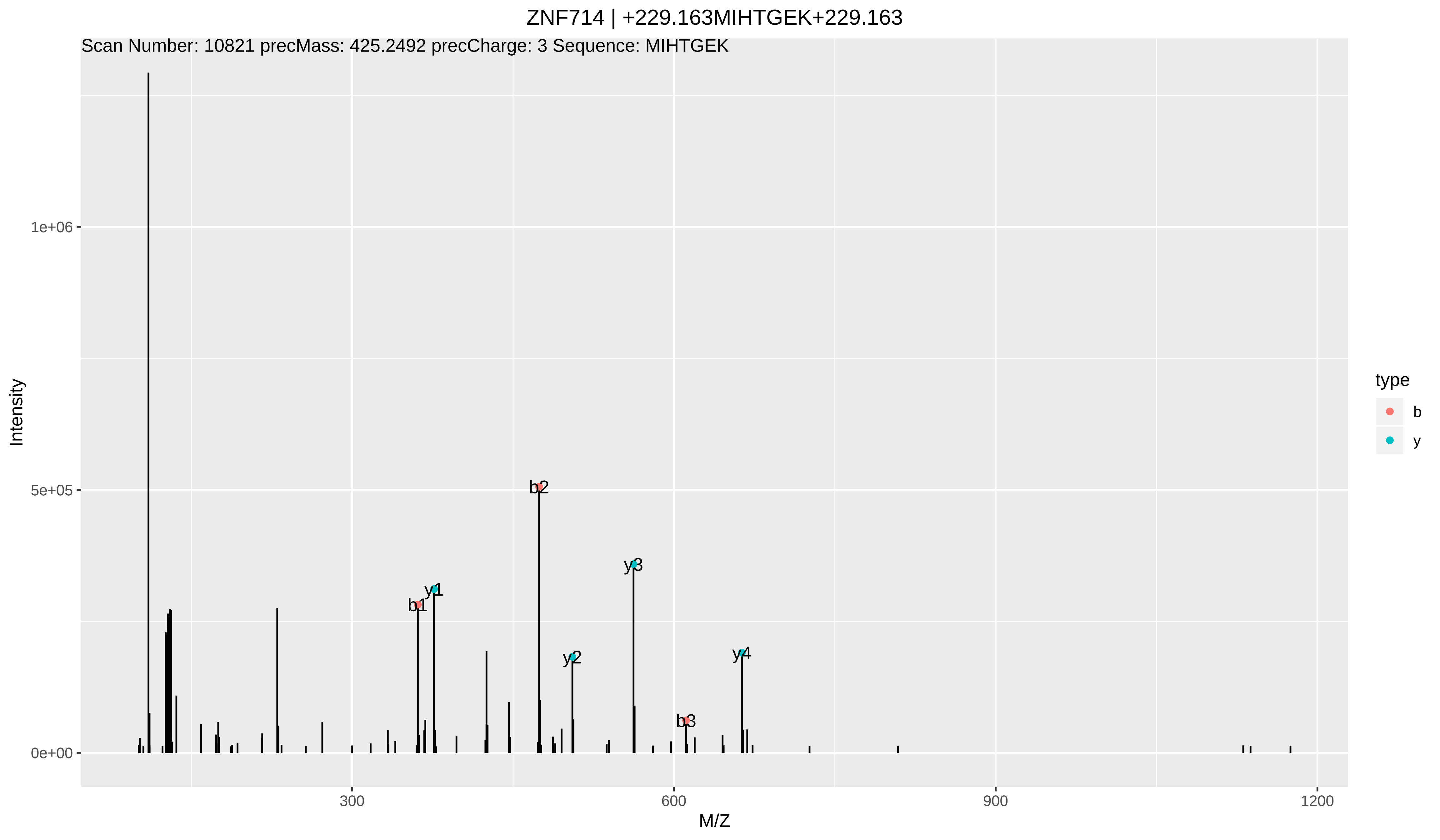

ZNF714 | +229.163MIHTGEK+229.163
Scan Number: 10821 precMass: 425.2492 precCharge: 3 Sequence: MIHTGEK
Intensity
1e+06
5e+05
0e+00
M/Z
300
600
900
1200
b1
y1
b2
y2
y3
b3
y4
type
b
y

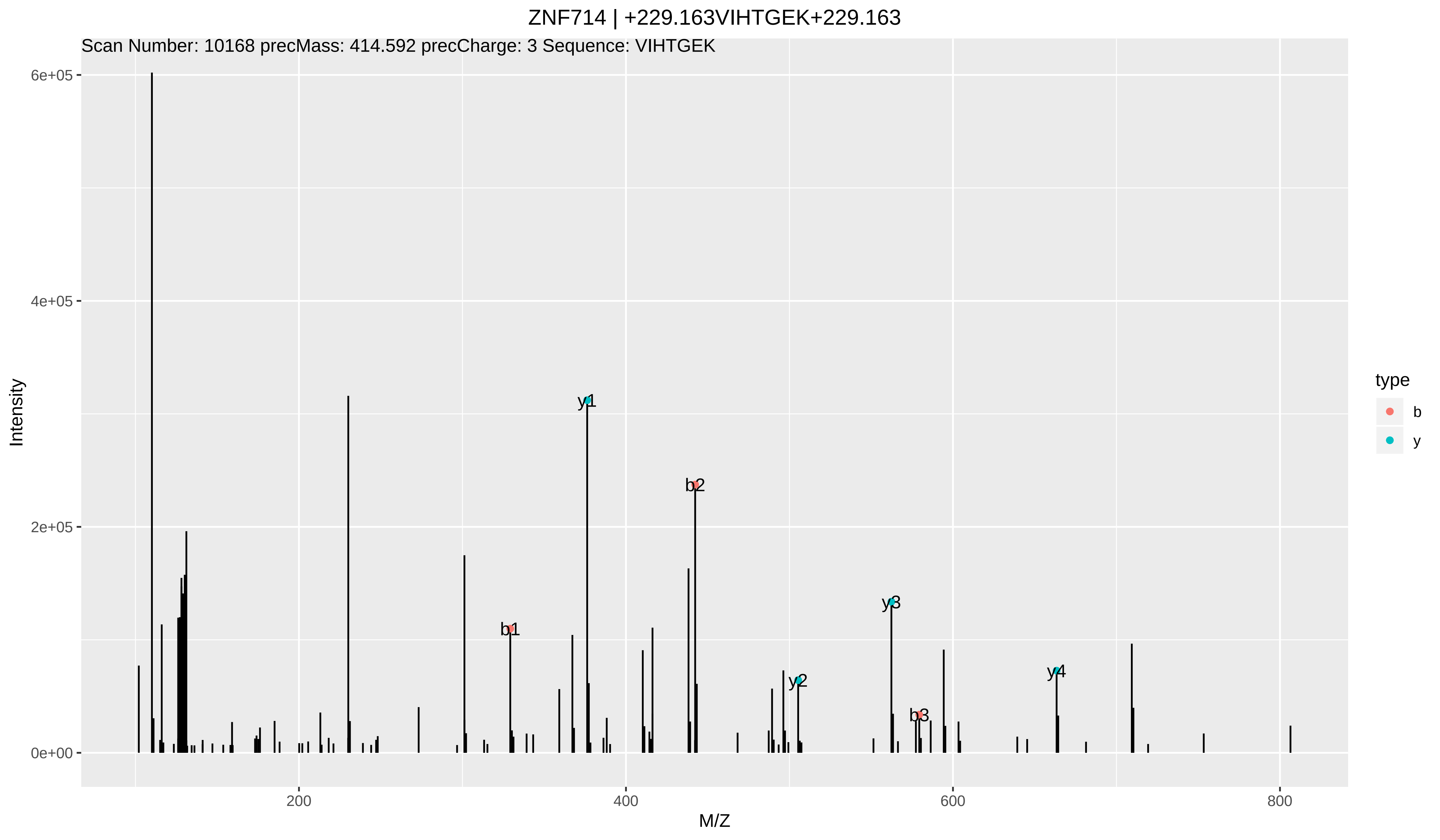

ZNF714 | +229.163VIHTGEK+229.163
Scan Number: 10168 precMass: 414.592 precCharge: 3 Sequence: VIHTGEK
Intensity
M/Z
6e+05
4e+05
2e+05
0e+00
200
400
600
800
y1
b2
b1
y3
y2
y4
b3
type
b
y

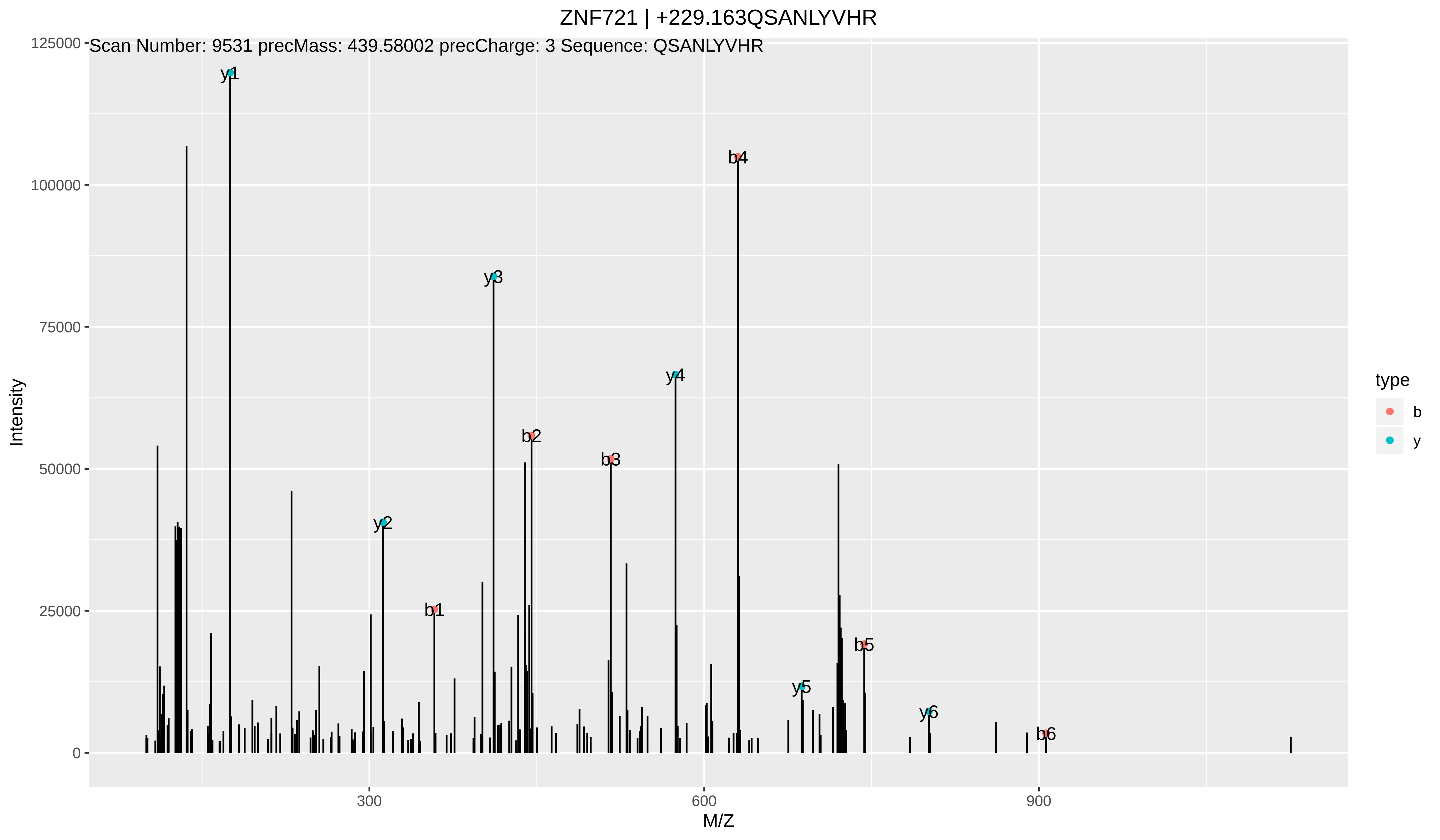

ZNF721 | +229.163QSANLYVHR
Scan Number: 9531 precMass: 439.58002 precCharge: 3 Sequence: QSANLYVHR
Intensity
M/Z
125000
100000
75000
50000
25000
0
300
600
900
y1
b4
y3
y4
b2
b3
y2
b1
b5
y5
y6
b6
type
b
y

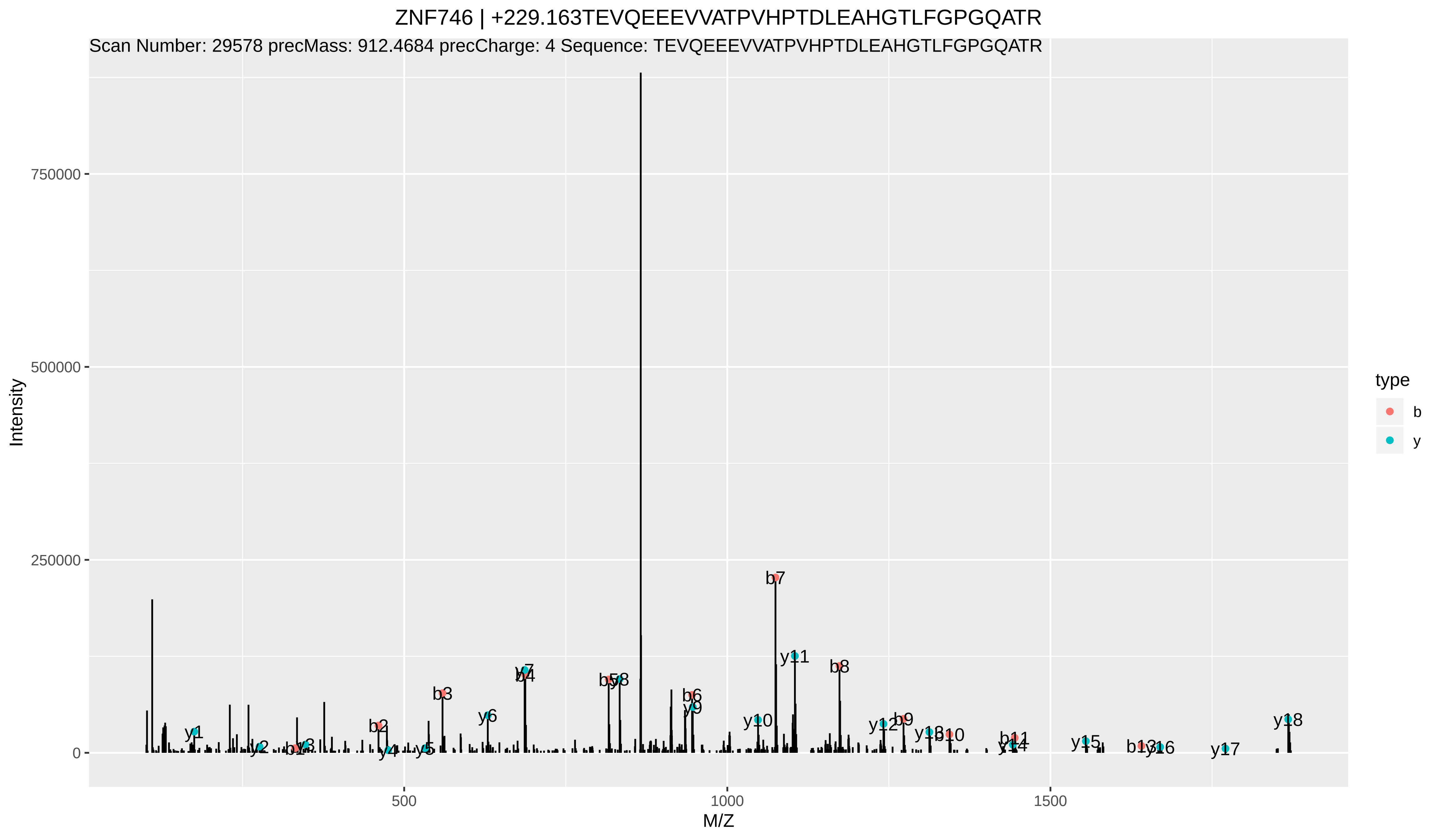
ZNF746 | +229.163TEVQEEEVVATPVHPTDLEAHGTLFGPGQATR
Scan Number: 29578 precMass: 912.4684 precCharge: 4 Sequence: TEVQEEEVVATPVHPTDLEAHGTLFGPGQATR
Intensity
M/Z
type
b
y

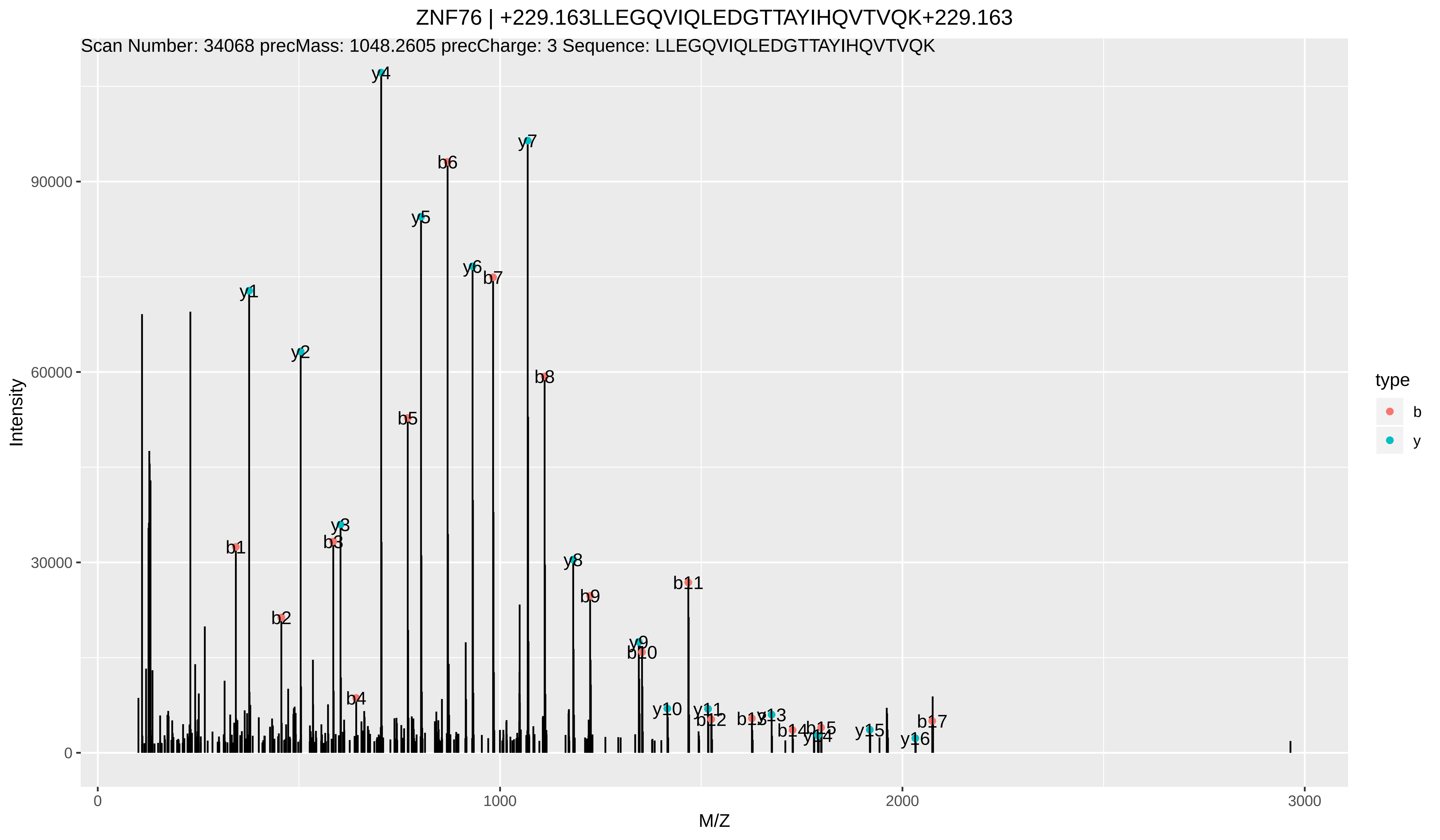

ZNF76 | +229.163LLEGQVIQLEDGTTAYIHQVTVQK+229.163
Scan Number: 34068 precMass: 1048.2605 precCharge: 3 Sequence: LLEGQVIQLEDGTTAYIHQVTVQK
Intensity
M/Z
type
b
y
y4
y7
b6
y5
y6
b7
y1
y2
b8
b5
y3
b3
b1
y8
b11
b9
b2
y9
b10
b4
y10
y11
b12
b13
y13
b14
b15
y14
y15
y16
b17
0
30000
60000
90000
1000
2000
3000

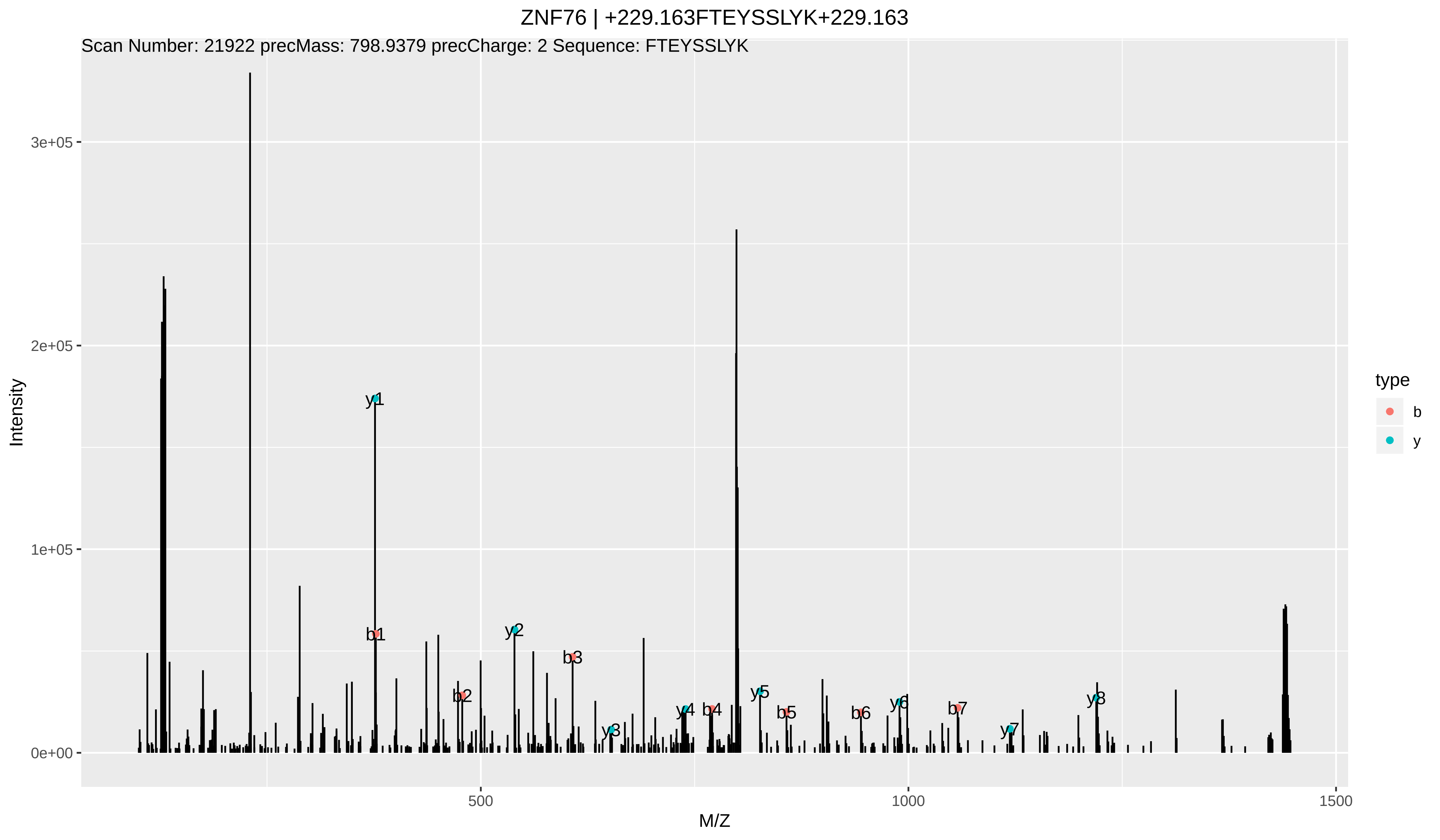

ZNF76 | +229.163FTEYSSLYK+229.163
Scan Number: 21922 precMass: 798.9379 precCharge: 2 Sequence: FTEYSSLYK
Intensity
3e+05
2e+05
1e+05
0e+00
M/Z
500
1000
1500
y1
b1
b2
y2
b3
y3
y4
b4
y5
b5
b6
y6
b7
y7
y8
type
b
y

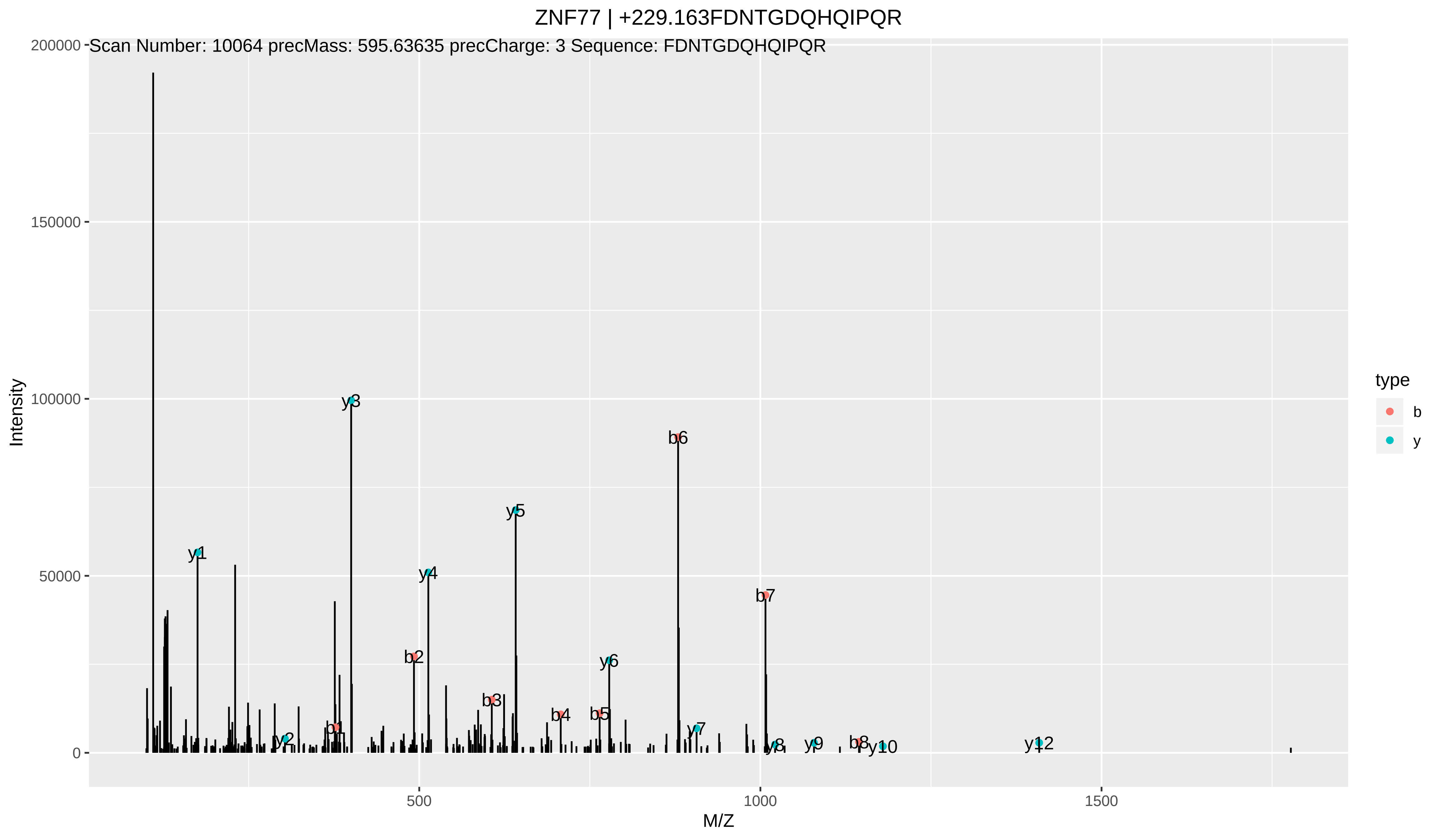
ZNF77 | +229.163FDNTGDQHQIPQR
Scan Number: 10064 precMass: 595.63635 precCharge: 3 Sequence: FDNTGDQHQIPQR
Intensity
M/Z
200000
150000
100000
50000
0
500
1000
1500
y1
y2
y3
y4
y5
y6
y7
y8
y9
y10
y12
b1
b2
b3
b4
b5
b6
b7
b8
type
b
y

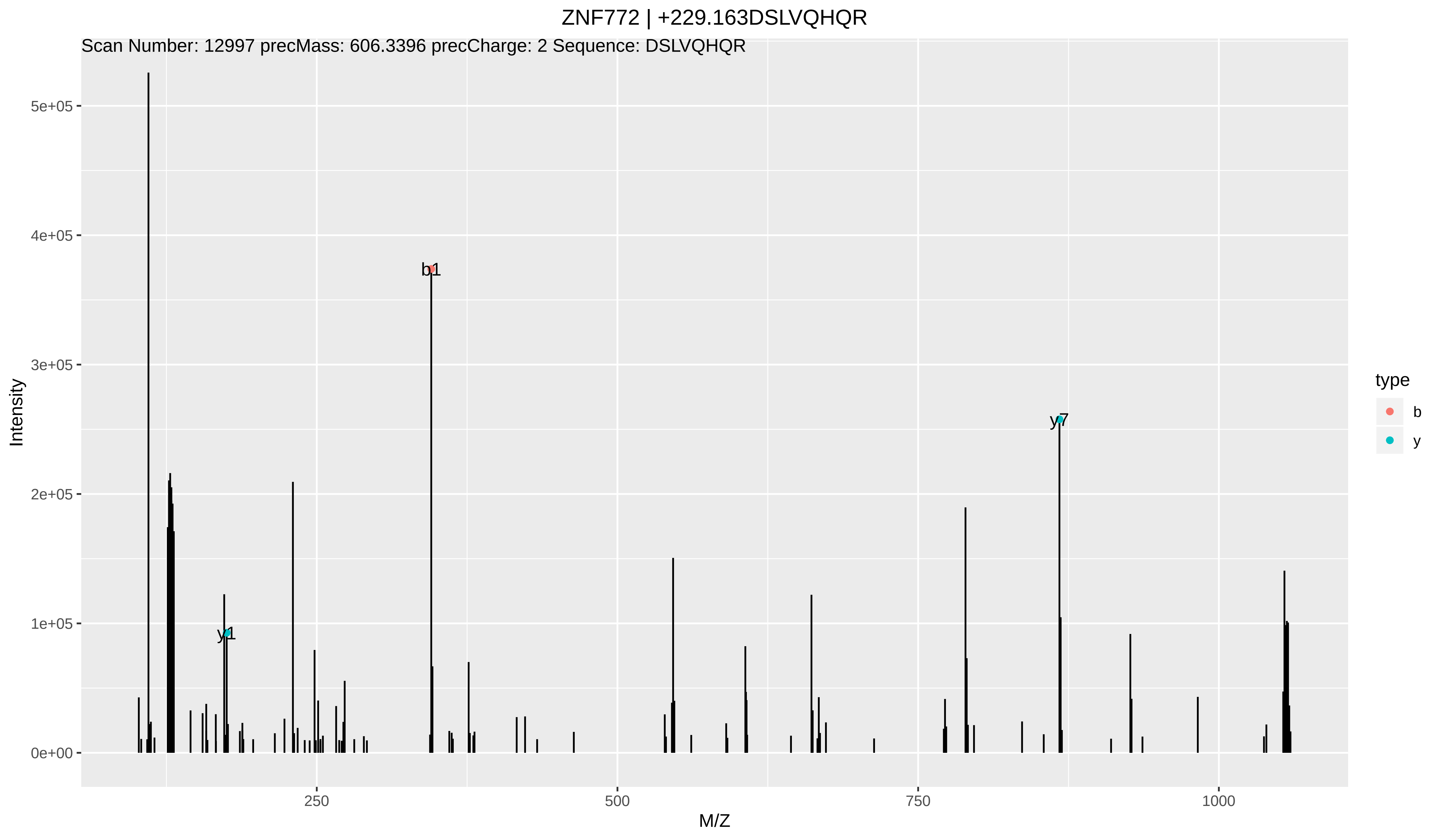

ZNF772 | +229.163DSLVQHQR
Scan Number: 12997 precMass: 606.3396 precCharge: 2 Sequence: DSLVQHQR
Intensity
5e+05
4e+05
3e+05
2e+05
1e+05
0e+00
M/Z
250
500
750
1000
b1
y1
y7
type
b
y

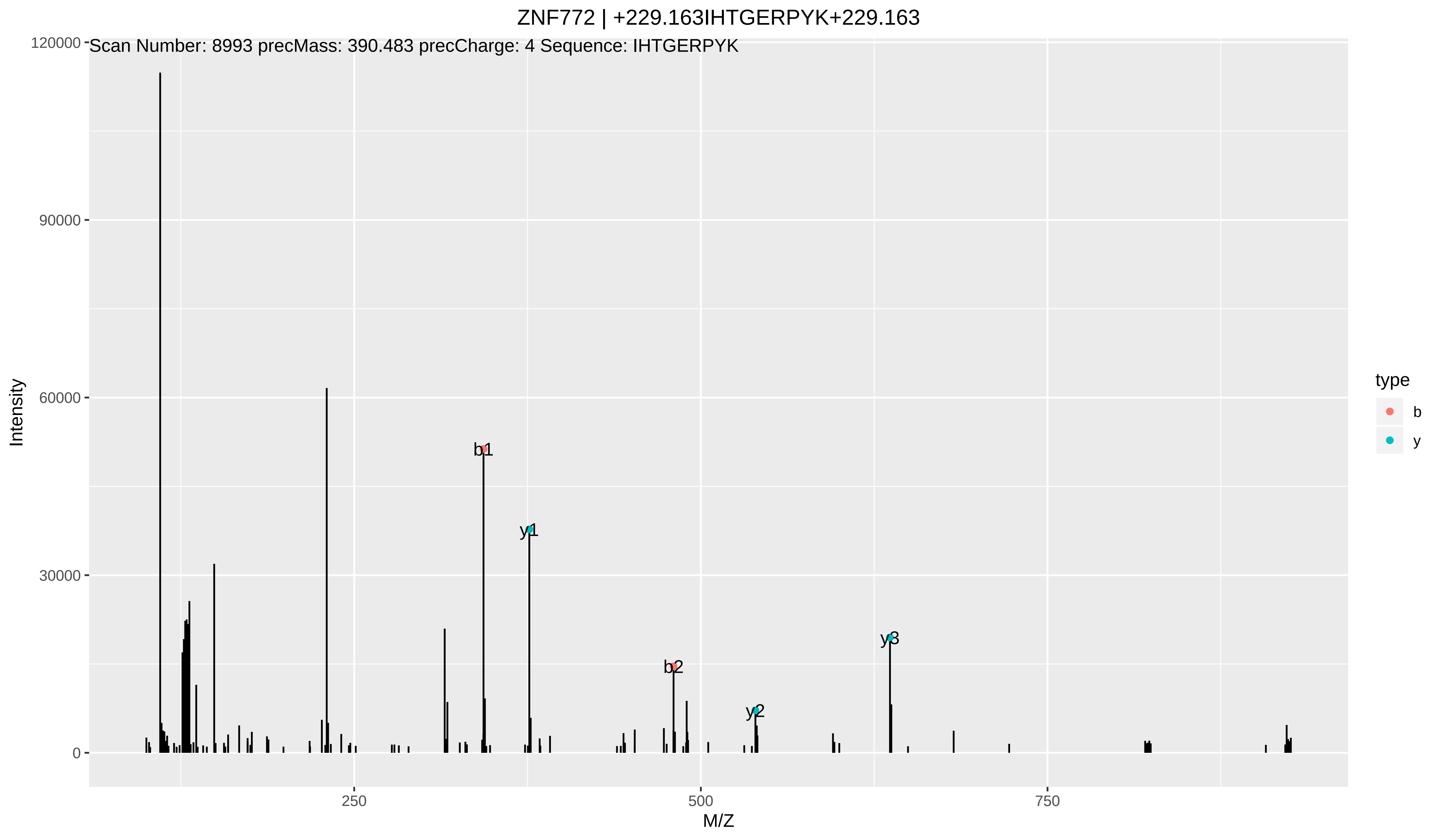

ZNF772 | +229.163IHTGERPYK+229.163
Scan Number: 8993 precMass: 390.483 precCharge: 4 Sequence: IHTGERPYK
Intensity
120000
90000
60000
30000
0
M/Z
250
500
750
b1
y1
b2
y2
y3
type
b
y

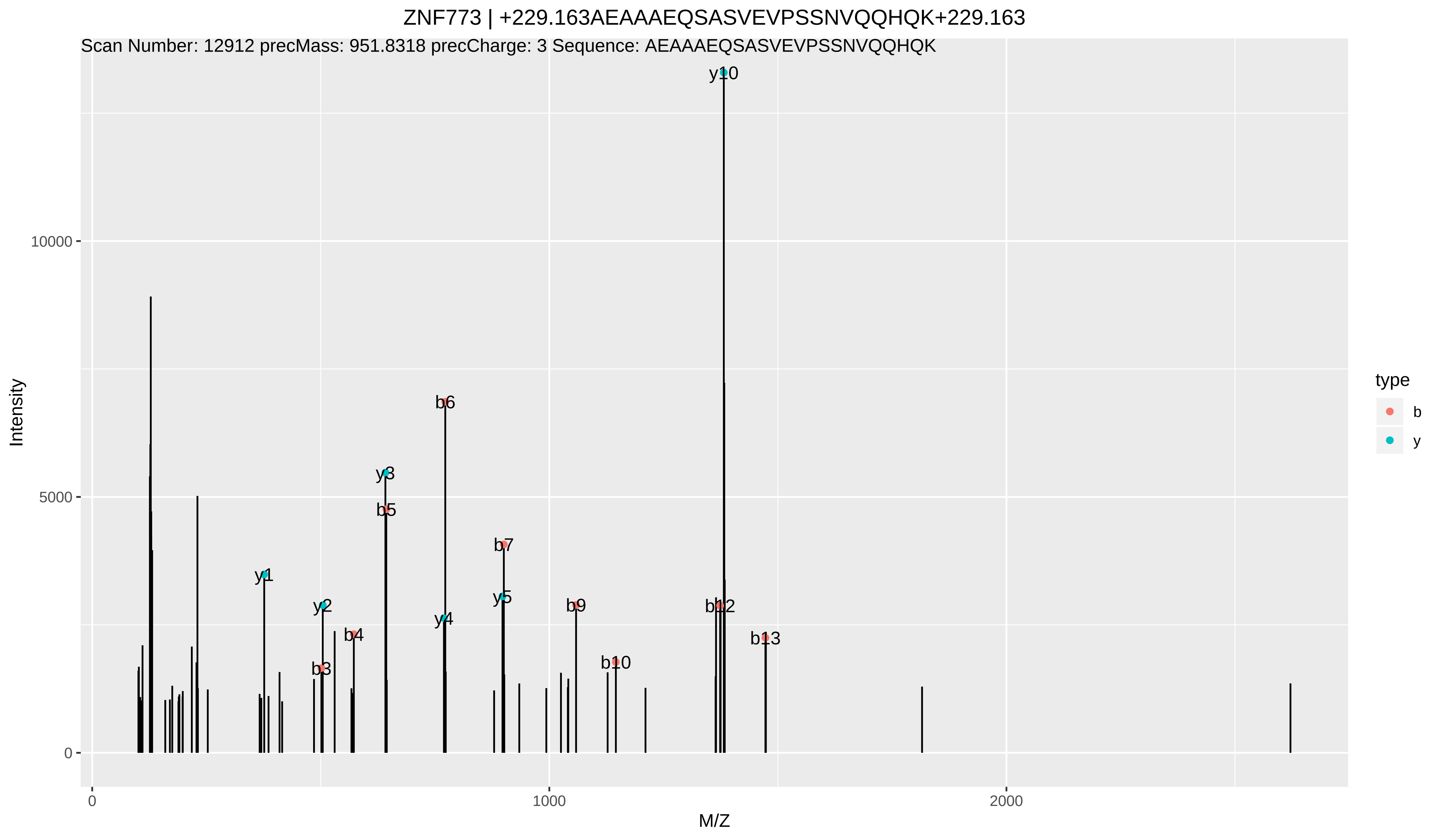

ZNF773 | +229.163AEAAAEQSASVEVPSSNVQQHQK+229.163
Scan Number: 12912 precMass: 951.8318 precCharge: 3 Sequence: AEAAAEQSASVEVPSSNVQQHQK
y10
b6
y3
b5
b7
y1
y5
y2
b9
b12
y4
b4
b13
b10
b3
Intensity
10000
5000
0
0
1000
2000
M/Z
type
b
y

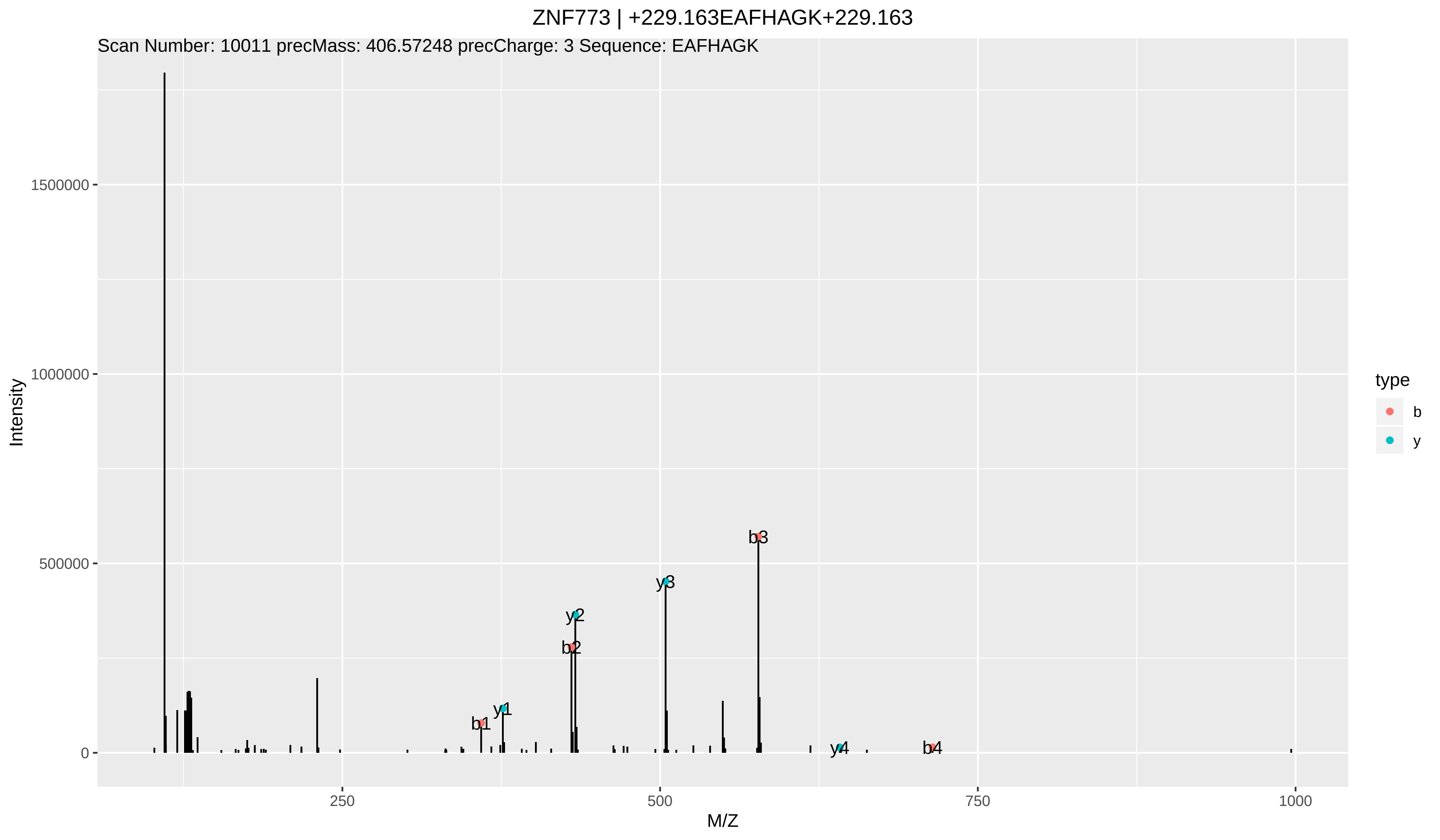
ZNF773 | +229.163EAFHAGK+229.163
Scan Number: 10011 precMass: 406.57248 precCharge: 3 Sequence: EAFHAGK
Intensity
1500000
1000000
500000
0
250
500
750
1000
M/Z
b1
y1
b2
y2
y3
b3
y4
b4
type
b
y

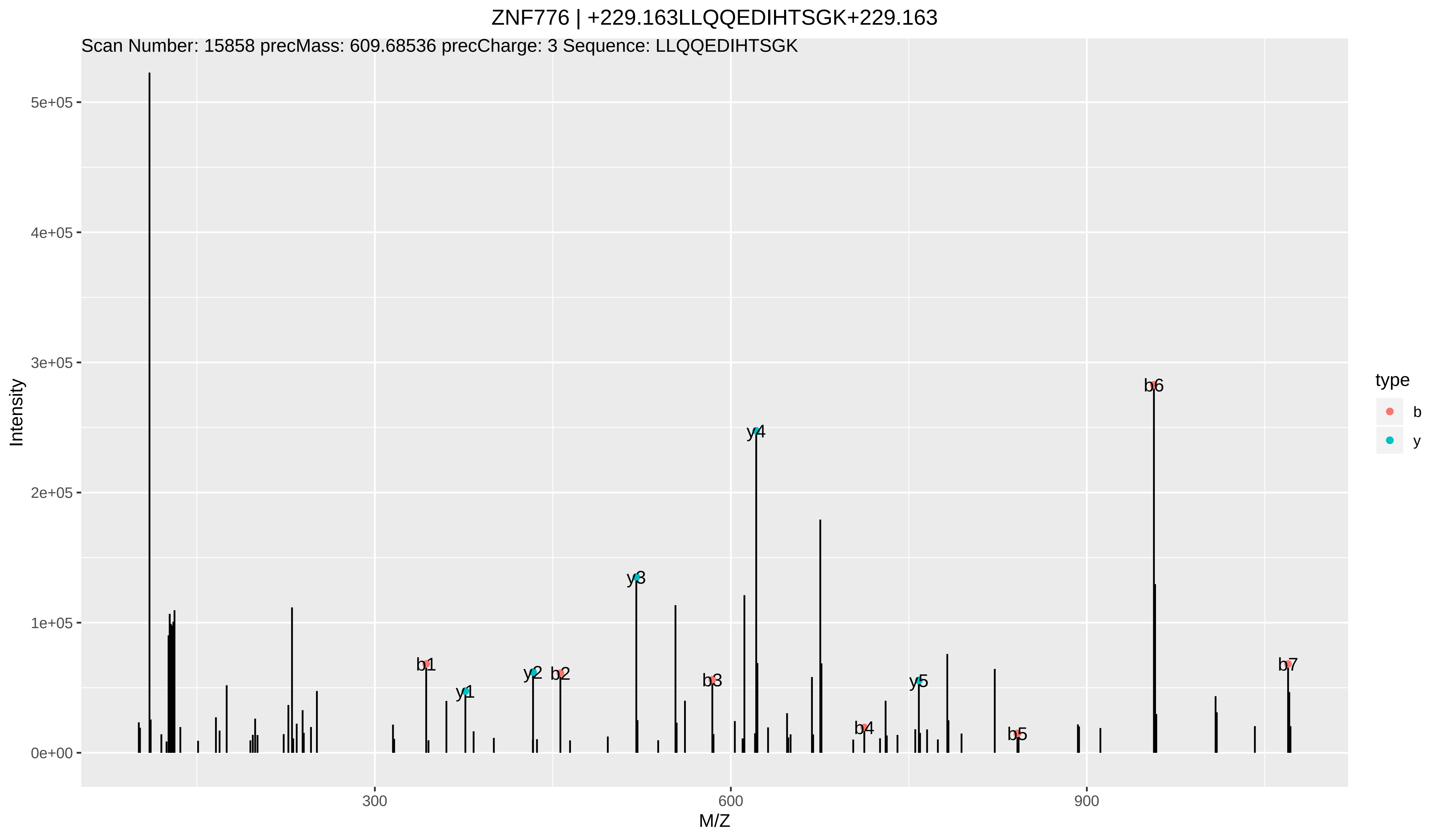

ZNF776 | +229.163LLQQEDIHTSGK+229.163
Scan Number: 15858 precMass: 609.68536 precCharge: 3 Sequence: LLQQEDIHTSGK
Intensity
5e+05
4e+05
3e+05
2e+05
1e+05
0e+00
M/Z
300
600
900
b1
y1
y2
b2
y3
b3
y4
b4
y5
b5
b6
b7
type
b
y

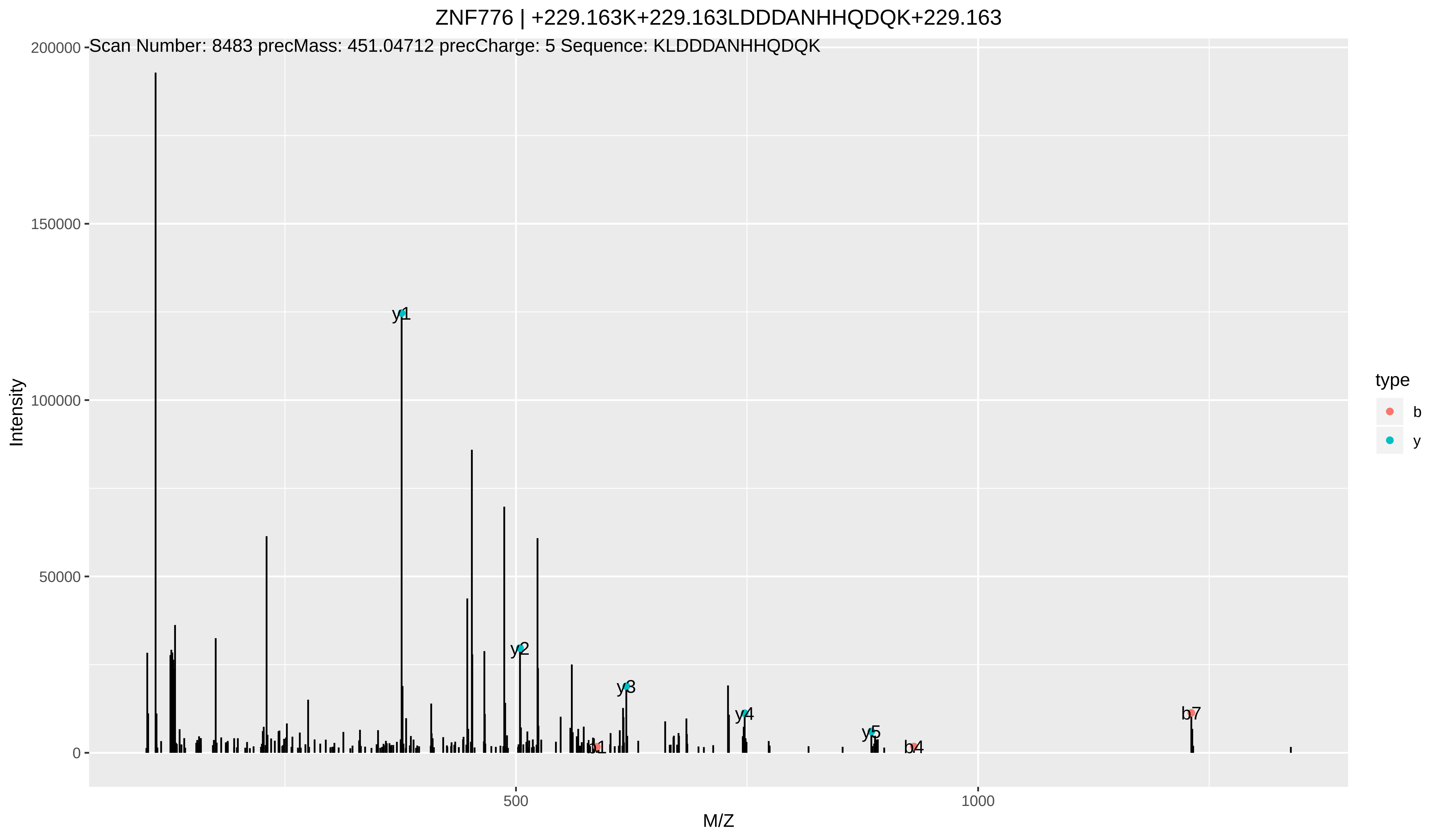

ZNF776 | +229.163K+229.163LDDDANHHQDQK+229.163
Scan Number: 8483 precMass: 451.04712 precCharge: 5 Sequence: KLDDDANHHQDQK
Intensity
M/Z
200000
150000
100000
50000
0
500
1000
y1
y2
y3
y4
y5
b1
b4
b7
type
b
y

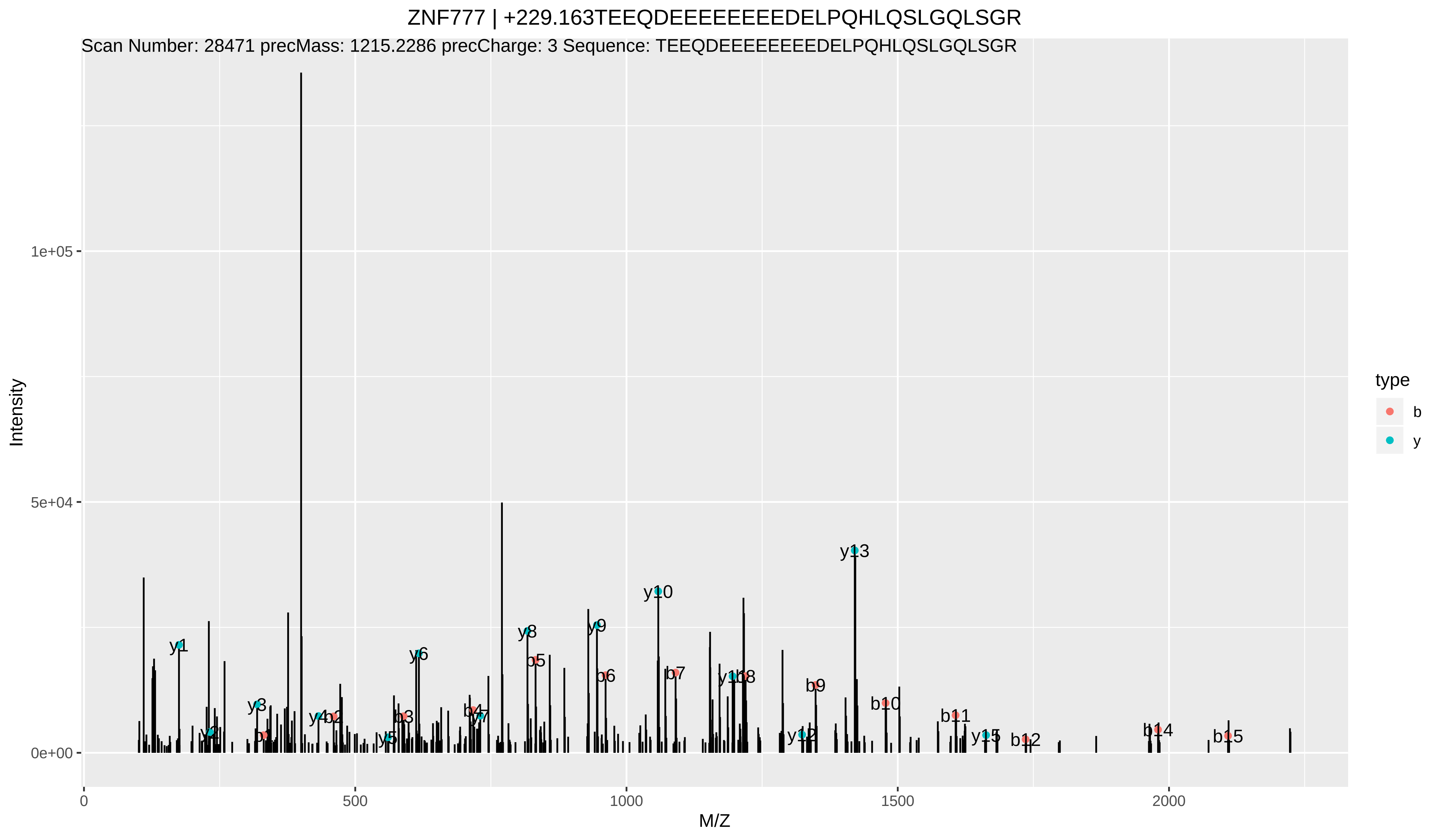

ZNF777 | +229.163TEEQDEEEEEEEEDELPQHLQSLGQLSGR
Scan Number: 28471 precMass: 1215.2286 precCharge: 3 Sequence: TEEQDEEEEEEEEDELPQHLQSLGQLSGR
Intensity
M/Z
0e+00
5e+04
1e+05
0
500
1000
1500
2000
type
b
y
y1
y2
y3
y4
y5
y6
y7
y8
y9
y10
y13
y15
b2
b3
b4
b5
b6
b7
b8
b9
b10
b11
b12
b14
b15

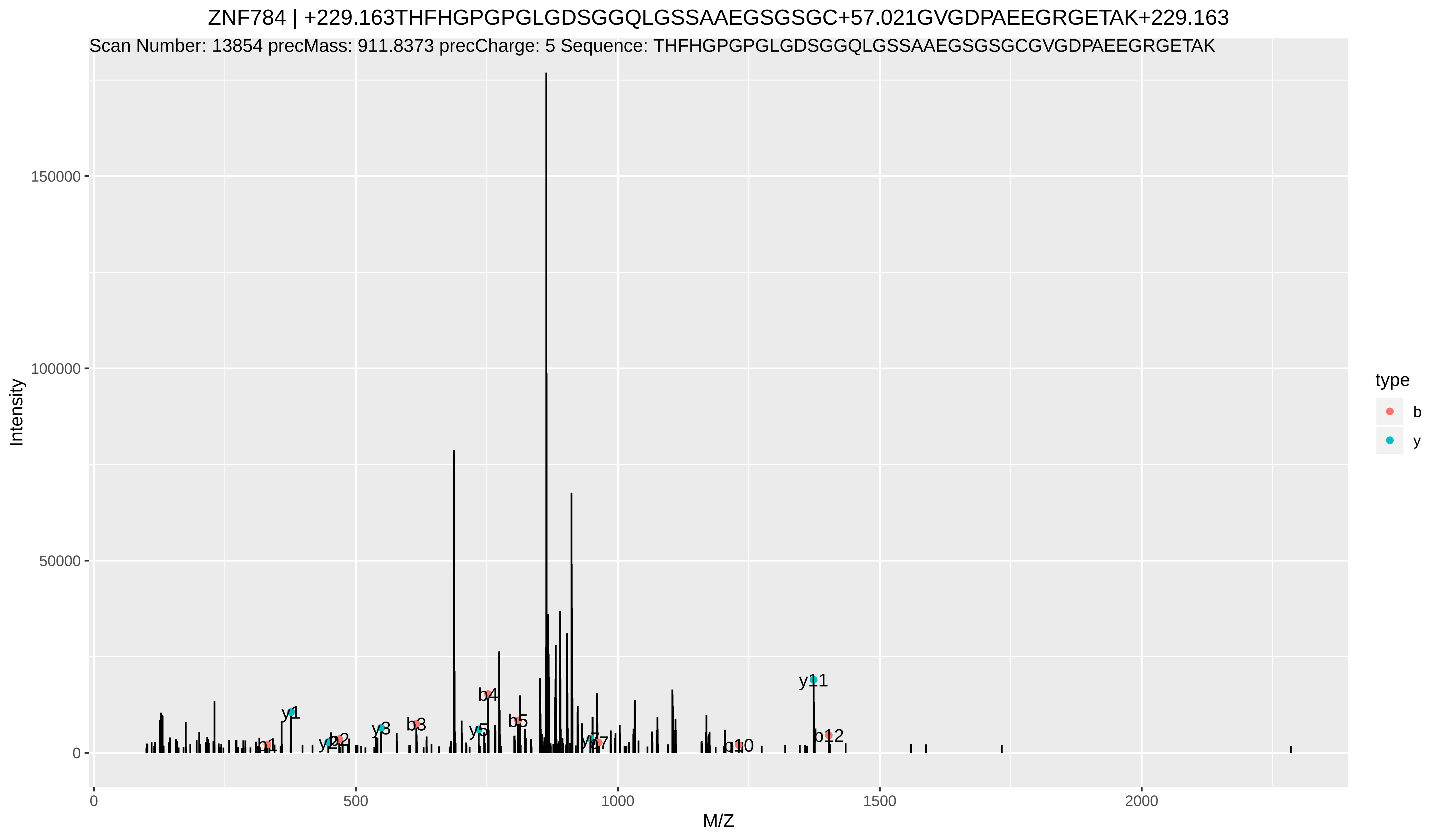
ZNF784 | +229.163THFHGPGPGLGDSGGQLGSSAAEGSGSGC+57.021GVGDPAEEGRGETAK+229.163
Scan Number: 13854 precMass: 911.8373 precCharge: 5 Sequence: THFHGPGPGLGDSGGQLGSSAAEGSGSGCGVGDPAEEGRGETAK
Intensity
M/Z
type
b
y
y1
b1
y2
b2
y3
b3
y5
b4
b5
b7
b10
y11
b12
0
50000
100000
150000
500
1000
1500
2000

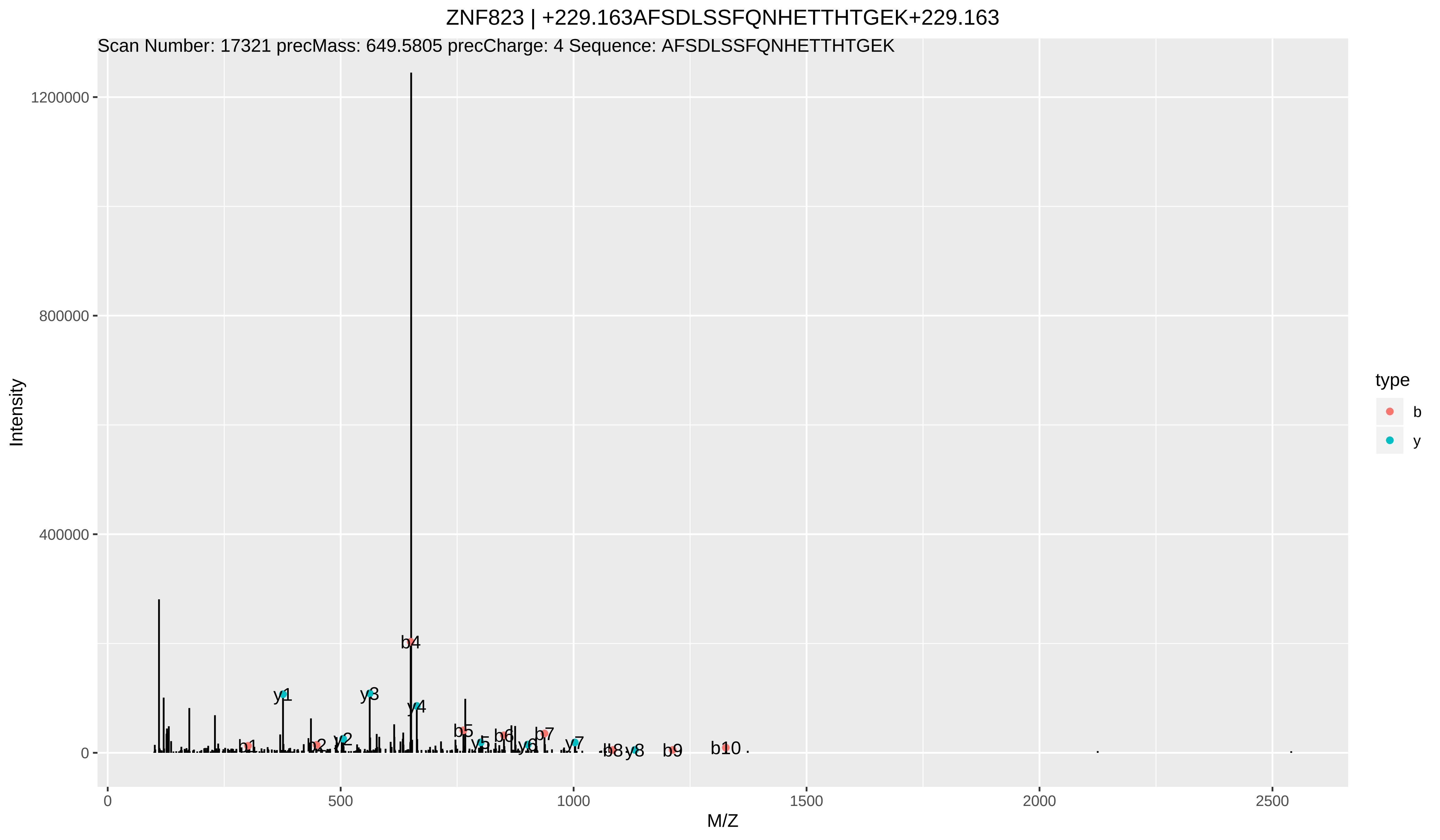

ZNF823 | +229.163AFSDLSSFQNHETTHTGEK+229.163
Scan Number: 17321 precMass: 649.5805 precCharge: 4 Sequence: AFSDLSSFQNHETTHTGEK
Intensity
M/Z
type
b
y

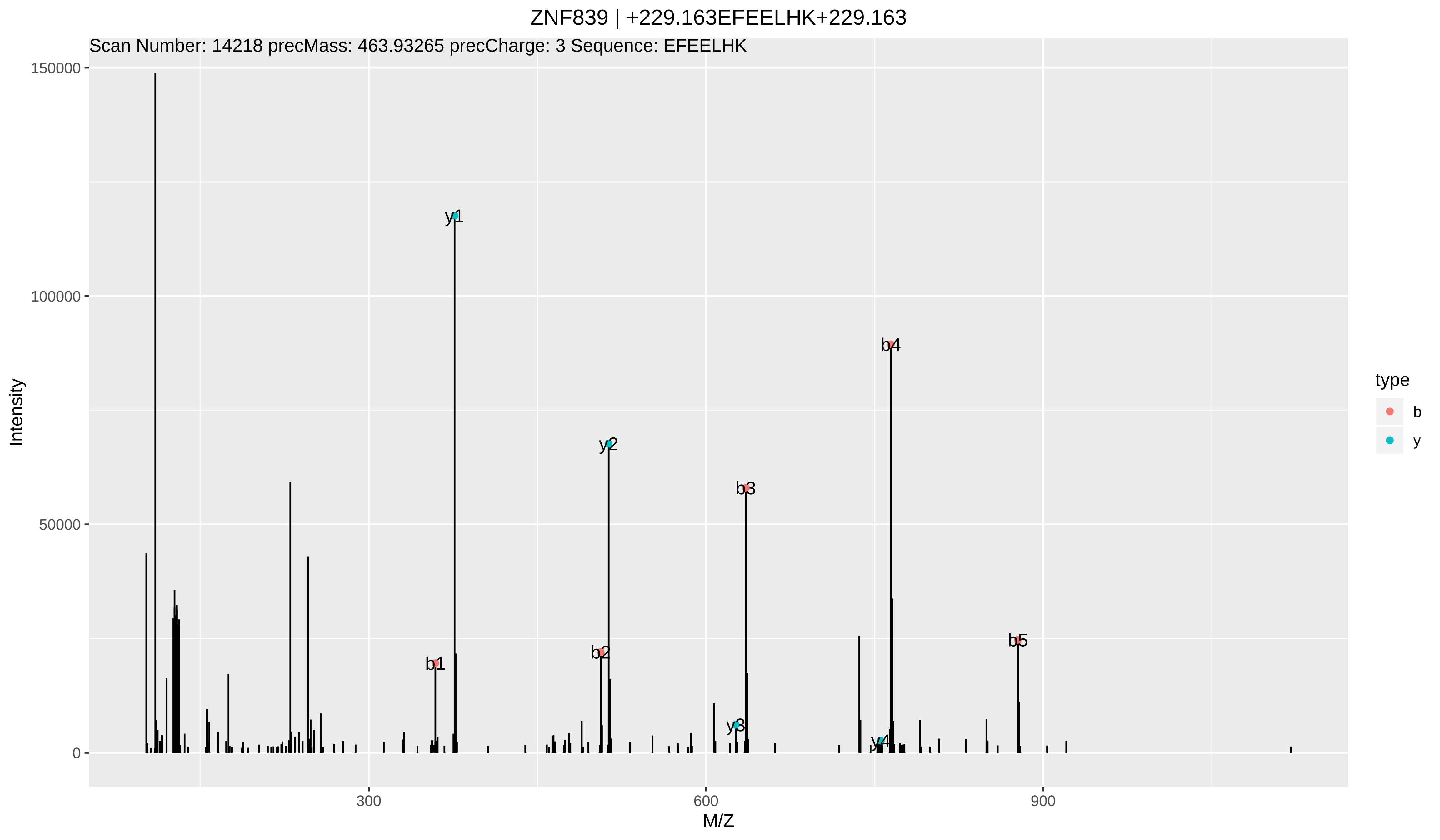

ZNF839 | +229.163EFEELHK+229.163
Scan Number: 14218 precMass: 463.93265 precCharge: 3 Sequence: EFEELHK
Intensity
M/Z
type
b
y
y1
y2
b1
b2
b3
b4
b5
y3
y4
150000
100000
50000
0
300
600
900

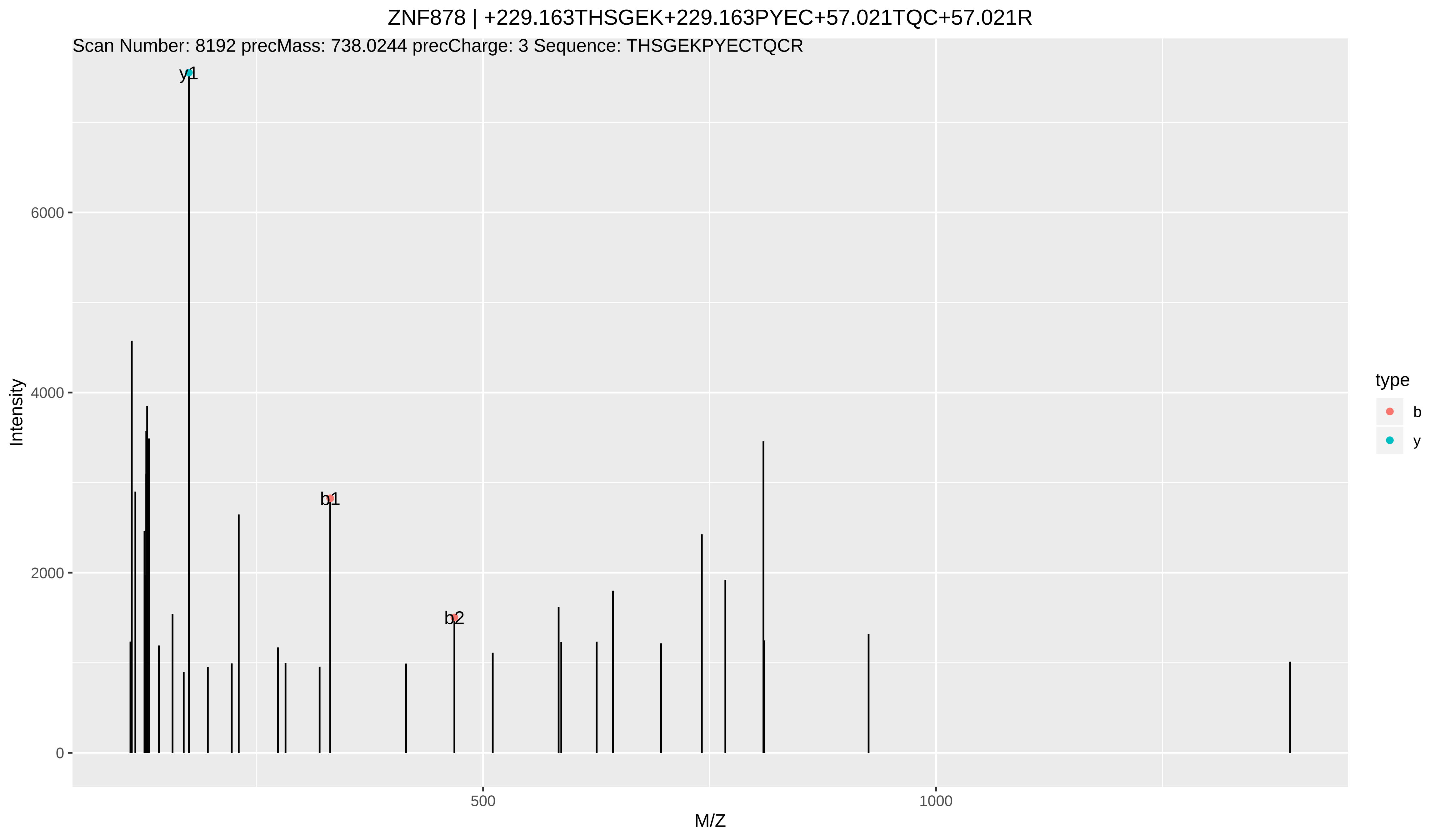

ZNF878 | +229.163THSGEK+229.163PYEC+57.021TQC+57.021R
Scan Number: 8192 precMass: 738.0244 precCharge: 3 Sequence: THSGEKPYECTQCR
y1
b1
b2
Intensity
6000
4000
2000
0
500
1000
M/Z
type
b
y

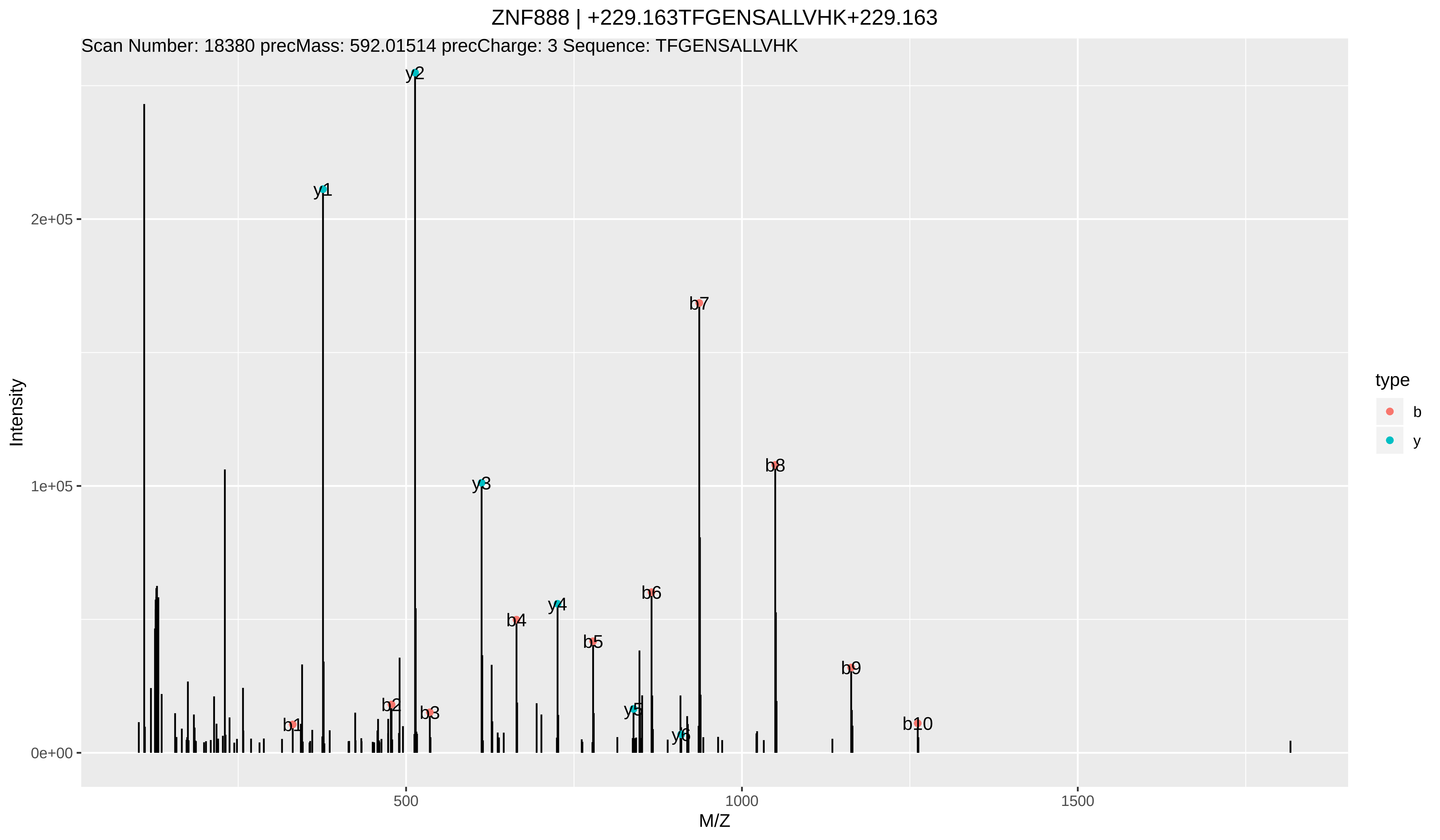

ZNF888 | +229.163TFGENSALLVHK+229.163
Scan Number: 18380 precMass: 592.01514 precCharge: 3 Sequence: TFGENSALLVHK
Intensity
M/Z
2e+05
1e+05
0e+00
500
1000
1500
type
b
y
y1
y2
y3
y4
y5
y6
b1
b2
b3
b4
b5
b6
b7
b8
b9
b10

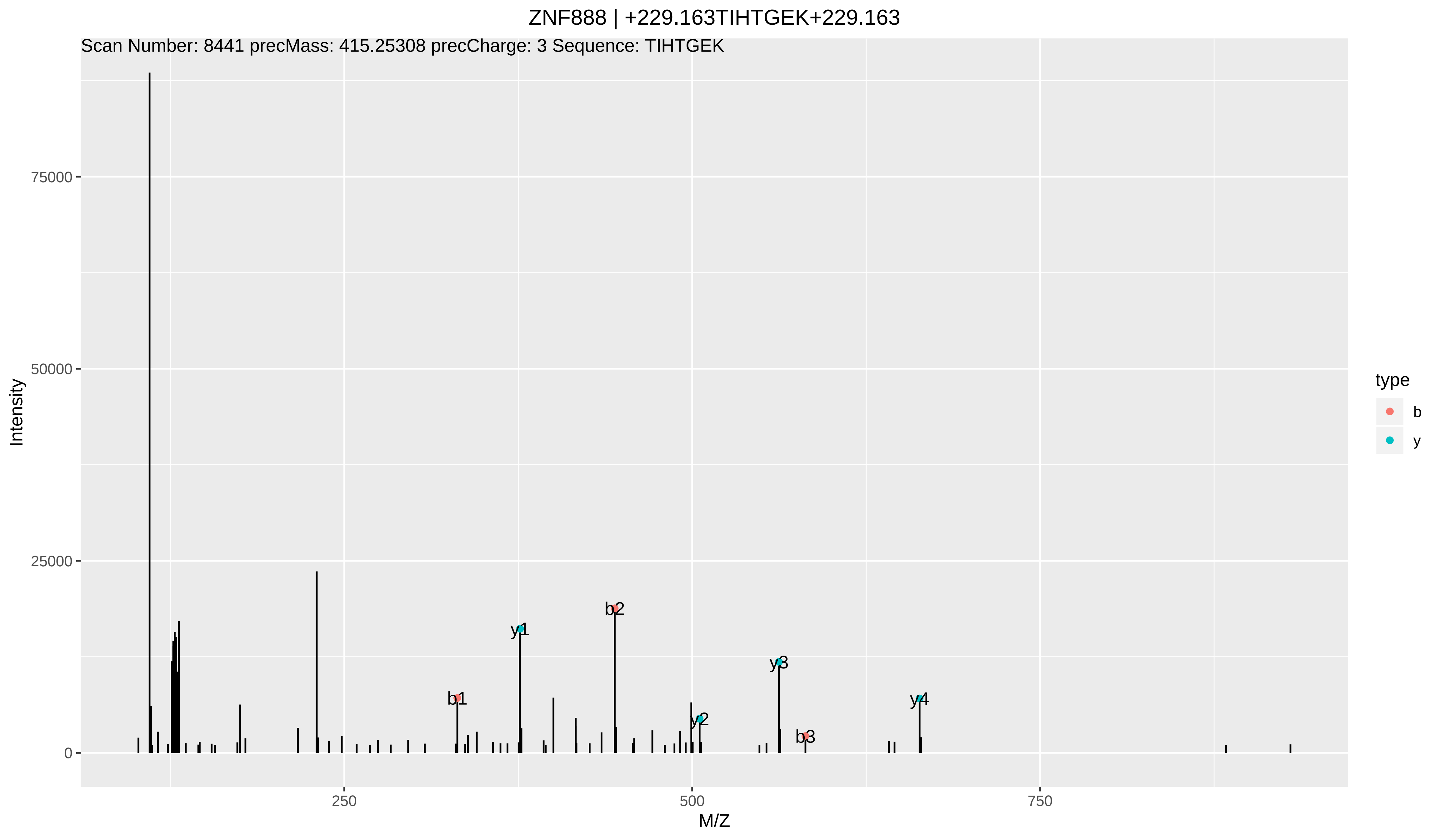
ZNF888 | +229.163TIHTGEK+229.163
Scan Number: 8441 precMass: 415.25308 precCharge: 3 Sequence: TIHTGEK
Intensity
75000
50000
25000
0
M/Z
250
500
750
b1
y1
b2
y2
y3
b3
y4
type
b
y

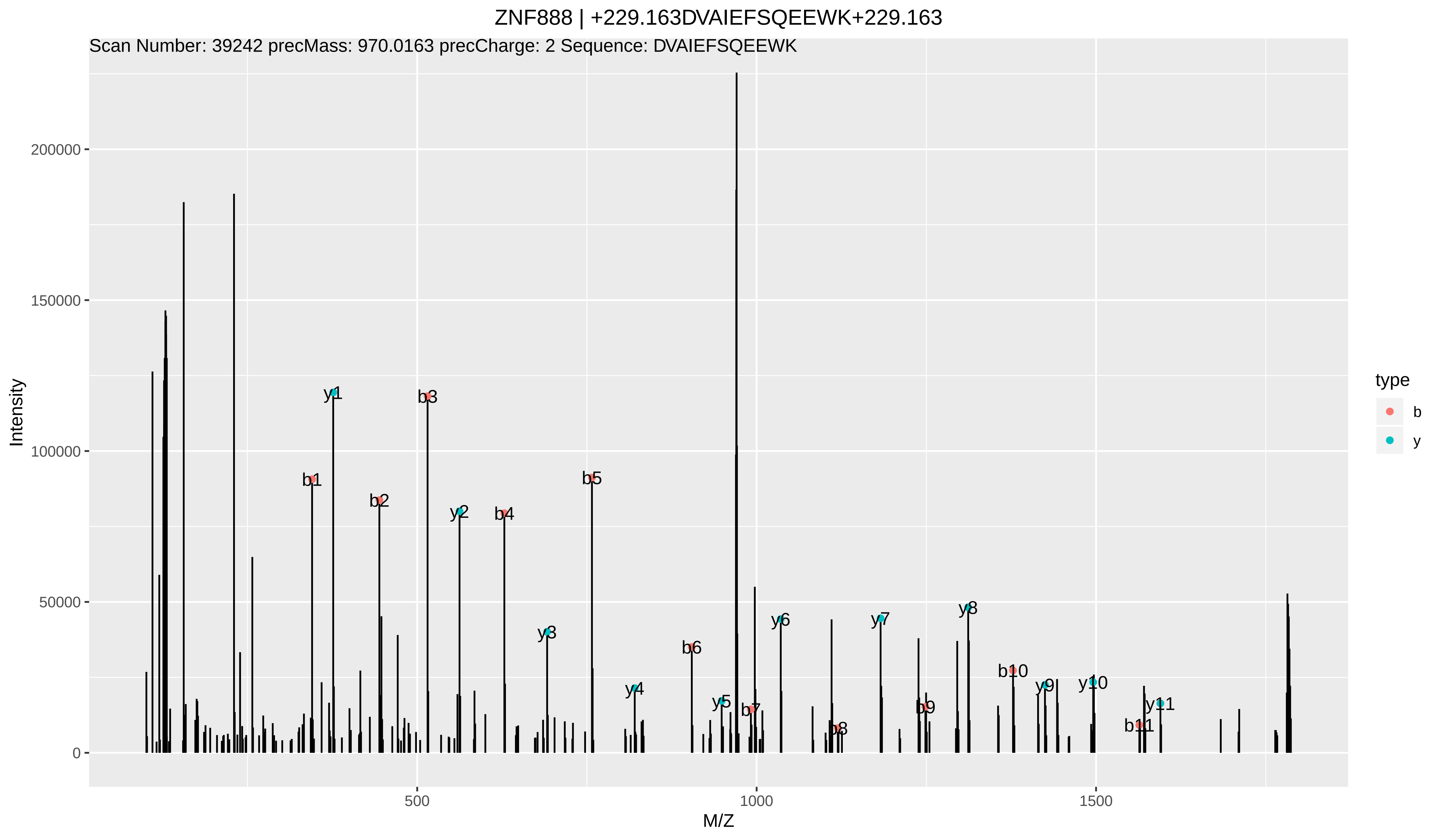

ZNF888 | +229.163DVAIEFSQEEWK+229.163
Scan Number: 39242 precMass: 970.0163 precCharge: 2 Sequence: DVAIEFSQEEWK
Intensity
M/Z
type
b
y
y1
b3
b1
b2
y2
b4
b5
y3
b6
y4
y5
b7
y6
b8
y7
b9
y8
b10
y9
y10
b11
y11
0
50000
100000
150000
200000
500
1000
1500

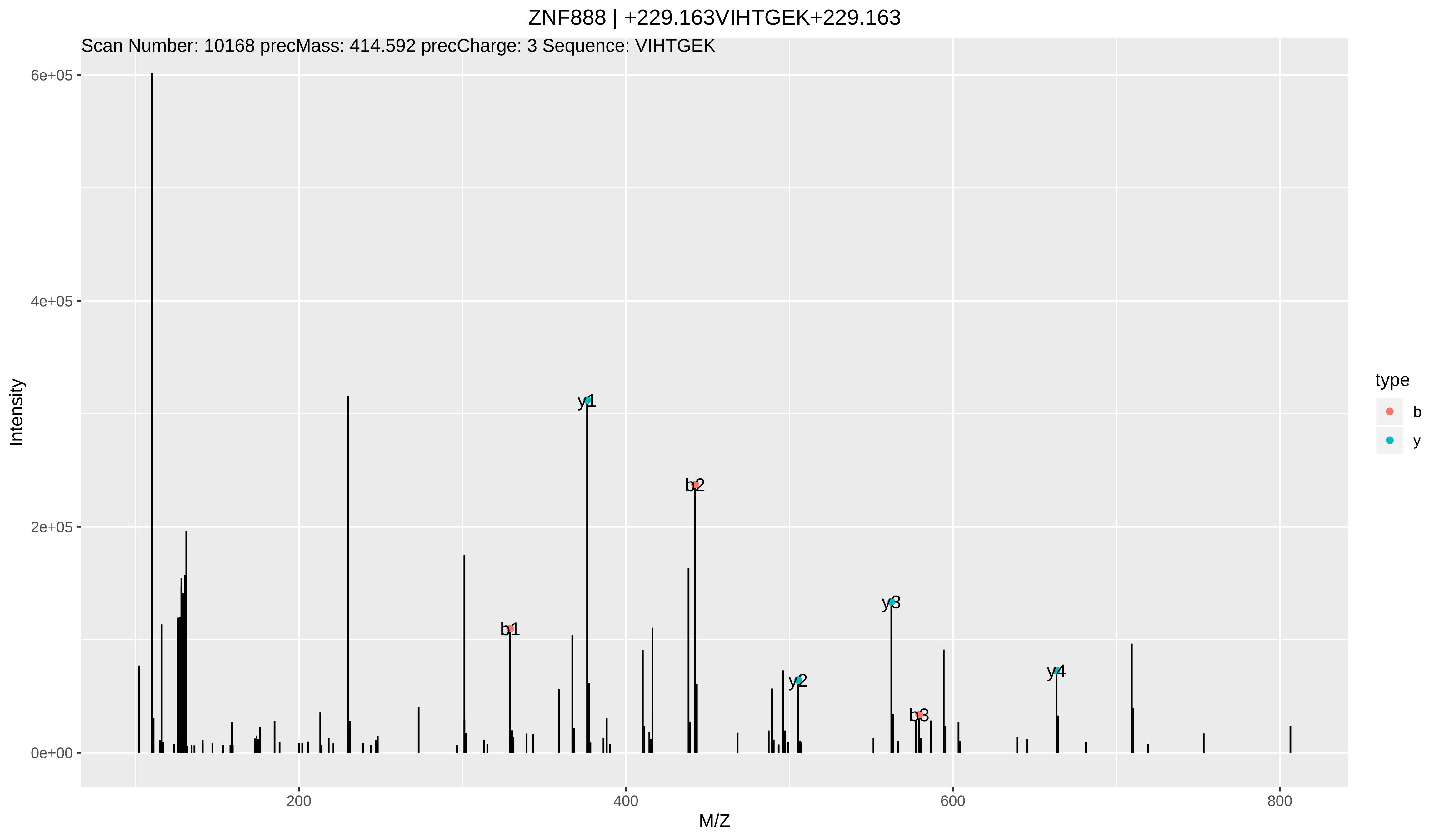
ZNF888 | +229.163VIHTGEK+229.163
Scan Number: 10168 precMass: 414.592 precCharge: 3 Sequence: VIHTGEK
Intensity
6e+05
4e+05
2e+05
0e+00
M/Z
200
400
600
800
y1
b2
b1
y3
y2
y4
b3
type
b
y

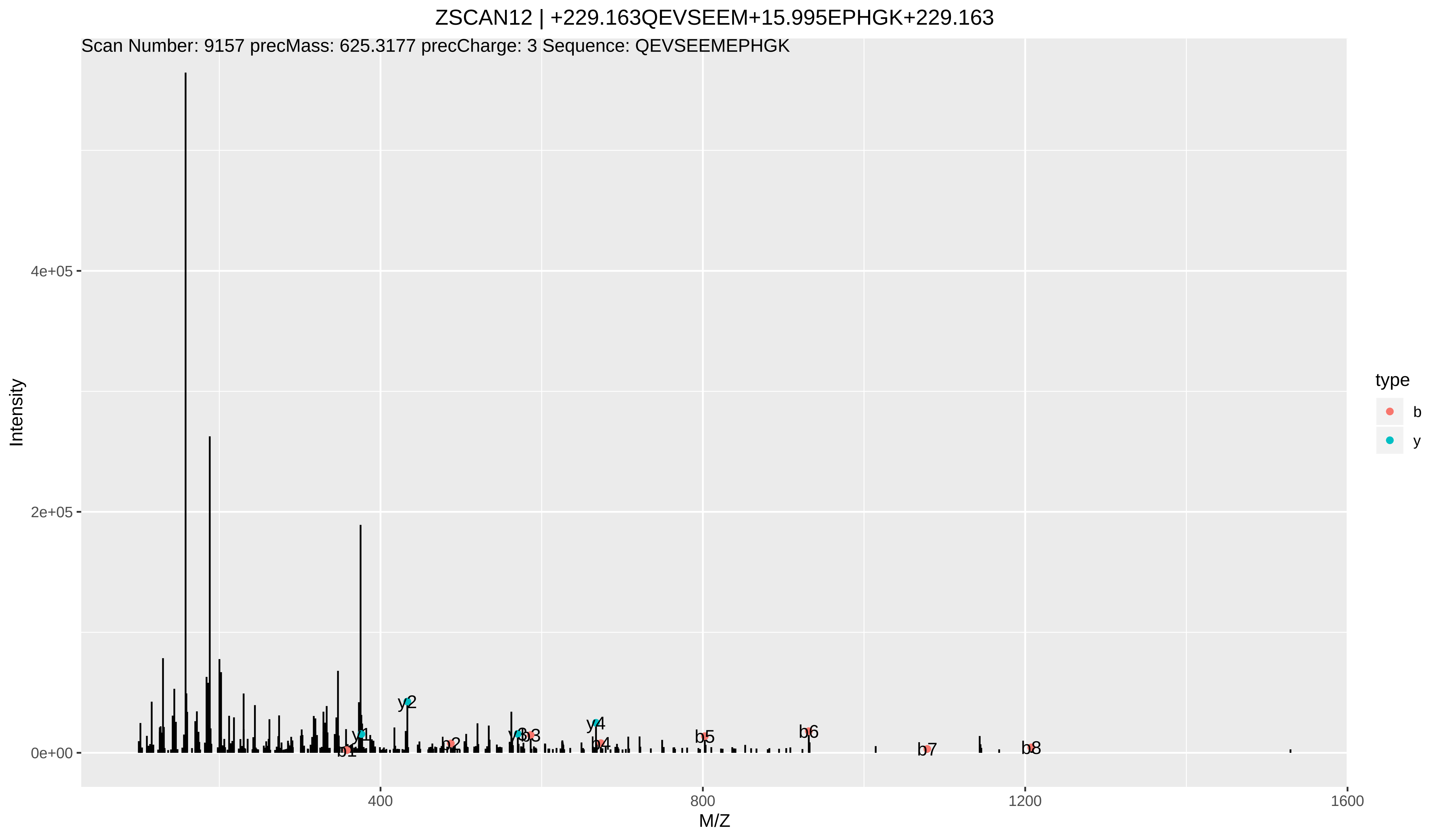
ZSCAN12 | +229.163QEVSEEM+15.995EPHGK+229.163
Scan Number: 9157 precMass: 625.3177 precCharge: 3 Sequence: QEVSEEMEPHGK
Intensity
4e+05
2e+05
0e+00
M/Z
400
800
1200
1600
type
b
y
y1
y2
y3
y4
b1
b2
b3
b4
b5
b6
b7
b8

ZSCAN12 | +229.163IHTGDRPYK+229.163

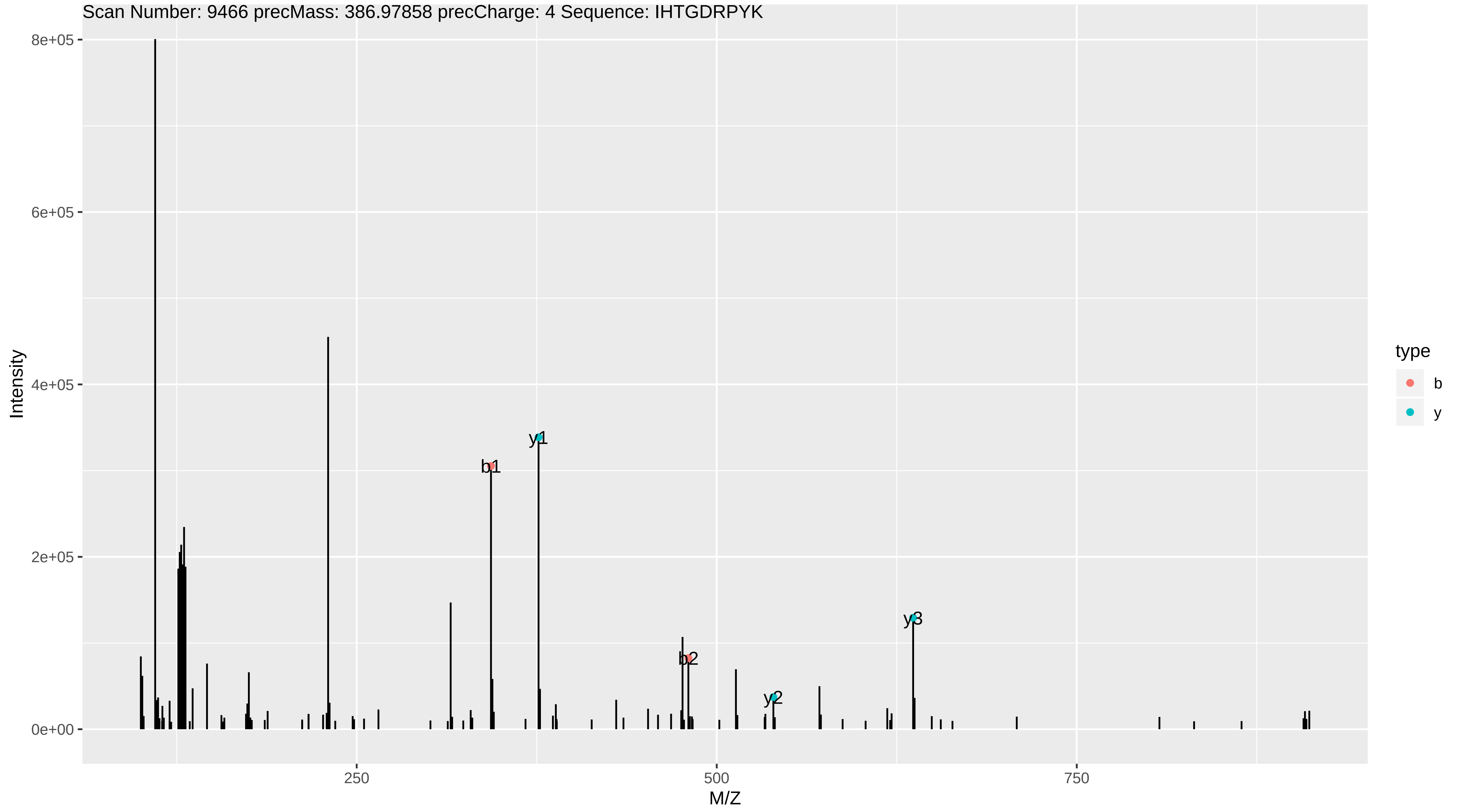

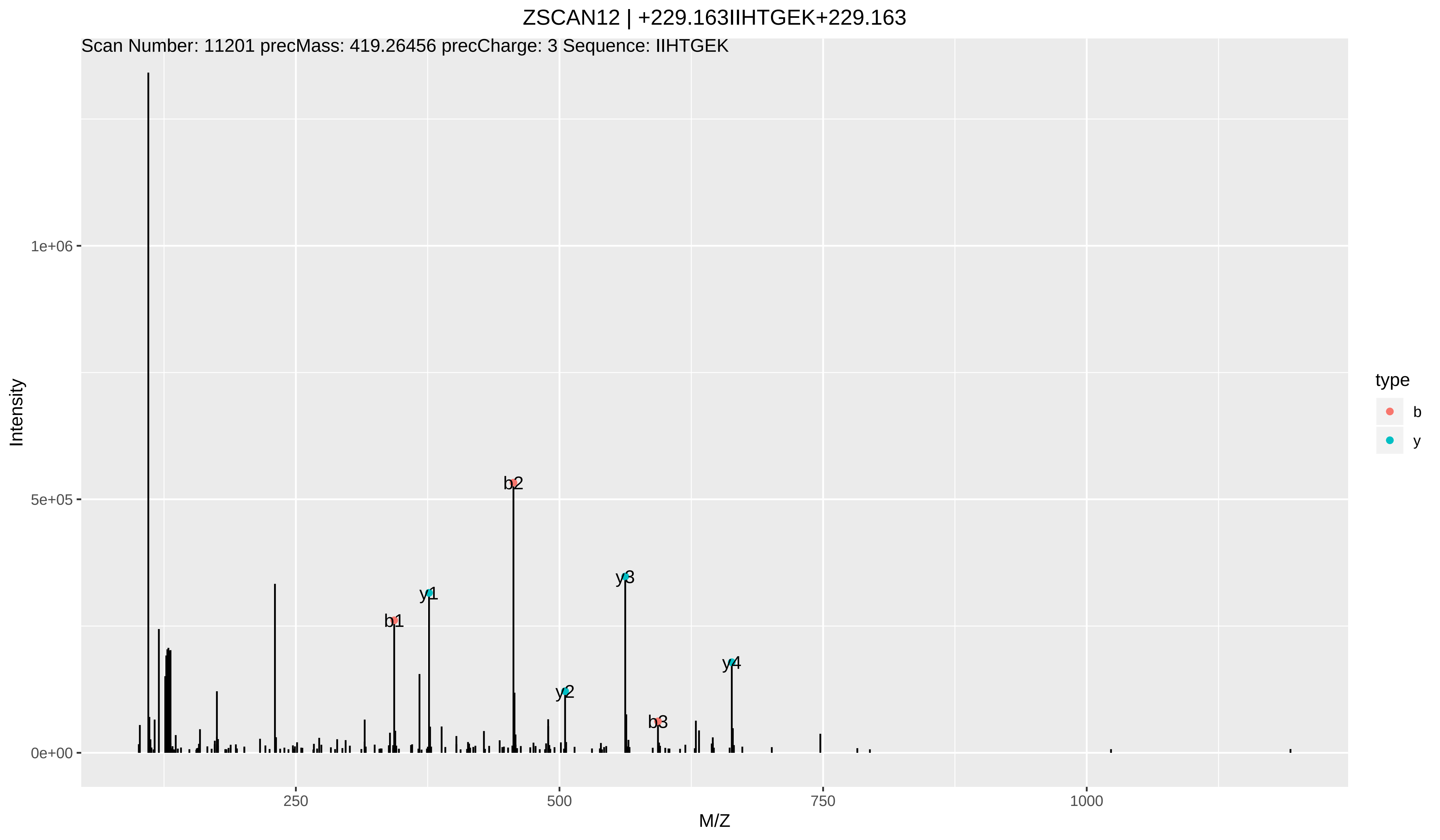
ZSCAN12 | +229.163IIHTGEK+229.163
Scan Number: 11201 precMass: 419.26456 precCharge: 3 Sequence: IIHTGEK
Intensity
1e+06
5e+05
0e+00
M/Z
250
500
750
1000
b1
y1
b2
y2
y3
b3
y4
type
b
y

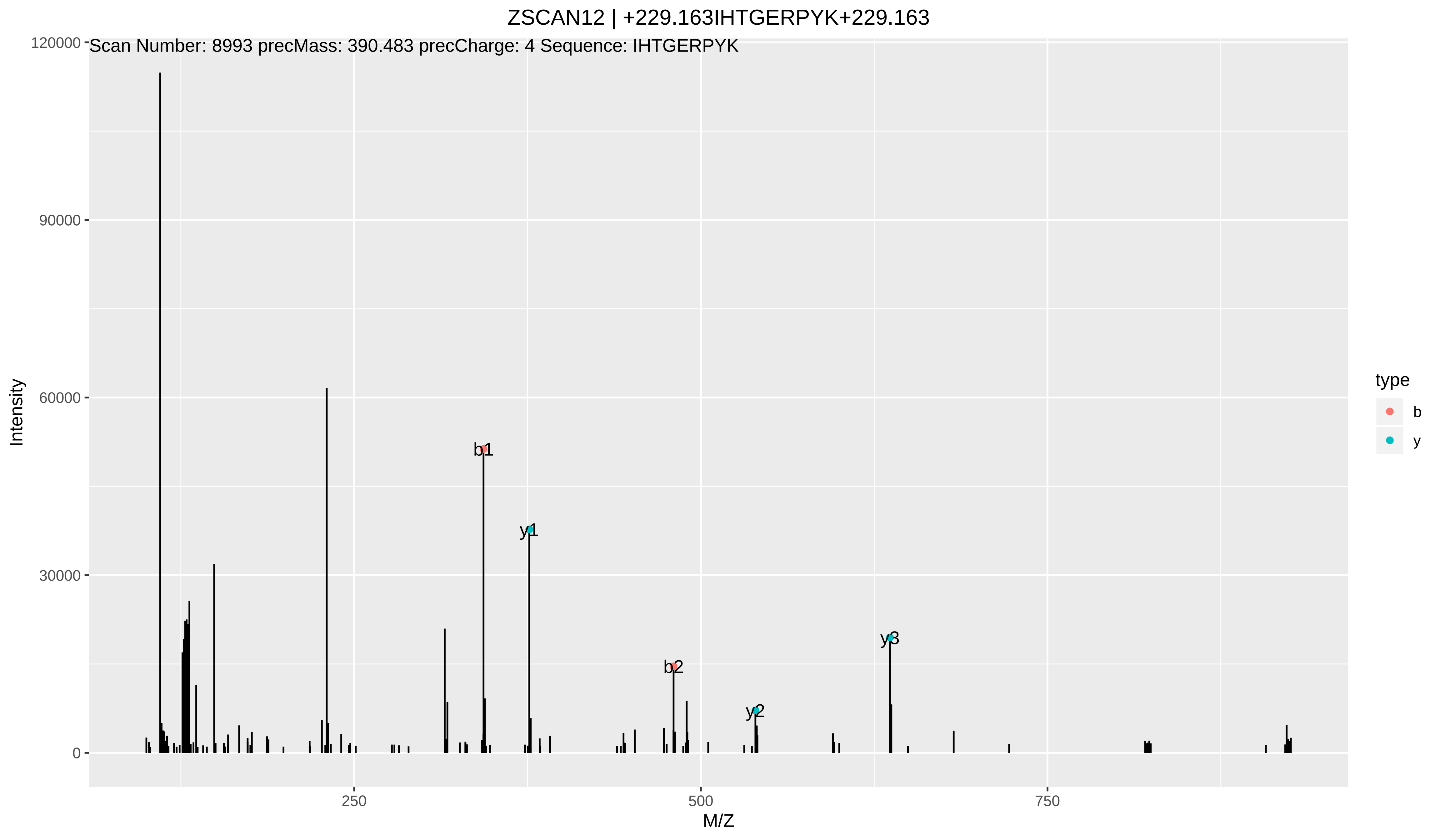
ZSCAN12 | +229.163IHTGERPYK+229.163
Scan Number: 8993 precMass: 390.483 precCharge: 4 Sequence: IHTGERPYK
Intensity
120000
90000
60000
30000
0
b1
y1
b2
y2
y3
250
500
750
M/Z
type
b
y

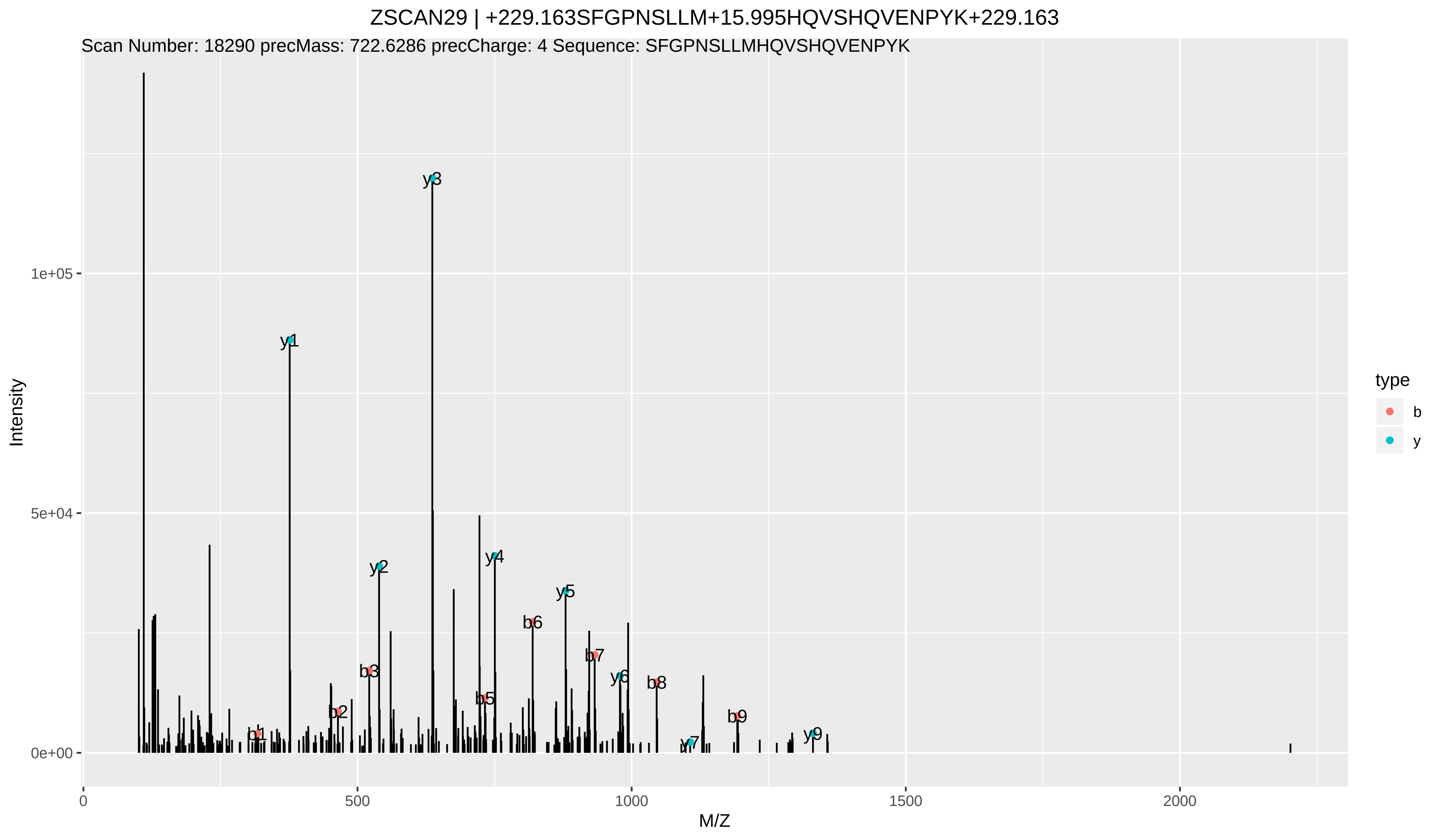
ZSCAN29 | +229.163SFGPNSLLM+15.995HQVSHQVENPYK+229.163
Scan Number: 18290 precMass: 722.6286 precCharge: 4 Sequence: SFGPNSLLMHQVSHQVENPYK
Intensity
M/Z
type
b
y
y1
y2
y3
y4
y5
y6
y7
y9
b1
b2
b3
b5
b6
b7
b8
b9
0e+00
5e+04
1e+05
0
500
1000
1500
2000

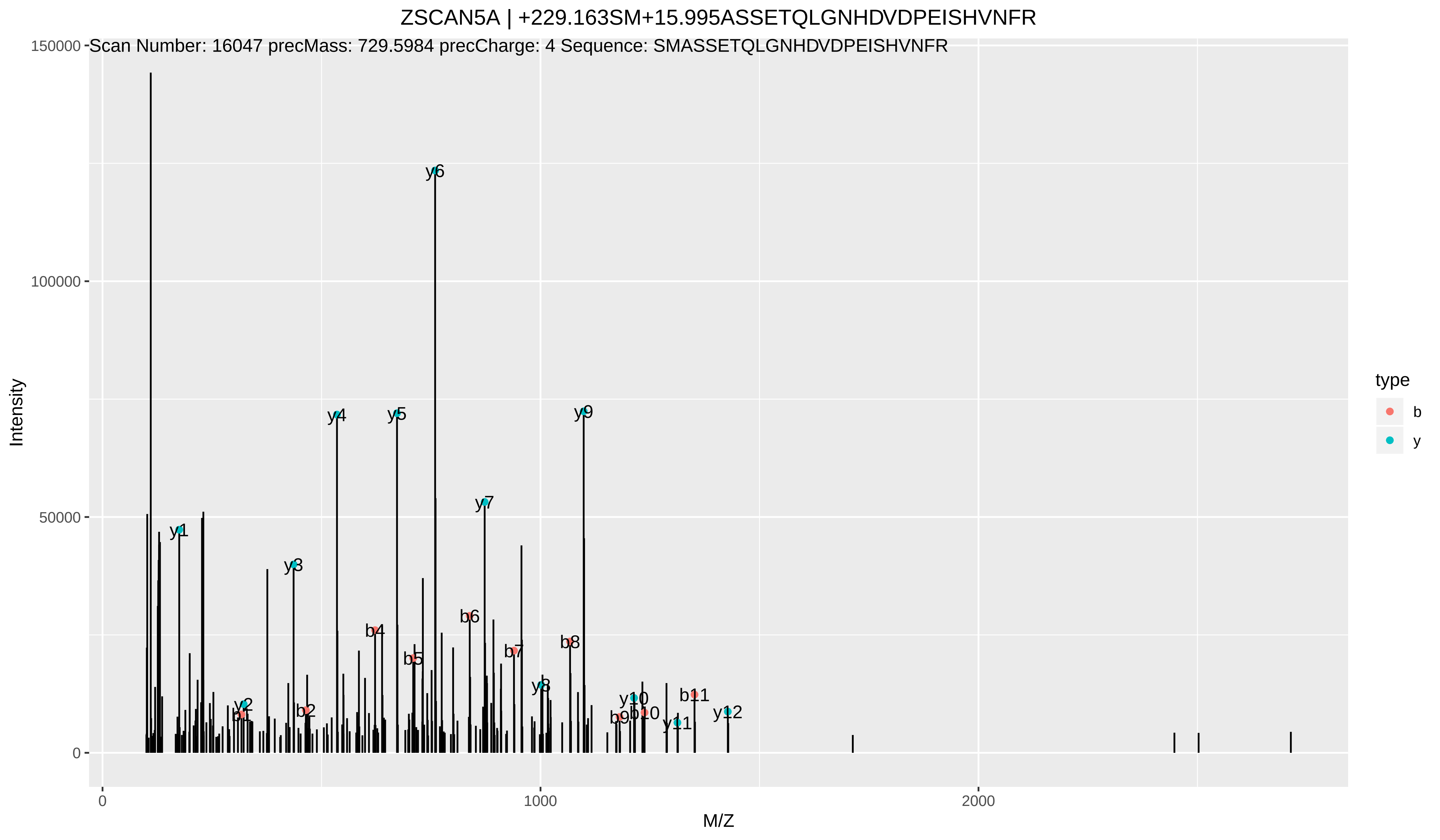
ZSCAN5A | +229.163SM+15.995ASSETQLGNHDVDPEISHVNFR
Scan Number: 16047 precMass: 729.5984 precCharge: 4 Sequence: SMASSETQLGNHDVDPEISHVNFR
Intensity
M/Z
0
50000
100000
150000
1000
2000
y1
y2
b1
y3
b2
y4
b4
y5
b5
y6
b6
y7
b7
y8
b8
y9
y10
b9
b10
y11
b11
y12
type
b
y

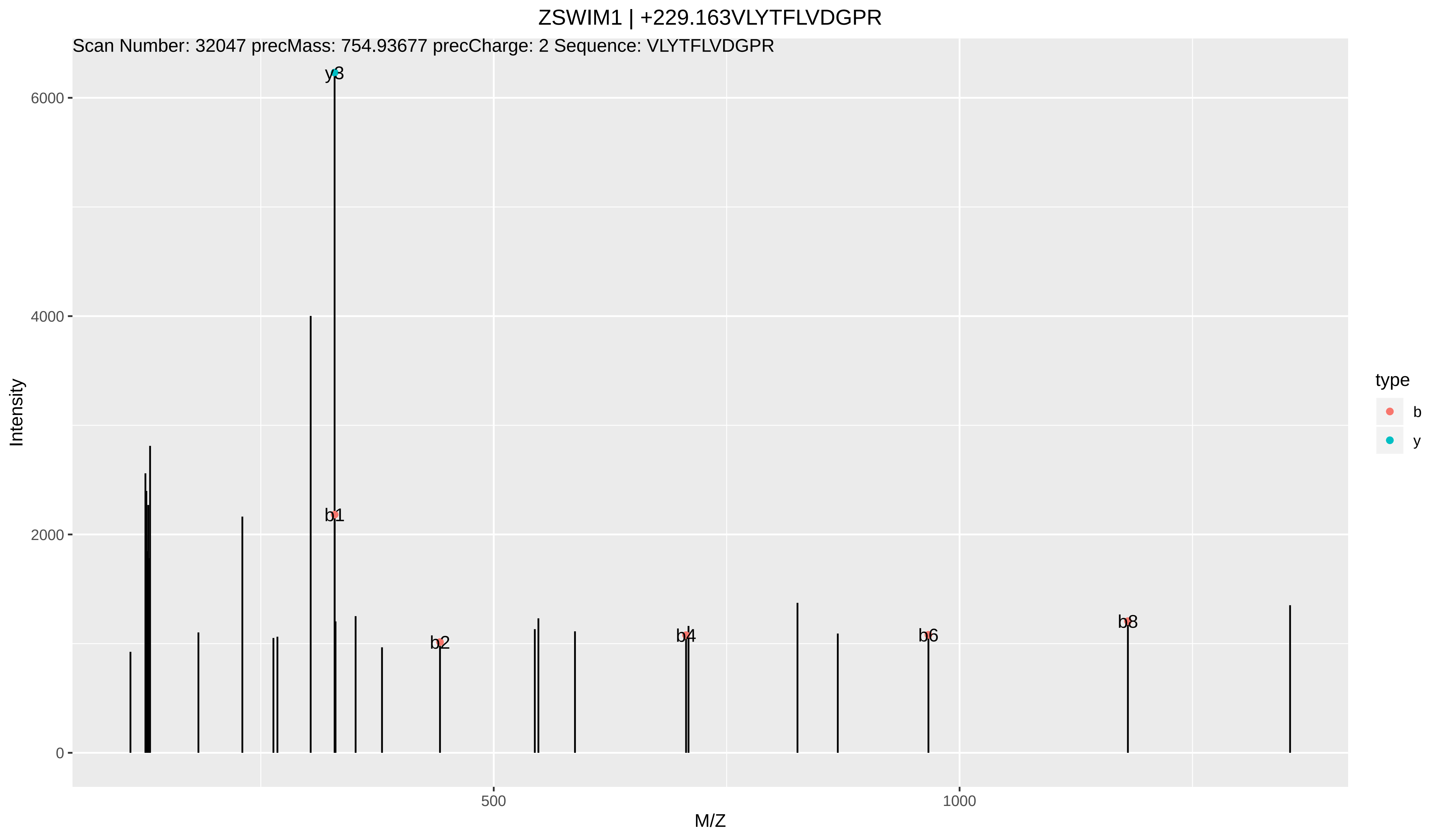

ZSWIM1 | +229.163VLYTFLVDGPR
Scan Number: 32047 precMass: 754.93677 precCharge: 2 Sequence: VLYTFLVDGPR
y3
b1
b2
b4
b6
b8
Intensity
0
2000
4000
6000
M/Z
500
1000
type
b
y

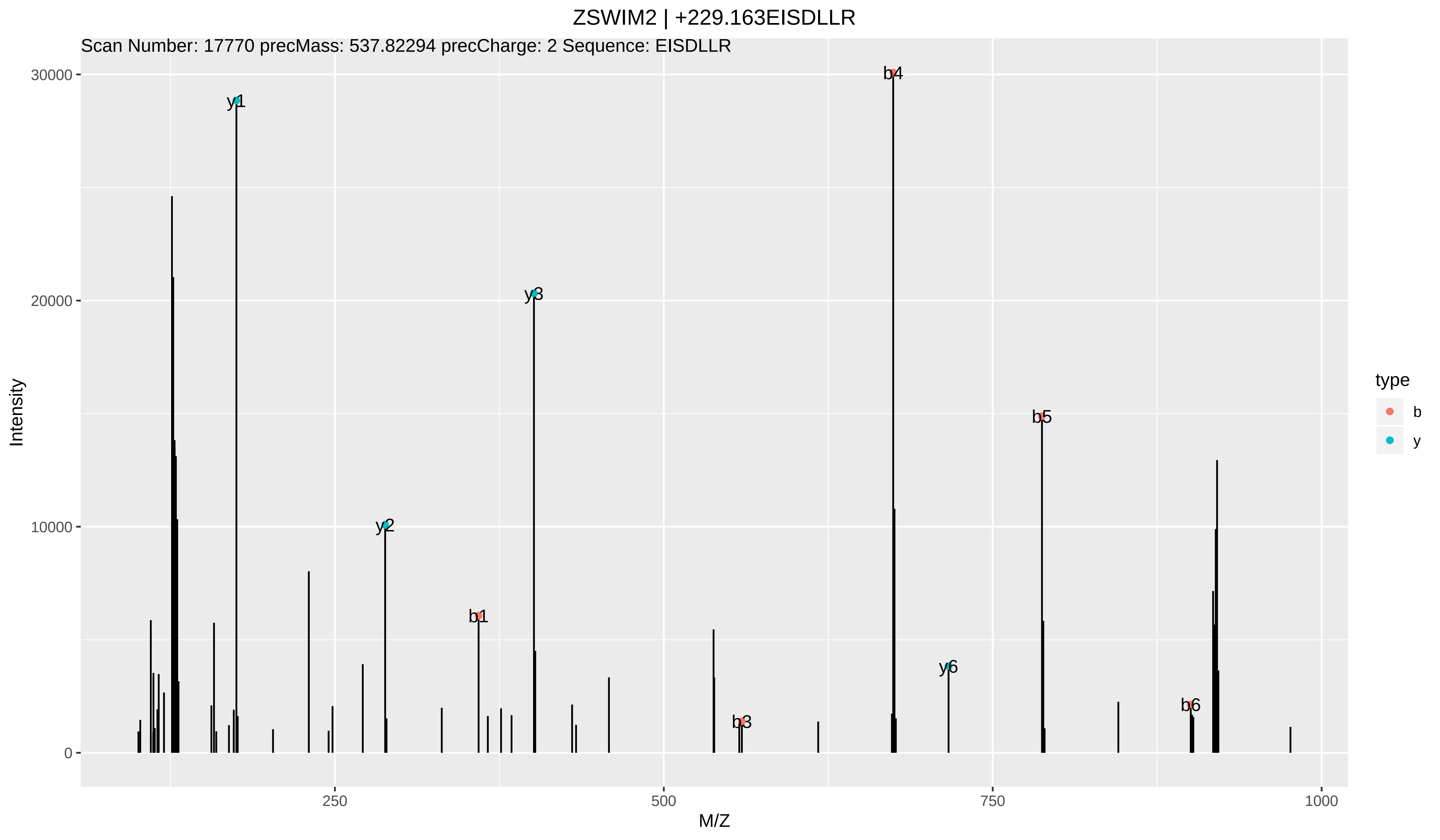
ZSWIM2 | +229.163EISDLLR
Scan Number: 17770 precMass: 537.82294 precCharge: 2 Sequence: EISDLLR
Intensity
M/Z
0
10000
20000
30000
250
500
750
1000
y1
y2
y3
y6
b1
b3
b4
b5
b6
type
b
y

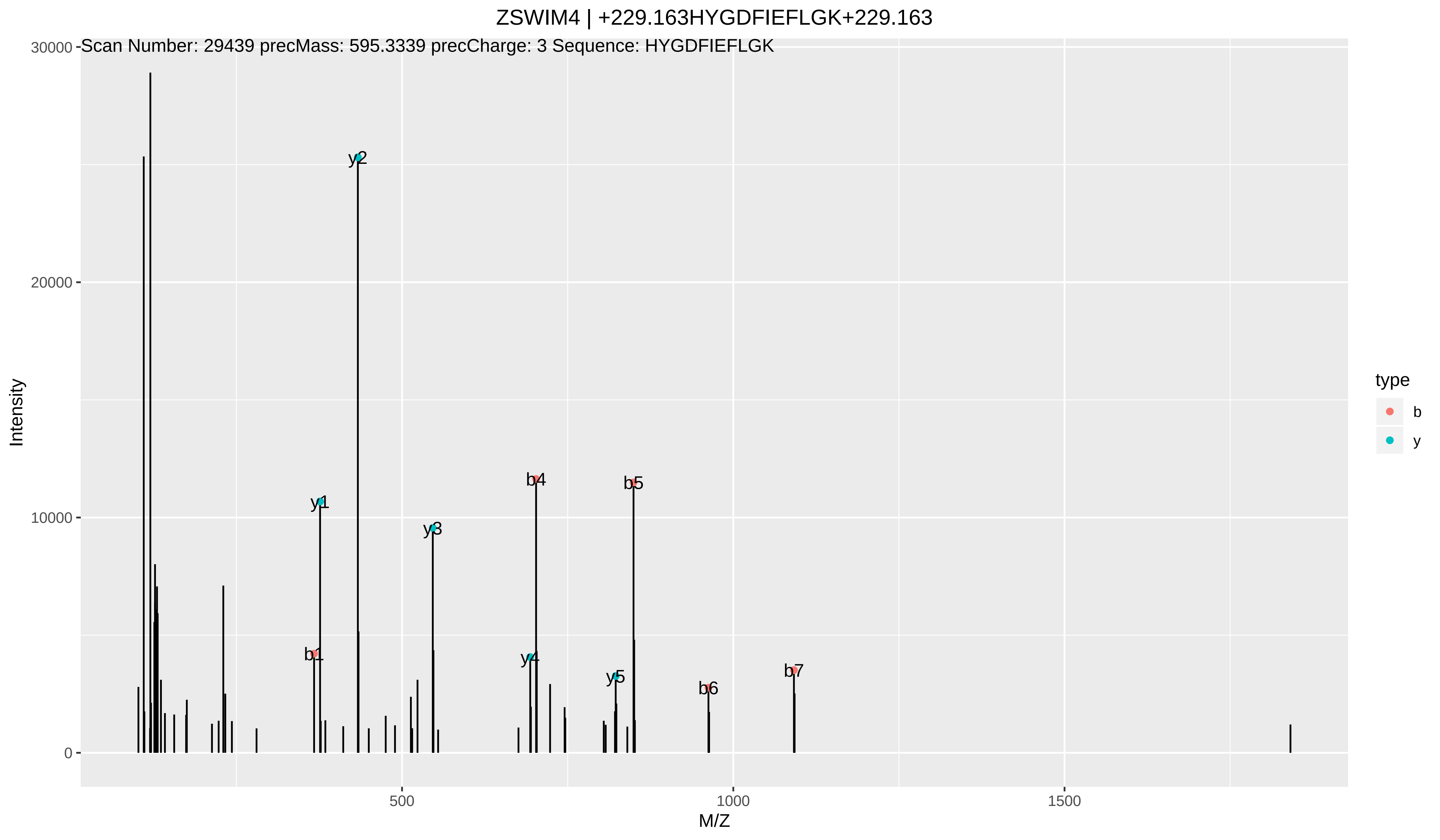
ZSWIM4 | +229.163HYGDFIEFLGK+229.163
Scan Number: 29439 precMass: 595.3339 precCharge: 3 Sequence: HYGDFIEFLGK
Intensity
M/Z
30000
20000
10000
0
500
1000
1500
y2
y1
y3
b4
b5
b1
y4
y5
b6
b7
type
b
y
